# Brain imaging derived phenotypes: a biomarker for the onset of inflammatory bowel disease and a potential mediator of mental complications

**DOI:** 10.3389/fimmu.2024.1359540

**Published:** 2024-02-26

**Authors:** Fan Li, Qi Zhao, Tongyu Tang, Yuyuan Liu, Zhaodi Wang, Zhi Wang, Xiaoping Han, Zifeng Xu, Yu Chang, Yuqin Li

**Affiliations:** ^1^ Department of Gastroenterology, The First Hospital of Jilin University, Changchun, China; ^2^ Norman Bethune Health Science Center, Jilin University, Changchun, China

**Keywords:** Mendelian randomization, imaging derived phenotypes, ulcerative colitis, Crohn’s disease, inflammatory bowel disease

## Abstract

**Background and aims:**

Inflammatory bowel disease (IBD), mainly categorized into Crohn’s disease (CD) and ulcerative colitis (UC), is a chronic relapsing gastrointestinal disorder that significantly impairs patients’ quality of life. IBD patients often experience comorbidities such as anxiety and depression, and the underlying mechanisms and treatment strategies remain areas of investigation.

**Methods:**

We conducted a Mendelian randomization(MR) analysis utilizing brain image derived phenotypes (IDP) from the UK Biobank database to investigate the causal relationships between IBD and alterations in brain structural morphology and connectivity of neural tracts. This study aimed to identify biological evidence linking IBD to psychiatric disorders such as anxiety and depression.

**Results:**

Specifically, the volume of grey matter in the Left Frontal Orbital Cortex exhibited a negative association with the onset of Crohn’s disease (odds ratio (OR) [95% confidence interval (CI)]: 0.315[0.180~0.551], adjusted P=0.001), while the volume of the superior frontal cortex in the right hemisphere showed a positive correlation with the development of Ulcerative colitis (OR [95% CI]: 2.285[1.793~2.911], adjusted P<0.001), and the volume of lateral occipital cortex in the left hemisphere demonstrated a positive relationship with Crohn’s disease onset (OR [95% CI]: 1.709[1.671~1.747], adjusted P<0.001). In the context of reverse causality, the onset of UC or CD has led to alterations in imaging derived phenotypes associated with five disorders (anxiety, depression, schizophrenia, bipolar disorder, pain) and three functions (memory, emotion, language).

**Conclusion:**

Our study has demonstrated a causal relationship between IBD and IDPs. IDPs may serve as potential biomarkers for the progression of IBD and as predictive intermediaries for the development of neurological diseases in IBD patients.

## 1 Introduction

Inflammatory Bowel Disease (IBD), encompassing ulcerative colitis (UC) and Crohn’s disease (CD), represents a chronic, relapsing autoimmune gastrointestinal disorder that has now emerged as a global health concern. Over the past 30 years, there has been a global rise in the incidence, prevalence, and mortality rates of IBD. In the next two decades, the burden of IBD is projected to persistently increase due to population growth and aging, particularly in developing countries (1).. The primary clinical manifestations of the disease include fever, abdominal pain, bloody stools, fatigue, malnutrition, as well as extraintestinal manifestations such as arthritis and uveitis, significantly impacting the quality of life. Furthermore, due to the prolonged suffering from the disease, IBD patients often have a higher susceptibility to mental disorders, including anxiety and depression (2). Current research on the gut-brain axis involves the study of IBD’s clinical presentations, such as pain, stress states, and emotional changes, inducing alterations in brain structure (3). Some voxel-based morphometry studies have demonstrated differences in certain brain structures, such as the frontal and temporal lobes and the amygdala, between patients with CD and UC and healthy individuals (4). However, due to variations in the delineation of brain regions and the different stages of disease chosen in previous research, it remains challenging to precisely determine how the disease affects brain structure. Therefore, we employed a Mendelian randomization approach to investigate the causal relationships between CD and UC and brain imaging derived phenotypes (IDPs) at the genetic level.

Mendelian randomization (MR) is an epidemiological approach that uses summary data from existing genome-wide association studies (GWAS) to identify eligible genetic variants as instrumental variables for inferring causal relationships between exposures and outcomes (5). Randomized controlled trials (RCTs) are often considered the gold standard for establishing causal relationships in observational study conclusions. However, due to the complexity of experimental design, financial constraints, ethical considerations, and other factors, RCTs are not always feasible to conduct (6). In such cases, MR serves as a valuable method for simulating RCT studies. This method is based on Mendel’s law, in which each trait is randomly allocated to independent individuals with allele variants, similar to the random allocation of individuals into control and exposure groups in an RCT. As genetic factors determine traits, the selection of appropriate instrumental variables can help mitigate the influence of common confounders on study outcomes and establish causal relationships (7).

In this study, we employed publicly available GWAS data to conduct two-sample bidirectional MR analyses to investigate the impact of UC and CD on brain IDPs. This study investigates the causal relationship between IBD and alterations in brain IDP, aiming to explore potential factors contributing to the occurrence of psychiatric symptoms in IBD patients. It emphasizes the significance of MRI in clinical practice, providing new evidence for the diagnosis of IBD and the prevention of neuropsychiatric complications.

## 2 Methods

### 2.1 Data sources

The GWAS summary data for UC and CD, stratified after inflammatory bowel disease, were obtained from the International IBD Genetics Consortium (IIBDGC). This study analyzed data from 86,640 European participants and 9,846 individuals of non-European ancestry (8), employing quality control, genotype data imputation, meta-analysis, and trans-ethnic analysis to investigate the genetic basis and heterogeneity of Crohn’s disease and ulcerative colitis in-depth. The diagnosis of IBD was based on recognized radiological, endoscopic, and histopathological evaluations. All included cases met the clinical criteria for IBD. Recruitment of study subjects was approved by the ethics committees or institutional review boards of all individual participating centers or countries. All study participants provided written informed consent. In our study, we exclusively utilized datasets of European ancestry for UC and CD. Both datasets included both male and female participants, with 6,968 UC cases and 20,464 controls in the UC dataset, and 5,956 CD cases and 14,927 controls in the CD dataset.

The GWAS summary data for brain imaging-derived phenotypes were obtained from a meta-analysis of 3,144 IDPs in 8,428 participants from the UK Biobank, including both male and female individuals (9). High-throughput sequencing technologies were employed, with filters applied to remove variants with a minor allele frequency (MAF) below 0.1% and imputation information scores below 0.3, excluding variants with Hardy-Weinberg equilibrium P-values <10^-7^ and MAF <0.1%. They identified 148 significant association clusters between single nucleotide polymorphisms (SNPs) and IDPs, with particularly notable and interpretable clusters encompassing genes related to iron transport and storage, white matter microstructure and pathology, brain development, and plasticity. For the definition of each IDP, segmentation was conducted using FreeSurfer v6.0.0 on the Desikan-Killiany-Tourville Atlas (referred to as DKT) and the Destrieux Atlas (referred to as a2009s). The UK Biobank has received approval from the Northwest Multi-center Research Ethics Committee (MREC) to acquire and disseminate participant data and samples (http://www.ukbiobank.ac.uk/ethics/), regulations that encompass the work in this study. All participants provided written informed consent. We included IDP phenotypes encompassing regional and tissue volumes (such as Cortex, thalamus, hypothalamus, basal ganglia, hippocampus and other structures), tfMRI activation, and white matter hyperintensity volume (see **Supplementary Table 1**). Our study solely extracted GWAS statistical data from previously published research, which is publicly accessible and does not require additional ethical approvals or informed consent. Based on the information from the two data set sources, there is no sample overlap in the MR analysis of this study.

### 2.2 Study approach

In this study, we utilized a European population GWAS summary datasets, conducted stratified analyses for two subtypes of inflammatory bowel disease, and performed reverse analyses by altering exposures and outcomes. We applied three MR hypotheses to select eligible instrumental variables and employed various analytical methods, including inverse variance weighting (IVW), MR-Egger regression, weighted median, weighted mode, and MR-RAPS. Sensitivity analyses, such as testing for heterogeneity, horizontal pleiotropy, and leave-one-out analysis, were performed to ensure the robustness of our results. We defined the causal direction from imaging derived phenotypes to inflammatory bowel disease as forward, and the opposite as reverse.

### 2.3 Genetic instrument selection

We selected single nucleotide polymorphisms as instrumental variables and randomized exposure factors indicated by genetic variations into groups to validate the association between exposure and outcomes.

Initially, we extracted genetic variations significantly associated with exposures at a genome-wide significance level (5×10^-8^). To ensure the retrieval of an adequate number of SNPs for further investigation, we lowered the significance level to 1×10^-6^ in forward batches. To ensure the independence of SNPs and reduce the risk of multicollinearity in the model, we filtered out SNPs with linkage disequilibrium (LD) by setting the parameters to r^2^ < 0.01 and a physical distance of 10,000 base pairs. We estimated the strength of the SNP-exposure association using the formula F = beta^2^/se^2^ and defined SNPs with F > 10 as non-weak instrumental variables, which were retained (10). When harmonizing data, we excluded unmatched ambiguous SNPs and palindromic SNPs. We queried each SNP for its phenotypic associations using PhenoScanner at a significance level of P < 5×10^-8^ and removed SNPs related to confounding factors (11). We applied MR-PRESSO to test and exclude the horizontal pleiotropy outliers.

We used MR-Steiger to verify the causal direction represented by all SNPs, and SNPs indicating the incorrect direction were removed. Finally, we applied Bonferroni multiple testing correction (P < 0.05/n, where n refers to the number of remaining SNPs) to eliminate SNPs directly associated with the outcome.

**Figure 1:**
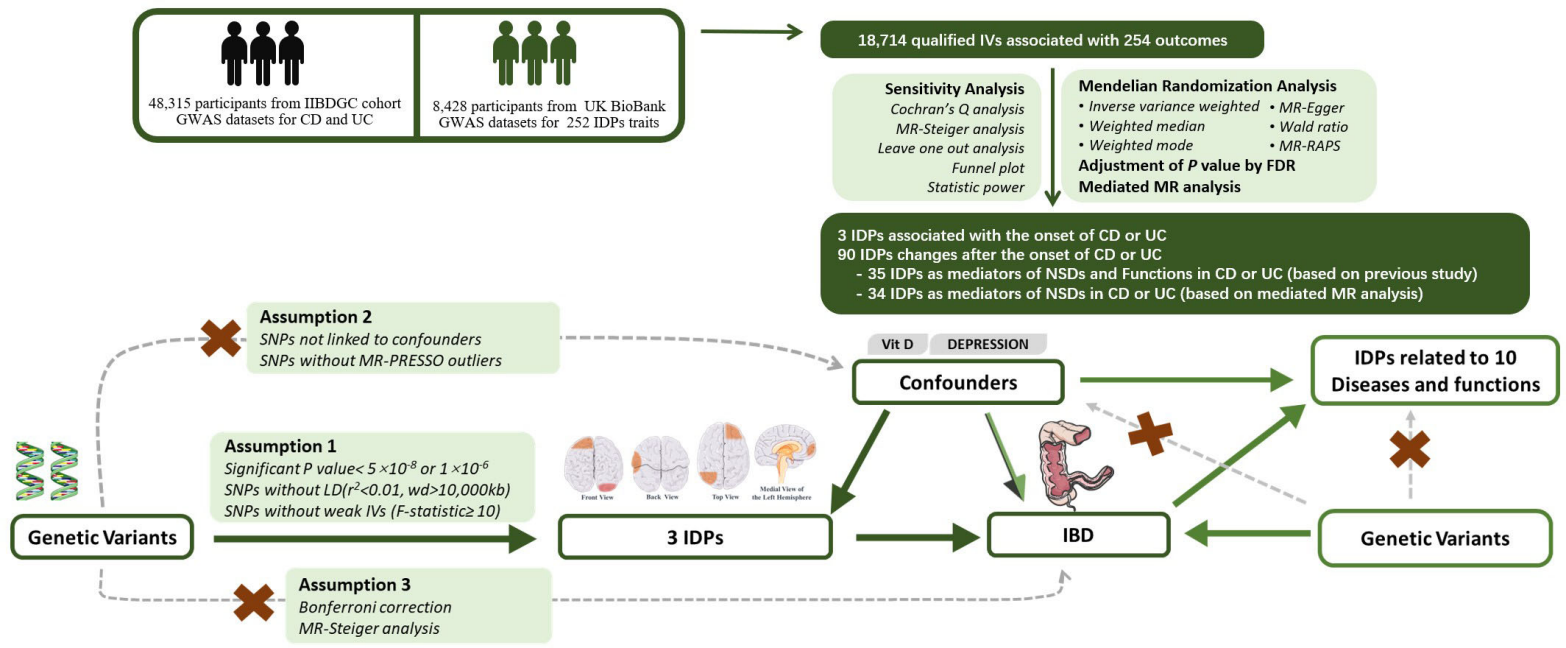
Experimental Design and Bidirectional Mendelian Randomization Directed Acyclic Graph. IDP, Imaging-derived phenotype;, IBD, Inflammatory Bowel Disease; UC, Ulcerative Colitis; CD, Crohn’s Disease; GWAS, Genome-wide Association Study; FDR, False Discovery Rate; RAPS, Randomized and Mendelian Randomization.

Through the instrumental-variable selection process described above, the eligible SNPs included in the study met the following criteria: 1) The SNP should exhibit a strong correlation with the exposure factor; 2) The SNP should only influence the outcome through the exposure factor; 3) The SNP should not be associated with confounding factors (**Figure 1**).

### 2.4 Mendelian randomization analysis

We conducted analyses using five different MR methods, and for each batch of studies, we determined a primary analytical method conclusion based on the results of sensitivity analyses, which served as the final conclusion. Inverse variance weighted is a weighted linear regression without an intercept term. It derives the final causal estimate by analyzing the Wald ratio results for each SNP (12, 13). The weights in IVW are the inverse of the genetic association variance and the outcome. Using first-order weights, IVW estimation is equivalent to the two-stage least squares (2SLS) method. According to guidelines (14), in the absence of horizontal pleiotropy and heterogeneity, we employ the IVW method with a multiplicative random-effects model as the primary analysis approach. Weighted median and weighted mode methods calculate causal effects based on the majority valid assumption and plurality valid assumption, respectively (14, 15). As long as over 50% of genetic variants are valid IVs, the median of the weighted median ratio estimate will tend towards the true causal effect. When less than 50% of variants are valid instrumental variables, weighted mode can determine the true causal effect as long as there is no larger group of invalid instrumental variables with the same ratio estimate. When there is heterogeneity among SNPs, both the weighted median method and the IVW method are required to jointly support a significant conclusion. The MR-Egger method inherently assumes the presence of horizontal pleiotropy and estimates this effect using the intercept term in the model (16). When the intercept term in the MR-Egger regression exhibits statistical significance, we consider the existence of horizontal pleiotropy, and employ MR-Egger as the primary analytical approach. The MR-RAPS method is a common modeling approach that estimates causal effects using the likelihood-based probability profile under the assumption of pleiotropy following a normal distribution centered at zero with position variance (17). The null hypothesis (H_0_) for all the aforementioned MR methods is that there is no causal relationship between exposure and outcome, whereas the alternative hypothesis (H_1_) posits a causal relationship between exposure and outcome. We applied FDR multiple testing correction to the significance P-values of MR results, using a threshold of corrected P < 0.05 to infer the likelihood of causal relationships.

### 2.5 Sensitivity analysis

To ensure the robustness of Mendelian randomization analysis results, we conducted various sensitivity analyses. We assessed heterogeneity among instrumental variables by calculating the weighted sum of squared differences between each variant estimate and the overall estimate using Cochran’s Q test. SNP with a Q test P-value < 0.05 were considered heterogeneous. We estimated the overall causal direction using MR-Steiger (18). To determine if any strongly influential SNPs were present, we conducted a leave-one-out sensitivity test. The MR-Egger regression assumes adherence to the InSIDE (Instrument Strength Independent of Direct Effect) assumption and the NOME (No Measurement Error) hypothesis. We generated a funnel plot and calculated the I2 statistic to validate the adherence to these assumptions. When I2 is less than 90% and the primary analytical method is MR-Egger, a correction for causal estimates is necessary (16, 19). Finally, we computed the statistical power for each batch of studies and excluded positive conclusions with power less than 80% to reduce the occurrence of statistical Type II errors (20).

### 2.6 Two-step mediation analysis

Following recommendations, we employed the neuropsychiatric GWAS dataset from FinnGen to conduct a two-step mediation analysis, which investigated the mediating effects of various IDPs on six neuropsychiatric disorders caused by UC or CD, and calculated the mediation effect ratio.

### 2.7 Visualization and statistical software

For each batch of MR analyses in this study, scatterplots, regression curve graphs, and forest plots of SNP effects were generated, which will be presented in the results. Circular heatmaps and forest plots for the overall results were also created. Some of the figures were created using Servier Medical Art (smart.servier.com), provided by Servier, and are licensed under a Creative Commons Attribution 3.0 unported license. Statistical analysis and visualization in this study were performed using R (version 4.1.2), with the application of R packages such as “TwoSampleMR,” “MR-PRESSO,” “mr.raps,” “forestploter,” and several foundational R packages. Flowcharts were created using Microsoft Office Powerpoint (version 2312).

## 3 Results

### 3.1 Selection of instrumental variables

In the forward MR analysis, we initially screened a total of 916 SNPs related to the IDPs. No weak instrument variables were identified. 8 SNPs were removed due to missing data in the outcome datasets, 115 SNPs were excluded as ambiguous or palindromic SNPs when harmonizing two datasets, and 7 SNPs, identified through PhenoScanner, were found to be related to confounding factors such as serum vitamin D levels (21) and depression (2), and were subsequently excluded (see **Supplementary Table 4**). MR-PRESSO test revealed 8 SNPs with horizontal pleiotropy. MR-Steiger test did not identify any SNPs with erroneous causal direction. After Bonferroni correction, 26 SNPs directly related to the outcome were removed. Ultimately, 752 eligible SNPs were included in the study (for detailed SNP data, see **Supplementary Table 3**).

In the reverse MR analysis, we initially screened a total of 23,000 SNPs related to the IDPs. No weak instrument variables were identified. 252 SNPs were removed due to missing data in the outcome database, 3,499 SNPs were excluded as ambiguous or palindromic SNPs when harmonizing two datasets, and 1,250 SNPs were excluded after a PhenoScanner search, as they were associated with confounding factors. MR-PRESSO test identified 10 SNPs with horizontal pleiotropy. MR-Steiger test found no SNPs with erroneous causal directions. After Bonferroni correction, 27 SNPs directly related to the outcome were removed. Ultimately, 17,962 eligible SNPs were included in the study.

### 3.2 IDPs as the cause of IBD onset

**Figure 2:**
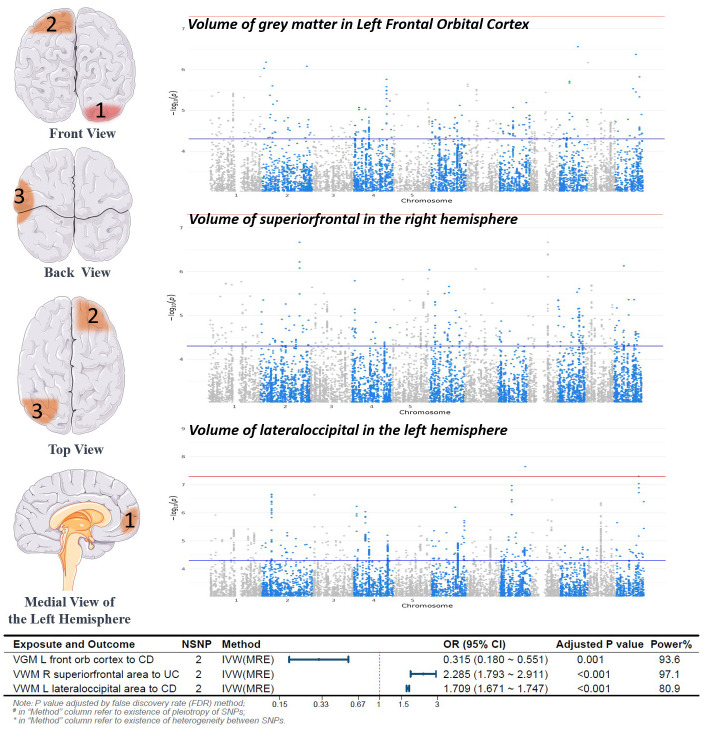
Forward Mendelian Randomization Analysis Results and Brain Region Schematic. Notes: Top left panel, Brain Region Schematic; Top right panel, Manhattan plot of positive IDP-associated GWAS data; Bottom panel, Forest plot of positive MR analysis. VGM, Volume of Gray Matter; VWM, Volume of White Matter; L, Left; R, Right; CD, Crohn’s Disease; UC, Ulcerative Colitis; IVW, Inverse Variance Weighted; MRE, Multiplicative Random Effects Model; OR, Odds Ratio; CI, Confidence Interval; NSNP, Number of SNPs included in the study.

In this study, 252 IDPs were included in the analysis, including regional and tissue volume, tfMRI activation, and white matter hyperintensity volume. The number of SNPs for each IDP ranged from 1 to 9, and the detailed information for the IVs of the 252 IDPs is provided in the **Supplementary Table**. Through MR analysis, we identified 3 types of IDPs associated with the causality of CD or UC subtypes. Analyses for these batches could not calculate heterogeneity and pleiotropy due to fewer than 3 SNPs. Specifically, for each 1 standard deviation (SD) increase in the Volume of grey matter (VGM) in the Left Frontal Orbital Cortex, the risk of CD decreased by 68% (odds ratio (OR) [95% confidence interval (CI)]: 0.315[0.180~0.551], adjusted P=0.001). For each 1 SD increase in the Volume of superior frontal in the right hemisphere, the risk of UC increased by 129% (OR [95% CI]: 2.285[1.793~2.911], adjusted P<0.001). Moreover, for each 1 SD increase in the Volume of lateral occipital in the left hemisphere, the risk of CD increased by 71% (OR [95% CI]: 1.709[1.671~1.747], adjusted P<0.001). The results of the MR analysis are depicted in **Figure 2**. Detailed information on the other batches is shown in the **Supplementary Materials**.

### 3.3 IBD promotes changes in mental complications and function-related IDPs

**Figure 3:**
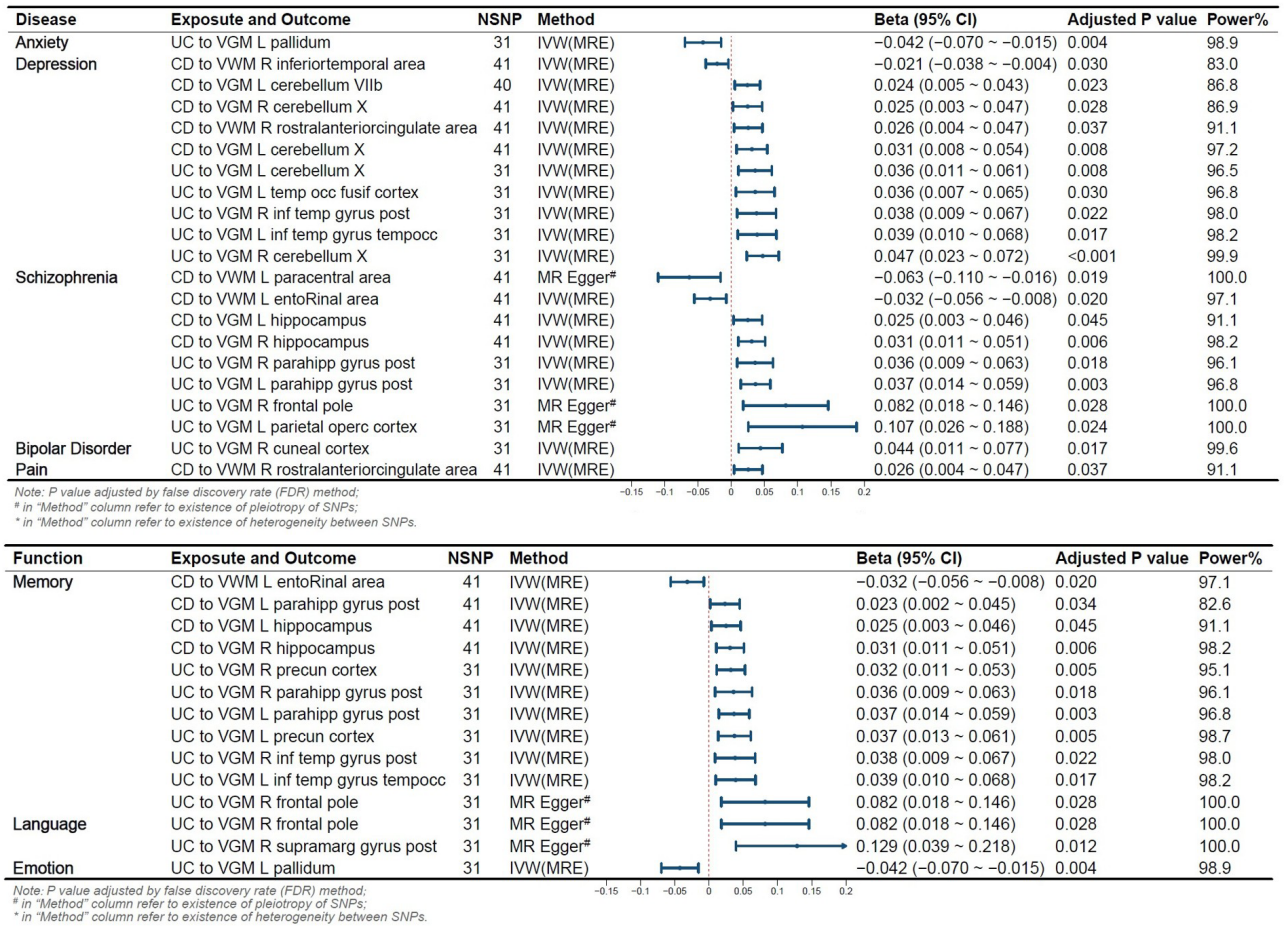
Reverse Mendelian Randomization Forest Plot. Annotation: Several IDPs are associated with two or more functions or diseases. VGM, Gray Matter Volume; VWM, White Matter Volume; L, Left; R, Right; CD, Crohn’s Disease; UC, Ulcerative Colitis; IVW, Inverse Variance Weighted; MRE, Multiplicative Random Effects Model; OR, Odds Ratio; CI, Confidence Interval; NSNP, Number of SNPs included in the study.

We conducted a functional query in the PubMed database for IDPs significantly altered due to the onset of IBD and found that the onset of UC or CD led to changes in IDPs related to five disorders (anxiety, depression, schizophrenia, bipolar disorder, pain) and three functions (memory, emotion, language)(See in **Figure 3**).

#### 3.3.1 IDPs classified by mental complications

The pallidum is associated with emotion regulation and contributes to the pathogenesis of anxiety disorders (22), with its volume changing in relation to the onset risk of UC. We identified 10 IDPs located within 3 brain regions (temporal lobe, cerebellum, anterior cingulate cortex) associated with CD and UC. Prior studies have shown alterations in low-frequency oscillatory amplitudes of the cerebellum and inferior temporal lobe, an index reflecting spontaneous brain activity, in patients with depression (23). We observed that 8 IDPs located in the brain regions of the parietal lobe, frontal lobe, paracentral lobule, olfactory-insular-hippocampus, and hippocampal parahippocampus were affected by the onset of CD and UC. The parietal lobe is associated with visual information processing, the frontal lobe is linked to decision-making and emotion processing, the olfactory-insular-hippocampus region is involved in memory, and the paracentral lobule is implicated in sensory and motor control. Previous research suggests their potential involvement in the onset of schizophrenia (24, 25). We also observed that UC onset altered 1 IDP associated with Bipolar Disorder, and CD onset altered 1 IDP related to pain.

#### 3.3.2 IDPs classified by function

Eleven IDPs located in 5 brain structures (wedge-shaped lobe, inferior temporal lobe, olfactory-hippocampal cortex, subhippocampal cortex, frontal lobe) are influenced by the risk of IBD onset. The olfactory-hippocampal cortex is a recognized vital component involved in memory formation (26). The frontal lobe, inferior temporal lobe, and wedge-shaped lobe have also been shown to play a role in visual memory formation (27). CD altered 4 IDPs, such as Volume of entorhinal in the left hemisphere, and UC modified 7 IDPs, such as VGM in Right Precuneous Cortex. Two IDPs located in 2 brain structures (supramarg gyrus post, frontal lobe) are influenced by UC onset, and these areas are considered as functional regions for language (28), specifically, VGM in the Right supramarg gyrus post and the right frontal pole. The pallidum, as a component of the limbic system, is involved in the regulation of emotions. Our results revealed that UC altered the VGM in the Left Pallidum.

According to Cochran’s Q test and MR-Egger interception test, some batches exhibited heterogeneity, with no evidence of potential heterogeneity or multiple effects biasing our findings (see **Table 1**). For the functional origins of brain volume, refer to **Supplementary Table 8**.

**Table 1:** Sensitivity analysis results.

Batch	Exposures and Outcomes	NSNP	Q from IVW	Pval_Q from IVW	Q from MR-Egger	Pval_Q from MR-Egger	I2 for MR-Egger	Pval of pleotropy	Dirction from MR-Steiger	LOO
66	UC to VGM L pallidum	31	22.071	20.336	0.851	0.882	0.985	0.198	TRUE	TRUE
777	CD to VWM R inferiortemporal area	41	34.649	34.366	0.709	0.681	0.980	0.598	TRUE	TRUE
115	CD to VGM L cerebellum VIIb	40	30.007	30.007	0.849	0.819	0.982	0.977	TRUE	TRUE
145	CD to VGM R cerebellum X	41	38.627	38.600	0.532	0.488	0.977	0.869	TRUE	TRUE
815	CD to VWM R rostralanteriorcingulate area	41	41.580	40.376	0.402	0.409	0.980	0.288	TRUE	TRUE
141	CD to VGM L cerebellum X	41	42.373	42.368	0.369	0.328	0.979	0.944	TRUE	TRUE
142	UC to VGM L cerebellum X	31	22.805	21.520	0.823	0.839	0.984	0.266	TRUE	TRUE
10	UC to VGM L temp occ fusif cortex	31	31.680	31.427	0.383	0.346	0.988	0.633	TRUE	TRUE
1994	UC to VGM R inf temp gyrus post	31	31.875	31.179	0.373	0.357	0.988	0.428	TRUE	TRUE
2016	UC to VGM L inf temp gyrus tempocc	31	29.705	28.759	0.481	0.478	0.989	0.339	TRUE	TRUE
146	UC to VGM R cerebellum X	31	21.093	20.767	0.885	0.867	0.988	0.573	TRUE	TRUE
559	CD to VWM L paracentral area	41	41.390	34.856	0.410	0.659	0.987	0.015	TRUE	TRUE
535	CD to VWM L entoRinal area	41	42.154	39.029	0.378	0.469	0.990	0.085	TRUE	TRUE
71	CD to VGM L hippocampus	41	47.030	46.911	0.207	0.180	0.990	0.755	TRUE	TRUE
73	CD to VGM R hippocampus	41	40.871	40.680	0.432	0.396	0.990	0.671	TRUE	TRUE
2724	UC to VGM R parahipp gyrus post	31	25.244	23.767	0.713	0.740	0.987	0.234	TRUE	TRUE
2722	UC to VGM L parahipp gyrus post	31	17.564	17.445	0.965	0.955	0.976	0.733	TRUE	TRUE
496	UC to VGM R frontal pole	31	25.818	20.749	0.684	0.868	0.988	0.032	TRUE	TRUE
28	UC to VGM L parietal operc cortex	31	34.214	29.482	0.272	0.440	0.990	0.039	TRUE	TRUE
2710	UC to VGM R cuneal cortex	31	39.940	34.912	0.106	0.207	0.992	0.050	TRUE	TRUE
2721	CD to VGM L parahipp gyrus post	41	36.594	35.536	0.624	0.629	0.984	0.310	TRUE	TRUE
2704	UC to VGM R precun cortex	31	21.482	21.163	0.872	0.853	0.982	0.577	TRUE	TRUE
2682	UC to VGM L precun cortex	31	27.799	24.819	0.581	0.688	0.989	0.095	TRUE	TRUE
2216	UC to VGM R supramarg gyrus post	31	39.002	30.882	0.126	0.371	0.990	0.010	TRUE	TRUE

Batches with fewer than 3 included SNPs are not eligible for heterogeneity and pleiotropy analysis. VGM, Gray Matter Volume; VWM, White Matter Volume; L, Left; R, Right; CD, Crohn’s Disease; UC, Ulcerative Colitis; IVW, Inverse Variance Weighted; MRE, Multiplicative Random Effects Model; OR, Odds Ratio; CI, Confidence Interval; NSNP, Number of SNPs included in the study; LOO, Leave-One-Out test.

### 3.4 Two-step mediation analysis

Through Mendelian randomization mediation analysis, we identified 34 IDPs as mediators for CD or UC in relation to six neuropsychiatric disorders. Among these mediators, the majority were complete mediating effects, with only MO Superior fronto-occipital fasciculus R and T1 FAST ROIsR parahippocampal gyrus post exhibiting partial mediating effects for UC-mediated pain (see **Table 2**).

**Table 2:** Results of two-step Mendelian randomization mediation analysis.

Exposure	Mediator	Outcome	X-Y		X-M		M-Y		Type	Effect rate
			OR 95%CI	P	OR 95%CI	P	OR 95%CI	P		
CD	T1 FAST ROIs L ventral striatum	Alzheimer’s disease	0.992(0.949~1.037)	0.717	1.021(1.003~1.040)	0.023	2.162(1.390~3.365)	0.027	TM	100%
CD	T1 FAST ROIs L cerebellum VIIb	Alzheimer’s disease	0.992(0.949~1.037)	0.717	1.025(1.005~1.044)	0.012	1.194(1.021~1.396)	0.027	TM	100%
CD	L2 Sagittal stratum L	Alzheimer’s disease	0.992(0.949~1.037)	0.717	0.972(0.951~0.992)	0.007	0.862(0.750~0.992)	0.038	TM	100%
UC	T1 SIENAX peripheral grey normalised volume	Alzheimer’s disease	0.974(0.940~1.010)	0.160	1.142(1.069~1.221)	0.001	1.591(1.062~2.383)	0.024	TM	100%
UC	T1 FAST ROIs L temp occ fusif cortex	Alzheimer’s disease	0.974(0.940~1.010)	0.160	1.037(1.007~1.067)	0.015	1.612(1.523~1.707)	<0.001	TM	100%
UC	T1 SIENAX peripheral grey unnormalised volume	Alzheimer’s disease	0.974(0.940~1.010)	0.160	1.096(1.047~1.147)	<0.001	1.969(1.089~3.558)	0.025	TM	100%
UC	ProbtrackX FA ml r	Alzheimer’s disease	0.974(0.940~1.010)	0.160	1.050(1.018~1.083)	0.002	1.244(1.040~1.489)	0.017	TM	100%
UC	ProbtrackX OD slf l	Alzheimer’s disease	0.974(0.940~1.010)	0.160	0.966(0.938~0.995)	0.020	0.844(0.718~0.991)	0.039	TM	100%
CD	L G&S paracentral area	Anxiety disorders	0.999(0.984~1.014)	0.889	0.940(0.894~0.987)	0.018	0.811(0.748~0.879)	<0.001	TM	100%
CD	MO Corticospinal tract R	Anxiety disorders	0.999(0.984~1.014)	0.889	0.968(0.943~0.993)	0.014	0.963(0.953~0.972)	<0.001	TM	100%
UC	T1 FAST ROIs L temp occ fusif cortex	Anxiety disorders	1.002(0.982~1.022)	0.847	1.037(1.007~1.067)	0.015	1.196(1.062~1.346)	0.003	TM	100%
CD	FA Sagittal stratum L	Bipolar disorders	1.087(0.999~1.183)	0.060	1.032(1.010~1.055)	0.005	1.329(1.278~1.382)	<0.001	TM	100%
CD	L2 Sagittal stratum L	Bipolar disorders	1.087(0.999~1.183)	0.060	0.972(0.951~0.992)	0.007	0.799(0.723~0.883)	<0.001	TM	100%
CD	ProbtrackX FA mcp	Bipolar disorders	1.087(0.999~1.183)	0.060	1.029(1.004~1.054)	0.023	1.237(1.107~1.382)	<0.001	TM	100%
CD	R S collat transv ant area	Pain	1.005(0.995~1.015)	0.327	0.936(0.888~0.986)	0.017	0.922(0.913~0.931)	<0.001	TM	100%
CD	ProbtrackX MO unc r	Pain	1.005(0.995~1.015)	0.327	0.924(0.871~0.980)	0.012	0.963(0.929~0.997)	0.034	TM	100%
UC	MO Superior fronto-occipital fasciculus R	Pain	1.019(1.008~1.030)	0.001	1.142(1.035~1.260)	0.013	1.062(1.025~1.101)	0.001	PM	42.43%
UC	T1 FAST ROIsR parahipp gyrus post	Pain	1.019(1.008~1.030)	0.001	1.037(1.009~1.065)	0.009	1.066(1.053~1.079)	<0.001	PM	12.33%
CD	T1 FAST ROIs L cerebellum VIIb	depression	1.007(0.987~1.028)	0.493	1.025(1.005~1.044)	0.012	1.090(1.004~1.183)	0.041	TM	100%
CD	volume Right-Lateral-Ventricle	depression	1.007(0.987~1.028)	0.493	0.971(0.951~0.992)	0.006	0.805(0.719~0.902)	<0.001	TM	100%
CD	L paracentral area	depression	1.007(0.987~1.028)	0.493	0.939(0.897~0.983)	0.010	0.958(0.927~0.990)	0.010	TM	100%
CD	L G&S paracentral area	depression	1.007(0.987~1.028)	0.493	0.940(0.894~0.987)	0.018	0.901(0.820~0.989)	0.028	TM	100%
CD	R S collat transv ant area	depression	1.007(0.987~1.028)	0.493	0.936(0.888~0.986)	0.017	0.827(0.714~0.959)	0.012	TM	100%
CD	ProbtrackX FA mcp	depression	1.007(0.987~1.028)	0.493	1.029(1.004~1.054)	0.023	1.160(1.016~1.325)	0.029	TM	100%
CD	ProbtrackX MO unc r	depression	1.007(0.987~1.028)	0.493	0.924(0.871~0.980)	0.012	0.894(0.847~0.944)	<0.001	TM	100%
UC	T1 FAST ROIs L temp occ fusif cortex	depression	1.011(0.979~1.044)	0.495	1.037(1.007~1.067)	0.015	1.181(1.062~1.313)	0.002	TM	100%
UC	L S front inf thickness	depression	1.011(0.979~1.044)	0.495	1.033(1.011~1.056)	0.004	1.187(1.001~1.408)	0.049	TM	100%
CD	T1 FAST ROIs L ventral striatum	Schizophrenia	1.036(0.991~1.084)	0.118	1.021(1.003~1.040)	0.023	1.373(1.037~1.819)	0.027	TM	100%
CD	L G&S paracentral area	Schizophrenia	1.036(0.991~1.084)	0.118	0.940(0.894~0.987)	0.018	0.802(0.762~0.844)	<0.001	TM	100%
CD	R inferiortemporal area	Schizophrenia	1.036(0.991~1.084)	0.118	0.979(0.962~0.996)	0.015	0.676(0.625~0.731)	<0.001	TM	100%
UC	L superiorparietal thickness	Schizophrenia	1.050(0.990~1.113)	0.105	1.041(1.006~1.077)	0.020	1.053(1.040~1.066)	<0.001	TM	100%
UC	L G temporal inf thickness	Schizophrenia	1.050(0.990~1.113)	0.105	1.047(1.019~1.076)	0.001	1.771(1.497~2.094)	<0.001	TM	100%
UC	L S precentral-sup-part thickness	Schizophrenia	1.050(0.990~1.113)	0.105	1.046(1.011~1.082)	0.010	1.481(1.442~1.520)	<0.001	TM	100%
UC	OD Middle cerebellar peduncle	Schizophrenia	1.050(0.990~1.113)	0.105	0.964(0.936~0.992)	0.014	0.728(0.541~0.979)	0.036	TM	100%

Mediator Type includes TM, Total Mediation; PM, Partial Mediation; L, Left; R, Right; CD, Crohn’s Disease; UC, Ulcerative Colitis; OR, Odds Ratio; CI, Confidence Interval.

## 4 Discussion

Inflammatory bowel disease is a chronic, relapsing autoimmune disorder. Previous observational studies suggest a relationship between IBD and changes in brain imaging derived phenotypes. However, whether due to confounding factors and the unclear direction of causality, the existence of a causal relationship between them remains inconclusive (29–31). In this study, we employed Mendelian randomization to investigate bidirectional causal relationships between IBD and IDPs, exploring the potential links between IBD onset and changes in brain structure. This provides new insights for the diagnosis and treatment of IBD.

Corticotropin-Releasing Hormone-Binding Protein (CRH-BP), a secreted glycoprotein, binds to CRH with very high affinity, modulating the signal transduction of CRH receptors. It suppresses adrenocorticotropic Hormone (ACTH) release mediated by CRH-Receptor 1 (CRHR1) while potentially facilitating it in CRHR2 (32). Additionally, it inhibits CRH-induced anterior pituitary cell ACTH release *in vitro (* 33). Research by Ketchesin KD et al. confirms the specific expression of CRH-BP in GABAergic neurons in the prefrontal cortex, particularly in interneurons expressing somatostatin (32). Our research findings suggest an increase in VGM in the frontal lobe is associated with a reduced risk of Crohn’s disease. This could be attributed to an upregulation of CRH-BP, which suppresses the activity of the neuroendocrine axis (i.e., the hypothalamus-pituitary-adrenal axis) and lowers the levels of CRH. Simultaneously it participates in the regulation of CRHR, lowering the occurrence of intestinal inflammation through multiple pathways. Elevated levels of CRH can damage the intestinal mucosal barrier, increase intestinal permeability, enhance autophagy in the gut, induce dysbiosis in the gut microbiota, and promote an M1/M2 polarization, consequently facilitating intestinal inflammation (34, 35). Furthermore, Li et al.’s study suggests that CRHR1 mediates intestinal injury through the mentioned pathway. Conversely, CRHR2 activates intestinal stem cells, promoting intestinal repair (36).

Additionally, our study suggests that an increase in gray matter volume in the prefrontal and extra-frontal lobes may increase the risk of UC and CD, although research on whether these structural changes trigger intestinal inflammation is yet to be discovered, and the underlying mechanisms require further exploration.

In a reverse causal relationship, we found that an increased risk of IBD onset leads to alterations in certain brain structures. This may be associated with the complications of neurocognitive disorders in IBD. In addition to common gastrointestinal clinical manifestations such as diarrhea and abdominal pain, IBD patients also present neuropsychiatric complications, with central nervous system involvement including cerebrovascular diseases (37) and psychiatric disorders such as cognitive impairments, anxiety, and depression (2, 38). Peripheral nervous system manifestations include peripheral neuropathy and demyelinating diseases (39). These clinical symptoms may be associated with the observed changes in IDP in this study. In this study, an increased risk of UC or CD was found to be associated with changes in the volume of the pallidum, anterior cingulate gyrus, frontal lobe, temporal lobe, inferior temporal gyrus, temporal-occipital fusiform gyrus, hippocampus, parahippocampal gyrus, and cerebellum, which is consistent with the observations made by Bao, Chun Hui et al. (40).

Human perception, memory, and emotional regulation are associated with the prefrontal-limbic system (41), which comprises the cingulate cortex, insular cortex, parahippocampal gyrus, hippocampus, prefrontal cortex, amygdala, temporal lobe, hypothalamus, and thalamus. These regions are interconnected by structures such as the fornix, corpus callosum, and superior longitudinal fasciculus, forming various functional circuits (42). Research has shown that IBD patients are more susceptible to developing mental and psychological disorders, including anxiety, depression, schizophrenia, bipolar affective disorder, and cognitive impairment (43, 44). The olfactory-hippocampal circuit is considered to be associated with human episodic memory (26), and structural defects in the olfactory-hippocampal cortex can lead to cognitive inflexibility and pattern separation impairments, consequently resulting in depression (45). Experiments conducted by He, Xiao-Fei et al. and others suggest that in DSS-induced colitis mice, microglial cell activation, an increase in A1-like astrocytes, impairment of the lymphatic clearance pathway, and the induction of hippocampal cortical neuron loss ultimately lead to cognitive dysfunction and neuronal damage (46). This aligns with the findings of our study, providing anatomical evidence to some extent for the association of IBD with the development of psychiatric disorders.

The frontal, temporal, and occipital lobes are central to processes related to visual and auditory perception, attention, and language (47–49). The encoding and perception of facial features occur in the occipitotemporal region, while the matching of newly encoded facial features with previously stored ones takes place in the temporal and frontal lobes (50, 51). Observational studies have suggested that in comparison to healthy individuals, patients with schizophrenia exhibit significant reductions in the volume of certain brain regions, including the temporal, frontal, occipital, and parietal lobes (52, 53). Our findings may offer an explanation for the increased susceptibility of IBD patients to develop disorders such as schizophrenia.

Chronic abdominal pain is a primary clinical manifestation of IBD, with pain signals transmitted through the spinal thalamocortical pathway, spinal amygdala pathway, and spinal thalamocortical pathway, among others, to the cortical edge region (54). The central processing of visceral sensory/pain signals occurs in the orbitofrontal cortex, insula, and anterior cingulate cortex (54), with a notable increase in glutamate concentration, especially enhanced activity in the anterior cingulate cortex. Glutamate is involved in the energy metabolism of astrocytes and serves as an excitatory neurotransmitter in signal transduction. However, excessive glutamate leads to excitotoxicity, causing damage to neurons and axons (55). During acute inflammation in IBD, there is an increased release of inflammatory cytokines (such as TNF-α) and an elevated blood-brain barrier permeability (56). Circulating cytokines, due to the compromised blood-brain barrier, impact neurotransmitter metabolism and induce apoptosis in astrocytes and oligodendrocytes, disrupting the conduction of brain structures and neural fiber bundles (57). Therefore, proactive management of intestinal inflammation, reducing the release of inflammatory factors, and alleviating chronic pain may potentially decrease the risk of IBD-associated mental health disorders.

Concurrently referencing previous studies, we also selected 6 neuropsychiatric disorder datasets for an additional mediation analysis of IDPs. Although the Mendelian randomization mediation effects differed from the mediating IDPs found in observational studies, both demonstrated that CD or UC promotes the onset of neuropsychiatric symptoms by mediating different brain IDPs.

Our research has unveiled a causal relationship between IBD and brain volume, offering anatomical and endocrine-related mechanisms for IBD-induced neurological disorders such as anxiety, depression, and schizophrenia. Therefore, medical doctors may consider early disease management by actively controlling intestinal inflammation, reducing systemic inflammatory factor release, preventing central nervous system damage, and reducing the incidence of long-term mental health disorders. Furthermore, certain changes in the volume of specific brain structures may serve as potential biomarkers for predicting the course of IBD. However, further research is required to substantiate this.

Prior neuroimaging studies on the impact of IBD on brain structure and function have been observational in nature. While they can establish an association between the disease and brain structure, they cannot confirm a causal relationship between the two. Our study innovatively applied Mendelian randomization, utilizing a selection of genetically associated variables significantly related to exposure factors, thereby mitigating confounding factors. This approach unveiled a bidirectional causal relationship between IBD and IDPs, with rigorous FDR correction, power calculations, and multiple sensitivity analyses effectively mitigating statistical Type I and Type II errors, as well as errors by abnormal SNP data, ensuring robust and reliable results. Finally, this paper provides an explanation of potential mechanisms, emphasizes the significance of neuroimaging applications, and offers a prospect for potential therapeutic approaches in the future.

Our study does have limitations. The population in this study consisted exclusively of individuals from European backgrounds, necessitating further exploration for populations from other regions. The brain imaging data used in this study were derived from a cross-sectional study, which precluded our investigation into the impact of the duration of inflammatory bowel disease on brain IDPs. Additionally, due to the utilization of aggregated data rather than individual data, stratified analysis of variables such as gender was not possible.

Given that the method of Wald ratio estimation assumes a linear causal relationship, this study cannot exclude the possibility of a non-linear relationship between IDPs and the onset of IBD. Lastly, it is inevitable that individual channelization exist in MR studies.

In conclusion, our study has unveiled a causal relationship between intestinal inflammation and changes in brain morphology through Mendelian randomization analysis, thereby emphasizing the role of distinct IDPs in patients with IBD. We believe that persistent inflammatory stimulation and continuous input of pain signals can lead to neuronal and neural pathway damage, consequently resulting in structural changes in the brain. Similarly, changes in brain structure can trigger the exacerbation of intestinal inflammation through regulatory mechanisms involving the nervous system and endocrine system. IDPs may serve as biomarkers for screening, monitoring, and identifying complications in clinical practice for IBD. This holds the potential to predict the progression of IBD disease by conducting and revisiting head MRI examinations, assessing changes in brain structure and neural fiber conductivity, and taking early therapeutic measures. Furthermore, proactive IBD treatment and inflammation control can reduce the risk of developing mental and psychological disorders associated with IBD.

## 5 Conclusion

Through Mendelian randomization analysis, we have revealed a causal relationship between inflammatory bowel disease and 252 Brain Imaging Derived Phenotypes. This underscores the role of brain IDPs in IBD patients. Three IDPs have been identified to have a close association with the onset of IBD, and we have also discovered some IDPs alterations related to anxiety, depression, and other 8 other disorders and functions that are attributable to IBD onset. These findings hold the promise of becoming valuable biomarkers in clinical practice for IBD screening and complication monitoring.

## Data availability statement

The original contributions presented in the study are included in the article/**Supplementary Material**. Further inquiries can be directed to the corresponding author.

## Author contributions

FL: Conceptualization, Data curation, Methodology, Writing – original draft. QZ: Conceptualization, Investigation, Writing – original draft, Writing – review & editing, Methodology. TT: Conceptualization, Writing – review & editing. YYL: Conceptualization, Funding acquisition, Writing – review & editing. ZDW: Visualization, Writing – review & editing. YQL: Investigation, Project administration, Writing – review & editing. ZW: Visualization, Writing – review & editing. XH: Project administration, Writing – review & editing. ZX: Project administration, Writing – review & editing. YC: Visualization, Writing – review & editing.

## References

[1] Wang S Dong Z Wan X. Global, regional, and national burden of inflammatory bowel disease and its associated anemia, 1990 to 2019 and predictions to 2050: An analysis of the global burden of disease study 2019. Autoimmun Rev. (2023) 23:103498. doi: 10.1016/j.autrev.2023.103498 38052263

[2] Bisgaard TH Allin KH Keefer L Ananthakrishnan AN Jess T. Depression and anxiety in inflammatory bowel disease: epidemiology, mechanisms and treatment. Nat Rev Gastroenterol Hepatol. (2022) 19:717–26. doi: 10.1038/s41575-022-00634-6 35732730

[3] Socala K Doboszewska U Szopa A Serefko A Wlodarczyk M Zielinska A. The role of microbiota-gut-brain axis in neuropsychiatric and neurological disorders. Pharmacol Res. (2021) 172:105840. doi: 10.1016/j.phrs.2021.105840 34450312

[4] Zikou AK Kosmidou M Astrakas LG Tzarouchi LC Tsianos E Argyropoulou MI. Brain involvement in patients with inflammatory bowel disease: a voxel-based morphometry and diffusion tensor imaging study. Eur Radiol. (2014) 24:2499–506. doi: 10.1007/s00330-014-3242-6 25001084

[5] Bowden J Holmes MV. Meta-analysis and Mendelian randomization: A review. Res Synth Methods. (2019) 10:486–96. doi: 10.1002/jrsm.1346 PMC6973275 30861319

[6] Gupta V Walia GK Sachdeva MP. 'Mendelian randomization': an approach for exploring causal relations in epidemiology. Public Health. (2017) 145:113–9. doi: 10.1016/j.puhe.2016.12.033 28359378

[7] Sekula P Del Greco MF Pattaro C Kottgen A. Mendelian randomization as an approach to assess causality using observational data. J Am Soc Nephrol. (2016) 27:3253–65. doi: 10.1681/ASN.2016010098 PMC5084898 27486138

[8] Liu JZ van Sommeren S Huang H Ng SC Alberts R Takahashi A. Association analyses identify 38 susceptibility loci for inflammatory bowel disease and highlight shared genetic risk across populations. Nat Genet. (2015) 47:979–86. doi: 10.1038/ng.3359 PMC4881818 26192919

[9] Elliott LT Sharp K Alfaro-Almagro F Shi S Miller KL Douaud G. Genome-wide association studies of brain imaging phenotypes in UK Biobank. Nature. (2018) 562:210–6. doi: 10.1038/s41586-018-0571-7 PMC6786974 30305740

[10] Burgess S Small DS Thompson SG. A review of instrumental variable estimators for Mendelian randomization. Stat Methods Med Res. (2017) 26:2333–55. doi: 10.1177/0962280215597579 PMC5642006 26282889

[11] Staley JR Blackshaw J Kamat MA Ellis S Surendran P Sun BB. PhenoScanner: a database of human genotype-phenotype associations. Bioinformatics. (2016) 32:3207–9. doi: 10.1093/bioinformatics/btw373 PMC5048068 27318201

[12] Thomas DC Conti DV. Commentary: the concept of 'Mendelian randomization'. Int J Epidemiol. (2004) 33:21–5. doi: 10.1093/ije/dyh048 15075141

[13] Burgess S Butterworth A Thompson SG. Mendelian randomization analysis with multiple genetic variants using summarized data. Genet Epidemiol. (2013) 37:658–65. doi: 10.1002/gepi.21758 PMC4377079 24114802

[14] Burgess S Davey Smith G Davies NM Dudbridge F Gill D Glymour MM. Guidelines for performing Mendelian randomization investigations: update for summer 2023. Wellcome Open Res. (2019) 4:186. doi: 10.12688/wellcomeopenres 32760811 PMC7384151

[15] Bowden J Davey Smith G Haycock PC Burgess S. Consistent estimation in Mendelian randomization with some invalid instruments using a weighted median estimator. Genet Epidemiol. (2016) 40:304–14. doi: 10.1002/gepi.21965 PMC4849733 27061298

[16] Burgess S Thompson SG. Interpreting findings from Mendelian randomization using the MR-Egger method. Eur J Epidemiol. (2017) 32:377–89. doi: 10.1007/s10654-017-0255-x PMC5506233 28527048

[17] Zhao Q Wang J Hemani G Bowden J Small DS. Statistical inference in two-sample summary-data Mendelian randomization using robust adjusted profile score. (2020). doi: 10.1214/19-AOS1866

[18] Hemani G Tilling K Smith GD. Orienting the causal relationship between imprecisely measured traits using GWAS summary data. PloS Genet. (2017) 13:e1007081. doi: 10.1371/journal.pgen.1007081 29149188 PMC5711033

[19] Bowden J Del Greco MF Minelli C Davey Smith G Sheehan NA Thompson JR. Assessing the suitability of summary data for two-sample Mendelian randomization analyses using MR-Egger regression: the role of the I2 statistic. Int J Epidemiol. (2016) 45:1961–74. doi: 10.1093/ije/dyw220 PMC5446088 27616674

[20] Burgess S. Sample size and power calculations in Mendelian randomization with a single instrumental variable and a binary outcome. Int J Epidemiol. (2014) 43:922–9. doi: 10.1093/ije/dyu005 PMC4052137 24608958

[21] Infantino C Francavilla R Vella A Cenni S Principi N Strisciuglio C. Role of vitamin D in celiac disease and inflammatory bowel diseases. Nutrients. (2022) 14. doi: 10.3390/nu14235154 PMC9735899 36501183

[22] Luo YJ Ge J Chen ZK Liu ZL Lazarus M Qu WM. Ventral pallidal glutamatergic neurons regulate wakefulness and emotion through separated projections. iScience. (2023) 26:107385. doi: 10.1016/j.isci.2023.107385 37609631 PMC10440712

[23] Wang L Dai W Su Y Wang G Tan Y Jin Z. Amplitude of low-frequency oscillations in first-episode, treatment-naive patients with major depressive disorder: a resting-state functional MRI study. PloS One. (2012) 7:e48658. doi: 10.1371/journal.pone.0048658 23119084 PMC3485382

[24] Takayanagi Y Sasabayashi D Takahashi T Furuichi A Kido M Nishikawa Y. Reduced cortical thickness in schizophrenia and schizotypal disorder. Schizophr Bull. (2020) 46:387–94. doi: 10.1093/schbul/sbz051 PMC7406196 31167030

[25] Hamazaki K Hamazaki T Inadera H. Abnormalities in the fatty acid composition of the postmortem entorhinal cortex of patients with schizophrenia, bipolar disorder, and major depressive disorder. Psychiatry Res. (2013) 210:346–50. doi: 10.1016/j.psychres.2013.05.006 23731984

[26] Kim IB Park SC. The entorhinal cortex and adult neurogenesis in major depression. Int J Mol Sci. (2021) 22. doi: 10.3390/ijms222111725 PMC8583901 34769155

[27] Tanglay O Young IM Dadario NB Briggs RG Fonseka RD Dhanaraj V. Anatomy and white-matter connections of the precuneus. Brain Imaging Behav. (2022) 16:574–86. doi: 10.1007/s11682-021-00529-1 34448064

[28] Hartwigsen G Weigel A Schuschan P Siebner HR Weise D Classen J. Dissociating parieto-frontal networks for phonological and semantic word decisions: A condition-and-perturb TMS study. Cereb Cortex. (2016) 26:2590–601. doi: 10.1093/cercor/bhv092 25953770

[29] Goodyear BG Heidari F Ingram RJM Cortese F Sharifi N Kaplan GG. Multimodal brain MRI of deep gray matter changes associated with inflammatory bowel disease. Inflamm Bowel Dis. (2023) 29:405–16. doi: 10.1093/ibd/izac089 PMC9977255 35590449

[30] Zhang S Chen F Wu J Liu C Yang G Piao R. Regional gray matter volume changes in brains of patients with ulcerative colitis. Inflamm Bowel Dis. (2022) 28:599–610. doi: 10.1093/ibd/izab252 34734248

[31] Agostini A Benuzzi F Filippini N Bertani A Scarcelli A Farinelli V. New insights into the brain involvement in patients with Crohn's disease: a voxel-based morphometry study. Neurogastroenterol Motil. (2013) 25:147–e82. doi: 10.1111/nmo.12017 22998431

[32] Ketchesin KD Huang NS Seasholtz AF. Cell type-specific expression of corticotropin-releasing hormone-binding protein in GABAergic interneurons in the prefrontal cortex. Front Neuroanat. (2017) 11:90. doi: 10.3389/fnana.2017.00090 29066956 PMC5641307

[33] Stinnett GS Westphal NJ Seasholtz AF. Pituitary CRH-binding protein and stress in female mice. Physiol Behav. (2015) 150:16–23. doi: 10.1016/j.physbeh.2015.02.050 25731977 PMC4546865

[34] Wallon C Yang PC Keita AV Ericson AC McKay DM Sherman PM. Corticotropin-releasing hormone (CRH) regulates macromolecular permeability *via* mast cells in normal human colonic biopsies in vitro. Gut. (2008) 57:50–8. doi: 10.1136/gut.2006.117549 17525093

[35] Wang SL Shao BZ Zhao SB Chang X Wang P Miao CY. Intestinal autophagy links psychosocial stress with gut microbiota to promote inflammatory bowel disease. Cell Death Dis. (2019) 10:391. doi: 10.1038/s41419-019-1634-x 31564717 PMC6766473

[36] Li B Lee C Filler T Hock A Wu RY Li Q. Inhibition of corticotropin-releasing hormone receptor 1 and activation of receptor 2 protect against colonic injury and promote epithelium repair. Sci Rep. (2017) 7:46616. doi: 10.1038/srep46616 28492284 PMC5425914

[37] Singh S Singh H Loftus EV Jr. Pardi DS. Risk of cerebrovascular accidents and ischemic heart disease in patients with inflammatory bowel disease: a systematic review and meta-analysis. Clin Gastroenterol Hepatol. (2014) 12:382–93 e1. doi: 10.1016/j.cgh.2013.08.023 23978350

[38] Zhang B Wang HE Bai YM Tsai SJ Su TP Chen TJ. Inflammatory bowel disease is associated with higher dementia risk: a nationwide longitudinal study. Gut. (2021) 70:85–91. doi: 10.1136/gutjnl-2020-320789 32576641

[39] Ferro JM Oliveira Santos M. Neurology of inflammatory bowel disease. J Neurol Sci. (2021) 424:117426. doi: 10.1016/j.jns.2021.117426 33810878

[40] Bao CH Liu P Liu HR Wu LY Shi Y Chen WF. Alterations in brain grey matter structures in patients with crohn's disease and their correlation with psychological distress. J Crohns Colitis. (2015) 9:532–40. doi: 10.1093/ecco-jcc/jjv057 25895879

[41] Kebets V Favre P Houenou J Polosan M Perroud N Aubry JM. Fronto-limbic neural variability as a transdiagnostic correlate of emotion dysregulation. Transl Psychiatry. (2021) 11:545. doi: 10.1038/s41398-021-01666-3 34675186 PMC8530999

[42] Kamali A Milosavljevic S Gandhi A Lano KR Shobeiri P Sherbaf FG. The cortico-limbo-thalamo-cortical circuits: an update to the original papez circuit of the human limbic system. Brain Topogr. (2023) 36:371–89. doi: 10.1007/s10548-023-00955-y PMC10164017 37148369

[43] Barberio B Zamani M Black CJ Savarino EV Ford AC. Prevalence of symptoms of anxiety and depression in patients with inflammatory bowel disease: a systematic review and meta-analysis. Lancet Gastroenterol Hepatol. (2021) 6:359–70. doi: 10.1016/S2468-1253(21)00014-5 33721557

[44] Bernstein CN Hitchon CA Walld R Bolton JM Sareen J Walker JR. Managing the effects of psychiatric comorbidity in chronic immunoinflammatory, increased burden of psychiatric disorders in inflammatory bowel disease. Inflamm Bowel Dis. (2019) 25:360–8. doi: 10.1093/ibd/izy235 PMC6391845 29986021

[45] Anacker C Hen R. Adult hippocampal neurogenesis and cognitive flexibility - linking memory and mood. Nat Rev Neurosci. (2017) 18:335–46. doi: 10.1038/nrn.2017.45 PMC6261347 28469276

[46] He XF Li LL Xian WB Li MY Zhang LY Xu JH. Chronic colitis exacerbates NLRP3-dependent neuroinflammation and cognitive impairment in middle-aged brain. J Neuroinflamm. (2021) 18:153. doi: 10.1186/s12974-021-02199-8 PMC8262017 34229722

[47] Teixeira S MaChado S Velasques B Sanfim A Minc D Peressutti C. Integrative parietal cortex processes: neurological and psychiatric aspects. J Neurol Sci. (2014) 338:12–22. doi: 10.1016/j.jns.2013.12.025 24398346

[48] Knopman AA Wong CH Stevenson RJ Homewood J Mohamed A Somerville E. The cognitive profile of occipital lobe epilepsy and the selective association of left temporal lobe hypometabolism with verbal memory impairment. Epilepsia. (2014) 55:e80–4. doi: 10.1111/epi.12623 24725141

[49] Catani M. The clinical anatomy of the temporal and parietal lobes. Cortex. (2017) 97:160–3. doi: 10.1016/j.cortex.2017.11.006 29203031

[50] Nestor A Plaut DC Behrmann M. Unraveling the distributed neural code of facial identity through spatiotemporal pattern analysis. Proc Natl Acad Sci USA. (2011) 108:9998–10003. doi: 10.1073/pnas.1102433108 21628569 PMC3116398

[51] Yun JY Hur JW Jung WH Jang JH Youn T Kang DH. Dysfunctional role of parietal lobe during self-face recognition in schizophrenia. Schizophr Res. (2014) 152:81–8. doi: 10.1016/j.schres.2013.07.010 23916187

[52] Van Rheenen TE Cropley V Zalesky A Bousman C Wells R Bruggemann J. Widespread volumetric reductions in schizophrenia and schizoaffective patients displaying compromised cognitive abilities. Schizophr Bull. (2018) 44:560–74. doi: 10.1093/schbul/sbx109 PMC5890481 28981831

[53] Zhao C Zhu J Liu X Pu C Lai Y Chen L. Structural and functional brain abnormalities in schizophrenia: A cross-sectional study at different stages of the disease. Prog Neuropsychopharmacol Biol Psychiatry. (2018) 83:27–32. doi: 10.1016/j.pnpbp.2017.12.017 29292241

[54] Price DD. Psychological and neural mechanisms of the affective dimension of pain. Science. (2000) 288:1769–72. doi: 10.1126/science.288.5472.1769 10846154

[55] Lv K Song W Tang R Pan Z Zhang Y Xu Y. Neurotransmitter alterations in the anterior cingulate cortex in Crohn's disease patients with abdominal pain: A preliminary MR spectroscopy study. NeuroImage Clin. (2018) 20:793–9. doi: 10.1016/j.nicl.2018.09.008 PMC6169252 30268988

[56] Abautret-Daly A Dempsey E Parra-Blanco A Medina C Harkin A. Gut-brain actions underlying comorbid anxiety and depression associated with inflammatory bowel disease. Acta Neuropsychiatr. (2018) 30:275–96. doi: 10.1017/neu.2017.3 28270247

[57] Stellwagen D Malenka RC. Synaptic scaling mediated by glial TNF-alpha. Nature. (2006) 440:1054–9. doi: 10.1038/nature04671 16547515

