# Supplementary material for: Brain imaging derived phenotypes: a biomarker for the onset of inflammatory bowel disease and a potential mediator of mental complications
Source: Front Immunol. 2024 Feb 26;15:1359540. doi: 10.3389/fimmu.2024.1359540 (PMC10925669; doi:10.3389/fimmu.2024.1359540)
Supplement: Supplementary file 2 [file Table_9.docx]

**Supplementary Table 9. STROBE-MR checklist of recommended items to address in reports of Mendelian randomization studies**^1^ ^2^

| **Item No.** | **Section** | **Checklist item** | **Position** | **Relevant text from manuscript** |
| --- | --- | --- | --- | --- |
| 1 | **TITLE and ABSTRACT** | Indicate Mendelian randomization (MR) as the study’s design in the title and/or the abstract if that is a main purpose of the study | Abstract | We conducted a Mendelian randomization analysis utilizing brain-derived image phenotypes from the UK Biobank database to investigate the causal relationships between IBD and alterations in brain structural morphology and connectivity of neural tracts. |
|  | **INTRODUCTION** |  |  |  |
| 2 | **Background** | Explain the scientific background and rationale for the reported study. What is the exposure? Is a potential causal relationship between exposure and outcome plausible? Justify why MR is a helpful method to address the study question | 1. Introduction | Inflammatory Bowel Disease (IBD), encompassing ulcerative colitis (UC) and Crohn's disease (CD), represents a chronic, relapsing autoimmune gastrointestinal disorder that can be diagnosed in young individuals…. |
| 3 | **Objectives** | State specific objectives clearly, including pre-specified causal hypotheses (if any). State that MR is a method that, under specific assumptions, intends to estimate causal effects | 1. Introduction | This study investigates the causal relationship between IBD and alterations in brain IDP, aiming to explore potential factors contributing to the occurrence of psychiatric symptoms in IBD patients…. |
|  | **METHODS** |  |  |  |
| 4 | **Study design and data sources** | Present key elements of the study design early in the article. Consider including a table listing sources of data for all phases of the study. For each data source contributing to the analysis, describe the following: | Supplementary Table 1 | Supplementary Table 1. Dataset information included in MR studies |
|  | a) | Setting: Describe the study design and the underlying population, if possible. Describe the setting, locations, and relevant dates, including periods of recruitment, exposure, follow-up, and data collection, when available. | 2.1 Data Sources | This study analyzed data from 86,640 European participants and 9,846 individuals of non-European ancestry…. |
|  | b) | Participants: Give the eligibility criteria, and the sources and methods of selection of participants. Report the sample size, and whether any power or sample size calculations were carried out prior to the main analysis | 2.1 Data Sources | In our study, we exclusively utilized datasets of European ancestry for UC and CD. Both datasets included both male and female participants, with 6,968 UC cases and 20,464 controls in the UC dataset, and 5,956 CD cases and 14,927 controls in the CD dataset…. |
|  | c) | Describe measurement, quality control and selection of genetic variants | 2.1 Data Sources | High-throughput sequencing technologies were employed, with filters applied to remove variants with a minor allele frequency (MAF) below 0.1% and imputation information scores below 0.3, excluding variants with Hardy-Weinberg equilibrium P-values <10-7 and MAF <0.1%... |
|  | d) | For each exposure, outcome, and other relevant variables, describe methods of assessment and diagnostic criteria for diseases | 2.1 Data Sources | For the definition of each IDP, segmentation was conducted using FreeSurfer v6.0.0 on the Desikan-Killiany-Tourville Atlas (referred to as DKT) and the Destrieux Atlas (referred to as a2009s)… |
|  | e) | Provide details of ethics committee approval and participant informed consent, if relevant | 2.1 Data Sources | The UK Biobank has received approval from the Northwest Multi-centre Research Ethics Committee (MREC) to acquire and disseminate participant data and samples (http://www.ukbiobank.ac.uk/ethics/), regulations that encompass the work in this study… |
| 5 | **Assumptions** | Explicitly state the three core IV assumptions for the main analysis (relevance, independence and exclusion restriction) as well assumptions for any additional or sensitivity analysis | 2.3 Genetic Instrument Selection | Through the instrumental-variable selection process described above, the eligible SNPs included in the study met the following criteria: 1) The SNP should exhibit a strong correlation with the exposure factor; 2) The SNP should only influence the outcome through the exposure factor; 3) The SNP should not be associated with confounding factors. |
| 6 | **Statistical methods: main analysis** | Describe statistical methods and statistics used |  |  |
|  | a) | Describe how quantitative variables were handled in the analyses (i.e., scale, units, model) | 2.4 Mendelian Randomization Analysis | We conducted analyses using five different MR methods, and for each batch of studies, we determined a primary analytical method conclusion based on the results of sensitivity analyses, which served as the final conclusion… |
|  | b) | Describe how genetic variants were handled in the analyses and, if applicable, how their weights were selected | 2.3 Genetic Instrument Selection | Initially, we extracted genetic variations significantly associated with exposures at a genome-wide significance level (5×10^-8^)… |
|  | c) | Describe the MR estimator (e.g. two-stage least squares, Wald ratio) and related statistics. Detail the included covariates and, in case of two-sample MR, whether the same covariate set was used for adjustment in the two samples | 2.4 Mendelian Randomization Analysis | Inverse variance weighted is a weighted linear regression without an intercept term. It derives the final causal estimate by analyzing the Wald ratio results for each SNP… |
|  | d) | Explain how missing data were addressed | 2.3 Genetic Instrument Selection | When harmonizing data, we excluded unmatched ambiguous SNPs and palindromic SNPs. |
|  | e) | If applicable, indicate how multiple testing was addressed | Not available | Not available |
| 7 | **Assessment of assumptions** | Describe any methods or prior knowledge used to assess the assumptions or justify their validity | 2.4 Mendelian Randomization Analysis | The null hypothesis (H0) for all the aforementioned MR methods is that there is no causal relationship between exposure and outcome, whereas the alternative hypothesis (H1) posits a causal relationship between exposure and outcome… |
| 8 | **Sensitivity analyses and additional analyses** | Describe any sensitivity analyses or additional analyses performed (e.g. comparison of effect estimates from different approaches, independent replication, bias analytic techniques, validation of instruments, simulations) | 2.5 Sensitivity Analysis | To ensure the robustness of Mendelian randomization analysis results, we conducted various sensitivity analyses… |
| 9 | **Software and pre-registration** |  |  |  |
|  | a) | Name statistical software and package(s), including version and settings used | 2.7 Visualization and Statistical Software | Statistical analysis and visualization in this study were performed using R (version 4.1.2), with the application of R packages such as "TwoSampleMR," "MR-PRESSO," "mr.raps," "forestploter," and several foundational R packages. Flowcharts were created using Microsoft Office Powerpoint (version 2312). |
|  | b) | State whether the study protocol and details were pre-registered (as well as when and where) | Not available | Not available |
|  | **RESULTS** |  |  |  |
| 10 | **Descriptive data** |  |  |  |
|  | a) | Report the numbers of individuals at each stage of included studies and reasons for exclusion. Consider use of a flow diagram | Figure 1 | Figure 1 |
|  | b) | Report summary statistics for phenotypic exposure(s), outcome(s), and other relevant variables (e.g. means, SDs, proportions) | Supplementary Table 1 | Supplementary Table 1 |
|  | c) | If the data sources include meta-analyses of previous studies, provide the assessments of heterogeneity across these studies | Supplementary Table 1 | Supplementary Table 1 |
|  | d) | For two-sample MR:  i.  Provide justification of the similarity of the genetic variant-exposure associations between the exposure and outcome samples  ii.  Provide information on the number of individuals who overlap between the exposure and outcome studies | i. Supplementary Table 3  ii. 2.1 Data Sources | i. Supplementary Table 3  ii. Based on the information from the two data set sources, there is no sample overlap in the MR analysis of this study. |
| 11 | **Main results** |  |  |  |
|  | a) | Report the associations between genetic variant and exposure, and between genetic variant and outcome, preferably on an interpretable scale | 3.2-3.4 | Specifically, for each 1 standard deviation (SD) increase in the Volume of grey matter (VGM) in the Left Frontal Orbital Cortex, the risk of CD decreased by 68% (odds ratio (OR) [95% confidence interval (CI)]: 0.315[0.180~0.551], adjusted P=0.001)… |
|  | b) | Report MR estimates of the relationship between exposure and outcome, and the measures of uncertainty from the MR analysis, on an interpretable scale, such as odds ratio or relative risk per SD difference | 3.2-3.4 | Specifically, for each 1 standard deviation (SD) increase in the Volume of grey matter (VGM) in the Left Frontal Orbital Cortex, the risk of CD decreased by 68% (odds ratio (OR) [95% confidence interval (CI)]: 0.315[0.180~0.551], adjusted P=0.001)… |
|  | c) | If relevant, consider translating estimates of relative risk into absolute risk for a meaningful time period | 3.2-3.4 | Specifically, for each 1 standard deviation (SD) increase in the Volume of grey matter (VGM) in the Left Frontal Orbital Cortex, the risk of CD decreased by 68% (odds ratio (OR) [95% confidence interval (CI)]: 0.315[0.180~0.551], adjusted P=0.001)… |
|  | d) | Consider plots to visualize results (e.g. forest plot, scatterplot of associations between genetic variants and outcome versus between genetic variants and exposure) | Figure 2-3, Supplementary Figure 1-4 | Figure 2-3, Supplementary Figure 1-4 |
| 12 | **Assessment of assumptions** |  |  |  |
|  | a) | Report the assessment of the validity of the assumptions | 3.2-3.4 | Specifically, for each 1 standard deviation (SD) increase in the Volume of grey matter (VGM) in the Left Frontal Orbital Cortex, the risk of CD decreased by 68% (odds ratio (OR) [95% confidence interval (CI)]: 0.315[0.180~0.551], adjusted P=0.001)… |
|  | b) | Report any additional statistics (e.g., assessments of heterogeneity across genetic variants, such as *I^2^*, Q statistic or E-value) | Table 1 | Table 1 |
| 13 | **Sensitivity analyses and additional analyses** |  |  |  |
|  | a) | Report any sensitivity analyses to assess the robustness of the main results to violations of the assumptions | Table 1 | Table 1 |
|  | b) | Report results from other sensitivity analyses or additional analyses | Supplementary Table 5-7 | Supplementary Table 5-7 |
|  | c) | Report any assessment of direction of causal relationship (e.g., bidirectional MR) | 3.2-3.4 | 3.2 IDPs as the Cause of IBD Onset; 3.3 IBD Promotes Changes in mental Complications and Function-Related IDPs; 3.4 Two-step Mediation Analysis. |
|  | d) | When relevant, report and compare with estimates from non-MR analyses | Not available | Not available |
|  | e) | Consider additional plots to visualize results (e.g., leave-one-out analyses) | Figure 2-3, Supplementary Figure 1-4 | Figure 2-3, Supplementary Figure 1-4 |
|  | **DISCUSSION** |  |  |  |
| 14 | **Key results** | Summarize key results with reference to study objectives | 5 Conclusion | Through Mendelian randomization analysis, we have revealed a causal relationship between inflammatory bowel disease and 252 Brain Imaging Derived Phenotypes… |
| 15 | **Limitations** | Discuss limitations of the study, taking into account the validity of the IV assumptions, other sources of potential bias, and imprecision. Discuss both direction and magnitude of any potential bias and any efforts to address them | 4 Discussion | Our study does have limitations. The population in this study consisted exclusively of individuals from European backgrounds, necessitating further exploration for populations from other regions. Additionally, due to the utilization of aggregated data rather than individual data, stratified analysis of variables such as gender was not possible. |
| 16 | **Interpretation** |  |  |  |
|  | a) | Meaning: Give a cautious overall interpretation of results in the context of their limitations and in comparison with other studies | 4 Discussion | Our study innovatively applied Mendelian randomization, utilizing a selection of genetically associated variables significantly related to exposure factors, thereby mitigating confounding factors. This approach unveiled a bidirectional causal relationship between IBD and IDPs, with rigorous FDR correction, power calculations, and multiple sensitivity analyses effectively mitigating statistical Type I and Type II errors, as well as errors by abnormal SNP data, ensuring robust and reliable results. |
|  | b) | Mechanism: Discuss underlying biological mechanisms that could drive a potential causal relationship between the investigated exposure and the outcome, and whether the gene-environment equivalence assumption is reasonable. Use causal language carefully, clarifying that IV estimates may provide causal effects only under certain assumptions | 4 Discussion | Corticotropin-Releasing Hormone-Binding Protein(CRH-BP), a secreted glycoprotein, binds to CRH with very high affinity, modulating the signal transduction of CRH receptors. It suppresses adrenocorticotropic Hormone (ACTH) release mediated by CRH-Receptor 1 (CRHR1) while potentially facilitating it in CRHR2. Additionally, it inhibits CRH-induced anterior pituitary cell ACTH release in vitro… |
|  | c) | Clinical relevance: Discuss whether the results have clinical or public policy relevance, and to what extent they inform effect sizes of possible interventions | 4 Discussion | IDPs may serve as biomarkers for screening, monitoring, and identifying complications in clinical practice for IBD. This holds the potential to predict the progression of IBD disease by conducting and revisiting head MRI examinations, assessing changes in brain structure and neural fiber conductivity, and taking early therapeutic measures. Furthermore, proactive IBD treatment and inflammation control can reduce the risk of developing mental and psychological disorders associated with IBD. |
| 17 | **Generalizability** | Discuss the generalizability of the study results (a) to other populations, (b) across other exposure periods/timings, and (c) across other levels of exposure | 4 Discussion | The population in this study consisted exclusively of individuals from European backgrounds, necessitating further exploration for populations from other regions…. |
|  | **OTHER INFORMATION** |  |  |  |
| 18 | **Funding** | Describe sources of funding and the role of funders in the present study and, if applicable, sources of funding for the databases and original study or studies on which the present study is based | 8 Funding | This work was supported by The Science and Technology Agency Jilin Province (grant nos. 20210402013GH and 20200201343JC). |
| 19 | **Data and data sharing** | Provide the data used to perform all analyses or report where and how the data can be accessed, and reference these sources in the article. Provide the statistical code needed to reproduce the results in the article, or report whether the code is publicly accessible and if so, where | 10 Data Availability Statement | All data involve in this study are from publicly available UK biobank (website: https://www.ukbiobank.ac.uk/), and IIBDGC (website: https://www.ibdgenetics.org/). |
| 20 | **Conflicts of Interest** | All authors should declare all potential conflicts of interest | 6 Conflict of Interest | The authors declare that the research was conducted in the absence of any commercial or financial relationships that could be construed as a potential conflict of interest. |

This checklist is copyrighted by the Equator Network under the Creative Commons Attribution 3.0 Unported (CC BY 3.0) license.

1. Skrivankova VW, Richmond RC, Woolf BAR, Yarmolinsky J, Davies NM, Swanson SA, et al. Strengthening the Reporting of Observational Studies in Epidemiology using Mendelian Randomization (STROBE-MR) Statement. JAMA. 2021;under review.

2. Skrivankova VW, Richmond RC, Woolf BAR, Davies NM, Swanson SA, VanderWeele TJ, et al. Strengthening the Reporting of Observational Studies in Epidemiology using Mendelian Randomisation (STROBE-MR): Explanation and Elaboration. BMJ. 2021;375:n2233.
