# Supplementary material for: Brain imaging derived phenotypes: a biomarker for the onset of inflammatory bowel disease and a potential mediator of mental complications
Source: Front Immunol. 2024 Feb 26;15:1359540. doi: 10.3389/fimmu.2024.1359540 (PMC10925669; doi:10.3389/fimmu.2024.1359540)

Batch 1 : Crohn's disease on IDP T1 SIENAX peripheral grey normalised volume

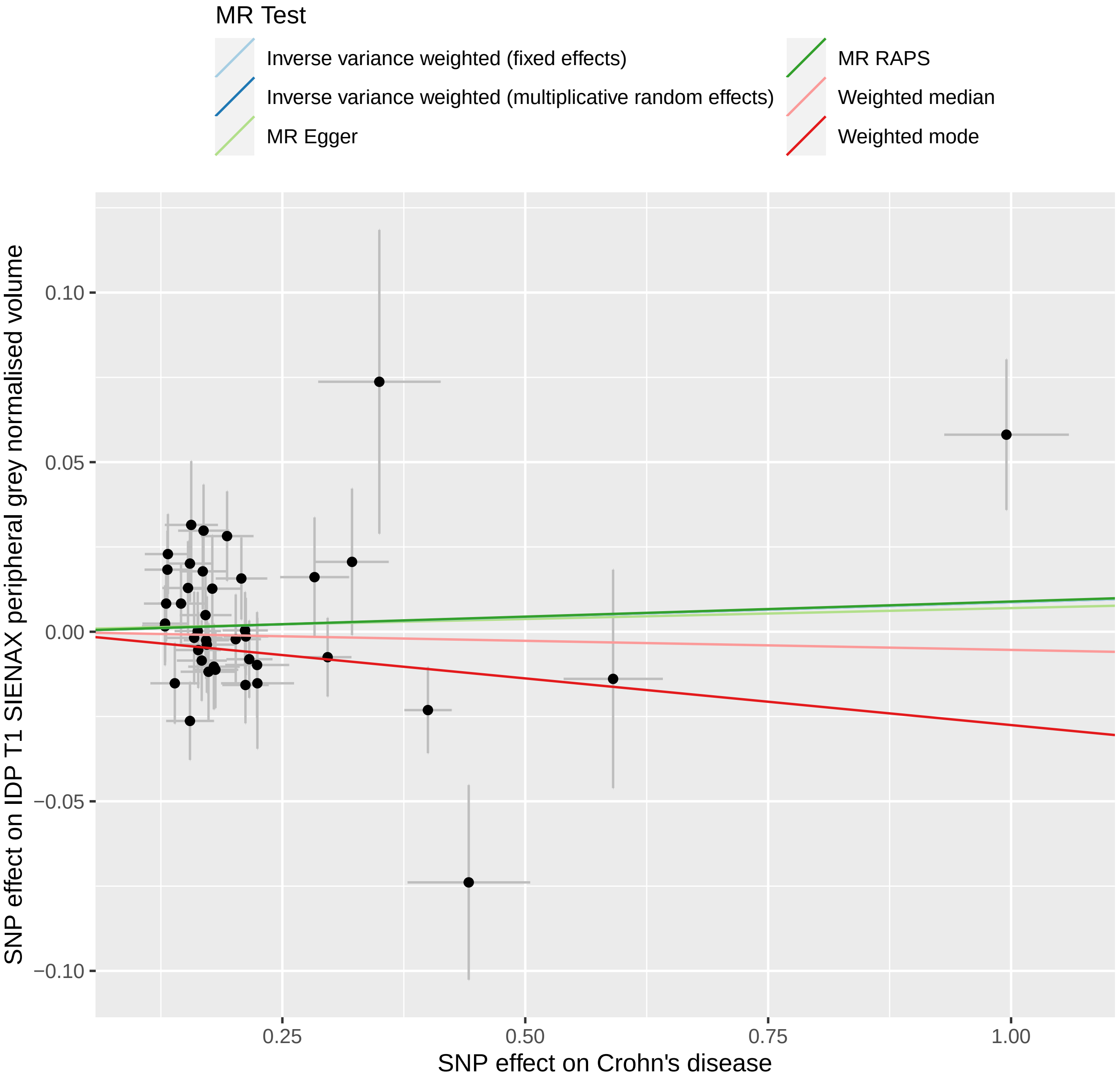

Batch 2 : Ulcerative colitis on IDP T1 SIENAX peripheral grey normalised volume

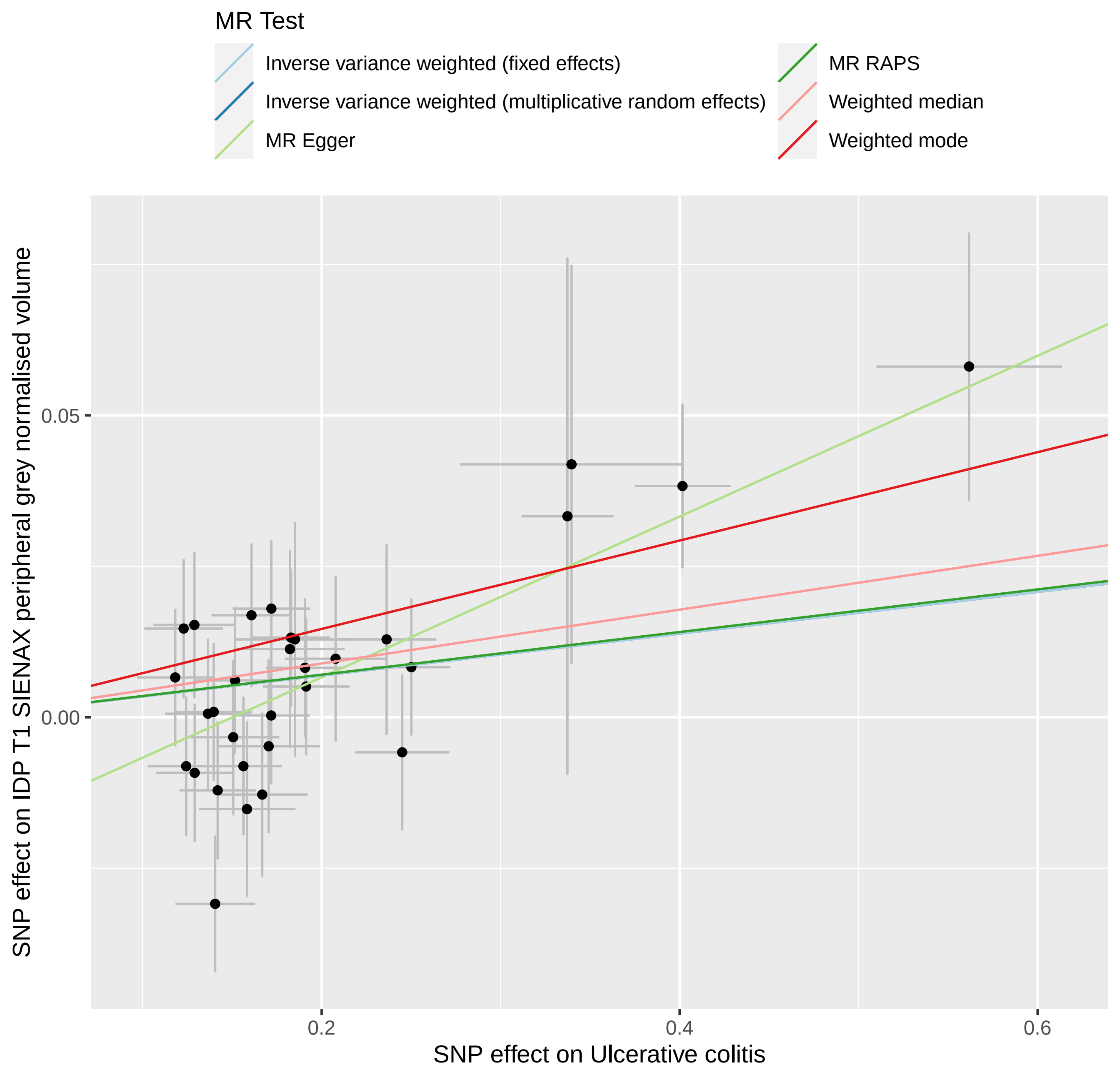

Batch 3 : Crohn's disease on IDP T1 SIENAX brain−unnormalised volume

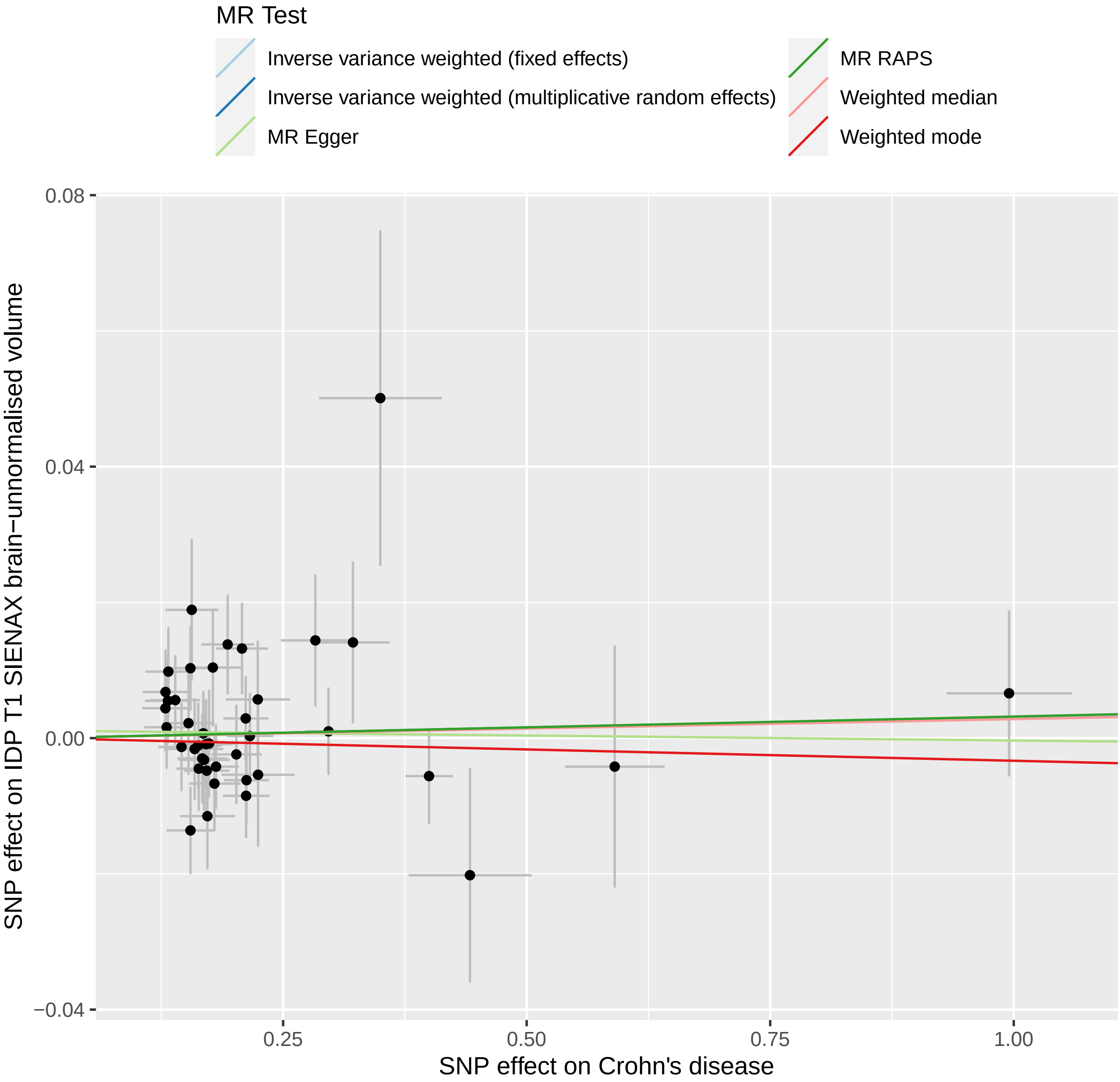

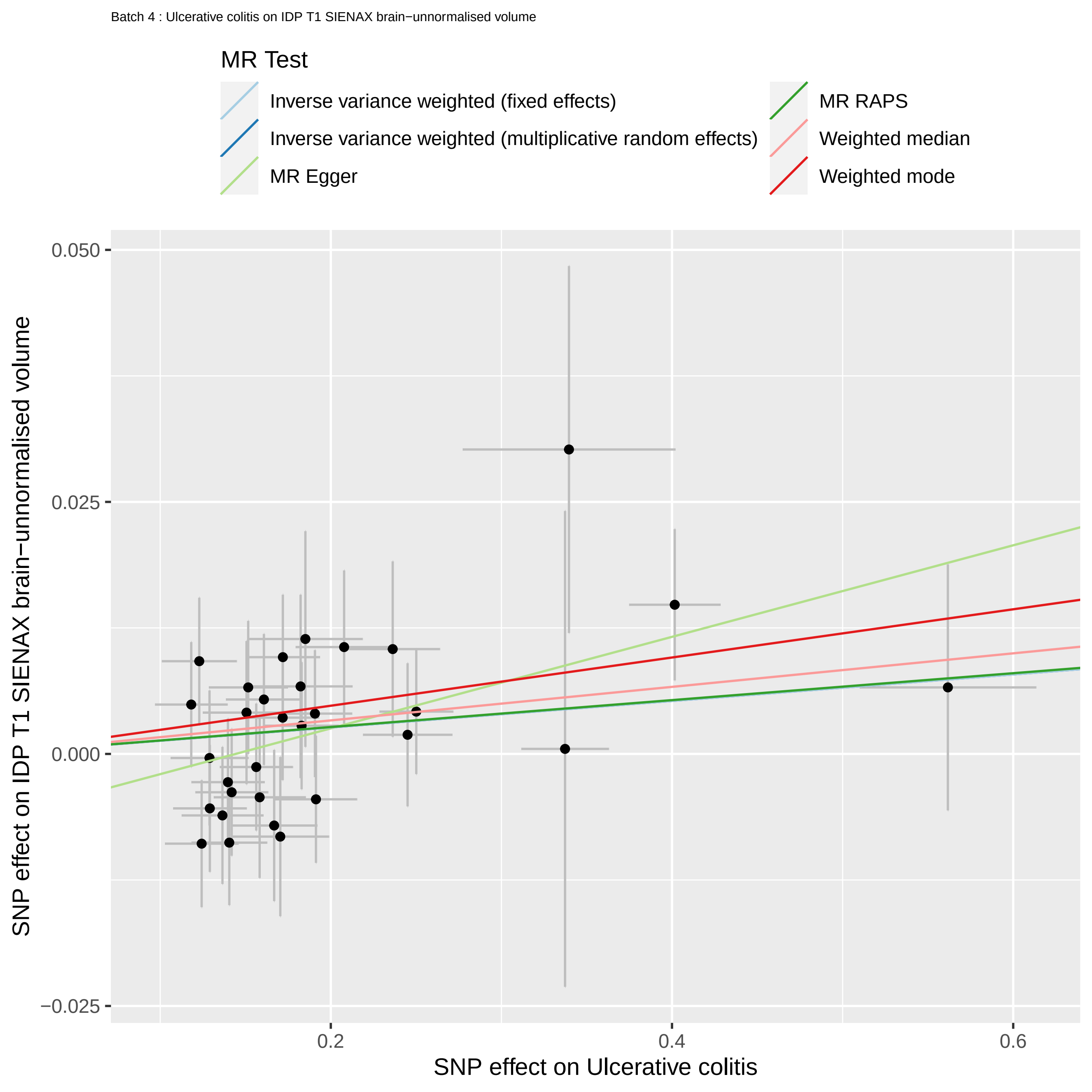
Batch 4 : Ulcerative colitis on IDP T1 SIENAX brain−unnormalised volume
MR Test
Inverse variance weighted (fixed effects)
Inverse variance weighted (multiplicative random effects)
MR Egger
MR RAPS
Weighted median
Weighted mode
0.050
0.025
0.000
−0.025
0.2
0.4
0.6
SNP effect on IDP T1 SIENAX brain−unnormalised volume
SNP effect on Ulcerative colitis

Batch 5 : Crohn's disease on IDP T1 FAST ROIs L temp fusif cortex post

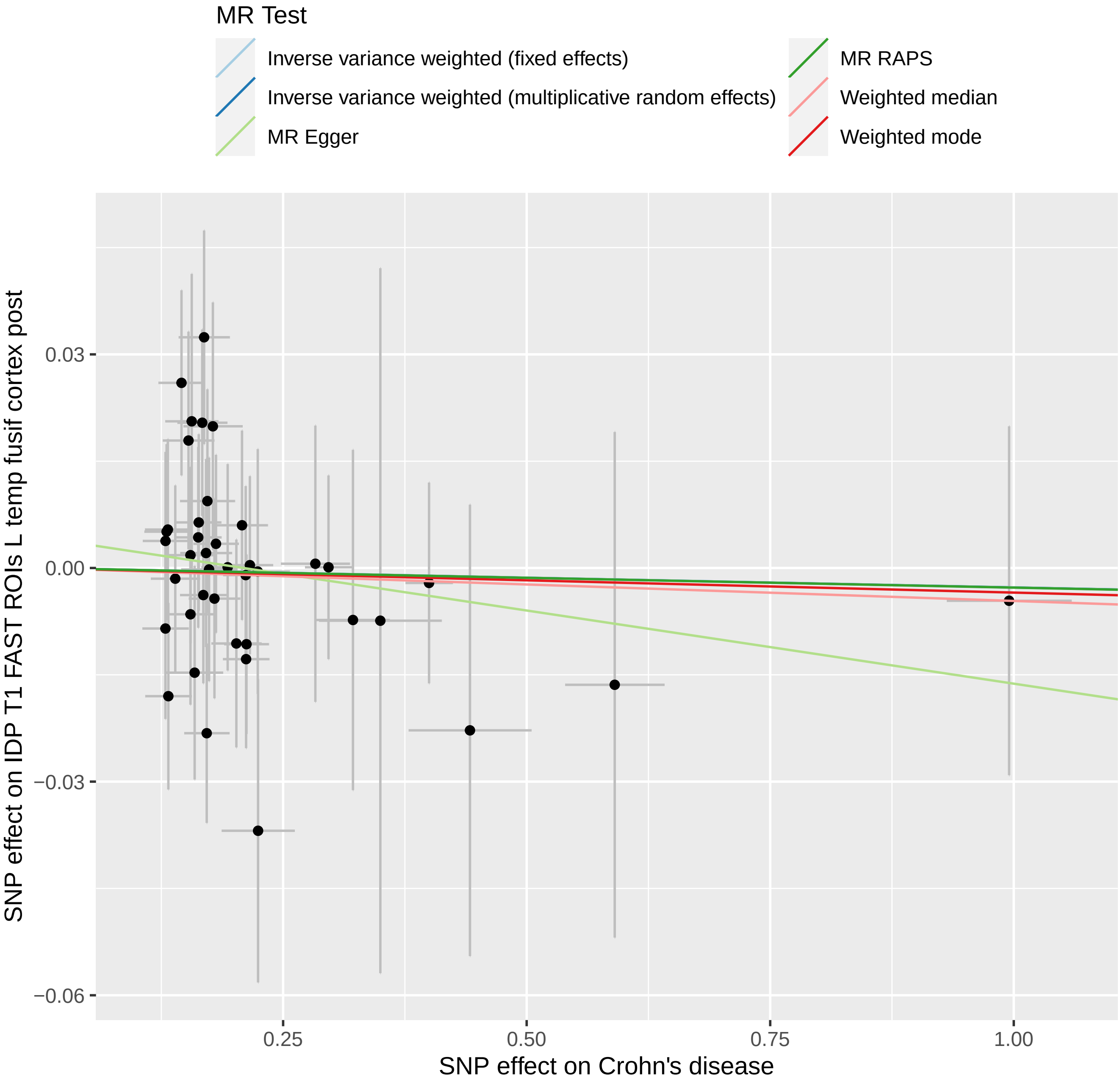

Batch 6 : Ulcerative colitis on IDP T1 FAST ROIs L temp fusif cortex post

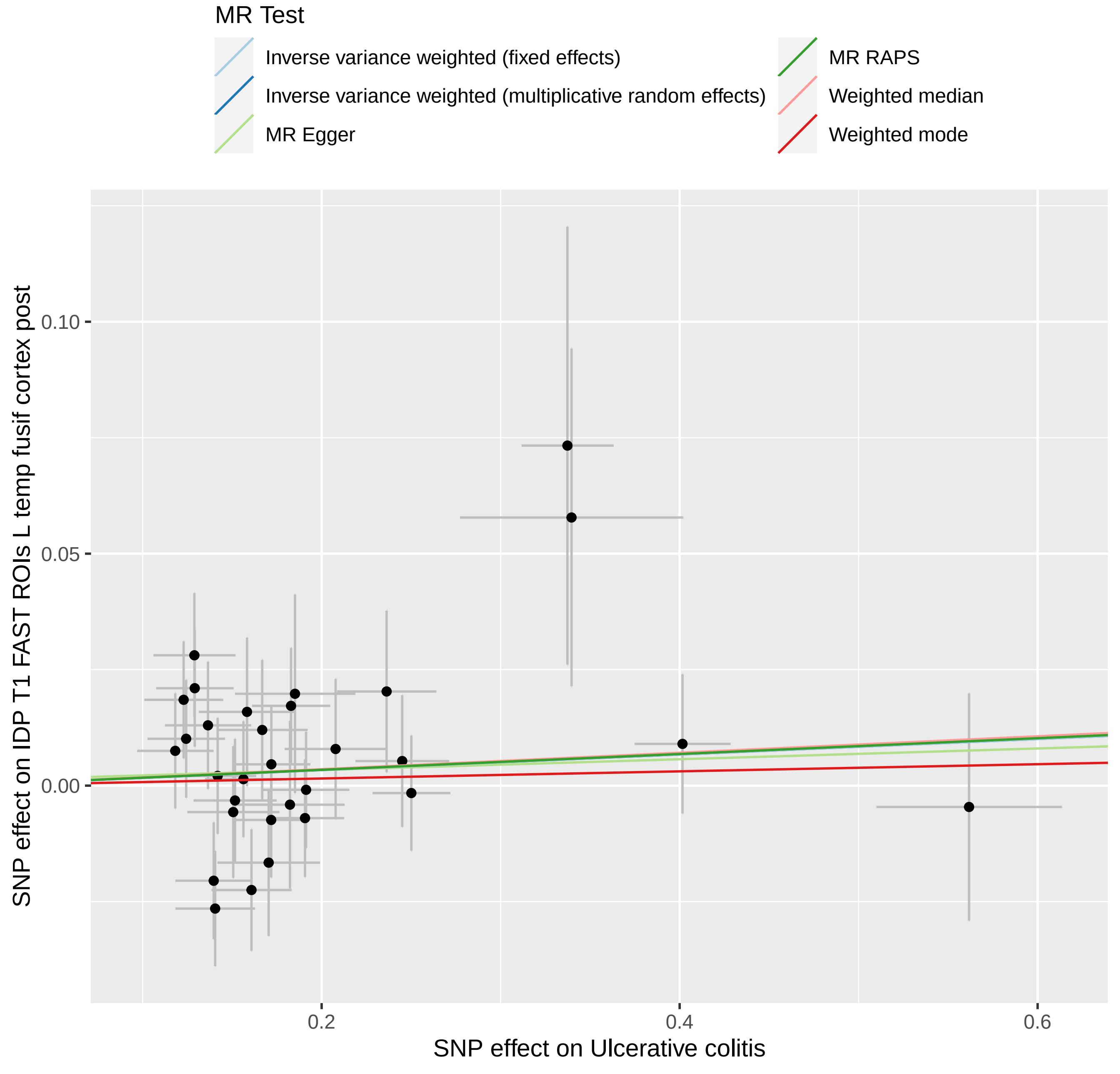

Batch 7 : Crohn's disease on IDP T1 FAST ROIs R temp fusif cortex post

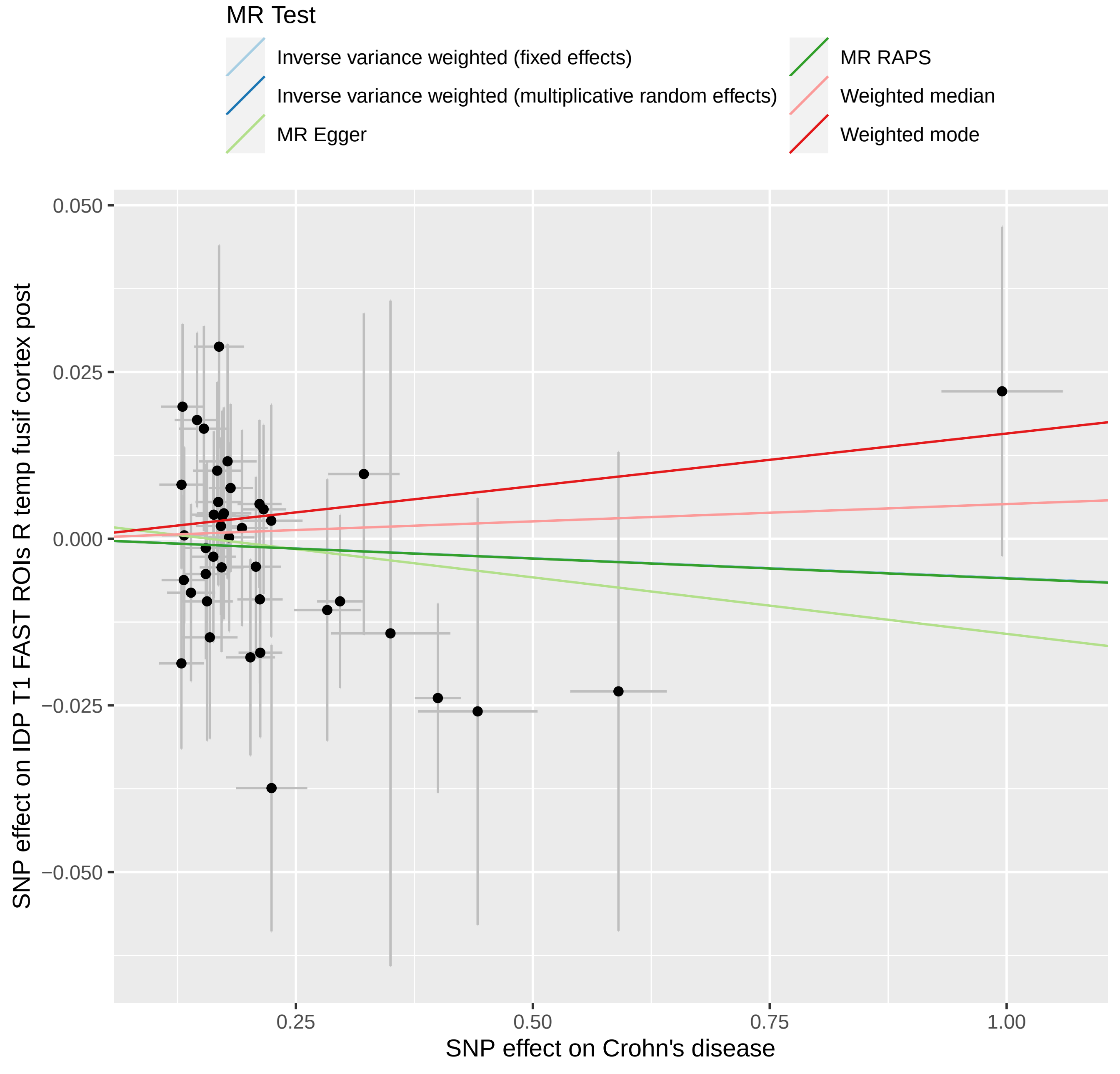

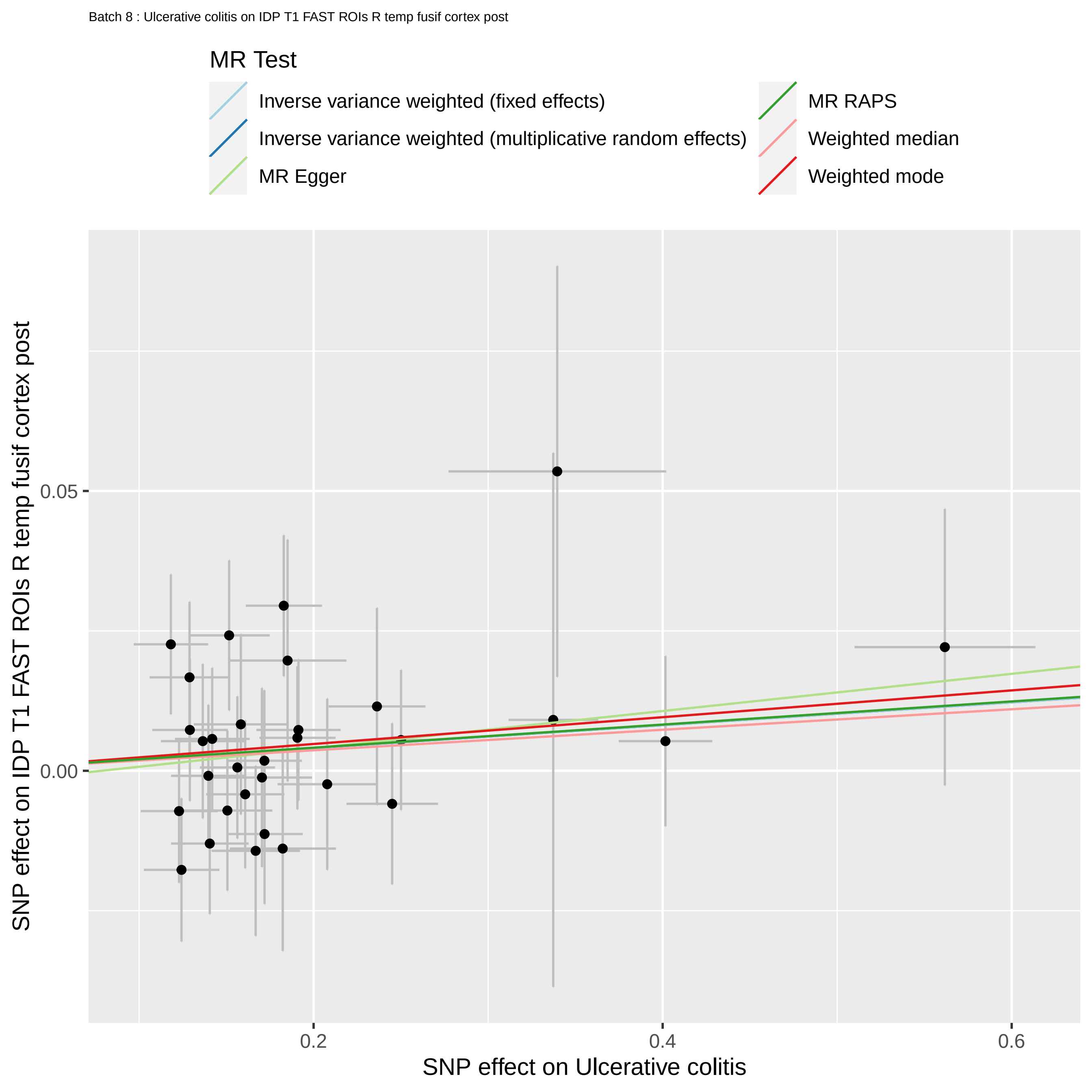
Batch 8 : Ulcerative colitis on IDP T1 FAST ROIs R temp fusif cortex post
MR Test
Inverse variance weighted (fixed effects)
Inverse variance weighted (multiplicative random effects)
MR Egger
MR RAPS
Weighted median
Weighted mode
0.05
0.00
0.2
0.4
0.6
SNP effect on Ulcerative colitis
SNP effect on IDP T1 FAST ROIs R temp fusif cortex post

Batch 9 : Crohn's disease on IDP T1 FAST ROIs L temp occ fusif cortex

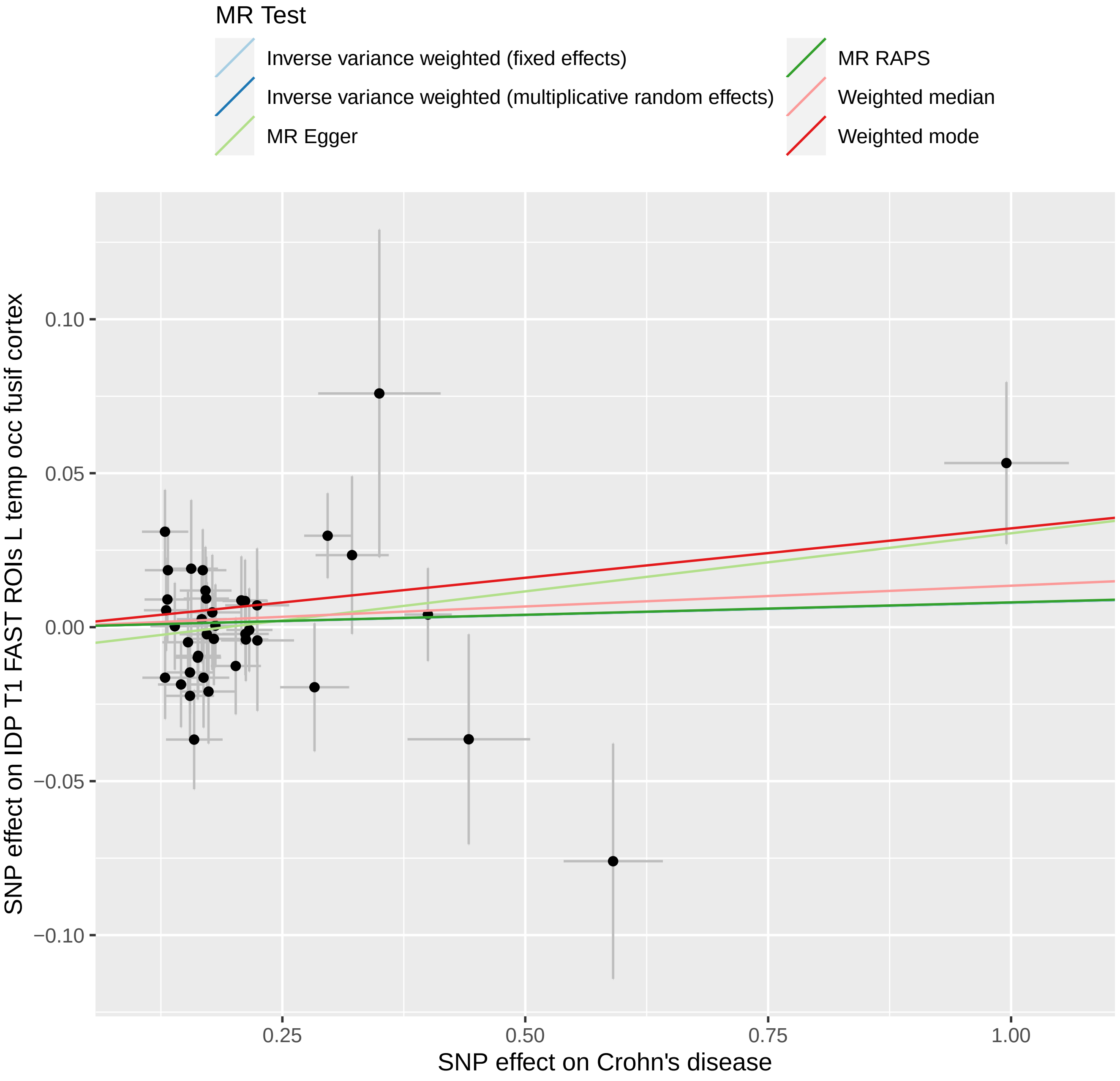

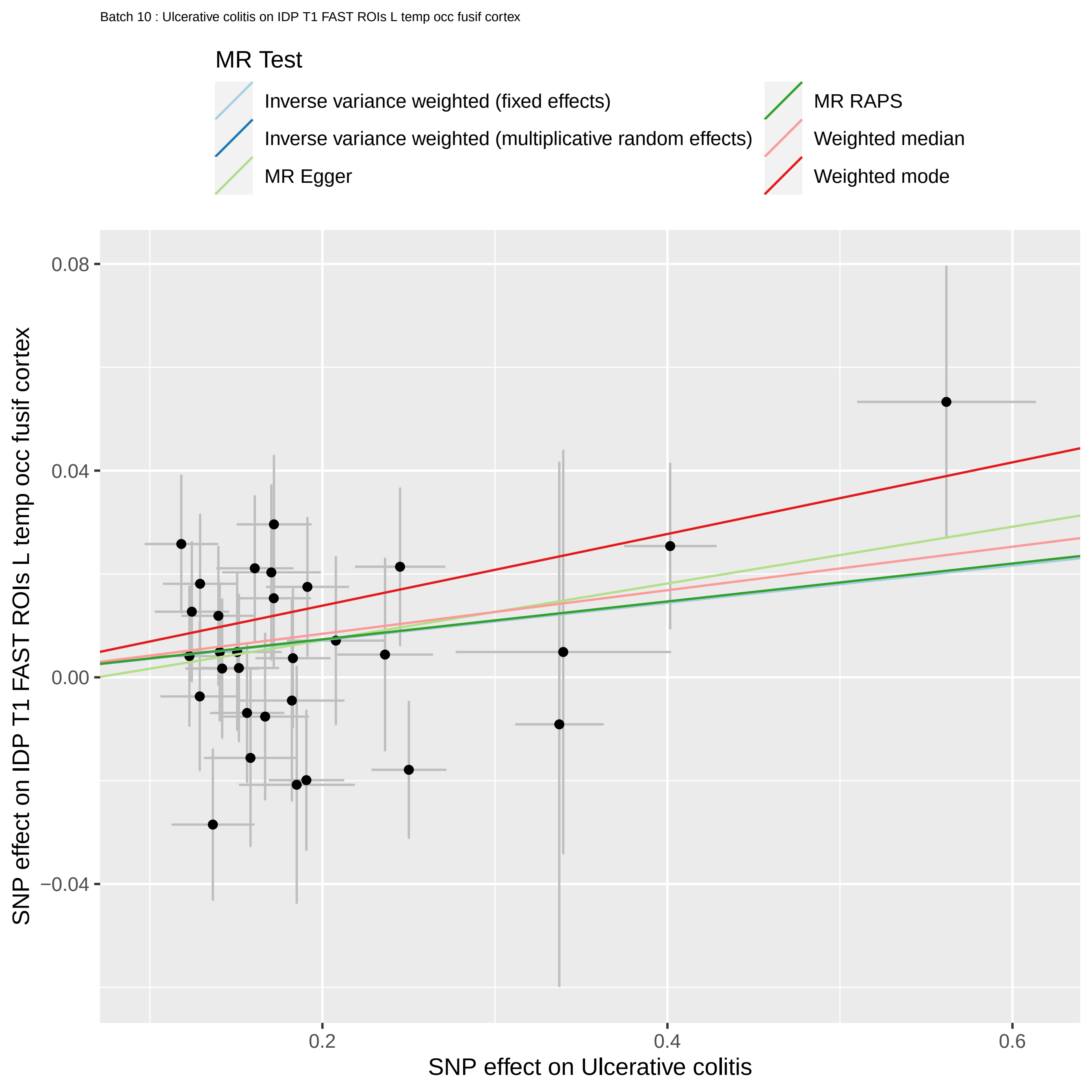

Batch 10 : Ulcerative colitis on IDP T1 FAST ROIs L temp occ fusif cortex
MR Test
Inverse variance weighted (fixed effects)
Inverse variance weighted (multiplicative random effects)
MR Egger
MR RAPS
Weighted median
Weighted mode
0.08
0.04
0.00
−0.04
0.2
0.4
0.6
SNP effect on IDP T1 FAST ROIs L temp occ fusif cortex
SNP effect on Ulcerative colitis

Batch 11 : Crohn's disease on IDP T1 FAST ROIs R temp occ fusif cortex

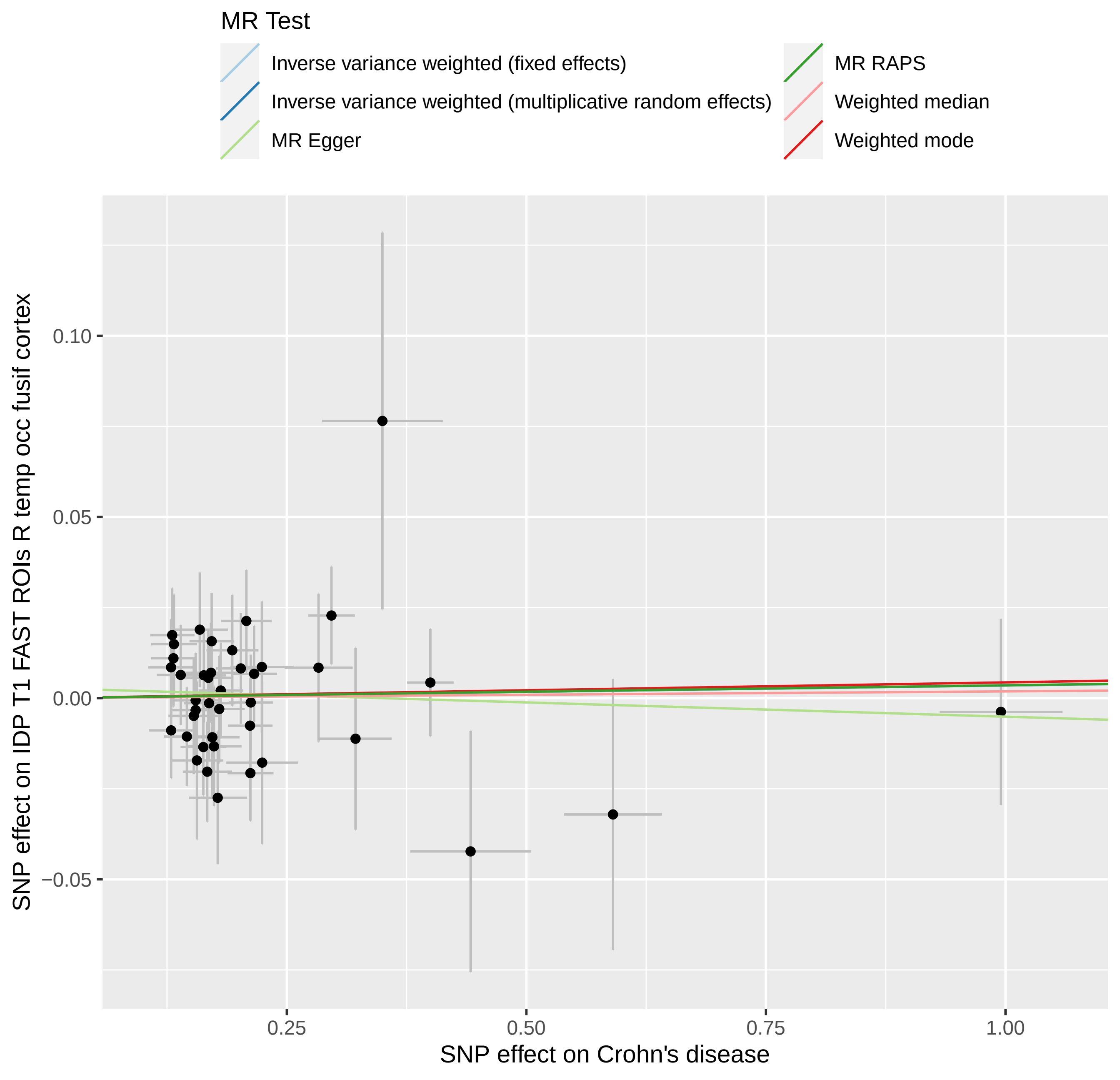

Batch 12 : Ulcerative colitis on IDP T1 FAST ROIs R temp occ fusif cortex

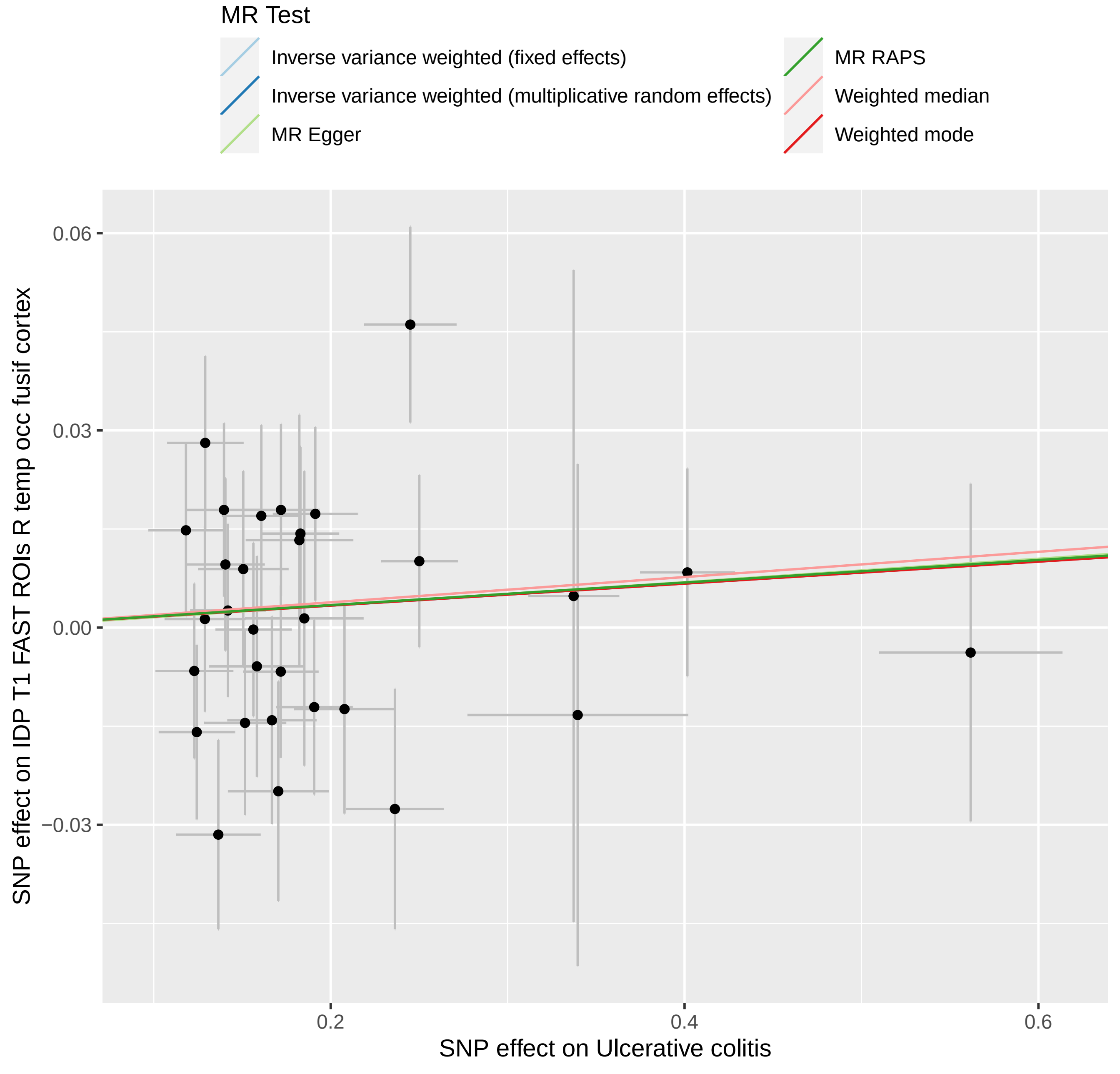

Batch 13 : Crohn's disease on IDP T1 FAST ROIs L occ fusif gyrus

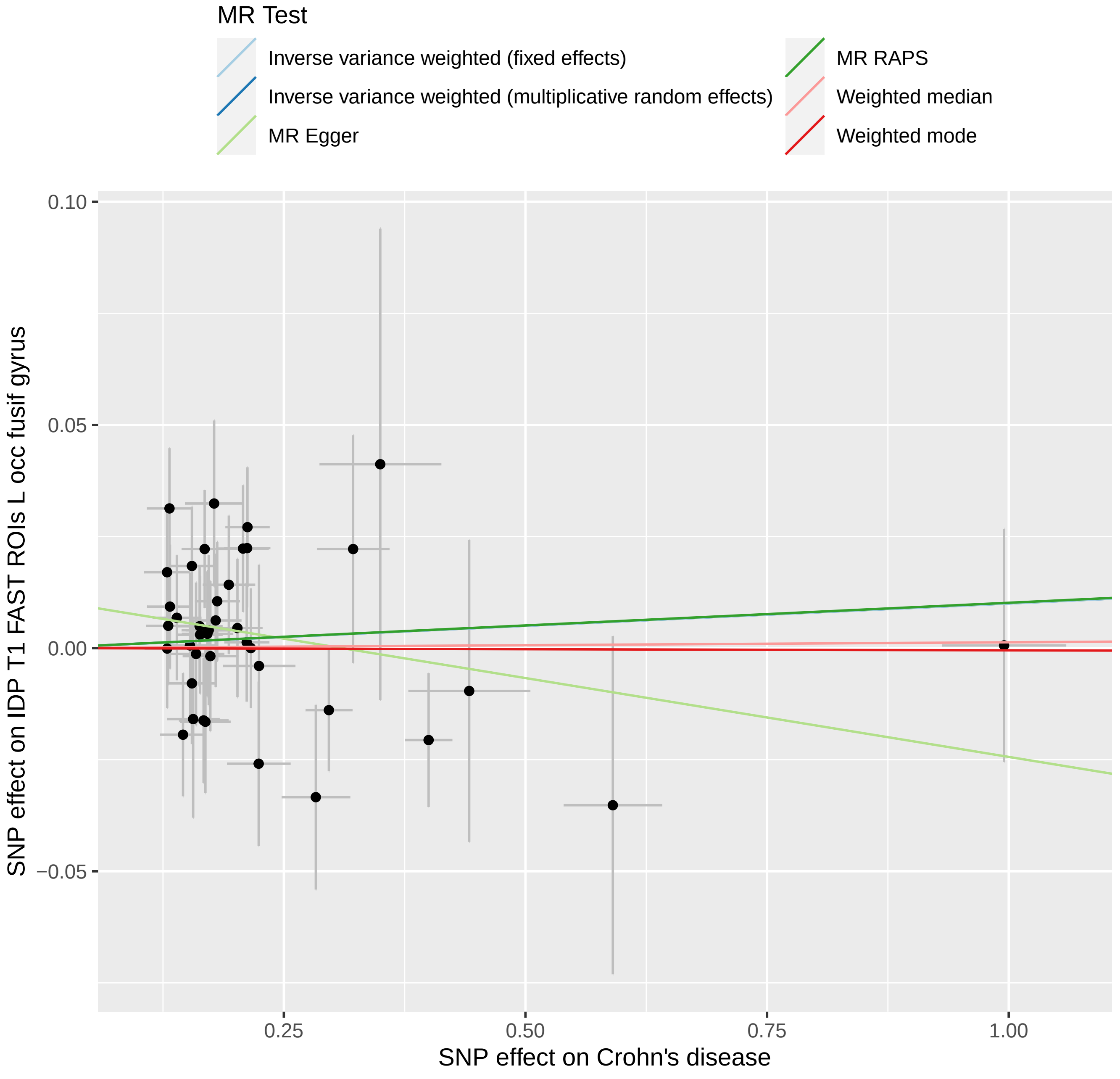

Batch 14 : Ulcerative colitis on IDP T1 FAST ROIs L occ fusif gyrus

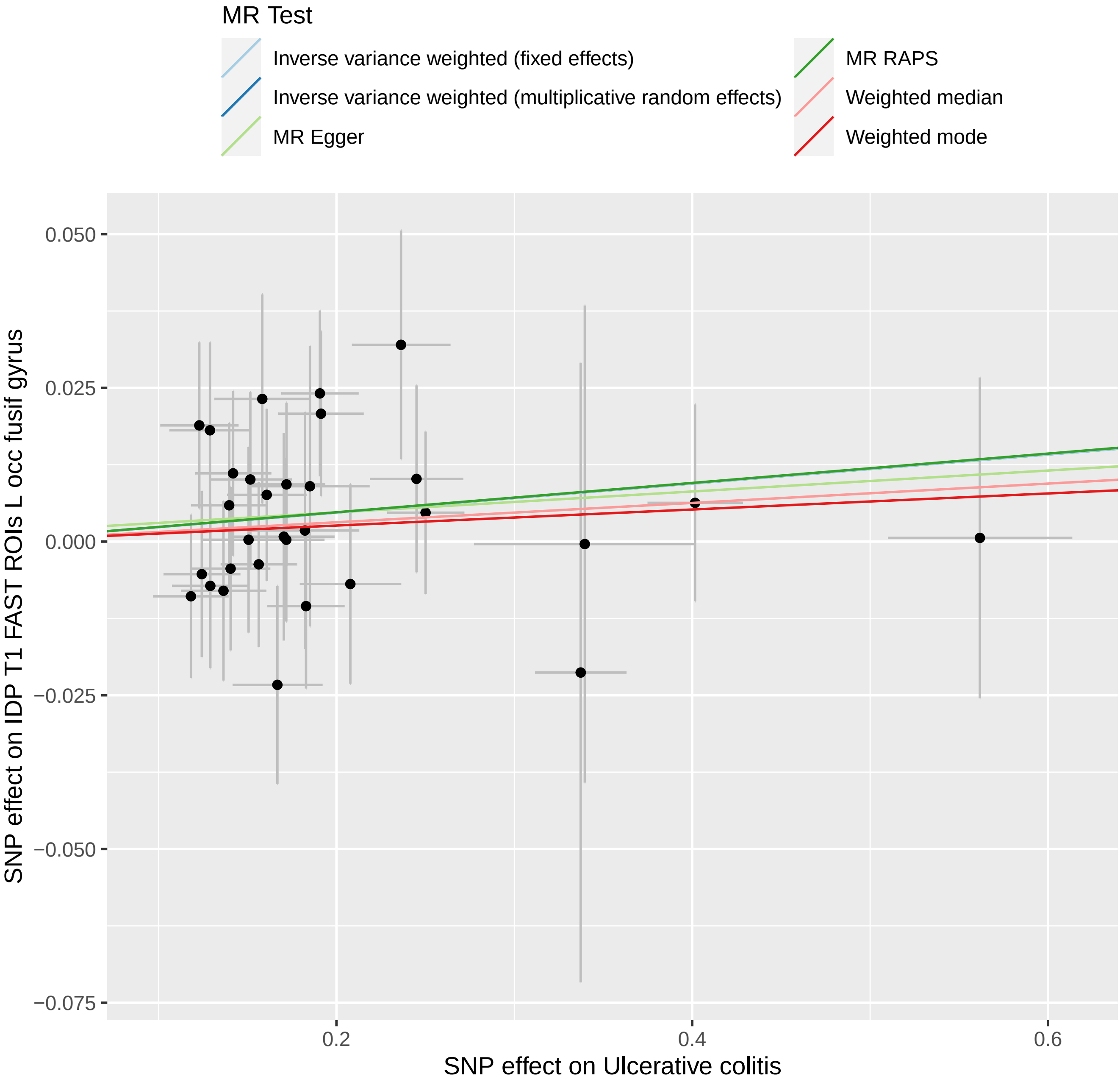

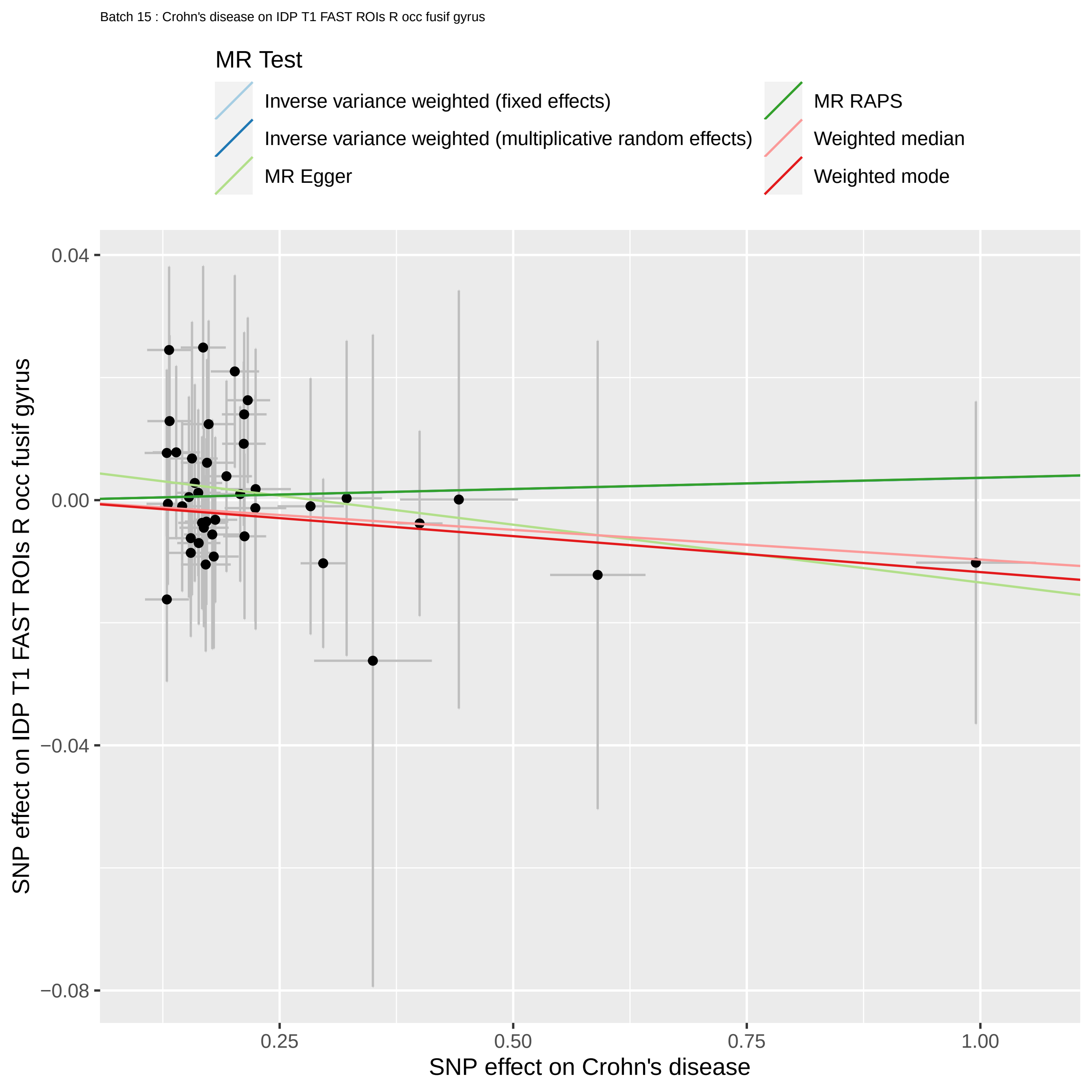
Batch 15 : Crohn's disease on IDP T1 FAST ROIs R occ fusif gyrus
MR Test
Inverse variance weighted (fixed effects)
Inverse variance weighted (multiplicative random effects)
MR Egger
MR RAPS
Weighted median
Weighted mode
SNP effect on IDP T1 FAST ROIs R occ fusif gyrus
0.04
0.00
−0.04
−0.08
0.25
0.50
0.75
1.00
SNP effect on Crohn's disease

Batch 16 : Ulcerative colitis on IDP T1 FAST ROIs R occ fusif gyrus

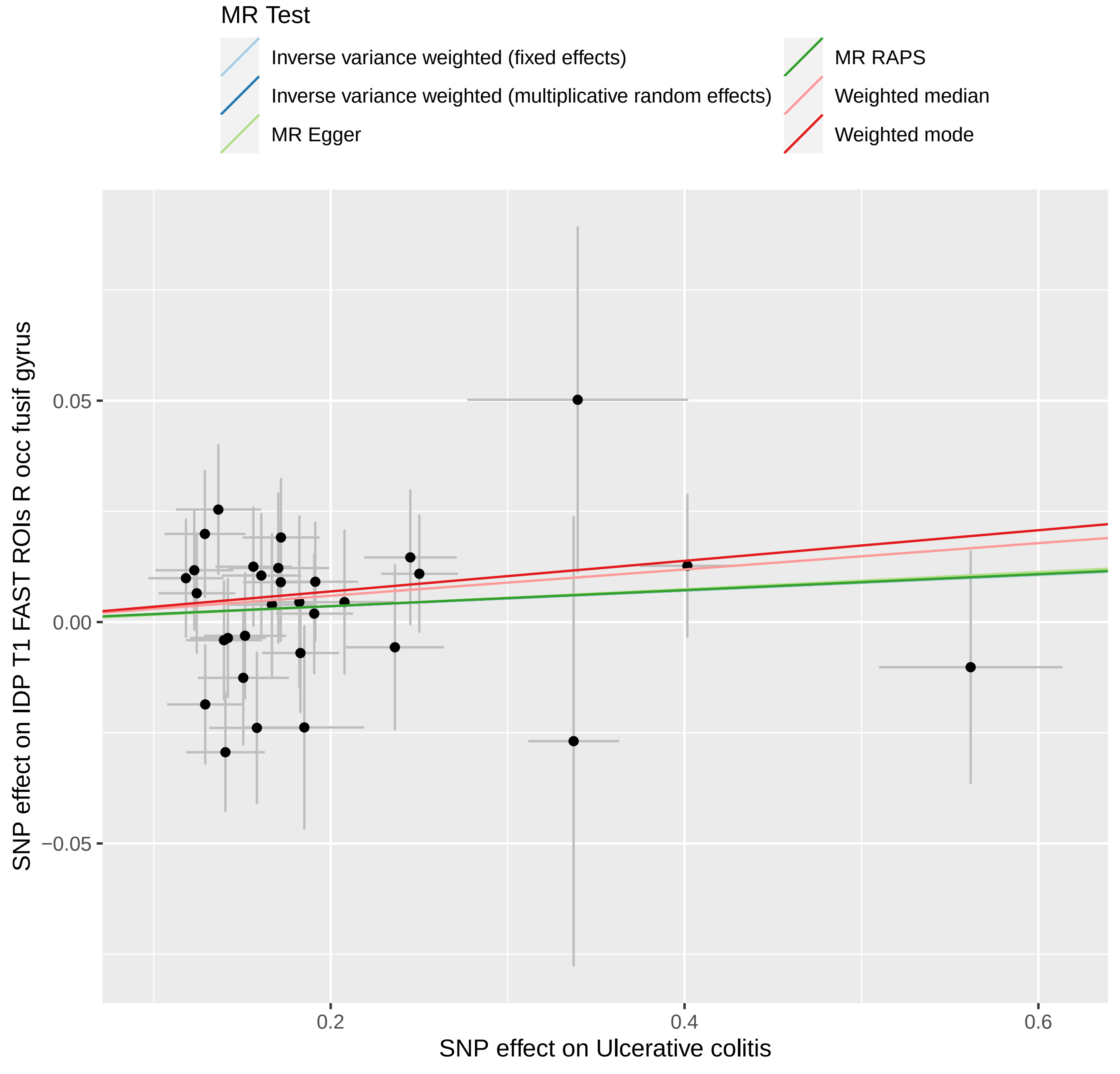

Batch 17 : Crohn's disease on IDP T1 FAST ROIs L front operc cortex

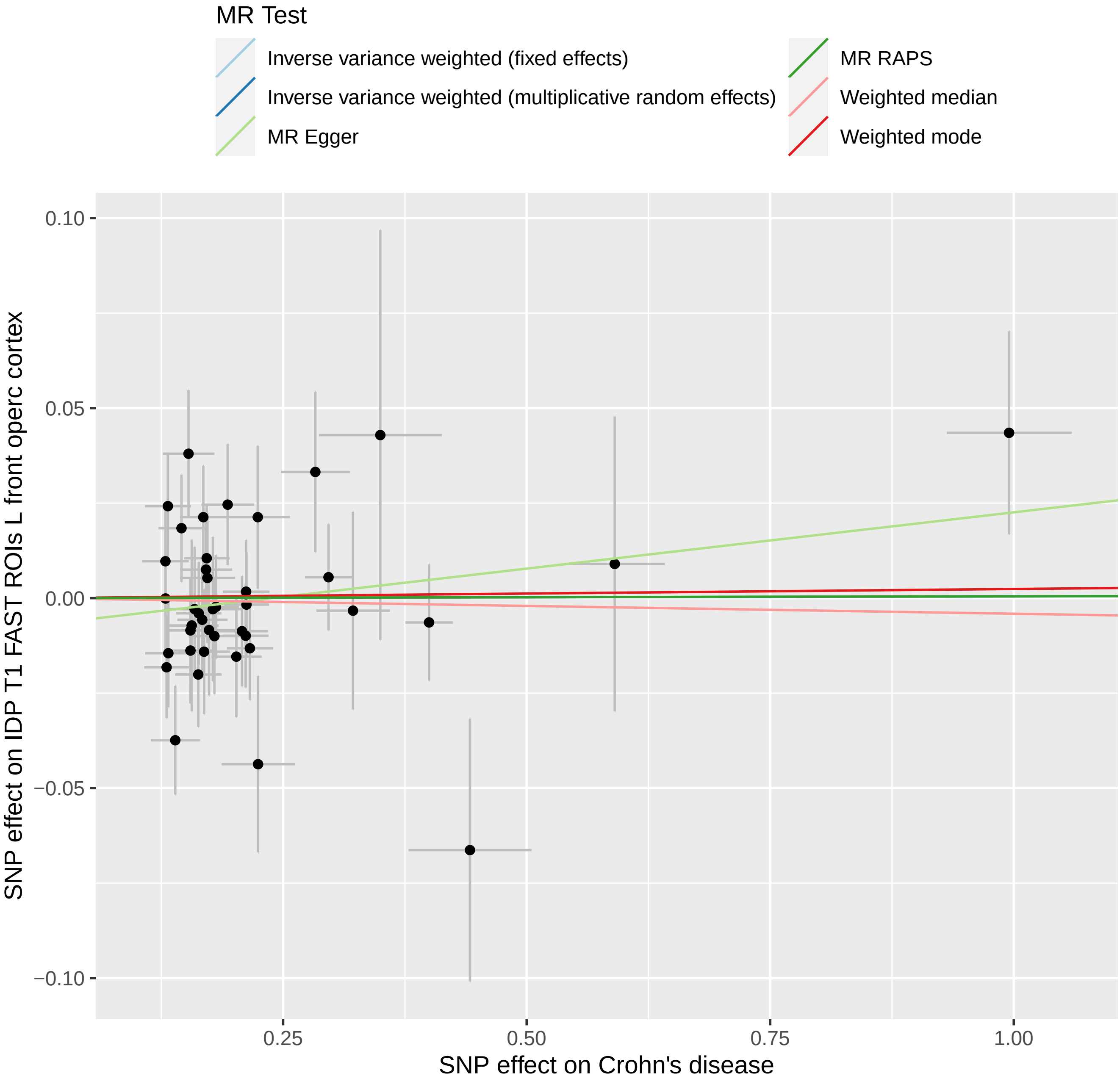

Batch 18 : Ulcerative colitis on IDP T1 FAST ROIs L front operc cortex

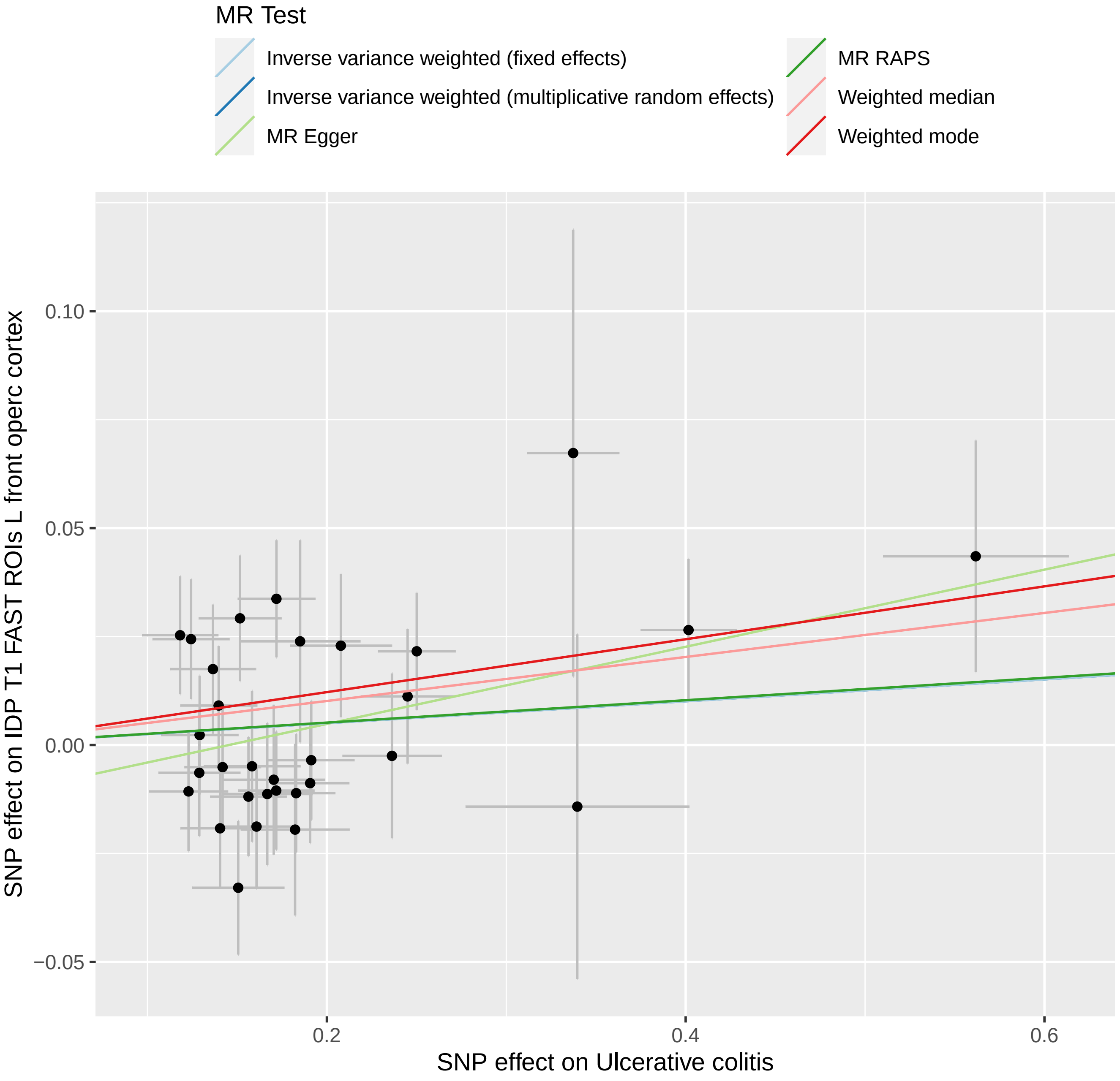

Batch 19 : Crohn's disease on IDP T1 FAST ROIs R front operc cortex

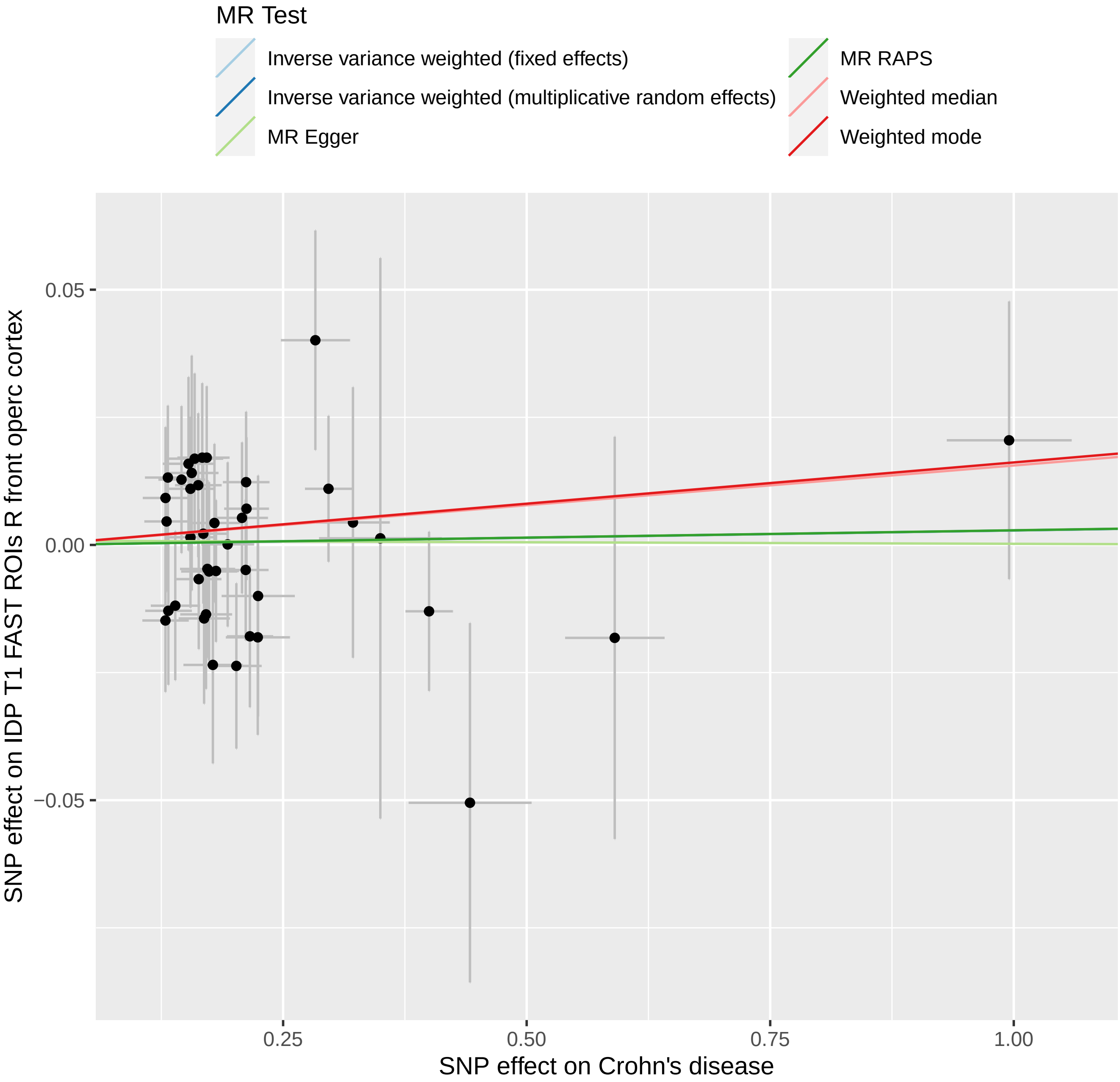

Batch 20 : Ulcerative colitis on IDP T1 FAST ROIs R front operc cortex

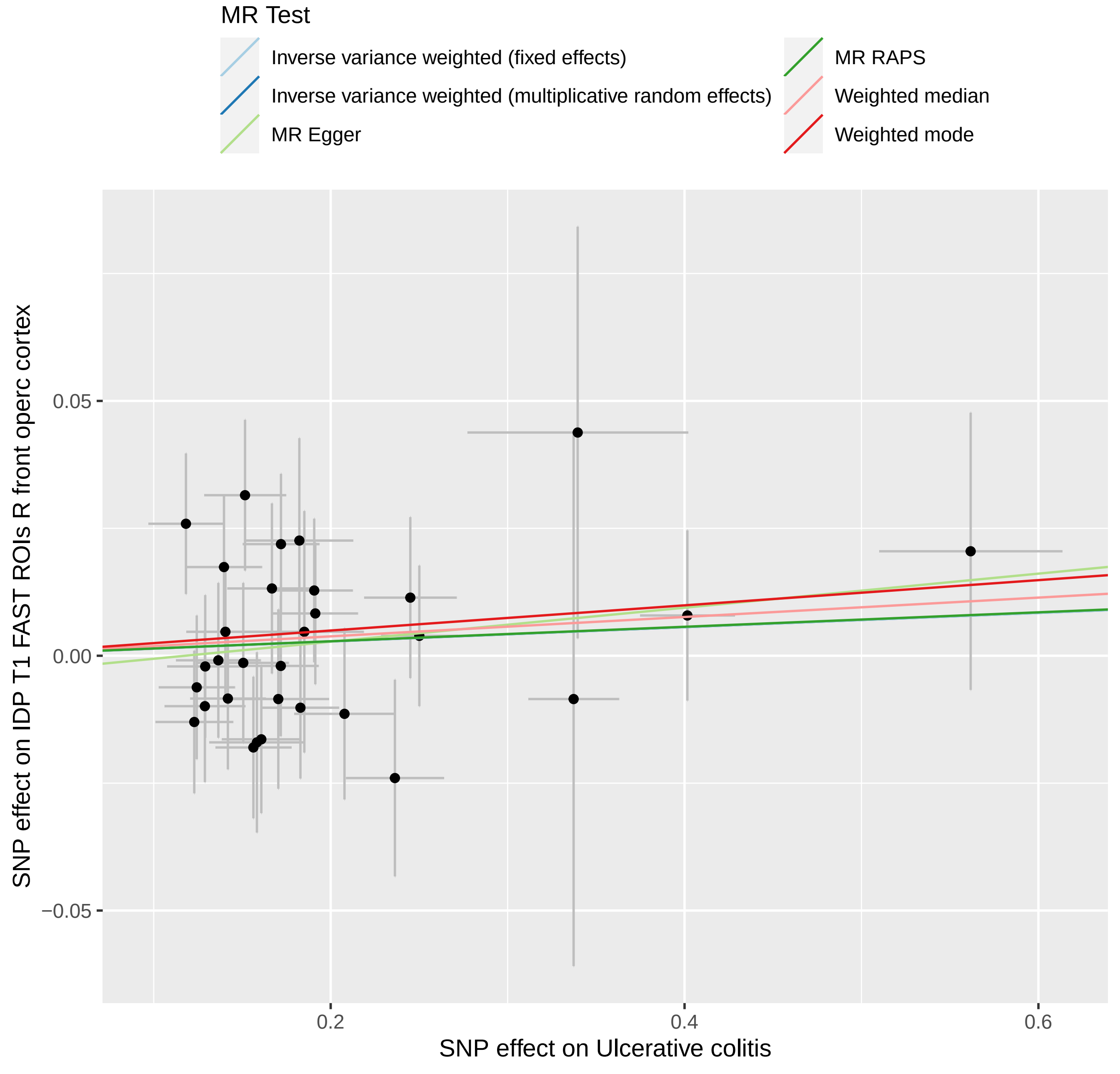

Batch 21 : Crohn's disease on IDP T1 FAST ROIs L cent operc cortex

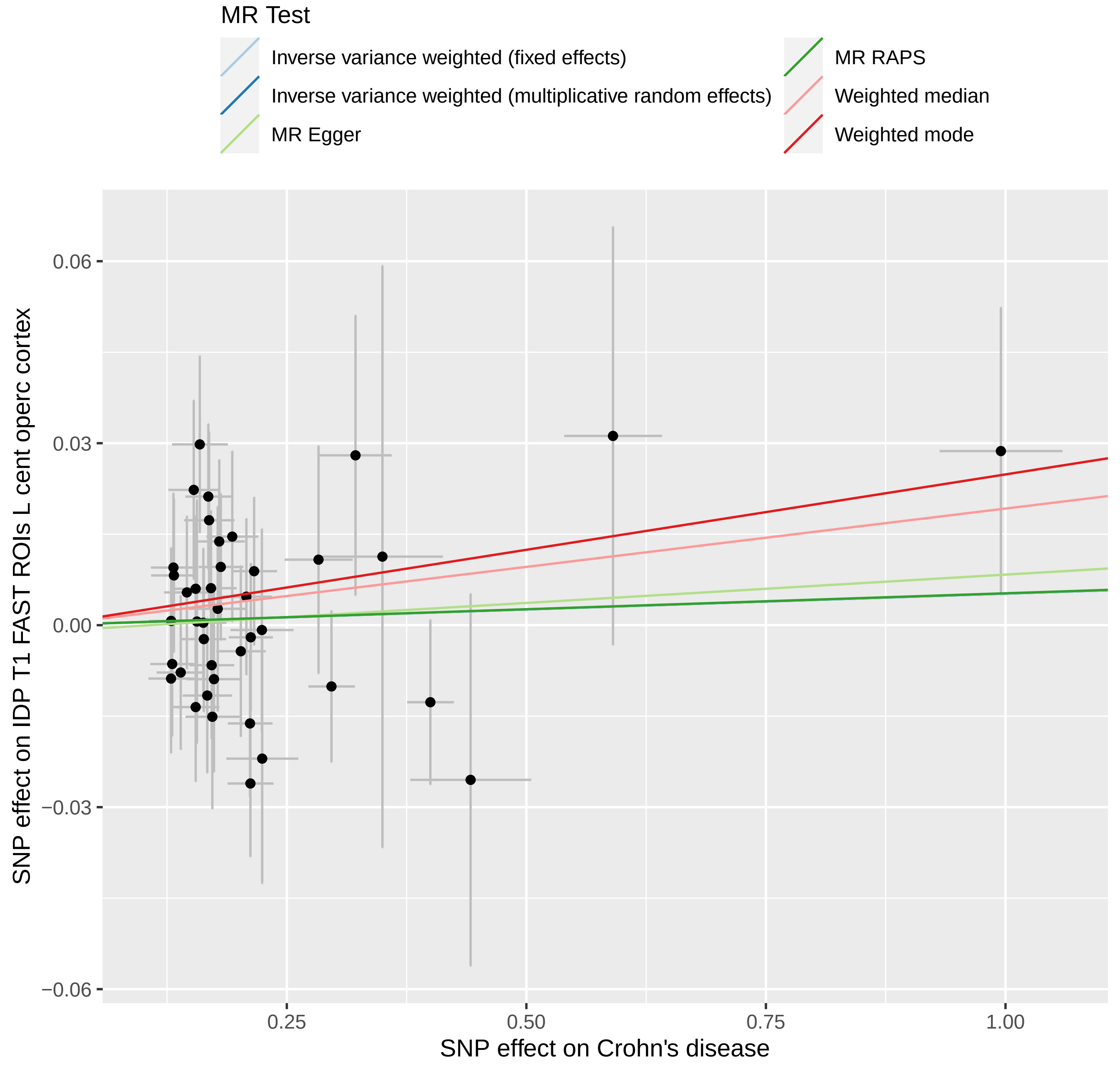

Batch 22 : Ulcerative colitis on IDP T1 FAST ROIs L cent operc cortex

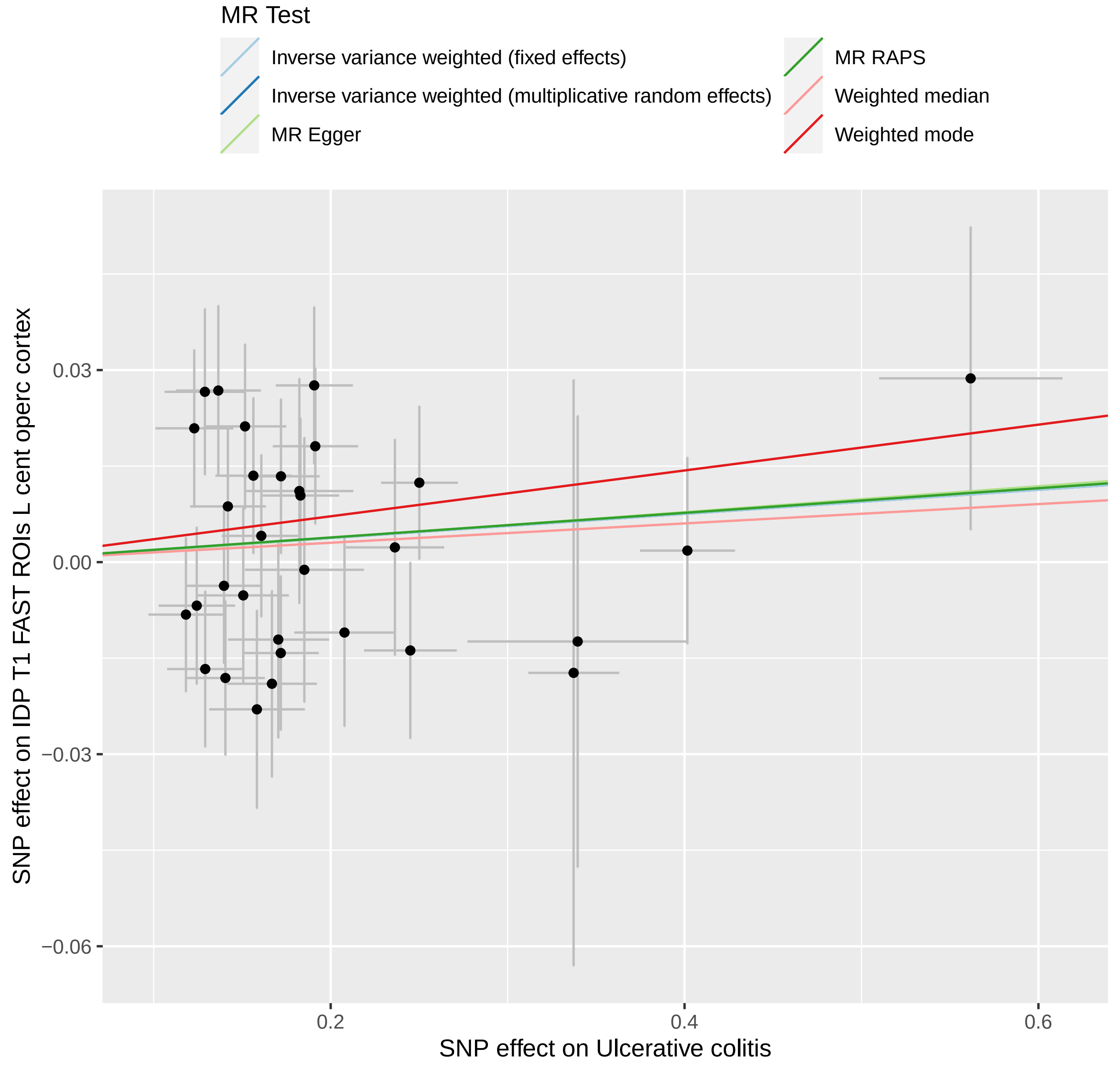

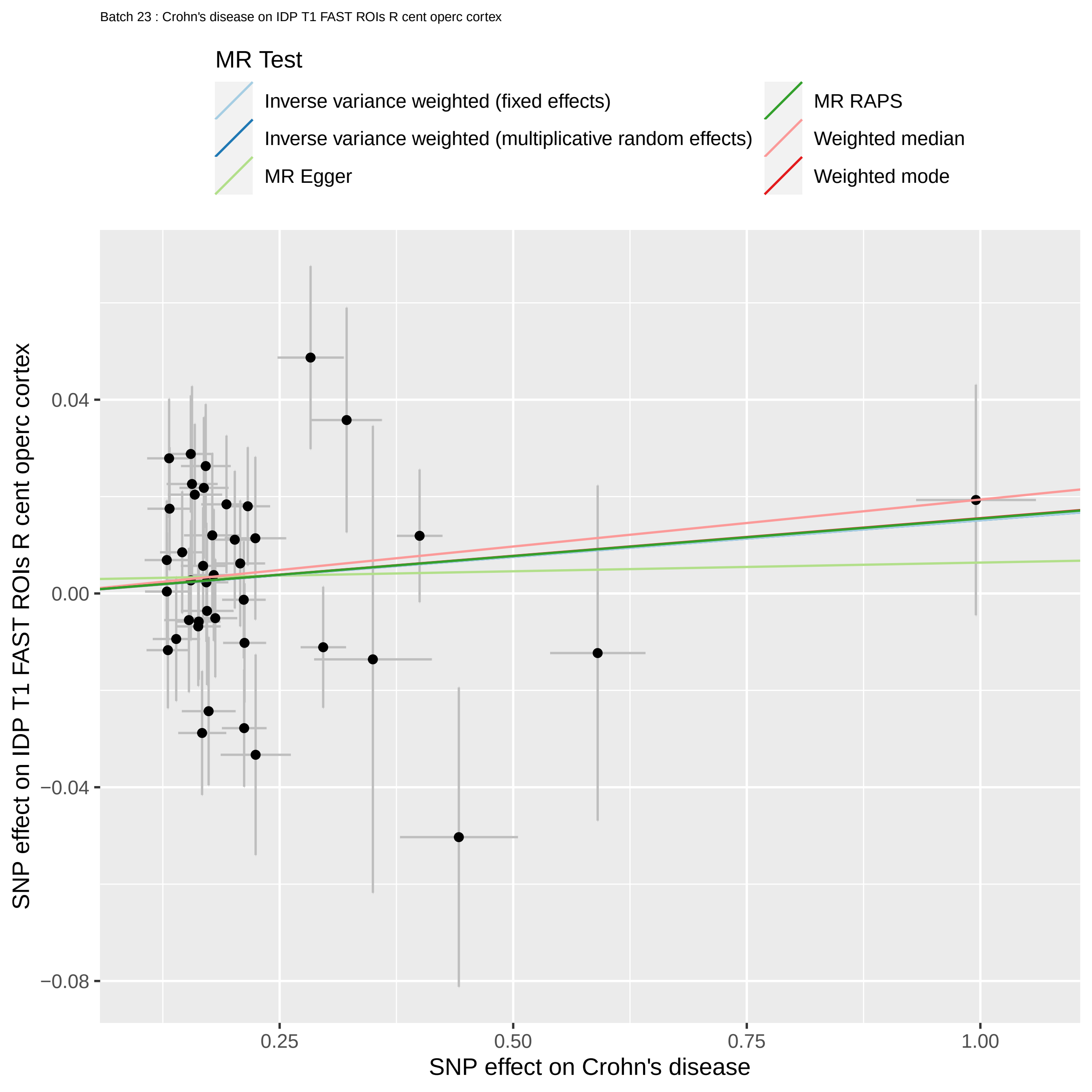
Batch 23 : Crohn's disease on IDP T1 FAST ROIs R cent operc cortex
MR Test
Inverse variance weighted (fixed effects)
Inverse variance weighted (multiplicative random effects)
MR Egger
MR RAPS
Weighted median
Weighted mode
SNP effect on IDP T1 FAST ROIs R cent operc cortex
0.04
0.00
−0.04
−0.08
0.25
0.50
0.75
1.00
SNP effect on Crohn's disease

Batch 24 : Ulcerative colitis on IDP T1 FAST ROIs R cent operc cortex

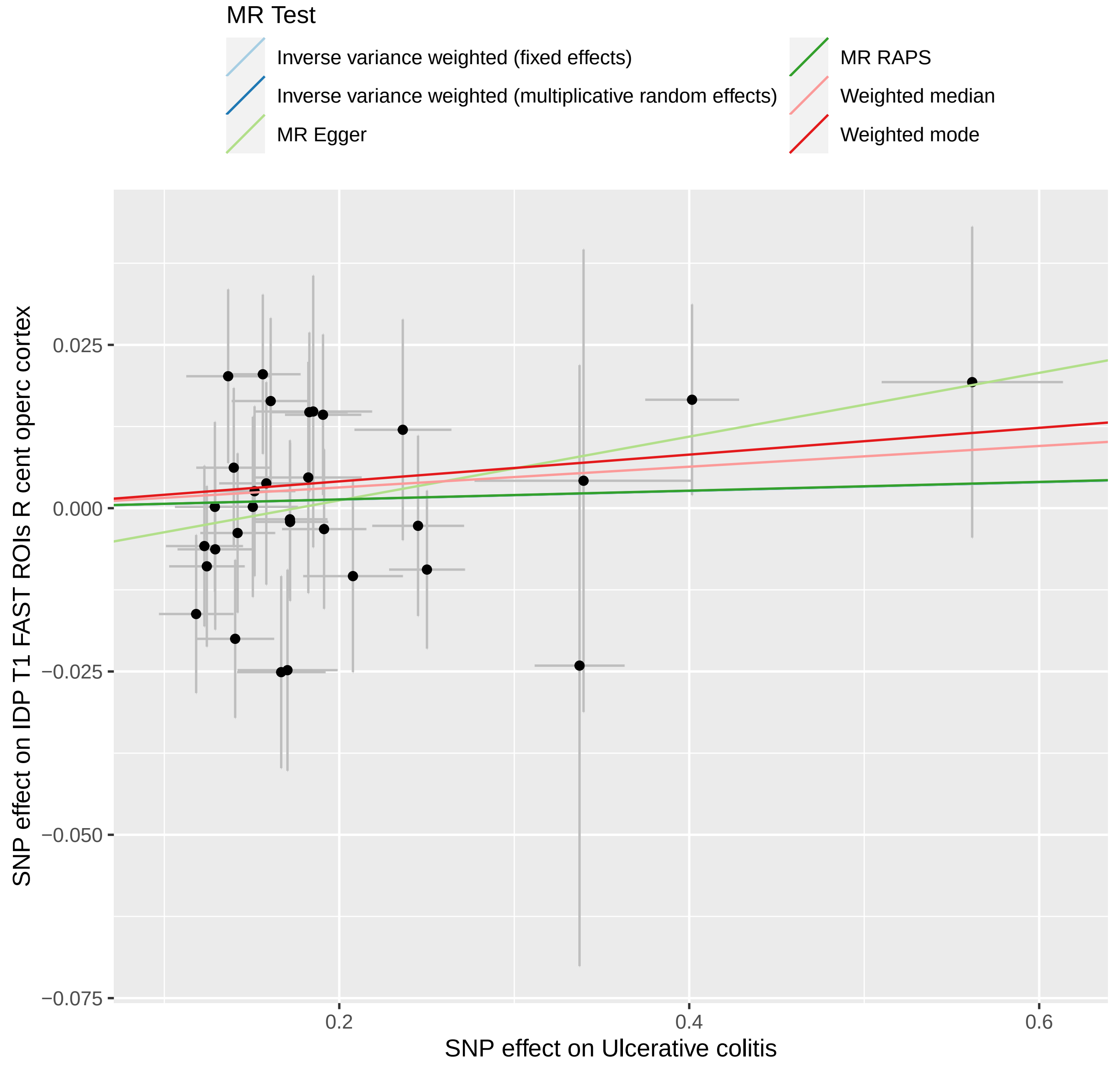

Batch 25 : Crohn's disease on IDP T1 FIRST left thalamus volume

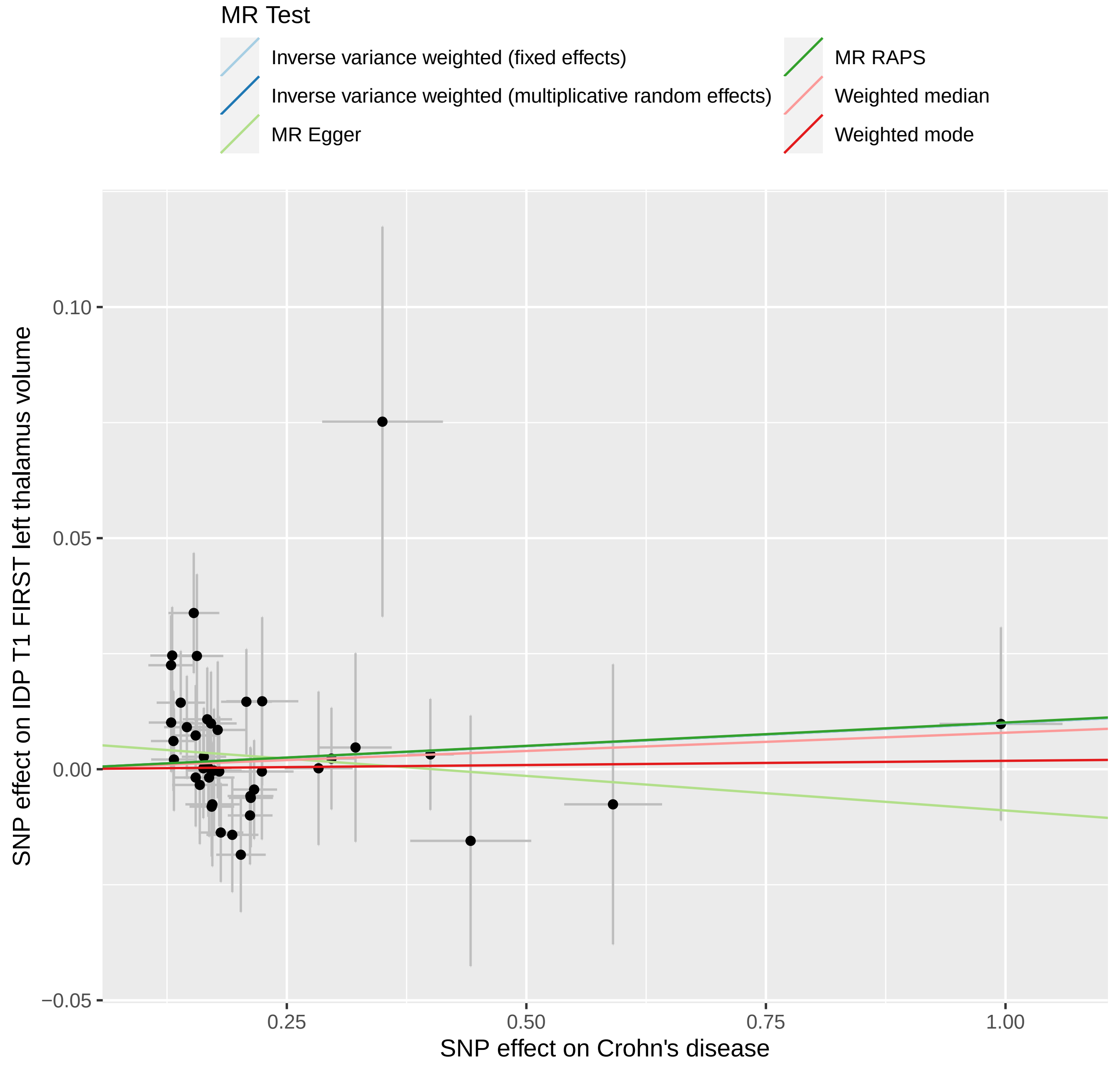

Batch 26 : Ulcerative colitis on IDP T1 FIRST left thalamus volume

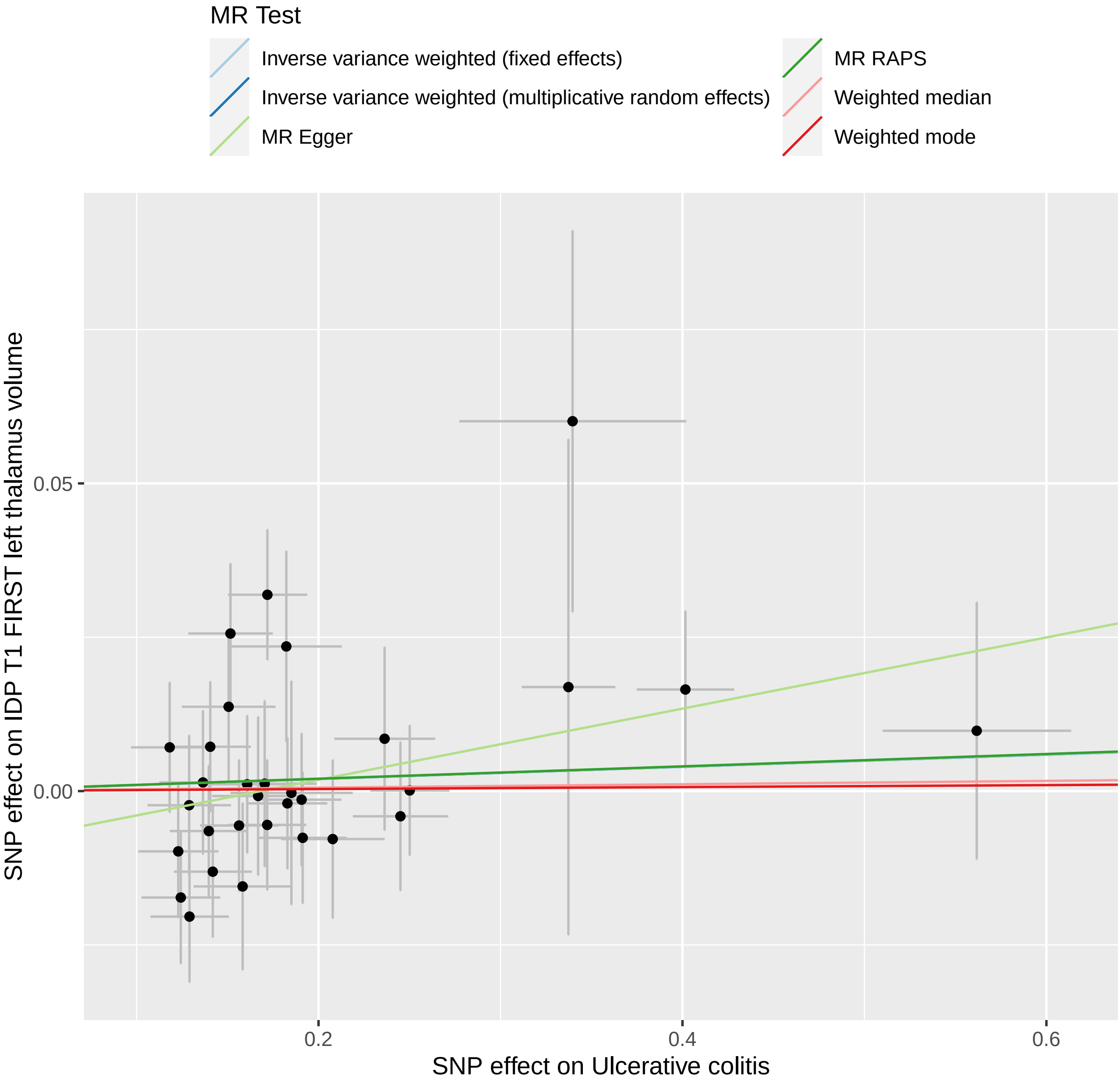

Batch 27 : Crohn's disease on IDP T1 FAST ROIs L parietal operc cortex

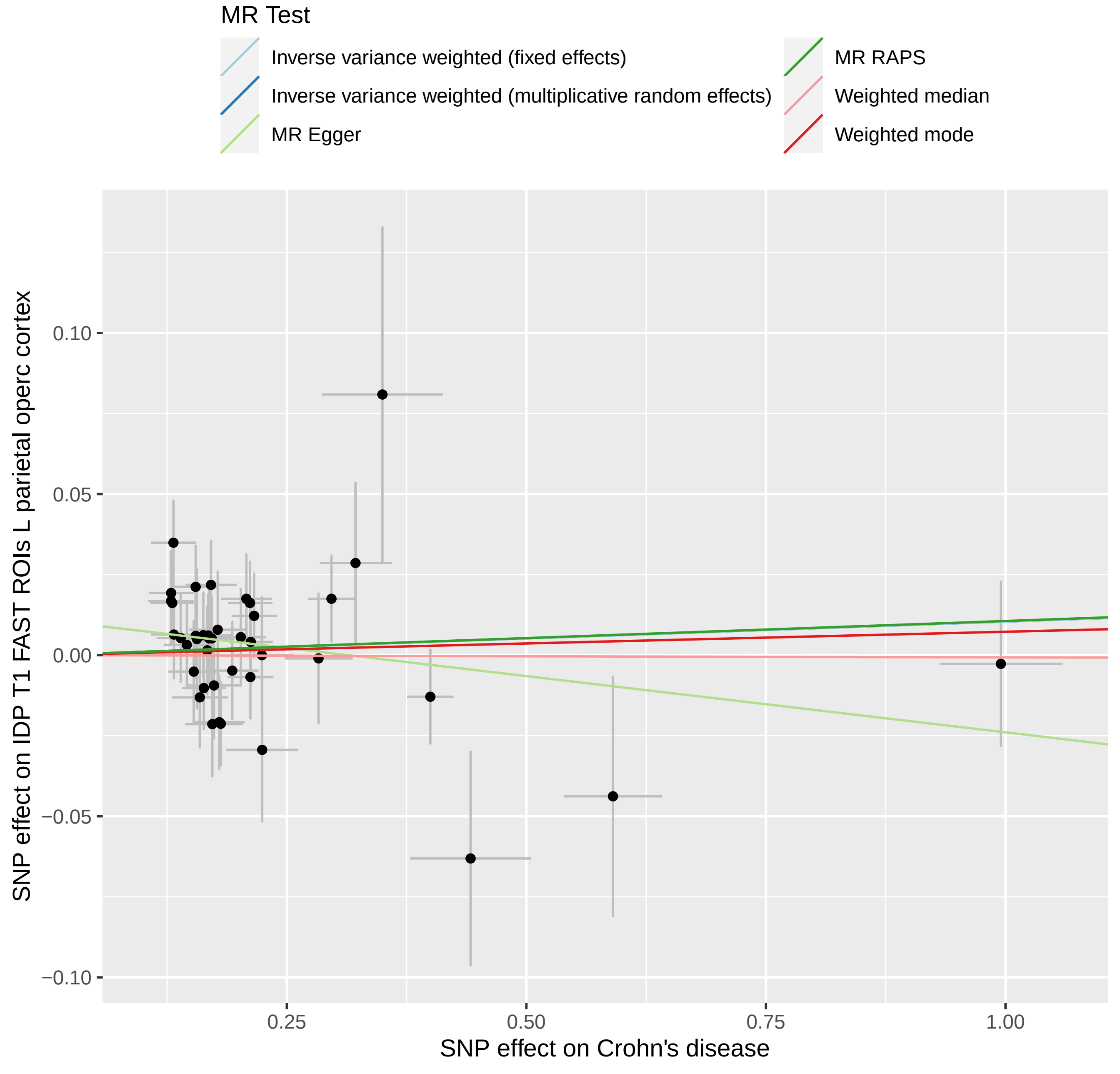

Batch 28 : Ulcerative colitis on IDP T1 FAST ROIs L parietal operc cortex

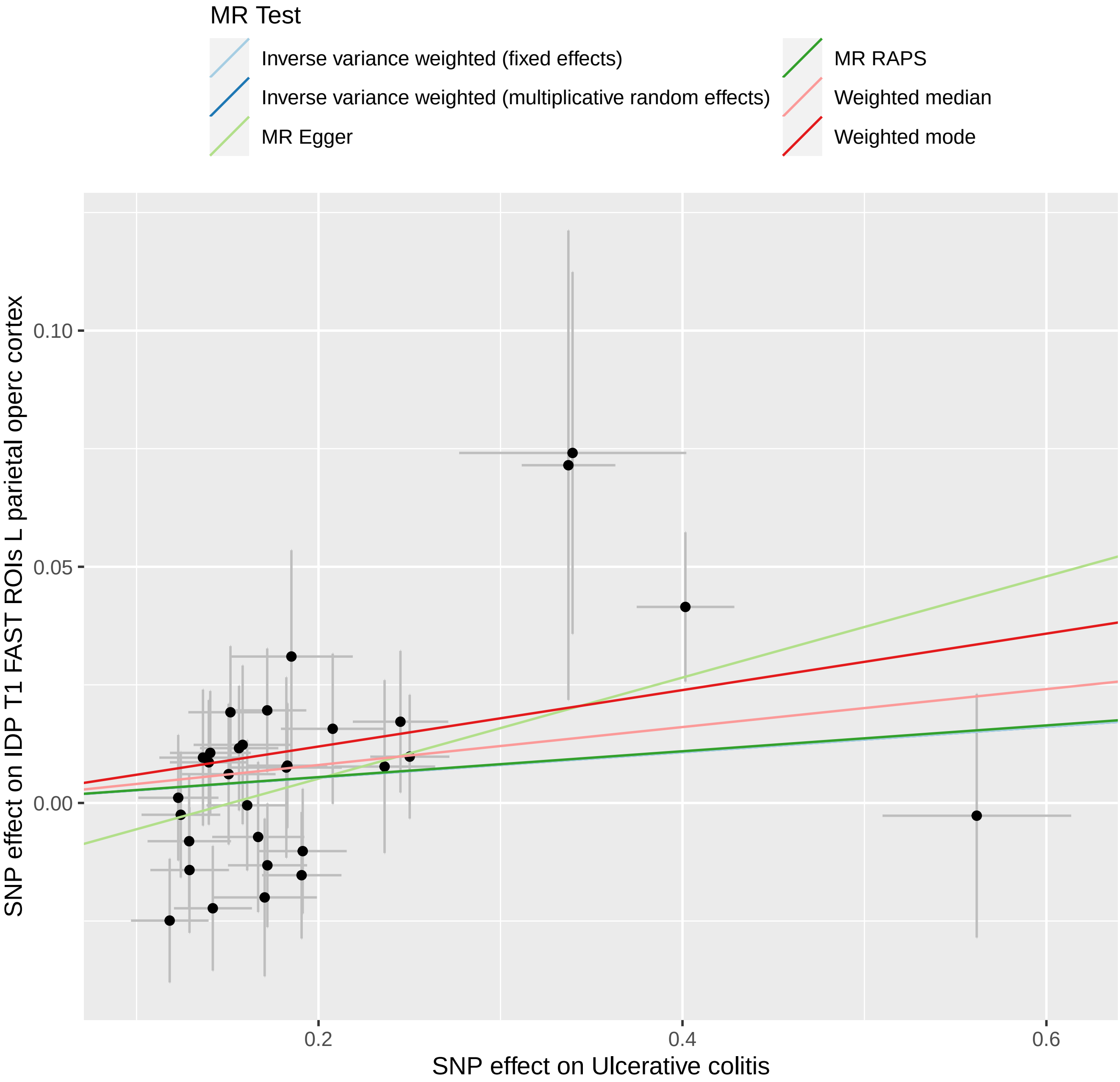

Batch 29 : Crohn's disease on IDP T1 FAST ROIs R parietal operc cortex

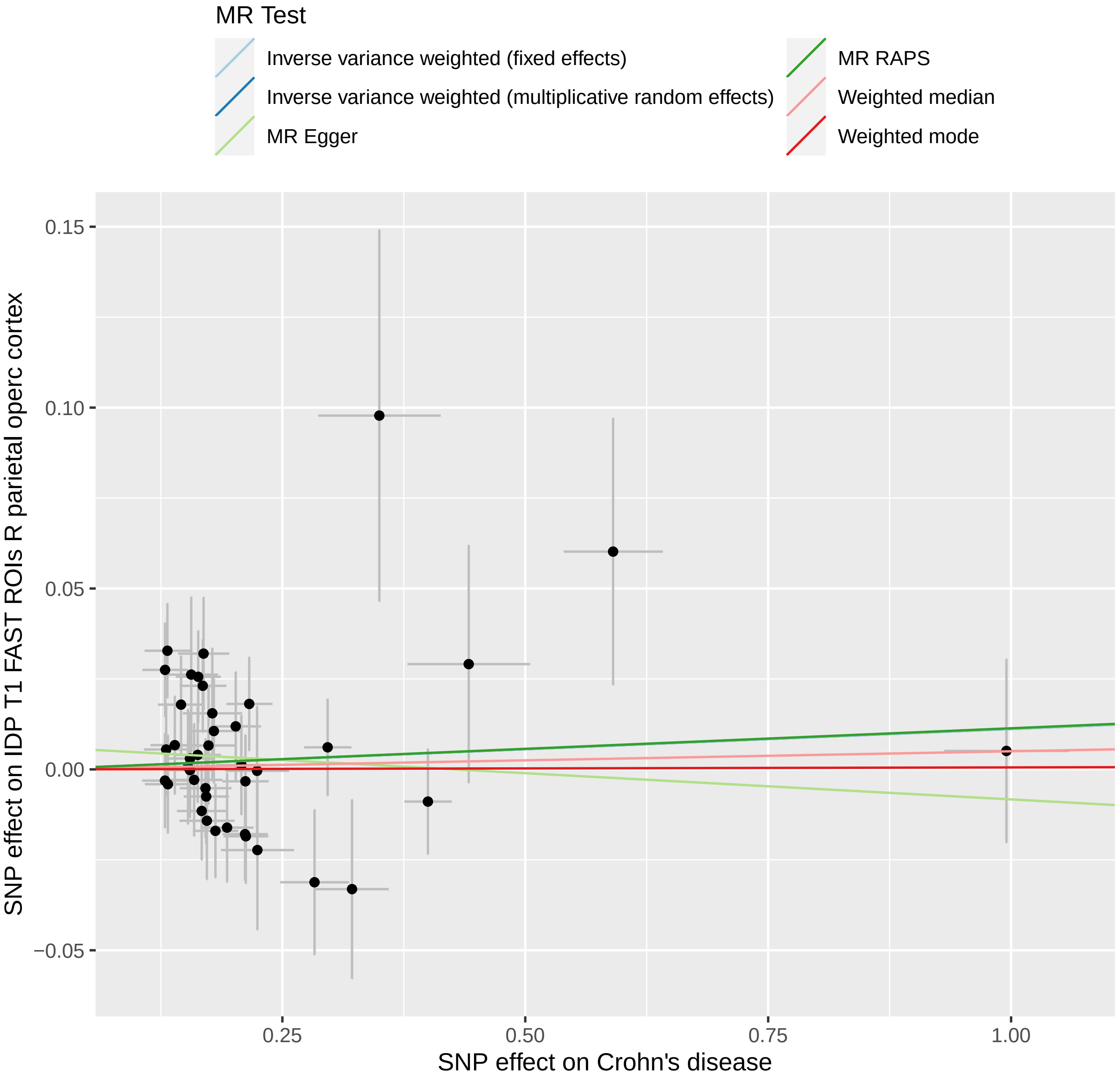

Batch 30 : Ulcerative colitis on IDP T1 FAST ROIs R parietal operc cortex

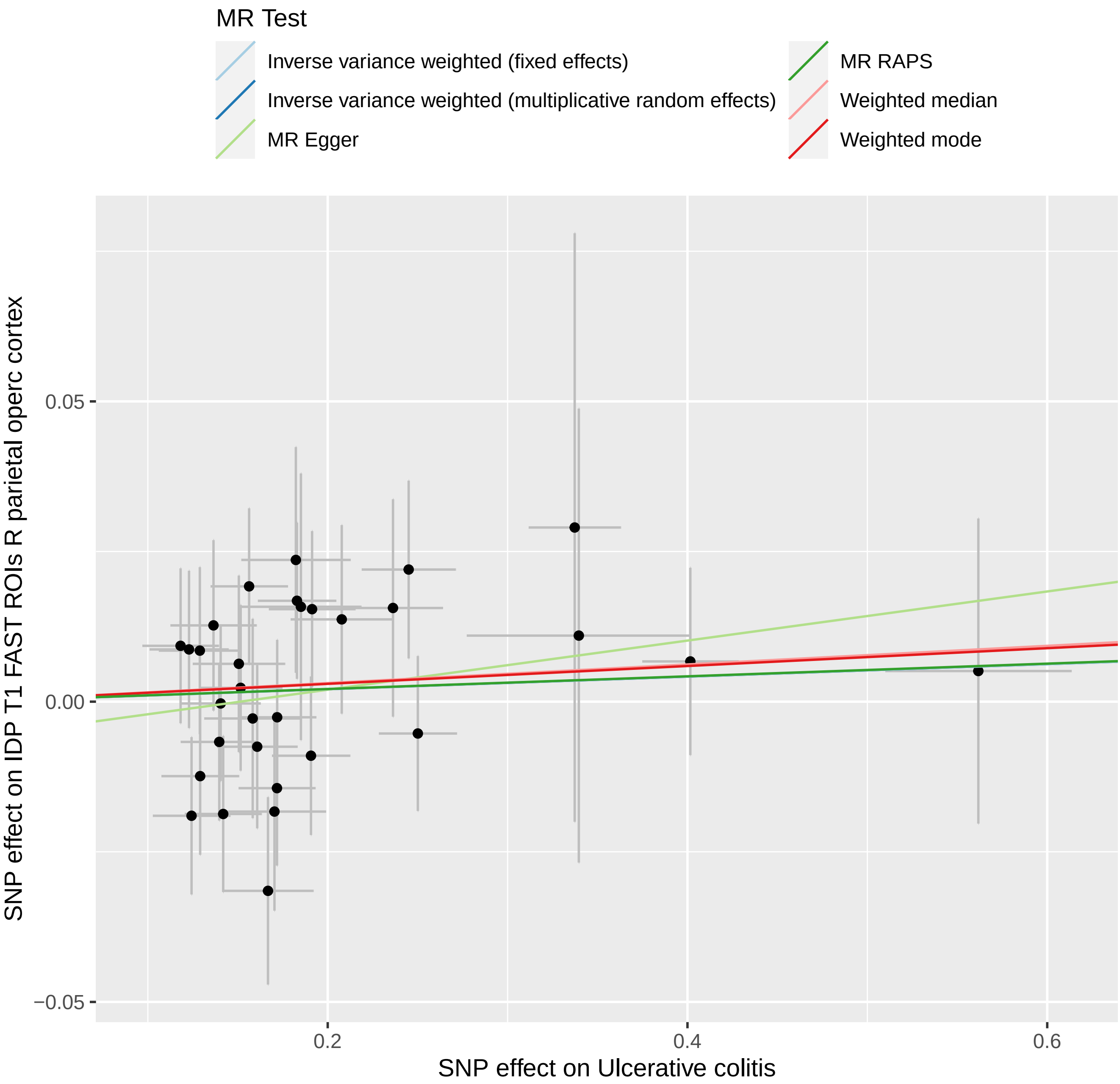

Batch 31 : Crohn's disease on IDP T1 FAST ROIs L planum polare

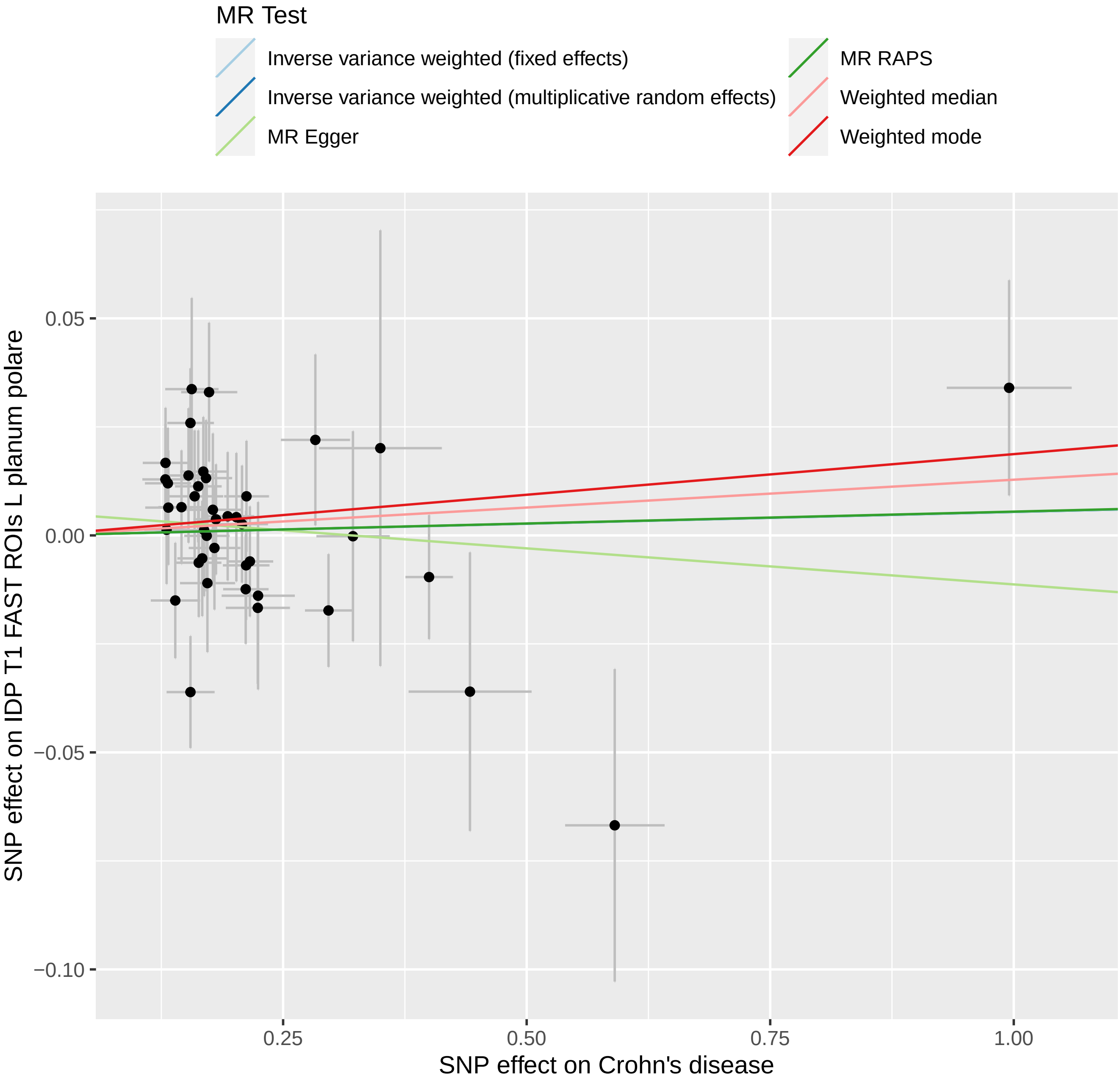

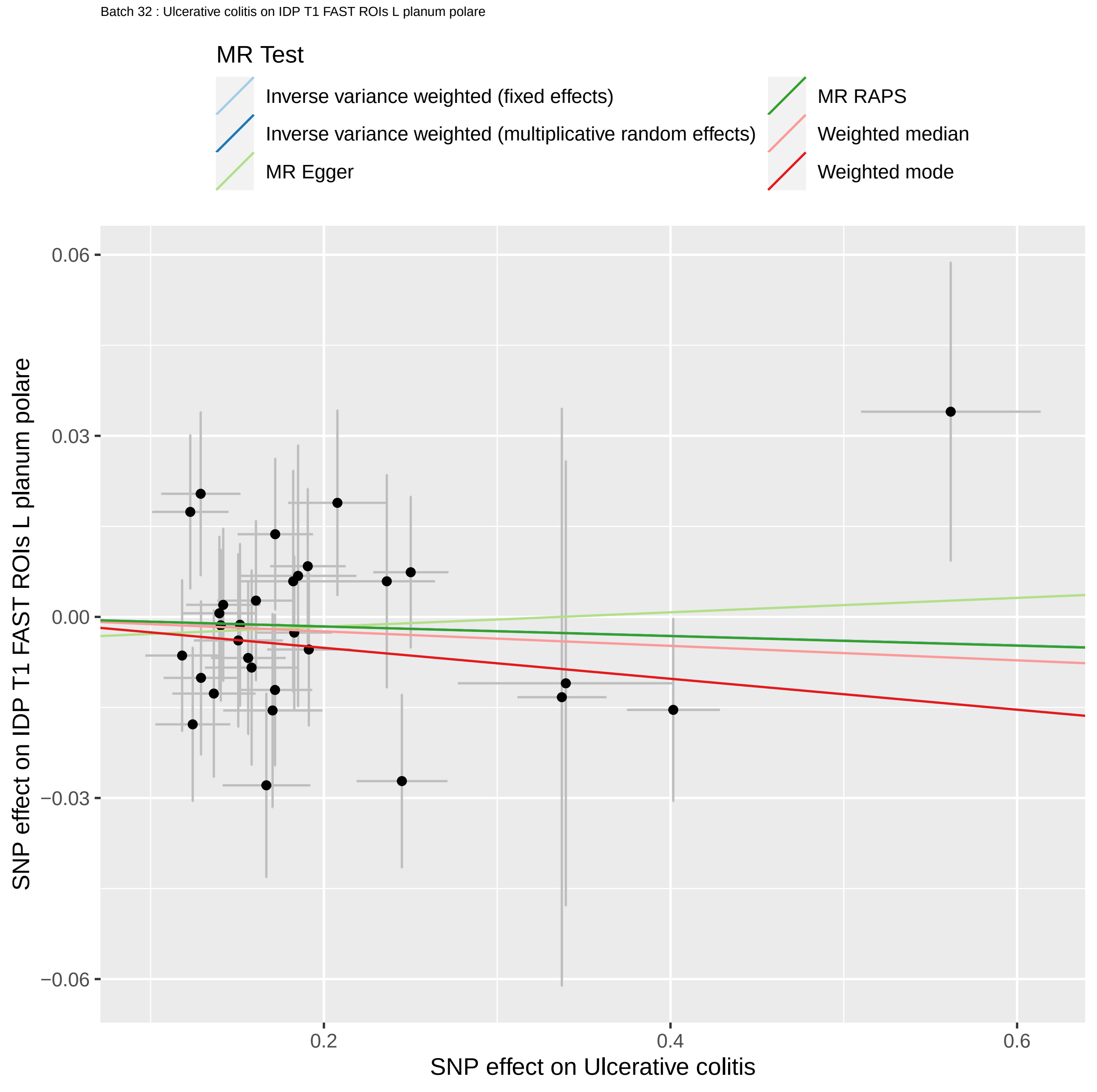
Batch 32 : Ulcerative colitis on IDP T1 FAST ROIs L planum polare
MR Test
Inverse variance weighted (fixed effects)
Inverse variance weighted (multiplicative random effects)
MR Egger
MR RAPS
Weighted median
Weighted mode
0.06
0.03
0.00
−0.03
−0.06
0.2
0.4
0.6
SNP effect on IDP T1 FAST ROIs L planum polare
SNP effect on Ulcerative colitis

Batch 33 : Crohn's disease on IDP T1 FAST ROIs R planum polare

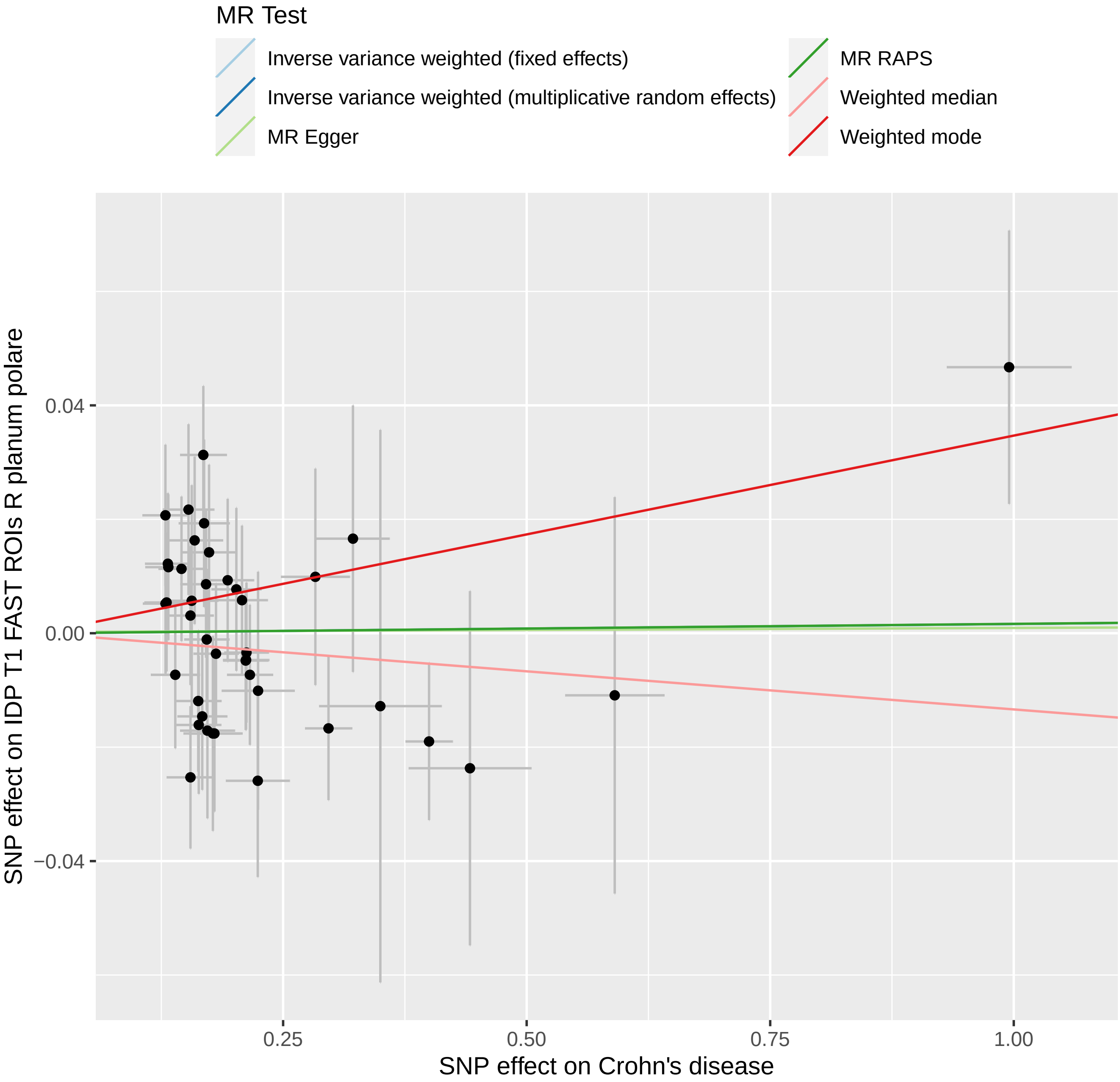

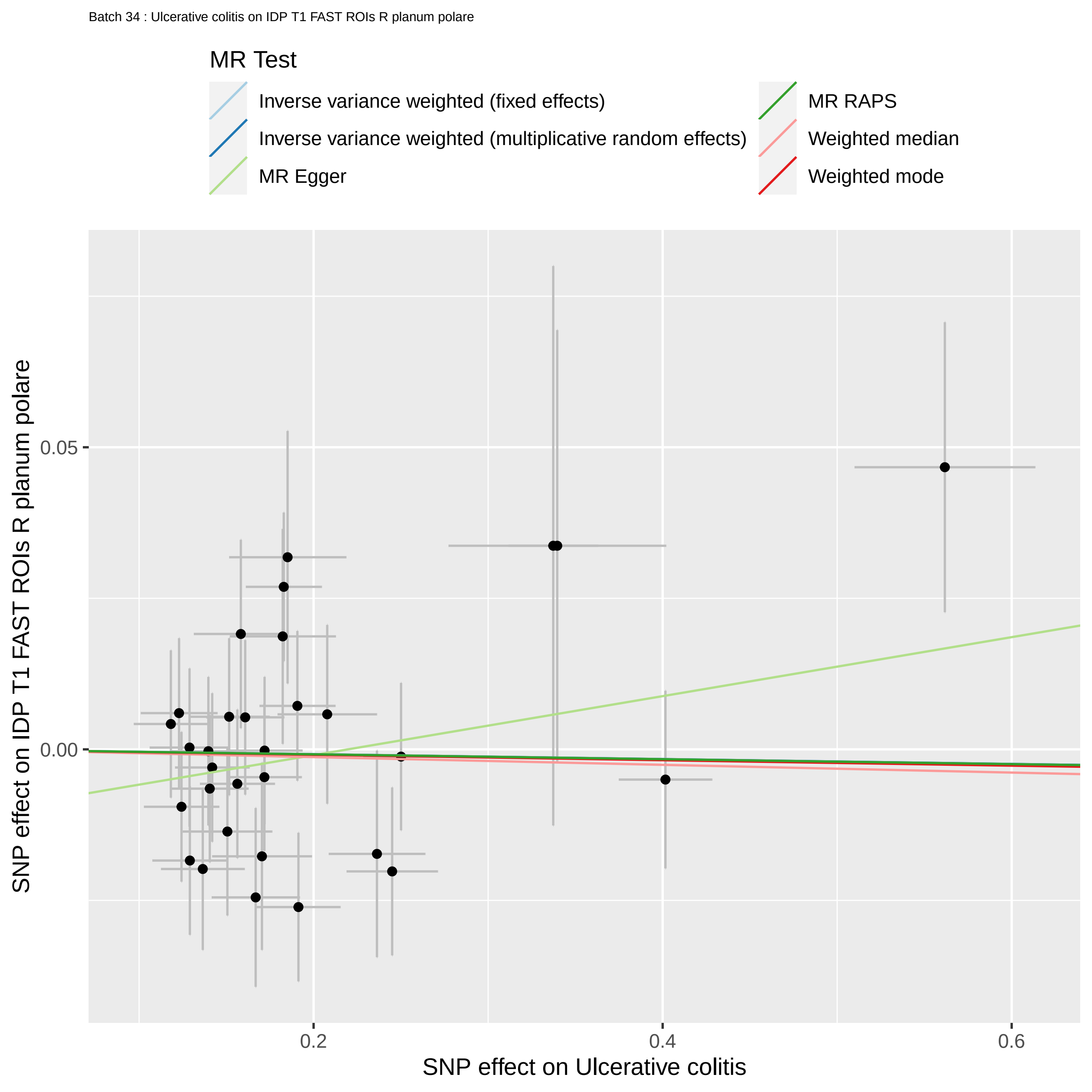

Batch 34 : Ulcerative colitis on IDP T1 FAST ROIs R planum polare
MR Test
Inverse variance weighted (fixed effects)
Inverse variance weighted (multiplicative random effects)
MR Egger
MR RAPS
Weighted median
Weighted mode
SNP effect on IDP T1 FAST ROIs R planum polare
0.05
0.00
0.2
0.4
0.6
SNP effect on Ulcerative colitis

Batch 35 : Crohn's disease on IDP T1 FAST ROIs L heschl gyrus

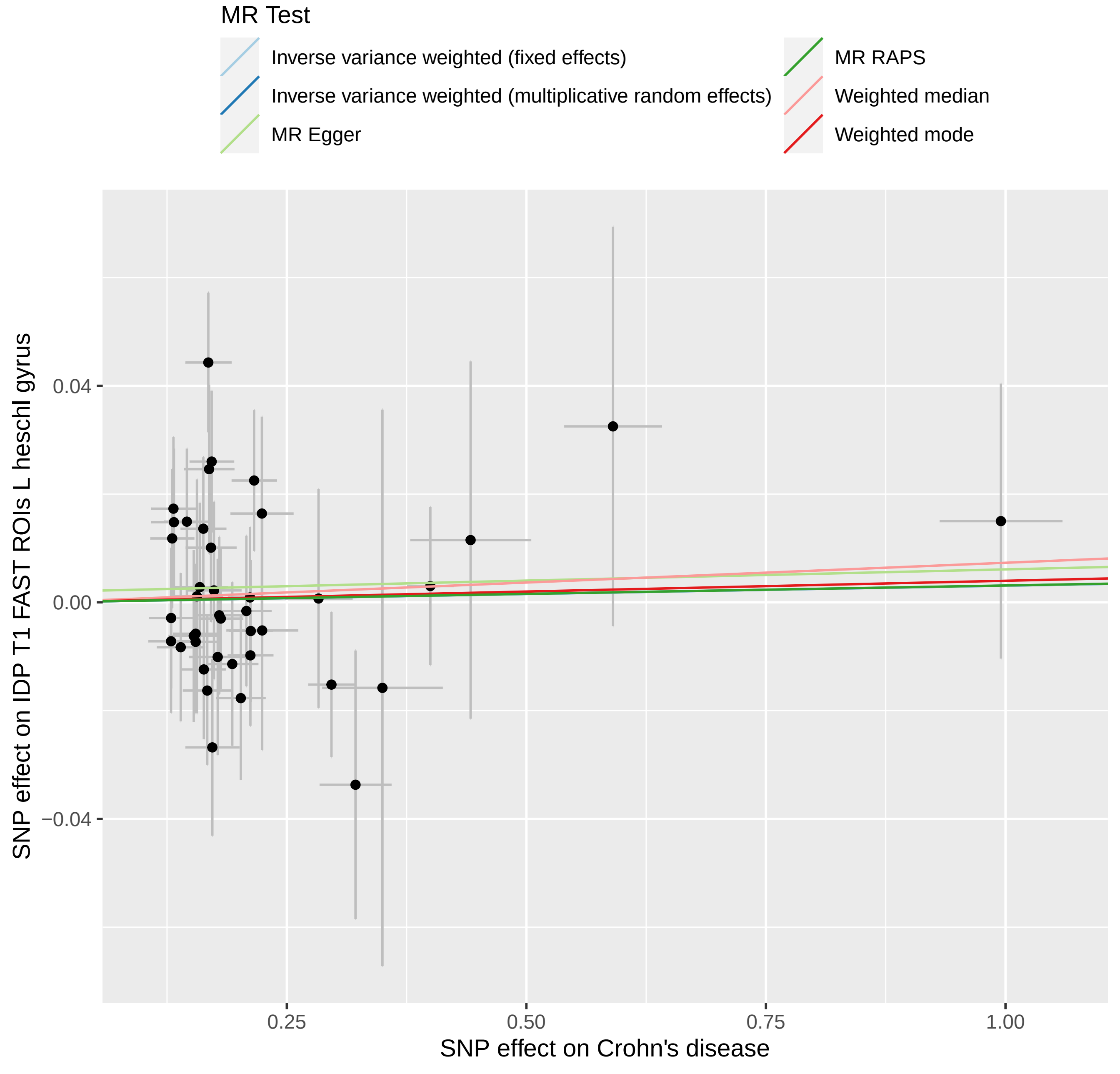

Batch 36 : Ulcerative colitis on IDP T1 FAST ROIs L heschl gyrus

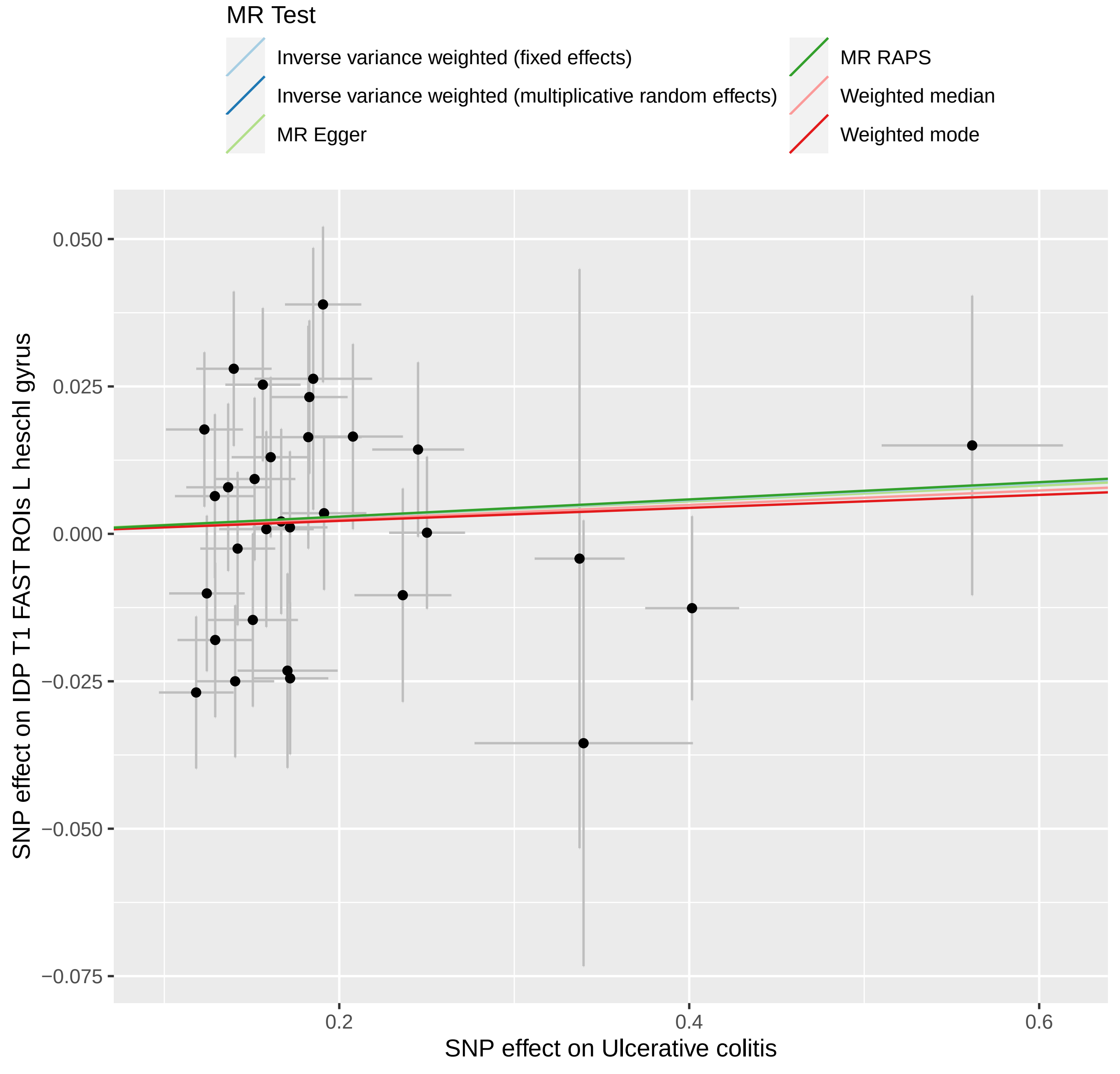

Batch 37 : Crohn's disease on IDP T1 FAST ROIs R heschl gyrus

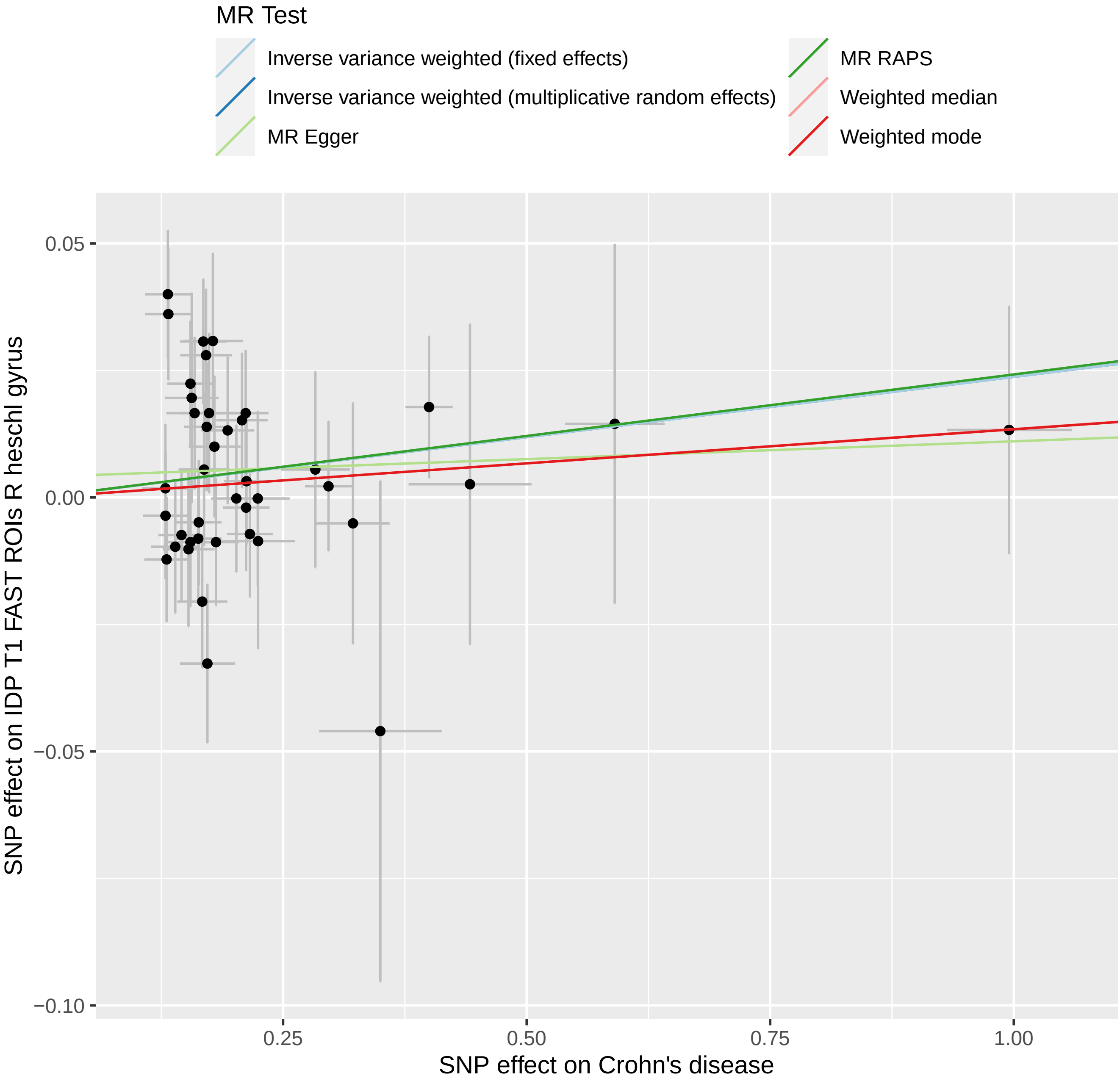

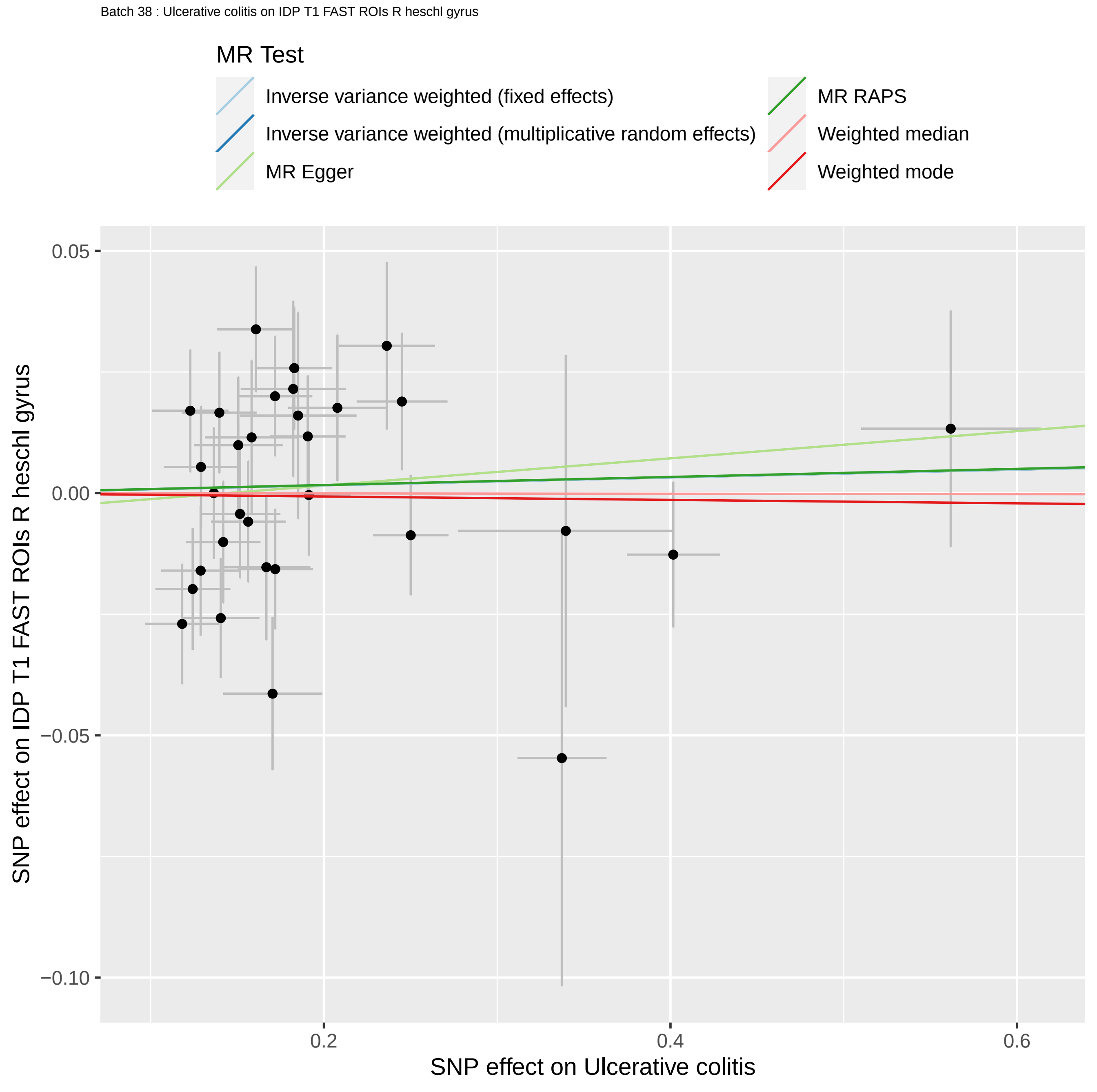

Batch 38 : Ulcerative colitis on IDP T1 FAST ROIs R heschl gyrus
MR Test
Inverse variance weighted (fixed effects)
Inverse variance weighted (multiplicative random effects)
MR Egger
MR RAPS
Weighted median
Weighted mode
0.05
0.00
−0.05
−0.10
0.2
0.4
0.6
SNP effect on IDP T1 FAST ROIs R heschl gyrus
SNP effect on Ulcerative colitis

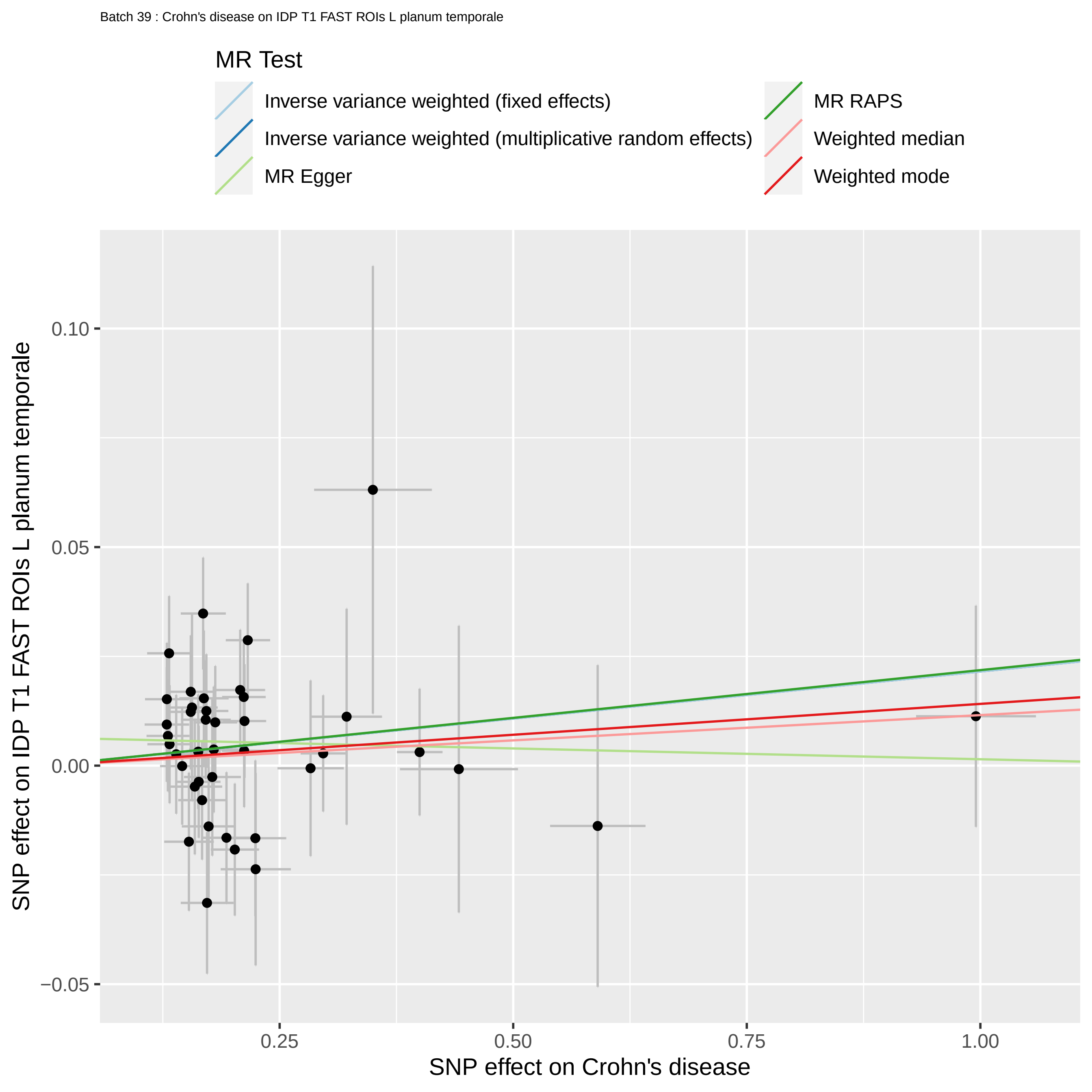
Batch 39 : Crohn's disease on IDP T1 FAST ROIs L planum temporale
MR Test
Inverse variance weighted (fixed effects)
Inverse variance weighted (multiplicative random effects)
MR Egger
MR RAPS
Weighted median
Weighted mode
0.10
0.05
0.00
−0.05
0.25
0.50
0.75
1.00
SNP effect on IDP T1 FAST ROIs L planum temporale
SNP effect on Crohn's disease

Batch 40 : Ulcerative colitis on IDP T1 FAST ROIs L planum temporale

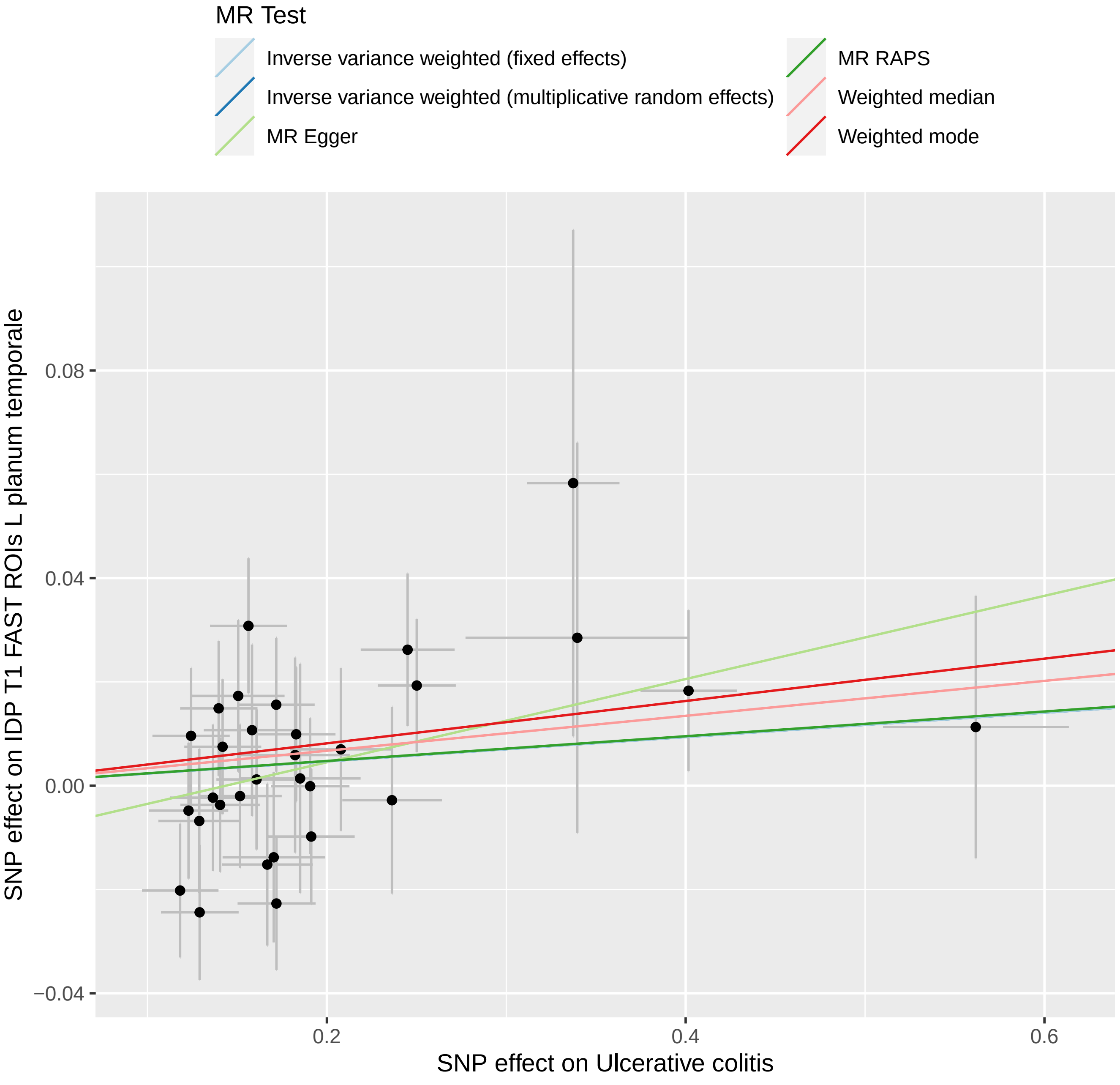

Batch 41 : Crohn's disease on IDP T1 FAST ROIs R planum temporale

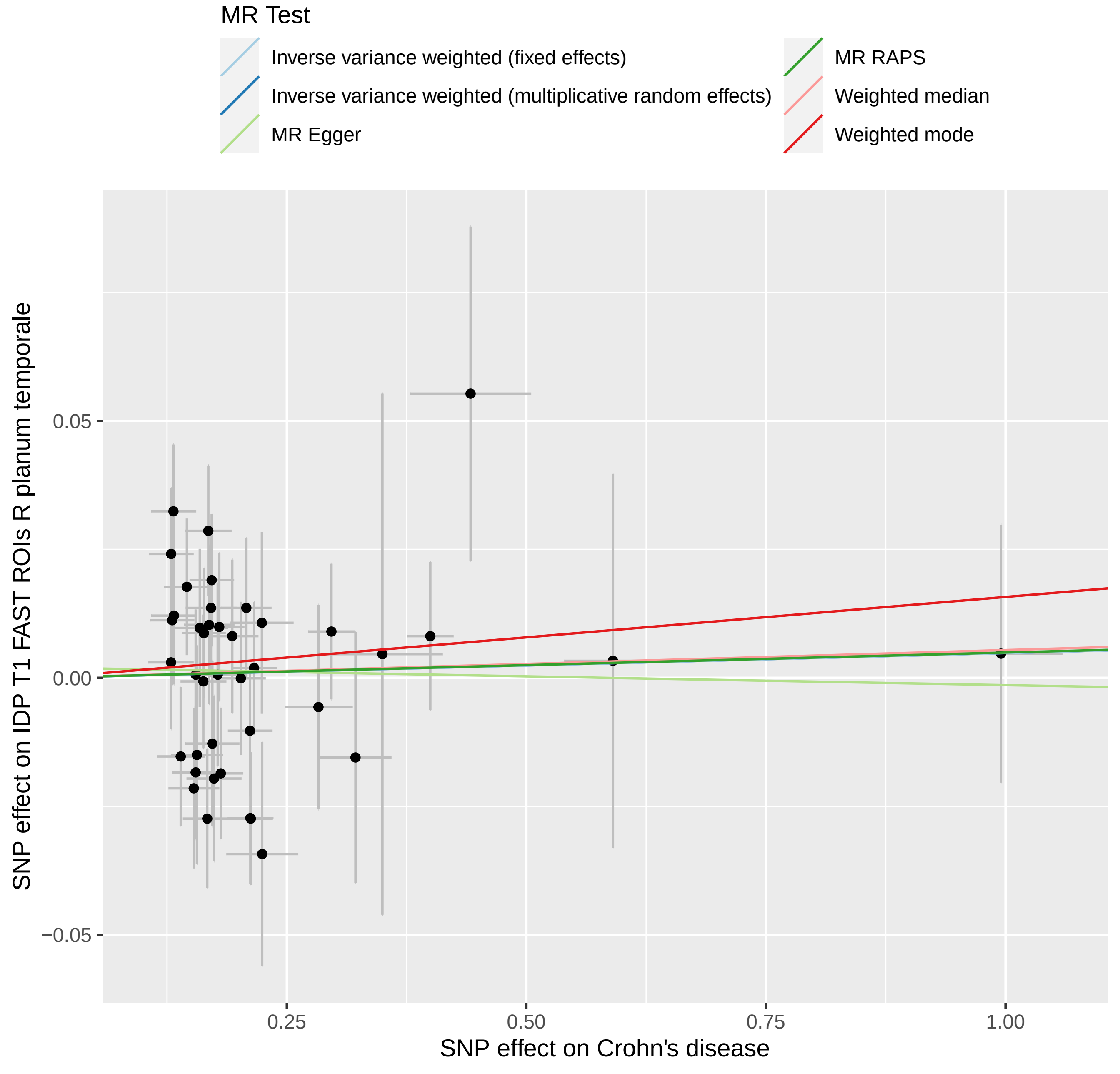

Batch 42 : Ulcerative colitis on IDP T1 FAST ROIs R planum temporale

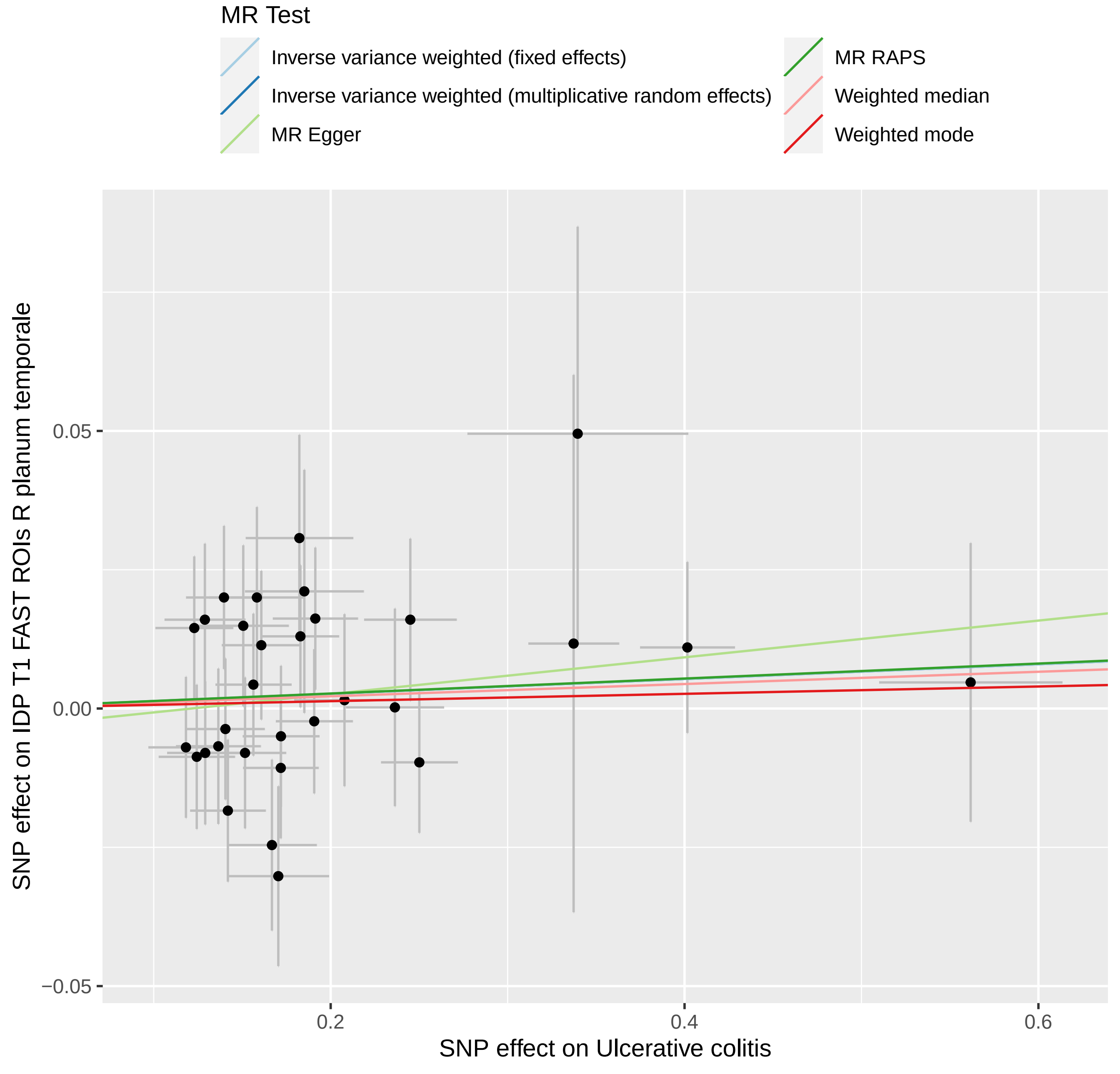

Batch 43 : Crohn's disease on IDP T1 FAST ROIs L supracalc cortex

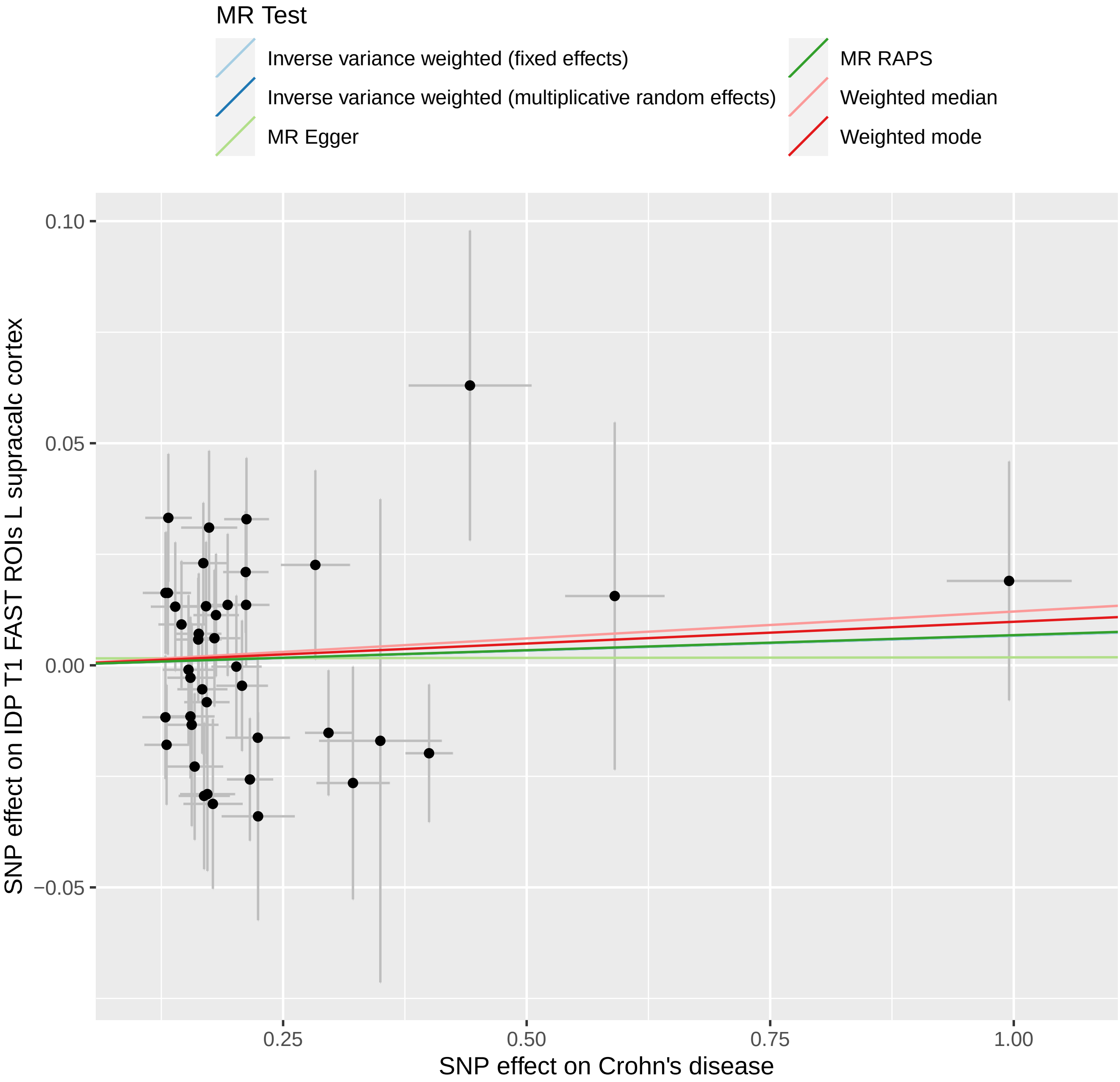

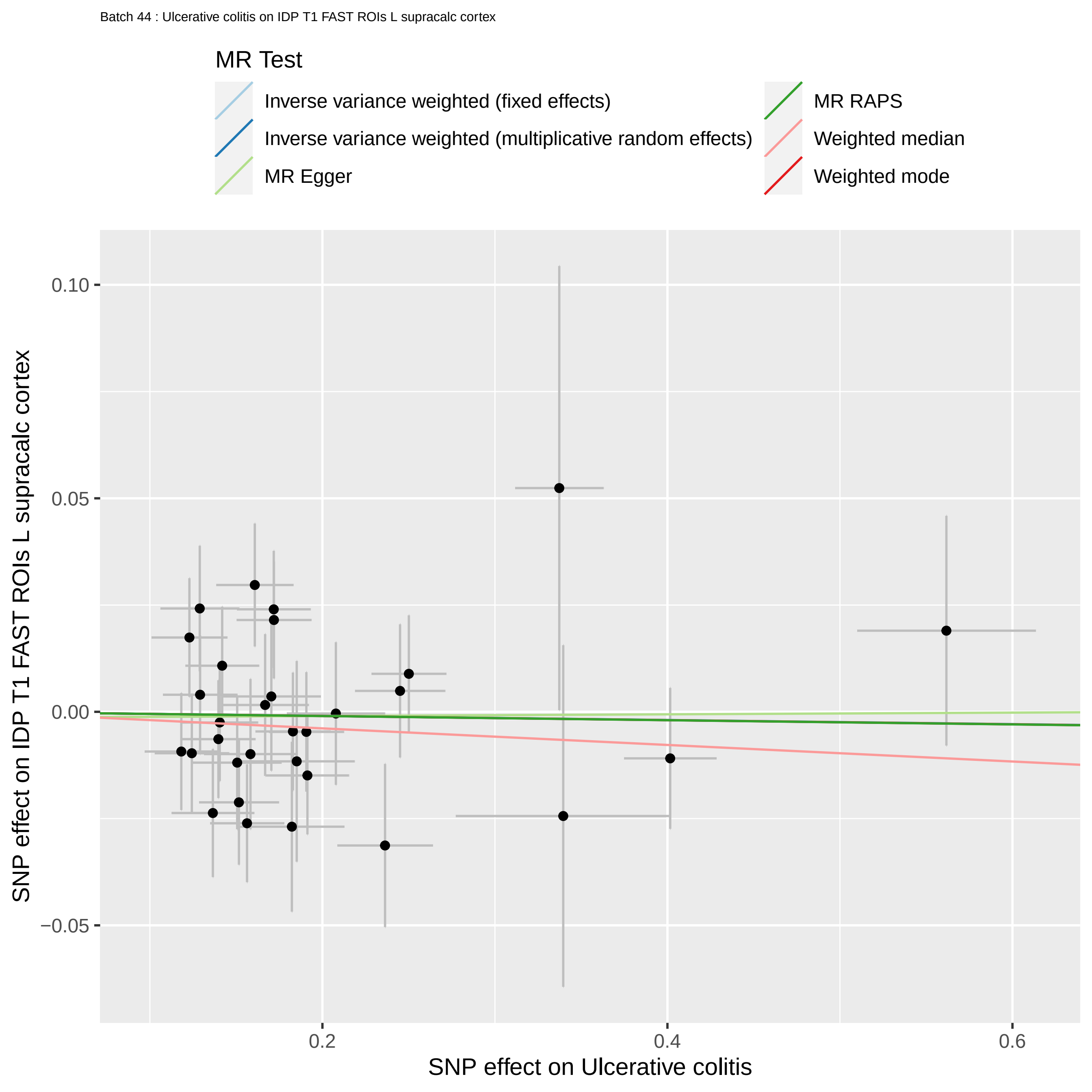

Batch 44 : Ulcerative colitis on IDP T1 FAST ROIs L supracalc cortex
MR Test
Inverse variance weighted (fixed effects)
Inverse variance weighted (multiplicative random effects)
MR Egger
MR RAPS
Weighted median
Weighted mode
0.10
0.05
0.00
−0.05
0.2
0.4
0.6
SNP effect on IDP T1 FAST ROIs L supracalc cortex
SNP effect on Ulcerative colitis

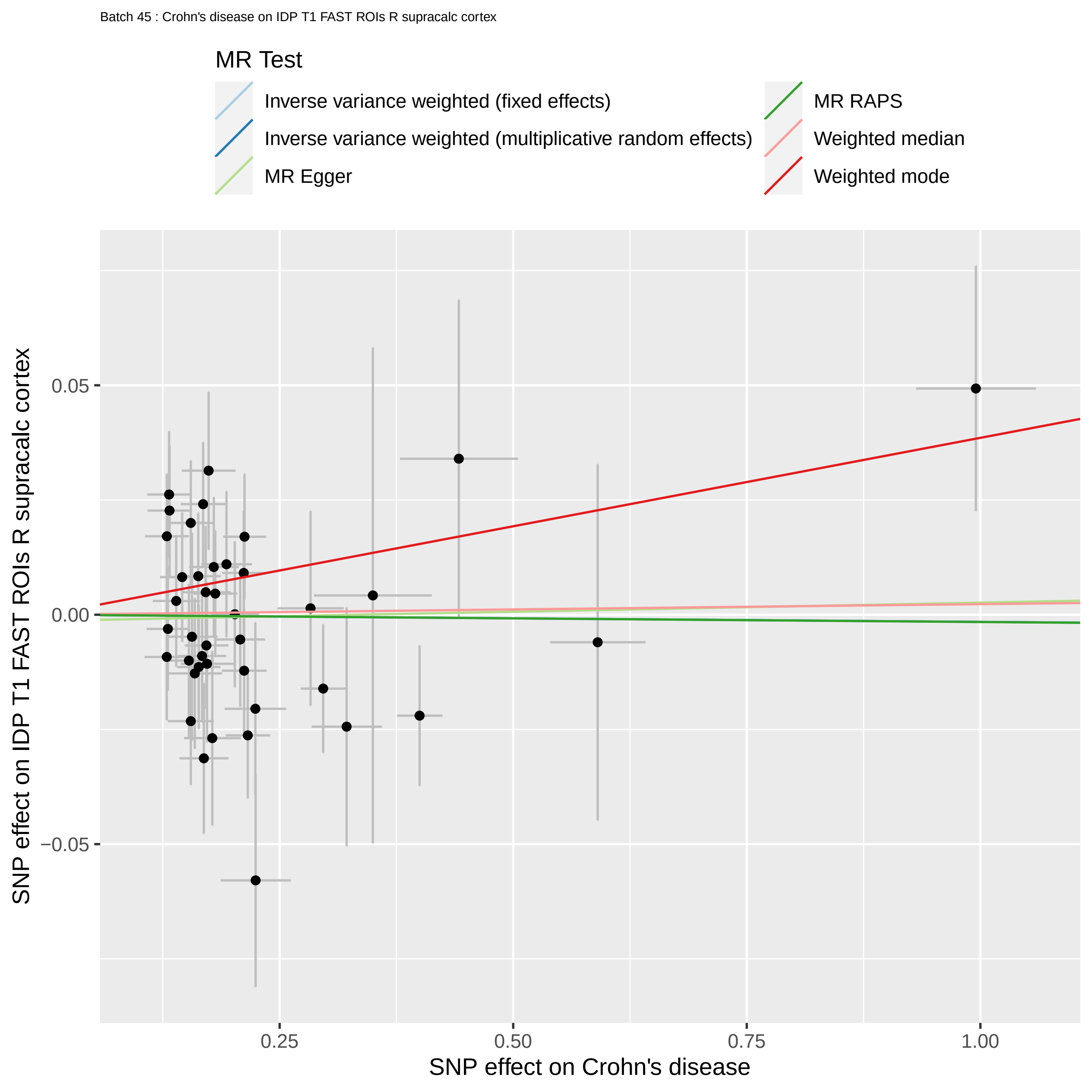
Batch 45 : Crohn's disease on IDP T1 FAST ROIs R supracalc cortex
MR Test
Inverse variance weighted (fixed effects)
Inverse variance weighted (multiplicative random effects)
MR Egger
MR RAPS
Weighted median
Weighted mode
SNP effect on IDP T1 FAST ROIs R supracalc cortex
0.05
0.00
−0.05
0.25
0.50
0.75
1.00
SNP effect on Crohn's disease

Batch 46 : Ulcerative colitis on IDP T1 FAST ROIs R supracalc cortex

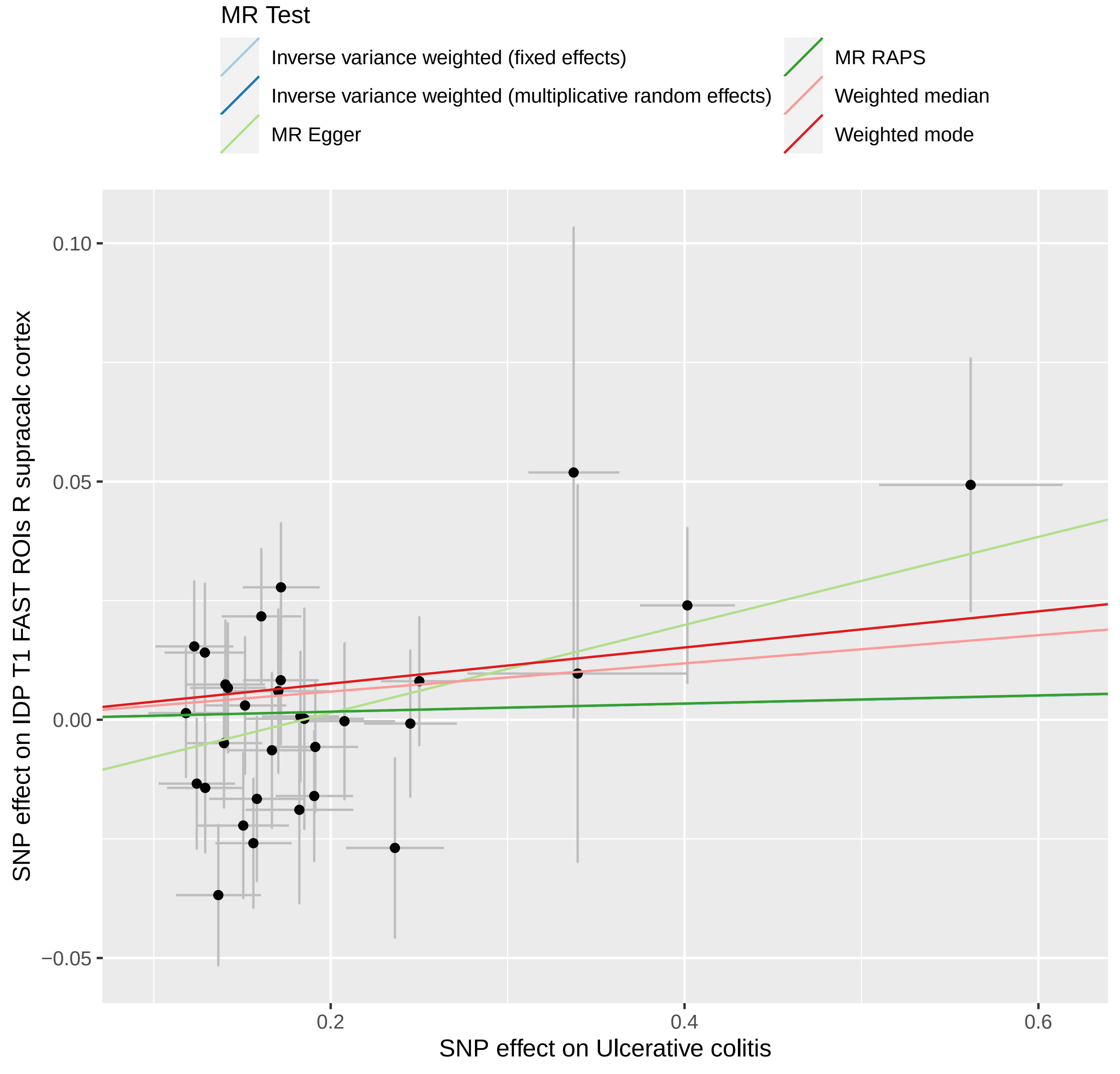

Batch 47 : Crohn's disease on IDP T1 FIRST right thalamus volume

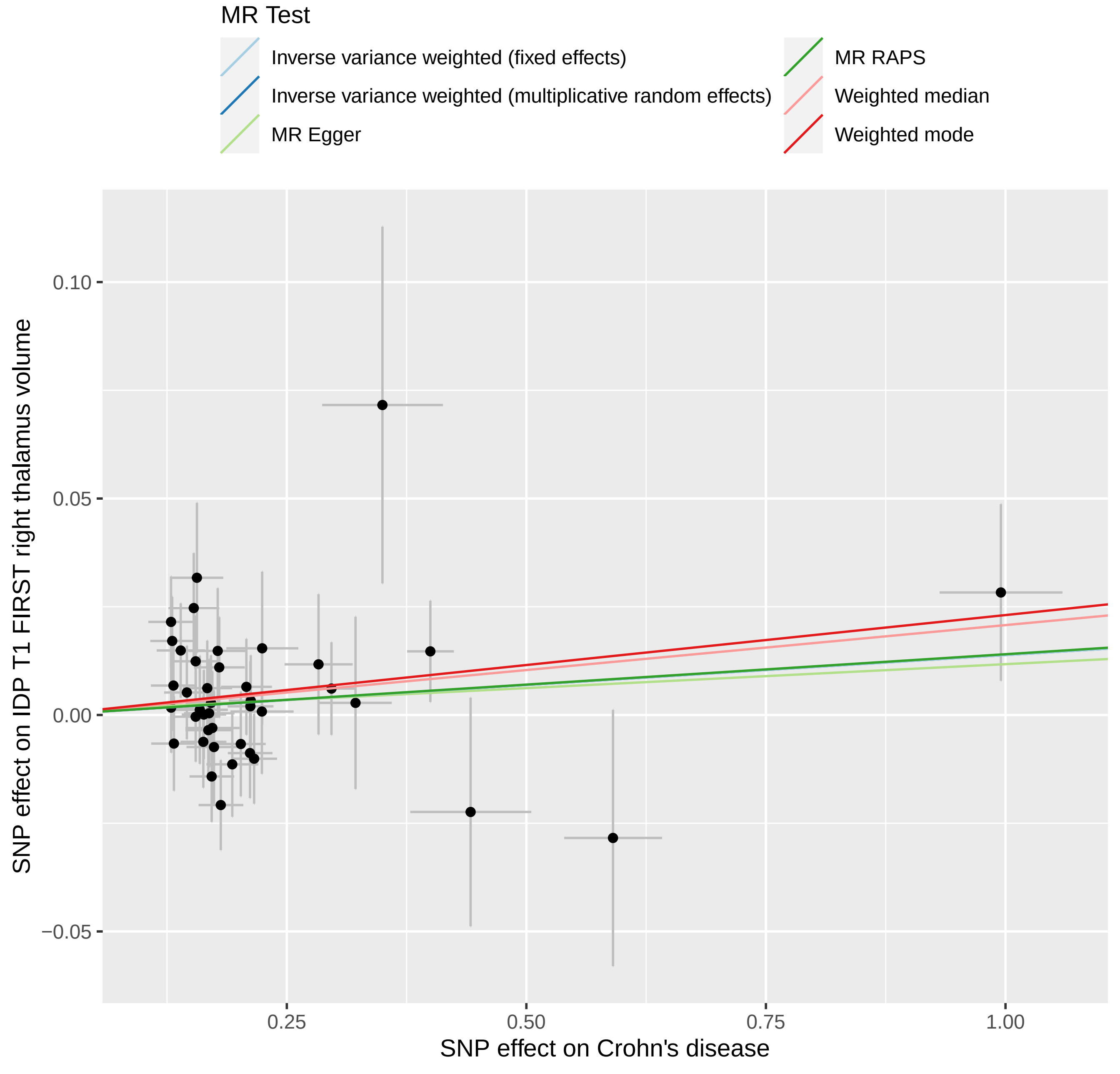

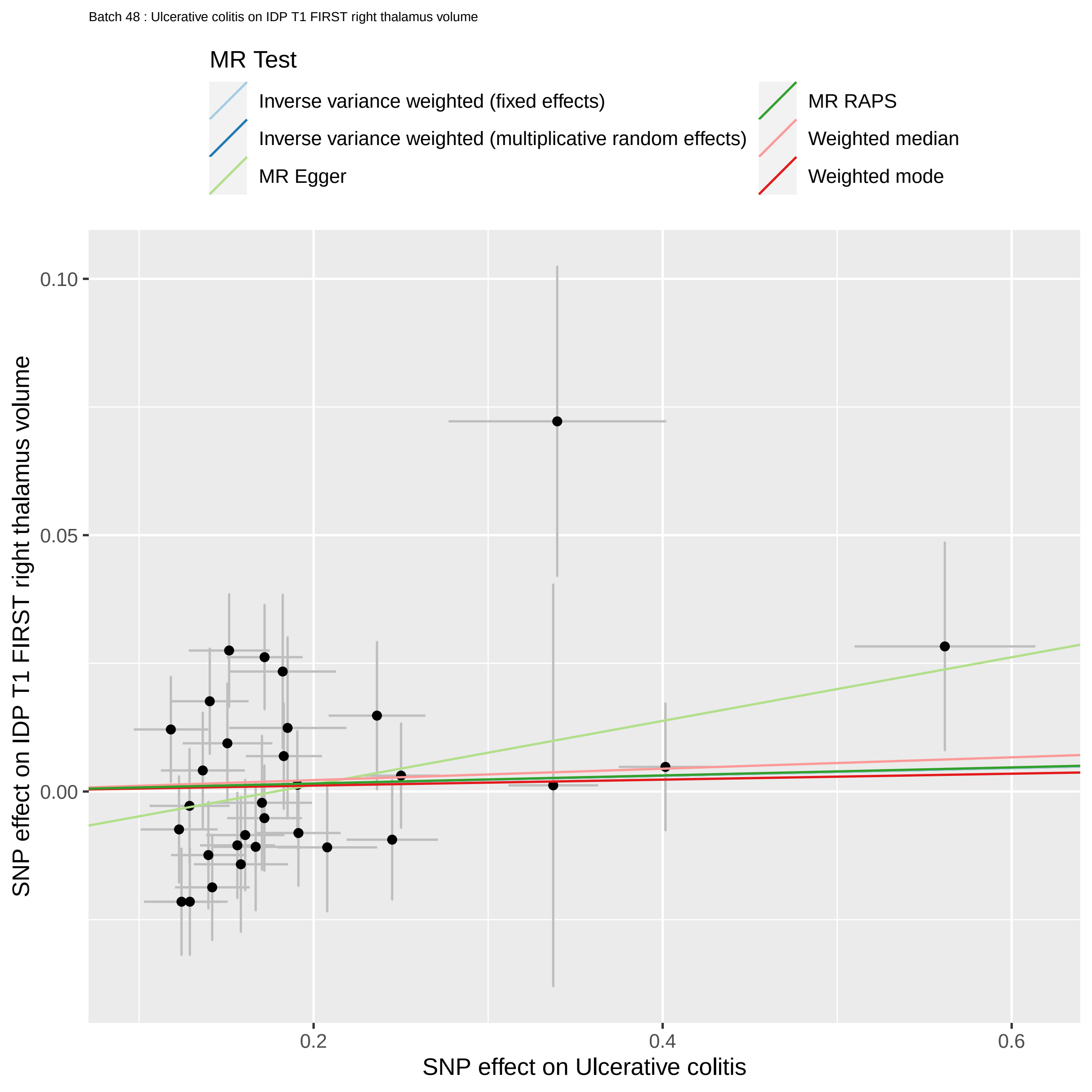

Batch 48 : Ulcerative colitis on IDP T1 FIRST right thalamus volume
MR Test
Inverse variance weighted (fixed effects)
Inverse variance weighted (multiplicative random effects)
MR Egger
MR RAPS
Weighted median
Weighted mode
0.10
0.05
0.00
0.2
0.4
0.6
SNP effect on IDP T1 FIRST right thalamus volume
SNP effect on Ulcerative colitis

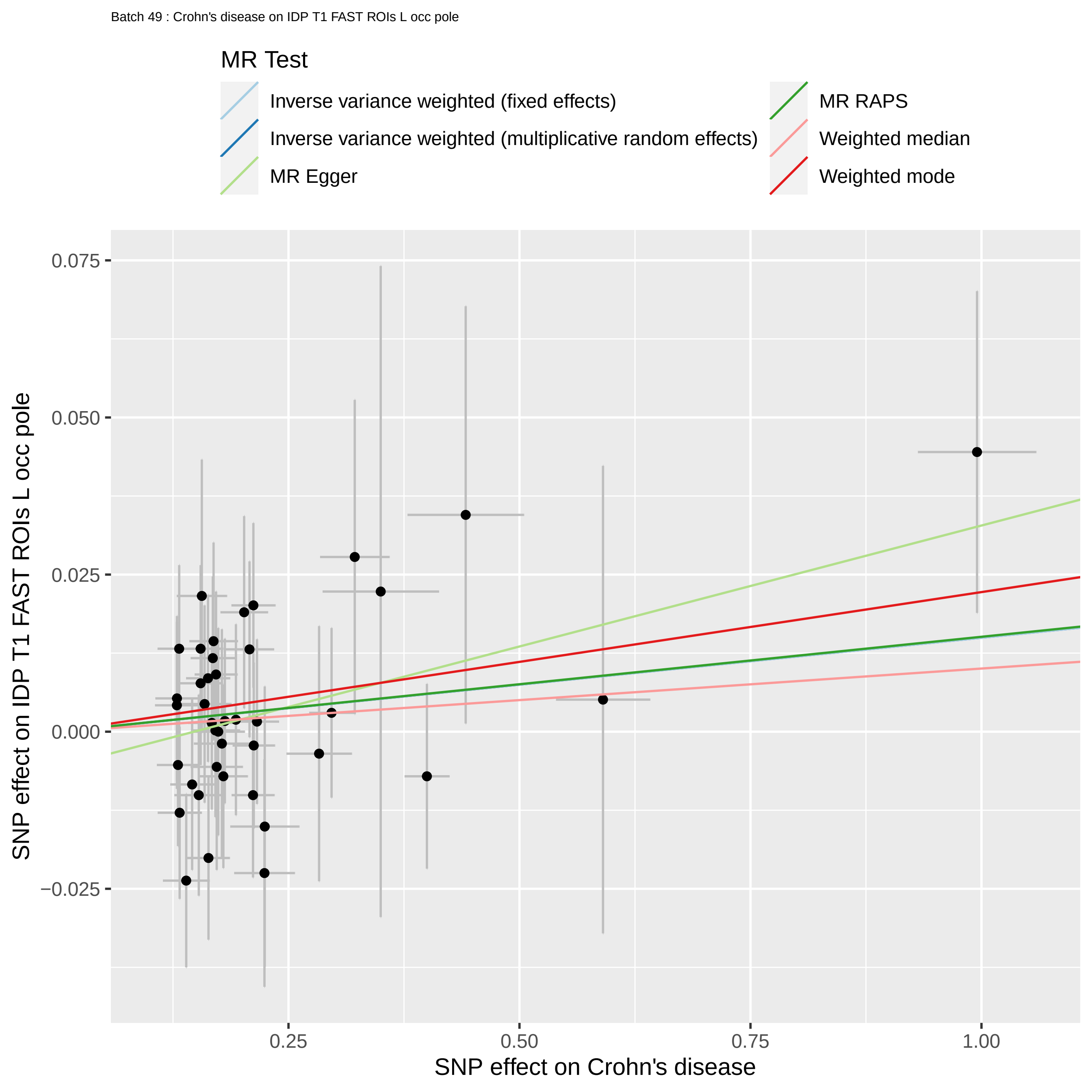

Batch 49 : Crohn's disease on IDP T1 FAST ROIs L occ pole
MR Test
Inverse variance weighted (fixed effects)
Inverse variance weighted (multiplicative random effects)
MR Egger
MR RAPS
Weighted median
Weighted mode
0.075
0.050
0.025
0.000
−0.025
0.25
0.50
0.75
1.00
SNP effect on IDP T1 FAST ROIs L occ pole
SNP effect on Crohn's disease

Batch 50 : Ulcerative colitis on IDP T1 FAST ROIs L occ pole

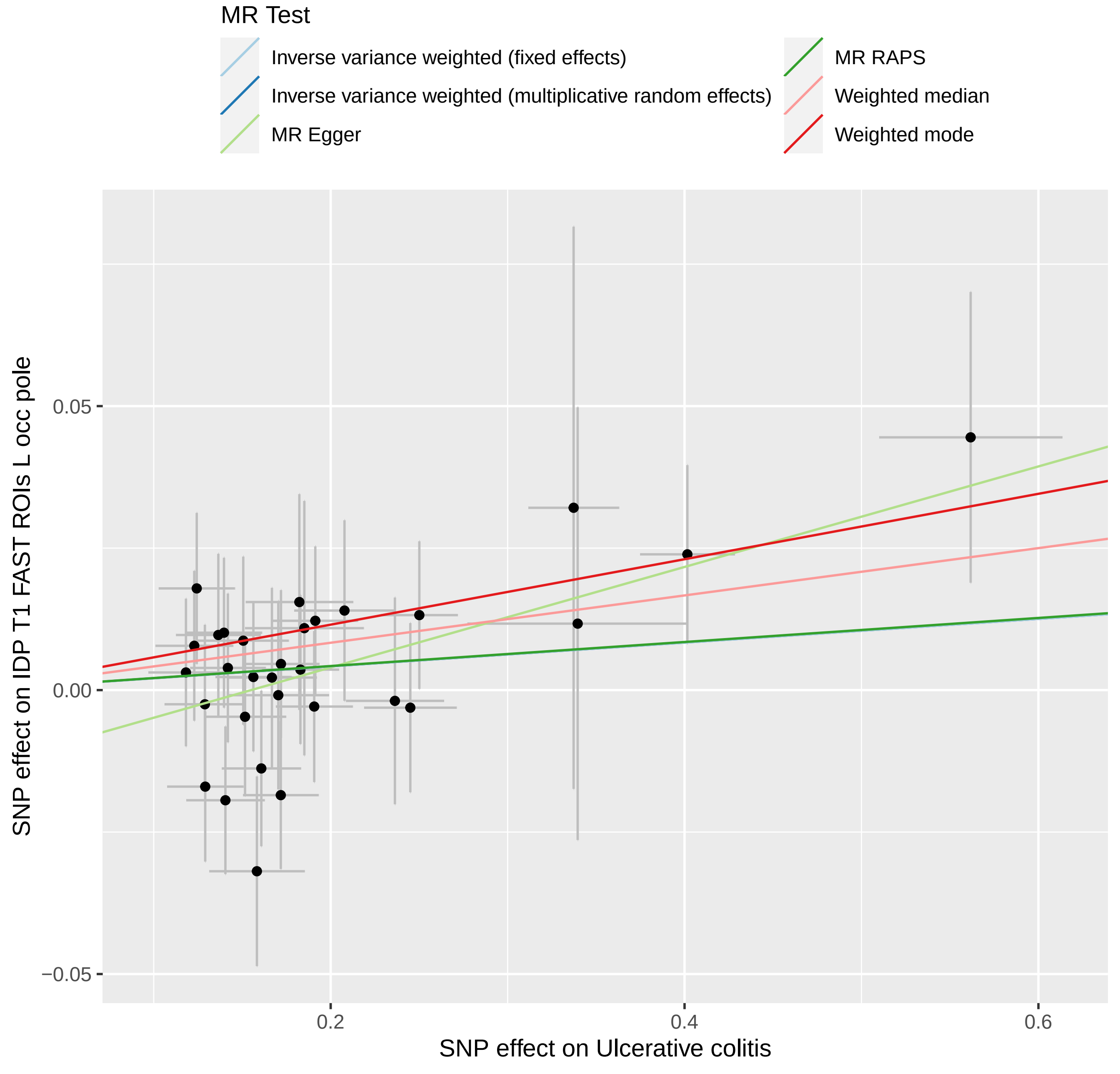

Batch 51 : Crohn's disease on IDP T1 FAST ROIs R occ pole

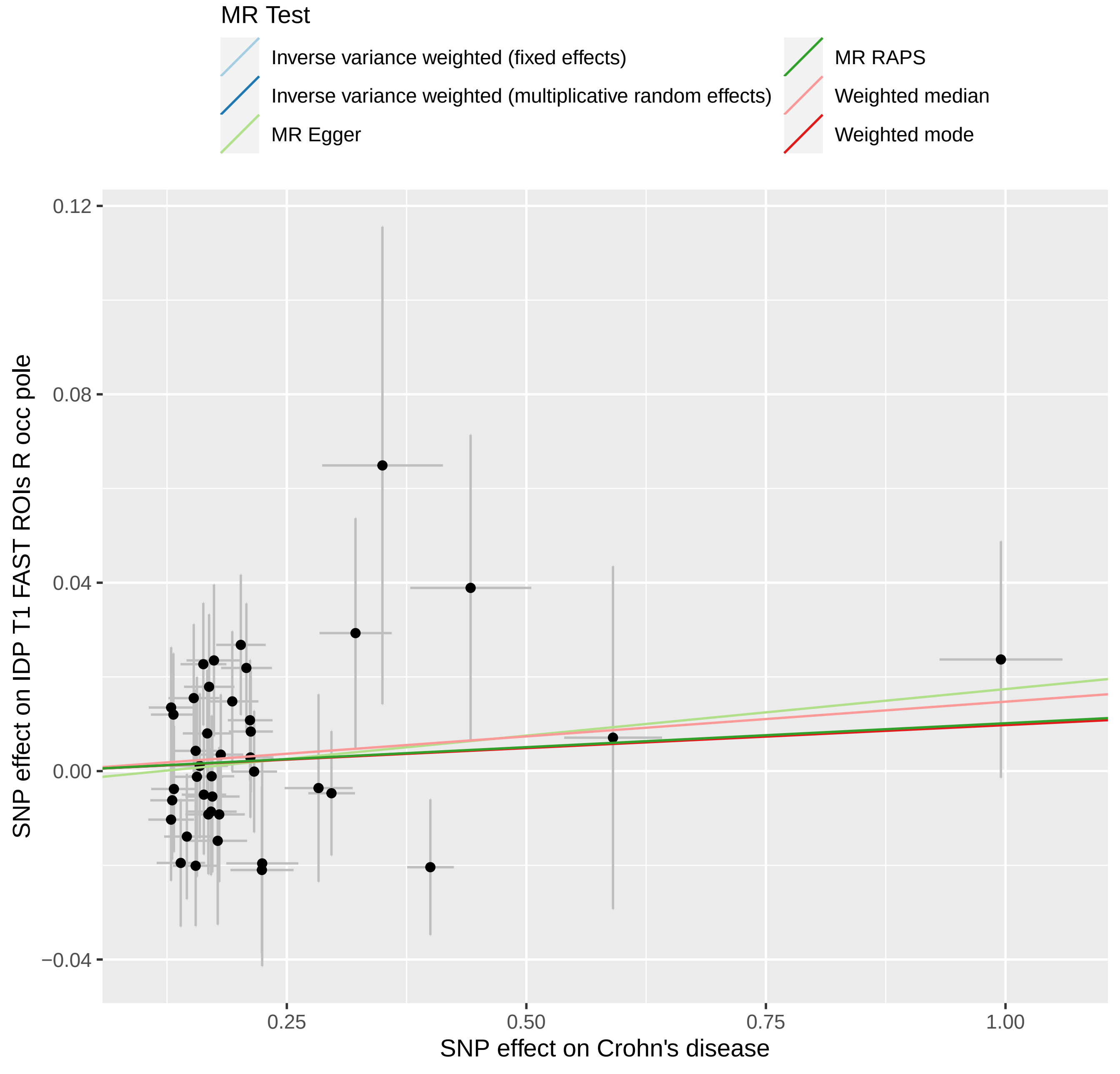

Batch 52 : Ulcerative colitis on IDP T1 FAST ROIs R occ pole

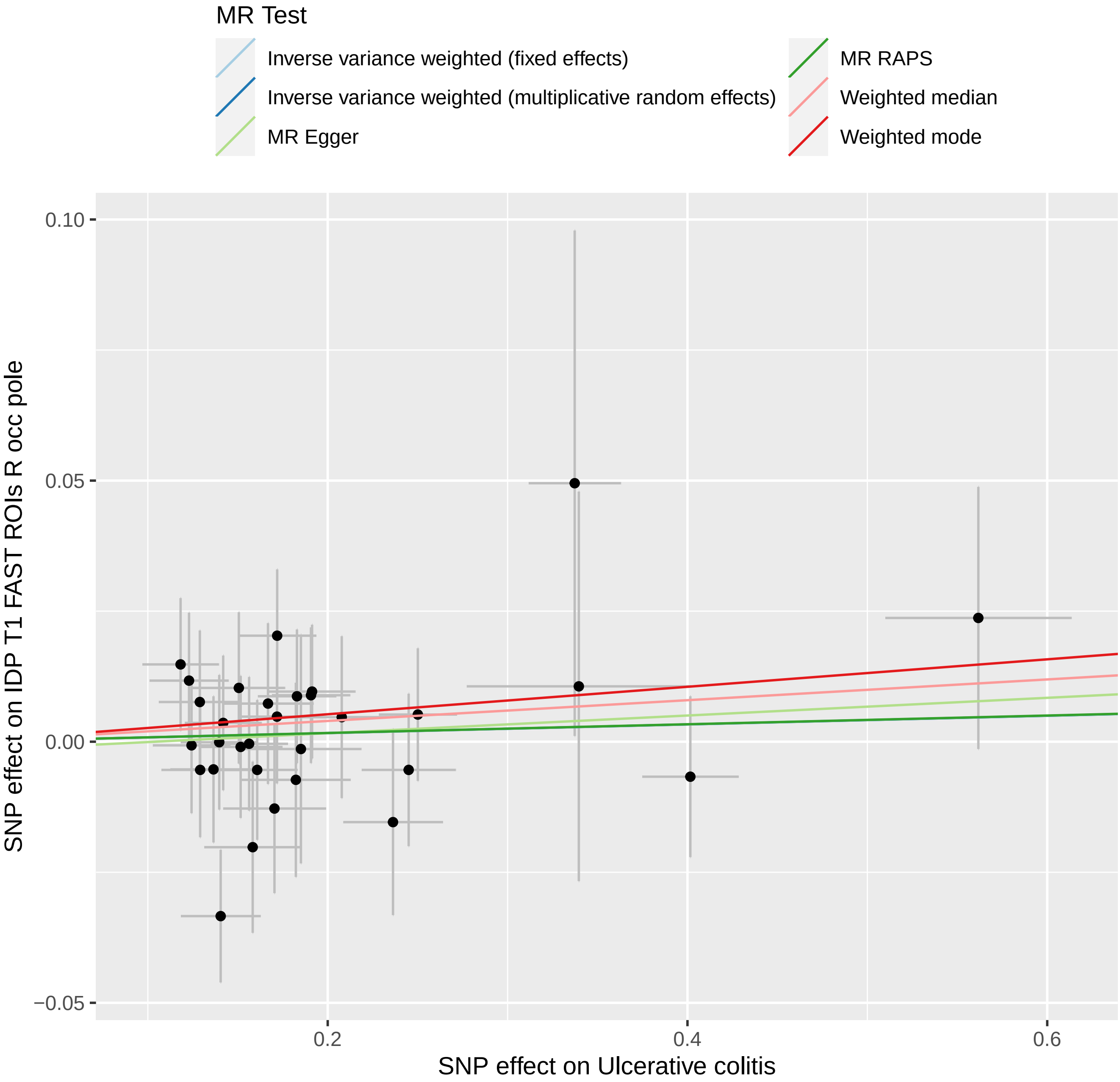

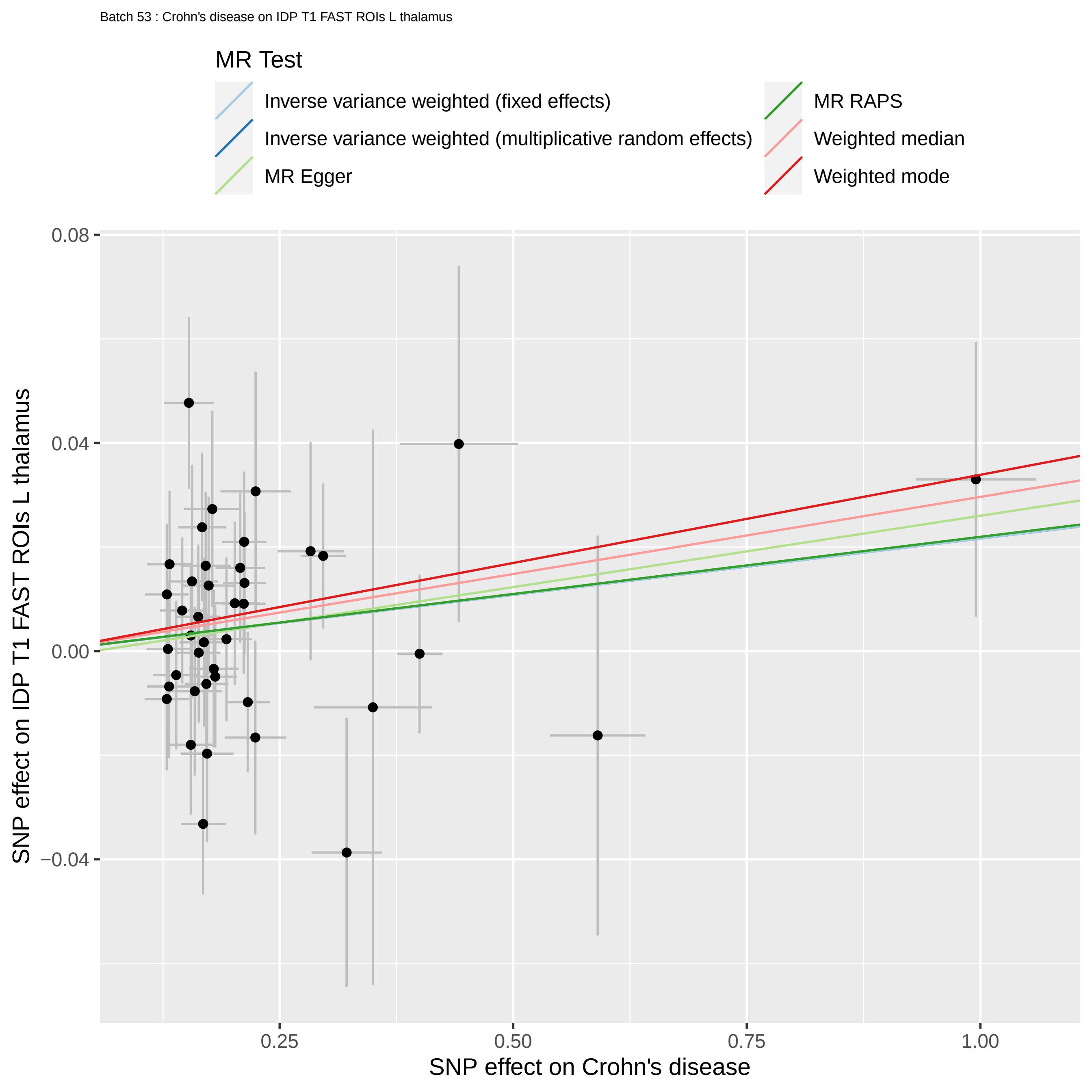

Batch 53 : Crohn's disease on IDP T1 FAST ROIs L thalamus
MR Test
Inverse variance weighted (fixed effects)
Inverse variance weighted (multiplicative random effects)
MR Egger
MR RAPS
Weighted median
Weighted mode
0.08
0.04
0.00
−0.04
0.25
0.50
0.75
1.00
SNP effect on IDP T1 FAST ROIs L thalamus
SNP effect on Crohn's disease

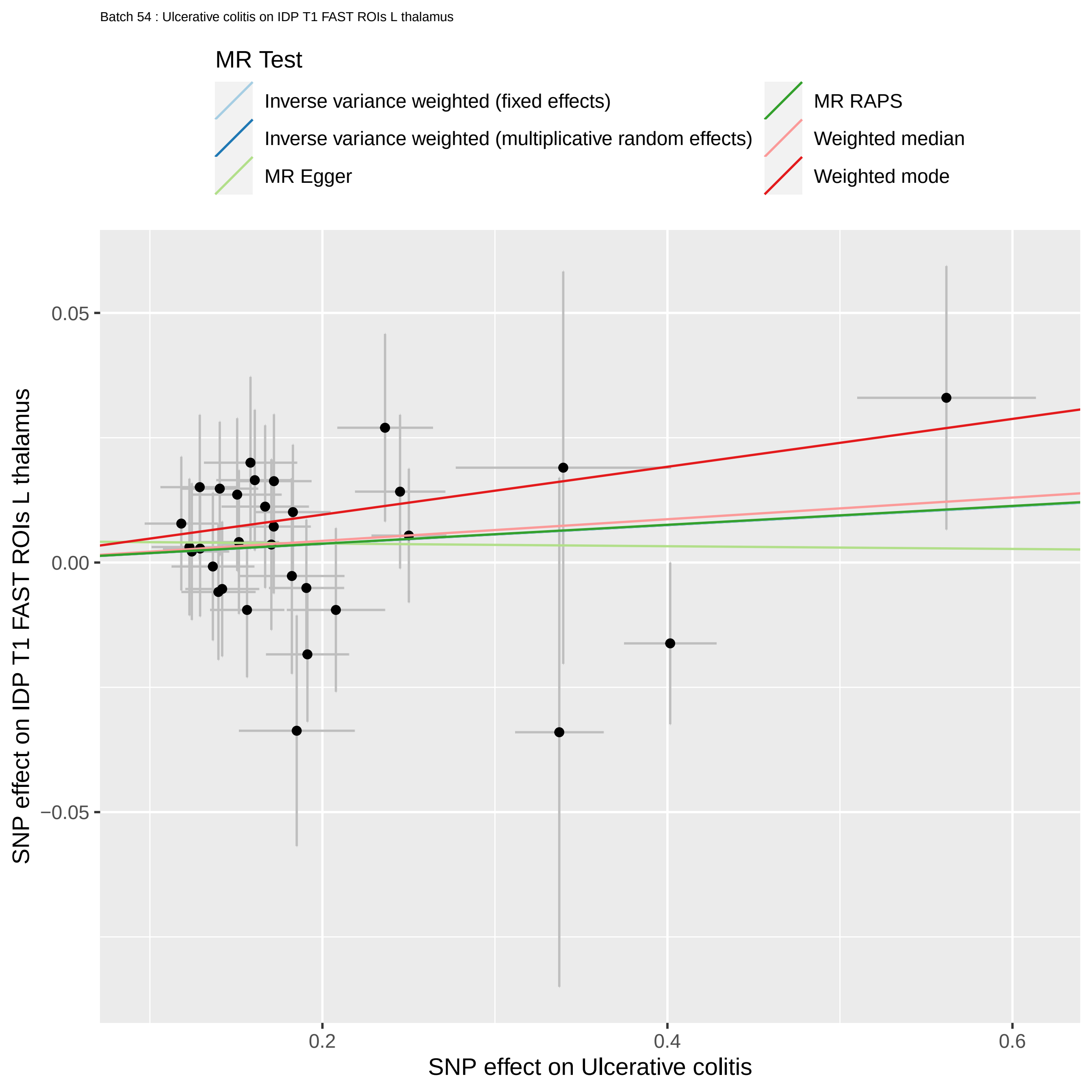
Batch 54 : Ulcerative colitis on IDP T1 FAST ROIs L thalamus
MR Test
Inverse variance weighted (fixed effects)
Inverse variance weighted (multiplicative random effects)
MR Egger
MR RAPS
Weighted median
Weighted mode
0.05
0.00
−0.05
0.2
0.4
0.6
SNP effect on IDP T1 FAST ROIs L thalamus
SNP effect on Ulcerative colitis

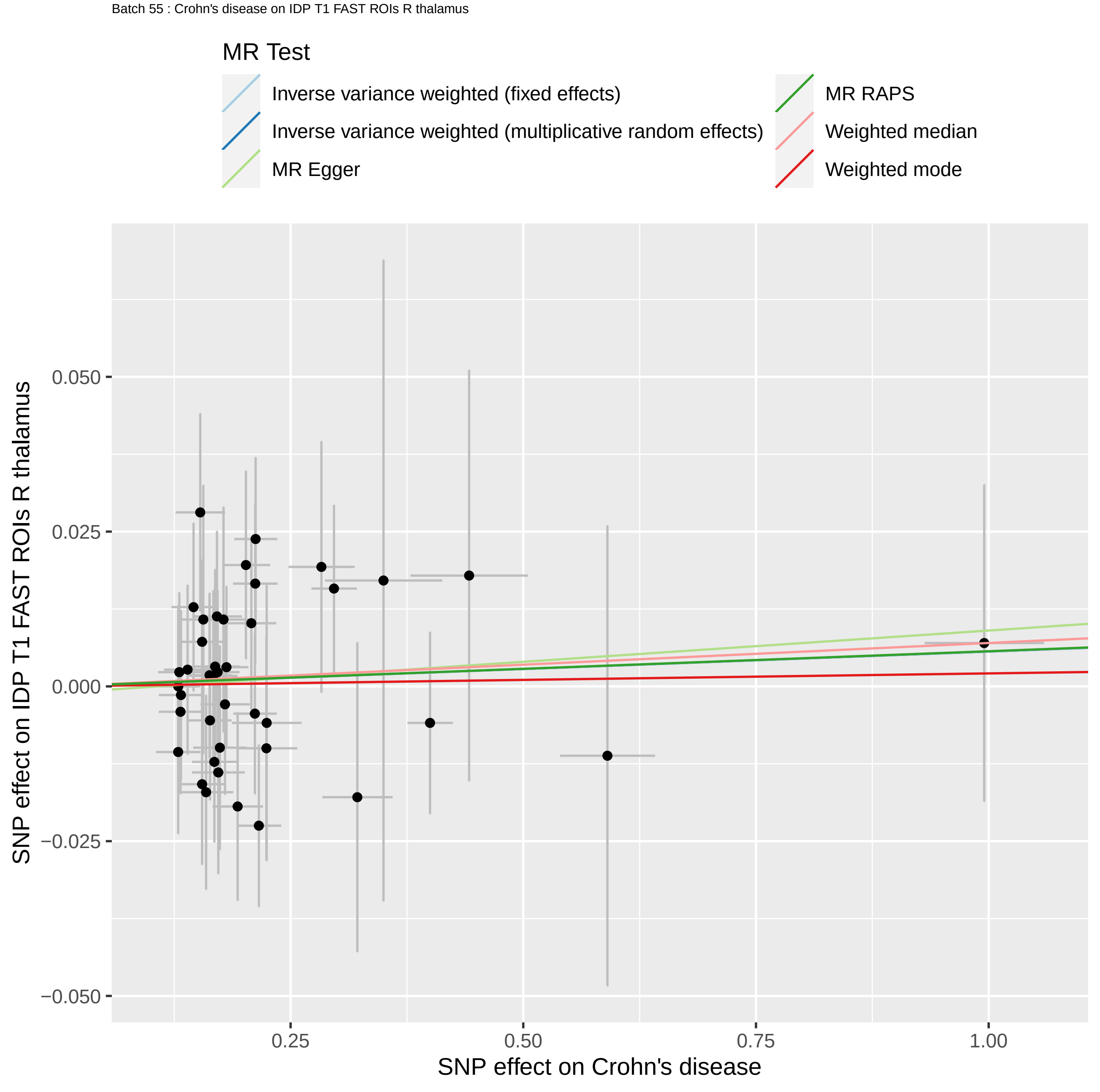

Batch 55 : Crohn's disease on IDP T1 FAST ROIs R thalamus
MR Test
Inverse variance weighted (fixed effects)
Inverse variance weighted (multiplicative random effects)
MR Egger
MR RAPS
Weighted median
Weighted mode
SNP effect on IDP T1 FAST ROIs R thalamus
0.050
0.025
0.000
−0.025
−0.050
0.25
0.50
0.75
1.00
SNP effect on Crohn's disease

Batch 56 : Ulcerative colitis on IDP T1 FAST ROIs R thalamus

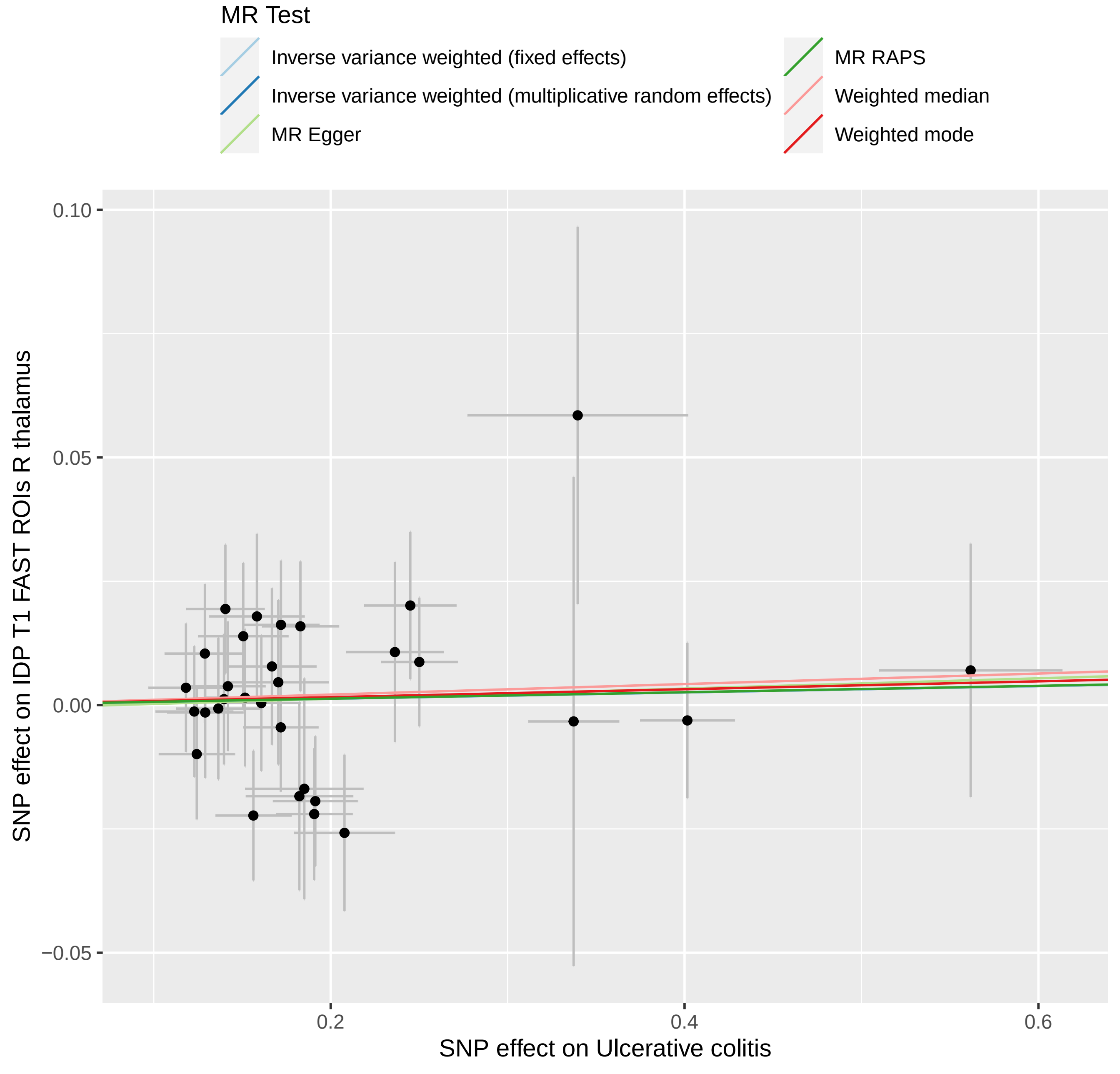

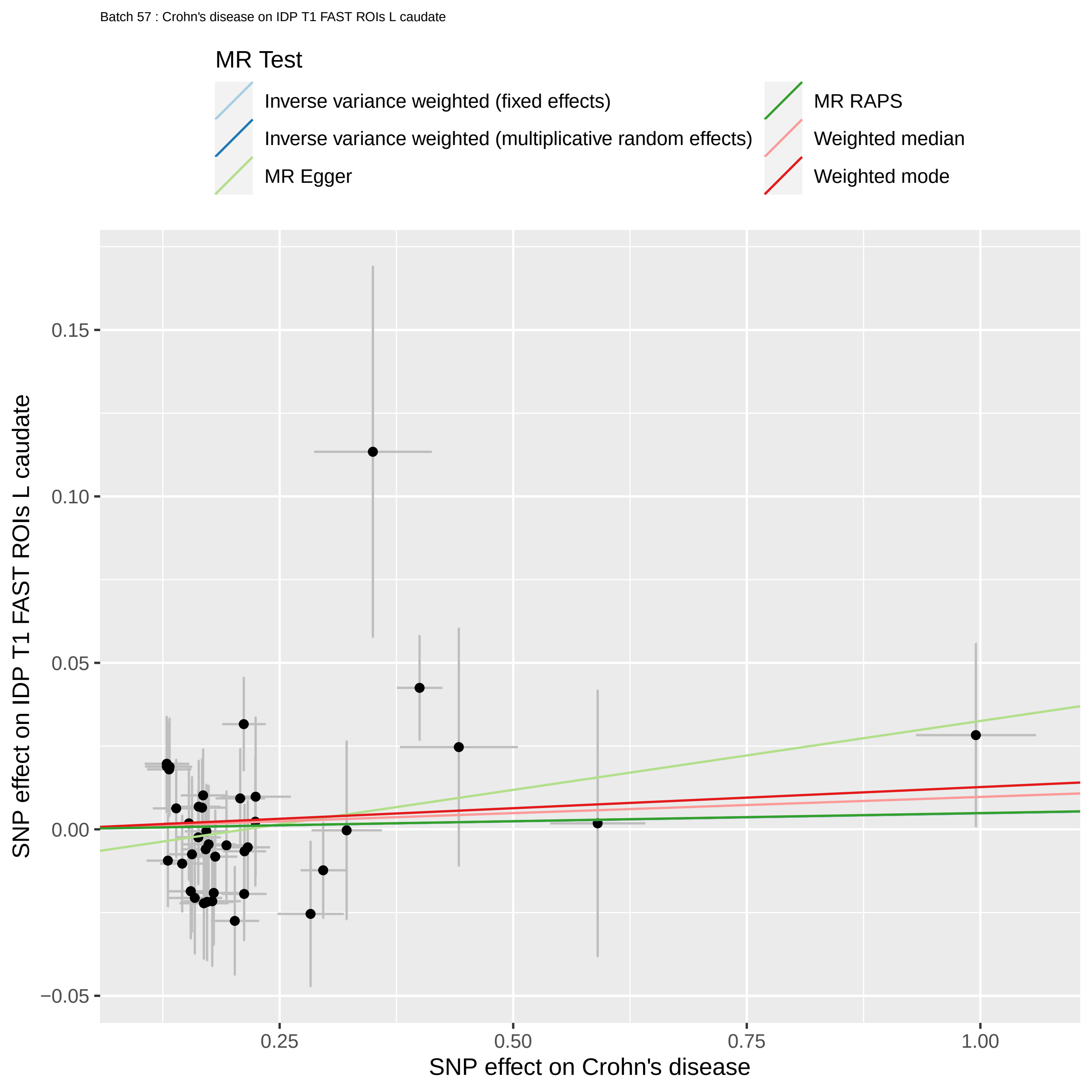

Batch 57 : Crohn's disease on IDP T1 FAST ROIs L caudate
MR Test
Inverse variance weighted (fixed effects)
Inverse variance weighted (multiplicative random effects)
MR Egger
MR RAPS
Weighted median
Weighted mode
0.15
0.10
0.05
0.00
−0.05
0.25
0.50
0.75
1.00
SNP effect on IDP T1 FAST ROIs L caudate
SNP effect on Crohn's disease

Batch 58 : Ulcerative colitis on IDP T1 FAST ROIs L caudate

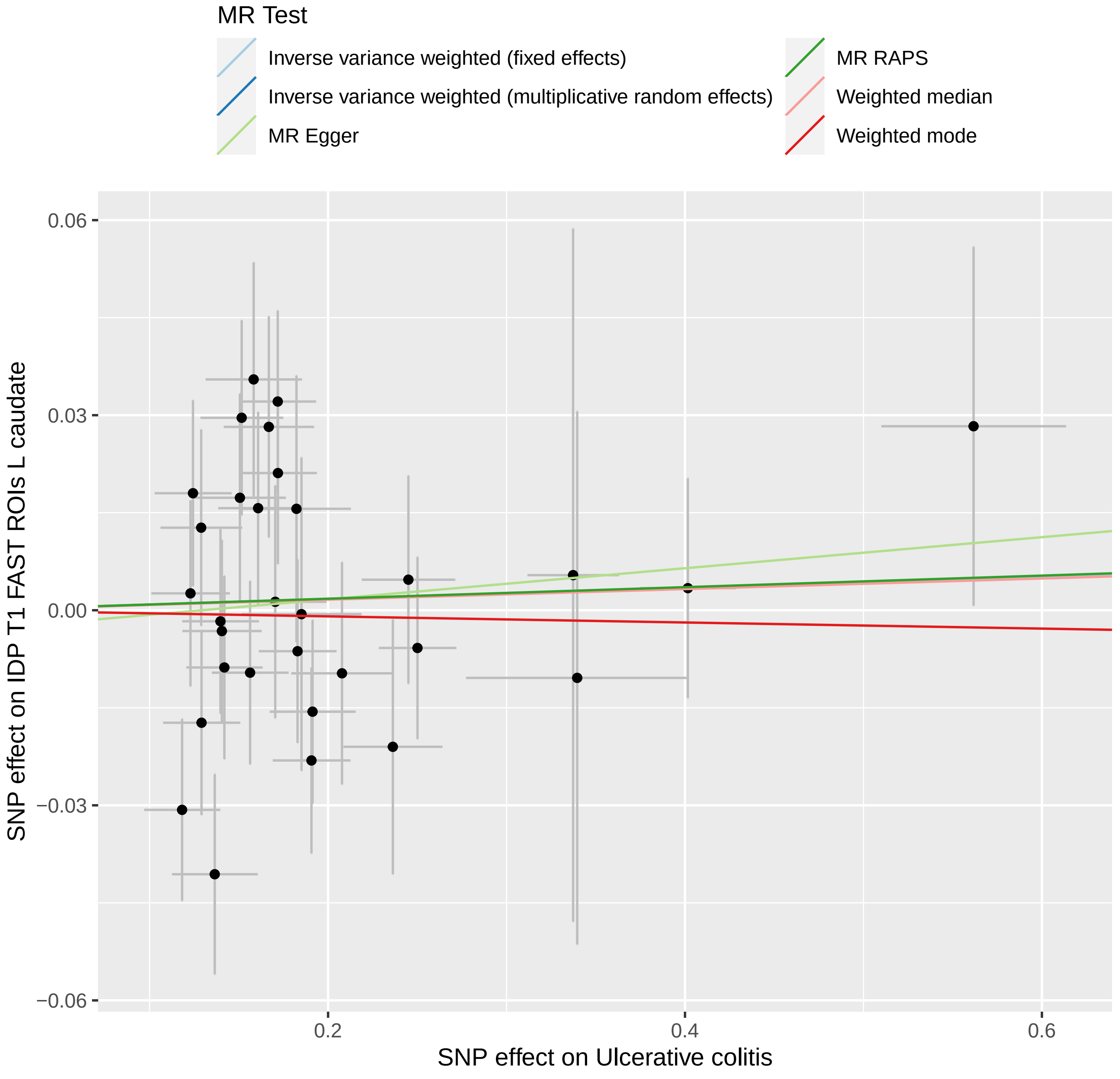

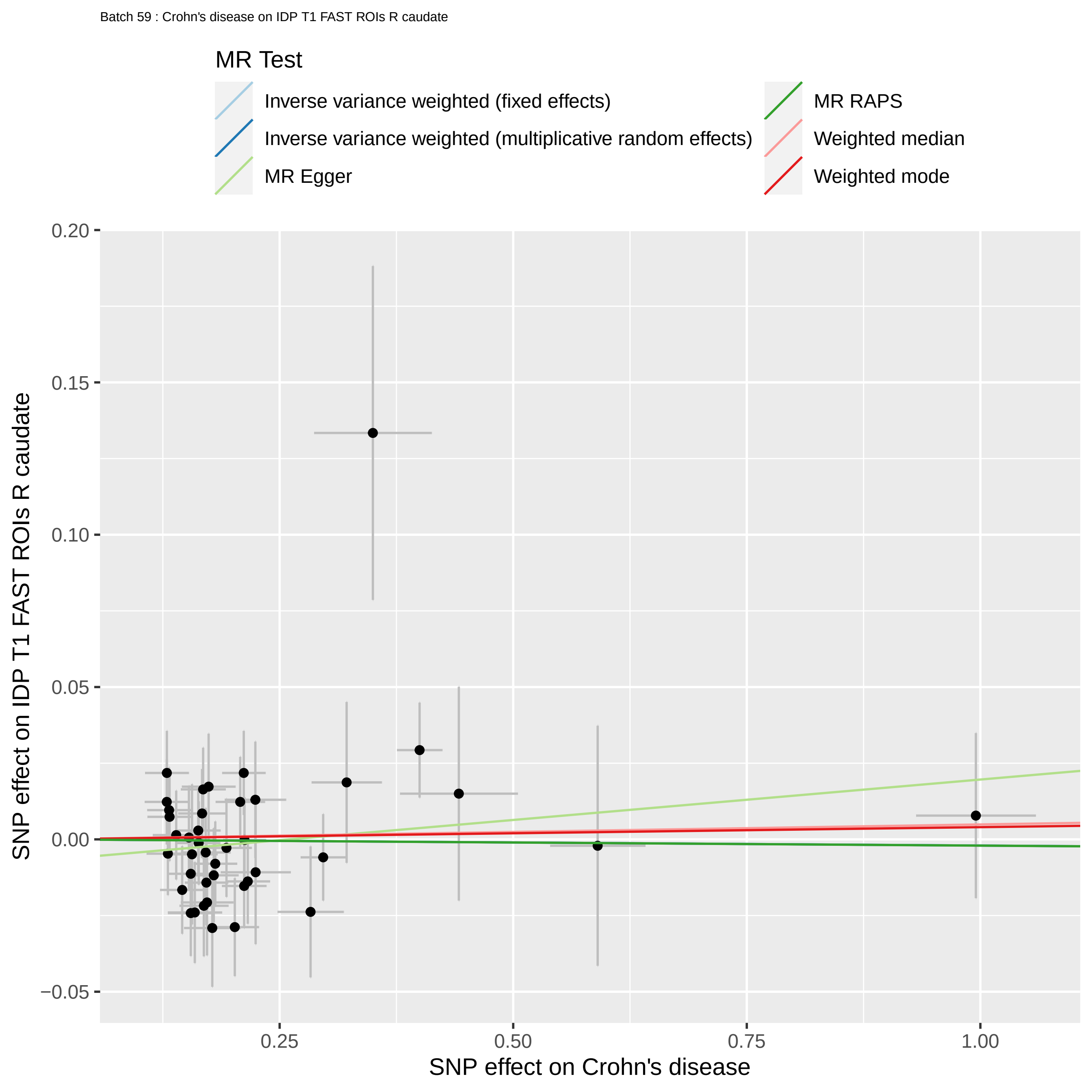

Batch 59 : Crohn's disease on IDP T1 FAST ROIs R caudate
MR Test
Inverse variance weighted (fixed effects)
Inverse variance weighted (multiplicative random effects)
MR Egger
MR RAPS
Weighted median
Weighted mode
SNP effect on IDP T1 FAST ROIs R caudate
SNP effect on Crohn's disease
0.20
0.15
0.10
0.05
0.00
−0.05
0.25
0.50
0.75
1.00

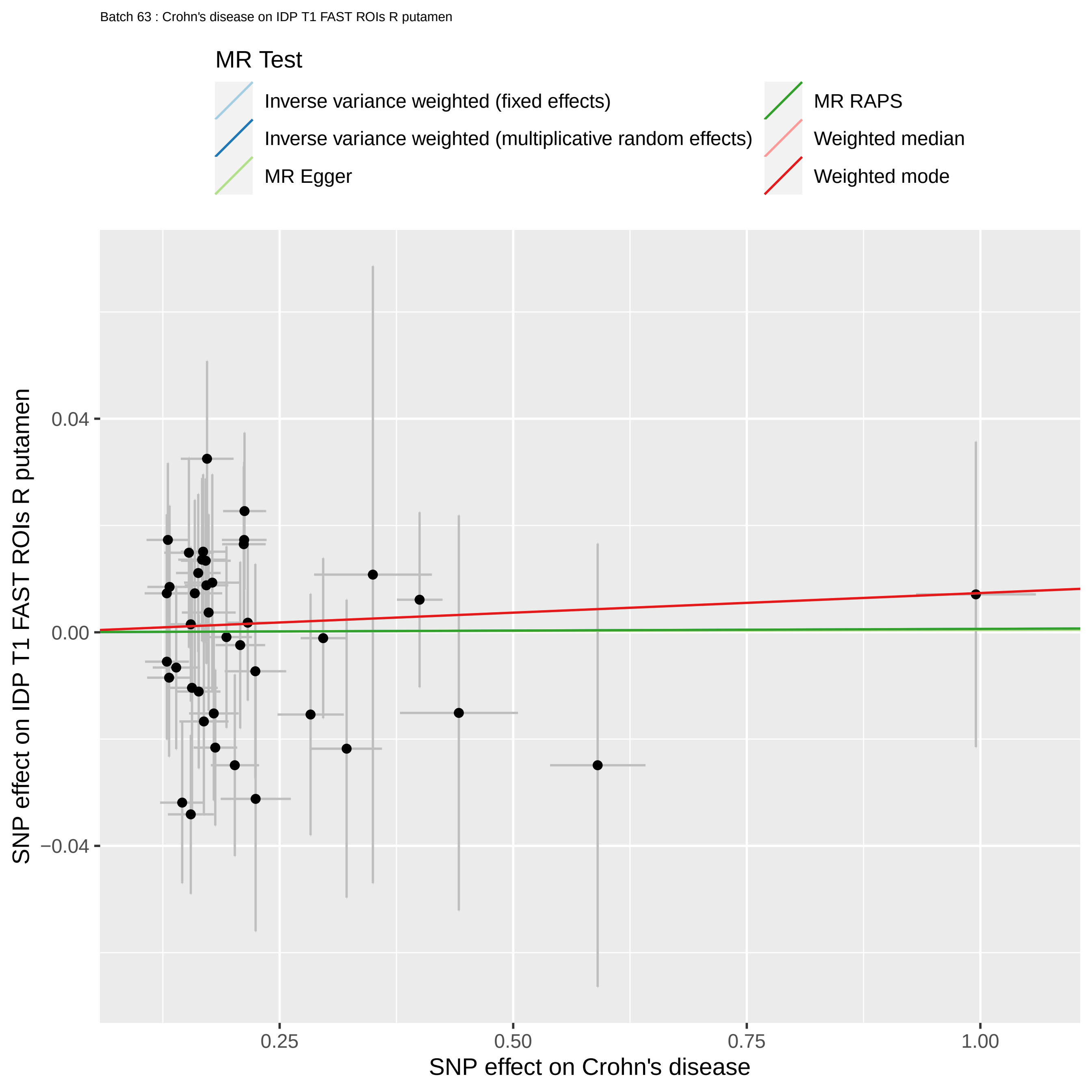
Batch 63 : Crohn's disease on IDP T1 FAST ROIs R putamen
MR Test
Inverse variance weighted (fixed effects)
Inverse variance weighted (multiplicative random effects)
MR Egger
MR RAPS
Weighted median
Weighted mode
0.04
0.00
−0.04
0.25
0.50
0.75
1.00
SNP effect on IDP T1 FAST ROIs R putamen
SNP effect on Crohn's disease

Batch 64 : Ulcerative colitis on IDP T1 FAST ROIs R putamen

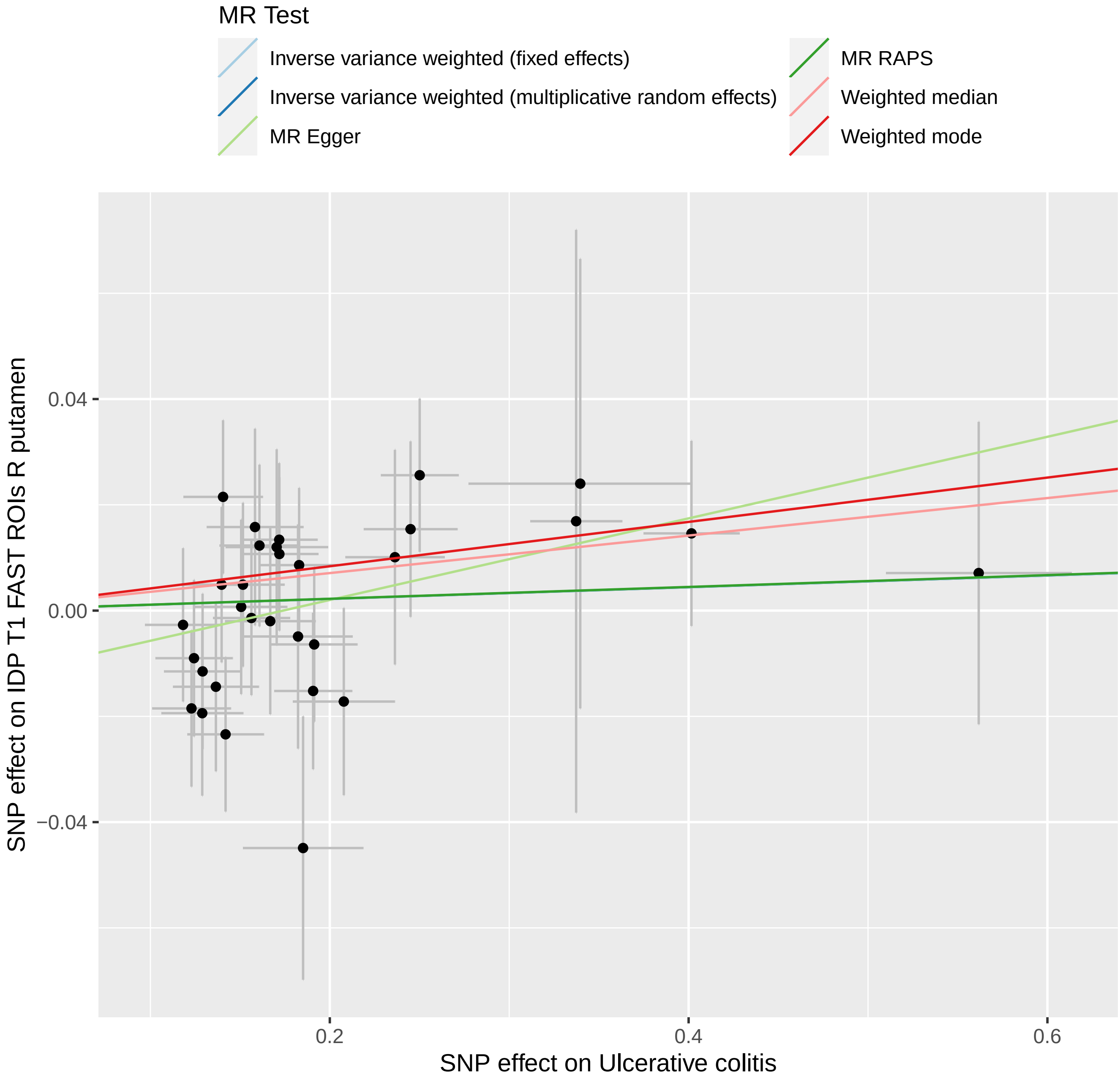

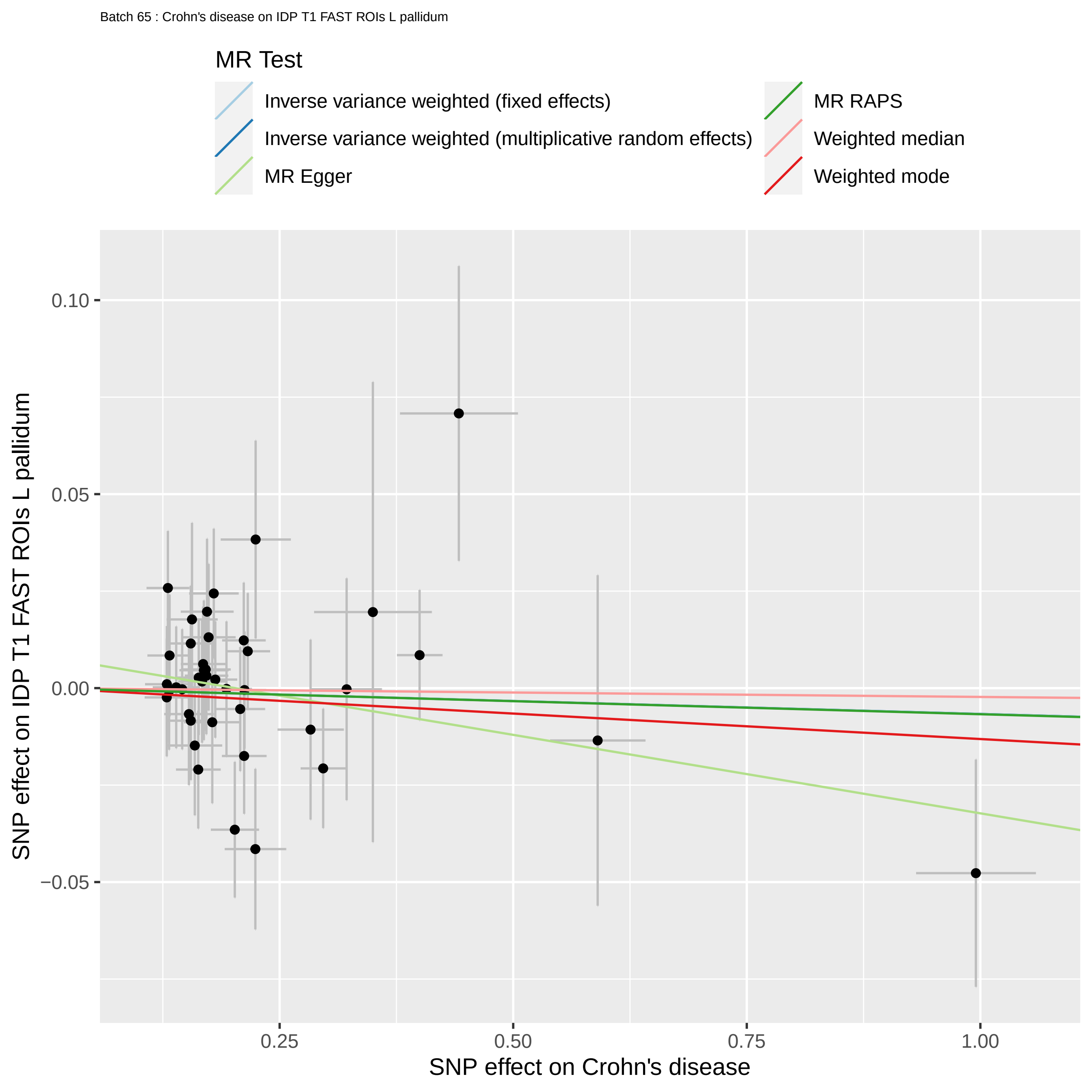
Batch 65 : Crohn's disease on IDP T1 FAST ROIs L pallidum
MR Test
Inverse variance weighted (fixed effects)
Inverse variance weighted (multiplicative random effects)
MR Egger
MR RAPS
Weighted median
Weighted mode
0.10
0.05
0.00
−0.05
0.25
0.50
0.75
1.00
SNP effect on IDP T1 FAST ROIs L pallidum
SNP effect on Crohn's disease

Batch 66 : Ulcerative colitis on IDP T1 FAST ROIs L pallidum

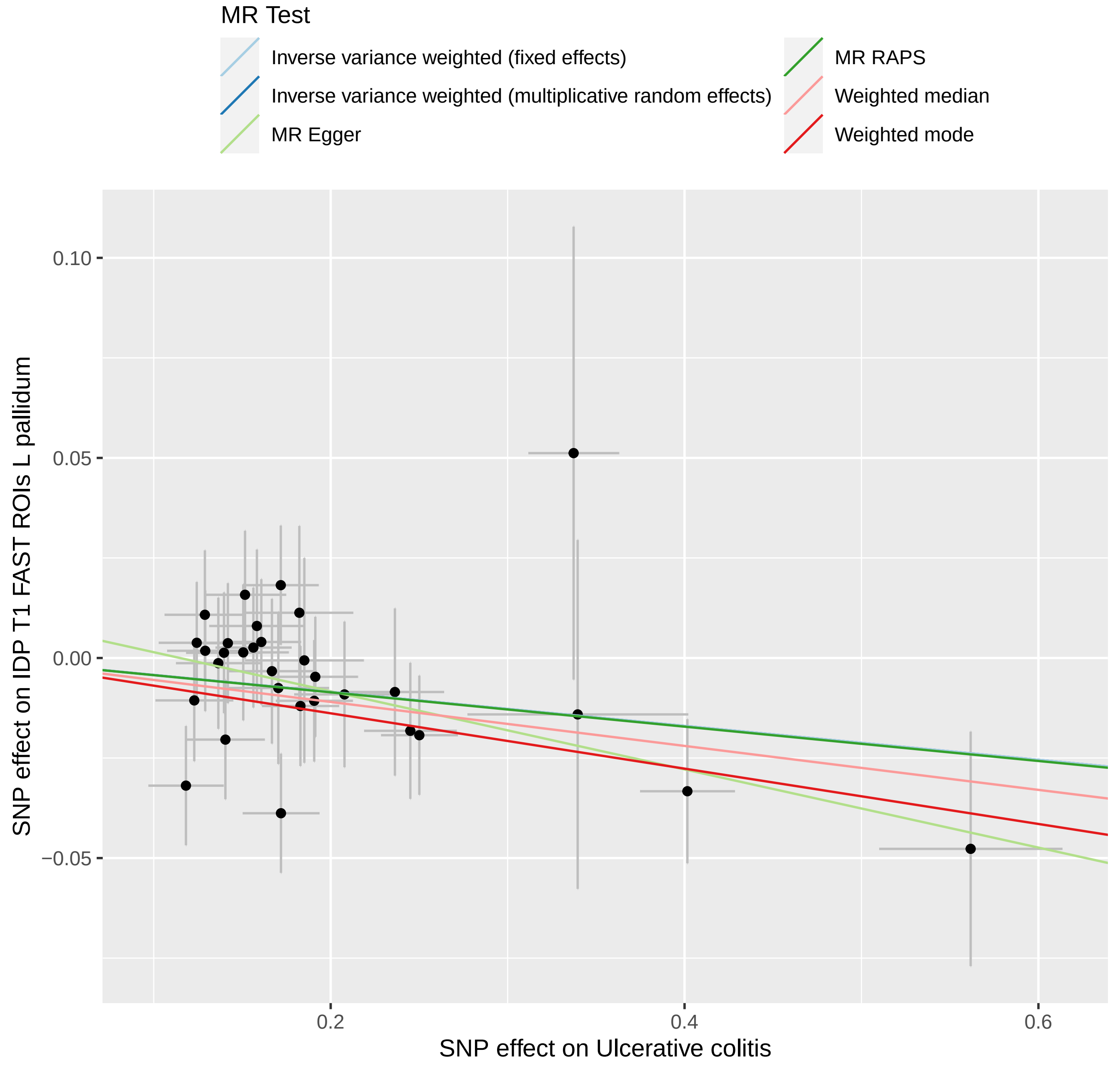

Batch 67 : Crohn's disease on IDP T1 FAST ROIs R pallidum

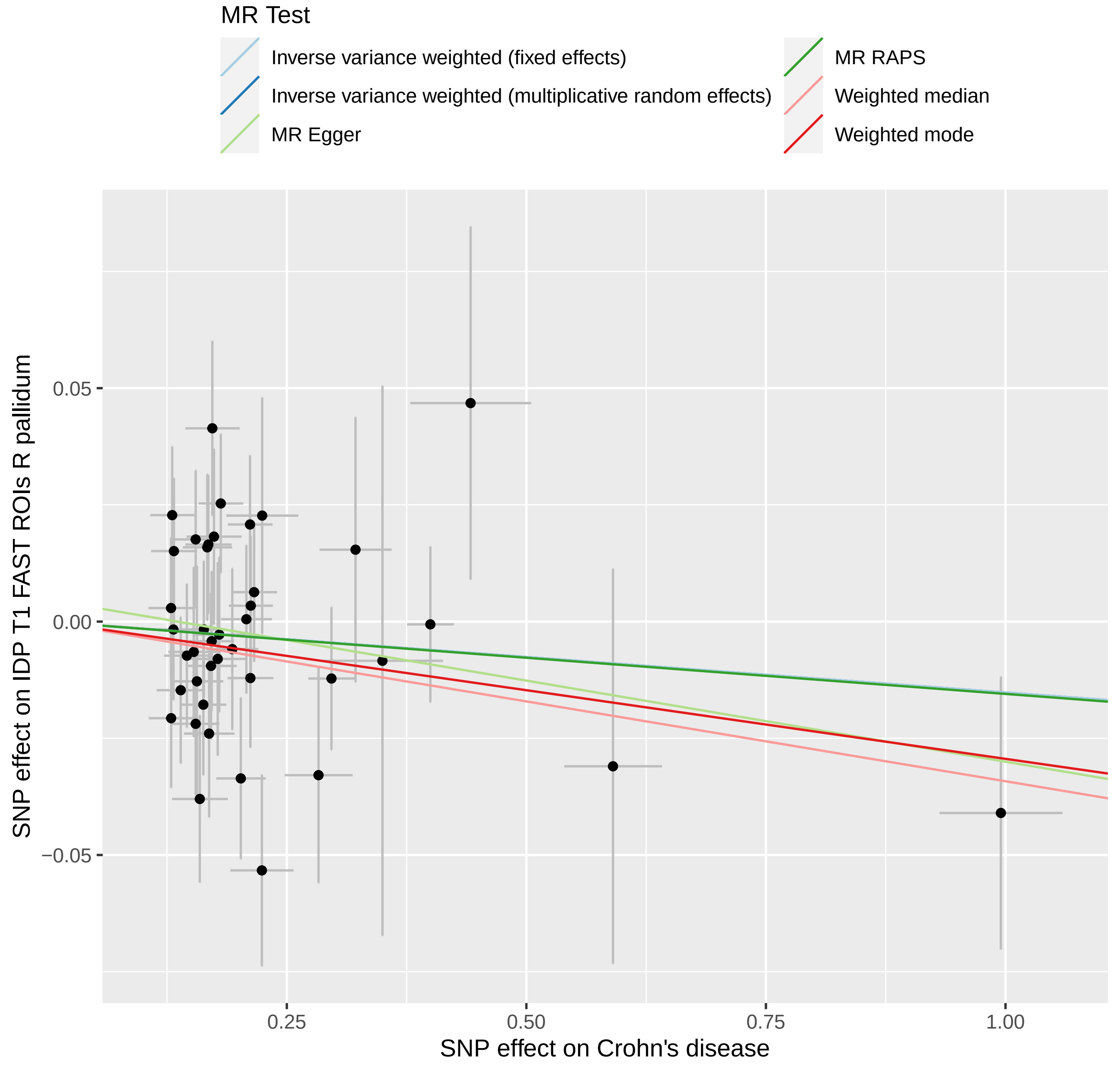

Batch 68 : Ulcerative colitis on IDP T1 FAST ROIs R pallidum

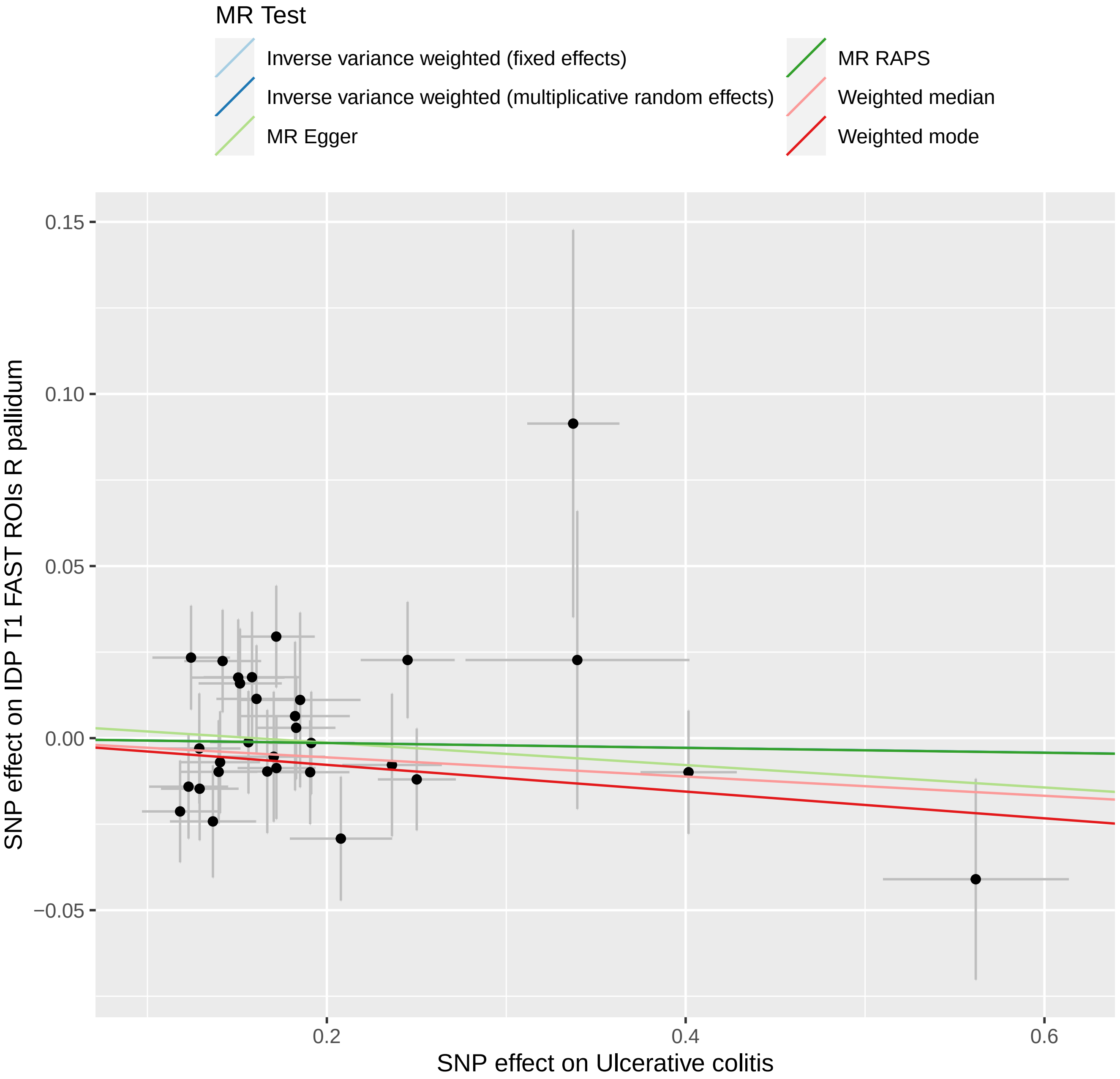

Batch 69 : Crohn's disease on IDP T1 FIRST left caudate volume

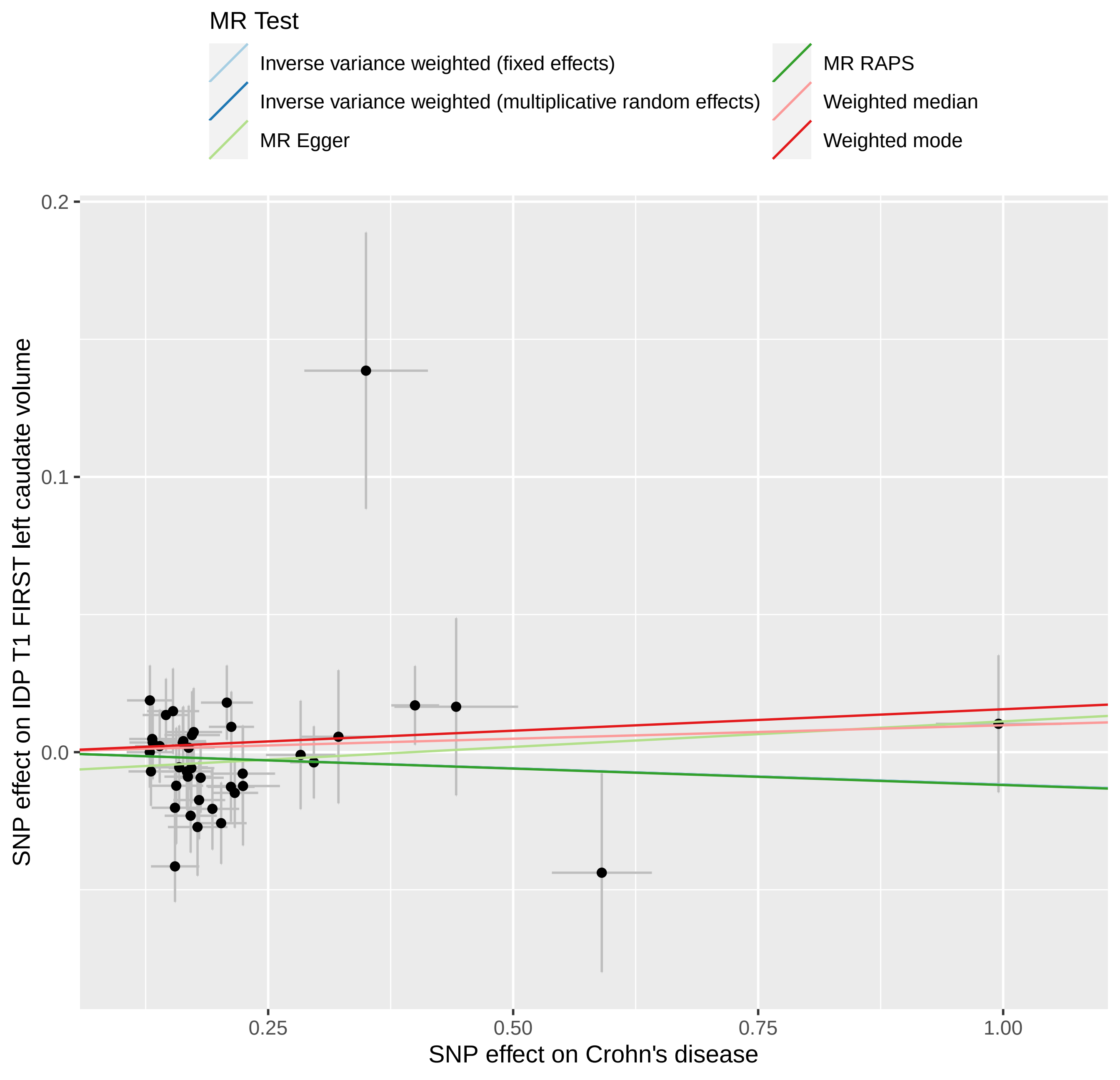

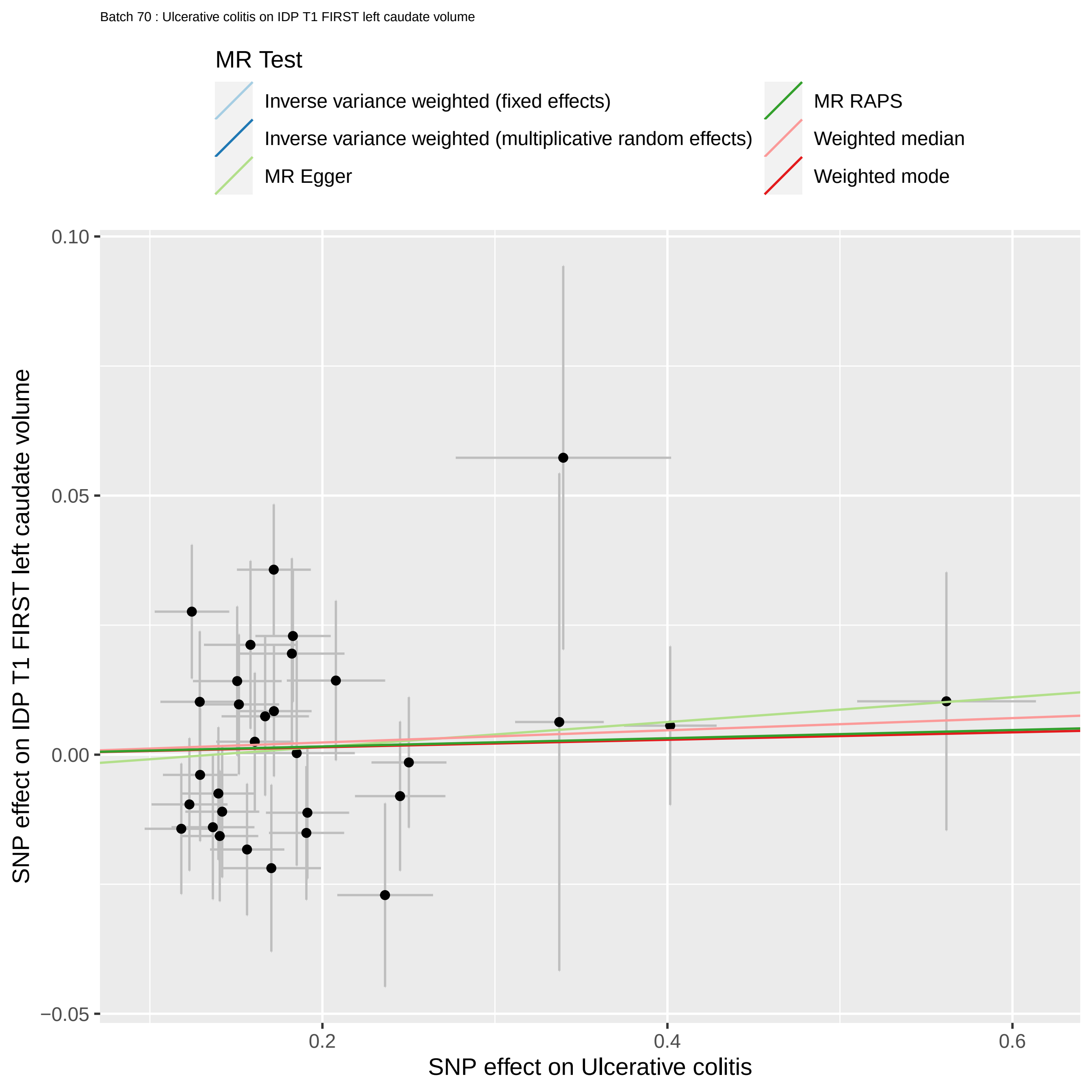

Batch 70 : Ulcerative colitis on IDP T1 FIRST left caudate volume
MR Test
Inverse variance weighted (fixed effects)
Inverse variance weighted (multiplicative random effects)
MR Egger
MR RAPS
Weighted median
Weighted mode
0.10
0.05
0.00
−0.05
0.2
0.4
0.6
SNP effect on IDP T1 FIRST left caudate volume
SNP effect on Ulcerative colitis

Batch 71 : Crohn's disease on IDP T1 FAST ROIs L hippocampus

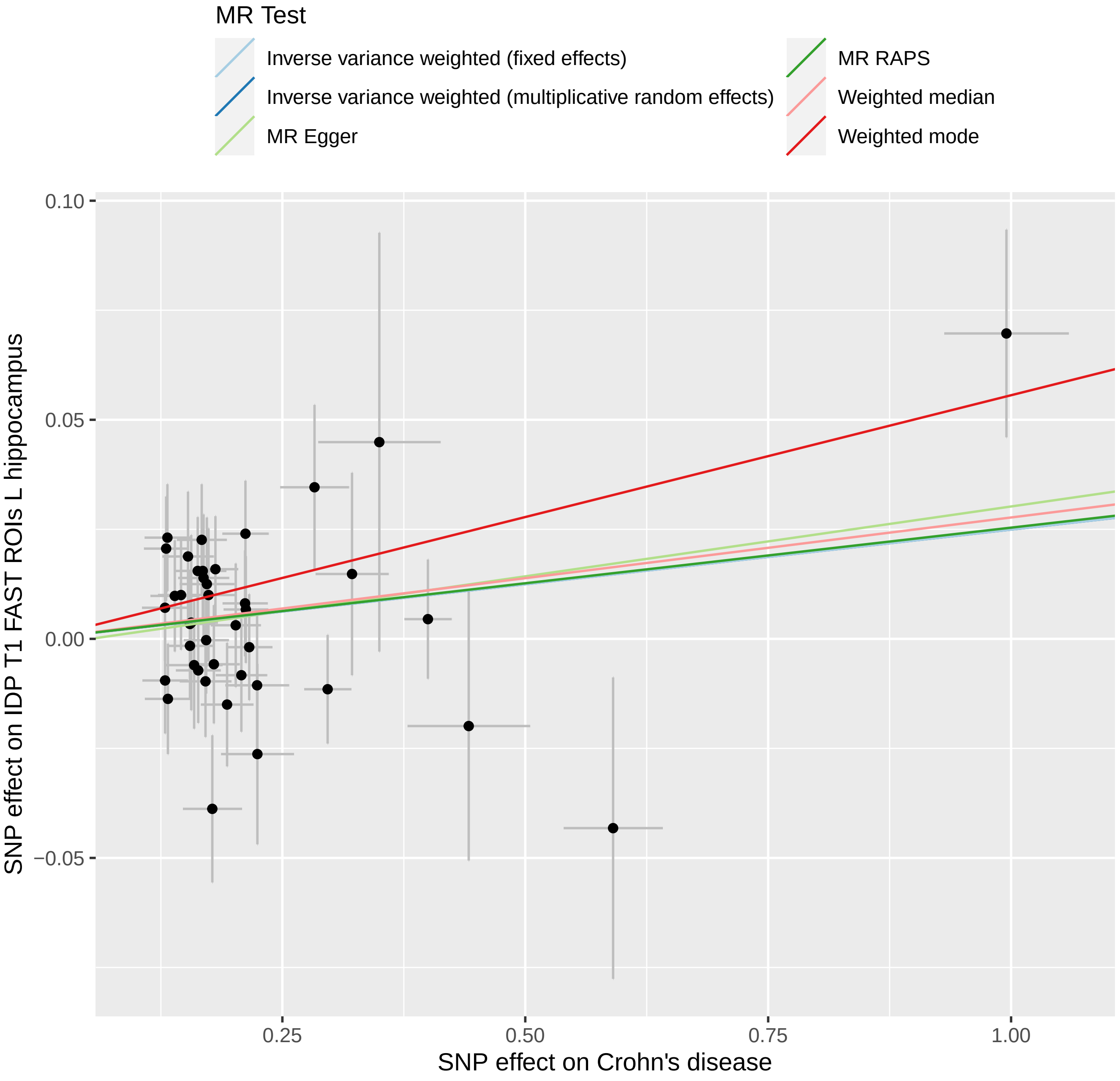

Batch 72 : Ulcerative colitis on IDP T1 FAST ROIs L hippocampus

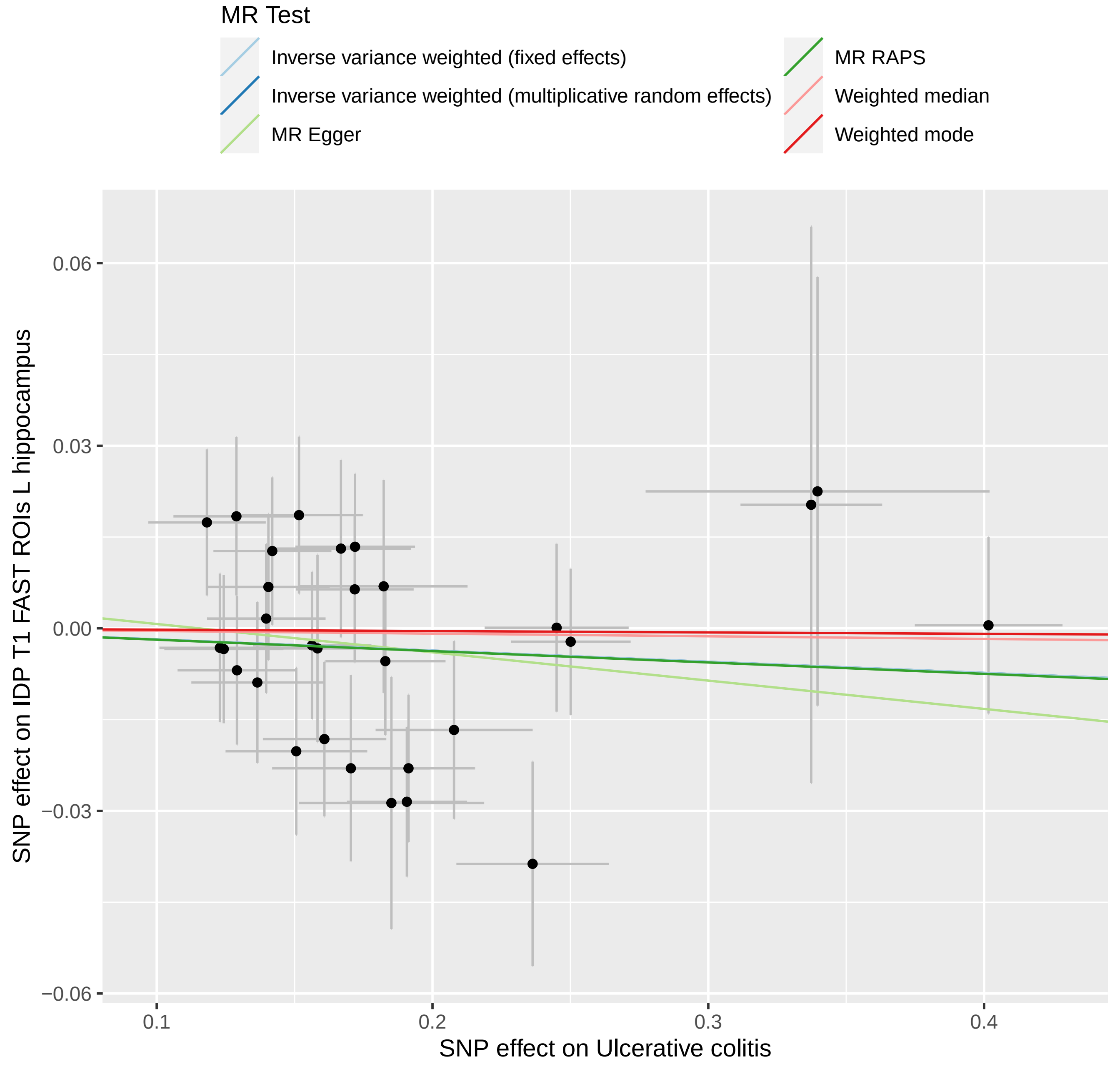

Batch 73 : Crohn's disease on IDP T1 FAST ROIs R hippocampus

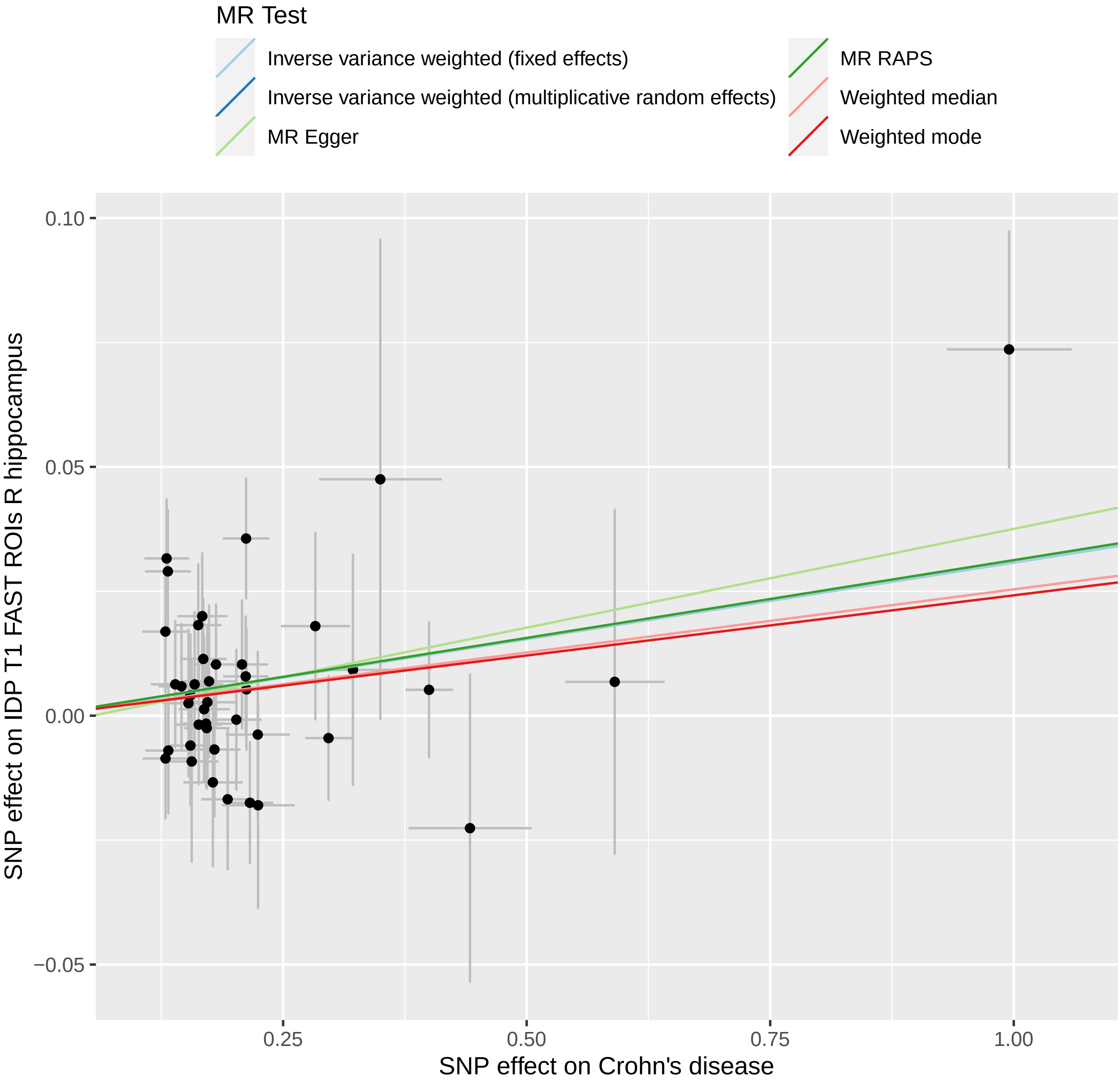

Batch 74 : Ulcerative colitis on IDP T1 FAST ROIs R hippocampus

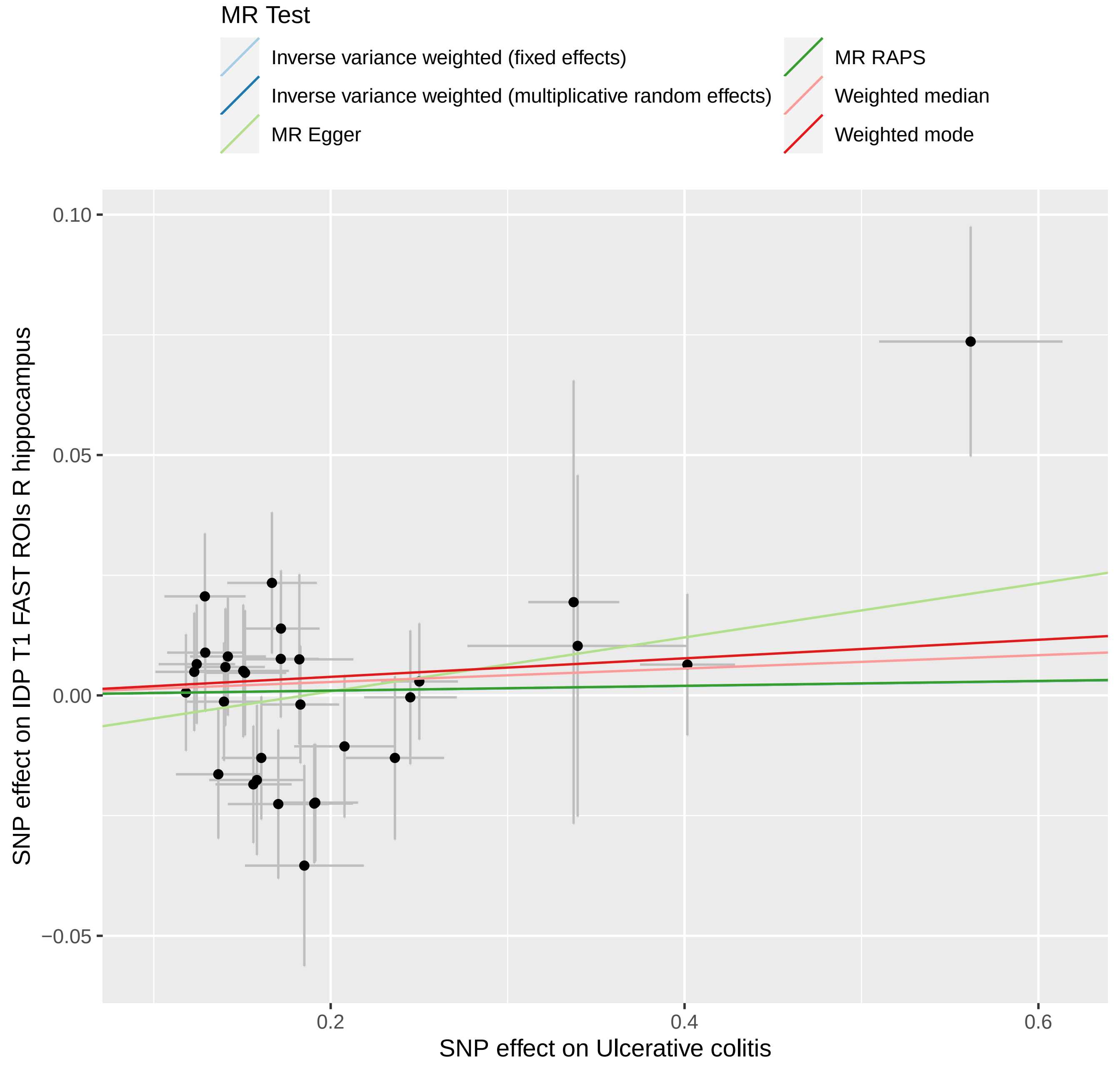

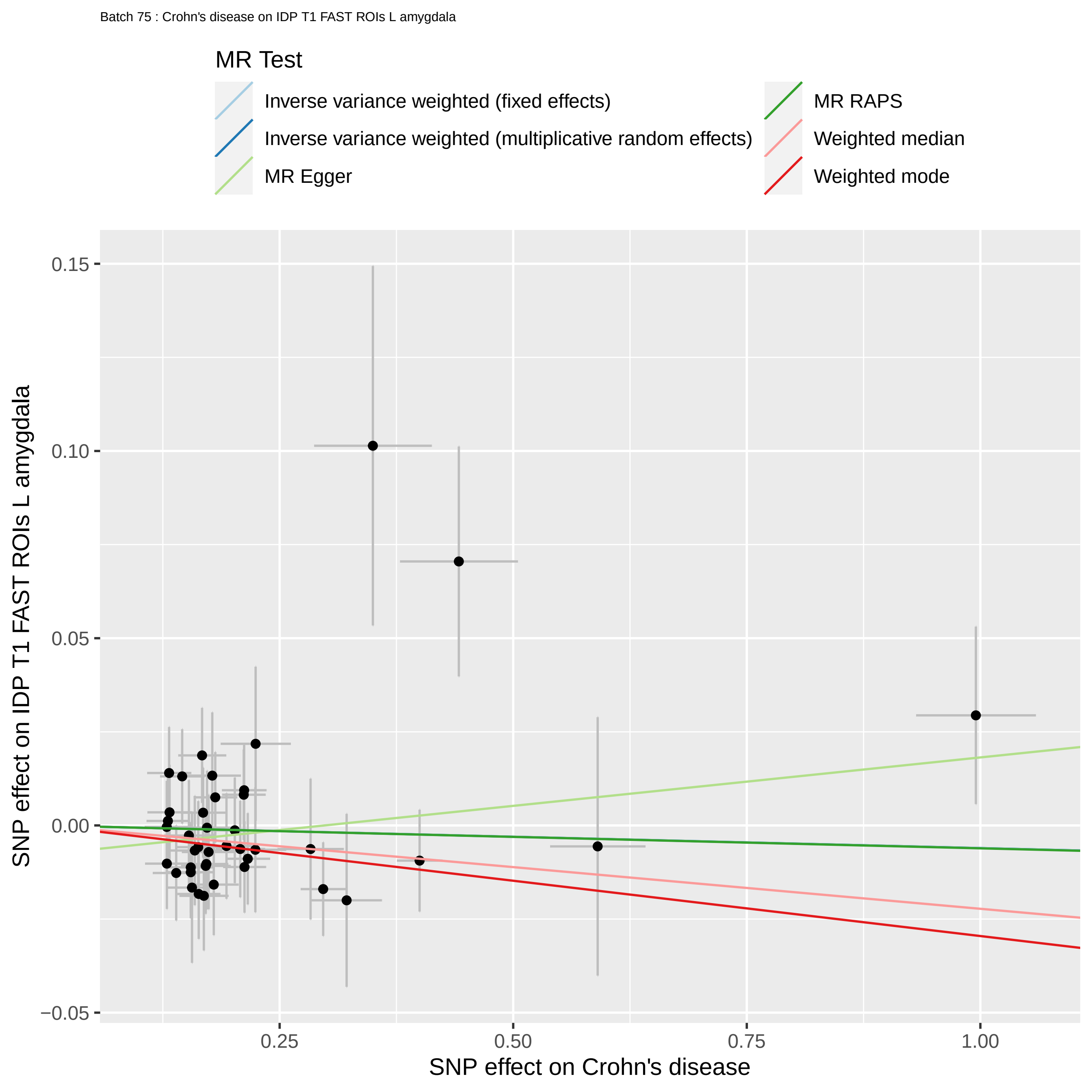
Batch 75 : Crohn's disease on IDP T1 FAST ROIs L amygdala
MR Test
Inverse variance weighted (fixed effects)
Inverse variance weighted (multiplicative random effects)
MR Egger
MR RAPS
Weighted median
Weighted mode
0.15
0.10
0.05
0.00
−0.05
0.25
0.50
0.75
1.00
SNP effect on IDP T1 FAST ROIs L amygdala
SNP effect on Crohn's disease

Batch 76 : Ulcerative colitis on IDP T1 FAST ROIs L amygdala

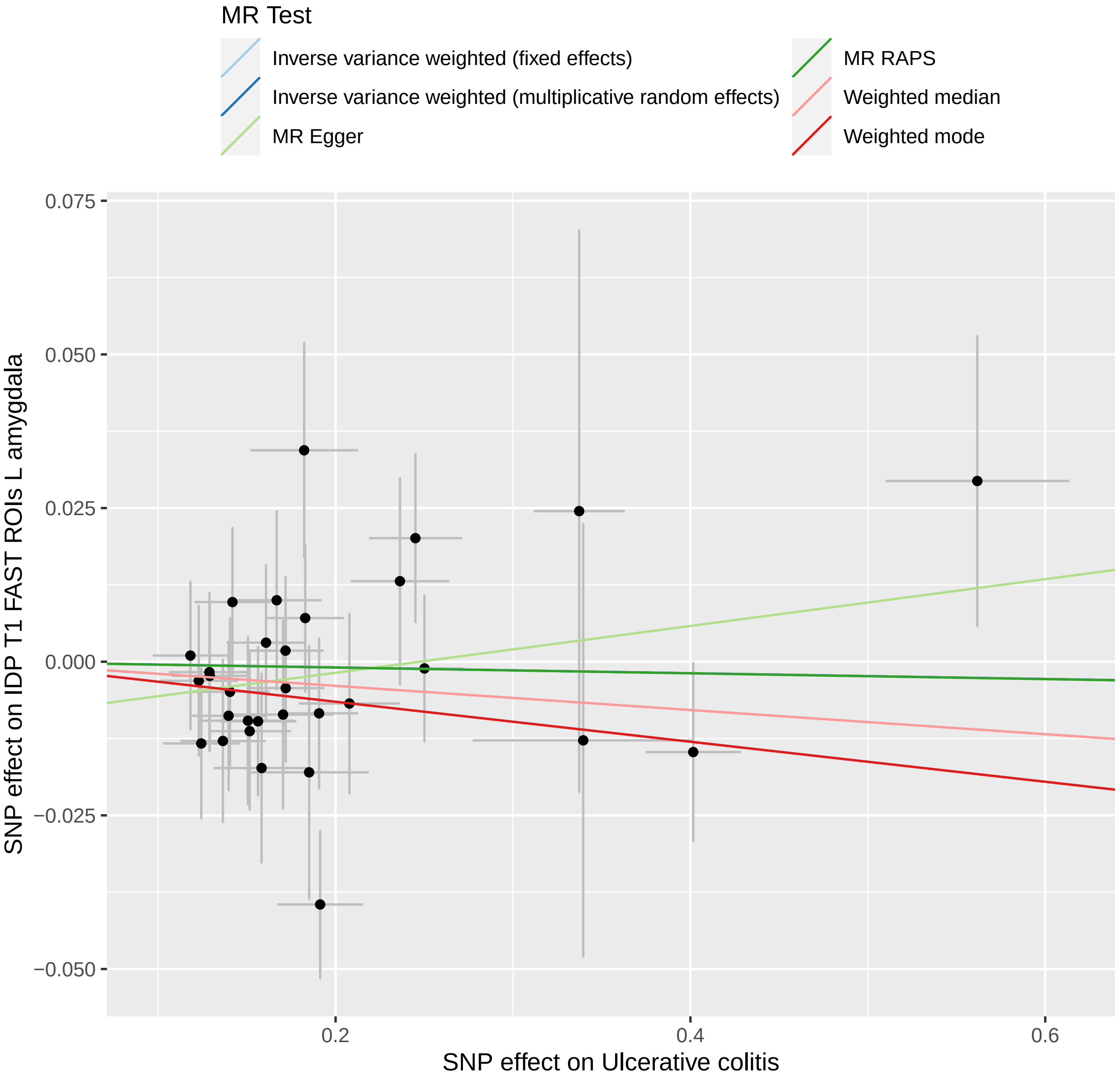

Batch 77 : Crohn's disease on IDP T1 FAST ROIs R amygdala

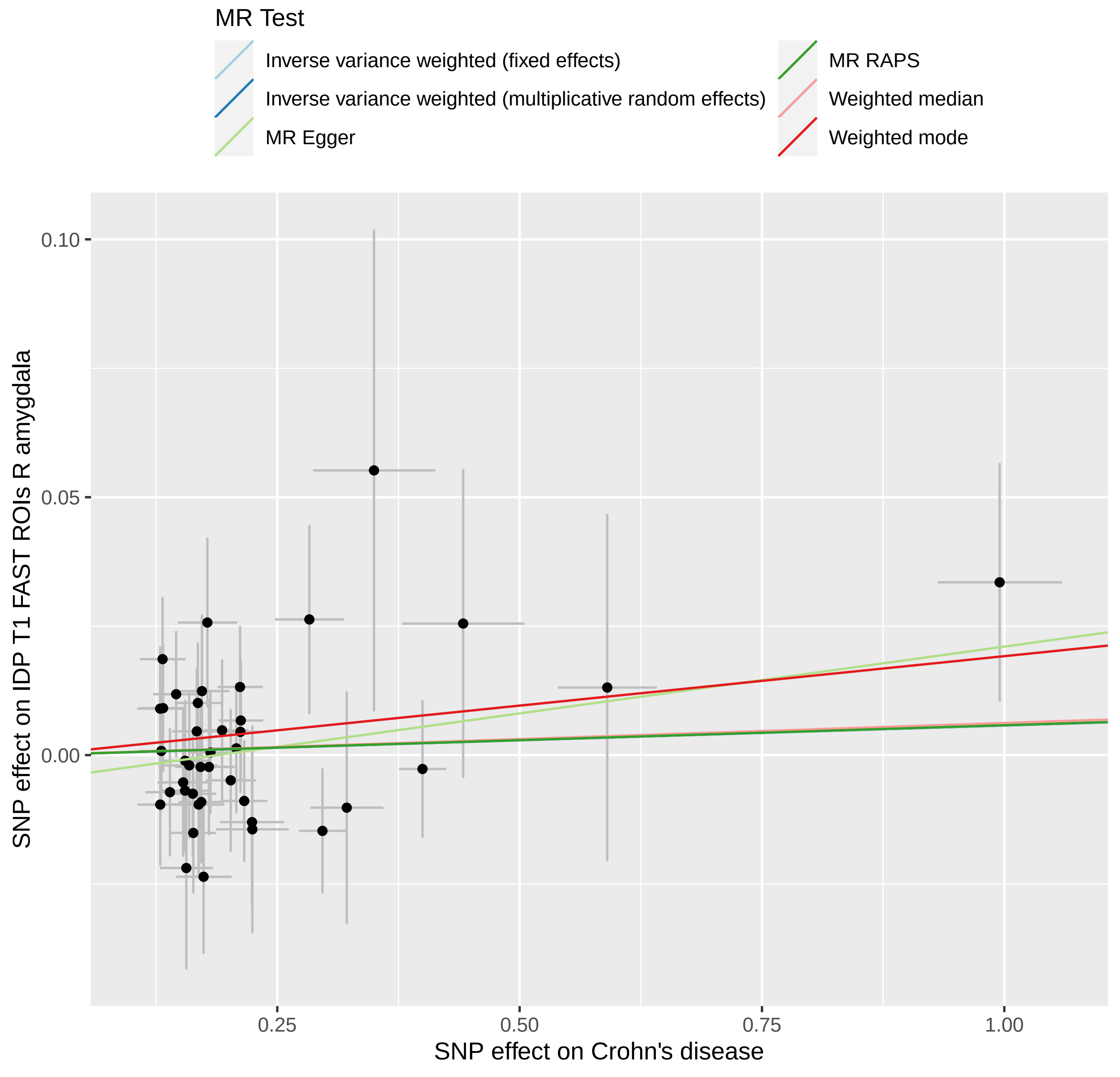

Batch 78 : Ulcerative colitis on IDP T1 FAST ROIs R amygdala

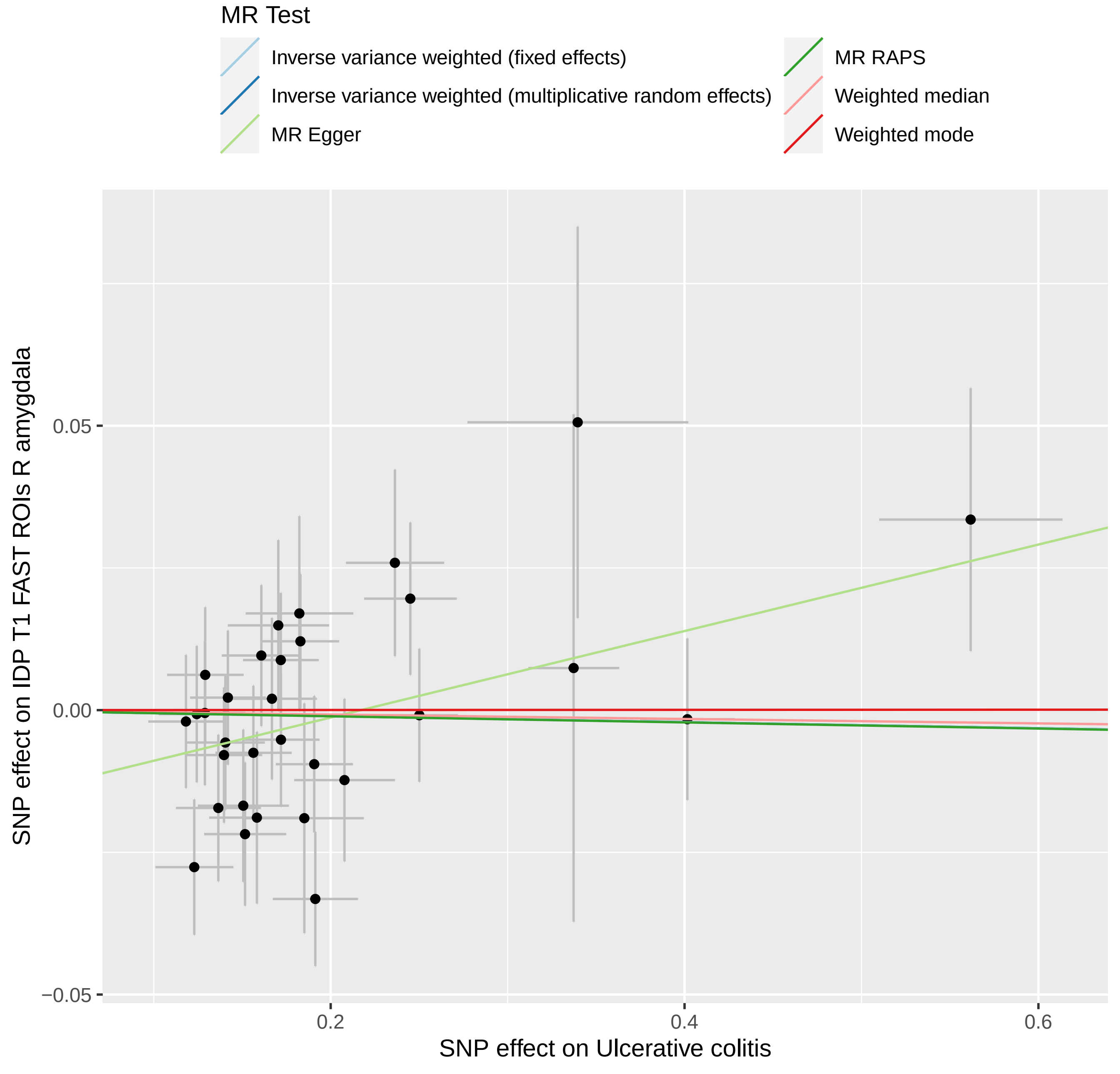

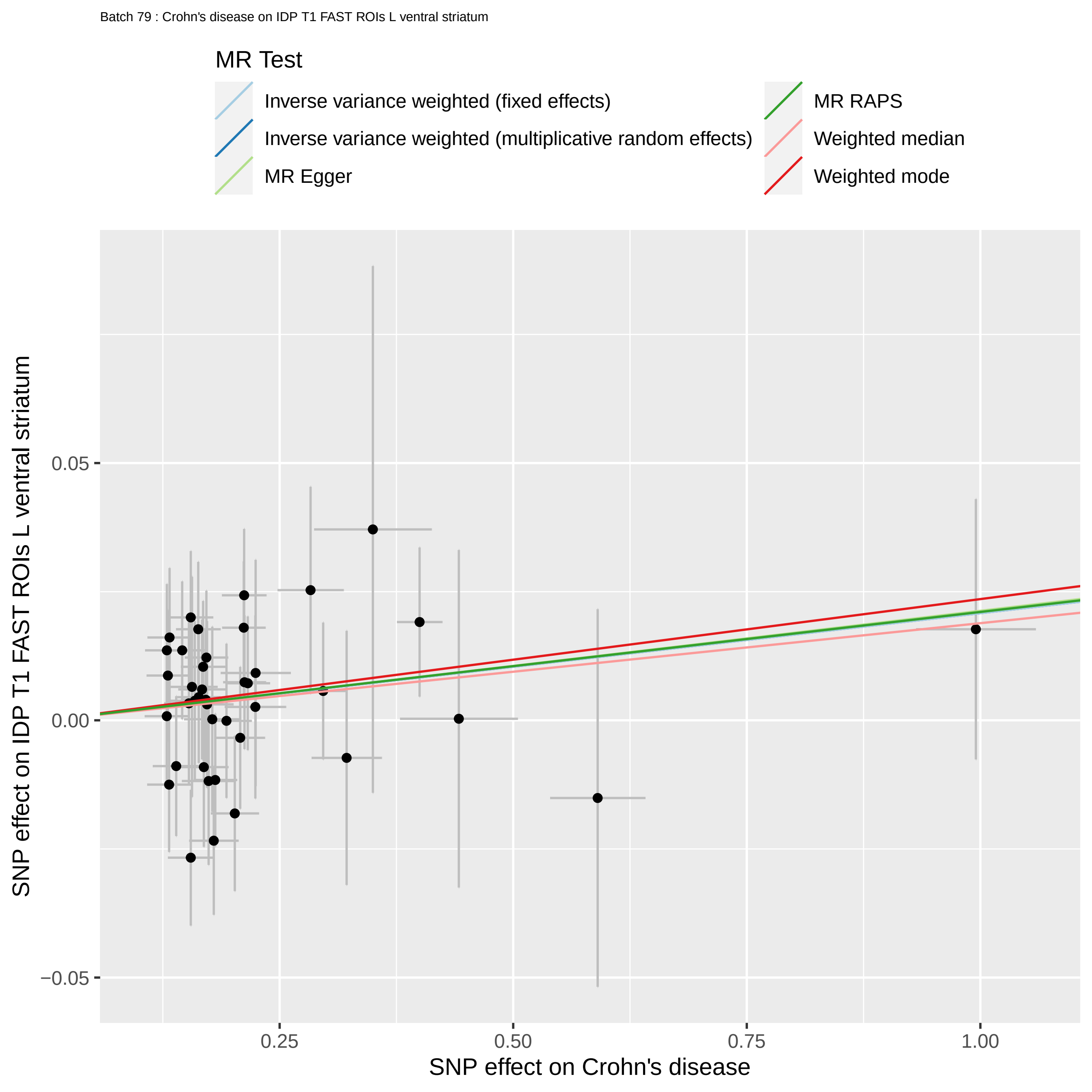
Batch 79 : Crohn's disease on IDP T1 FAST ROIs L ventral striatum
MR Test
Inverse variance weighted (fixed effects)
Inverse variance weighted (multiplicative random effects)
MR Egger
MR RAPS
Weighted median
Weighted mode
SNP effect on IDP T1 FAST ROIs L ventral striatum
0.05
0.00
−0.05
SNP effect on Crohn's disease
0.25
0.50
0.75
1.00

Batch 80 : Ulcerative colitis on IDP T1 FAST ROIs L ventral striatum

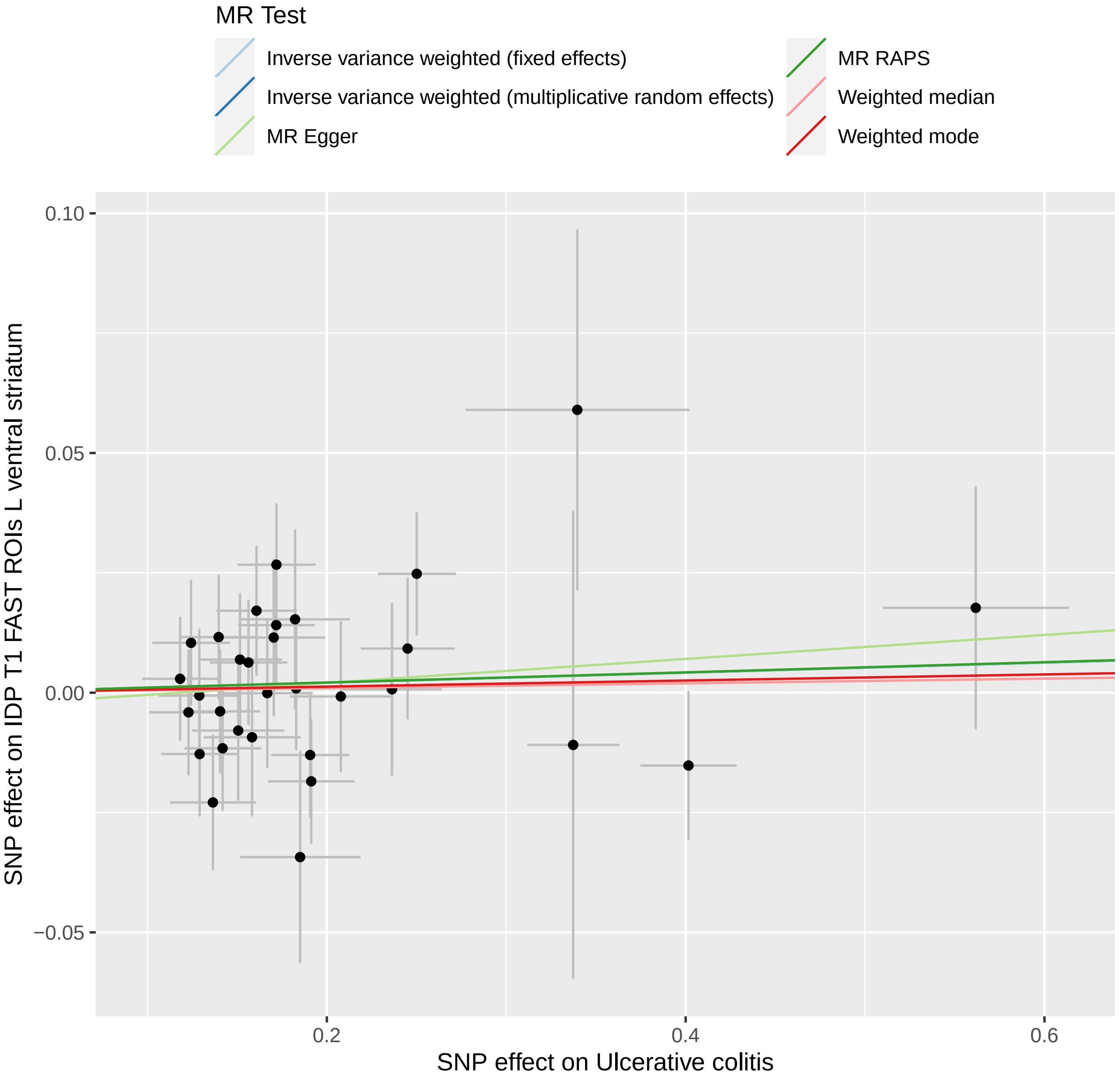

Batch 81 : Crohn's disease on IDP T1 FAST ROIs R ventral striatum

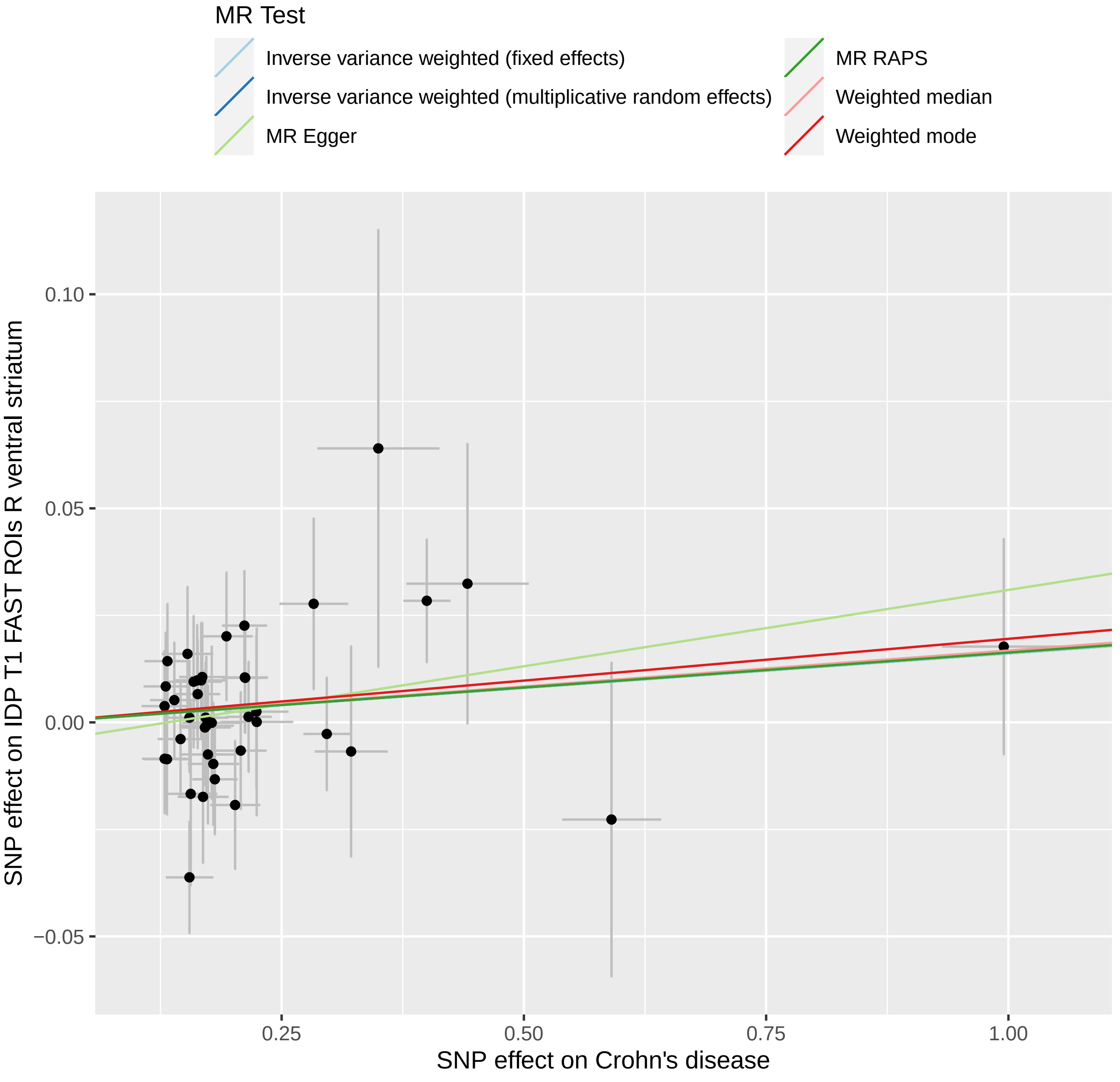

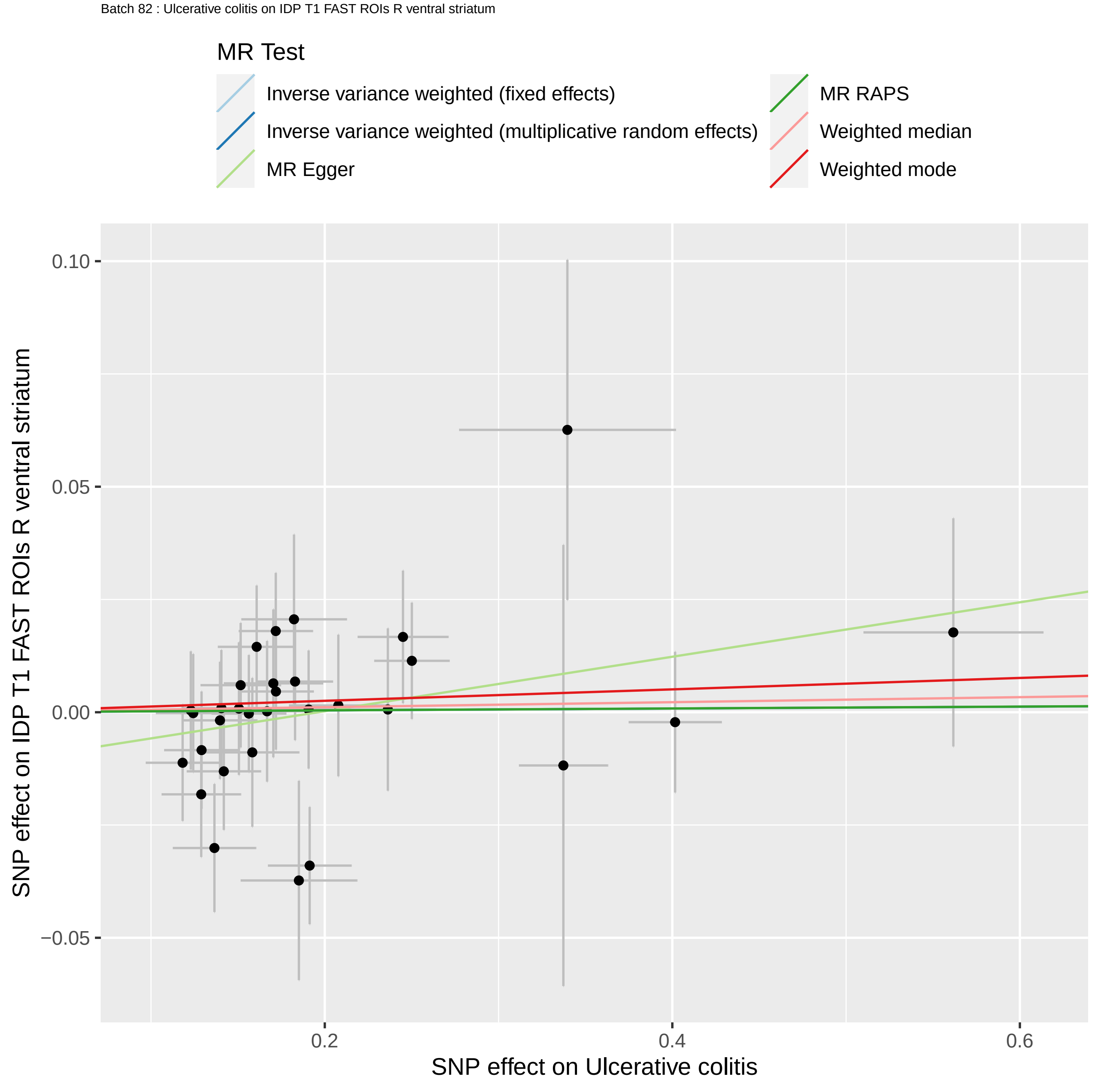

Batch 82 : Ulcerative colitis on IDP T1 FAST ROIs R ventral striatum
MR Test
Inverse variance weighted (fixed effects)
Inverse variance weighted (multiplicative random effects)
MR Egger
MR RAPS
Weighted median
Weighted mode
0.10
0.05
0.00
−0.05
0.2
0.4
0.6
SNP effect on IDP T1 FAST ROIs R ventral striatum
SNP effect on Ulcerative colitis

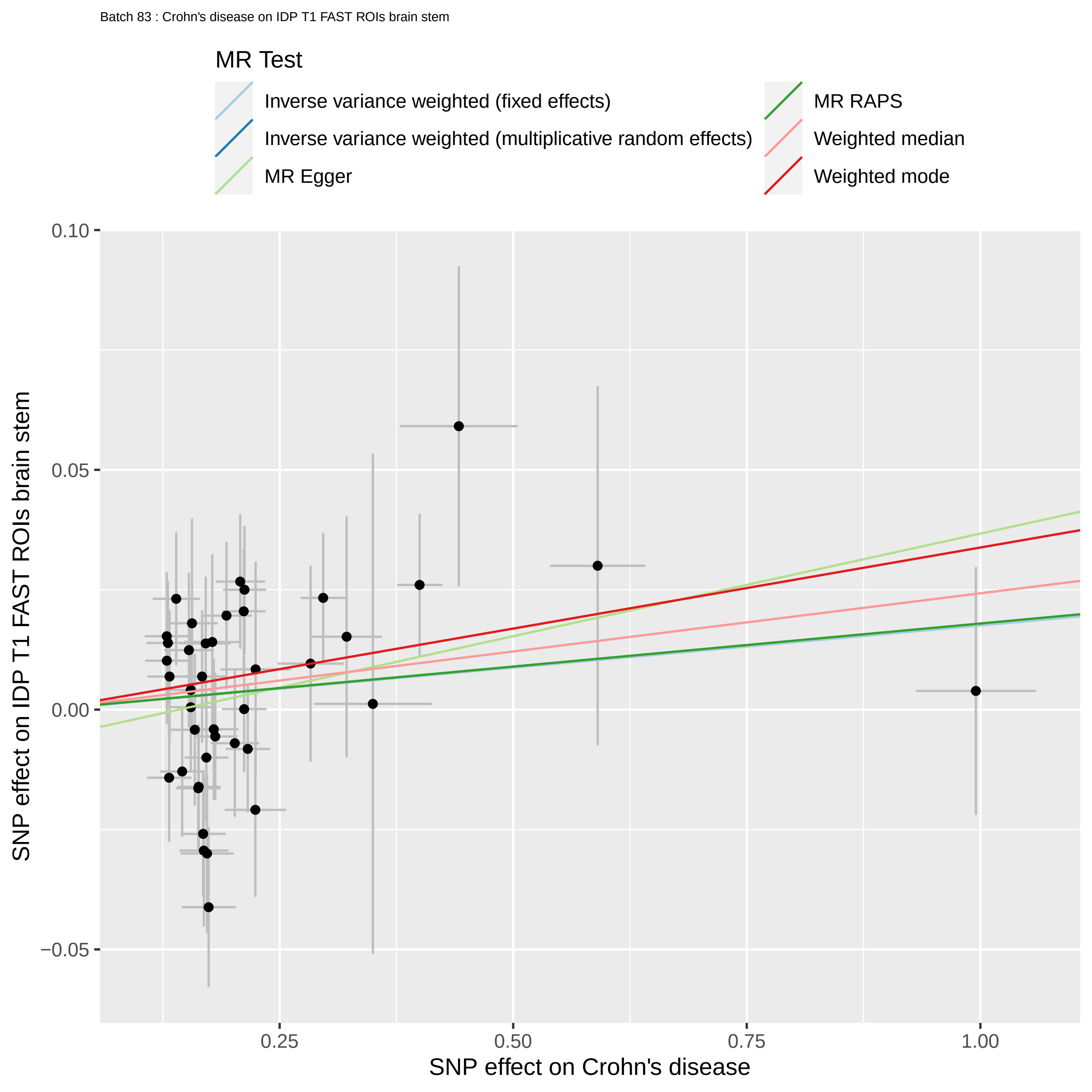

Batch 83 : Crohn's disease on IDP T1 FAST ROIs brain stem
MR Test
Inverse variance weighted (fixed effects)
Inverse variance weighted (multiplicative random effects)
MR Egger
MR RAPS
Weighted median
Weighted mode
0.10
0.05
0.00
−0.05
0.25
0.50
0.75
1.00
SNP effect on IDP T1 FAST ROIs brain stem
SNP effect on Crohn's disease

Batch 84 : Ulcerative colitis on IDP T1 FAST ROIs brain stem

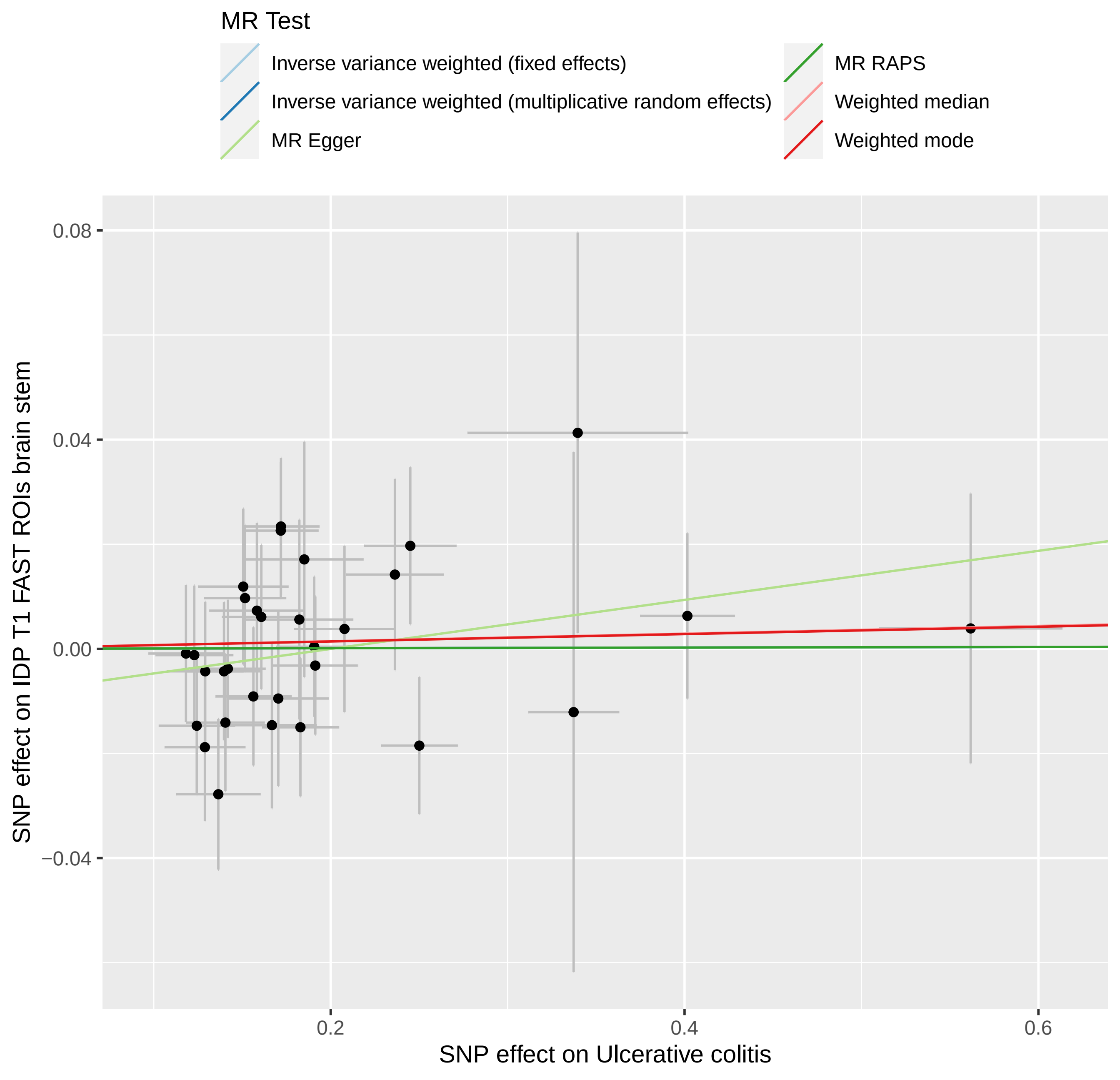

Batch 85 : Crohn's disease on IDP T1 FAST ROIs L cerebellum I−IV

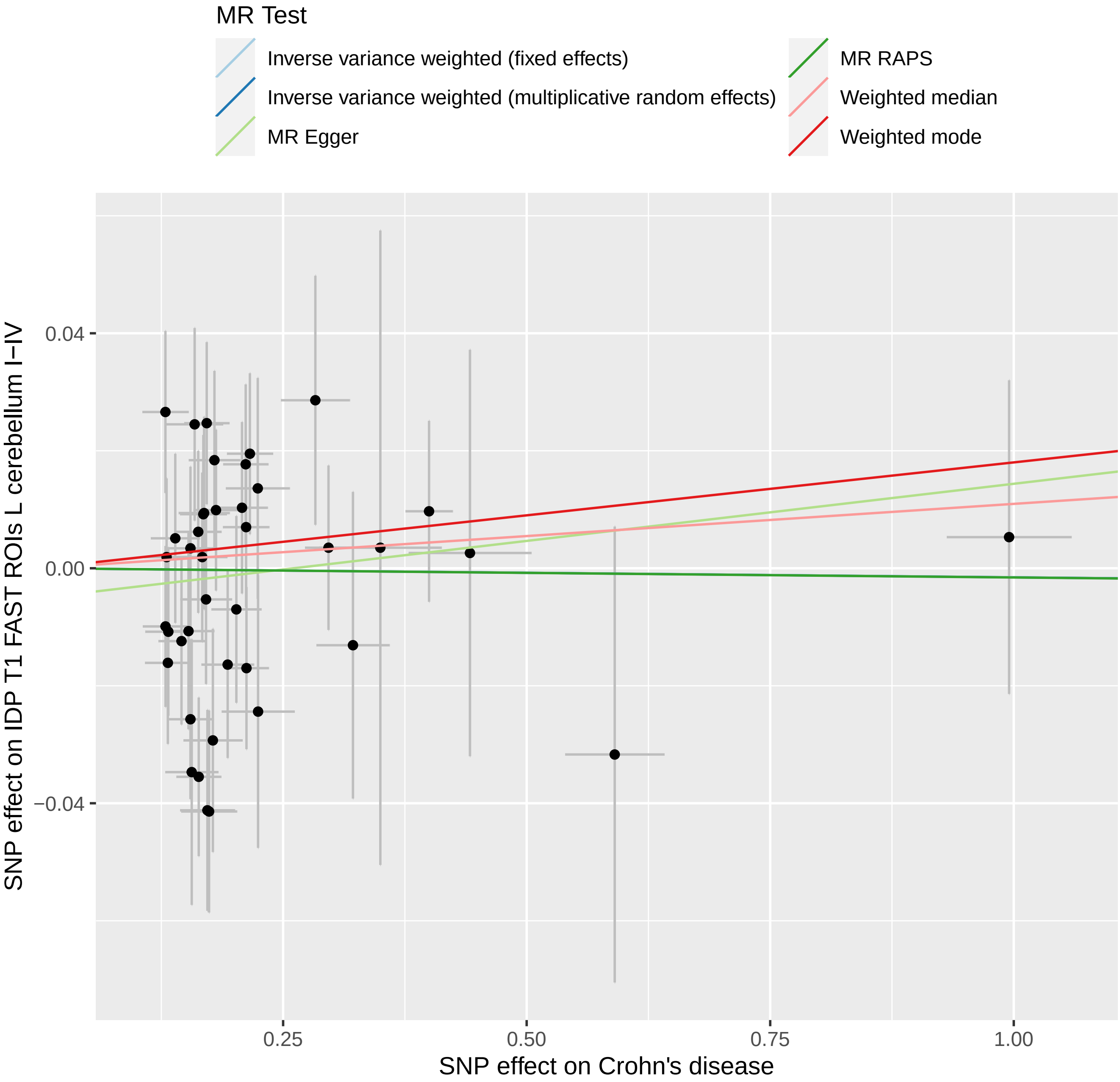

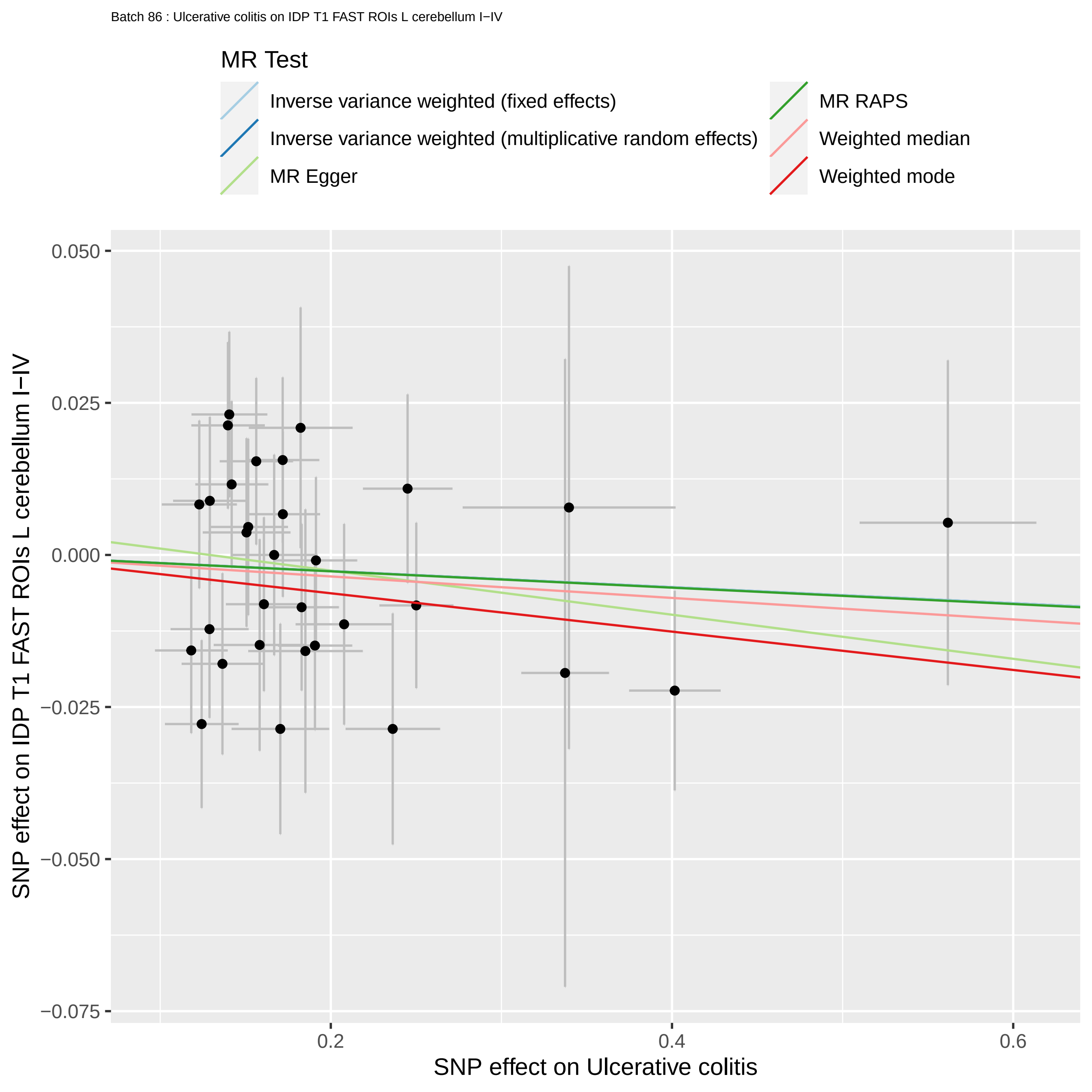

Batch 86 : Ulcerative colitis on IDP T1 FAST ROIs L cerebellum I−IV
MR Test
Inverse variance weighted (fixed effects)
Inverse variance weighted (multiplicative random effects)
MR Egger
MR RAPS
Weighted median
Weighted mode
0.050
0.025
0.000
−0.025
−0.050
−0.075
0.2
0.4
0.6
SNP effect on IDP T1 FAST ROIs L cerebellum I−IV
SNP effect on Ulcerative colitis

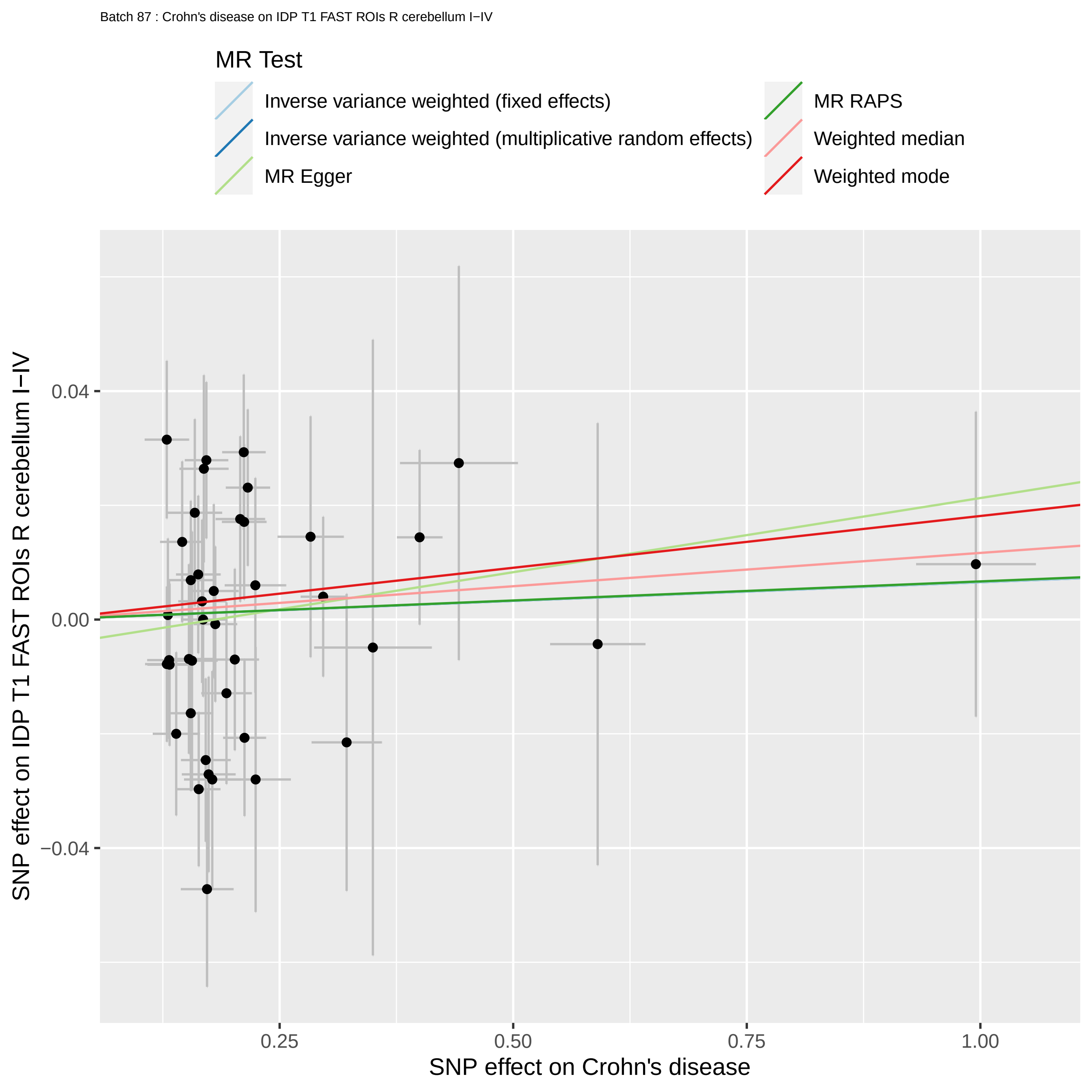
Batch 87 : Crohn's disease on IDP T1 FAST ROIs R cerebellum I−IV
MR Test
Inverse variance weighted (fixed effects)
Inverse variance weighted (multiplicative random effects)
MR Egger
MR RAPS
Weighted median
Weighted mode
SNP effect on IDP T1 FAST ROIs R cerebellum I−IV
0.04
0.00
−0.04
0.25
0.50
0.75
1.00
SNP effect on Crohn's disease

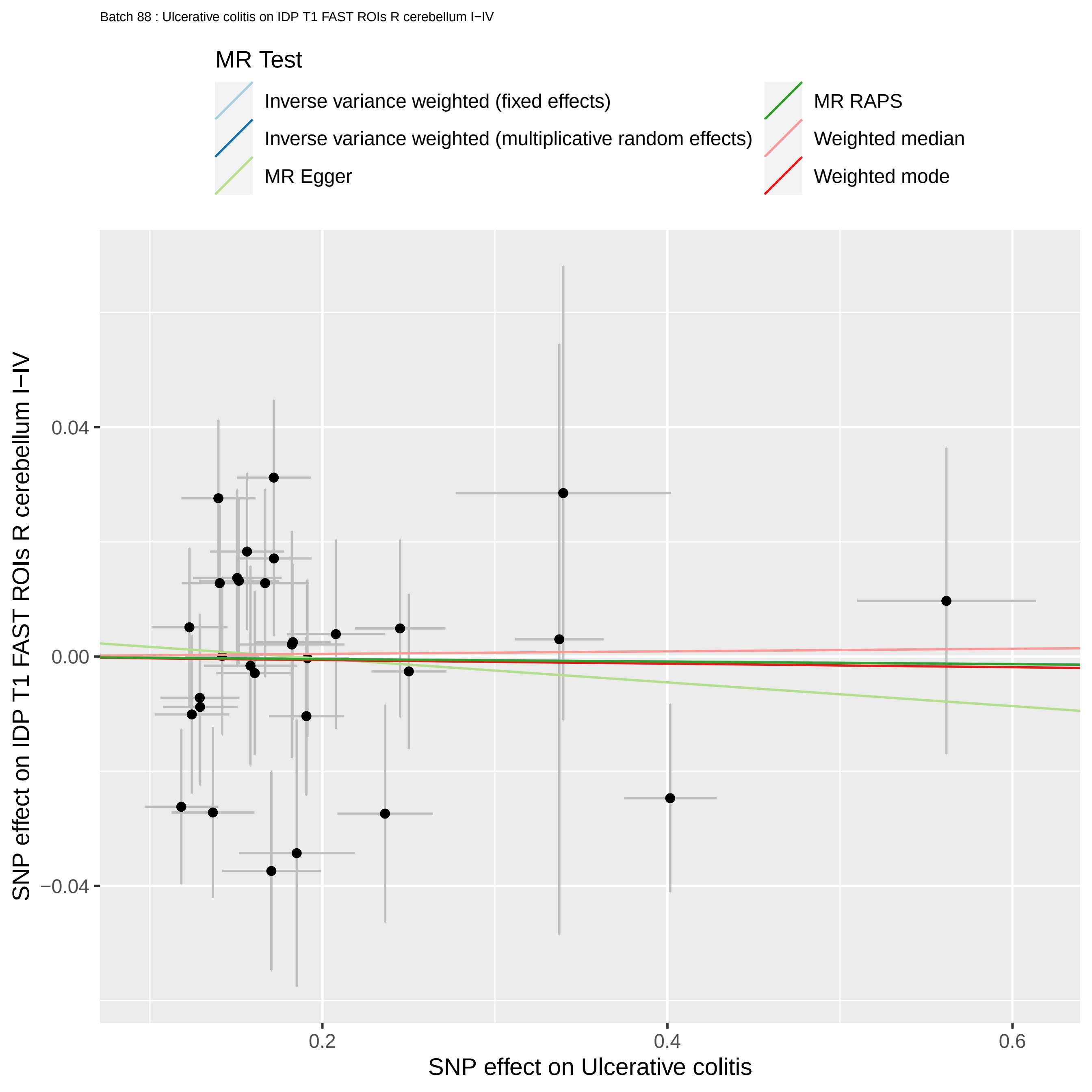

Batch 88 : Ulcerative colitis on IDP T1 FAST ROIs R cerebellum I−IV
MR Test
Inverse variance weighted (fixed effects)
Inverse variance weighted (multiplicative random effects)
MR Egger
MR RAPS
Weighted median
Weighted mode
SNP effect on IDP T1 FAST ROIs R cerebellum I−IV
0.04
0.00
−0.04
0.2
0.4
0.6
SNP effect on Ulcerative colitis

Batch 89 : Crohn's disease on IDP T1 FAST ROIs L cerebellum V

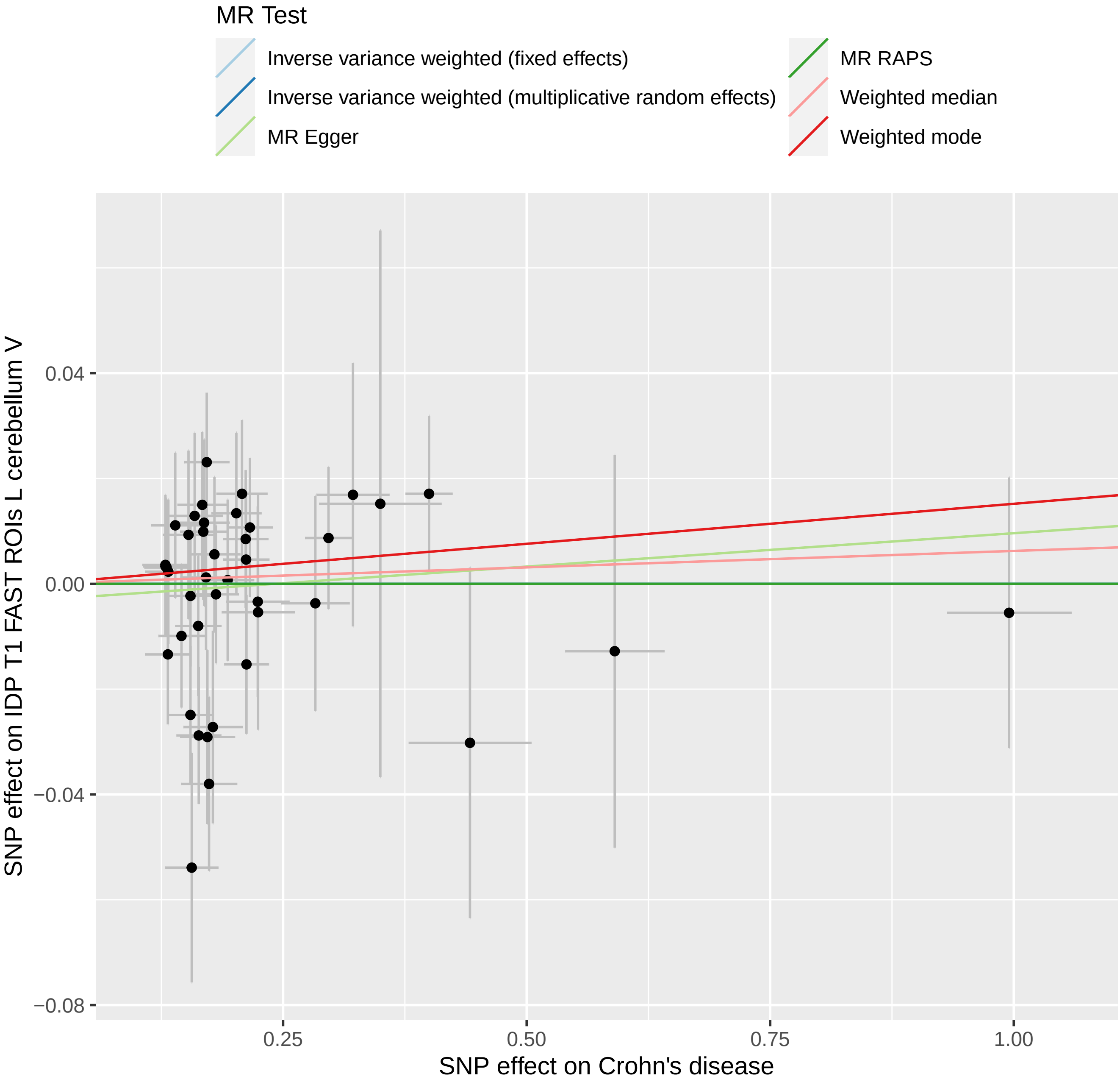

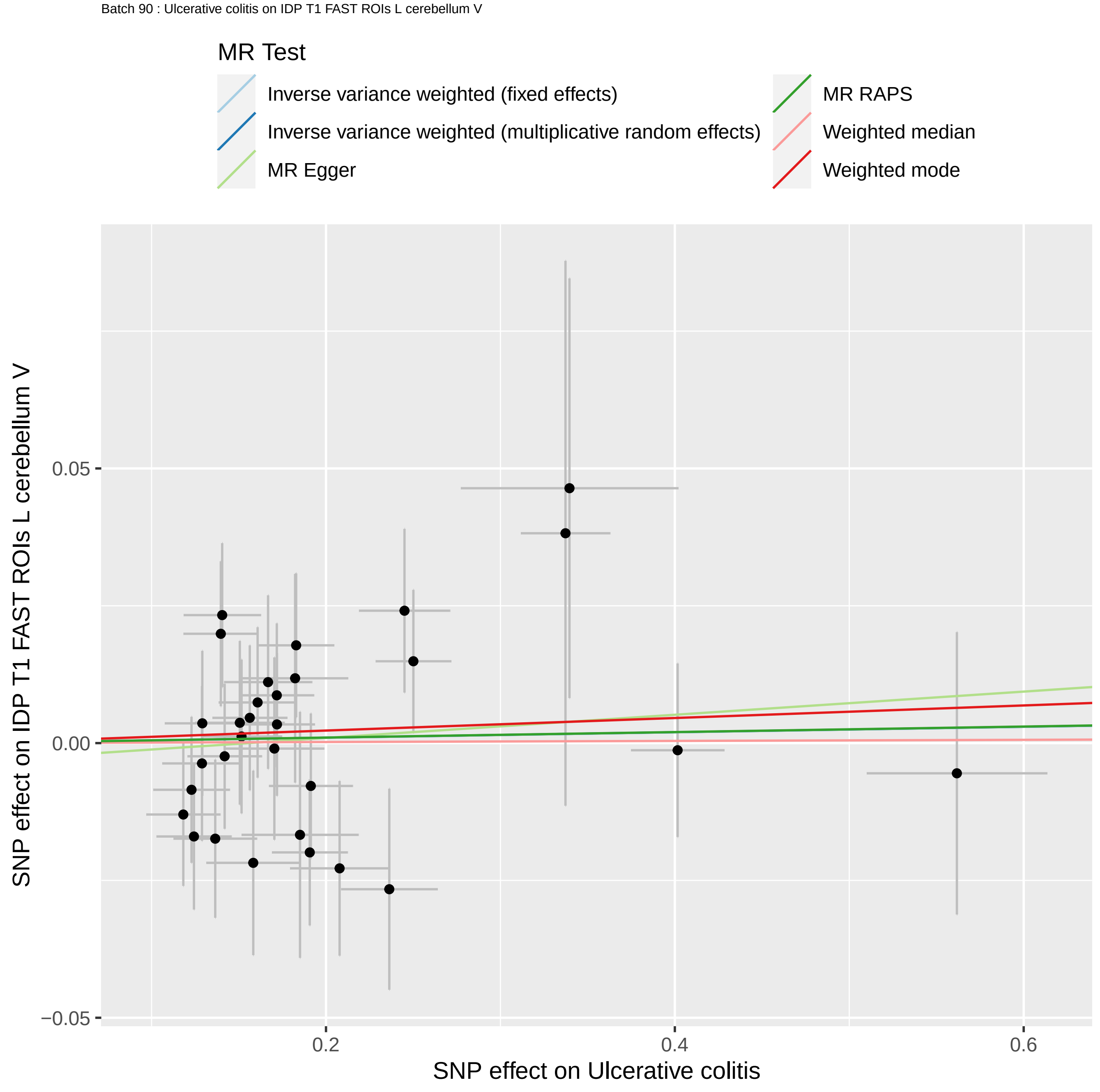
Batch 90 : Ulcerative colitis on IDP T1 FAST ROIs L cerebellum V
MR Test
Inverse variance weighted (fixed effects)
Inverse variance weighted (multiplicative random effects)
MR Egger
MR RAPS
Weighted median
Weighted mode
0.05
0.00
−0.05
0.2
0.4
0.6
SNP effect on IDP T1 FAST ROIs L cerebellum V
SNP effect on Ulcerative colitis

Batch 91 : Crohn's disease on IDP T1 FIRST right caudate volume

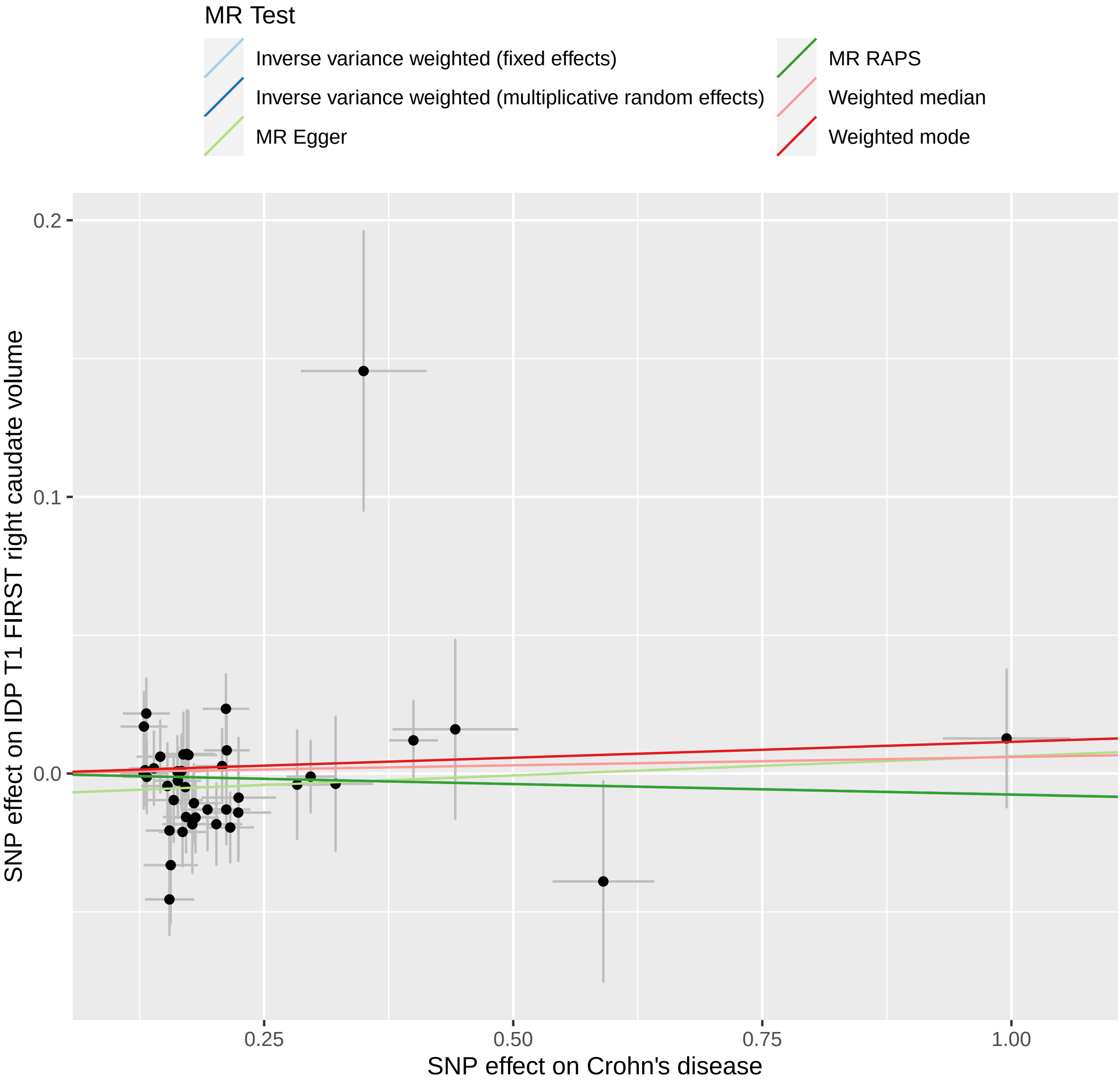

Batch 92 : Ulcerative colitis on IDP T1 FIRST right caudate volume

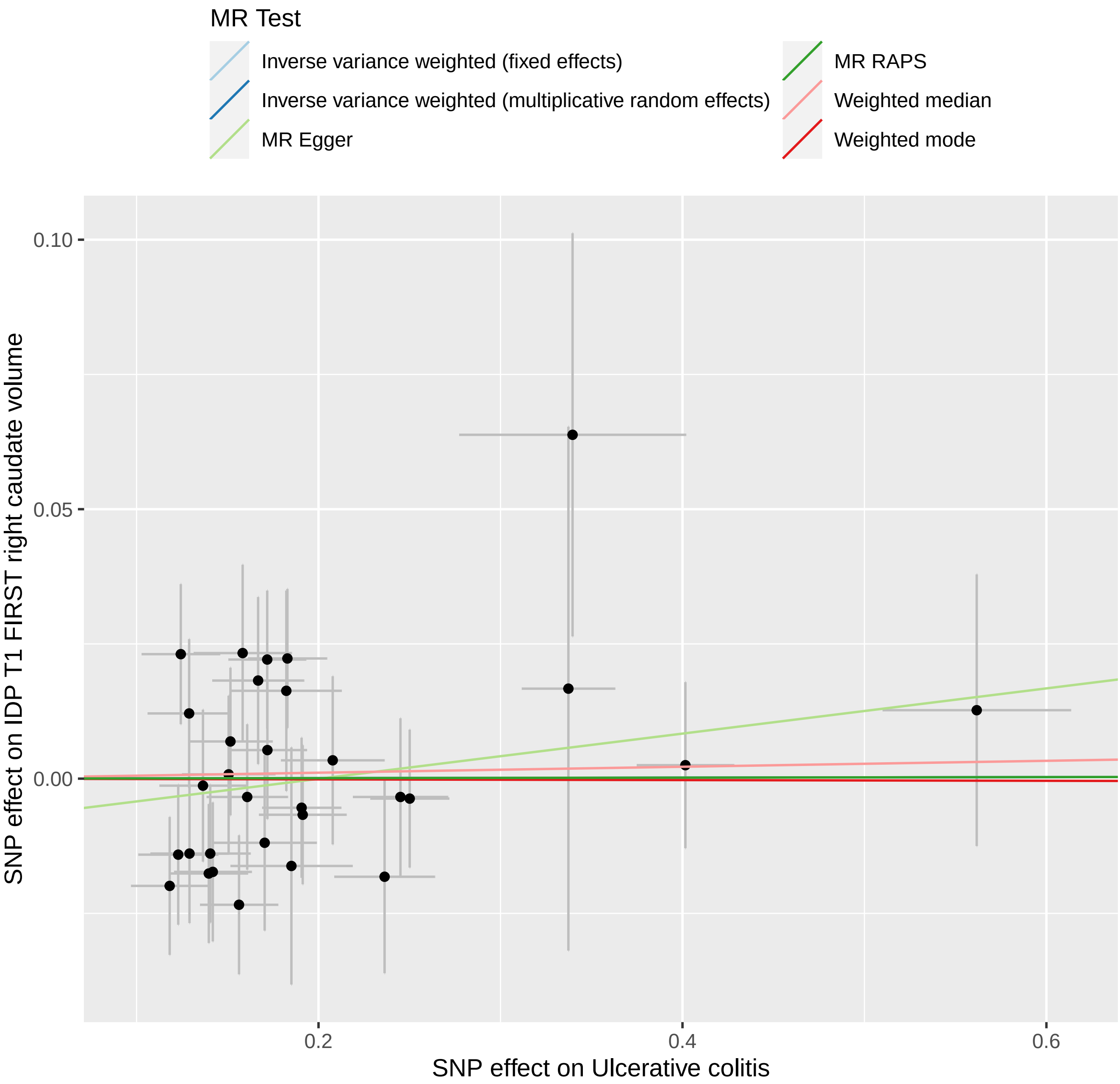

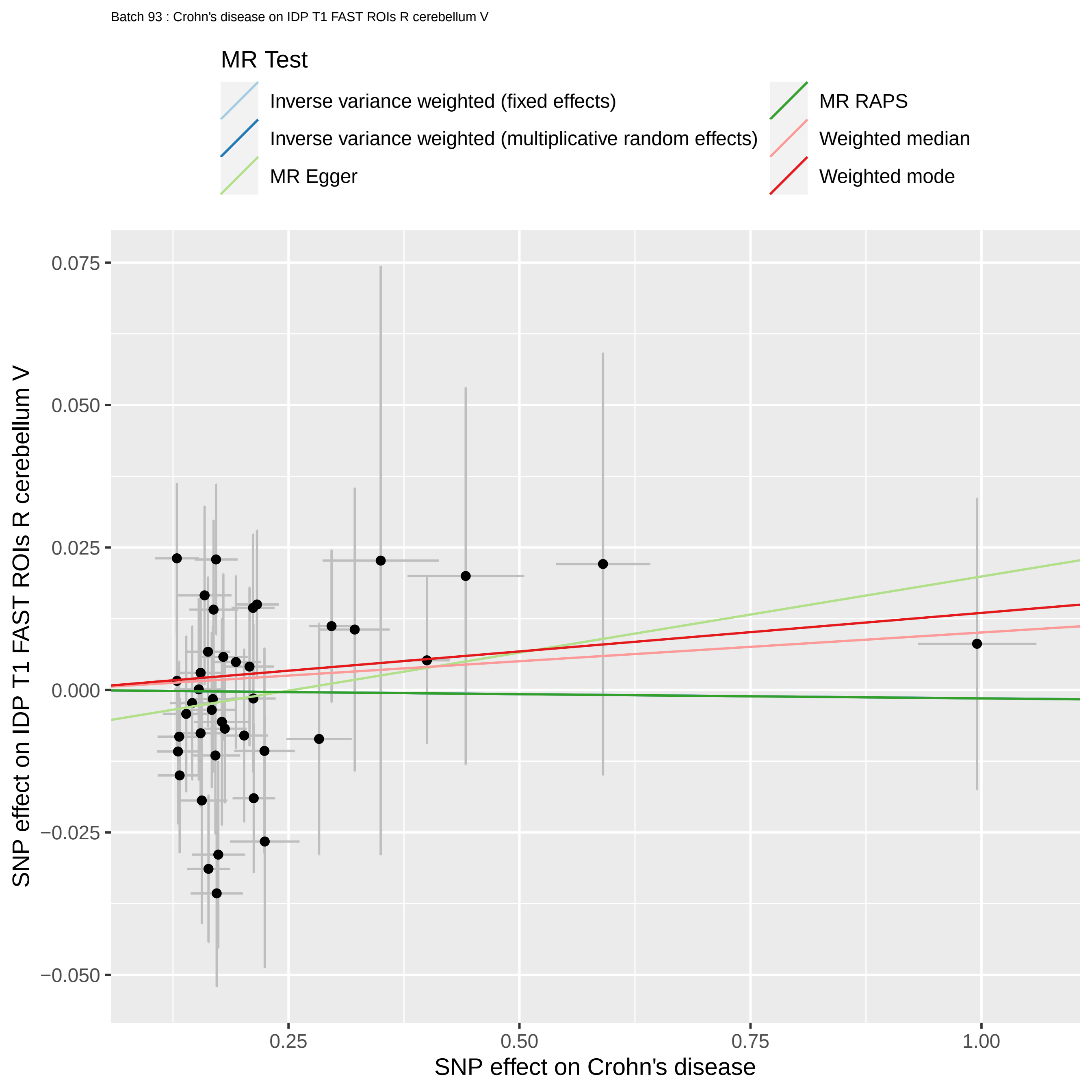

Batch 93 : Crohn's disease on IDP T1 FAST ROIs R cerebellum V
MR Test
Inverse variance weighted (fixed effects)
Inverse variance weighted (multiplicative random effects)
MR Egger
MR RAPS
Weighted median
Weighted mode
0.075
0.050
0.025
0.000
−0.025
−0.050
0.25
0.50
0.75
1.00
SNP effect on IDP T1 FAST ROIs R cerebellum V
SNP effect on Crohn's disease

Batch 94 : Ulcerative colitis on IDP T1 FAST ROIs R cerebellum V

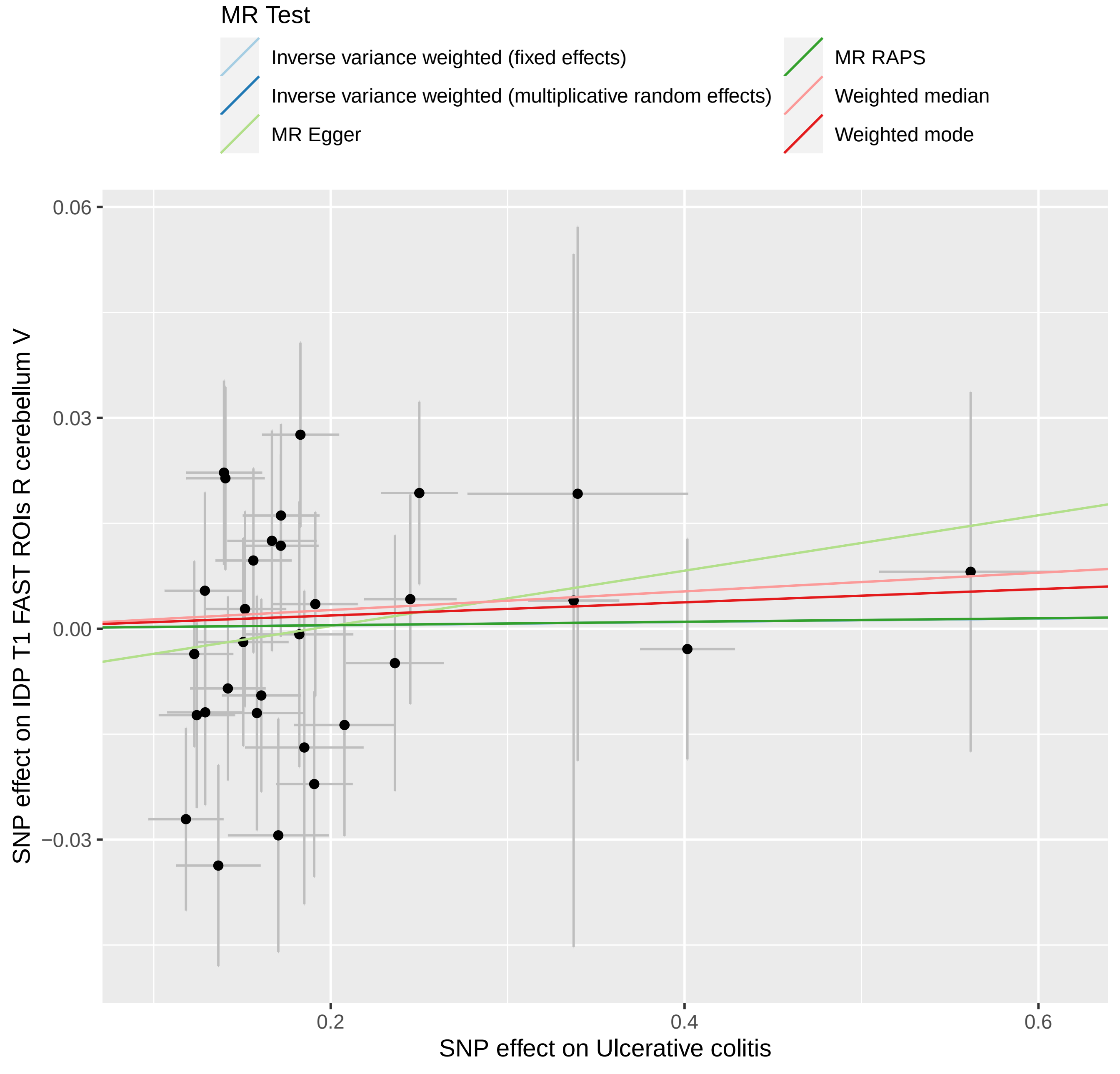

Batch 95 : Crohn's disease on IDP T1 FAST ROIs L cerebellum VI

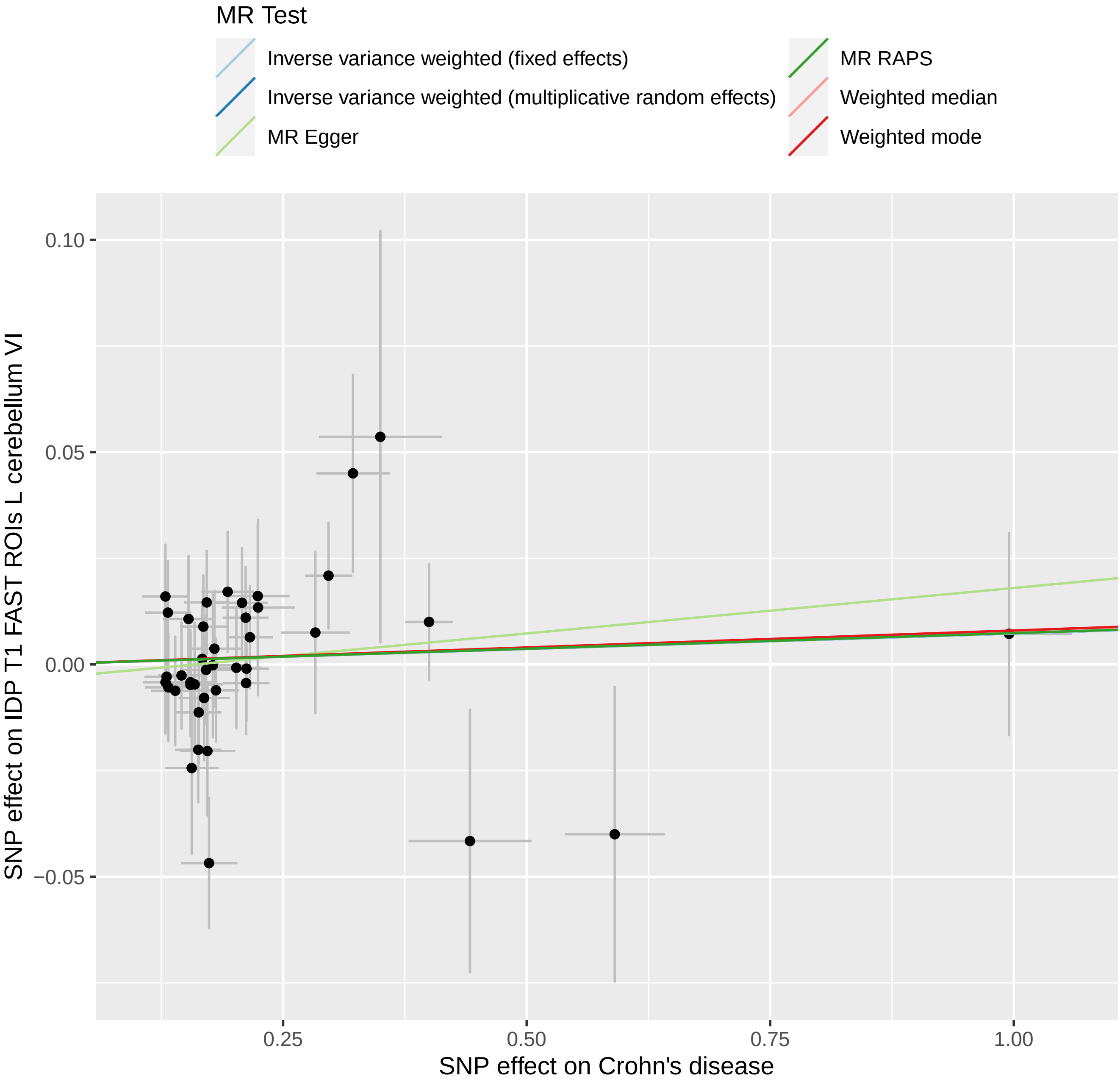

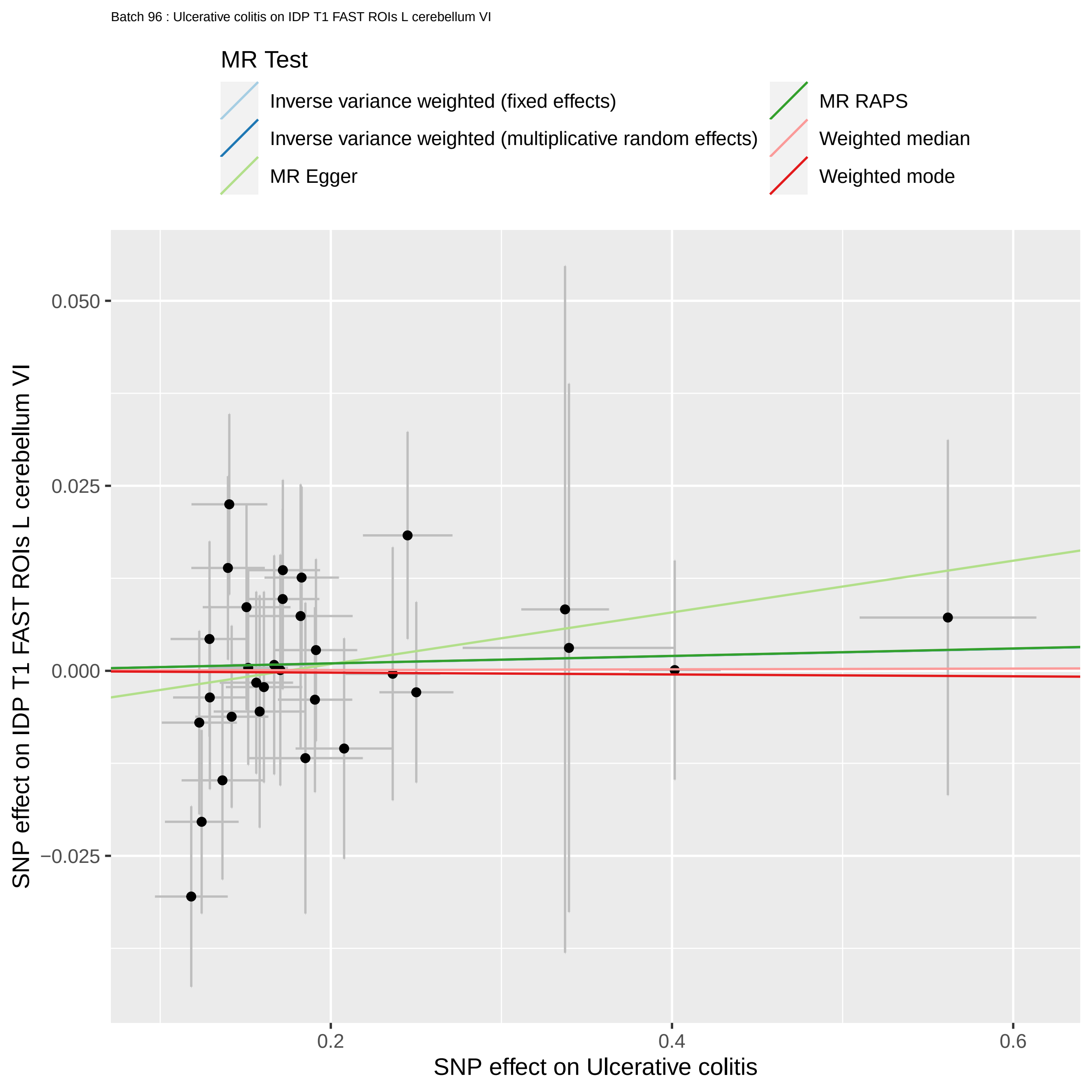

Batch 96 : Ulcerative colitis on IDP T1 FAST ROIs L cerebellum VI
MR Test
Inverse variance weighted (fixed effects)
Inverse variance weighted (multiplicative random effects)
MR Egger
MR RAPS
Weighted median
Weighted mode
0.050
0.025
0.000
−0.025
0.2
0.4
0.6
SNP effect on IDP T1 FAST ROIs L cerebellum VI
SNP effect on Ulcerative colitis

Batch 97 : Crohn's disease on IDP T1 FAST ROIs V cerebellum VI

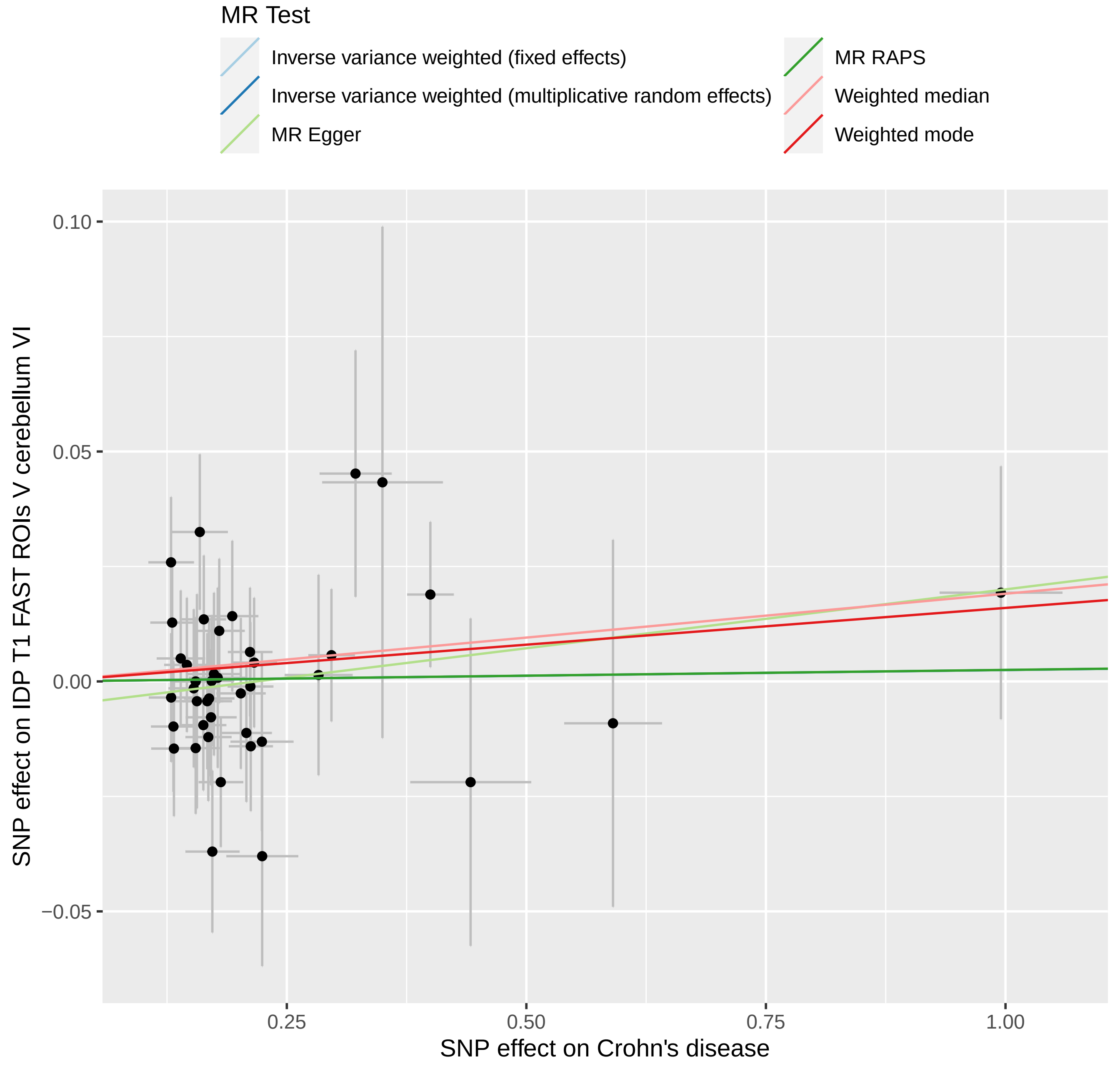

Batch 98 : Ulcerative colitis on IDP T1 FAST ROIs V cerebellum VI

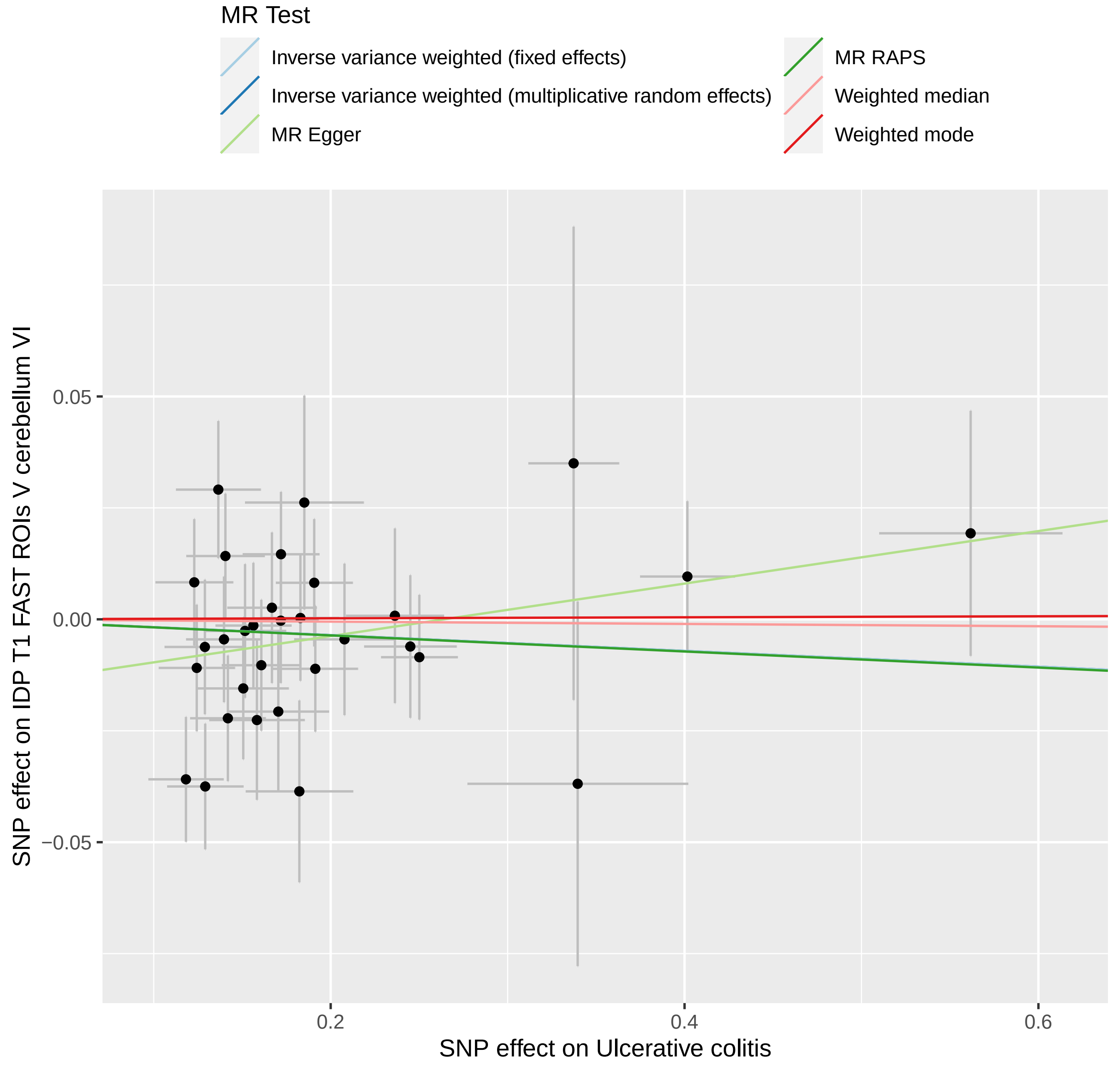

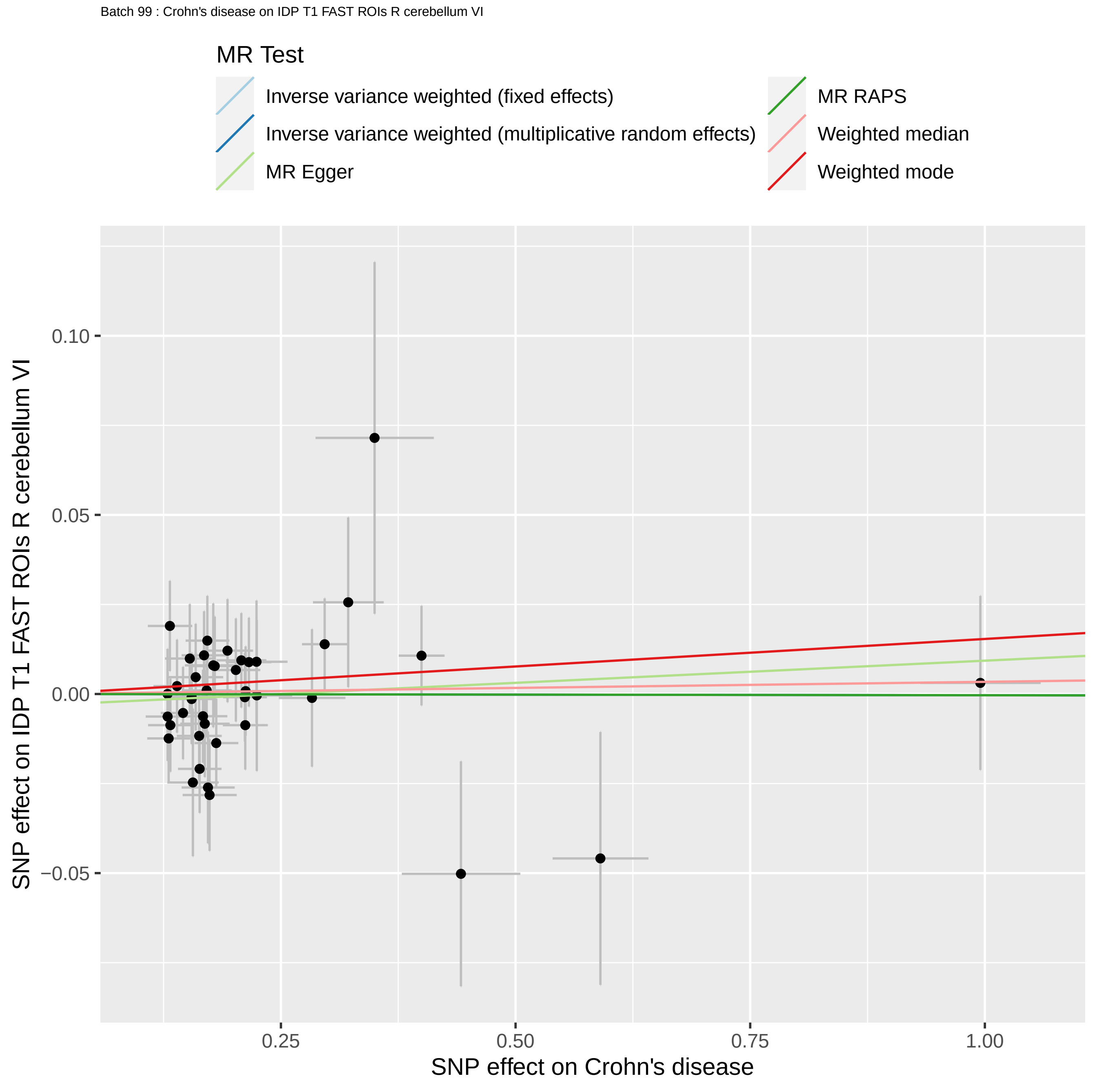

Batch 99 : Crohn's disease on IDP T1 FAST ROIs R cerebellum VI
MR Test
Inverse variance weighted (fixed effects)
Inverse variance weighted (multiplicative random effects)
MR Egger
MR RAPS
Weighted median
Weighted mode
0.10
0.05
0.00
−0.05
0.25
0.50
0.75
1.00
SNP effect on IDP T1 FAST ROIs R cerebellum VI
SNP effect on Crohn's disease

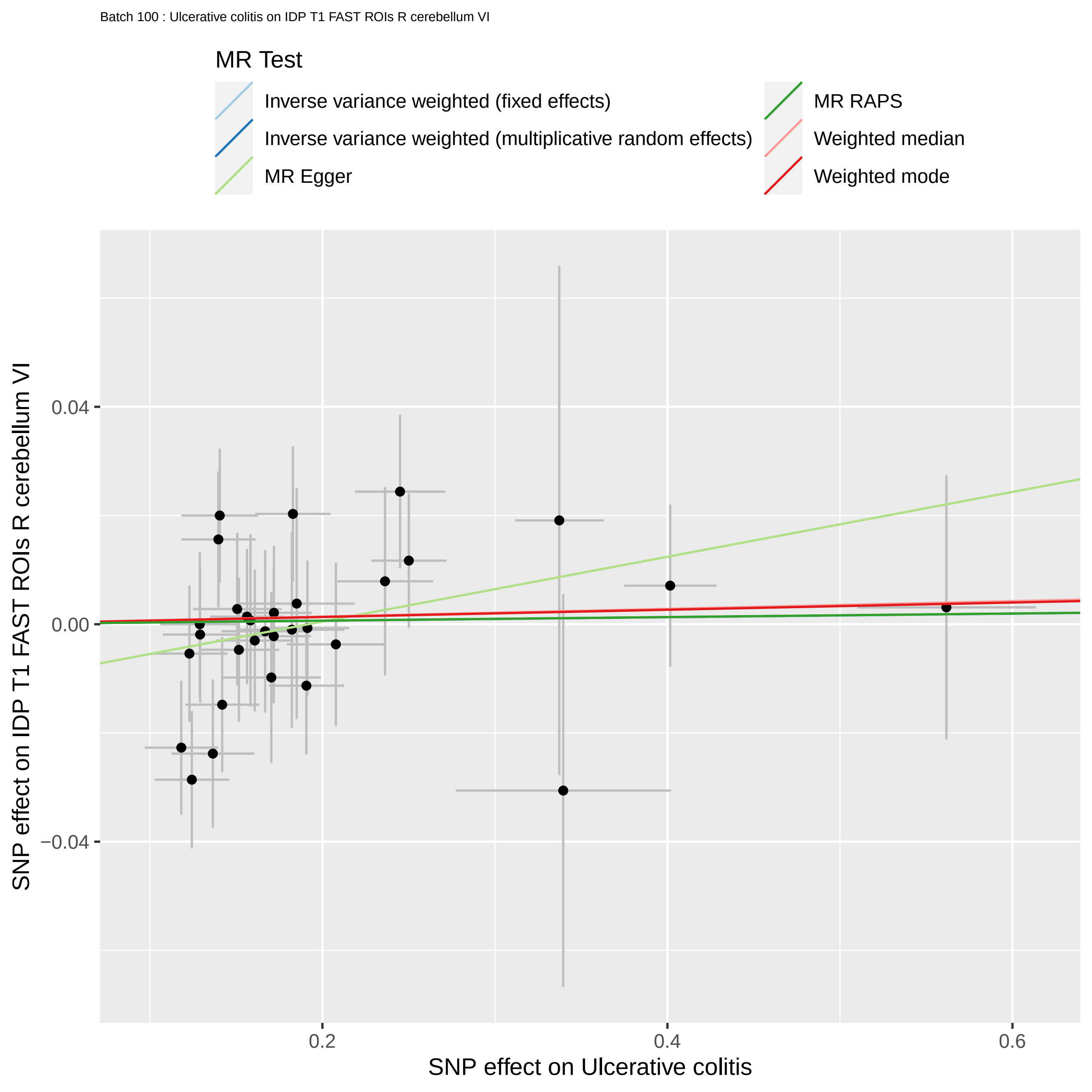

Batch 100 : Ulcerative colitis on IDP T1 FAST ROIs R cerebellum VI
MR Test
Inverse variance weighted (fixed effects)
Inverse variance weighted (multiplicative random effects)
MR Egger
MR RAPS
Weighted median
Weighted mode
0.04
0.00
−0.04
0.2
0.4
0.6
SNP effect on IDP T1 FAST ROIs R cerebellum VI
SNP effect on Ulcerative colitis

Batch 101 : Crohn's disease on IDP T1 FAST ROIs L cerebellum crus I

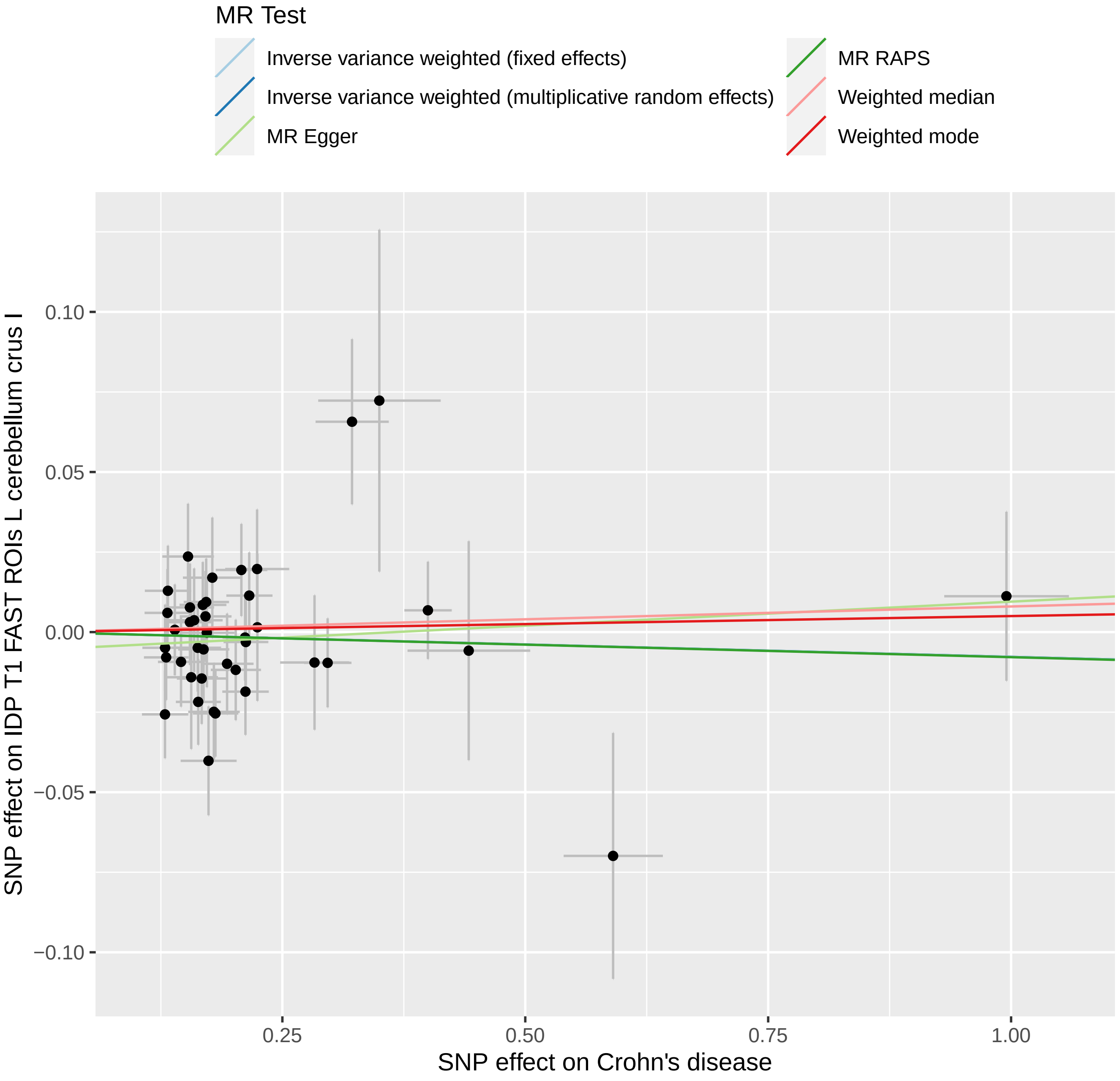

Batch 102 : Ulcerative colitis on IDP T1 FAST ROIs L cerebellum crus I

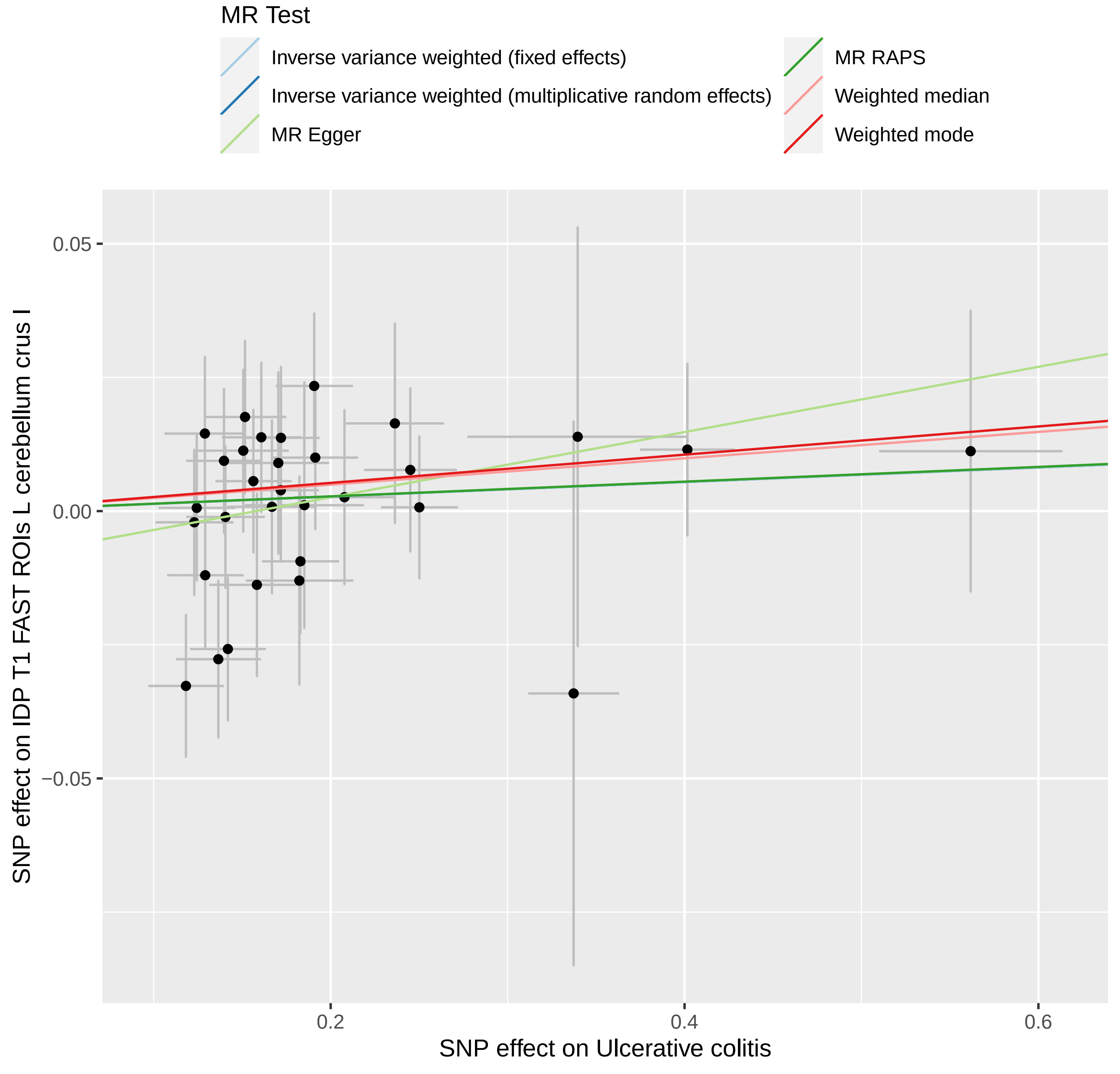

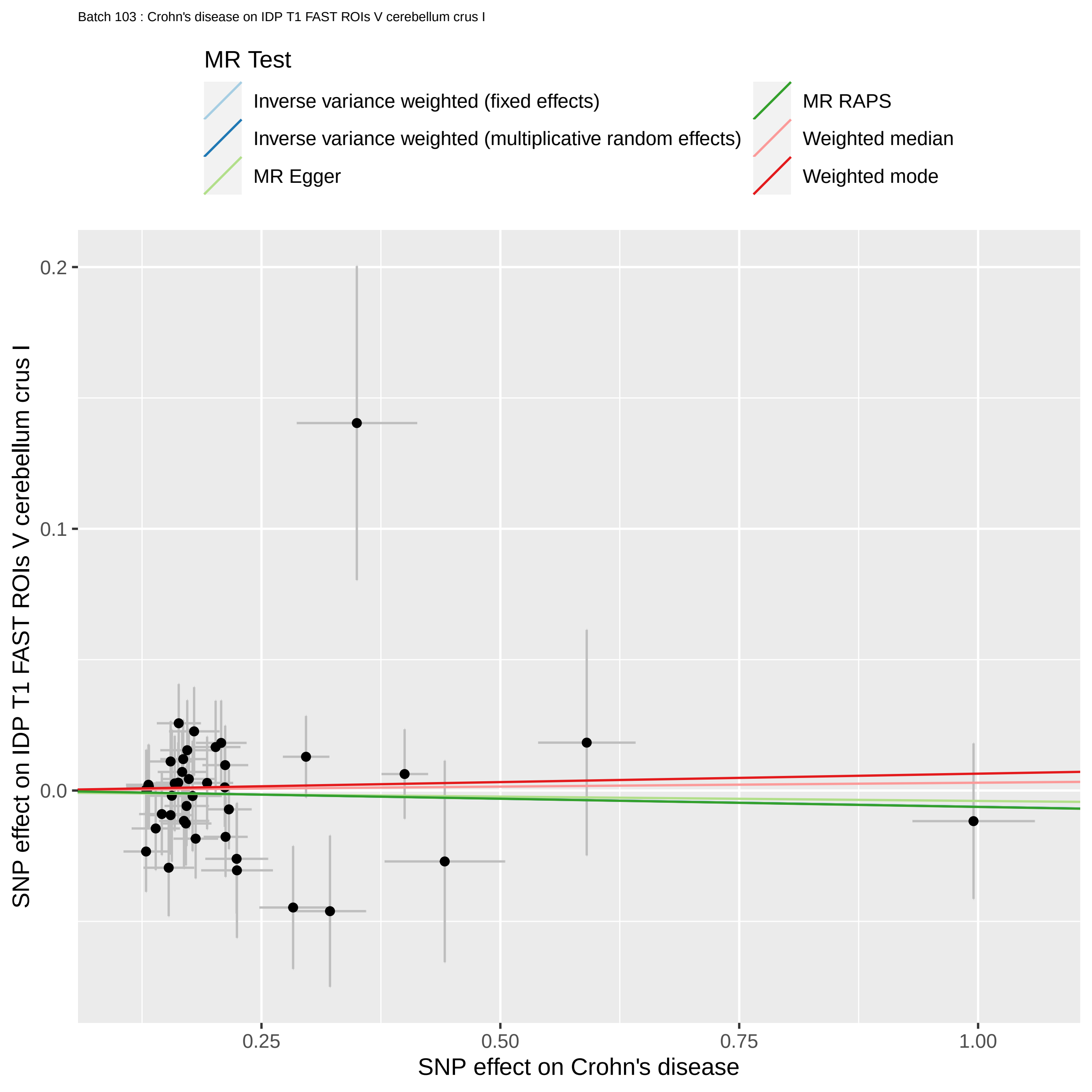

Batch 103 : Crohn's disease on IDP T1 FAST ROIs V cerebellum crus I
MR Test
Inverse variance weighted (fixed effects)
Inverse variance weighted (multiplicative random effects)
MR Egger
MR RAPS
Weighted median
Weighted mode
SNP effect on IDP T1 FAST ROIs V cerebellum crus I
0.2
0.1
0.0
0.25
0.50
0.75
1.00
SNP effect on Crohn's disease

Batch 104 : Ulcerative colitis on IDP T1 FAST ROIs V cerebellum crus I

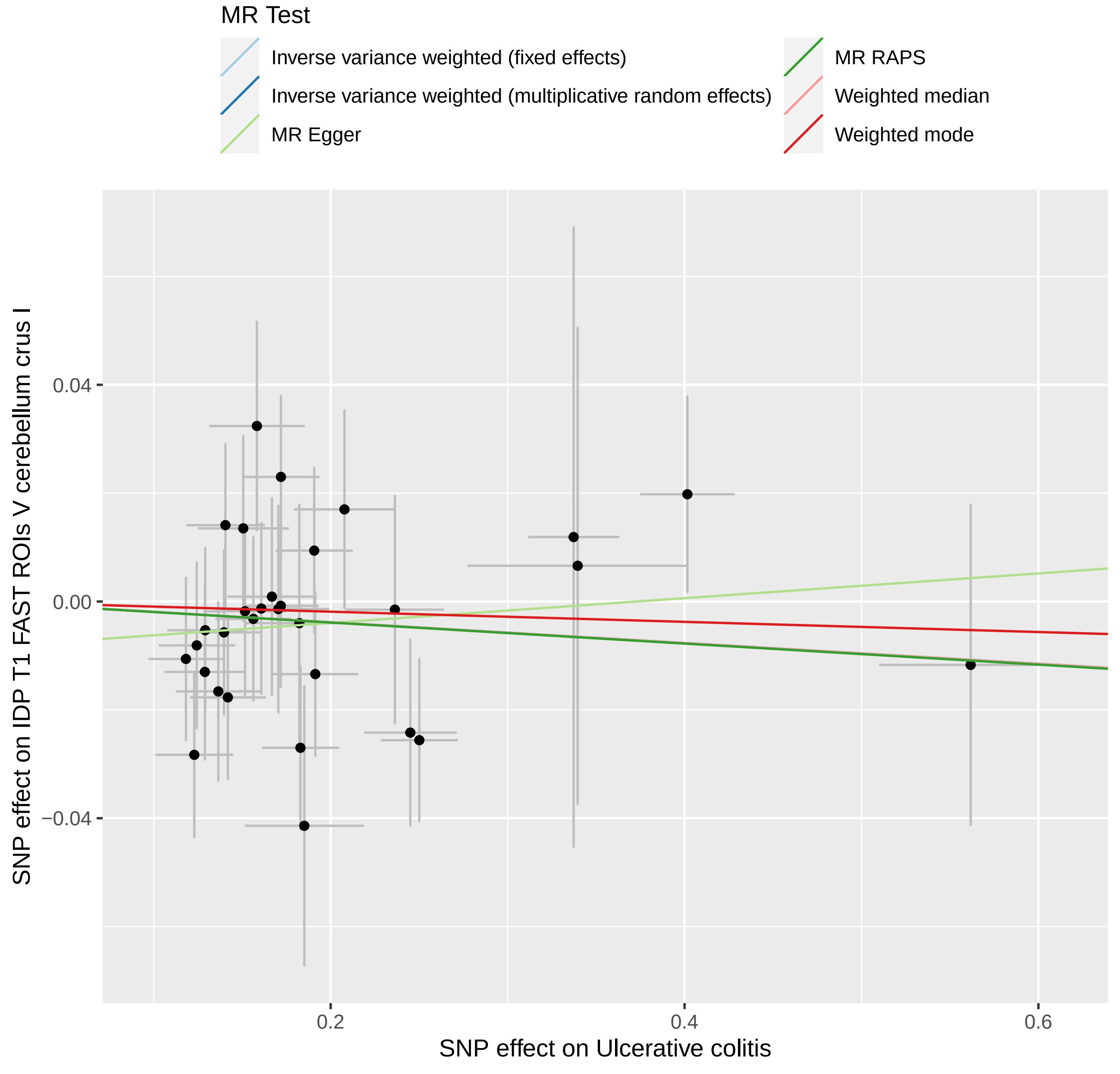

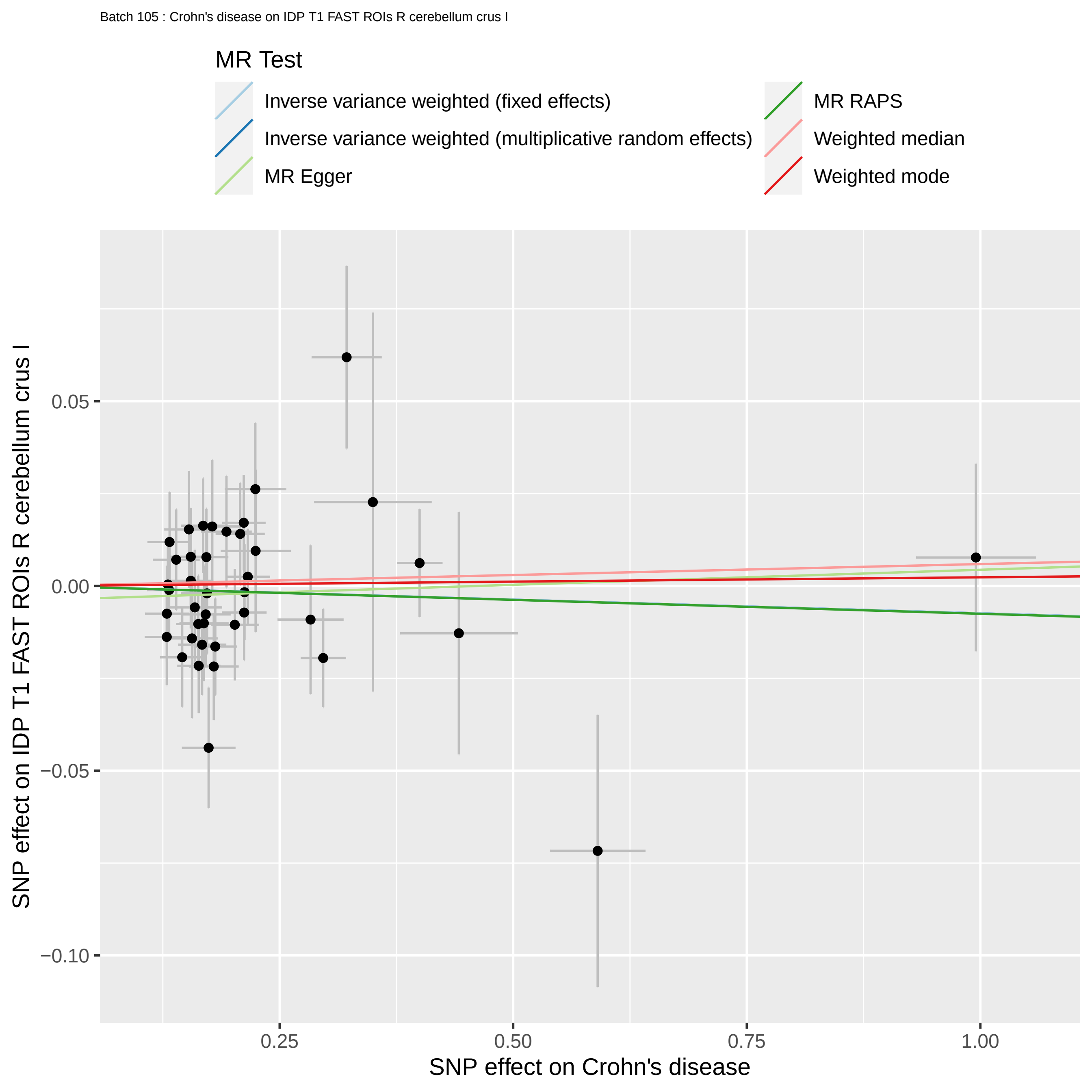

Batch 105 : Crohn's disease on IDP T1 FAST ROIs R cerebellum crus I
MR Test
Inverse variance weighted (fixed effects)
Inverse variance weighted (multiplicative random effects)
MR Egger
MR RAPS
Weighted median
Weighted mode
SNP effect on IDP T1 FAST ROIs R cerebellum crus I
0.05
0.00
−0.05
−0.10
0.25
0.50
0.75
1.00
SNP effect on Crohn's disease

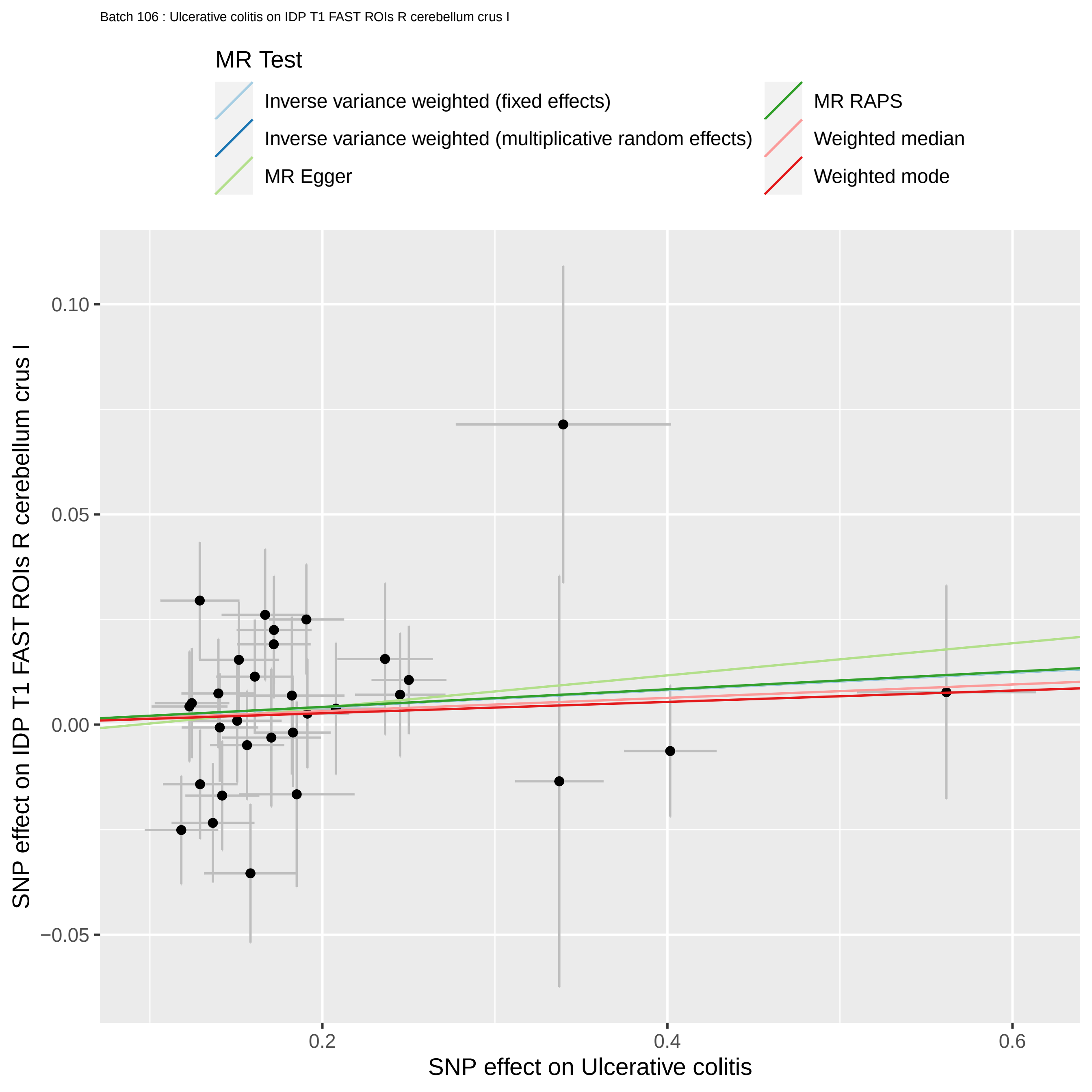
Batch 106 : Ulcerative colitis on IDP T1 FAST ROIs R cerebellum crus I
MR Test
Inverse variance weighted (fixed effects)
Inverse variance weighted (multiplicative random effects)
MR Egger
MR RAPS
Weighted median
Weighted mode
0.10
0.05
0.00
−0.05
0.2
0.4
0.6
SNP effect on IDP T1 FAST ROIs R cerebellum crus I
SNP effect on Ulcerative colitis

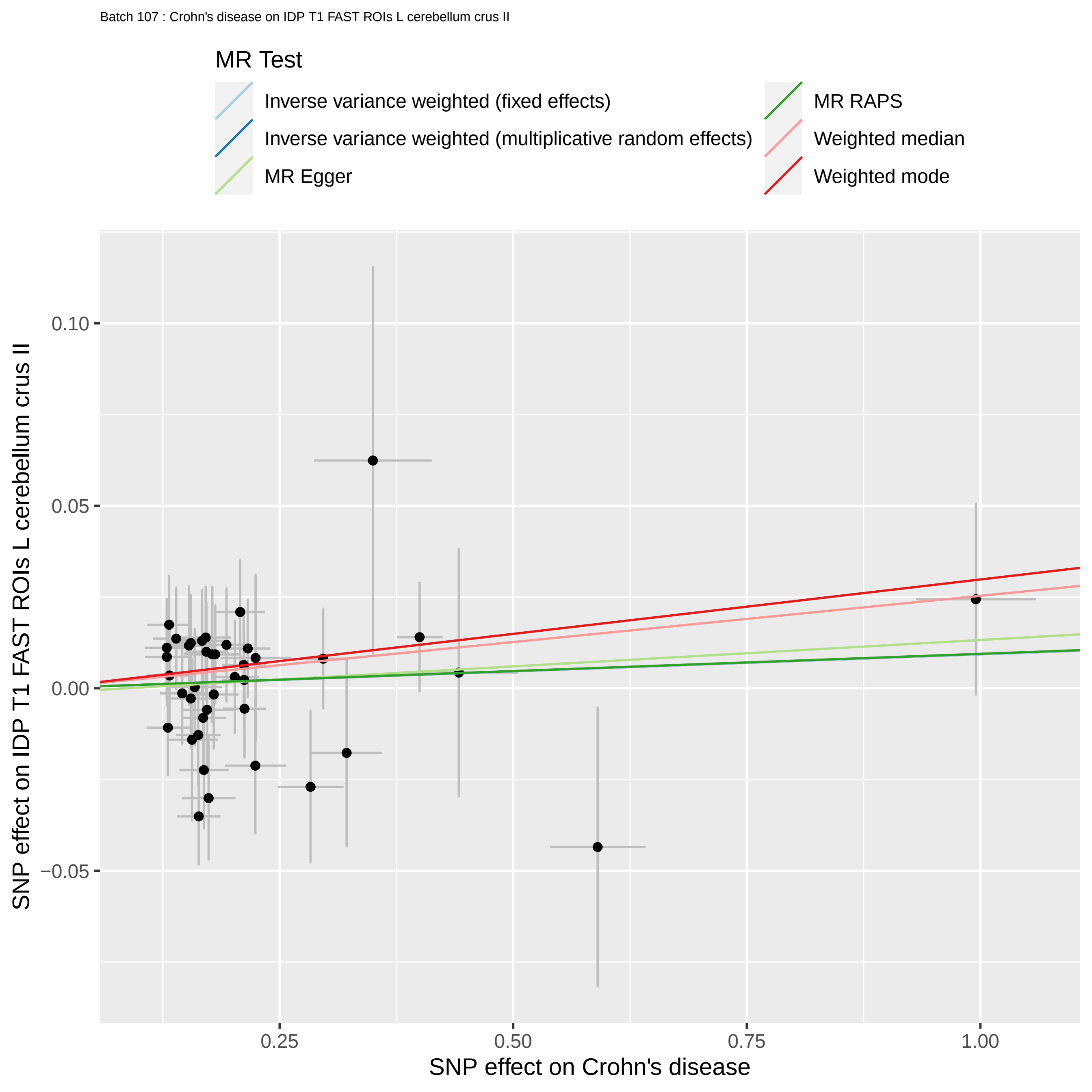
Batch 107 : Crohn's disease on IDP T1 FAST ROIs L cerebellum crus II
MR Test
Inverse variance weighted (fixed effects)
Inverse variance weighted (multiplicative random effects)
MR Egger
MR RAPS
Weighted median
Weighted mode
0.10
0.05
0.00
−0.05
0.25
0.50
0.75
1.00
SNP effect on IDP T1 FAST ROIs L cerebellum crus II
SNP effect on Crohn's disease

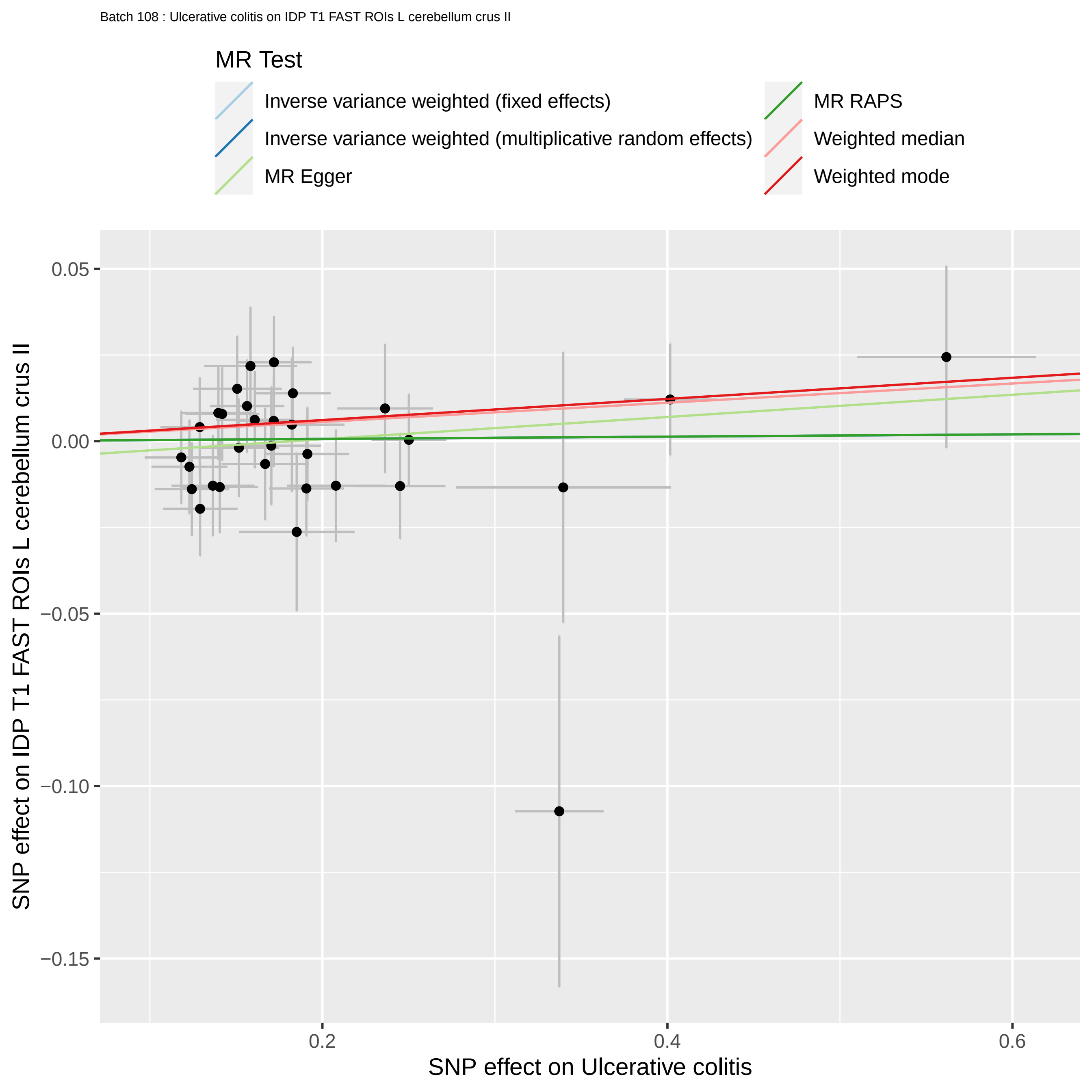
Batch 108 : Ulcerative colitis on IDP T1 FAST ROIs L cerebellum crus II
MR Test
Inverse variance weighted (fixed effects)
Inverse variance weighted (multiplicative random effects)
MR Egger
MR RAPS
Weighted median
Weighted mode
0.05
0.00
−0.05
−0.10
−0.15
0.2
0.4
0.6
SNP effect on IDP T1 FAST ROIs L cerebellum crus II
SNP effect on Ulcerative colitis

Batch 109 : Crohn's disease on IDP T1 FAST ROIs V cerebellum crus II

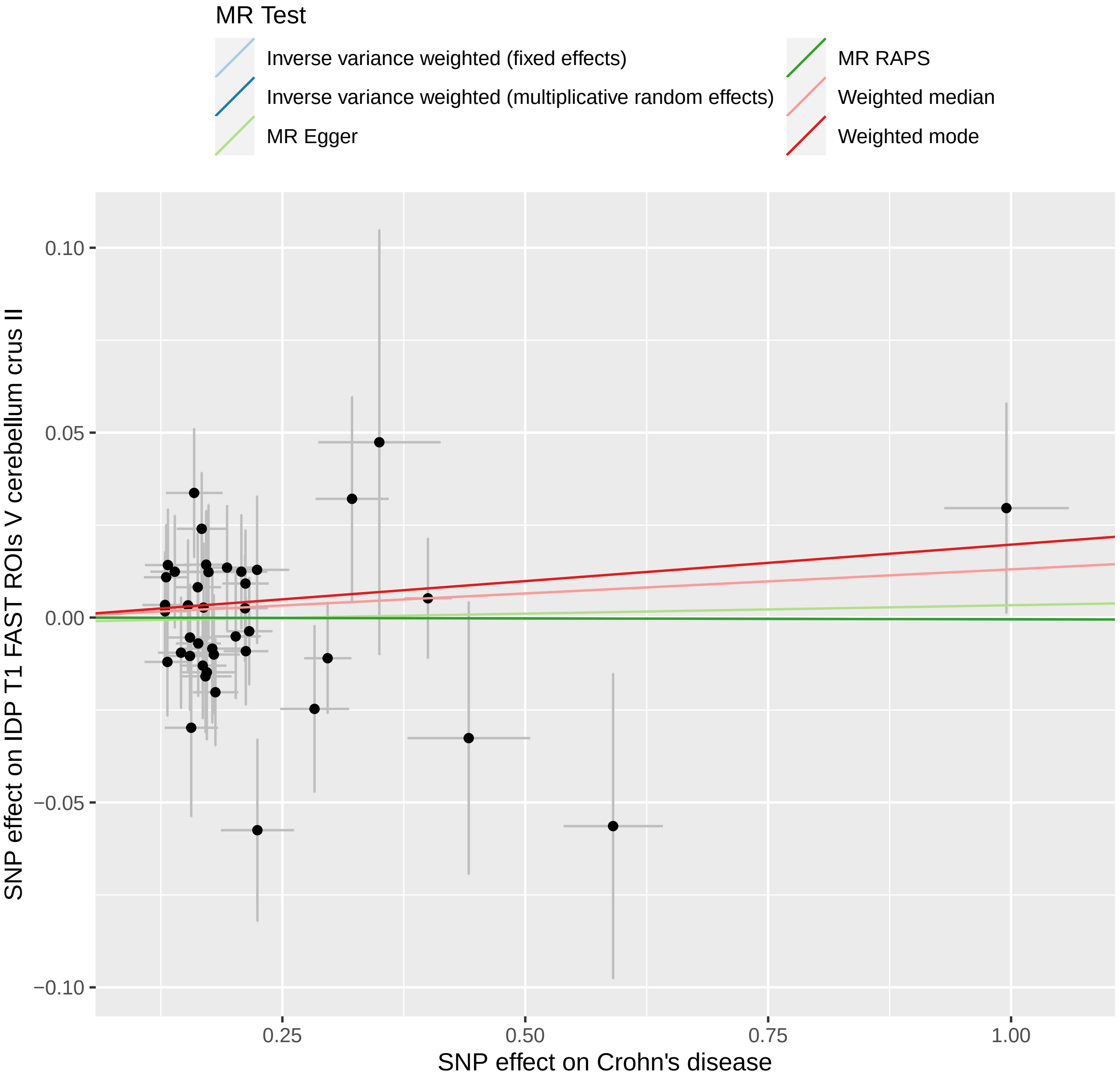

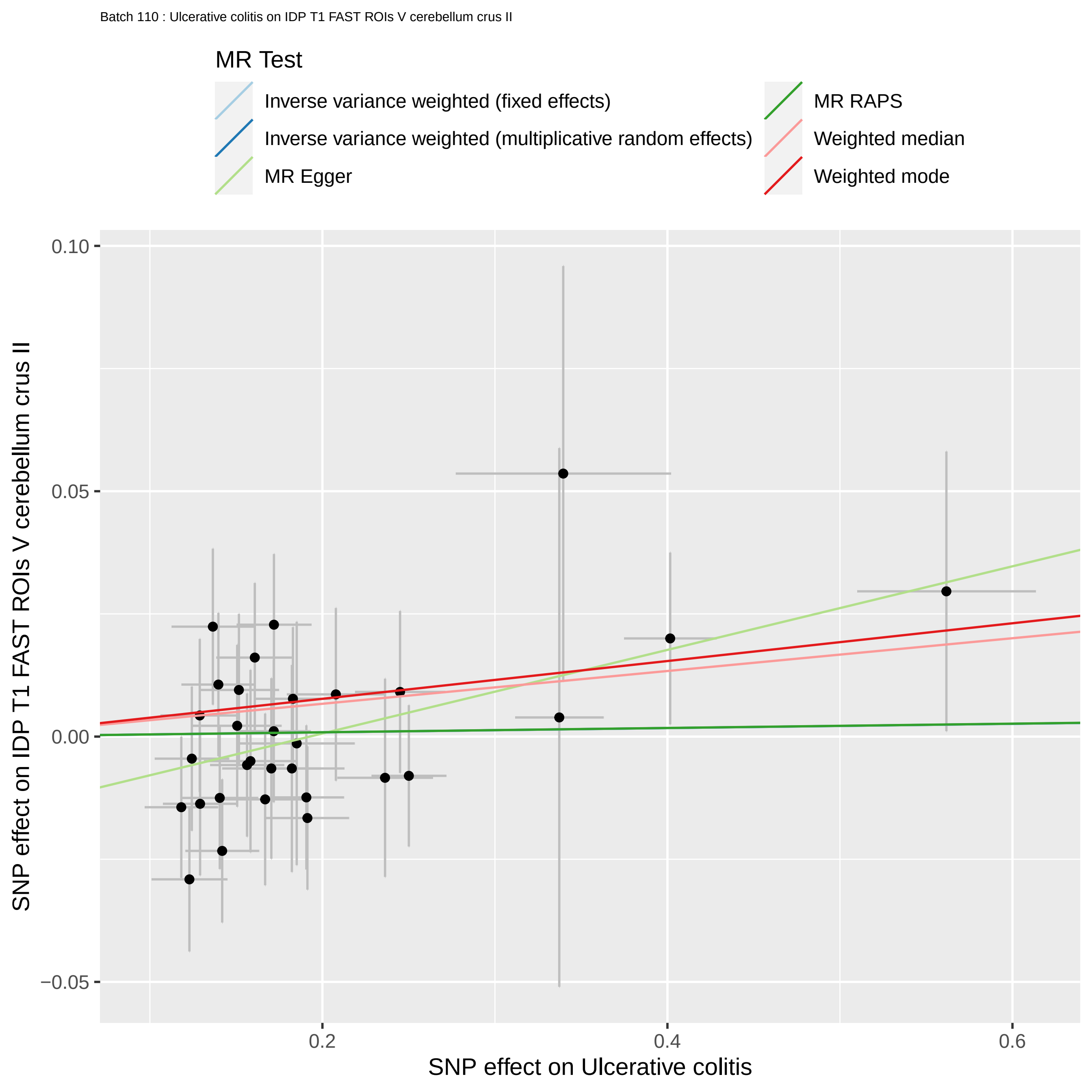
Batch 110 : Ulcerative colitis on IDP T1 FAST ROIs V cerebellum crus II
MR Test
Inverse variance weighted (fixed effects)
Inverse variance weighted (multiplicative random effects)
MR Egger
MR RAPS
Weighted median
Weighted mode
0.10
0.05
0.00
−0.05
0.2
0.4
0.6
SNP effect on IDP T1 FAST ROIs V cerebellum crus II
SNP effect on Ulcerative colitis

Batch 111 : Crohn's disease on IDP T1 FAST ROIs R cerebellum crus II

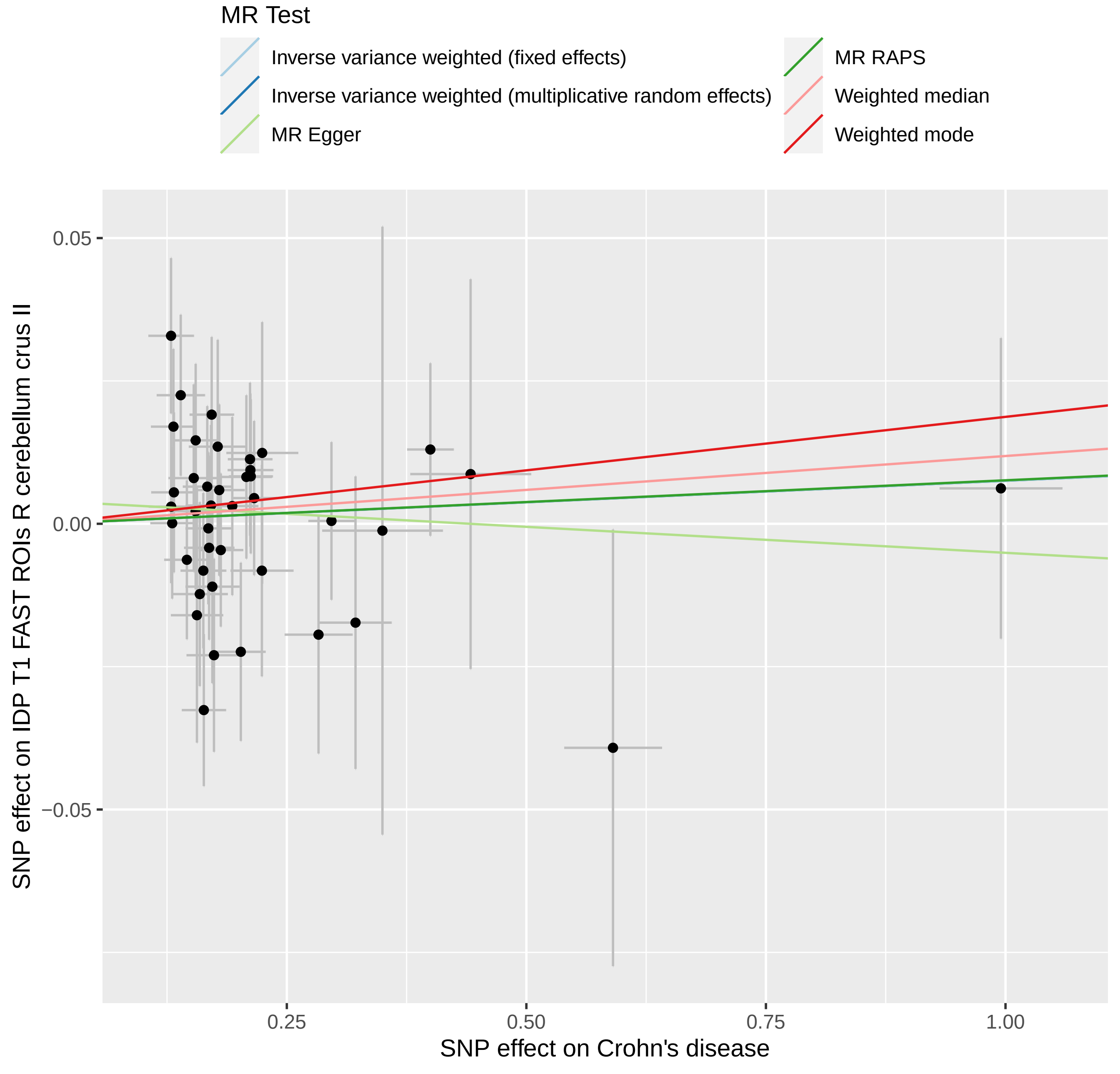

Batch 112 : Ulcerative colitis on IDP T1 FAST ROIs R cerebellum crus II

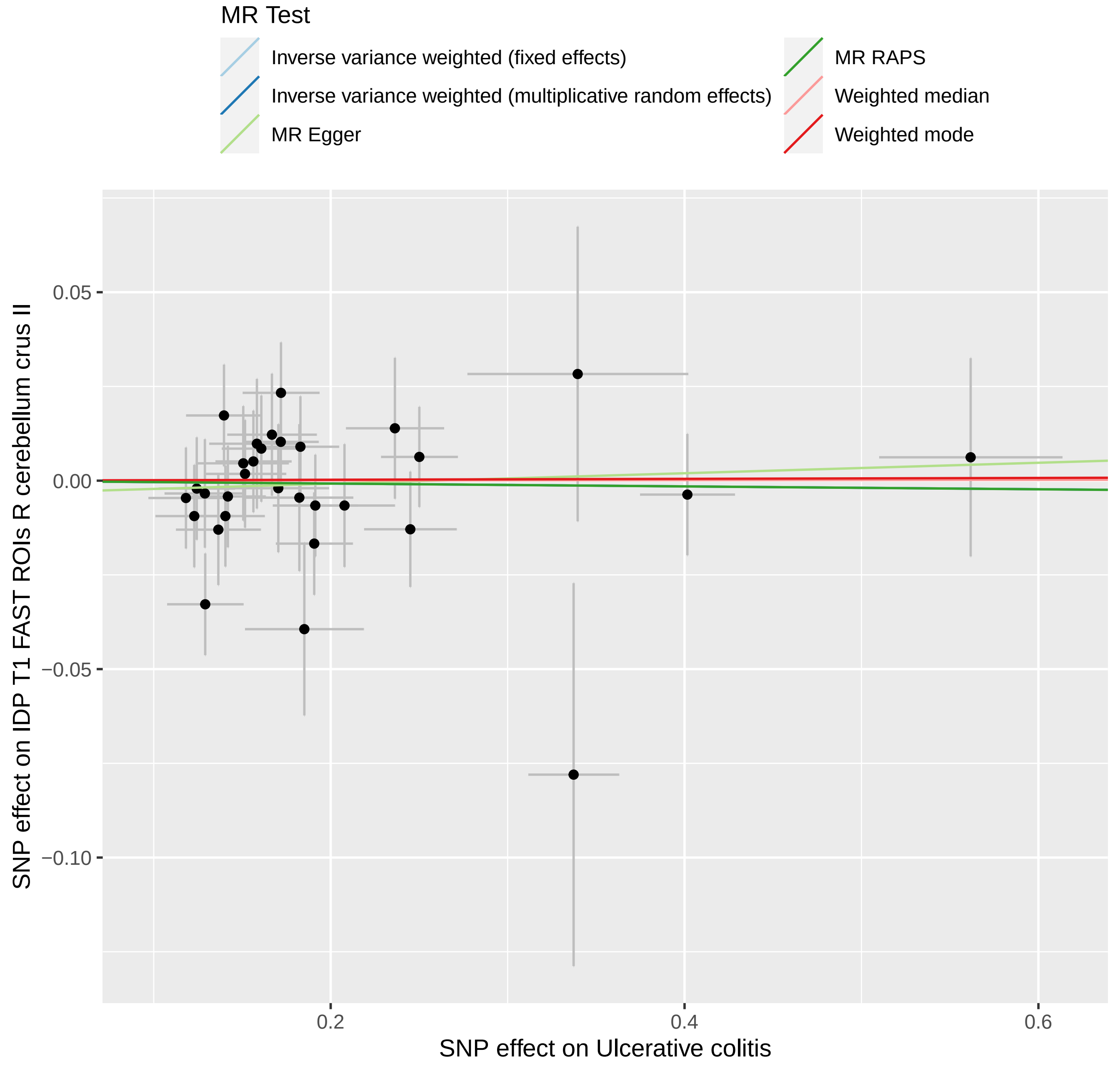

Batch 113 : Crohn's disease on IDP T1 FIRST left putamen volume

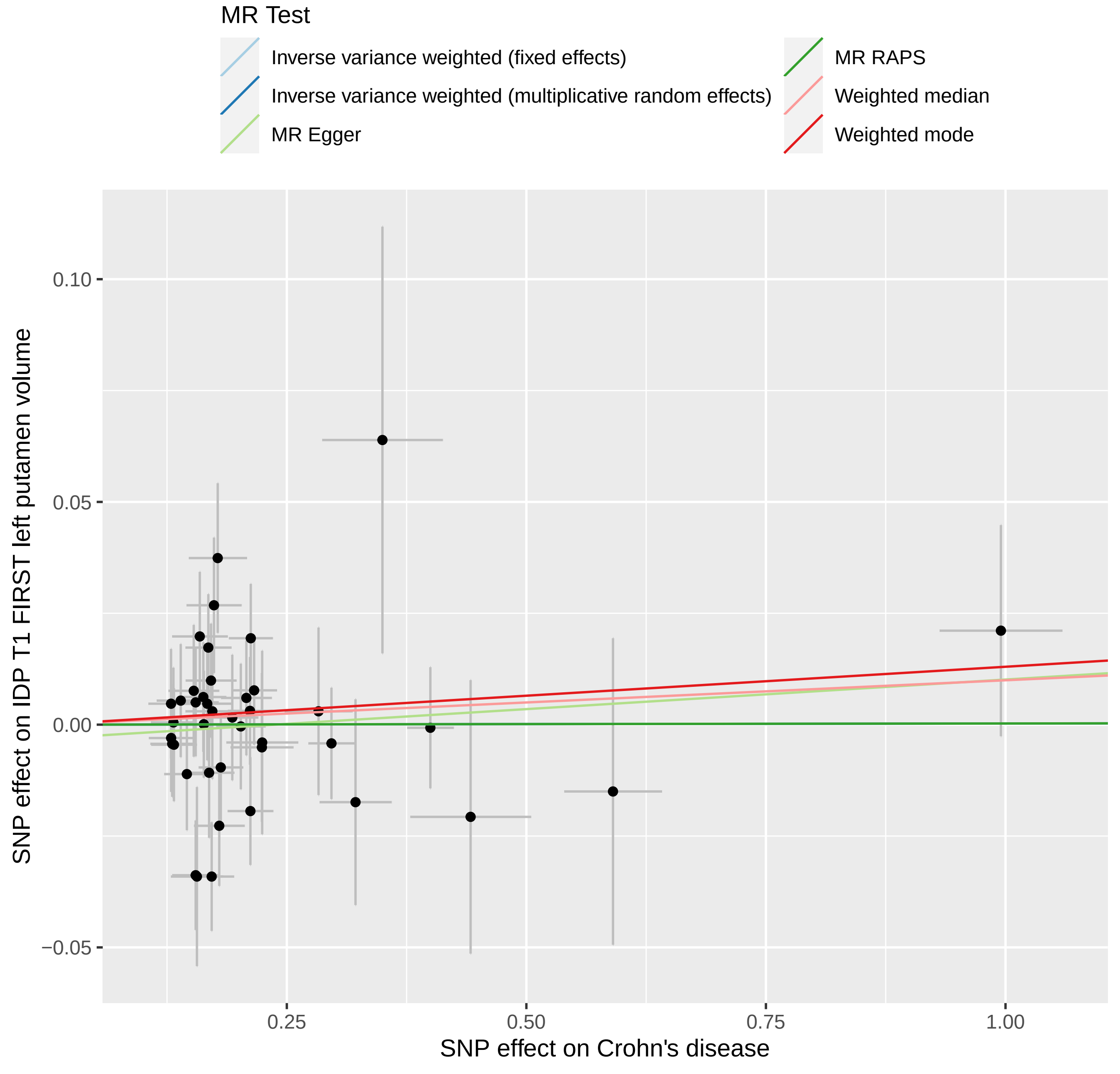

Batch 114 : Ulcerative colitis on IDP T1 FIRST left putamen volume

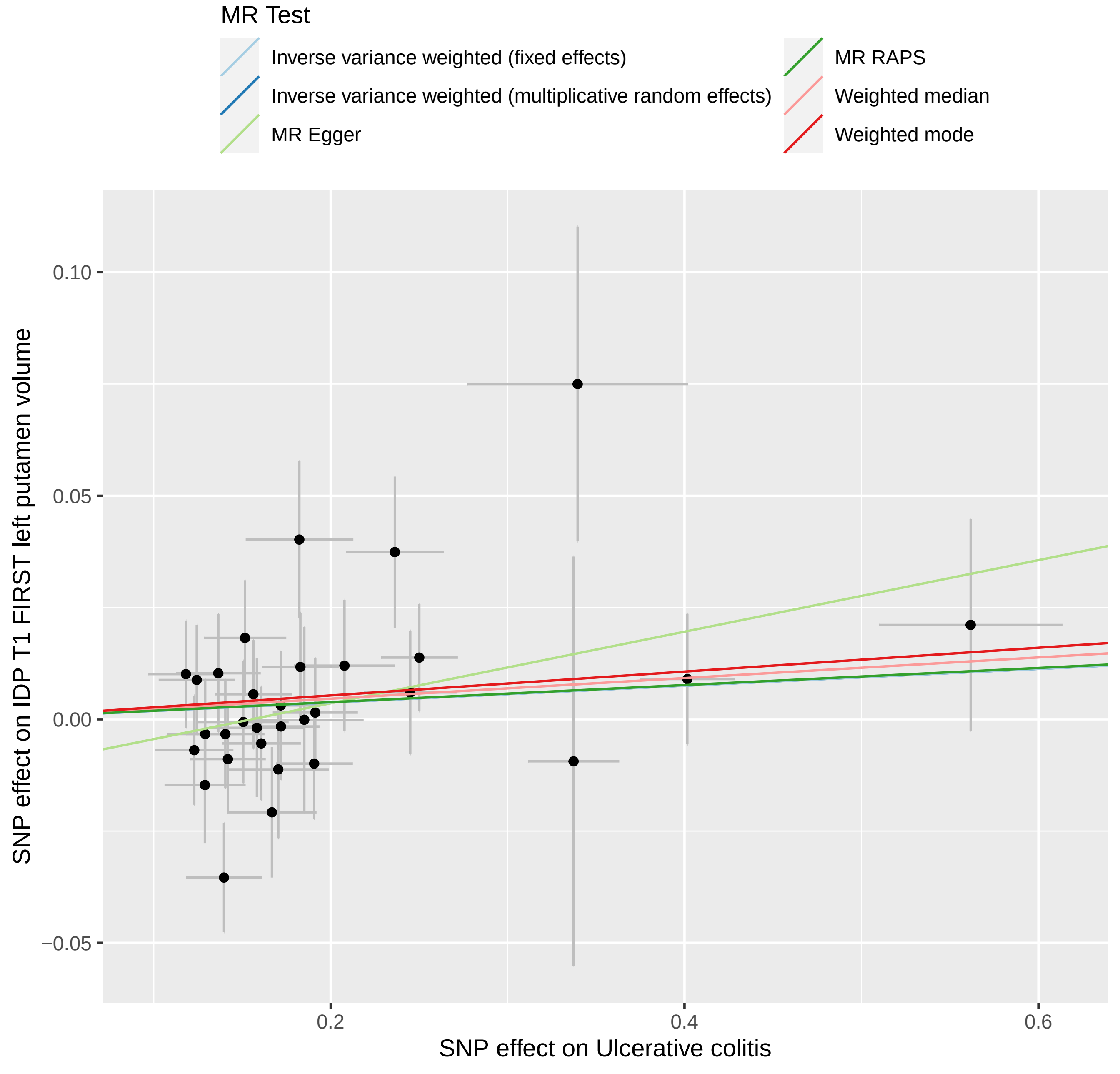

Batch 115 : Crohn's disease on IDP T1 FAST ROIs L cerebellum VIIb

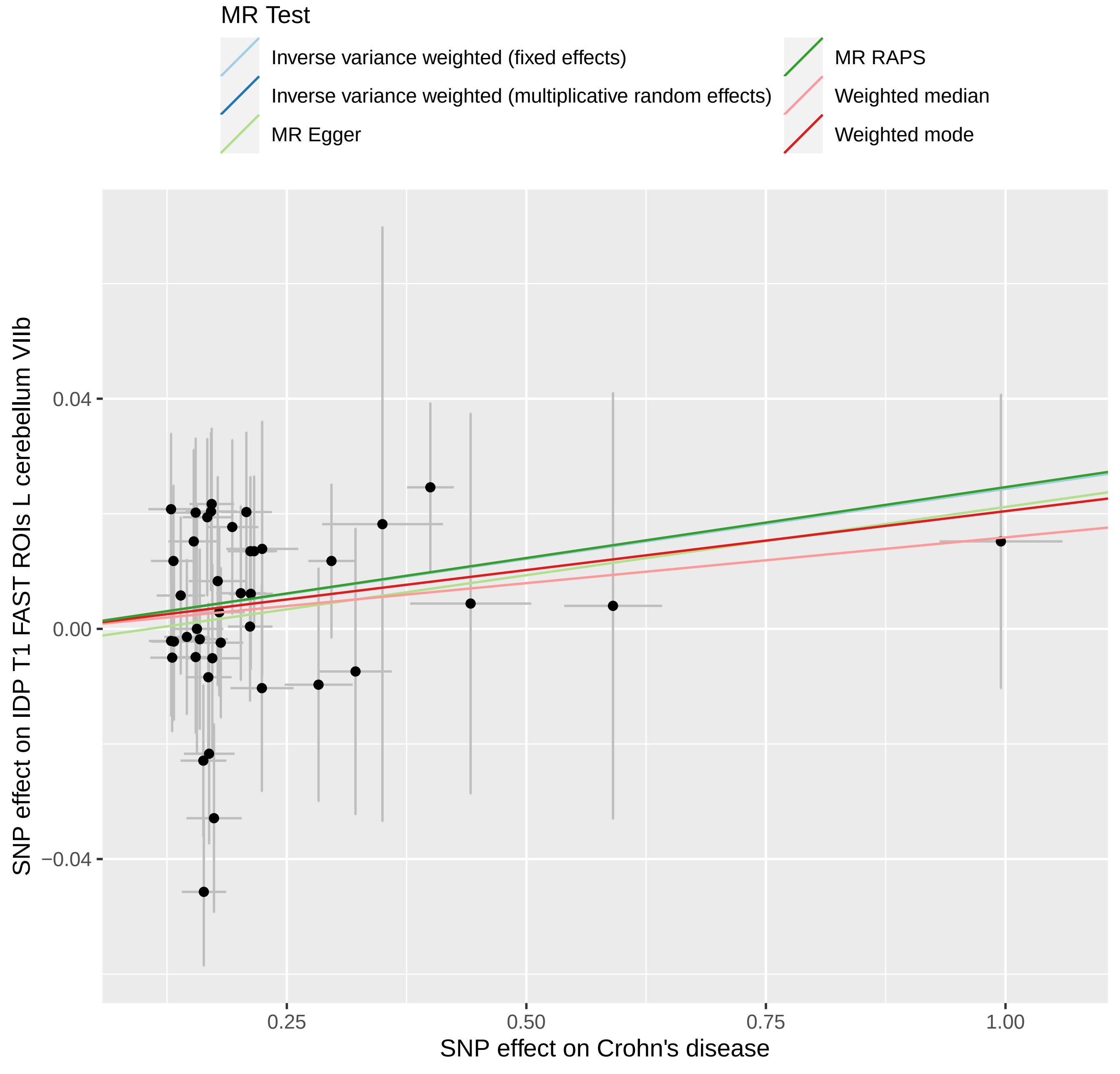

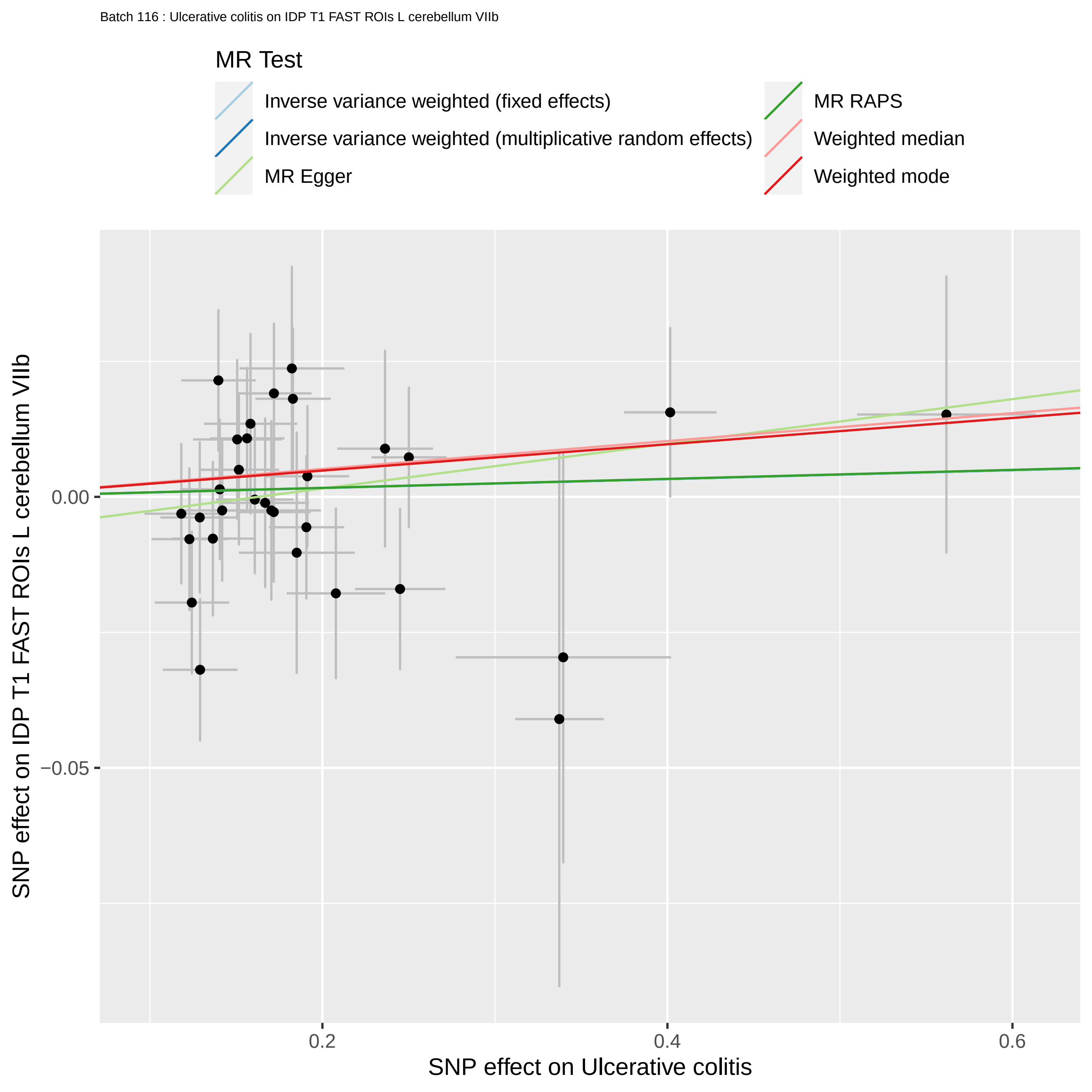
Batch 116 : Ulcerative colitis on IDP T1 FAST ROIs L cerebellum VIIb
MR Test
Inverse variance weighted (fixed effects)
Inverse variance weighted (multiplicative random effects)
MR Egger
MR RAPS
Weighted median
Weighted mode
SNP effect on IDP T1 FAST ROIs L cerebellum VIIb
0.00
−0.05
0.2
0.4
0.6
SNP effect on Ulcerative colitis

Batch 117 : Crohn's disease on IDP T1 FAST ROIs V cerebellum VIIb

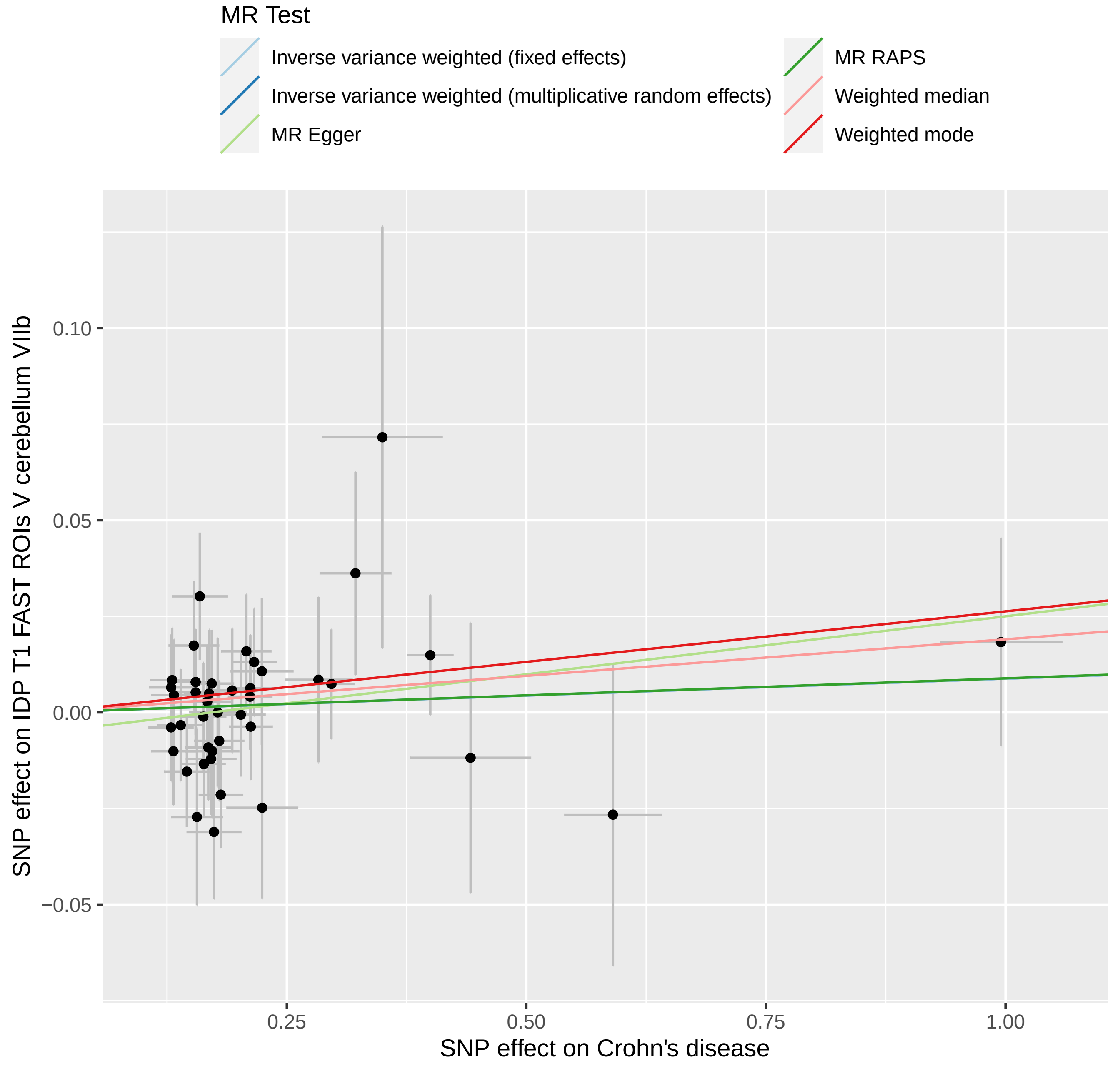

Batch 118 : Ulcerative colitis on IDP T1 FAST ROIs V cerebellum VIIb

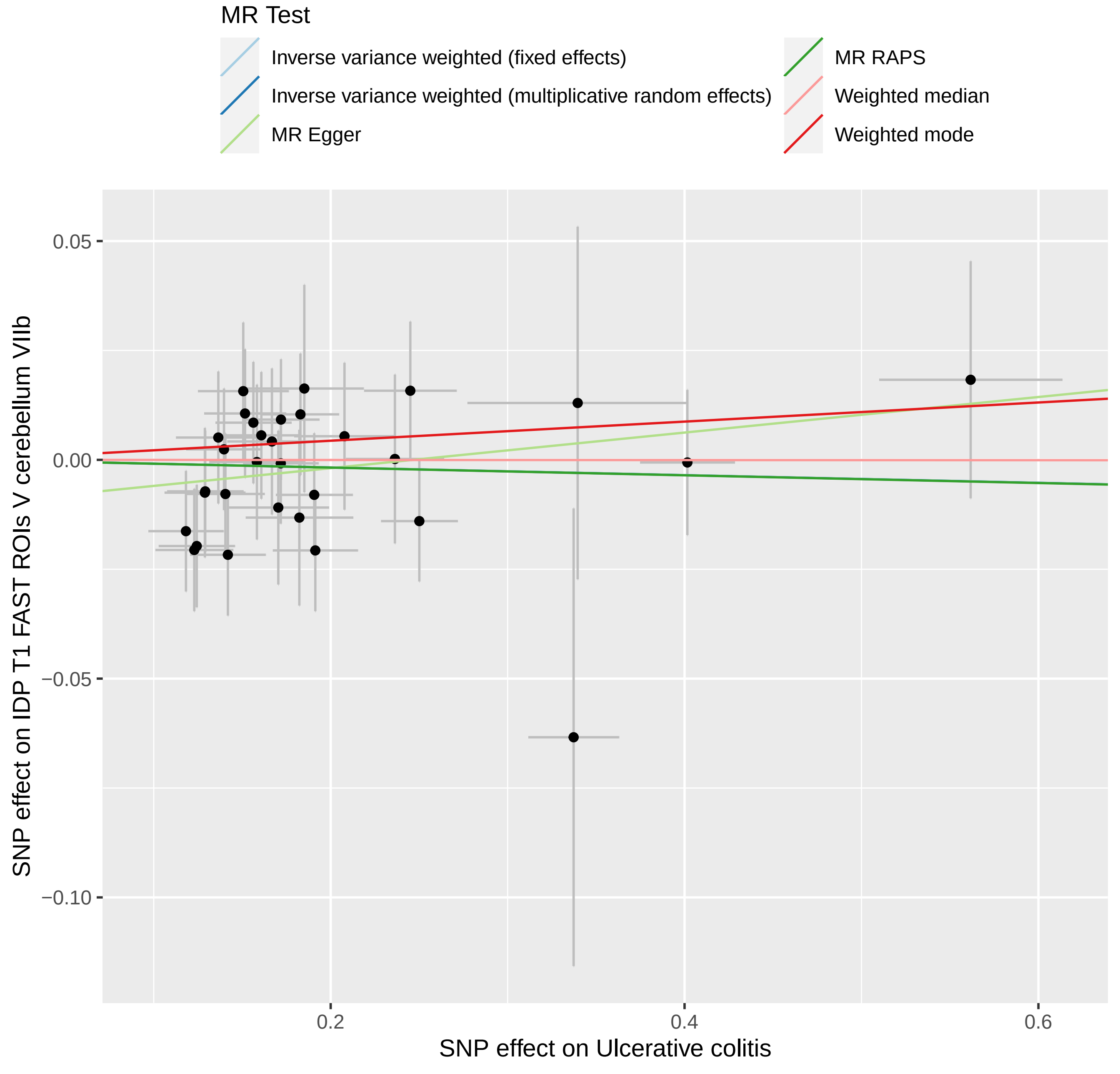

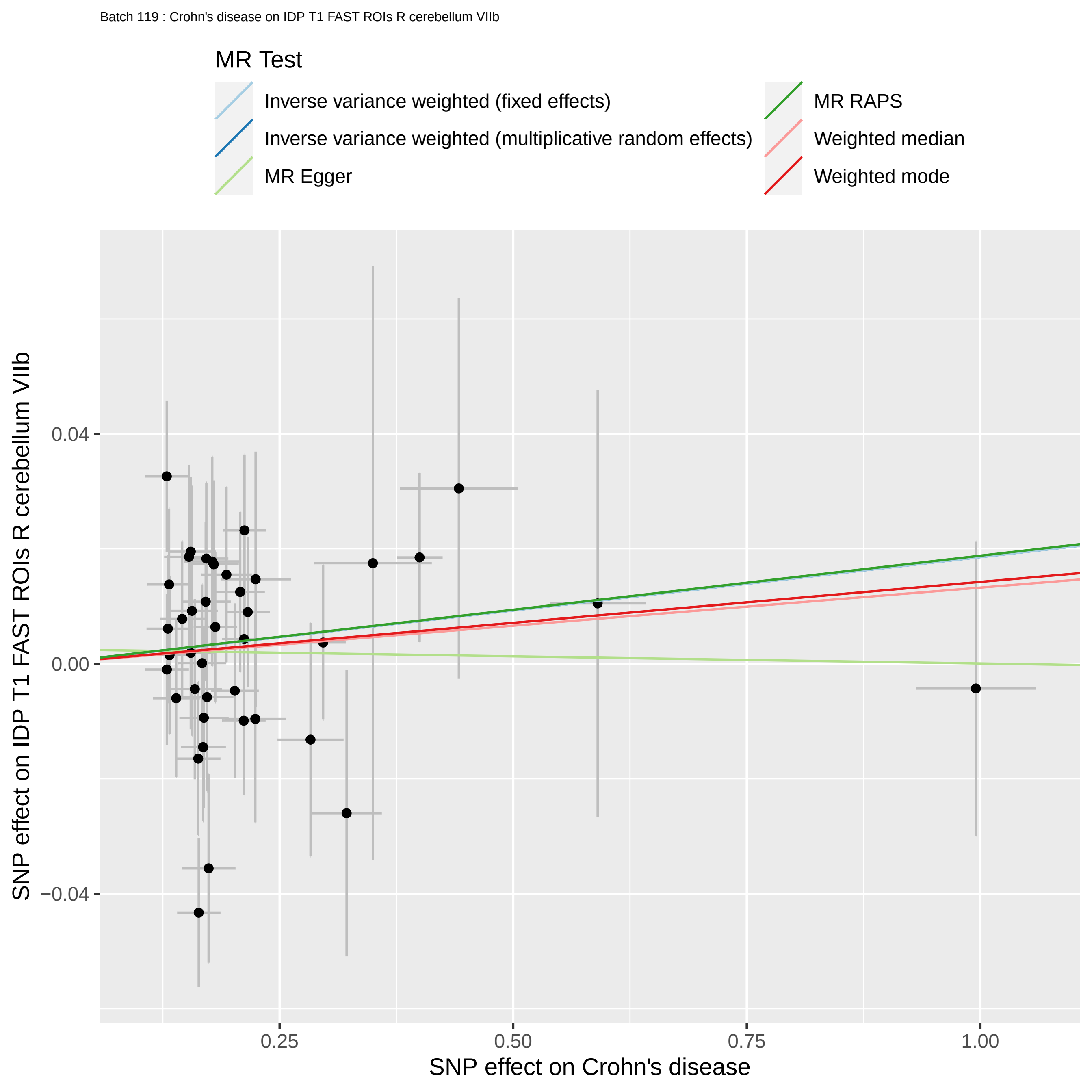

Batch 119 : Crohn's disease on IDP T1 FAST ROIs R cerebellum VIIb
MR Test
Inverse variance weighted (fixed effects)
Inverse variance weighted (multiplicative random effects)
MR Egger
MR RAPS
Weighted median
Weighted mode
SNP effect on IDP T1 FAST ROIs R cerebellum VIIb
0.04
0.00
−0.04
0.25
0.50
0.75
1.00
SNP effect on Crohn's disease

Batch 120 : Ulcerative colitis on IDP T1 FAST ROIs R cerebellum VIIb

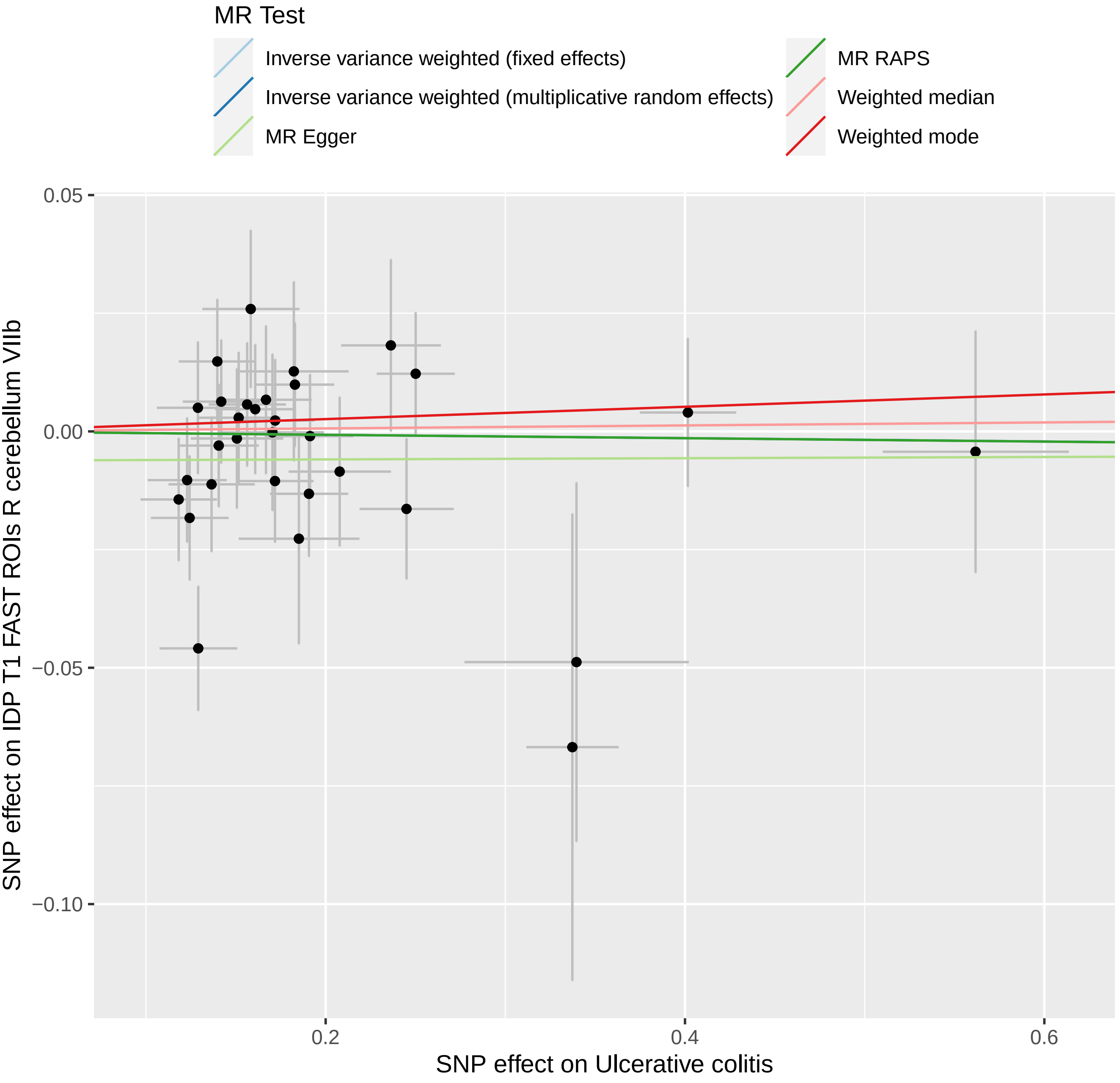

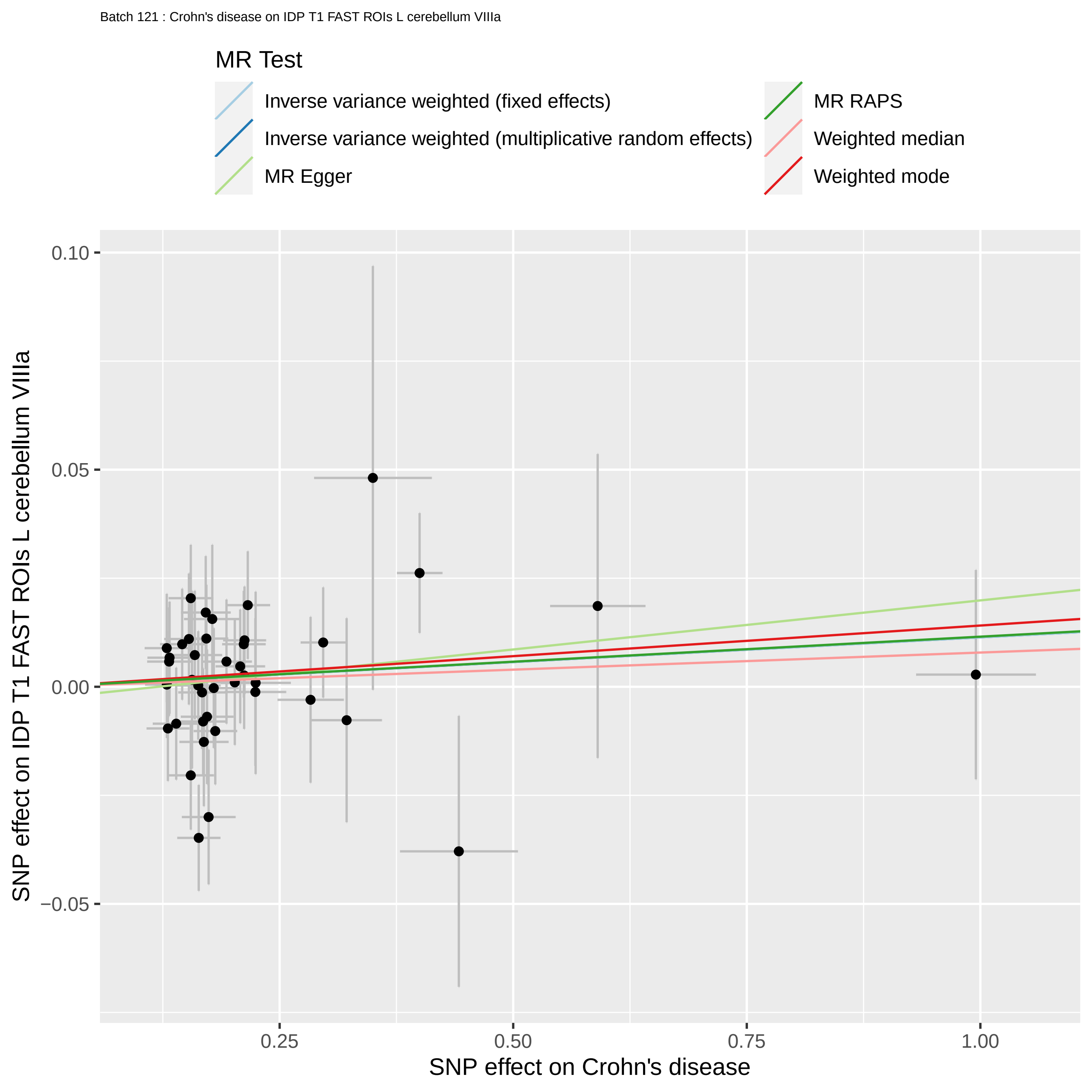
Batch 121 : Crohn's disease on IDP T1 FAST ROIs L cerebellum VIIIa
MR Test
Inverse variance weighted (fixed effects)
Inverse variance weighted (multiplicative random effects)
MR Egger
MR RAPS
Weighted median
Weighted mode
0.10
0.05
0.00
−0.05
0.25
0.50
0.75
1.00
SNP effect on IDP T1 FAST ROIs L cerebellum VIIIa
SNP effect on Crohn's disease

Batch 122 : Ulcerative colitis on IDP T1 FAST ROIs L cerebellum VIIIa

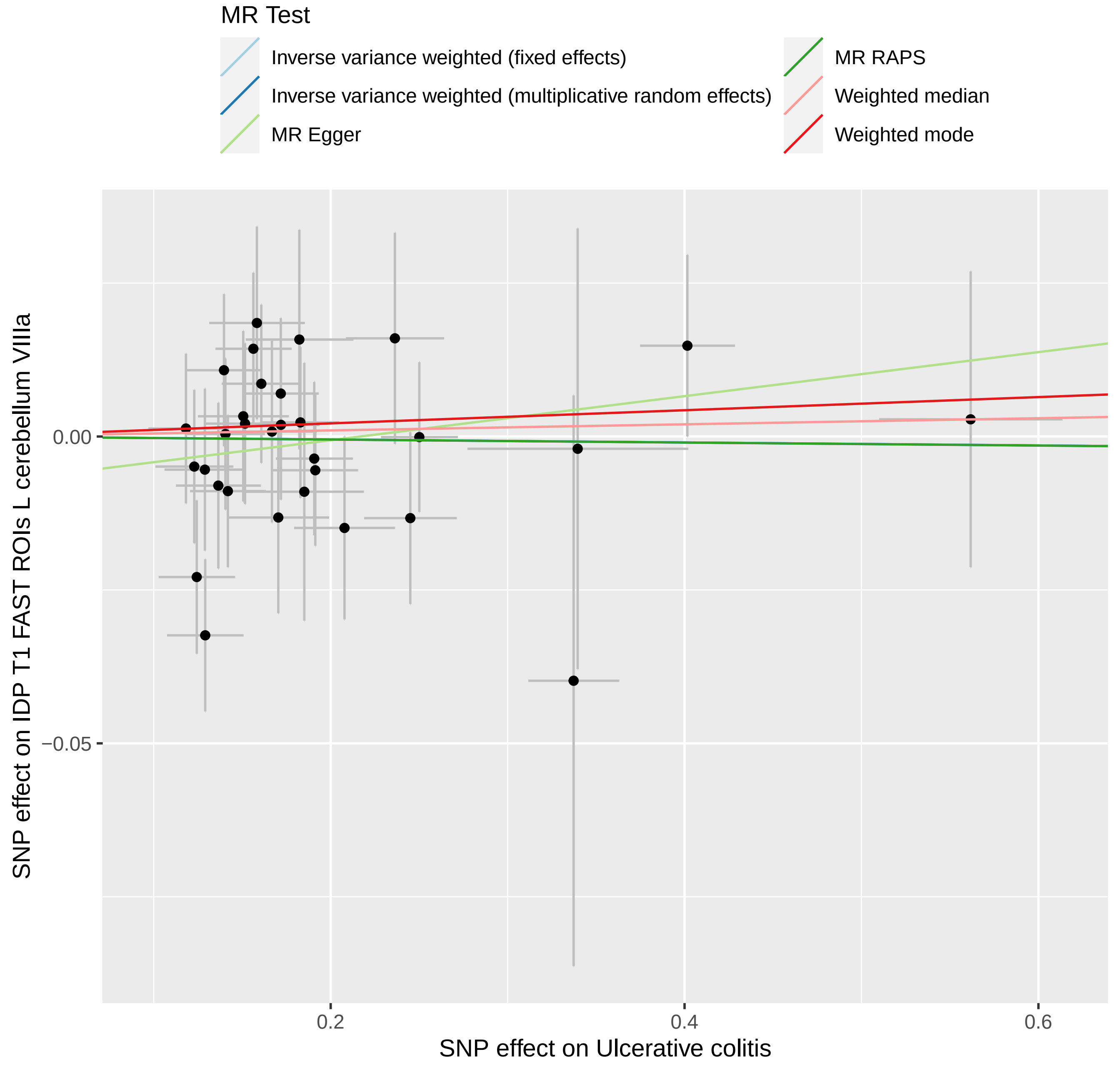

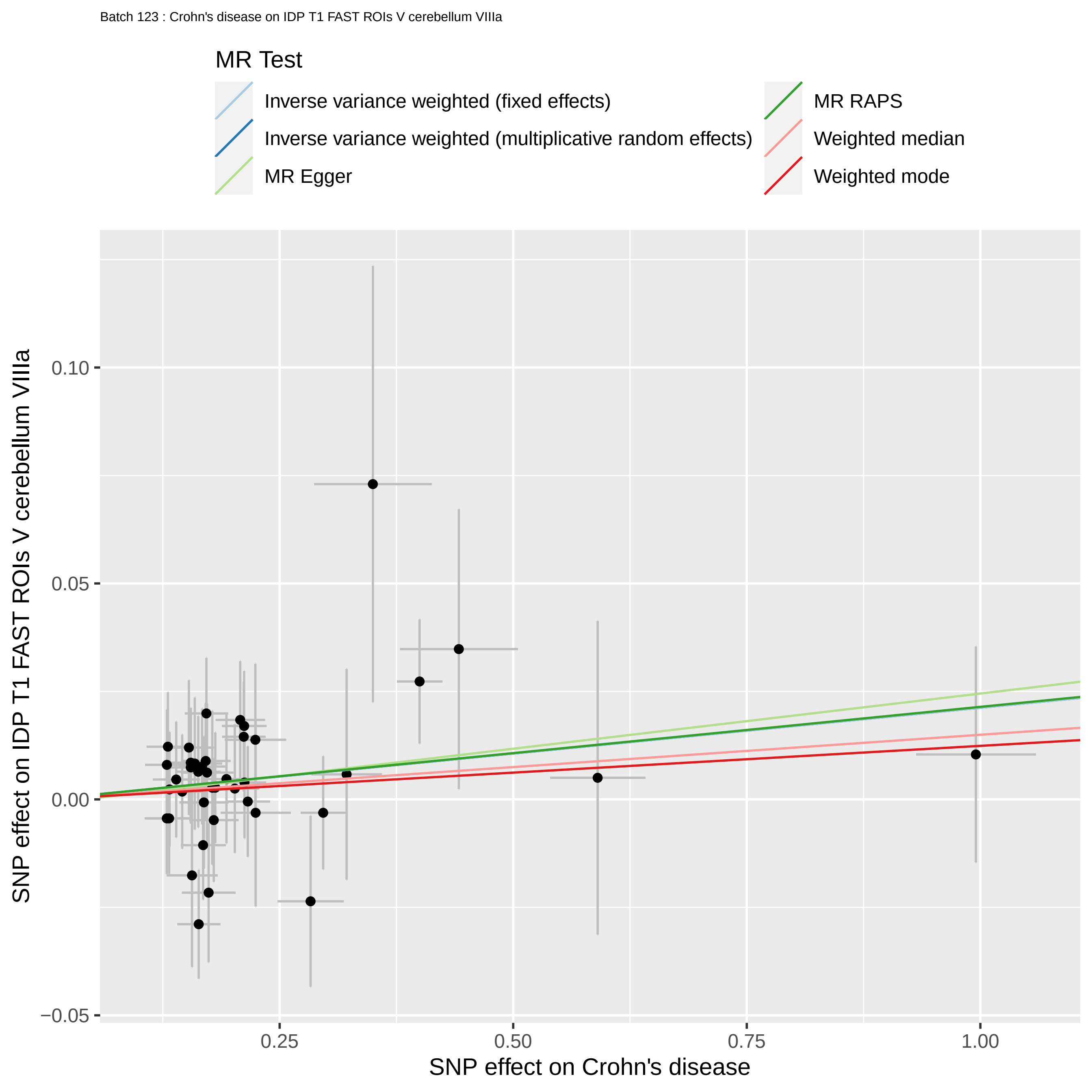

Batch 123 : Crohn's disease on IDP T1 FAST ROIs V cerebellum VIIIa
MR Test
Inverse variance weighted (fixed effects)
Inverse variance weighted (multiplicative random effects)
MR Egger
MR RAPS
Weighted median
Weighted mode
SNP effect on IDP T1 FAST ROIs V cerebellum VIIIa
0.10
0.05
0.00
−0.05
0.25
0.50
0.75
1.00
SNP effect on Crohn's disease

Batch 124 : Ulcerative colitis on IDP T1 FAST ROIs V cerebellum VIIIa

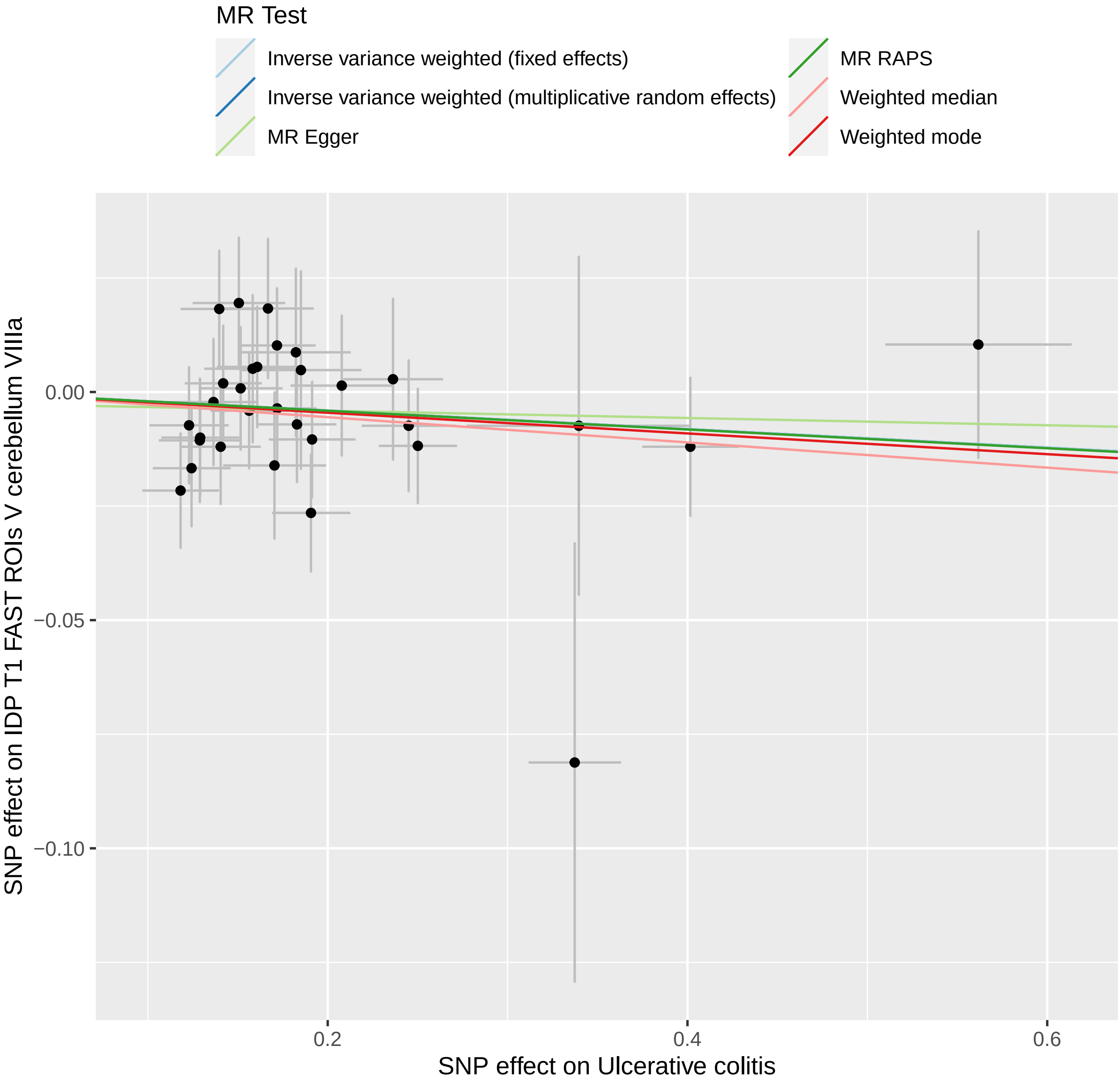

Batch 125 : Crohn's disease on IDP T1 FAST ROIs R cerebellum VIIIa

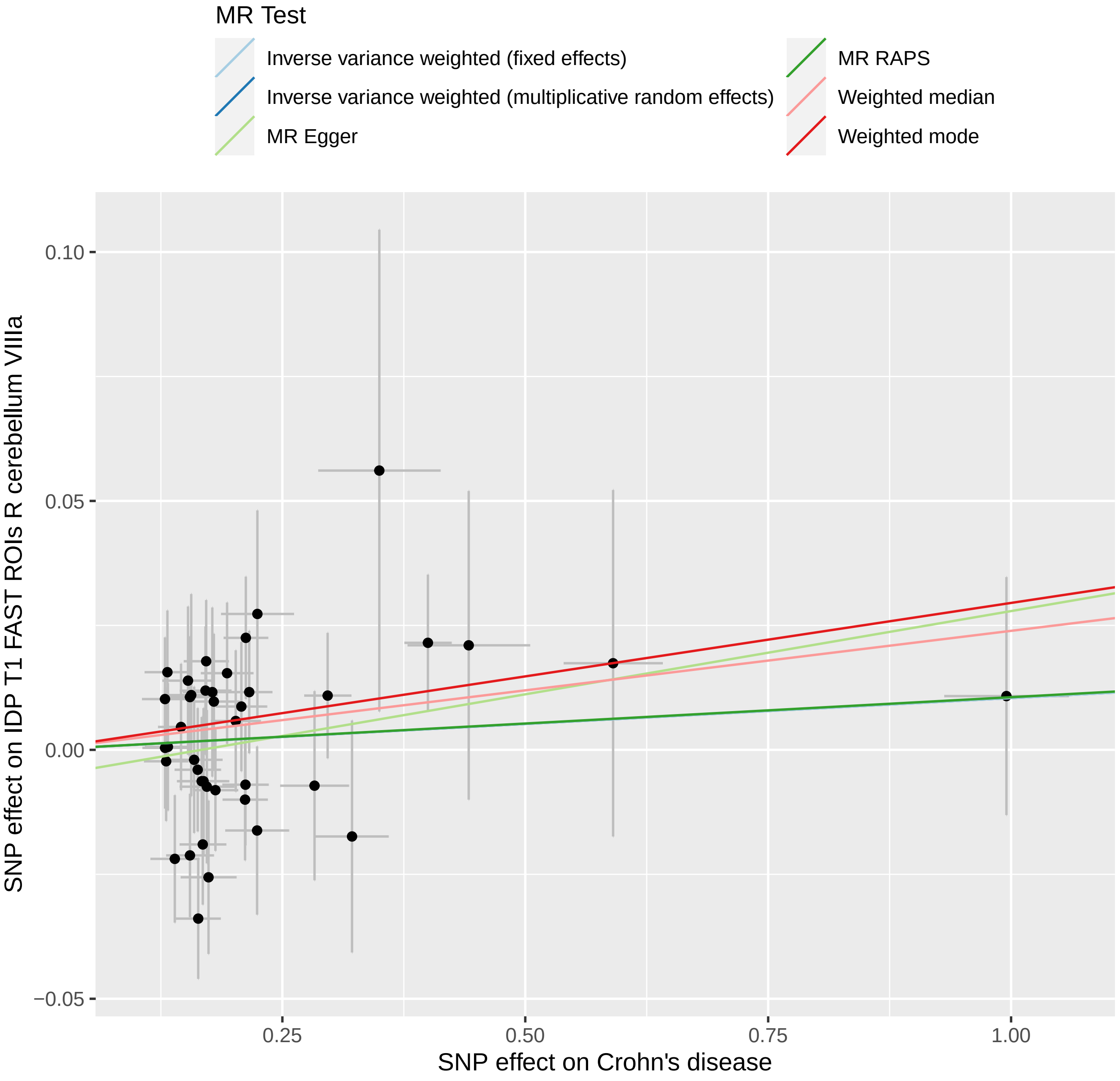

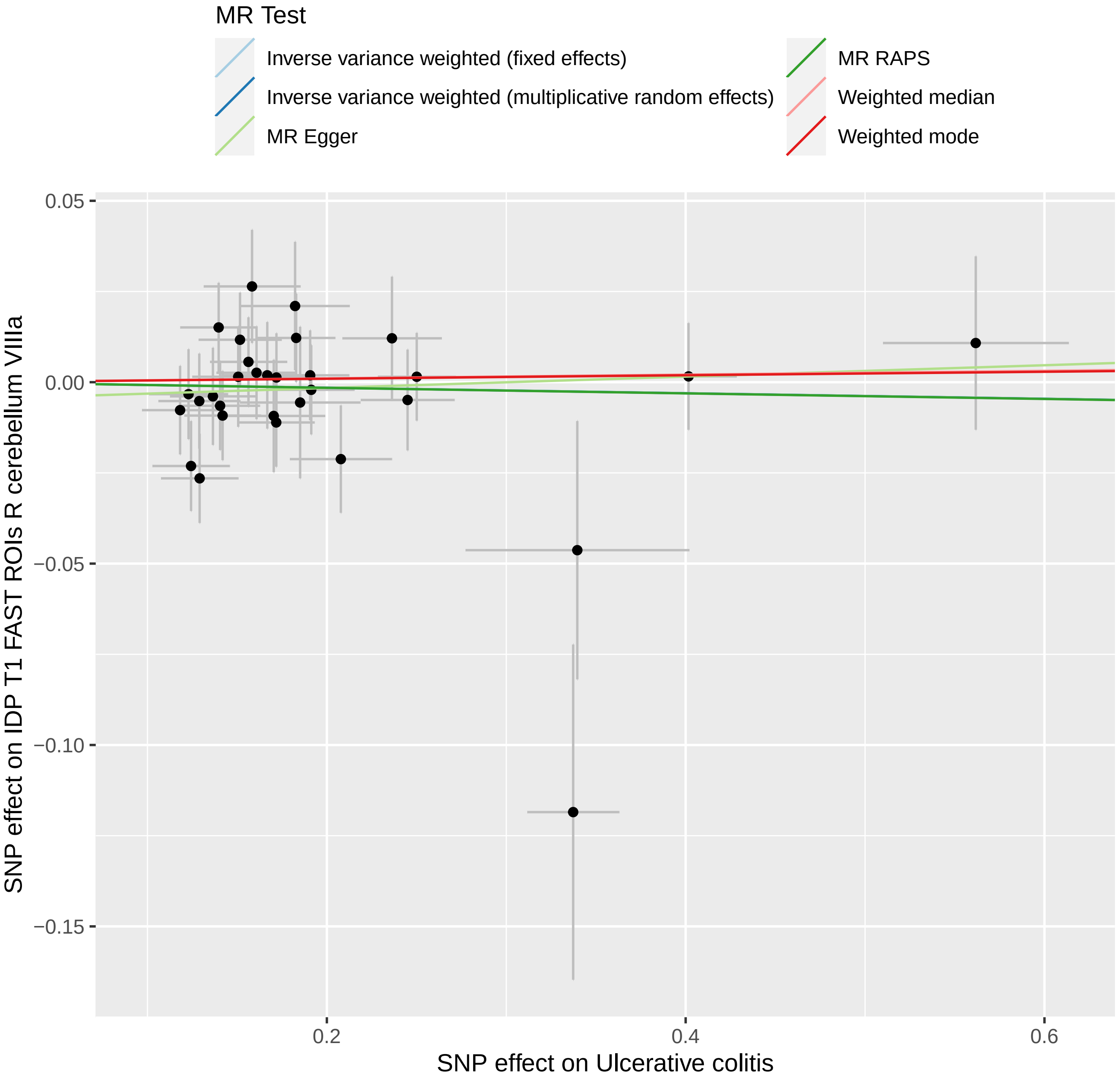
MR Test
Inverse variance weighted (fixed effects)
Inverse variance weighted (multiplicative random effects)
MR Egger
MR RAPS
Weighted median
Weighted mode
0.05
0.00
−0.05
−0.10
−0.15
0.2
0.4
0.6
SNP effect on IDP T1 FAST ROIs R cerebellum VIIIa
SNP effect on Ulcerative colitis

Batch 127 : Crohn's disease on IDP T1 FAST ROIs L cerebellum VIIIb

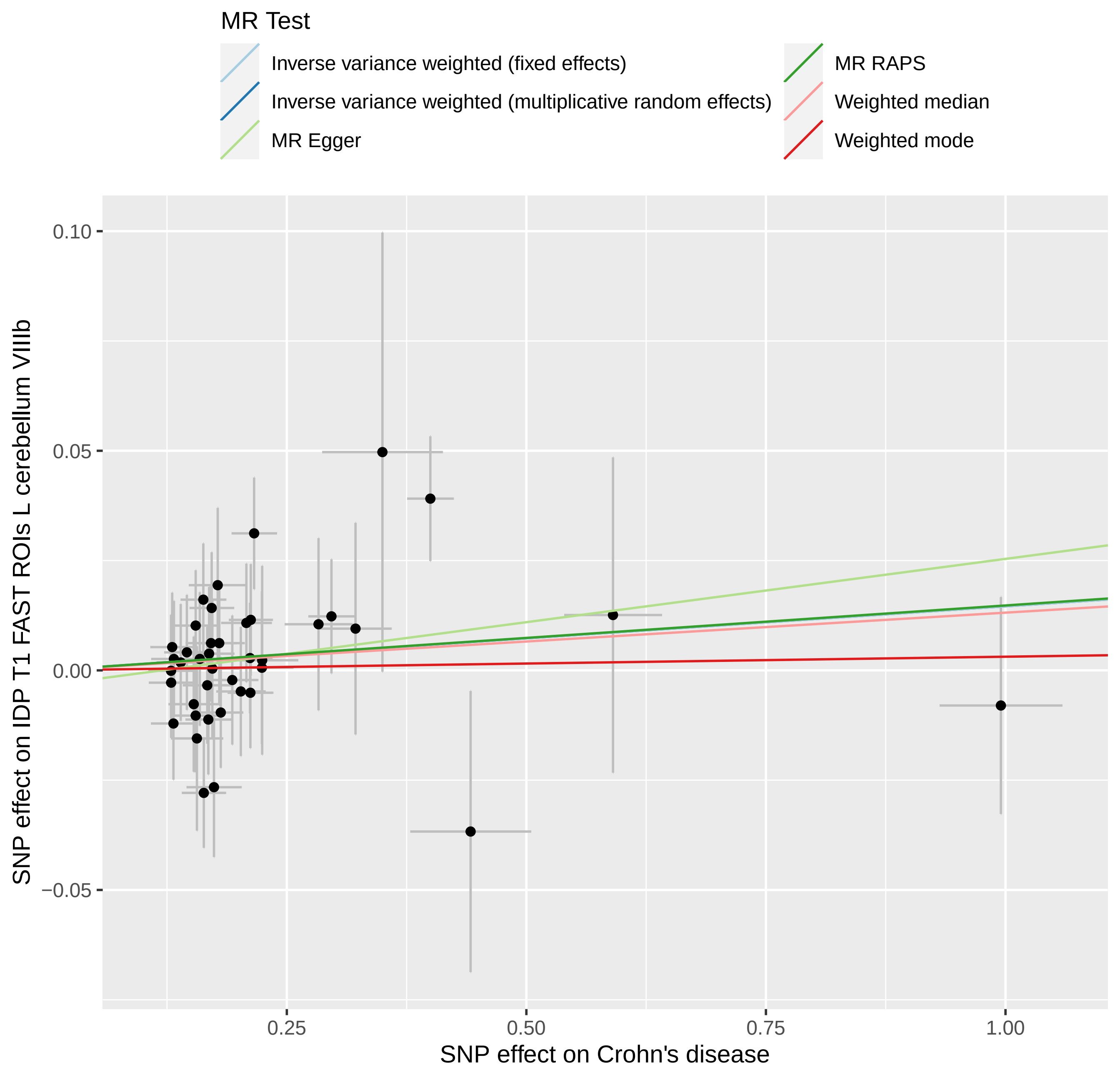

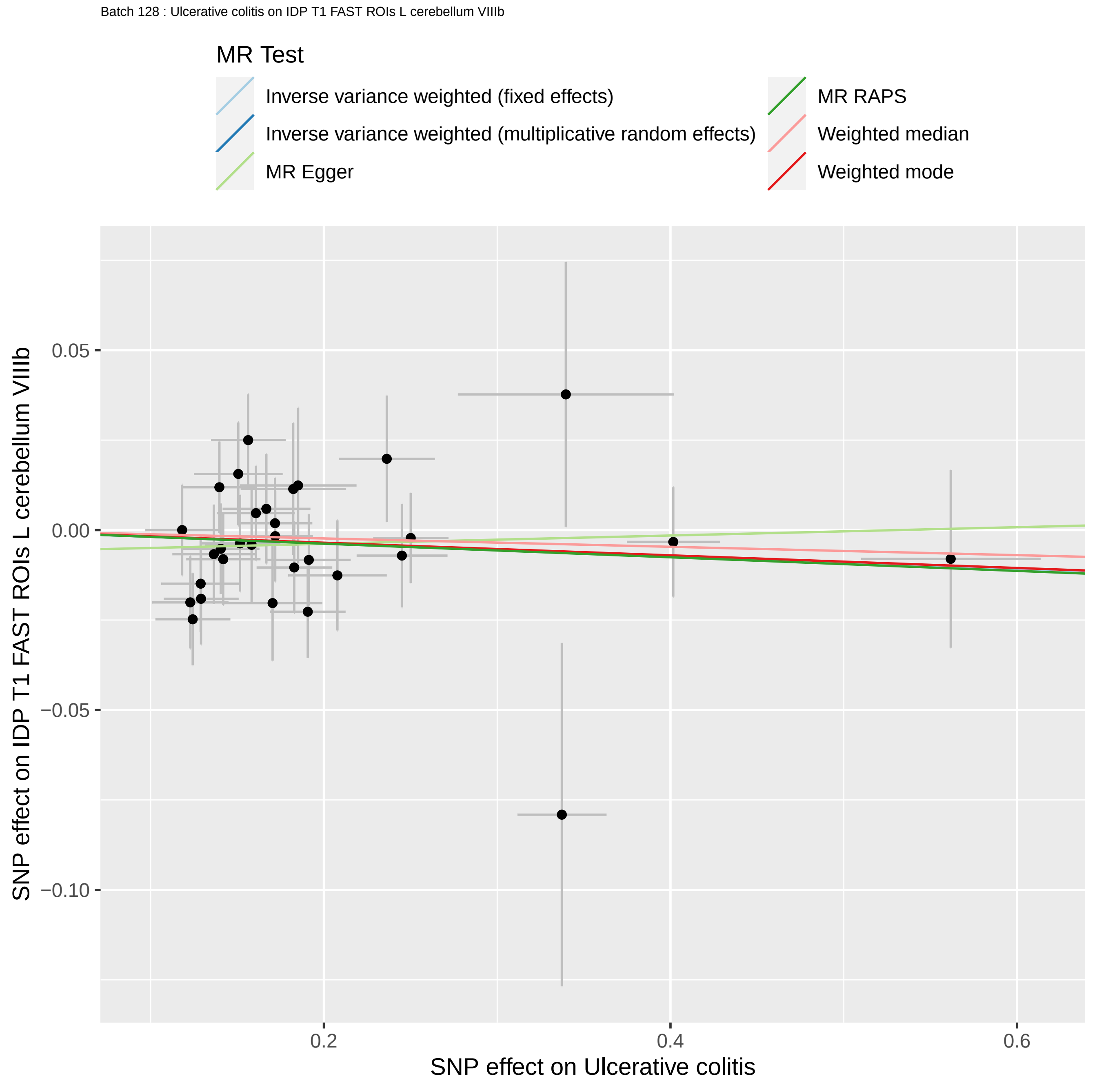

Batch 128 : Ulcerative colitis on IDP T1 FAST ROIs L cerebellum VIIIb
MR Test
Inverse variance weighted (fixed effects)
Inverse variance weighted (multiplicative random effects)
MR Egger
MR RAPS
Weighted median
Weighted mode
SNP effect on IDP T1 FAST ROIs L cerebellum VIIIb
0.05
0.00
−0.05
−0.10
0.2
0.4
0.6
SNP effect on Ulcerative colitis

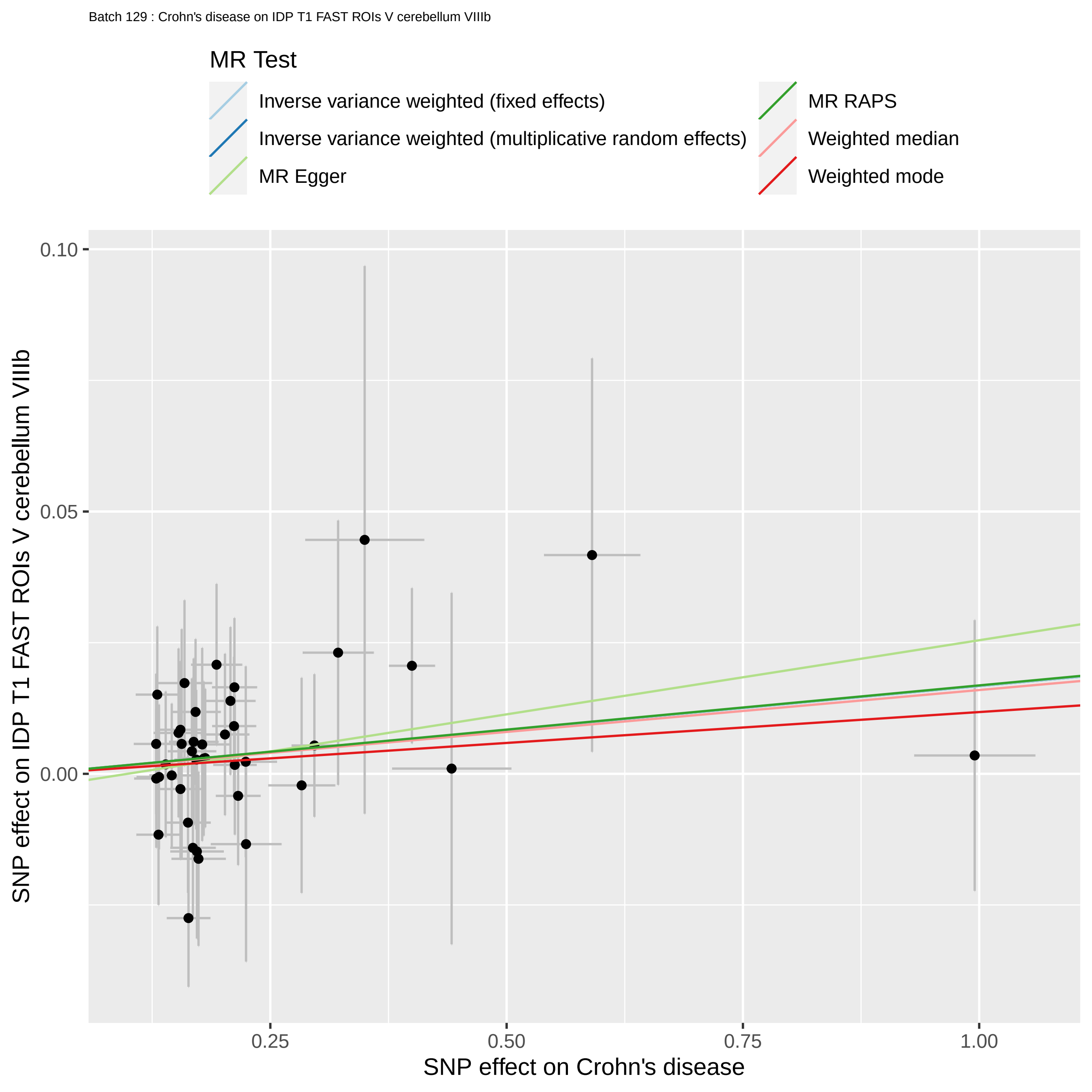
Batch 129 : Crohn's disease on IDP T1 FAST ROIs V cerebellum VIIIb
MR Test
Inverse variance weighted (fixed effects)
Inverse variance weighted (multiplicative random effects)
MR Egger
MR RAPS
Weighted median
Weighted mode
0.10
0.05
0.00
SNP effect on IDP T1 FAST ROIs V cerebellum VIIIb
0.25
0.50
0.75
1.00
SNP effect on Crohn's disease

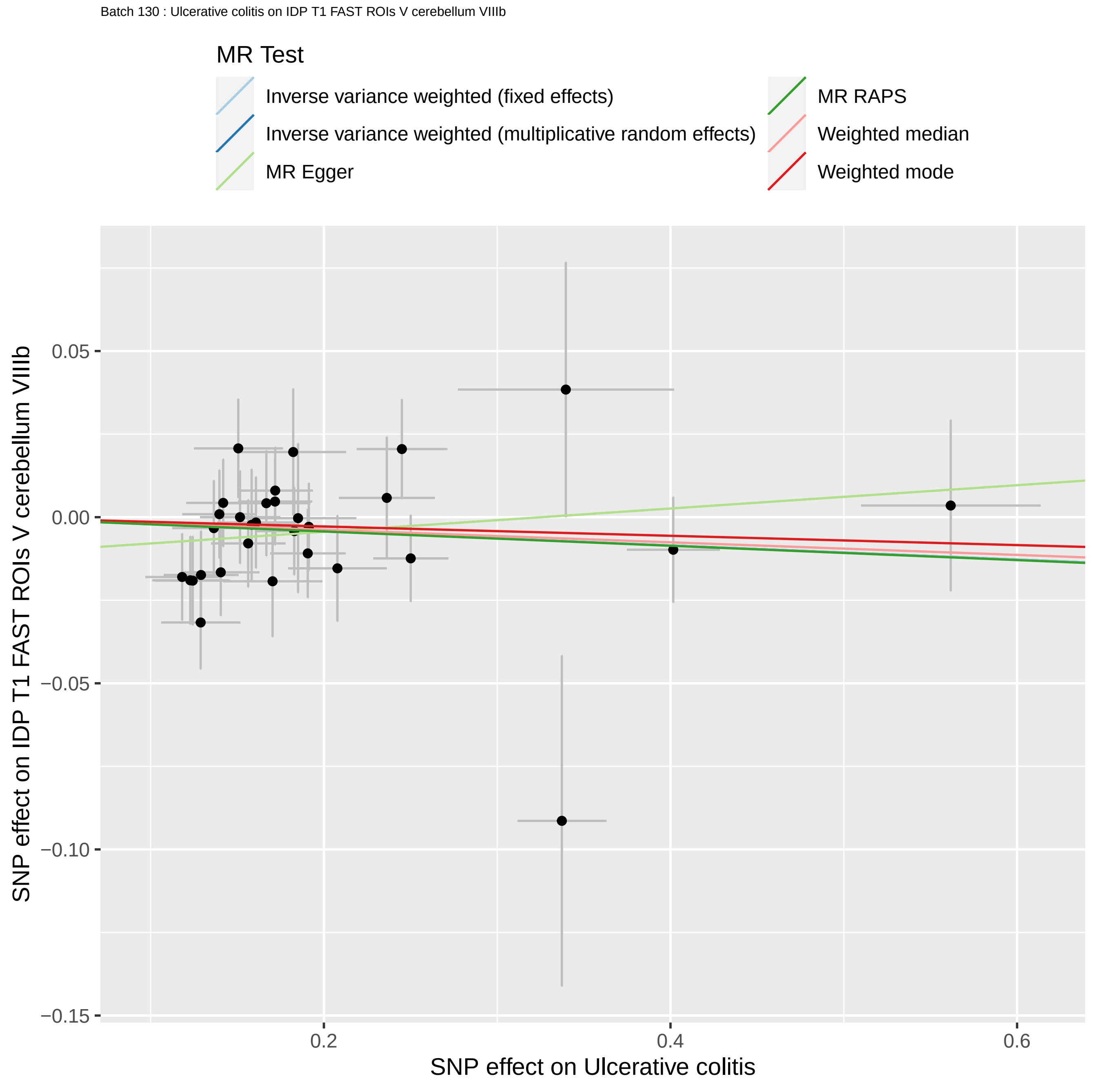

Batch 130 : Ulcerative colitis on IDP T1 FAST ROIs V cerebellum VIIIb
MR Test
Inverse variance weighted (fixed effects)
Inverse variance weighted (multiplicative random effects)
MR Egger
MR RAPS
Weighted median
Weighted mode
0.05
0.00
−0.05
−0.10
−0.15
0.2
0.4
0.6
SNP effect on IDP T1 FAST ROIs V cerebellum VIIIb
SNP effect on Ulcerative colitis

Batch 131 : Crohn's disease on IDP T1 FAST ROIs R cerebellum VIIIb

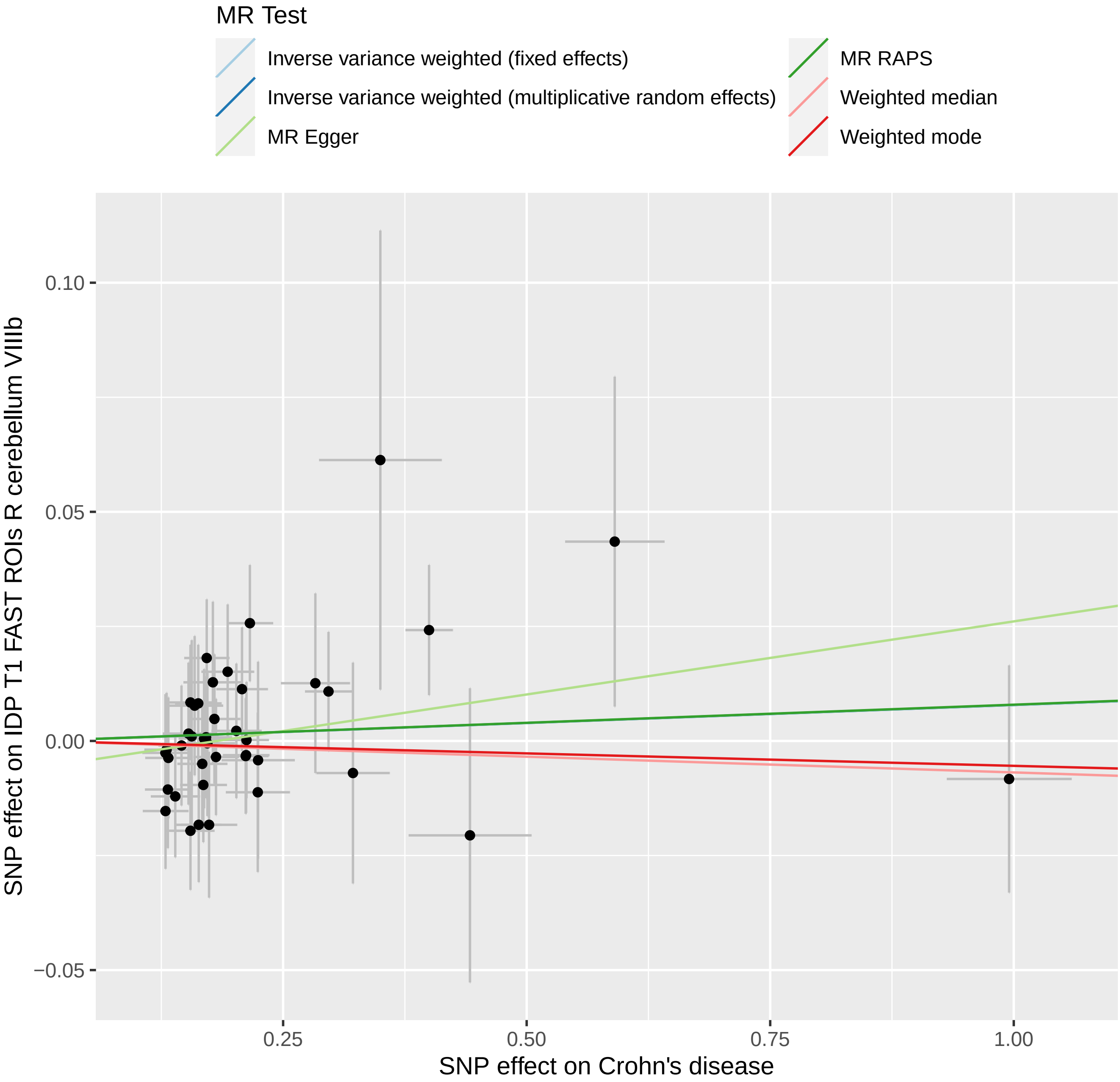

Batch 132 : Ulcerative colitis on IDP T1 FAST ROIs R cerebellum VIIIb

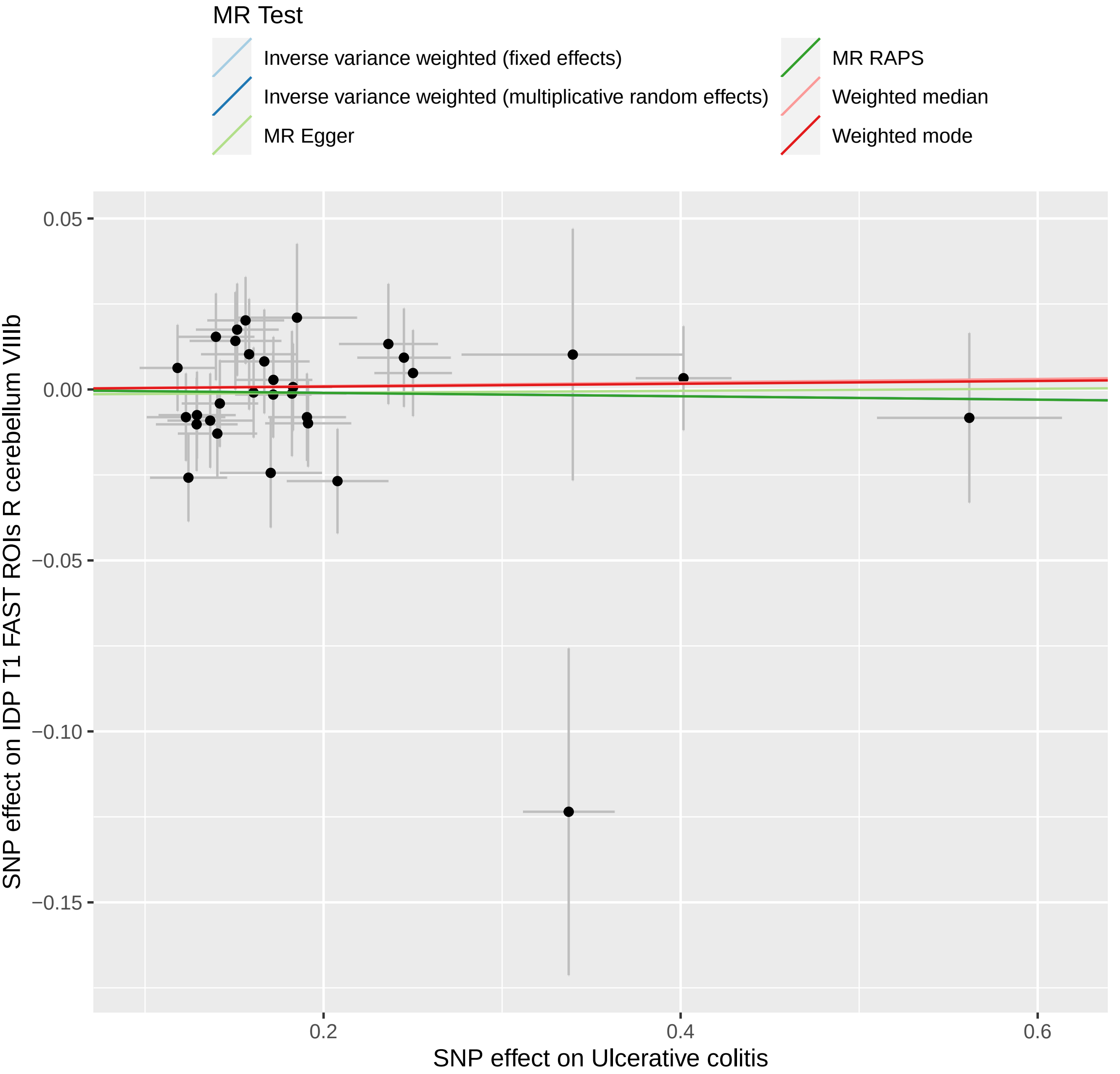

Batch 133 : Crohn's disease on IDP T1 FAST ROIs L cerebellum IX

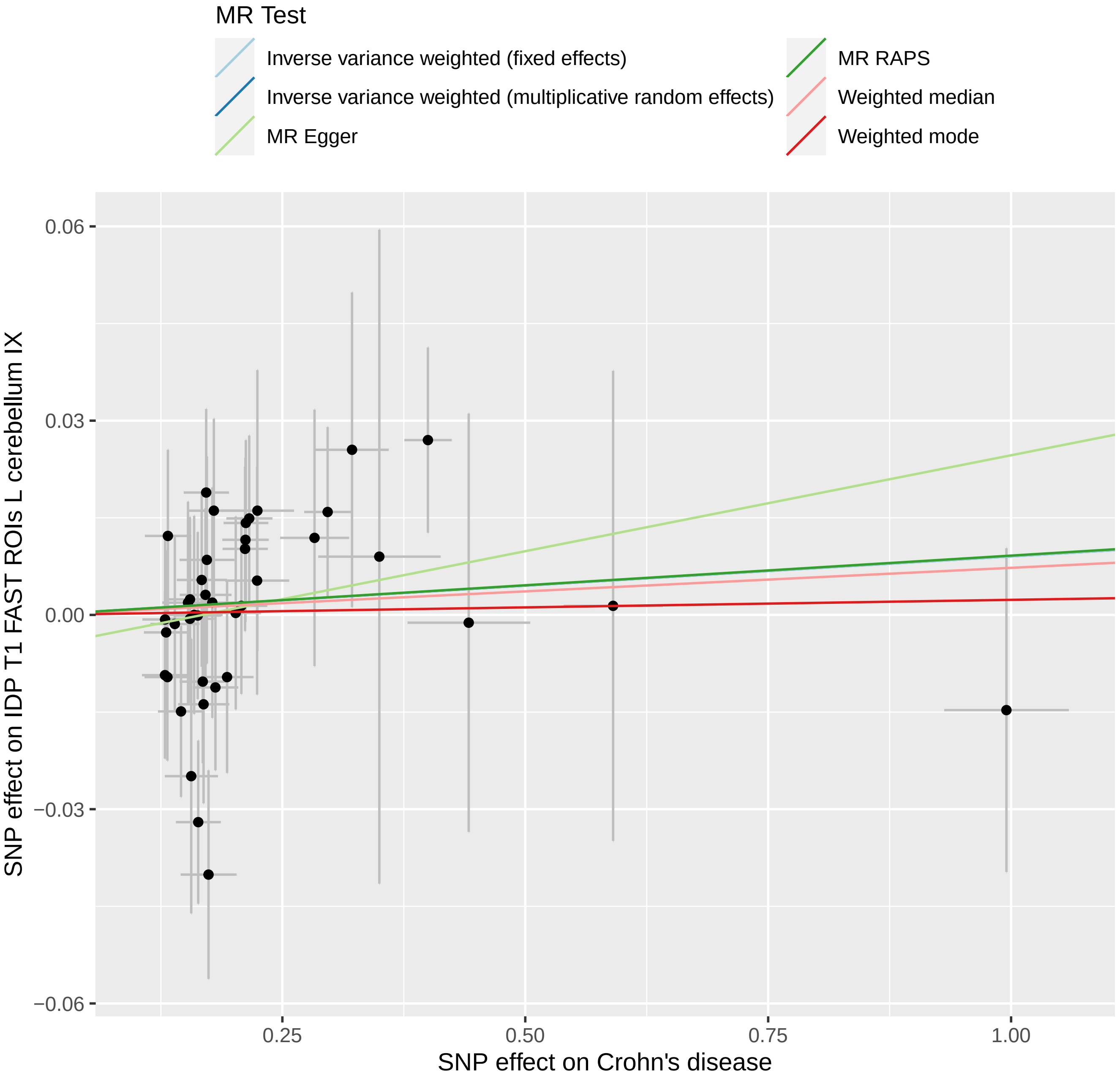

Batch 134 : Ulcerative colitis on IDP T1 FAST ROIs L cerebellum IX

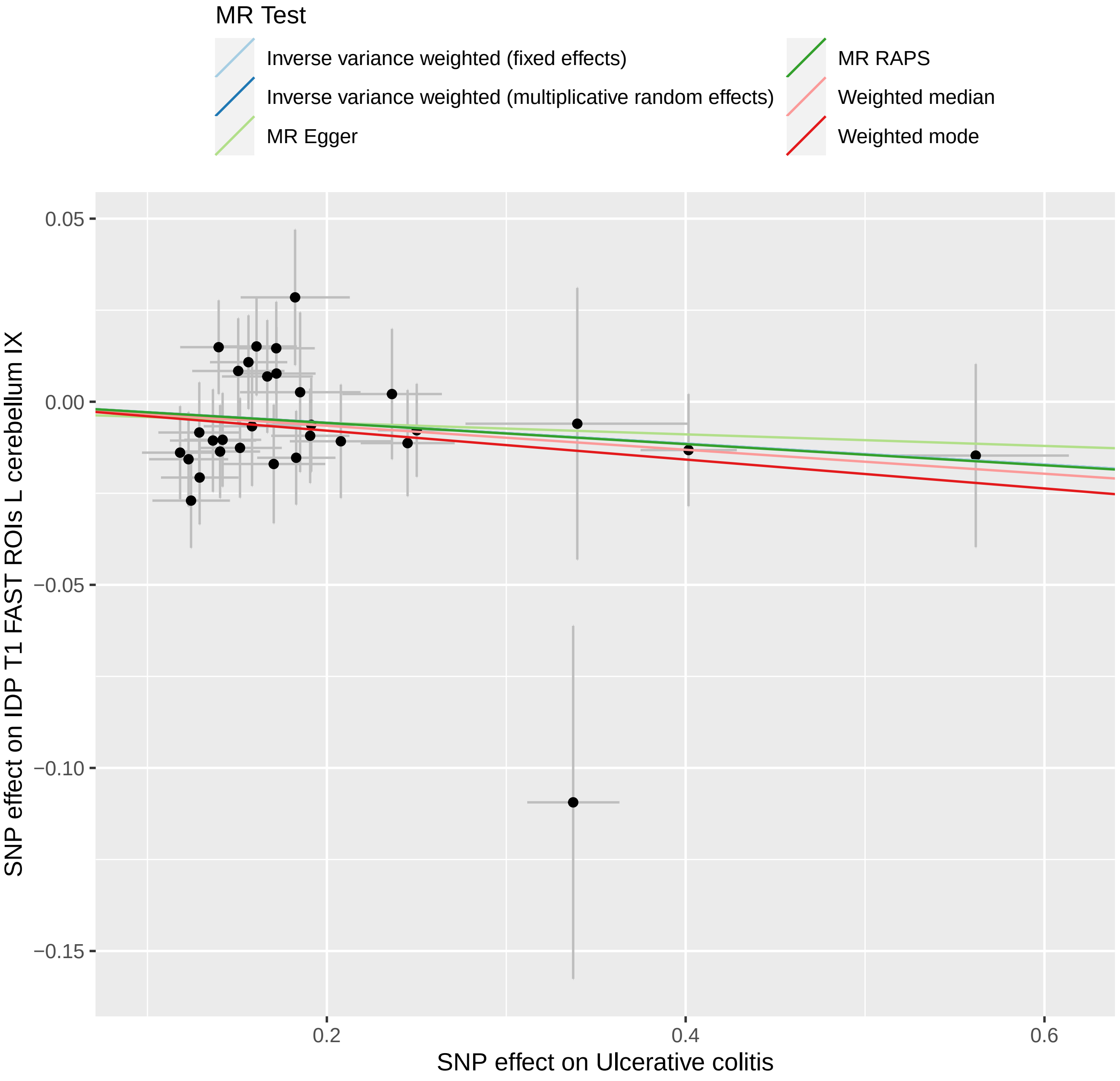

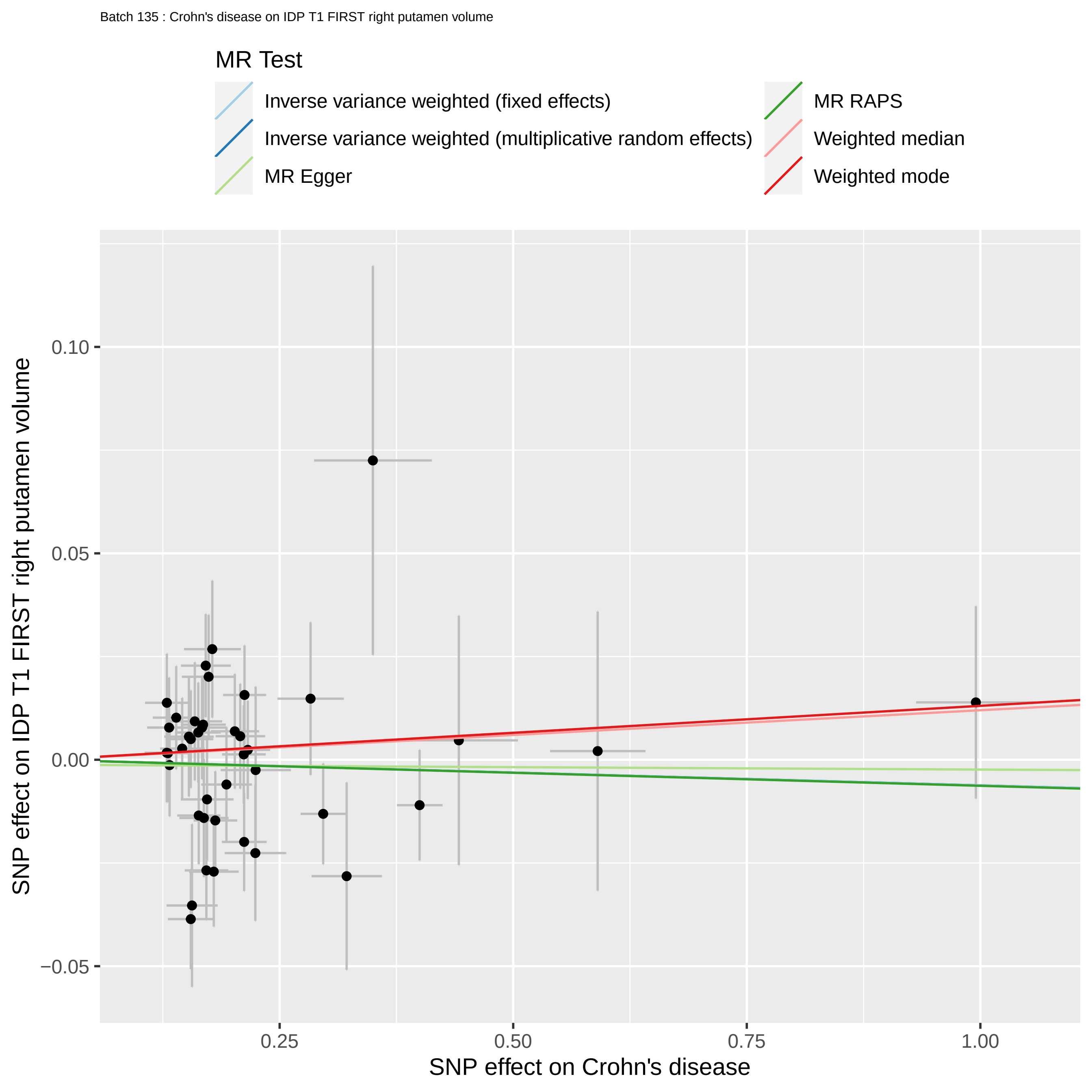
Batch 135 : Crohn's disease on IDP T1 FIRST right putamen volume
MR Test
Inverse variance weighted (fixed effects)
Inverse variance weighted (multiplicative random effects)
MR Egger
MR RAPS
Weighted median
Weighted mode
SNP effect on IDP T1 FIRST right putamen volume
0.10
0.05
0.00
−0.05
0.25
0.50
0.75
1.00
SNP effect on Crohn's disease

Batch 136 : Ulcerative colitis on IDP T1 FIRST right putamen volume

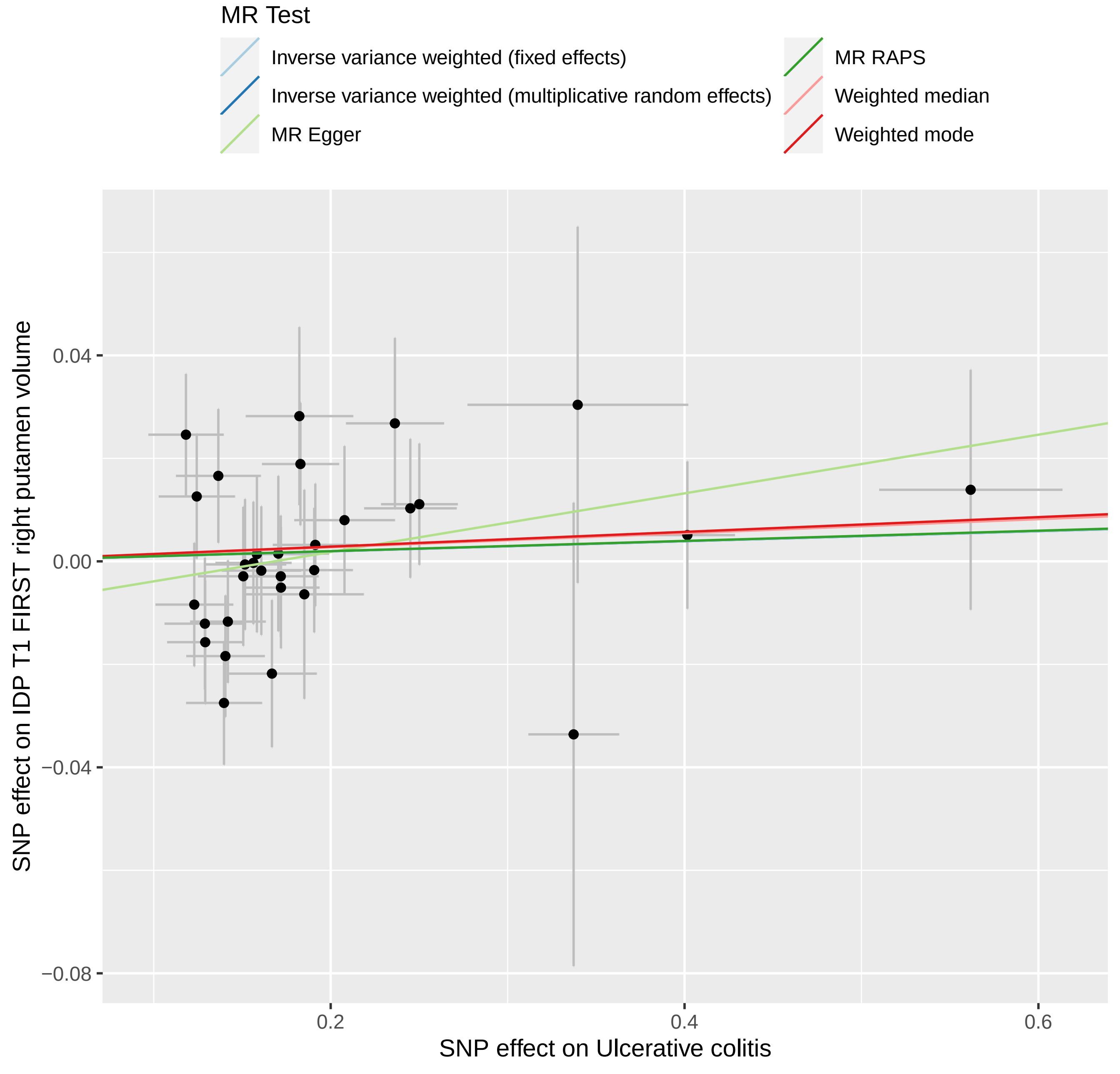

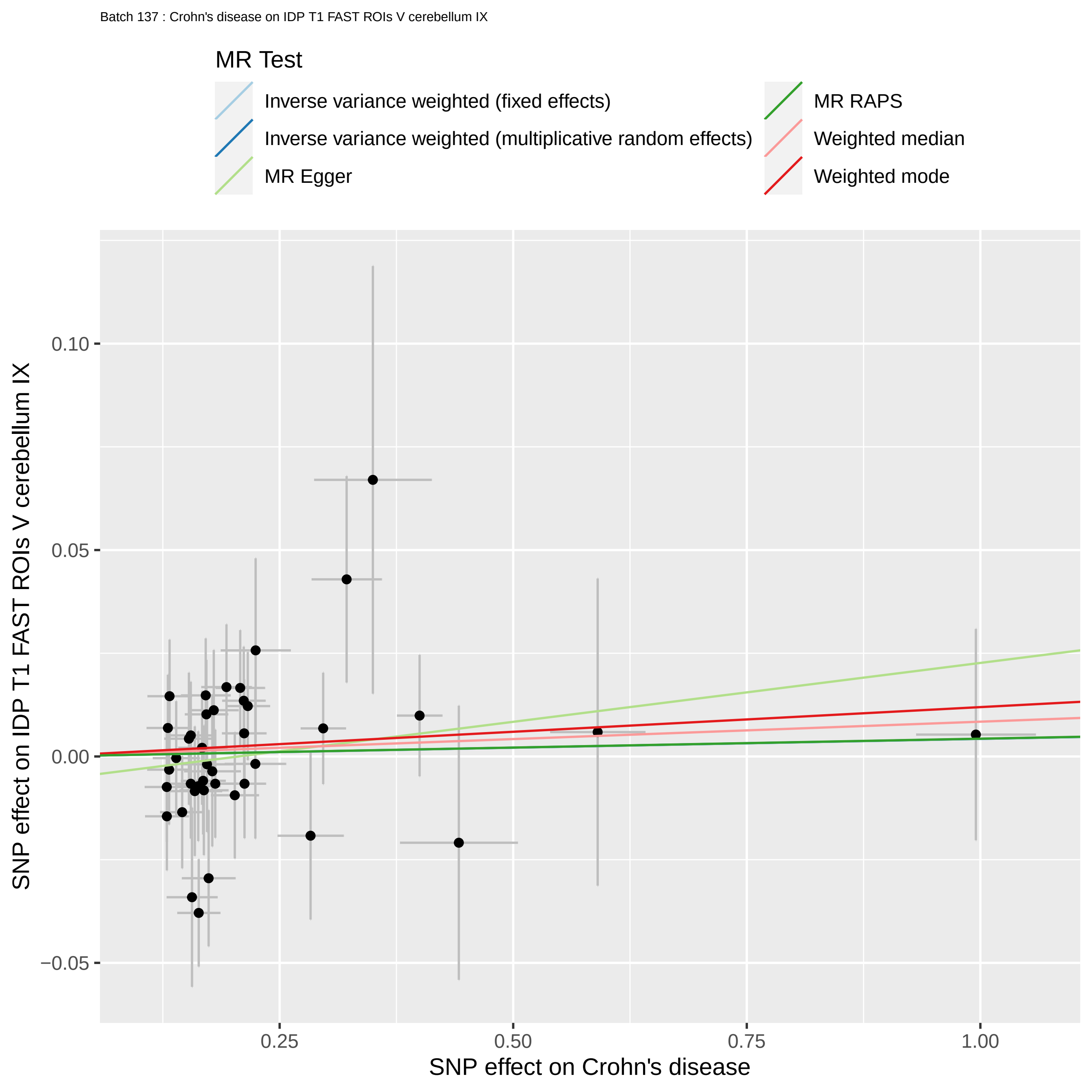
Batch 137 : Crohn's disease on IDP T1 FAST ROIs V cerebellum IX
MR Test
Inverse variance weighted (fixed effects)
Inverse variance weighted (multiplicative random effects)
MR Egger
MR RAPS
Weighted median
Weighted mode
0.10
0.05
0.00
−0.05
0.25
0.50
0.75
1.00
SNP effect on IDP T1 FAST ROIs V cerebellum IX
SNP effect on Crohn's disease

Batch 138 : Ulcerative colitis on IDP T1 FAST ROIs V cerebellum IX

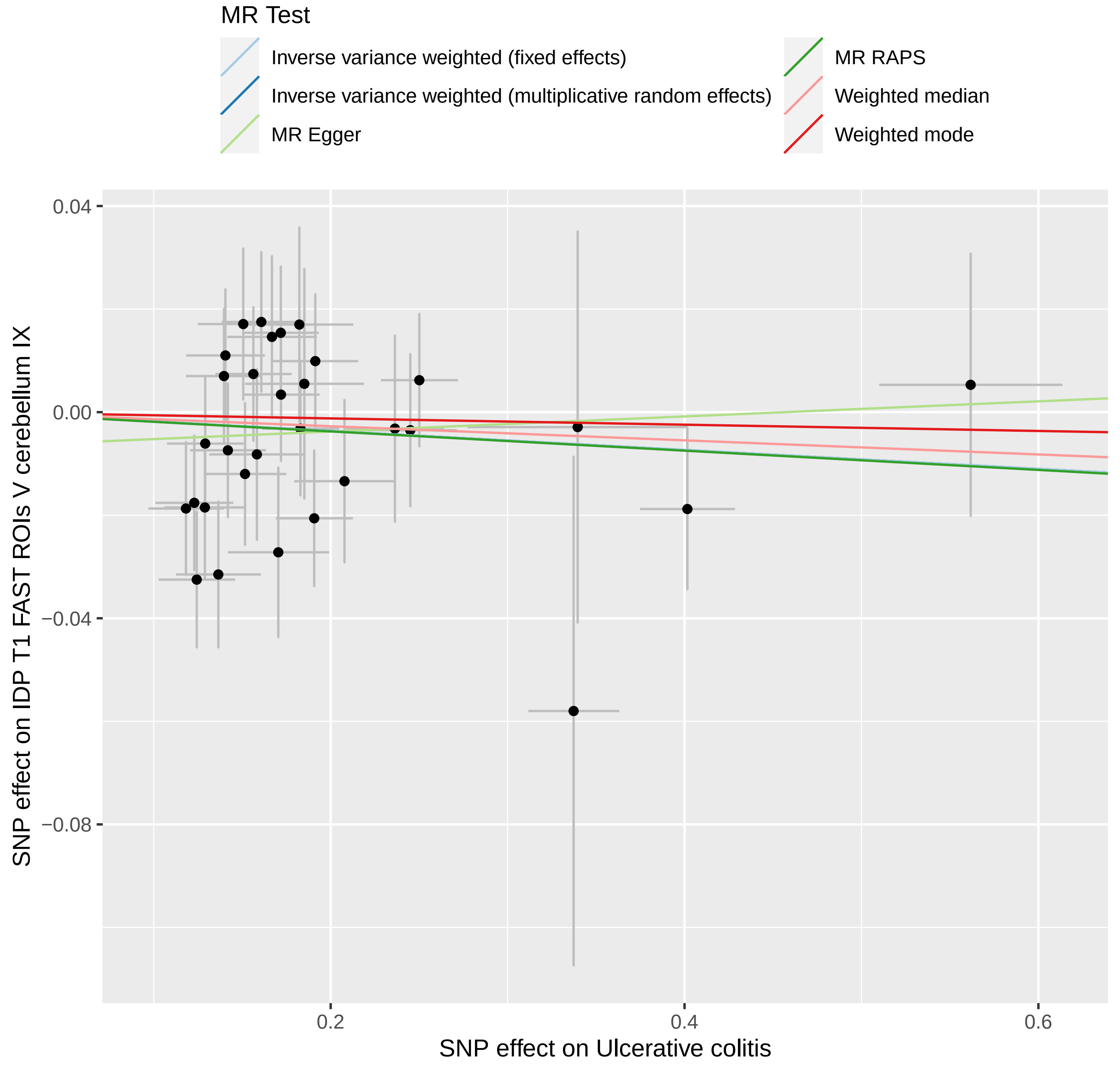

Batch 139 : Crohn's disease on IDP T1 FAST ROIs R cerebellum IX

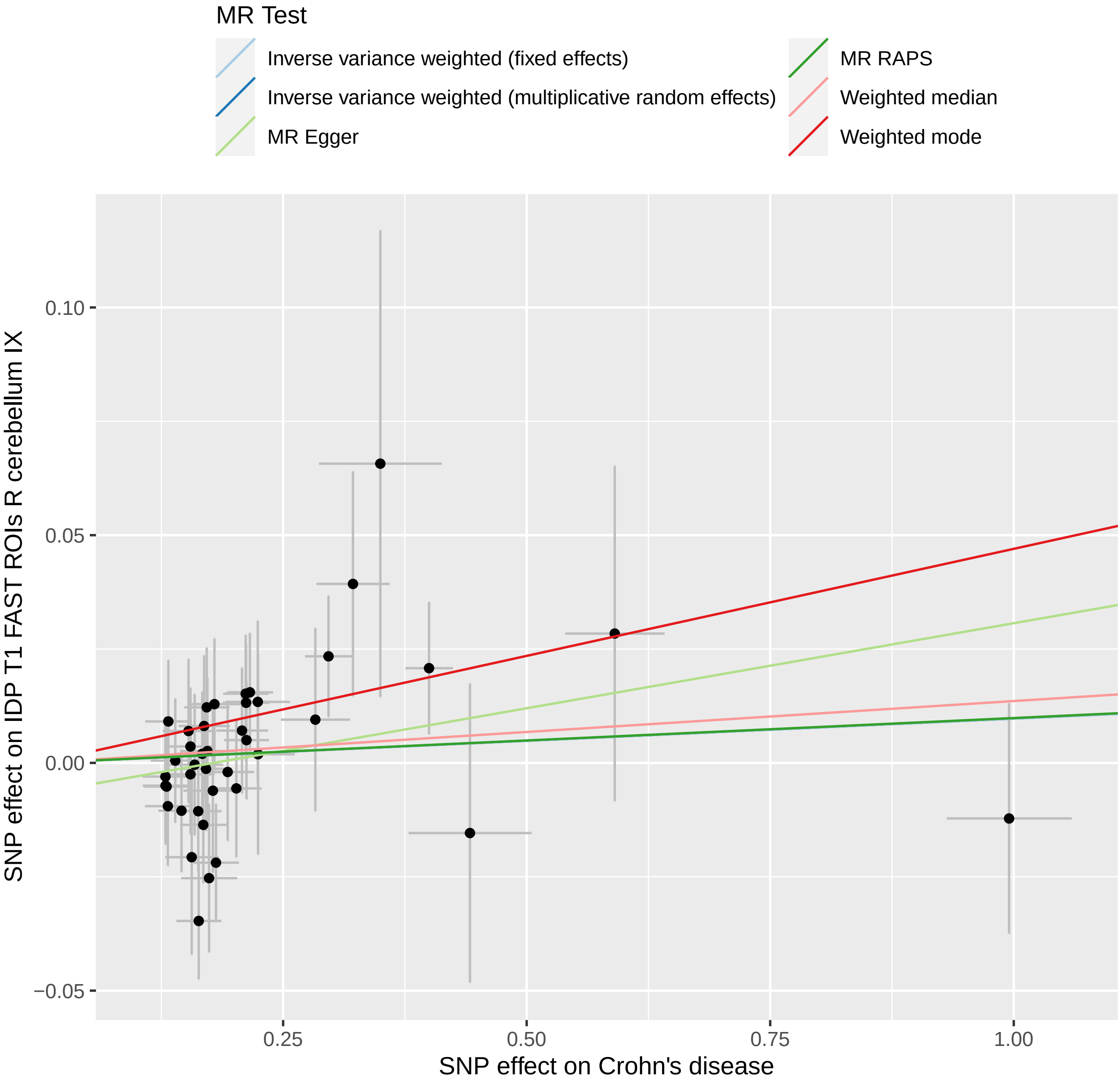

Batch 140 : Ulcerative colitis on IDP T1 FAST ROIs R cerebellum IX

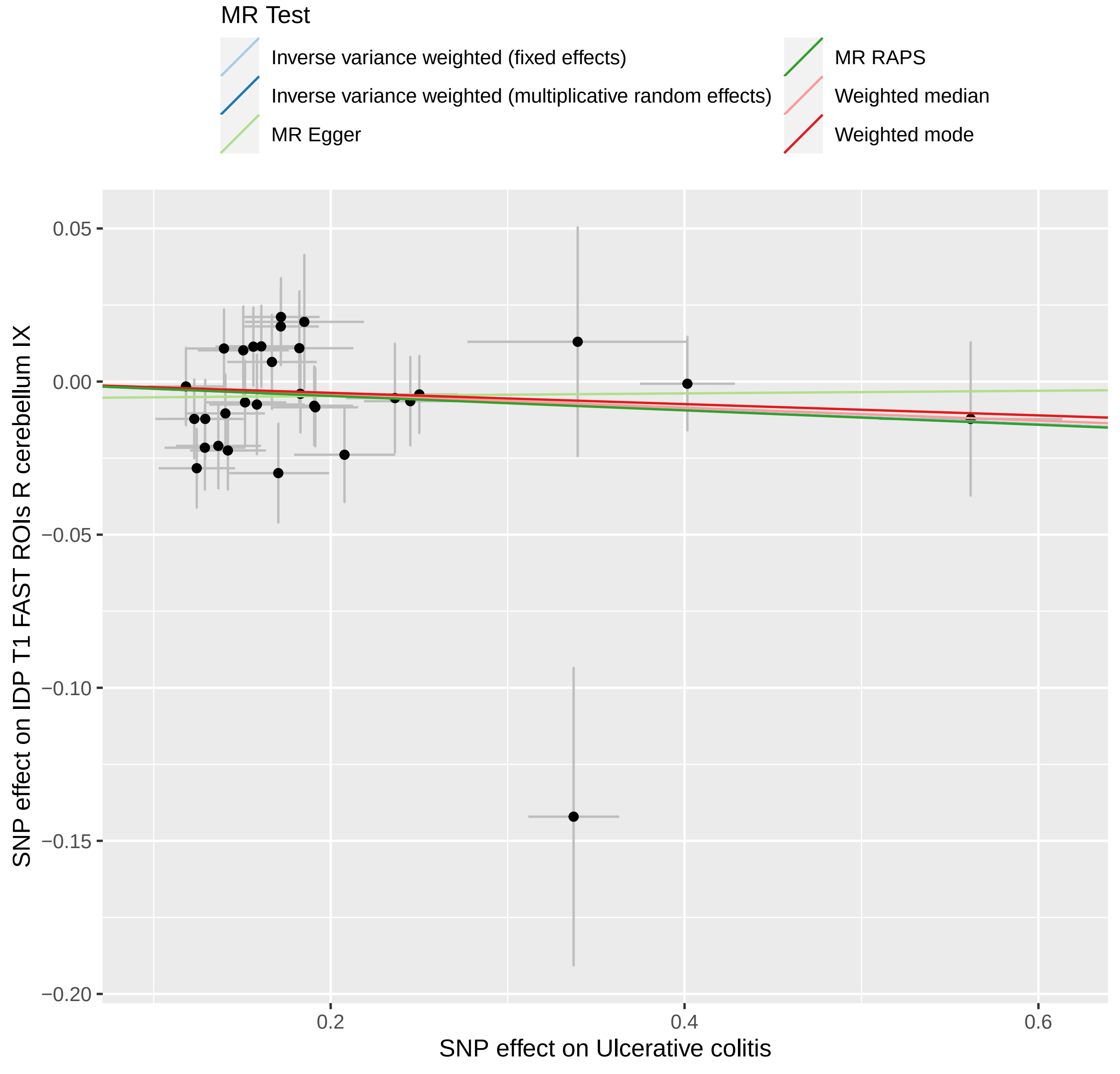

Batch 141 : Crohn's disease on IDP T1 FAST ROIs L cerebellum X

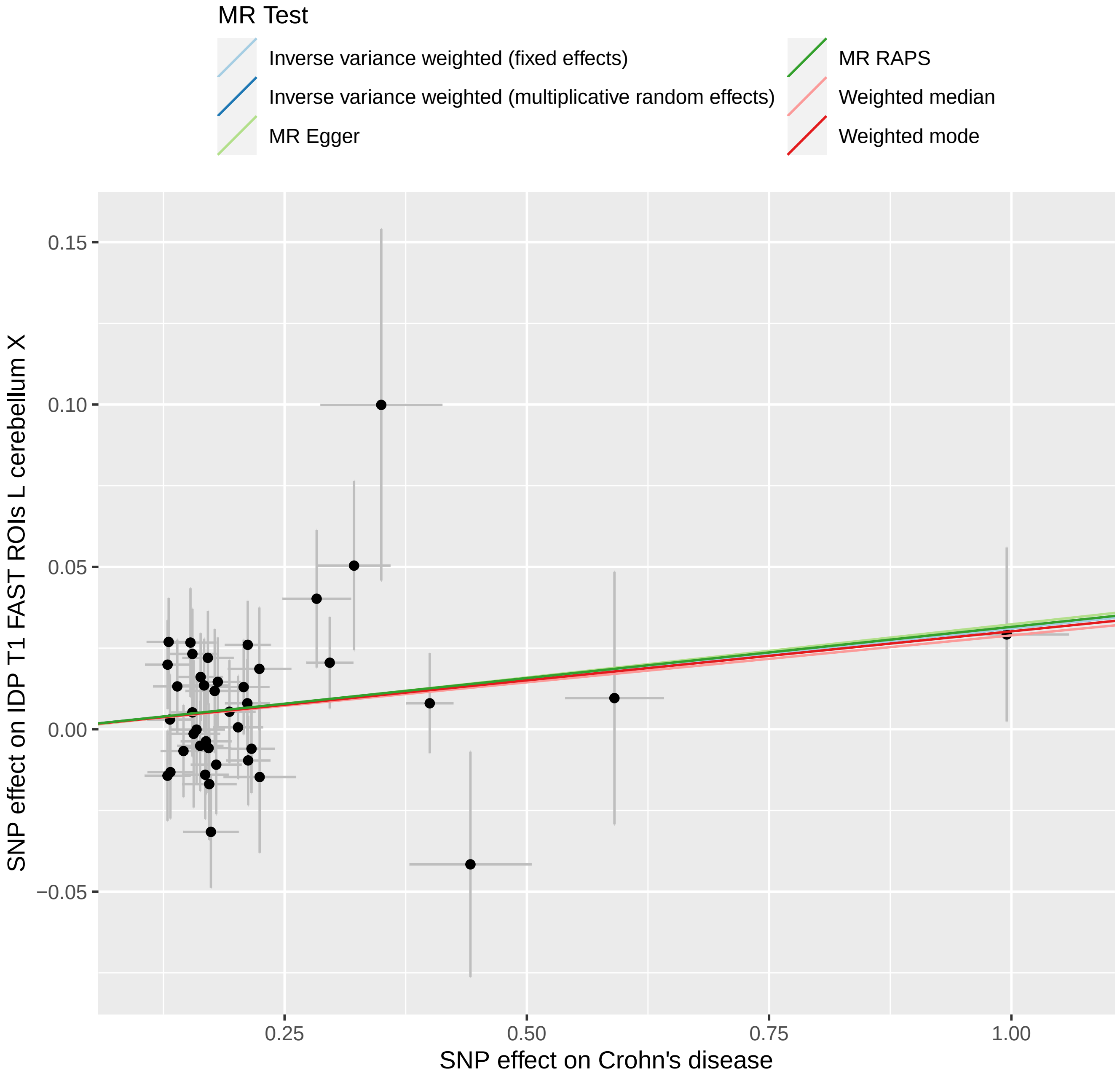

Batch 142 : Ulcerative colitis on IDP T1 FAST ROIs L cerebellum X

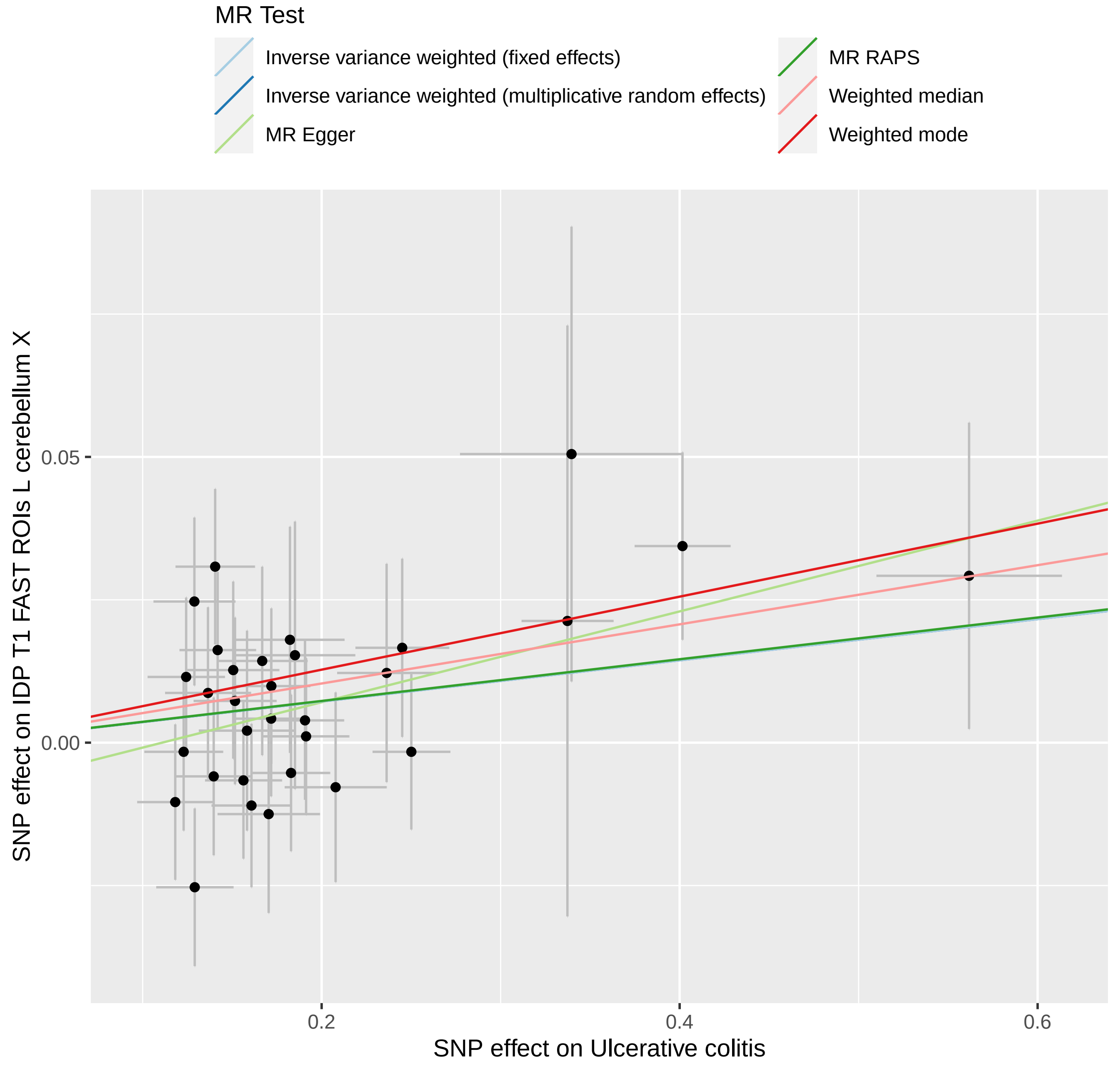

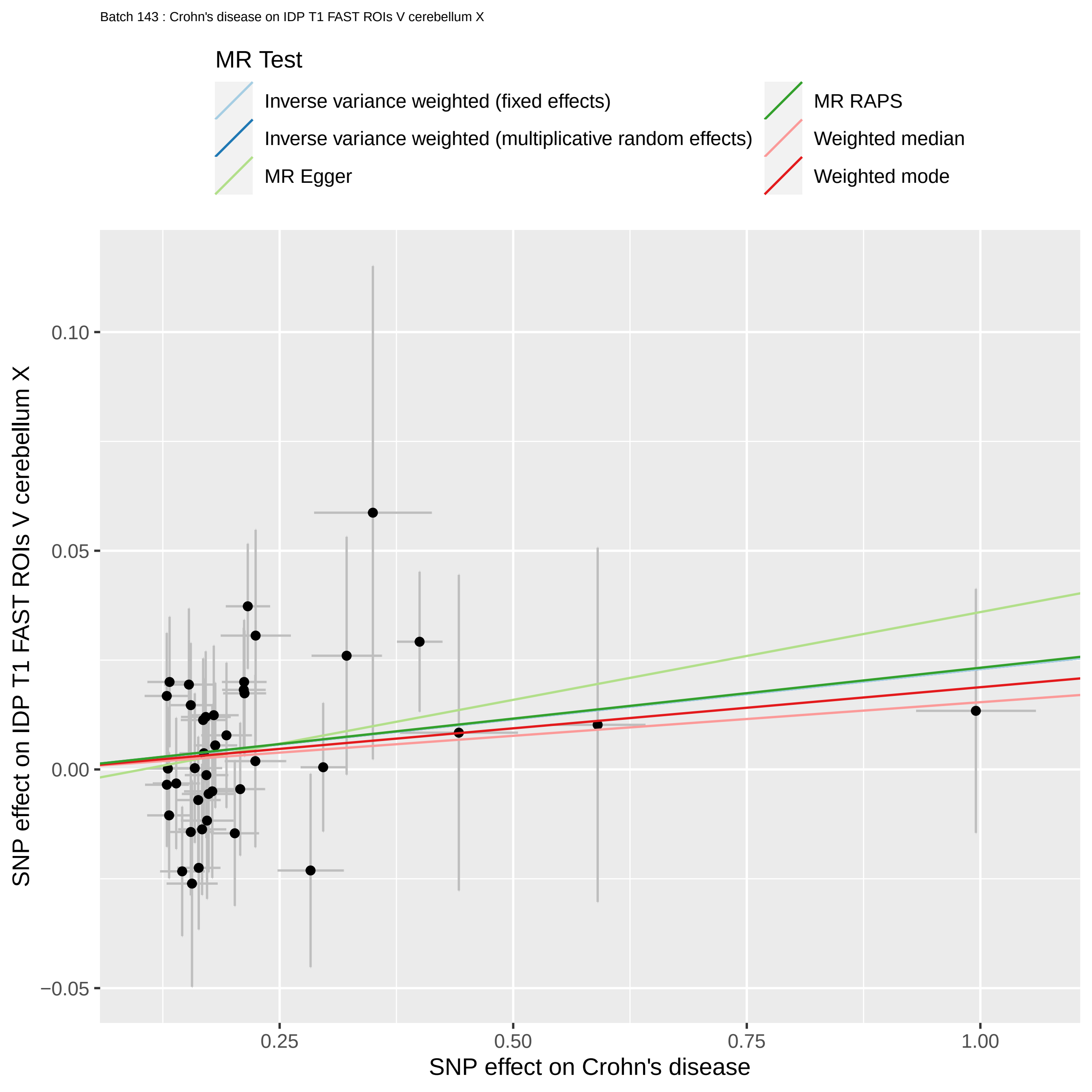
Batch 143 : Crohn's disease on IDP T1 FAST ROIs V cerebellum X
MR Test
Inverse variance weighted (fixed effects)
Inverse variance weighted (multiplicative random effects)
MR Egger
MR RAPS
Weighted median
Weighted mode
0.10
0.05
0.00
−0.05
0.25
0.50
0.75
1.00
SNP effect on IDP T1 FAST ROIs V cerebellum X
SNP effect on Crohn's disease

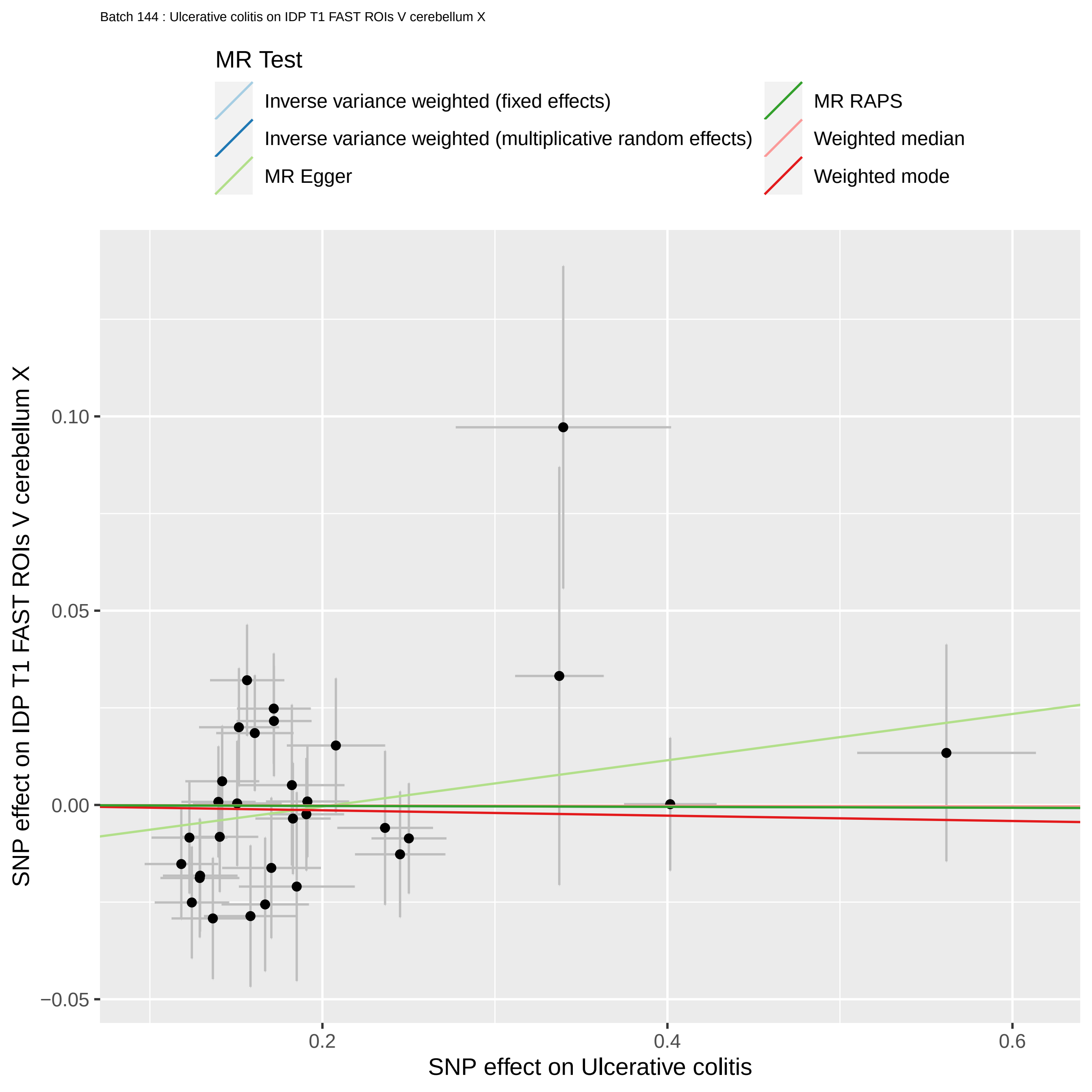

Batch 144 : Ulcerative colitis on IDP T1 FAST ROIs V cerebellum X
MR Test
Inverse variance weighted (fixed effects)
Inverse variance weighted (multiplicative random effects)
MR Egger
MR RAPS
Weighted median
Weighted mode
SNP effect on IDP T1 FAST ROIs V cerebellum X
0.10
0.05
0.00
−0.05
0.2
0.4
0.6
SNP effect on Ulcerative colitis

Batch 145 : Crohn's disease on IDP T1 FAST ROIs R cerebellum X

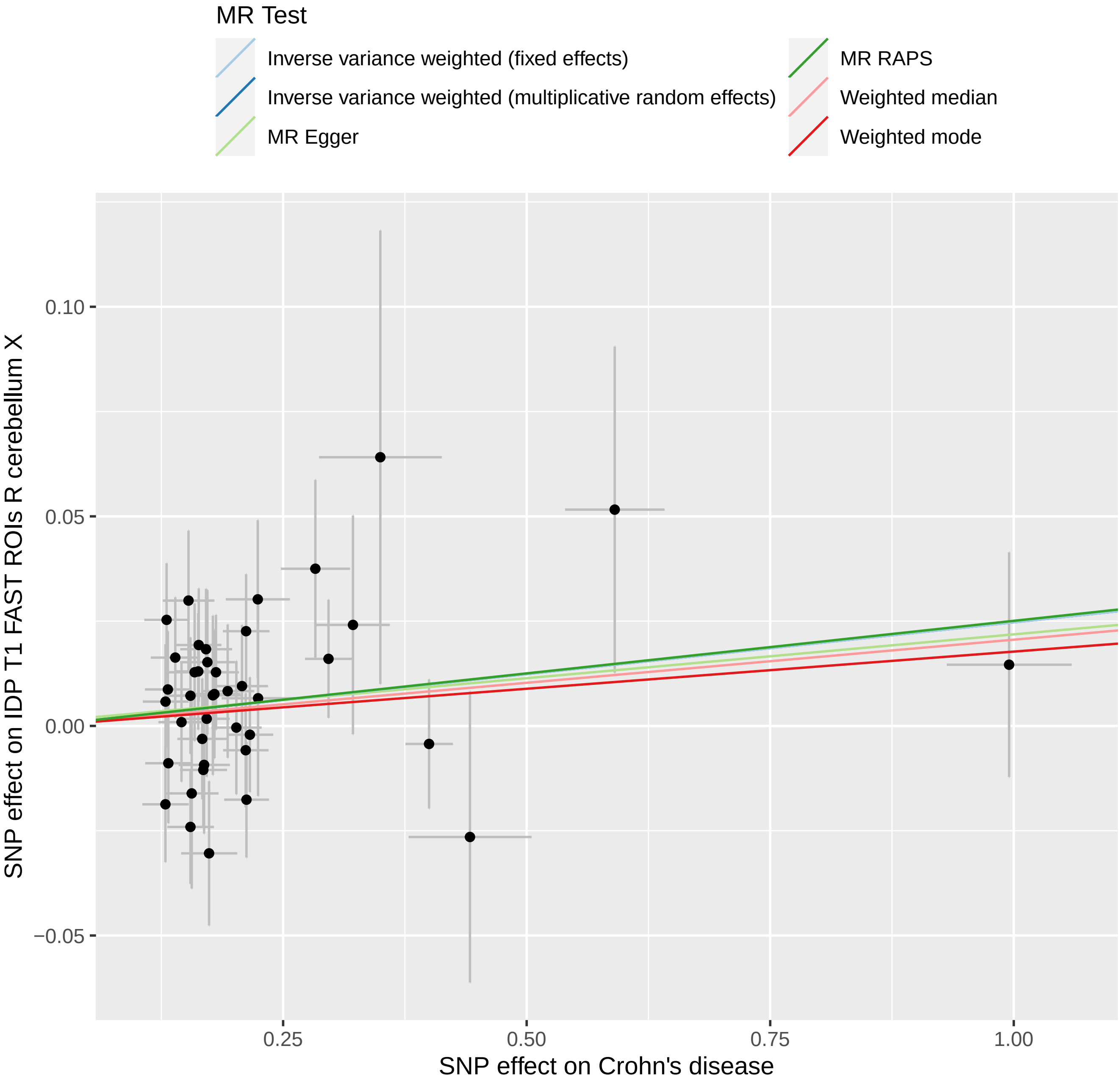

Batch 146 : Ulcerative colitis on IDP T1 FAST ROIs R cerebellum X

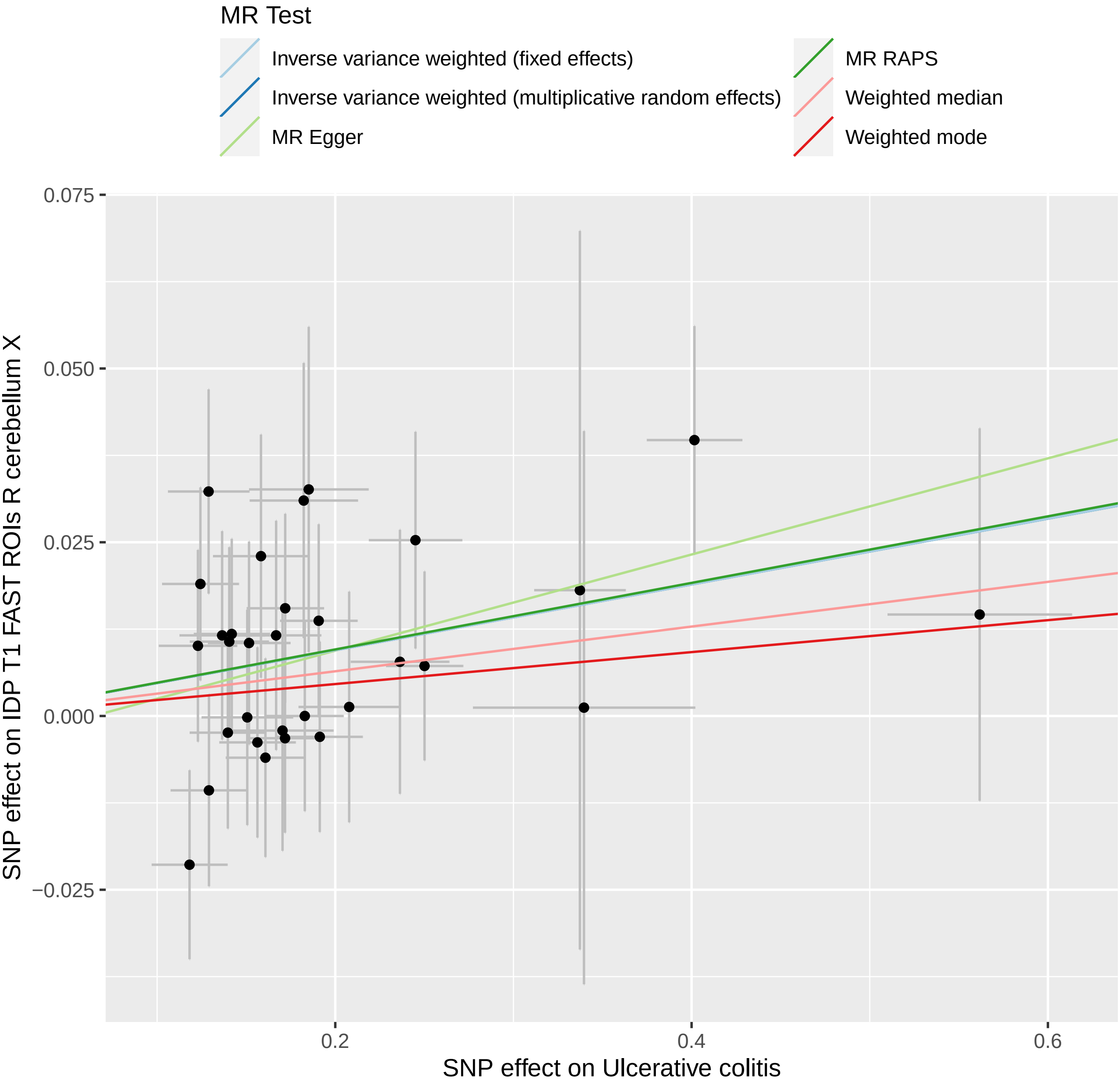

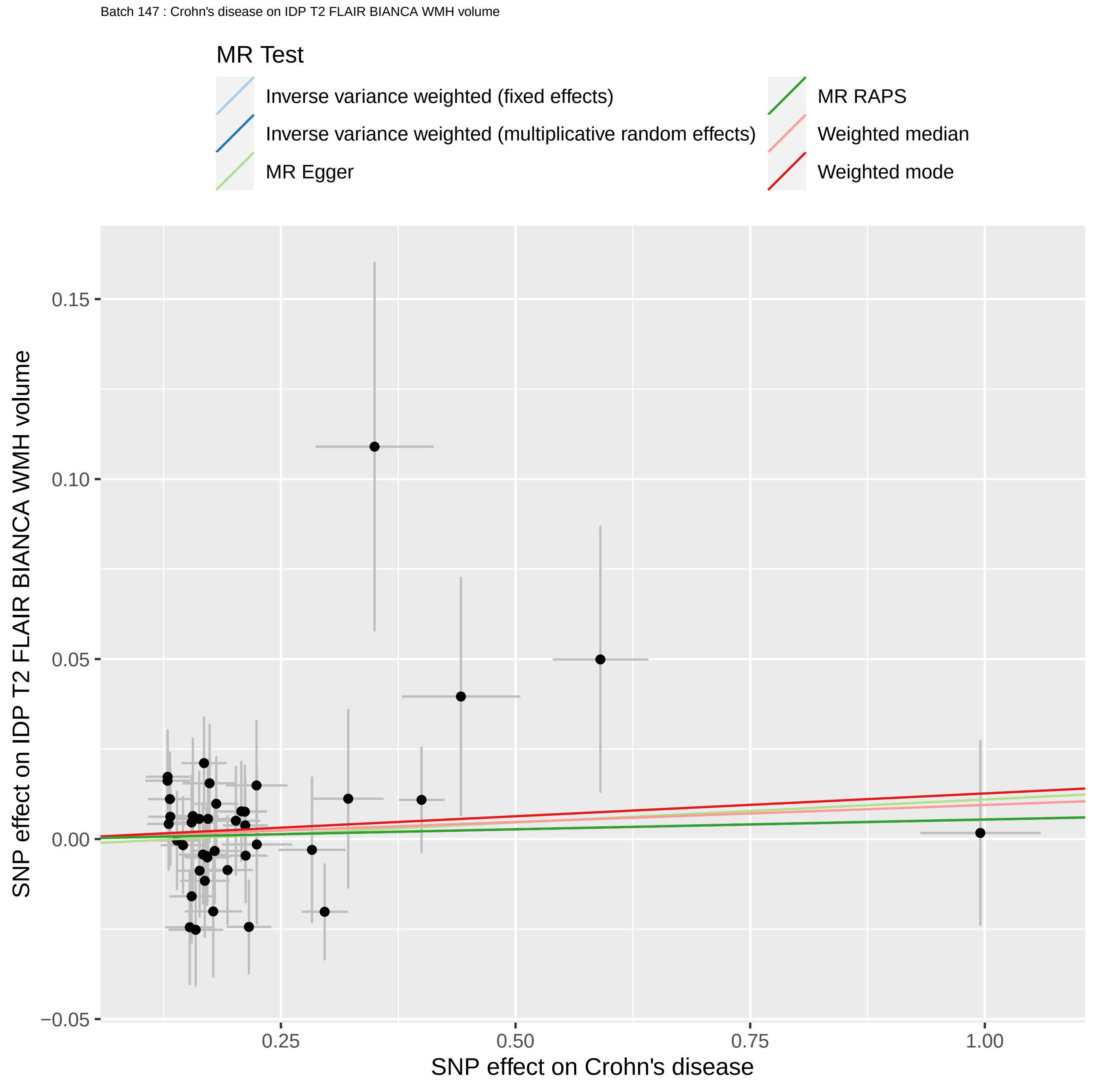
Batch 147 : Crohn's disease on IDP T2 FLAIR BIANCA WMH volume
MR Test
Inverse variance weighted (fixed effects)
Inverse variance weighted (multiplicative random effects)
MR Egger
MR RAPS
Weighted median
Weighted mode
SNP effect on IDP T2 FLAIR BIANCA WMH volume
0.15
0.10
0.05
0.00
−0.05
0.25
0.50
0.75
1.00
SNP effect on Crohn's disease

Batch 148 : Ulcerative colitis on IDP T2 FLAIR BIANCA WMH volume

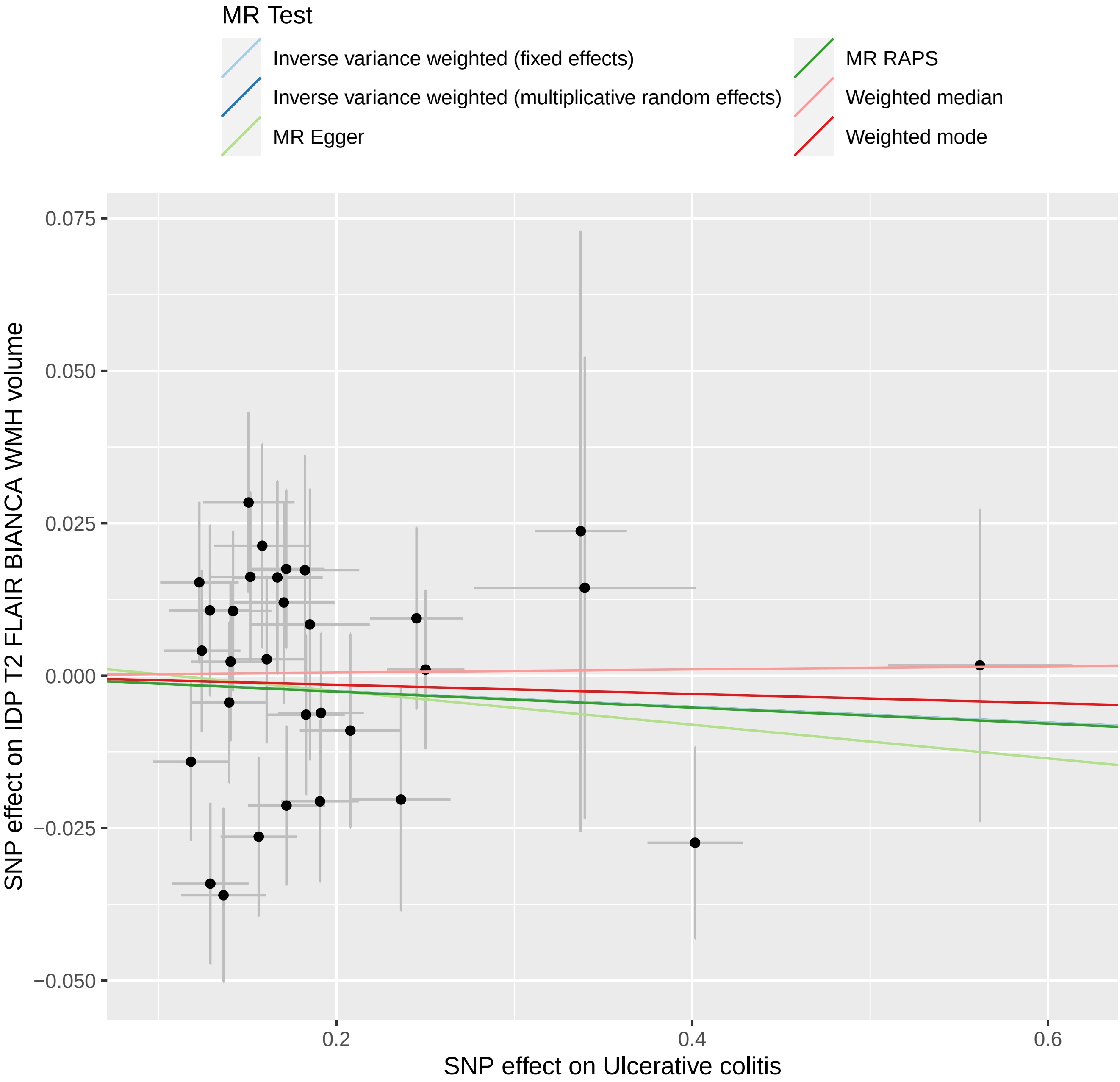

Batch 157 : Crohn's disease on IDP T1 FIRST left pallidum volume

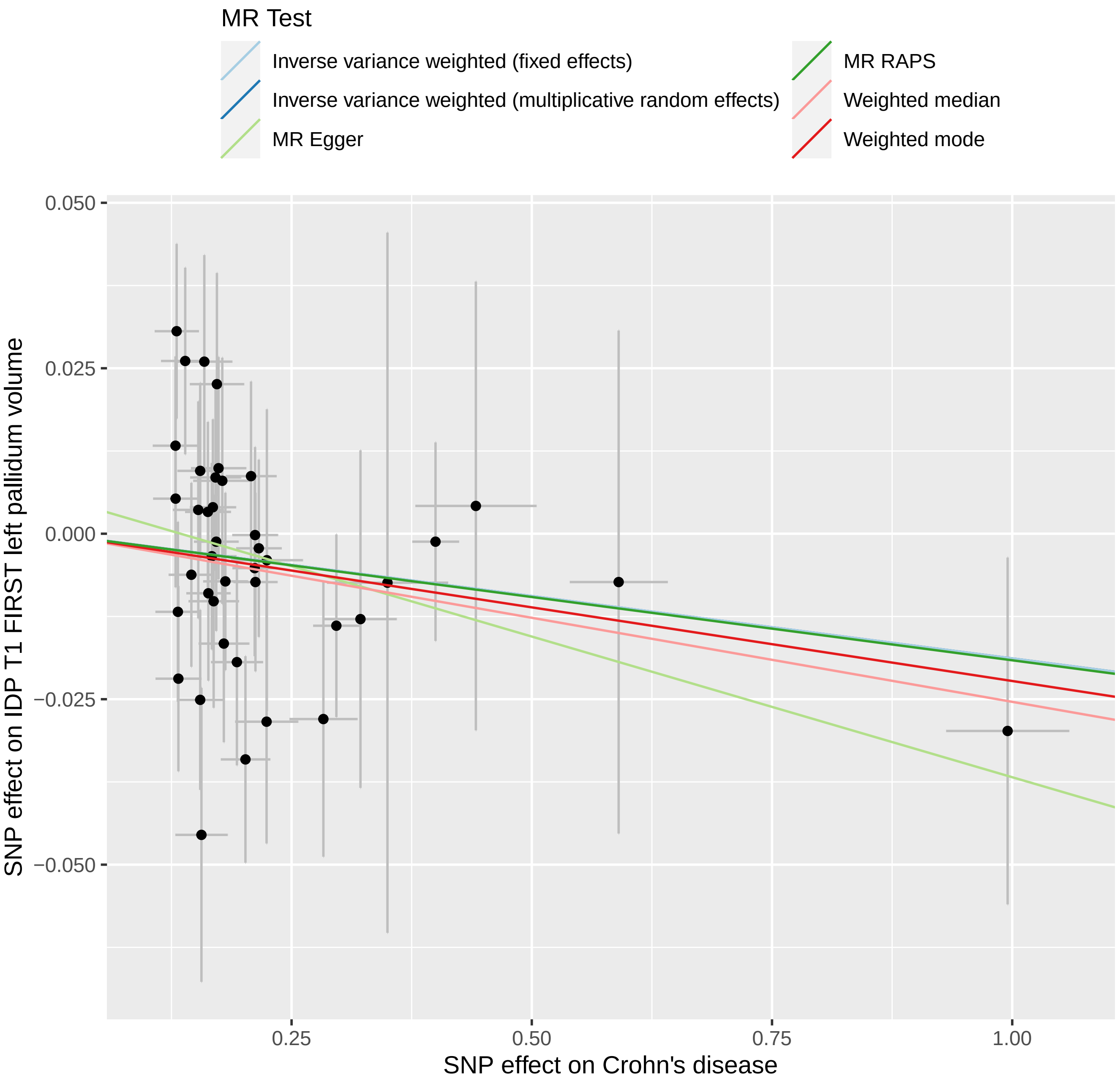

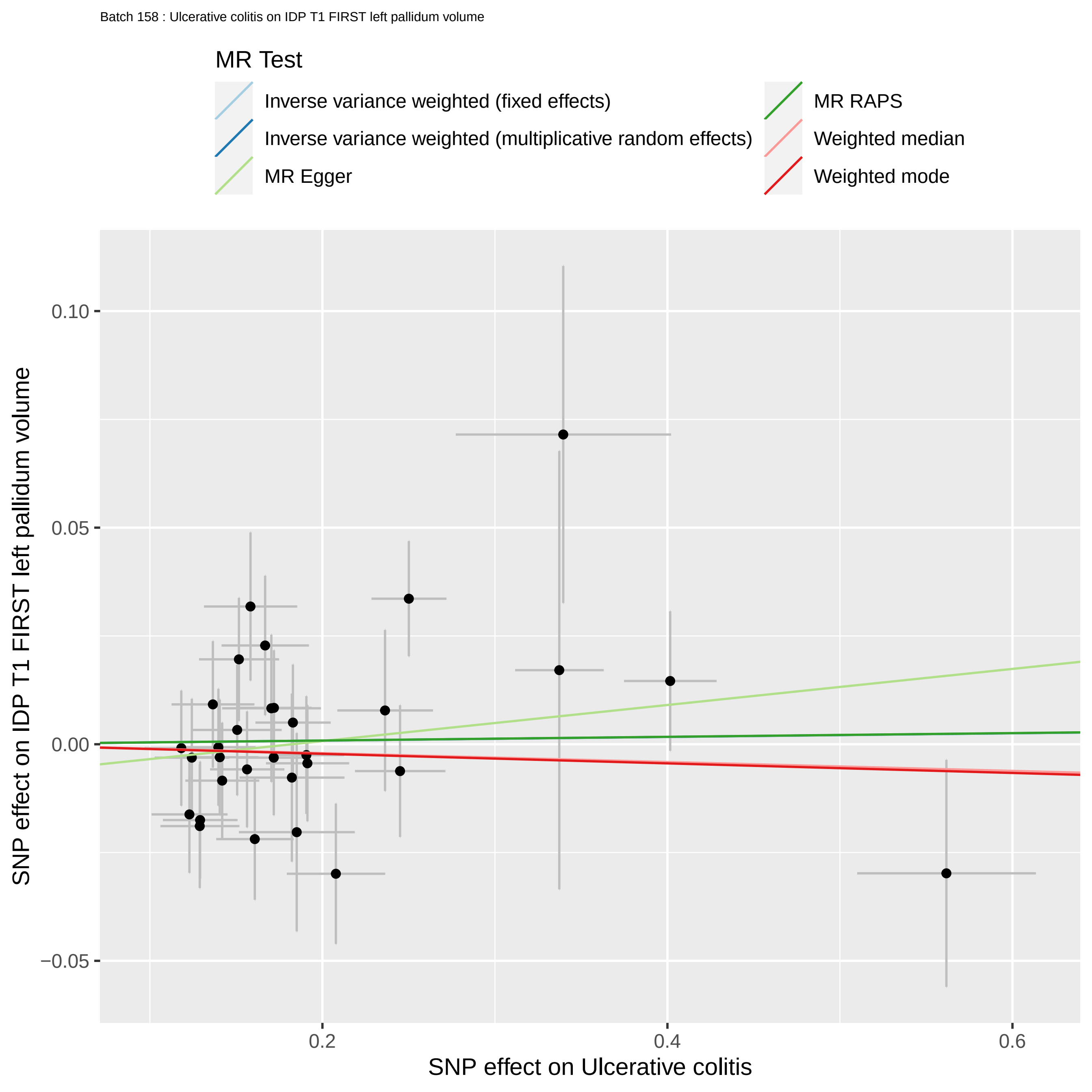
Batch 158 : Ulcerative colitis on IDP T1 FIRST left pallidum volume
MR Test
Inverse variance weighted (fixed effects)
Inverse variance weighted (multiplicative random effects)
MR Egger
MR RAPS
Weighted median
Weighted mode
0.10
0.05
0.00
−0.05
0.2
0.4
0.6
SNP effect on IDP T1 FIRST left pallidum volume
SNP effect on Ulcerative colitis

Batch 179 : Crohn's disease on IDP T1 FIRST right pallidum volume

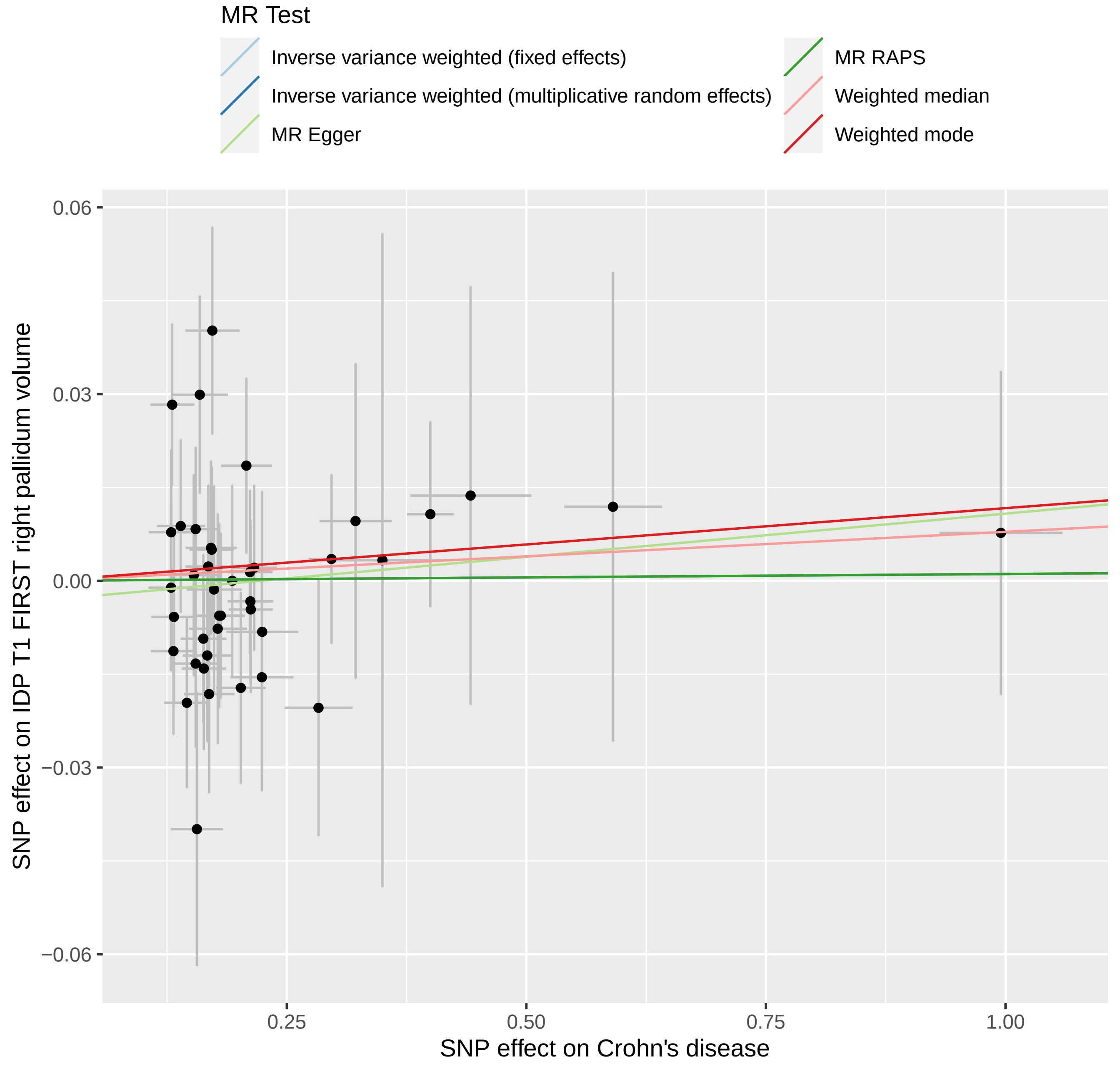

Batch 180 : Ulcerative colitis on IDP T1 FIRST right pallidum volume

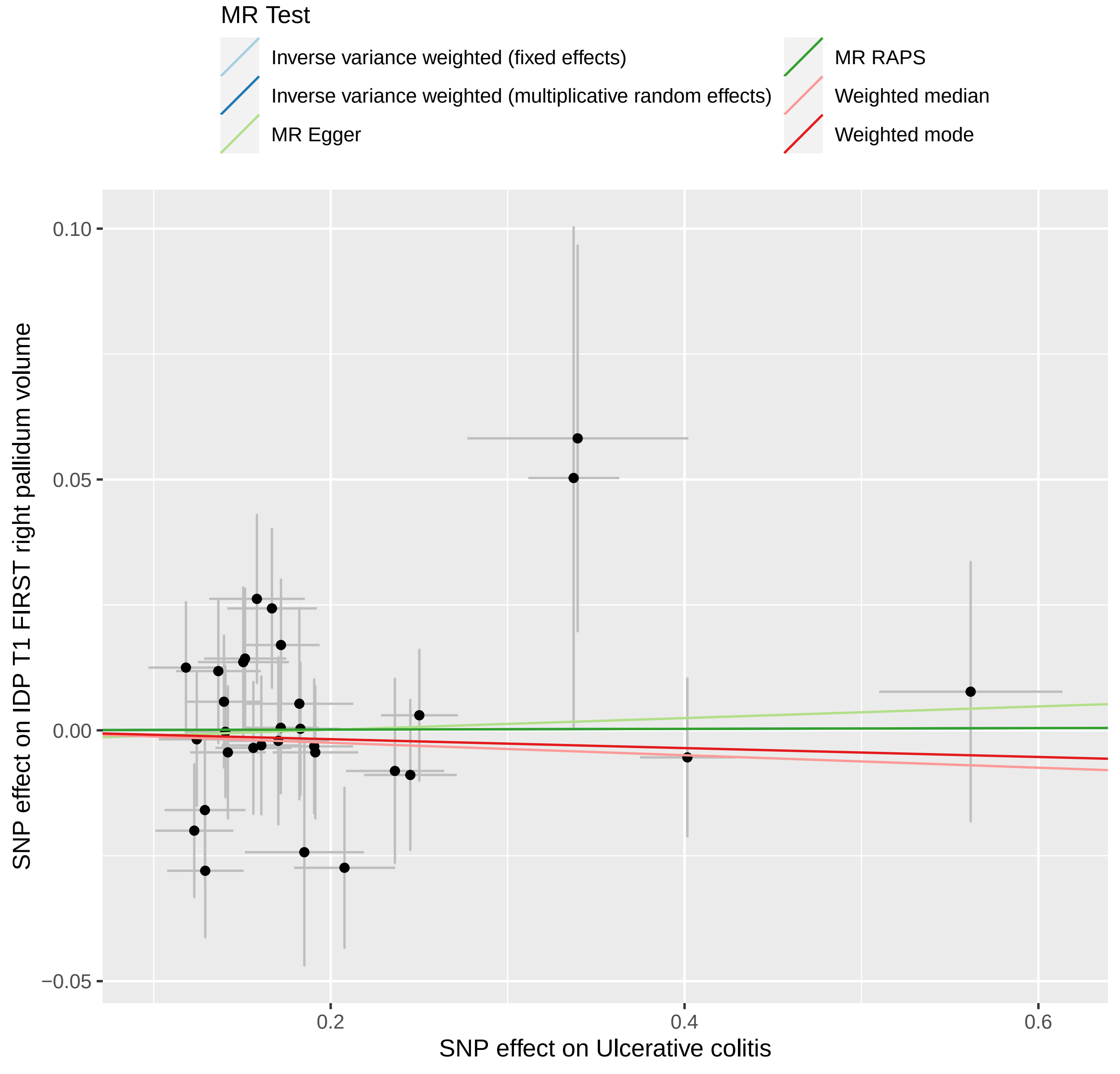

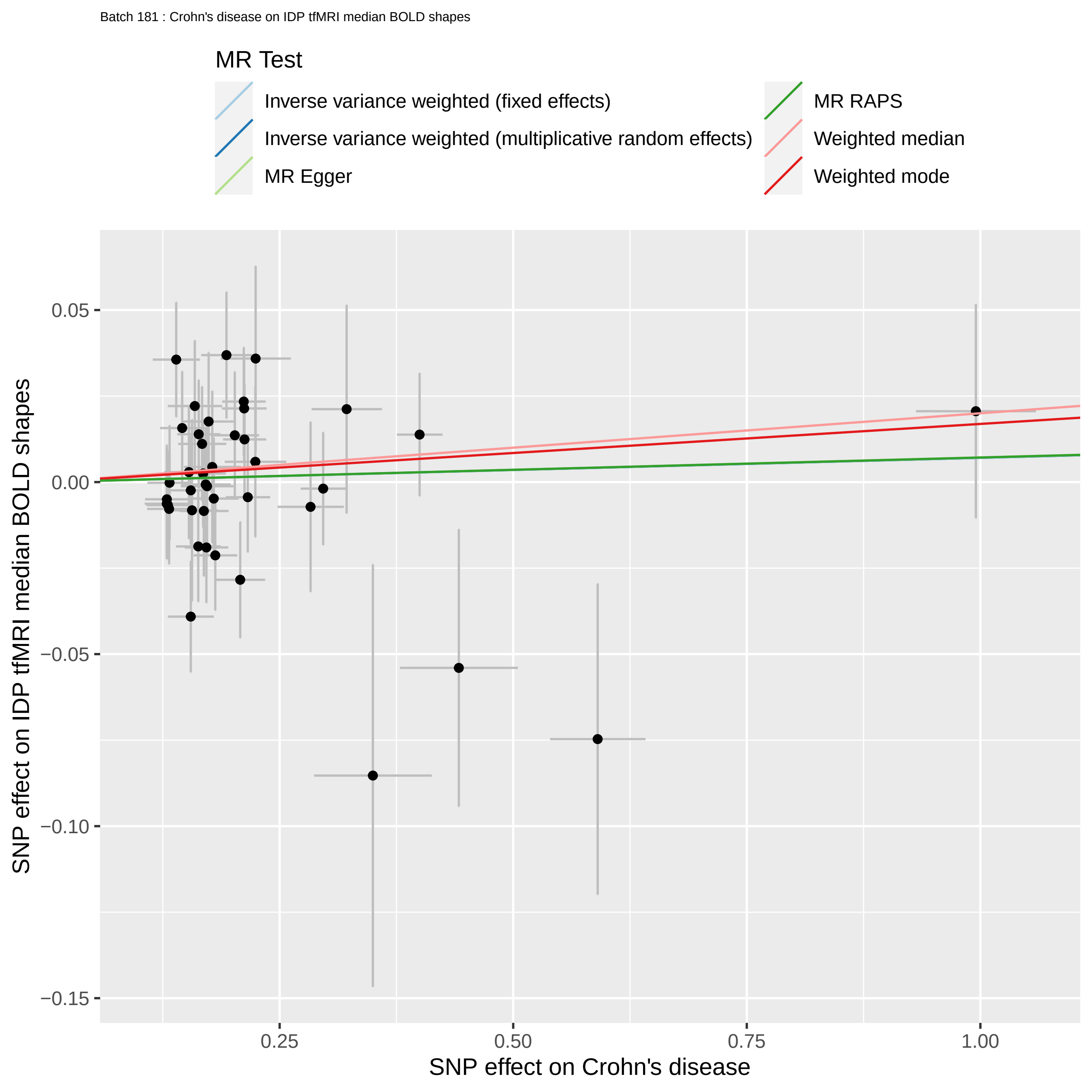

Batch 181 : Crohn's disease on IDP tfMRI median BOLD shapes
MR Test
Inverse variance weighted (fixed effects)
Inverse variance weighted (multiplicative random effects)
MR Egger
MR RAPS
Weighted median
Weighted mode
SNP effect on IDP tfMRI median BOLD shapes
0.05
0.00
−0.05
−0.10
−0.15
0.25
0.50
0.75
1.00
SNP effect on Crohn's disease

Batch 182 : Ulcerative colitis on IDP tfMRI median BOLD shapes

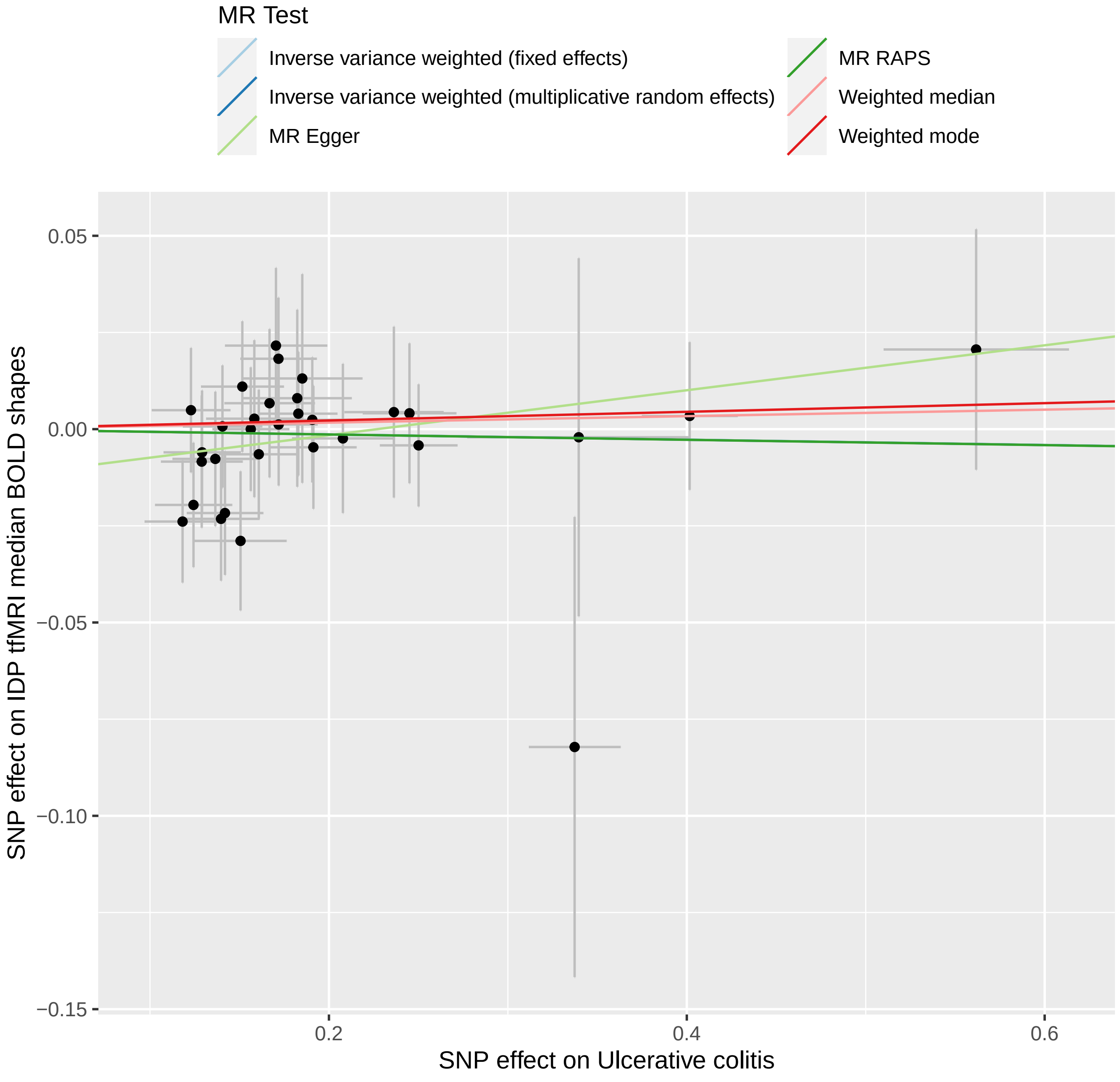

Batch 183 : Crohn's disease on IDP tfMRI 90th−percentile BOLD shapes

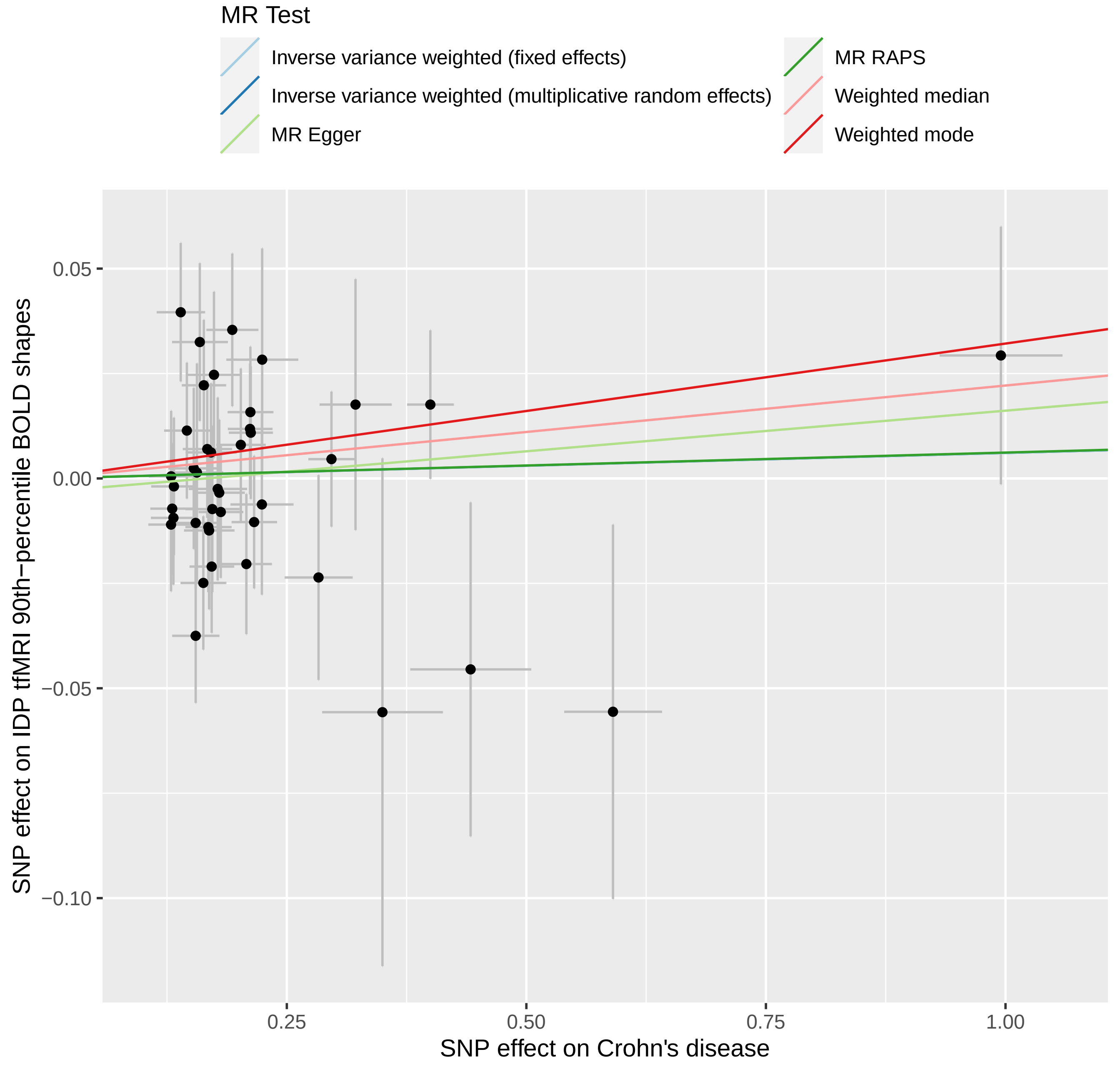

Batch 184 : Ulcerative colitis on IDP tfMRI 90th−percentile BOLD shapes

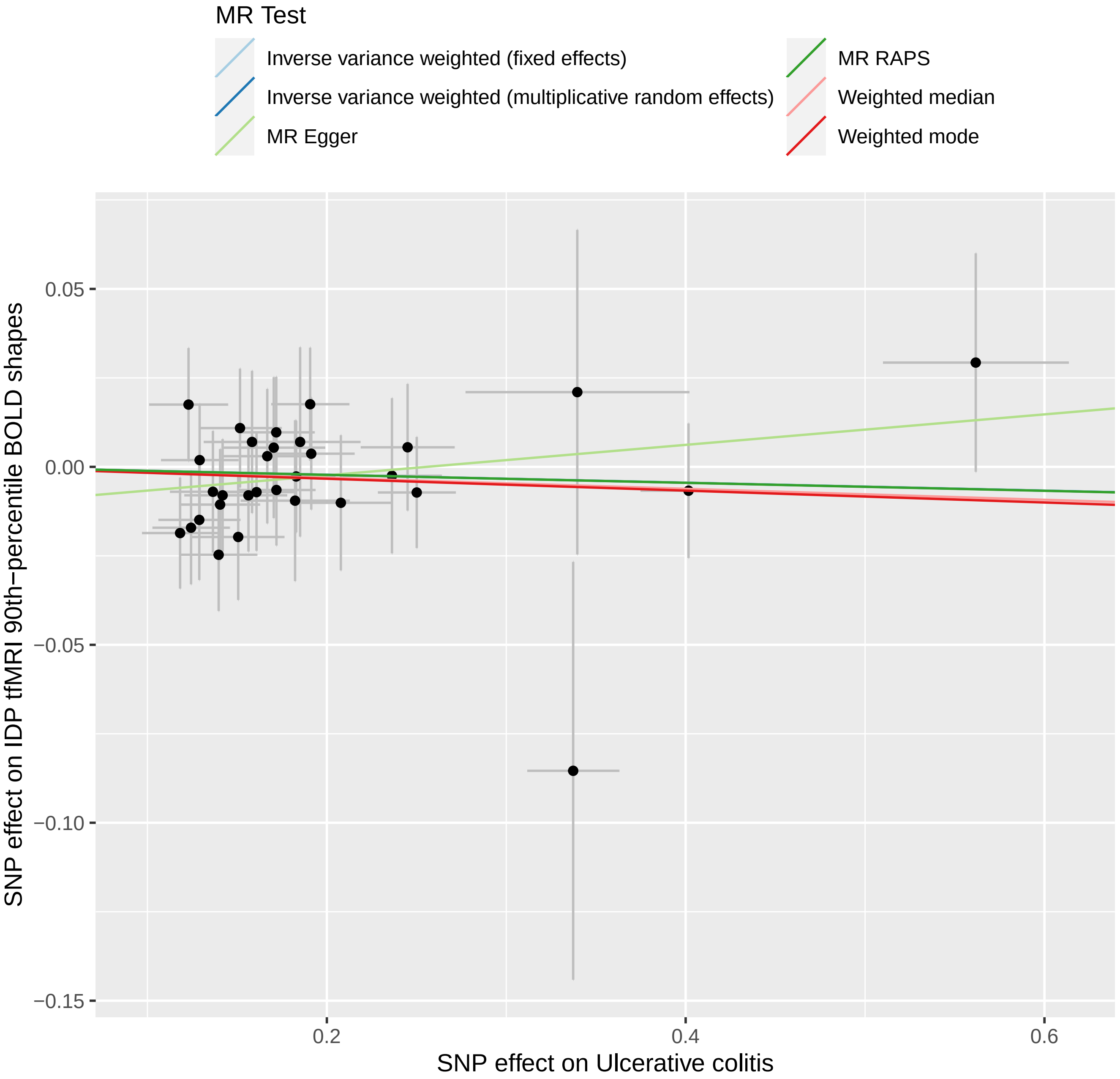

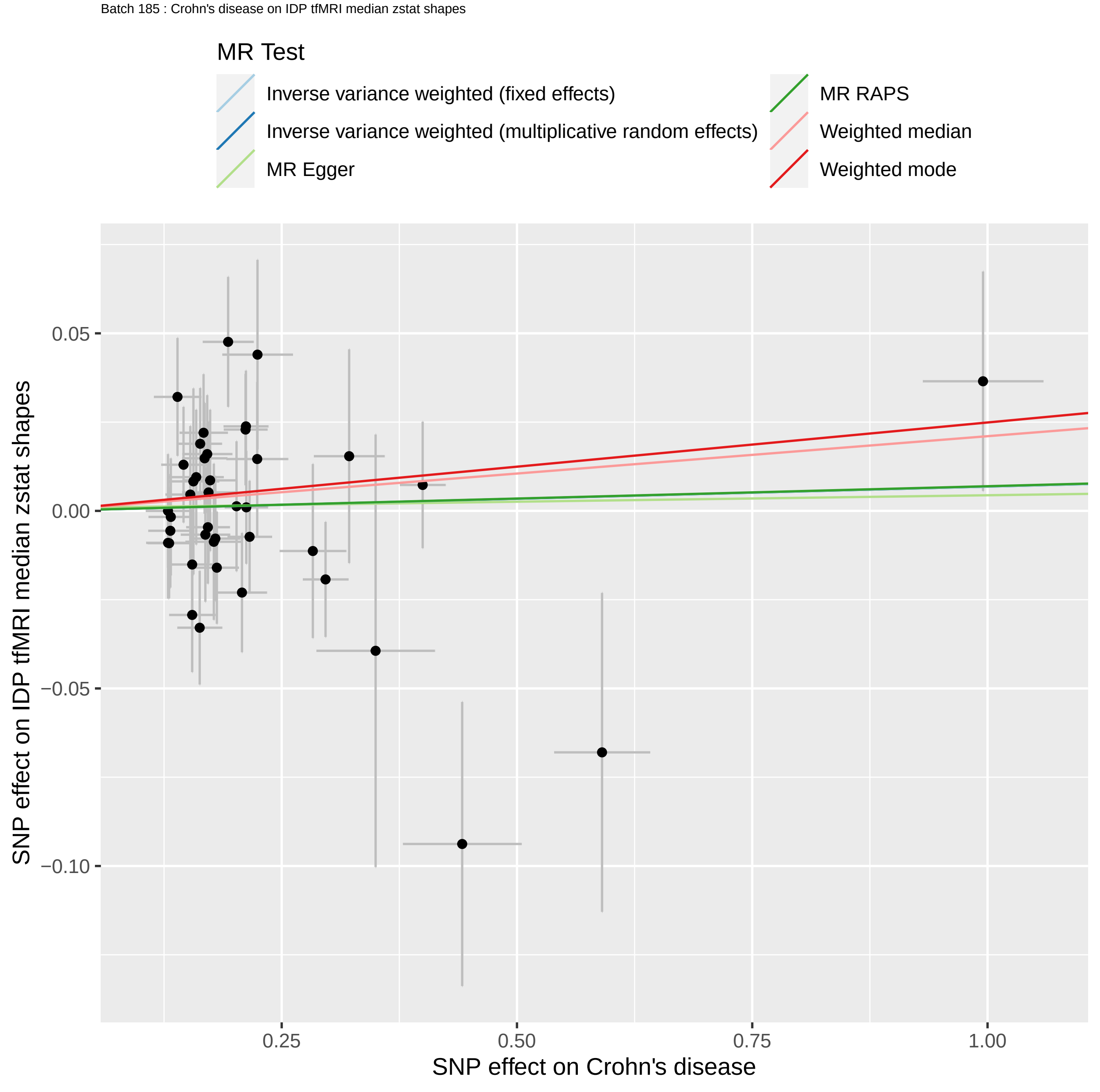
Batch 185 : Crohn's disease on IDP tfMRI median zstat shapes
MR Test
Inverse variance weighted (fixed effects)
Inverse variance weighted (multiplicative random effects)
MR Egger
MR RAPS
Weighted median
Weighted mode
0.05
0.00
−0.05
−0.10
0.25
0.50
0.75
1.00
SNP effect on IDP tfMRI median zstat shapes
SNP effect on Crohn's disease

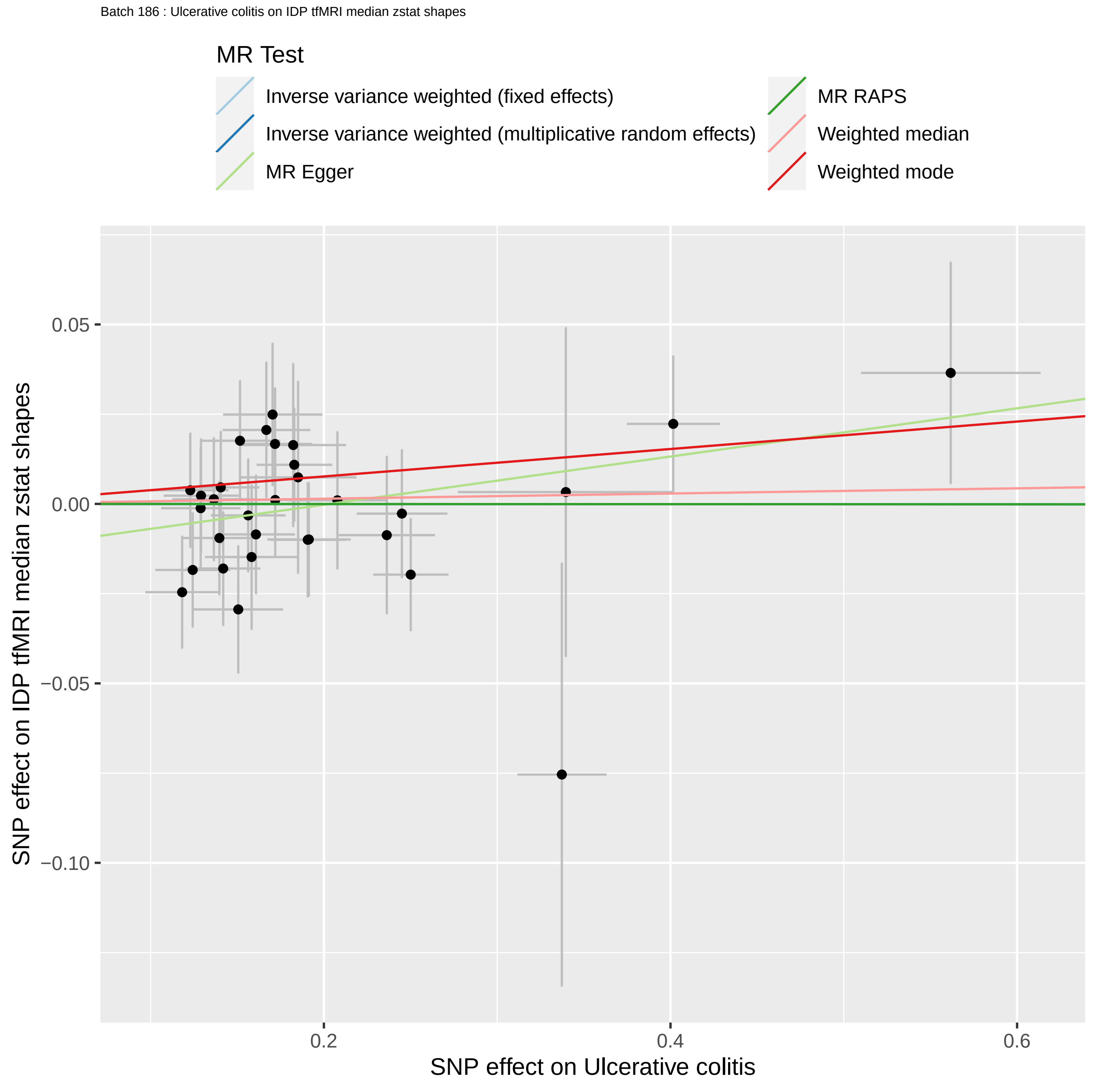
Batch 186 : Ulcerative colitis on IDP tfMRI median zstat shapes
MR Test
Inverse variance weighted (fixed effects)
Inverse variance weighted (multiplicative random effects)
MR Egger
MR RAPS
Weighted median
Weighted mode
SNP effect on IDP tfMRI median zstat shapes
0.05
0.00
−0.05
−0.10
SNP effect on Ulcerative colitis
0.2
0.4
0.6

Batch 187 : Crohn's disease on IDP tfMRI 90th−percentile zstat shapes

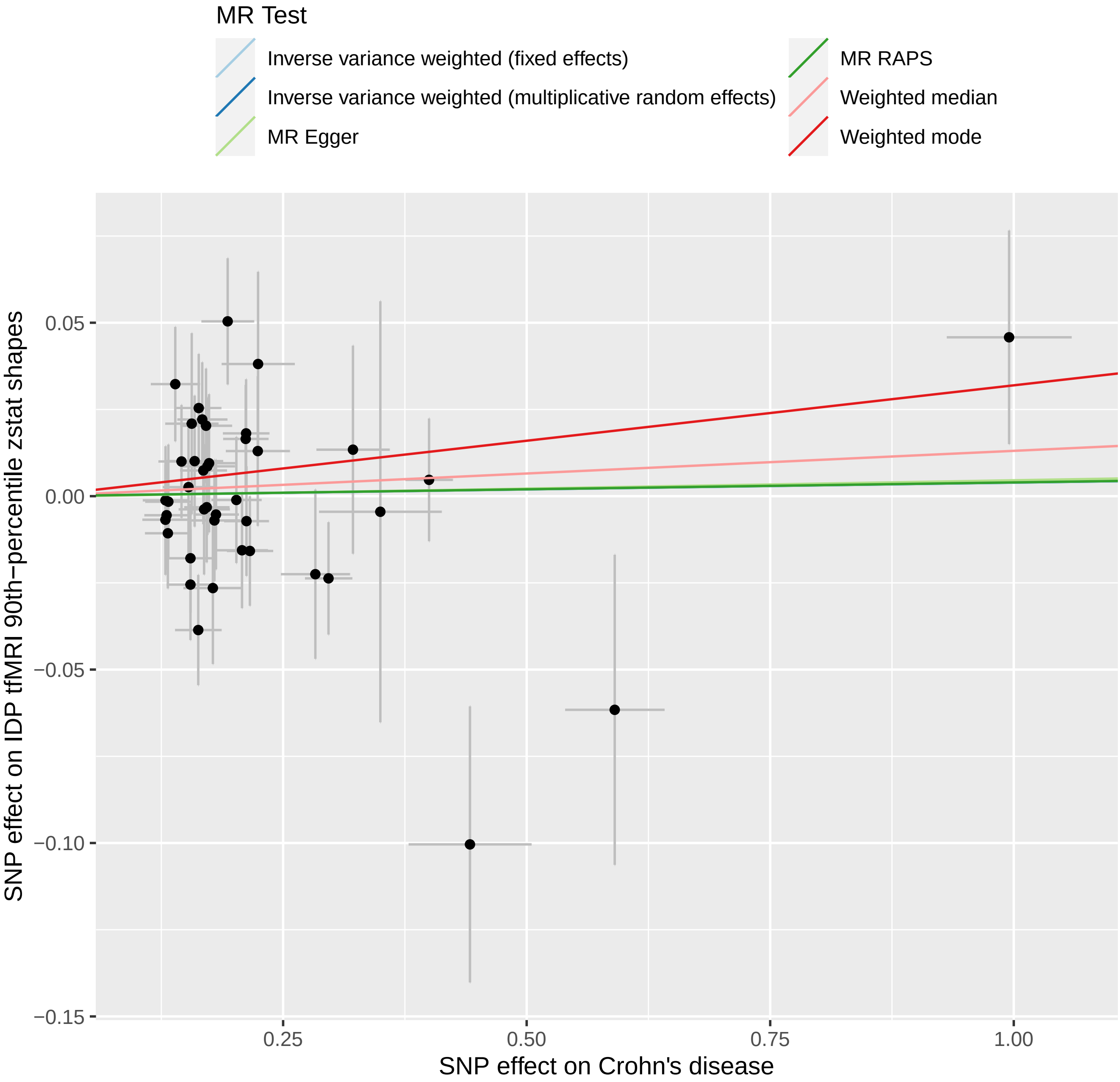

Batch 188 : Ulcerative colitis on IDP tfMRI 90th−percentile zstat shapes

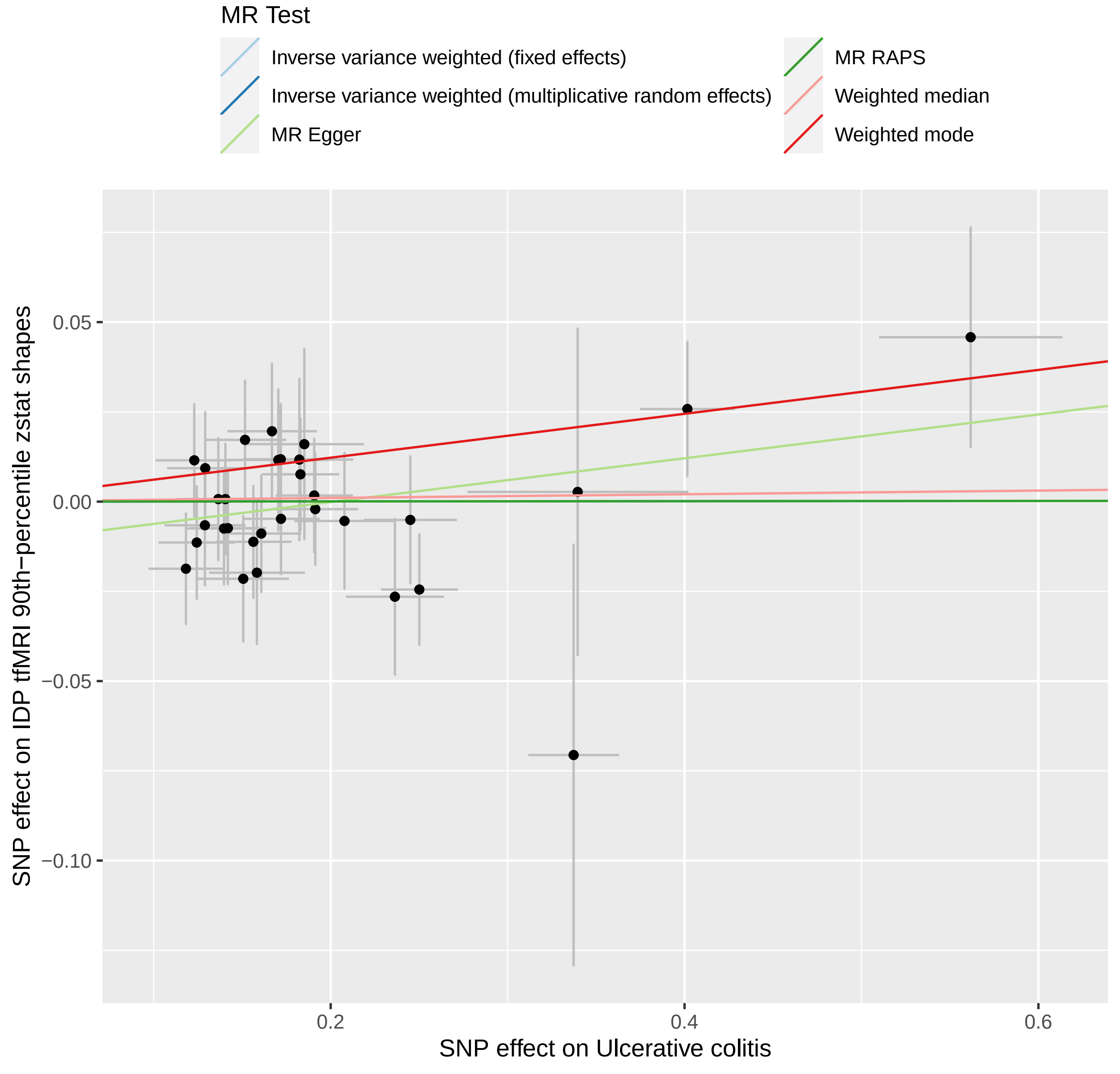

Batch 189 : Crohn's disease on IDP tfMRI median BOLD faces

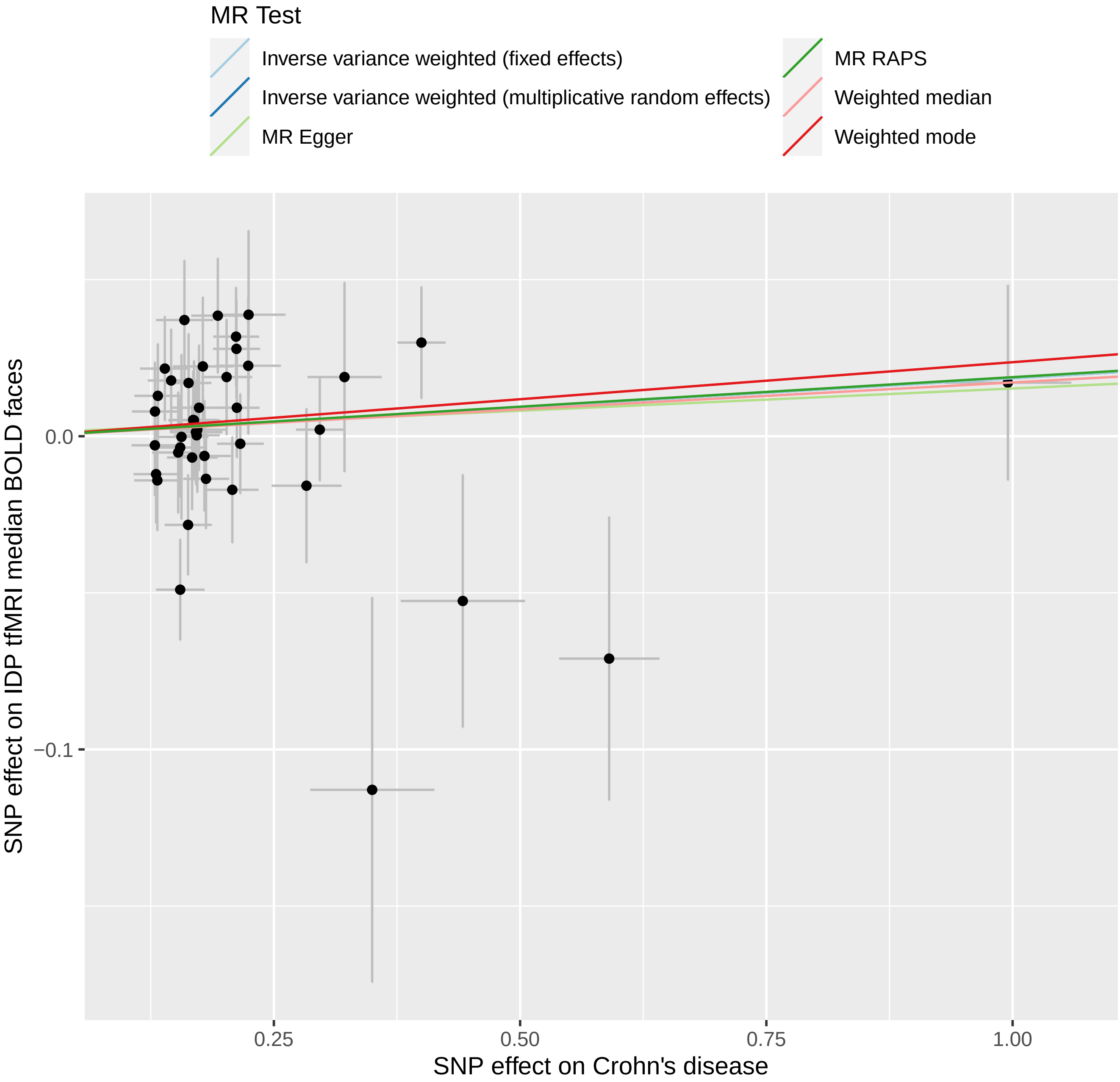

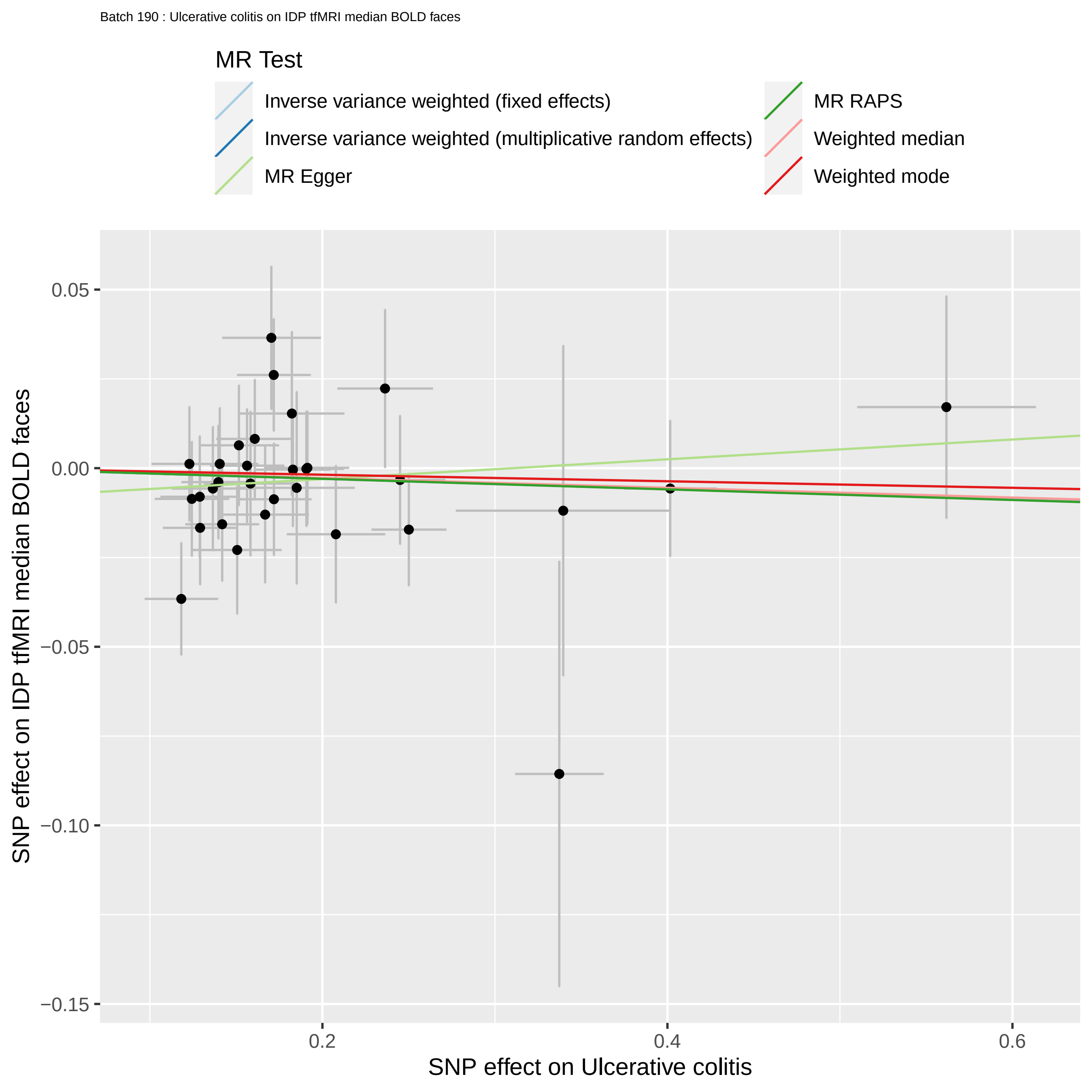

Batch 190 : Ulcerative colitis on IDP tfMRI median BOLD faces
MR Test
Inverse variance weighted (fixed effects)
Inverse variance weighted (multiplicative random effects)
MR Egger
MR RAPS
Weighted median
Weighted mode
0.05
0.00
−0.05
−0.10
−0.15
SNP effect on IDP tfMRI median BOLD faces
0.2
0.4
0.6
SNP effect on Ulcerative colitis

Batch 191 : Crohn's disease on IDP tfMRI 90th−percentile BOLD faces

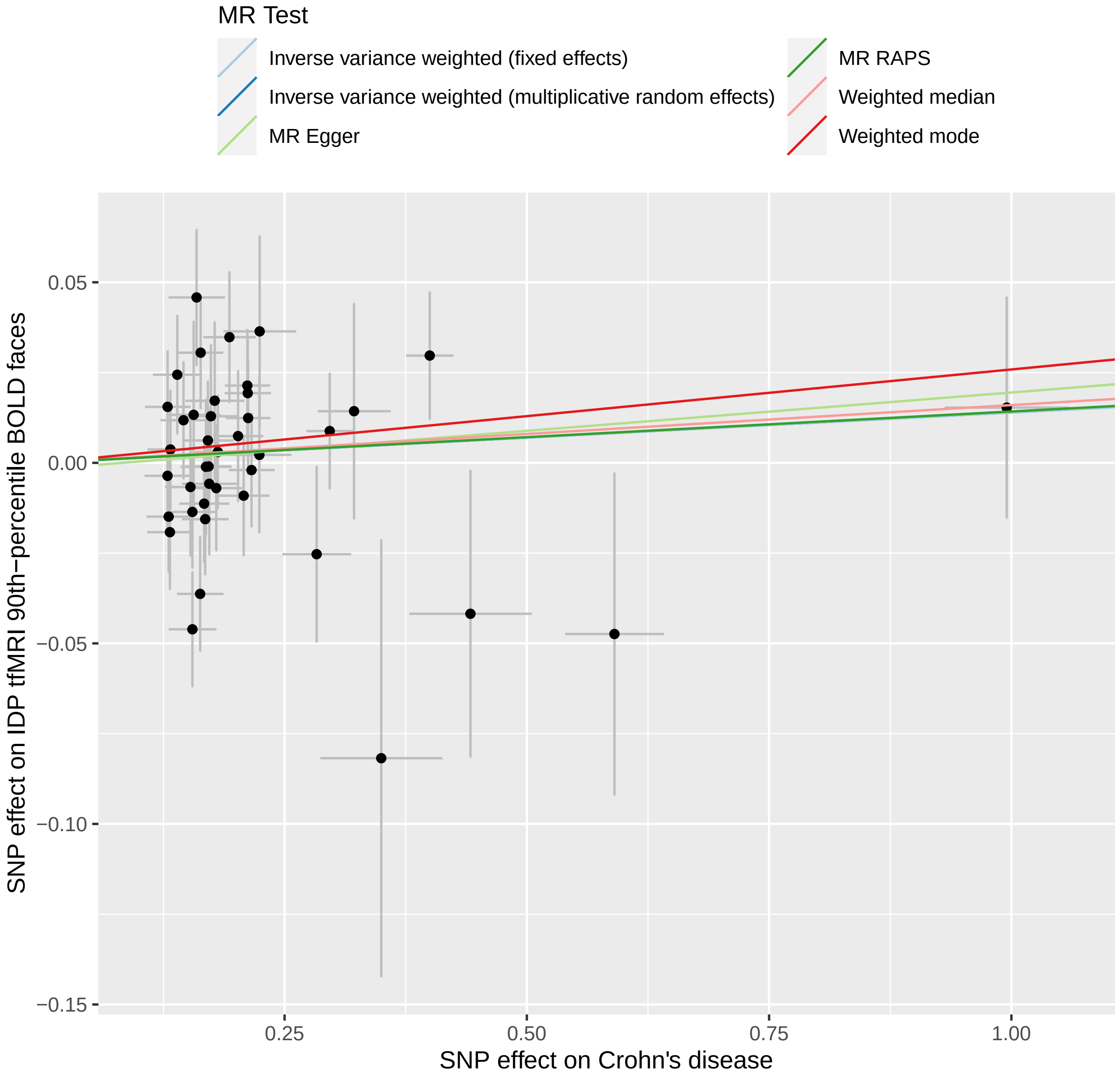

Batch 192 : Ulcerative colitis on IDP tfMRI 90th−percentile BOLD faces

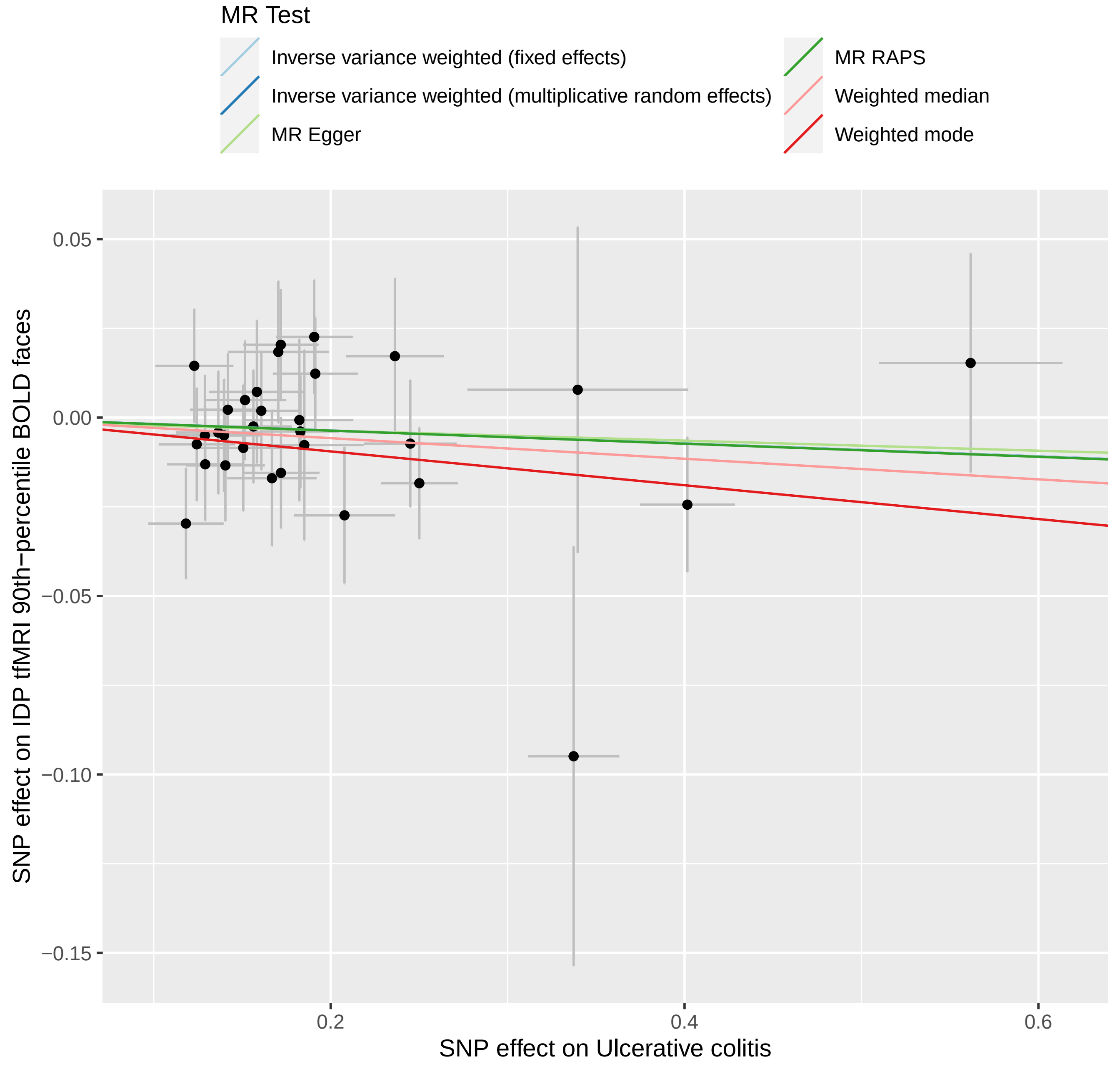

Batch 193 : Crohn's disease on IDP tfMRI median zstat faces

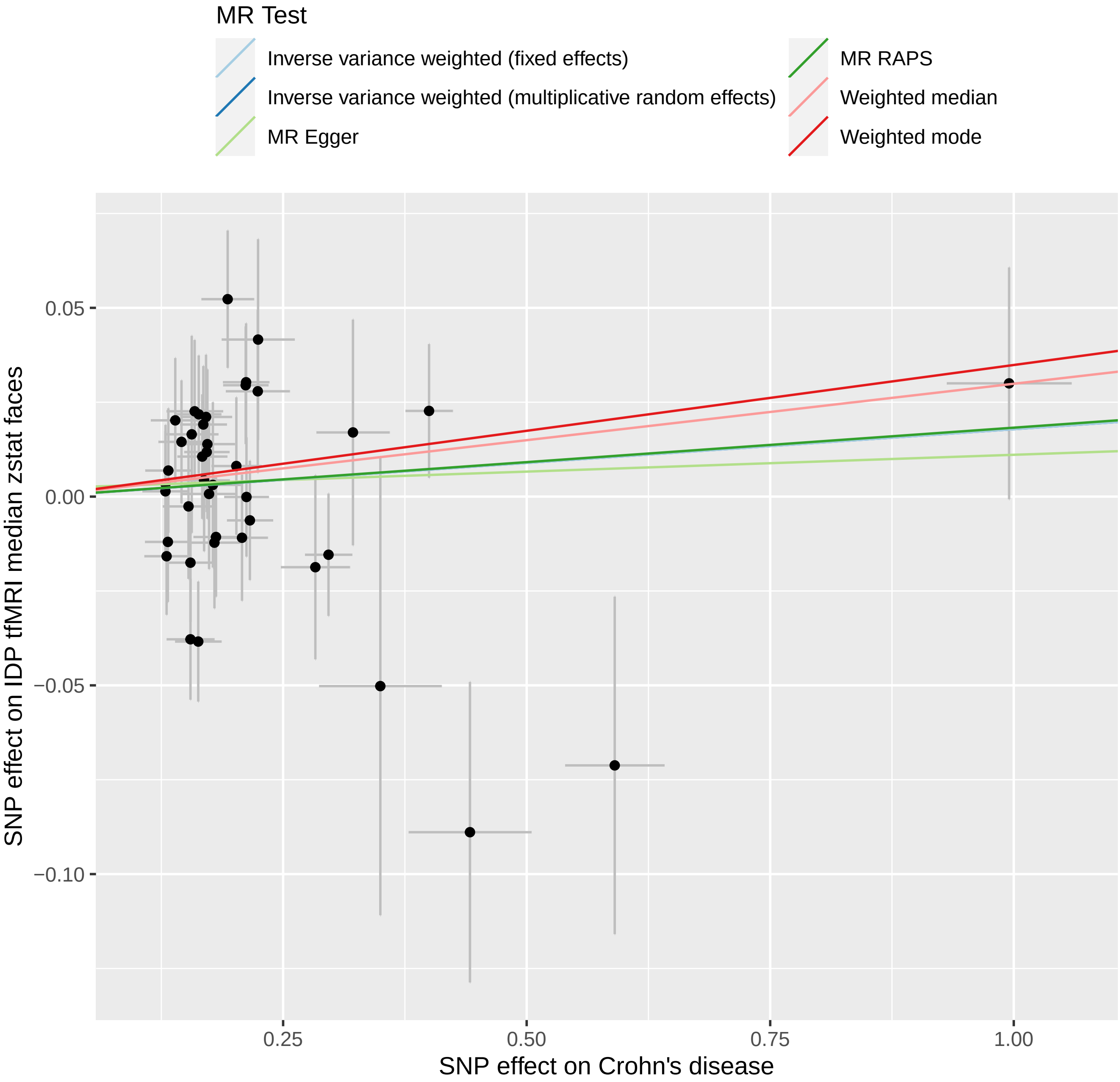

Batch 194 : Ulcerative colitis on IDP tfMRI median zstat faces

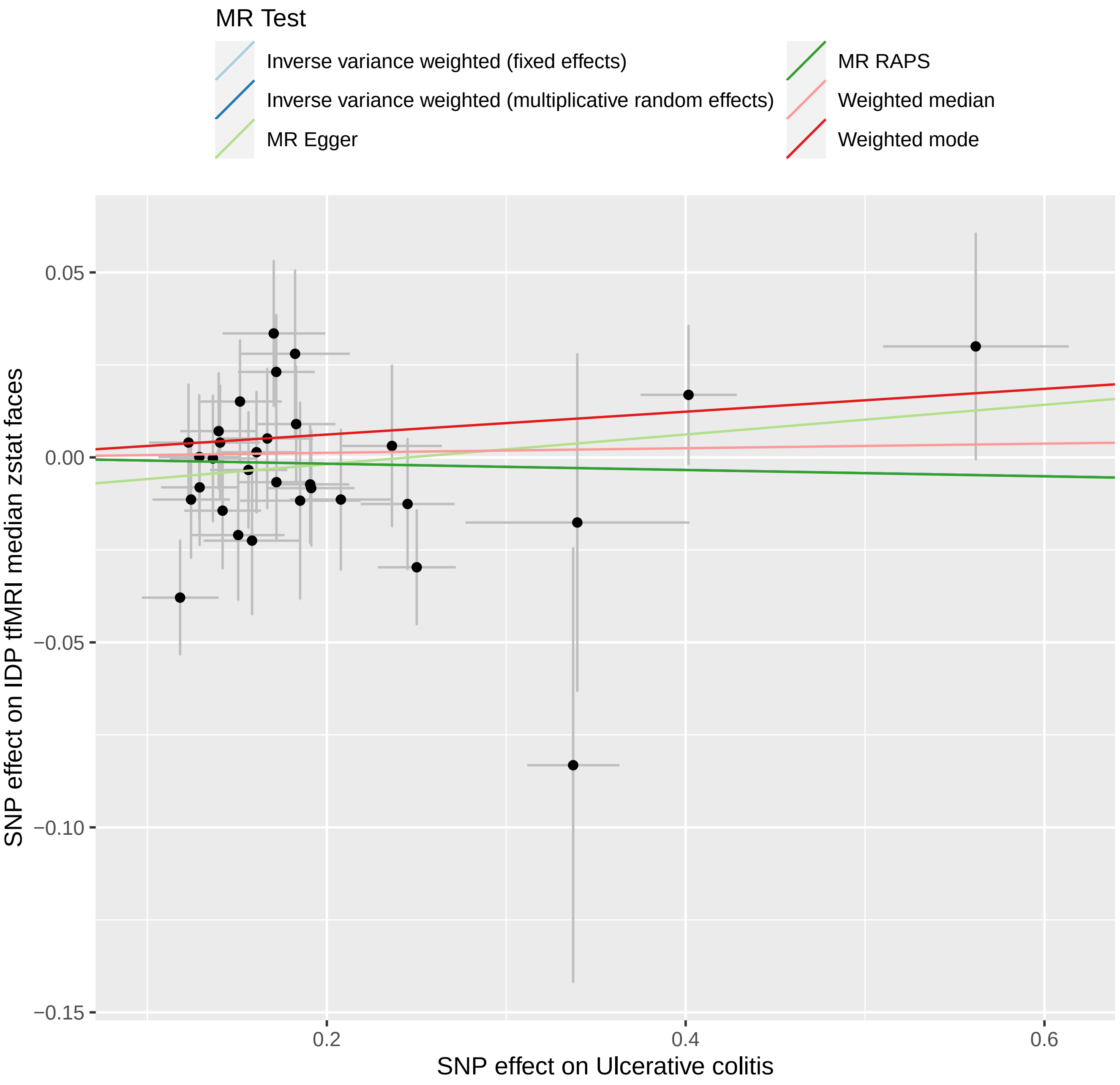

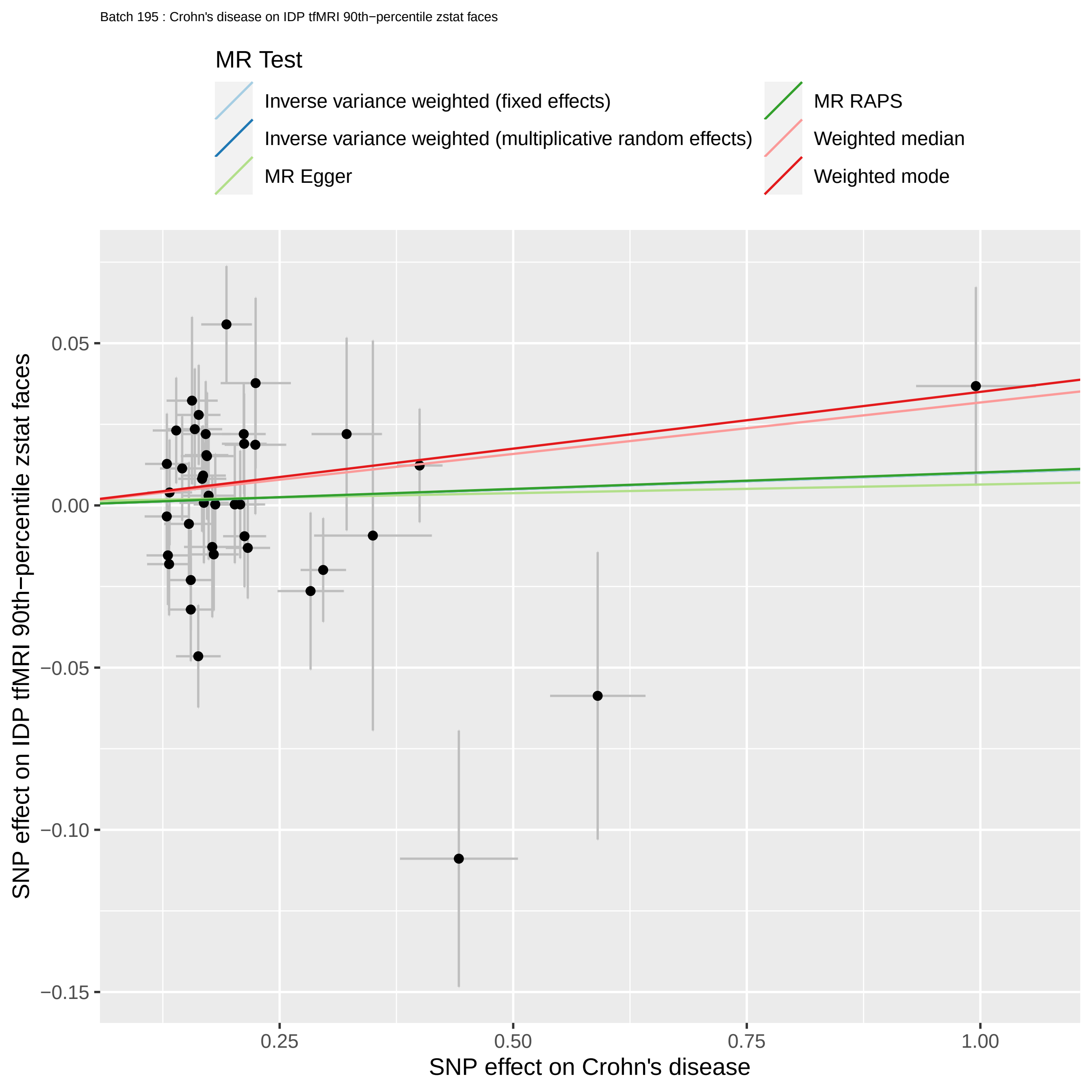

Batch 195 : Crohn's disease on IDP tfMRI 90th−percentile zstat faces
MR Test
Inverse variance weighted (fixed effects)
Inverse variance weighted (multiplicative random effects)
MR Egger
MR RAPS
Weighted median
Weighted mode
0.05
0.00
−0.05
−0.10
−0.15
0.25
0.50
0.75
1.00
SNP effect on IDP tfMRI 90th−percentile zstat faces
SNP effect on Crohn's disease

Batch 196 : Ulcerative colitis on IDP tfMRI 90th−percentile zstat faces

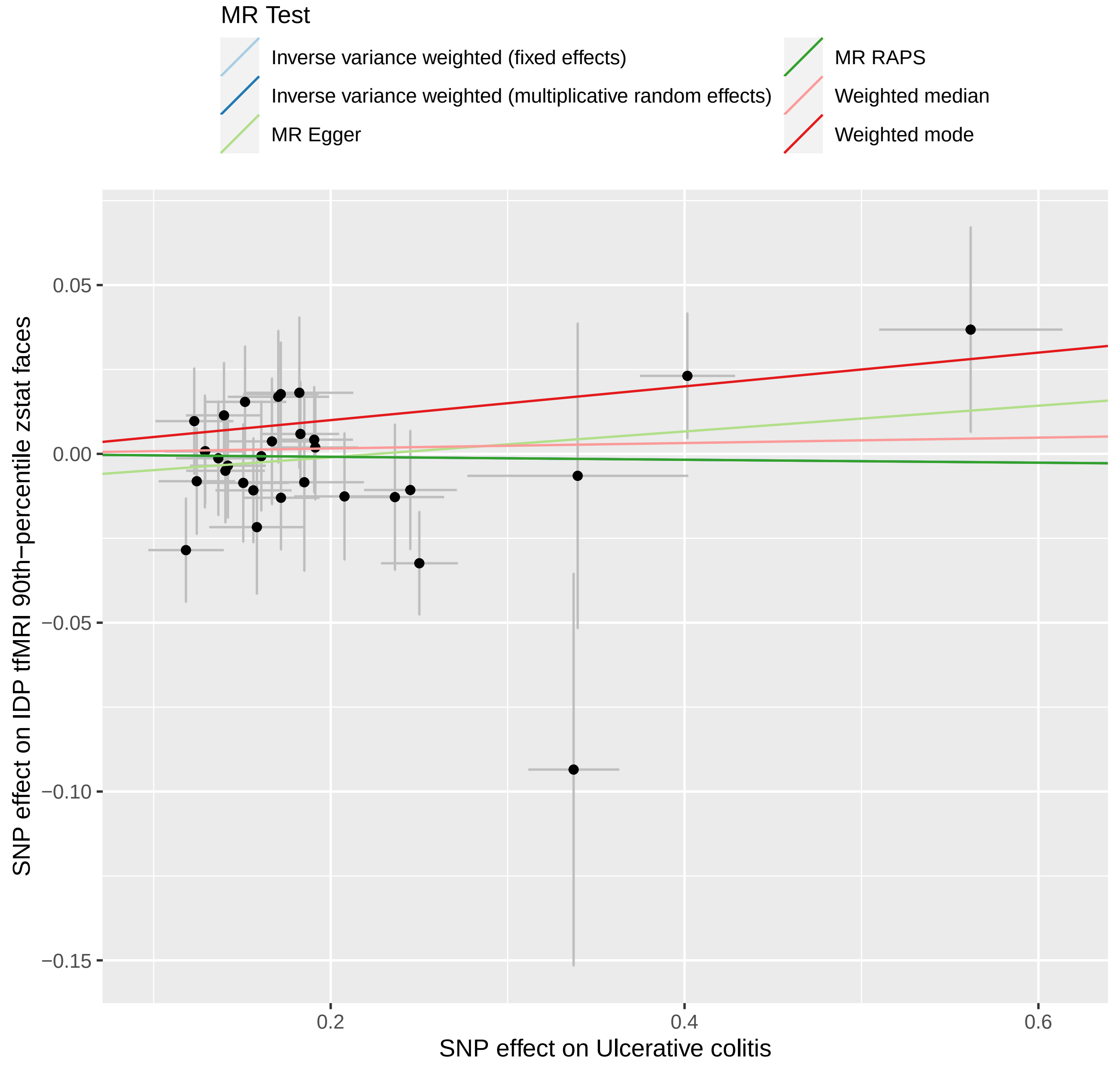

Batch 197 : Crohn's disease on IDP tfMRI median BOLD faces−shapes

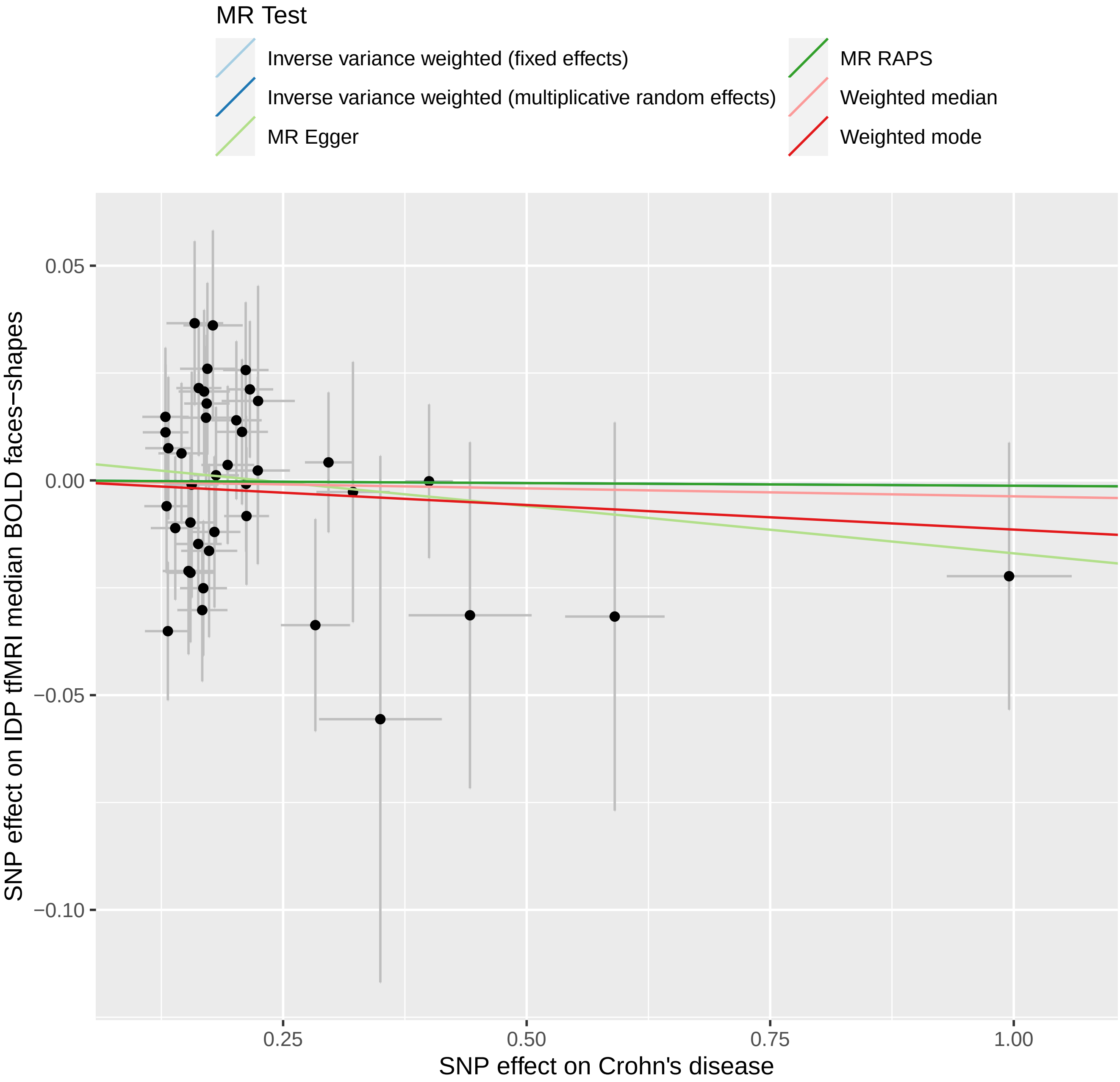

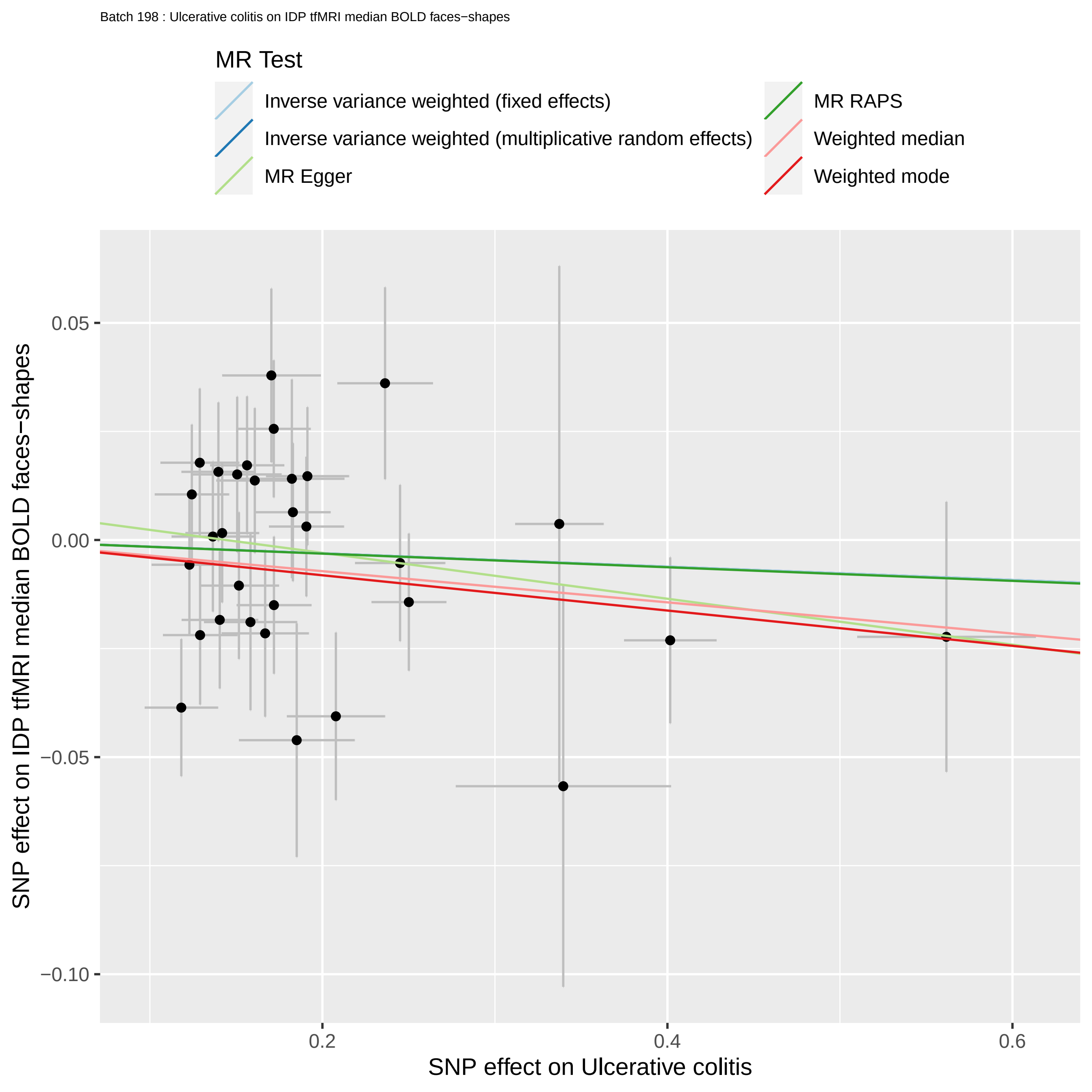
Batch 198 : Ulcerative colitis on IDP tfMRI median BOLD faces−shapes
MR Test
Inverse variance weighted (fixed effects)
Inverse variance weighted (multiplicative random effects)
MR Egger
MR RAPS
Weighted median
Weighted mode
0.05
0.00
−0.05
−0.10
0.2
0.4
0.6
SNP effect on IDP tfMRI median BOLD faces−shapes
SNP effect on Ulcerative colitis

Batch 199 : Crohn's disease on IDP tfMRI 90th−percentile BOLD faces−shapes

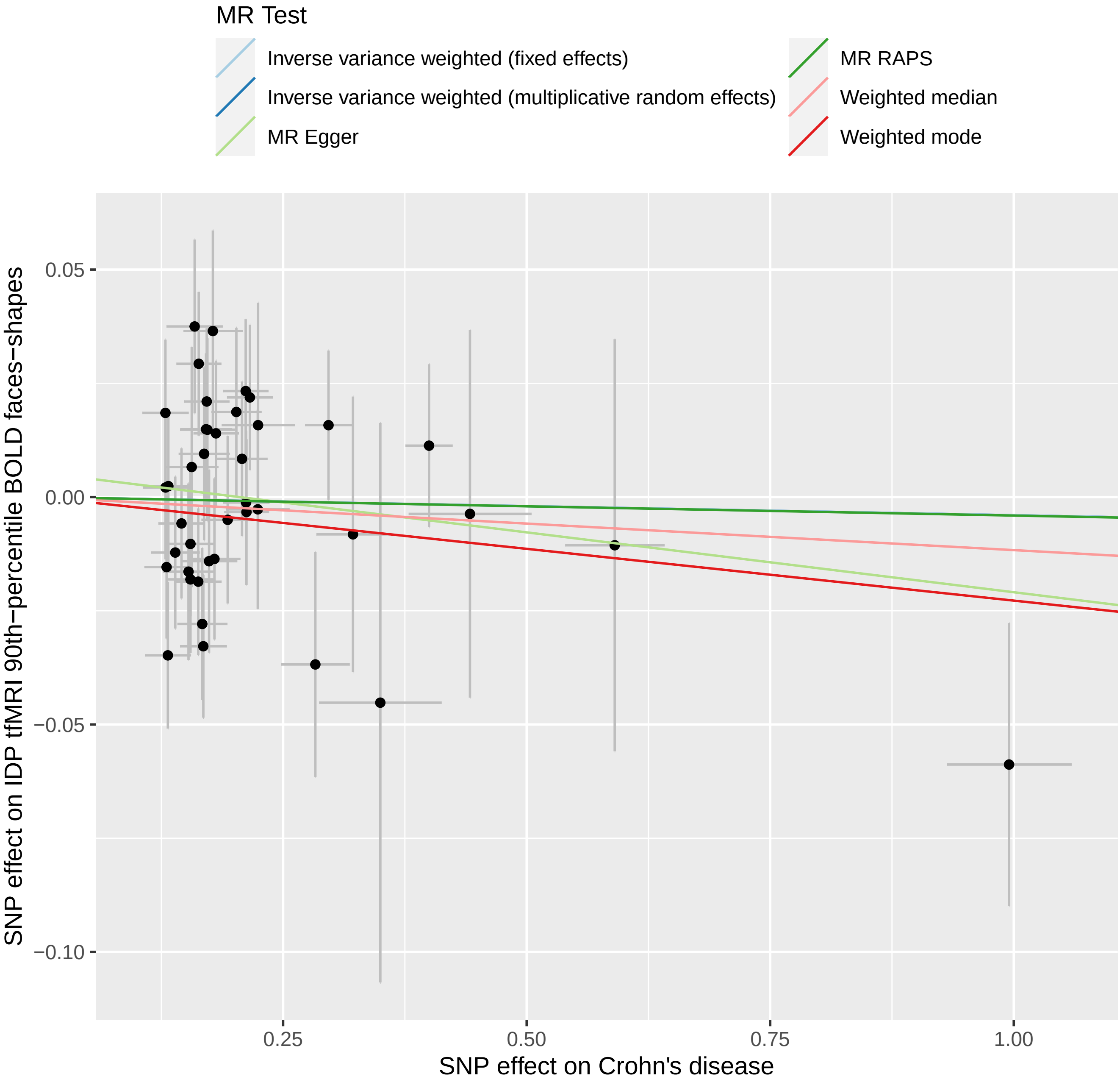

Batch 200 : Ulcerative colitis on IDP tfMRI 90th−percentile BOLD faces−shapes

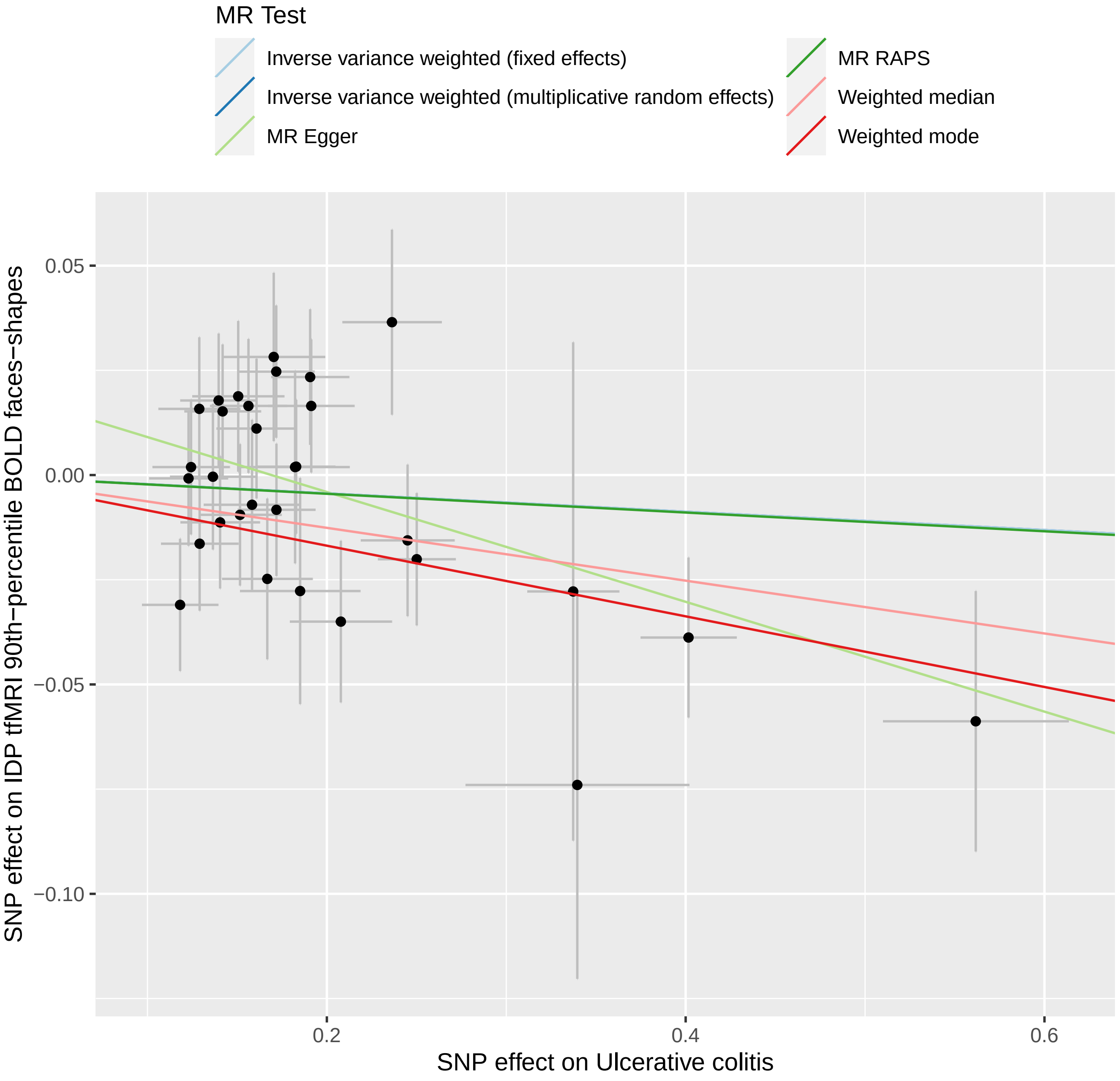

Batch 201 : Crohn's disease on IDP T1 FIRST left hippocampus volume

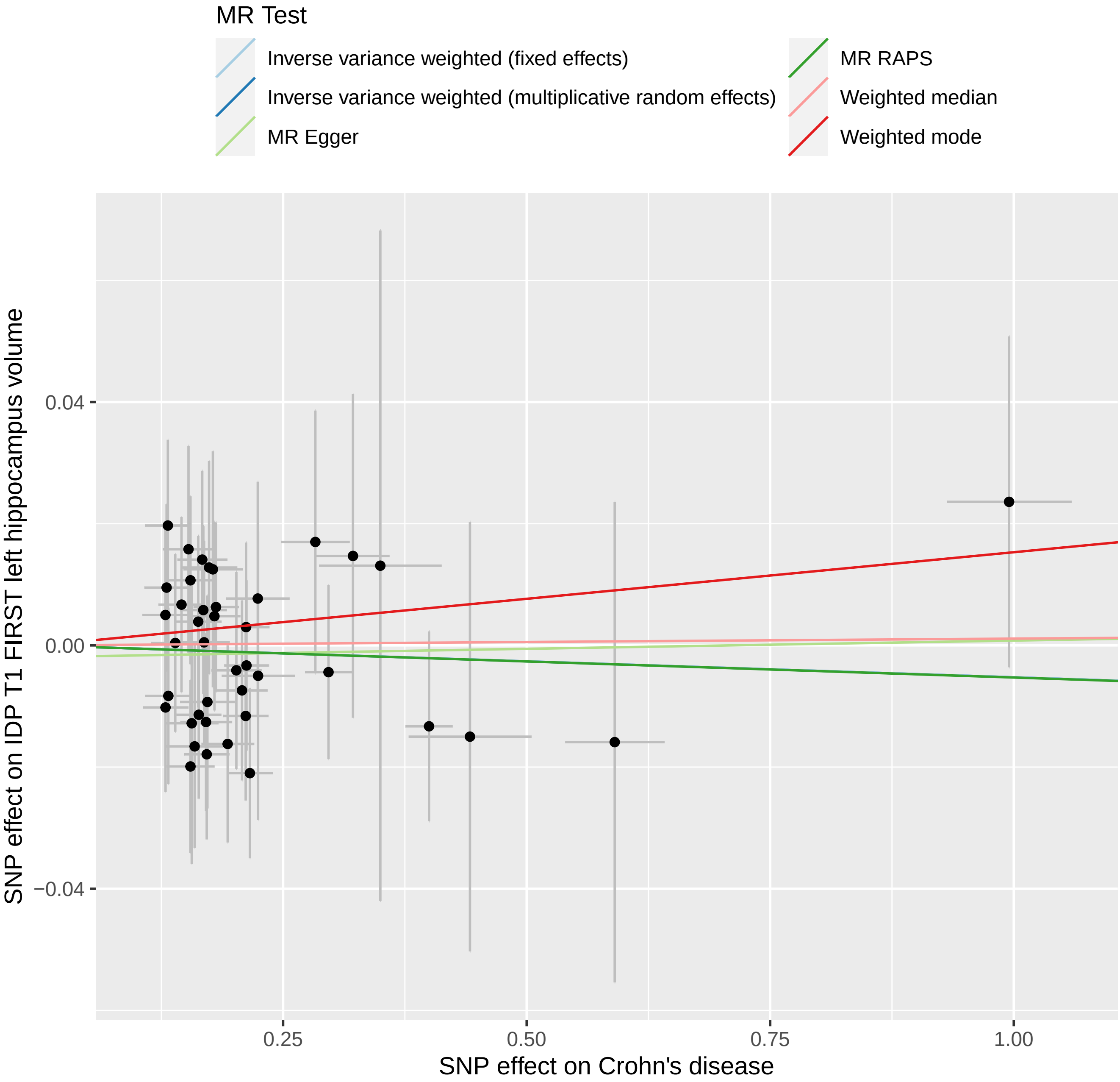

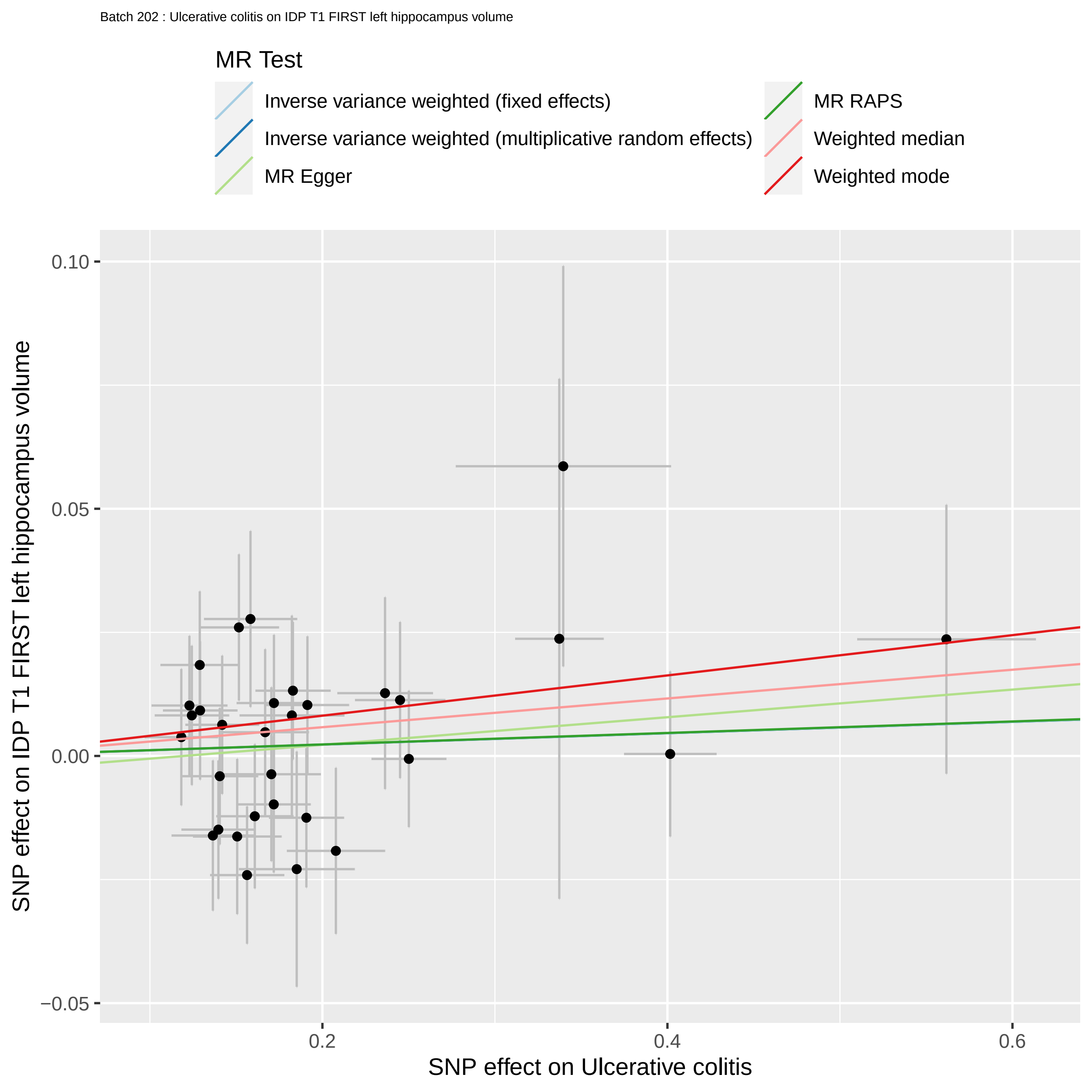
Batch 202 : Ulcerative colitis on IDP T1 FIRST left hippocampus volume
MR Test
Inverse variance weighted (fixed effects)
Inverse variance weighted (multiplicative random effects)
MR Egger
MR RAPS
Weighted median
Weighted mode
0.10
0.05
0.00
−0.05
0.2
0.4
0.6
SNP effect on IDP T1 FIRST left hippocampus volume
SNP effect on Ulcerative colitis

Batch 203 : Crohn's disease on IDP tfMRI median zstat faces−shapes

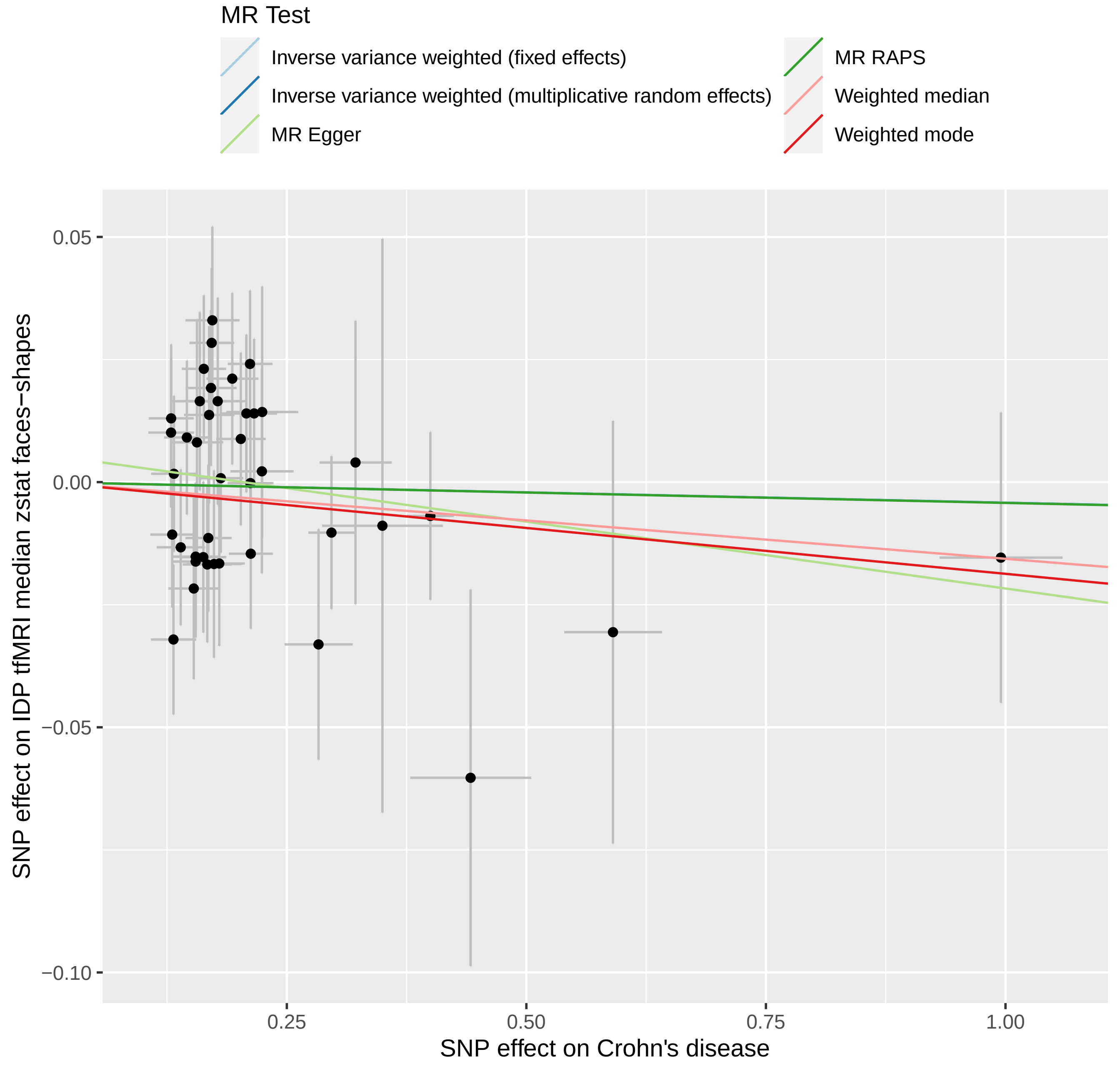

Batch 204 : Ulcerative colitis on IDP tfMRI median zstat faces−shapes

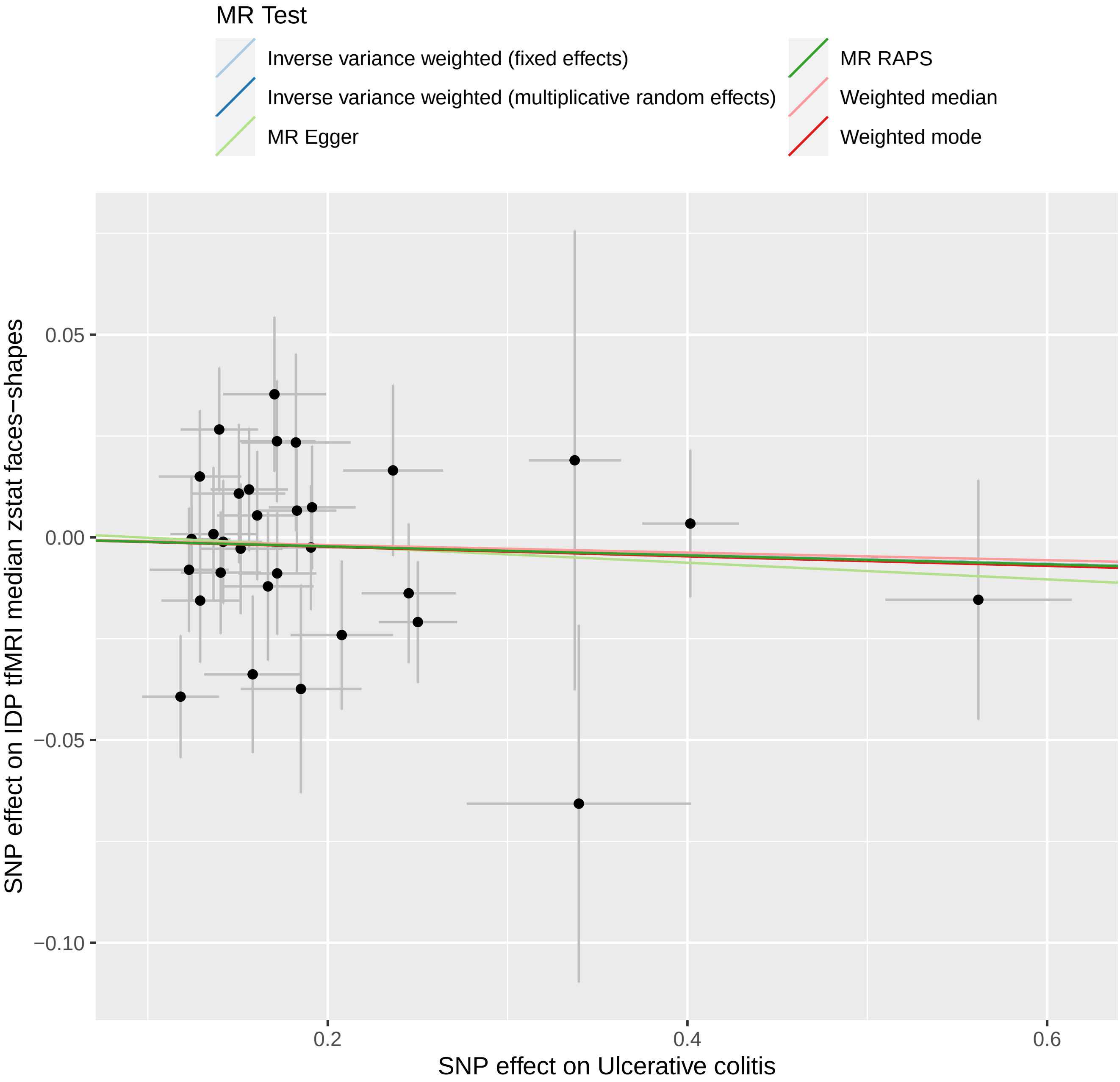

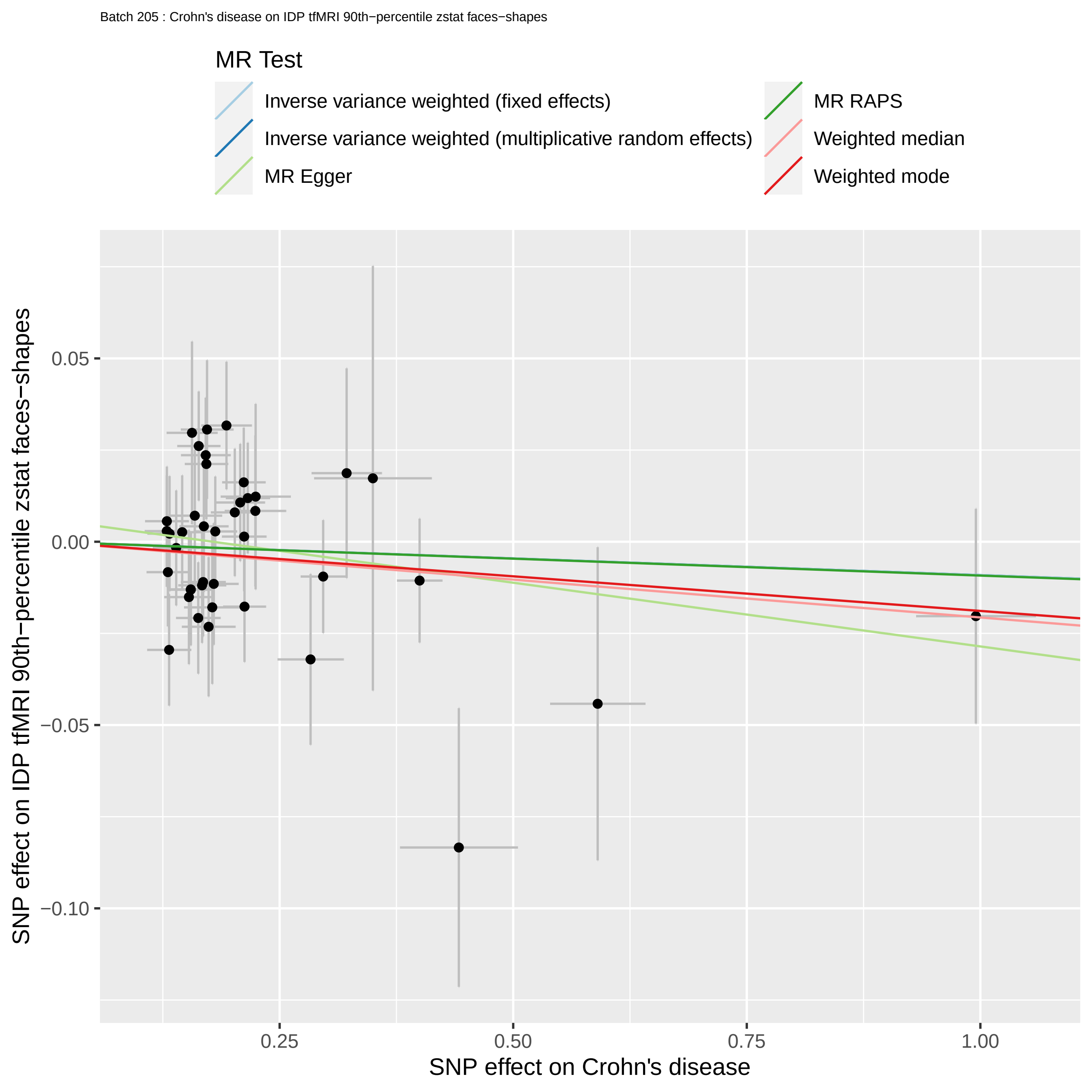
Batch 205 : Crohn's disease on IDP tfMRI 90th−percentile zstat faces−shapes
MR Test
Inverse variance weighted (fixed effects)
Inverse variance weighted (multiplicative random effects)
MR Egger
MR RAPS
Weighted median
Weighted mode
0.05
0.00
−0.05
−0.10
0.25
0.50
0.75
1.00
SNP effect on Crohn's disease
SNP effect on IDP tfMRI 90th−percentile zstat faces−shapes

Batch 206 : Ulcerative colitis on IDP tfMRI 90th−percentile zstat faces−shapes

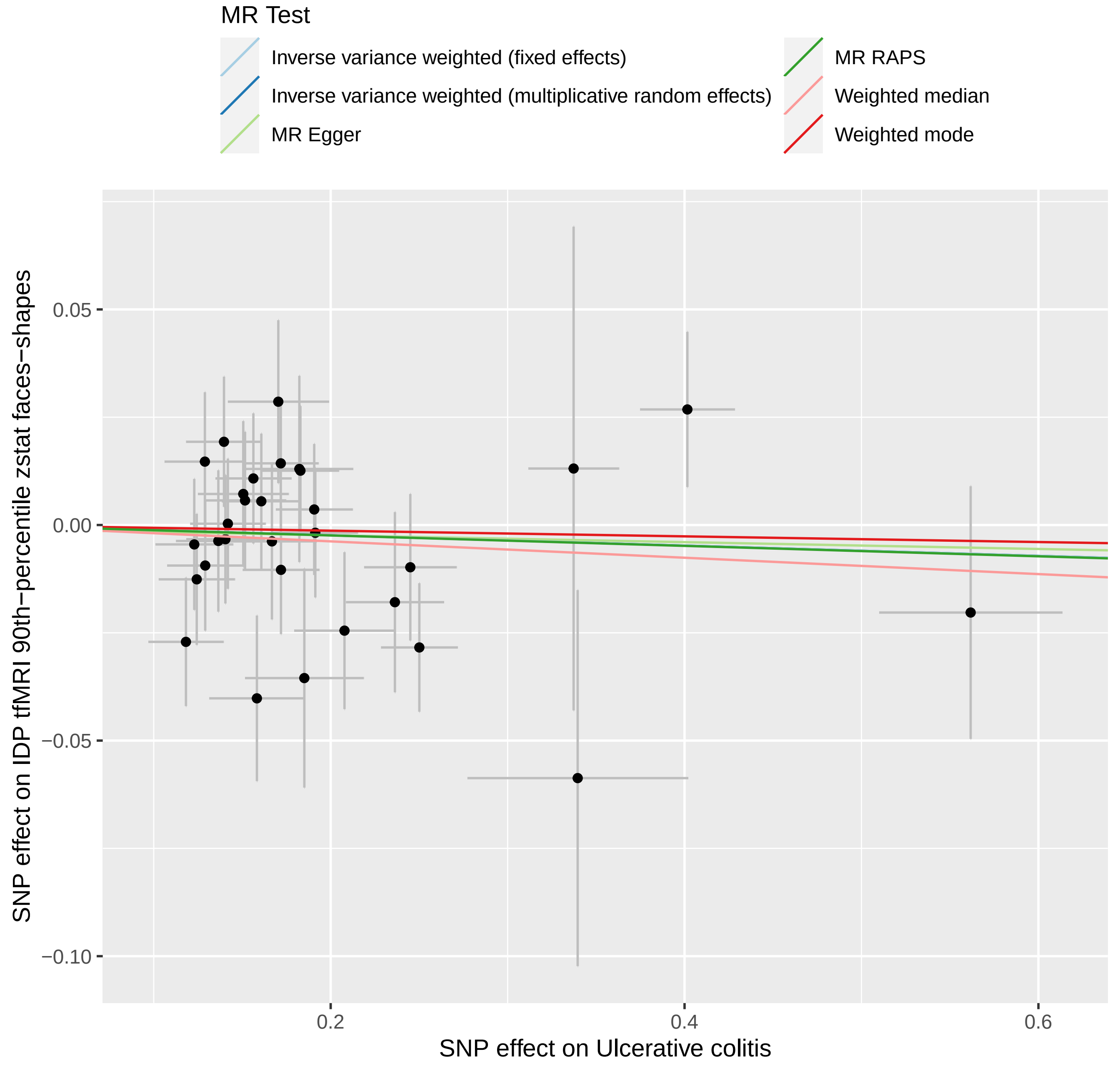

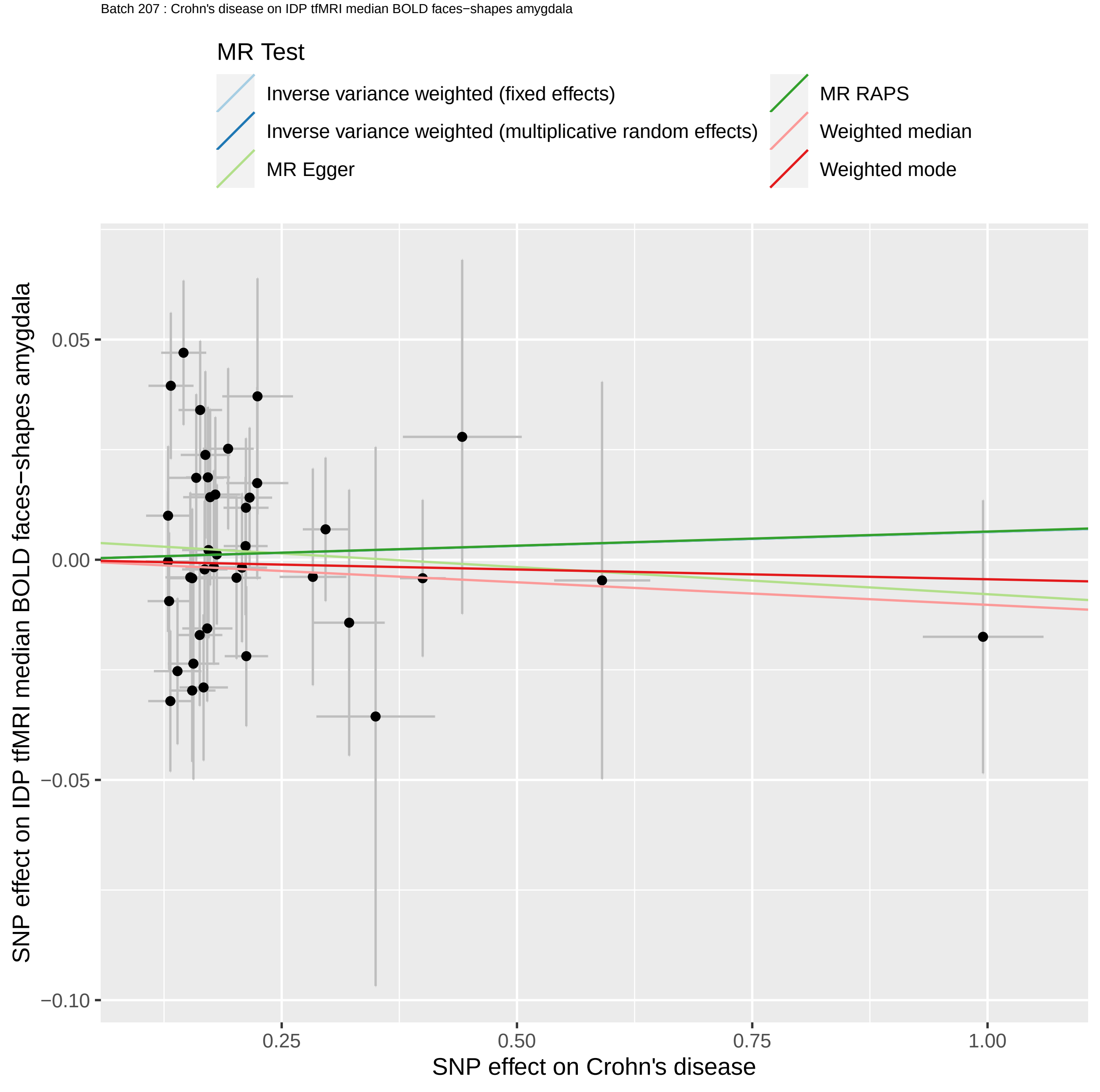

Batch 207 : Crohn's disease on IDP tfMRI median BOLD faces−shapes amygdala
MR Test
Inverse variance weighted (fixed effects)
Inverse variance weighted (multiplicative random effects)
MR Egger
MR RAPS
Weighted median
Weighted mode
SNP effect on IDP tfMRI median BOLD faces−shapes amygdala
0.05
0.00
−0.05
−0.10
0.25
0.50
0.75
1.00
SNP effect on Crohn's disease

Batch 208 : Ulcerative colitis on IDP tfMRI median BOLD faces−shapes amygdala

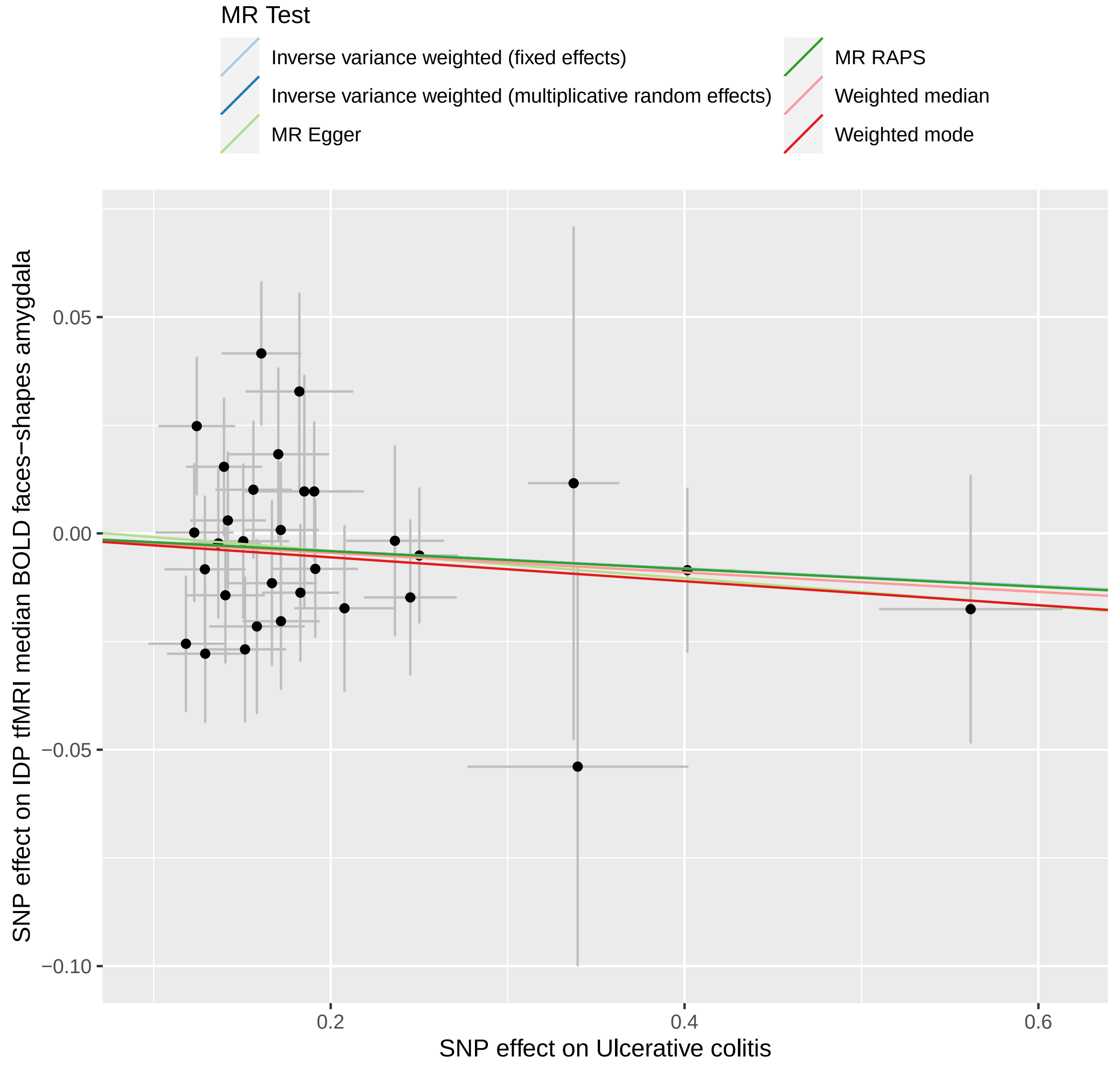

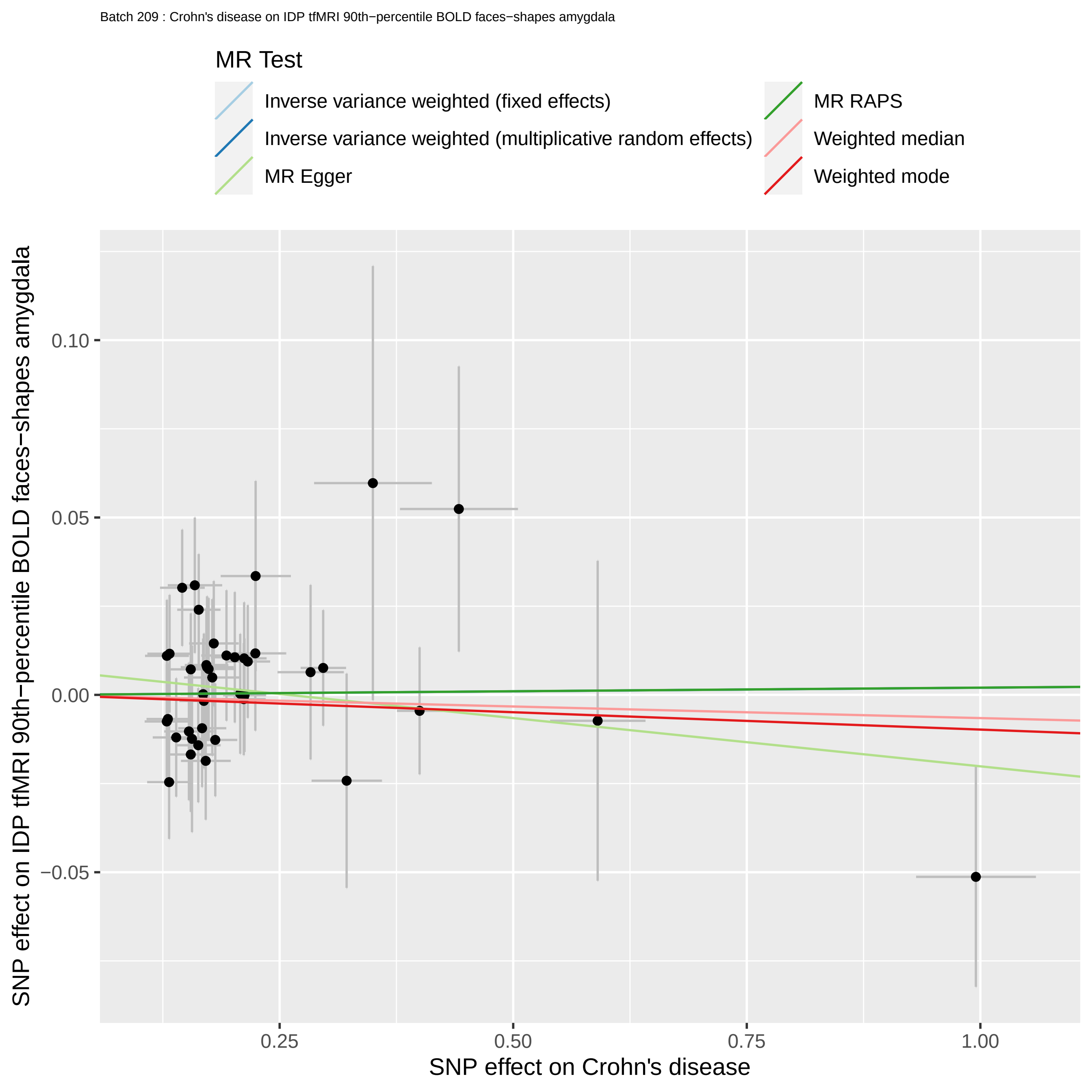
Batch 209 : Crohn's disease on IDP tfMRI 90th−percentile BOLD faces−shapes amygdala
MR Test
Inverse variance weighted (fixed effects)
Inverse variance weighted (multiplicative random effects)
MR Egger
MR RAPS
Weighted median
Weighted mode
0.10
0.05
0.00
−0.05
0.25
0.50
0.75
1.00
SNP effect on IDP tfMRI 90th−percentile BOLD faces−shapes amygdala
SNP effect on Crohn's disease

Batch 210 : Ulcerative colitis on IDP tfMRI 90th−percentile BOLD faces−shapes amygdala

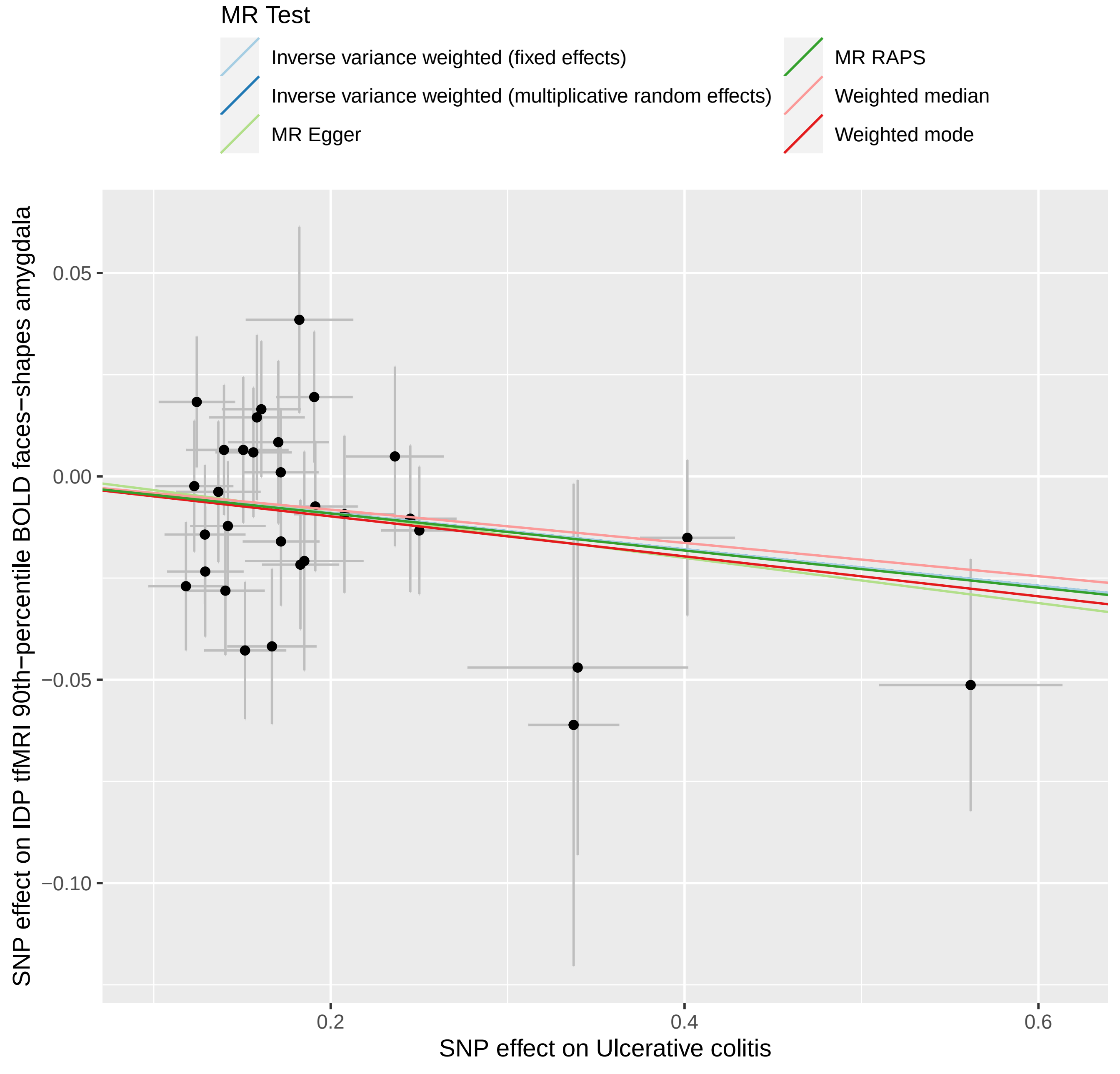

Batch 211 : Crohn's disease on IDP tfMRI median zstat faces−shapes amygdala

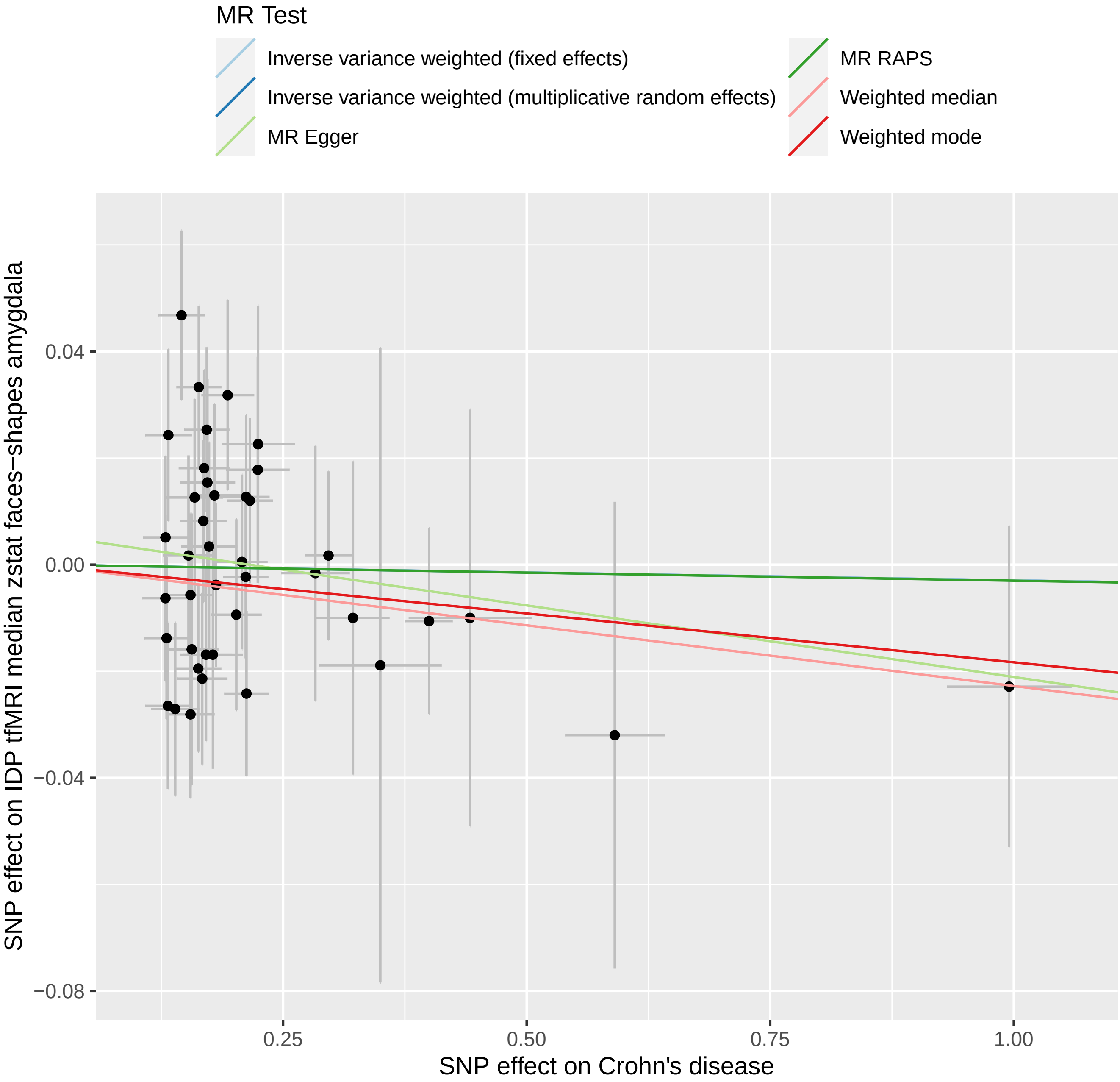

Batch 212 : Ulcerative colitis on IDP tfMRI median zstat faces−shapes amygdala

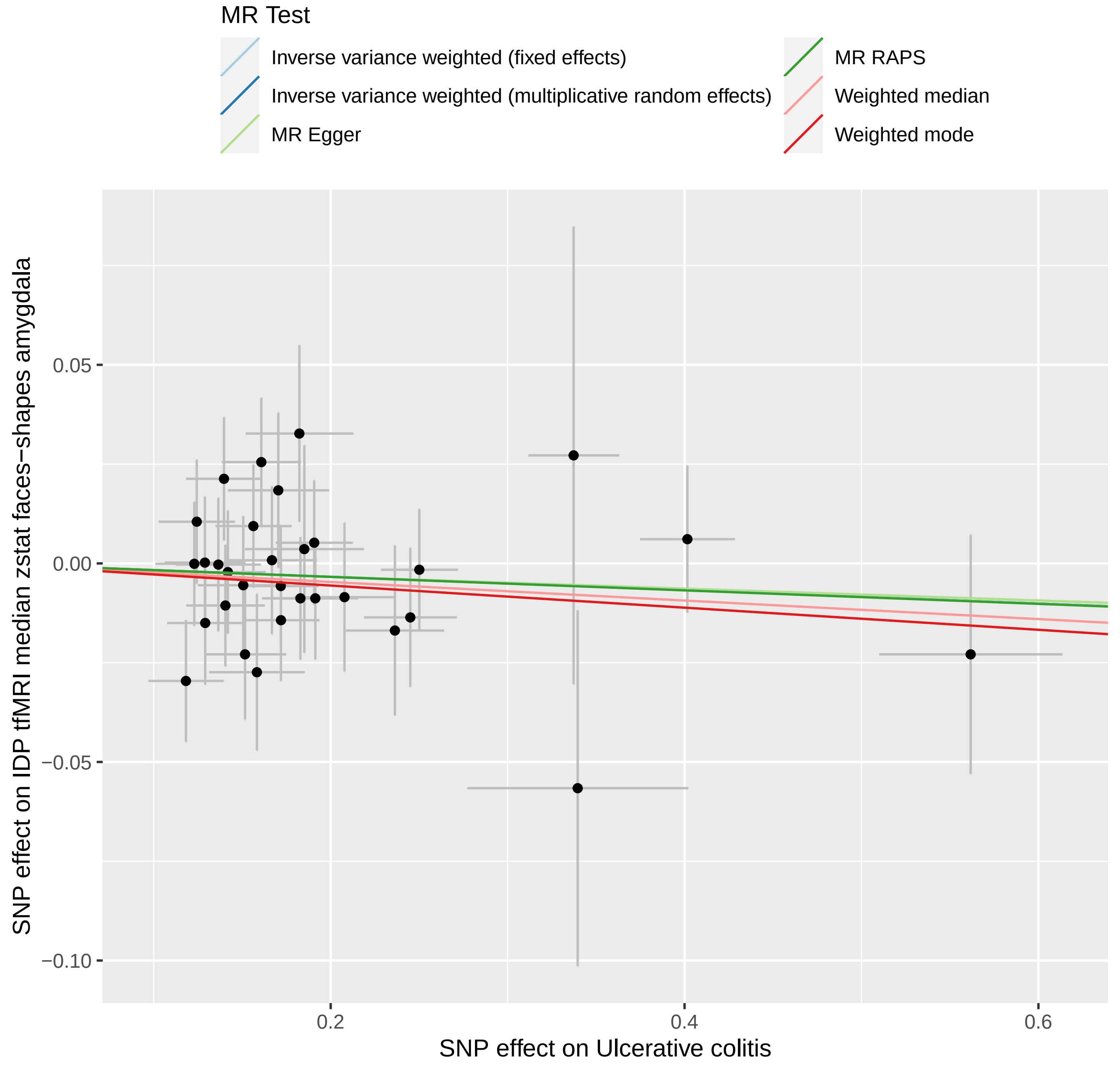

Batch 213 : Crohn's disease on IDP tfMRI 90th−percentile zstat faces−shapes amygdala

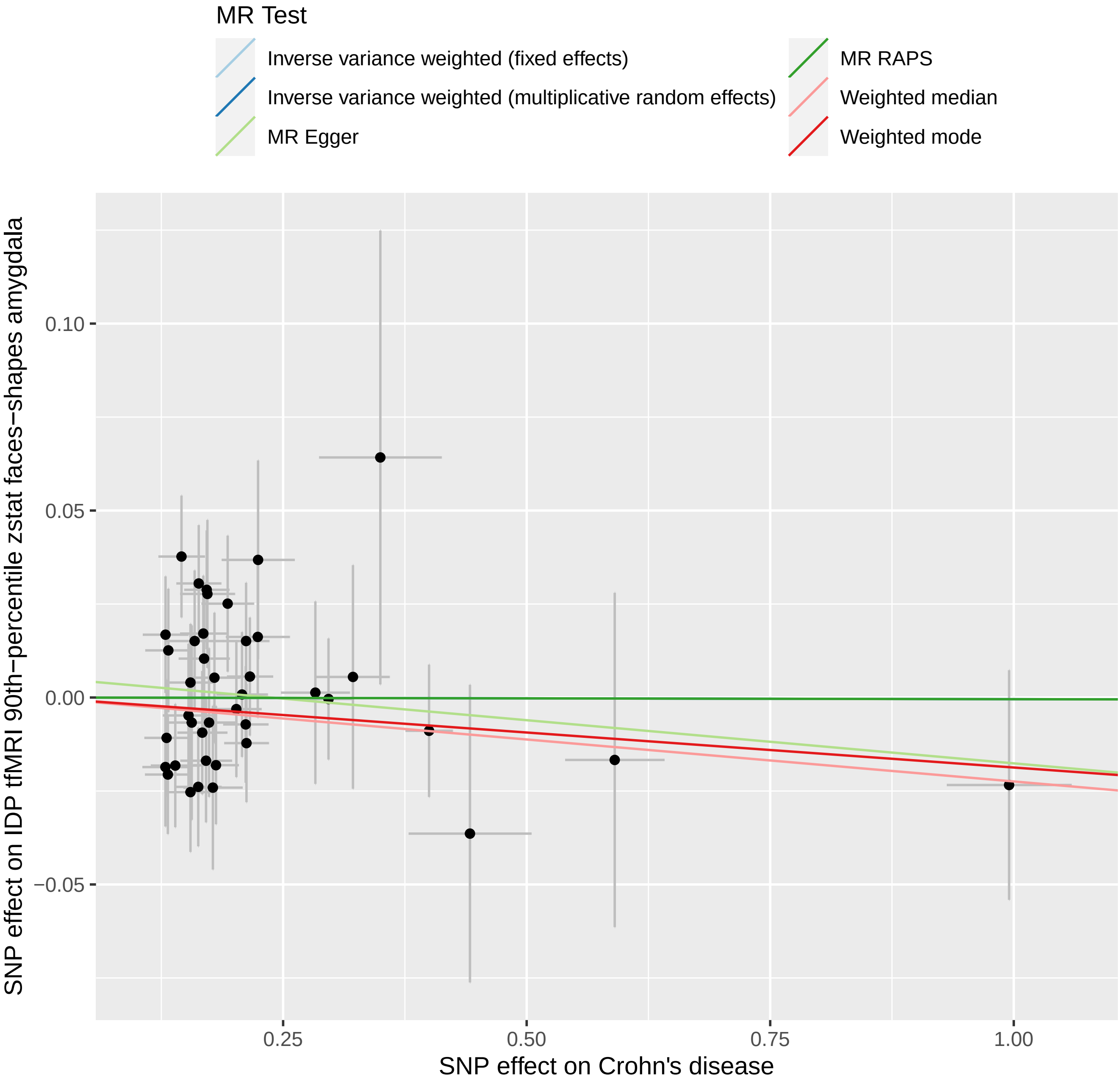

Batch 214 : Ulcerative colitis on IDP tfMRI 90th−percentile zstat faces−shapes amygdala

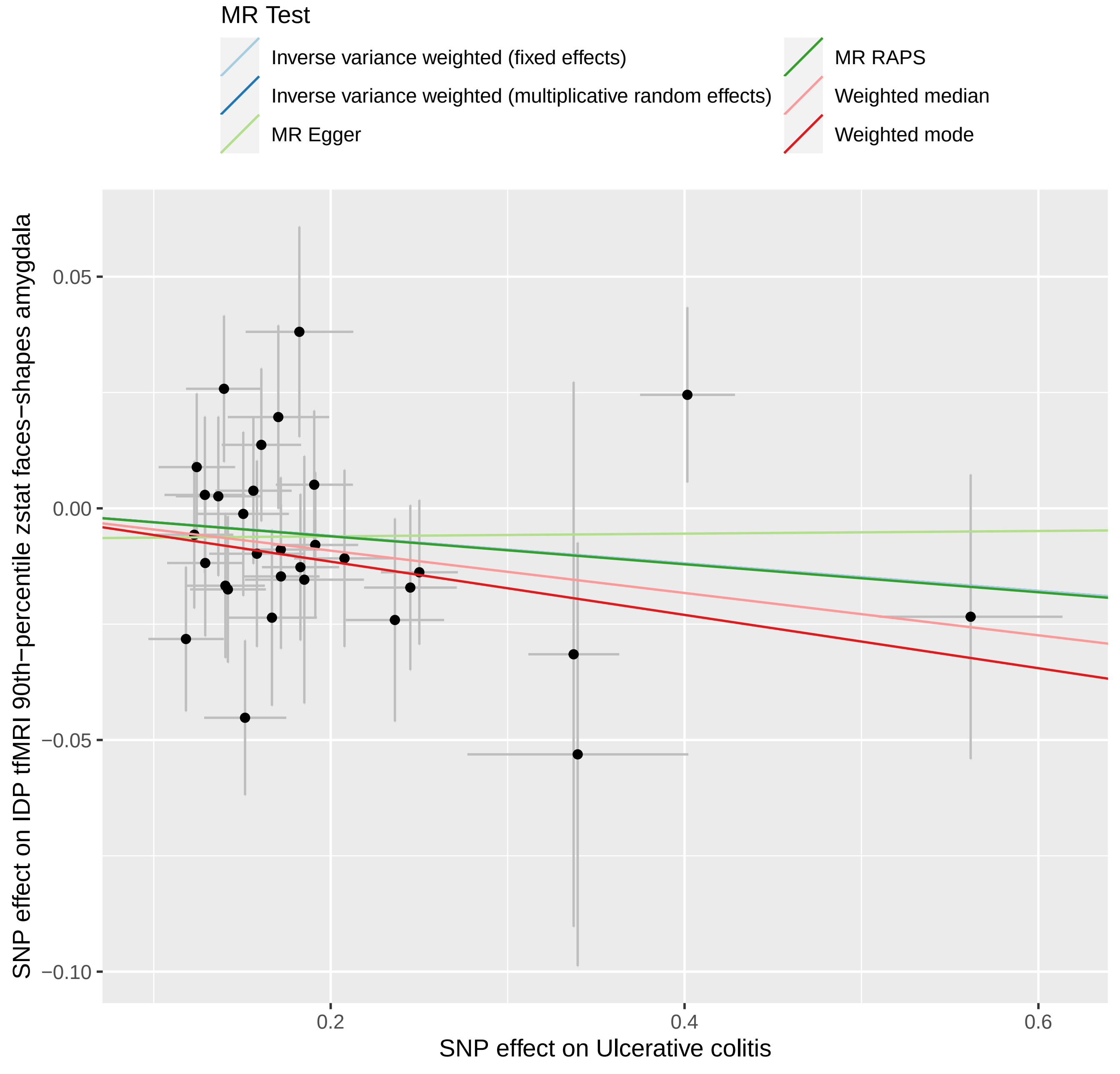

Batch 223 : Crohn's disease on IDP T1 SIENAX peripheral grey unnormalised volume

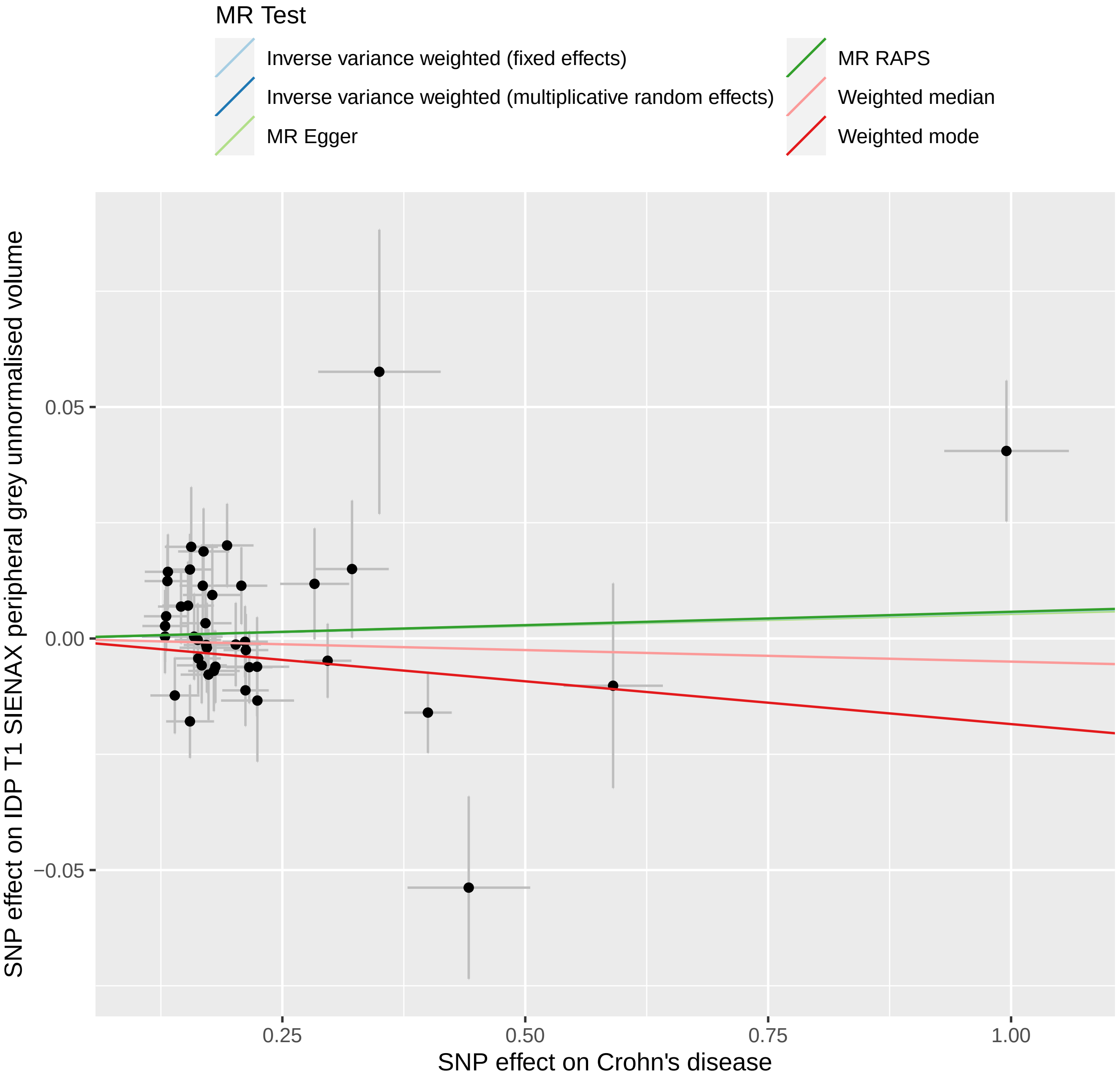

Batch 224 : Ulcerative colitis on IDP T1 SIENAX peripheral grey unnormalised volume

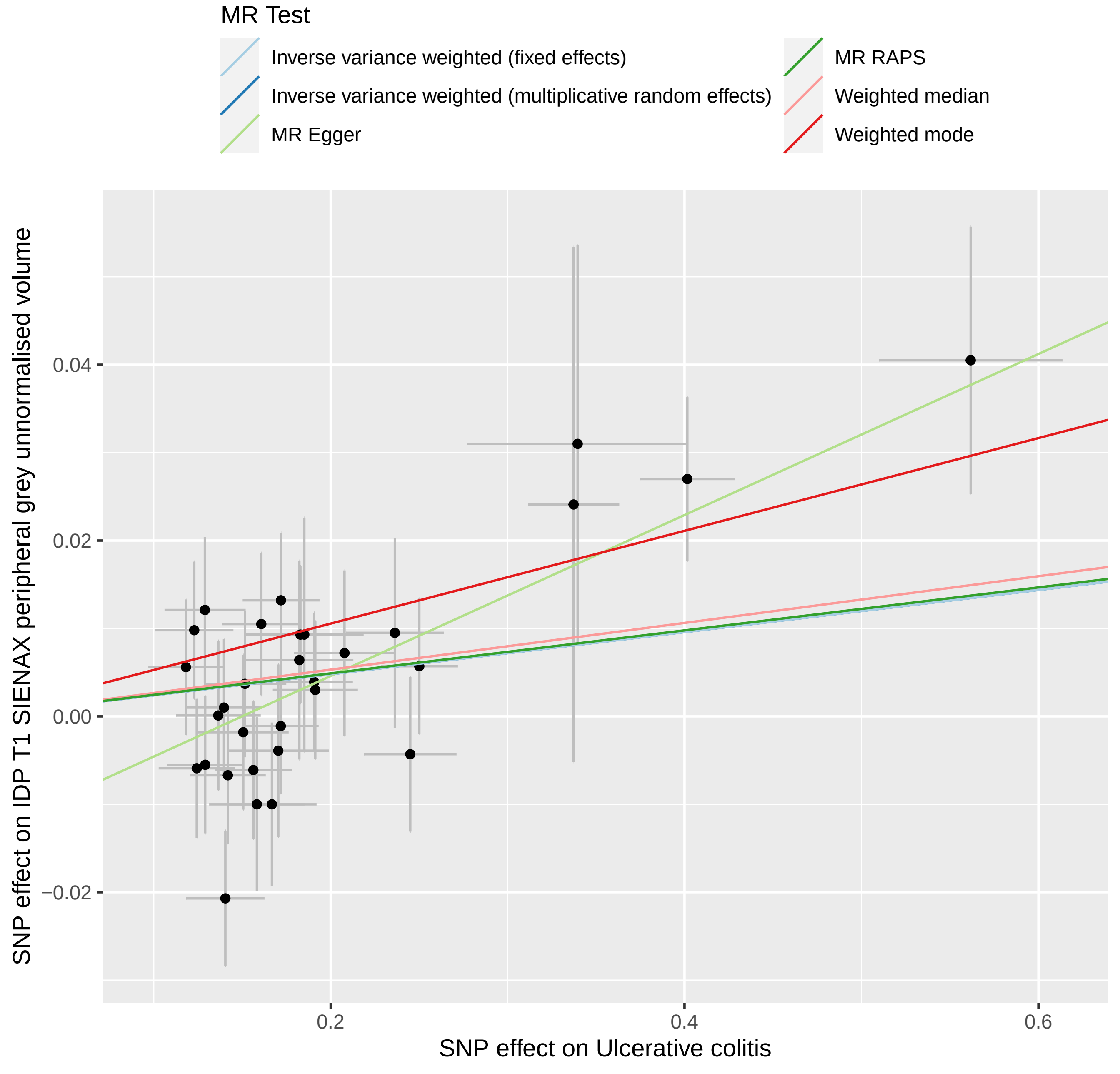

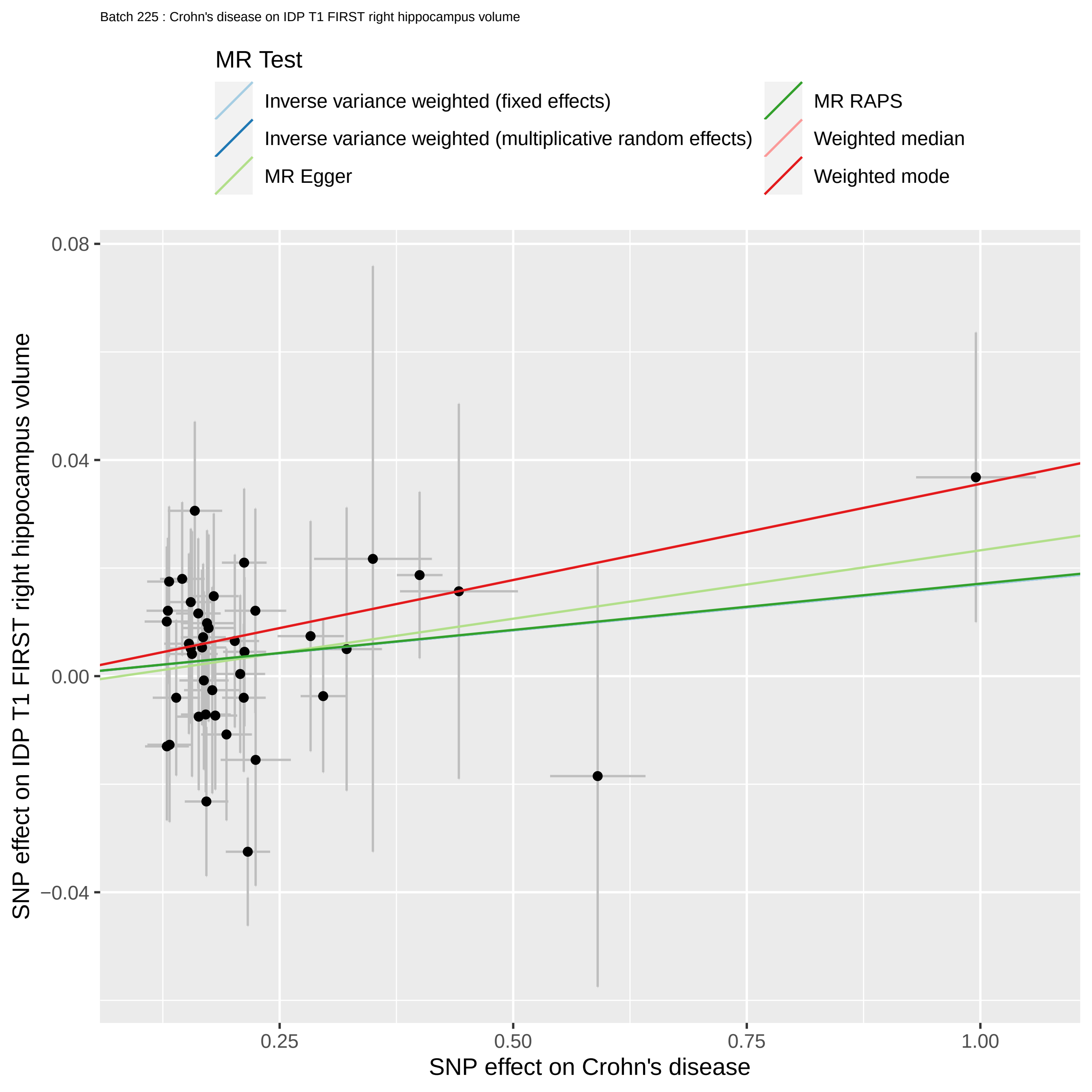

Batch 225 : Crohn's disease on IDP T1 FIRST right hippocampus volume
MR Test
Inverse variance weighted (fixed effects)
Inverse variance weighted (multiplicative random effects)
MR Egger
MR RAPS
Weighted median
Weighted mode
0.08
0.04
0.00
−0.04
0.25
0.50
0.75
1.00
SNP effect on IDP T1 FIRST right hippocampus volume
SNP effect on Crohn's disease

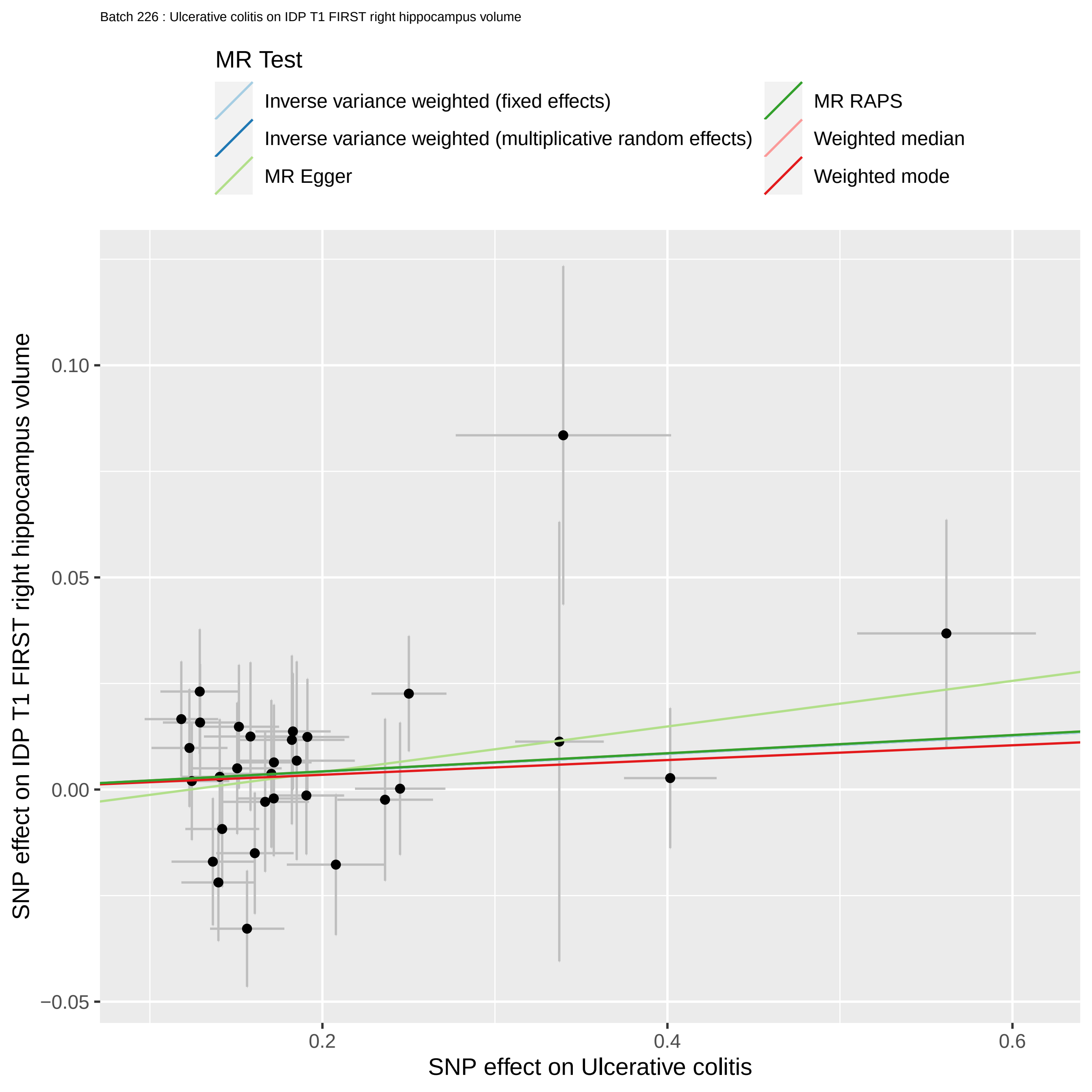
Batch 226 : Ulcerative colitis on IDP T1 FIRST right hippocampus volume
MR Test
Inverse variance weighted (fixed effects)
Inverse variance weighted (multiplicative random effects)
MR Egger
MR RAPS
Weighted median
Weighted mode
SNP effect on IDP T1 FIRST right hippocampus volume
0.10
0.05
0.00
−0.05
0.2
0.4
0.6
SNP effect on Ulcerative colitis

Batch 247 : Crohn's disease on IDP T1 FIRST left amygdala volume

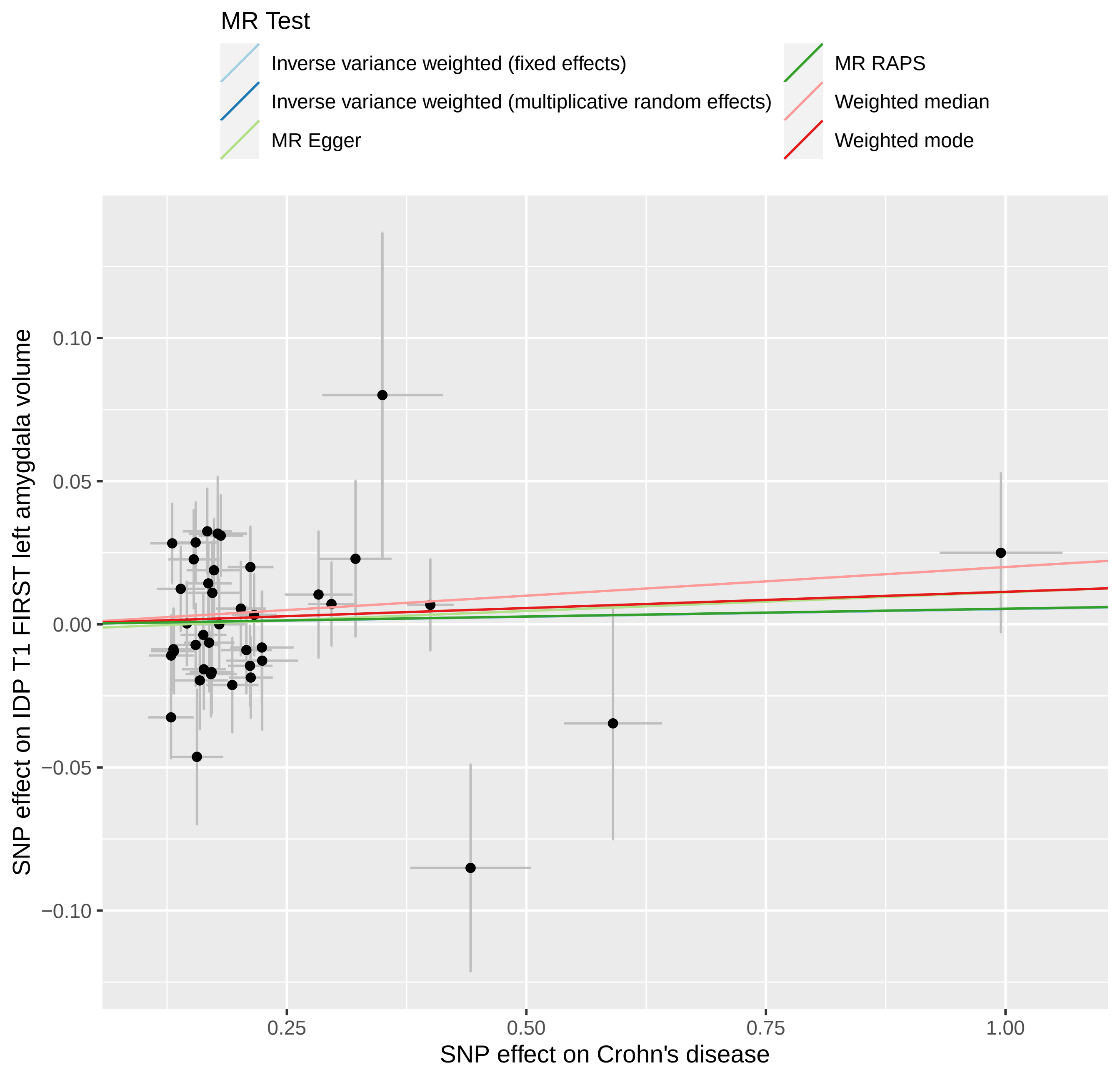

Batch 248 : Ulcerative colitis on IDP T1 FIRST left amygdala volume

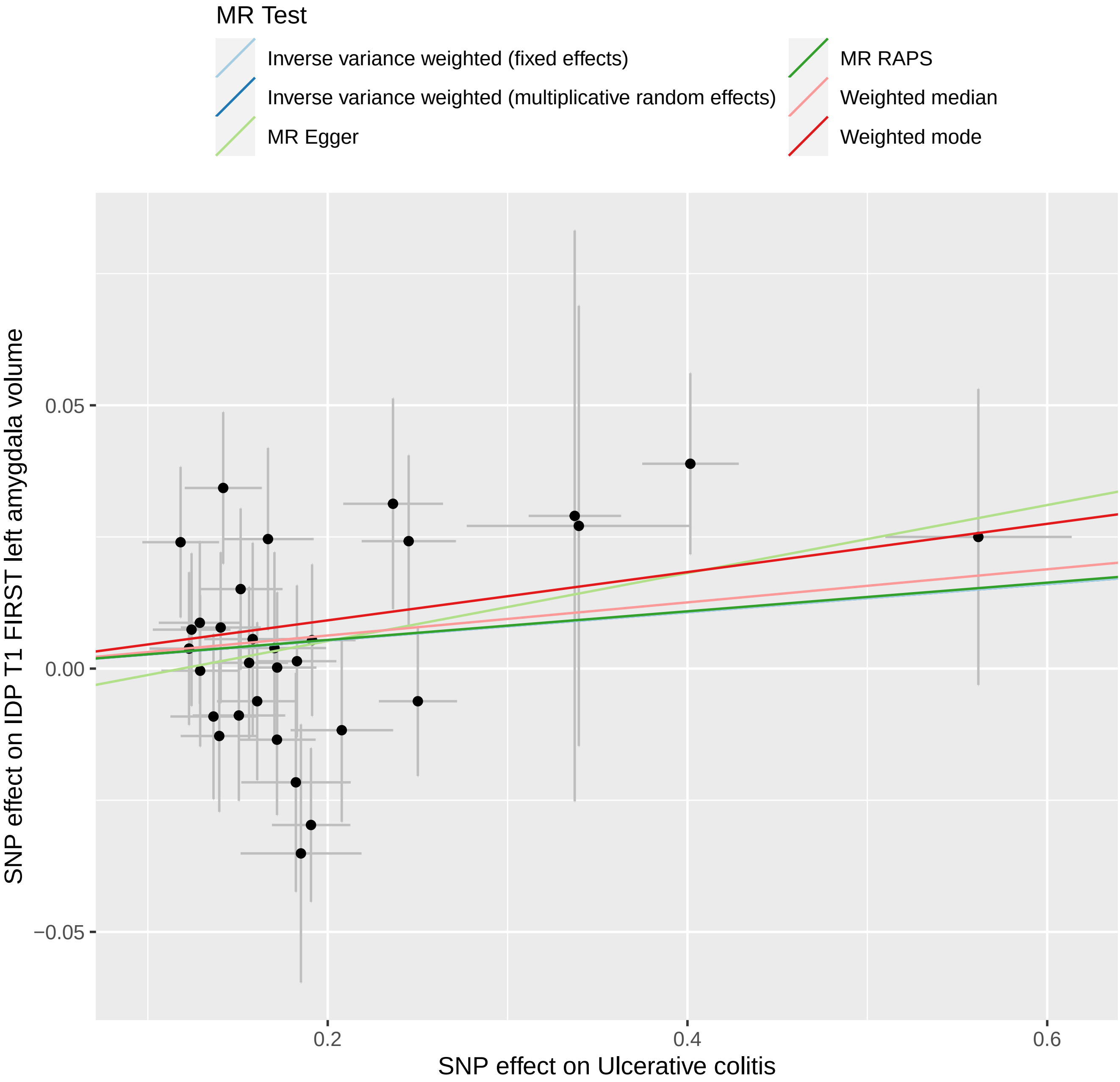

Batch 269 : Crohn's disease on IDP T1 FIRST right amygdala volume

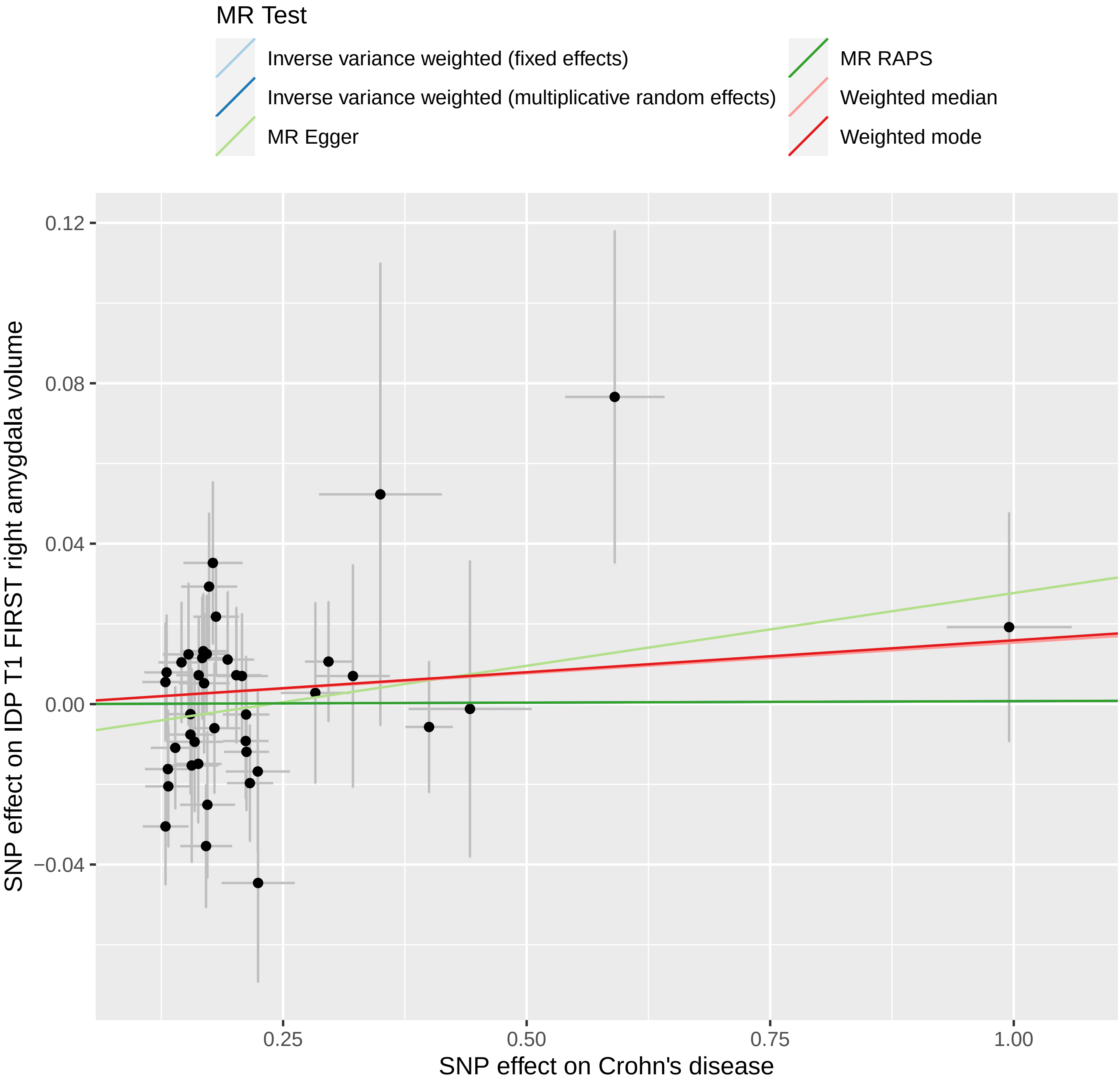

Batch 270 : Ulcerative colitis on IDP T1 FIRST right amygdala volume

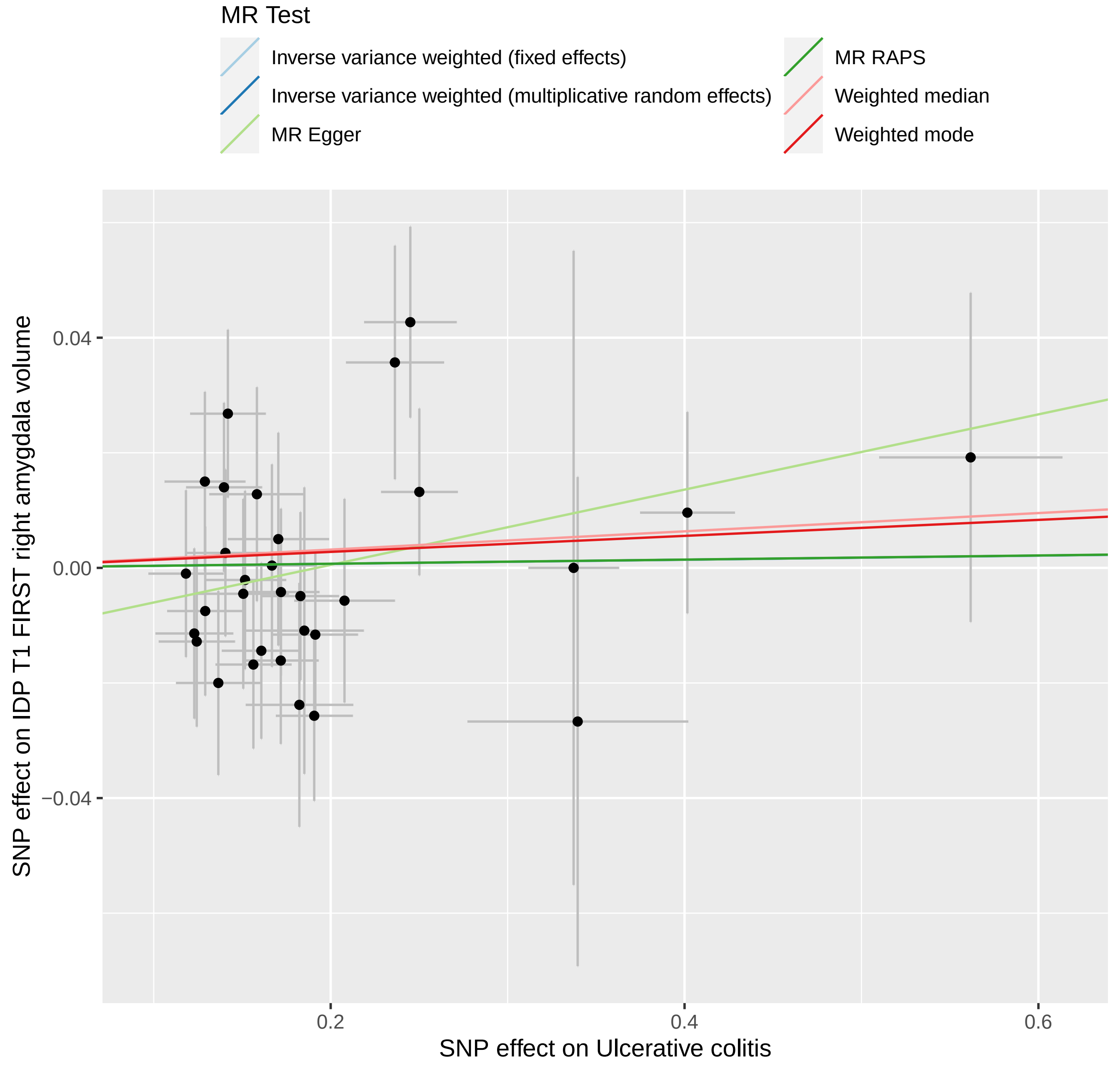

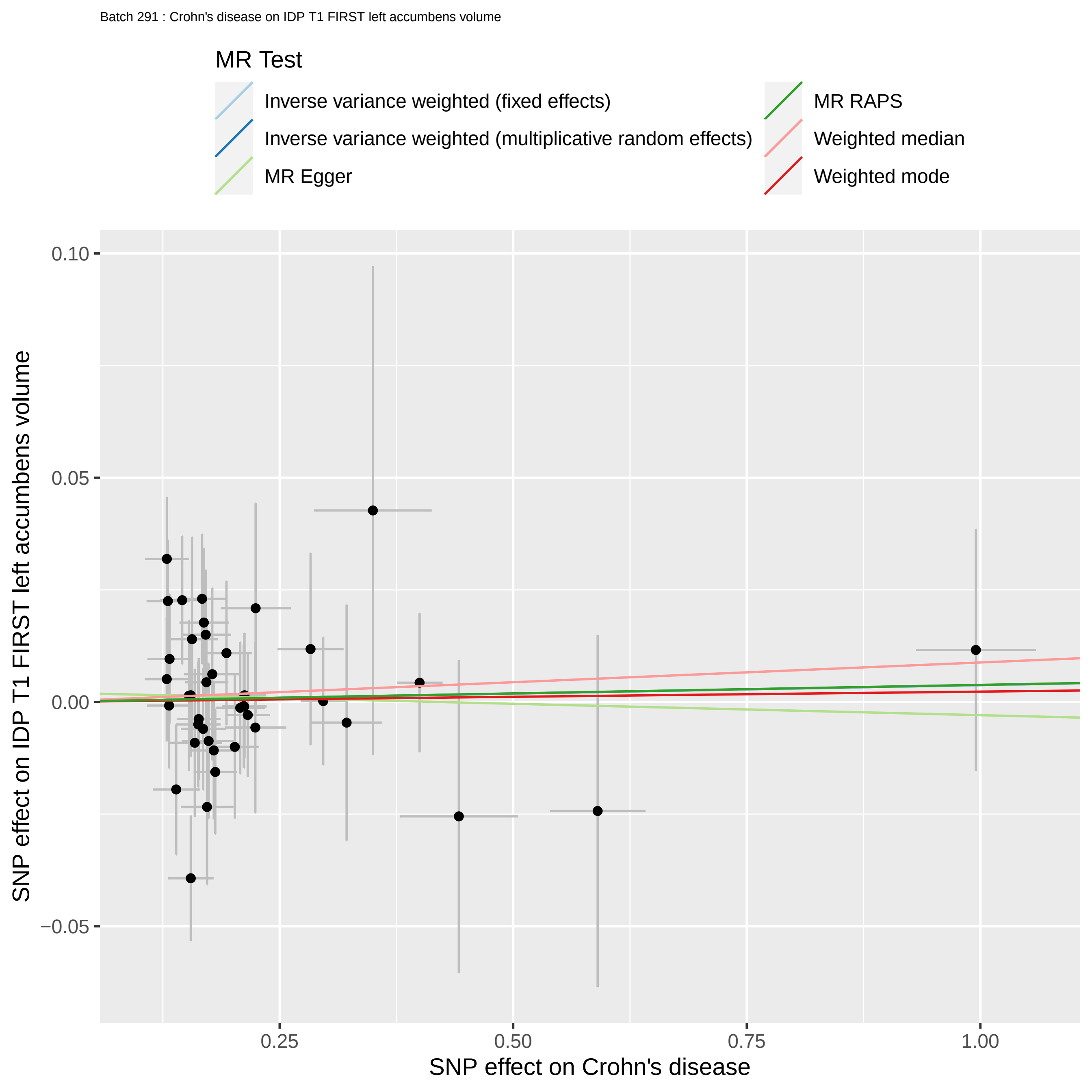
Batch 291 : Crohn's disease on IDP T1 FIRST left accumbens volume
MR Test
Inverse variance weighted (fixed effects)
Inverse variance weighted (multiplicative random effects)
MR Egger
MR RAPS
Weighted median
Weighted mode
0.10
0.05
0.00
−0.05
0.25
0.50
0.75
1.00
SNP effect on IDP T1 FIRST left accumbens volume
SNP effect on Crohn's disease

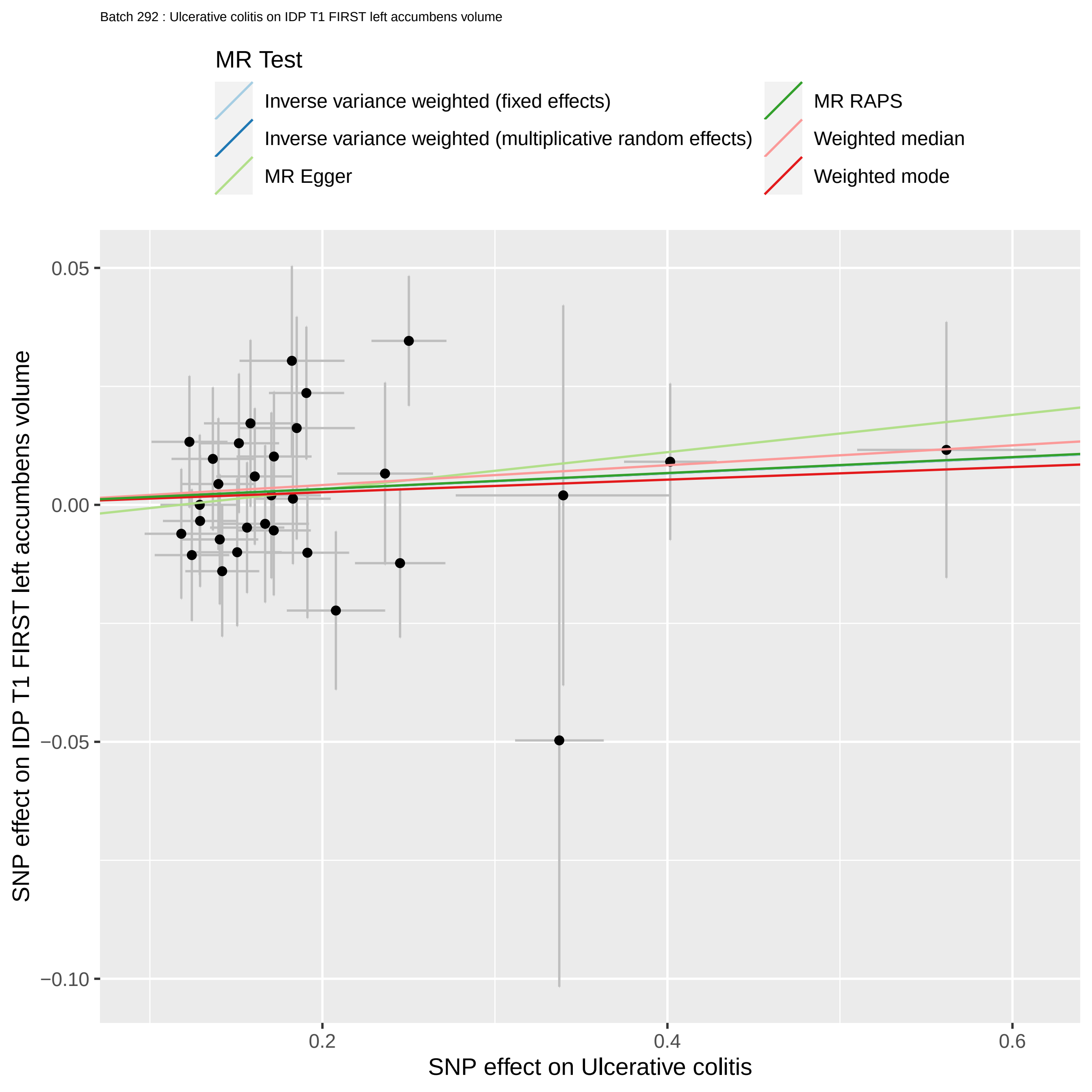

Batch 292 : Ulcerative colitis on IDP T1 FIRST left accumbens volume
MR Test
Inverse variance weighted (fixed effects)
Inverse variance weighted (multiplicative random effects)
MR Egger
MR RAPS
Weighted median
Weighted mode
0.05
0.00
−0.05
−0.10
0.2
0.4
0.6
SNP effect on IDP T1 FIRST left accumbens volume
SNP effect on Ulcerative colitis

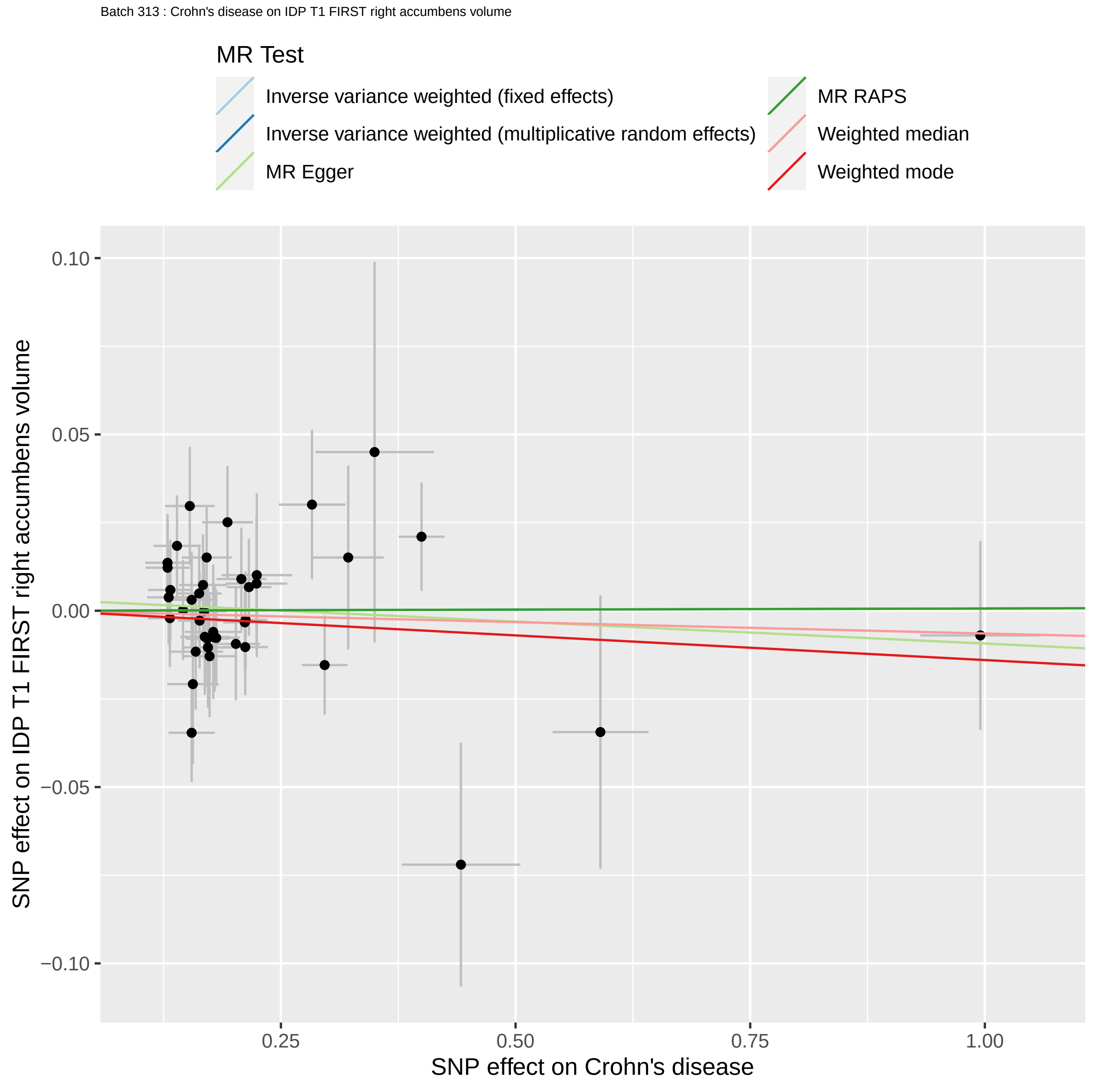

Batch 313 : Crohn's disease on IDP T1 FIRST right accumbens volume
MR Test
Inverse variance weighted (fixed effects)
Inverse variance weighted (multiplicative random effects)
MR Egger
MR RAPS
Weighted median
Weighted mode
0.10
0.05
0.00
−0.05
−0.10
0.25
0.50
0.75
1.00
SNP effect on IDP T1 FIRST right accumbens volume
SNP effect on Crohn's disease

Batch 314 : Ulcerative colitis on IDP T1 FIRST right accumbens volume

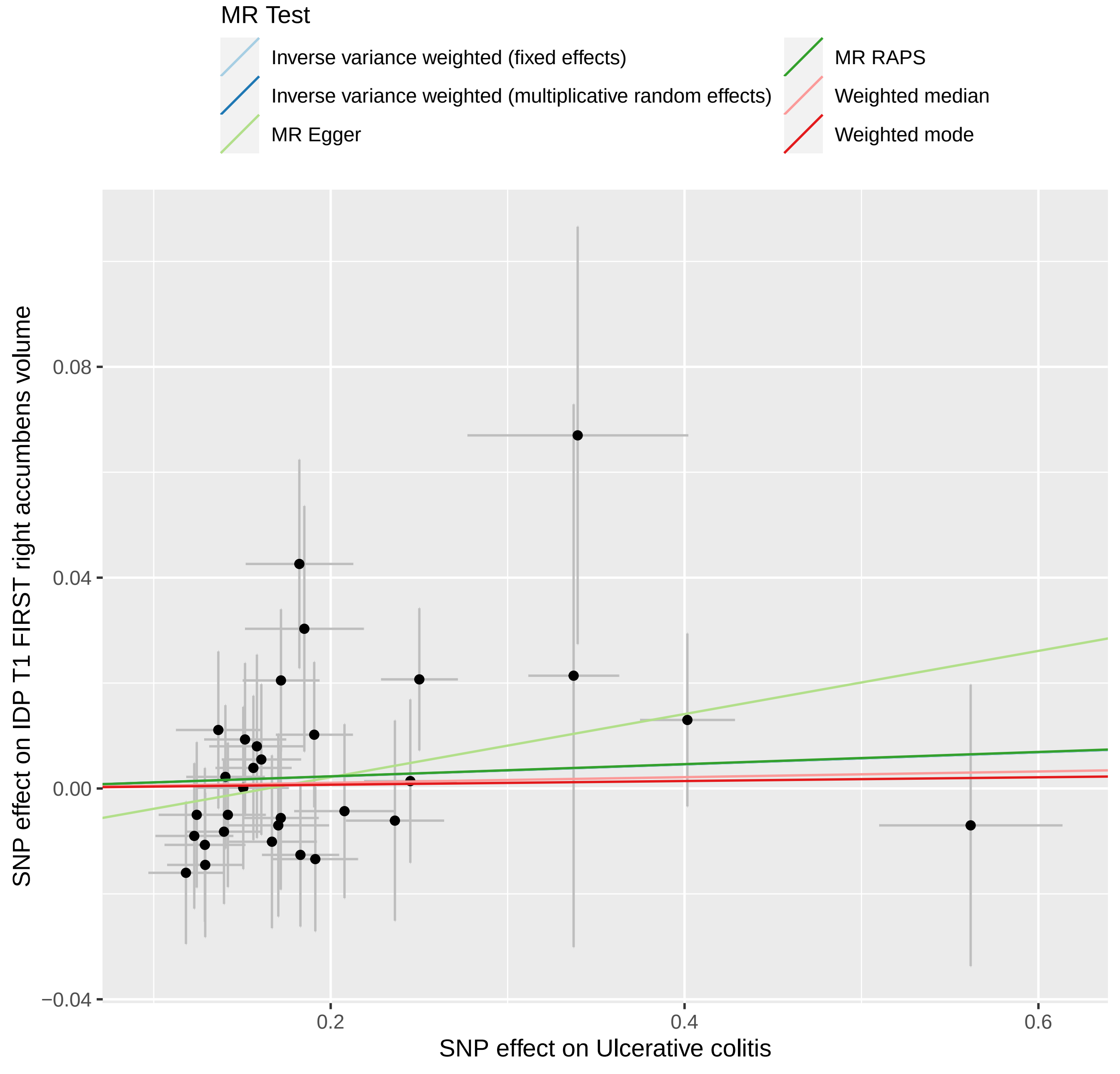

Batch 335 : Crohn's disease on IDP T1 FIRST brain stem+4th ventricle volume

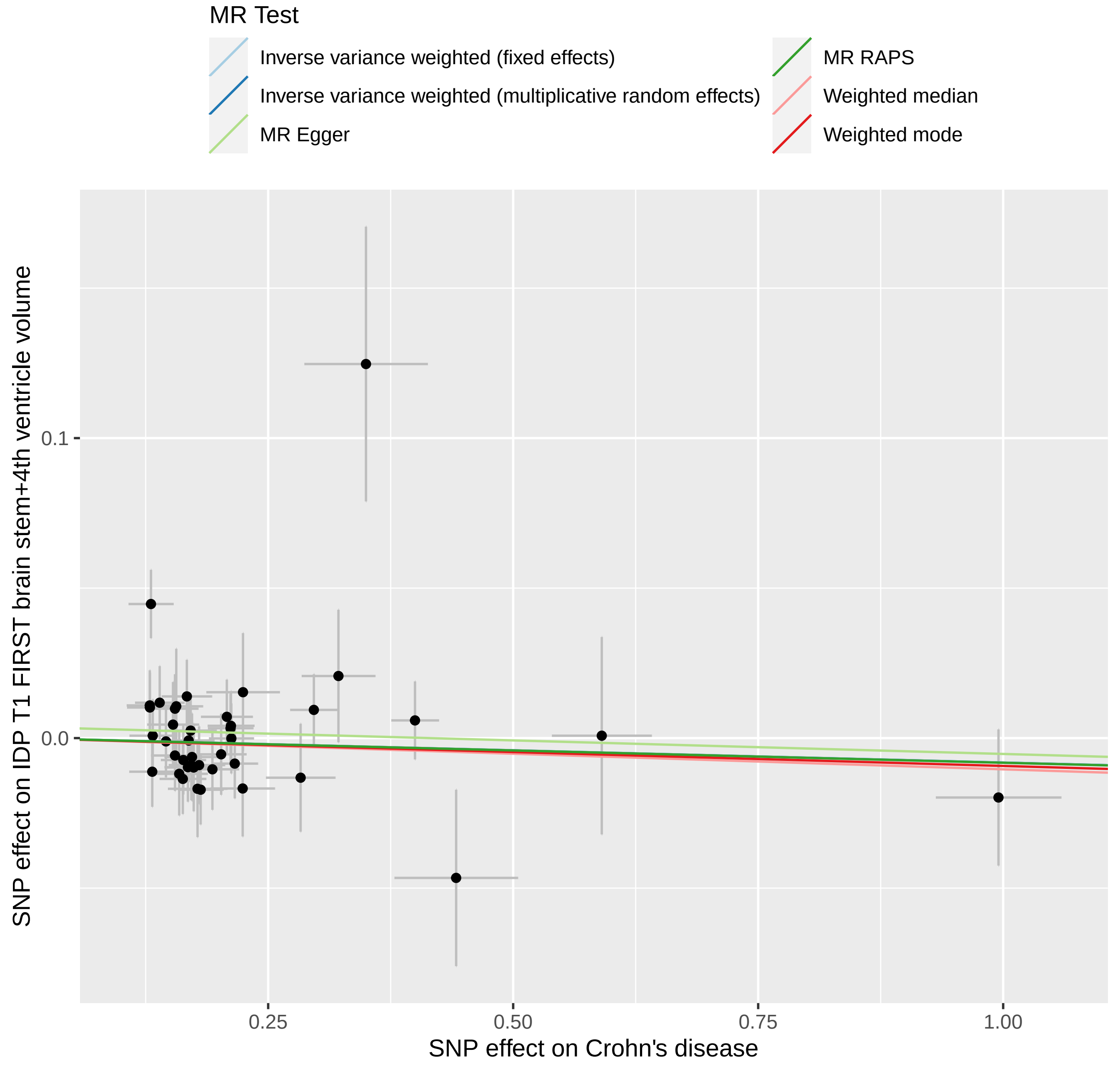

Batch 336 : Ulcerative colitis on IDP T1 FIRST brain stem+4th ventricle volume

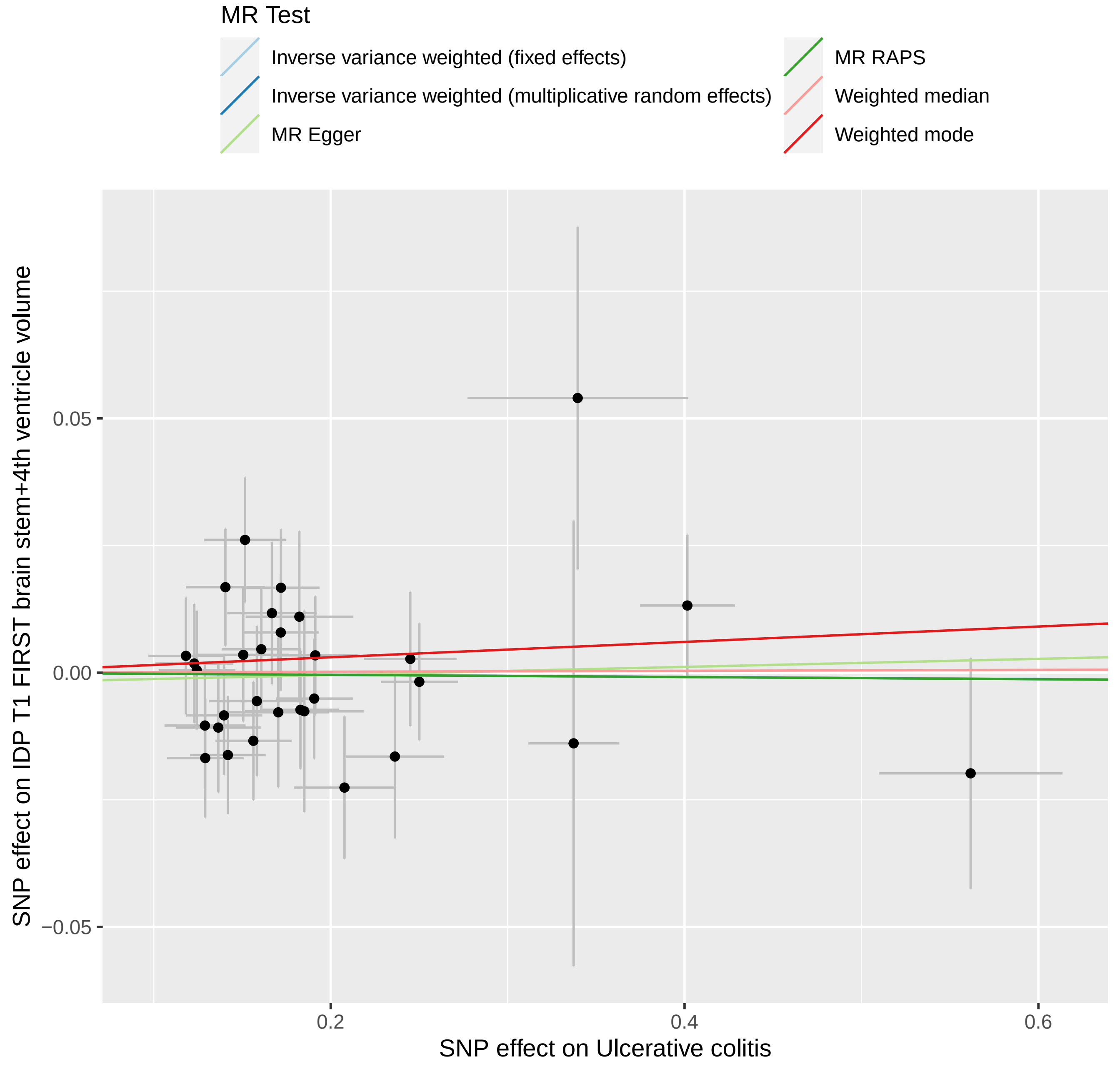

Batch 357 : Crohn's disease on IDP T1 FAST ROIs L frontal pole

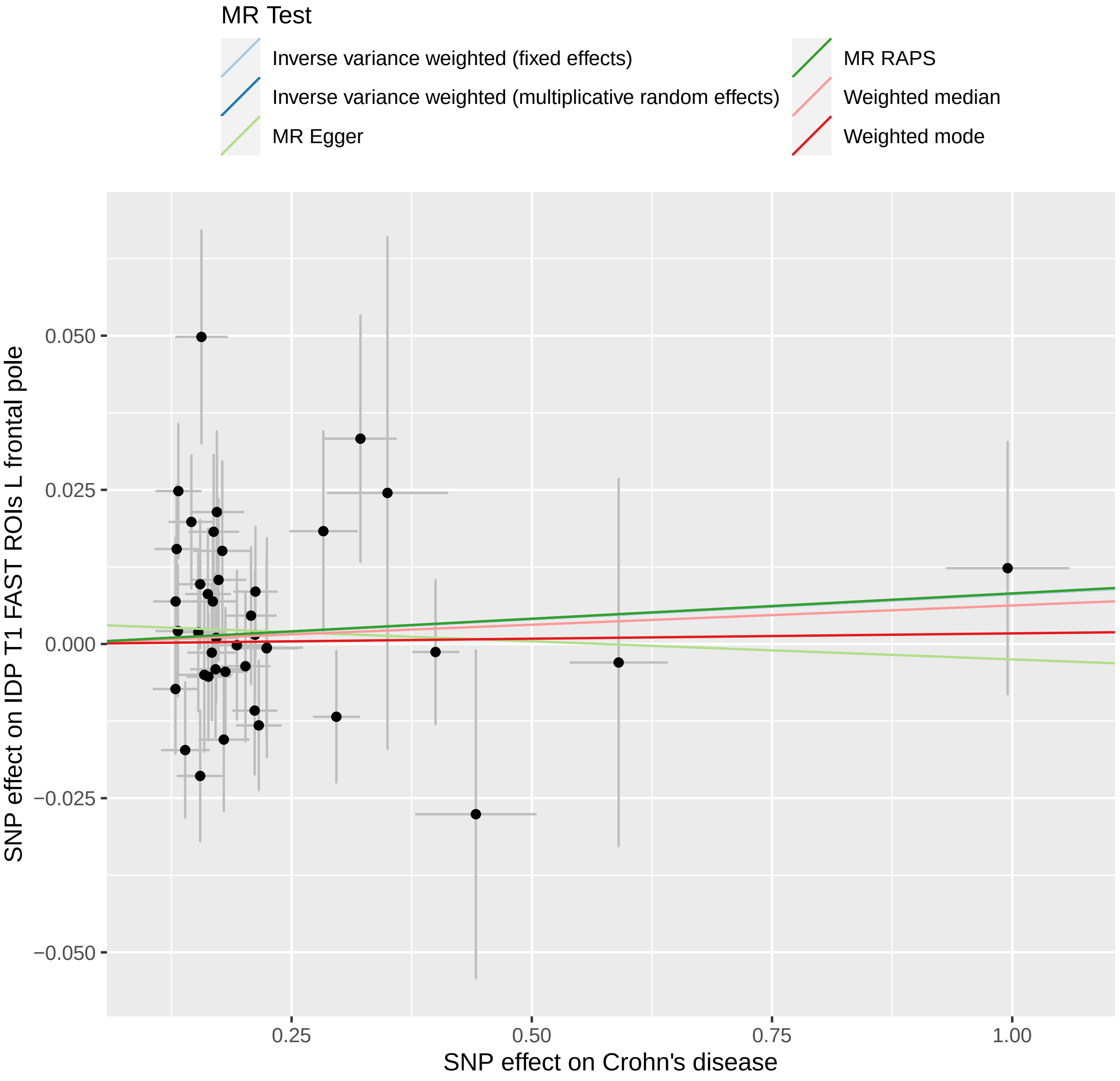

Batch 358 : Ulcerative colitis on IDP T1 FAST ROIs L frontal pole

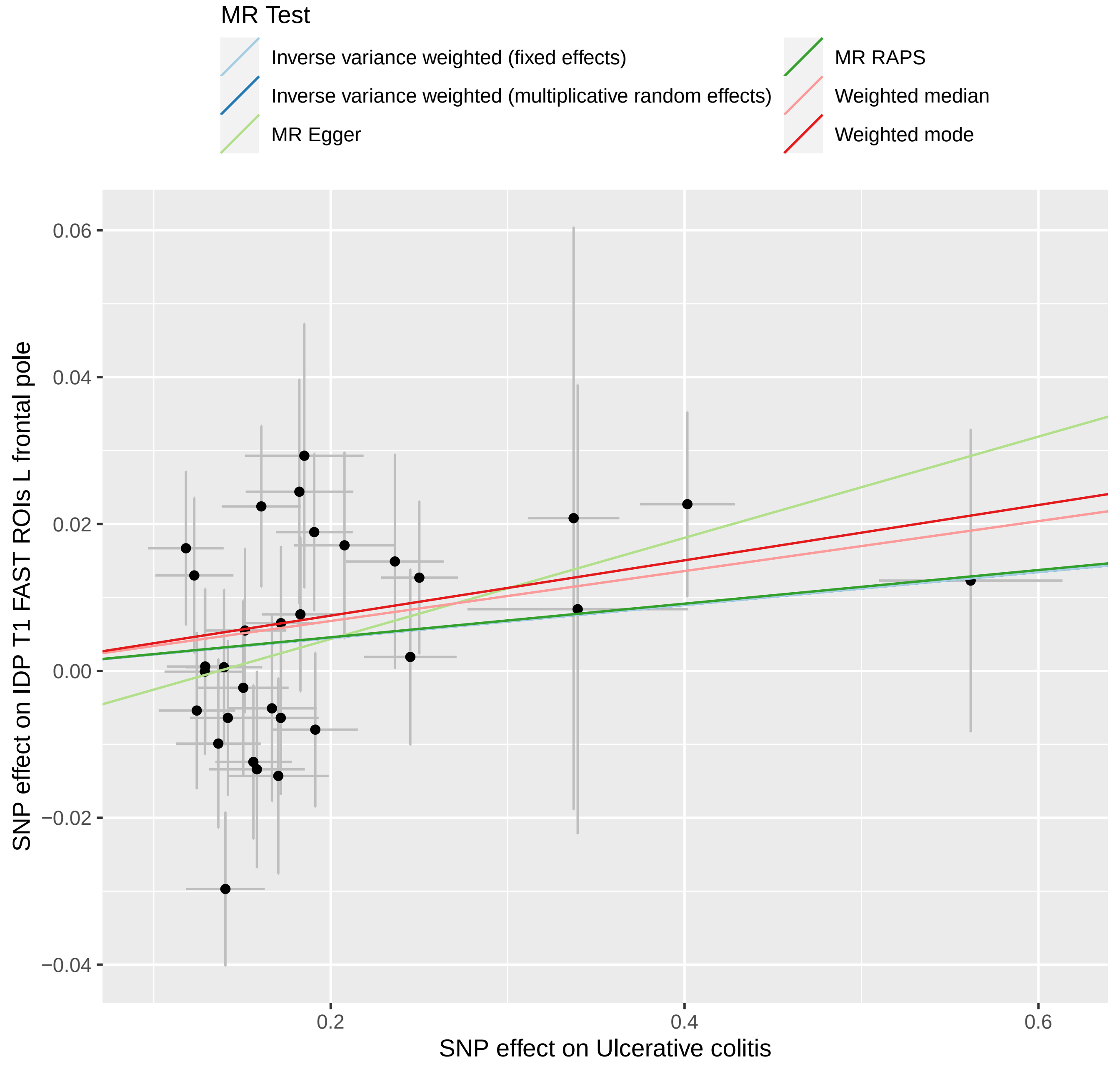

Batch 369 : Crohn's disease on IDP T1 FIRST left thalamus volume plus IDP T1 FIRST right thalamus volume

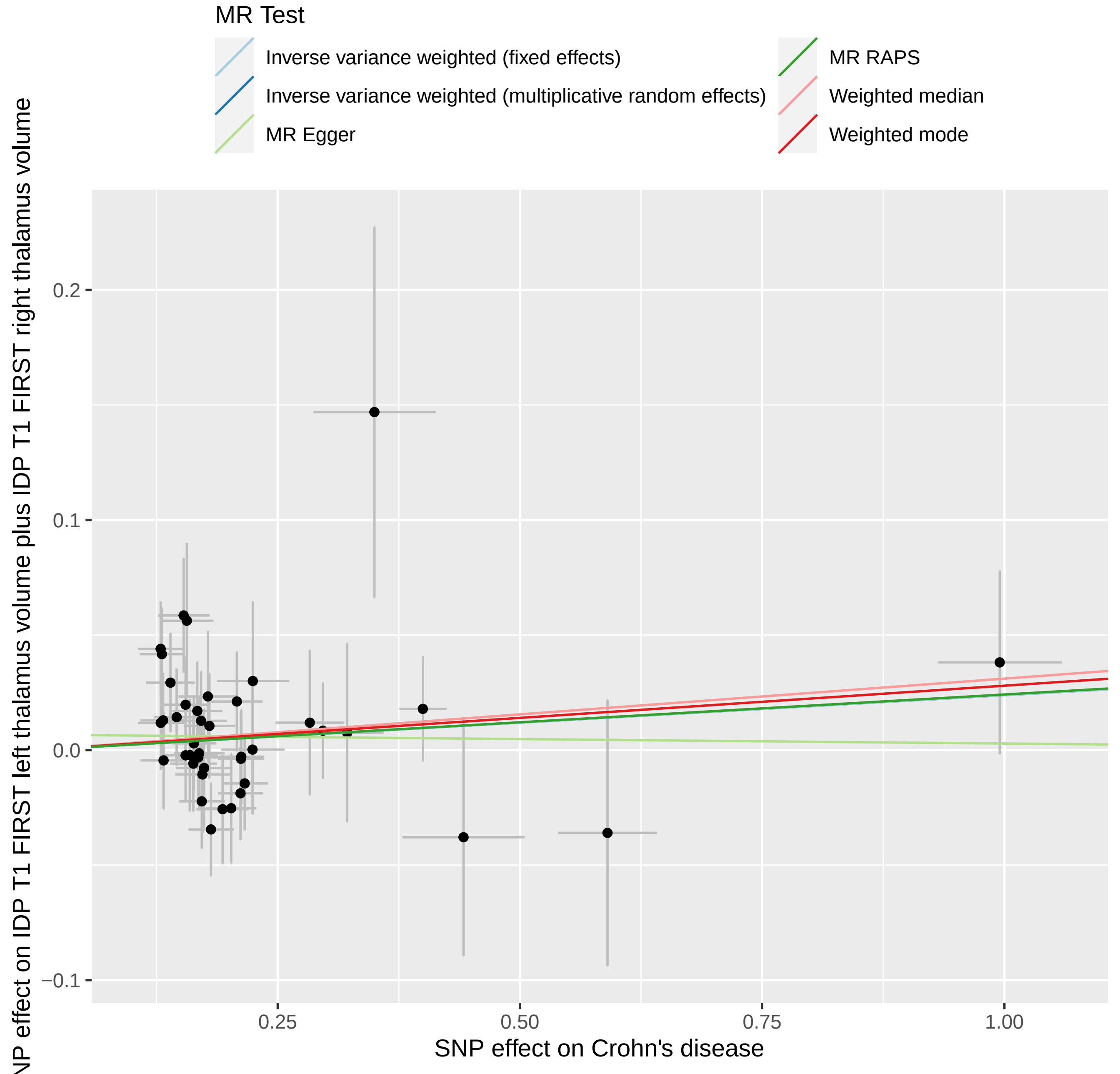

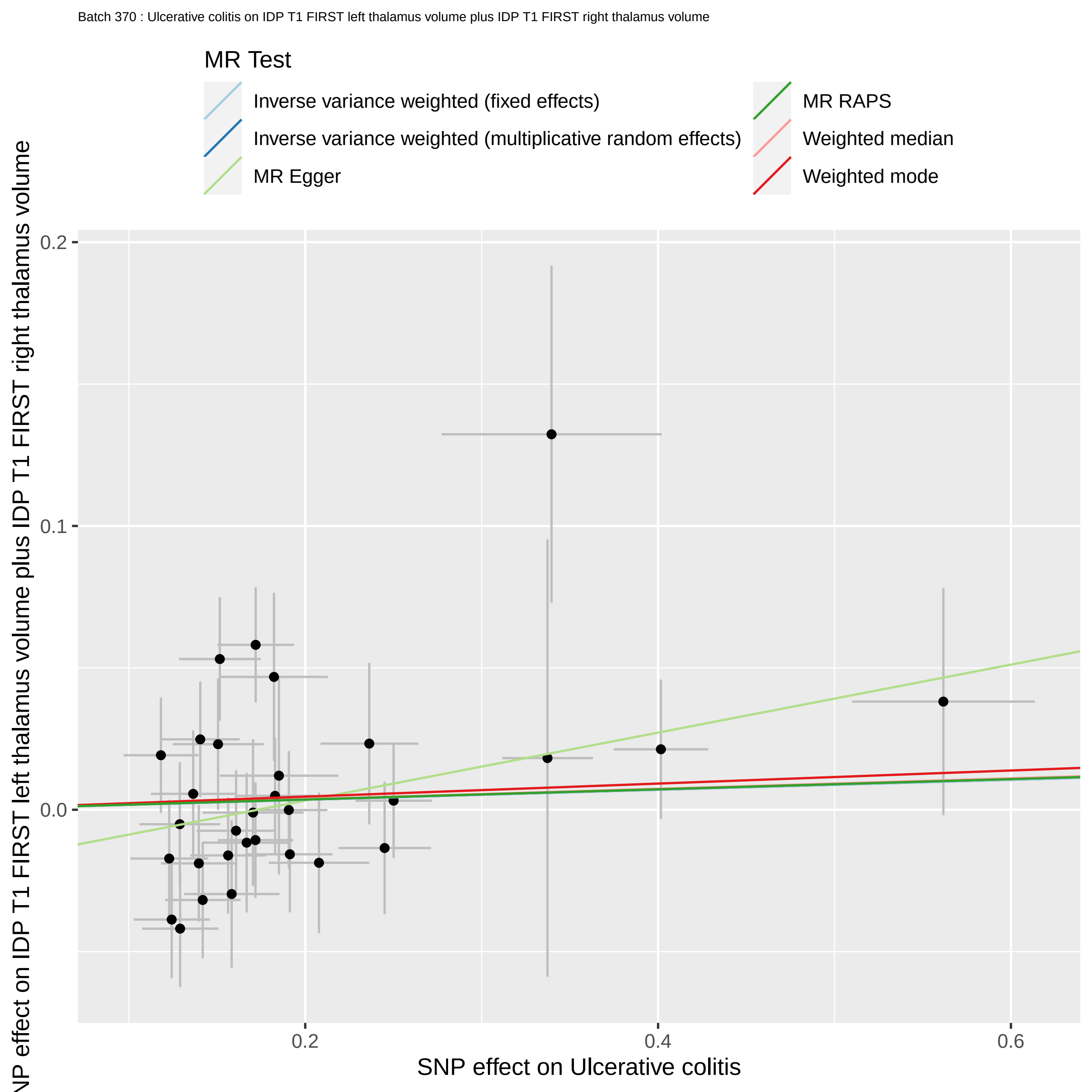

Batch 370 : Ulcerative colitis on IDP T1 FIRST left thalamus volume plus IDP T1 FIRST right thalamus volume
MR Test
Inverse variance weighted (fixed effects)
Inverse variance weighted (multiplicative random effects)
MR Egger
MR RAPS
Weighted median
Weighted mode
0.2
0.1
0.0
0.2
0.4
0.6
SNP effect on Ulcerative colitis
NP effect on IDP T1 FIRST left thalamus volume plus IDP T1 FIRST right thalamus volume

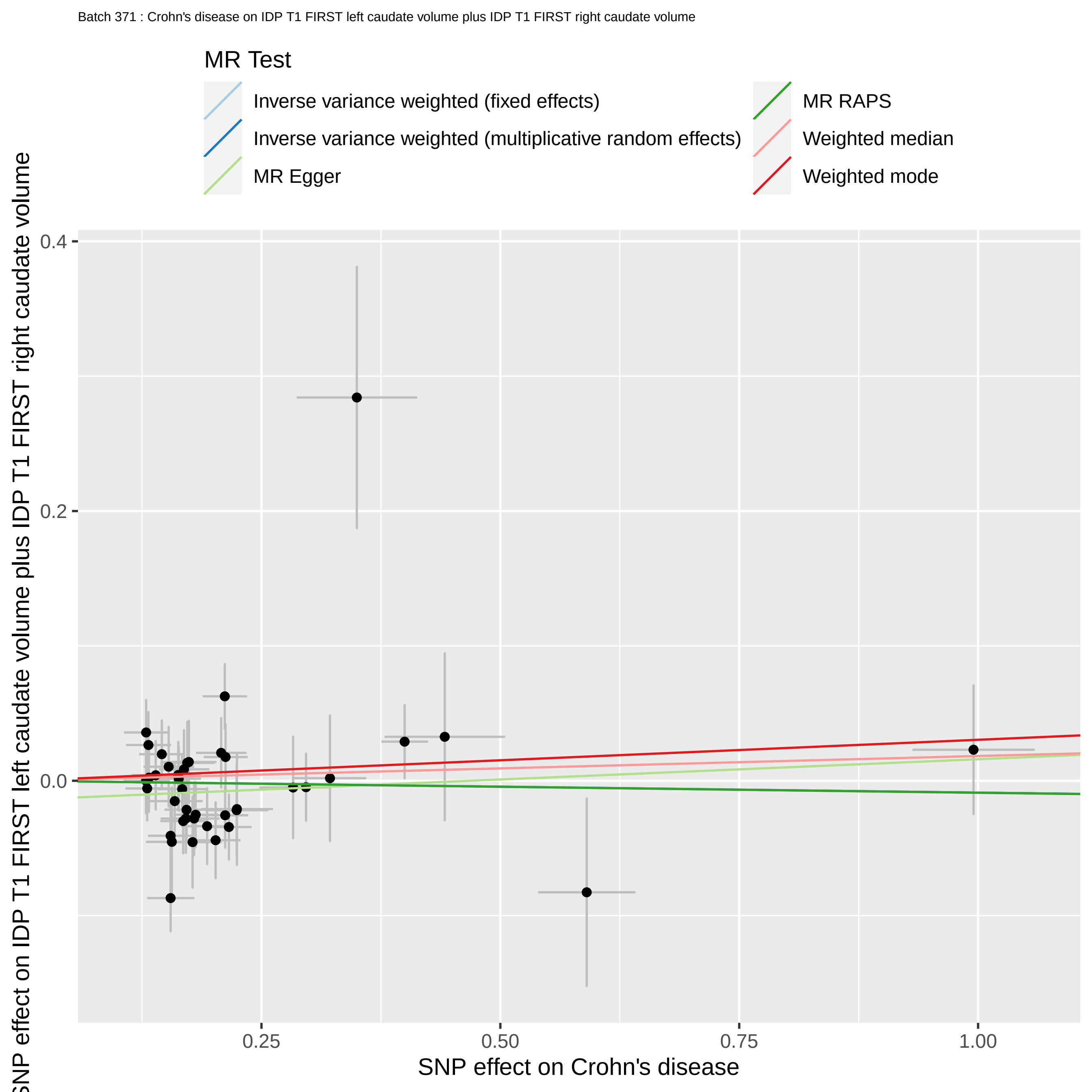

Batch 371 : Crohn's disease on IDP T1 FIRST left caudate volume plus IDP T1 FIRST right caudate volume
MR Test
Inverse variance weighted (fixed effects)
Inverse variance weighted (multiplicative random effects)
MR Egger
MR RAPS
Weighted median
Weighted mode
0.4
0.2
0.0
0.25
0.50
0.75
1.00
SNP effect on Crohn's disease
SNP effect on IDP T1 FIRST left caudate volume plus IDP T1 FIRST right caudate volume

Batch 372 : Ulcerative colitis on IDP T1 FIRST left caudate volume plus IDP T1 FIRST right caudate volume

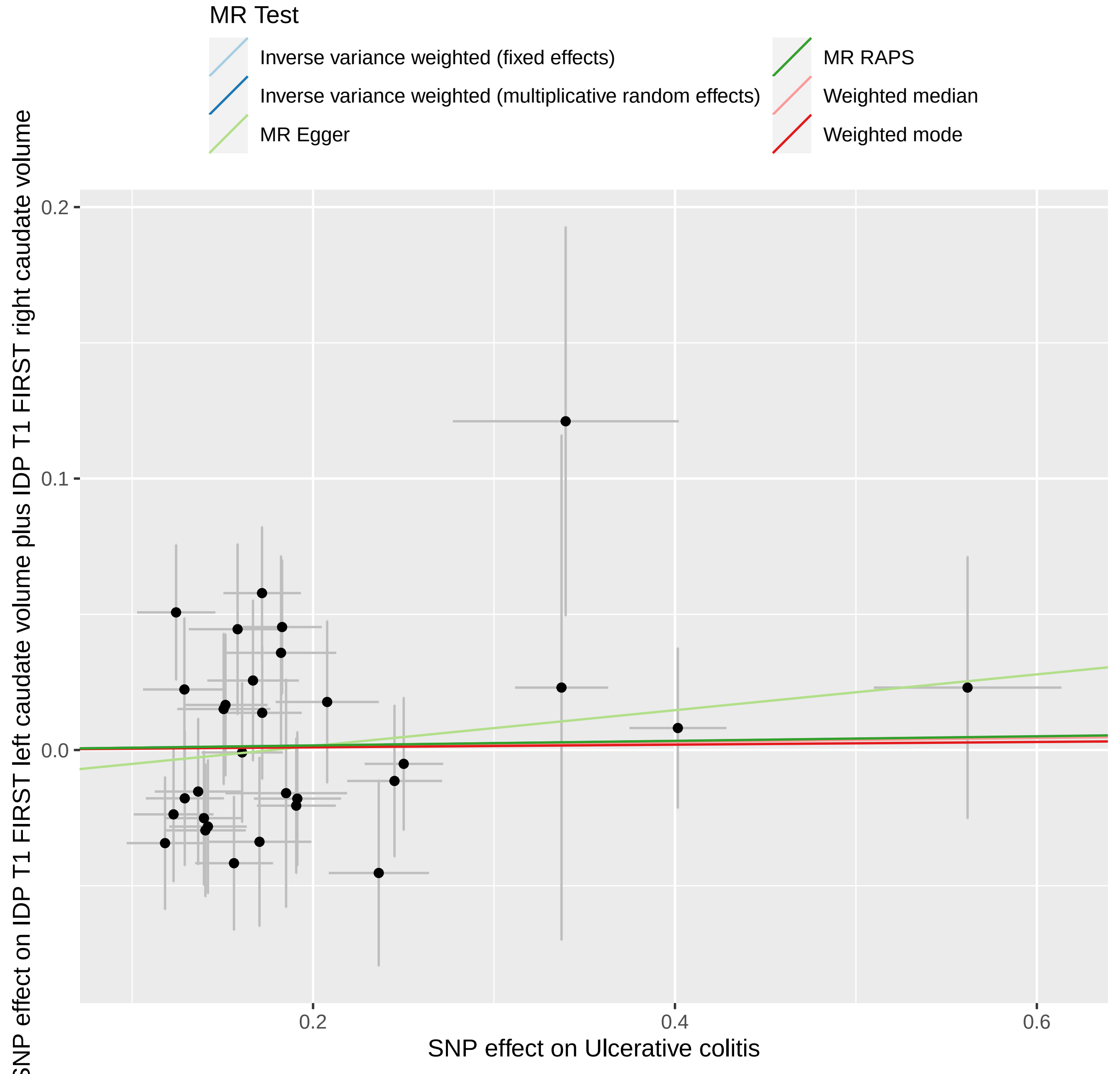

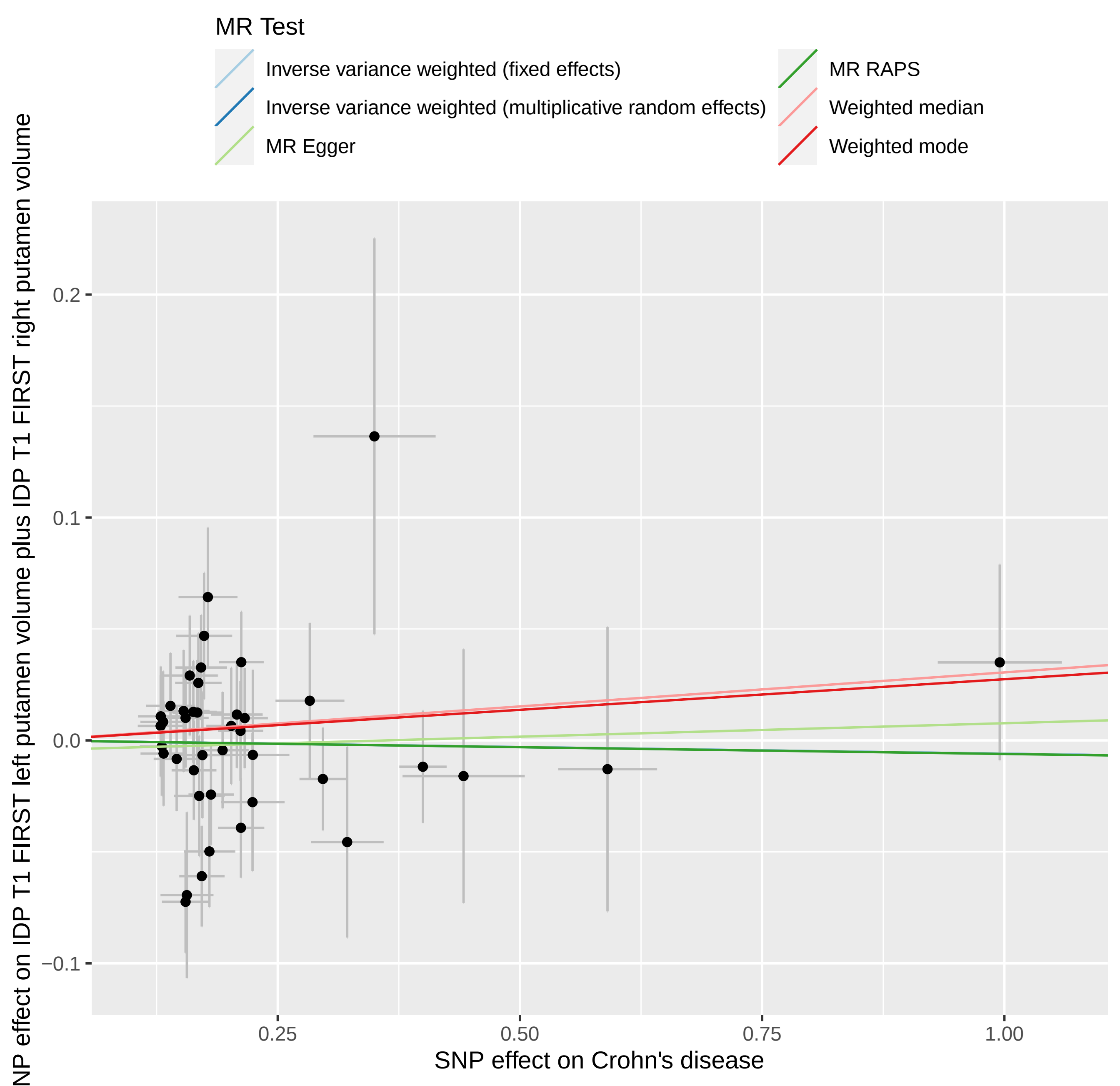
Batch 373 : Crohn's disease on IDP T1 FIRST left putamen volume plus IDP T1 FIRST right putamen volume
MR Test
Inverse variance weighted (fixed effects)
Inverse variance weighted (multiplicative random effects)
MR Egger
MR RAPS
Weighted median
Weighted mode
0.2
0.1
0.0
−0.1
0.25
0.50
0.75
1.00
SNP effect on Crohn's disease
NP effect on IDP T1 FIRST left putamen volume plus IDP T1 FIRST right putamen volume

Batch 374 : Ulcerative colitis on IDP T1 FIRST left putamen volume plus IDP T1 FIRST right putamen volume

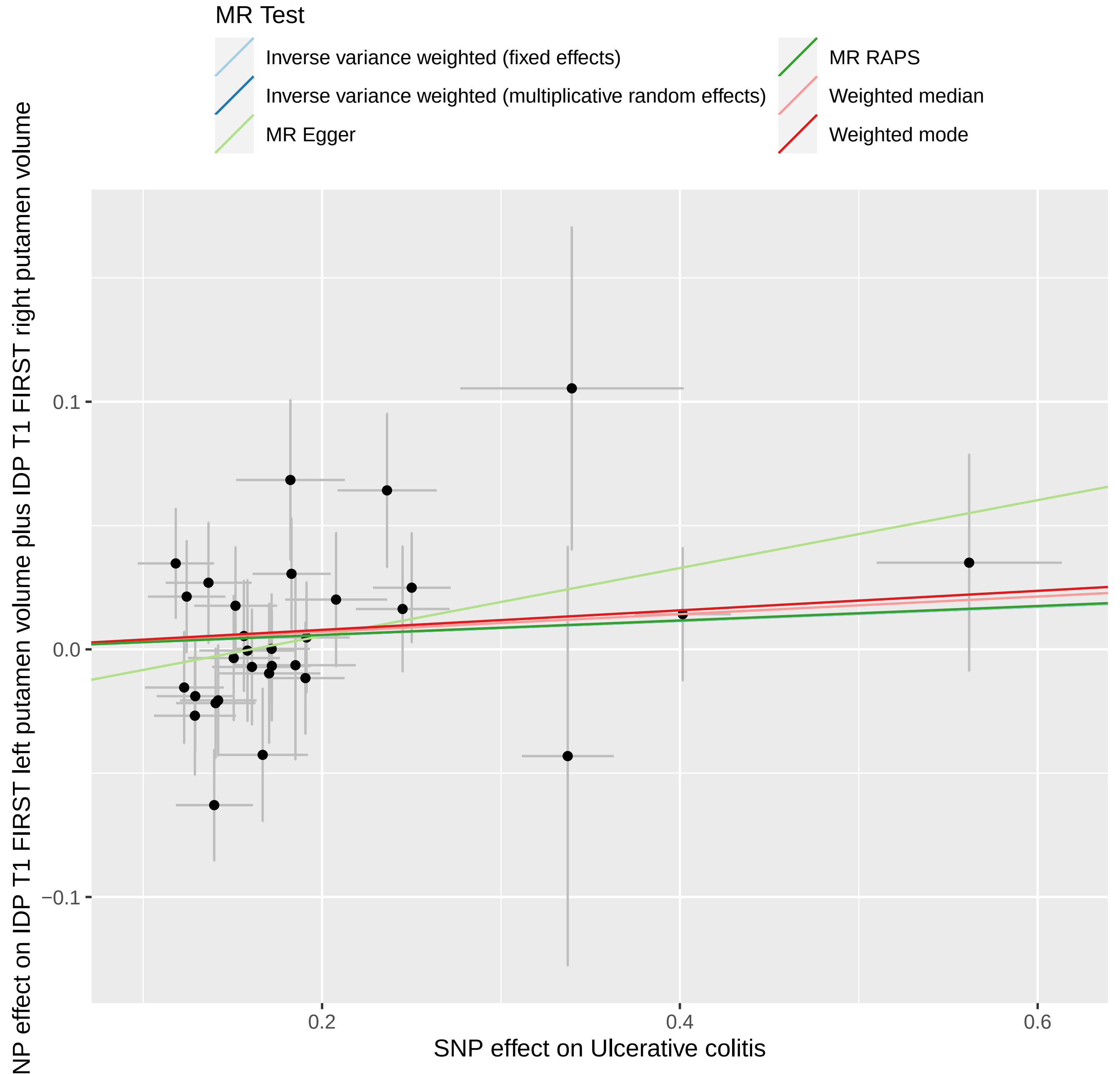

Batch 375 : Crohn's disease on IDP T1 FIRST left pallidum volume plus IDP T1 FIRST right pallidum volume

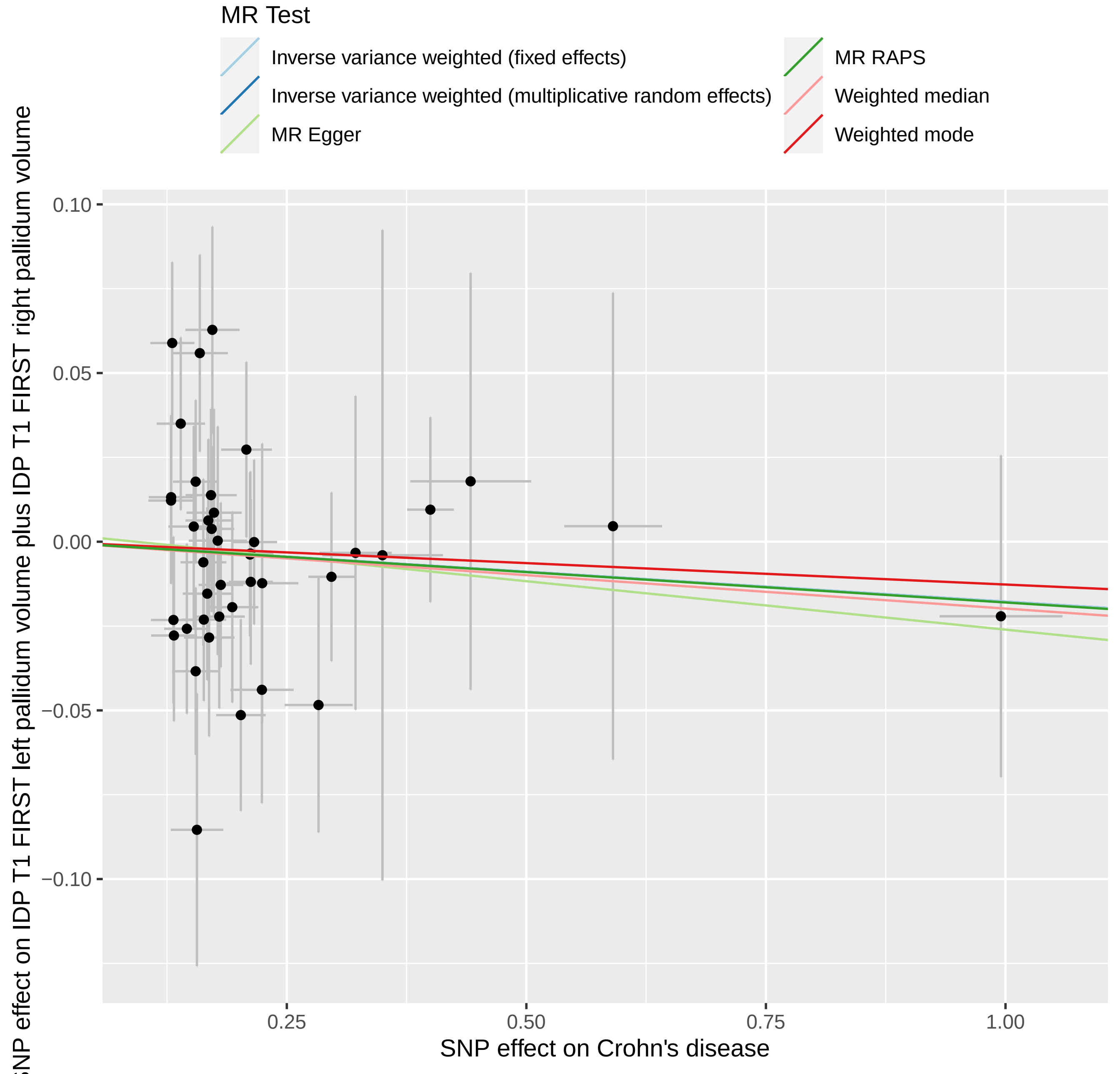

Batch 376 : Ulcerative colitis on IDP T1 FIRST left pallidum volume plus IDP T1 FIRST right pallidum volume

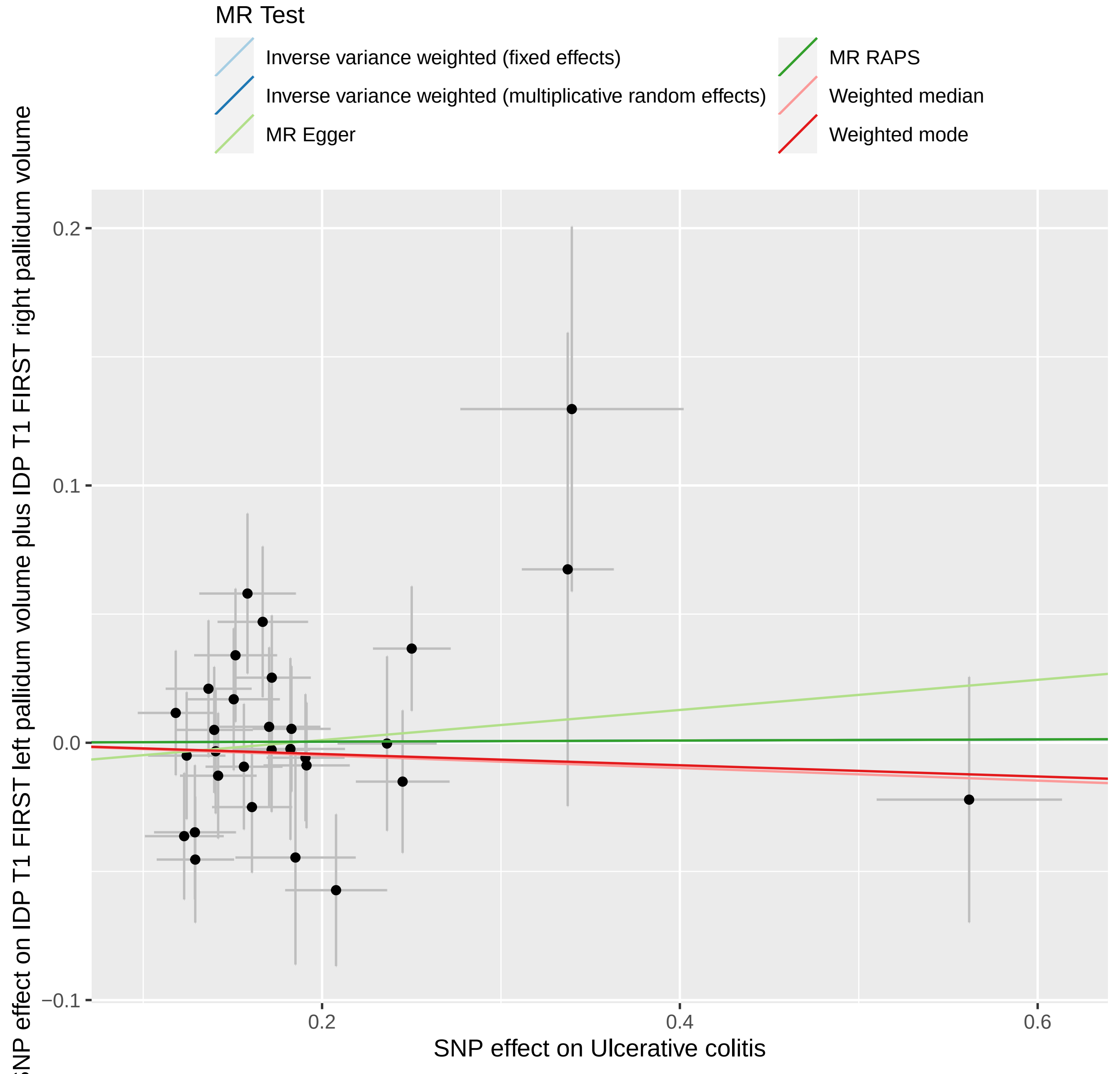

Batch 377 : Crohn's disease on IDP T1 FIRST left hippocampus volume plus IDP T1 FIRST right hippocampus volume

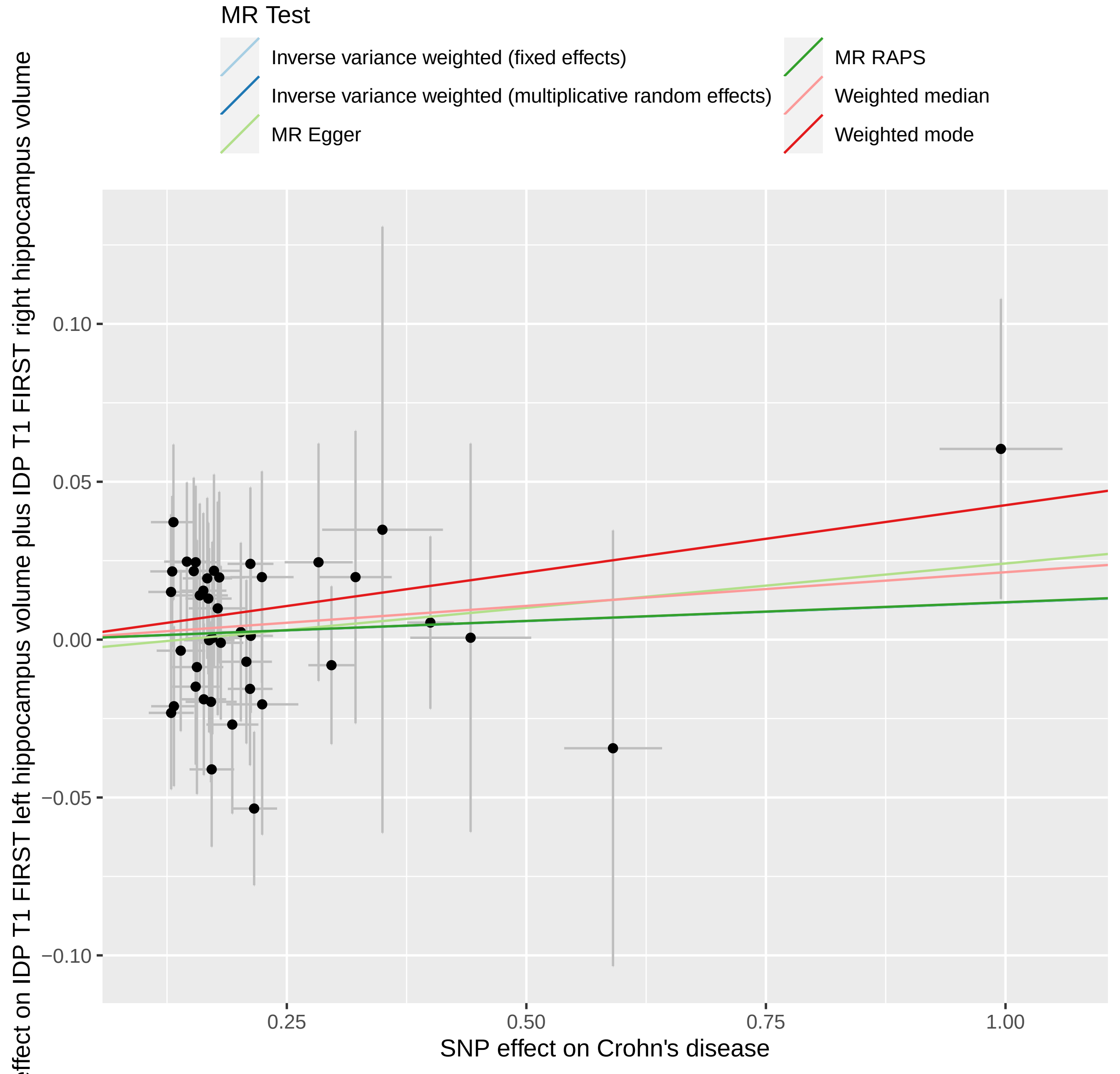

Batch 378 : Ulcerative colitis on IDP T1 FIRST left hippocampus volume plus IDP T1 FIRST right hippocampus volume

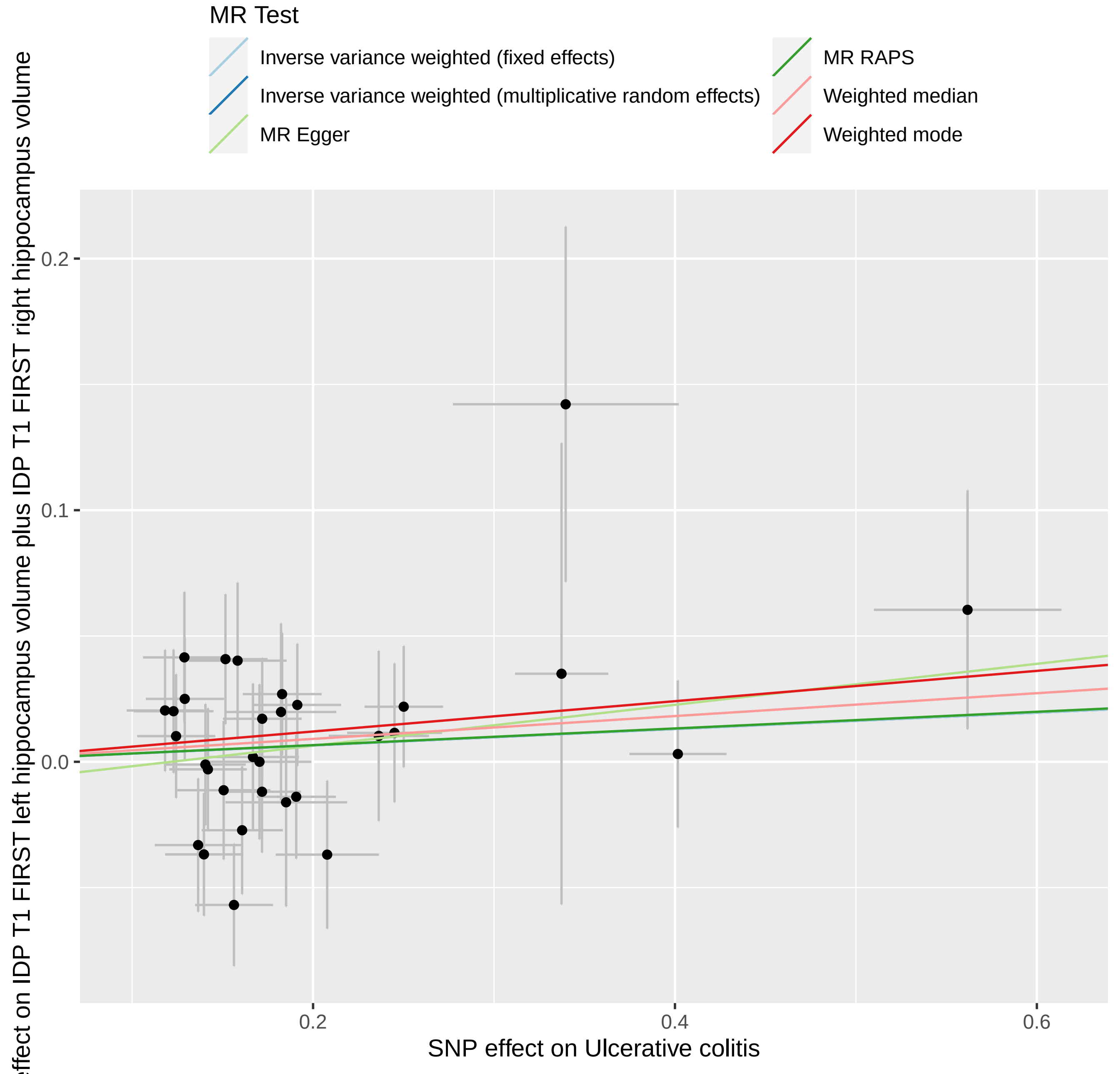

Batch 379 : Crohn's disease on IDP T1 FIRST left amygdala volume plus IDP T1 FIRST right amygdala volume

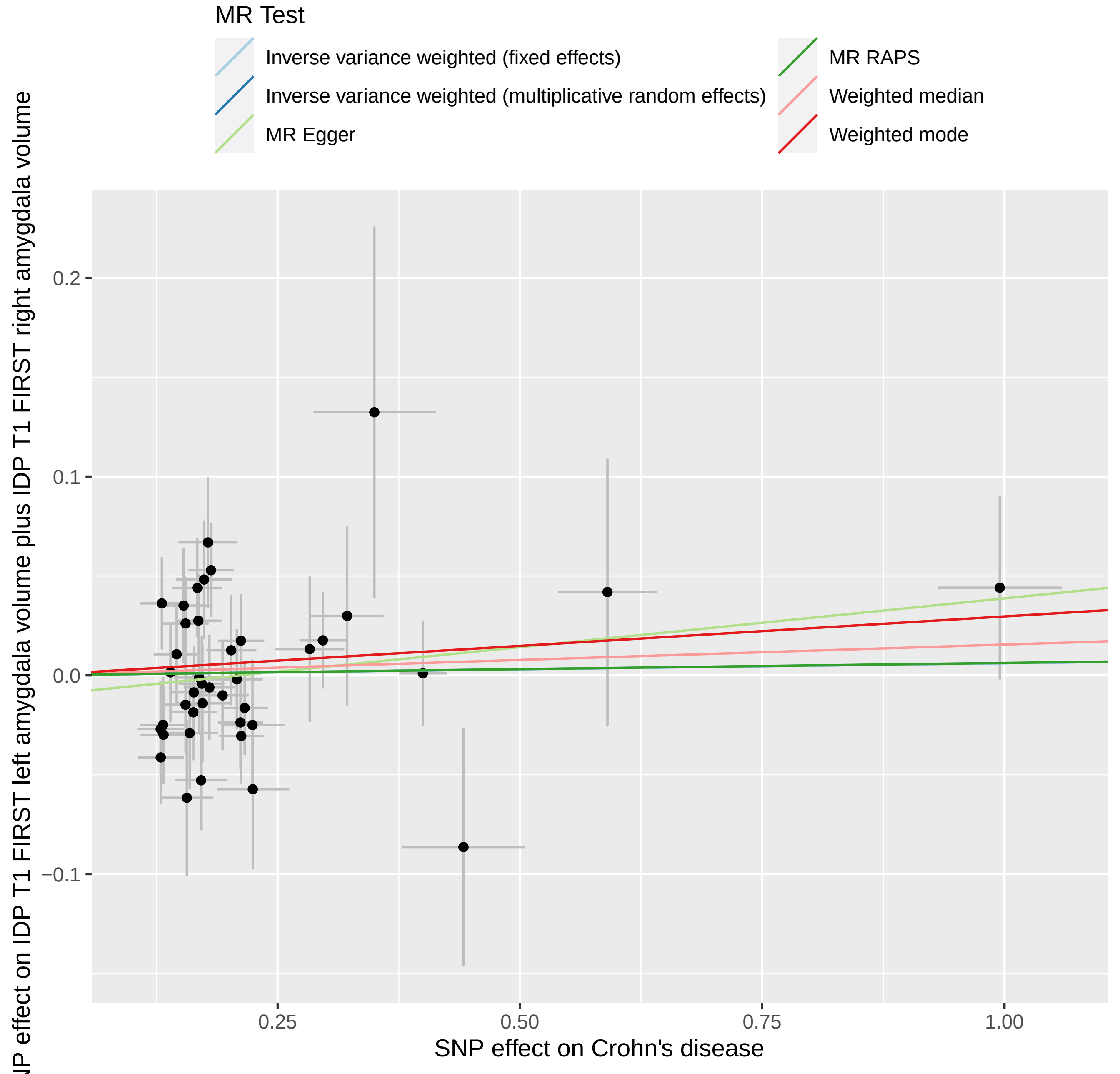

Batch 380 : Ulcerative colitis on IDP T1 FIRST left amygdala volume plus IDP T1 FIRST right amygdala volume

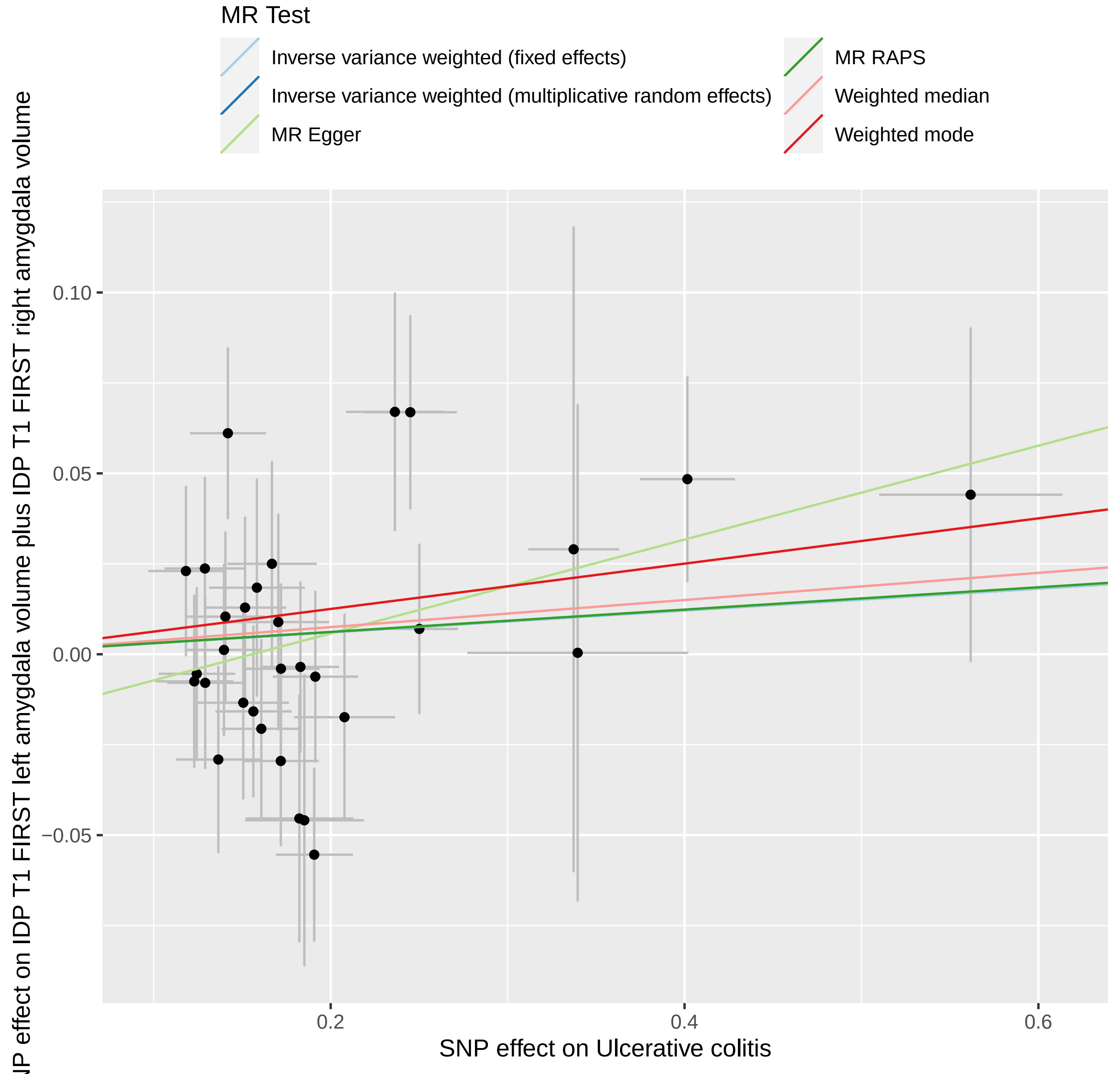

Batch 381 : Crohn's disease on IDP T1 FIRST left accumbens volume plus IDP T1 FIRST right accumbens volume

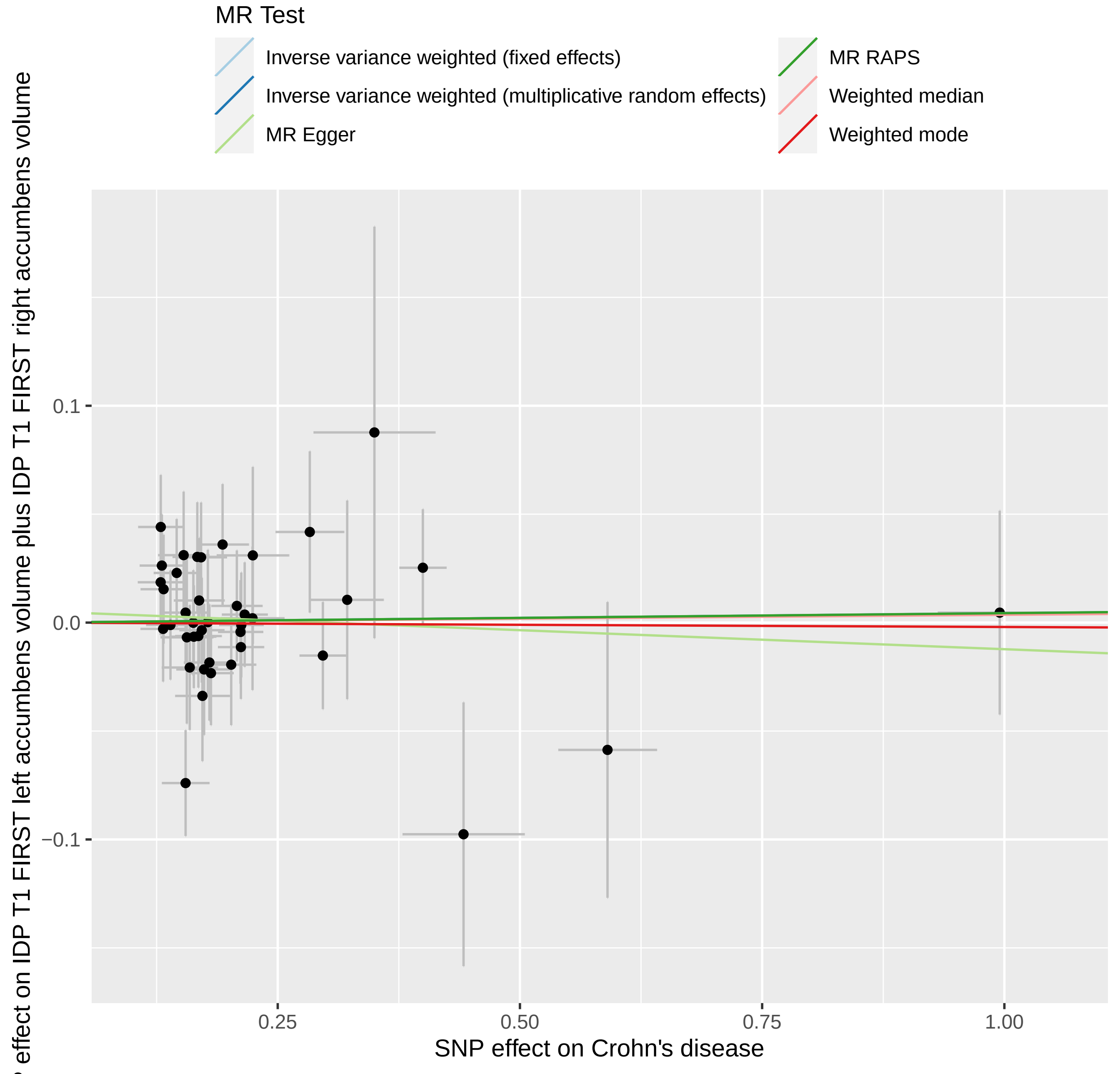

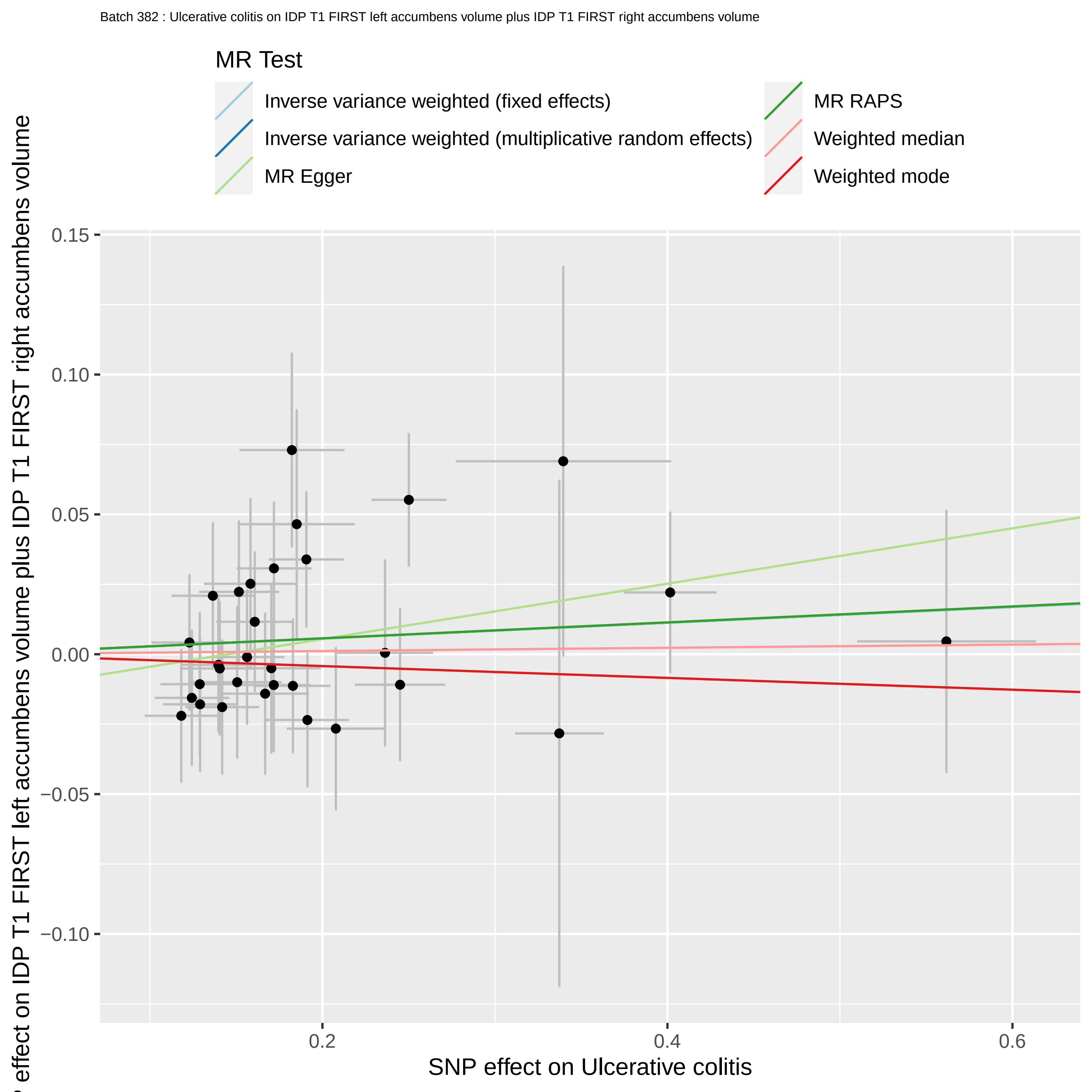
Batch 382 : Ulcerative colitis on IDP T1 FIRST left accumbens volume plus IDP T1 FIRST right accumbens volume
MR Test
Inverse variance weighted (fixed effects)
Inverse variance weighted (multiplicative random effects)
MR Egger
MR RAPS
Weighted median
Weighted mode
0.15
0.10
0.05
0.00
−0.05
−0.10
0.2
0.4
0.6
SNP effect on Ulcerative colitis
effect on IDP T1 FIRST left accumbens volume plus IDP T1 FIRST right accumbens volume

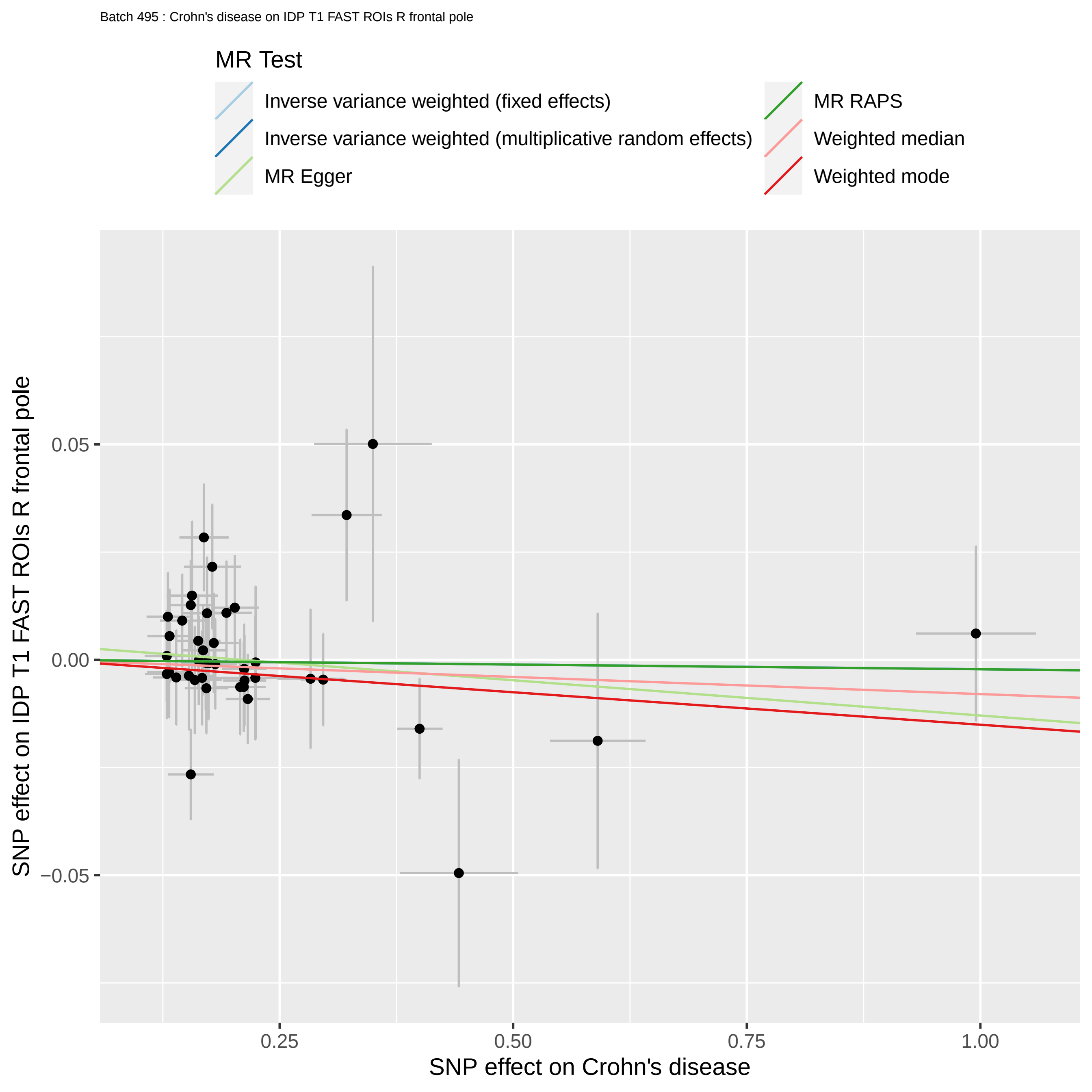
Batch 495 : Crohn's disease on IDP T1 FAST ROIs R frontal pole
MR Test
Inverse variance weighted (fixed effects)
Inverse variance weighted (multiplicative random effects)
MR Egger
MR RAPS
Weighted median
Weighted mode
SNP effect on IDP T1 FAST ROIs R frontal pole
0.05
0.00
−0.05
0.25
0.50
0.75
1.00
SNP effect on Crohn's disease

Batch 496 : Ulcerative colitis on IDP T1 FAST ROIs R frontal pole

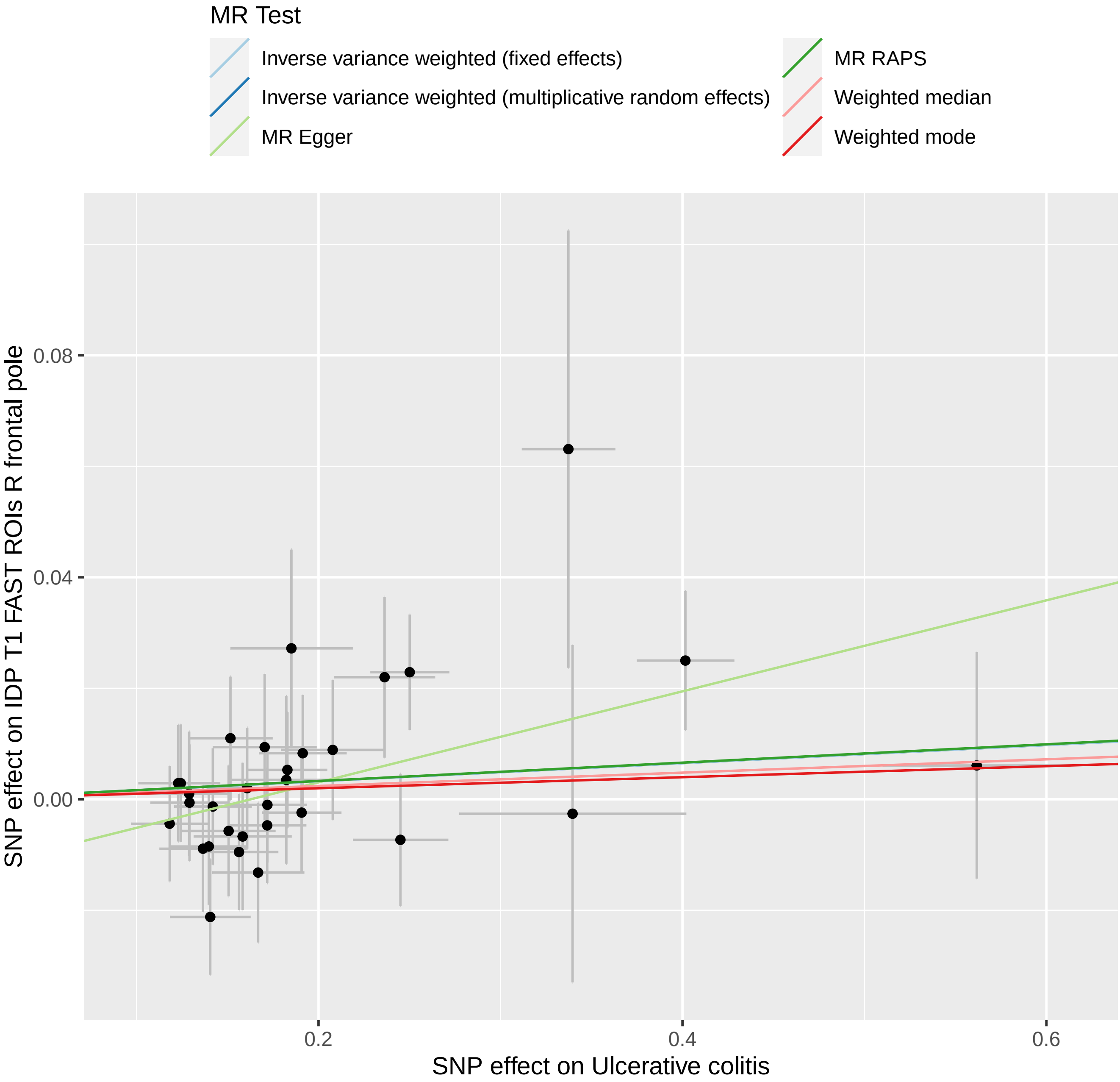

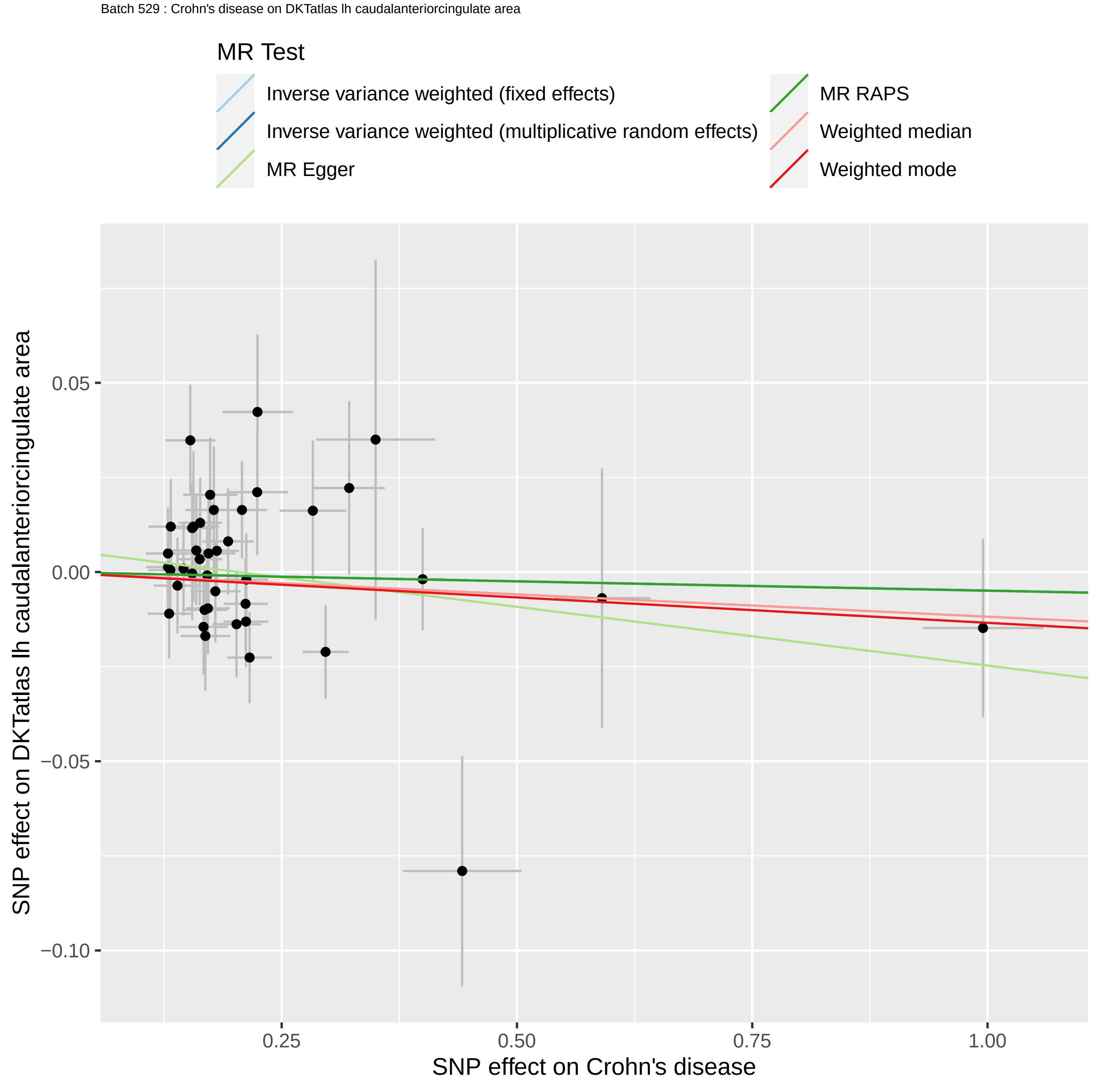

Batch 529 : Crohn's disease on DKTatlas lh caudalanteriorcingulate area
MR Test
Inverse variance weighted (fixed effects)
Inverse variance weighted (multiplicative random effects)
MR Egger
MR RAPS
Weighted median
Weighted mode
SNP effect on DKTatlas lh caudalanteriorcingulate area
0.05
0.00
−0.05
−0.10
0.25
0.50
0.75
1.00
SNP effect on Crohn's disease

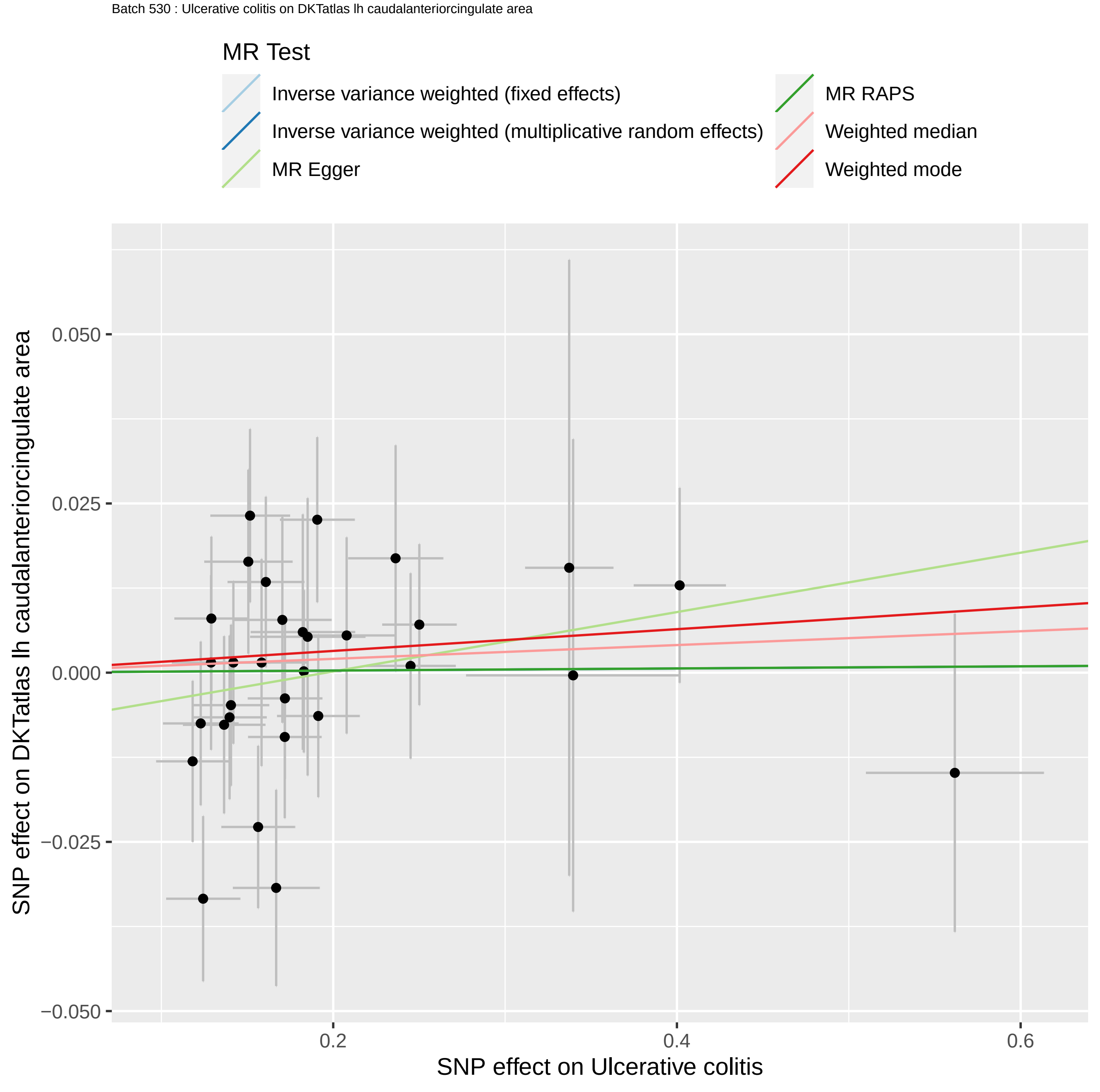

Batch 530 : Ulcerative colitis on DKTatlas lh caudalanteriorcingulate area
MR Test
Inverse variance weighted (fixed effects)
Inverse variance weighted (multiplicative random effects)
MR Egger
MR RAPS
Weighted median
Weighted mode
0.050
0.025
0.000
−0.025
−0.050
0.2
0.4
0.6
SNP effect on DKTatlas lh caudalanteriorcingulate area
SNP effect on Ulcerative colitis

Batch 531 : Crohn's disease on DKTatlas lh caudalmiddlefrontal area

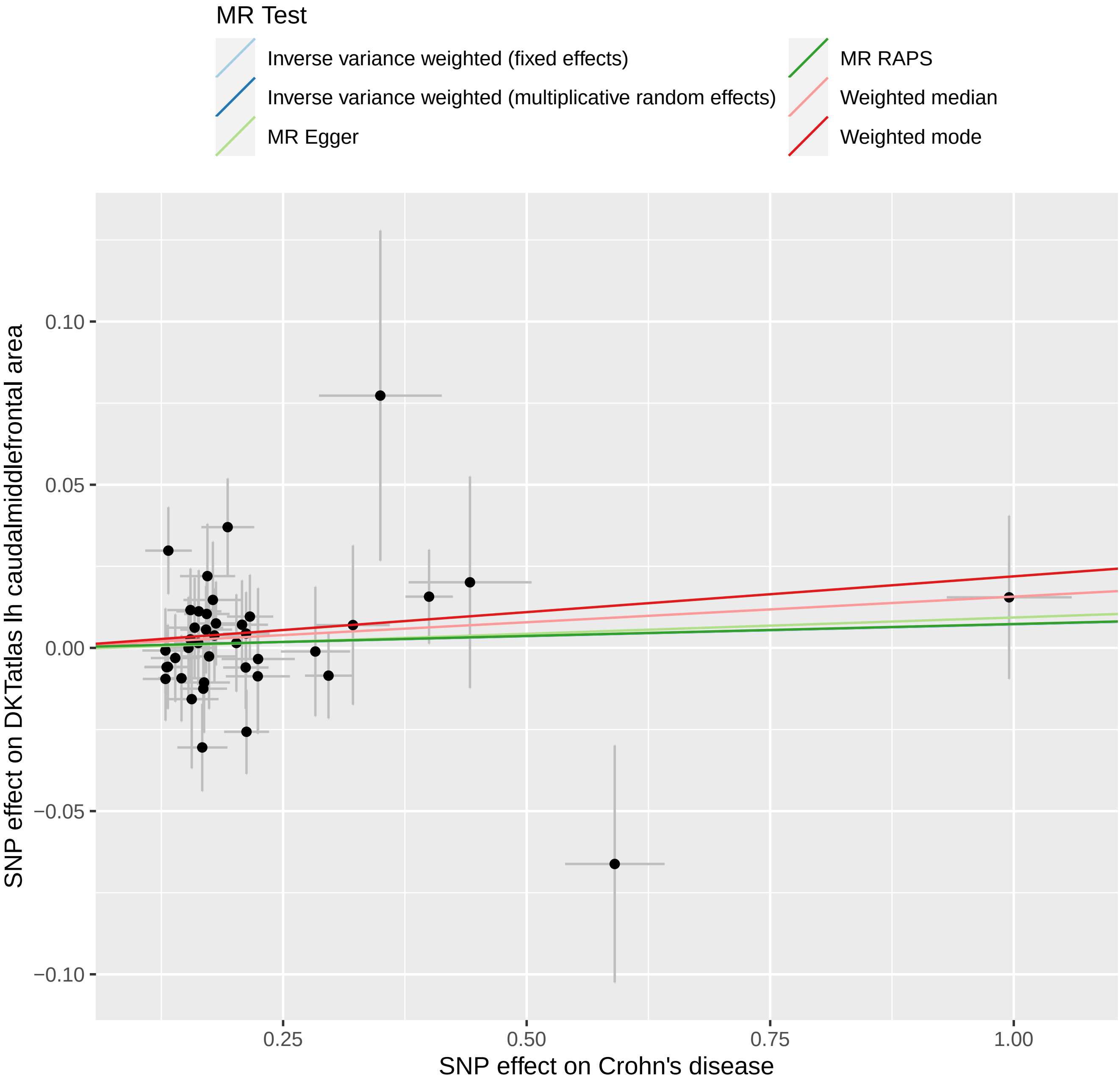

Batch 532 : Ulcerative colitis on DKTatlas lh caudalmiddlefrontal area

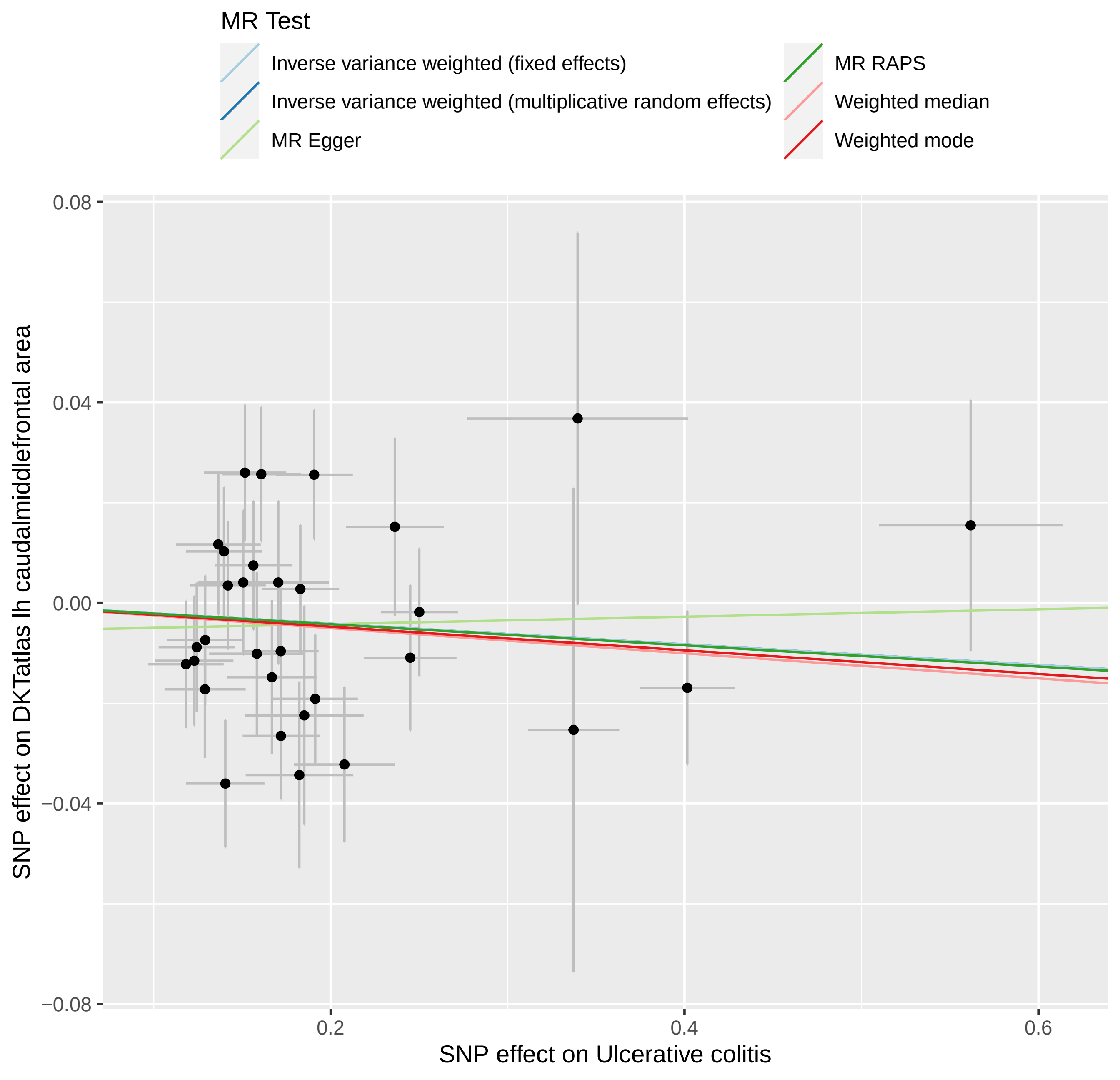

Batch 533 : Crohn's disease on DKTatlas lh cuneus area

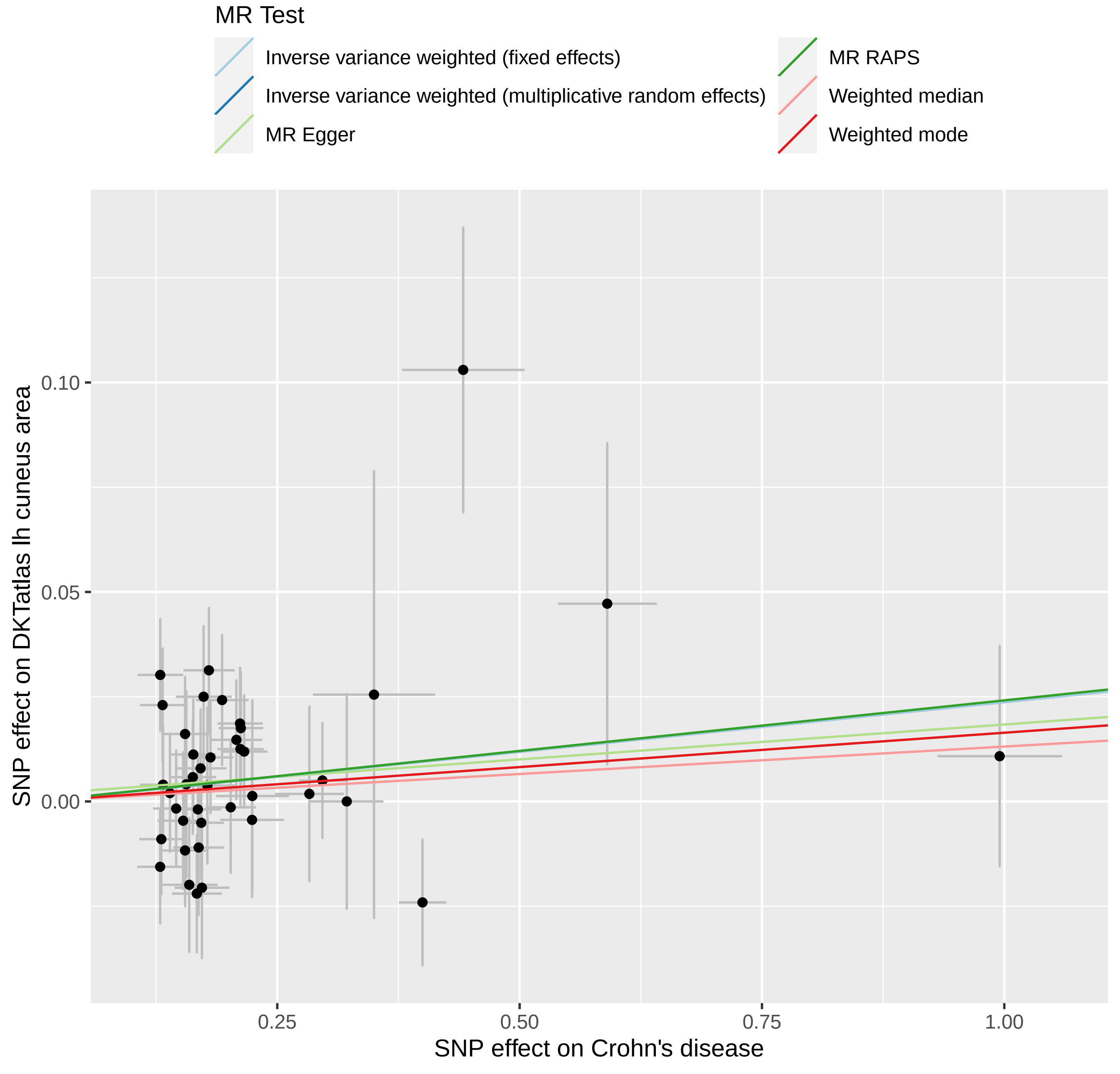

Batch 534 : Ulcerative colitis on DKTatlas lh cuneus area

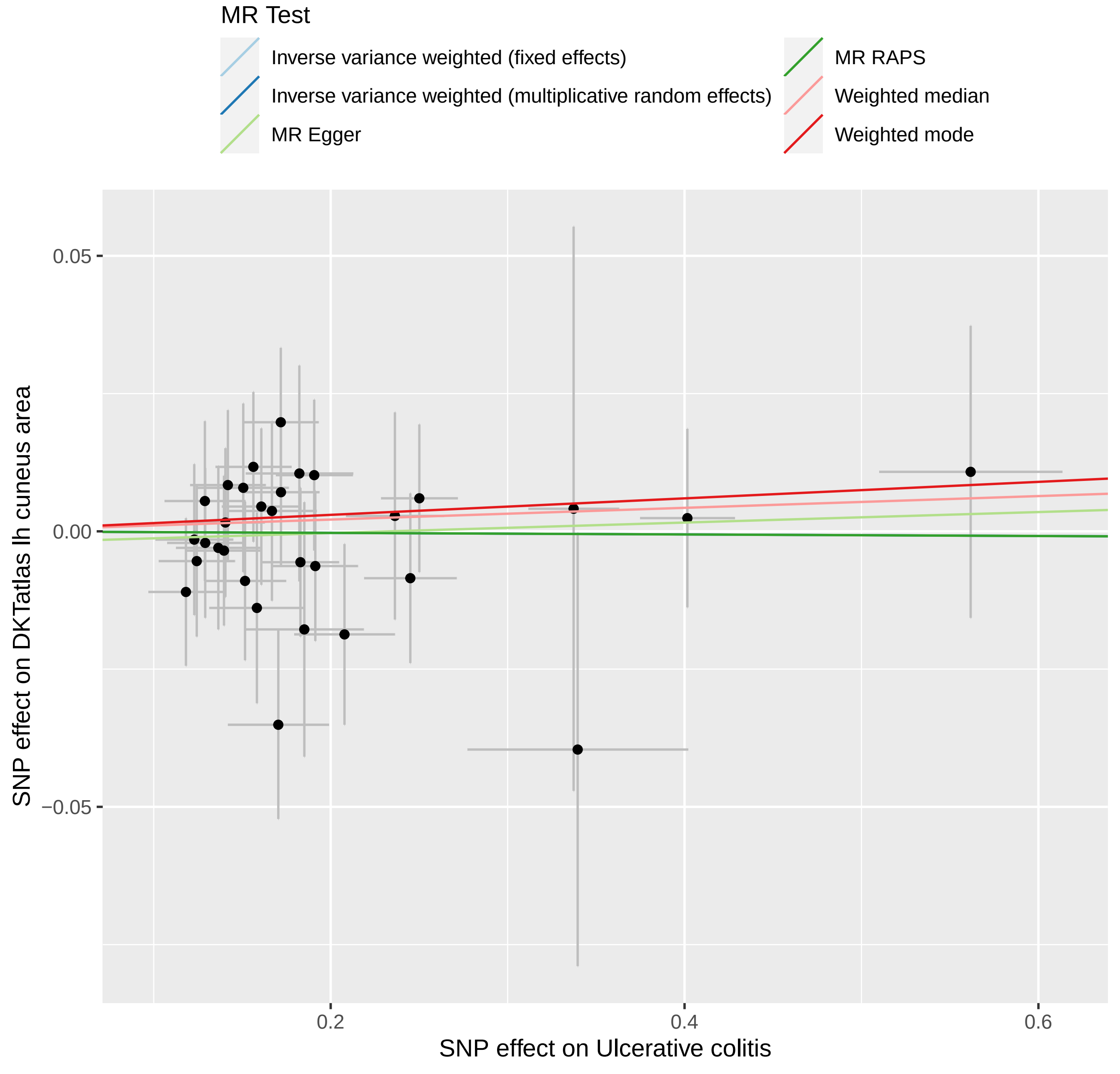

Batch 535 : Crohn's disease on DKTatlas lh entorhinal area

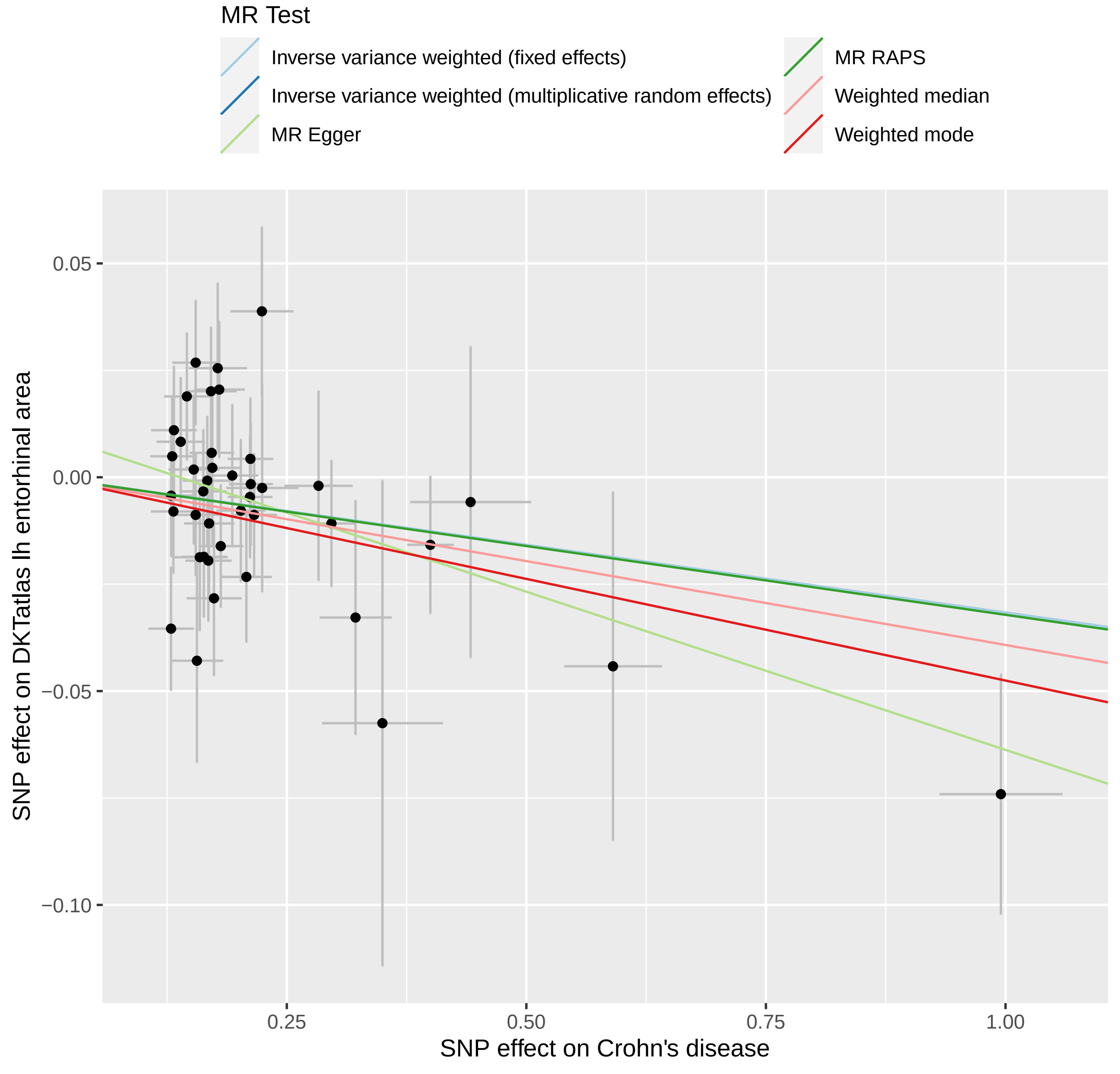

Batch 536 : Ulcerative colitis on DKTatlas lh entorhinal area

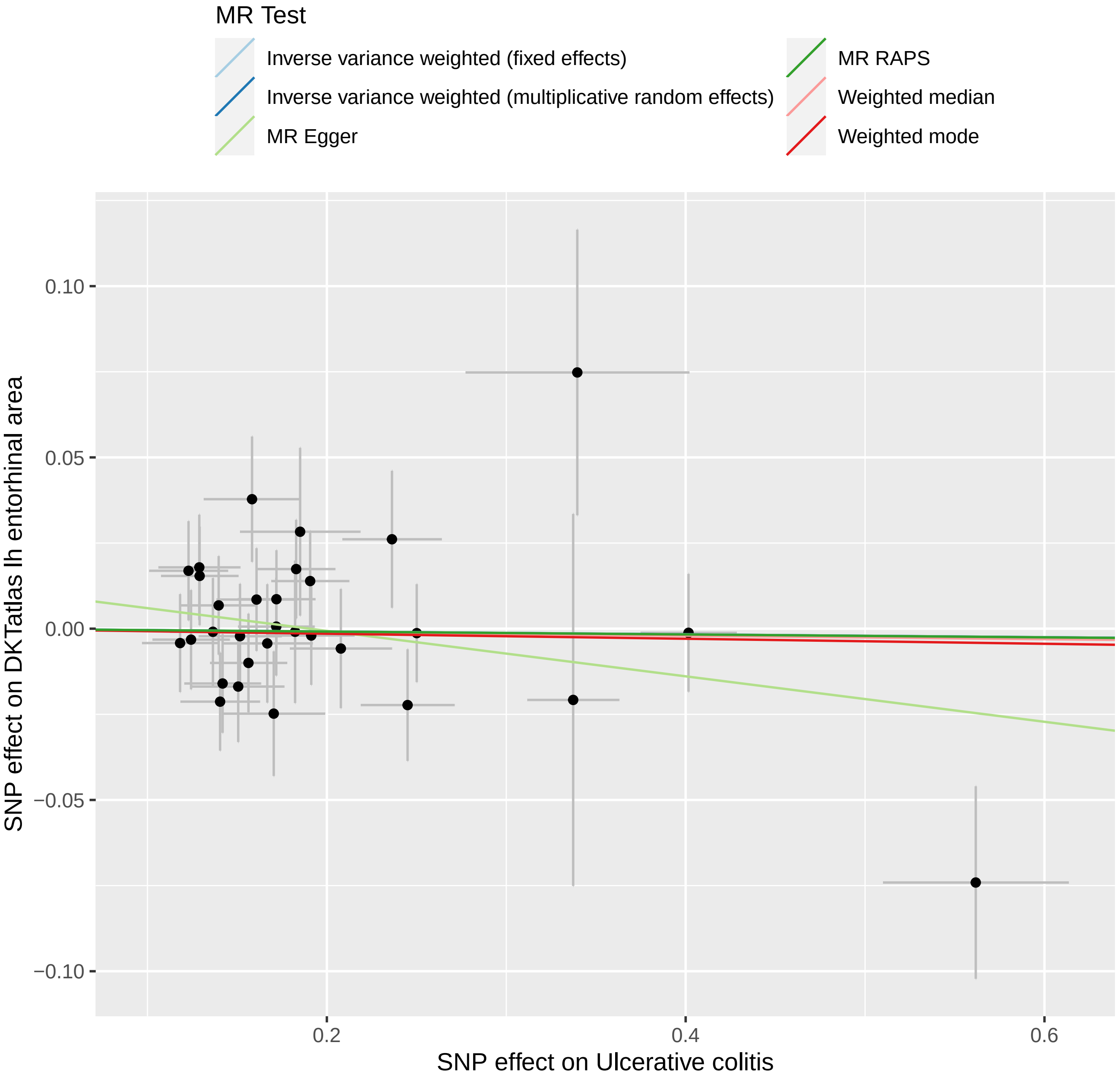

Batch 537 : Crohn's disease on DKTatlas lh fusiform area

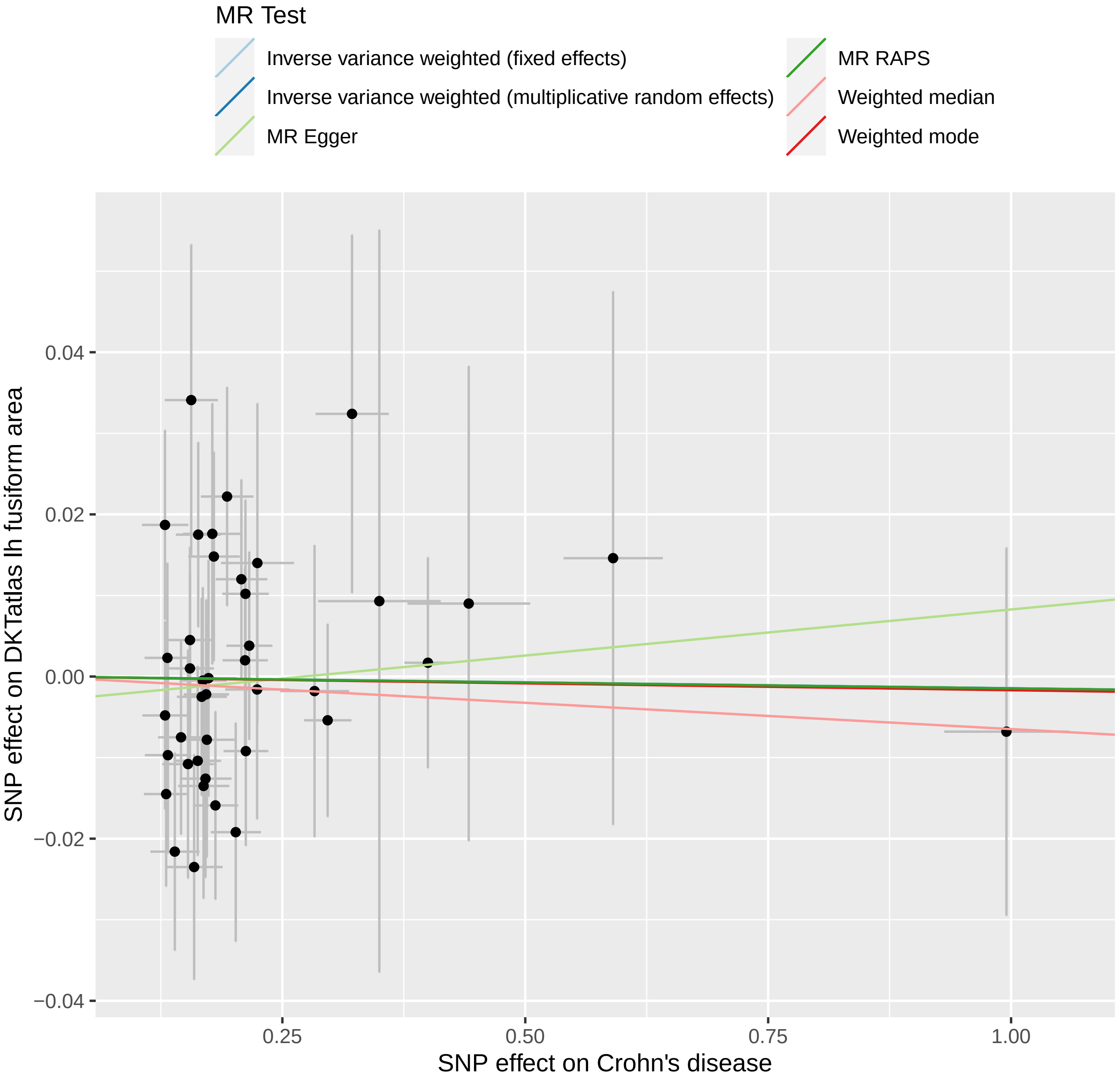

Batch 538 : Ulcerative colitis on DKTatlas lh fusiform area

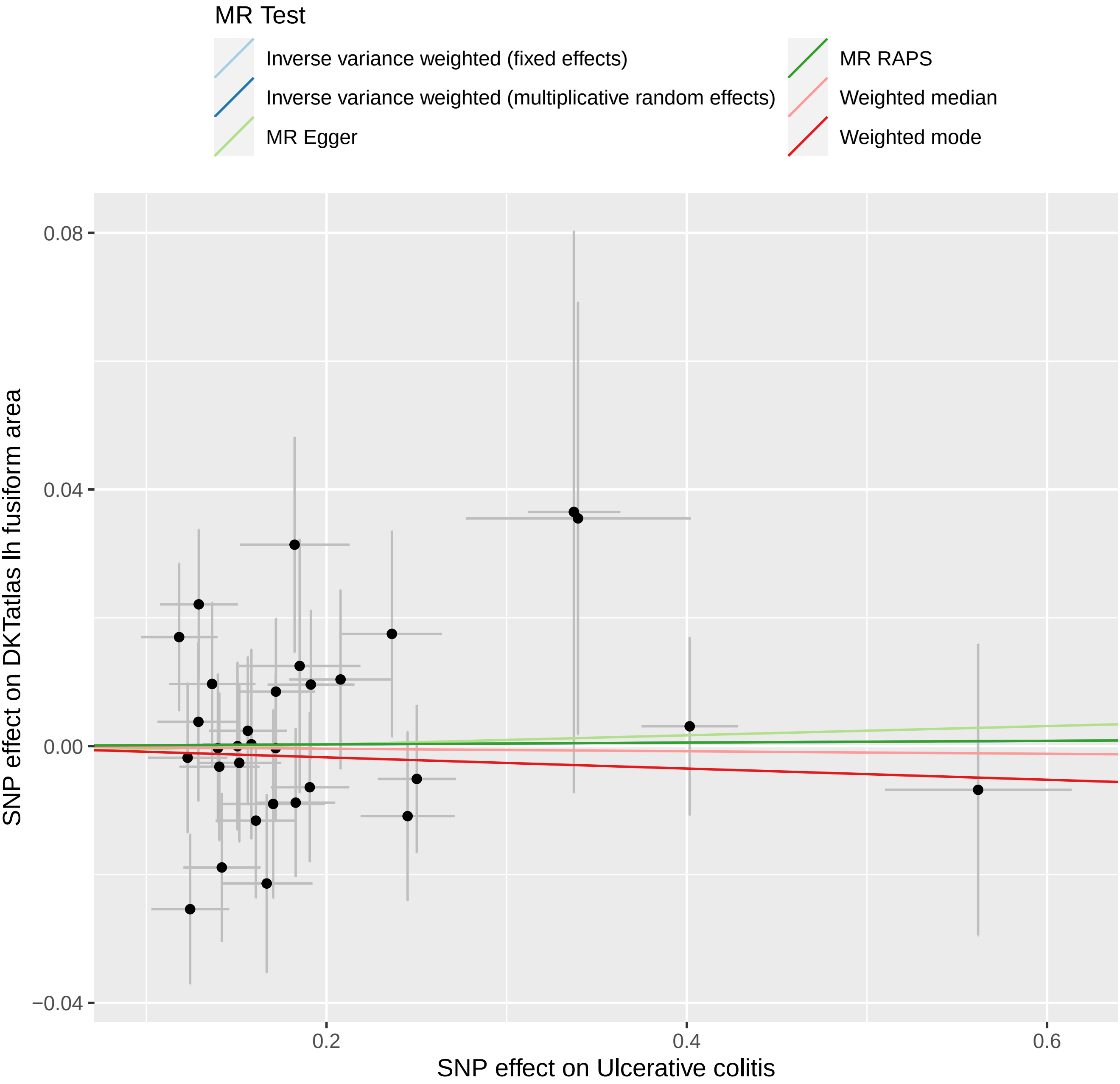

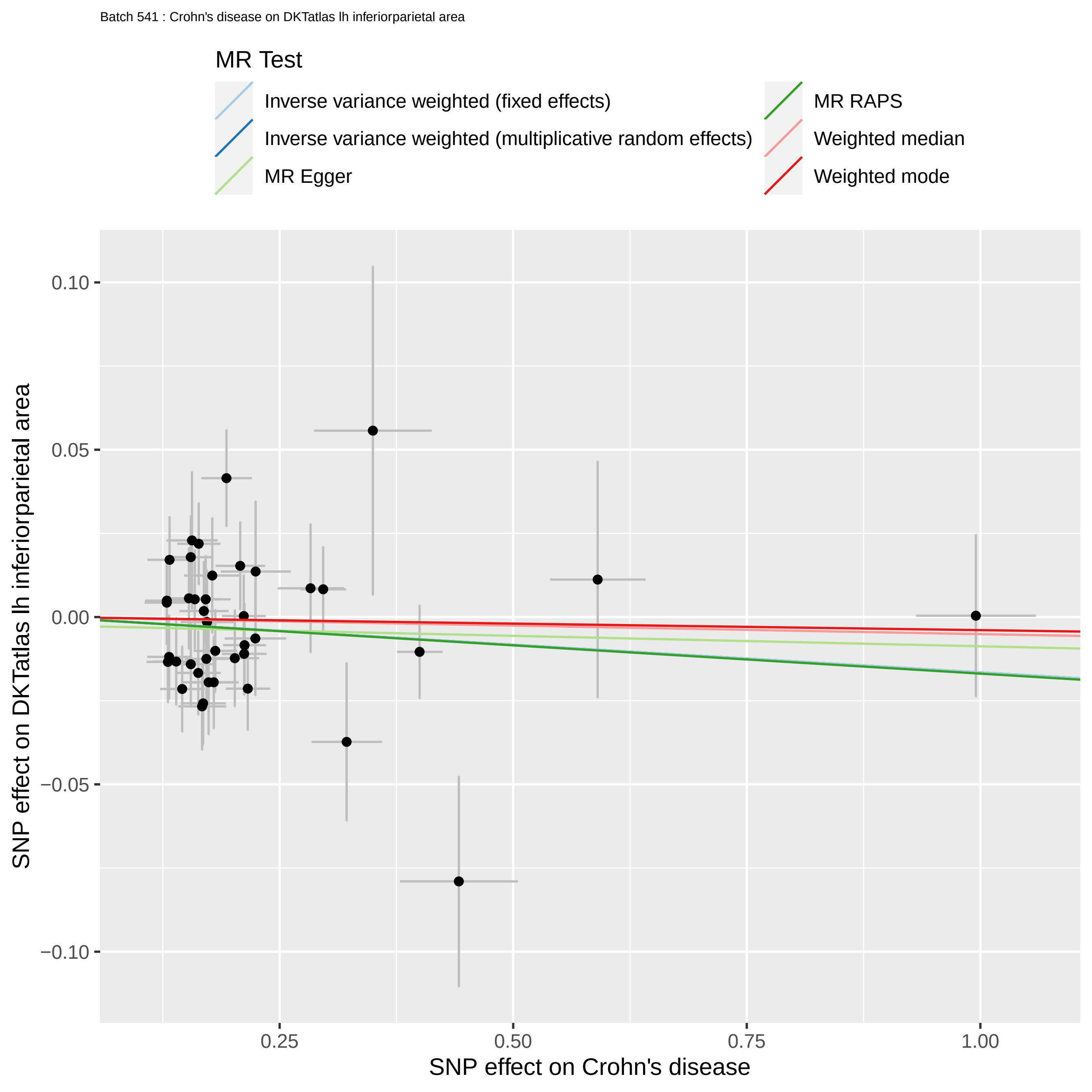

Batch 541 : Crohn's disease on DKTatlas lh inferiorparietal area
MR Test
Inverse variance weighted (fixed effects)
Inverse variance weighted (multiplicative random effects)
MR Egger
MR RAPS
Weighted median
Weighted mode
0.10
0.05
0.00
−0.05
−0.10
0.25
0.50
0.75
1.00
SNP effect on Crohn's disease
SNP effect on DKTatlas lh inferiorparietal area

Batch 542 : Ulcerative colitis on DKTatlas lh inferiorparietal area

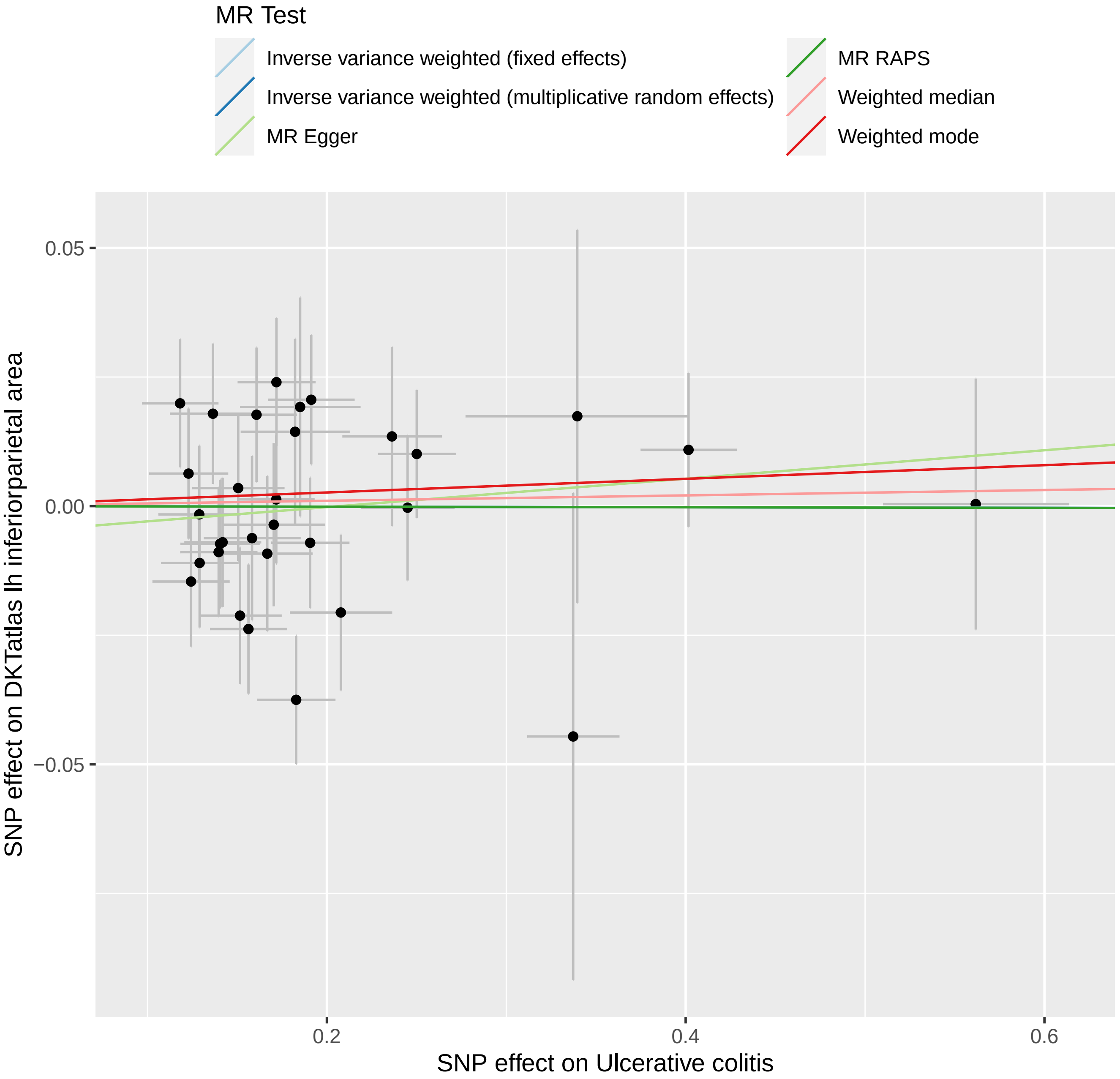

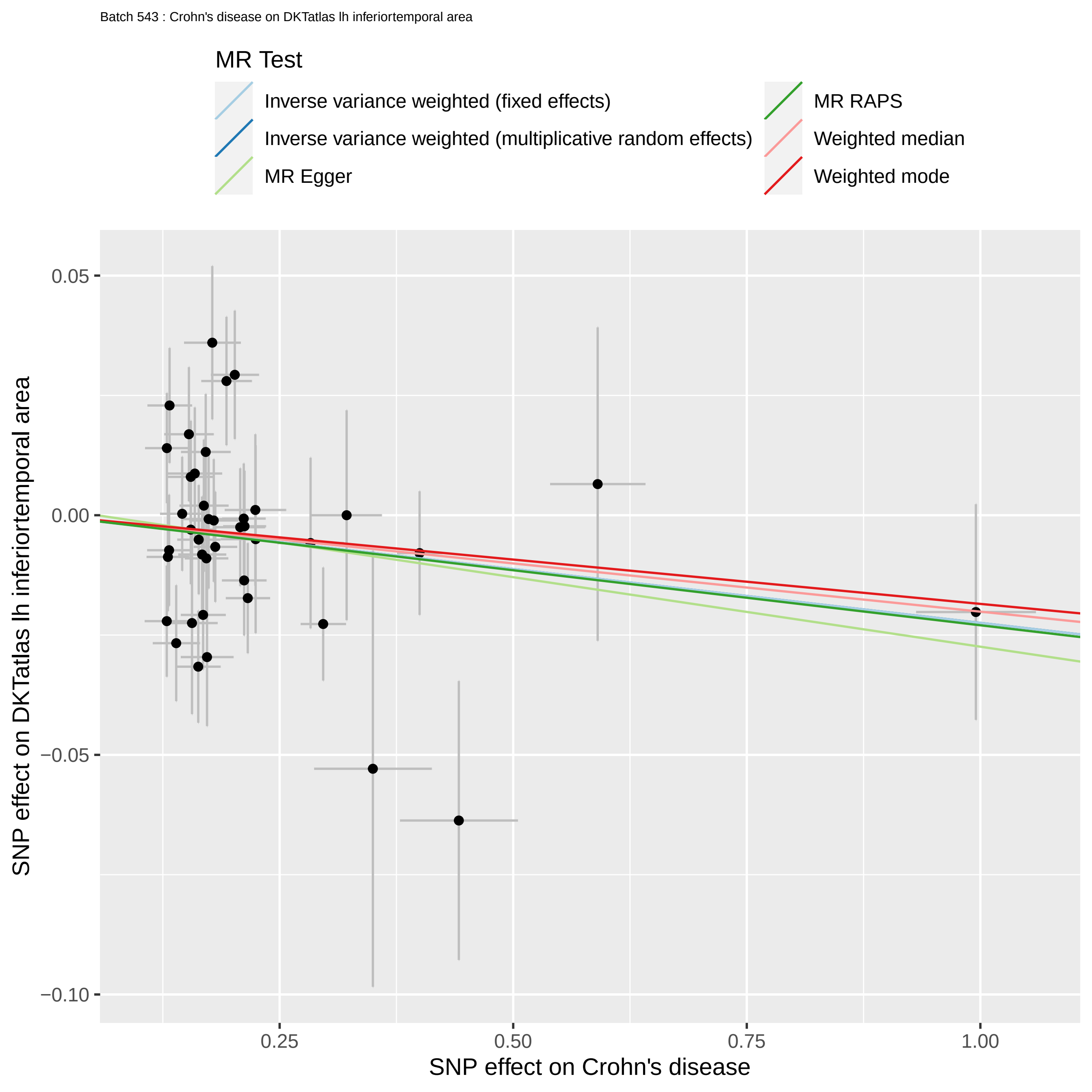

Batch 543 : Crohn's disease on DKTatlas lh inferiortemporal area
MR Test
Inverse variance weighted (fixed effects)
Inverse variance weighted (multiplicative random effects)
MR Egger
MR RAPS
Weighted median
Weighted mode
0.05
0.00
−0.05
−0.10
0.25
0.50
0.75
1.00
SNP effect on DKTatlas lh inferiortemporal area
SNP effect on Crohn's disease

Batch 544 : Ulcerative colitis on DKTatlas lh inferiortemporal area

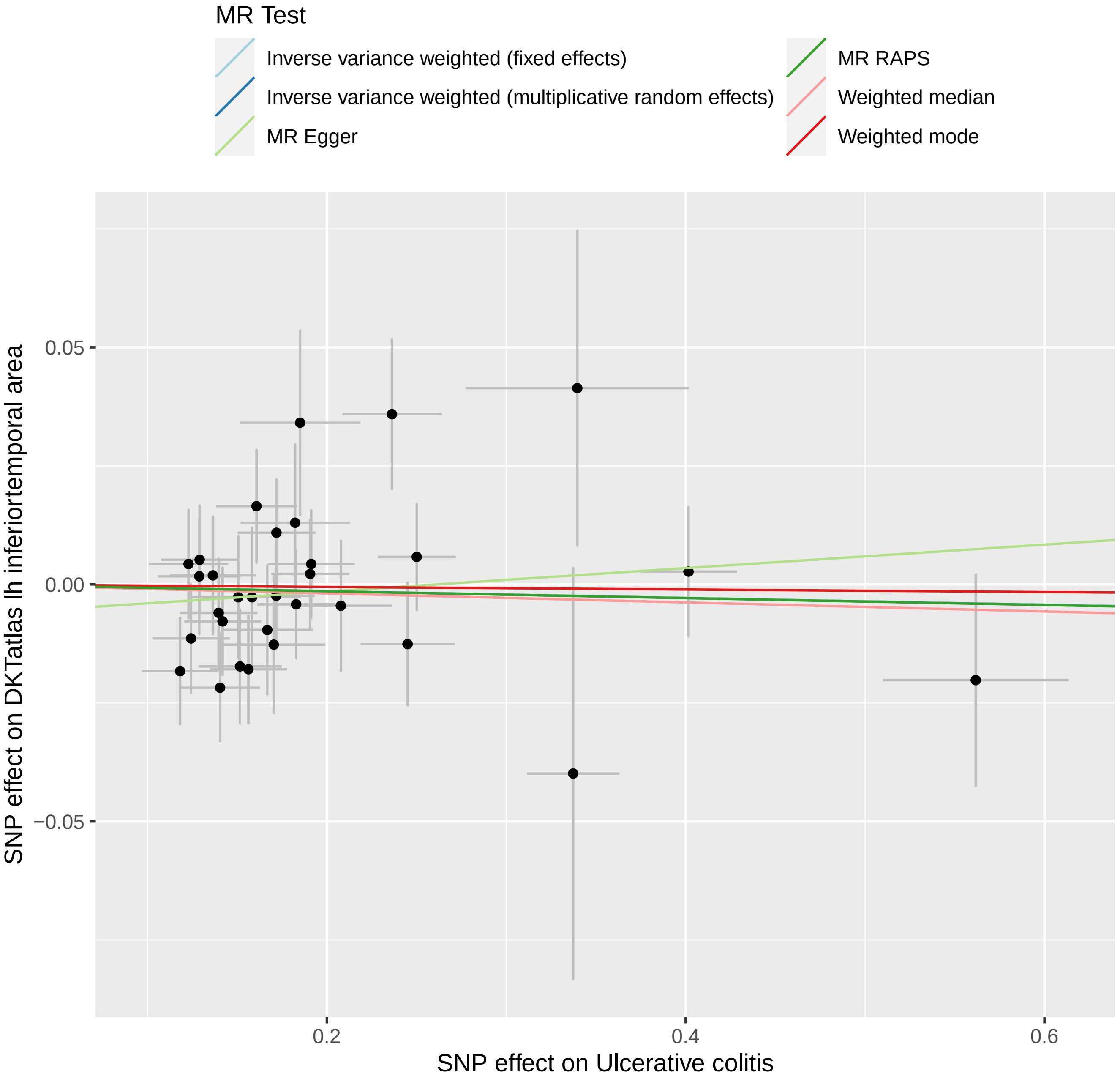

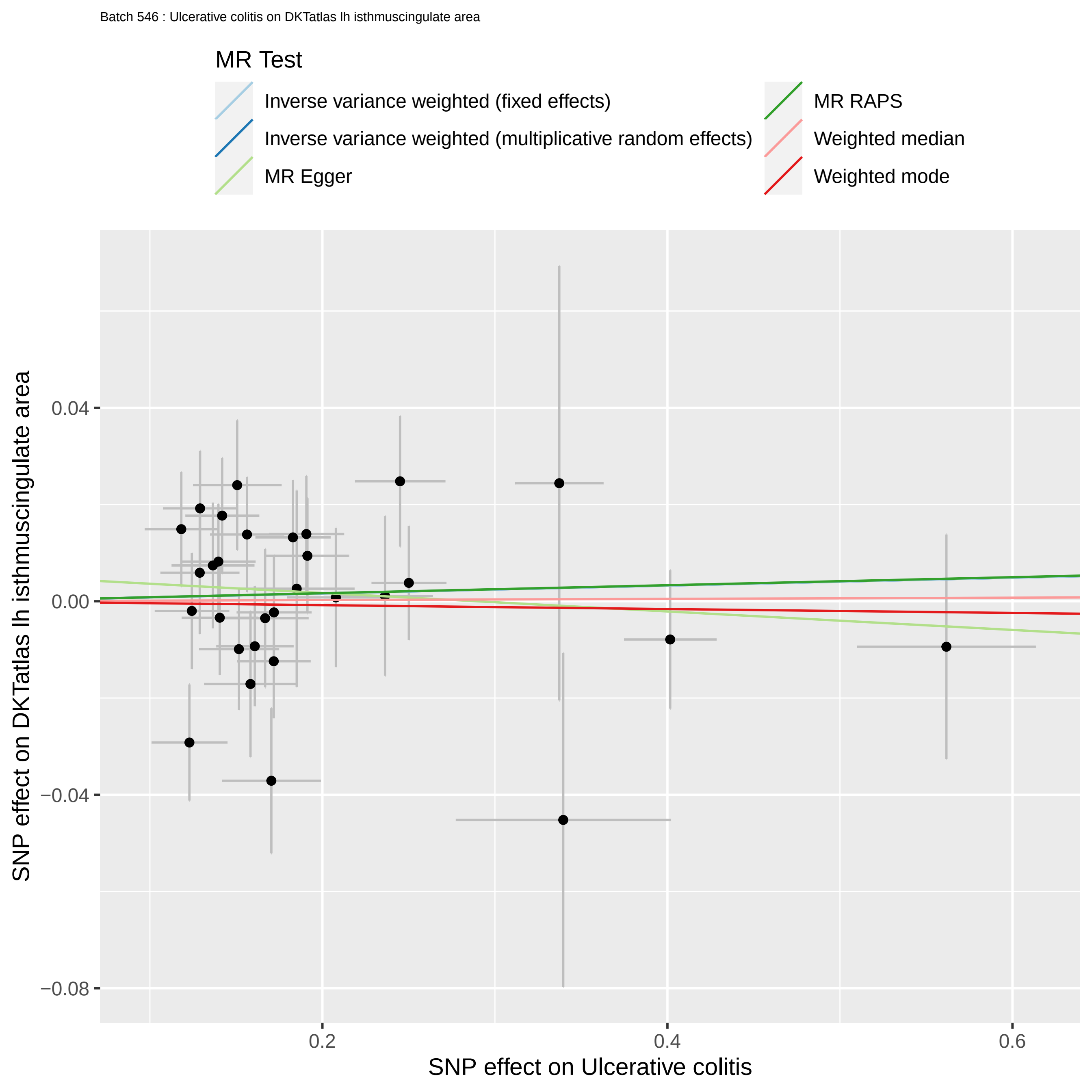

Batch 546 : Ulcerative colitis on DKTatlas lh isthmuscingulate area
MR Test
Inverse variance weighted (fixed effects)
Inverse variance weighted (multiplicative random effects)
MR Egger
MR RAPS
Weighted median
Weighted mode
0.04
0.00
−0.04
−0.08
0.2
0.4
0.6
SNP effect on DKTatlas lh isthmuscingulate area
SNP effect on Ulcerative colitis

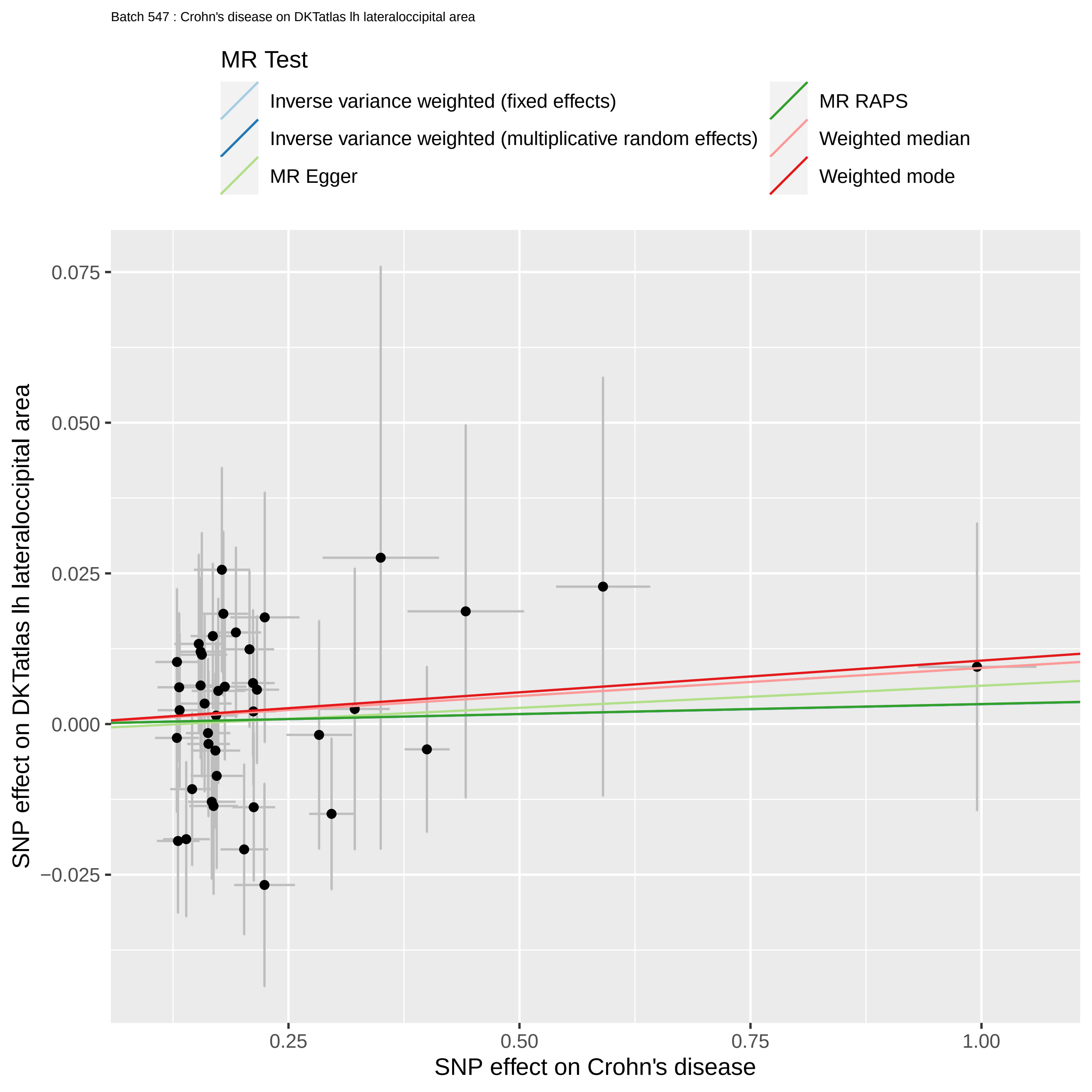
Batch 547 : Crohn's disease on DKTatlas lh lateraloccipital area
MR Test
Inverse variance weighted (fixed effects)
Inverse variance weighted (multiplicative random effects)
MR Egger
MR RAPS
Weighted median
Weighted mode
0.075
0.050
0.025
0.000
−0.025
0.25
0.50
0.75
1.00
SNP effect on DKTatlas lh lateraloccipital area
SNP effect on Crohn's disease

Batch 548 : Ulcerative colitis on DKTatlas lh lateraloccipital area

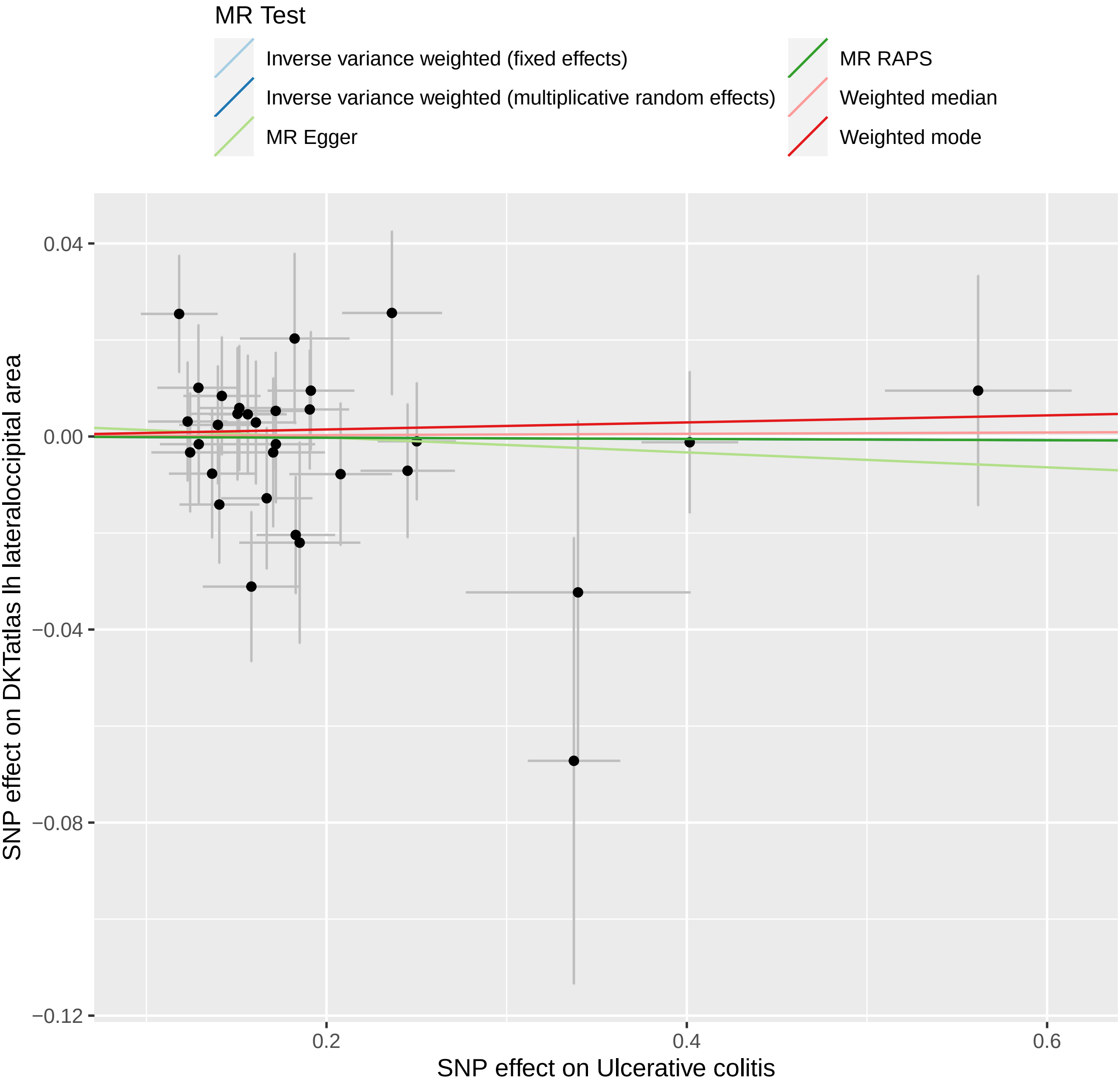

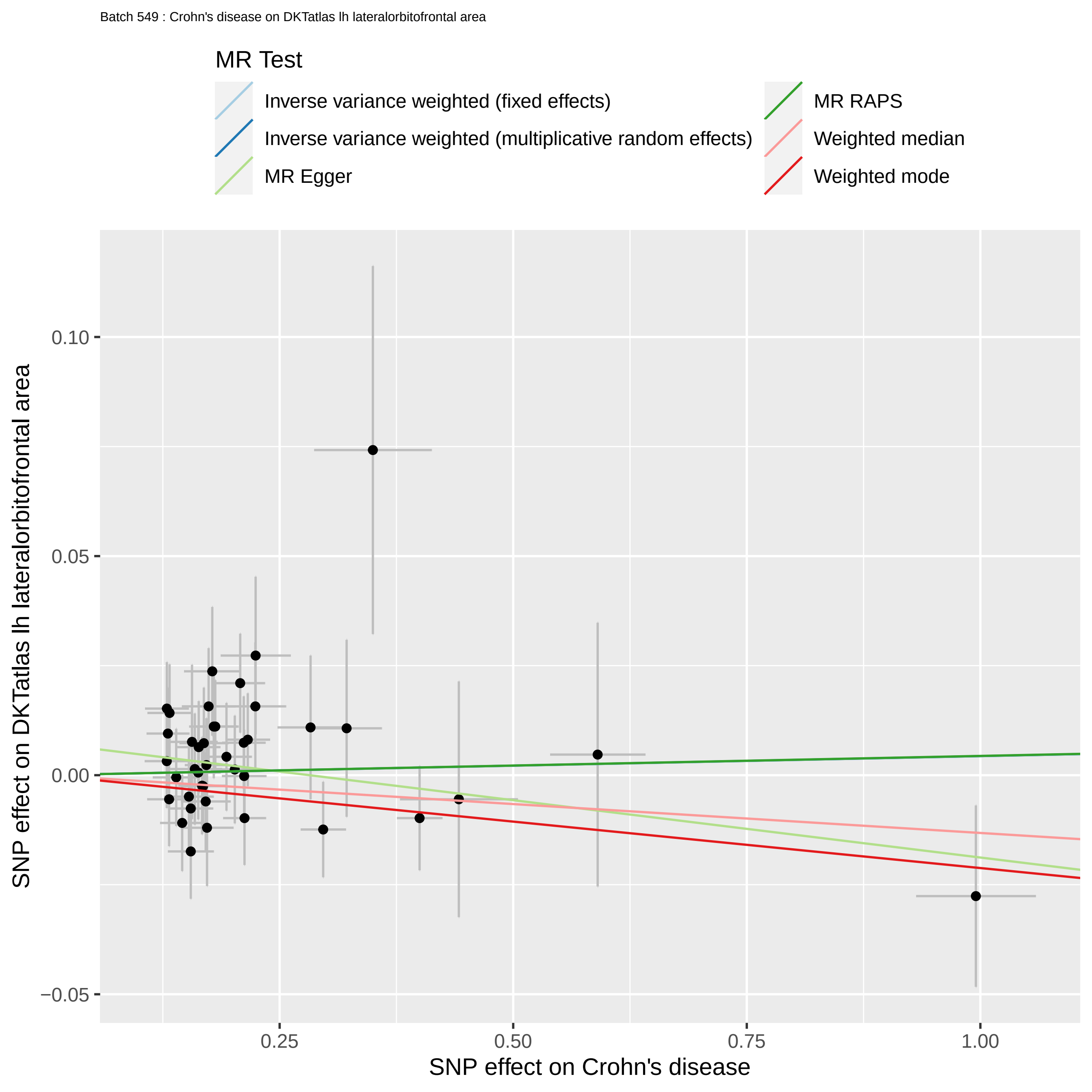

Batch 549 : Crohn's disease on DKTatlas lh lateralorbitofrontal area
MR Test
Inverse variance weighted (fixed effects)
Inverse variance weighted (multiplicative random effects)
MR Egger
MR RAPS
Weighted median
Weighted mode
0.10
0.05
0.00
−0.05
0.25
0.50
0.75
1.00
SNP effect on DKTatlas lh lateralorbitofrontal area
SNP effect on Crohn's disease

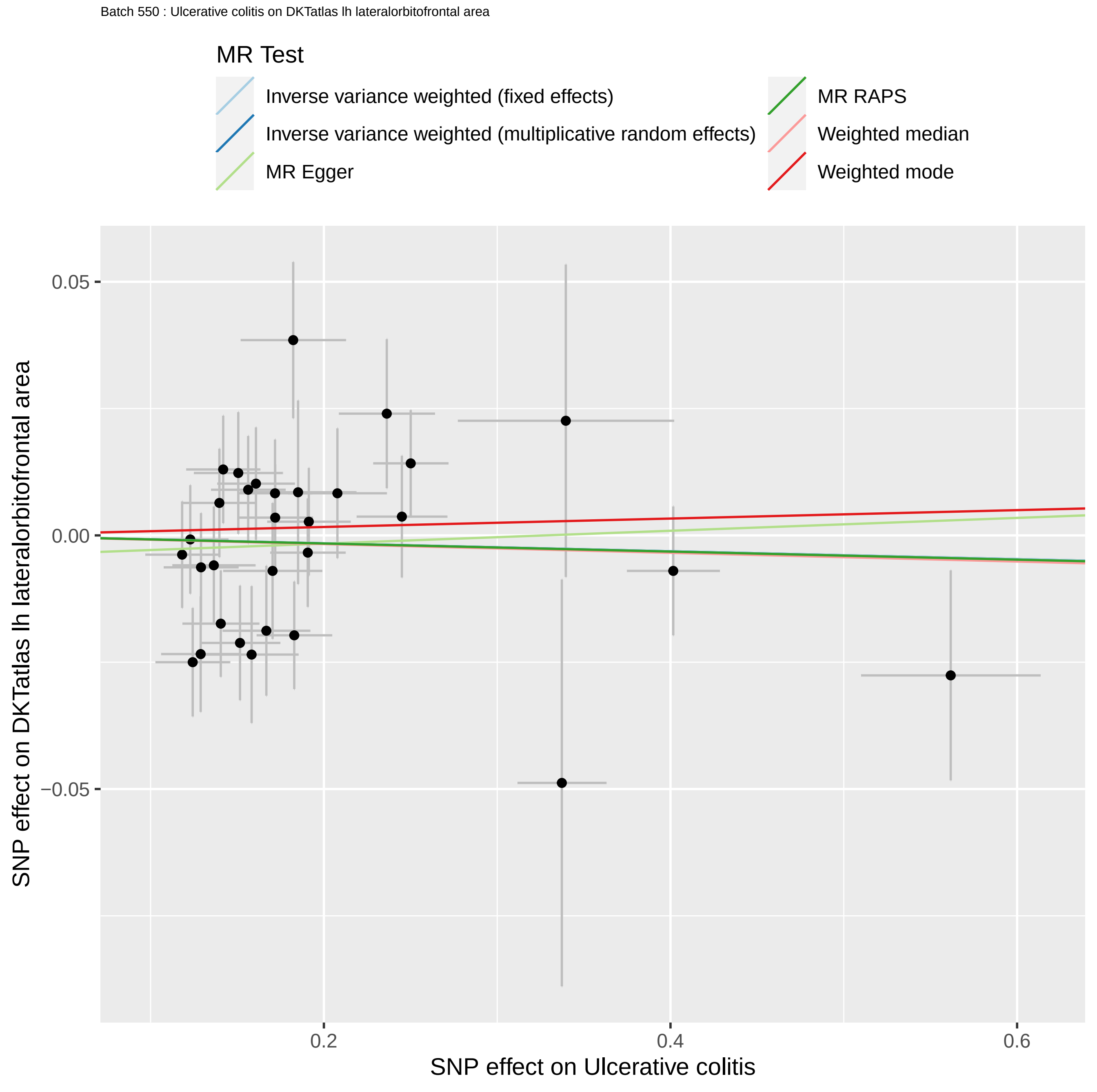

Batch 550 : Ulcerative colitis on DKTatlas lh lateralorbitofrontal area
MR Test
Inverse variance weighted (fixed effects)
Inverse variance weighted (multiplicative random effects)
MR Egger
MR RAPS
Weighted median
Weighted mode
0.05
0.00
−0.05
0.2
0.4
0.6
SNP effect on Ulcerative colitis
SNP effect on DKTatlas lh lateralorbitofrontal area

Batch 551 : Crohn's disease on DKTatlas lh lingual area

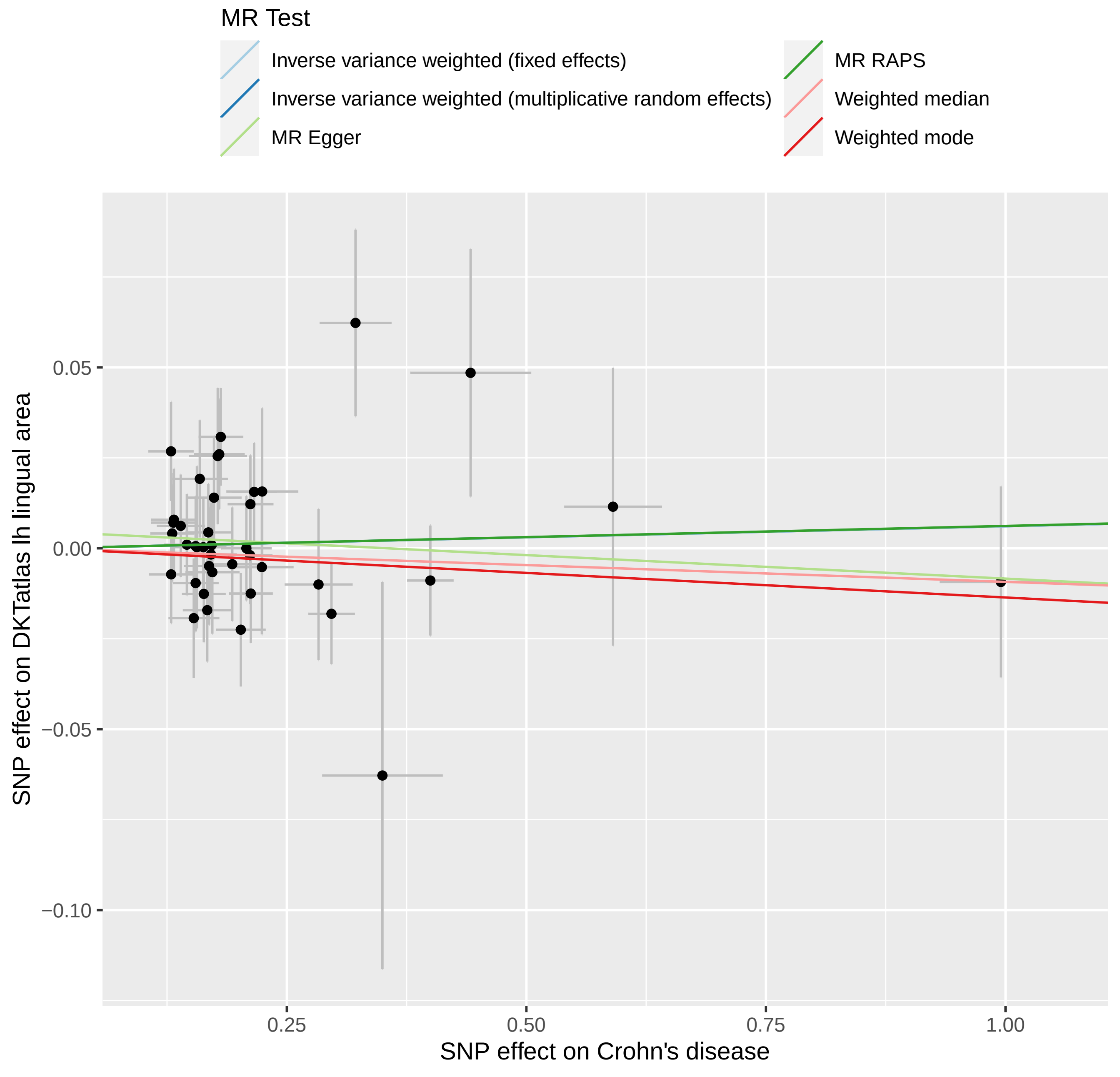

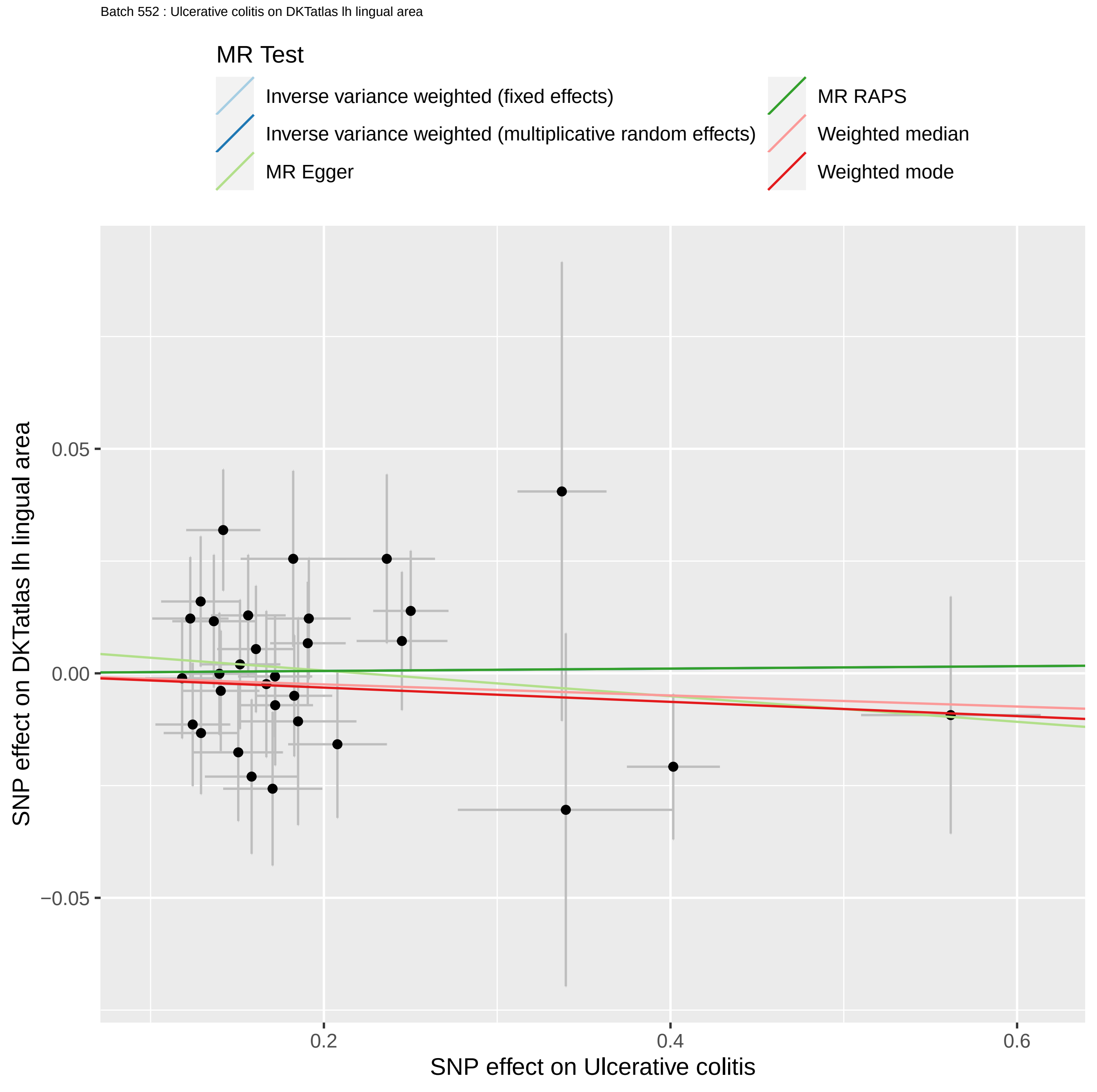
Batch 552 : Ulcerative colitis on DKTatlas lh lingual area
MR Test
Inverse variance weighted (fixed effects)
Inverse variance weighted (multiplicative random effects)
MR Egger
MR RAPS
Weighted median
Weighted mode
0.05
0.00
−0.05
0.2
0.4
0.6
SNP effect on DKTatlas lh lingual area
SNP effect on Ulcerative colitis

Batch 553 : Crohn's disease on DKTatlas lh medialorbitofrontal area

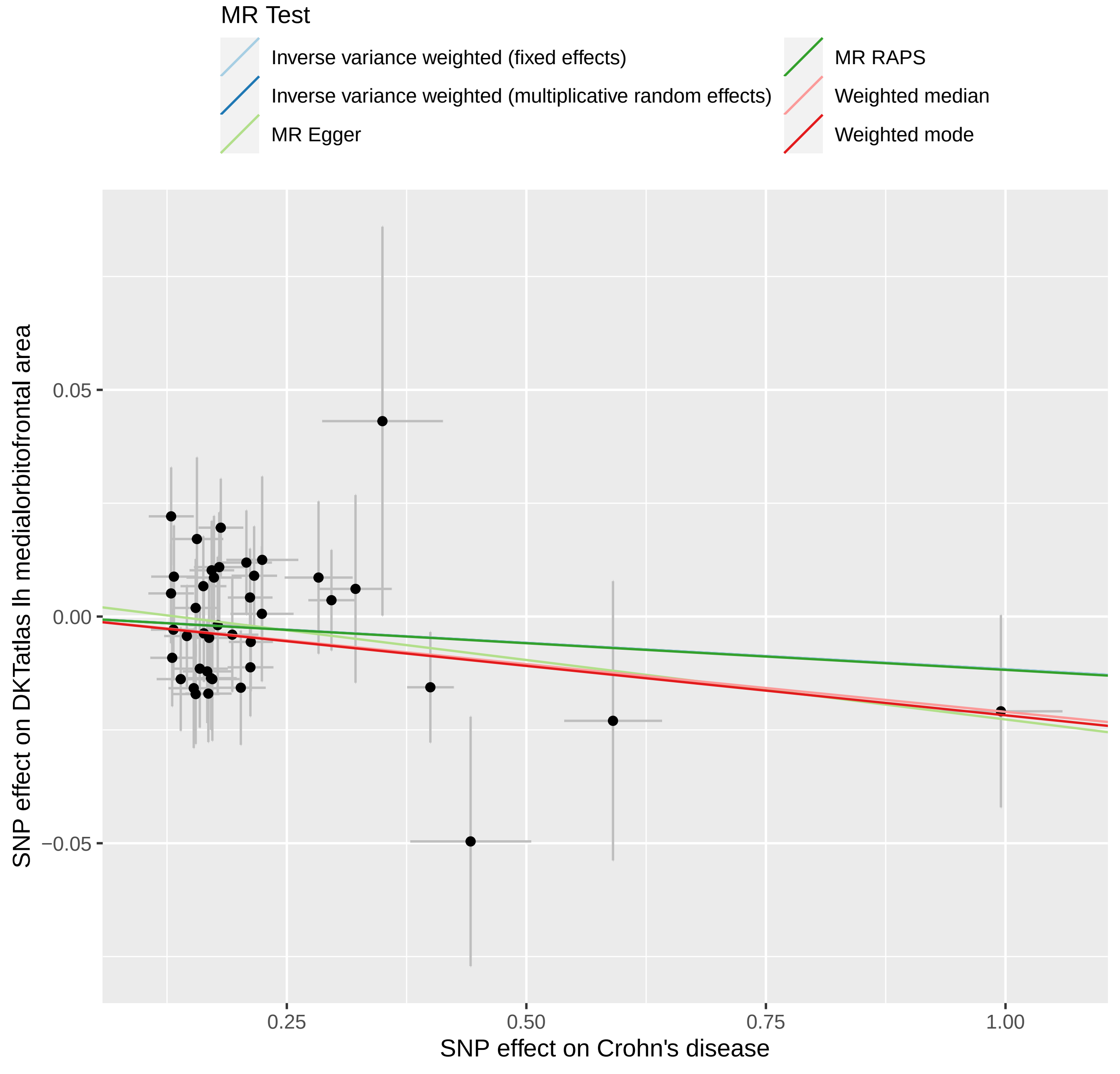

Batch 554 : Ulcerative colitis on DKTatlas lh medialorbitofrontal area

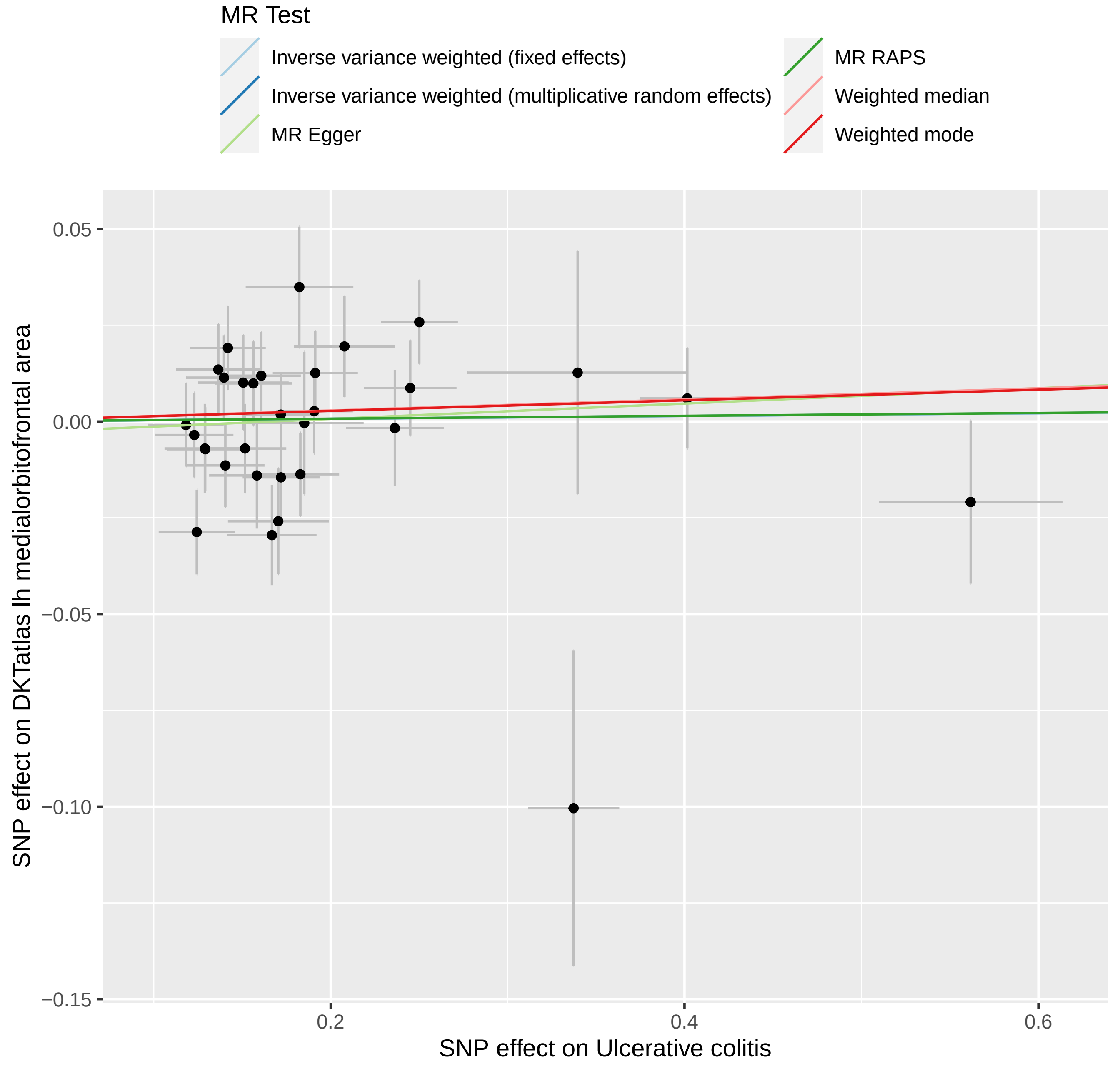

Batch 555 : Crohn's disease on DKTatlas lh middletemporal area

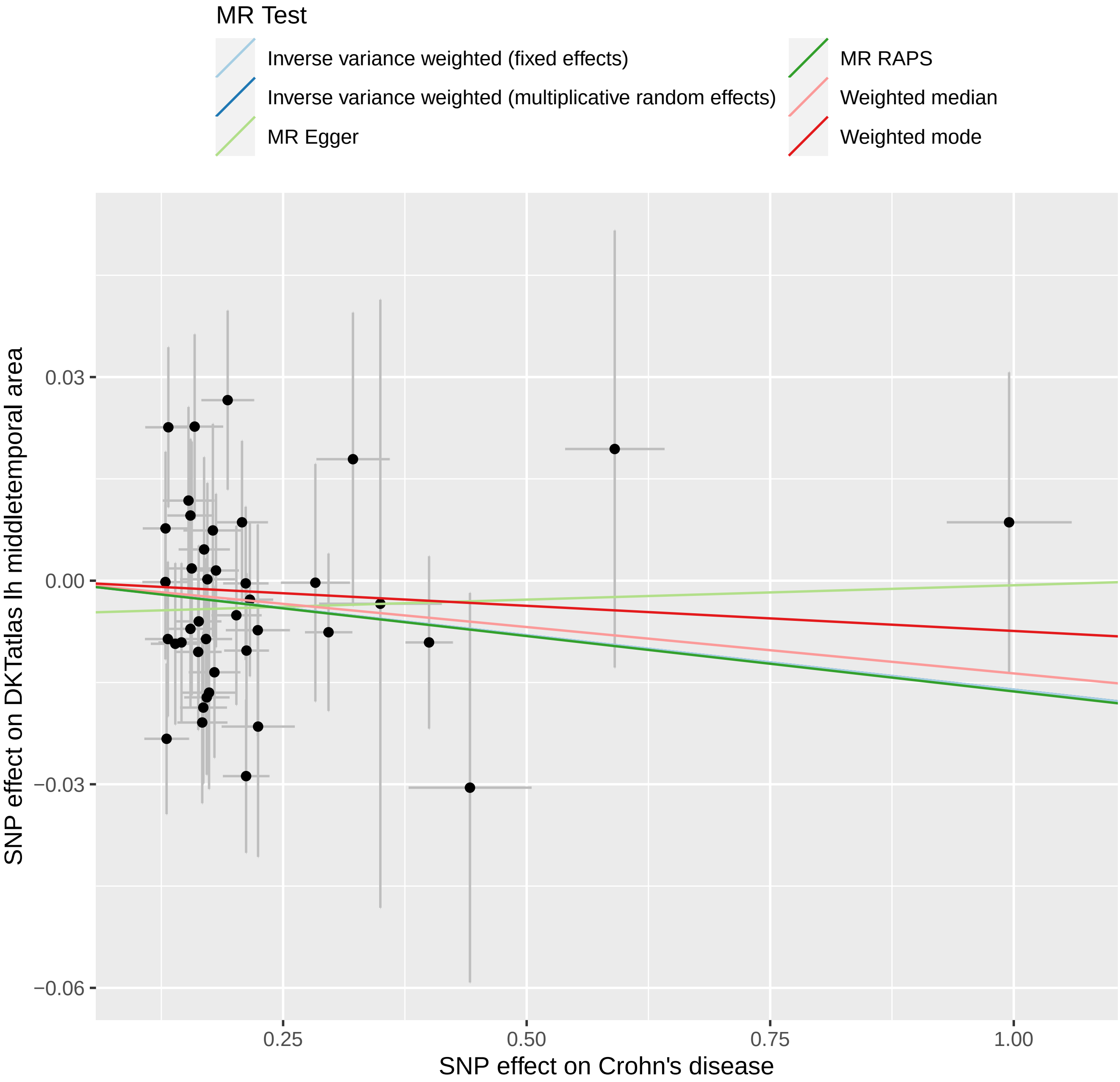

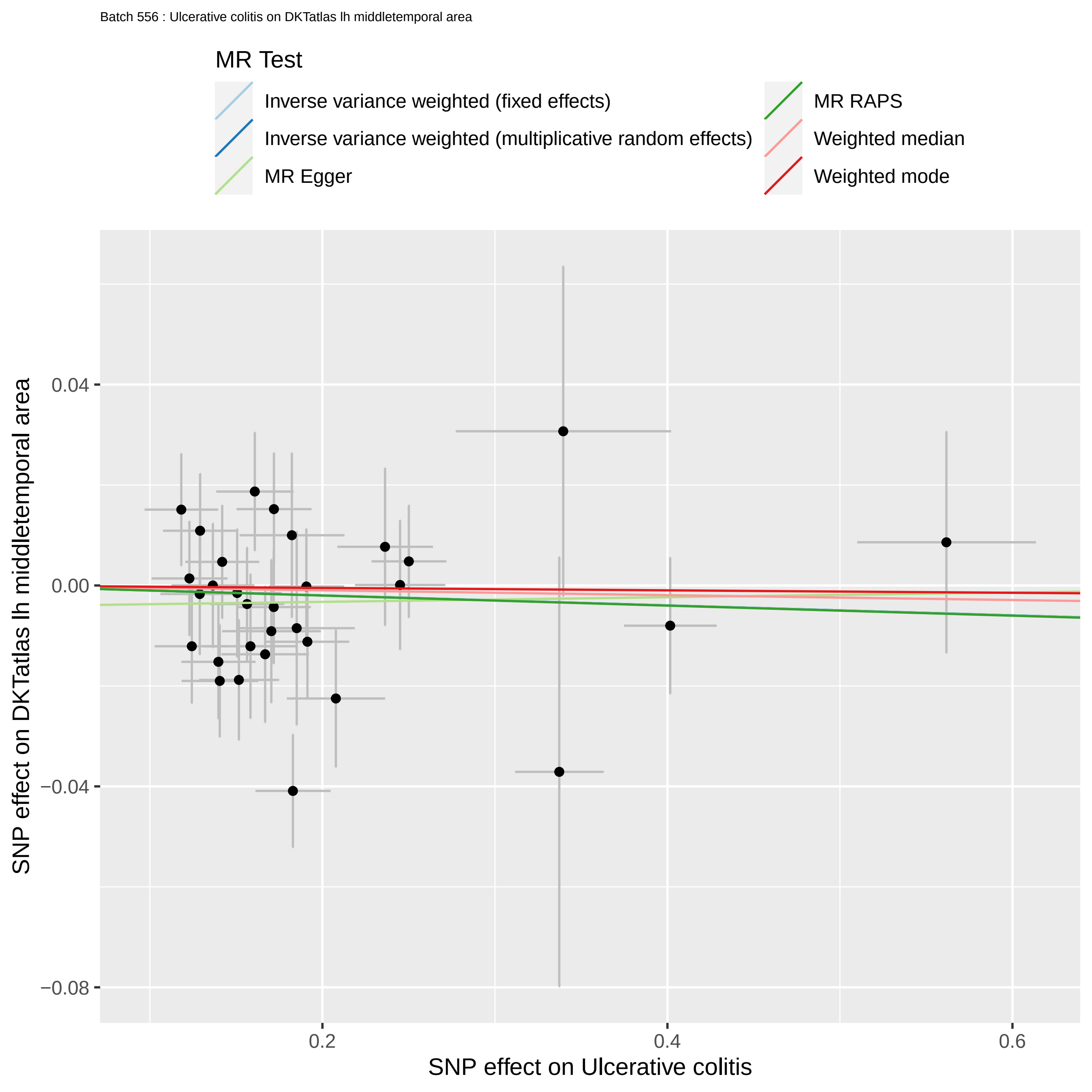

Batch 556 : Ulcerative colitis on DKTatlas lh middletemporal area
MR Test
Inverse variance weighted (fixed effects)
Inverse variance weighted (multiplicative random effects)
MR Egger
MR RAPS
Weighted median
Weighted mode
SNP effect on DKTatlas lh middletemporal area
0.04
0.00
−0.04
−0.08
0.2
0.4
0.6
SNP effect on Ulcerative colitis

Batch 557 : Crohn's disease on DKTatlas lh parahippocampal area

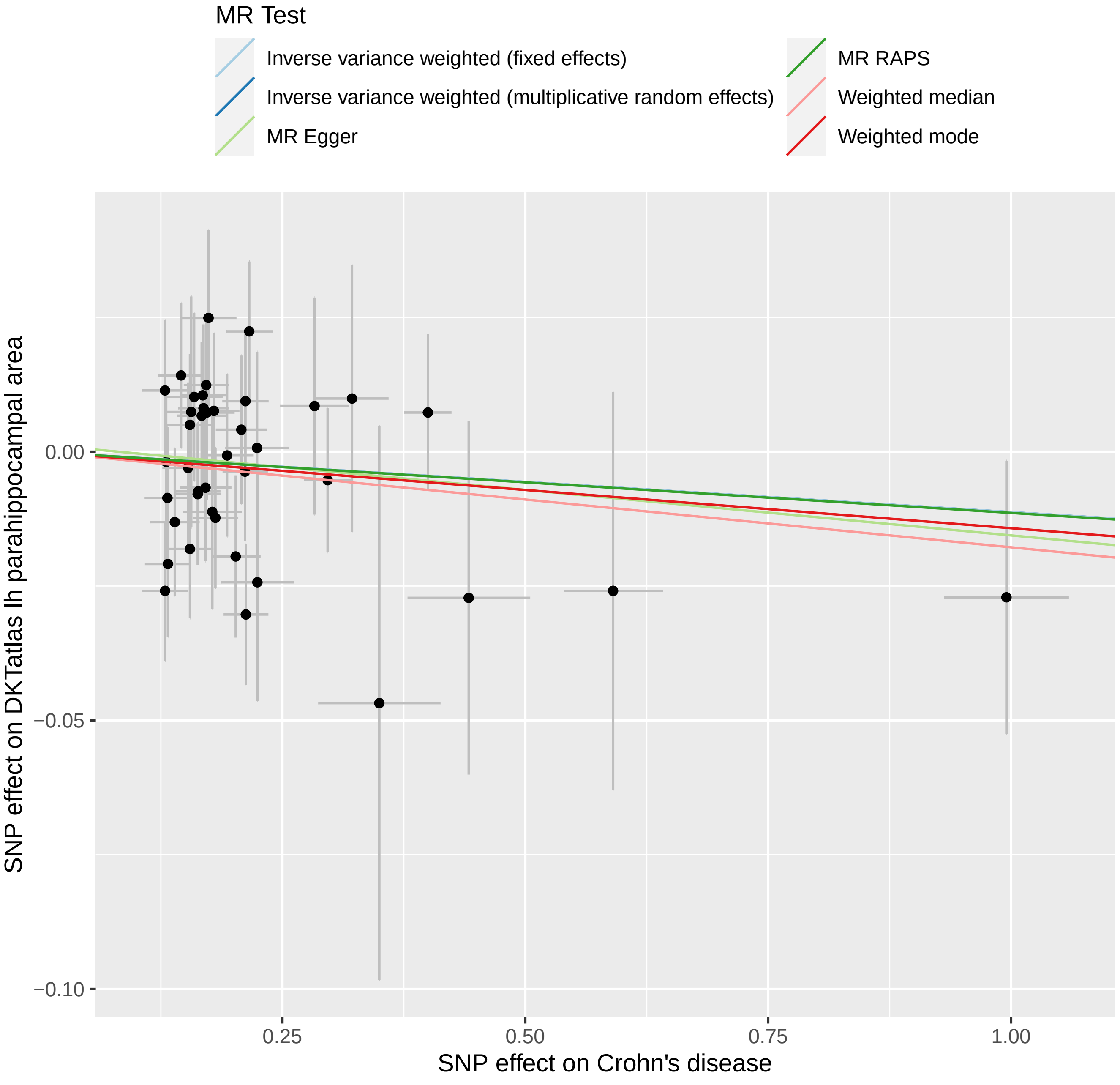

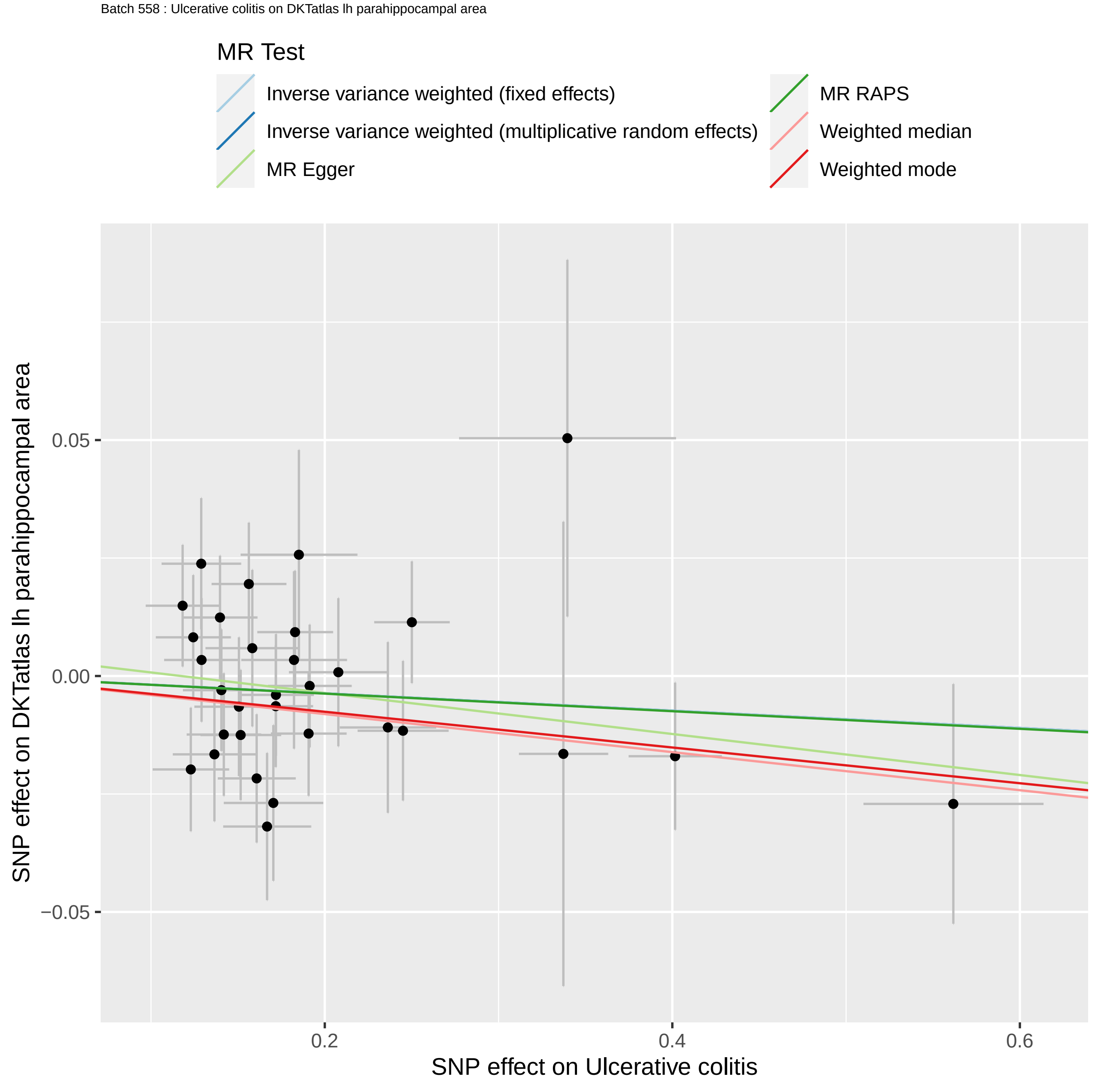

Batch 558 : Ulcerative colitis on DKTatlas lh parahippocampal area
MR Test
Inverse variance weighted (fixed effects)
Inverse variance weighted (multiplicative random effects)
MR Egger
MR RAPS
Weighted median
Weighted mode
0.05
0.00
−0.05
0.2
0.4
0.6
SNP effect on DKTatlas lh parahippocampal area
SNP effect on Ulcerative colitis

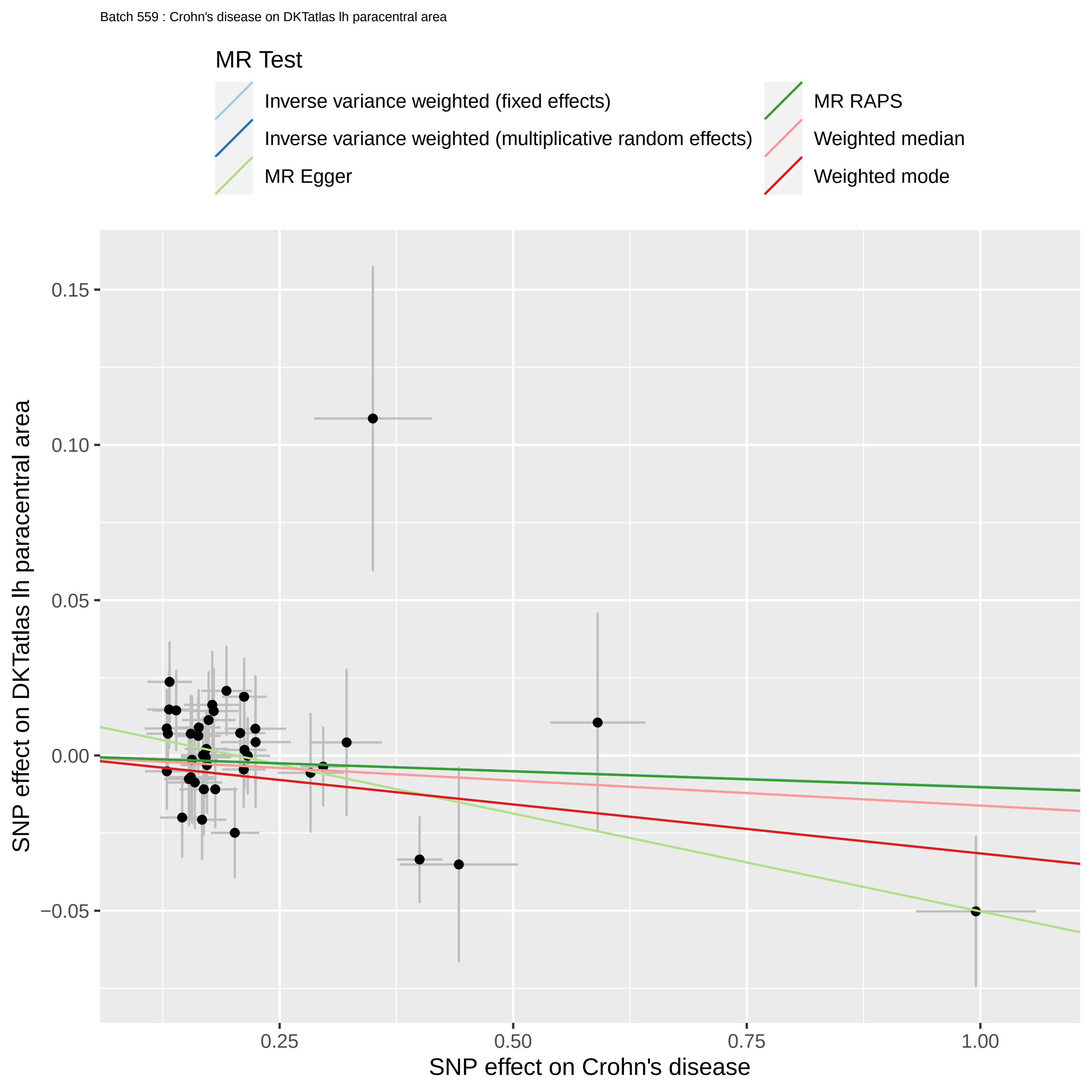
Batch 559 : Crohn's disease on DKTatlas lh paracentral area
MR Test
Inverse variance weighted (fixed effects)
Inverse variance weighted (multiplicative random effects)
MR Egger
MR RAPS
Weighted median
Weighted mode
SNP effect on DKTatlas lh paracentral area
0.15
0.10
0.05
0.00
−0.05
0.25
0.50
0.75
1.00
SNP effect on Crohn's disease

Batch 560 : Ulcerative colitis on DKTatlas lh paracentral area

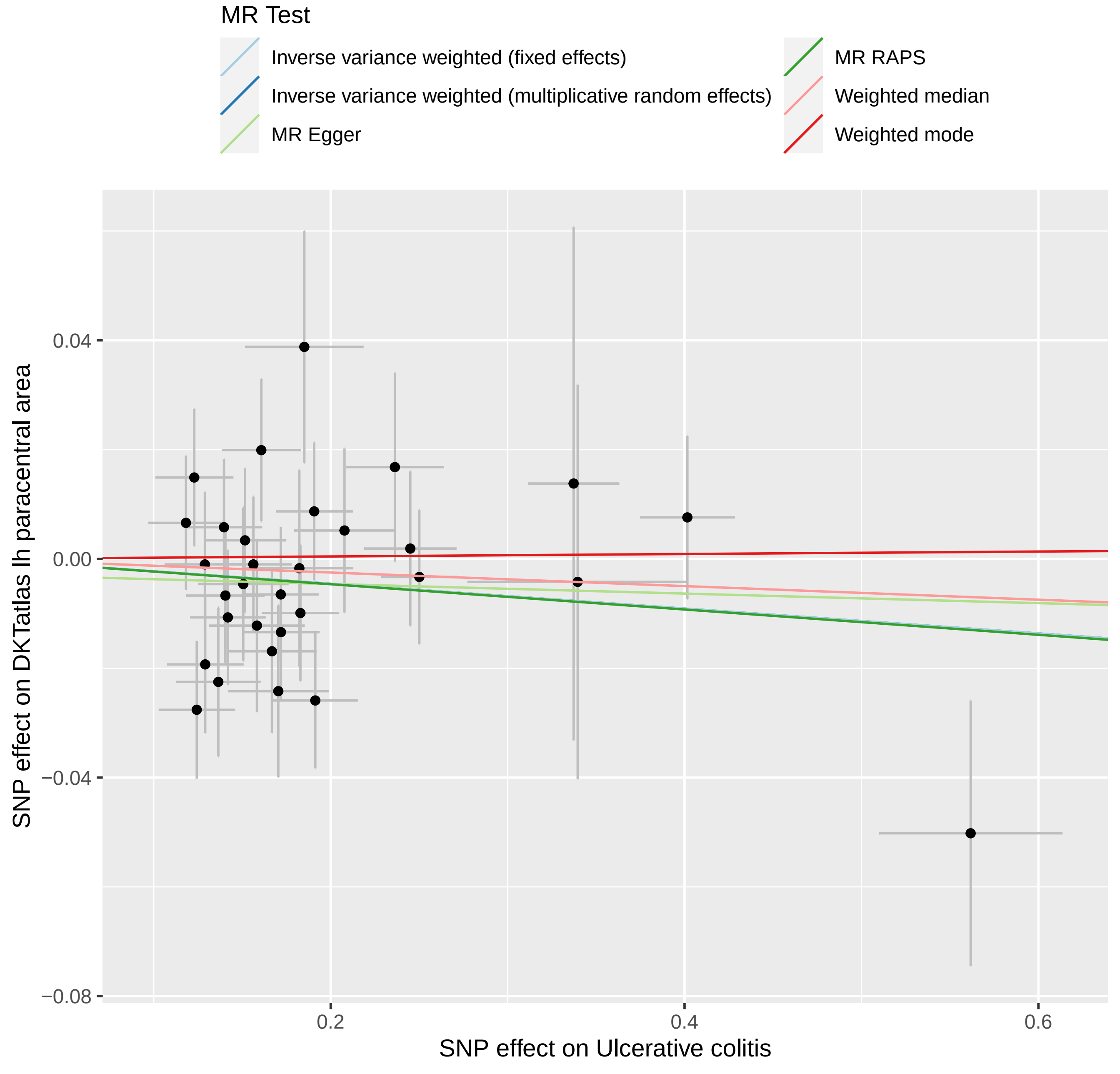

Batch 563 : Crohn's disease on DKTatlas lh parsopercularis area

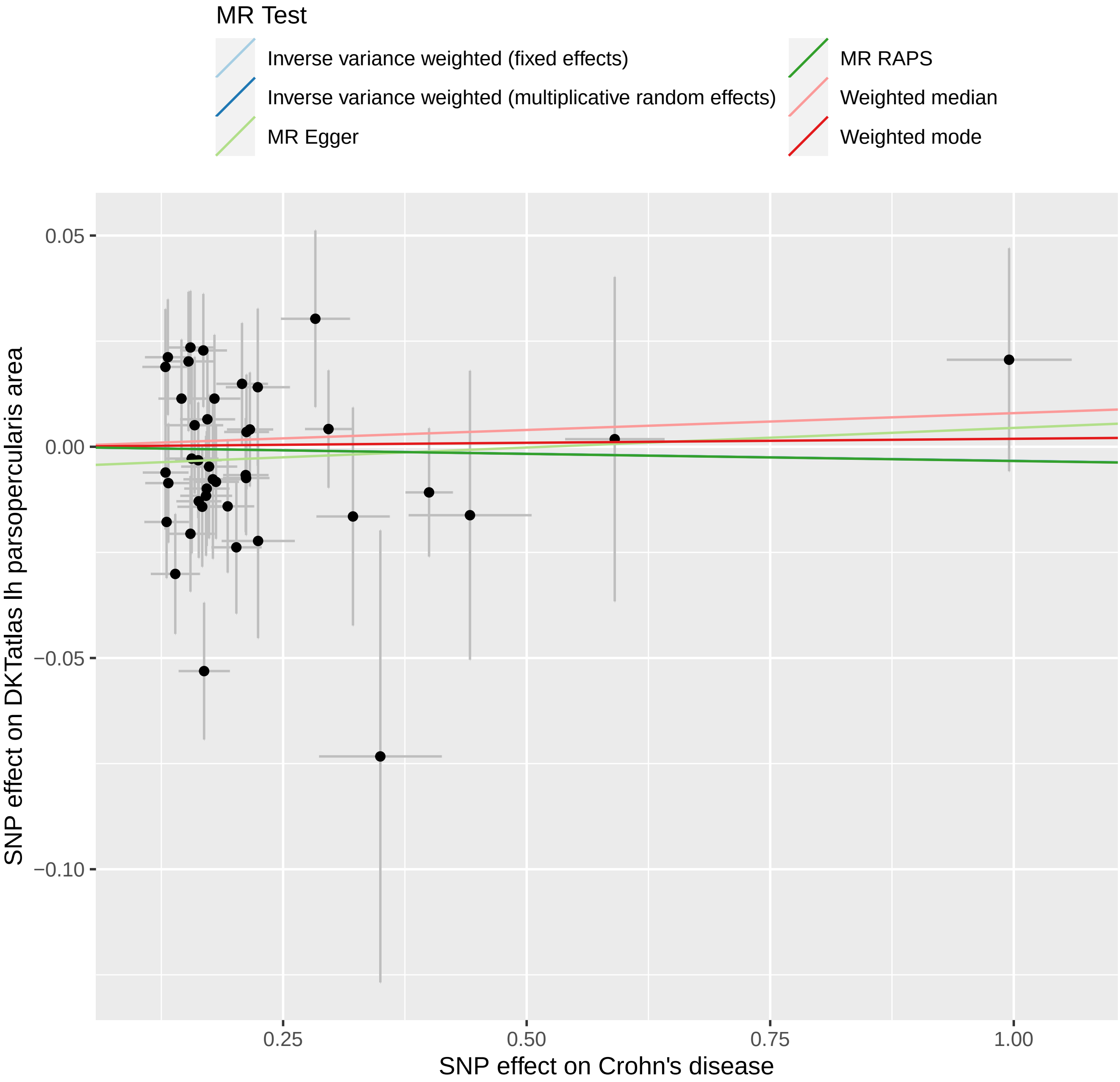

Batch 564 : Ulcerative colitis on DKTatlas lh parsopercularis area

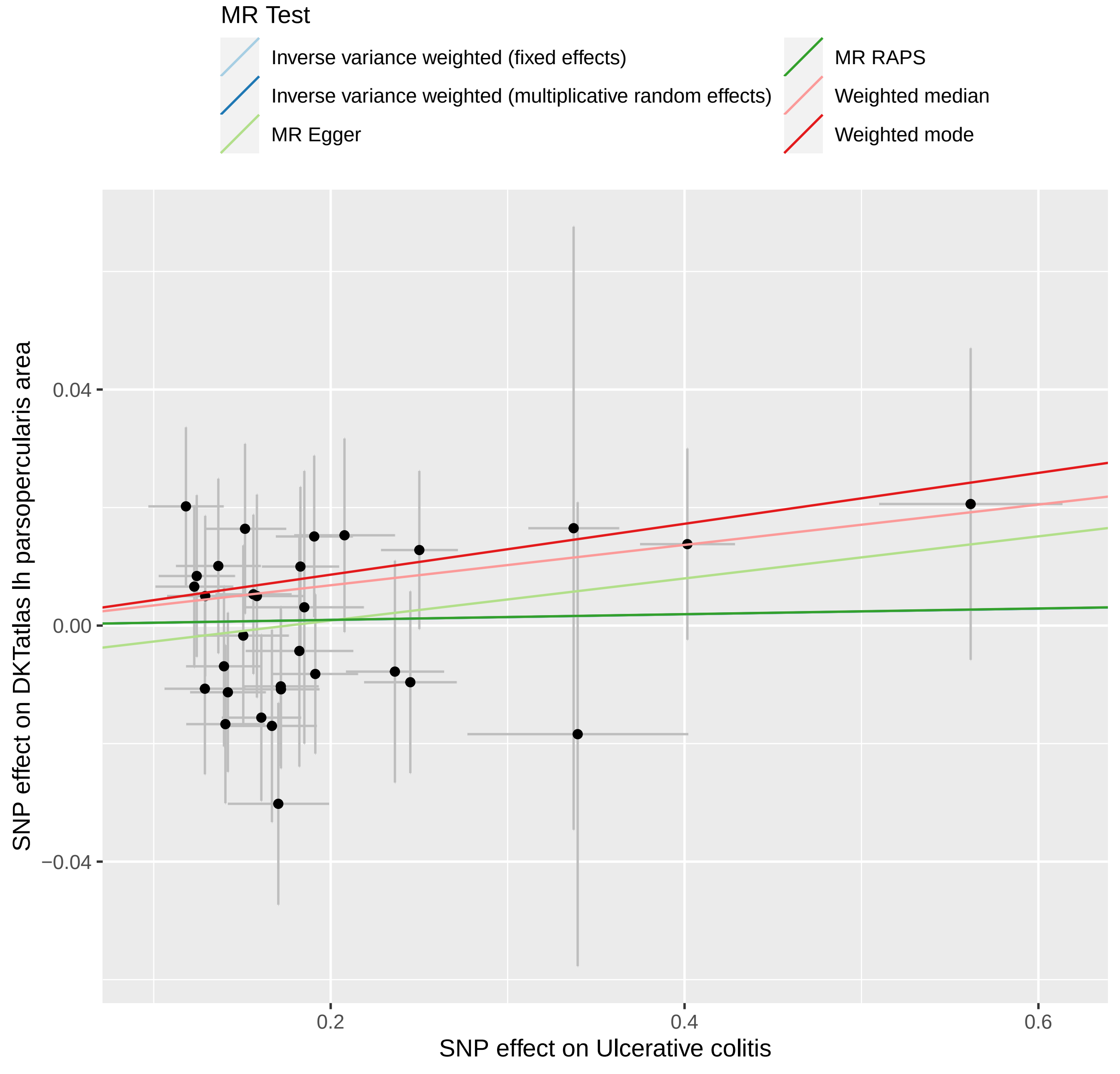

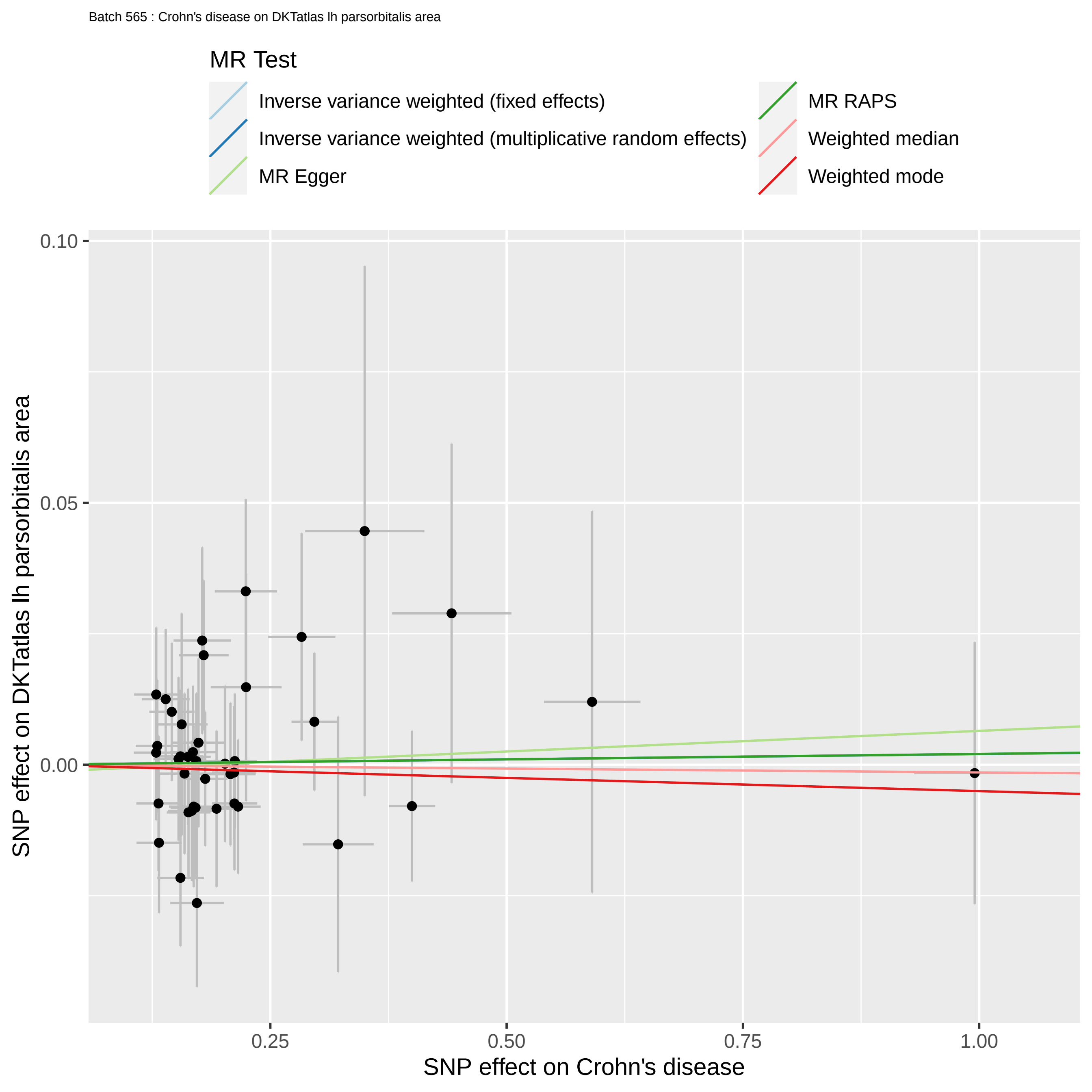
Batch 565 : Crohn's disease on DKTatlas lh parsorbitalis area
MR Test
Inverse variance weighted (fixed effects)
Inverse variance weighted (multiplicative random effects)
MR Egger
MR RAPS
Weighted median
Weighted mode
0.10
0.05
0.00
0.25
0.50
0.75
1.00
SNP effect on DKTatlas lh parsorbitalis area
SNP effect on Crohn's disease

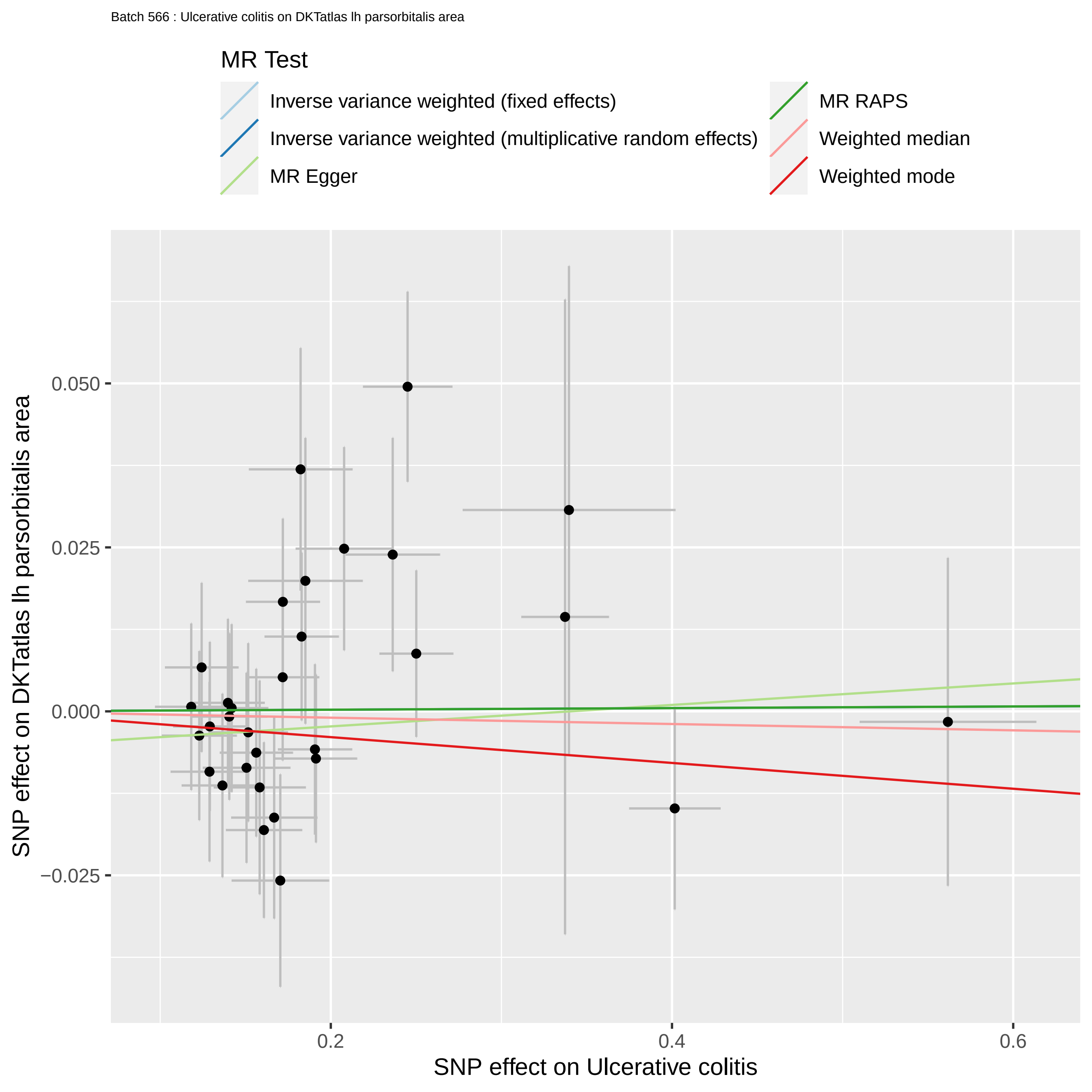

Batch 566 : Ulcerative colitis on DKTatlas lh parsorbitalis area
MR Test
Inverse variance weighted (fixed effects)
Inverse variance weighted (multiplicative random effects)
MR Egger
MR RAPS
Weighted median
Weighted mode
0.050
0.025
0.000
−0.025
0.2
0.4
0.6
SNP effect on DKTatlas lh parsorbitalis area
SNP effect on Ulcerative colitis

Batch 567 : Crohn's disease on DKTatlas lh parstriangularis area

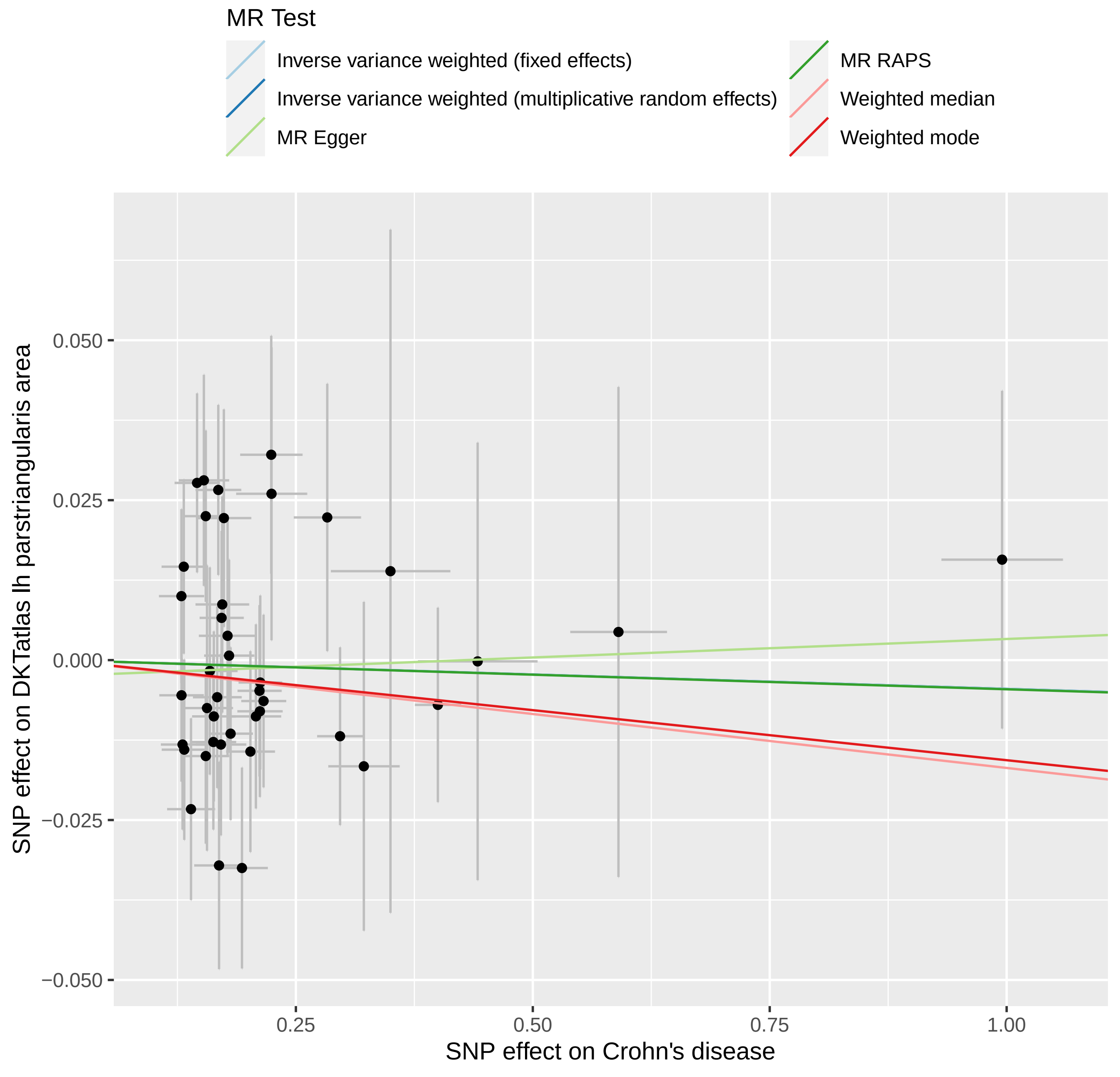

Batch 568 : Ulcerative colitis on DKTatlas lh parstriangularis area

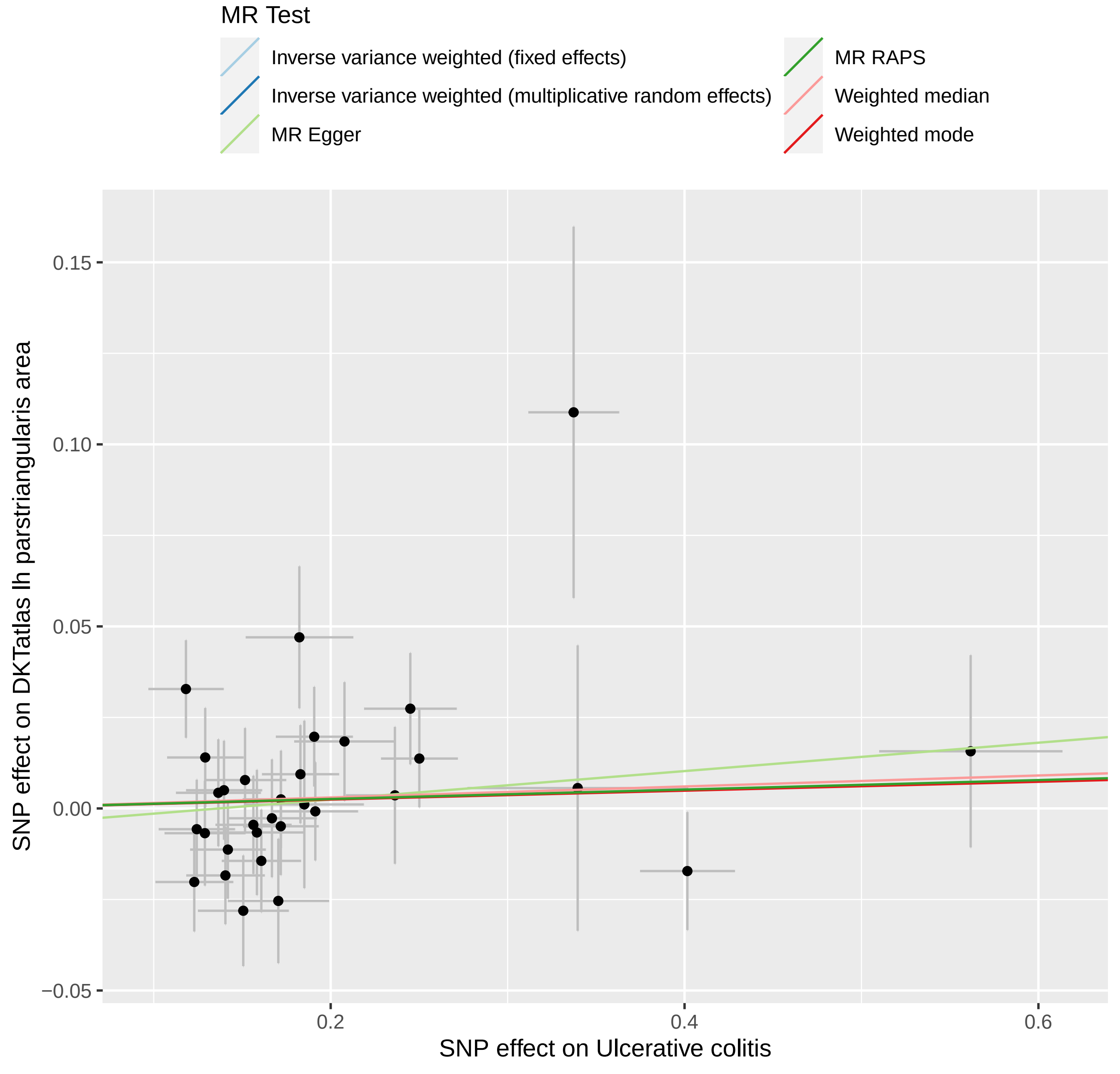

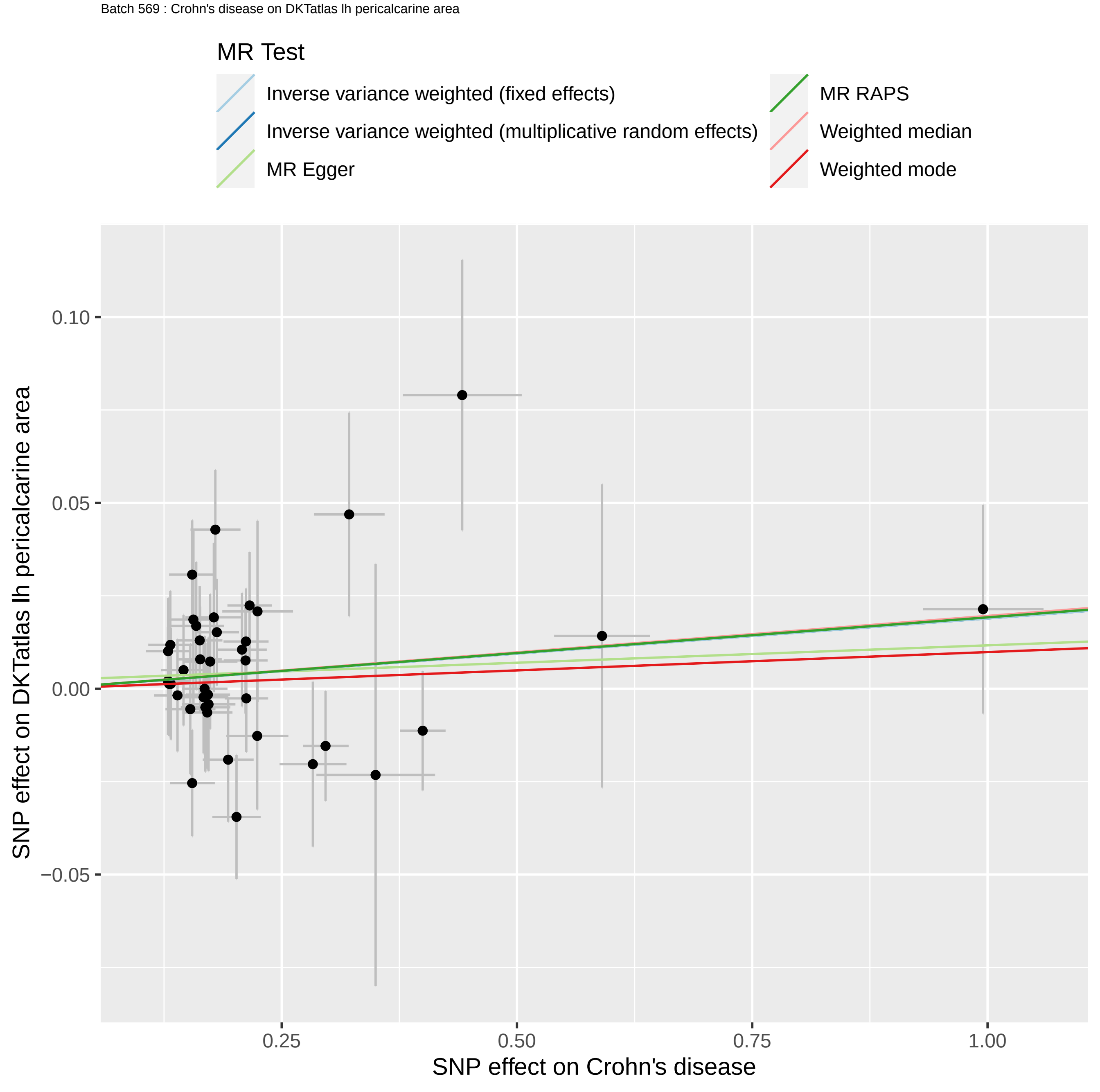

Batch 569 : Crohn's disease on DKTatlas lh pericalcarine area
MR Test
Inverse variance weighted (fixed effects)
Inverse variance weighted (multiplicative random effects)
MR Egger
MR RAPS
Weighted median
Weighted mode
0.10
0.05
0.00
−0.05
0.25
0.50
0.75
1.00
SNP effect on DKTatlas lh pericalcarine area
SNP effect on Crohn's disease

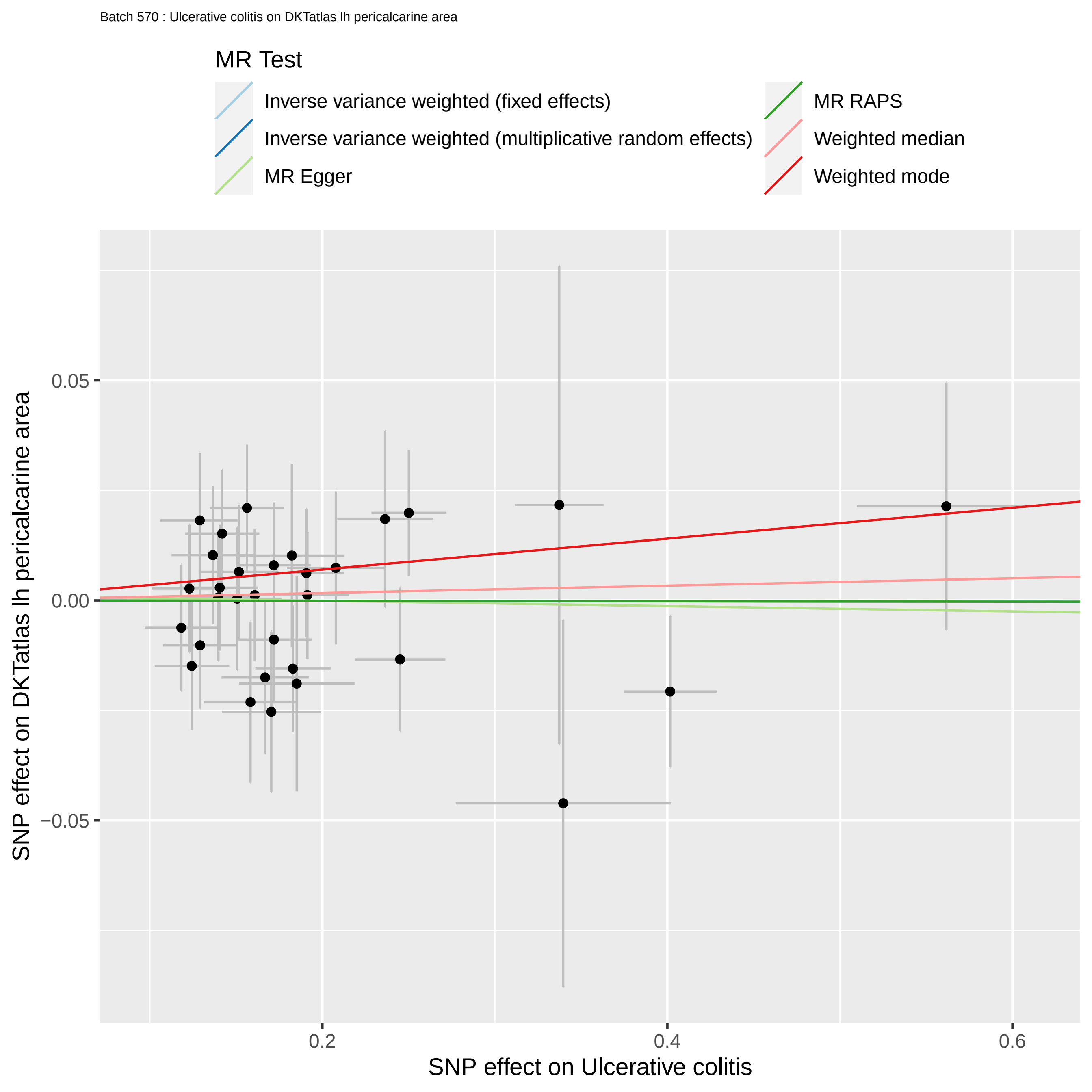
Batch 570 : Ulcerative colitis on DKTatlas lh pericalcarine area
MR Test
Inverse variance weighted (fixed effects)
Inverse variance weighted (multiplicative random effects)
MR Egger
MR RAPS
Weighted median
Weighted mode
0.05
0.00
−0.05
0.2
0.4
0.6
SNP effect on DKTatlas lh pericalcarine area
SNP effect on Ulcerative colitis

Batch 571 : Crohn's disease on DKTatlas lh postcentral area

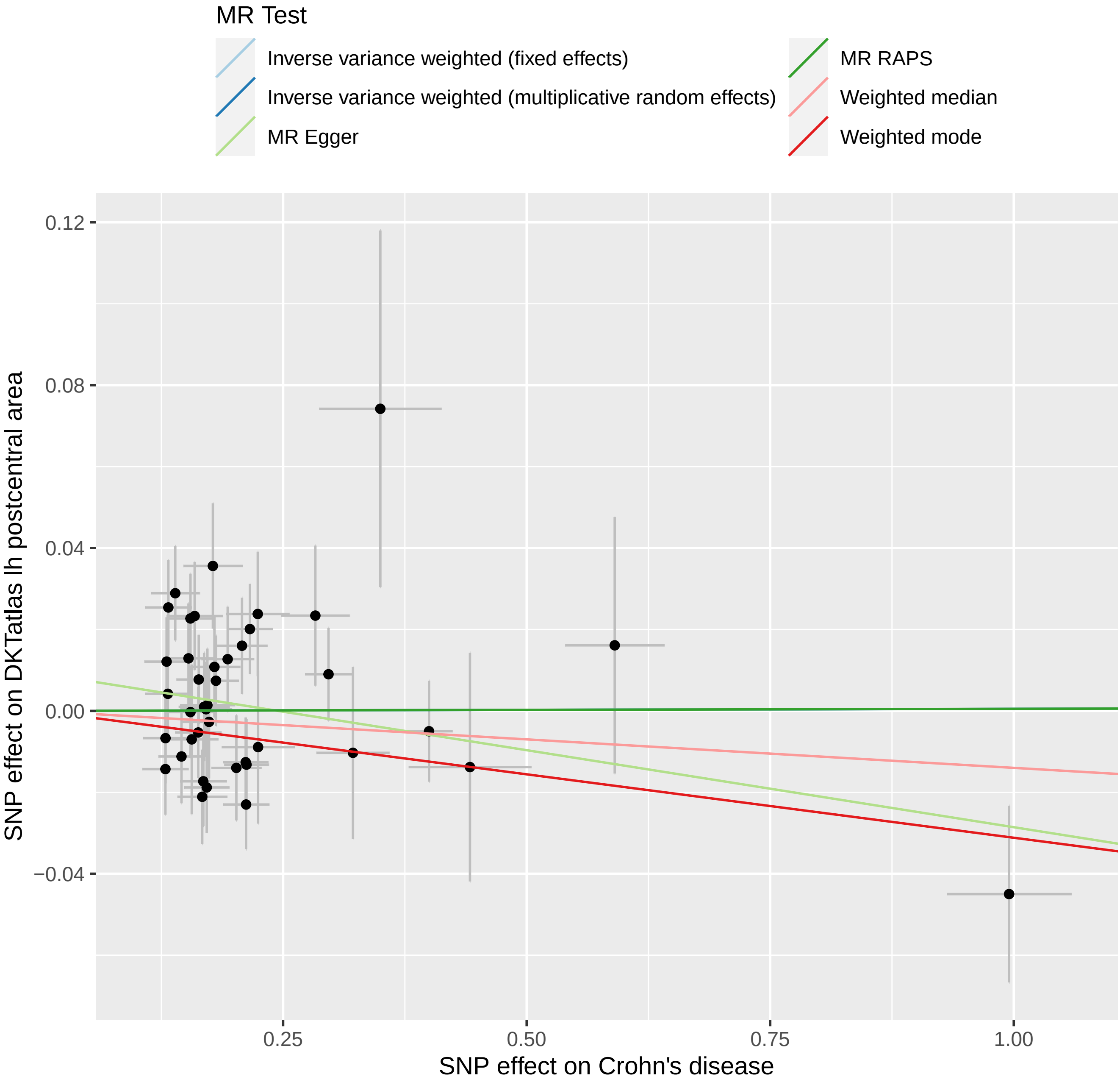

Batch 572 : Ulcerative colitis on DKTatlas lh postcentral area

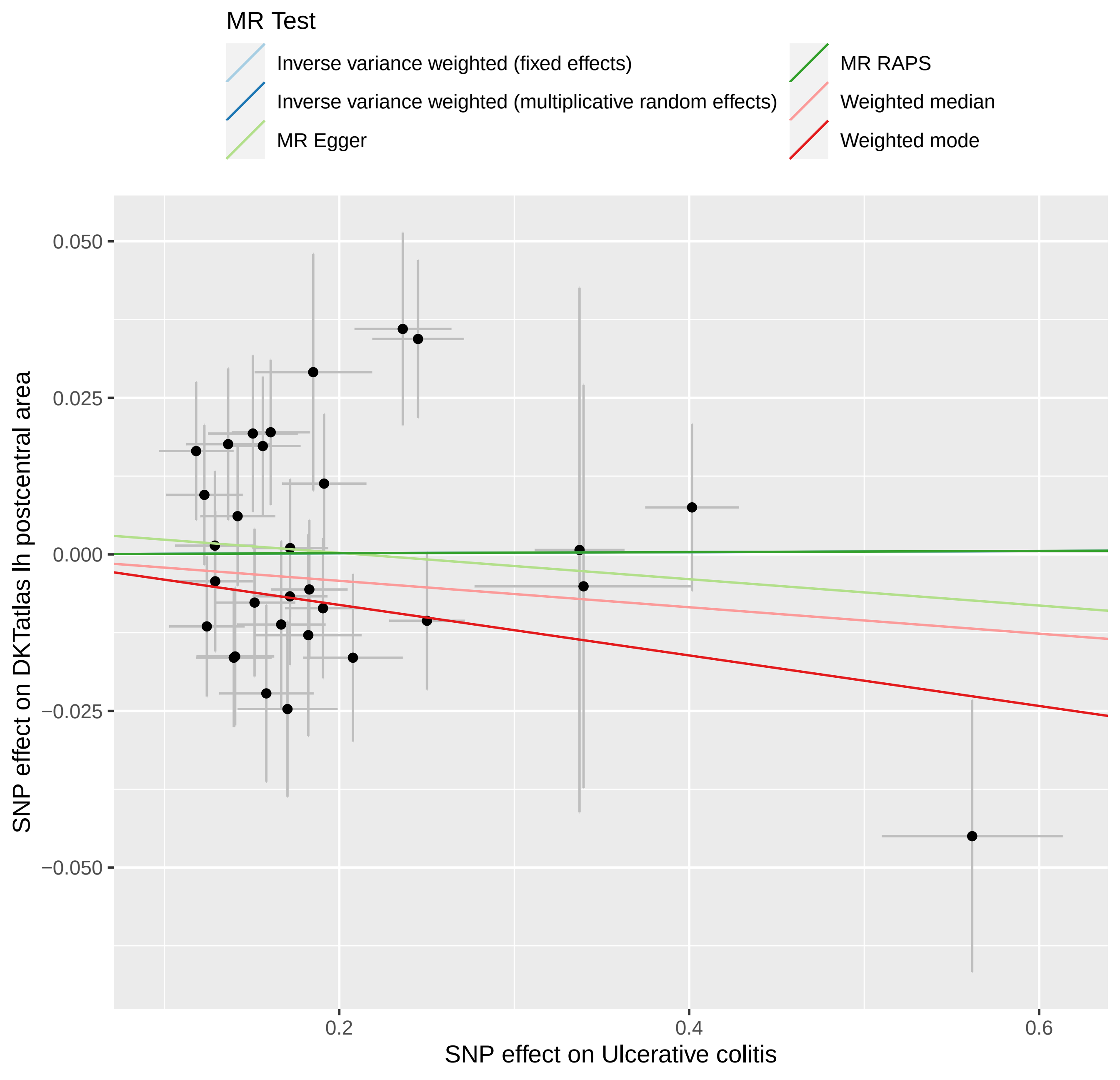

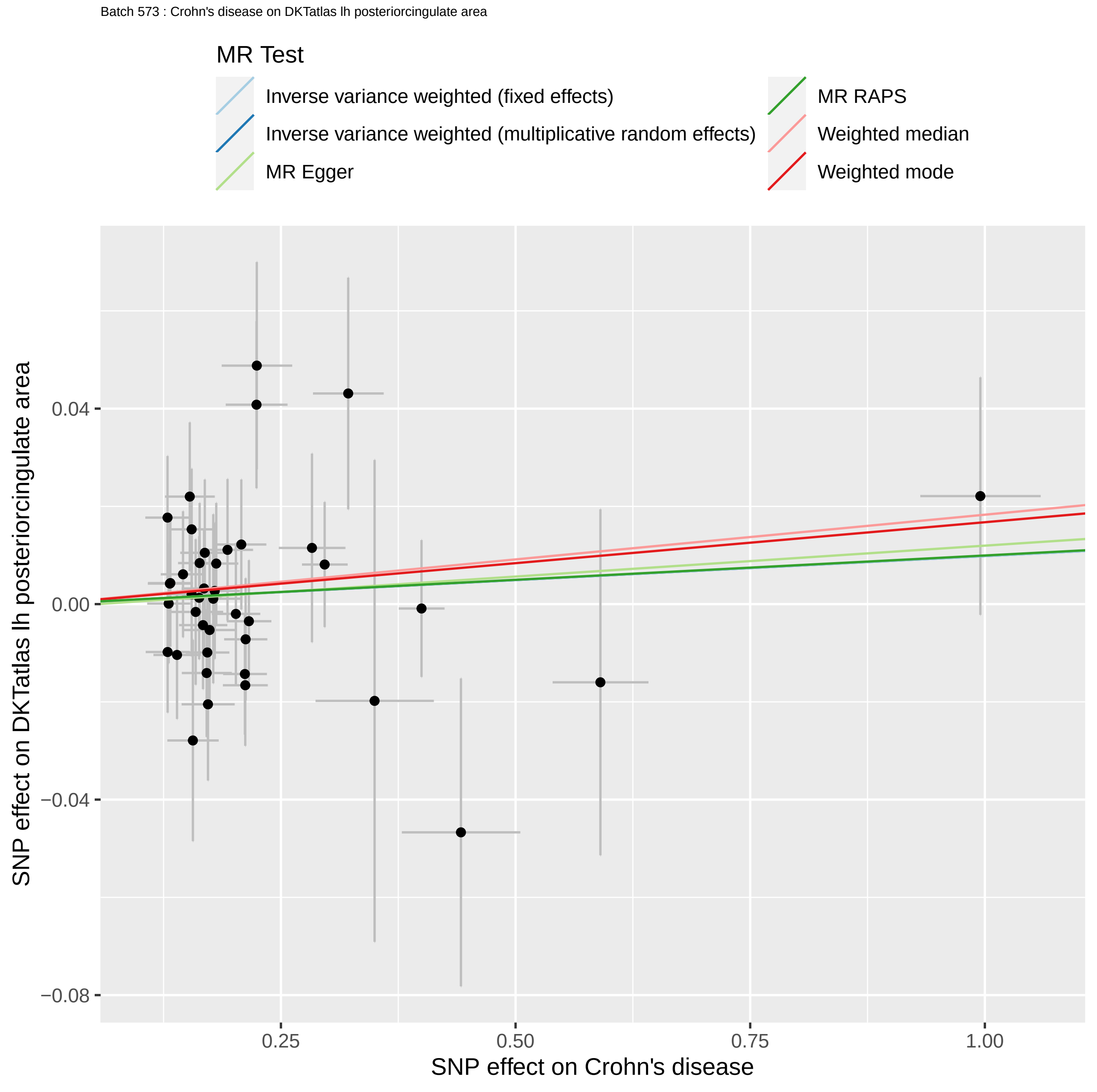
Batch 573 : Crohn's disease on DKTatlas lh posteriorcingulate area
MR Test
Inverse variance weighted (fixed effects)
Inverse variance weighted (multiplicative random effects)
MR Egger
MR RAPS
Weighted median
Weighted mode
SNP effect on DKTatlas lh posteriorcingulate area
0.04
0.00
−0.04
−0.08
0.25
0.50
0.75
1.00
SNP effect on Crohn's disease

Batch 574 : Ulcerative colitis on DKTatlas lh posteriorcingulate area

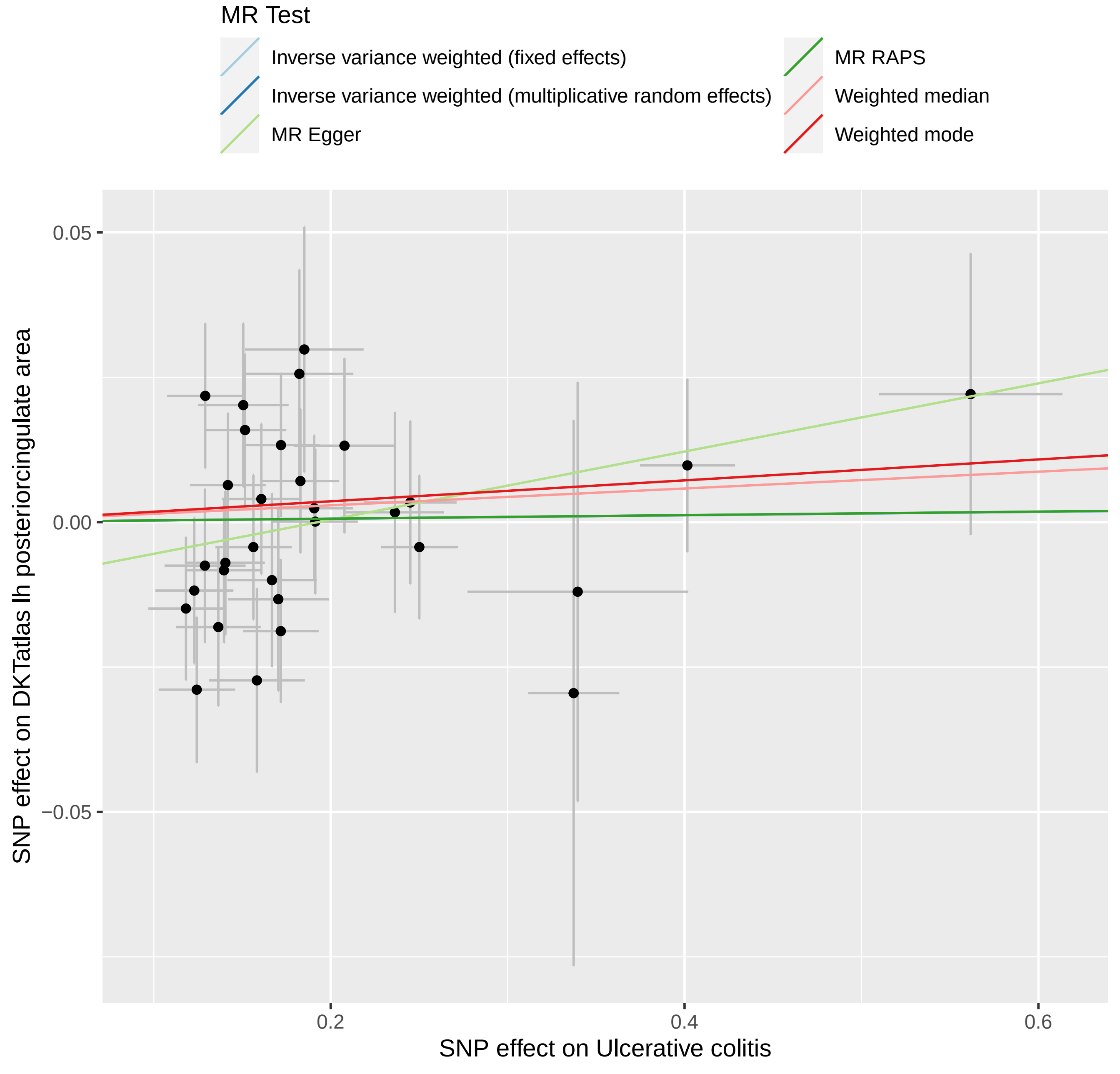

Batch 575 : Crohn's disease on DKTatlas lh precentral area

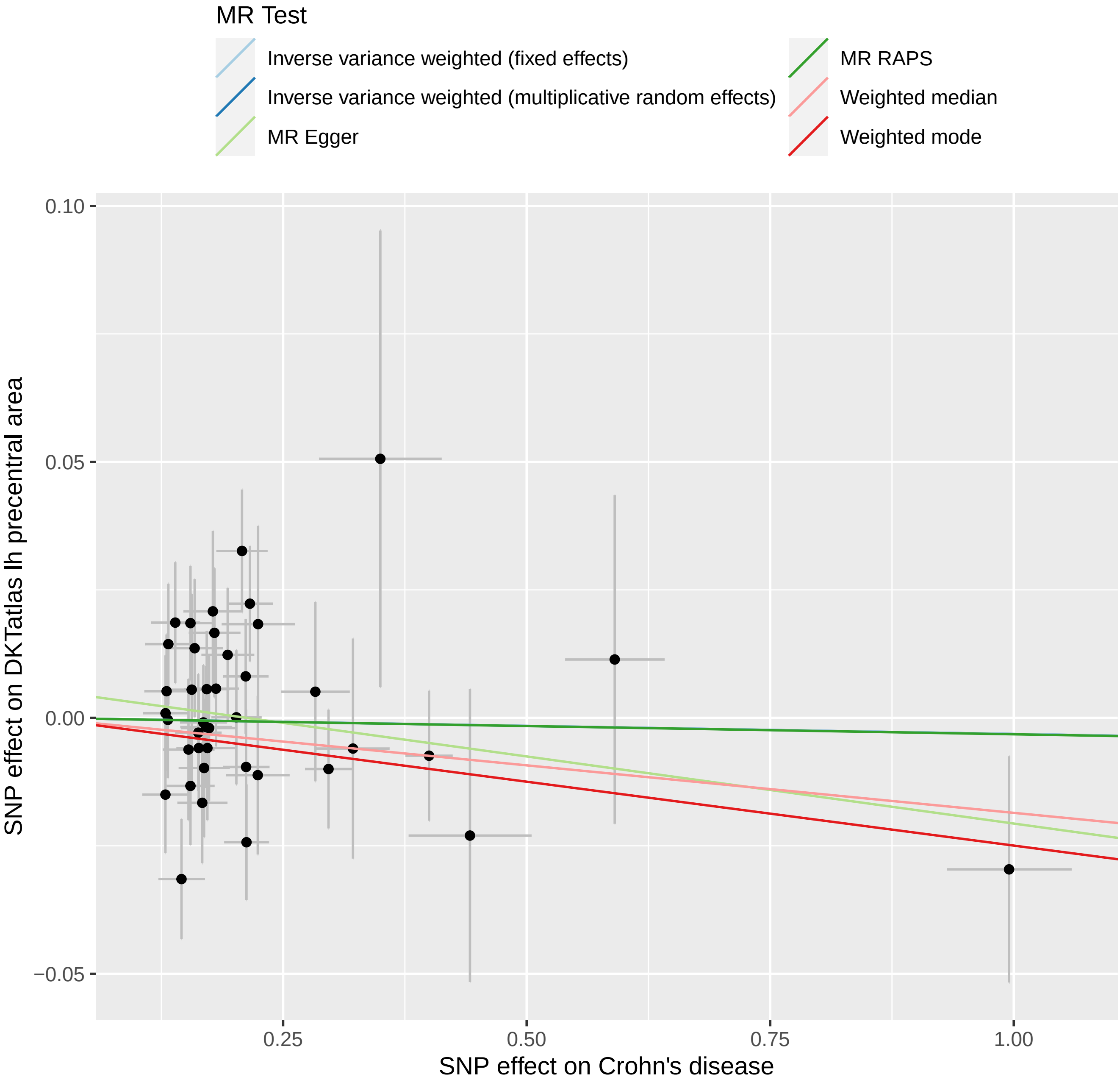

Batch 576 : Ulcerative colitis on DKTatlas lh precentral area

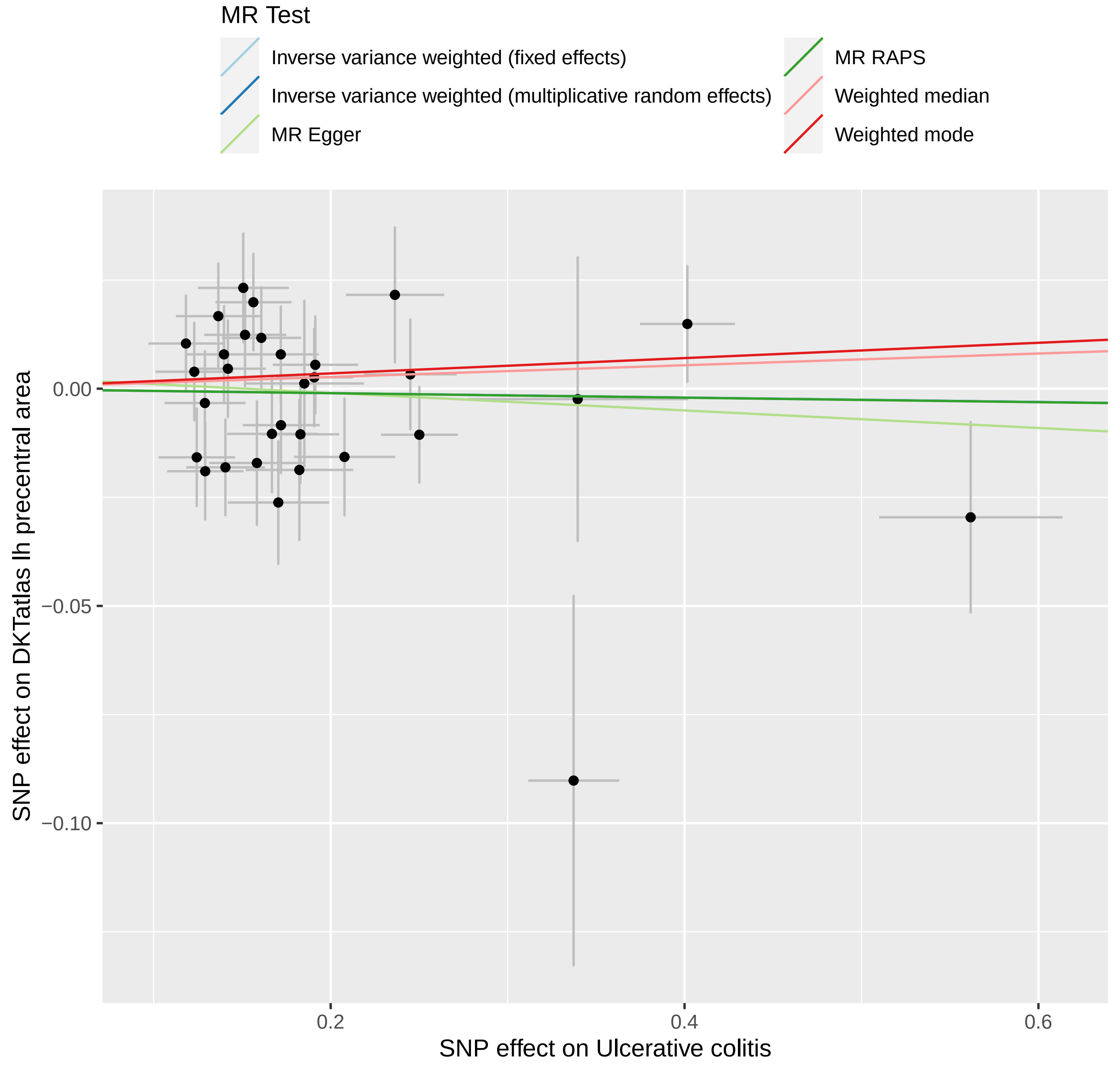

Batch 577 : Crohn's disease on DKTatlas lh precuneus area

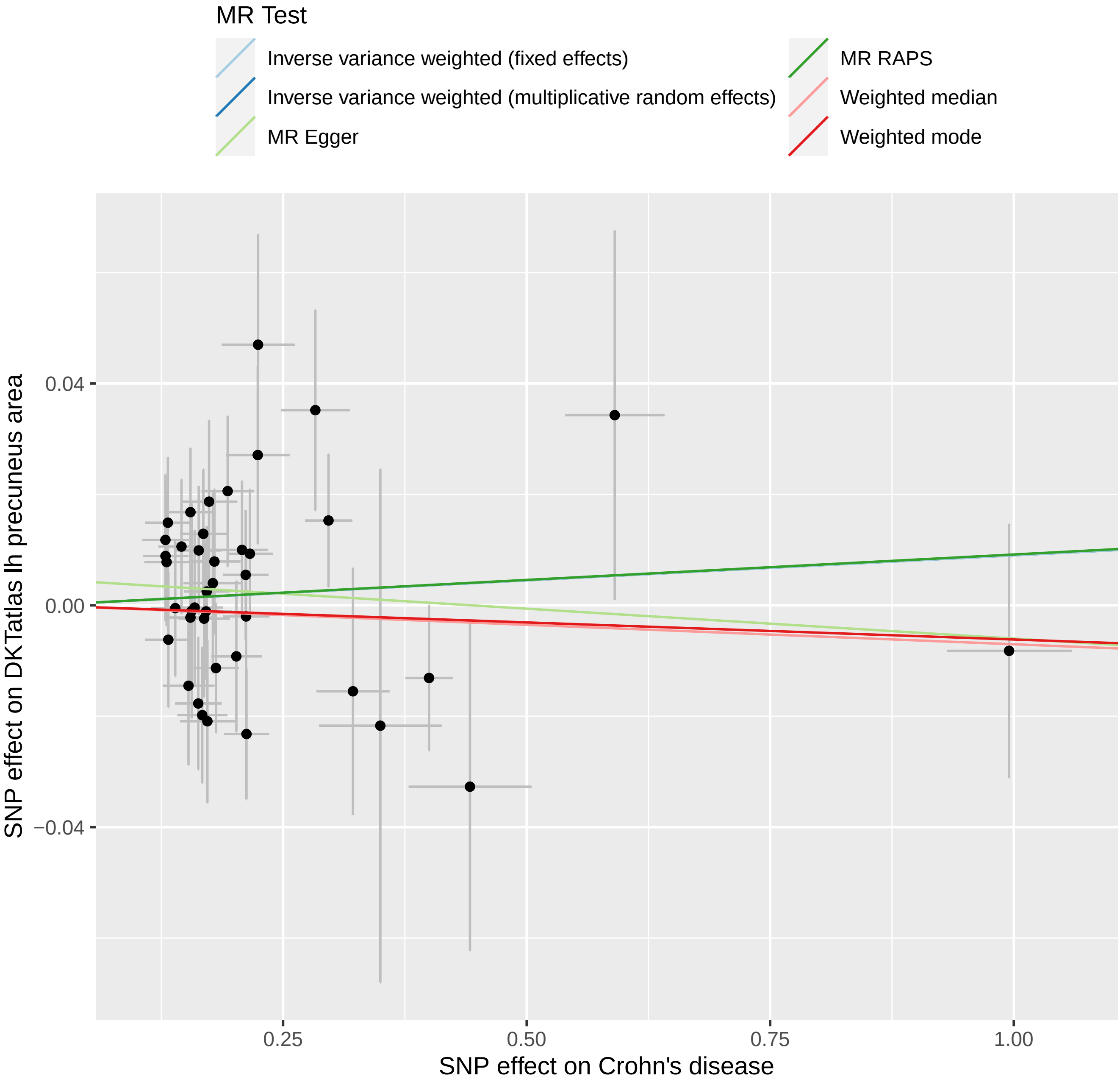

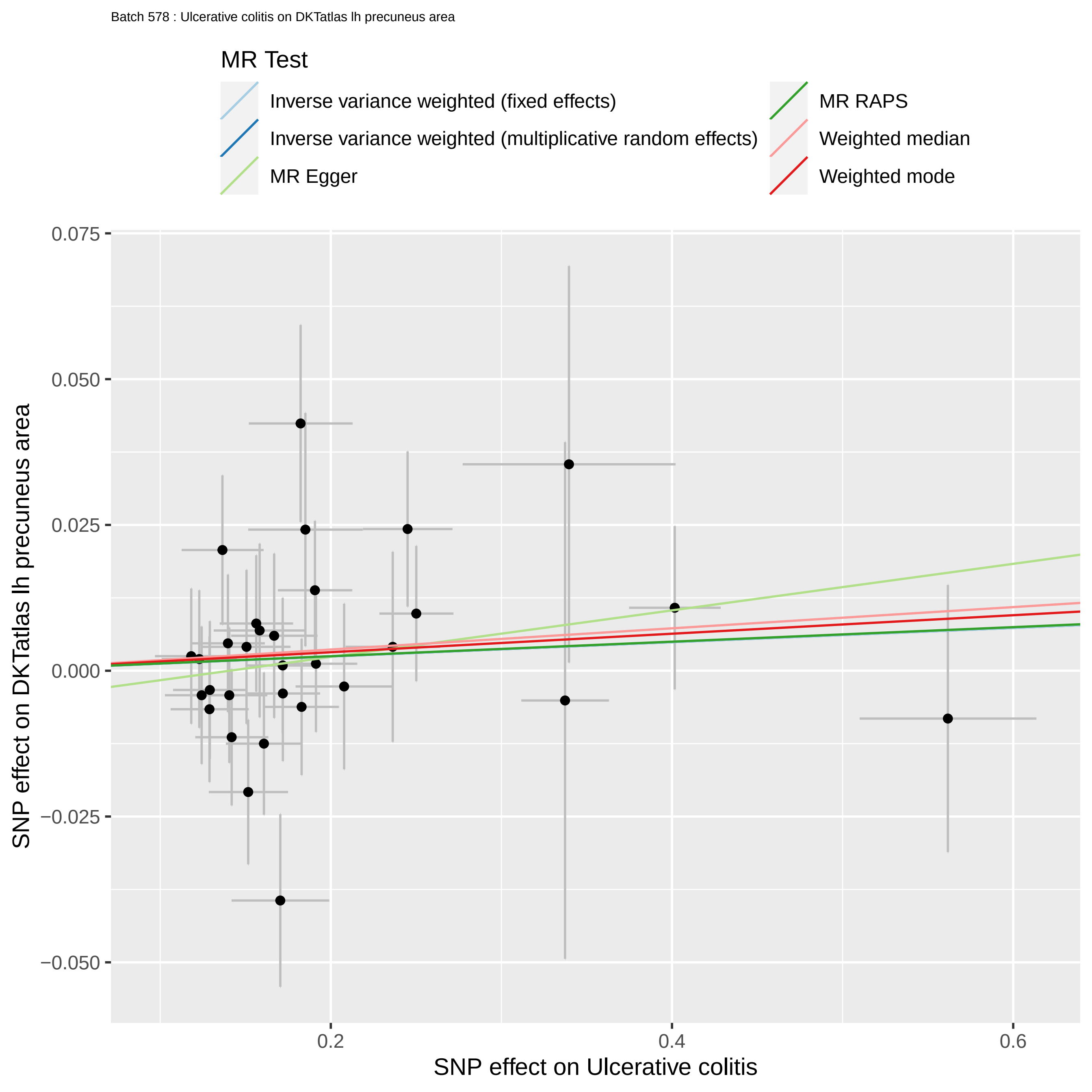

Batch 578 : Ulcerative colitis on DKTatlas lh precuneus area
MR Test
Inverse variance weighted (fixed effects)
Inverse variance weighted (multiplicative random effects)
MR Egger
MR RAPS
Weighted median
Weighted mode
0.075
0.050
0.025
0.000
−0.025
−0.050
SNP effect on DKTatlas lh precuneus area
0.2
0.4
0.6
SNP effect on Ulcerative colitis

Batch 579 : Crohn's disease on DKTatlas lh rostralanteriorcingulate area

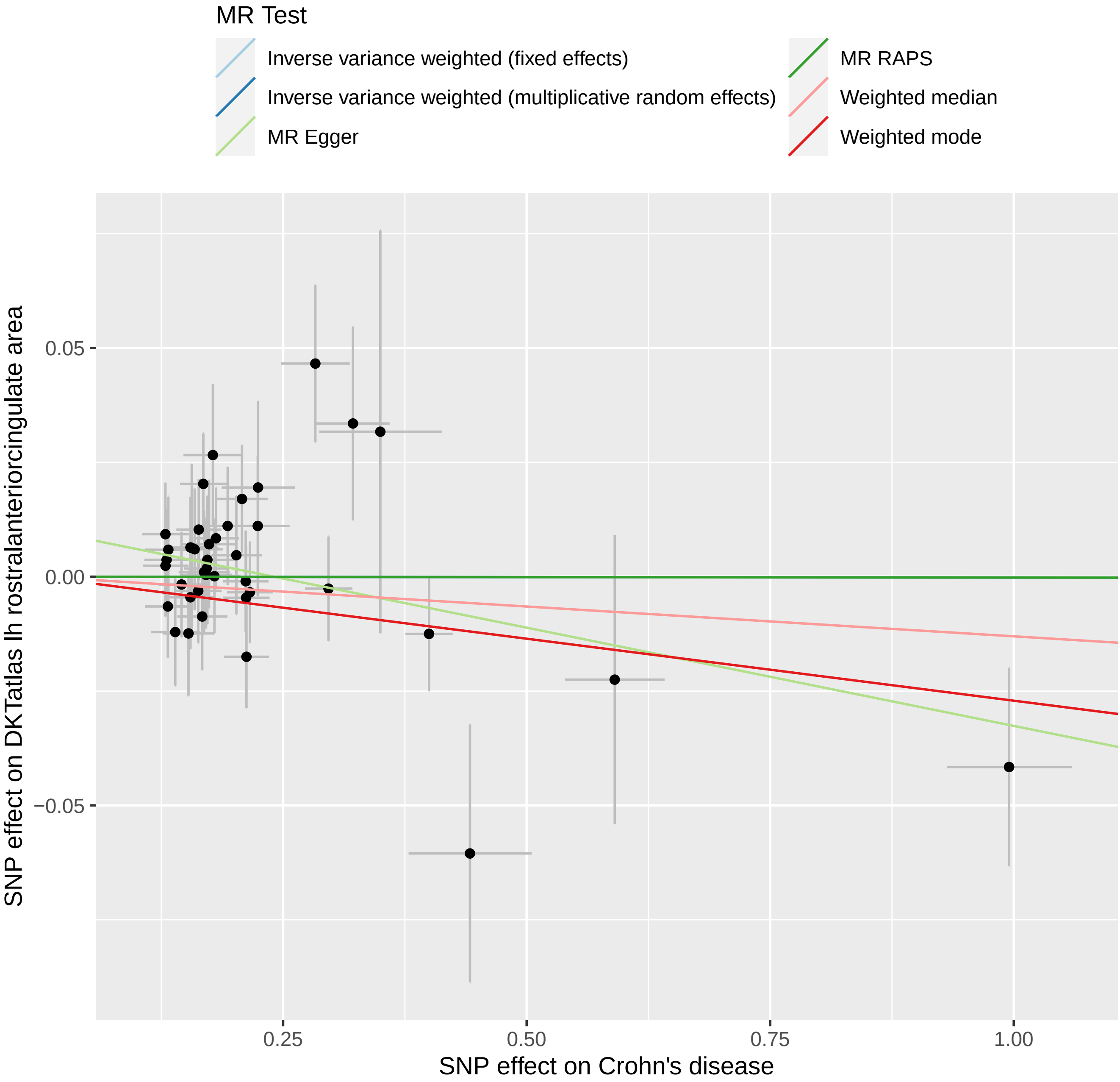

Batch 580 : Ulcerative colitis on DKTatlas lh rostralanteriorcingulate area

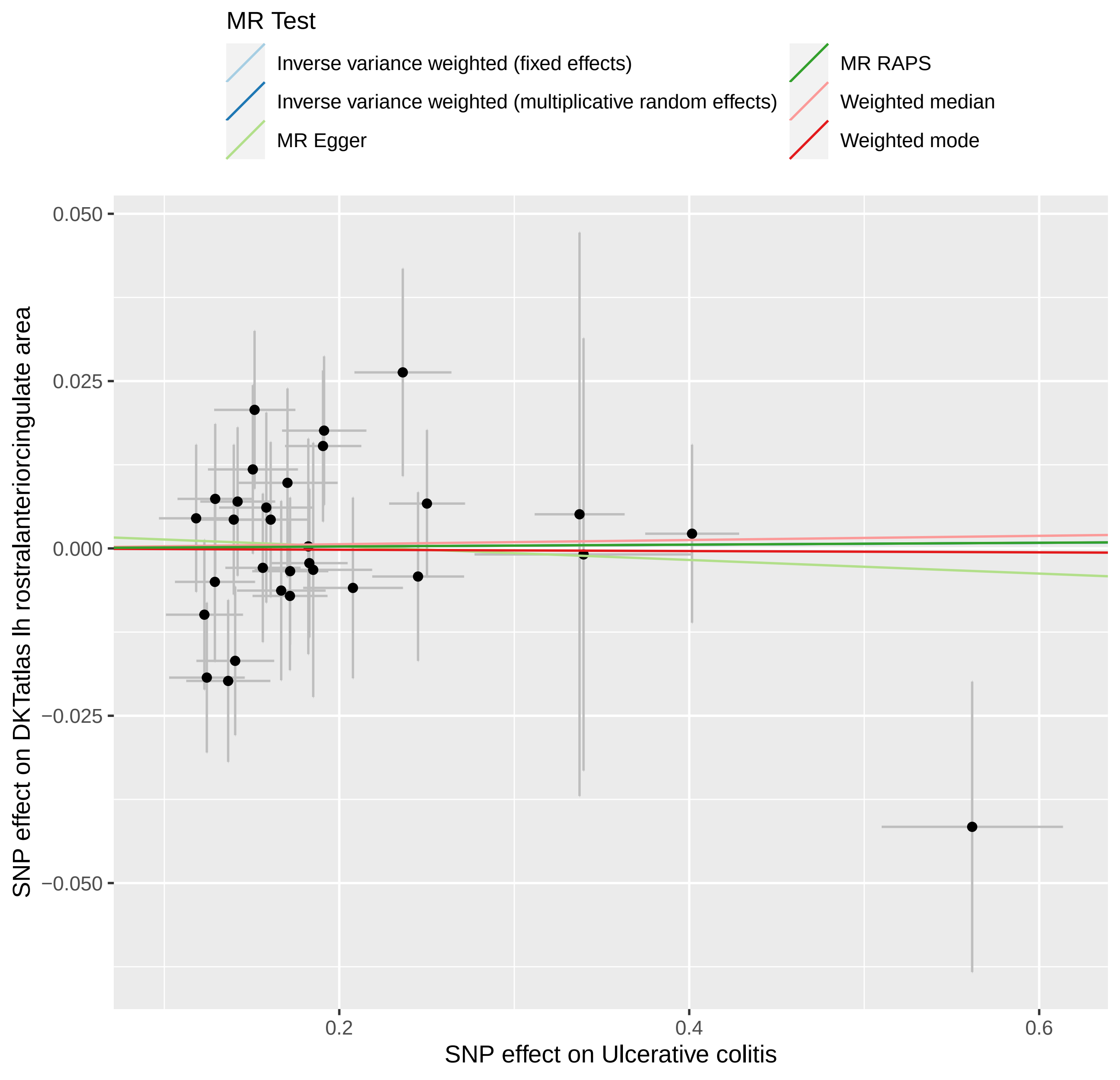

Batch 581 : Crohn's disease on DKTatlas lh rostralmiddlefrontal area

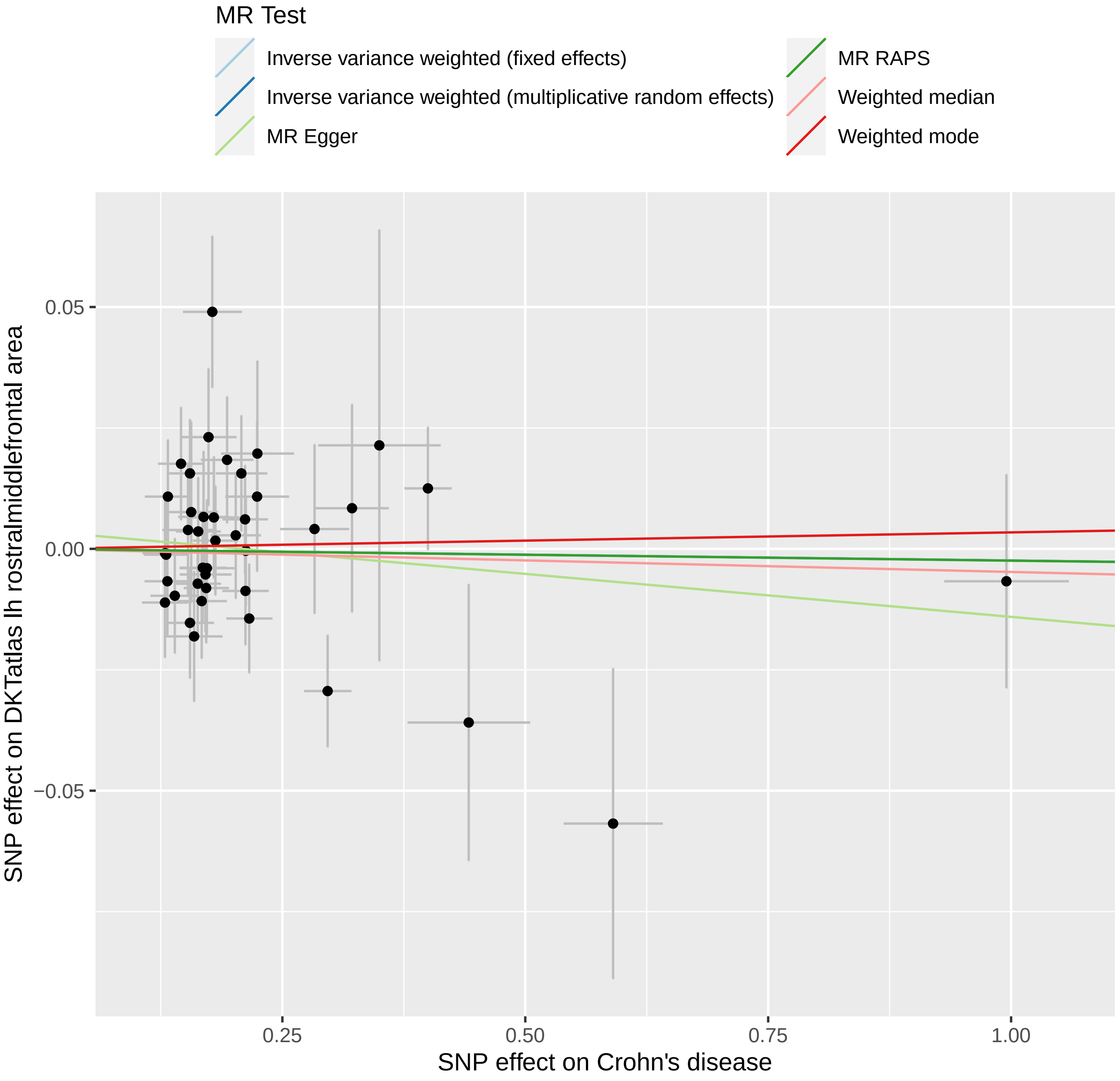

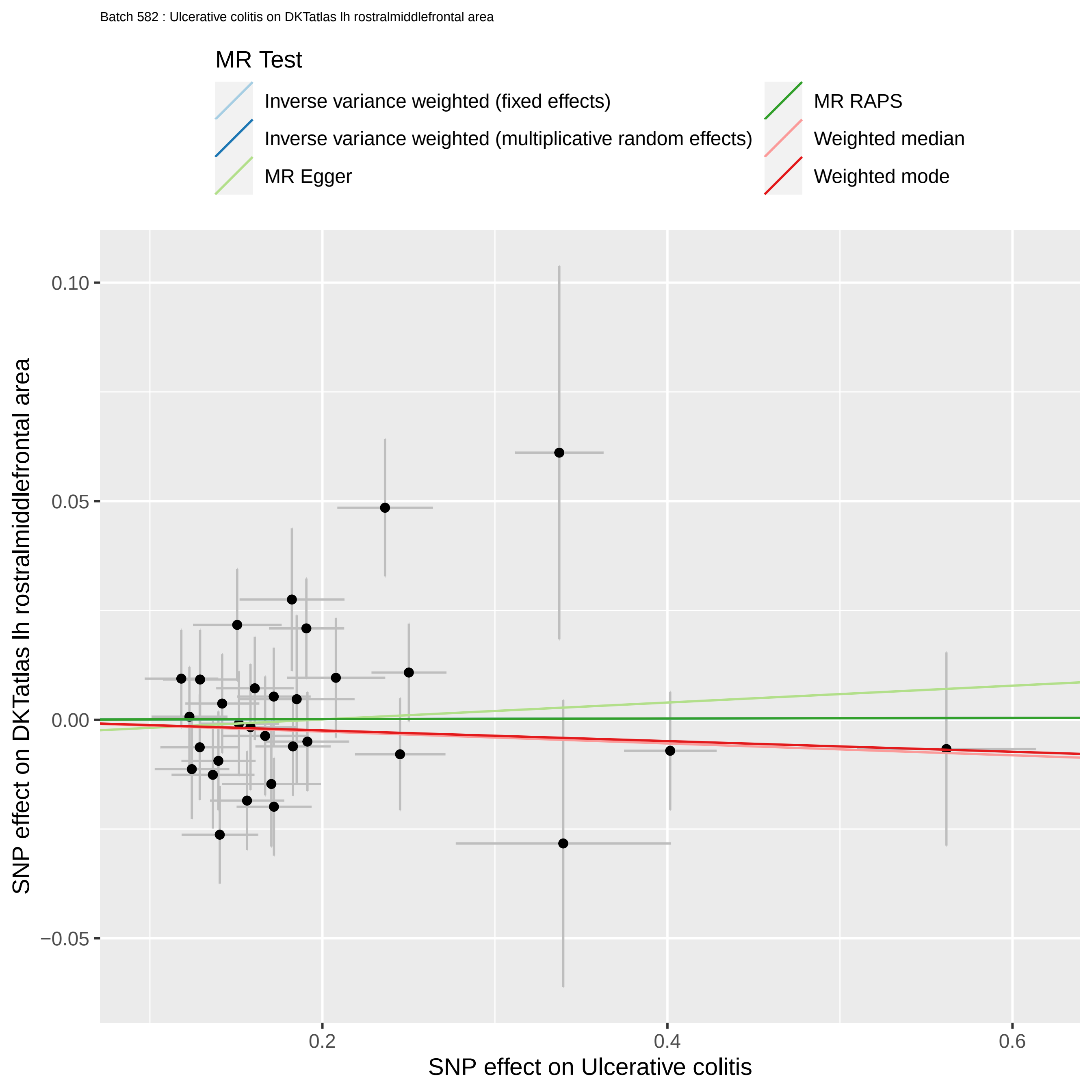
Batch 582 : Ulcerative colitis on DKTatlas lh rostralmiddlefrontal area
MR Test
Inverse variance weighted (fixed effects)
Inverse variance weighted (multiplicative random effects)
MR Egger
MR RAPS
Weighted median
Weighted mode
0.10
0.05
0.00
−0.05
0.2
0.4
0.6
SNP effect on DKTatlas lh rostralmiddlefrontal area
SNP effect on Ulcerative colitis

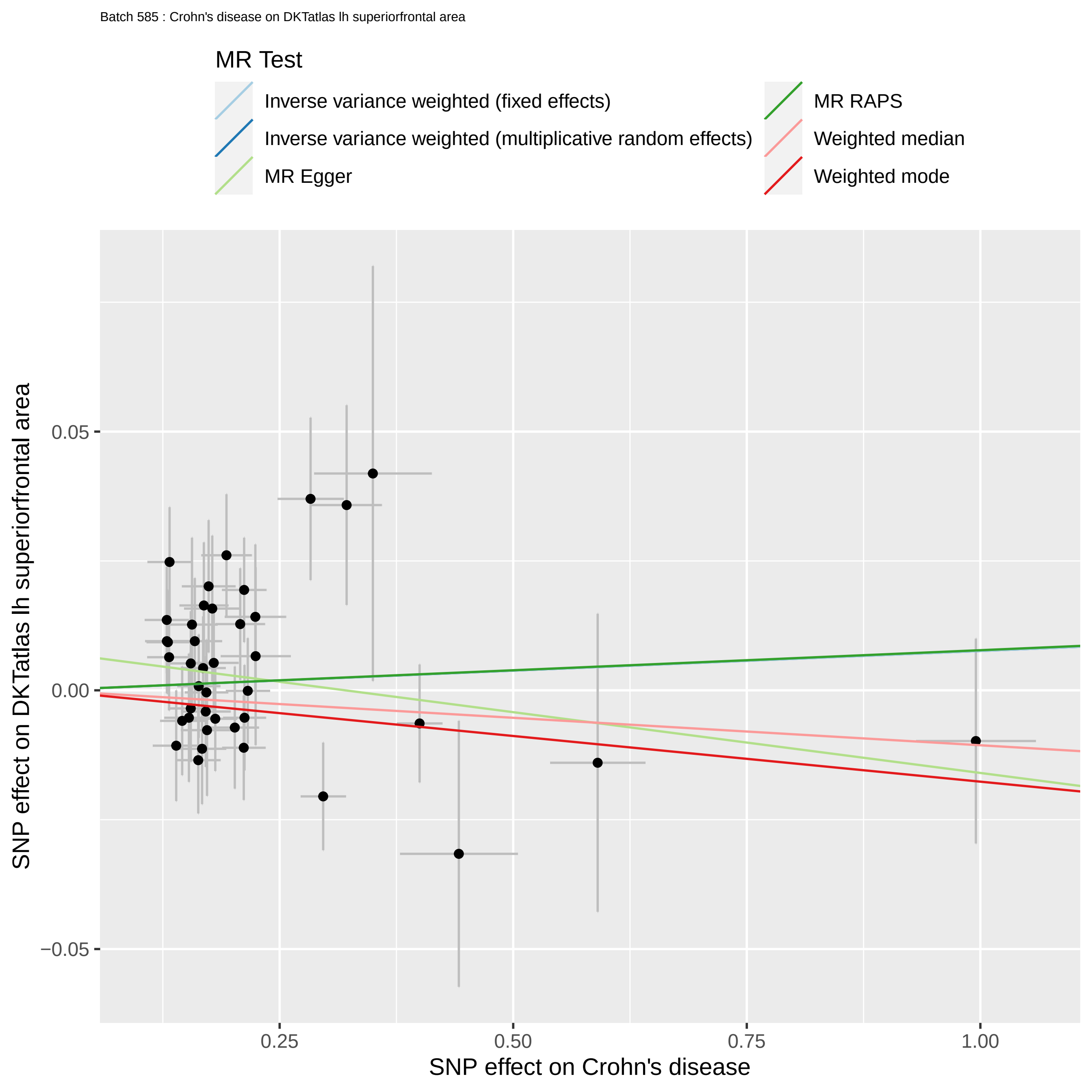
Batch 585 : Crohn's disease on DKTatlas lh superiorfrontal area
MR Test
Inverse variance weighted (fixed effects)
Inverse variance weighted (multiplicative random effects)
MR Egger
MR RAPS
Weighted median
Weighted mode
0.05
0.00
−0.05
0.25
0.50
0.75
1.00
SNP effect on DKTatlas lh superiorfrontal area
SNP effect on Crohn's disease

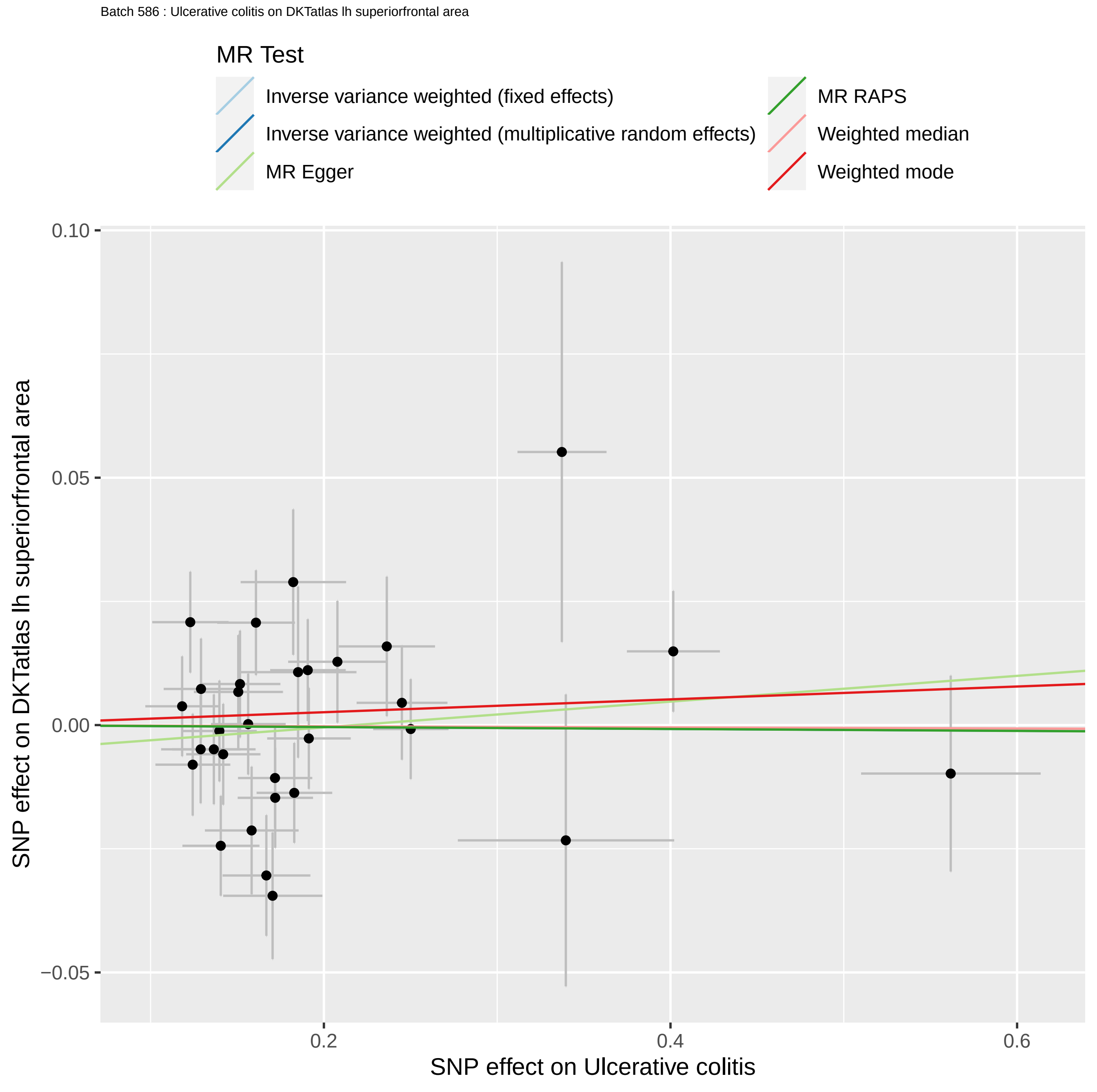

Batch 586 : Ulcerative colitis on DKTatlas lh superiorfrontal area
MR Test
Inverse variance weighted (fixed effects)
Inverse variance weighted (multiplicative random effects)
MR Egger
MR RAPS
Weighted median
Weighted mode
0.10
0.05
0.00
−0.05
0.2
0.4
0.6
SNP effect on DKTatlas lh superiorfrontal area
SNP effect on Ulcerative colitis

Batch 587 : Crohn's disease on DKTatlas lh superiorparietal area

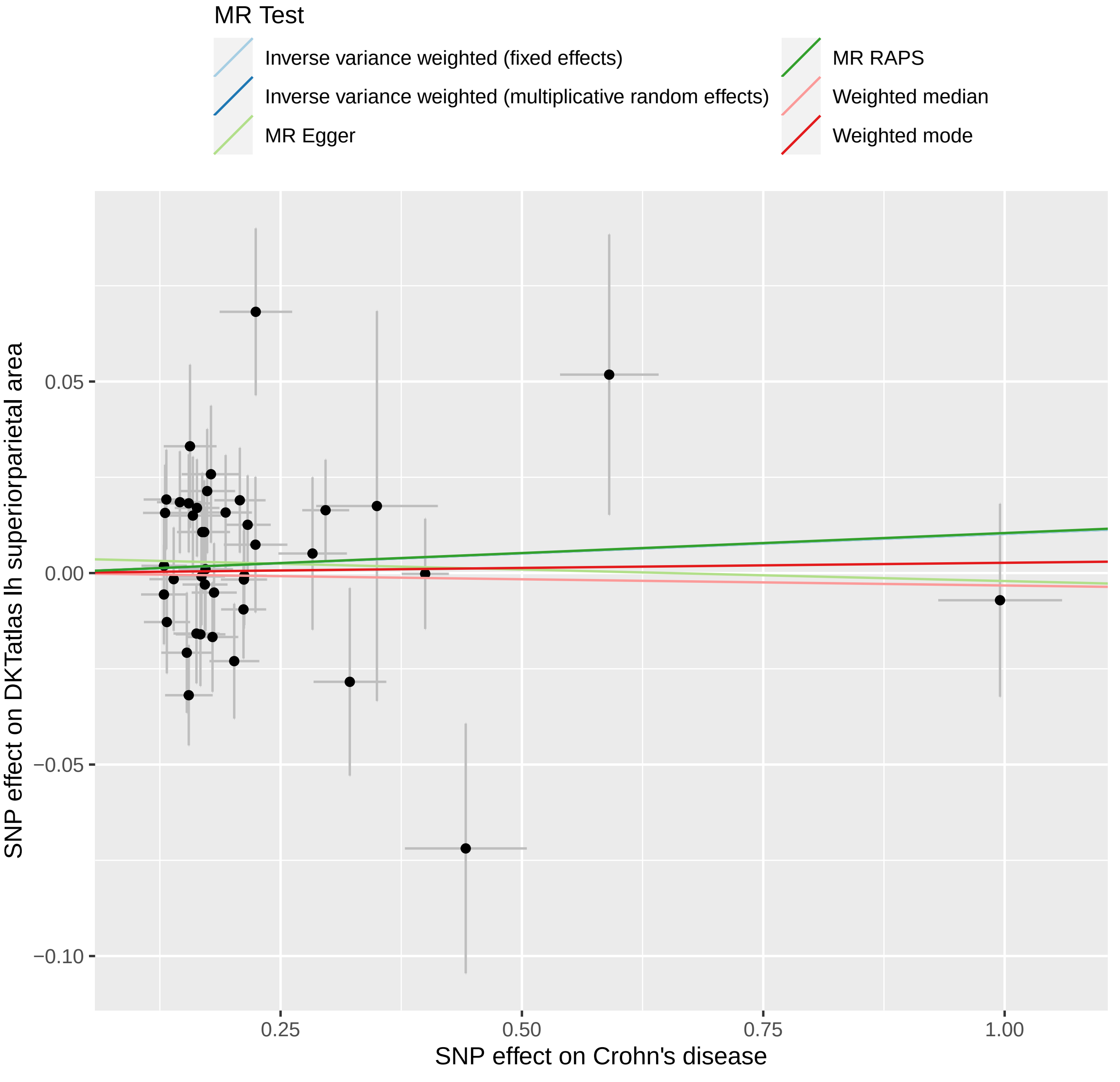

Batch 588 : Ulcerative colitis on DKTatlas lh superiorparietal area

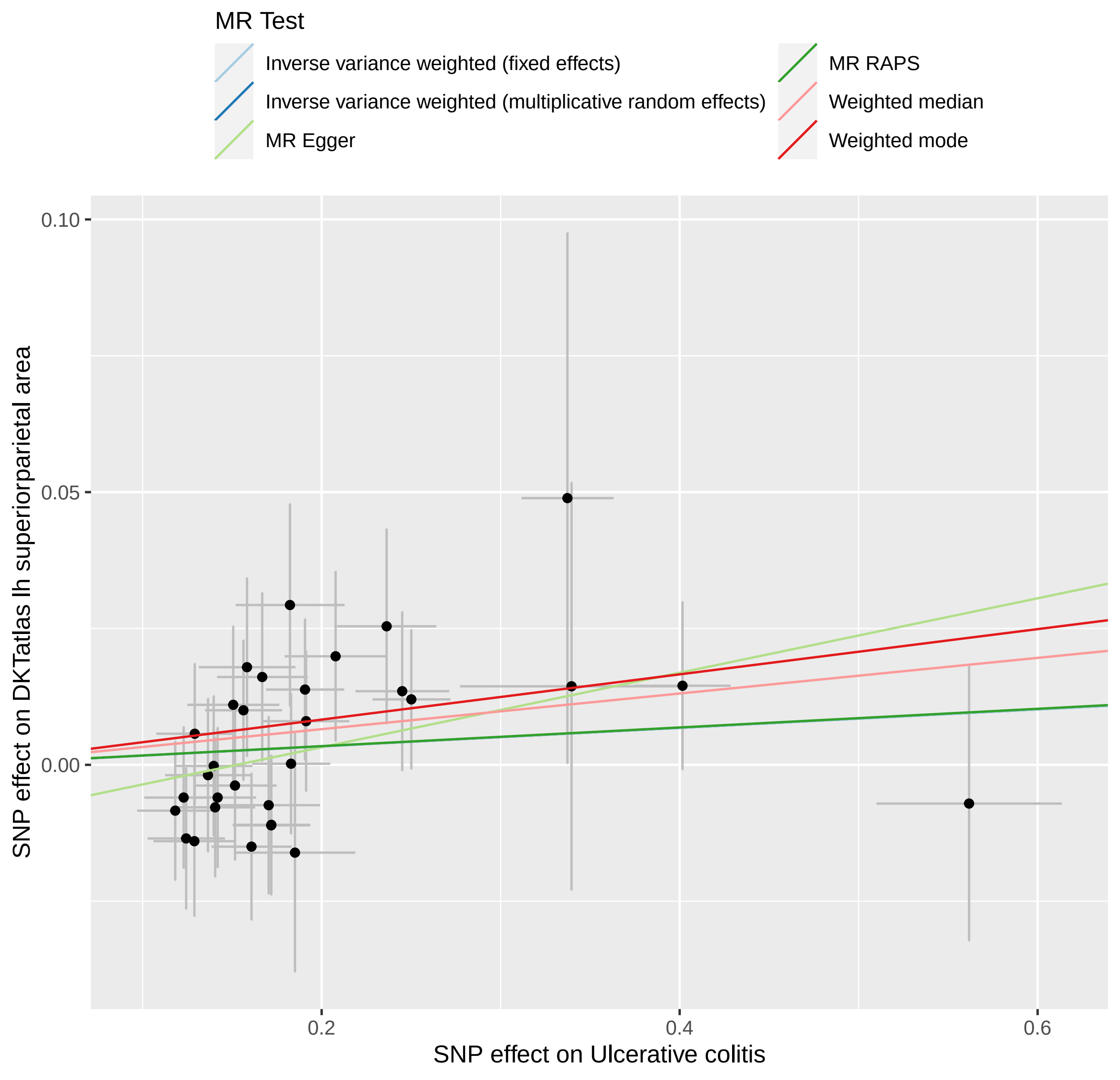

Batch 589 : Crohn's disease on DKTatlas lh superiortemporal area

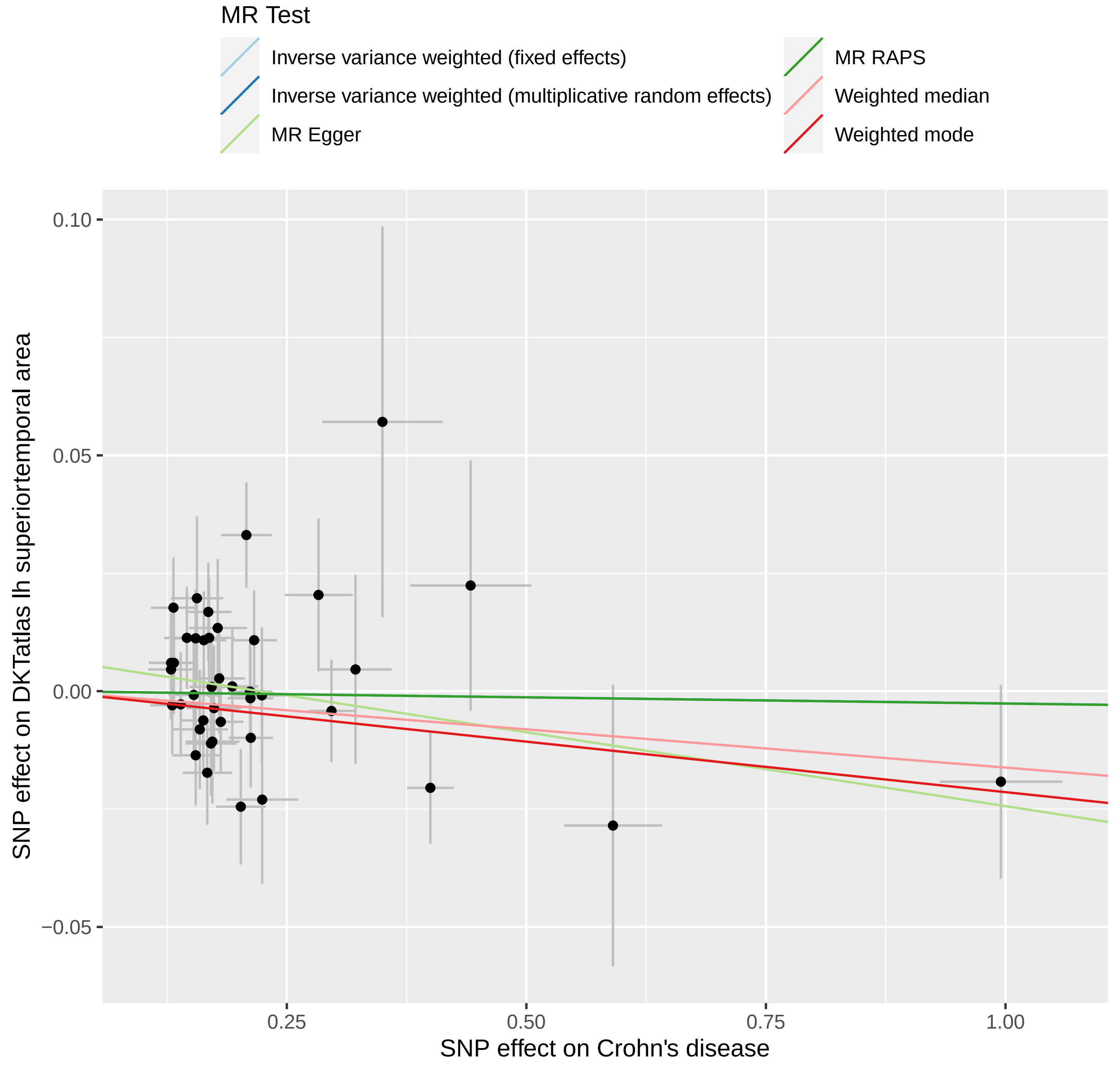

Batch 590 : Ulcerative colitis on DKTatlas lh superiortemporal area

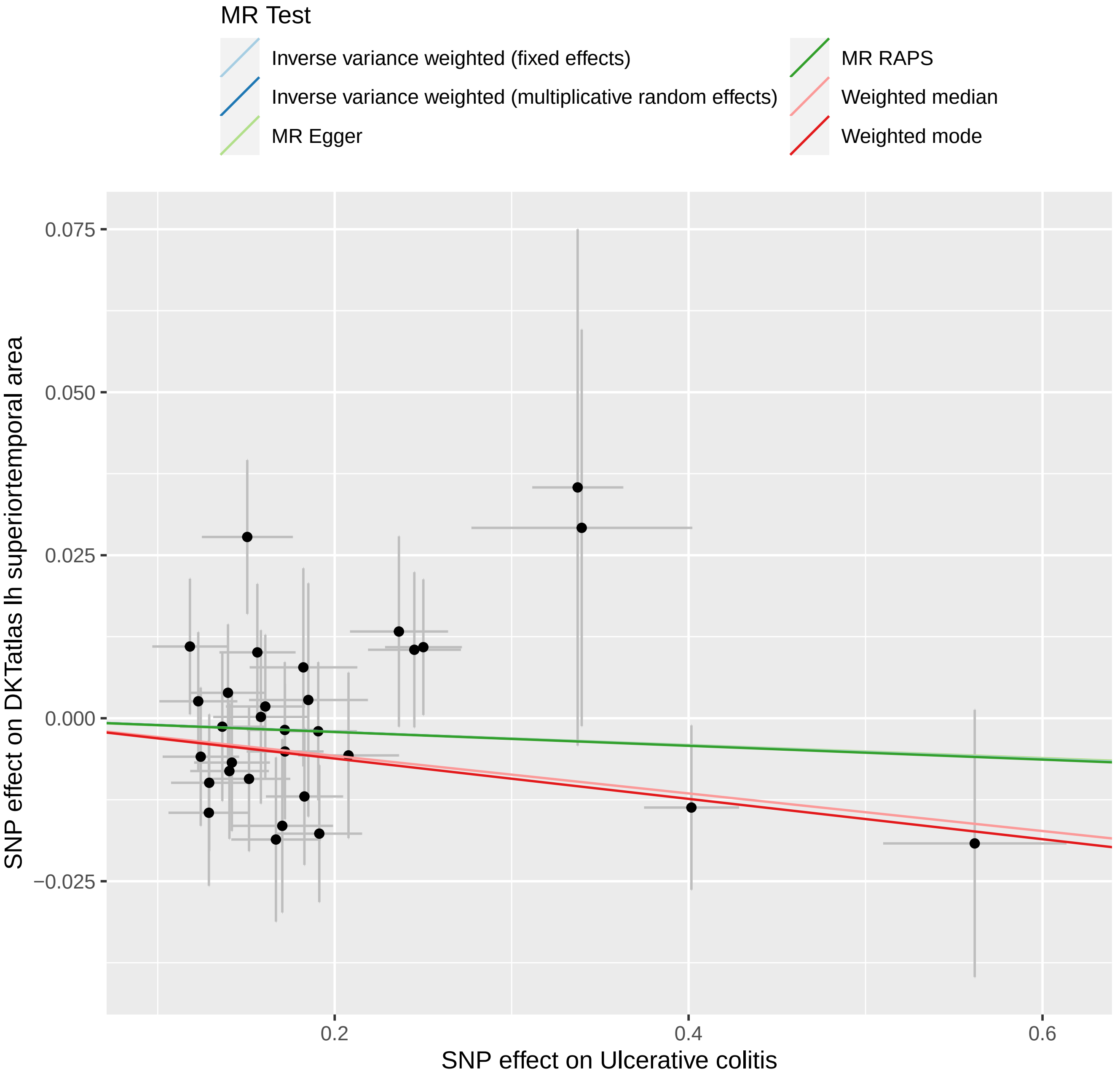

Batch 591 : Crohn's disease on DKTatlas lh supramarginal area

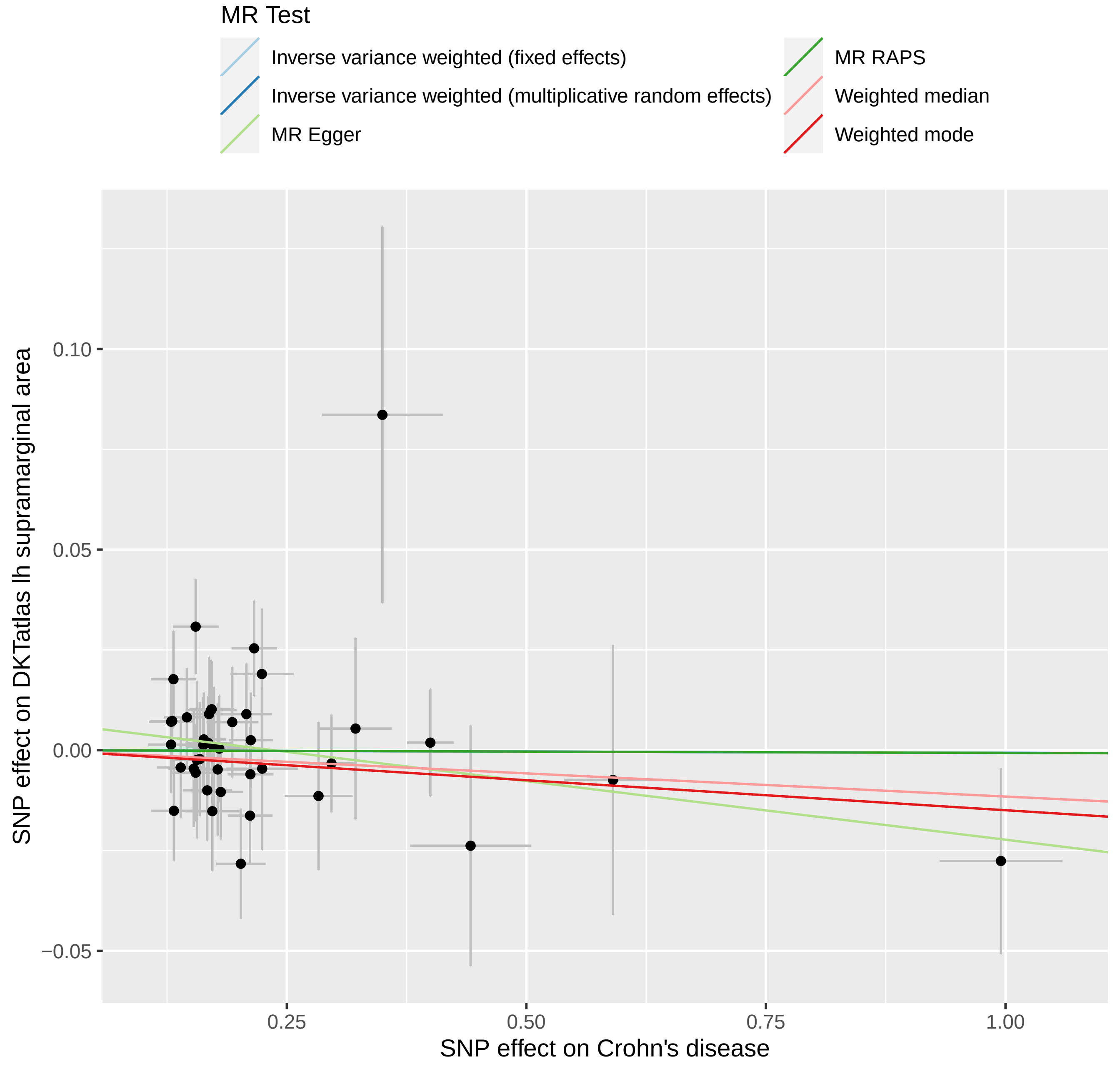

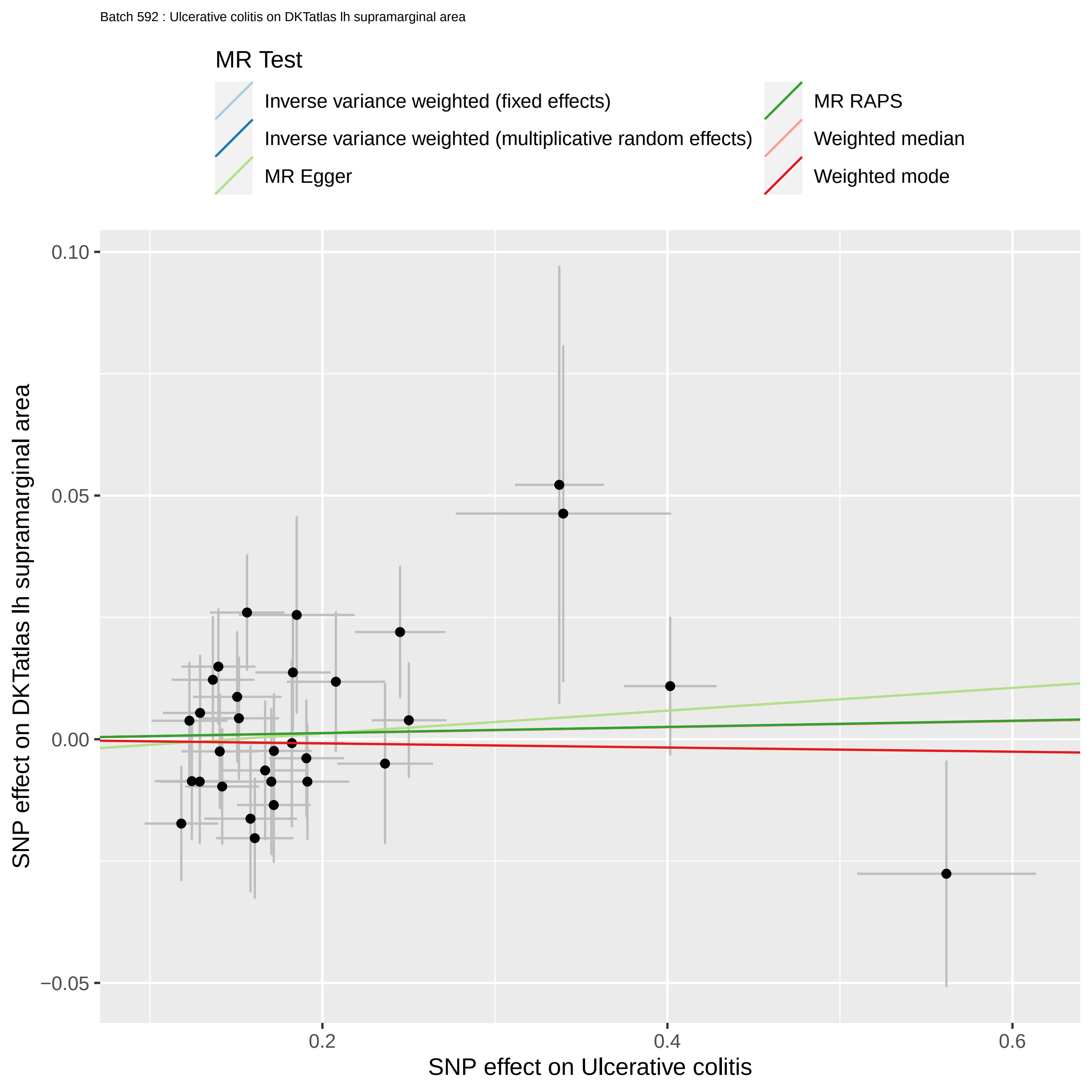

Batch 592 : Ulcerative colitis on DKTatlas lh supramarginal area
MR Test
Inverse variance weighted (fixed effects)
Inverse variance weighted (multiplicative random effects)
MR Egger
MR RAPS
Weighted median
Weighted mode
0.10
0.05
0.00
−0.05
0.2
0.4
0.6
SNP effect on DKTatlas lh supramarginal area
SNP effect on Ulcerative colitis

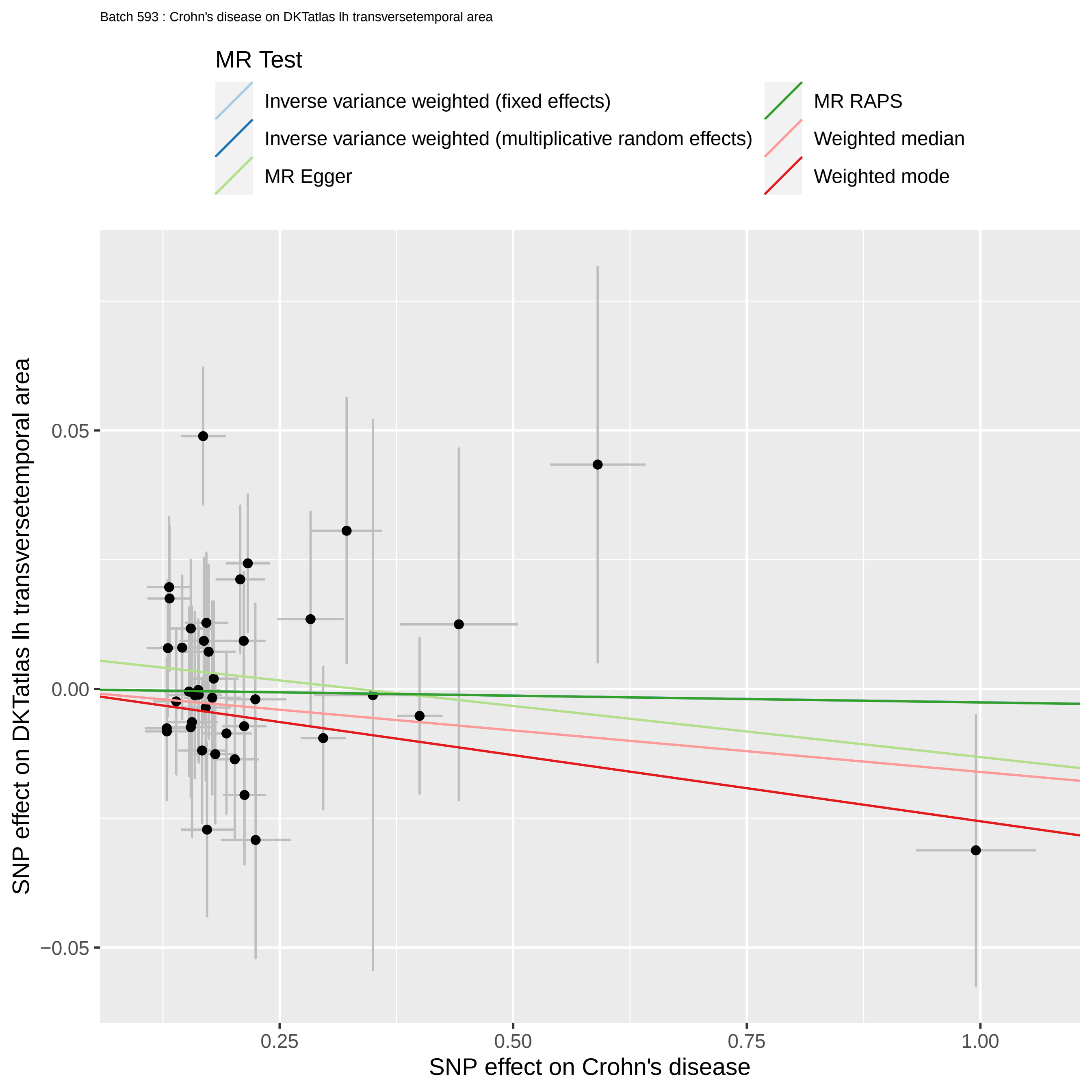
Batch 593 : Crohn's disease on DKTatlas lh transversetemporal area
MR Test
Inverse variance weighted (fixed effects)
Inverse variance weighted (multiplicative random effects)
MR Egger
MR RAPS
Weighted median
Weighted mode
SNP effect on DKTatlas lh transversetemporal area
0.05
0.00
−0.05
0.25
0.50
0.75
1.00
SNP effect on Crohn's disease

Batch 594 : Ulcerative colitis on DKTatlas lh transversetemporal area

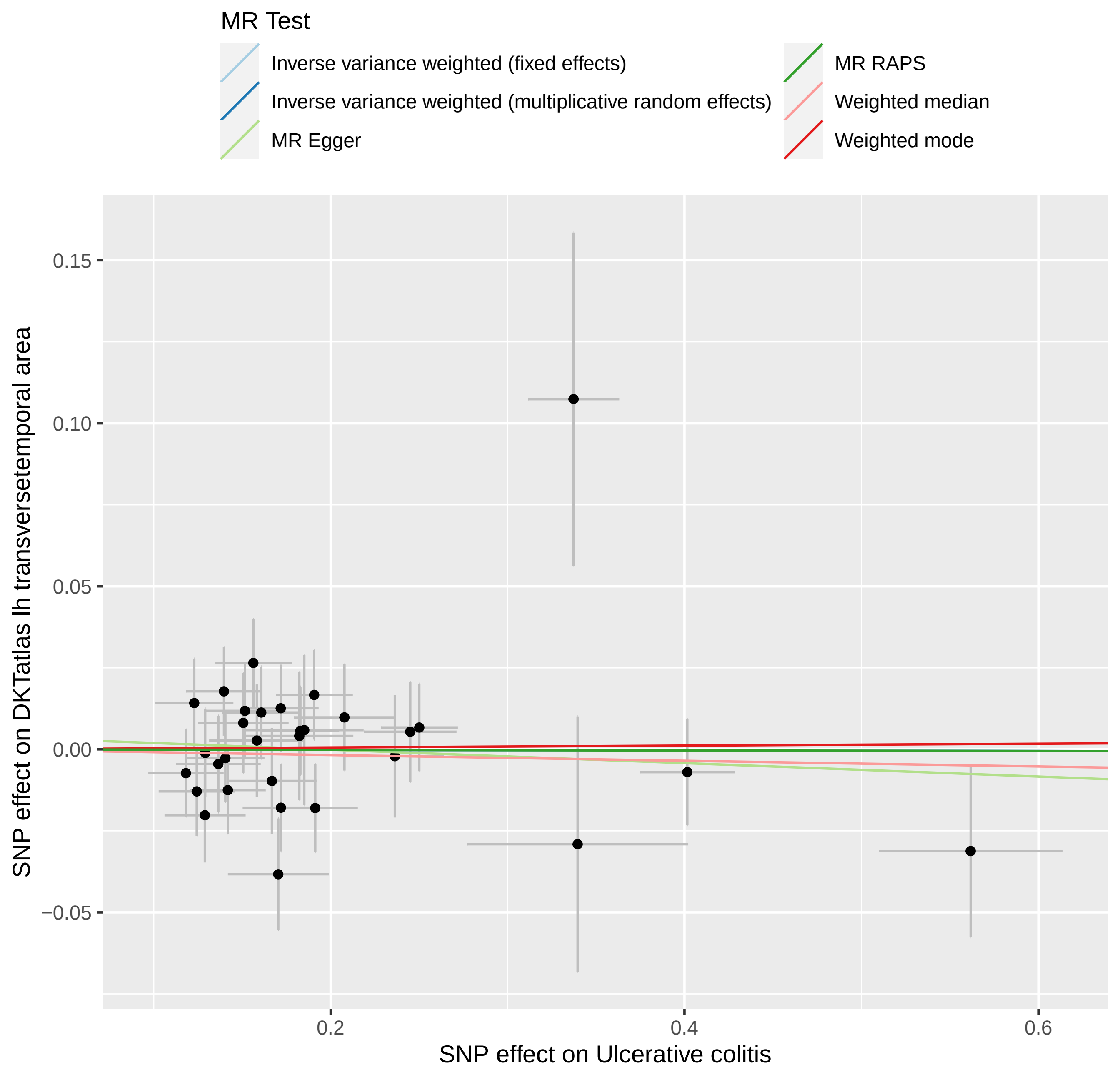

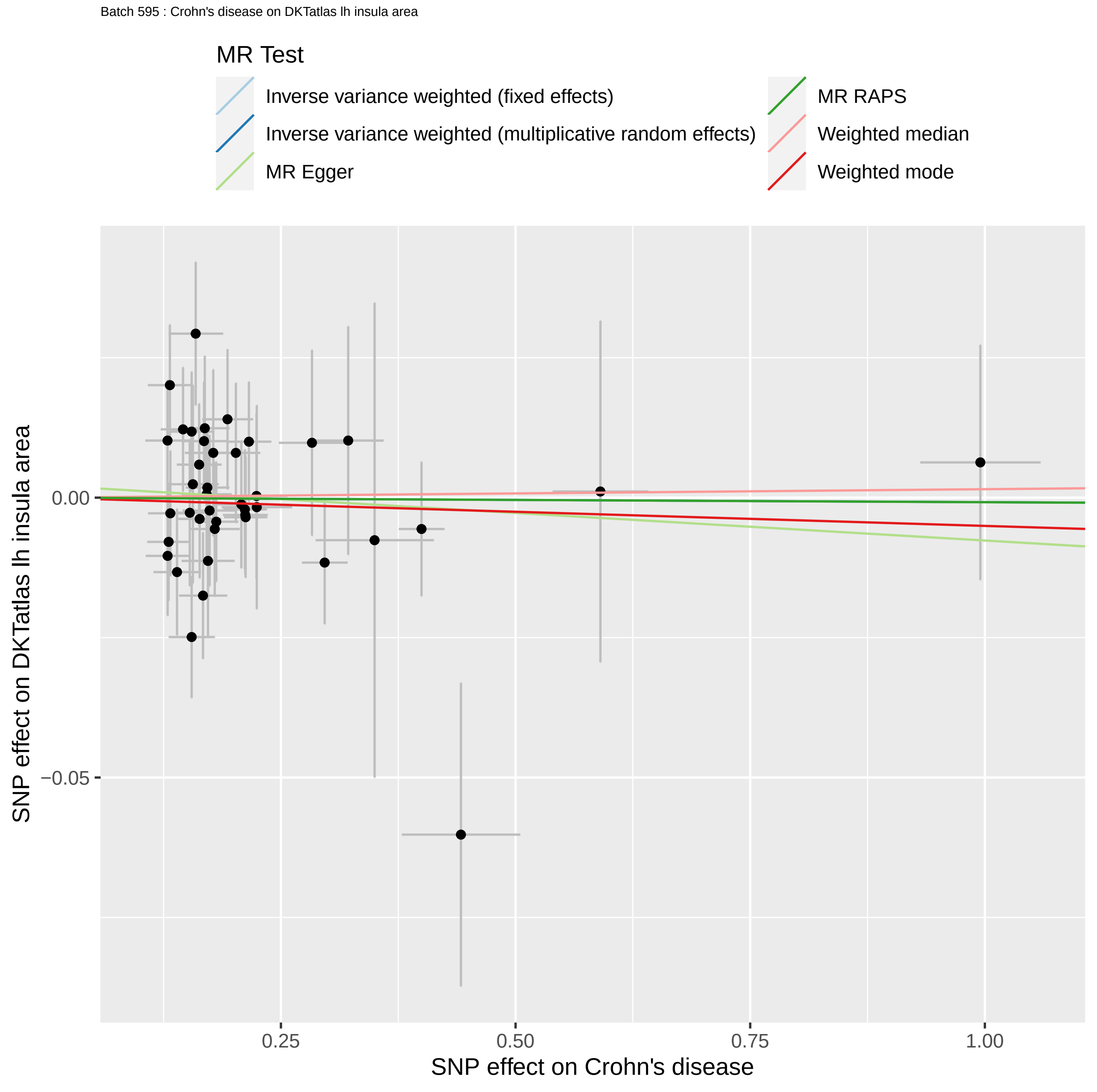

Batch 595 : Crohn's disease on DKTatlas lh insula area
MR Test
Inverse variance weighted (fixed effects)
Inverse variance weighted (multiplicative random effects)
MR Egger
MR RAPS
Weighted median
Weighted mode
0.00
−0.05
0.25
0.50
0.75
1.00
SNP effect on DKTatlas lh insula area
SNP effect on Crohn's disease

Batch 596 : Ulcerative colitis on DKTatlas lh insula area

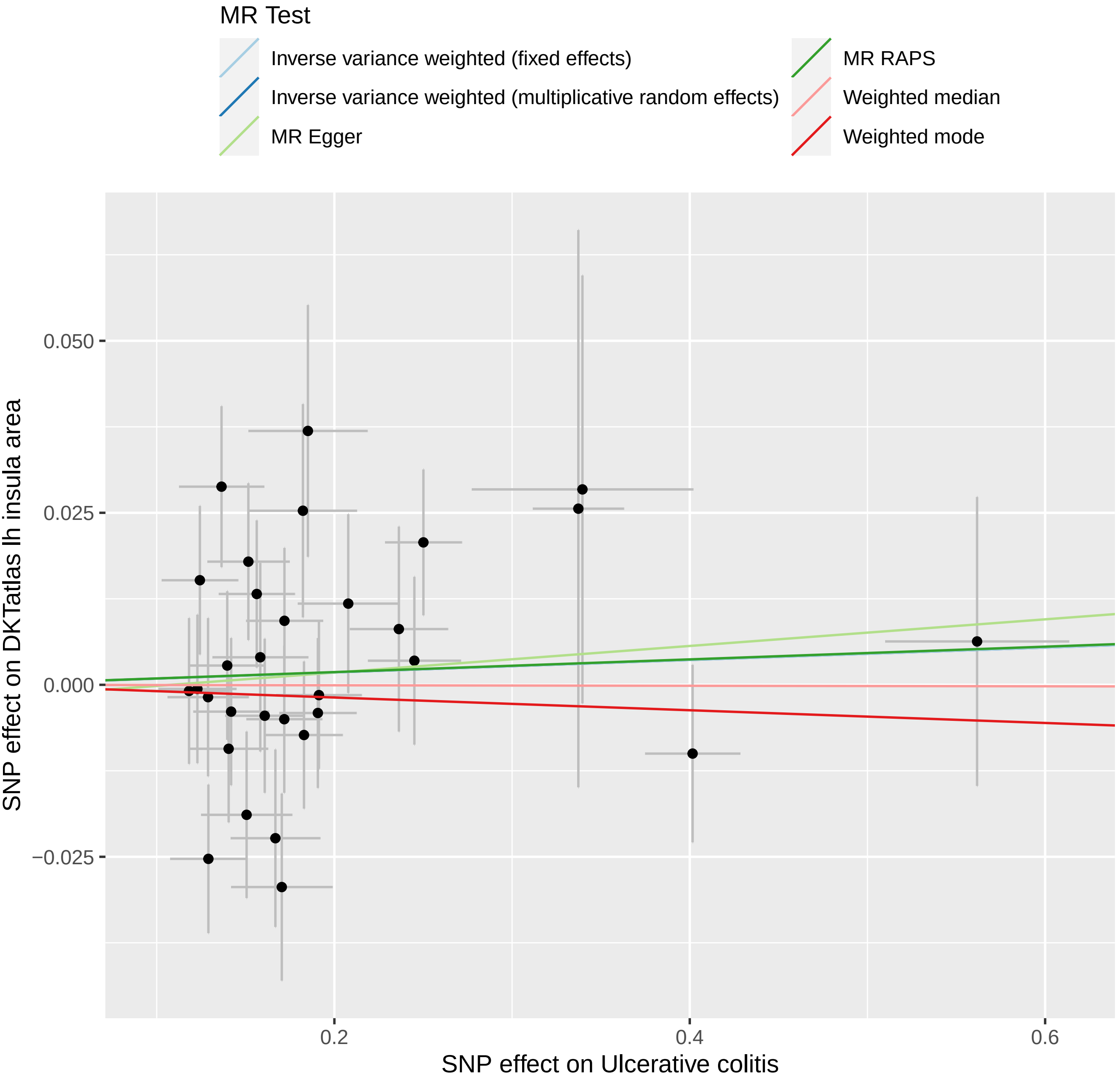

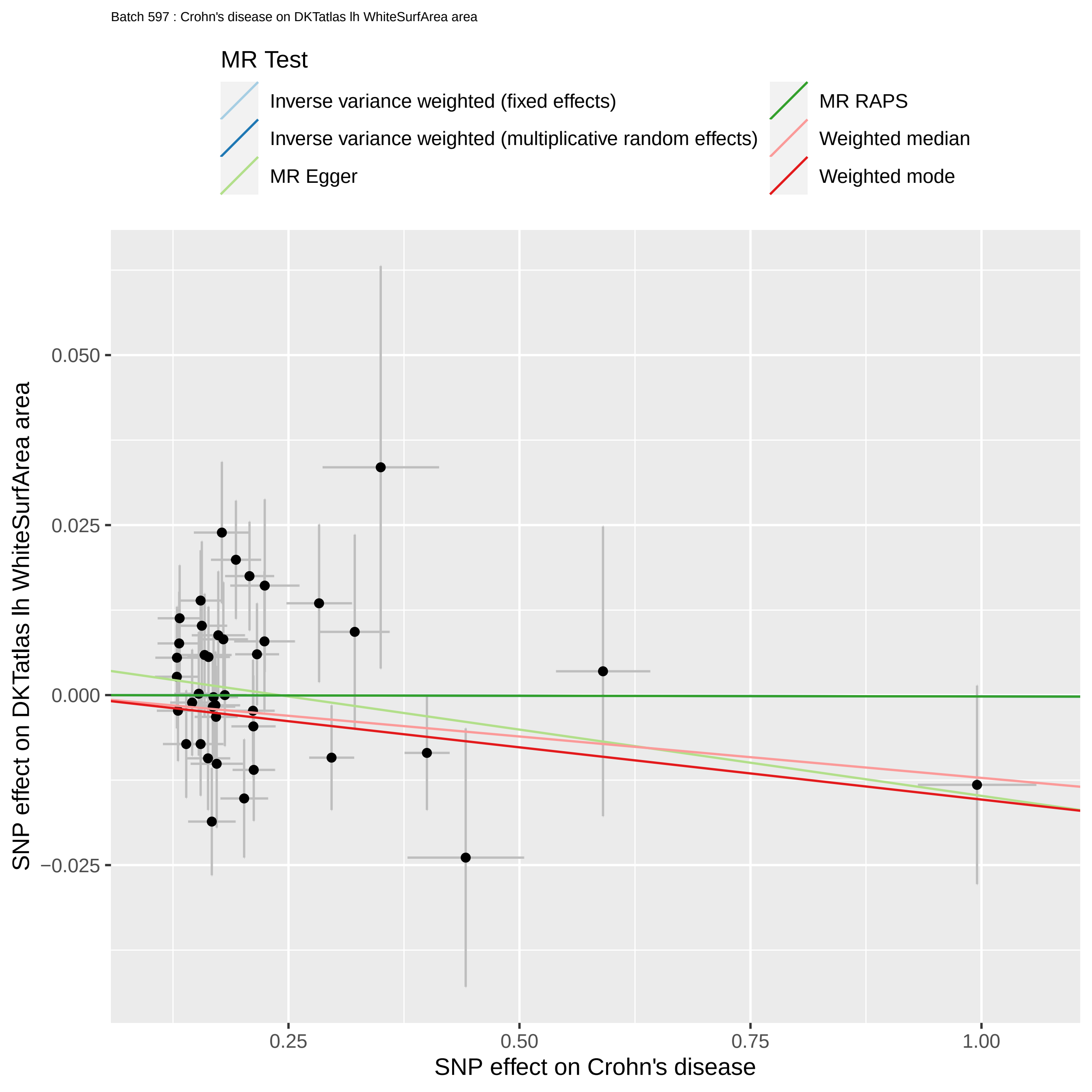
Batch 597 : Crohn's disease on DKTatlas lh WhiteSurfArea area
MR Test
Inverse variance weighted (fixed effects)
Inverse variance weighted (multiplicative random effects)
MR Egger
MR RAPS
Weighted median
Weighted mode
0.050
0.025
0.000
−0.025
0.25
0.50
0.75
1.00
SNP effect on DKTatlas lh WhiteSurfArea area
SNP effect on Crohn's disease

Batch 598 : Ulcerative colitis on DKTatlas lh WhiteSurfArea area

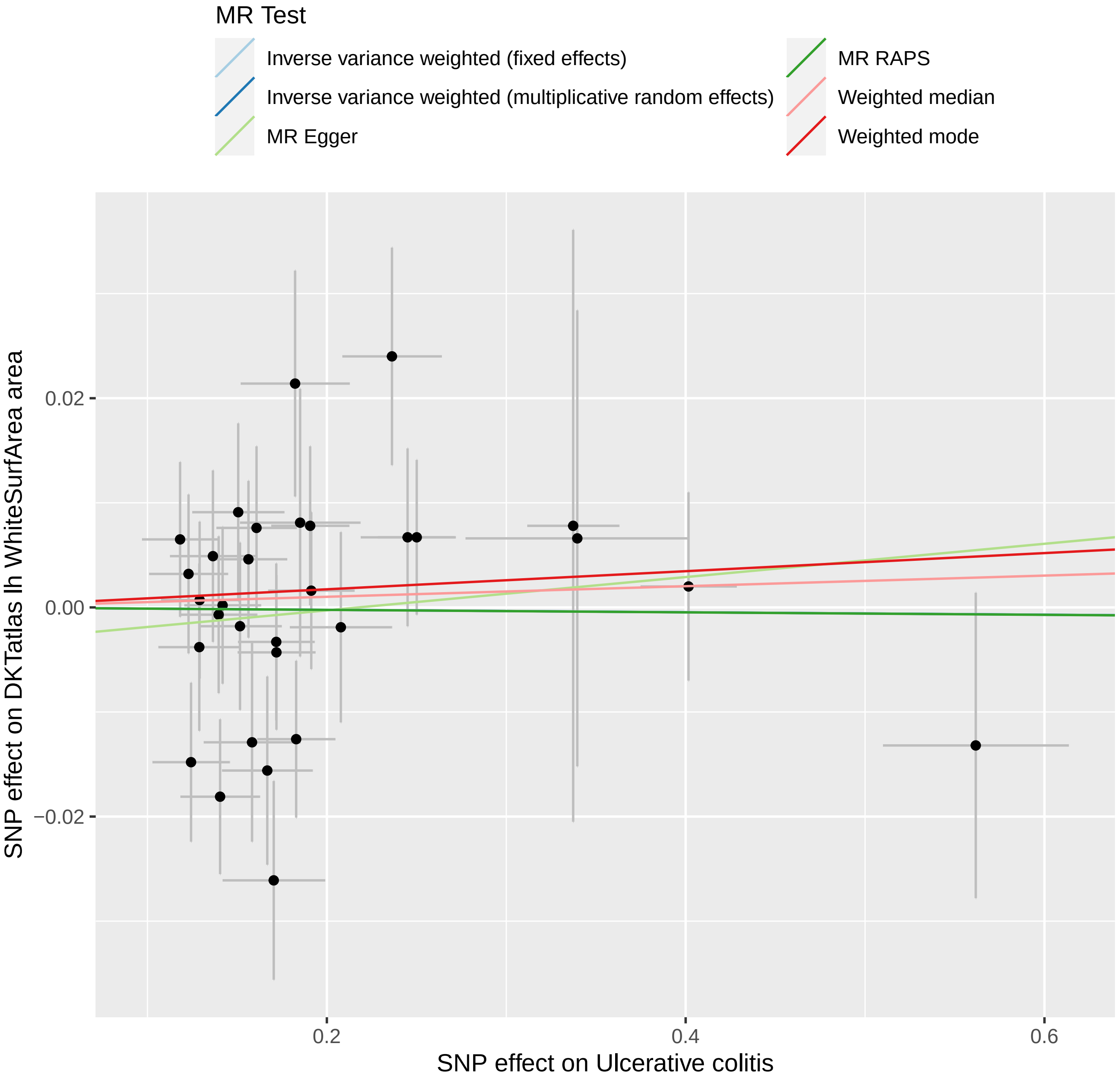

Batch 715 : Crohn's disease on IDP T1 FAST ROIs L insular cortex

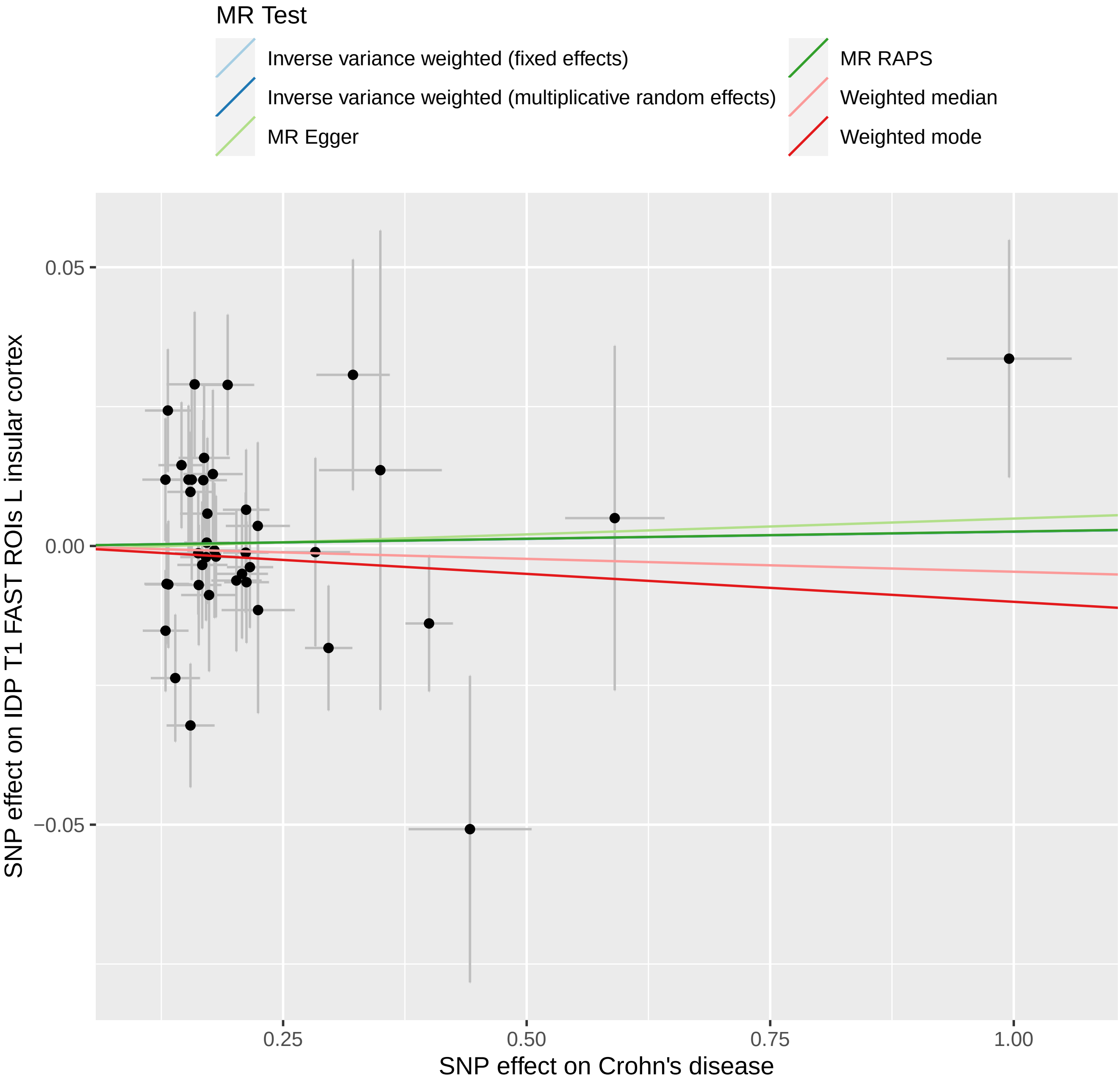

Batch 716 : Ulcerative colitis on IDP T1 FAST ROIs L insular cortex

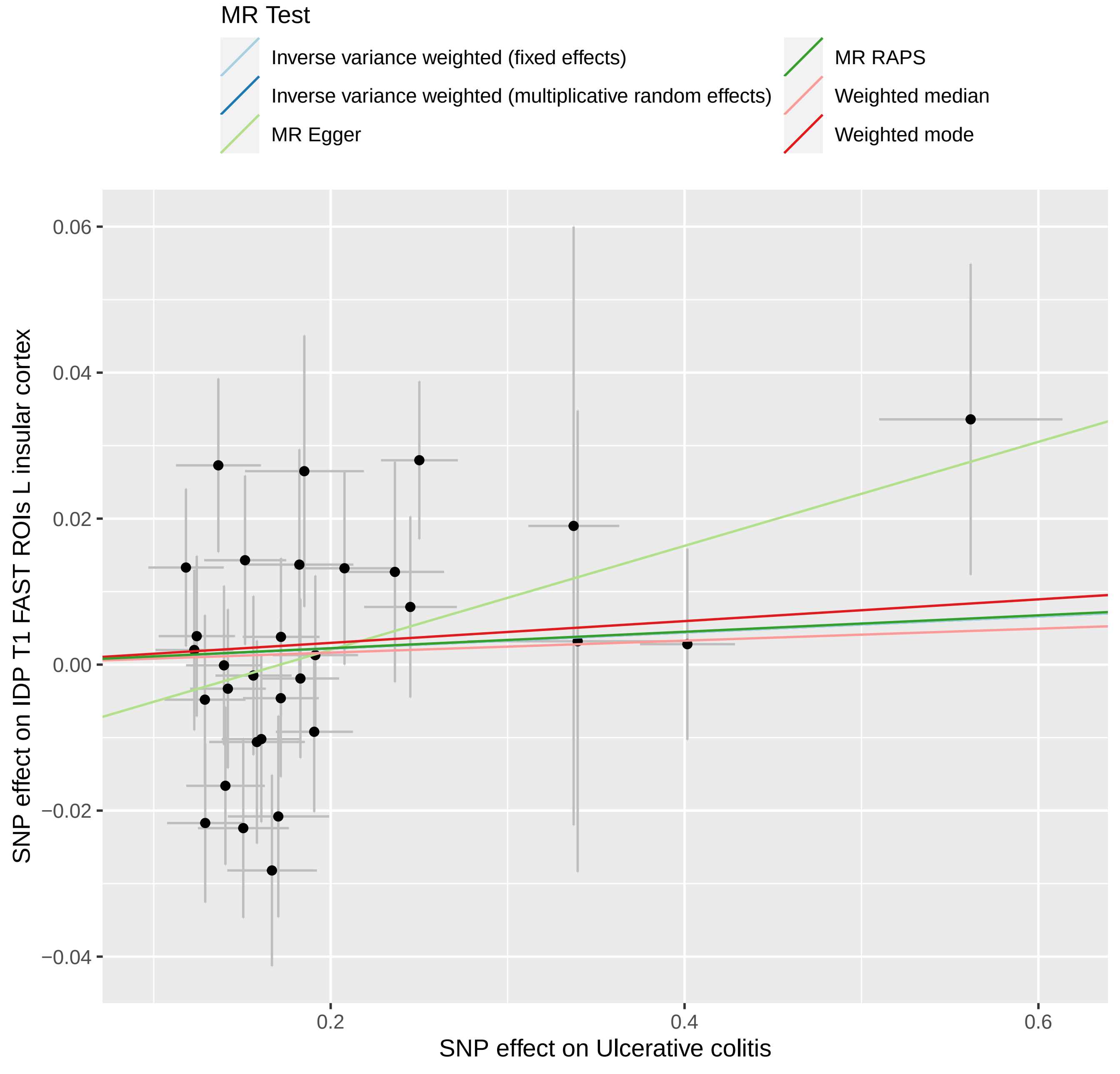

Batch 765 : Crohn's disease on DKTatlas rh caudalanteriorcingulate area

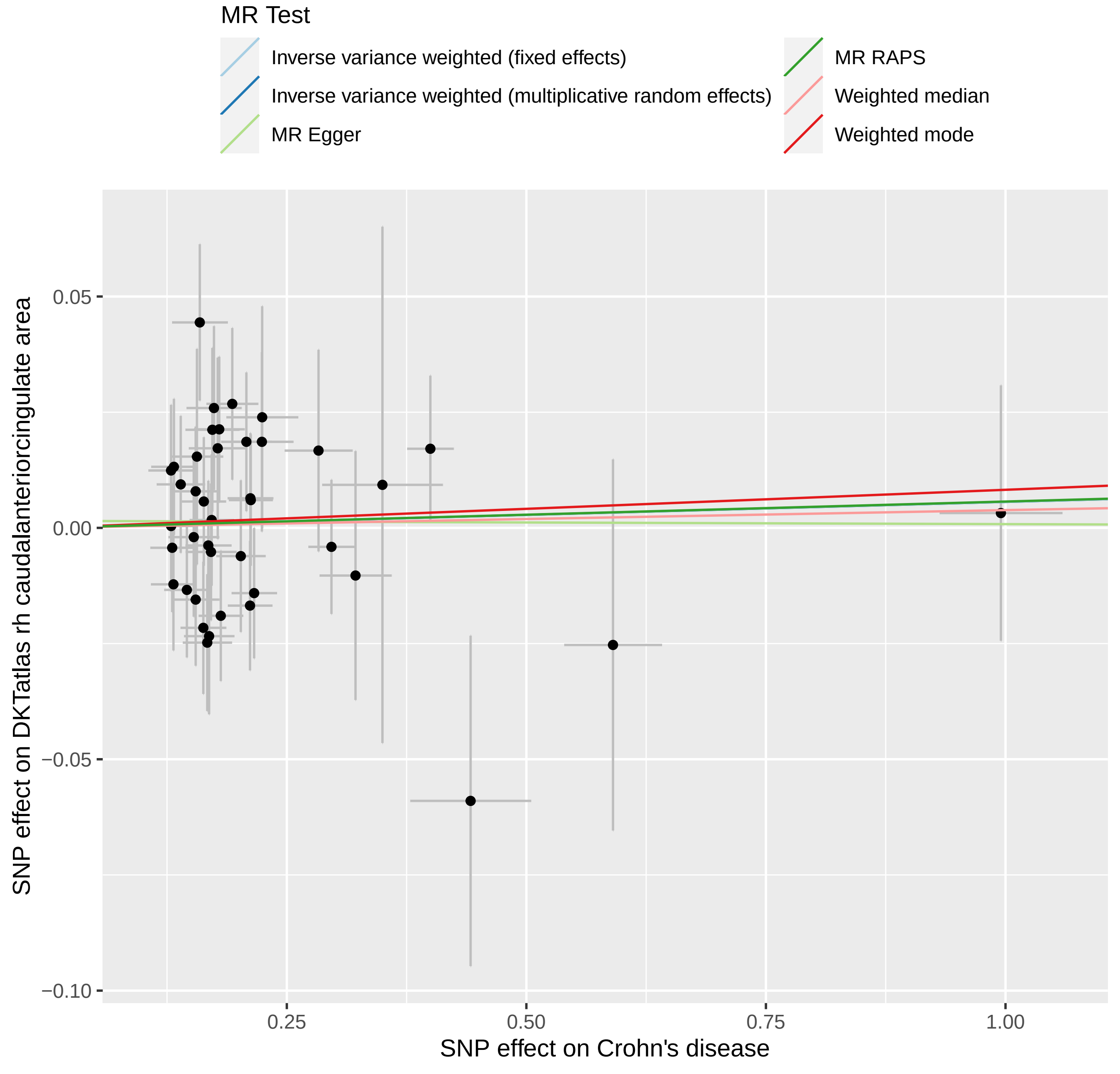

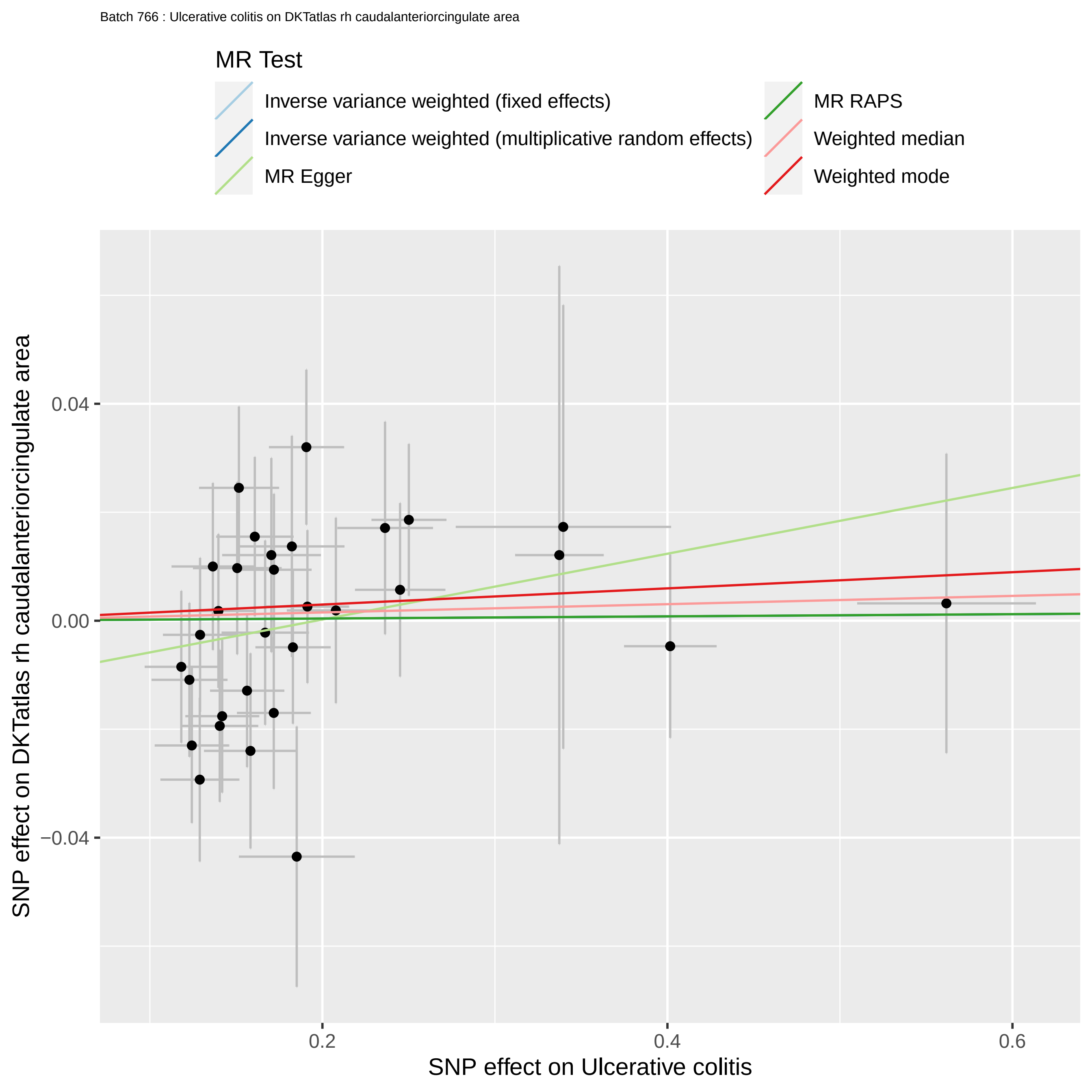

Batch 766 : Ulcerative colitis on DKTatlas rh caudalanteriorcingulate area
MR Test
Inverse variance weighted (fixed effects)
Inverse variance weighted (multiplicative random effects)
MR Egger
MR RAPS
Weighted median
Weighted mode
SNP effect on DKTatlas rh caudalanteriorcingulate area
0.04
0.00
−0.04
0.2
0.4
0.6
SNP effect on Ulcerative colitis

Batch 767 : Crohn's disease on DKTatlas rh caudalmiddlefrontal area

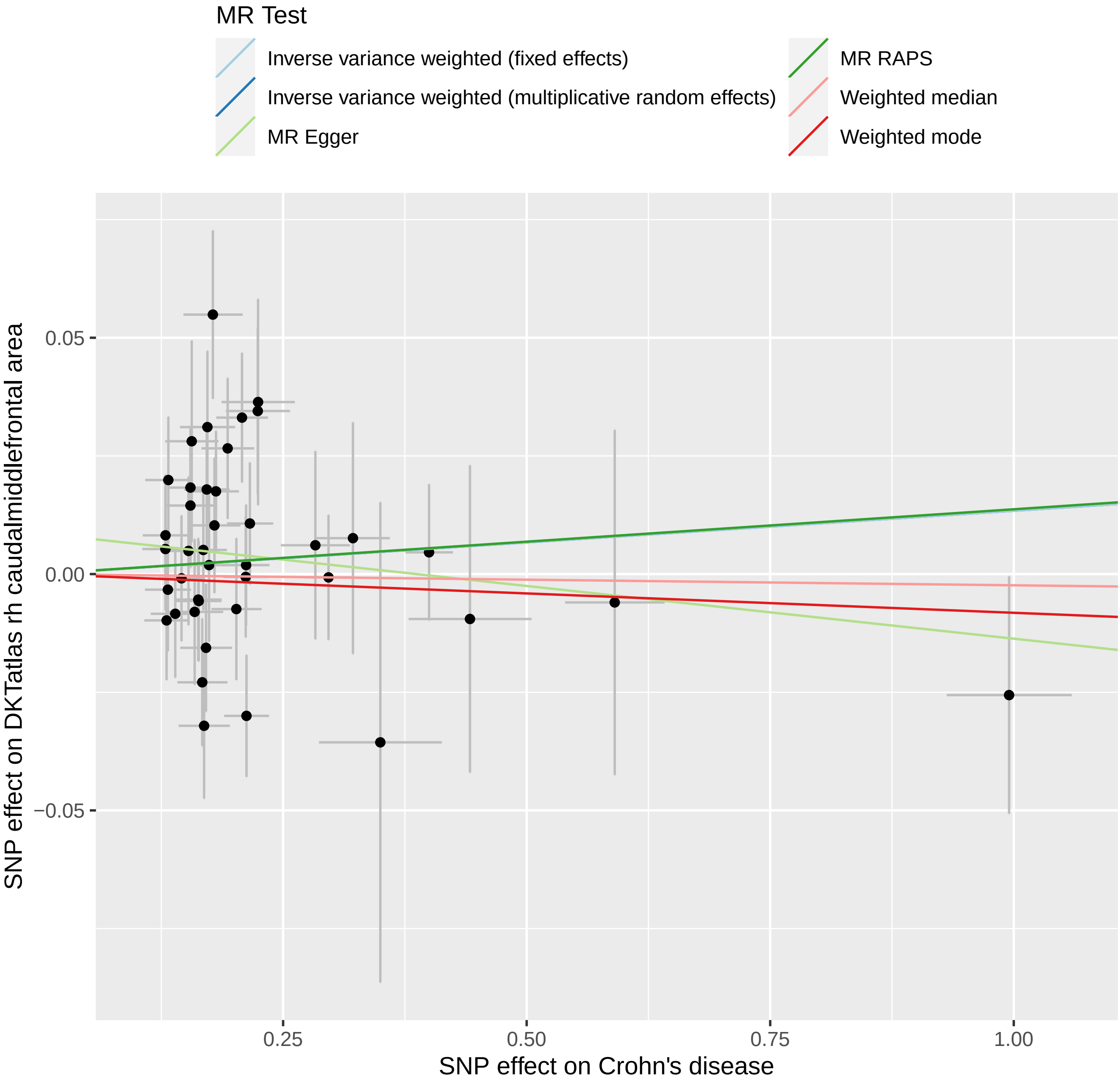

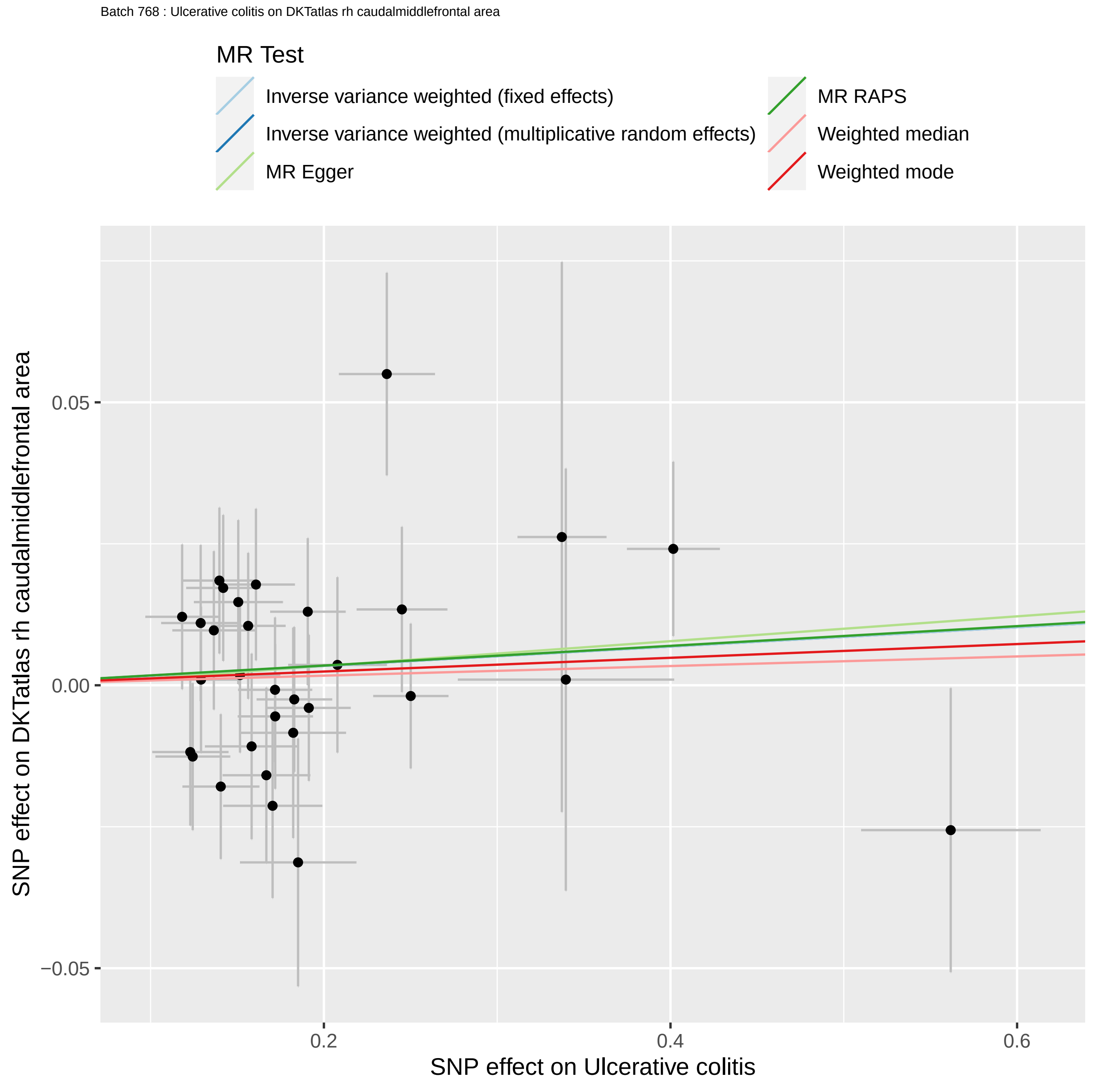

Batch 768 : Ulcerative colitis on DKTatlas rh caudalmiddlefrontal area
MR Test
Inverse variance weighted (fixed effects)
Inverse variance weighted (multiplicative random effects)
MR Egger
MR RAPS
Weighted median
Weighted mode
0.05
0.00
−0.05
0.2
0.4
0.6
SNP effect on DKTatlas rh caudalmiddlefrontal area
SNP effect on Ulcerative colitis

Batch 769 : Crohn's disease on DKTatlas rh cuneus area

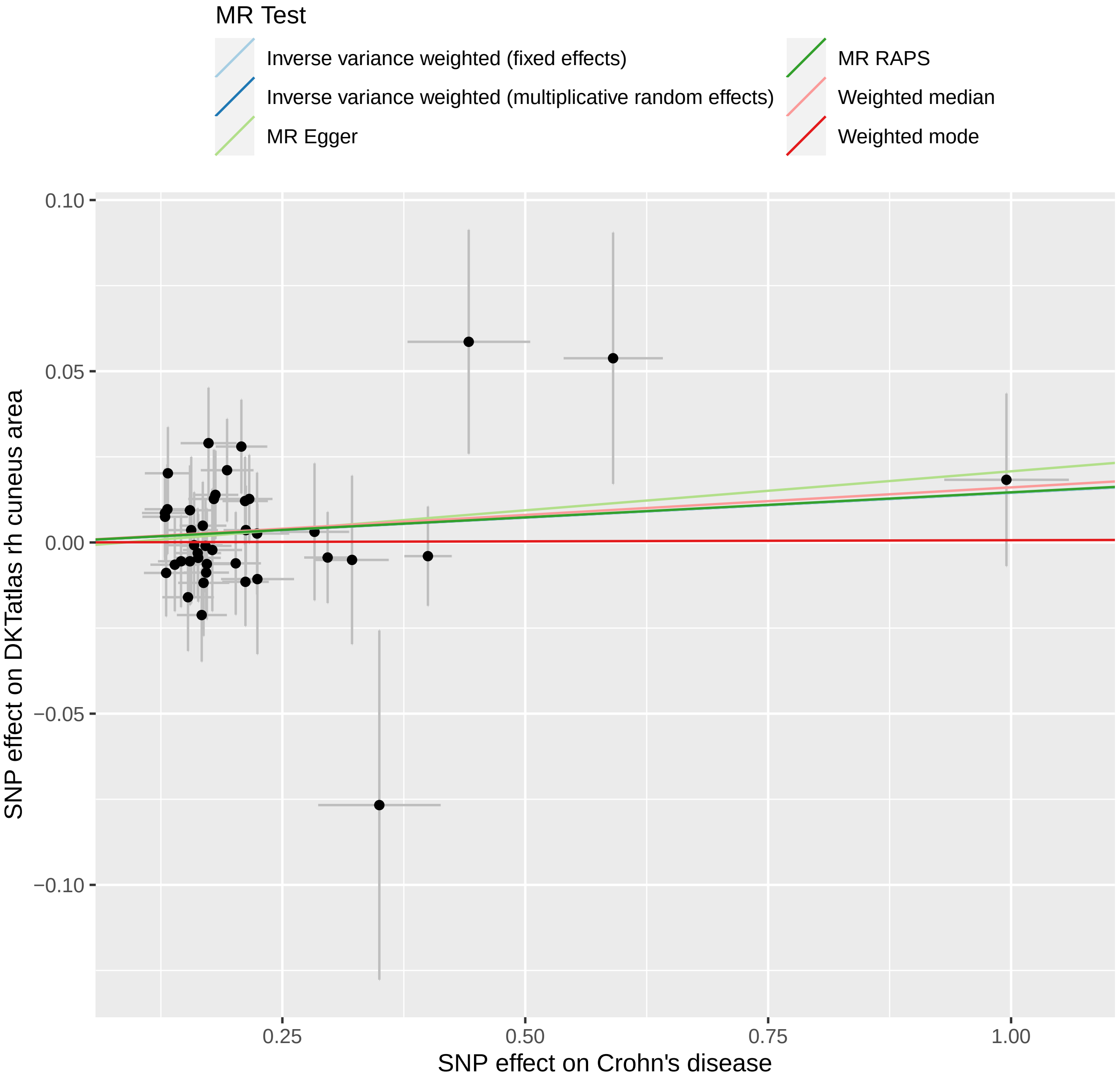

Batch 770 : Ulcerative colitis on DKTatlas rh cuneus area

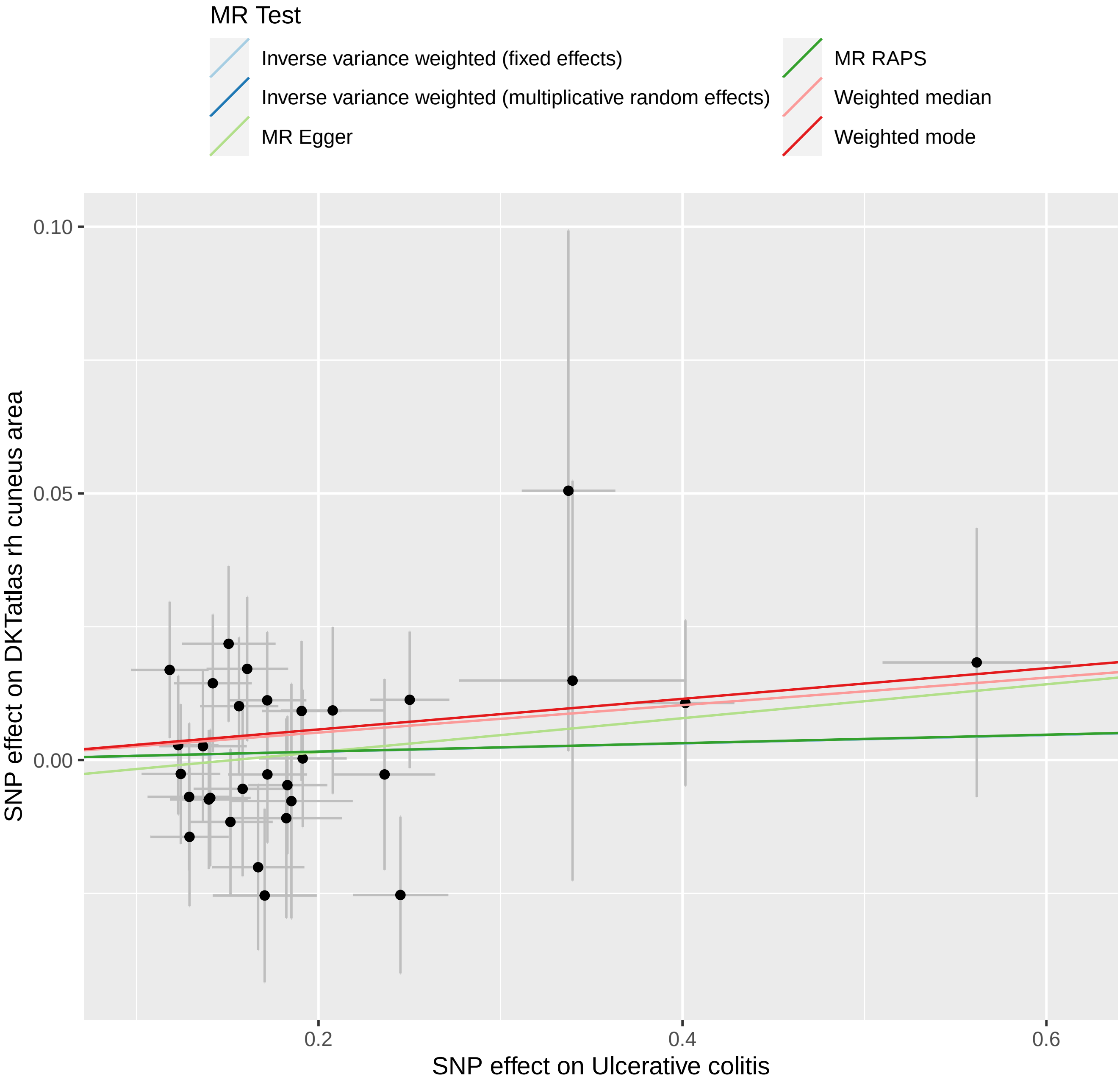

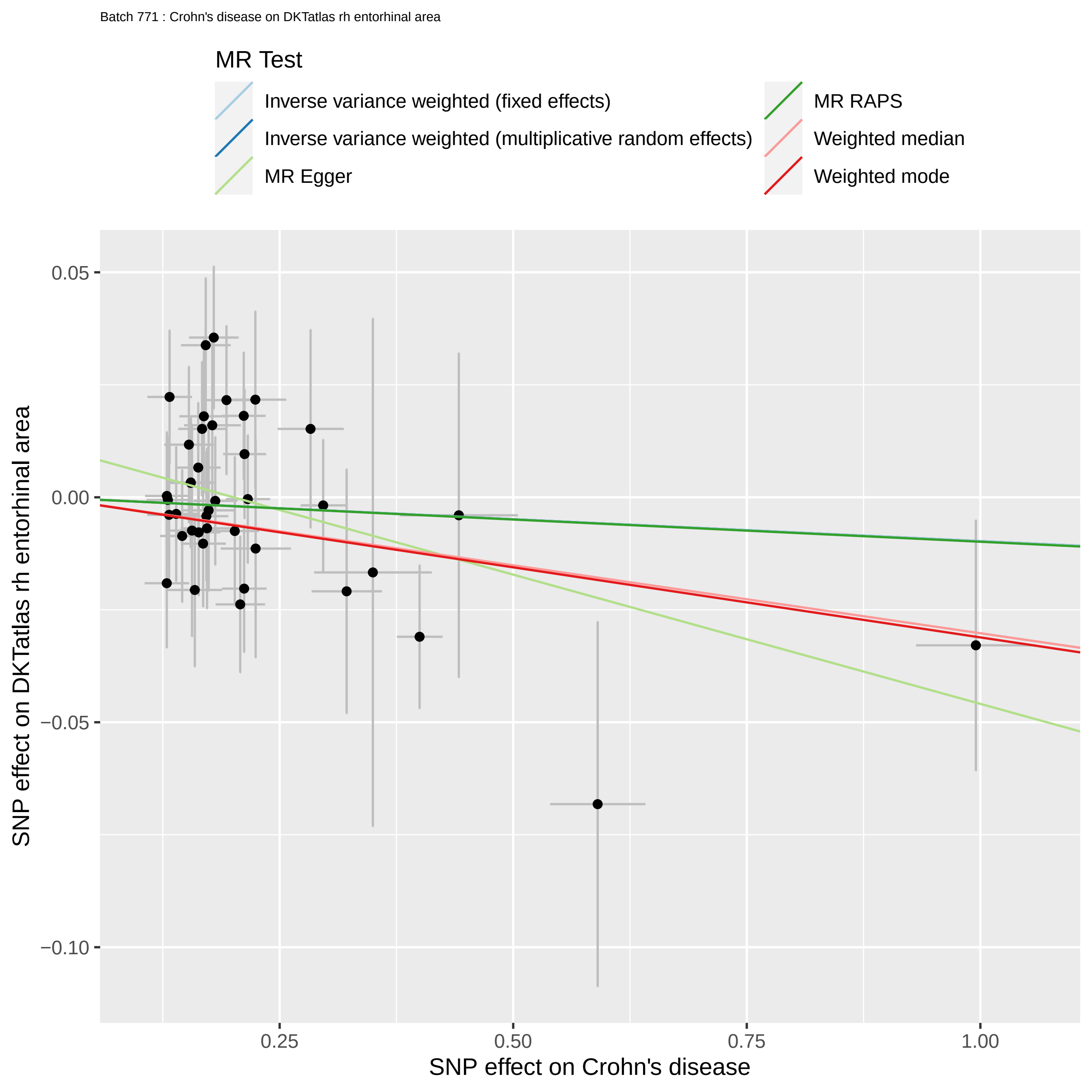
Batch 771 : Crohn's disease on DKTatlas rh entorhinal area
MR Test
Inverse variance weighted (fixed effects)
Inverse variance weighted (multiplicative random effects)
MR Egger
MR RAPS
Weighted median
Weighted mode
0.05
0.00
−0.05
−0.10
0.25
0.50
0.75
1.00
SNP effect on DKTatlas rh entorhinal area
SNP effect on Crohn's disease

Batch 772 : Ulcerative colitis on DKTatlas rh entorhinal area

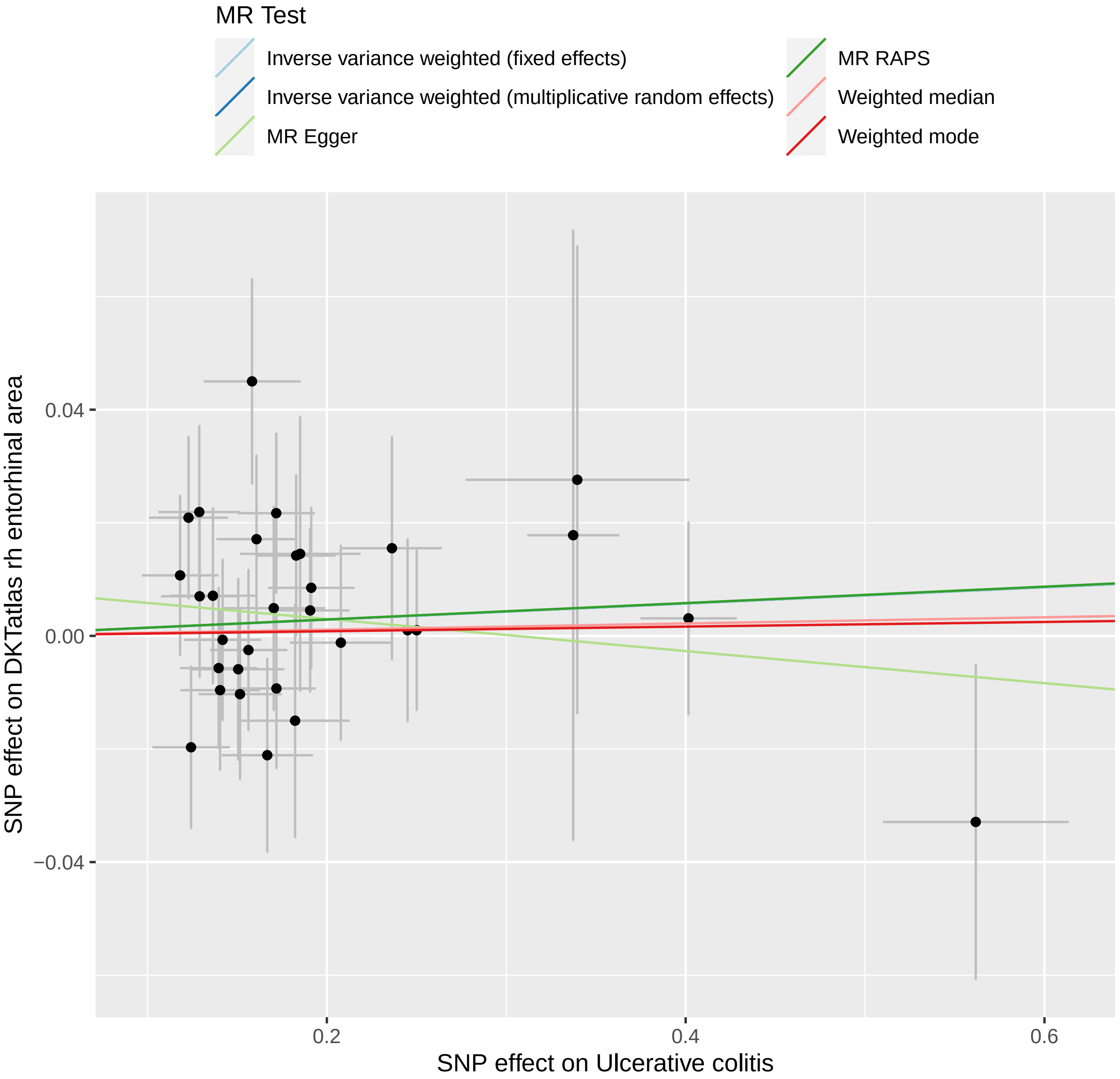

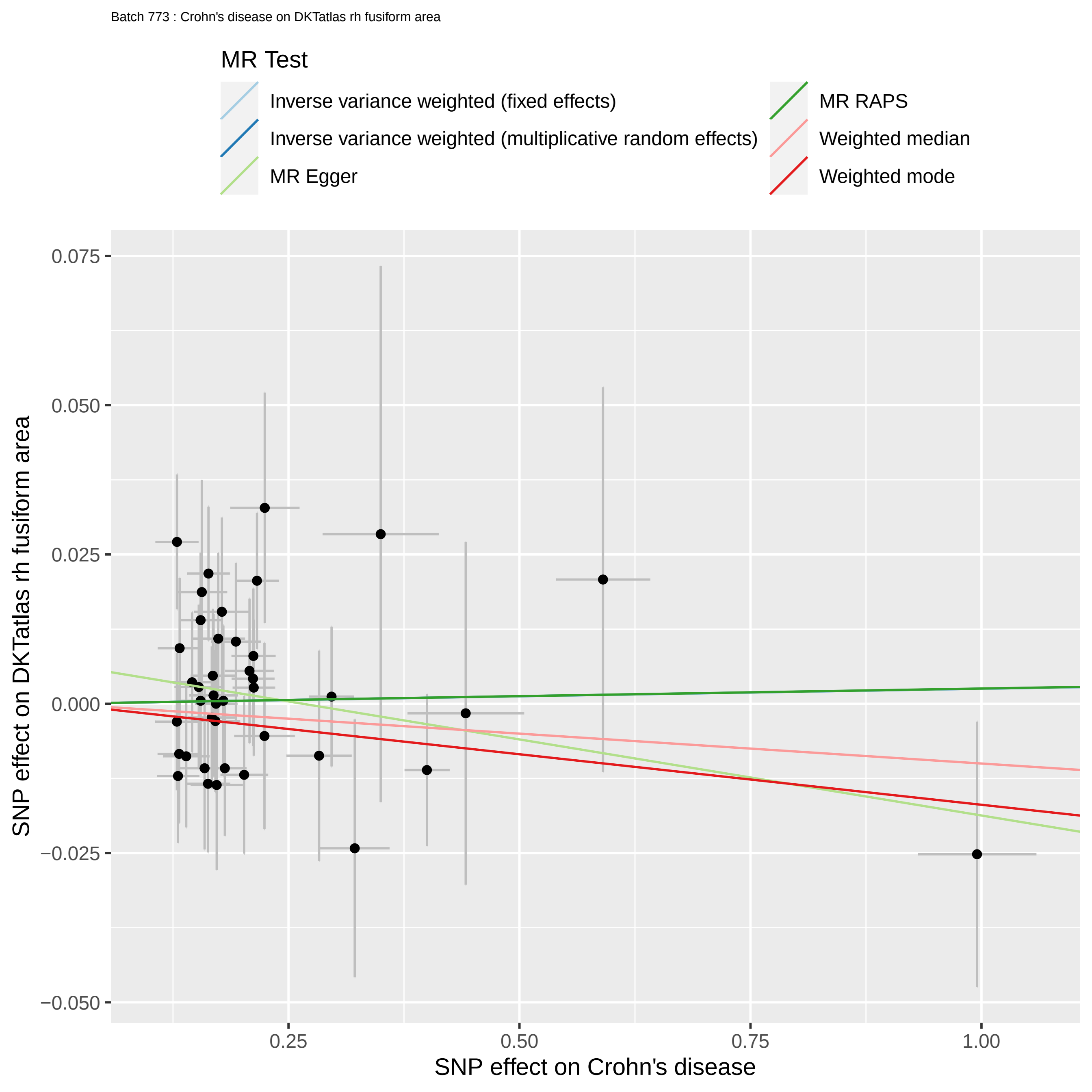

Batch 773 : Crohn's disease on DKTatlas rh fusiform area
MR Test
Inverse variance weighted (fixed effects)
Inverse variance weighted (multiplicative random effects)
MR Egger
MR RAPS
Weighted median
Weighted mode
0.075
0.050
0.025
0.000
−0.025
−0.050
0.25
0.50
0.75
1.00
SNP effect on DKTatlas rh fusiform area
SNP effect on Crohn's disease

Batch 774 : Ulcerative colitis on DKTatlas rh fusiform area

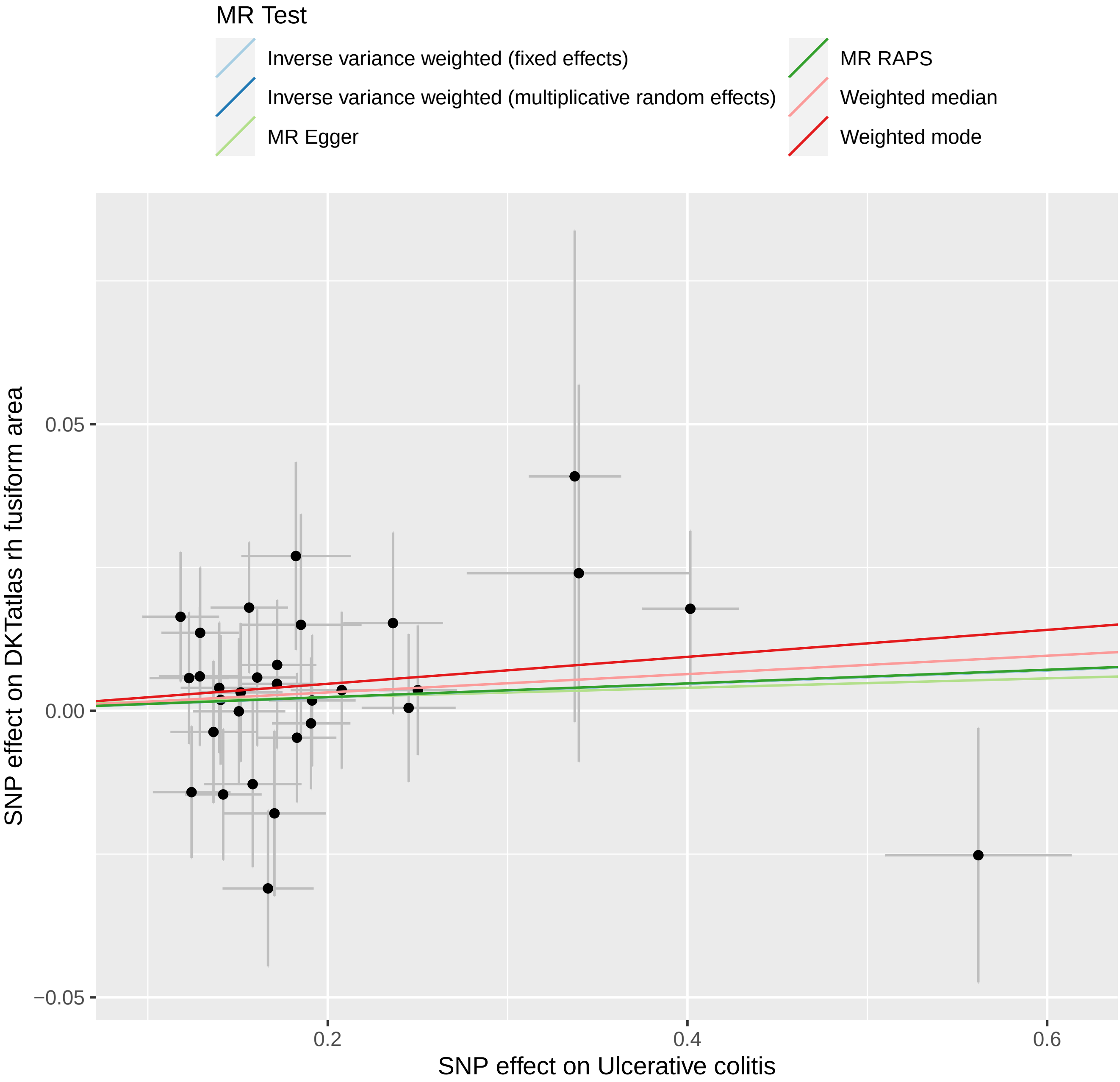

Batch 775 : Crohn's disease on DKTatlas rh inferiorparietal area

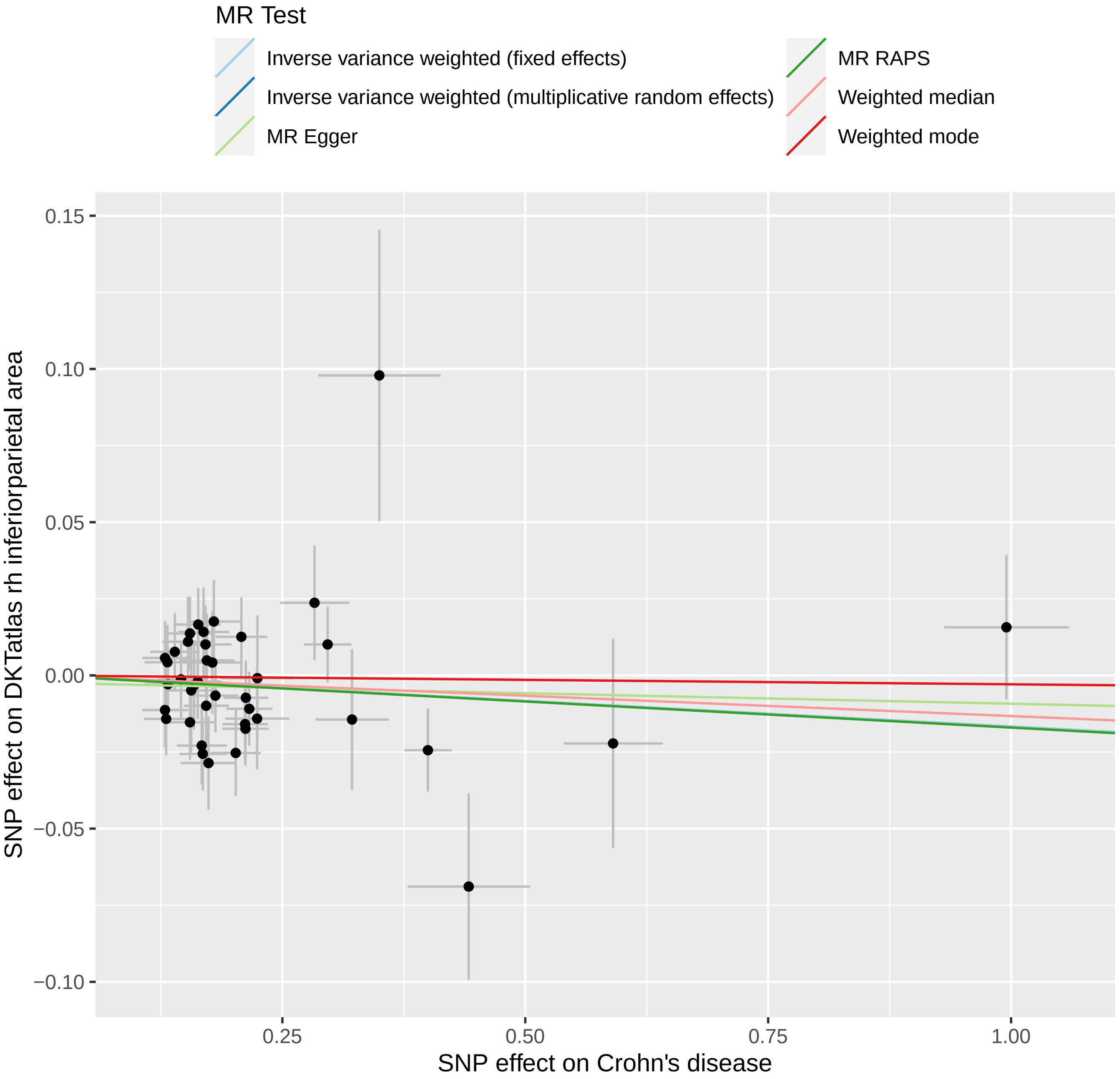

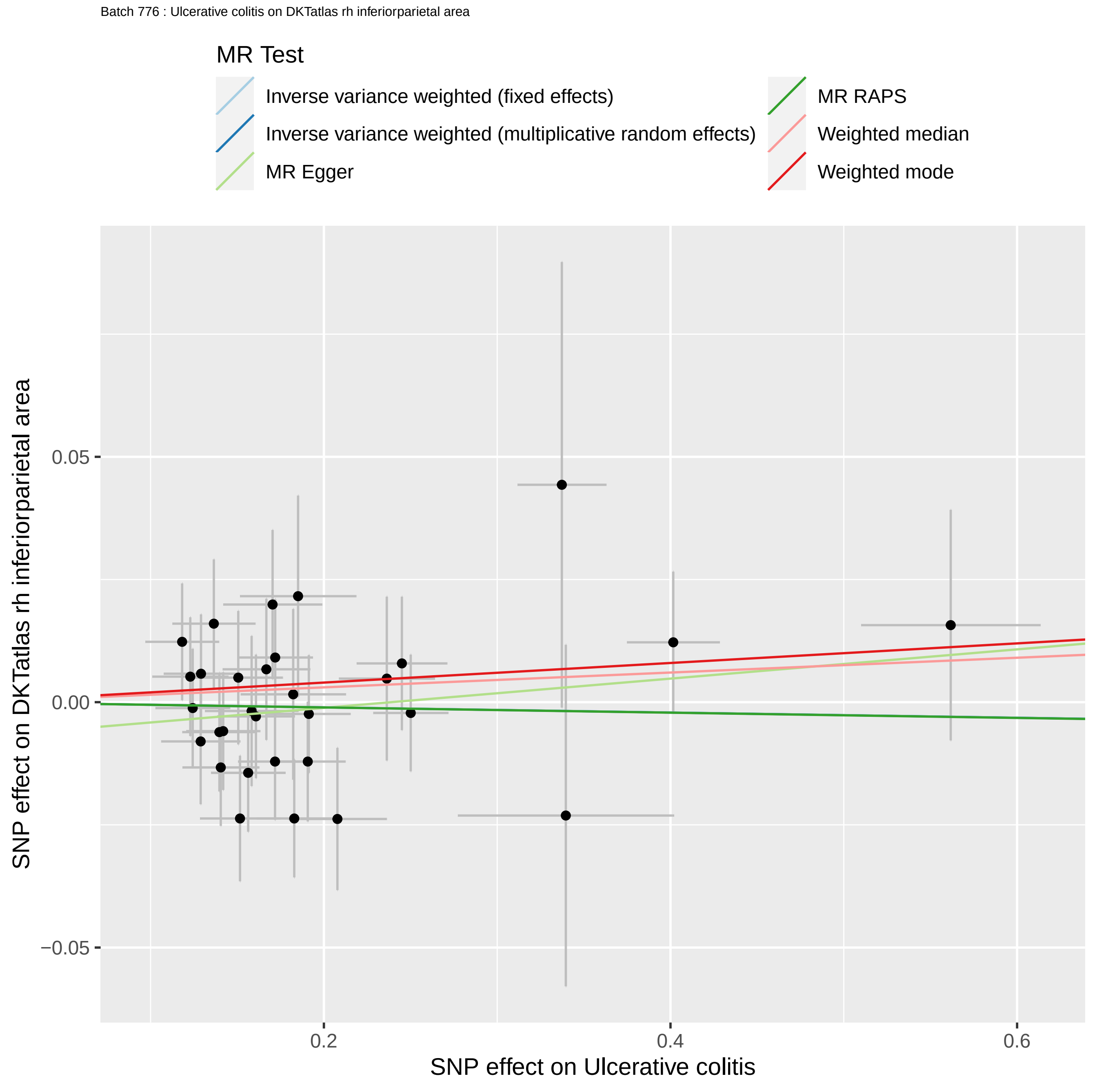

Batch 776 : Ulcerative colitis on DKTatlas rh inferiorparietal area
MR Test
Inverse variance weighted (fixed effects)
Inverse variance weighted (multiplicative random effects)
MR Egger
MR RAPS
Weighted median
Weighted mode
SNP effect on DKTatlas rh inferiorparietal area
0.05
0.00
−0.05
0.2
0.4
0.6
SNP effect on Ulcerative colitis

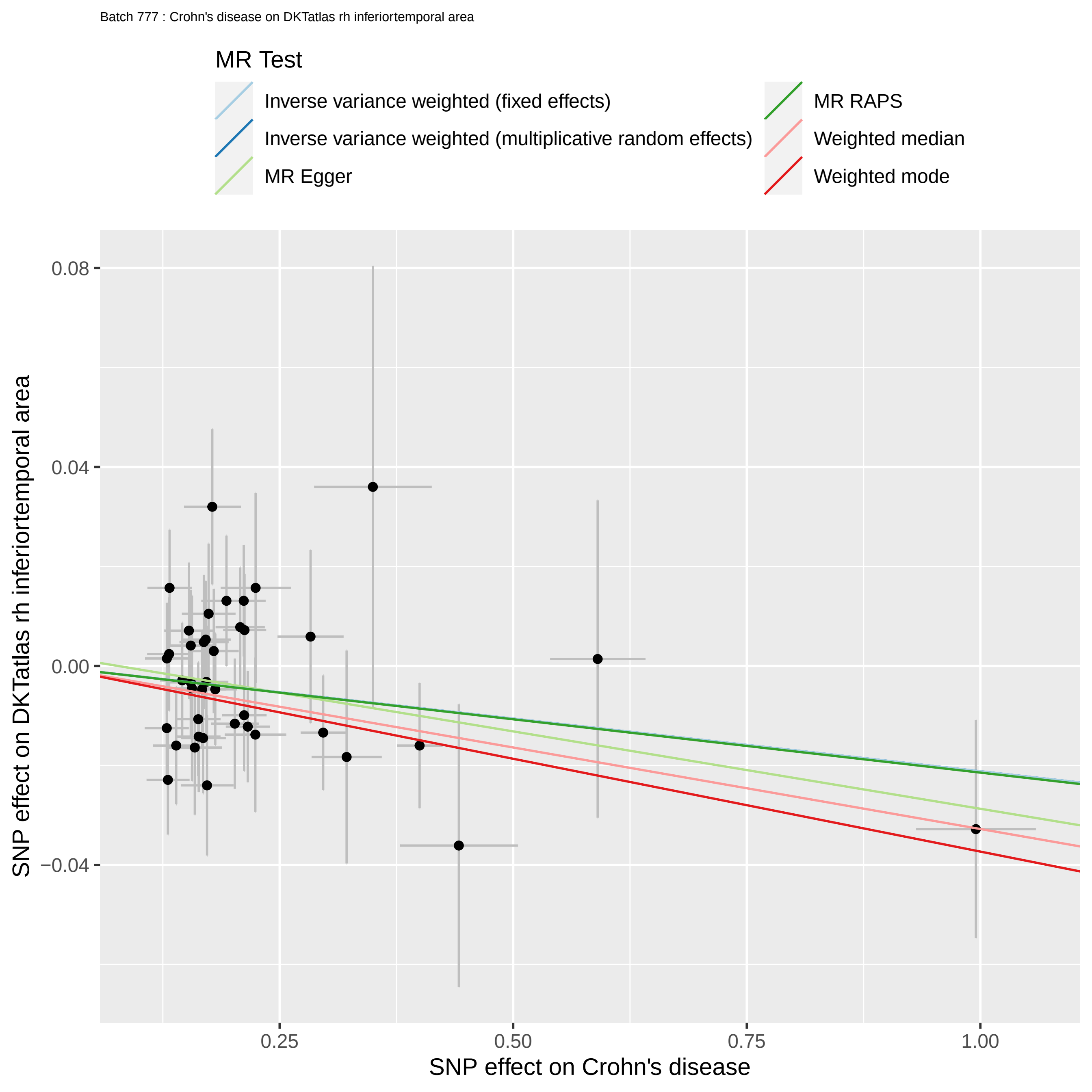
Batch 777 : Crohn's disease on DKTatlas rh inferiortemporal area
MR Test
Inverse variance weighted (fixed effects)
Inverse variance weighted (multiplicative random effects)
MR Egger
MR RAPS
Weighted median
Weighted mode
0.08
0.04
0.00
−0.04
0.25
0.50
0.75
1.00
SNP effect on DKTatlas rh inferiortemporal area
SNP effect on Crohn's disease

Batch 778 : Ulcerative colitis on DKTatlas rh inferiortemporal area

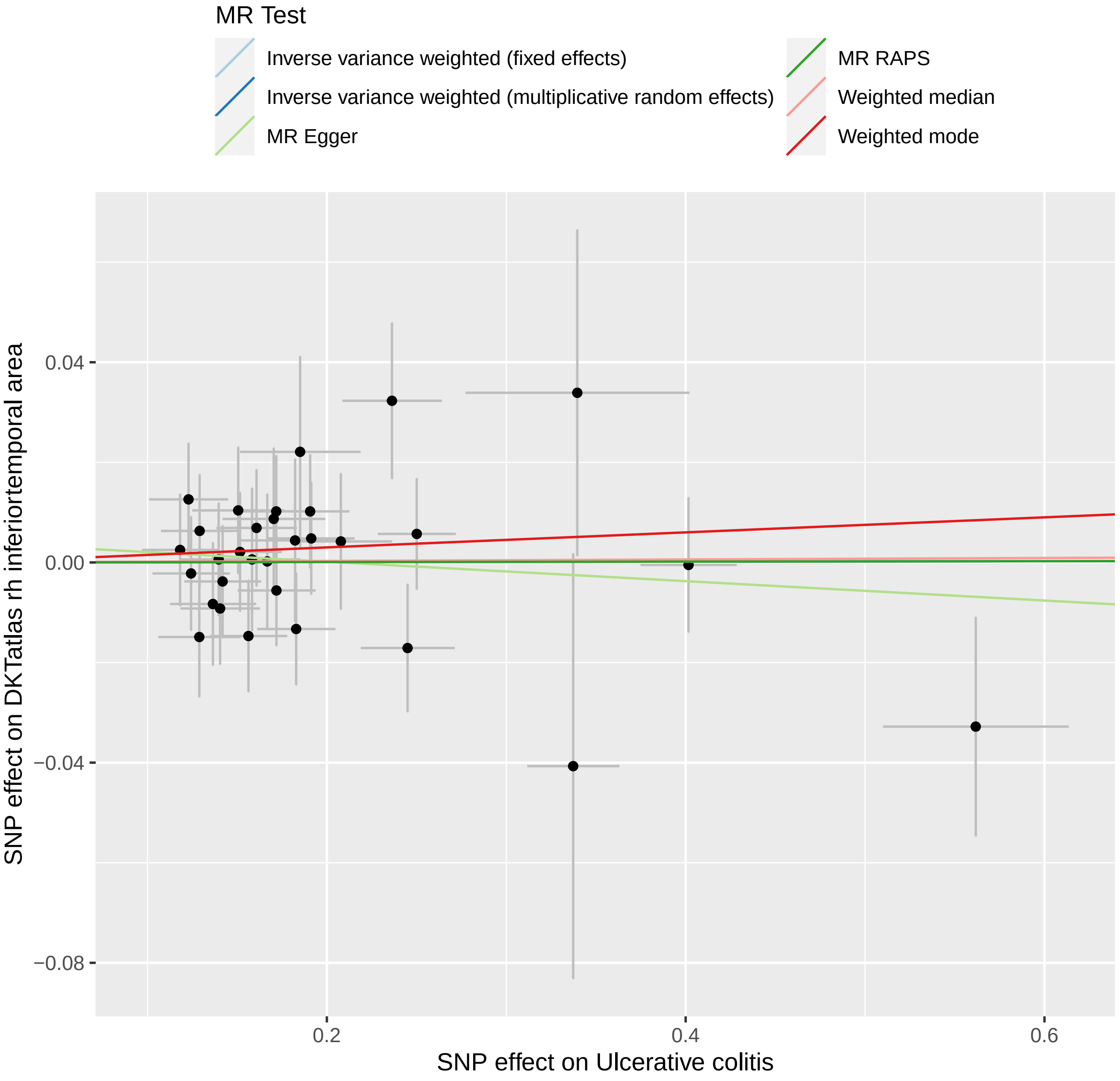

Batch 779 : Crohn's disease on DKTatlas rh isthmuscingulate area

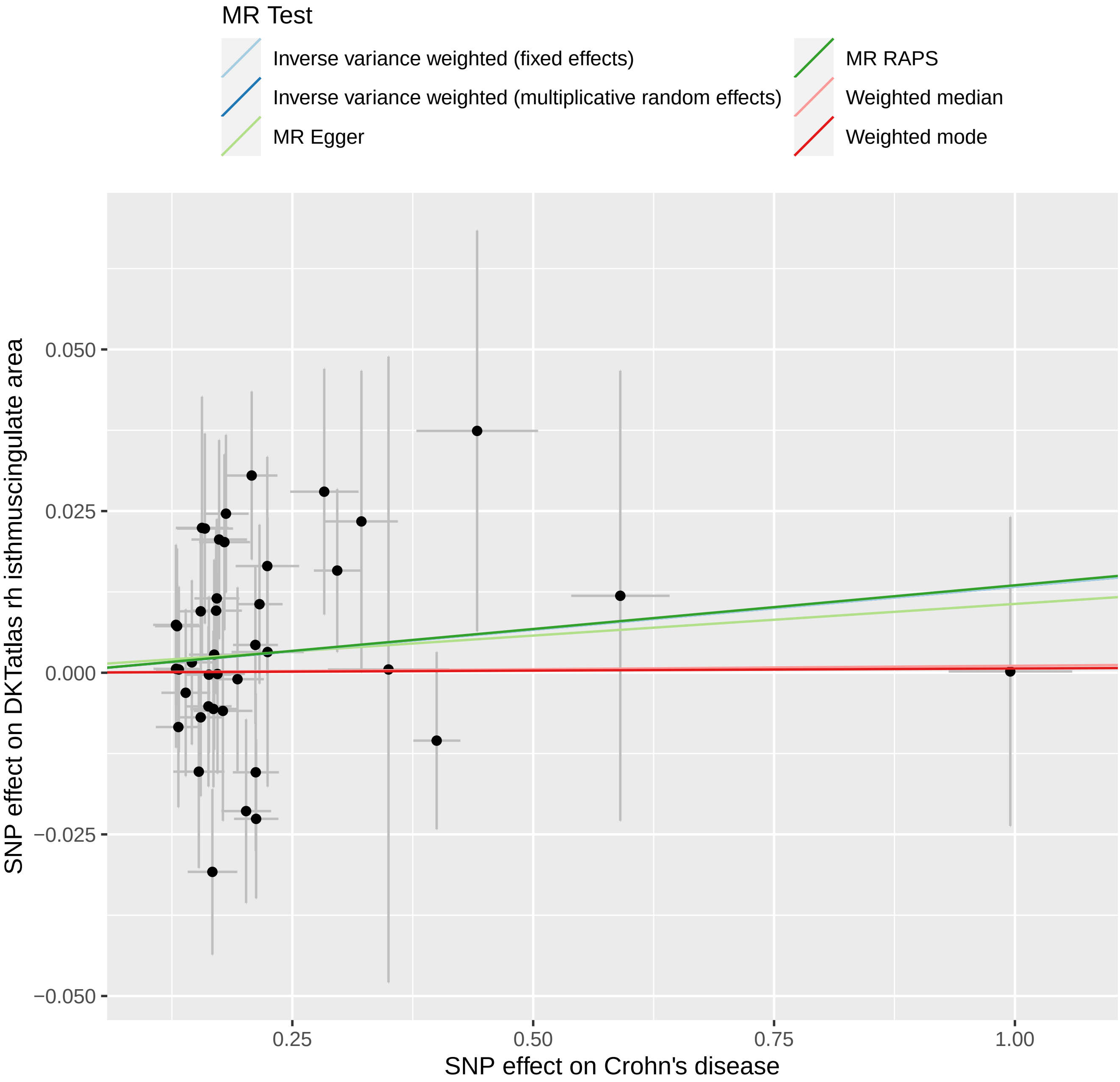

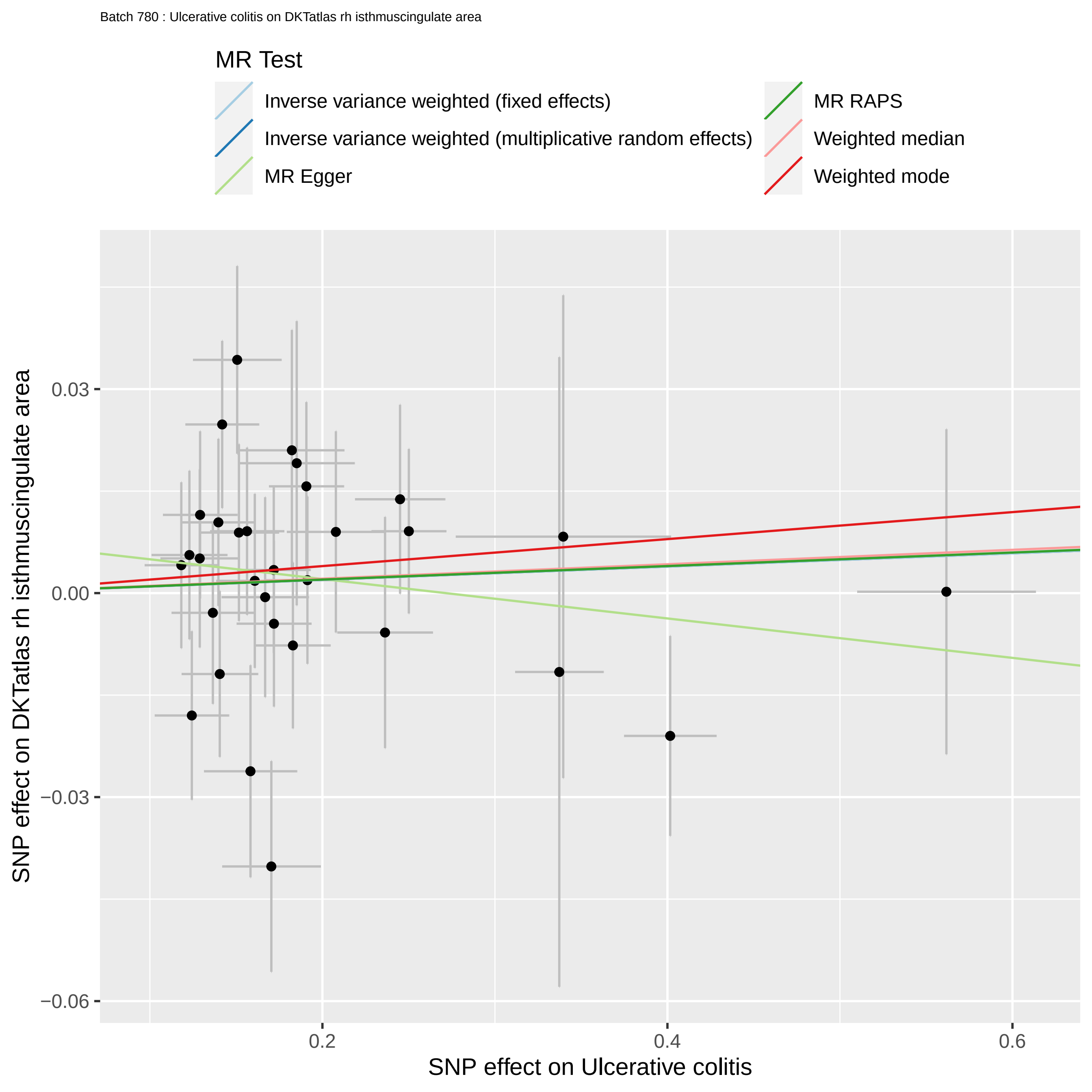

Batch 780 : Ulcerative colitis on DKTatlas rh isthmuscingulate area
MR Test
Inverse variance weighted (fixed effects)
Inverse variance weighted (multiplicative random effects)
MR Egger
MR RAPS
Weighted median
Weighted mode
0.03
0.00
−0.03
−0.06
0.2
0.4
0.6
SNP effect on DKTatlas rh isthmuscingulate area
SNP effect on Ulcerative colitis

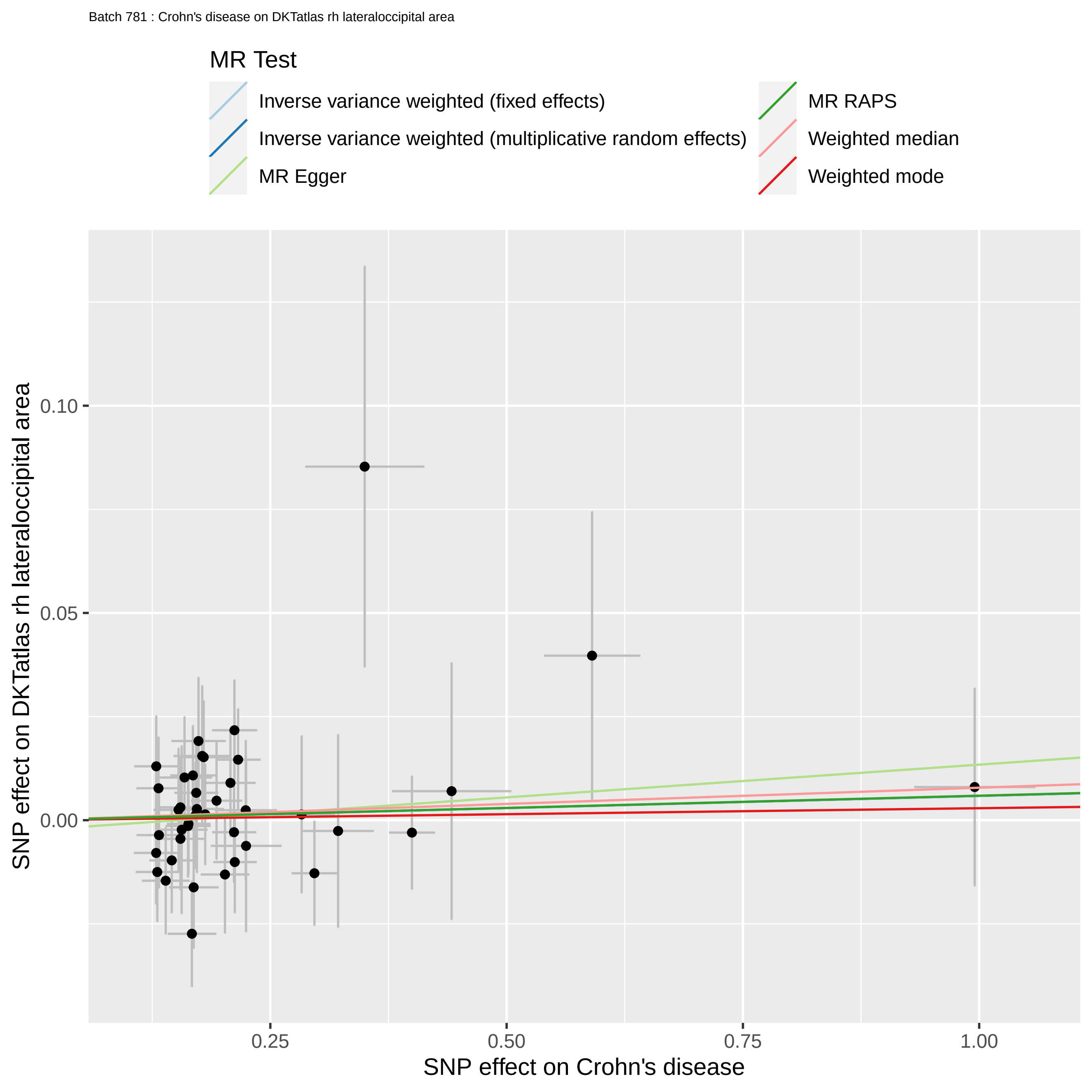

Batch 781 : Crohn's disease on DKTatlas rh lateraloccipital area
MR Test
Inverse variance weighted (fixed effects)
Inverse variance weighted (multiplicative random effects)
MR Egger
MR RAPS
Weighted median
Weighted mode
0.10
0.05
0.00
0.25
0.50
0.75
1.00
SNP effect on DKTatlas rh lateraloccipital area
SNP effect on Crohn's disease

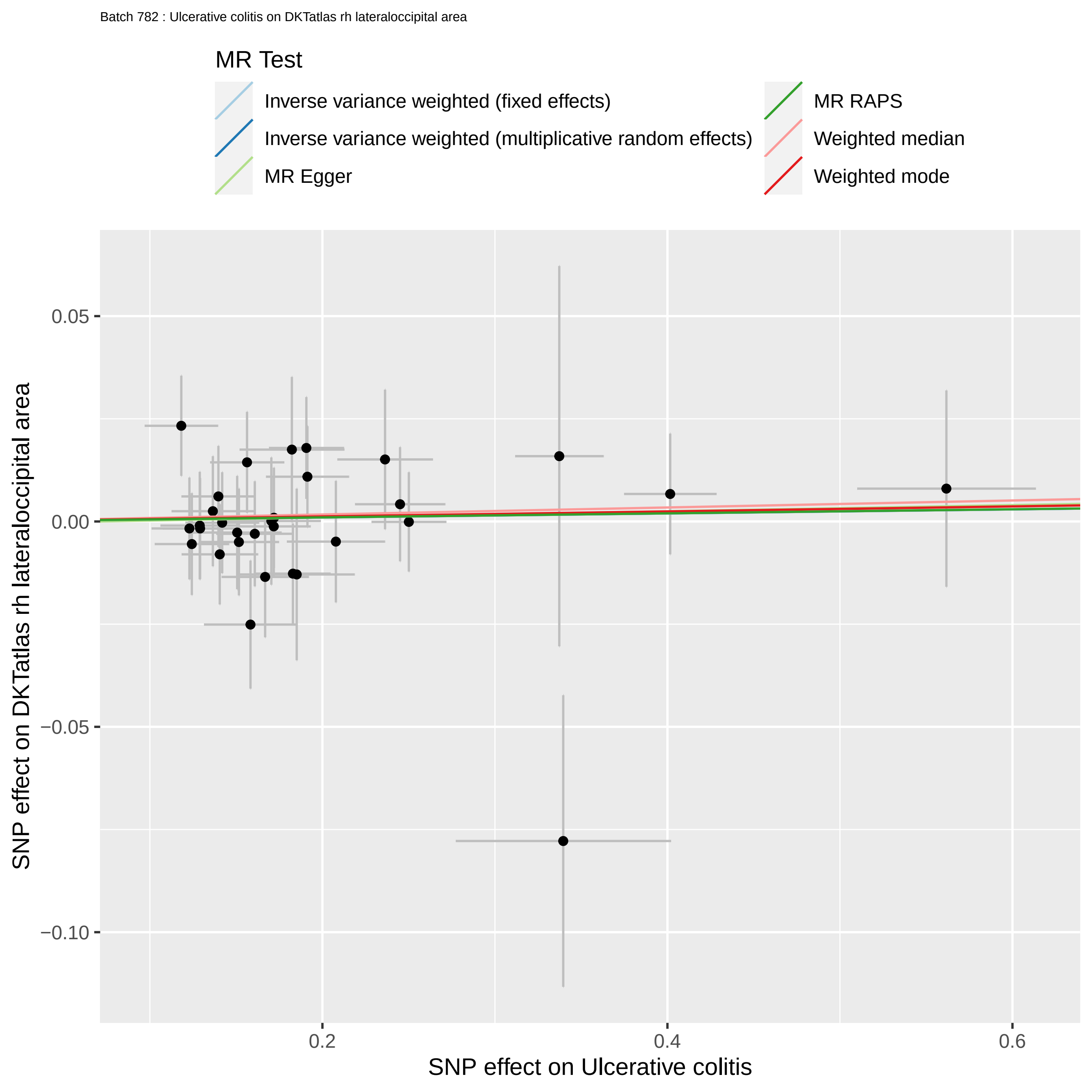

Batch 782 : Ulcerative colitis on DKTatlas rh lateraloccipital area
MR Test
Inverse variance weighted (fixed effects)
Inverse variance weighted (multiplicative random effects)
MR Egger
MR RAPS
Weighted median
Weighted mode
0.05
0.00
−0.05
−0.10
0.2
0.4
0.6
SNP effect on DKTatlas rh lateraloccipital area
SNP effect on Ulcerative colitis

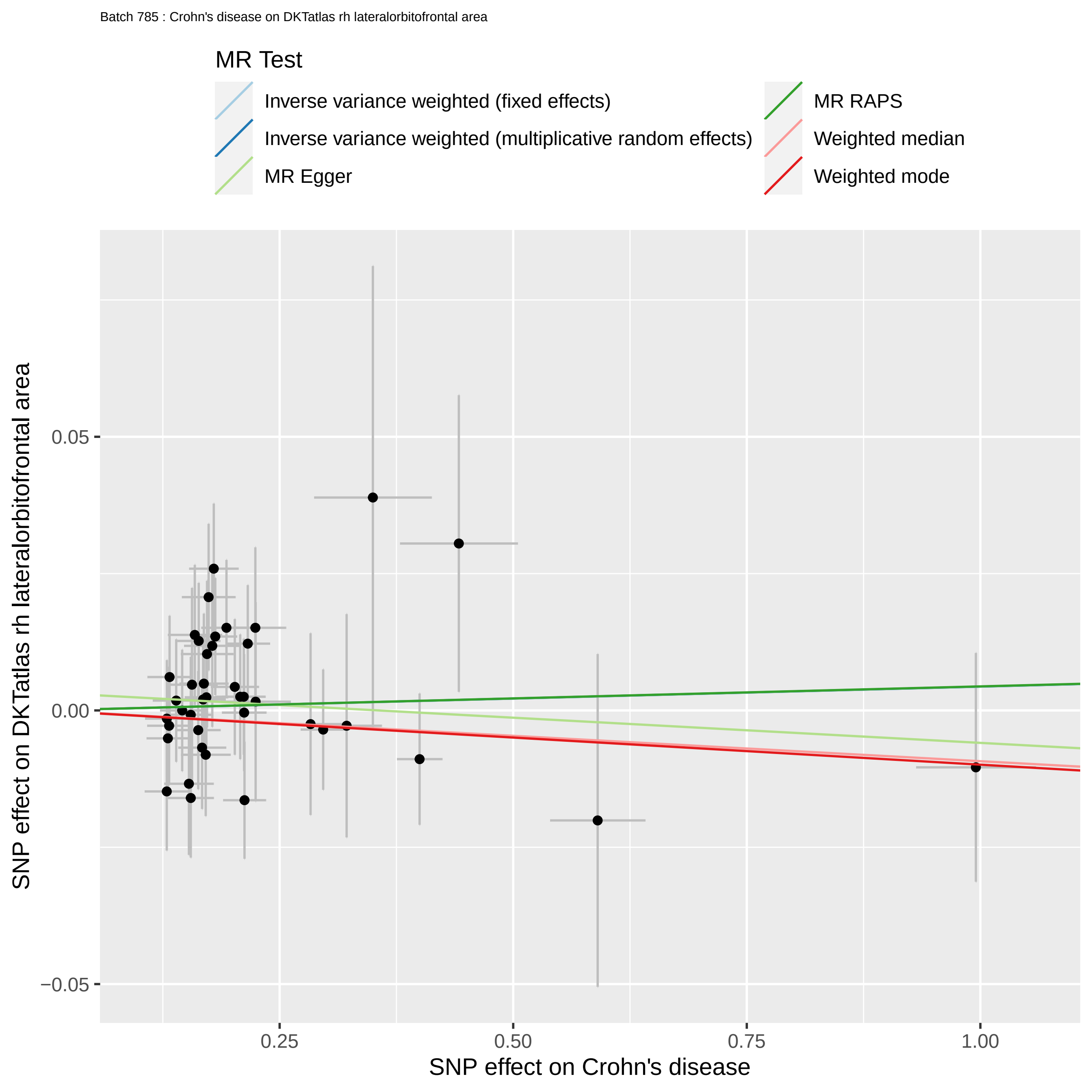

Batch 785 : Crohn's disease on DKTatlas rh lateralorbitofrontal area
MR Test
Inverse variance weighted (fixed effects)
Inverse variance weighted (multiplicative random effects)
MR Egger
MR RAPS
Weighted median
Weighted mode
0.05
0.00
−0.05
0.25
0.50
0.75
1.00
SNP effect on DKTatlas rh lateralorbitofrontal area
SNP effect on Crohn's disease

Batch 786 : Ulcerative colitis on DKTatlas rh lateralorbitofrontal area

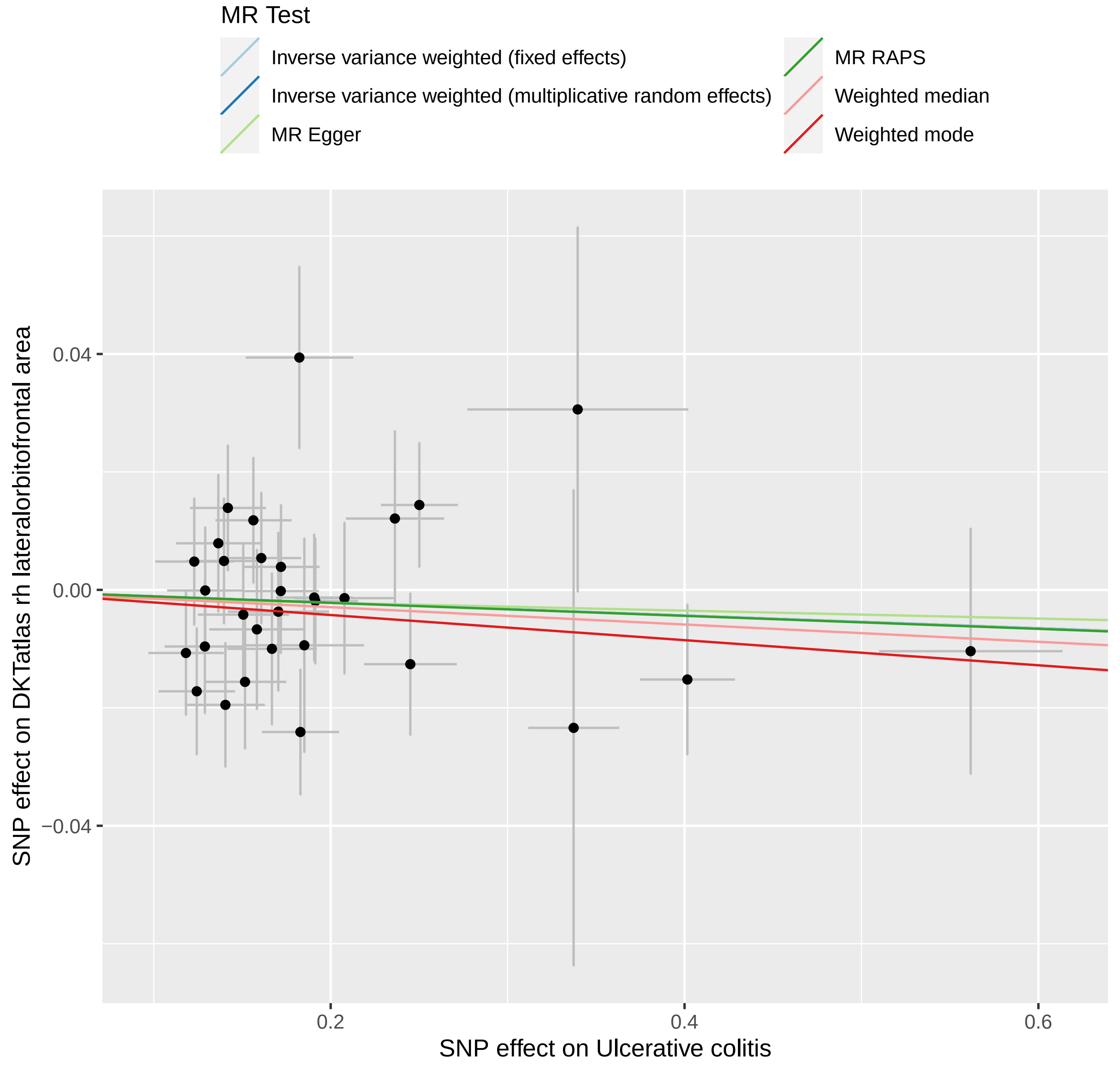

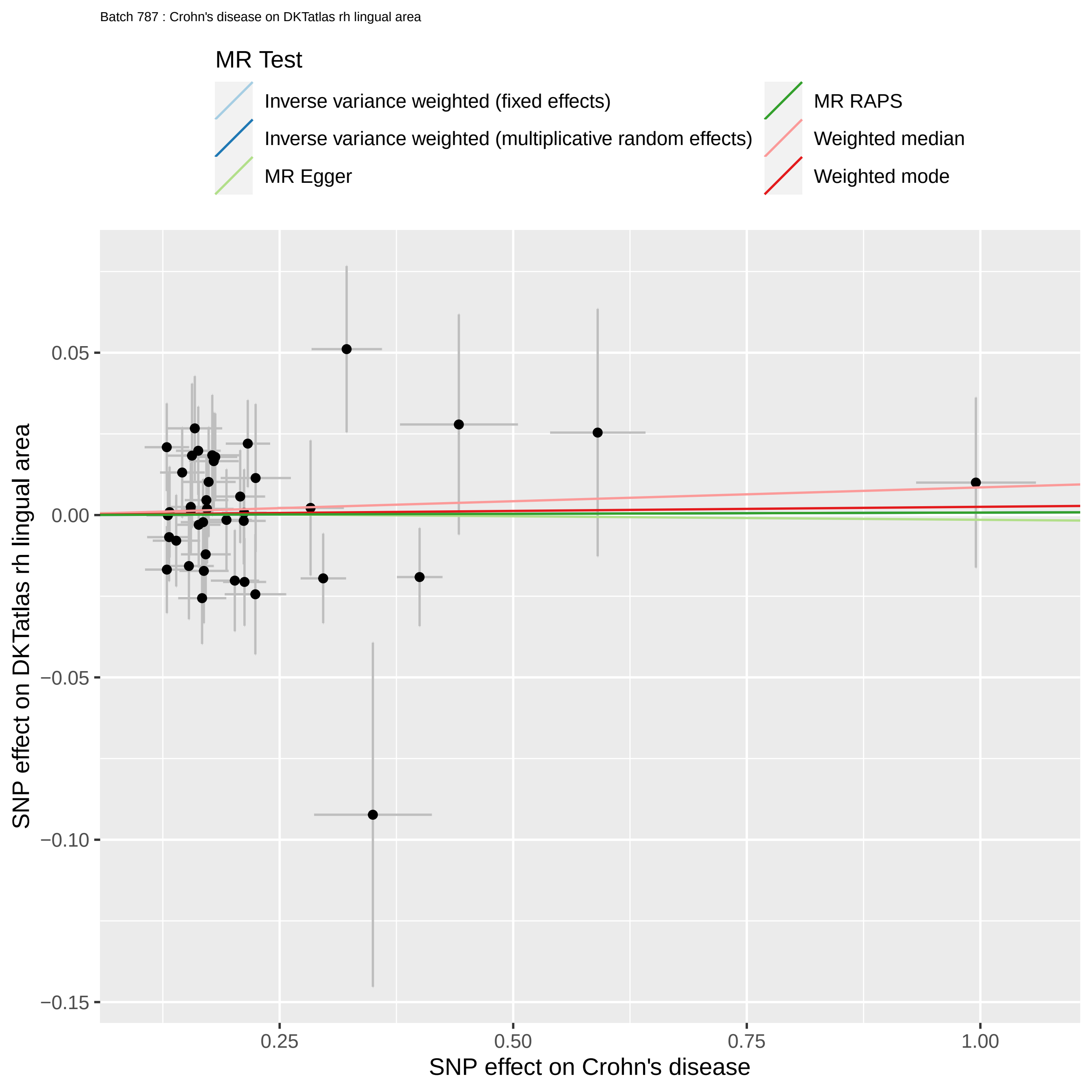

Batch 787 : Crohn's disease on DKTatlas rh lingual area
MR Test
Inverse variance weighted (fixed effects)
Inverse variance weighted (multiplicative random effects)
MR Egger
MR RAPS
Weighted median
Weighted mode
SNP effect on DKTatlas rh lingual area
0.05
0.00
−0.05
−0.10
−0.15
0.25
0.50
0.75
1.00
SNP effect on Crohn's disease

Batch 788 : Ulcerative colitis on DKTatlas rh lingual area

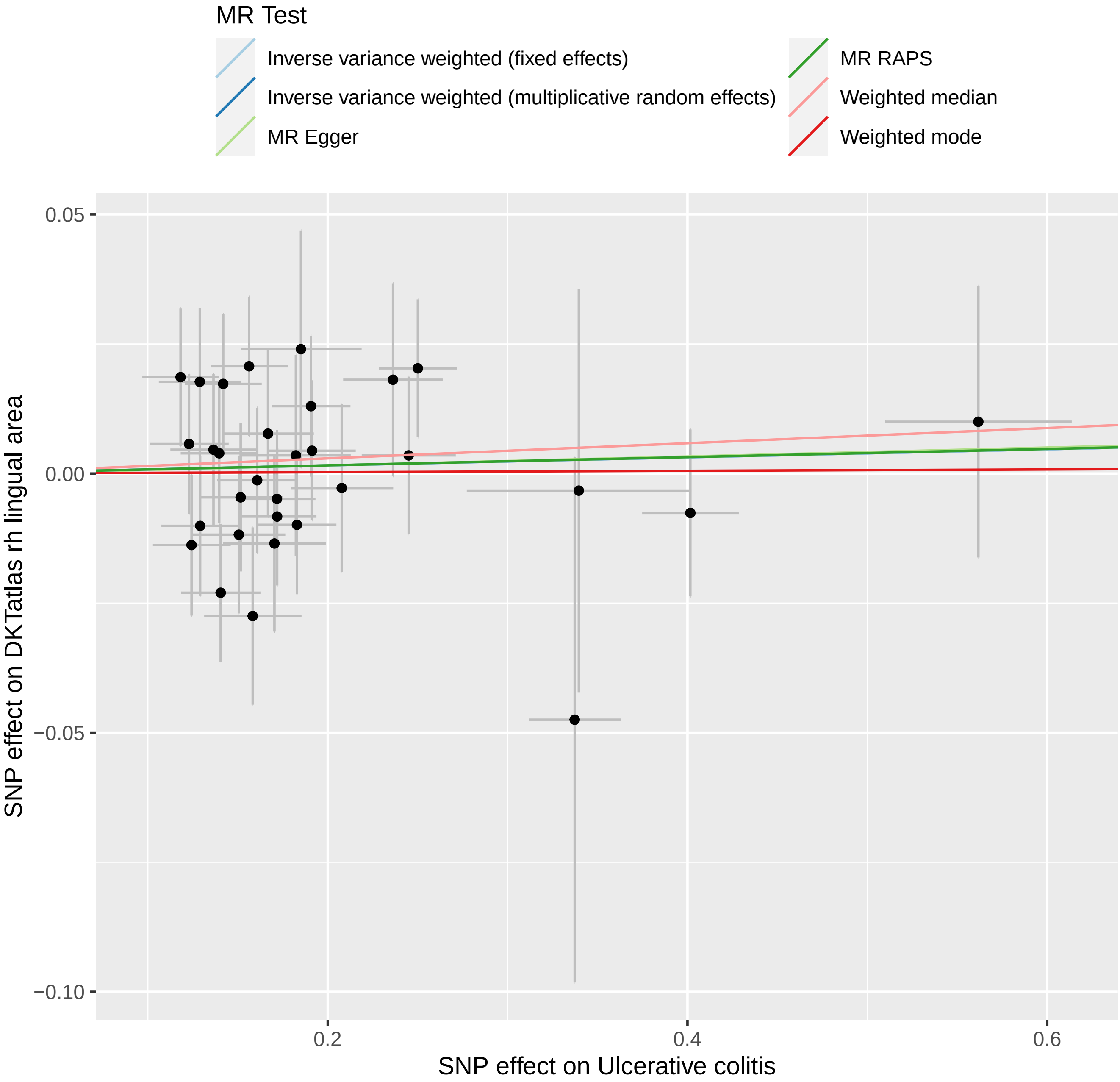

Batch 789 : Crohn's disease on DKTatlas rh medialorbitofrontal area

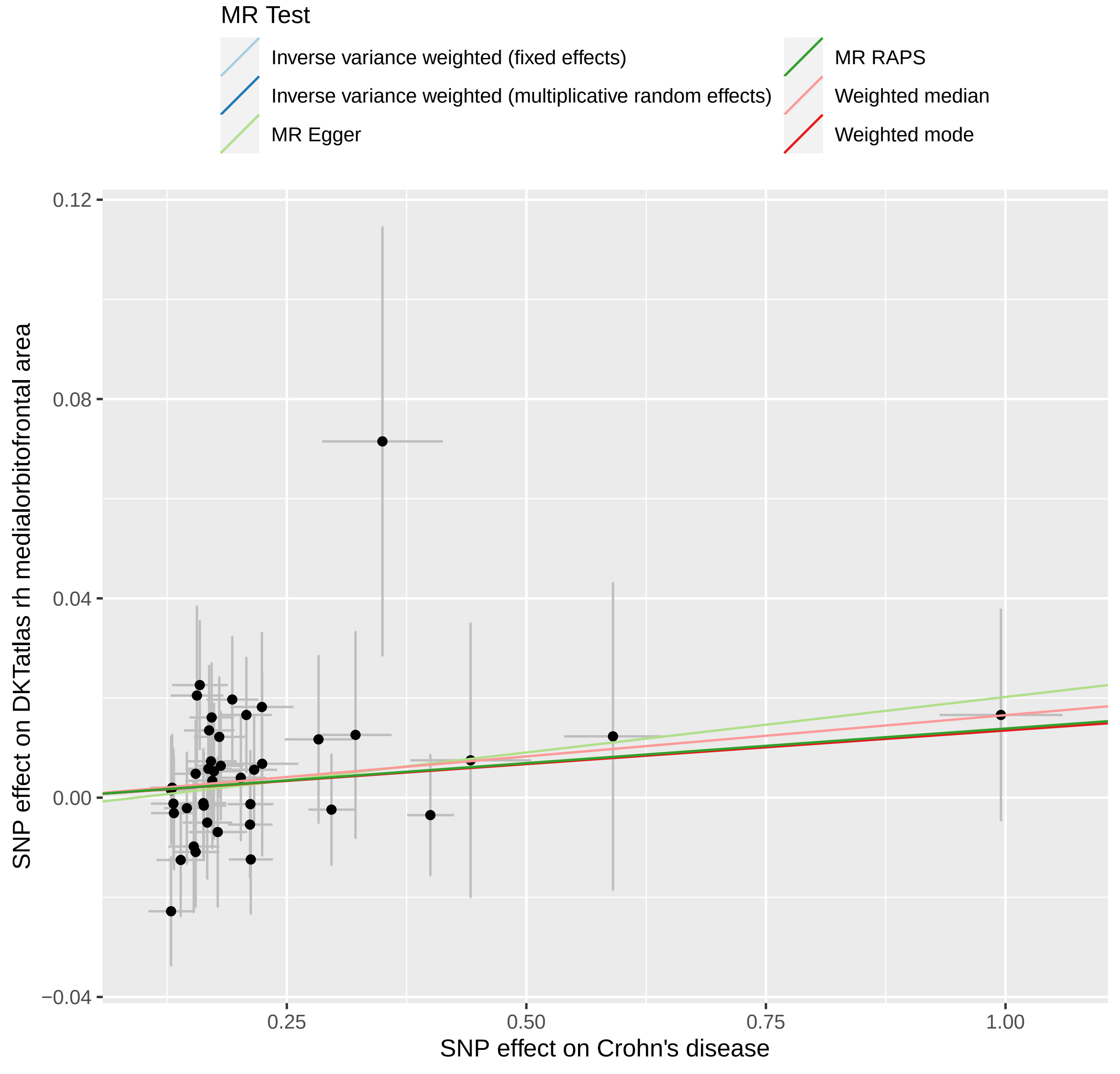

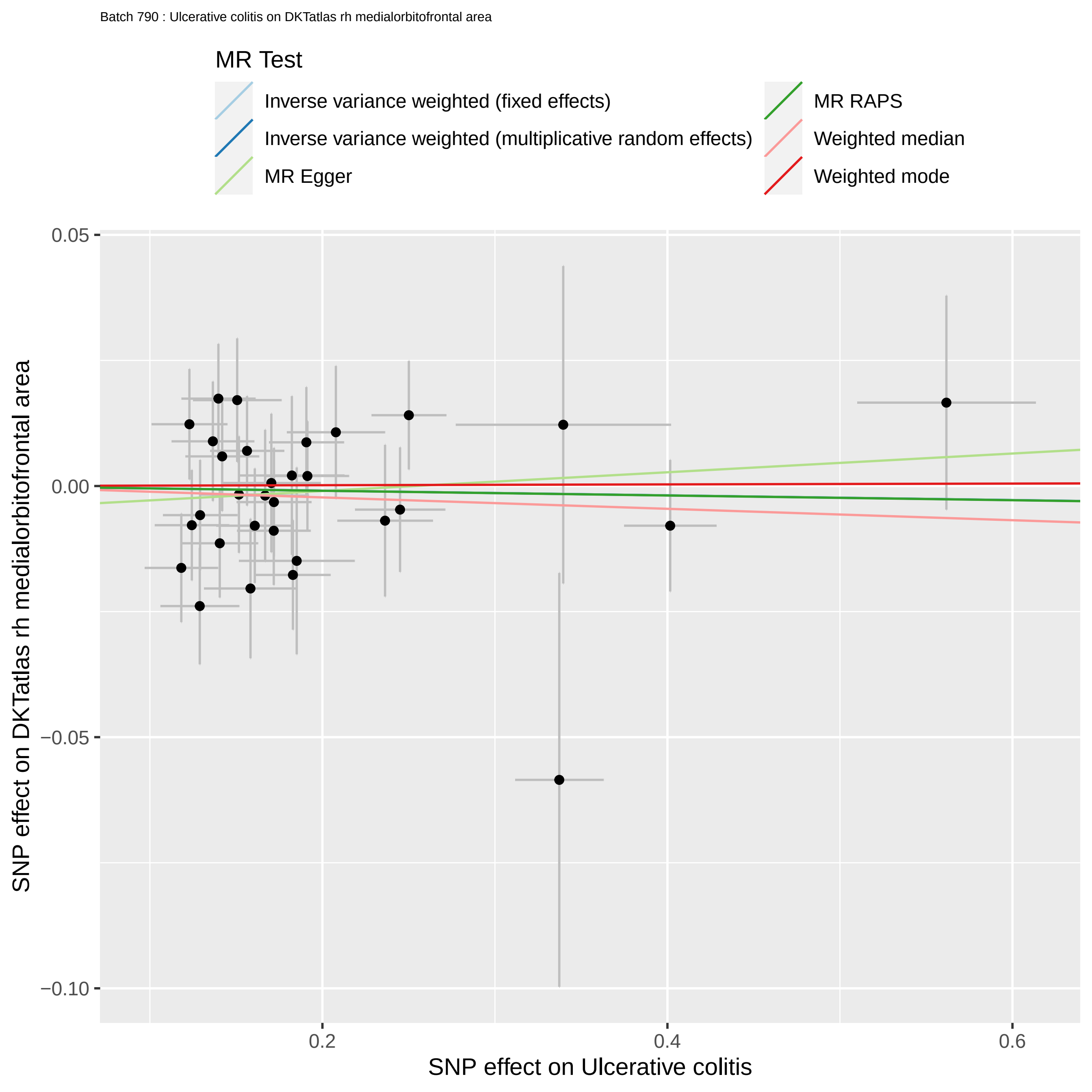
Batch 790 : Ulcerative colitis on DKTatlas rh medialorbitofrontal area
MR Test
Inverse variance weighted (fixed effects)
Inverse variance weighted (multiplicative random effects)
MR Egger
MR RAPS
Weighted median
Weighted mode
0.05
0.00
−0.05
−0.10
0.2
0.4
0.6
SNP effect on DKTatlas rh medialorbitofrontal area
SNP effect on Ulcerative colitis

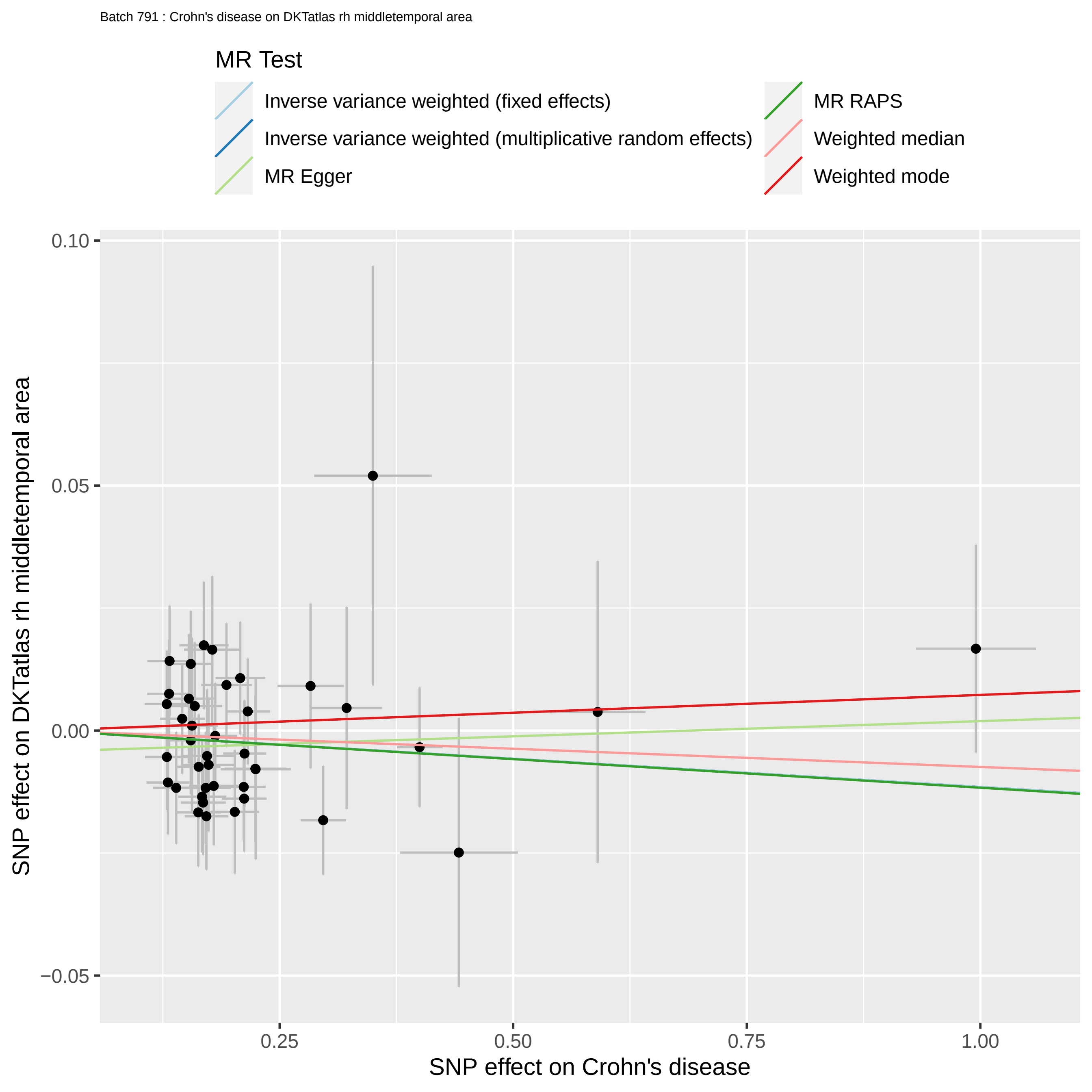

Batch 791 : Crohn's disease on DKTatlas rh middletemporal area
MR Test
Inverse variance weighted (fixed effects)
Inverse variance weighted (multiplicative random effects)
MR Egger
MR RAPS
Weighted median
Weighted mode
0.10
0.05
0.00
−0.05
0.25
0.50
0.75
1.00
SNP effect on DKTatlas rh middletemporal area
SNP effect on Crohn's disease

Batch 792 : Ulcerative colitis on DKTatlas rh middletemporal area

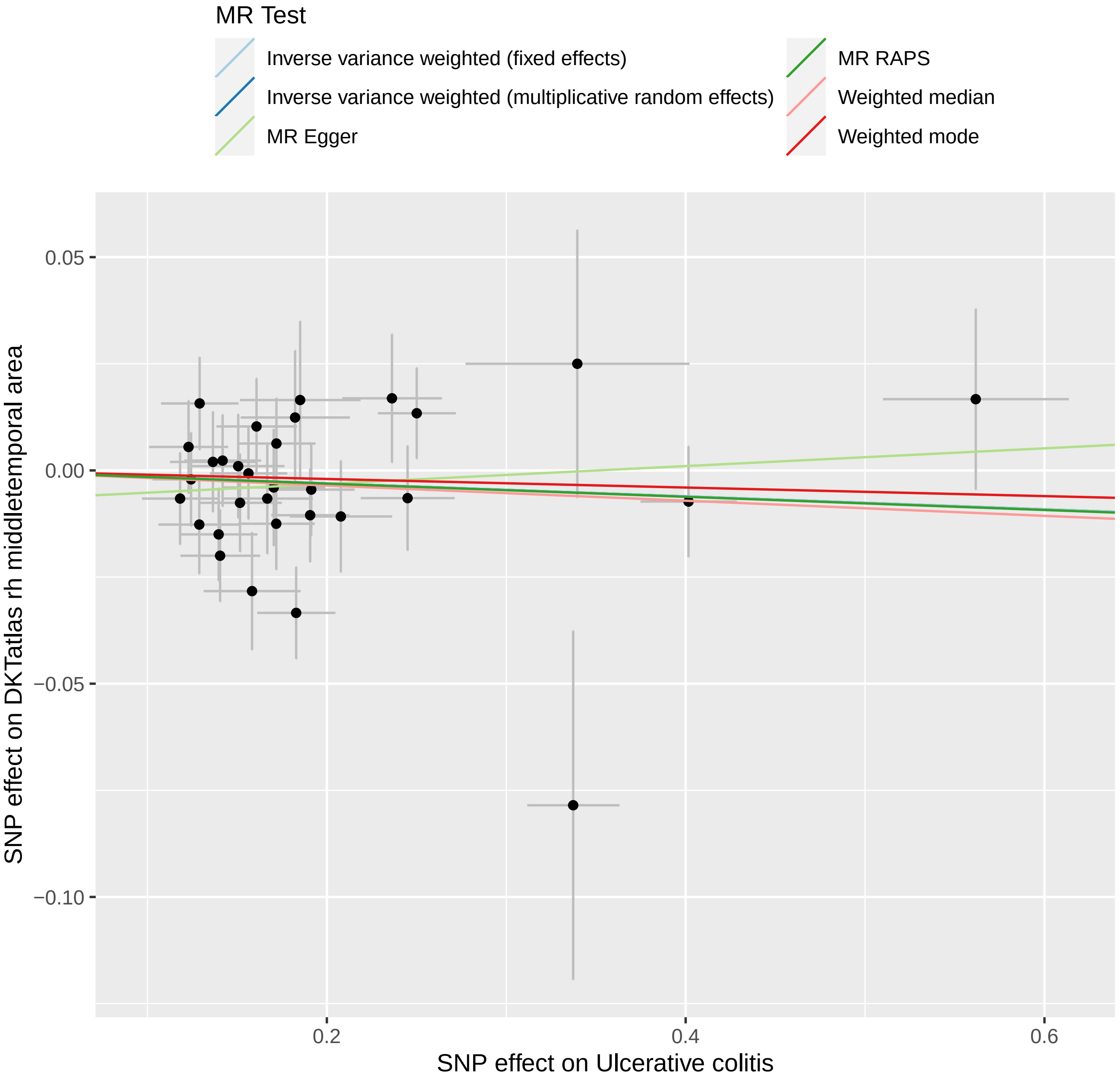

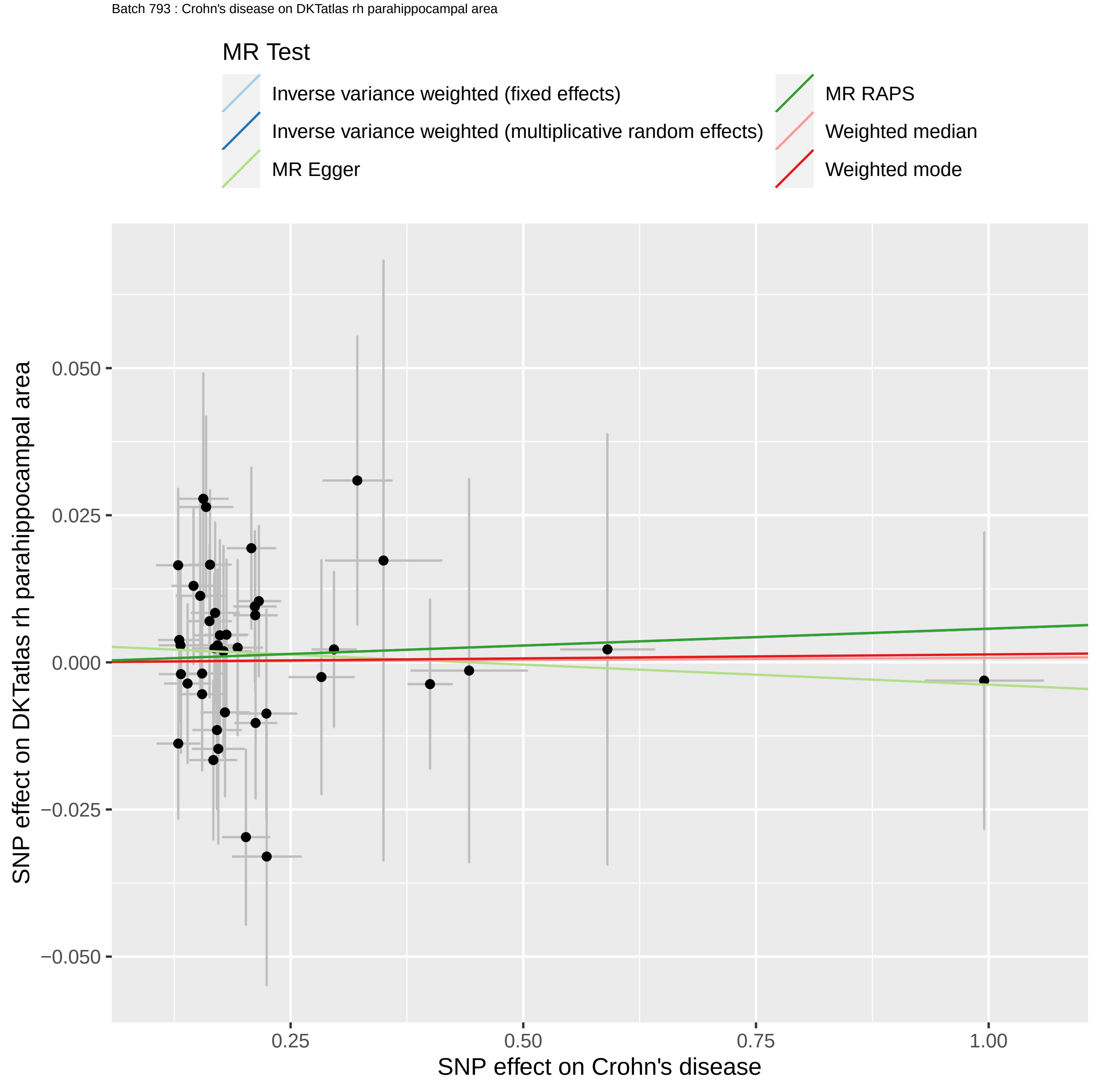
Batch 793 : Crohn's disease on DKTatlas rh parahippocampal area
MR Test
Inverse variance weighted (fixed effects)
Inverse variance weighted (multiplicative random effects)
MR Egger
MR RAPS
Weighted median
Weighted mode
0.050
0.025
0.000
−0.025
−0.050
0.25
0.50
0.75
1.00
SNP effect on DKTatlas rh parahippocampal area
SNP effect on Crohn's disease

Batch 794 : Ulcerative colitis on DKTatlas rh parahippocampal area

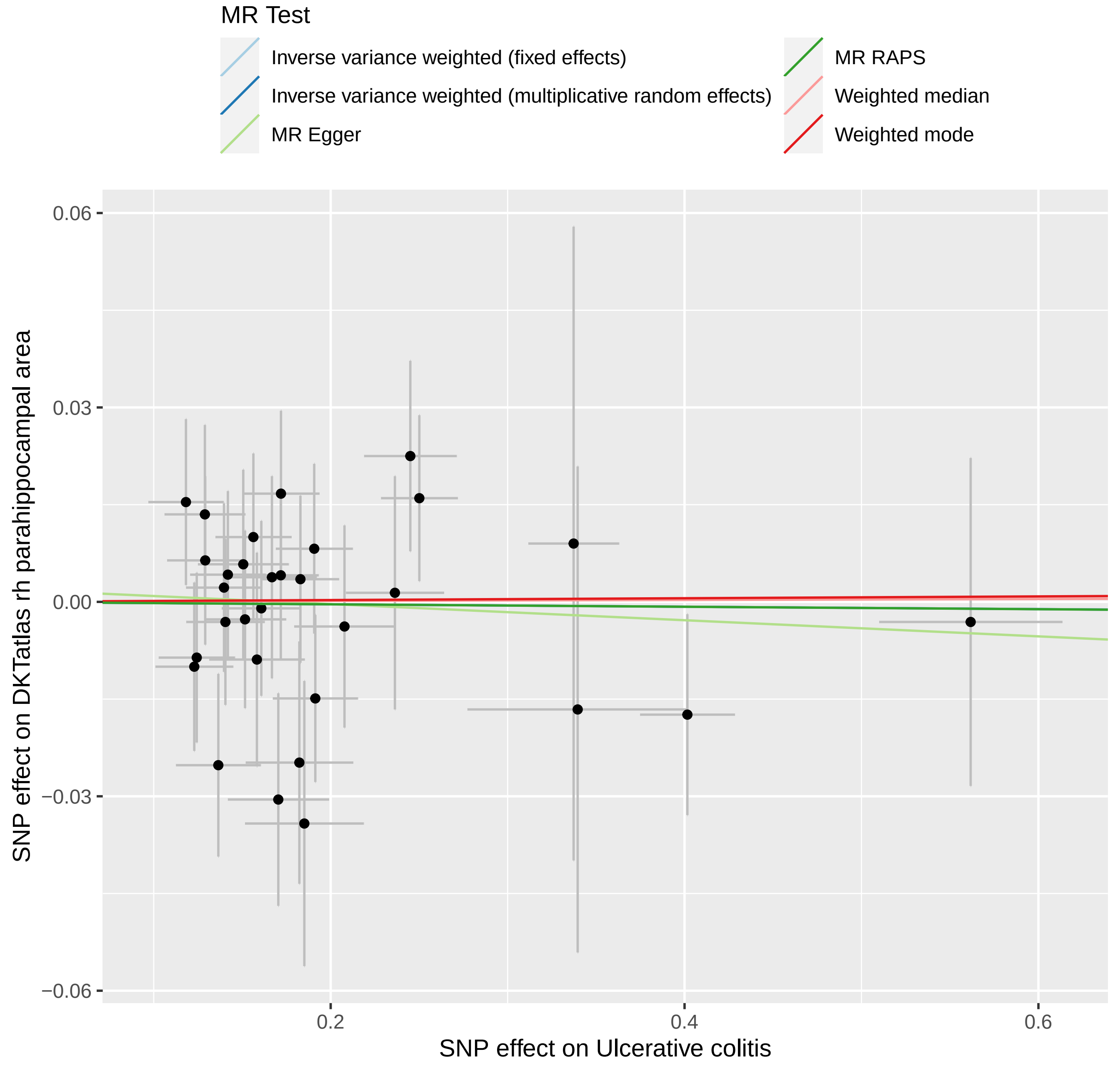

Batch 795 : Crohn's disease on DKTatlas rh paracentral area

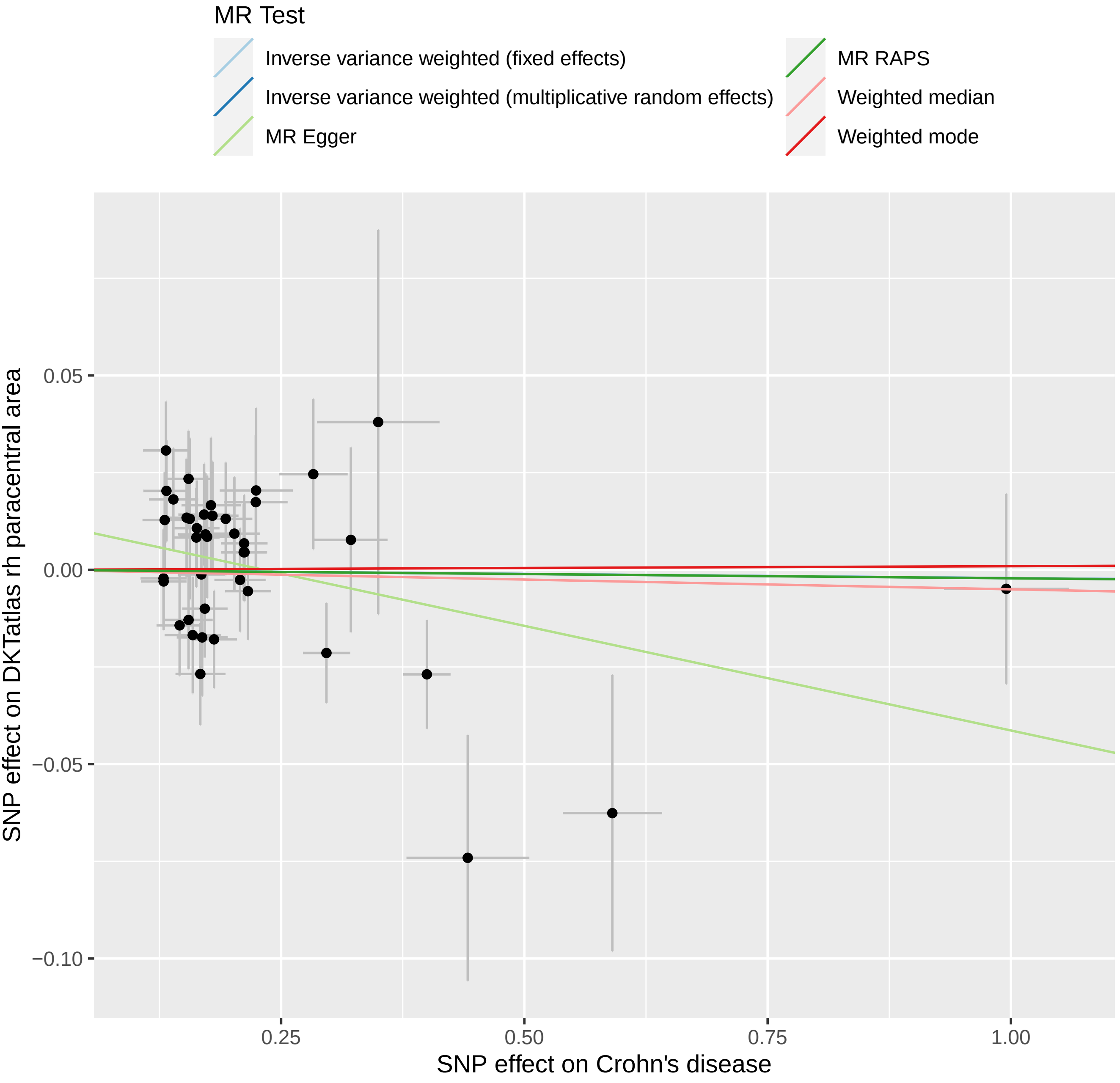

Batch 796 : Ulcerative colitis on DKTatlas rh paracentral area

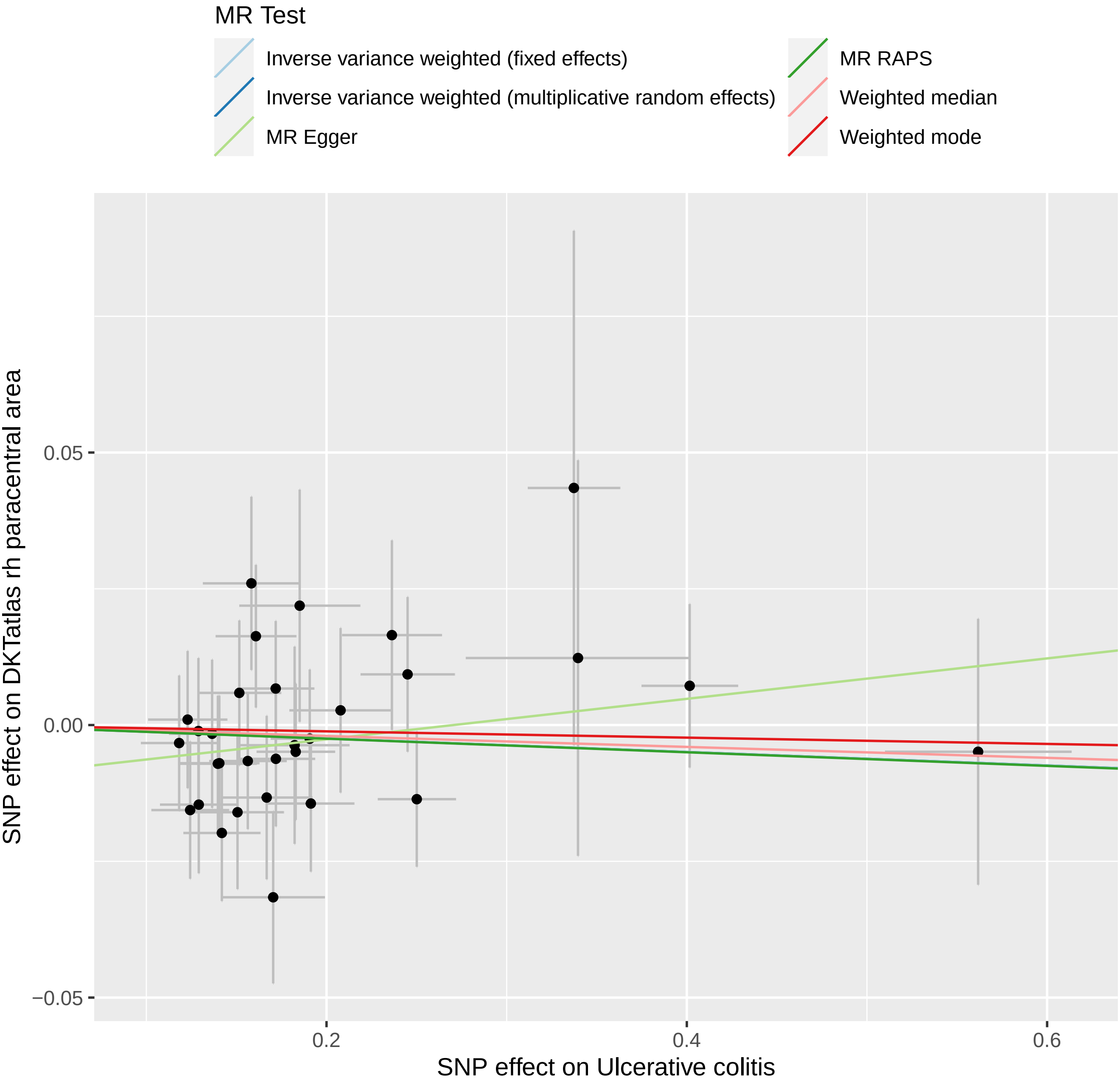

Batch 797 : Crohn's disease on DKTatlas rh parsopercularis area

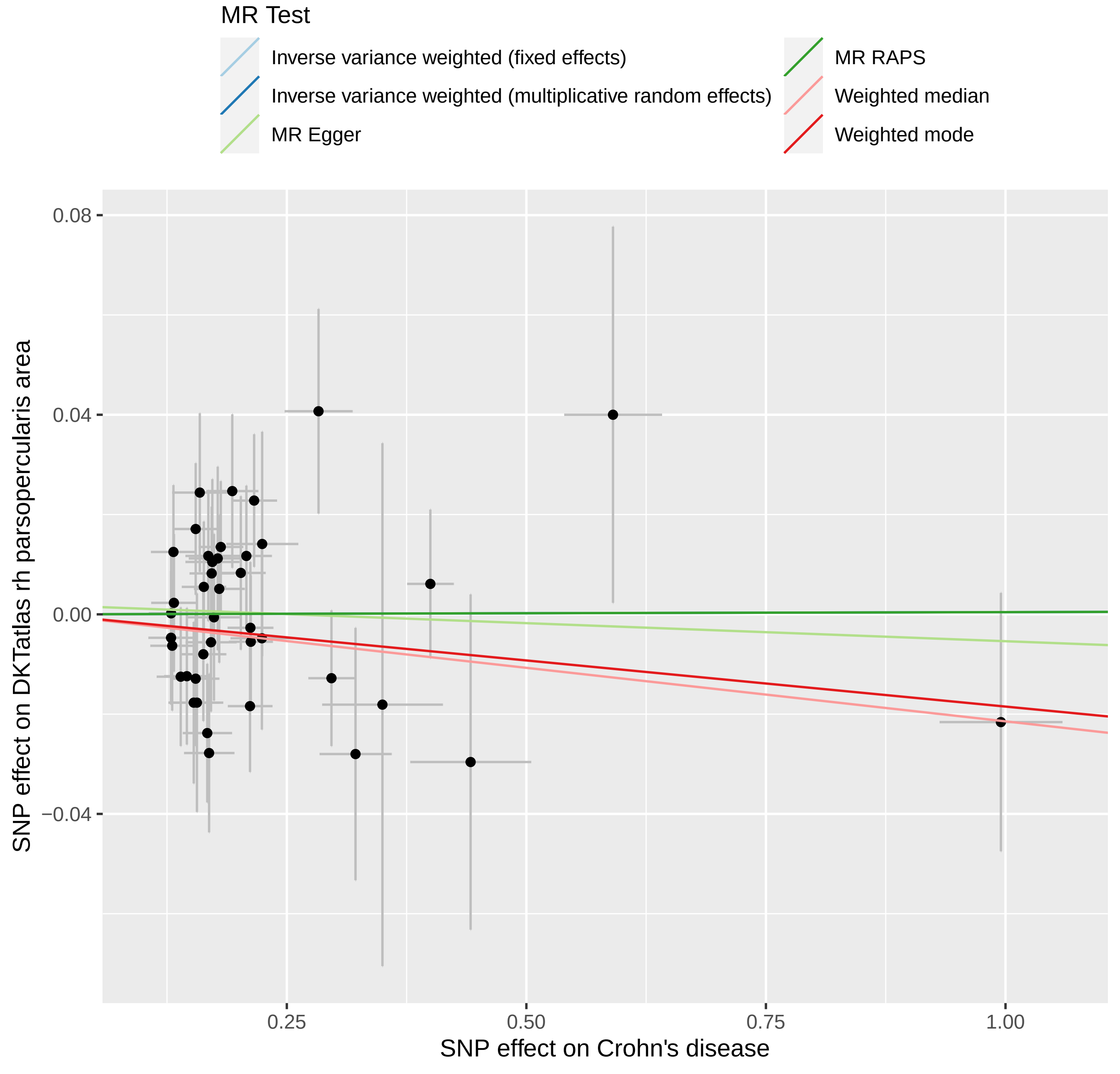

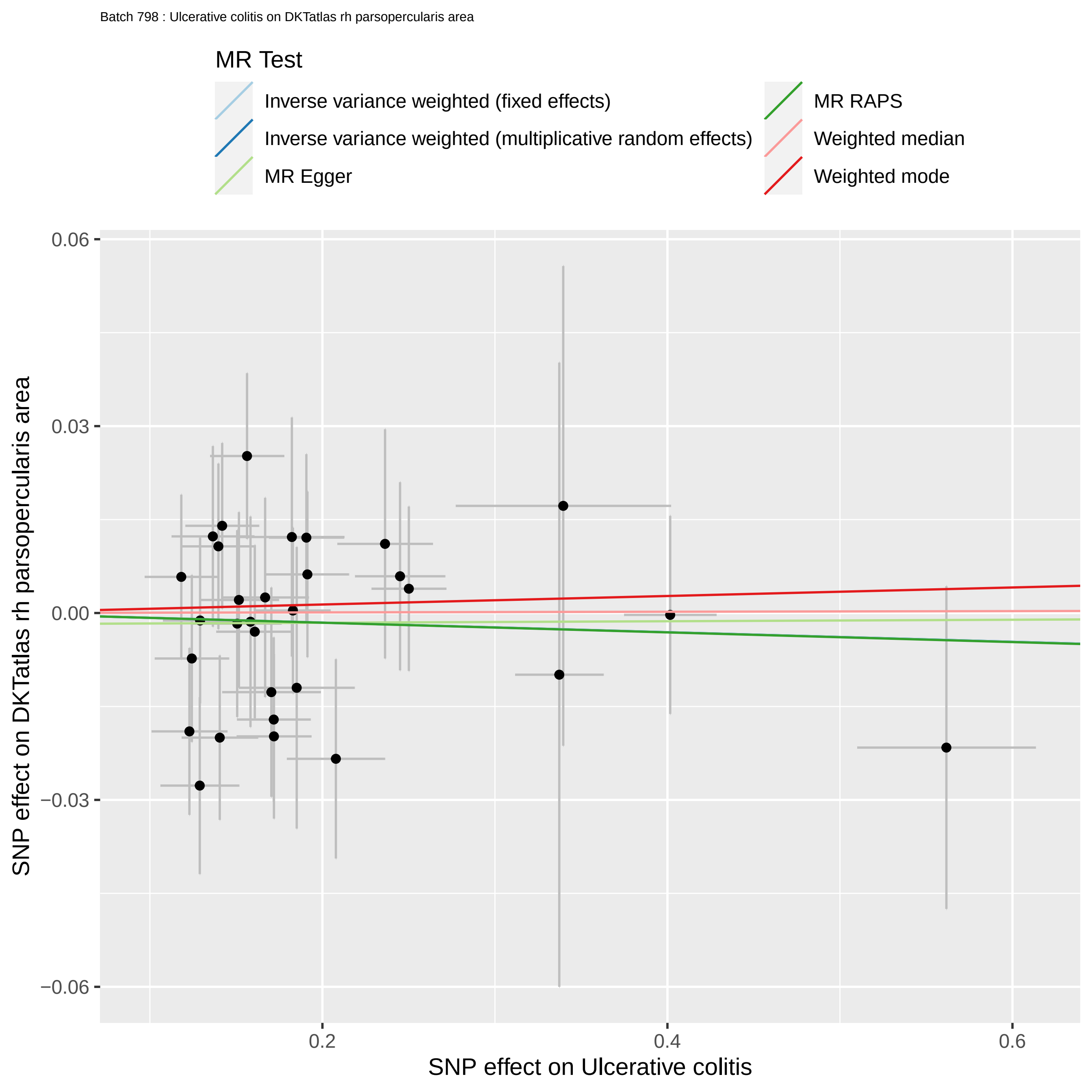
Batch 798 : Ulcerative colitis on DKTatlas rh parsopercularis area
MR Test
Inverse variance weighted (fixed effects)
Inverse variance weighted (multiplicative random effects)
MR Egger
MR RAPS
Weighted median
Weighted mode
0.06
0.03
0.00
−0.03
−0.06
0.2
0.4
0.6
SNP effect on DKTatlas rh parsopercularis area
SNP effect on Ulcerative colitis

Batch 799 : Crohn's disease on DKTatlas rh parsorbitalis area

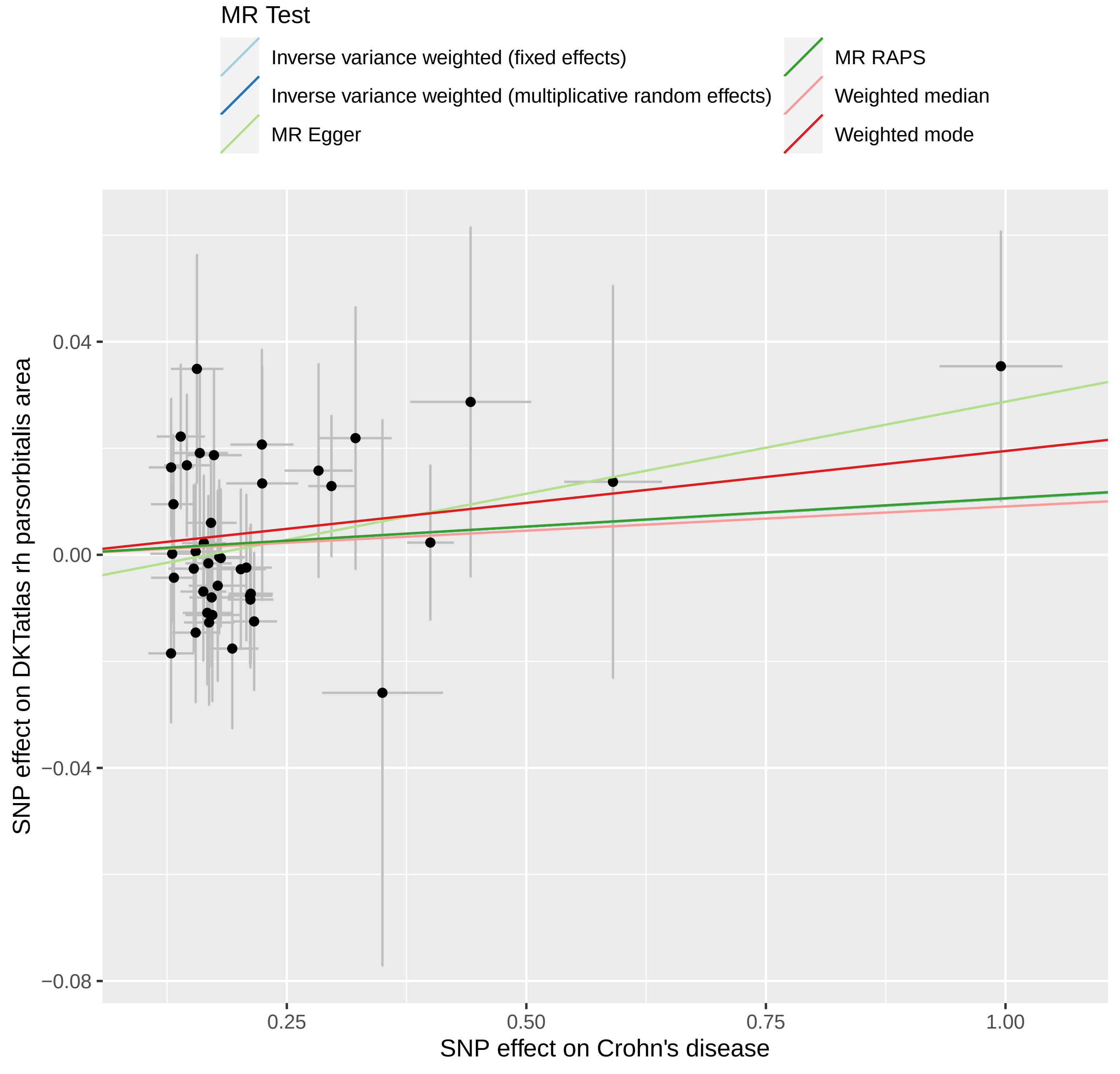

Batch 800 : Ulcerative colitis on DKTatlas rh parsorbitalis area

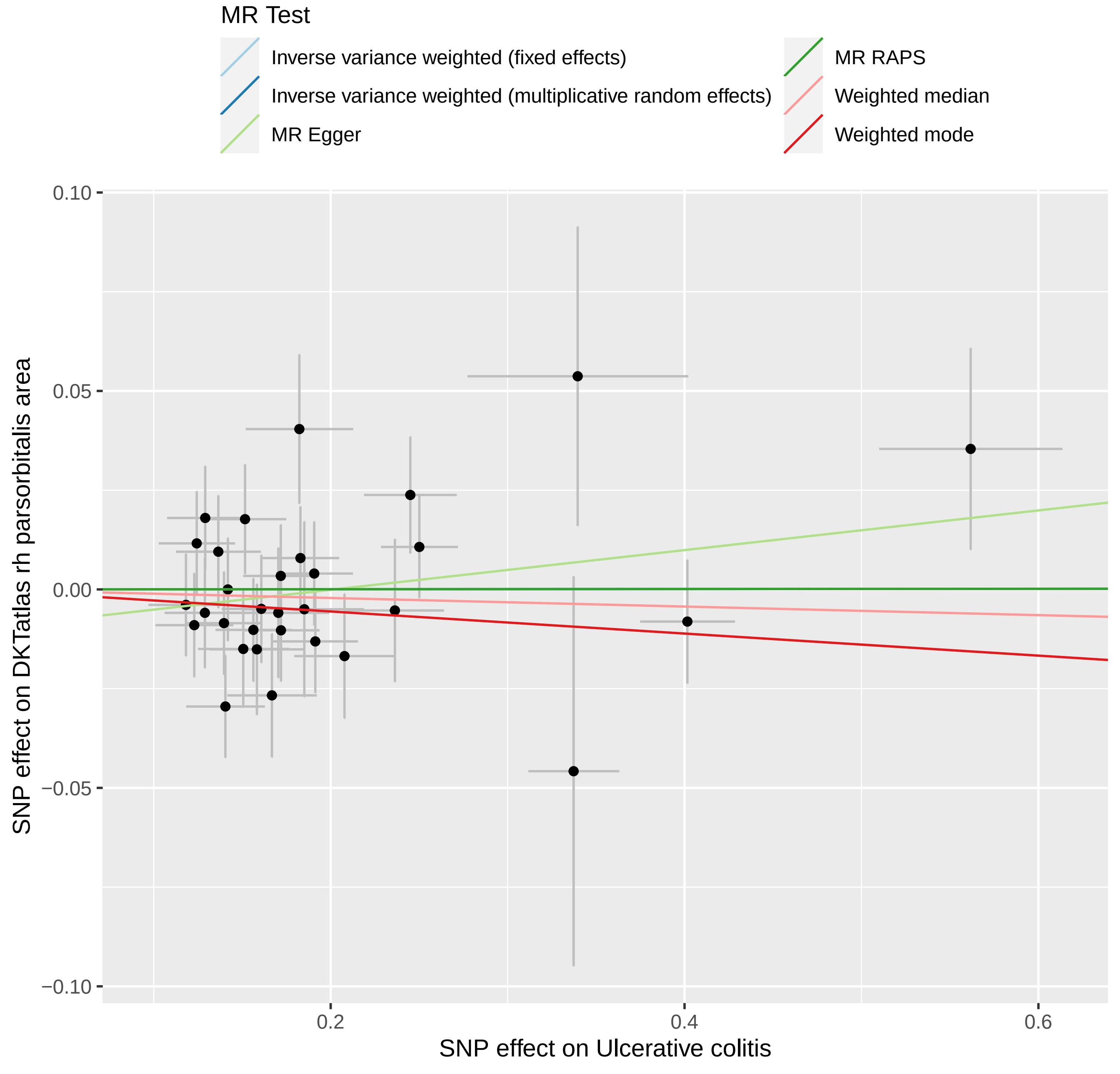

Batch 801 : Crohn's disease on DKTatlas rh parstriangularis area

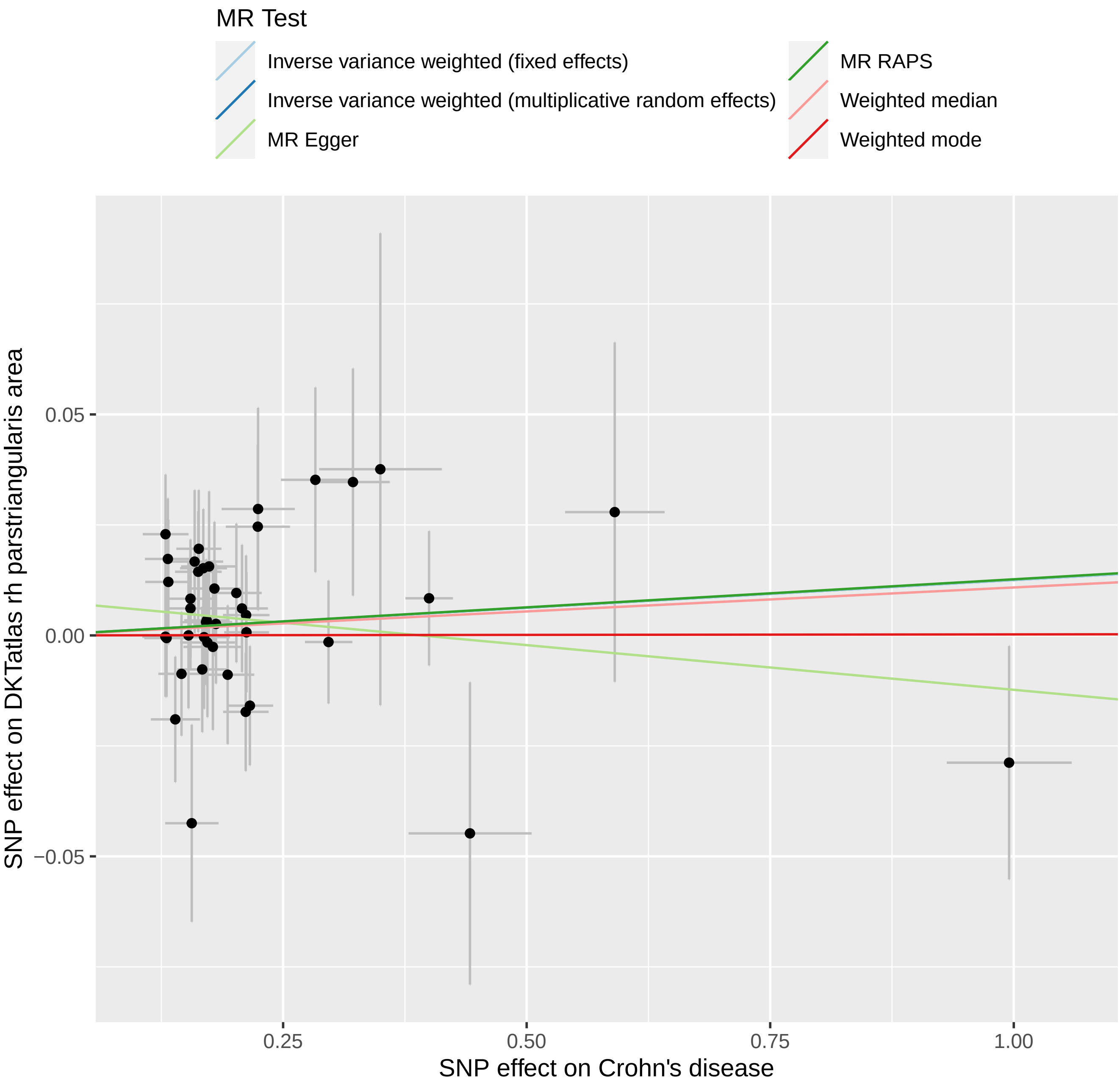

Batch 802 : Ulcerative colitis on DKTatlas rh parstriangularis area

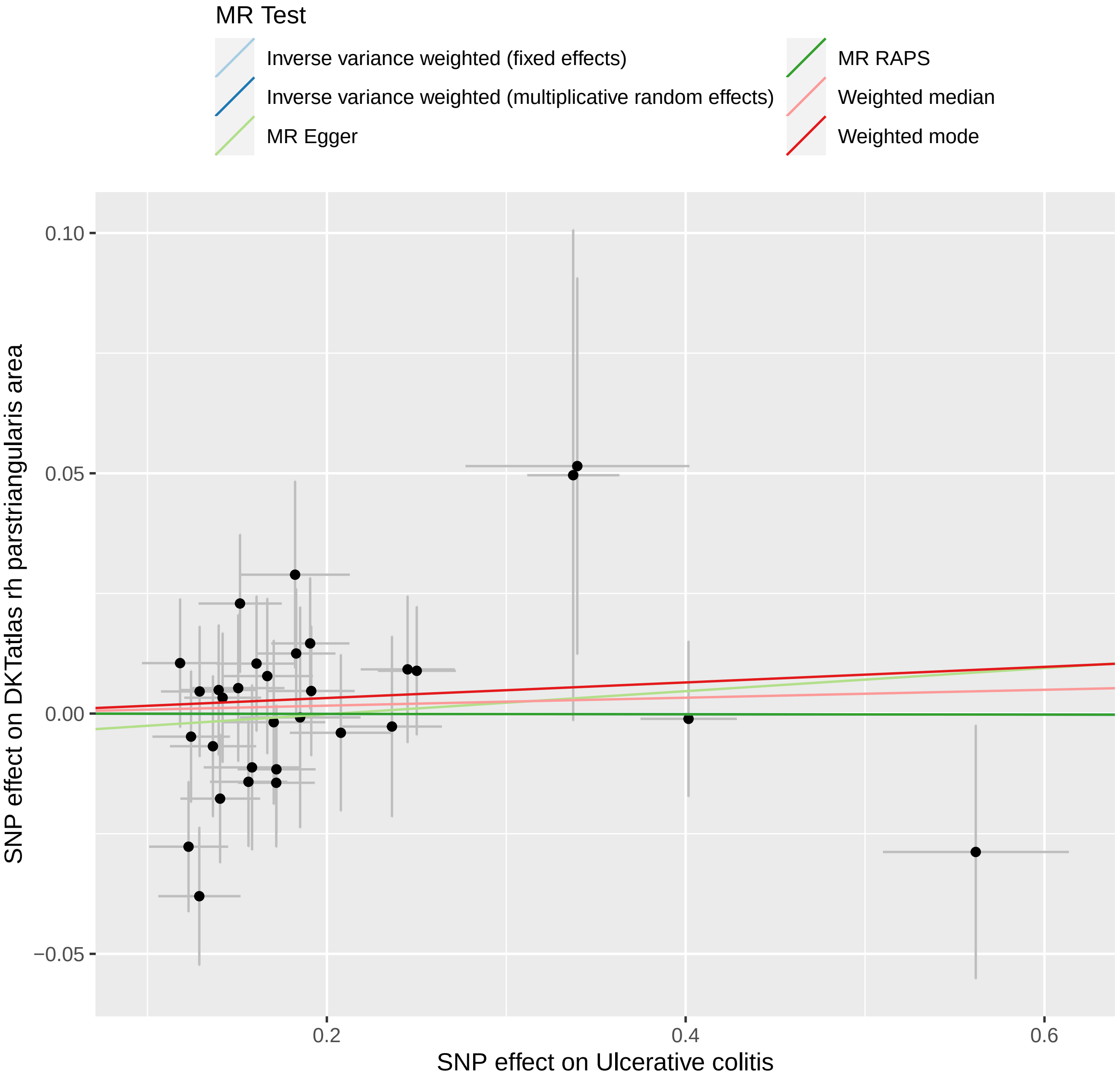

Batch 803 : Crohn's disease on DKTatlas rh pericalcarine area

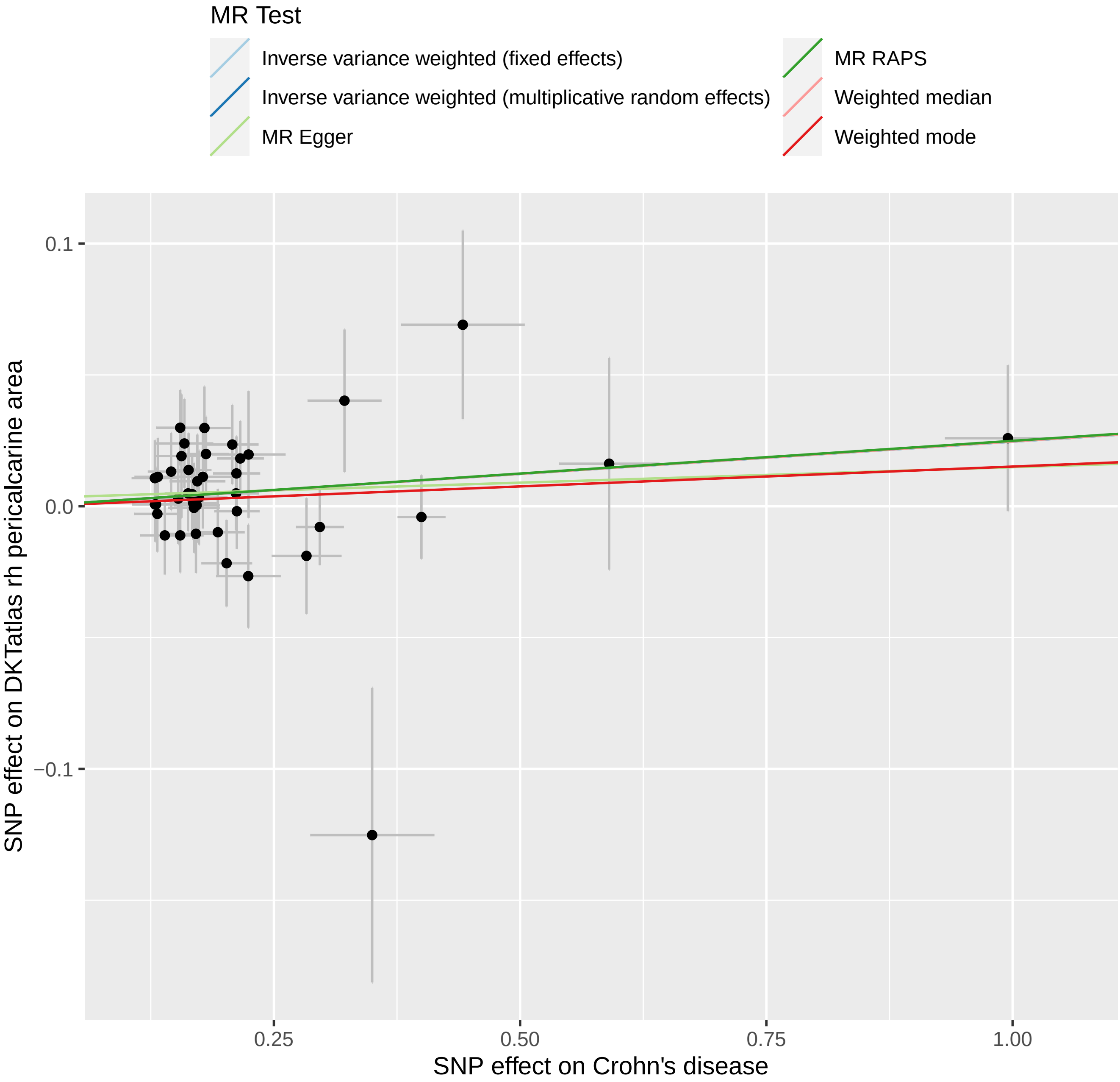

Batch 804 : Ulcerative colitis on DKTatlas rh pericalcarine area

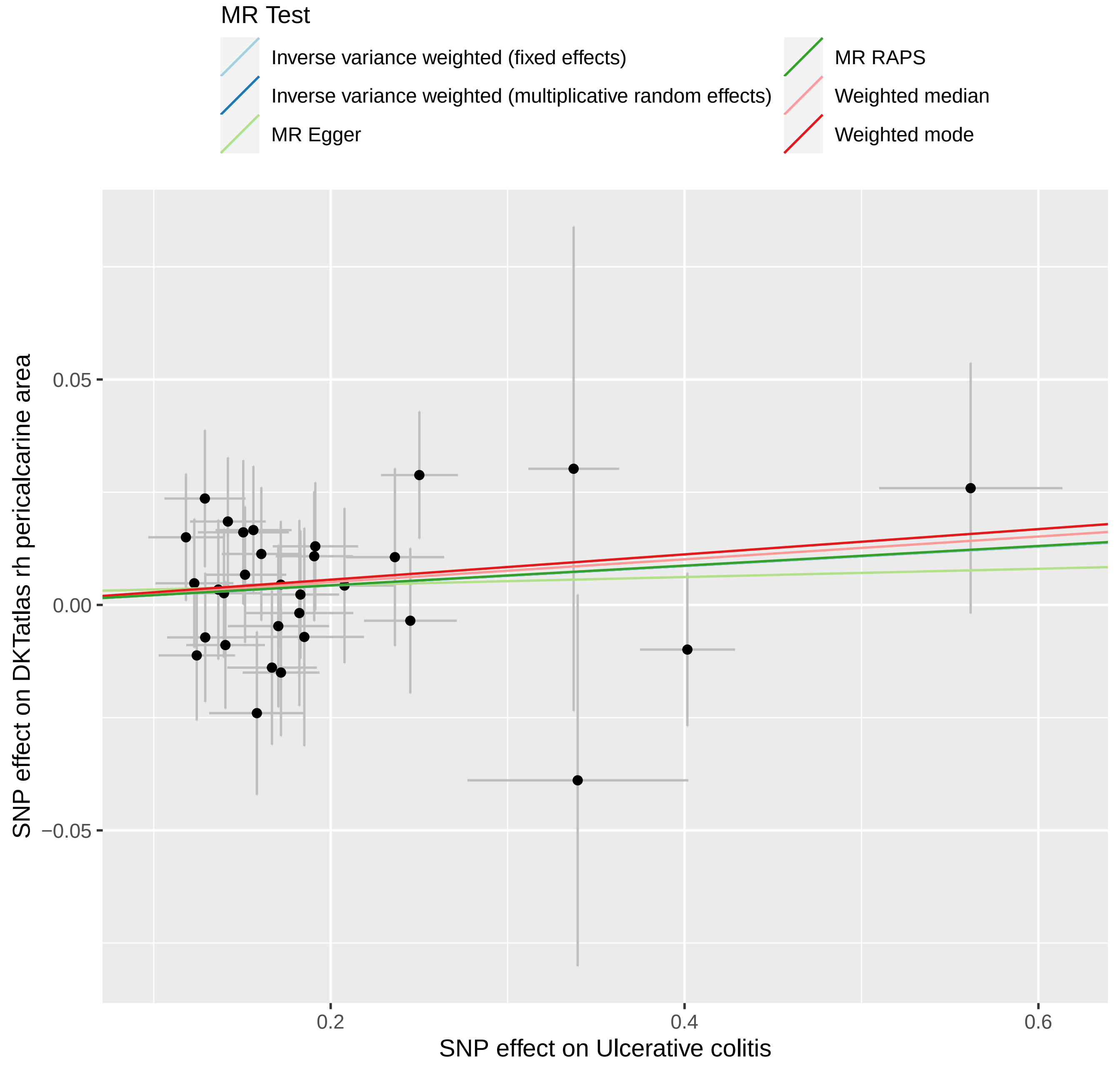

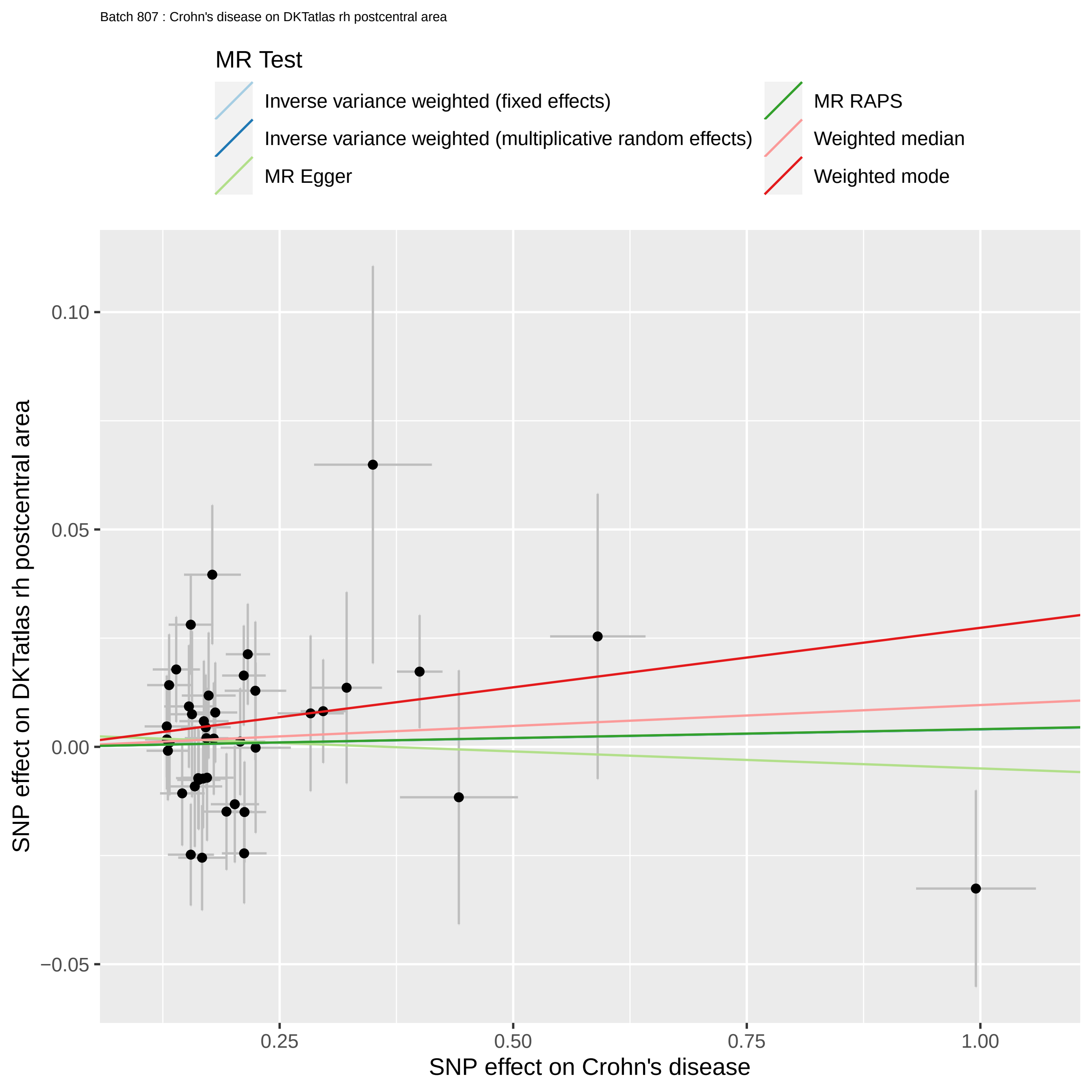
Batch 807 : Crohn's disease on DKTatlas rh postcentral area
MR Test
Inverse variance weighted (fixed effects)
Inverse variance weighted (multiplicative random effects)
MR Egger
MR RAPS
Weighted median
Weighted mode
SNP effect on DKTatlas rh postcentral area
0.10
0.05
0.00
−0.05
0.25
0.50
0.75
1.00
SNP effect on Crohn's disease

Batch 808 : Ulcerative colitis on DKTatlas rh postcentral area

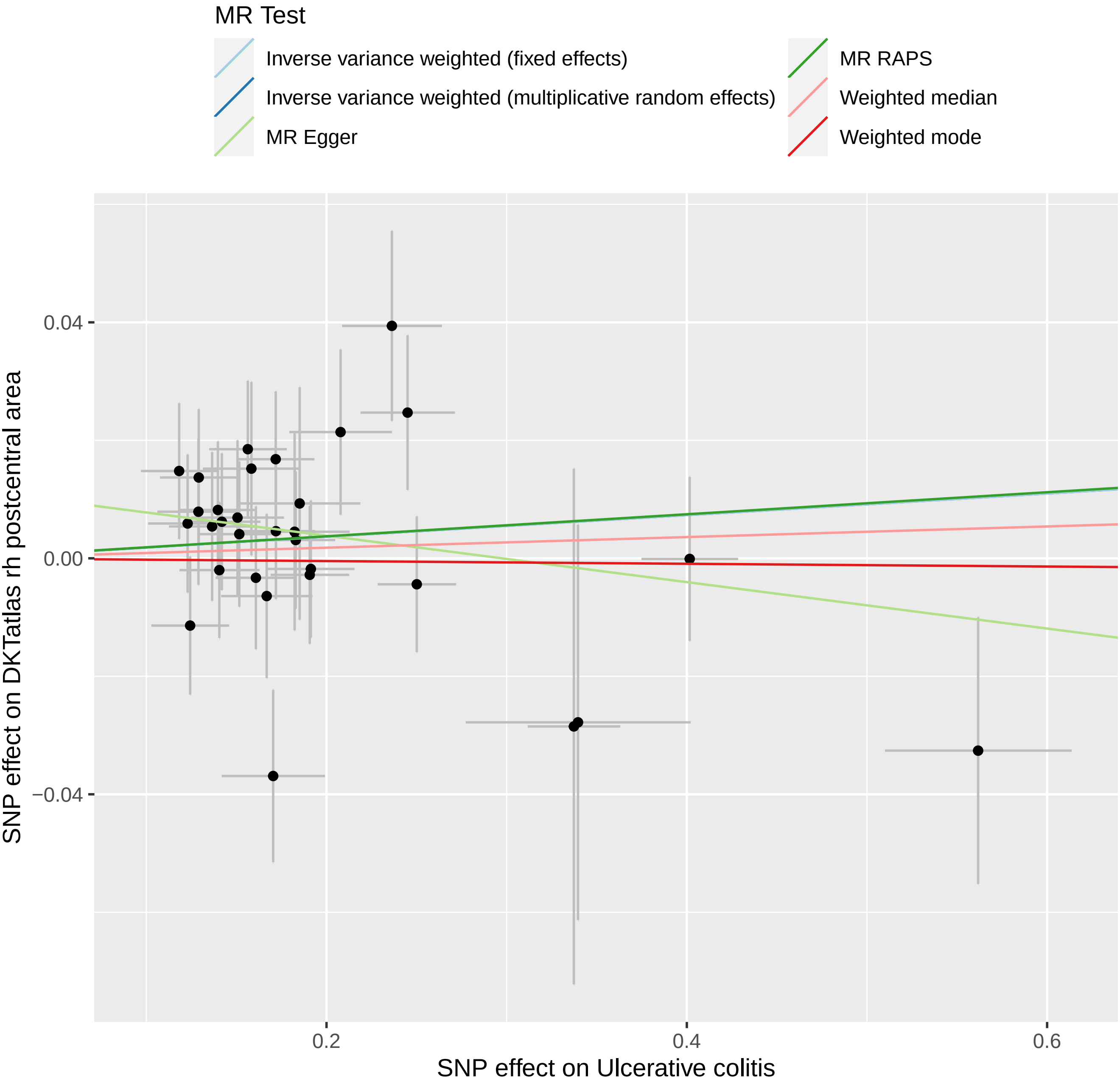

Batch 809 : Crohn's disease on DKTatlas rh posteriorcingulate area

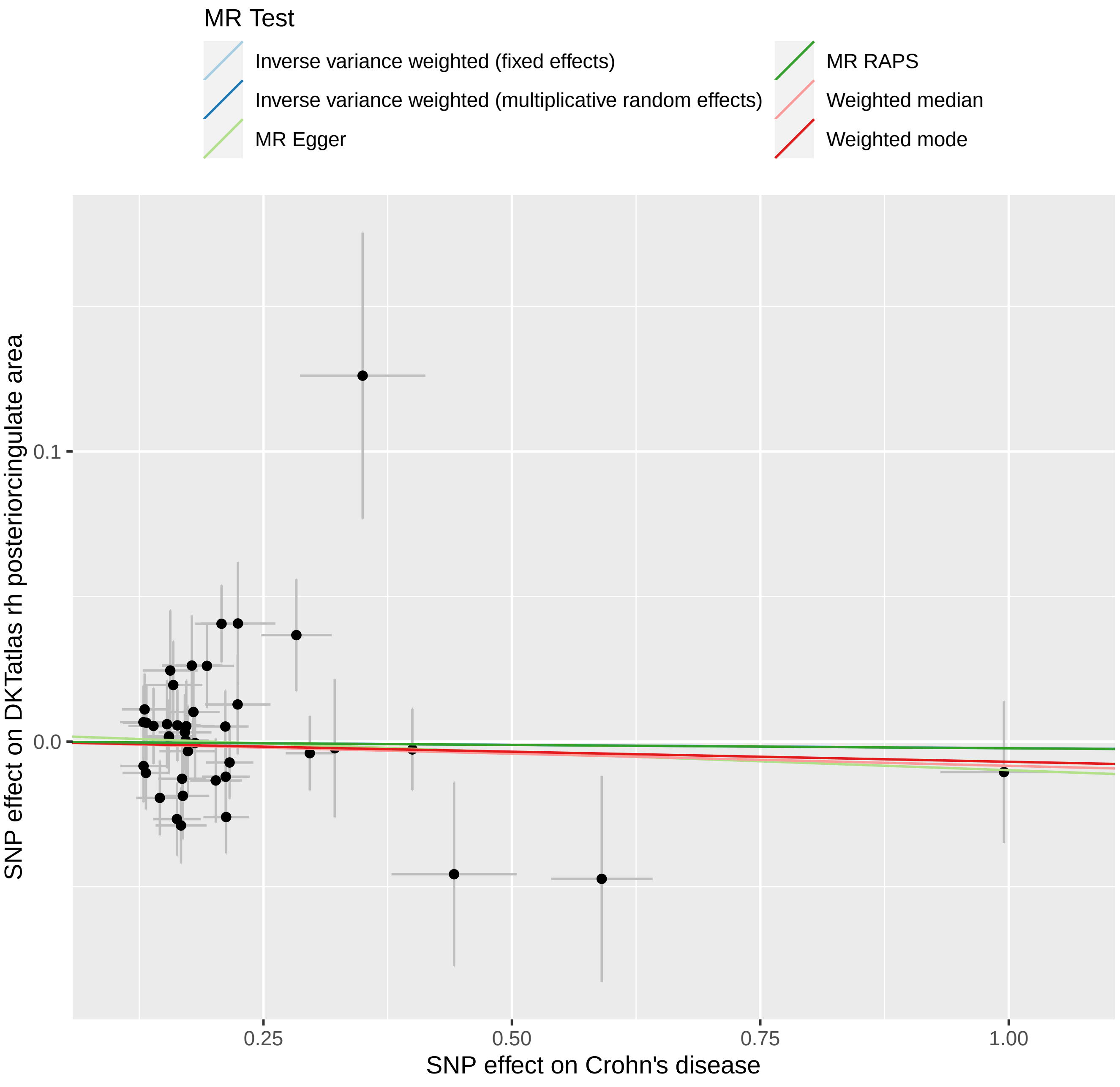

Batch 810 : Ulcerative colitis on DKTatlas rh posteriorcingulate area

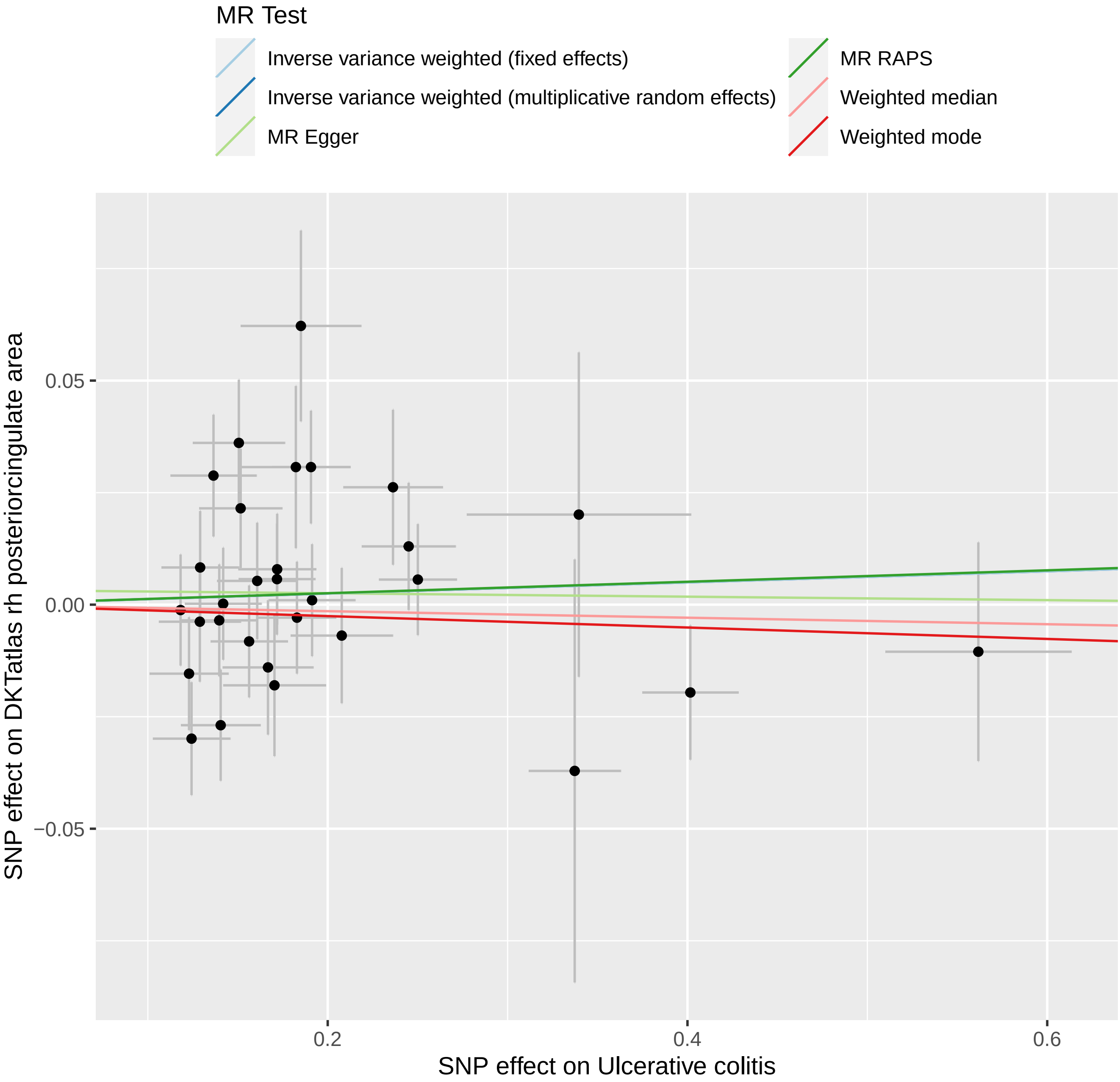

Batch 811 : Crohn's disease on DKTatlas rh precentral area

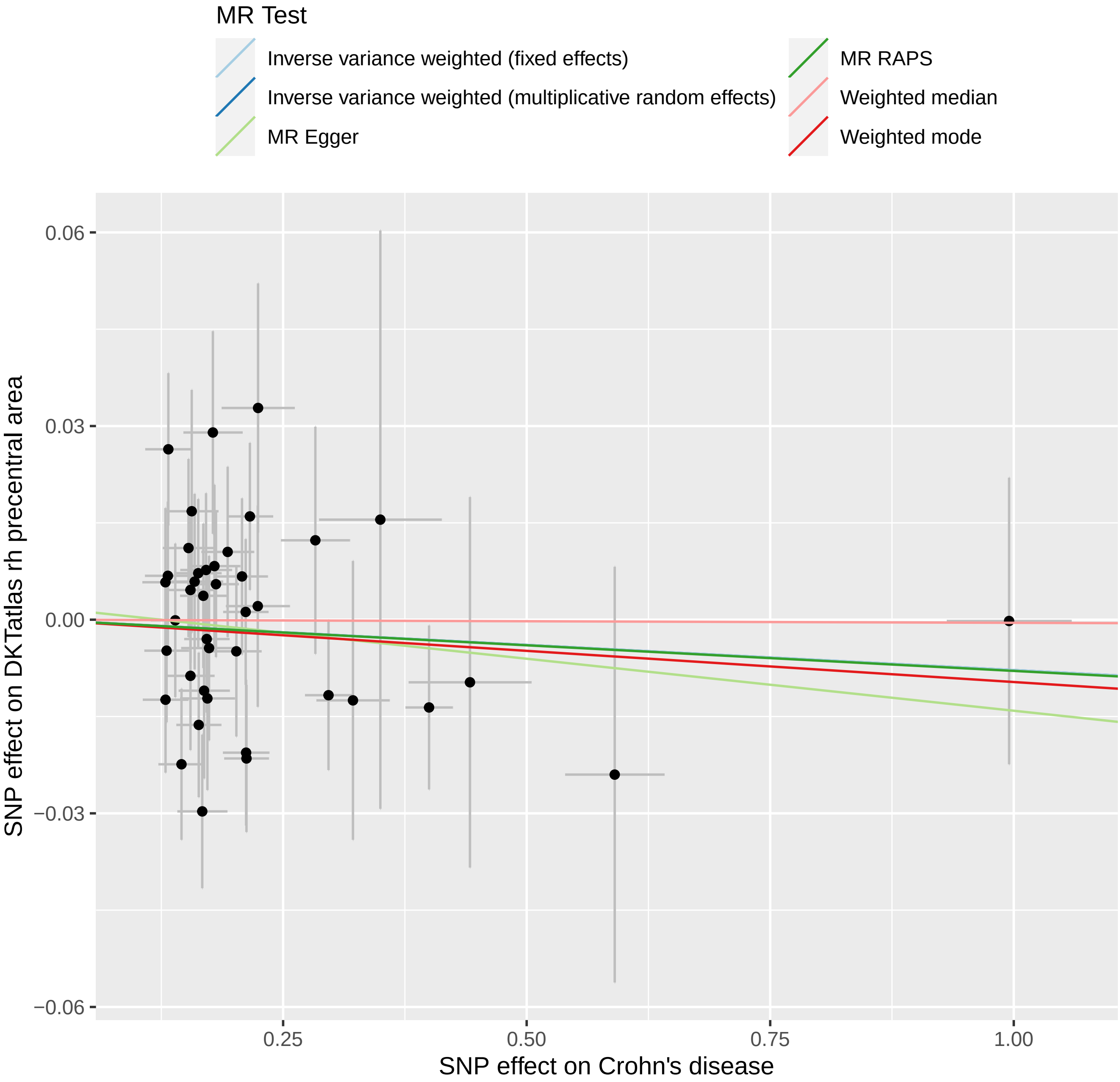

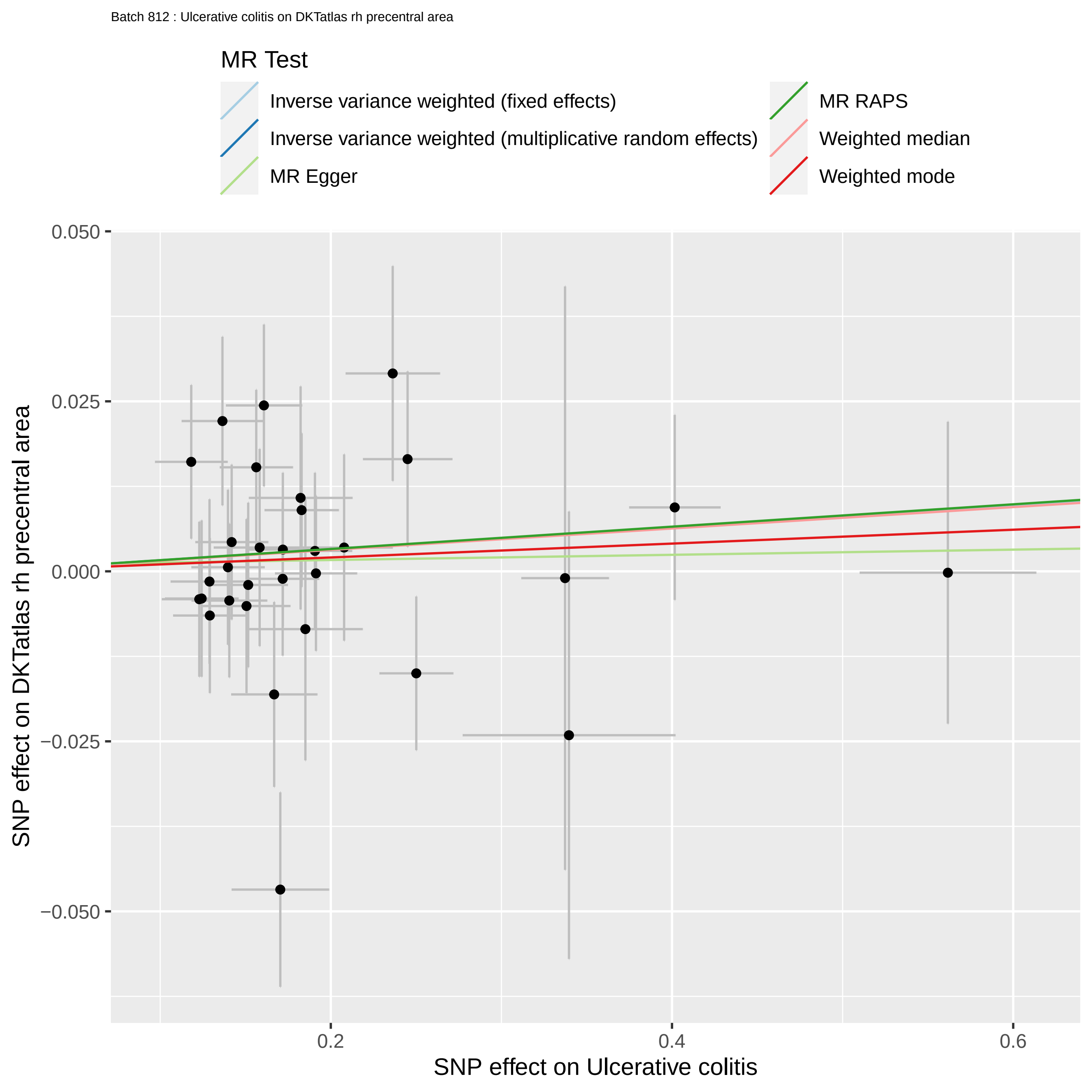

Batch 812 : Ulcerative colitis on DKTatlas rh precentral area
MR Test
Inverse variance weighted (fixed effects)
Inverse variance weighted (multiplicative random effects)
MR Egger
MR RAPS
Weighted median
Weighted mode
0.050
0.025
0.000
−0.025
−0.050
0.2
0.4
0.6
SNP effect on DKTatlas rh precentral area
SNP effect on Ulcerative colitis

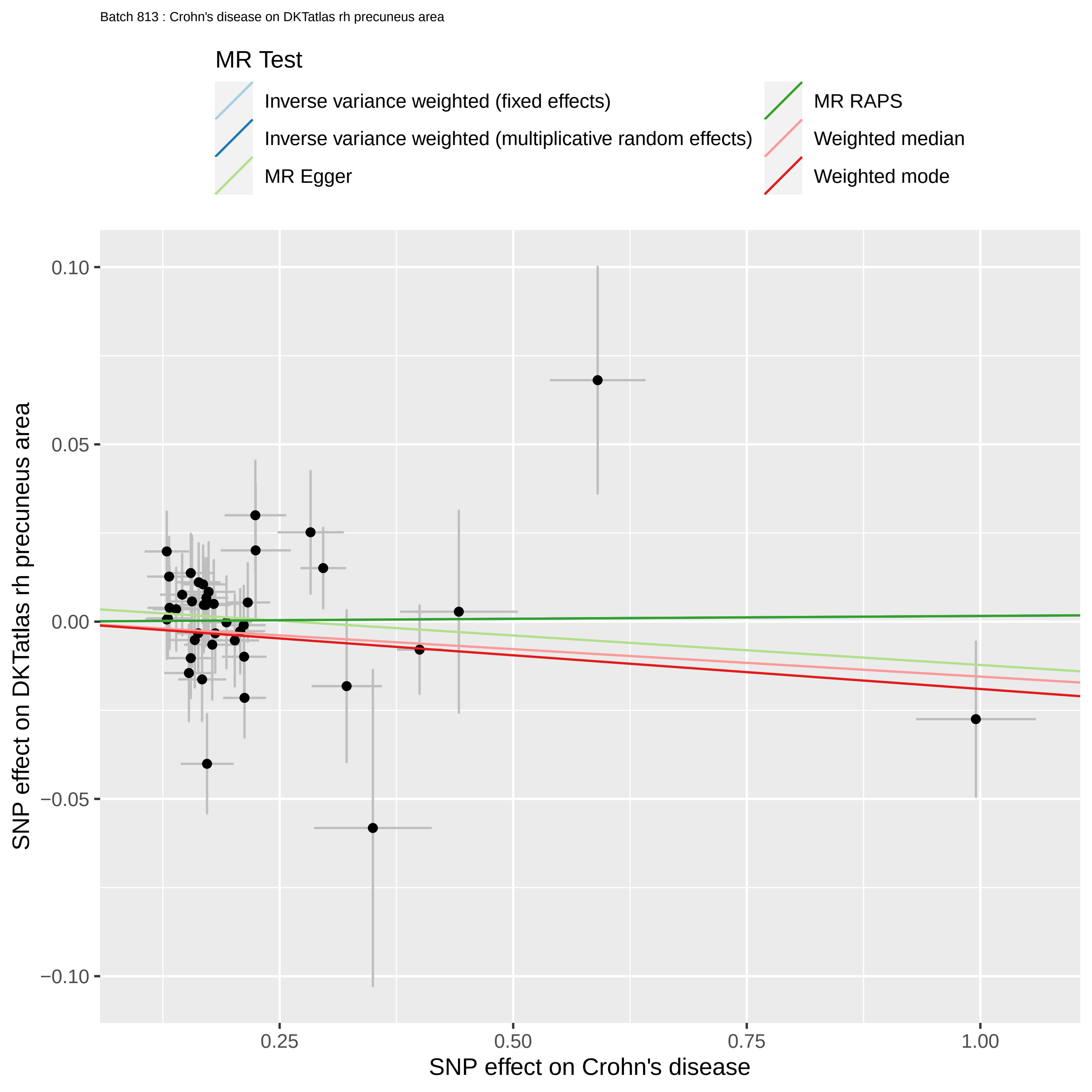

Batch 813 : Crohn's disease on DKTatlas rh precuneus area
MR Test
Inverse variance weighted (fixed effects)
Inverse variance weighted (multiplicative random effects)
MR Egger
MR RAPS
Weighted median
Weighted mode
SNP effect on DKTatlas rh precuneus area
0.10
0.05
0.00
−0.05
−0.10
0.25
0.50
0.75
1.00
SNP effect on Crohn's disease

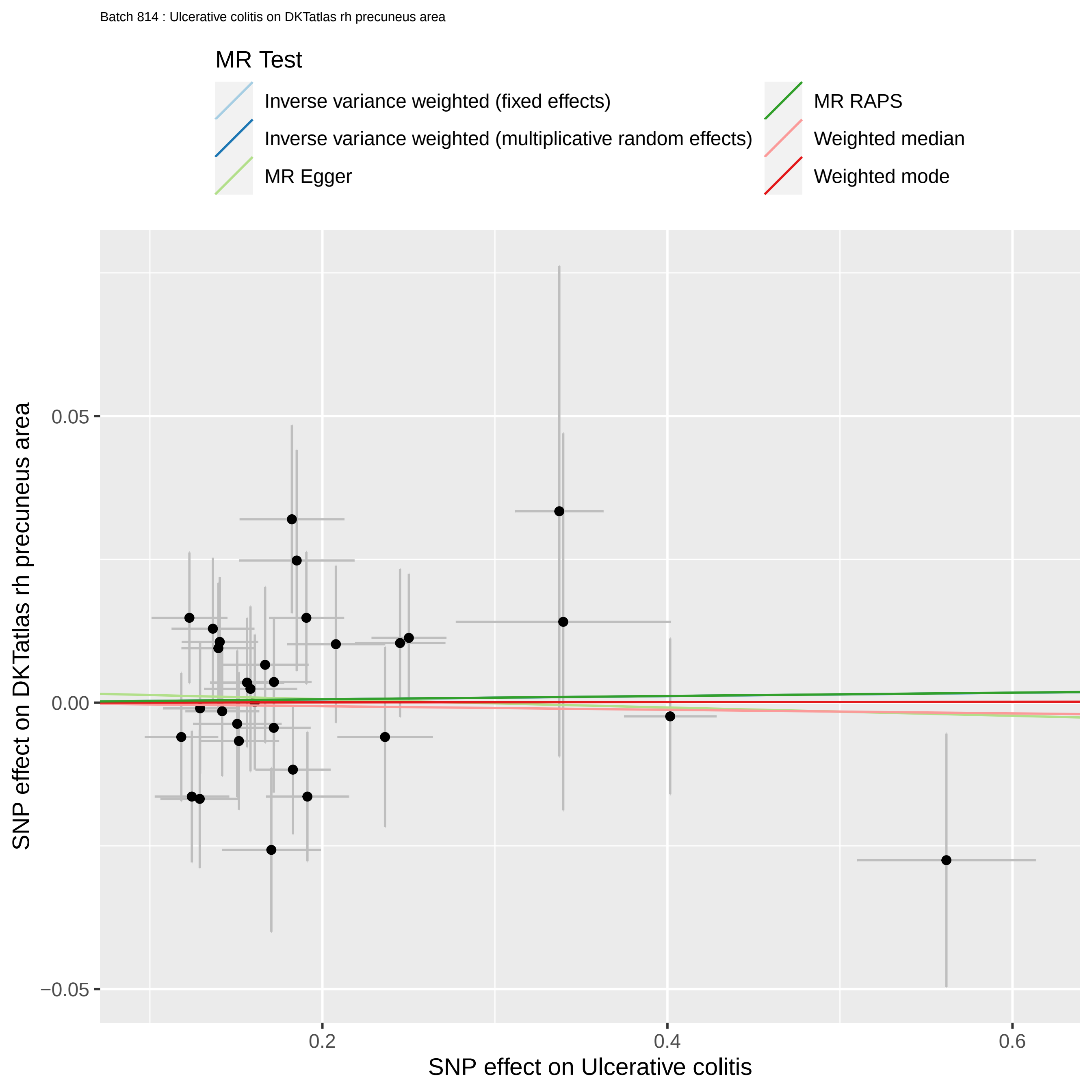

Batch 814 : Ulcerative colitis on DKTatlas rh precuneus area
MR Test
Inverse variance weighted (fixed effects)
Inverse variance weighted (multiplicative random effects)
MR Egger
MR RAPS
Weighted median
Weighted mode
SNP effect on DKTatlas rh precuneus area
0.05
0.00
−0.05
0.2
0.4
0.6
SNP effect on Ulcerative colitis

Batch 815 : Crohn's disease on DKTatlas rh rostralanteriorcingulate area

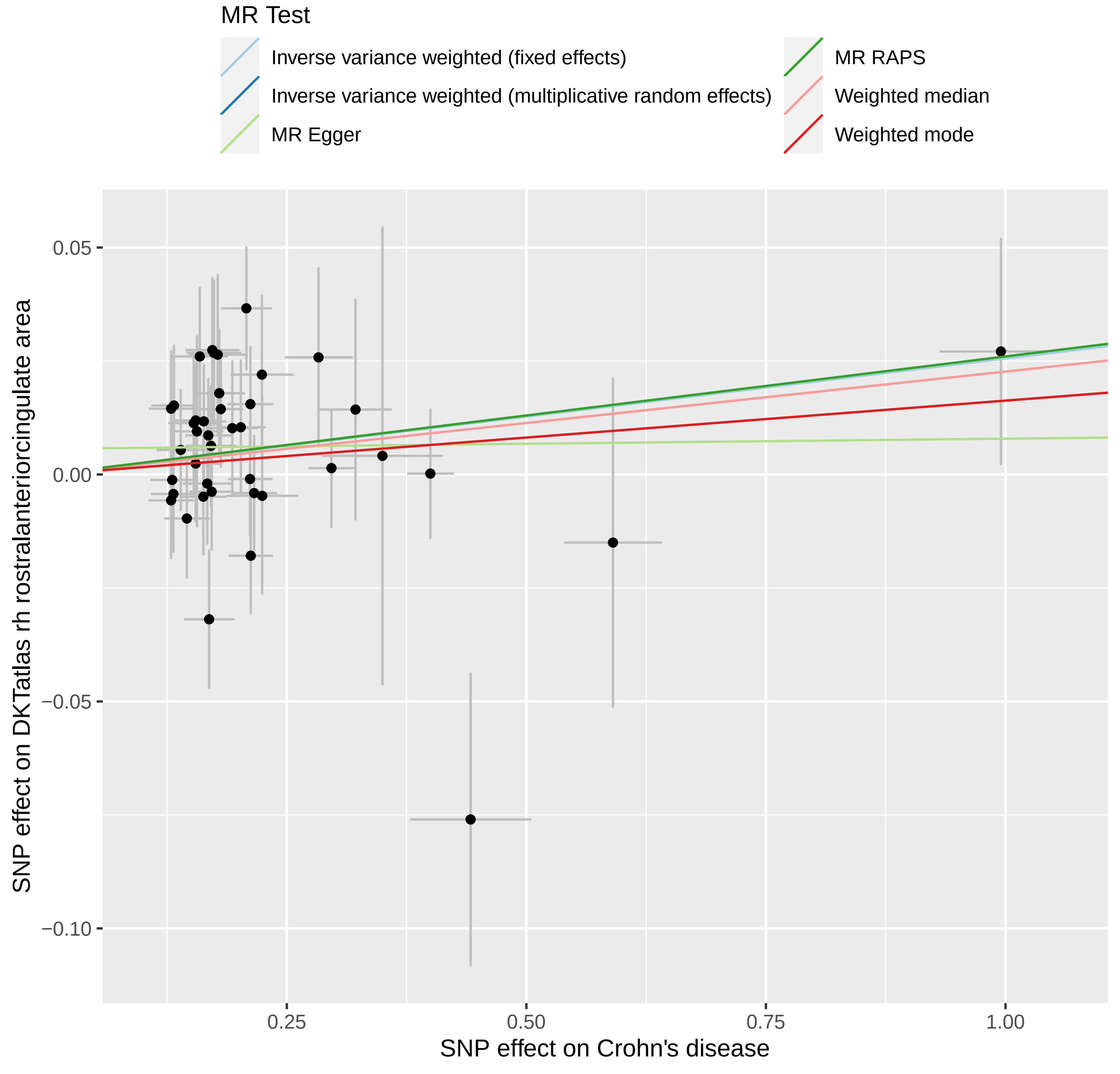

Batch 816 : Ulcerative colitis on DKTatlas rh rostralanteriorcingulate area

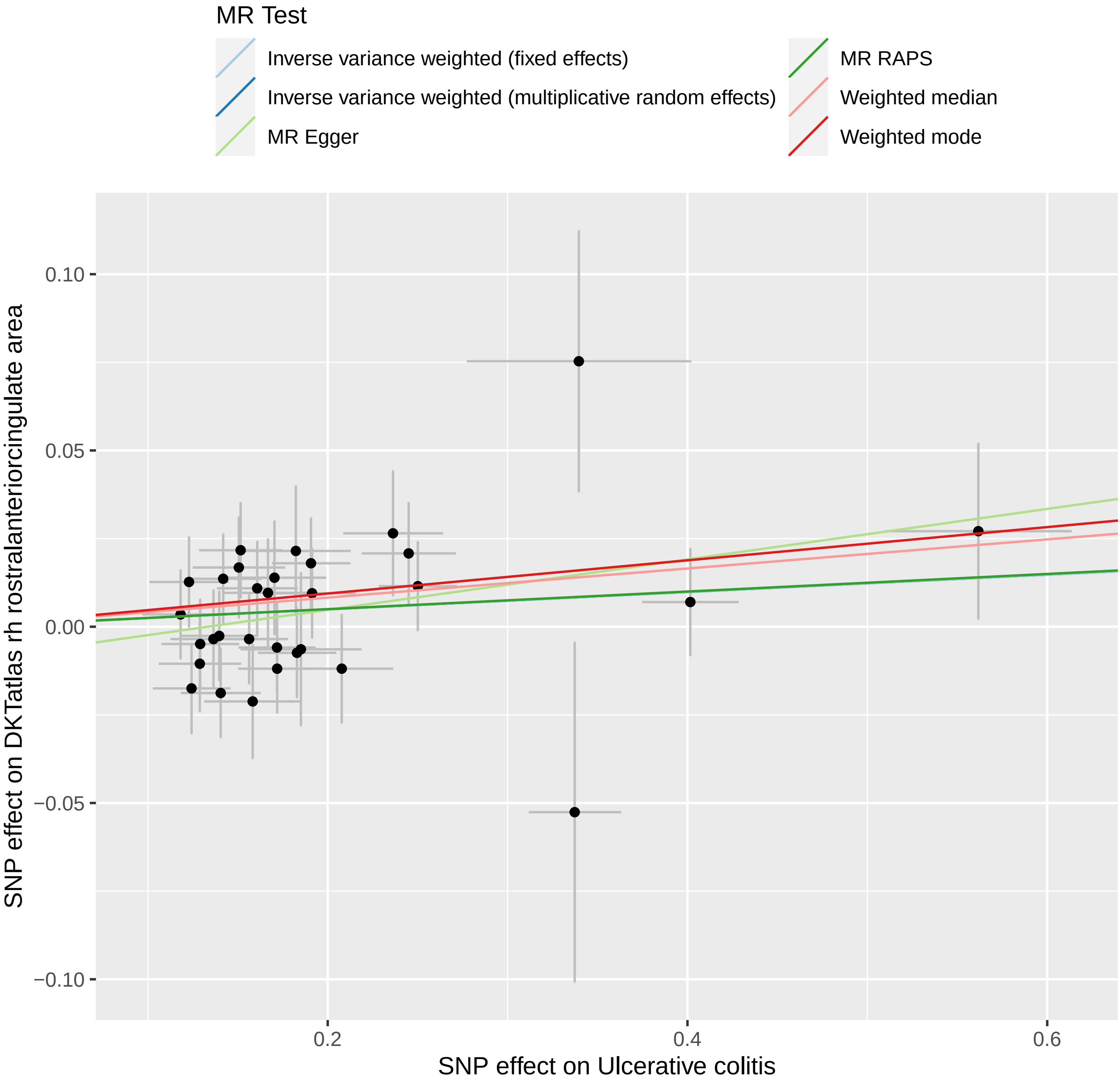

Batch 817 : Crohn's disease on DKTatlas rh rostralmiddlefrontal area

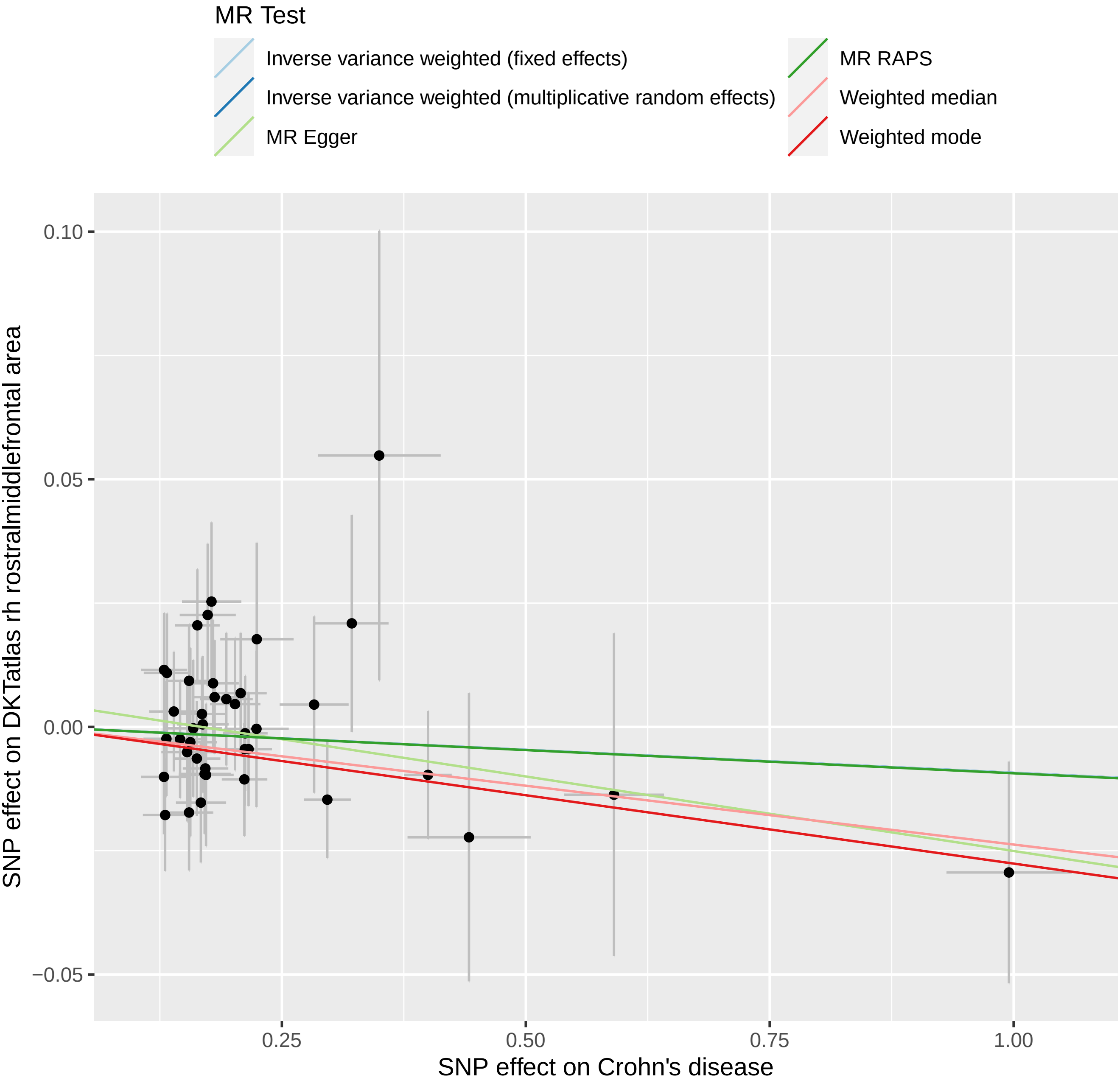

Batch 818 : Ulcerative colitis on DKTatlas rh rostralmiddlefrontal area

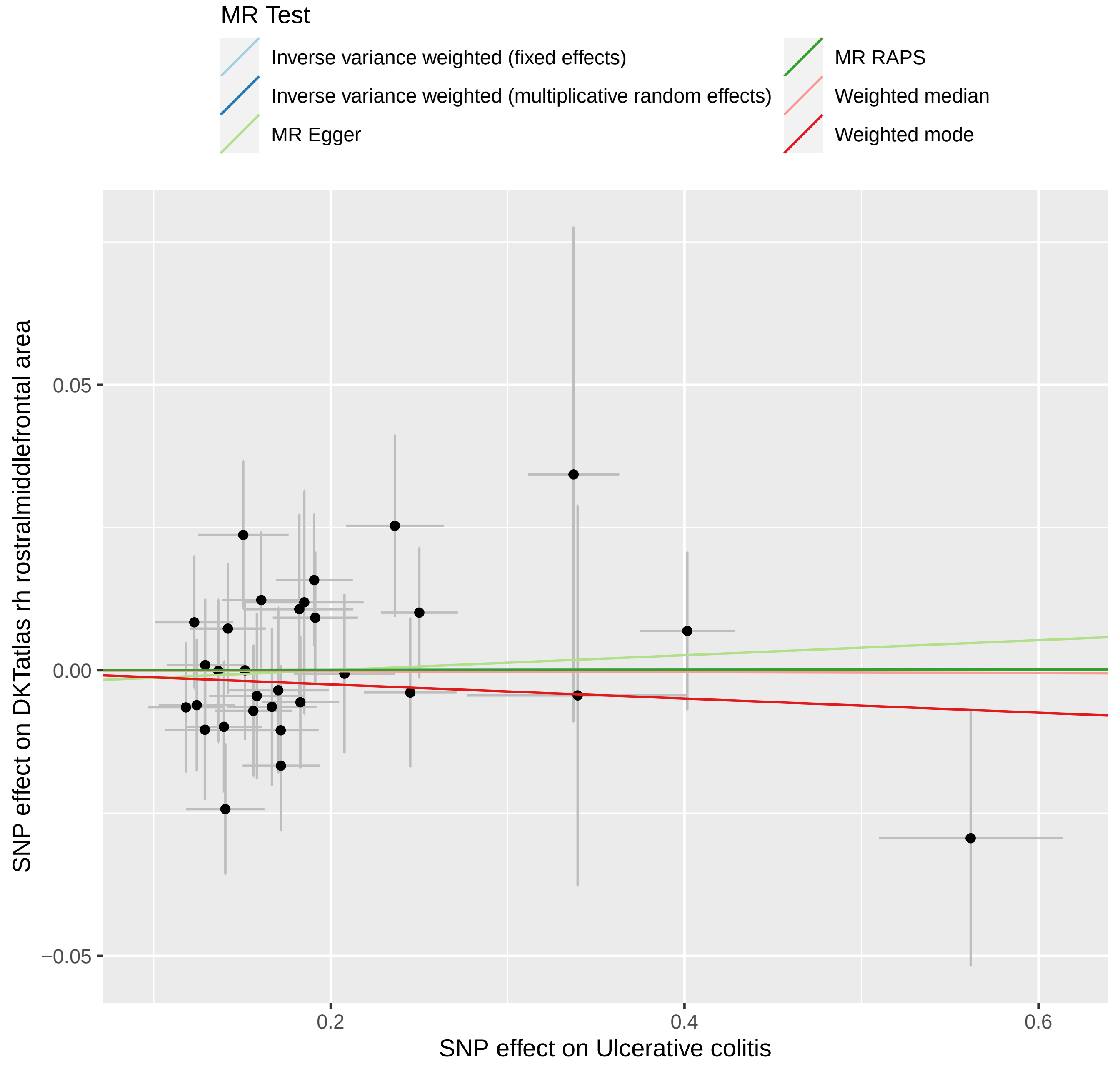

Batch 819 : Crohn's disease on DKTatlas rh superiorfrontal area

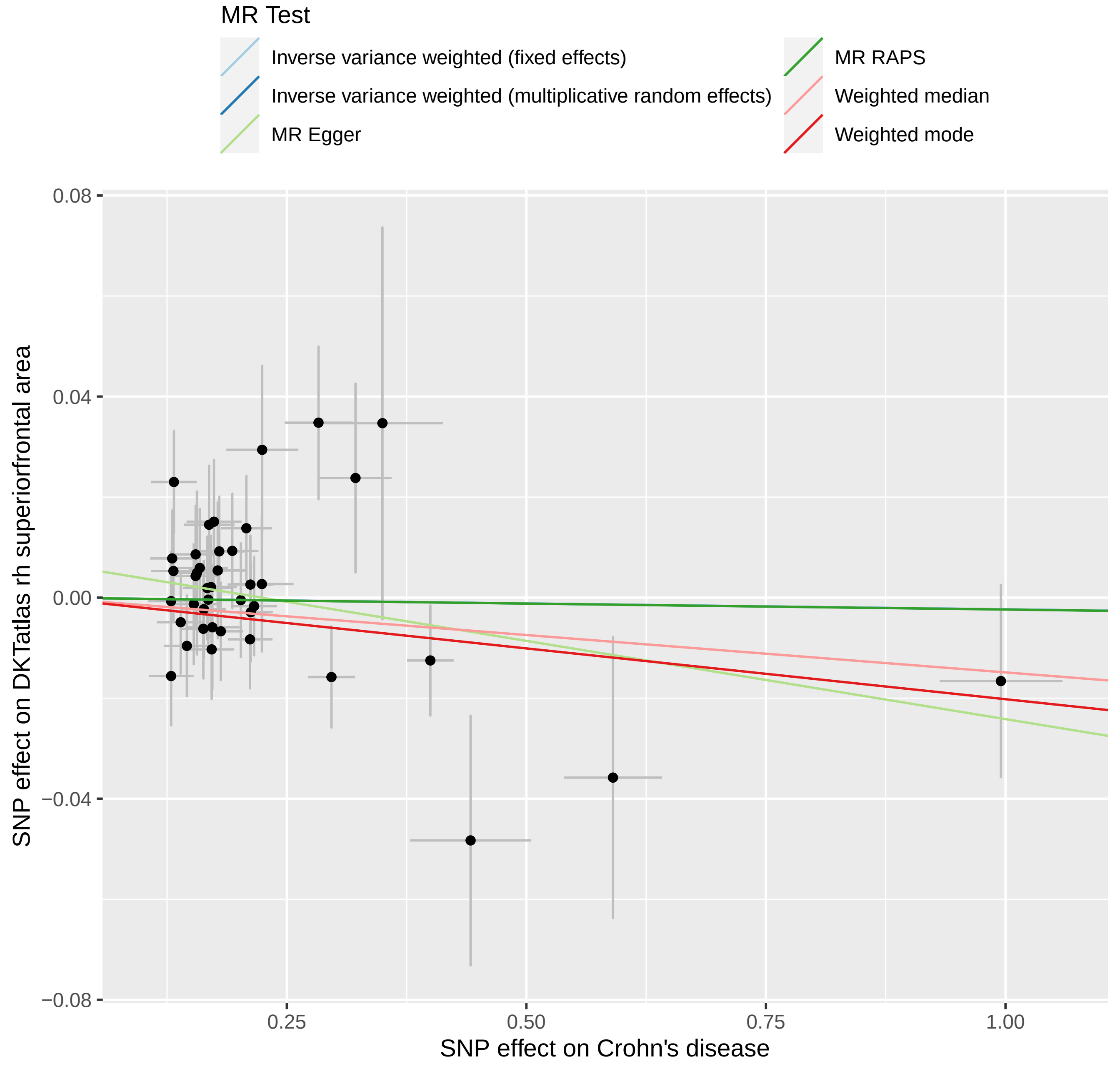

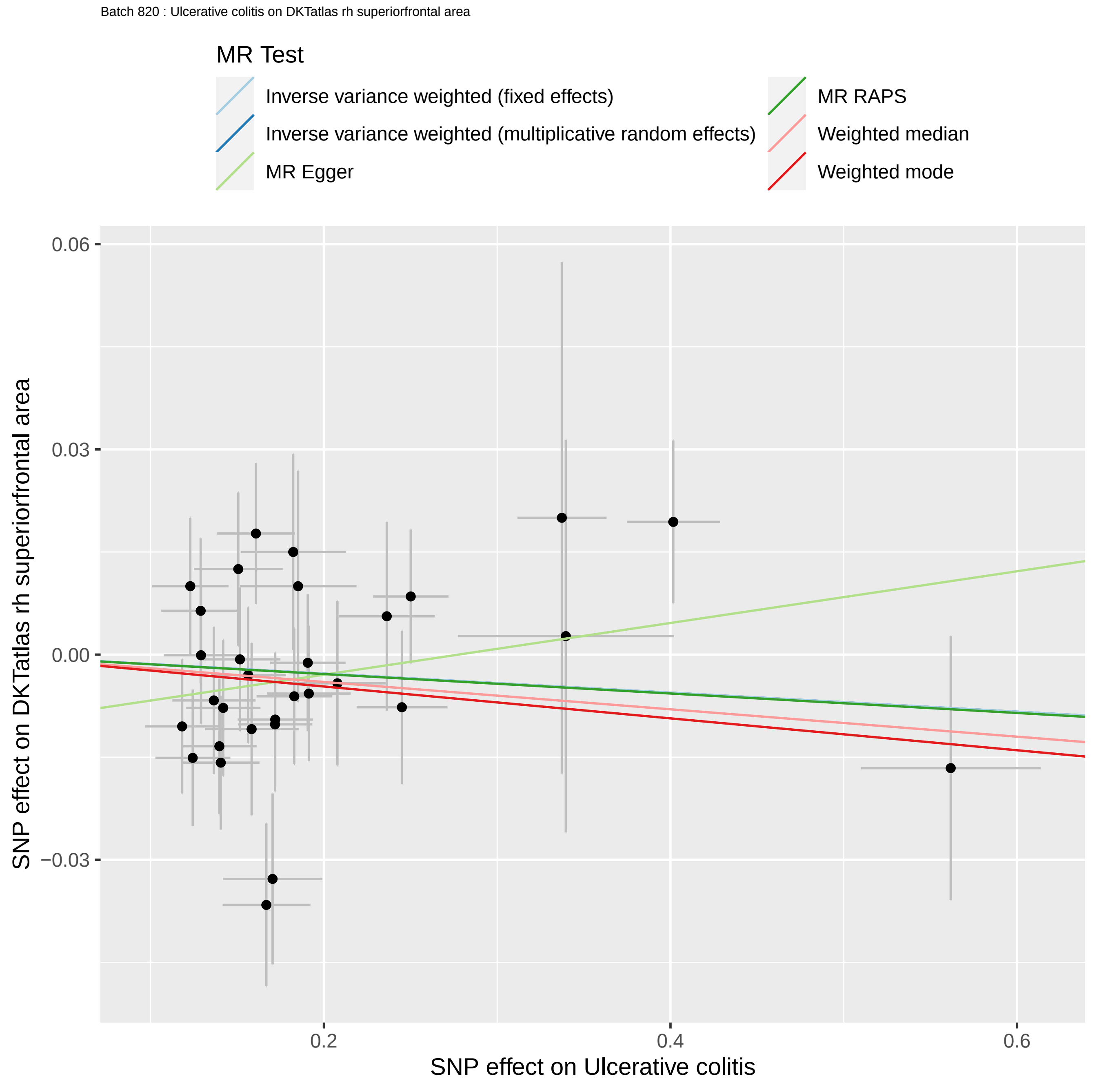

Batch 820 : Ulcerative colitis on DKTatlas rh superiorfrontal area
MR Test
Inverse variance weighted (fixed effects)
Inverse variance weighted (multiplicative random effects)
MR Egger
MR RAPS
Weighted median
Weighted mode
0.06
0.03
0.00
−0.03
0.2
0.4
0.6
SNP effect on DKTatlas rh superiorfrontal area
SNP effect on Ulcerative colitis

Batch 821 : Crohn's disease on DKTatlas rh superiorparietal area

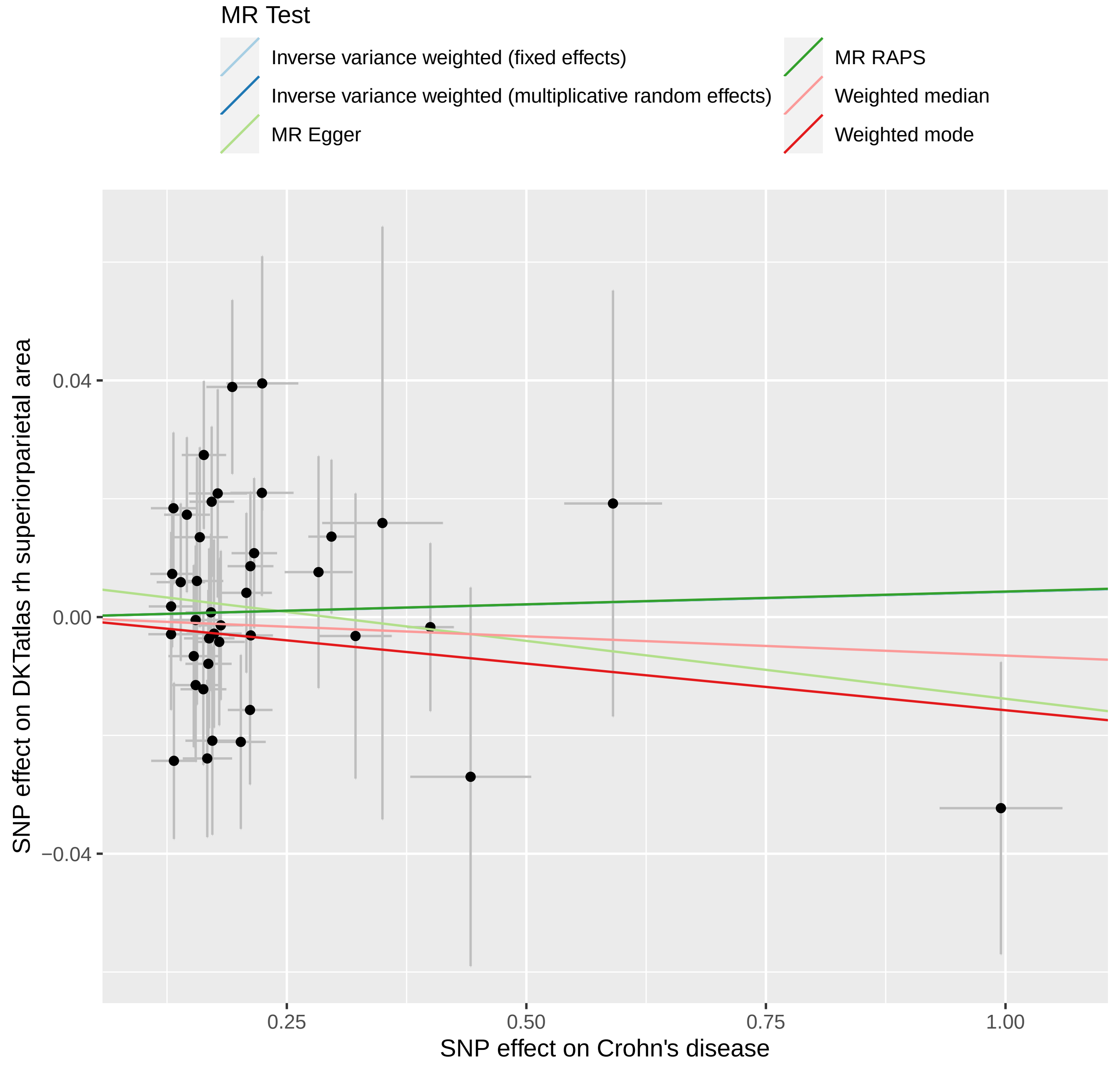

Batch 822 : Ulcerative colitis on DKTatlas rh superiorparietal area

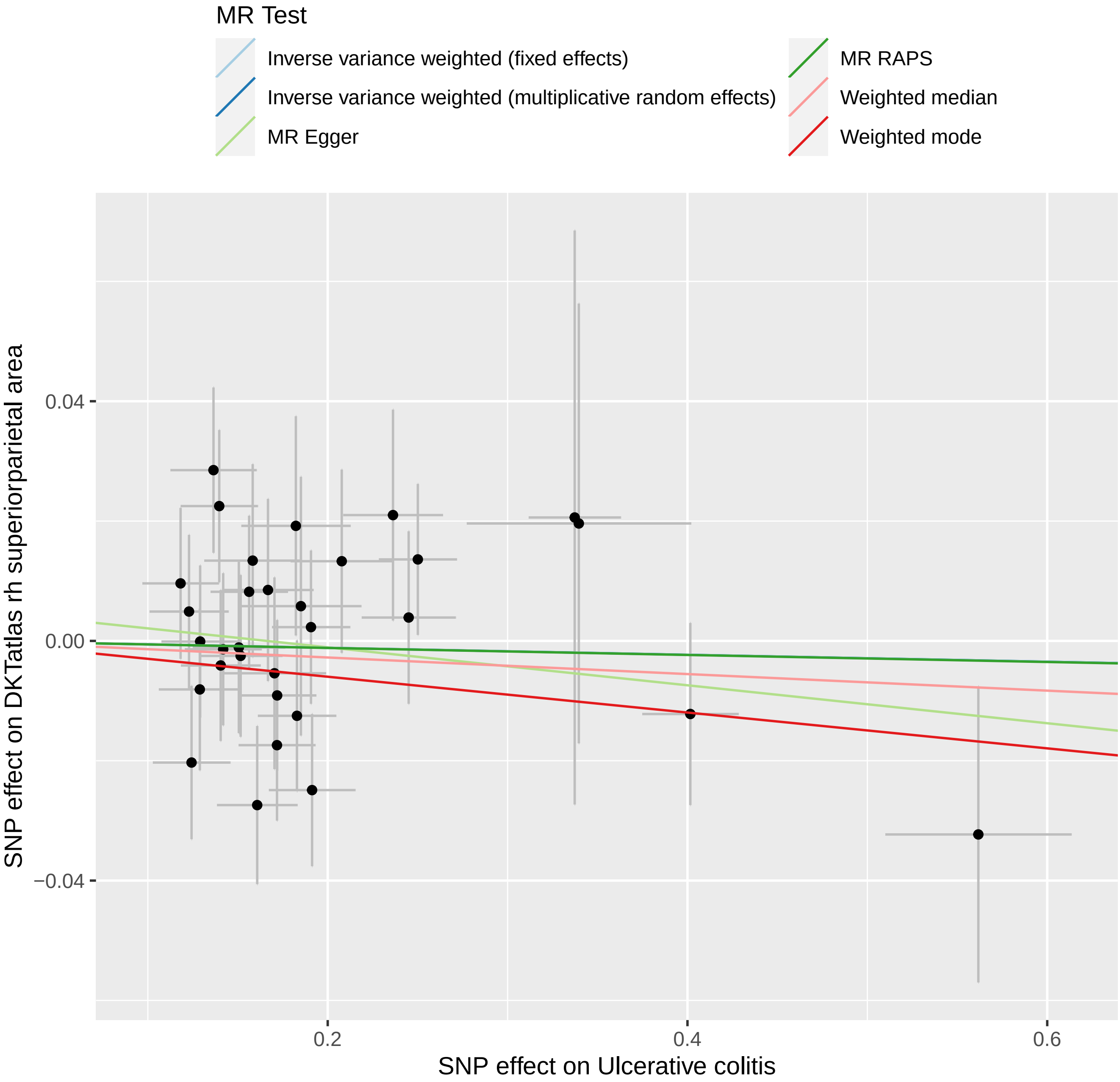

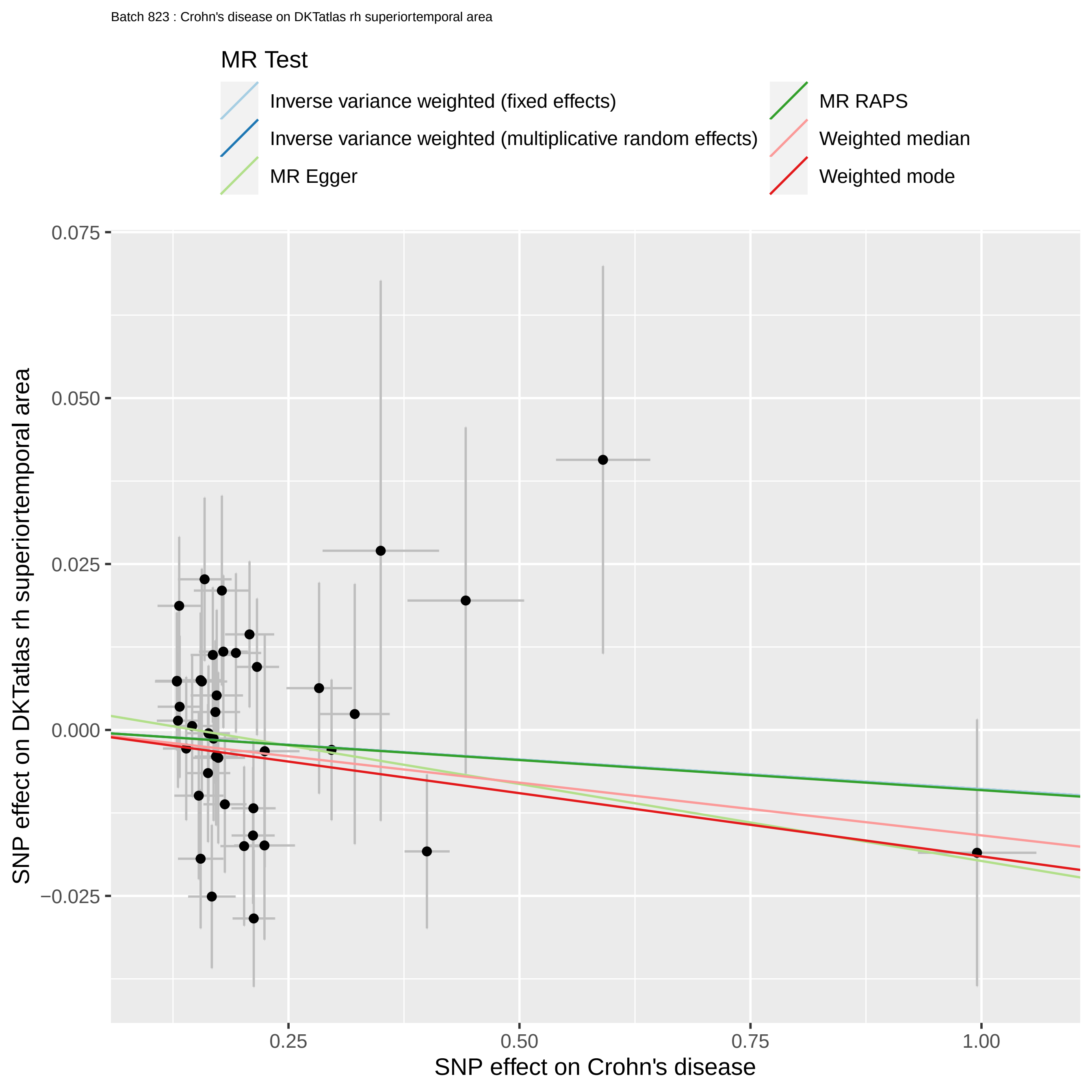
Batch 823 : Crohn's disease on DKTatlas rh superiortemporal area
MR Test
Inverse variance weighted (fixed effects)
Inverse variance weighted (multiplicative random effects)
MR Egger
MR RAPS
Weighted median
Weighted mode
0.075
0.050
0.025
0.000
−0.025
0.25
0.50
0.75
1.00
SNP effect on DKTatlas rh superiortemporal area
SNP effect on Crohn's disease

Batch 824 : Ulcerative colitis on DKTatlas rh superiortemporal area

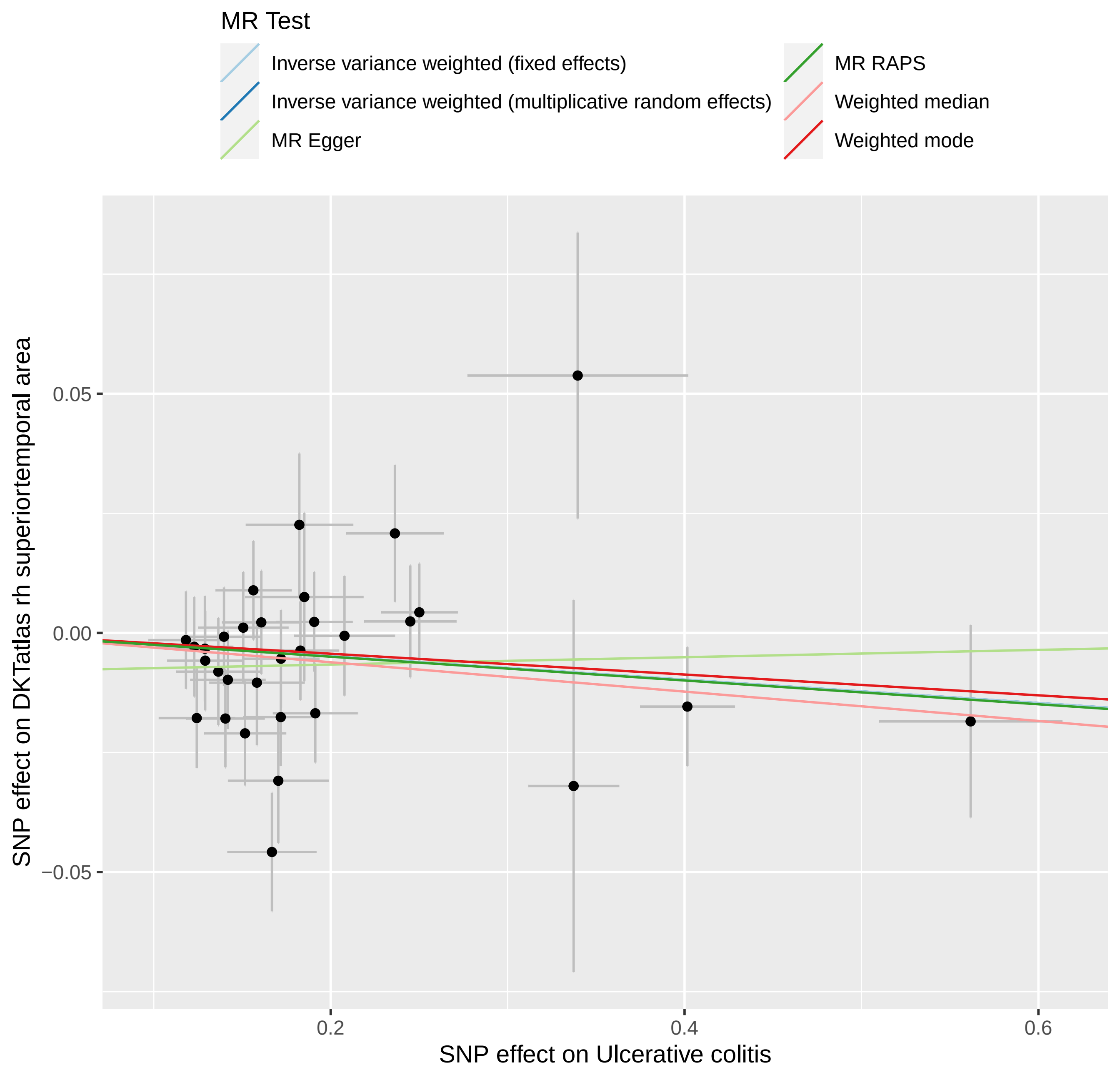

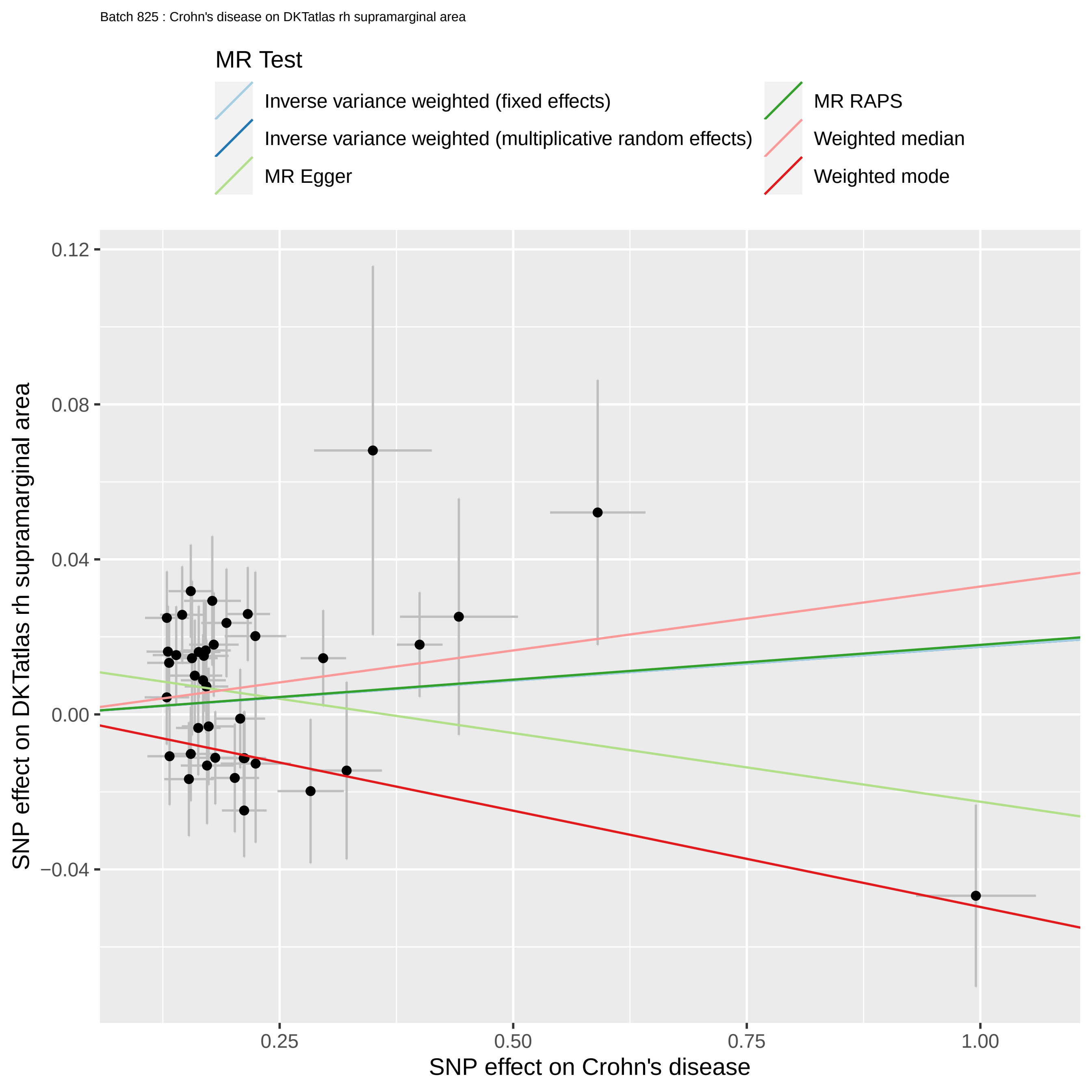
Batch 825 : Crohn's disease on DKTatlas rh supramarginal area
MR Test
Inverse variance weighted (fixed effects)
Inverse variance weighted (multiplicative random effects)
MR Egger
MR RAPS
Weighted median
Weighted mode
0.12
0.08
0.04
0.00
−0.04
0.25
0.50
0.75
1.00
SNP effect on DKTatlas rh supramarginal area
SNP effect on Crohn's disease

Batch 826 : Ulcerative colitis on DKTatlas rh supramarginal area

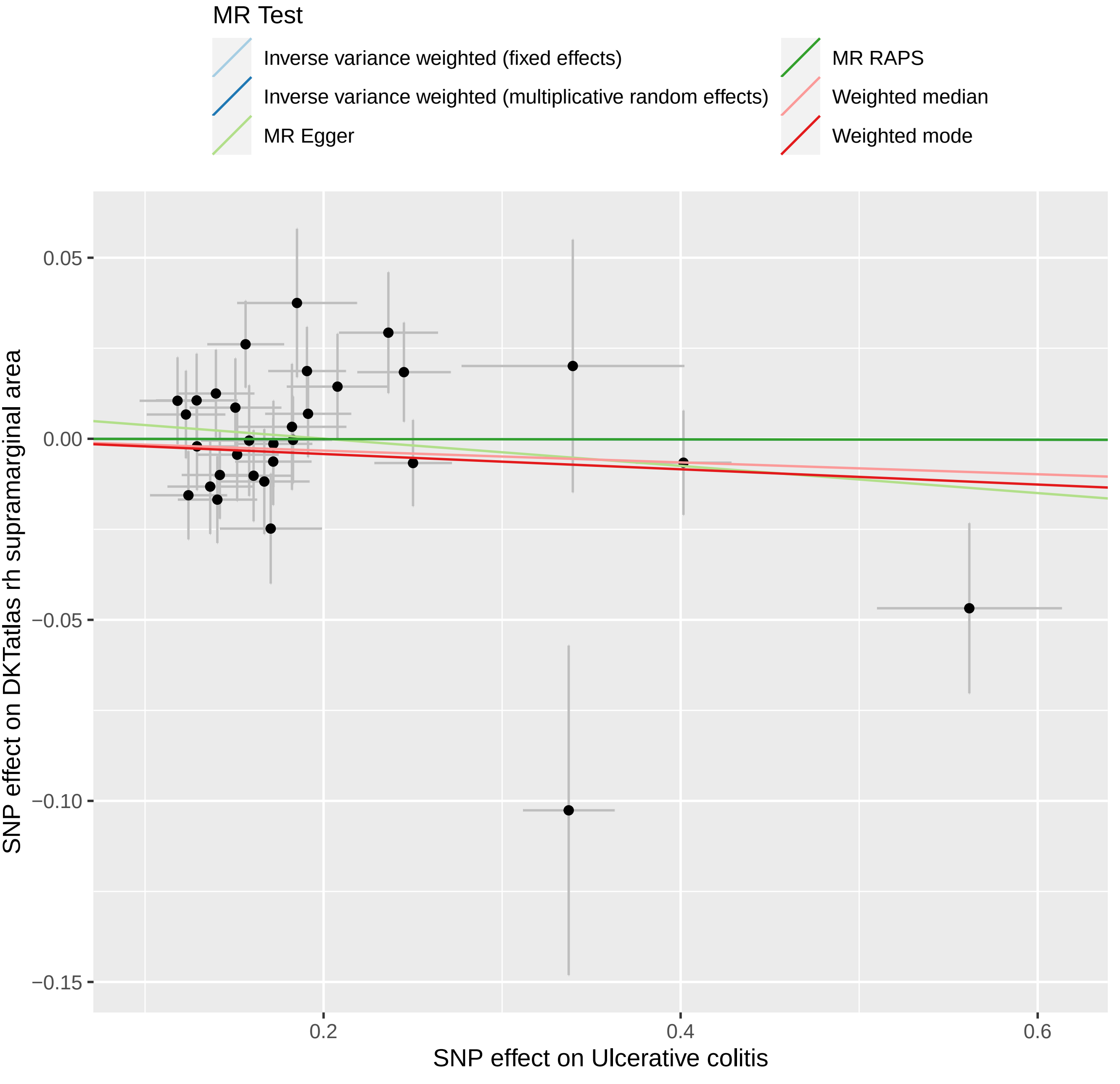

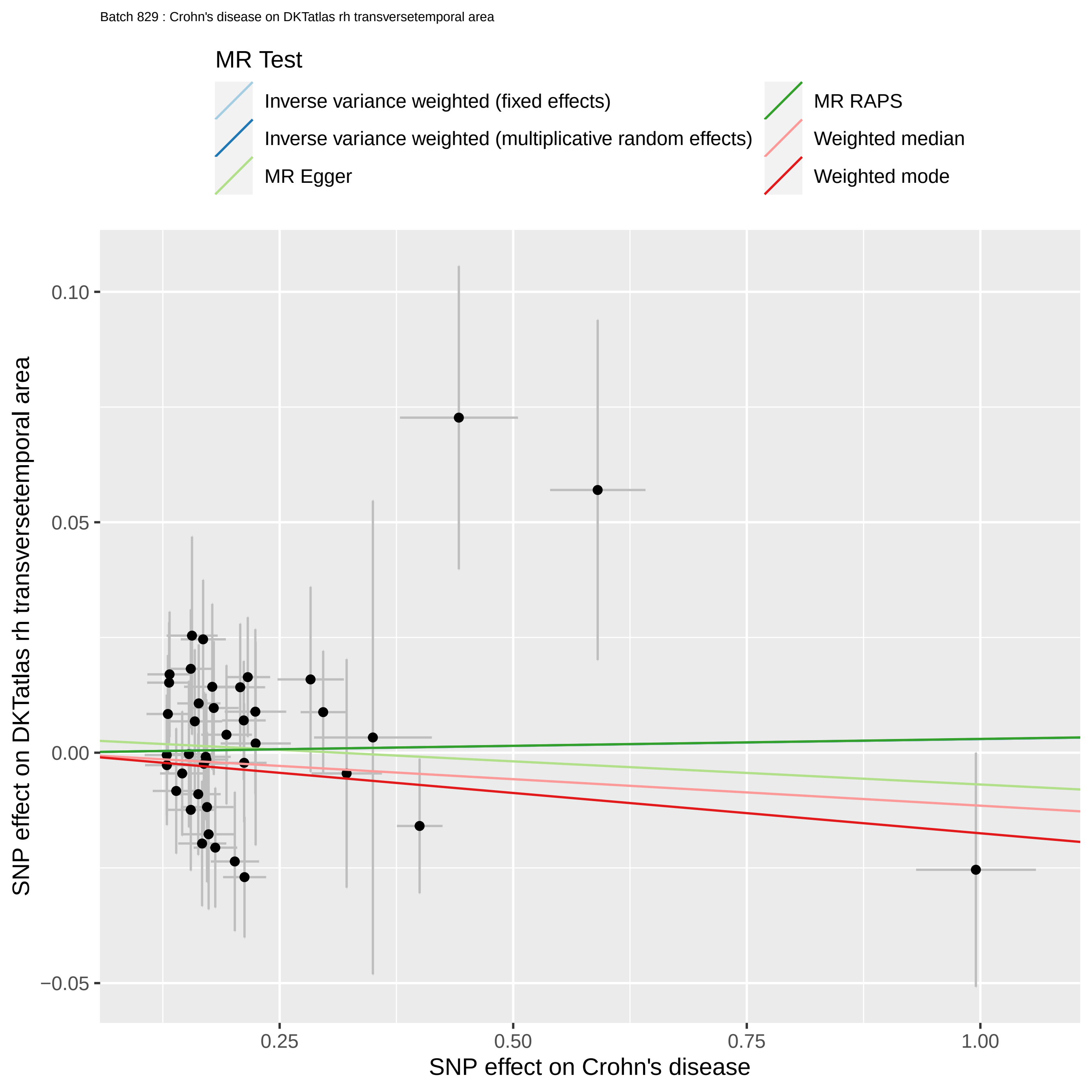
Batch 829 : Crohn's disease on DKTatlas rh transversetemporal area
MR Test
Inverse variance weighted (fixed effects)
Inverse variance weighted (multiplicative random effects)
MR Egger
MR RAPS
Weighted median
Weighted mode
0.10
0.05
0.00
−0.05
0.25
0.50
0.75
1.00
SNP effect on DKTatlas rh transversetemporal area
SNP effect on Crohn's disease

Batch 830 : Ulcerative colitis on DKTatlas rh transversetemporal area

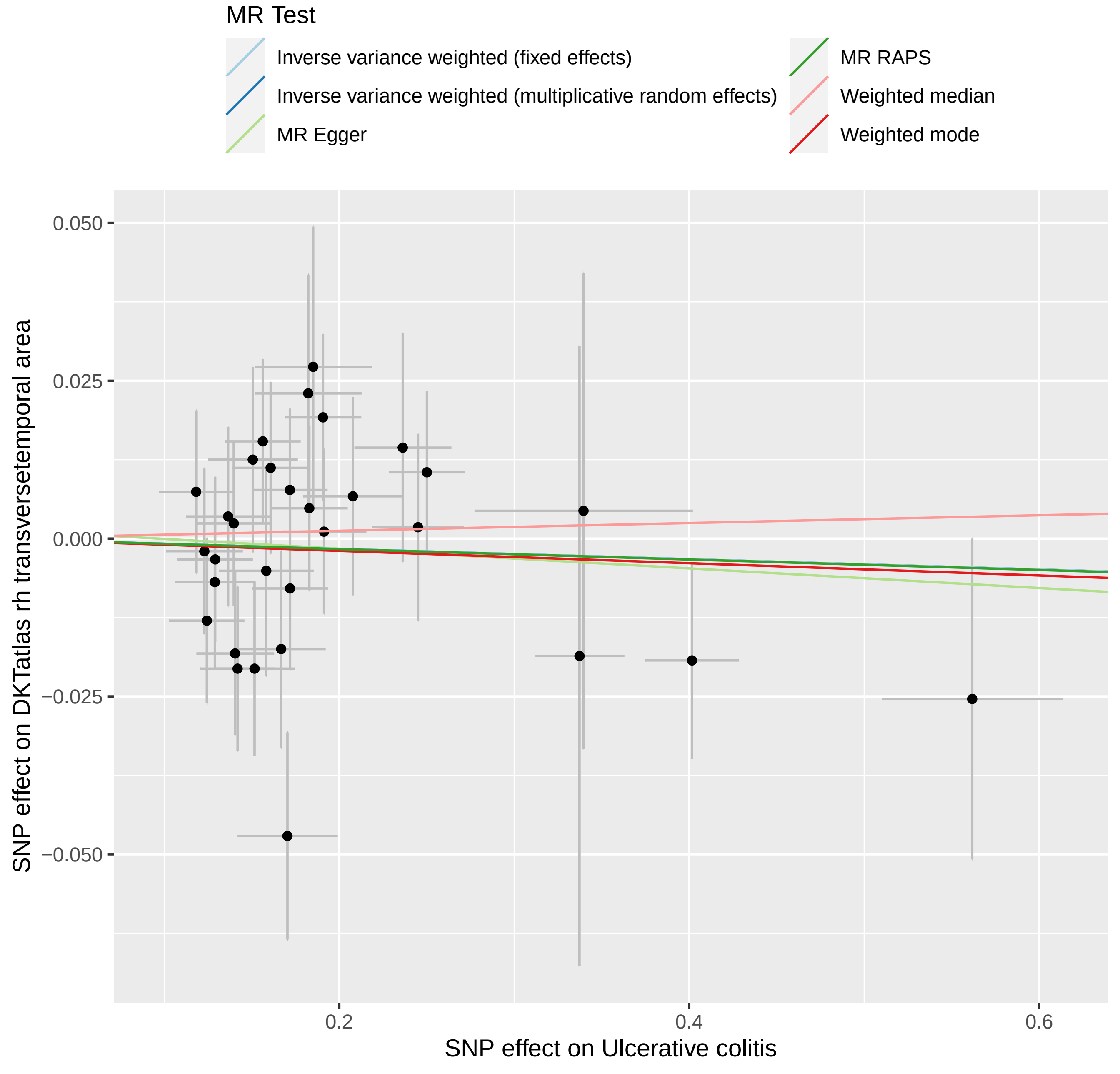

Batch 831 : Crohn's disease on DKTatlas rh insula area

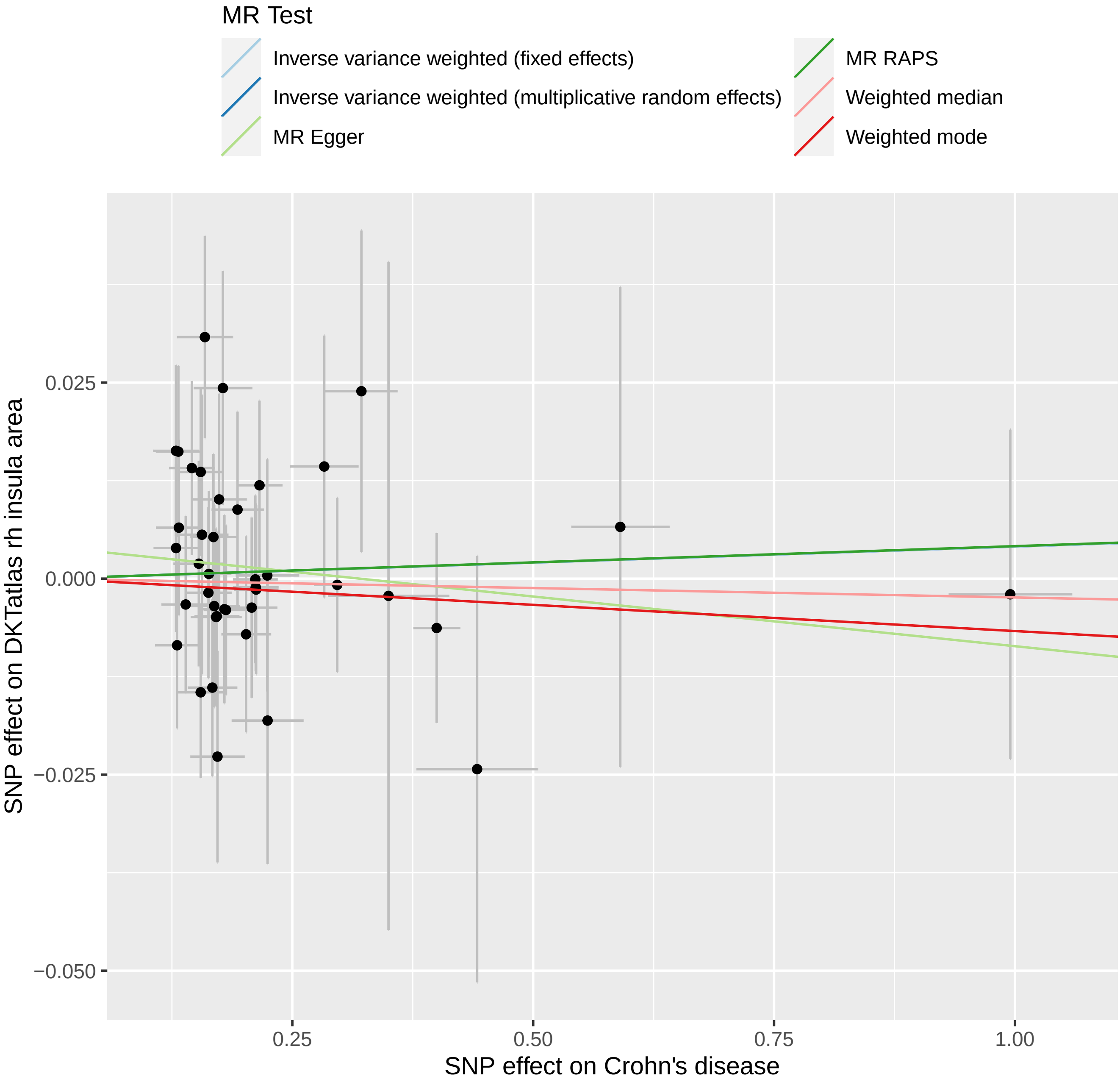

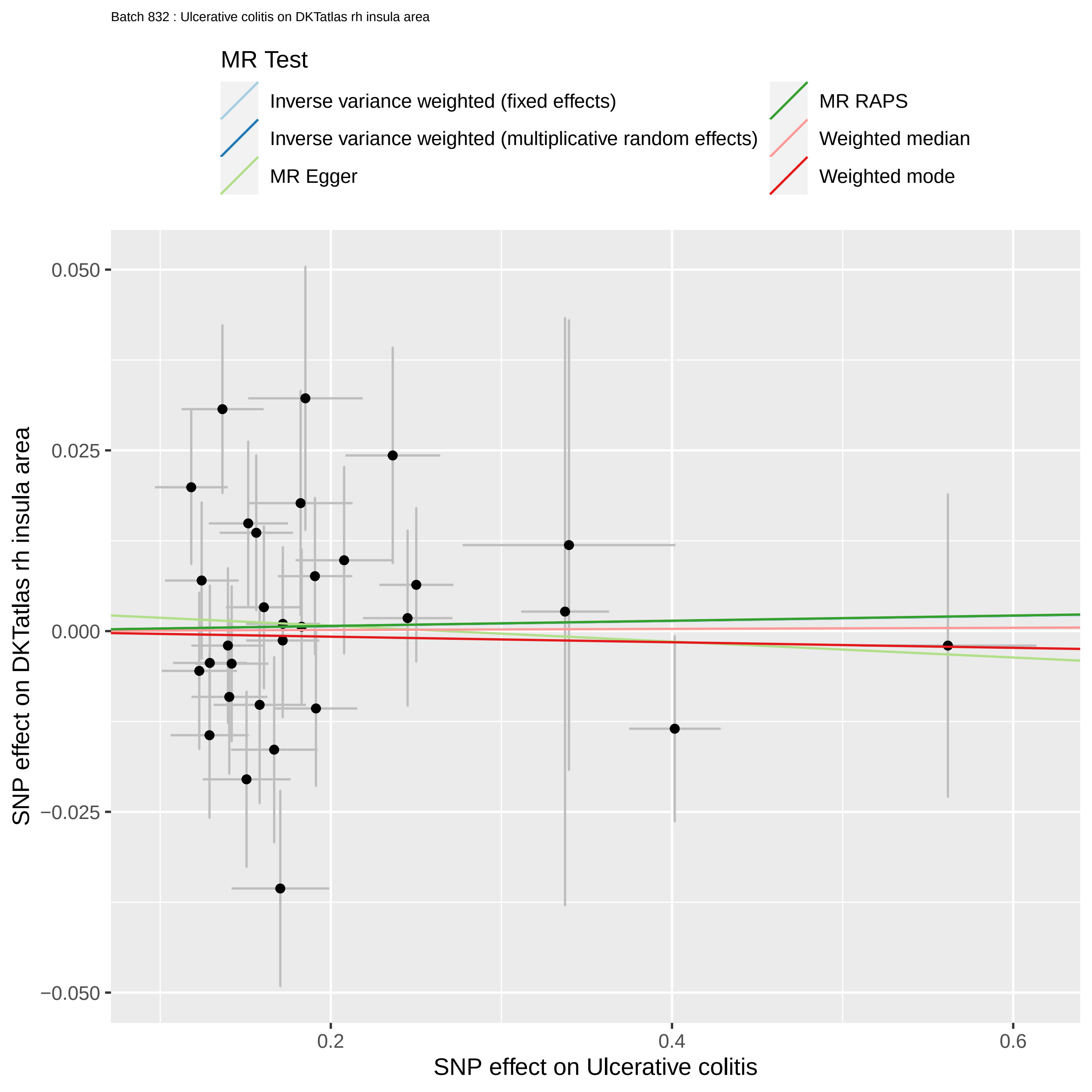

Batch 832 : Ulcerative colitis on DKTatlas rh insula area
MR Test
Inverse variance weighted (fixed effects)
Inverse variance weighted (multiplicative random effects)
MR Egger
MR RAPS
Weighted median
Weighted mode
SNP effect on DKTatlas rh insula area
0.050
0.025
0.000
−0.025
−0.050
0.2
0.4
0.6
SNP effect on Ulcerative colitis

Batch 833 : Crohn's disease on DKTatlas rh WhiteSurfArea area

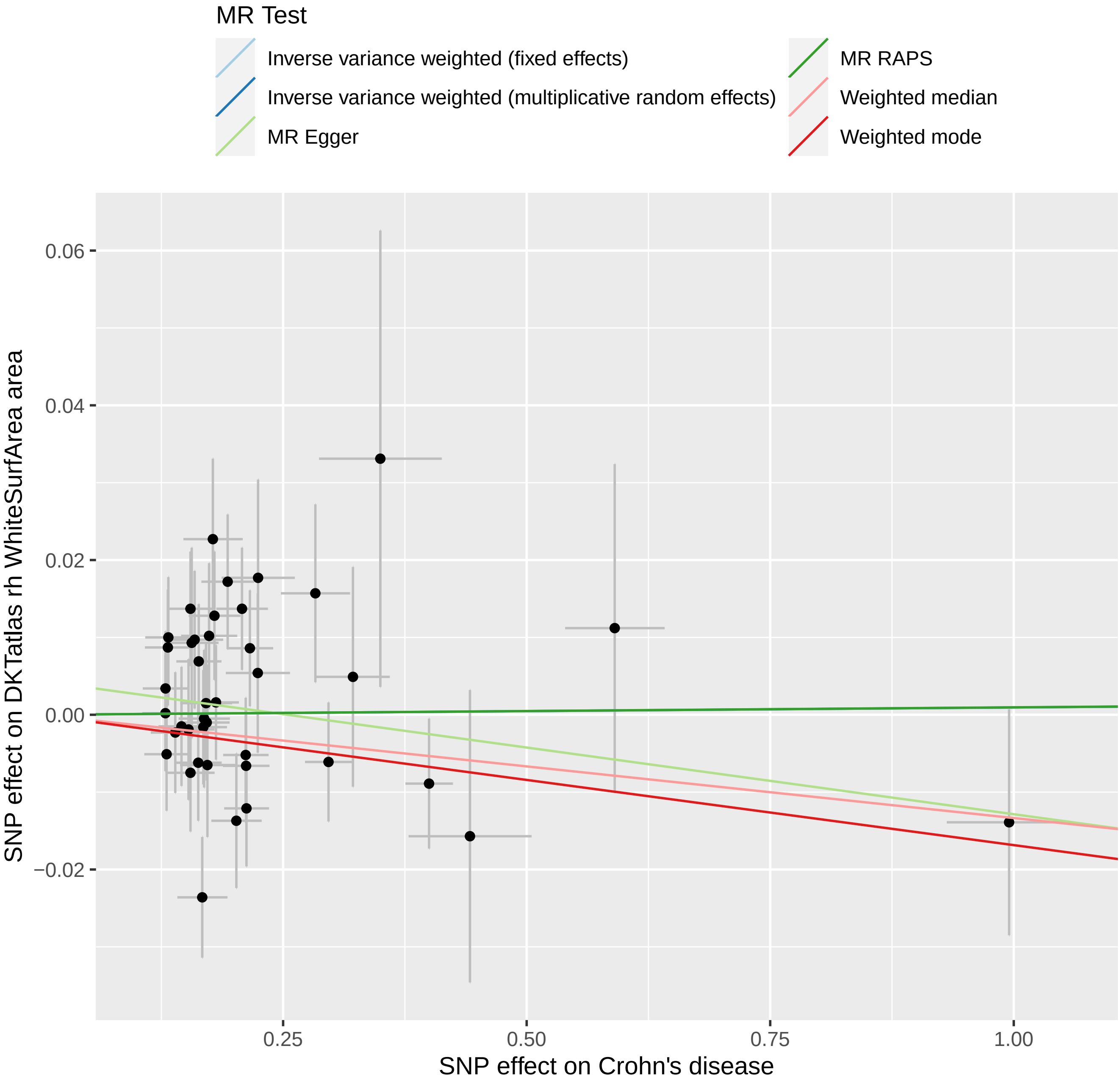

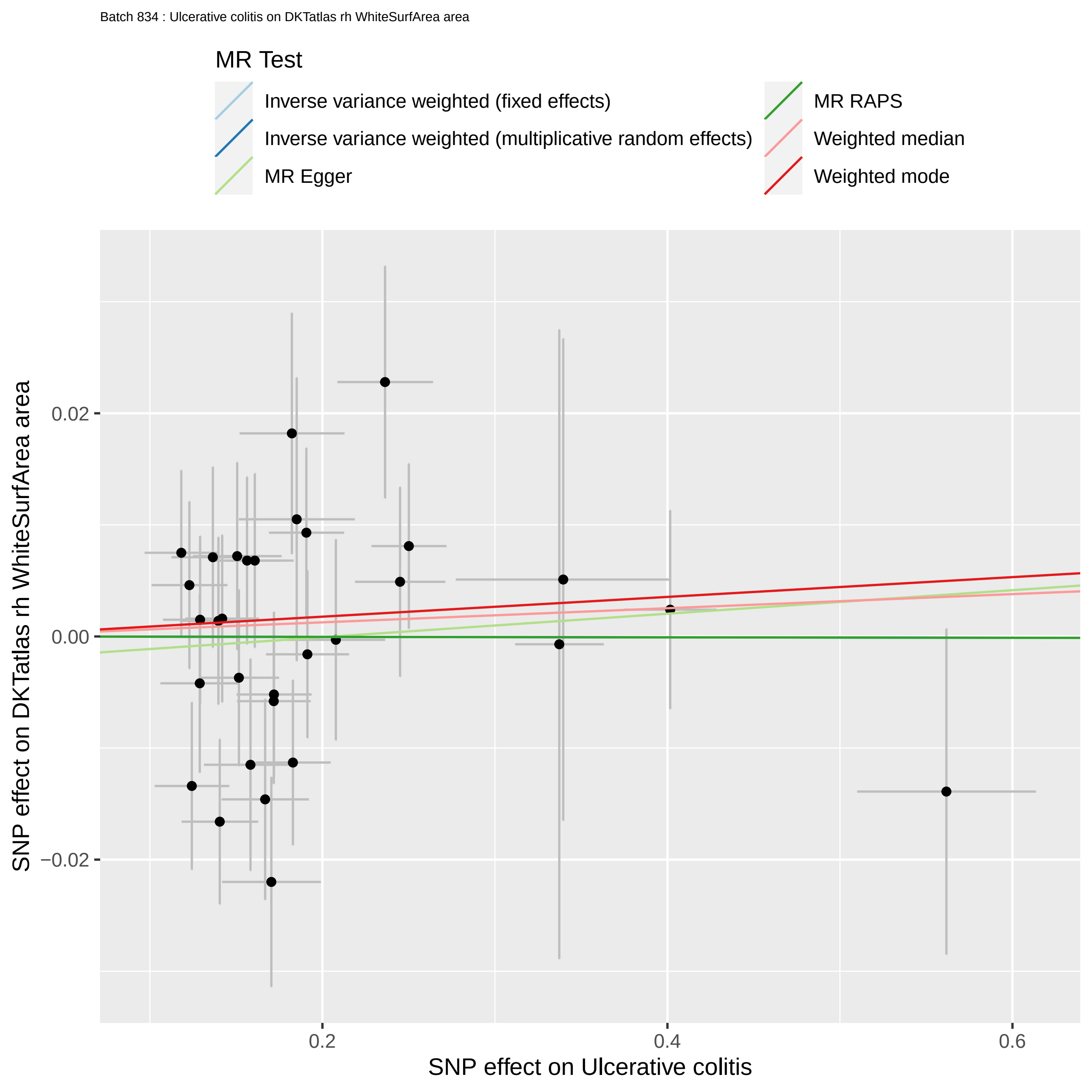
Batch 834 : Ulcerative colitis on DKTatlas rh WhiteSurfArea area
MR Test
Inverse variance weighted (fixed effects)
Inverse variance weighted (multiplicative random effects)
MR Egger
MR RAPS
Weighted median
Weighted mode
0.02
0.00
−0.02
0.2
0.4
0.6
SNP effect on DKTatlas rh WhiteSurfArea area
SNP effect on Ulcerative colitis

Batch 937 : Crohn's disease on IDP T1 FAST ROIs R insular cortex

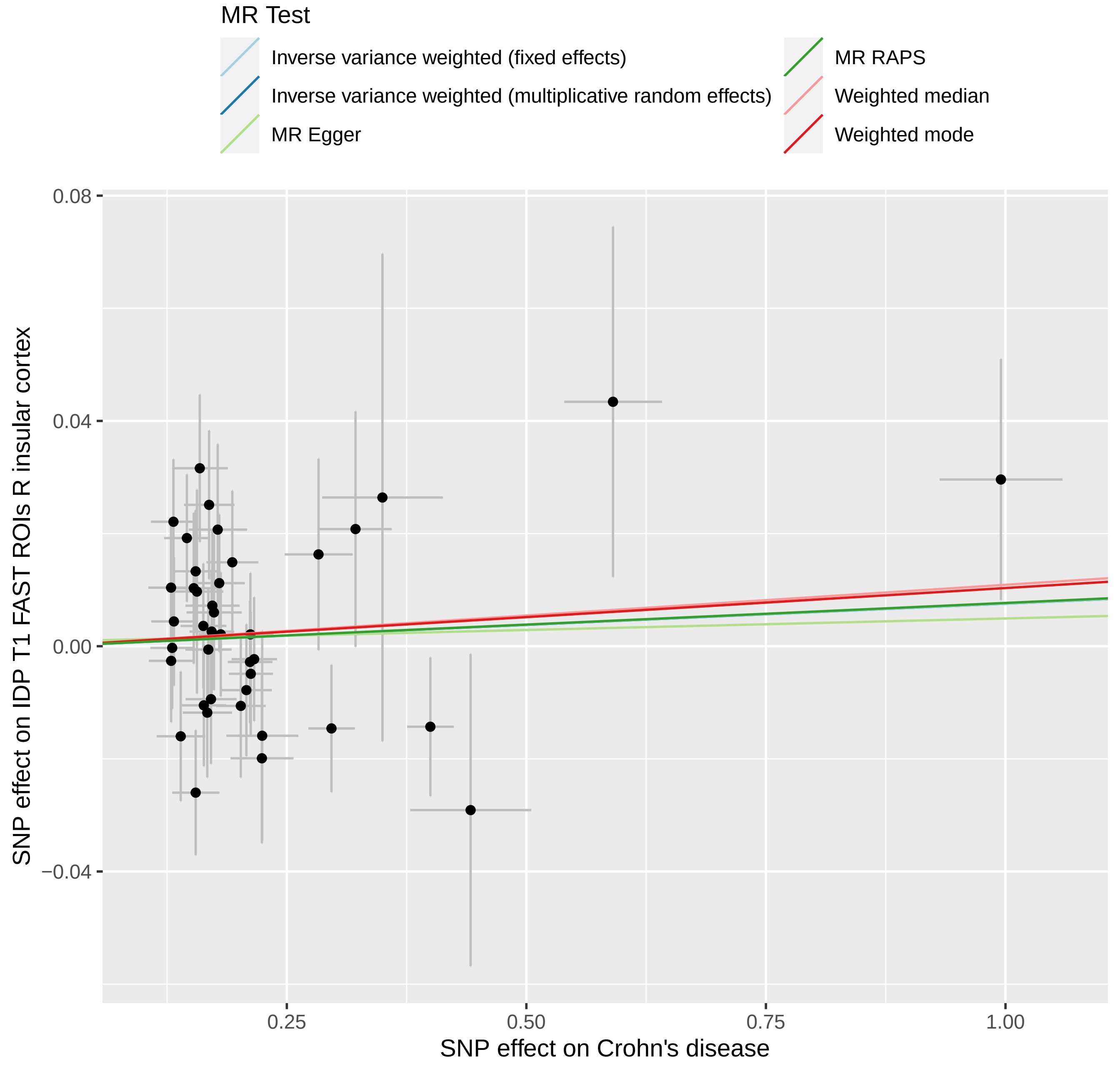

Batch 938 : Ulcerative colitis on IDP T1 FAST ROIs R insular cortex

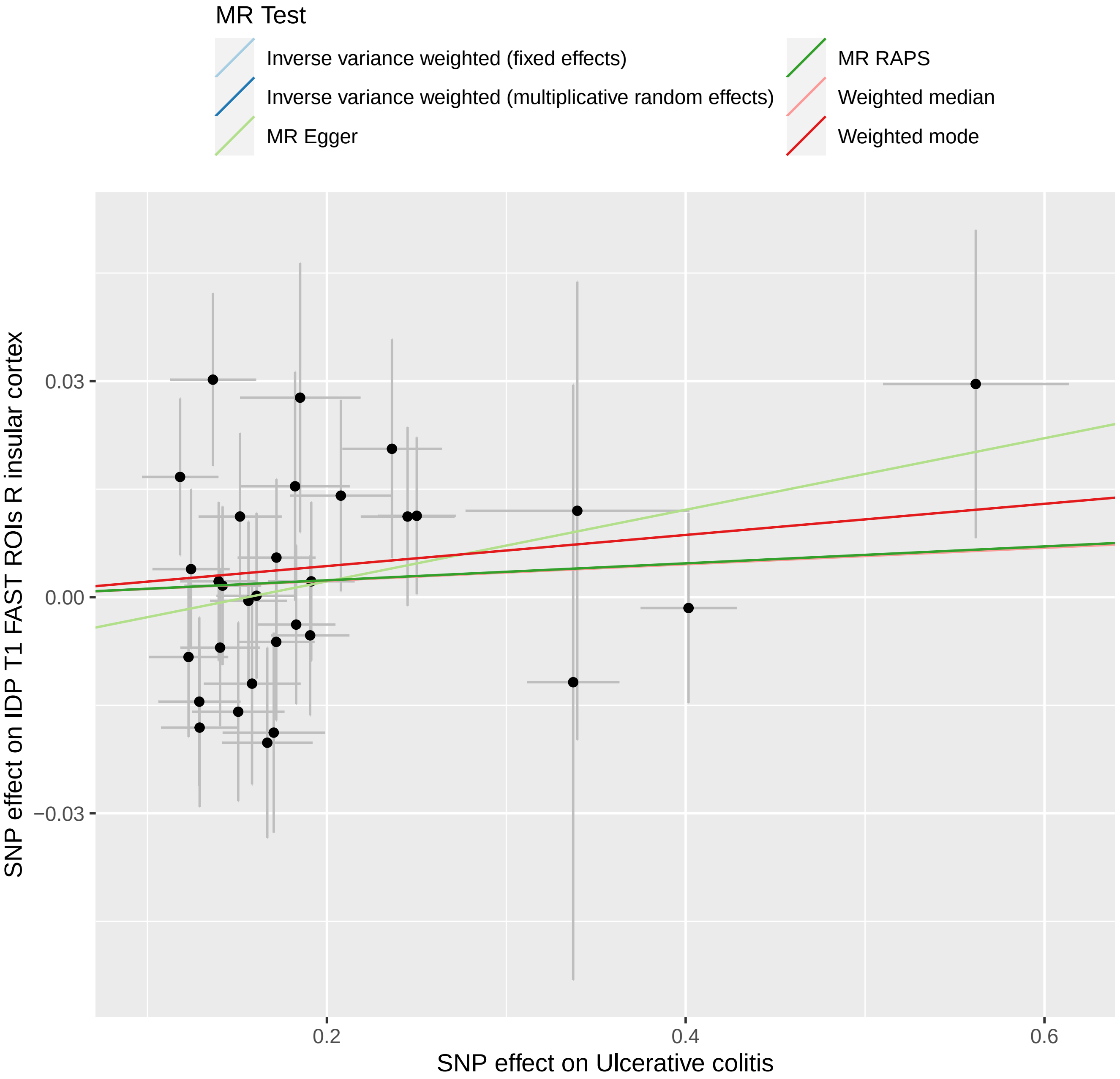

Batch 1159 : Crohn's disease on IDP T1 SIENAX CSF normalised volume

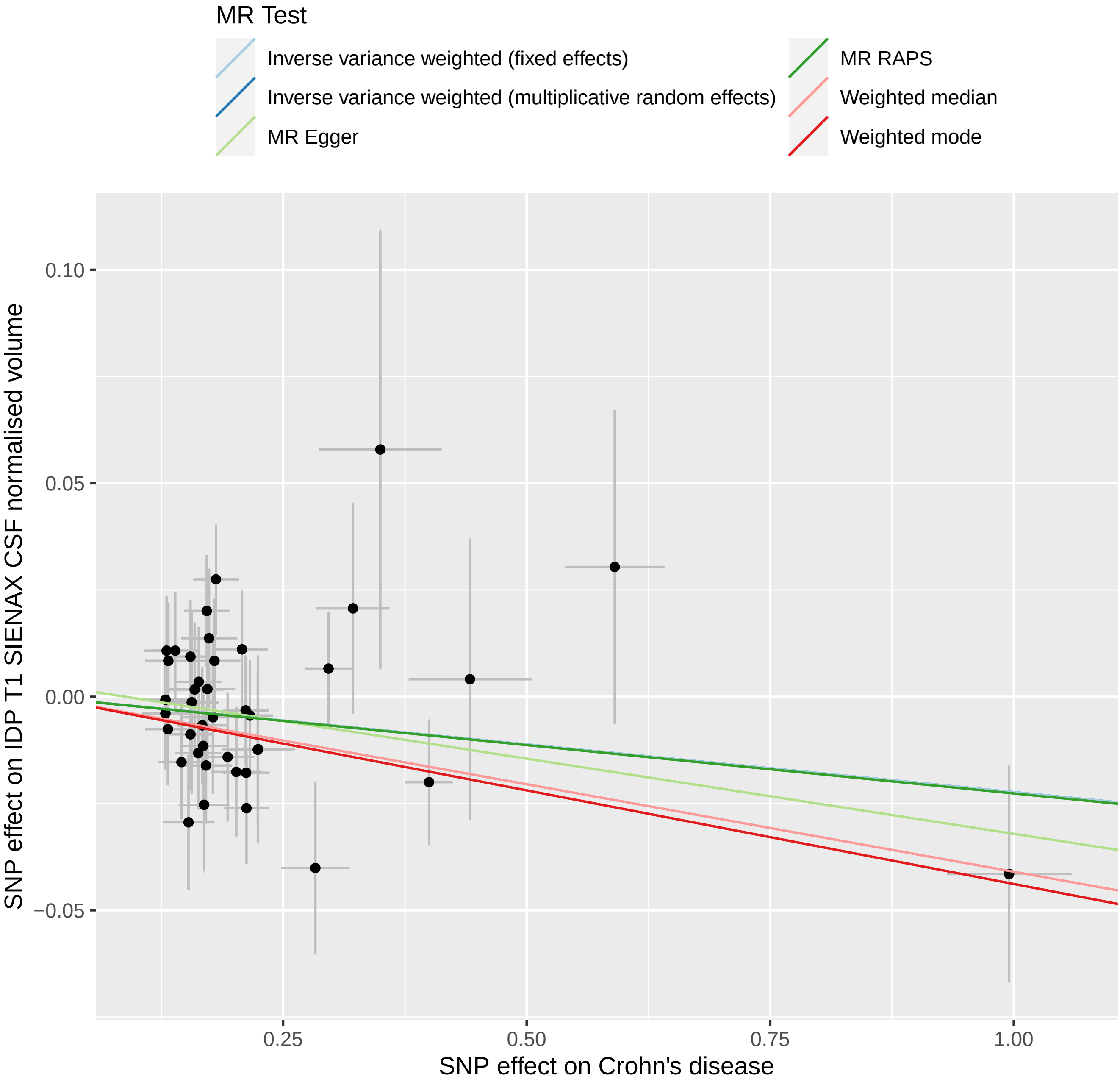

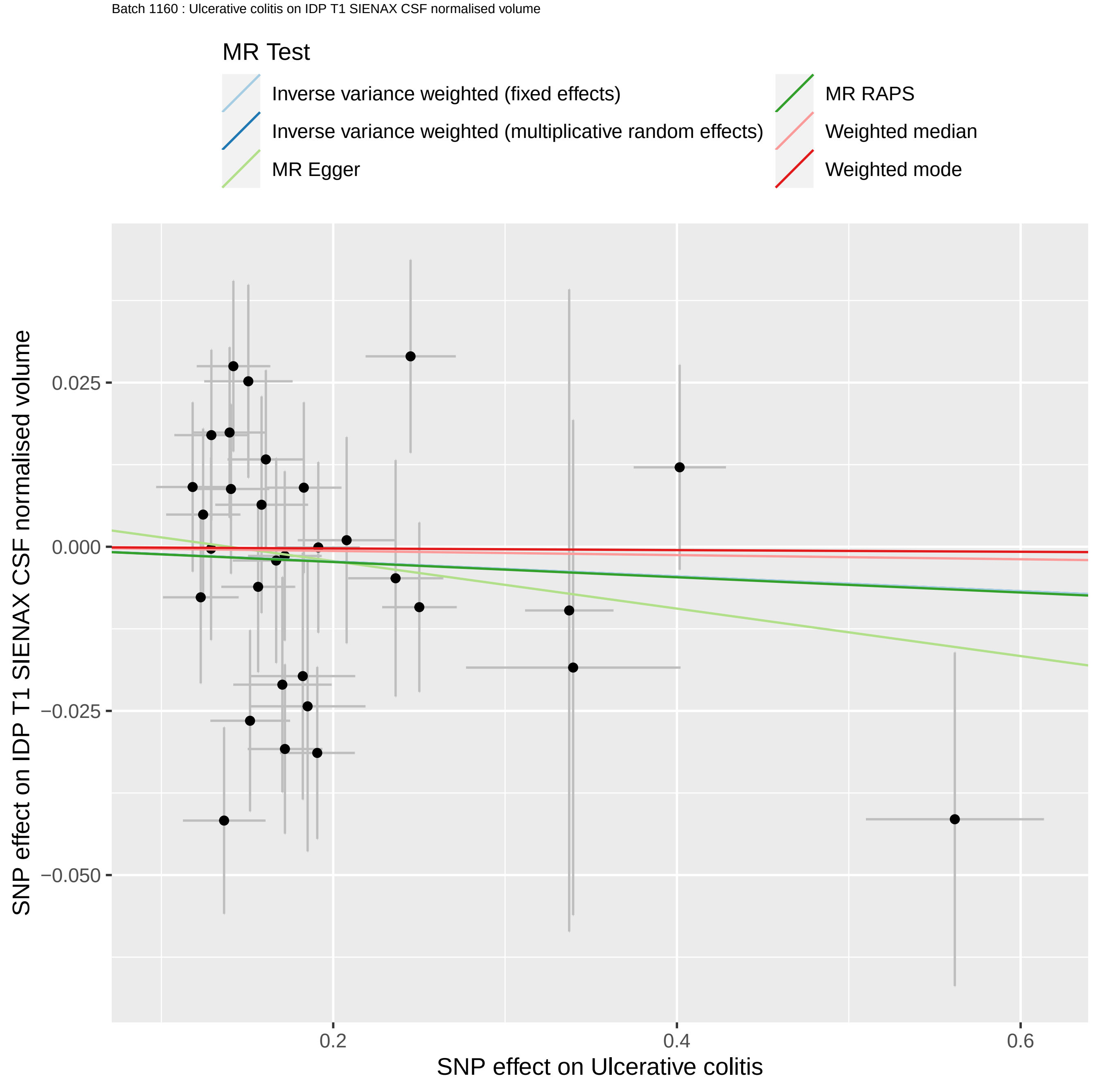
Batch 1160 : Ulcerative colitis on IDP T1 SIENAX CSF normalised volume
MR Test
Inverse variance weighted (fixed effects)
Inverse variance weighted (multiplicative random effects)
MR Egger
MR RAPS
Weighted median
Weighted mode
SNP effect on IDP T1 SIENAX CSF normalised volume
0.025
0.000
−0.025
−0.050
SNP effect on Ulcerative colitis
0.2
0.4
0.6

Batch 1161 : Crohn's disease on IDP T1 FAST ROIs L sup front gyrus

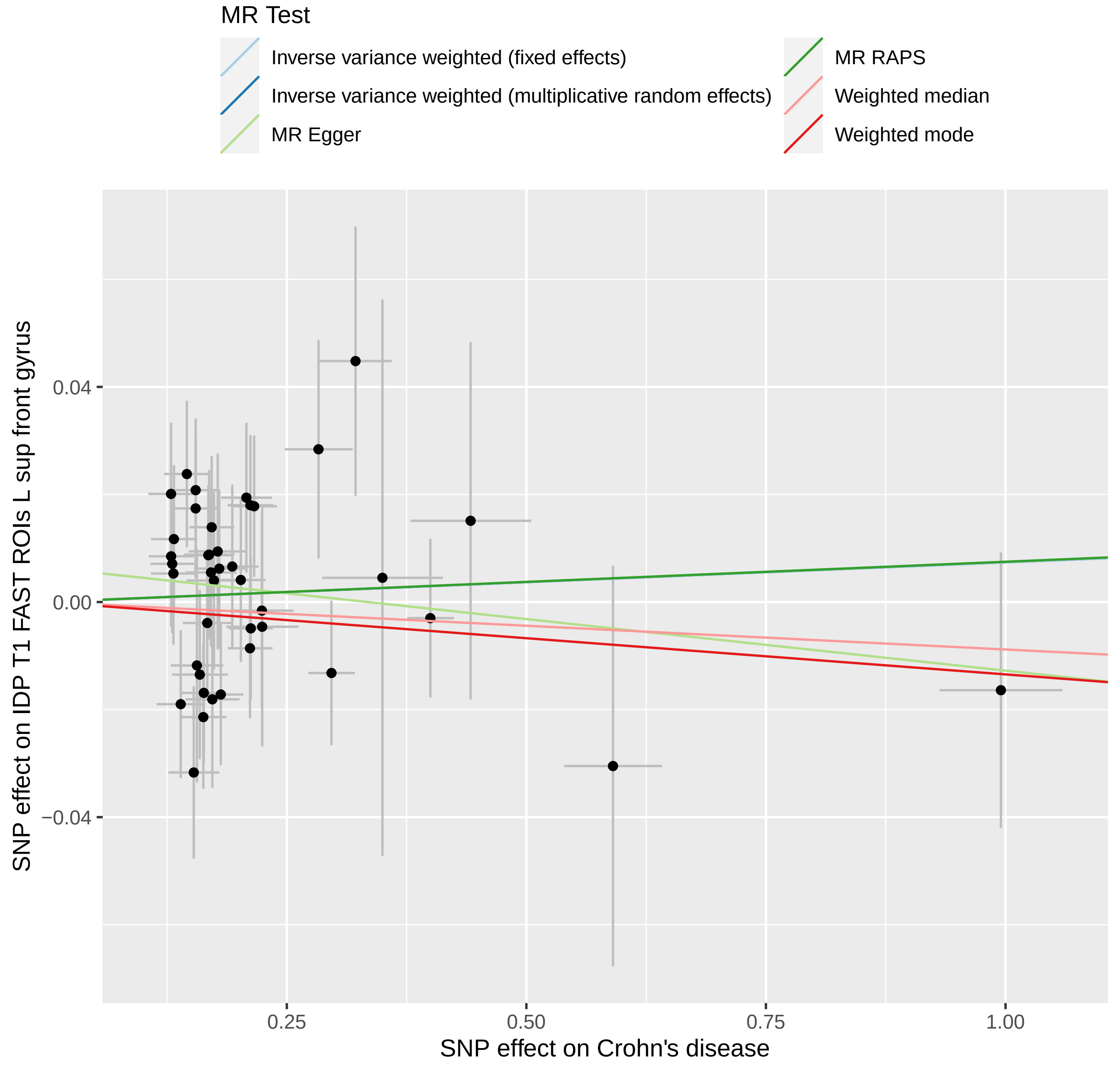

Batch 1162 : Ulcerative colitis on IDP T1 FAST ROIs L sup front gyrus

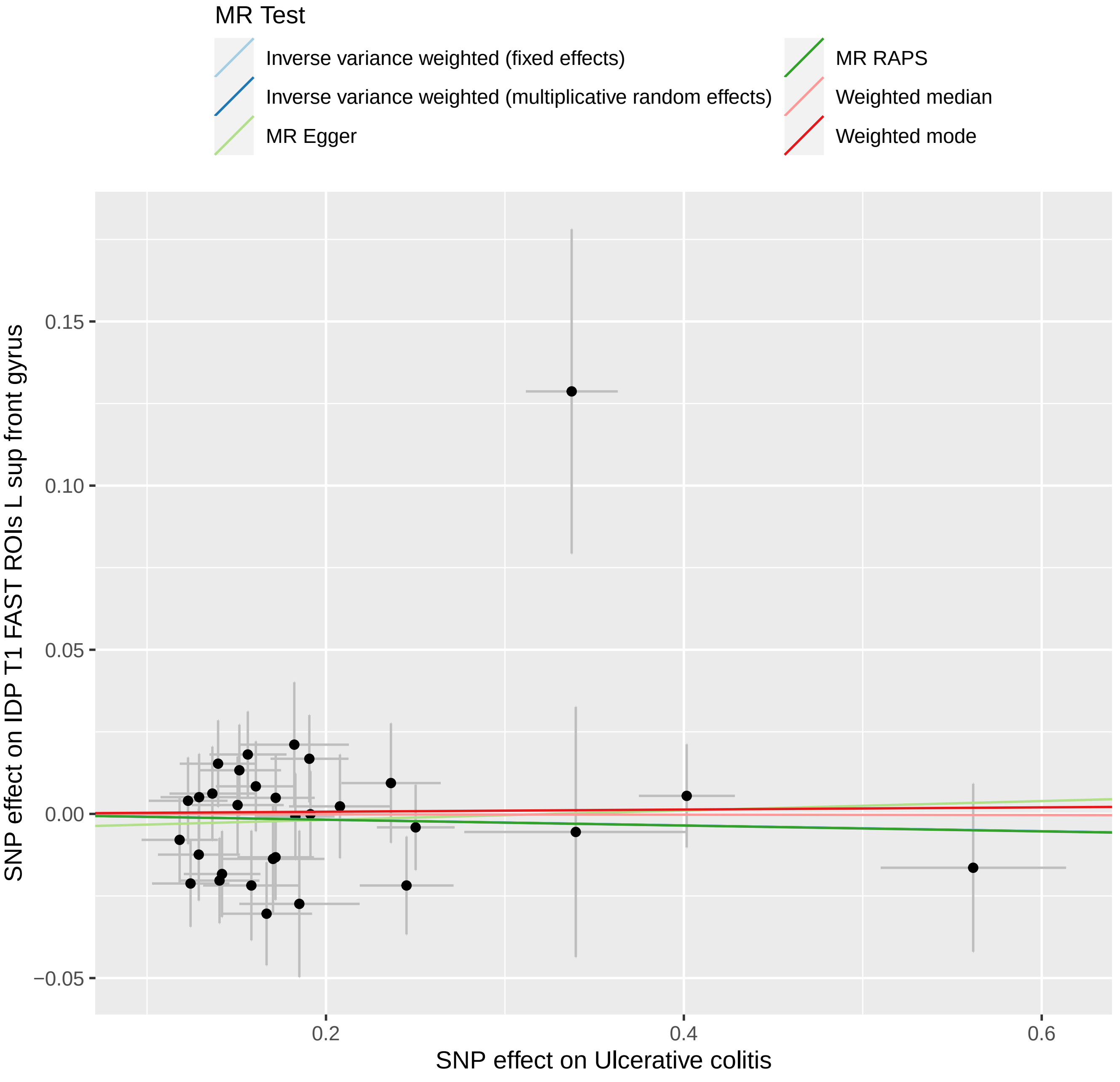

Batch 1383 : Crohn's disease on IDP T1 FAST ROIs R sup front gyrus

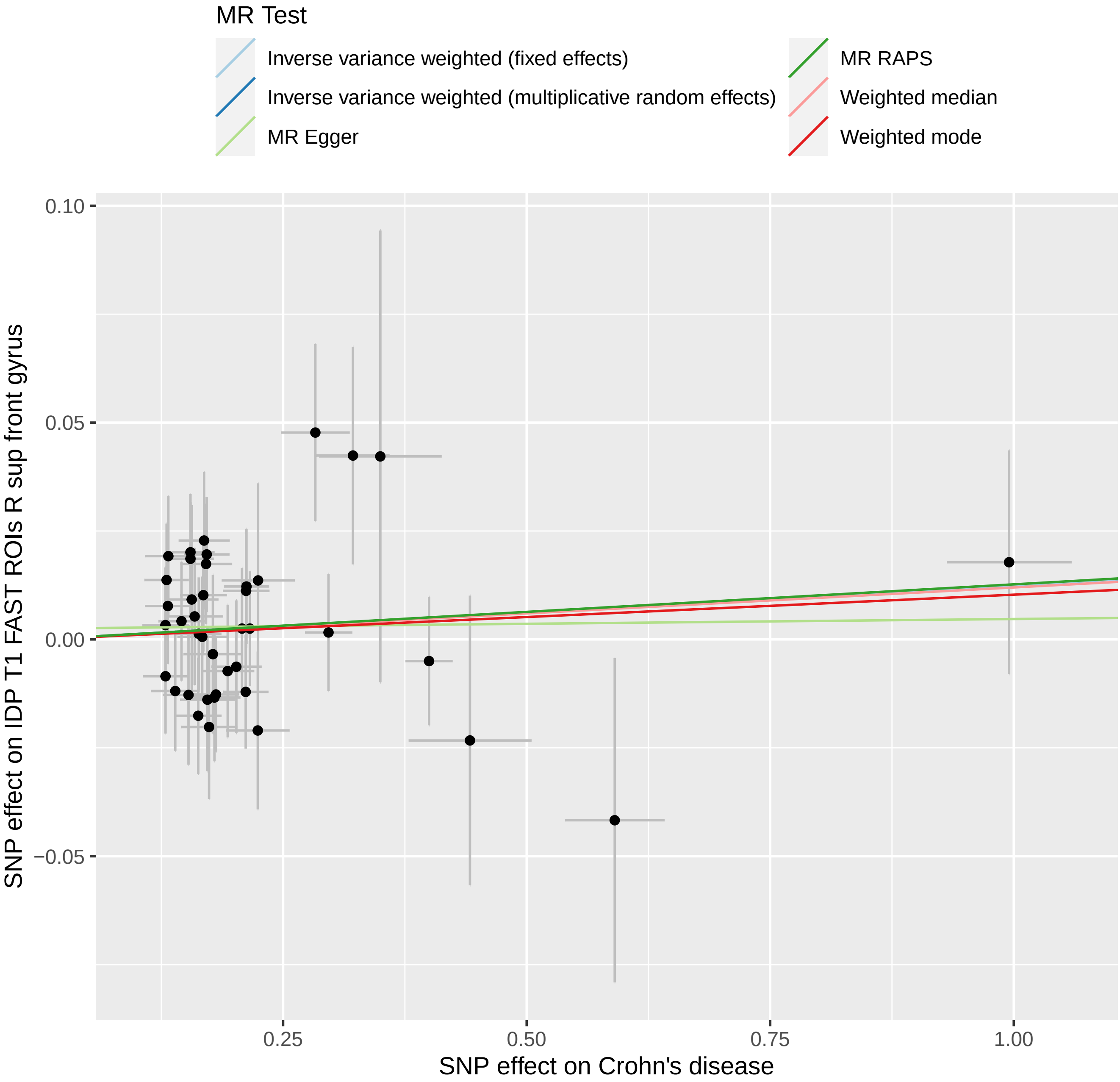

Batch 1384 : Ulcerative colitis on IDP T1 FAST ROIs R sup front gyrus

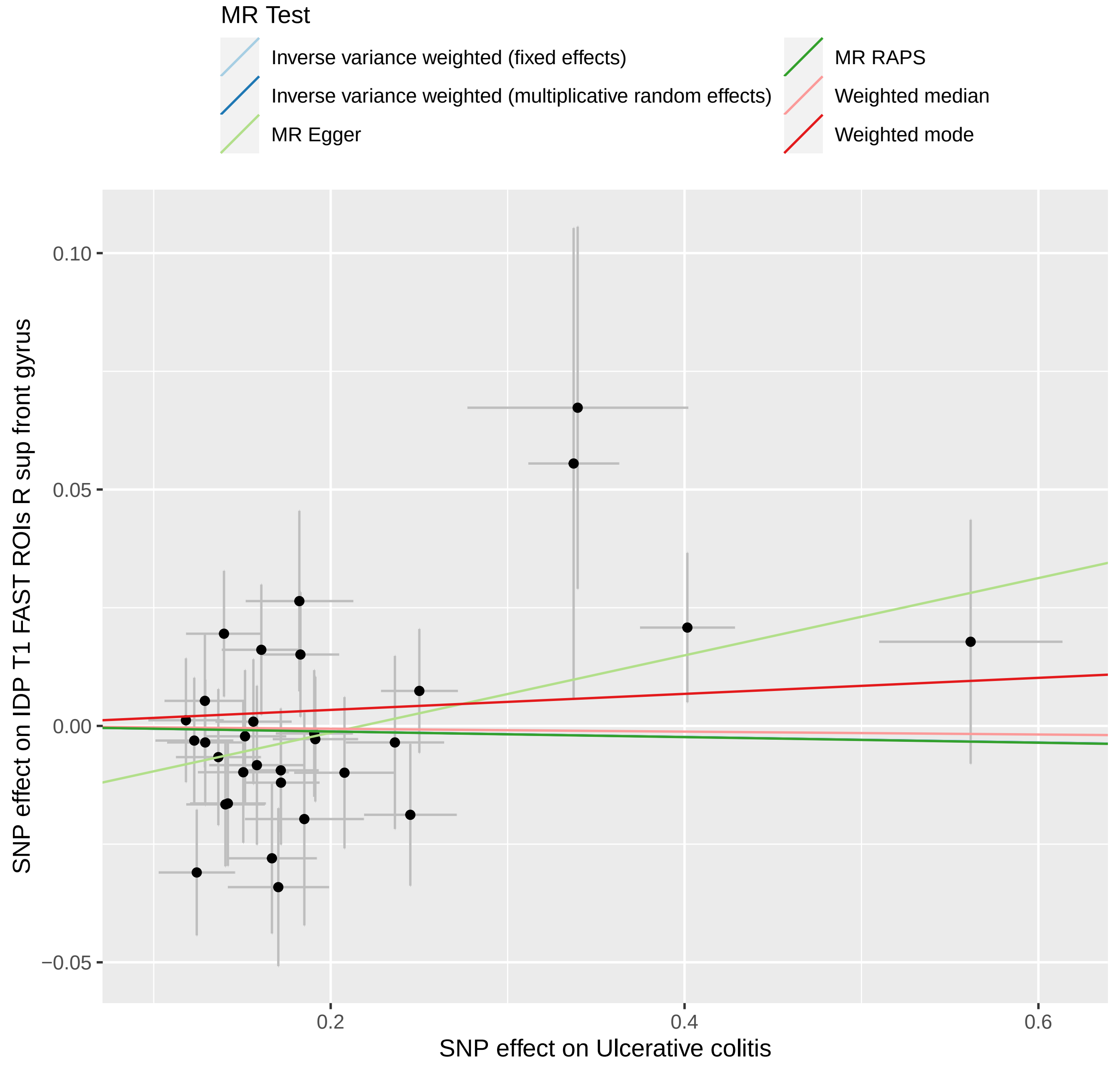

Batch 1483 : Crohn's disease on IDP T1 FAST ROIs L mid front gyrus

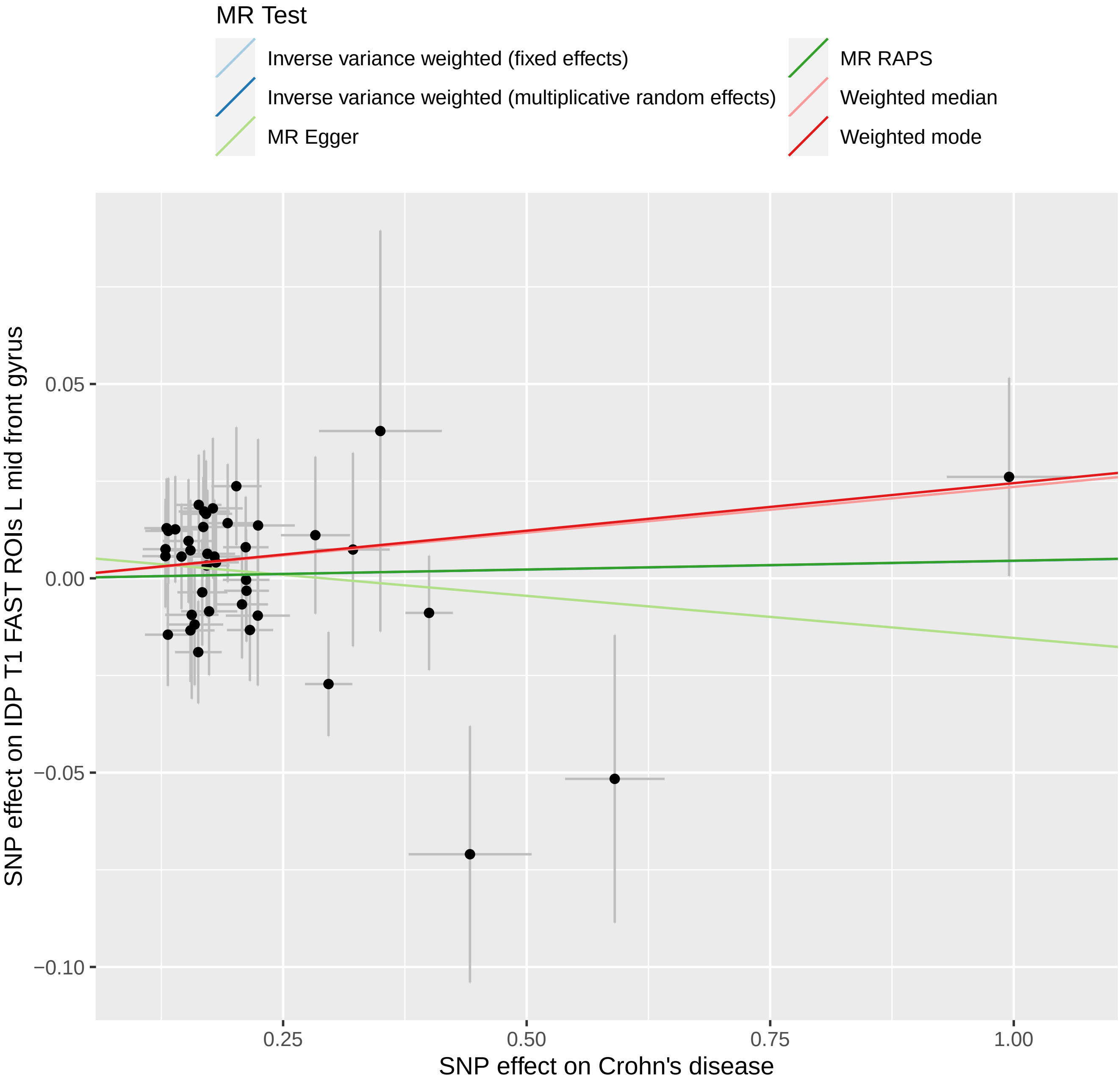

Batch 1484 : Ulcerative colitis on IDP T1 FAST ROIs L mid front gyrus

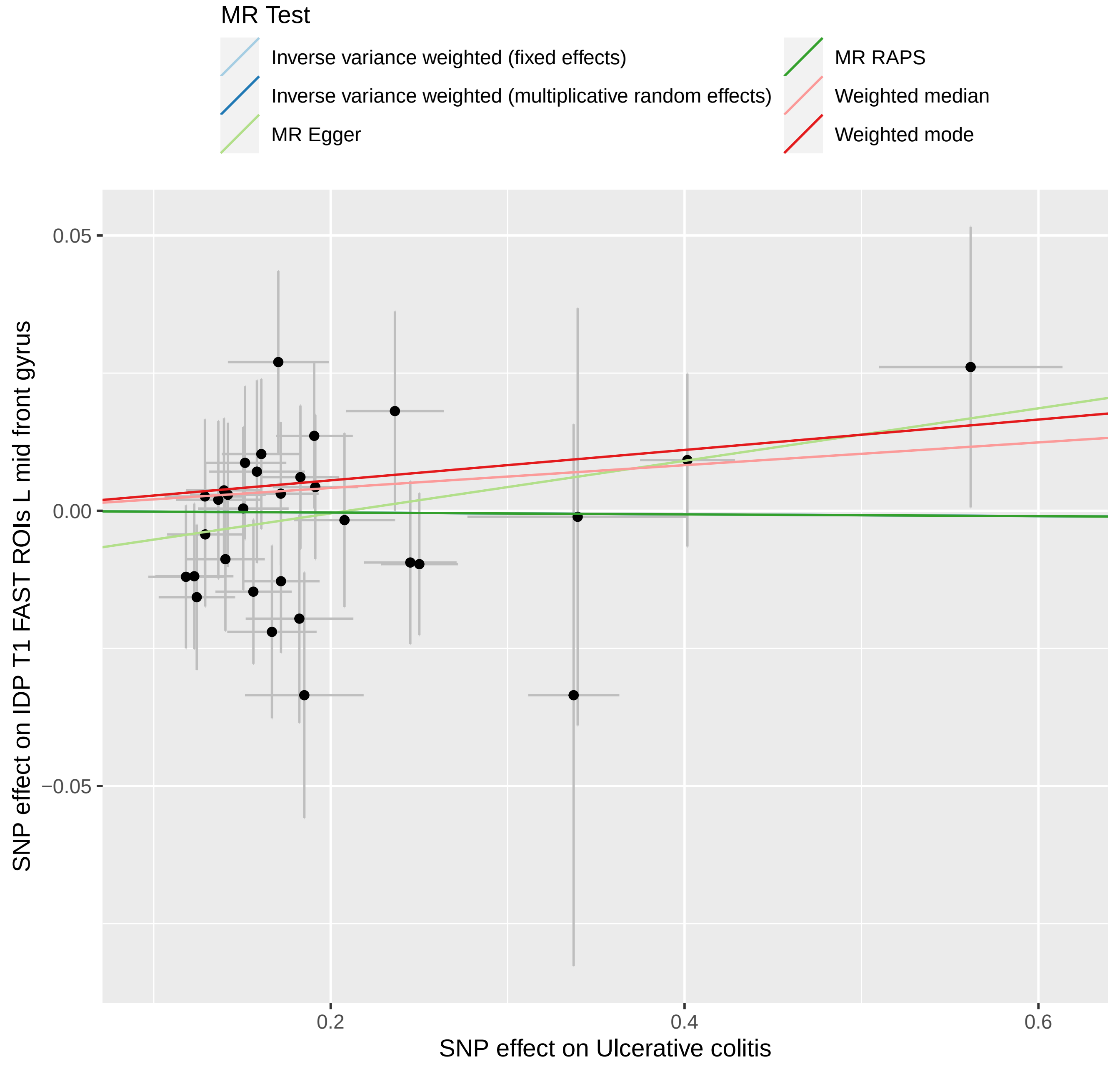

Batch 1505 : Crohn's disease on IDP T1 FAST ROIs R mid front gyrus

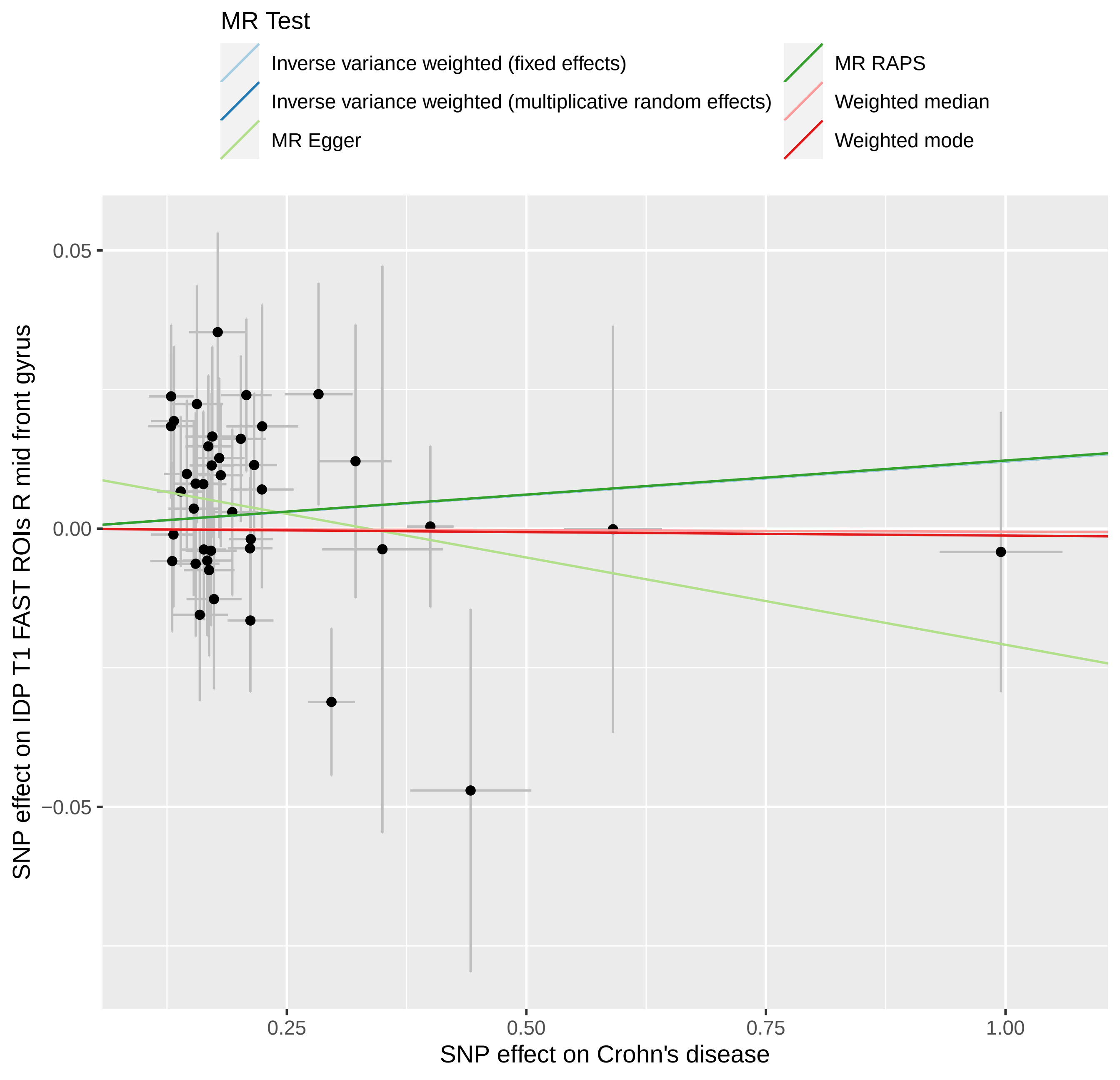

Batch 1506 : Ulcerative colitis on IDP T1 FAST ROIs R mid front gyrus

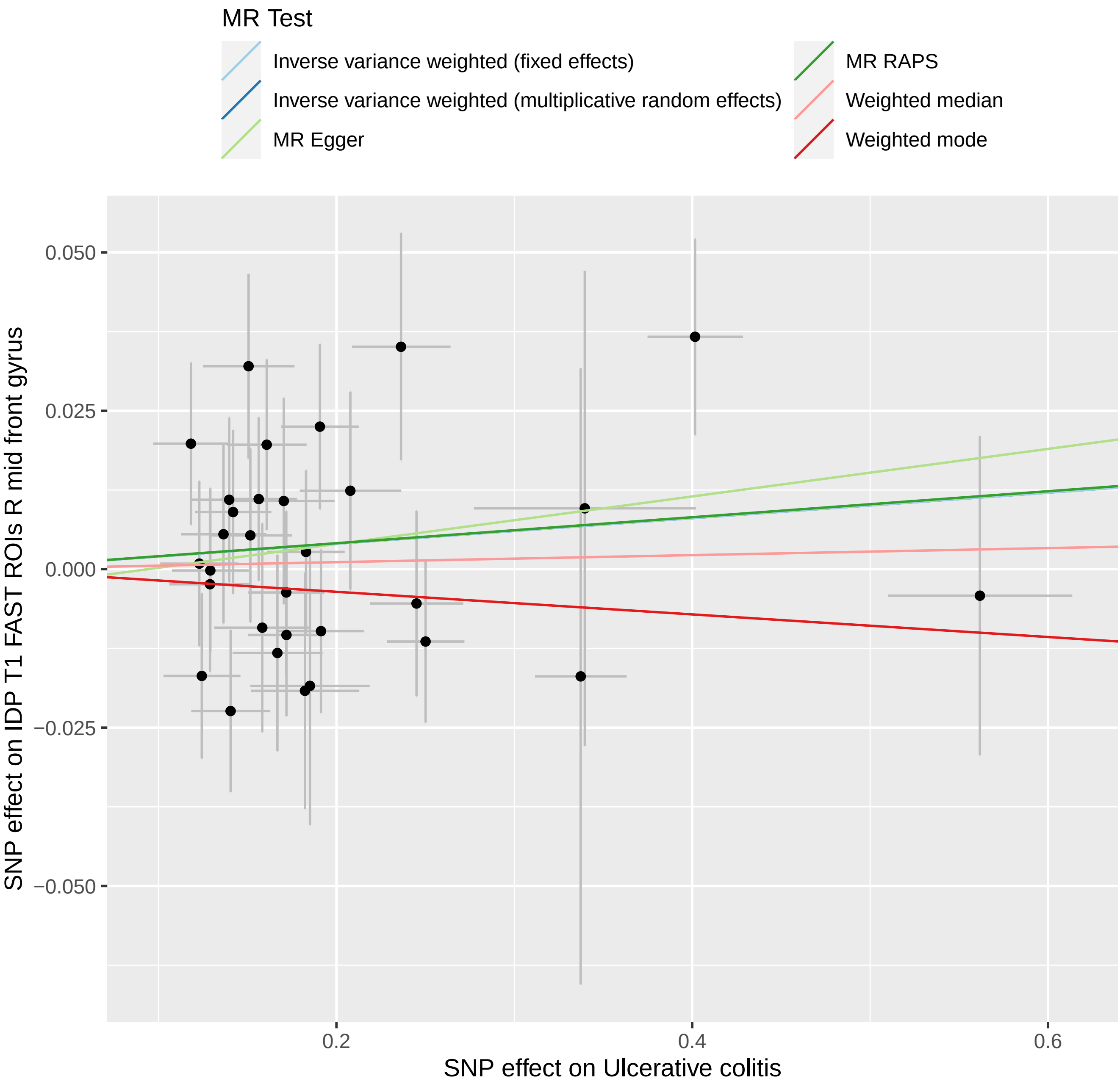

Batch 1527 : Crohn's disease on IDP T1 FAST ROIs L inf front gyrus parstri

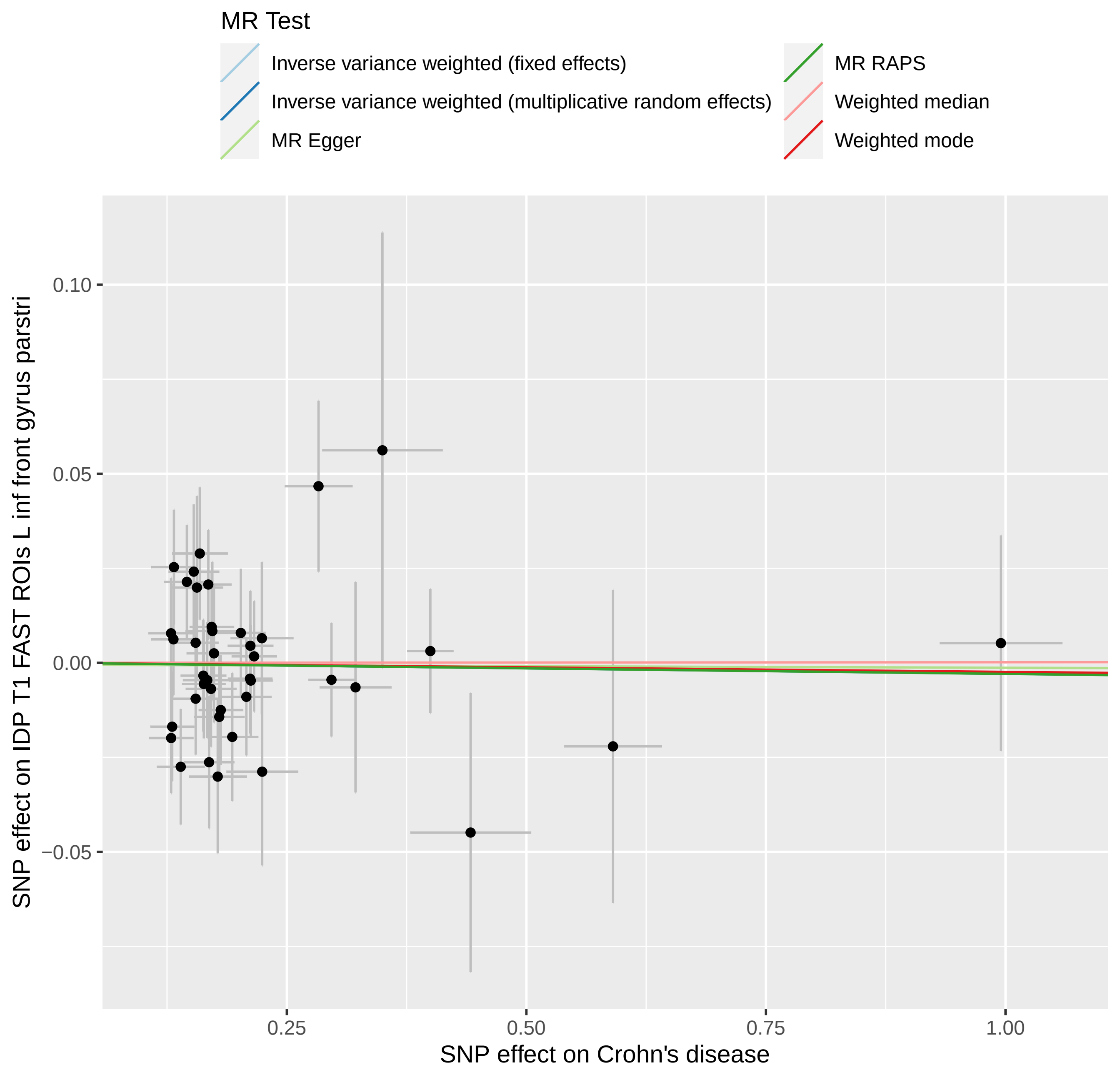

Batch 1528 : Ulcerative colitis on IDP T1 FAST ROIs L inf front gyrus parstri

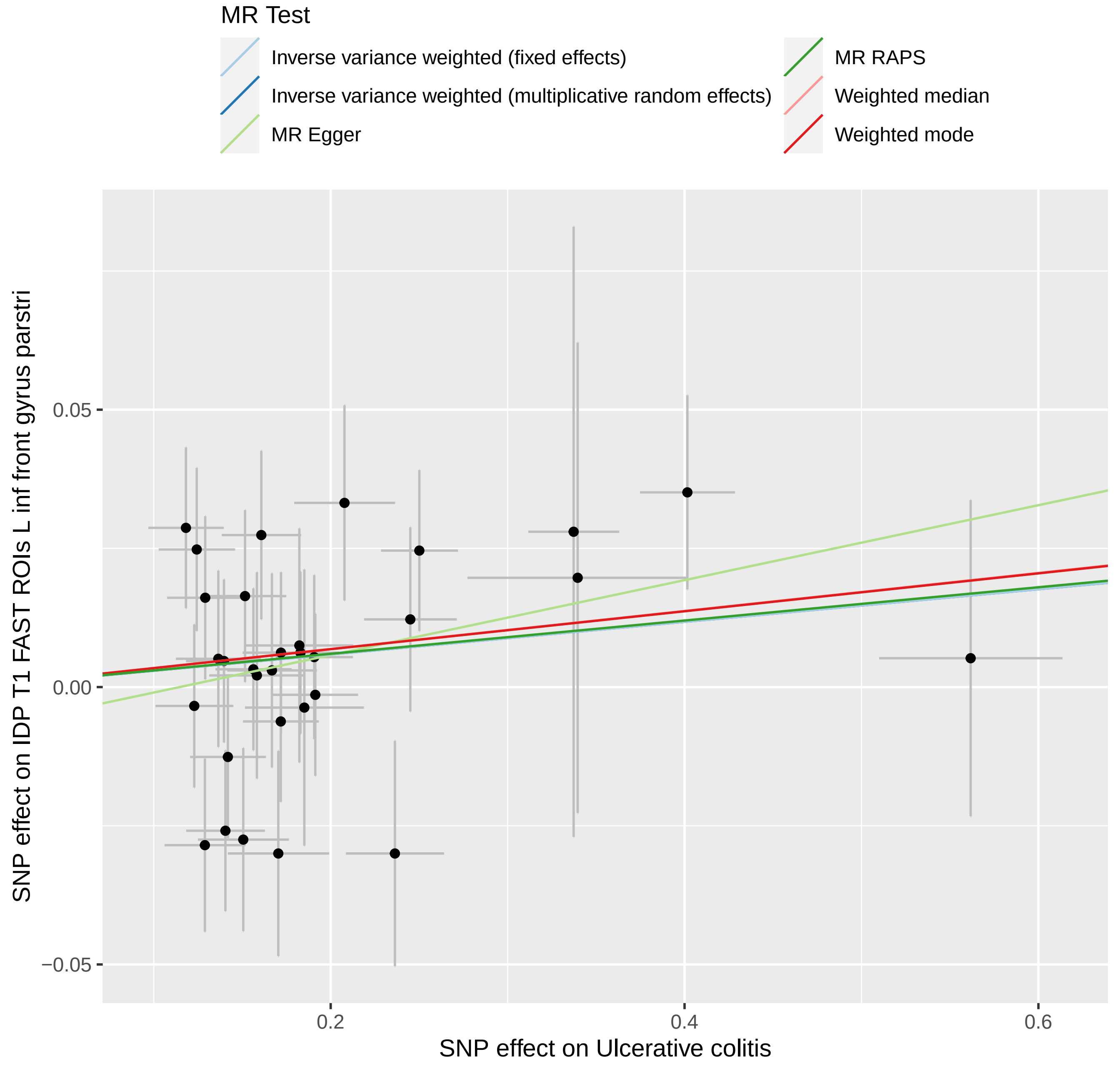

Batch 1549 : Crohn's disease on IDP T1 FAST ROIs R inf front gyrus parstri

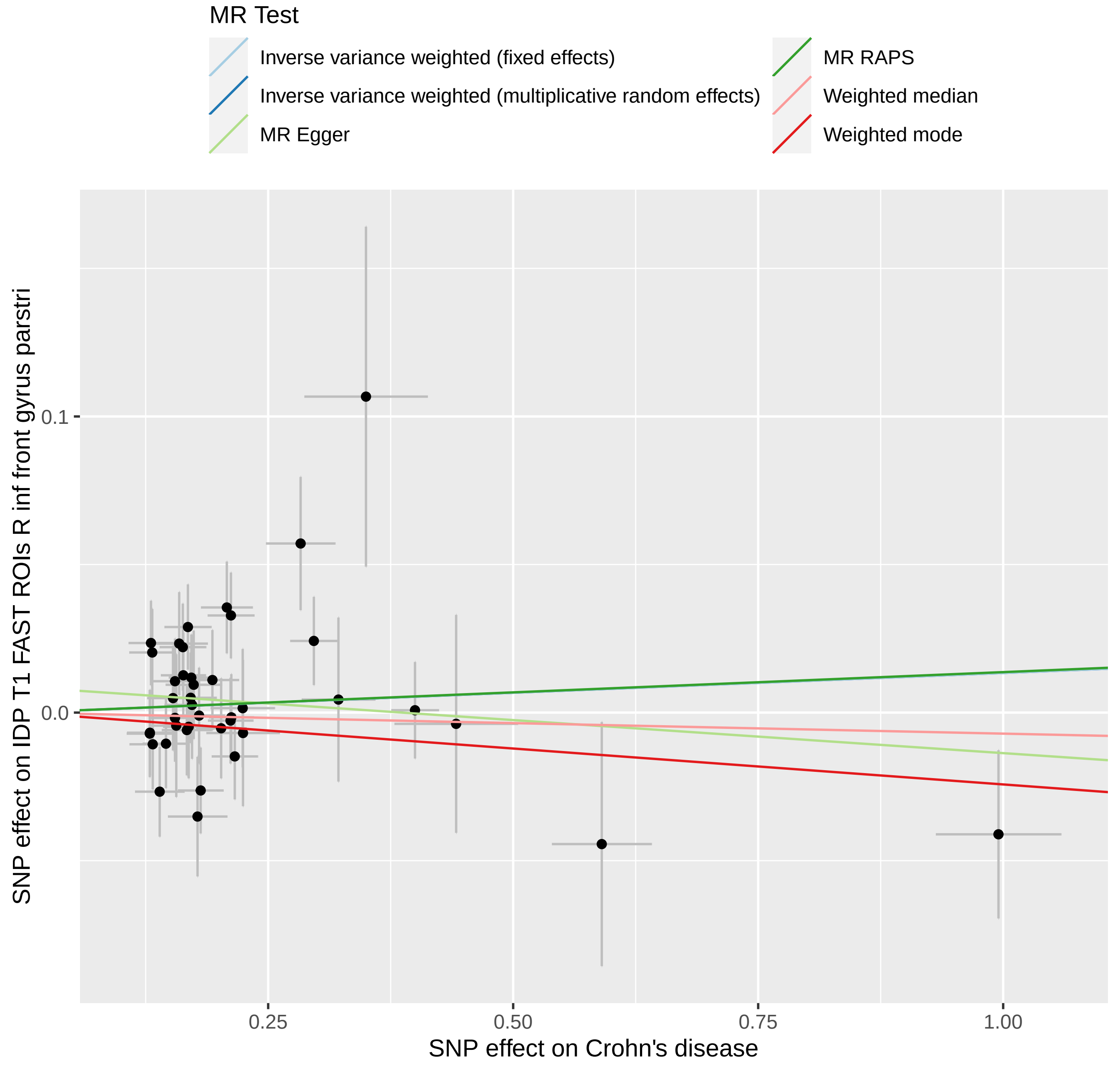

Batch 1550 : Ulcerative colitis on IDP T1 FAST ROIs R inf front gyrus parstri

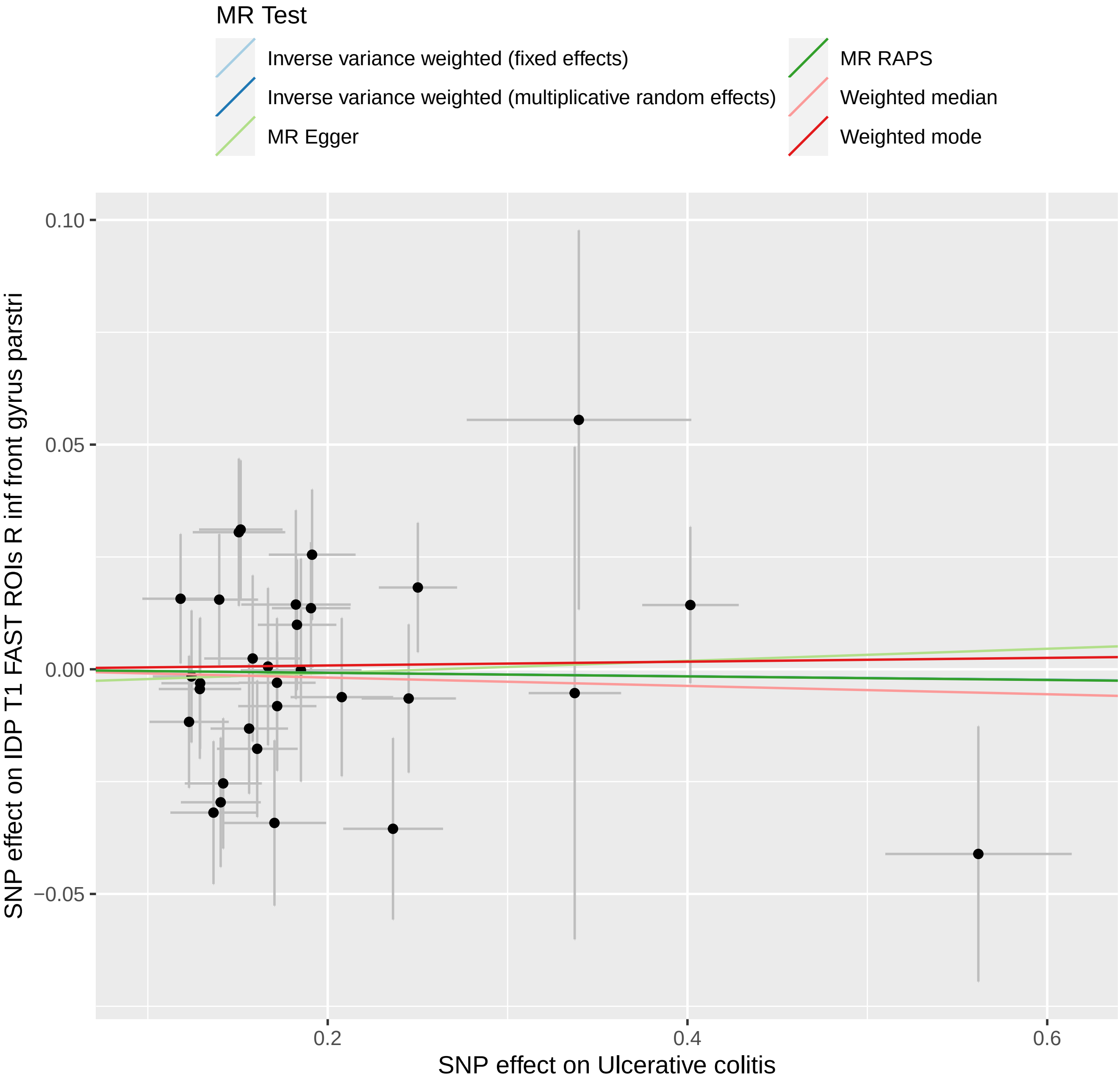

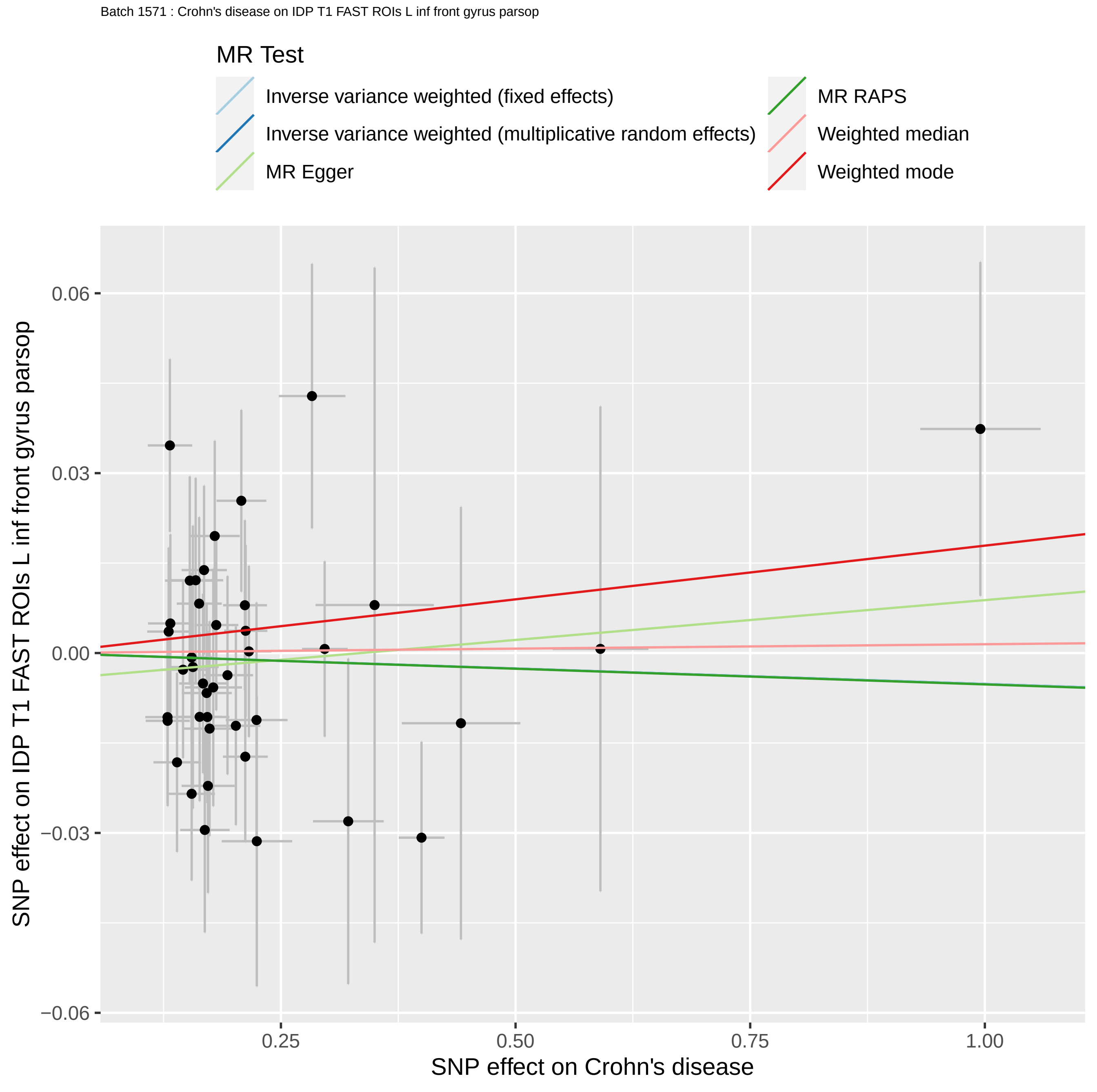
Batch 1571 : Crohn's disease on IDP T1 FAST ROIs L inf front gyrus parsop
MR Test
Inverse variance weighted (fixed effects)
Inverse variance weighted (multiplicative random effects)
MR Egger
MR RAPS
Weighted median
Weighted mode
SNP effect on IDP T1 FAST ROIs L inf front gyrus parsop
0.06
0.03
0.00
−0.03
−0.06
0.25
0.50
0.75
1.00
SNP effect on Crohn's disease

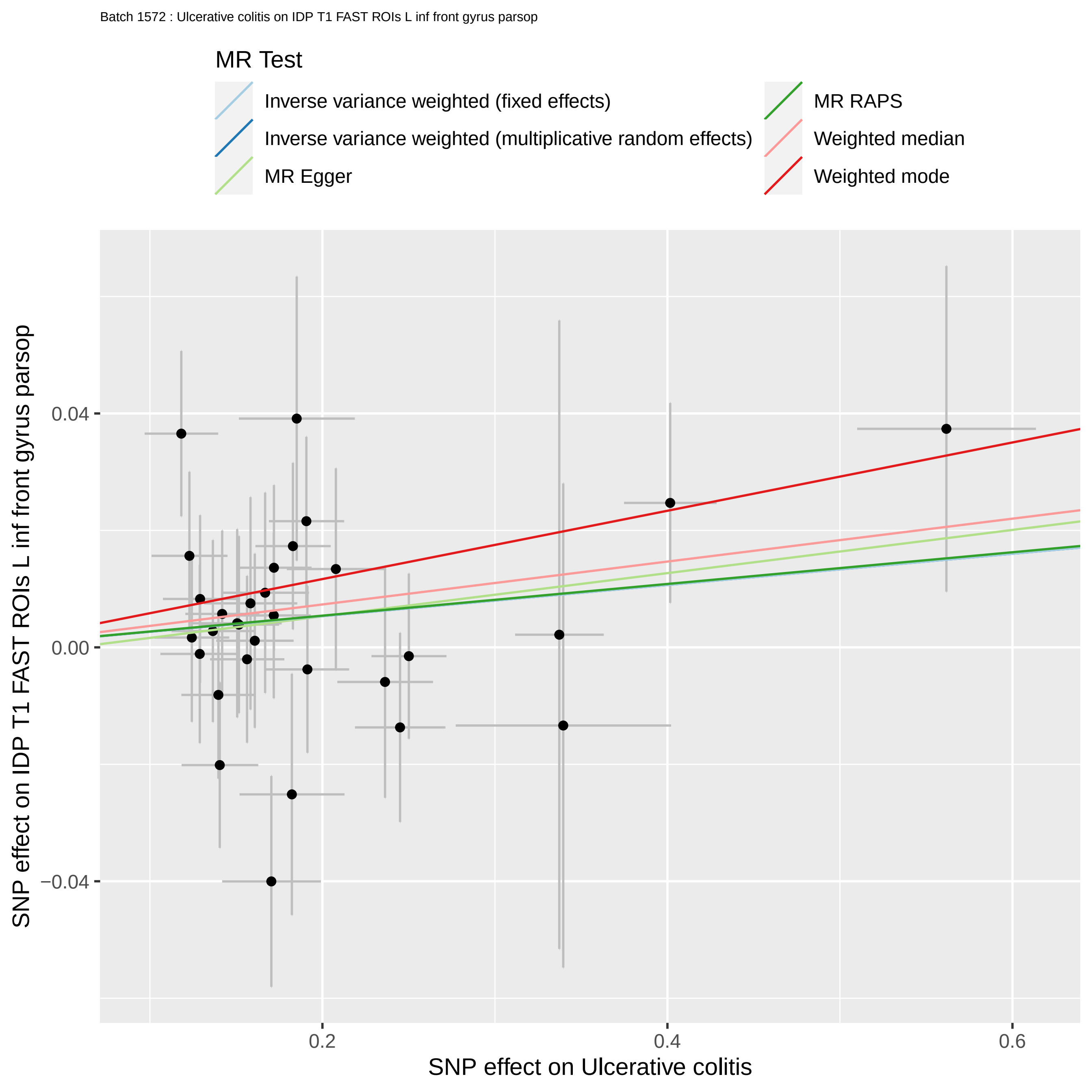
Batch 1572 : Ulcerative colitis on IDP T1 FAST ROIs L inf front gyrus parsop
MR Test
Inverse variance weighted (fixed effects)
Inverse variance weighted (multiplicative random effects)
MR Egger
MR RAPS
Weighted median
Weighted mode
0.04
0.00
−0.04
0.2
0.4
0.6
SNP effect on IDP T1 FAST ROIs L inf front gyrus parsop
SNP effect on Ulcerative colitis

Batch 1593 : Crohn's disease on IDP T1 FAST ROIs R inf front gyrus parsop

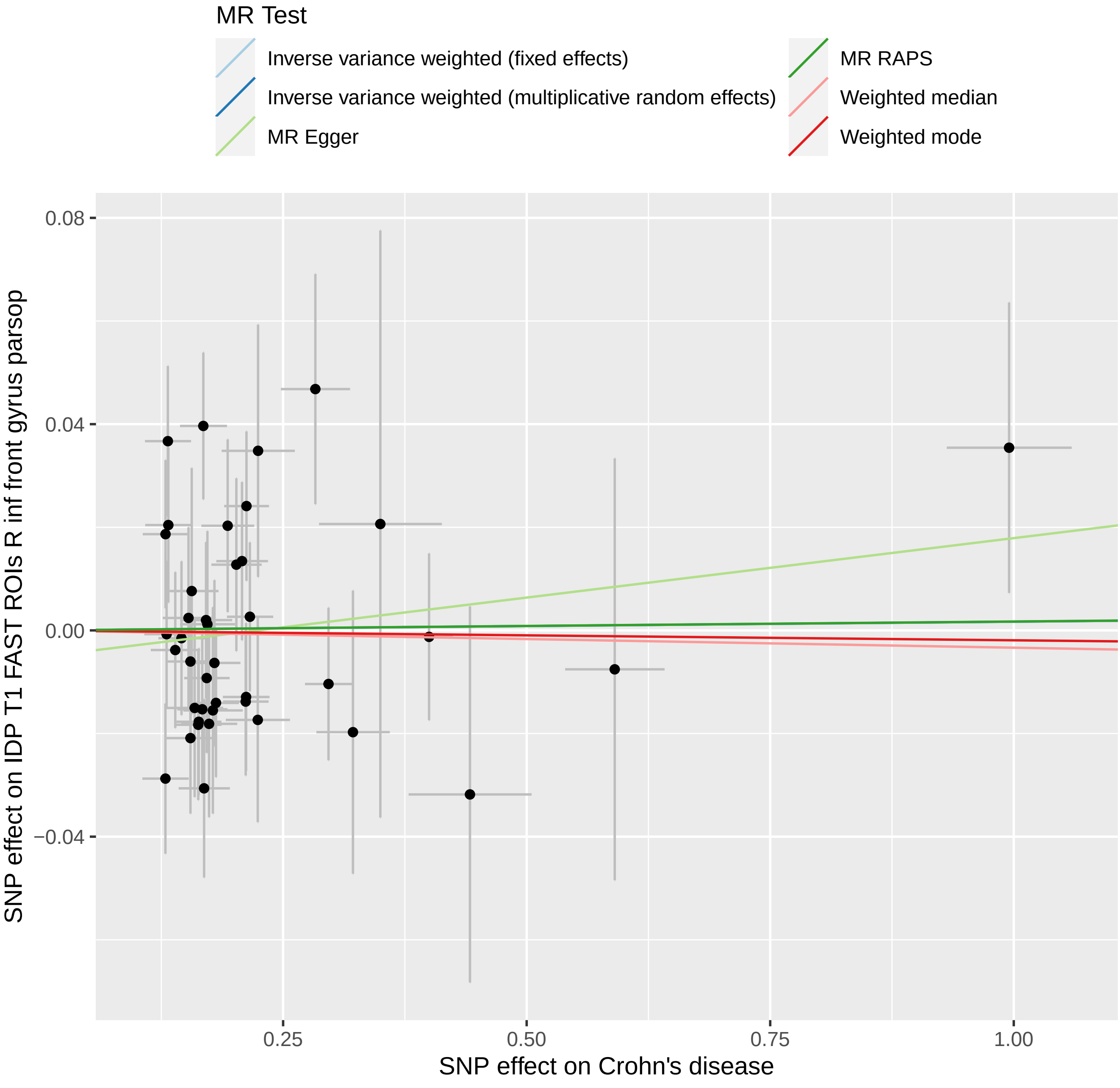

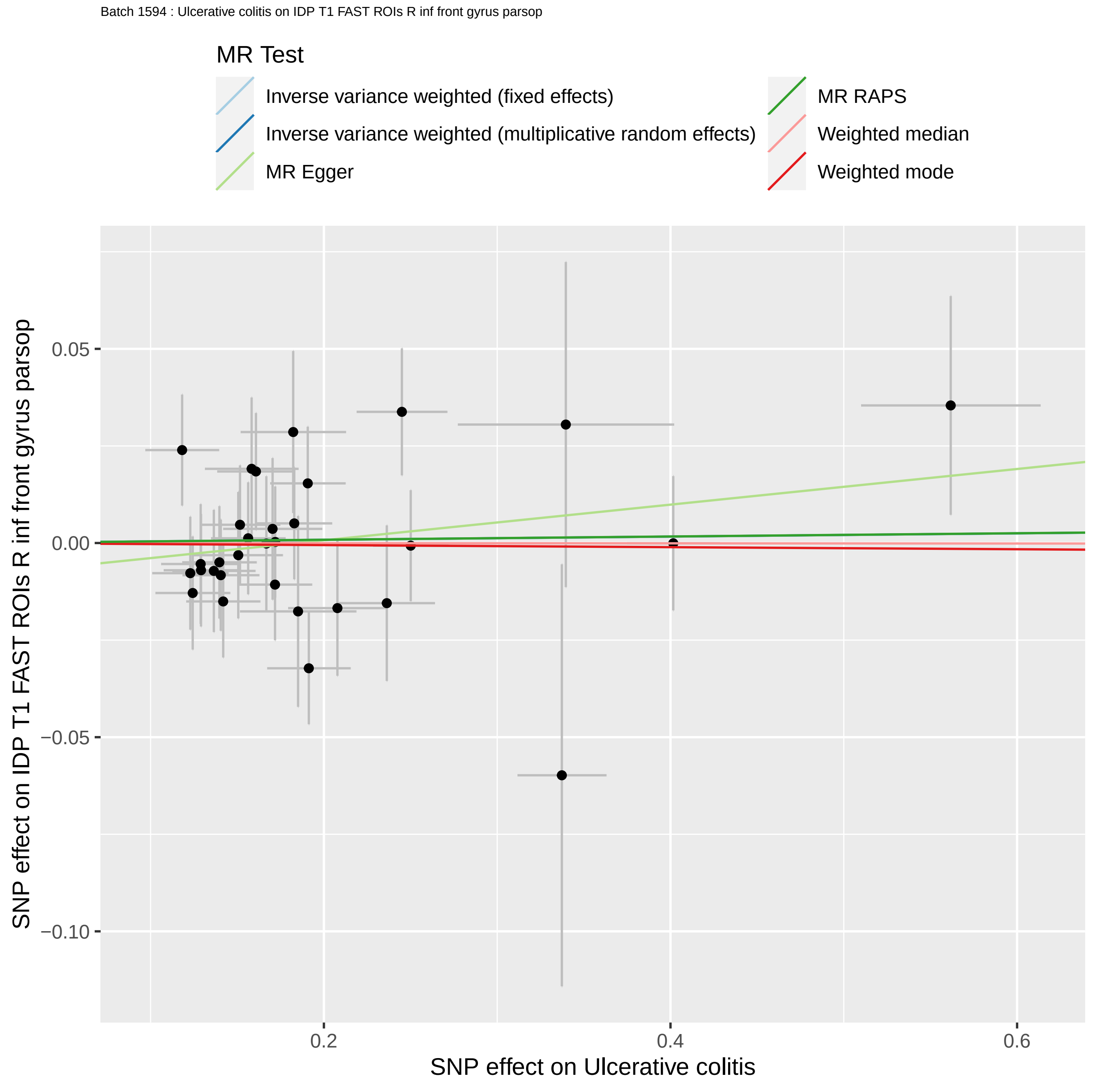

Batch 1594 : Ulcerative colitis on IDP T1 FAST ROIs R inf front gyrus parsop
MR Test
Inverse variance weighted (fixed effects)
Inverse variance weighted (multiplicative random effects)
MR Egger
MR RAPS
Weighted median
Weighted mode
SNP effect on IDP T1 FAST ROIs R inf front gyrus parsop
0.05
0.00
−0.05
−0.10
0.2
0.4
0.6
SNP effect on Ulcerative colitis

Batch 1615 : Crohn's disease on IDP T1 FAST ROIs L precentral gyrus

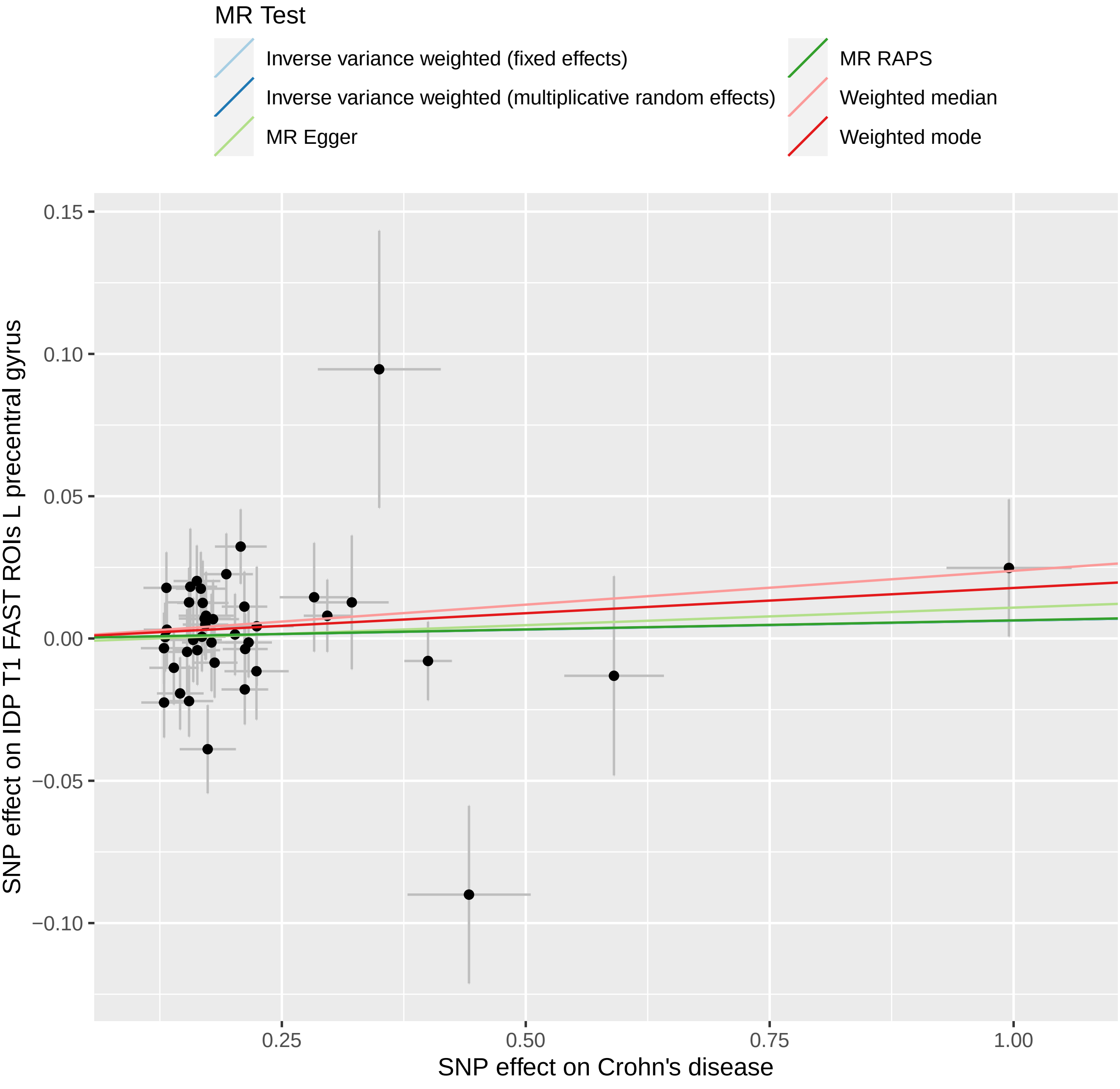

Batch 1616 : Ulcerative colitis on IDP T1 FAST ROIs L precentral gyrus

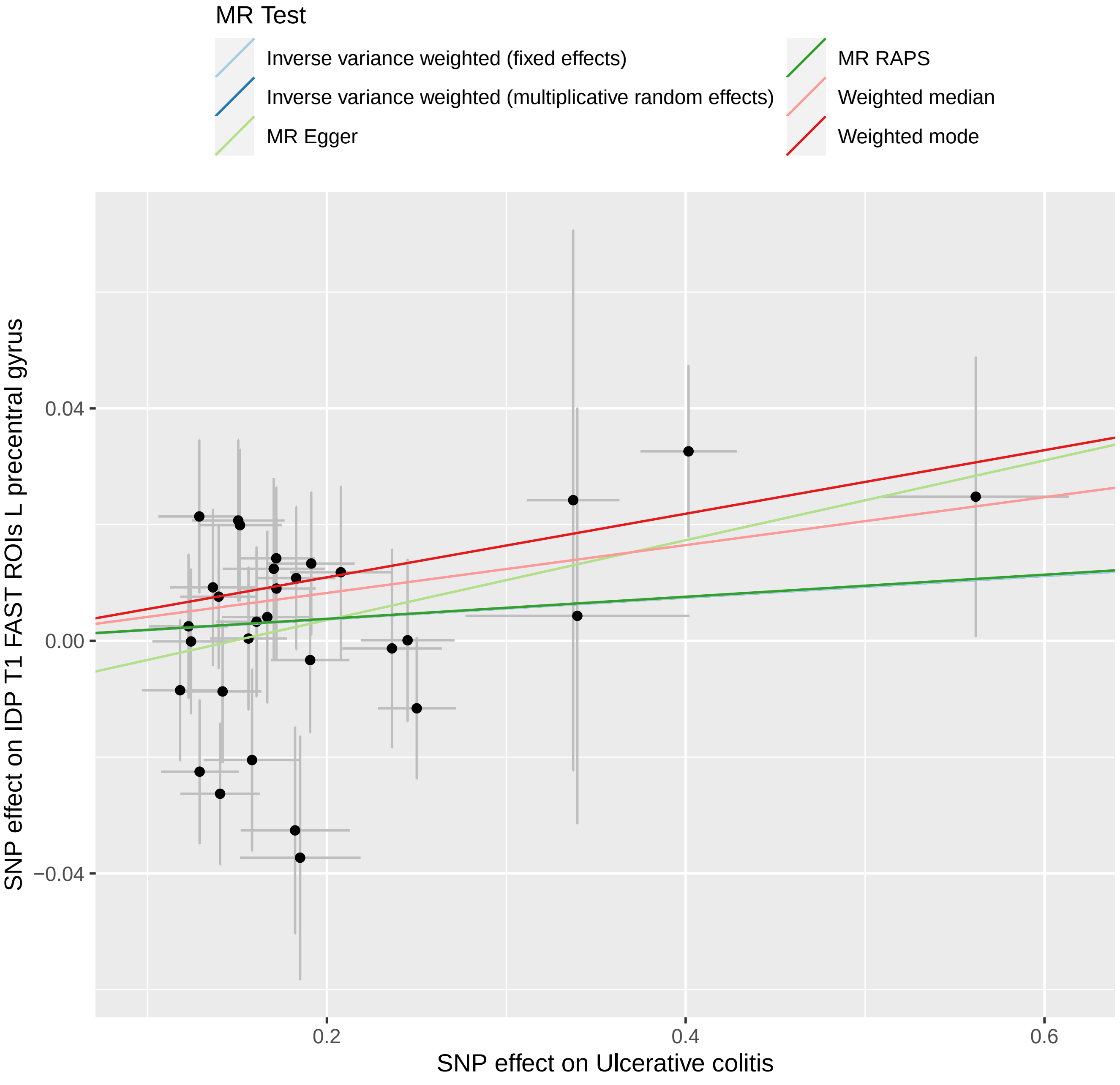

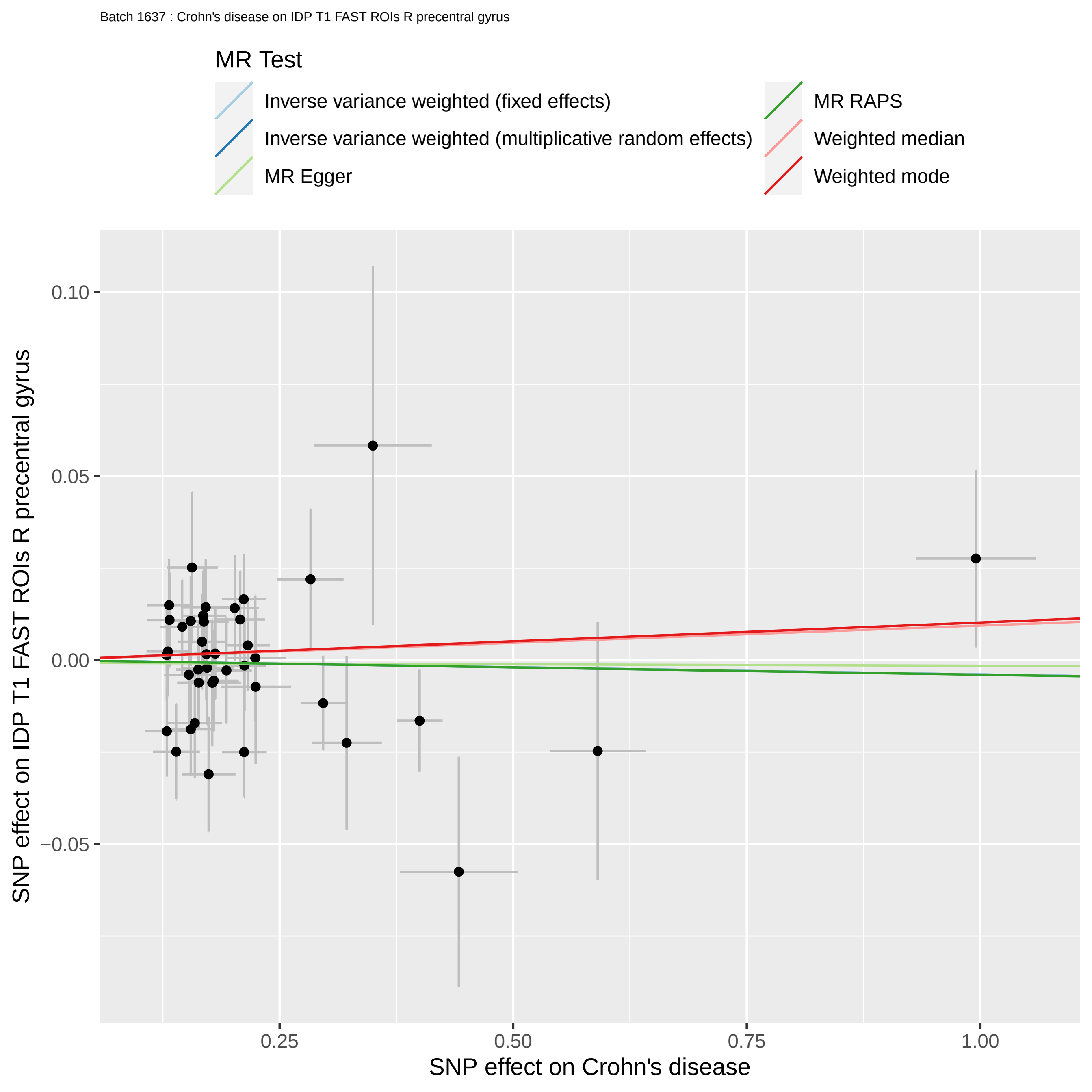
Batch 1637 : Crohn's disease on IDP T1 FAST ROIs R precentral gyrus
MR Test
Inverse variance weighted (fixed effects)
Inverse variance weighted (multiplicative random effects)
MR Egger
MR RAPS
Weighted median
Weighted mode
SNP effect on IDP T1 FAST ROIs R precentral gyrus
0.10
0.05
0.00
−0.05
0.25
0.50
0.75
1.00
SNP effect on Crohn's disease

Batch 1638 : Ulcerative colitis on IDP T1 FAST ROIs R precentral gyrus

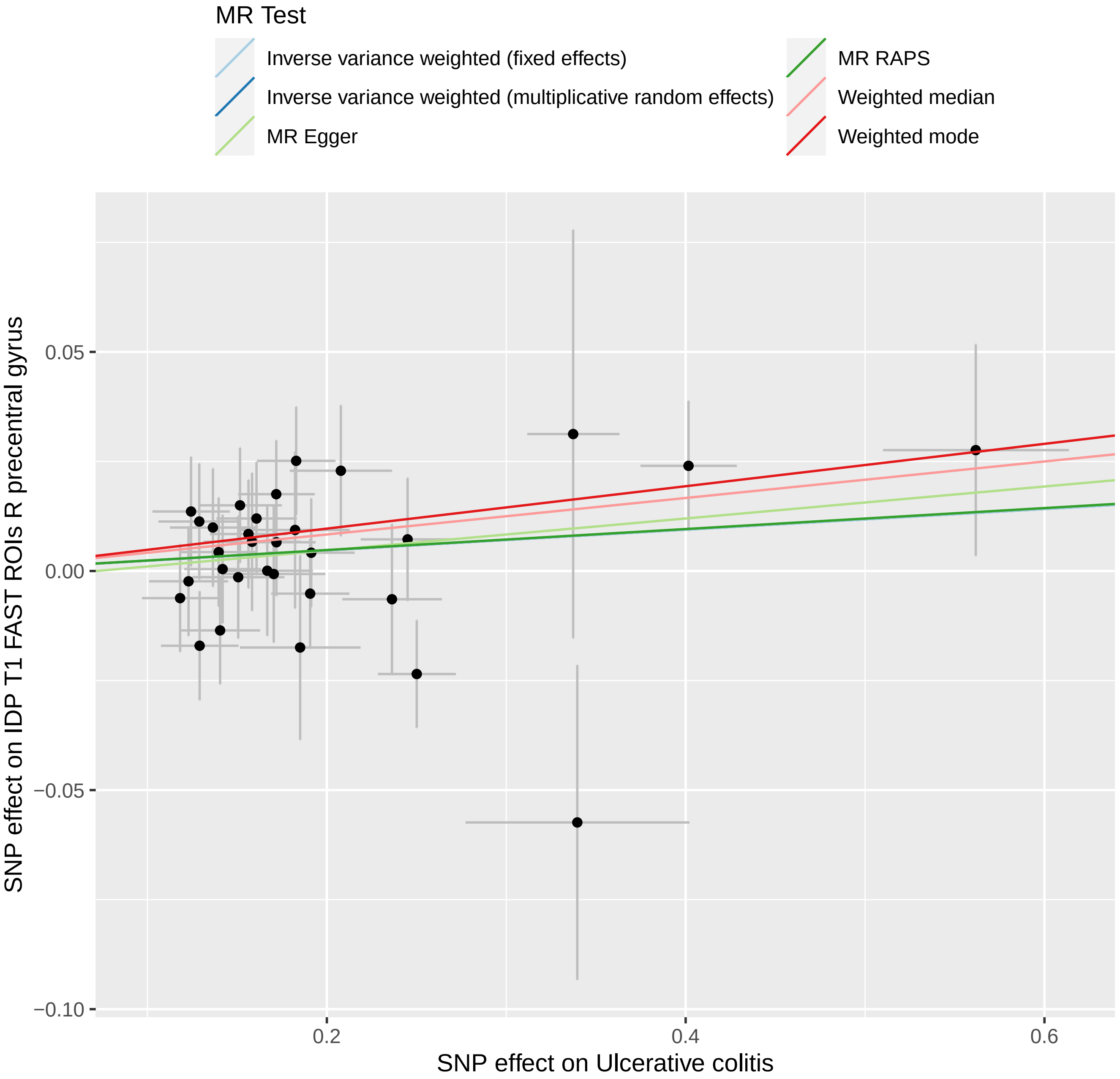

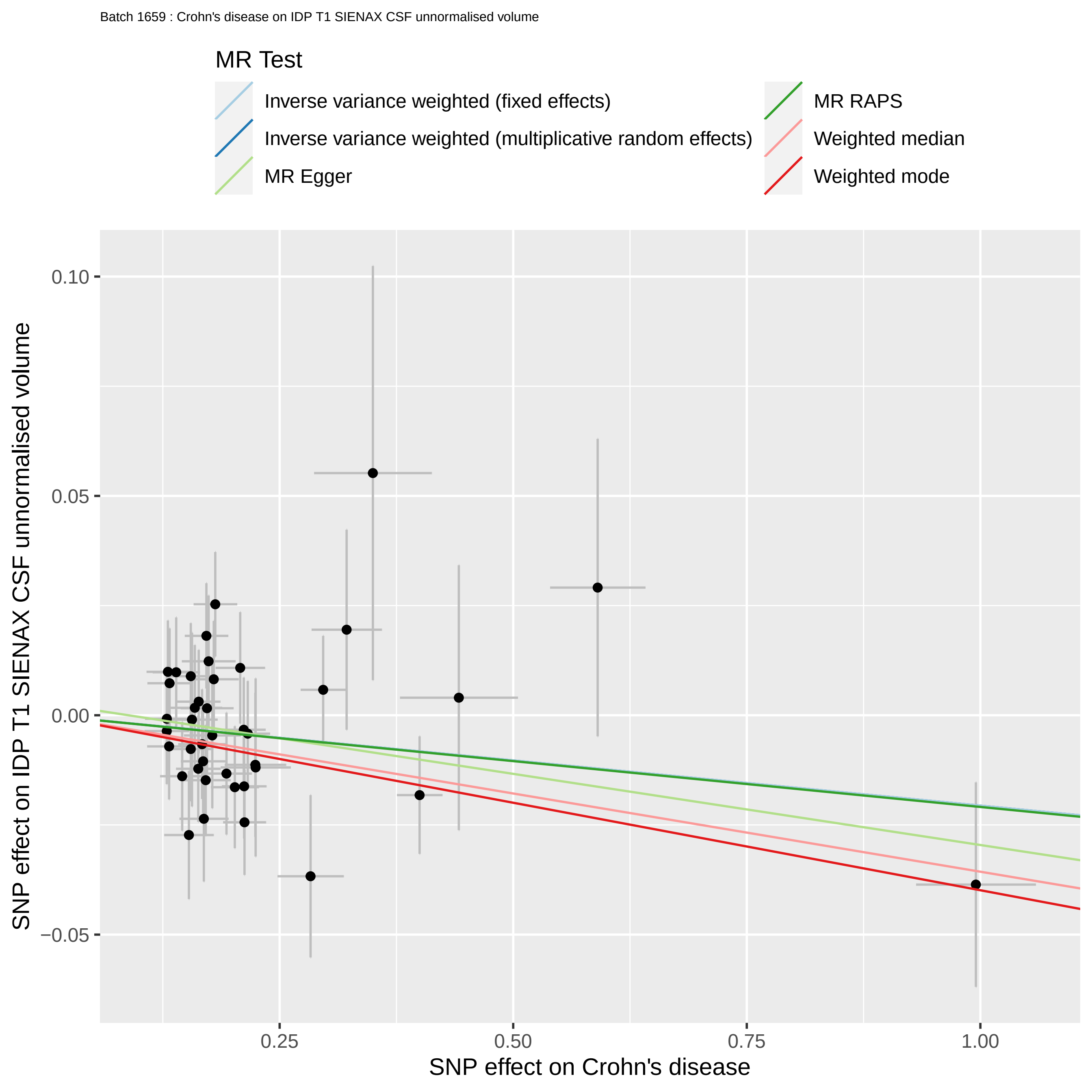
Batch 1659 : Crohn's disease on IDP T1 SIENAX CSF unnormalised volume
MR Test
Inverse variance weighted (fixed effects)
Inverse variance weighted (multiplicative random effects)
MR Egger
MR RAPS
Weighted median
Weighted mode
SNP effect on IDP T1 SIENAX CSF unnormalised volume
0.10
0.05
0.00
−0.05
SNP effect on Crohn's disease
0.25
0.50
0.75
1.00

Batch 1660 : Ulcerative colitis on IDP T1 SIENAX CSF unnormalised volume

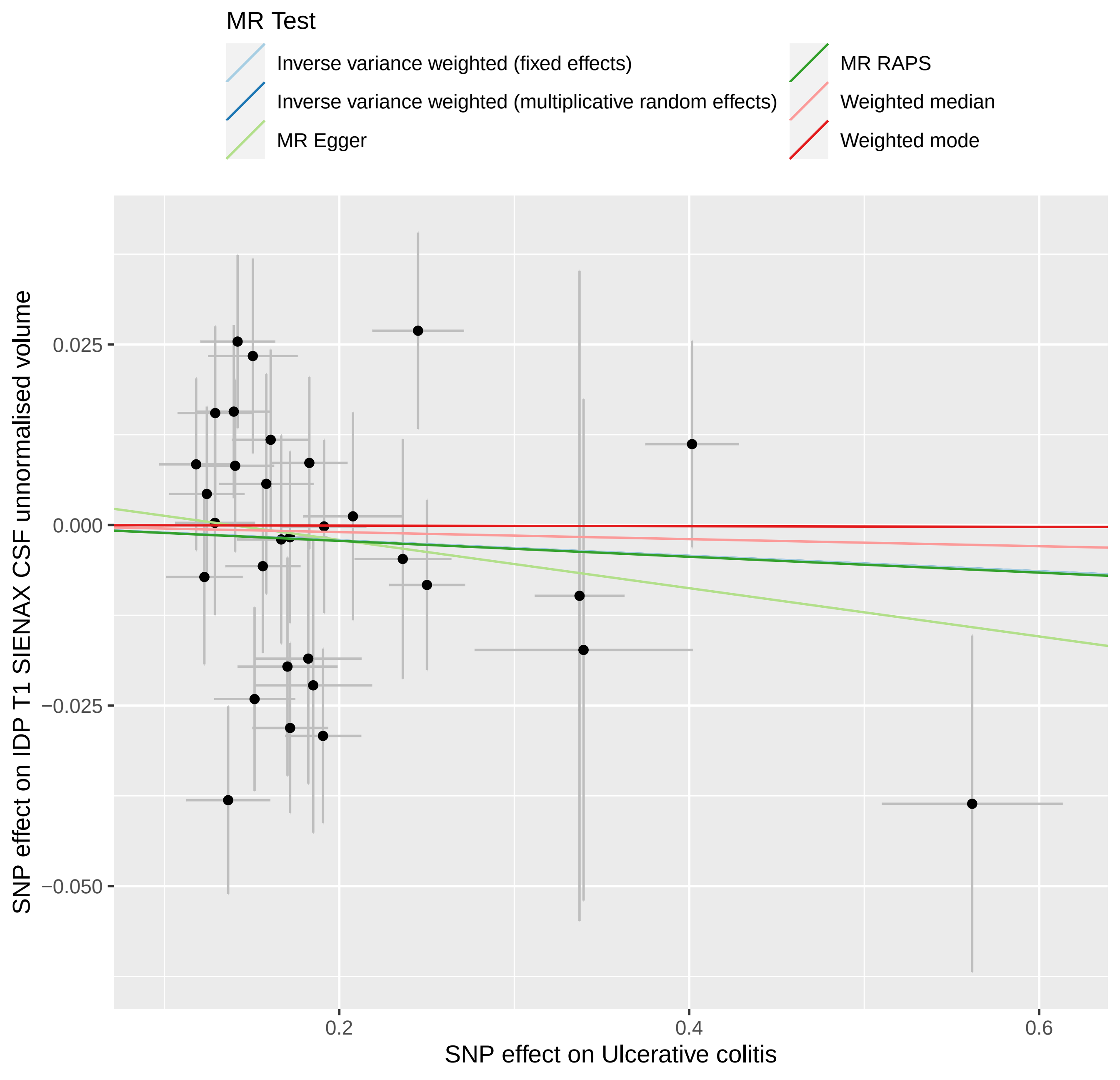

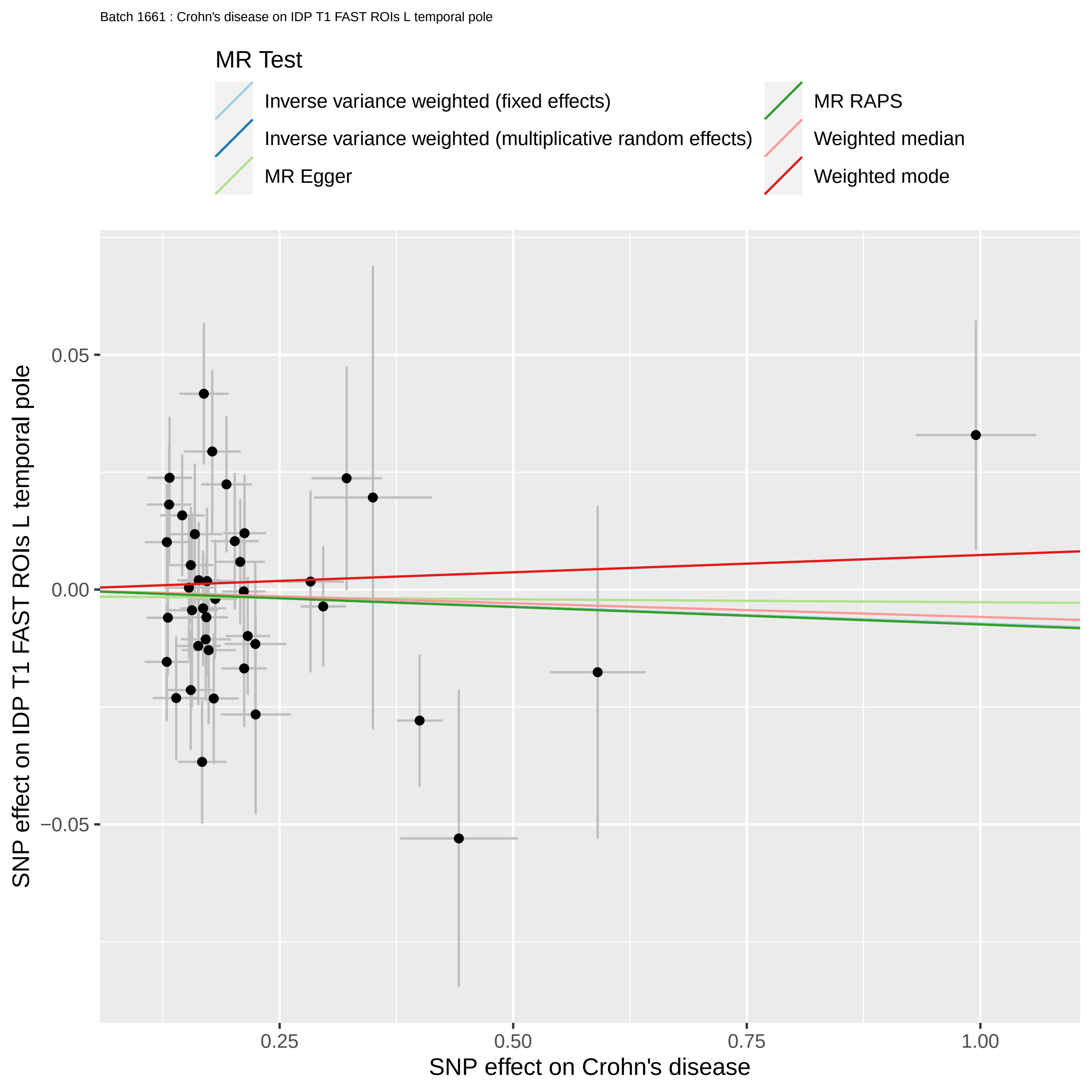

Batch 1661 : Crohn's disease on IDP T1 FAST ROIs L temporal pole
MR Test
Inverse variance weighted (fixed effects)
Inverse variance weighted (multiplicative random effects)
MR Egger
MR RAPS
Weighted median
Weighted mode
0.05
0.00
−0.05
0.25
0.50
0.75
1.00
SNP effect on IDP T1 FAST ROIs L temporal pole
SNP effect on Crohn's disease

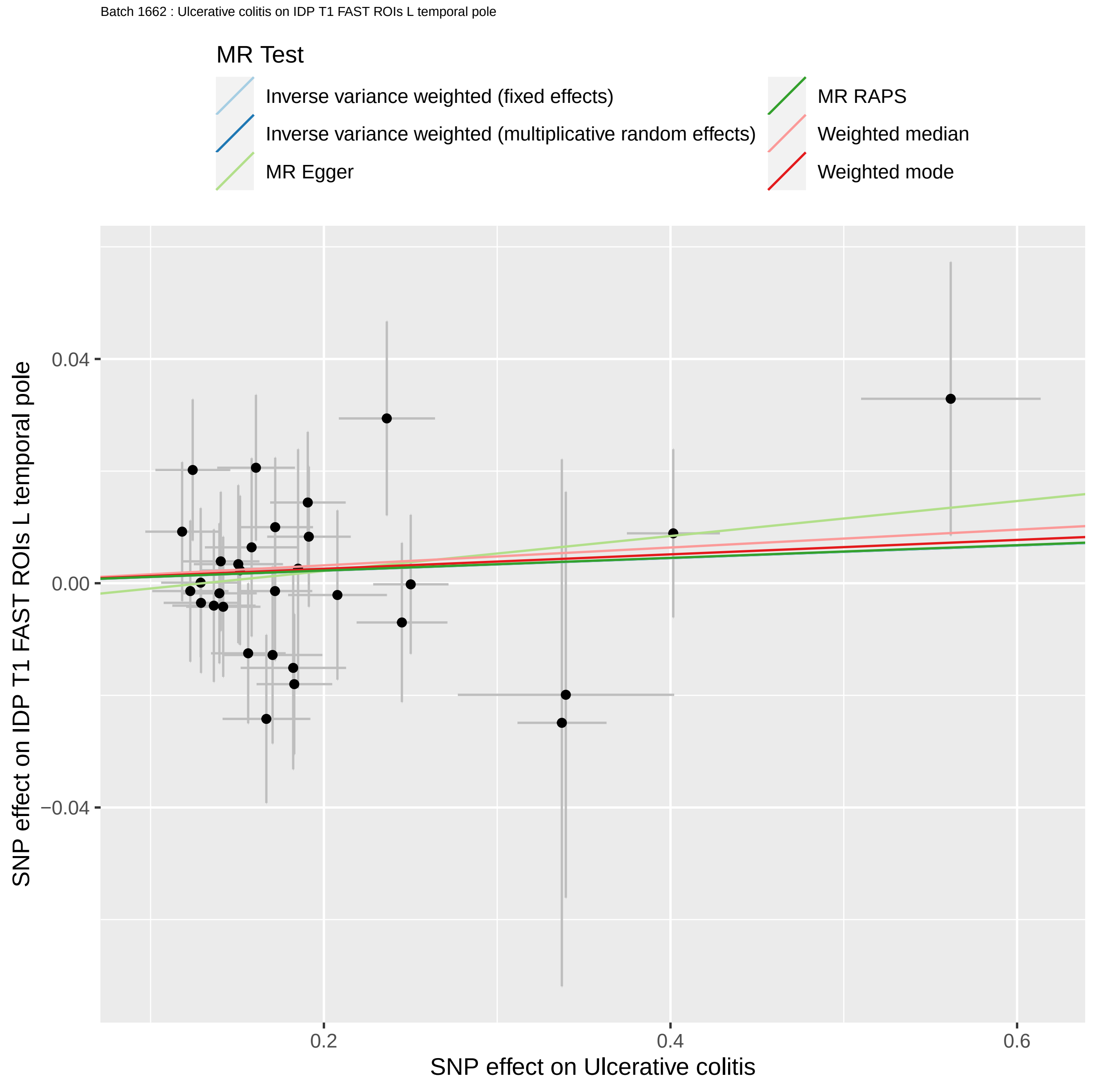
Batch 1662 : Ulcerative colitis on IDP T1 FAST ROIs L temporal pole
MR Test
Inverse variance weighted (fixed effects)
Inverse variance weighted (multiplicative random effects)
MR Egger
MR RAPS
Weighted median
Weighted mode
0.04
0.00
−0.04
0.2
0.4
0.6
SNP effect on IDP T1 FAST ROIs L temporal pole
SNP effect on Ulcerative colitis

Batch 1683 : Crohn's disease on IDP T1 FAST ROIs R temporal pole

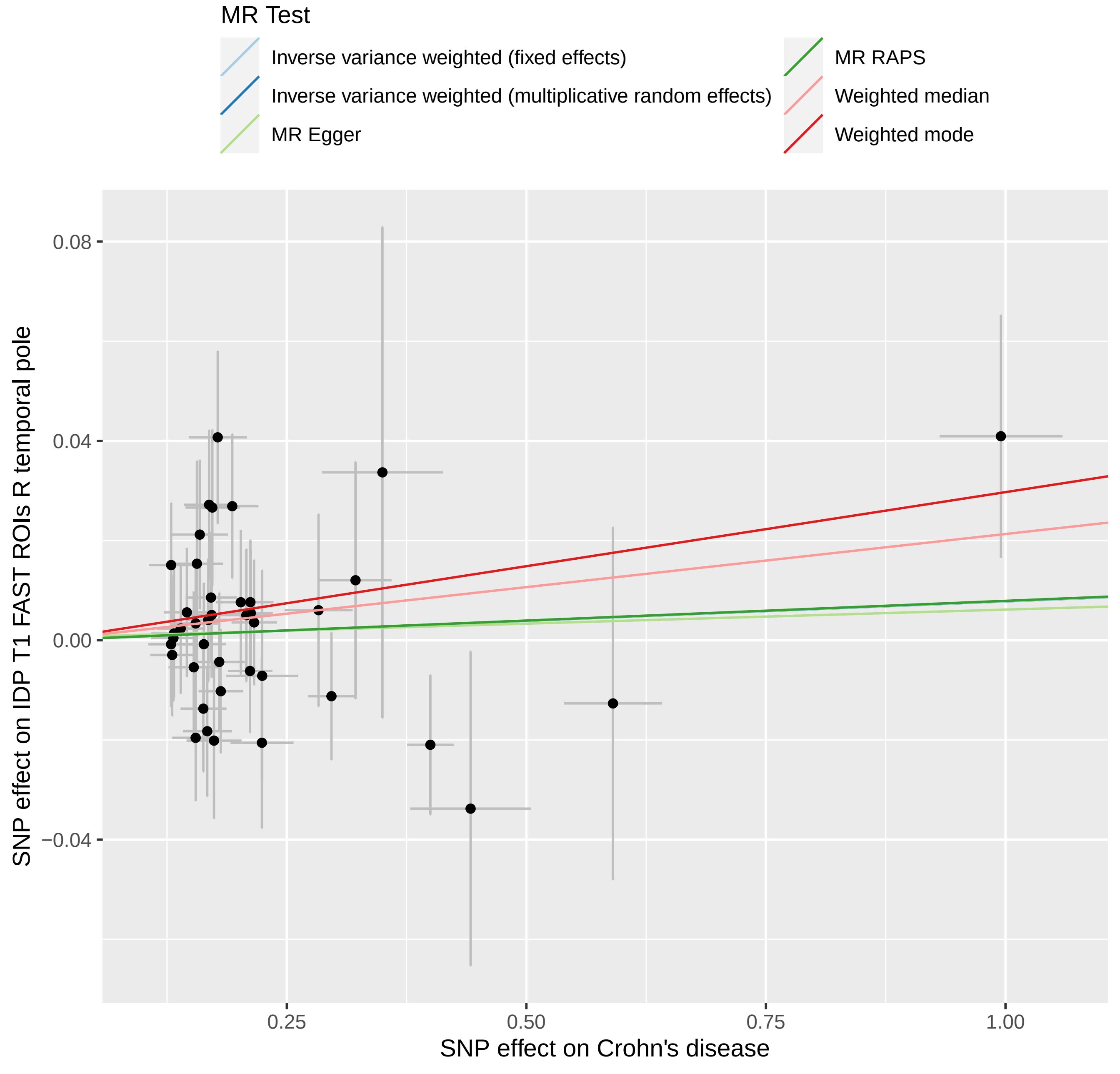

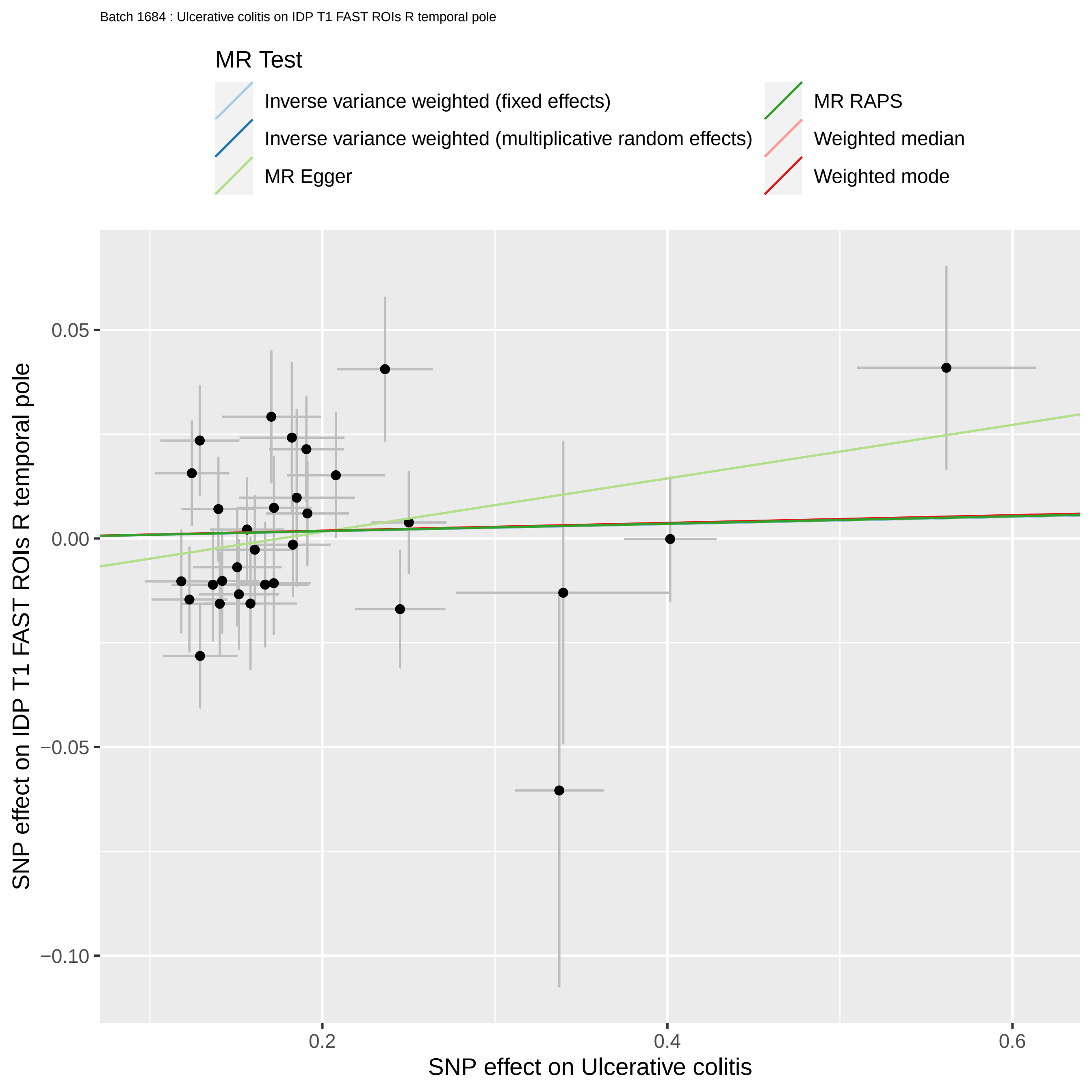

Batch 1684 : Ulcerative colitis on IDP T1 FAST ROIs R temporal pole
MR Test
Inverse variance weighted (fixed effects)
Inverse variance weighted (multiplicative random effects)
MR Egger
MR RAPS
Weighted median
Weighted mode
0.05
0.00
−0.05
−0.10
0.2
0.4
0.6
SNP effect on IDP T1 FAST ROIs R temporal pole
SNP effect on Ulcerative colitis

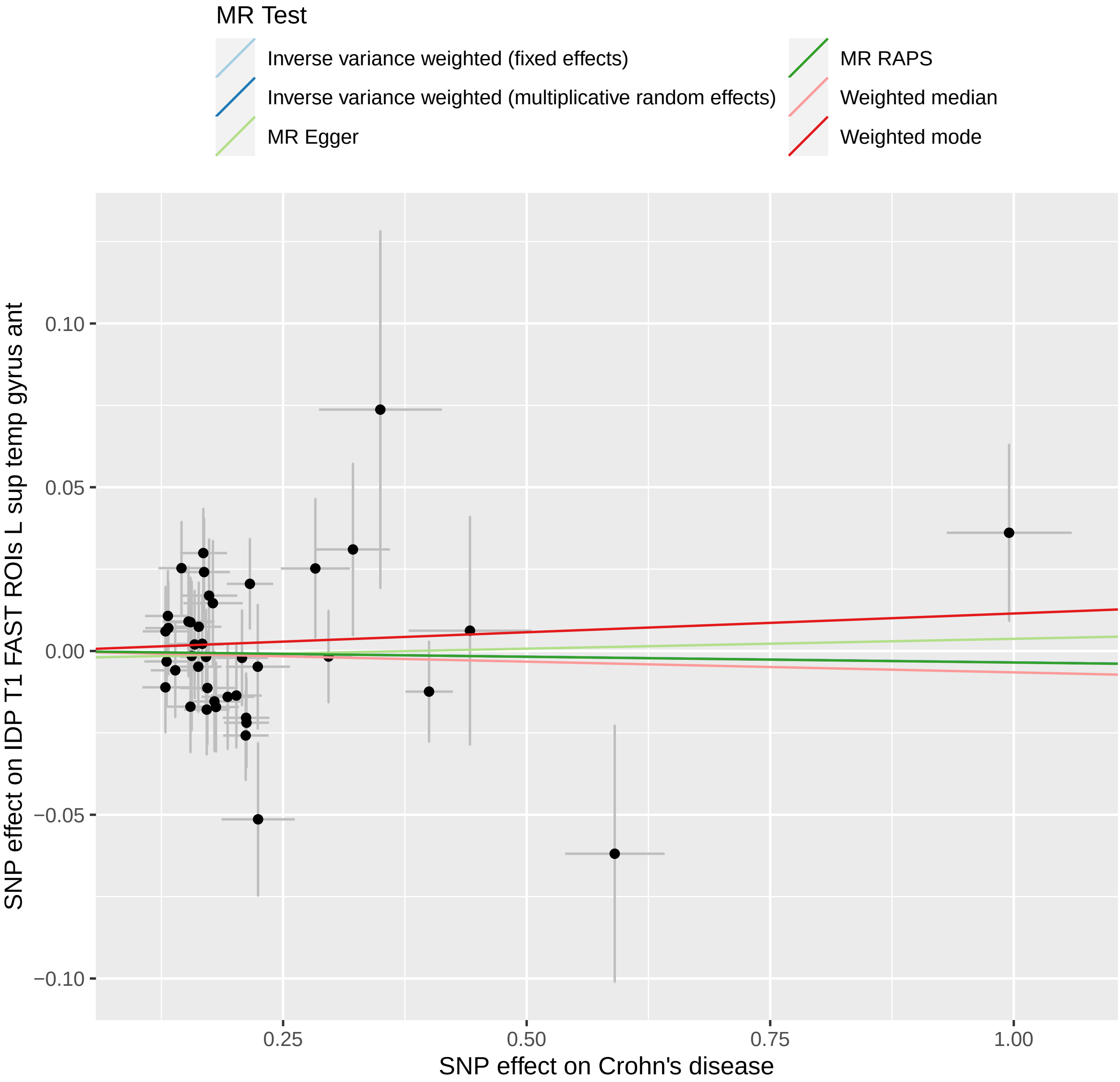
MR Test
Inverse variance weighted (fixed effects)
Inverse variance weighted (multiplicative random effects)
MR Egger
MR RAPS
Weighted median
Weighted mode
SNP effect on IDP T1 FAST ROIs L sup temp gyrus ant
0.10
0.05
0.00
−0.05
−0.10
0.25
0.50
0.75
1.00
SNP effect on Crohn's disease

Batch 1706 : Ulcerative colitis on IDP T1 FAST ROIs L sup temp gyrus ant

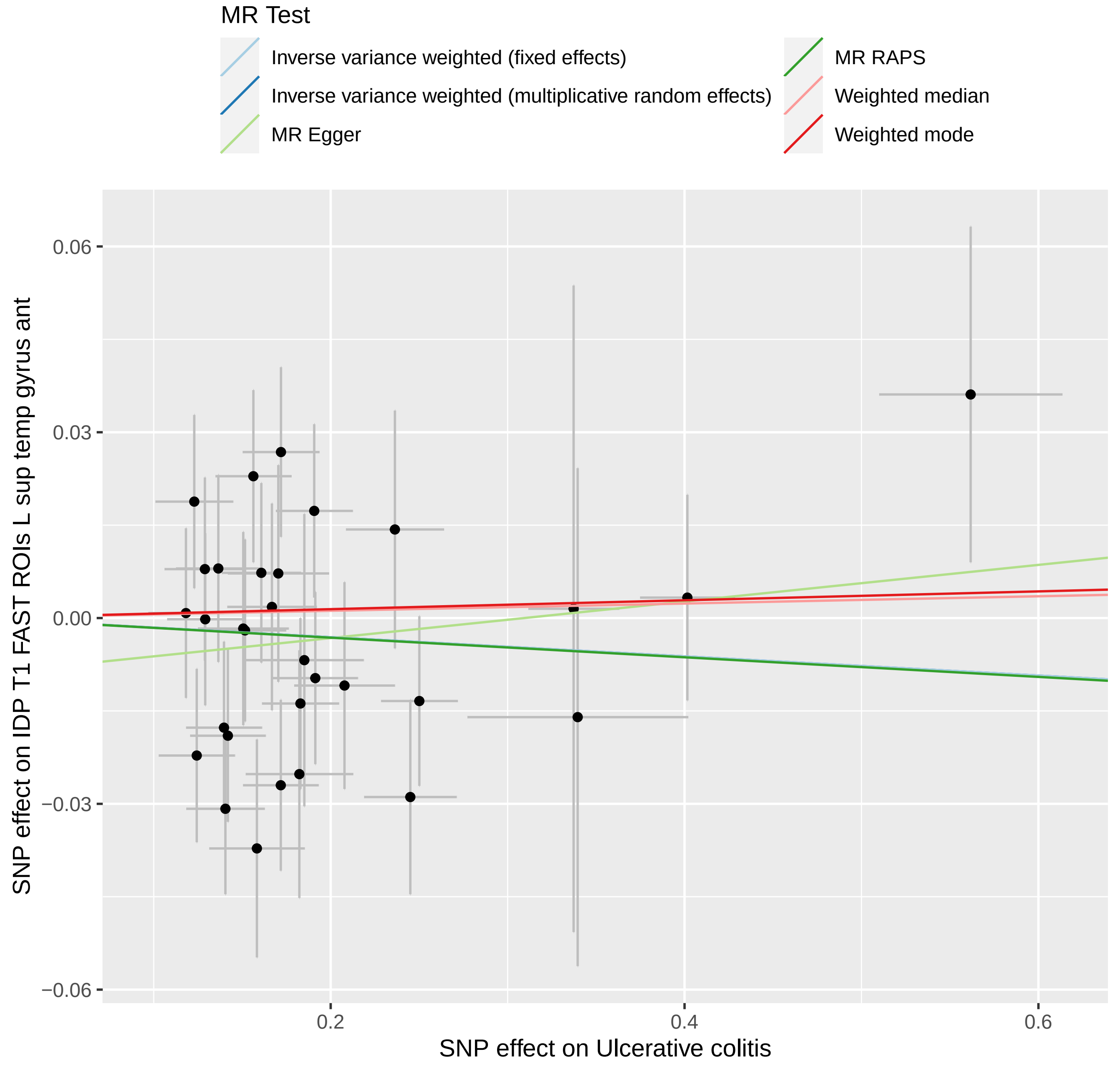

Batch 1727 : Crohn's disease on IDP T1 FAST ROIs R sup temp gyrus ant

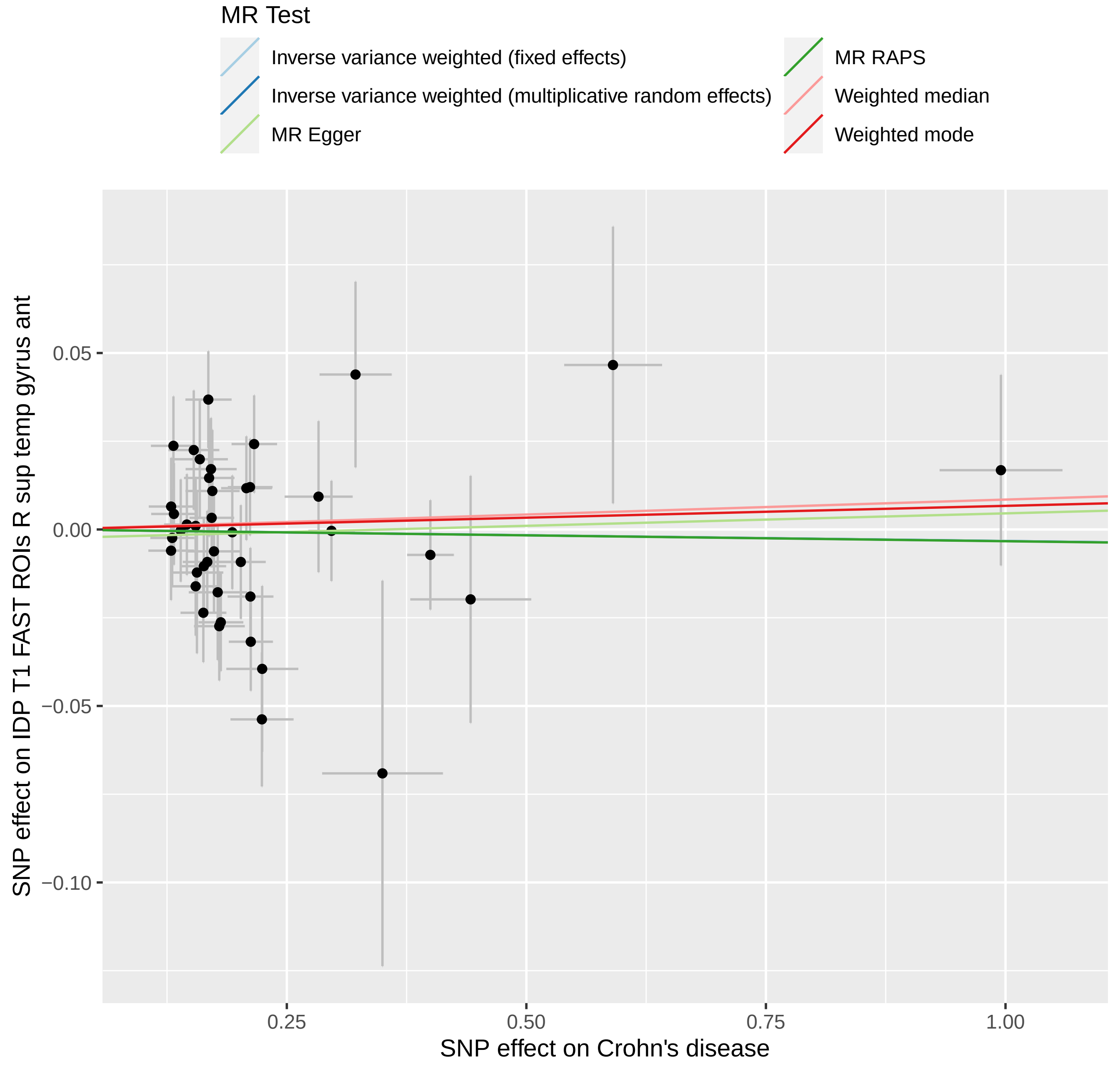

Batch 1728 : Ulcerative colitis on IDP T1 FAST ROIs R sup temp gyrus ant

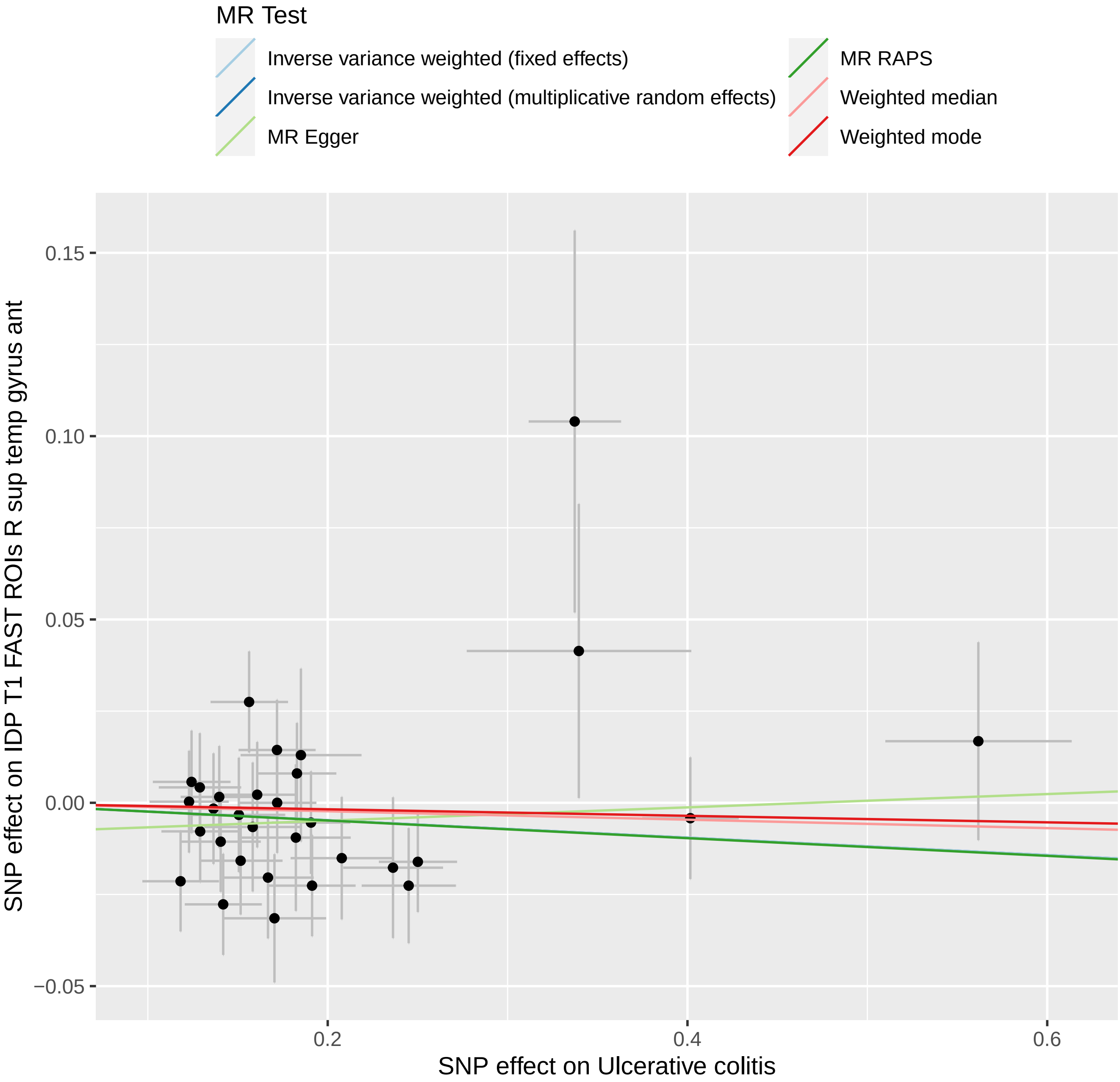

Batch 1749 : Crohn's disease on IDP T1 FAST ROIs L sup temp gyrus post

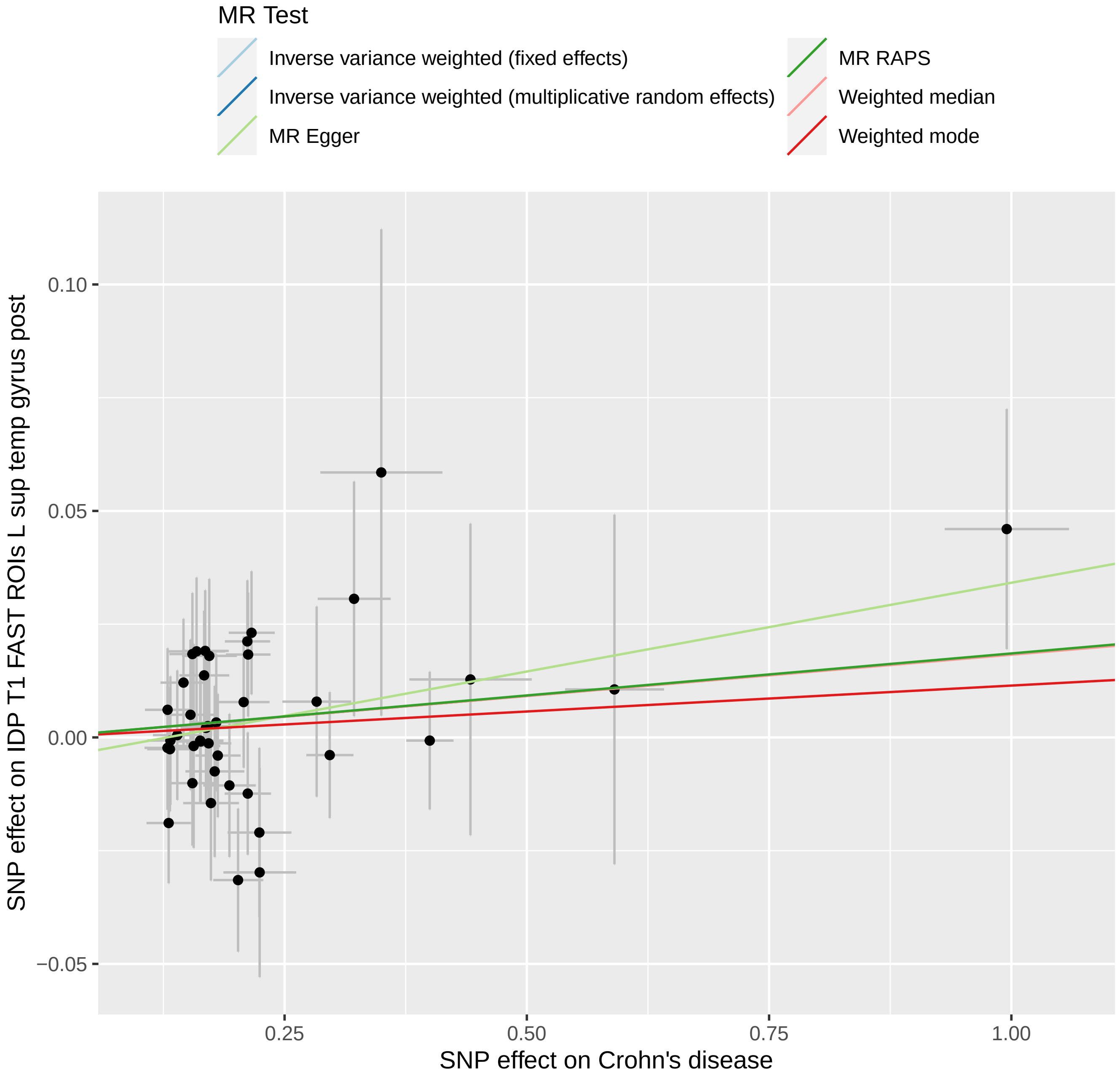

Batch 1750 : Ulcerative colitis on IDP T1 FAST ROIs L sup temp gyrus post

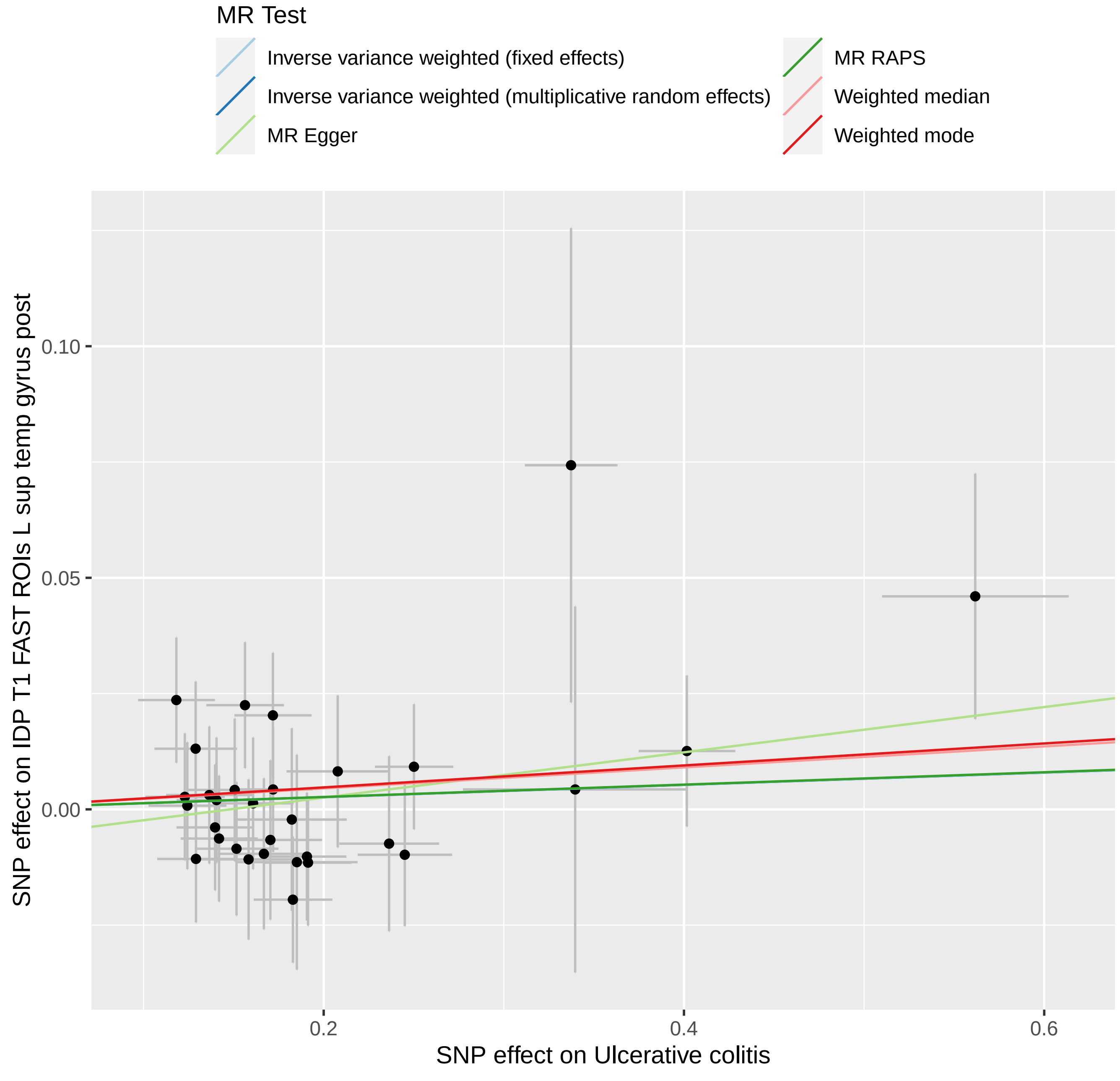

Batch 1771 : Crohn's disease on IDP T1 FAST ROIs R sup temp gyrus post

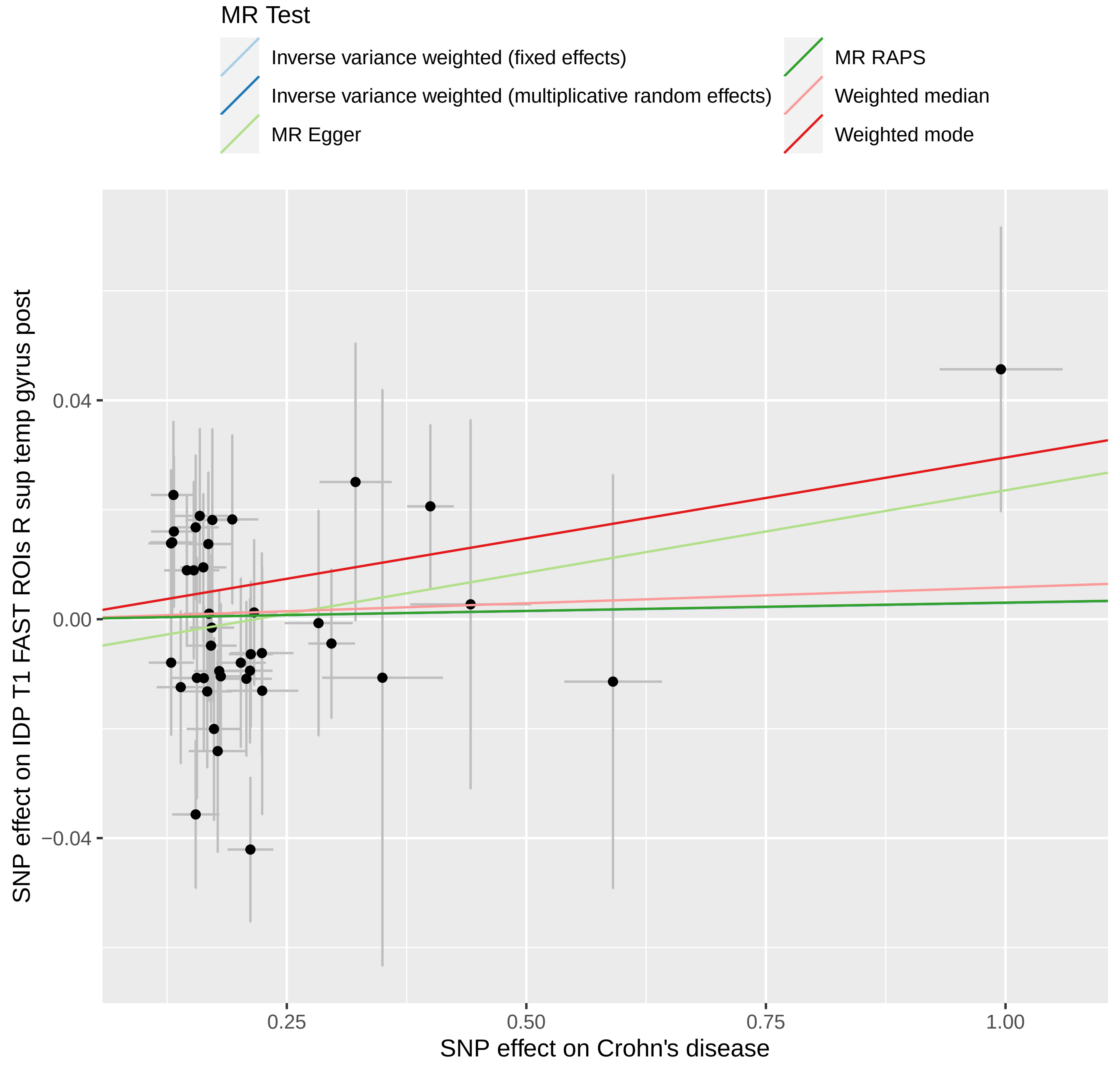

Batch 1772 : Ulcerative colitis on IDP T1 FAST ROIs R sup temp gyrus post

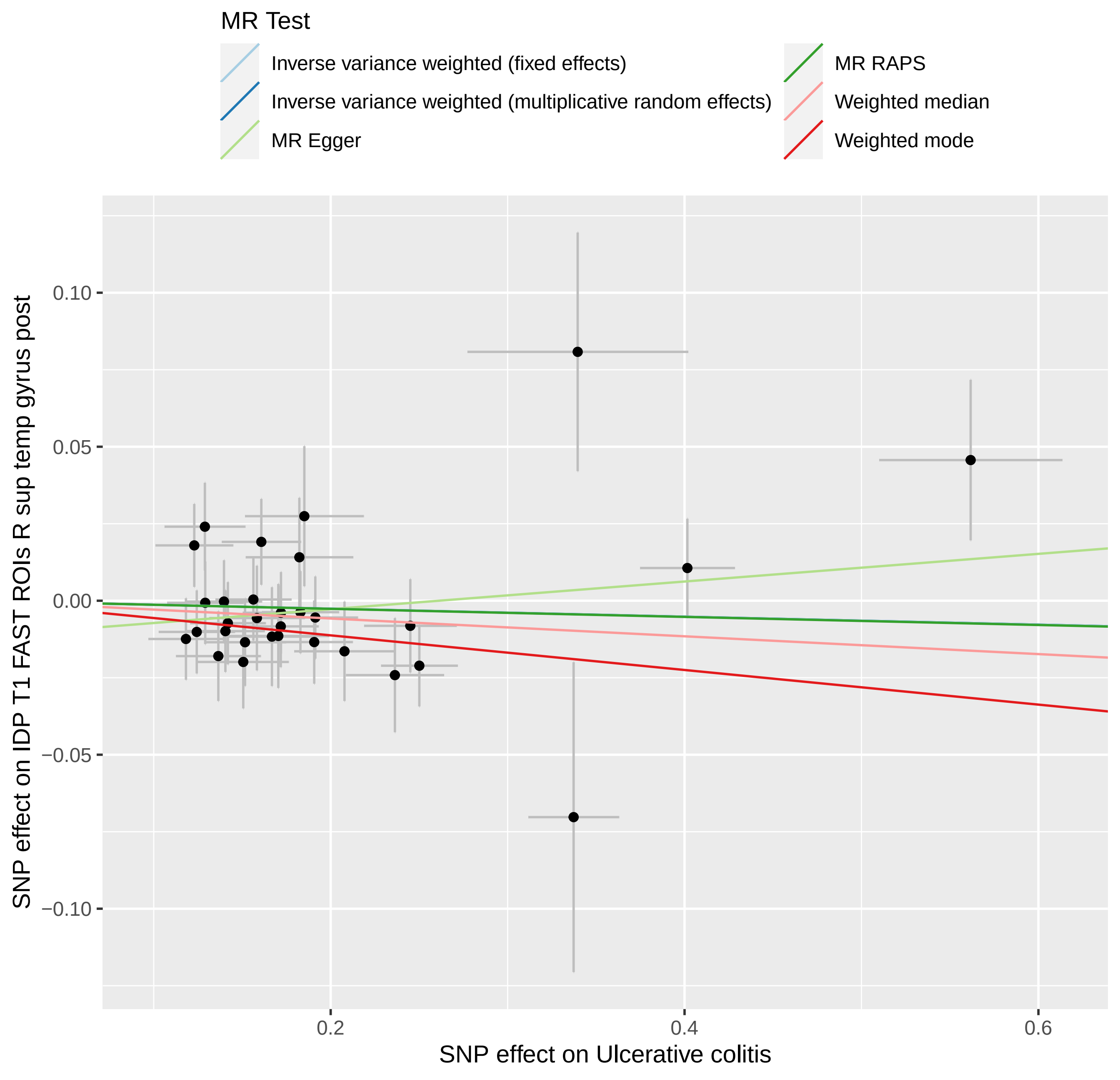

Batch 1793 : Crohn's disease on IDP T1 FAST ROIs L mid temp gyrus ant

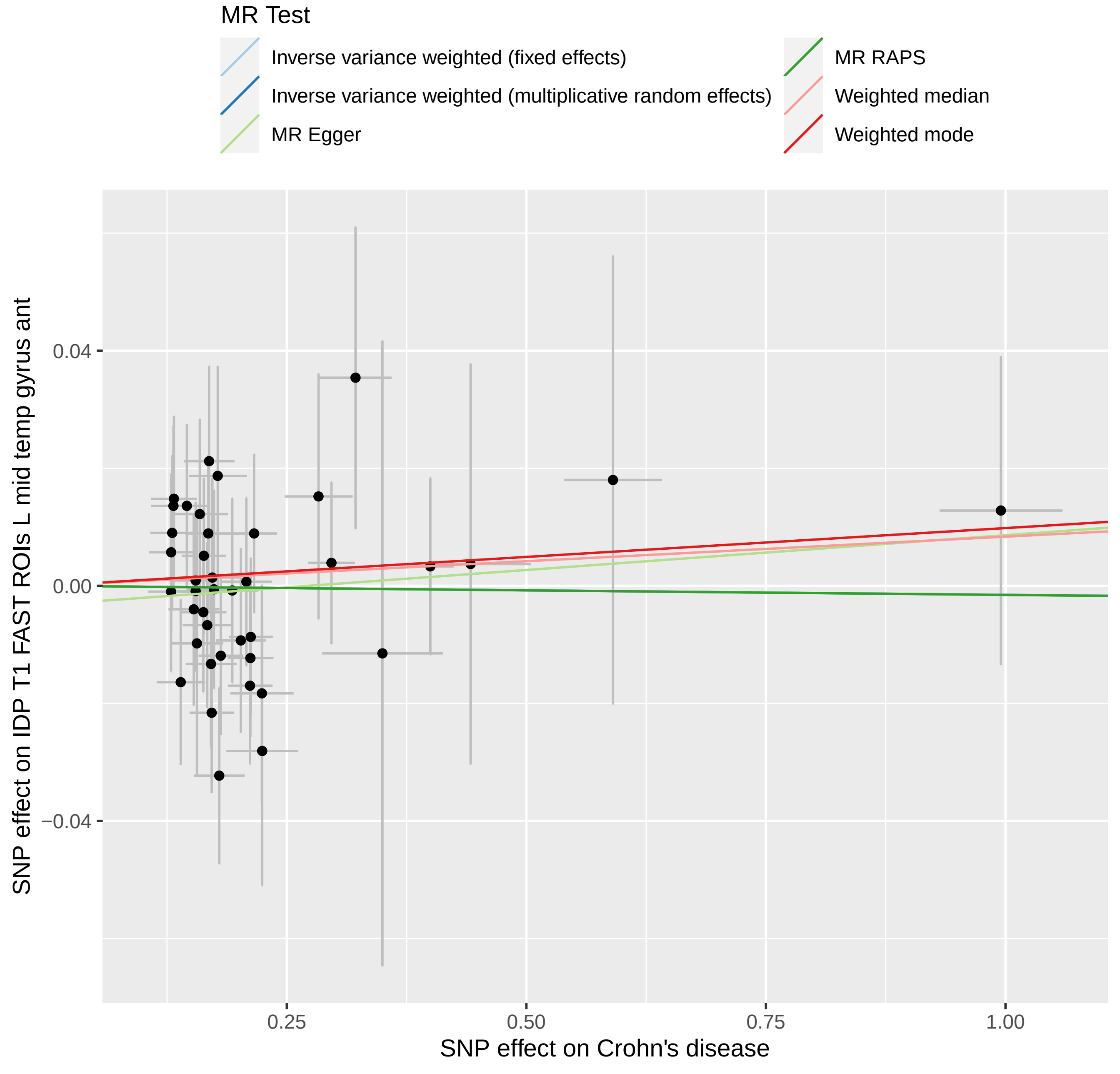

Batch 1794 : Ulcerative colitis on IDP T1 FAST ROIs L mid temp gyrus ant

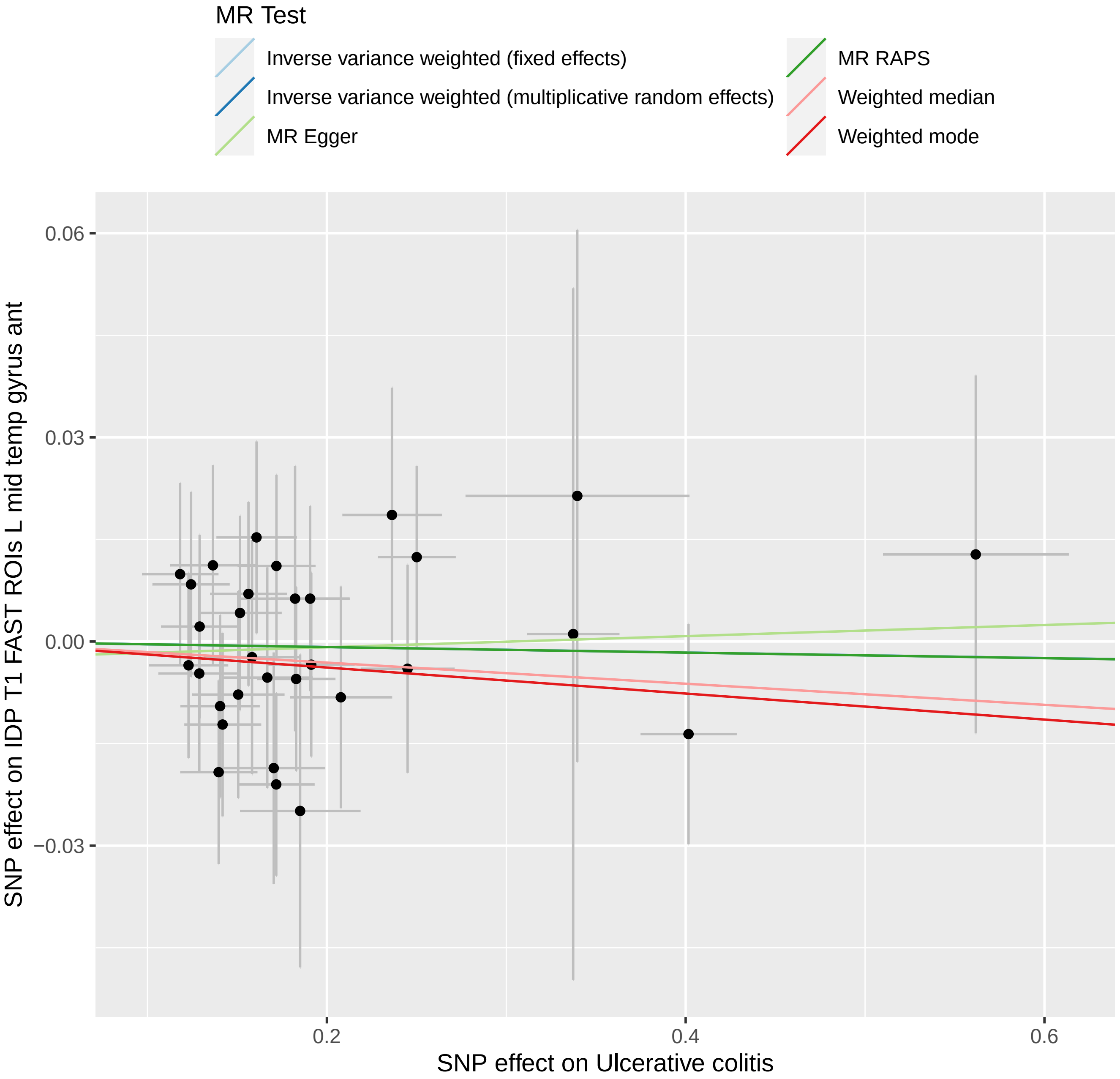

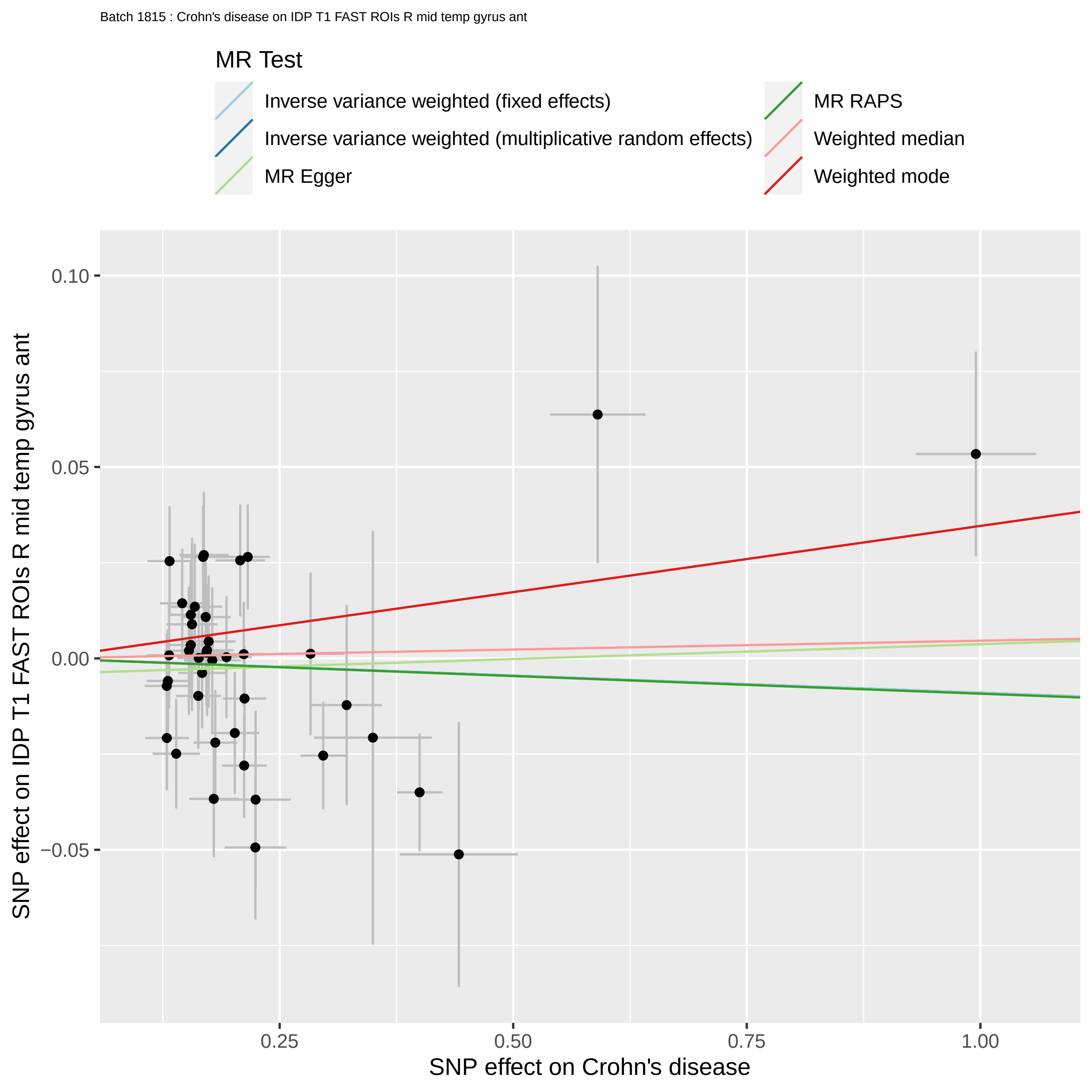
Batch 1815 : Crohn's disease on IDP T1 FAST ROIs R mid temp gyrus ant
MR Test
Inverse variance weighted (fixed effects)
Inverse variance weighted (multiplicative random effects)
MR Egger
MR RAPS
Weighted median
Weighted mode
0.10
0.05
0.00
−0.05
0.25
0.50
0.75
1.00
SNP effect on IDP T1 FAST ROIs R mid temp gyrus ant
SNP effect on Crohn's disease

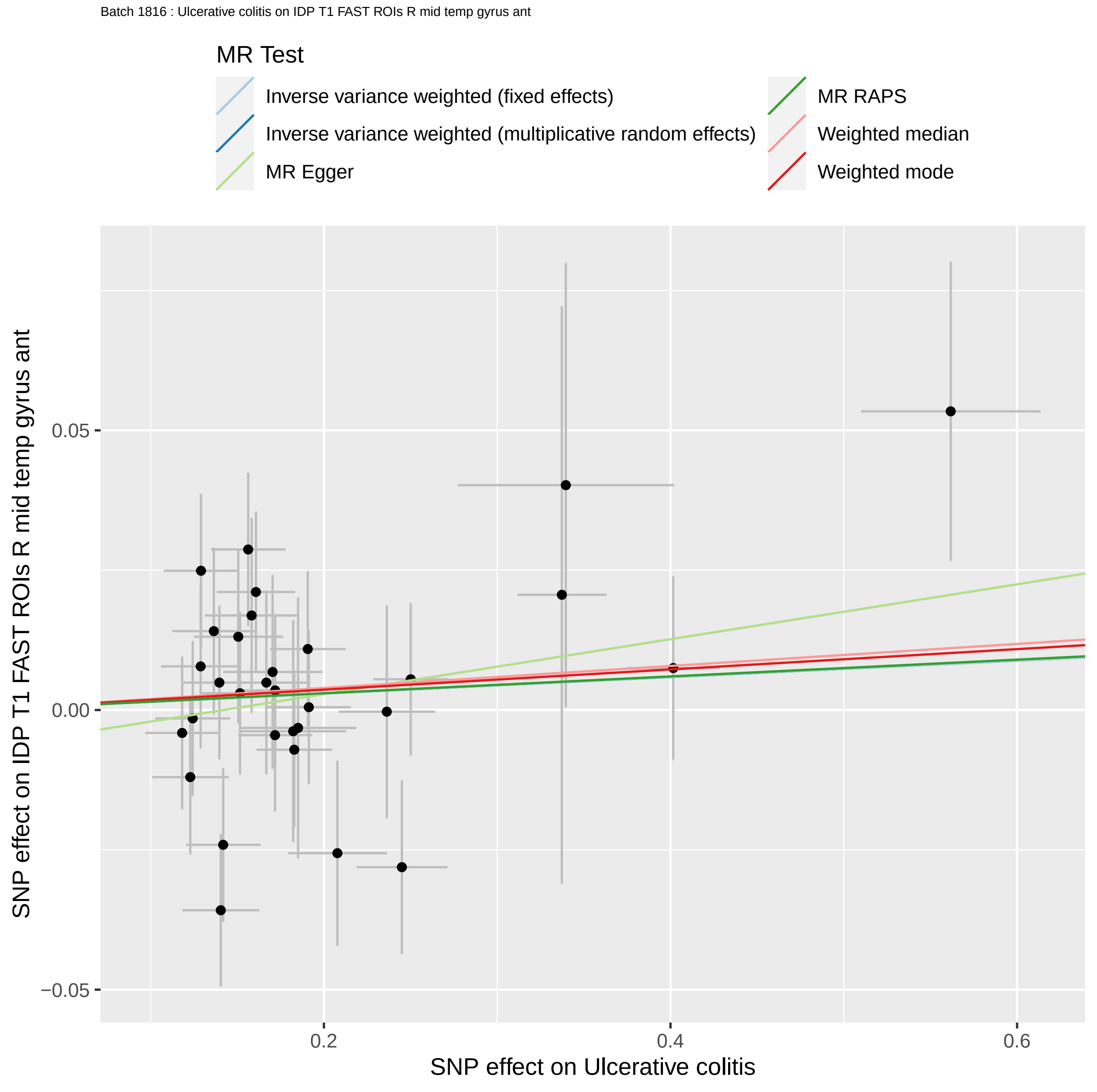

Batch 1816 : Ulcerative colitis on IDP T1 FAST ROIs R mid temp gyrus ant
MR Test
Inverse variance weighted (fixed effects)
Inverse variance weighted (multiplicative random effects)
MR Egger
MR RAPS
Weighted median
Weighted mode
SNP effect on IDP T1 FAST ROIs R mid temp gyrus ant
0.05
0.00
−0.05
0.2
0.4
0.6
SNP effect on Ulcerative colitis

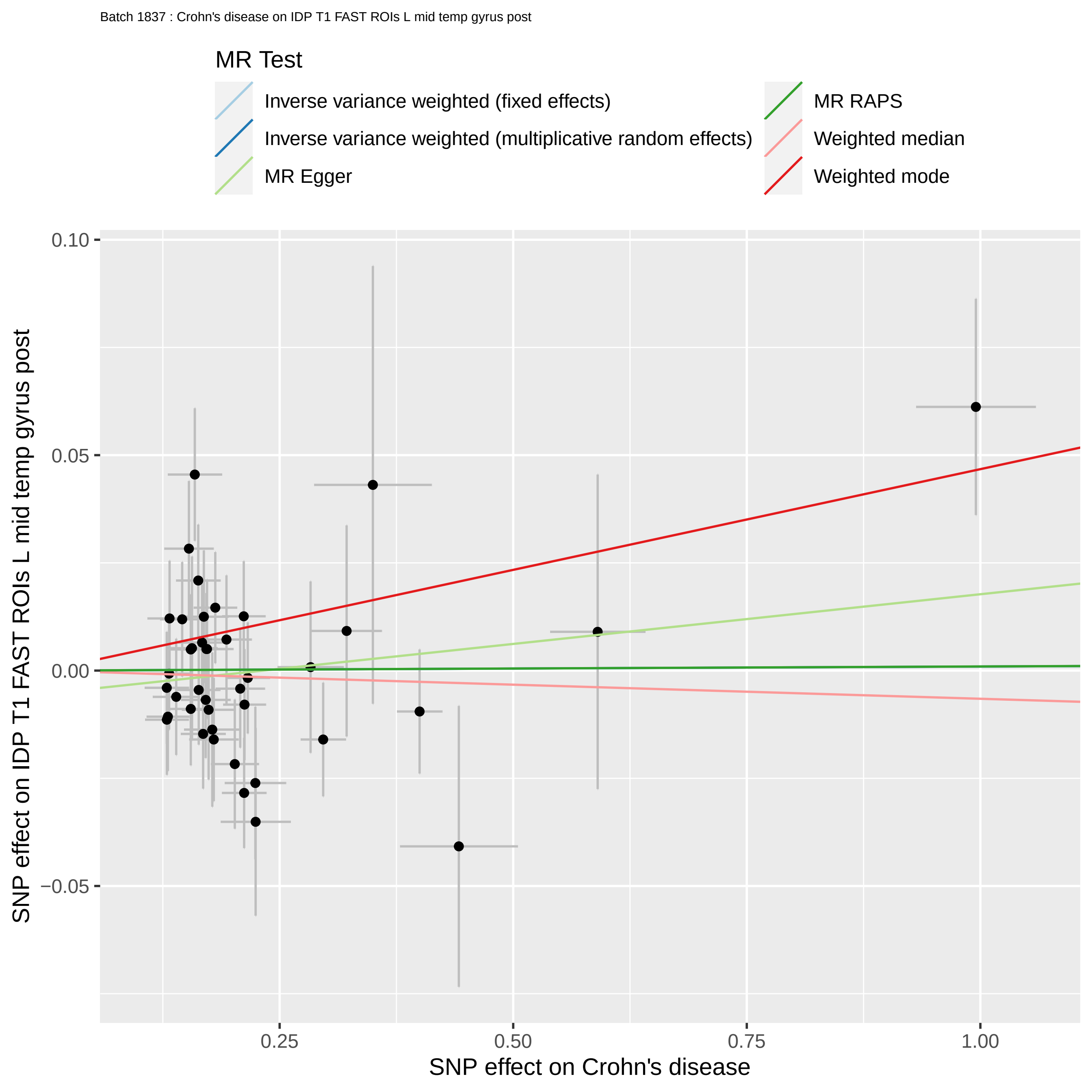
Batch 1837 : Crohn's disease on IDP T1 FAST ROIs L mid temp gyrus post
MR Test
Inverse variance weighted (fixed effects)
Inverse variance weighted (multiplicative random effects)
MR Egger
MR RAPS
Weighted median
Weighted mode
0.10
0.05
0.00
−0.05
0.25
0.50
0.75
1.00
SNP effect on Crohn's disease
SNP effect on IDP T1 FAST ROIs L mid temp gyrus post

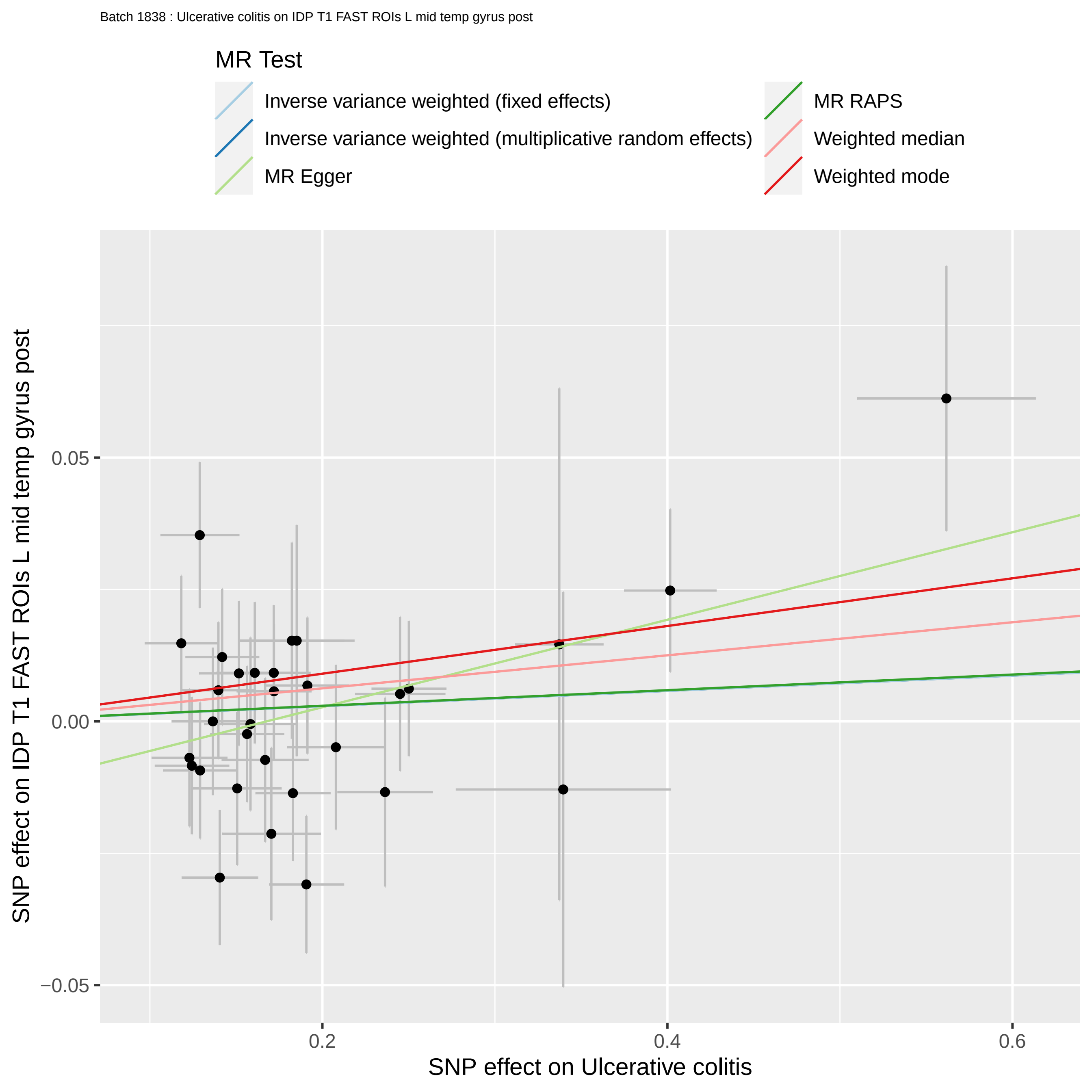

Batch 1838 : Ulcerative colitis on IDP T1 FAST ROIs L mid temp gyrus post
MR Test
Inverse variance weighted (fixed effects)
Inverse variance weighted (multiplicative random effects)
MR Egger
MR RAPS
Weighted median
Weighted mode
SNP effect on IDP T1 FAST ROIs L mid temp gyrus post
0.05
0.00
−0.05
0.2
0.4
0.6
SNP effect on Ulcerative colitis

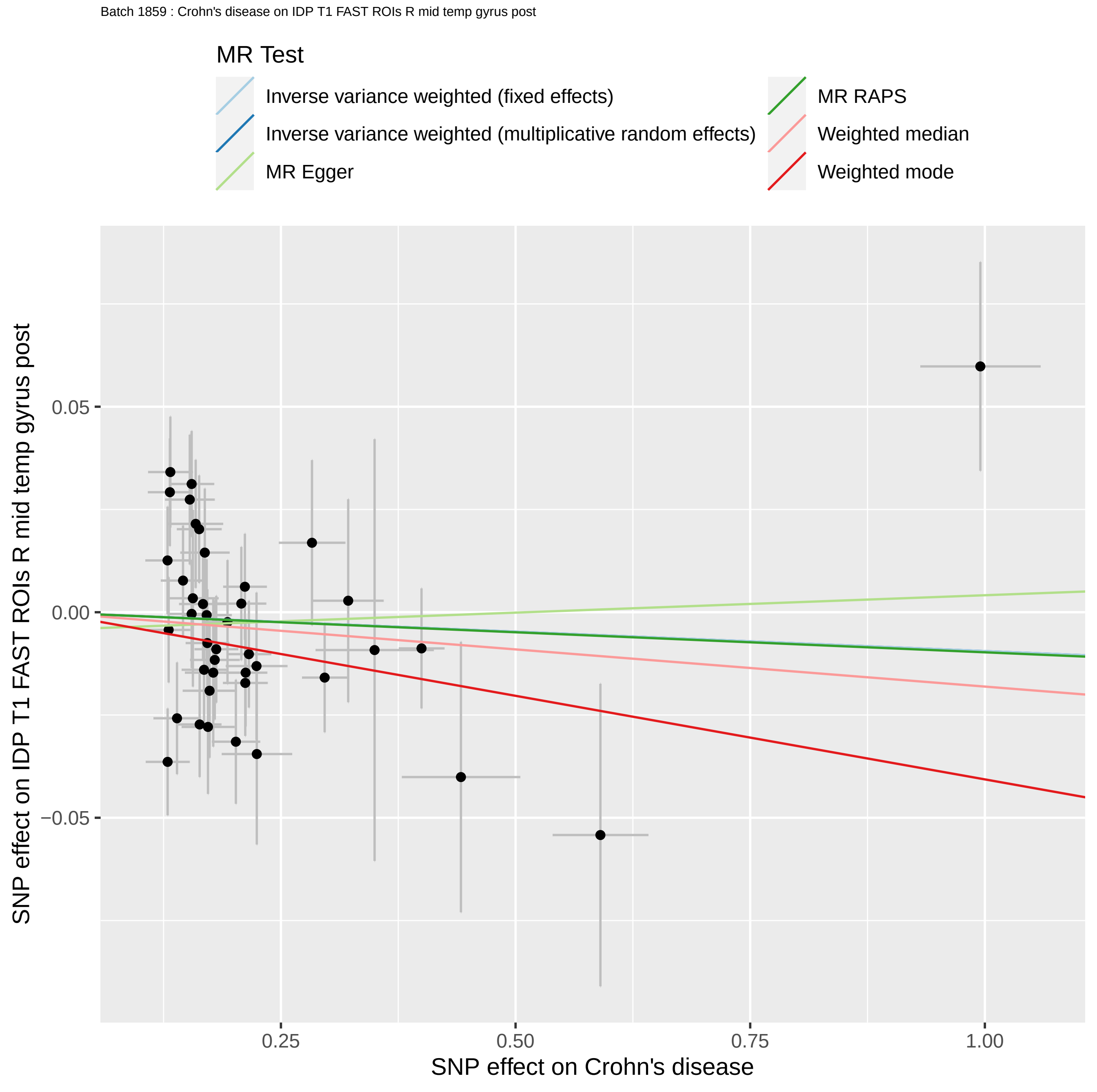
Batch 1859 : Crohn's disease on IDP T1 FAST ROIs R mid temp gyrus post
MR Test
Inverse variance weighted (fixed effects)
Inverse variance weighted (multiplicative random effects)
MR Egger
MR RAPS
Weighted median
Weighted mode
0.05
0.00
−0.05
0.25
0.50
0.75
1.00
SNP effect on IDP T1 FAST ROIs R mid temp gyrus post
SNP effect on Crohn's disease

Batch 1860 : Ulcerative colitis on IDP T1 FAST ROIs R mid temp gyrus post

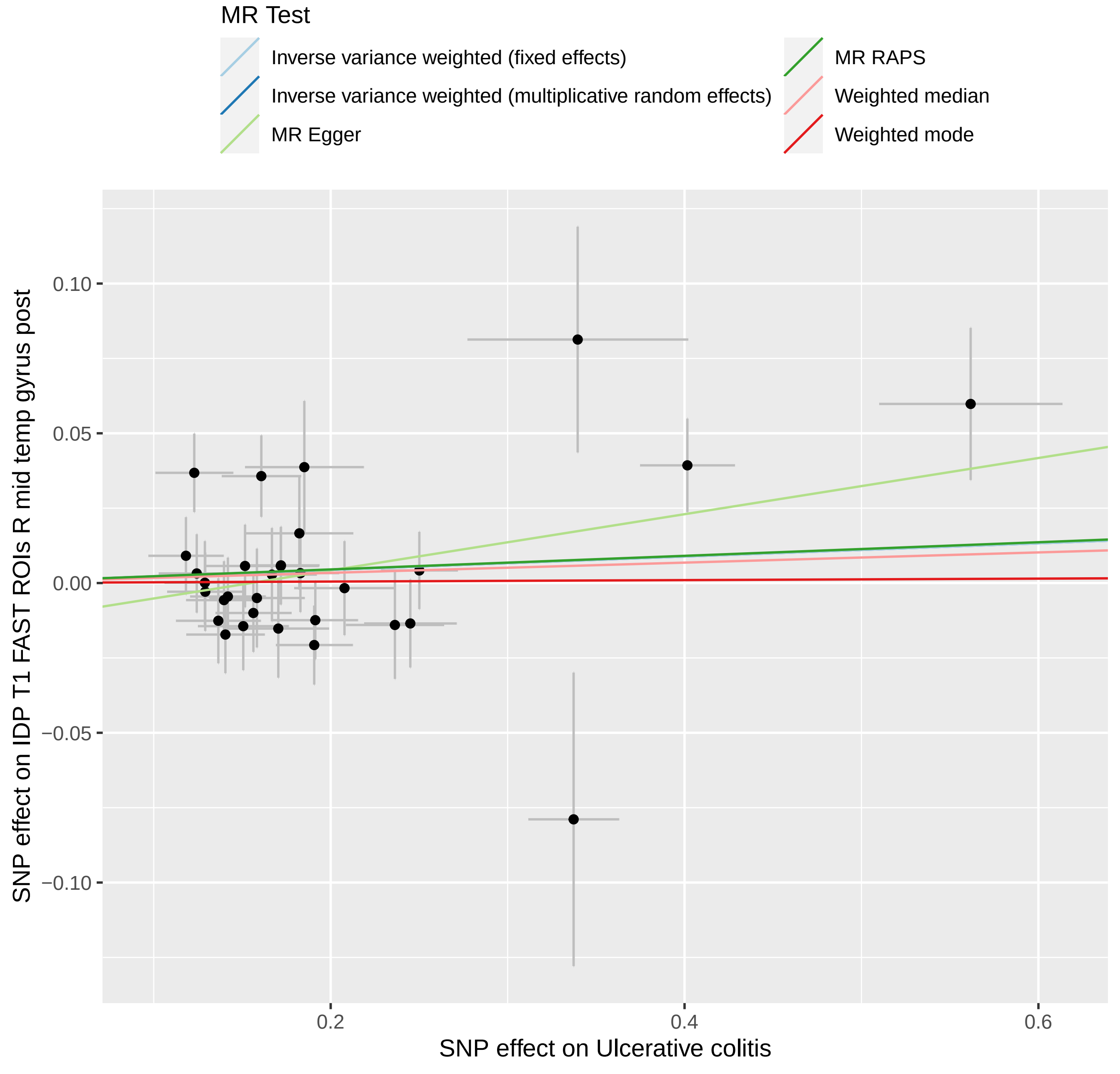

Batch 1881 : Crohn's disease on IDP T1 SIENAX grey normalised volume

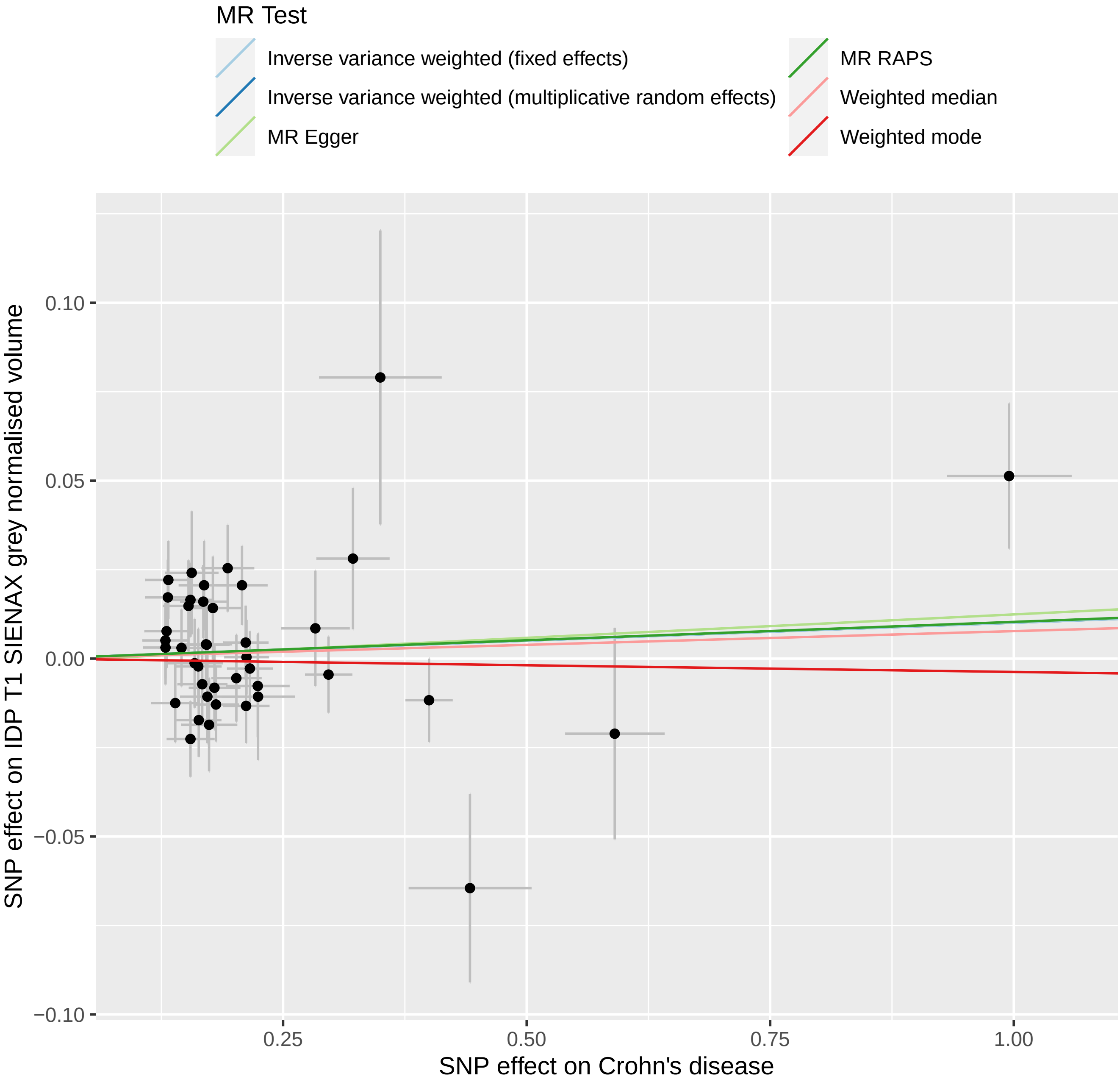

Batch 1882 : Ulcerative colitis on IDP T1 SIENAX grey normalised volume

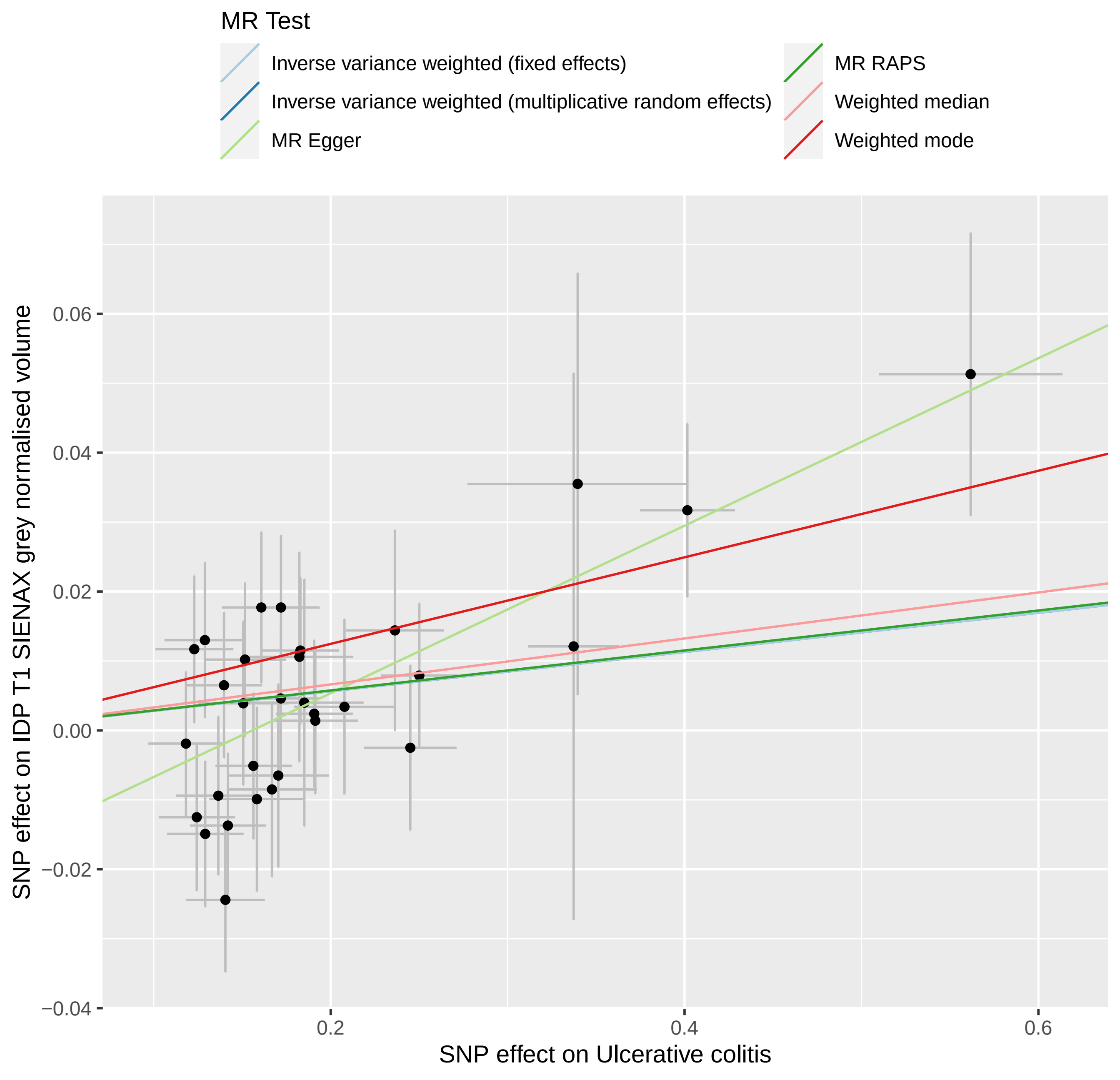

Batch 1883 : Crohn's disease on IDP T1 FAST ROIs L mid temp gyrus tempocc

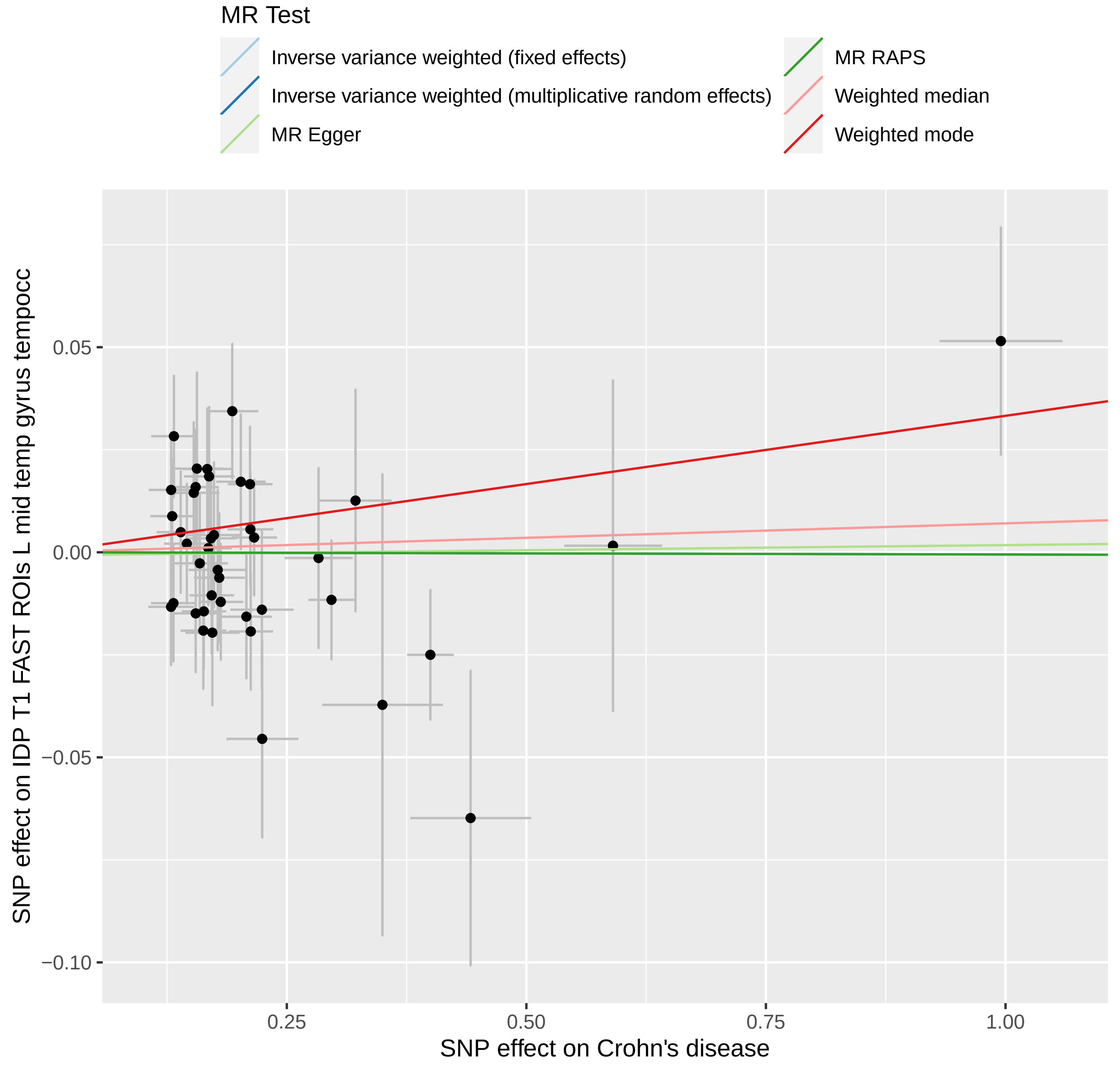

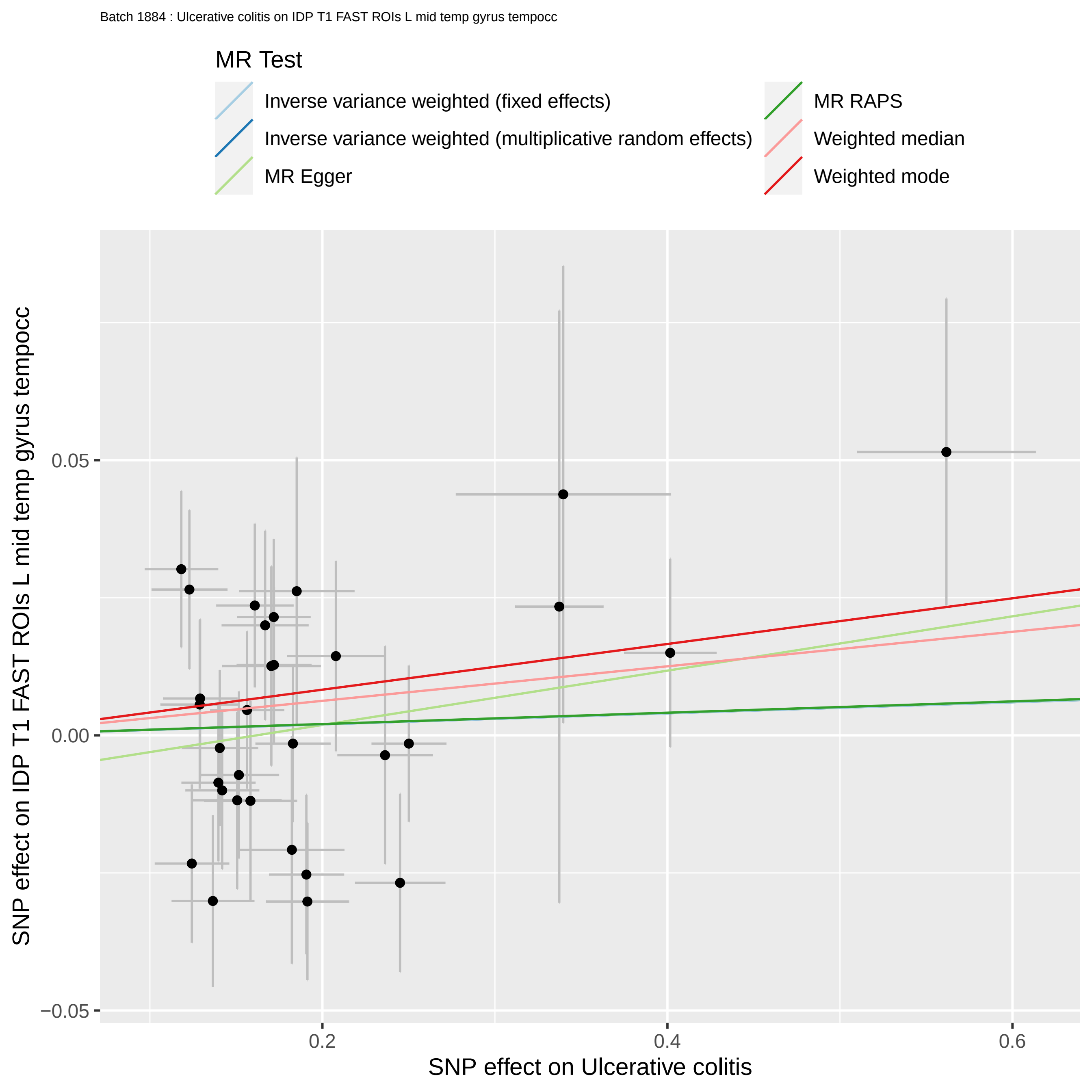

Batch 1884 : Ulcerative colitis on IDP T1 FAST ROIs L mid temp gyrus tempocc
MR Test
Inverse variance weighted (fixed effects)
Inverse variance weighted (multiplicative random effects)
MR Egger
MR RAPS
Weighted median
Weighted mode
SNP effect on IDP T1 FAST ROIs L mid temp gyrus tempocc
0.05
0.00
−0.05
0.2
0.4
0.6
SNP effect on Ulcerative colitis

Batch 1905 : Crohn's disease on IDP T1 FAST ROIs R mid temp gyrus tempocc

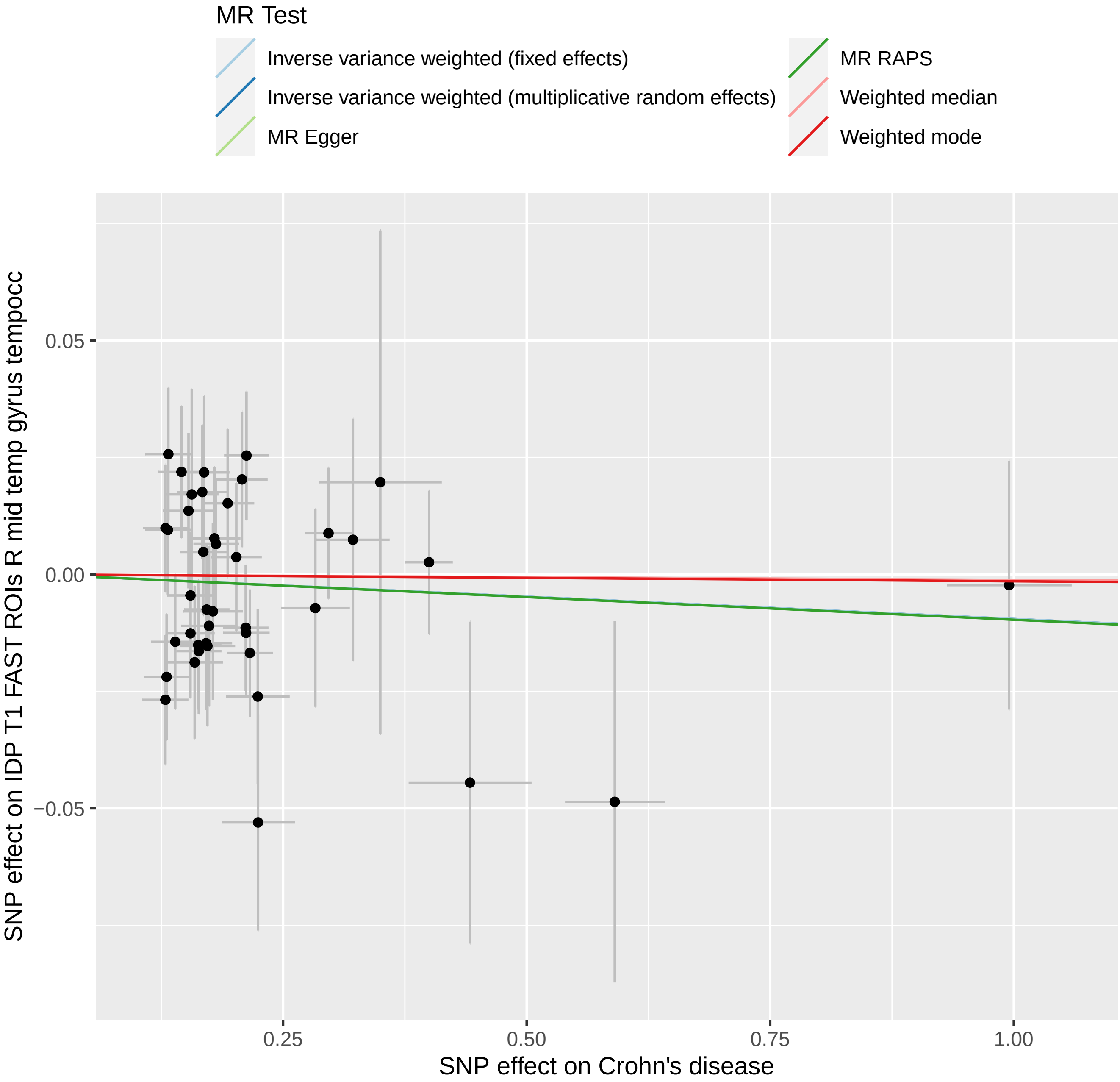

Batch 1906 : Ulcerative colitis on IDP T1 FAST ROIs R mid temp gyrus tempocc

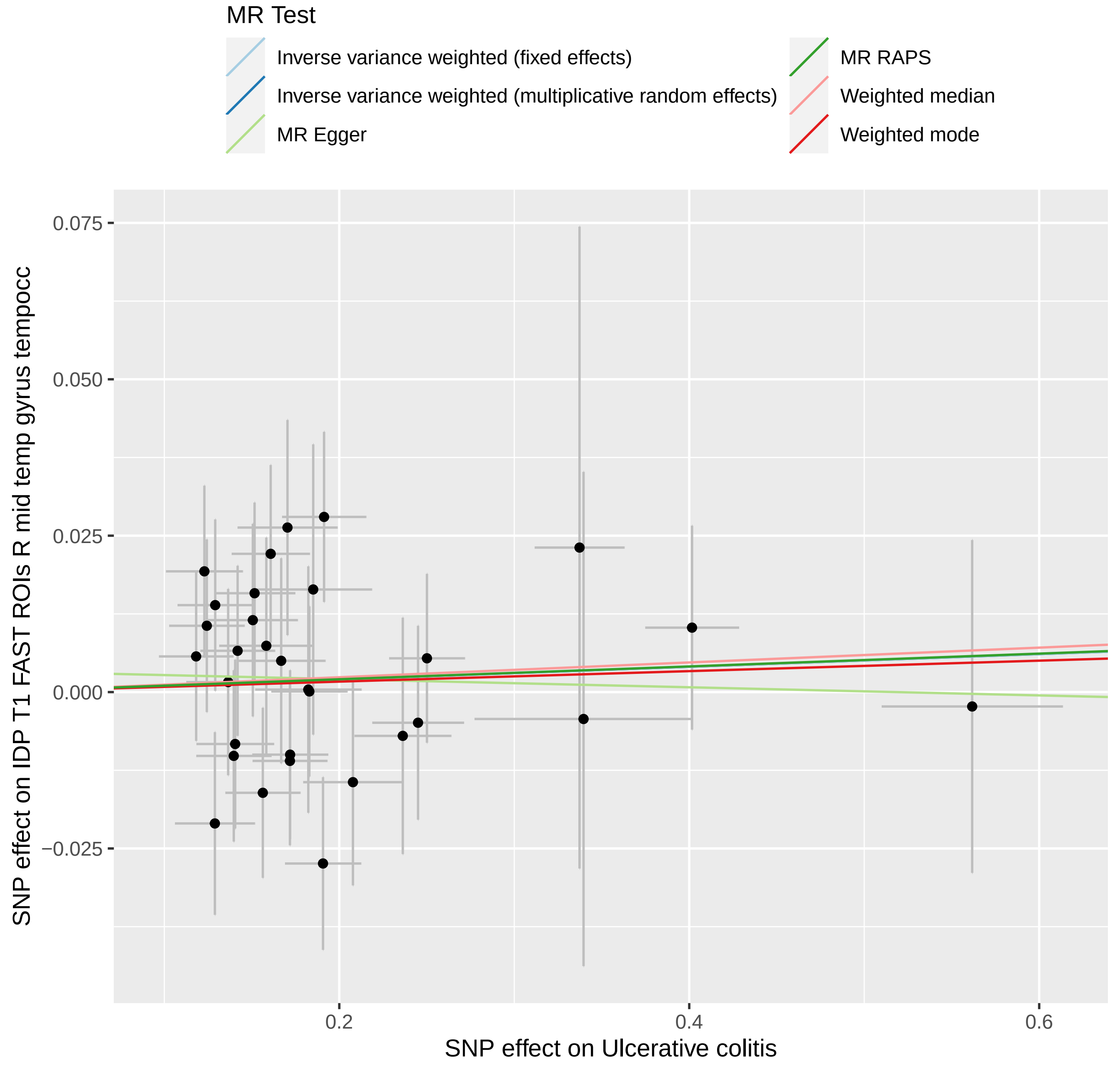

Batch 1927 : Crohn's disease on IDP T1 FAST ROIs L inf temp gyrus ant

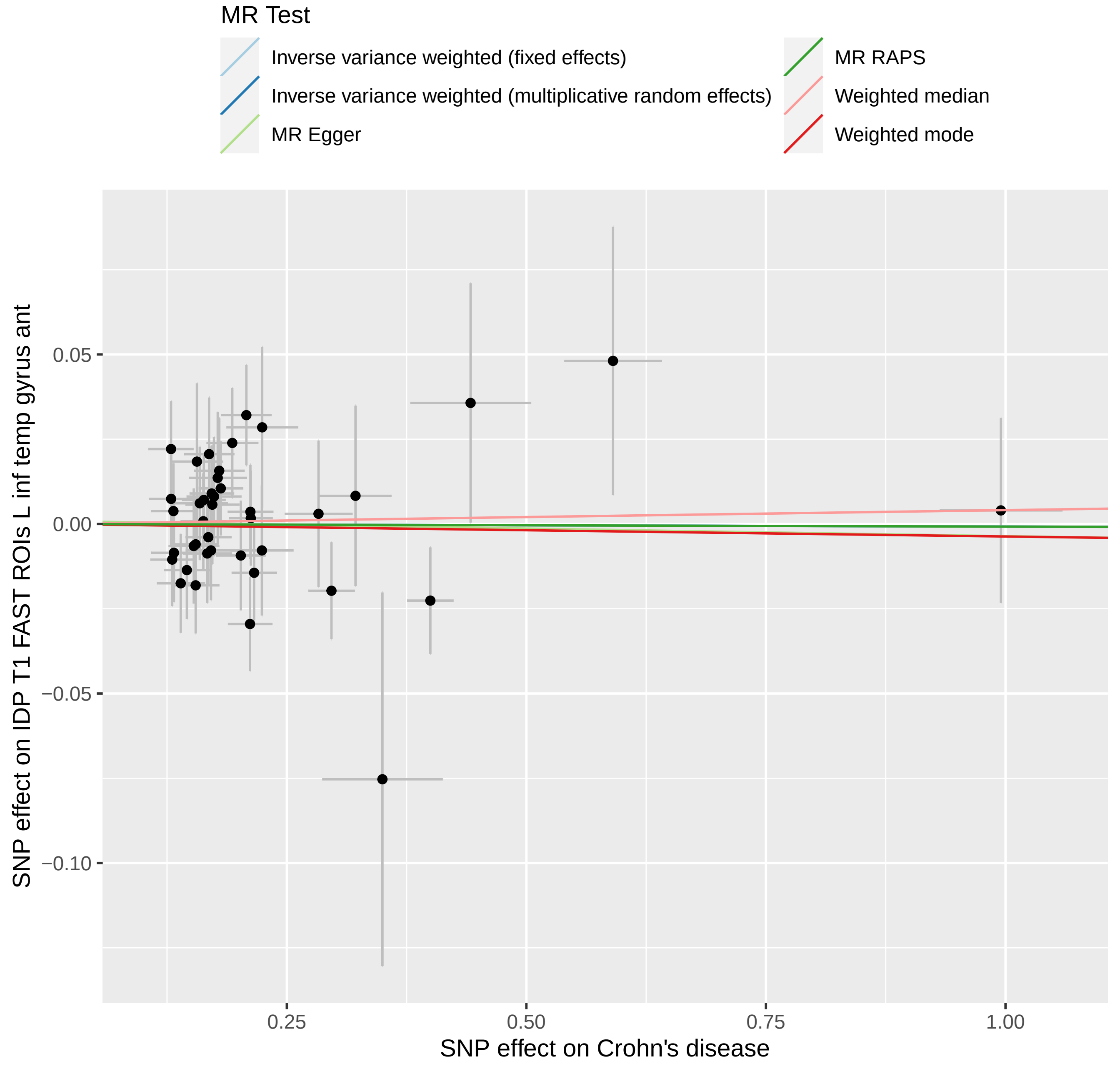

Batch 1928 : Ulcerative colitis on IDP T1 FAST ROIs L inf temp gyrus ant

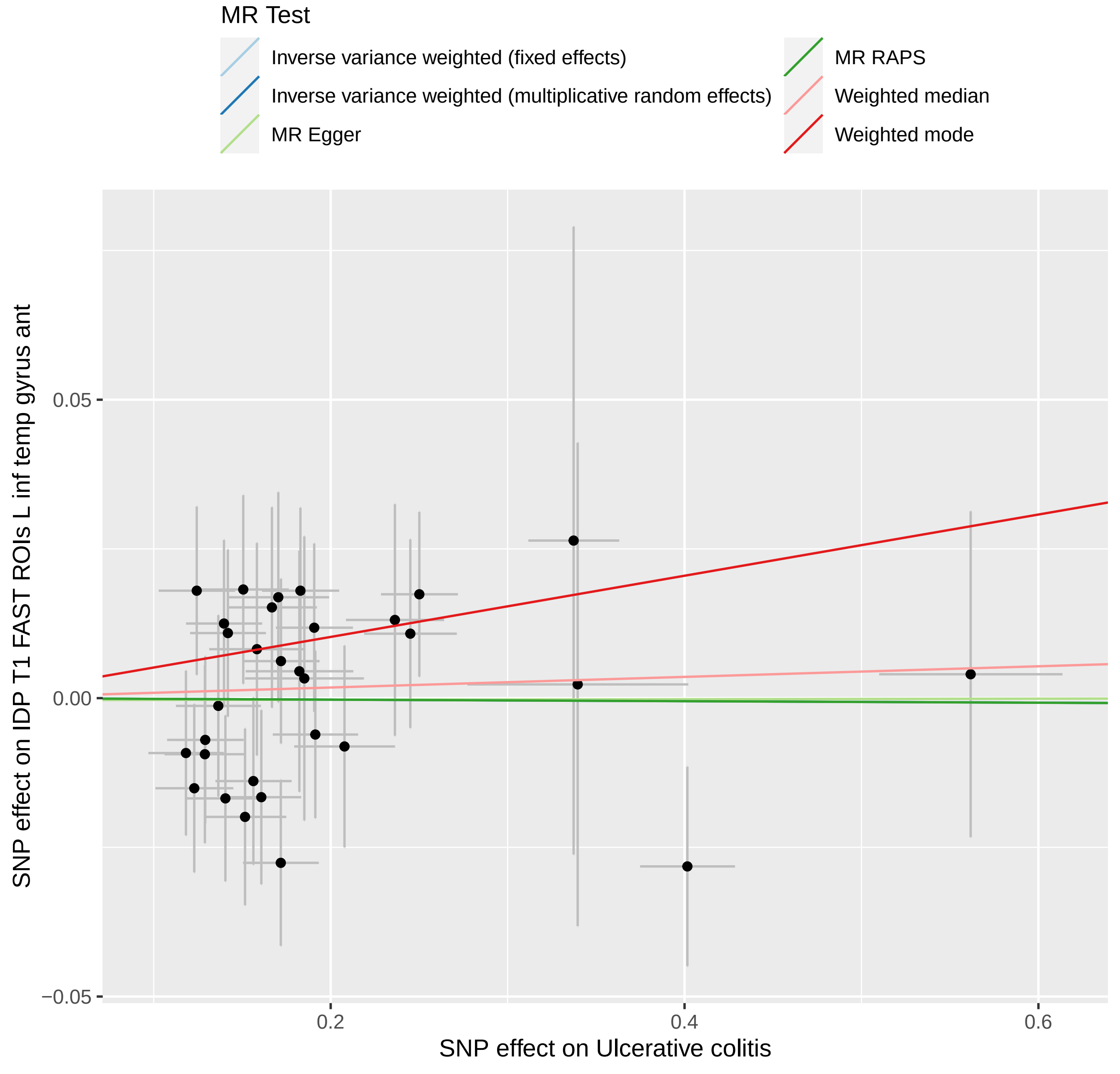

Batch 1949 : Crohn's disease on IDP T1 FAST ROIs R inf temp gyrus ant

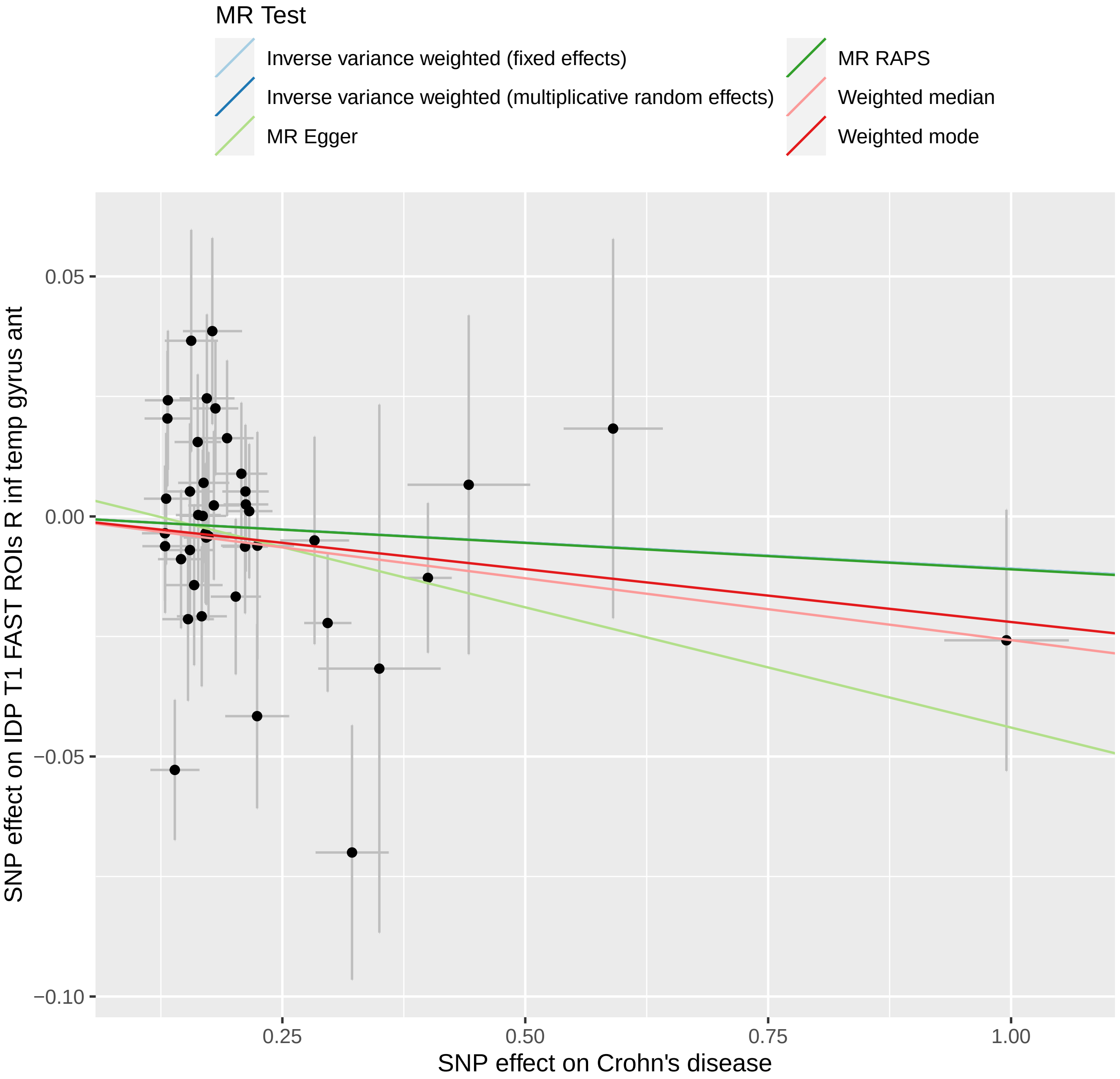

Batch 1950 : Ulcerative colitis on IDP T1 FAST ROIs R inf temp gyrus ant

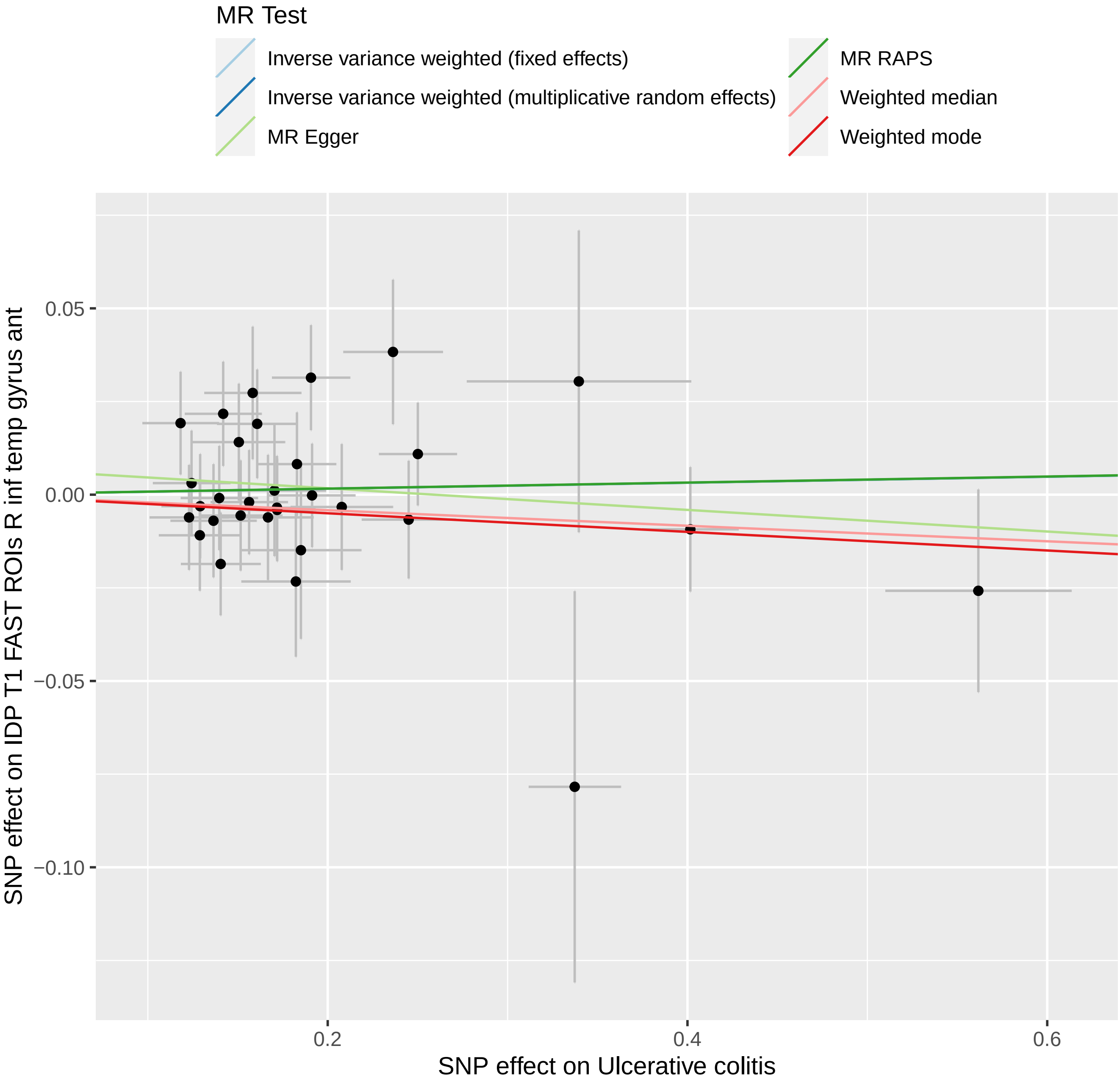

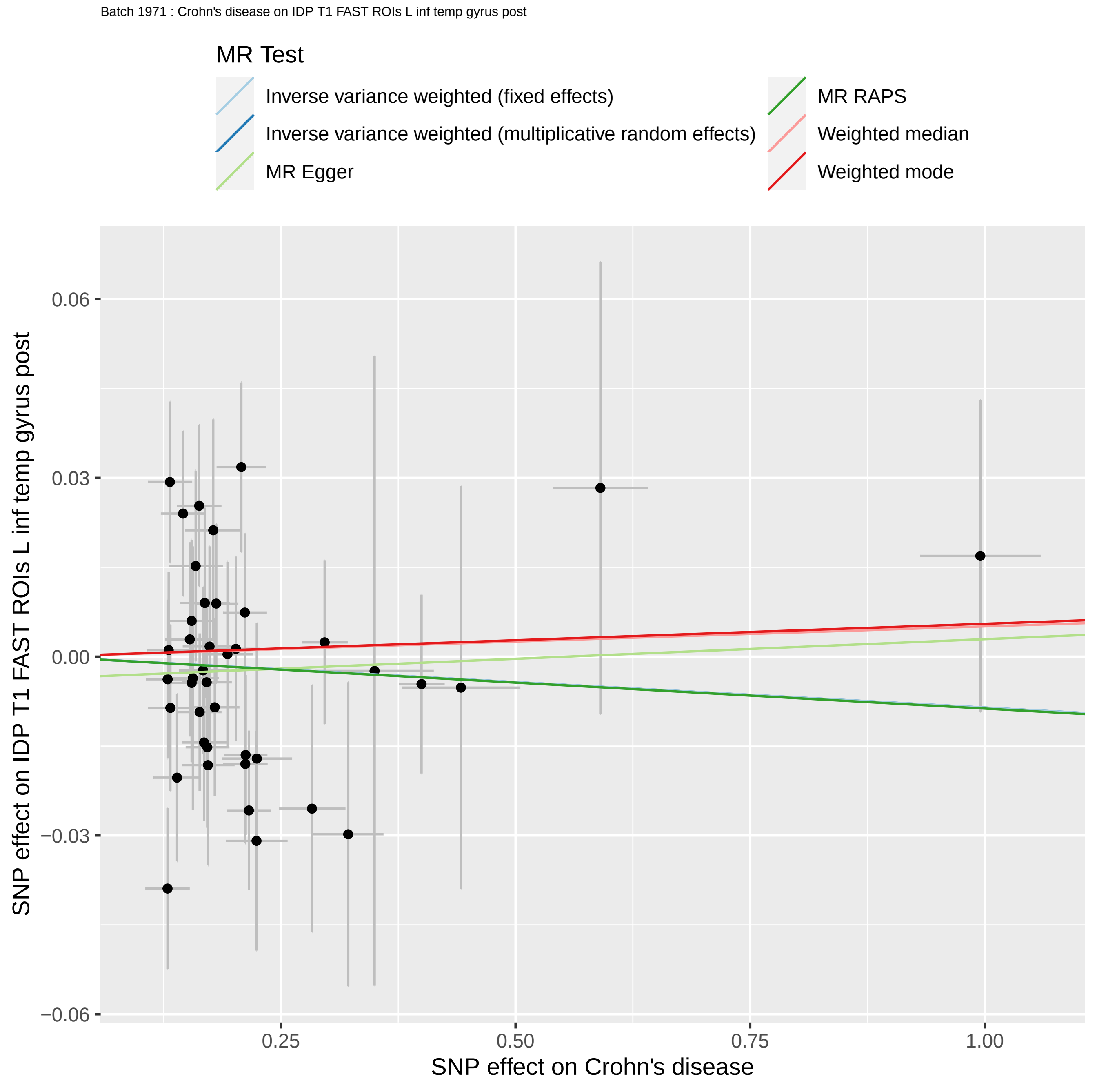

Batch 1971 : Crohn's disease on IDP T1 FAST ROIs L inf temp gyrus post
MR Test
Inverse variance weighted (fixed effects)
Inverse variance weighted (multiplicative random effects)
MR Egger
MR RAPS
Weighted median
Weighted mode
SNP effect on IDP T1 FAST ROIs L inf temp gyrus post
0.06
0.03
0.00
−0.03
−0.06
0.25
0.50
0.75
1.00
SNP effect on Crohn's disease

Batch 1972 : Ulcerative colitis on IDP T1 FAST ROIs L inf temp gyrus post

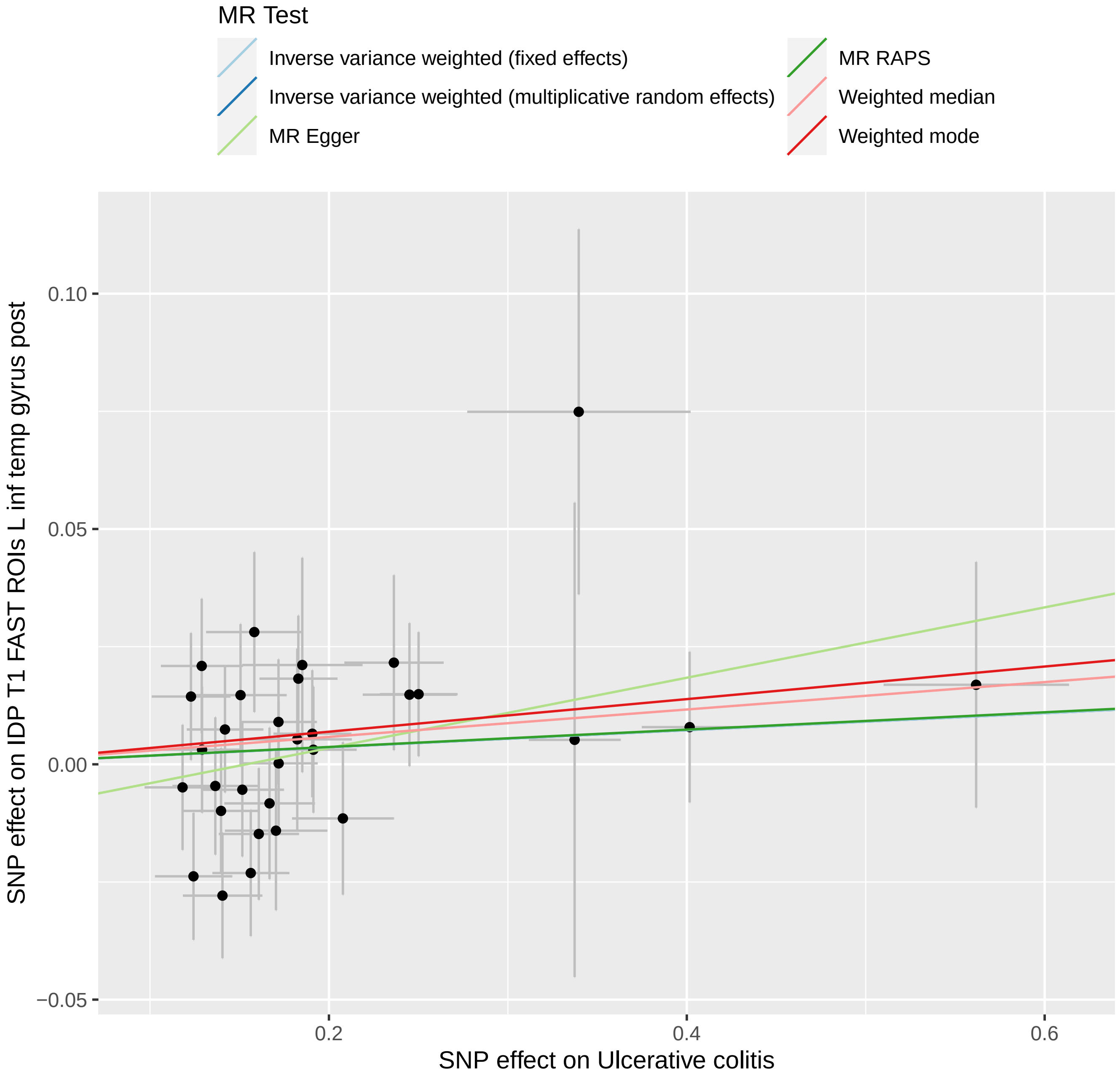

Batch 1993 : Crohn's disease on IDP T1 FAST ROIs R inf temp gyrus post

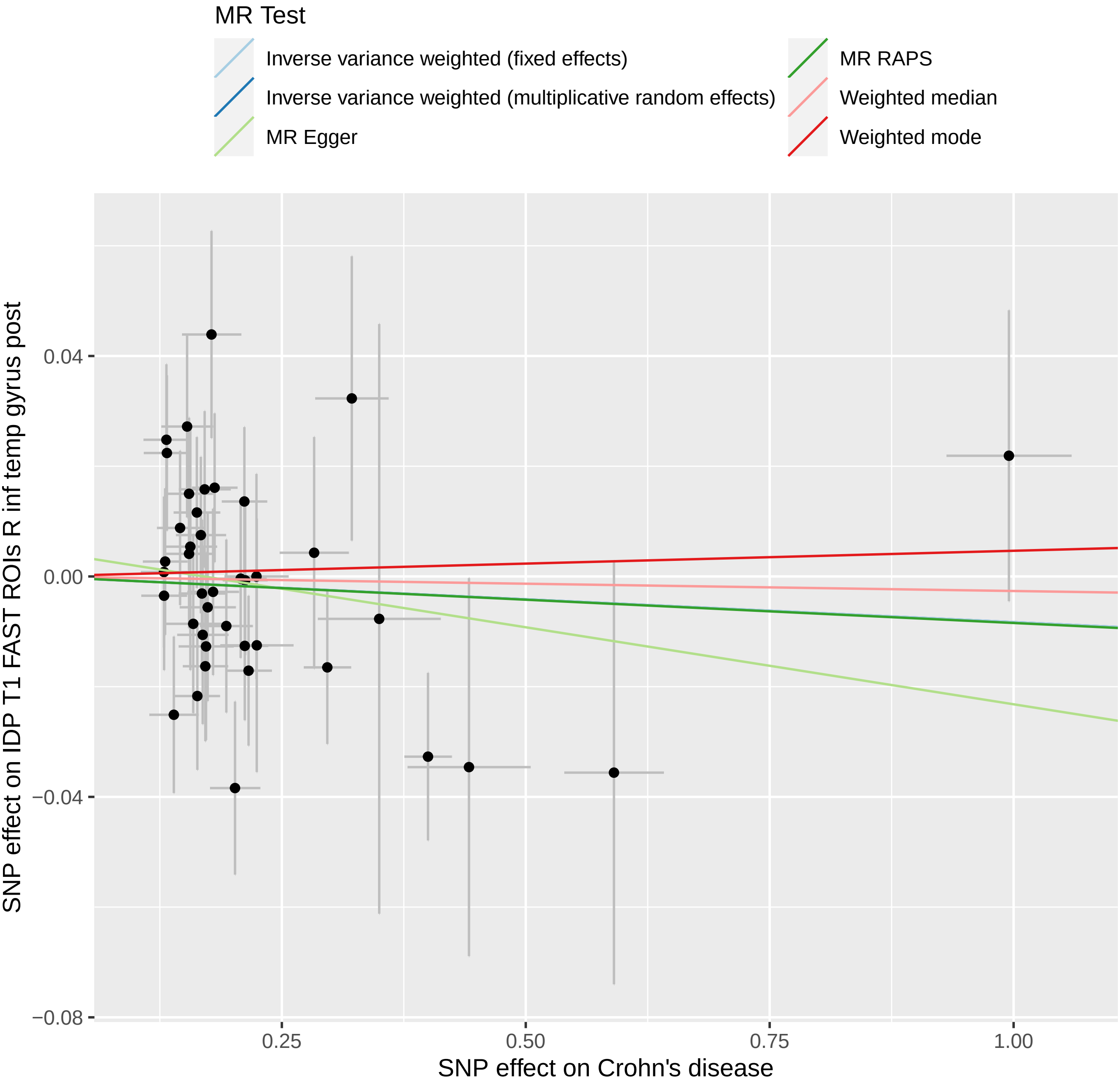

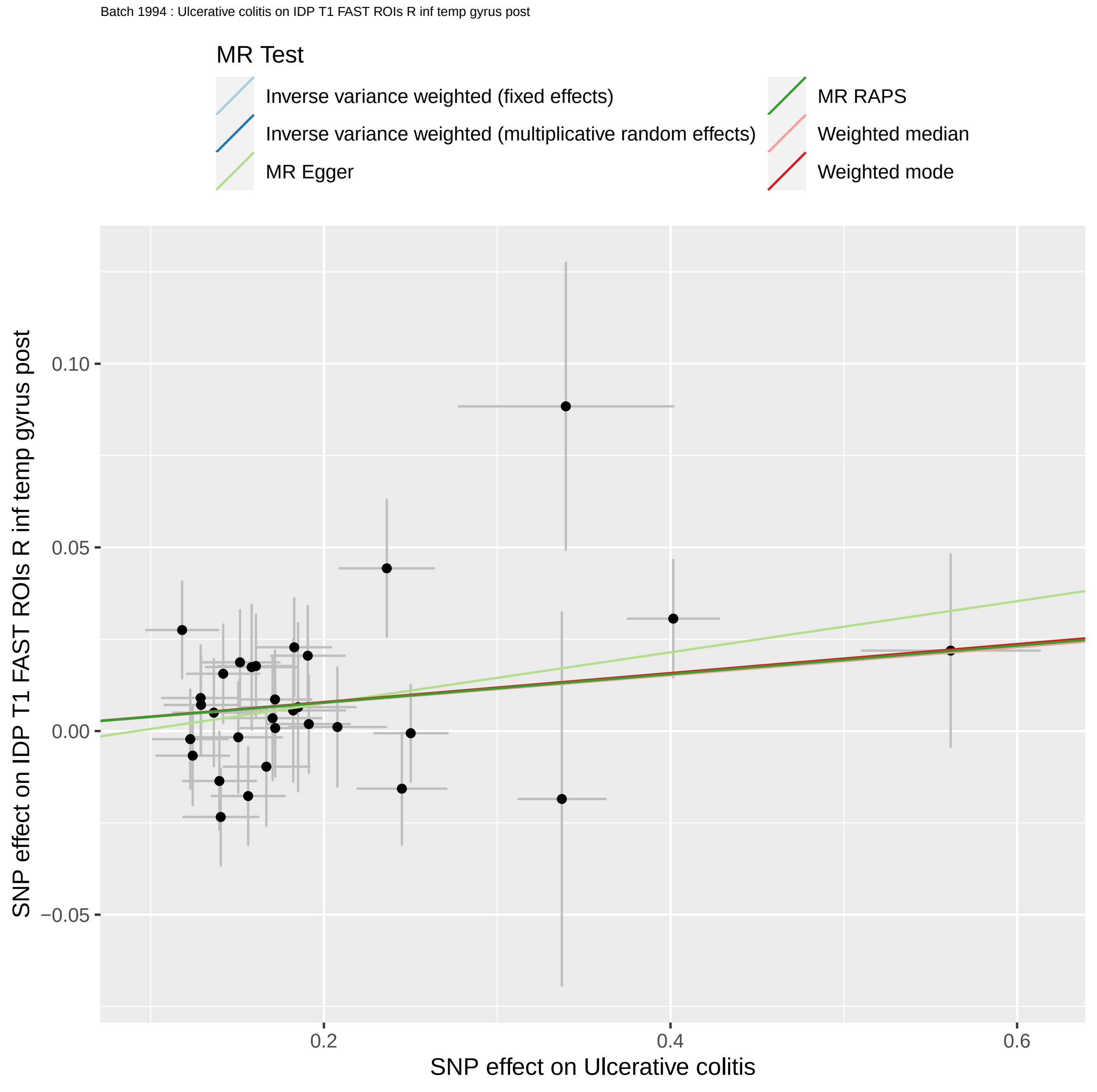

Batch 1994 : Ulcerative colitis on IDP T1 FAST ROIs R inf temp gyrus post
MR Test
Inverse variance weighted (fixed effects)
Inverse variance weighted (multiplicative random effects)
MR Egger
MR RAPS
Weighted median
Weighted mode
0.10
0.05
0.00
−0.05
0.2
0.4
0.6
SNP effect on IDP T1 FAST ROIs R inf temp gyrus post
SNP effect on Ulcerative colitis

Batch 2015 : Crohn's disease on IDP T1 FAST ROIs L inf temp gyrus tempocc

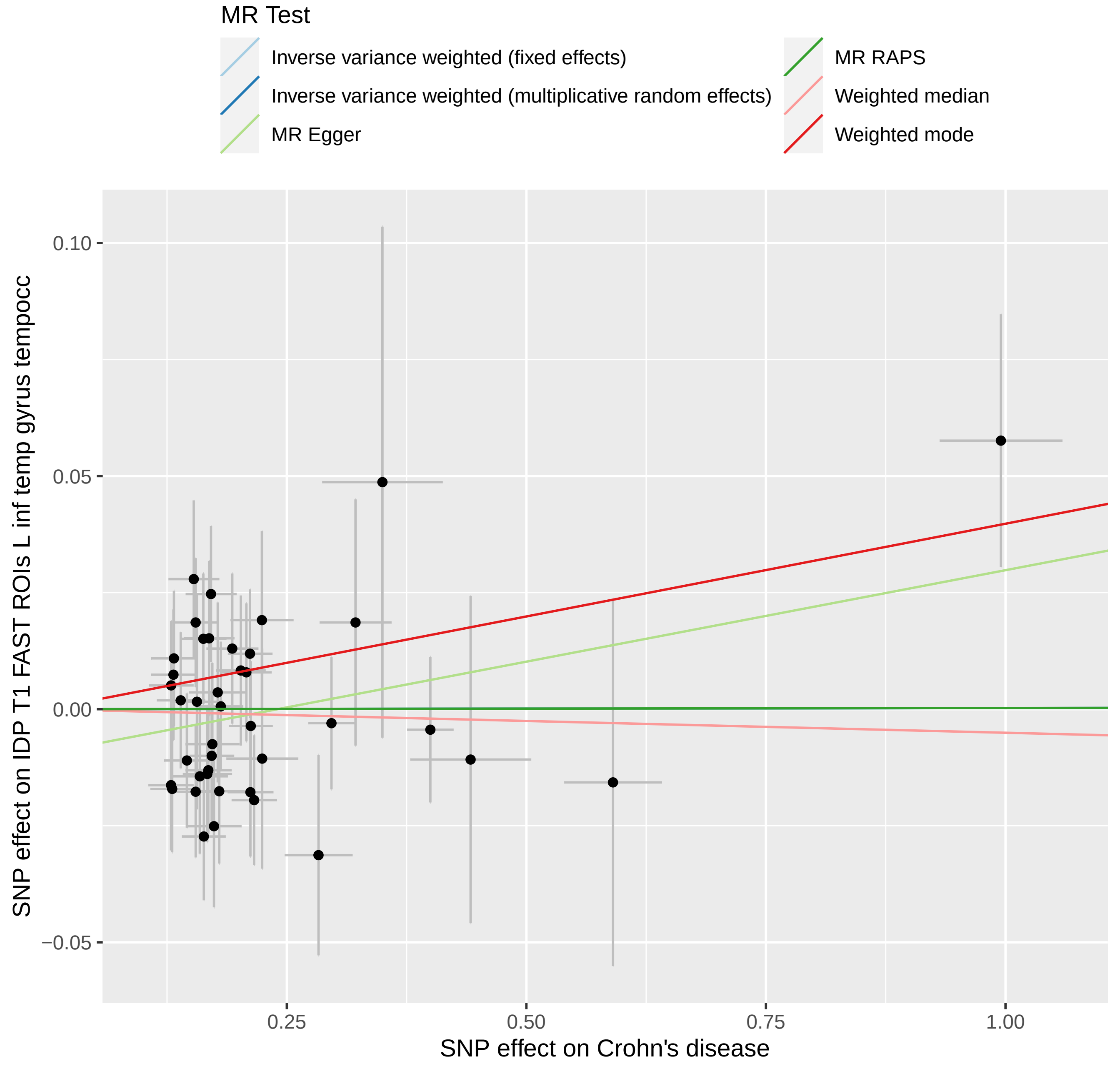

Batch 2016 : Ulcerative colitis on IDP T1 FAST ROIs L inf temp gyrus tempocc

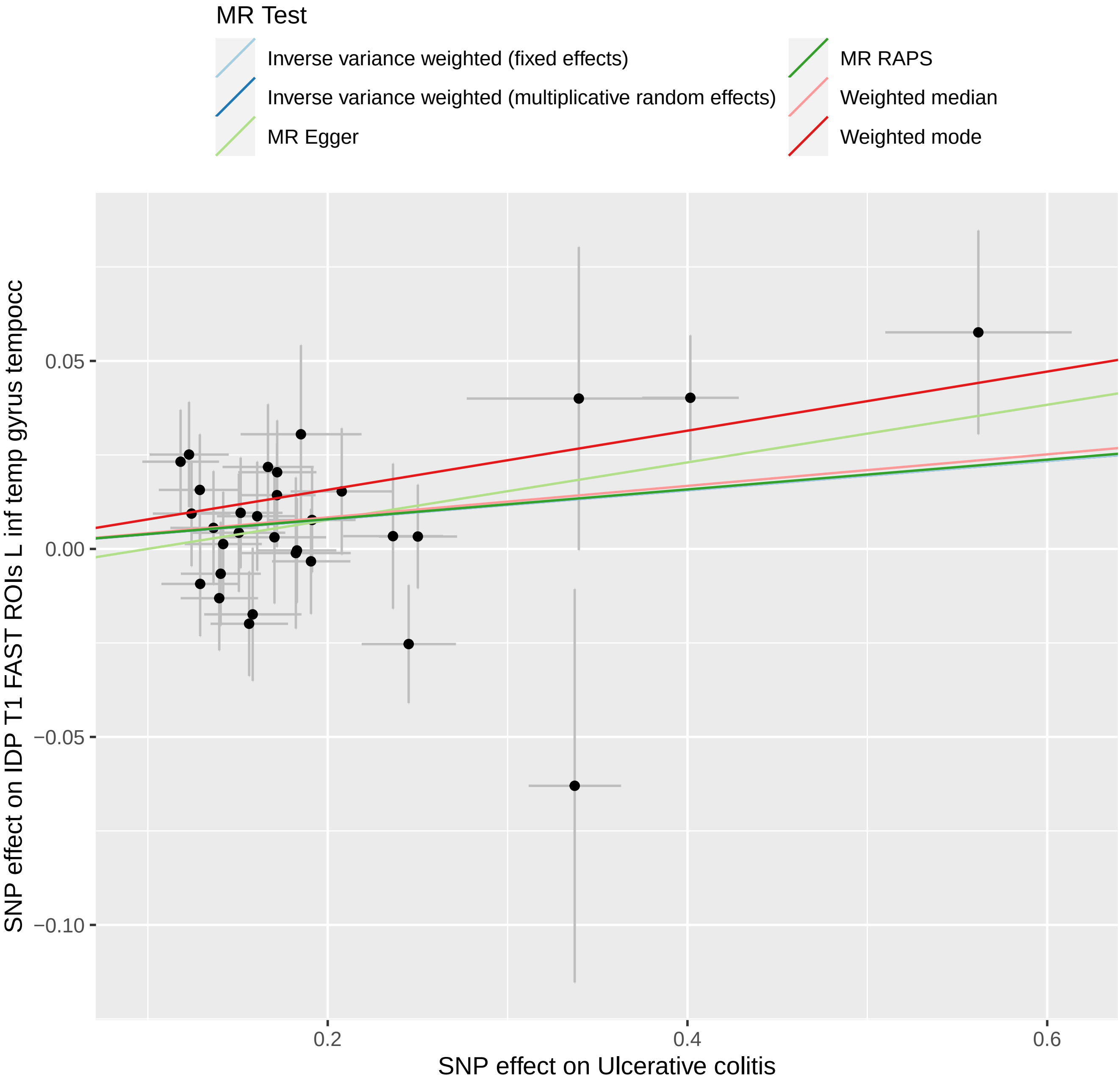

Batch 2037 : Crohn's disease on IDP T1 FAST ROIs R inf temp gyrus tempocc

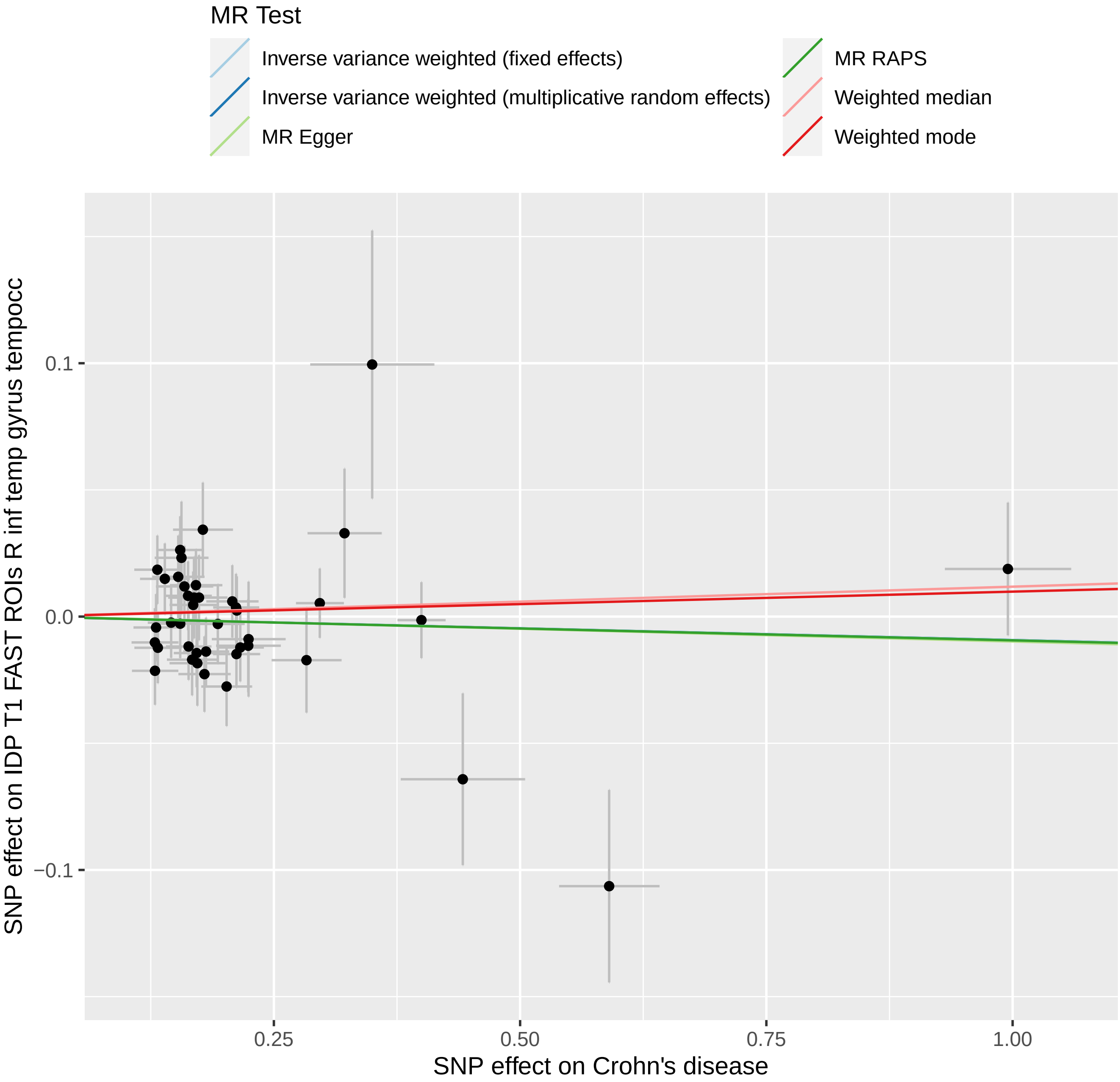

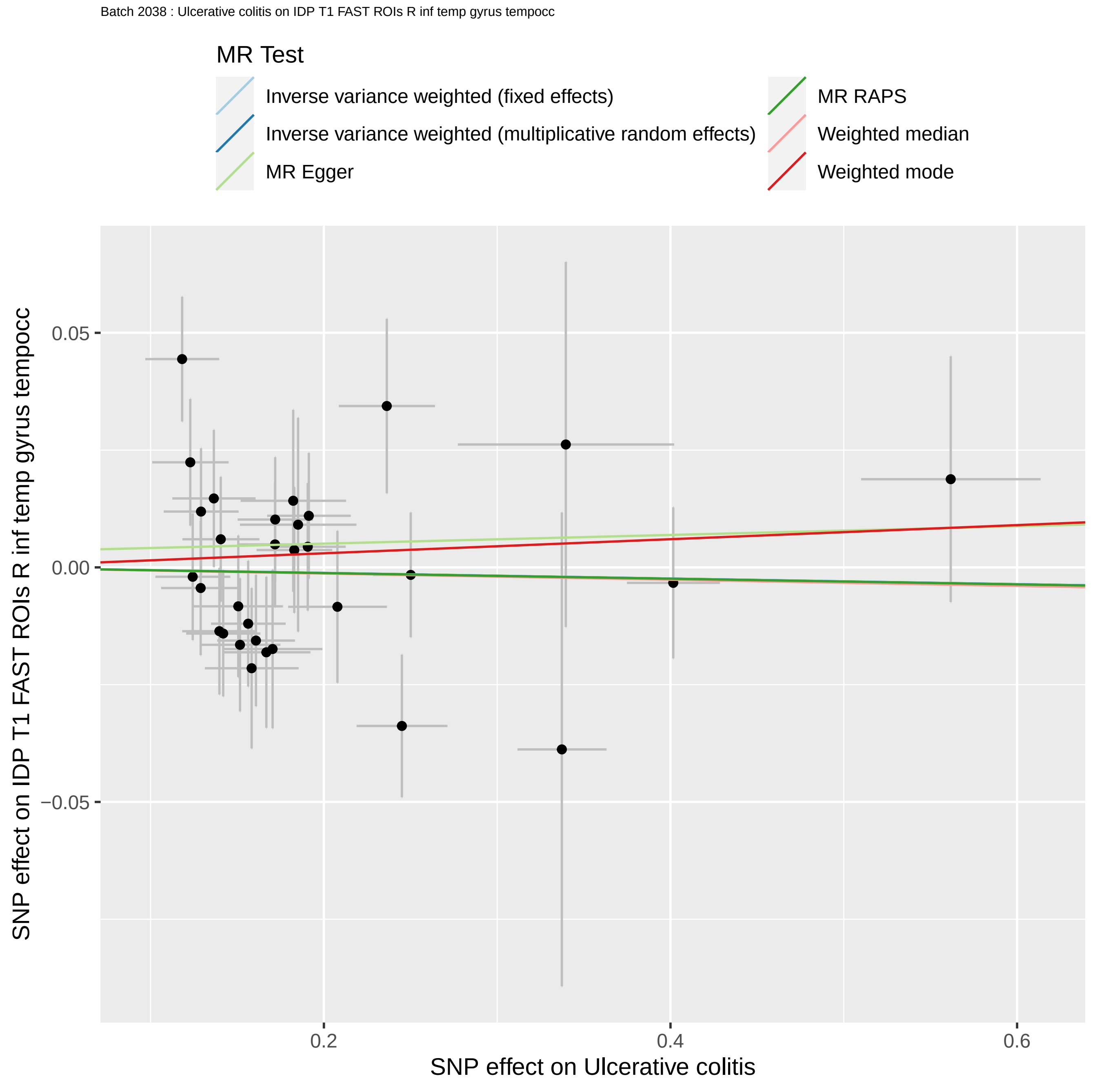

Batch 2038 : Ulcerative colitis on IDP T1 FAST ROIs R inf temp gyrus tempocc
MR Test
Inverse variance weighted (fixed effects)
Inverse variance weighted (multiplicative random effects)
MR Egger
MR RAPS
Weighted median
Weighted mode
0.05
0.00
−0.05
0.2
0.4
0.6
SNP effect on IDP T1 FAST ROIs R inf temp gyrus tempocc
SNP effect on Ulcerative colitis

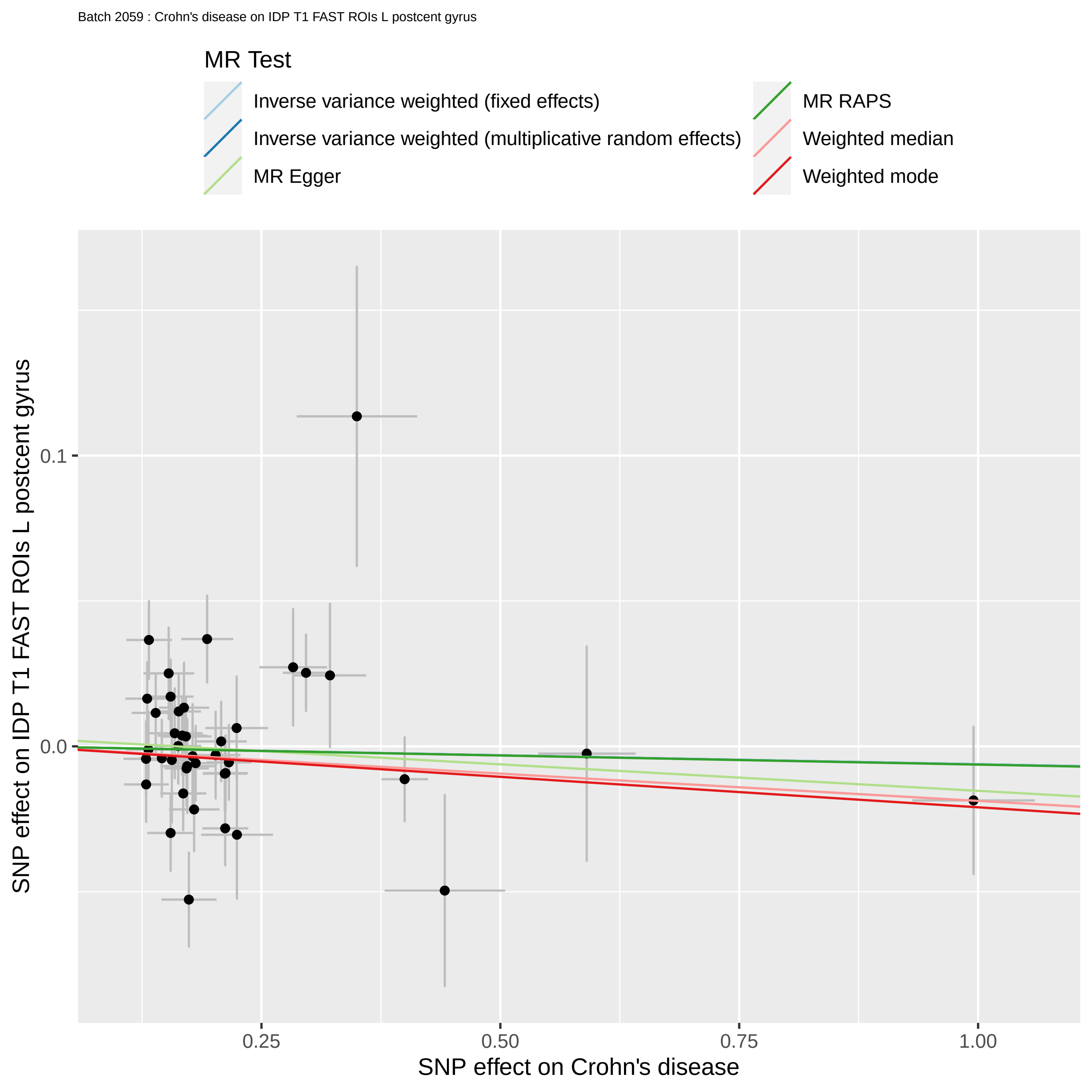
Batch 2059 : Crohn's disease on IDP T1 FAST ROIs L postcent gyrus
MR Test
Inverse variance weighted (fixed effects)
Inverse variance weighted (multiplicative random effects)
MR Egger
MR RAPS
Weighted median
Weighted mode
SNP effect on IDP T1 FAST ROIs L postcent gyrus
0.1
0.0
0.25
0.50
0.75
1.00
SNP effect on Crohn's disease

Batch 2060 : Ulcerative colitis on IDP T1 FAST ROIs L postcent gyrus

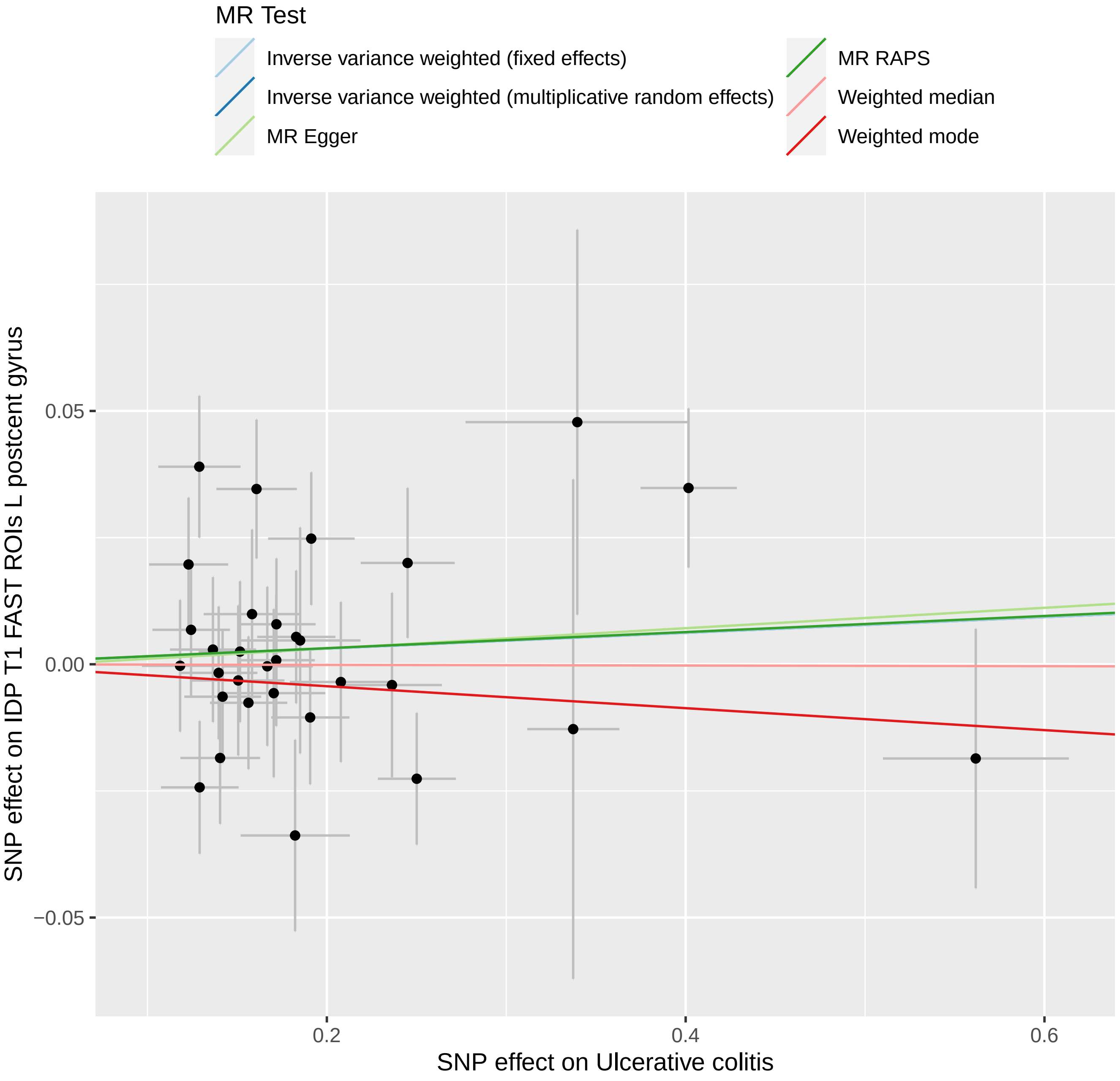

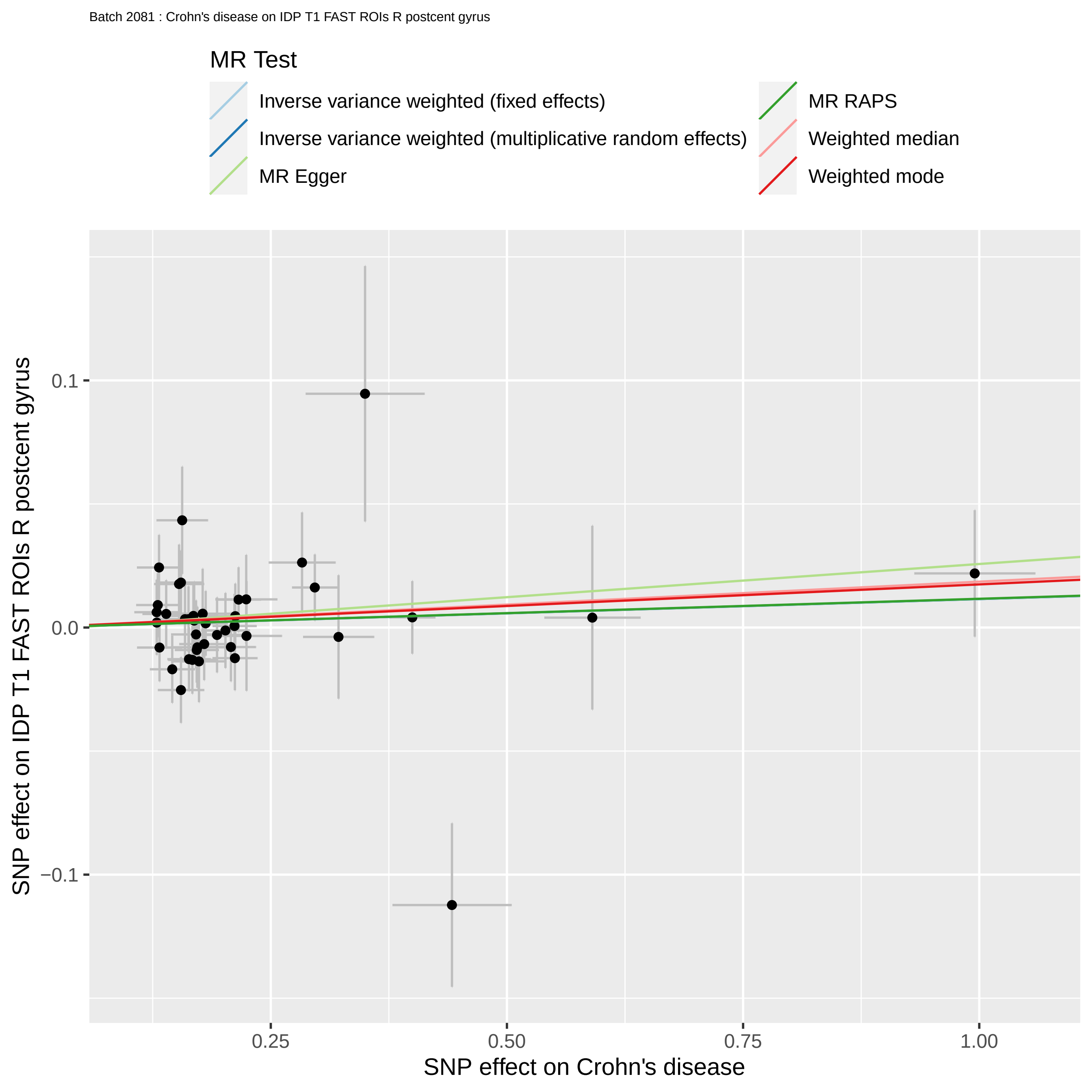

Batch 2081 : Crohn's disease on IDP T1 FAST ROIs R postcent gyrus
MR Test
Inverse variance weighted (fixed effects)
Inverse variance weighted (multiplicative random effects)
MR Egger
MR RAPS
Weighted median
Weighted mode
0.1
0.0
−0.1
0.25
0.50
0.75
1.00
SNP effect on IDP T1 FAST ROIs R postcent gyrus
SNP effect on Crohn's disease

Batch 2082 : Ulcerative colitis on IDP T1 FAST ROIs R postcent gyrus

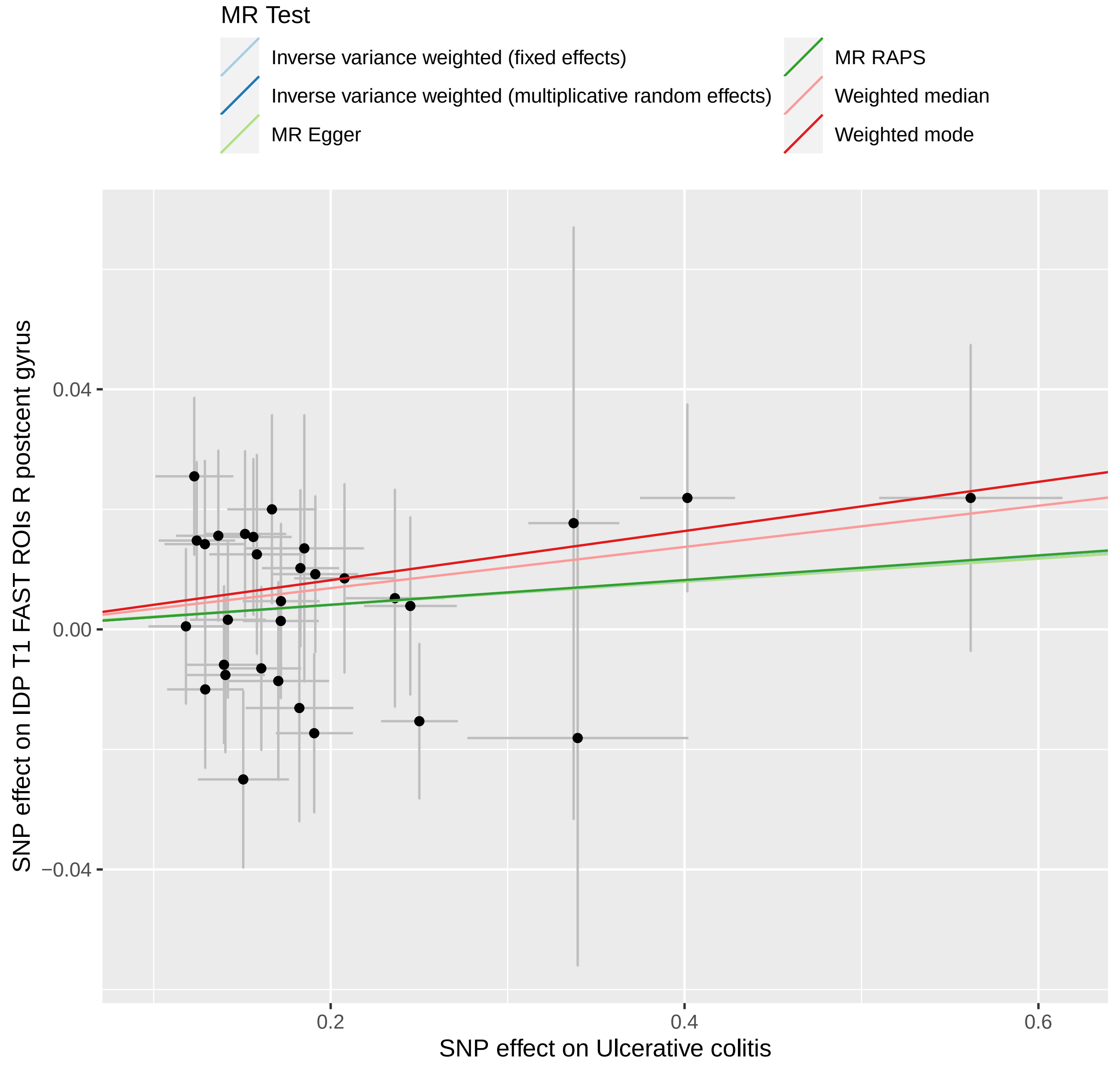

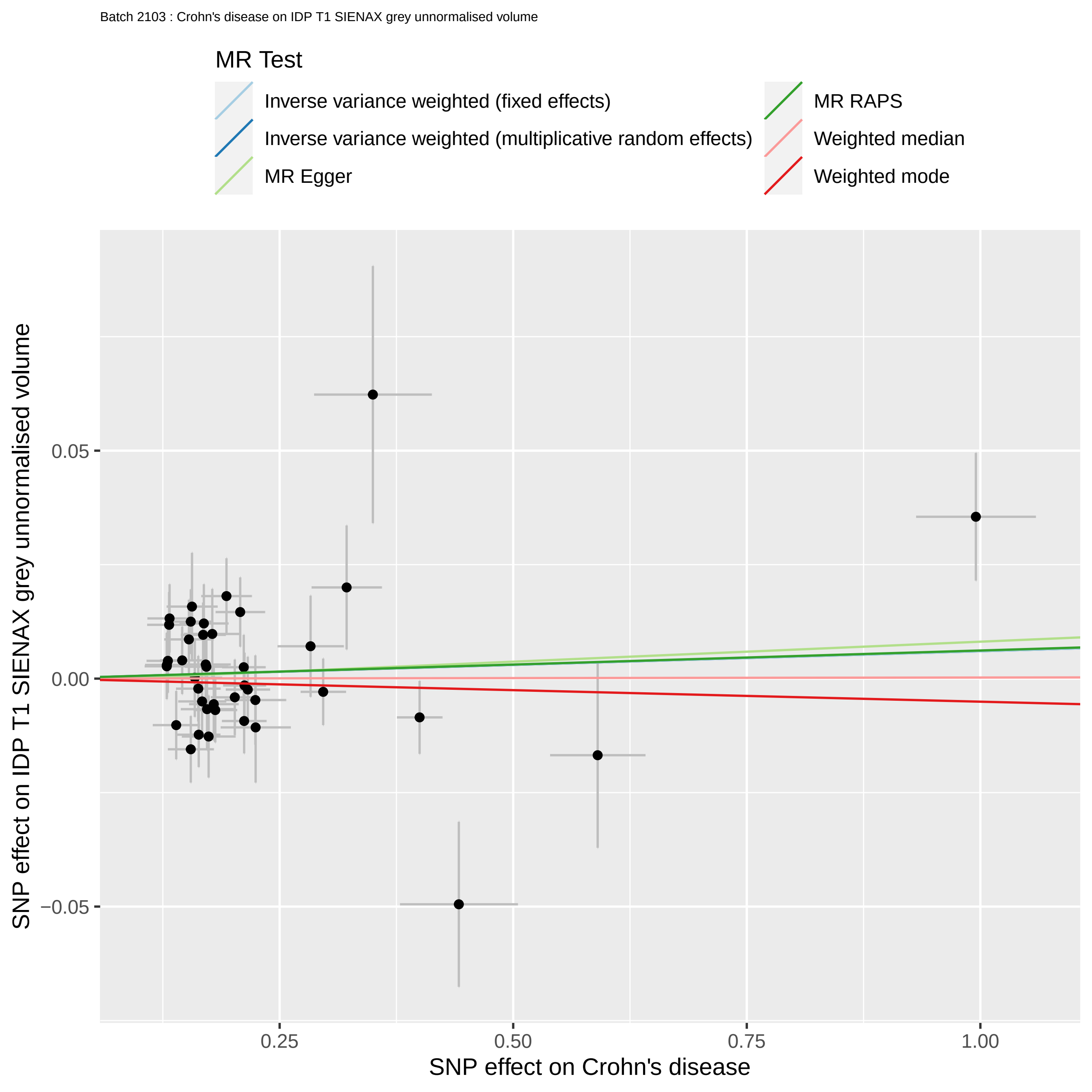

Batch 2103 : Crohn's disease on IDP T1 SIENAX grey unnormalised volume
MR Test
Inverse variance weighted (fixed effects)
Inverse variance weighted (multiplicative random effects)
MR Egger
MR RAPS
Weighted median
Weighted mode
SNP effect on IDP T1 SIENAX grey unnormalised volume
0.05
0.00
−0.05
0.25
0.50
0.75
1.00
SNP effect on Crohn's disease

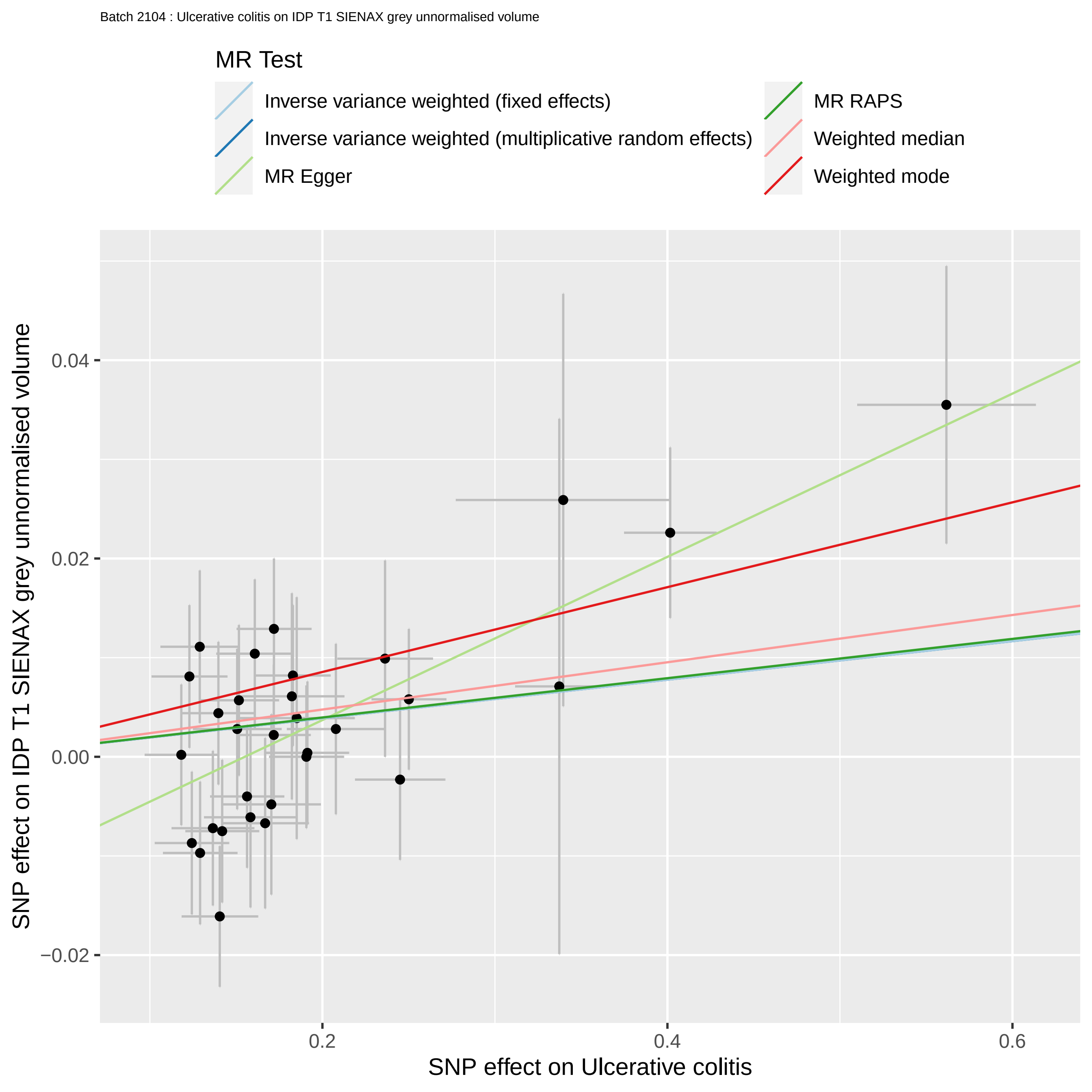

Batch 2104 : Ulcerative colitis on IDP T1 SIENAX grey unnormalised volume
MR Test
Inverse variance weighted (fixed effects)
Inverse variance weighted (multiplicative random effects)
MR Egger
MR RAPS
Weighted median
Weighted mode
SNP effect on IDP T1 SIENAX grey unnormalised volume
0.04
0.02
0.00
−0.02
0.2
0.4
0.6
SNP effect on Ulcerative colitis

Batch 2105 : Crohn's disease on IDP T1 FAST ROIs L sup parietal lobule

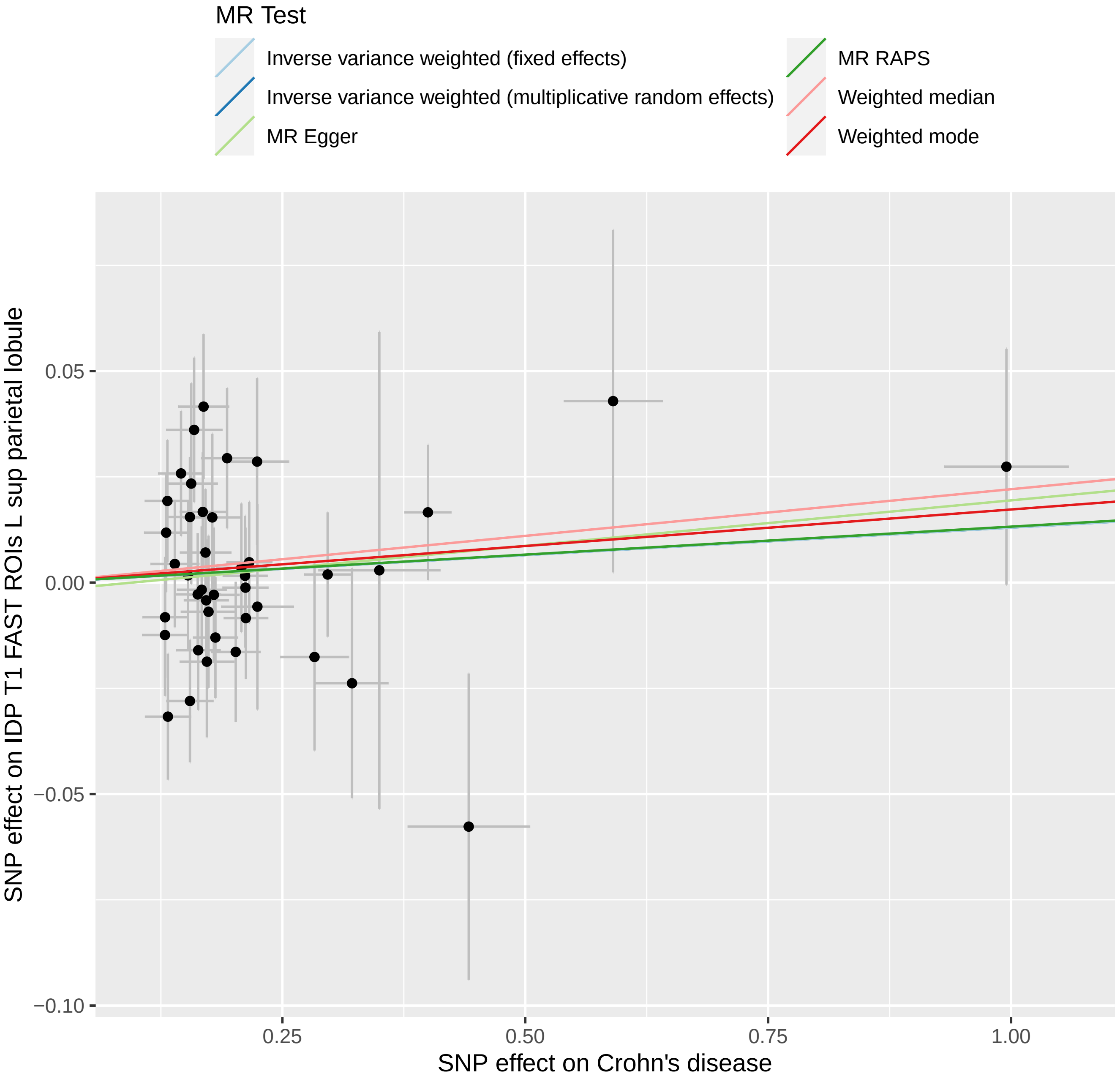

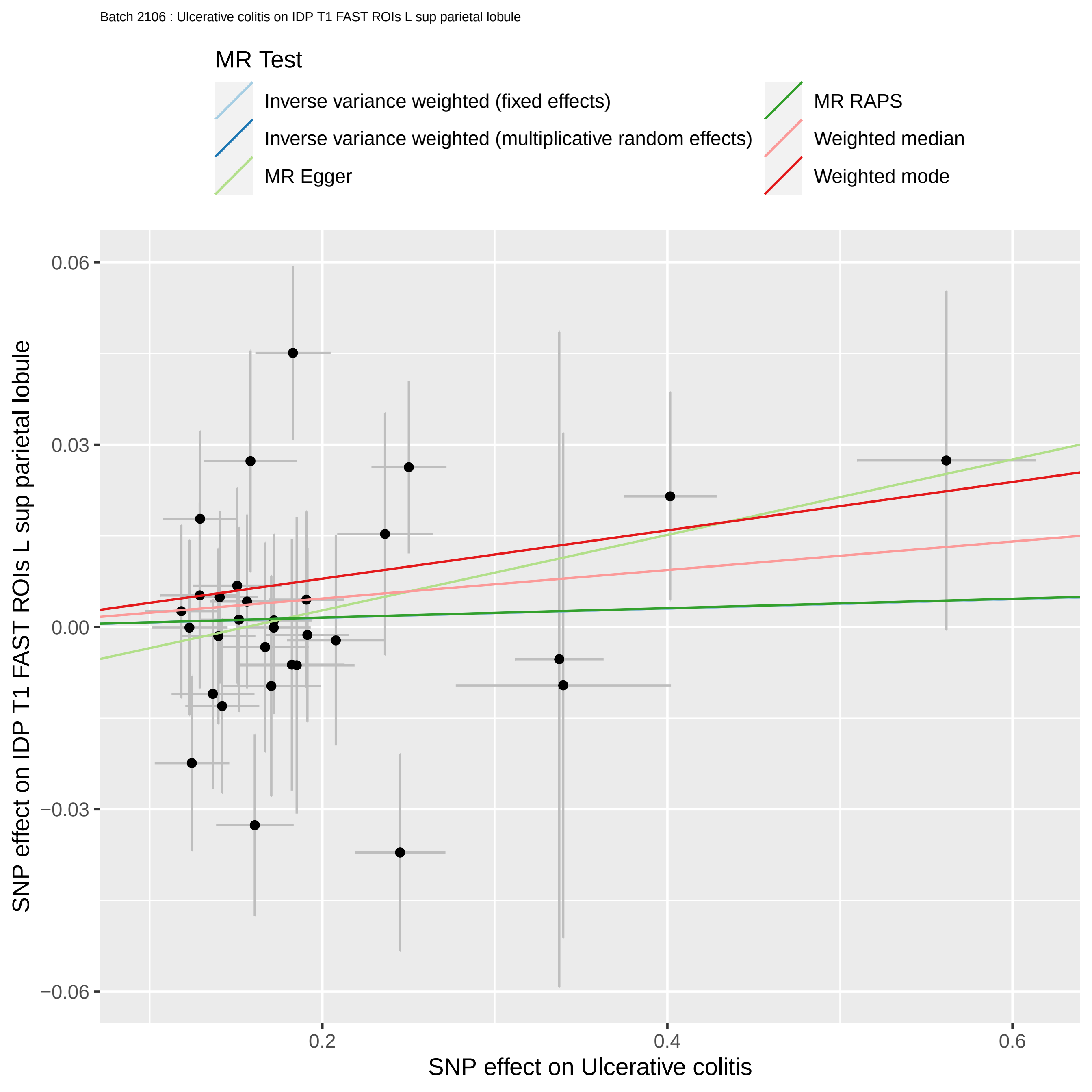

Batch 2106 : Ulcerative colitis on IDP T1 FAST ROIs L sup parietal lobule
MR Test
Inverse variance weighted (fixed effects)
Inverse variance weighted (multiplicative random effects)
MR Egger
MR RAPS
Weighted median
Weighted mode
0.06
0.03
0.00
−0.03
−0.06
0.2
0.4
0.6
SNP effect on IDP T1 FAST ROIs L sup parietal lobule
SNP effect on Ulcerative colitis

Batch 2127 : Crohn's disease on IDP T1 FAST ROIs R sup parietal lobule

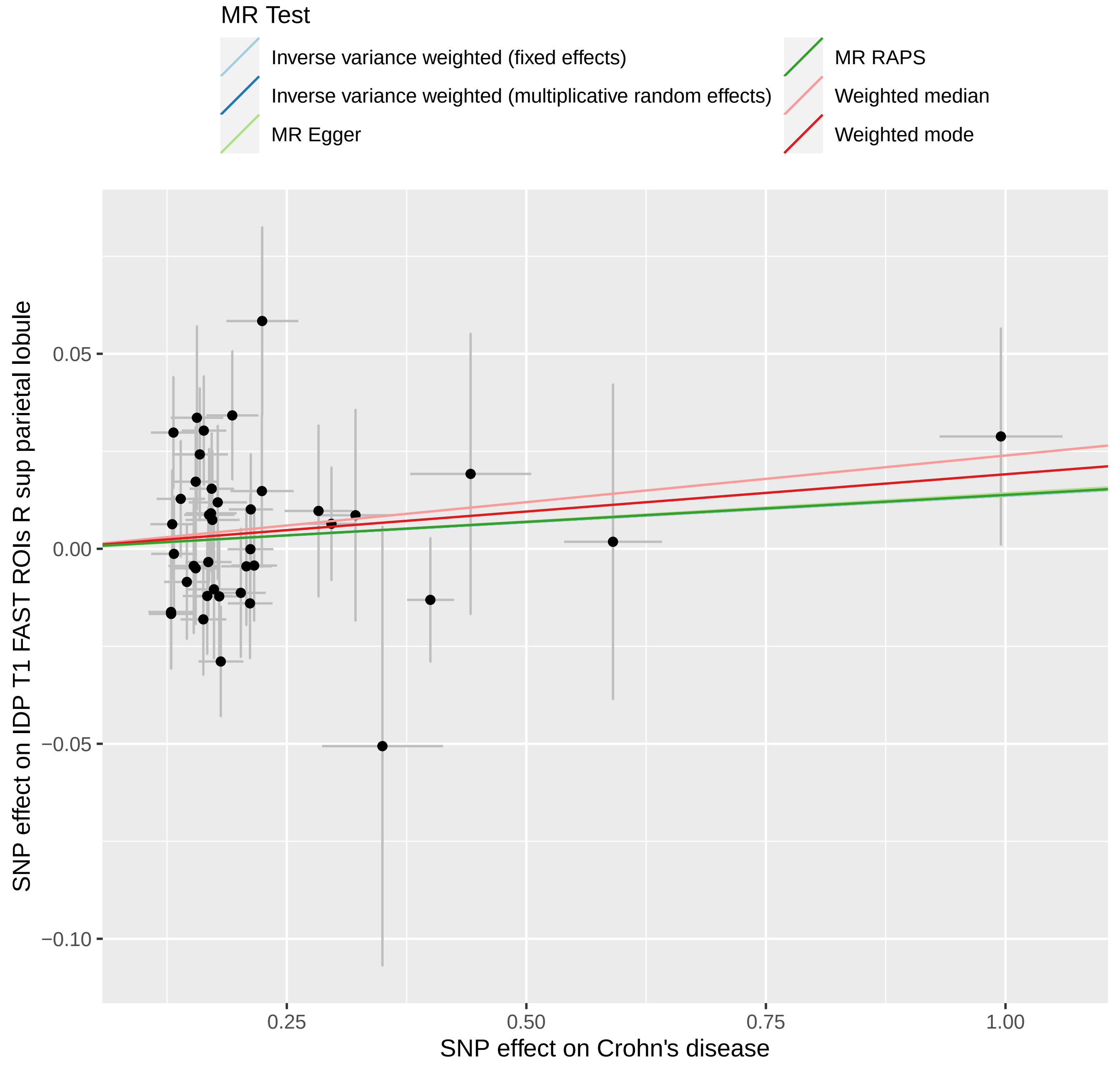

Batch 2128 : Ulcerative colitis on IDP T1 FAST ROIs R sup parietal lobule

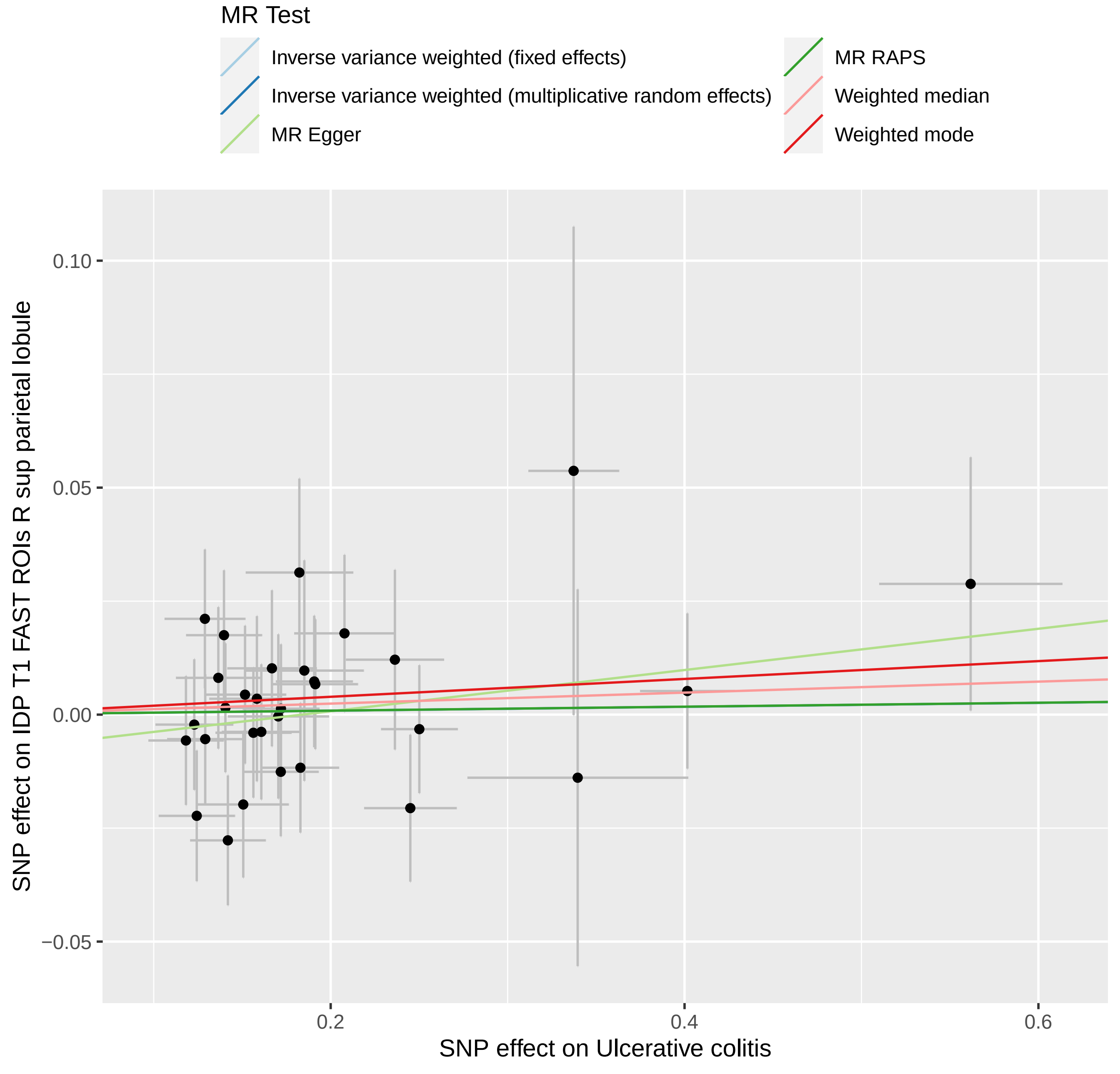

Batch 2149 : Crohn's disease on IDP T1 FAST ROIs L supramarg gyrus ant

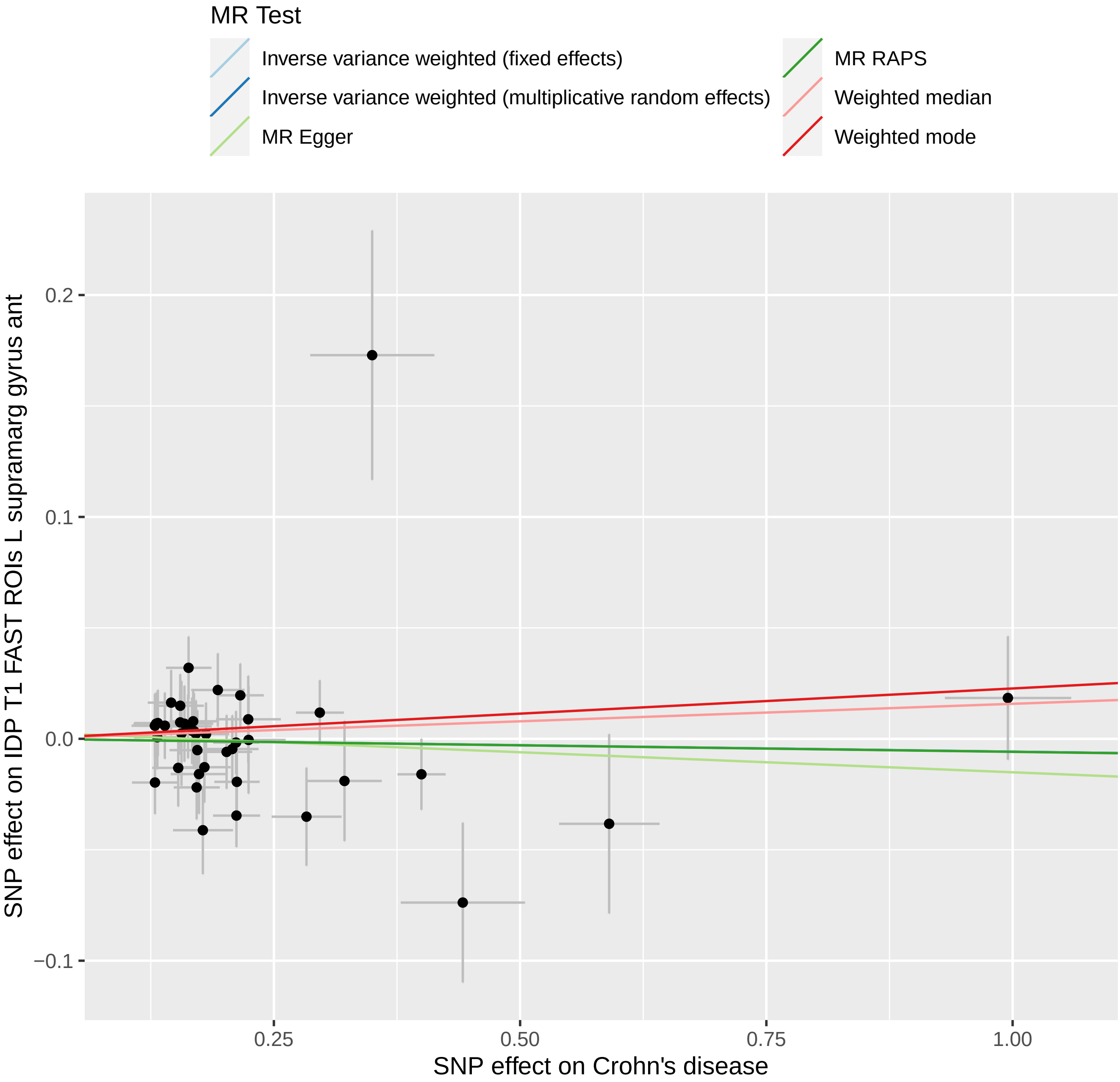

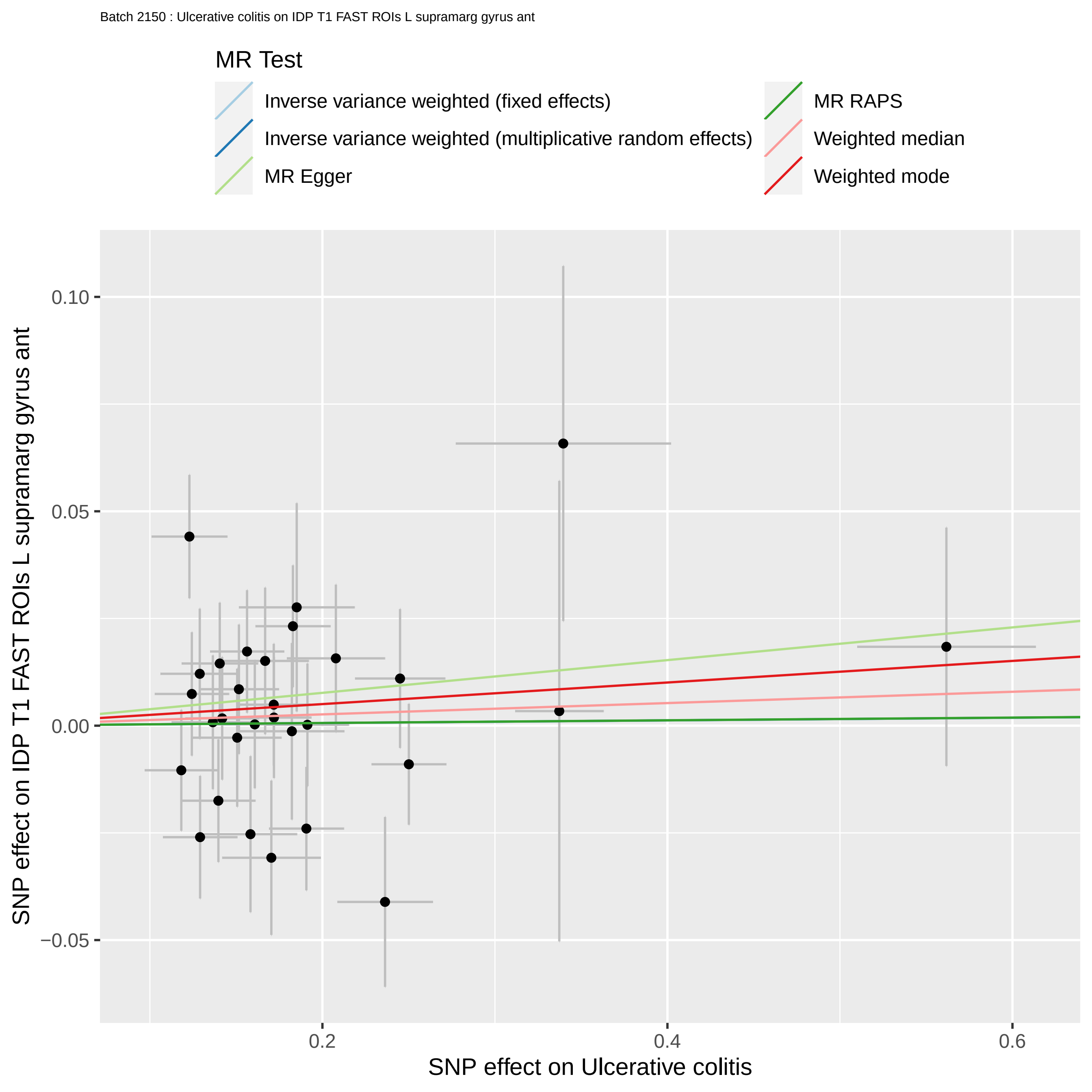

Batch 2150 : Ulcerative colitis on IDP T1 FAST ROIs L supramarg gyrus ant
MR Test
Inverse variance weighted (fixed effects)
Inverse variance weighted (multiplicative random effects)
MR Egger
MR RAPS
Weighted median
Weighted mode
0.10
0.05
0.00
−0.05
0.2
0.4
0.6
SNP effect on IDP T1 FAST ROIs L supramarg gyrus ant
SNP effect on Ulcerative colitis

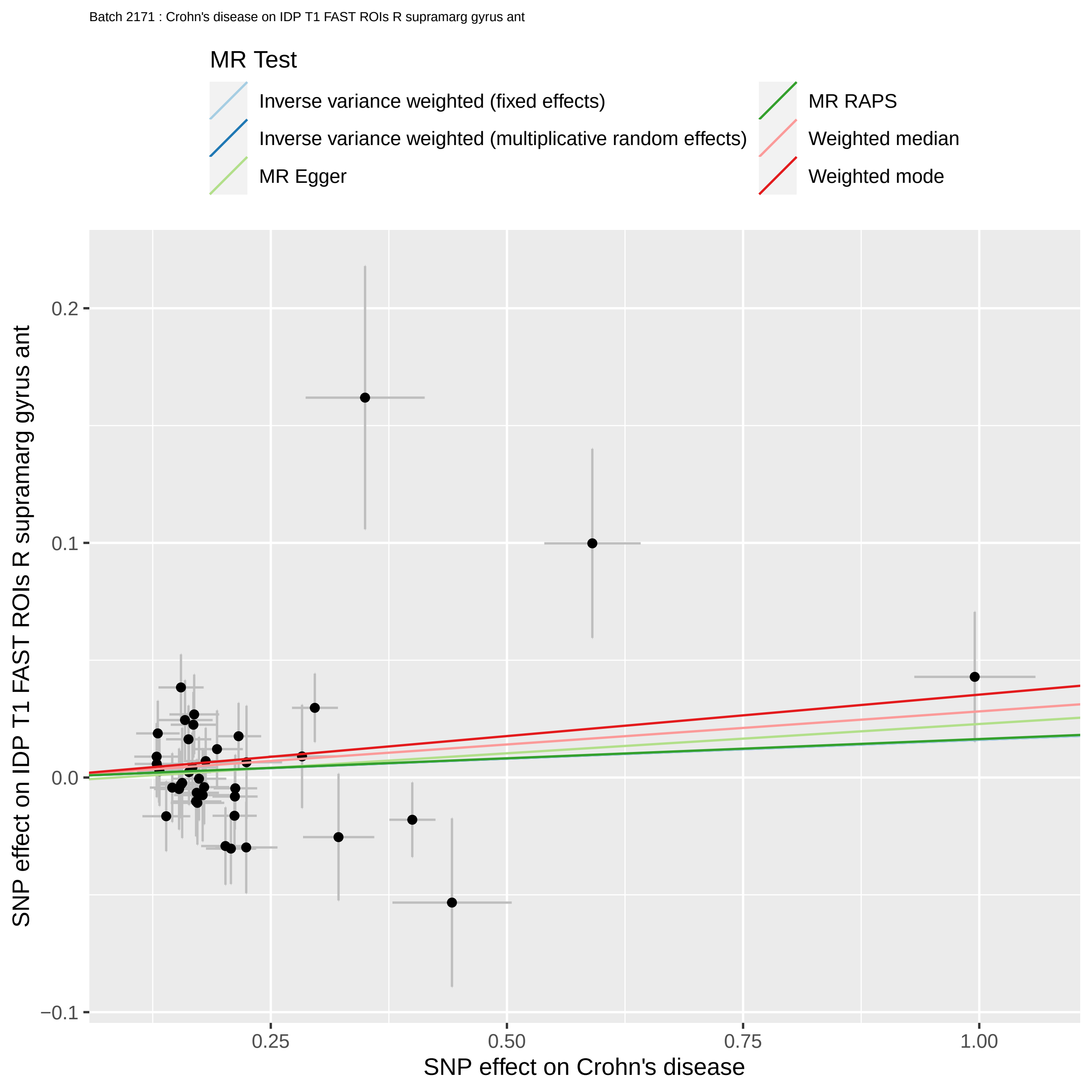

Batch 2171 : Crohn's disease on IDP T1 FAST ROIs R supramarg gyrus ant
MR Test
Inverse variance weighted (fixed effects)
Inverse variance weighted (multiplicative random effects)
MR Egger
MR RAPS
Weighted median
Weighted mode
SNP effect on IDP T1 FAST ROIs R supramarg gyrus ant
0.2
0.1
0.0
−0.1
0.25
0.50
0.75
1.00
SNP effect on Crohn's disease

Batch 2172 : Ulcerative colitis on IDP T1 FAST ROIs R supramarg gyrus ant

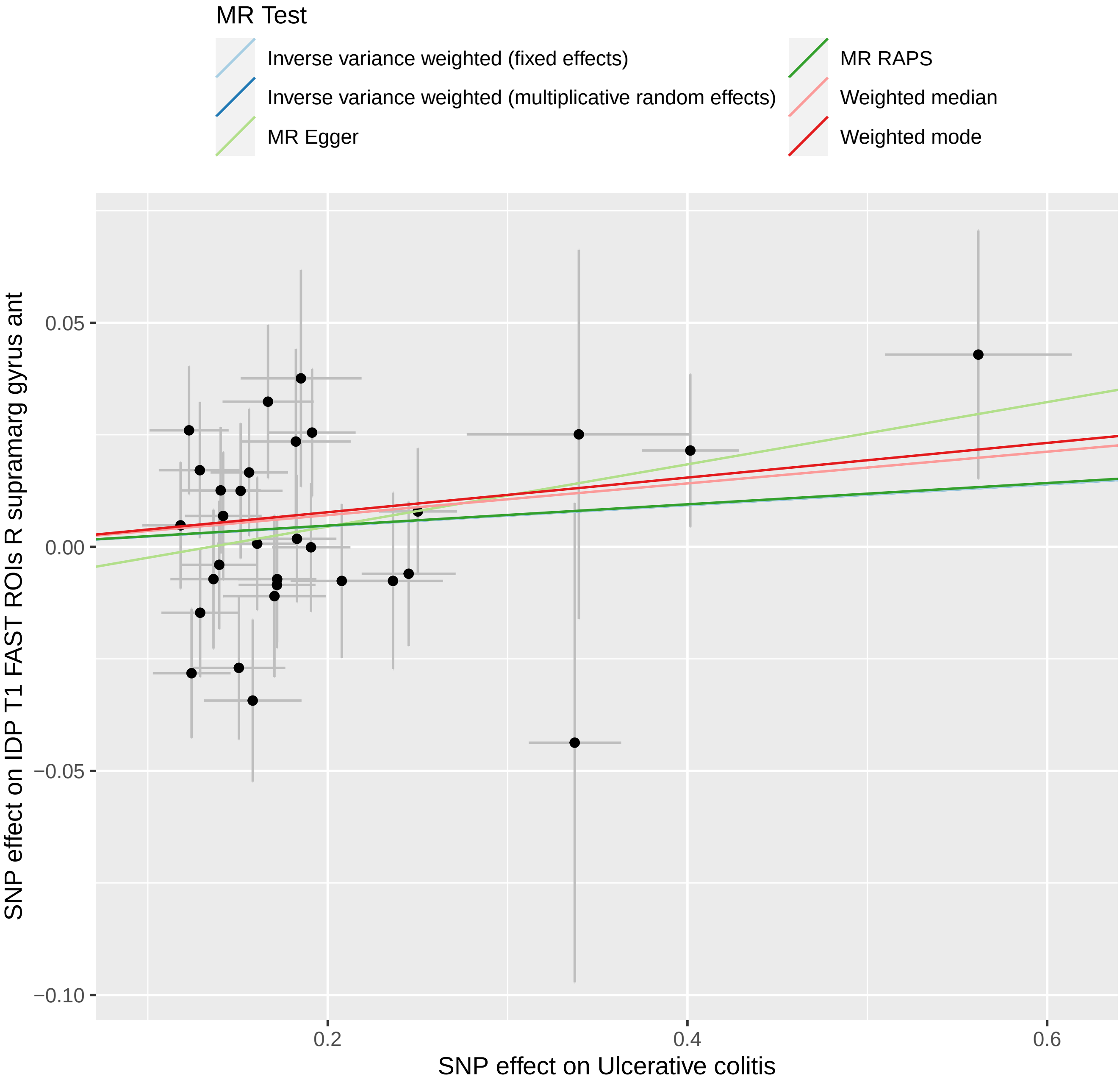

Batch 2193 : Crohn's disease on IDP T1 FAST ROIs L supramarg gyrus post

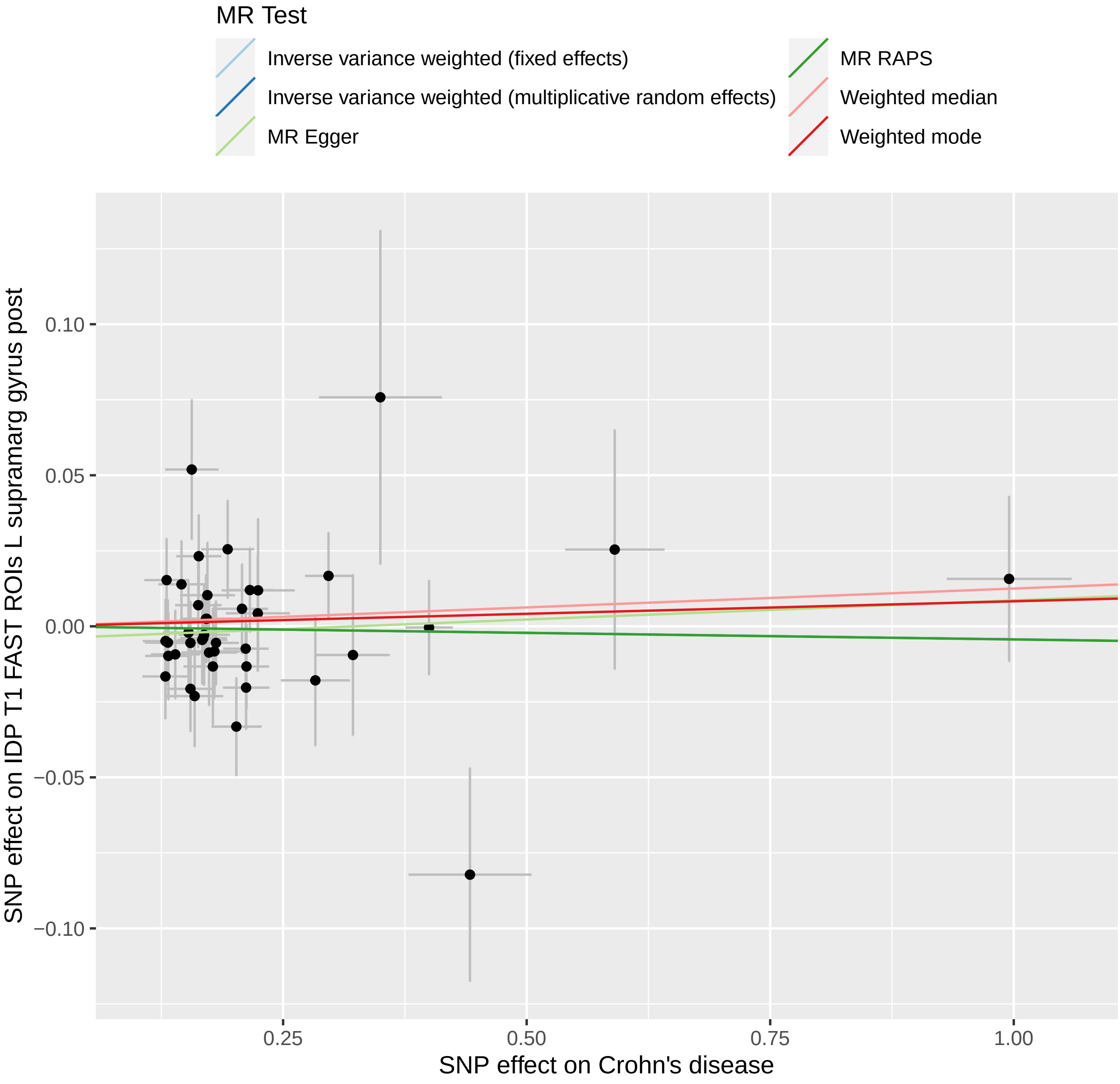

Batch 2194 : Ulcerative colitis on IDP T1 FAST ROIs L supramarg gyrus post

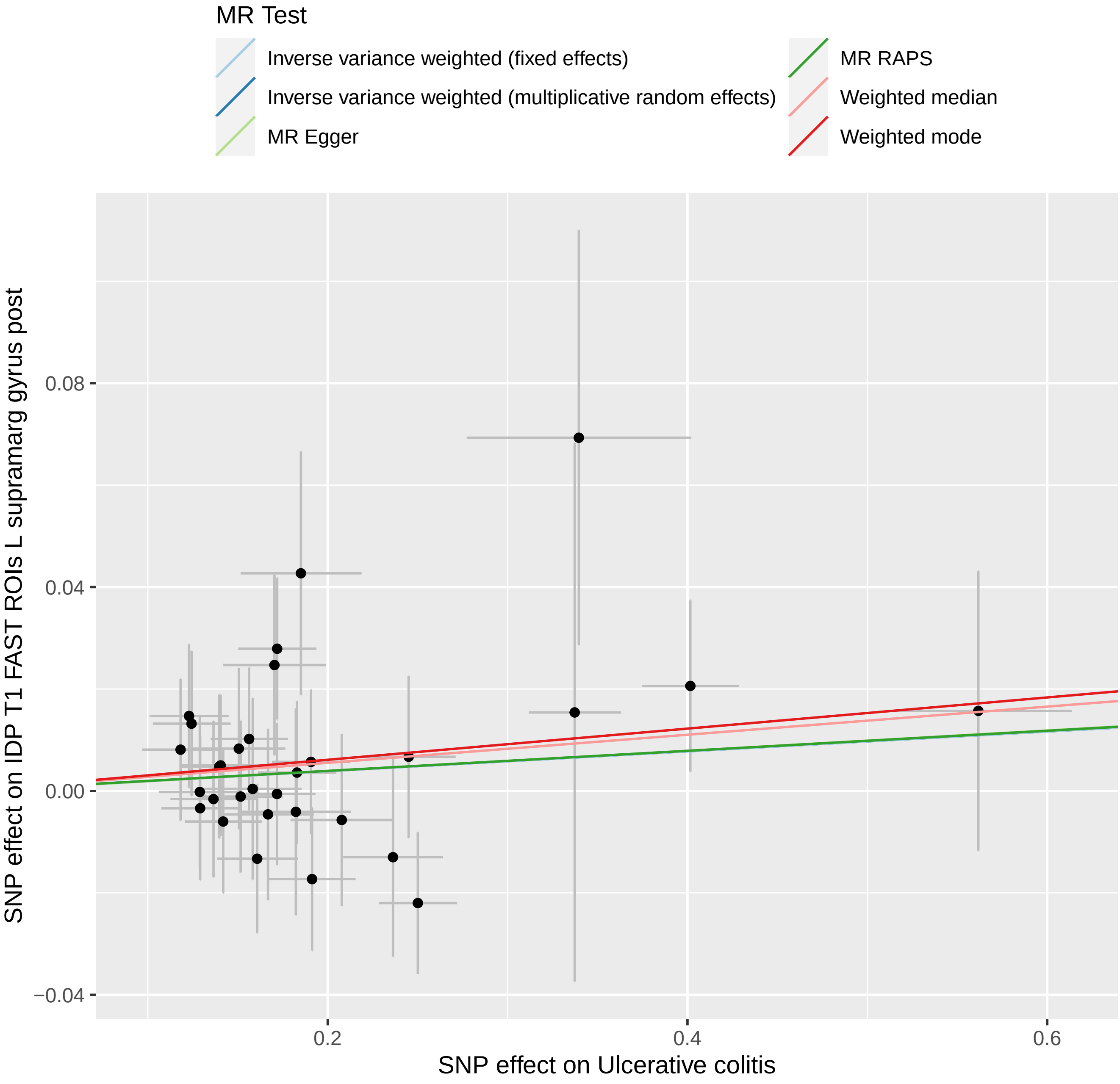

Batch 2215 : Crohn's disease on IDP T1 FAST ROIs R supramarg gyrus post

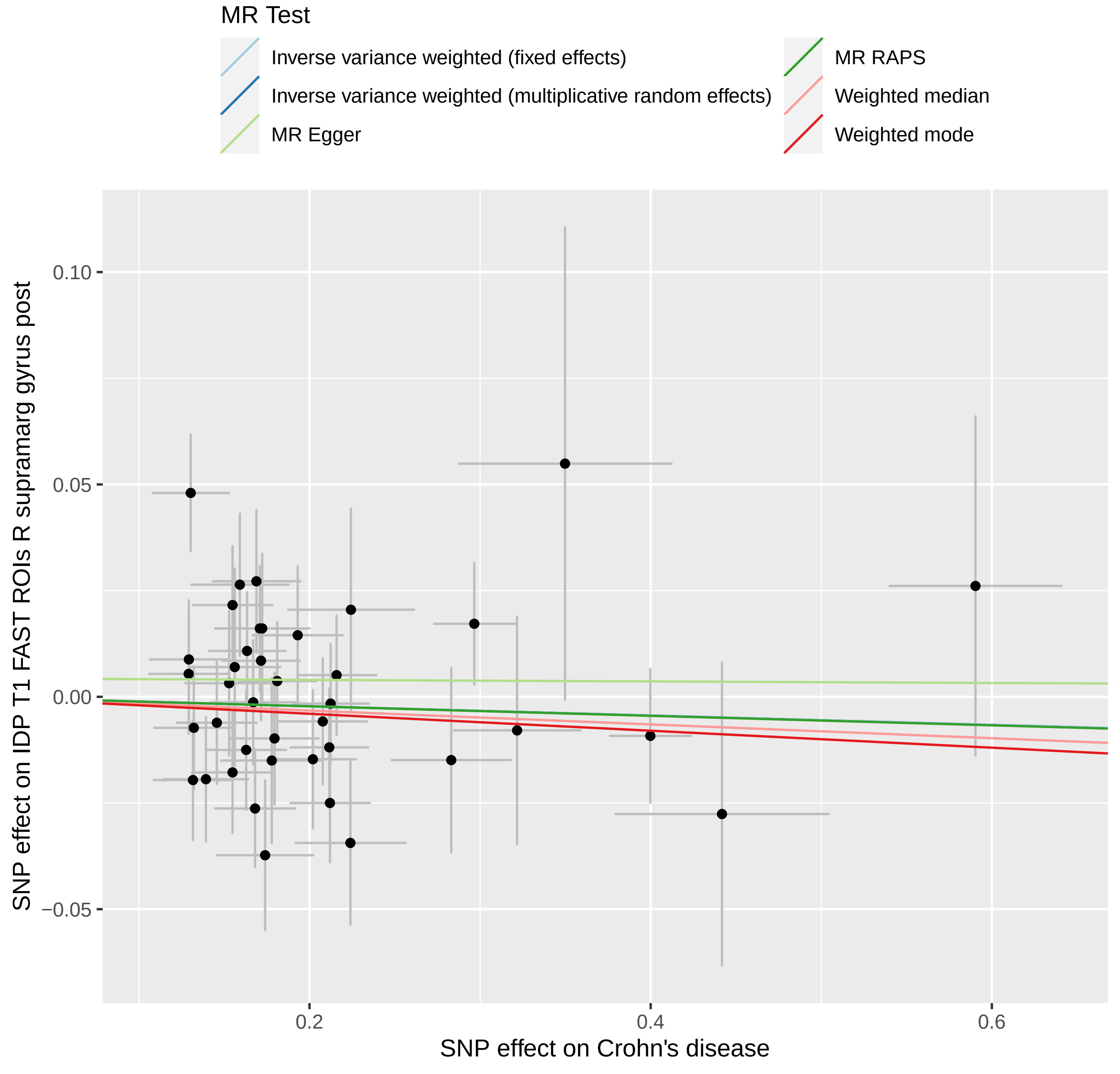

Batch 2216 : Ulcerative colitis on IDP T1 FAST ROIs R supramarg gyrus post

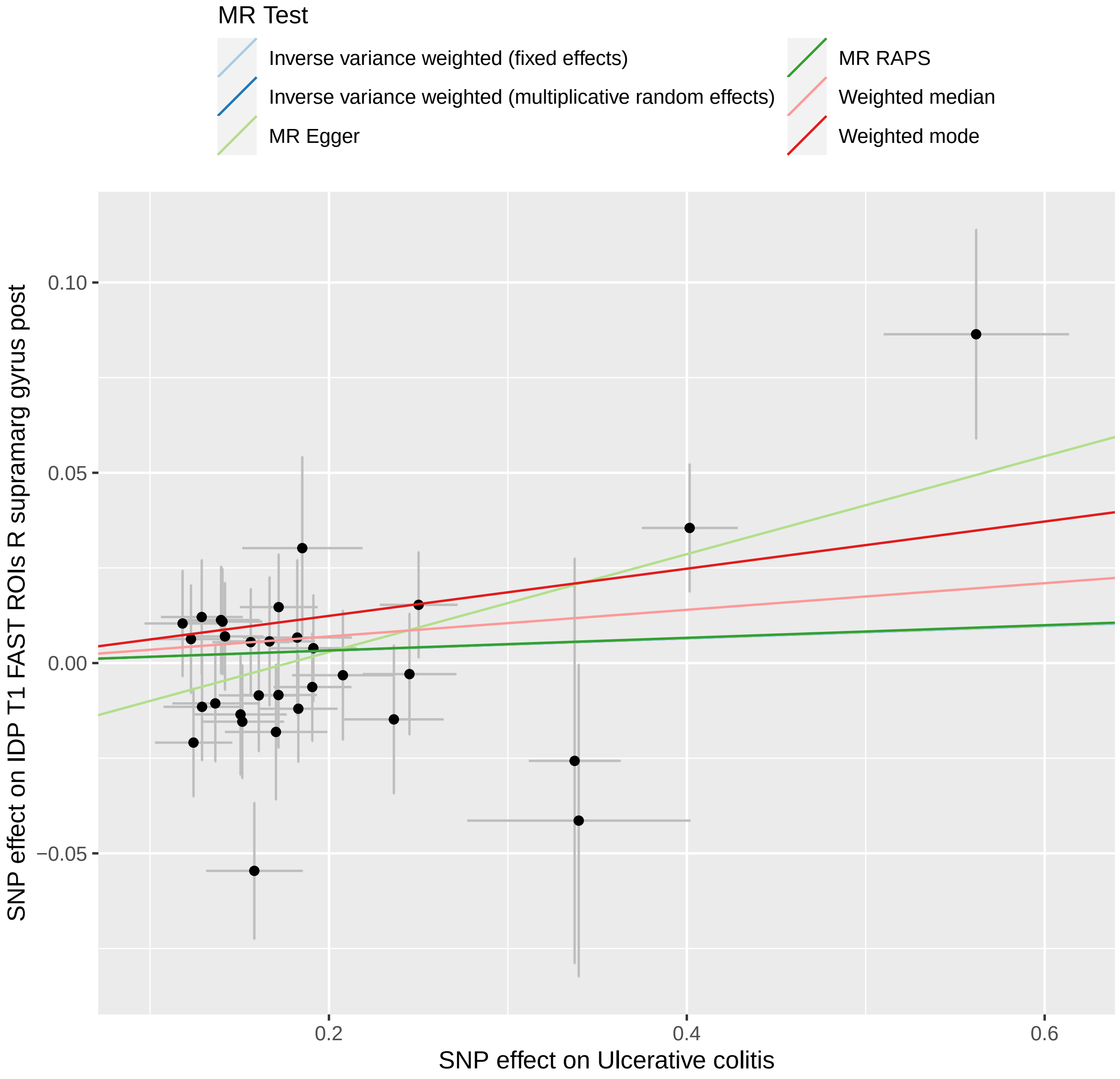

Batch 2237 : Crohn's disease on IDP T1 FAST ROIs L angular gyrus

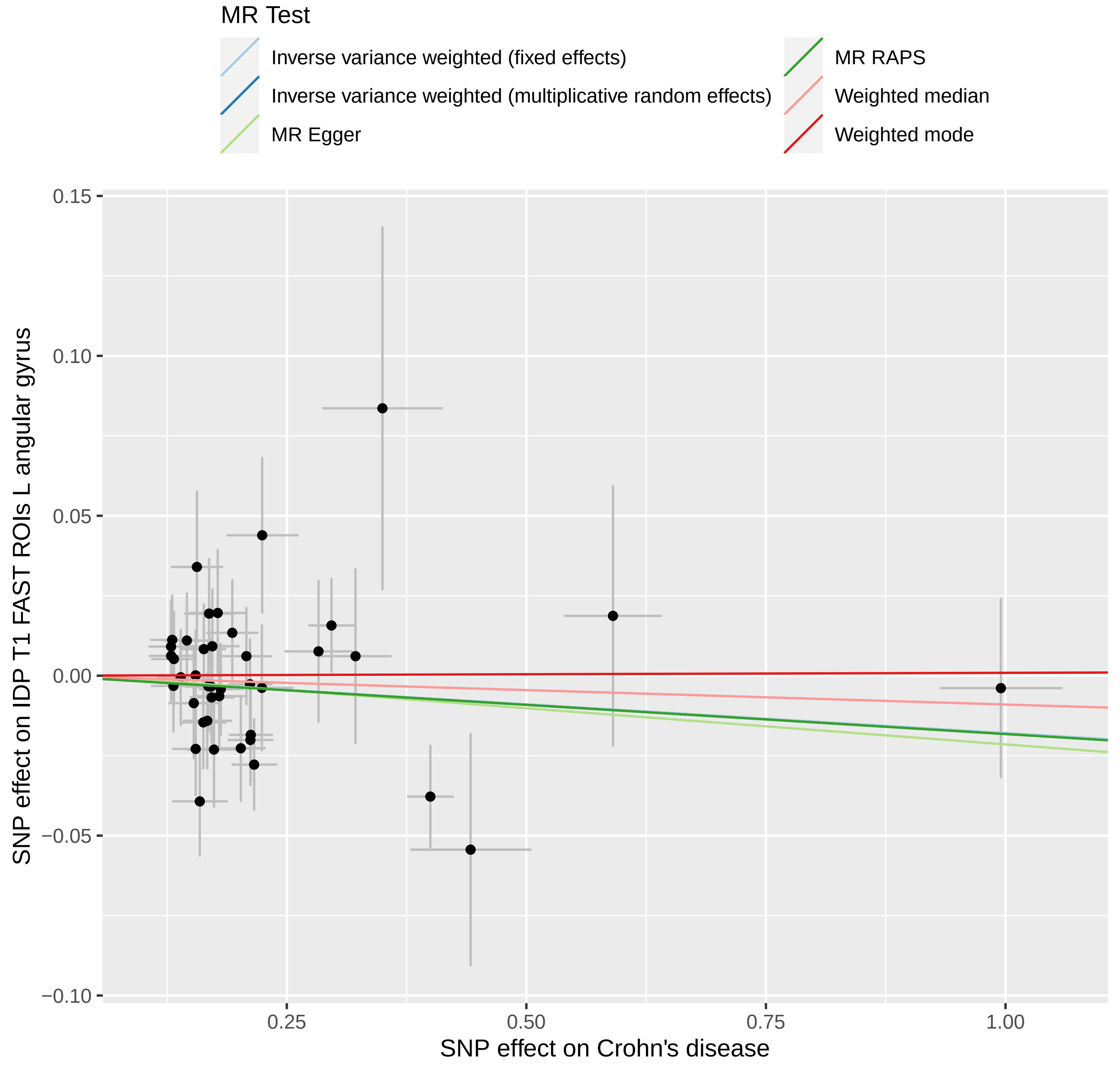

Batch 2238 : Ulcerative colitis on IDP T1 FAST ROIs L angular gyrus

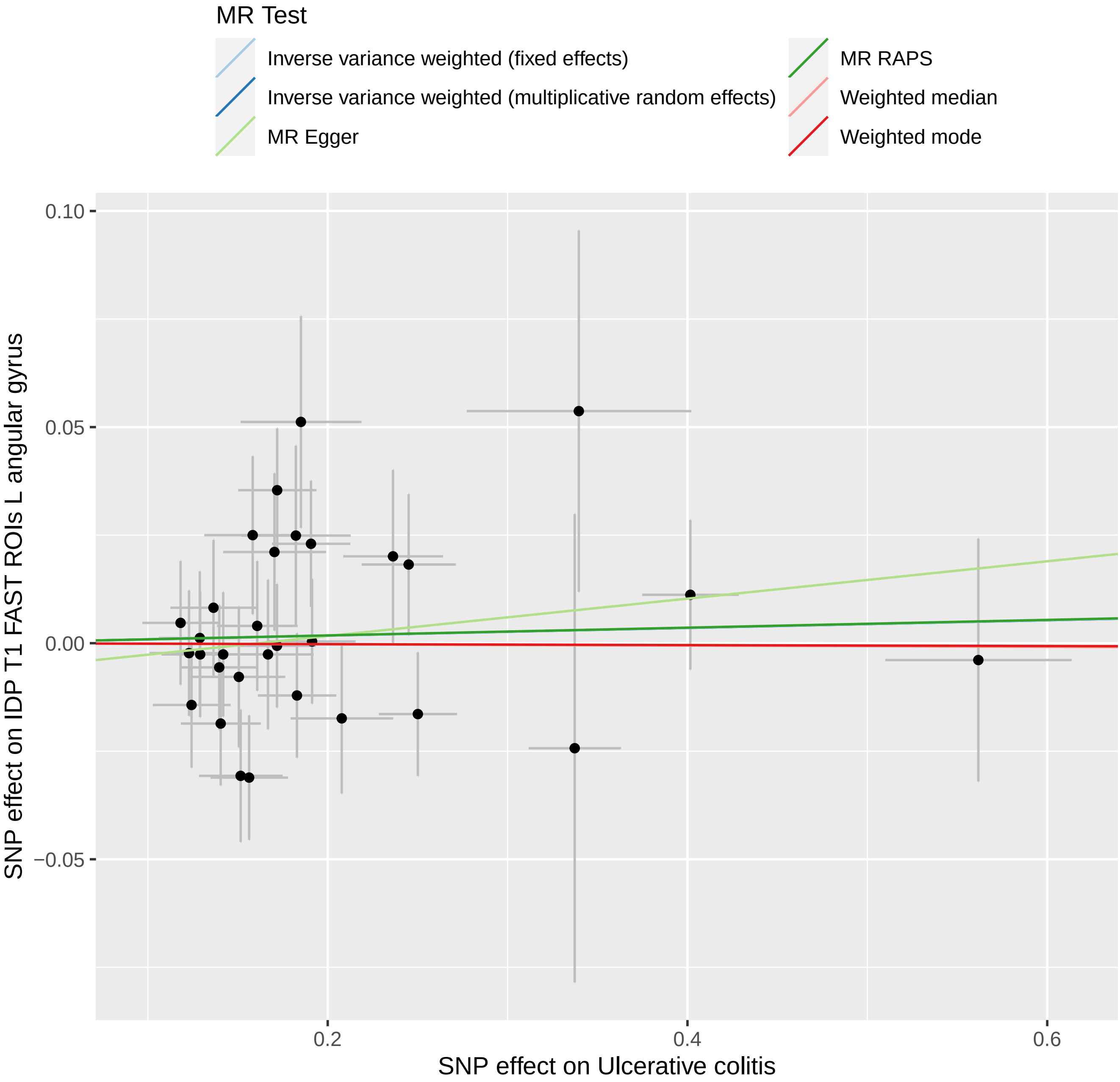

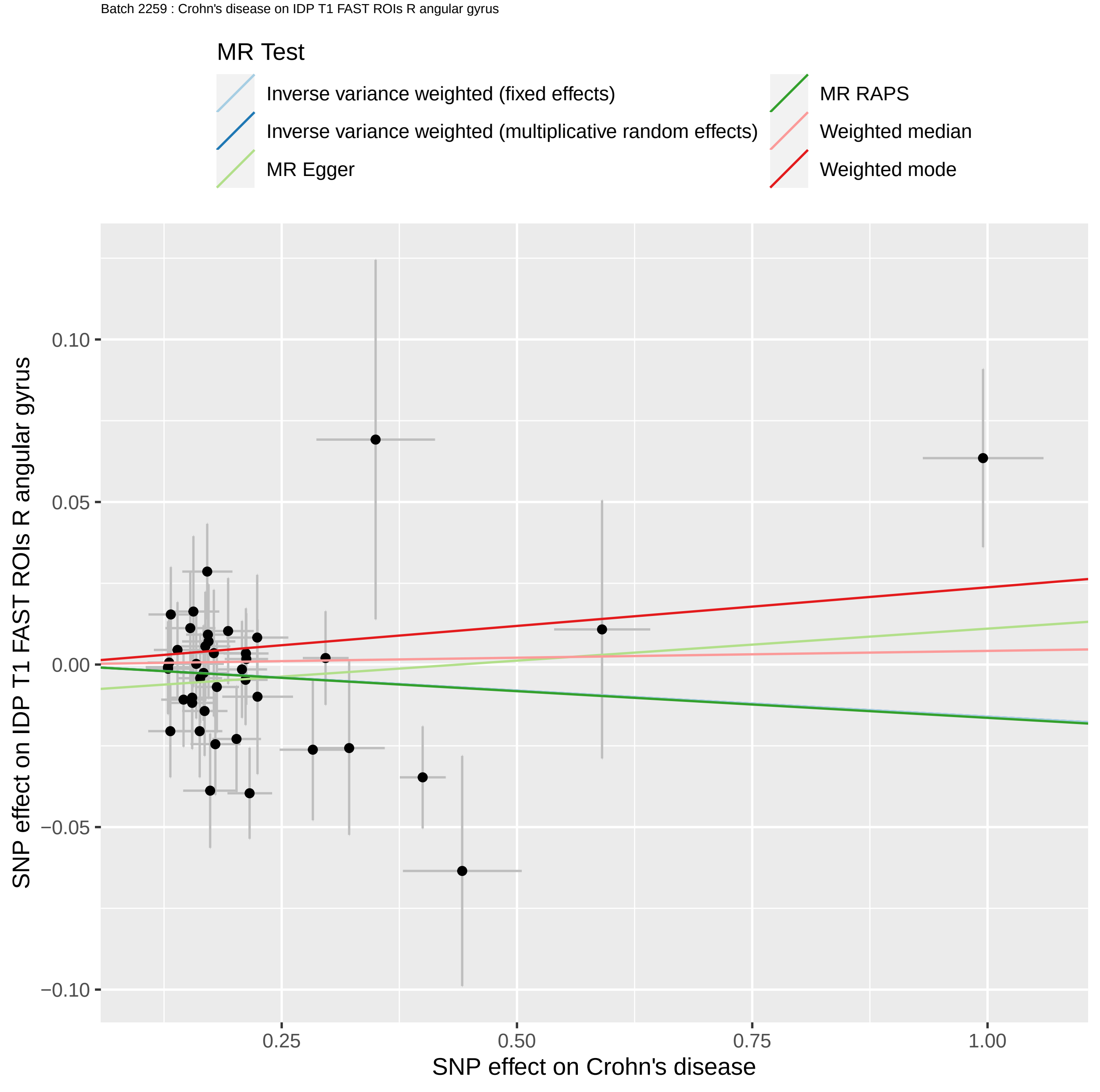

Batch 2259 : Crohn's disease on IDP T1 FAST ROIs R angular gyrus
MR Test
Inverse variance weighted (fixed effects)
Inverse variance weighted (multiplicative random effects)
MR Egger
MR RAPS
Weighted median
Weighted mode
SNP effect on IDP T1 FAST ROIs R angular gyrus
0.10
0.05
0.00
−0.05
−0.10
0.25
0.50
0.75
1.00
SNP effect on Crohn's disease

Batch 2260 : Ulcerative colitis on IDP T1 FAST ROIs R angular gyrus

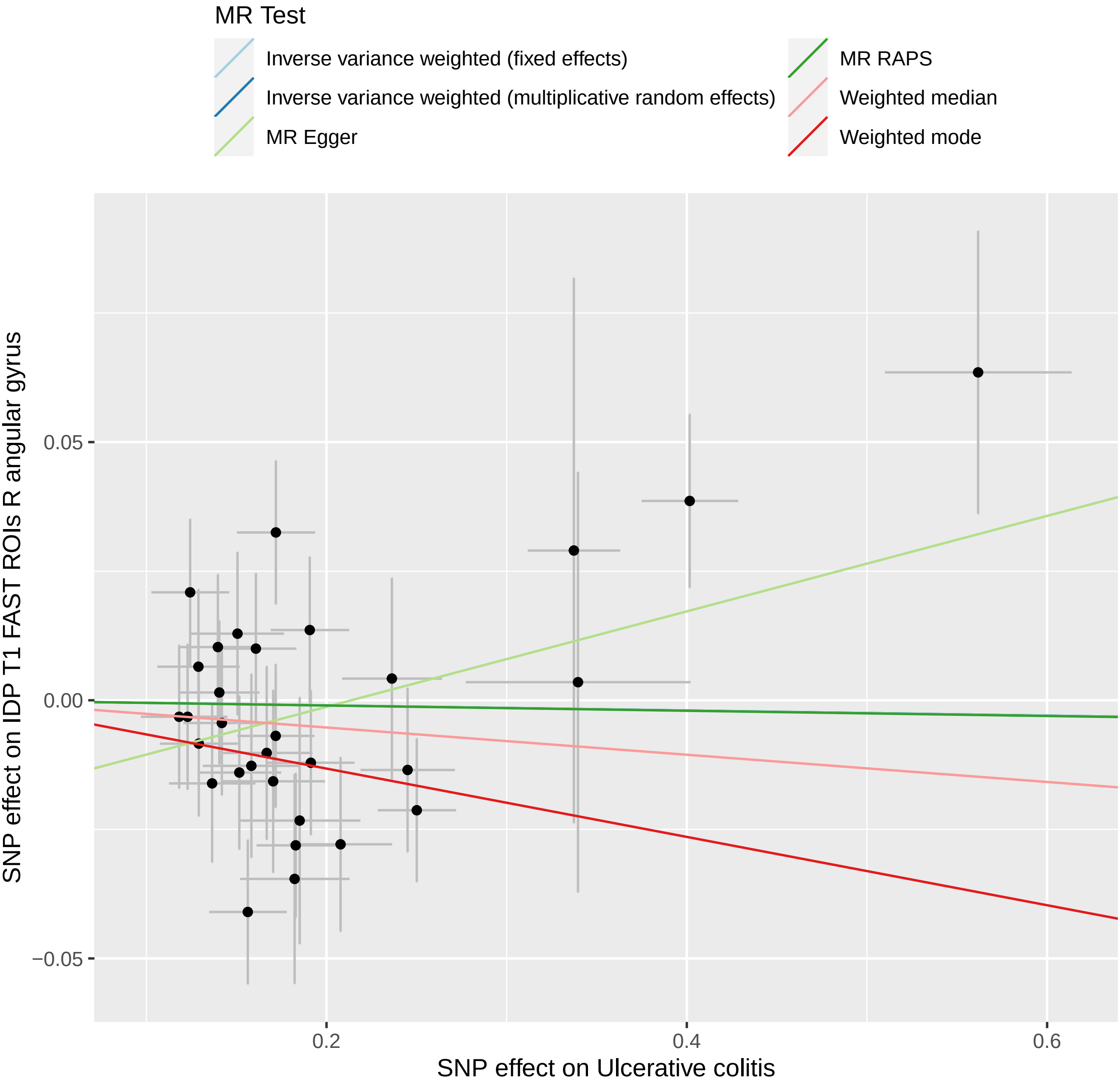

Batch 2281 : Crohn's disease on IDP T1 FAST ROIs L latocc cortex sup

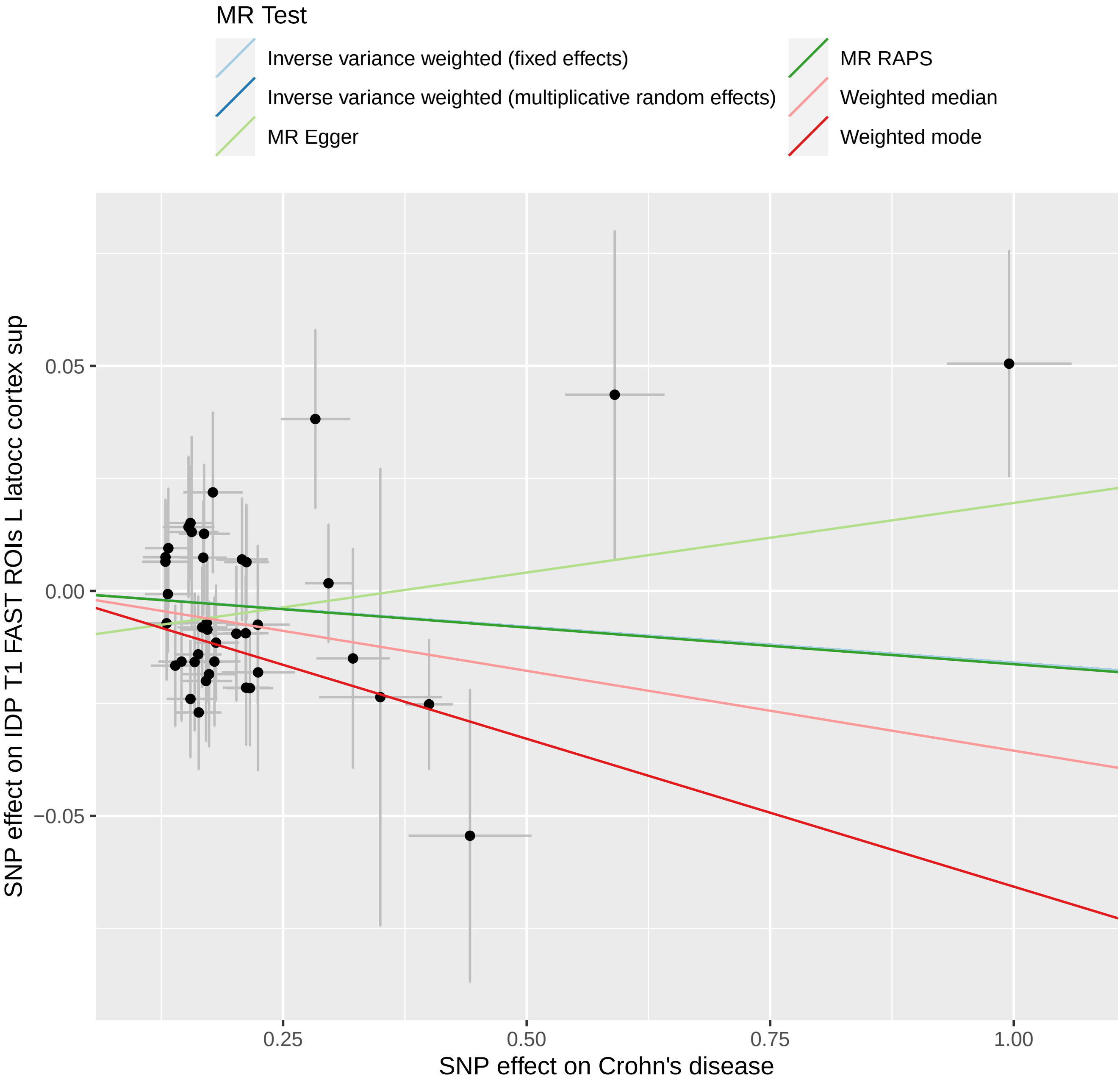

Batch 2282 : Ulcerative colitis on IDP T1 FAST ROIs L latocc cortex sup

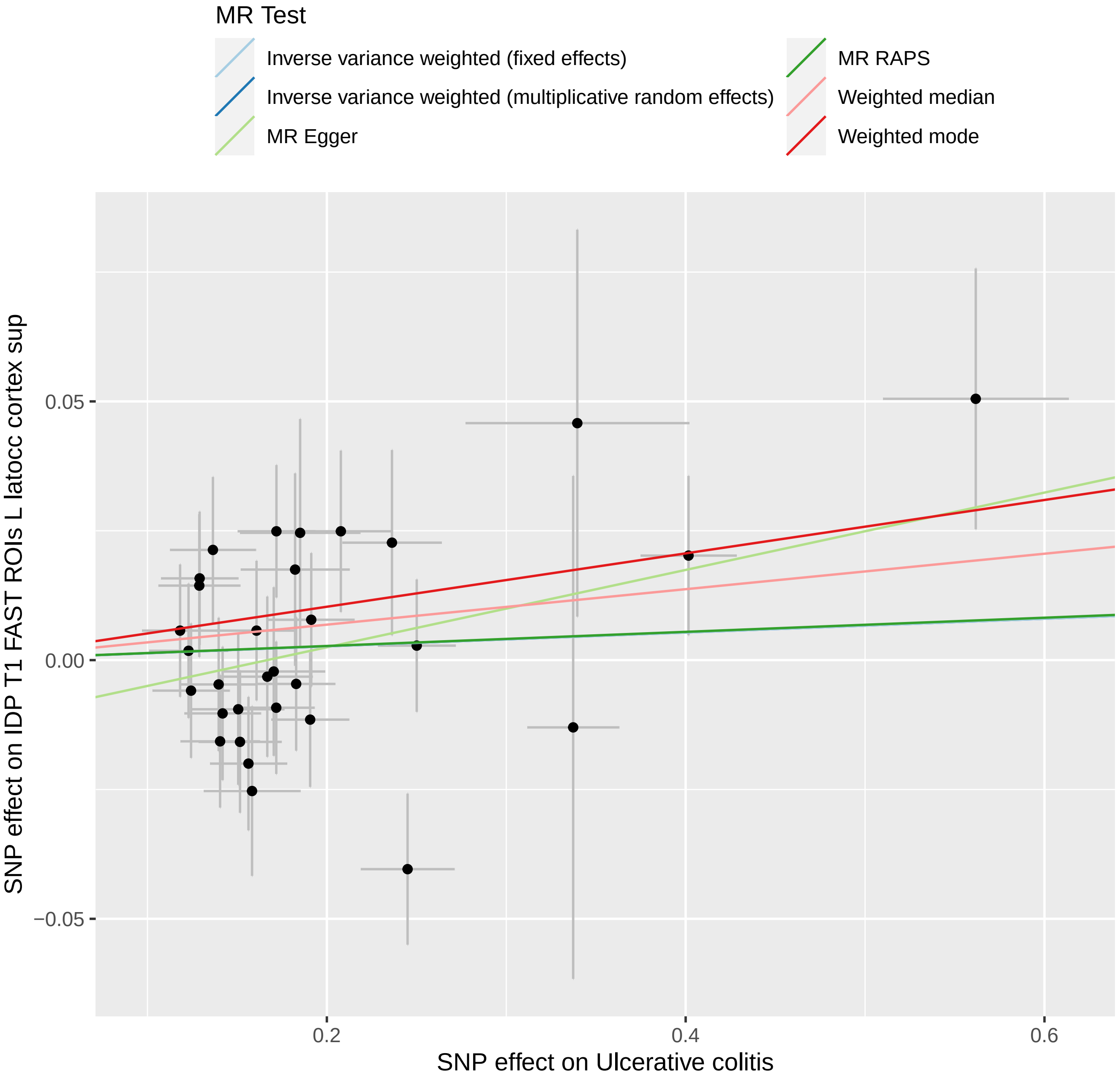

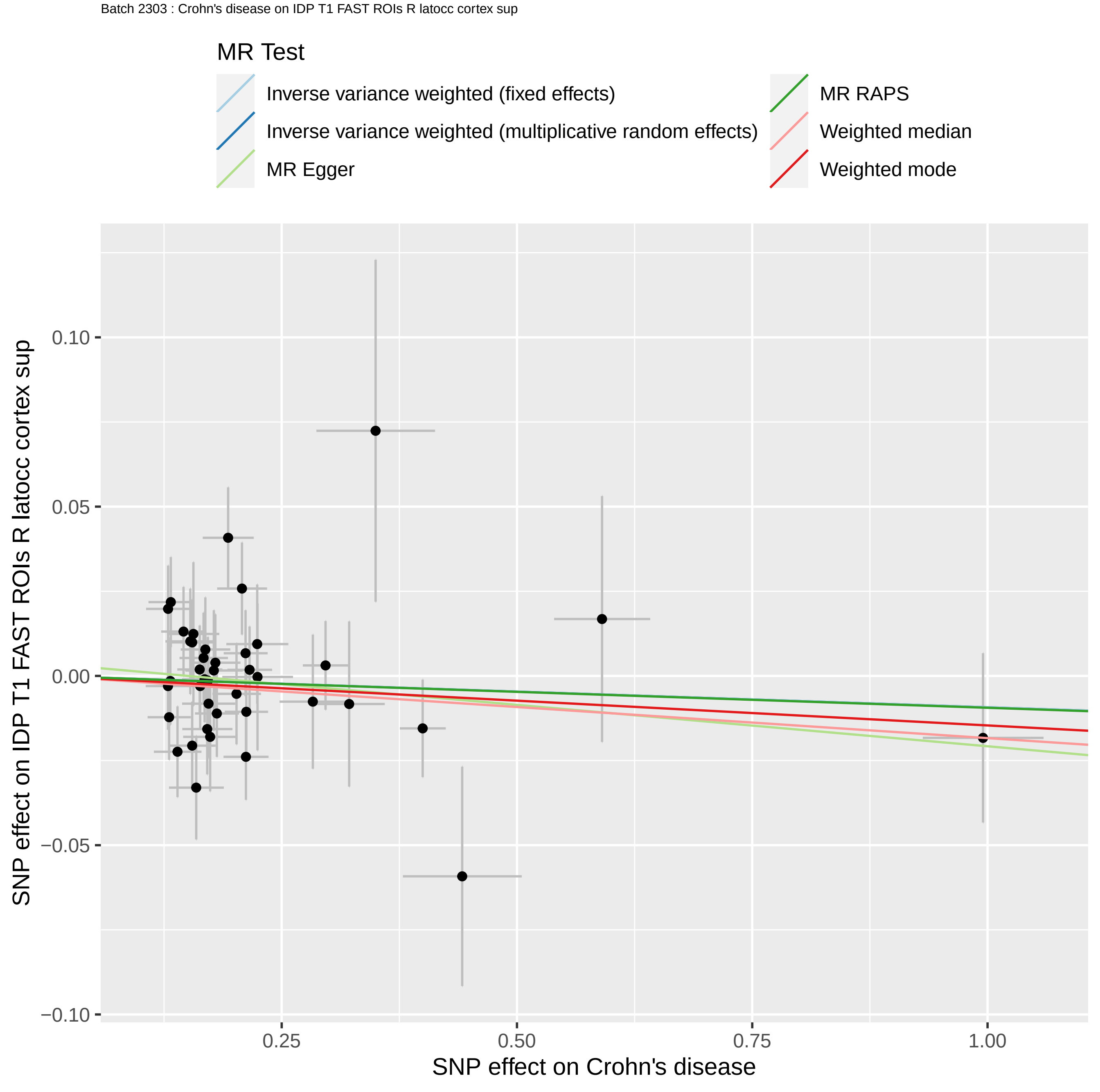
Batch 2303 : Crohn's disease on IDP T1 FAST ROIs R latocc cortex sup
MR Test
Inverse variance weighted (fixed effects)
Inverse variance weighted (multiplicative random effects)
MR Egger
MR RAPS
Weighted median
Weighted mode
SNP effect on IDP T1 FAST ROIs R latocc cortex sup
0.10
0.05
0.00
−0.05
−0.10
0.25
0.50
0.75
1.00
SNP effect on Crohn's disease

Batch 2304 : Ulcerative colitis on IDP T1 FAST ROIs R latocc cortex sup

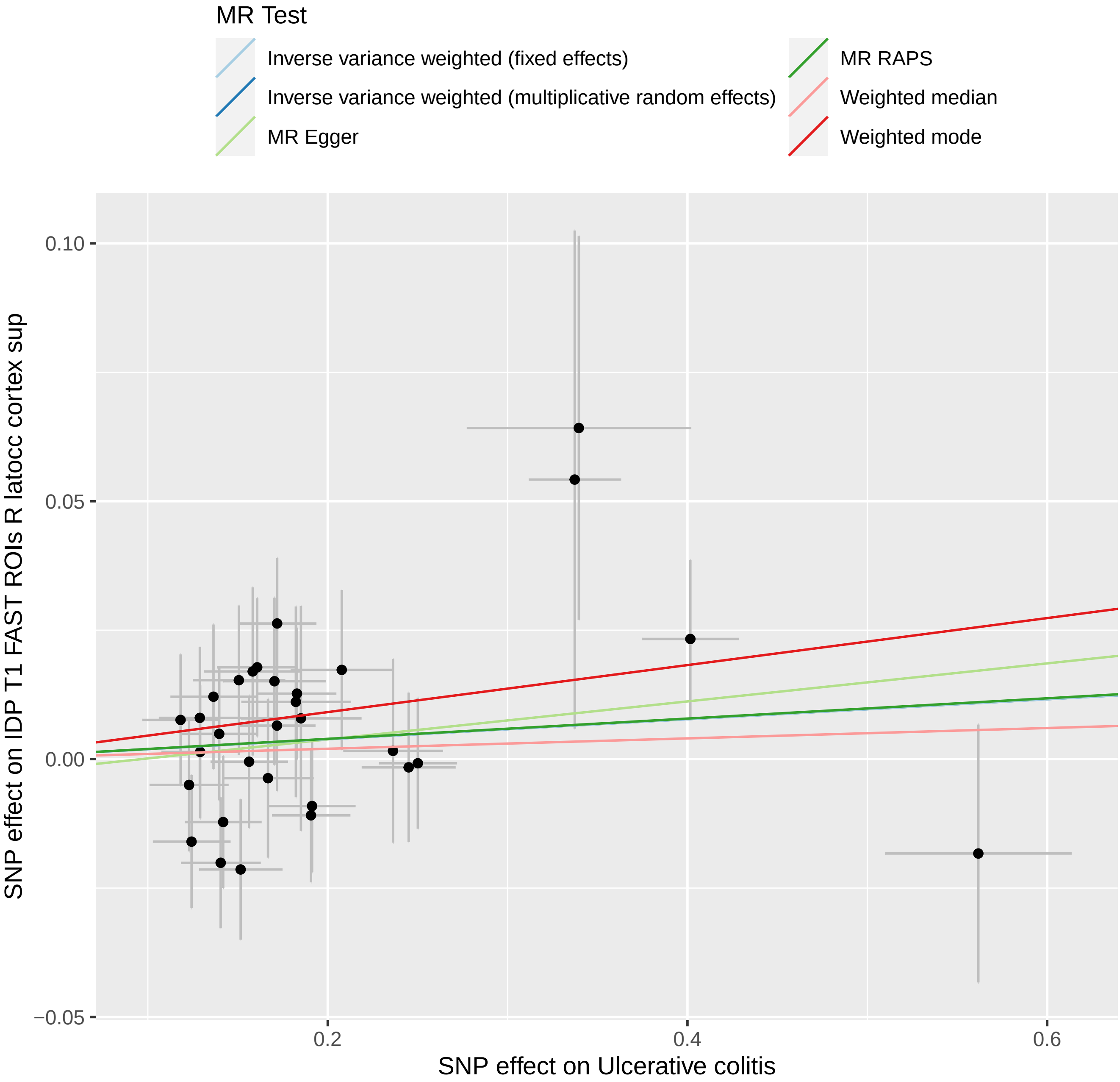

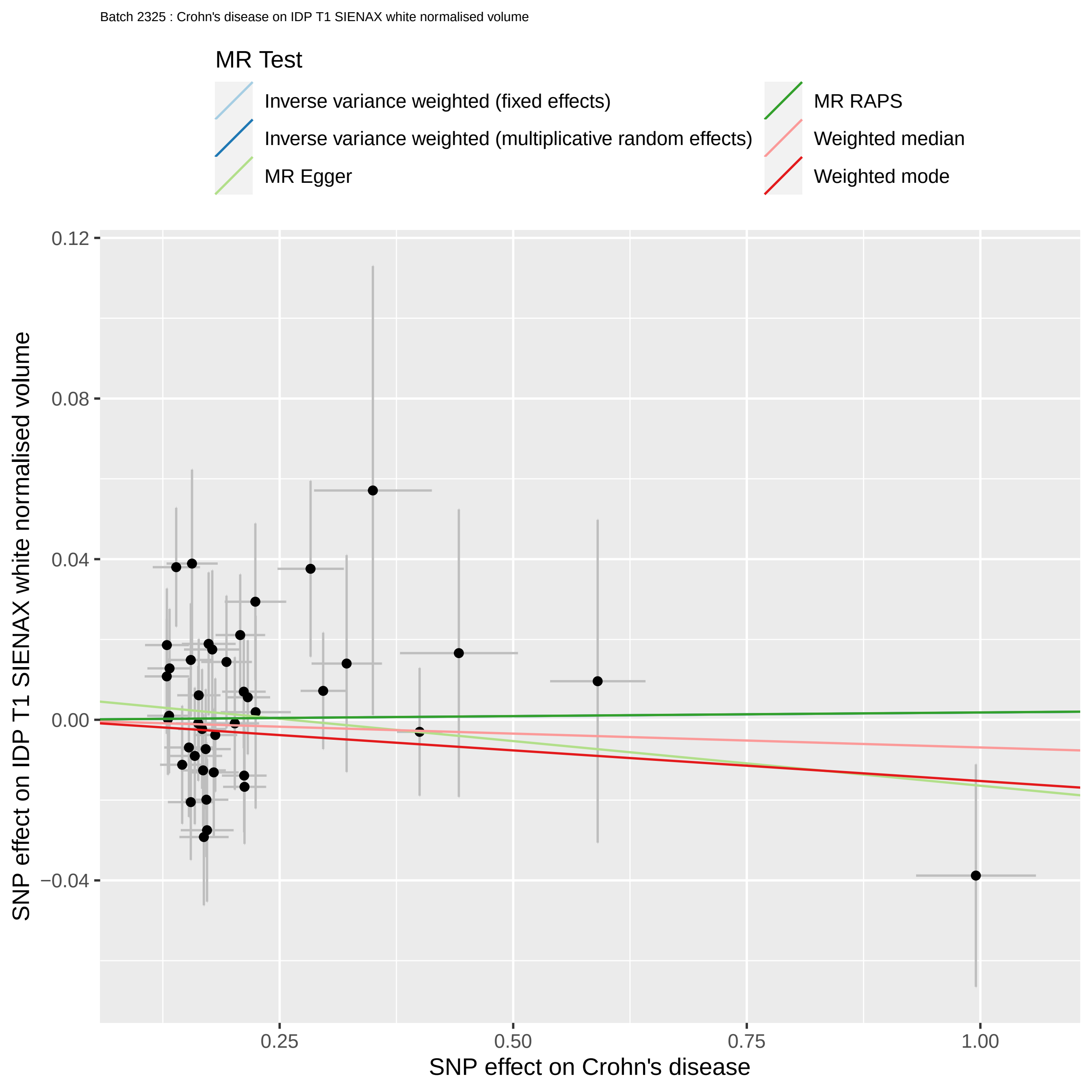

Batch 2325 : Crohn's disease on IDP T1 SIENAX white normalised volume
MR Test
Inverse variance weighted (fixed effects)
Inverse variance weighted (multiplicative random effects)
MR Egger
MR RAPS
Weighted median
Weighted mode
0.12
0.08
0.04
0.00
−0.04
0.25
0.50
0.75
1.00
SNP effect on IDP T1 SIENAX white normalised volume
SNP effect on Crohn's disease

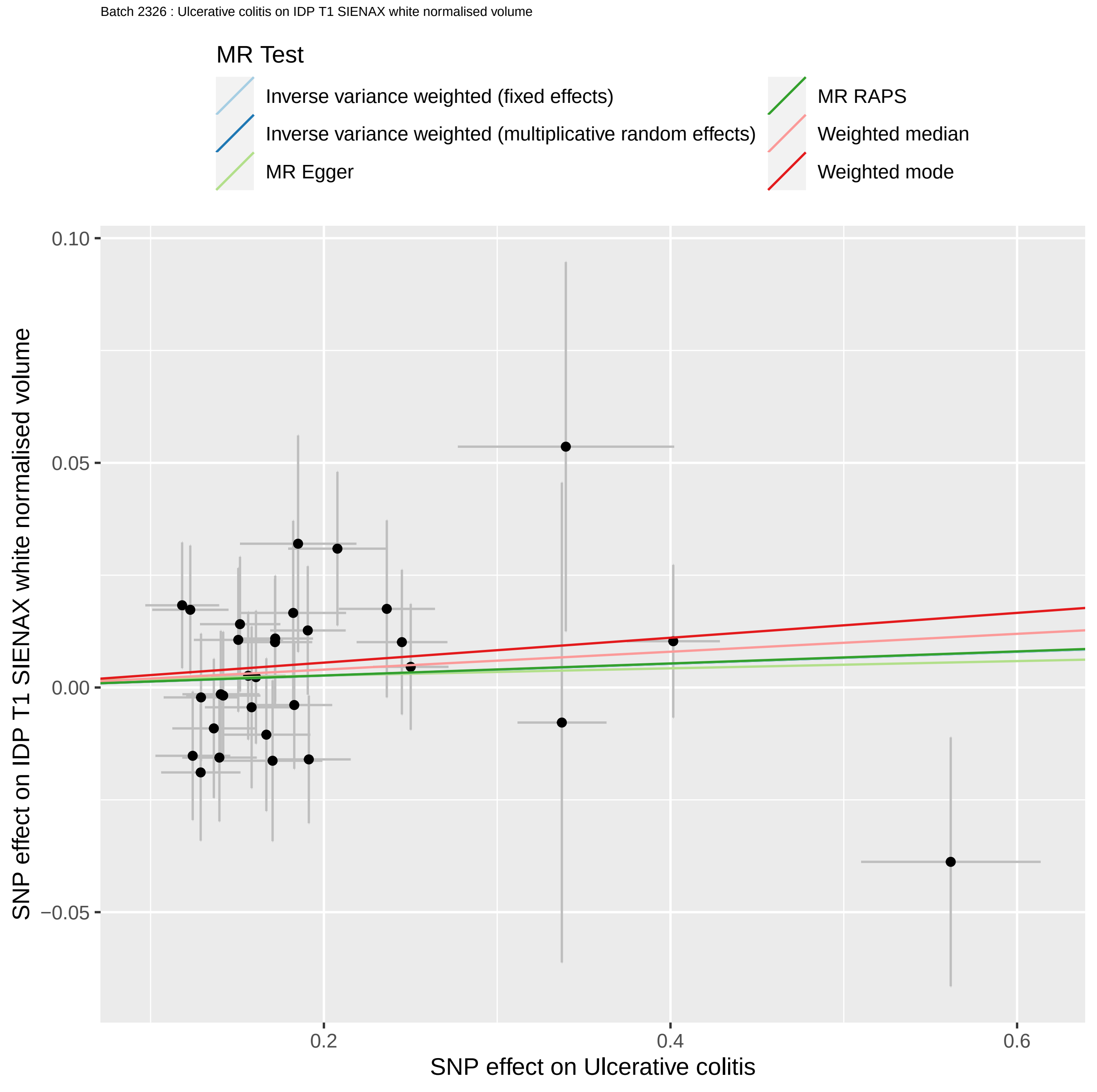
Batch 2326 : Ulcerative colitis on IDP T1 SIENAX white normalised volume
MR Test
Inverse variance weighted (fixed effects)
Inverse variance weighted (multiplicative random effects)
MR Egger
MR RAPS
Weighted median
Weighted mode
0.10
0.05
0.00
−0.05
0.2
0.4
0.6
SNP effect on IDP T1 SIENAX white normalised volume
SNP effect on Ulcerative colitis

Batch 2327 : Crohn's disease on IDP T1 FAST ROIs L latocc cortex inf

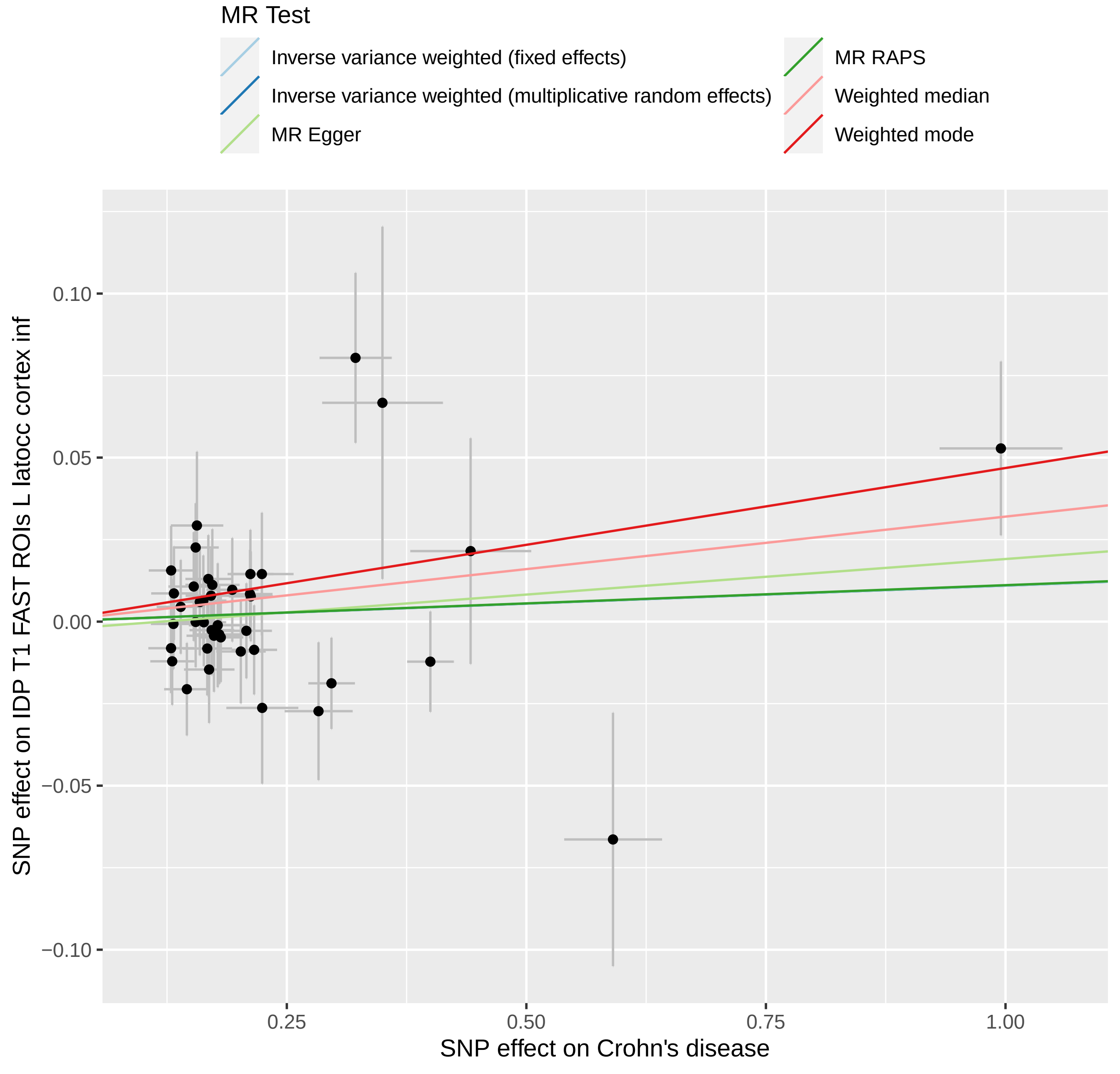

Batch 2328 : Ulcerative colitis on IDP T1 FAST ROIs L latocc cortex inf

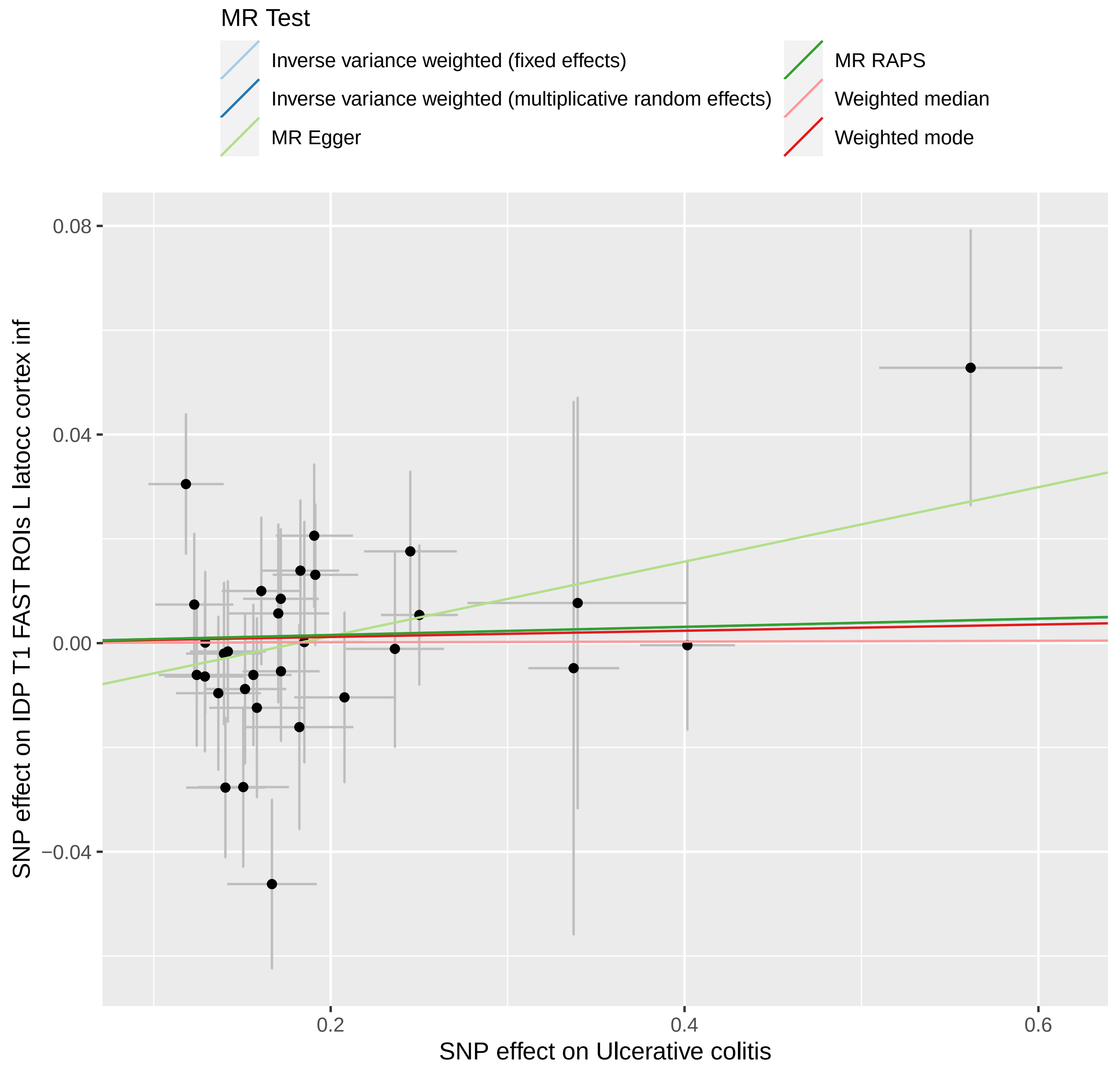

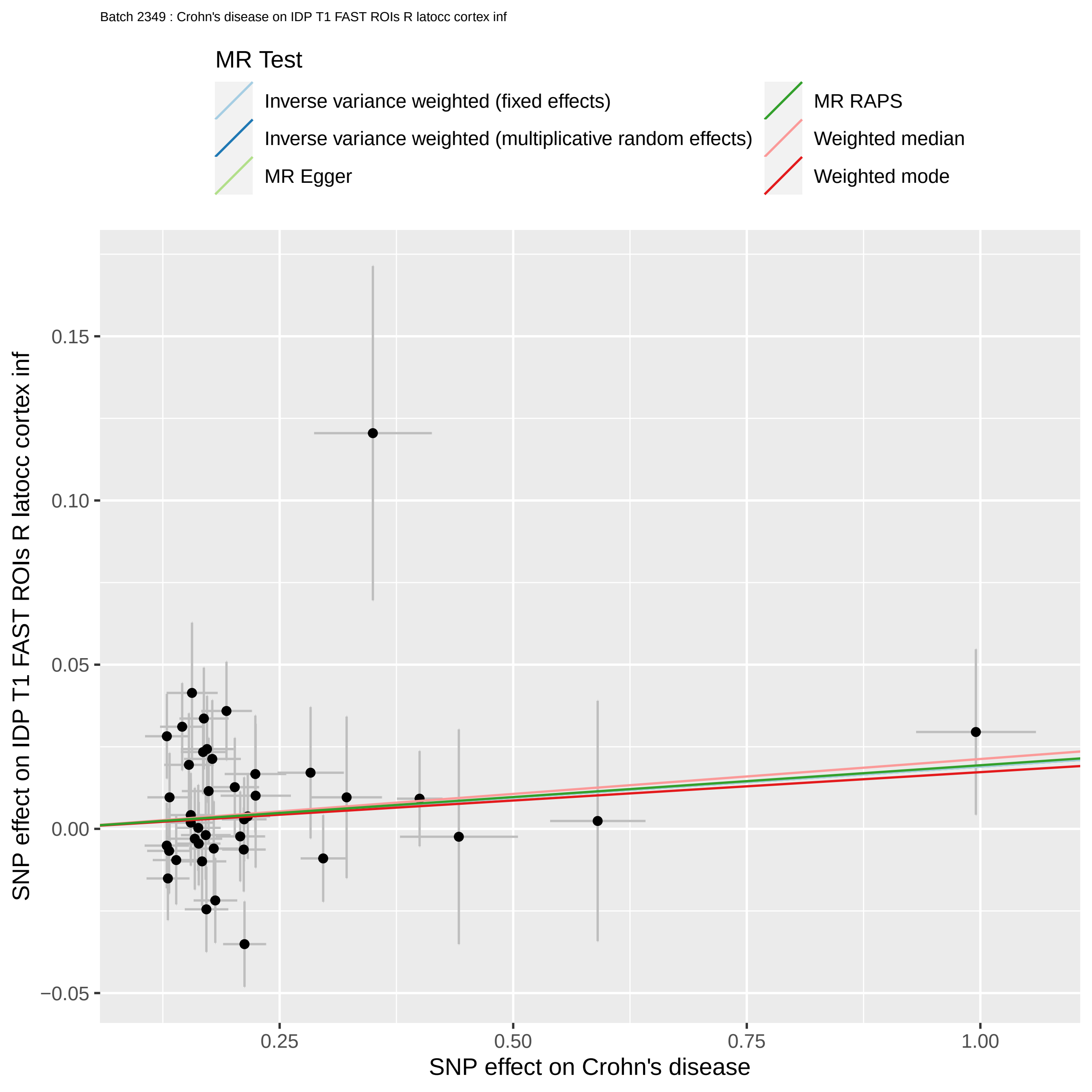

Batch 2349 : Crohn's disease on IDP T1 FAST ROIs R latocc cortex inf
MR Test
Inverse variance weighted (fixed effects)
Inverse variance weighted (multiplicative random effects)
MR Egger
MR RAPS
Weighted median
Weighted mode
SNP effect on IDP T1 FAST ROIs R latocc cortex inf
0.15
0.10
0.05
0.00
−0.05
0.25
0.50
0.75
1.00
SNP effect on Crohn's disease

Batch 2350 : Ulcerative colitis on IDP T1 FAST ROIs R latocc cortex inf

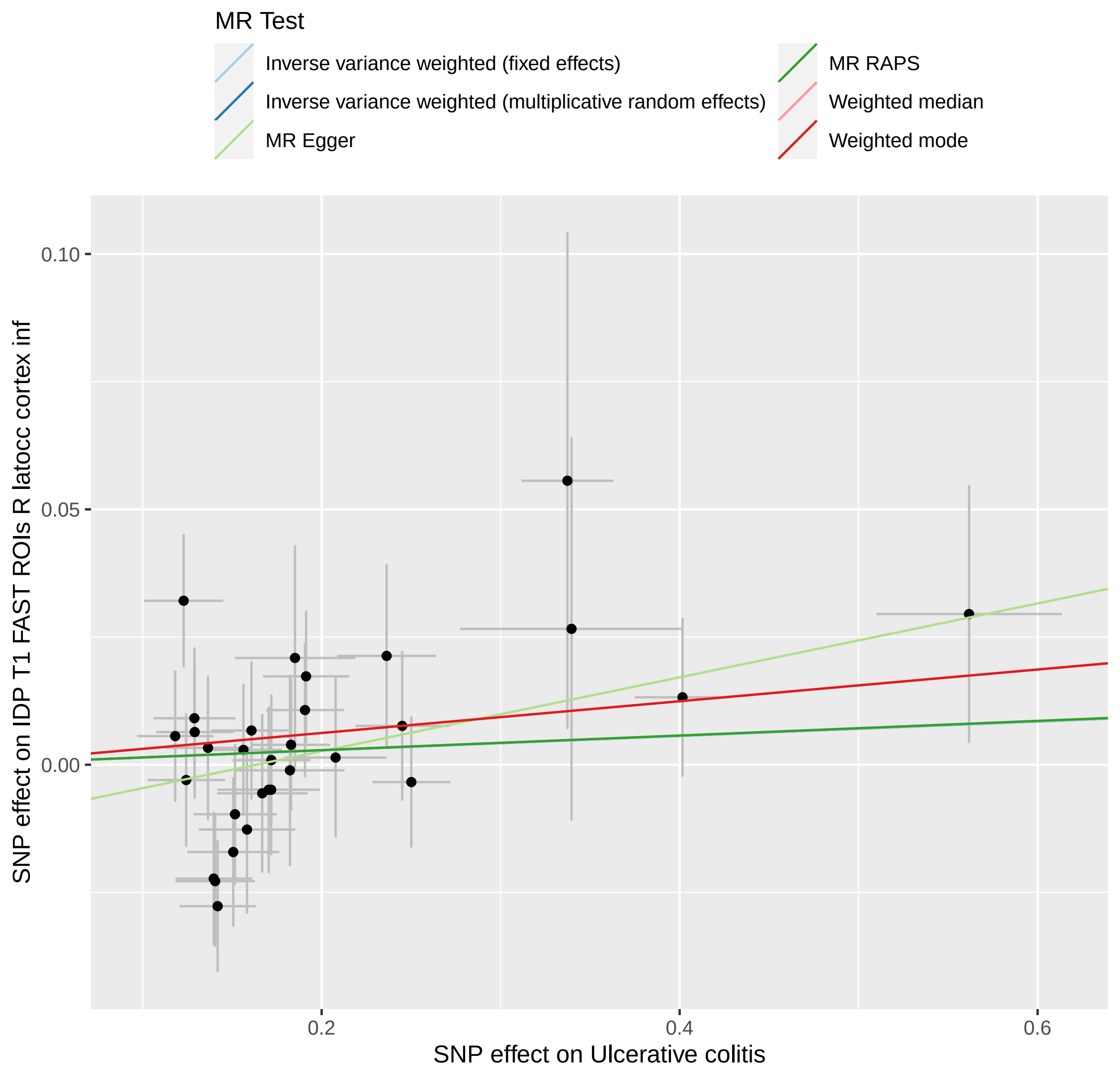

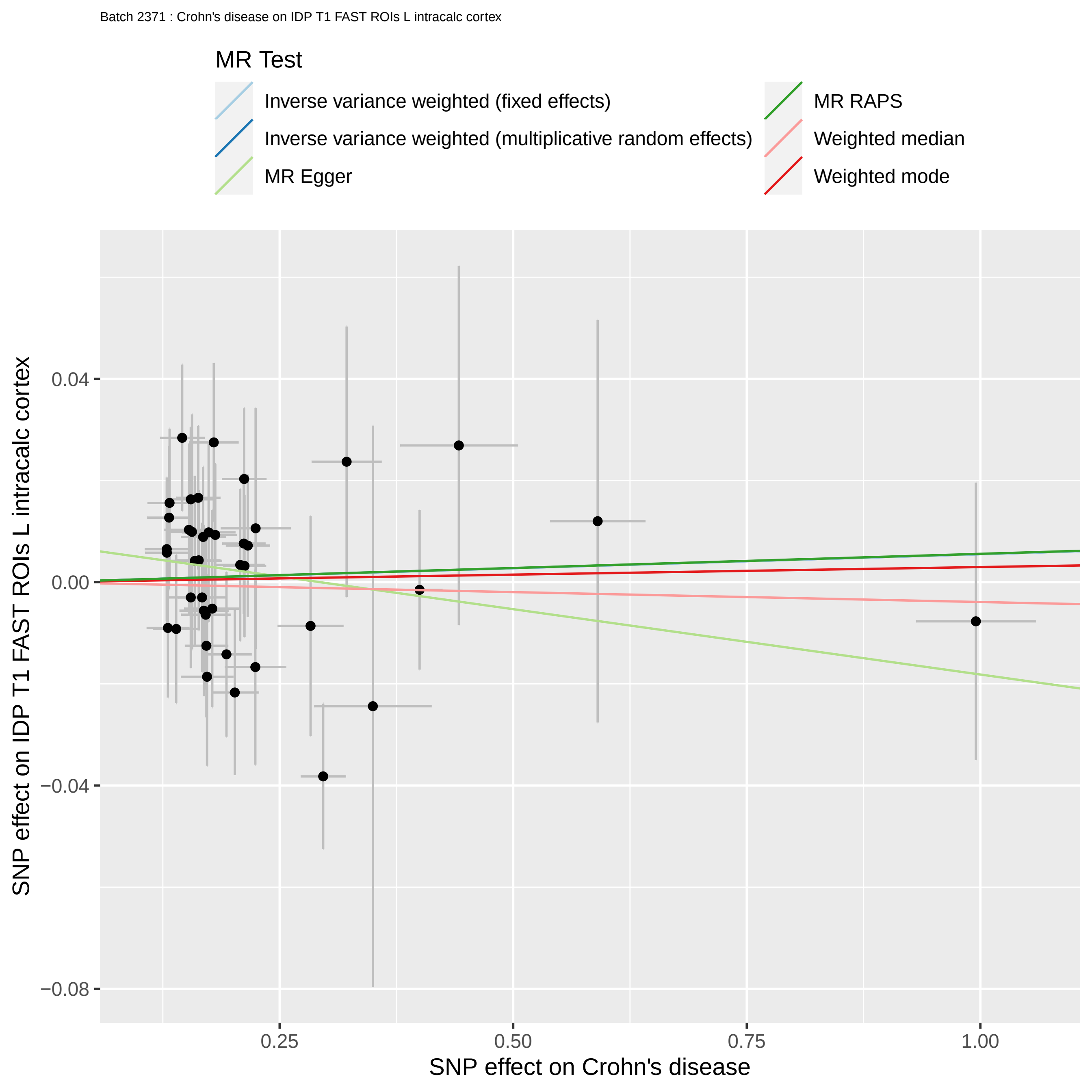

Batch 2371 : Crohn's disease on IDP T1 FAST ROIs L intracalc cortex
MR Test
Inverse variance weighted (fixed effects)
Inverse variance weighted (multiplicative random effects)
MR Egger
MR RAPS
Weighted median
Weighted mode
0.04
0.00
−0.04
−0.08
0.25
0.50
0.75
1.00
SNP effect on IDP T1 FAST ROIs L intracalc cortex
SNP effect on Crohn's disease

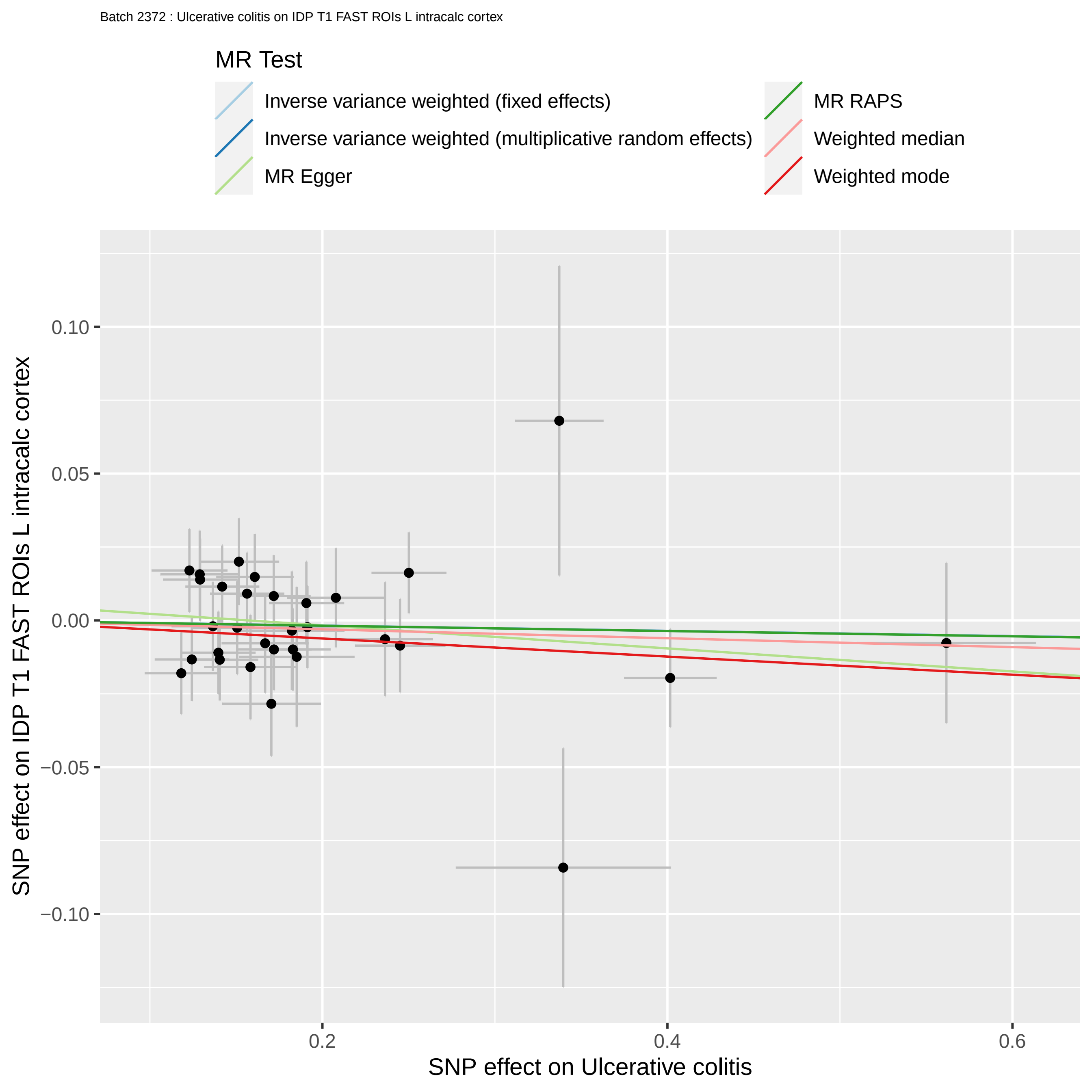

Batch 2372 : Ulcerative colitis on IDP T1 FAST ROIs L intracalc cortex
MR Test
Inverse variance weighted (fixed effects)
Inverse variance weighted (multiplicative random effects)
MR Egger
MR RAPS
Weighted median
Weighted mode
0.10
0.05
0.00
−0.05
−0.10
0.2
0.4
0.6
SNP effect on Ulcerative colitis
SNP effect on IDP T1 FAST ROIs L intracalc cortex

Batch 2393 : Crohn's disease on IDP T1 FAST ROIs R intracalc cortex

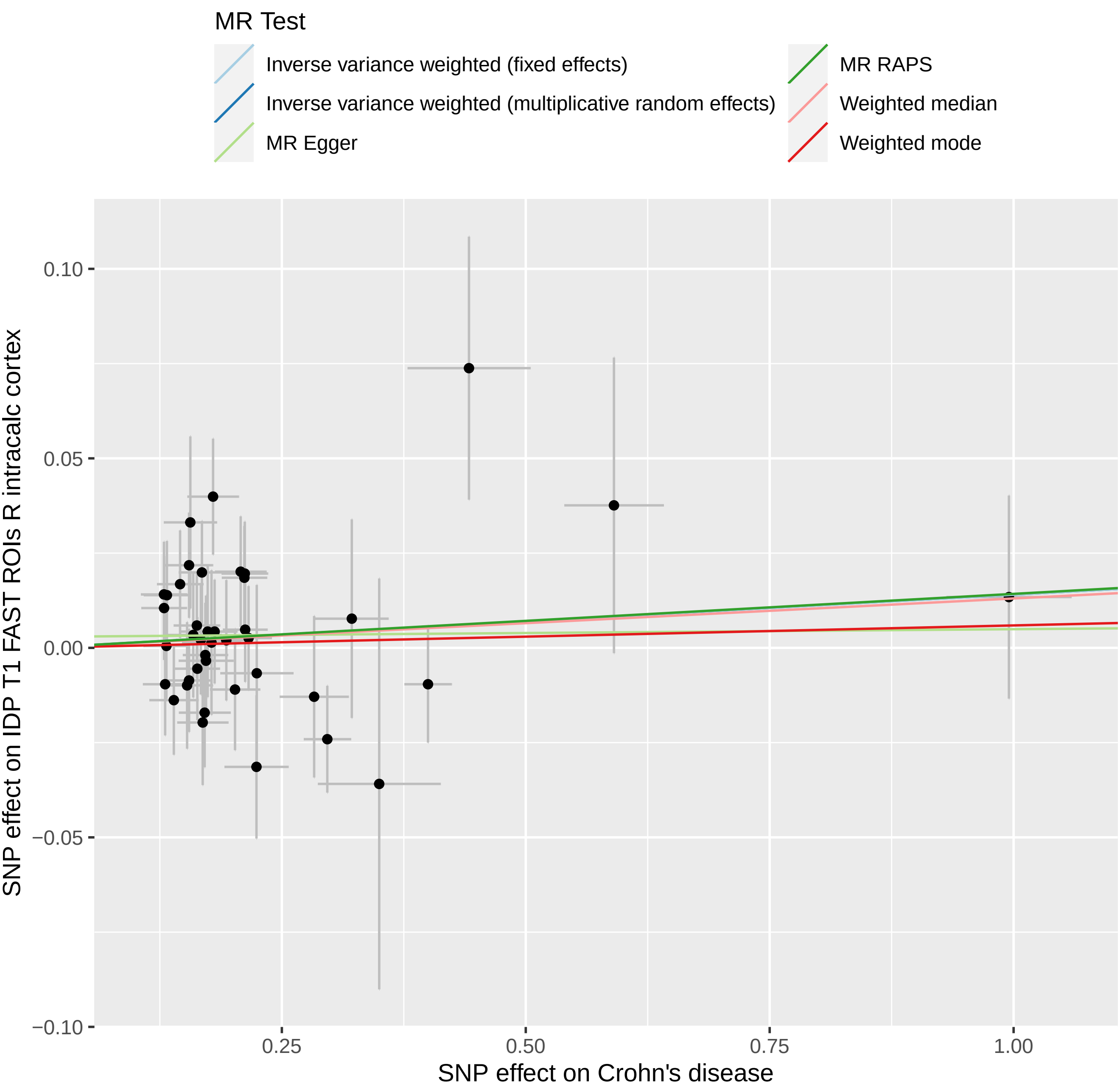

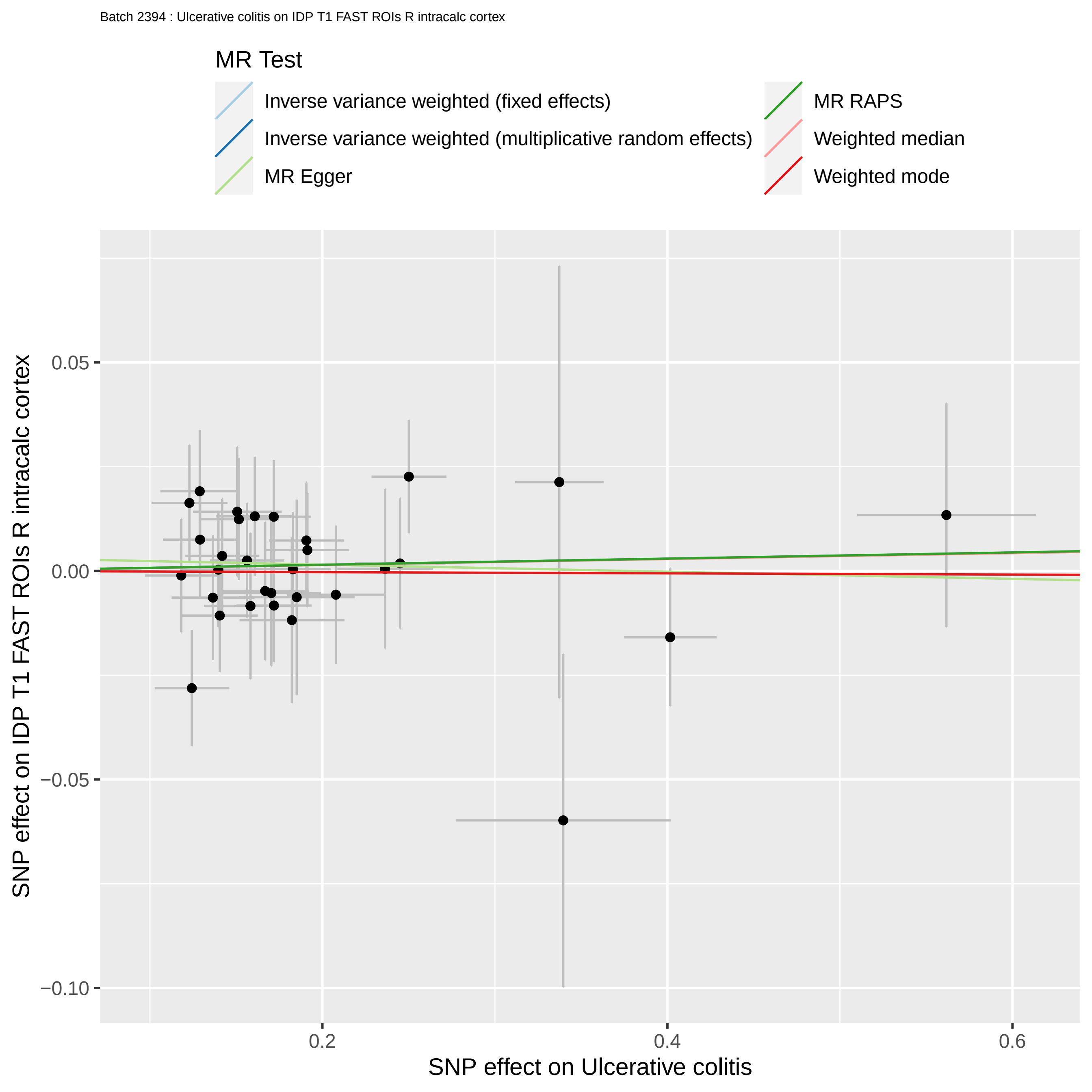

Batch 2394 : Ulcerative colitis on IDP T1 FAST ROIs R intracalc cortex
MR Test
Inverse variance weighted (fixed effects)
Inverse variance weighted (multiplicative random effects)
MR Egger
MR RAPS
Weighted median
Weighted mode
0.05
0.00
−0.05
−0.10
0.2
0.4
0.6
SNP effect on IDP T1 FAST ROIs R intracalc cortex
SNP effect on Ulcerative colitis

Batch 2415 : Crohn's disease on IDP T1 FAST ROIs L front med cortex

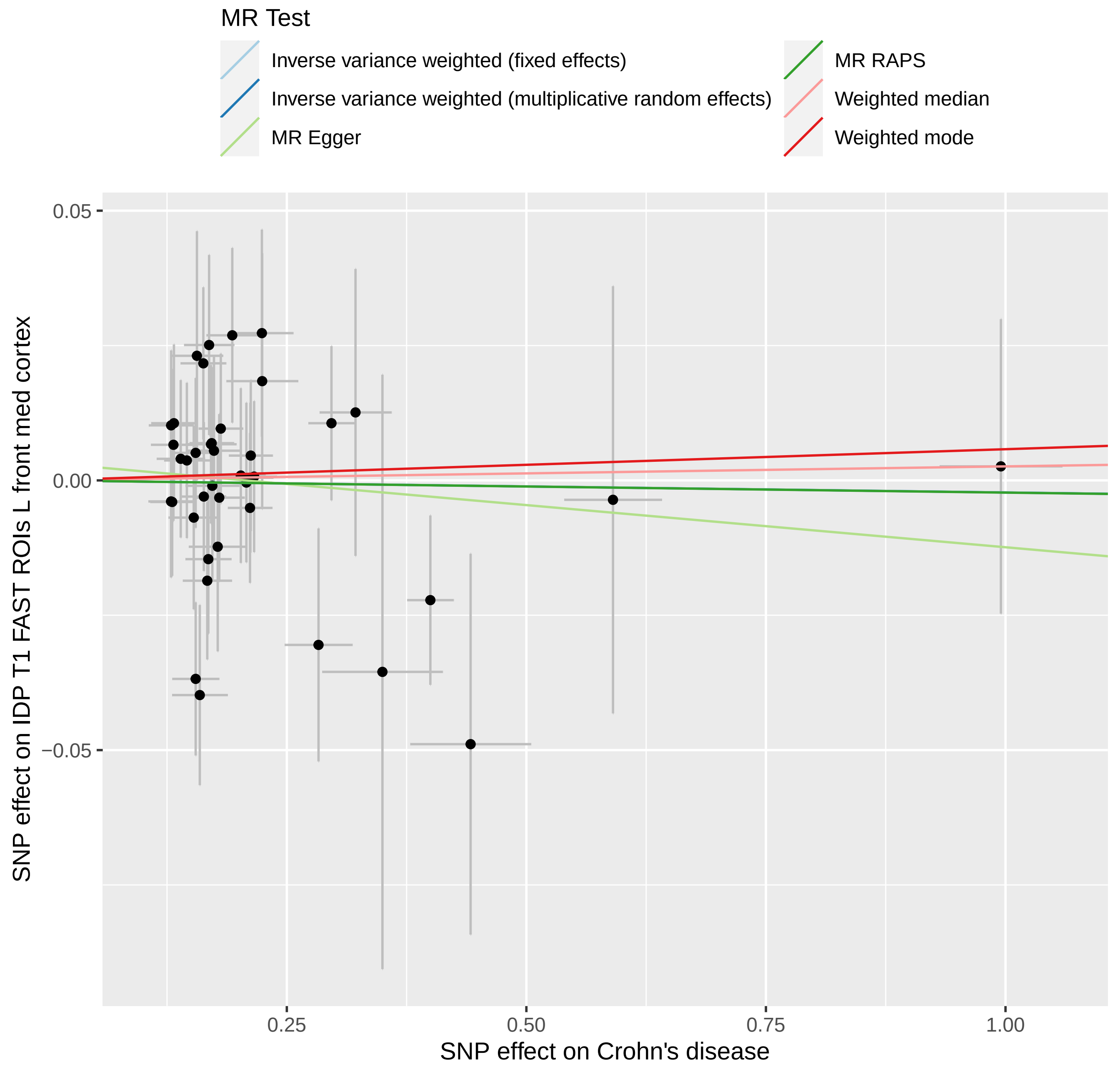

Batch 2416 : Ulcerative colitis on IDP T1 FAST ROIs L front med cortex

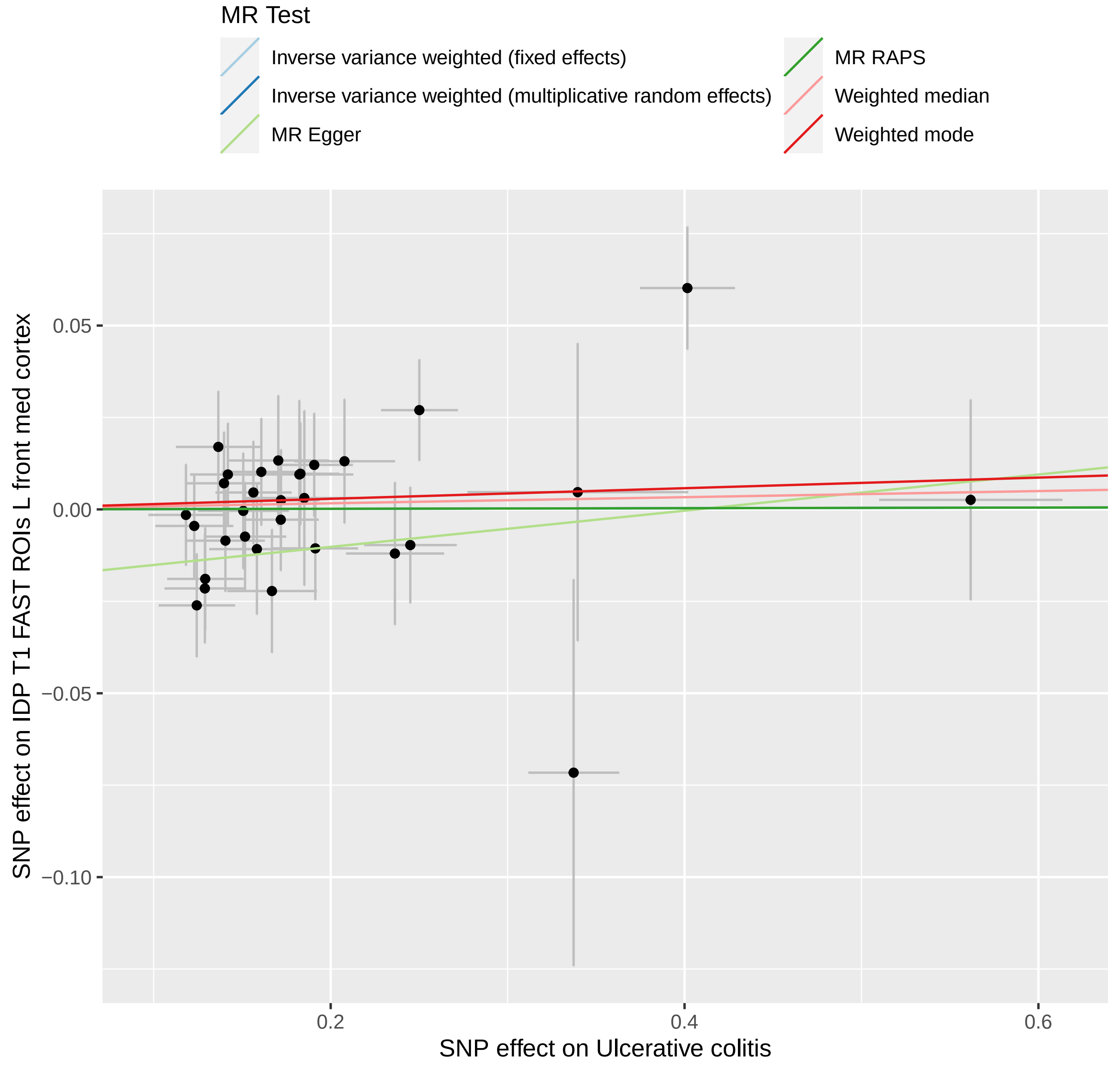

Batch 2437 : Crohn's disease on IDP T1 FAST ROIs R front med cortex

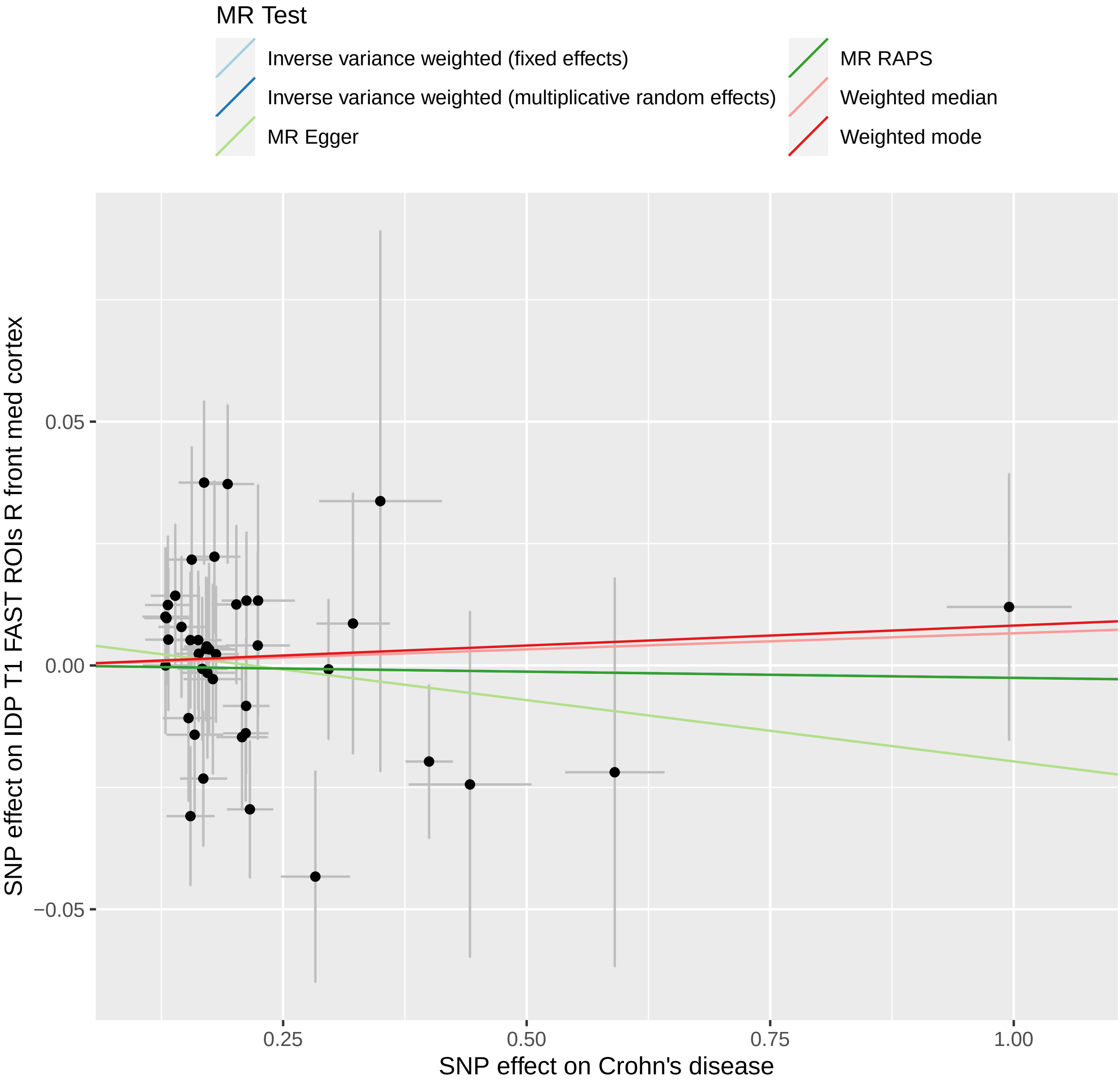

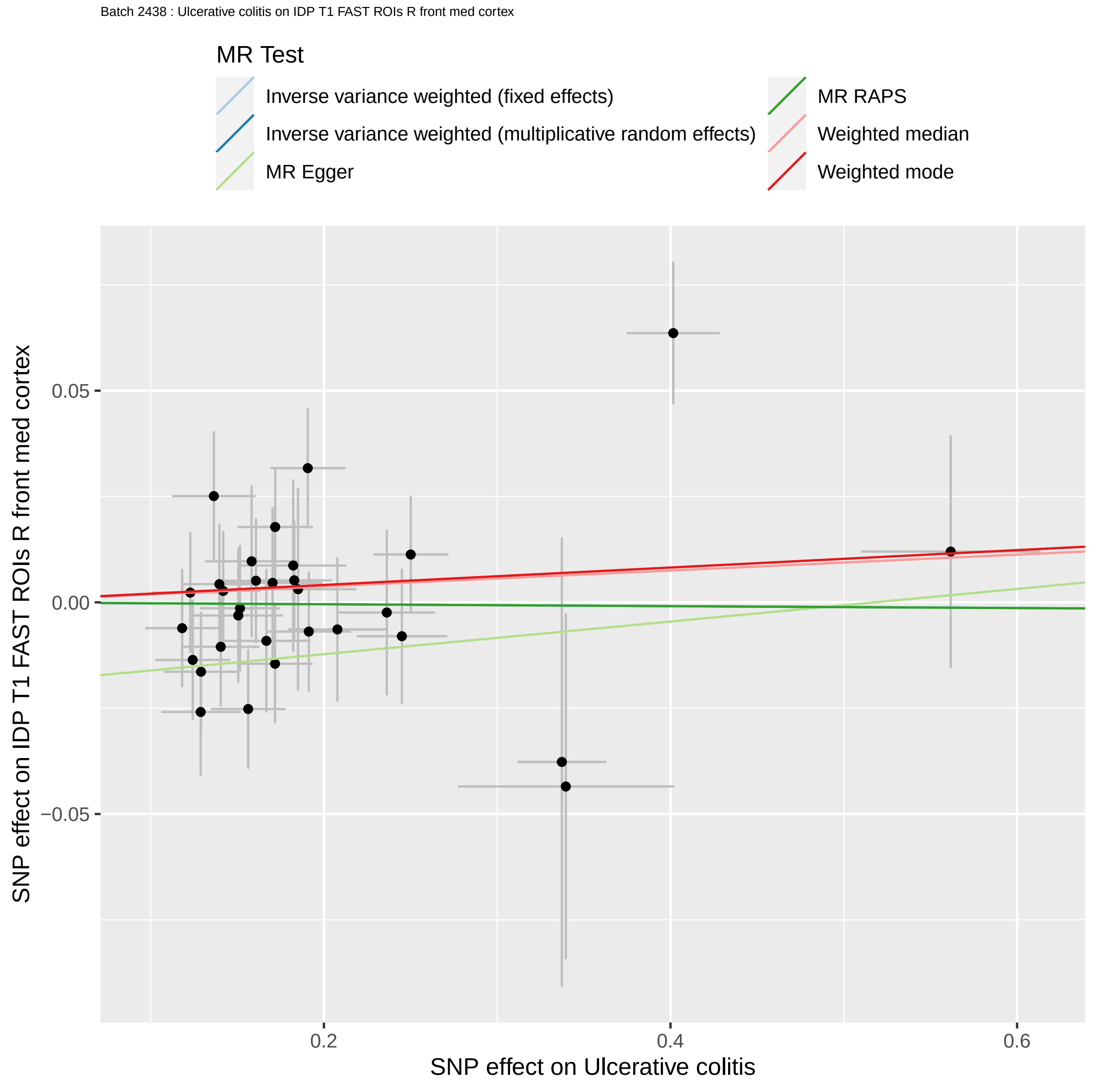
Batch 2438 : Ulcerative colitis on IDP T1 FAST ROIs R front med cortex
MR Test
Inverse variance weighted (fixed effects)
Inverse variance weighted (multiplicative random effects)
MR Egger
MR RAPS
Weighted median
Weighted mode
0.05
0.00
−0.05
0.2
0.4
0.6
SNP effect on Ulcerative colitis
SNP effect on IDP T1 FAST ROIs R front med cortex

Batch 2459 : Crohn's disease on IDP T1 FAST ROIs L juxtapos lobule cortex

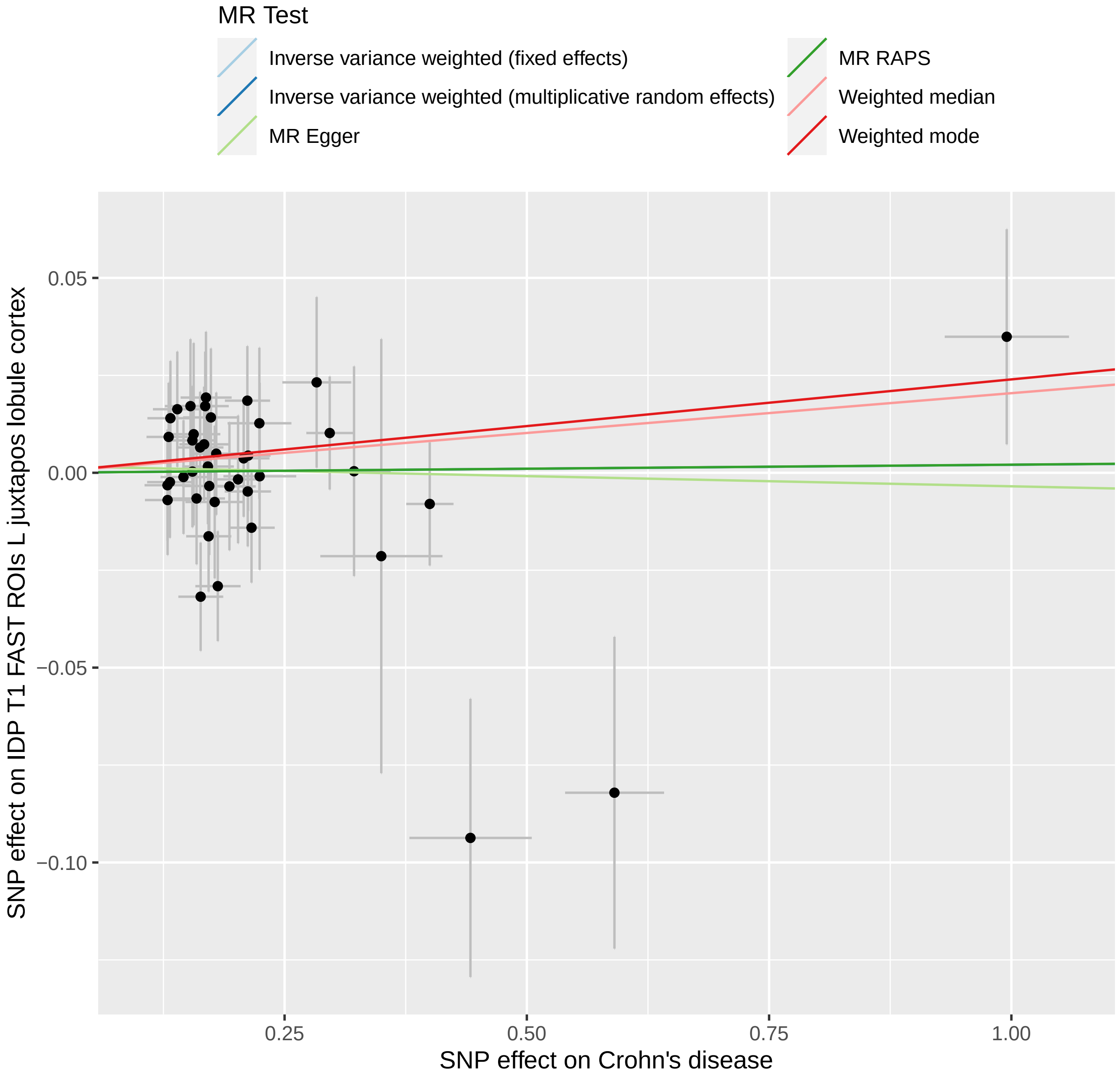

Batch 2460 : Ulcerative colitis on IDP T1 FAST ROIs L juxtapos lobule cortex

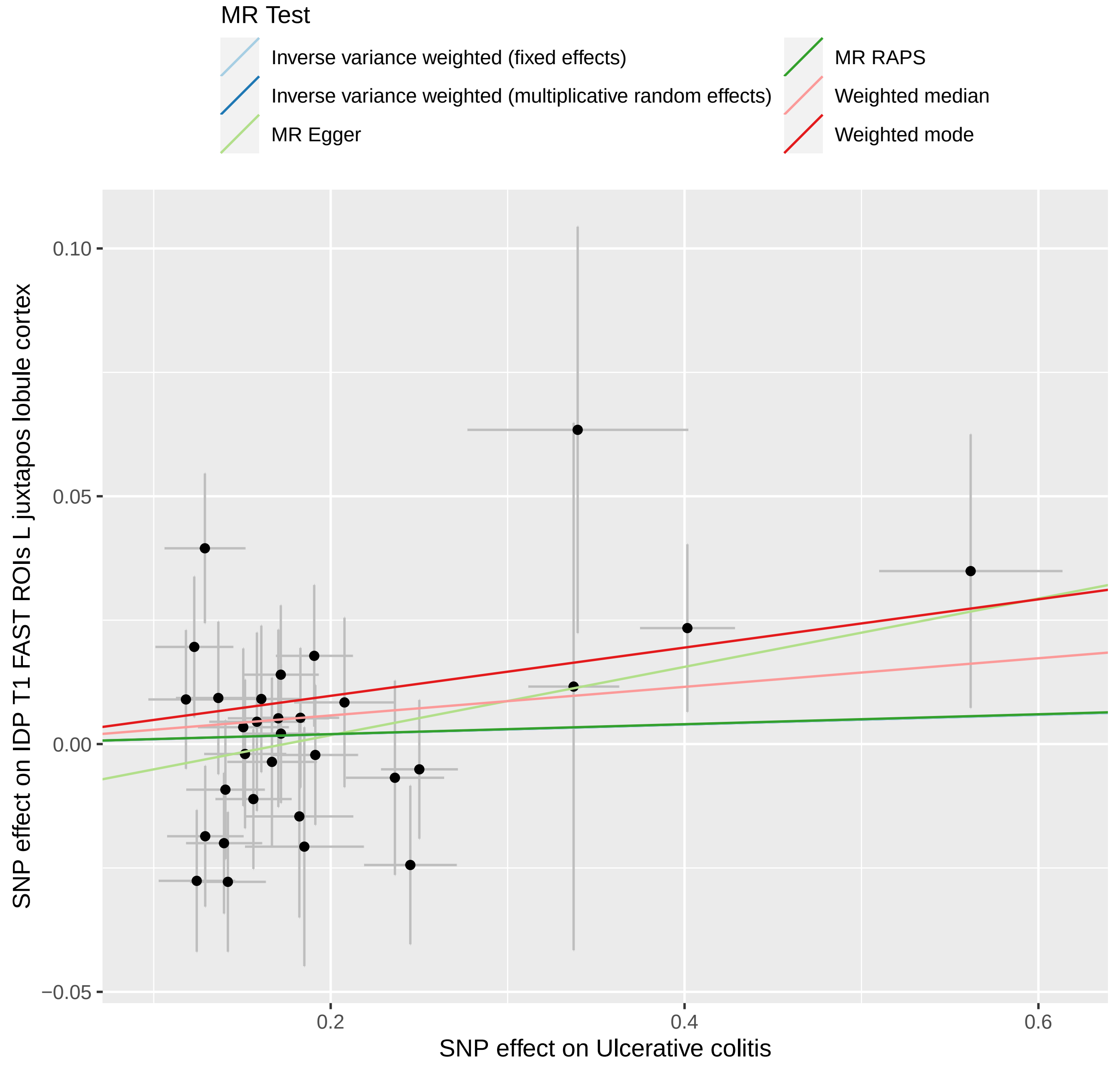

Batch 2481 : Crohn's disease on IDP T1 FAST ROIs R juxtapos lobule cortex

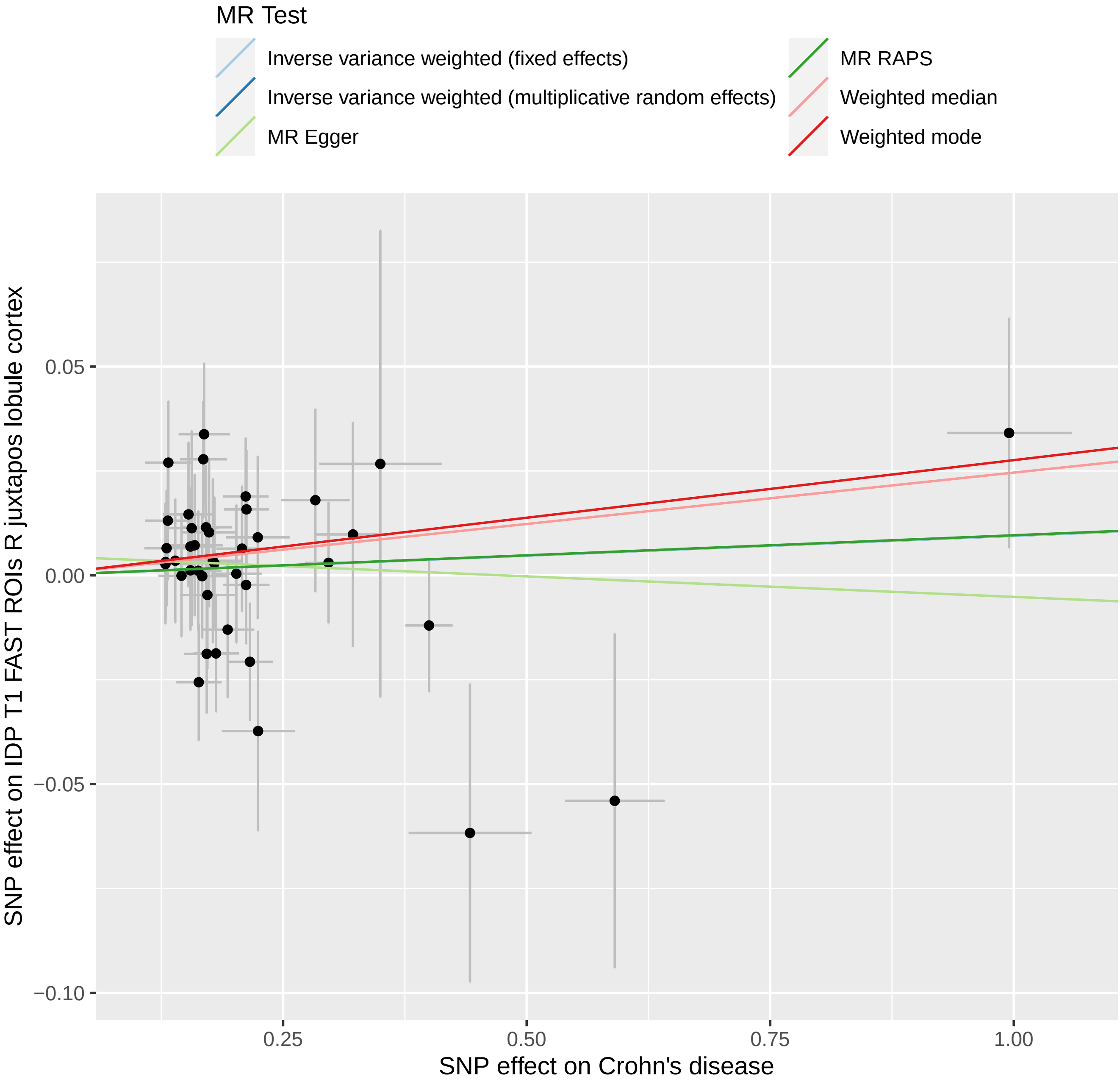

Batch 2482 : Ulcerative colitis on IDP T1 FAST ROIs R juxtapos lobule cortex

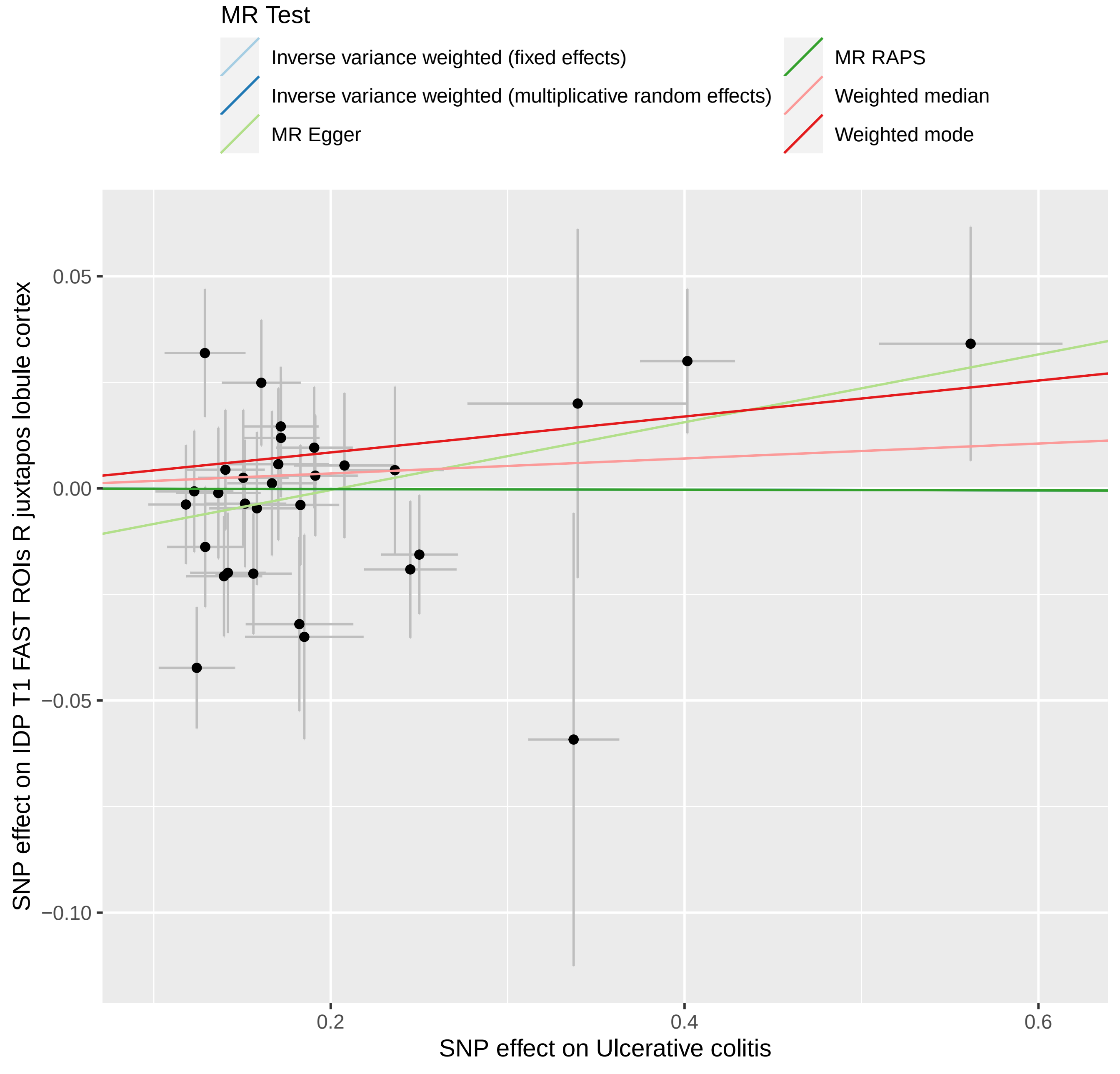

Batch 2503 : Crohn's disease on IDP T1 FAST ROIs L subcallosal cortex

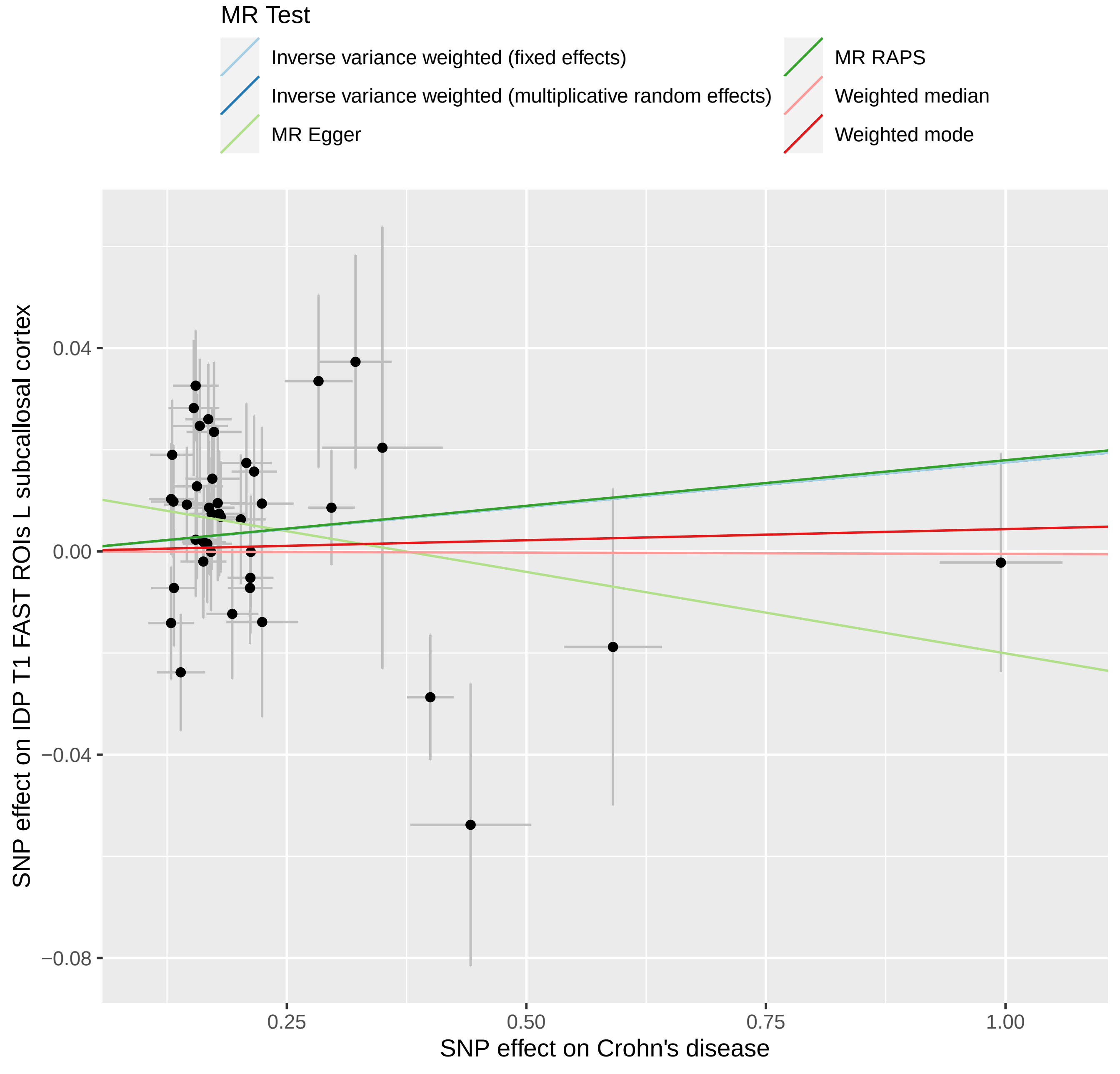

Batch 2504 : Ulcerative colitis on IDP T1 FAST ROIs L subcallosal cortex

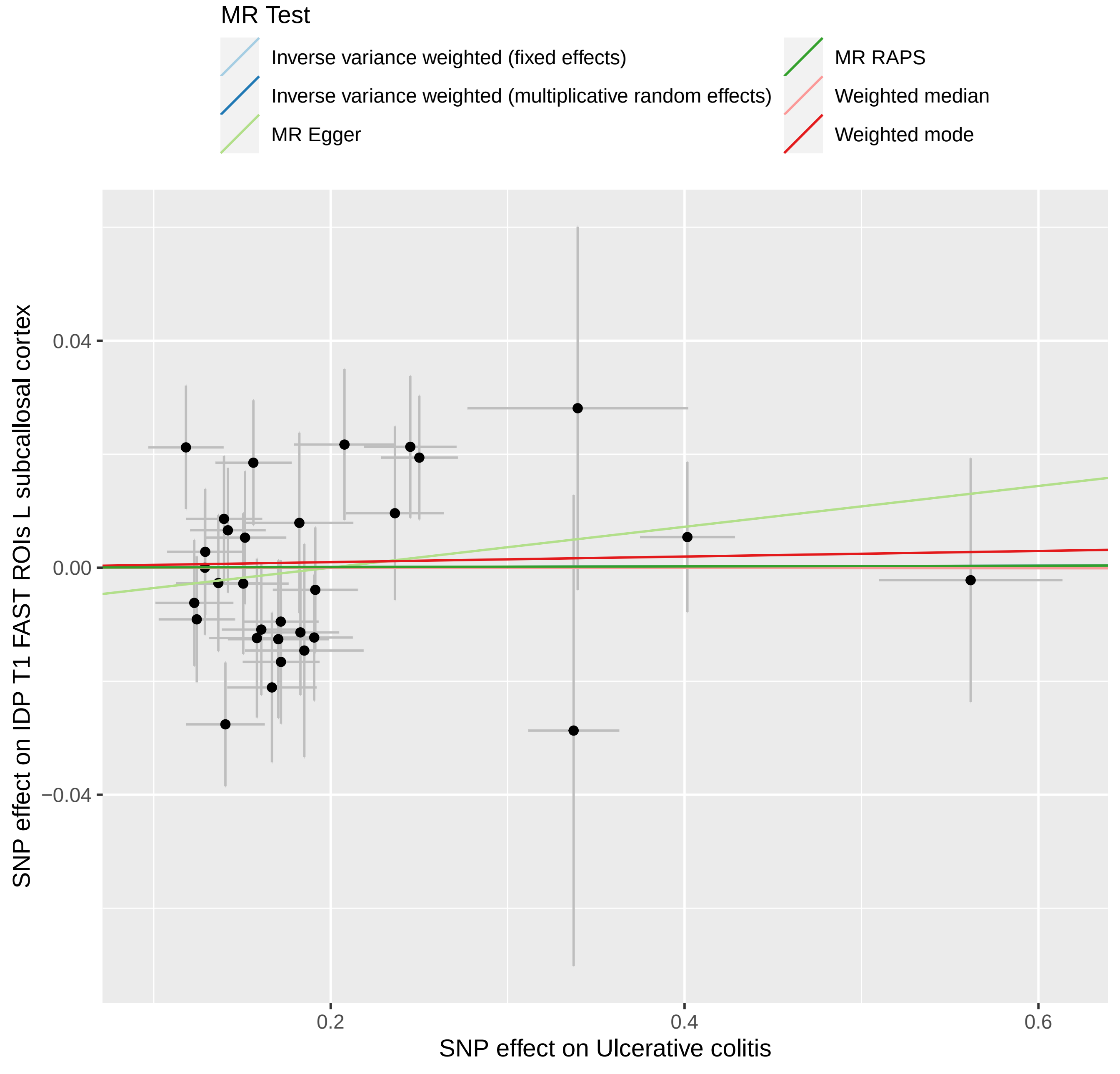

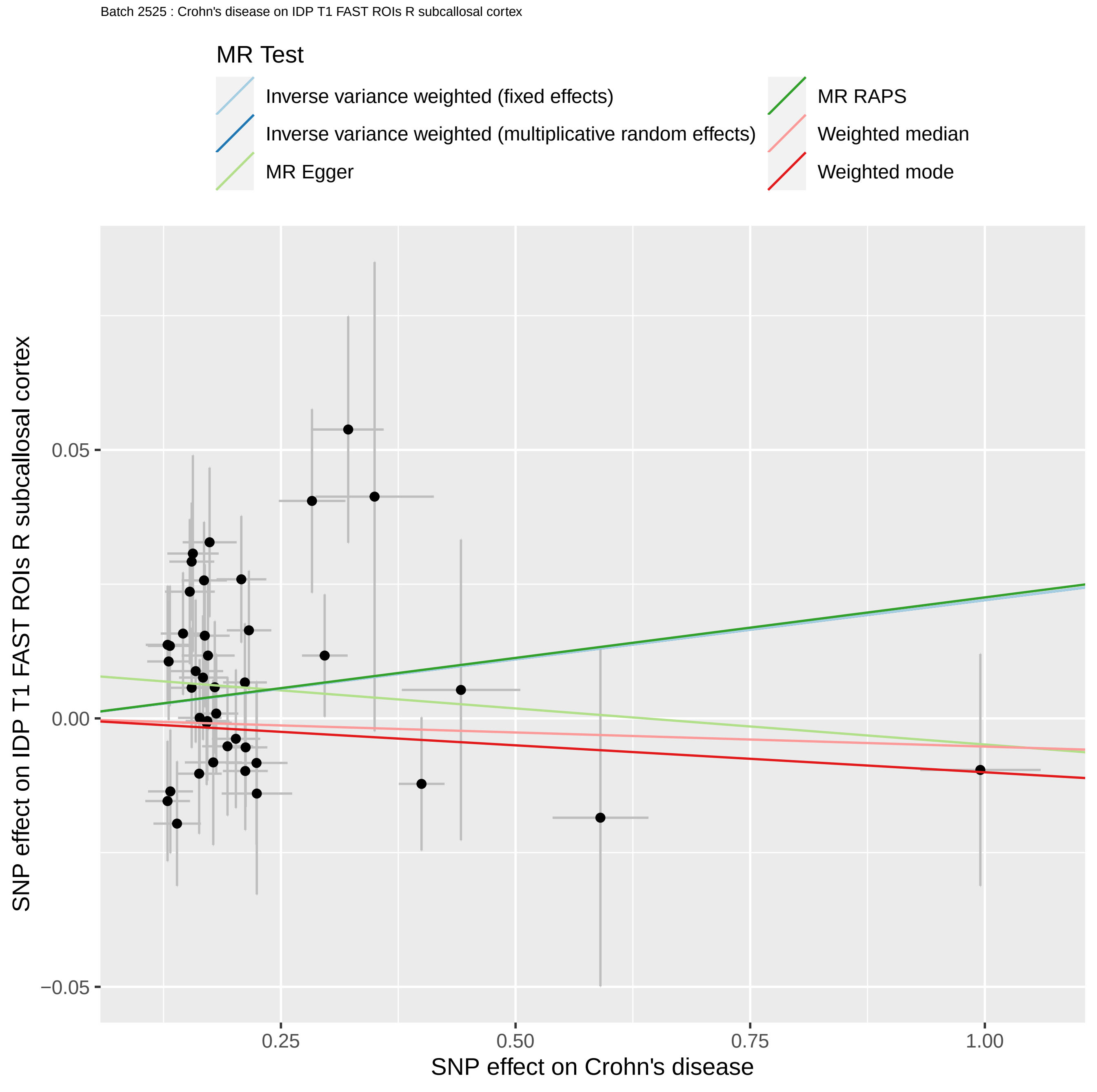

Batch 2525 : Crohn's disease on IDP T1 FAST ROIs R subcallosal cortex
MR Test
Inverse variance weighted (fixed effects)
Inverse variance weighted (multiplicative random effects)
MR Egger
MR RAPS
Weighted median
Weighted mode
0.05
0.00
−0.05
0.25
0.50
0.75
1.00
SNP effect on Crohn's disease
SNP effect on IDP T1 FAST ROIs R subcallosal cortex

Batch 2526 : Ulcerative colitis on IDP T1 FAST ROIs R subcallosal cortex

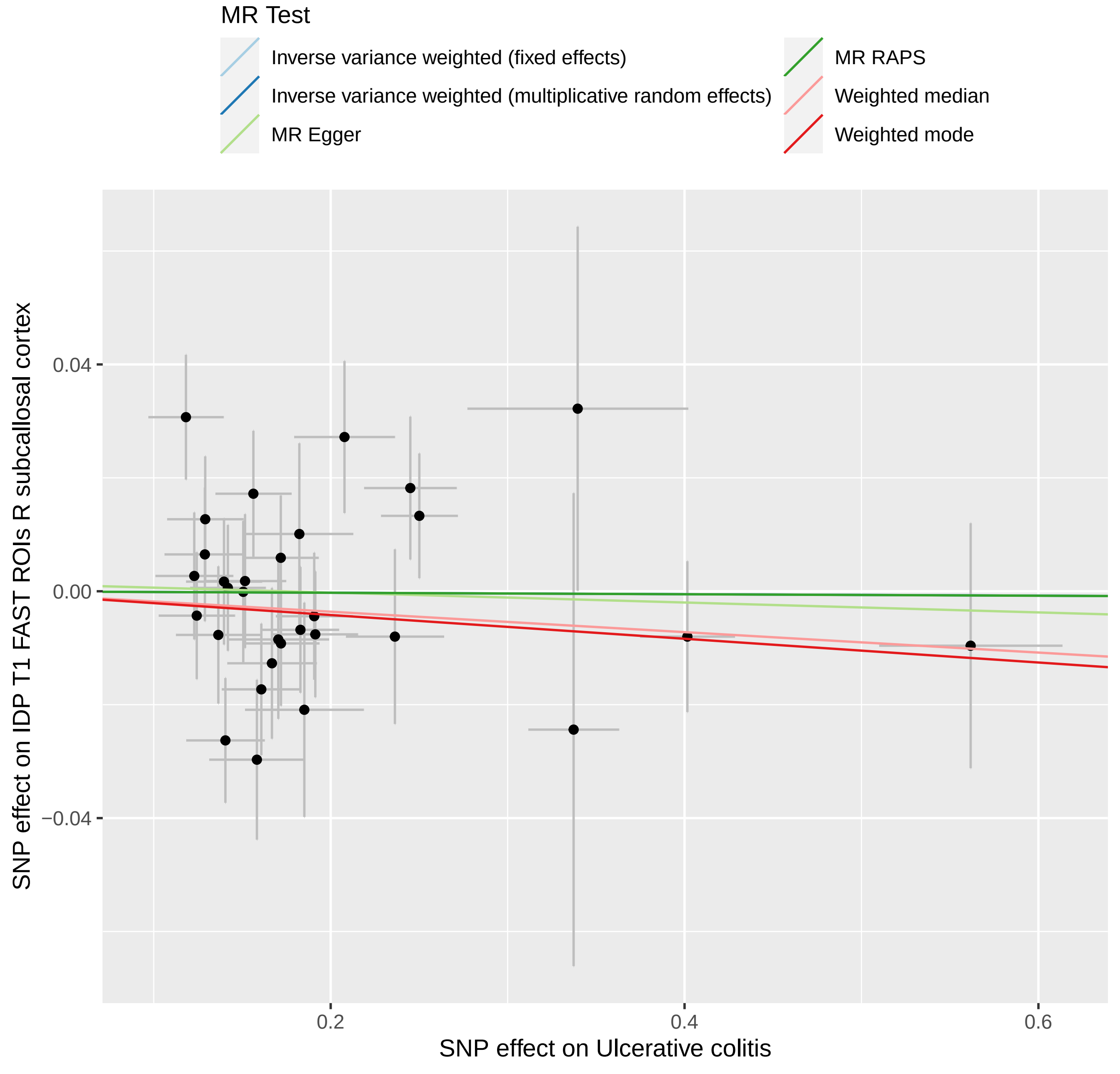

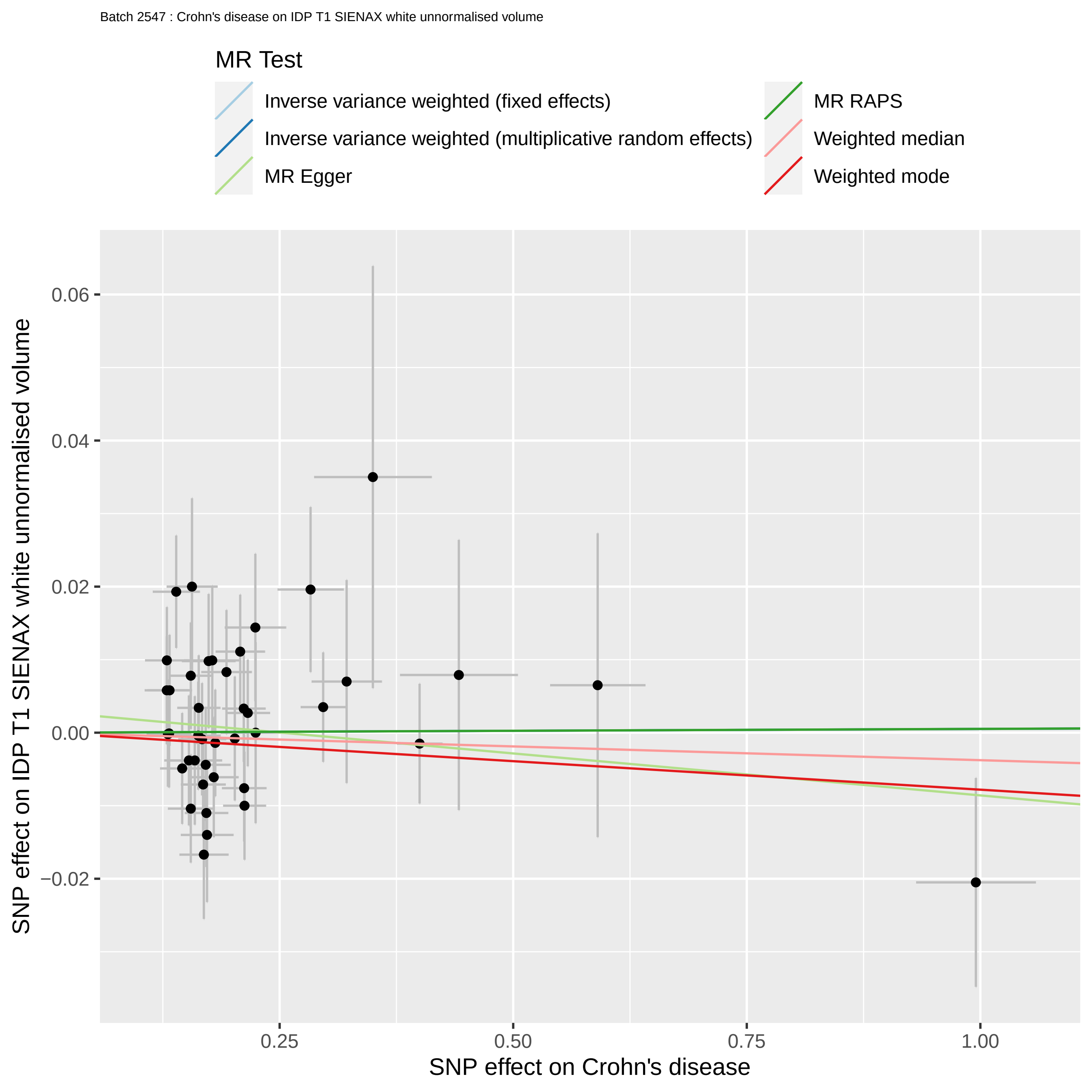

Batch 2547 : Crohn's disease on IDP T1 SIENAX white unnormalised volume
MR Test
Inverse variance weighted (fixed effects)
Inverse variance weighted (multiplicative random effects)
MR Egger
MR RAPS
Weighted median
Weighted mode
SNP effect on IDP T1 SIENAX white unnormalised volume
0.06
0.04
0.02
0.00
−0.02
0.25
0.50
0.75
1.00
SNP effect on Crohn's disease

Batch 2548 : Ulcerative colitis on IDP T1 SIENAX white unnormalised volume

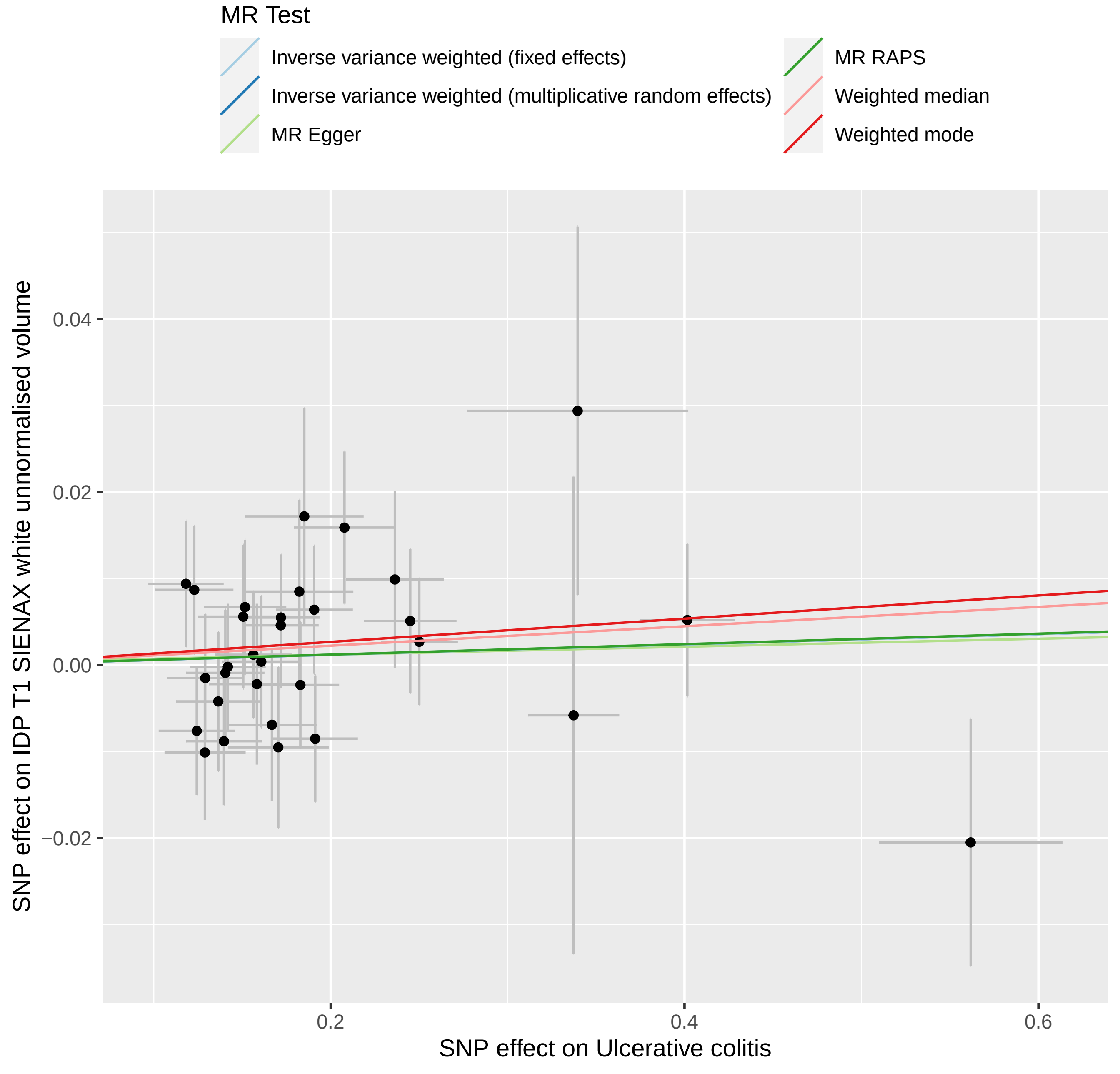

Batch 2549 : Crohn's disease on IDP T1 FAST ROIs L paracing gyrus

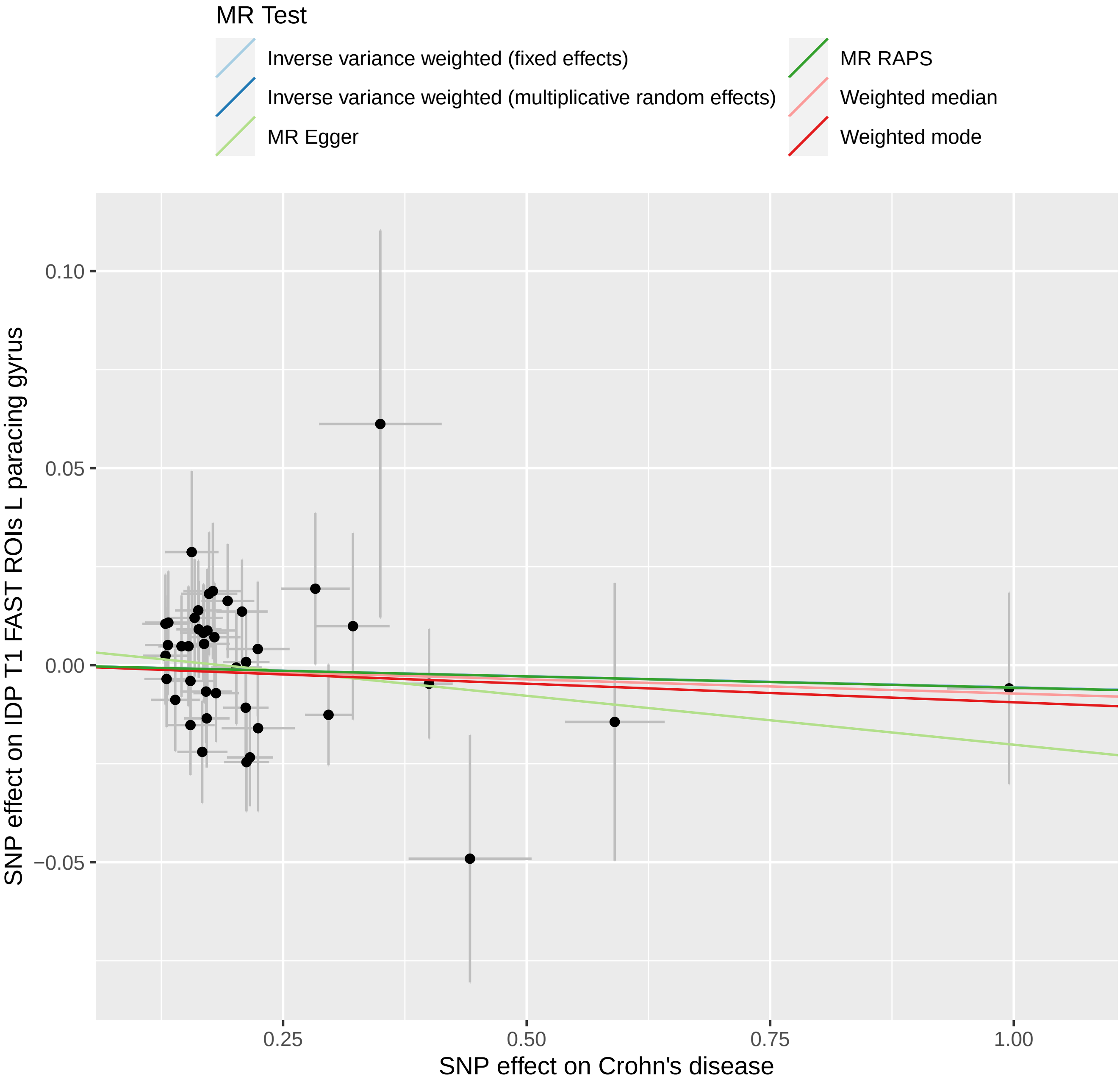

Batch 2550 : Ulcerative colitis on IDP T1 FAST ROIs L paracing gyrus

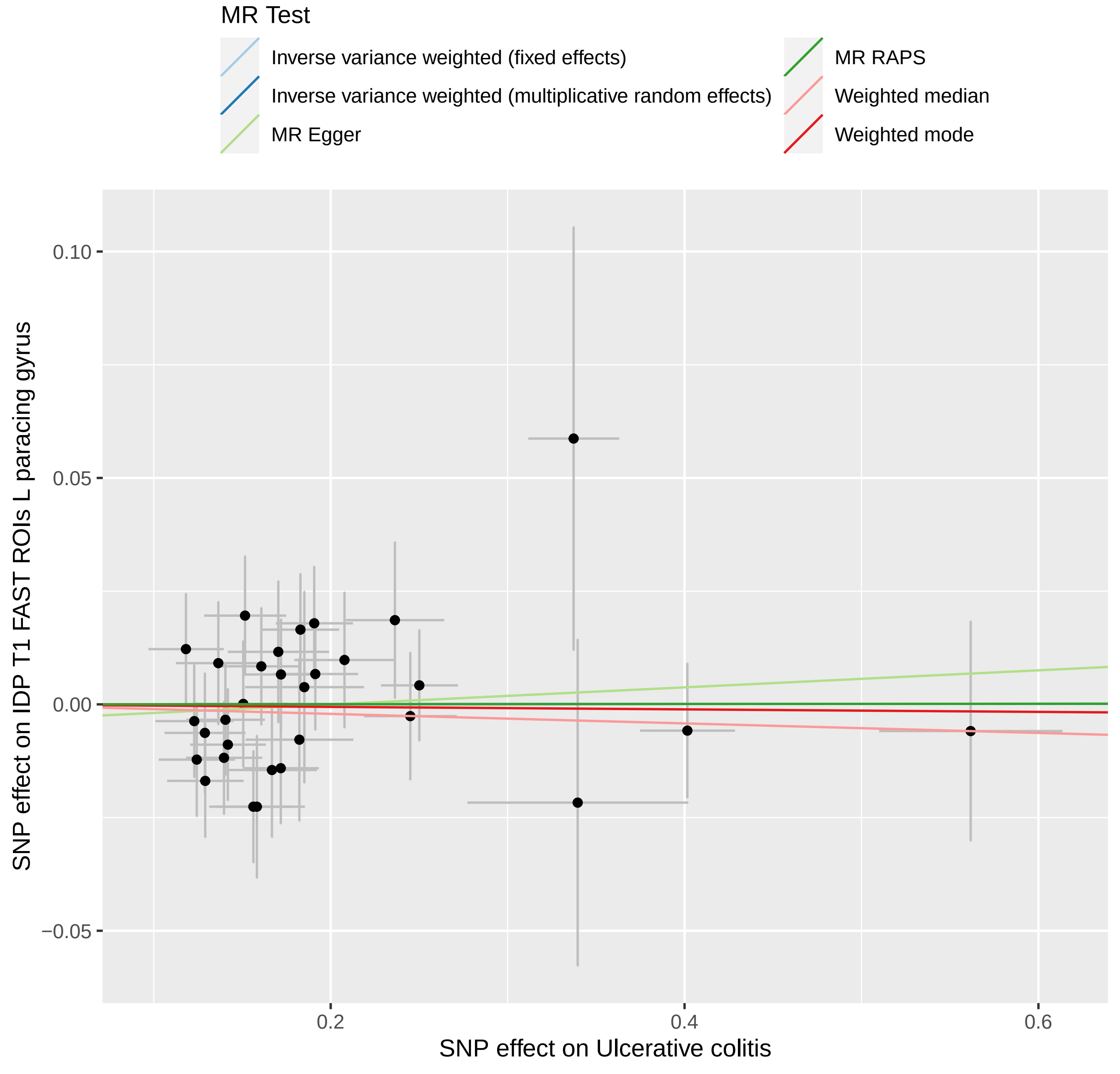

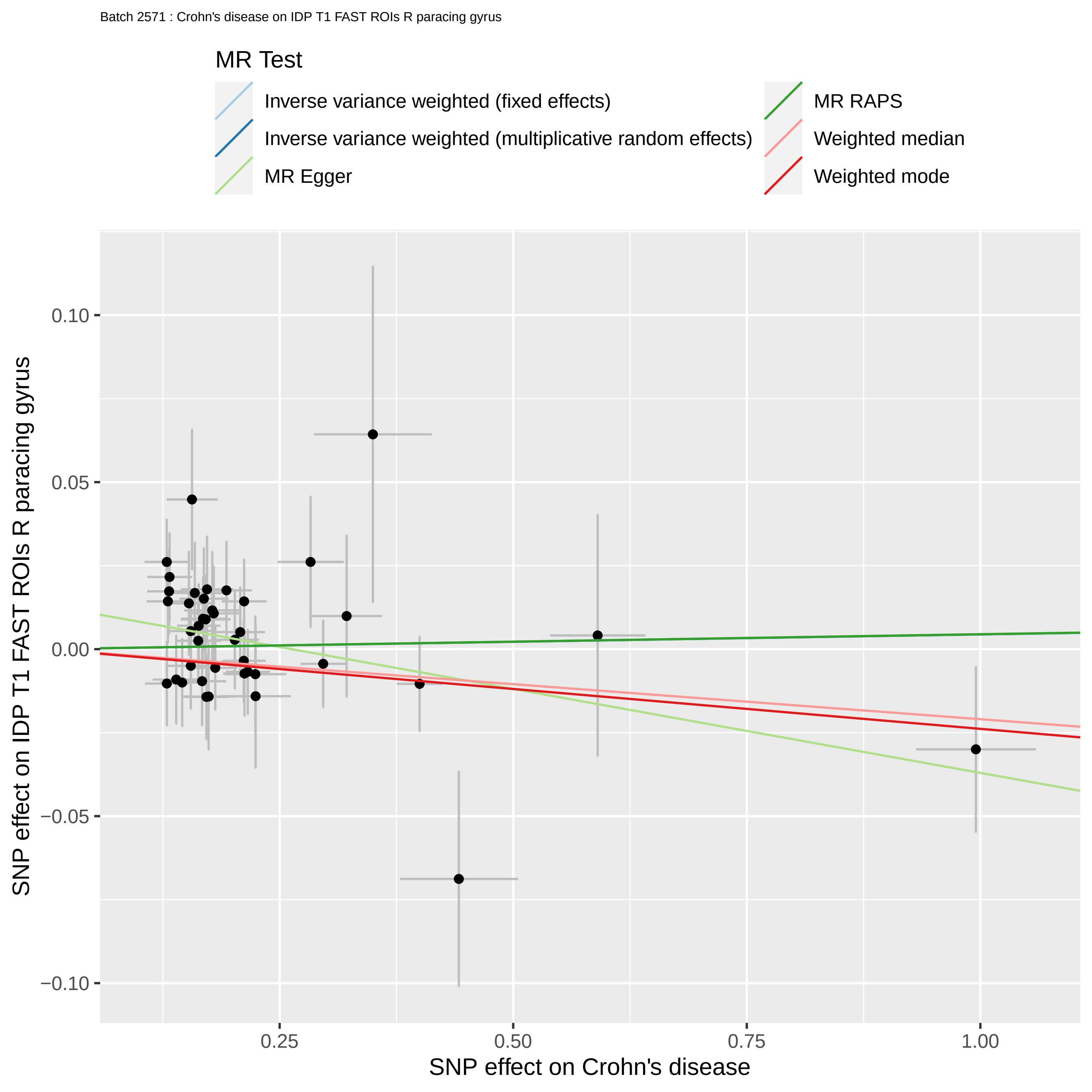

Batch 2571 : Crohn's disease on IDP T1 FAST ROIs R paracing gyrus
MR Test
Inverse variance weighted (fixed effects)
Inverse variance weighted (multiplicative random effects)
MR Egger
MR RAPS
Weighted median
Weighted mode
0.10
0.05
0.00
−0.05
−0.10
0.25
0.50
0.75
1.00
SNP effect on IDP T1 FAST ROIs R paracing gyrus
SNP effect on Crohn's disease

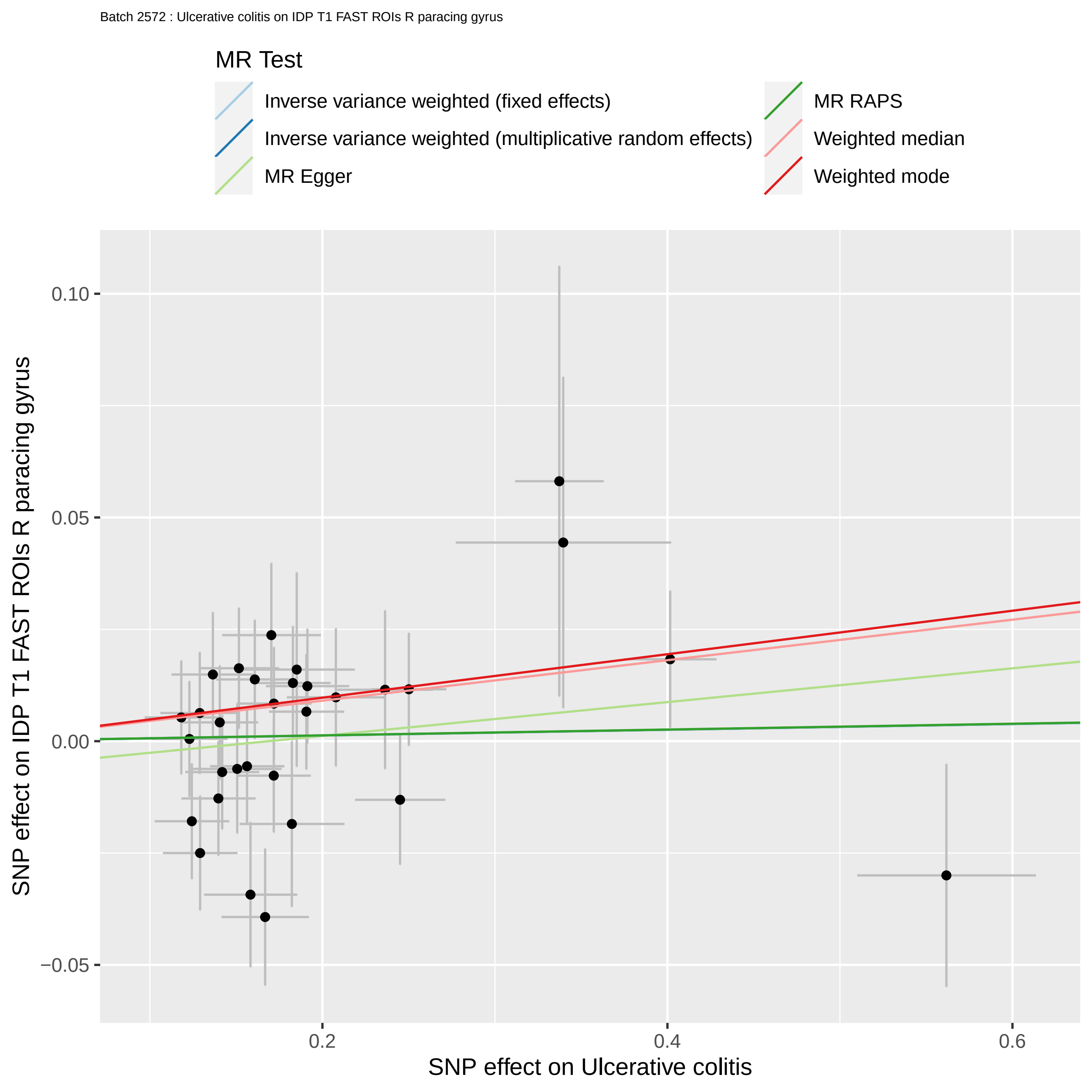
Batch 2572 : Ulcerative colitis on IDP T1 FAST ROIs R paracing gyrus
MR Test
Inverse variance weighted (fixed effects)
Inverse variance weighted (multiplicative random effects)
MR Egger
MR RAPS
Weighted median
Weighted mode
SNP effect on IDP T1 FAST ROIs R paracing gyrus
0.10
0.05
0.00
−0.05
0.2
0.4
0.6
SNP effect on Ulcerative colitis

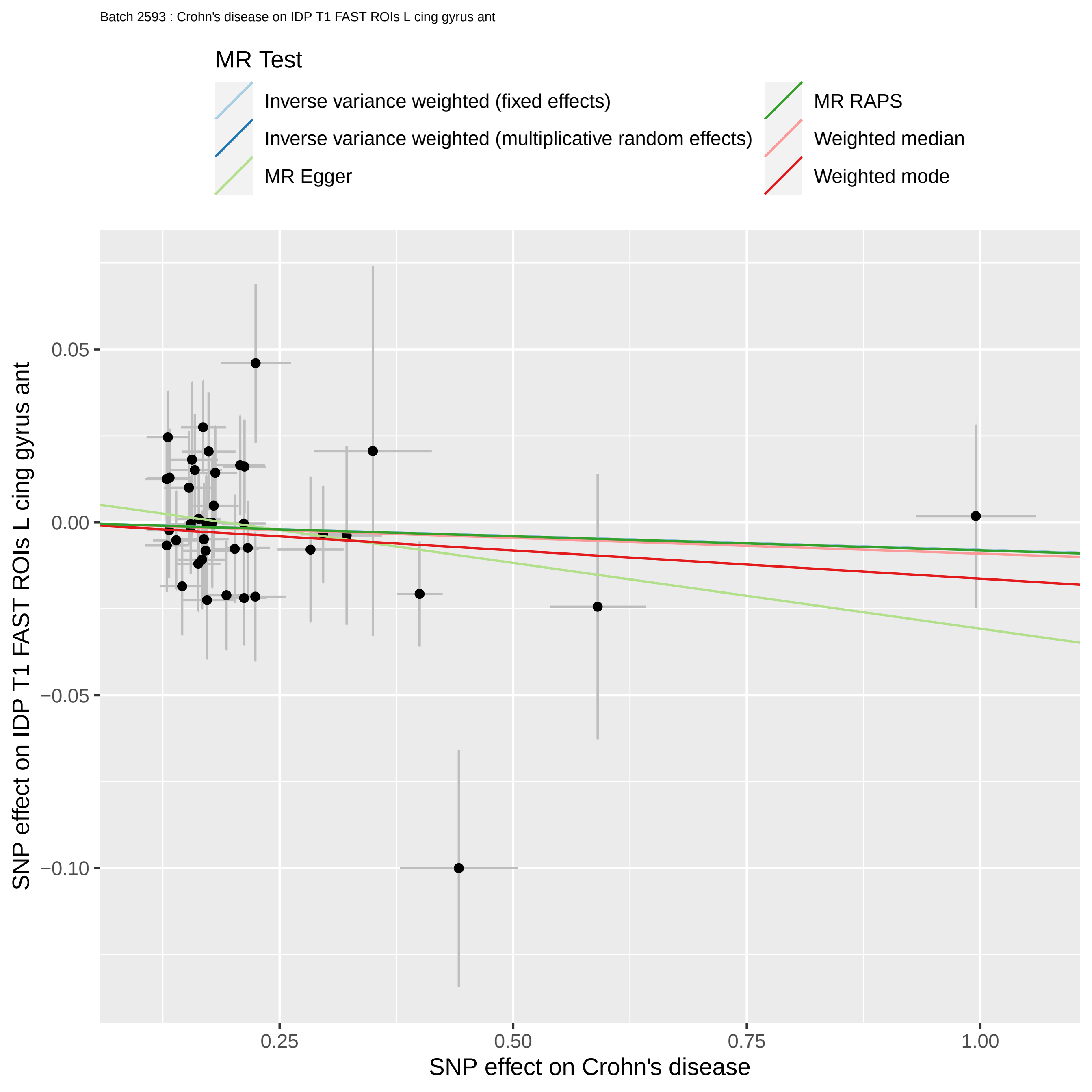

Batch 2593 : Crohn's disease on IDP T1 FAST ROIs L cing gyrus ant
MR Test
Inverse variance weighted (fixed effects)
Inverse variance weighted (multiplicative random effects)
MR Egger
MR RAPS
Weighted median
Weighted mode
0.05
0.00
−0.05
−0.10
0.25
0.50
0.75
1.00
SNP effect on IDP T1 FAST ROIs L cing gyrus ant
SNP effect on Crohn's disease

Batch 2594 : Ulcerative colitis on IDP T1 FAST ROIs L cing gyrus ant

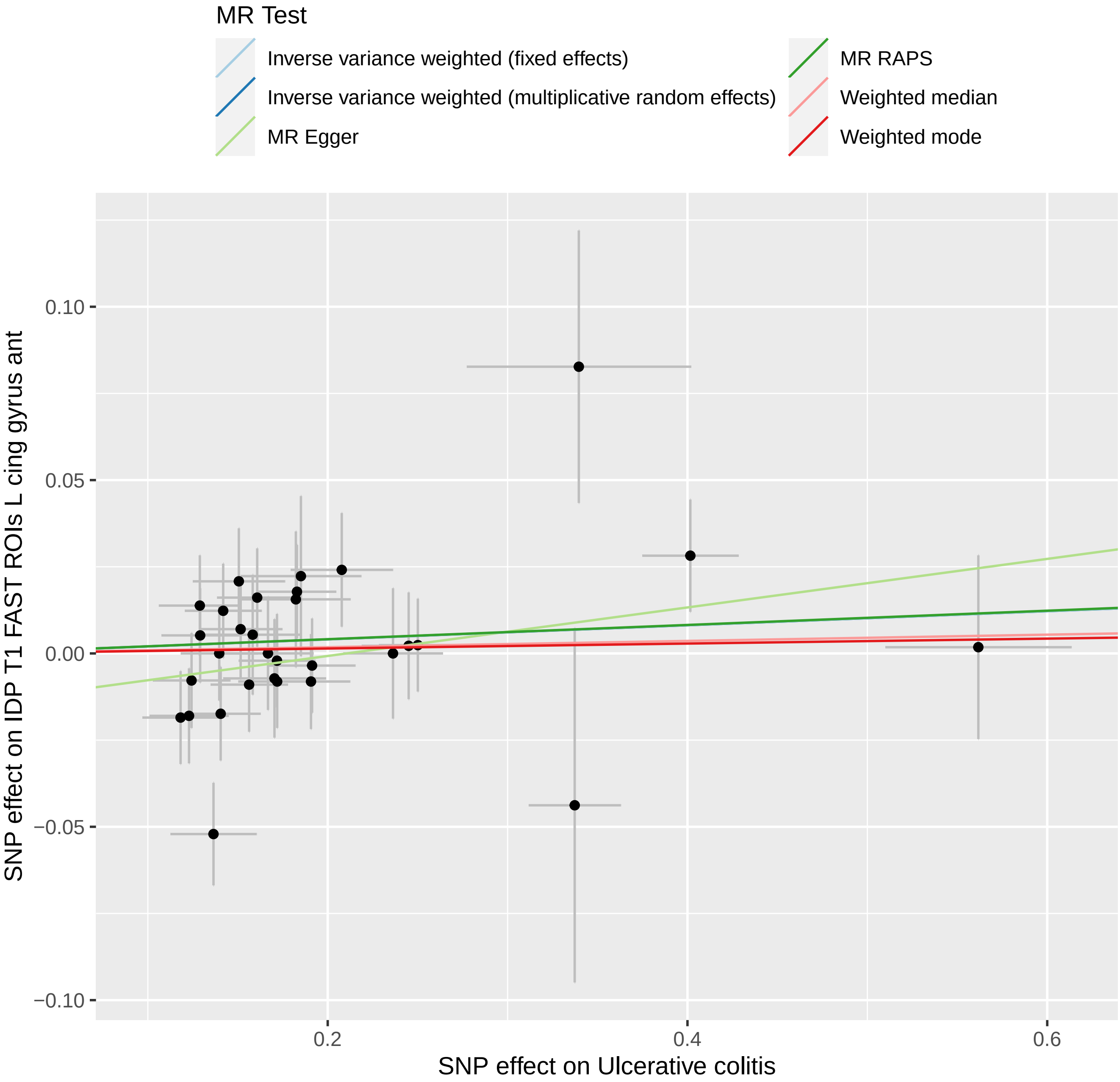

Batch 2615 : Crohn's disease on IDP T1 FAST ROIs R cing gyrus ant

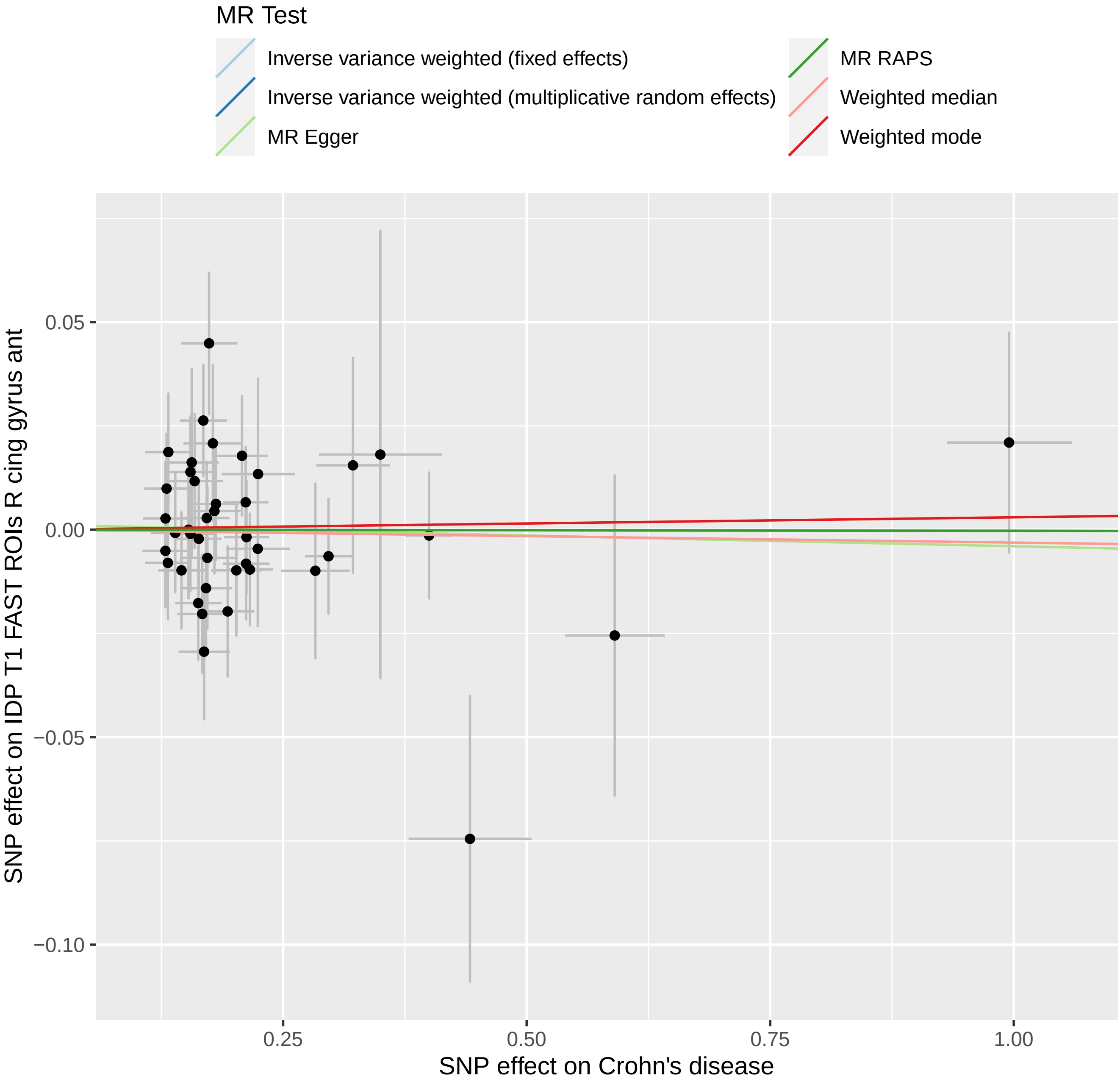

Batch 2616 : Ulcerative colitis on IDP T1 FAST ROIs R cing gyrus ant

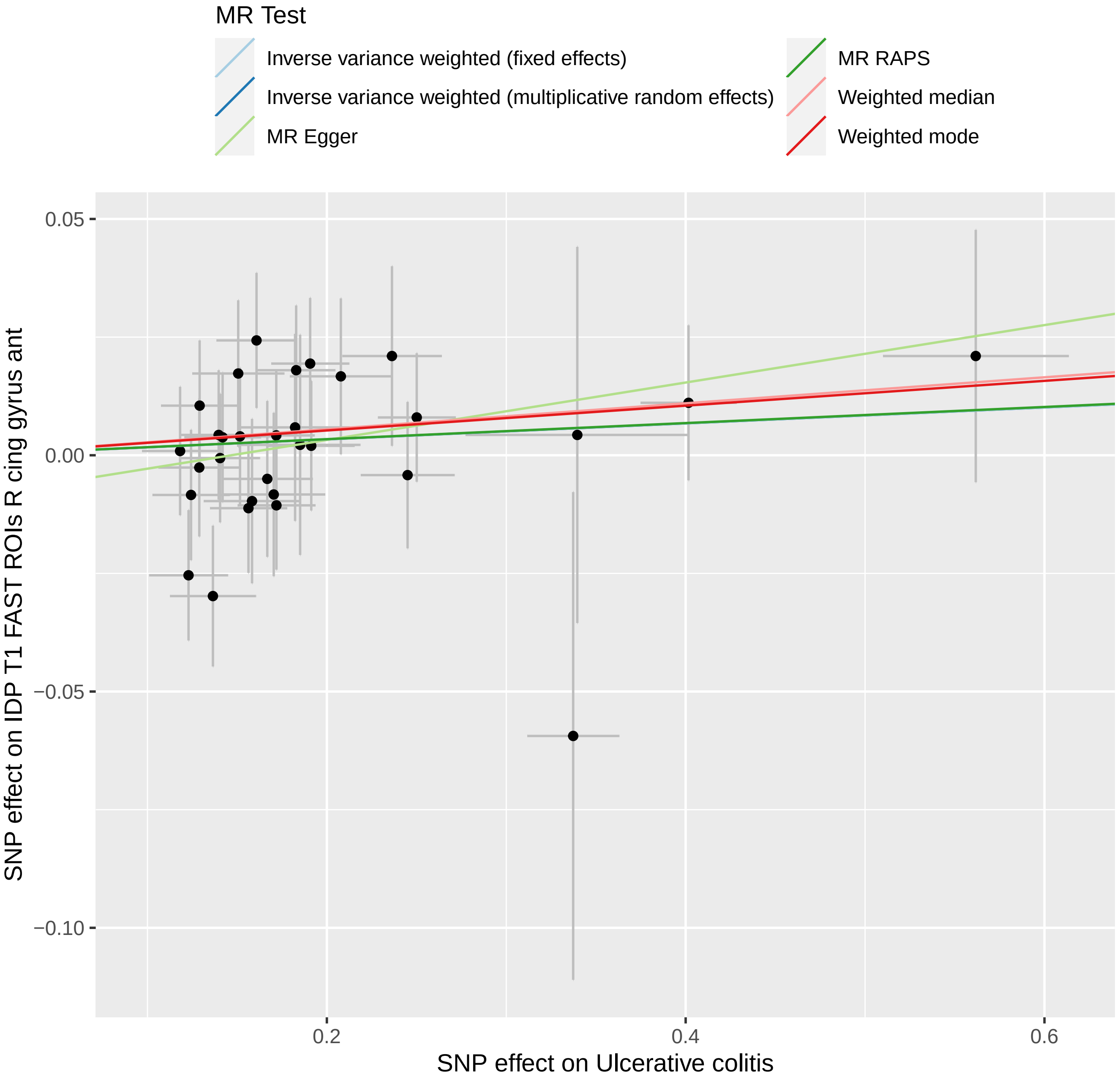

Batch 2637 : Crohn's disease on IDP T1 FAST ROIs L cing gyrus post

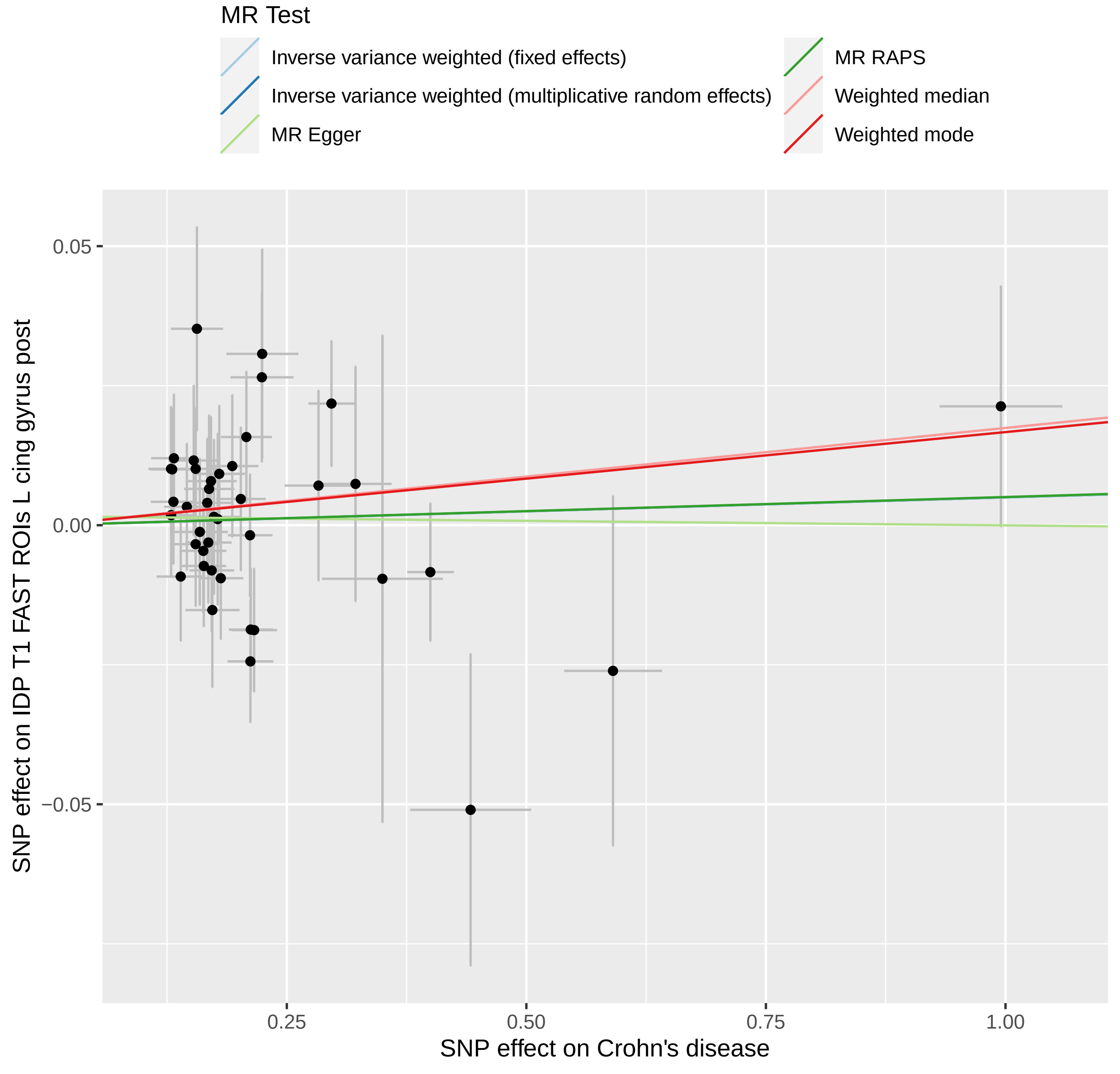

Batch 2638 : Ulcerative colitis on IDP T1 FAST ROIs L cing gyrus post

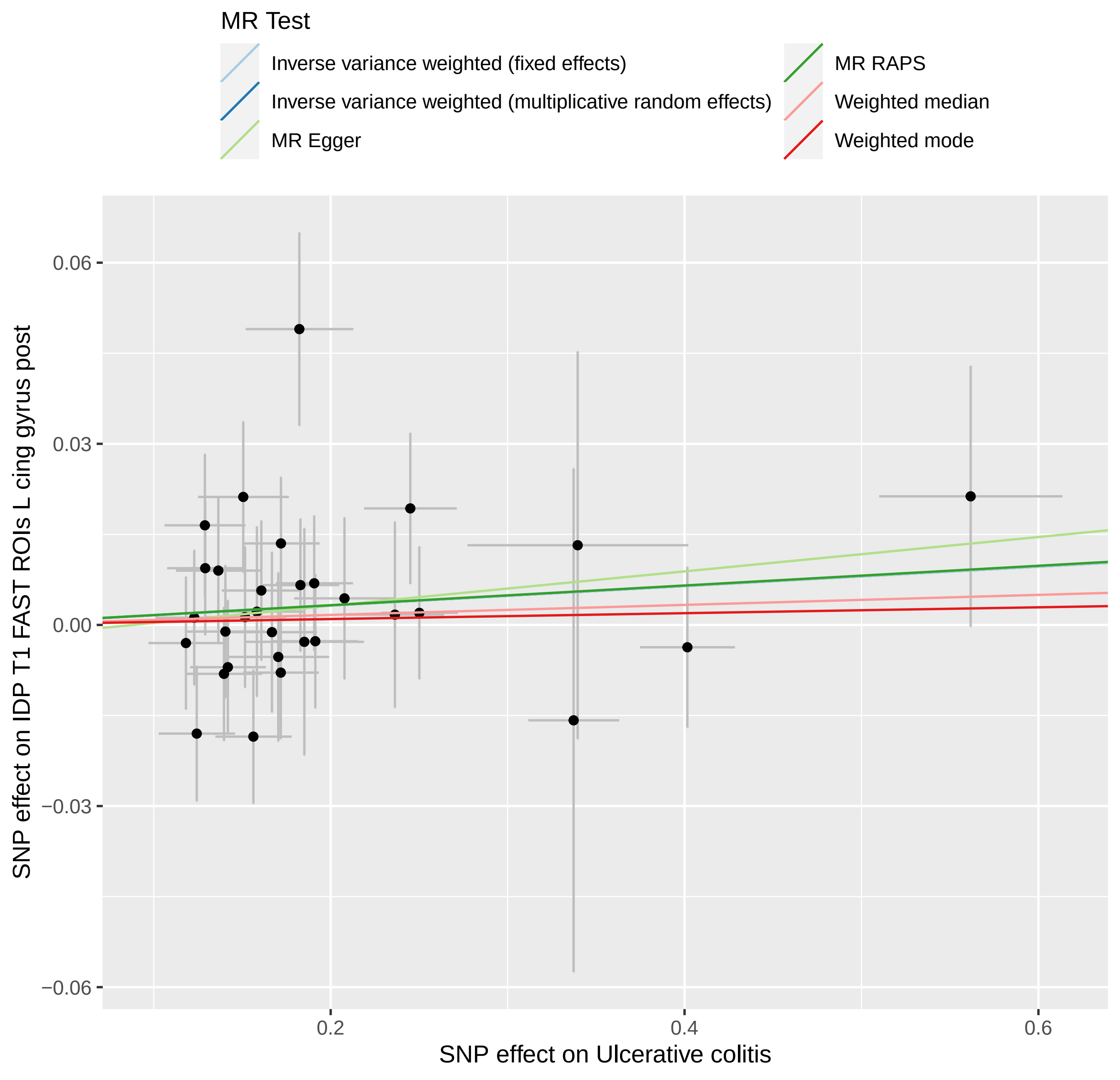

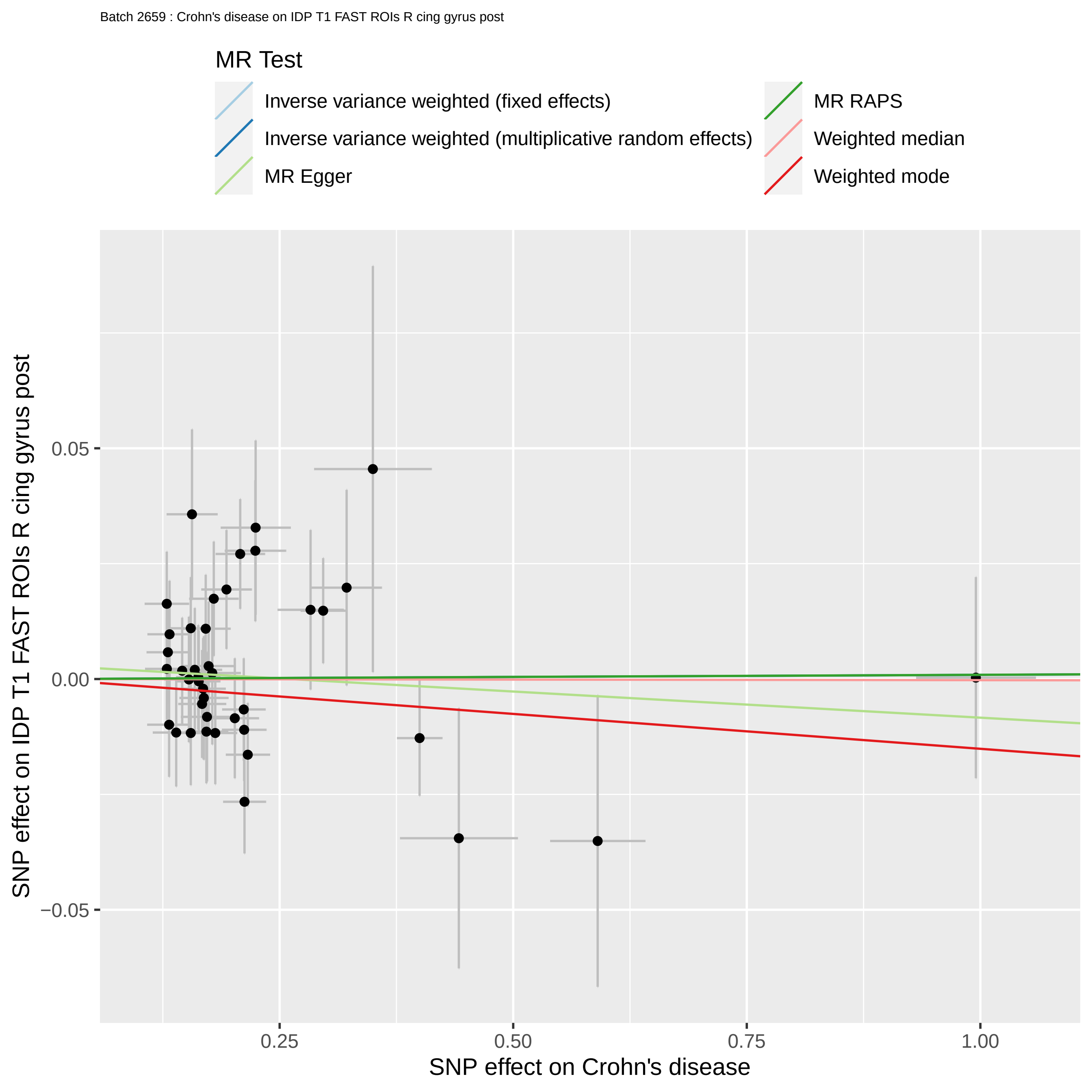
Batch 2659 : Crohn's disease on IDP T1 FAST ROIs R cing gyrus post
MR Test
Inverse variance weighted (fixed effects)
Inverse variance weighted (multiplicative random effects)
MR Egger
MR RAPS
Weighted median
Weighted mode
0.05
0.00
−0.05
0.25
0.50
0.75
1.00
SNP effect on Crohn's disease
SNP effect on IDP T1 FAST ROIs R cing gyrus post

Batch 2660 : Ulcerative colitis on IDP T1 FAST ROIs R cing gyrus post

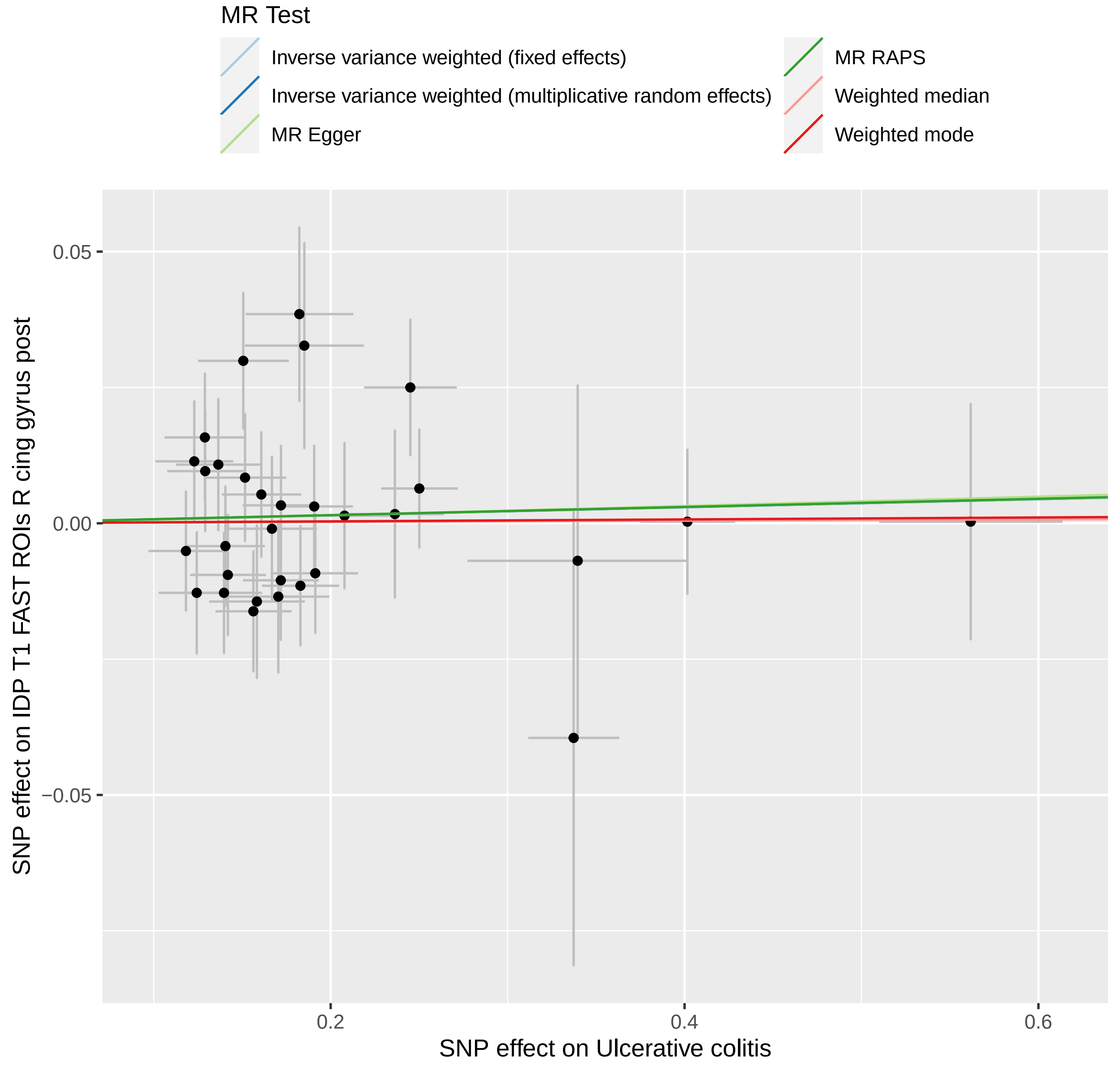

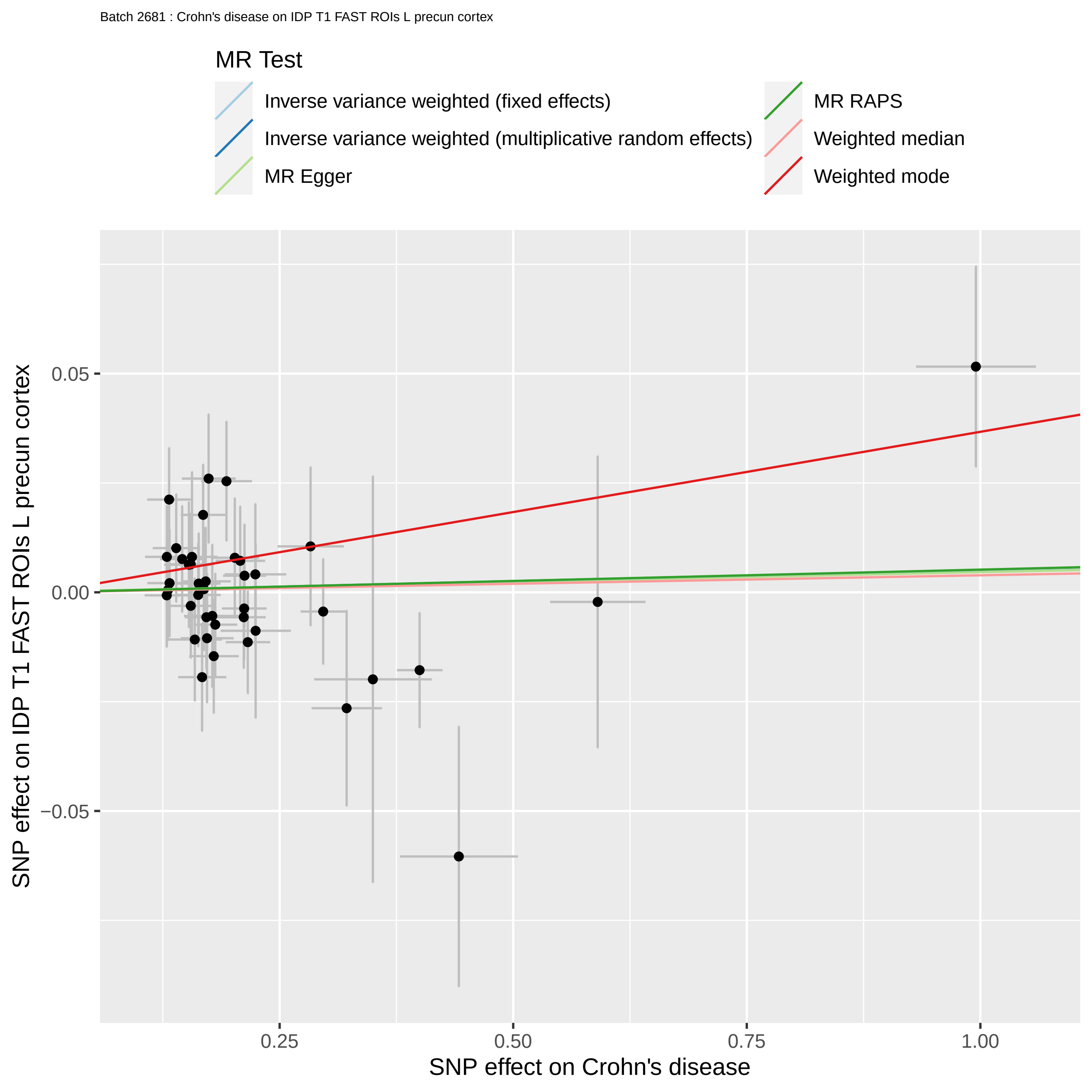

Batch 2681 : Crohn's disease on IDP T1 FAST ROIs L precun cortex
MR Test
Inverse variance weighted (fixed effects)
Inverse variance weighted (multiplicative random effects)
MR Egger
MR RAPS
Weighted median
Weighted mode
0.05
0.00
−0.05
0.25
0.50
0.75
1.00
SNP effect on Crohn's disease
SNP effect on IDP T1 FAST ROIs L precun cortex

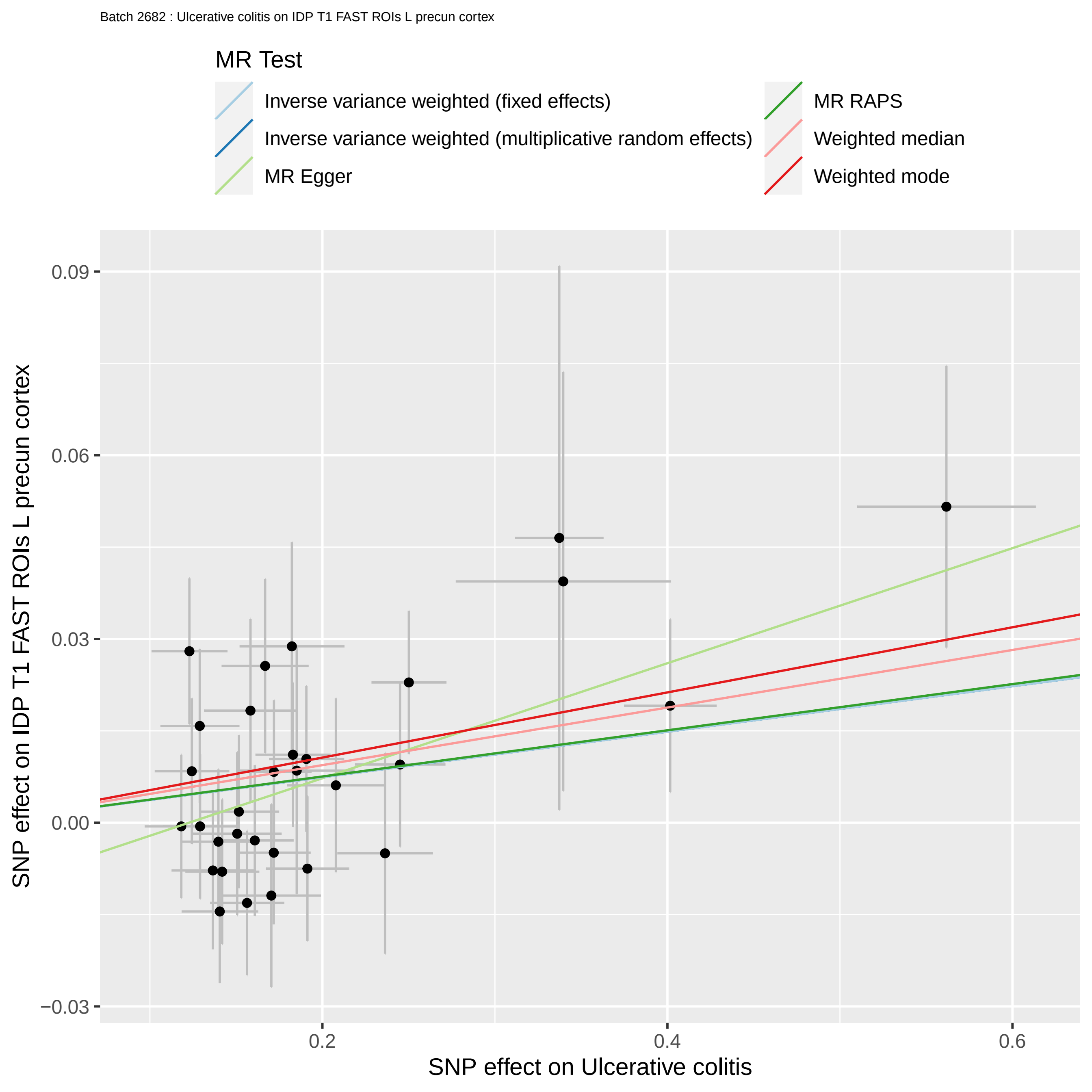
Batch 2682 : Ulcerative colitis on IDP T1 FAST ROIs L precun cortex
MR Test
Inverse variance weighted (fixed effects)
Inverse variance weighted (multiplicative random effects)
MR Egger
MR RAPS
Weighted median
Weighted mode
0.09
0.06
0.03
0.00
−0.03
0.2
0.4
0.6
SNP effect on Ulcerative colitis
SNP effect on IDP T1 FAST ROIs L precun cortex

Batch 2703 : Crohn's disease on IDP T1 FAST ROIs R precun cortex

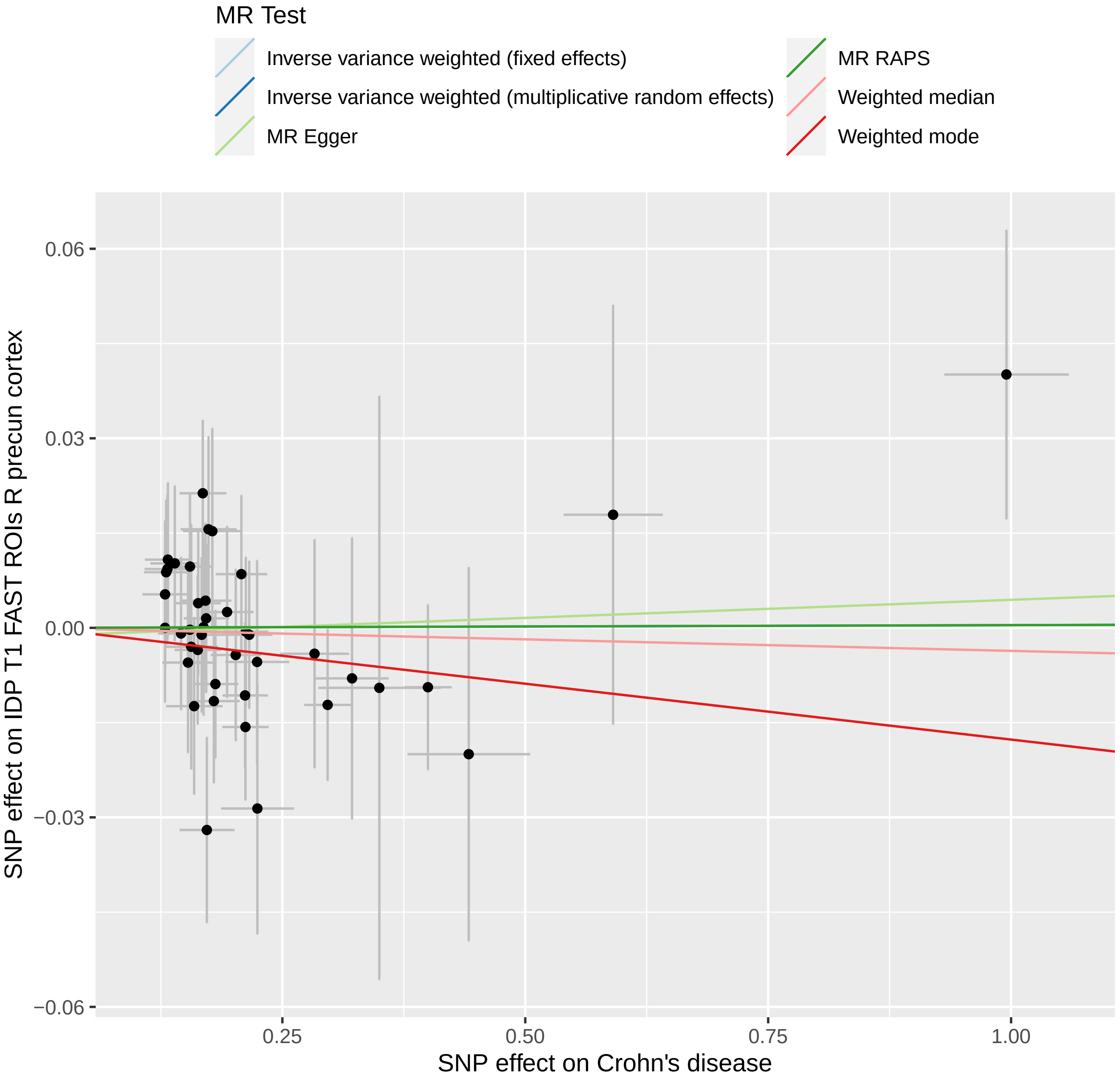

Batch 2704 : Ulcerative colitis on IDP T1 FAST ROIs R precun cortex

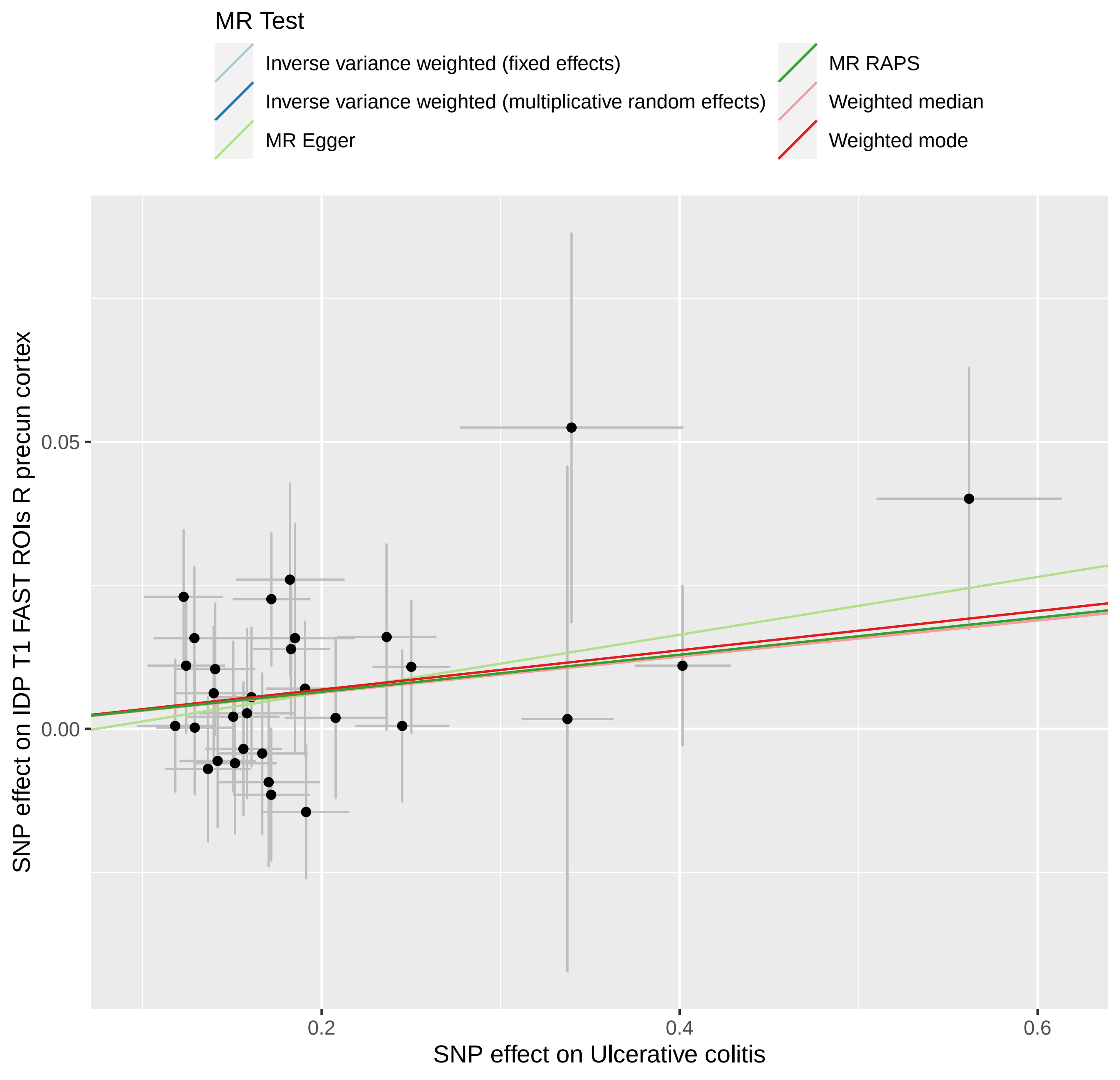

Batch 2707 : Crohn's disease on IDP T1 FAST ROIs L cuneal cortex

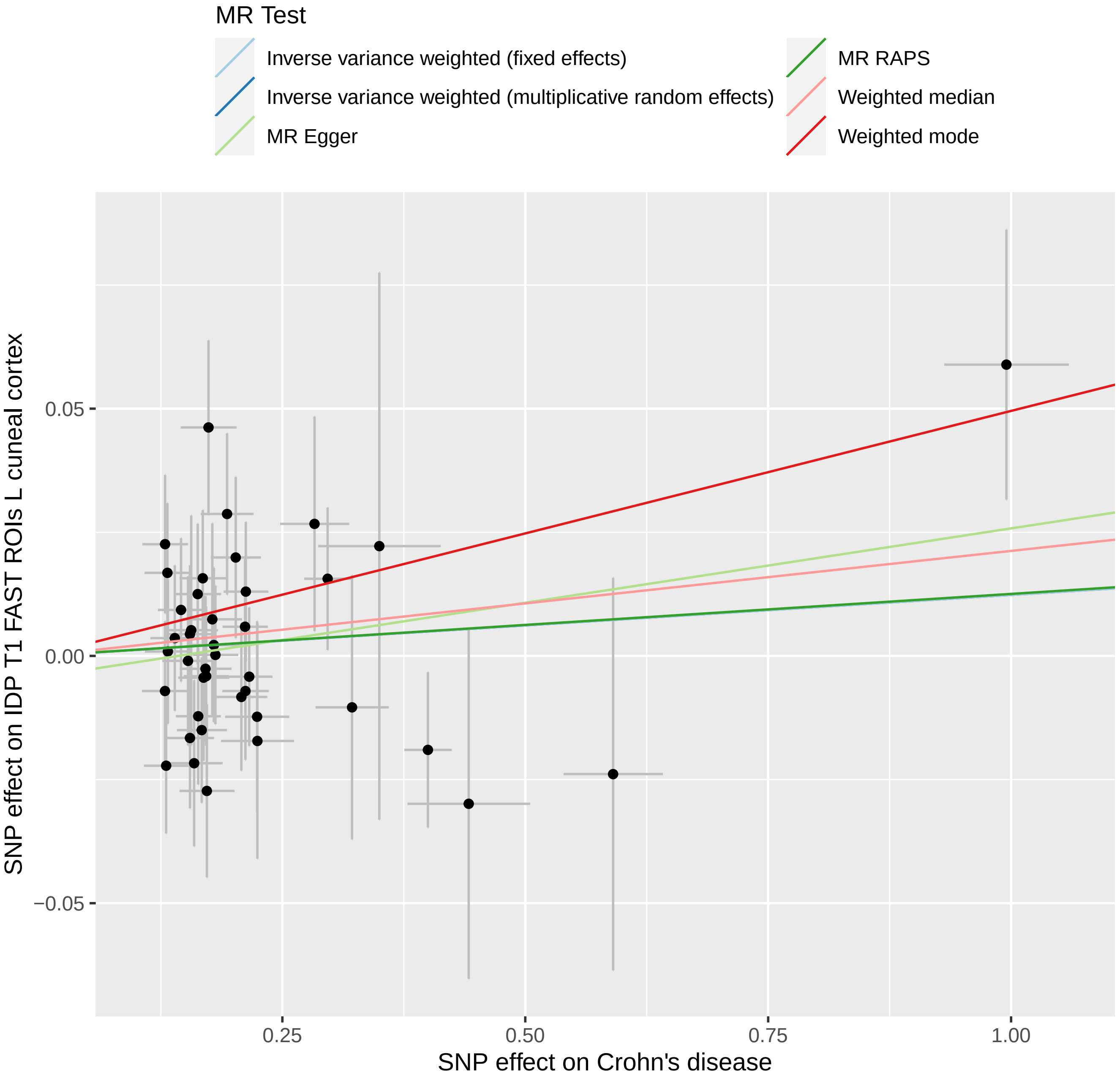

Batch 2708 : Ulcerative colitis on IDP T1 FAST ROIs L cuneal cortex

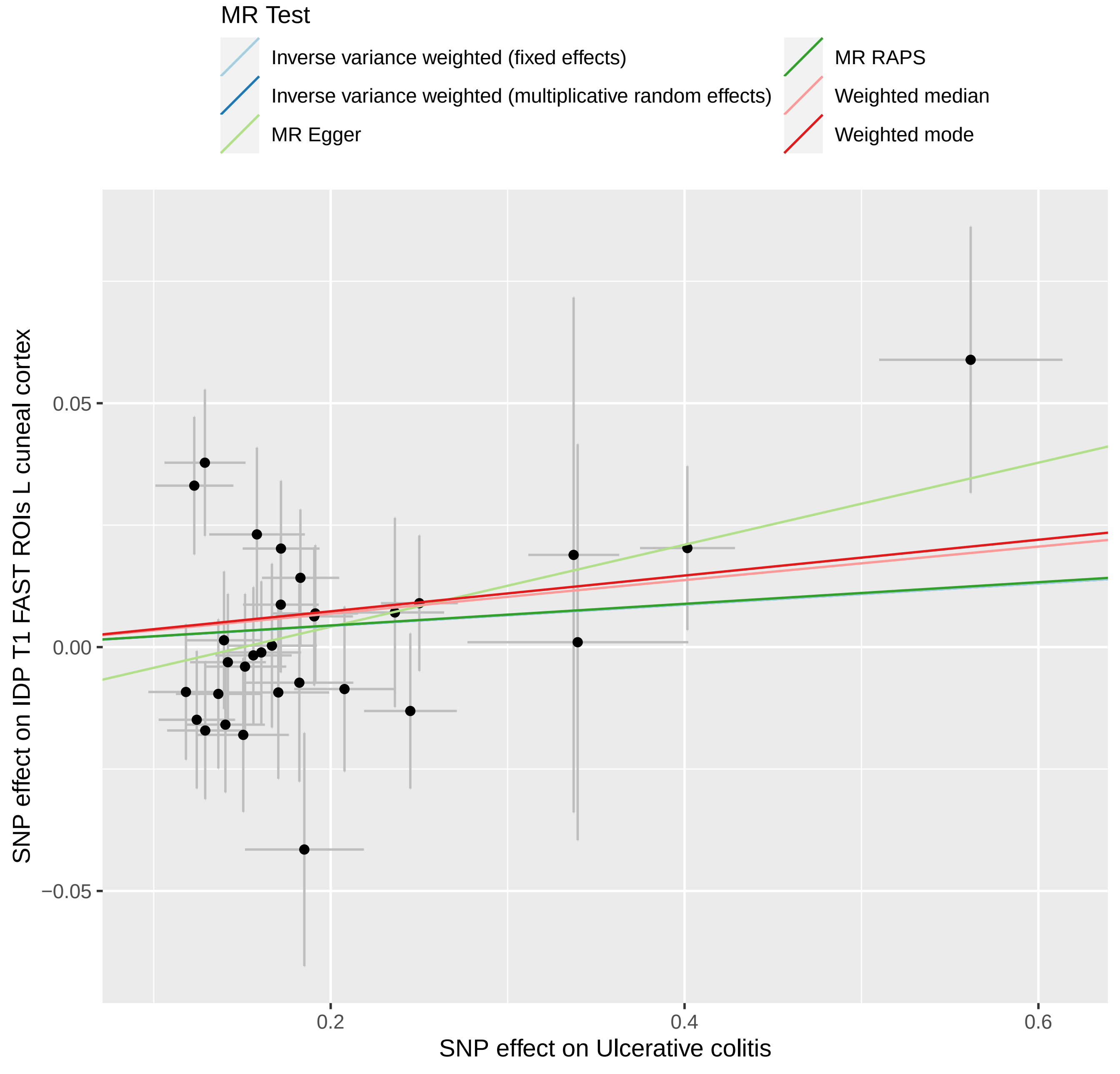

Batch 2709 : Crohn's disease on IDP T1 FAST ROIs R cuneal cortex

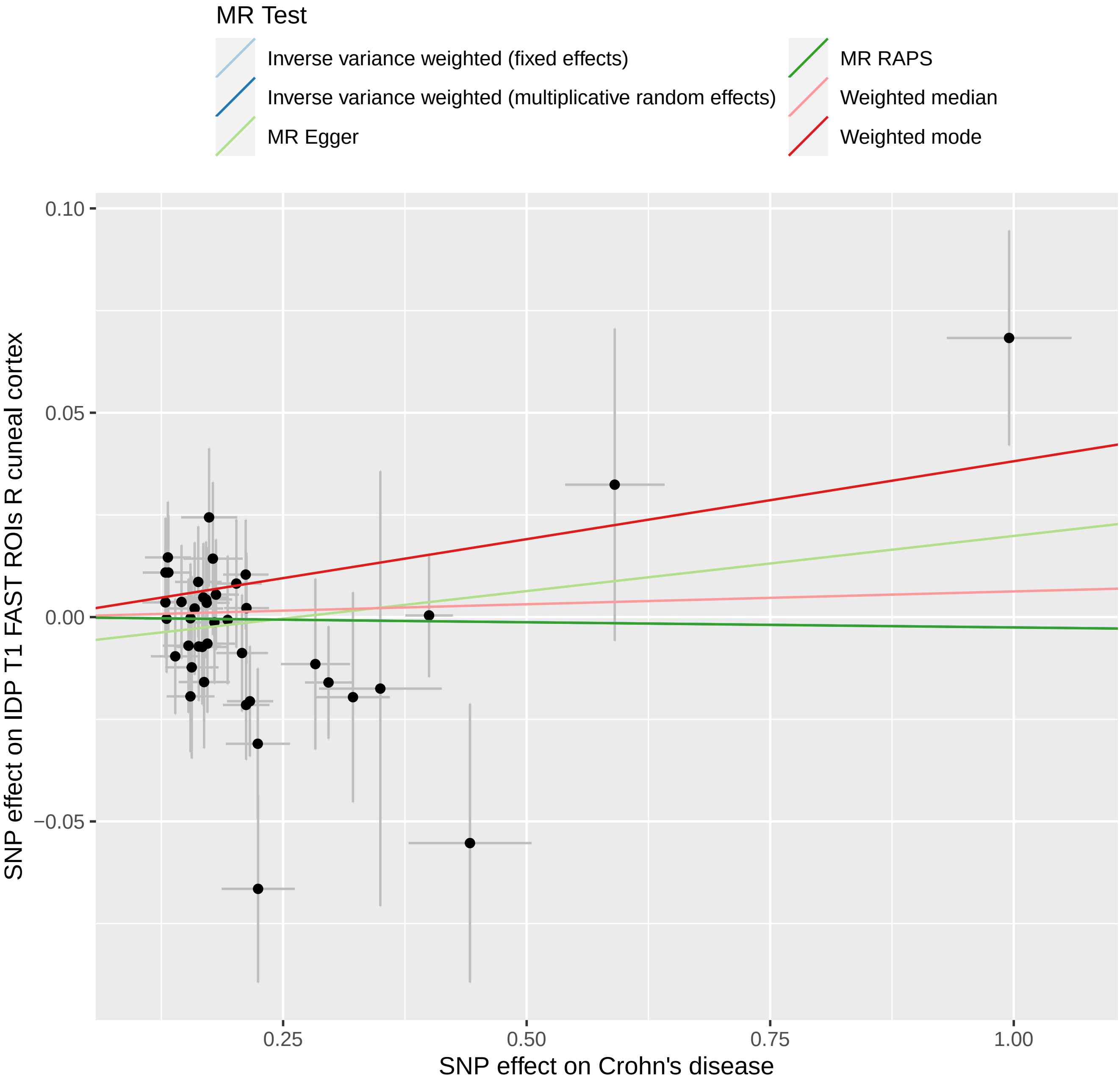

Batch 2710 : Ulcerative colitis on IDP T1 FAST ROIs R cuneal cortex

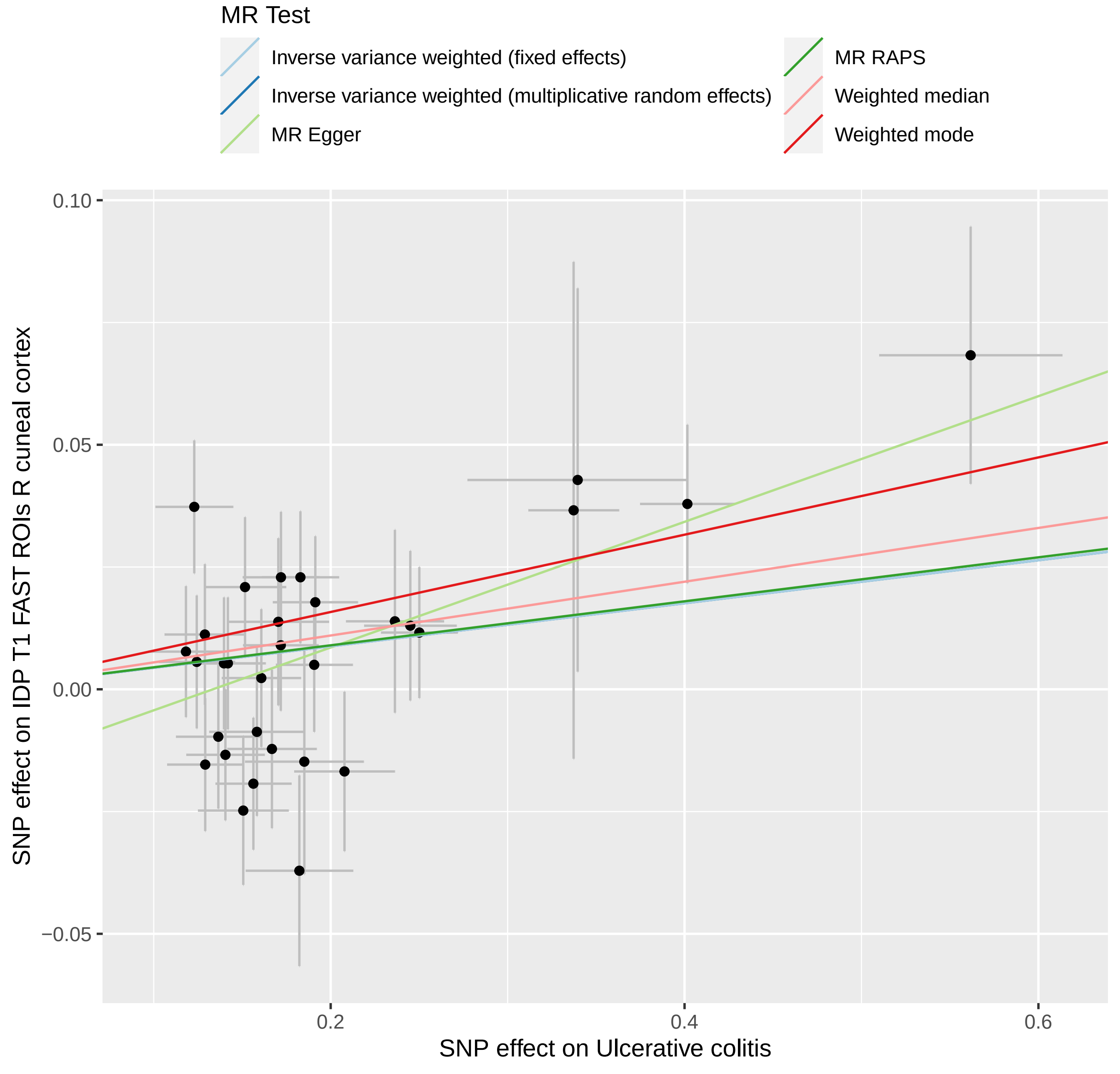

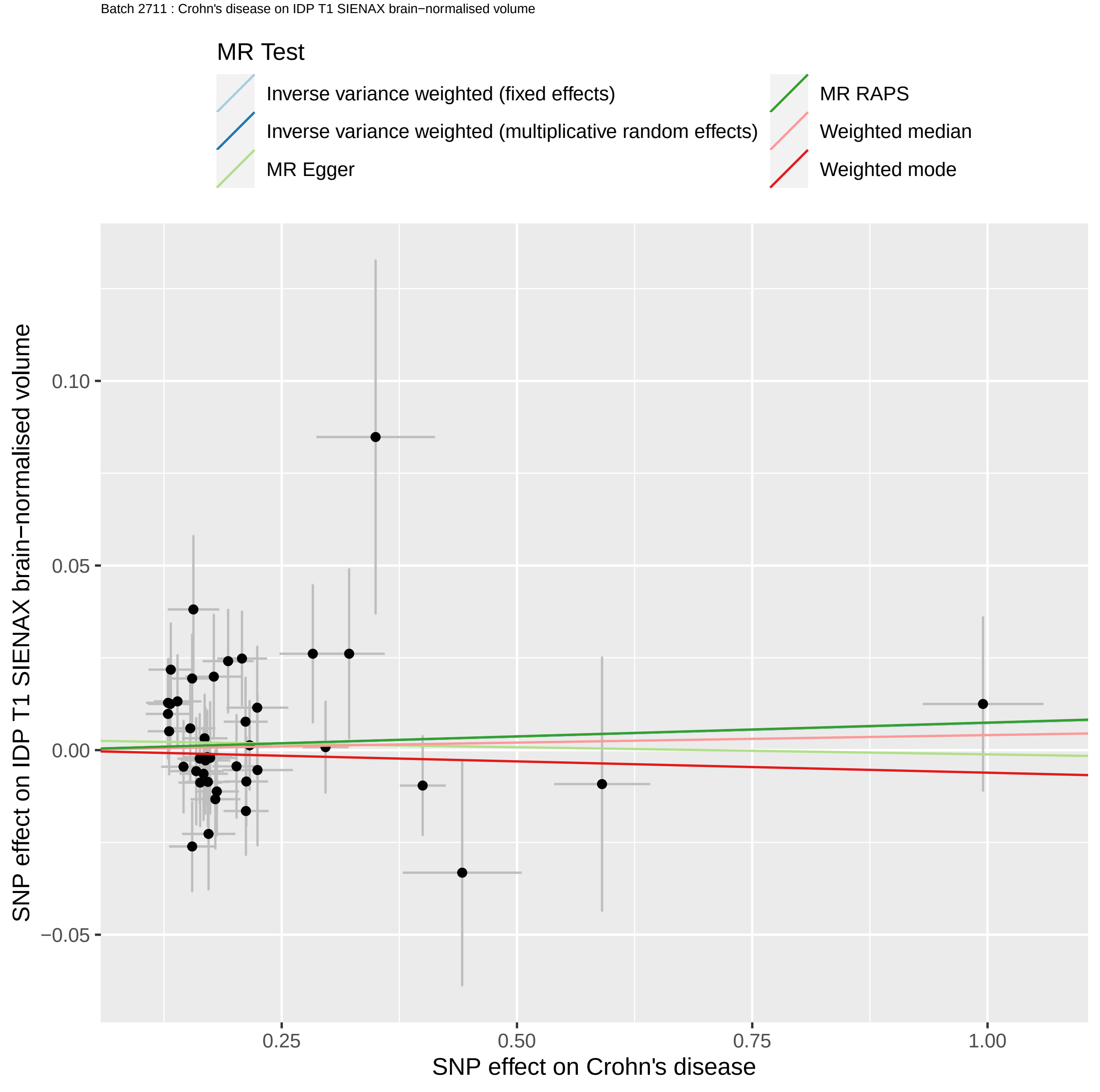

Batch 2711 : Crohn's disease on IDP T1 SIENAX brain−normalised volume
MR Test
Inverse variance weighted (fixed effects)
Inverse variance weighted (multiplicative random effects)
MR Egger
MR RAPS
Weighted median
Weighted mode
SNP effect on IDP T1 SIENAX brain−normalised volume
0.10
0.05
0.00
−0.05
0.25
0.50
0.75
1.00
SNP effect on Crohn's disease

Batch 2712 : Ulcerative colitis on IDP T1 SIENAX brain−normalised volume

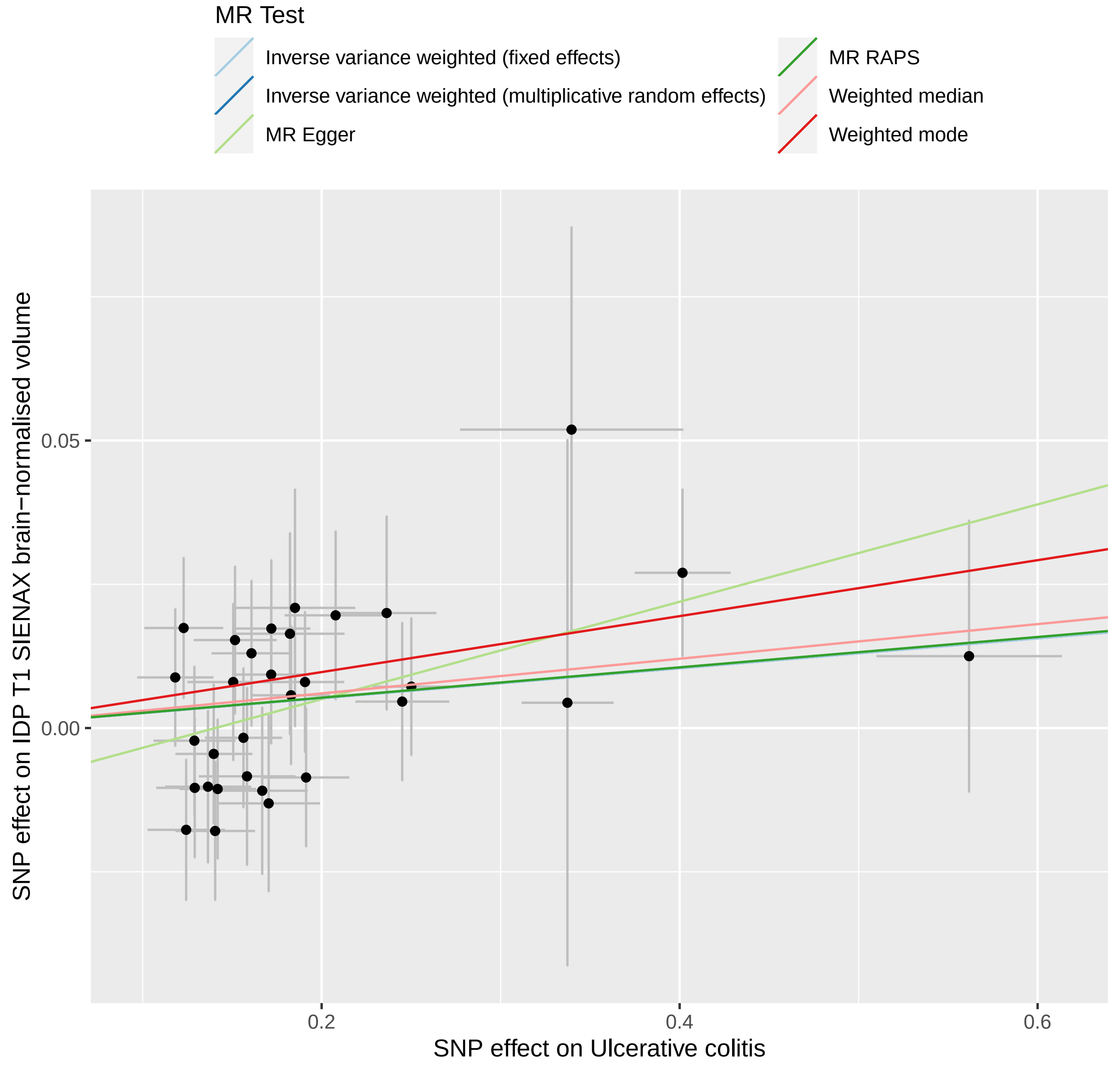

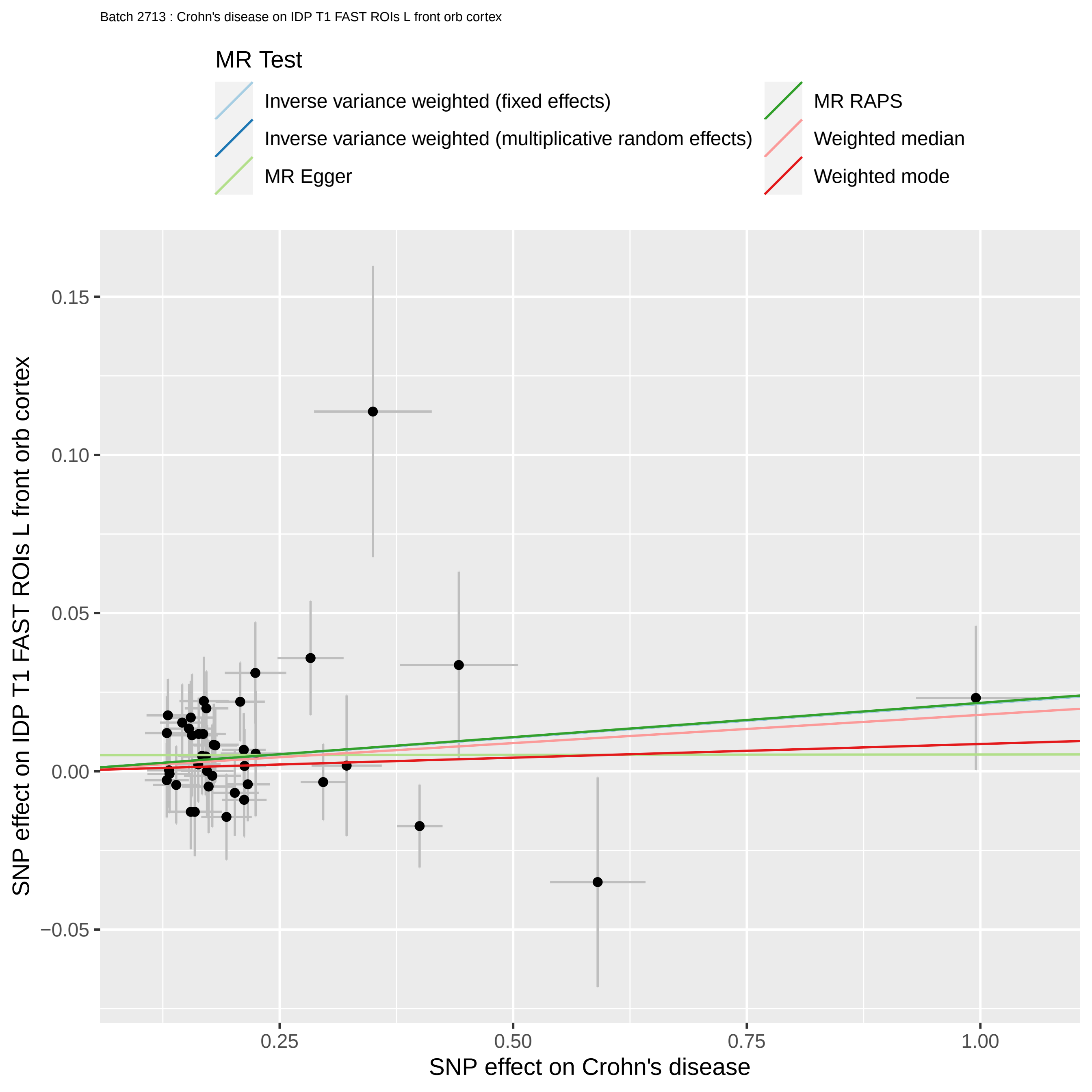
Batch 2713 : Crohn's disease on IDP T1 FAST ROIs L front orb cortex
MR Test
Inverse variance weighted (fixed effects)
Inverse variance weighted (multiplicative random effects)
MR Egger
MR RAPS
Weighted median
Weighted mode
SNP effect on IDP T1 FAST ROIs L front orb cortex
0.15
0.10
0.05
0.00
−0.05
0.25
0.50
0.75
1.00
SNP effect on Crohn's disease

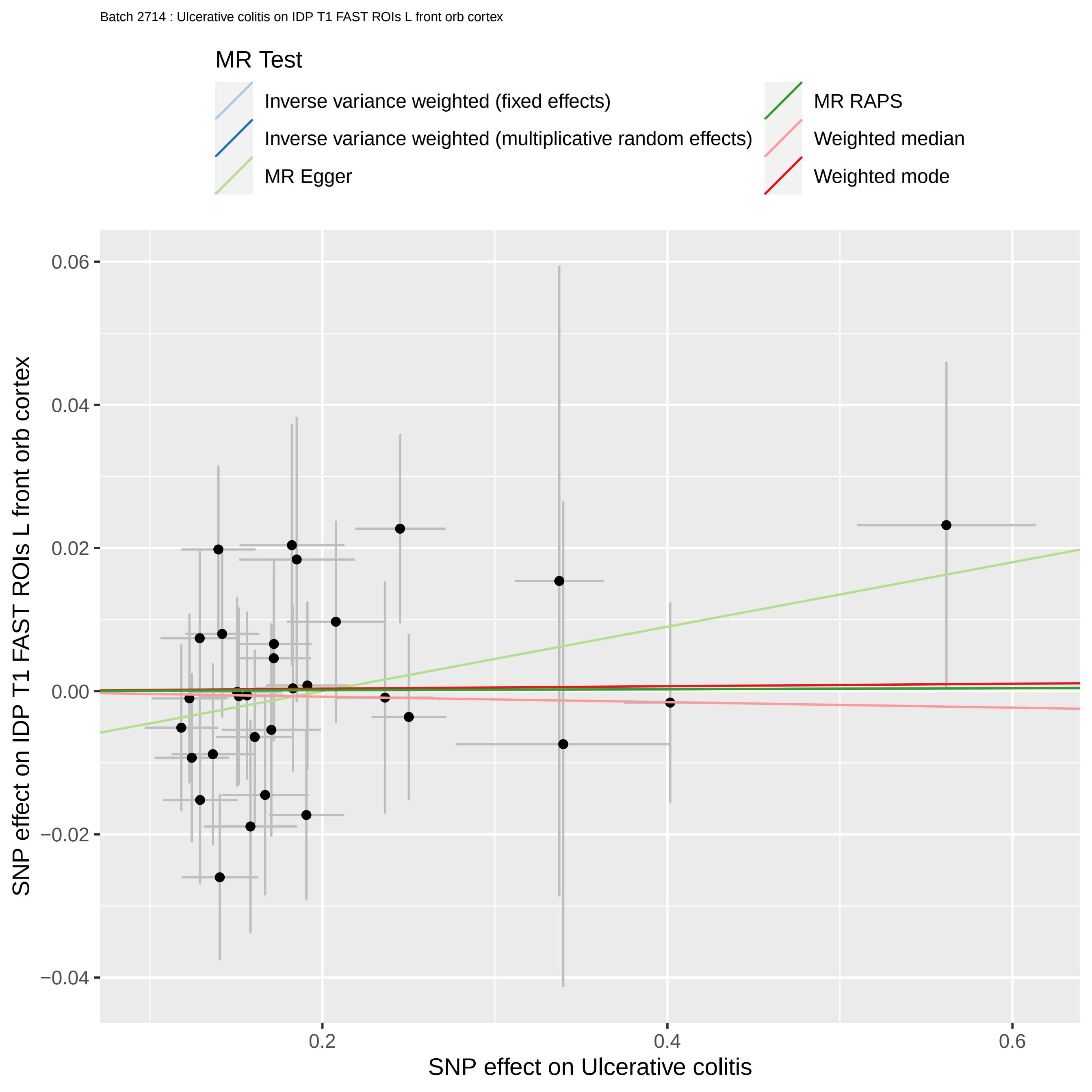

Batch 2714 : Ulcerative colitis on IDP T1 FAST ROIs L front orb cortex
MR Test
Inverse variance weighted (fixed effects)
Inverse variance weighted (multiplicative random effects)
MR Egger
MR RAPS
Weighted median
Weighted mode
0.06
0.04
0.02
0.00
−0.02
−0.04
0.2
0.4
0.6
SNP effect on IDP T1 FAST ROIs L front orb cortex
SNP effect on Ulcerative colitis

Batch 2715 : Crohn's disease on IDP T1 FAST ROIs R front orb cortex

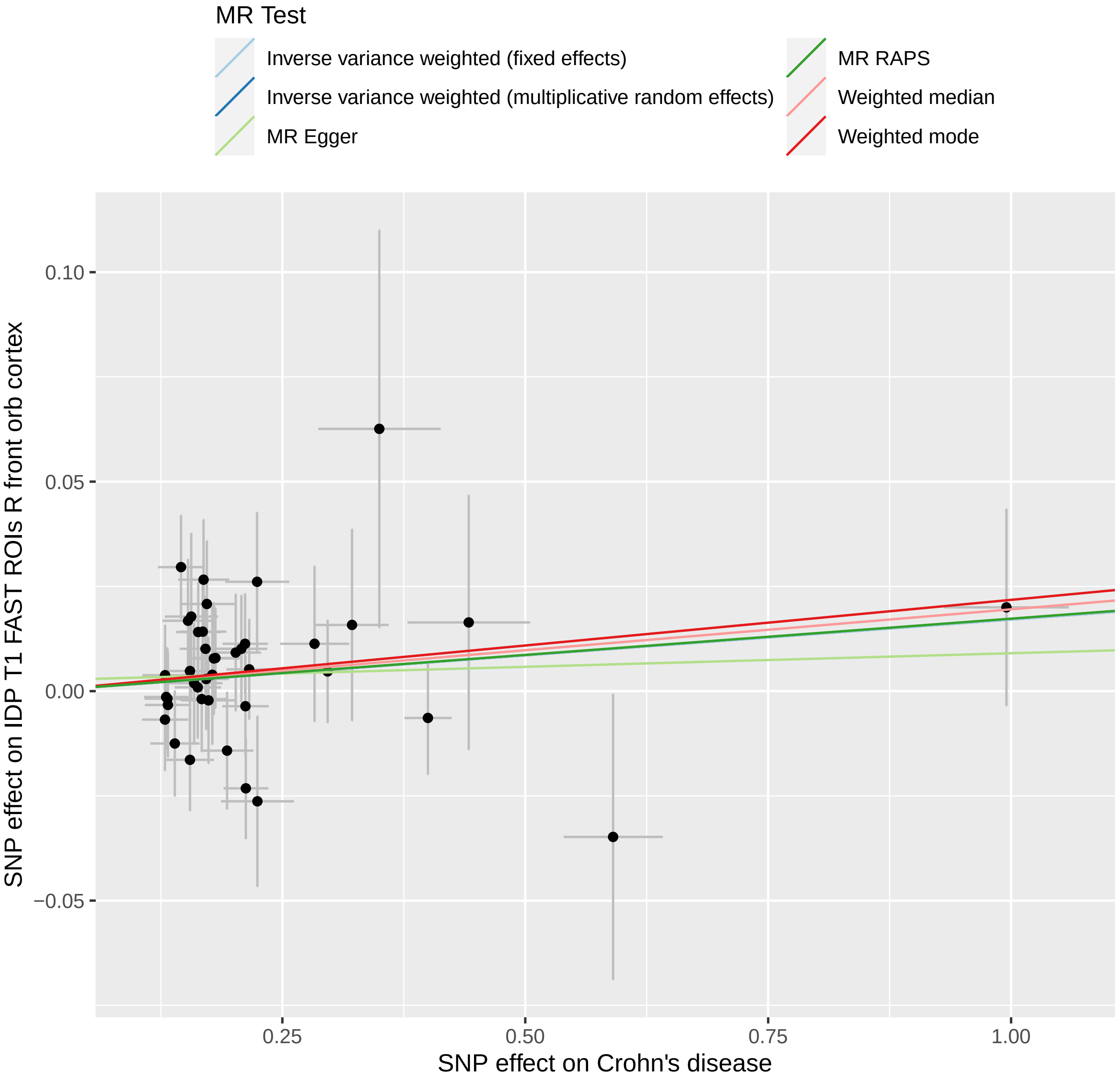

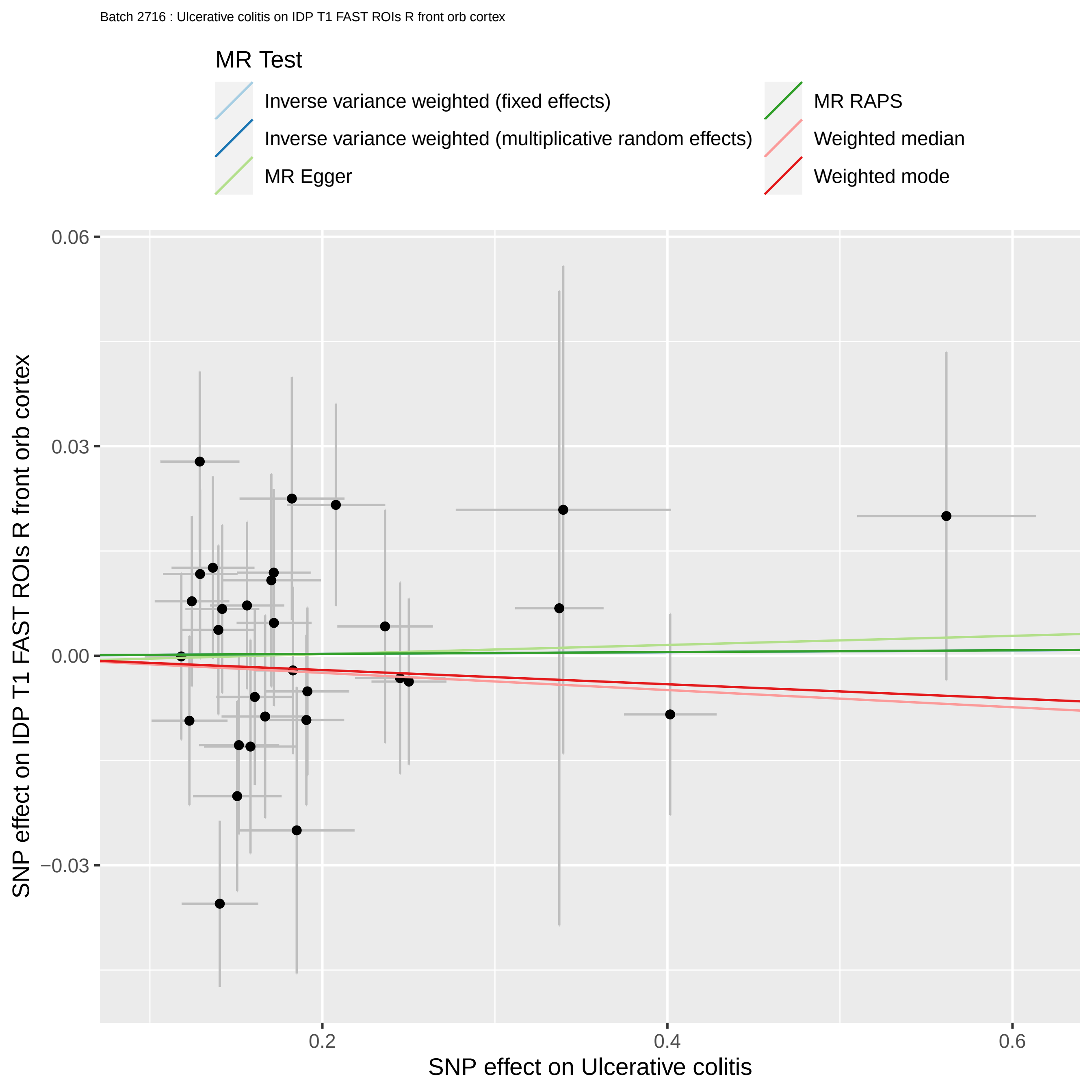

Batch 2716 : Ulcerative colitis on IDP T1 FAST ROIs R front orb cortex
MR Test
Inverse variance weighted (fixed effects)
Inverse variance weighted (multiplicative random effects)
MR Egger
MR RAPS
Weighted median
Weighted mode
0.06
0.03
0.00
−0.03
0.2
0.4
0.6
SNP effect on IDP T1 FAST ROIs R front orb cortex
SNP effect on Ulcerative colitis

Batch 2717 : Crohn's disease on IDP T1 FAST ROIs L parahipp gyrus ant

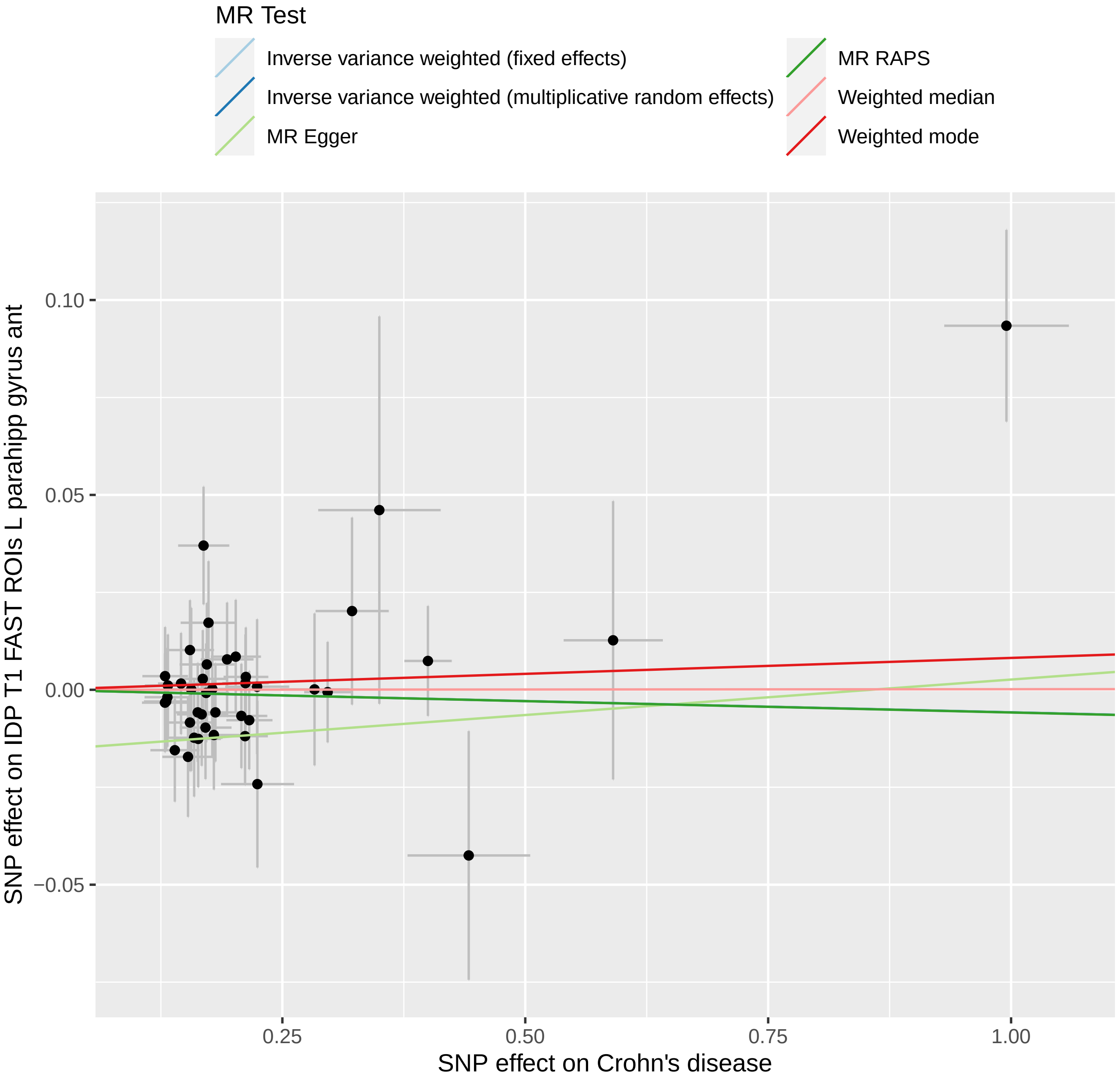

Batch 2718 : Ulcerative colitis on IDP T1 FAST ROIs L parahipp gyrus ant

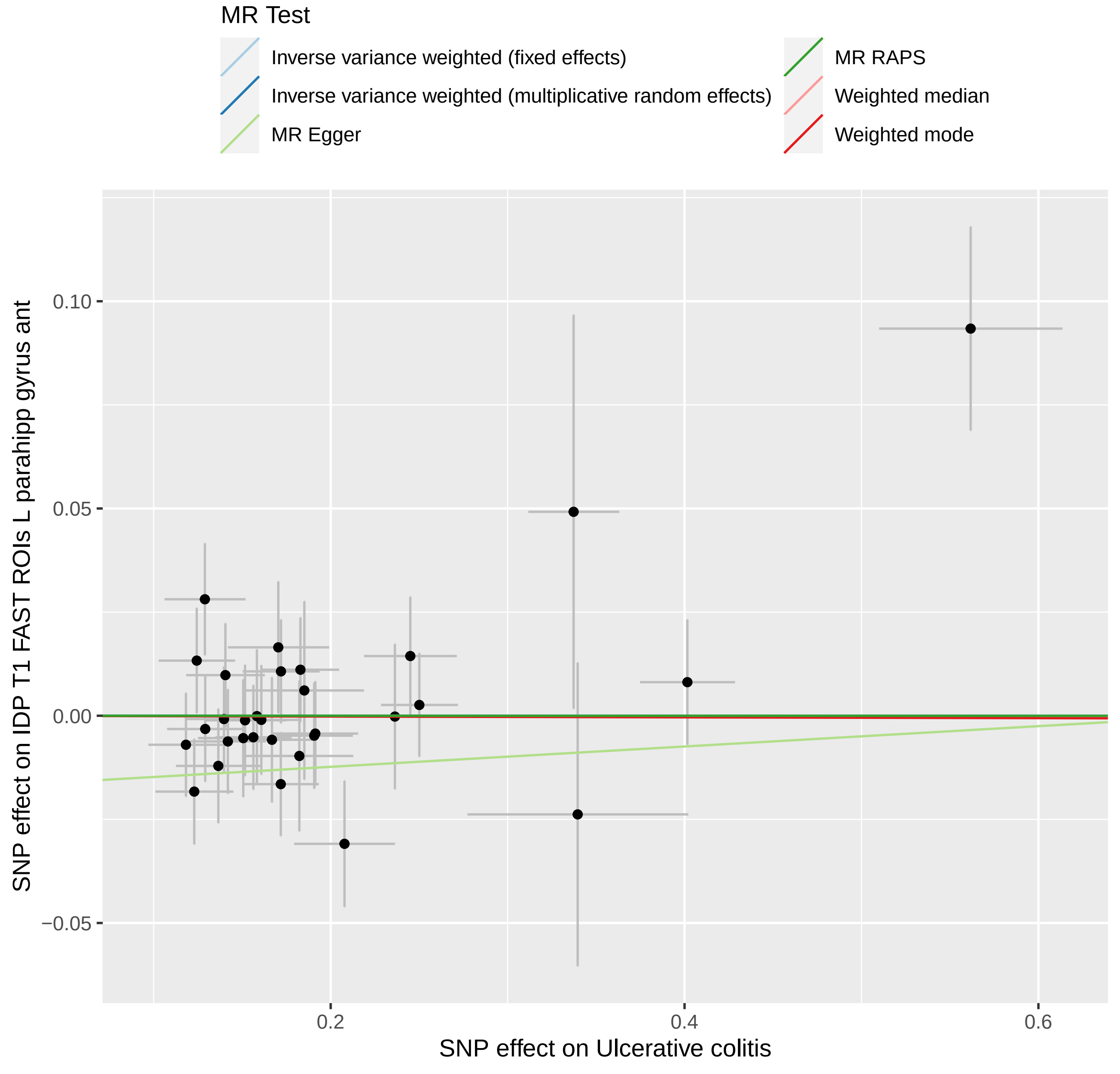

Batch 2719 : Crohn's disease on IDP T1 FAST ROIs R parahipp gyrus ant

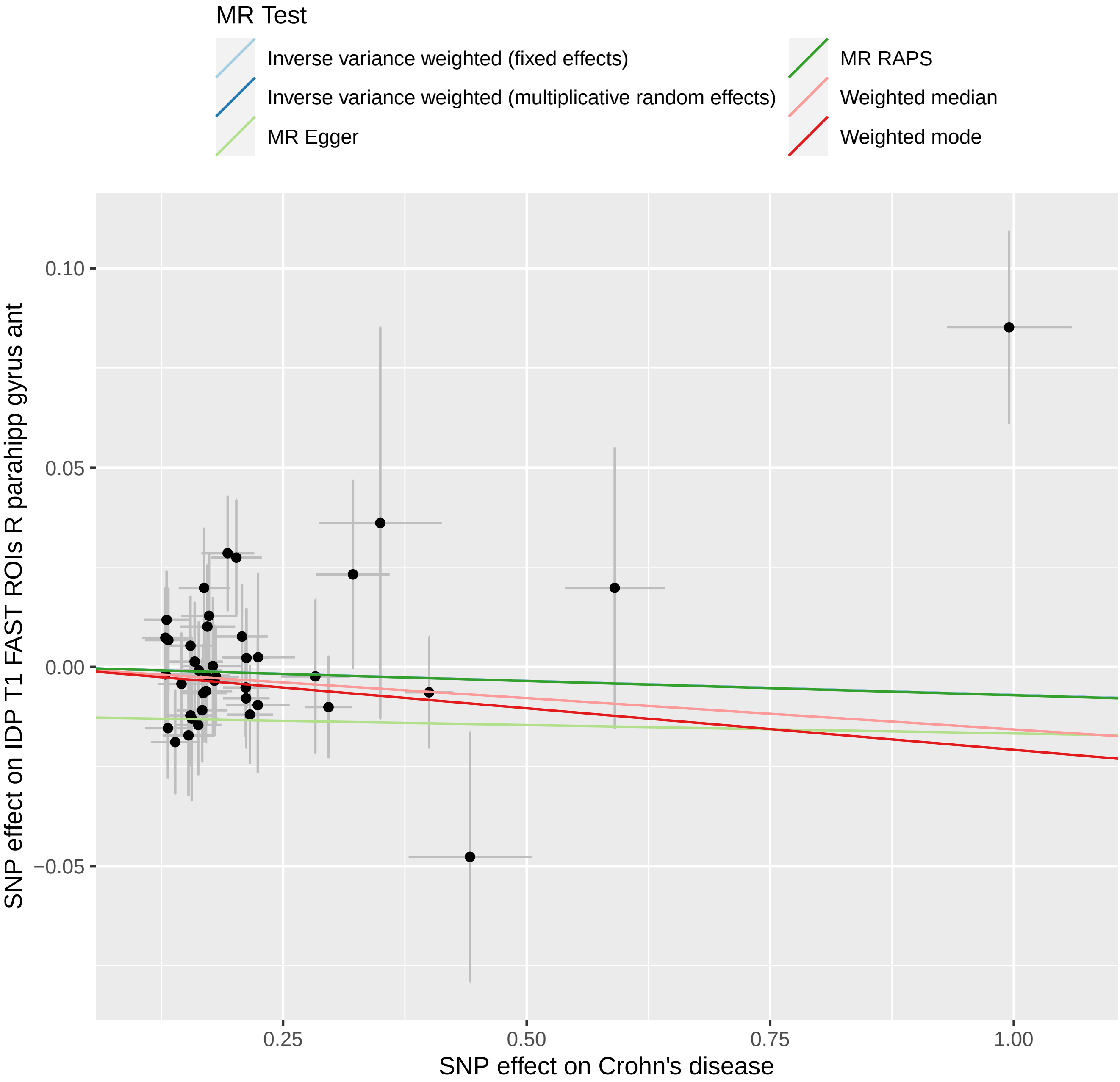

Batch 2720 : Ulcerative colitis on IDP T1 FAST ROIs R parahipp gyrus ant

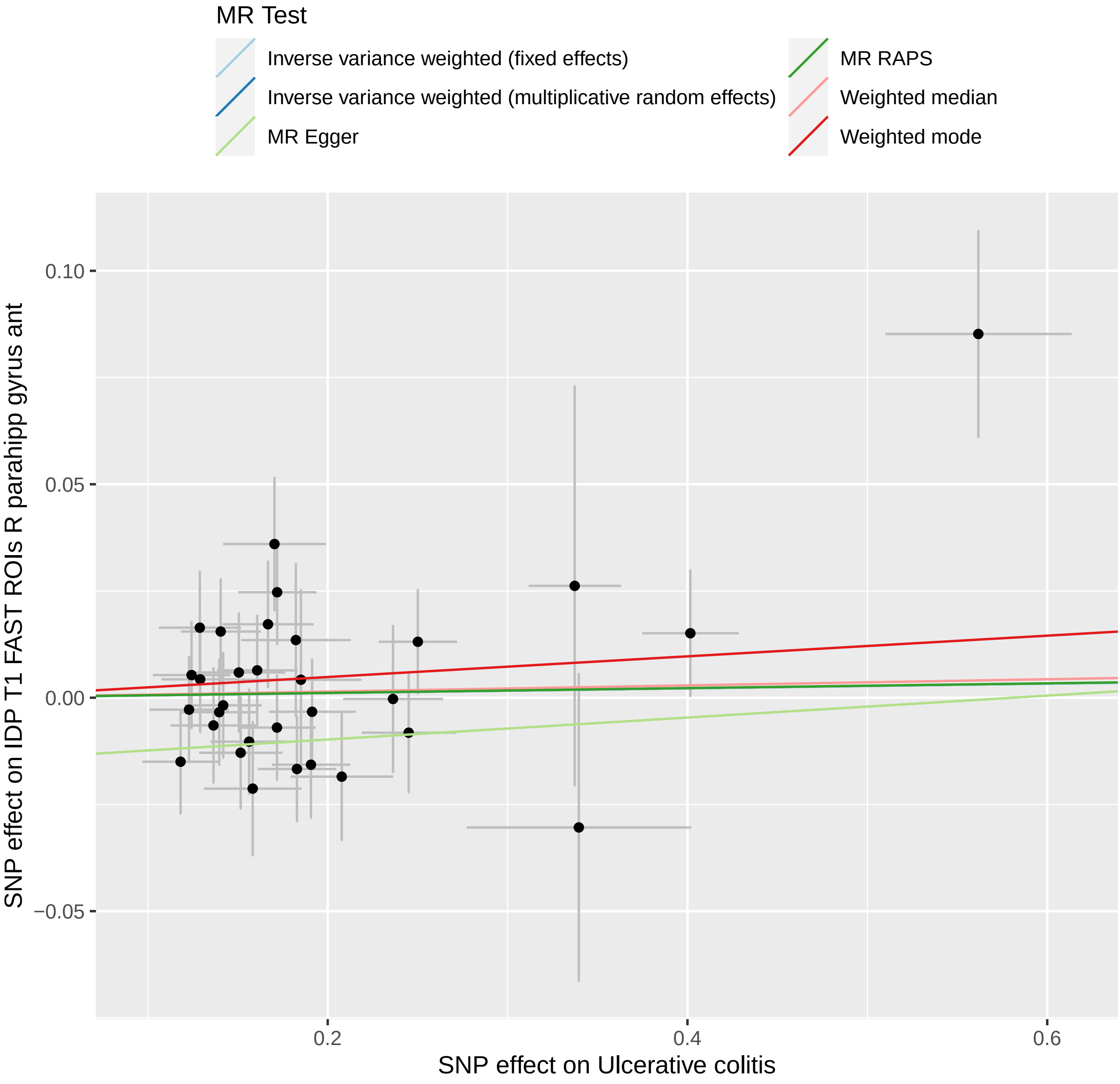

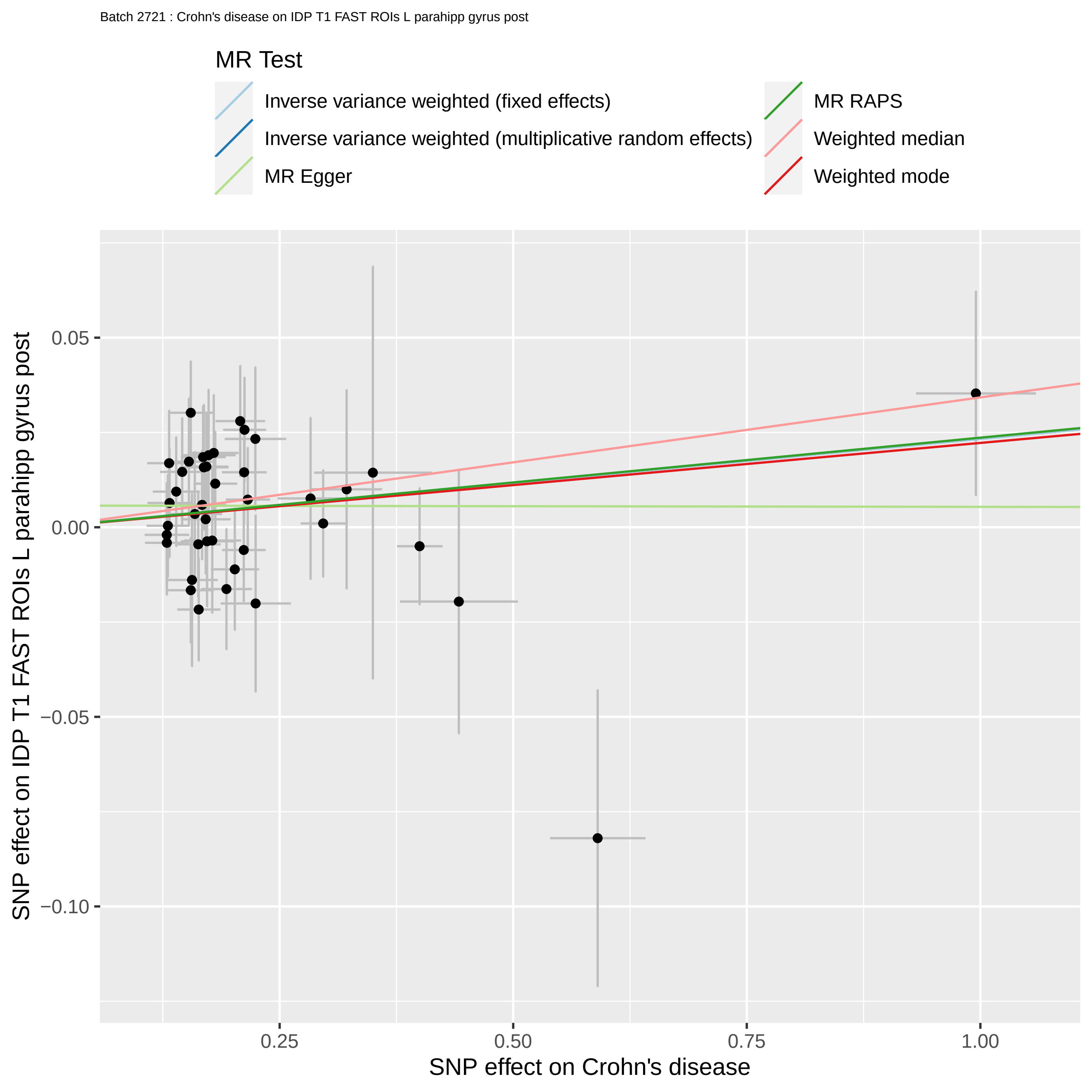
Batch 2721 : Crohn's disease on IDP T1 FAST ROIs L parahipp gyrus post
MR Test
Inverse variance weighted (fixed effects)
Inverse variance weighted (multiplicative random effects)
MR Egger
MR RAPS
Weighted median
Weighted mode
0.05
0.00
−0.05
−0.10
0.25
0.50
0.75
1.00
SNP effect on Crohn's disease
SNP effect on IDP T1 FAST ROIs L parahipp gyrus post

Batch 2722 : Ulcerative colitis on IDP T1 FAST ROIs L parahipp gyrus post

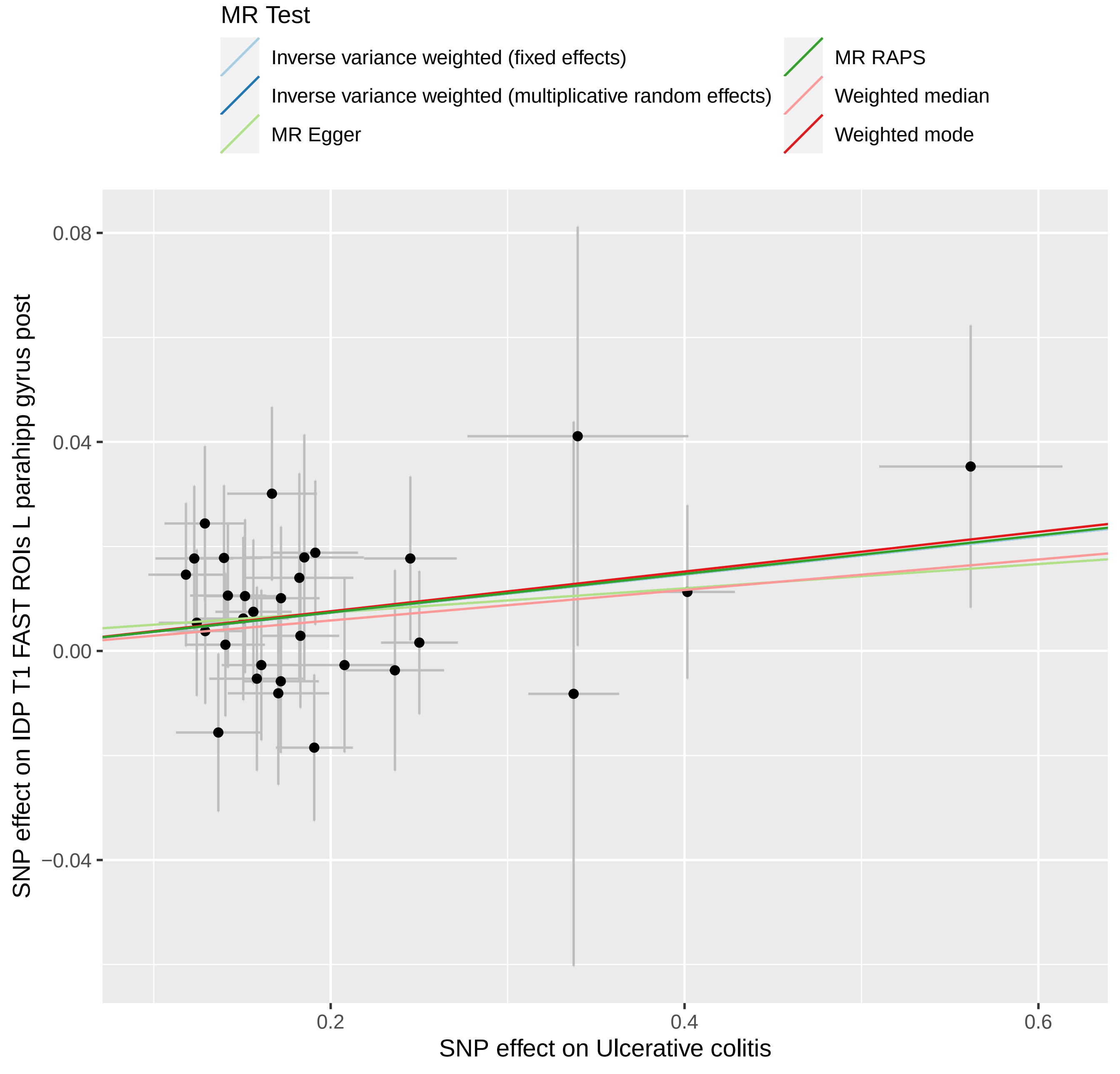

Batch 2723 : Crohn's disease on IDP T1 FAST ROIs R parahipp gyrus post

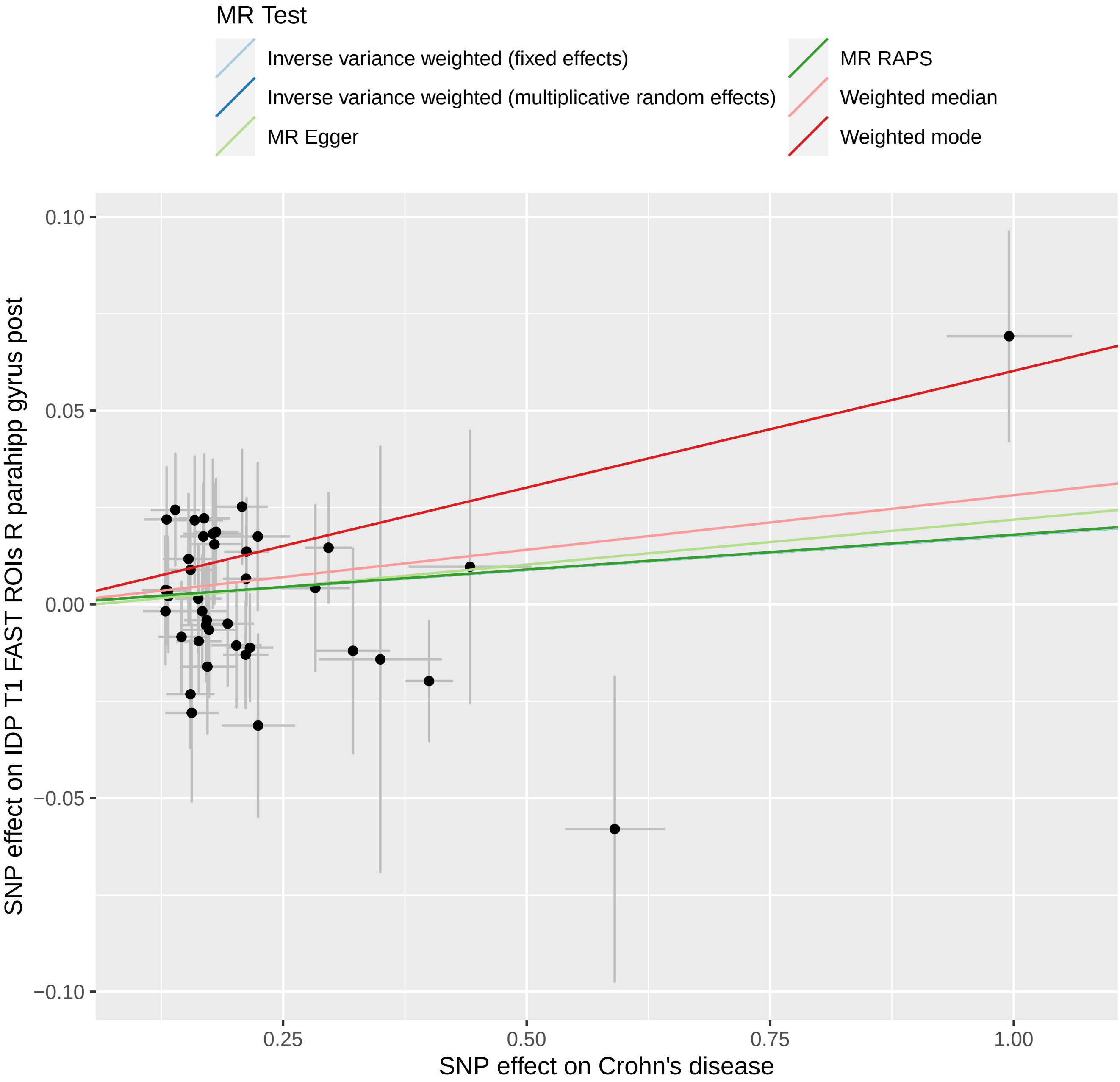

Batch 2724 : Ulcerative colitis on IDP T1 FAST ROIs R parahipp gyrus post

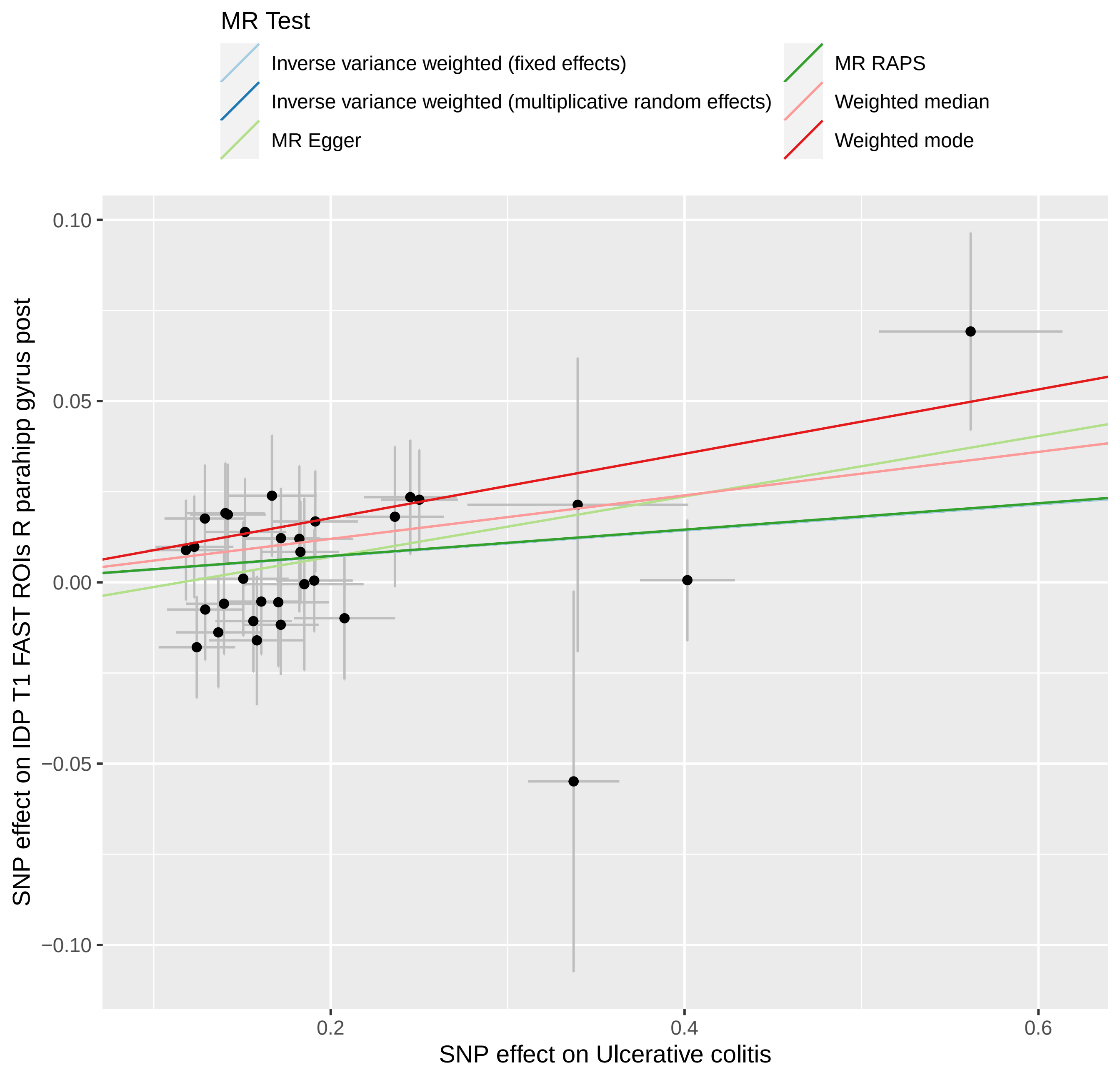

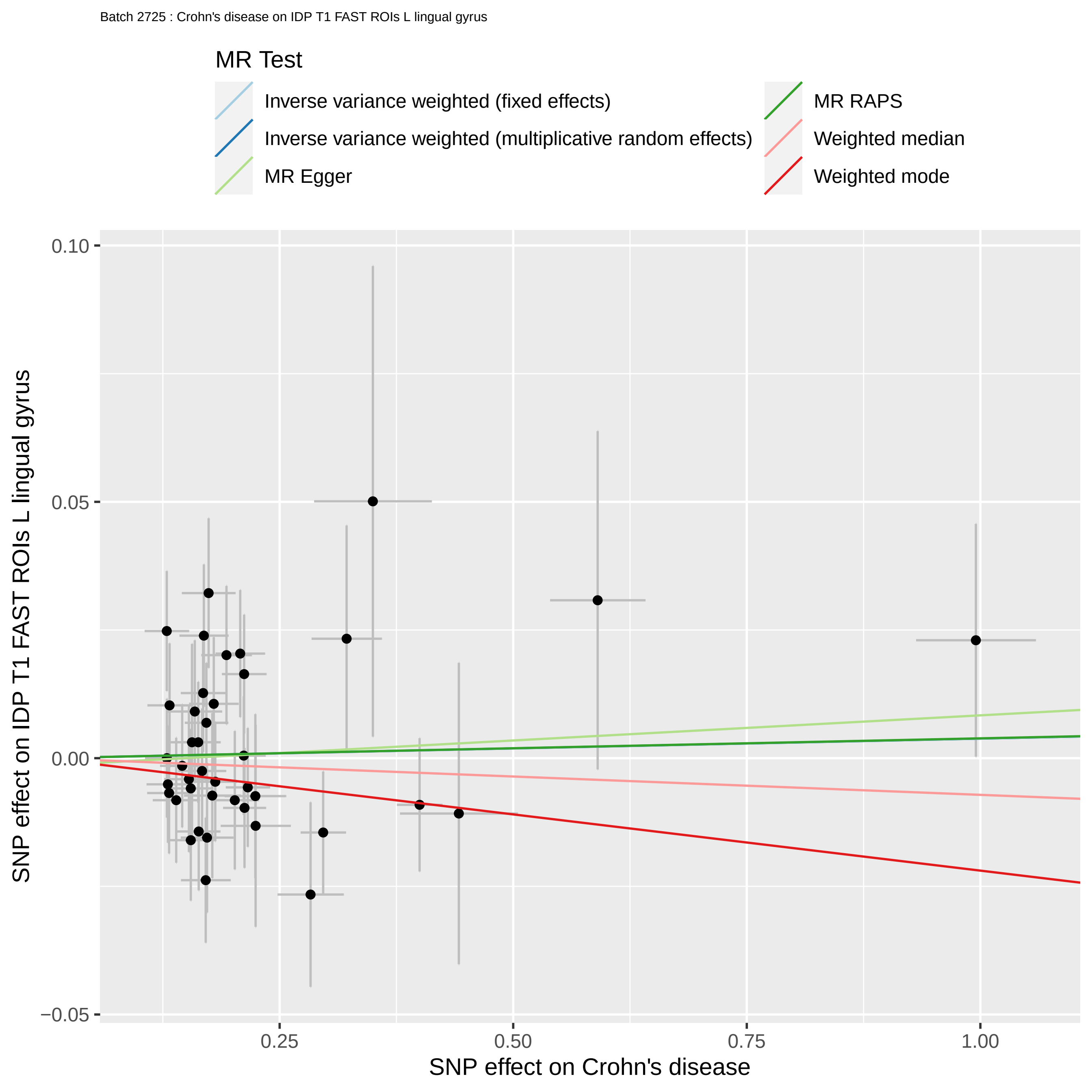
Batch 2725 : Crohn's disease on IDP T1 FAST ROIs L lingual gyrus
MR Test
Inverse variance weighted (fixed effects)
Inverse variance weighted (multiplicative random effects)
MR Egger
MR RAPS
Weighted median
Weighted mode
SNP effect on IDP T1 FAST ROIs L lingual gyrus
0.10
0.05
0.00
−0.05
0.25
0.50
0.75
1.00
SNP effect on Crohn's disease

Batch 2726 : Ulcerative colitis on IDP T1 FAST ROIs L lingual gyrus

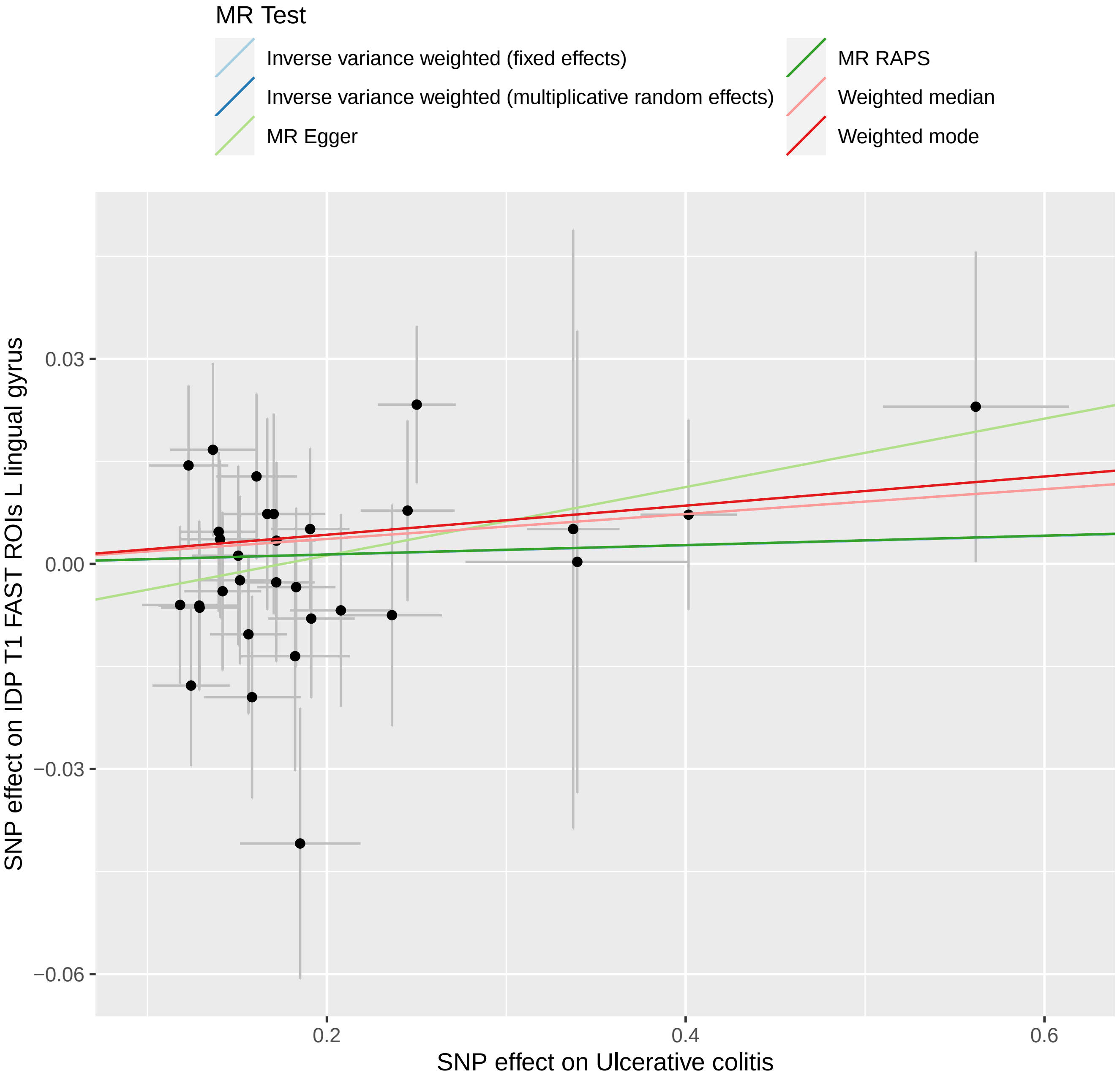

Batch 2727 : Crohn's disease on IDP T1 FAST ROIs R lingual gyrus

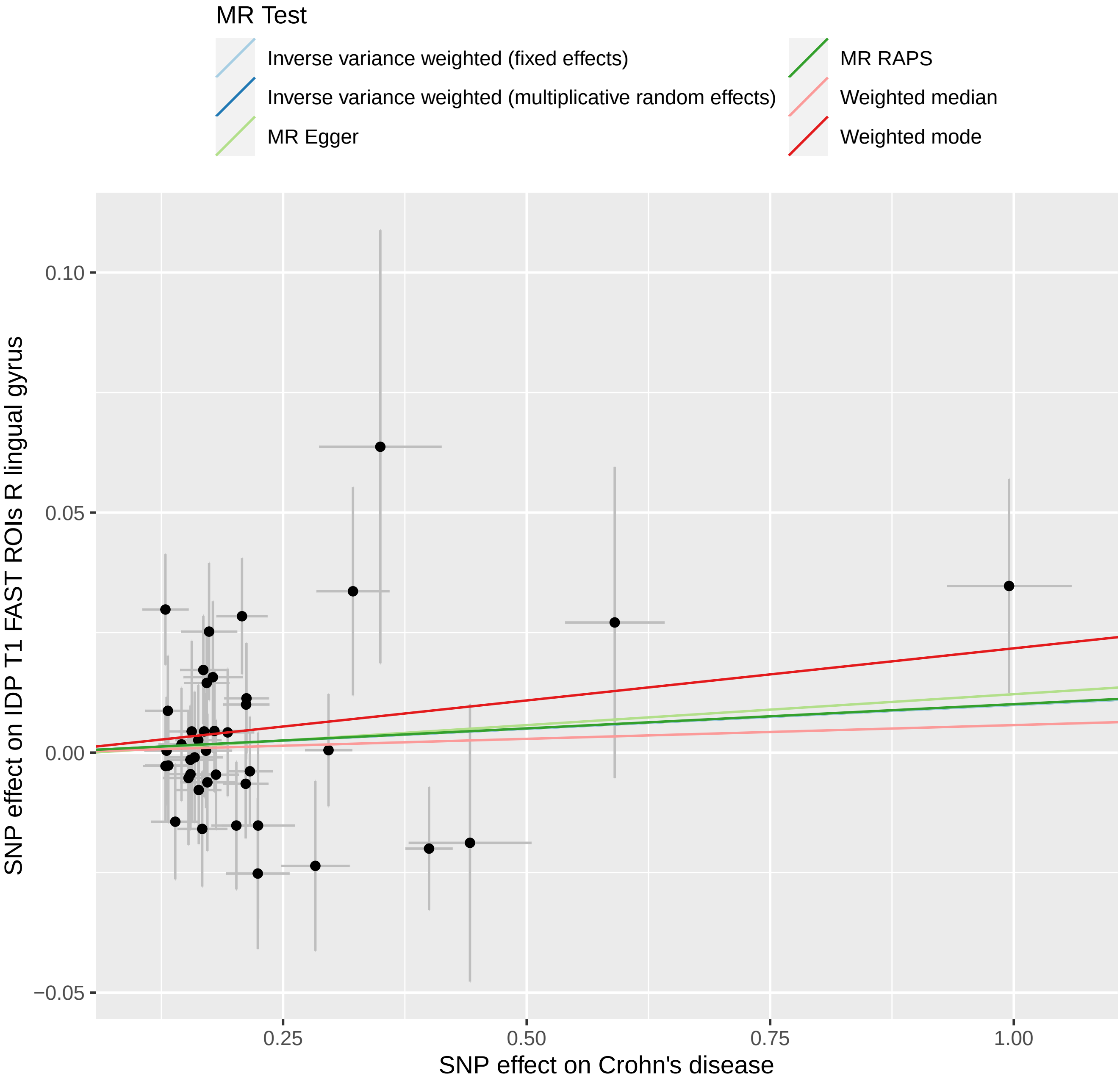

Batch 2728 : Ulcerative colitis on IDP T1 FAST ROIs R lingual gyrus

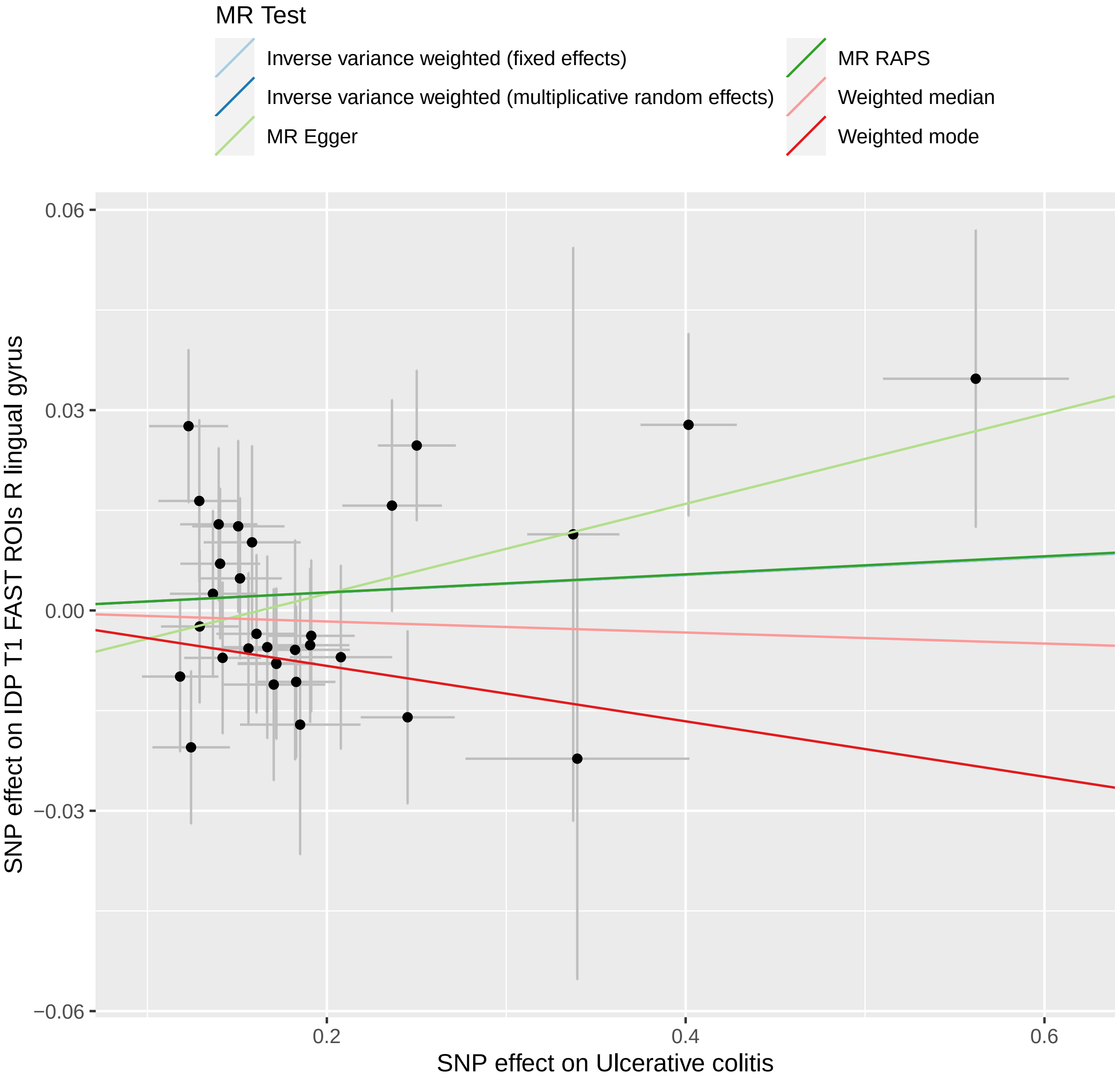

Batch 2729 : Crohn's disease on IDP T1 FAST ROIs L temp fusif cortex ant

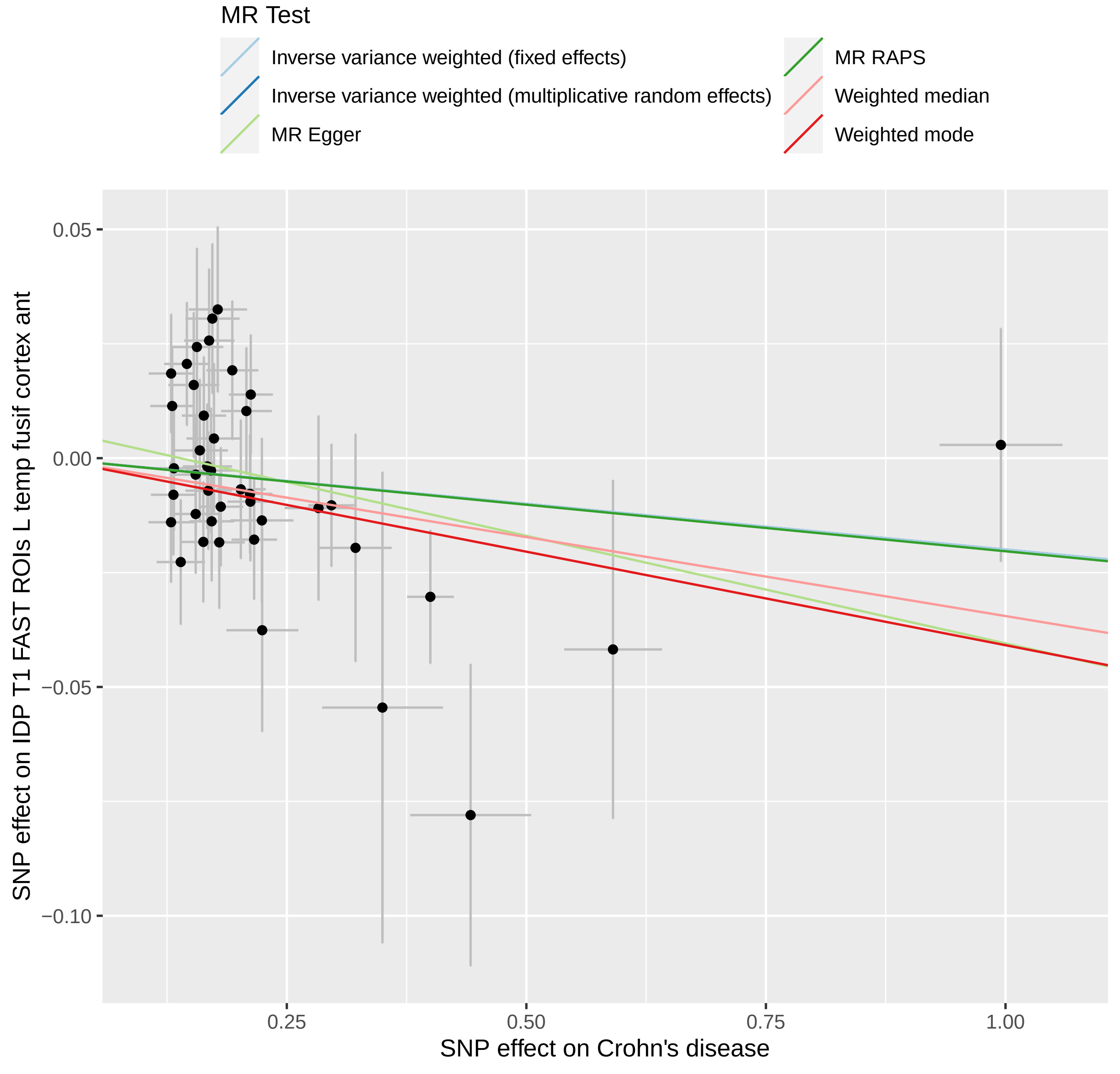

Batch 2730 : Ulcerative colitis on IDP T1 FAST ROIs L temp fusif cortex ant

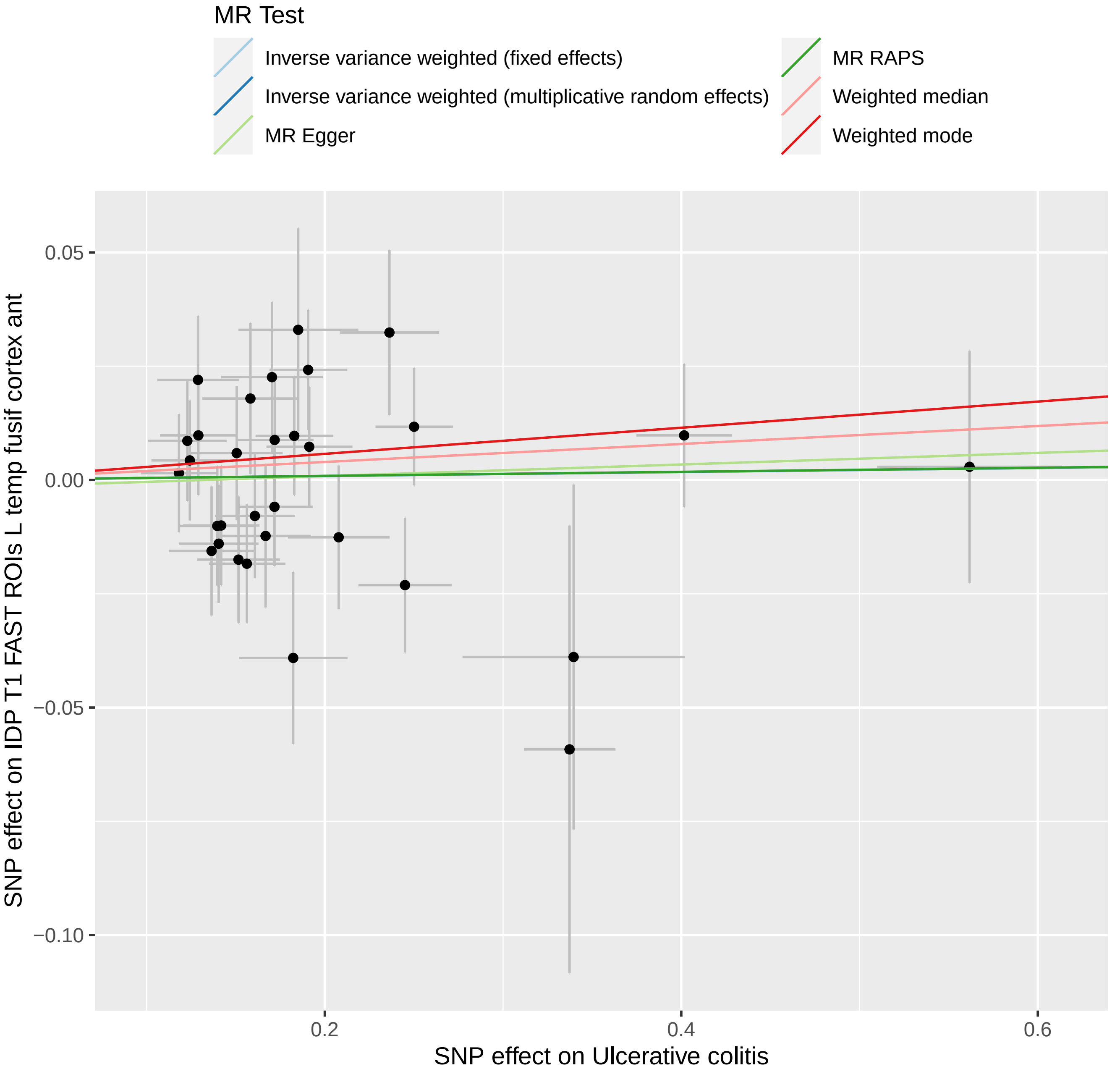

Batch 2731 : Crohn's disease on IDP T1 FAST ROIs R temp fusif cortex ant

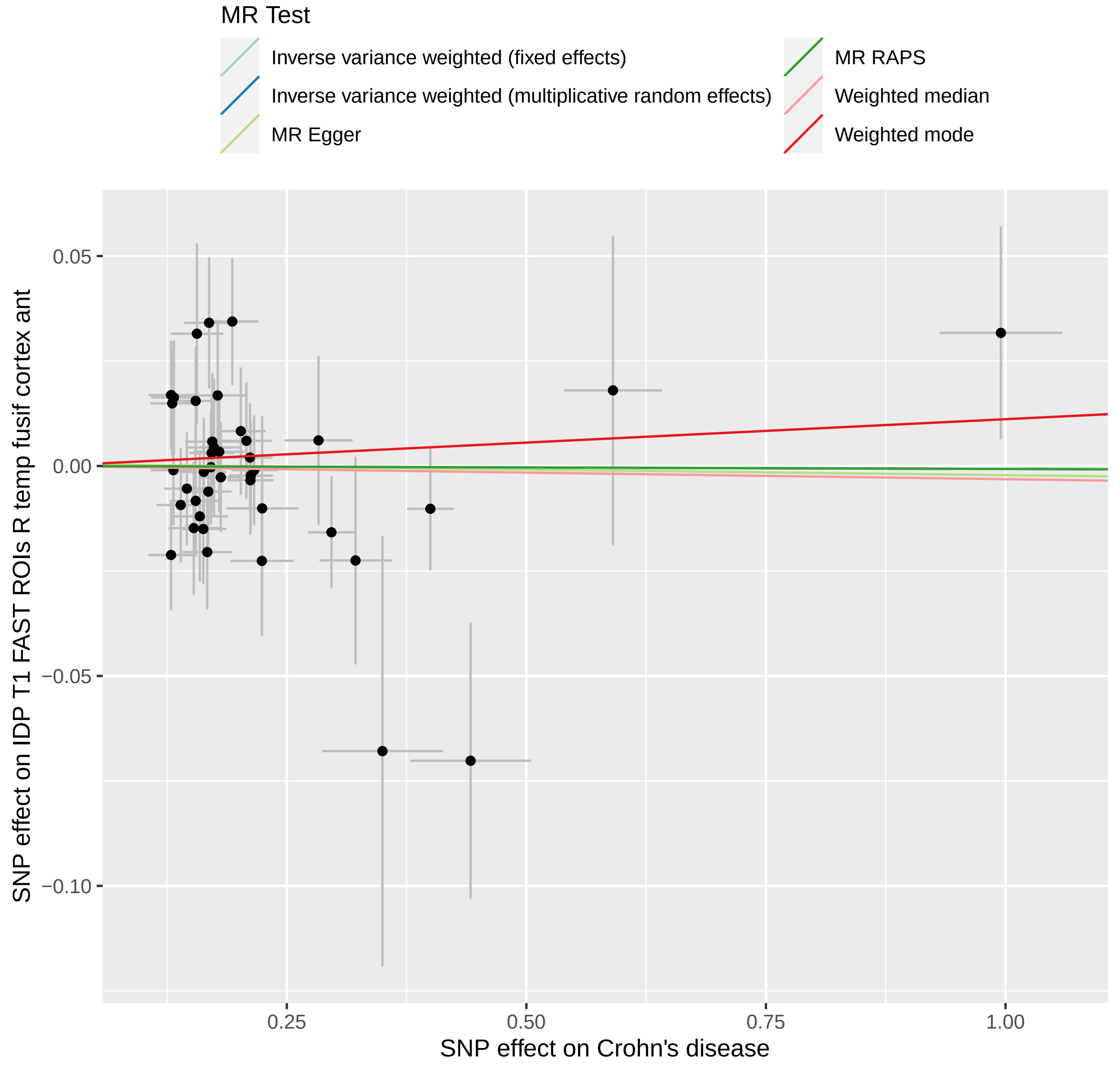

Batch 2732 : Ulcerative colitis on IDP T1 FAST ROIs R temp fusif cortex ant

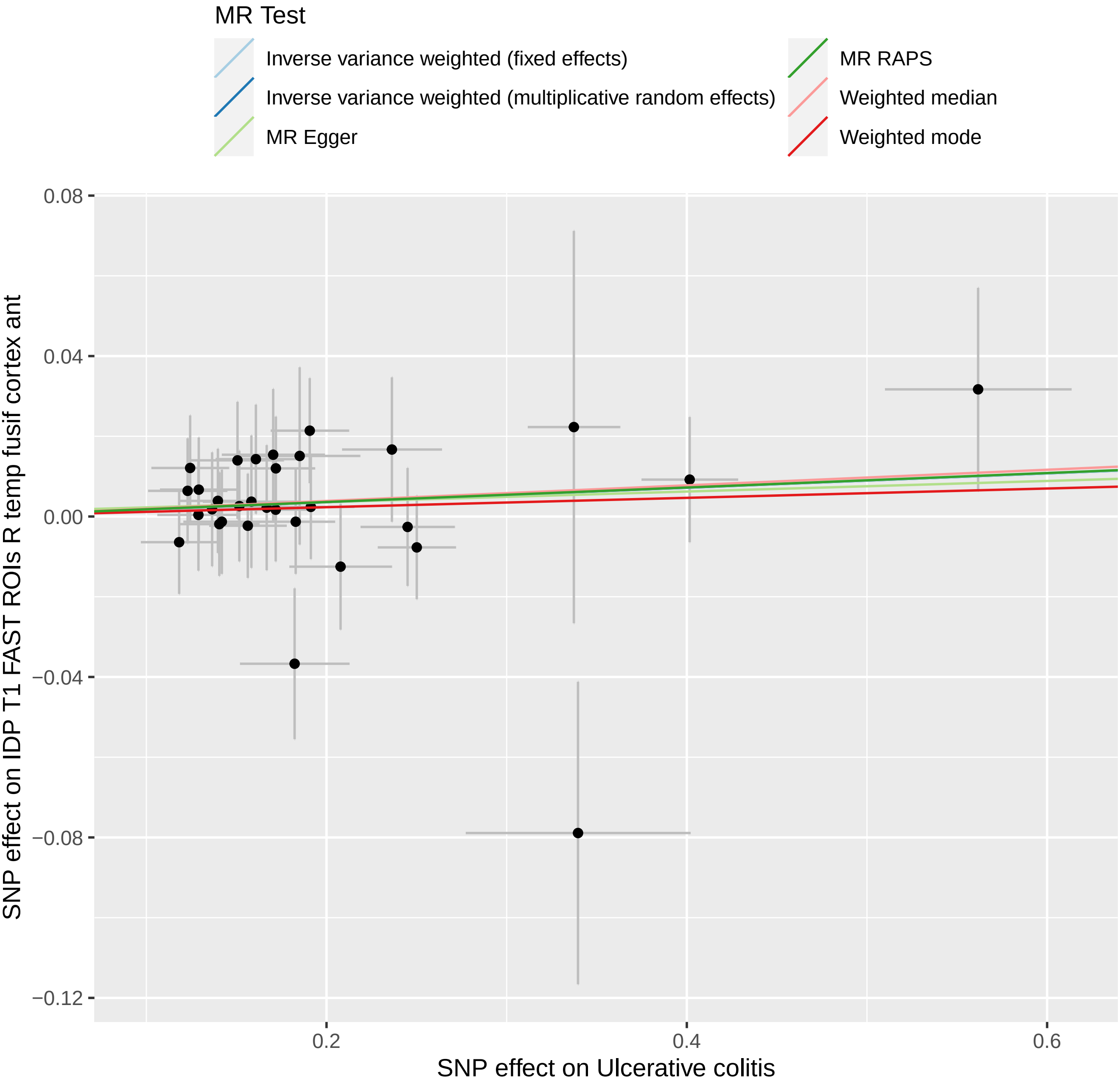

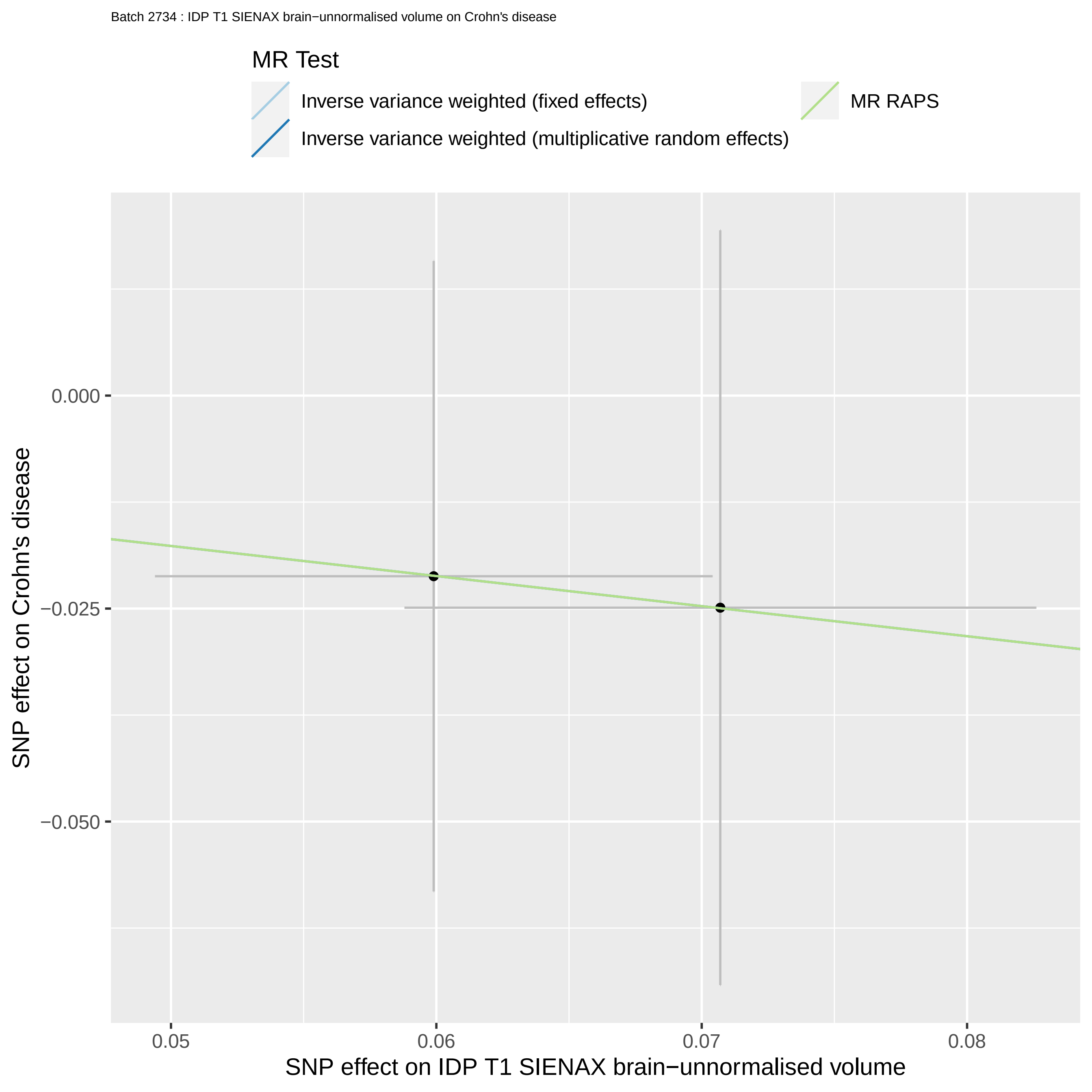
Batch 2734 : IDP T1 SIENAX brain−unnormalised volume on Crohn's disease
MR Test
Inverse variance weighted (fixed effects)
Inverse variance weighted (multiplicative random effects)
MR RAPS
0.000
−0.025
−0.050
0.05
0.06
0.07
0.08
SNP effect on Crohn's disease
SNP effect on IDP T1 SIENAX brain−unnormalised volume

Batch 2736 : IDP T1 FAST ROIs R temp fusif cortex post on Crohn's disease

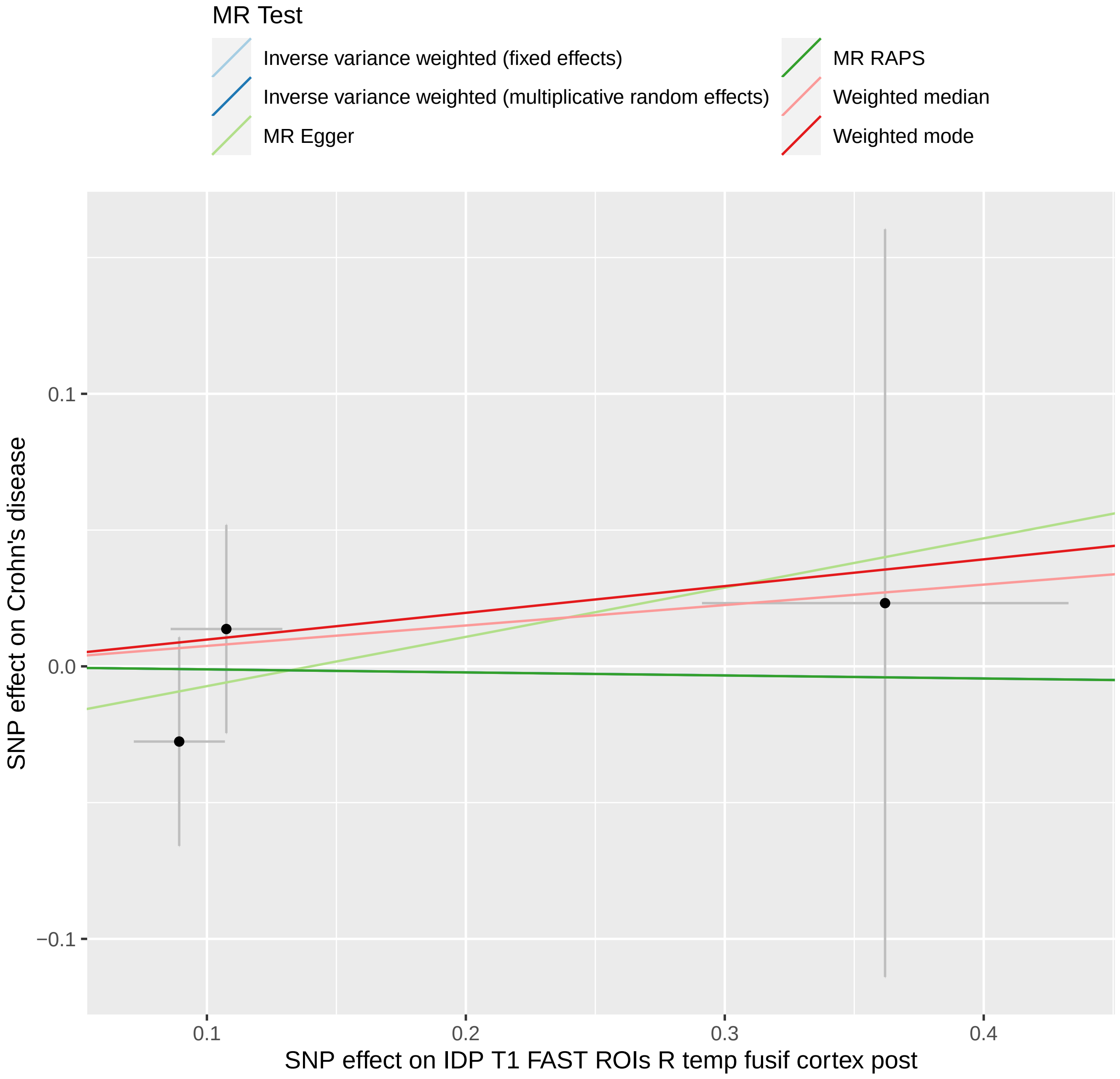

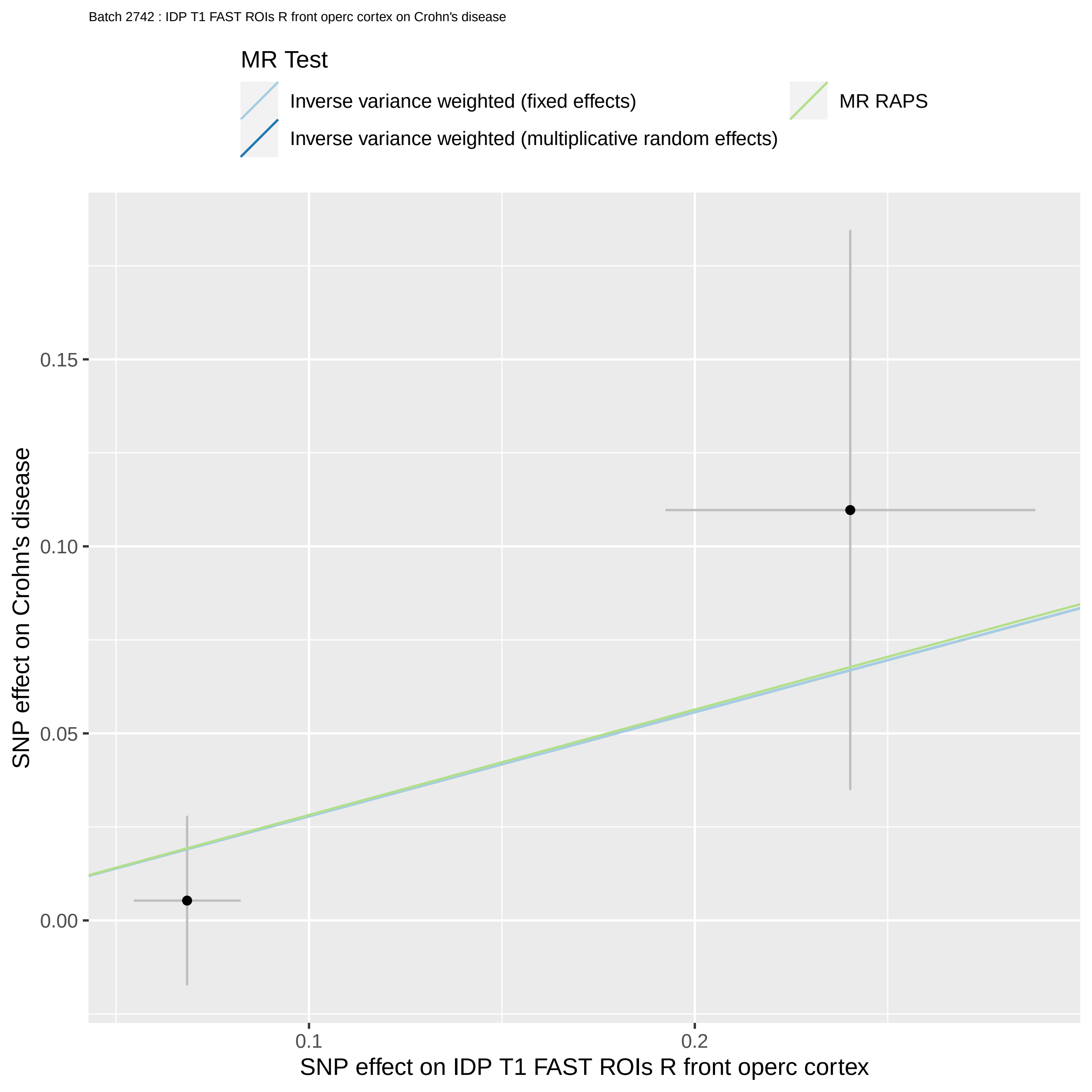
Batch 2742 : IDP T1 FAST ROIs R front operc cortex on Crohn's disease
MR Test
Inverse variance weighted (fixed effects)
Inverse variance weighted (multiplicative random effects)
MR RAPS
0.15
0.10
0.05
0.00
0.1
0.2
SNP effect on Crohn's disease
SNP effect on IDP T1 FAST ROIs R front operc cortex

Batch 2745 : IDP T1 FIRST left thalamus volume on Crohn's disease

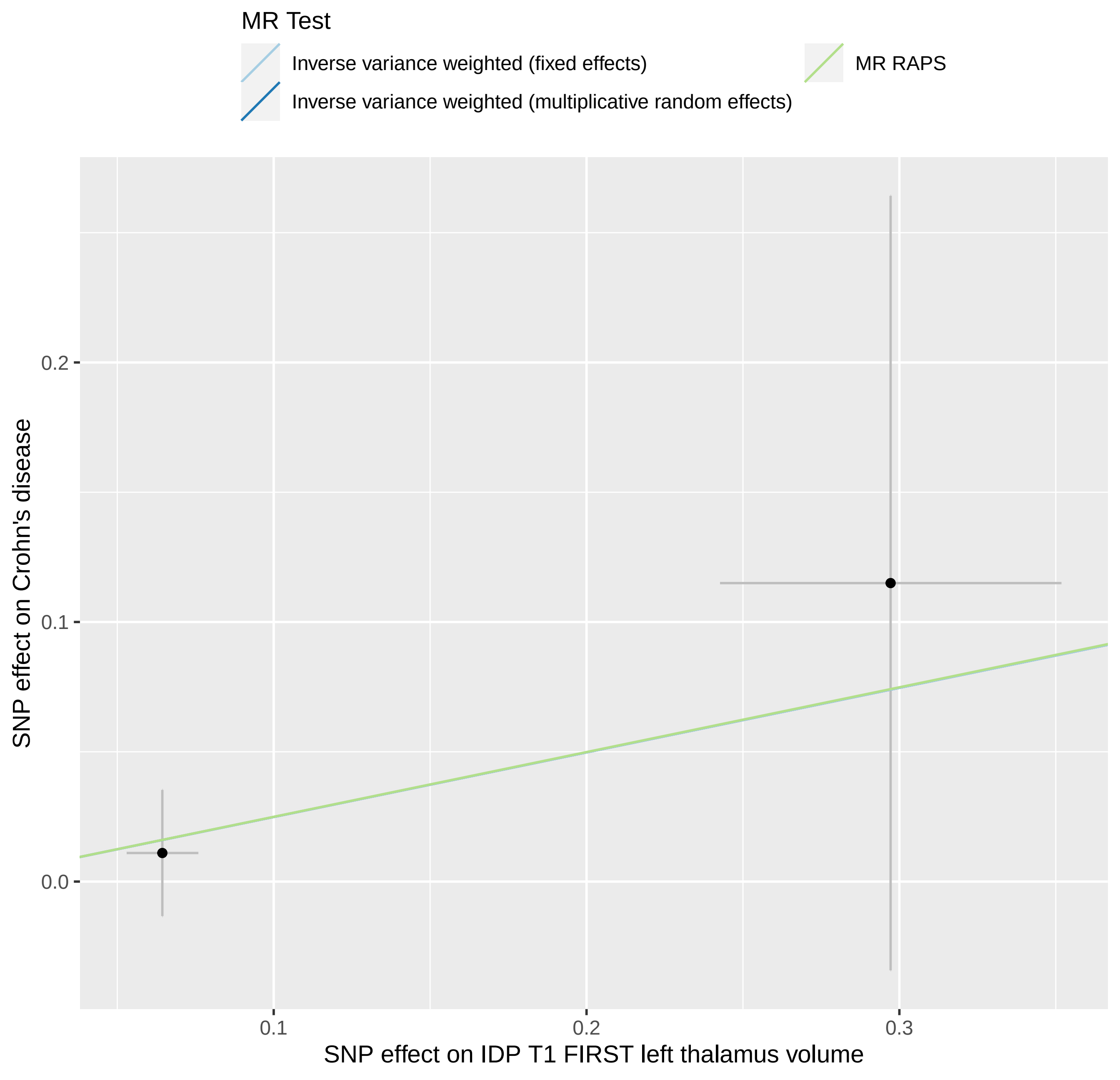

Batch 2746 : IDP T1 FAST ROIs L parietal operc cortex on Crohn's disease

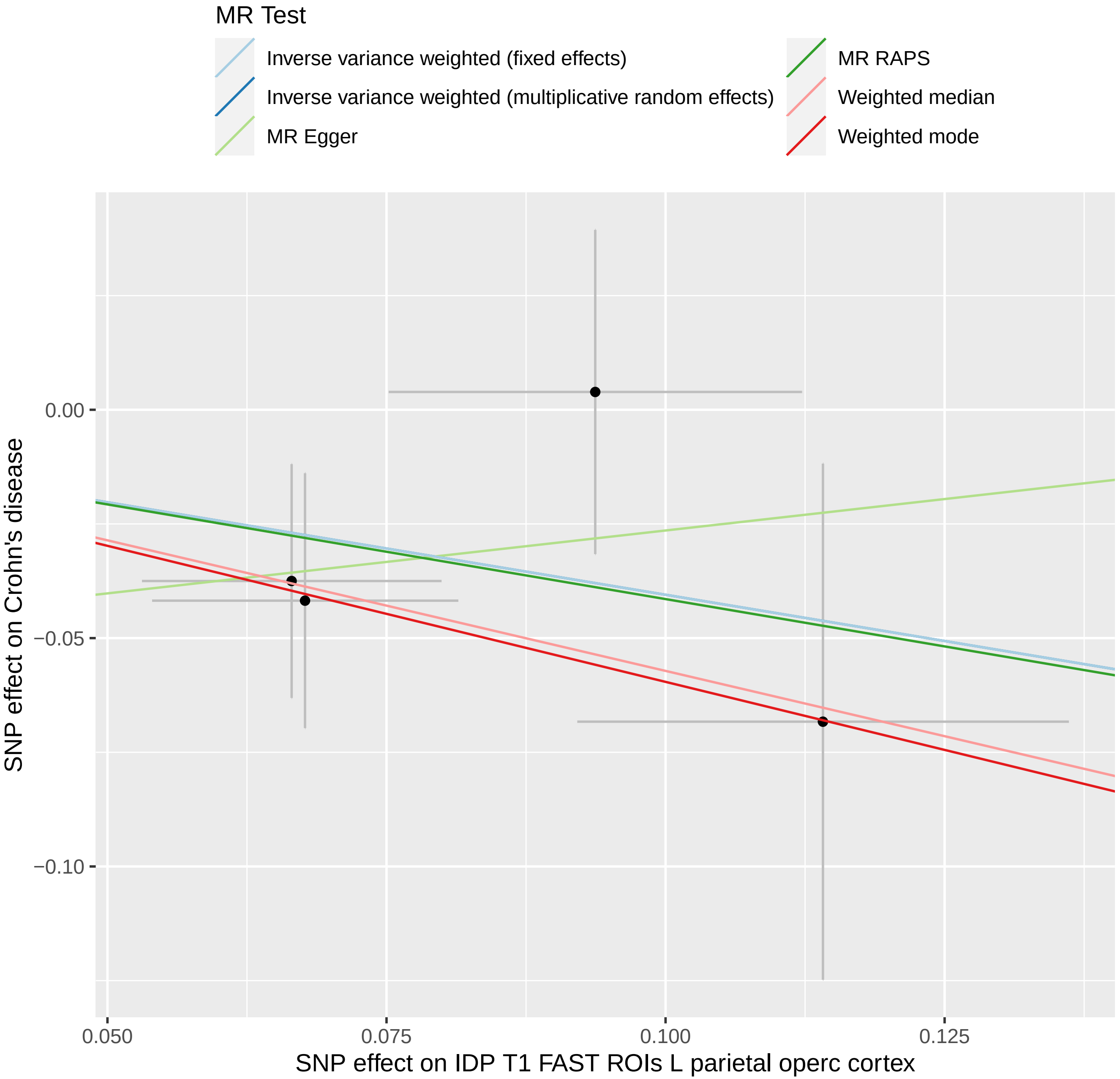

Batch 2747 : IDP T1 FAST ROIs R parietal operc cortex on Crohn's disease

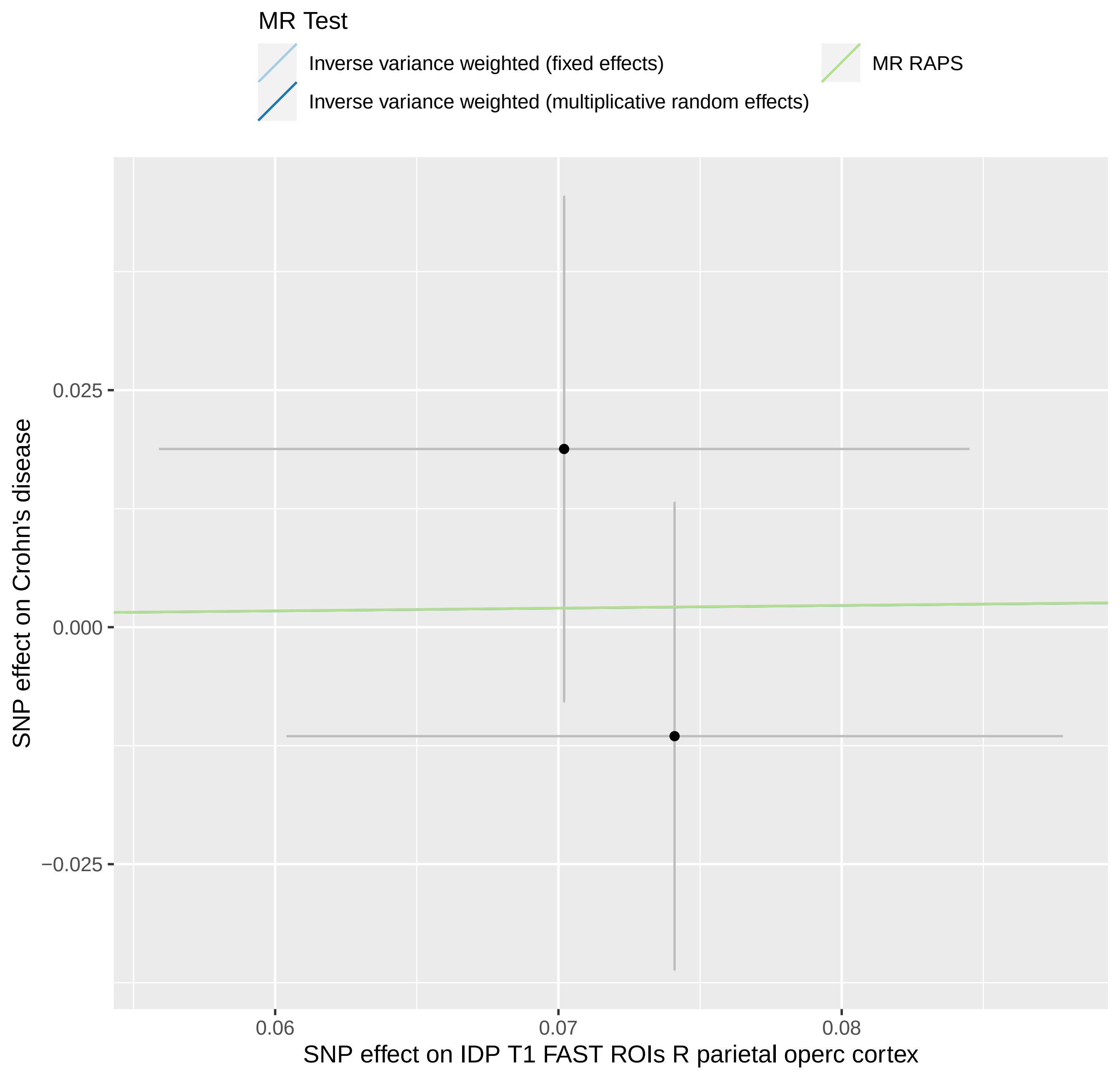

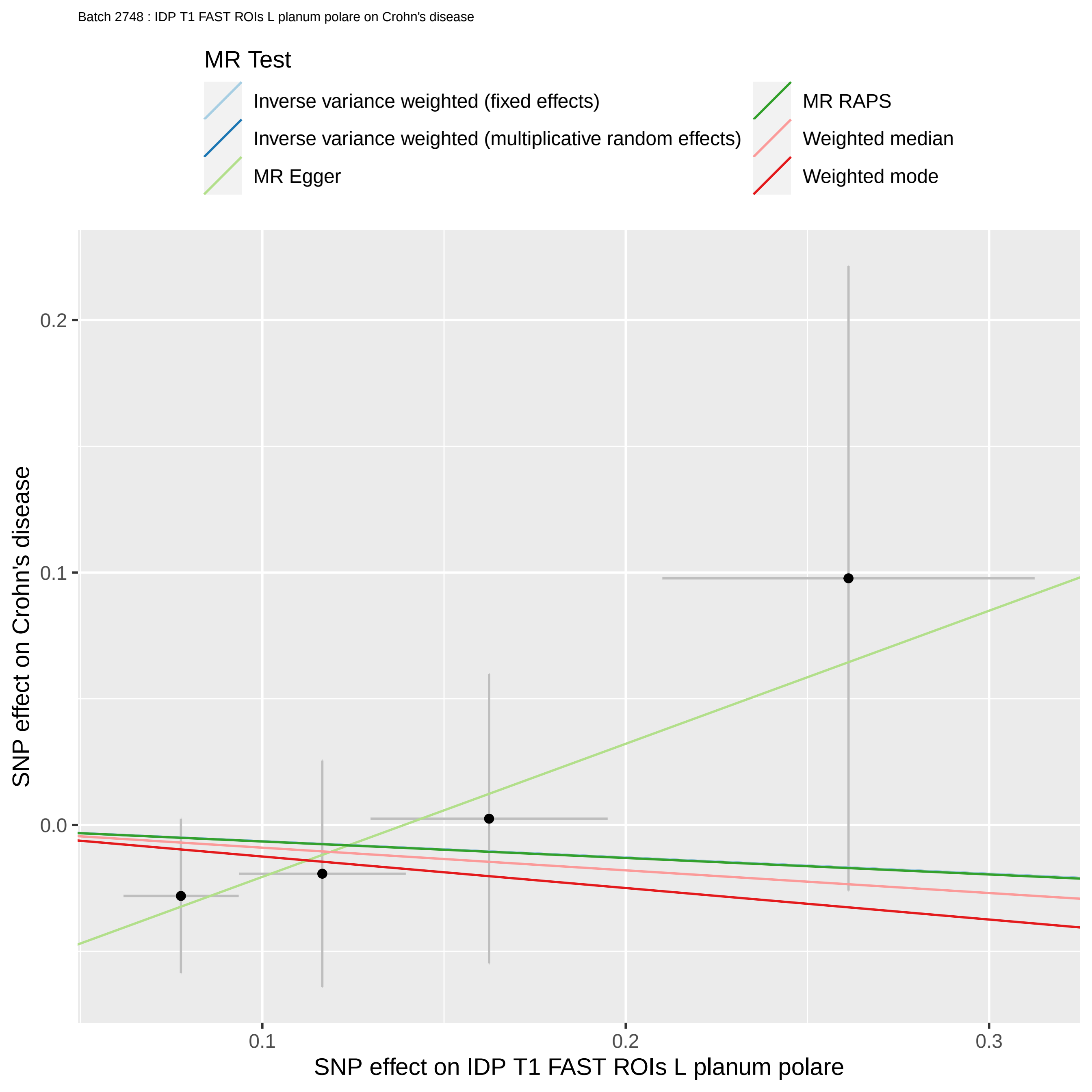
Batch 2748 : IDP T1 FAST ROIs L planum polare on Crohn's disease
MR Test
Inverse variance weighted (fixed effects)
Inverse variance weighted (multiplicative random effects)
MR Egger
MR RAPS
Weighted median
Weighted mode
0.2
0.1
0.0
0.1
0.2
0.3
SNP effect on Crohn's disease
SNP effect on IDP T1 FAST ROIs L planum polare

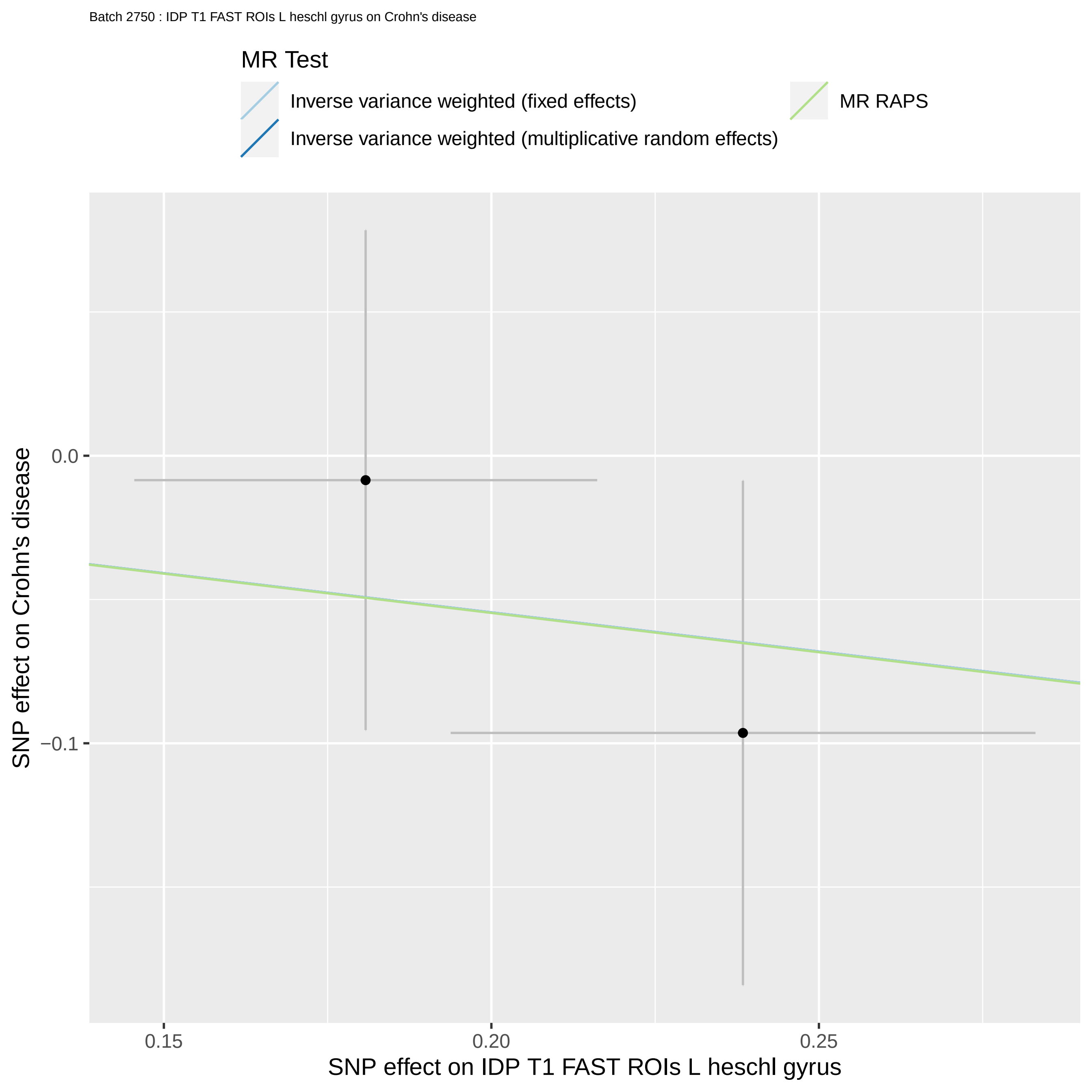
Batch 2750 : IDP T1 FAST ROIs L heschl gyrus on Crohn's disease
MR Test
Inverse variance weighted (fixed effects)
Inverse variance weighted (multiplicative random effects)
MR RAPS
0.0
−0.1
0.15
0.20
0.25
SNP effect on IDP T1 FAST ROIs L heschl gyrus
SNP effect on Crohn's disease

Batch 2751 : IDP T1 FAST ROIs R heschl gyrus on Crohn's disease

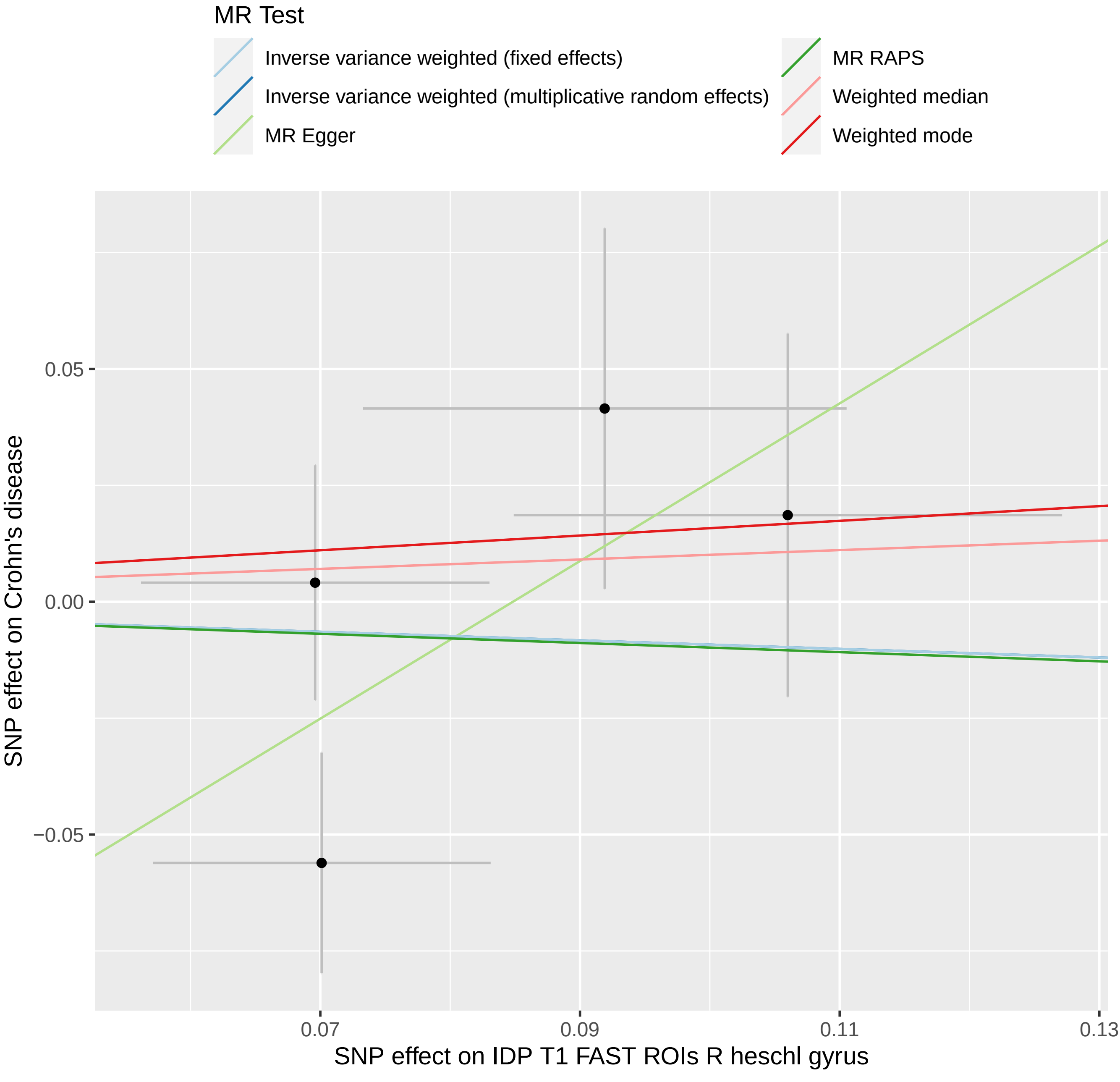

Batch 2753 : IDP T1 FAST ROIs R planum temporale on Crohn's disease

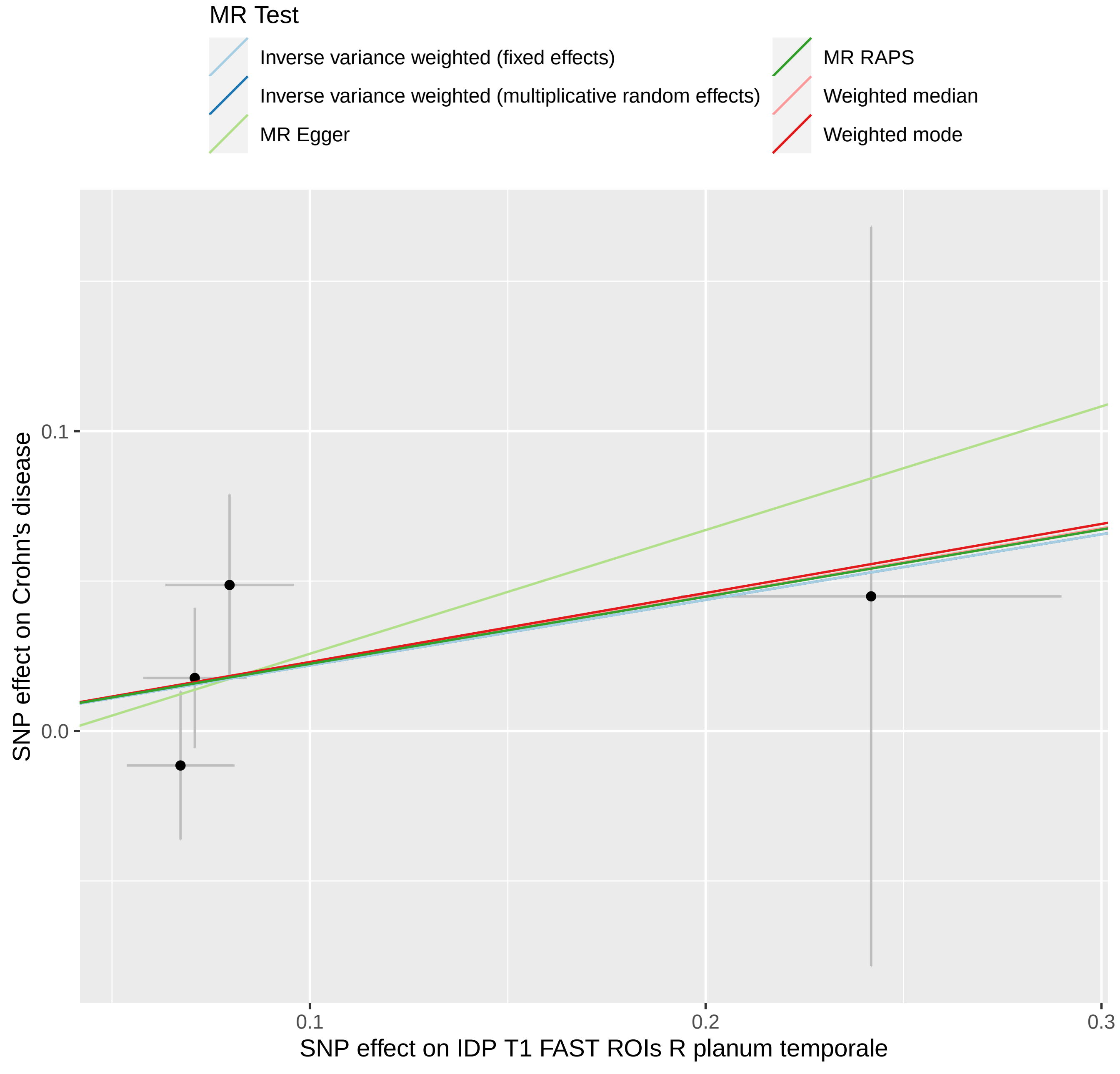

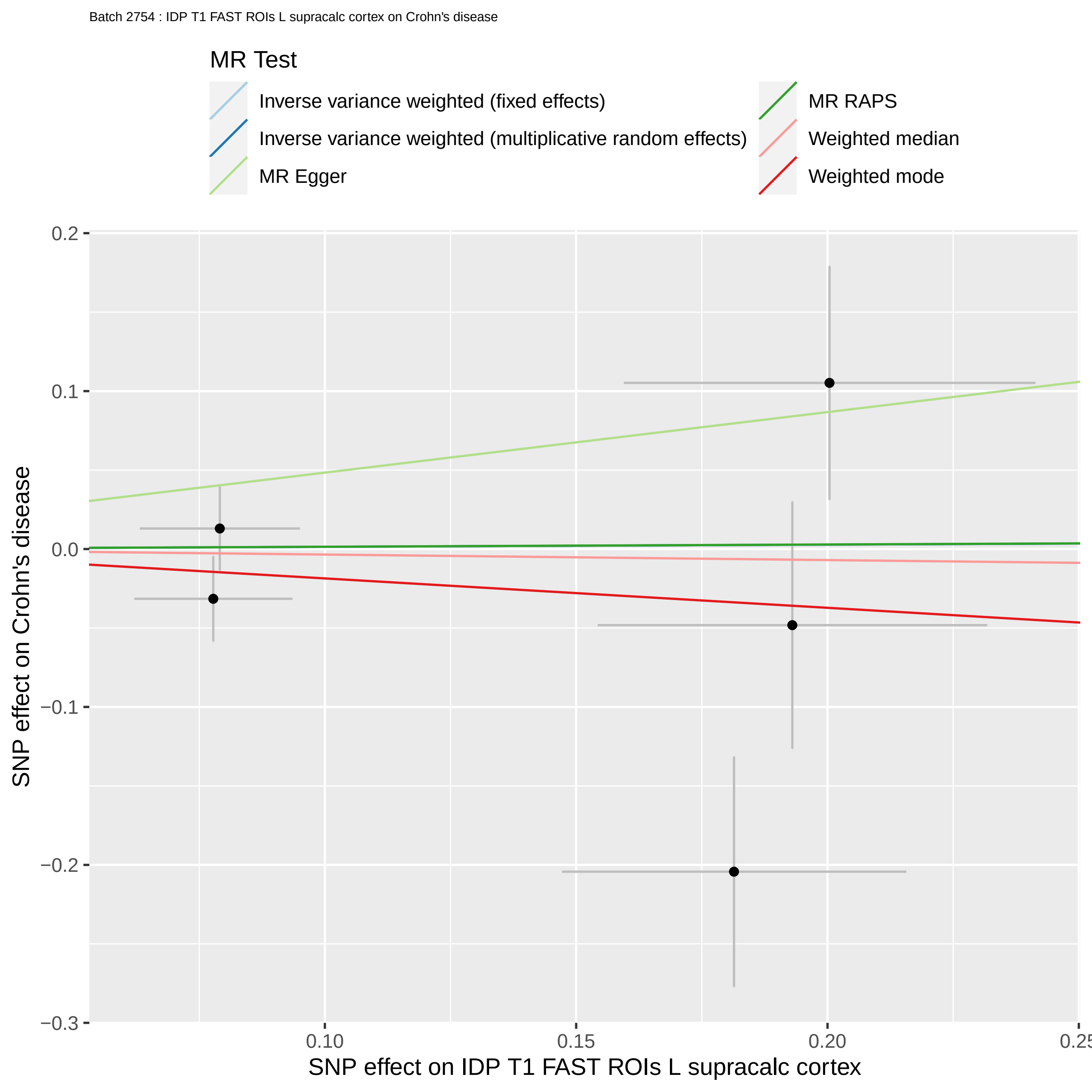

Batch 2754 : IDP T1 FAST ROIs L supracalc cortex on Crohn's disease
MR Test
Inverse variance weighted (fixed effects)
Inverse variance weighted (multiplicative random effects)
MR Egger
MR RAPS
Weighted median
Weighted mode
0.2
0.1
0.0
−0.1
−0.2
−0.3
0.10
0.15
0.20
0.25
SNP effect on Crohn's disease
SNP effect on IDP T1 FAST ROIs L supracalc cortex

Batch 2755 : IDP T1 FAST ROIs R supracalc cortex on Crohn's disease

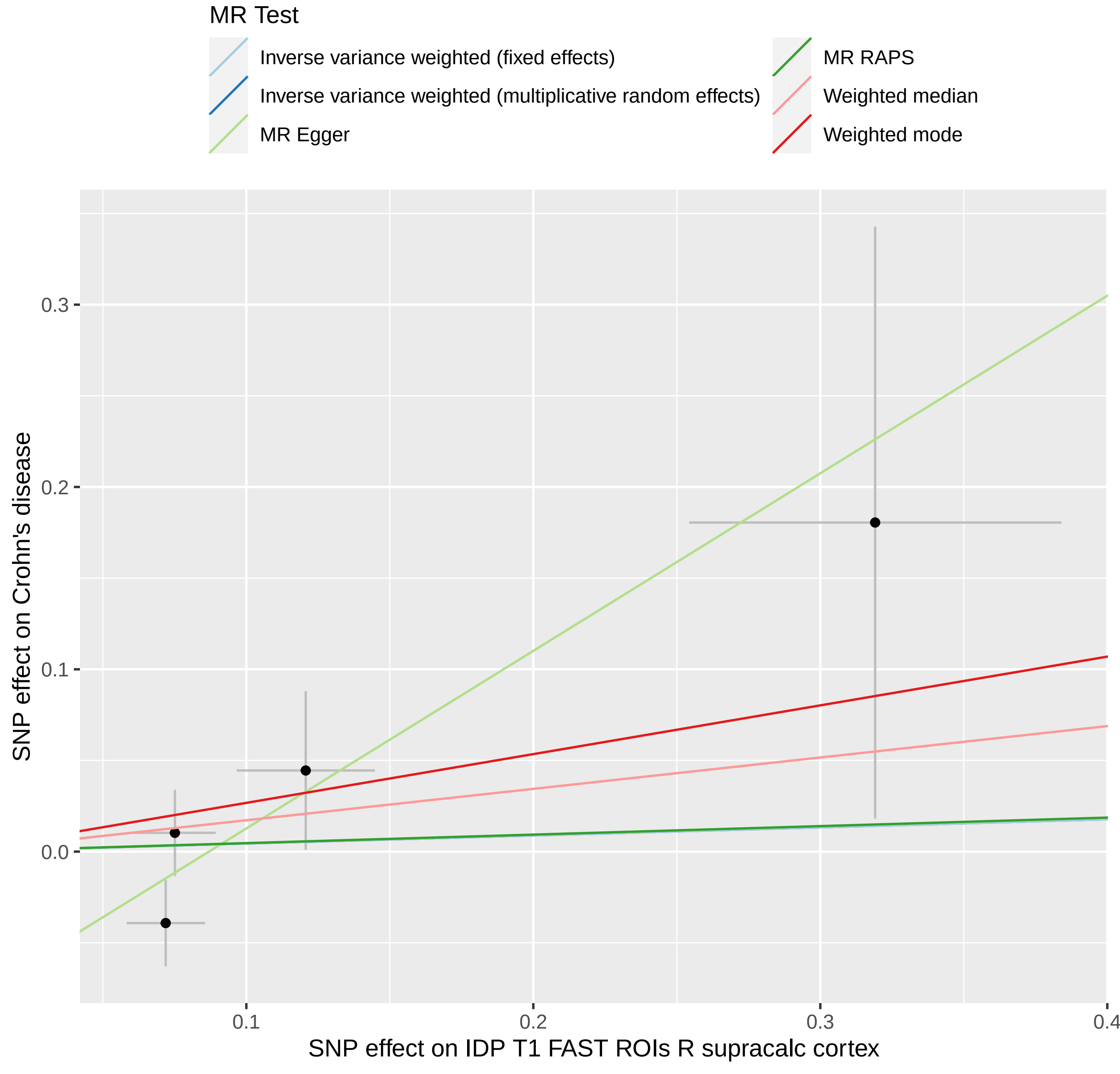

Batch 2757 : IDP T1 FAST ROIs L occ pole on Crohn's disease

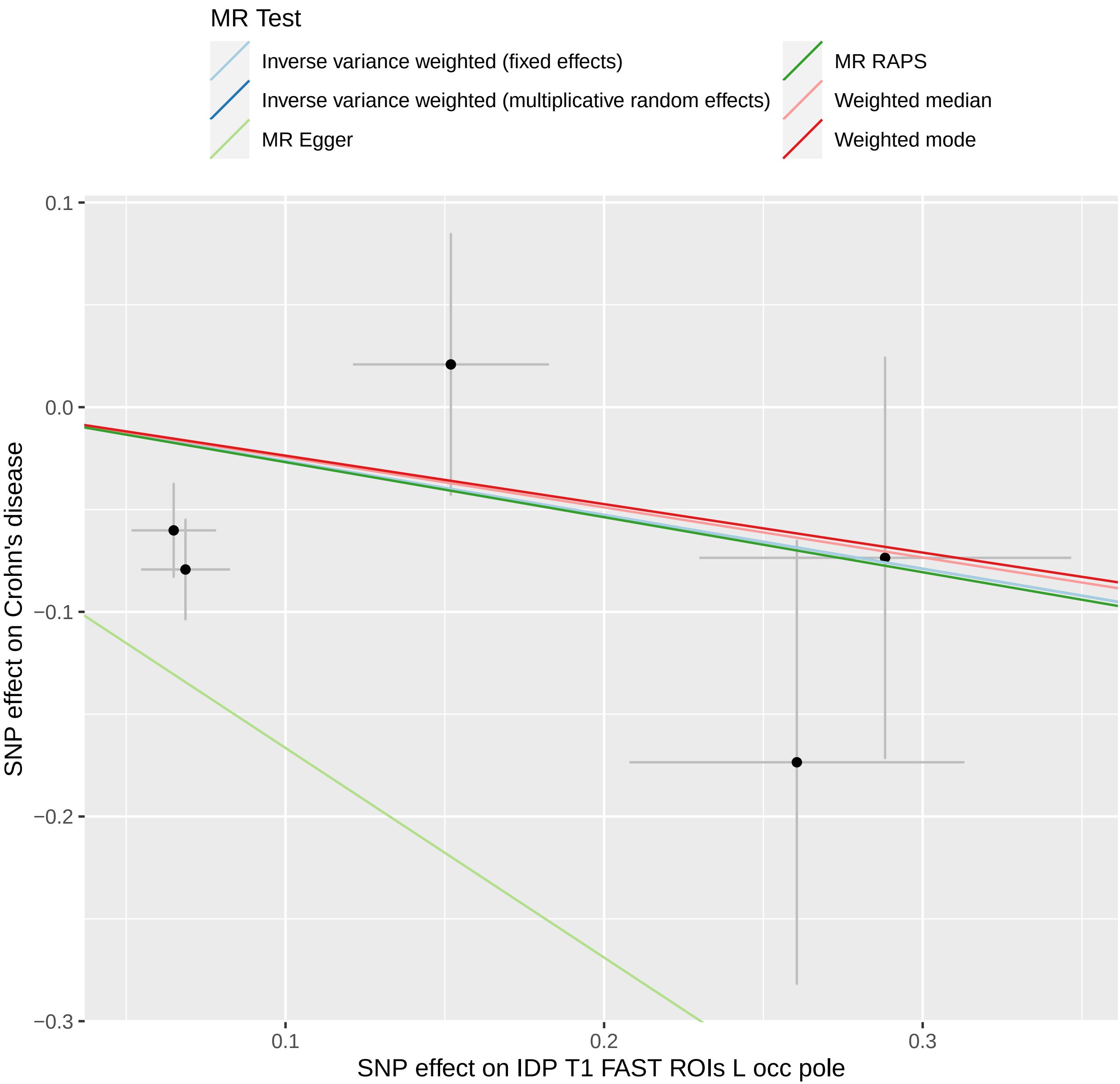

Batch 2759 : IDP T1 FAST ROIs L thalamus on Crohn's disease

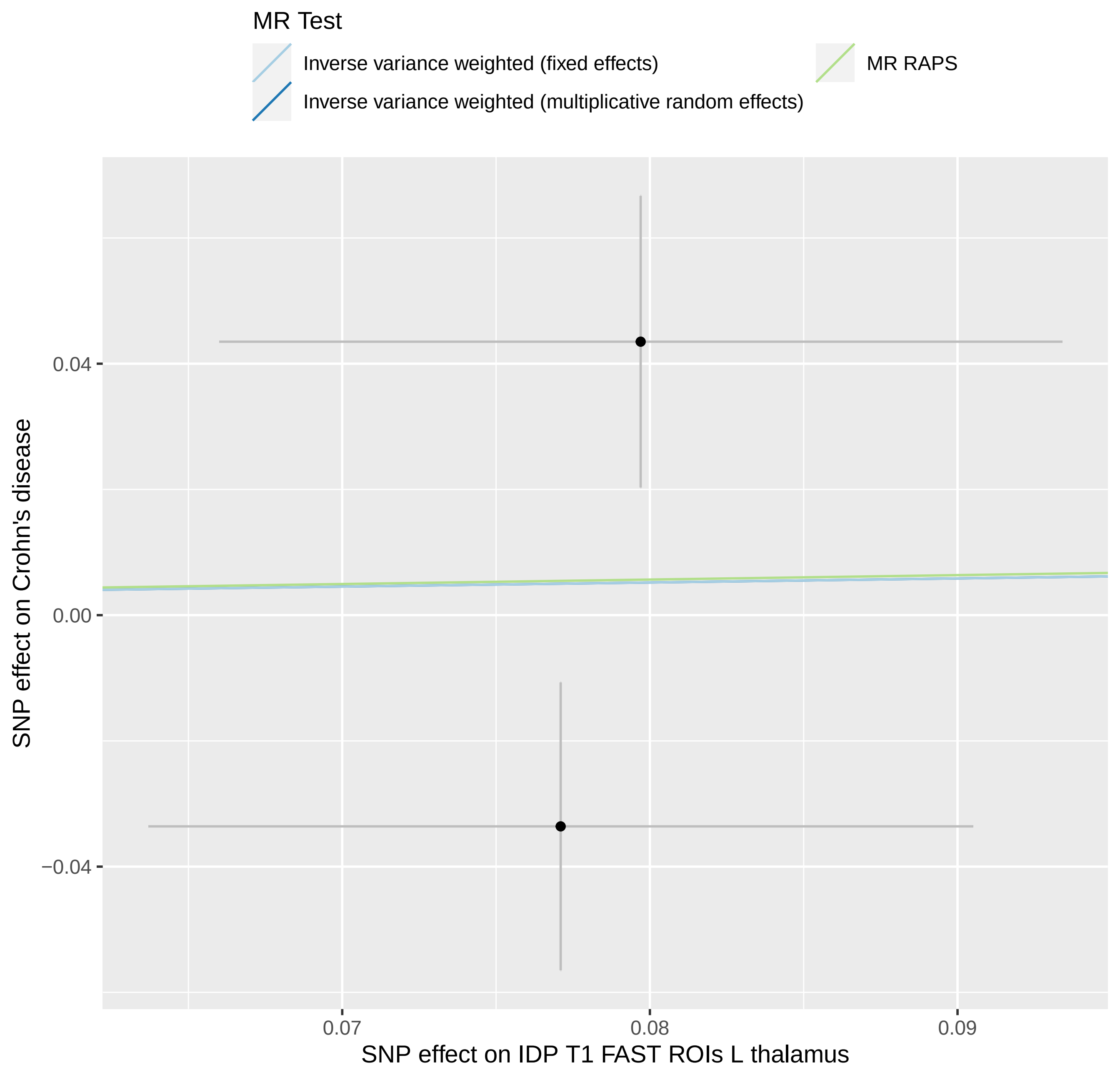

Batch 2766 : IDP T1 FAST ROIs R pallidum on Crohn's disease

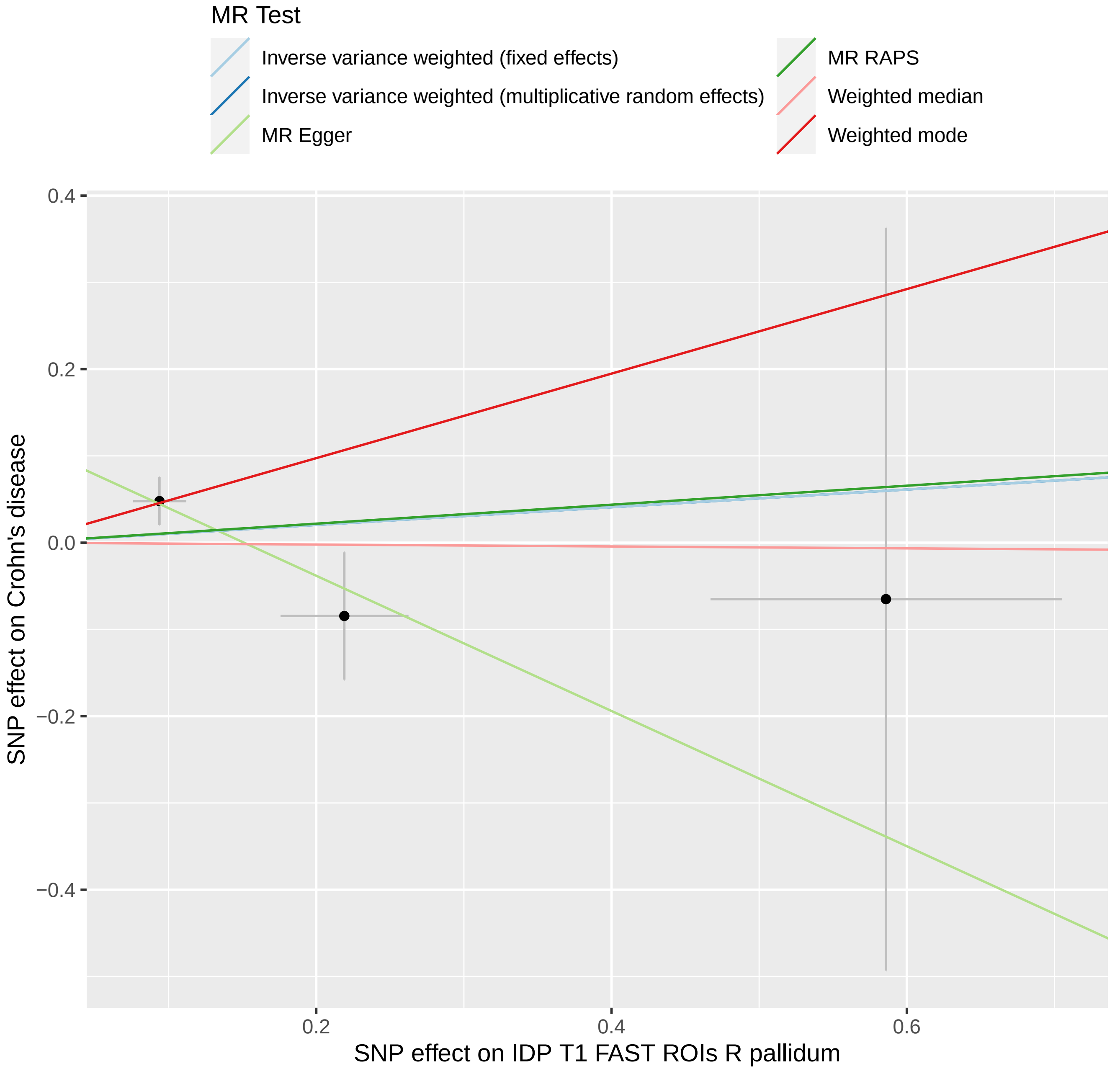

Batch 2768 : IDP T1 FAST ROIs L hippocampus on Crohn's disease

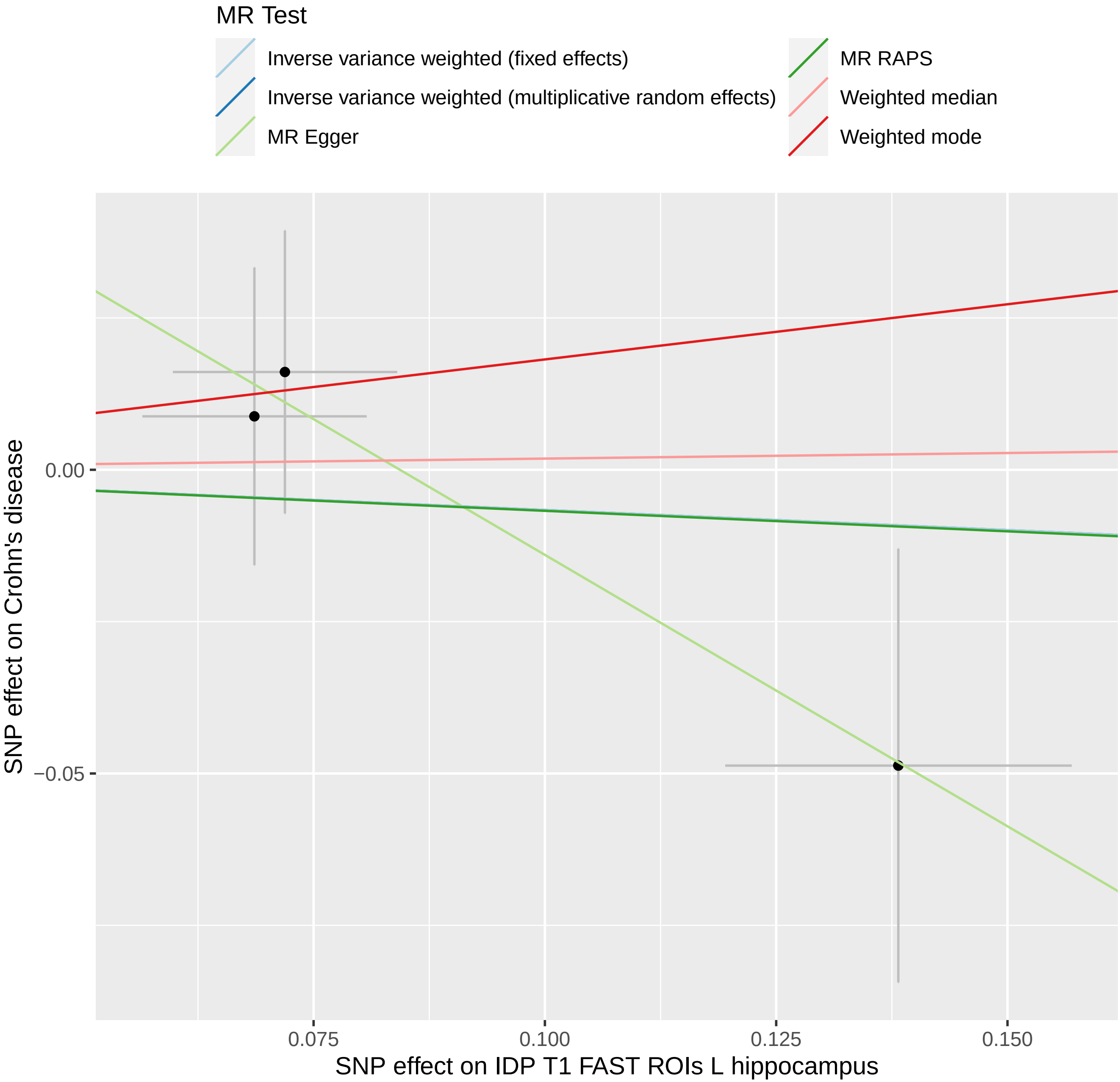

Batch 2769 : IDP T1 FAST ROIs R hippocampus on Crohn's disease

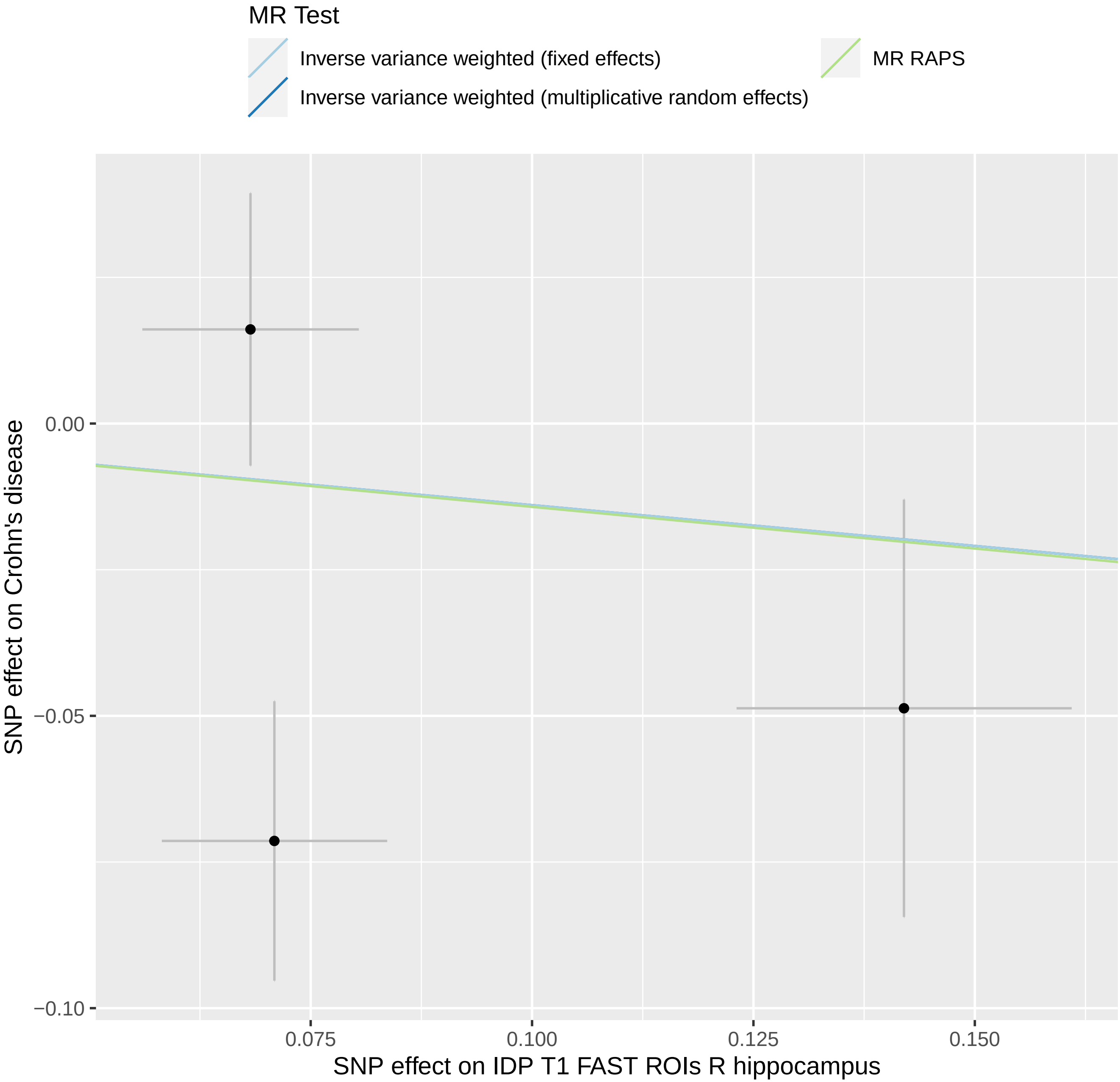

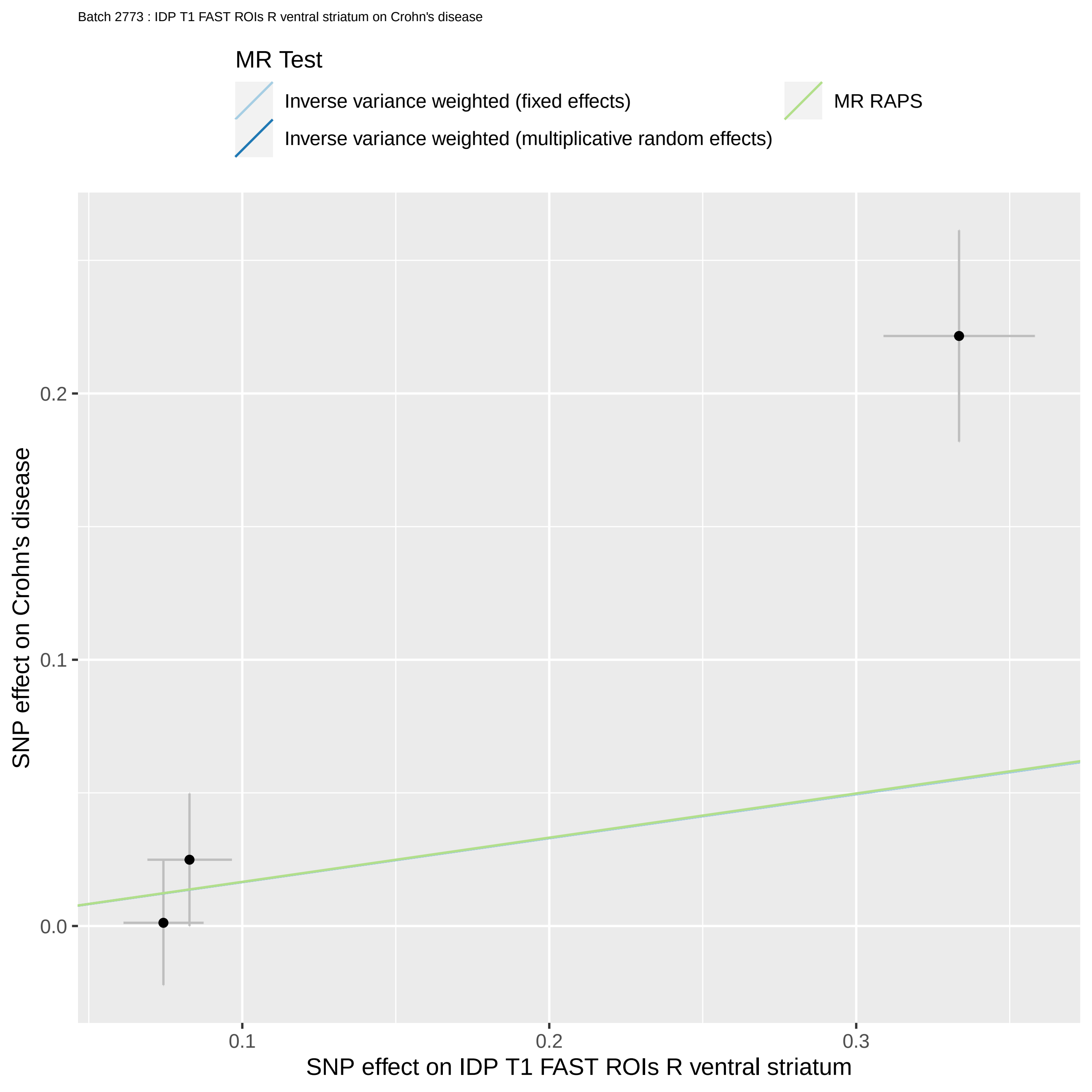
Batch 2773 : IDP T1 FAST ROIs R ventral striatum on Crohn's disease
MR Test
Inverse variance weighted (fixed effects)
Inverse variance weighted (multiplicative random effects)
MR RAPS
SNP effect on Crohn's disease
0.0
0.1
0.2
0.1
0.2
0.3
SNP effect on IDP T1 FAST ROIs R ventral striatum

Batch 2775 : IDP T1 FAST ROIs L cerebellum I−IV on Crohn's disease

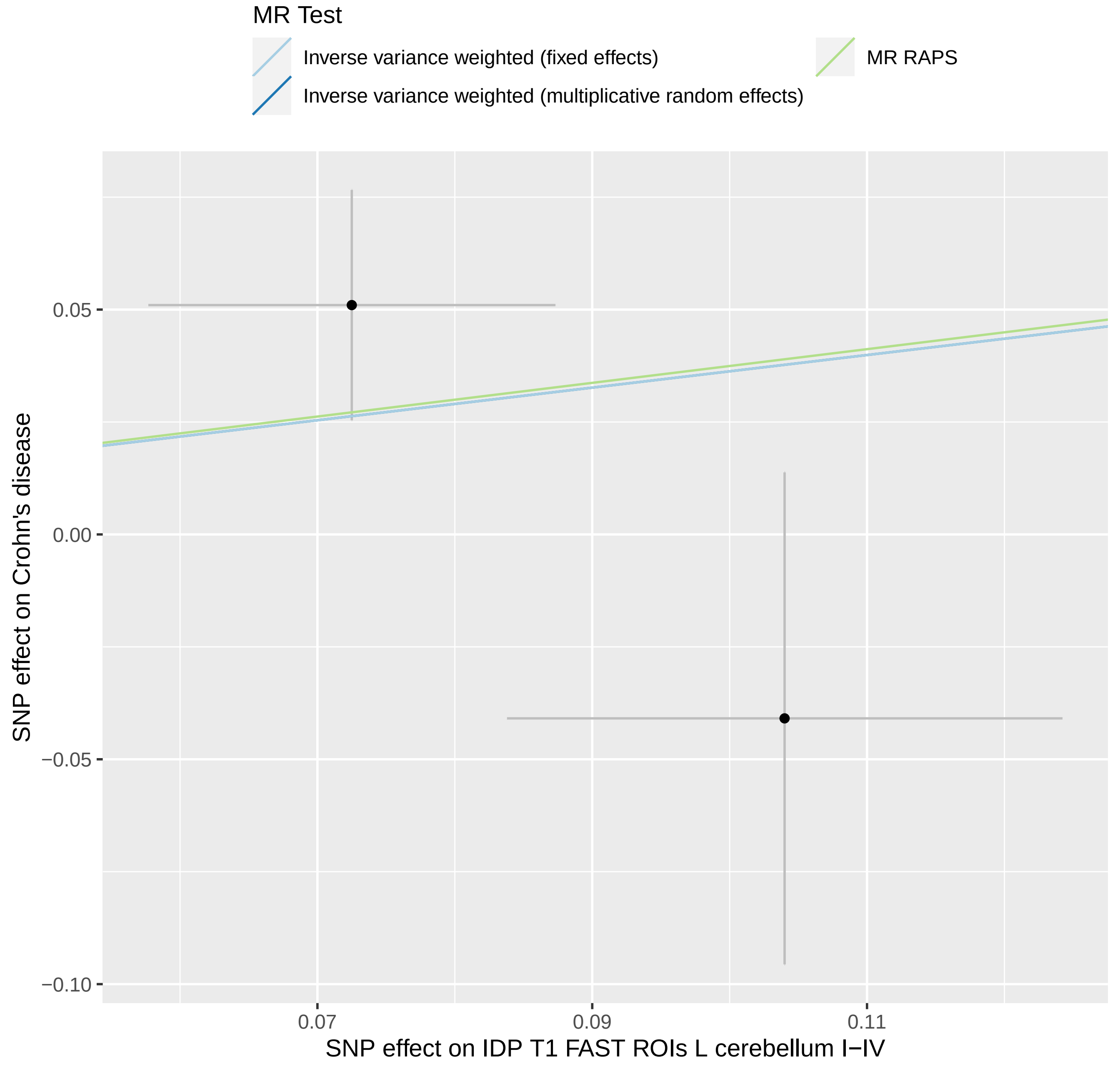

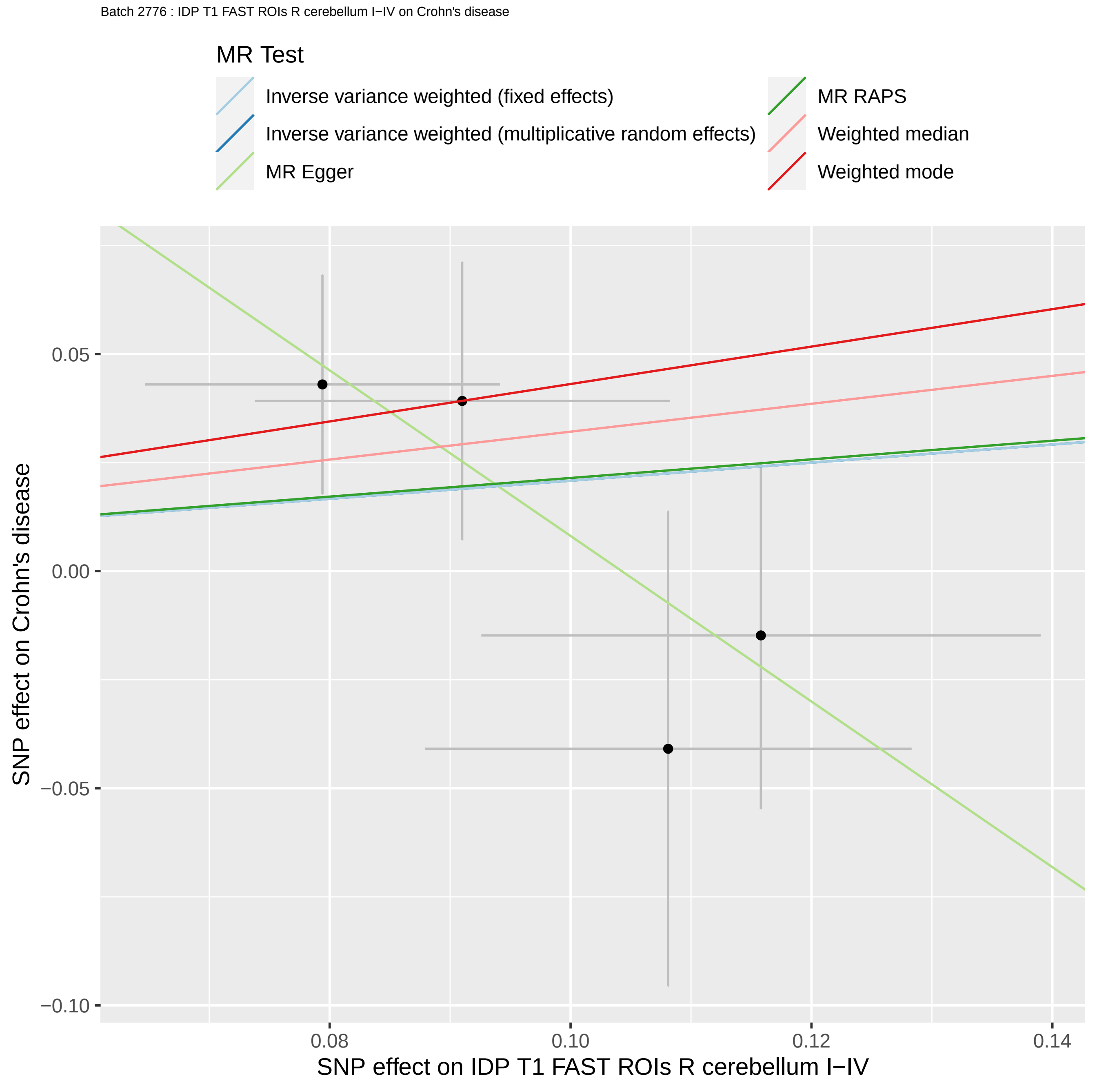

Batch 2776 : IDP T1 FAST ROIs R cerebellum I−IV on Crohn's disease
MR Test
Inverse variance weighted (fixed effects)
Inverse variance weighted (multiplicative random effects)
MR Egger
MR RAPS
Weighted median
Weighted mode
0.05
0.00
−0.05
−0.10
0.08
0.10
0.12
0.14
SNP effect on Crohn's disease
SNP effect on IDP T1 FAST ROIs R cerebellum I−IV

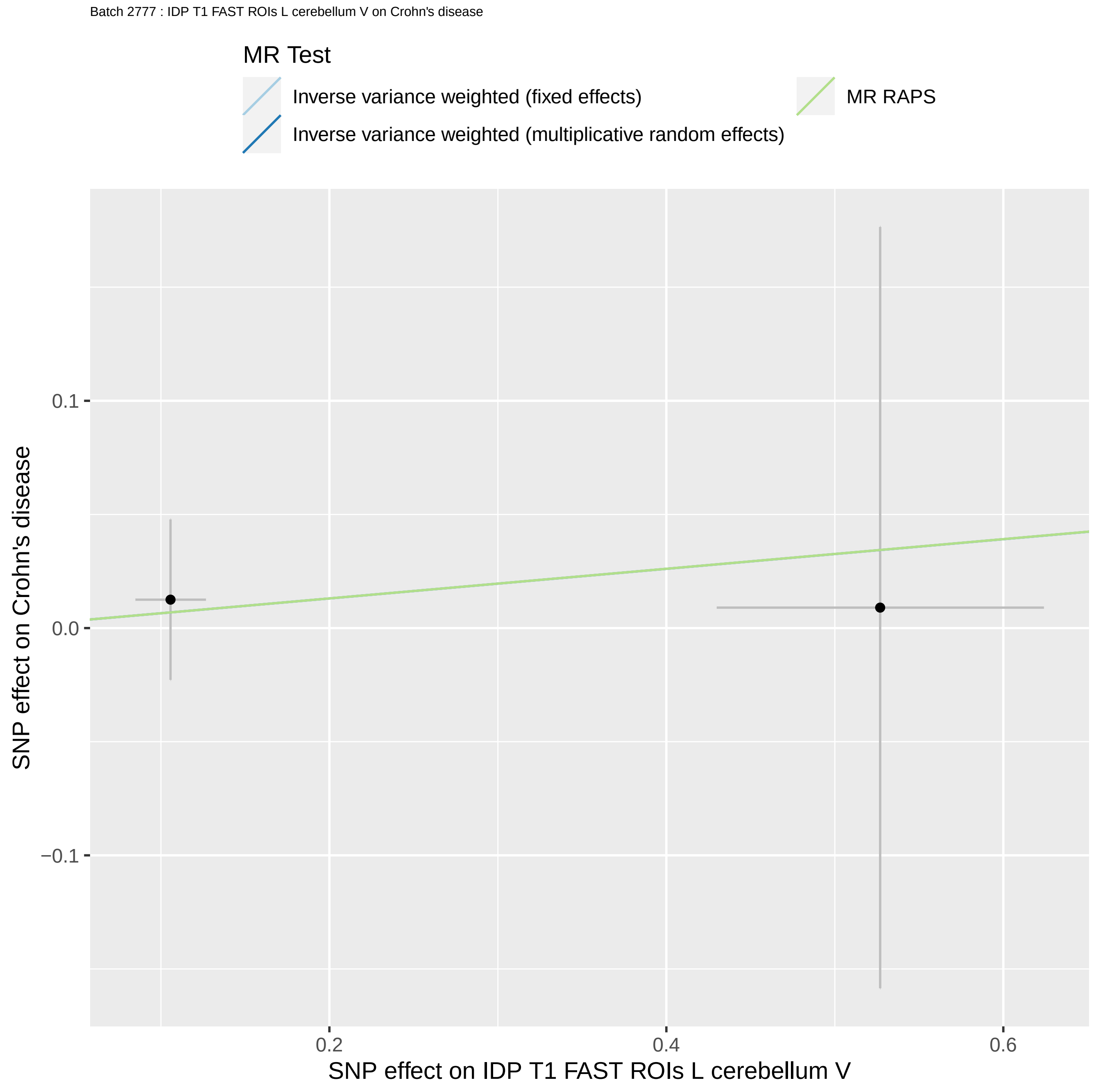
Batch 2777 : IDP T1 FAST ROIs L cerebellum V on Crohn's disease
MR Test
Inverse variance weighted (fixed effects)
Inverse variance weighted (multiplicative random effects)
MR RAPS
0.1
0.0
−0.1
0.2
0.4
0.6
SNP effect on Crohn's disease
SNP effect on IDP T1 FAST ROIs L cerebellum V

Batch 2779 : IDP T1 FAST ROIs R cerebellum V on Crohn's disease

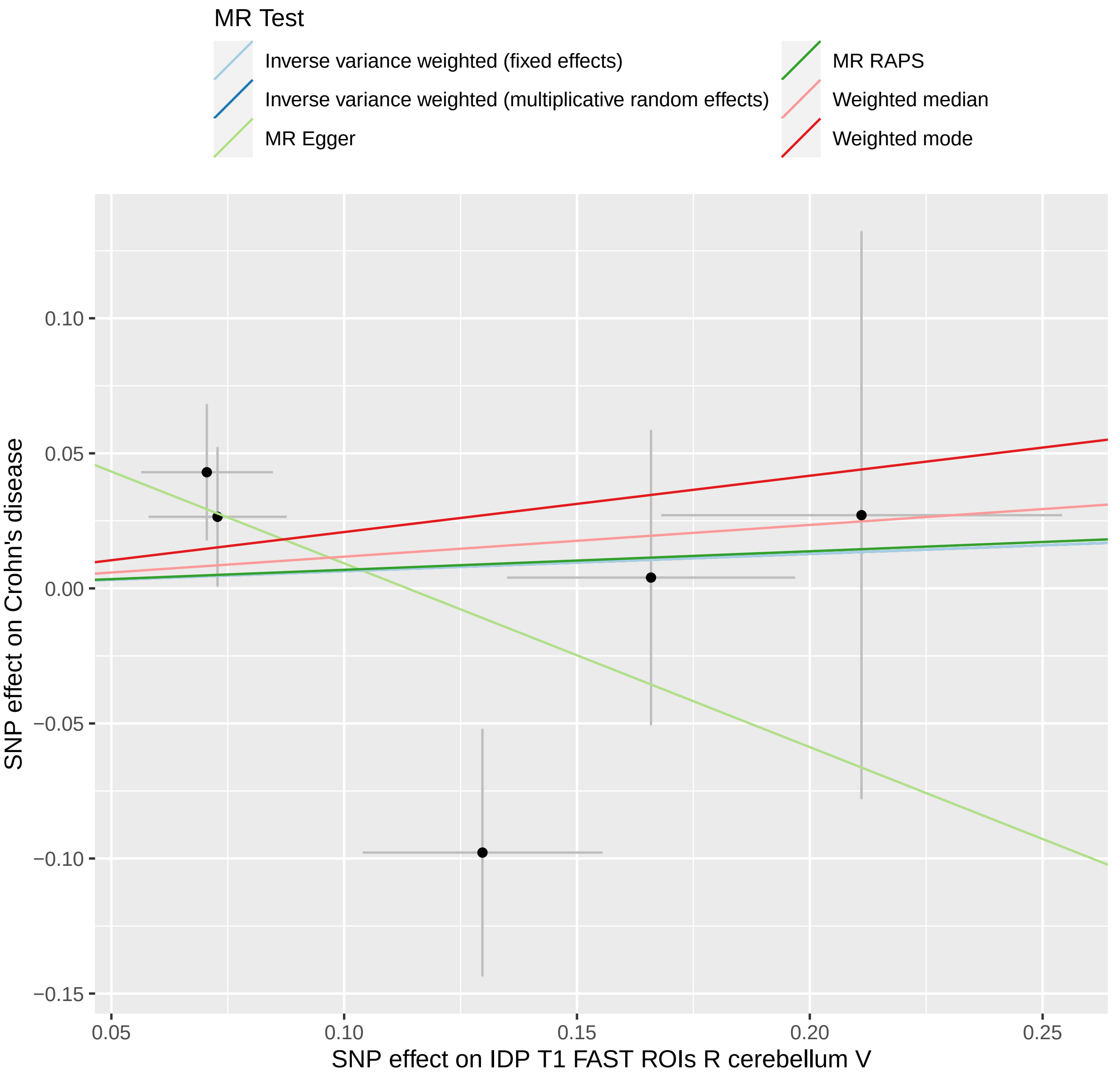

Batch 2781 : IDP T1 FAST ROIs V cerebellum VI on Crohn's disease

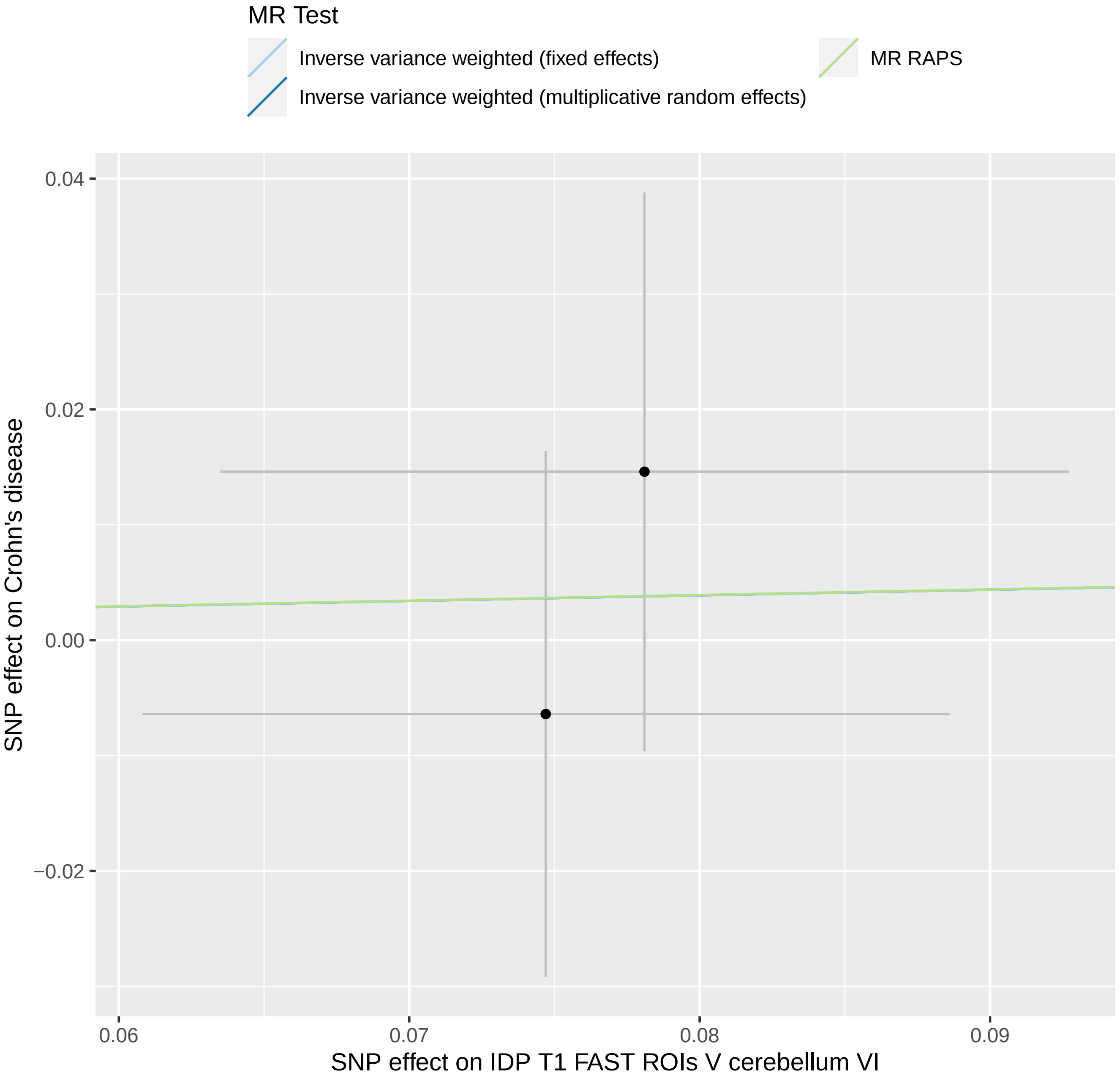

Batch 2783 : IDP T1 FAST ROIs L cerebellum crus I on Crohn's disease

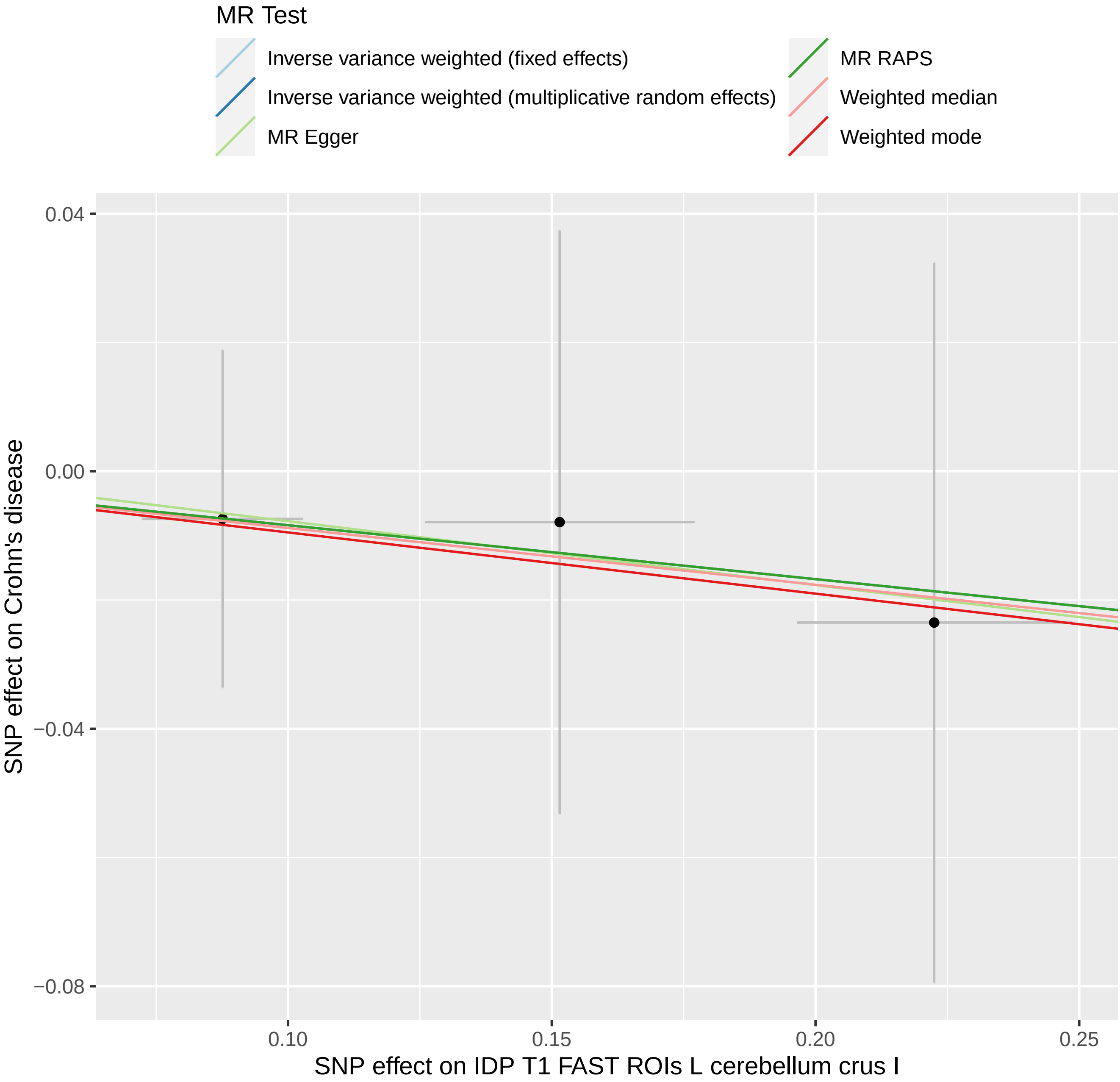

Batch 2785 : IDP T1 FAST ROIs R cerebellum crus I on Crohn's disease

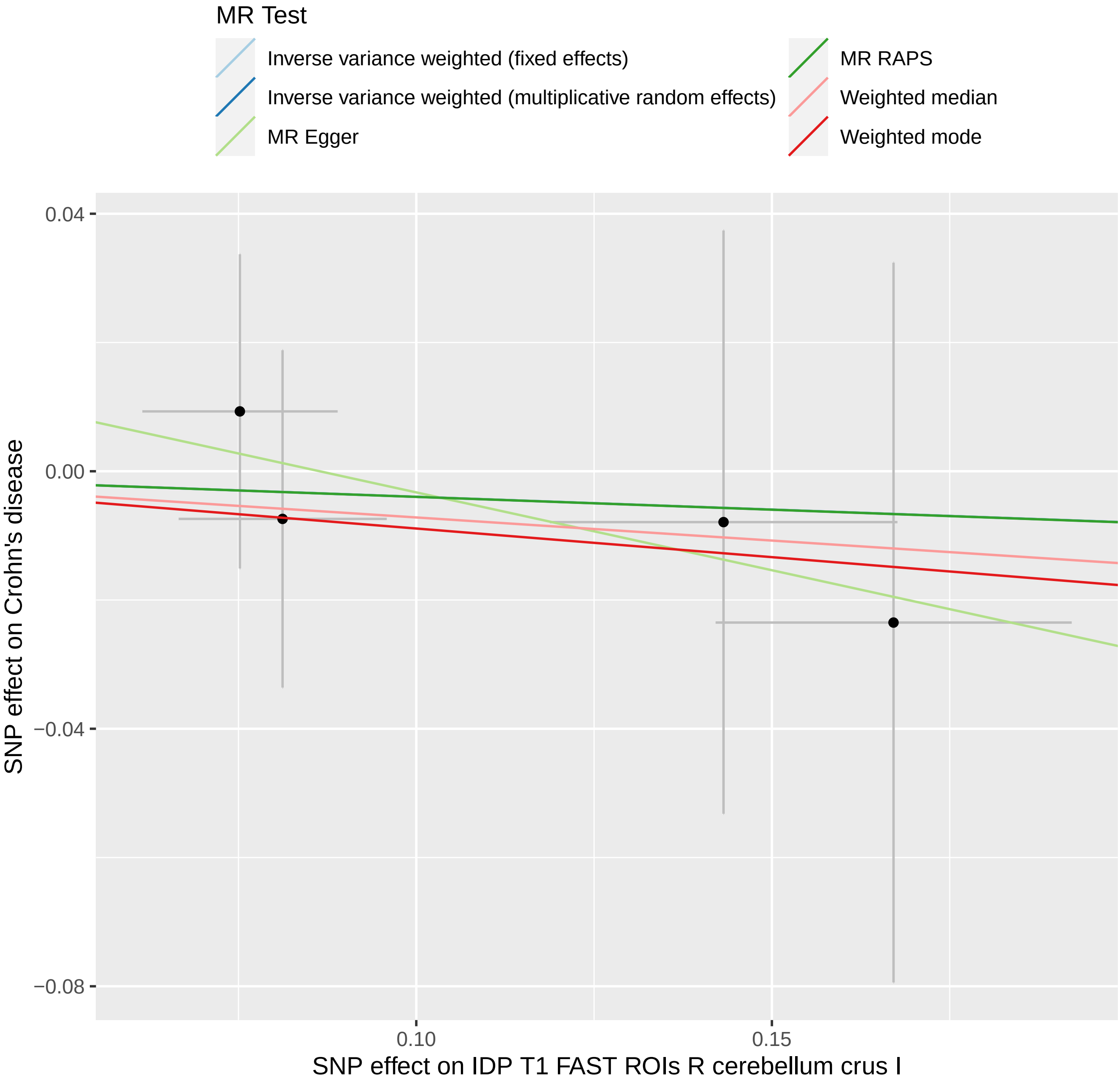

Batch 2786 : IDP T1 FAST ROIs L cerebellum crus II on Crohn's disease

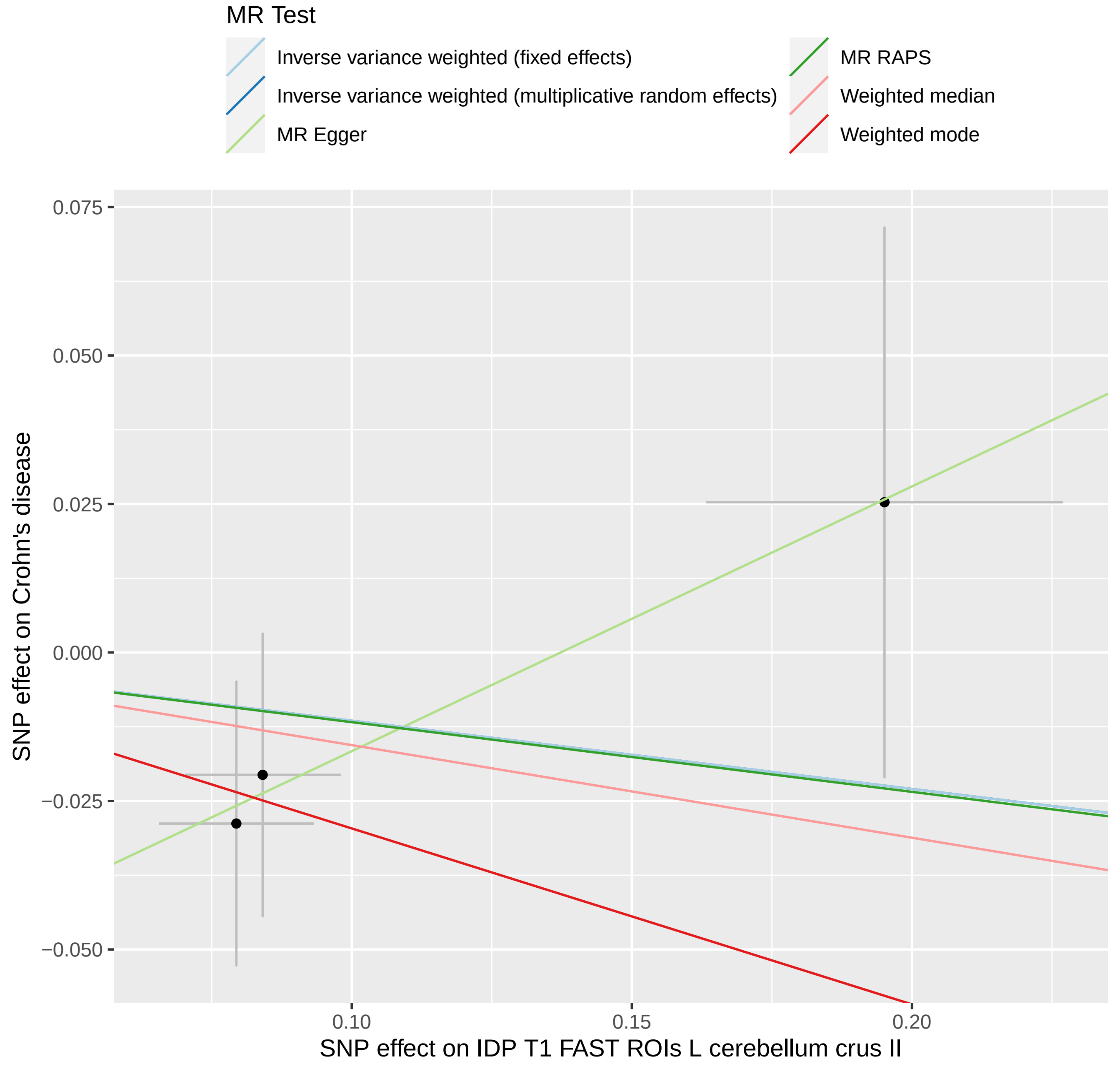

Batch 2787 : IDP T1 FAST ROIs V cerebellum crus II on Crohn's disease

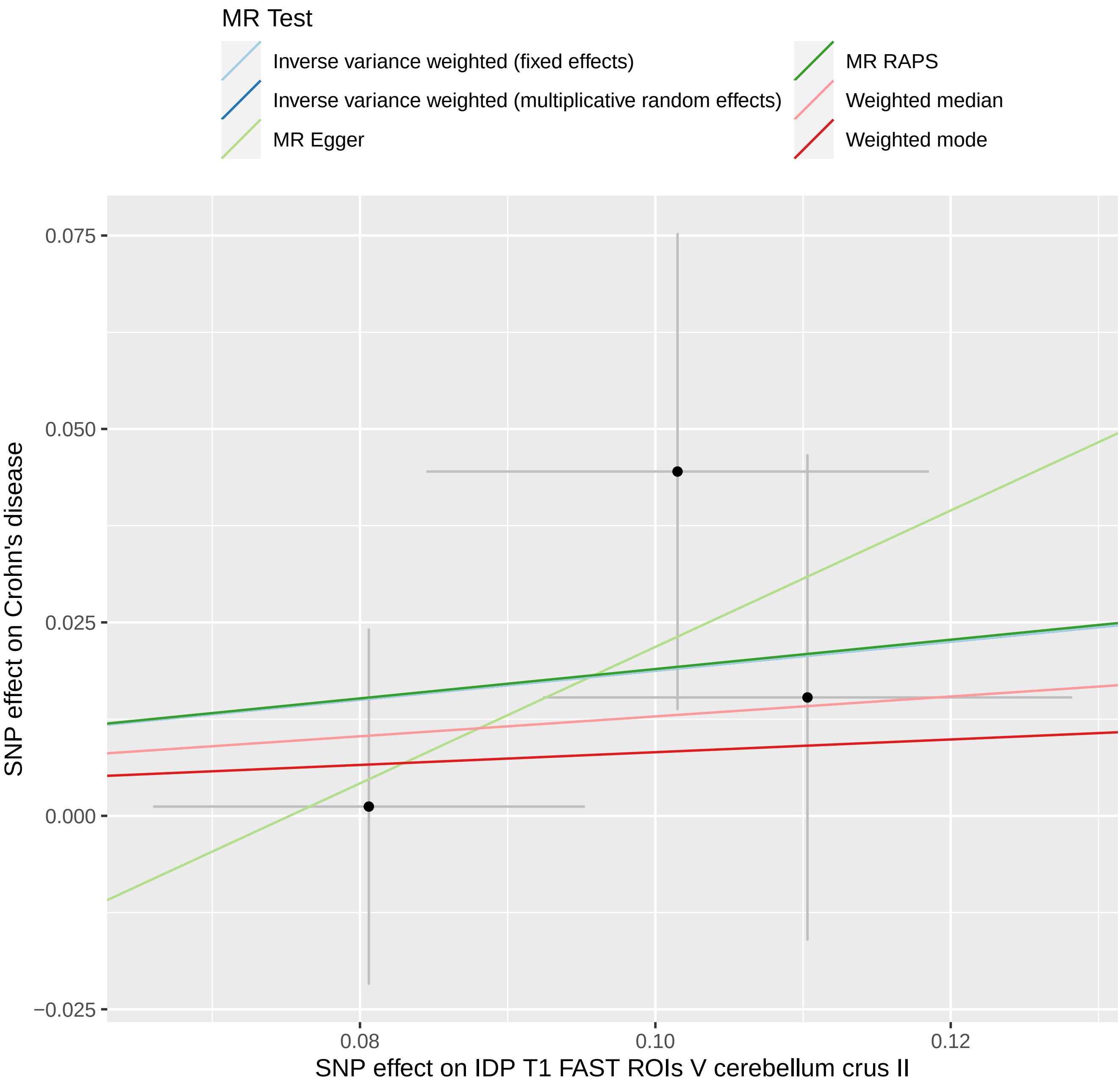

Batch 2788 : IDP T1 FAST ROIs R cerebellum crus II on Crohn's disease

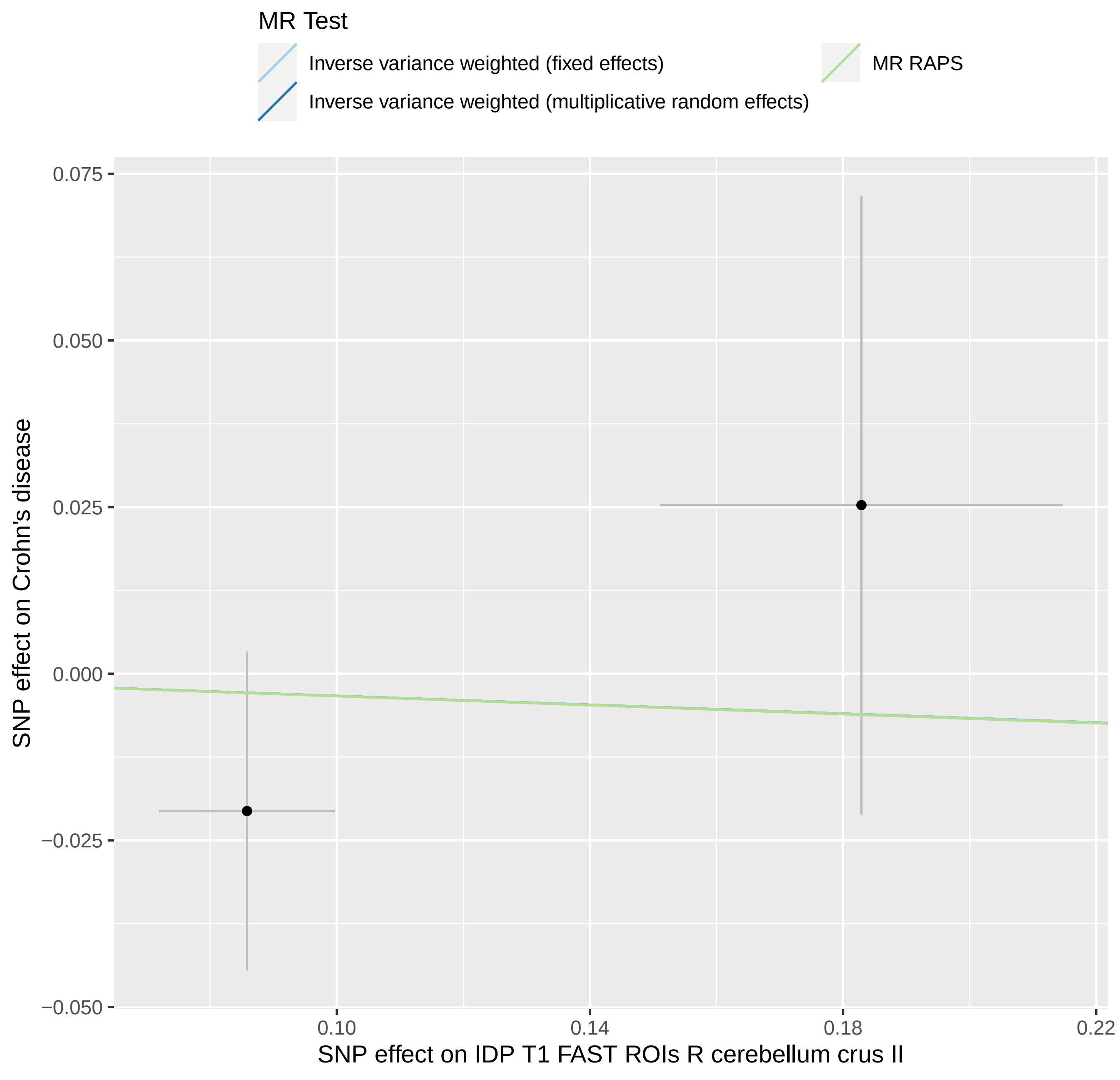

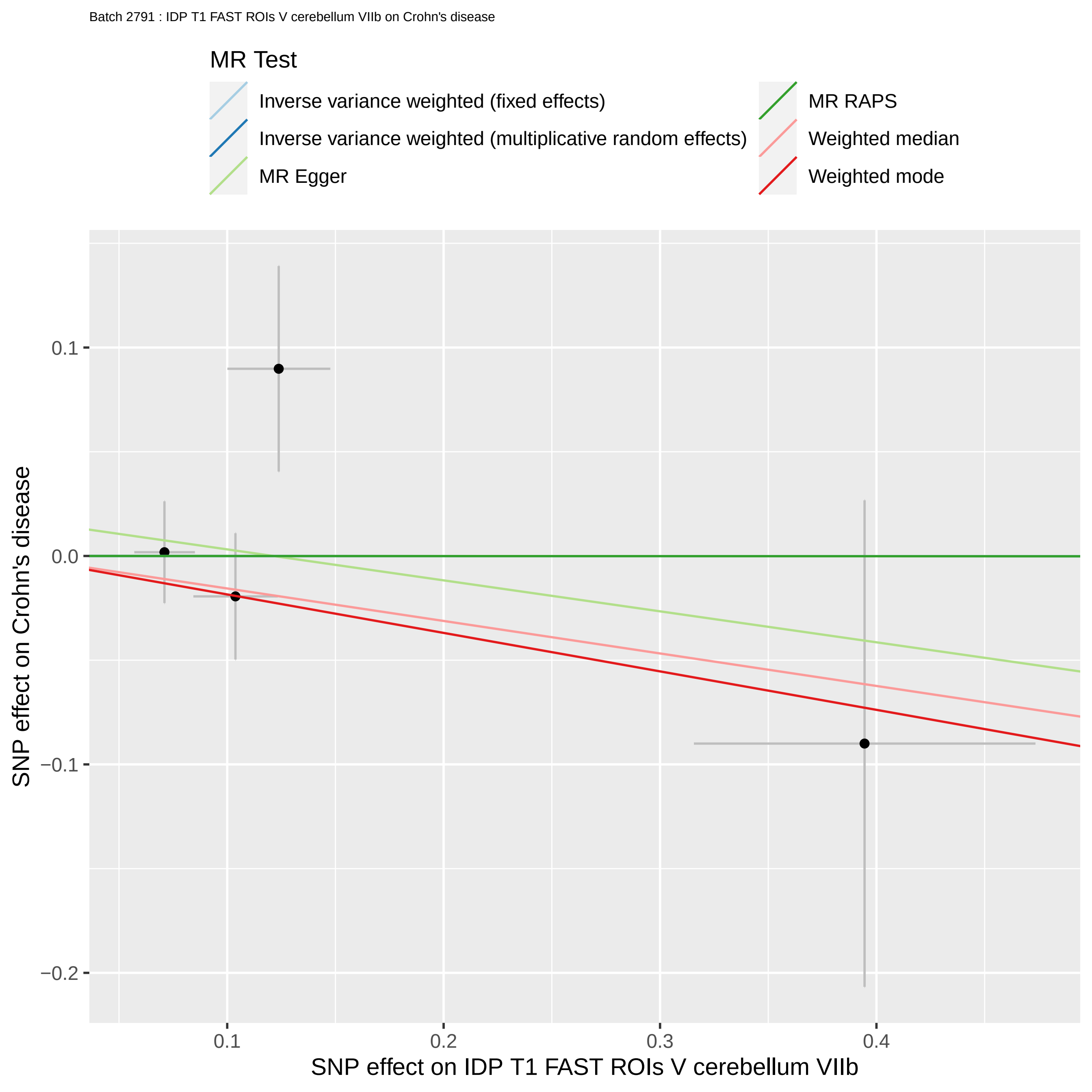
Batch 2791 : IDP T1 FAST ROIs V cerebellum VIIb on Crohn's disease
MR Test
Inverse variance weighted (fixed effects)
Inverse variance weighted (multiplicative random effects)
MR Egger
MR RAPS
Weighted median
Weighted mode
SNP effect on Crohn's disease
0.1
0.0
−0.1
−0.2
0.1
0.2
0.3
0.4
SNP effect on IDP T1 FAST ROIs V cerebellum VIIb

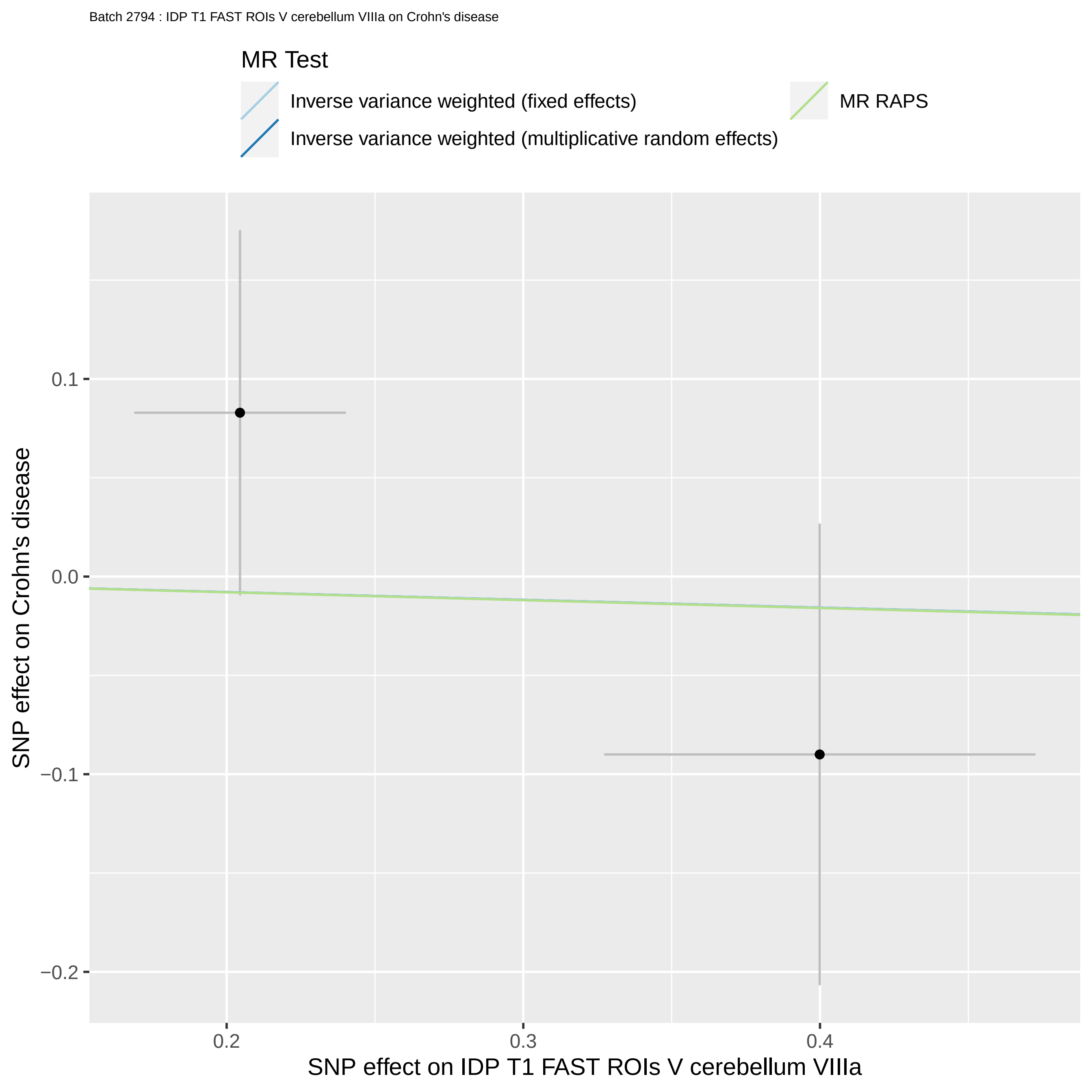
Batch 2794 : IDP T1 FAST ROIs V cerebellum VIIIa on Crohn's disease
MR Test
Inverse variance weighted (fixed effects)
Inverse variance weighted (multiplicative random effects)
MR RAPS
0.1
0.0
−0.1
−0.2
0.2
0.3
0.4
SNP effect on Crohn's disease
SNP effect on IDP T1 FAST ROIs V cerebellum VIIIa

Batch 2803 : IDP T1 FAST ROIs L cerebellum X on Crohn's disease

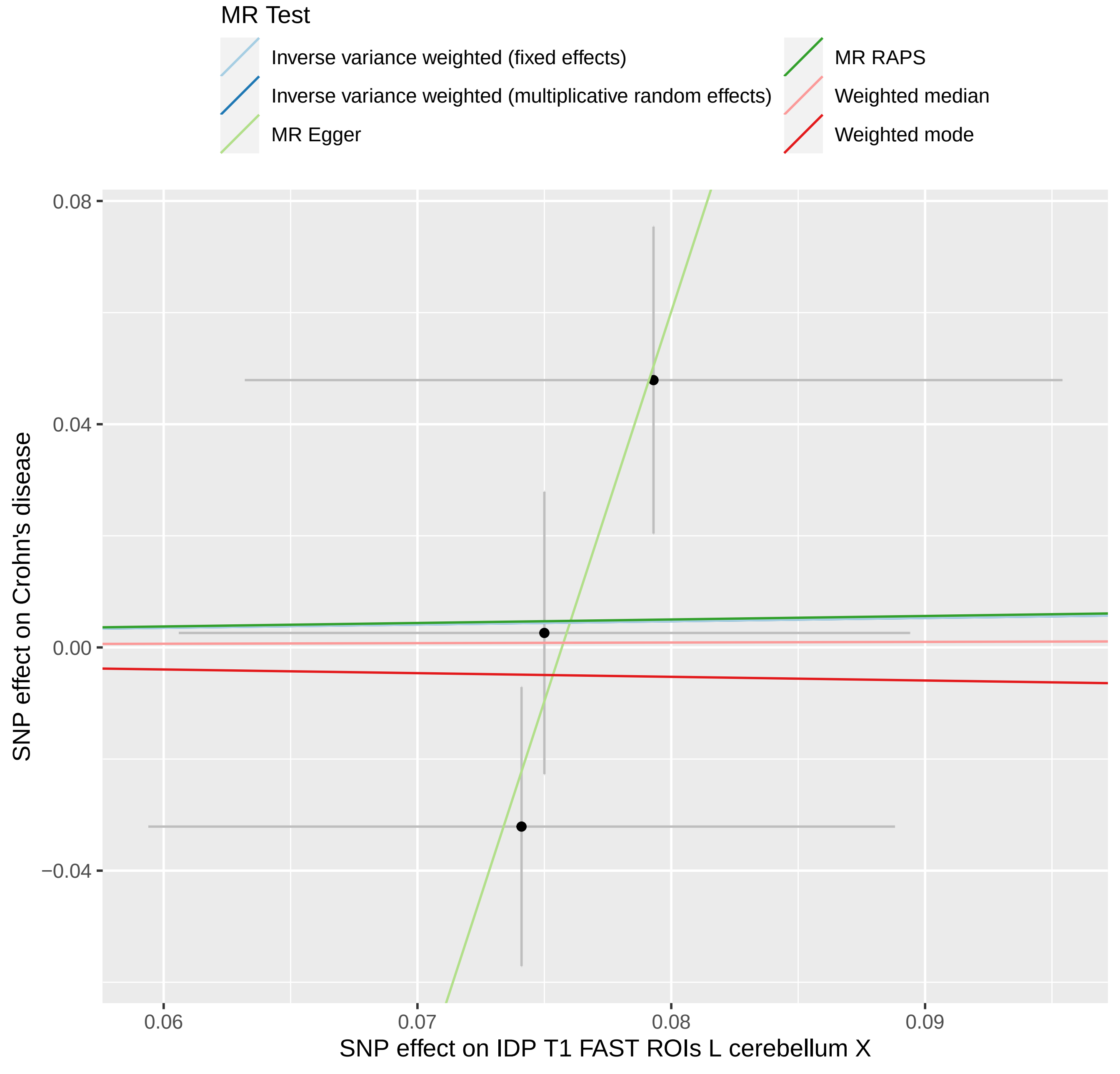

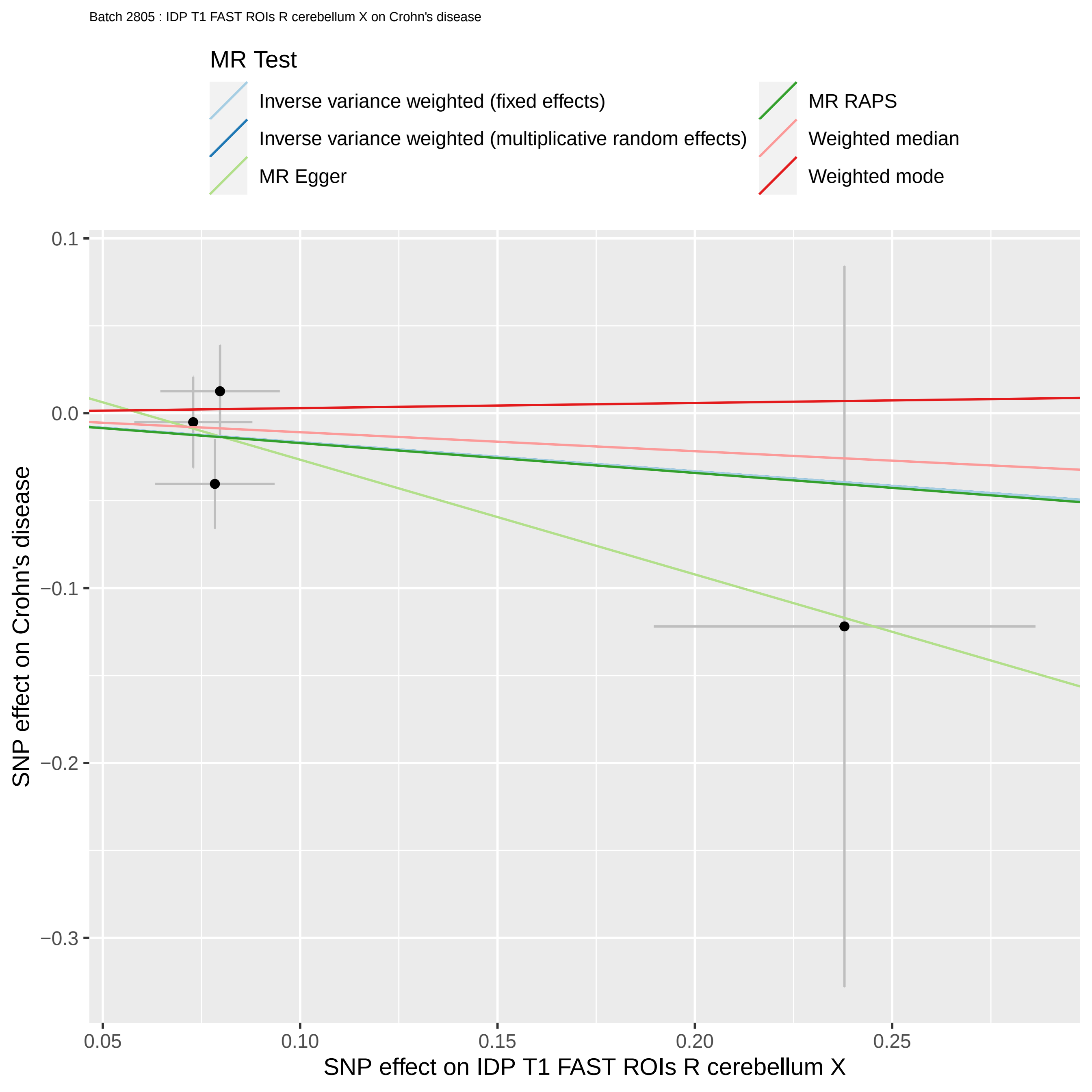

Batch 2805 : IDP T1 FAST ROIs R cerebellum X on Crohn's disease
MR Test
Inverse variance weighted (fixed effects)
Inverse variance weighted (multiplicative random effects)
MR Egger
MR RAPS
Weighted median
Weighted mode
0.1
0.0
−0.1
−0.2
−0.3
SNP effect on Crohn's disease
0.05
0.10
0.15
0.20
0.25
SNP effect on IDP T1 FAST ROIs R cerebellum X

Batch 2806 : IDP T2 FLAIR BIANCA WMH volume on Crohn's disease

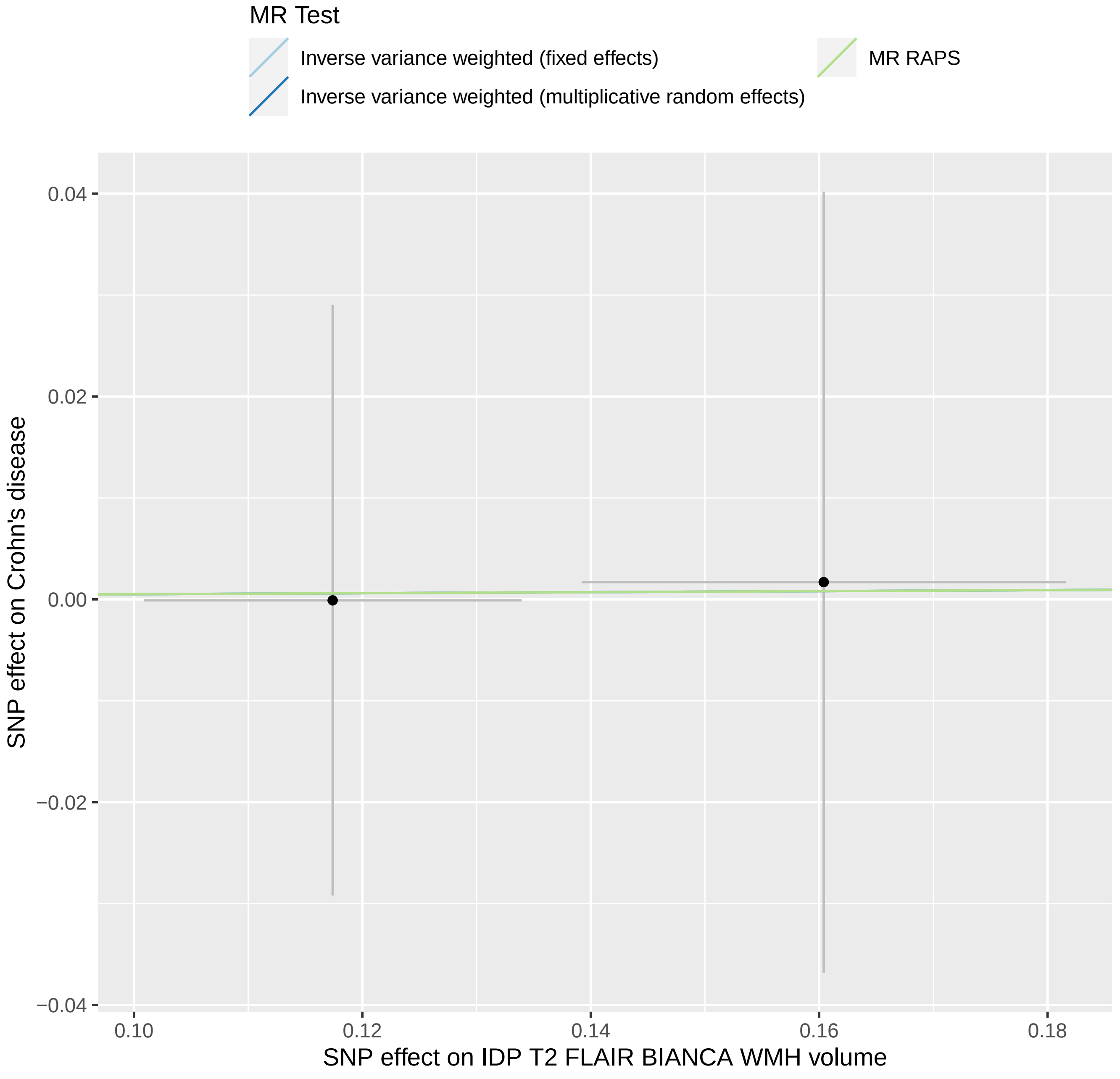

Batch 2824 : IDP tfMRI 90th−percentile BOLD shapes on Crohn's disease

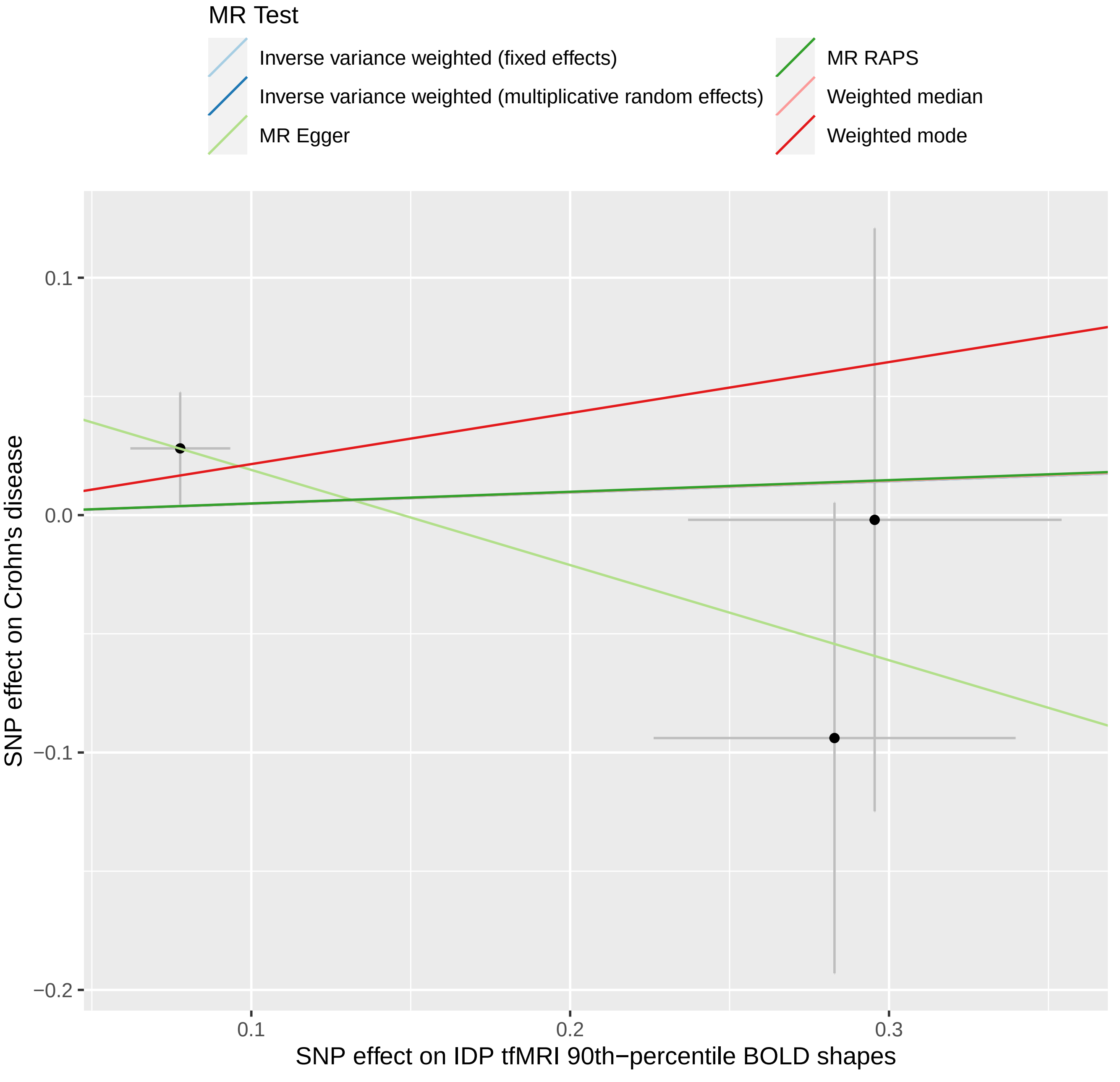

Batch 2825 : IDP tfMRI median zstat shapes on Crohn's disease

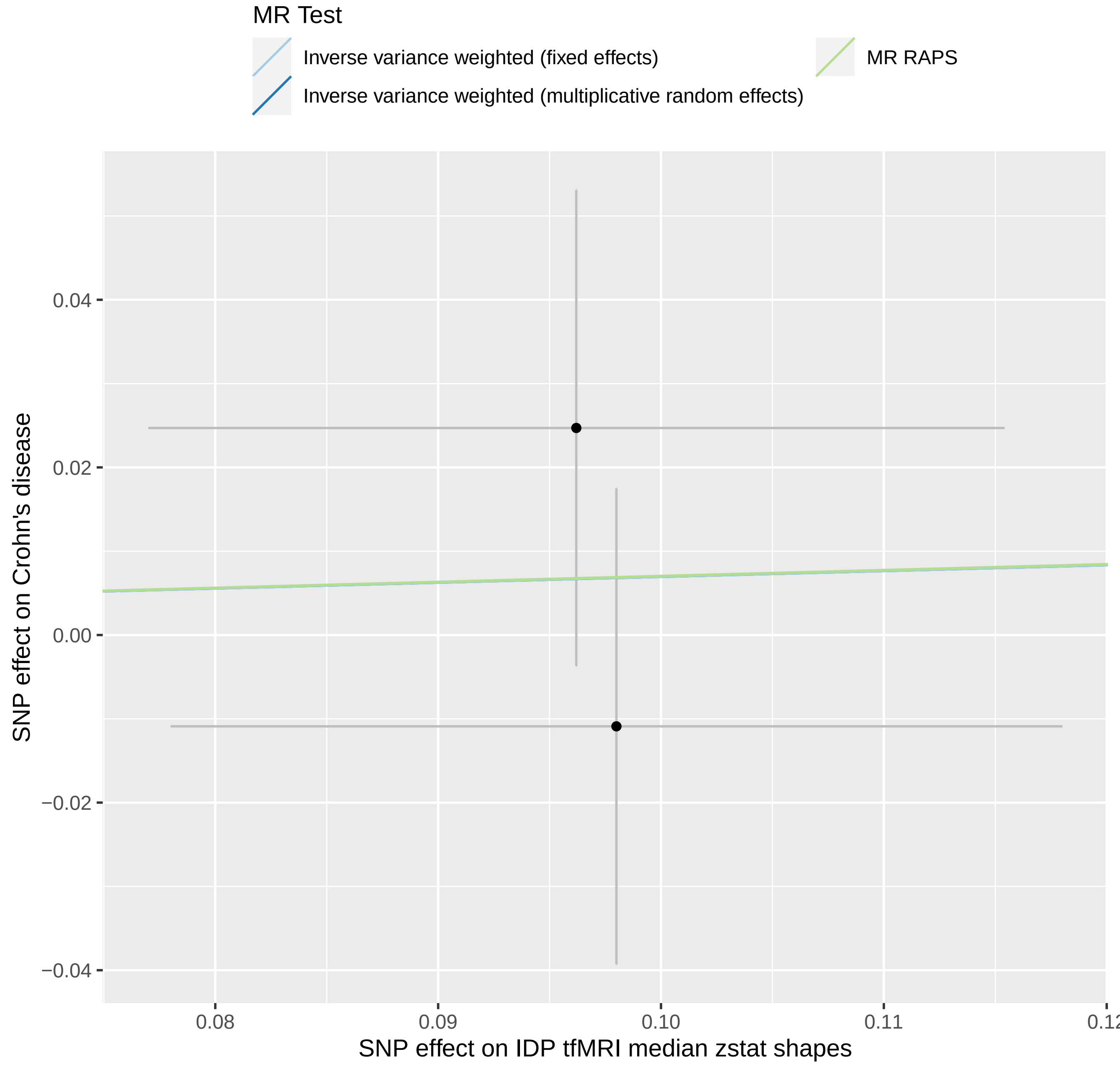

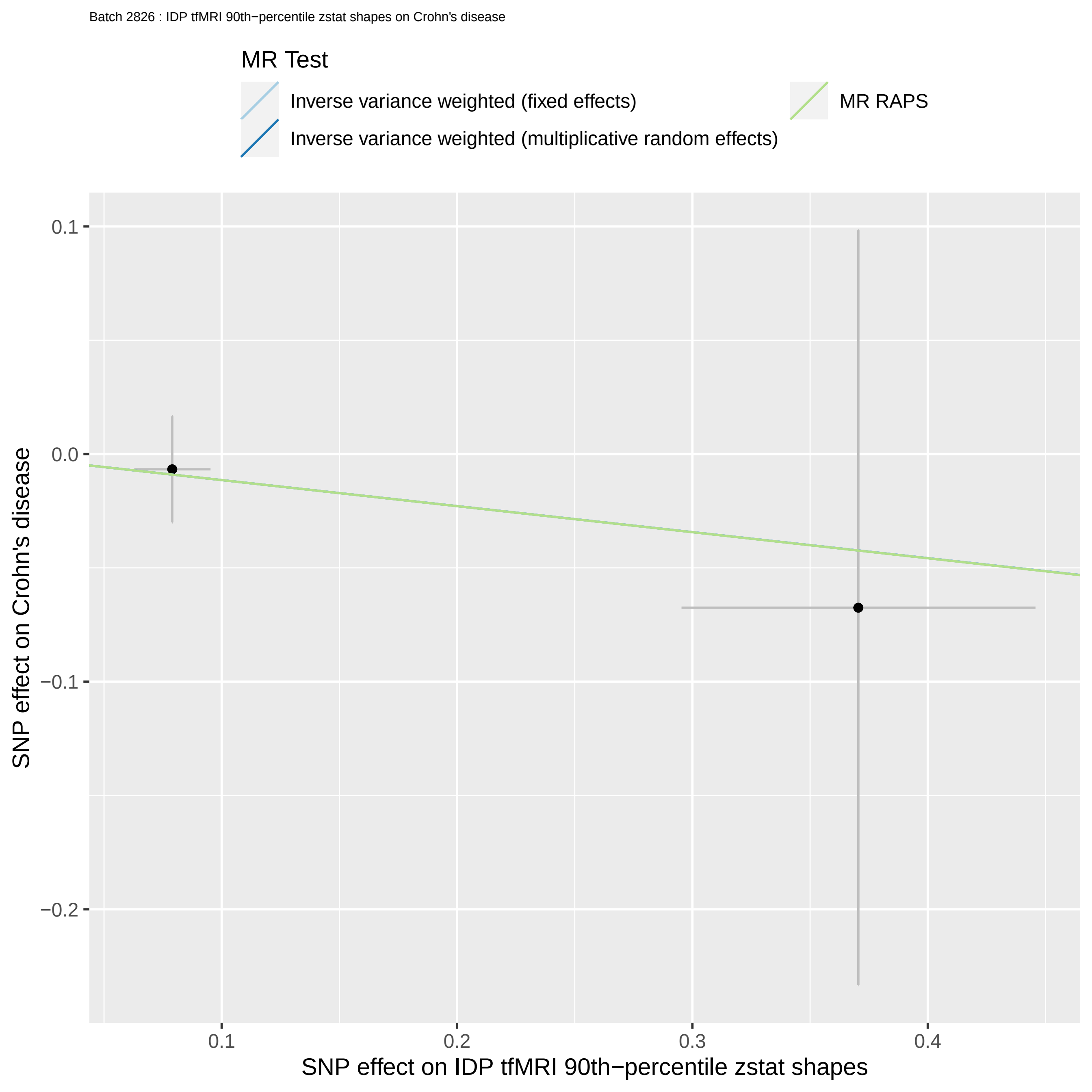

Batch 2826 : IDP tfMRI 90th−percentile zstat shapes on Crohn's disease
MR Test
Inverse variance weighted (fixed effects)
Inverse variance weighted (multiplicative random effects)
MR RAPS
SNP effect on Crohn's disease
SNP effect on IDP tfMRI 90th−percentile zstat shapes
0.1
0.0
−0.1
−0.2
0.1
0.2
0.3
0.4

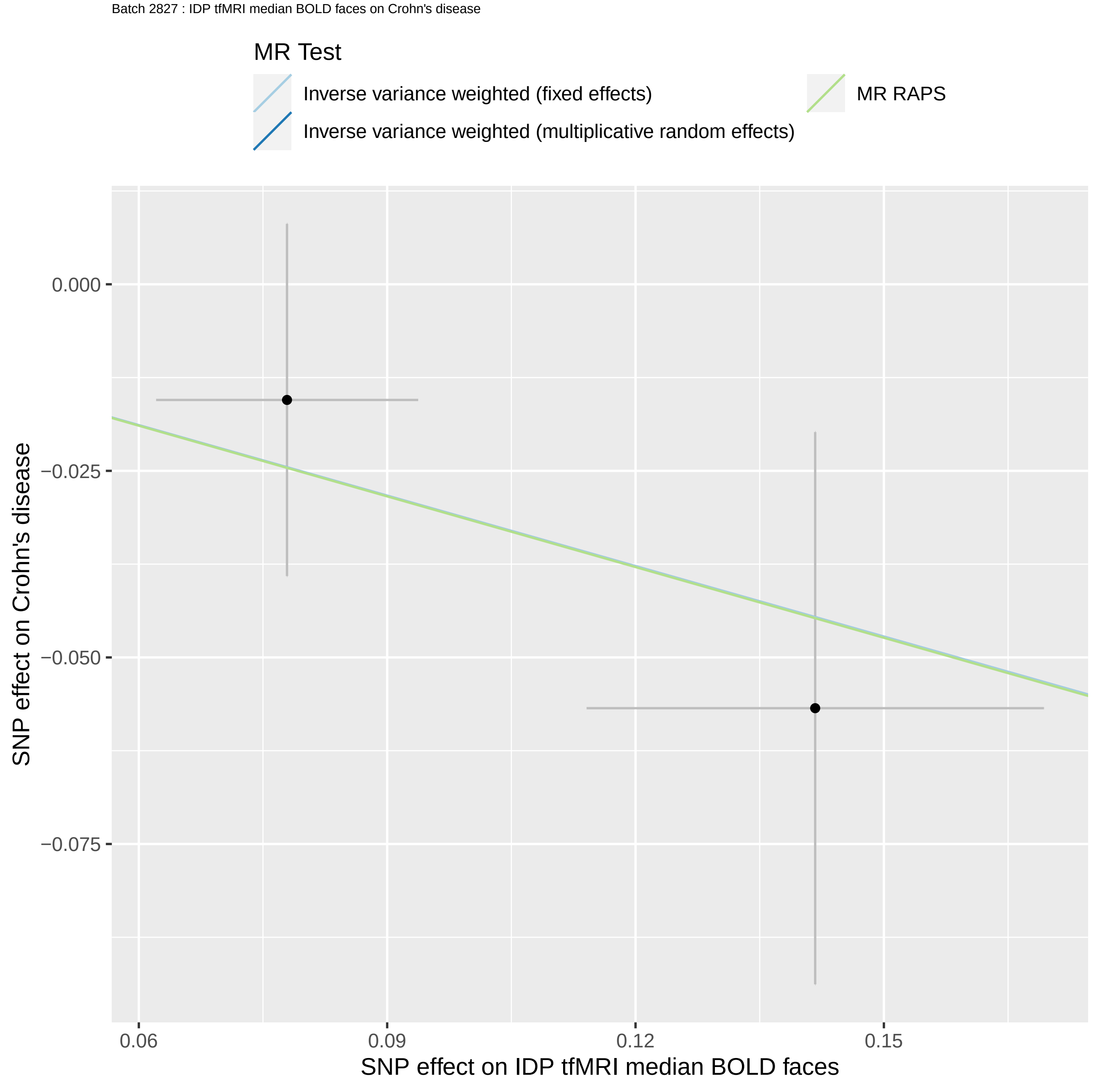

Batch 2827 : IDP tfMRI median BOLD faces on Crohn's disease
MR Test
Inverse variance weighted (fixed effects)
Inverse variance weighted (multiplicative random effects)
MR RAPS
0.000
−0.025
−0.050
−0.075
0.06
0.09
0.12
0.15
SNP effect on Crohn's disease
SNP effect on IDP tfMRI median BOLD faces

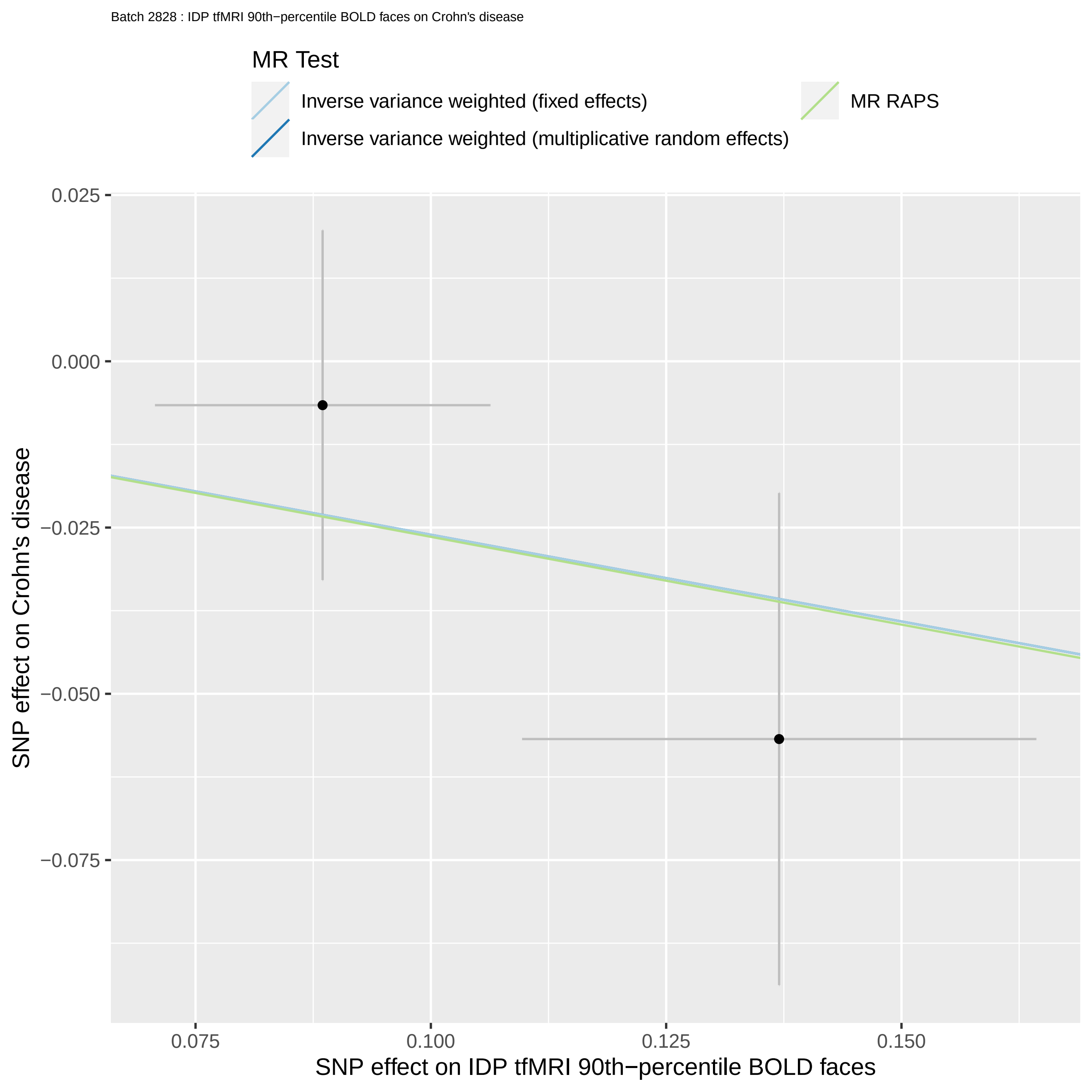
Batch 2828 : IDP tfMRI 90th−percentile BOLD faces on Crohn's disease
MR Test
Inverse variance weighted (fixed effects)
Inverse variance weighted (multiplicative random effects)
MR RAPS
0.025
0.000
−0.025
−0.050
−0.075
0.075
0.100
0.125
0.150
SNP effect on Crohn's disease
SNP effect on IDP tfMRI 90th−percentile BOLD faces

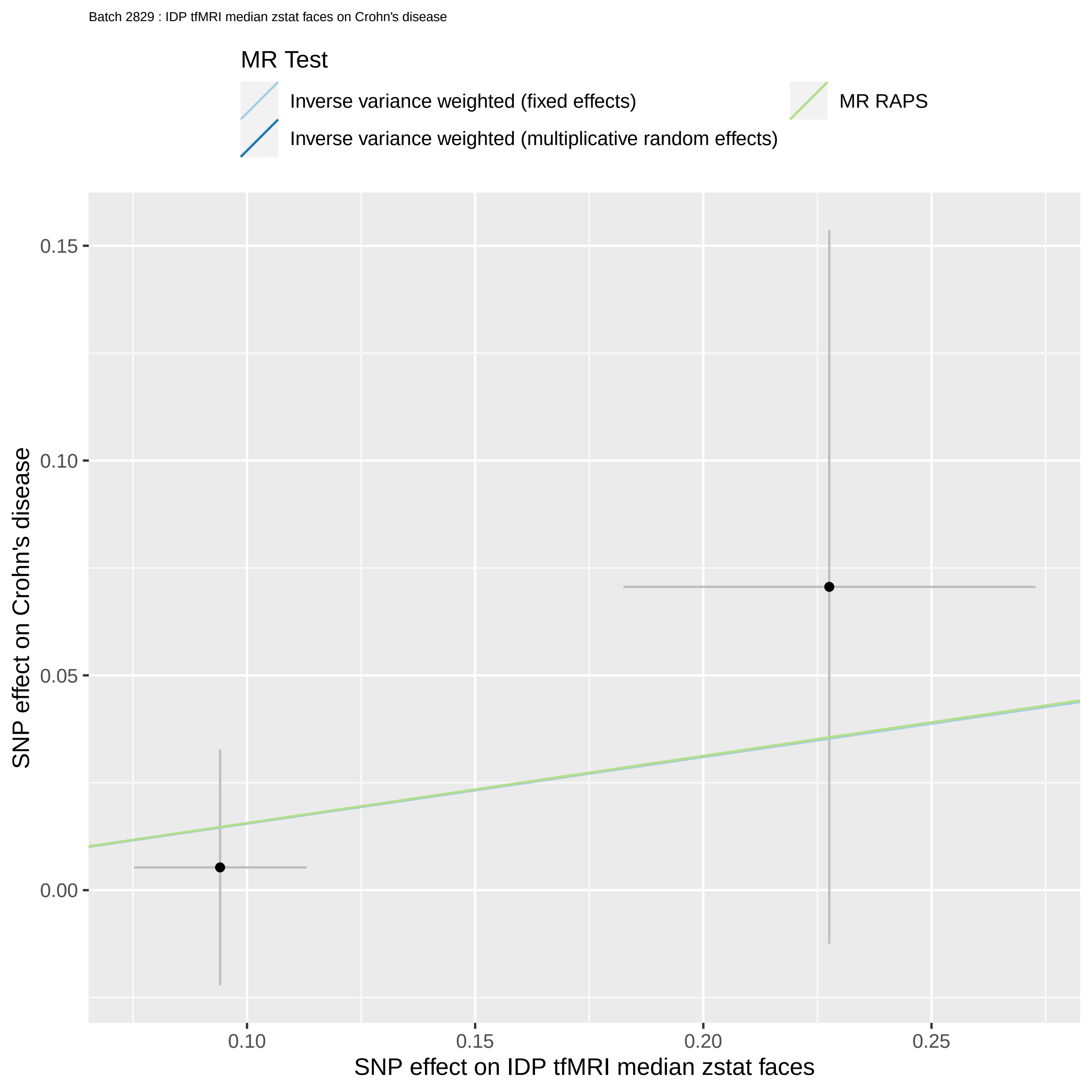
Batch 2829 : IDP tfMRI median zstat faces on Crohn's disease
MR Test
Inverse variance weighted (fixed effects)
Inverse variance weighted (multiplicative random effects)
MR RAPS
0.15
0.10
0.05
0.00
SNP effect on Crohn's disease
0.10
0.15
0.20
0.25
SNP effect on IDP tfMRI median zstat faces

Batch 2832 : IDP tfMRI 90th−percentile BOLD faces−shapes on Crohn's disease

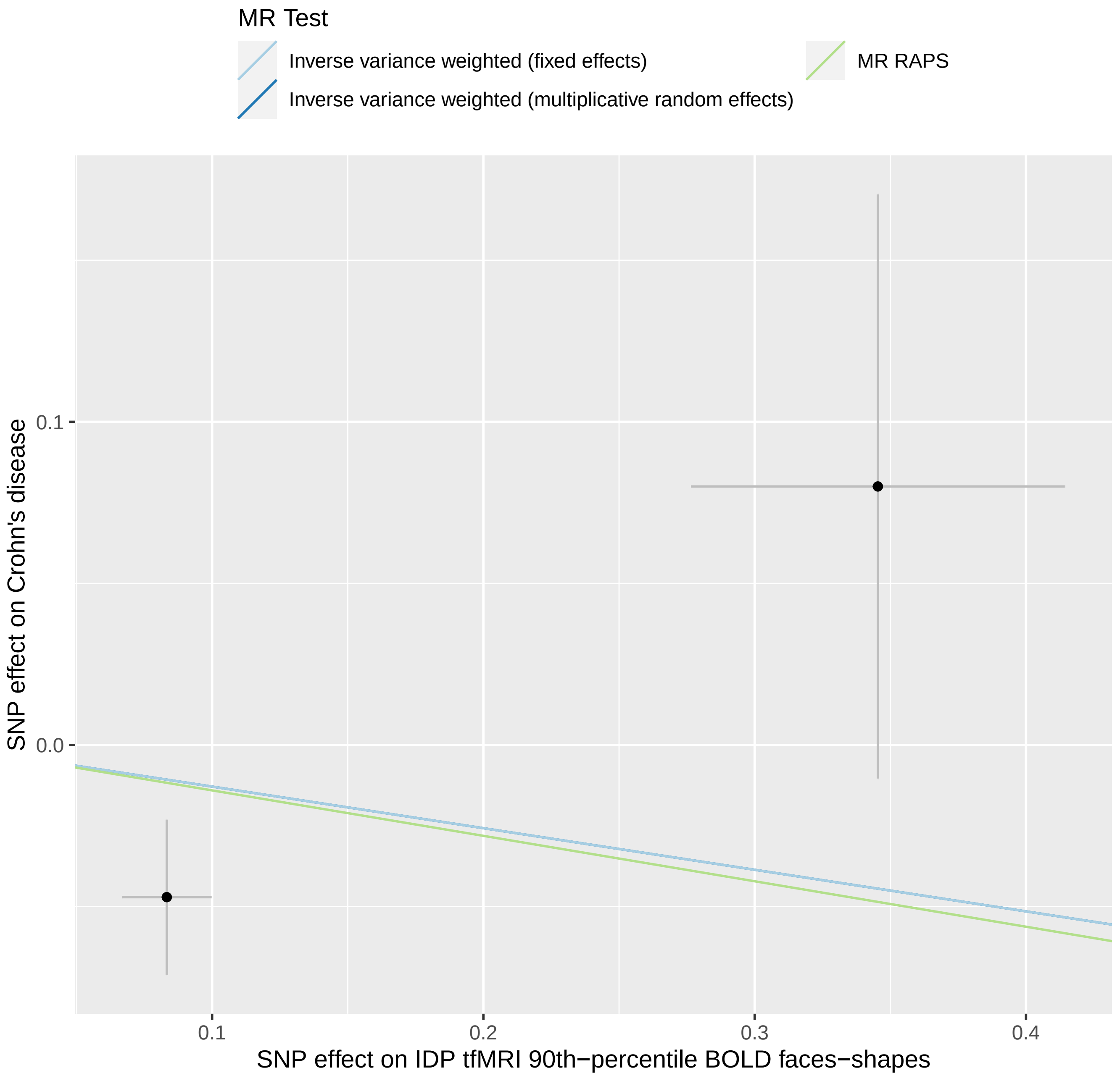

Batch 2833 : IDP T1 FIRST left hippocampus volume on Crohn's disease

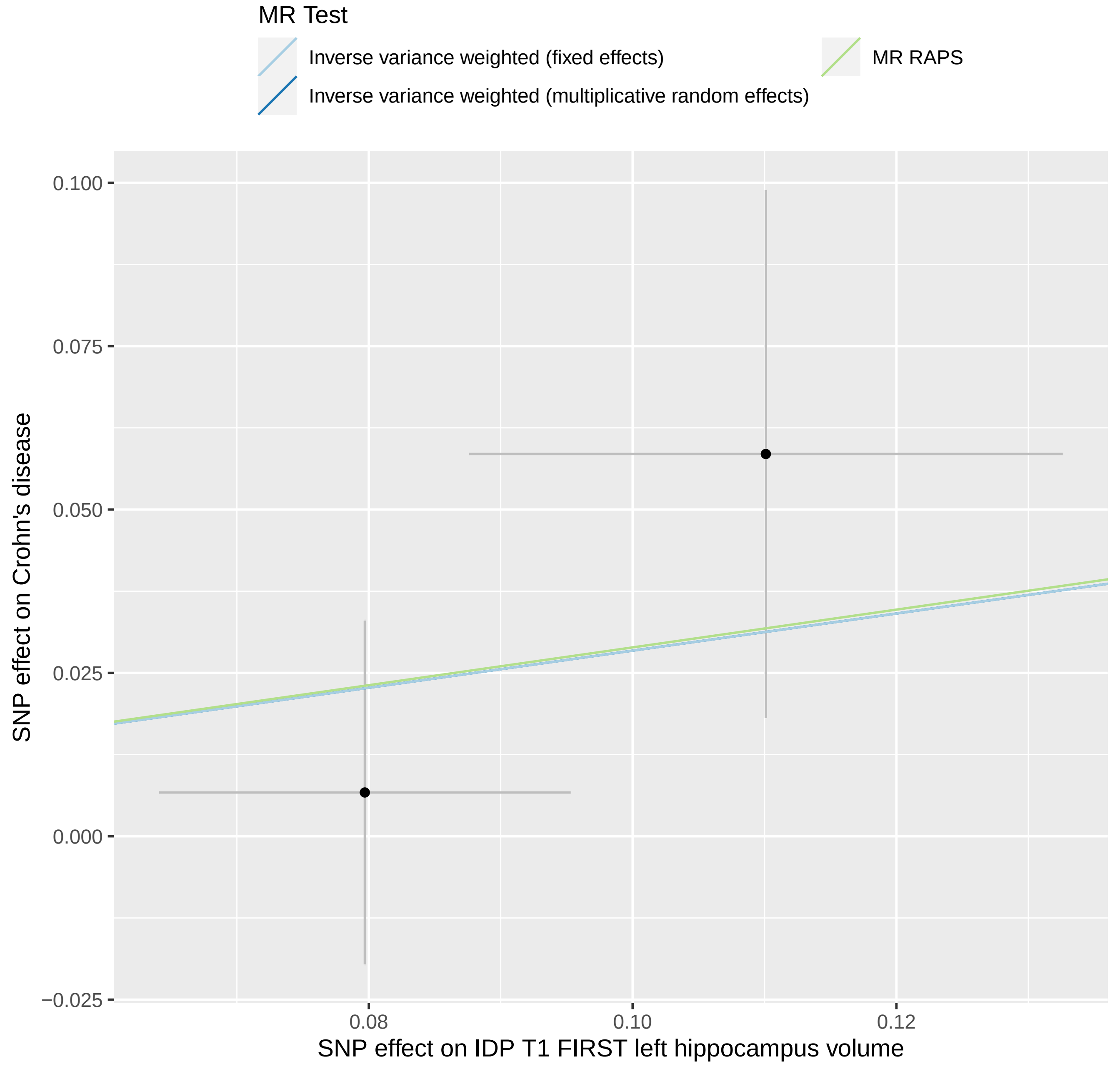

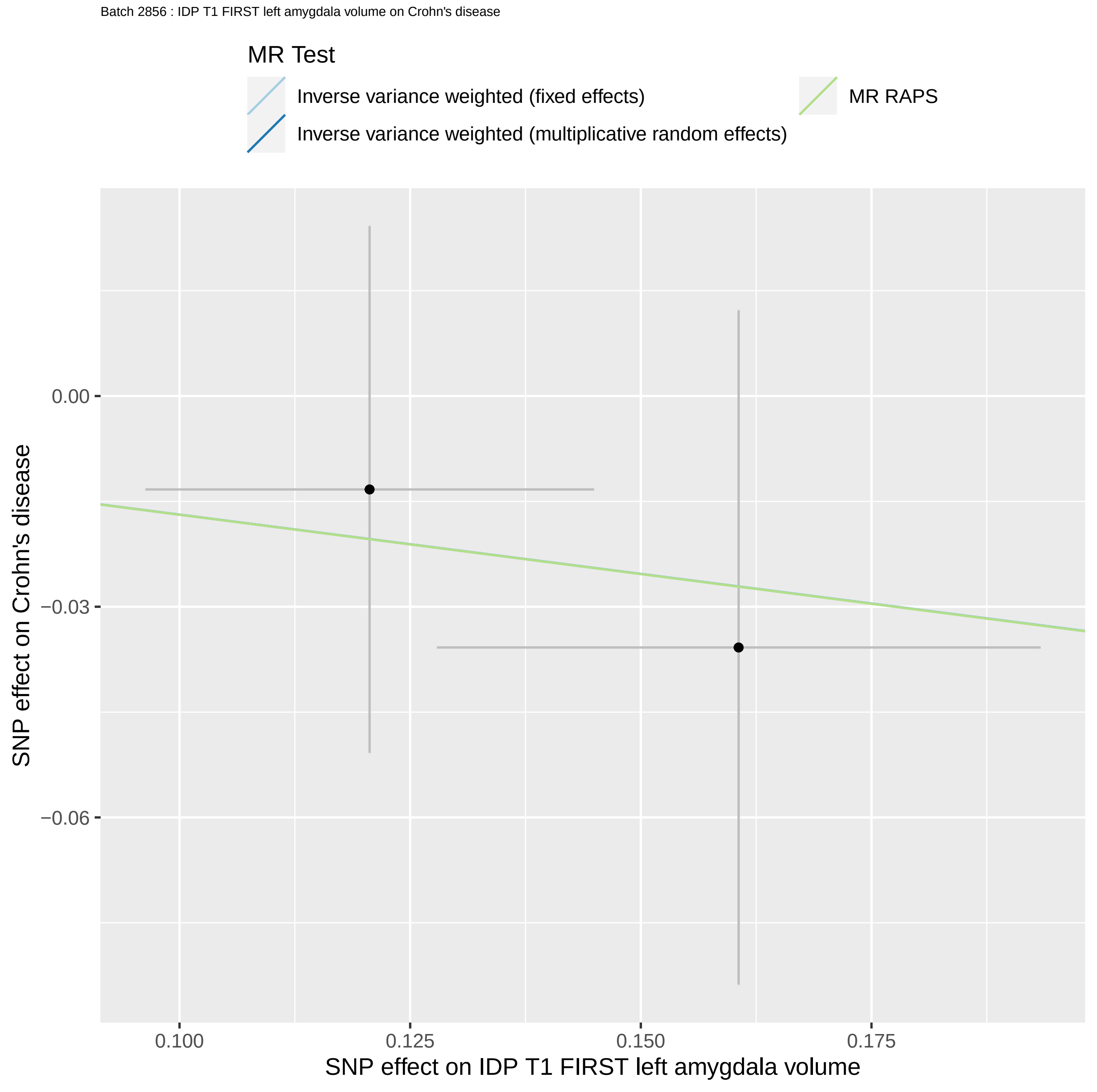
Batch 2856 : IDP T1 FIRST left amygdala volume on Crohn's disease
MR Test
Inverse variance weighted (fixed effects)
Inverse variance weighted (multiplicative random effects)
MR RAPS
0.00
−0.03
−0.06
0.100
0.125
0.150
0.175
SNP effect on Crohn's disease
SNP effect on IDP T1 FIRST left amygdala volume

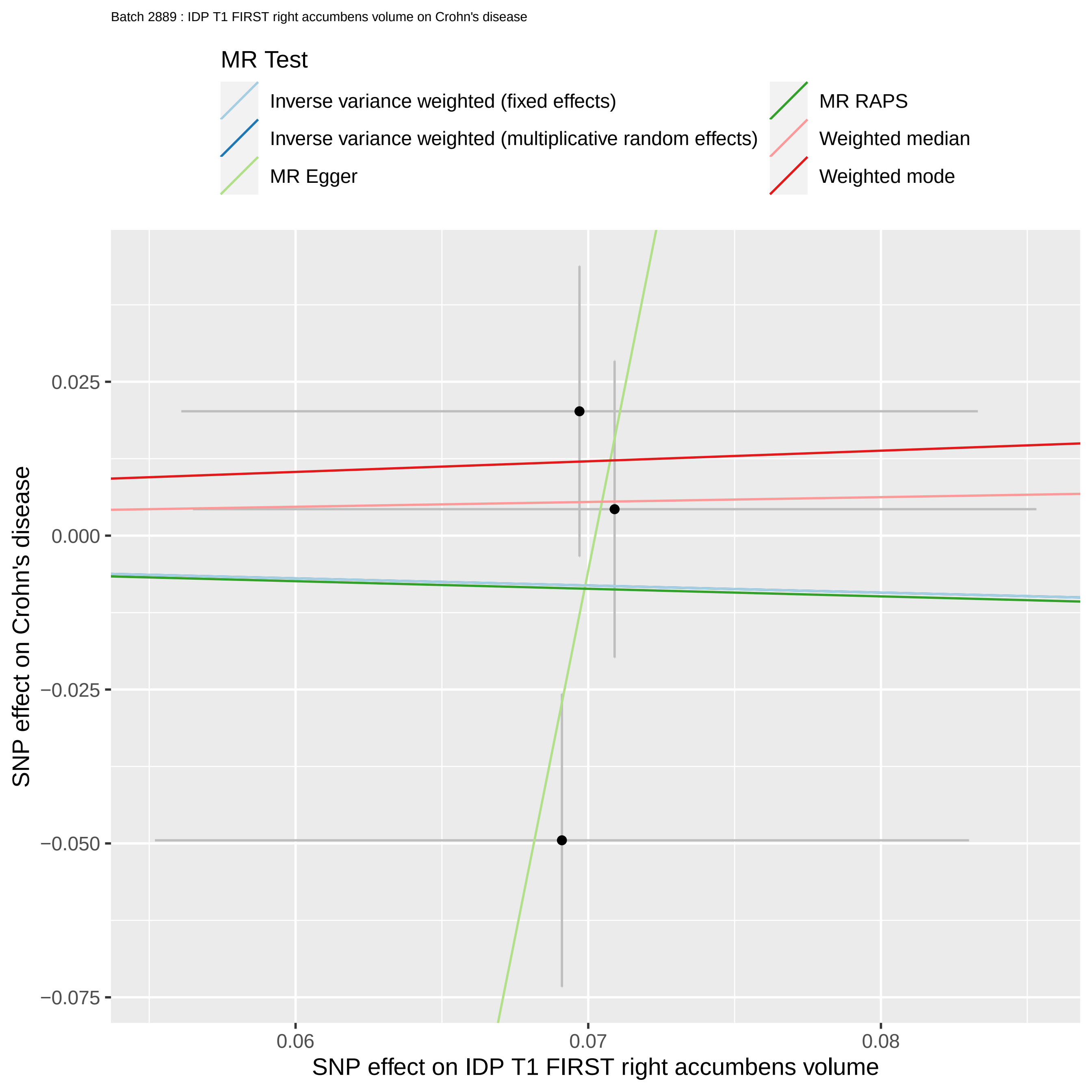
Batch 2889 : IDP T1 FIRST right accumbens volume on Crohn's disease
MR Test
Inverse variance weighted (fixed effects)
Inverse variance weighted (multiplicative random effects)
MR Egger
MR RAPS
Weighted median
Weighted mode
0.025
0.000
−0.025
−0.050
−0.075
SNP effect on Crohn's disease
0.06
0.07
0.08
SNP effect on IDP T1 FIRST right accumbens volume

Batch 2900 : IDP T1 FIRST brain stem+4th ventricle volume on Crohn's disease

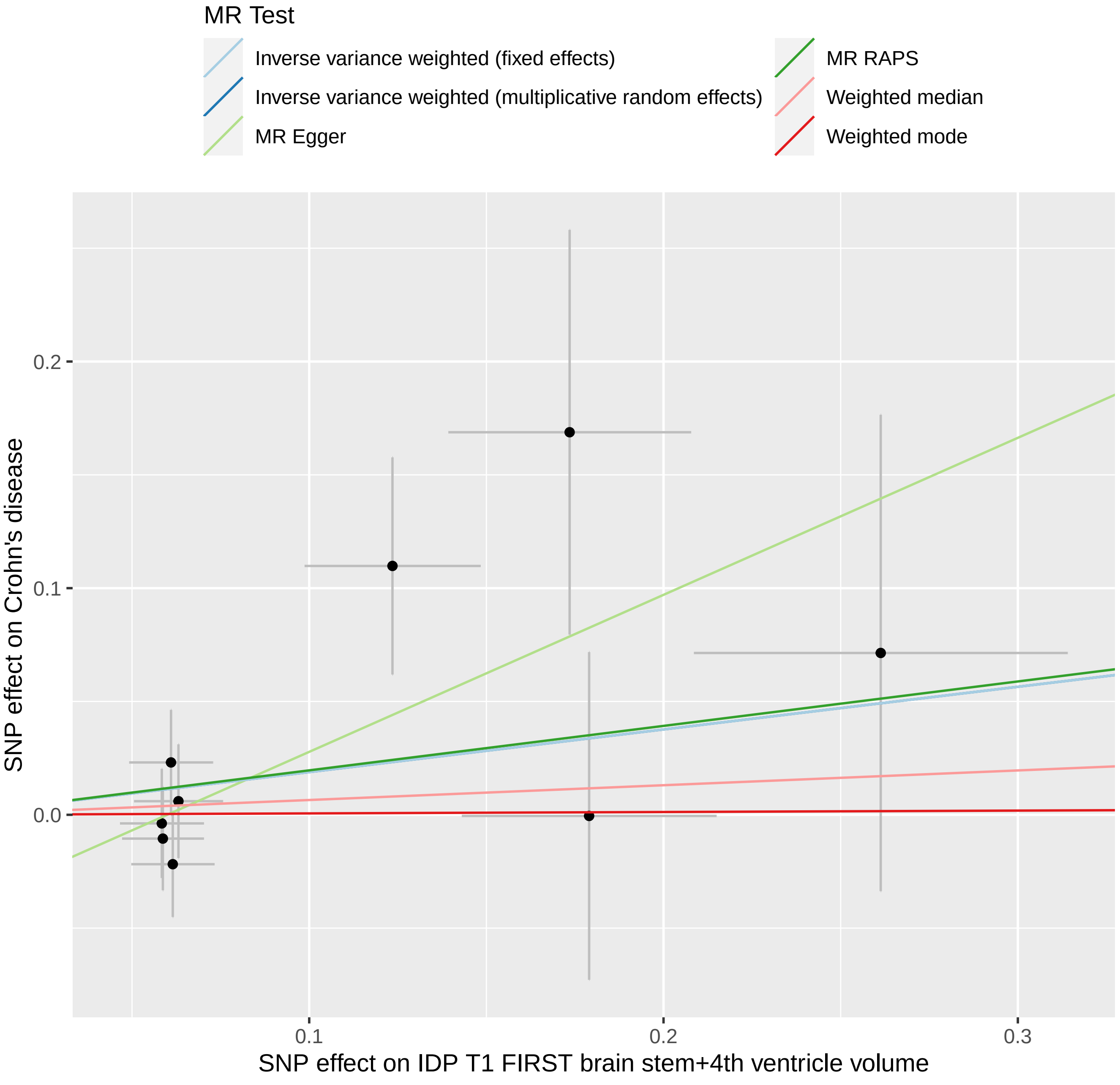

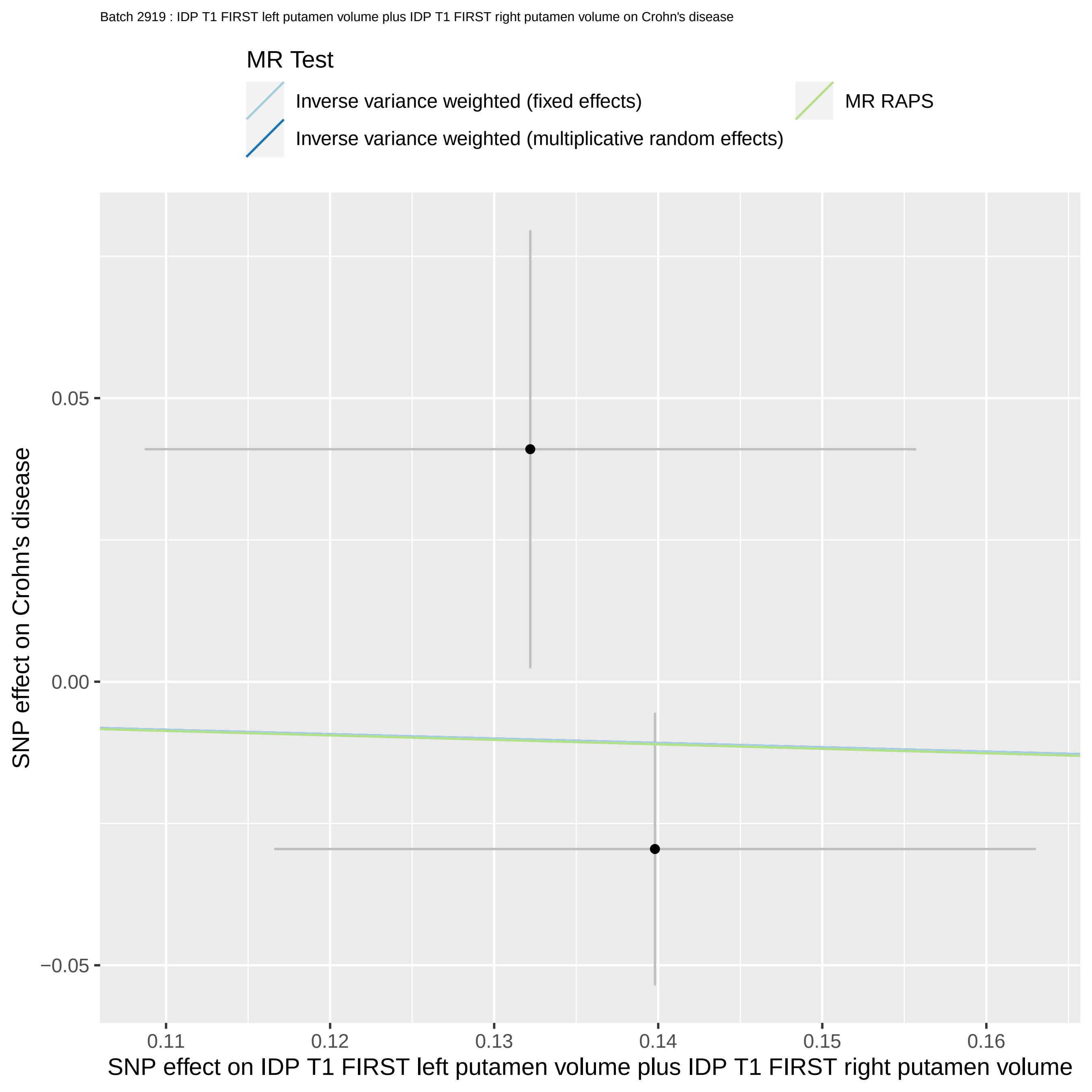
Batch 2919 : IDP T1 FIRST left putamen volume plus IDP T1 FIRST right putamen volume on Crohn's disease
MR Test
Inverse variance weighted (fixed effects)
Inverse variance weighted (multiplicative random effects)
MR RAPS
0.05
0.00
−0.05
0.11
0.12
0.13
0.14
0.15
0.16
SNP effect on Crohn's disease
SNP effect on IDP T1 FIRST left putamen volume plus IDP T1 FIRST right putamen volume

Batch 2921 : IDP T1 FIRST left hippocampus volume plus IDP T1 FIRST right hippocampus volume on Crohn's disease

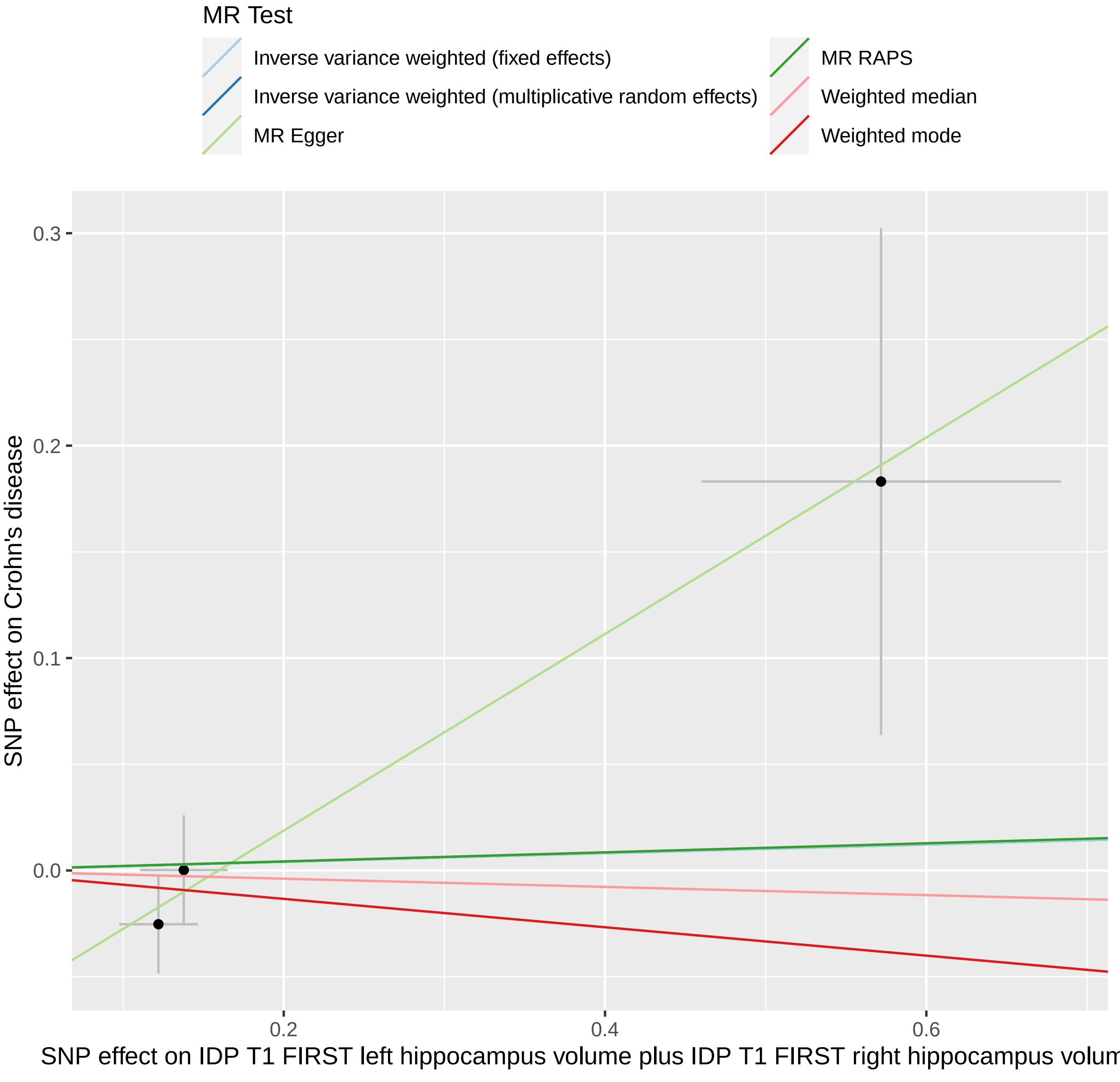

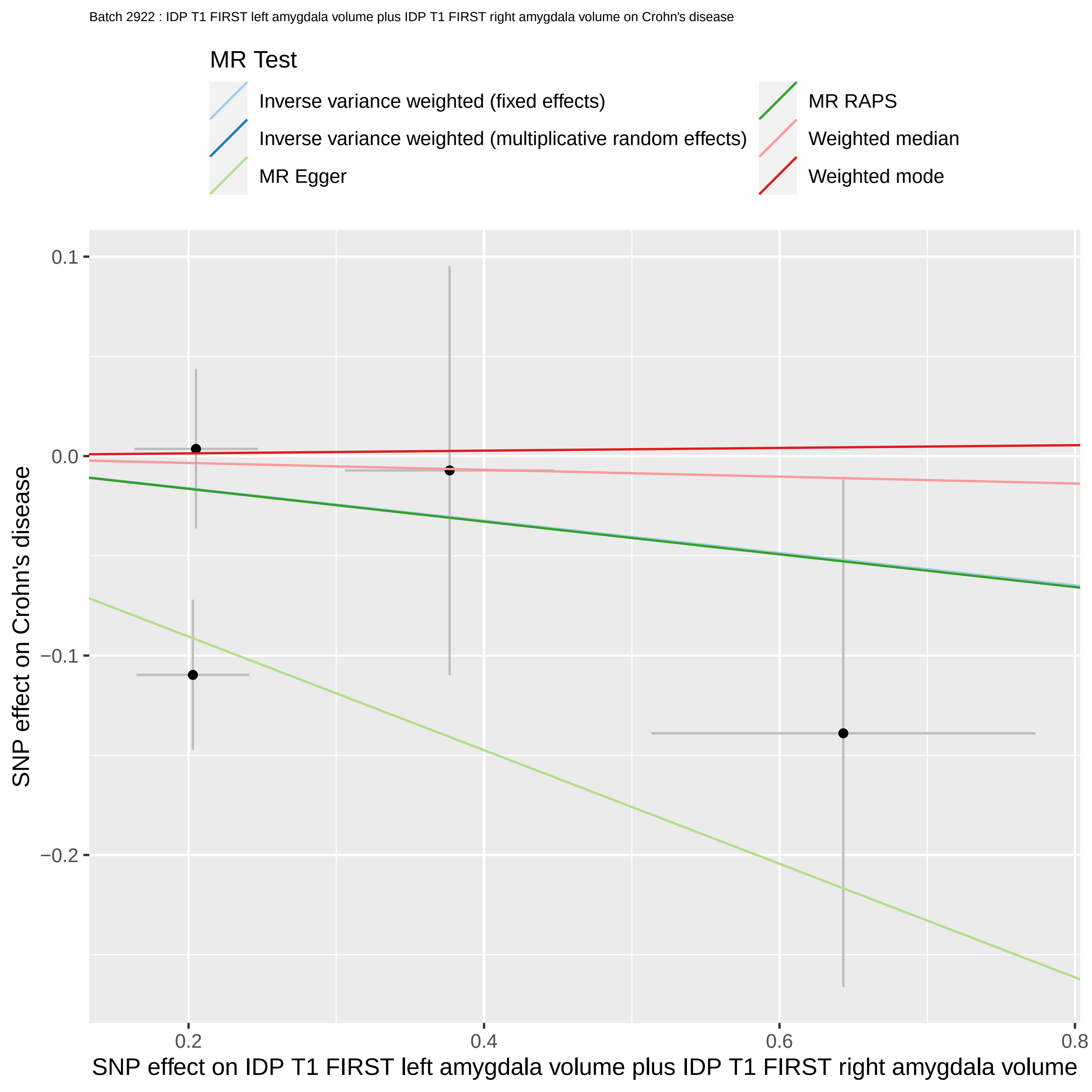
Batch 2922 : IDP T1 FIRST left amygdala volume plus IDP T1 FIRST right amygdala volume on Crohn's disease
MR Test
Inverse variance weighted (fixed effects)
Inverse variance weighted (multiplicative random effects)
MR Egger
MR RAPS
Weighted median
Weighted mode
SNP effect on Crohn's disease
0.1
0.0
−0.1
−0.2
0.2
0.4
0.6
0.8
SNP effect on IDP T1 FIRST left amygdala volume plus IDP T1 FIRST right amygdala volume

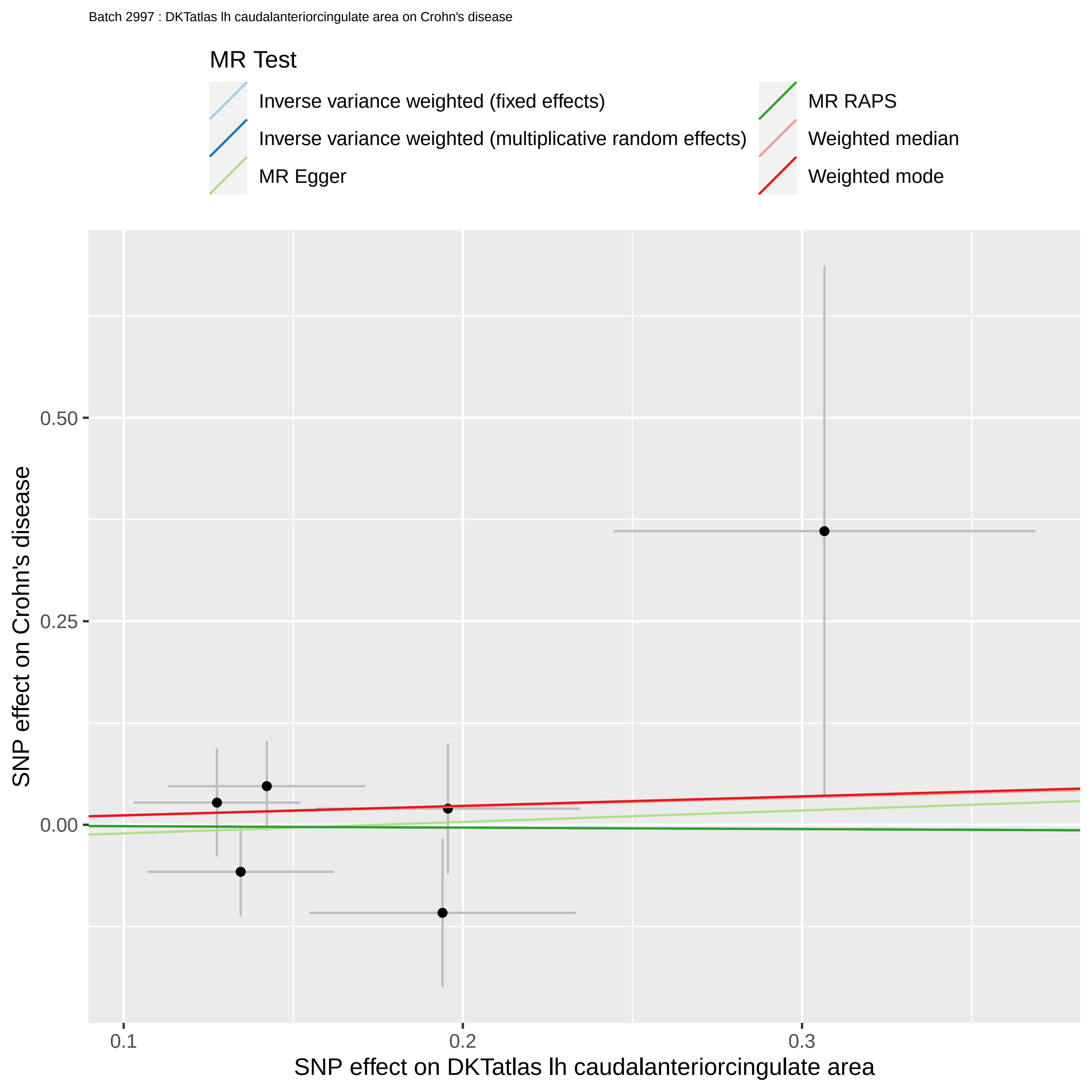

Batch 2997 : DKTatlas lh caudalanteriorcingulate area on Crohn's disease
MR Test
Inverse variance weighted (fixed effects)
Inverse variance weighted (multiplicative random effects)
MR Egger
MR RAPS
Weighted median
Weighted mode
0.50
0.25
0.00
0.1
0.2
0.3
SNP effect on Crohn's disease
SNP effect on DKTatlas lh caudalanteriorcingulate area

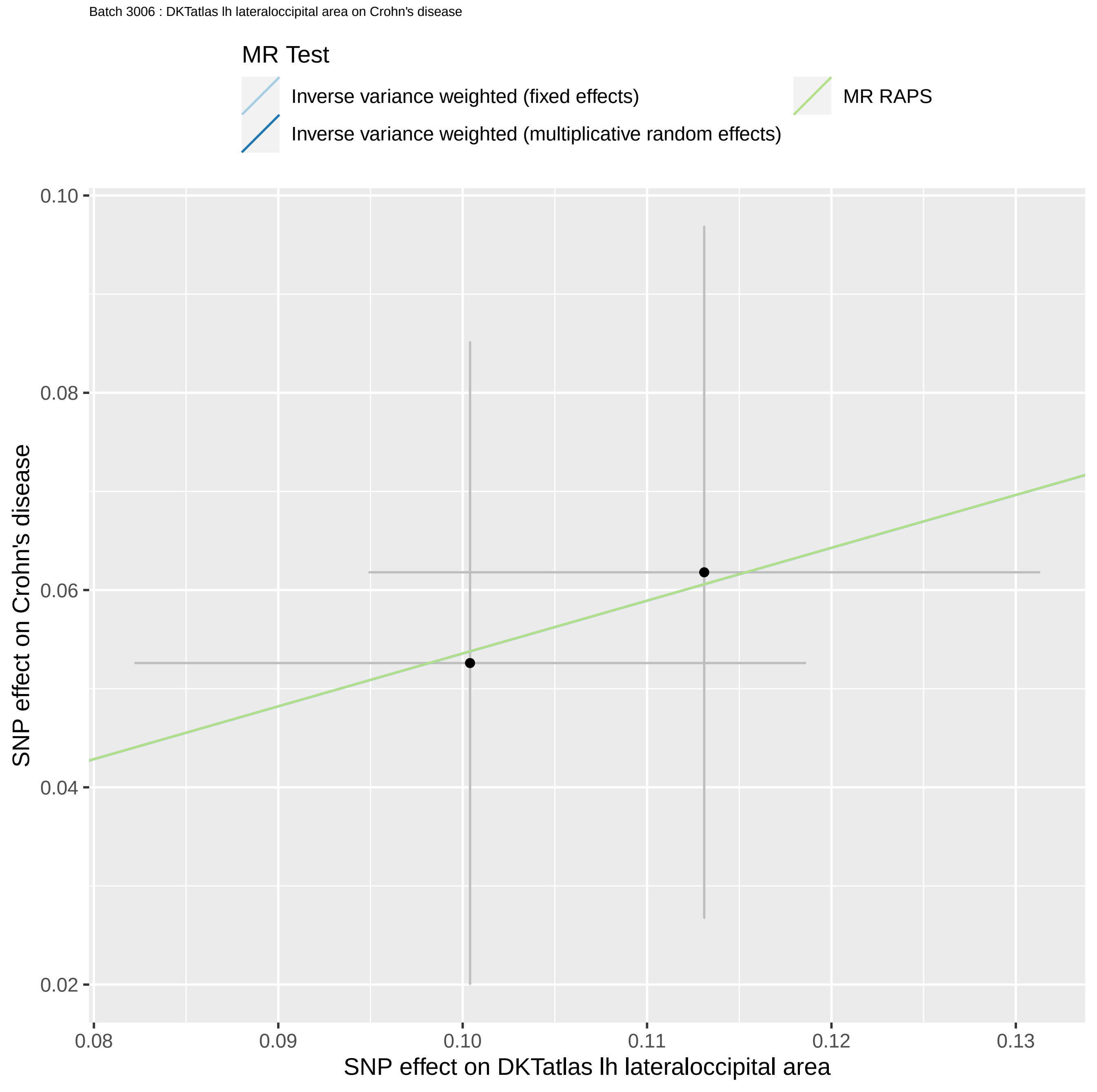
Batch 3006 : DKTatlas lh lateraloccipital area on Crohn's disease
MR Test
Inverse variance weighted (fixed effects)
Inverse variance weighted (multiplicative random effects)
MR RAPS
0.10
0.08
0.06
0.04
0.02
SNP effect on Crohn's disease
0.08
0.09
0.10
0.11
0.12
0.13
SNP effect on DKTatlas lh lateraloccipital area

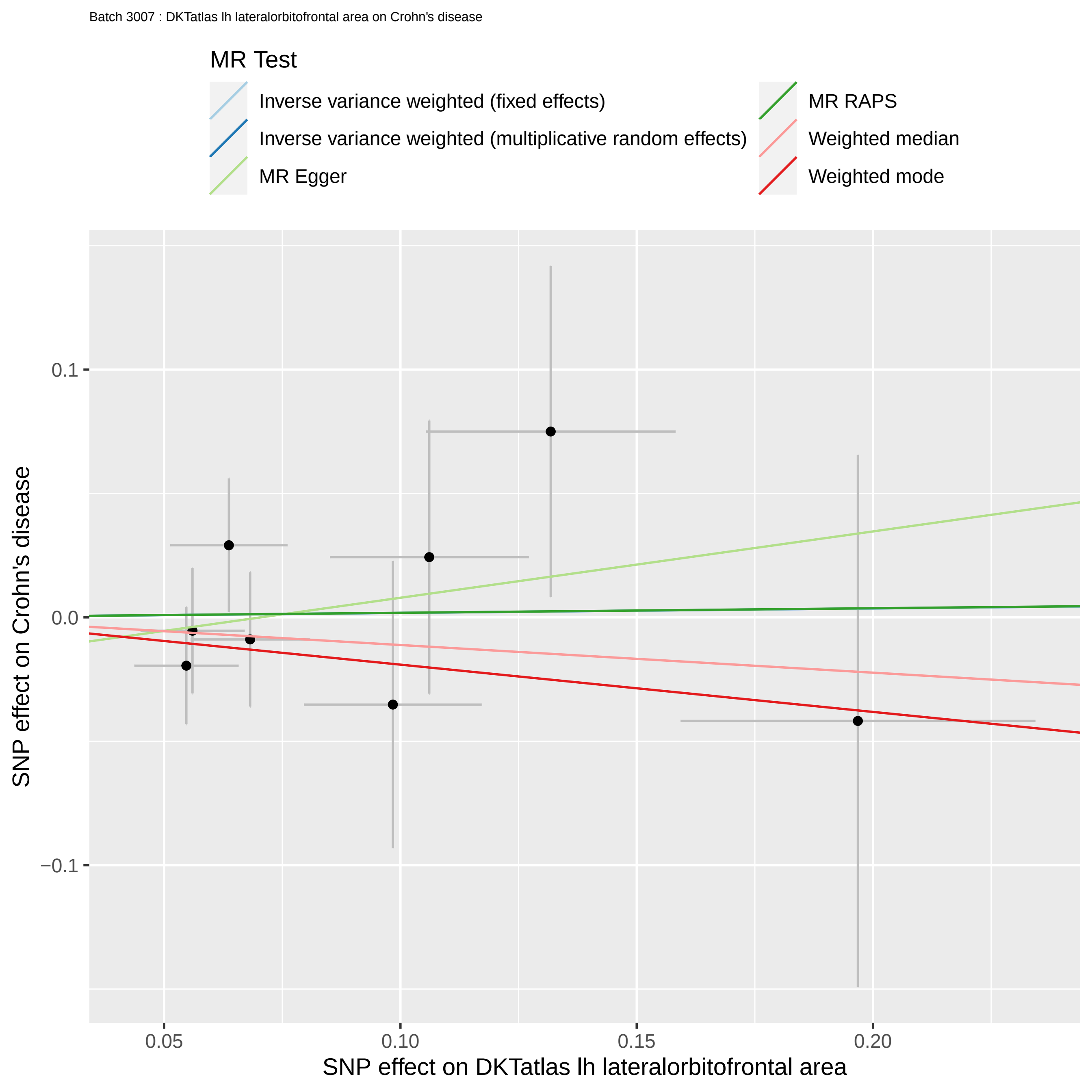

Batch 3007 : DKTatlas lh lateralorbitofrontal area on Crohn's disease
MR Test
Inverse variance weighted (fixed effects)
Inverse variance weighted (multiplicative random effects)
MR Egger
MR RAPS
Weighted median
Weighted mode
0.1
0.0
−0.1
0.05
0.10
0.15
0.20
SNP effect on Crohn's disease
SNP effect on DKTatlas lh lateralorbitofrontal area

Batch 3009 : DKTatlas lh medialorbitofrontal area on Crohn's disease

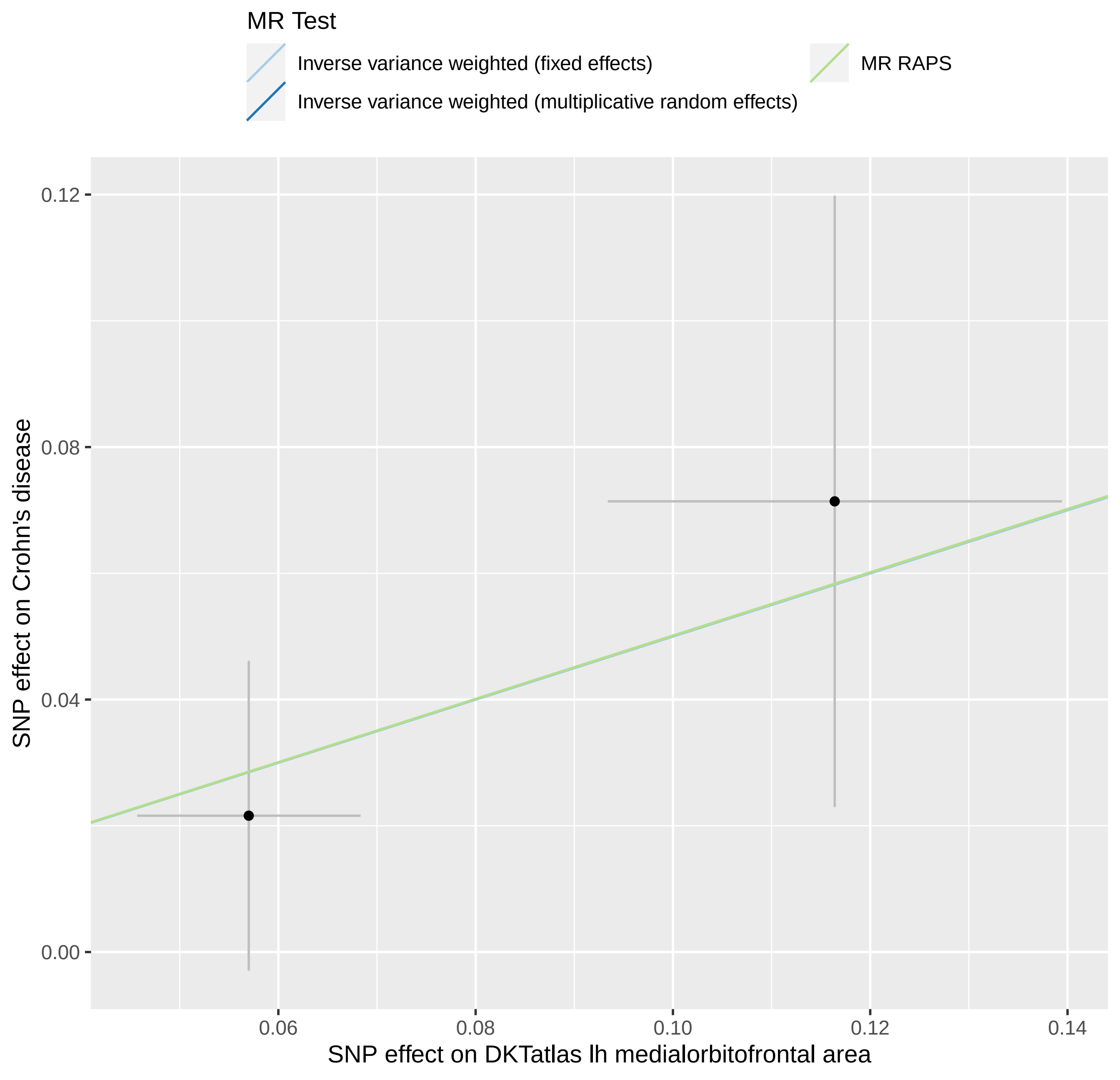

Batch 3011 : DKTatlas lh parahippocampal area on Crohn's disease

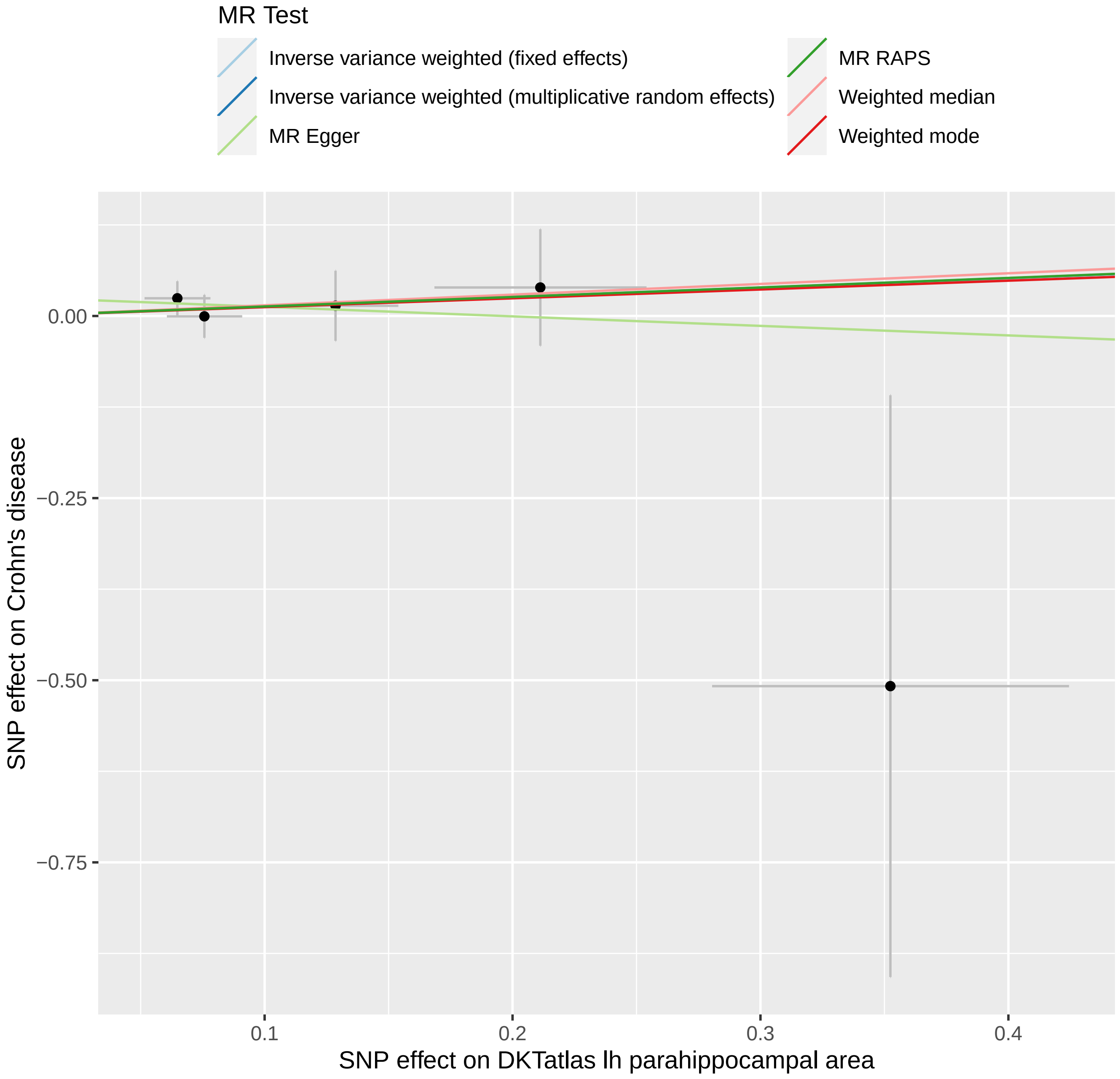

Batch 3012 : DKTatlas lh paracentral area on Crohn's disease

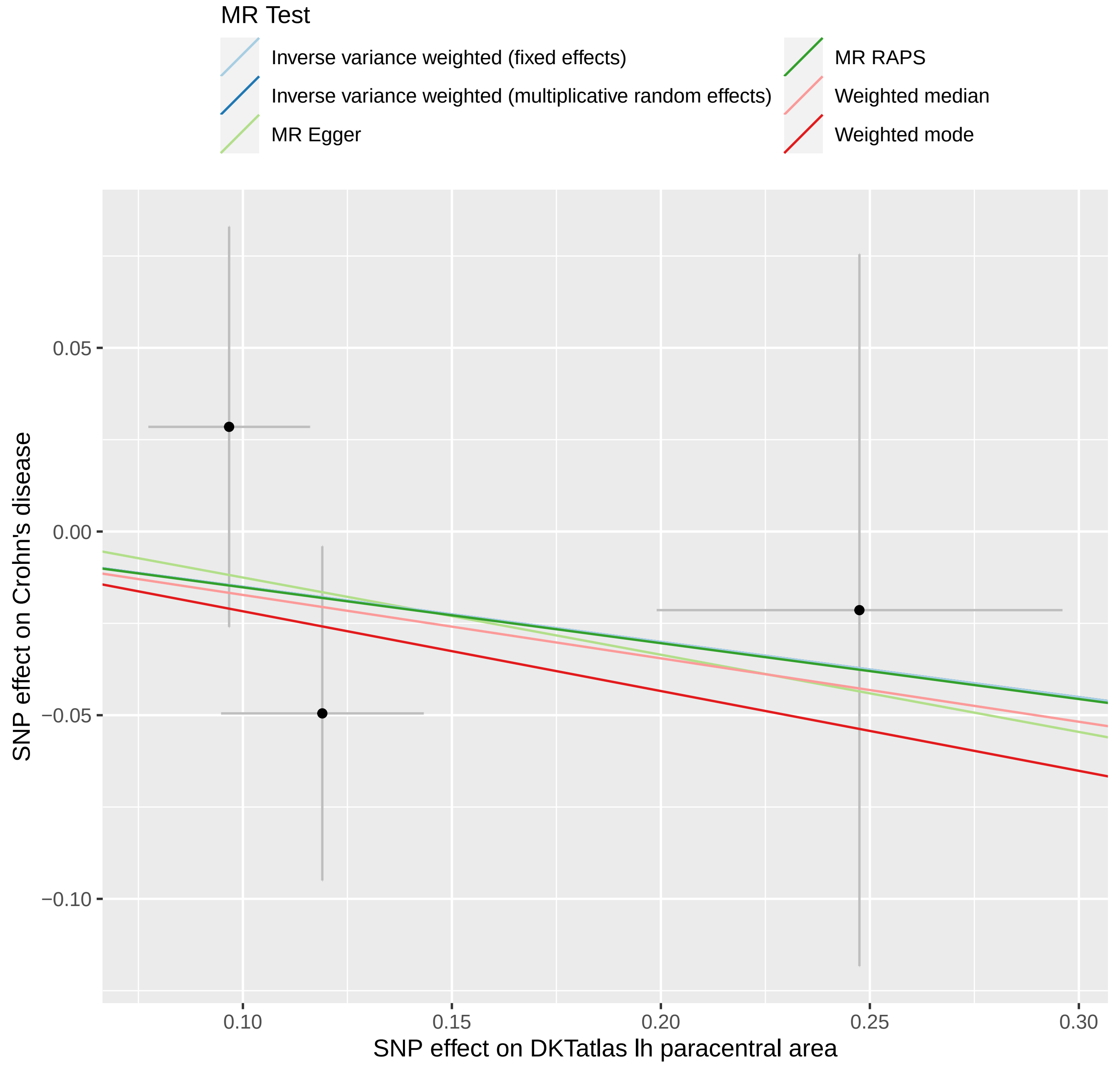

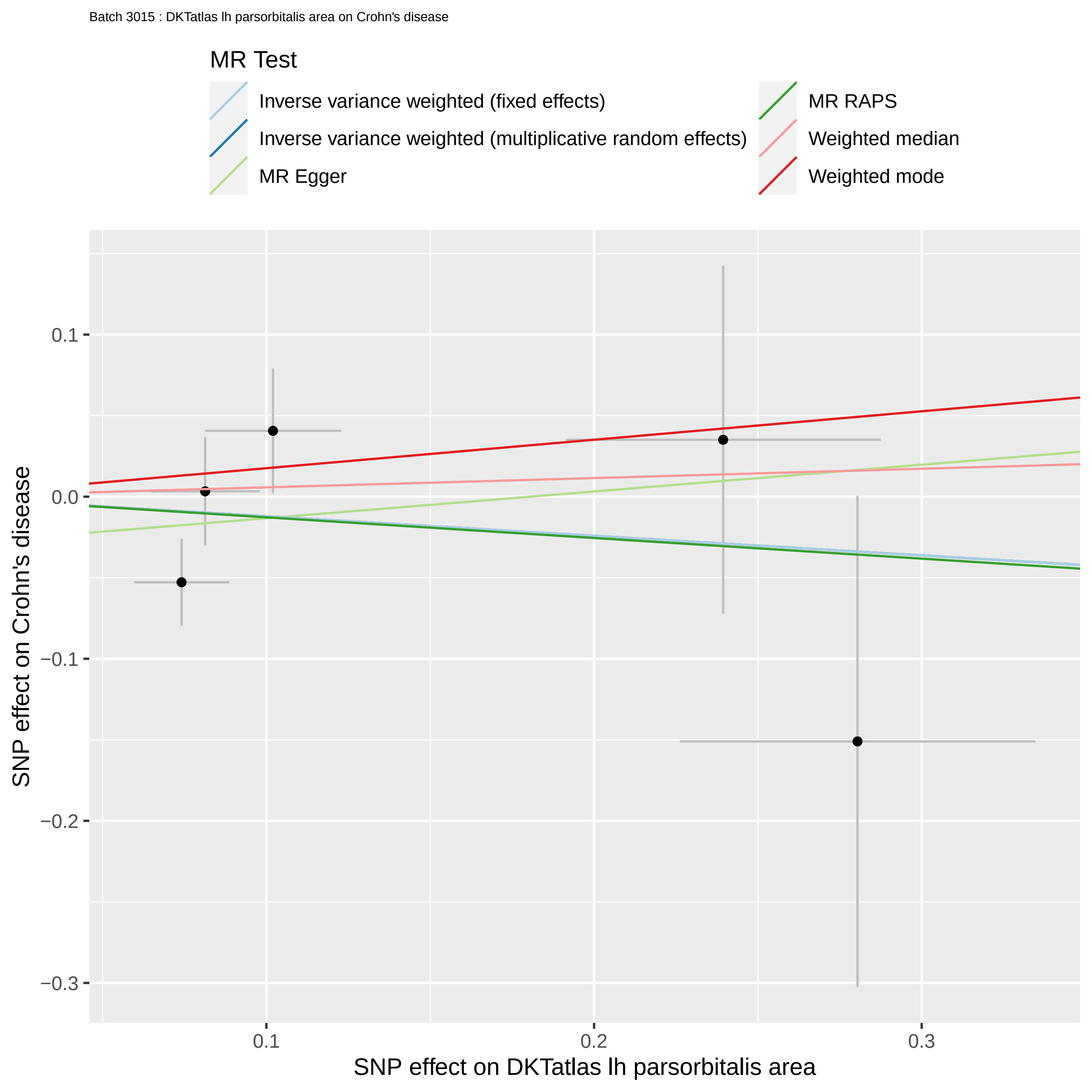

Batch 3015 : DKTatlas lh parsorbitalis area on Crohn's disease
MR Test
Inverse variance weighted (fixed effects)
Inverse variance weighted (multiplicative random effects)
MR Egger
MR RAPS
Weighted median
Weighted mode
0.1
0.0
−0.1
−0.2
−0.3
0.1
0.2
0.3
SNP effect on Crohn's disease
SNP effect on DKTatlas lh parsorbitalis area

Batch 3018 : DKTatlas lh postcentral area on Crohn's disease

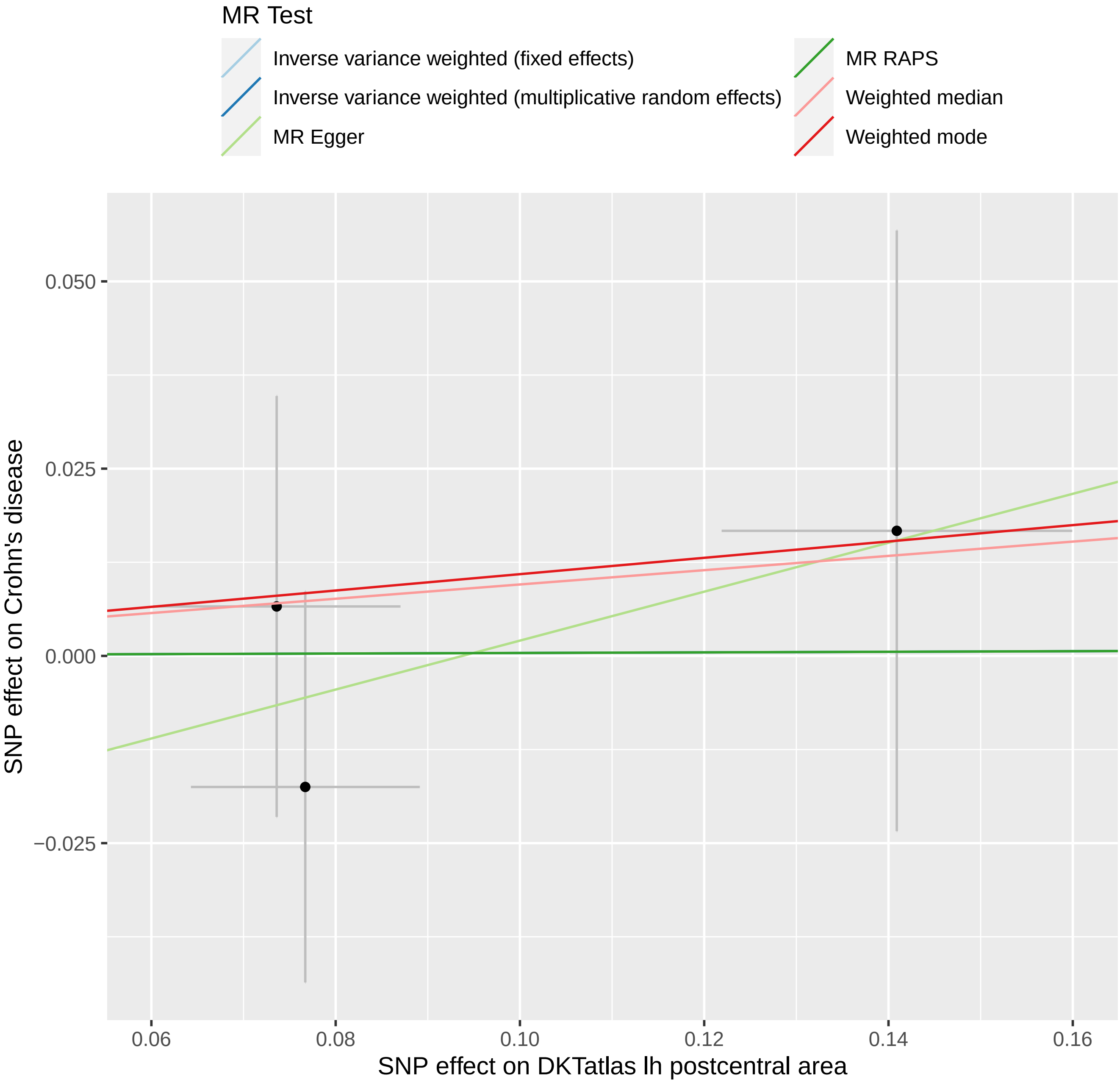

Batch 3021 : DKTatlas lh precuneus area on Crohn's disease

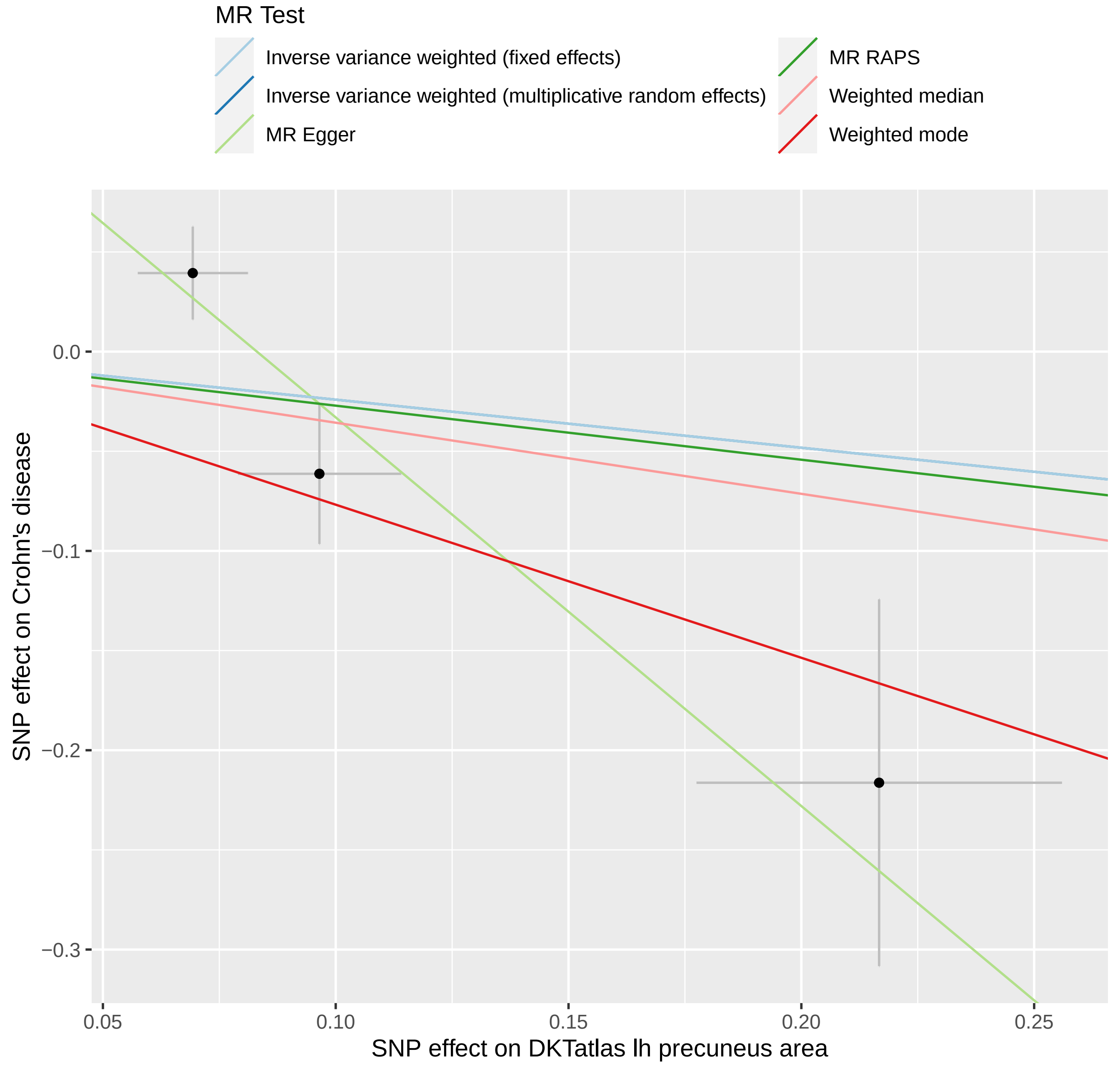

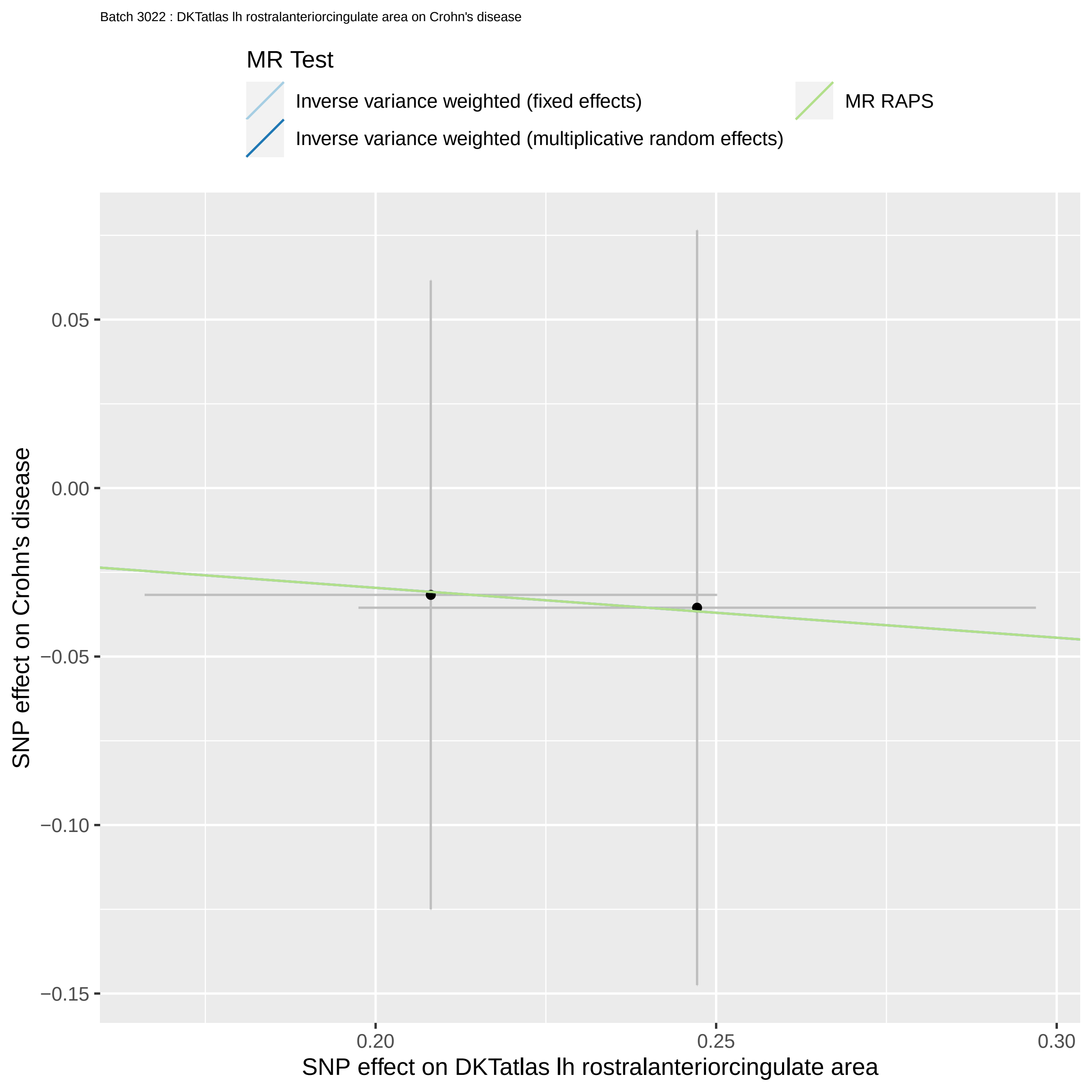
Batch 3022 : DKTatlas lh rostralanteriorcingulate area on Crohn's disease
MR Test
Inverse variance weighted (fixed effects)
Inverse variance weighted (multiplicative random effects)
MR RAPS
0.05
0.00
−0.05
−0.10
−0.15
0.20
0.25
0.30
SNP effect on Crohn's disease
SNP effect on DKTatlas lh rostralanteriorcingulate area

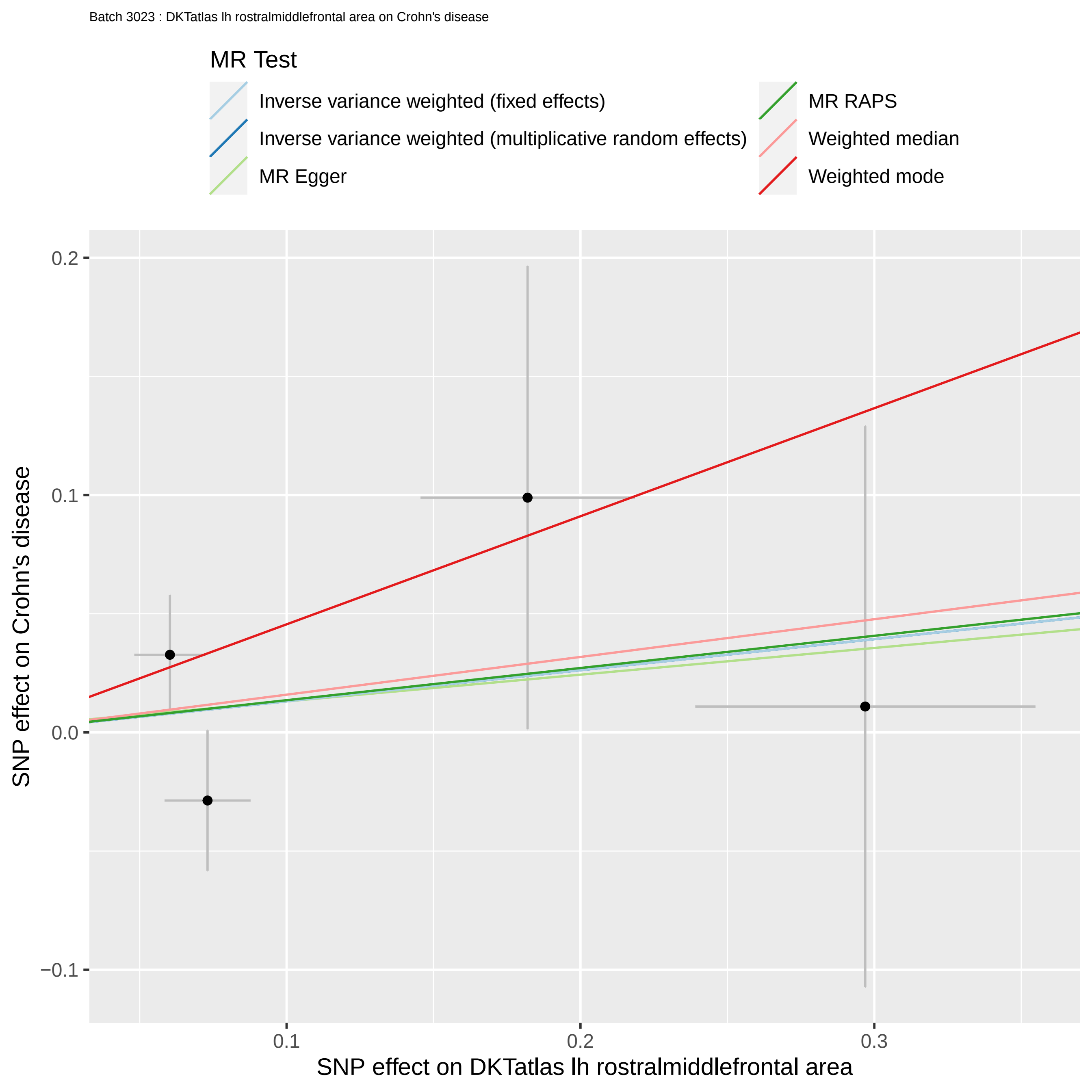
Batch 3023 : DKTatlas lh rostralmiddlefrontal area on Crohn's disease
MR Test
Inverse variance weighted (fixed effects)
Inverse variance weighted (multiplicative random effects)
MR Egger
MR RAPS
Weighted median
Weighted mode
0.2
0.1
0.0
−0.1
0.1
0.2
0.3
SNP effect on Crohn's disease
SNP effect on DKTatlas lh rostralmiddlefrontal area

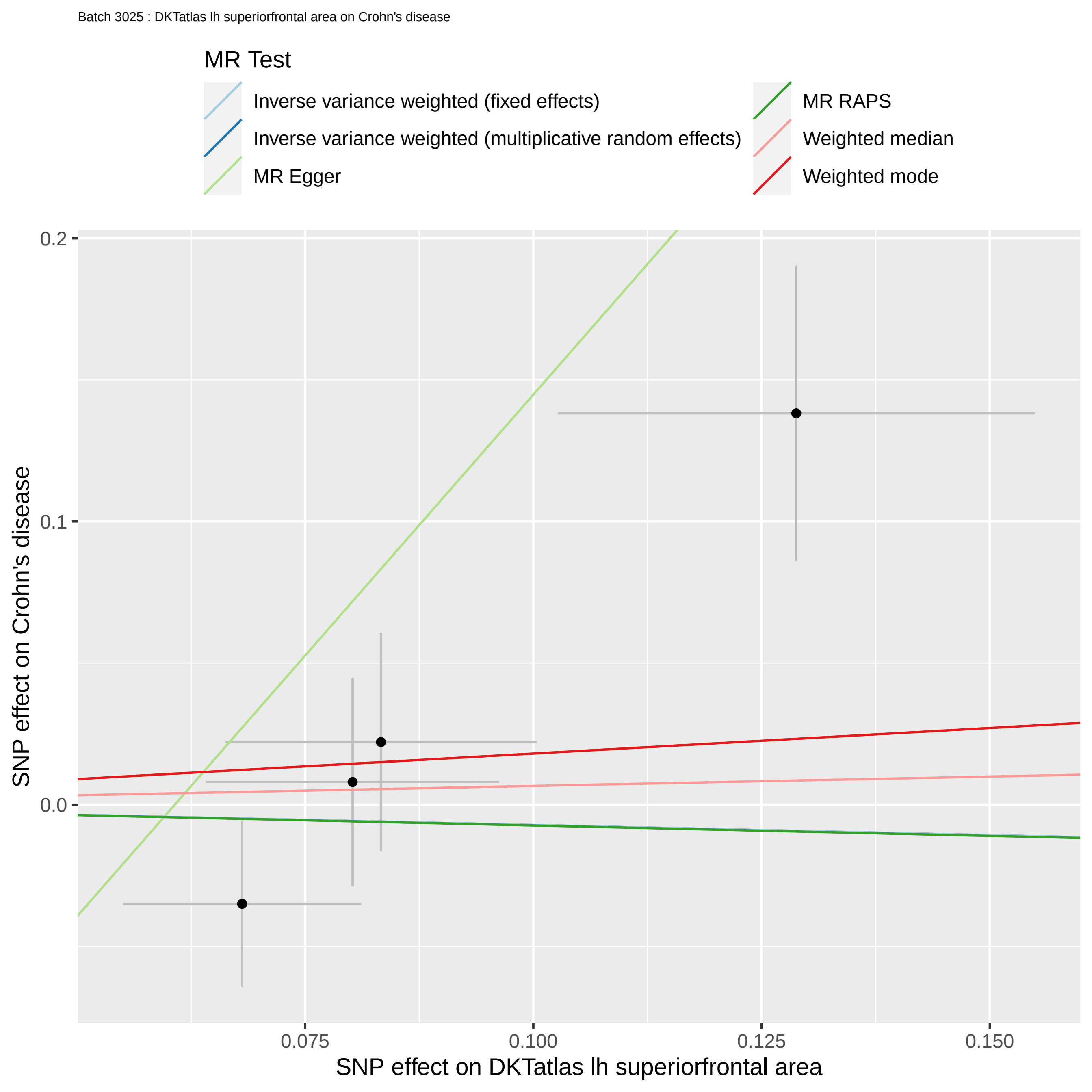
Batch 3025 : DKTatlas lh superiorfrontal area on Crohn's disease
MR Test
Inverse variance weighted (fixed effects)
Inverse variance weighted (multiplicative random effects)
MR Egger
MR RAPS
Weighted median
Weighted mode
0.2
0.1
0.0
0.075
0.100
0.125
0.150
SNP effect on Crohn's disease
SNP effect on DKTatlas lh superiorfrontal area

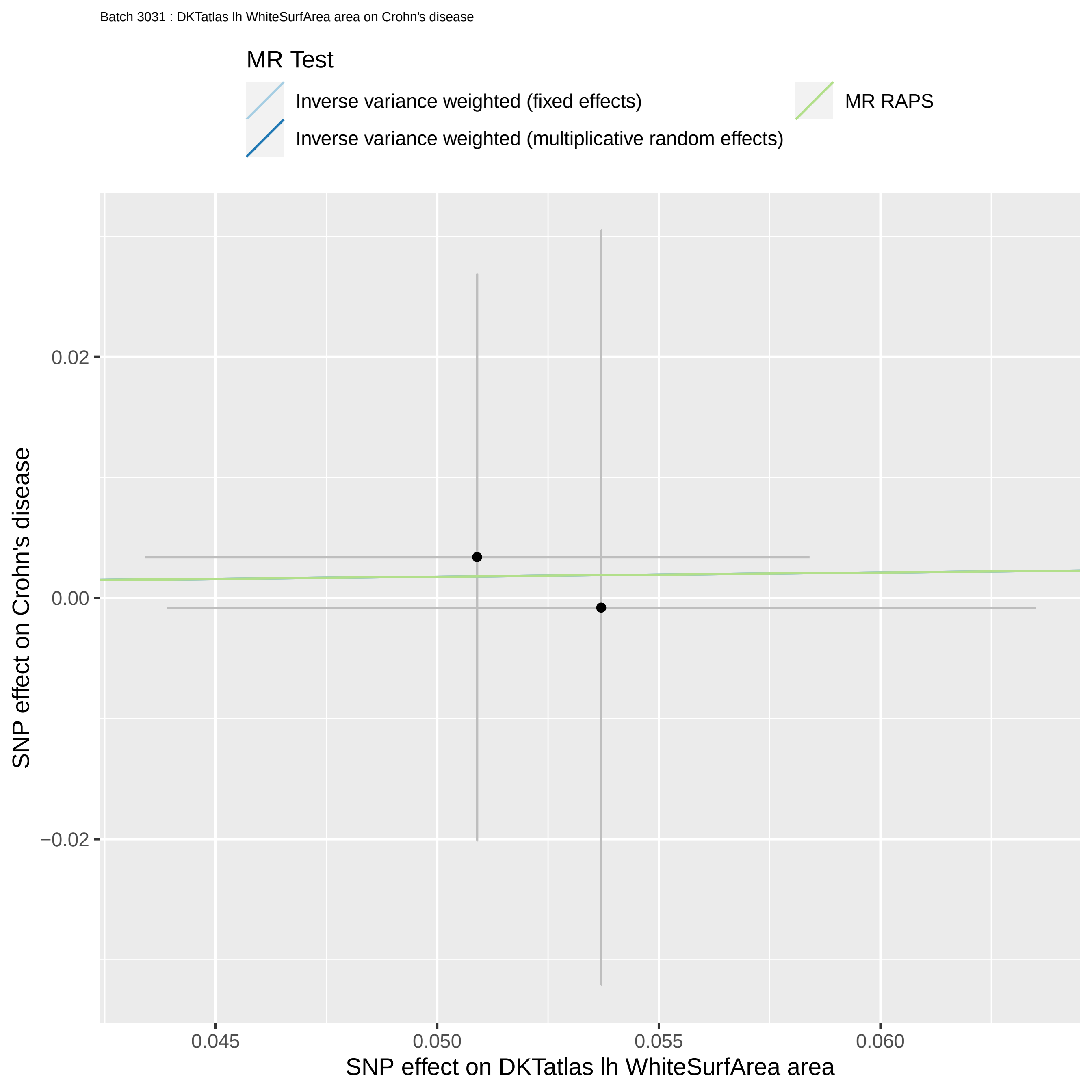
Batch 3031 : DKTatlas lh WhiteSurfArea area on Crohn's disease
MR Test
Inverse variance weighted (fixed effects)
Inverse variance weighted (multiplicative random effects)
MR RAPS
0.02
0.00
−0.02
0.045
0.050
0.055
0.060
SNP effect on Crohn's disease
SNP effect on DKTatlas lh WhiteSurfArea area

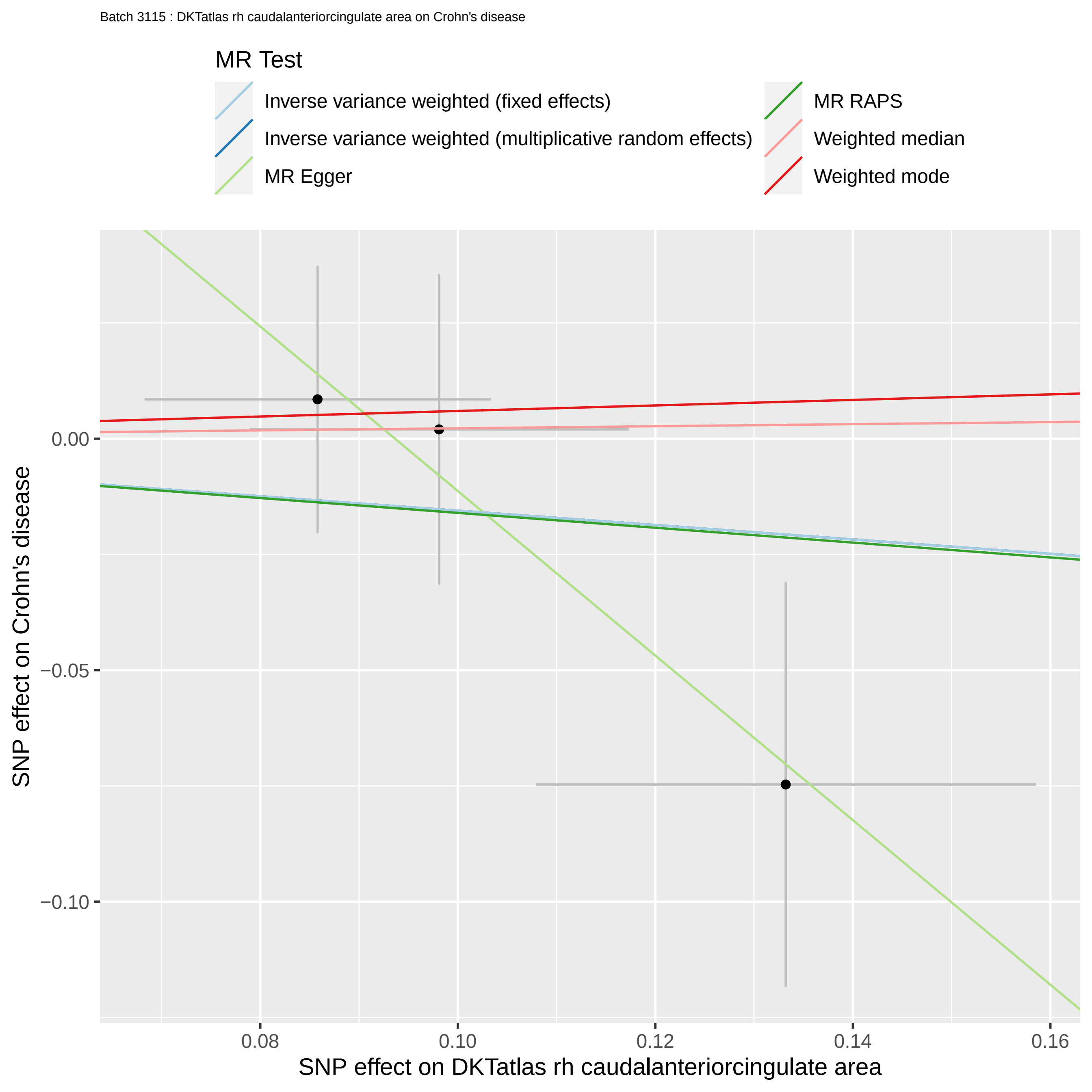

Batch 3115 : DKTatlas rh caudalanteriorcingulate area on Crohn's disease
MR Test
Inverse variance weighted (fixed effects)
Inverse variance weighted (multiplicative random effects)
MR Egger
MR RAPS
Weighted median
Weighted mode
0.00
−0.05
−0.10
SNP effect on Crohn's disease
0.08
0.10
0.12
0.14
0.16
SNP effect on DKTatlas rh caudalanteriorcingulate area

Batch 3118 : DKTatlas rh entorhinal area on Crohn's disease

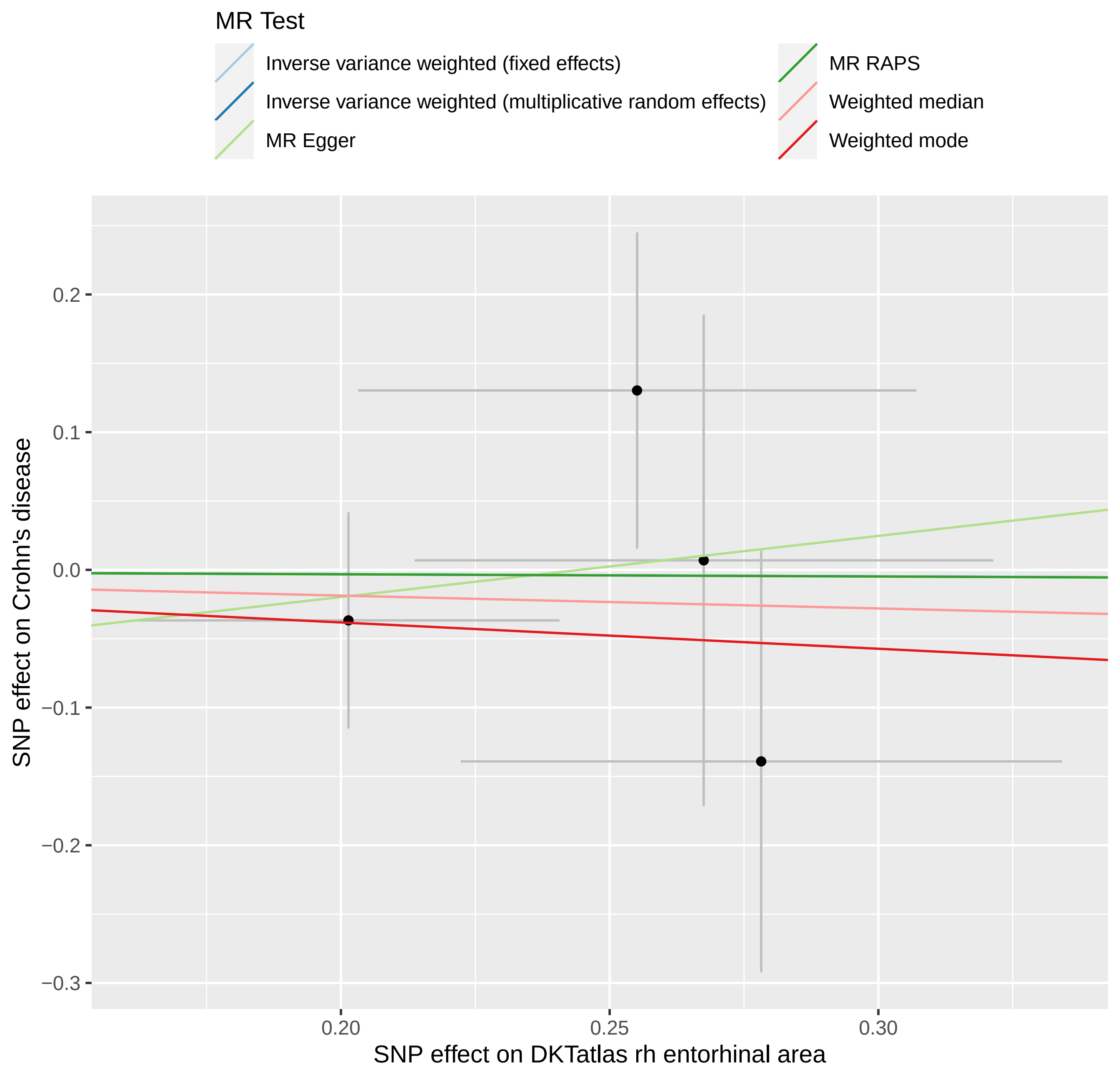

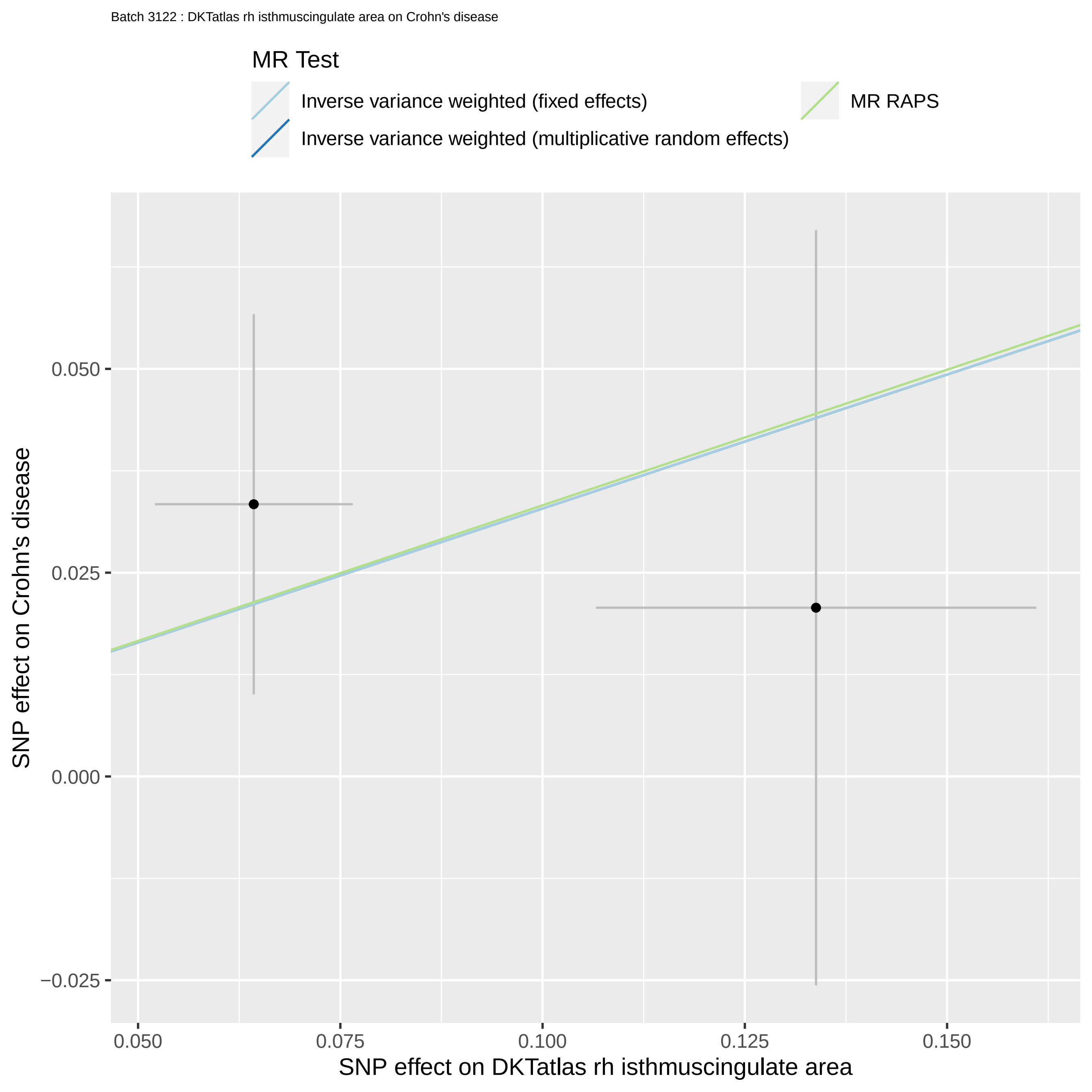

Batch 3122 : DKTatlas rh isthmuscingulate area on Crohn's disease
MR Test
Inverse variance weighted (fixed effects)
Inverse variance weighted (multiplicative random effects)
MR RAPS
SNP effect on Crohn's disease
0.050
0.025
0.000
−0.025
0.050
0.075
0.100
0.125
0.150
SNP effect on DKTatlas rh isthmuscingulate area

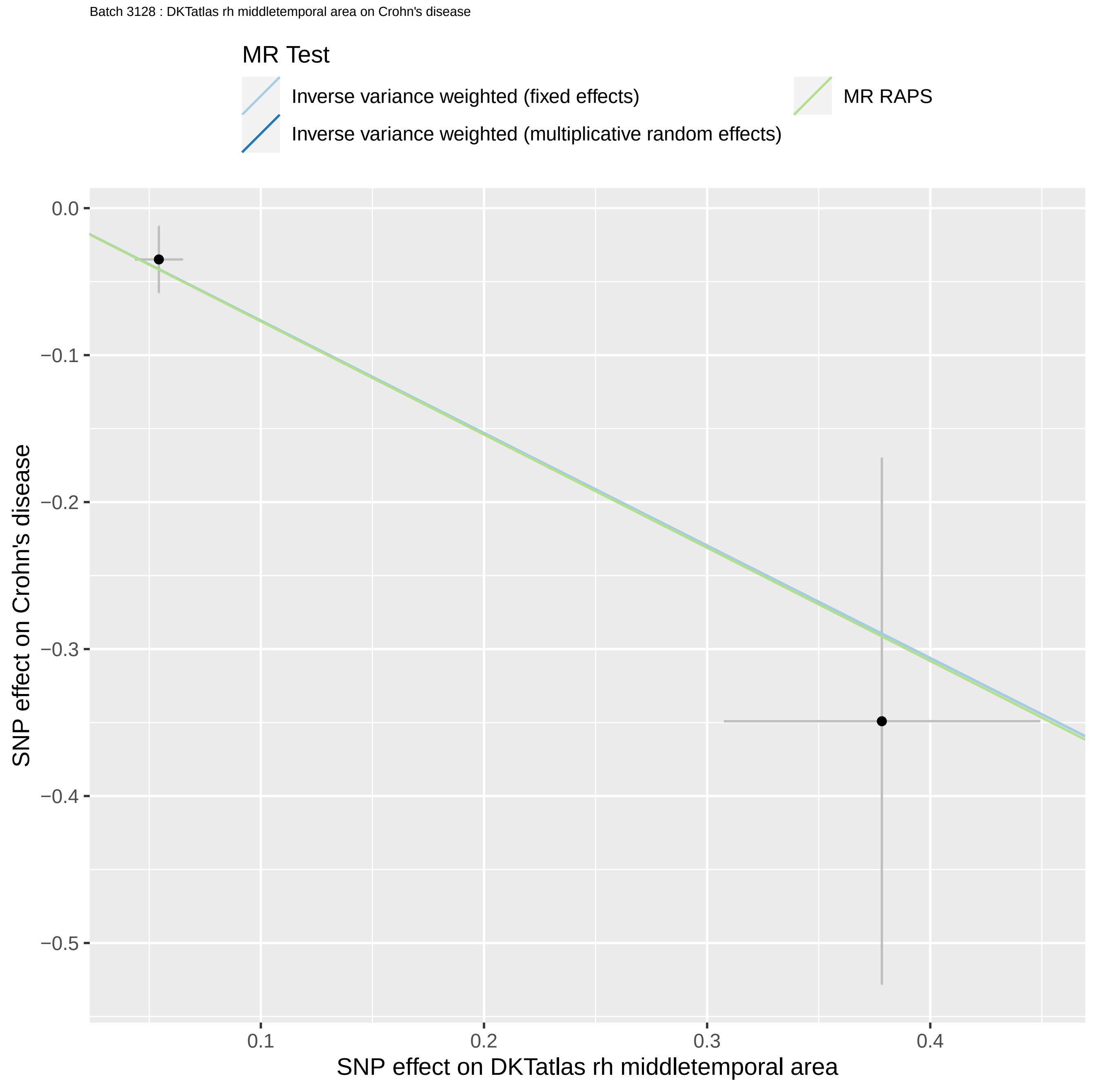

Batch 3128 : DKTatlas rh middletemporal area on Crohn's disease
MR Test
Inverse variance weighted (fixed effects)
Inverse variance weighted (multiplicative random effects)
MR RAPS
0.0
−0.1
−0.2
−0.3
−0.4
−0.5
0.1
0.2
0.3
0.4
SNP effect on Crohn's disease
SNP effect on DKTatlas rh middletemporal area

Batch 3131 : DKTatlas rh parsopercularis area on Crohn's disease

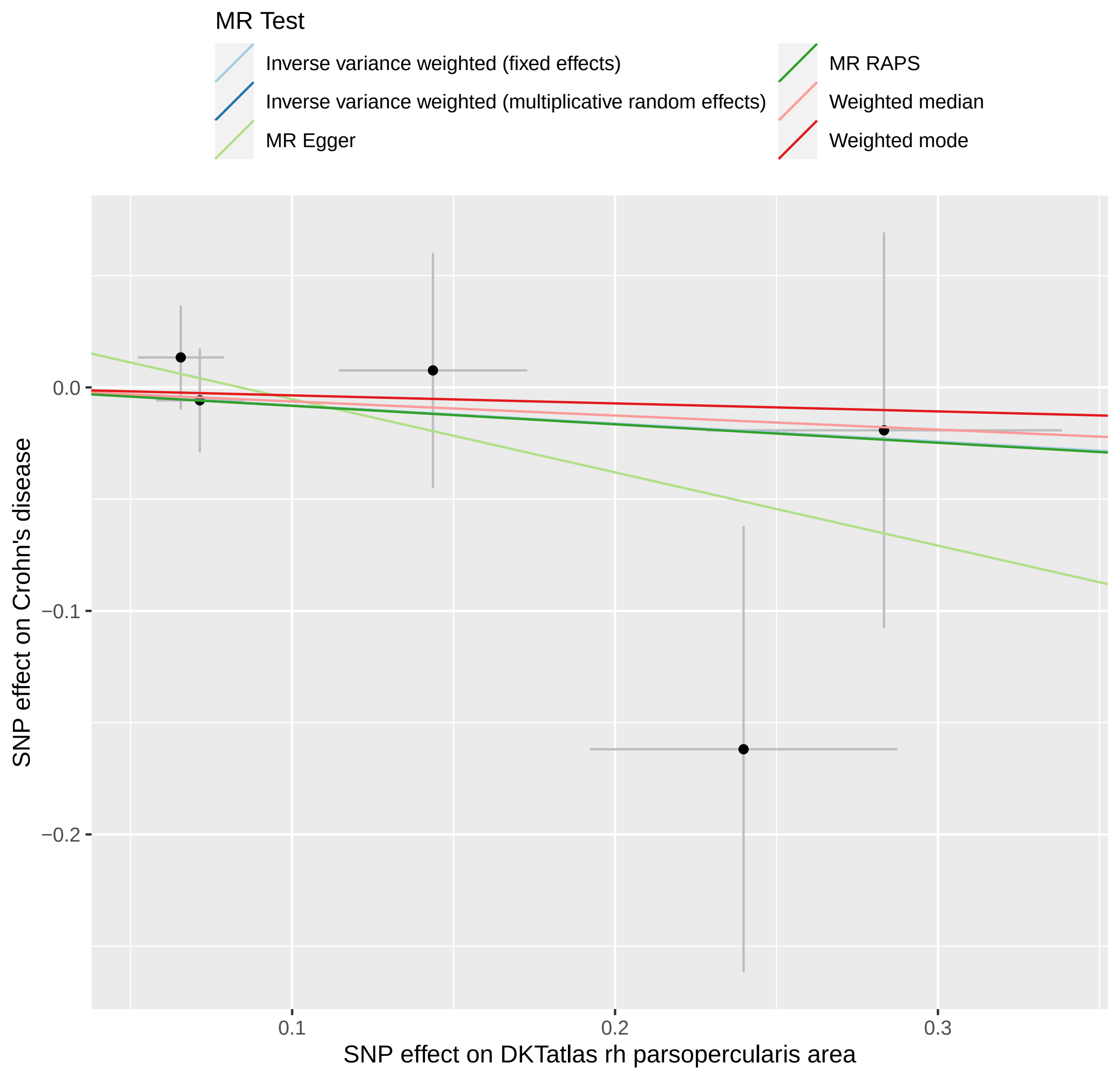

Batch 3134 : DKTatlas rh pericalcarine area on Crohn's disease

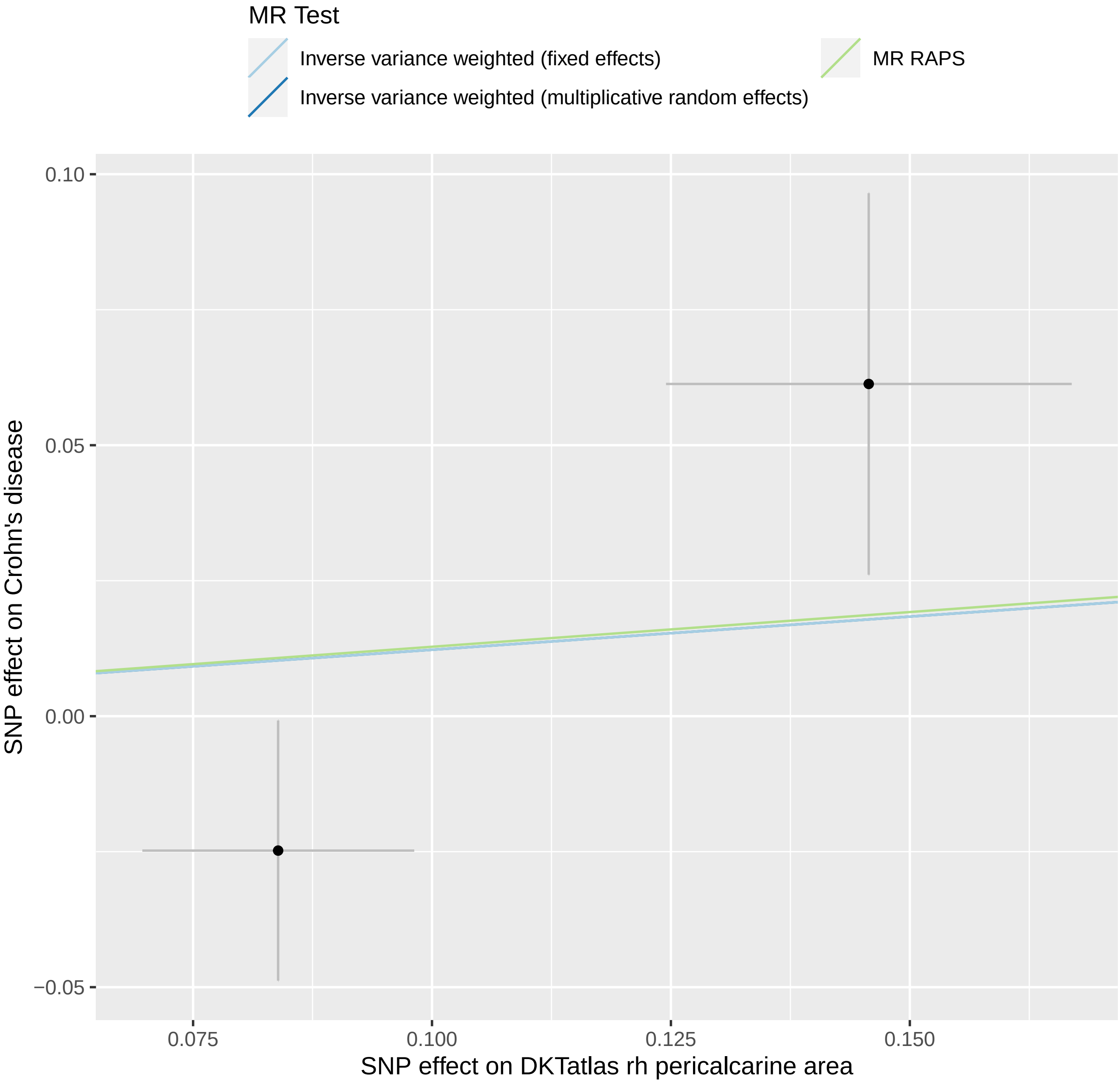

Batch 3136 : DKTatlas rh postcentral area on Crohn's disease

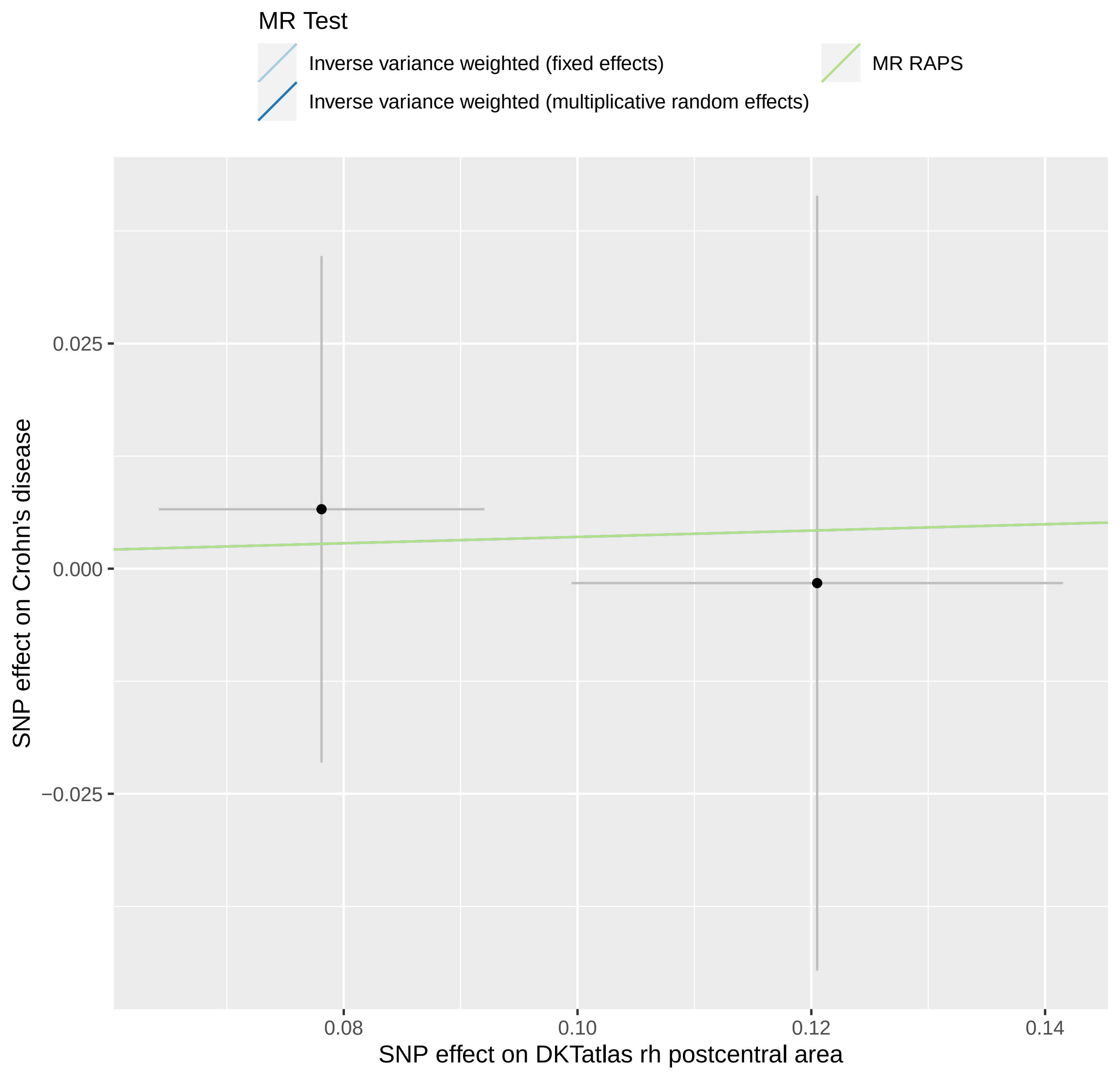

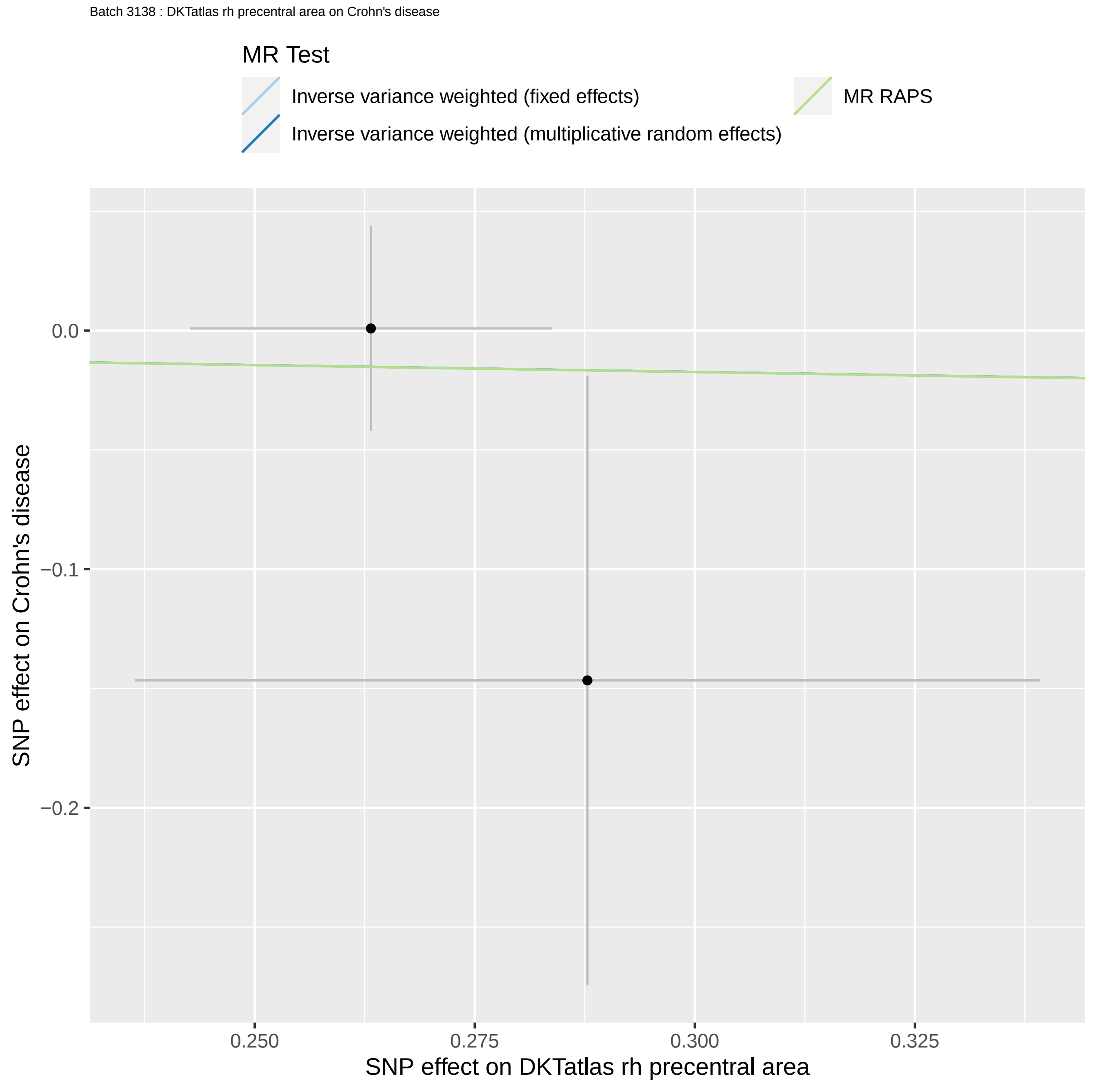
Batch 3138 : DKTatlas rh precentral area on Crohn's disease
MR Test
Inverse variance weighted (fixed effects)
Inverse variance weighted (multiplicative random effects)
MR RAPS
0.0
−0.1
−0.2
0.250
0.275
0.300
0.325
SNP effect on Crohn's disease
SNP effect on DKTatlas rh precentral area

Batch 3139 : DKTatlas rh precuneus area on Crohn's disease

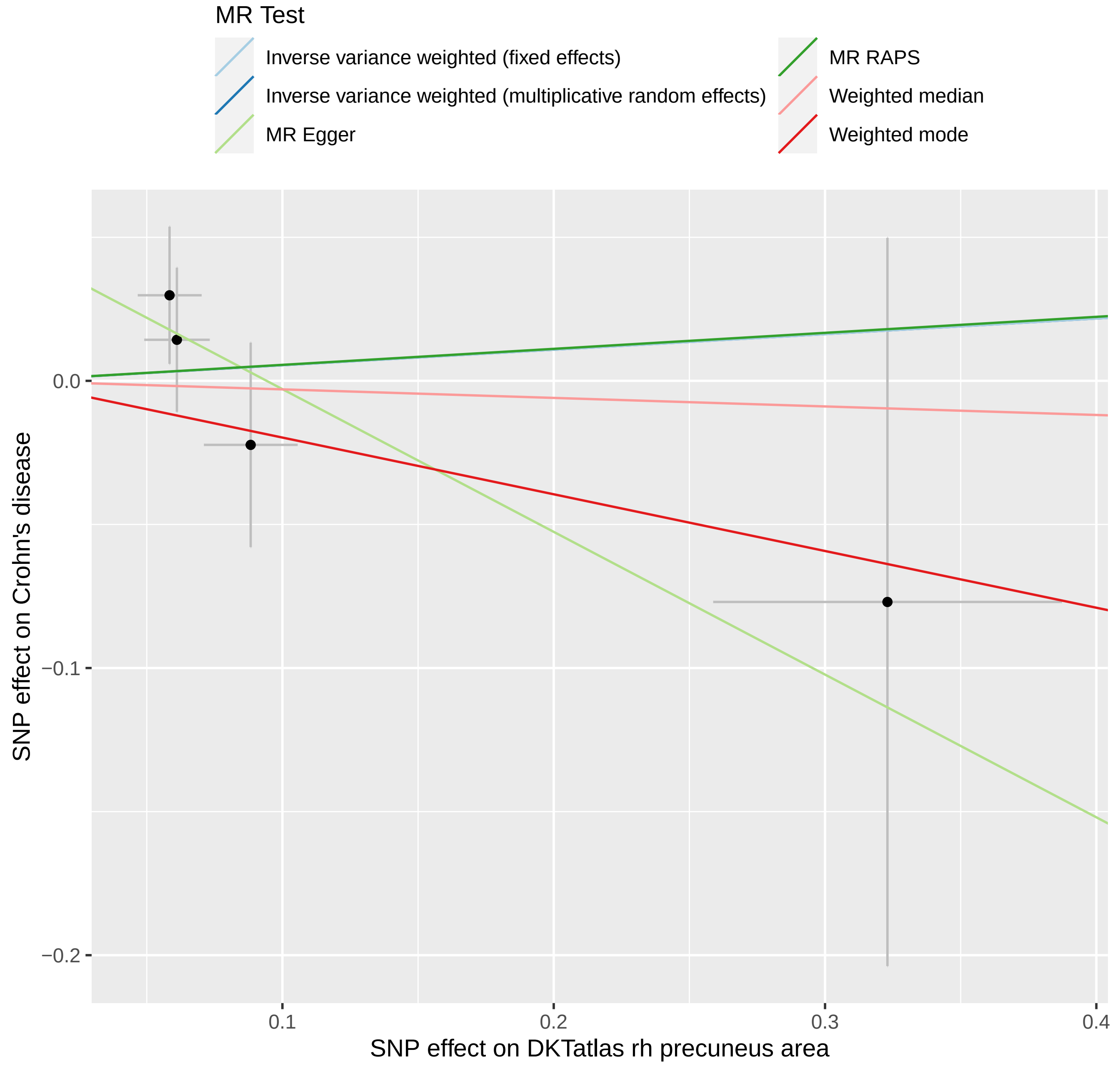

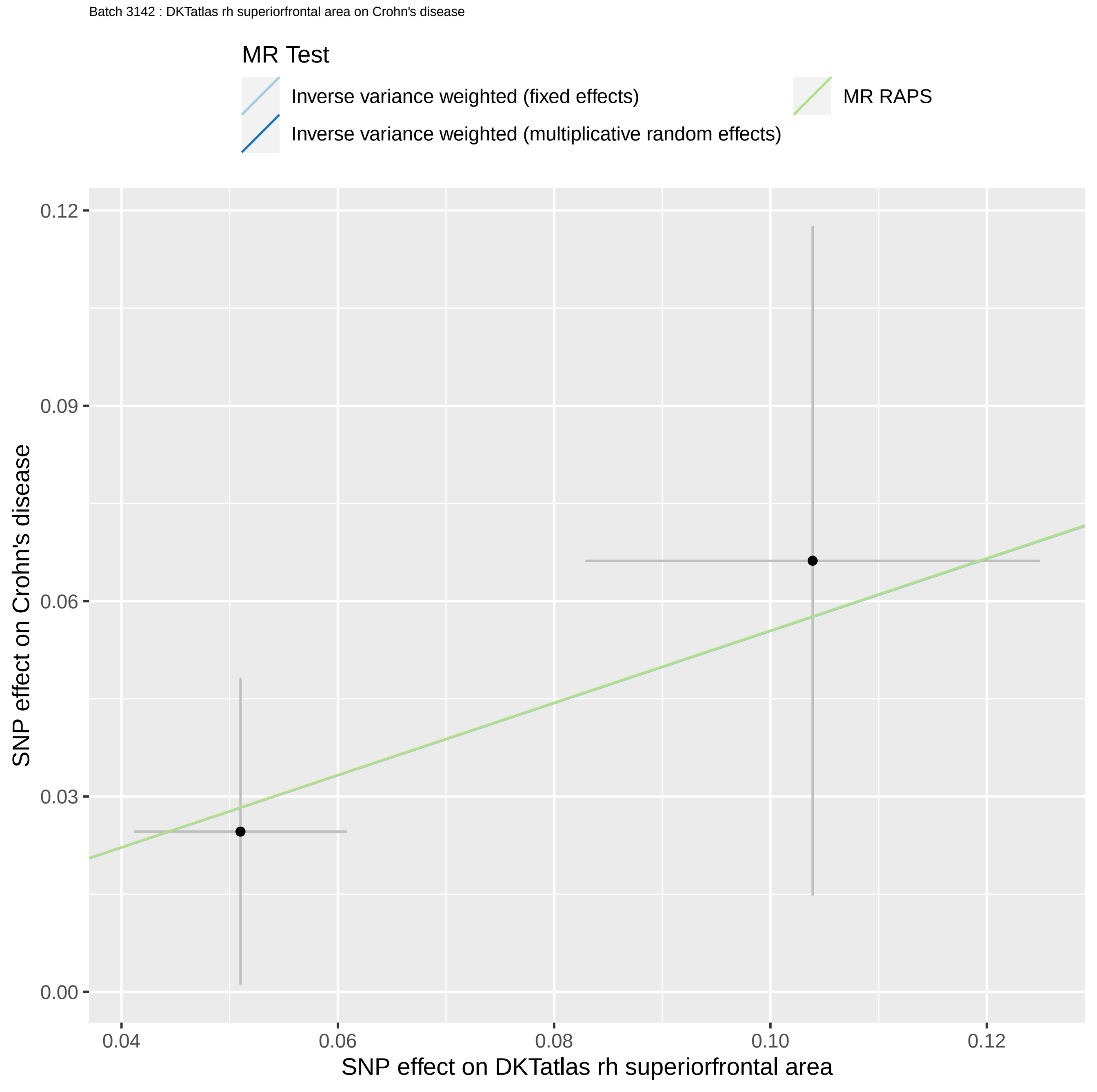
Batch 3142 : DKTatlas rh superiorfrontal area on Crohn's disease
MR Test
Inverse variance weighted (fixed effects)
Inverse variance weighted (multiplicative random effects)
MR RAPS
SNP effect on Crohn's disease
0.12
0.09
0.06
0.03
0.00
0.04
0.06
0.08
0.10
0.12
SNP effect on DKTatlas rh superiorfrontal area

Batch 3144 : DKTatlas rh superiortemporal area on Crohn's disease

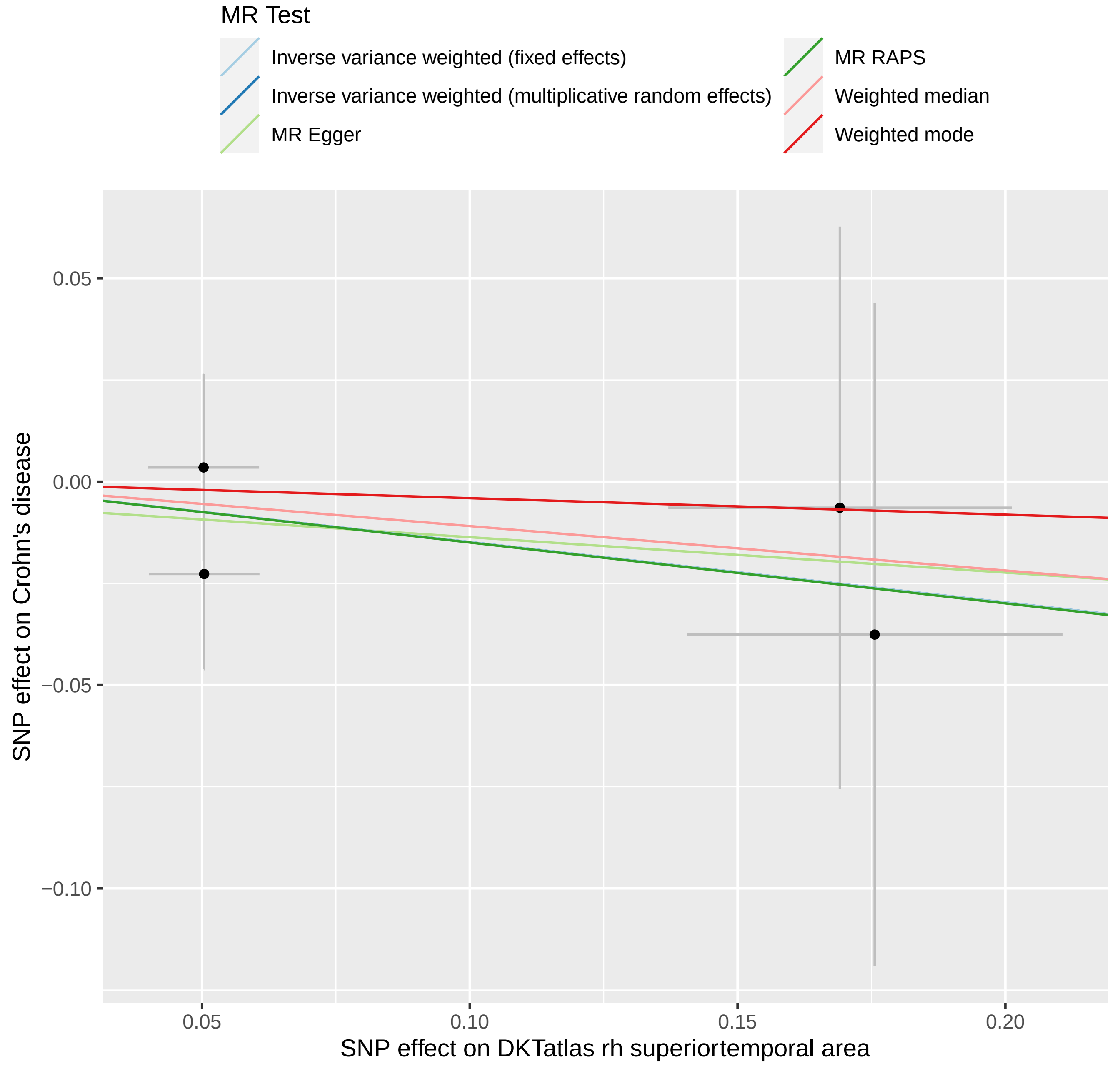

Batch 3147 : DKTatlas rh transversetemporal area on Crohn's disease

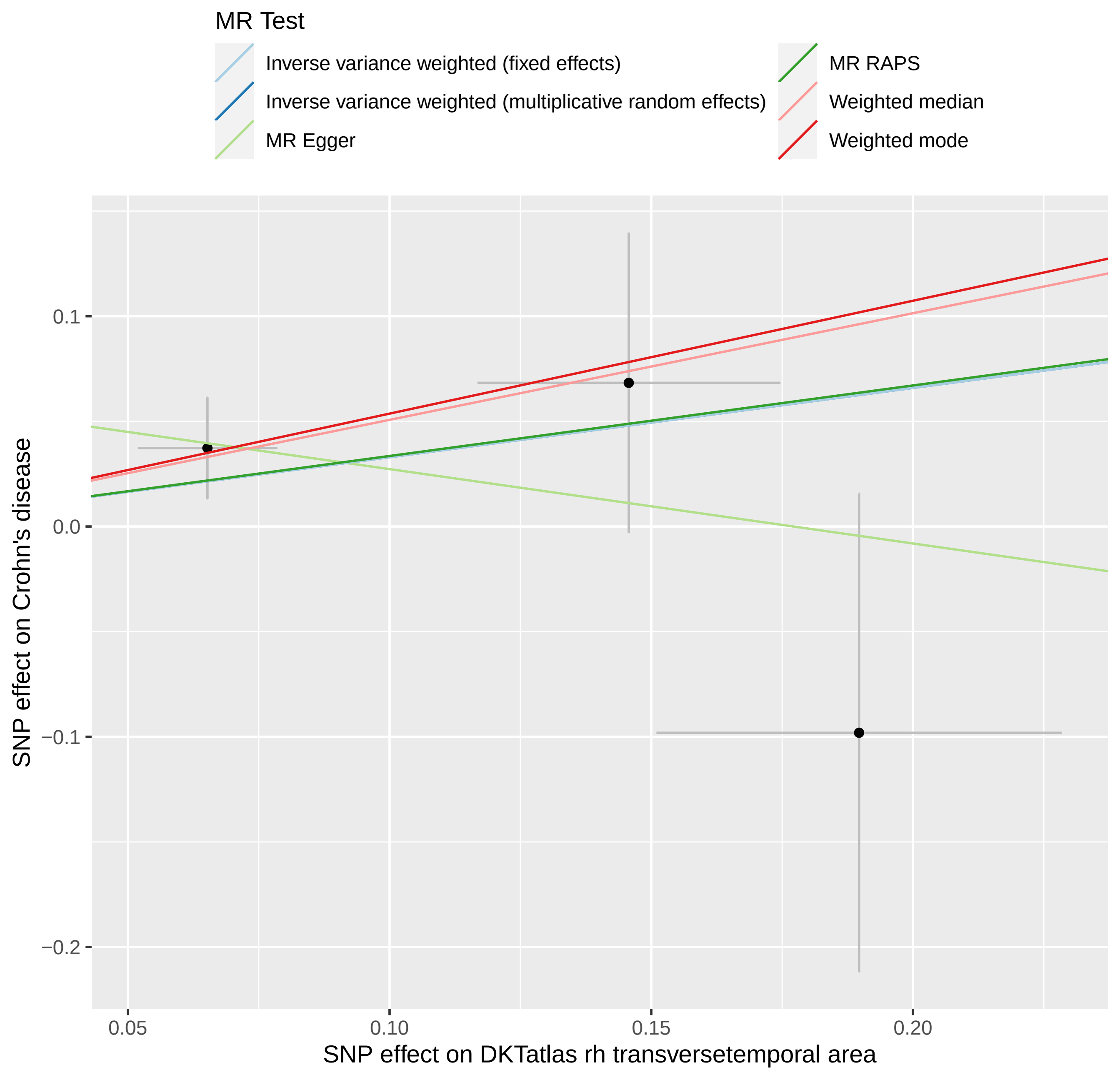

Batch 3148 : DKTatlas rh insula area on Crohn's disease

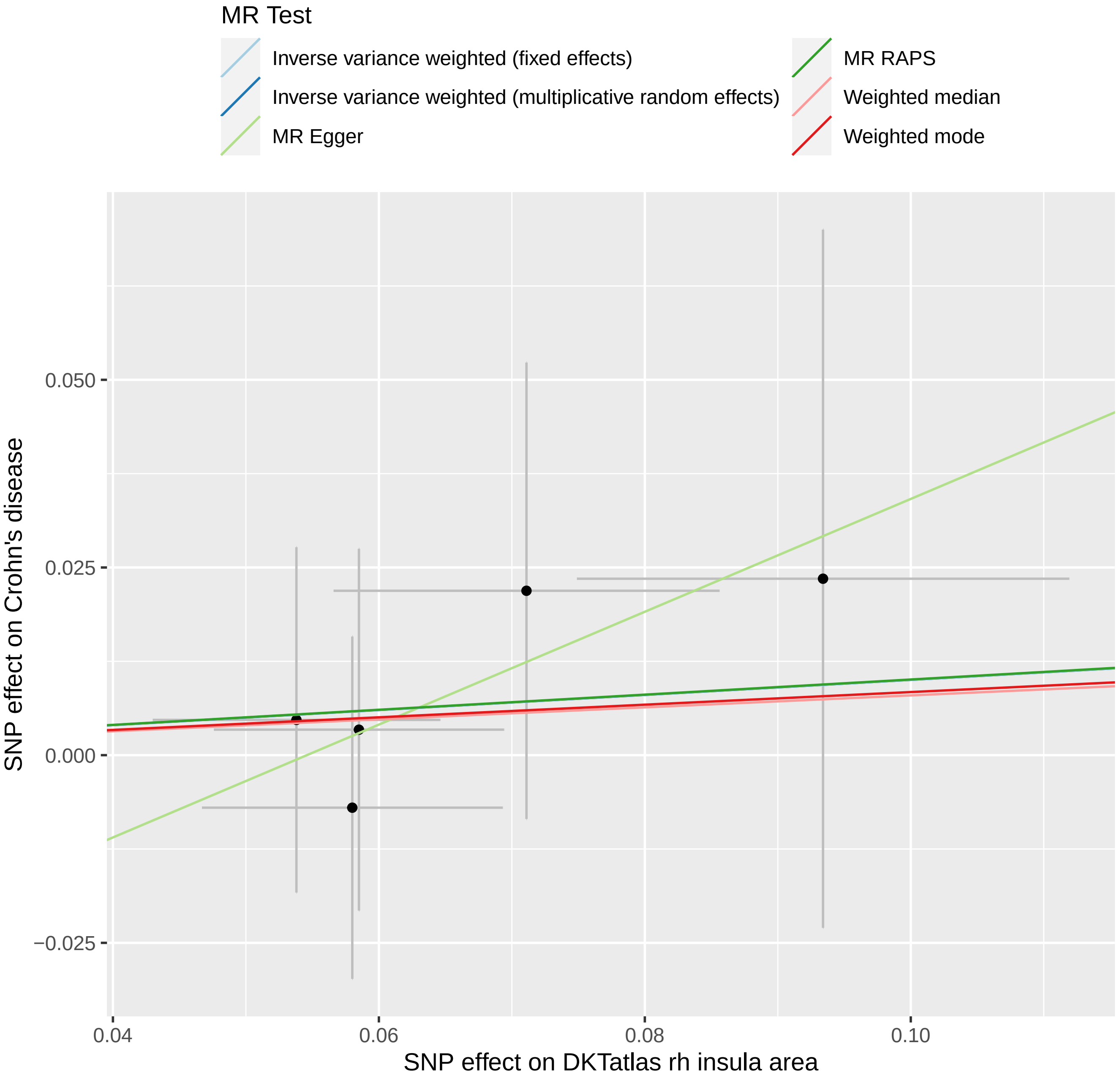

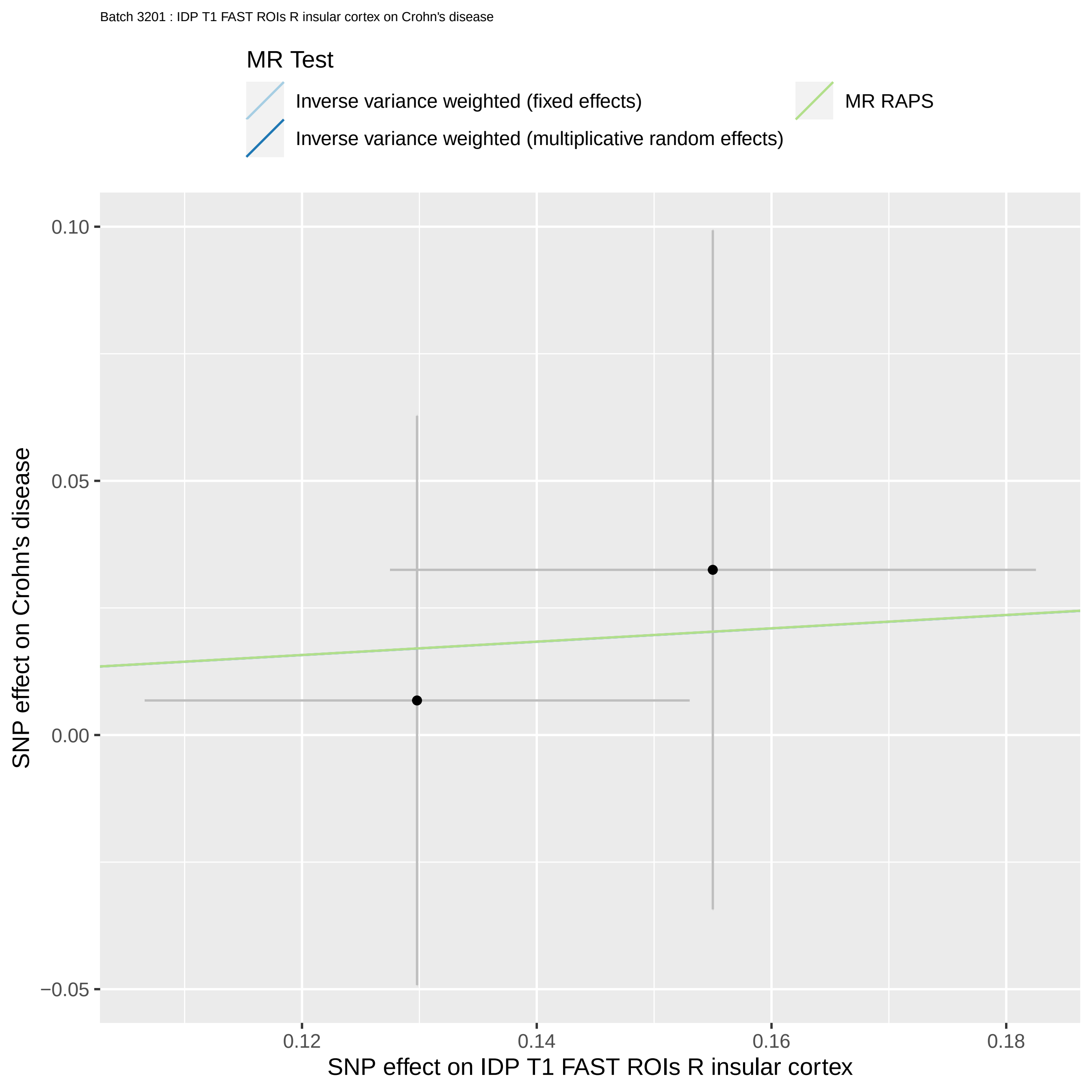

Batch 3201 : IDP T1 FAST ROIs R insular cortex on Crohn's disease
MR Test
Inverse variance weighted (fixed effects)
Inverse variance weighted (multiplicative random effects)
MR RAPS
0.10
0.05
0.00
−0.05
0.12
0.14
0.16
0.18
SNP effect on Crohn's disease
SNP effect on IDP T1 FAST ROIs R insular cortex

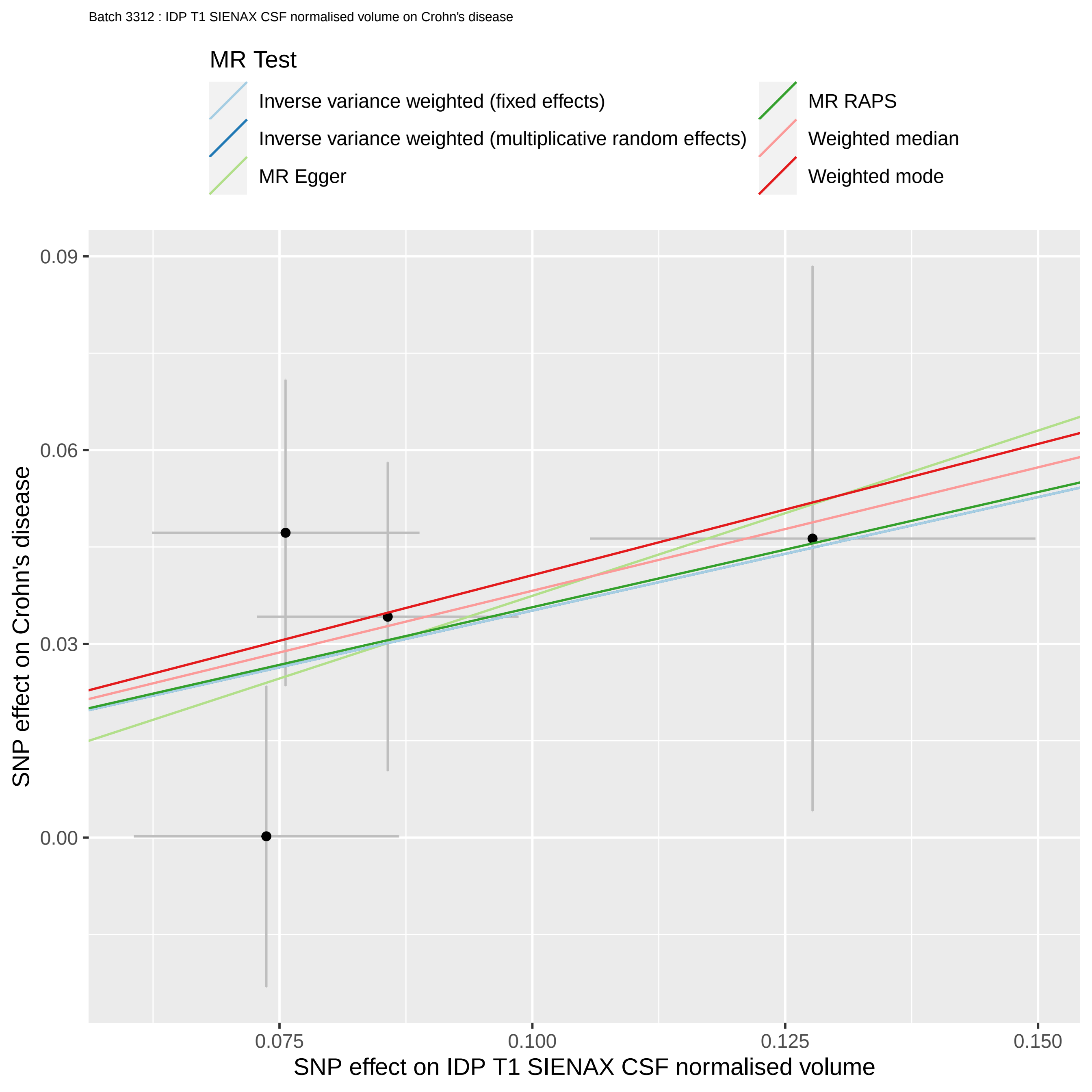

Batch 3312 : IDP T1 SIENAX CSF normalised volume on Crohn's disease
MR Test
Inverse variance weighted (fixed effects)
Inverse variance weighted (multiplicative random effects)
MR Egger
MR RAPS
Weighted median
Weighted mode
0.09
0.06
0.03
0.00
0.075
0.100
0.125
0.150
SNP effect on Crohn's disease
SNP effect on IDP T1 SIENAX CSF normalised volume

Batch 3424 : IDP T1 FAST ROIs R sup front gyrus on Crohn's disease

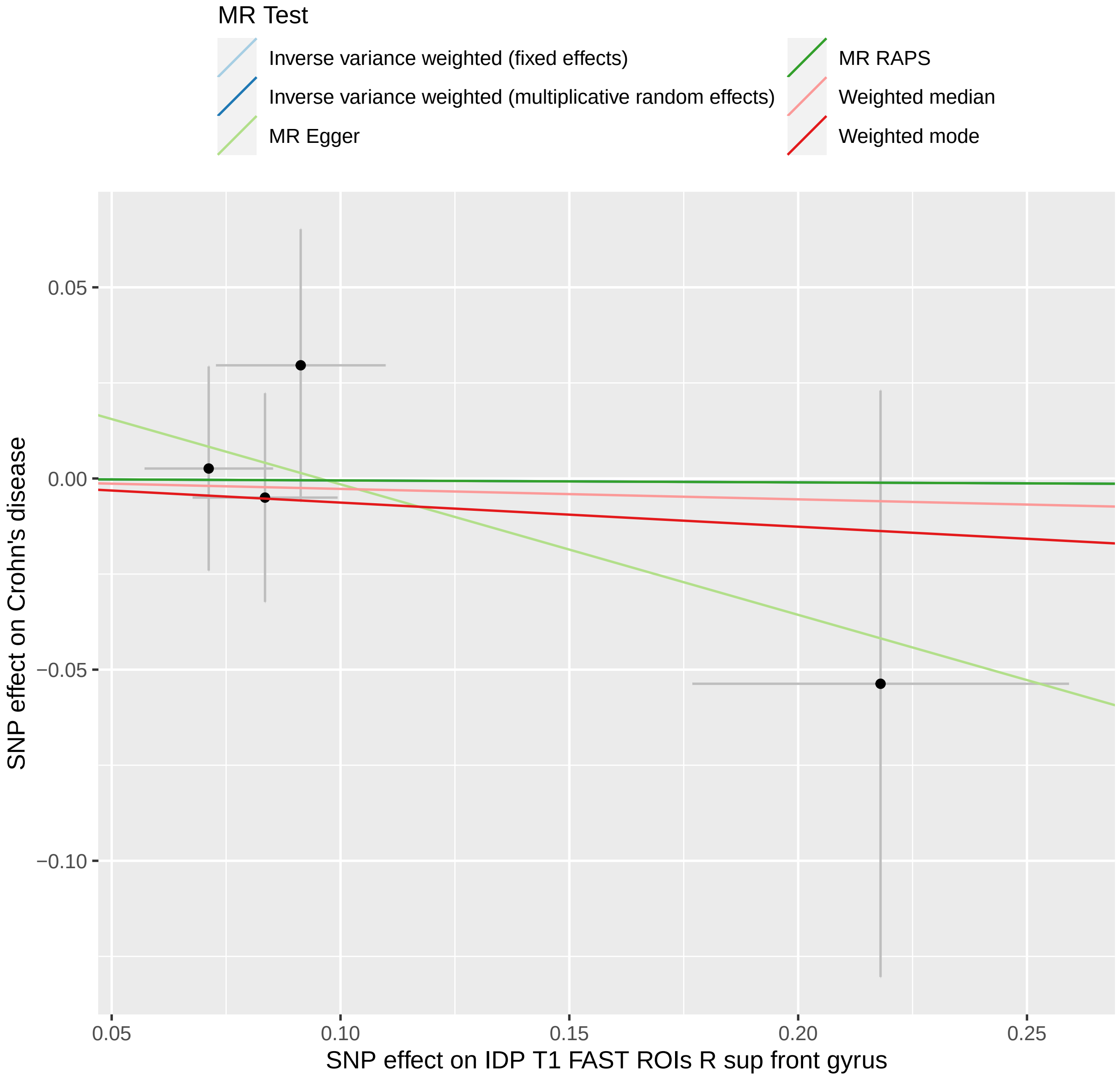

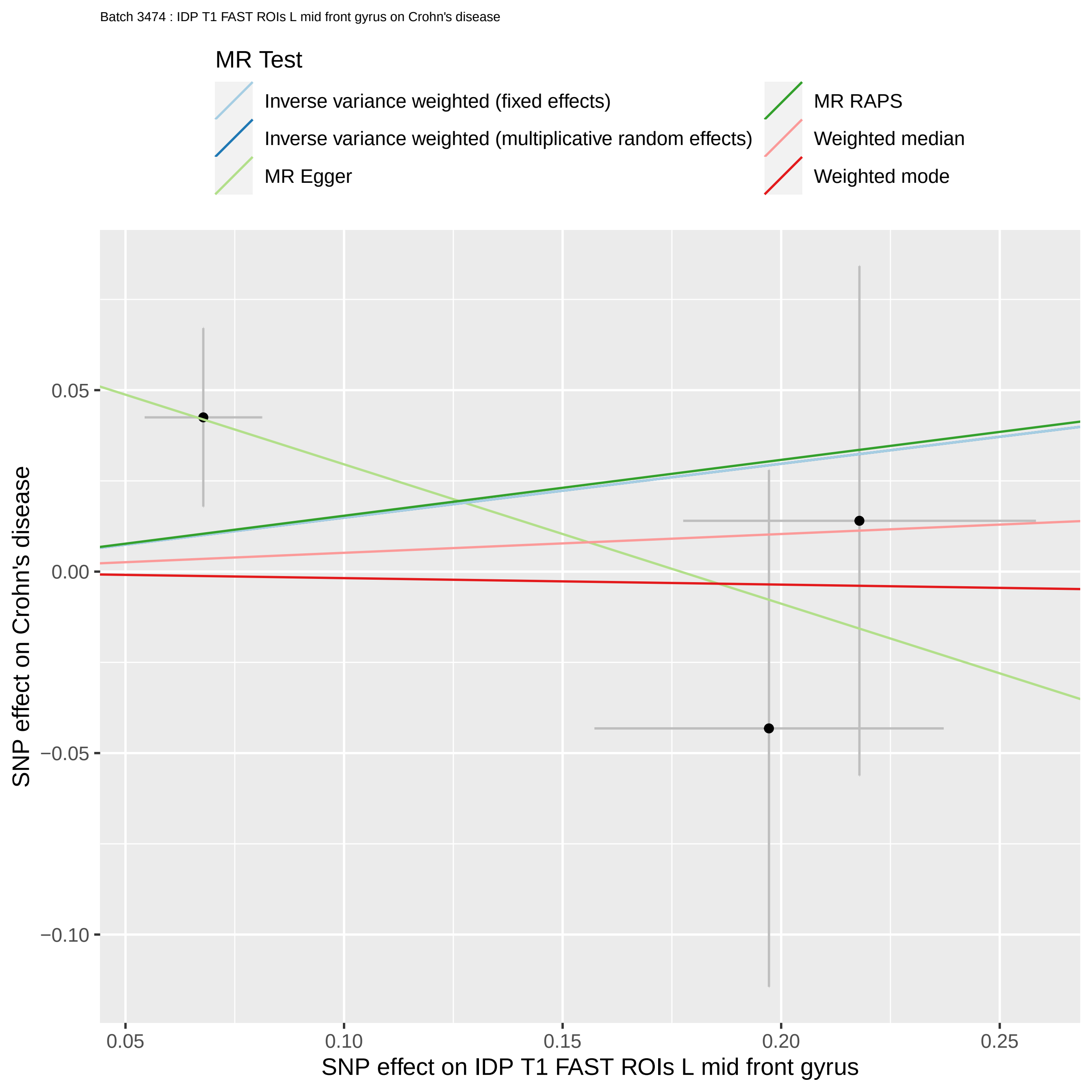
Batch 3474 : IDP T1 FAST ROIs L mid front gyrus on Crohn's disease
MR Test
Inverse variance weighted (fixed effects)
Inverse variance weighted (multiplicative random effects)
MR Egger
MR RAPS
Weighted median
Weighted mode
0.05
0.00
−0.05
−0.10
0.05
0.10
0.15
0.20
0.25
SNP effect on Crohn's disease
SNP effect on IDP T1 FAST ROIs L mid front gyrus

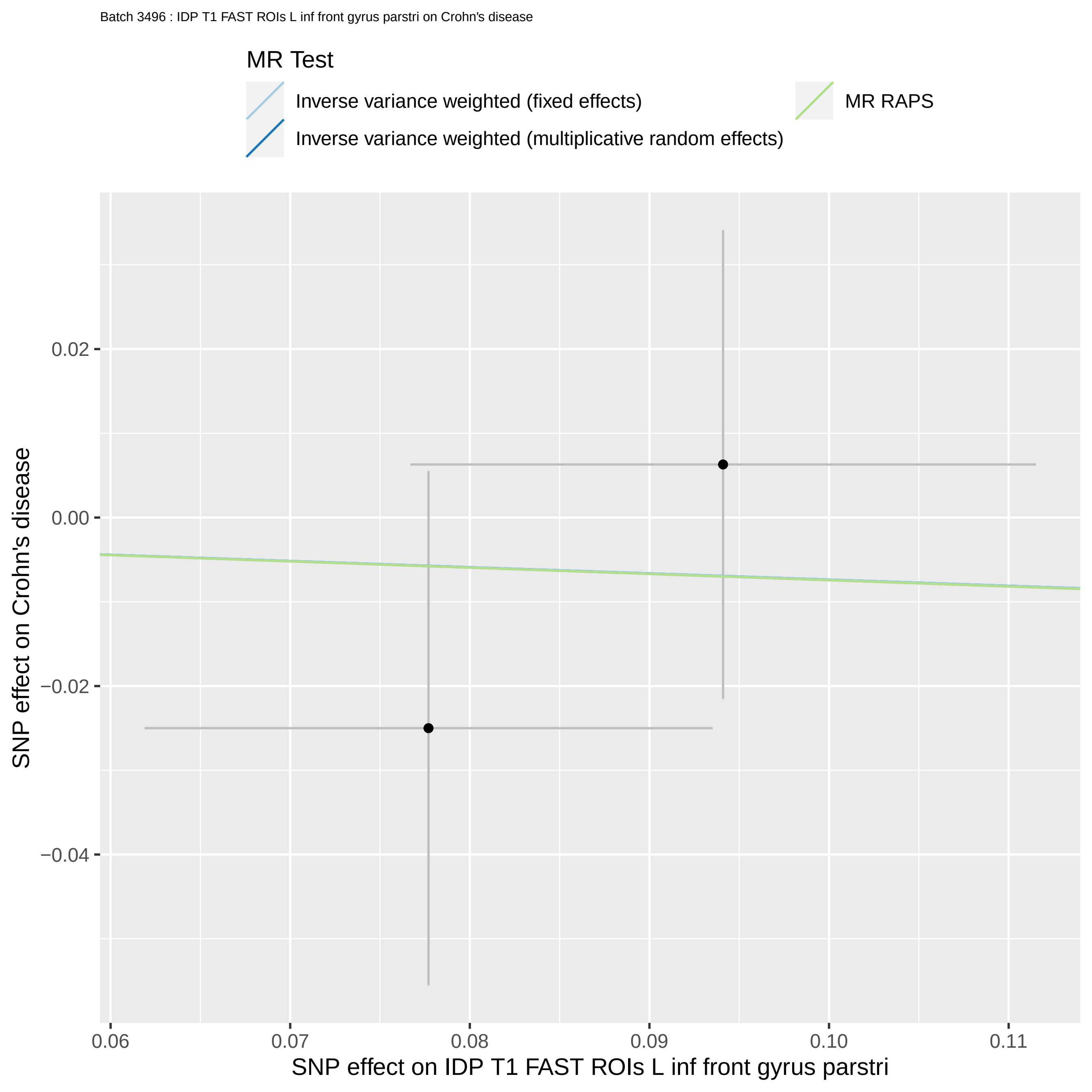

Batch 3496 : IDP T1 FAST ROIs L inf front gyrus parstri on Crohn's disease
MR Test
Inverse variance weighted (fixed effects)
Inverse variance weighted (multiplicative random effects)
MR RAPS
0.02
0.00
−0.02
−0.04
0.06
0.07
0.08
0.09
0.10
0.11
SNP effect on Crohn's disease
SNP effect on IDP T1 FAST ROIs L inf front gyrus parstri

Batch 3561 : IDP T1 SIENAX CSF unnormalised volume on Crohn's disease

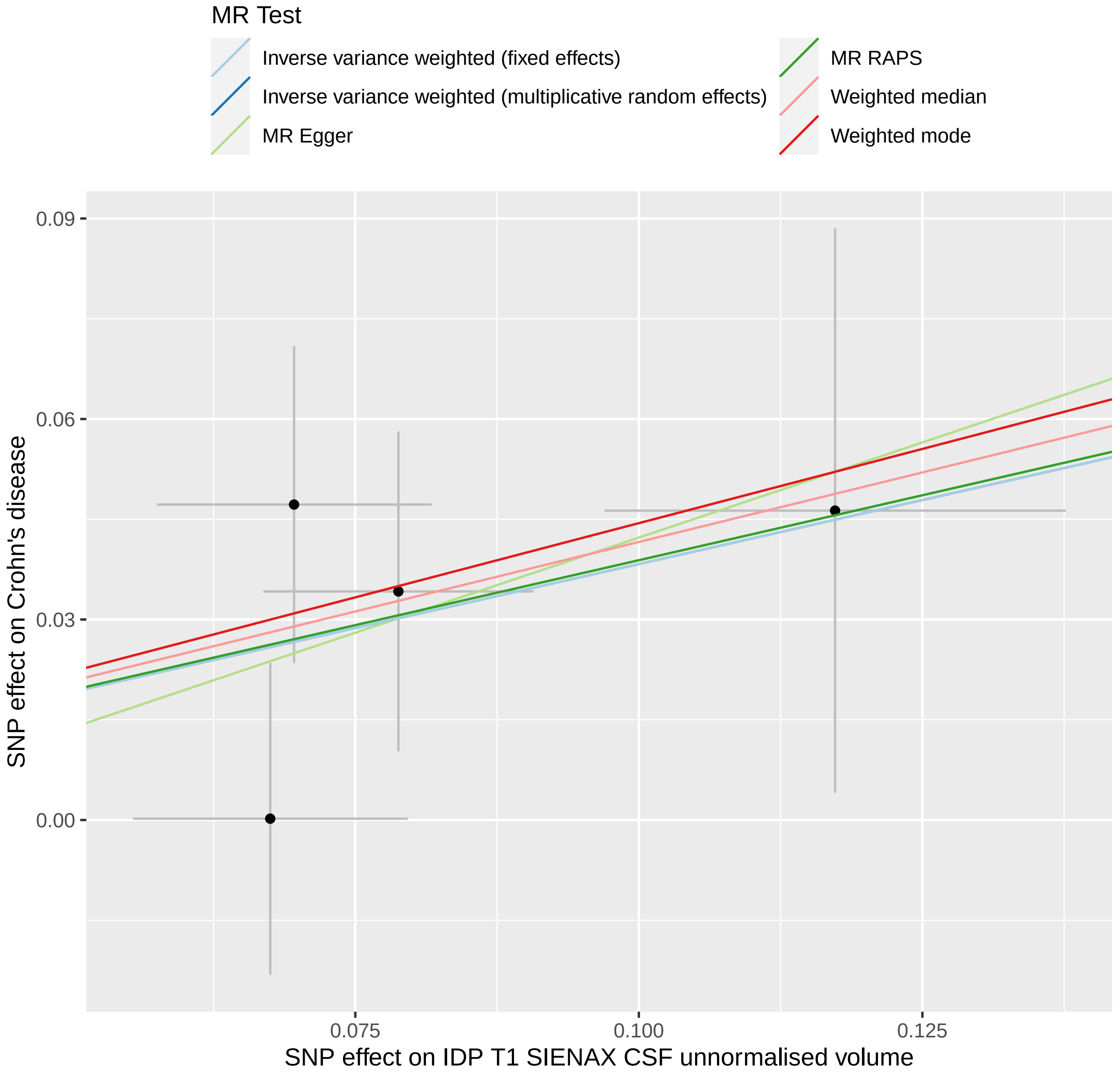

Batch 3583 : IDP T1 FAST ROIs L sup temp gyrus ant on Crohn's disease

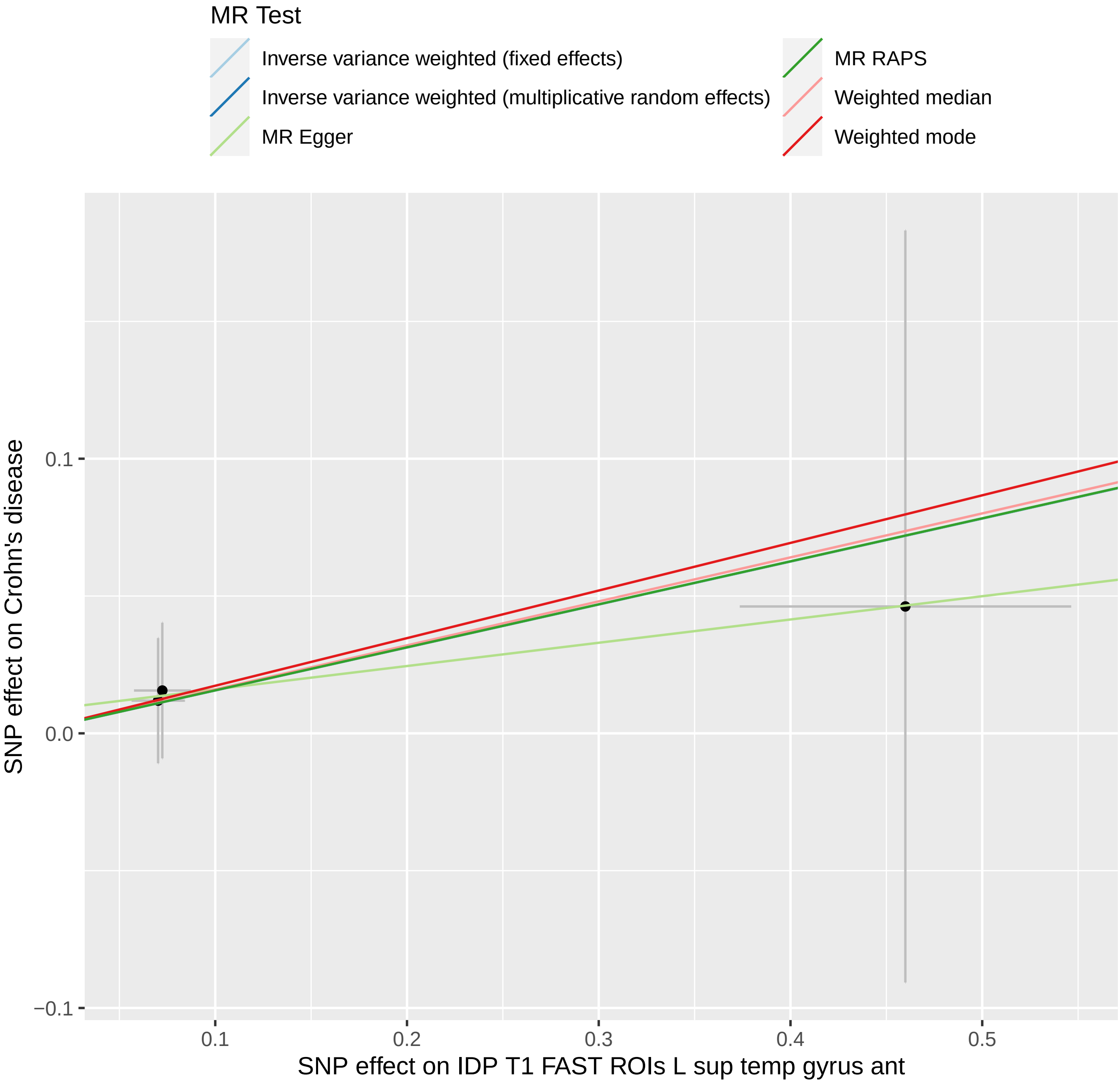

Batch 3594 : IDP T1 FAST ROIs R sup temp gyrus ant on Crohn's disease

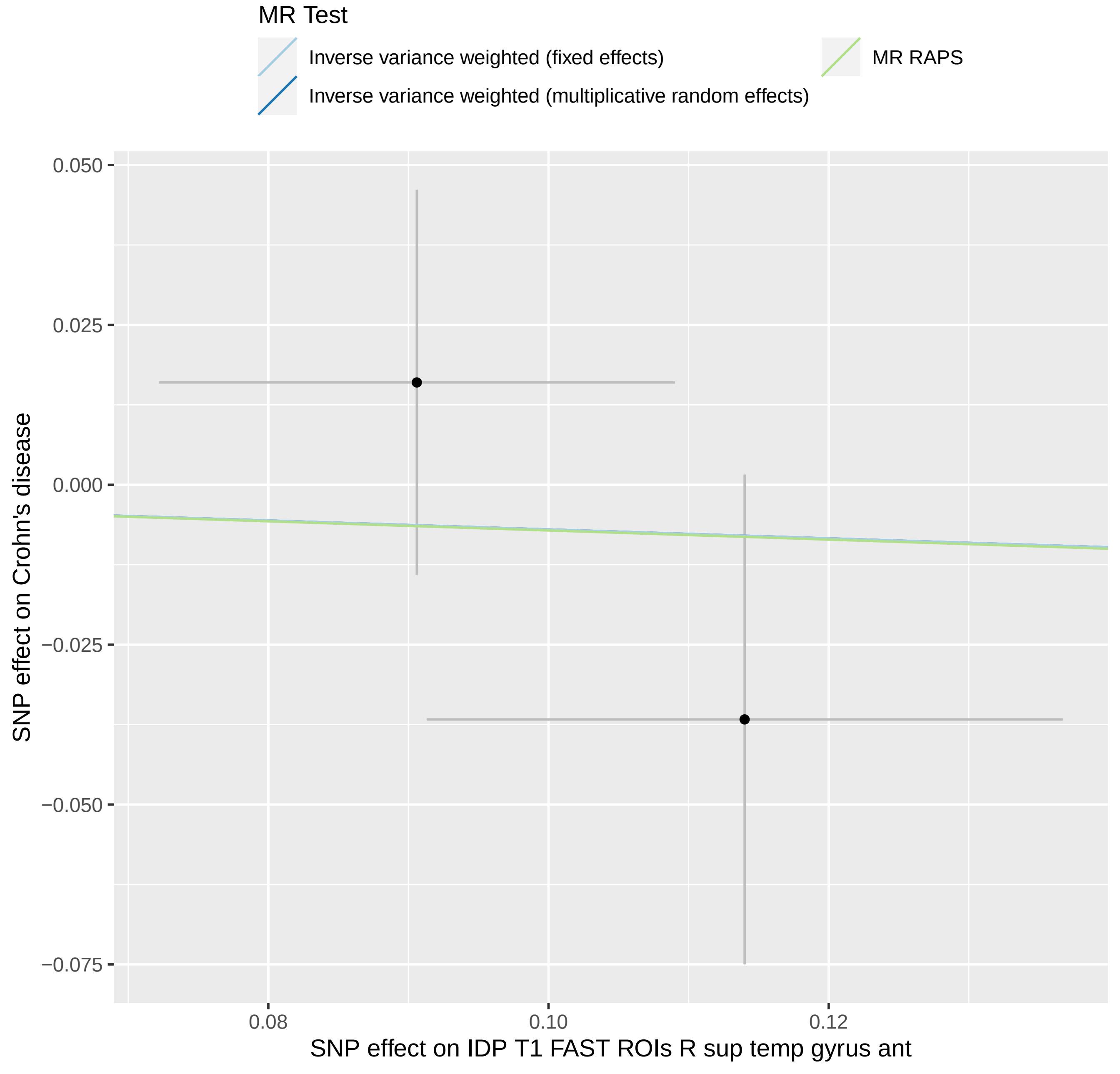

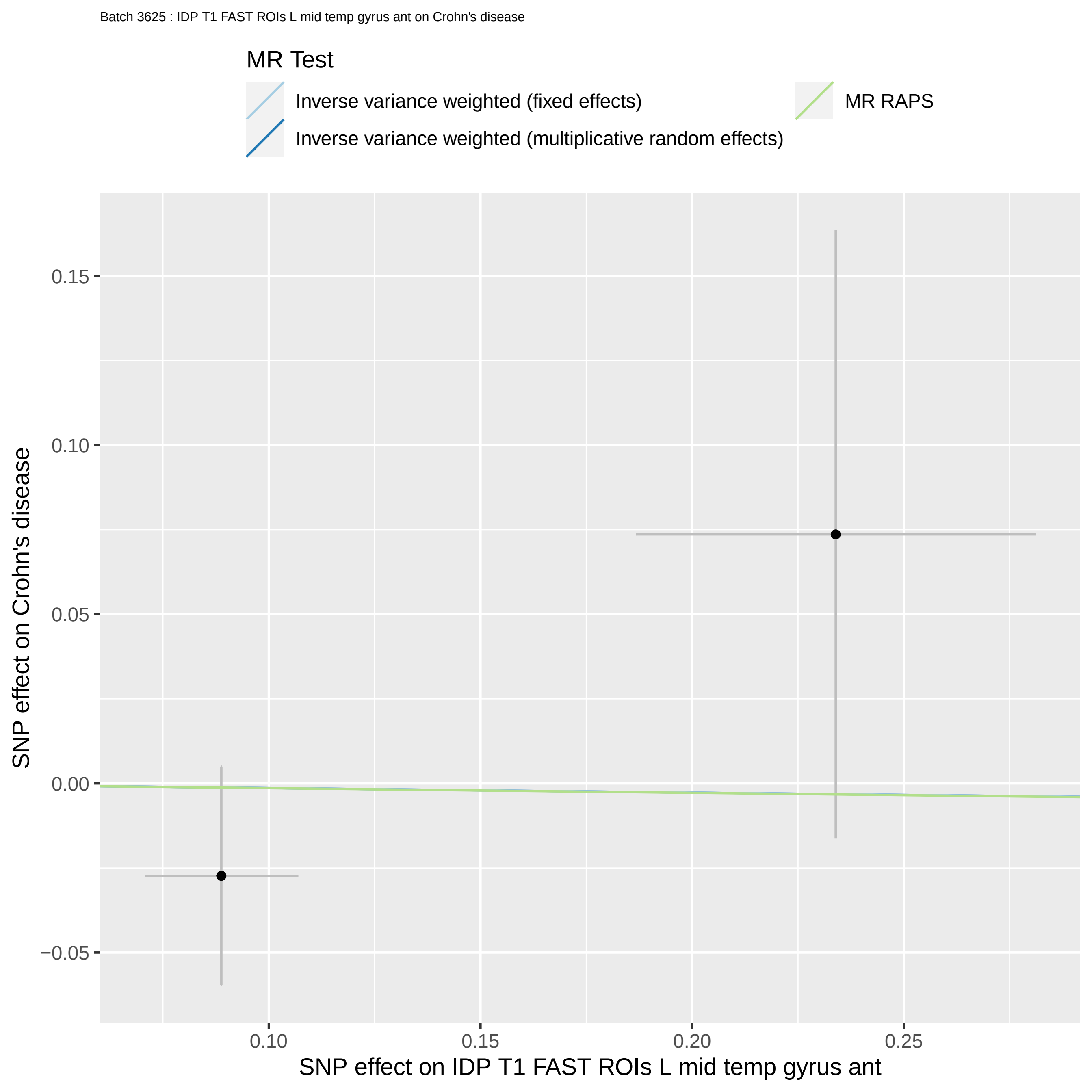

Batch 3625 : IDP T1 FAST ROIs L mid temp gyrus ant on Crohn's disease
MR Test
Inverse variance weighted (fixed effects)
Inverse variance weighted (multiplicative random effects)
MR RAPS
SNP effect on Crohn's disease
0.15
0.10
0.05
0.00
−0.05
0.10
0.15
0.20
0.25
SNP effect on IDP T1 FAST ROIs L mid temp gyrus ant

Batch 3669 : IDP T1 SIENAX grey normalised volume on Crohn's disease

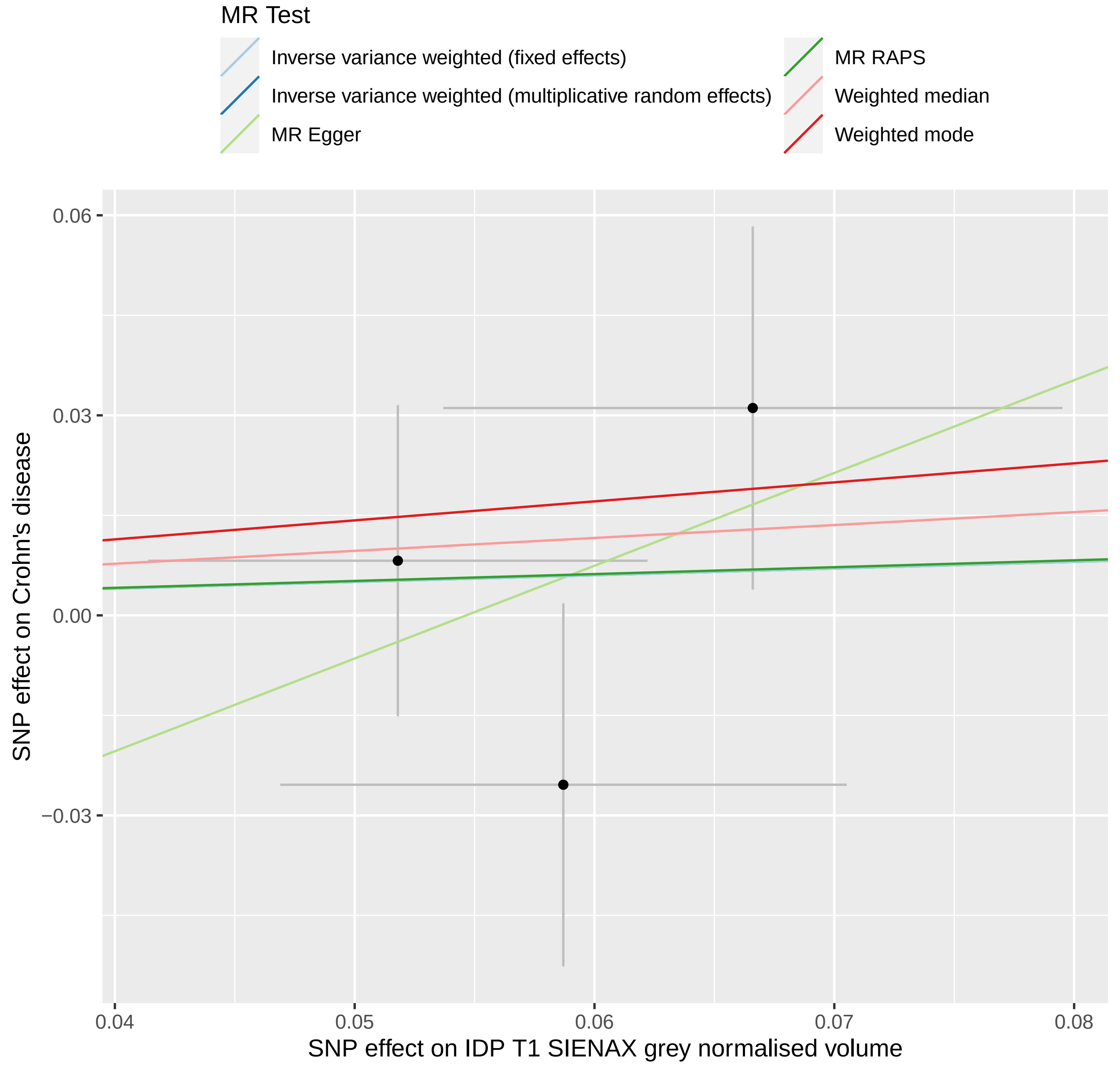

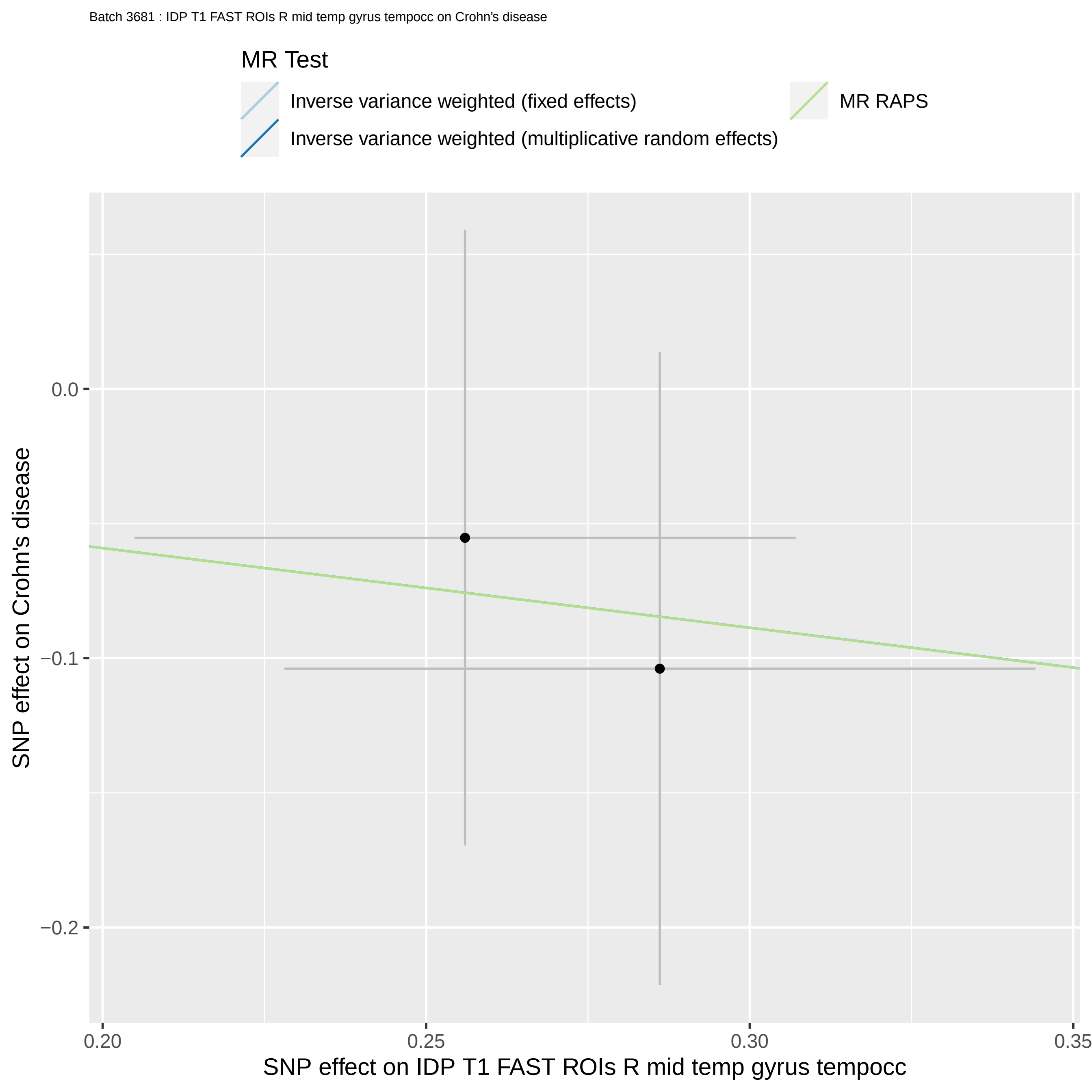
Batch 3681 : IDP T1 FAST ROIs R mid temp gyrus tempocc on Crohn's disease
MR Test
Inverse variance weighted (fixed effects)
Inverse variance weighted (multiplicative random effects)
MR RAPS
0.0
−0.1
−0.2
0.20
0.25
0.30
0.35
SNP effect on Crohn's disease
SNP effect on IDP T1 FAST ROIs R mid temp gyrus tempocc

Batch 3692 : IDP T1 FAST ROIs L inf temp gyrus ant on Crohn's disease

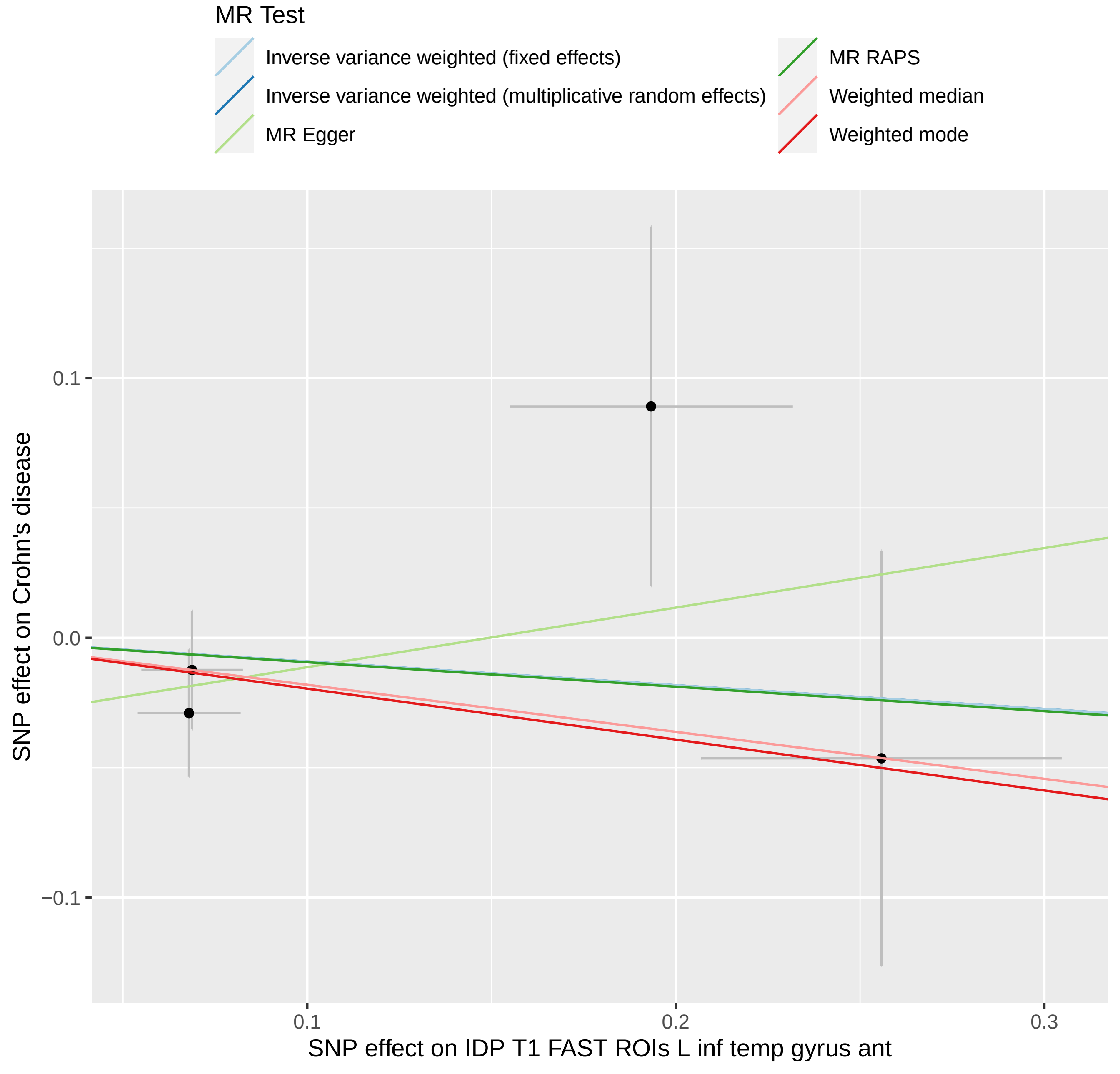

Batch 3703 : IDP T1 FAST ROIs R inf temp gyrus ant on Crohn's disease

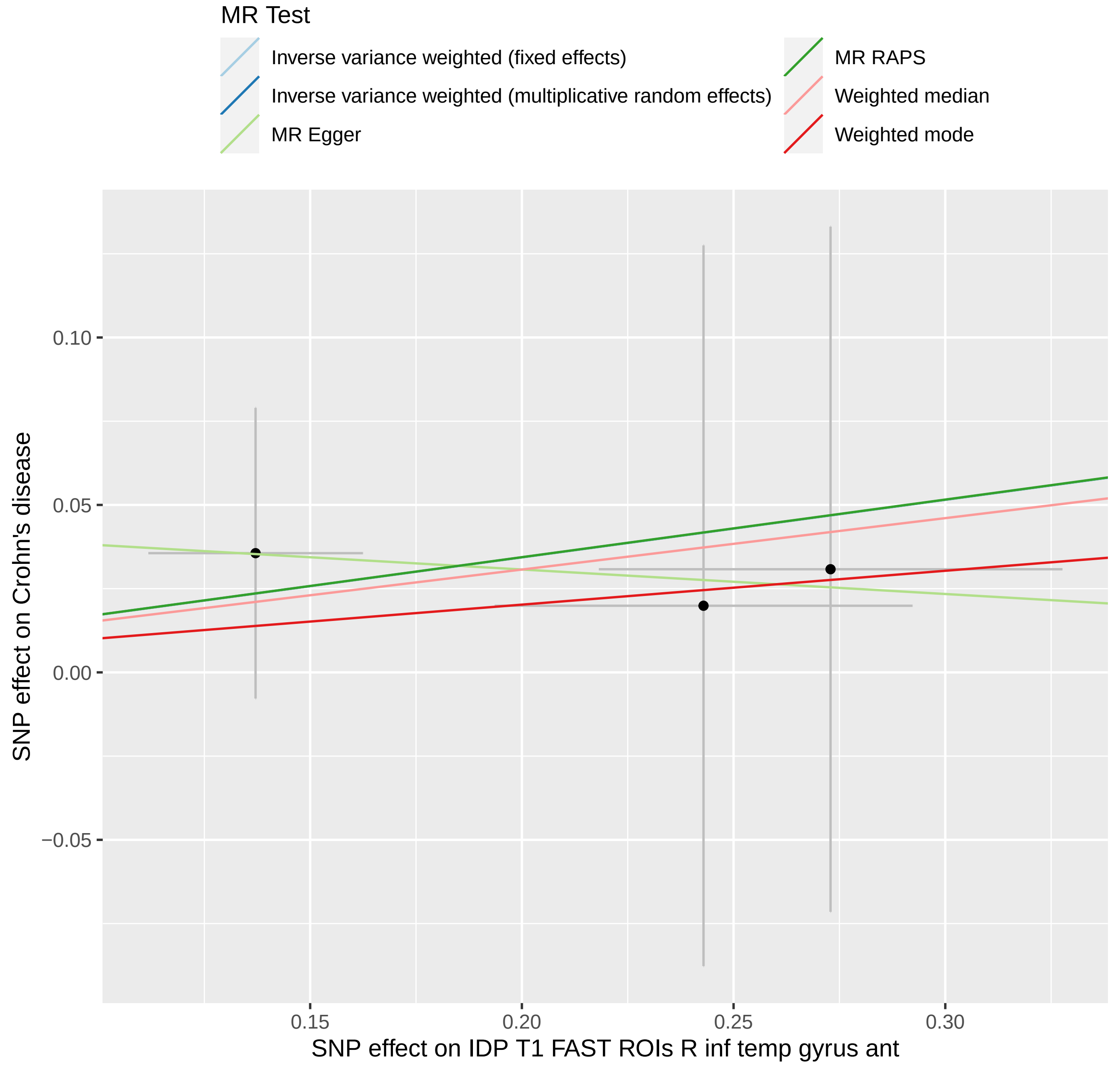

Batch 3714 : IDP T1 FAST ROIs L inf temp gyrus post on Crohn's disease

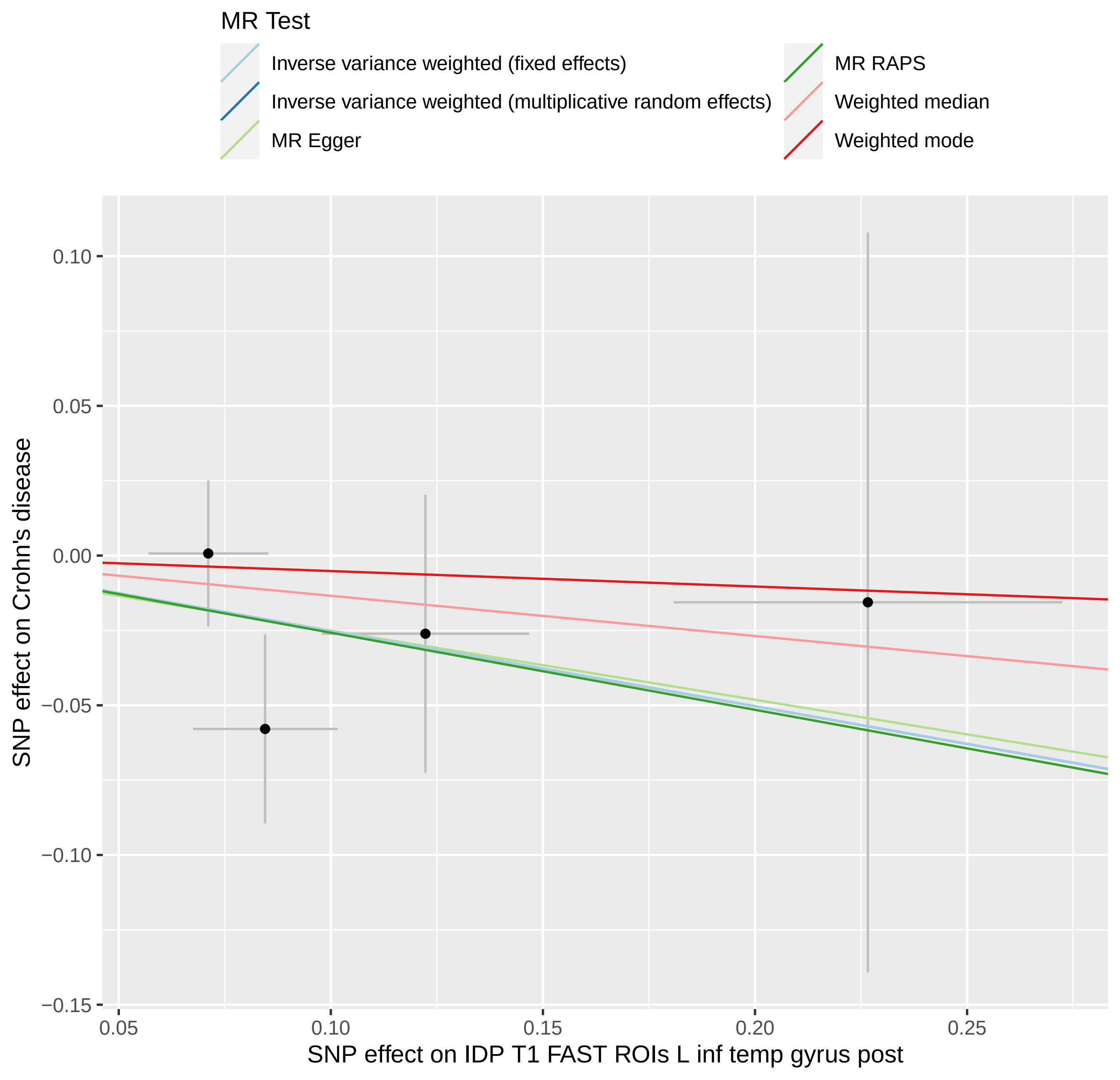

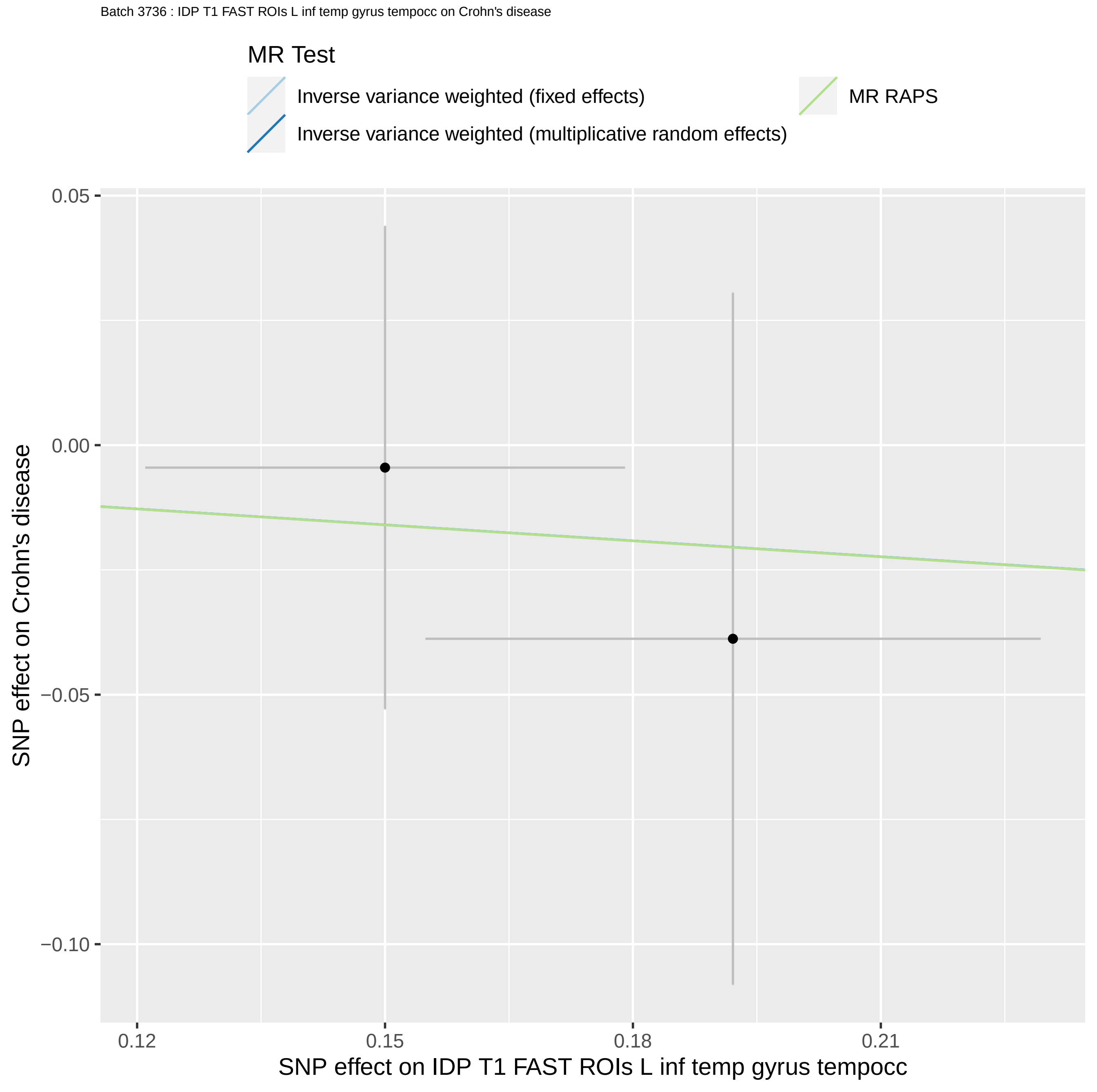
Batch 3736 : IDP T1 FAST ROIs L inf temp gyrus tempocc on Crohn's disease
MR Test
Inverse variance weighted (fixed effects)
Inverse variance weighted (multiplicative random effects)
MR RAPS
0.05
0.00
−0.05
−0.10
SNP effect on Crohn's disease
0.12
0.15
0.18
0.21
SNP effect on IDP T1 FAST ROIs L inf temp gyrus tempocc

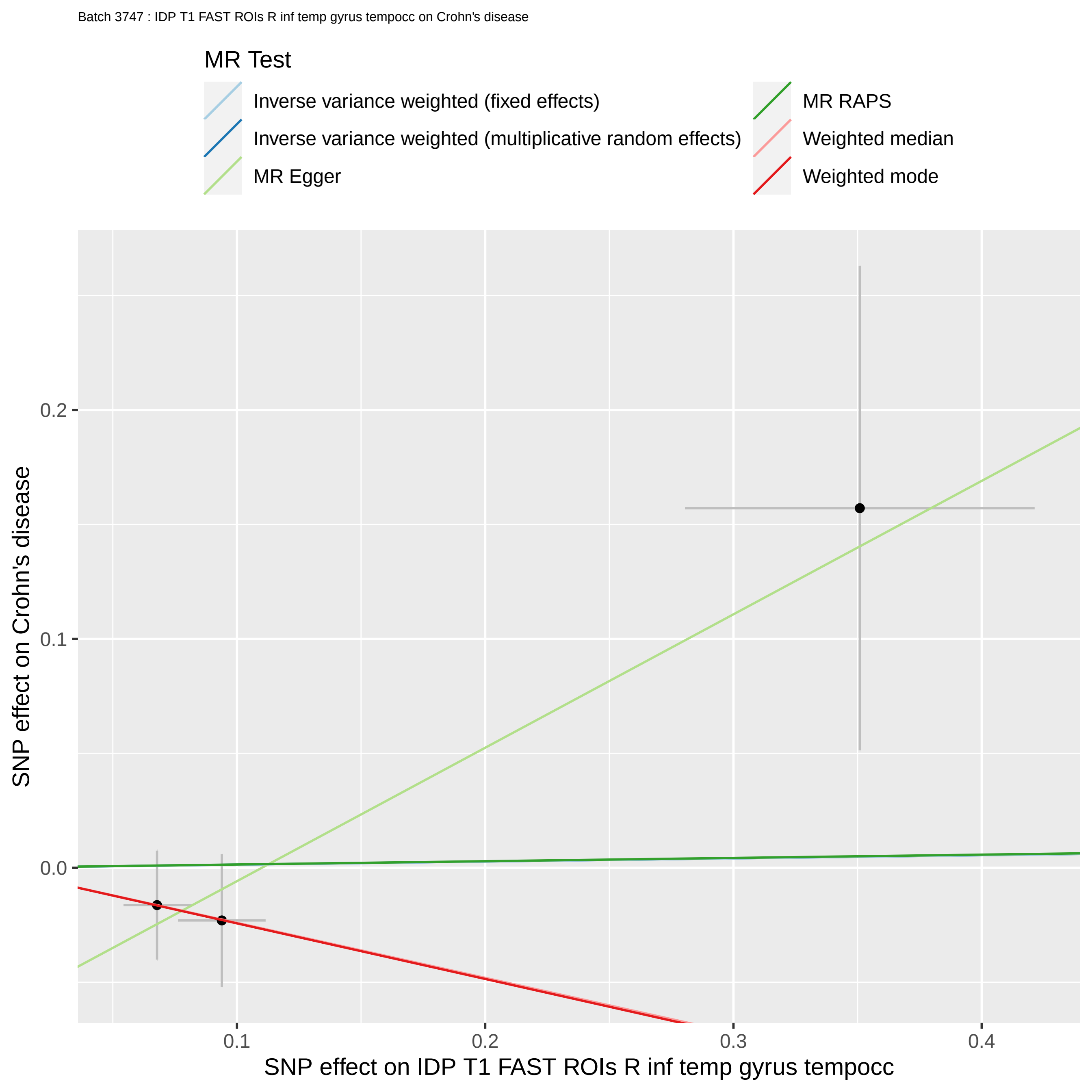
Batch 3747 : IDP T1 FAST ROIs R inf temp gyrus tempocc on Crohn's disease
MR Test
Inverse variance weighted (fixed effects)
Inverse variance weighted (multiplicative random effects)
MR Egger
MR RAPS
Weighted median
Weighted mode
SNP effect on Crohn's disease
0.2
0.1
0.0
0.1
0.2
0.3
0.4
SNP effect on IDP T1 FAST ROIs R inf temp gyrus tempocc

Batch 3758 : IDP T1 FAST ROIs L postcent gyrus on Crohn's disease

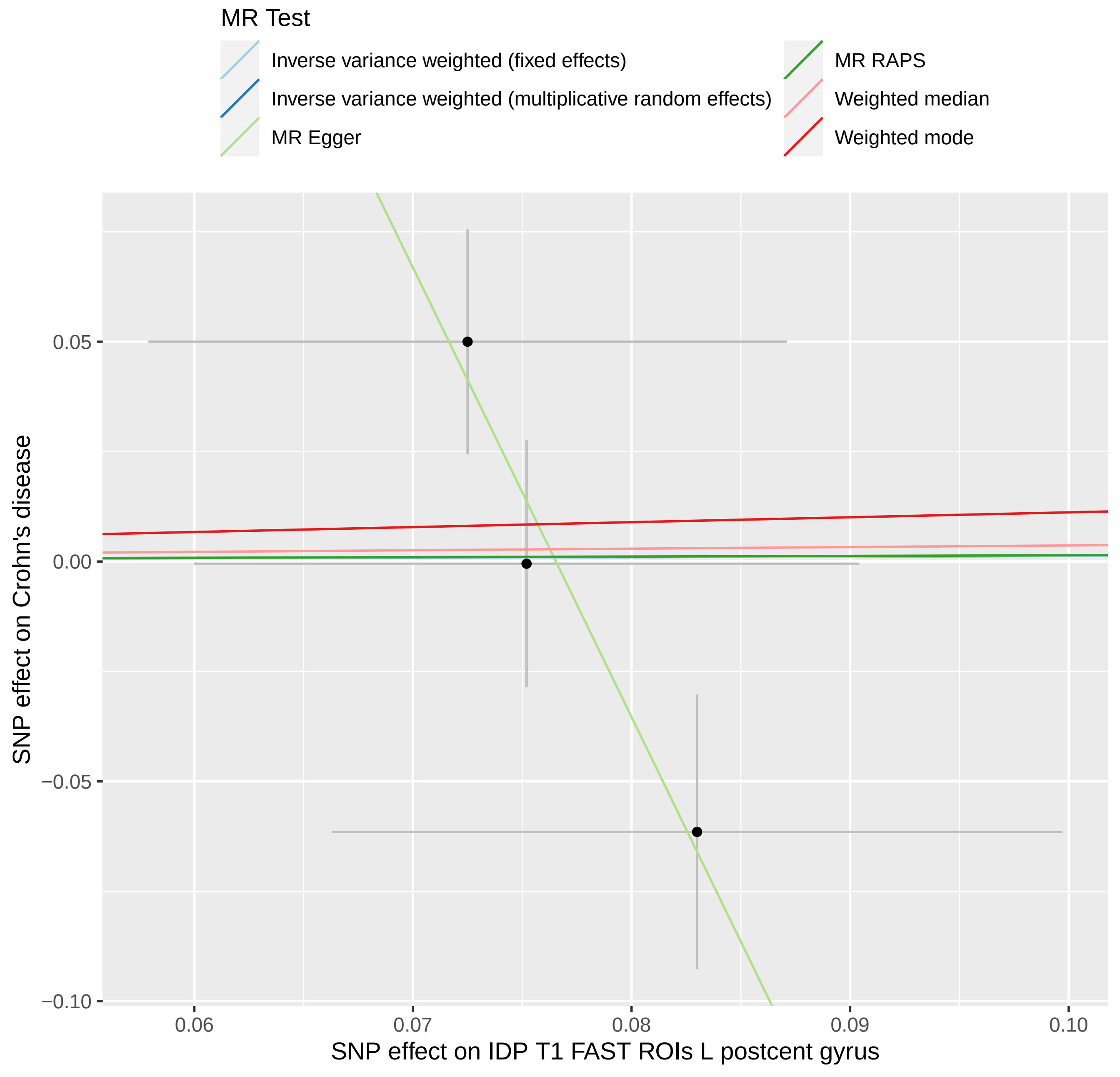

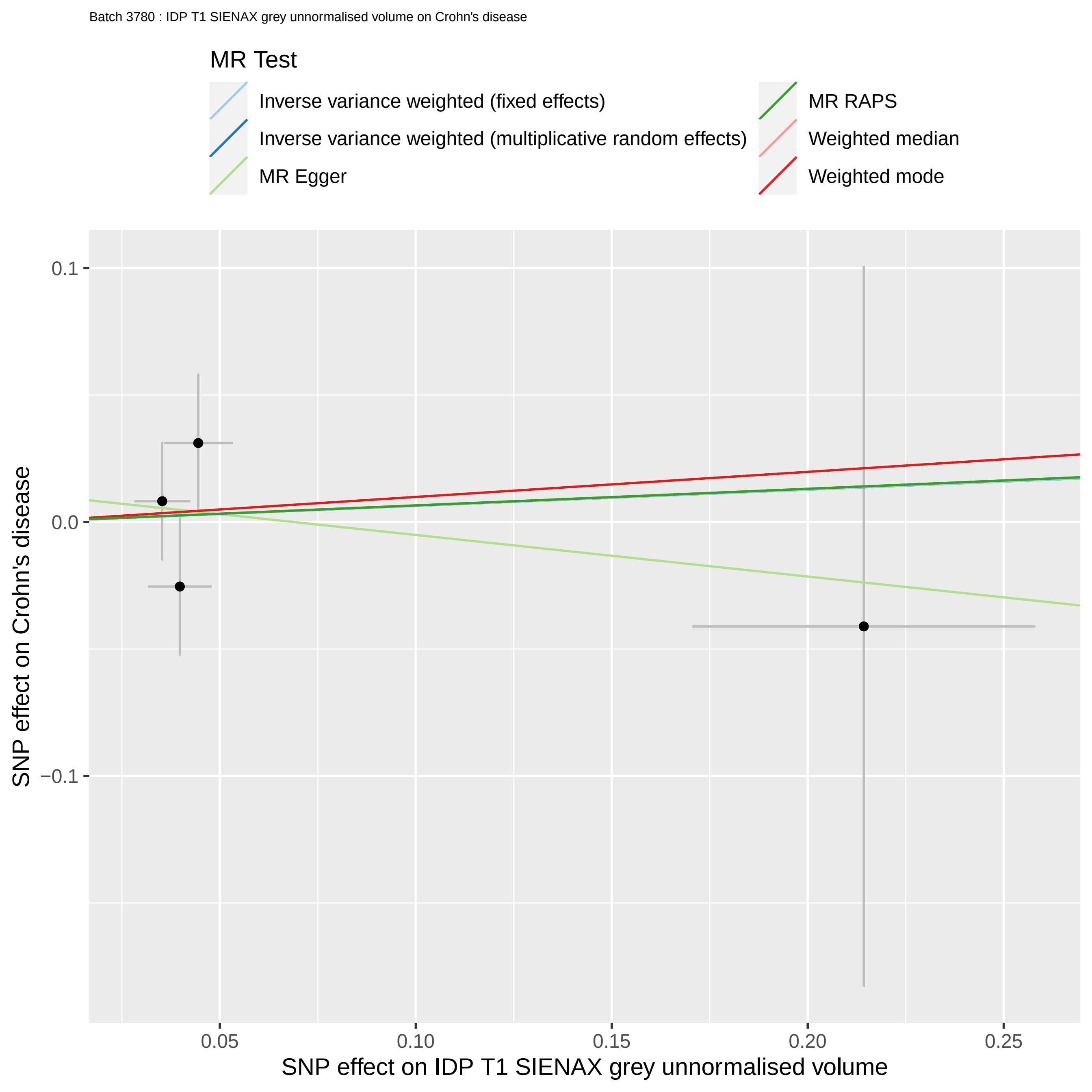

Batch 3780 : IDP T1 SIENAX grey unnormalised volume on Crohn's disease
MR Test
Inverse variance weighted (fixed effects)
Inverse variance weighted (multiplicative random effects)
MR Egger
MR RAPS
Weighted median
Weighted mode
0.1
0.0
−0.1
0.05
0.10
0.15
0.20
0.25
SNP effect on Crohn's disease
SNP effect on IDP T1 SIENAX grey unnormalised volume

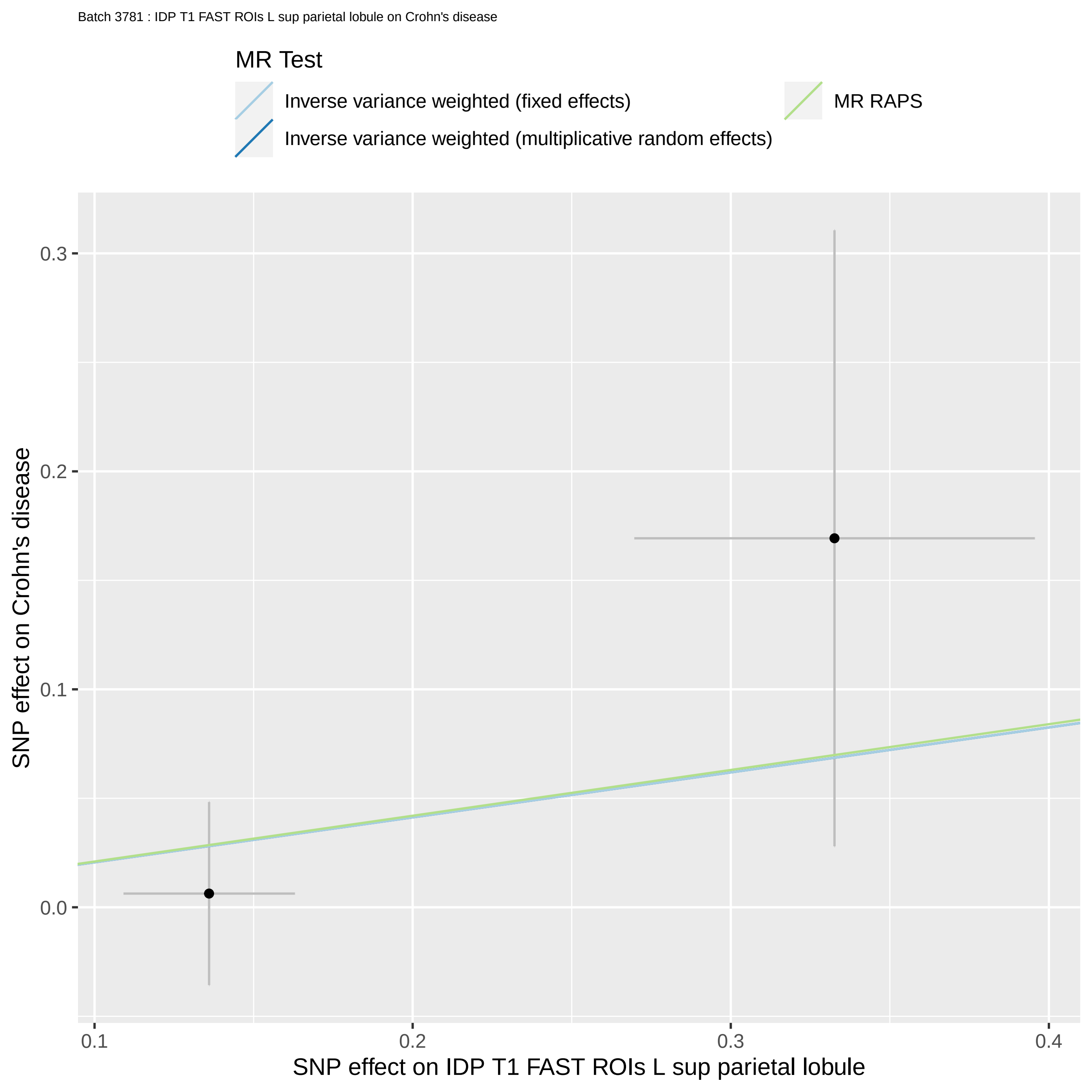
Batch 3781 : IDP T1 FAST ROIs L sup parietal lobule on Crohn's disease
MR Test
Inverse variance weighted (fixed effects)
Inverse variance weighted (multiplicative random effects)
MR RAPS
0.3
0.2
0.1
0.0
0.1
0.2
0.3
0.4
SNP effect on Crohn's disease
SNP effect on IDP T1 FAST ROIs L sup parietal lobule

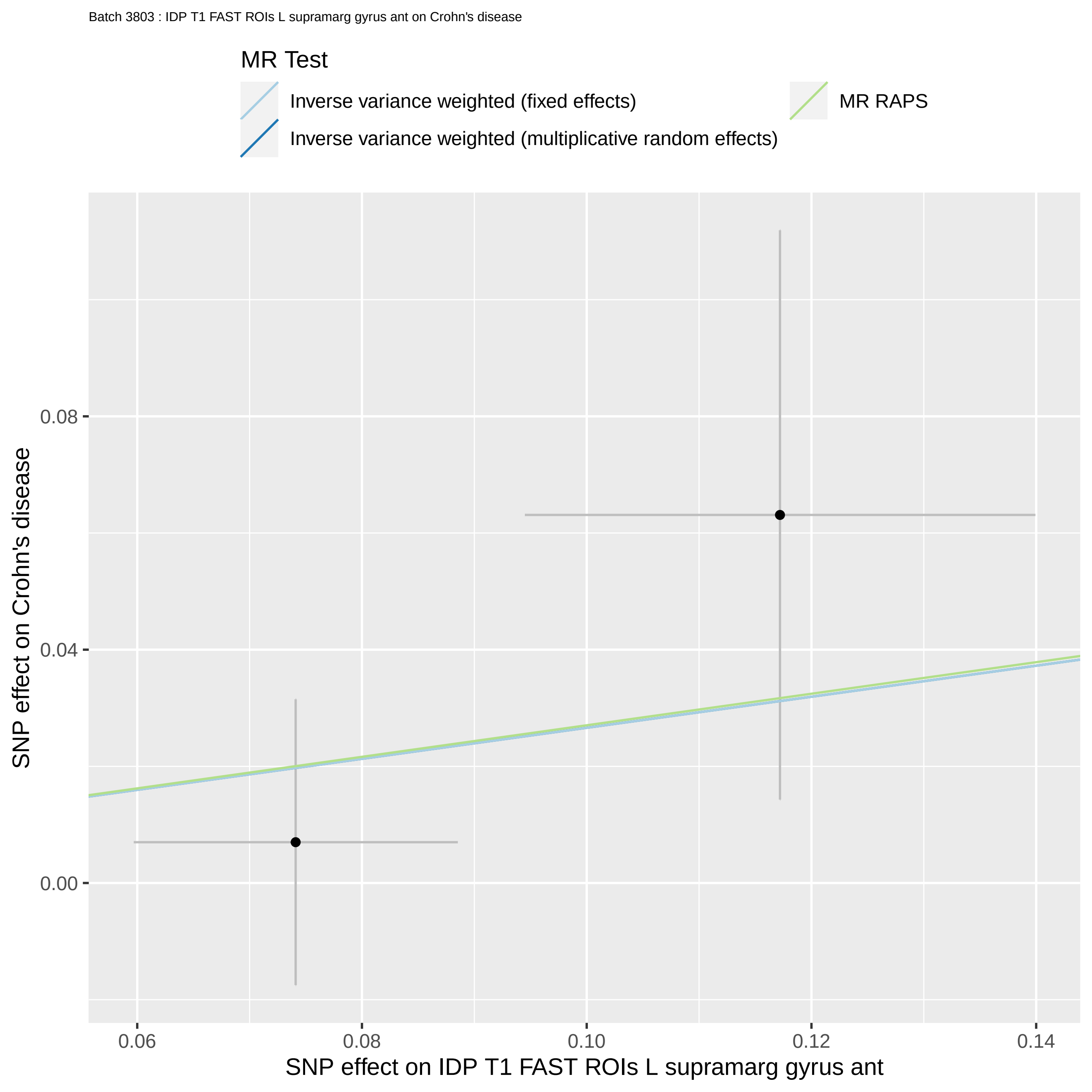

Batch 3803 : IDP T1 FAST ROIs L supramarg gyrus ant on Crohn's disease
MR Test
Inverse variance weighted (fixed effects)
Inverse variance weighted (multiplicative random effects)
MR RAPS
0.08
0.04
0.00
SNP effect on Crohn's disease
0.06
0.08
0.10
0.12
0.14
SNP effect on IDP T1 FAST ROIs L supramarg gyrus ant

Batch 3847 : IDP T1 FAST ROIs L angular gyrus on Crohn's disease

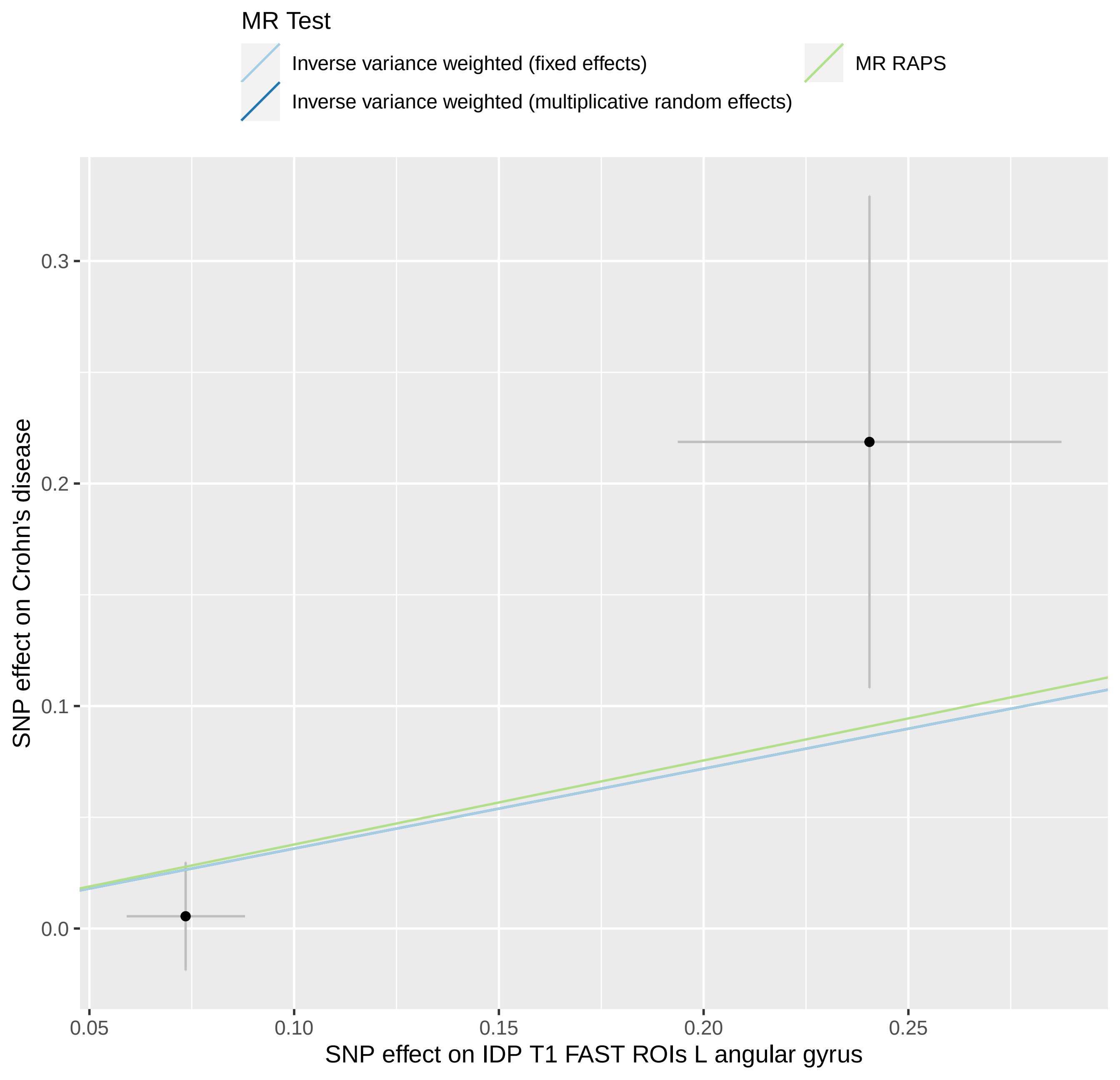

Batch 3869 : IDP T1 FAST ROIs L latocc cortex sup on Crohn's disease

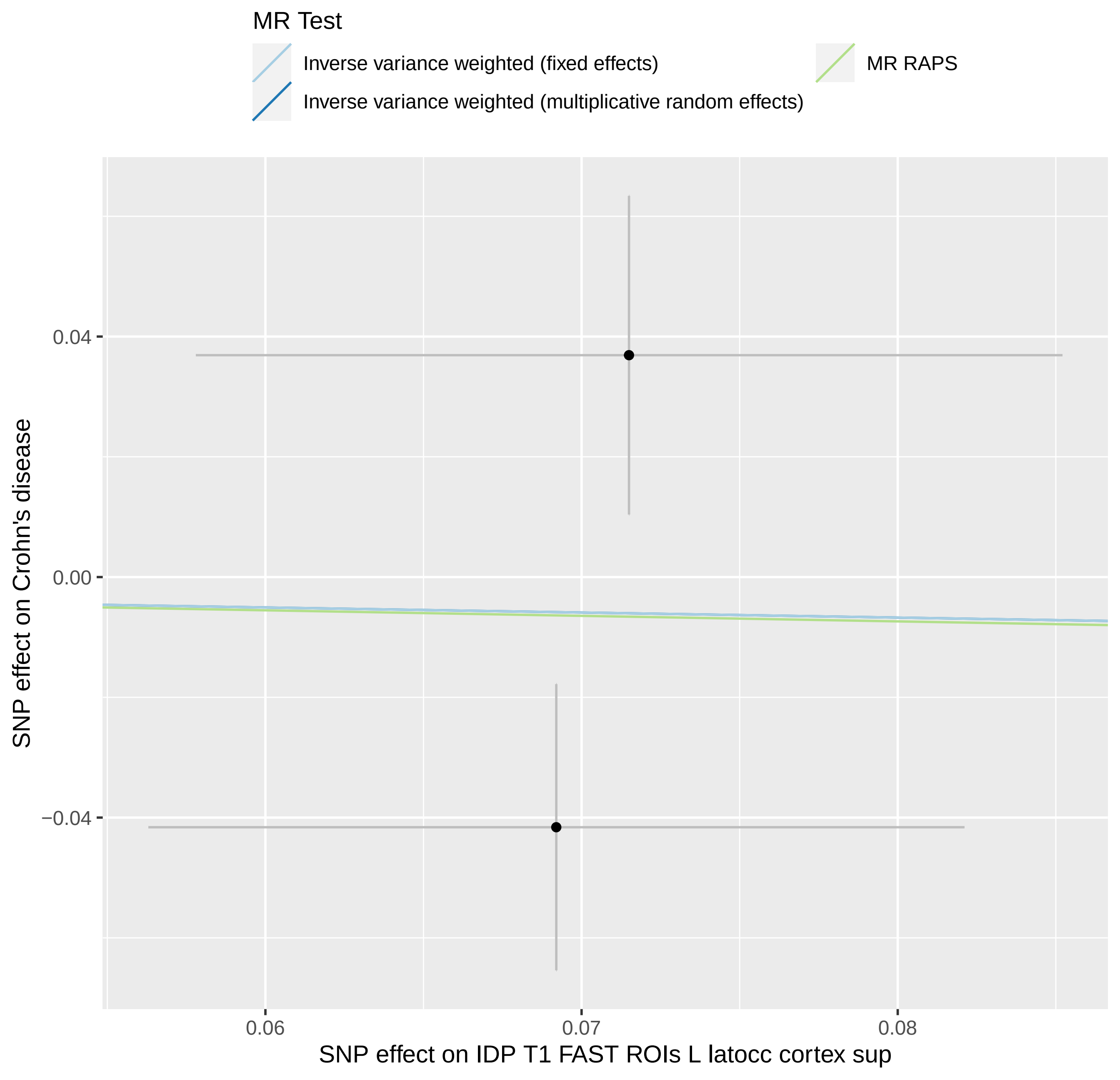

Batch 3902 : IDP T1 FAST ROIs R latocc cortex inf on Crohn's disease

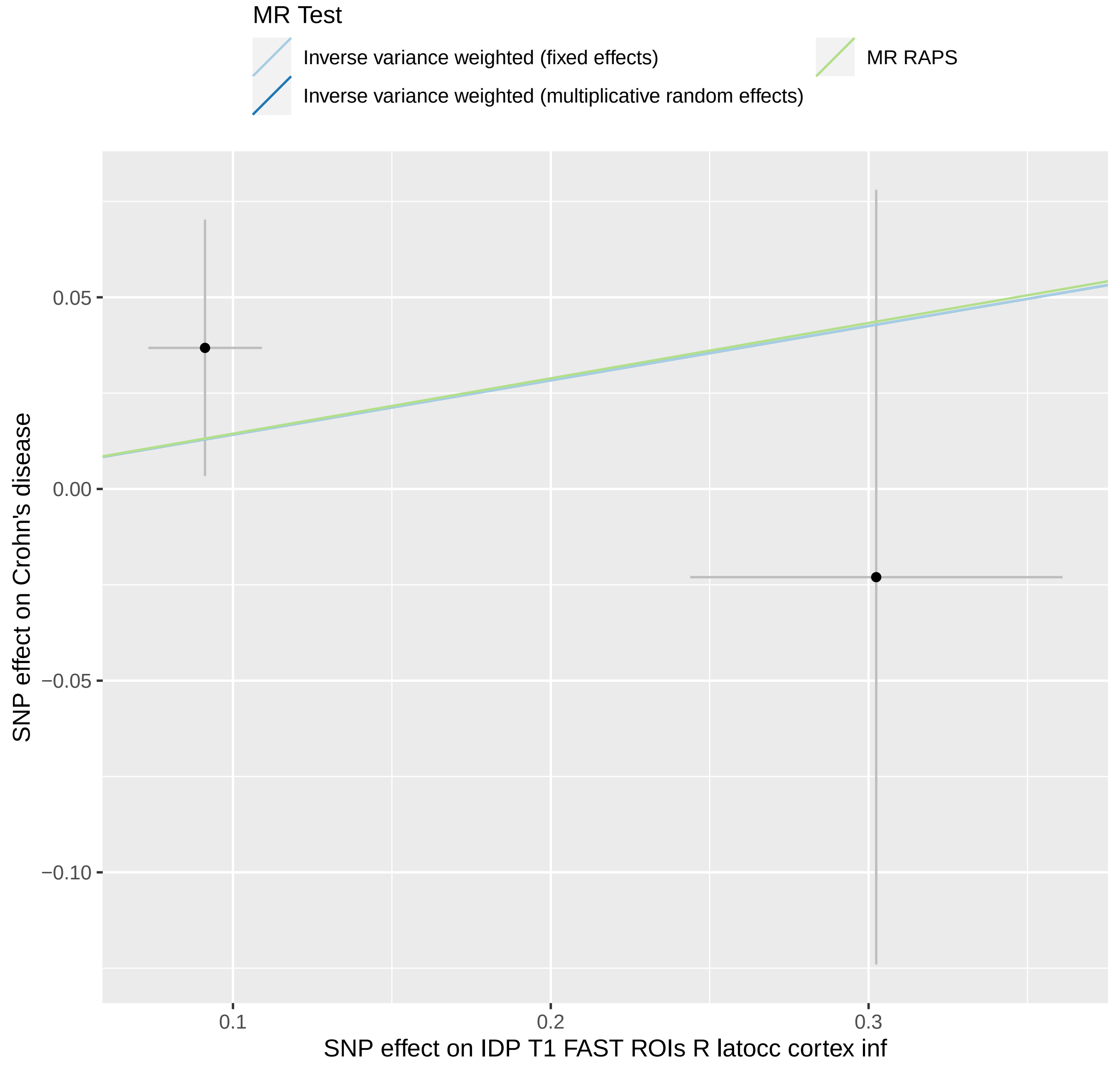

Batch 3968 : IDP T1 FAST ROIs R juxtapos lobule cortex on Crohn's disease

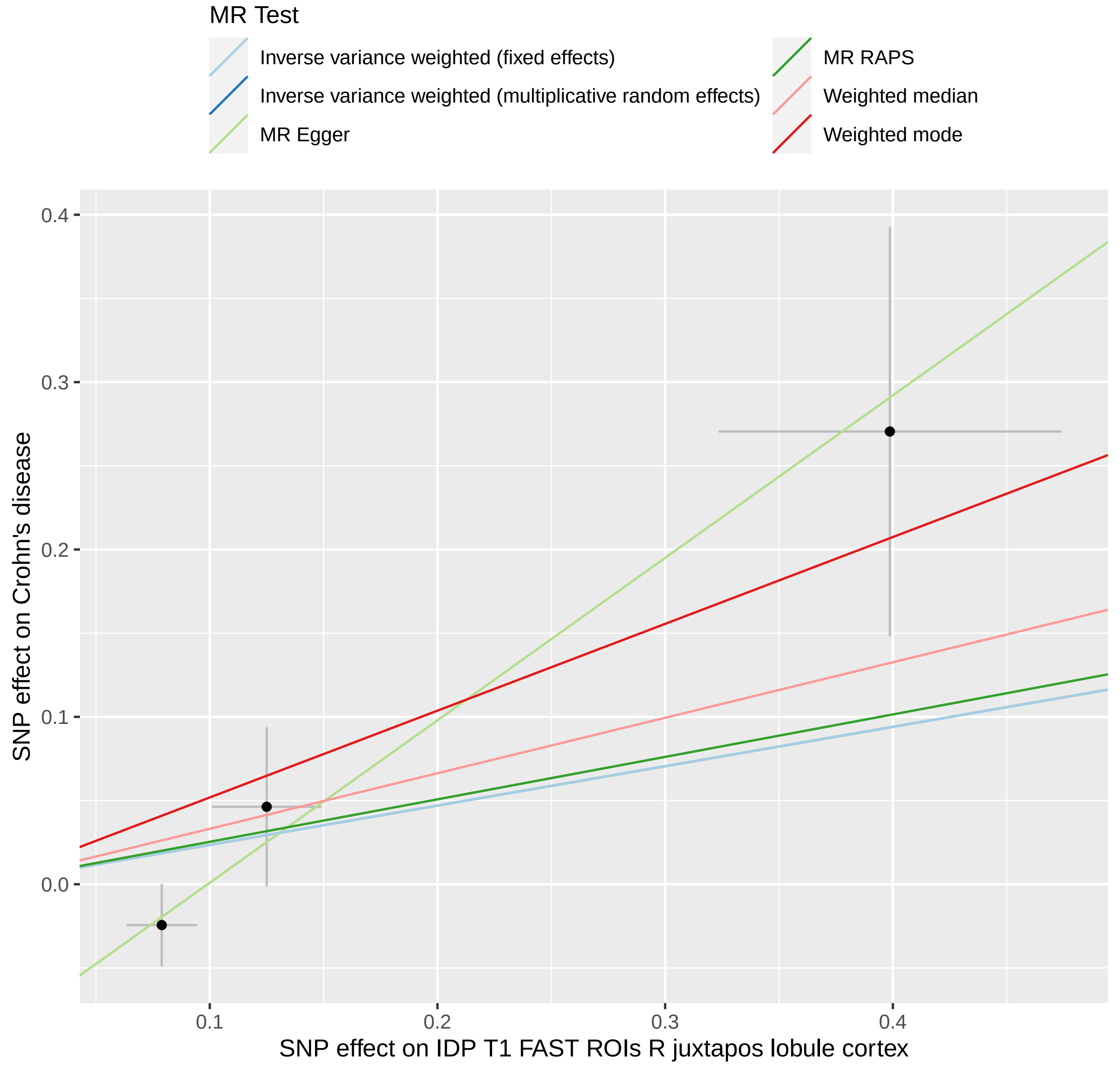

Batch 3979 : IDP T1 FAST ROIs L subcallosal cortex on Crohn's disease

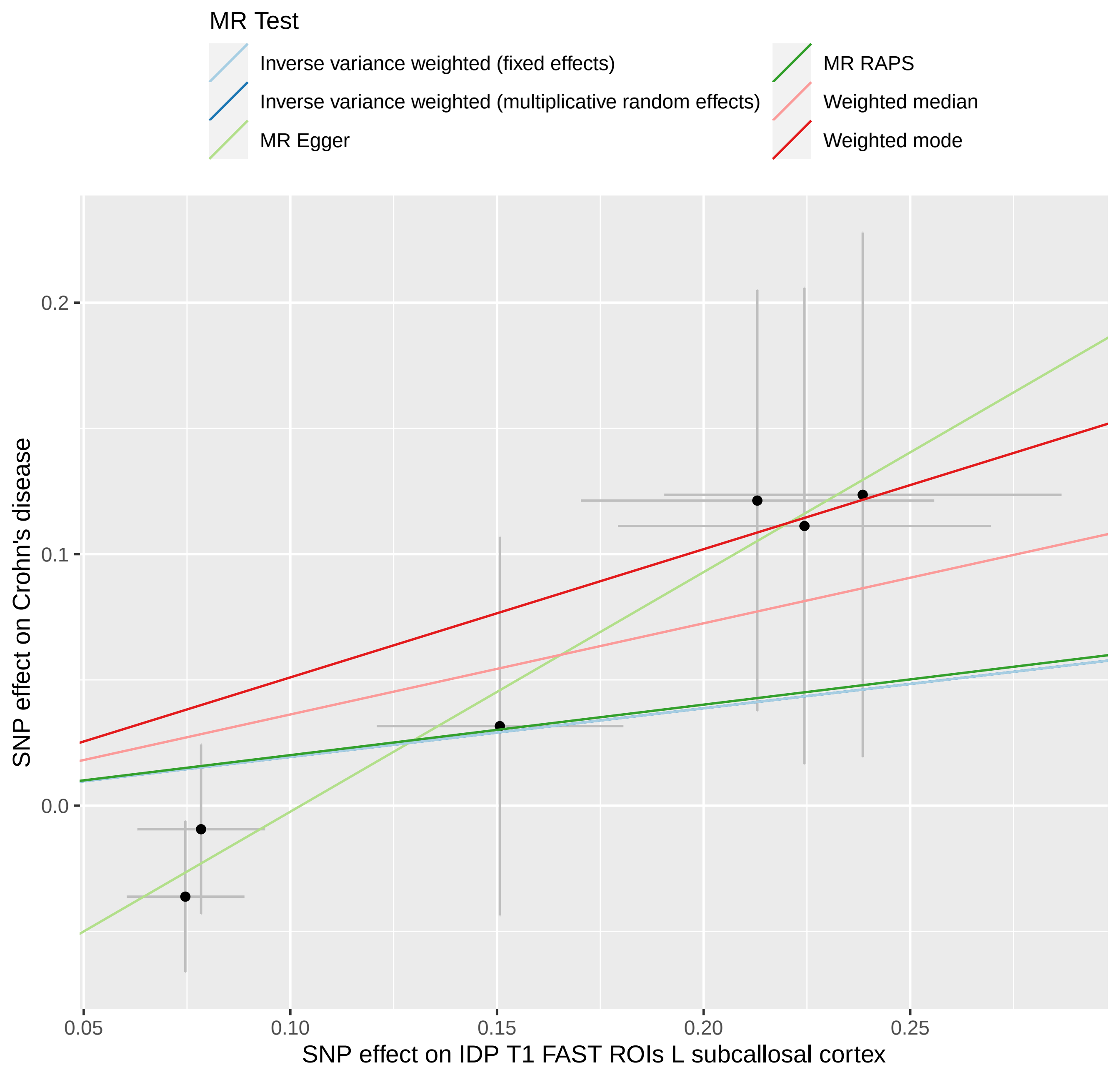

Batch 3990 : IDP T1 FAST ROIs R subcallosal cortex on Crohn's disease

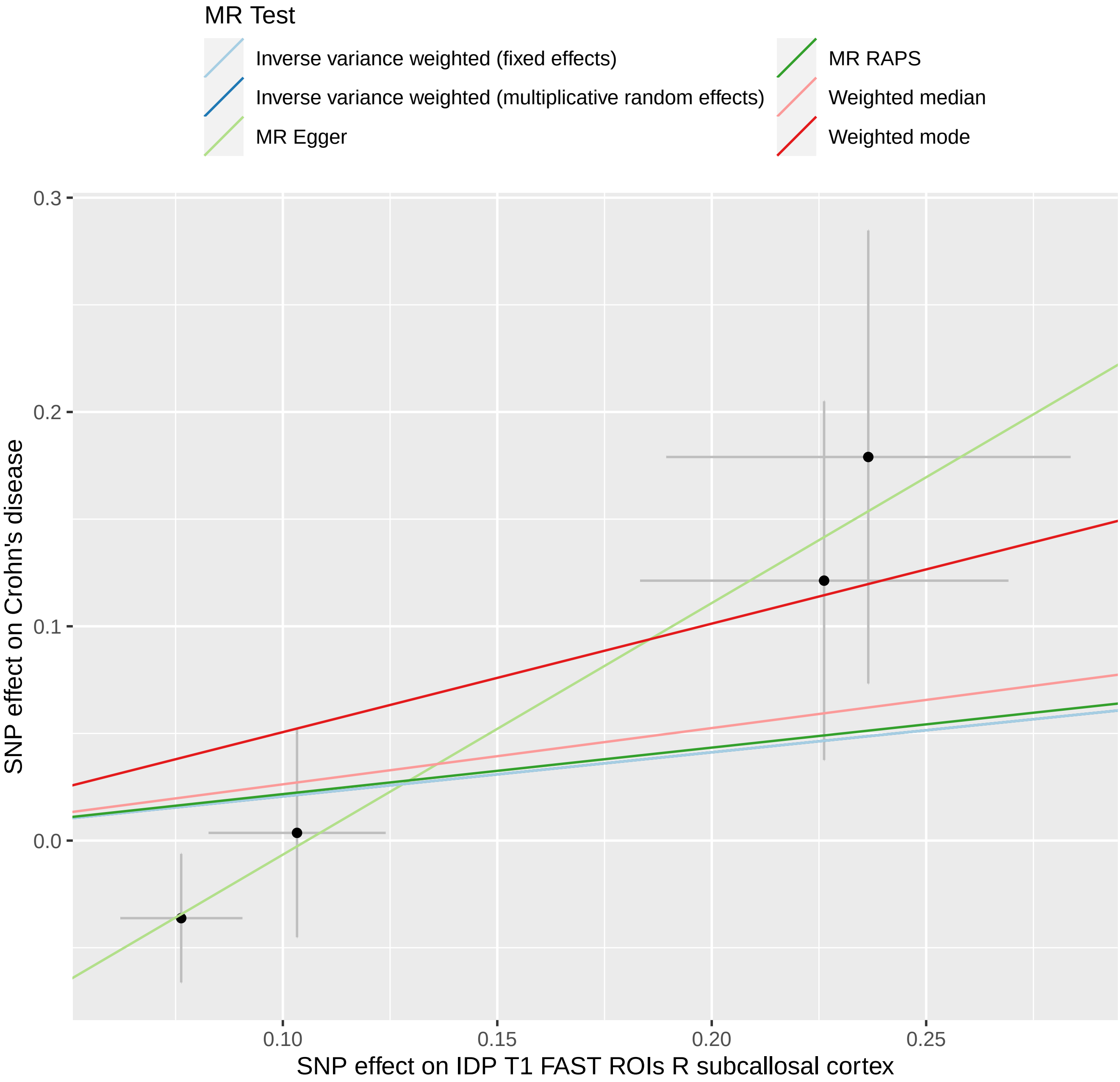

Batch 4035 : IDP T1 FAST ROIs R cing gyrus ant on Crohn's disease

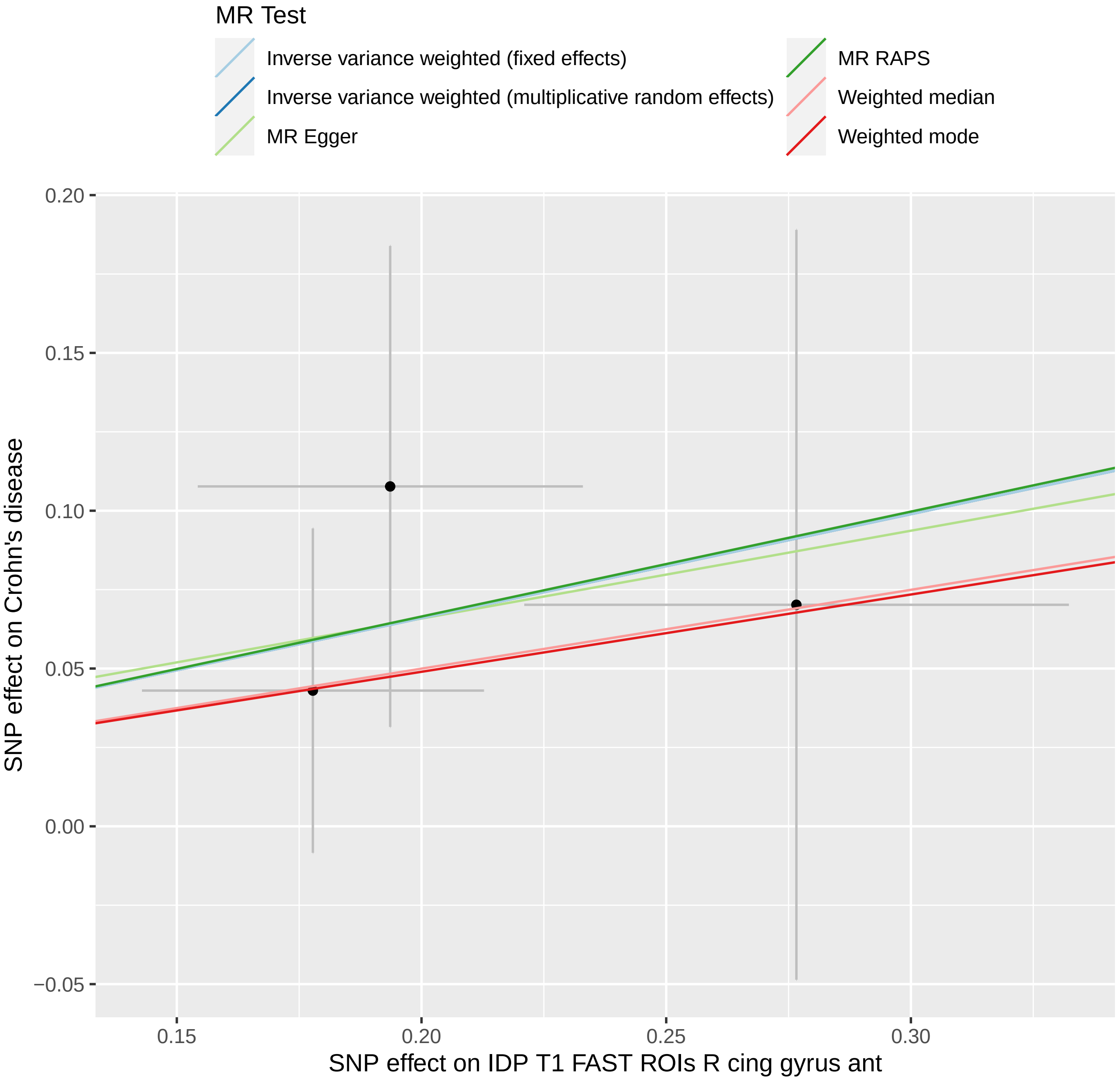

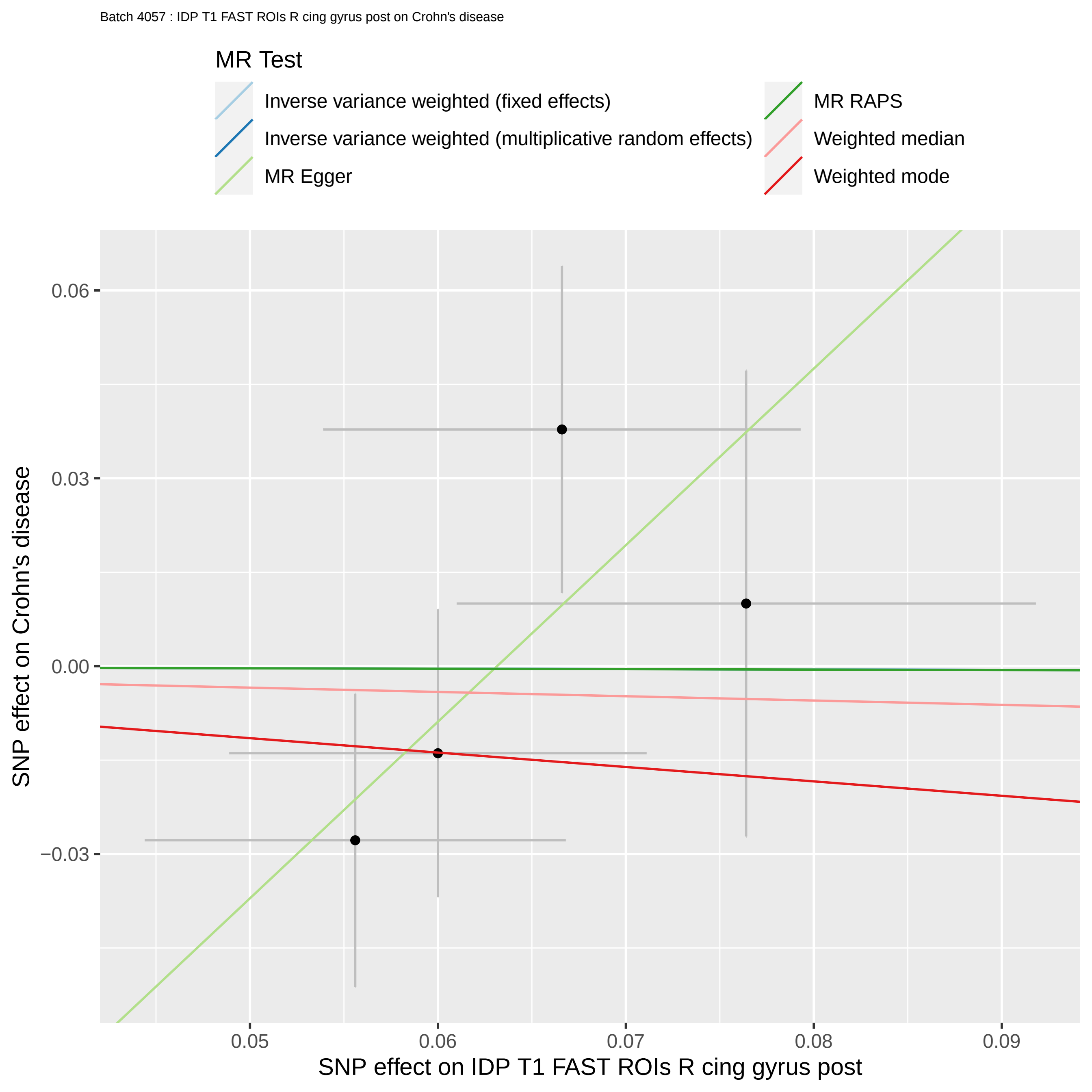

Batch 4057 : IDP T1 FAST ROIs R cing gyrus post on Crohn's disease
MR Test
Inverse variance weighted (fixed effects)
Inverse variance weighted (multiplicative random effects)
MR Egger
MR RAPS
Weighted median
Weighted mode
0.06
0.03
0.00
−0.03
SNP effect on Crohn's disease
0.05
0.06
0.07
0.08
0.09
SNP effect on IDP T1 FAST ROIs R cing gyrus post

Batch 4068 : IDP T1 FAST ROIs L precun cortex on Crohn's disease

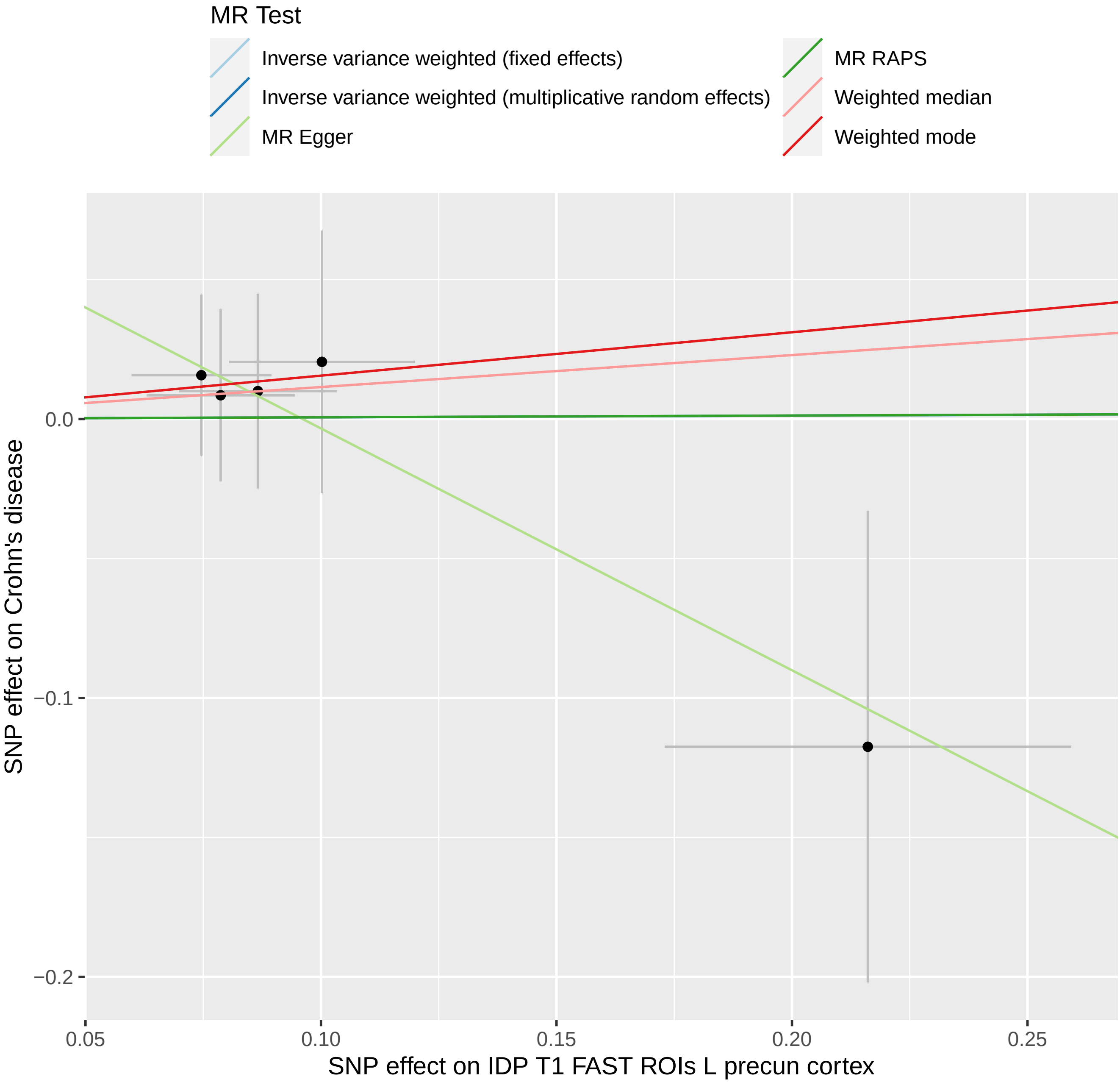

Batch 4081 : IDP T1 FAST ROIs L cuneal cortex on Crohn's disease

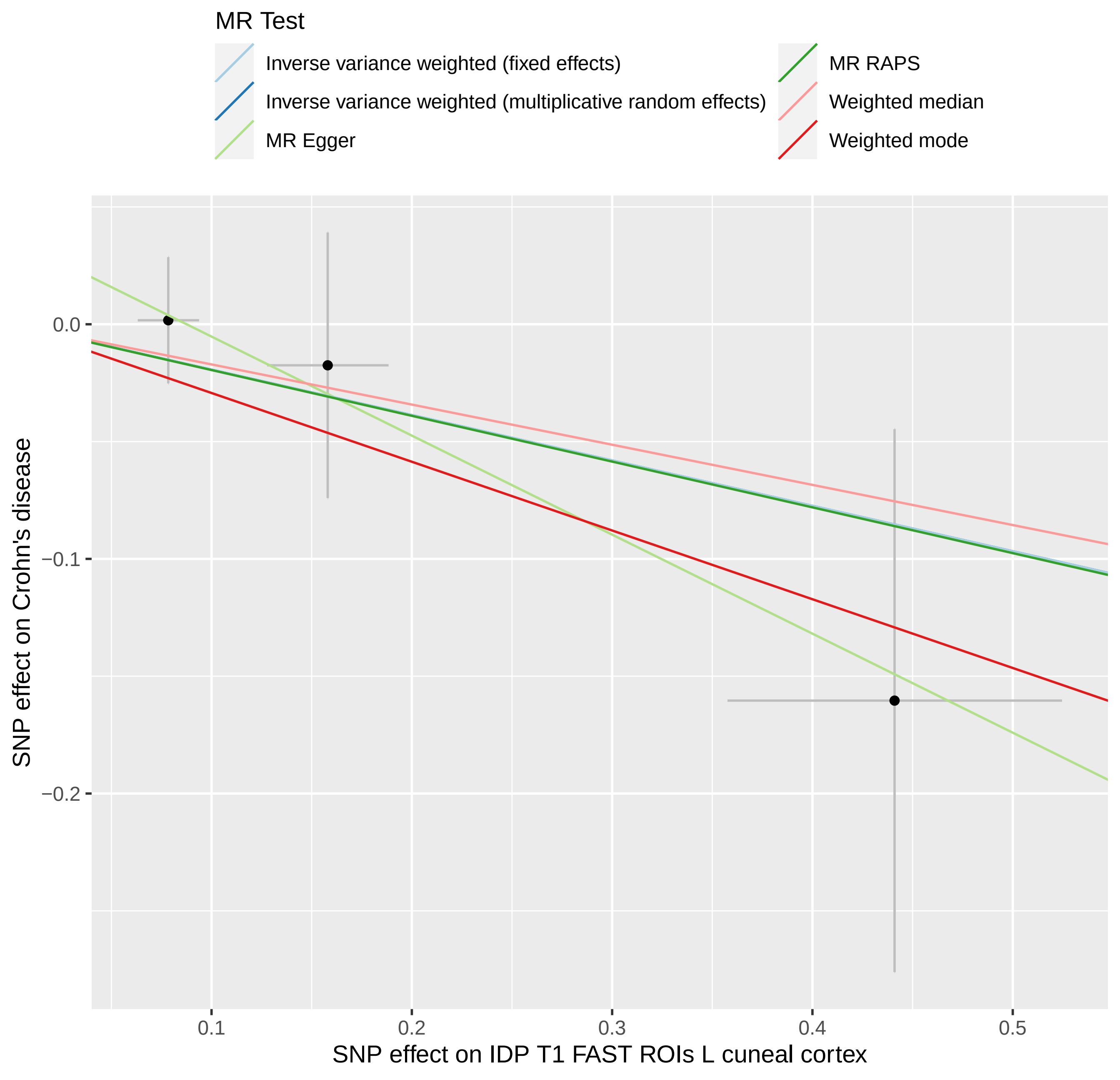

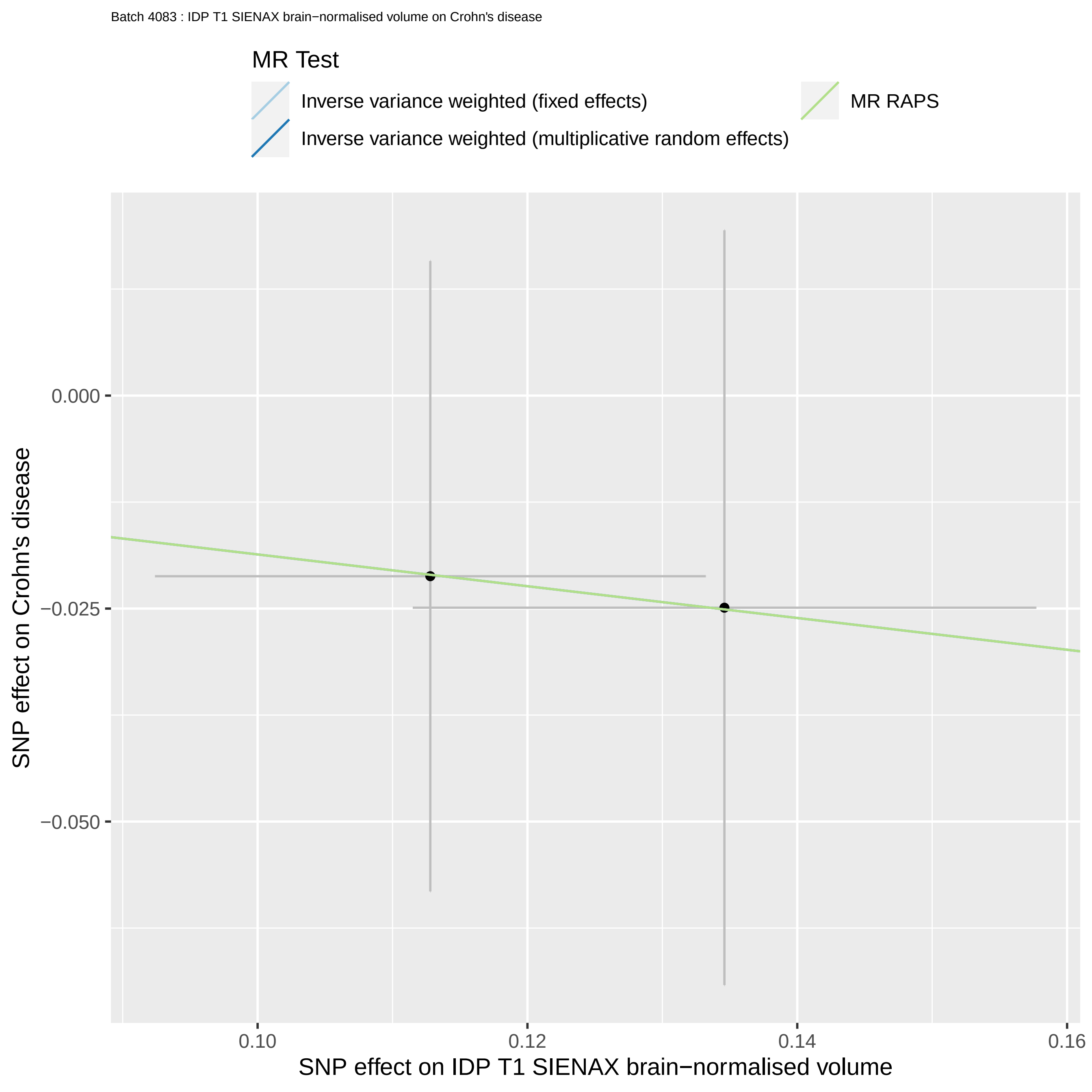
Batch 4083 : IDP T1 SIENAX brain−normalised volume on Crohn's disease
MR Test
Inverse variance weighted (fixed effects)
Inverse variance weighted (multiplicative random effects)
MR RAPS
0.000
−0.025
−0.050
SNP effect on Crohn's disease
0.10
0.12
0.14
0.16
SNP effect on IDP T1 SIENAX brain−normalised volume

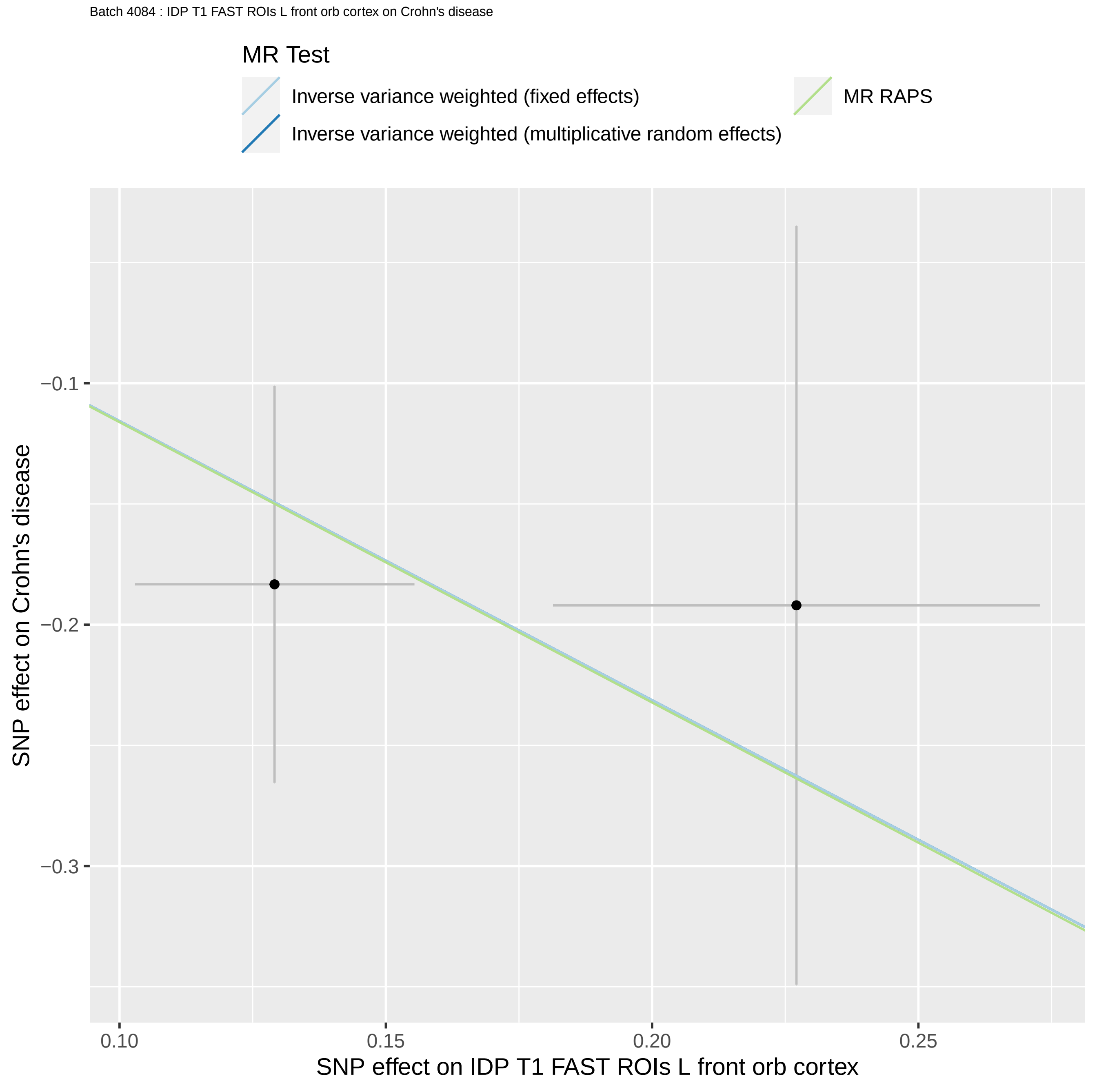

Batch 4084 : IDP T1 FAST ROIs L front orb cortex on Crohn's disease
MR Test
Inverse variance weighted (fixed effects)
Inverse variance weighted (multiplicative random effects)
MR RAPS
−0.1
−0.2
−0.3
0.10
0.15
0.20
0.25
SNP effect on Crohn's disease
SNP effect on IDP T1 FAST ROIs L front orb cortex

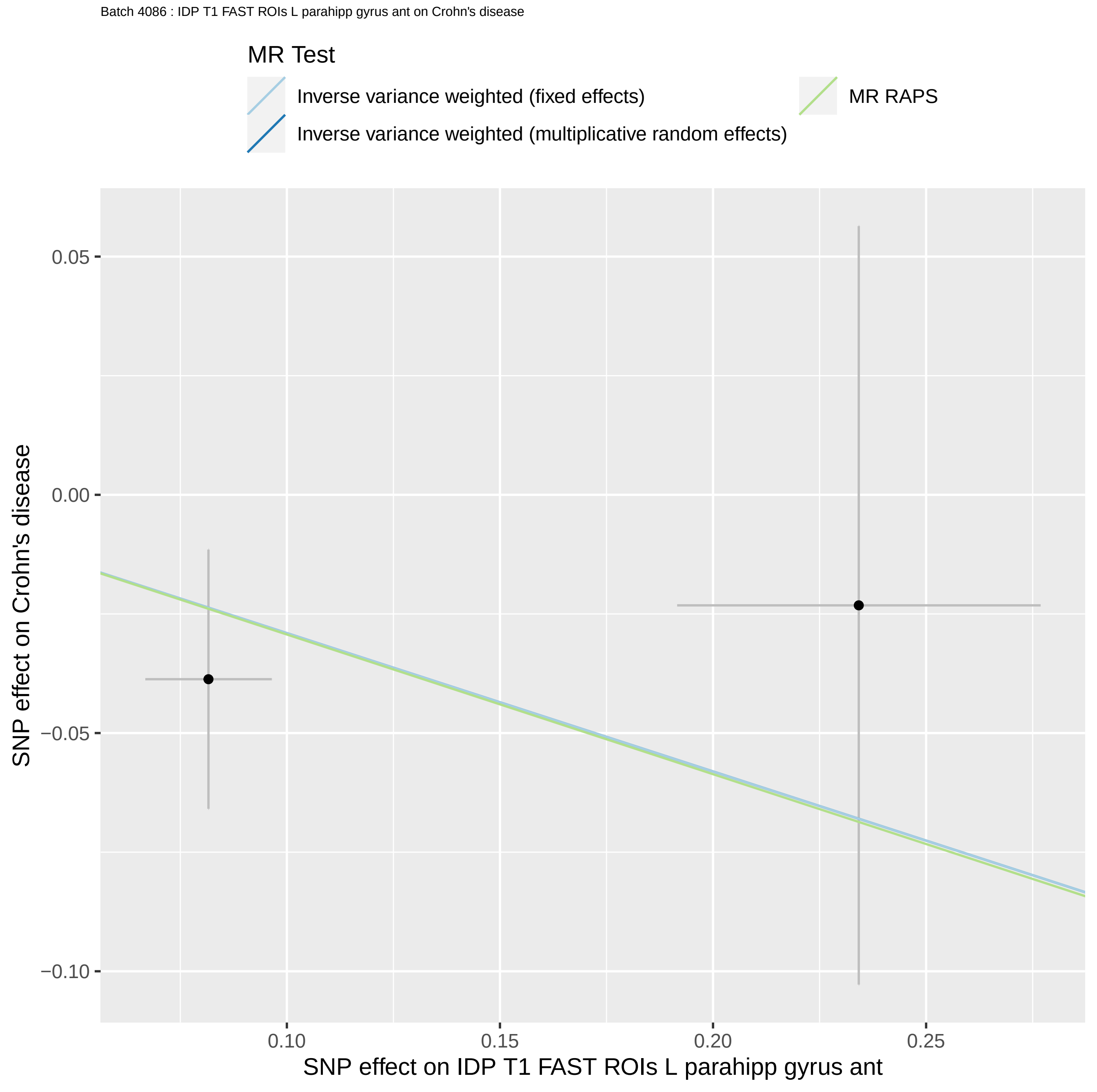
Batch 4086 : IDP T1 FAST ROIs L parahipp gyrus ant on Crohn's disease
MR Test
Inverse variance weighted (fixed effects)
Inverse variance weighted (multiplicative random effects)
MR RAPS
0.05
0.00
−0.05
−0.10
0.10
0.15
0.20
0.25
SNP effect on Crohn's disease
SNP effect on IDP T1 FAST ROIs L parahipp gyrus ant

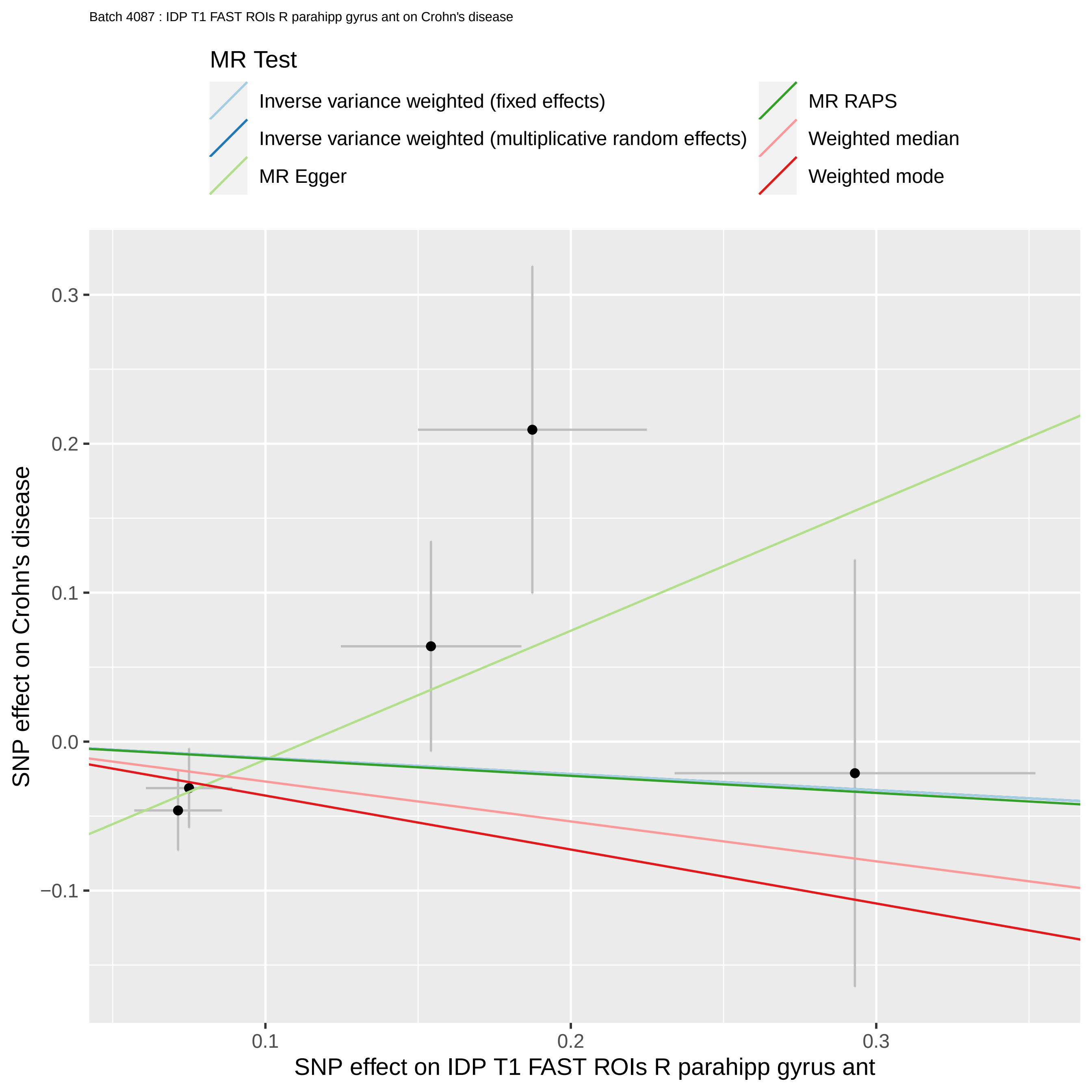
Batch 4087 : IDP T1 FAST ROIs R parahipp gyrus ant on Crohn's disease
MR Test
Inverse variance weighted (fixed effects)
Inverse variance weighted (multiplicative random effects)
MR Egger
MR RAPS
Weighted median
Weighted mode
0.3
0.2
0.1
0.0
−0.1
SNP effect on Crohn's disease
0.1
0.2
0.3
SNP effect on IDP T1 FAST ROIs R parahipp gyrus ant

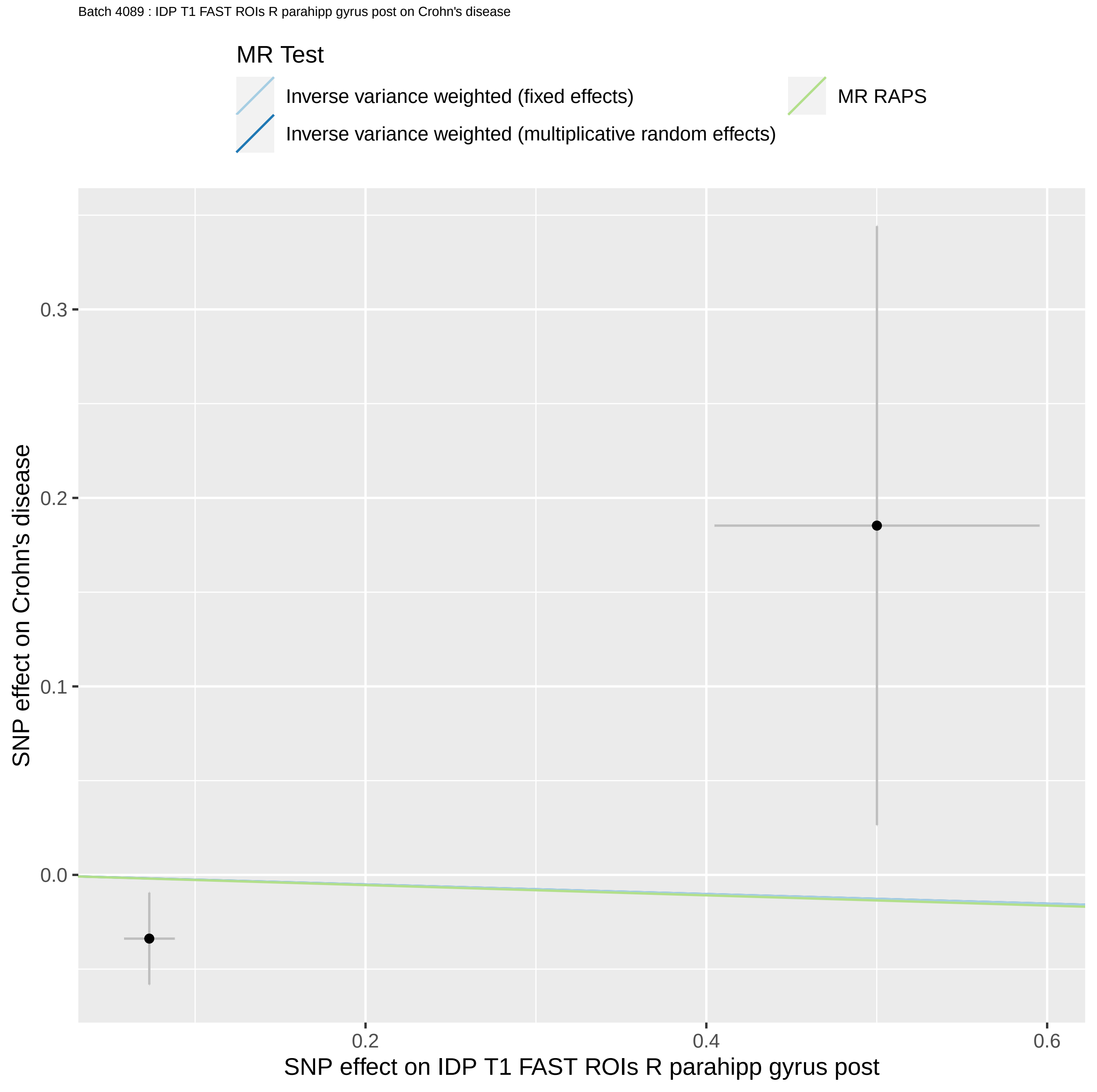

Batch 4089 : IDP T1 FAST ROIs R parahipp gyrus post on Crohn's disease
MR Test
Inverse variance weighted (fixed effects)
Inverse variance weighted (multiplicative random effects)
MR RAPS
0.3
0.2
0.1
0.0
SNP effect on Crohn's disease
0.2
0.4
0.6
SNP effect on IDP T1 FAST ROIs R parahipp gyrus post

Batch 4091 : IDP T1 FAST ROIs R lingual gyrus on Crohn's disease

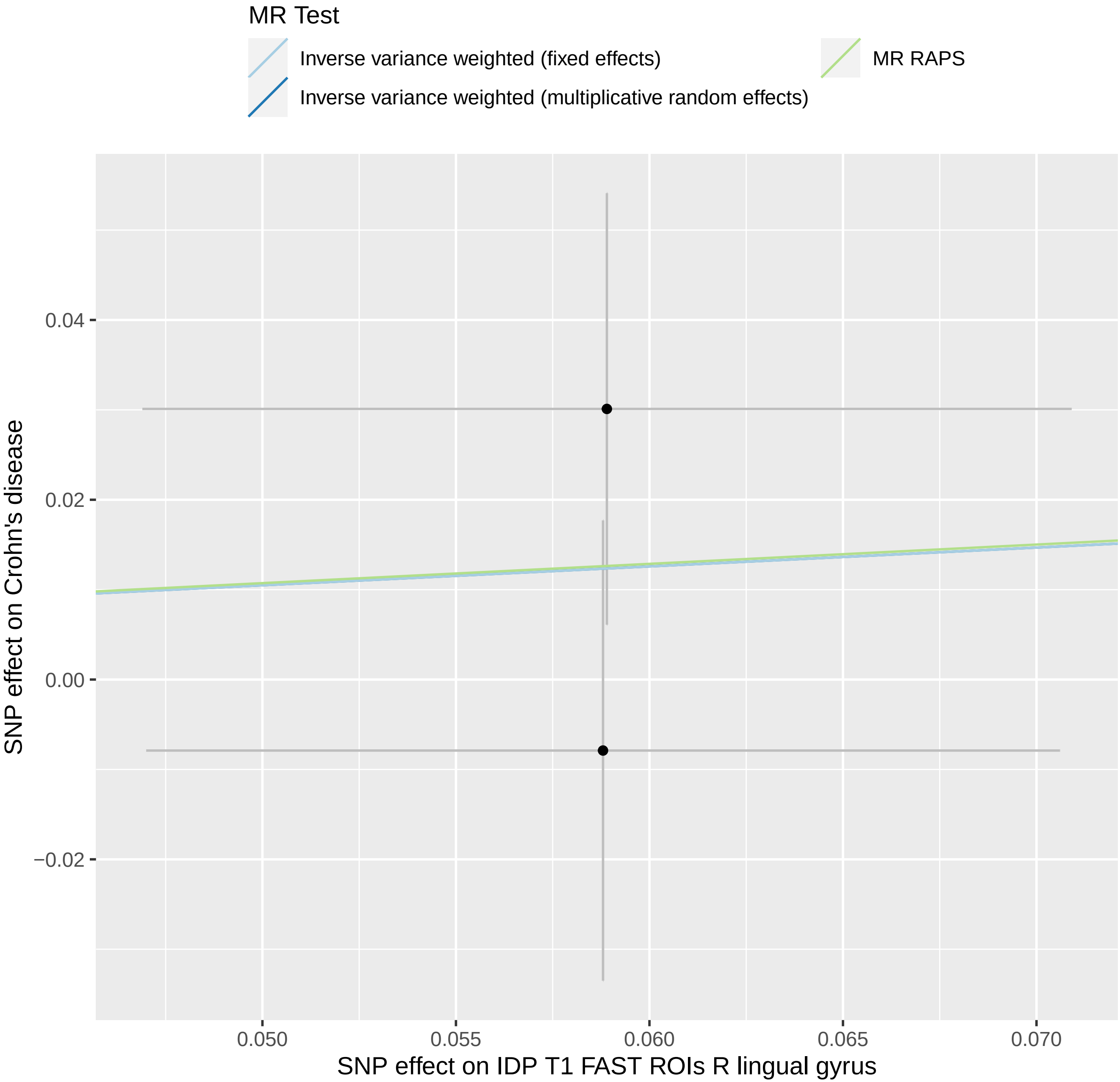

Batch 4095 : IDP T1 SIENAX brain−unnormalised volume on Ulcerative colitis

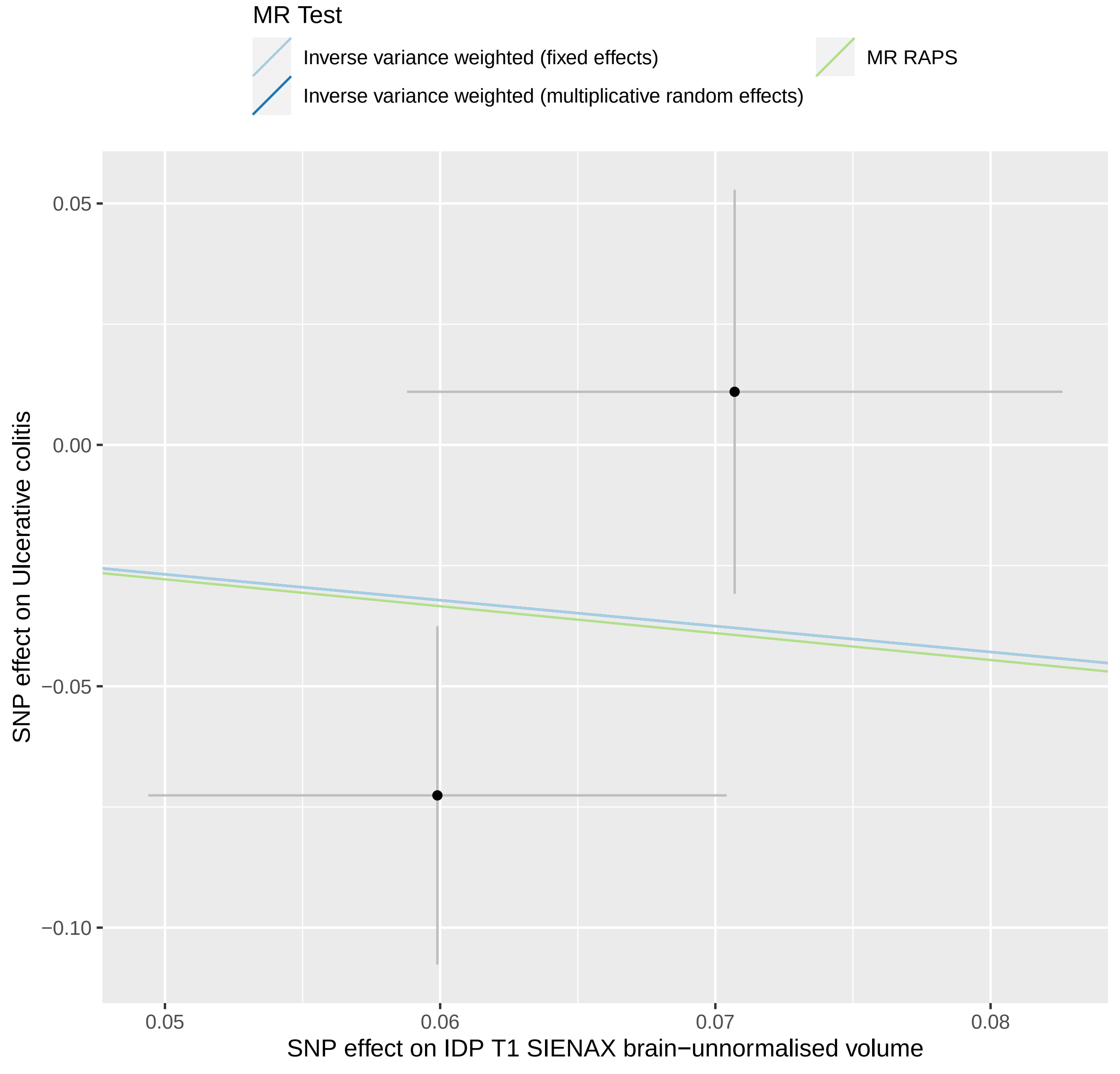

Batch 4097 : IDP T1 FAST ROIs R temp fusif cortex post on Ulcerative colitis

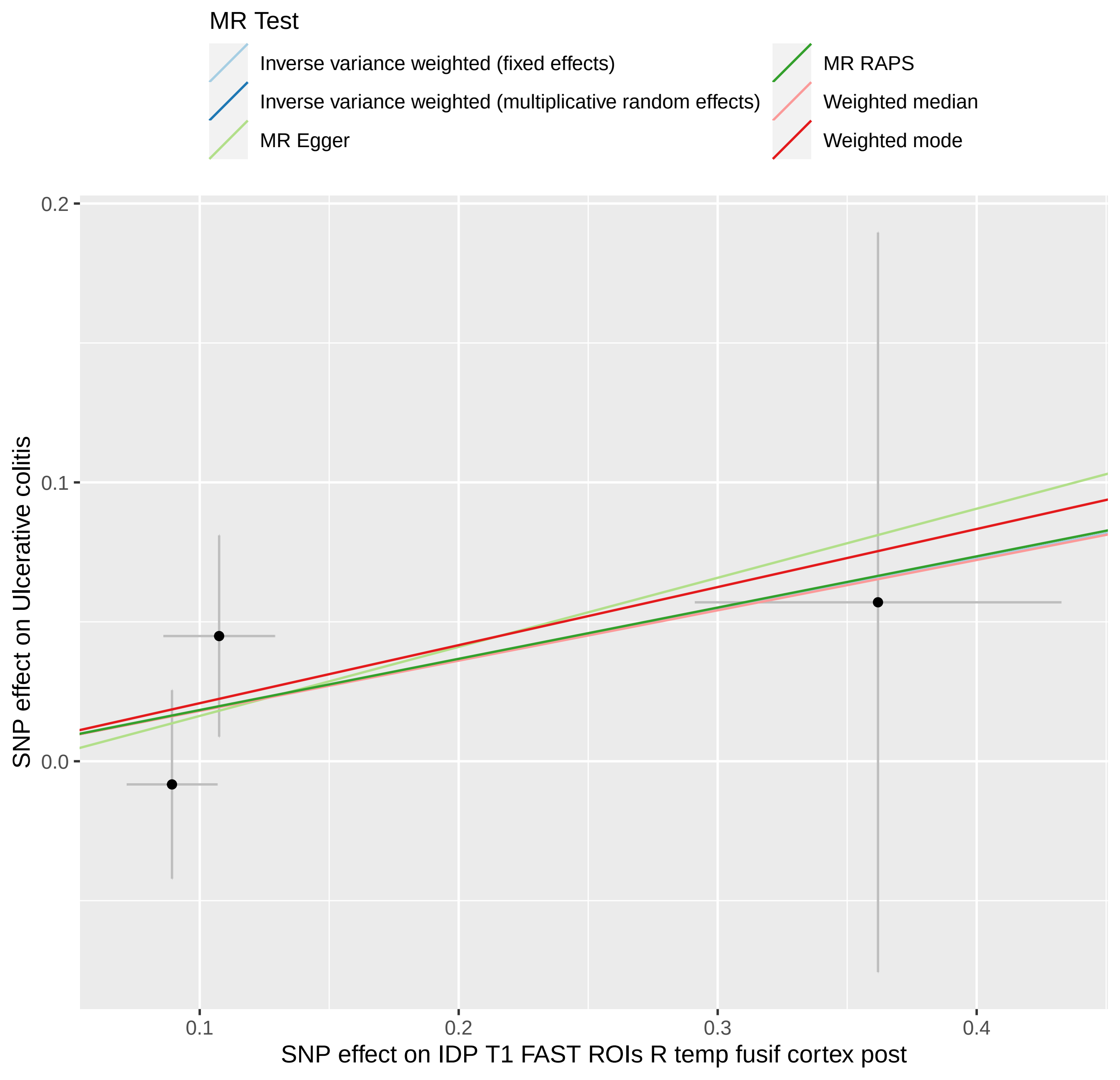

Batch 4103 : IDP T1 FAST ROIs R front operc cortex on Ulcerative colitis

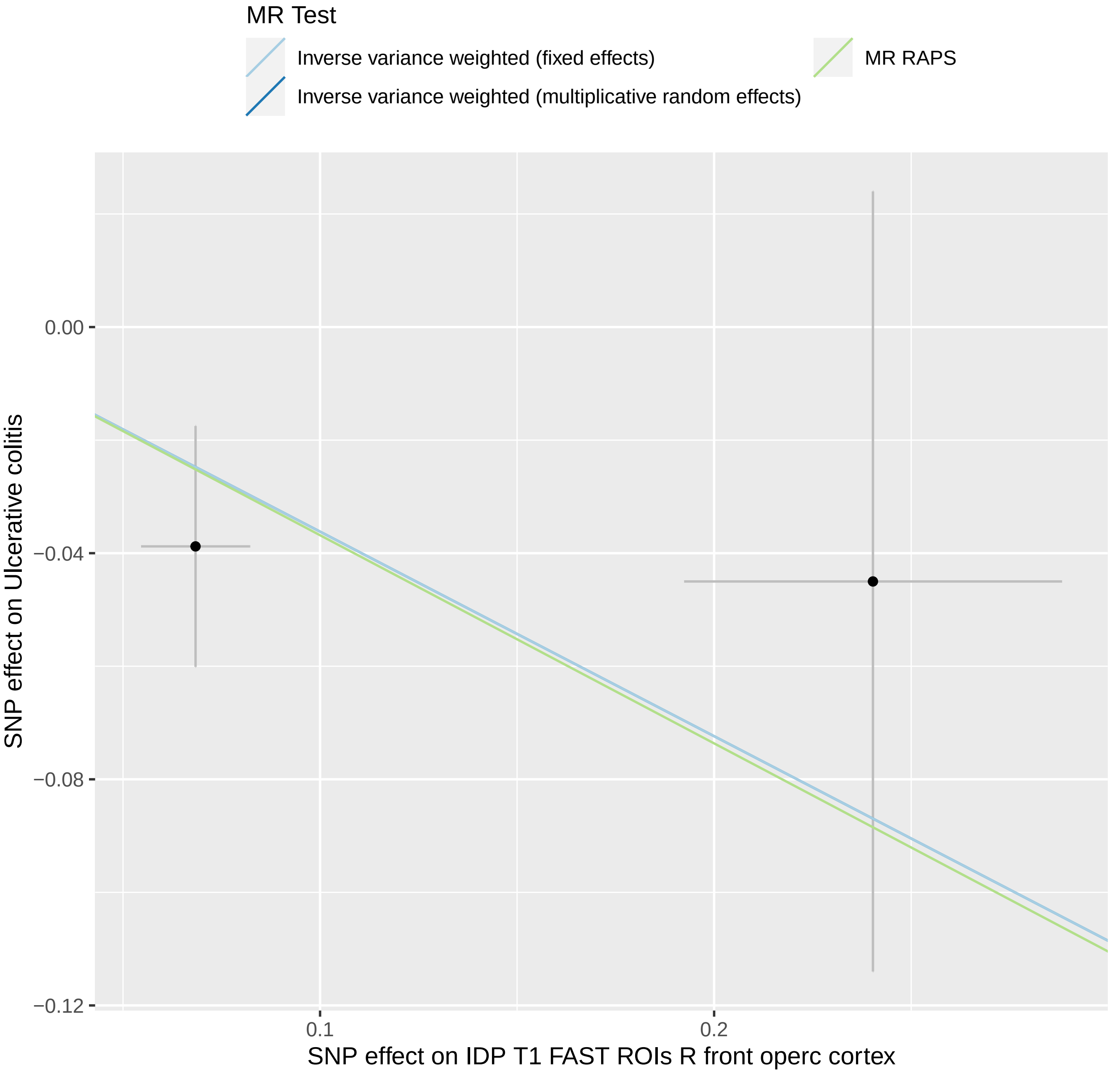

Batch 4106 : IDP T1 FIRST left thalamus volume on Ulcerative colitis

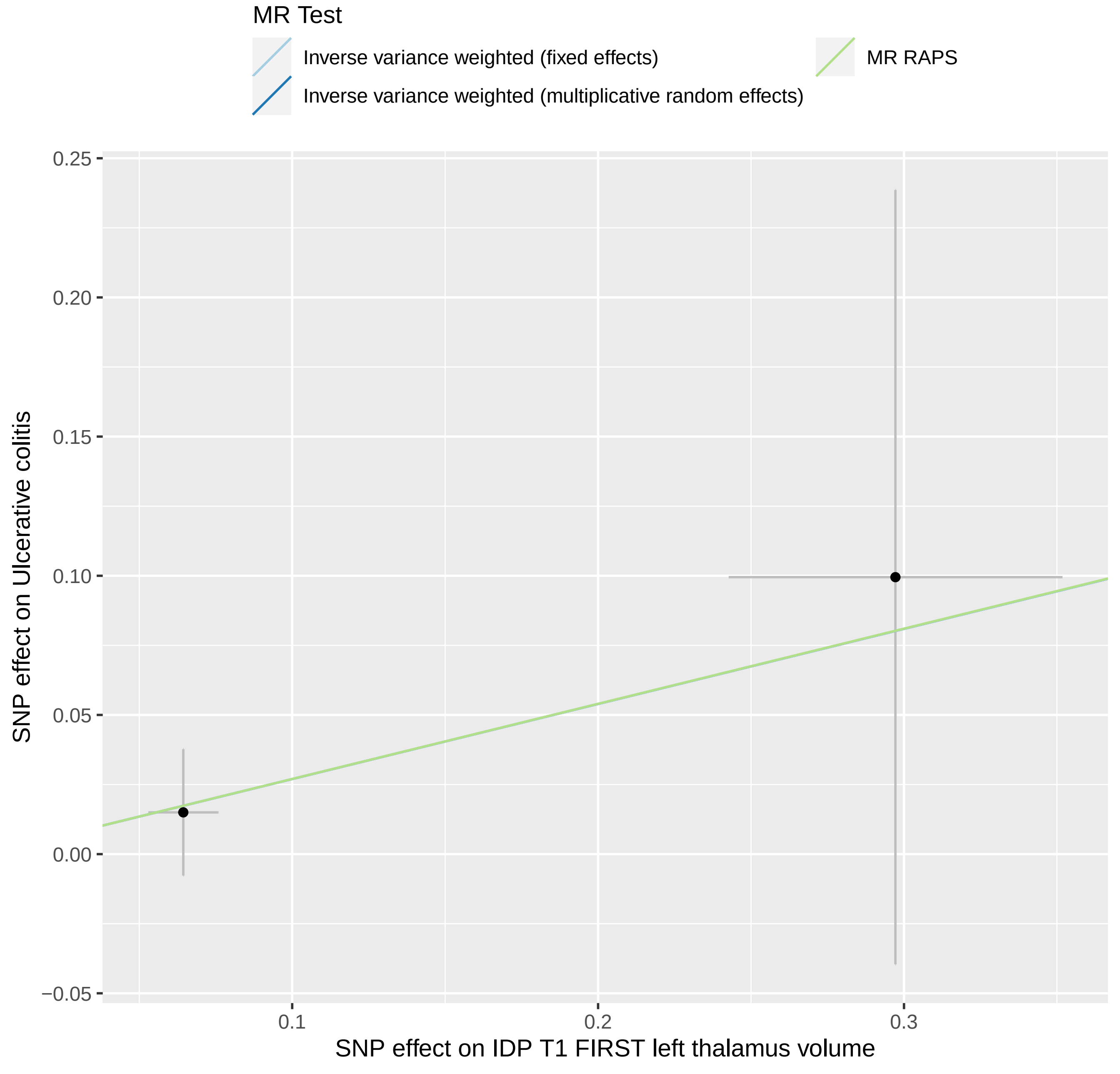

Batch 4107 : IDP T1 FAST ROIs L parietal operc cortex on Ulcerative colitis

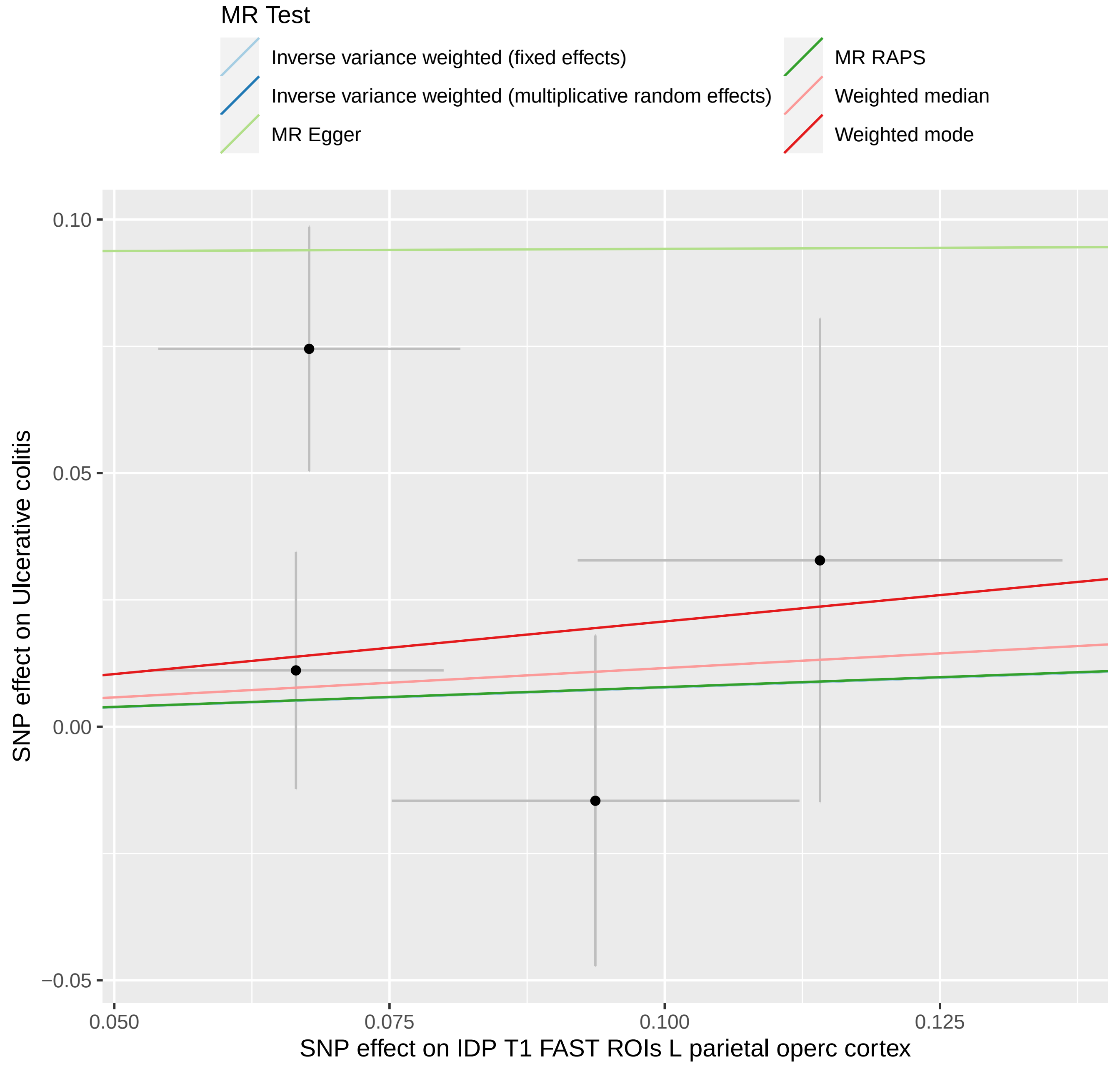

Batch 4108 : IDP T1 FAST ROIs R parietal operc cortex on Ulcerative colitis

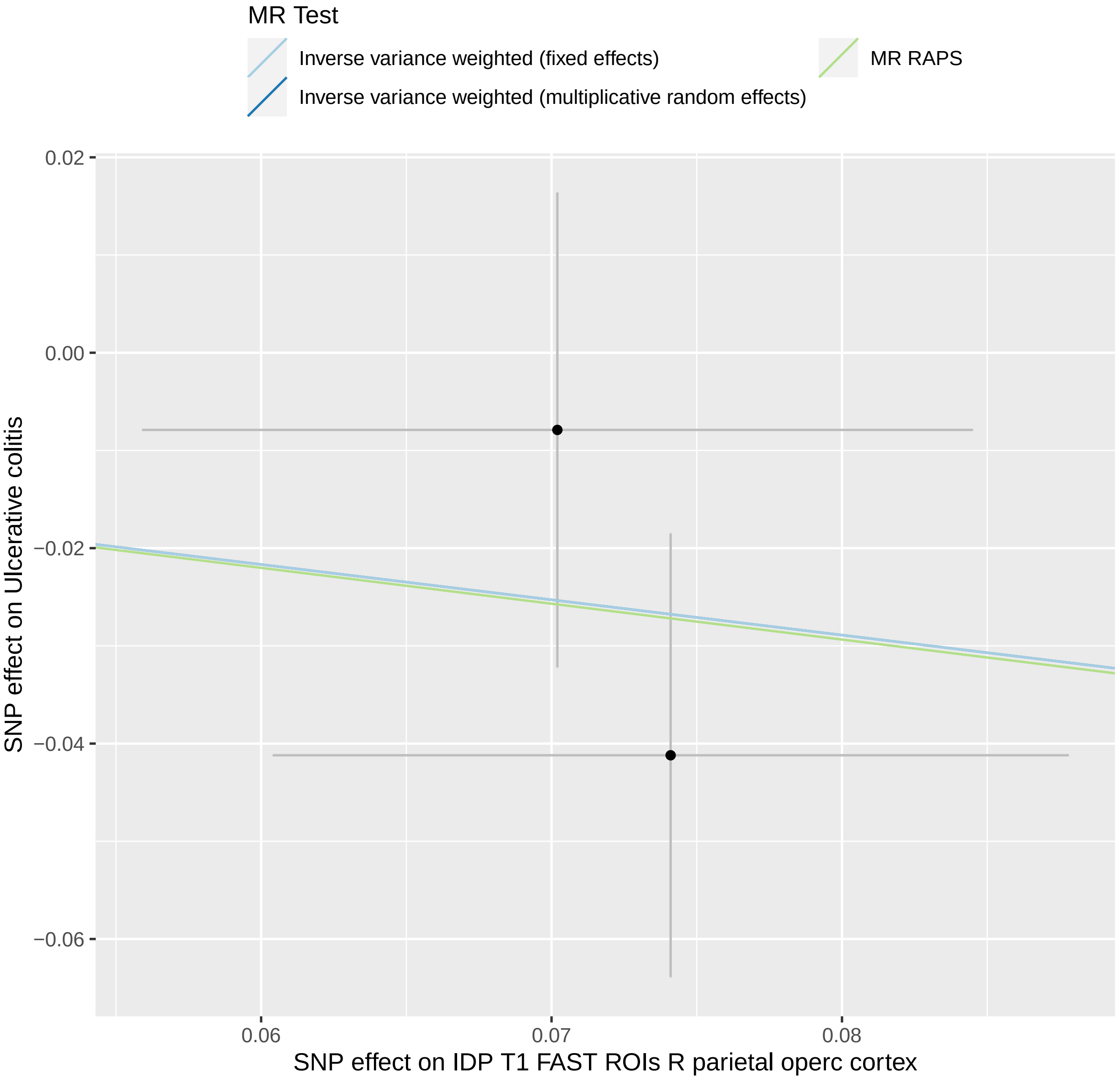

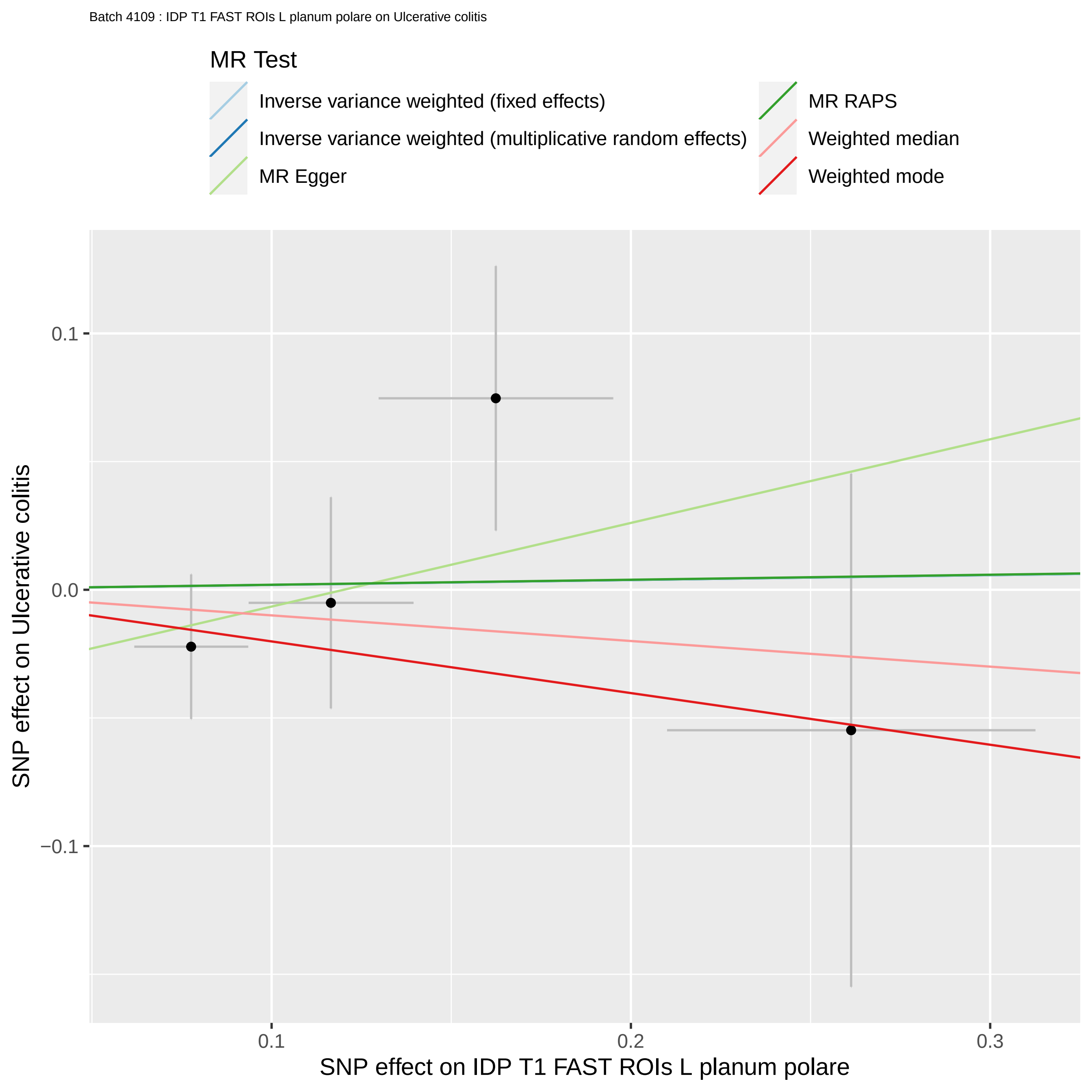

Batch 4109 : IDP T1 FAST ROIs L planum polare on Ulcerative colitis
MR Test
Inverse variance weighted (fixed effects)
Inverse variance weighted (multiplicative random effects)
MR Egger
MR RAPS
Weighted median
Weighted mode
0.1
0.0
−0.1
0.1
0.2
0.3
SNP effect on Ulcerative colitis
SNP effect on IDP T1 FAST ROIs L planum polare

Batch 4111 : IDP T1 FAST ROIs L heschl gyrus on Ulcerative colitis

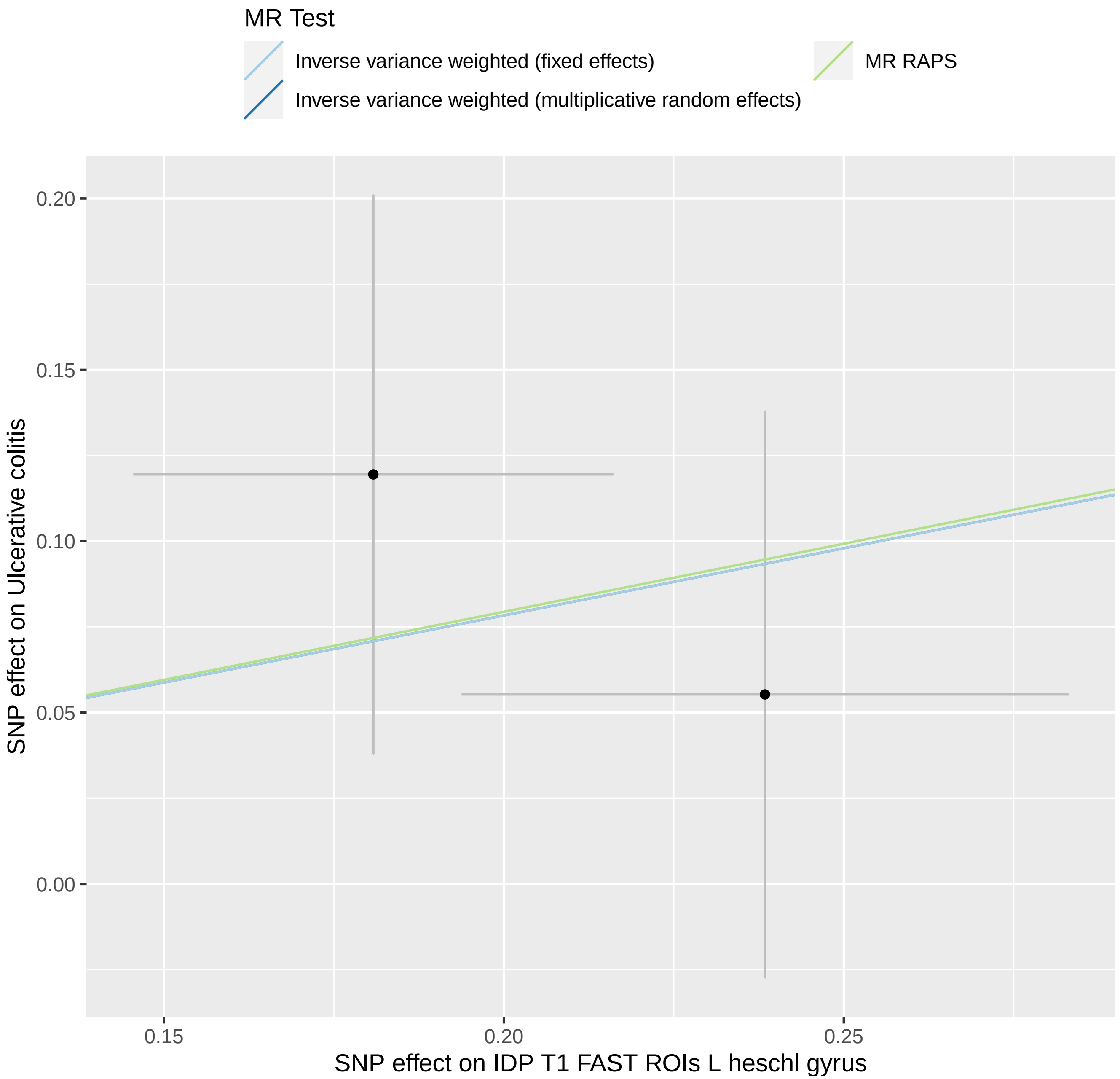

Batch 4112 : IDP T1 FAST ROIs R heschl gyrus on Ulcerative colitis

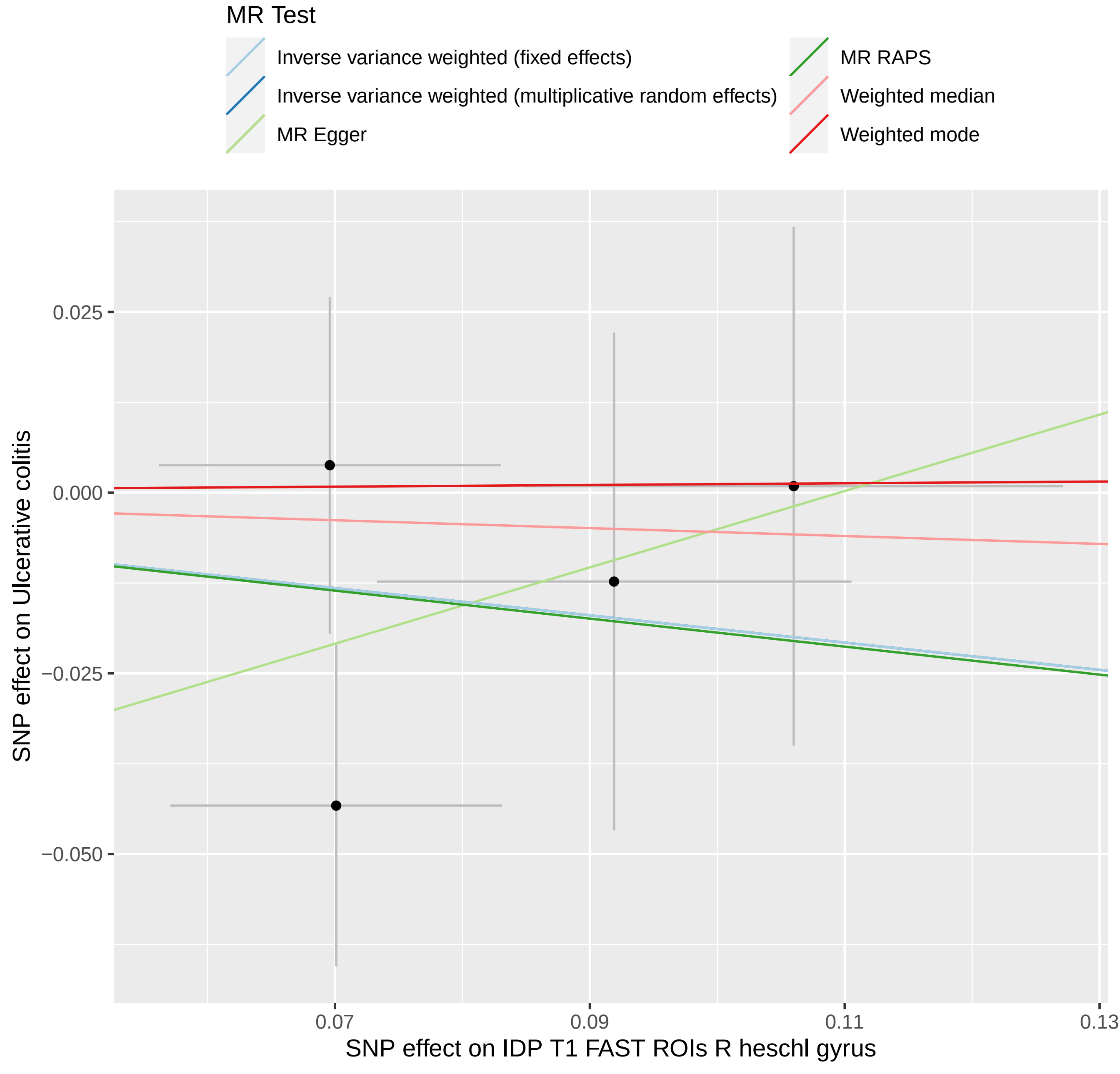

Batch 4114 : IDP T1 FAST ROIs R planum temporale on Ulcerative colitis

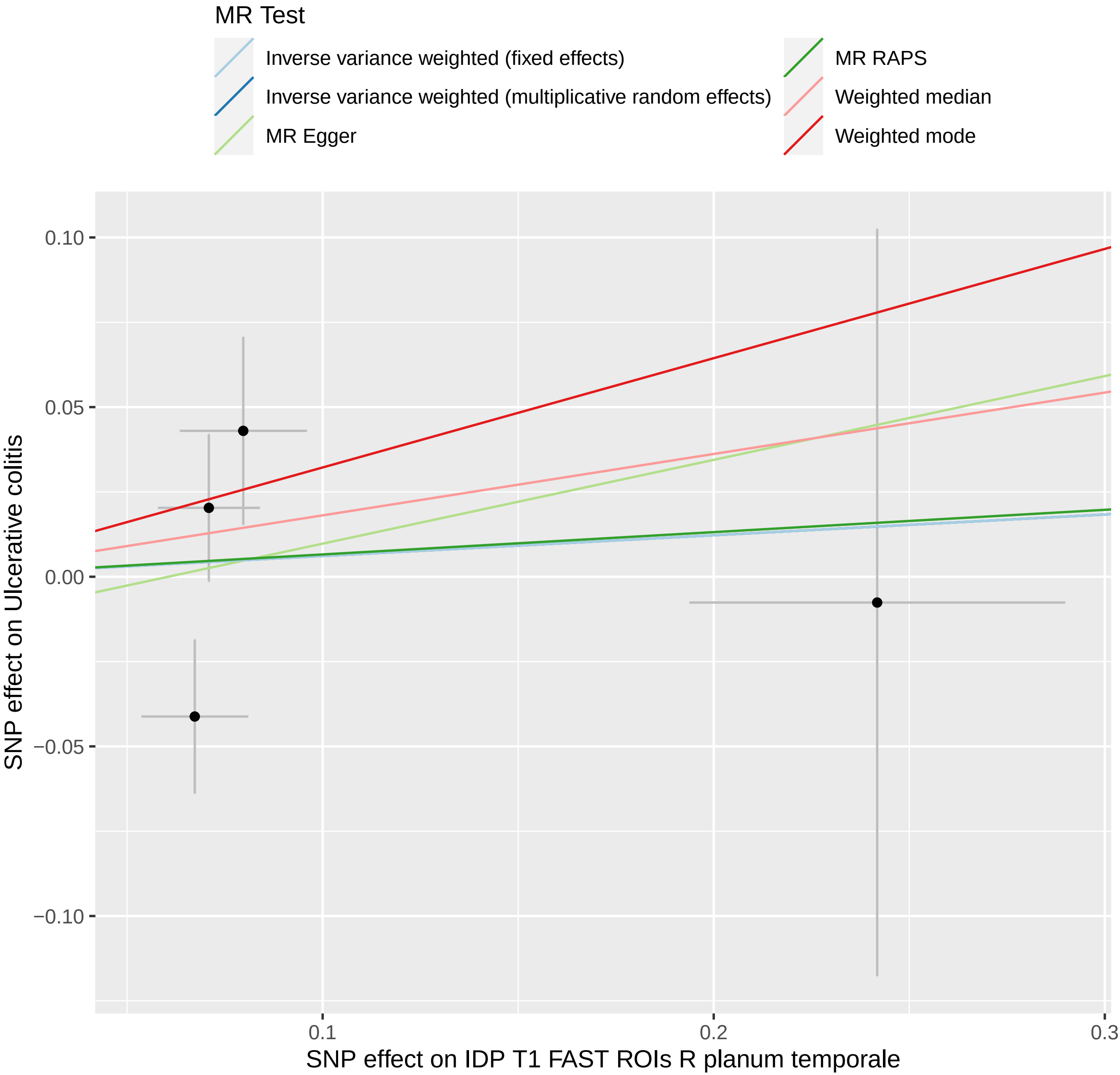

Batch 4115 : IDP T1 FAST ROIs L supracalc cortex on Ulcerative colitis

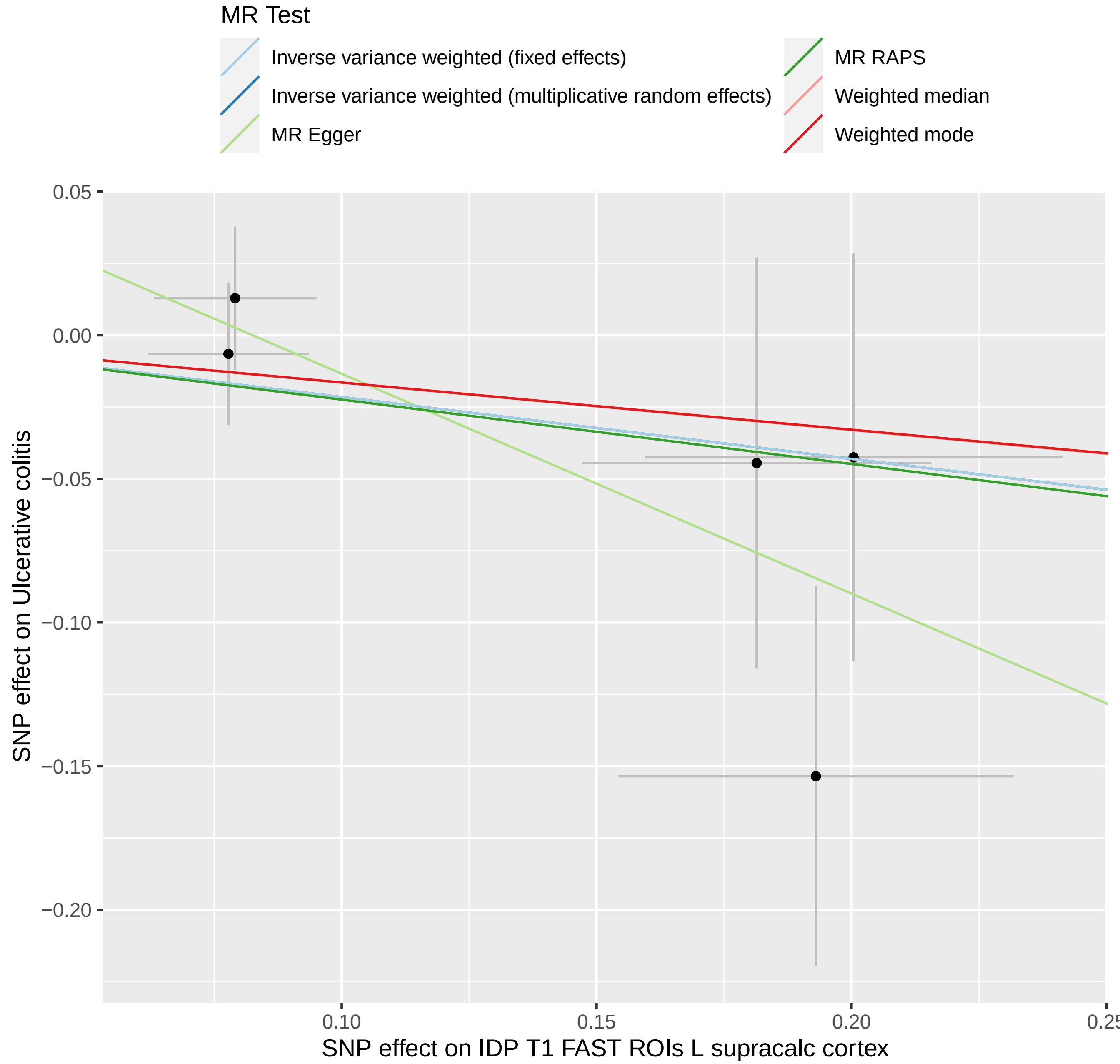

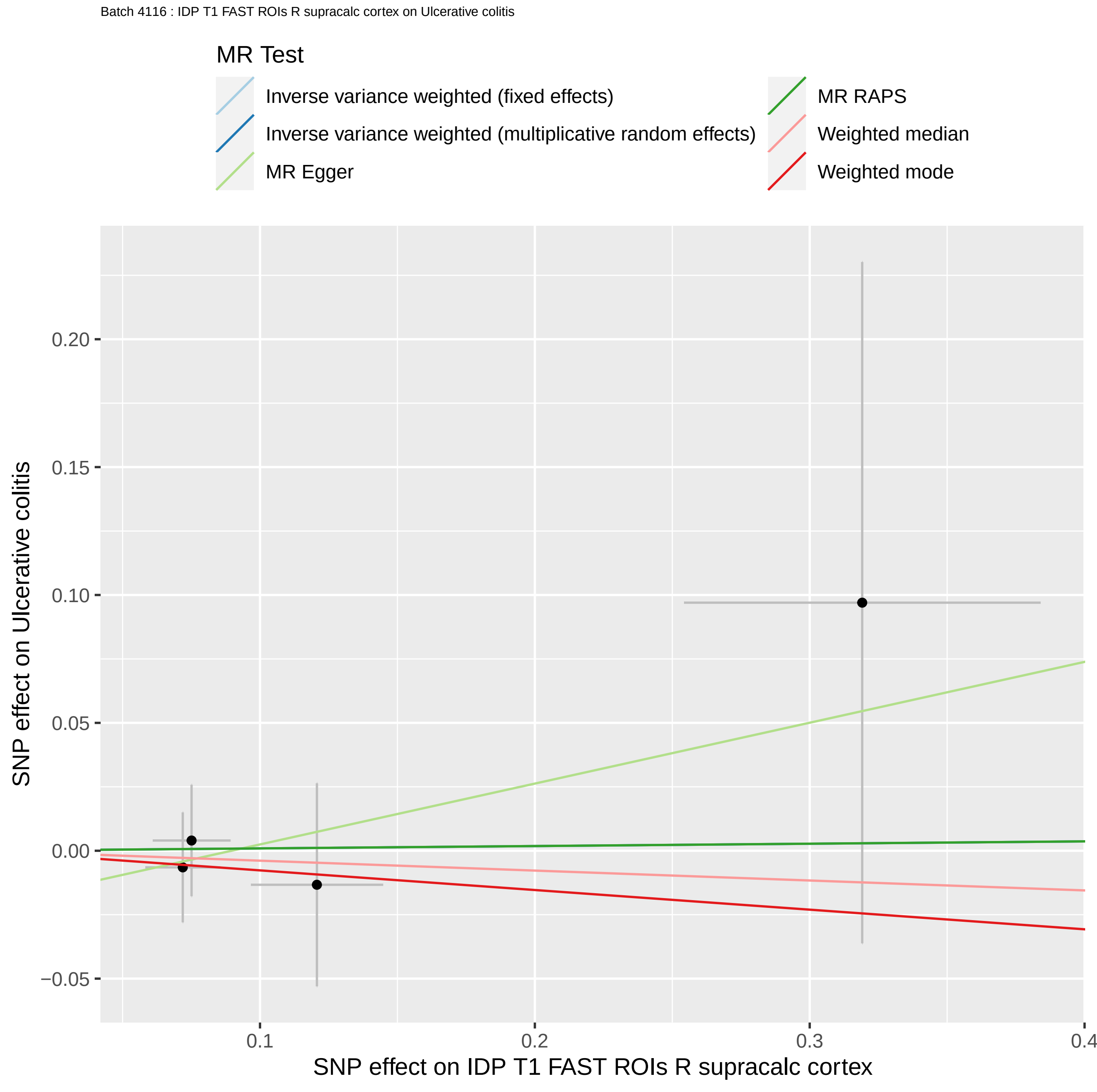

Batch 4116 : IDP T1 FAST ROIs R supracalc cortex on Ulcerative colitis
MR Test
Inverse variance weighted (fixed effects)
Inverse variance weighted (multiplicative random effects)
MR Egger
MR RAPS
Weighted median
Weighted mode
0.20
0.15
0.10
0.05
0.00
−0.05
SNP effect on Ulcerative colitis
0.1
0.2
0.3
0.4
SNP effect on IDP T1 FAST ROIs R supracalc cortex

Batch 4118 : IDP T1 FAST ROIs L occ pole on Ulcerative colitis

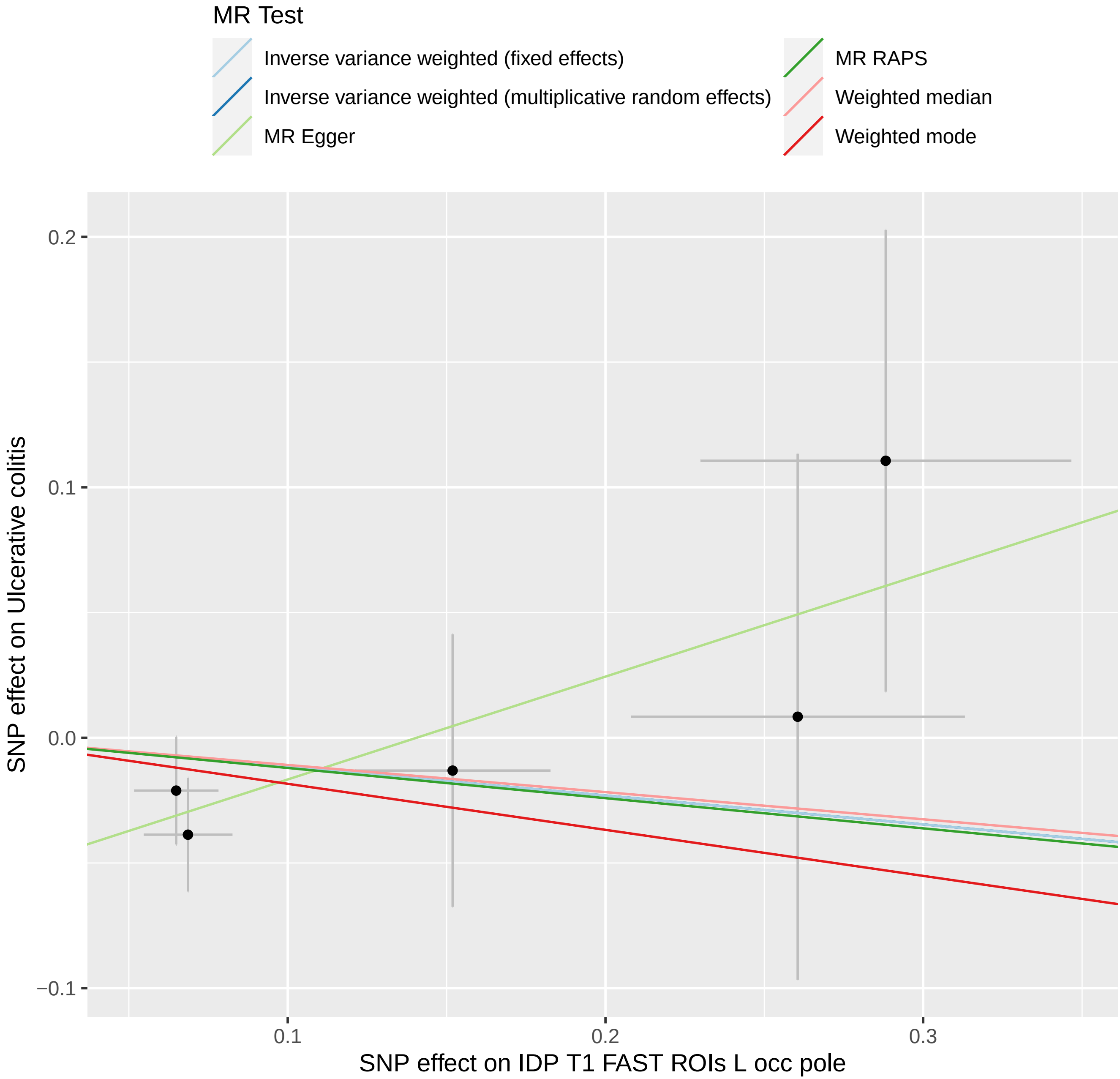

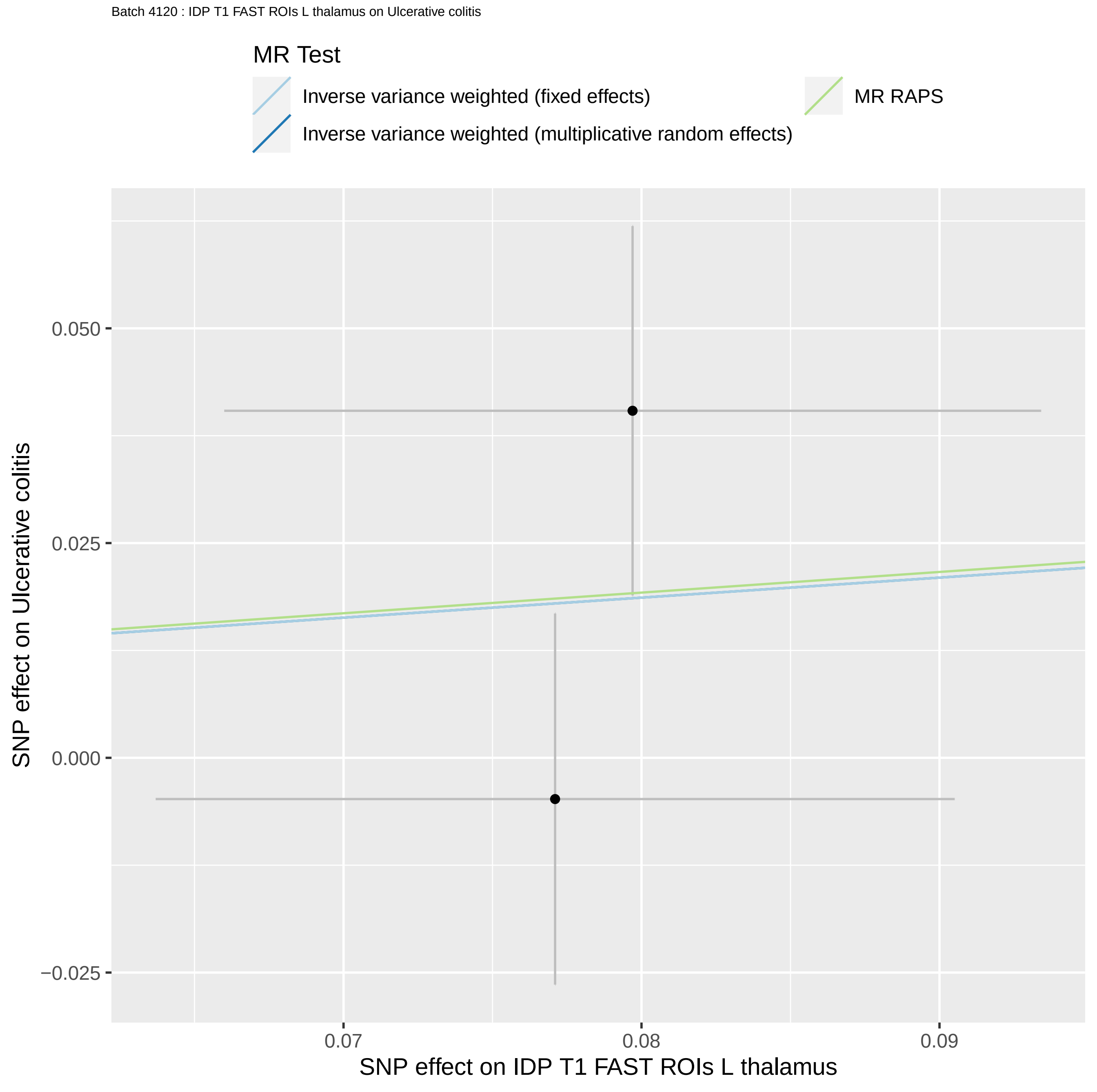
Batch 4120 : IDP T1 FAST ROIs L thalamus on Ulcerative colitis
MR Test
Inverse variance weighted (fixed effects)
Inverse variance weighted (multiplicative random effects)
MR RAPS
0.050
0.025
0.000
−0.025
0.07
0.08
0.09
SNP effect on Ulcerative colitis
SNP effect on IDP T1 FAST ROIs L thalamus

Batch 4124 : IDP T1 FAST ROIs L putamen on Ulcerative colitis

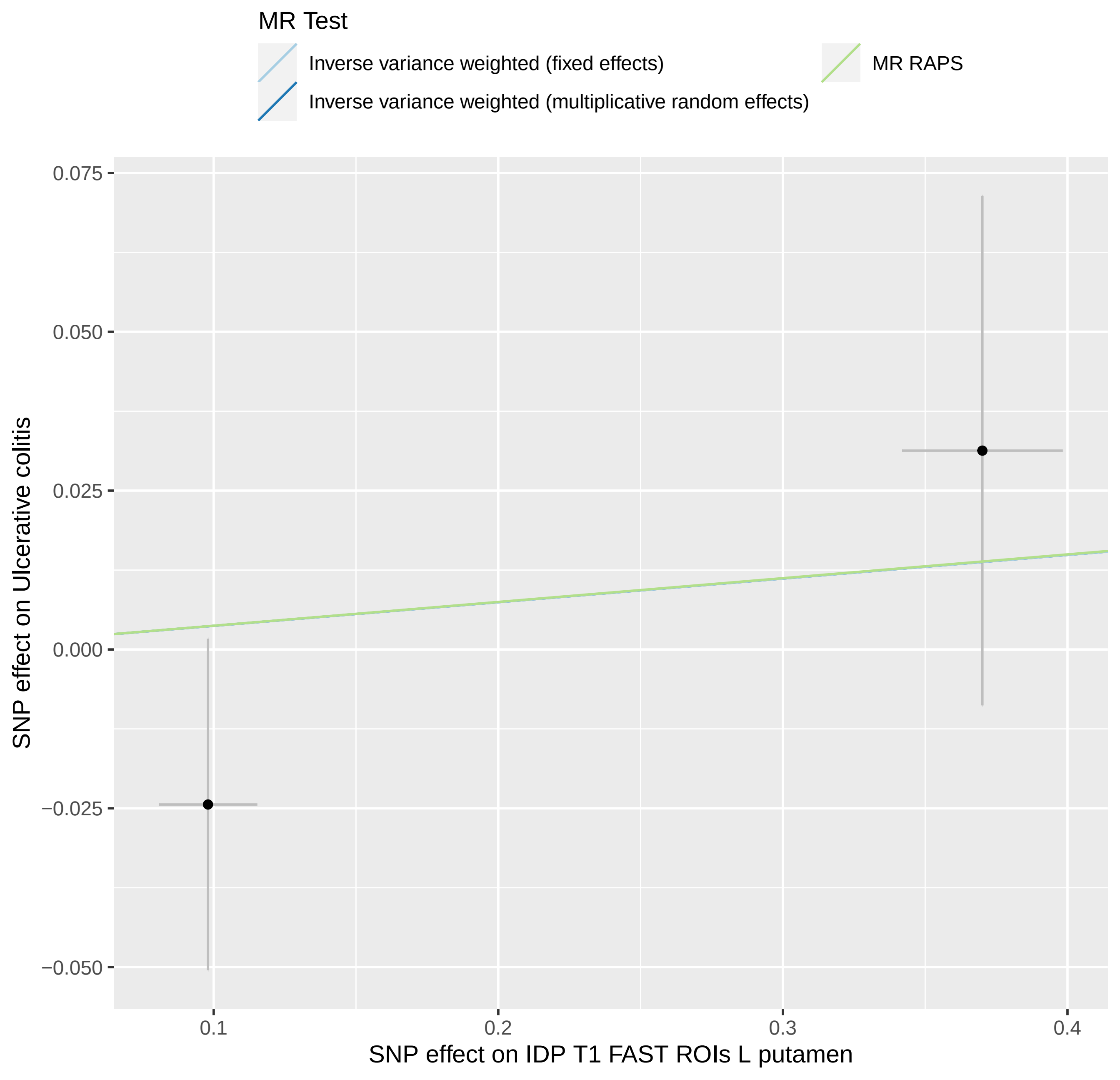

Batch 4125 : IDP T1 FAST ROIs R putamen on Ulcerative colitis

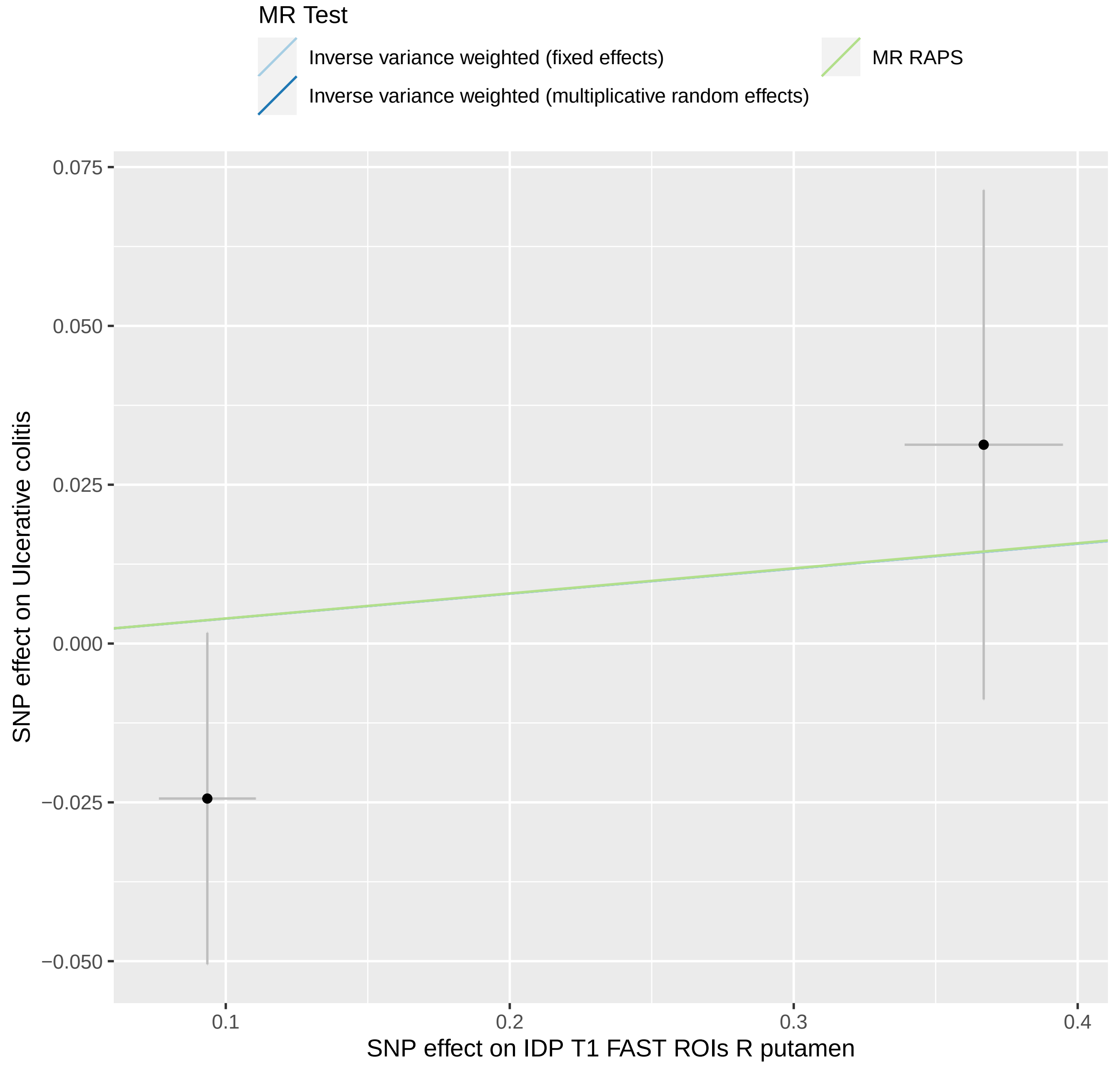

Batch 4127 : IDP T1 FAST ROIs R pallidum on Ulcerative colitis

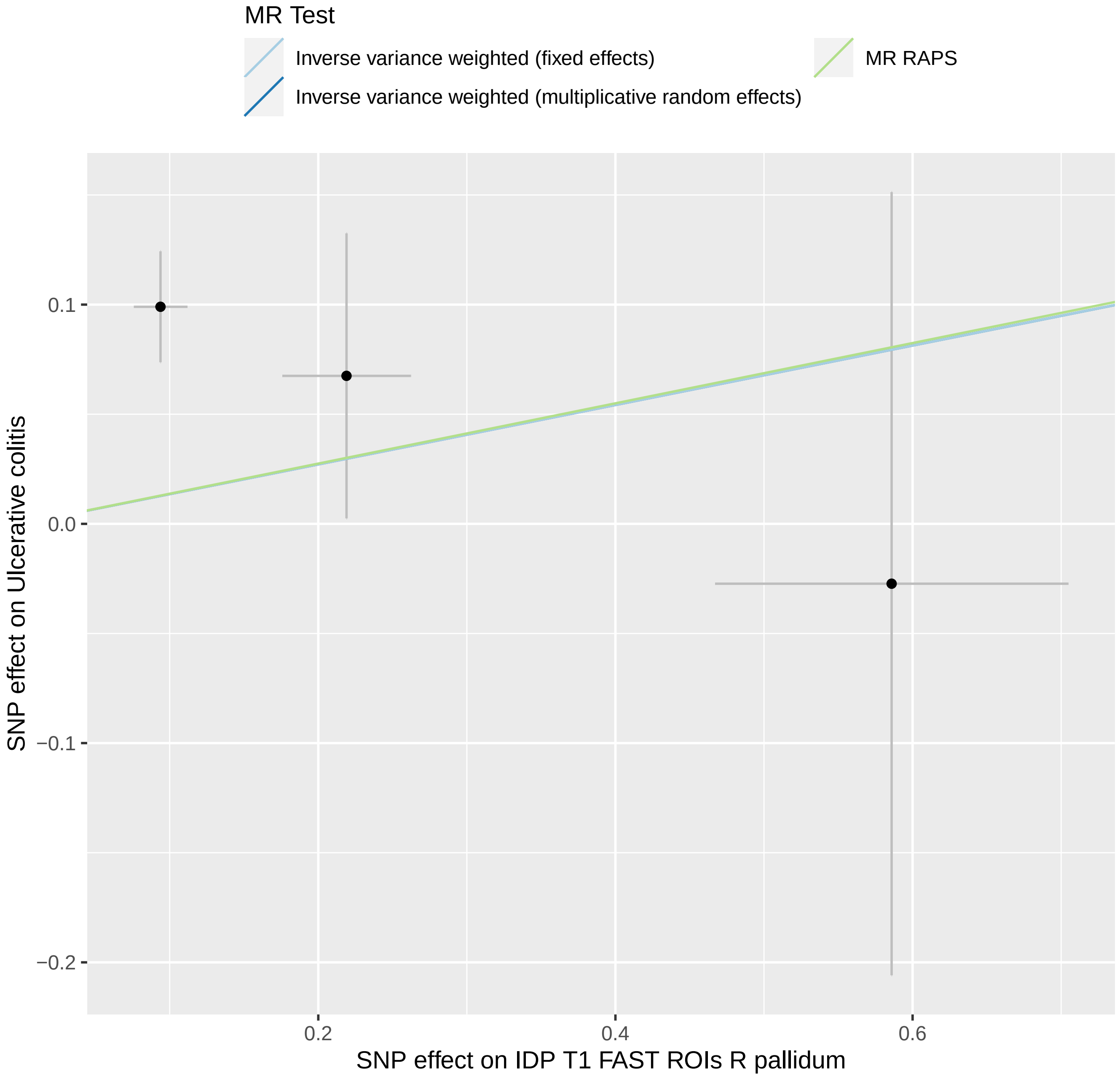

Batch 4128 : IDP T1 FIRST left caudate volume on Ulcerative colitis

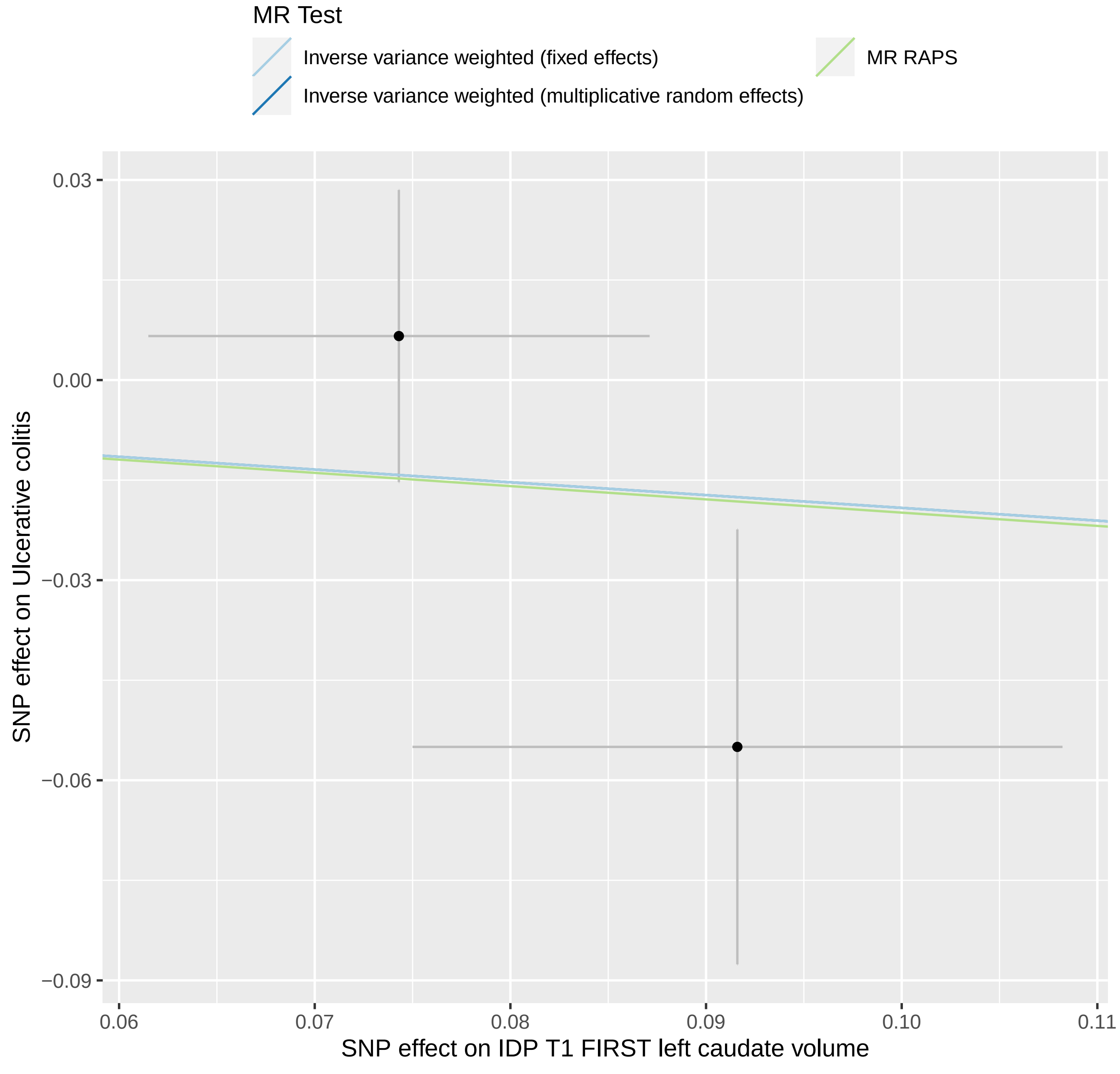

Batch 4129 : IDP T1 FAST ROIs L hippocampus on Ulcerative colitis

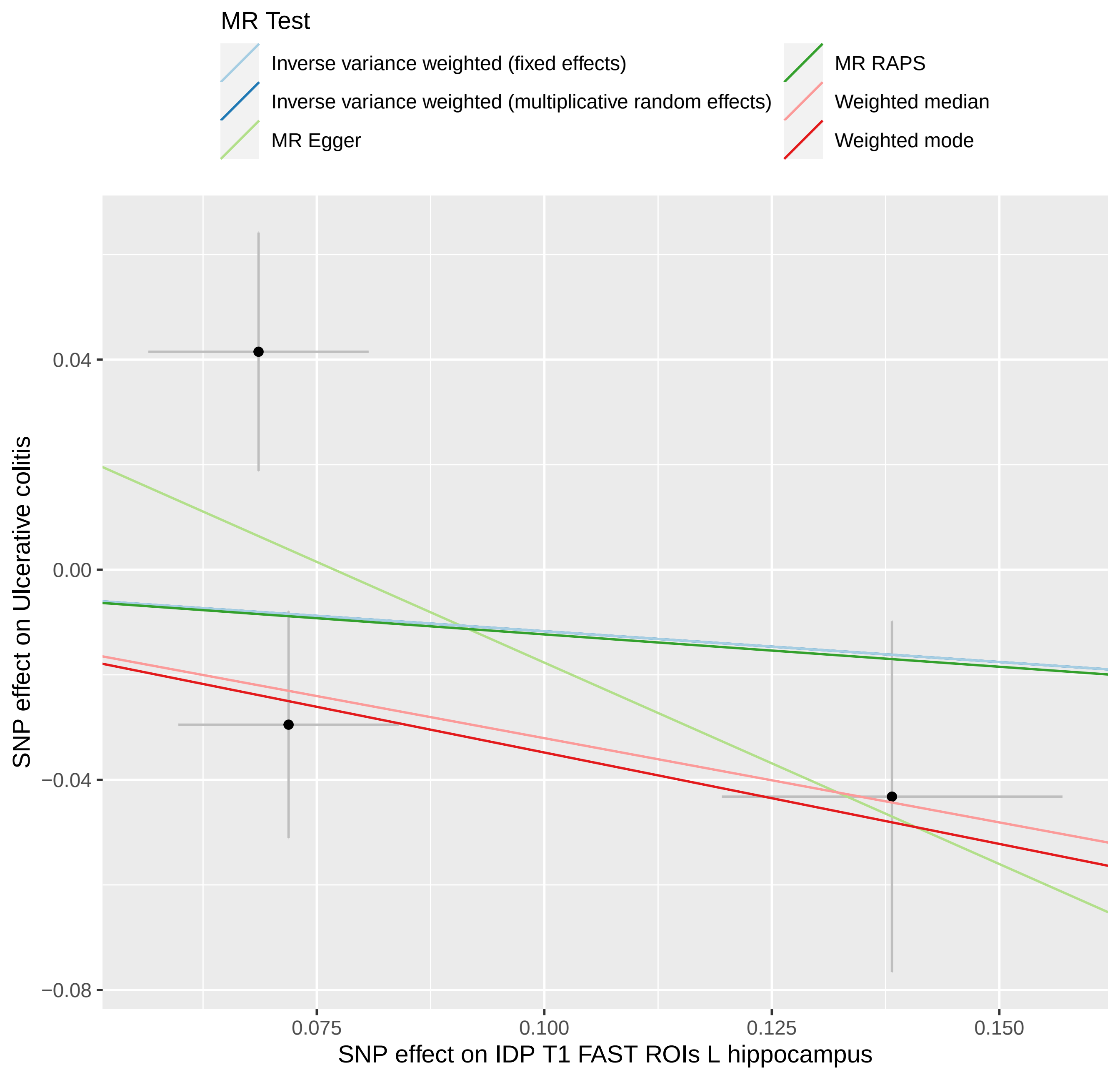

Batch 4130 : IDP T1 FAST ROIs R hippocampus on Ulcerative colitis

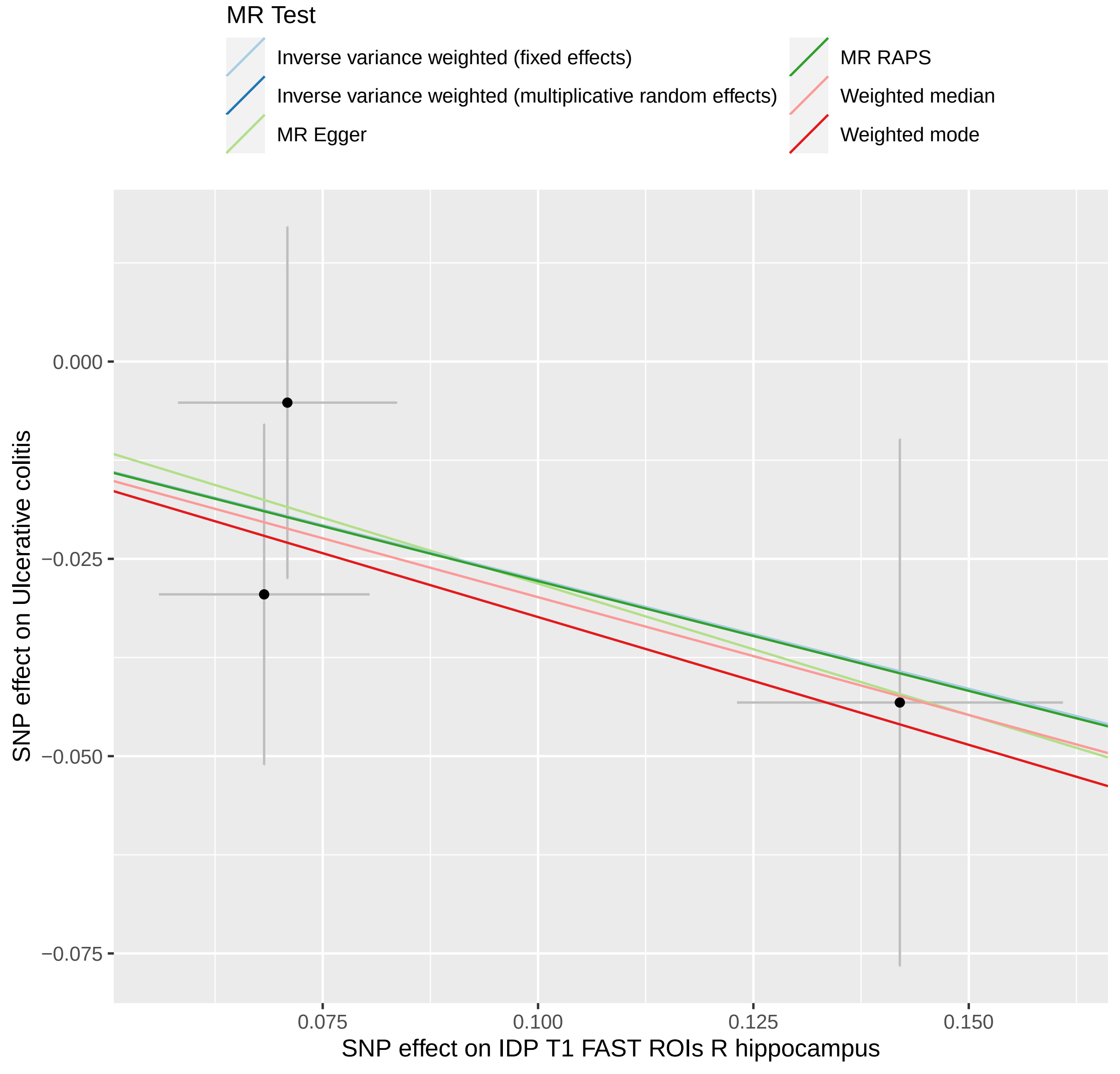

Batch 4133 : IDP T1 FAST ROIs L ventral striatum on Ulcerative colitis

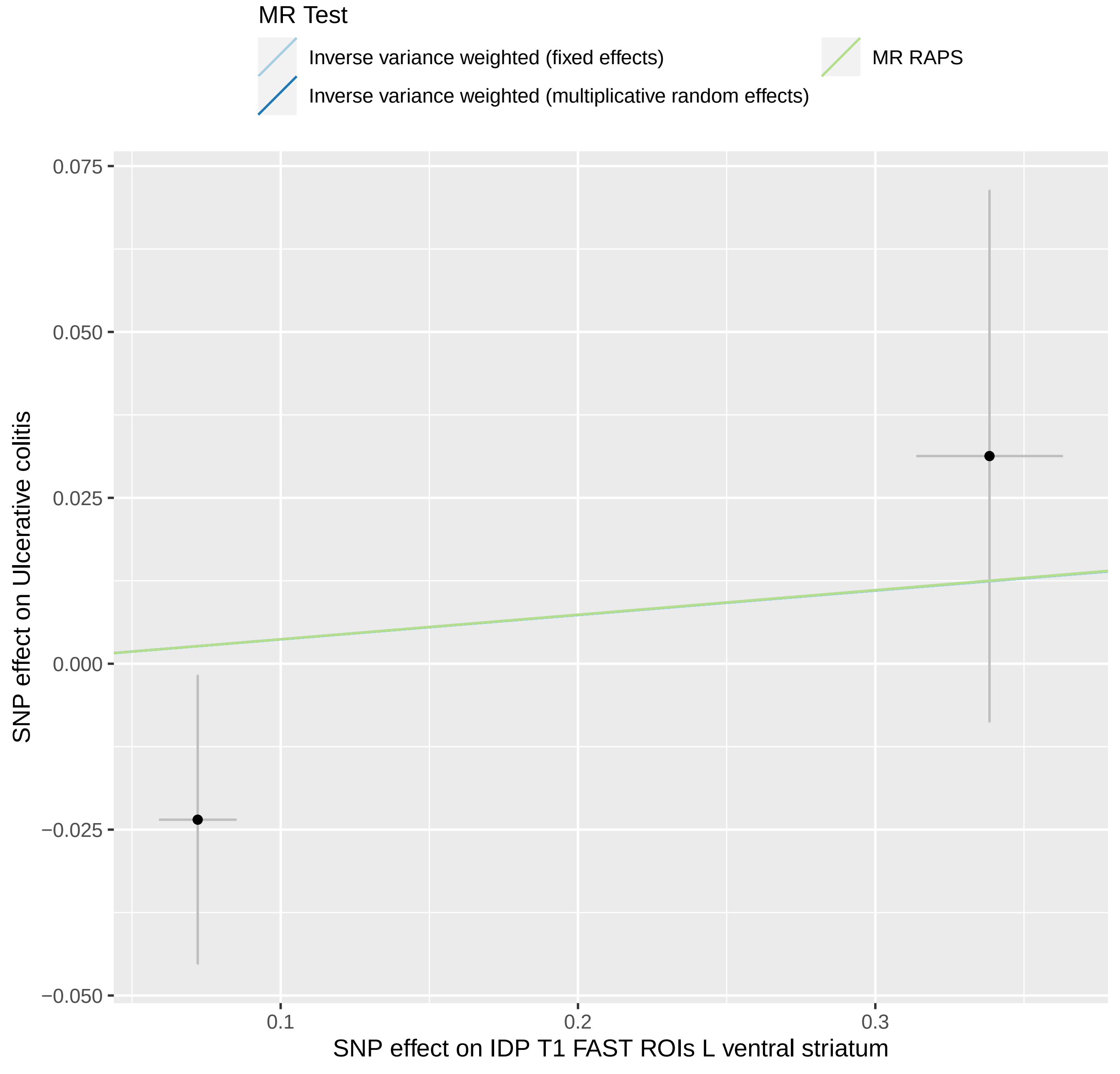

Batch 4134 : IDP T1 FAST ROIs R ventral striatum on Ulcerative colitis

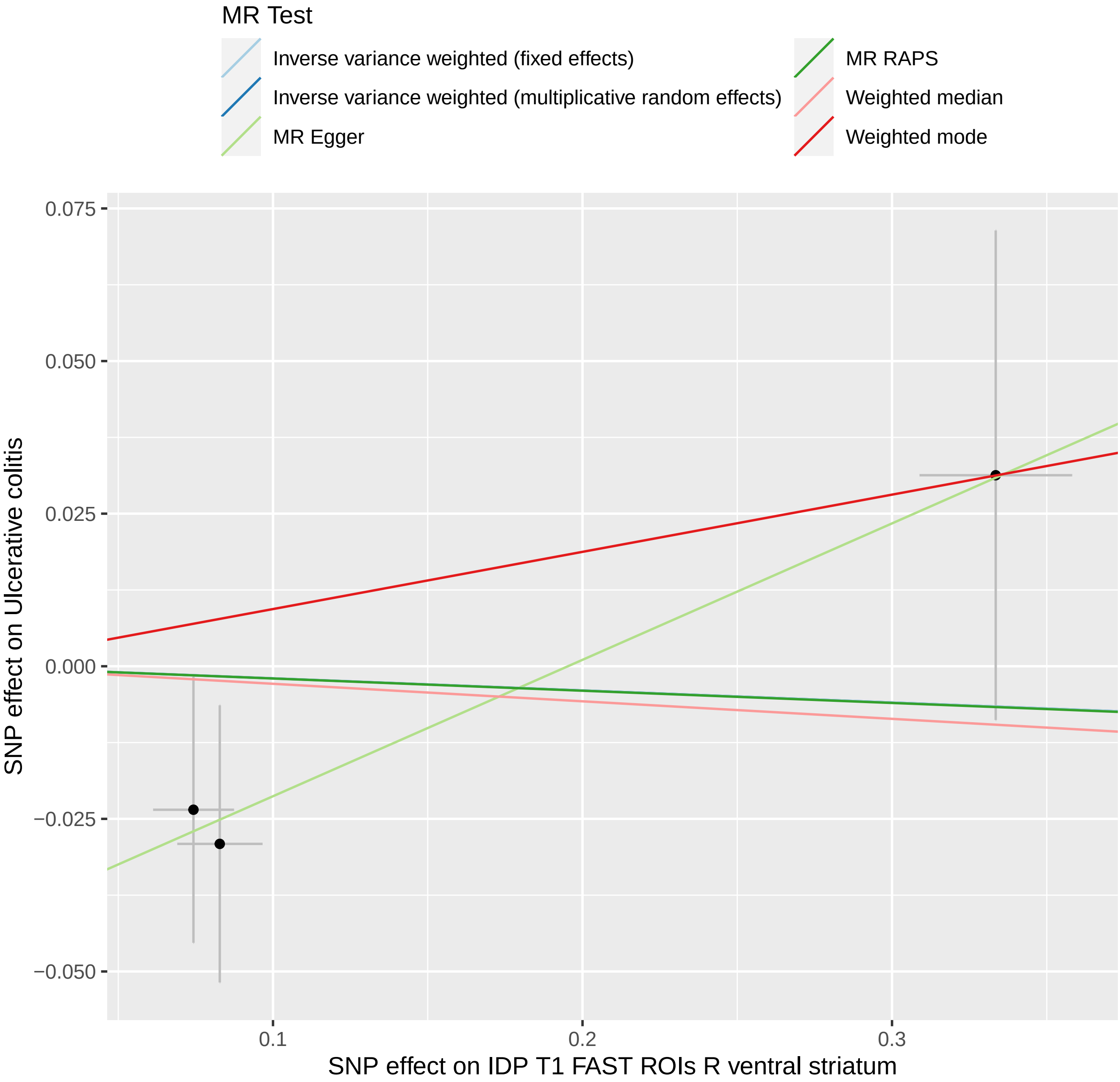

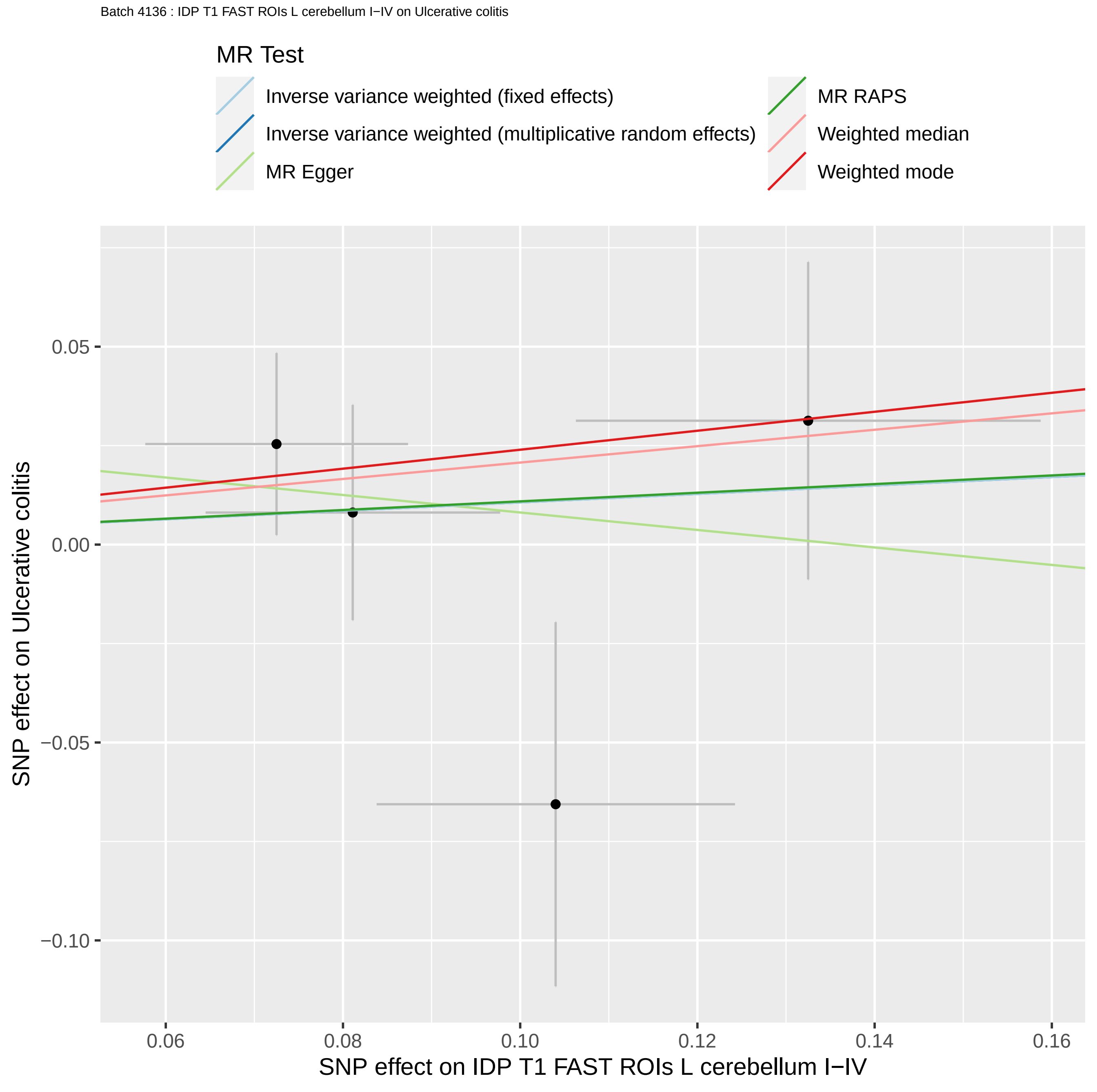
Batch 4136 : IDP T1 FAST ROIs L cerebellum I−IV on Ulcerative colitis
MR Test
Inverse variance weighted (fixed effects)
Inverse variance weighted (multiplicative random effects)
MR Egger
MR RAPS
Weighted median
Weighted mode
0.05
0.00
−0.05
−0.10
SNP effect on Ulcerative colitis
0.06
0.08
0.10
0.12
0.14
0.16
SNP effect on IDP T1 FAST ROIs L cerebellum I−IV

Batch 4137 : IDP T1 FAST ROIs R cerebellum I−IV on Ulcerative colitis

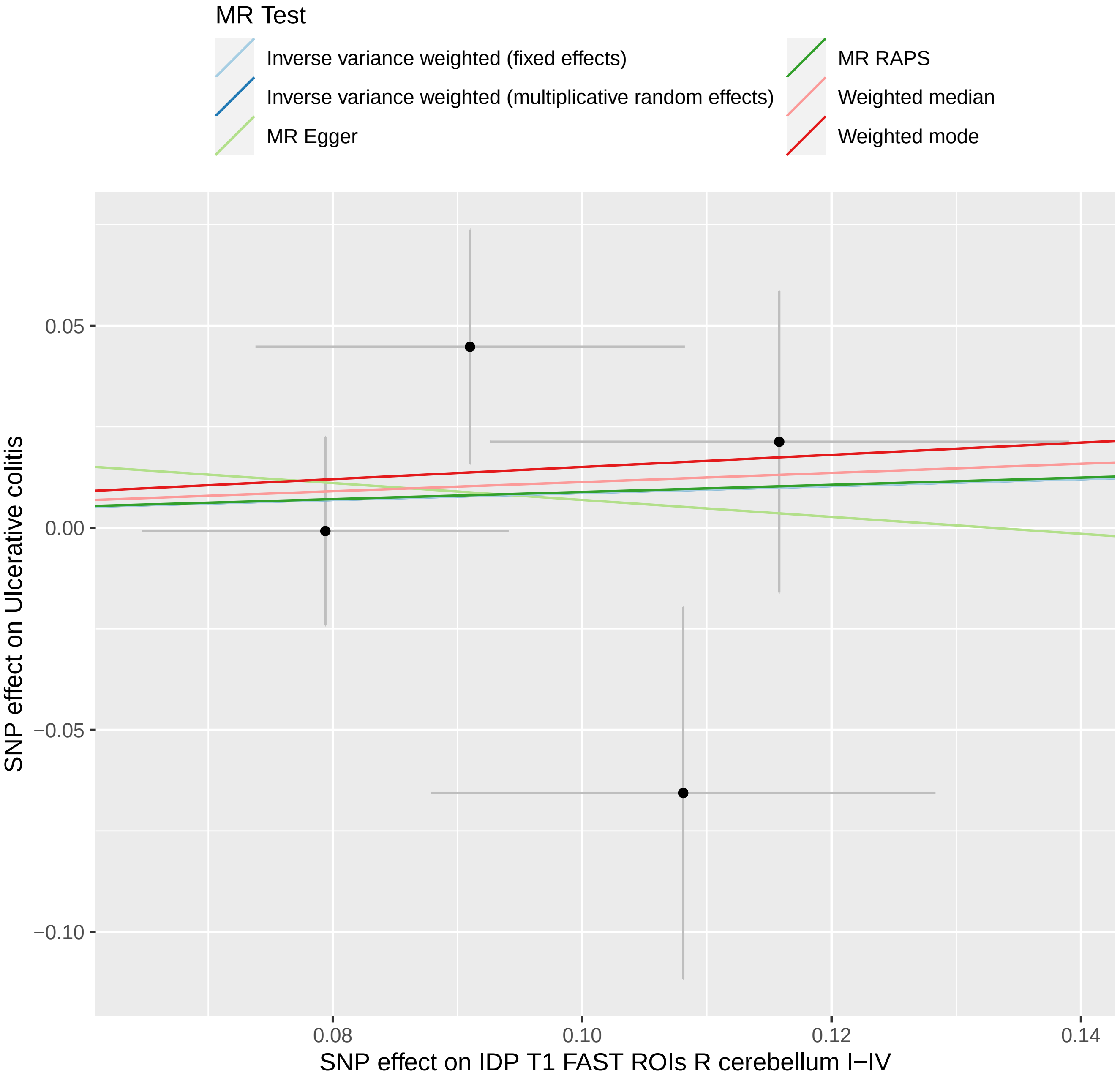

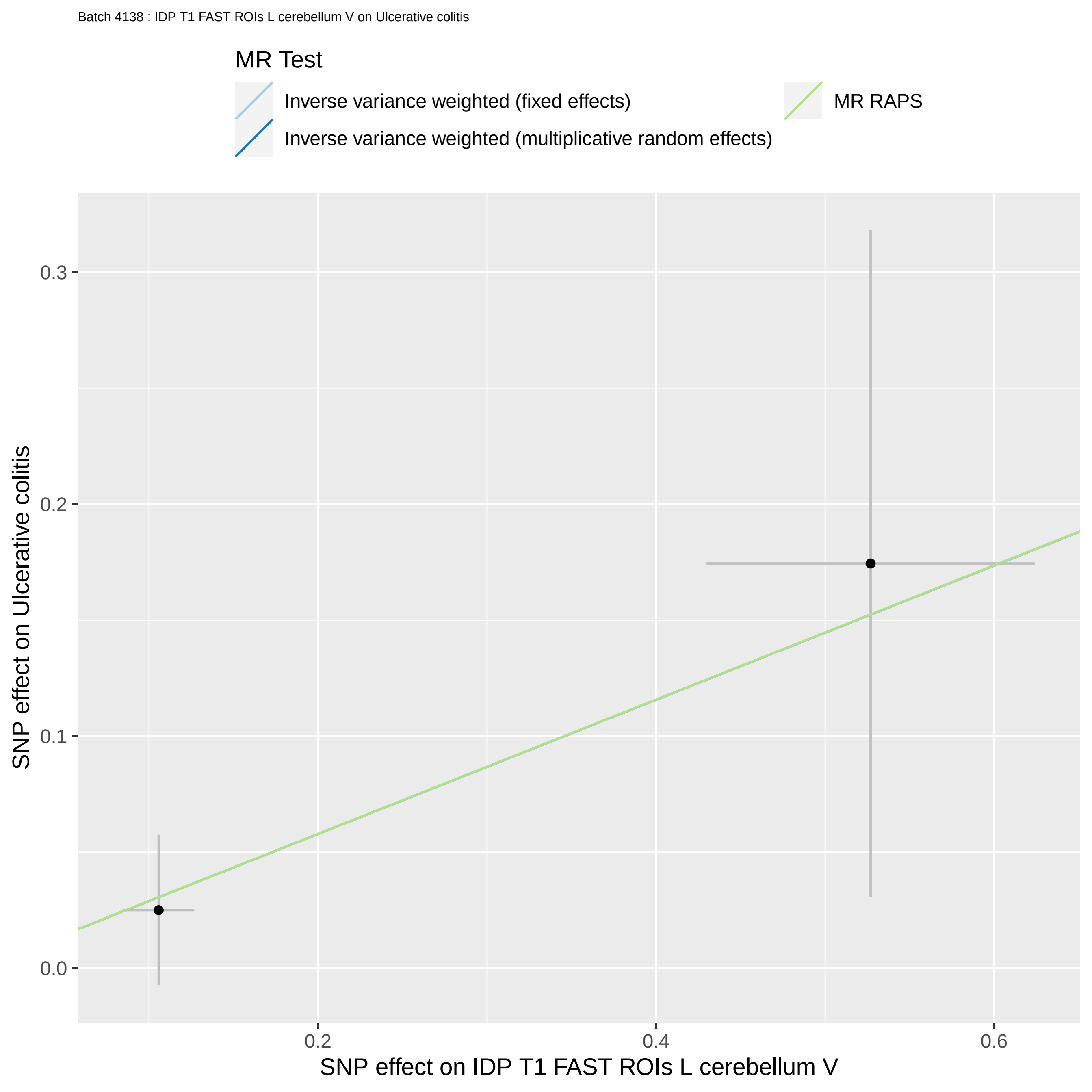

Batch 4138 : IDP T1 FAST ROIs L cerebellum V on Ulcerative colitis
MR Test
Inverse variance weighted (fixed effects)
Inverse variance weighted (multiplicative random effects)
MR RAPS
0.3
0.2
0.1
0.0
0.2
0.4
0.6
SNP effect on Ulcerative colitis
SNP effect on IDP T1 FAST ROIs L cerebellum V

Batch 4140 : IDP T1 FAST ROIs R cerebellum V on Ulcerative colitis

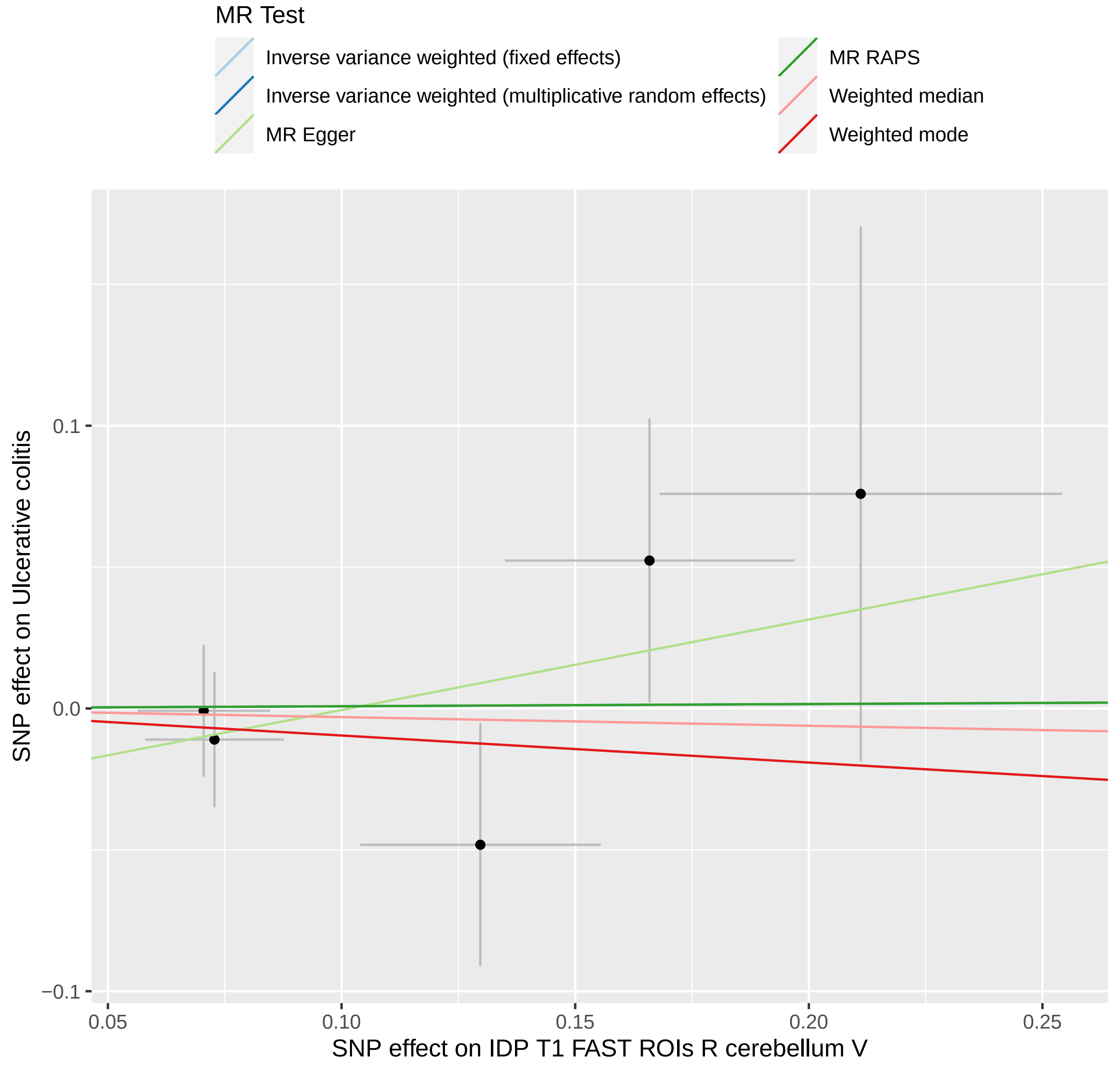

Batch 4142 : IDP T1 FAST ROIs V cerebellum VI on Ulcerative colitis

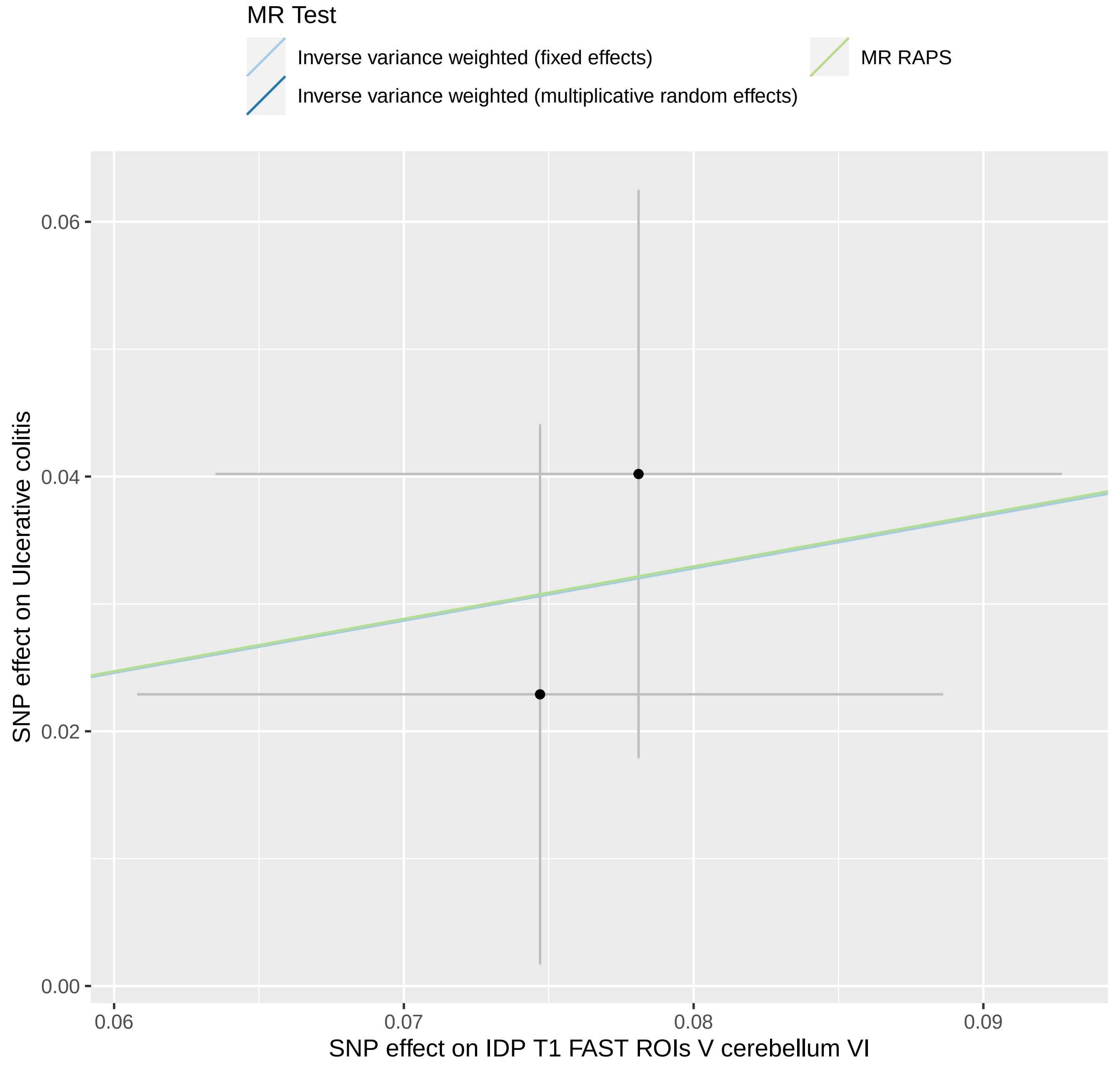

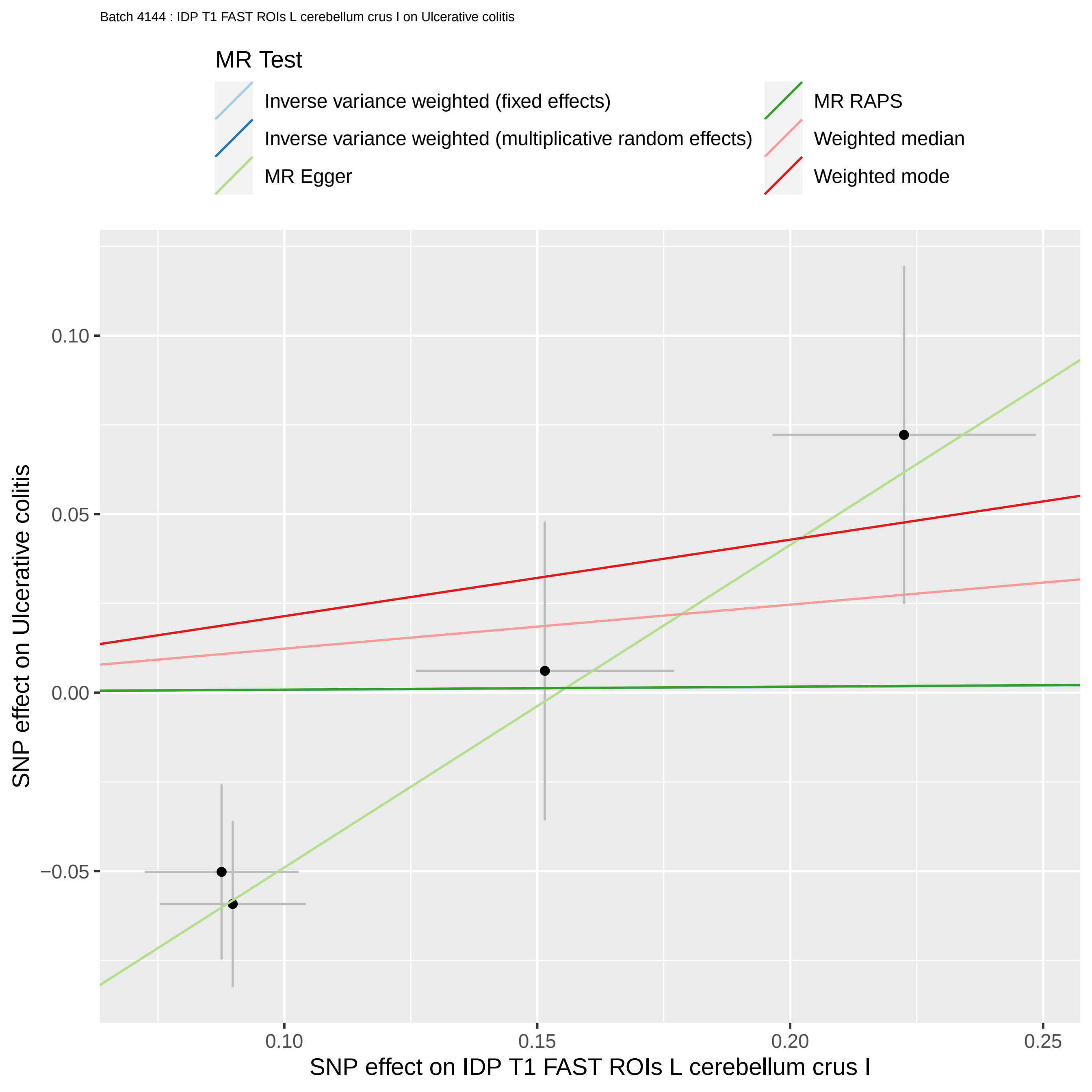
Batch 4144 : IDP T1 FAST ROIs L cerebellum crus I on Ulcerative colitis
MR Test
Inverse variance weighted (fixed effects)
Inverse variance weighted (multiplicative random effects)
MR Egger
MR RAPS
Weighted median
Weighted mode
0.10
0.05
0.00
−0.05
0.10
0.15
0.20
0.25
SNP effect on Ulcerative colitis
SNP effect on IDP T1 FAST ROIs L cerebellum crus I

Batch 4146 : IDP T1 FAST ROIs R cerebellum crus I on Ulcerative colitis

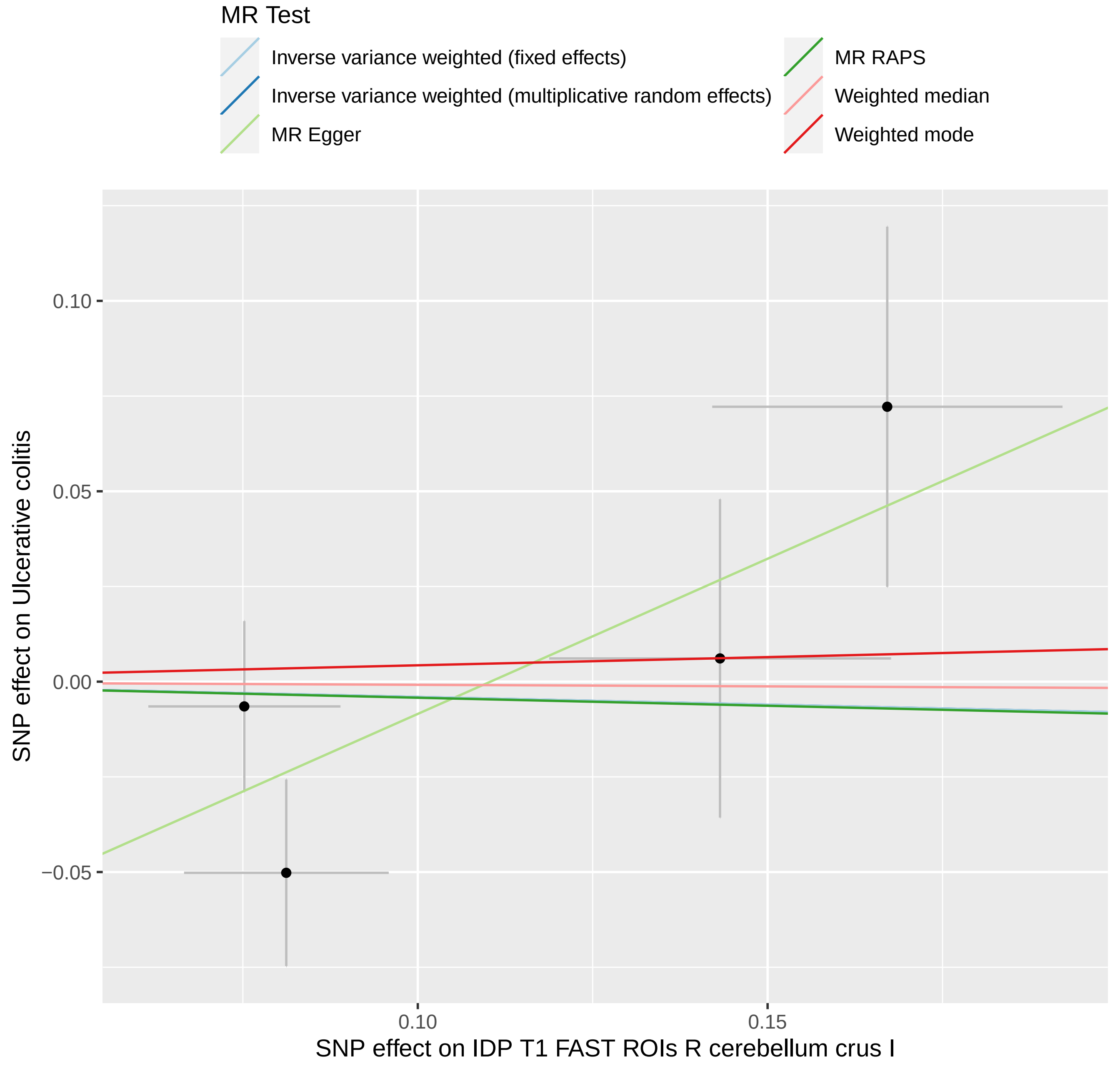

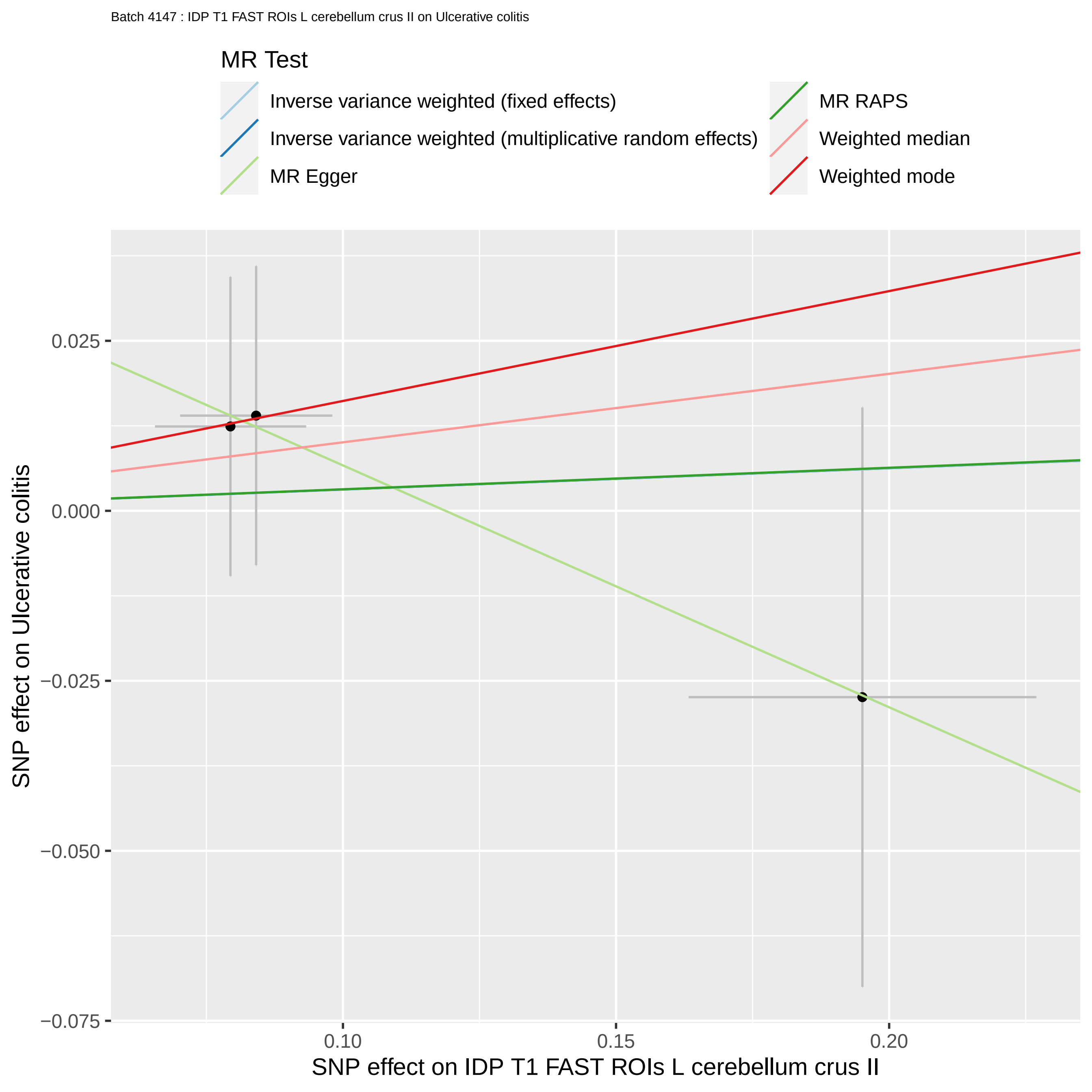
Batch 4147 : IDP T1 FAST ROIs L cerebellum crus II on Ulcerative colitis
MR Test
Inverse variance weighted (fixed effects)
Inverse variance weighted (multiplicative random effects)
MR Egger
MR RAPS
Weighted median
Weighted mode
0.025
0.000
−0.025
−0.050
−0.075
0.10
0.15
0.20
SNP effect on Ulcerative colitis
SNP effect on IDP T1 FAST ROIs L cerebellum crus II

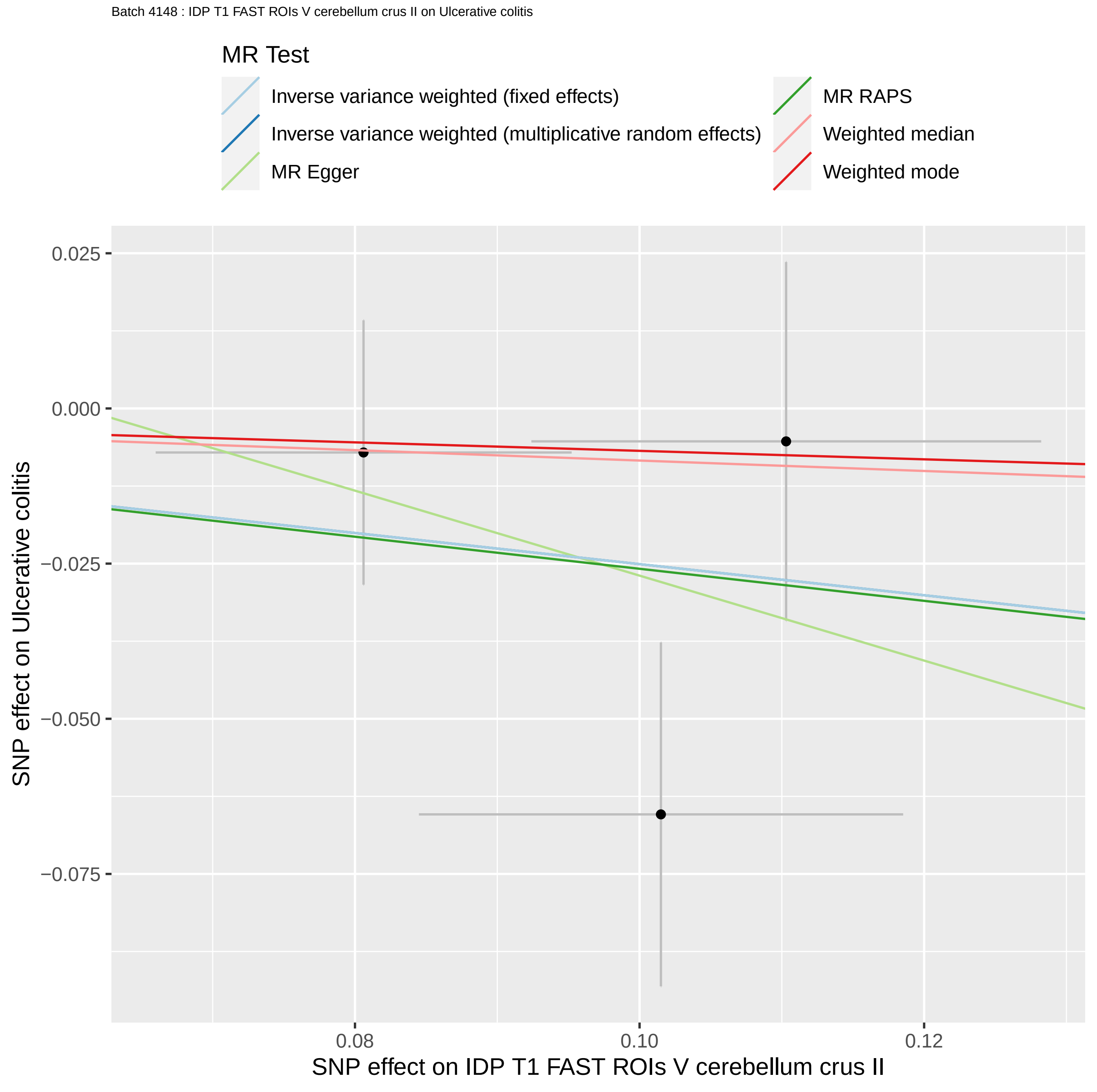

Batch 4148 : IDP T1 FAST ROIs V cerebellum crus II on Ulcerative colitis
MR Test
Inverse variance weighted (fixed effects)
Inverse variance weighted (multiplicative random effects)
MR Egger
MR RAPS
Weighted median
Weighted mode
0.025
0.000
−0.025
−0.050
−0.075
0.08
0.10
0.12
SNP effect on Ulcerative colitis
SNP effect on IDP T1 FAST ROIs V cerebellum crus II

Batch 4149 : IDP T1 FAST ROIs R cerebellum crus II on Ulcerative colitis

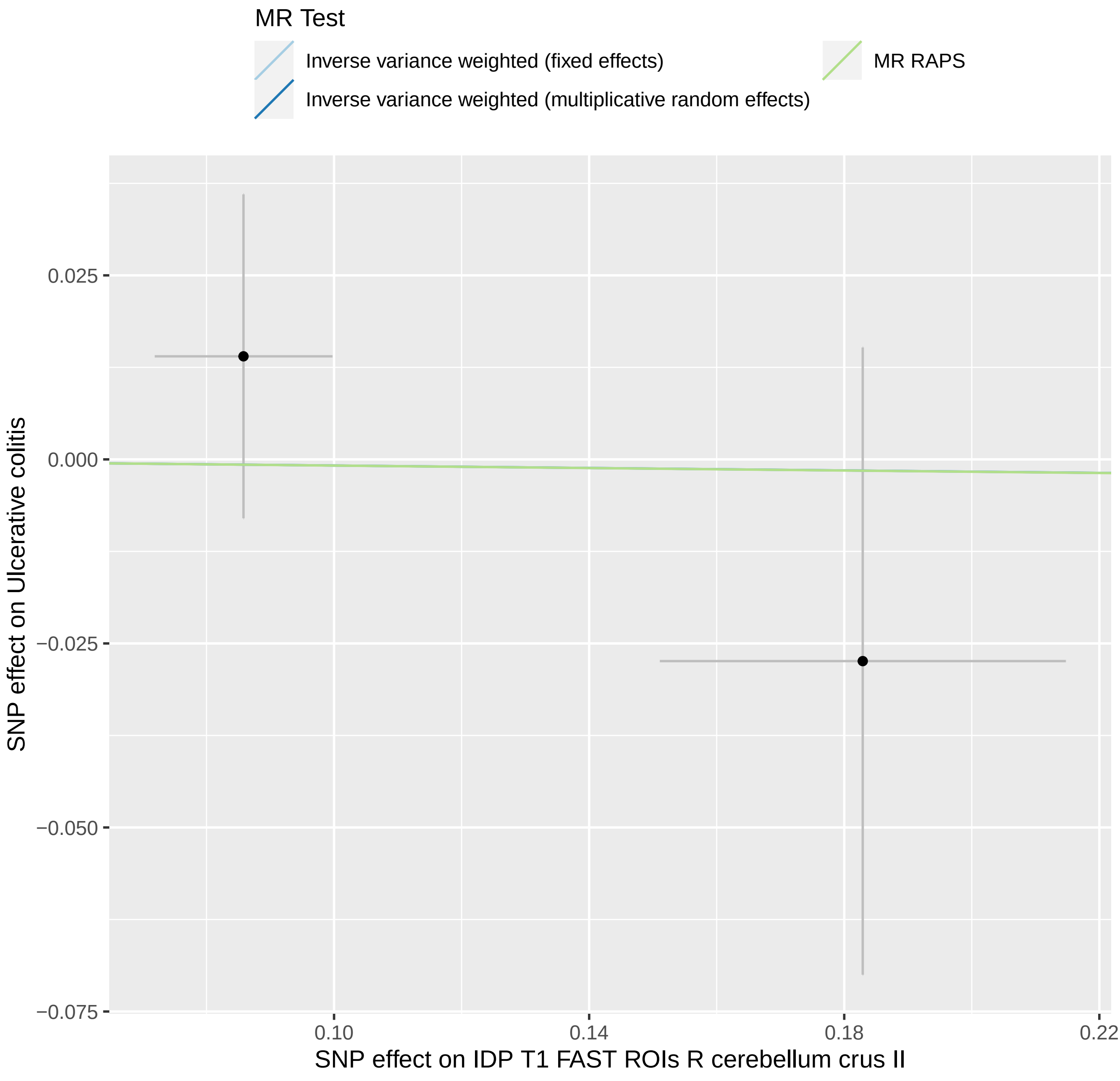

Batch 4152 : IDP T1 FAST ROIs V cerebellum VIIb on Ulcerative colitis

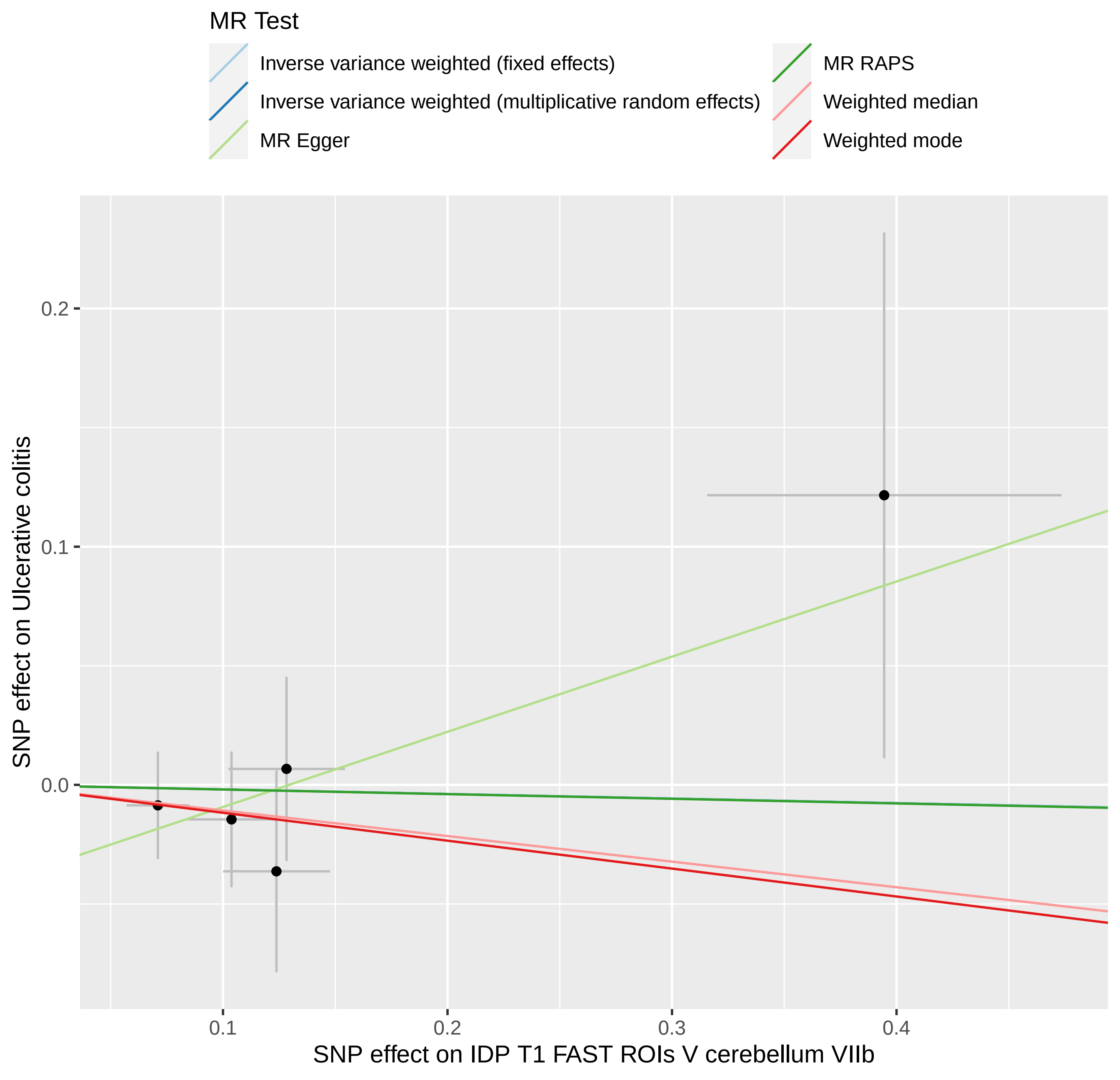

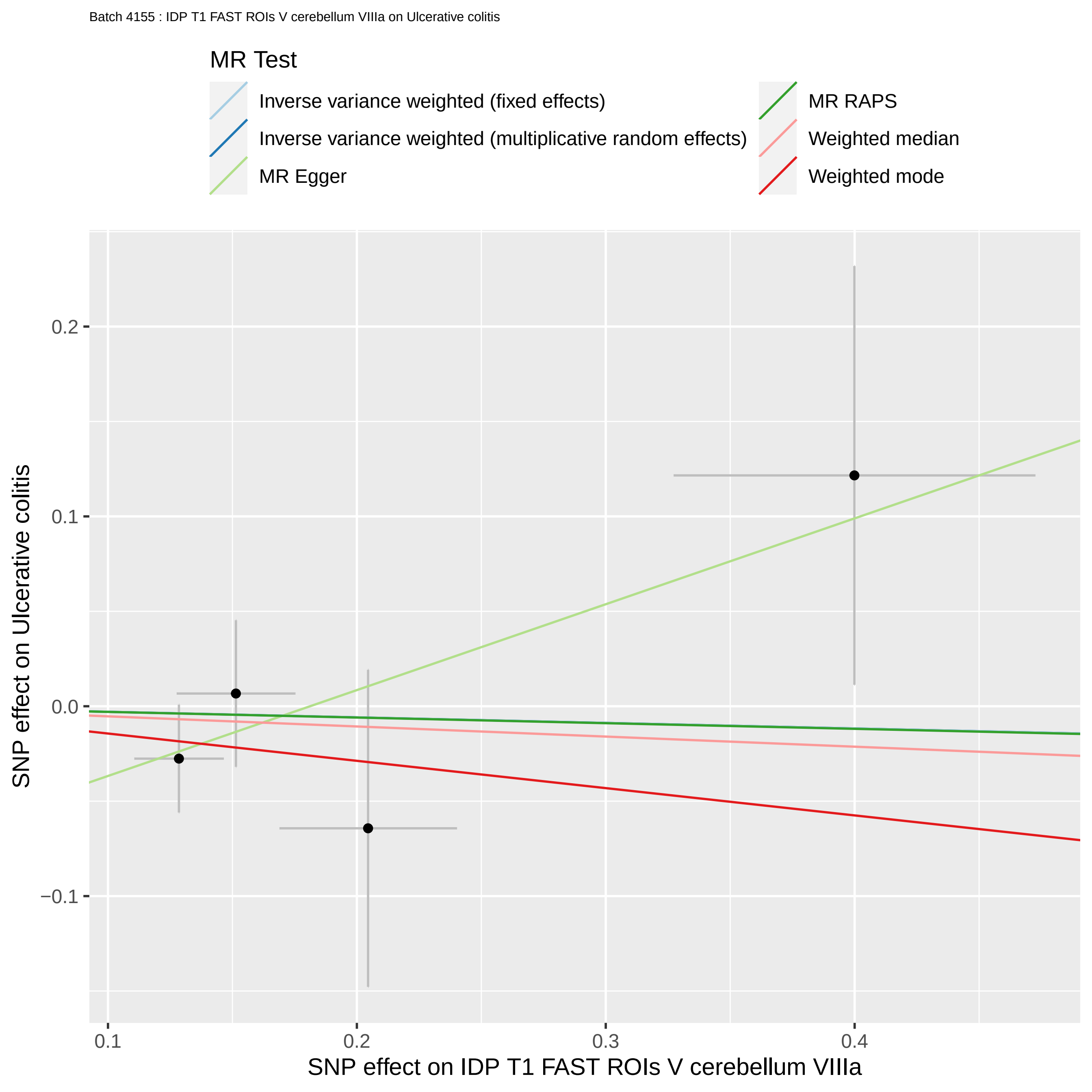
Batch 4155 : IDP T1 FAST ROIs V cerebellum VIIIa on Ulcerative colitis
MR Test
Inverse variance weighted (fixed effects)
Inverse variance weighted (multiplicative random effects)
MR Egger
MR RAPS
Weighted median
Weighted mode
0.2
0.1
0.0
−0.1
0.1
0.2
0.3
0.4
SNP effect on Ulcerative colitis
SNP effect on IDP T1 FAST ROIs V cerebellum VIIIa

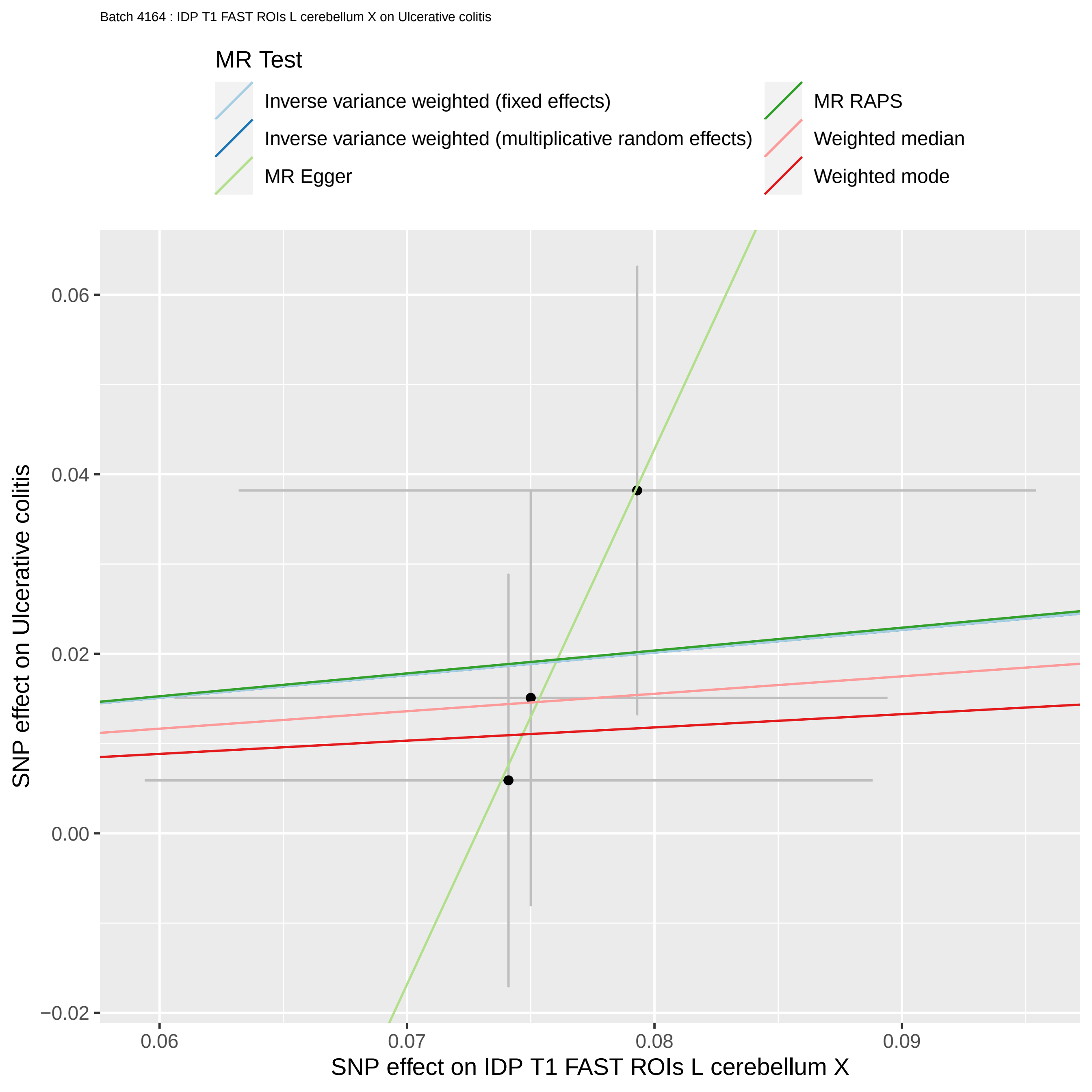
Batch 4164 : IDP T1 FAST ROIs L cerebellum X on Ulcerative colitis
MR Test
Inverse variance weighted (fixed effects)
Inverse variance weighted (multiplicative random effects)
MR Egger
MR RAPS
Weighted median
Weighted mode
SNP effect on Ulcerative colitis
0.06
0.04
0.02
0.00
−0.02
0.06
0.07
0.08
0.09
SNP effect on IDP T1 FAST ROIs L cerebellum X

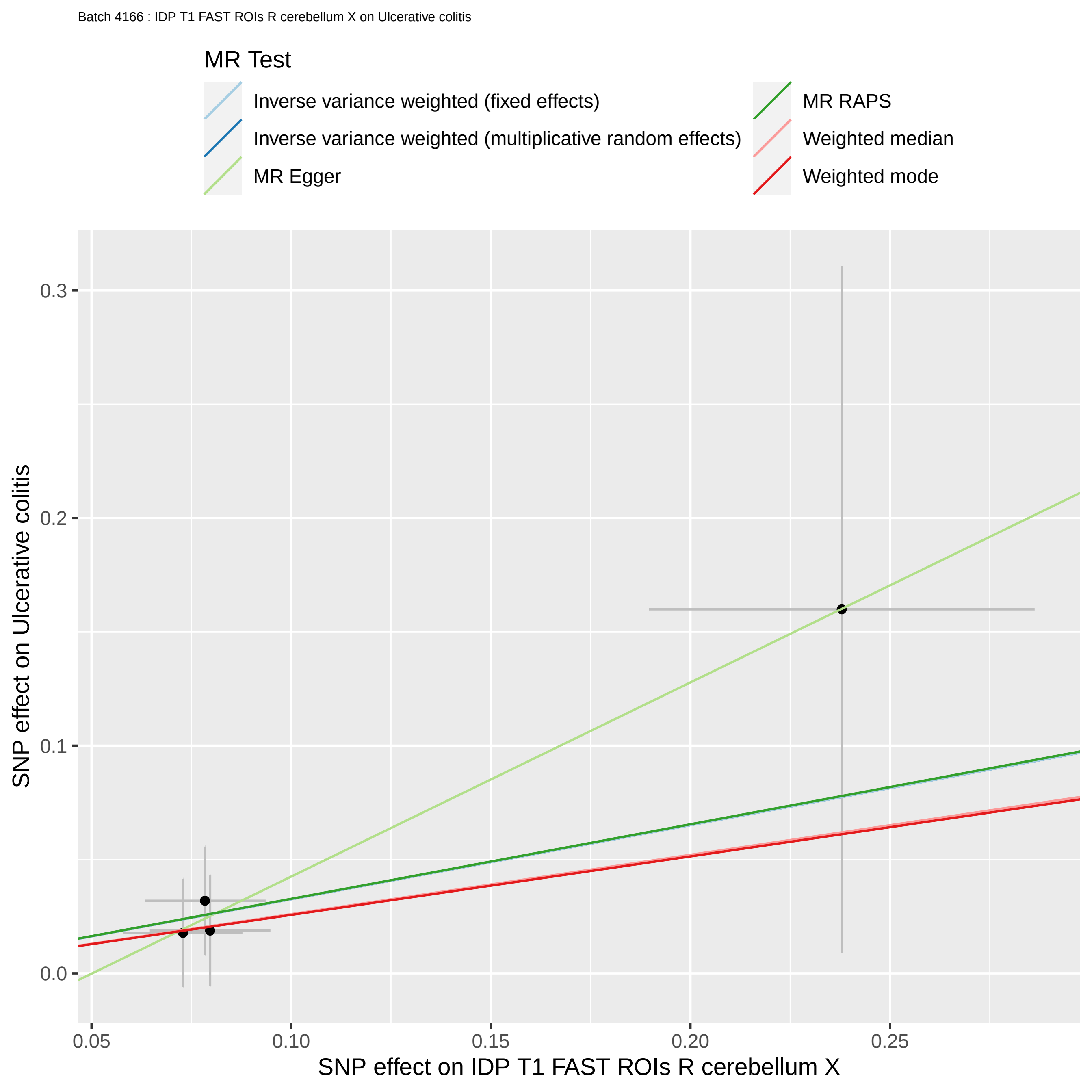
Batch 4166 : IDP T1 FAST ROIs R cerebellum X on Ulcerative colitis
MR Test
Inverse variance weighted (fixed effects)
Inverse variance weighted (multiplicative random effects)
MR Egger
MR RAPS
Weighted median
Weighted mode
0.3
0.2
0.1
0.0
0.05
0.10
0.15
0.20
0.25
SNP effect on Ulcerative colitis
SNP effect on IDP T1 FAST ROIs R cerebellum X

Batch 4167 : IDP T2 FLAIR BIANCA WMH volume on Ulcerative colitis

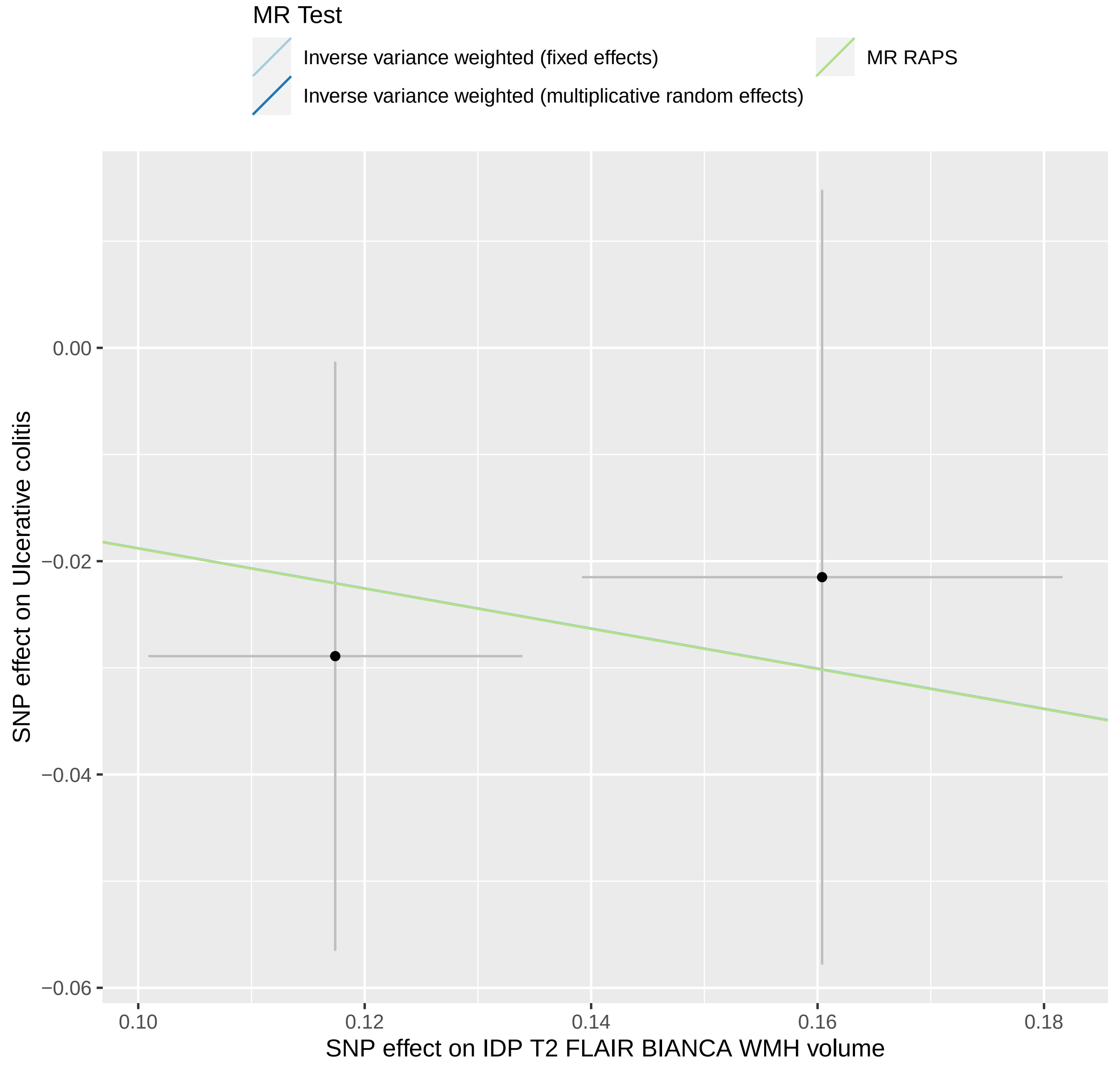

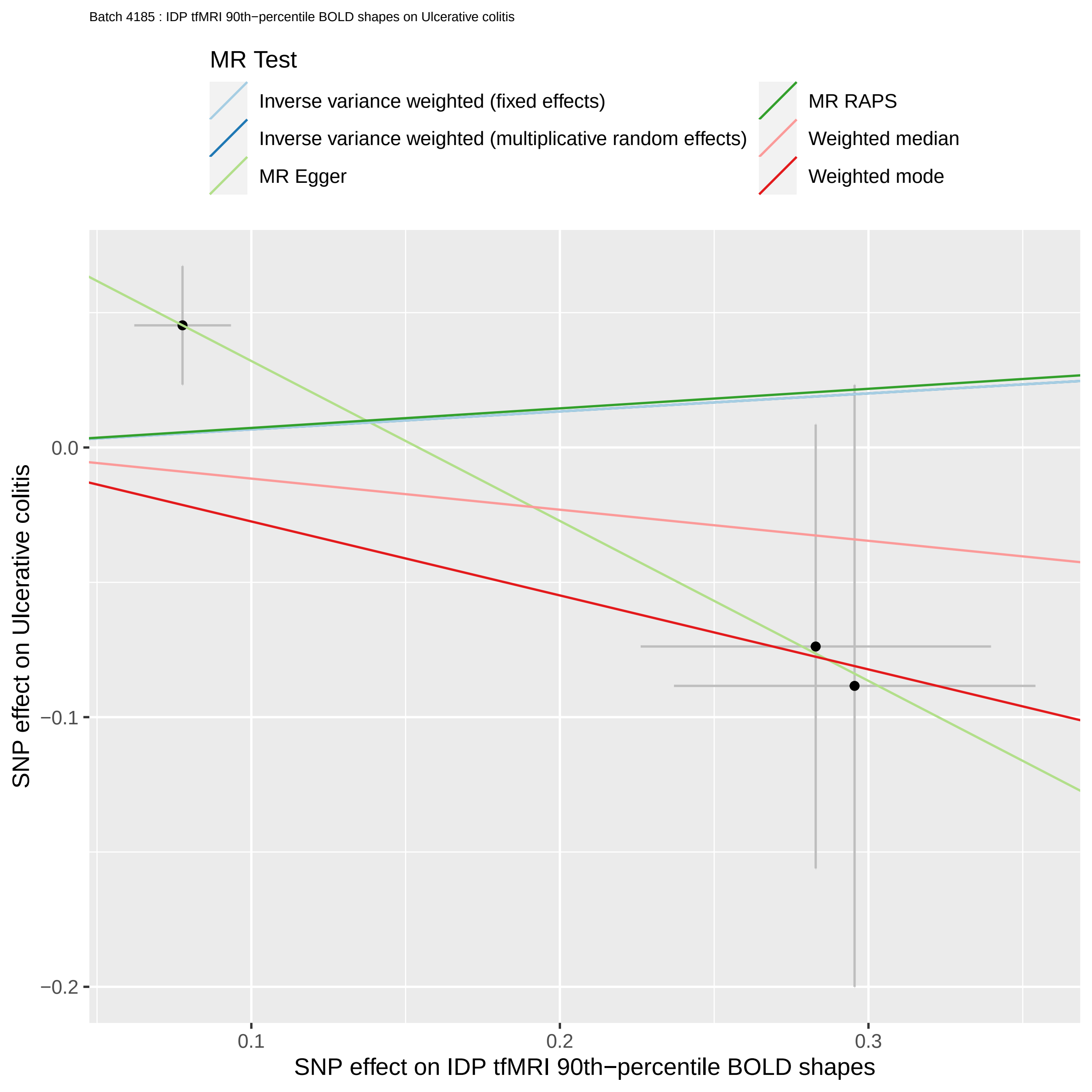
Batch 4185 : IDP tfMRI 90th−percentile BOLD shapes on Ulcerative colitis
MR Test
Inverse variance weighted (fixed effects)
Inverse variance weighted (multiplicative random effects)
MR Egger
MR RAPS
Weighted median
Weighted mode
0.0
−0.1
−0.2
0.1
0.2
0.3
SNP effect on IDP tfMRI 90th−percentile BOLD shapes
SNP effect on Ulcerative colitis

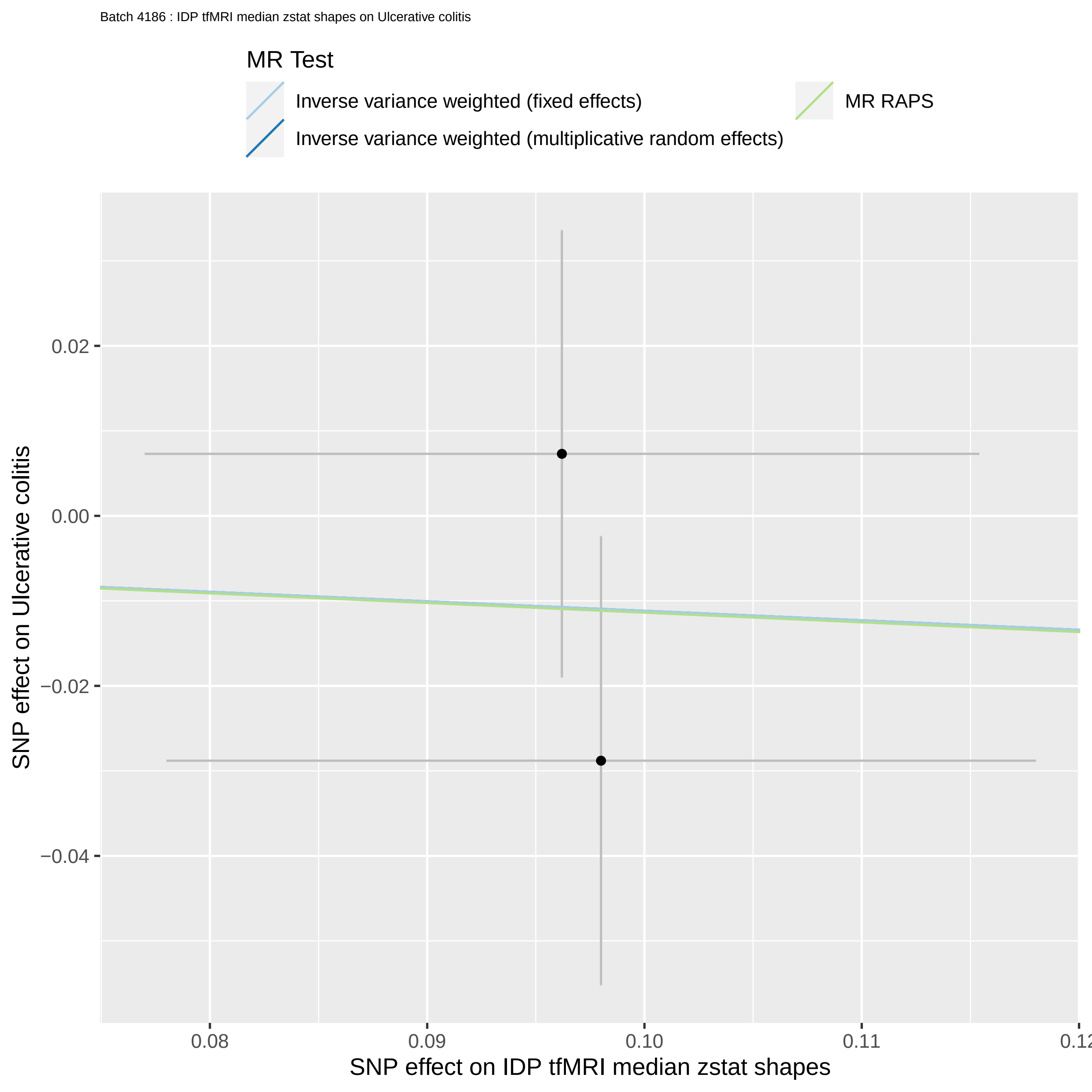
Batch 4186 : IDP tfMRI median zstat shapes on Ulcerative colitis
MR Test
Inverse variance weighted (fixed effects)
Inverse variance weighted (multiplicative random effects)
MR RAPS
0.02
0.00
−0.02
−0.04
0.08
0.09
0.10
0.11
0.12
SNP effect on Ulcerative colitis
SNP effect on IDP tfMRI median zstat shapes

Batch 4187 : IDP tfMRI 90th−percentile zstat shapes on Ulcerative colitis

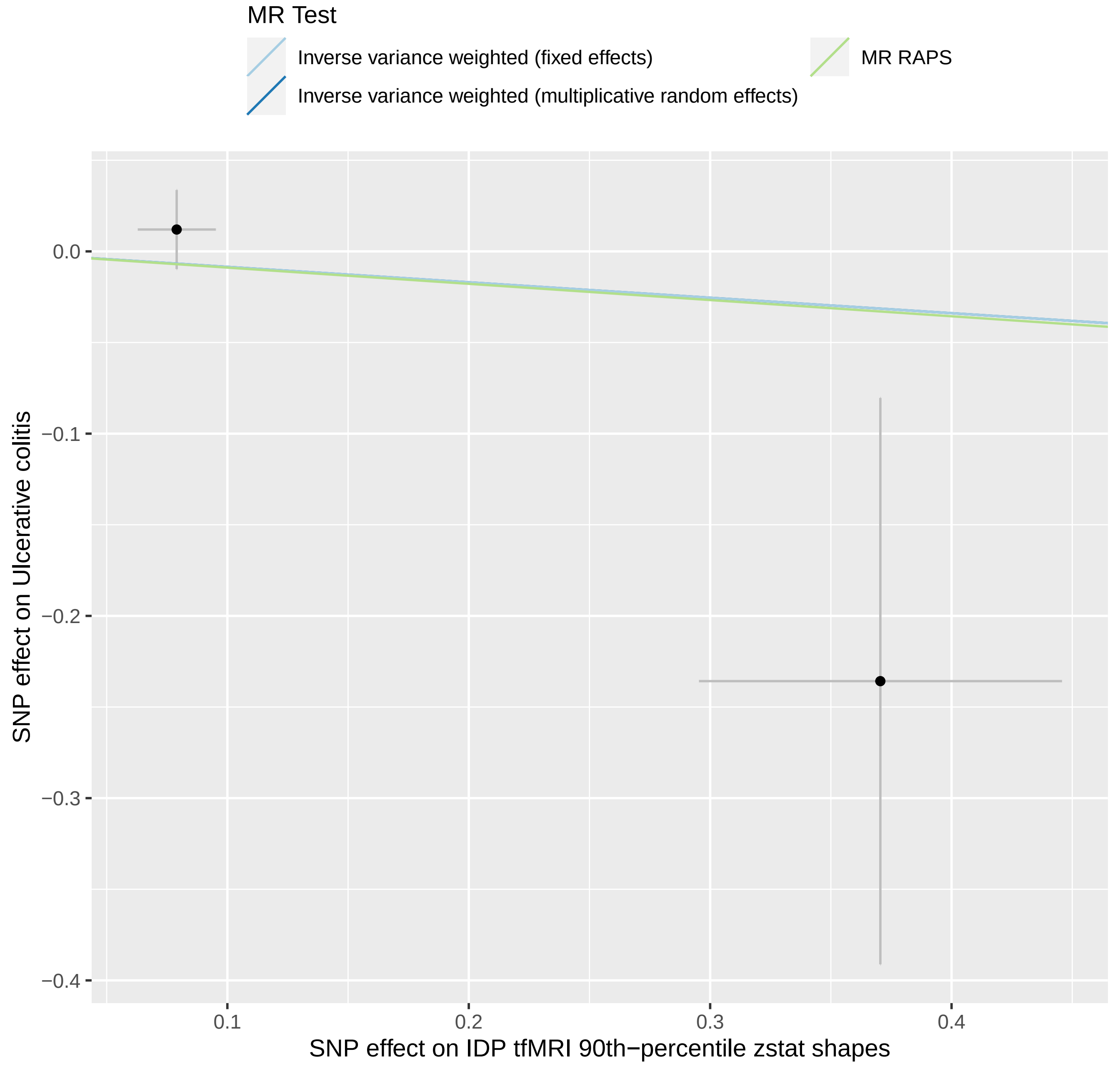

Batch 4188 : IDP tfMRI median BOLD faces on Ulcerative colitis

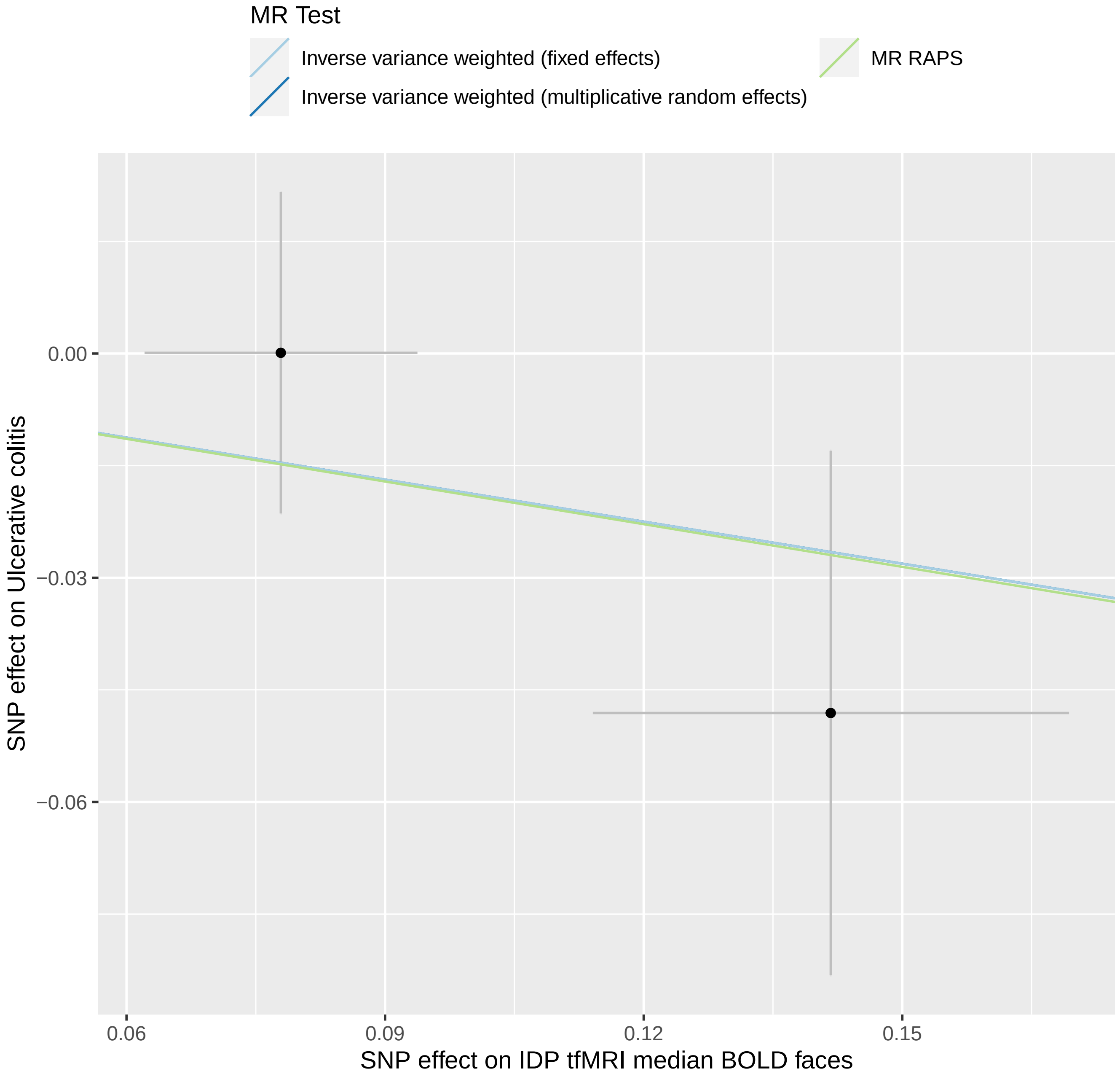

Batch 4189 : IDP tfMRI 90th−percentile BOLD faces on Ulcerative colitis

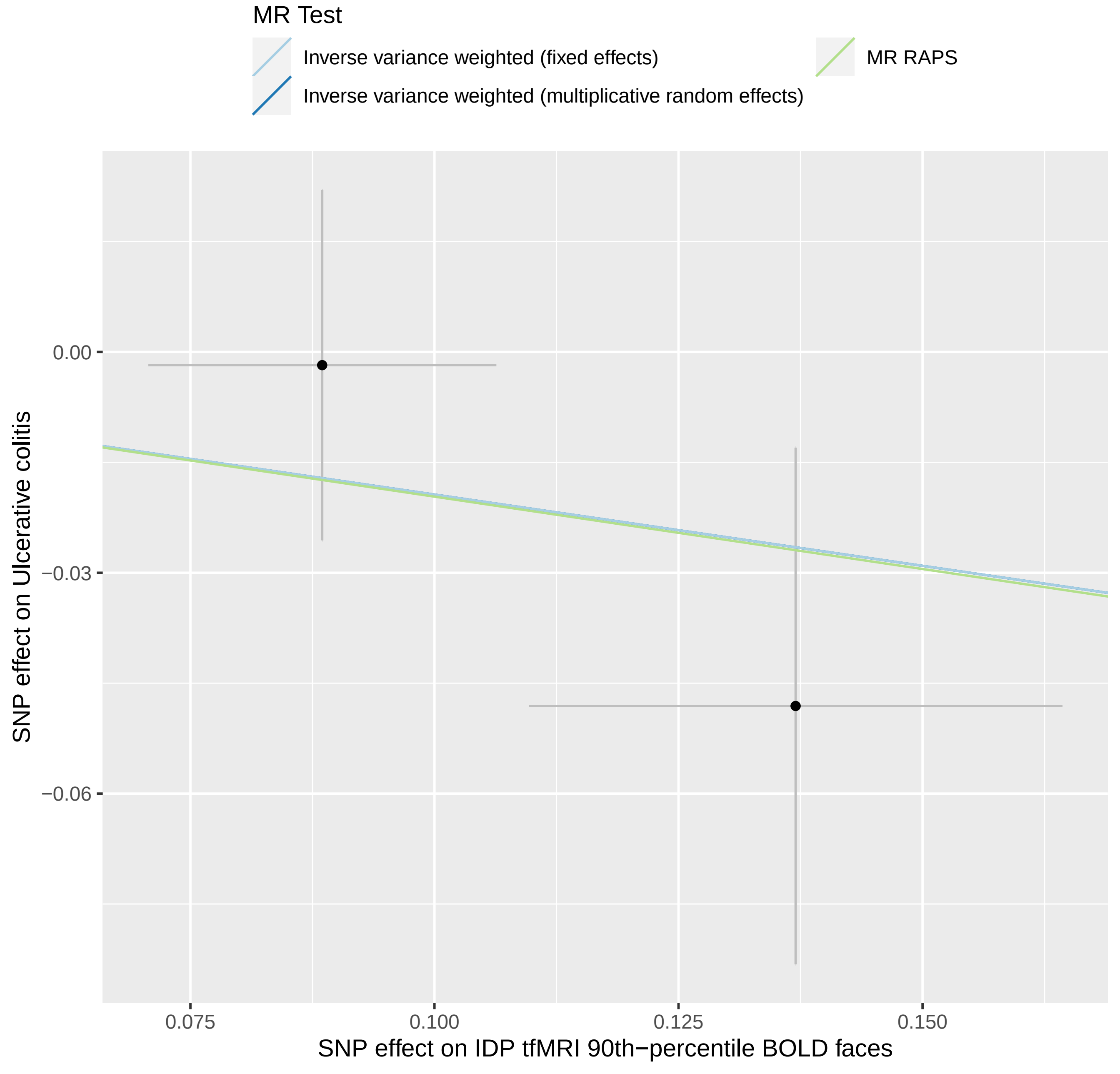

Batch 4190 : IDP tfMRI median zstat faces on Ulcerative colitis

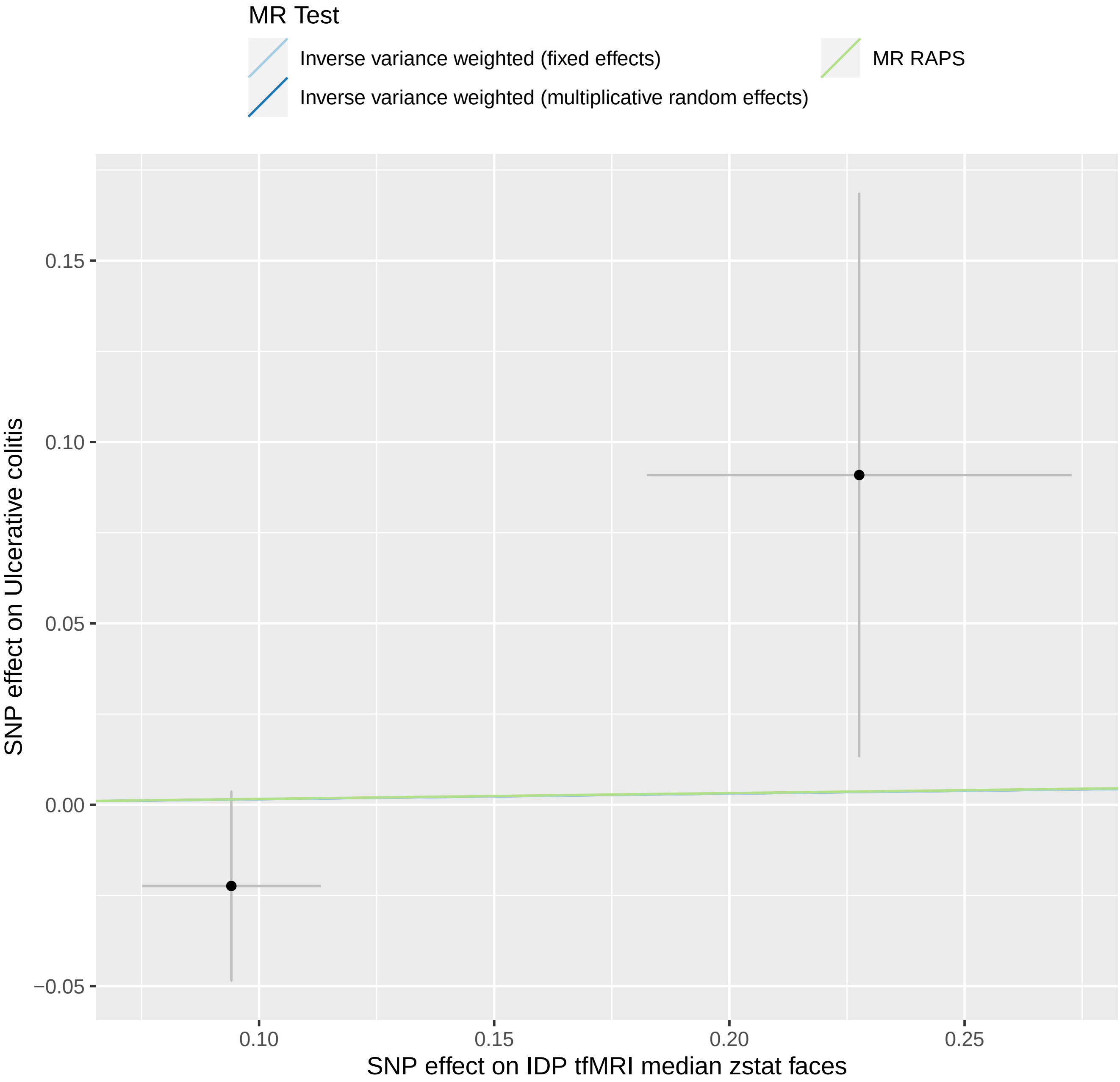

Batch 4193 : IDP tfMRI 90th−percentile BOLD faces−shapes on Ulcerative colitis

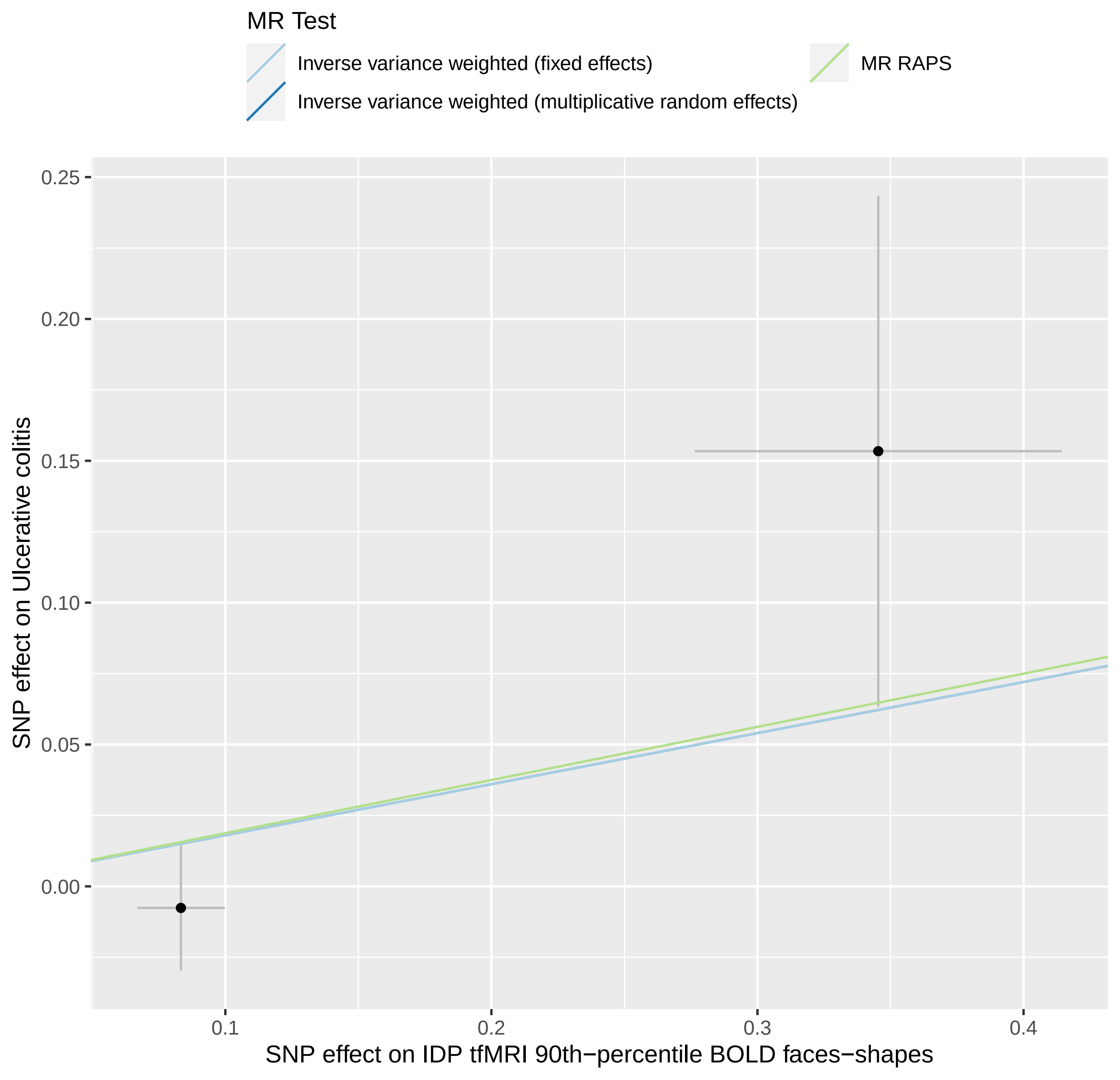

Batch 4194 : IDP T1 FIRST left hippocampus volume on Ulcerative colitis

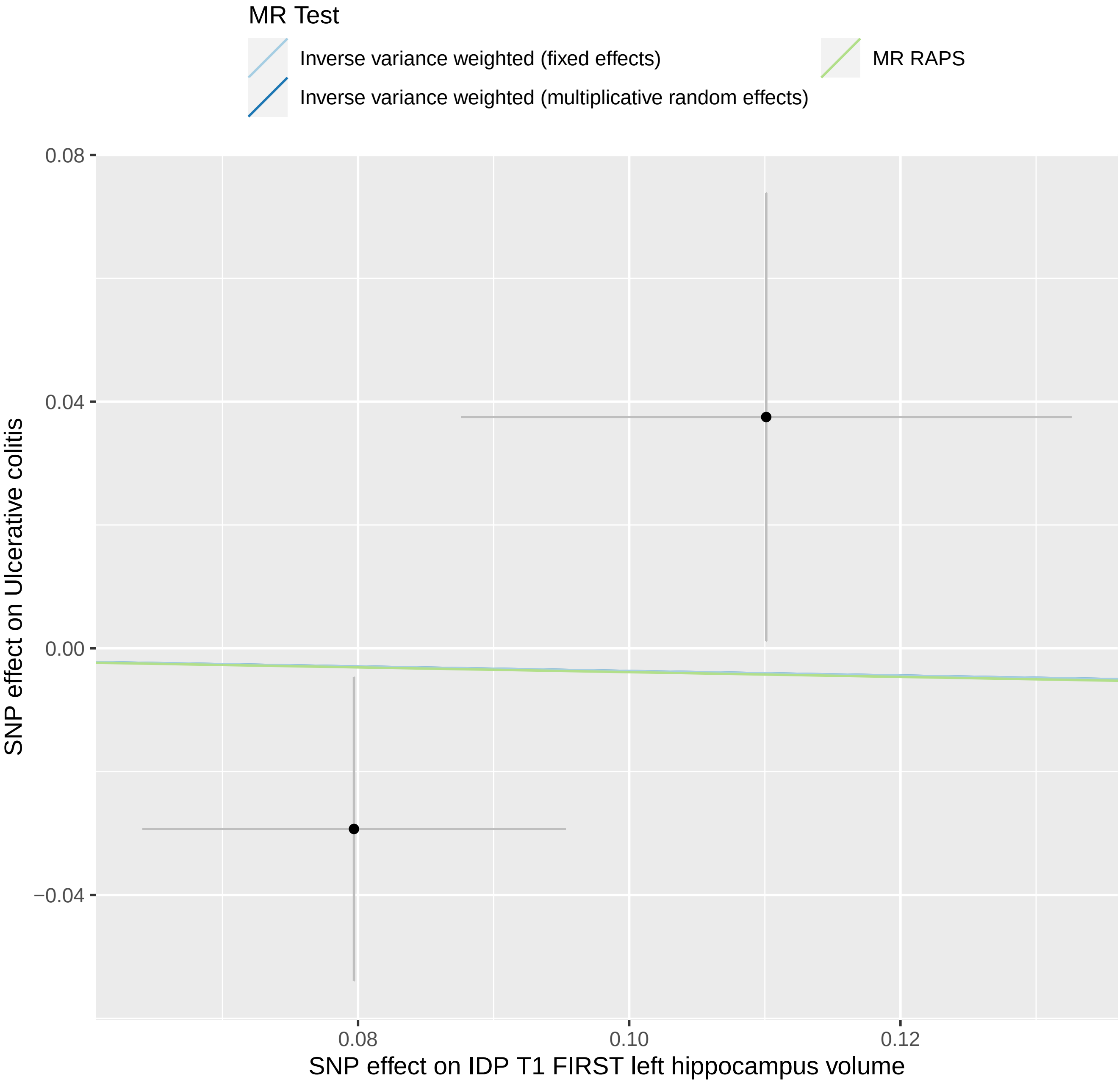

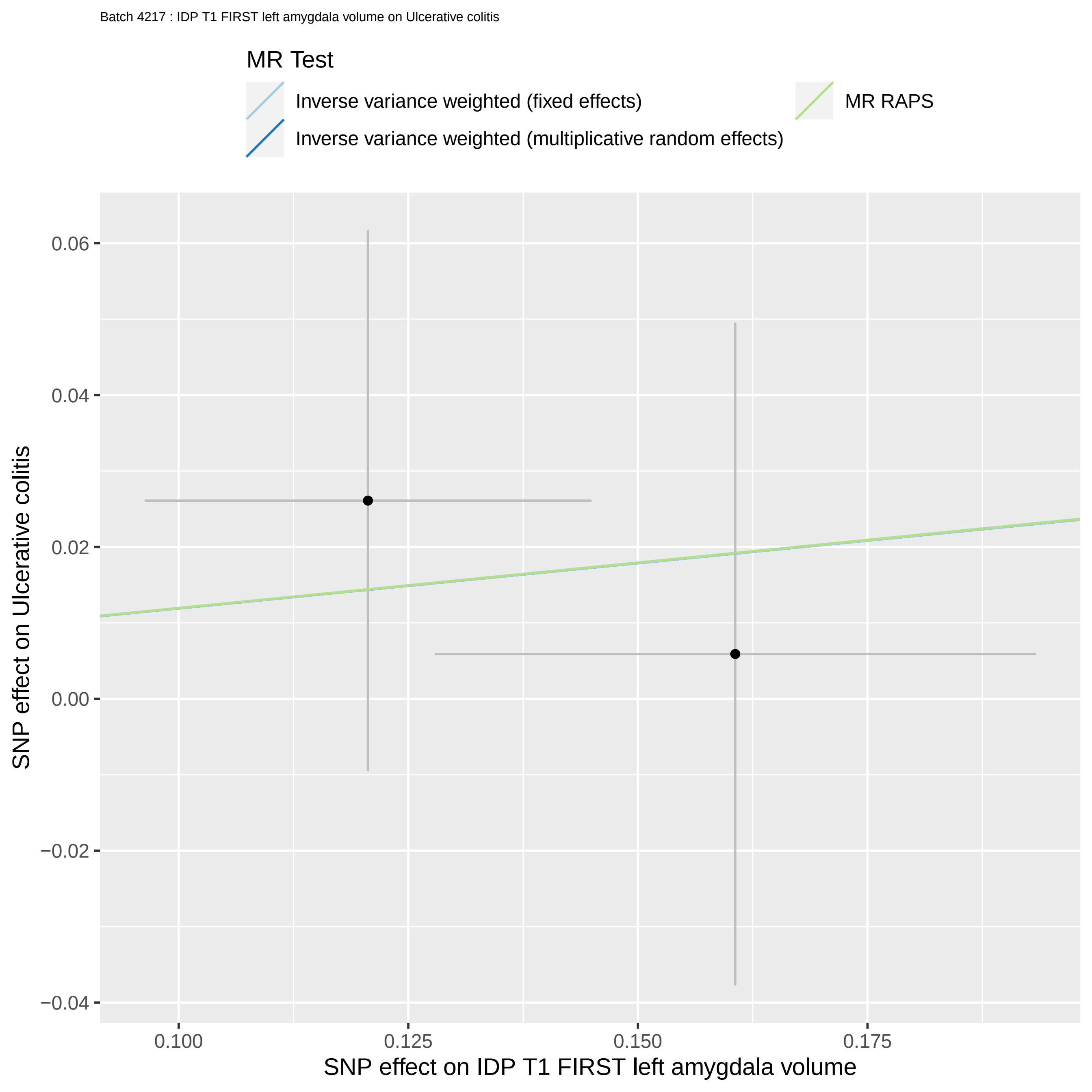

Batch 4217 : IDP T1 FIRST left amygdala volume on Ulcerative colitis
MR Test
Inverse variance weighted (fixed effects)
Inverse variance weighted (multiplicative random effects)
MR RAPS
0.06
0.04
0.02
0.00
−0.02
−0.04
0.100
0.125
0.150
0.175
SNP effect on Ulcerative colitis
SNP effect on IDP T1 FIRST left amygdala volume

Batch 4250 : IDP T1 FIRST right accumbens volume on Ulcerative colitis

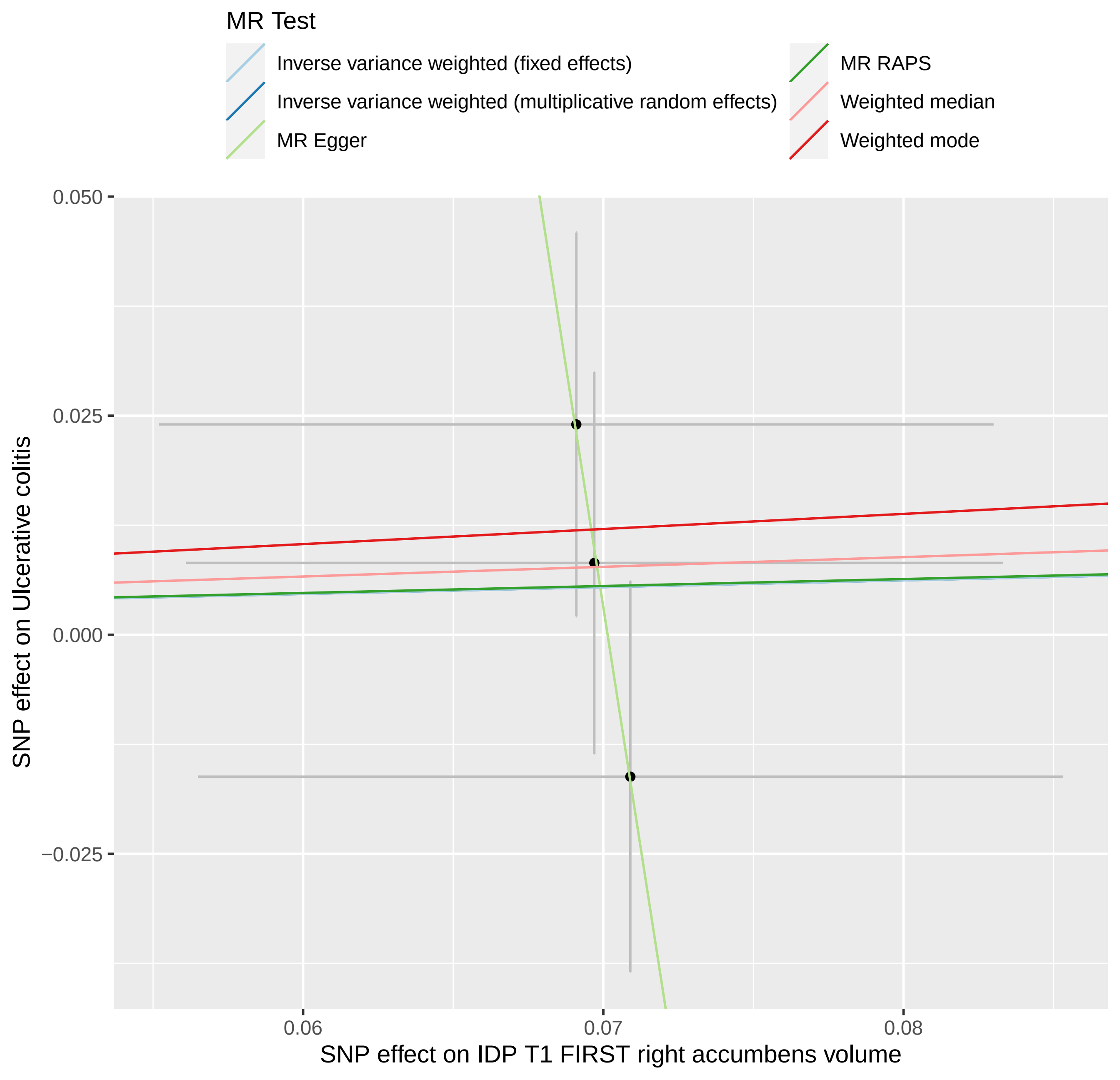

Batch 4261 : IDP T1 FIRST brain stem+4th ventricle volume on Ulcerative colitis

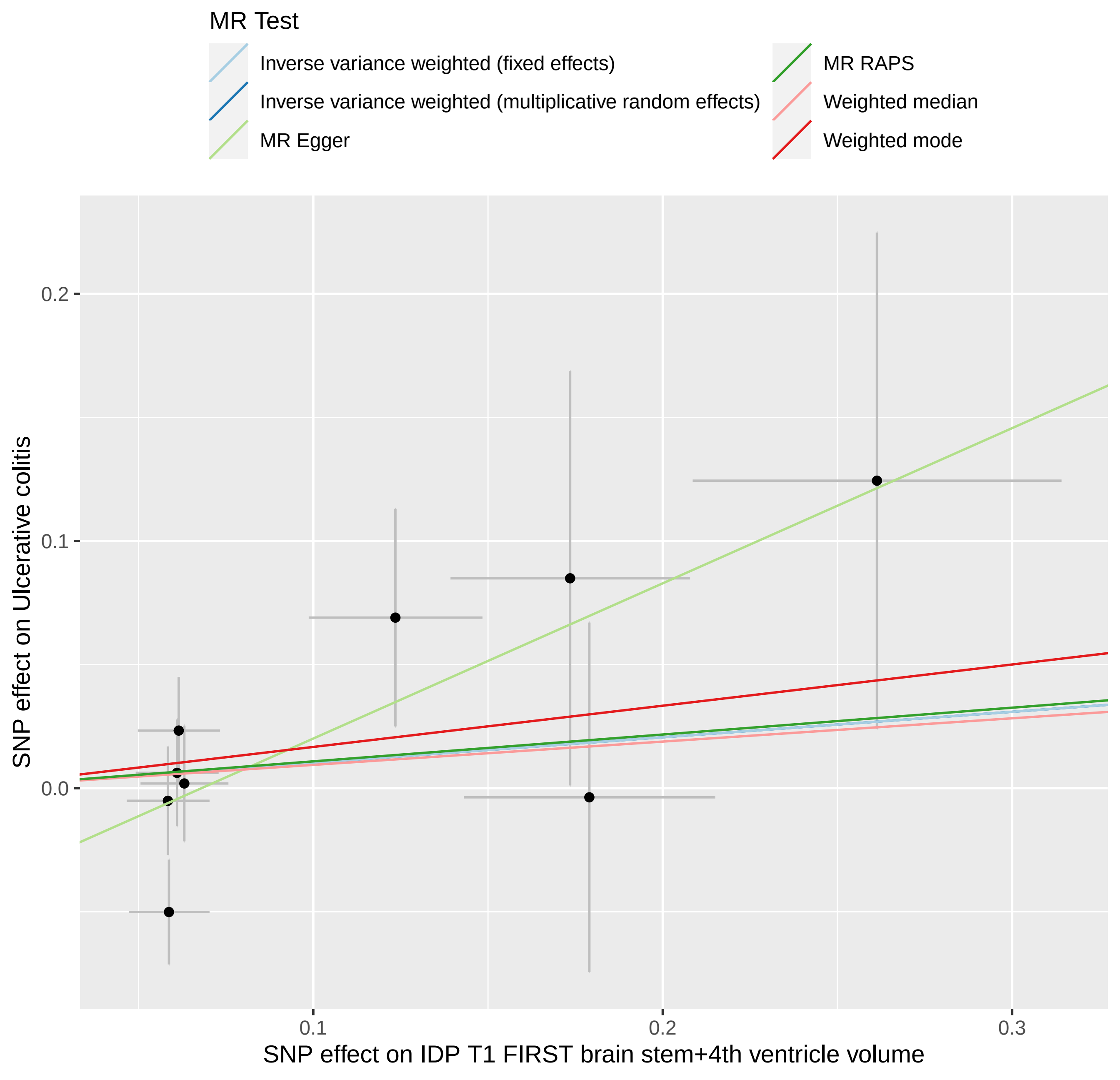

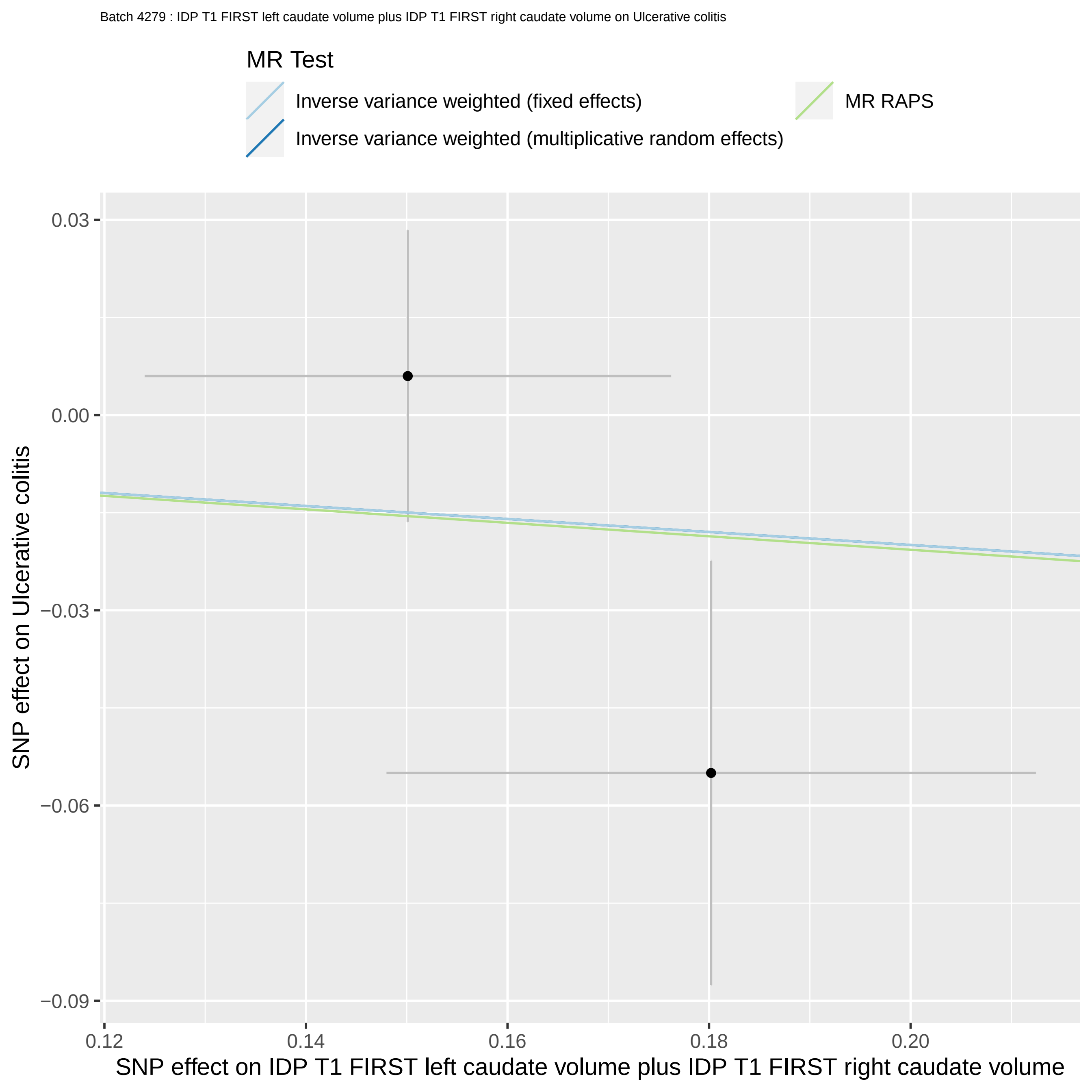

Batch 4279 : IDP T1 FIRST left caudate volume plus IDP T1 FIRST right caudate volume on Ulcerative colitis
MR Test
Inverse variance weighted (fixed effects)
Inverse variance weighted (multiplicative random effects)
MR RAPS
0.03
0.00
−0.03
−0.06
−0.09
0.12
0.14
0.16
0.18
0.20
SNP effect on Ulcerative colitis
SNP effect on IDP T1 FIRST left caudate volume plus IDP T1 FIRST right caudate volume

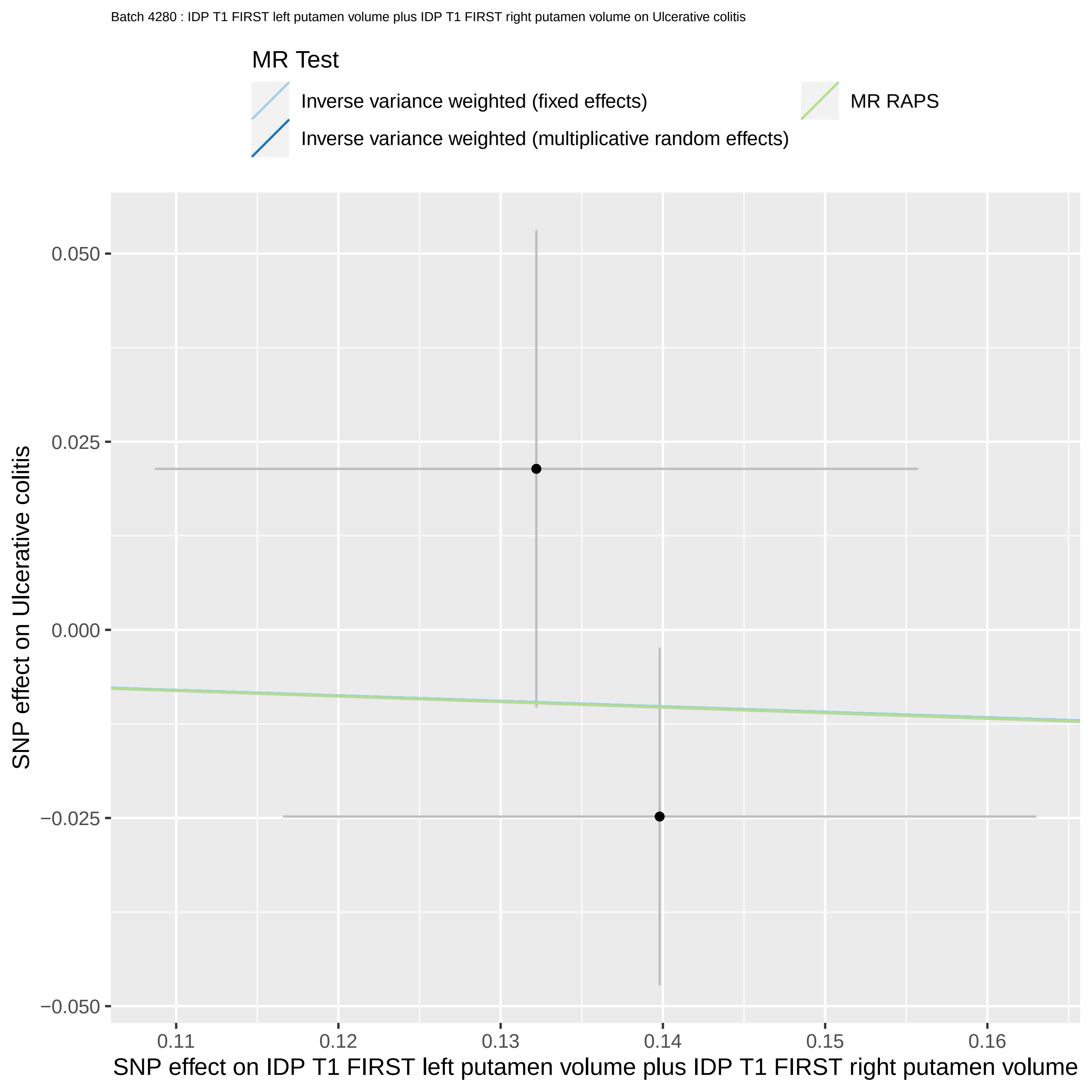

Batch 4280 : IDP T1 FIRST left putamen volume plus IDP T1 FIRST right putamen volume on Ulcerative colitis
MR Test
Inverse variance weighted (fixed effects)
Inverse variance weighted (multiplicative random effects)
MR RAPS
0.050
0.025
0.000
−0.025
−0.050
SNP effect on Ulcerative colitis
0.11
0.12
0.13
0.14
0.15
0.16
SNP effect on IDP T1 FIRST left putamen volume plus IDP T1 FIRST right putamen volume

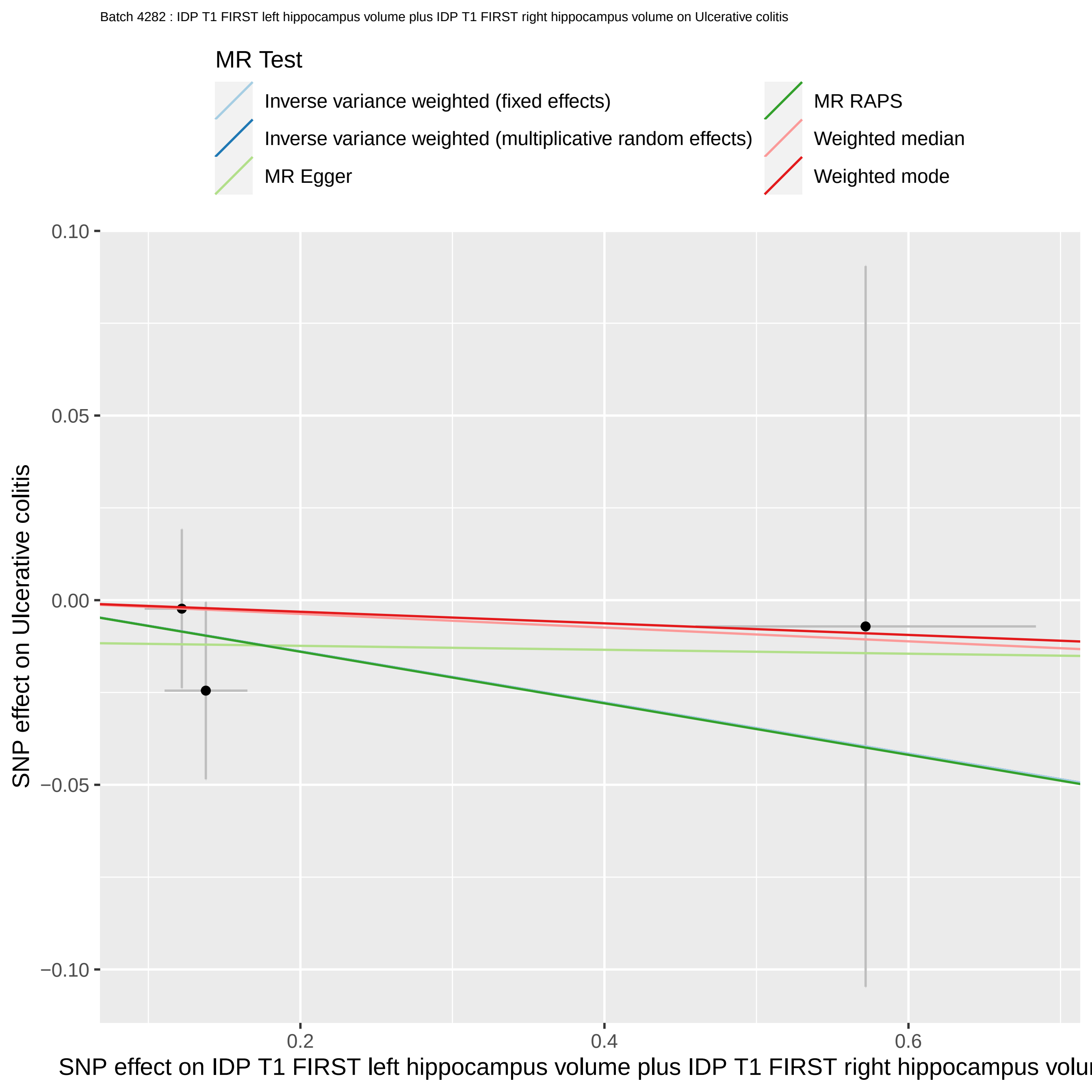

Batch 4282 : IDP T1 FIRST left hippocampus volume plus IDP T1 FIRST right hippocampus volume on Ulcerative colitis
MR Test
Inverse variance weighted (fixed effects)
Inverse variance weighted (multiplicative random effects)
MR Egger
MR RAPS
Weighted median
Weighted mode
0.10
0.05
0.00
−0.05
−0.10
0.2
0.4
0.6
SNP effect on Ulcerative colitis
SNP effect on IDP T1 FIRST left hippocampus volume plus IDP T1 FIRST right hippocampus volu

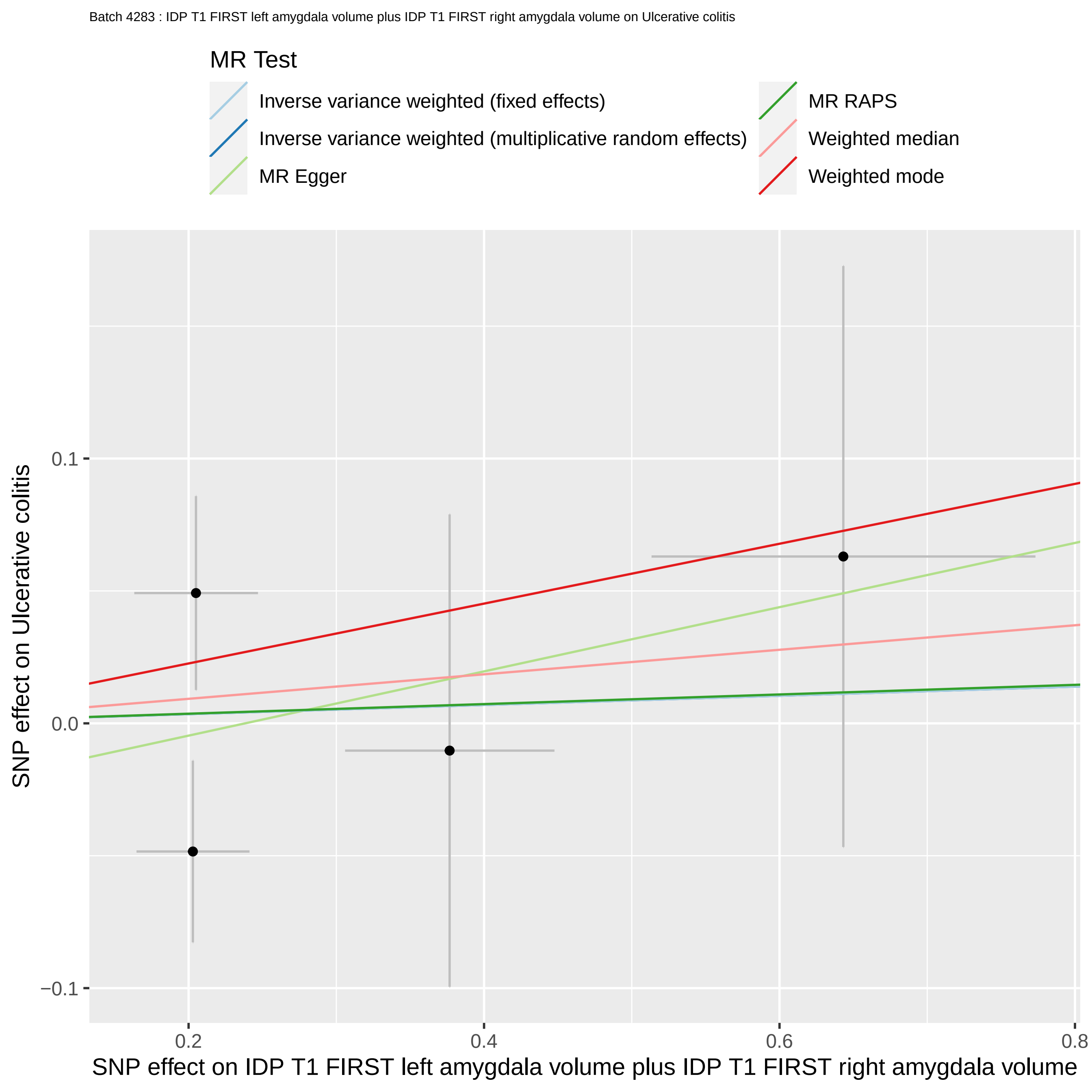

Batch 4283 : IDP T1 FIRST left amygdala volume plus IDP T1 FIRST right amygdala volume on Ulcerative colitis
MR Test
Inverse variance weighted (fixed effects)
Inverse variance weighted (multiplicative random effects)
MR Egger
MR RAPS
Weighted median
Weighted mode
0.1
0.0
−0.1
0.2
0.4
0.6
0.8
SNP effect on Ulcerative colitis
SNP effect on IDP T1 FIRST left amygdala volume plus IDP T1 FIRST right amygdala volume

Batch 4358 : DKTatlas lh caudalanteriorcingulate area on Ulcerative colitis

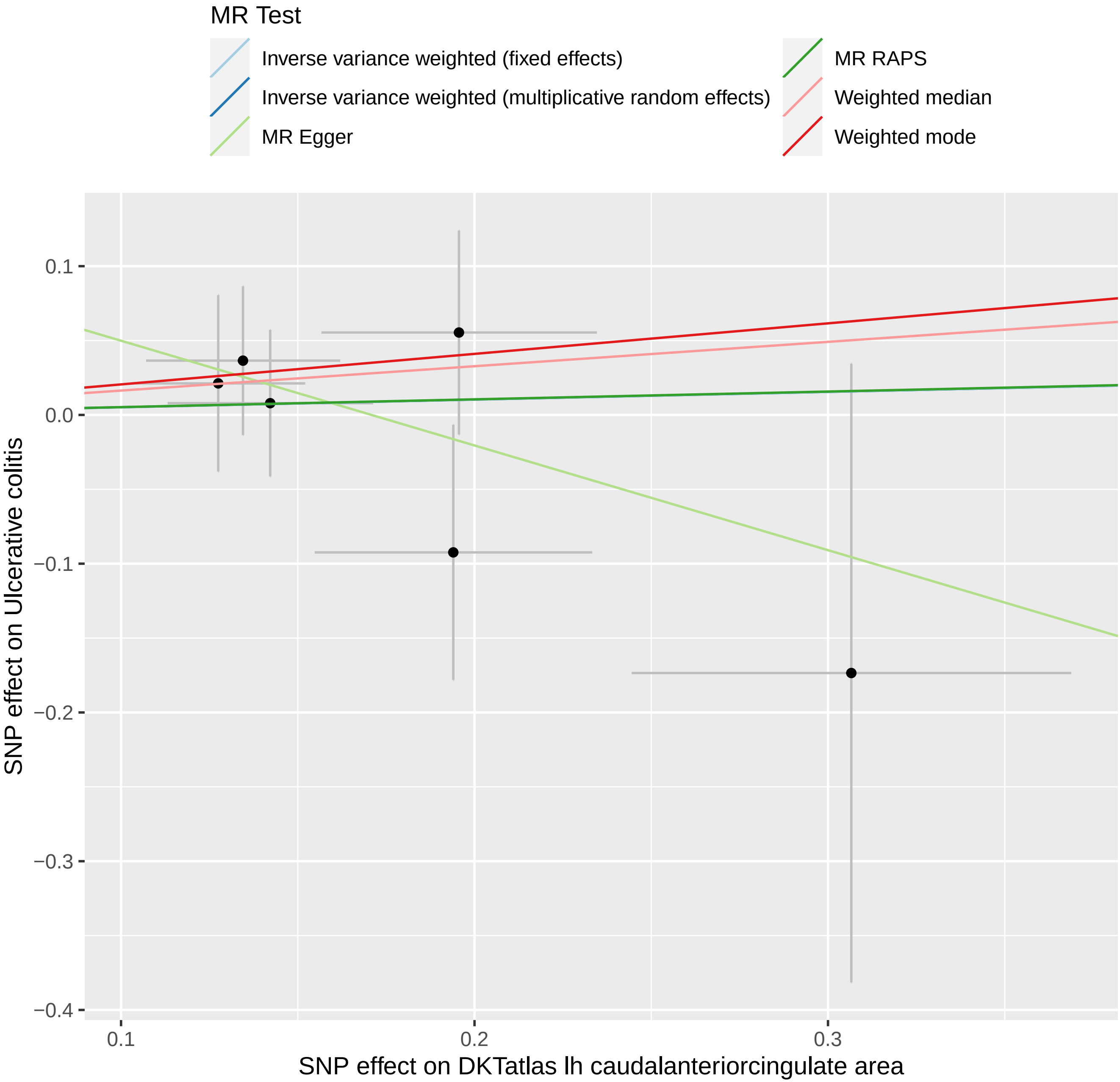

Batch 4367 : DKTatlas lh lateraloccipital area on Ulcerative colitis

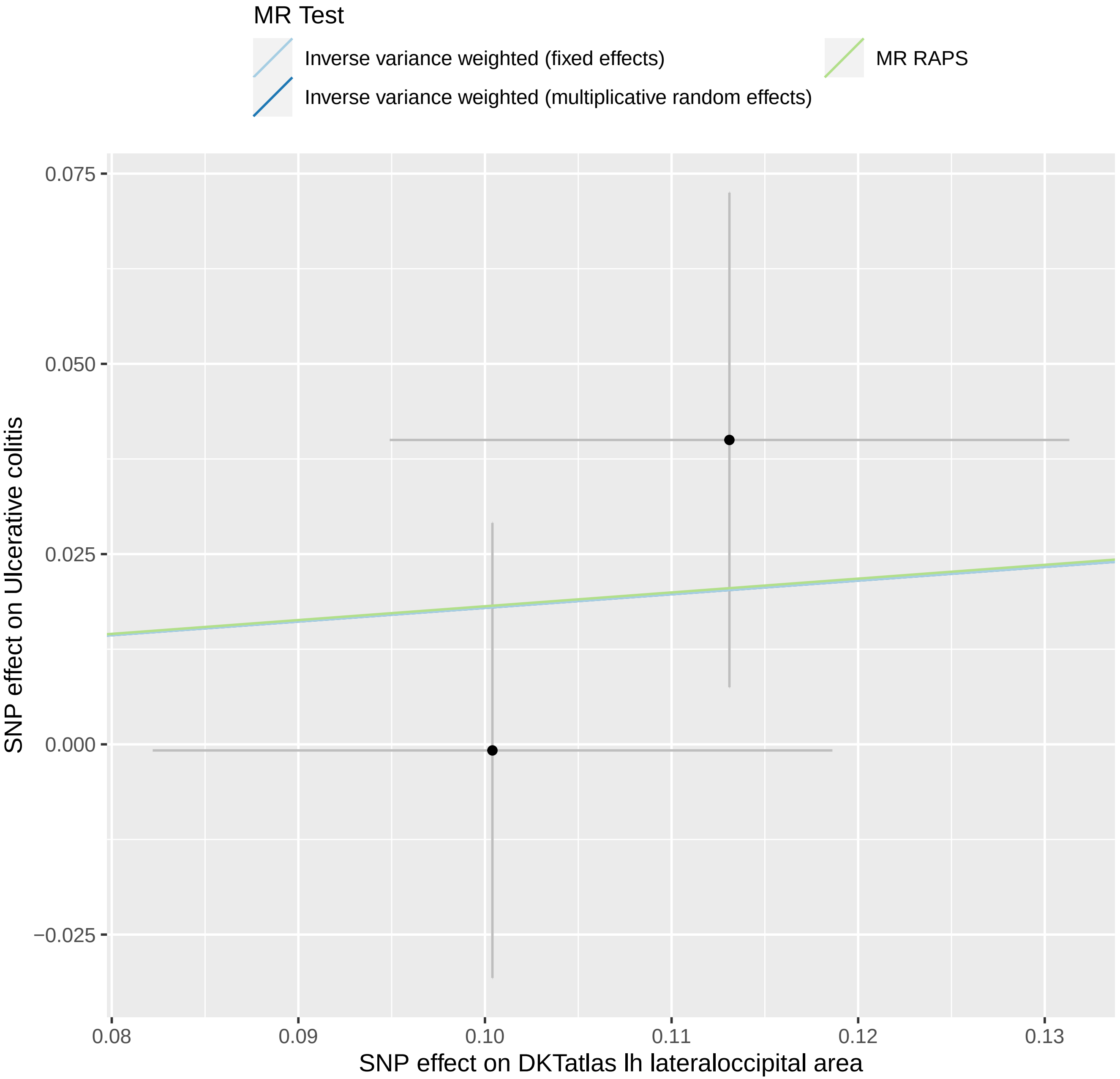

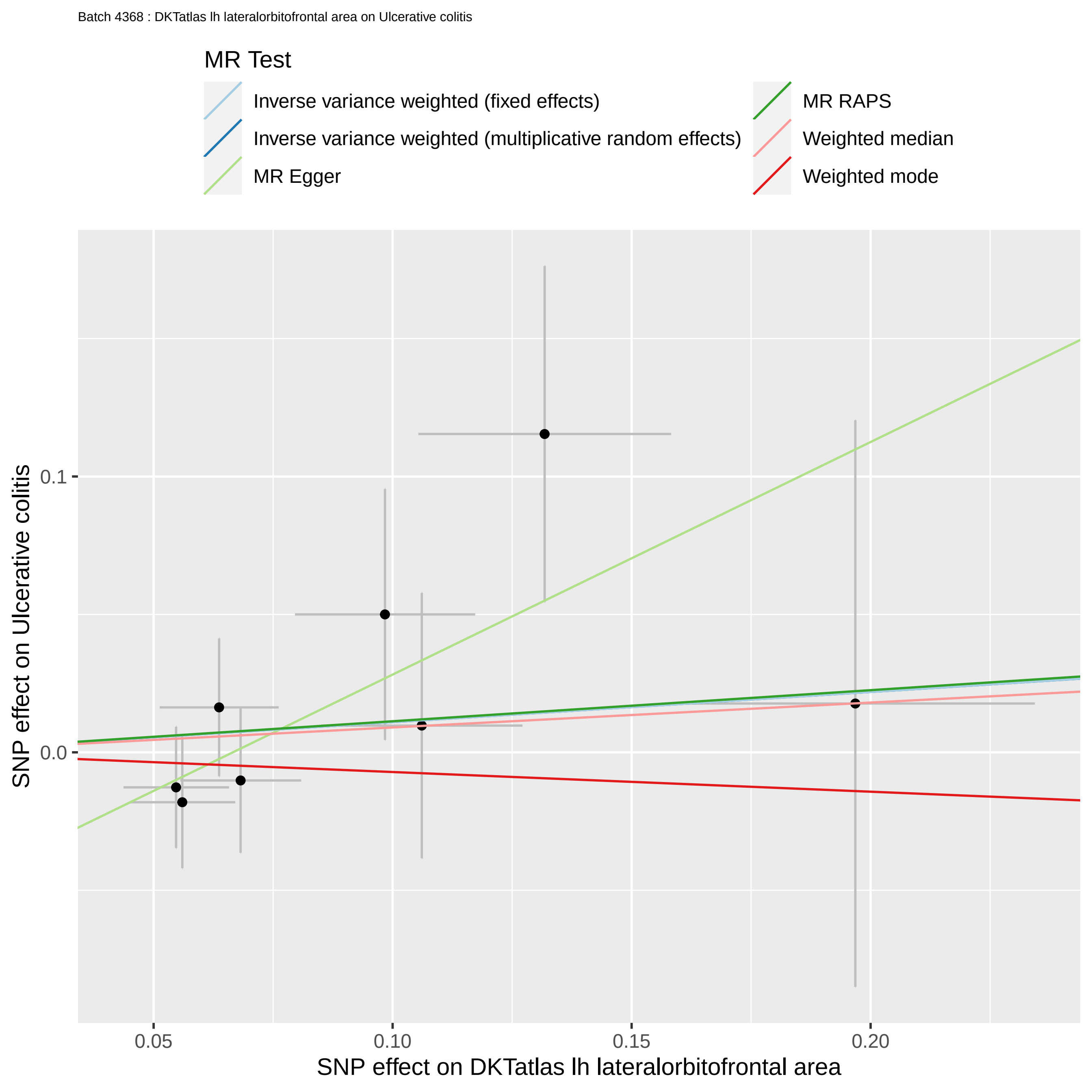
Batch 4368 : DKTatlas lh lateralorbitofrontal area on Ulcerative colitis
MR Test
Inverse variance weighted (fixed effects)
Inverse variance weighted (multiplicative random effects)
MR Egger
MR RAPS
Weighted median
Weighted mode
SNP effect on Ulcerative colitis
0.1
0.0
0.05
0.10
0.15
0.20
SNP effect on DKTatlas lh lateralorbitofrontal area

Batch 4370 : DKTatlas lh medialorbitofrontal area on Ulcerative colitis

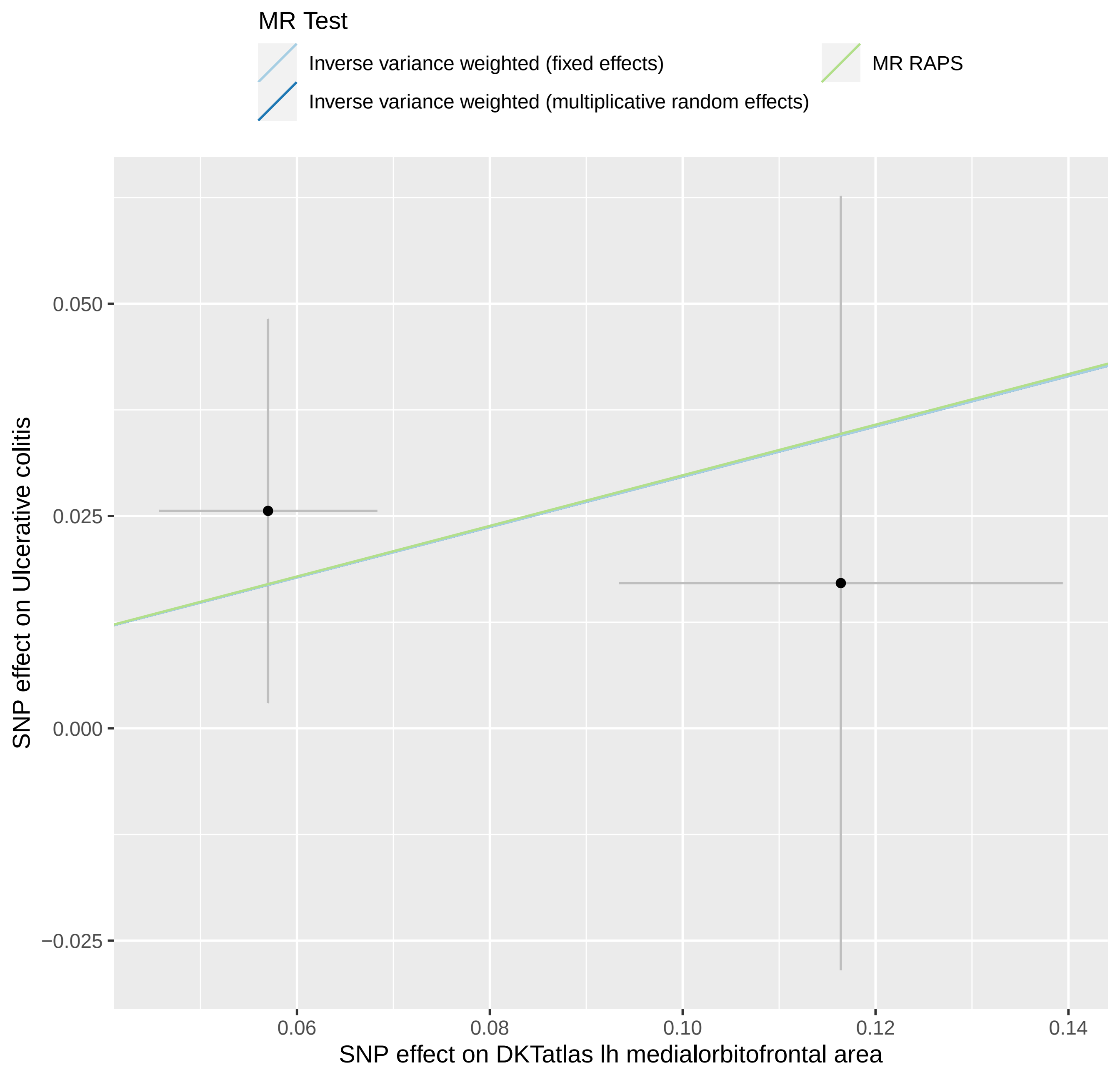

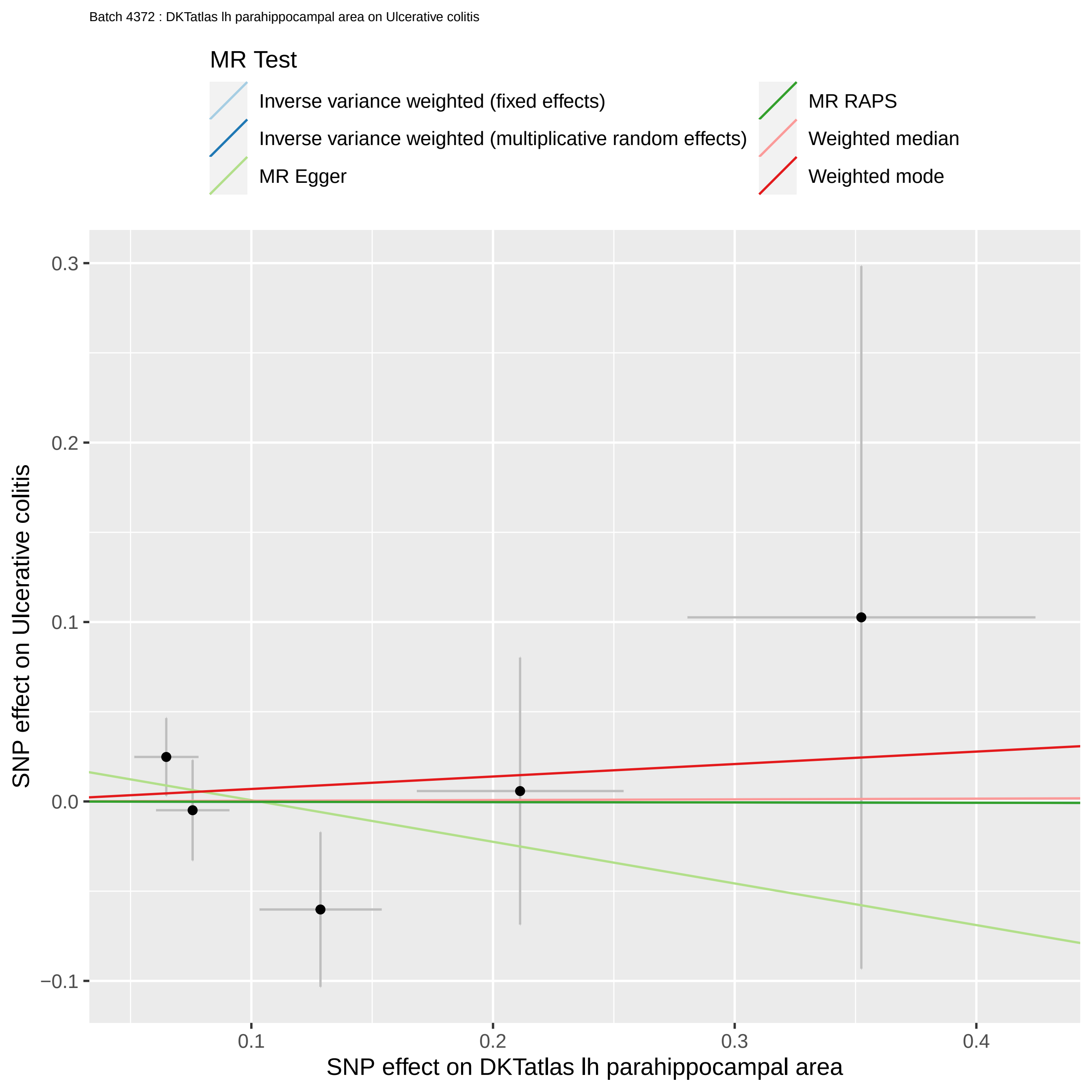

Batch 4372 : DKTatlas lh parahippocampal area on Ulcerative colitis
MR Test
Inverse variance weighted (fixed effects)
Inverse variance weighted (multiplicative random effects)
MR Egger
MR RAPS
Weighted median
Weighted mode
0.3
0.2
0.1
0.0
−0.1
0.1
0.2
0.3
0.4
SNP effect on Ulcerative colitis
SNP effect on DKTatlas lh parahippocampal area

Batch 4373 : DKTatlas lh paracentral area on Ulcerative colitis

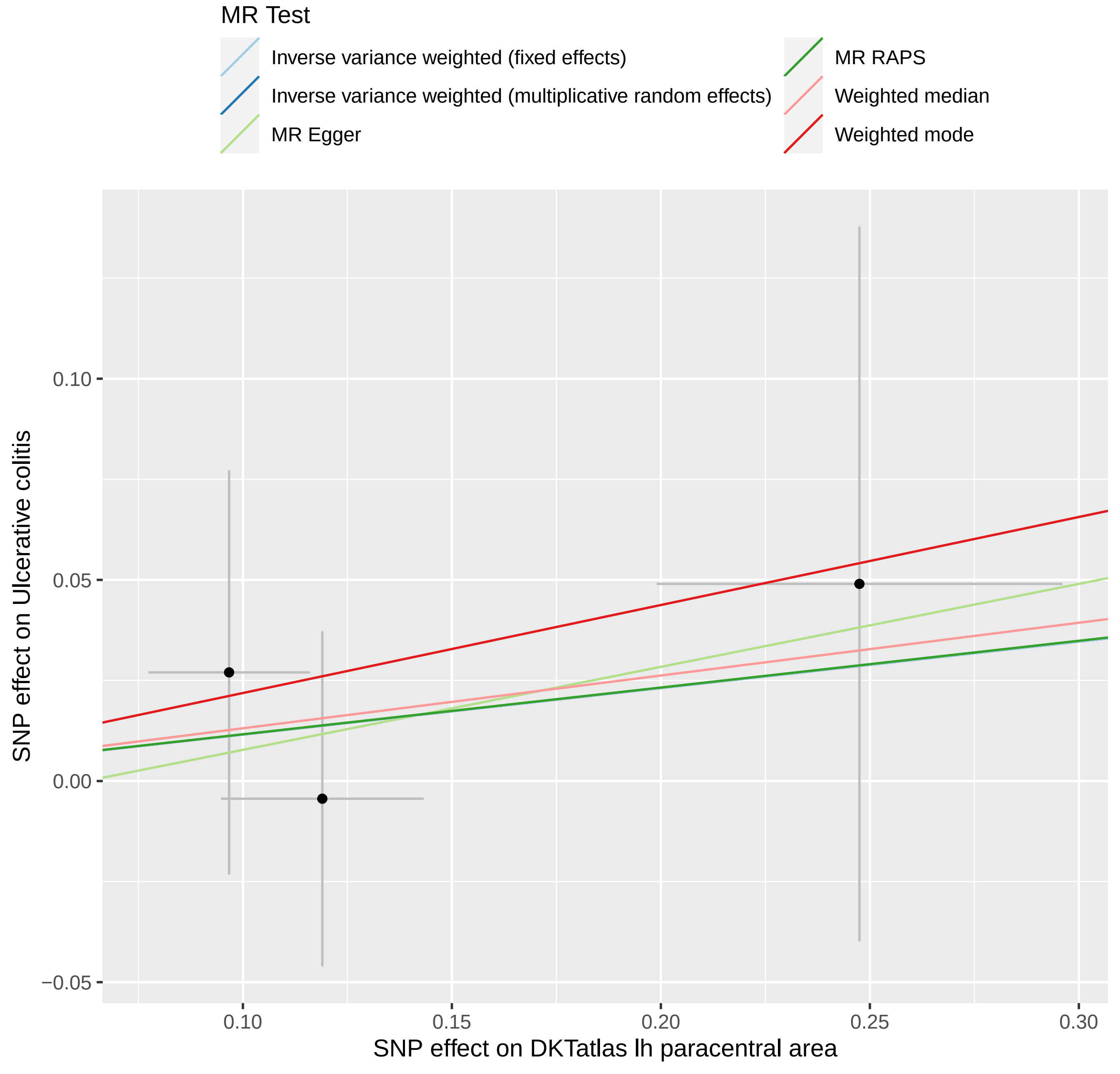

Batch 4376 : DKTatlas lh parsorbitalis area on Ulcerative colitis

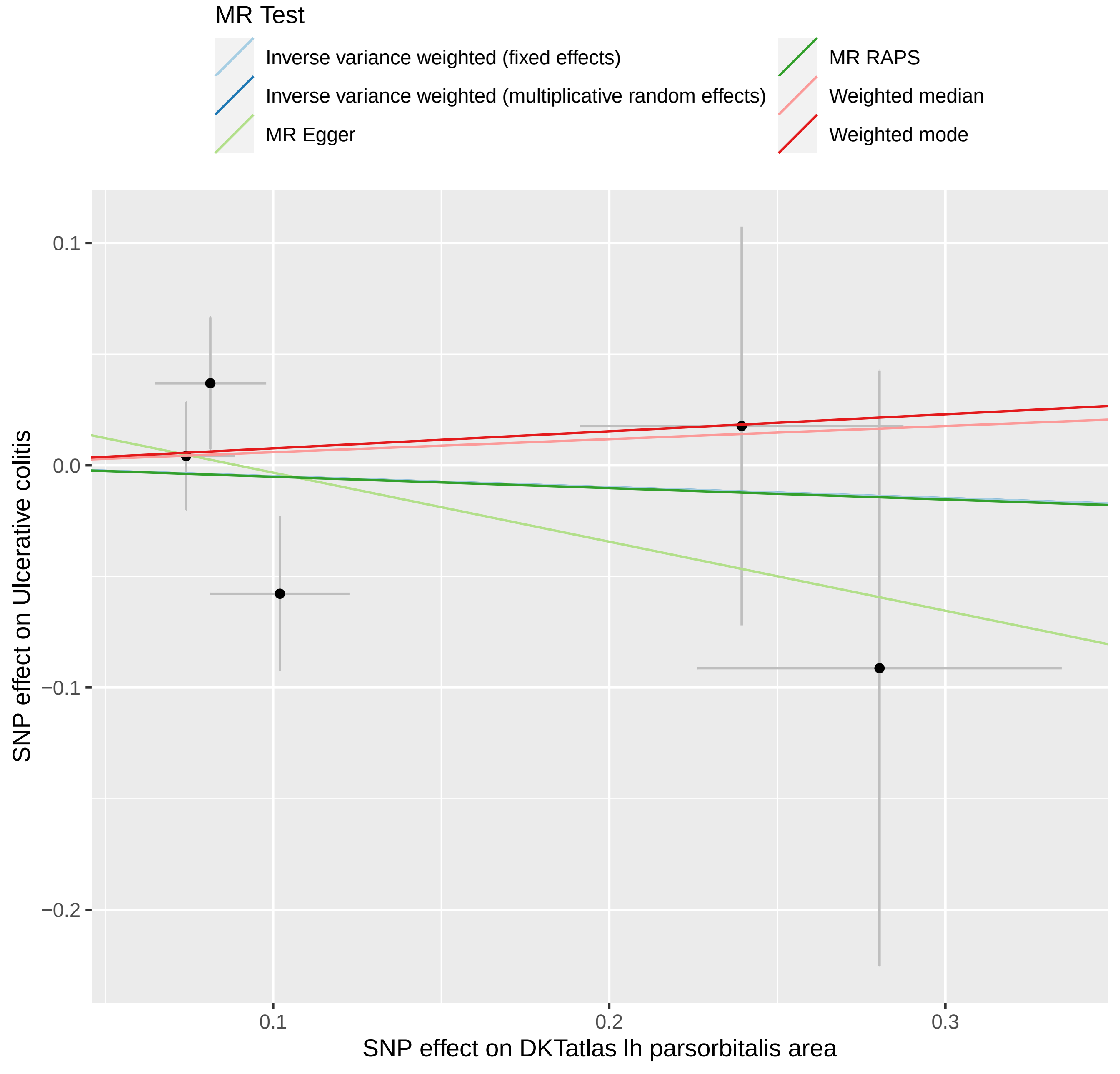

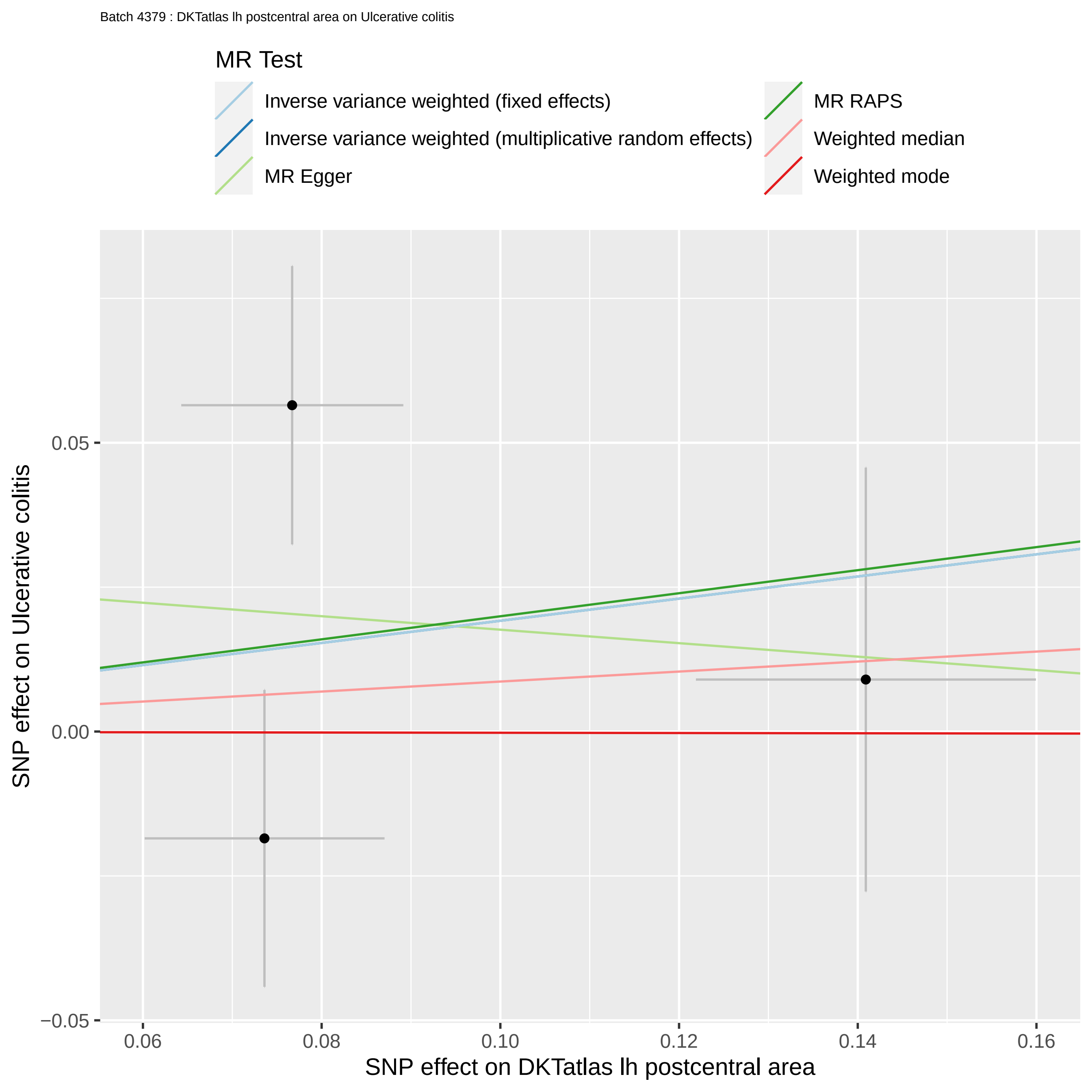

Batch 4379 : DKTatlas lh postcentral area on Ulcerative colitis
MR Test
Inverse variance weighted (fixed effects)
Inverse variance weighted (multiplicative random effects)
MR Egger
MR RAPS
Weighted median
Weighted mode
SNP effect on Ulcerative colitis
0.05
0.00
−0.05
0.06
0.08
0.10
0.12
0.14
0.16
SNP effect on DKTatlas lh postcentral area

Batch 4382 : DKTatlas lh precuneus area on Ulcerative colitis

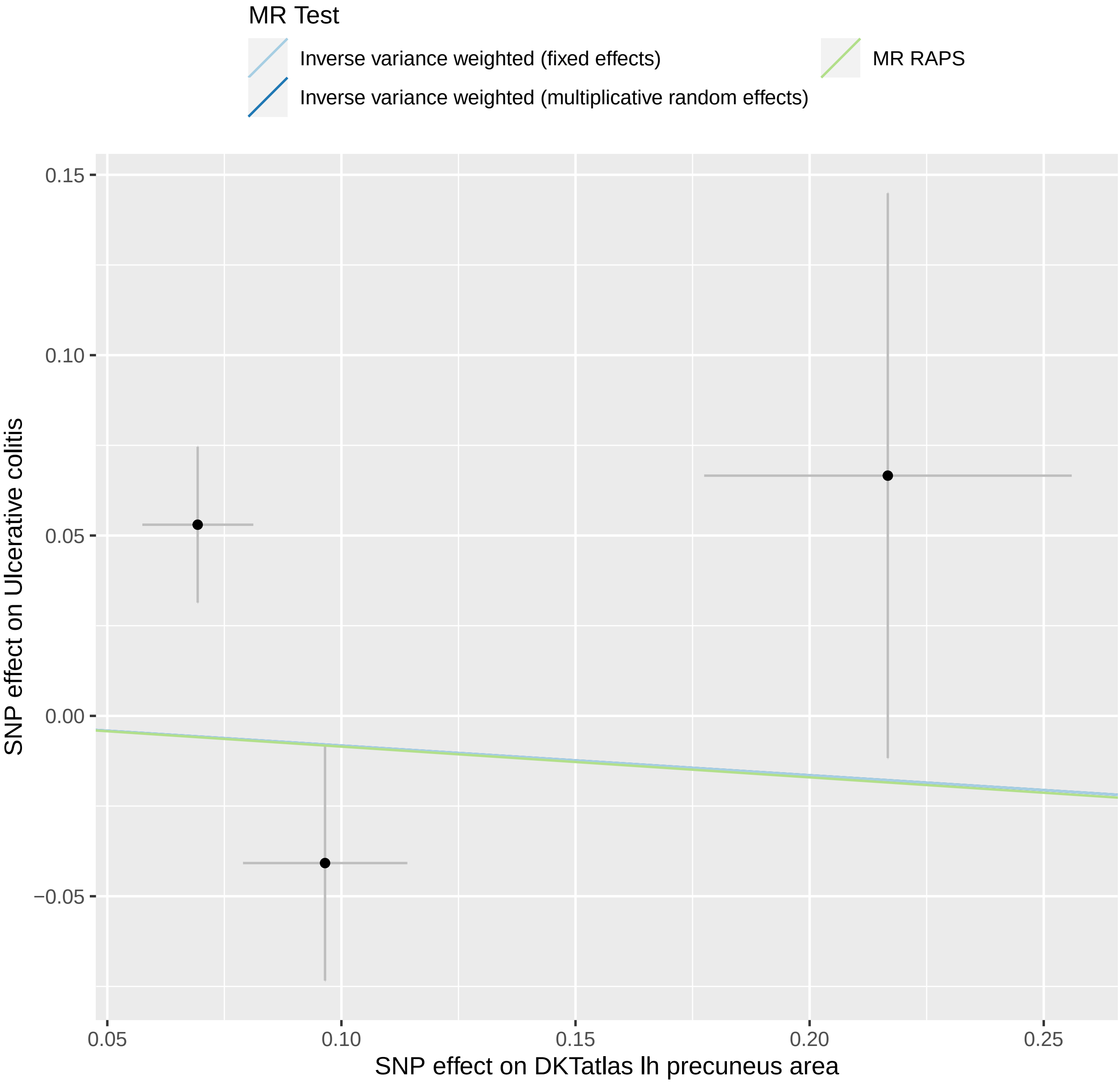

Batch 4383 : DKTatlas lh rostralanteriorcingulate area on Ulcerative colitis

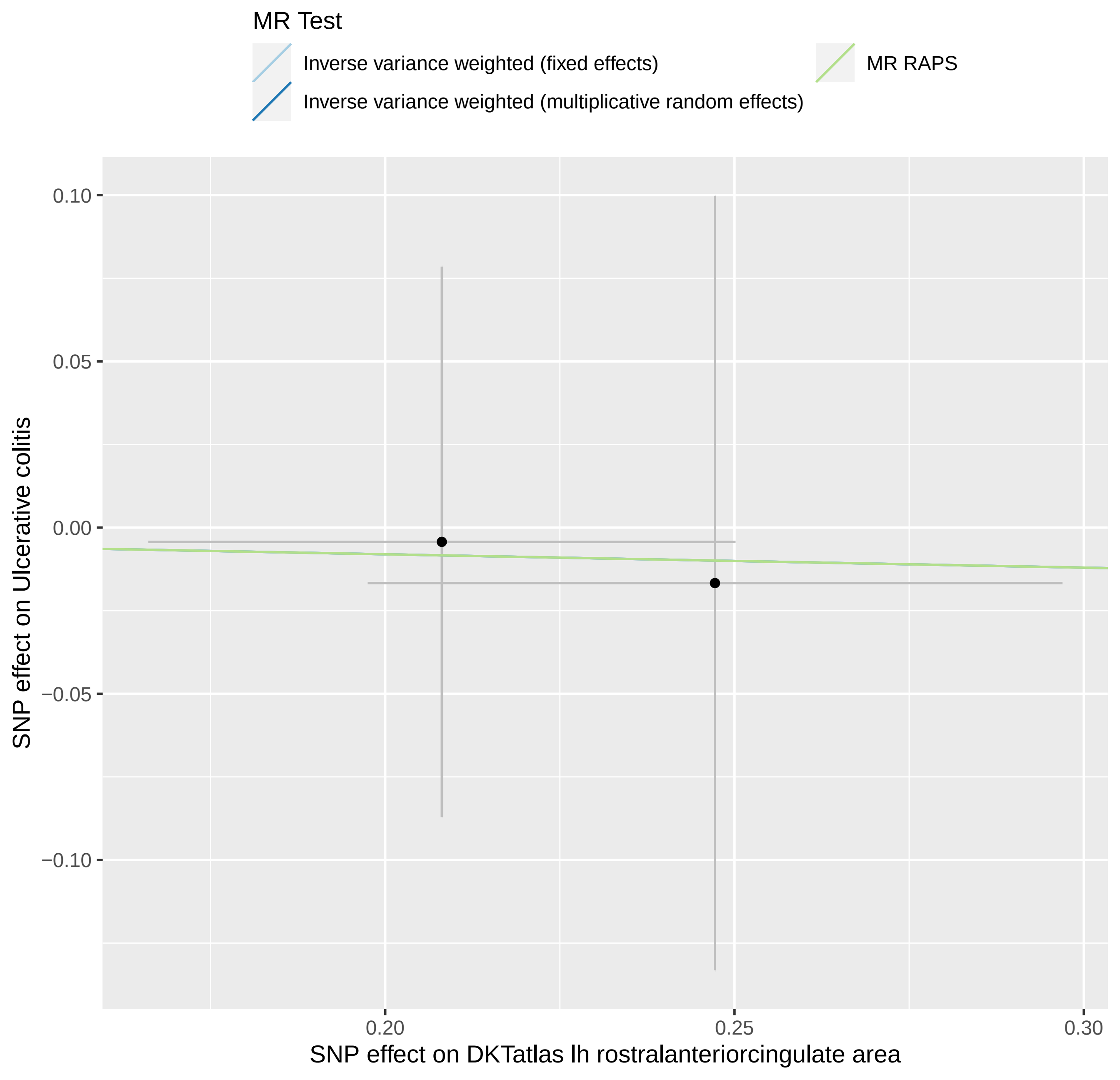

Batch 4384 : DKTatlas lh rostralmiddlefrontal area on Ulcerative colitis

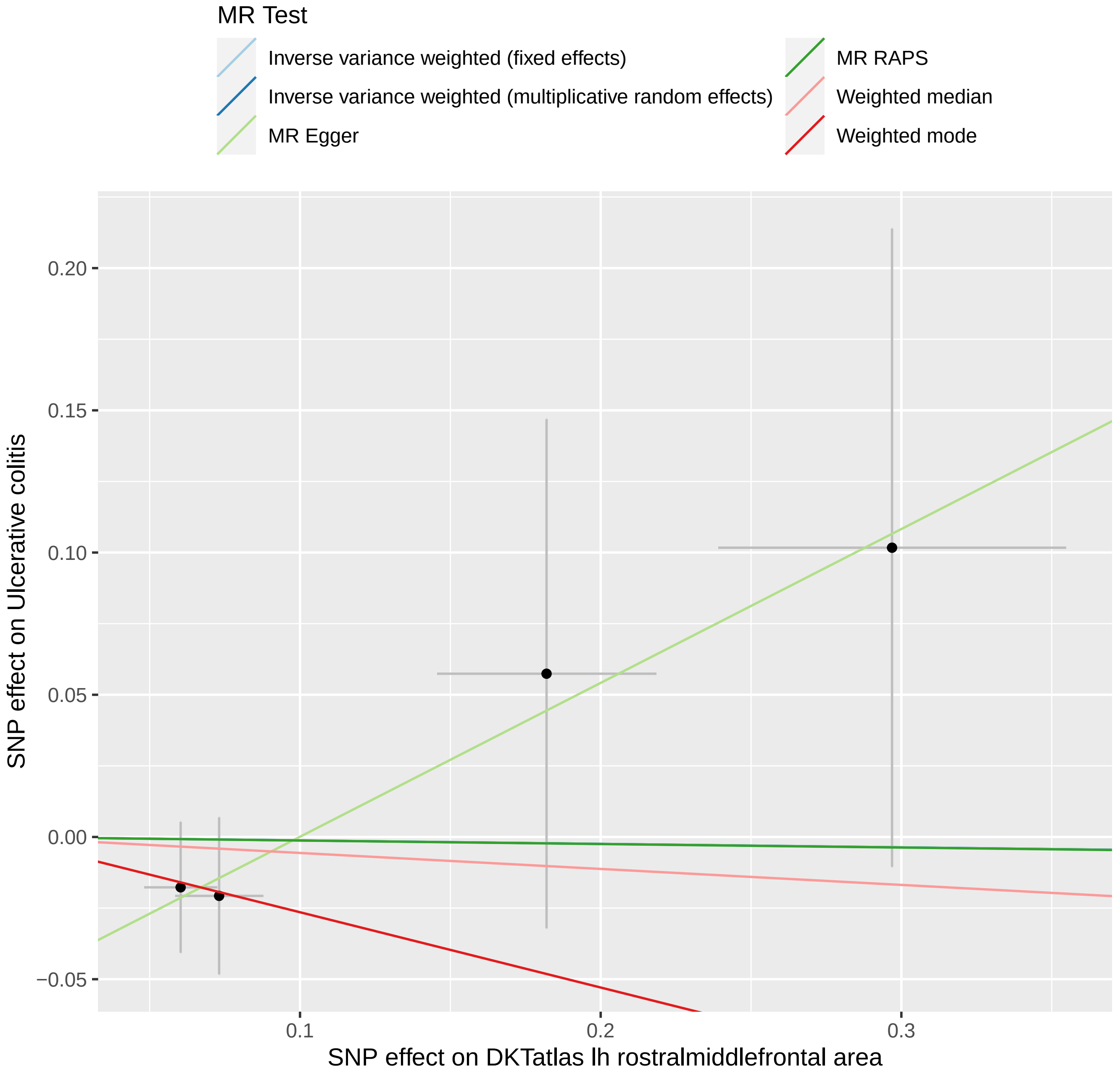

Batch 4386 : DKTatlas lh superiorfrontal area on Ulcerative colitis

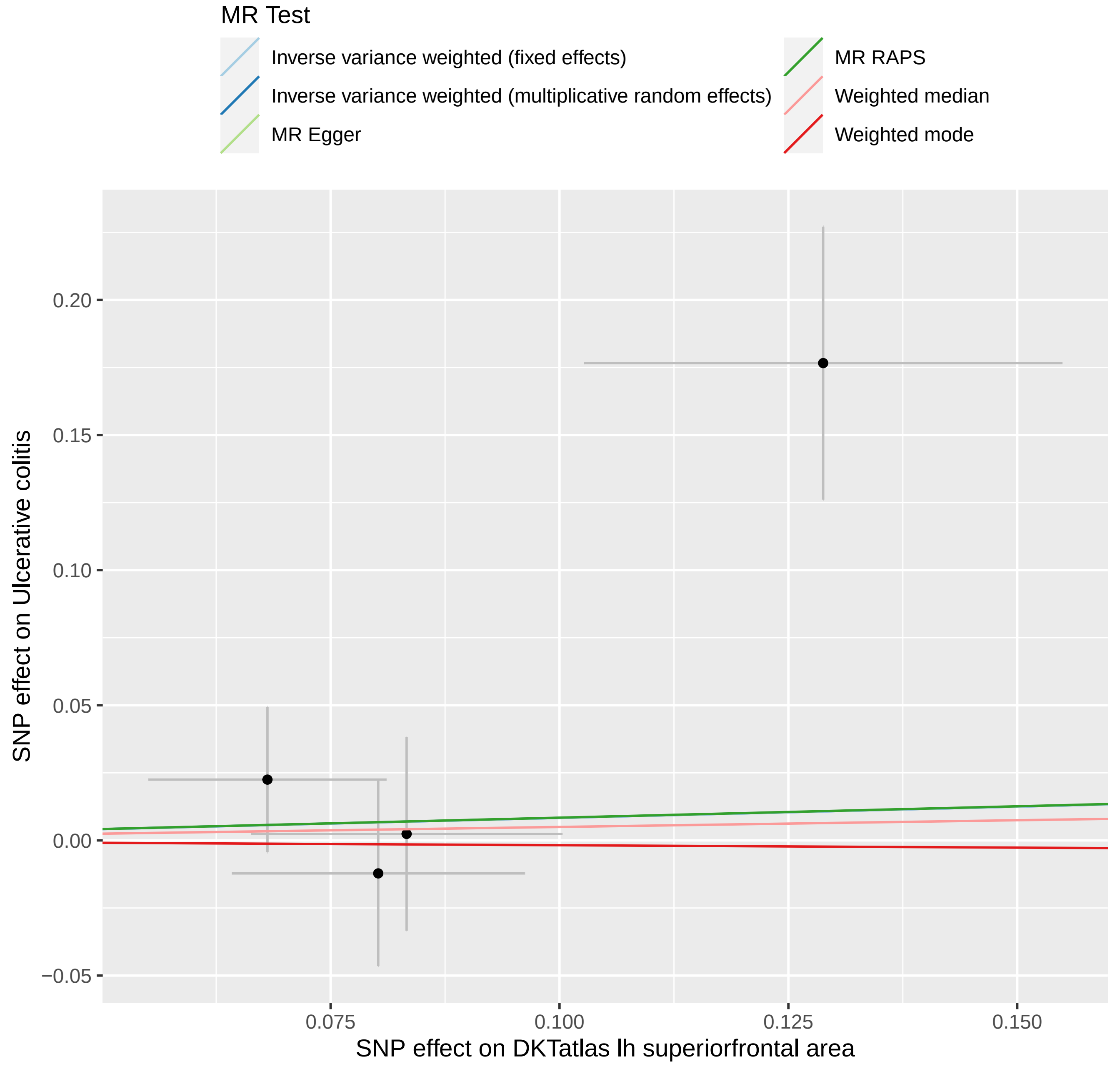

Batch 4392 : DKTatlas lh WhiteSurfArea area on Ulcerative colitis

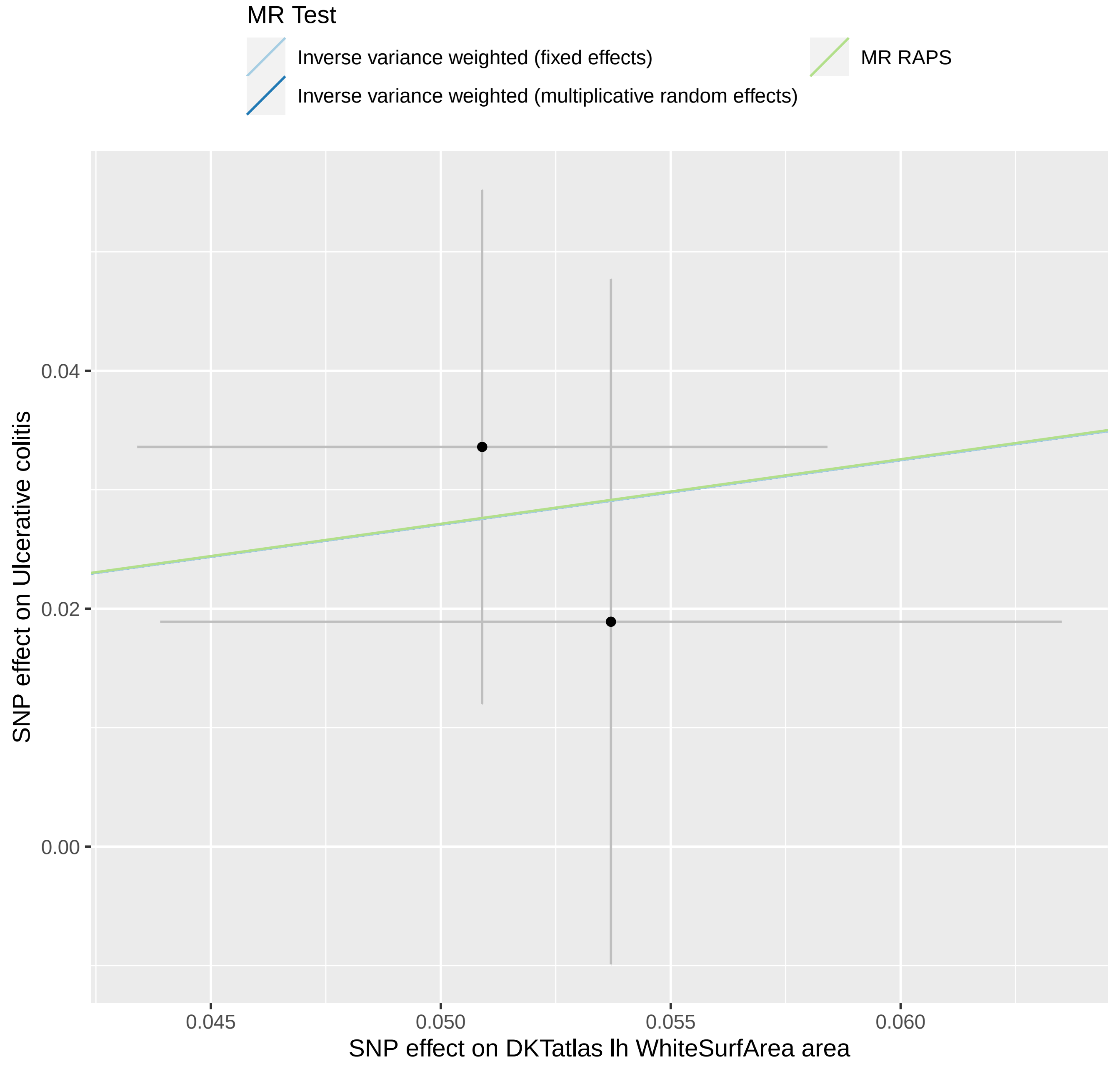

Batch 4476 : DKTatlas rh caudalanteriorcingulate area on Ulcerative colitis

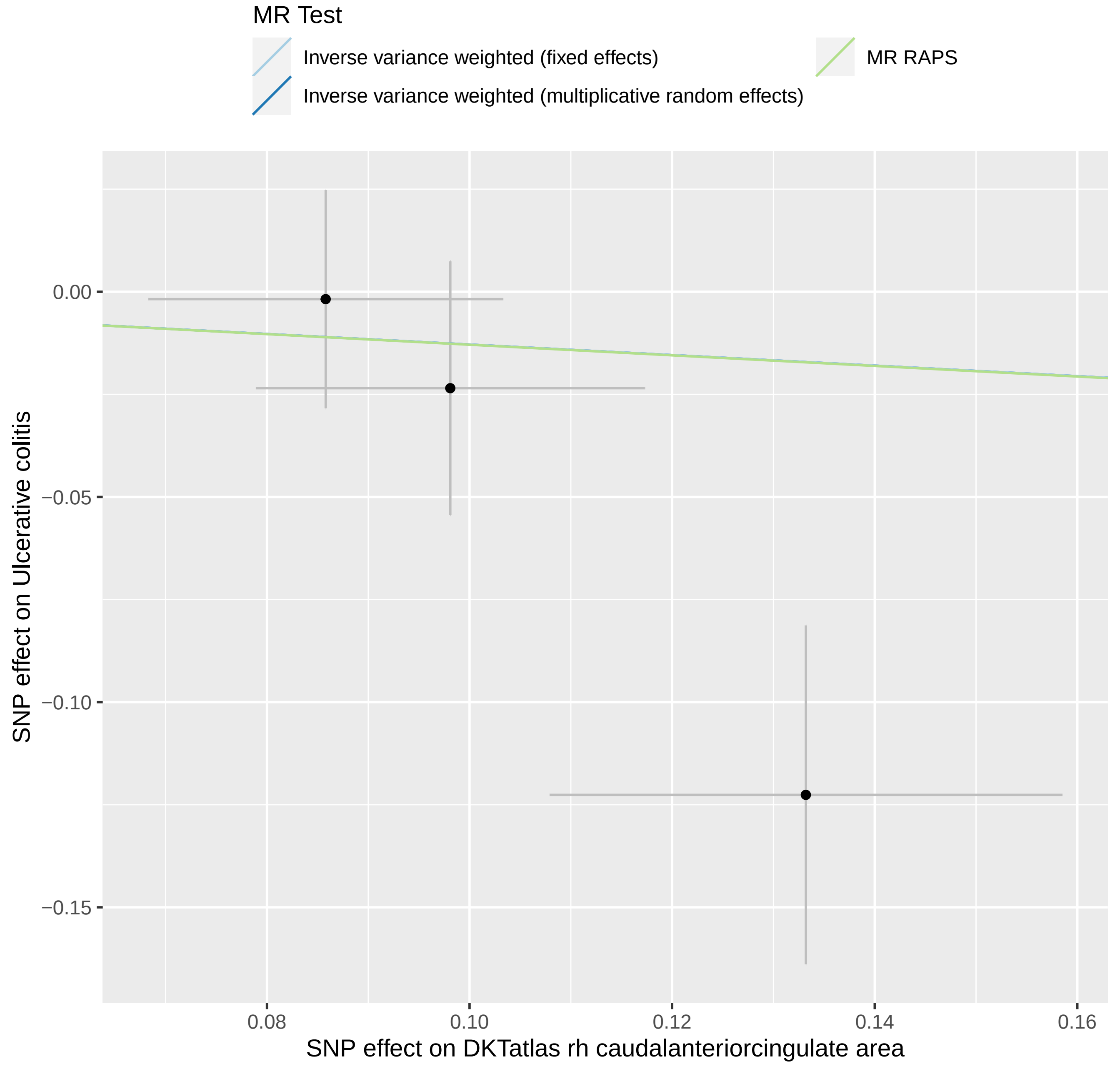

Batch 4479 : DKTatlas rh entorhinal area on Ulcerative colitis

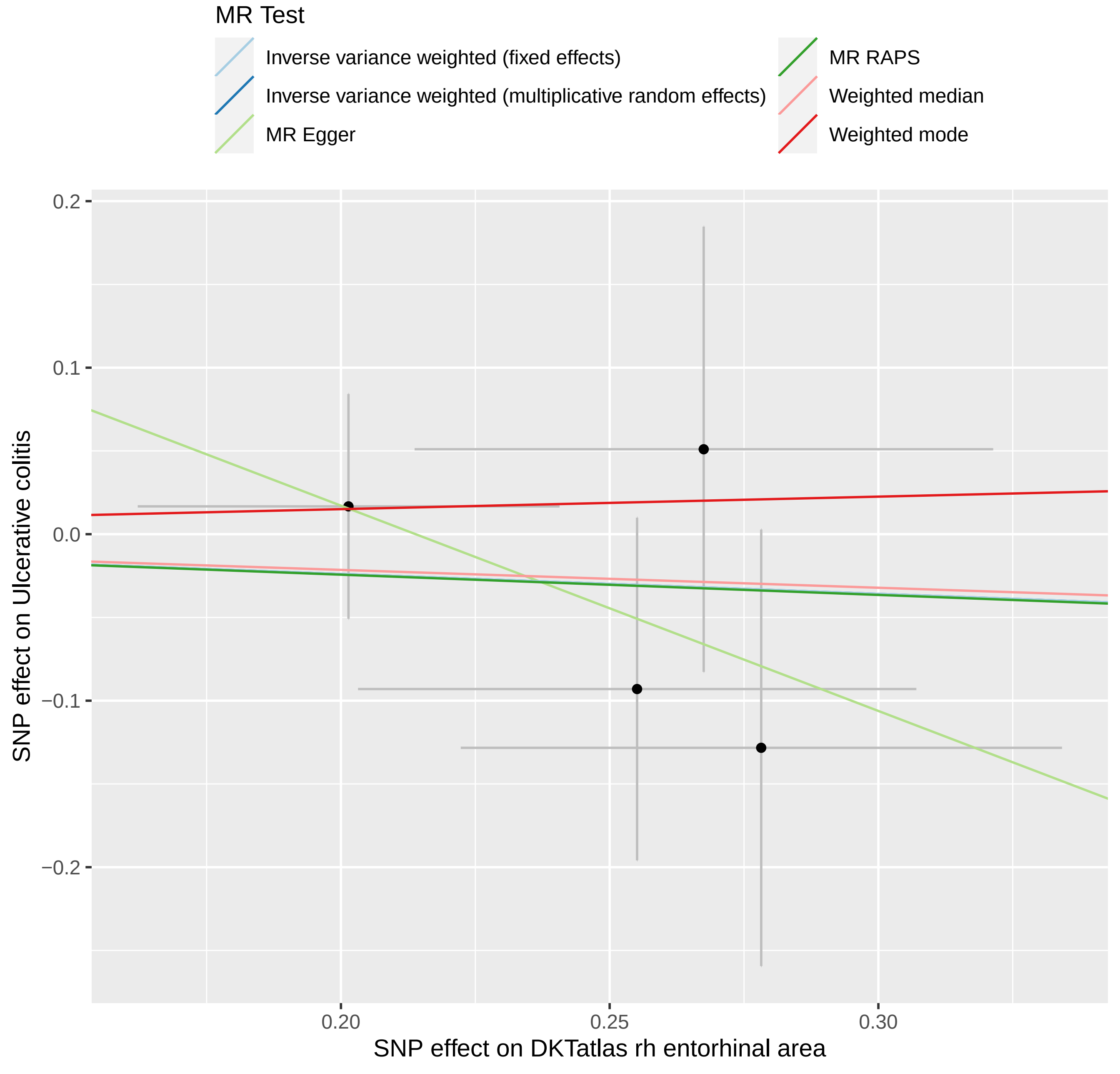

Batch 4483 : DKTatlas rh isthmuscingulate area on Ulcerative colitis

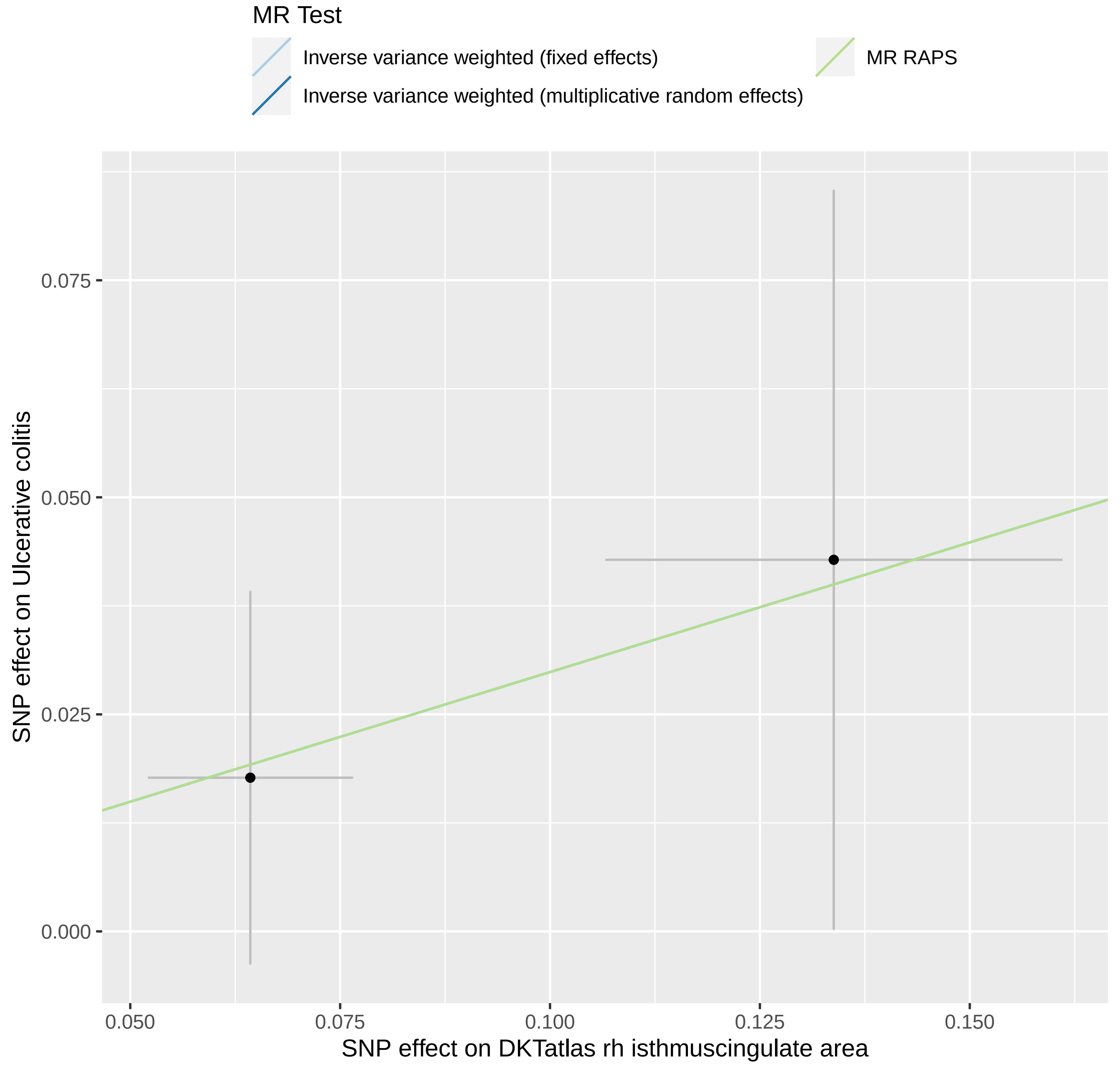

Batch 4484 : DKTatlas rh lateraloccipital area on Ulcerative colitis

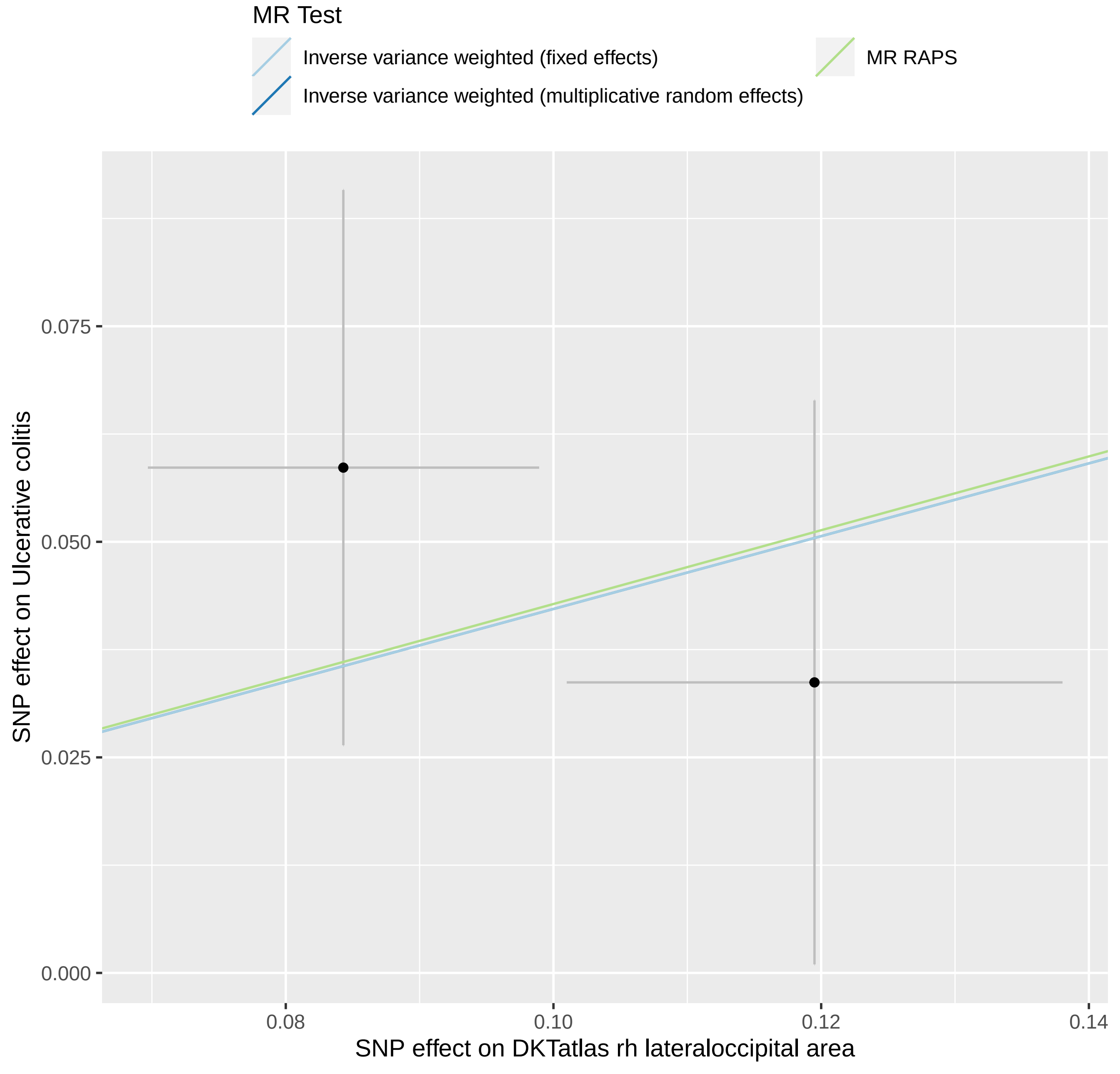

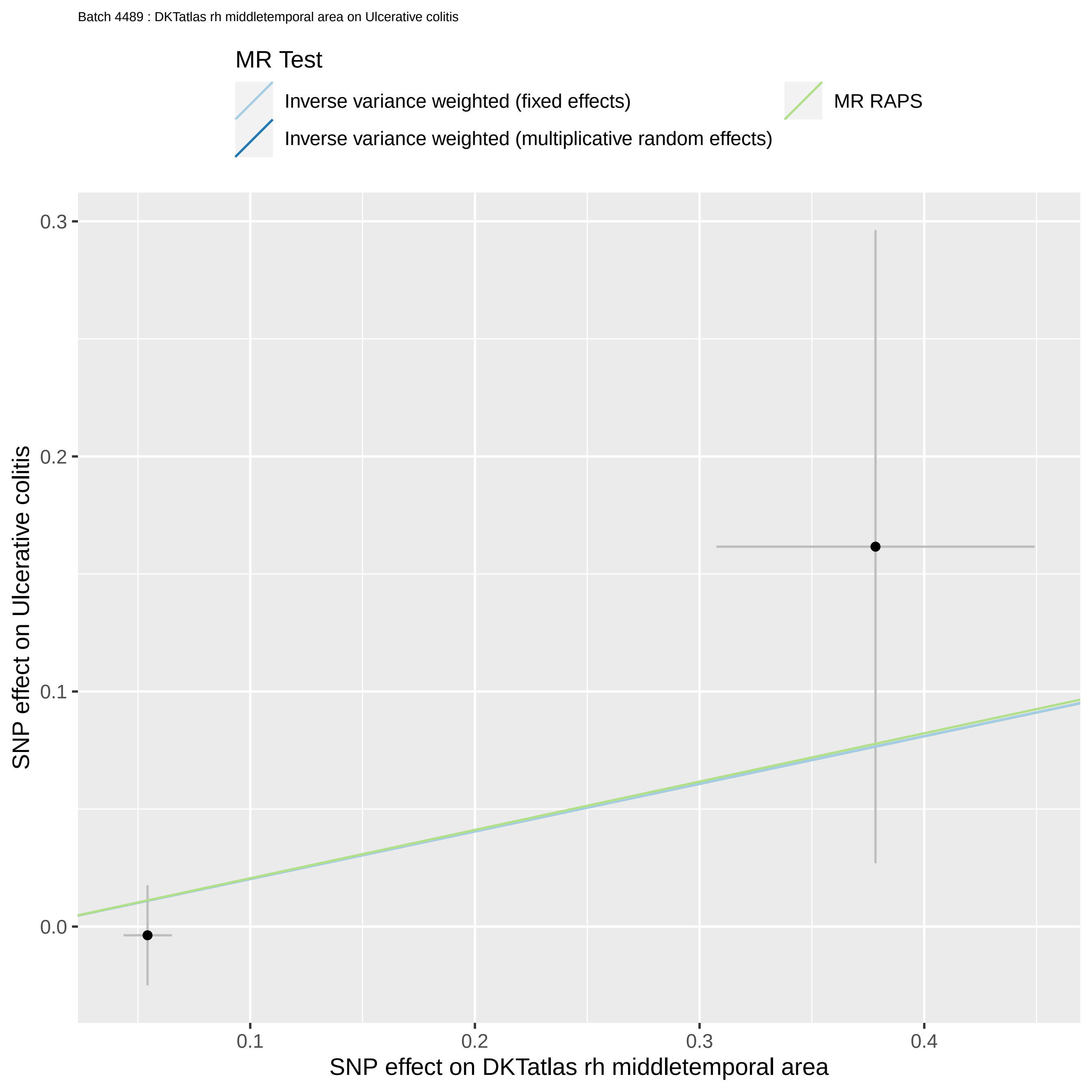
Batch 4489 : DKTatlas rh middletemporal area on Ulcerative colitis
MR Test
Inverse variance weighted (fixed effects)
Inverse variance weighted (multiplicative random effects)
MR RAPS
0.3
0.2
0.1
0.0
0.1
0.2
0.3
0.4
SNP effect on Ulcerative colitis
SNP effect on DKTatlas rh middletemporal area

Batch 4492 : DKTatlas rh parsopercularis area on Ulcerative colitis

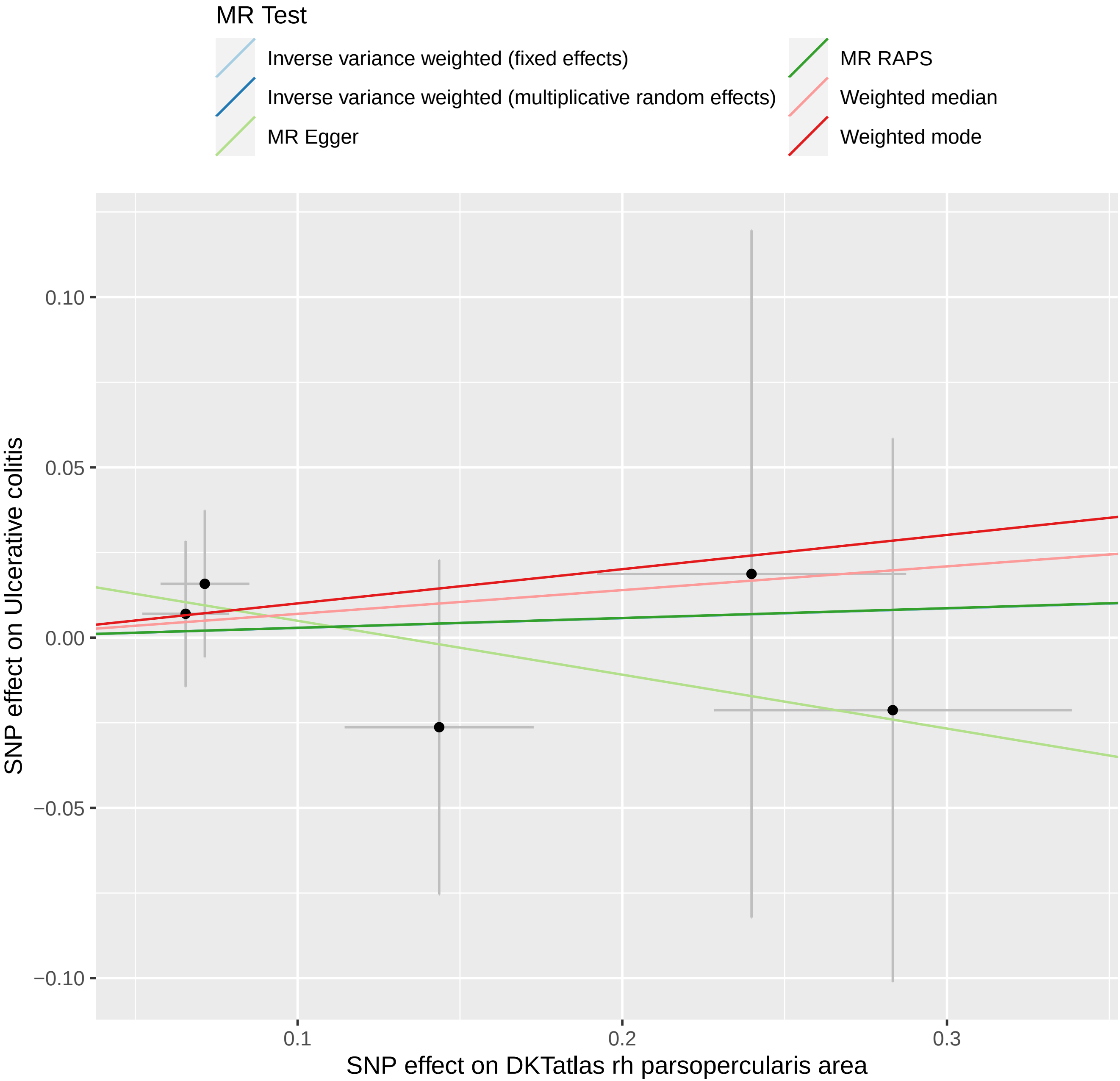

Batch 4495 : DKTatlas rh pericalcarine area on Ulcerative colitis

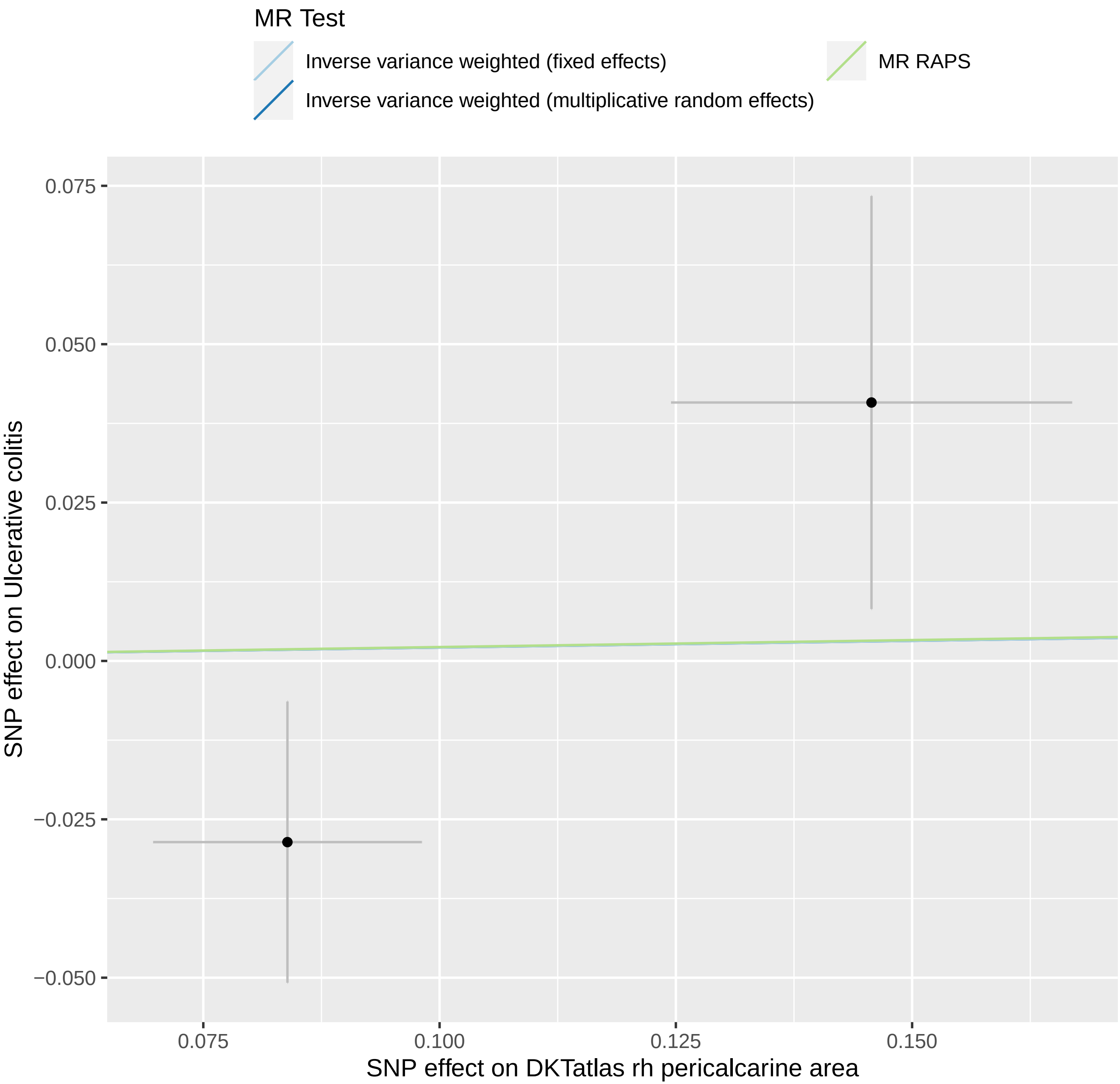

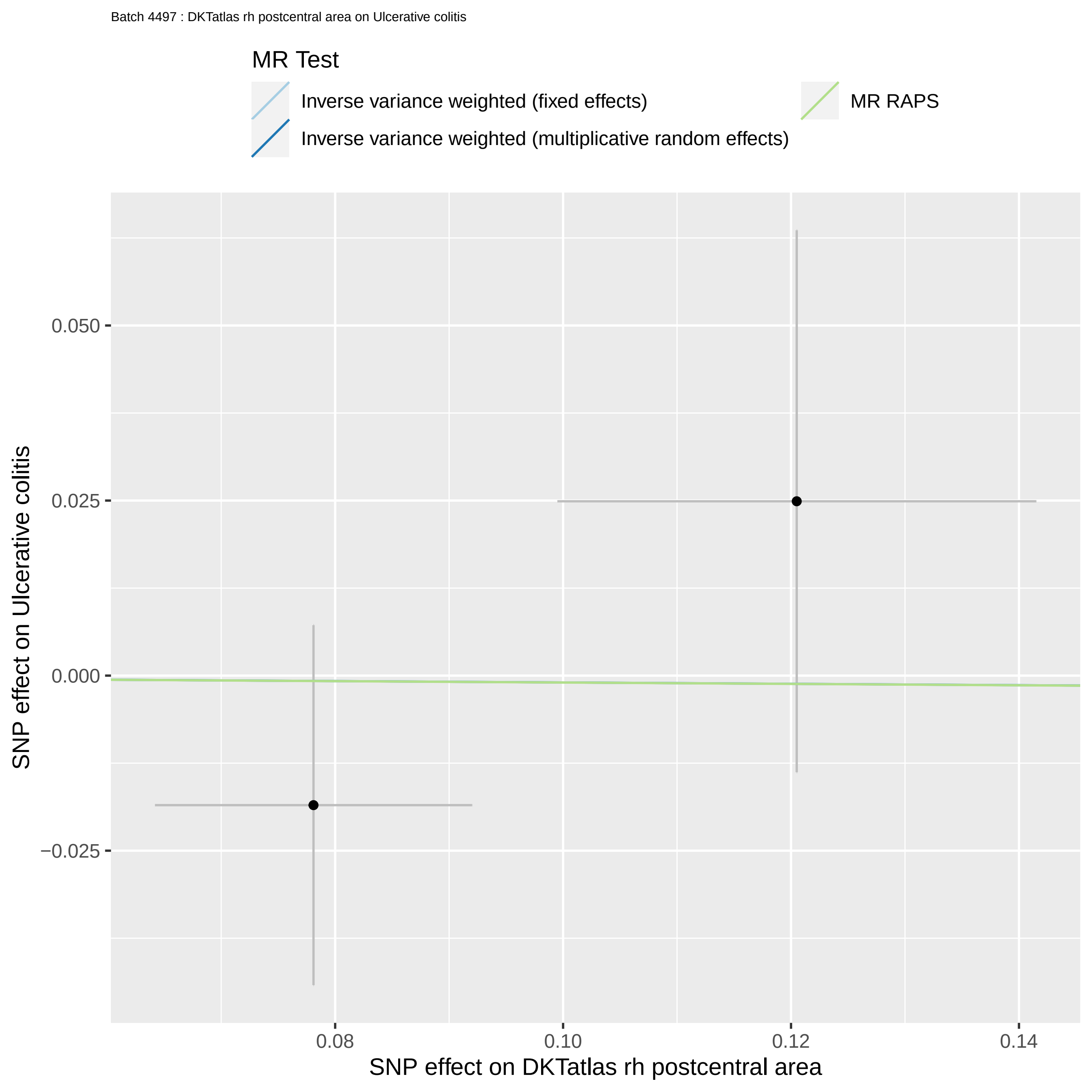
Batch 4497 : DKTatlas rh postcentral area on Ulcerative colitis
MR Test
Inverse variance weighted (fixed effects)
Inverse variance weighted (multiplicative random effects)
MR RAPS
0.050
0.025
0.000
−0.025
0.08
0.10
0.12
0.14
SNP effect on DKTatlas rh postcentral area
SNP effect on Ulcerative colitis

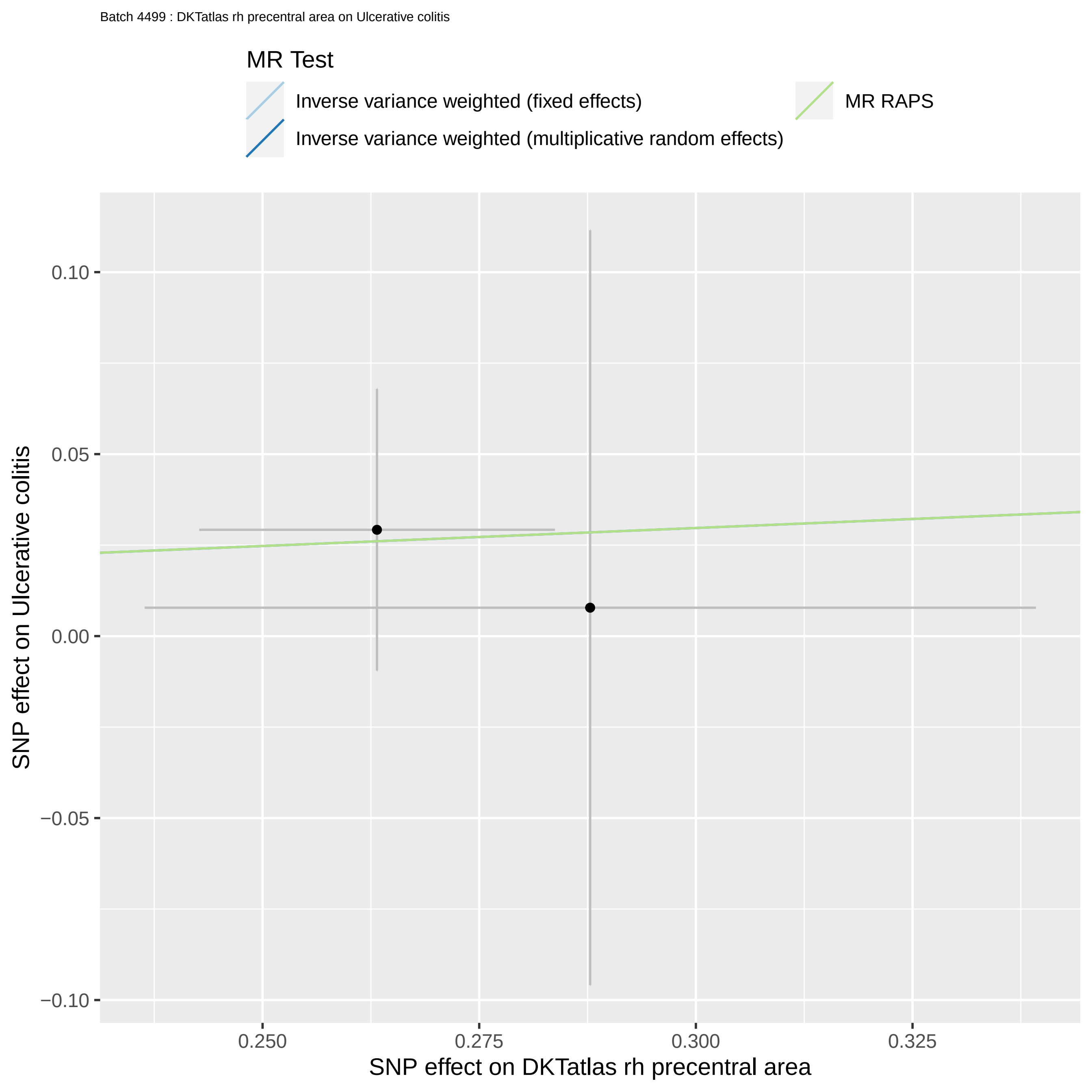
Batch 4499 : DKTatlas rh precentral area on Ulcerative colitis
MR Test
Inverse variance weighted (fixed effects)
Inverse variance weighted (multiplicative random effects)
MR RAPS
0.10
0.05
0.00
−0.05
−0.10
SNP effect on Ulcerative colitis
0.250
0.275
0.300
0.325
SNP effect on DKTatlas rh precentral area

Batch 4500 : DKTatlas rh precuneus area on Ulcerative colitis

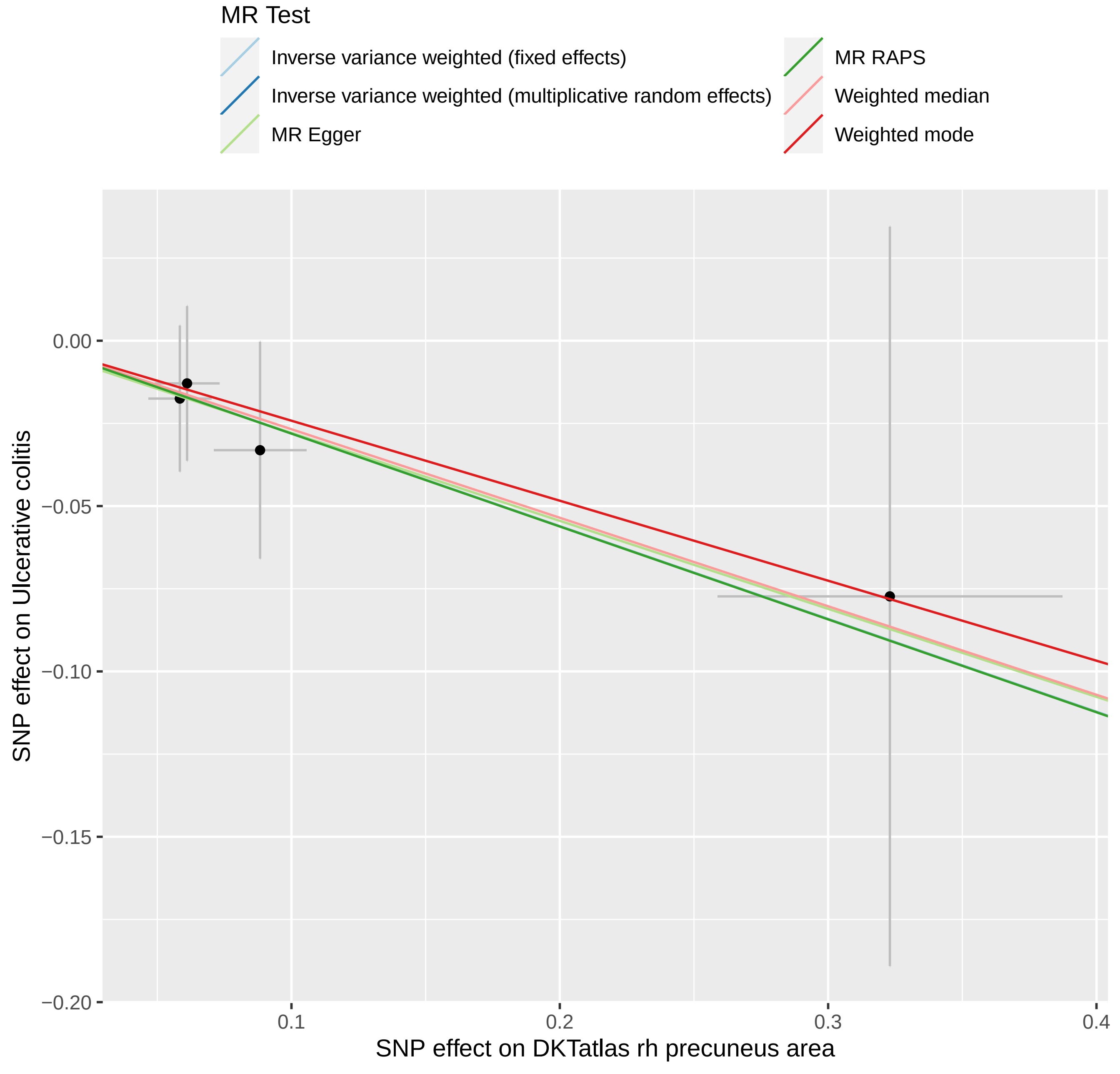

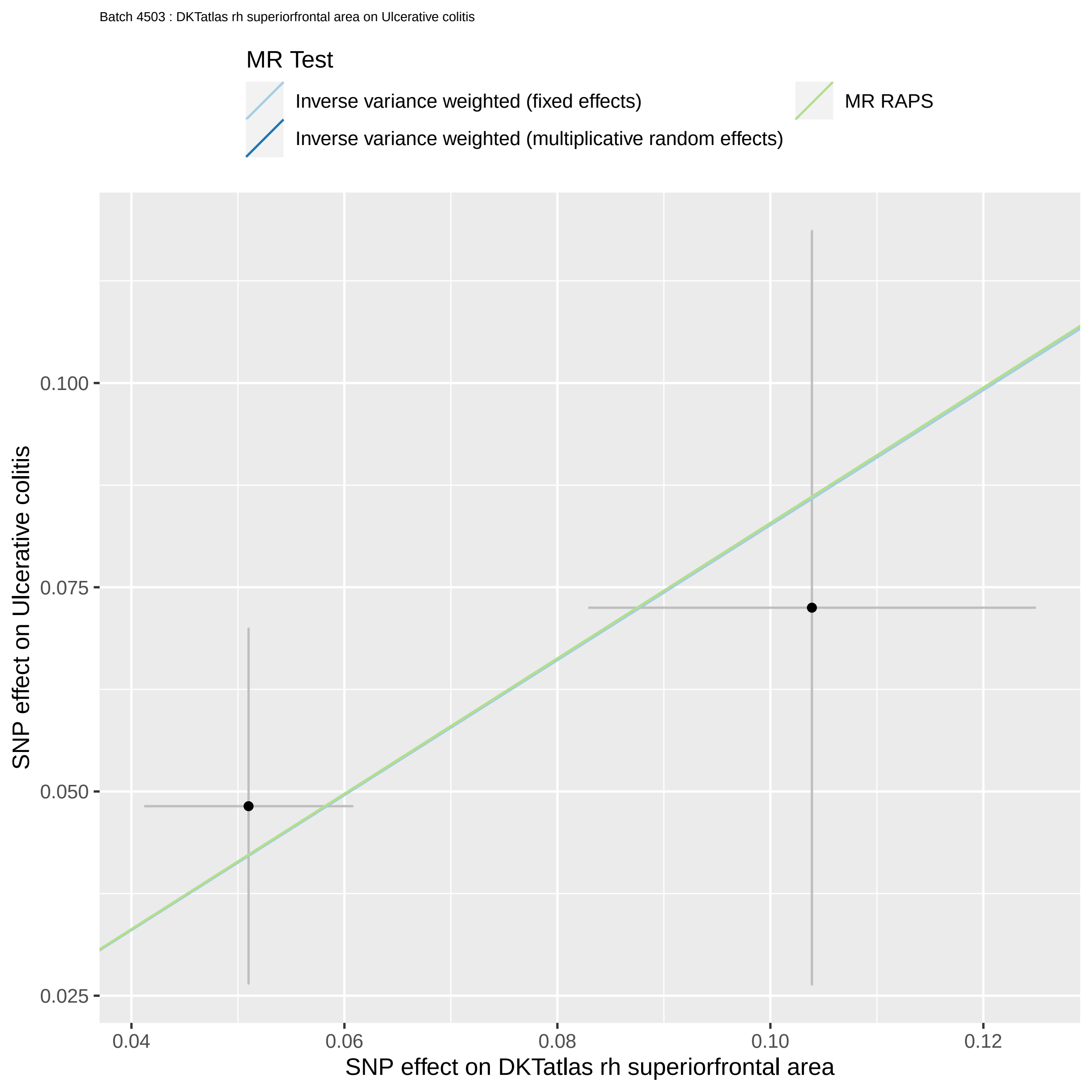

Batch 4503 : DKTatlas rh superiorfrontal area on Ulcerative colitis
MR Test
Inverse variance weighted (fixed effects)
Inverse variance weighted (multiplicative random effects)
MR RAPS
SNP effect on Ulcerative colitis
0.100
0.075
0.050
0.025
0.04
0.06
0.08
0.10
0.12
SNP effect on DKTatlas rh superiorfrontal area

Batch 4505 : DKTatlas rh superiortemporal area on Ulcerative colitis

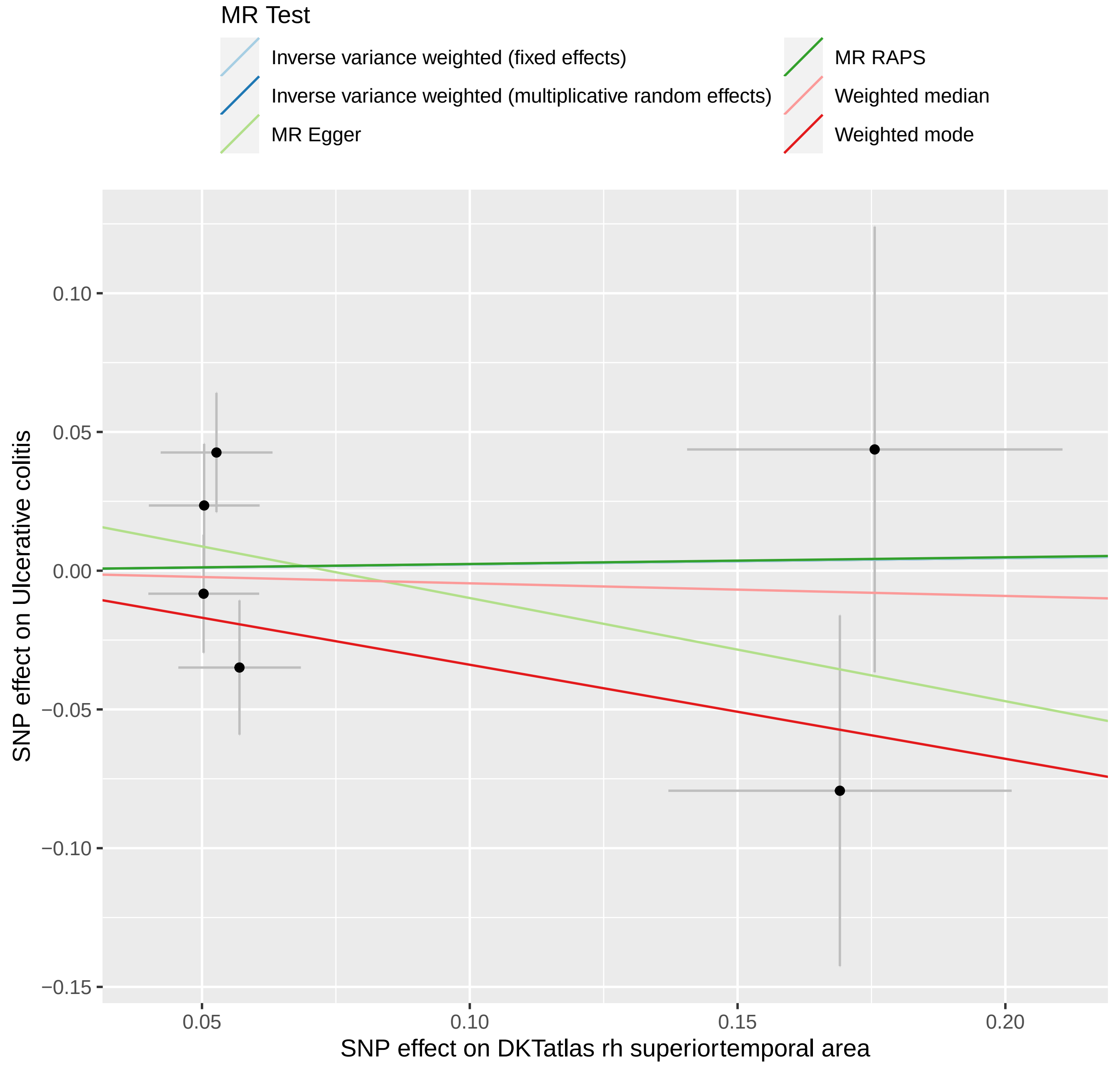

Batch 4508 : DKTatlas rh transversetemporal area on Ulcerative colitis

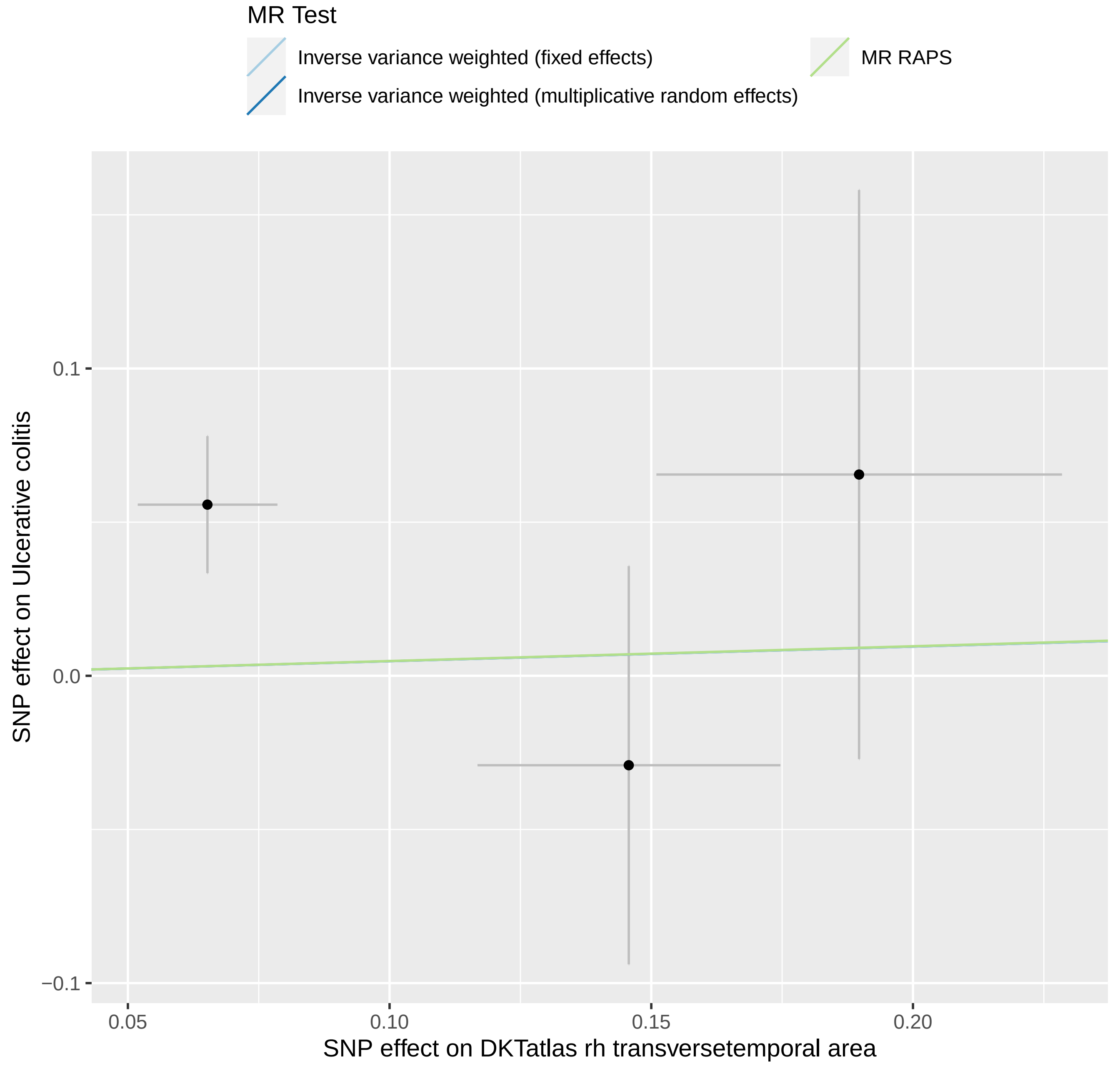

Batch 4509 : DKTatlas rh insula area on Ulcerative colitis

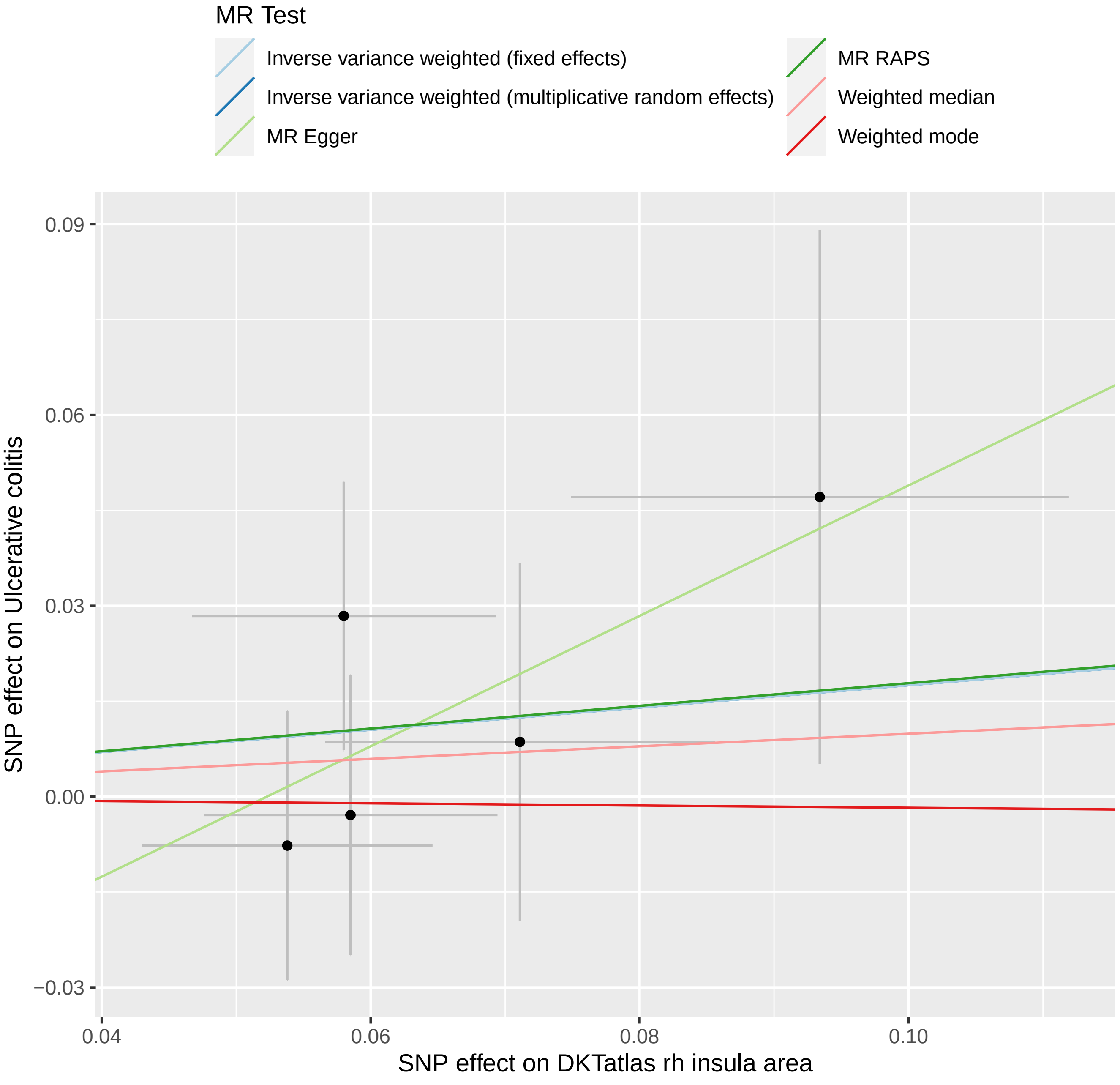

Batch 4510 : DKTatlas rh WhiteSurfArea area on Ulcerative colitis

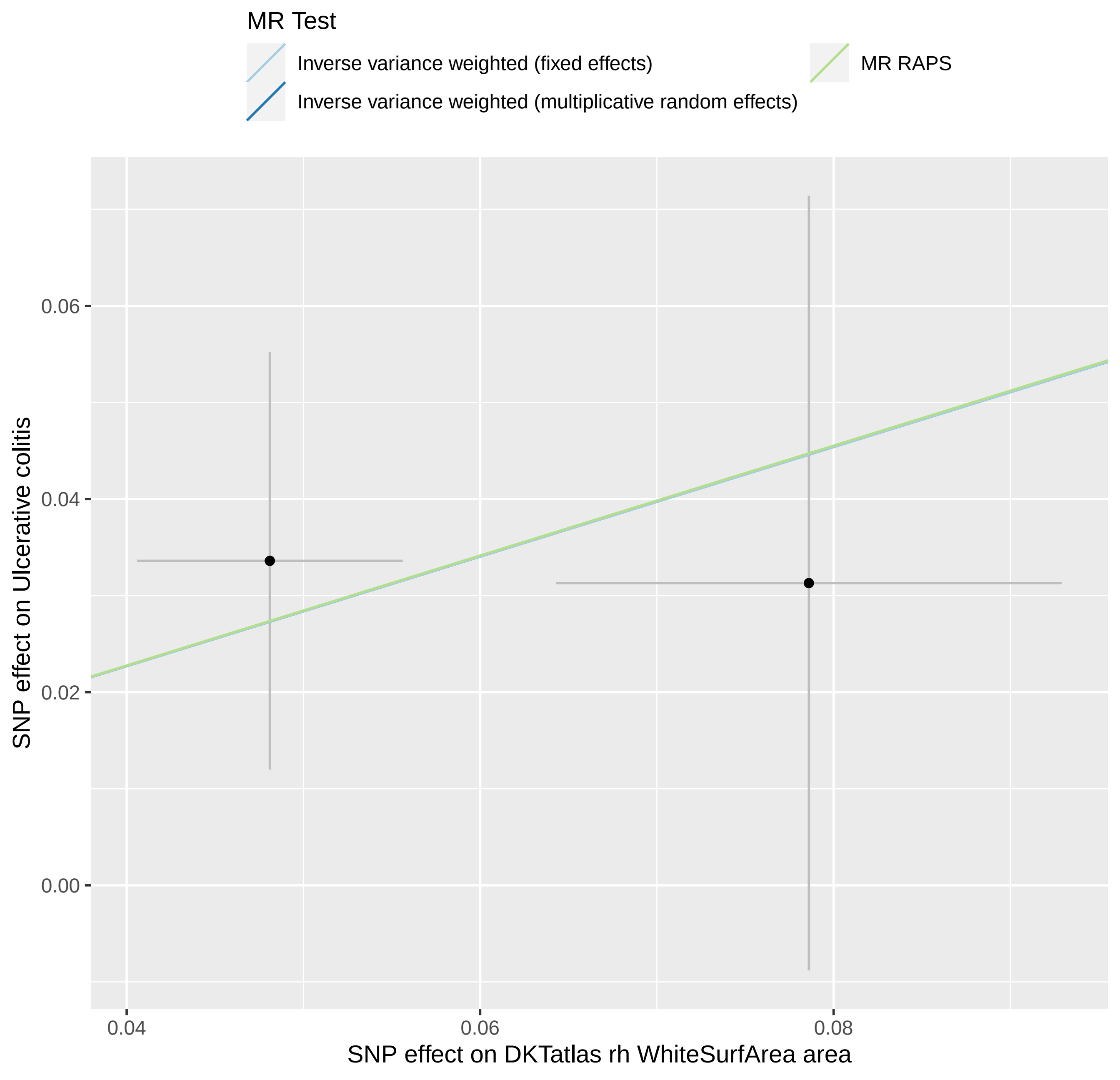

Batch 4562 : IDP T1 FAST ROIs R insular cortex on Ulcerative colitis

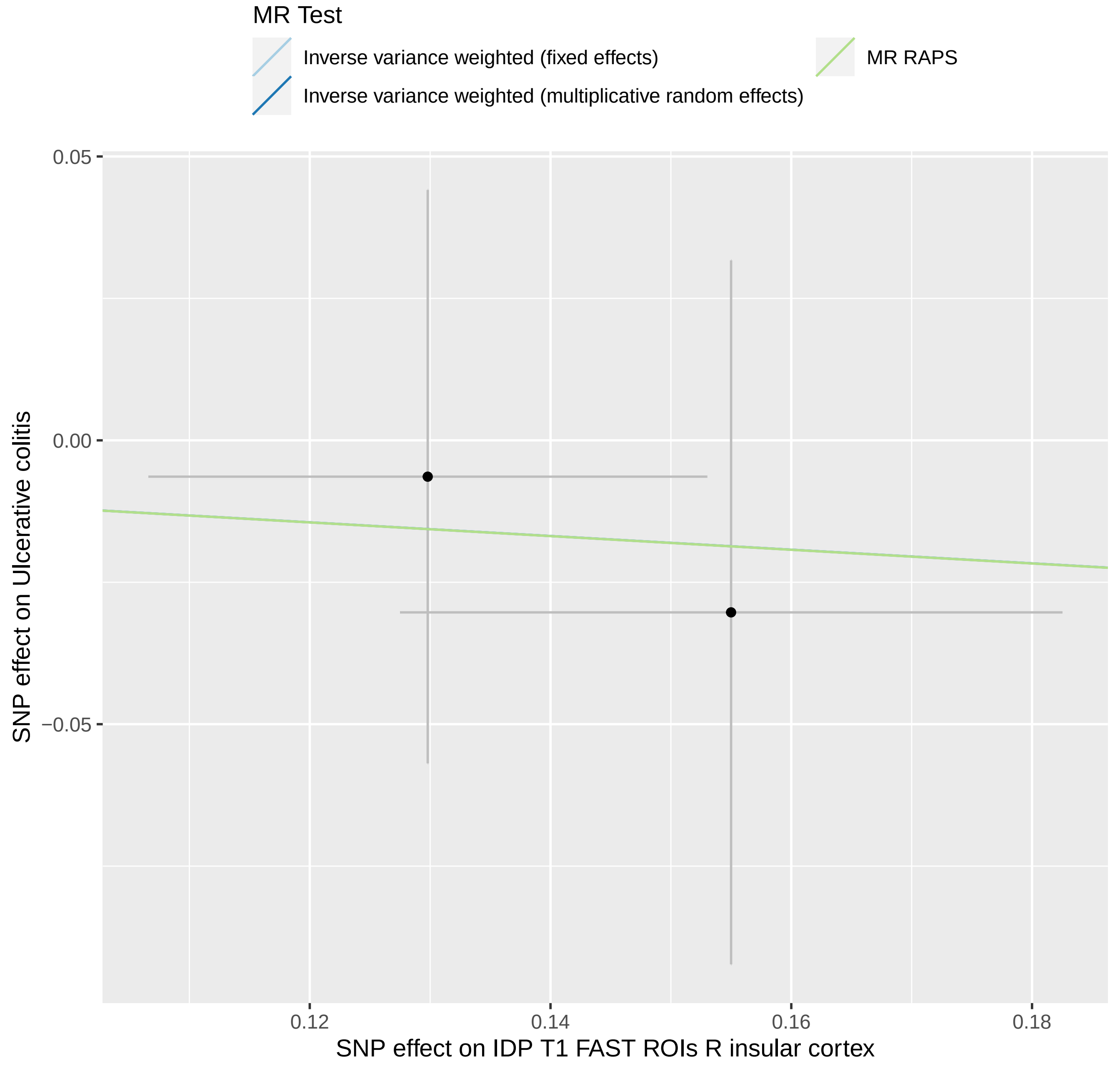

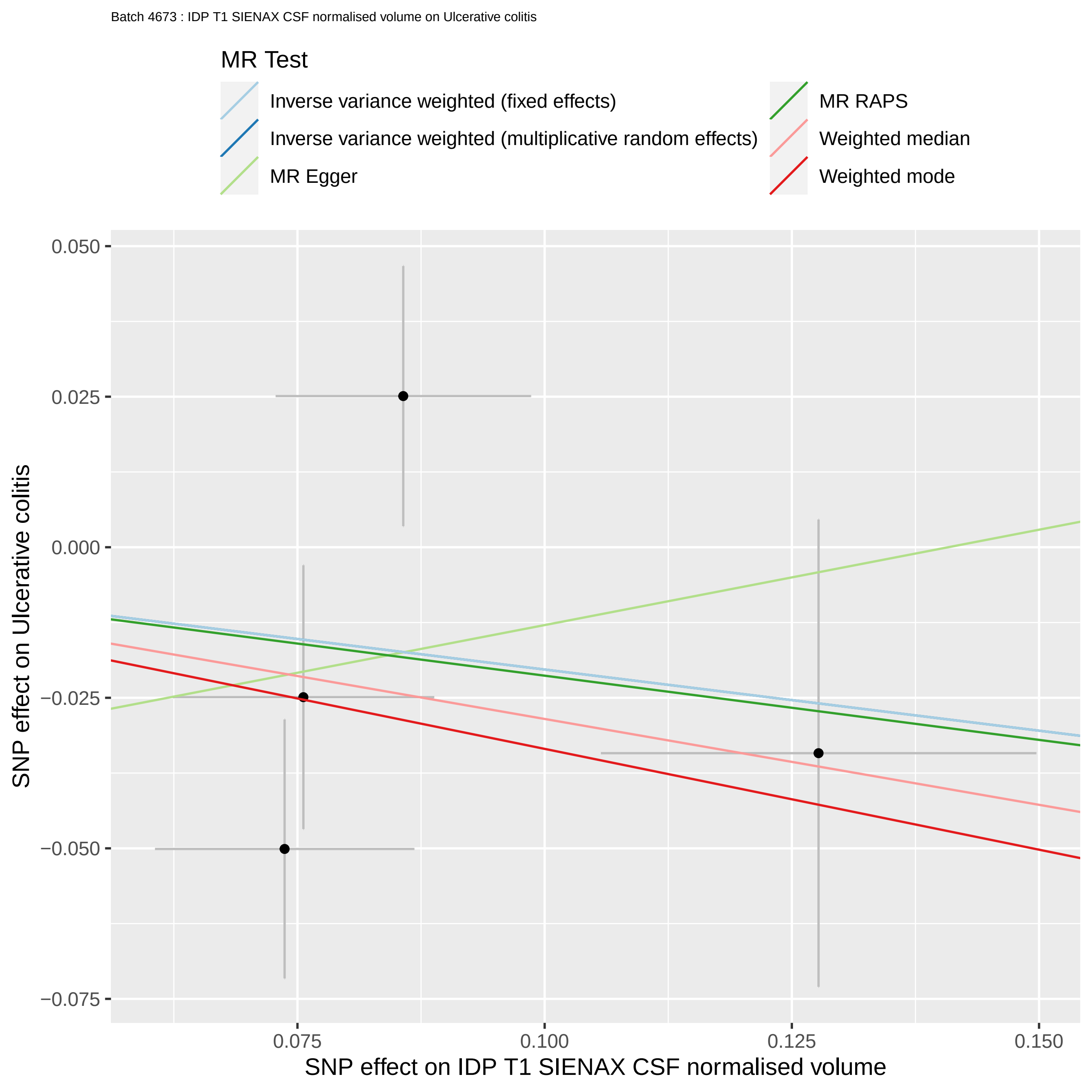

Batch 4673 : IDP T1 SIENAX CSF normalised volume on Ulcerative colitis
MR Test
Inverse variance weighted (fixed effects)
Inverse variance weighted (multiplicative random effects)
MR Egger
MR RAPS
Weighted median
Weighted mode
0.050
0.025
0.000
−0.025
−0.050
−0.075
SNP effect on Ulcerative colitis
0.075
0.100
0.125
0.150
SNP effect on IDP T1 SIENAX CSF normalised volume

Batch 4785 : IDP T1 FAST ROIs R sup front gyrus on Ulcerative colitis

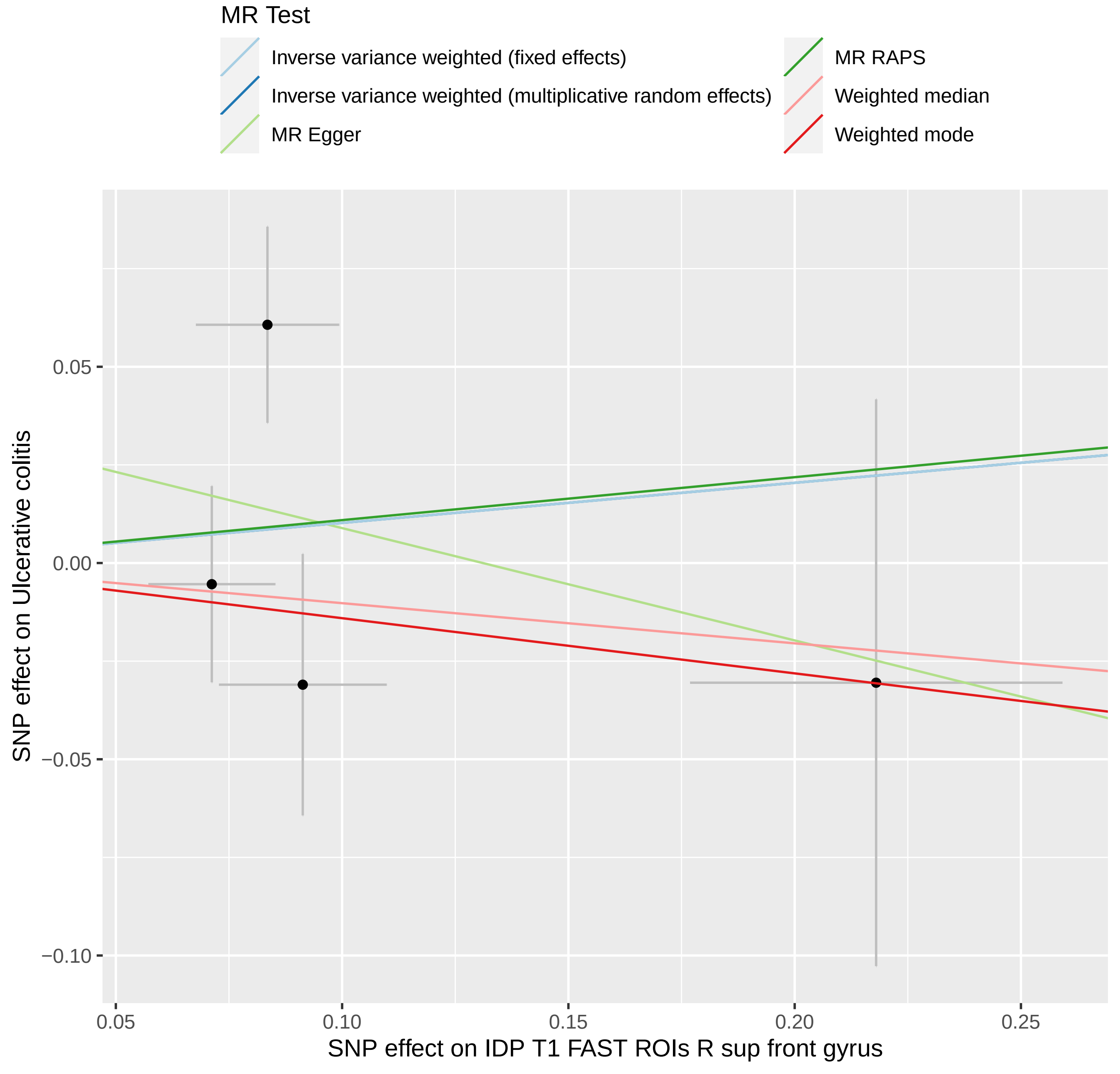

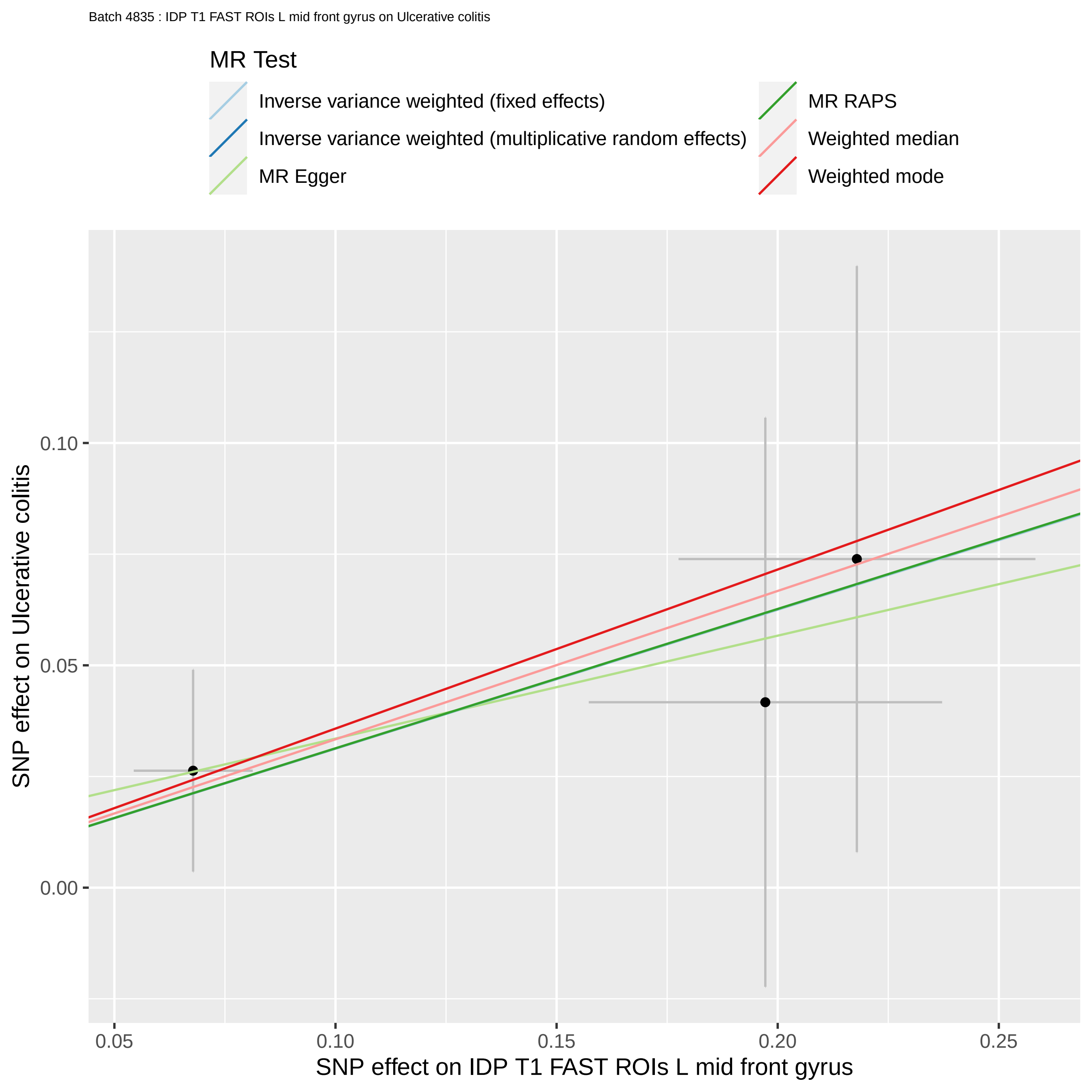

Batch 4835 : IDP T1 FAST ROIs L mid front gyrus on Ulcerative colitis
MR Test
Inverse variance weighted (fixed effects)
Inverse variance weighted (multiplicative random effects)
MR Egger
MR RAPS
Weighted median
Weighted mode
0.10
0.05
0.00
SNP effect on Ulcerative colitis
0.05
0.10
0.15
0.20
0.25
SNP effect on IDP T1 FAST ROIs L mid front gyrus

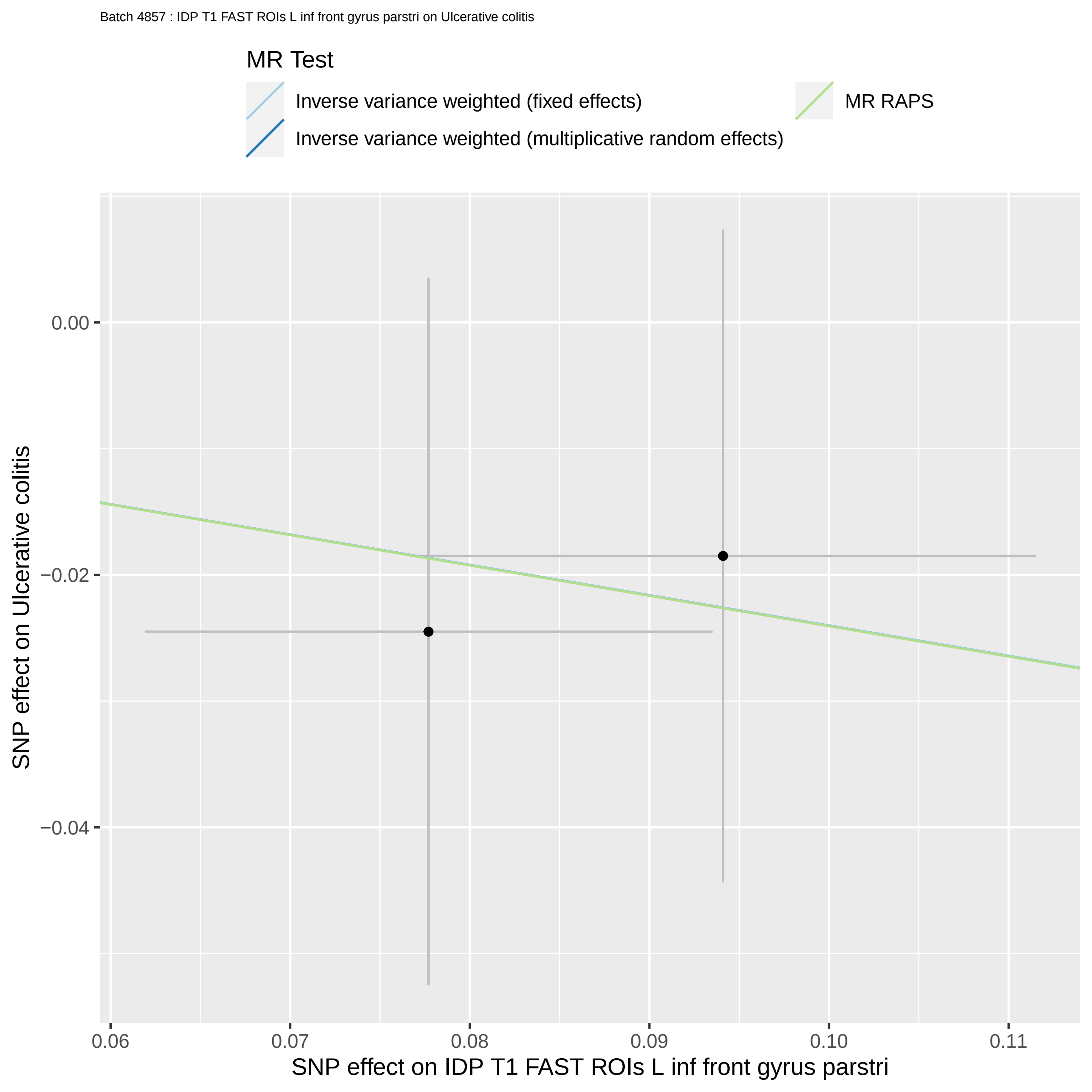

Batch 4857 : IDP T1 FAST ROIs L inf front gyrus parstri on Ulcerative colitis
MR Test
Inverse variance weighted (fixed effects)
Inverse variance weighted (multiplicative random effects)
MR RAPS
0.00
−0.02
−0.04
SNP effect on Ulcerative colitis
0.06
0.07
0.08
0.09
0.10
0.11
SNP effect on IDP T1 FAST ROIs L inf front gyrus parstri

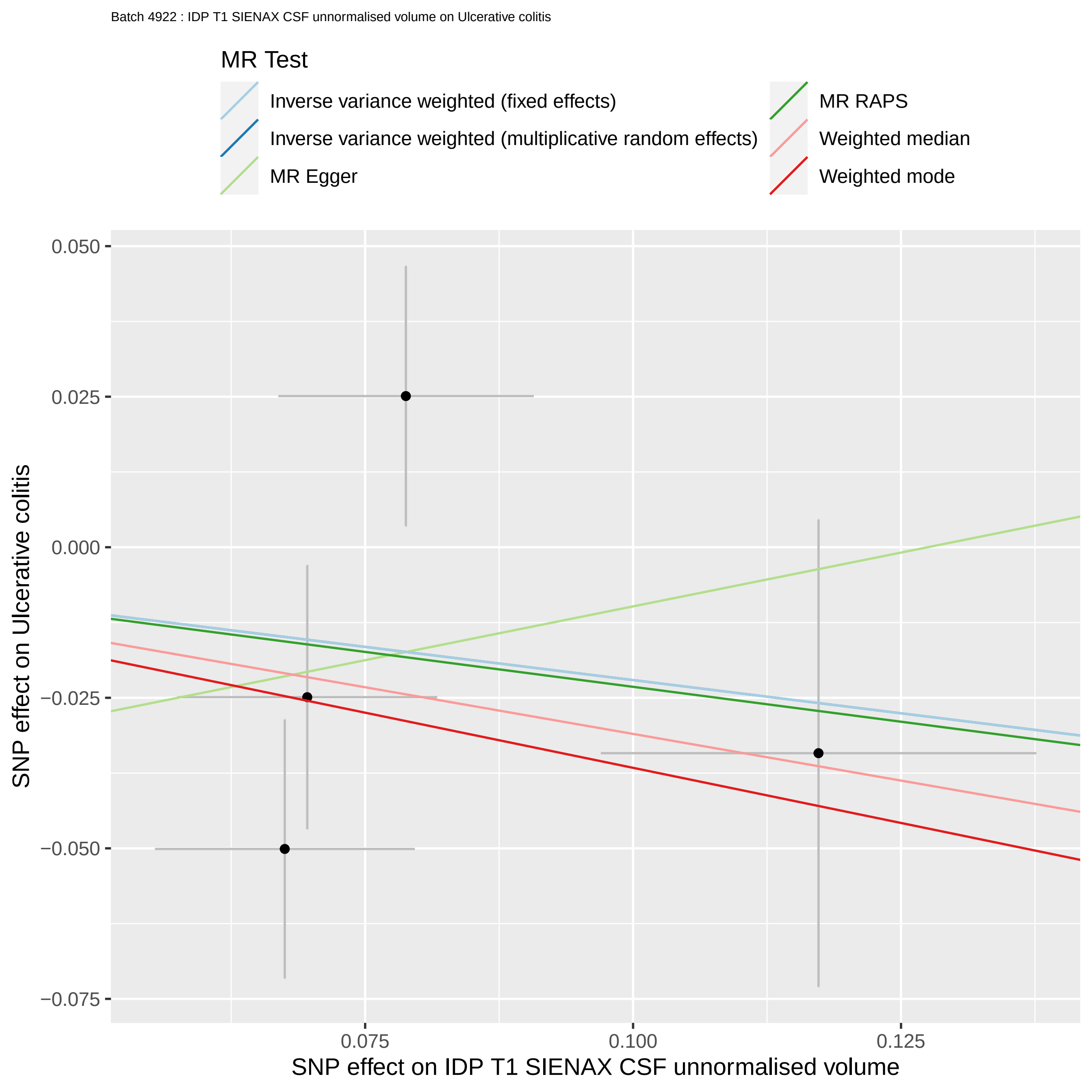

Batch 4922 : IDP T1 SIENAX CSF unnormalised volume on Ulcerative colitis
MR Test
Inverse variance weighted (fixed effects)
Inverse variance weighted (multiplicative random effects)
MR Egger
MR RAPS
Weighted median
Weighted mode
0.050
0.025
0.000
−0.025
−0.050
−0.075
SNP effect on Ulcerative colitis
0.075
0.100
0.125
SNP effect on IDP T1 SIENAX CSF unnormalised volume

Batch 4944 : IDP T1 FAST ROIs L sup temp gyrus ant on Ulcerative colitis

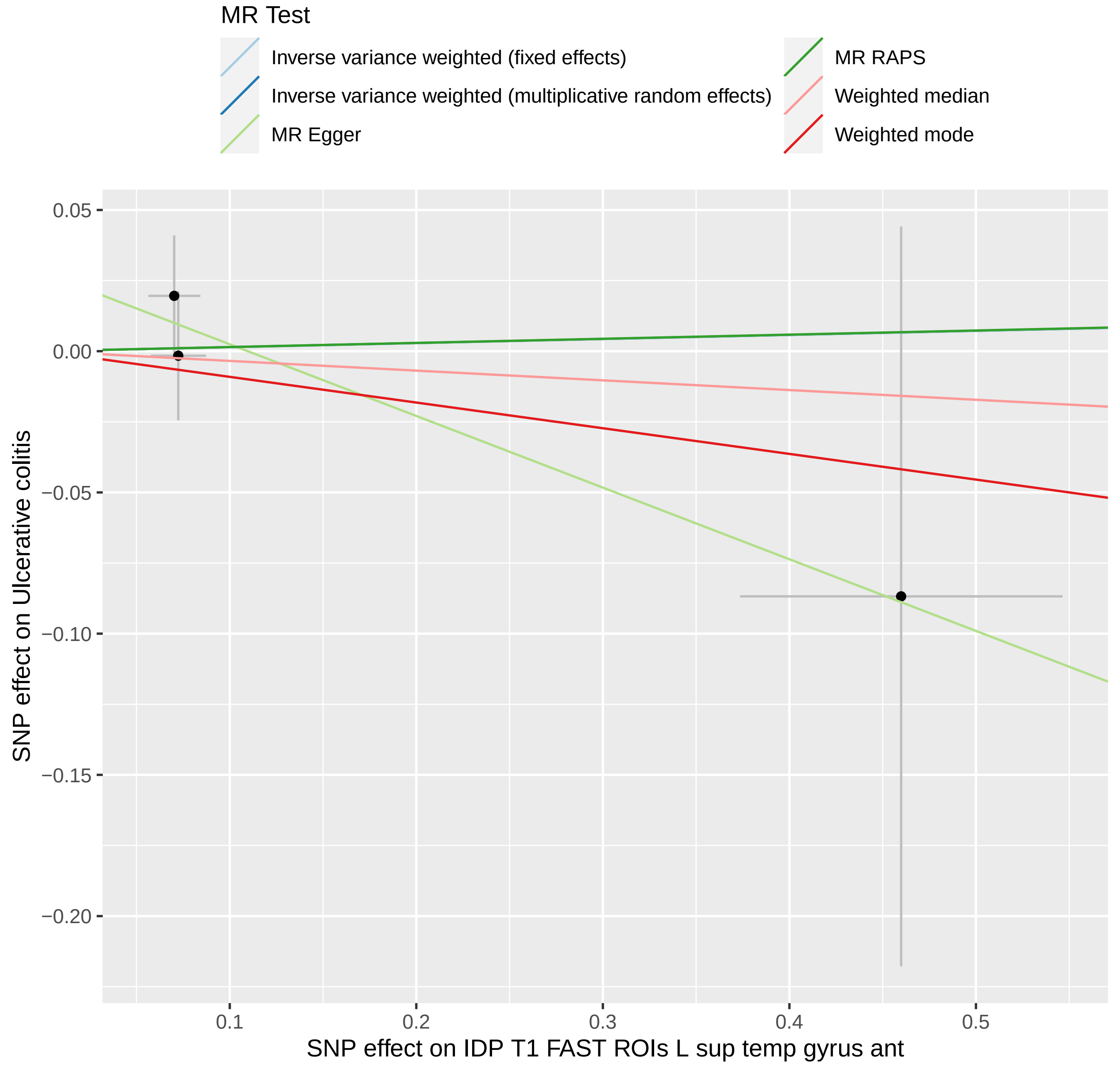

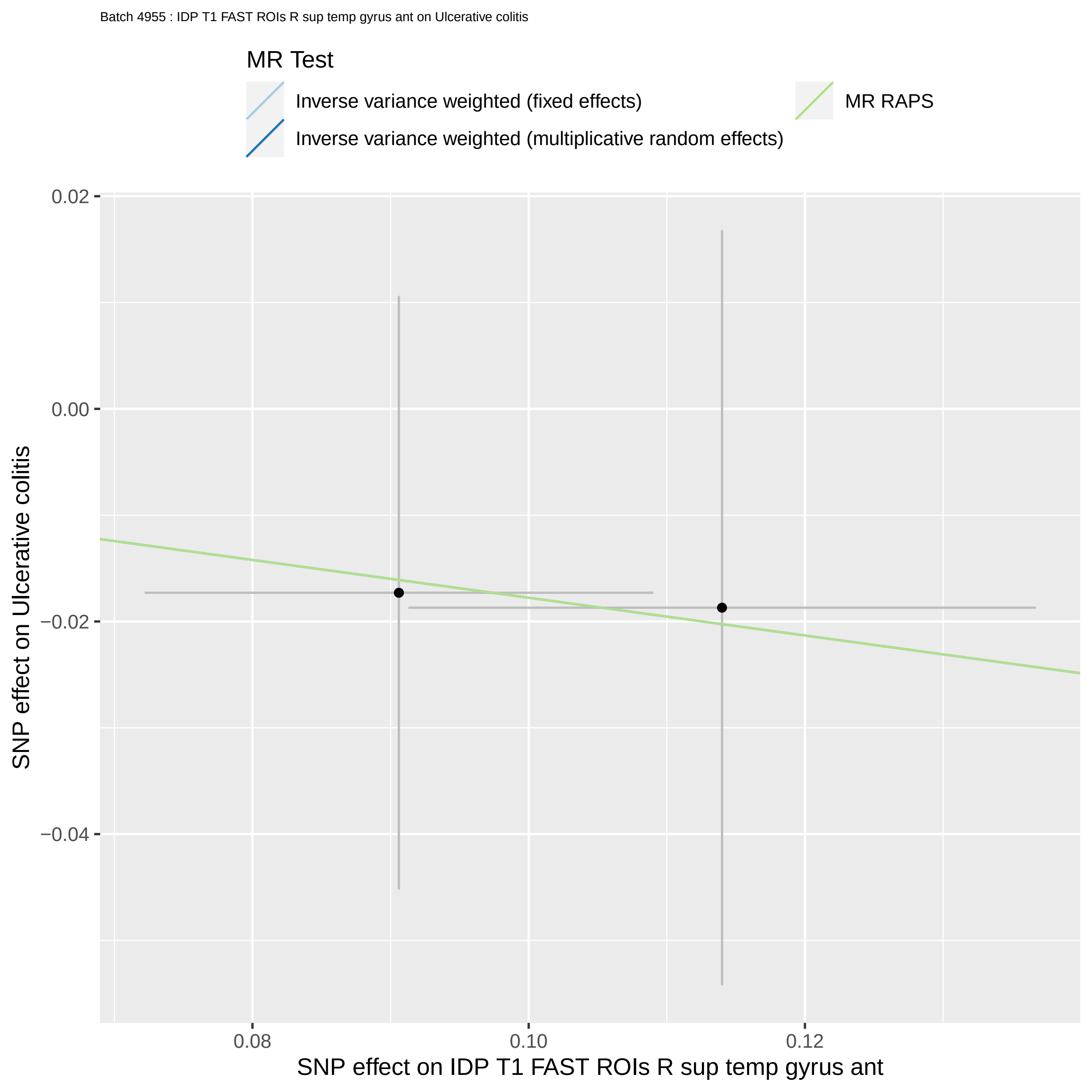

Batch 4955 : IDP T1 FAST ROIs R sup temp gyrus ant on Ulcerative colitis
MR Test
Inverse variance weighted (fixed effects)
Inverse variance weighted (multiplicative random effects)
MR RAPS
0.02
0.00
−0.02
−0.04
0.08
0.10
0.12
SNP effect on Ulcerative colitis
SNP effect on IDP T1 FAST ROIs R sup temp gyrus ant

Batch 4966 : IDP T1 FAST ROIs L sup temp gyrus post on Ulcerative colitis

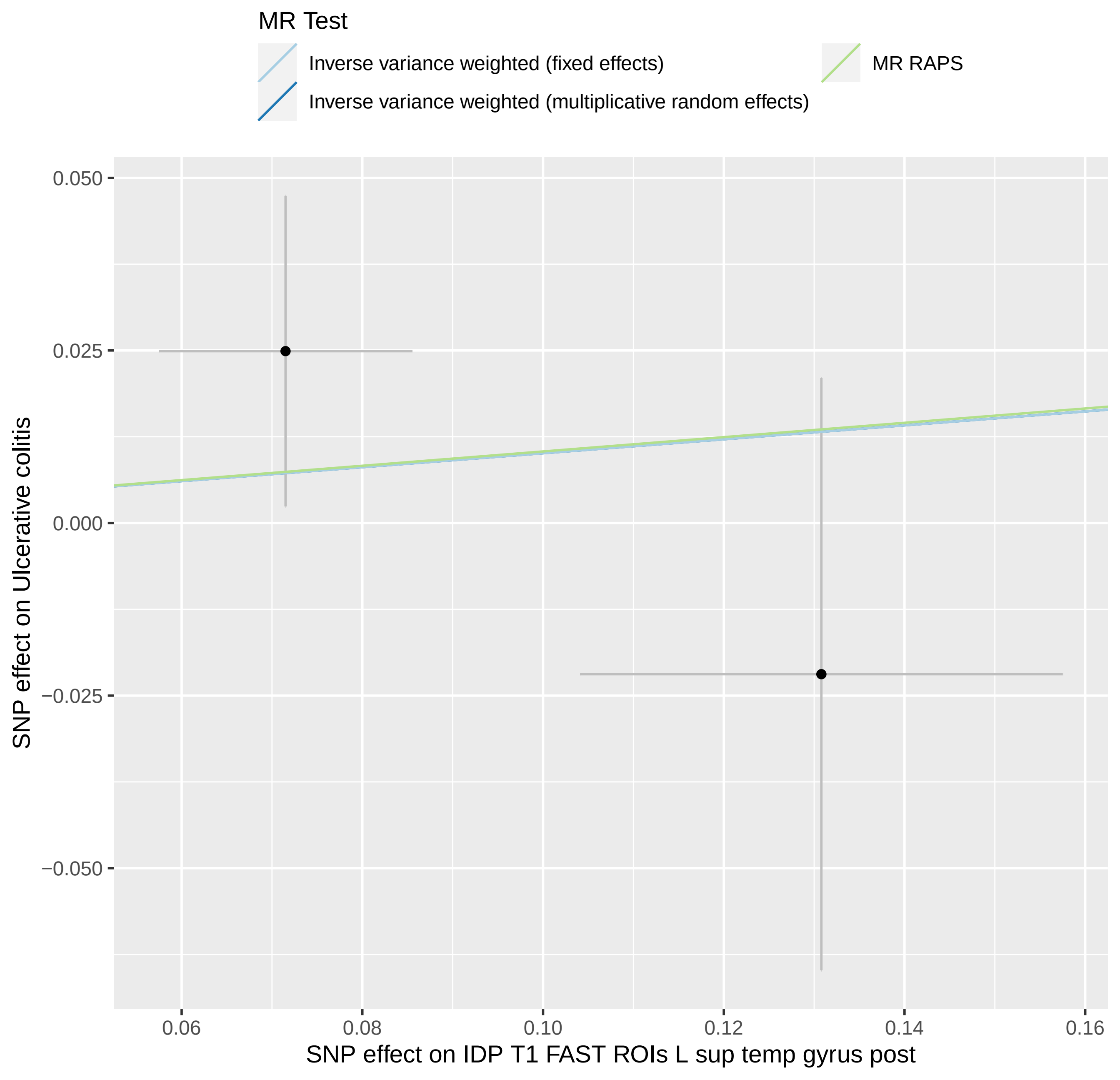

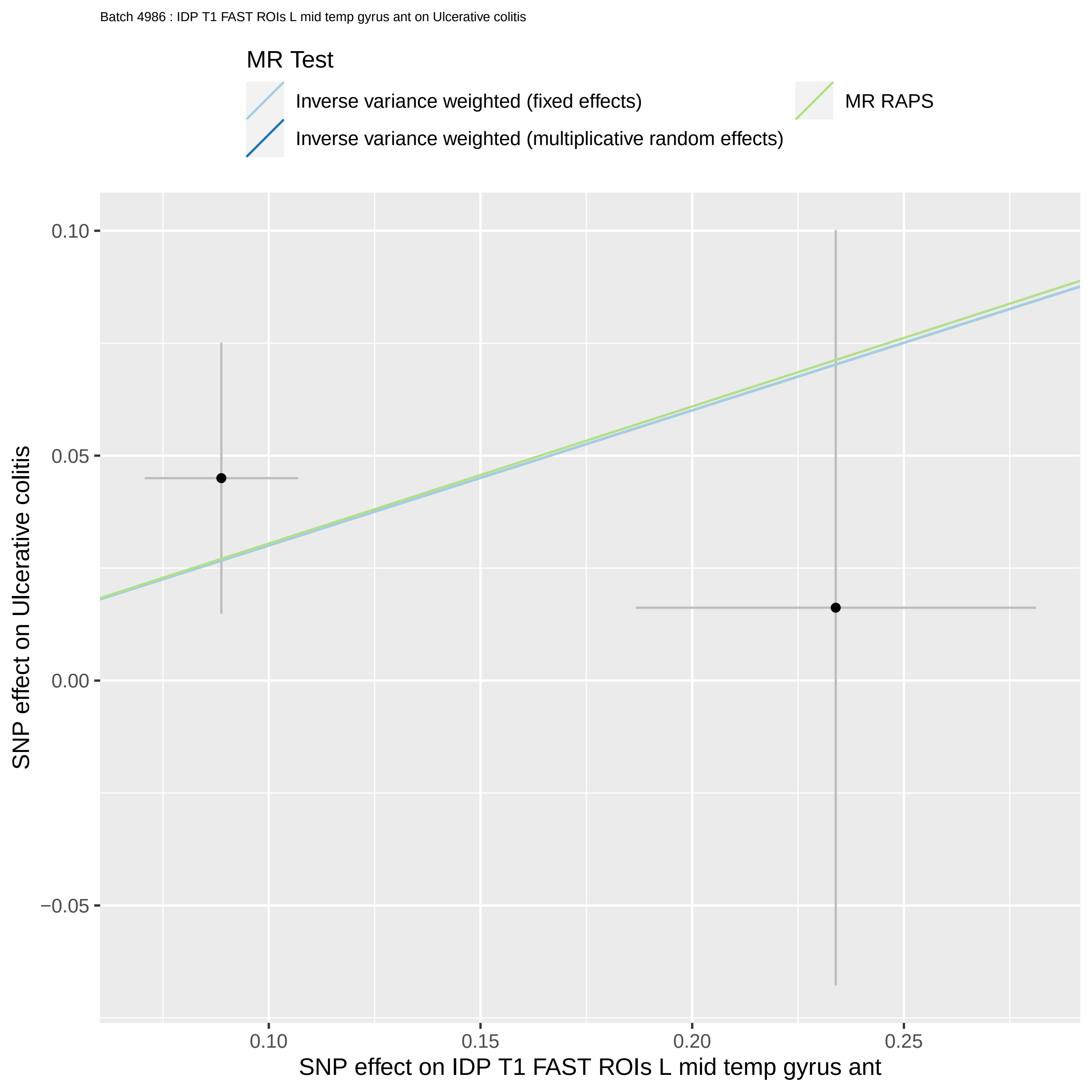

Batch 4986 : IDP T1 FAST ROIs L mid temp gyrus ant on Ulcerative colitis
MR Test
Inverse variance weighted (fixed effects)
Inverse variance weighted (multiplicative random effects)
MR RAPS
0.10
0.05
0.00
−0.05
SNP effect on Ulcerative colitis
0.10
0.15
0.20
0.25
SNP effect on IDP T1 FAST ROIs L mid temp gyrus ant

Batch 5030 : IDP T1 SIENAX grey normalised volume on Ulcerative colitis

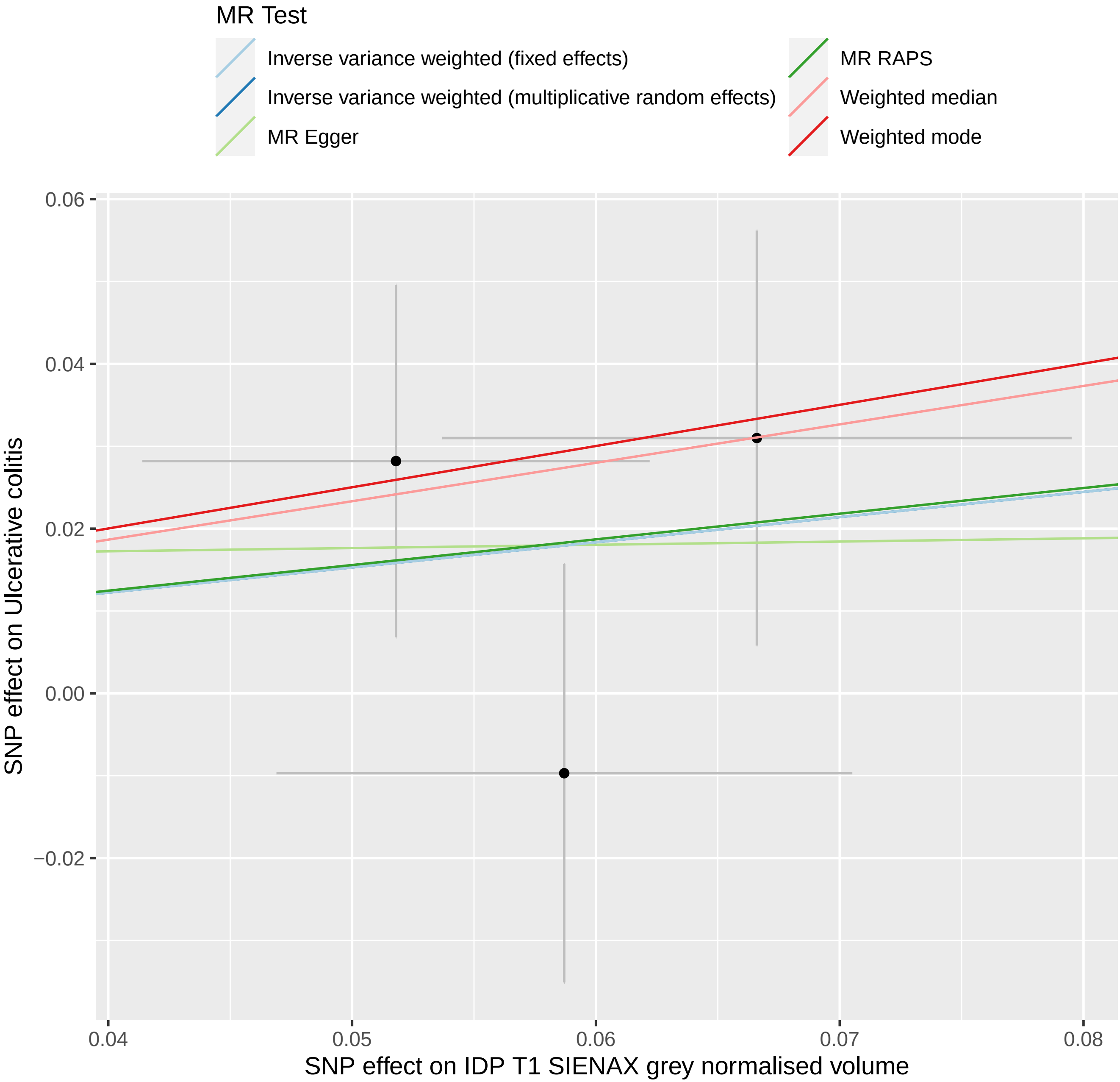

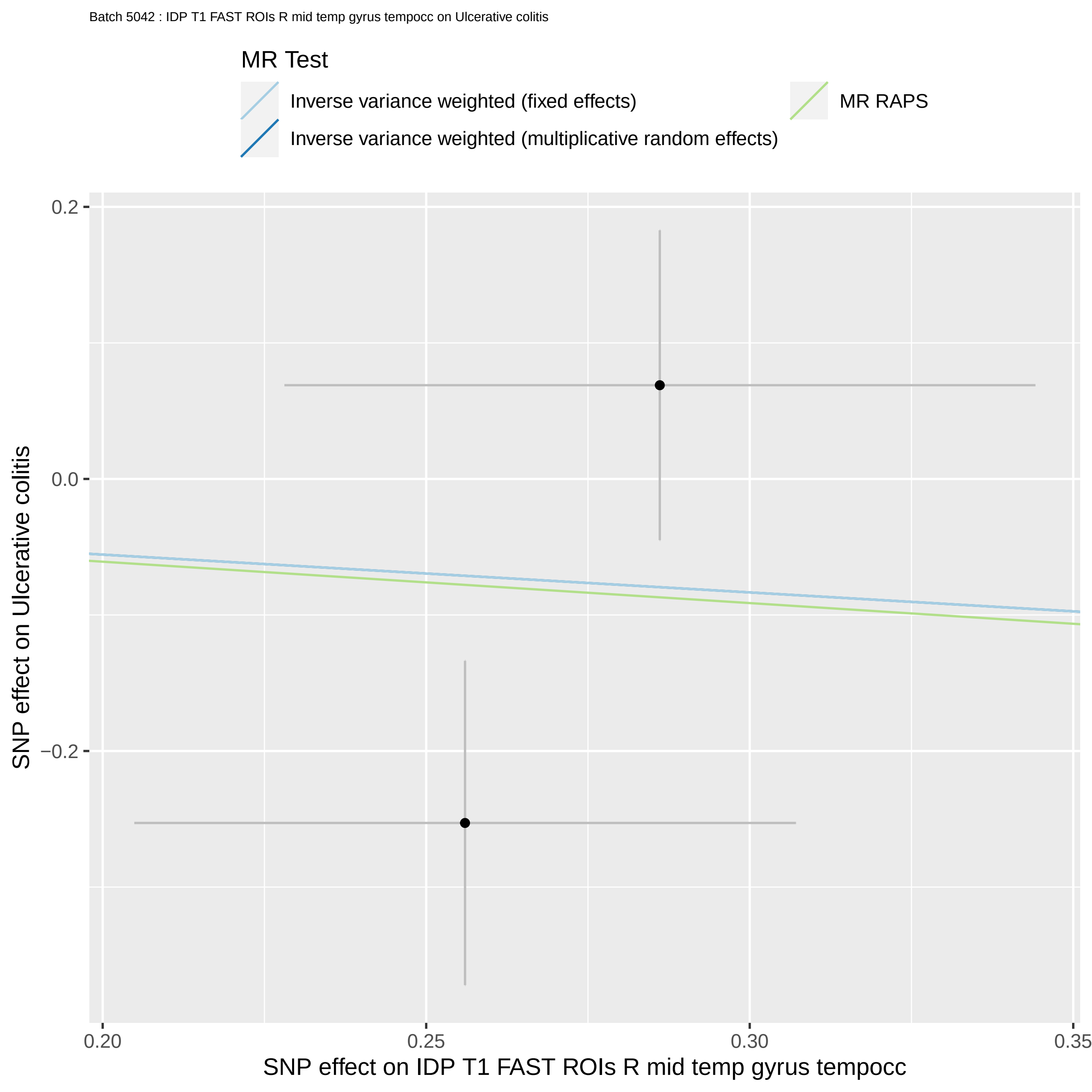

Batch 5042 : IDP T1 FAST ROIs R mid temp gyrus tempocc on Ulcerative colitis
MR Test
Inverse variance weighted (fixed effects)
Inverse variance weighted (multiplicative random effects)
MR RAPS
0.2
0.0
−0.2
0.20
0.25
0.30
0.35
SNP effect on Ulcerative colitis
SNP effect on IDP T1 FAST ROIs R mid temp gyrus tempocc

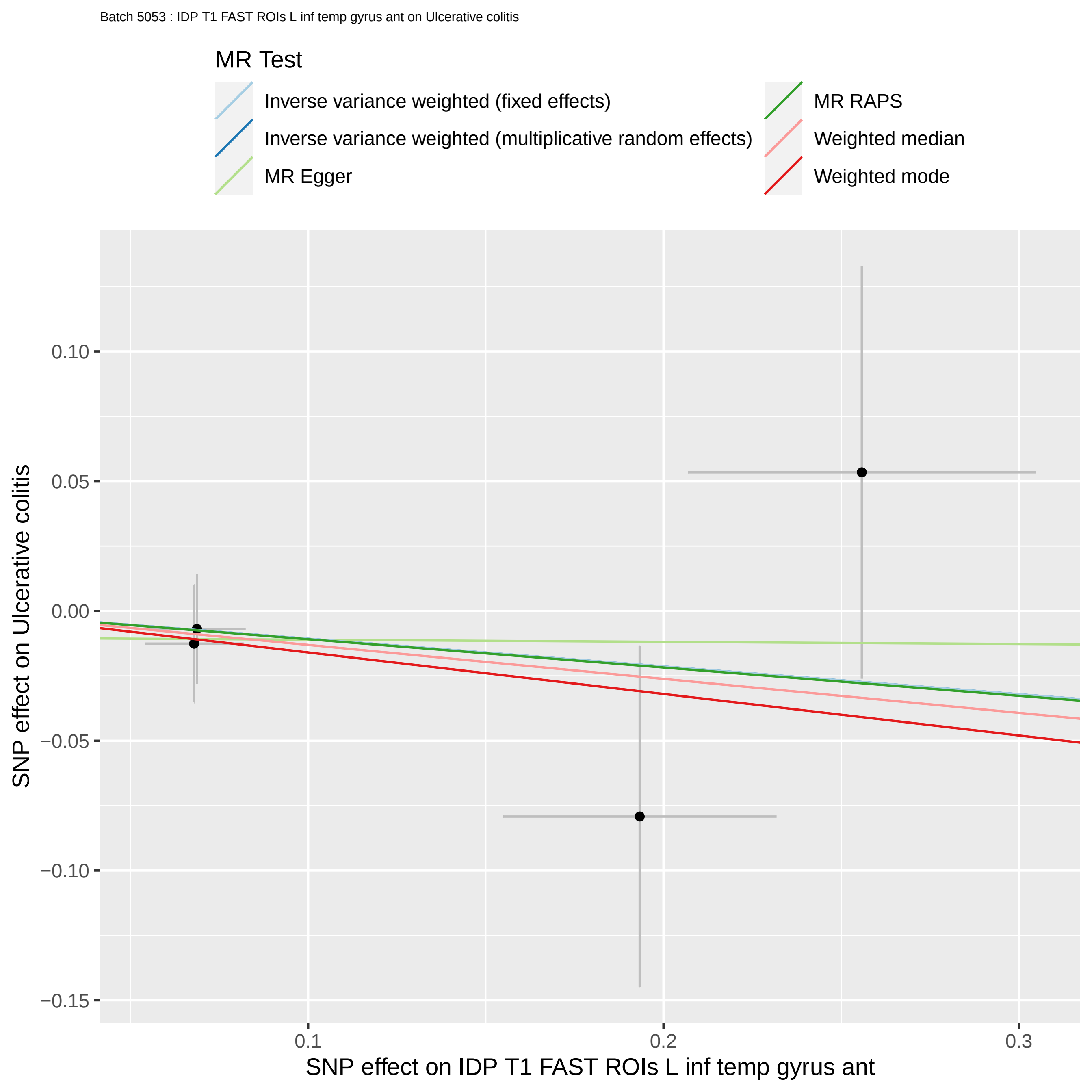
Batch 5053 : IDP T1 FAST ROIs L inf temp gyrus ant on Ulcerative colitis
MR Test
Inverse variance weighted (fixed effects)
Inverse variance weighted (multiplicative random effects)
MR Egger
MR RAPS
Weighted median
Weighted mode
0.10
0.05
0.00
−0.05
−0.10
−0.15
SNP effect on Ulcerative colitis
0.1
0.2
0.3
SNP effect on IDP T1 FAST ROIs L inf temp gyrus ant

Batch 5064 : IDP T1 FAST ROIs R inf temp gyrus ant on Ulcerative colitis

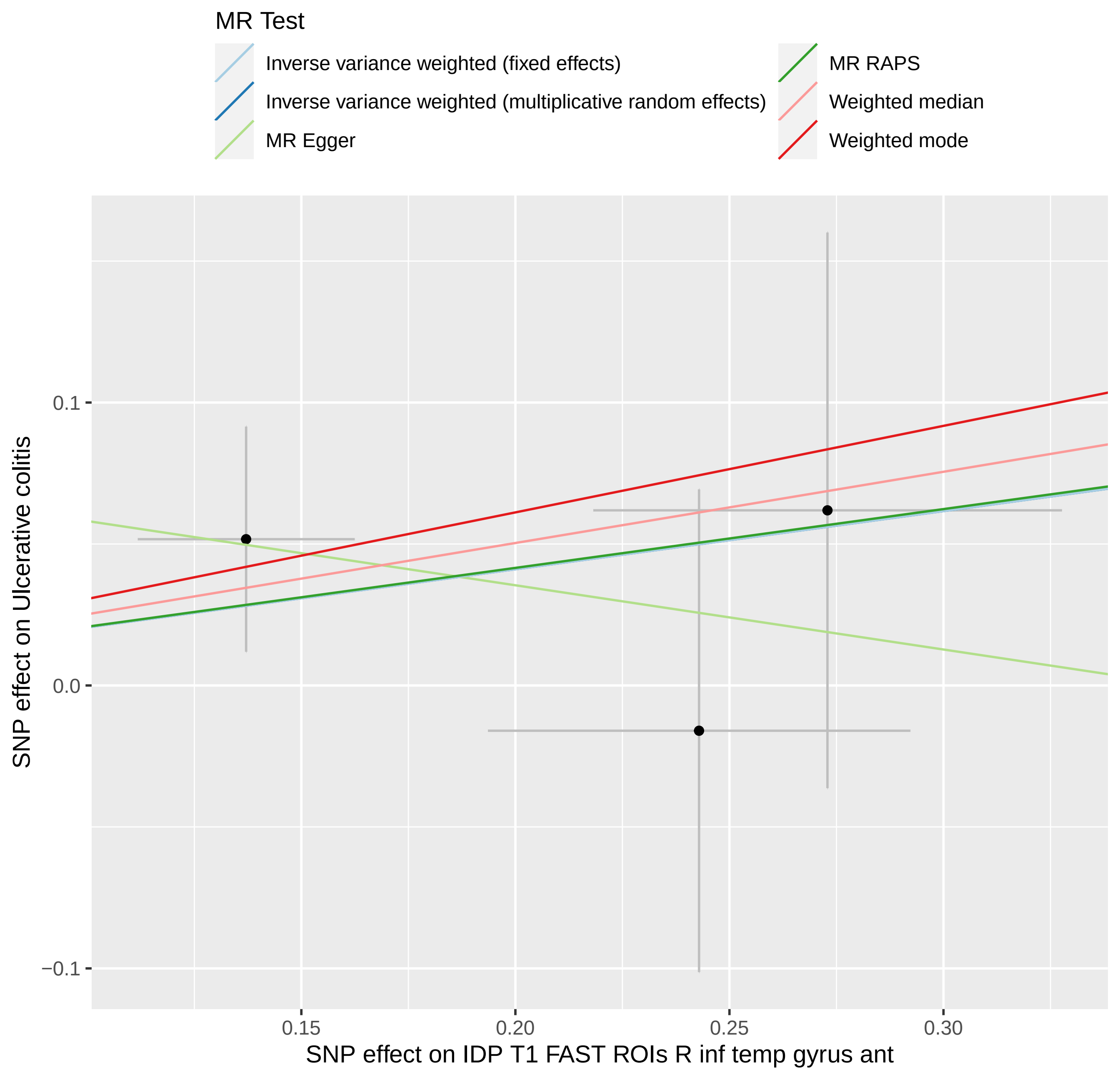

Batch 5075 : IDP T1 FAST ROIs L inf temp gyrus post on Ulcerative colitis

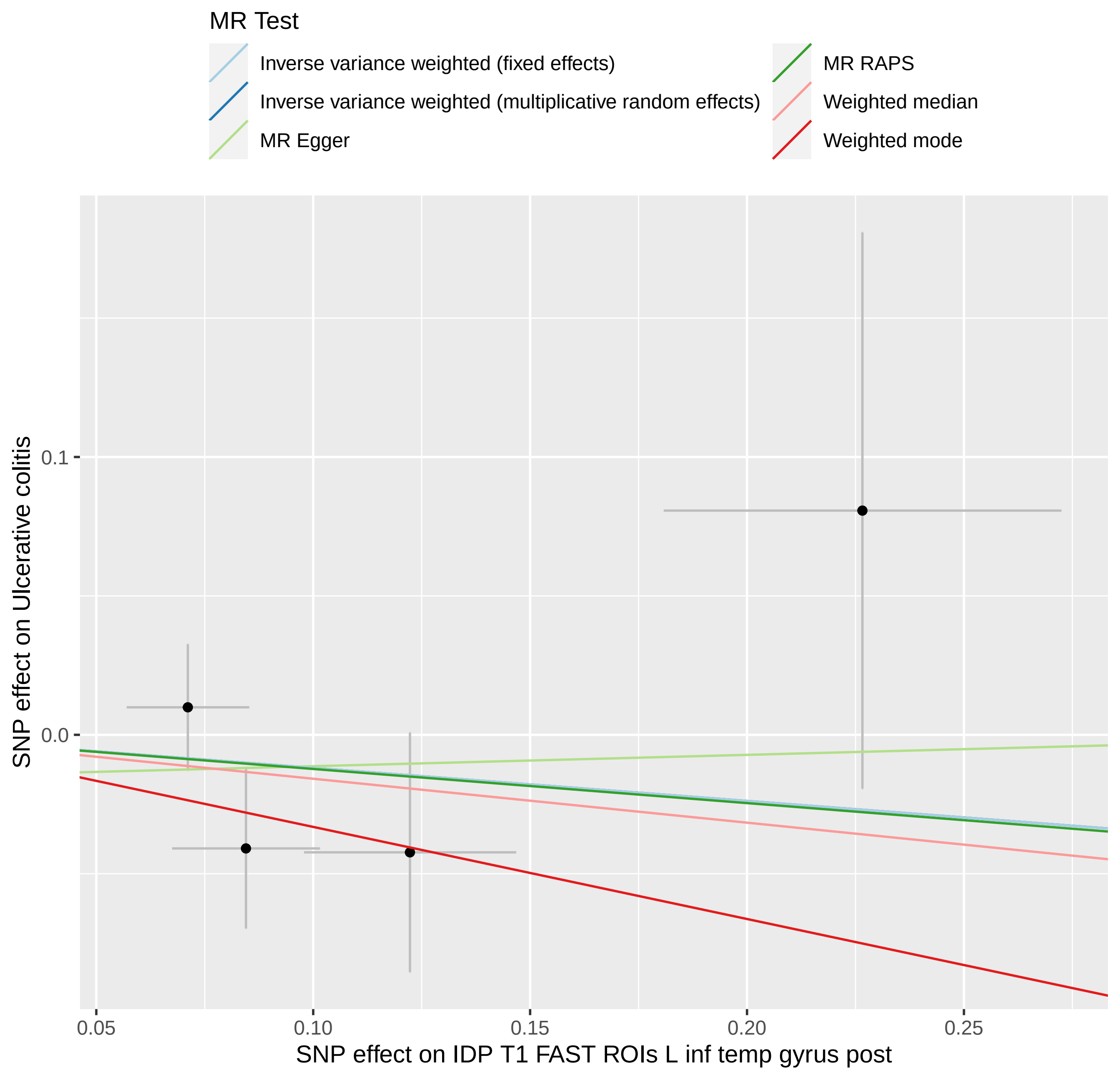

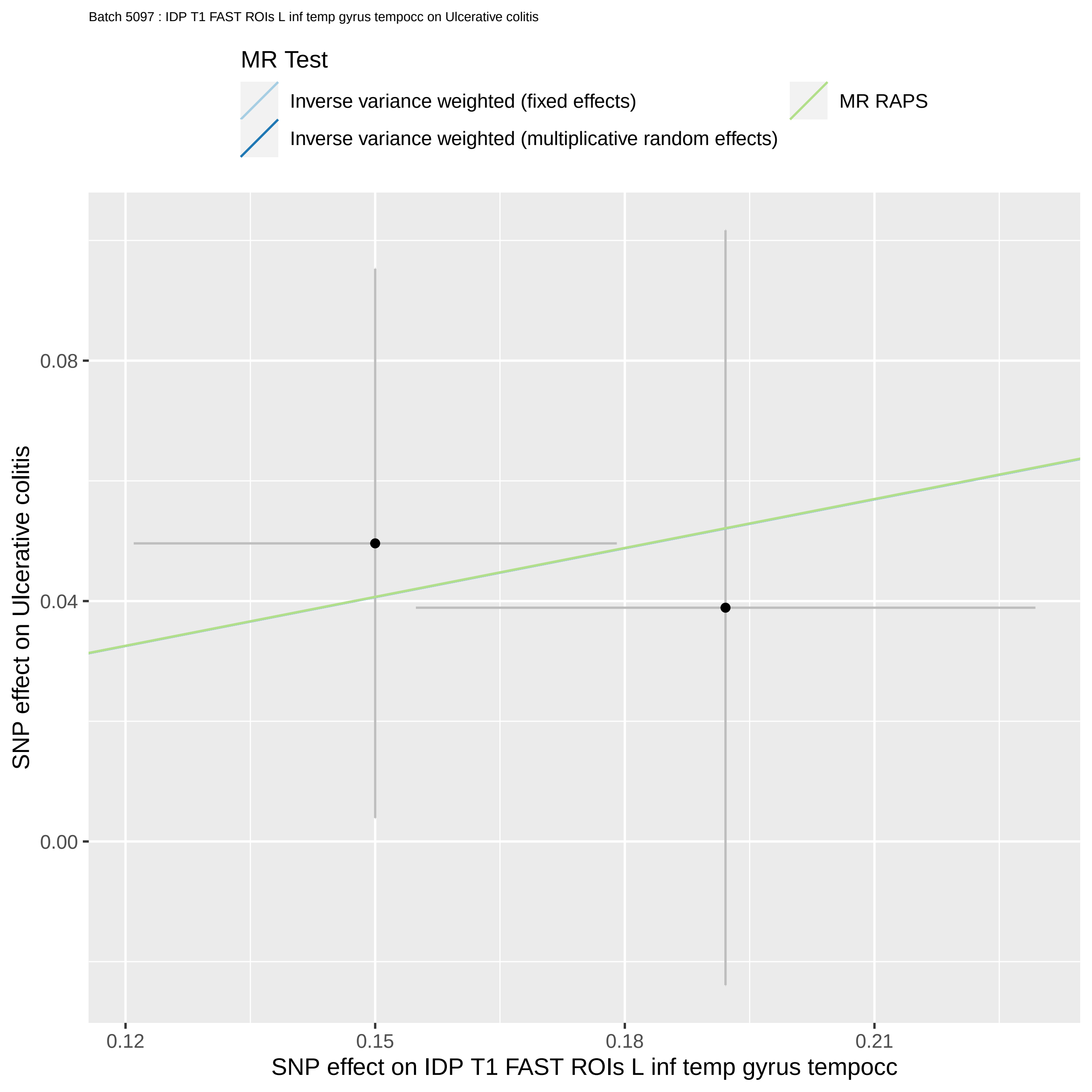

Batch 5097 : IDP T1 FAST ROIs L inf temp gyrus tempocc on Ulcerative colitis
MR Test
Inverse variance weighted (fixed effects)
Inverse variance weighted (multiplicative random effects)
MR RAPS
0.08
0.04
0.00
0.12
0.15
0.18
0.21
SNP effect on Ulcerative colitis
SNP effect on IDP T1 FAST ROIs L inf temp gyrus tempocc

Batch 5108 : IDP T1 FAST ROIs R inf temp gyrus tempocc on Ulcerative colitis

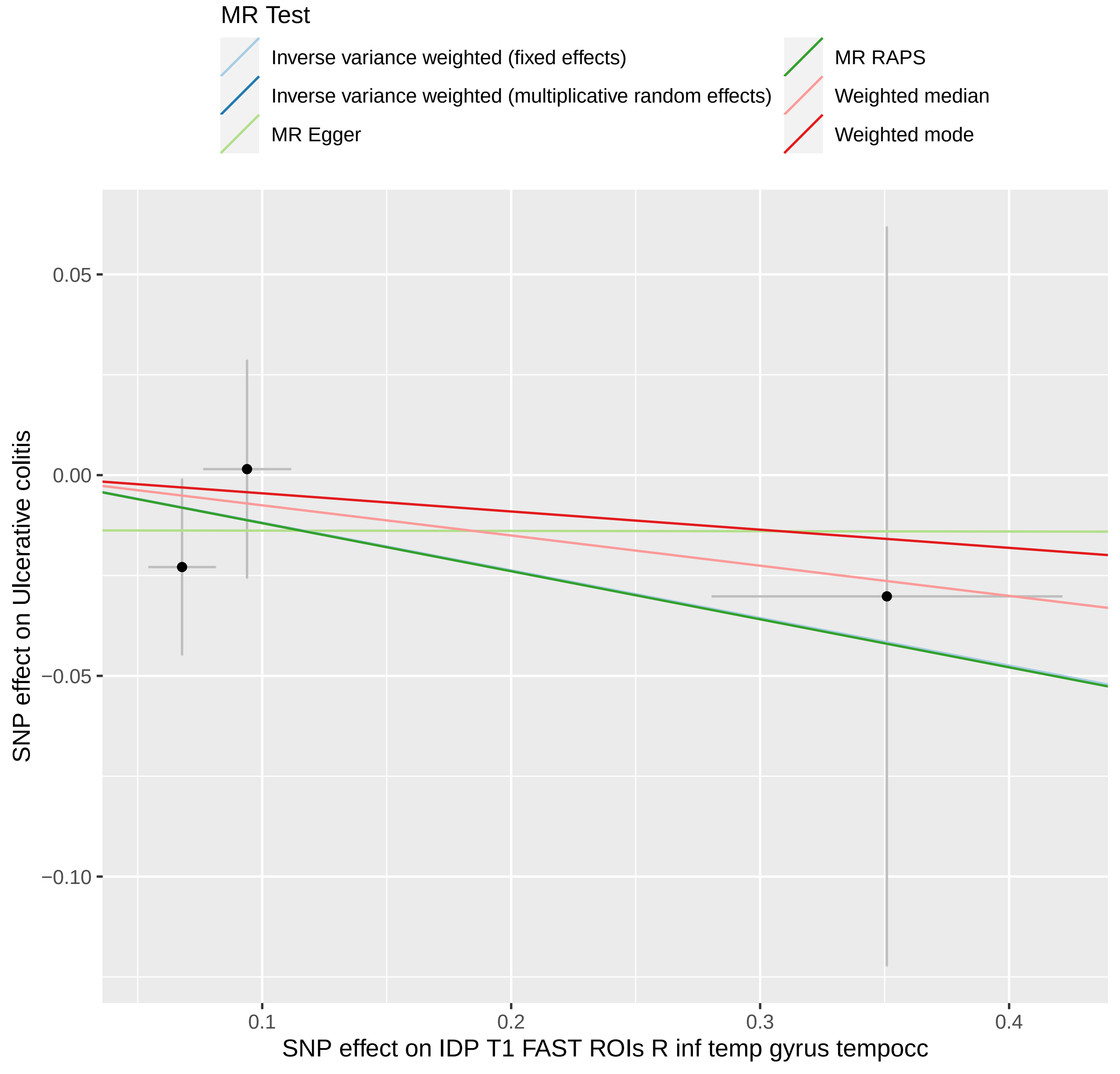

Batch 5119 : IDP T1 FAST ROIs L postcent gyrus on Ulcerative colitis

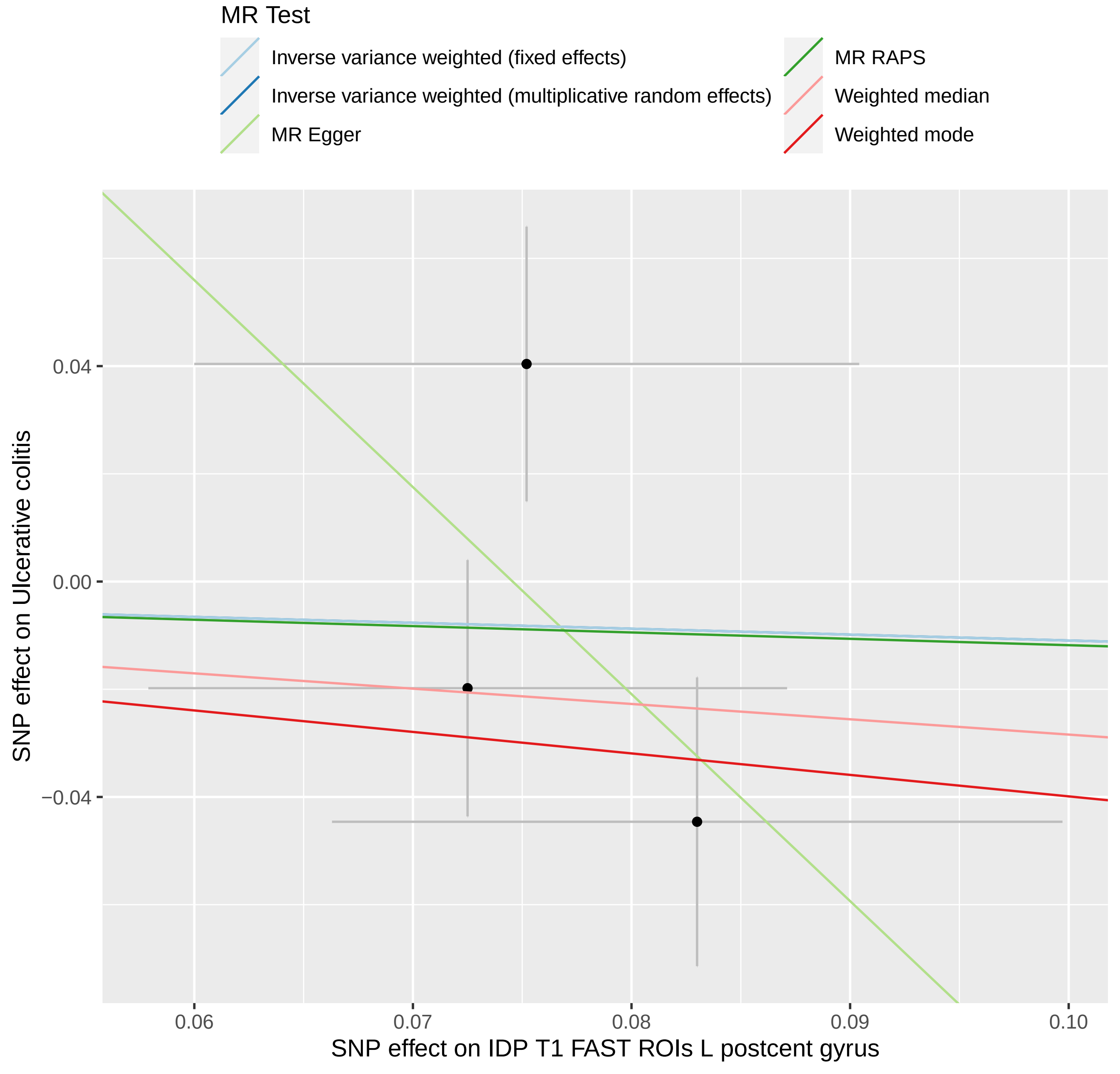

Batch 5141 : IDP T1 SIENAX grey unnormalised volume on Ulcerative colitis

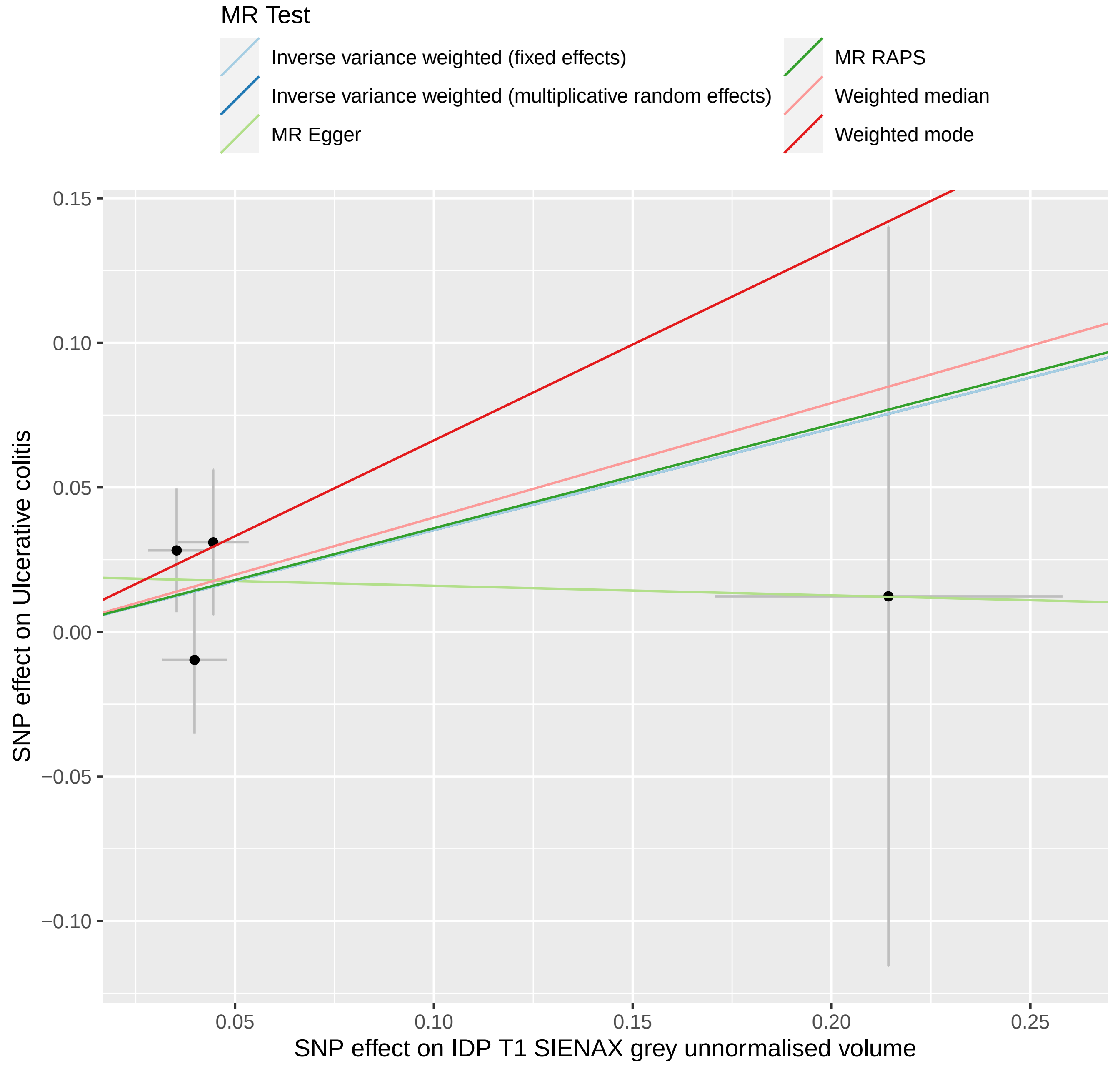

Batch 5142 : IDP T1 FAST ROIs L sup parietal lobule on Ulcerative colitis

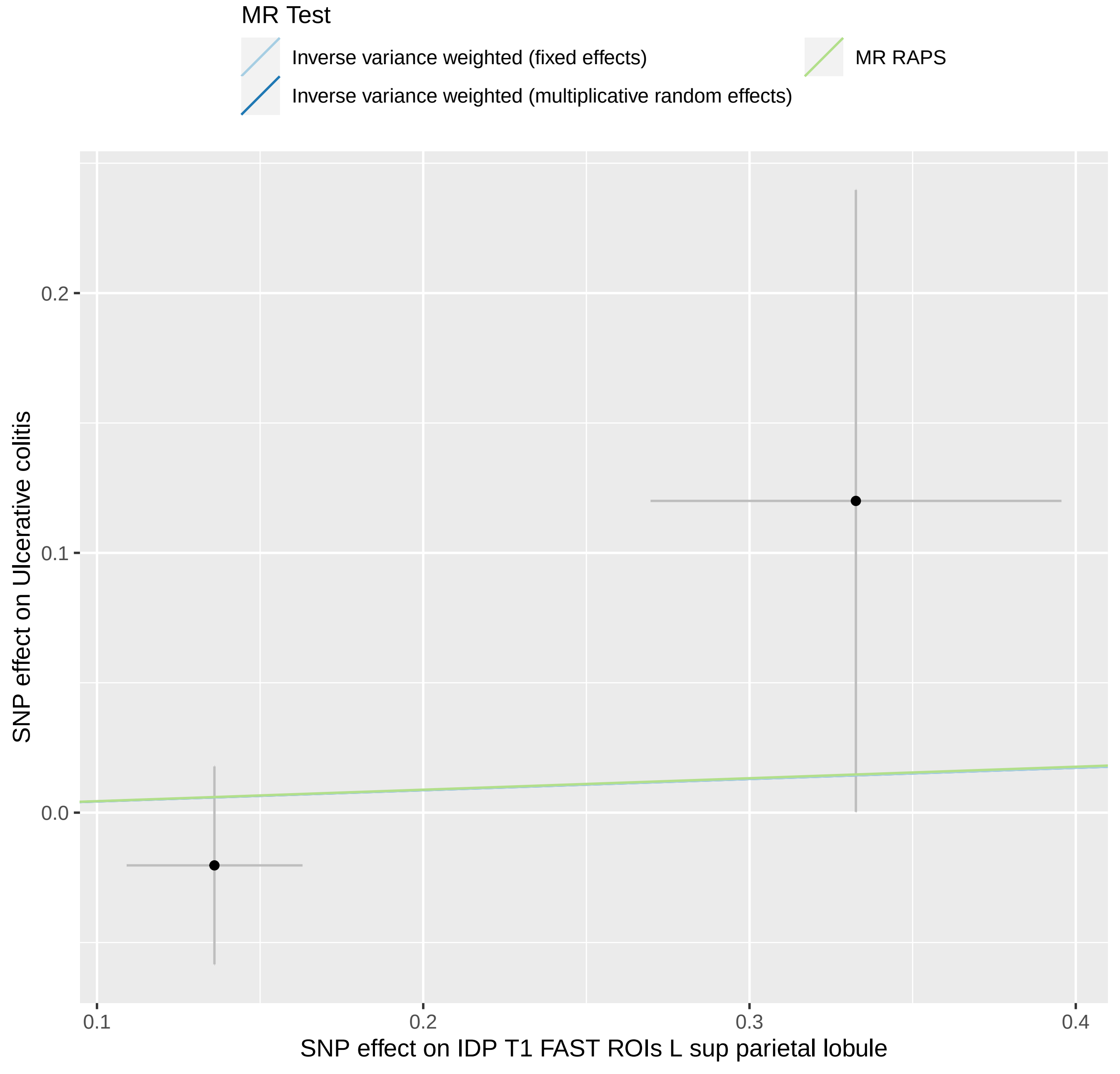

Batch 5164 : IDP T1 FAST ROIs L supramarg gyrus ant on Ulcerative colitis

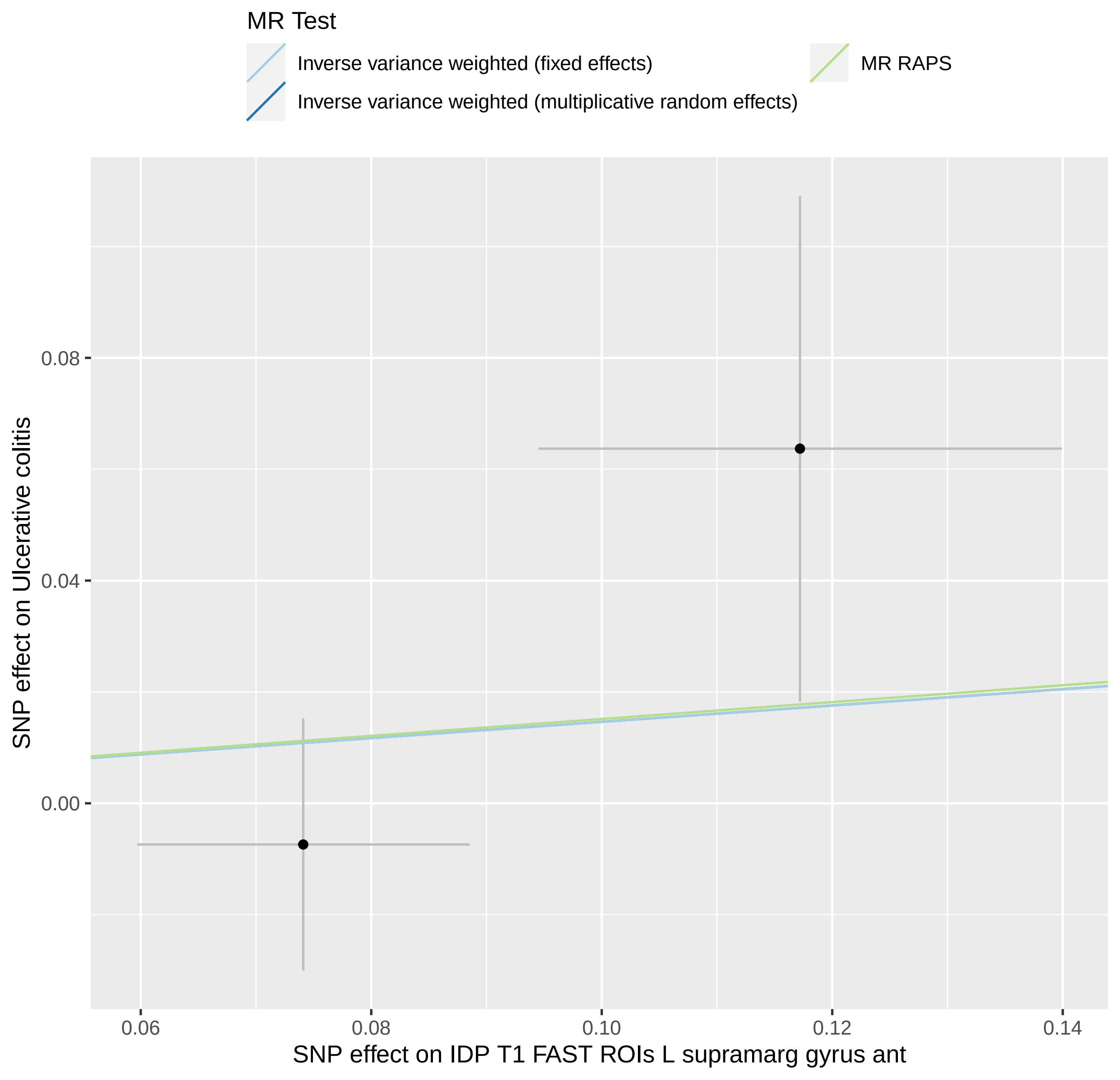

Batch 5208 : IDP T1 FAST ROIs L angular gyrus on Ulcerative colitis

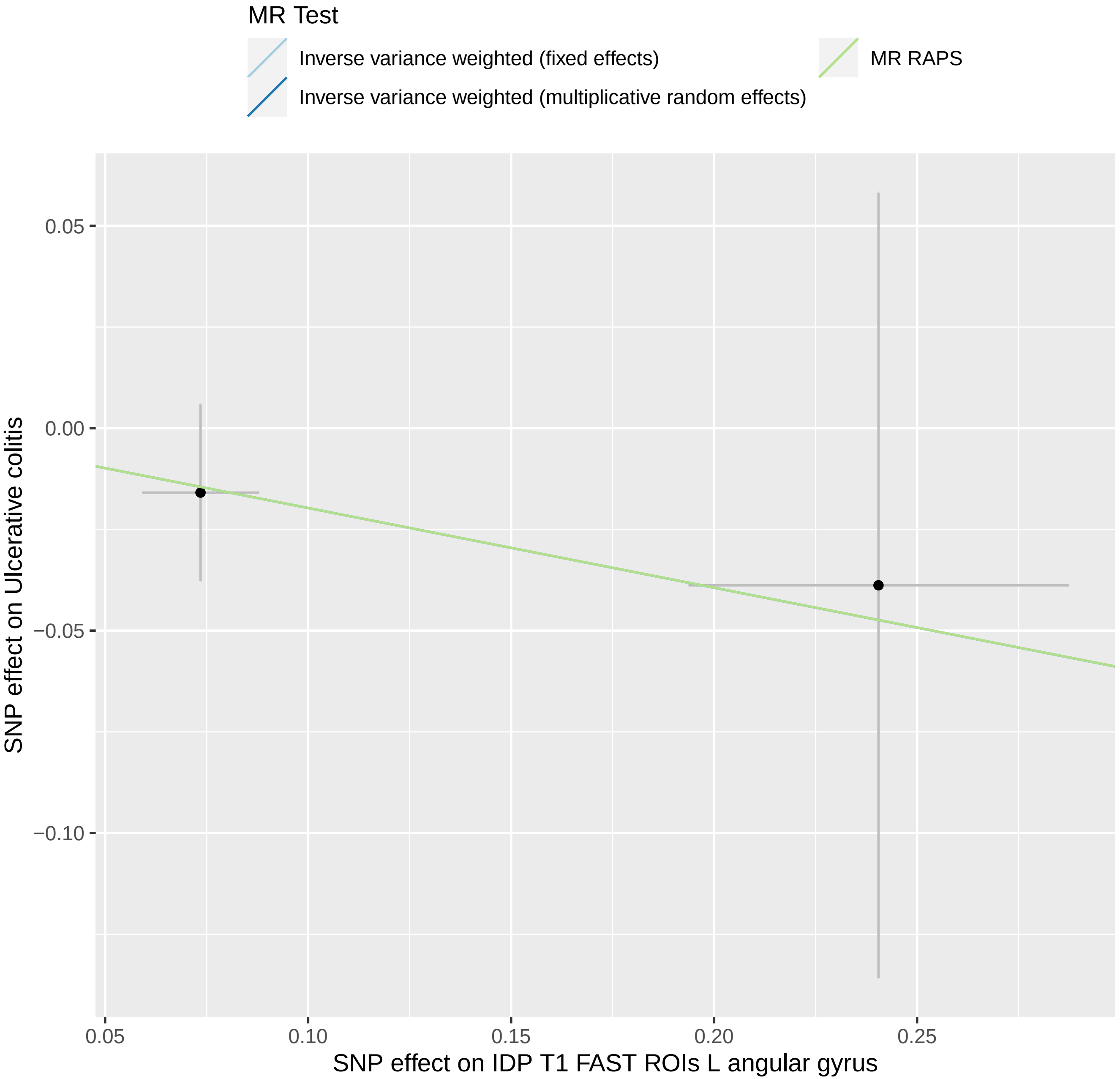

Batch 5230 : IDP T1 FAST ROIs L latocc cortex sup on Ulcerative colitis

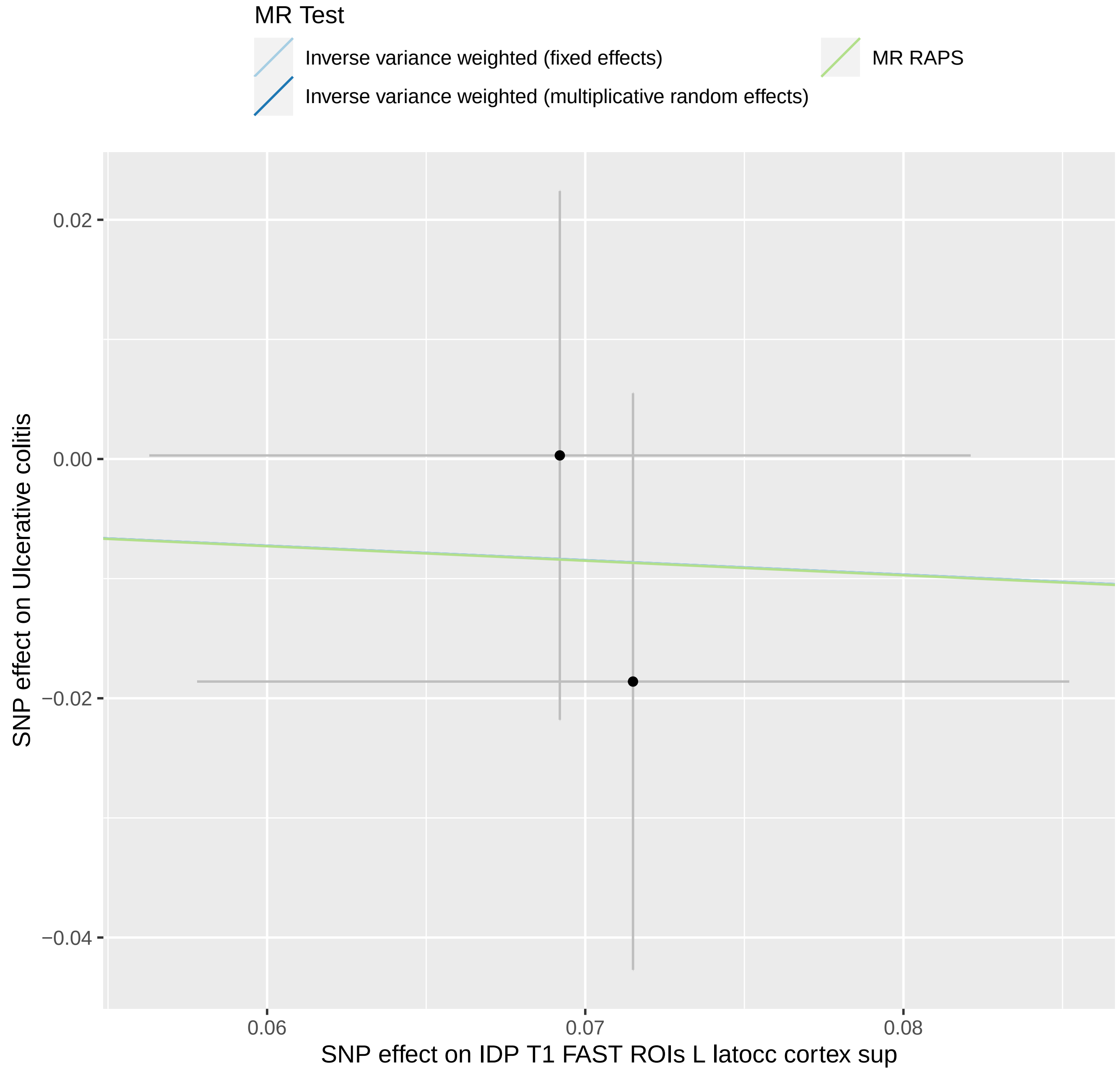

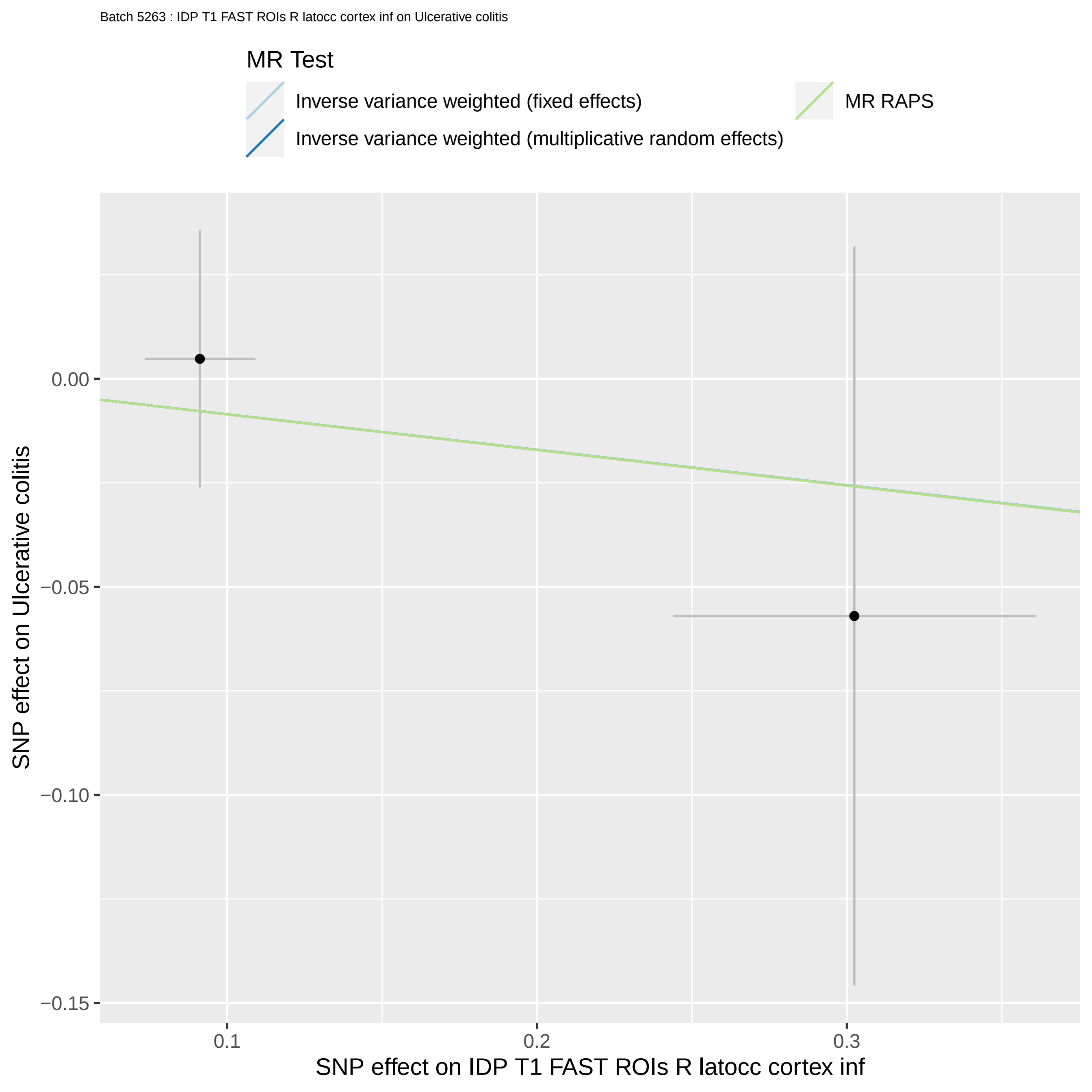
Batch 5263 : IDP T1 FAST ROIs R latocc cortex inf on Ulcerative colitis
MR Test
Inverse variance weighted (fixed effects)
Inverse variance weighted (multiplicative random effects)
MR RAPS
0.00
−0.05
−0.10
−0.15
0.1
0.2
0.3
SNP effect on Ulcerative colitis
SNP effect on IDP T1 FAST ROIs R latocc cortex inf

Batch 5274 : IDP T1 FAST ROIs L intracalc cortex on Ulcerative colitis

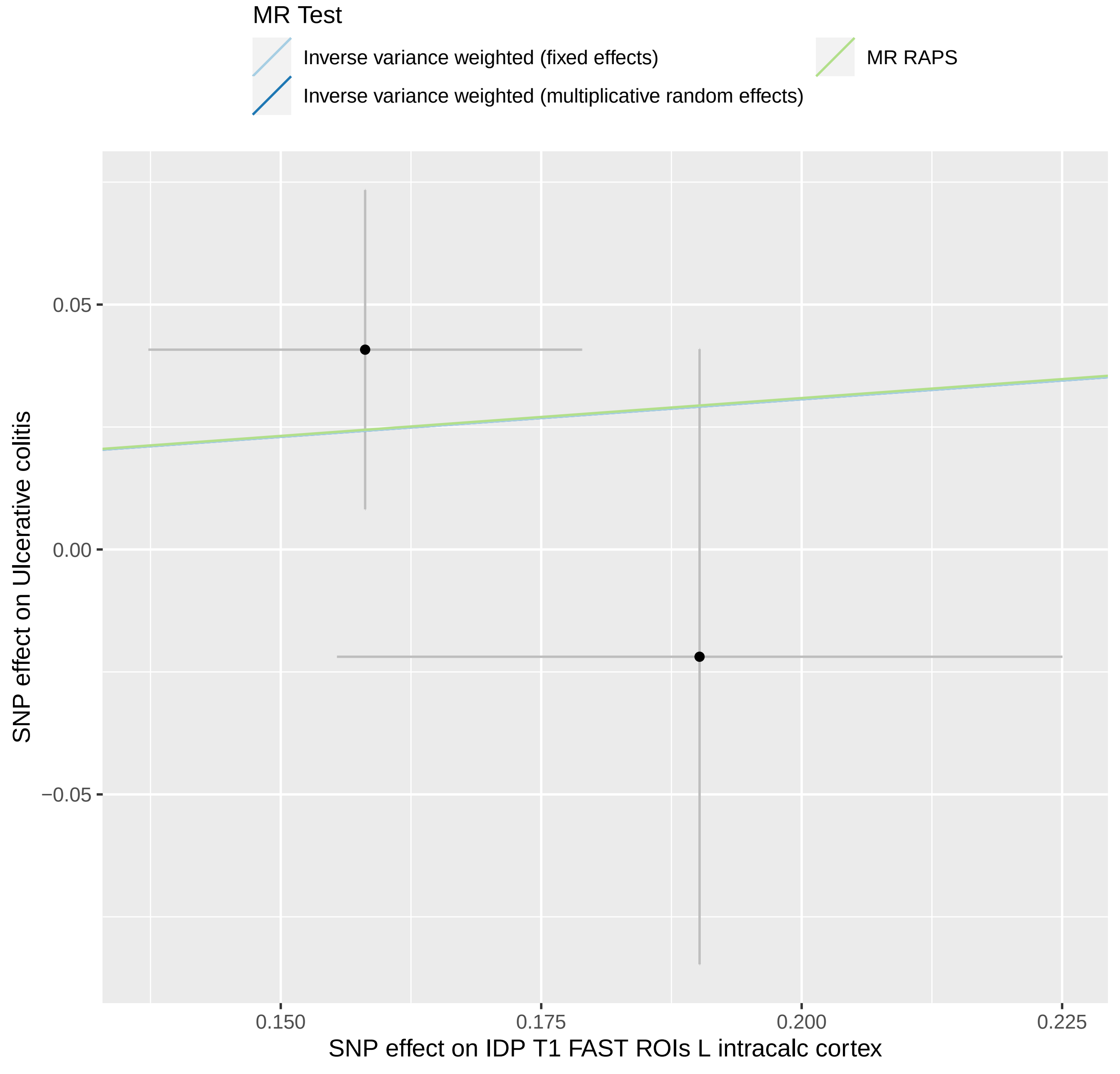

Batch 5329 : IDP T1 FAST ROIs R juxtapos lobule cortex on Ulcerative colitis

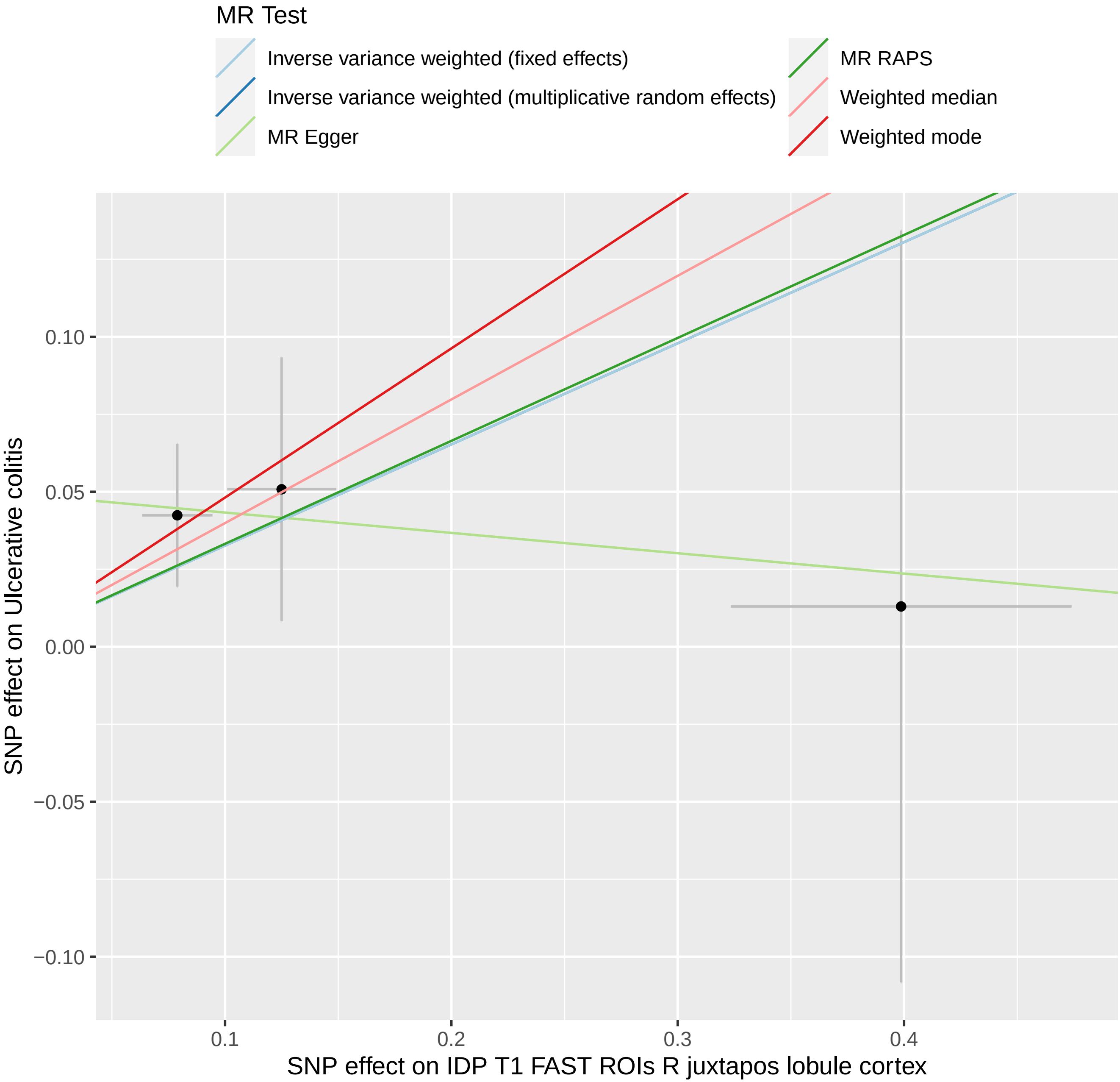

Batch 5340 : IDP T1 FAST ROIs L subcallosal cortex on Ulcerative colitis

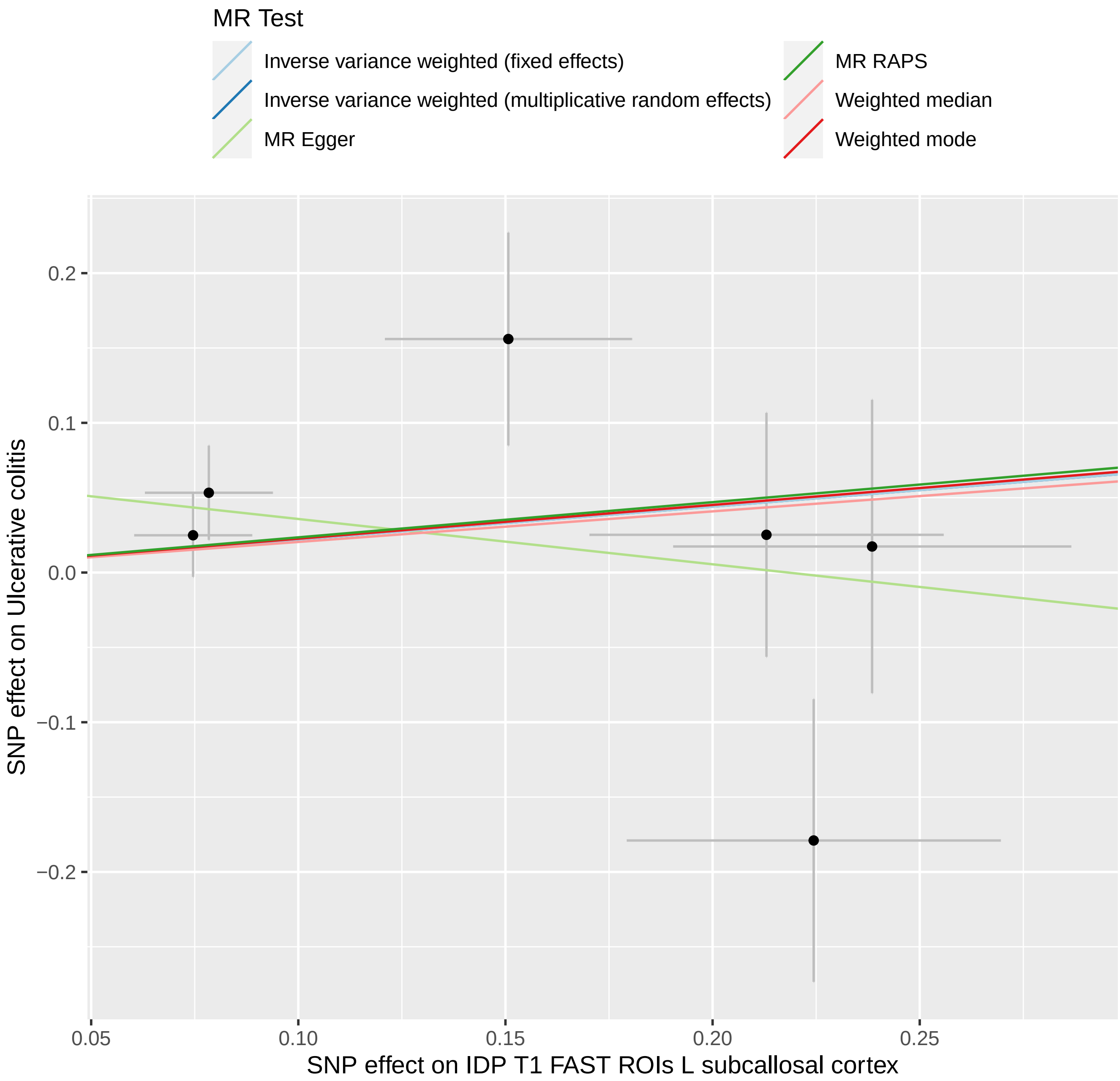

Batch 5351 : IDP T1 FAST ROIs R subcallosal cortex on Ulcerative colitis

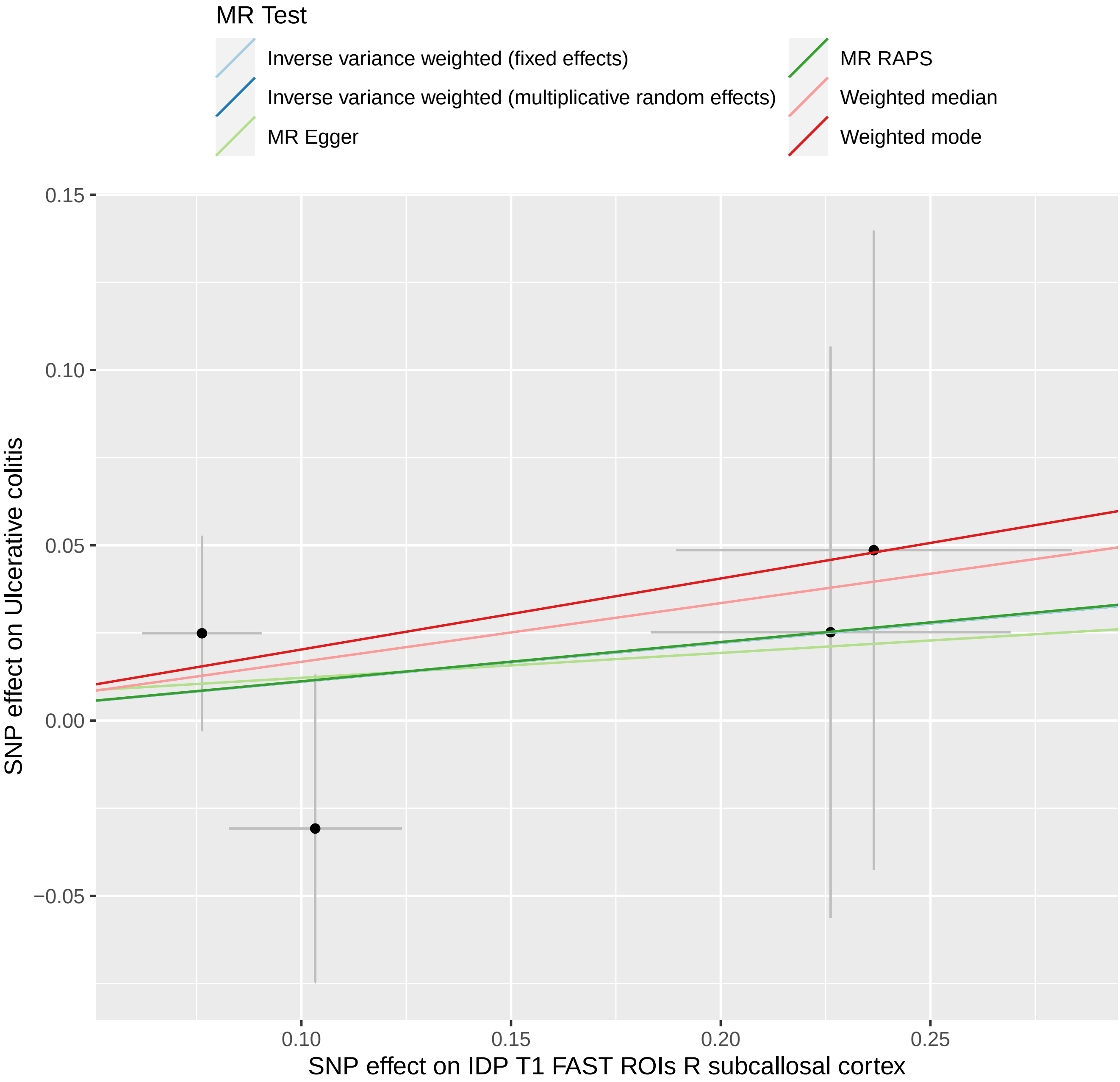

Batch 5396 : IDP T1 FAST ROIs R cing gyrus ant on Ulcerative colitis

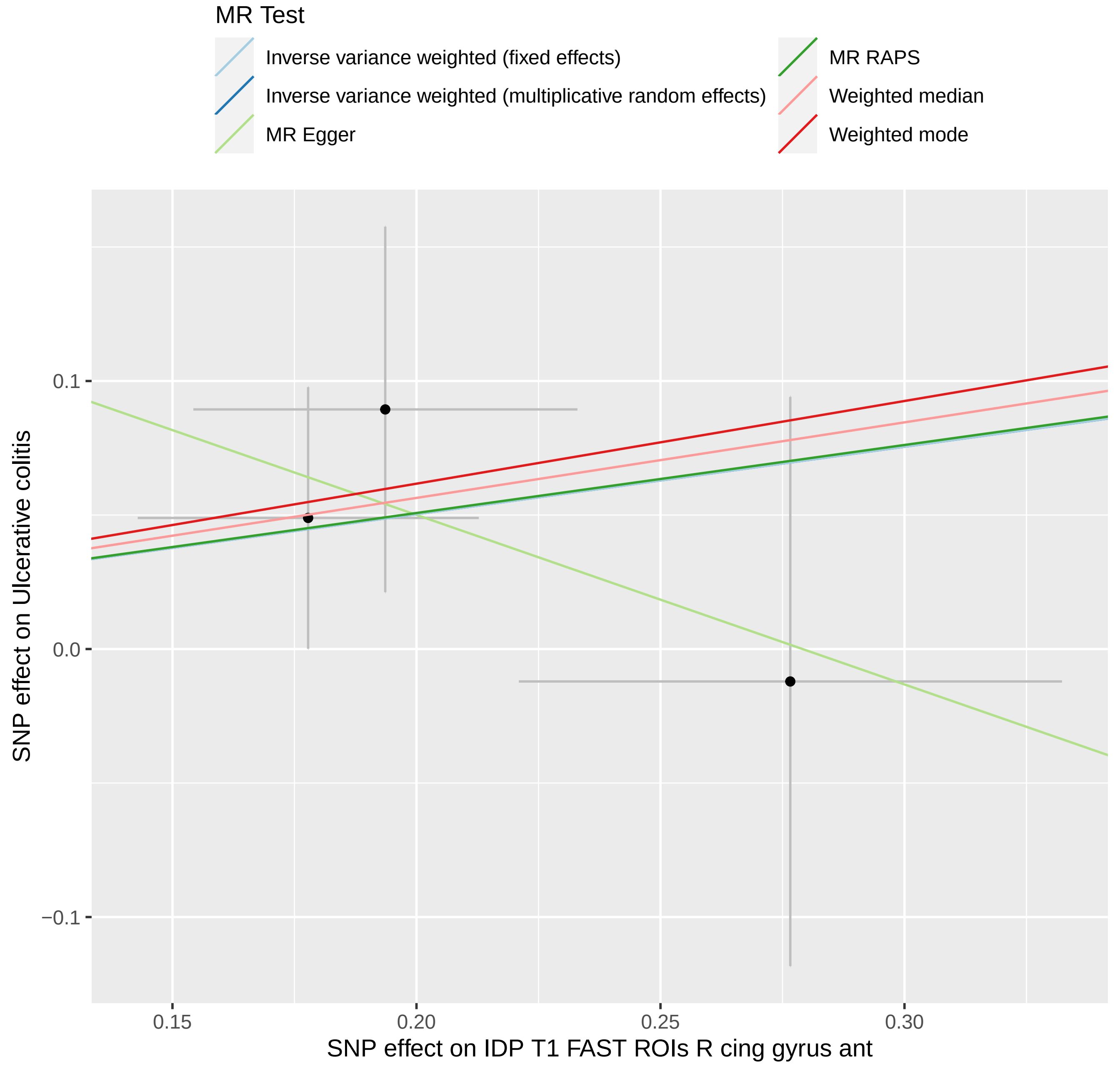

Batch 5418 : IDP T1 FAST ROIs R cing gyrus post on Ulcerative colitis

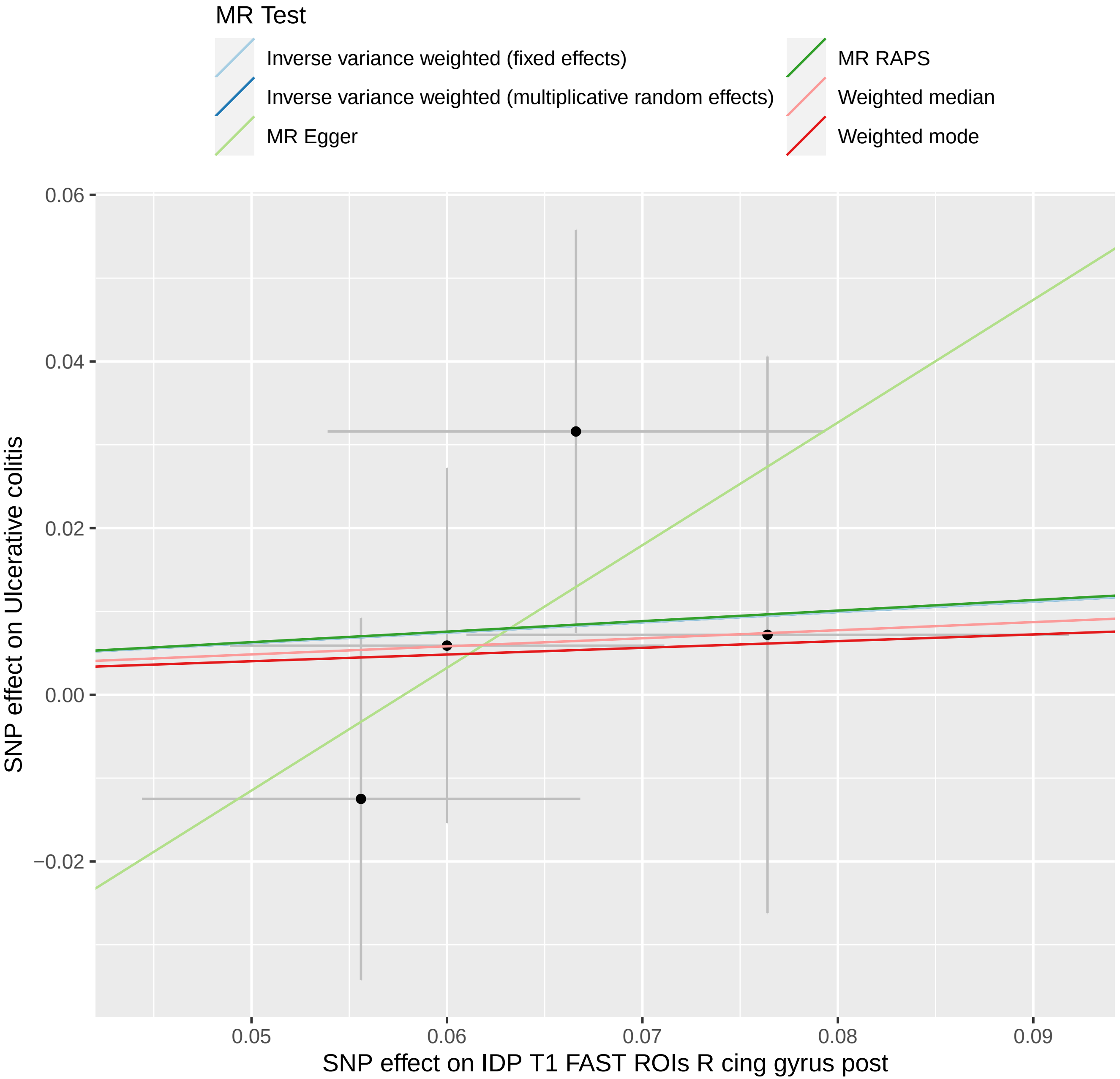

Batch 5429 : IDP T1 FAST ROIs L precun cortex on Ulcerative colitis

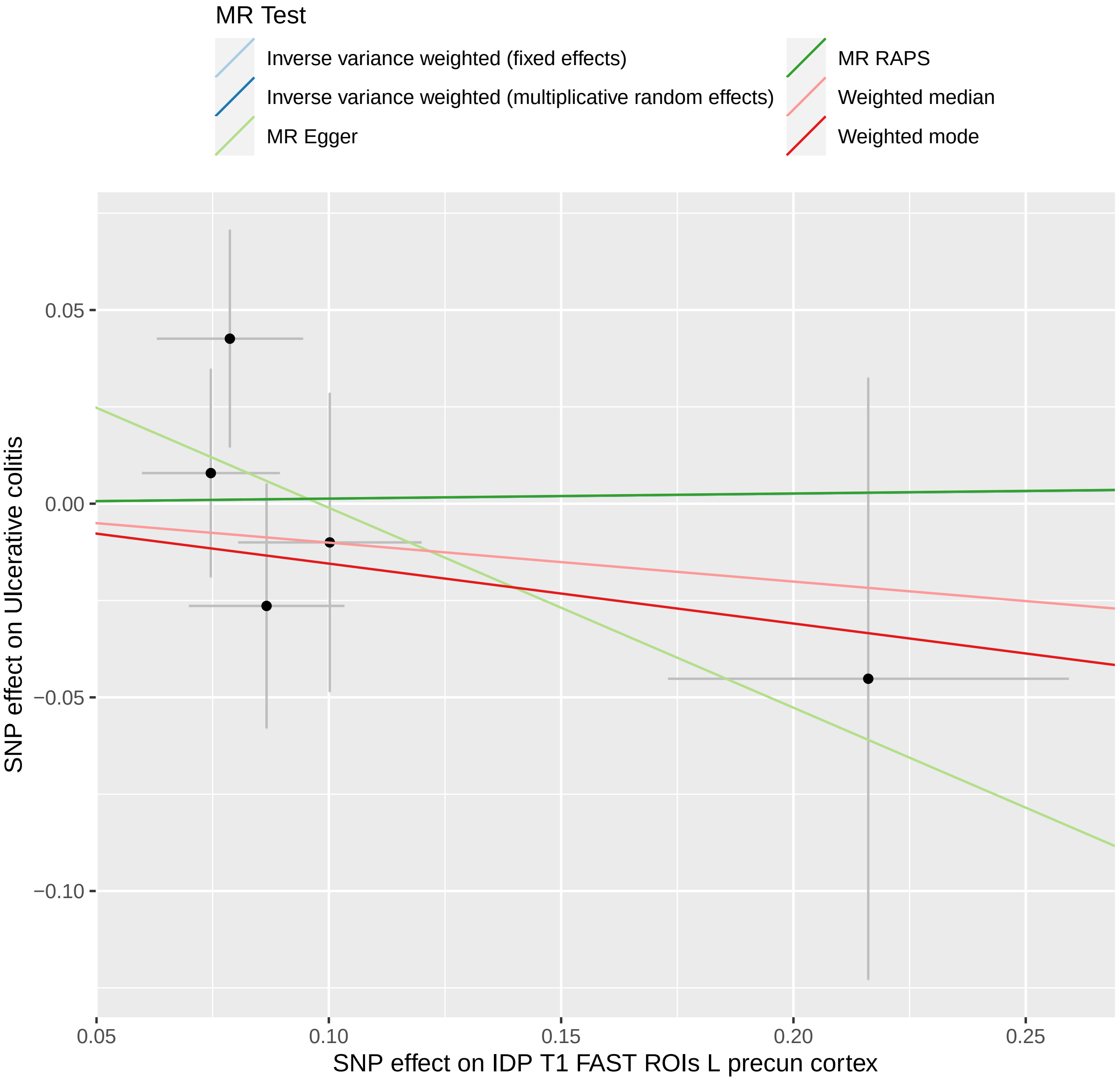

Batch 5442 : IDP T1 FAST ROIs L cuneal cortex on Ulcerative colitis

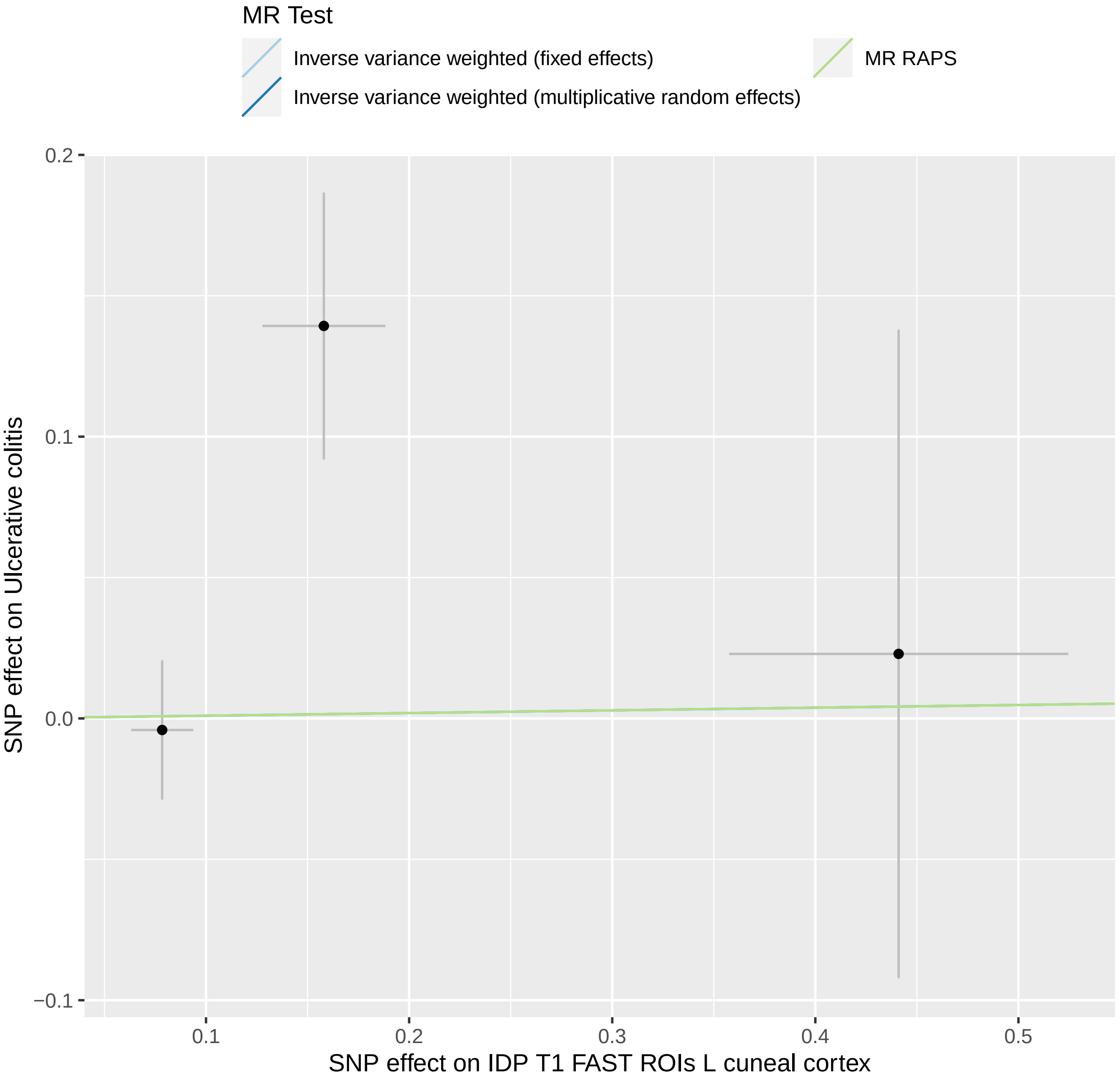

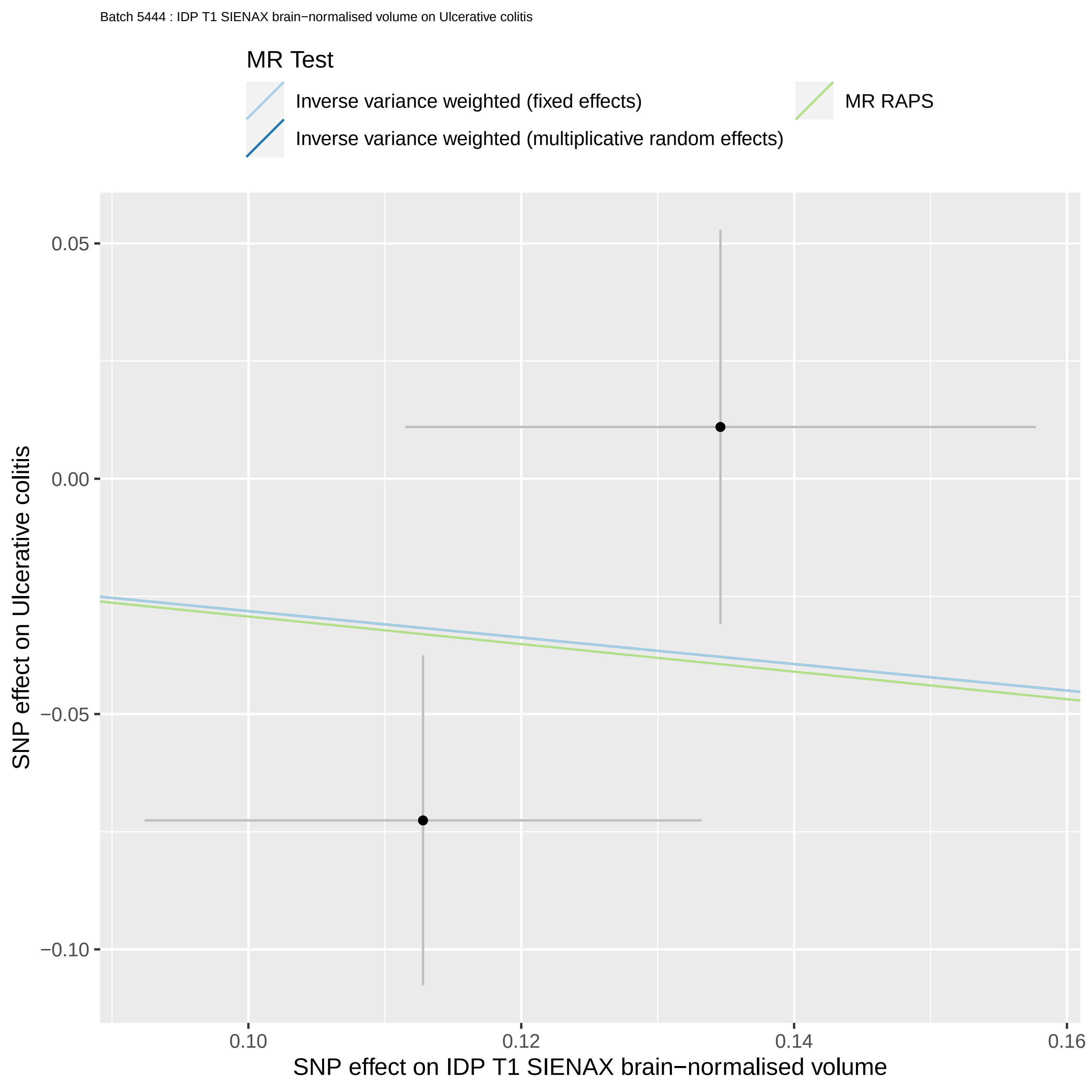
Batch 5444 : IDP T1 SIENAX brain−normalised volume on Ulcerative colitis
MR Test
Inverse variance weighted (fixed effects)
Inverse variance weighted (multiplicative random effects)
MR RAPS
0.05
0.00
−0.05
−0.10
0.10
0.12
0.14
0.16
SNP effect on IDP T1 SIENAX brain−normalised volume
SNP effect on Ulcerative colitis

Batch 5445 : IDP T1 FAST ROIs L front orb cortex on Ulcerative colitis

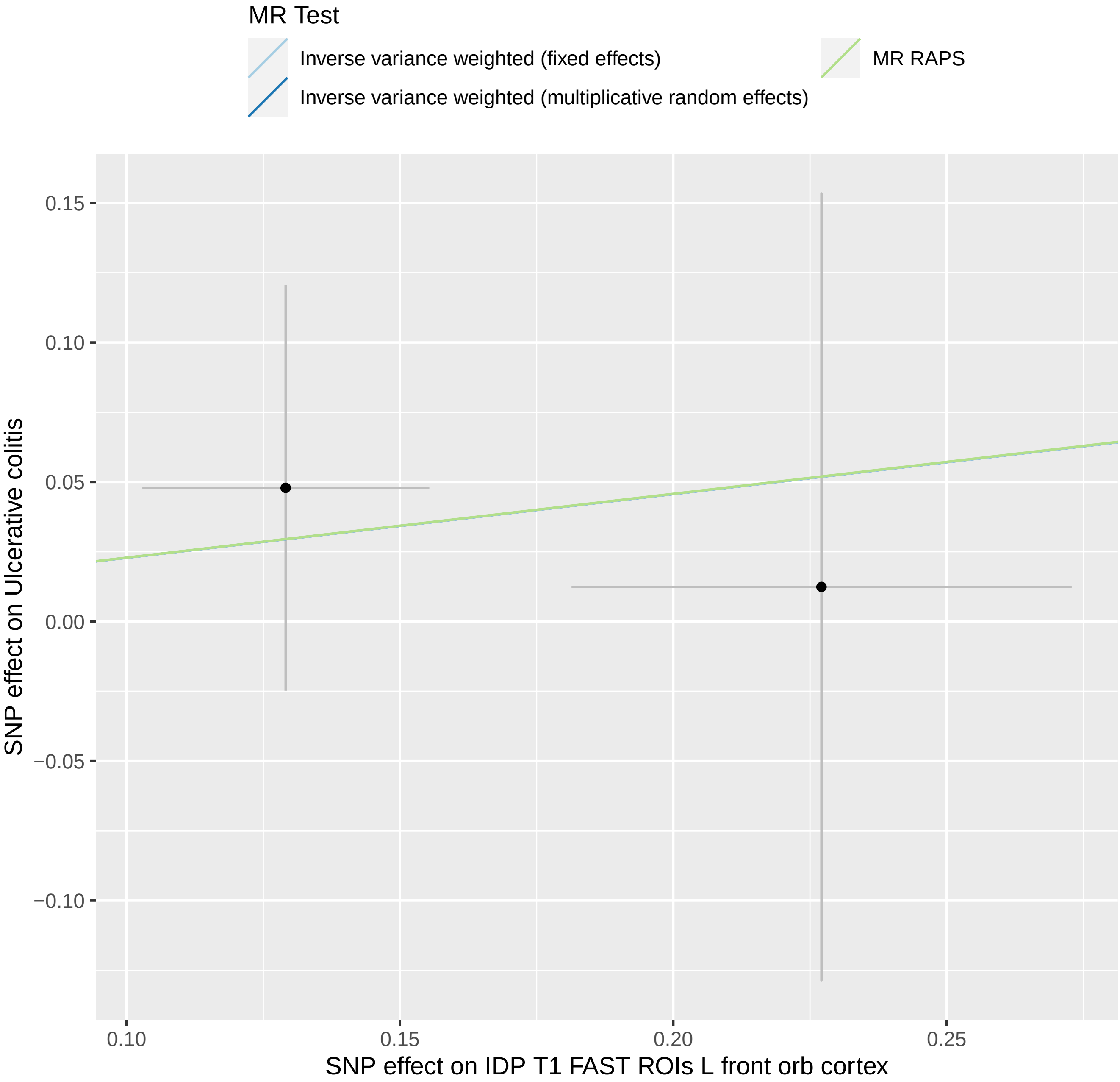

Batch 5447 : IDP T1 FAST ROIs L parahipp gyrus ant on Ulcerative colitis

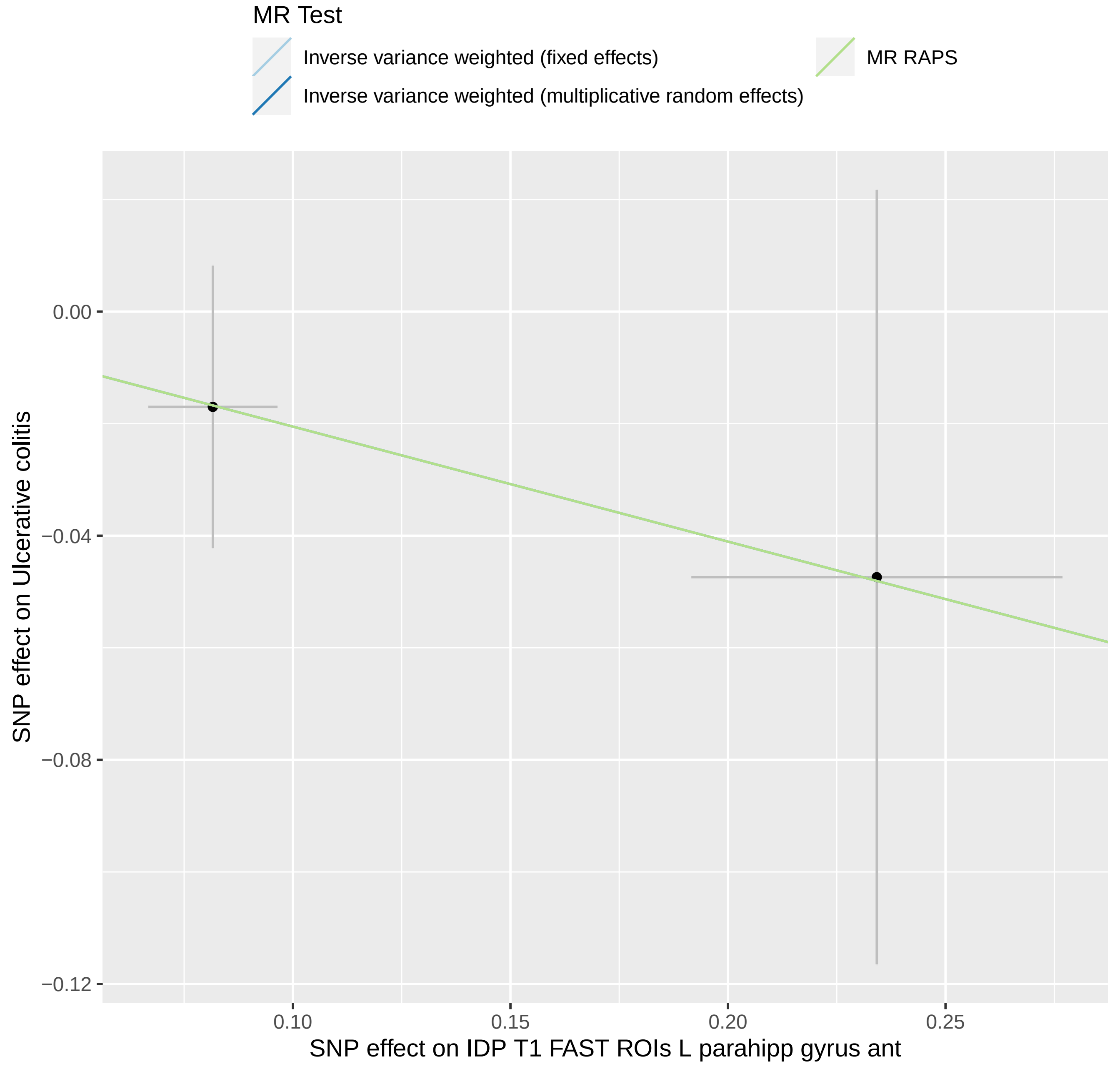

Batch 5448 : IDP T1 FAST ROIs R parahipp gyrus ant on Ulcerative colitis

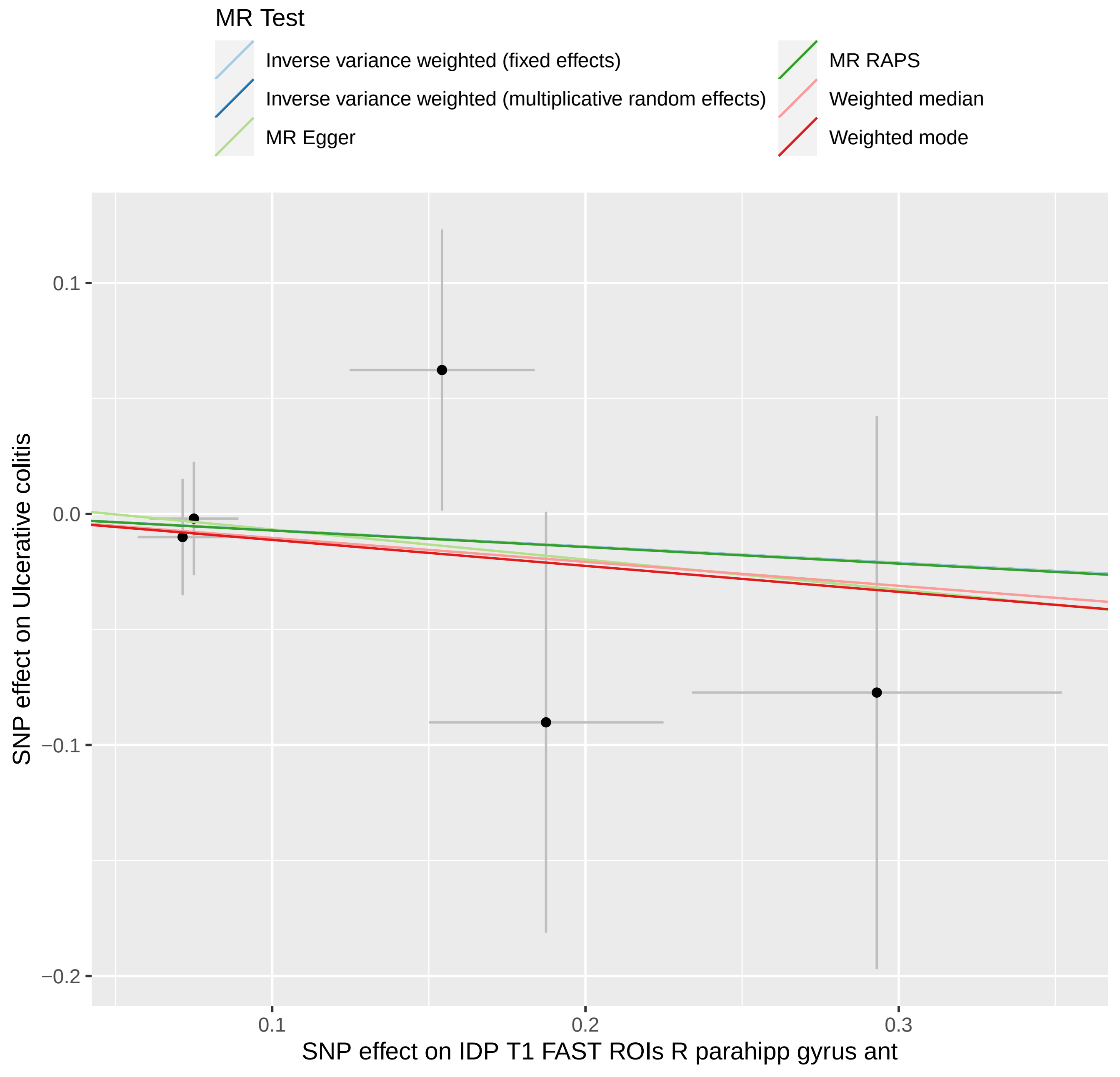

Batch 5450 : IDP T1 FAST ROIs R parahipp gyrus post on Ulcerative colitis

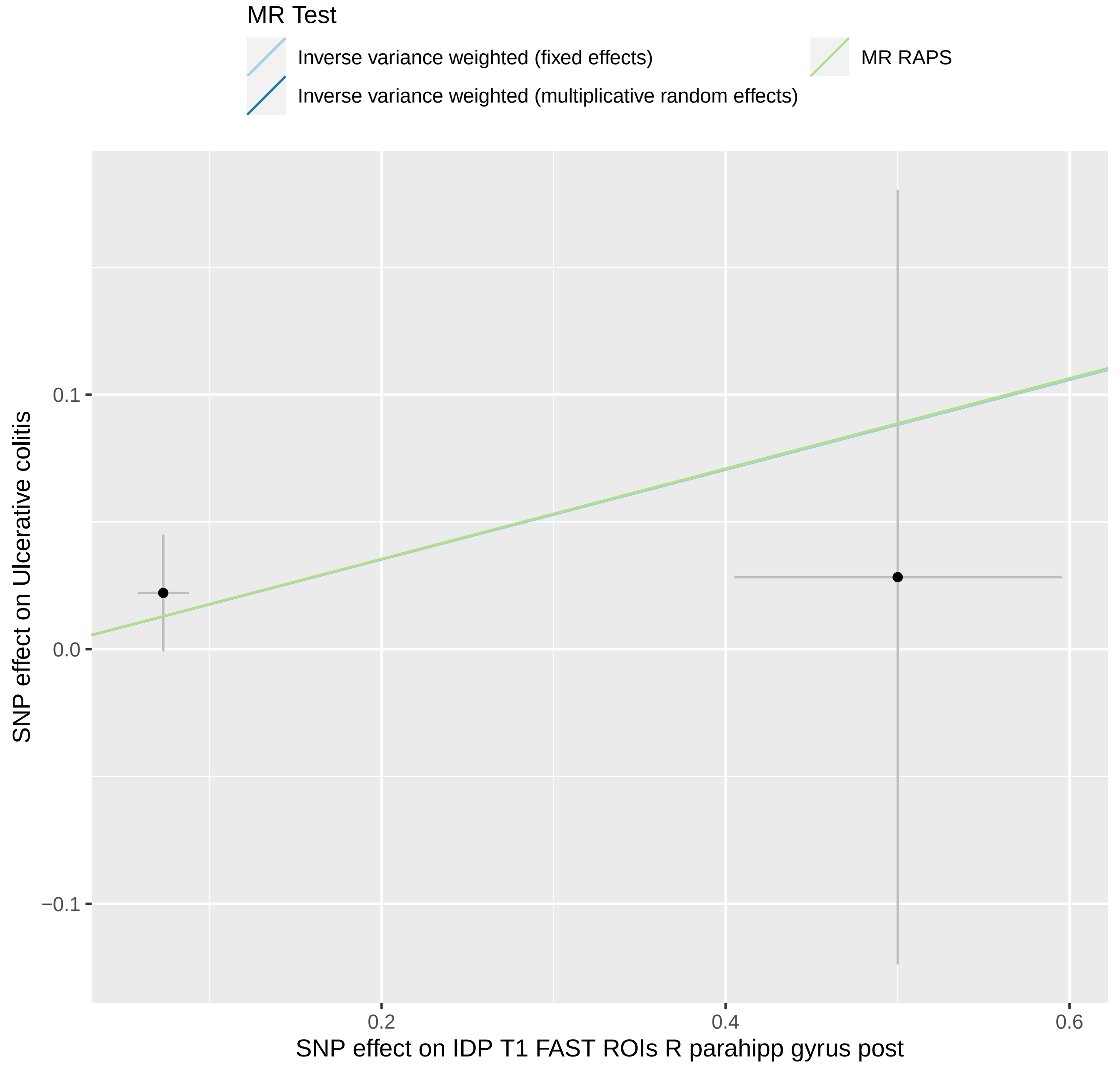

Batch 5452 : IDP T1 FAST ROIs R lingual gyrus on Ulcerative colitis

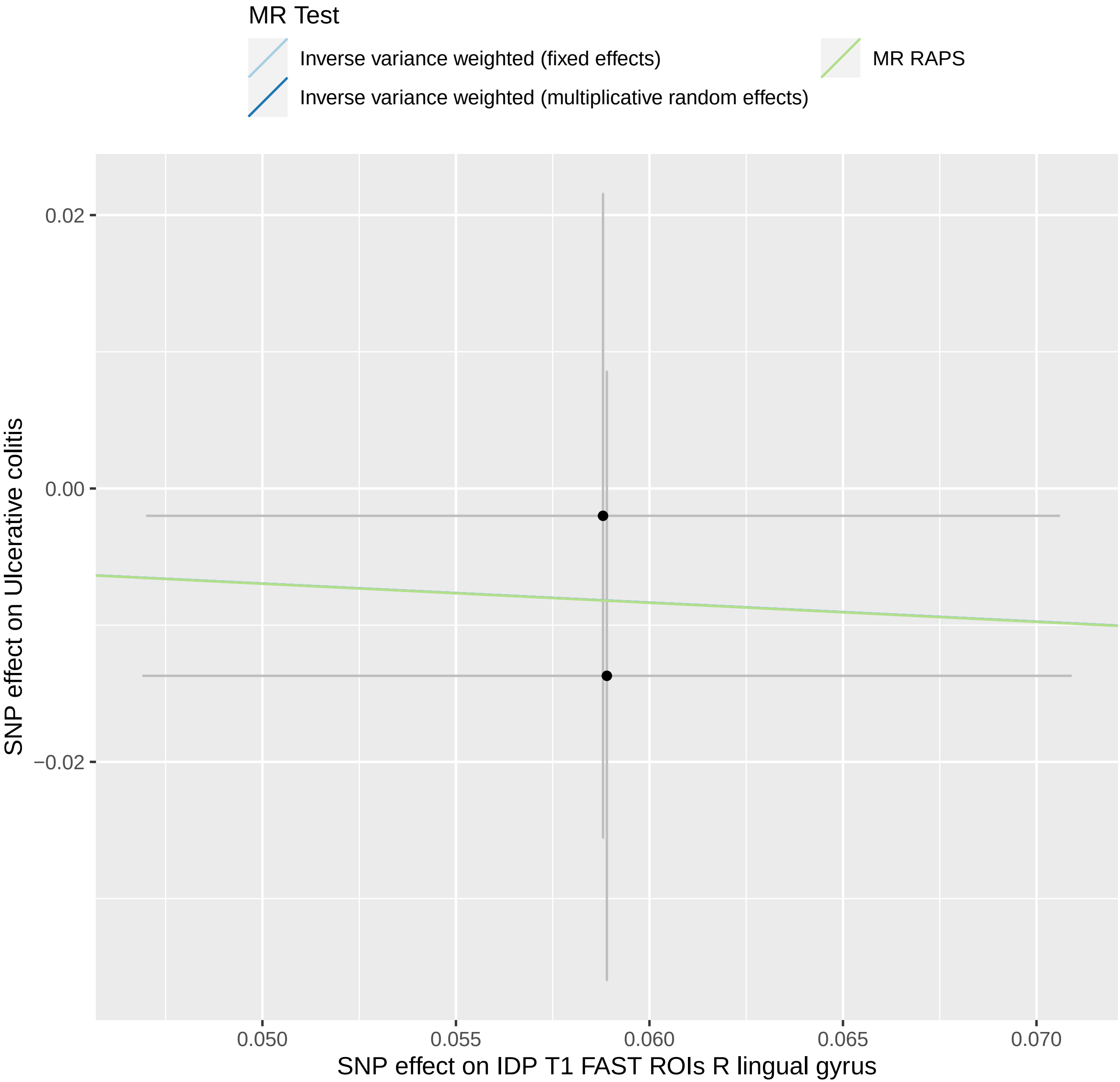

Supplement: Supplementary file 4 [file DataSheet_2.pdf]
