# Supplementary material for: Brain imaging derived phenotypes: a biomarker for the onset of inflammatory bowel disease and a potential mediator of mental complications
Source: Front Immunol. 2024 Feb 26;15:1359540. doi: 10.3389/fimmu.2024.1359540 (PMC10925669; doi:10.3389/fimmu.2024.1359540)

Batch 1 : Crohn's disease on IDP T1 SIENAX peripheral grey normalised volume

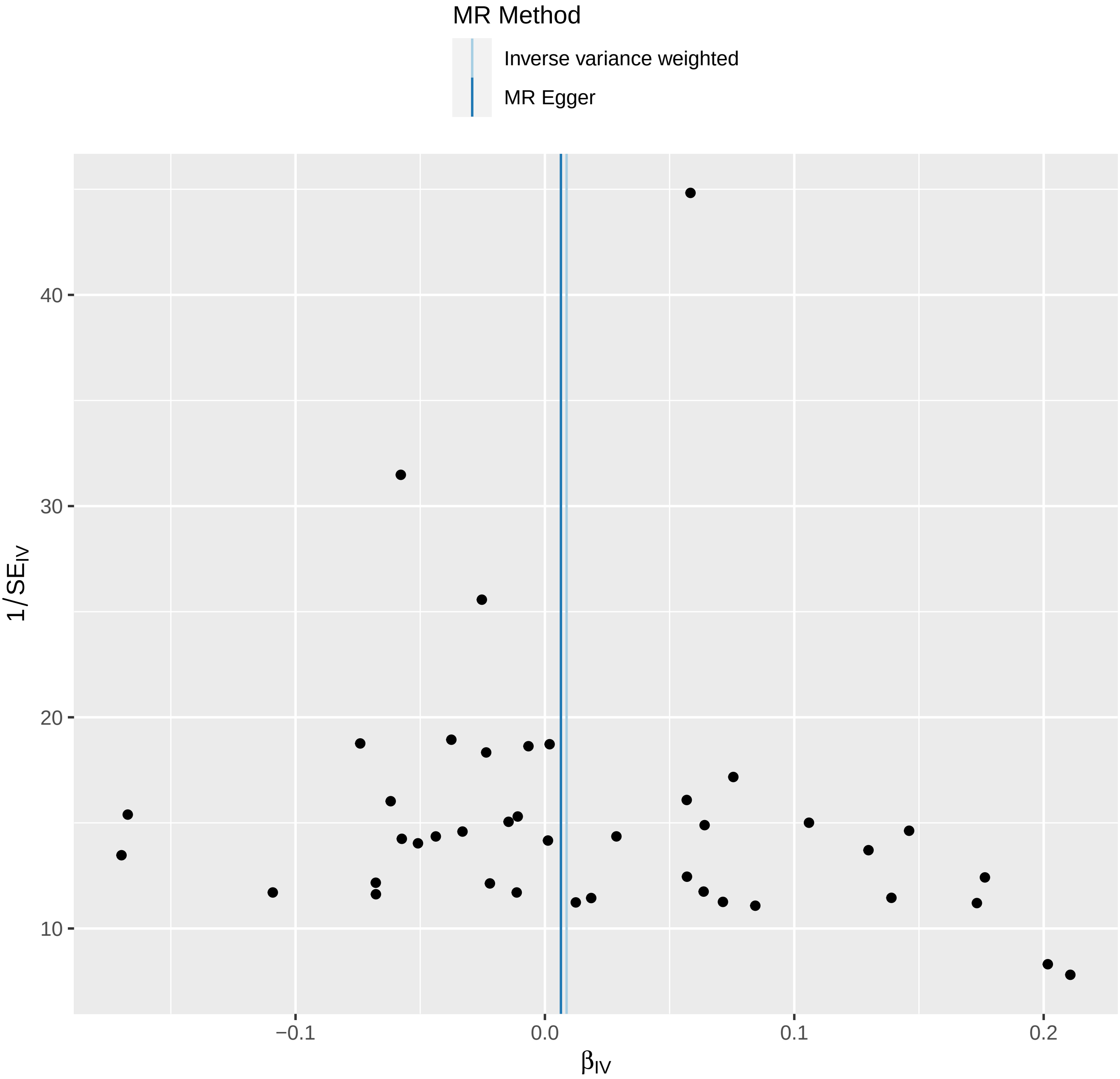

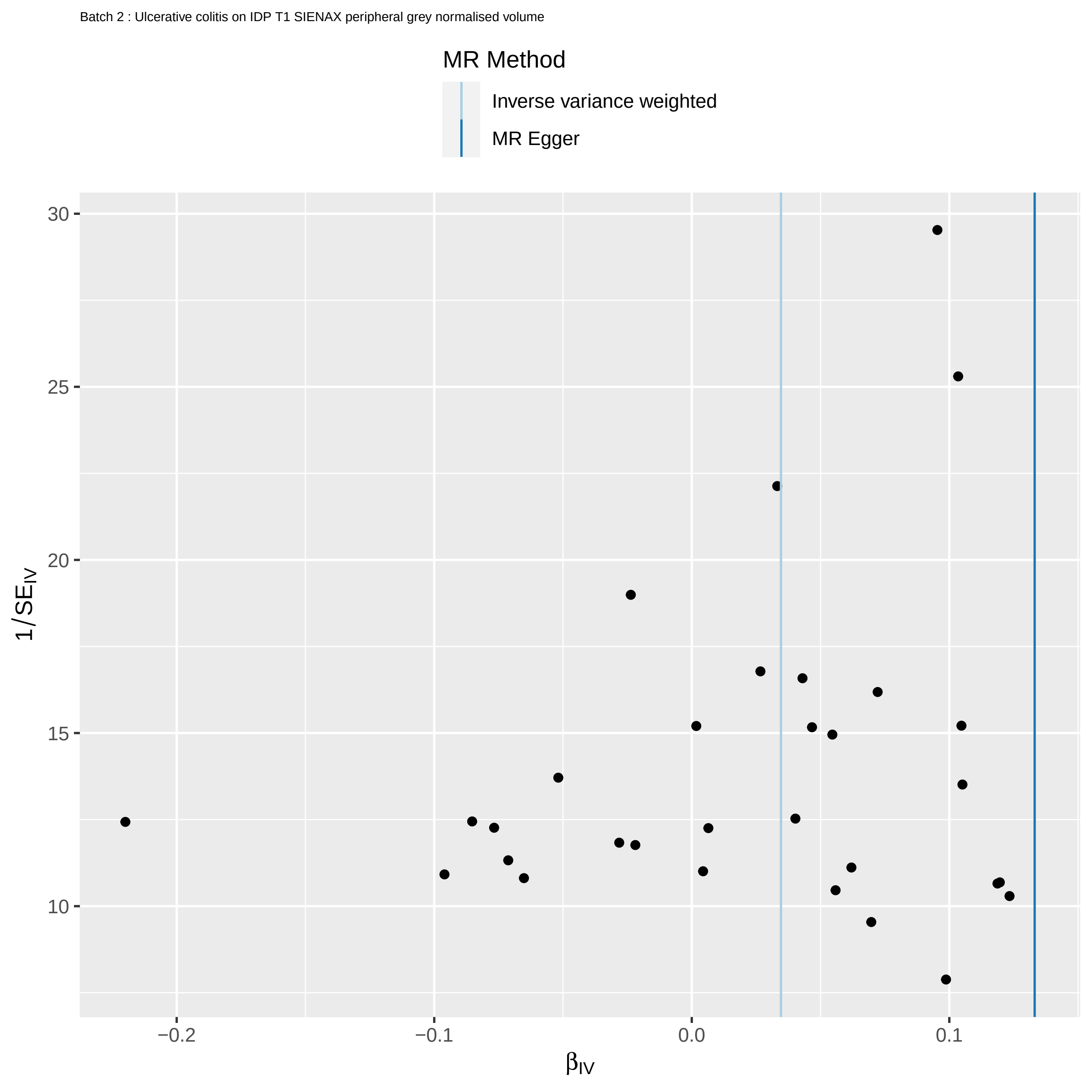
Batch 2 : Ulcerative colitis on IDP T1 SIENAX peripheral grey normalised volume
MR Method
Inverse variance weighted
MR Egger
30
25
20
15
10
1/SE_IV
−0.2
−0.1
0.0
0.1
β_IV

Batch 3 : Crohn's disease on IDP T1 SIENAX brain−unnormalised volume

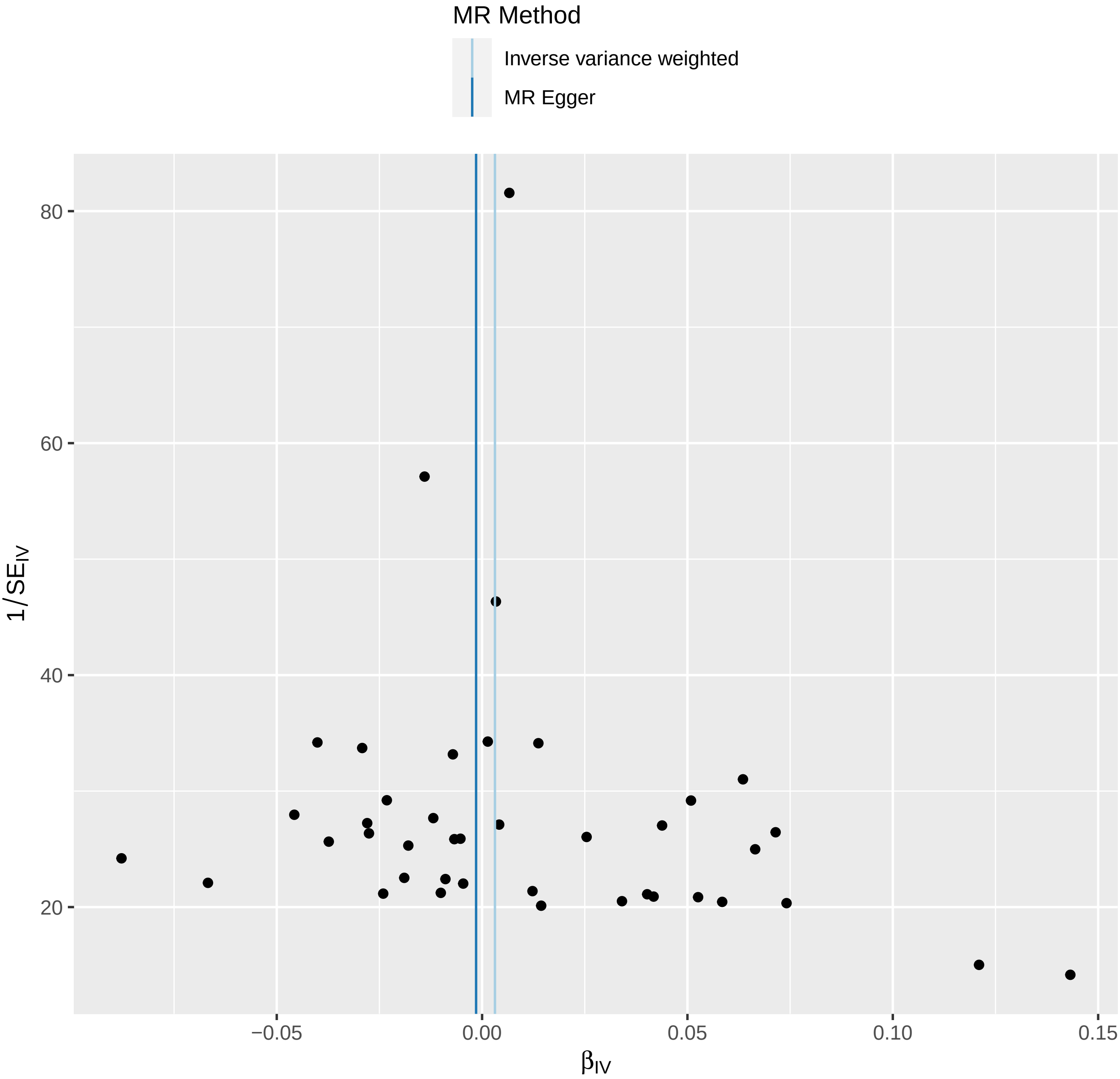

Batch 4 : Ulcerative colitis on IDP T1 SIENAX brain−unnormalised volume

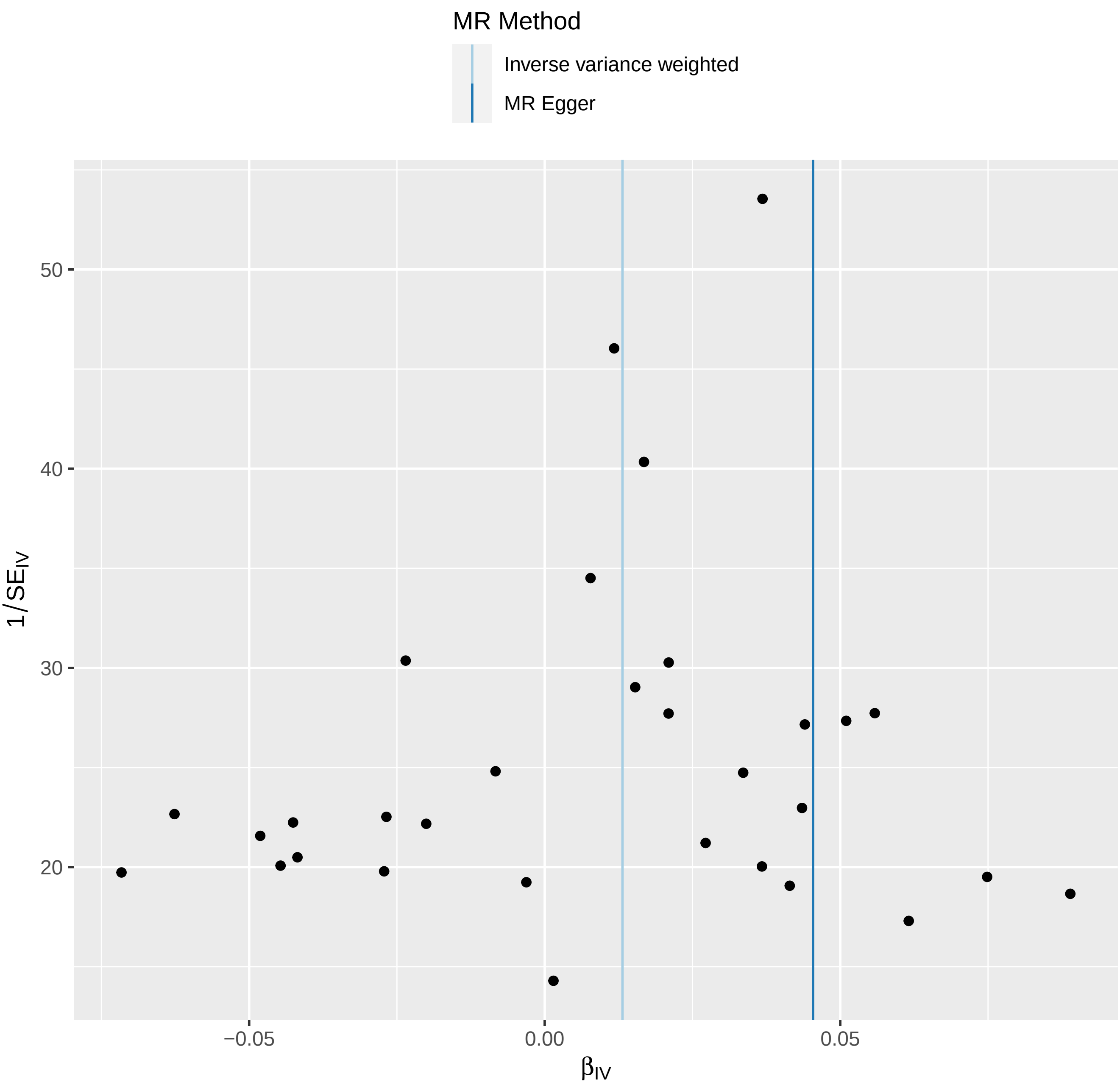

Batch 5 : Crohn's disease on IDP T1 FAST ROIs L temp fusif cortex post

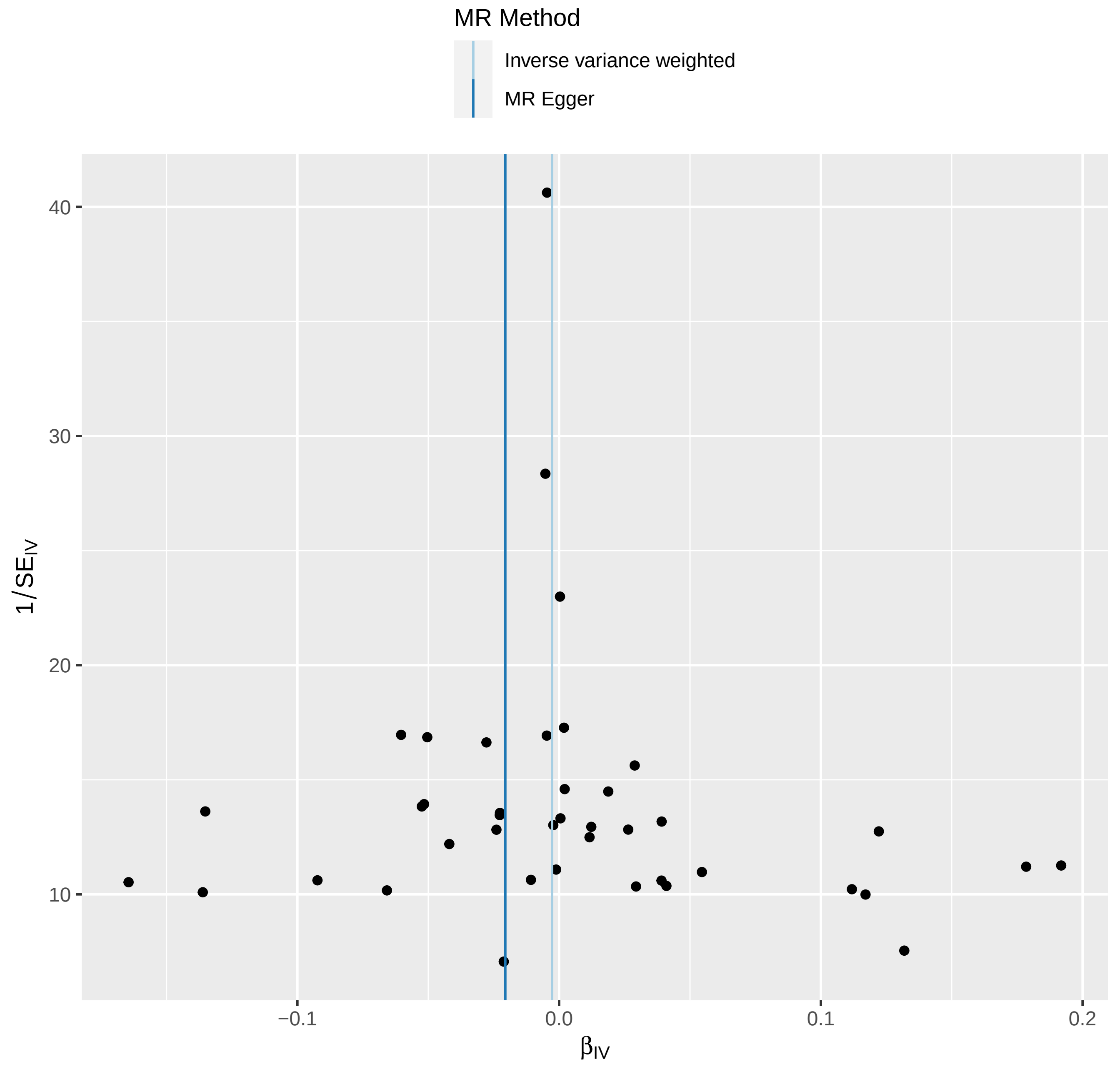

Batch 6 : Ulcerative colitis on IDP T1 FAST ROIs L temp fusif cortex post

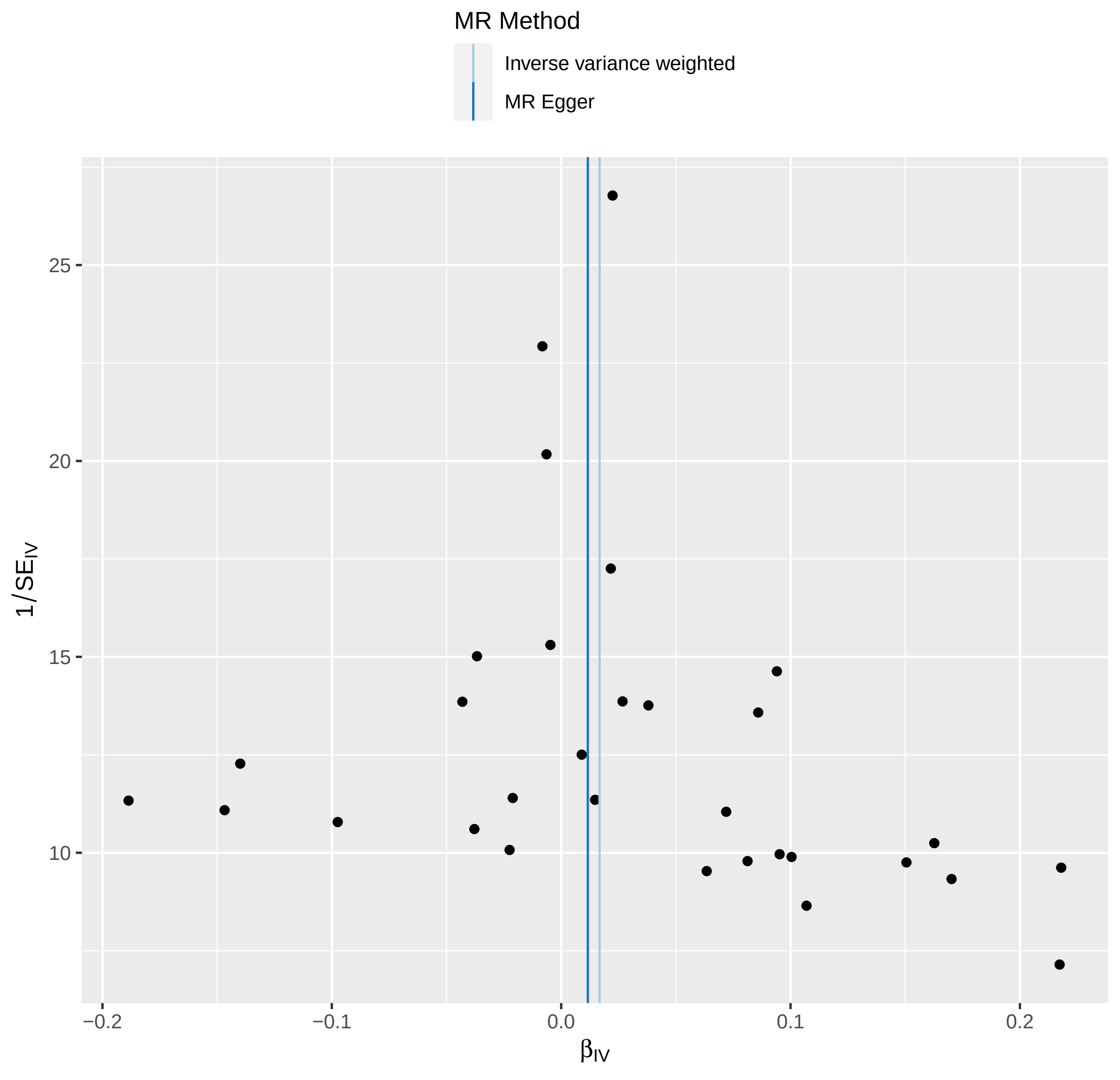

Batch 7 : Crohn's disease on IDP T1 FAST ROIs R temp fusif cortex post

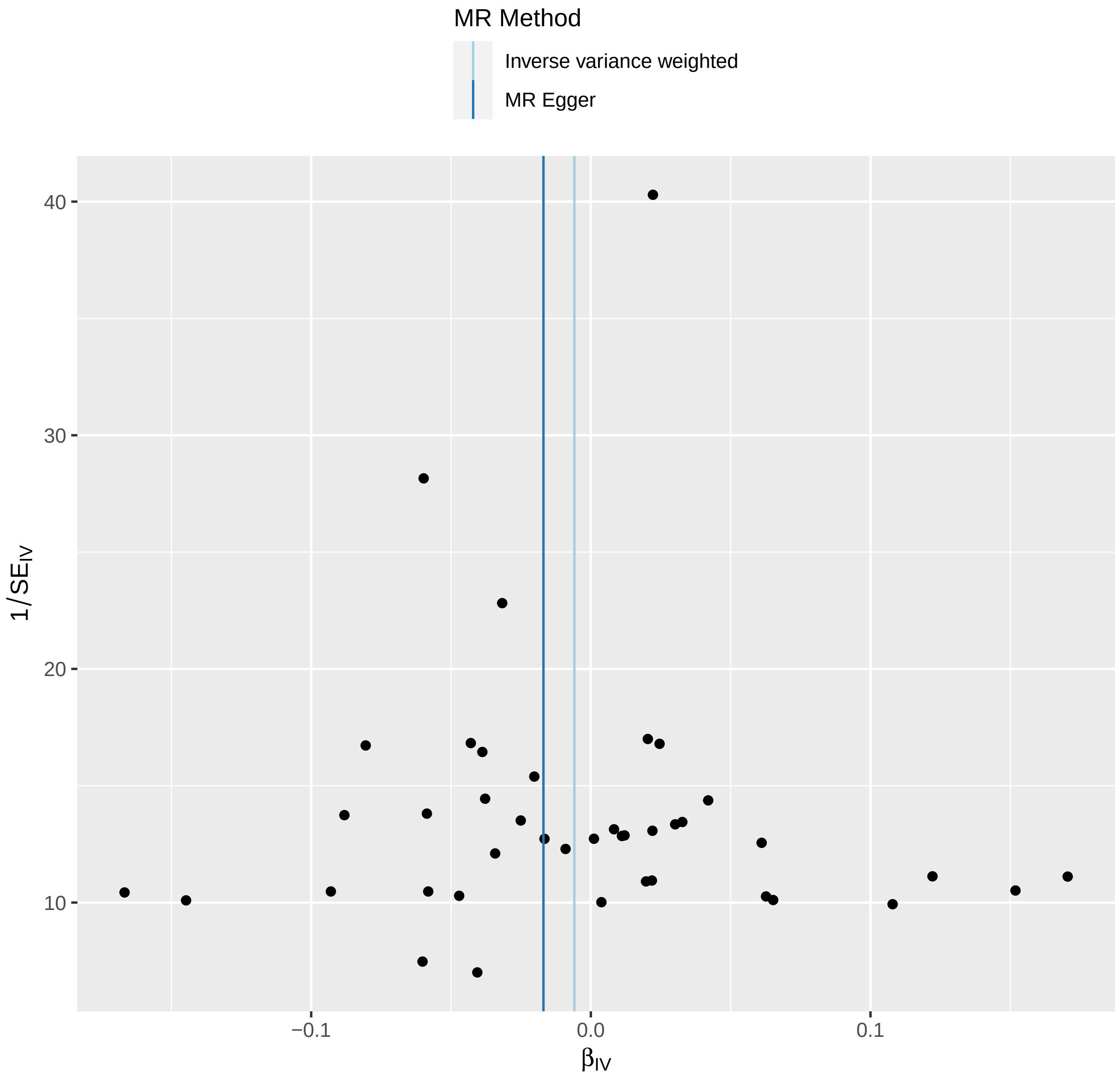

Batch 8 : Ulcerative colitis on IDP T1 FAST ROIs R temp fusif cortex post

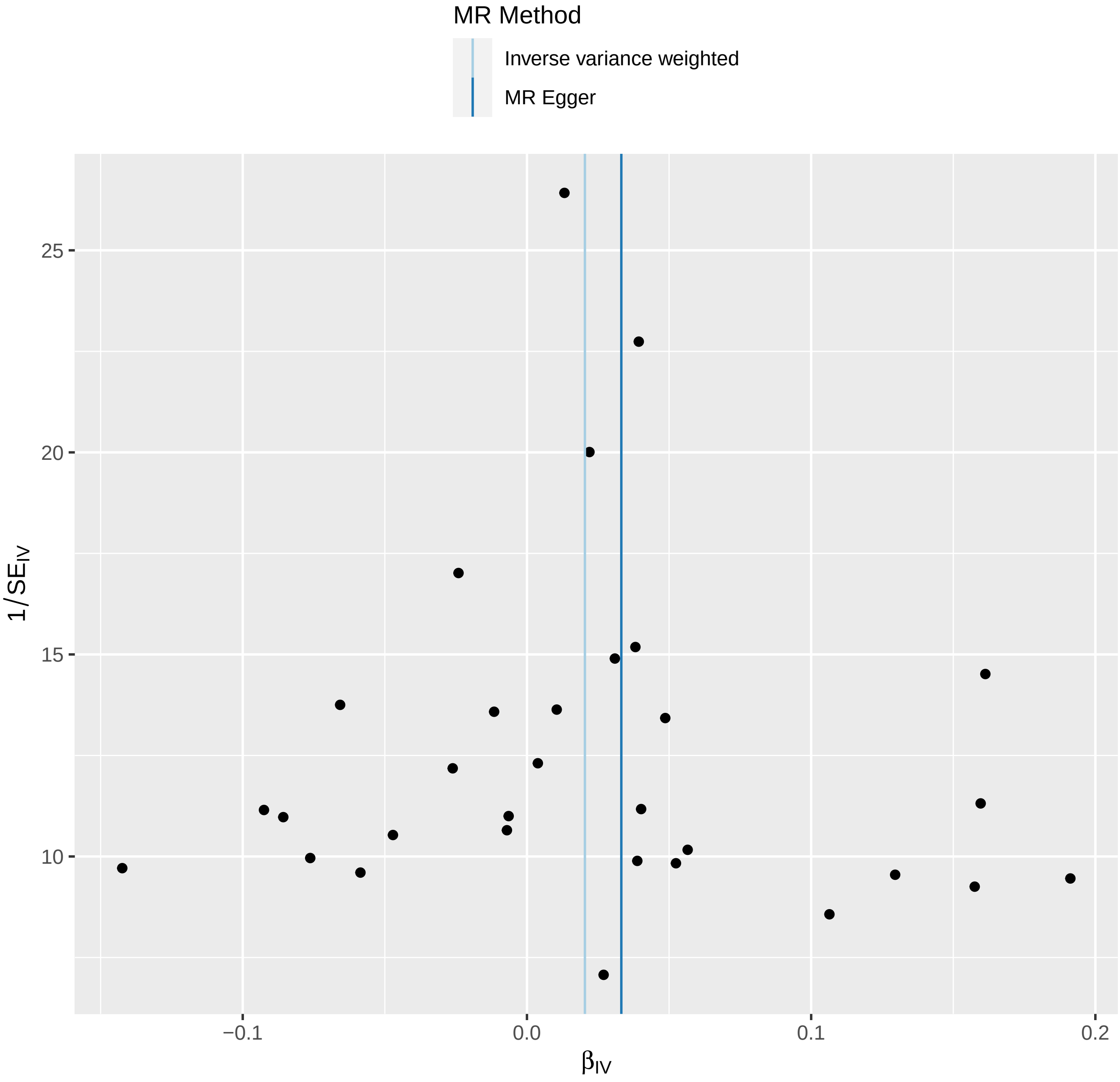

Batch 9 : Crohn's disease on IDP T1 FAST ROIs L temp occ fusif cortex

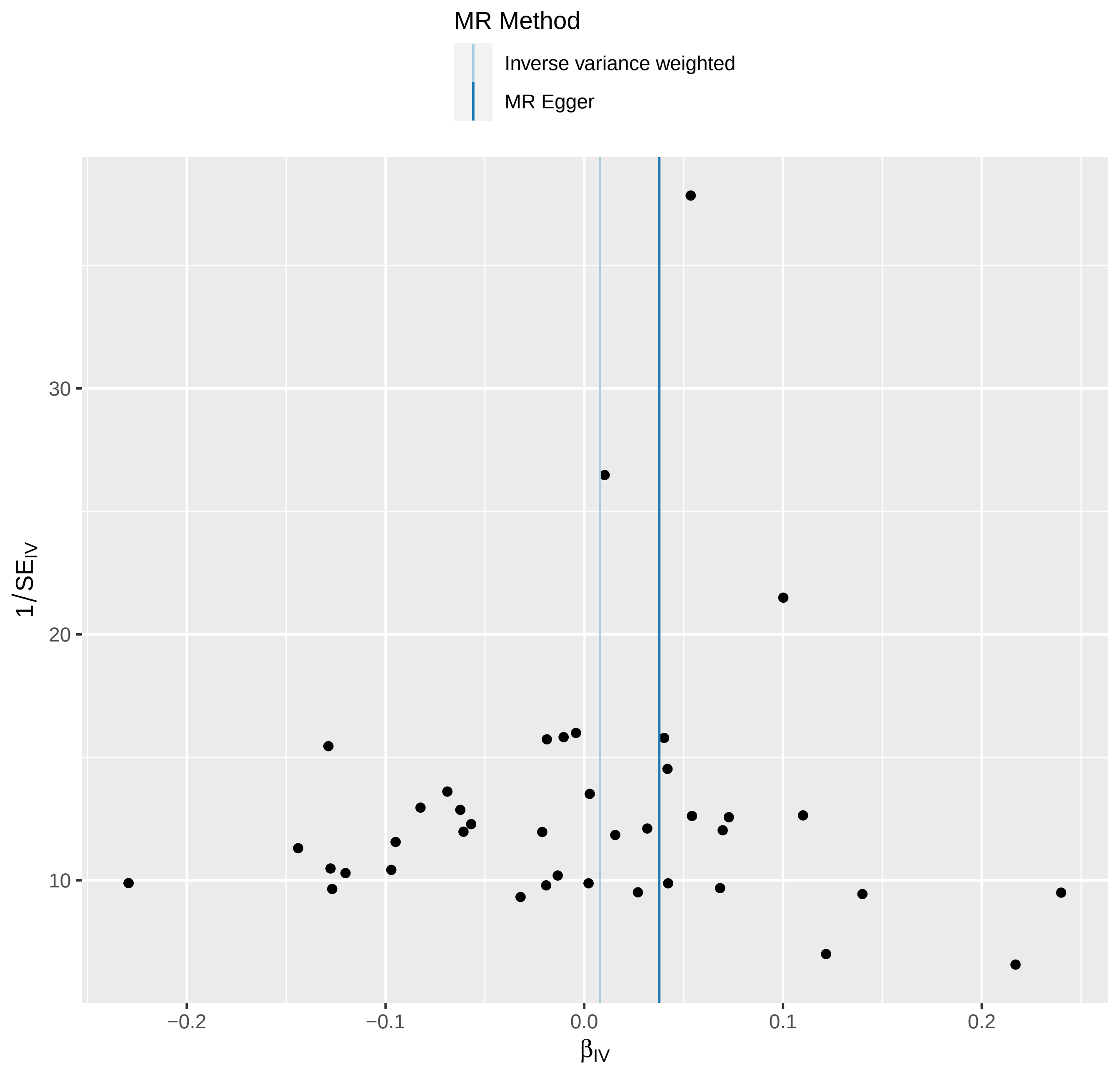

Batch 10 : Ulcerative colitis on IDP T1 FAST ROIs L temp occ fusif cortex

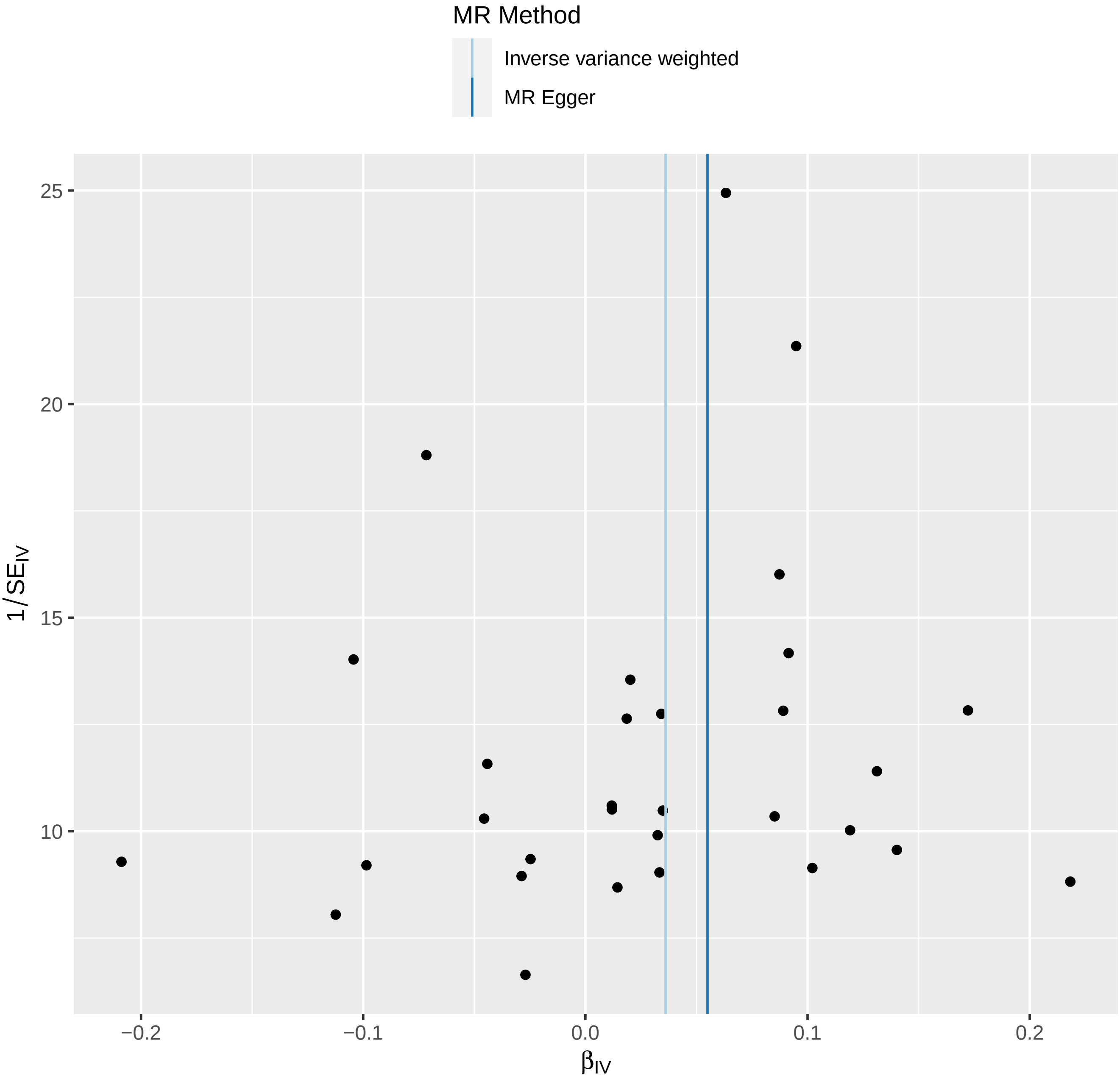

Batch 11 : Crohn's disease on IDP T1 FAST ROIs R temp occ fusif cortex

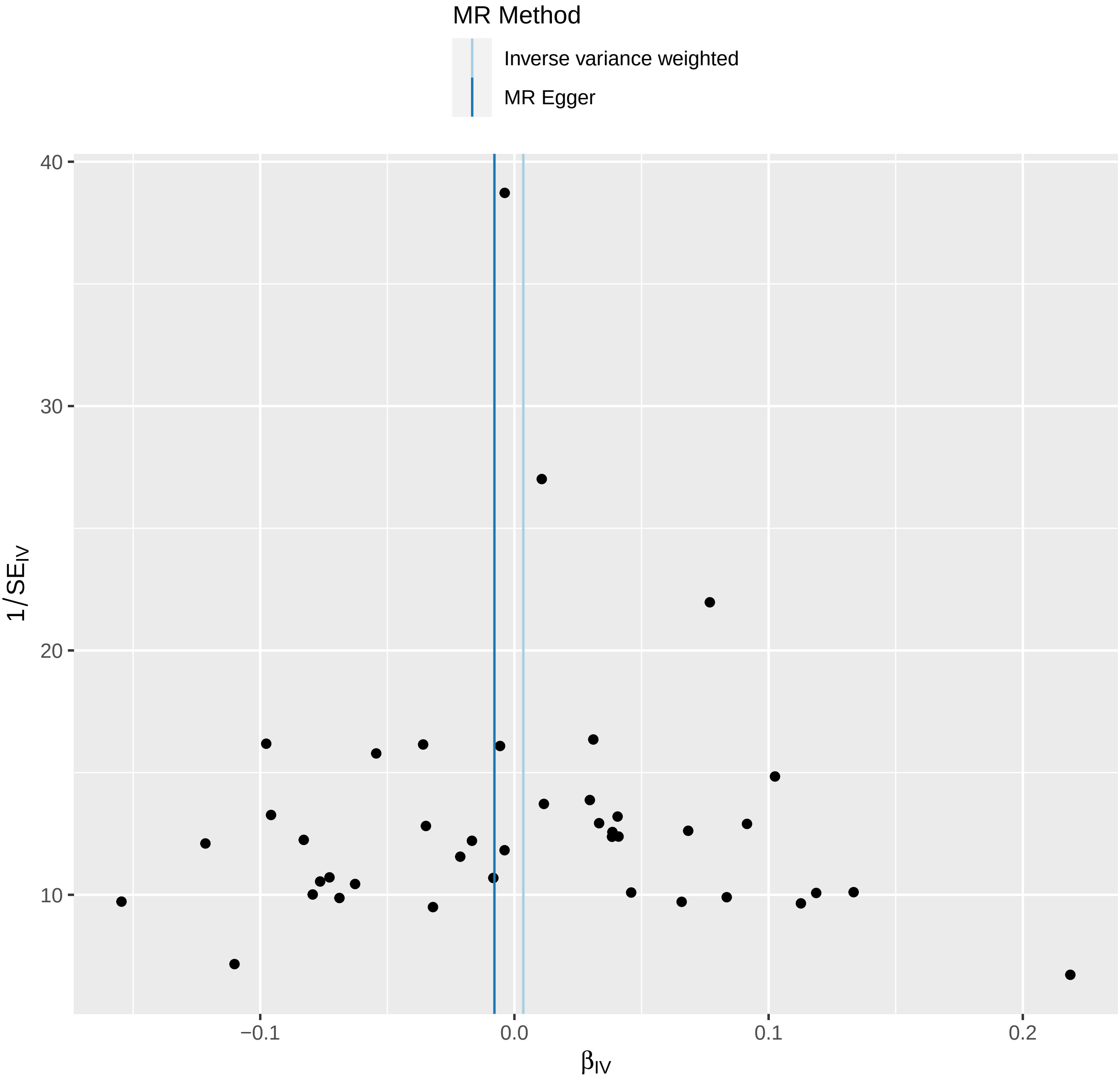

Batch 12 : Ulcerative colitis on IDP T1 FAST ROIs R temp occ fusif cortex

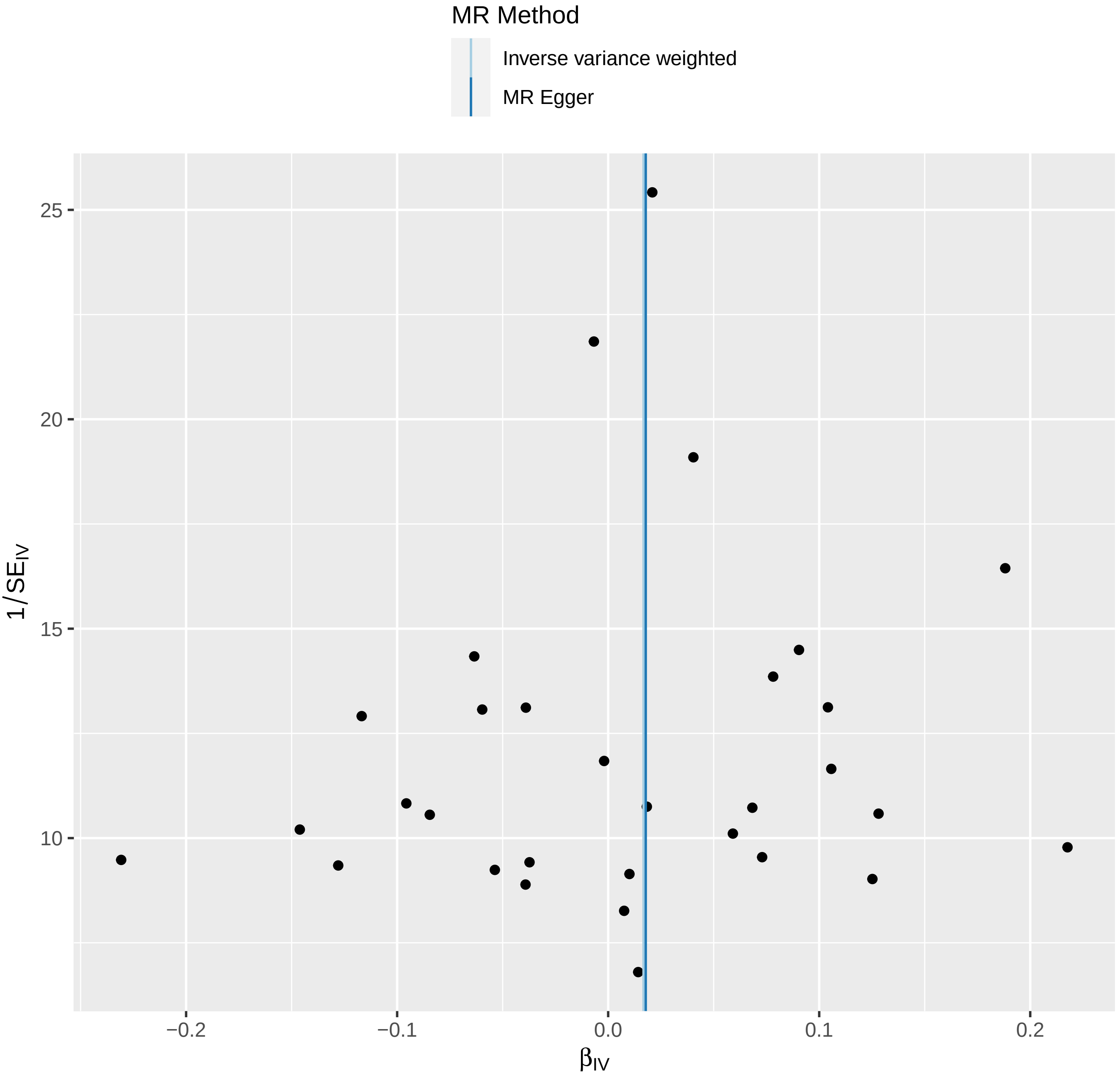

Batch 13 : Crohn's disease on IDP T1 FAST ROIs L occ fusif gyrus

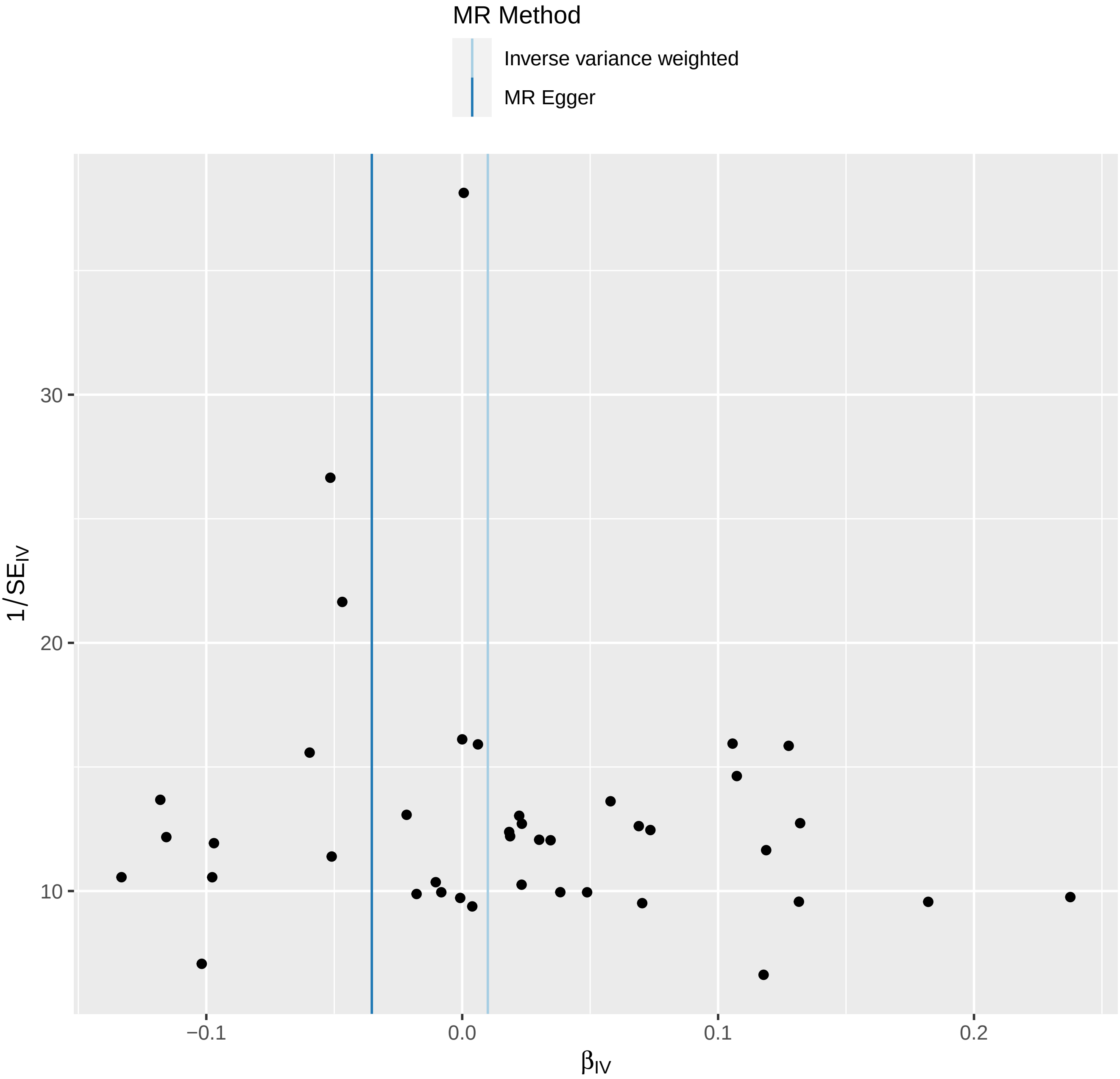

Batch 14 : Ulcerative colitis on IDP T1 FAST ROIs L occ fusif gyrus

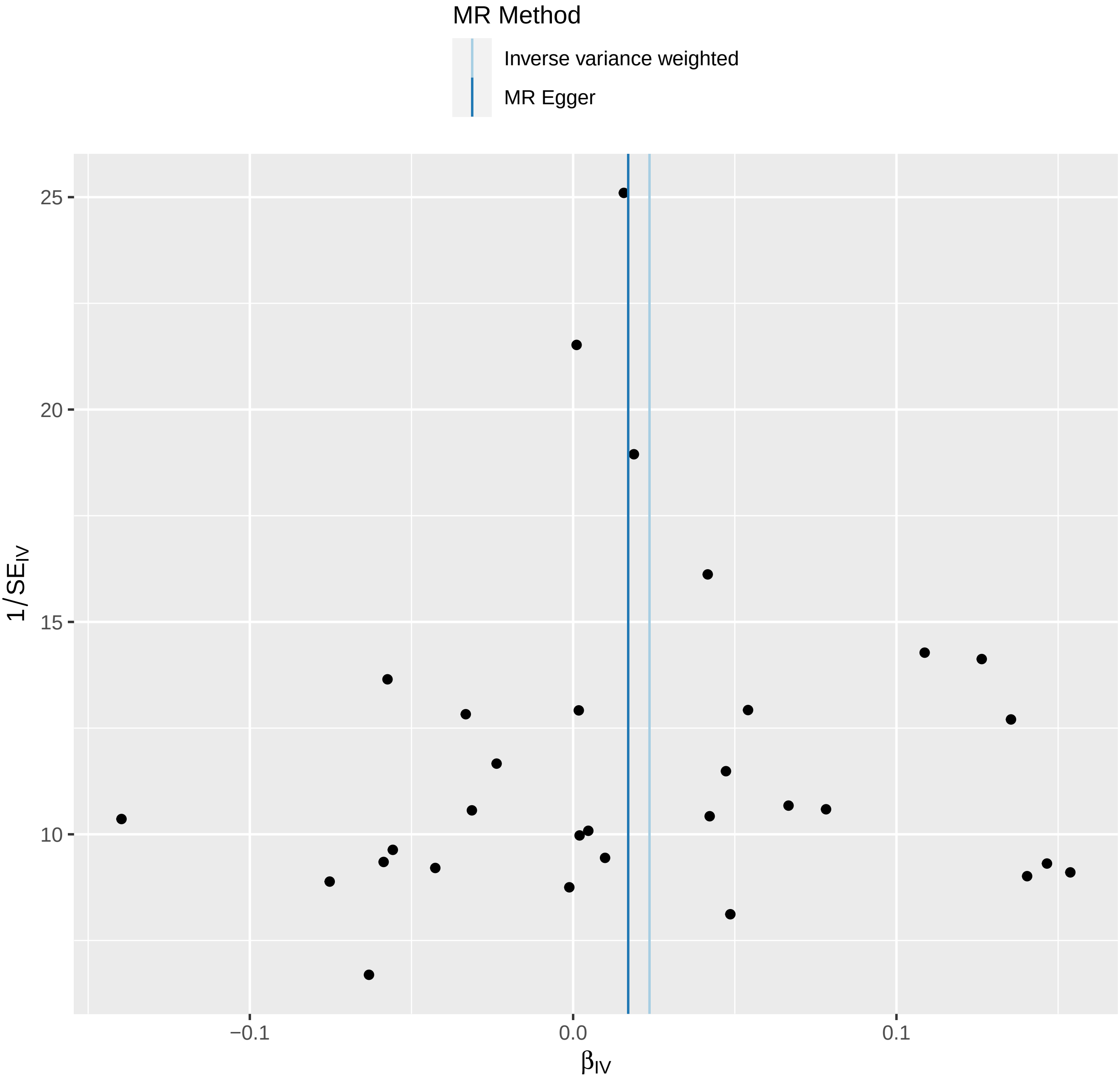

Batch 15 : Crohn's disease on IDP T1 FAST ROIs R occ fusif gyrus

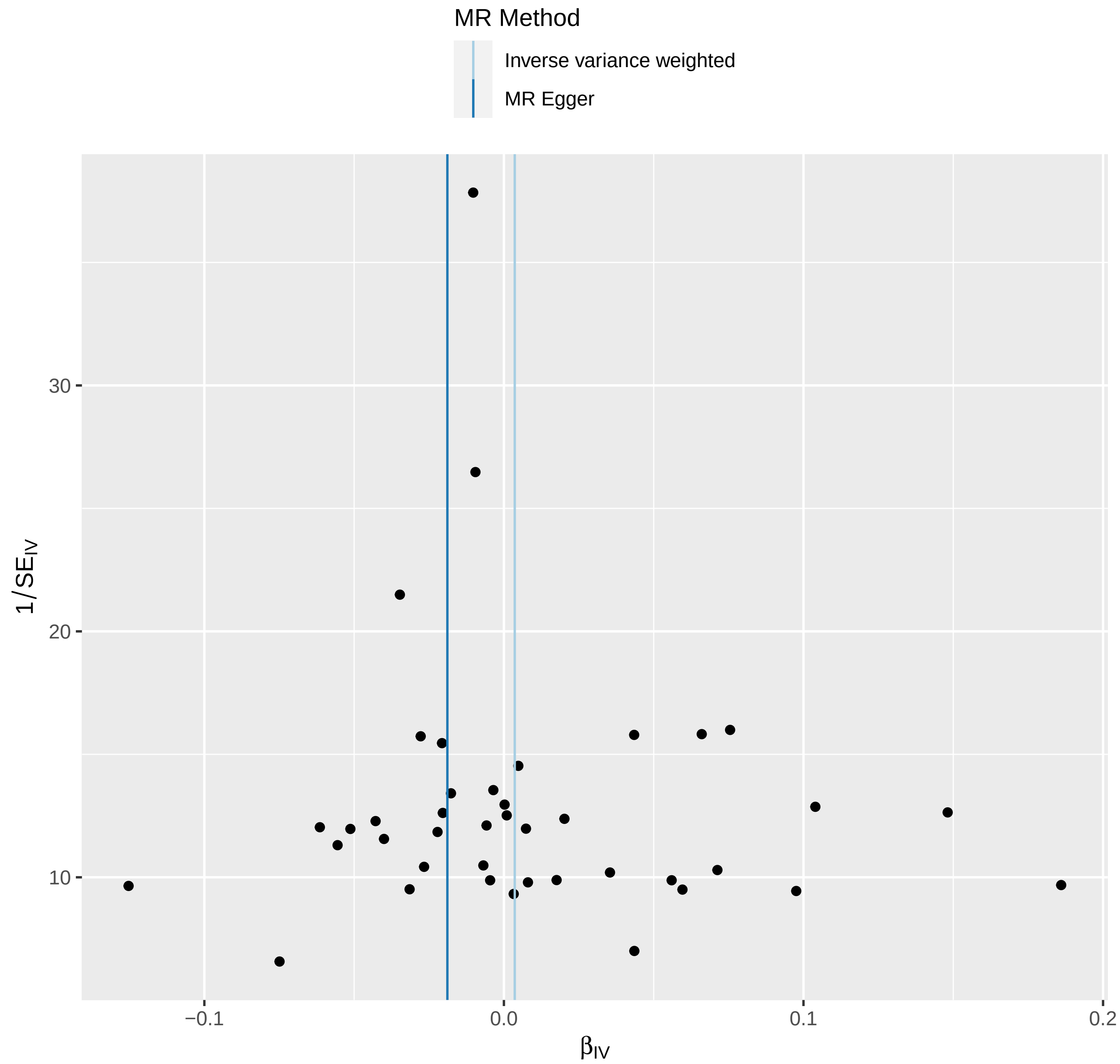

Batch 16 : Ulcerative colitis on IDP T1 FAST ROIs R occ fusif gyrus

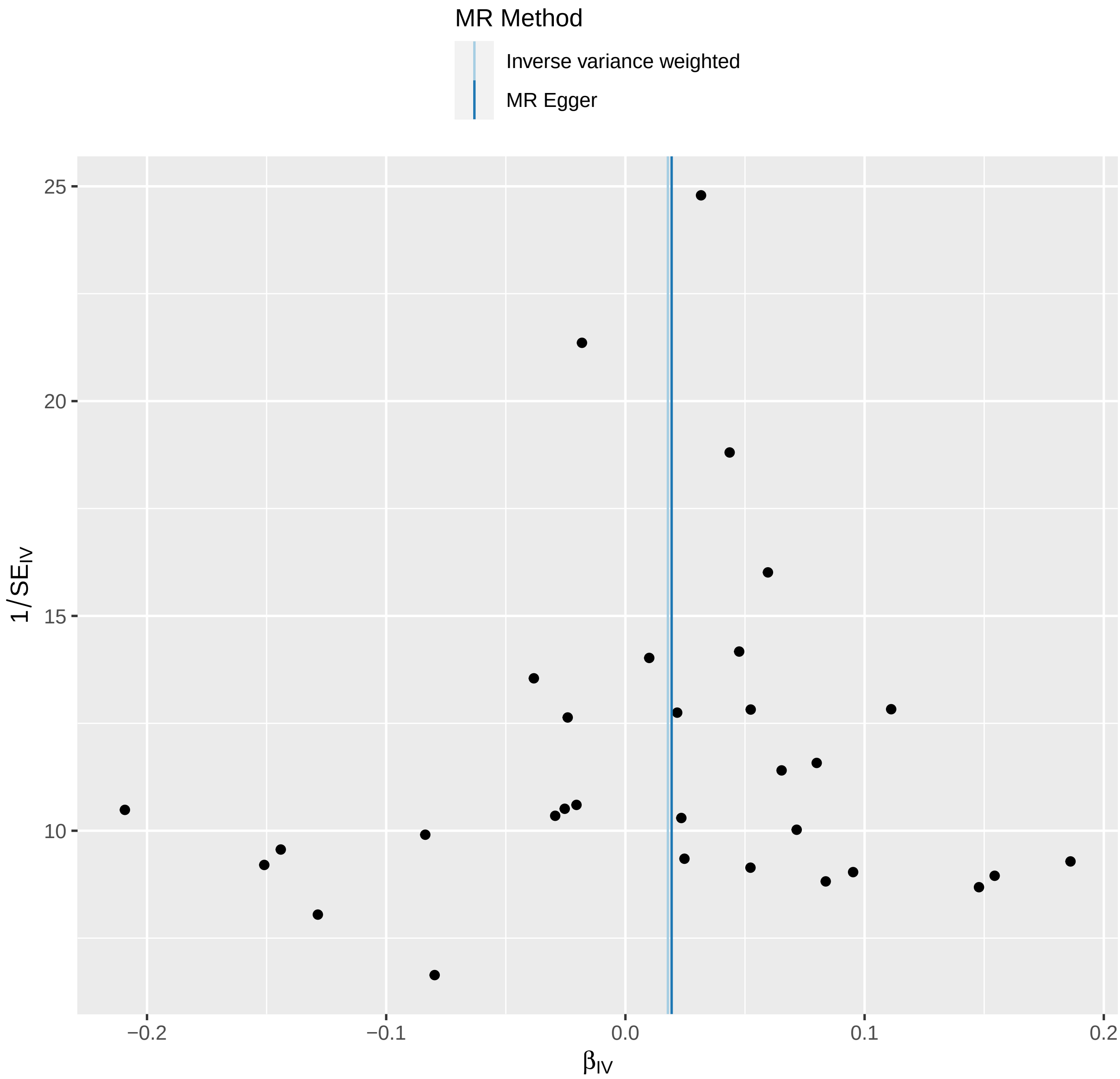

Batch 17 : Crohn's disease on IDP T1 FAST ROIs L front operc cortex

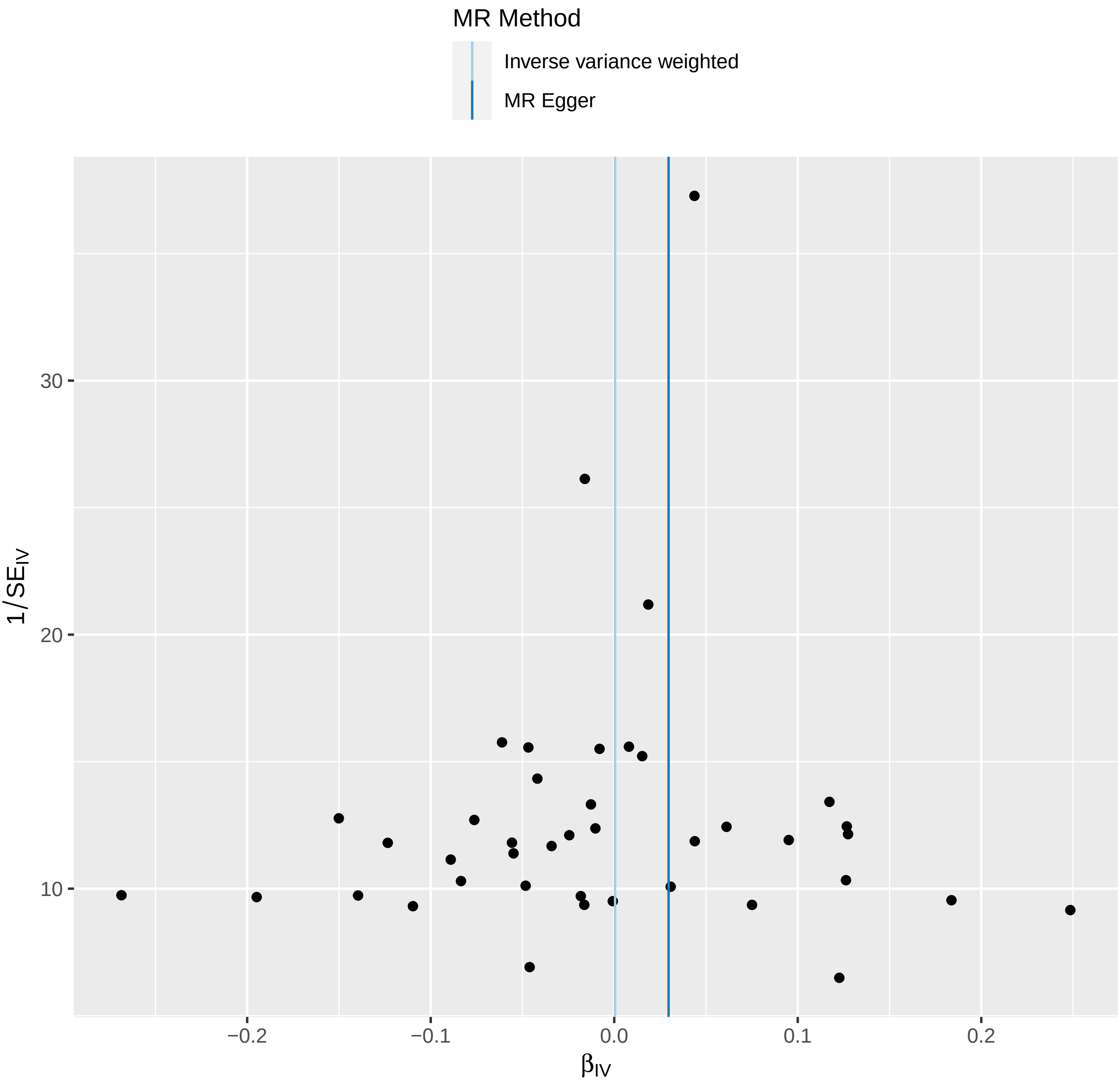

Batch 18 : Ulcerative colitis on IDP T1 FAST ROIs L front operc cortex

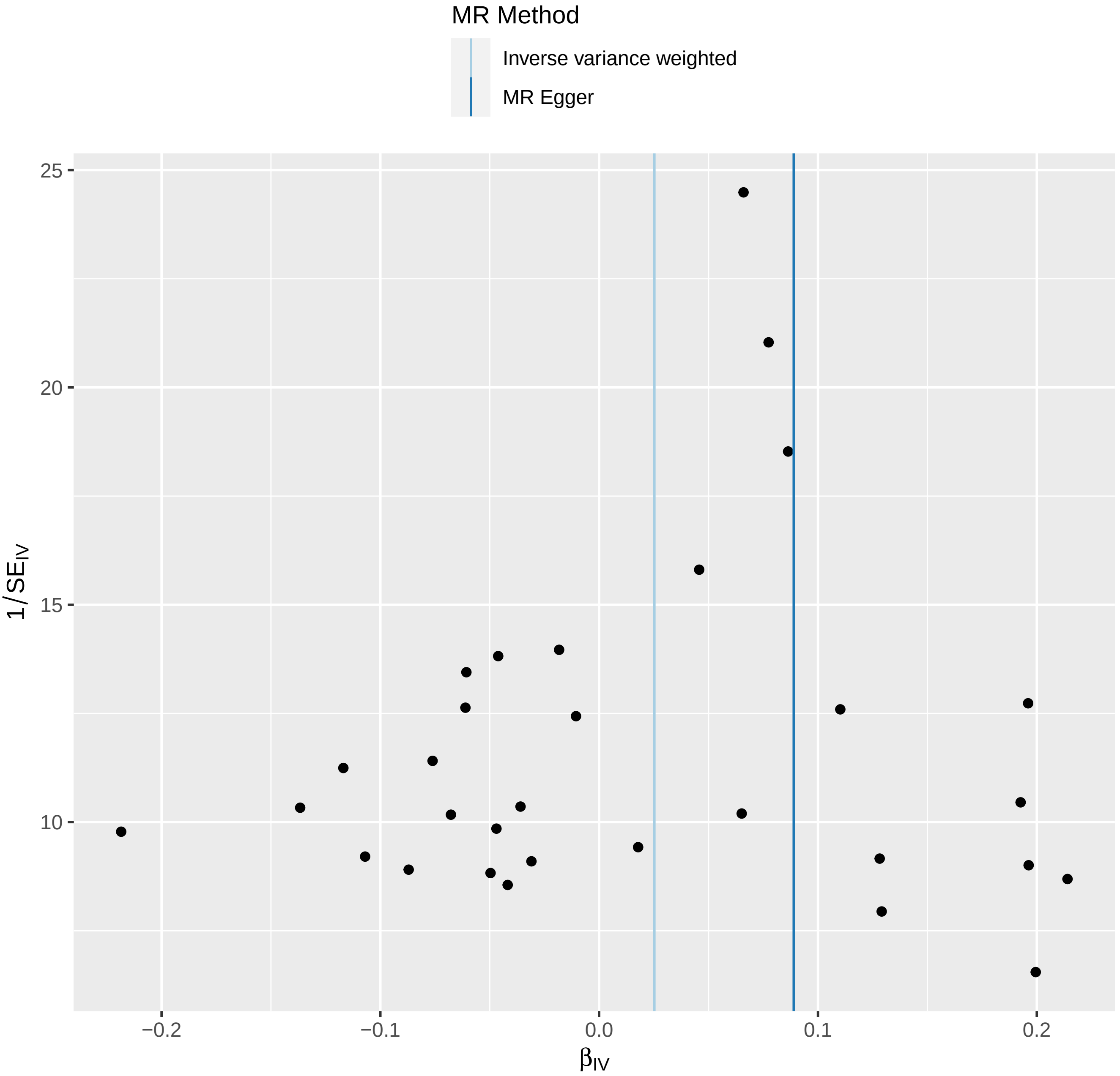

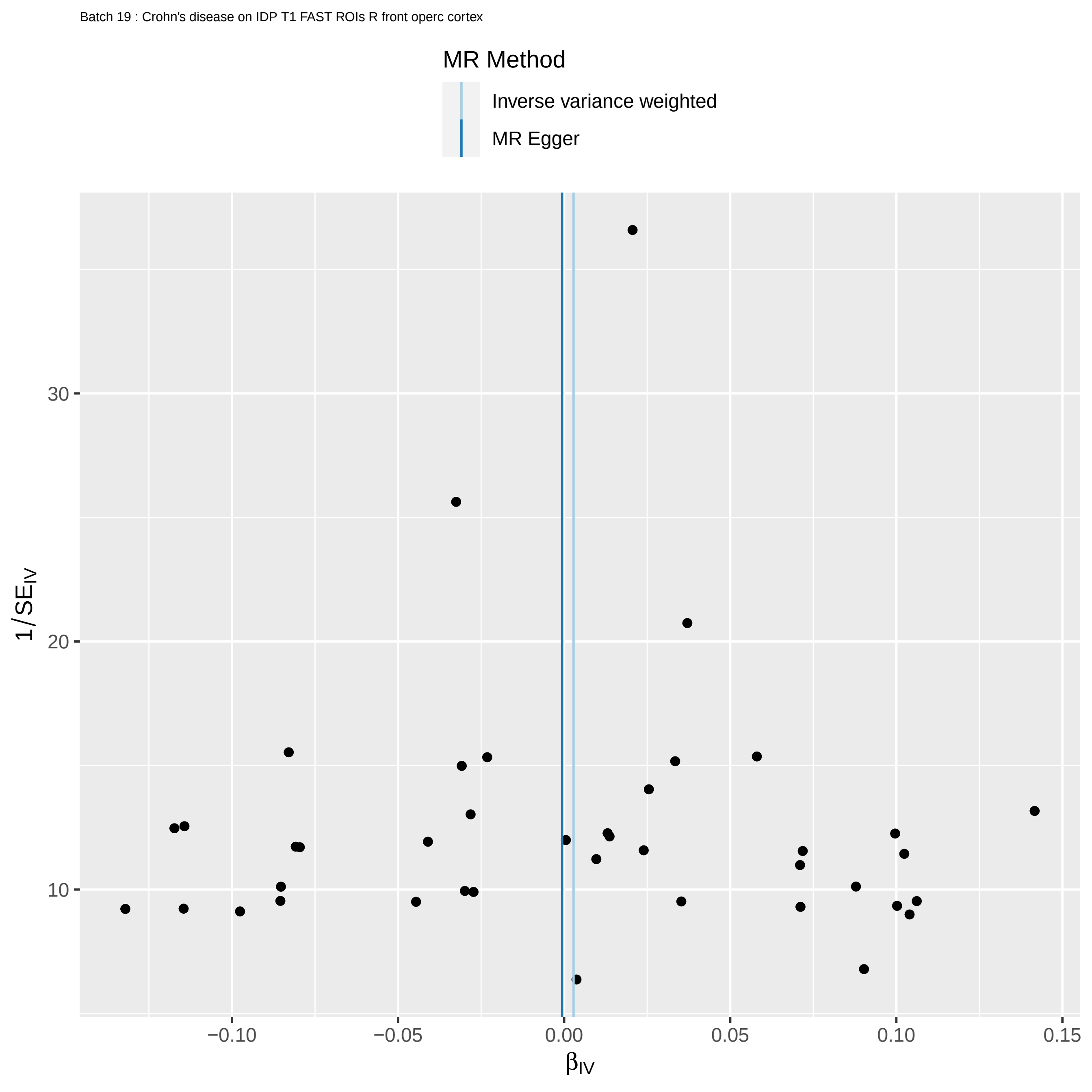
Batch 19 : Crohn's disease on IDP T1 FAST ROIs R front operc cortex
MR Method
Inverse variance weighted
MR Egger
30
20
10
$1/SE_{IV}$
−0.10
−0.05
0.00
0.05
0.10
0.15
β_{IV}

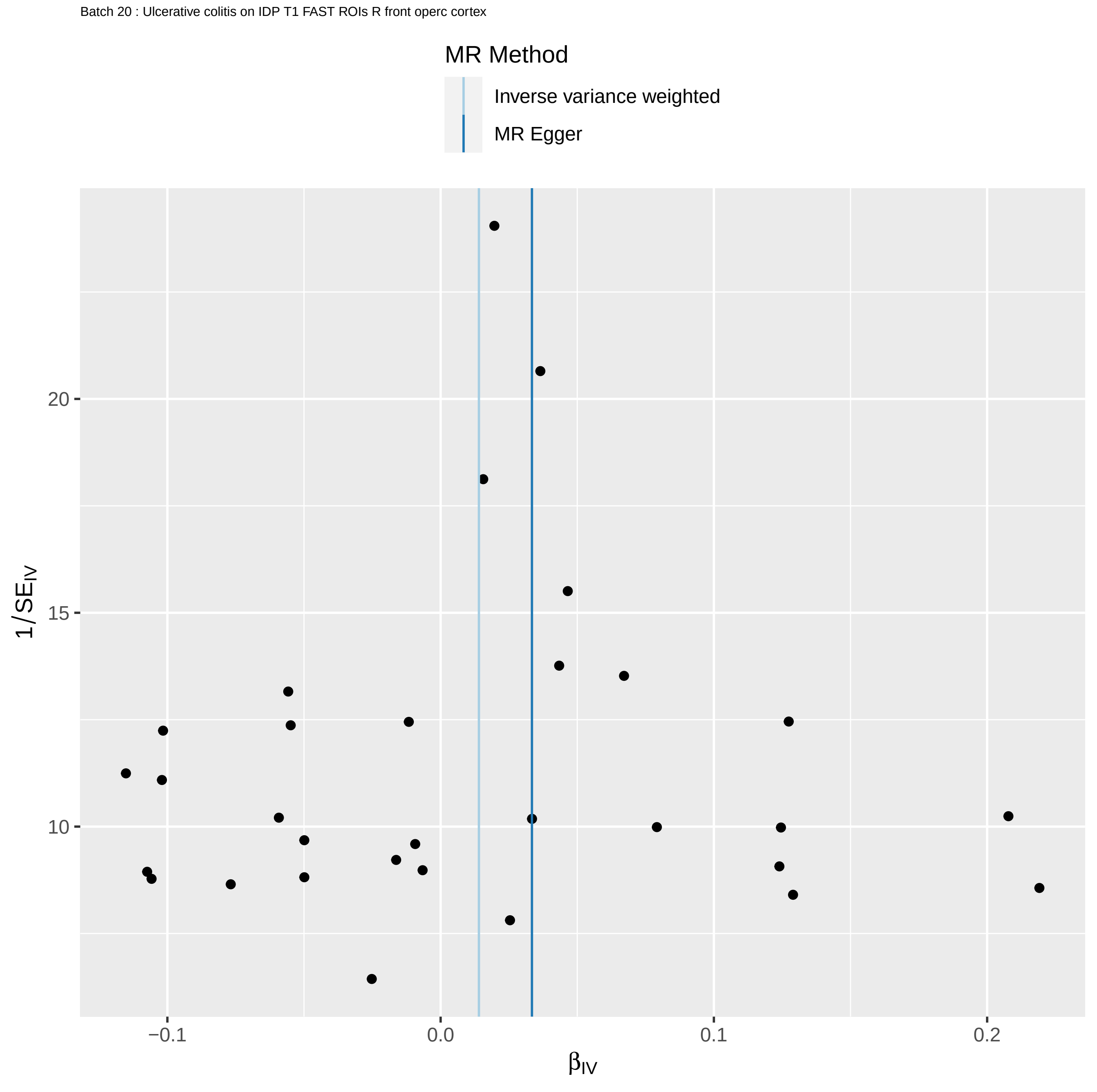
Batch 20 : Ulcerative colitis on IDP T1 FAST ROIs R front operc cortex
MR Method
Inverse variance weighted
MR Egger
20
15
10
1/SE_IV
−0.1
0.0
0.1
0.2
β_IV

Batch 21 : Crohn's disease on IDP T1 FAST ROIs L cent operc cortex

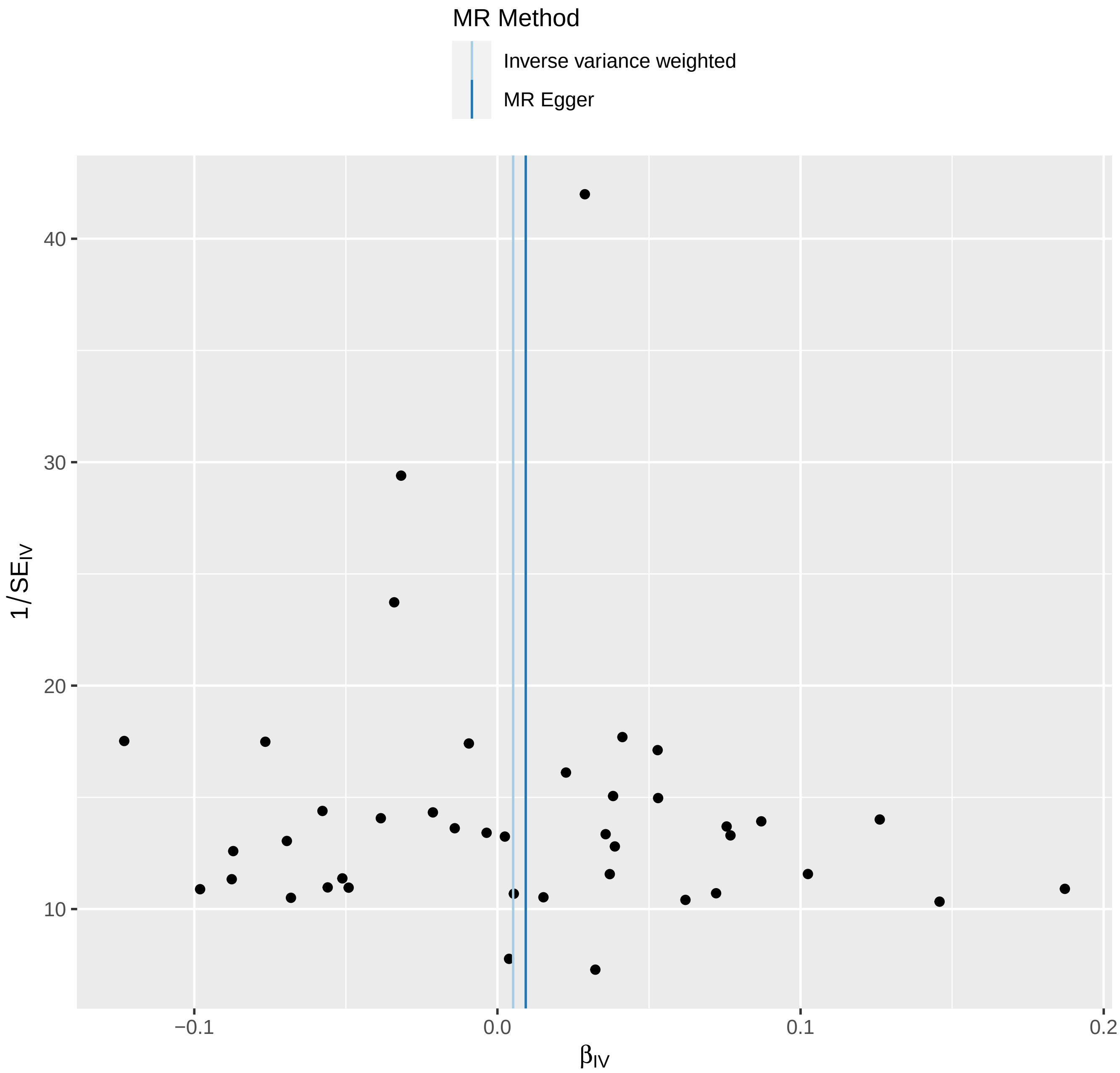

Batch 22 : Ulcerative colitis on IDP T1 FAST ROIs L cent operc cortex

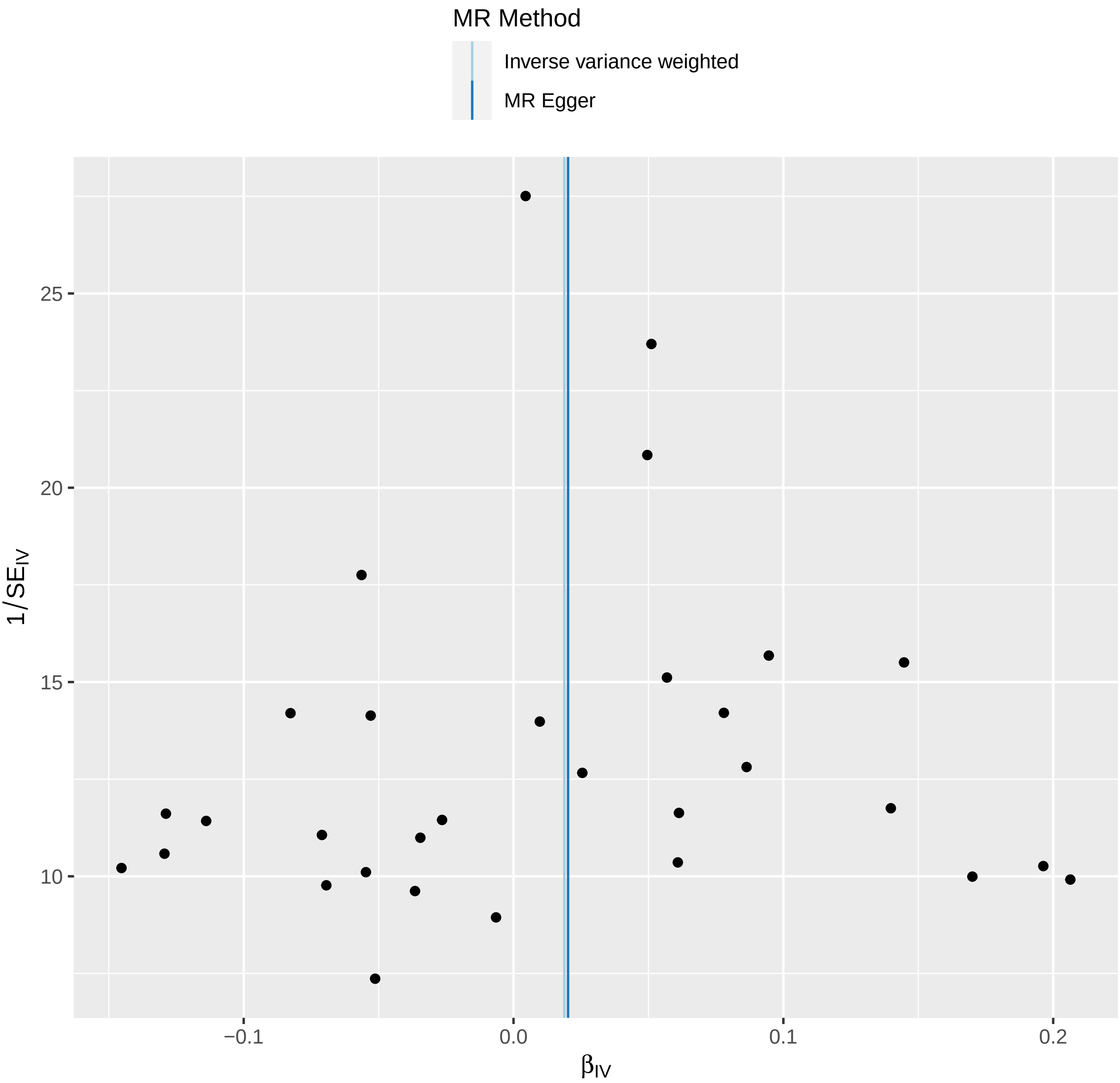

Batch 23 : Crohn's disease on IDP T1 FAST ROIs R cent operc cortex

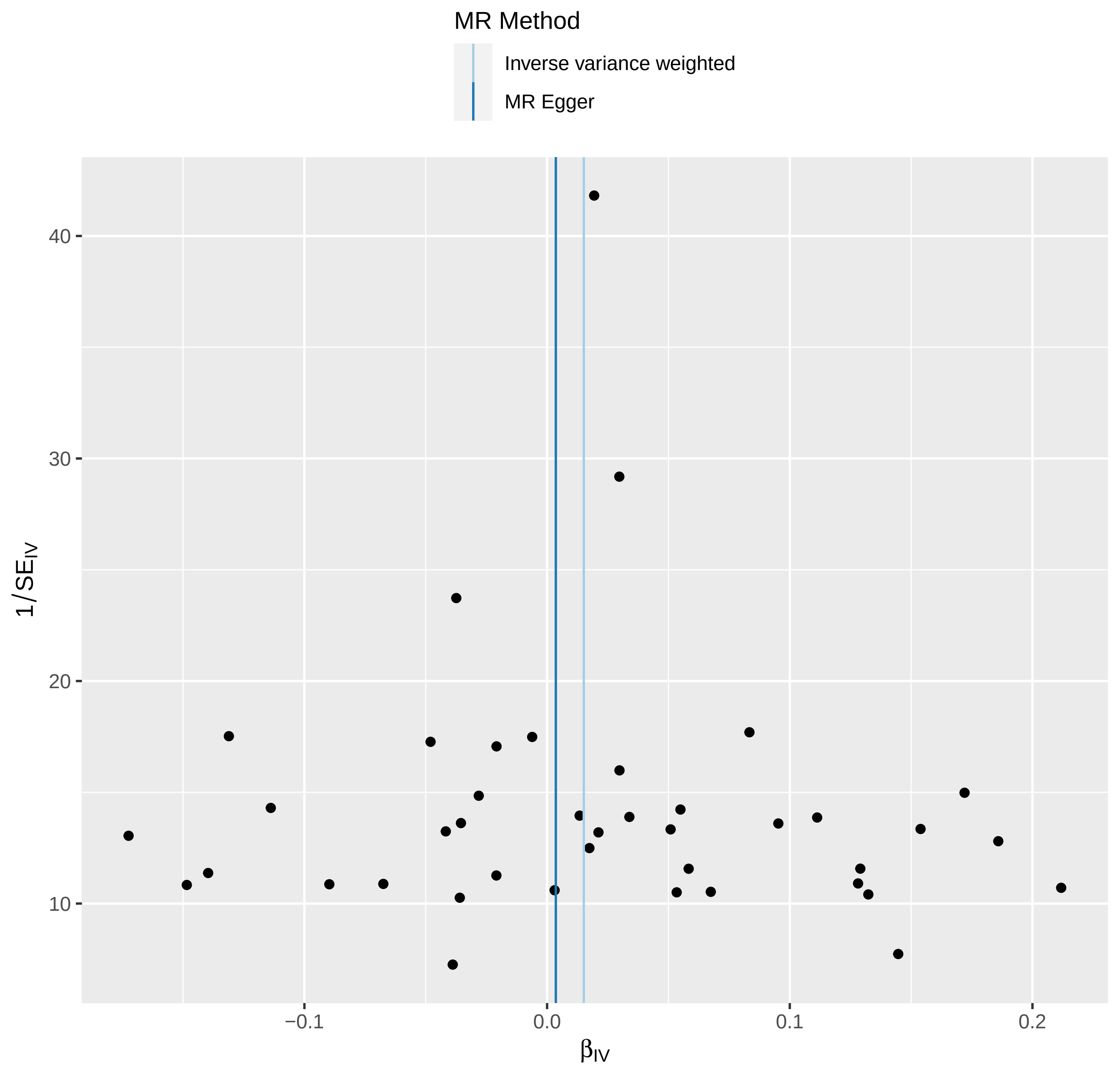

Batch 24 : Ulcerative colitis on IDP T1 FAST ROIs R cent operc cortex

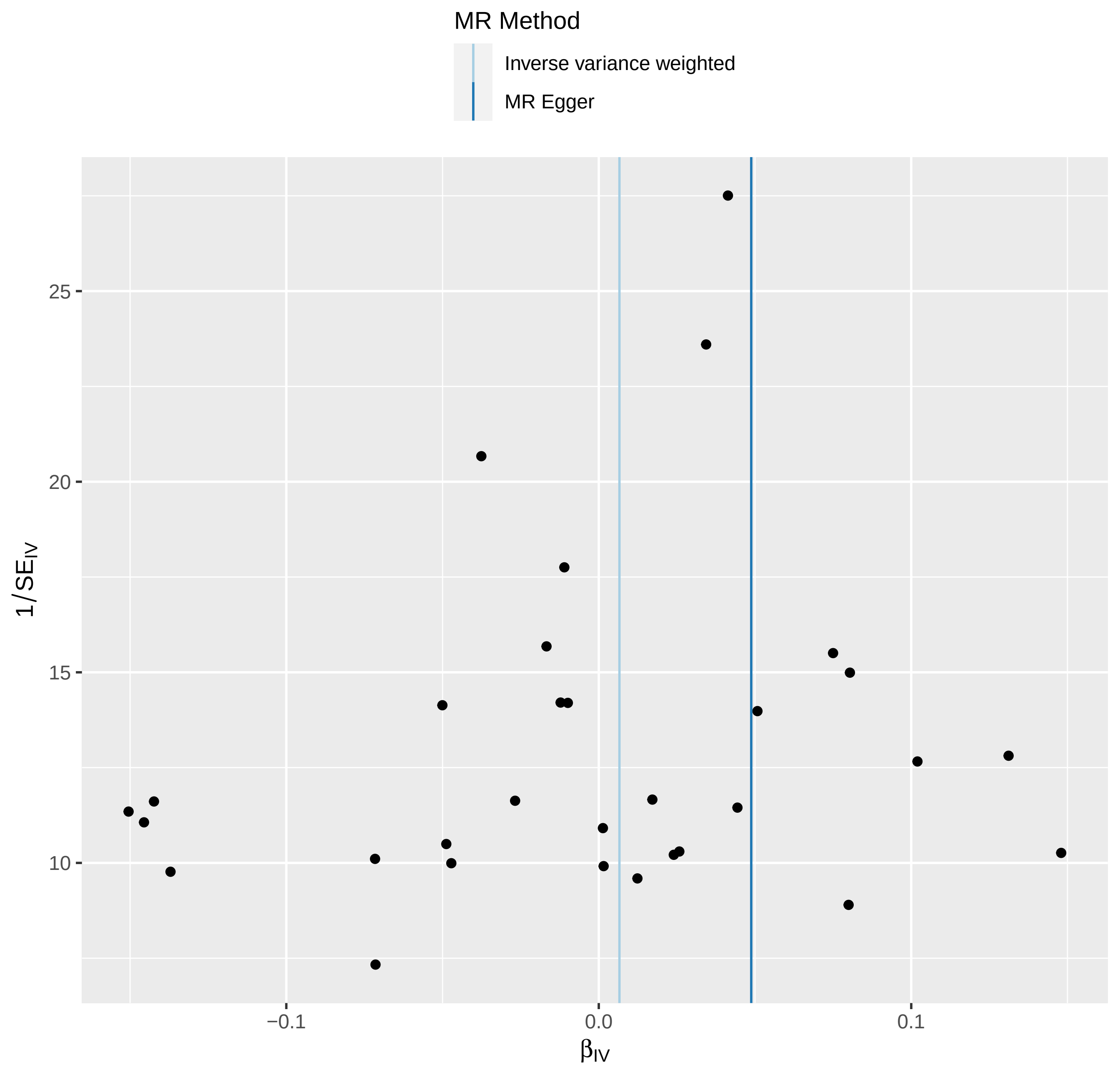

Batch 25 : Crohn's disease on IDP T1 FIRST left thalamus volume

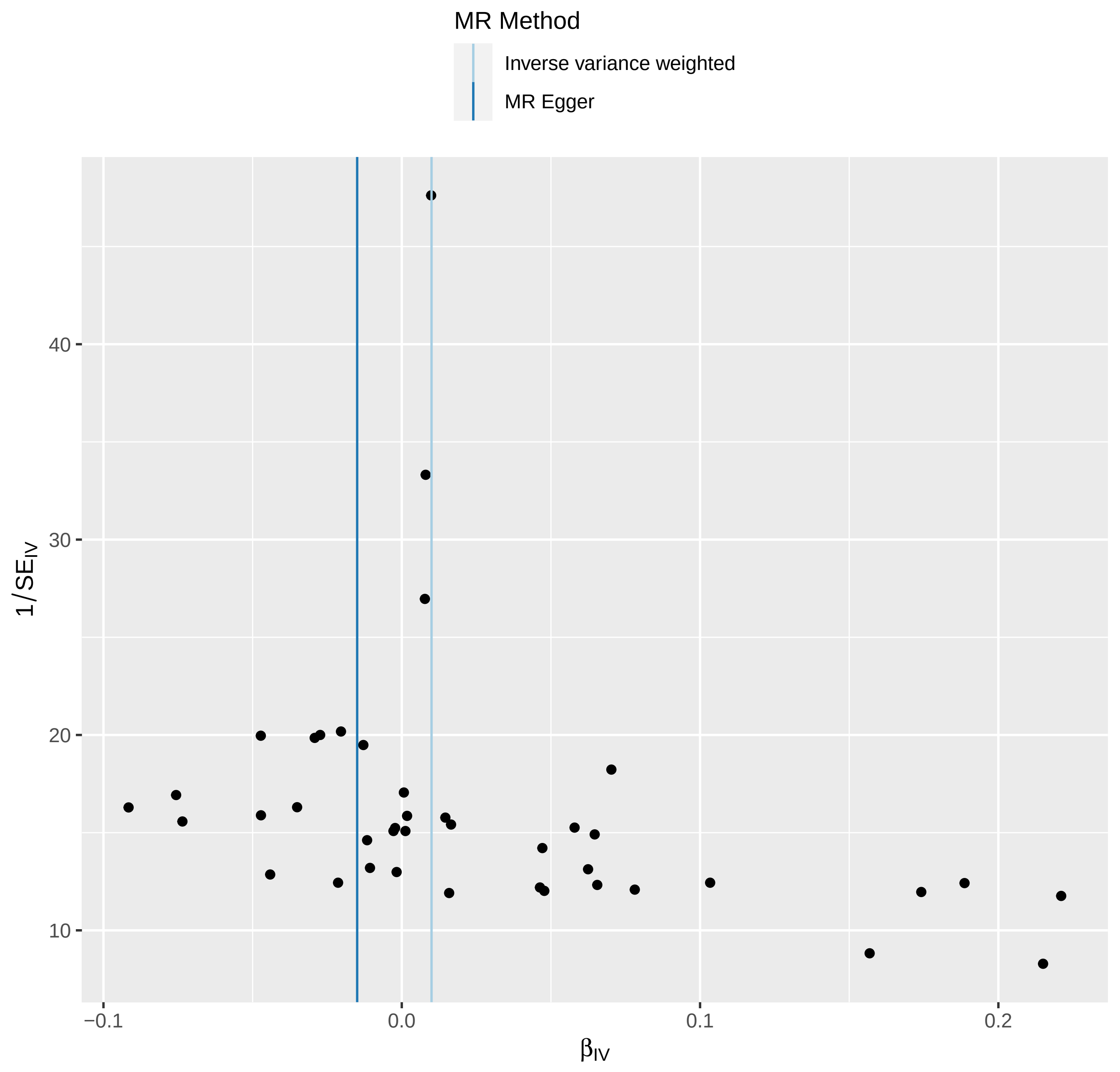

Batch 26 : Ulcerative colitis on IDP T1 FIRST left thalamus volume

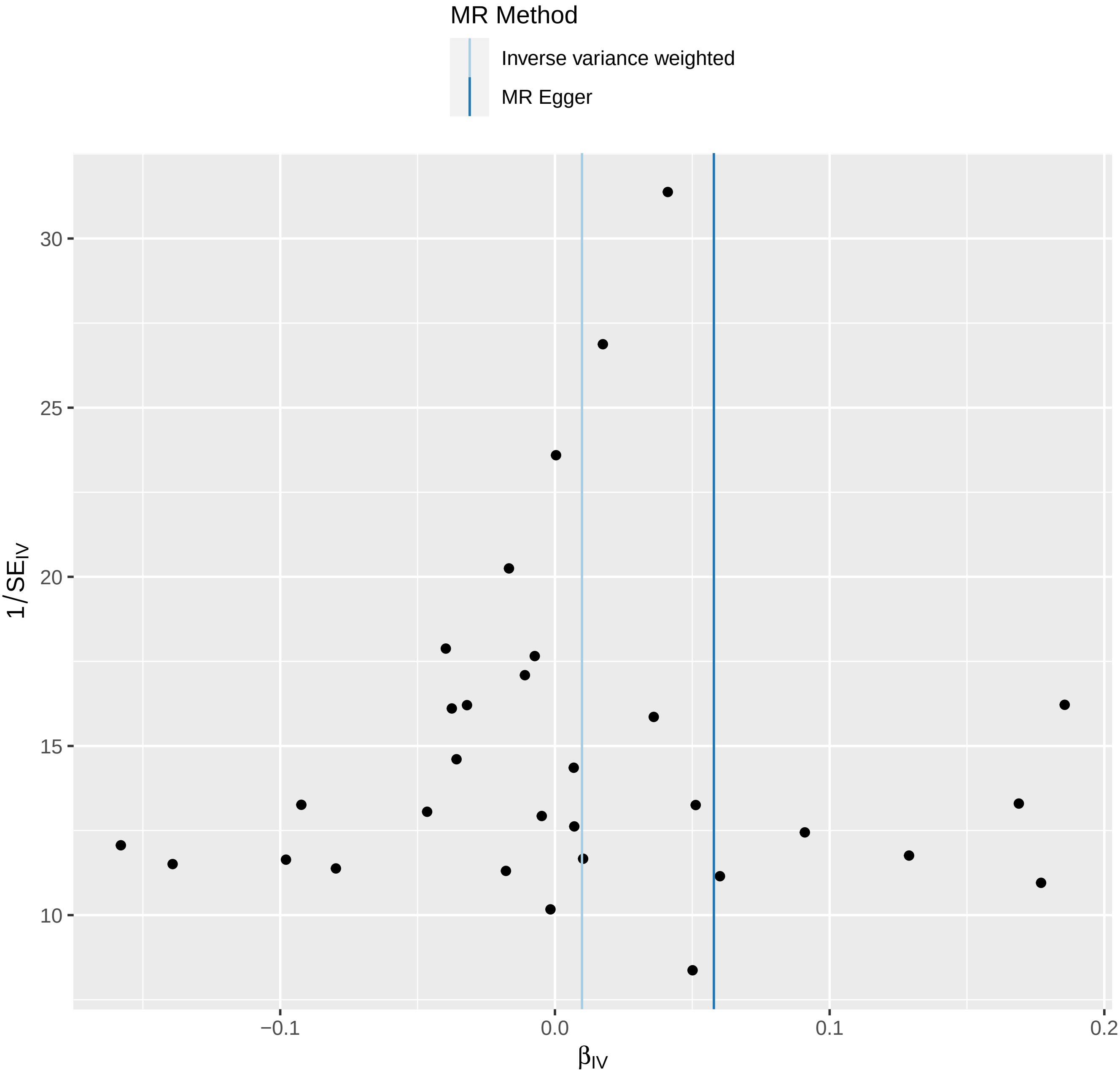

Batch 27 : Crohn's disease on IDP T1 FAST ROIs L parietal operc cortex

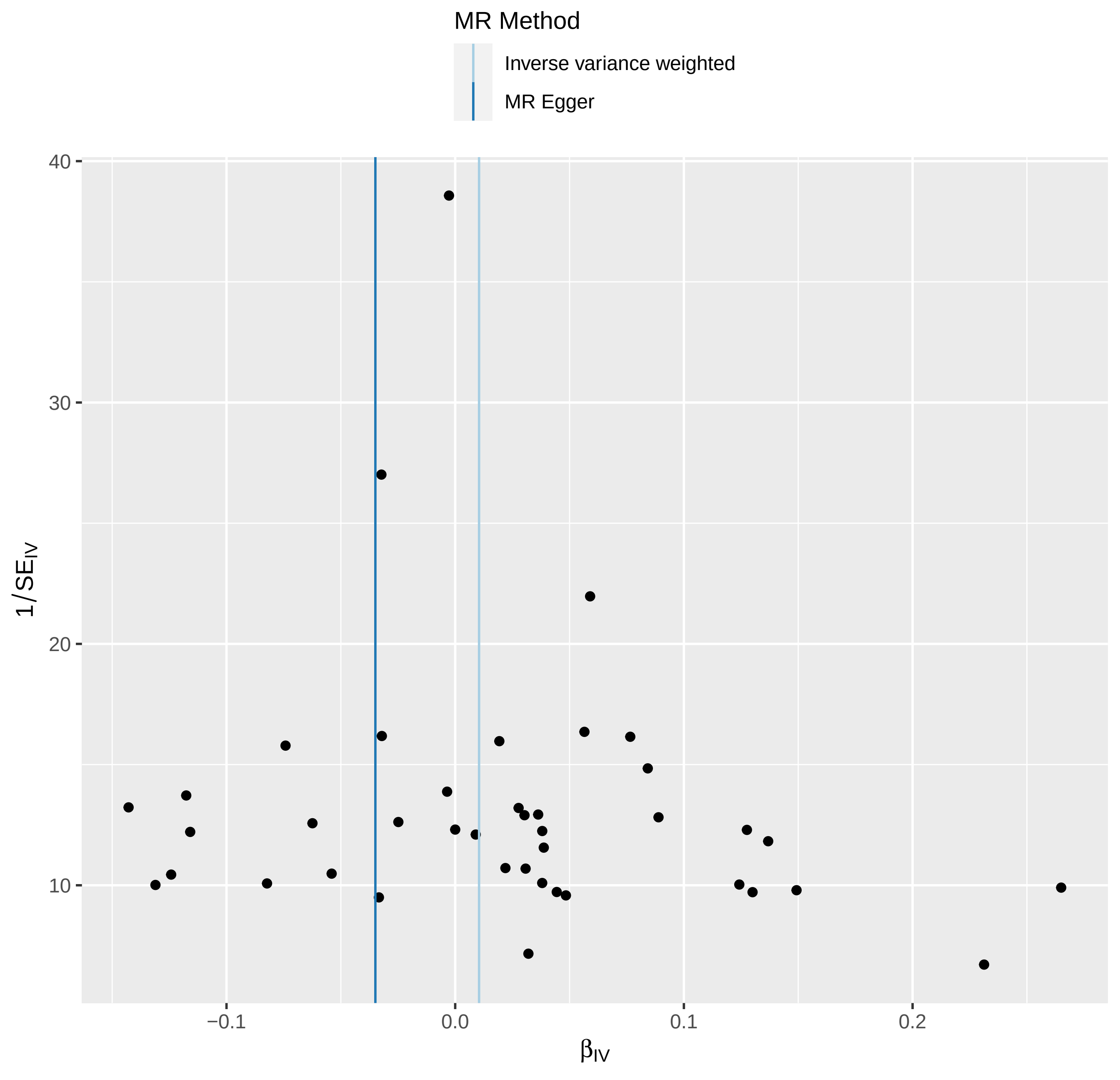

Batch 28 : Ulcerative colitis on IDP T1 FAST ROIs L parietal operc cortex

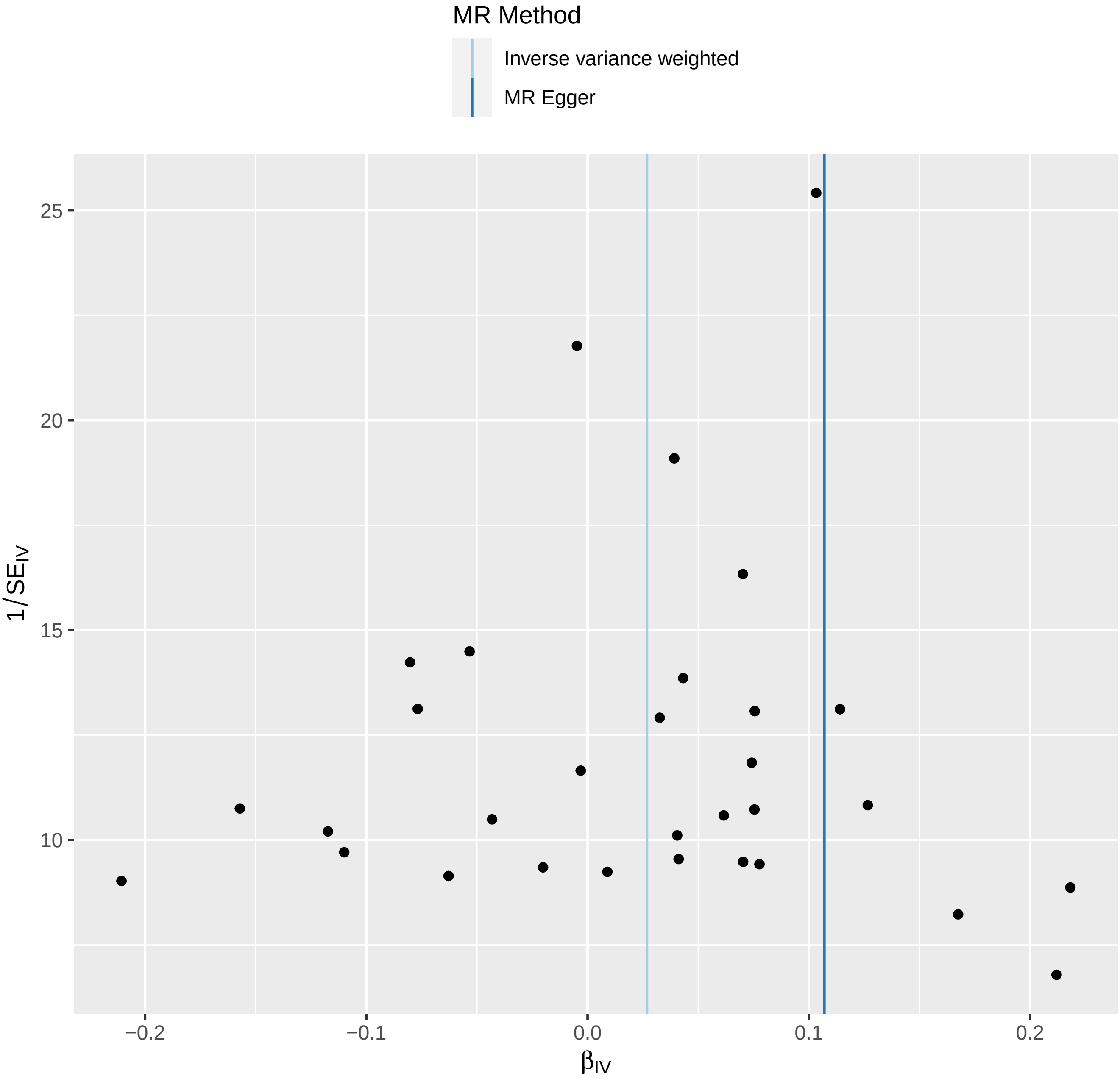

Batch 29 : Crohn's disease on IDP T1 FAST ROIs R parietal operc cortex

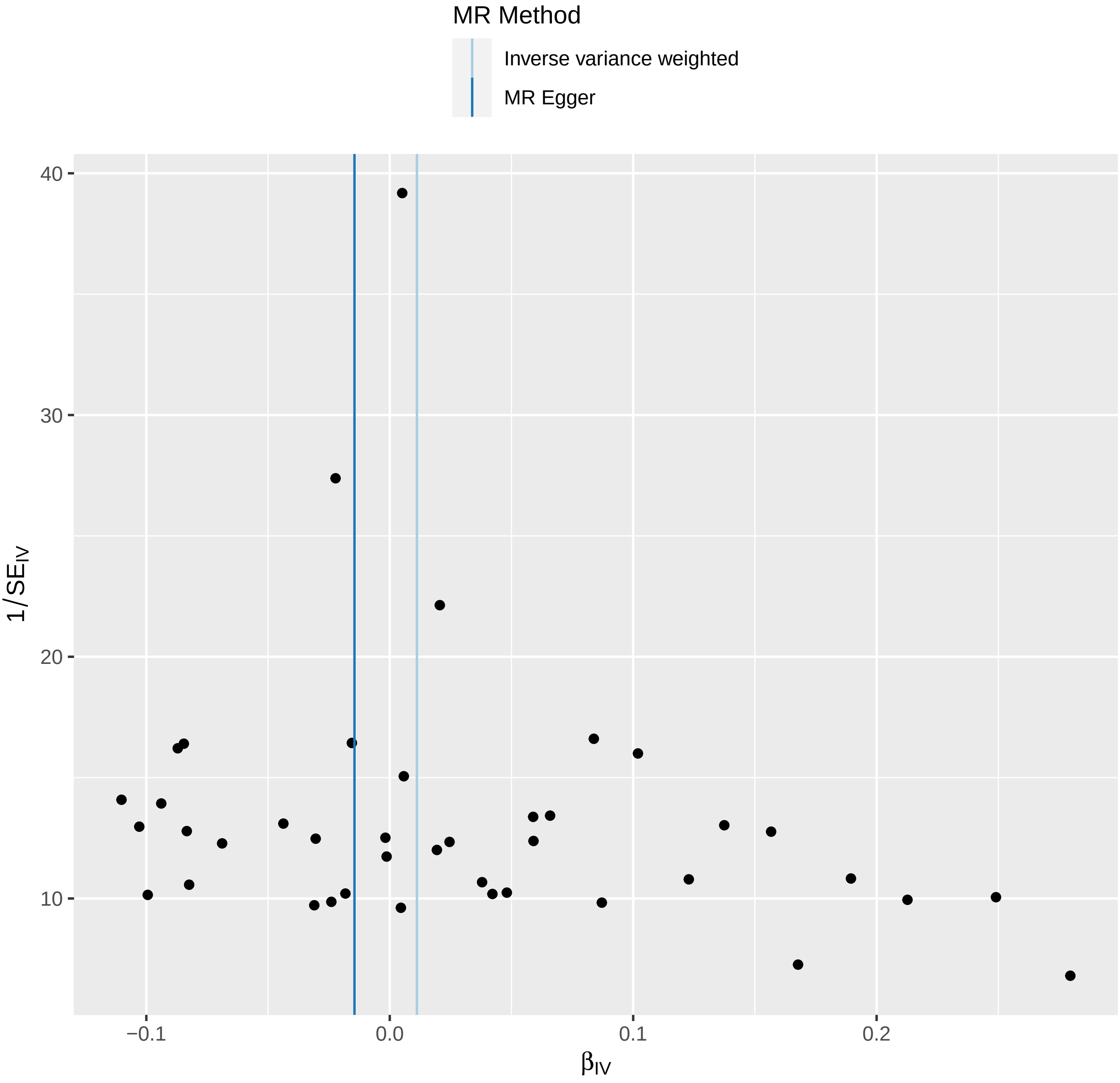

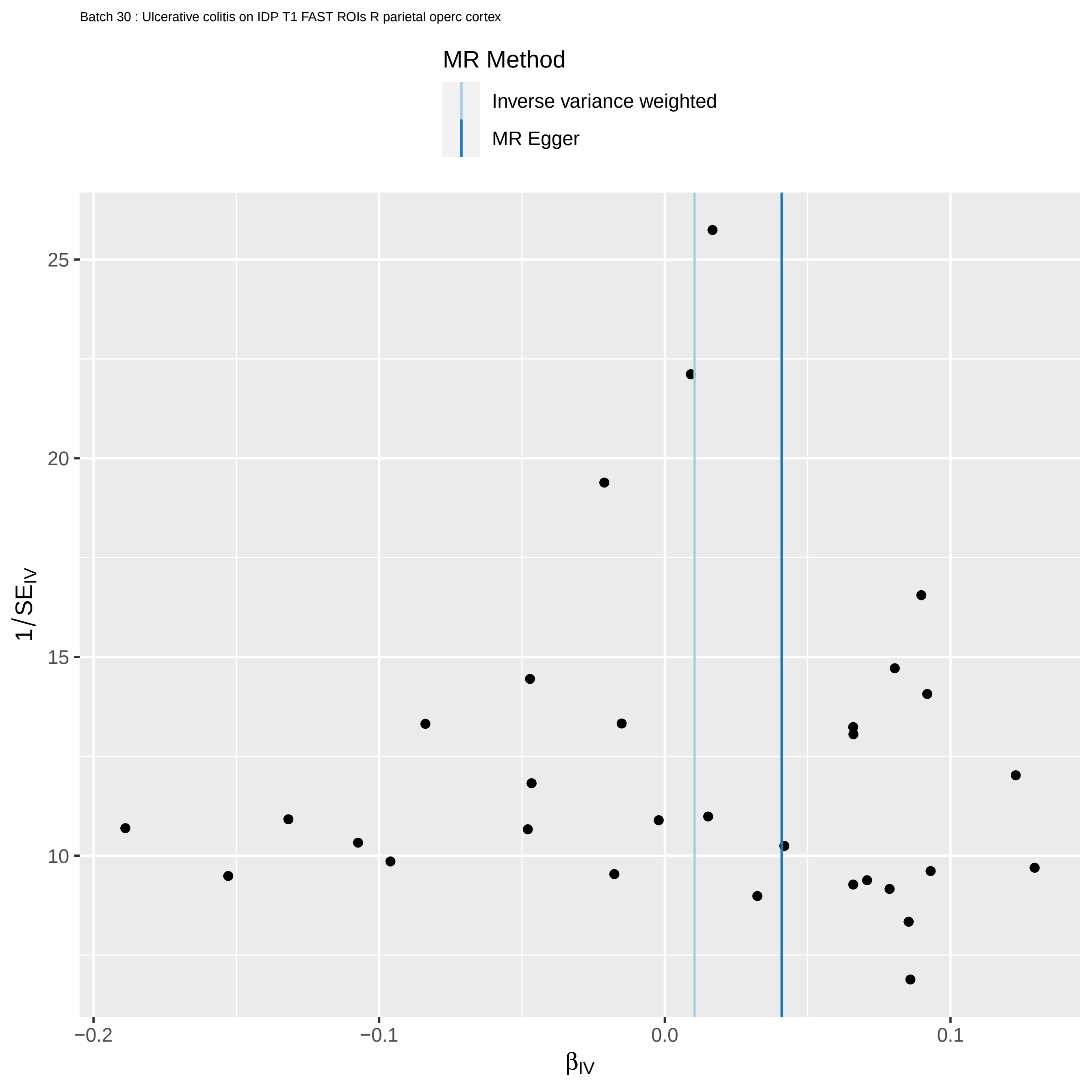
Batch 30 : Ulcerative colitis on IDP T1 FAST ROIs R parietal operc cortex
MR Method
Inverse variance weighted
MR Egger
25
20
15
10
$1/SE_{IV}$
−0.2
−0.1
0.0
0.1
β_{IV}

Batch 31 : Crohn's disease on IDP T1 FAST ROIs L planum polare

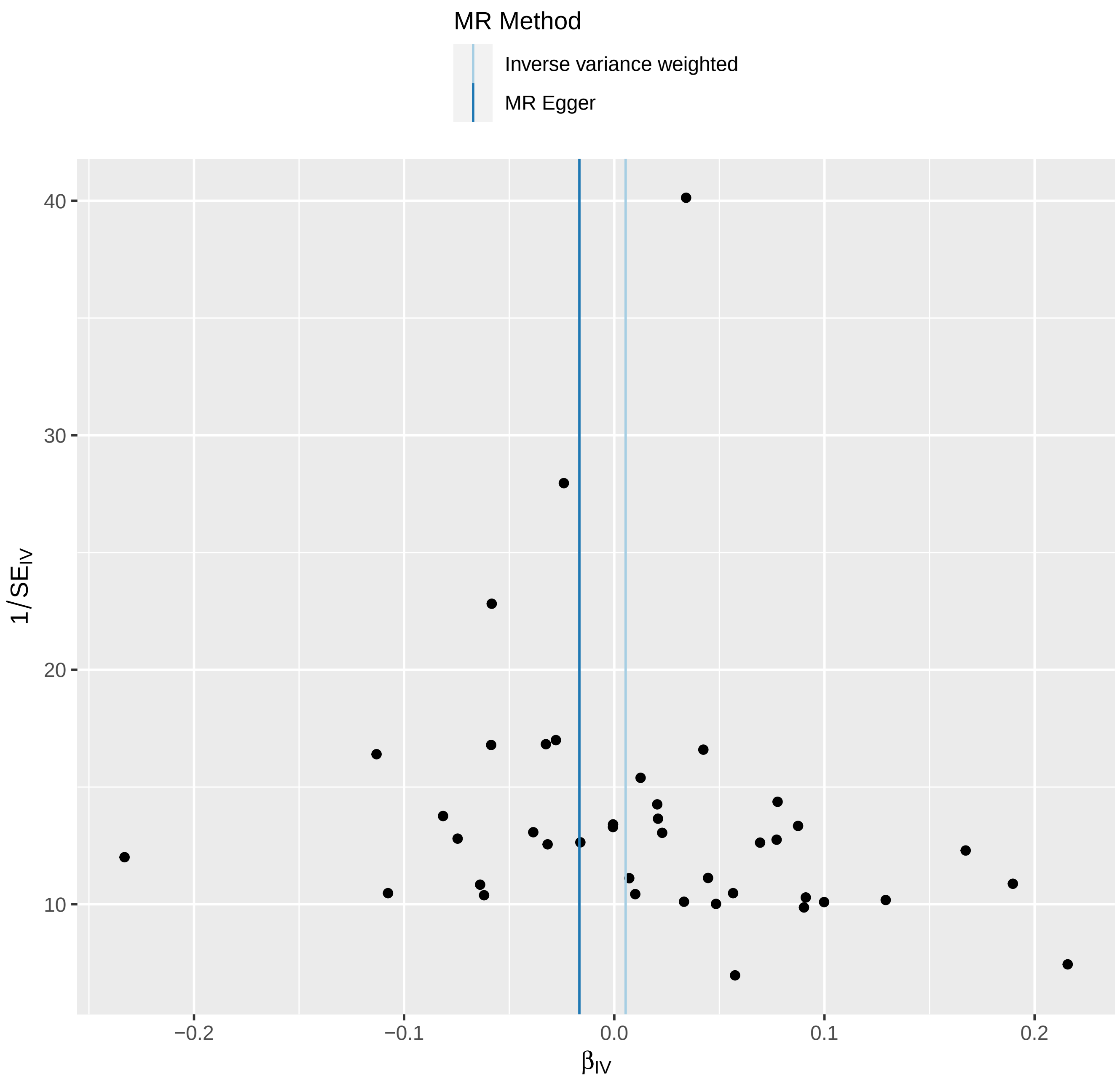

Batch 32 : Ulcerative colitis on IDP T1 FAST ROIs L planum polare

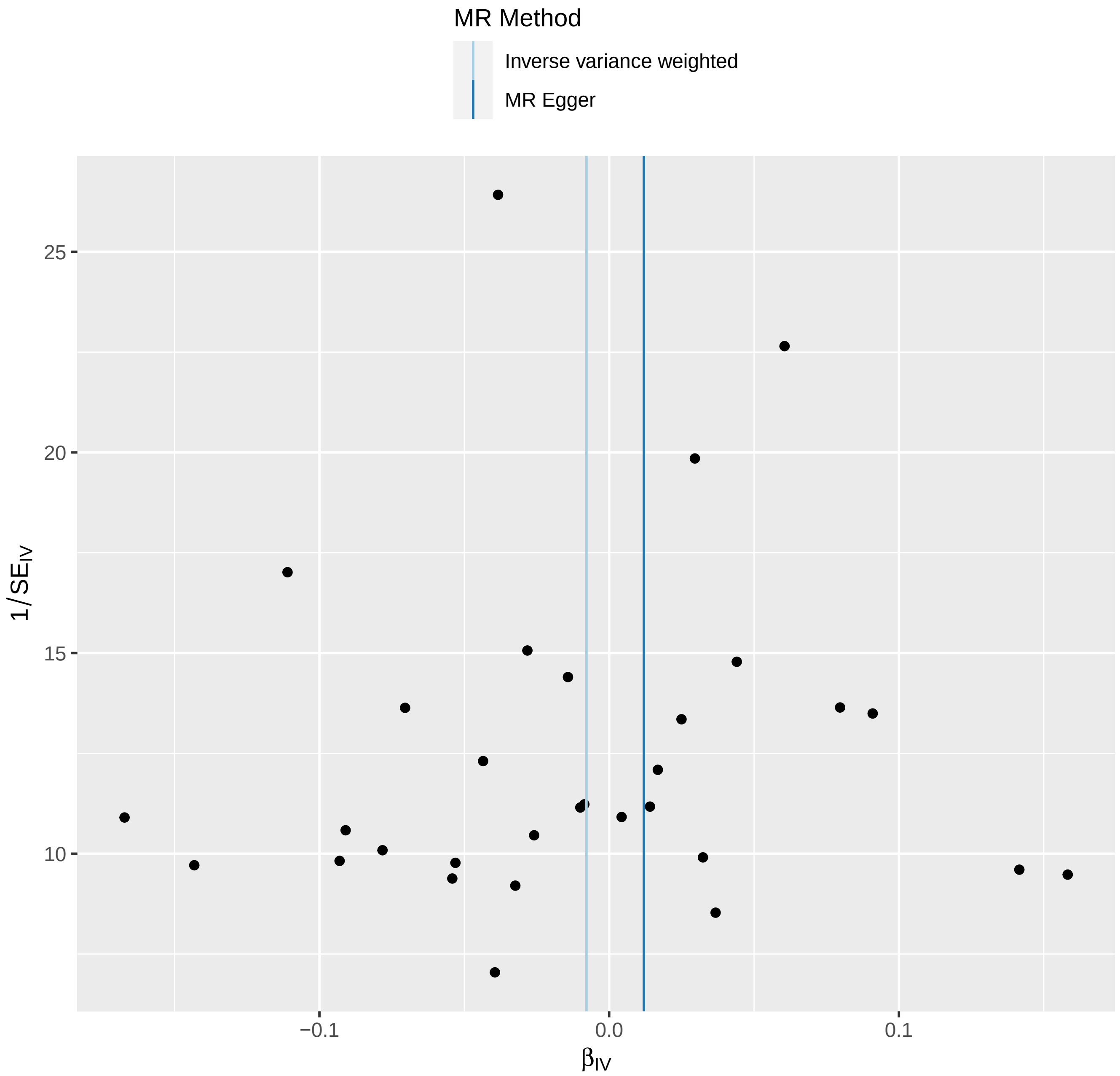

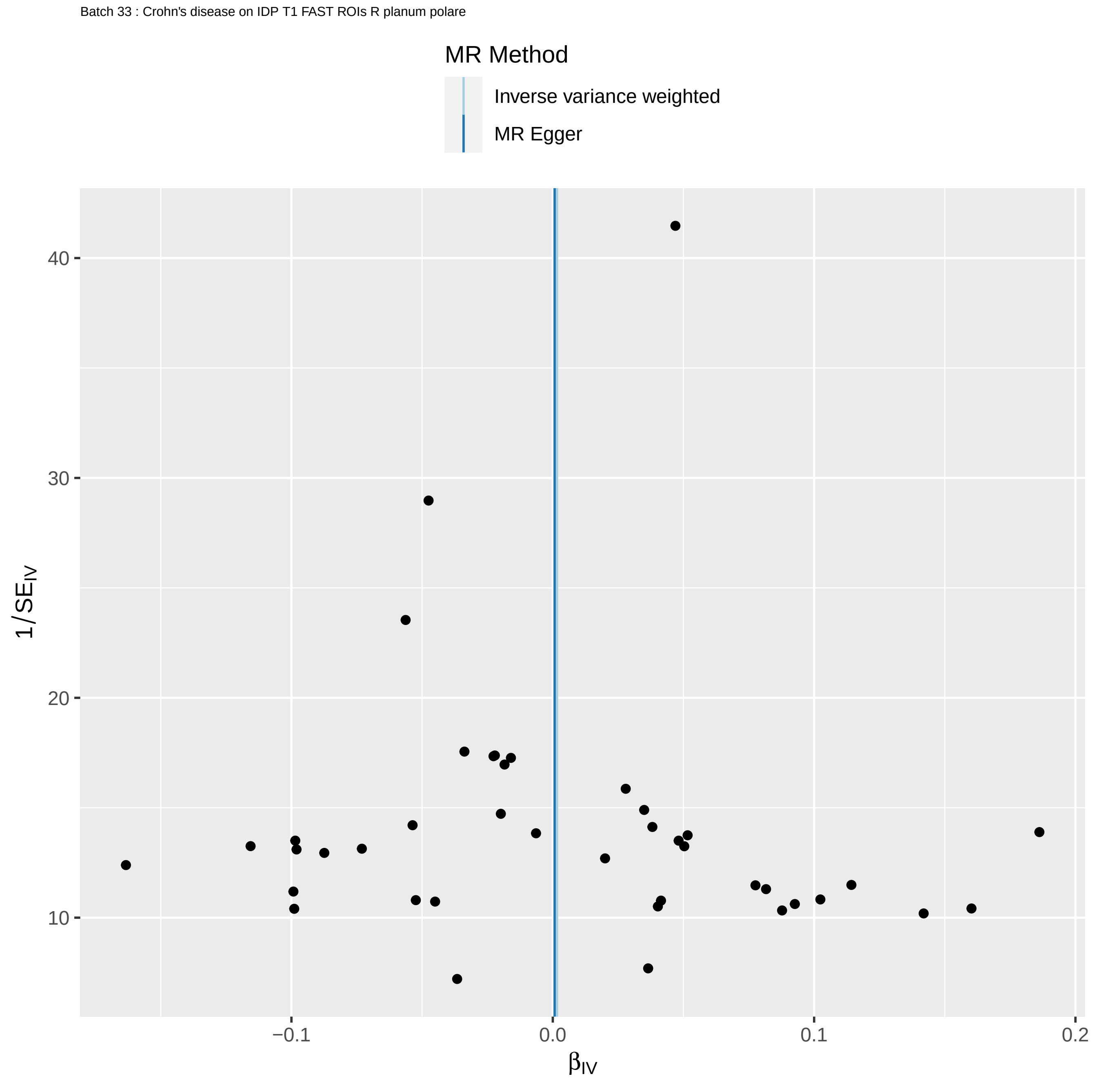
Batch 33 : Crohn's disease on IDP T1 FAST ROIs R planum polare
MR Method
Inverse variance weighted
MR Egger
1/SE_IV
β_IV
40
30
20
10
−0.1
0.0
0.1
0.2

Batch 34 : Ulcerative colitis on IDP T1 FAST ROIs R planum polare

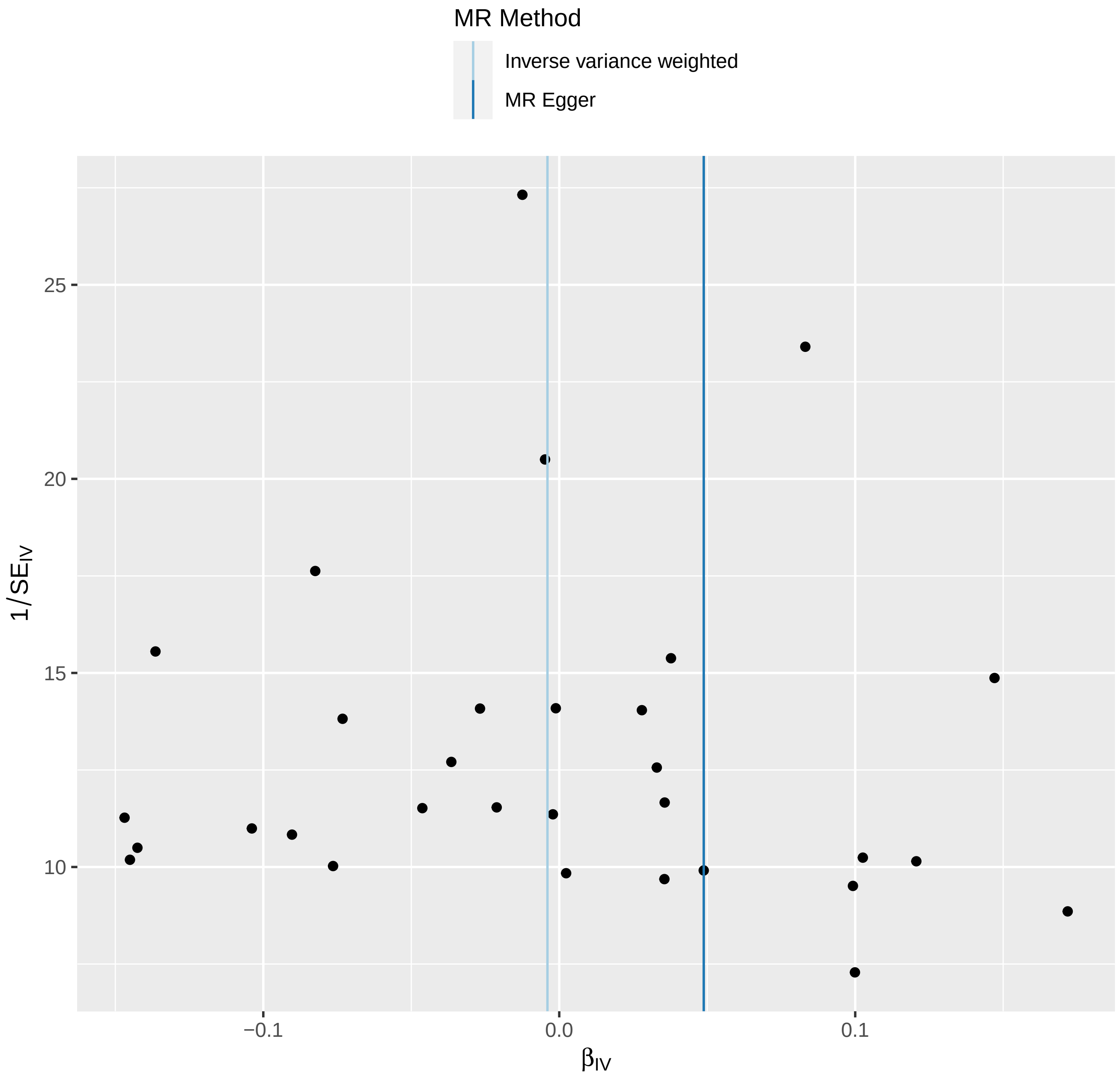

Batch 35 : Crohn's disease on IDP T1 FAST ROIs L heschl gyrus

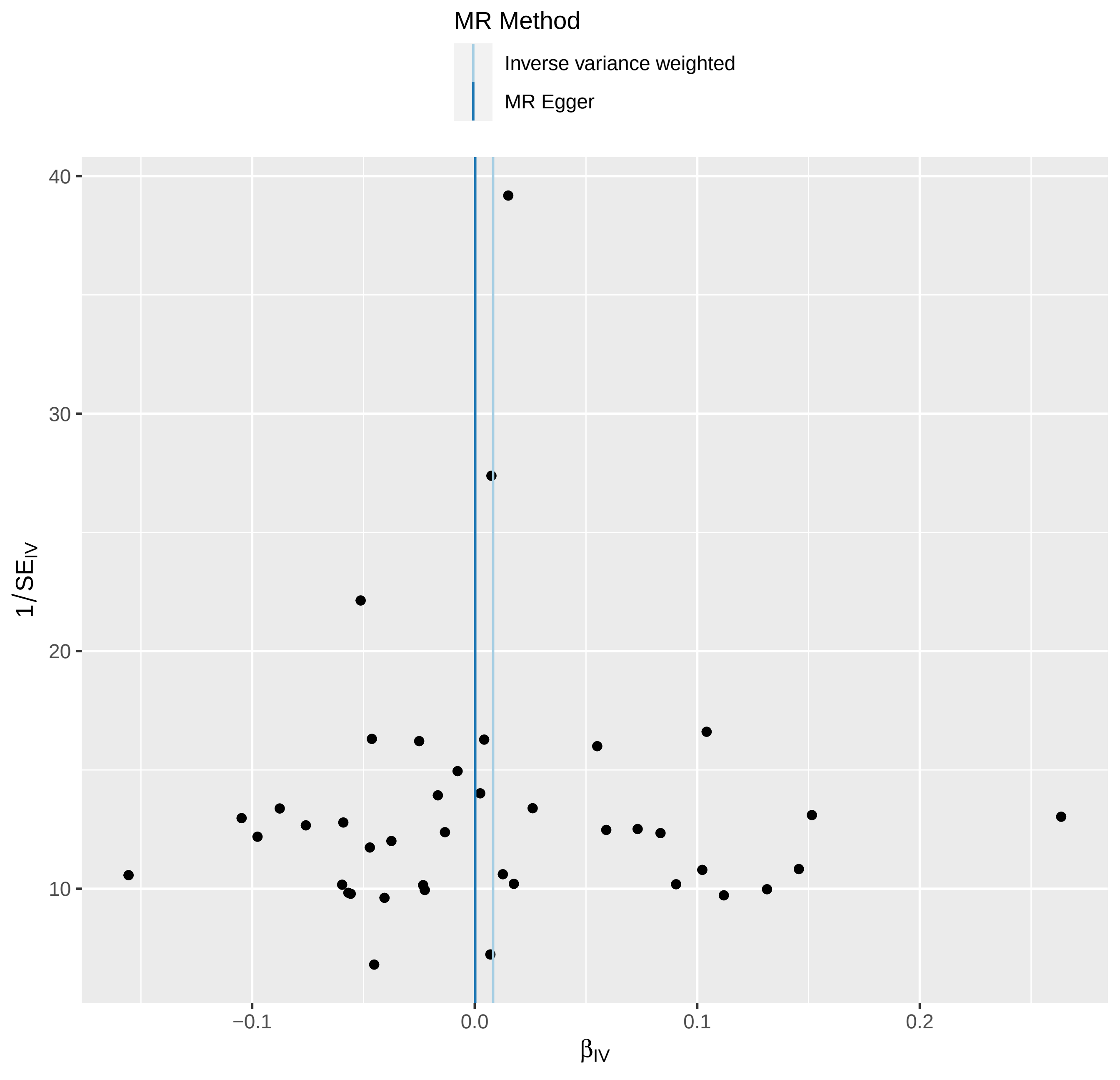

Batch 36 : Ulcerative colitis on IDP T1 FAST ROIs L heschl gyrus

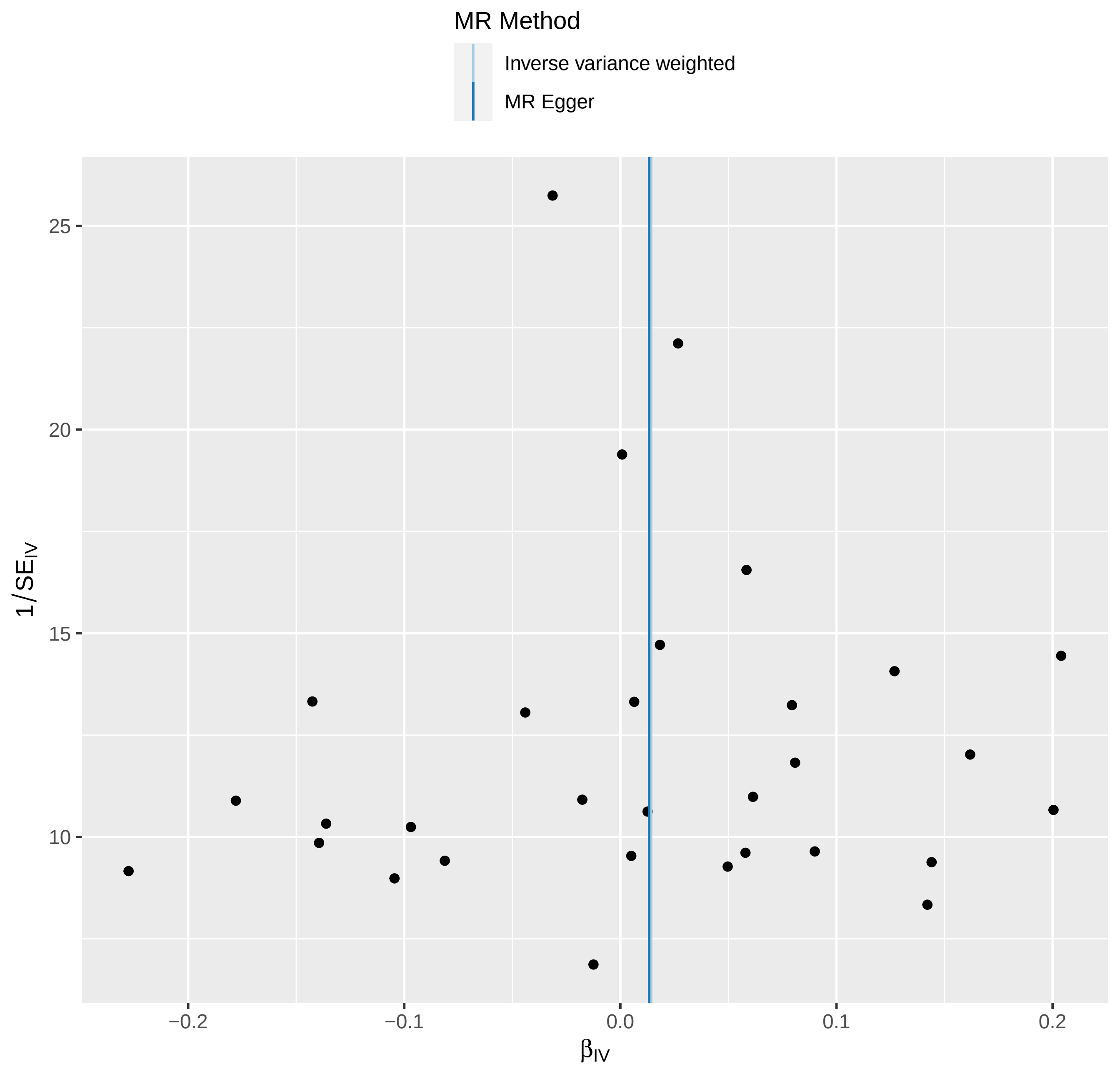

Batch 37 : Crohn's disease on IDP T1 FAST ROIs R heschl gyrus

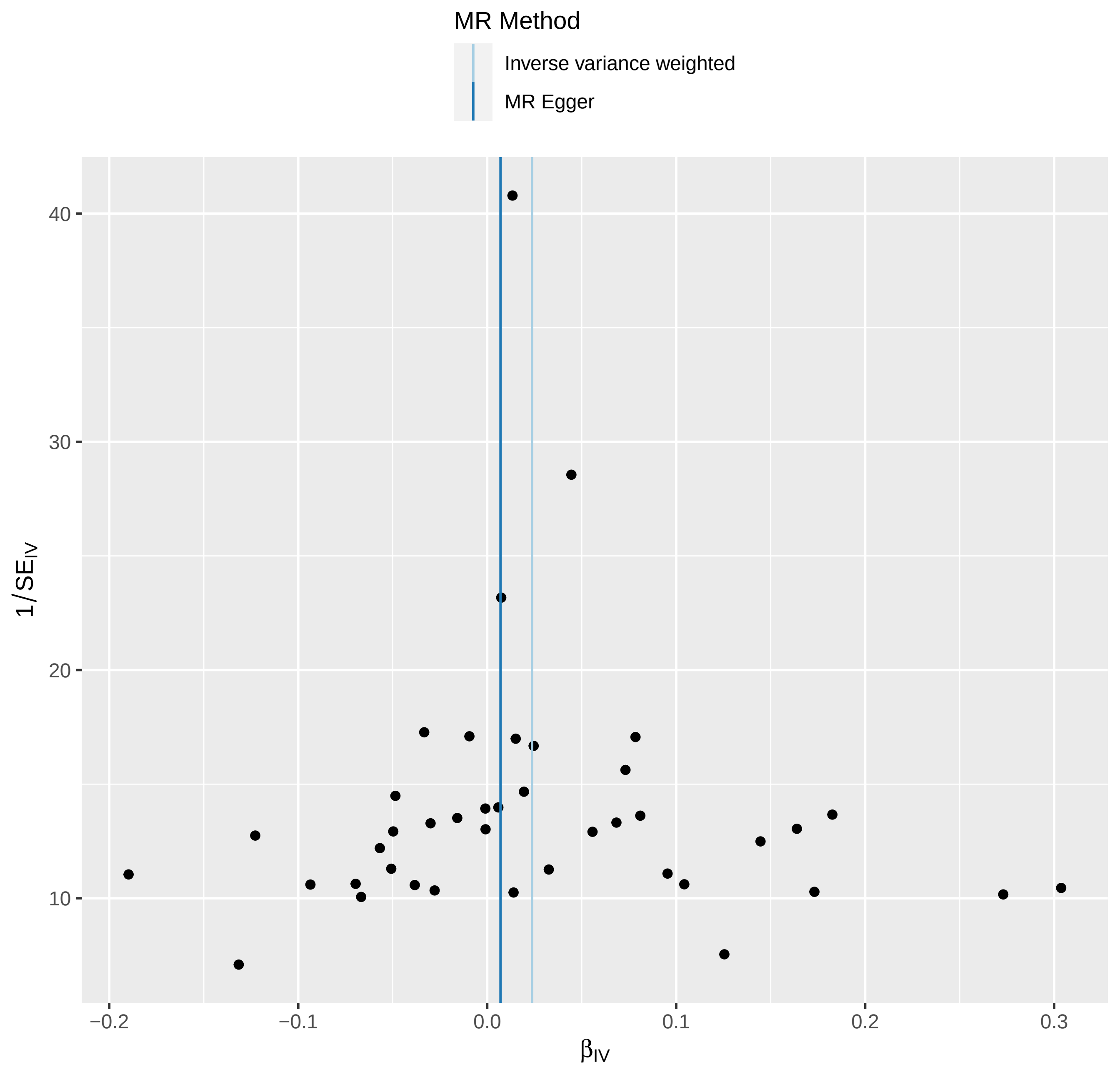

Batch 38 : Ulcerative colitis on IDP T1 FAST ROIs R heschl gyrus

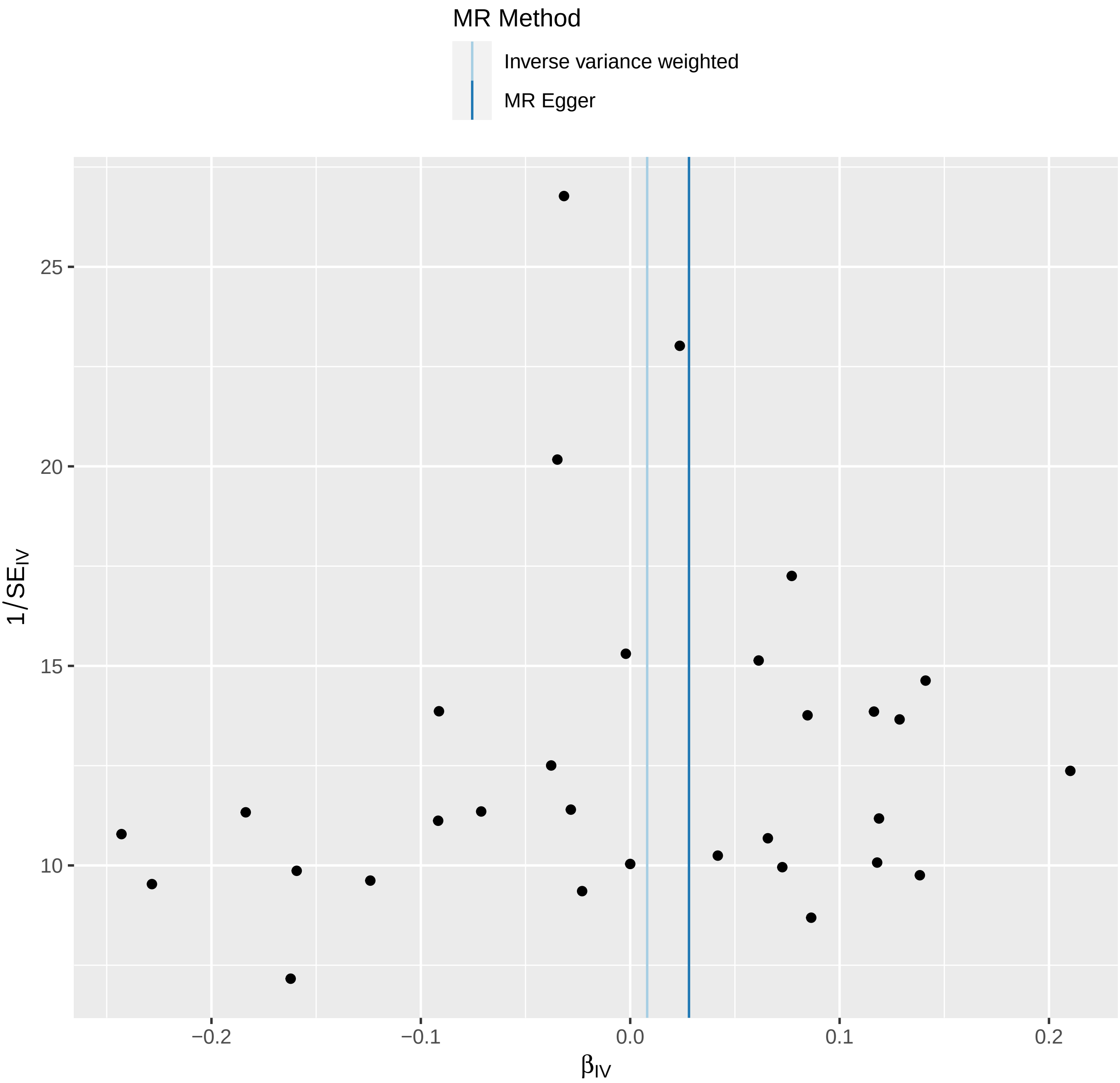

Batch 39 : Crohn's disease on IDP T1 FAST ROIs L planum temporale

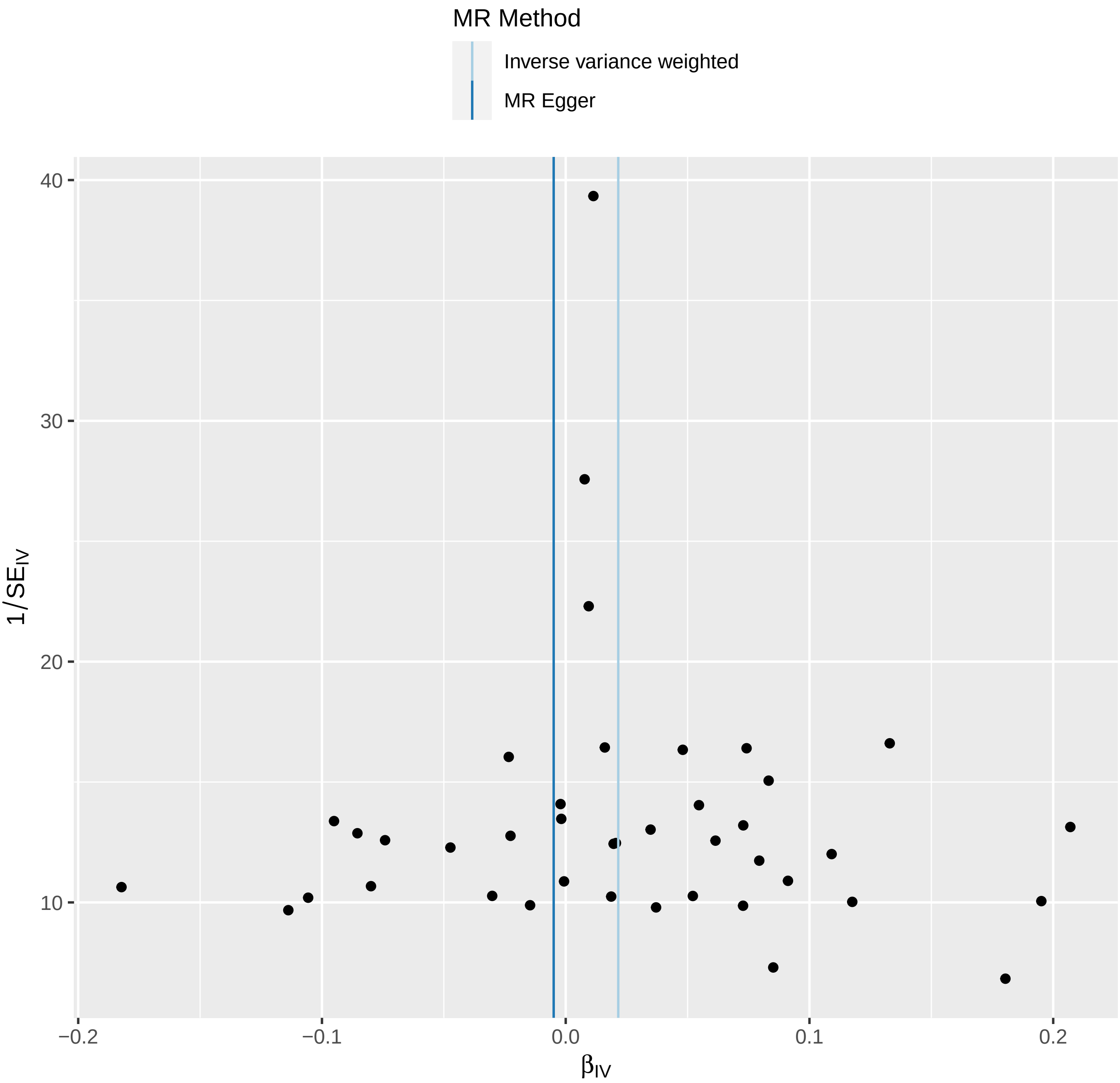

Batch 40 : Ulcerative colitis on IDP T1 FAST ROIs L planum temporale

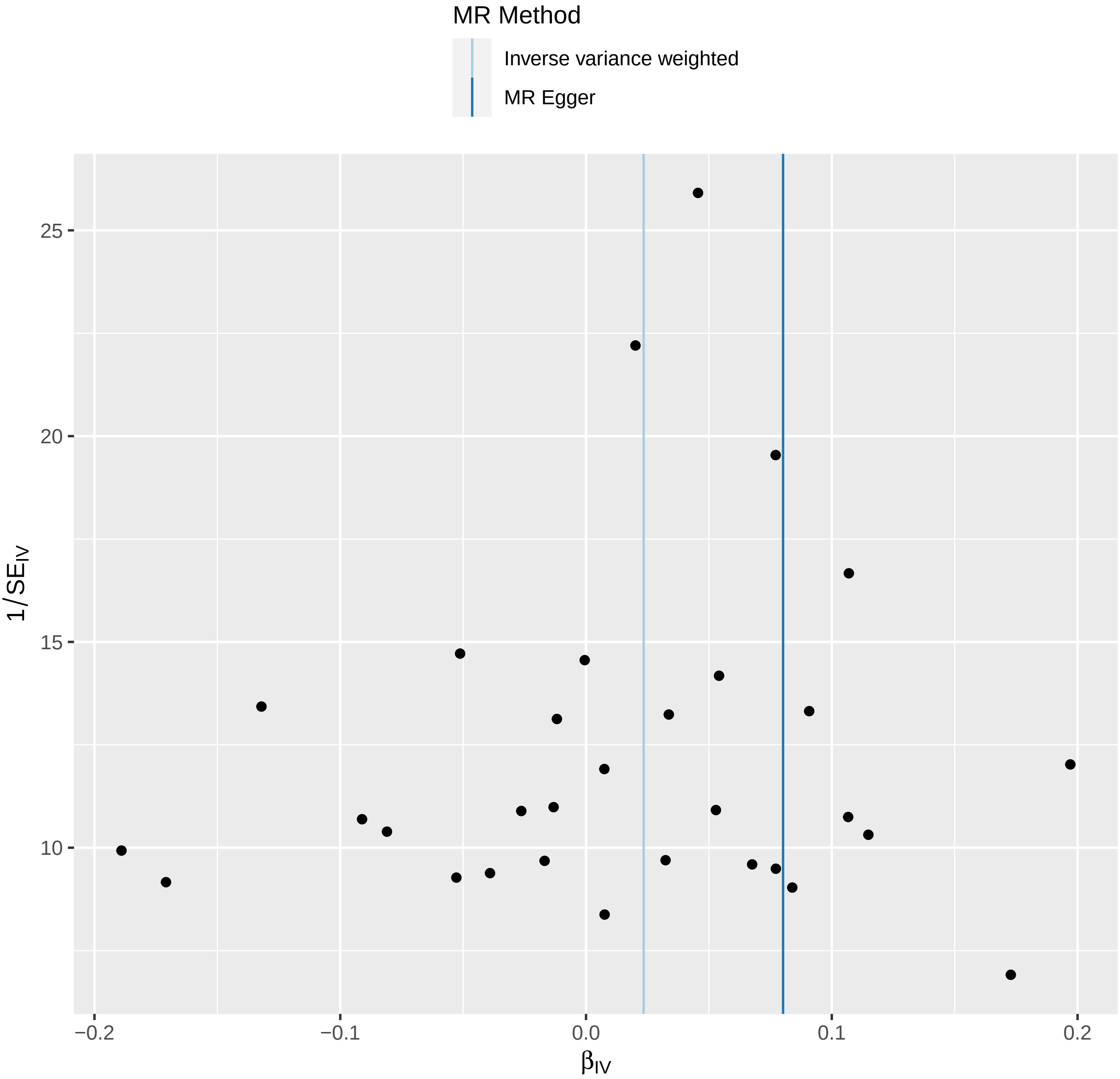

Batch 41 : Crohn's disease on IDP T1 FAST ROIs R planum temporale

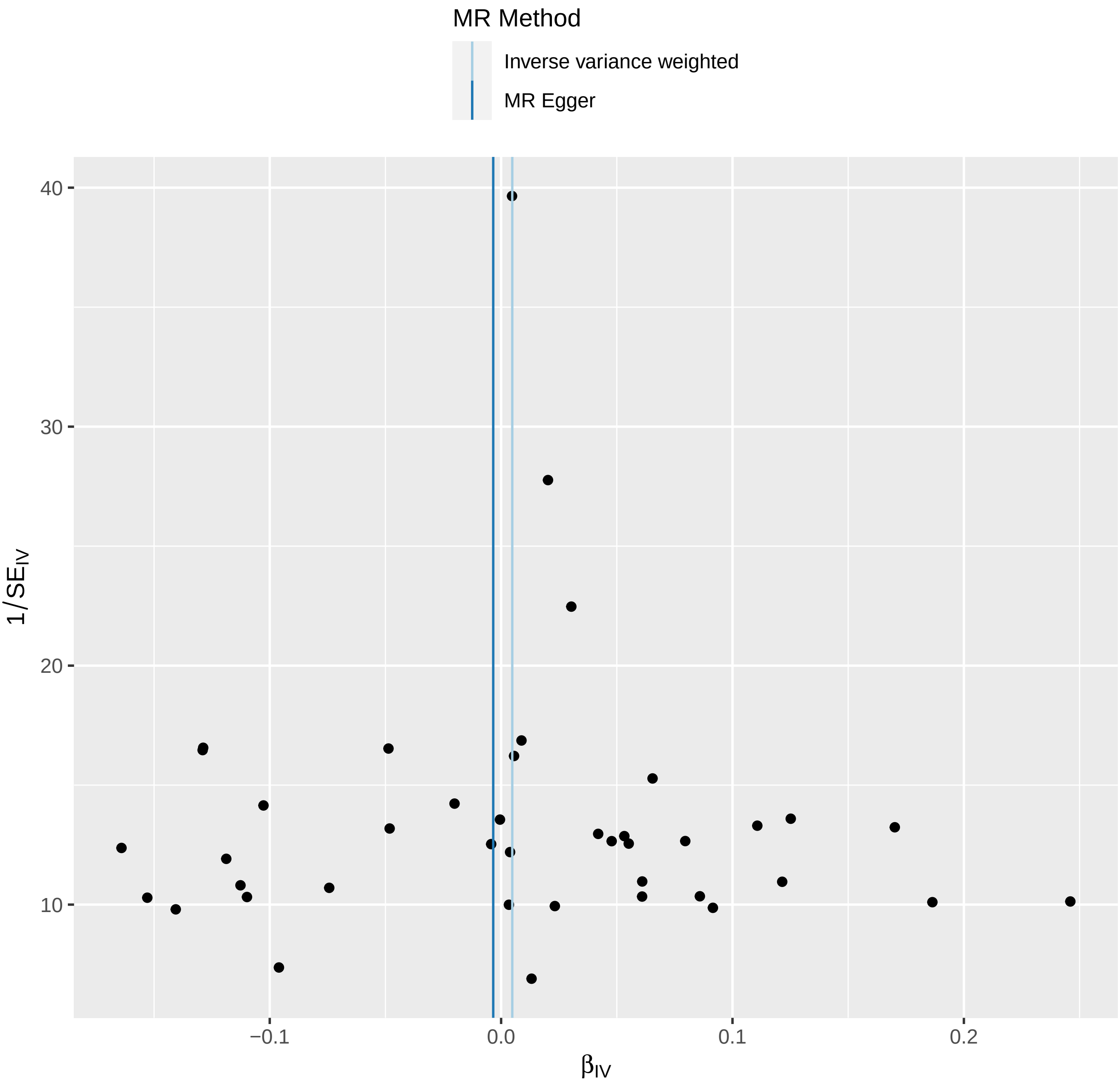

Batch 42 : Ulcerative colitis on IDP T1 FAST ROIs R planum temporale

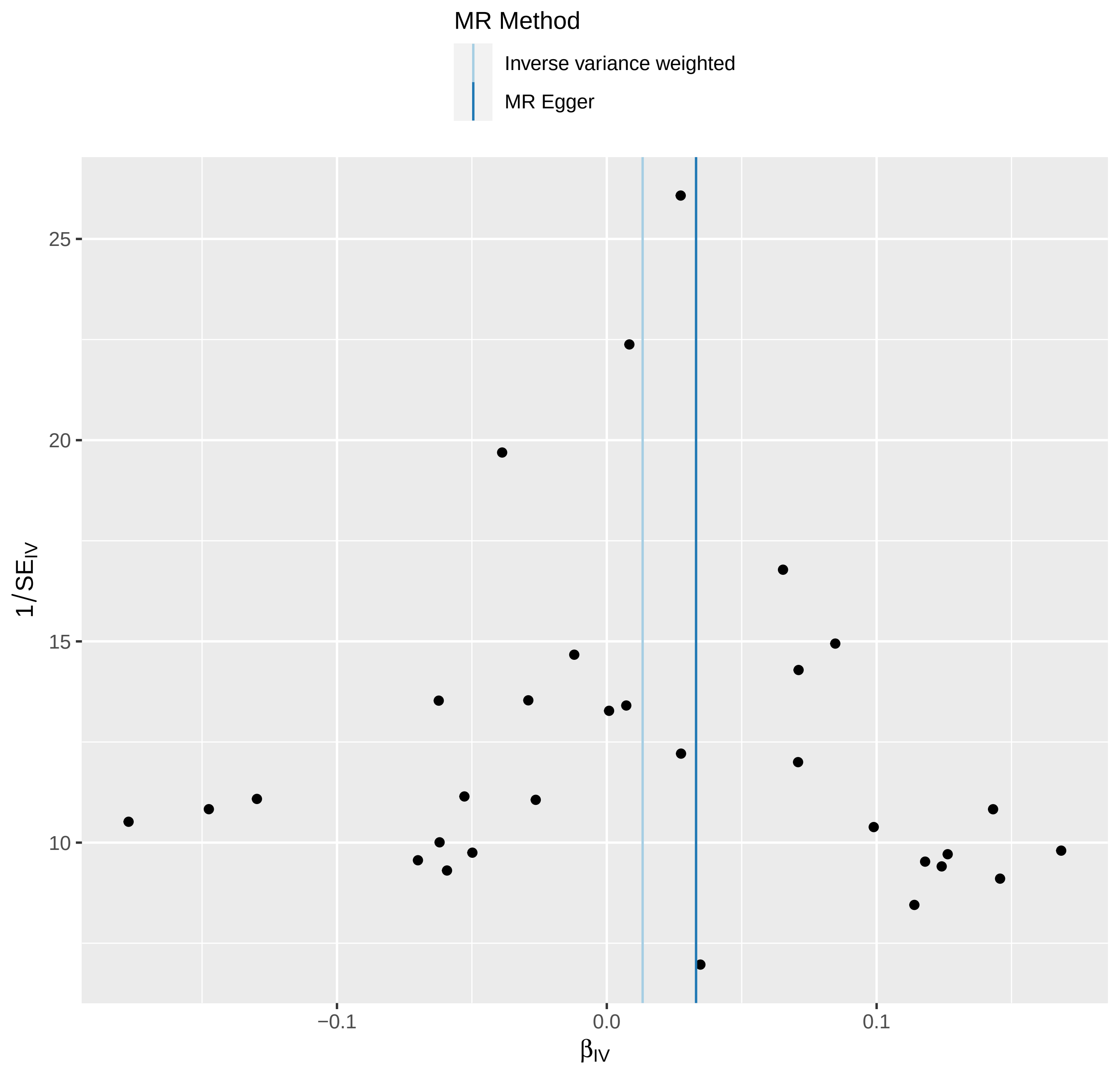

Batch 43 : Crohn's disease on IDP T1 FAST ROIs L supracalc cortex

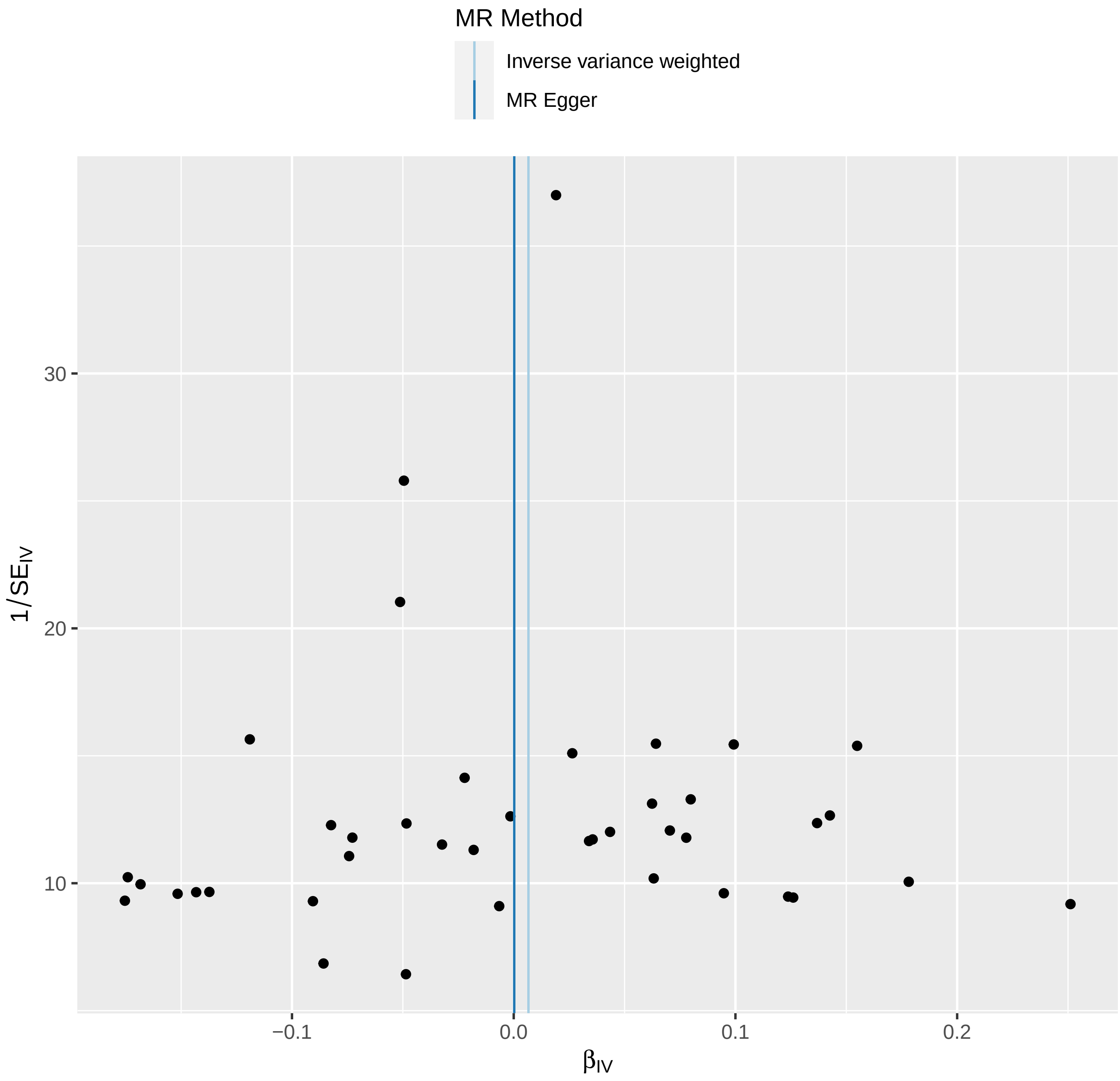

Batch 44 : Ulcerative colitis on IDP T1 FAST ROIs L supracalc cortex

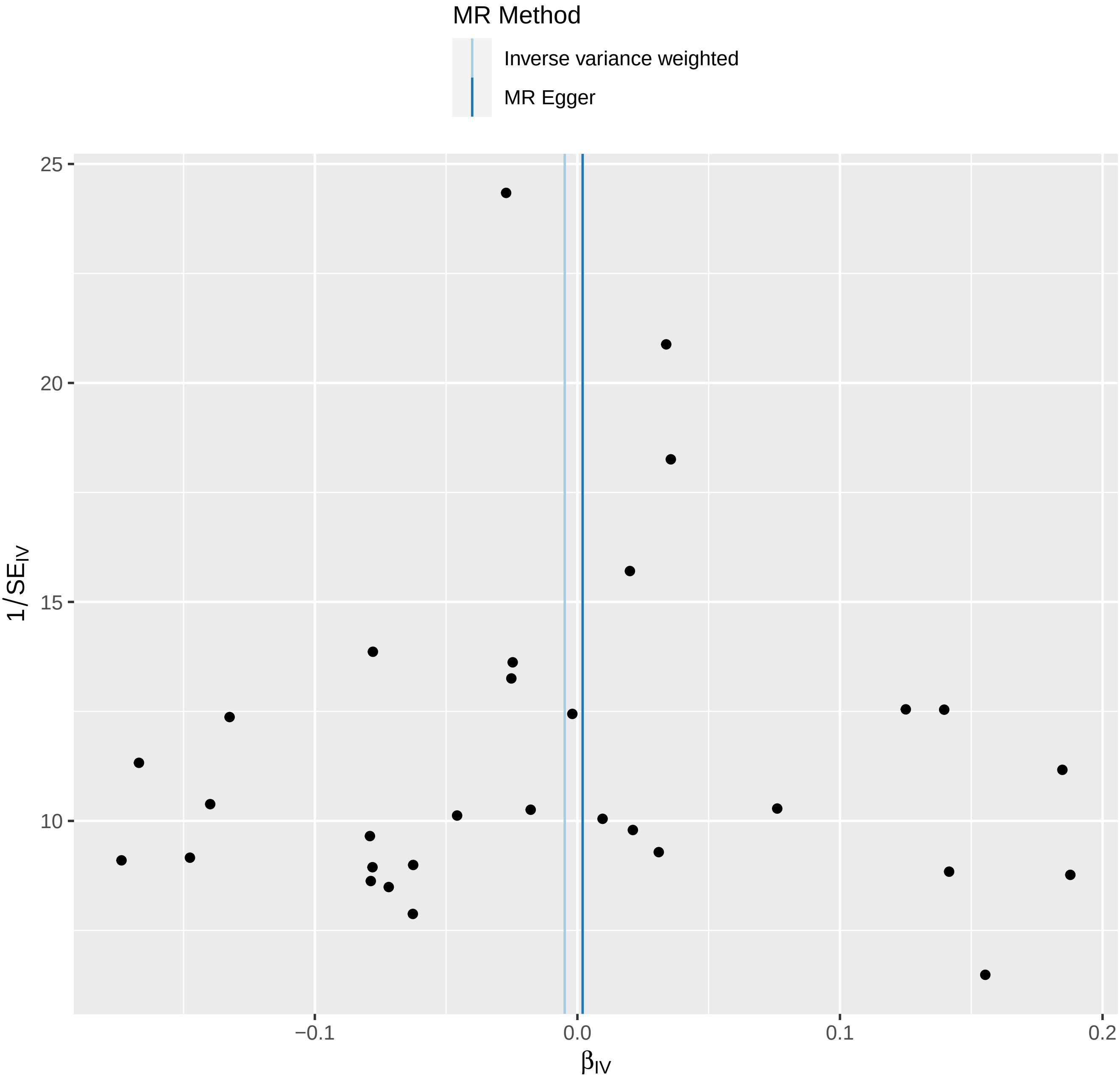

Batch 45 : Crohn's disease on IDP T1 FAST ROIs R supracalc cortex

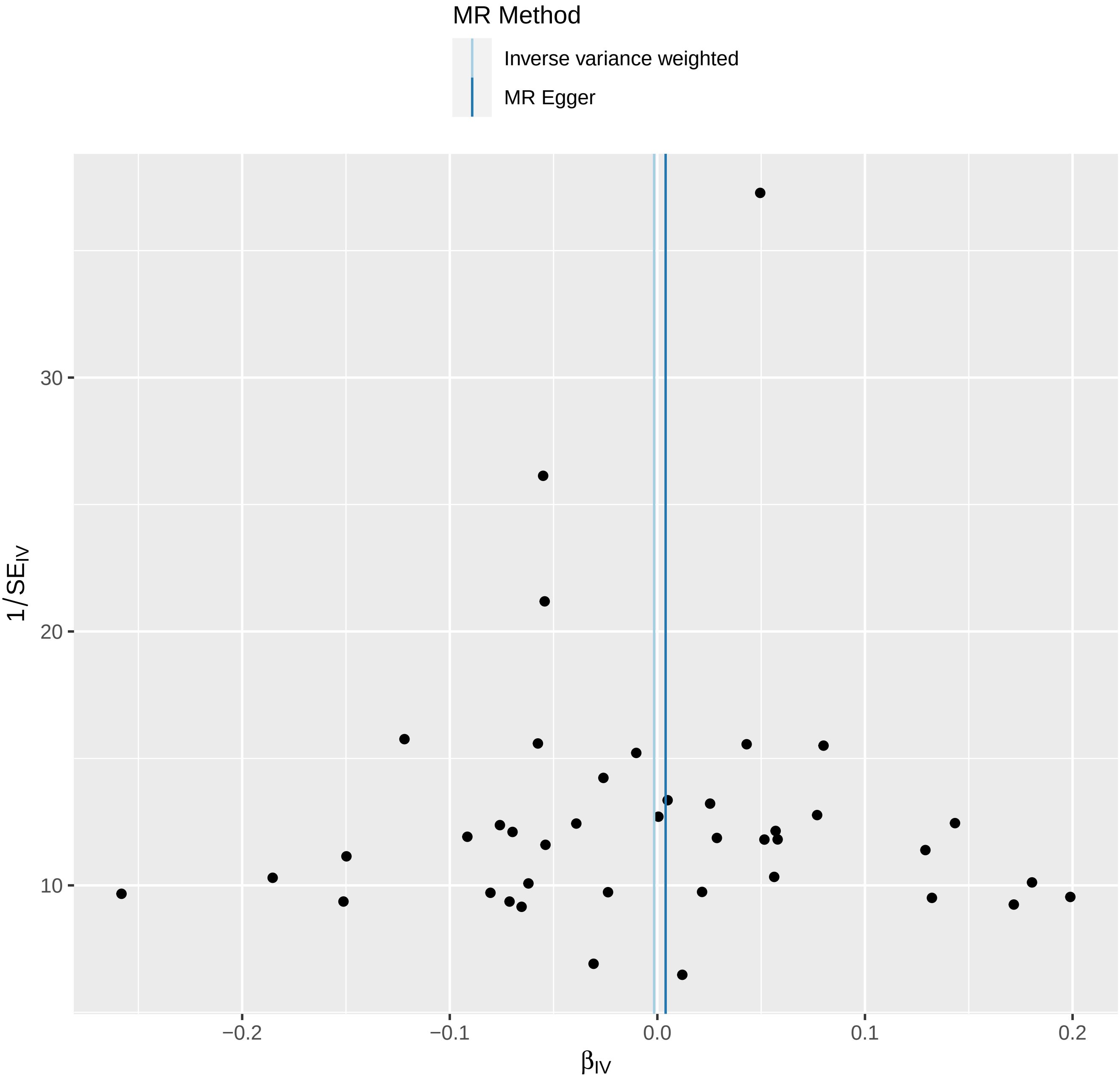

Batch 46 : Ulcerative colitis on IDP T1 FAST ROIs R supracalc cortex

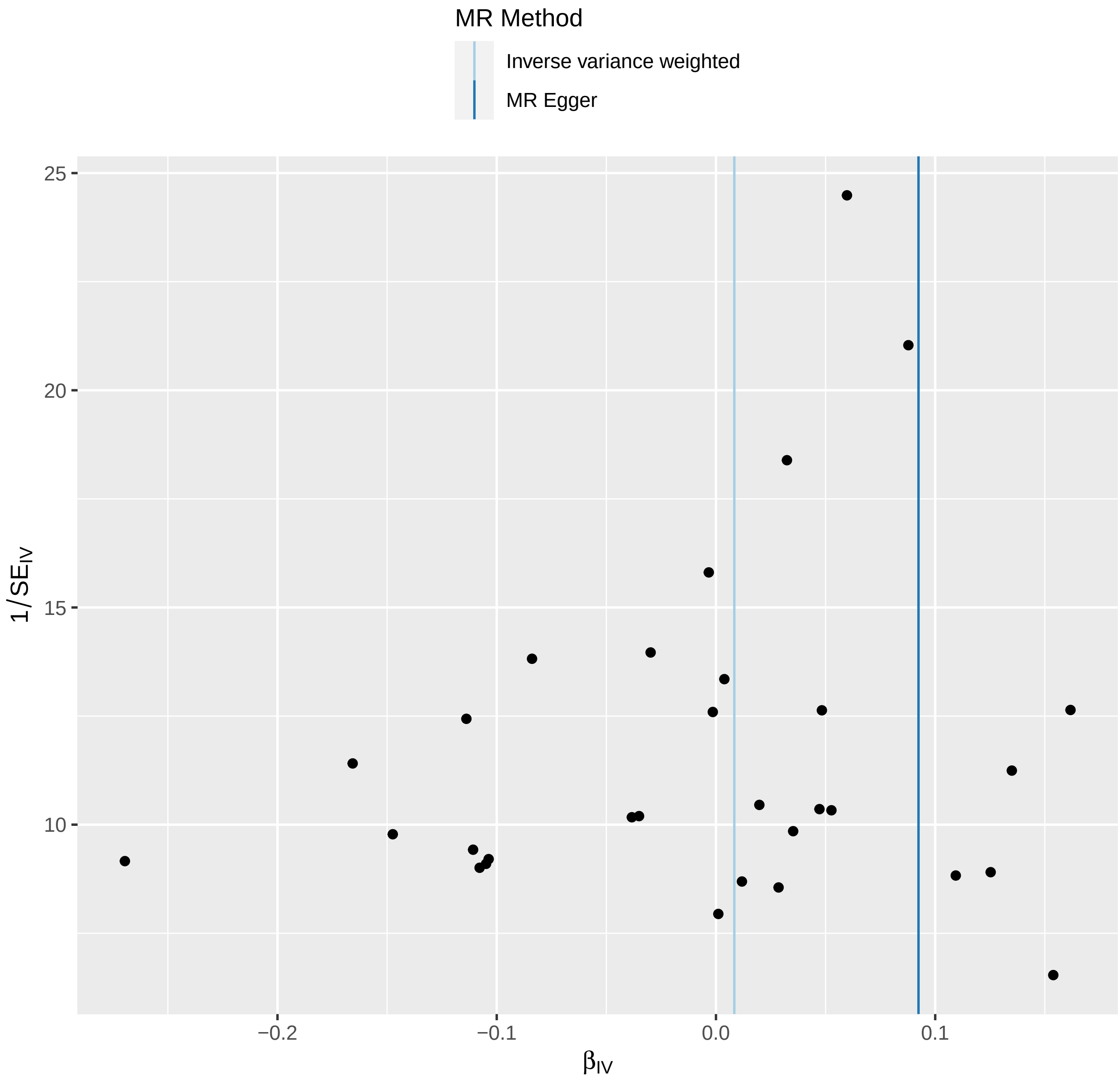

Batch 47 : Crohn's disease on IDP T1 FIRST right thalamus volume

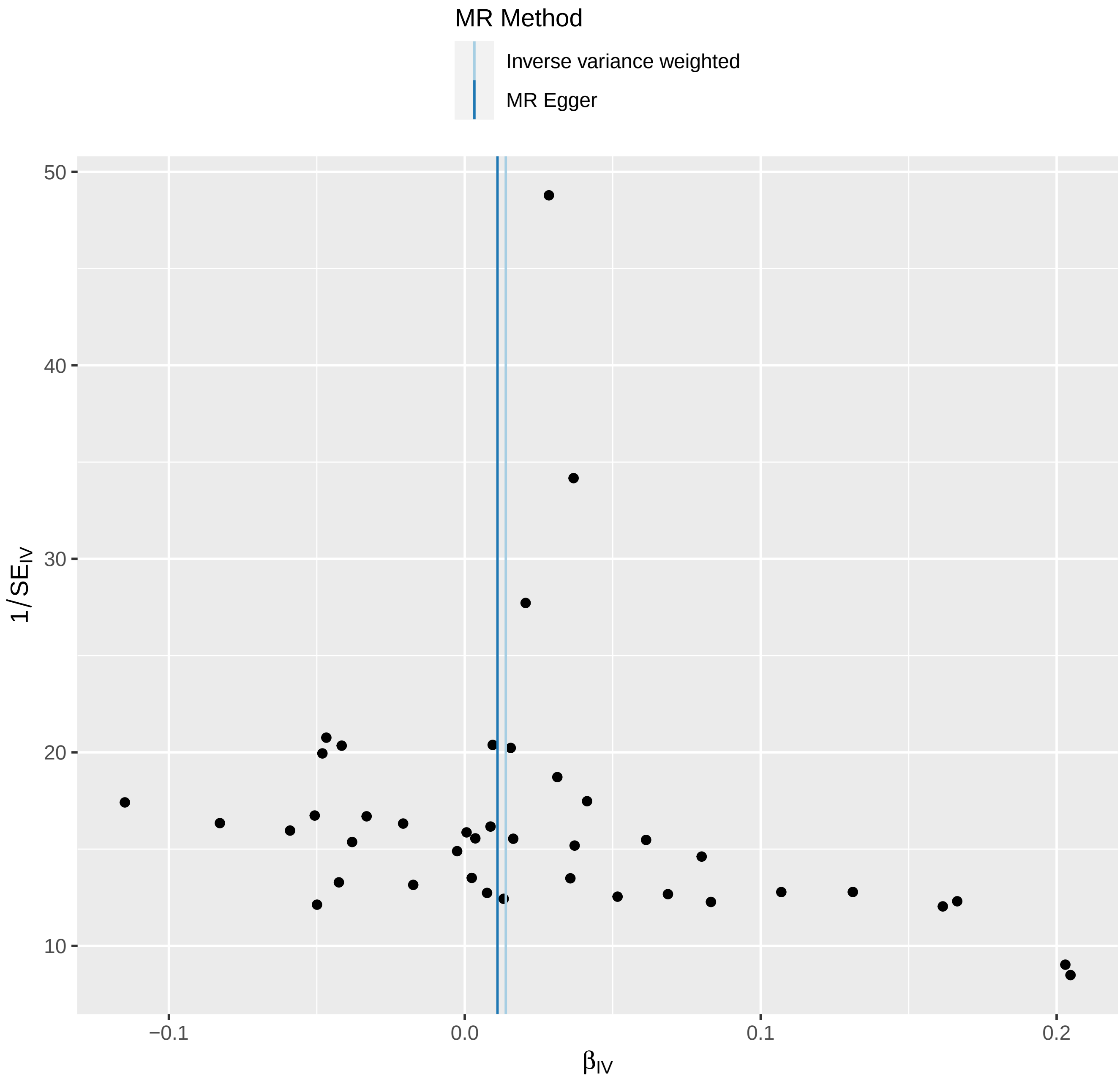

Batch 48 : Ulcerative colitis on IDP T1 FIRST right thalamus volume

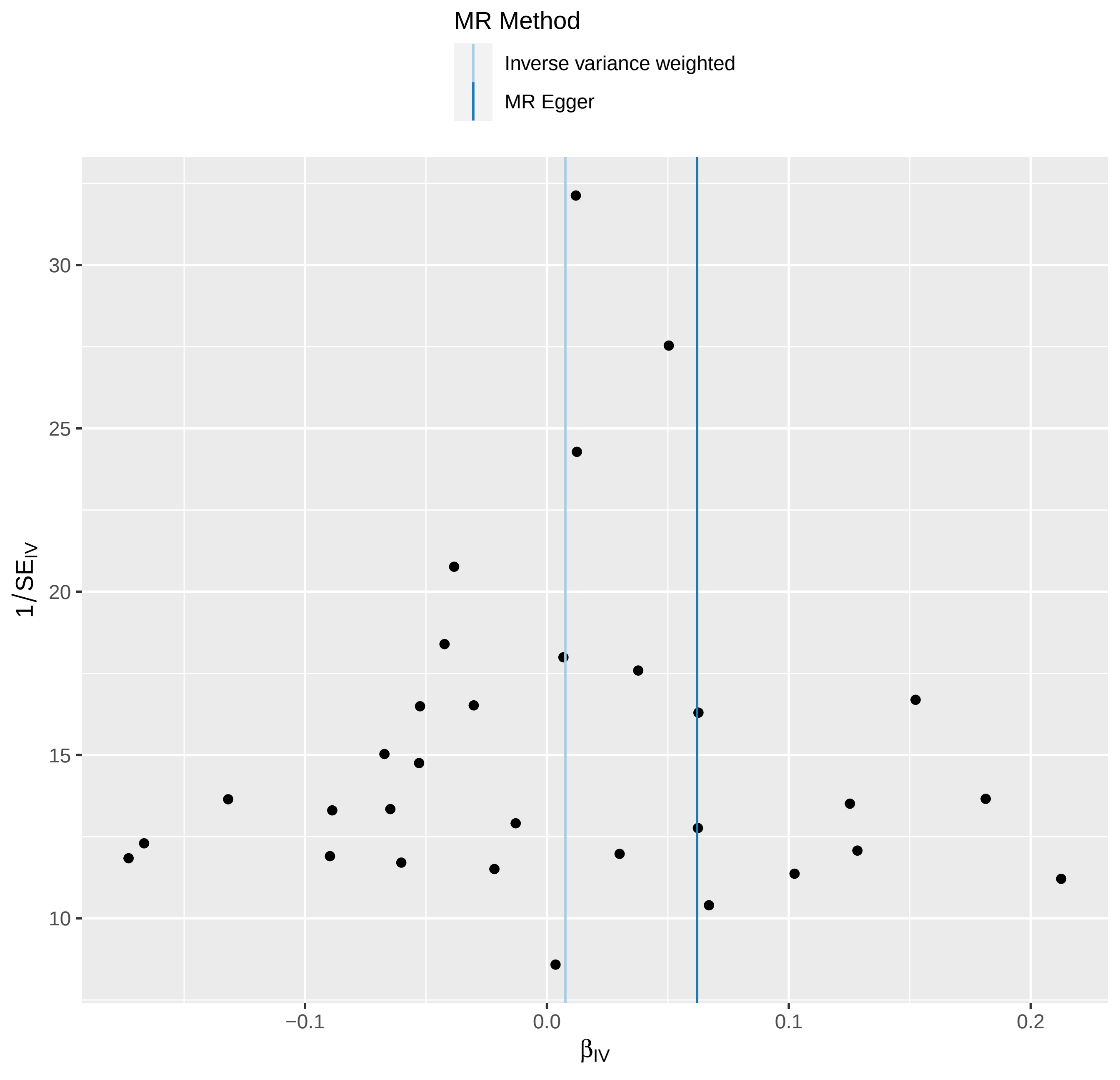

Batch 49 : Crohn's disease on IDP T1 FAST ROIs L occ pole

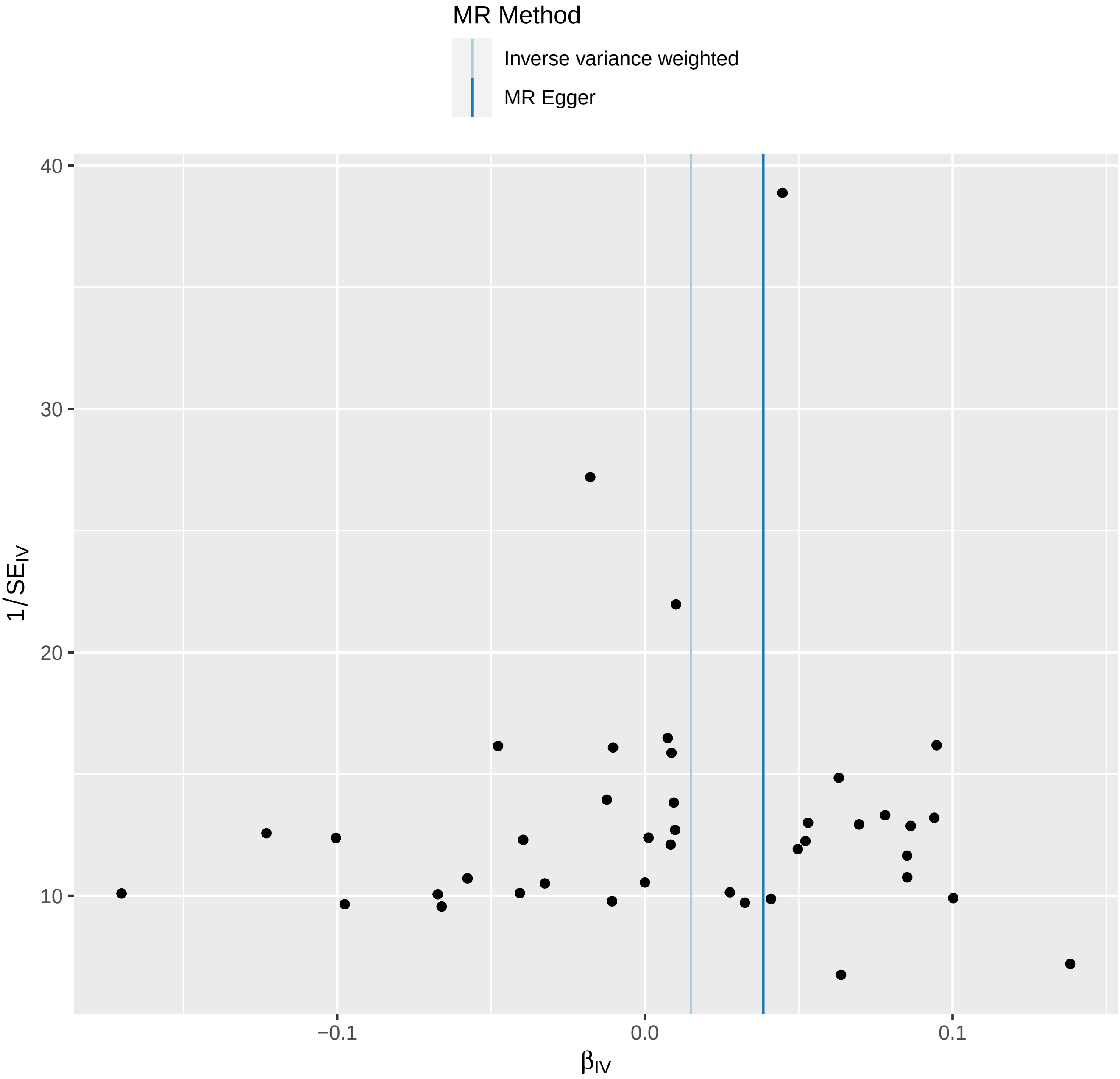

Batch 50 : Ulcerative colitis on IDP T1 FAST ROIs L occ pole

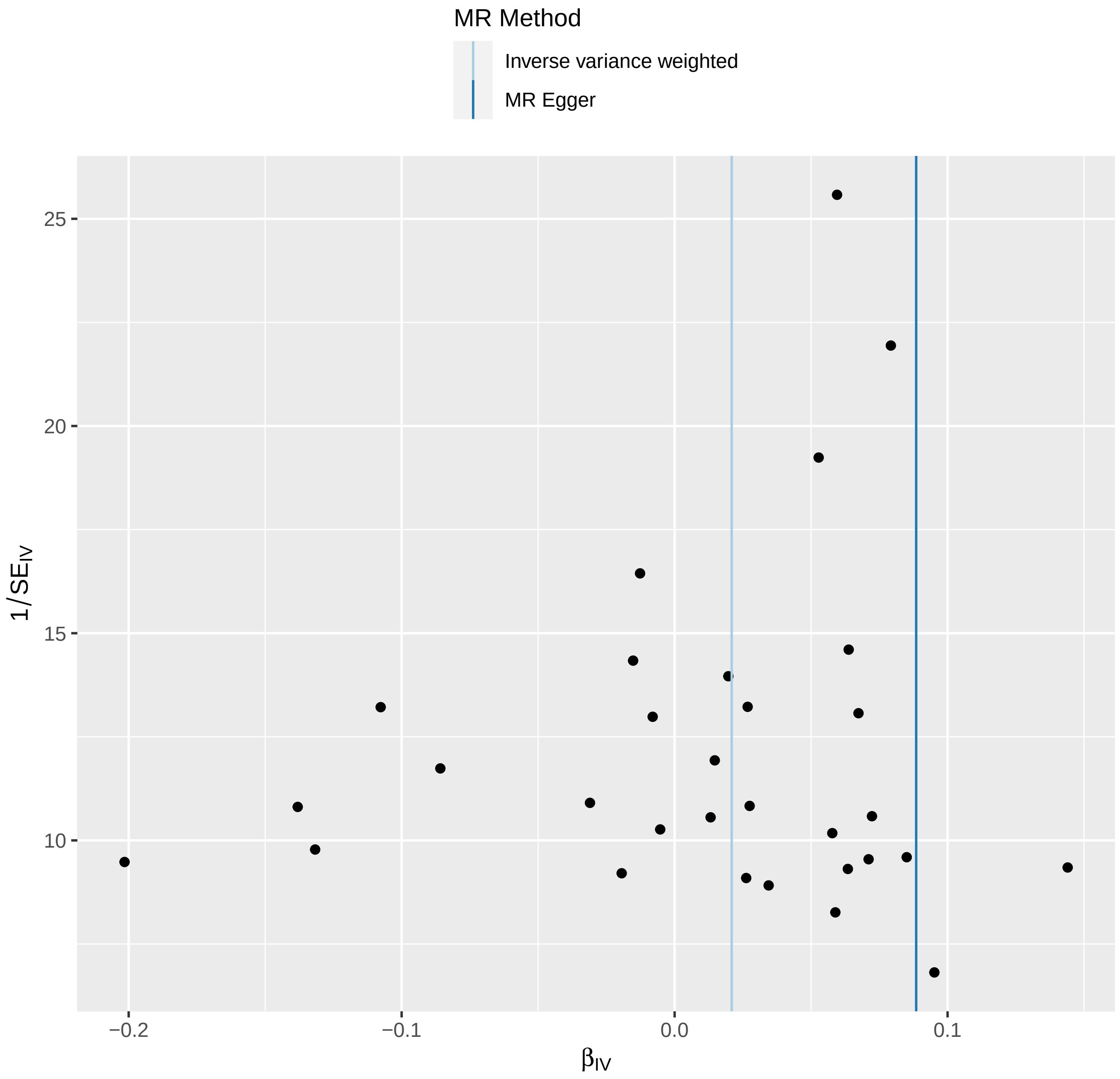

Batch 51 : Crohn's disease on IDP T1 FAST ROIs R occ pole

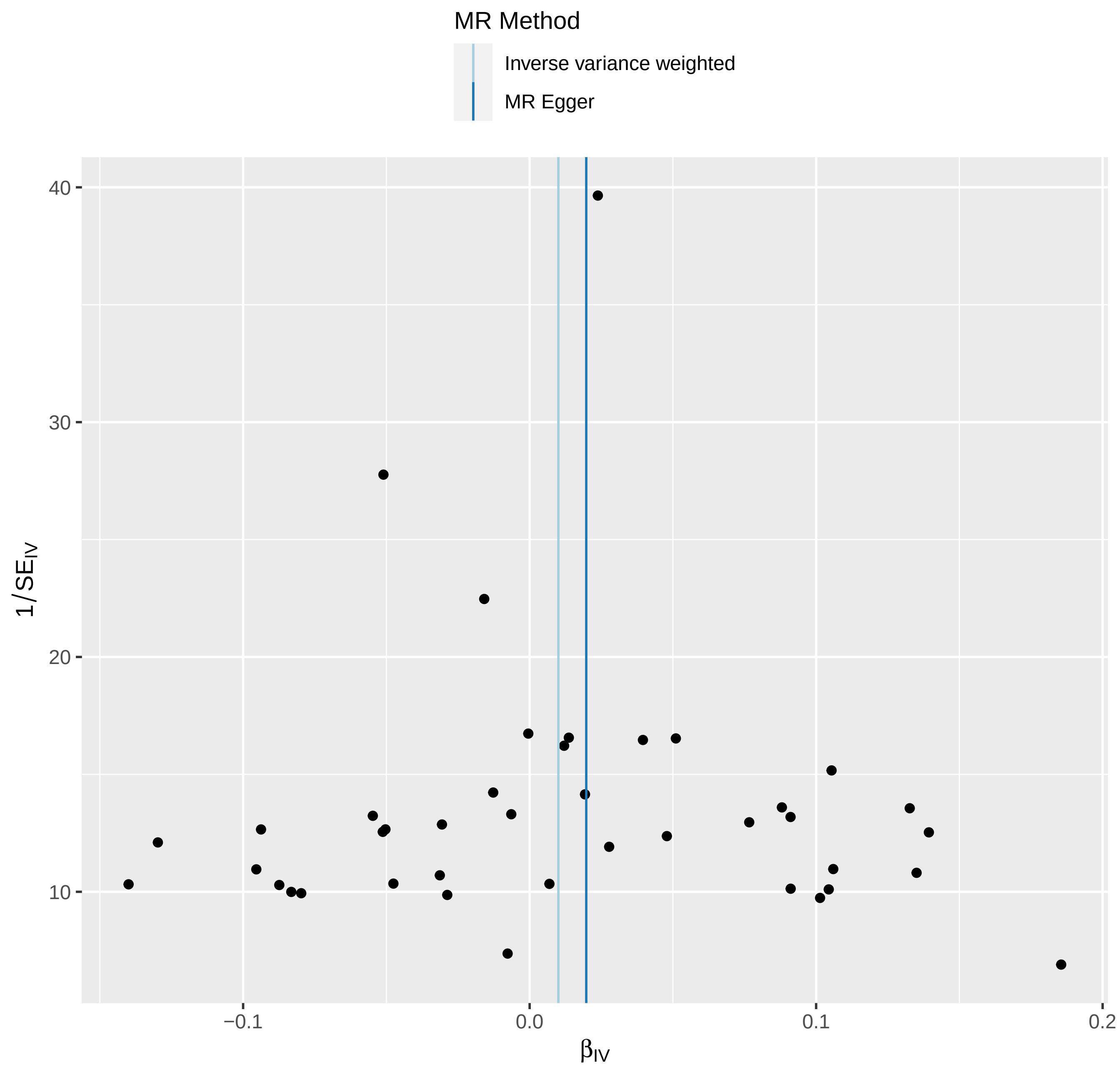

Batch 52 : Ulcerative colitis on IDP T1 FAST ROIs R occ pole

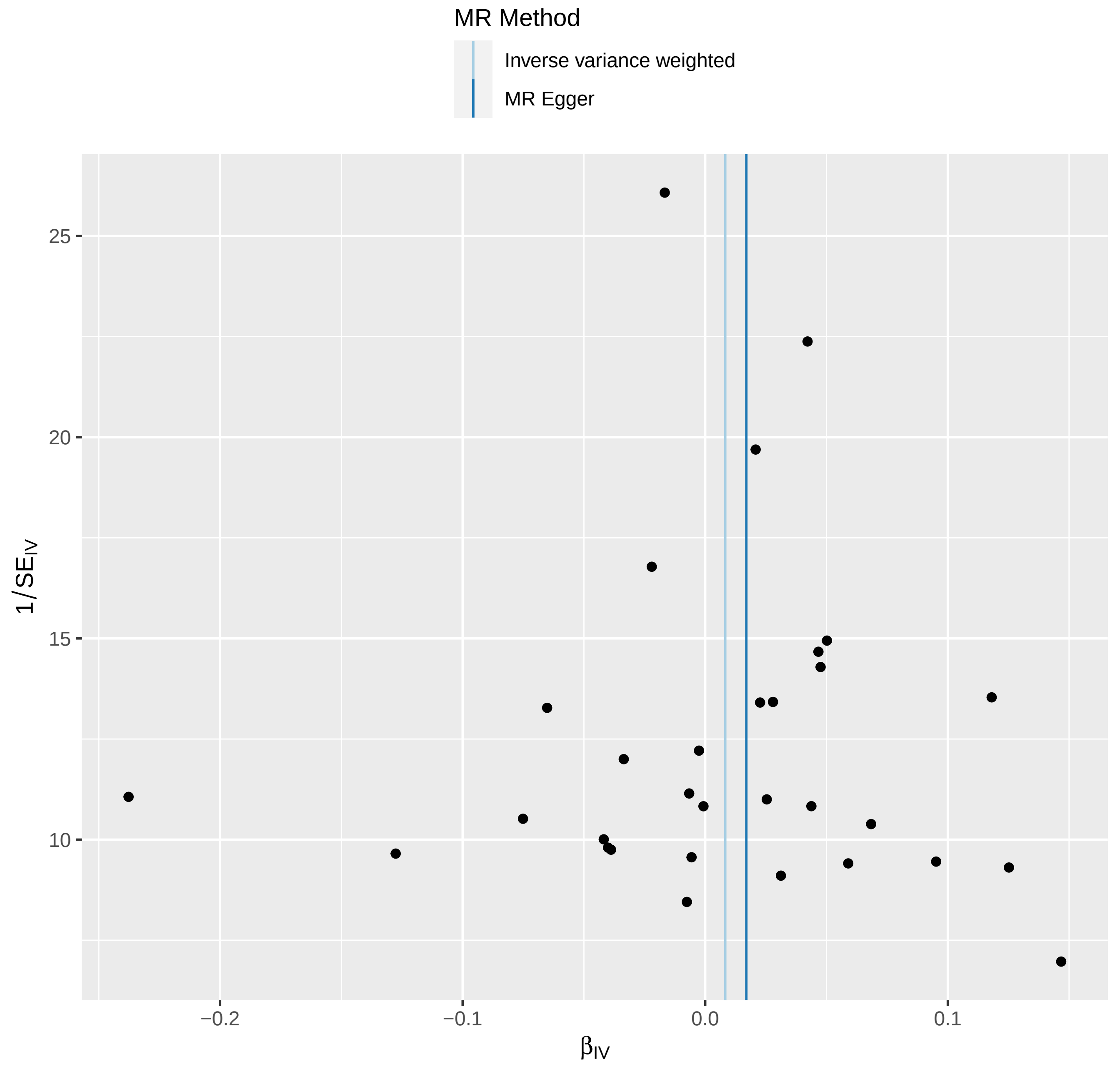

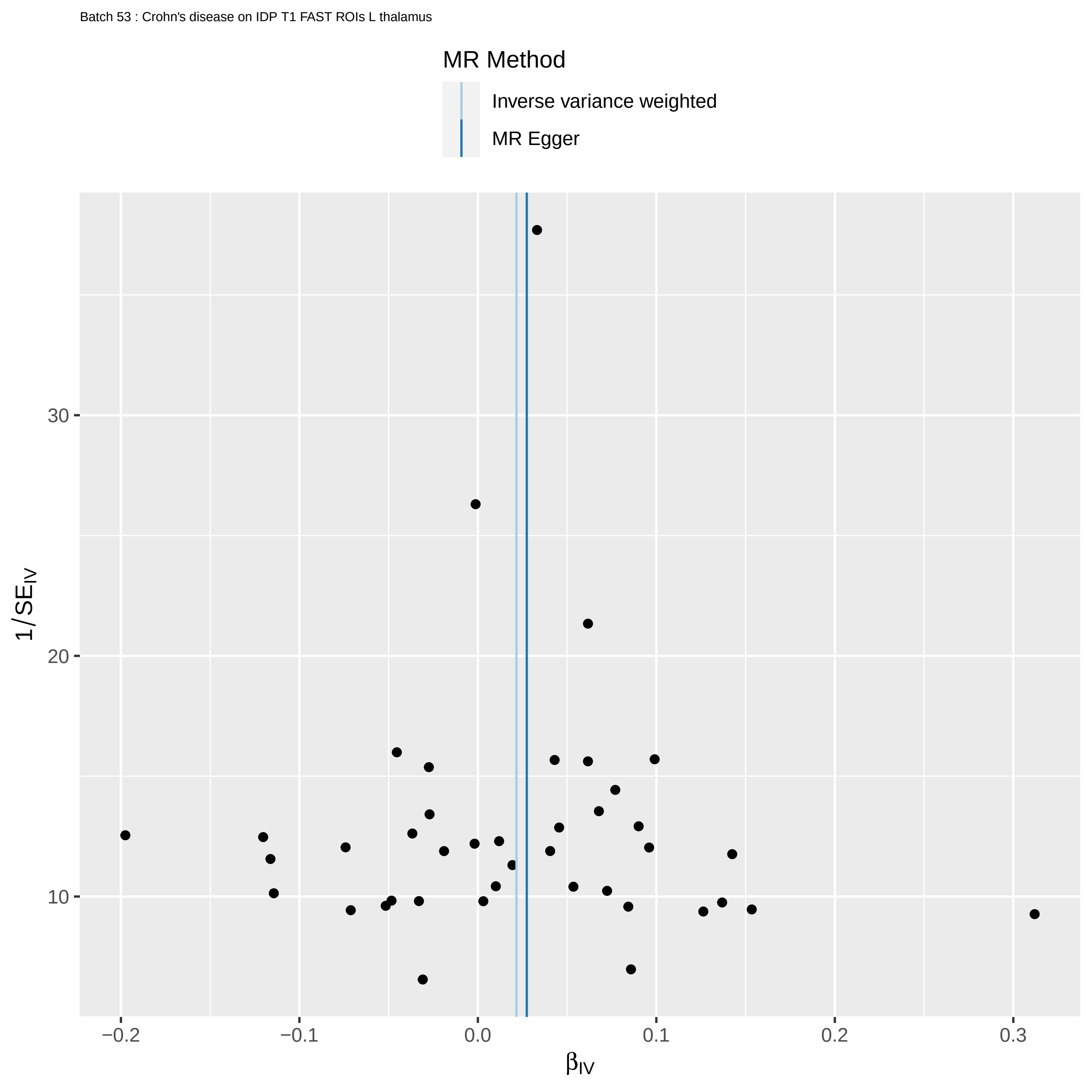
Batch 53 : Crohn's disease on IDP T1 FAST ROIs L thalamus
MR Method
Inverse variance weighted
MR Egger
30
20
10
1/SE_IV
−0.2
−0.1
0.0
0.1
0.2
0.3
β_IV

Batch 54 : Ulcerative colitis on IDP T1 FAST ROIs L thalamus

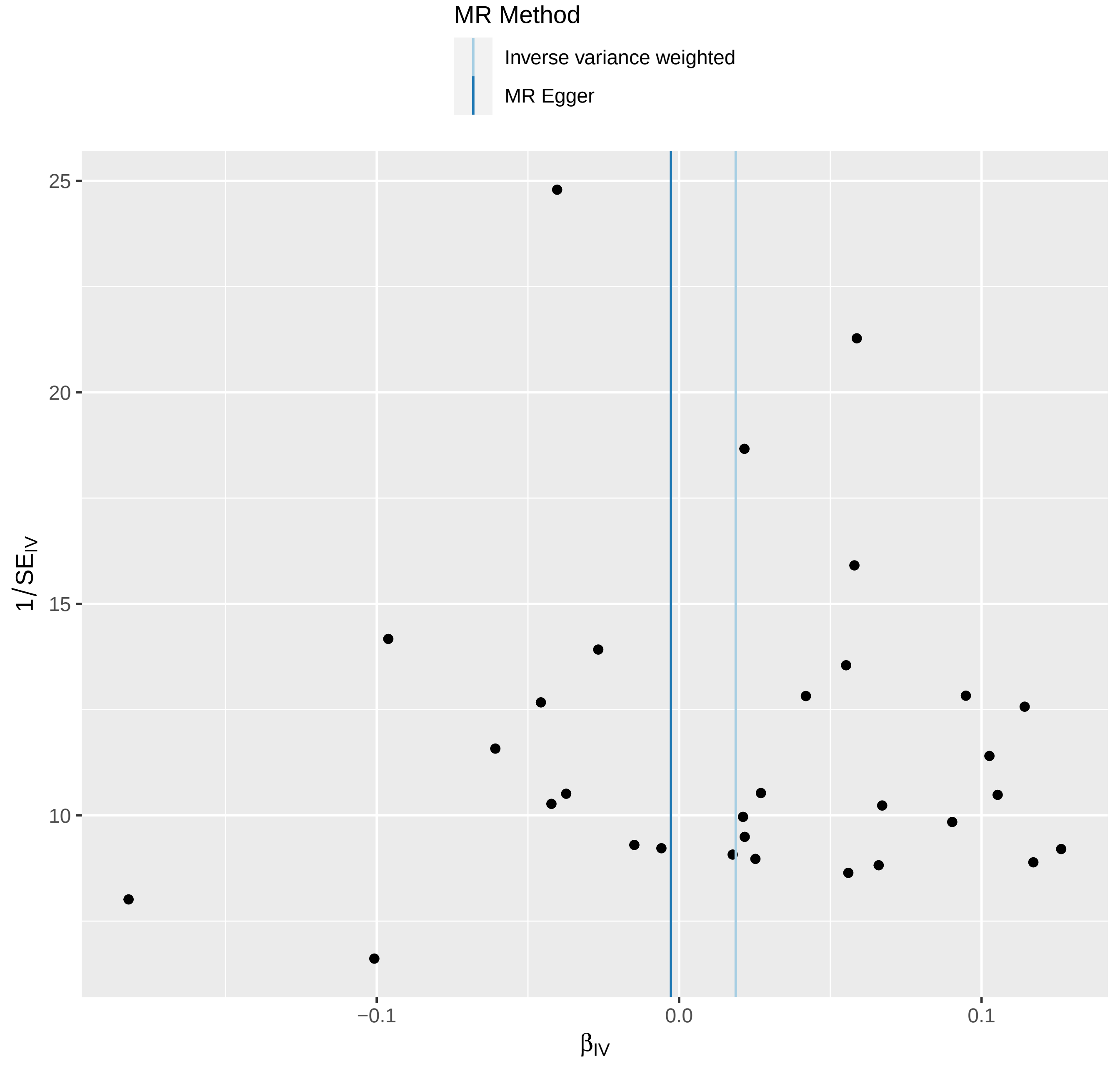

Batch 55 : Crohn's disease on IDP T1 FAST ROIs R thalamus

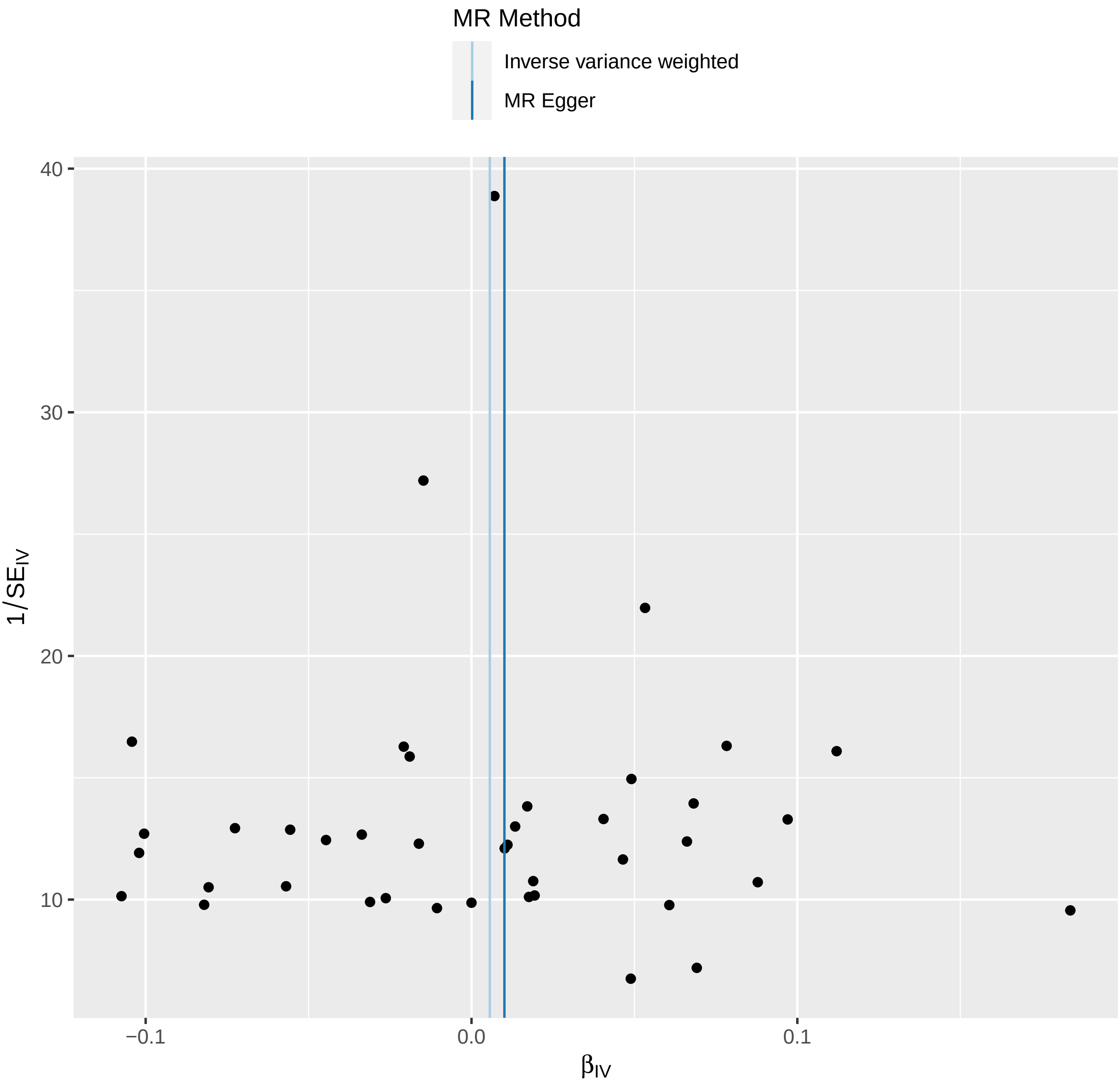

Batch 56 : Ulcerative colitis on IDP T1 FAST ROIs R thalamus

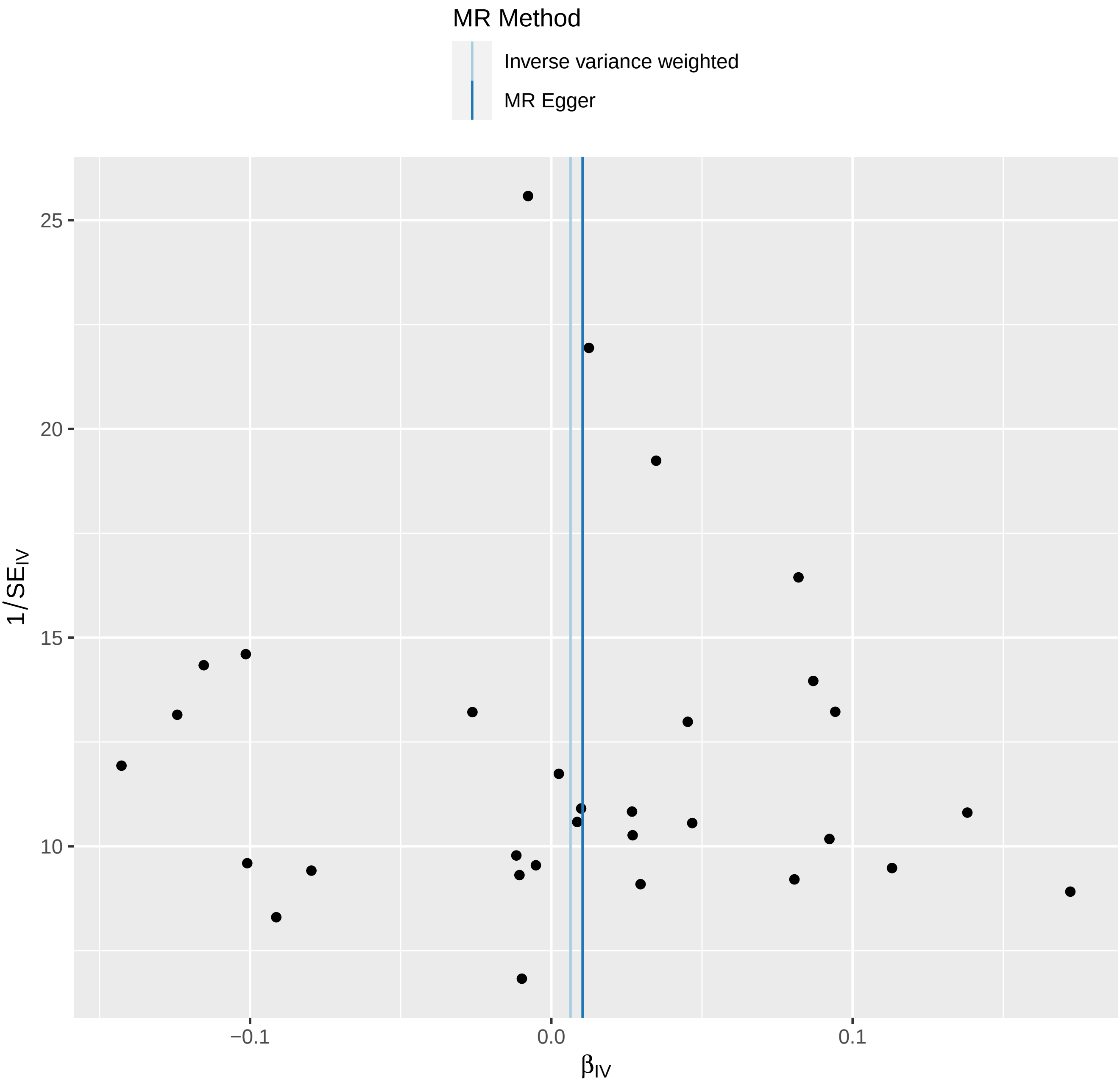

Batch 57 : Crohn's disease on IDP T1 FAST ROIs L caudate

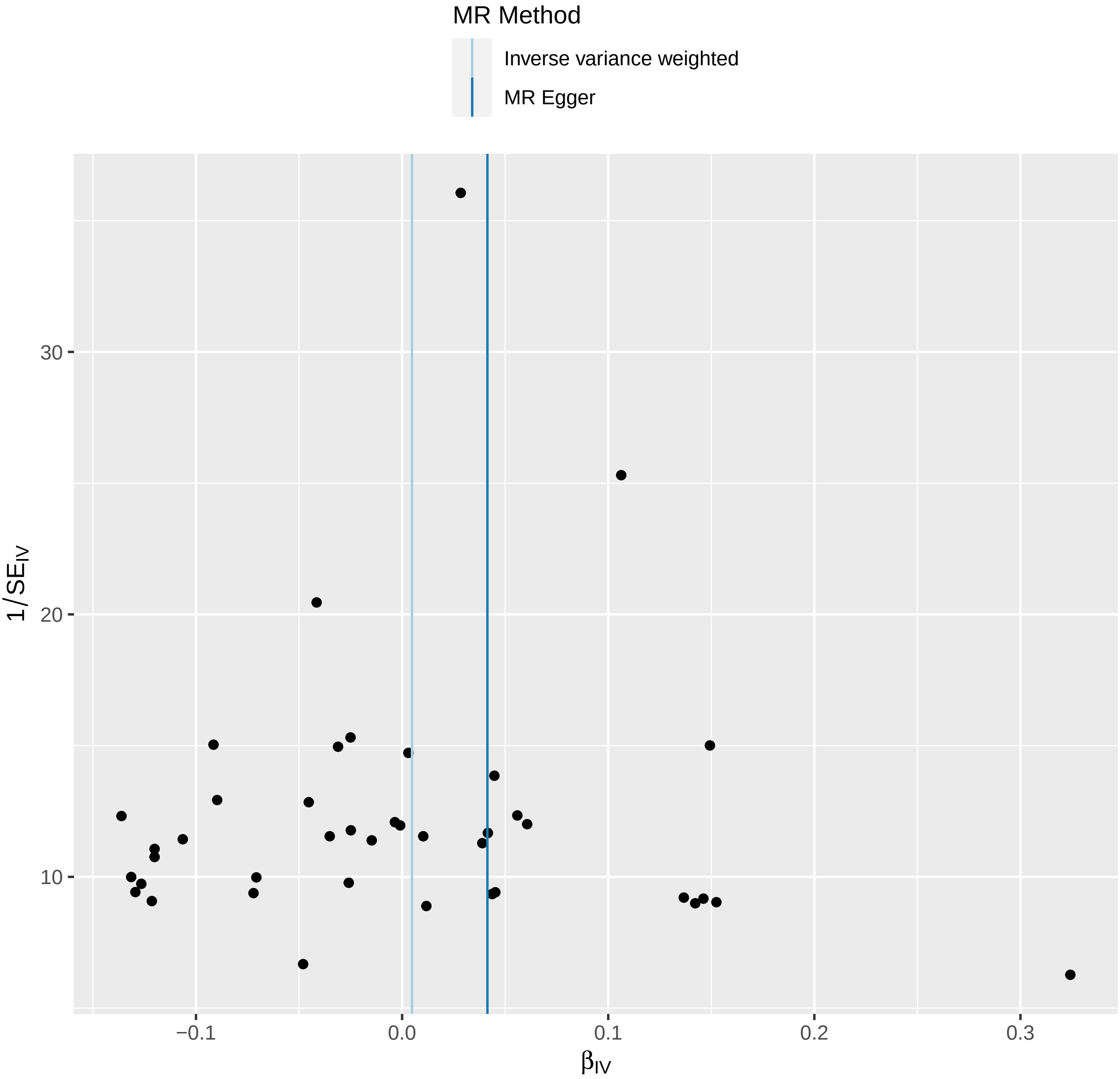

Batch 58 : Ulcerative colitis on IDP T1 FAST ROIs L caudate

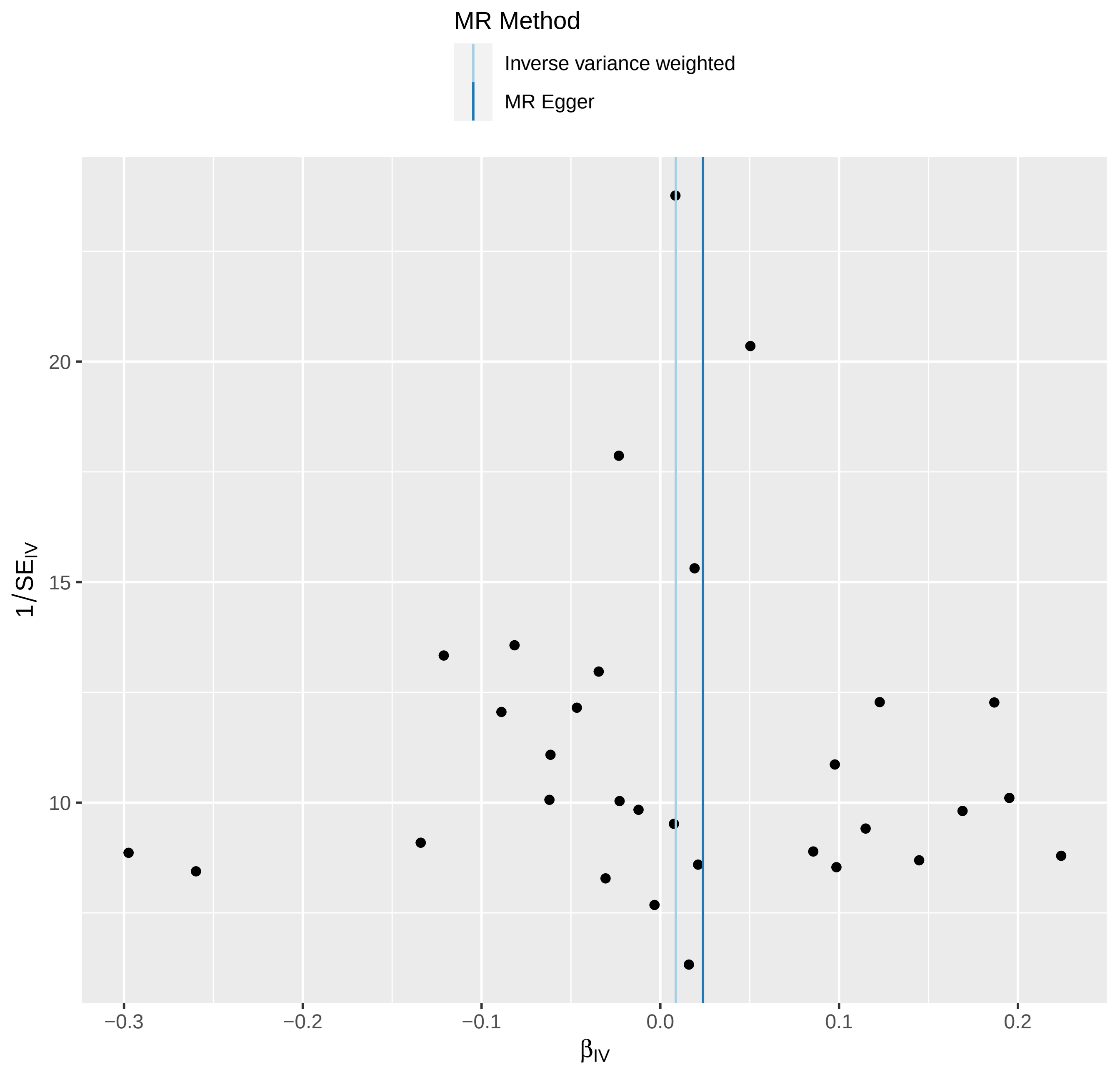

Batch 59 : Crohn's disease on IDP T1 FAST ROIs R caudate

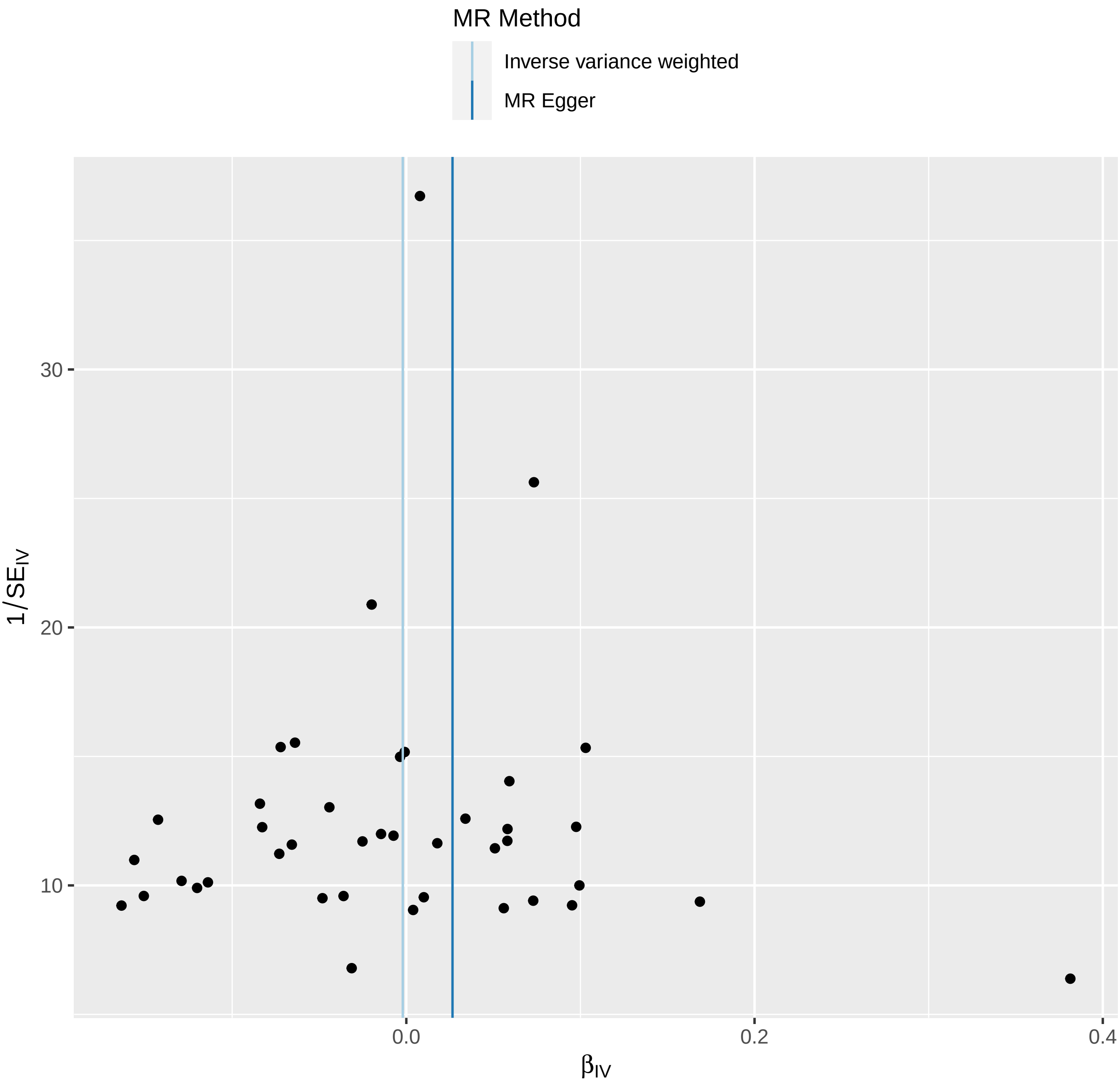

Batch 63 : Crohn's disease on IDP T1 FAST ROIs R putamen

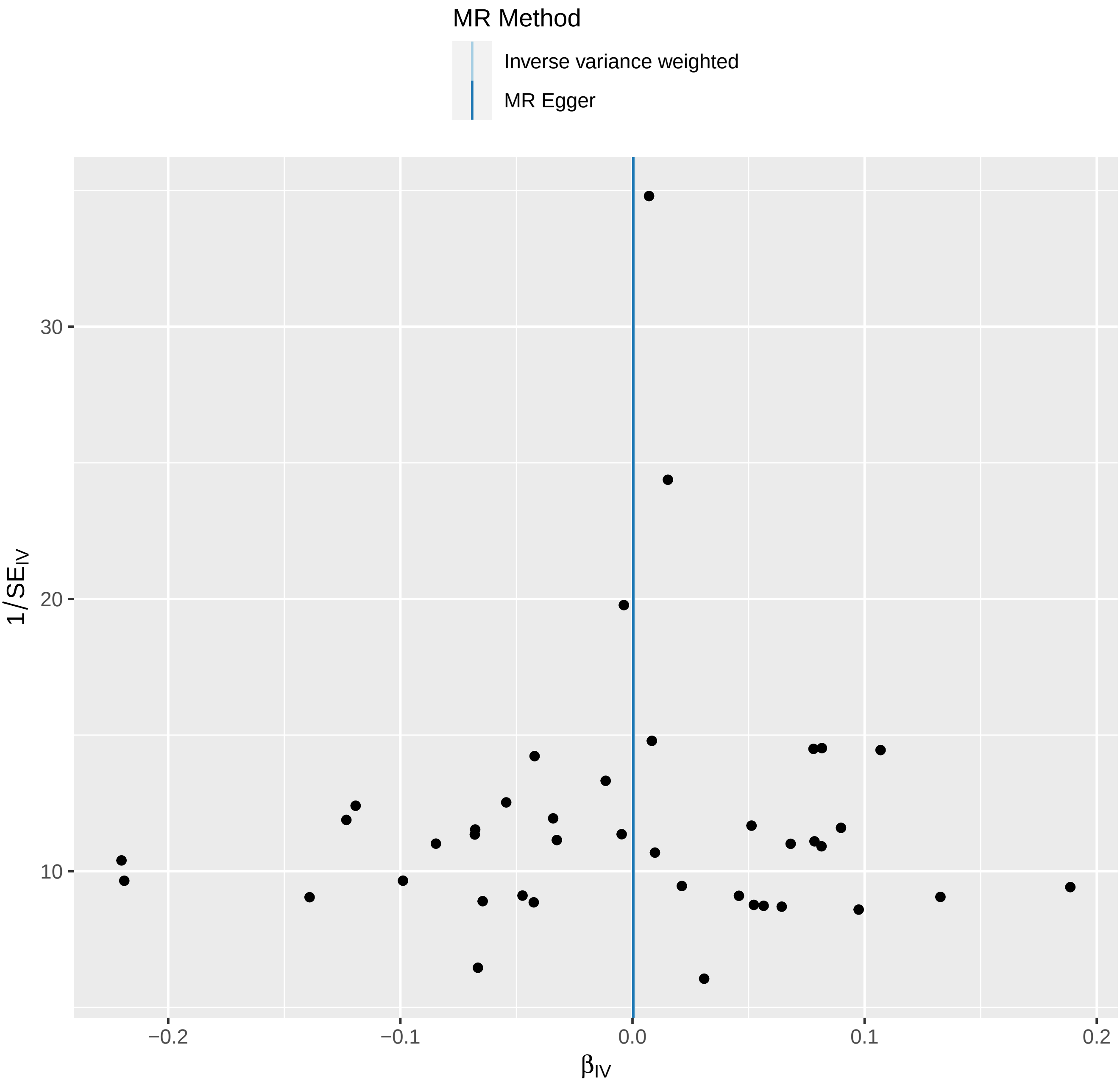

Batch 64 : Ulcerative colitis on IDP T1 FAST ROIs R putamen

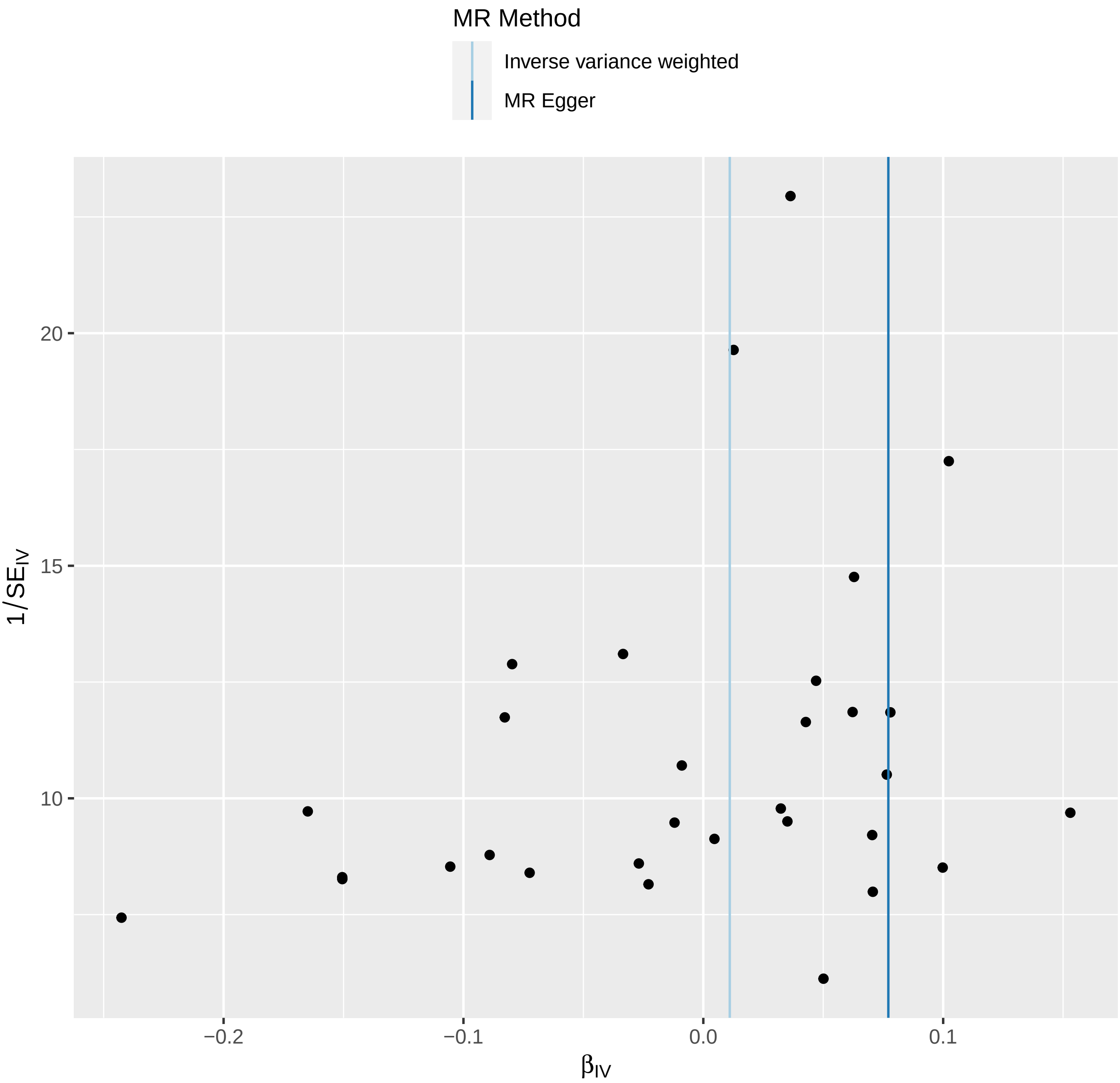

Batch 65 : Crohn's disease on IDP T1 FAST ROIs L pallidum

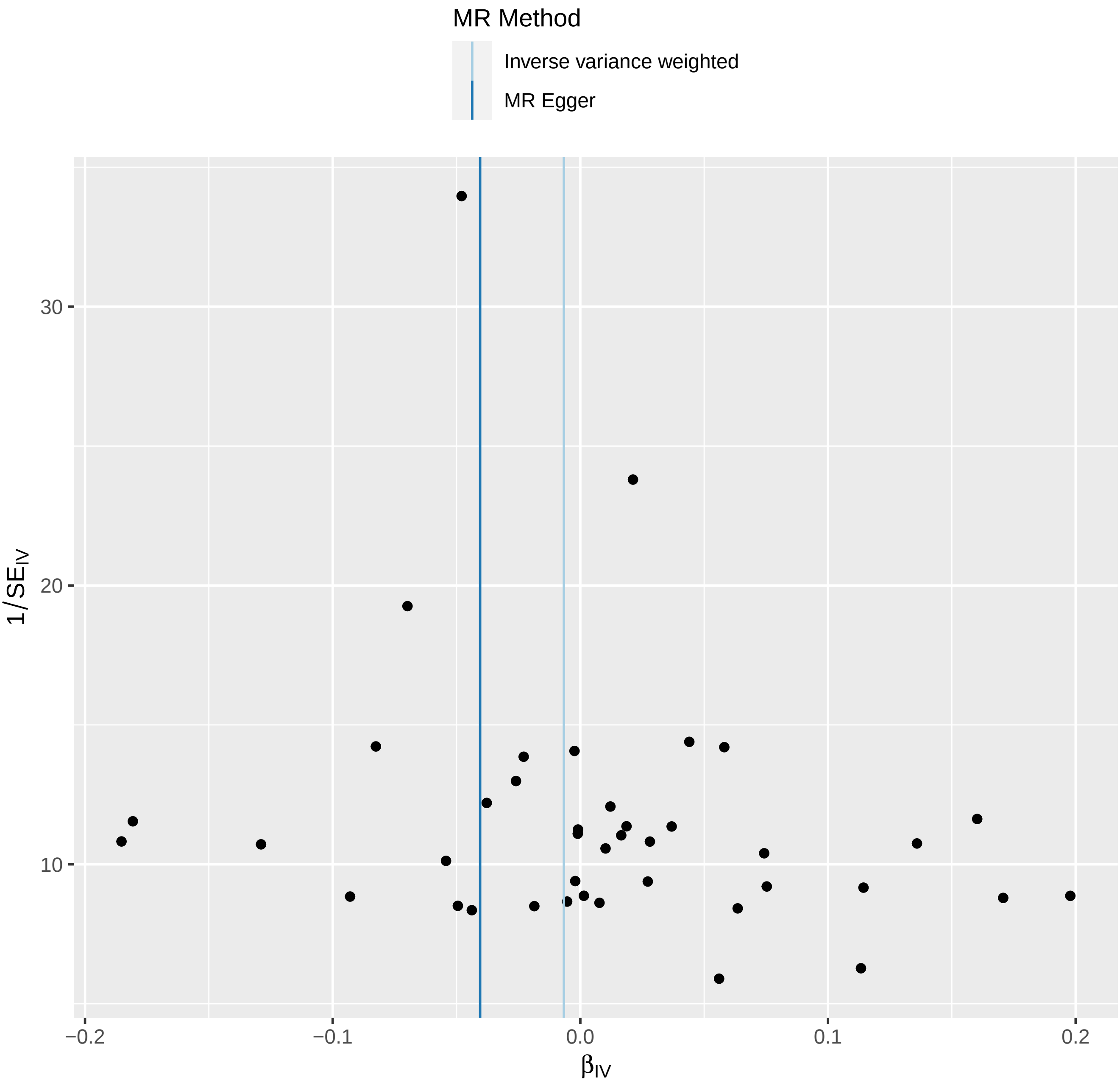

Batch 66 : Ulcerative colitis on IDP T1 FAST ROIs L pallidum

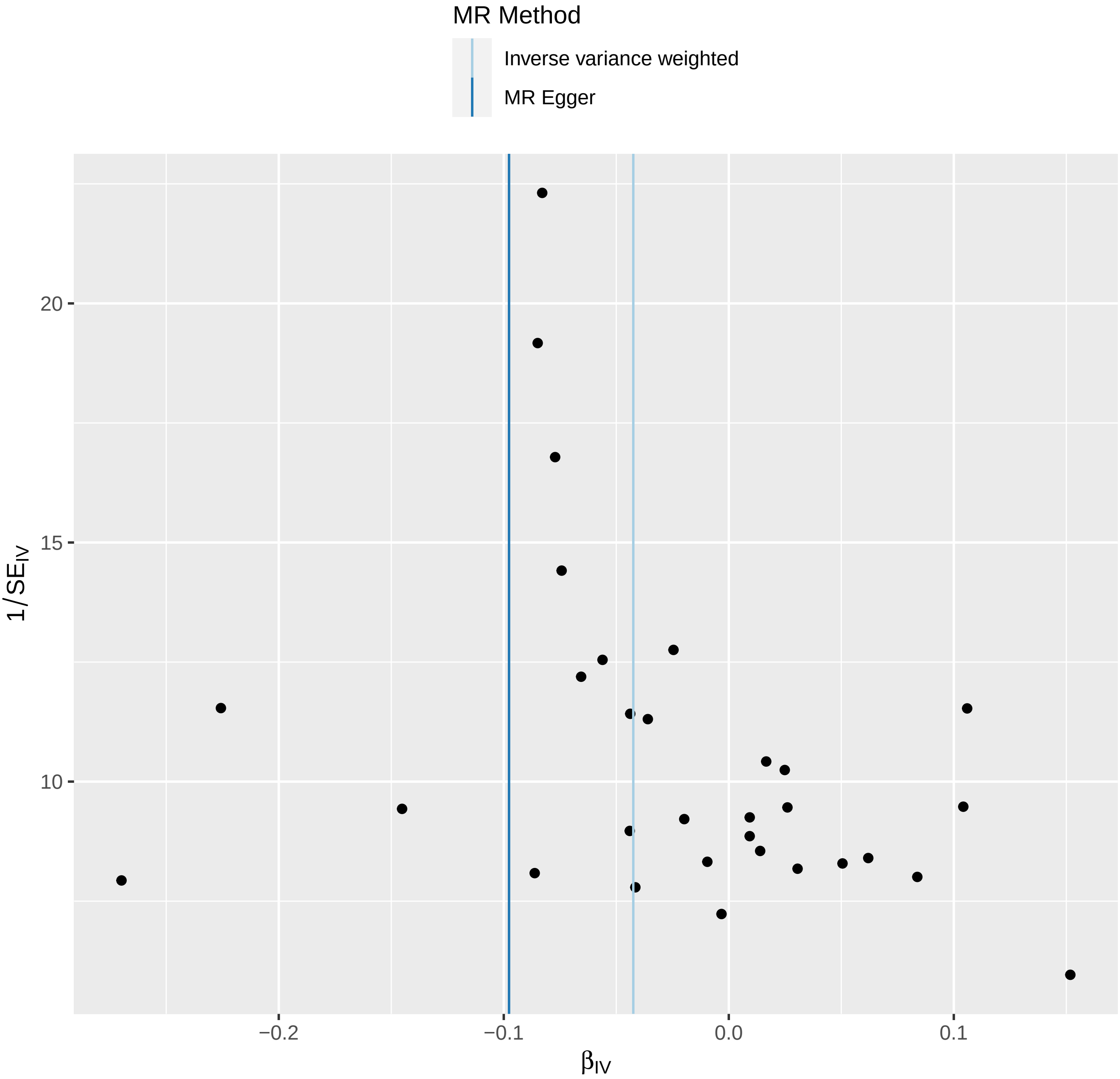

Batch 67 : Crohn's disease on IDP T1 FAST ROIs R pallidum

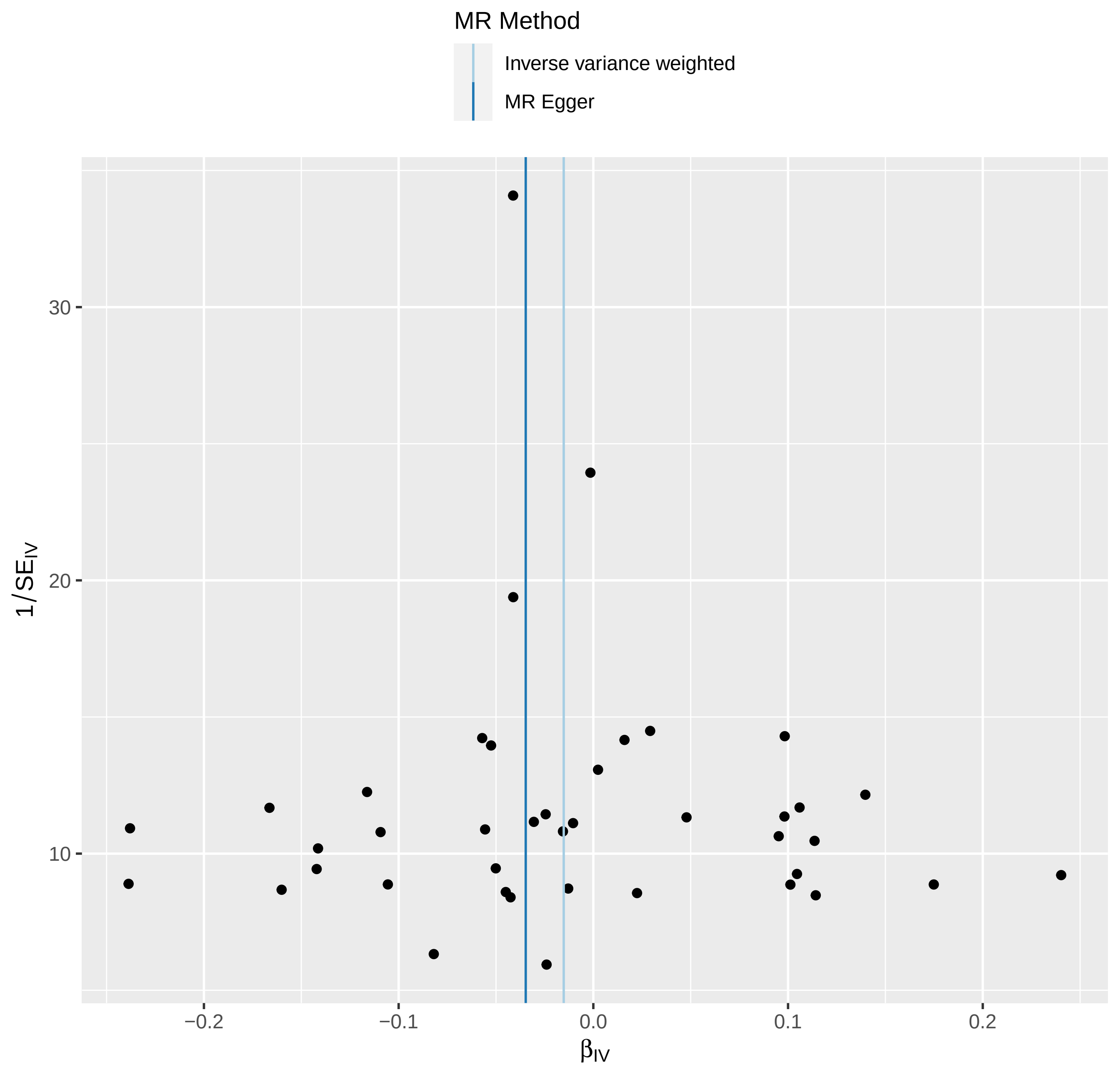

Batch 68 : Ulcerative colitis on IDP T1 FAST ROIs R pallidum

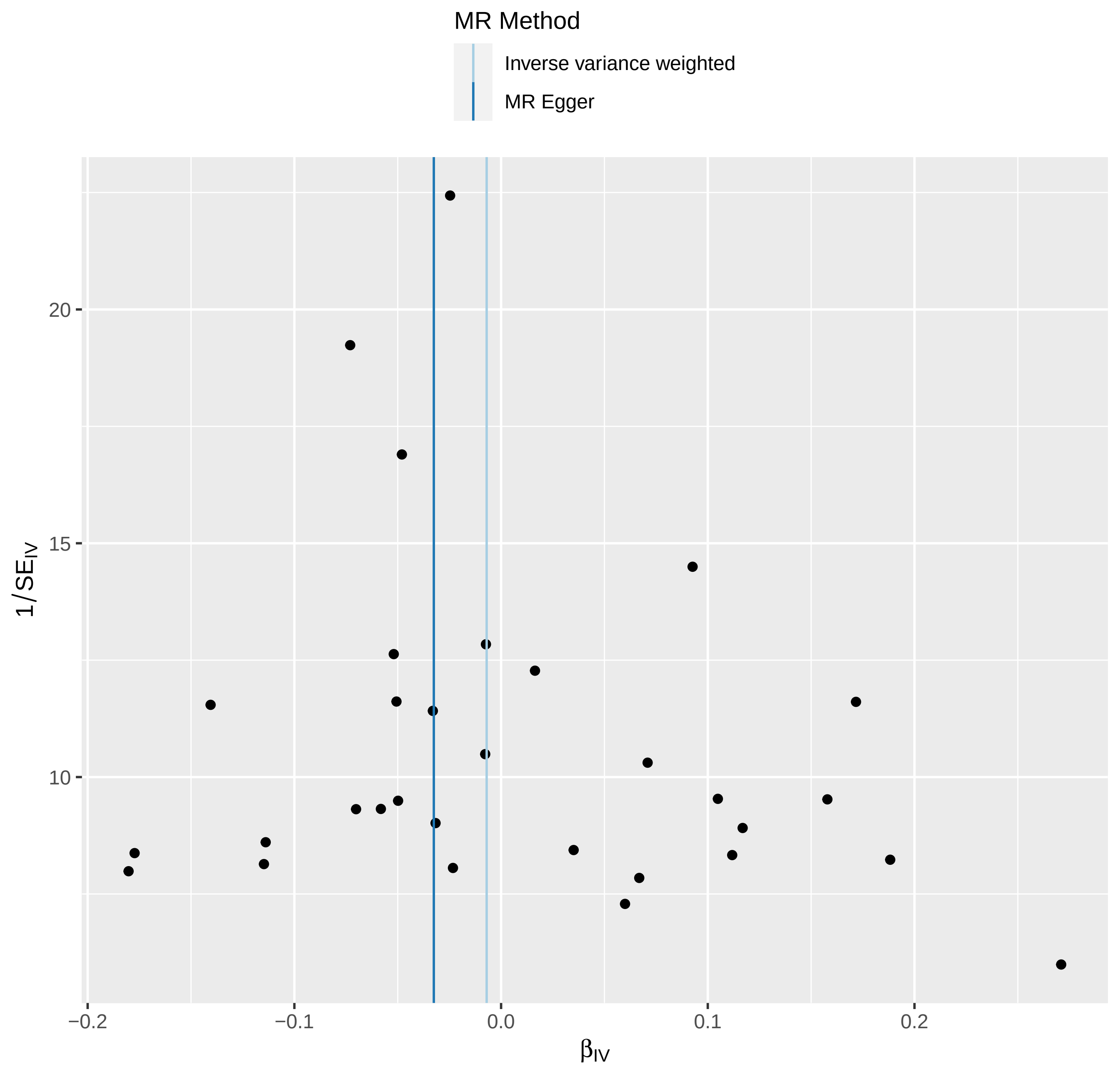

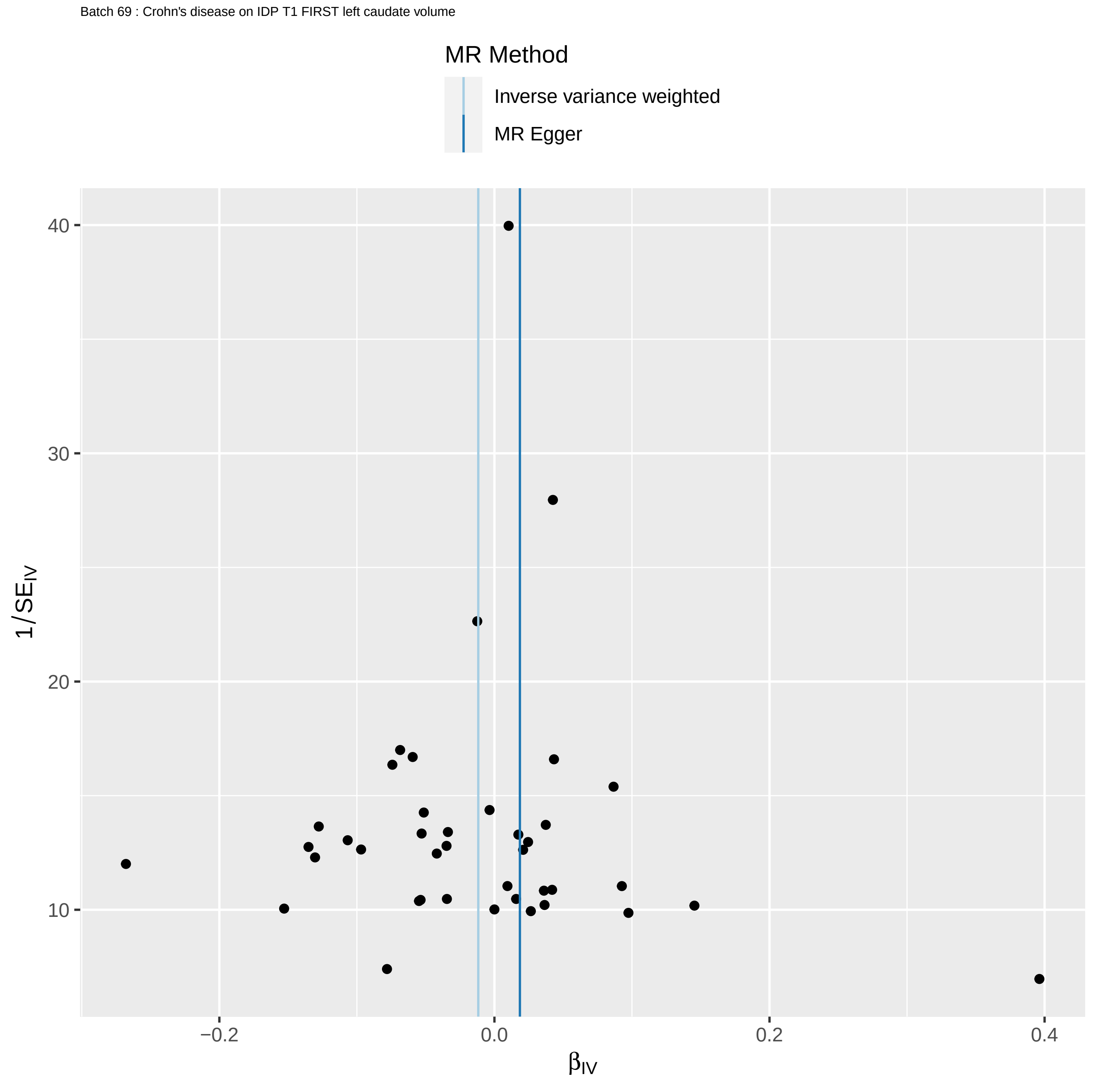

Batch 69 : Crohn's disease on IDP T1 FIRST left caudate volume
MR Method
Inverse variance weighted
MR Egger
40
30
20
10
1/SE$_{IV}$
−0.2
0.0
0.2
0.4
β$_{IV}$

Batch 70 : Ulcerative colitis on IDP T1 FIRST left caudate volume

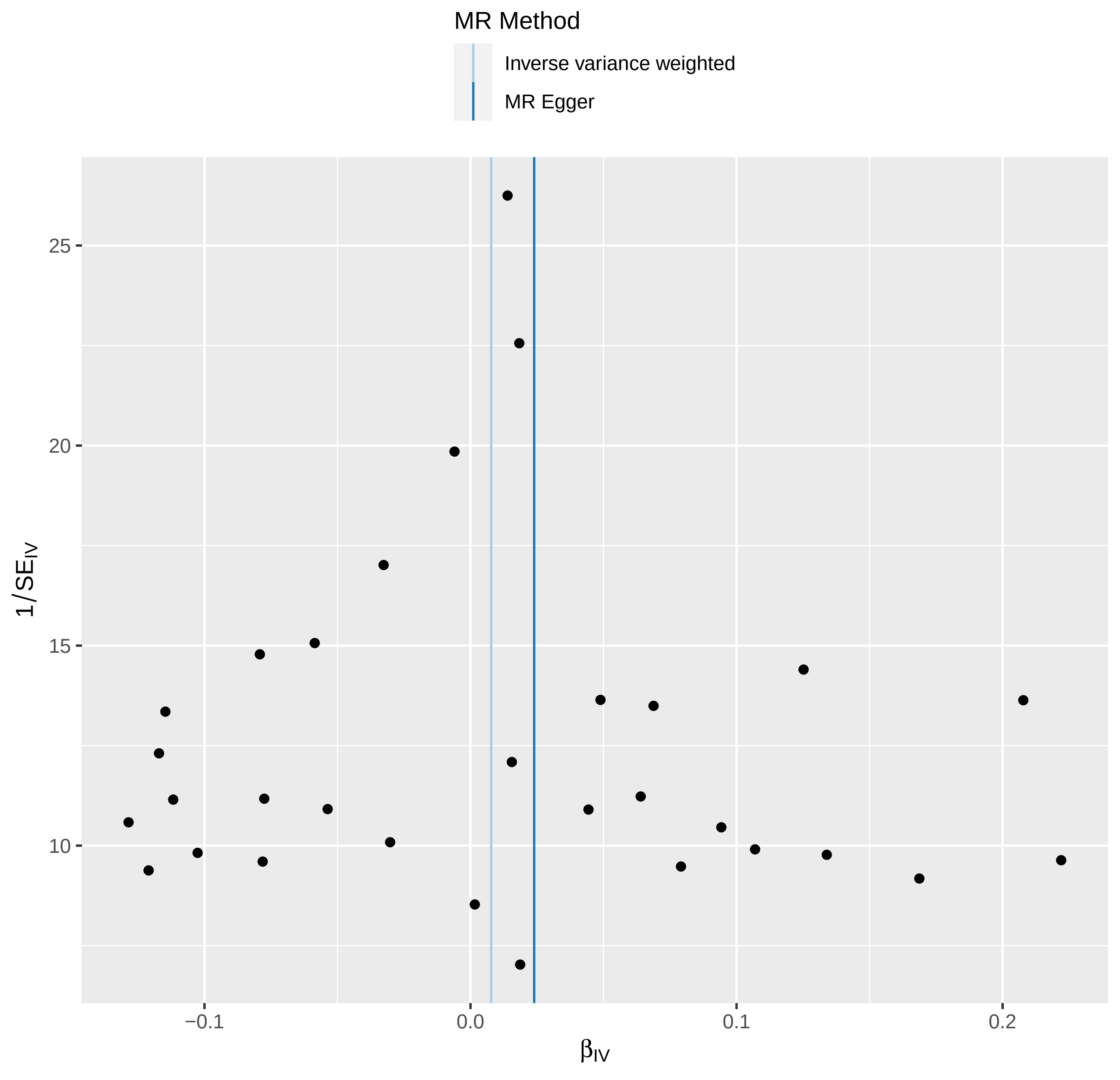

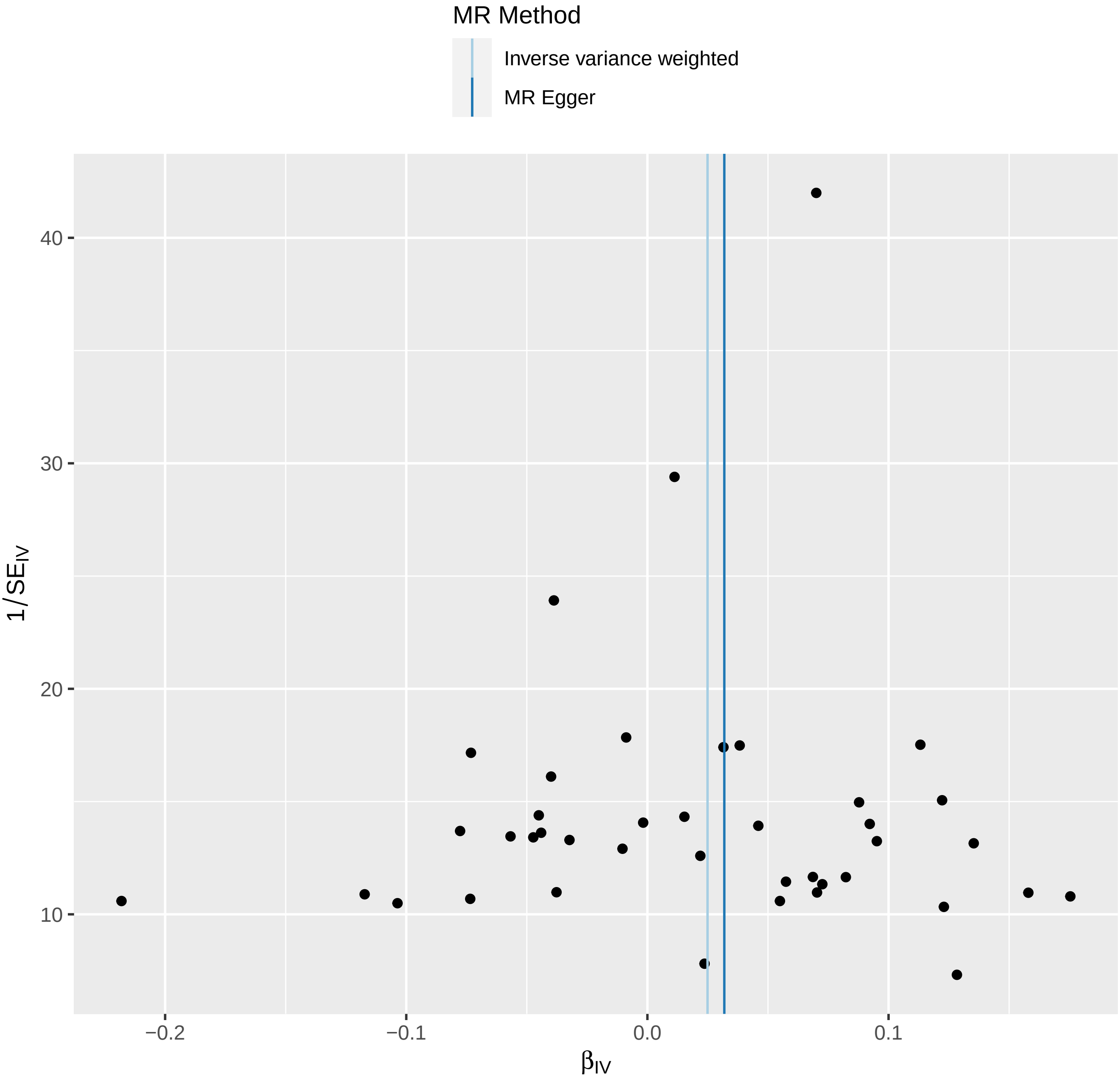
MR Method
Inverse variance weighted
MR Egger
1/SE_IV
β_IV
40
30
20
10
−0.2
−0.1
0.0
0.1

Batch 72 : Ulcerative colitis on IDP T1 FAST ROIs L hippocampus

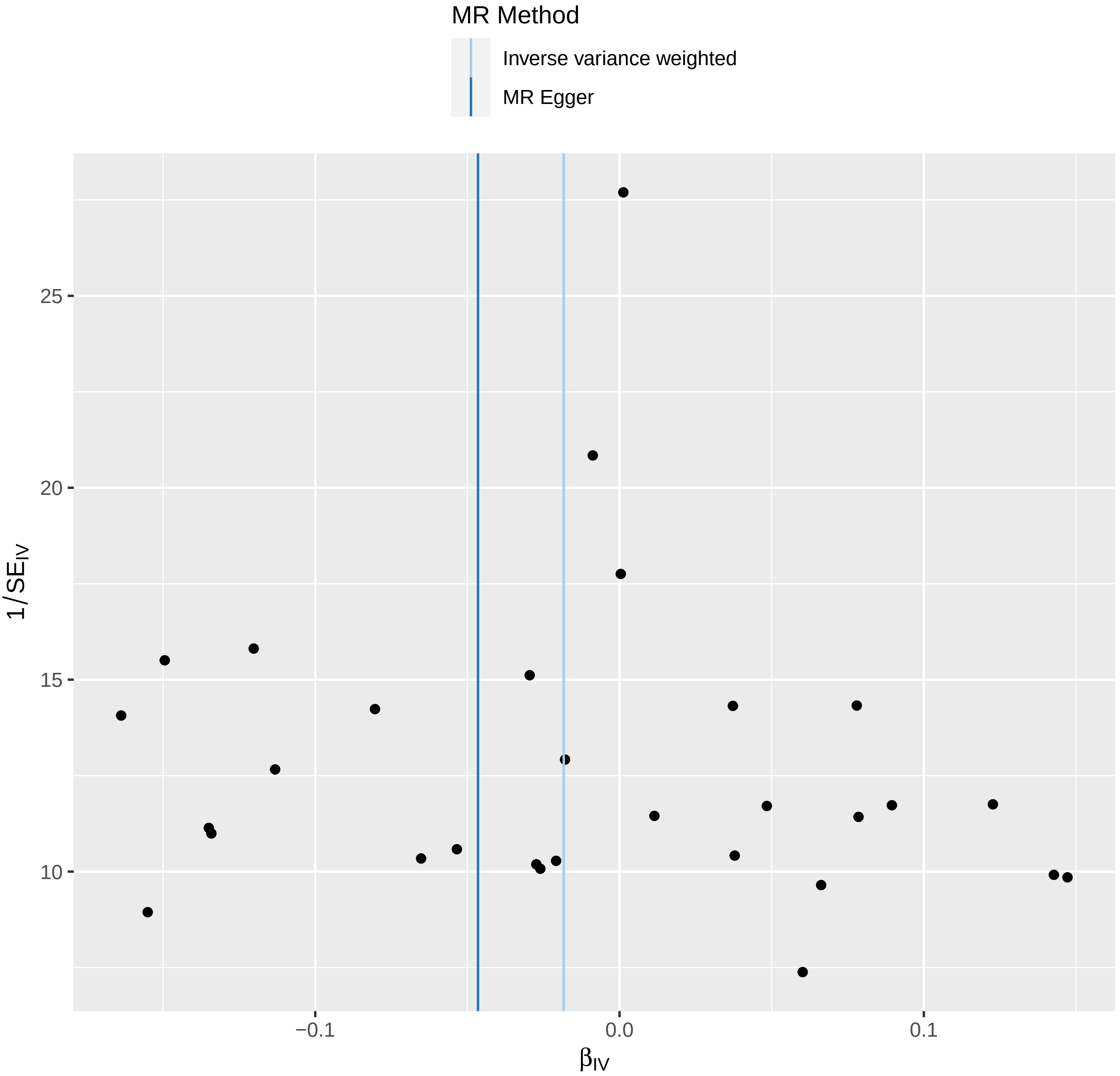

Batch 73 : Crohn's disease on IDP T1 FAST ROIs R hippocampus

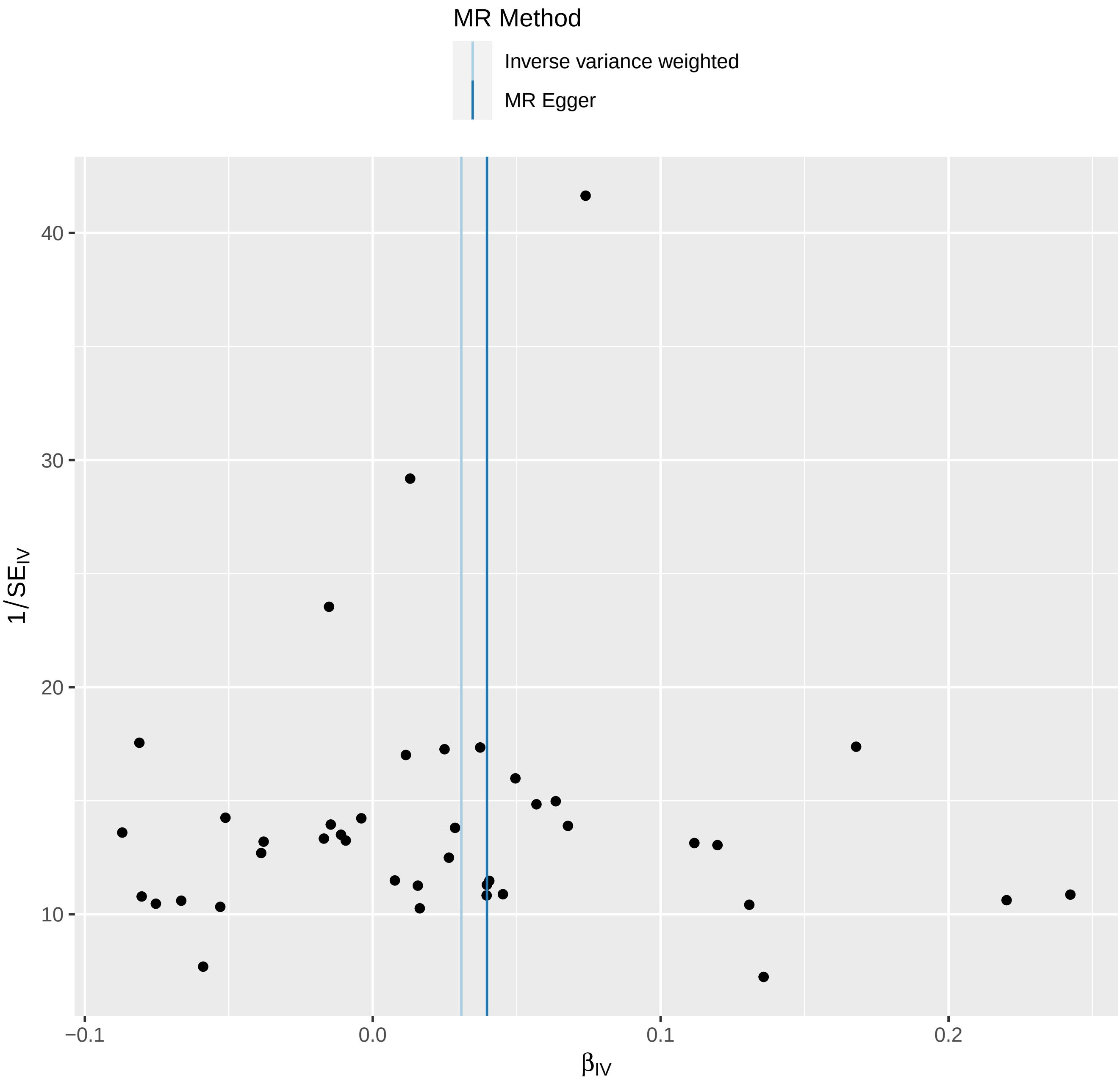

Batch 74 : Ulcerative colitis on IDP T1 FAST ROIs R hippocampus

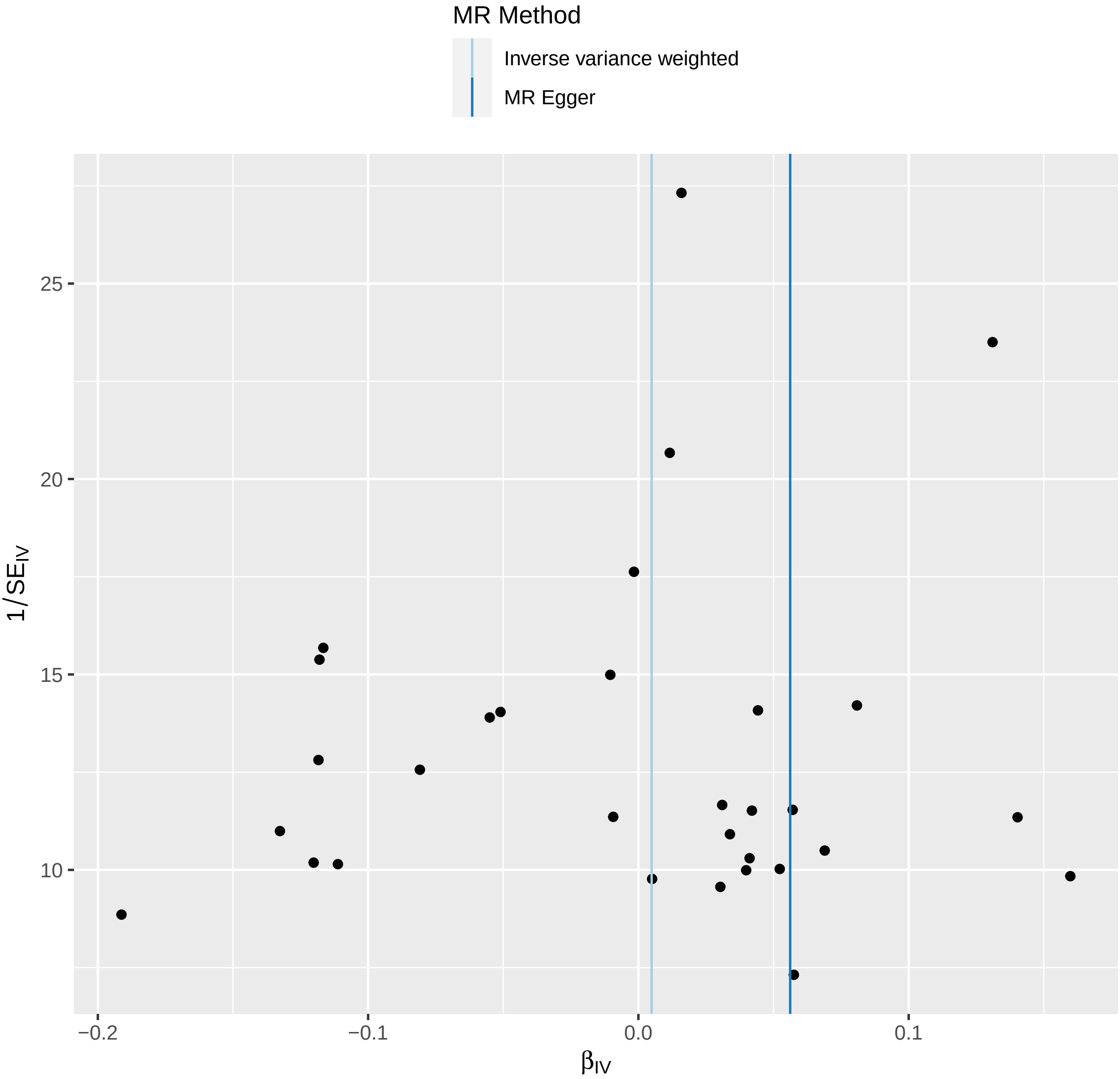

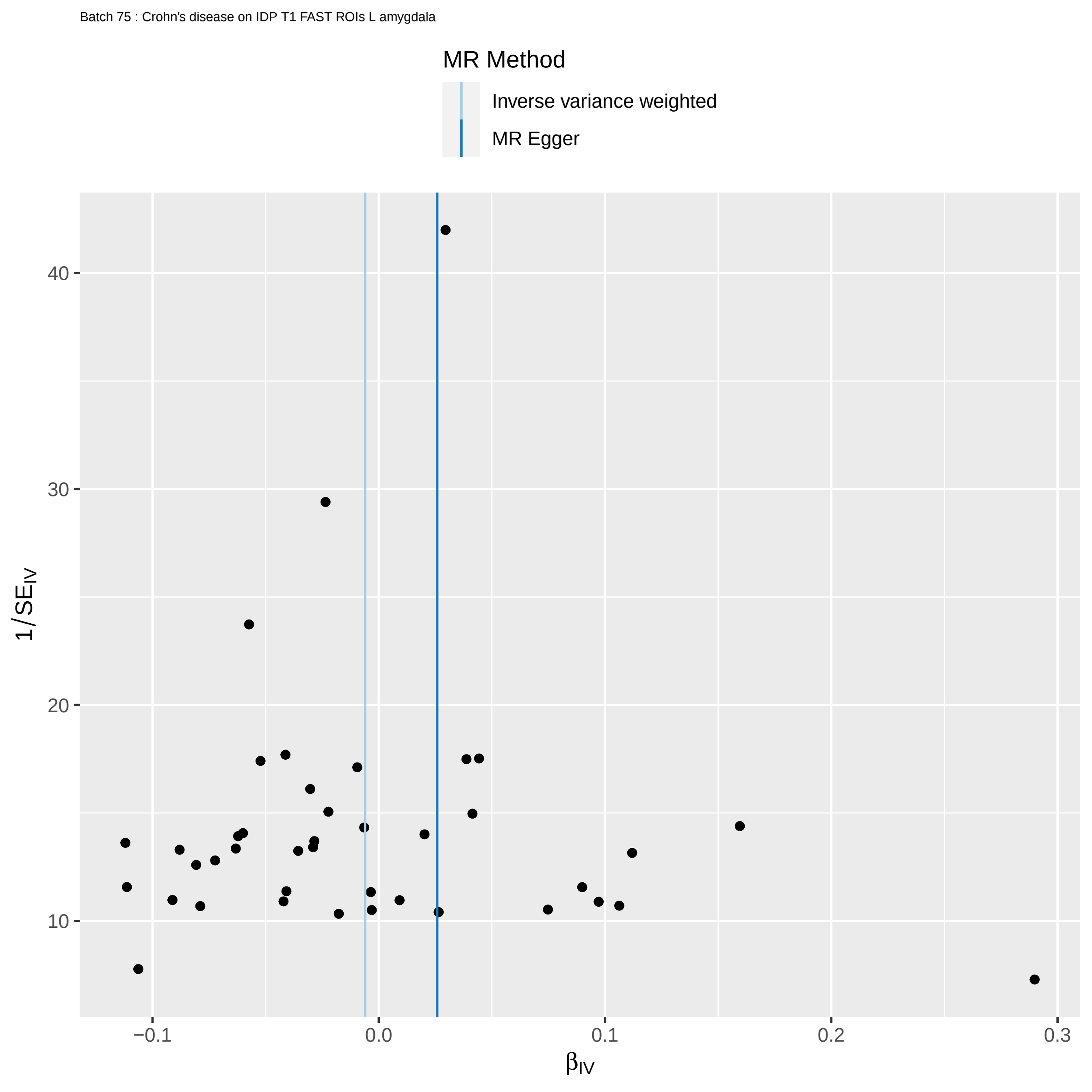

Batch 75 : Crohn's disease on IDP T1 FAST ROIs L amygdala
MR Method
Inverse variance weighted
MR Egger
40
30
20
10
1/SE$_{IV}$
−0.1
0.0
0.1
0.2
0.3
β$_{IV}$

Batch 76 : Ulcerative colitis on IDP T1 FAST ROIs L amygdala

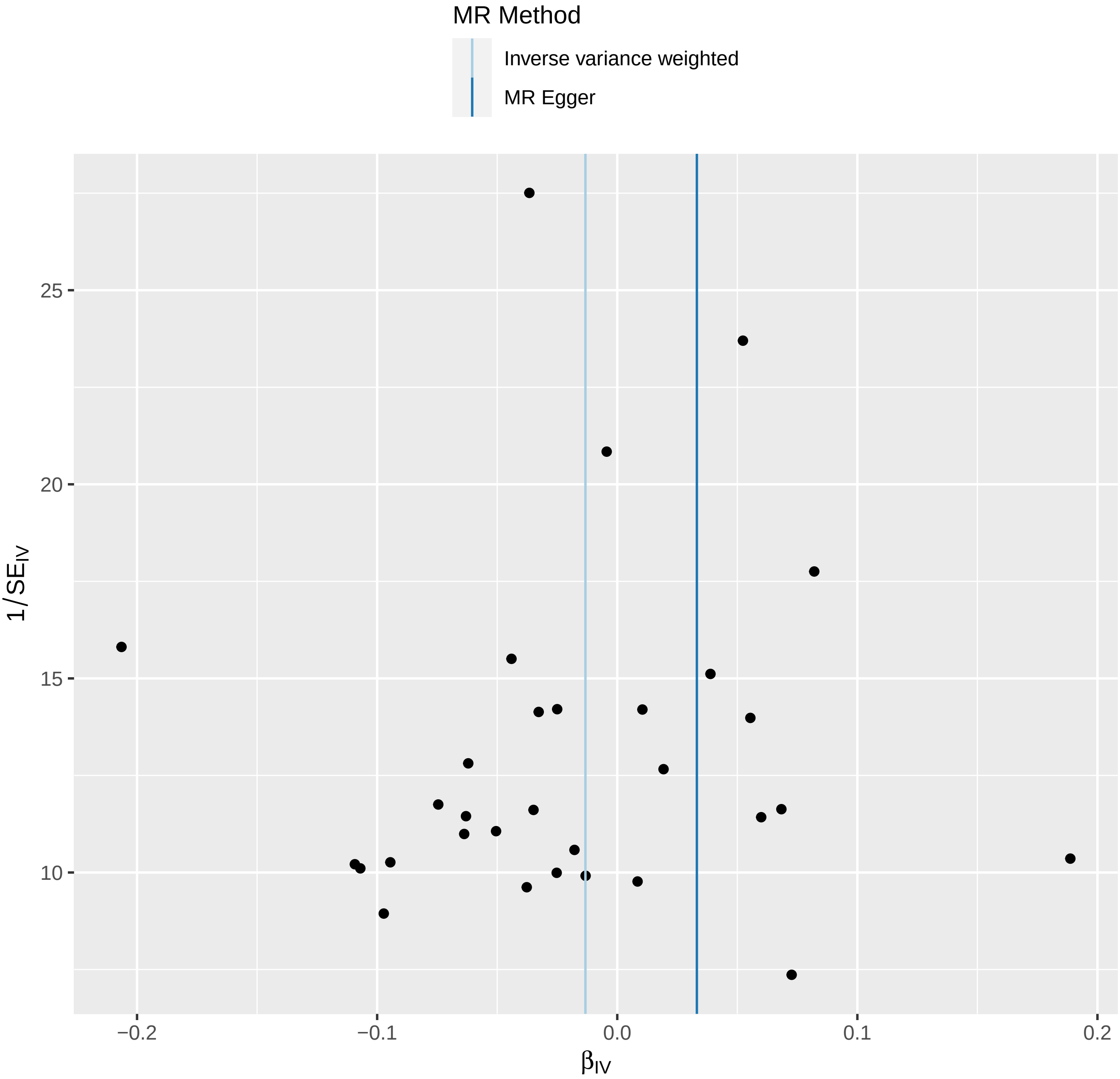

Batch 77 : Crohn's disease on IDP T1 FAST ROIs R amygdala

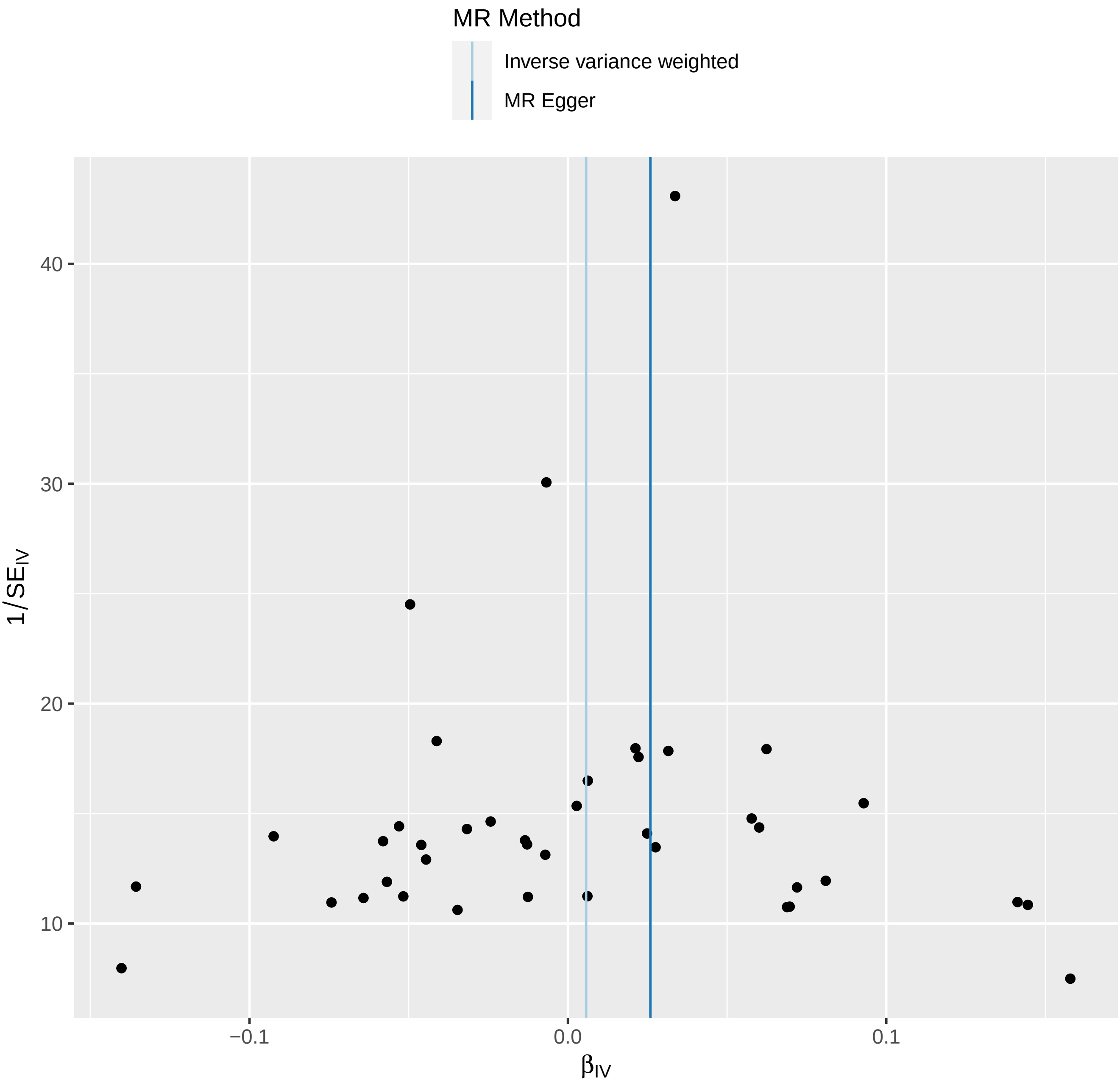

Batch 78 : Ulcerative colitis on IDP T1 FAST ROIs R amygdala

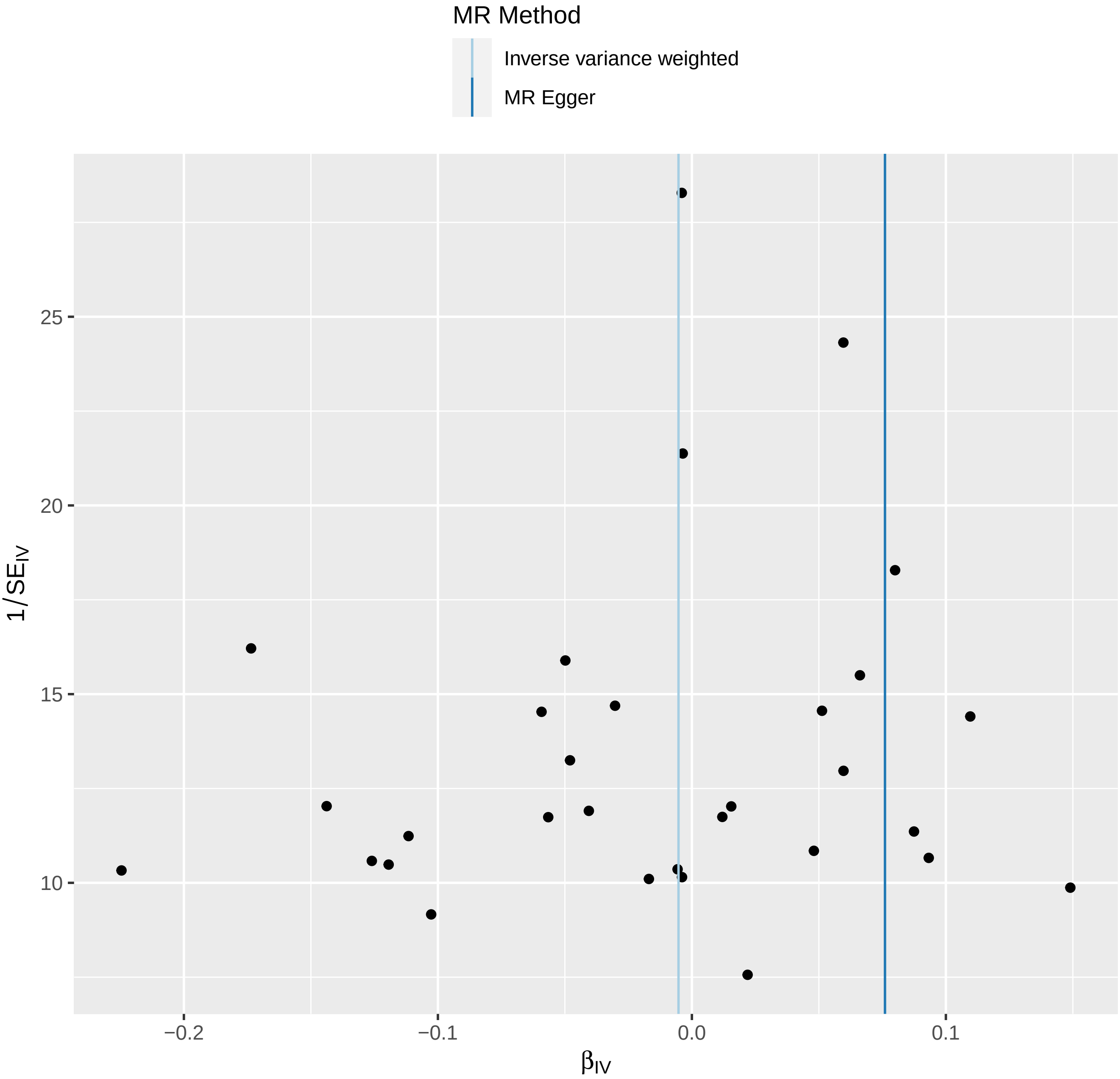

Batch 79 : Crohn's disease on IDP T1 FAST ROIs L ventral striatum

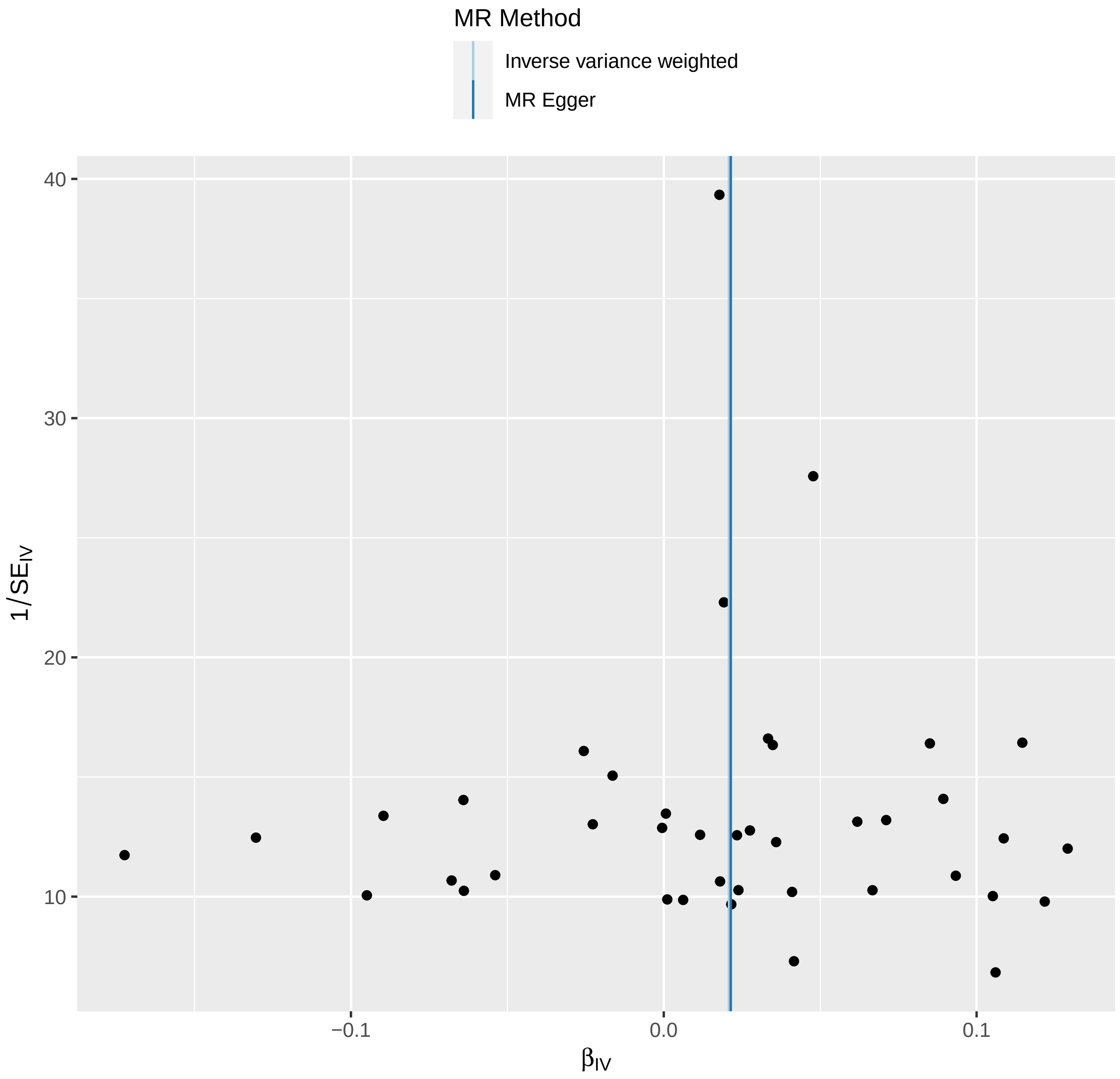

Batch 80 : Ulcerative colitis on IDP T1 FAST ROIs L ventral striatum

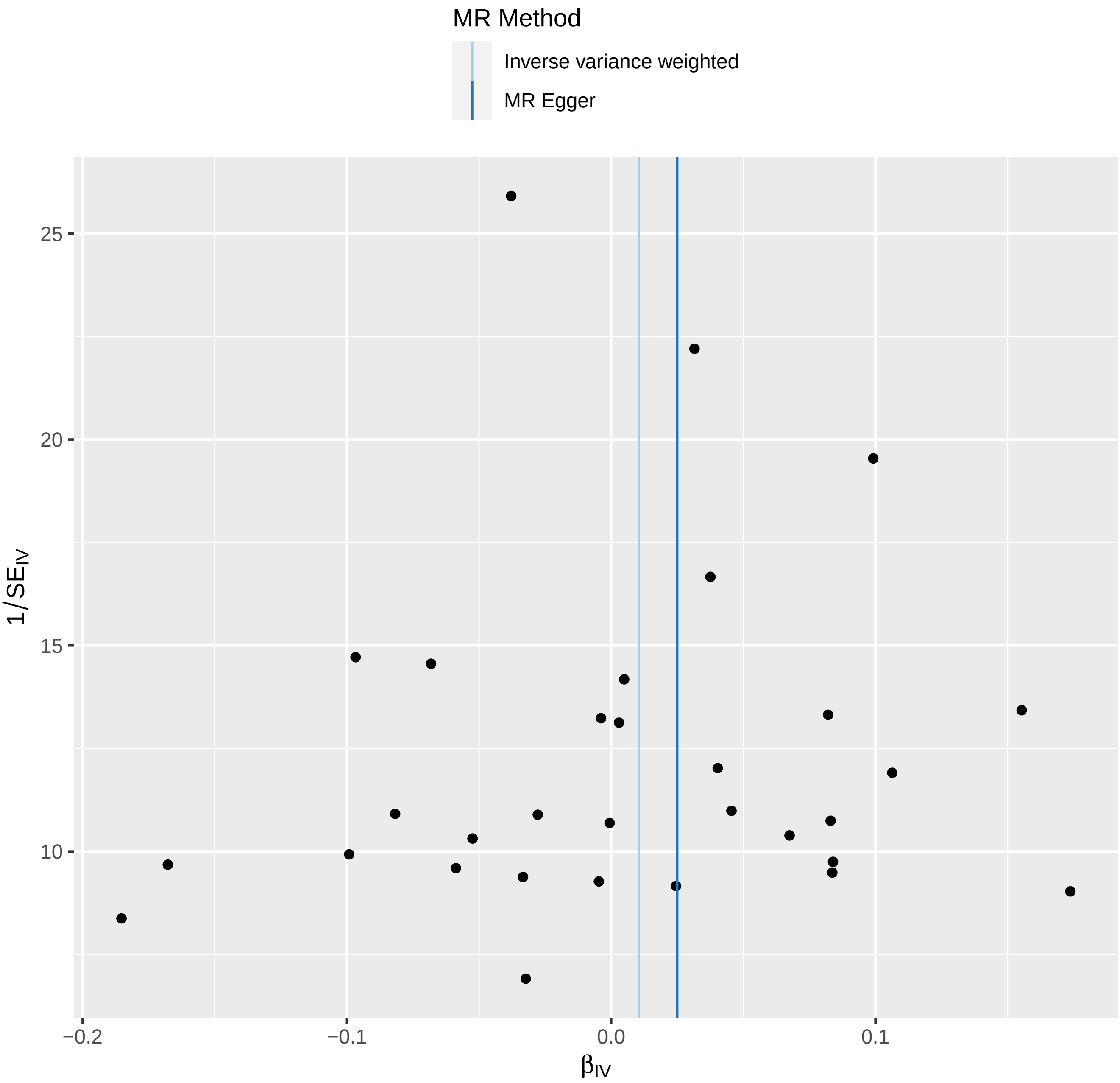

Batch 81 : Crohn's disease on IDP T1 FAST ROIs R ventral striatum

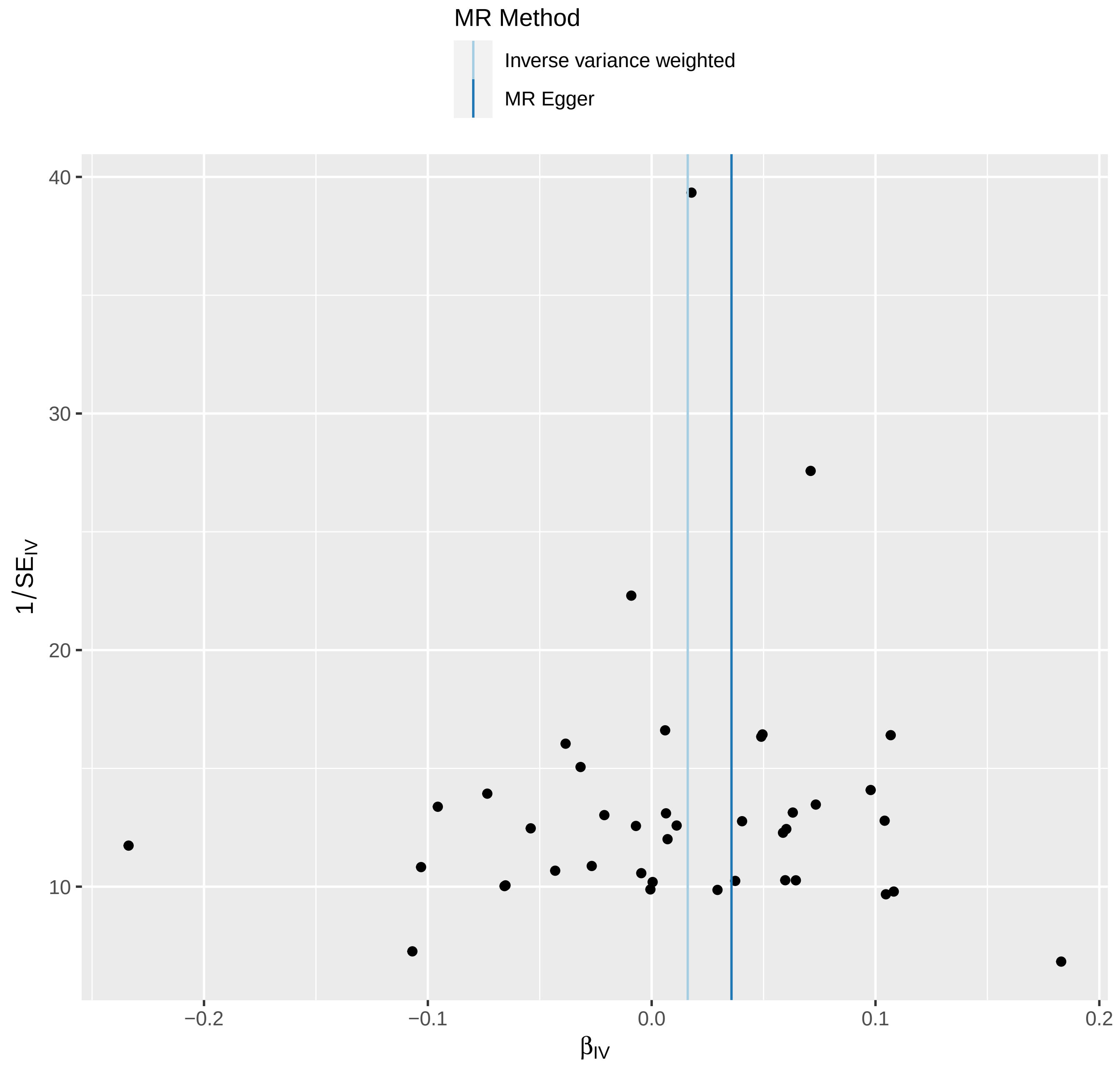

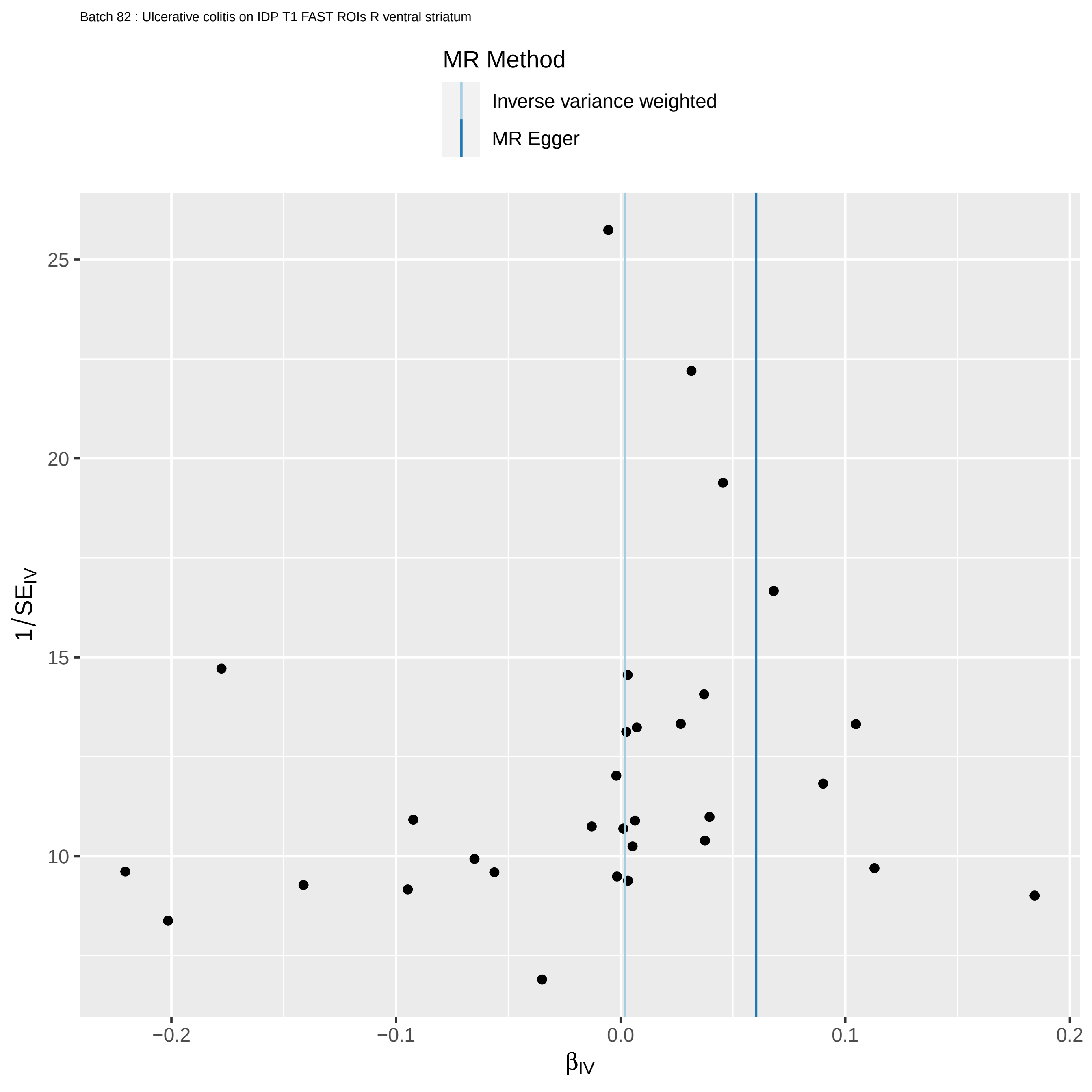

Batch 82 : Ulcerative colitis on IDP T1 FAST ROIs R ventral striatum
MR Method
Inverse variance weighted
MR Egger
25
20
15
10
1/SE$_{IV}$
−0.2
−0.1
0.0
0.1
0.2
β$_{IV}$

Batch 83 : Crohn's disease on IDP T1 FAST ROIs brain stem

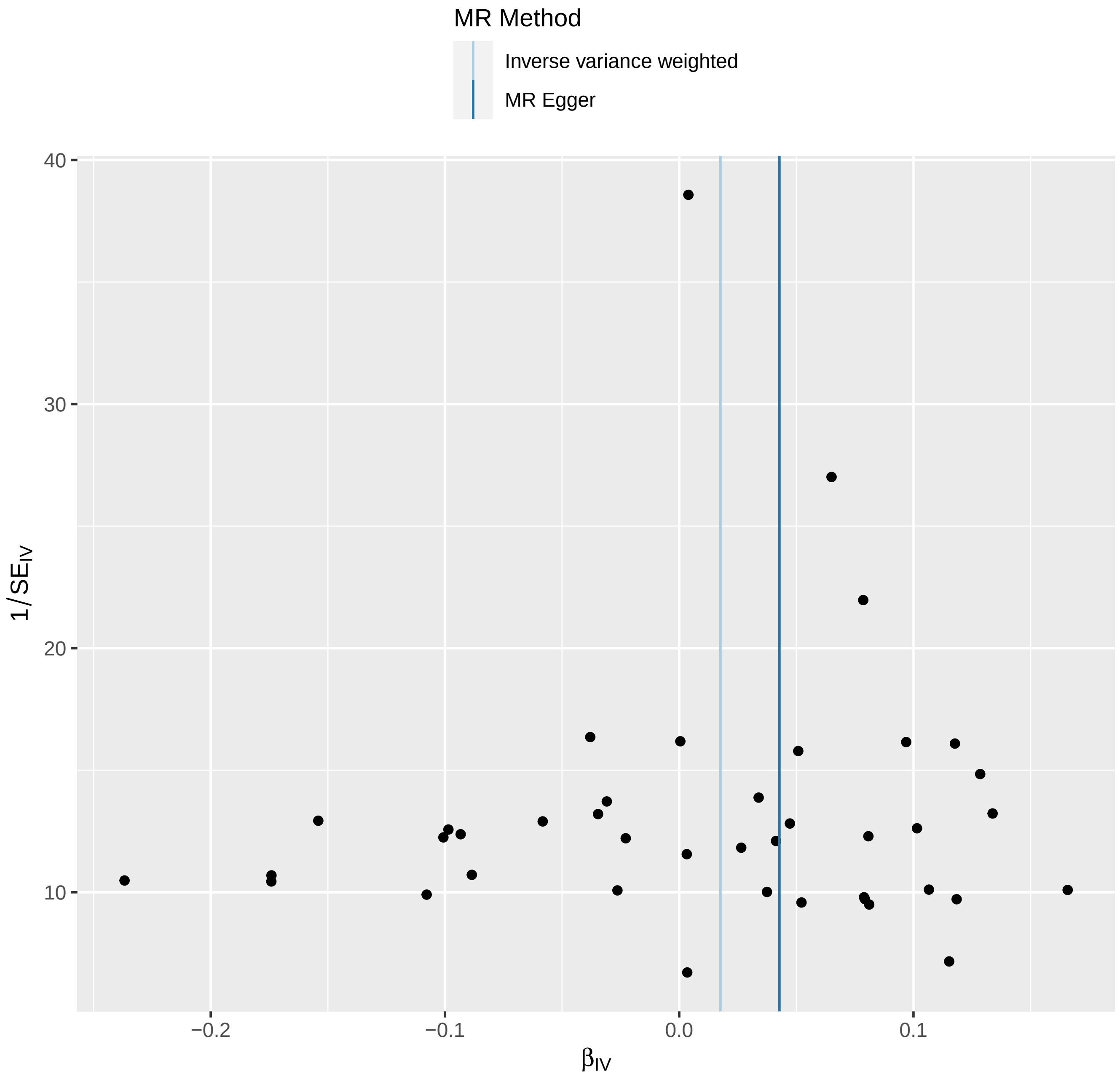

Batch 84 : Ulcerative colitis on IDP T1 FAST ROIs brain stem

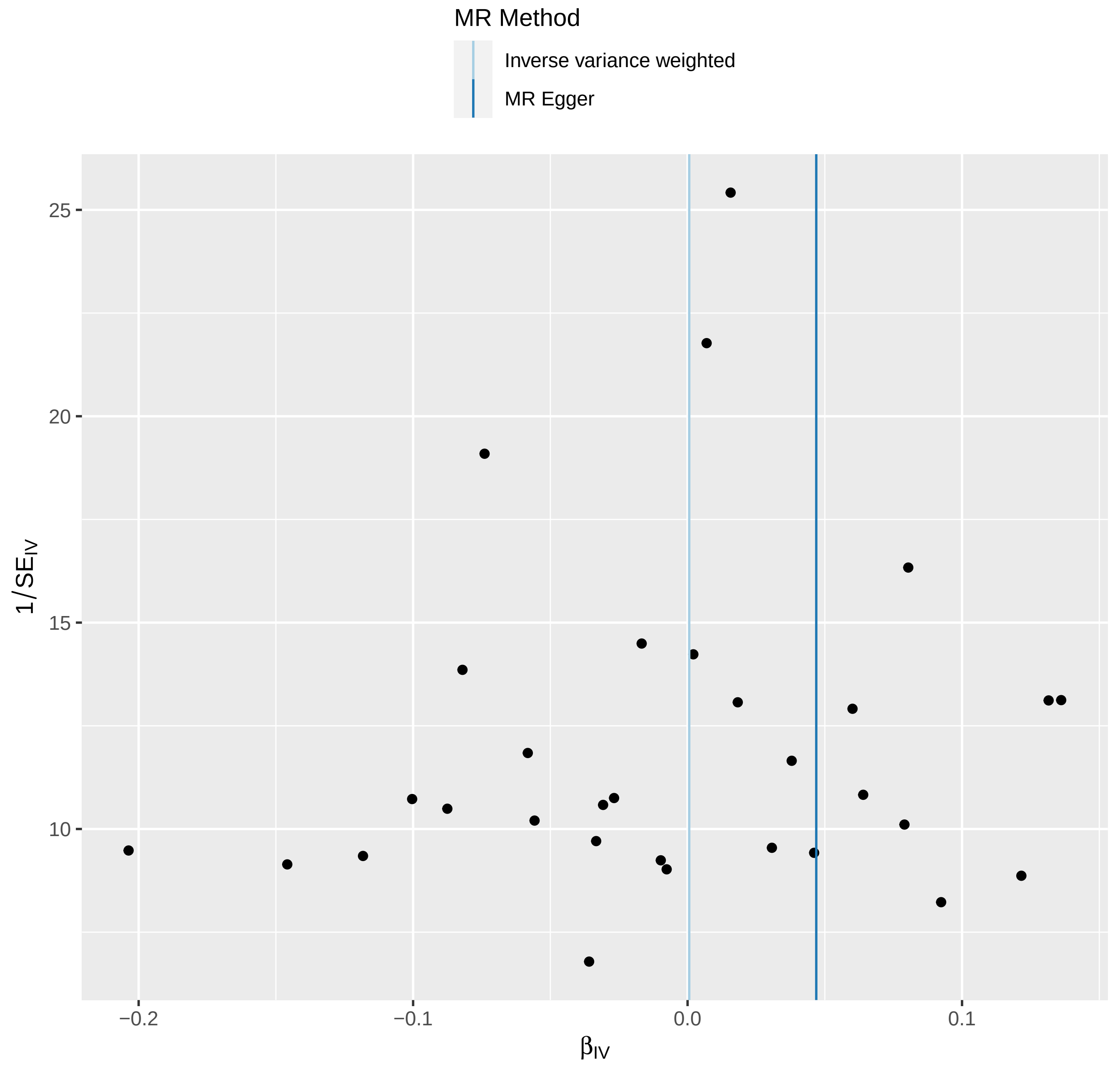

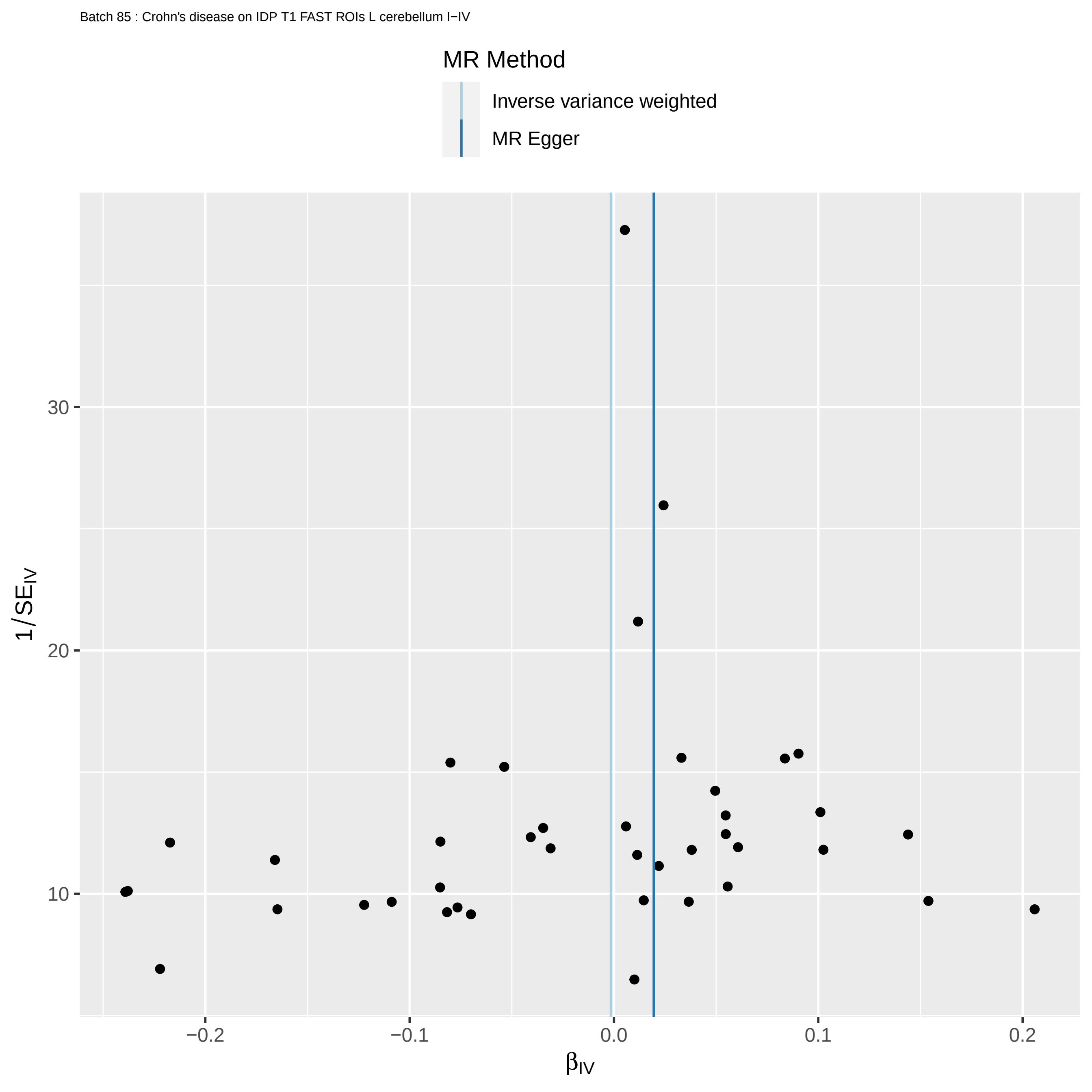

Batch 85 : Crohn's disease on IDP T1 FAST ROIs L cerebellum I−IV
MR Method
Inverse variance weighted
MR Egger
30
20
10
1/SE_IV
−0.2
−0.1
0.0
0.1
0.2
β_IV

Batch 86 : Ulcerative colitis on IDP T1 FAST ROIs L cerebellum I−IV

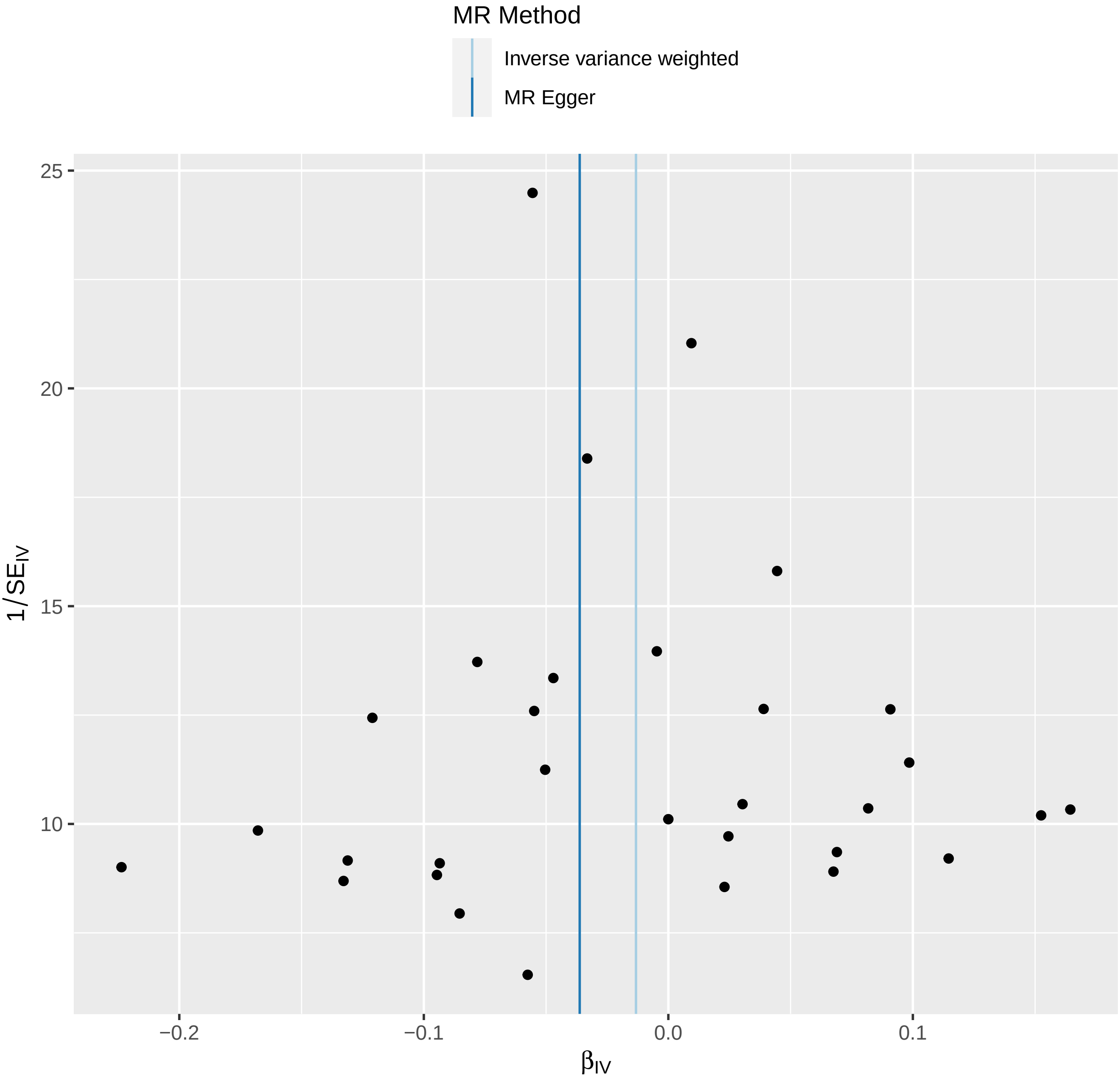

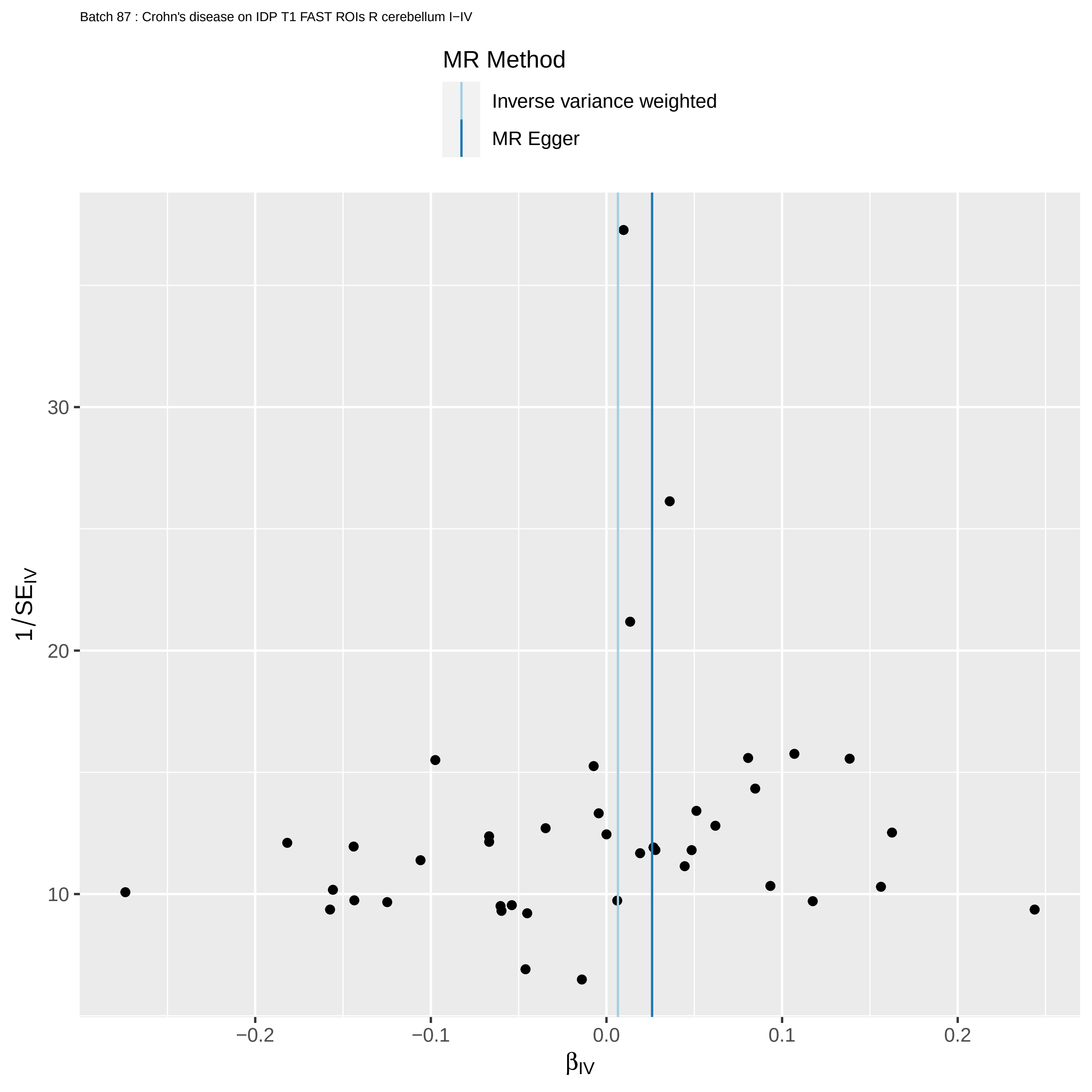
Batch 87 : Crohn's disease on IDP T1 FAST ROIs R cerebellum I−IV
MR Method
Inverse variance weighted
MR Egger
30
20
10
−0.2
−0.1
0.0
0.1
0.2
$1/SE_{IV}$
β_{IV}

Batch 88 : Ulcerative colitis on IDP T1 FAST ROIs R cerebellum I−IV

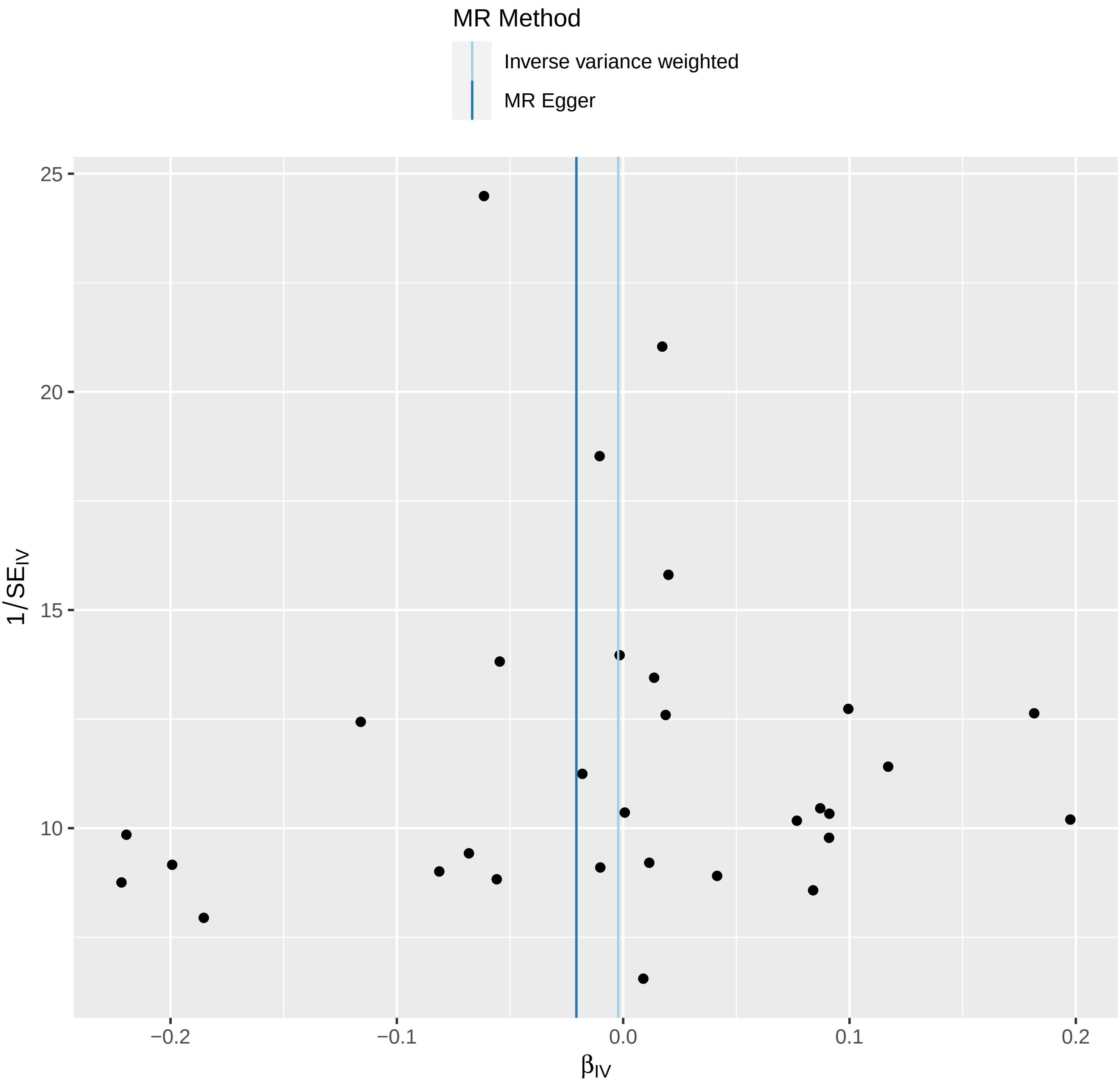

Batch 89 : Crohn's disease on IDP T1 FAST ROIs L cerebellum V

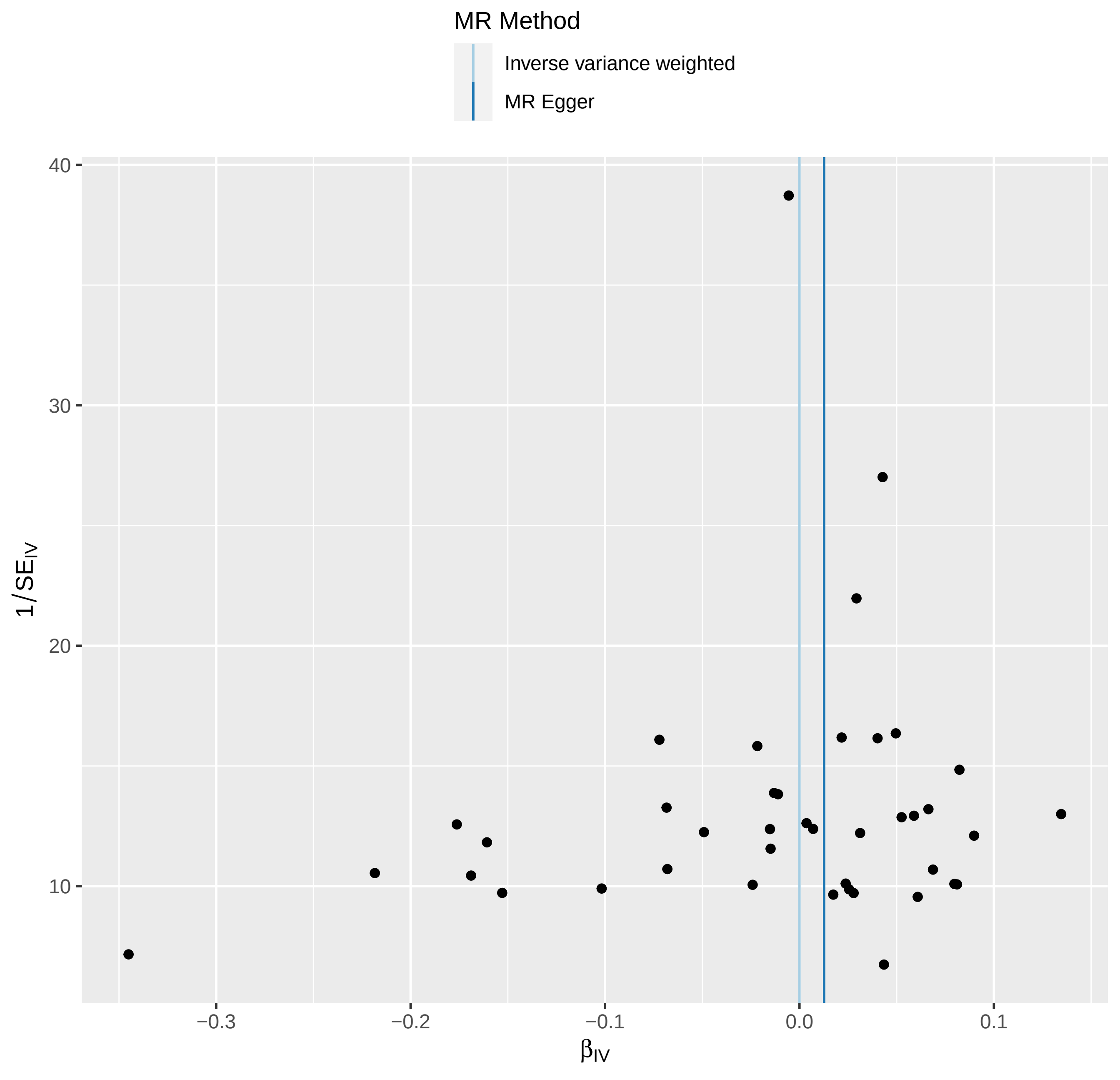

Batch 90 : Ulcerative colitis on IDP T1 FAST ROIs L cerebellum V

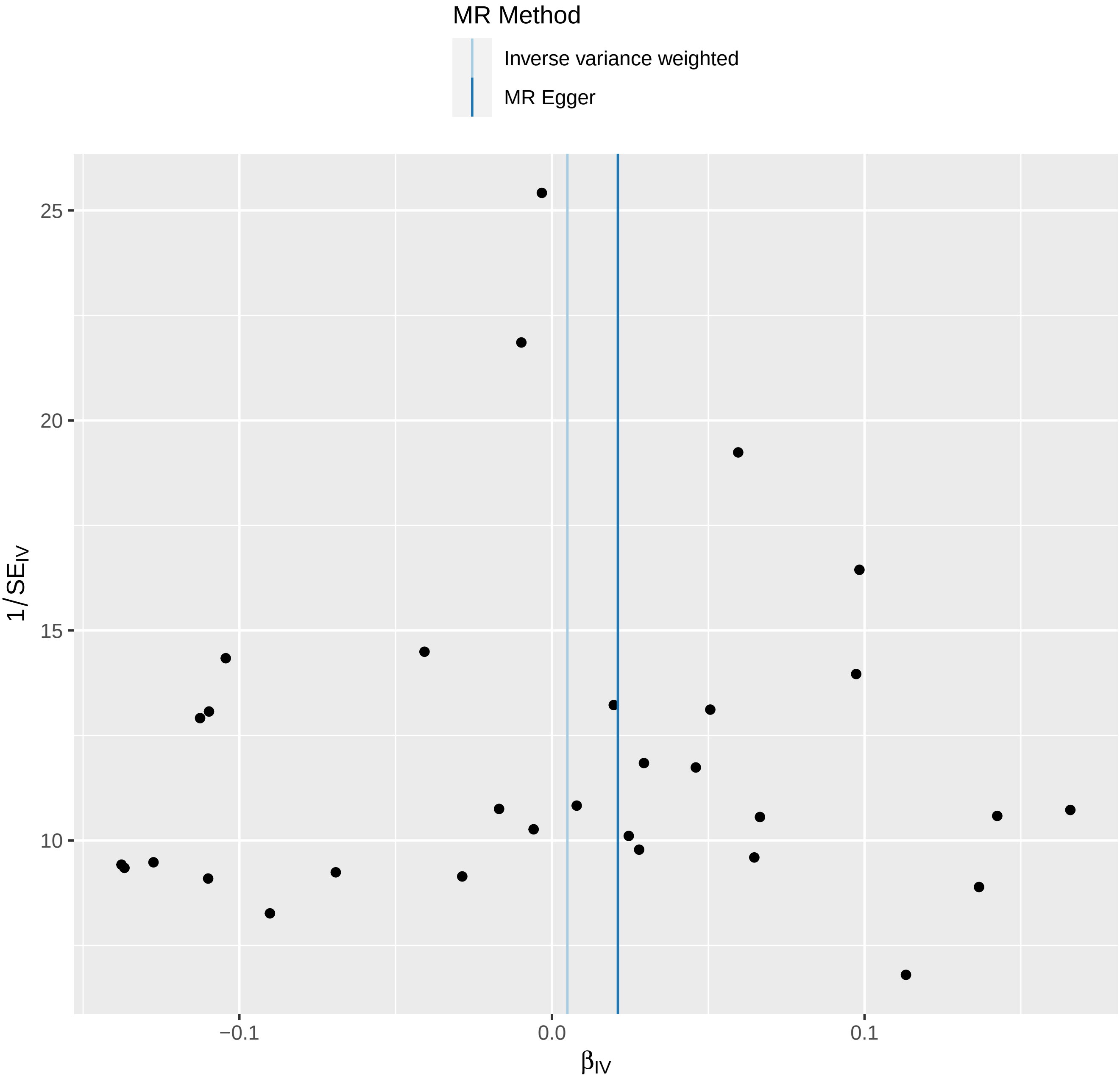

Batch 91 : Crohn's disease on IDP T1 FIRST right caudate volume

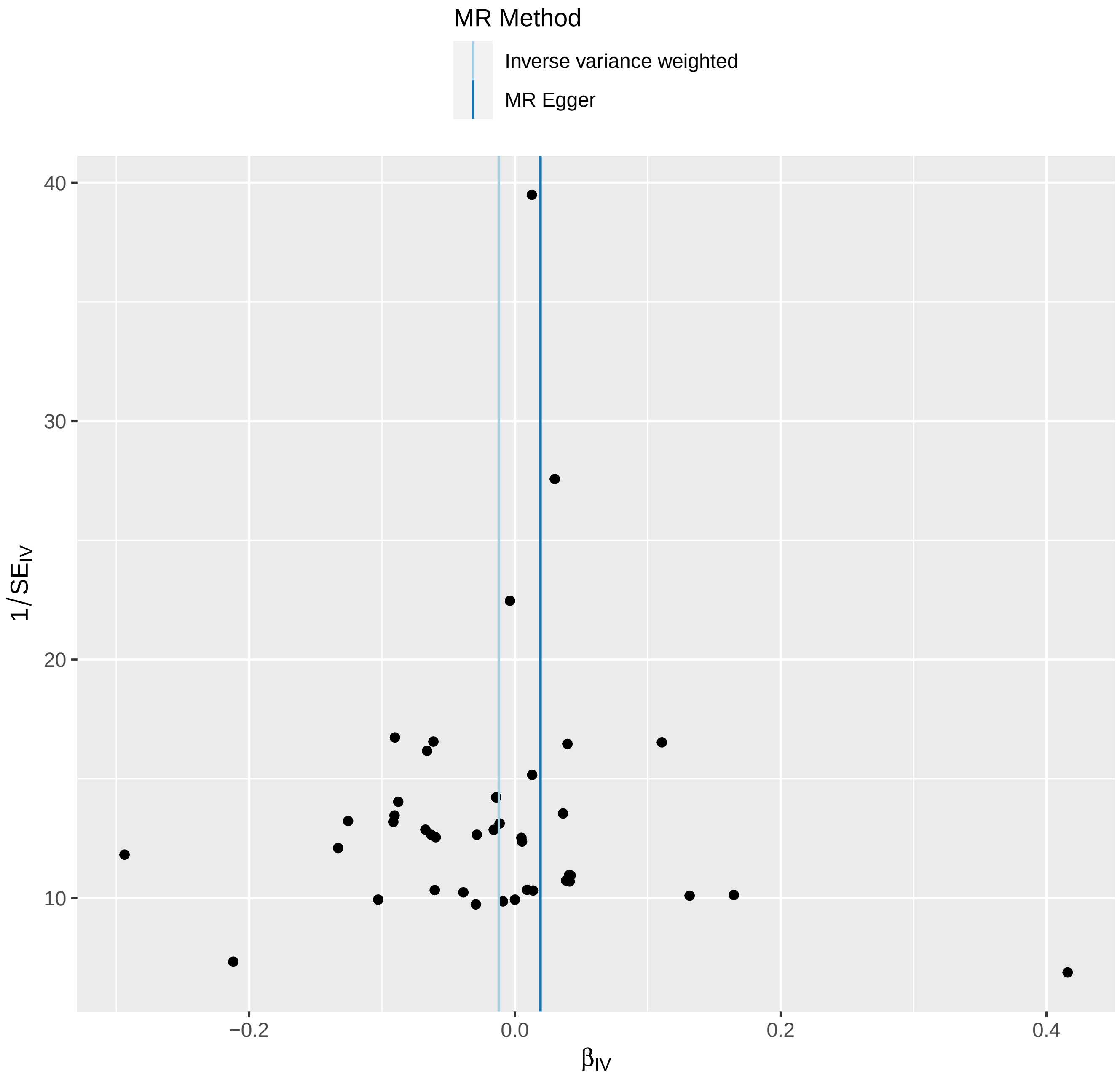

Batch 92 : Ulcerative colitis on IDP T1 FIRST right caudate volume

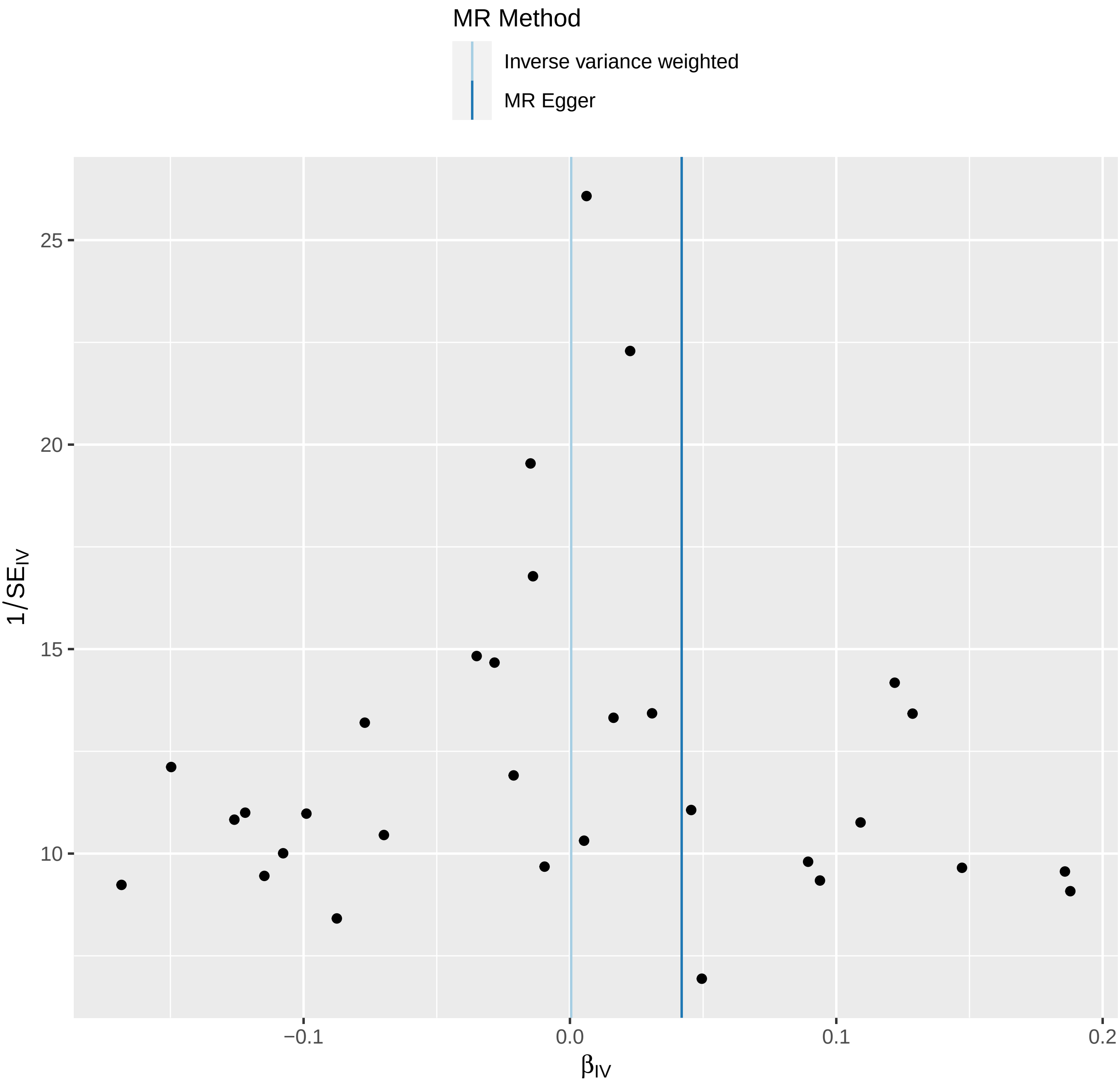

Batch 93 : Crohn's disease on IDP T1 FAST ROIs R cerebellum V

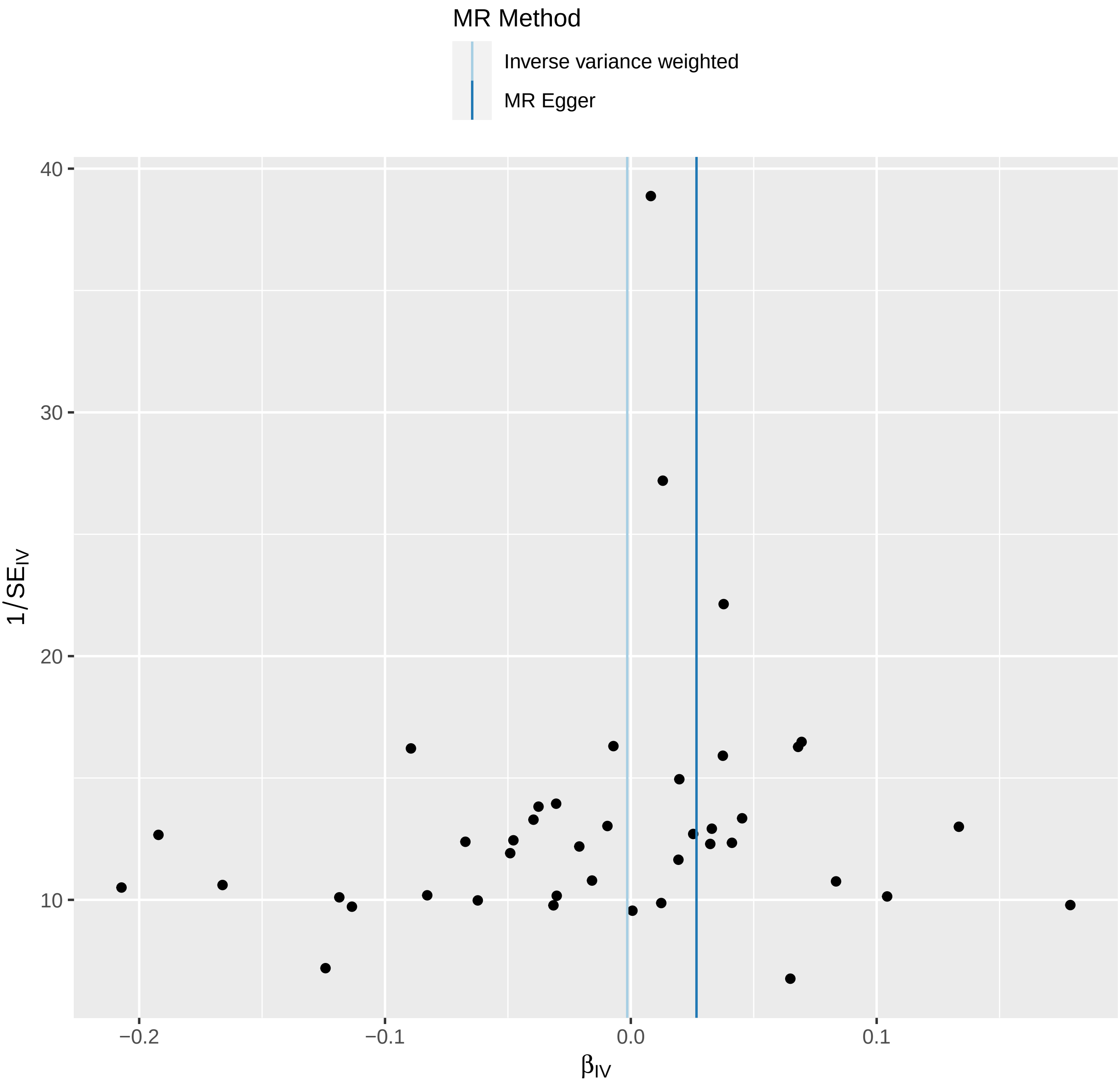

Batch 94 : Ulcerative colitis on IDP T1 FAST ROIs R cerebellum V

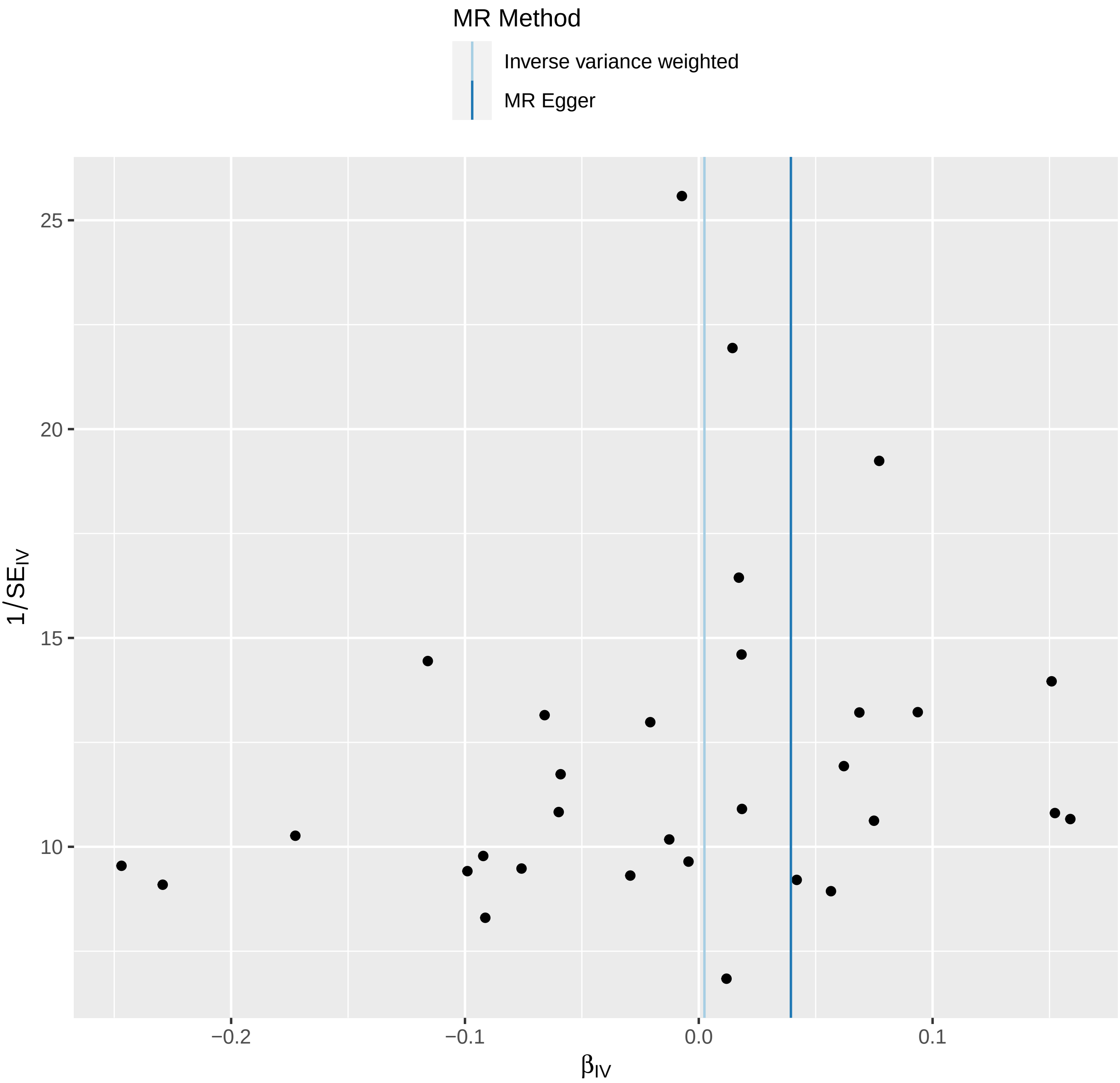

Batch 95 : Crohn's disease on IDP T1 FAST ROIs L cerebellum VI

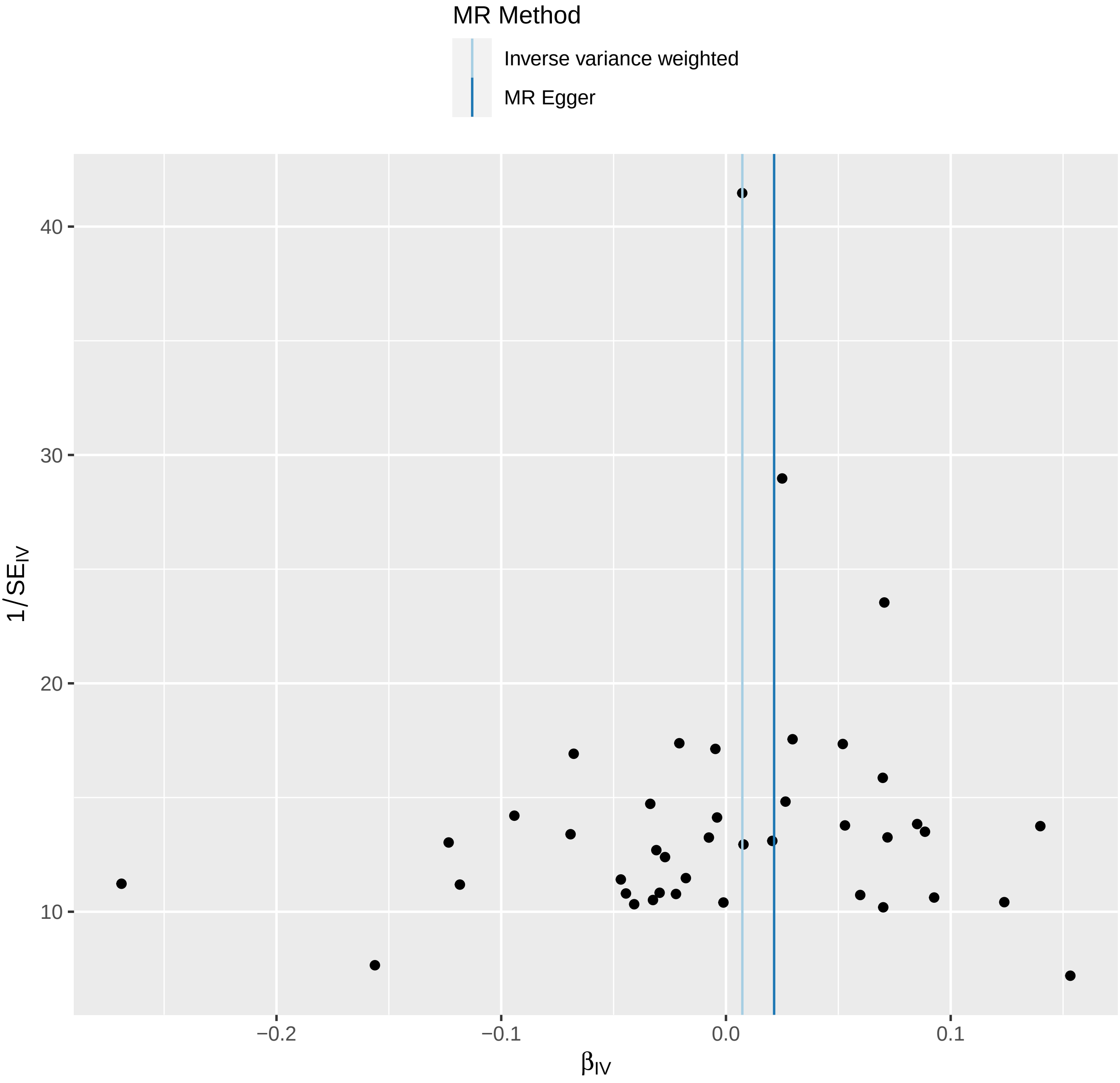

Batch 96 : Ulcerative colitis on IDP T1 FAST ROIs L cerebellum VI

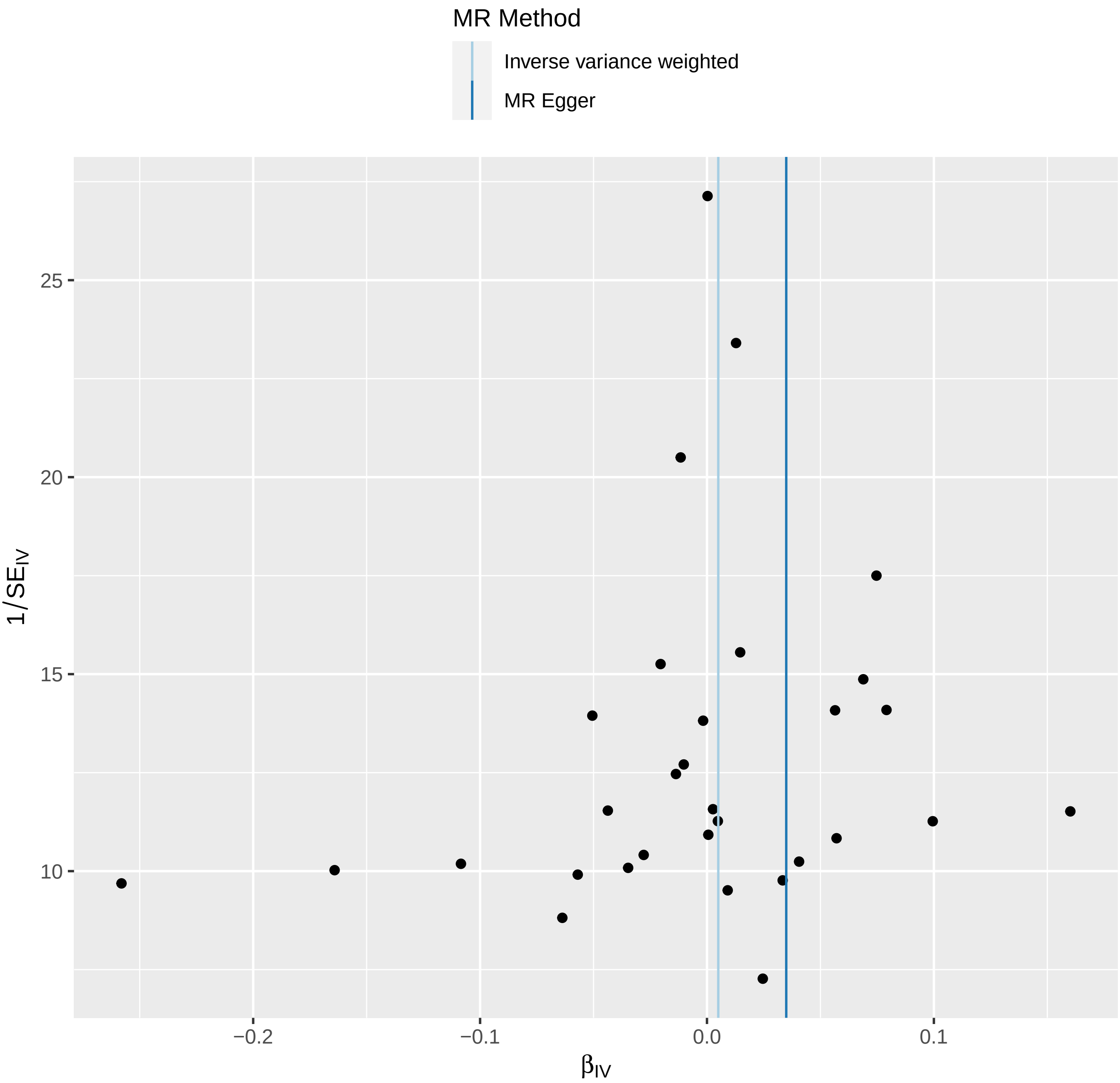

Batch 97 : Crohn's disease on IDP T1 FAST ROIs V cerebellum VI

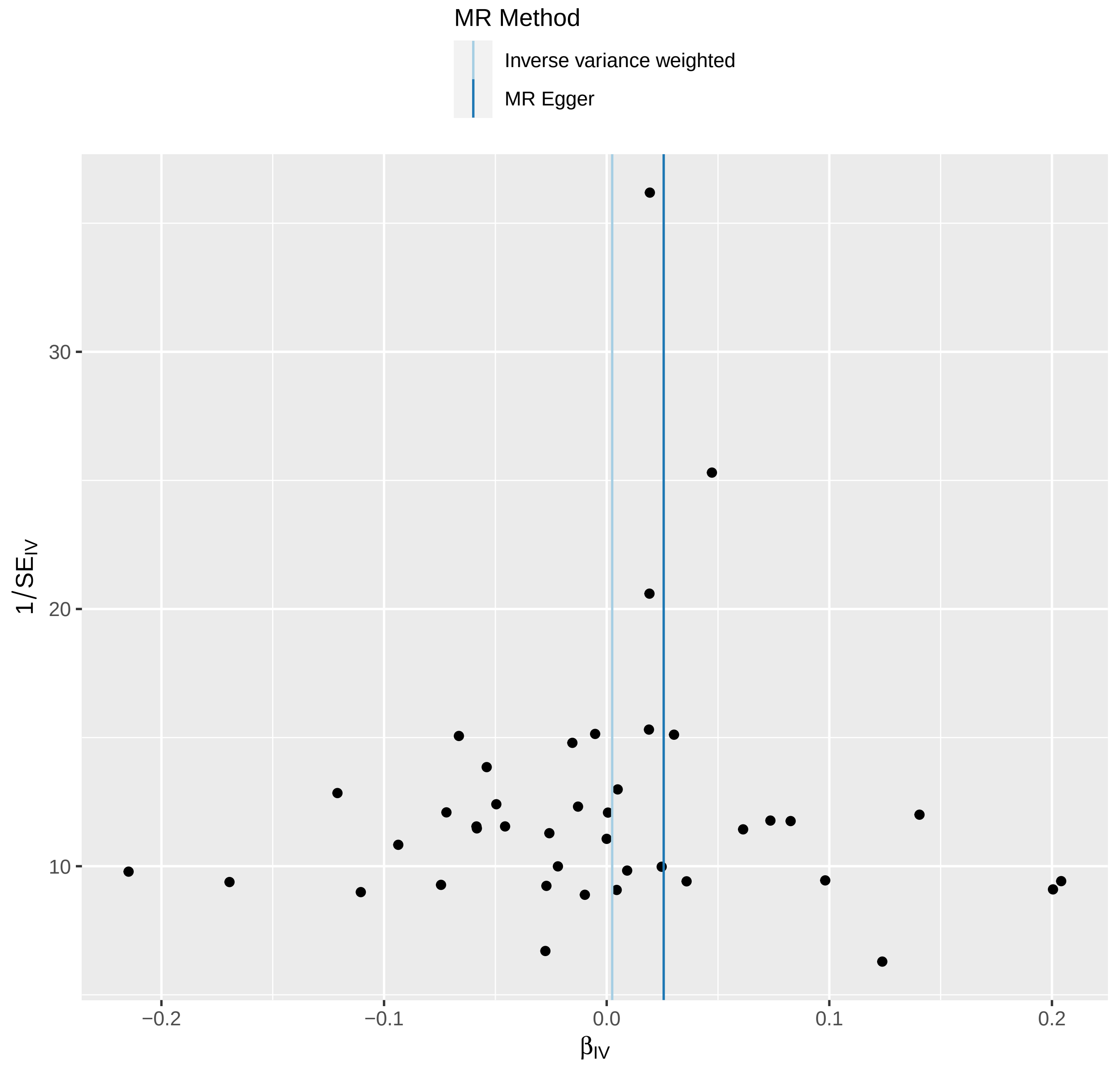

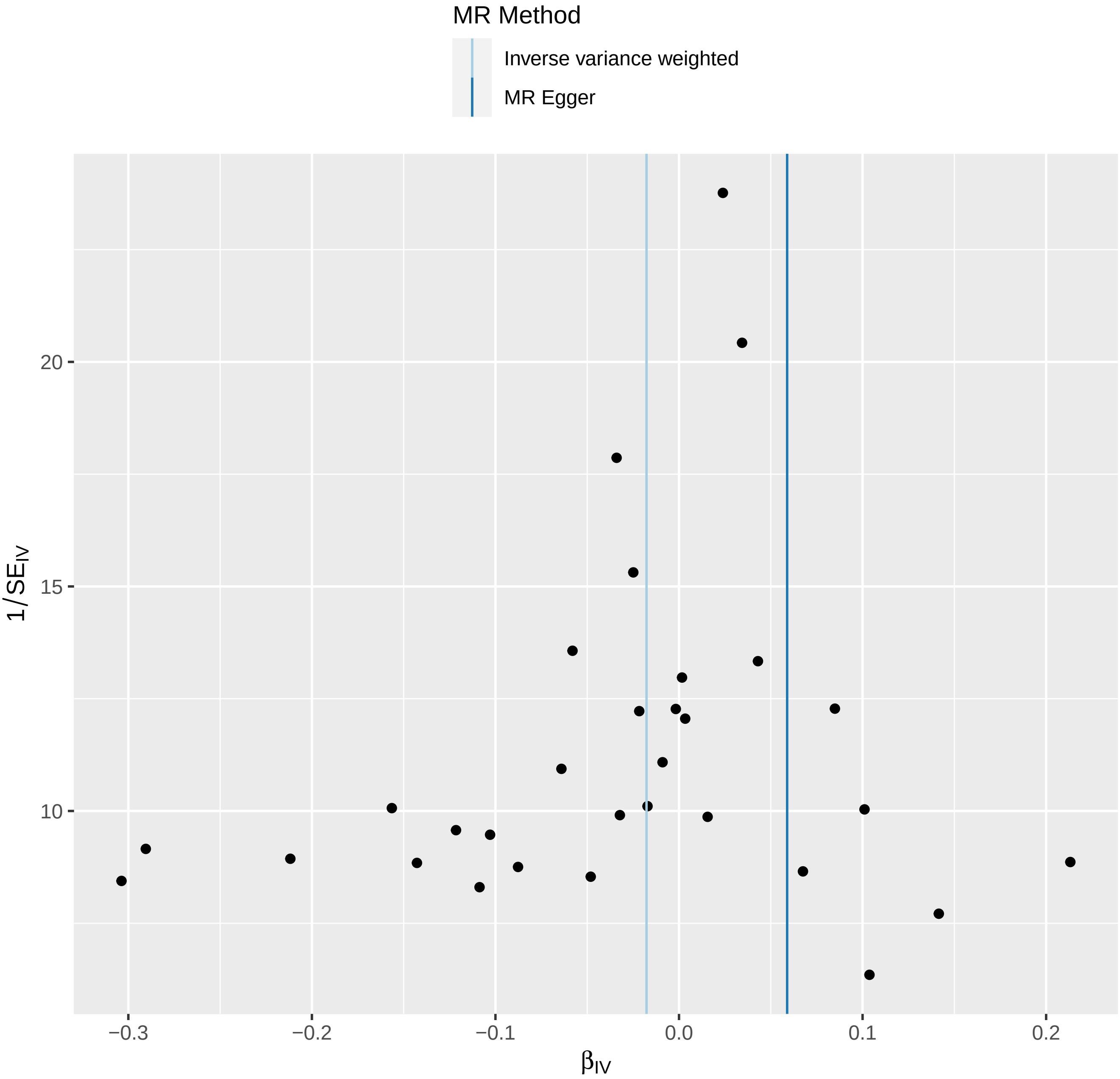
MR Method
Inverse variance weighted
MR Egger
20
15
10
1/SE_IV
−0.3
−0.2
−0.1
0.0
0.1
0.2
β_IV

Batch 99 : Crohn's disease on IDP T1 FAST ROIs R cerebellum VI

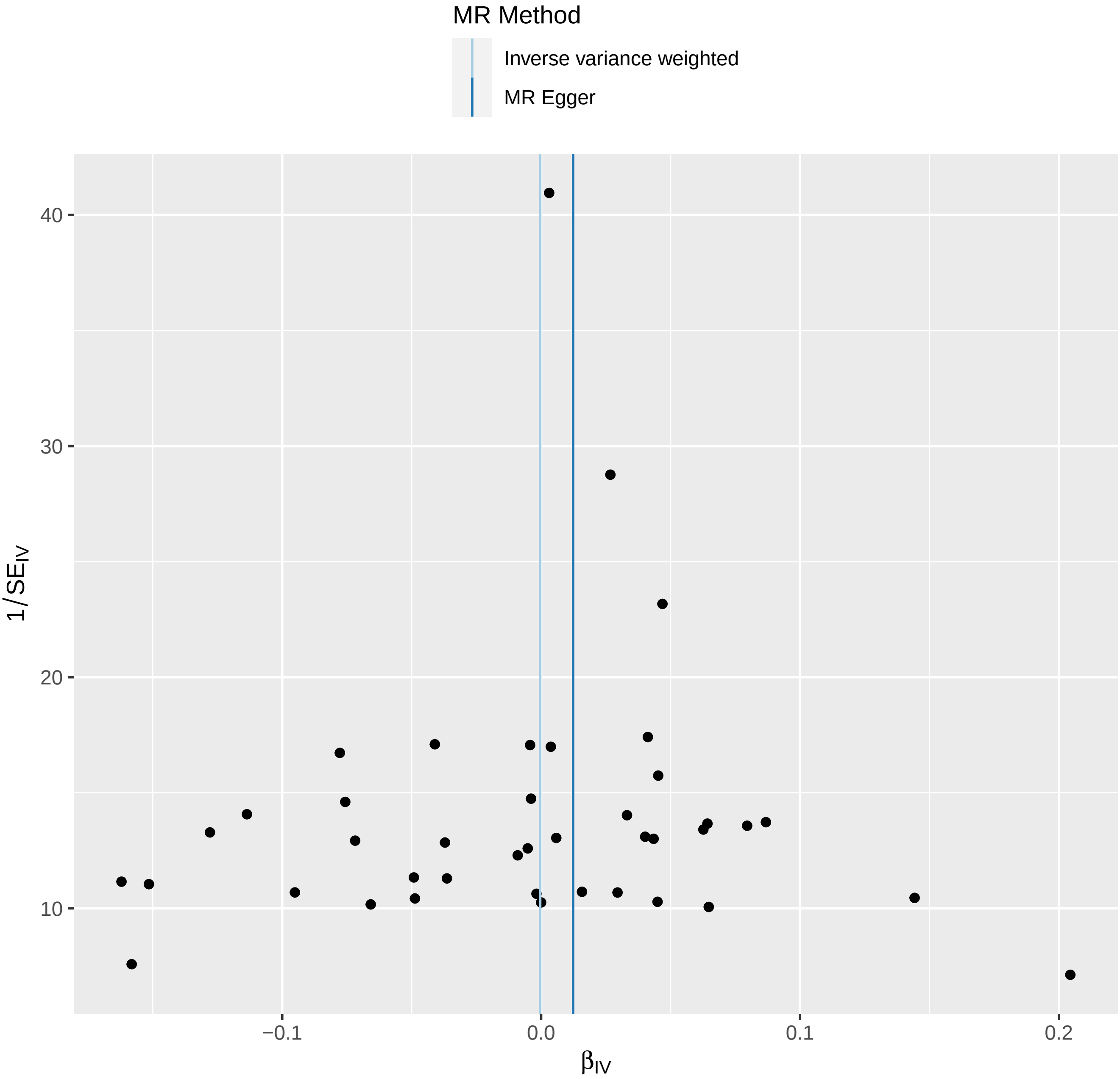

Batch 100 : Ulcerative colitis on IDP T1 FAST ROIs R cerebellum VI

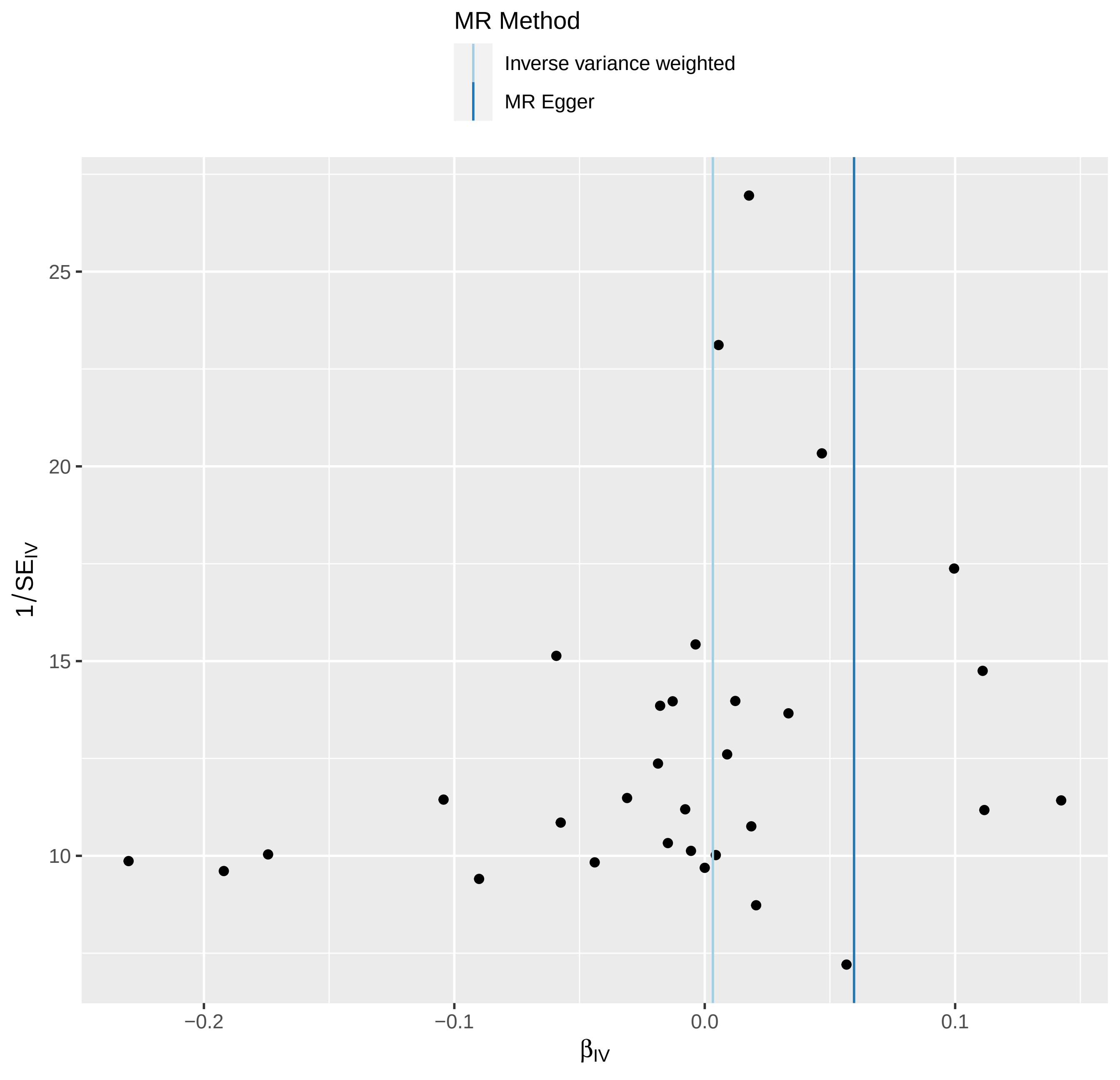

Batch 101 : Crohn's disease on IDP T1 FAST ROIs L cerebellum crus I

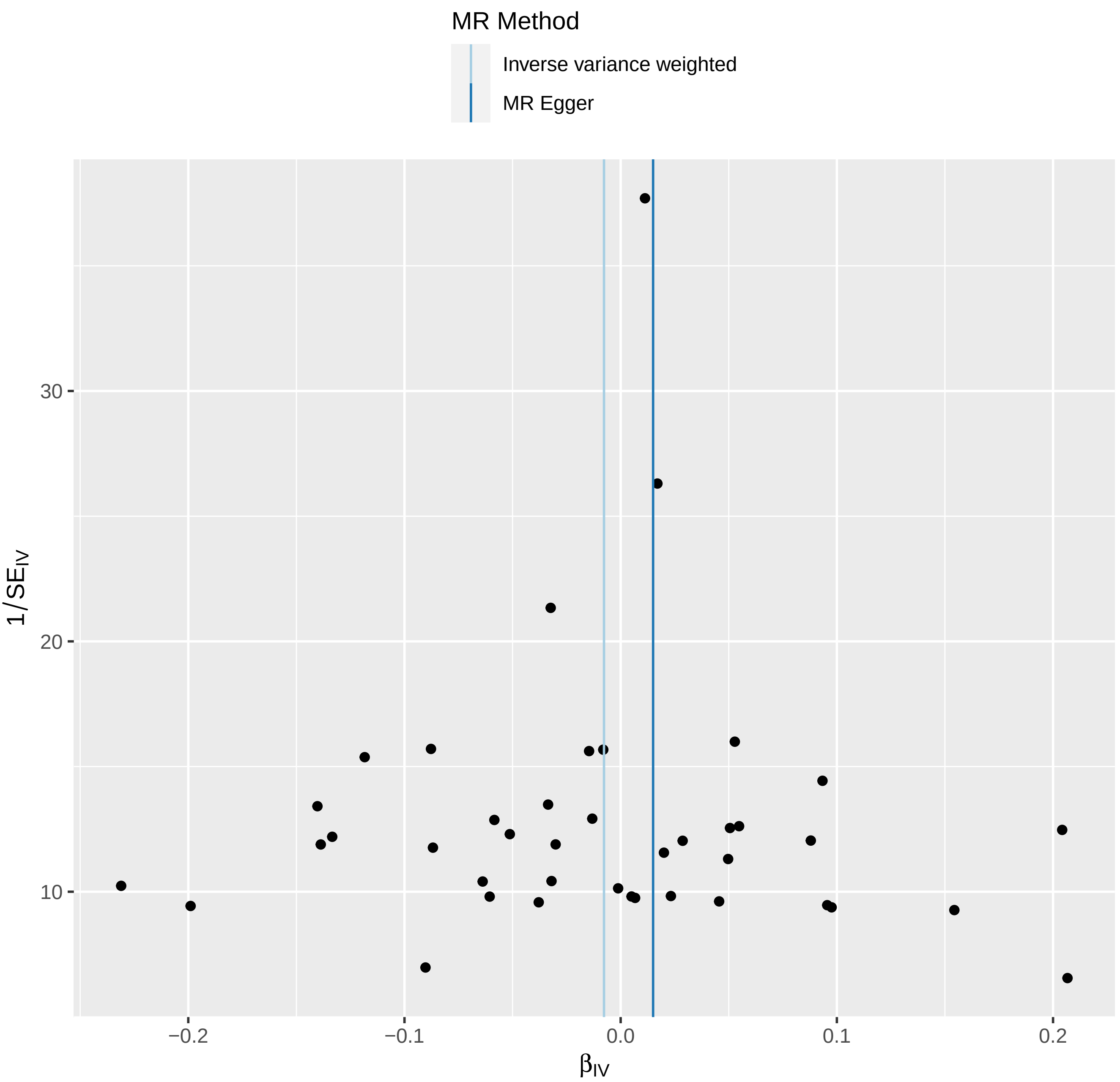

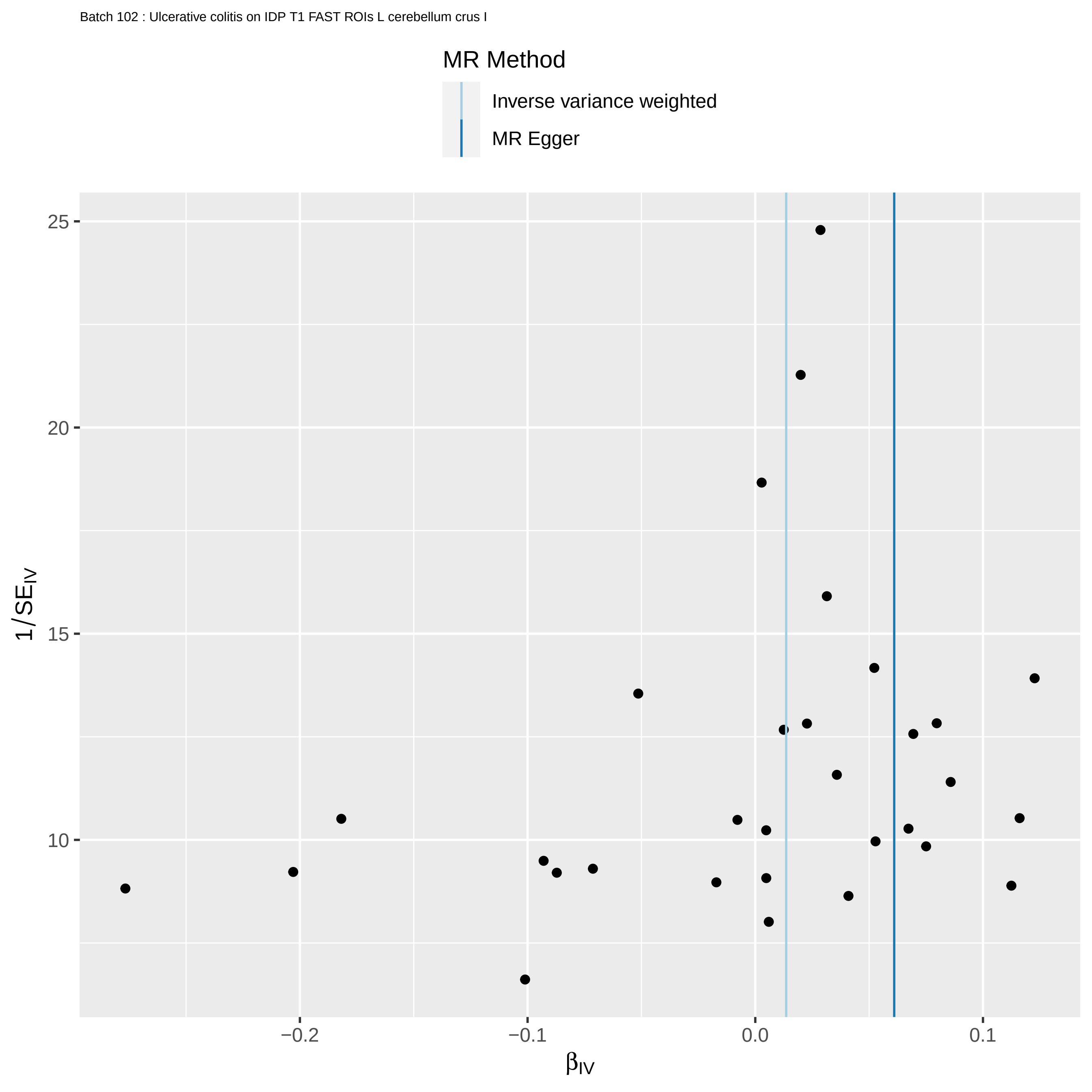
Batch 102 : Ulcerative colitis on IDP T1 FAST ROIs L cerebellum crus I
MR Method
Inverse variance weighted
MR Egger
25
20
15
10
1/SE_IV
−0.2
−0.1
0.0
0.1
β_IV

Batch 103 : Crohn's disease on IDP T1 FAST ROIs V cerebellum crus I

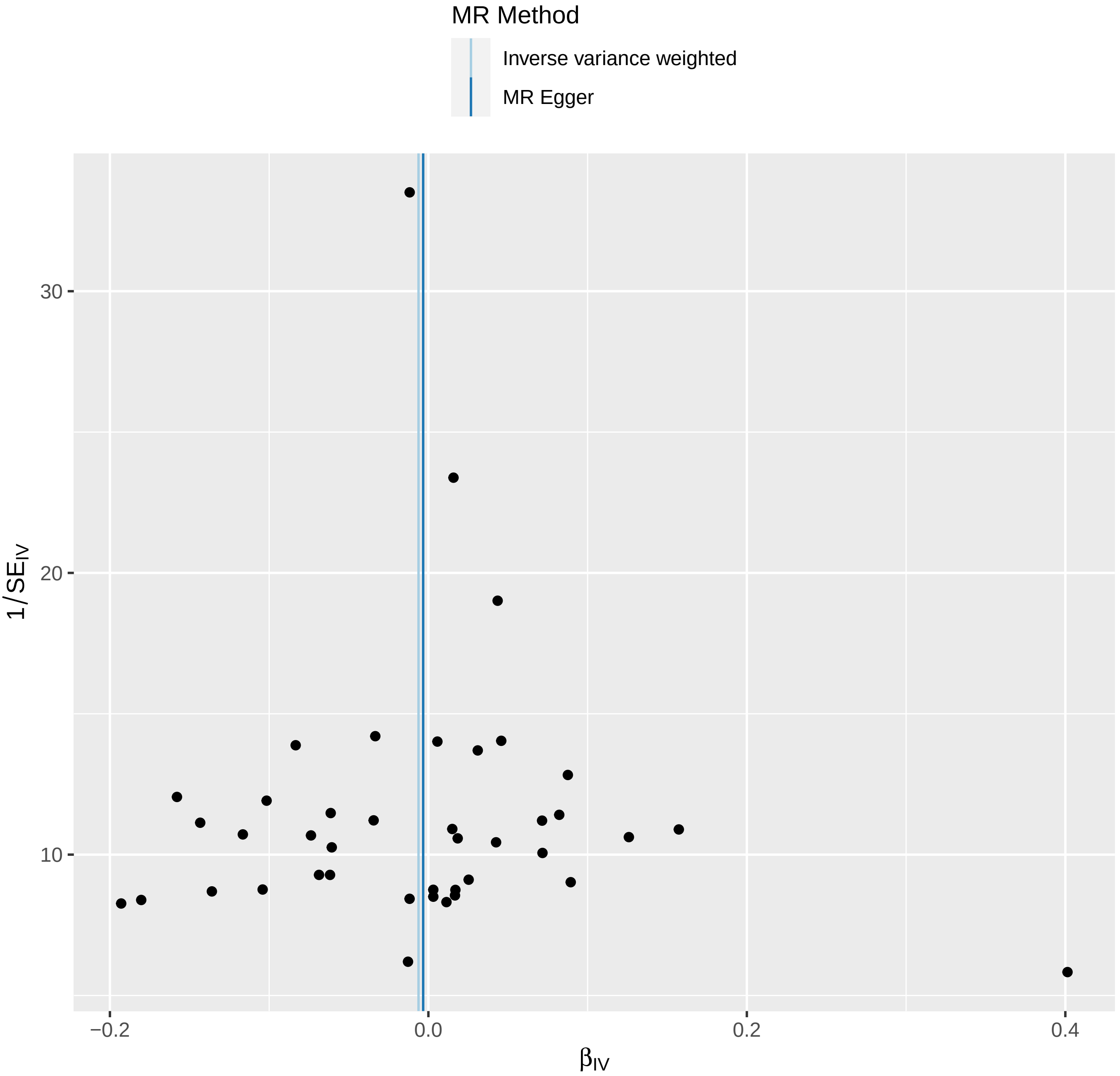

Batch 104 : Ulcerative colitis on IDP T1 FAST ROIs V cerebellum crus I

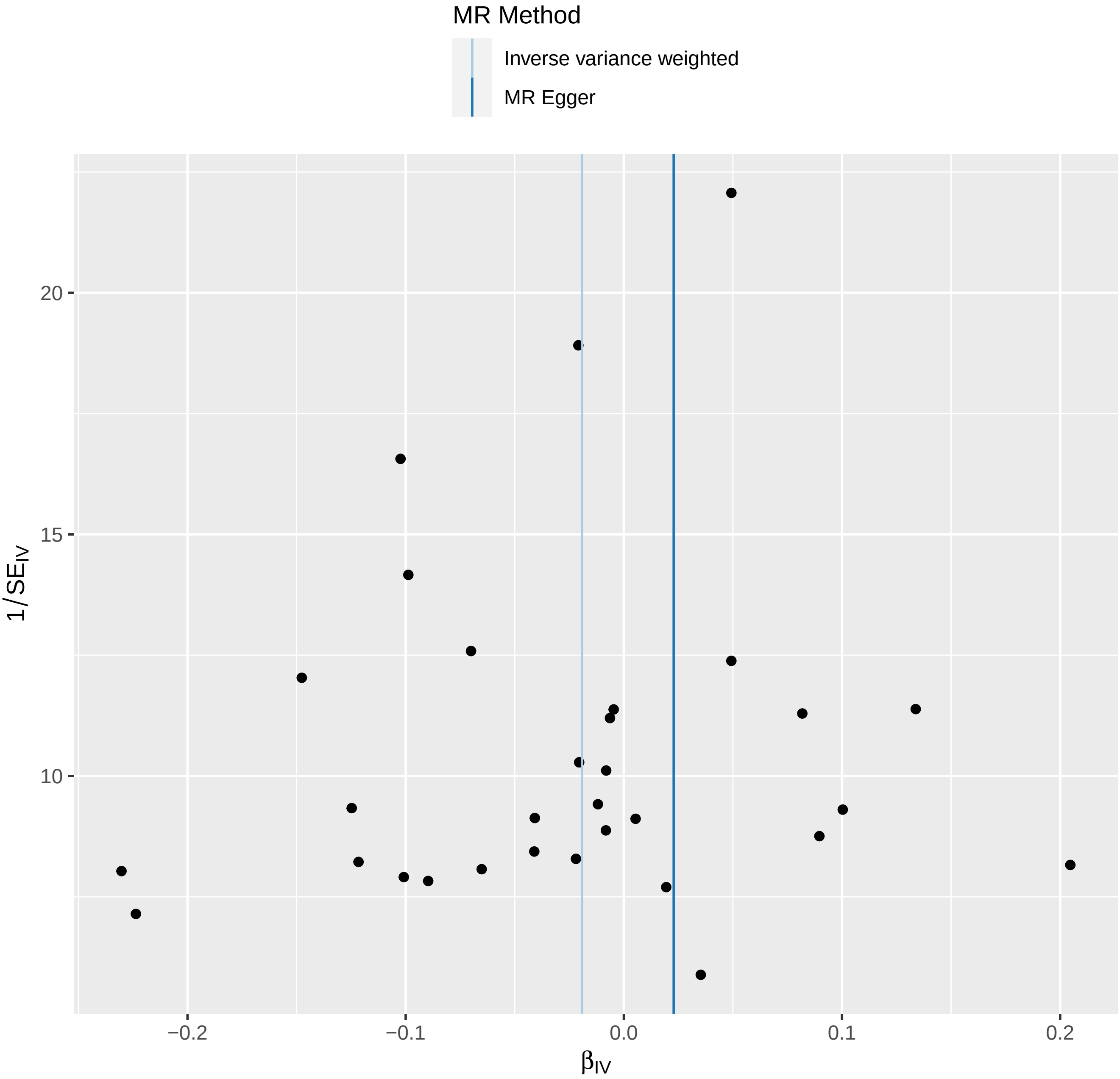

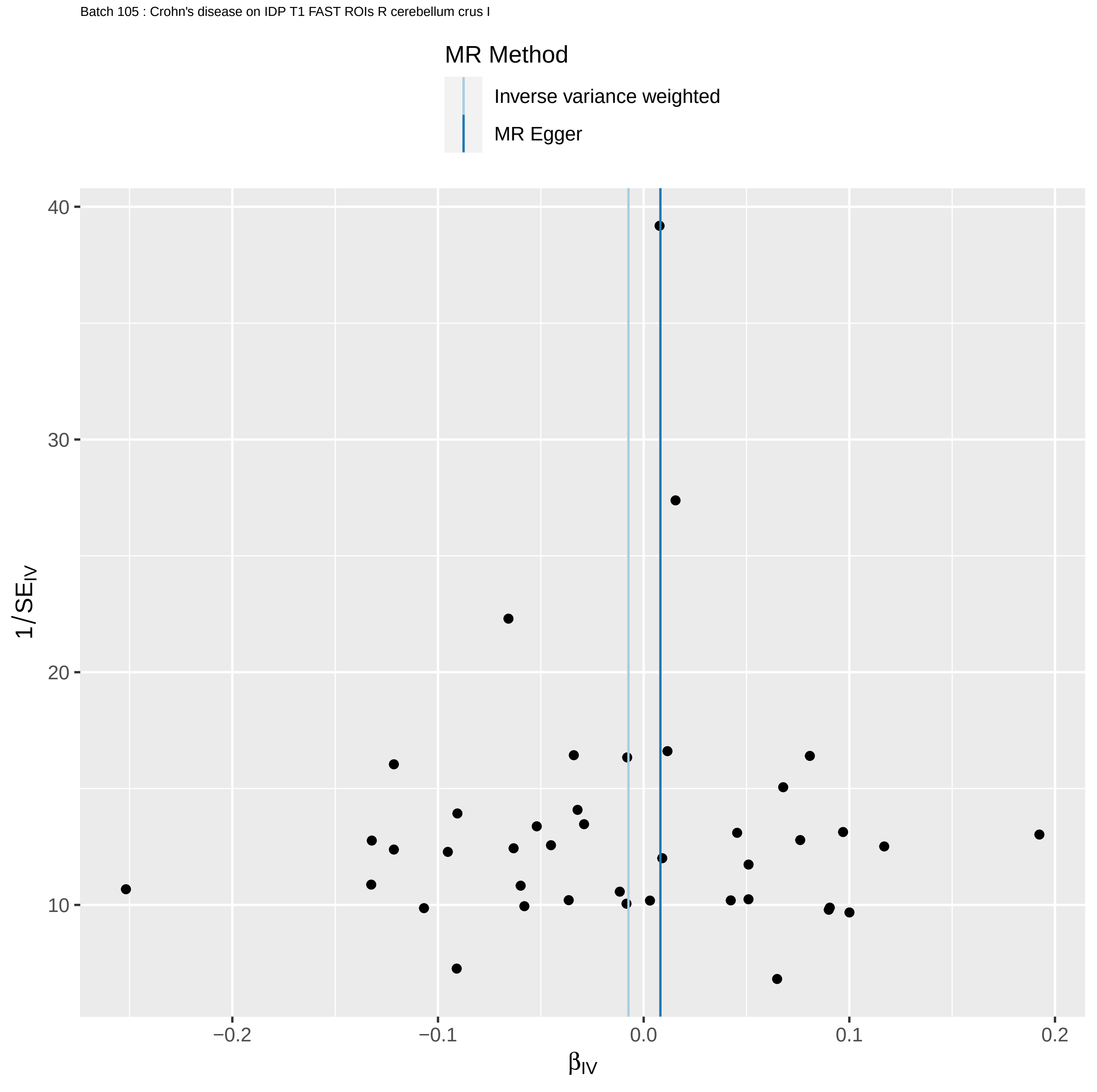
Batch 105 : Crohn's disease on IDP T1 FAST ROIs R cerebellum crus I
MR Method
Inverse variance weighted
MR Egger
40
30
20
10
1/SE$_{IV}$
−0.2
−0.1
0.0
0.1
0.2
β_{IV}

Batch 106 : Ulcerative colitis on IDP T1 FAST ROIs R cerebellum crus I

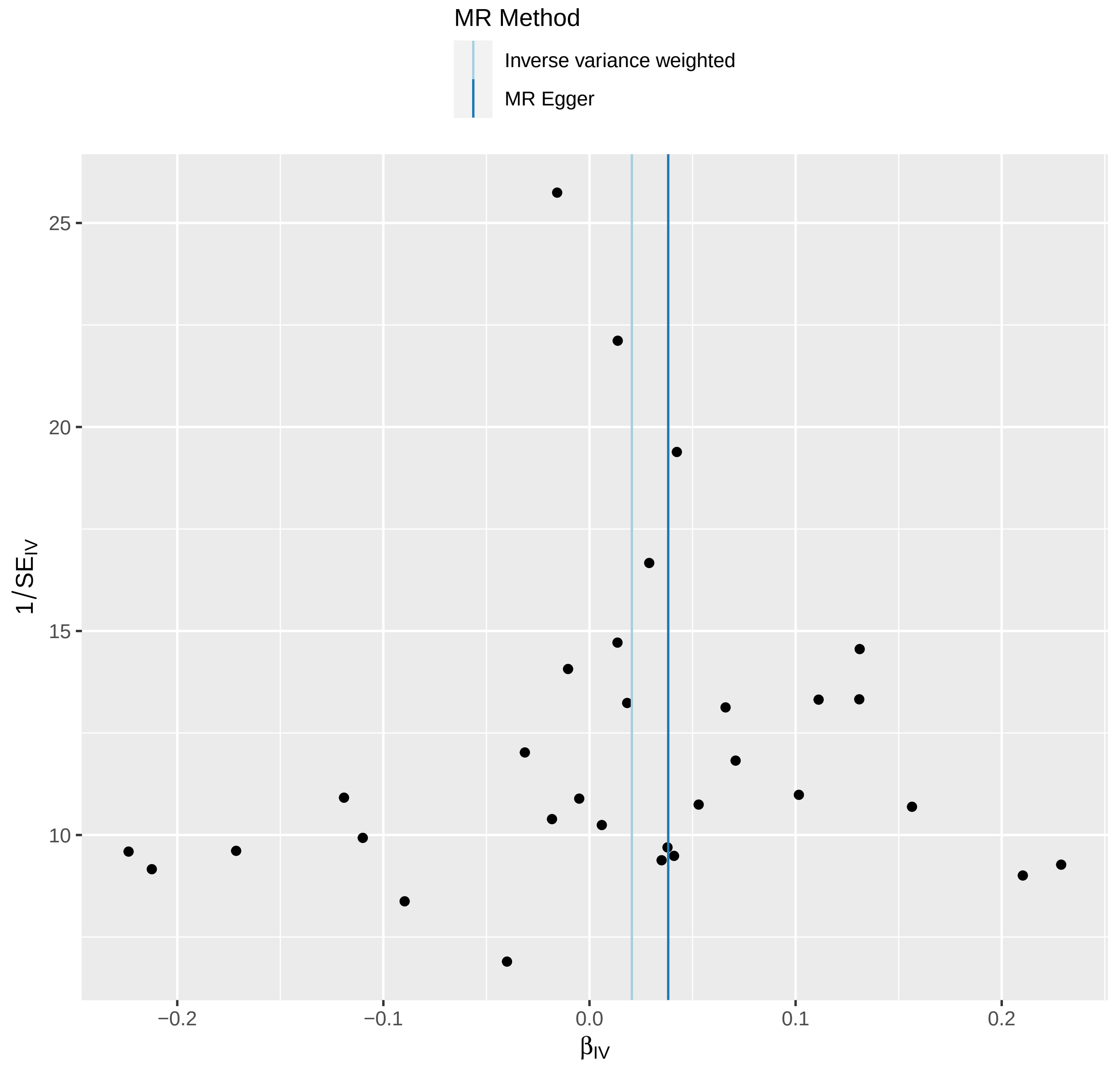

Batch 107 : Crohn's disease on IDP T1 FAST ROIs L cerebellum crus II

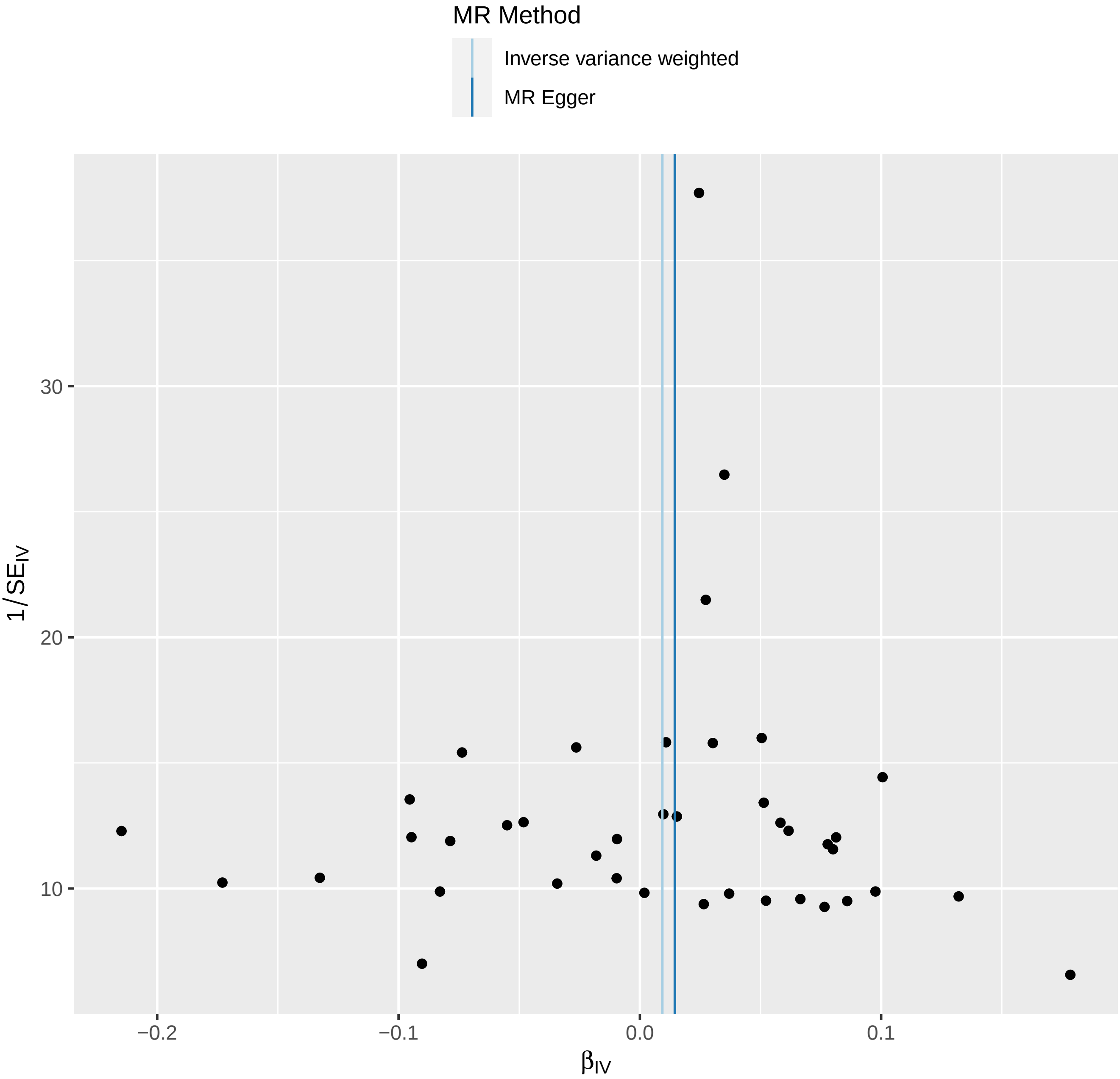

Batch 108 : Ulcerative colitis on IDP T1 FAST ROIs L cerebellum crus II

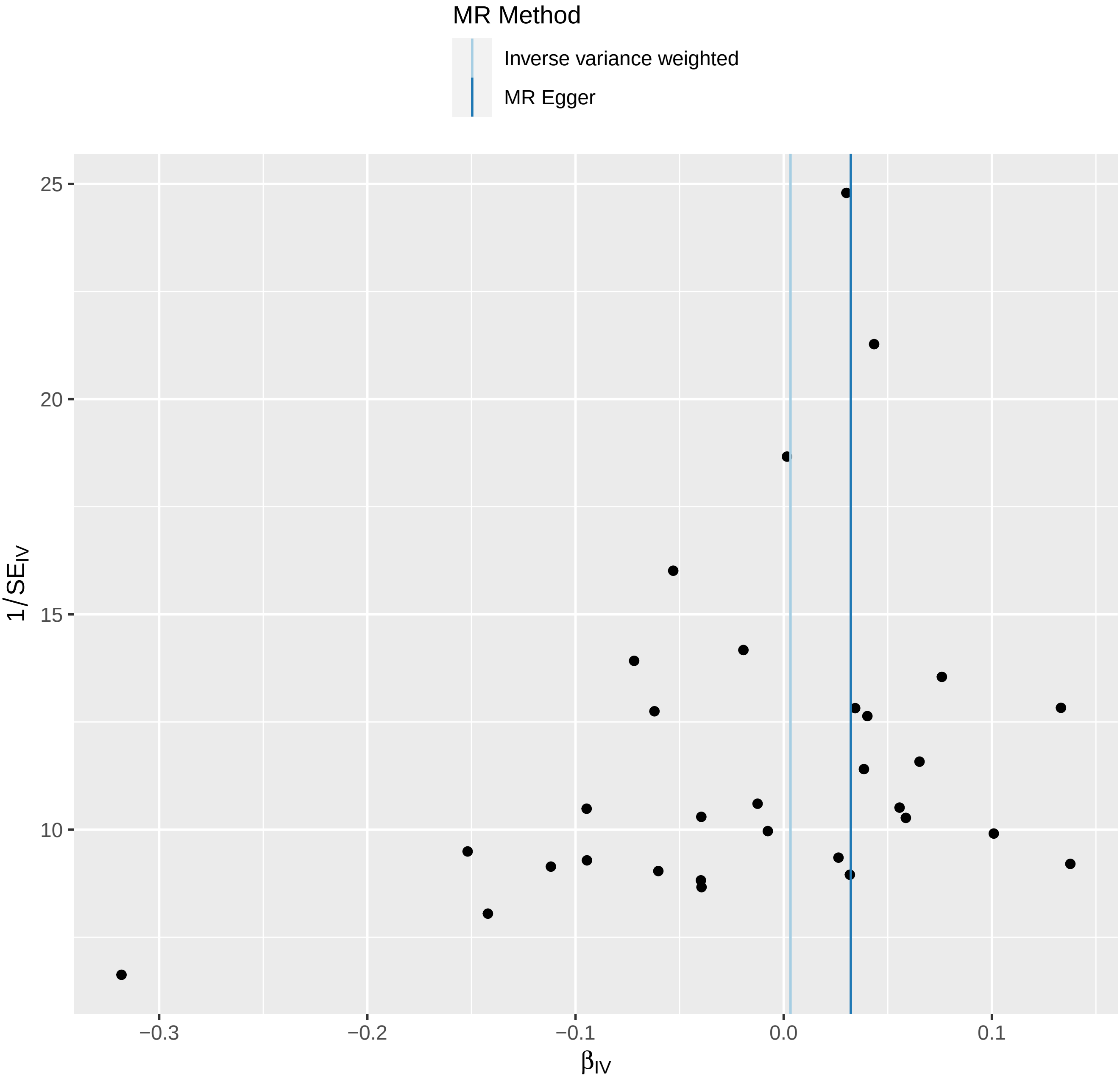

Batch 109 : Crohn's disease on IDP T1 FAST ROIs V cerebellum crus II

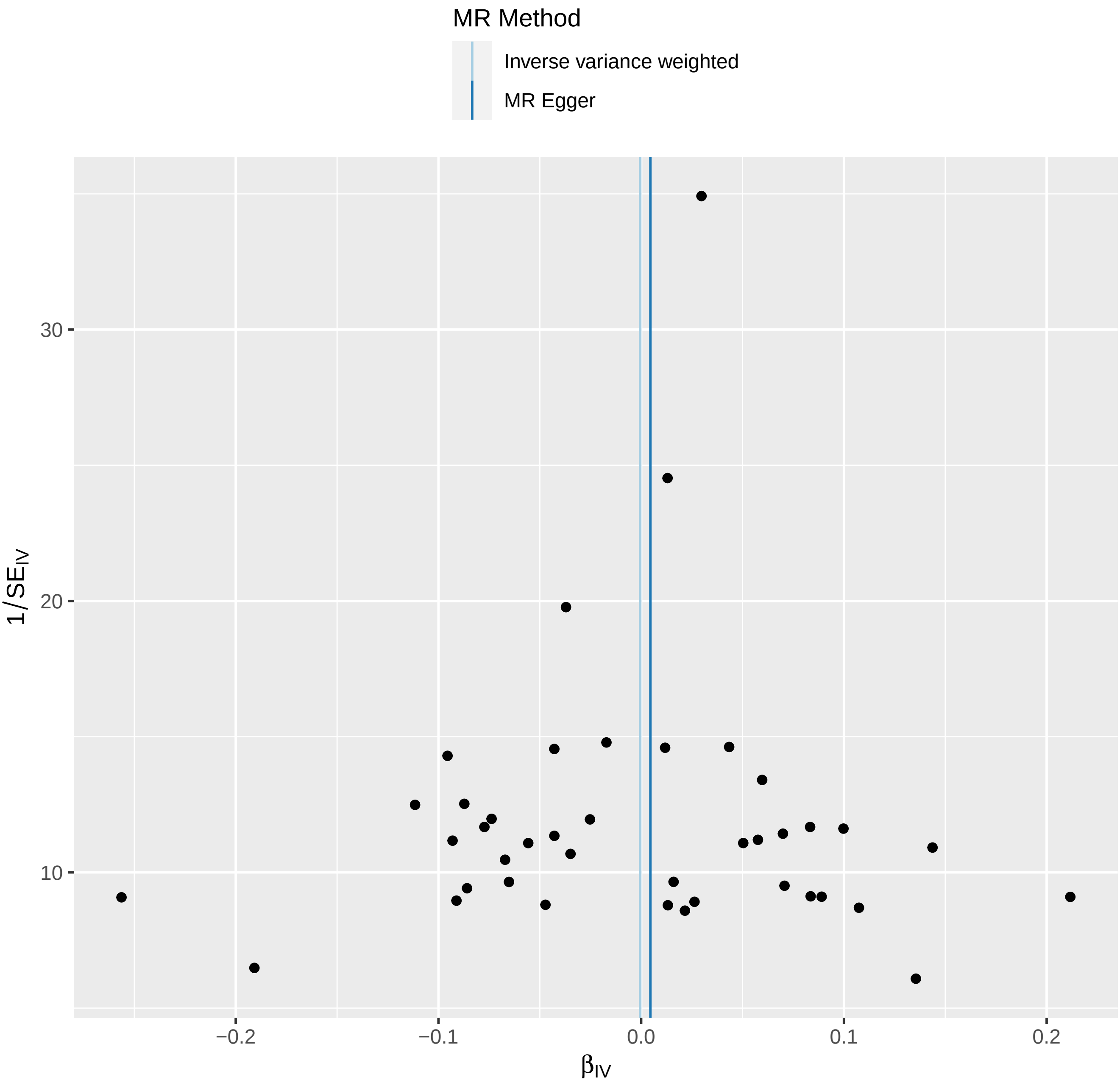

Batch 110 : Ulcerative colitis on IDP T1 FAST ROIs V cerebellum crus II

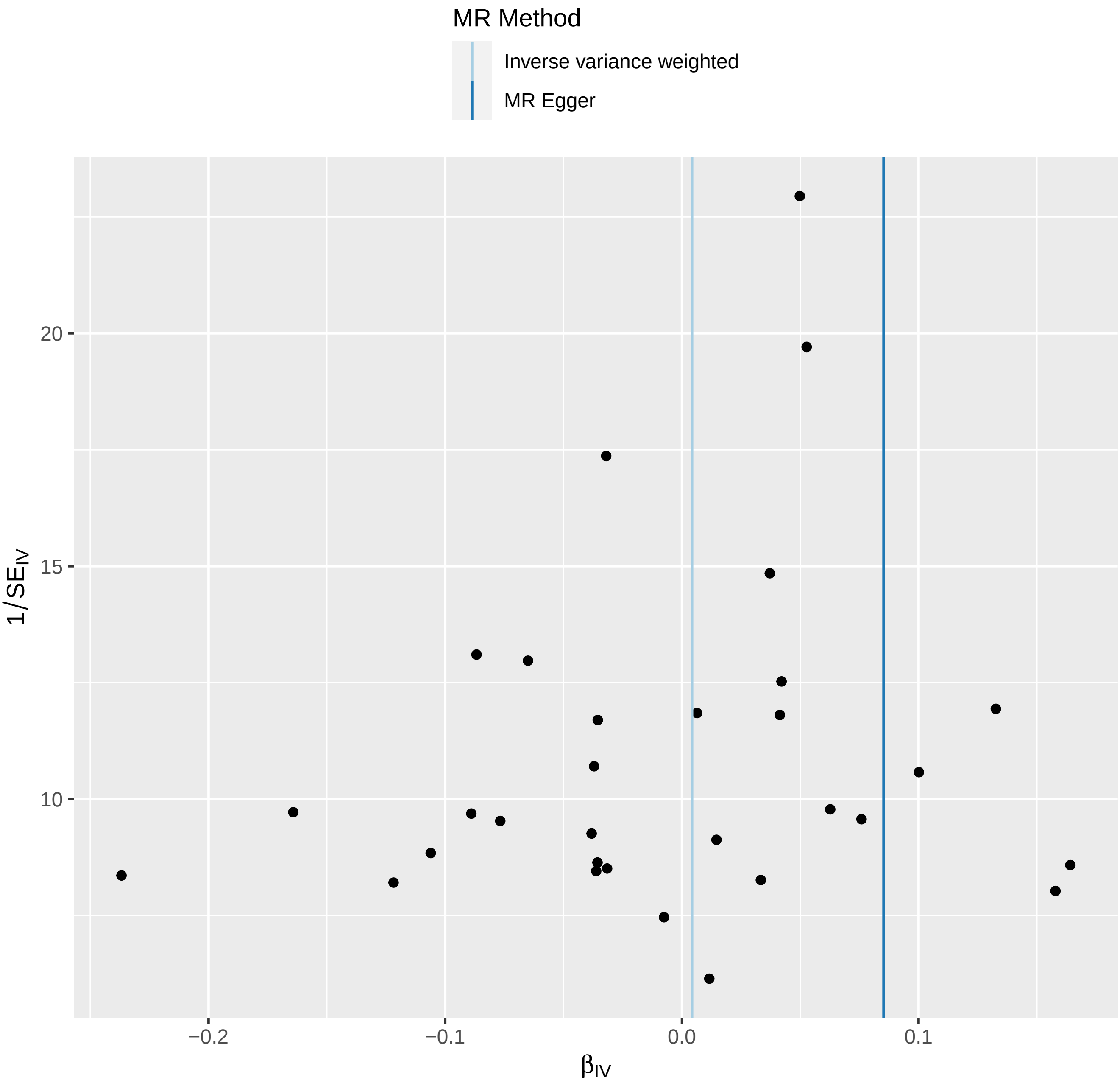

Batch 111 : Crohn's disease on IDP T1 FAST ROIs R cerebellum crus II

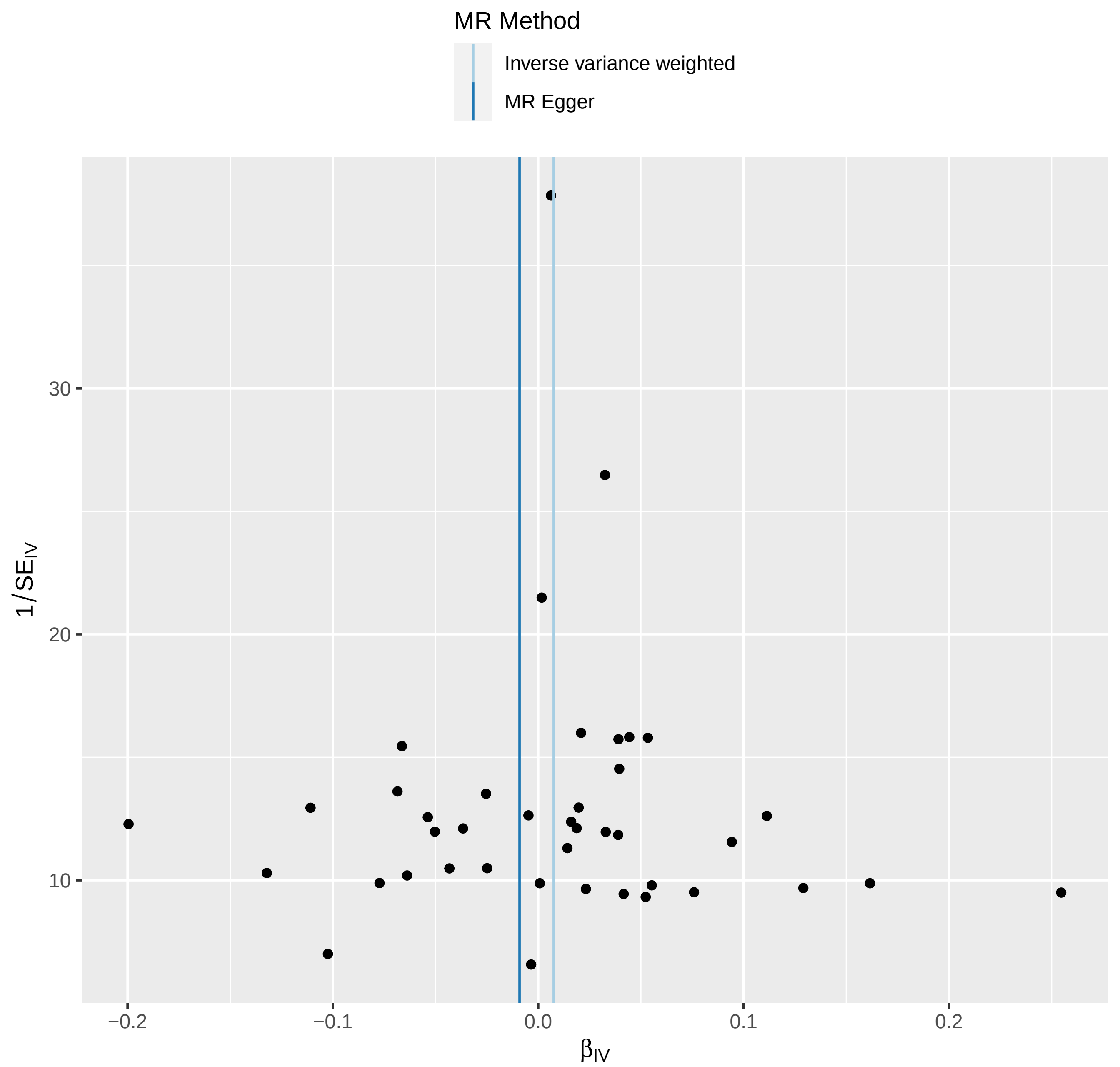

Batch 112 : Ulcerative colitis on IDP T1 FAST ROIs R cerebellum crus II

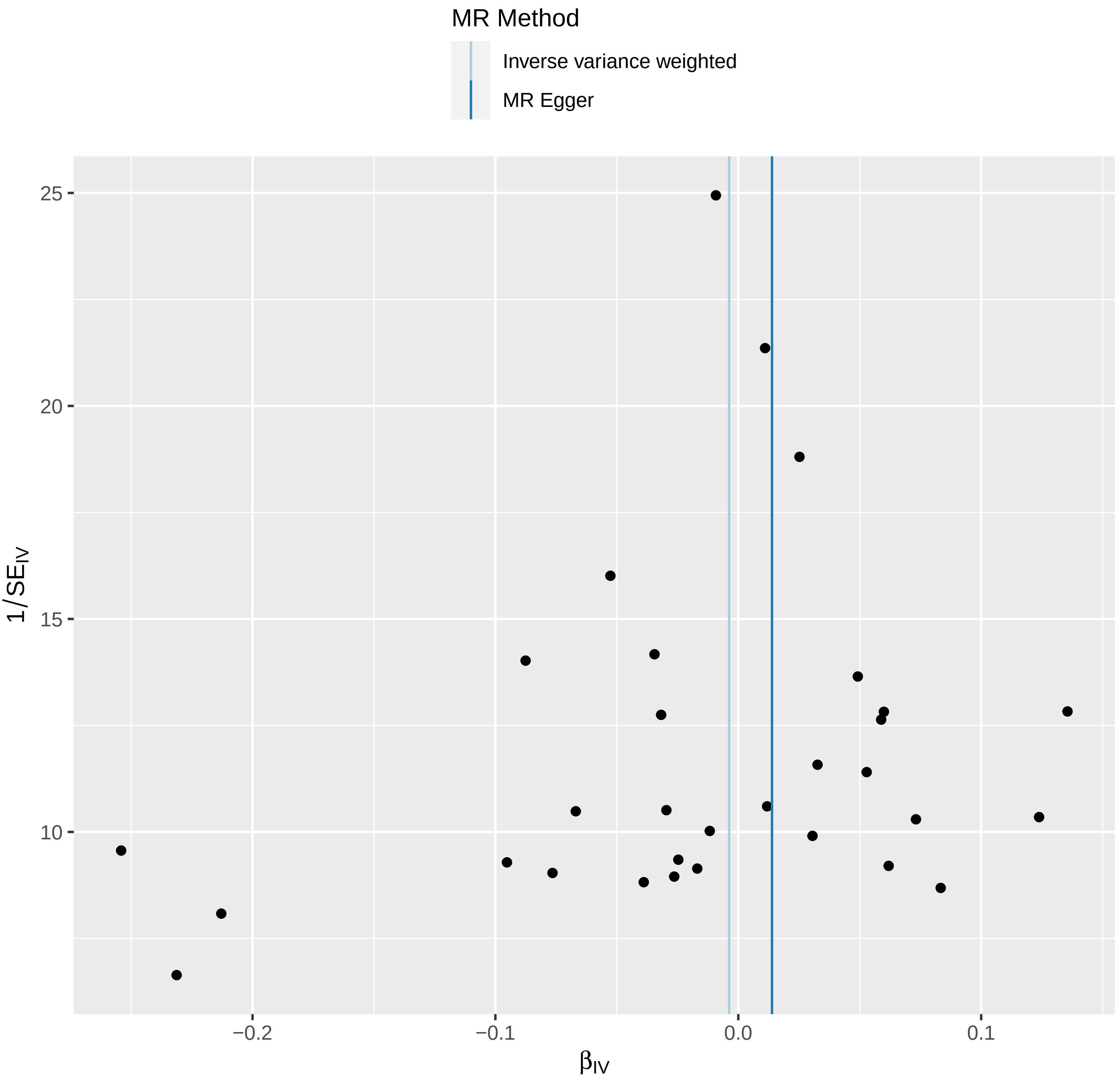

Batch 113 : Crohn's disease on IDP T1 FIRST left putamen volume

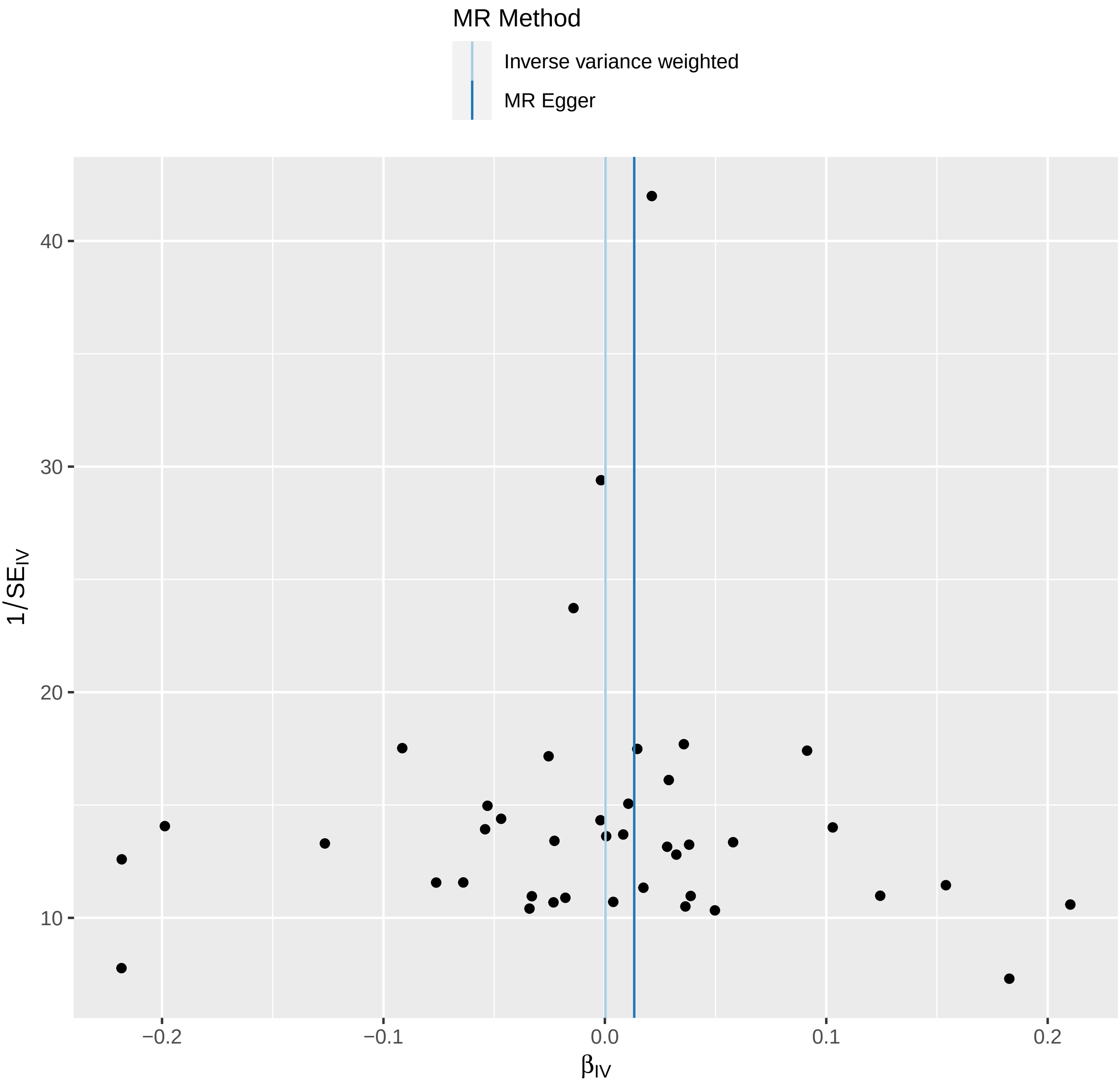

Batch 114 : Ulcerative colitis on IDP T1 FIRST left putamen volume

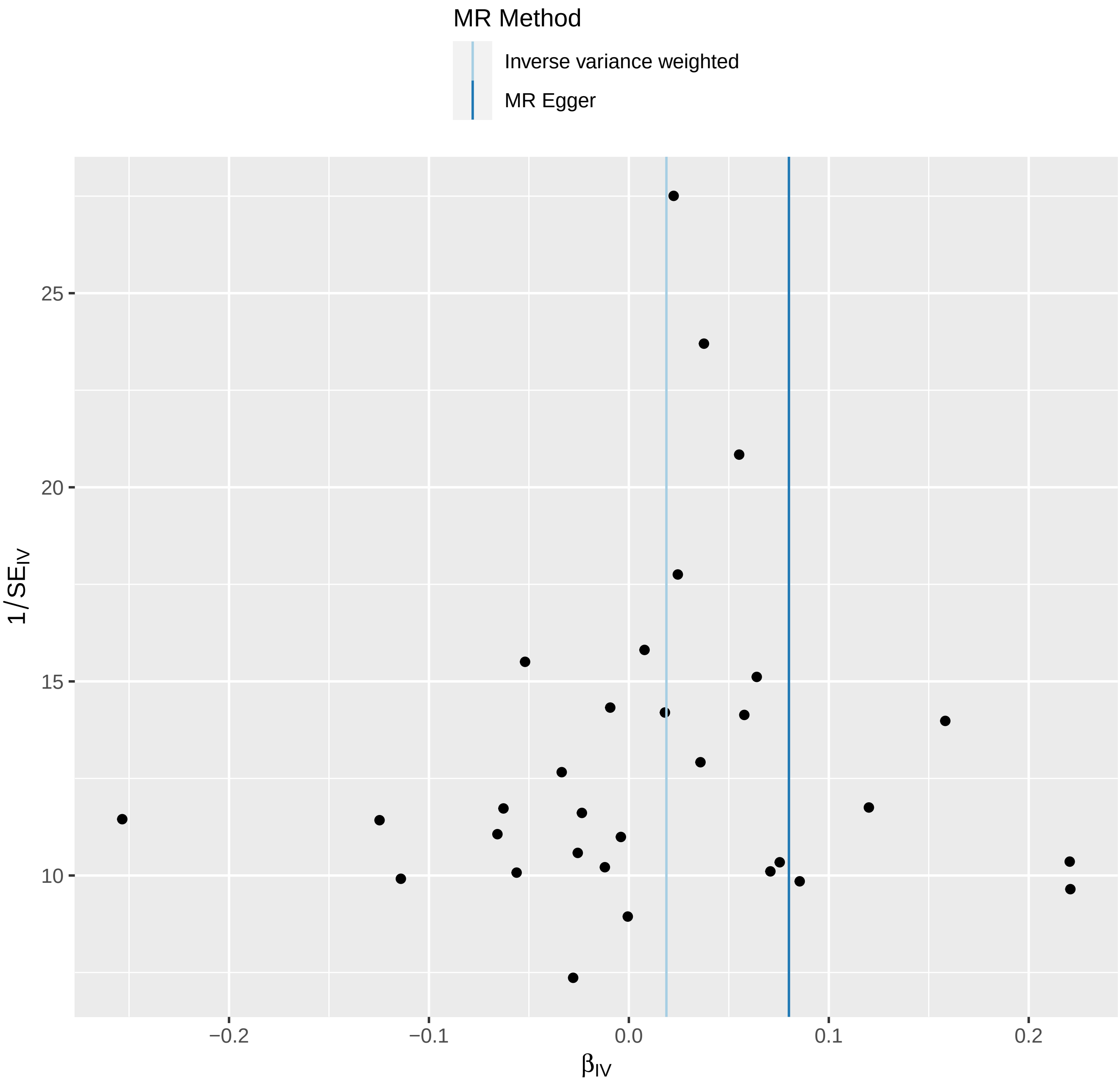

Batch 115 : Crohn's disease on IDP T1 FAST ROIs L cerebellum VIIb

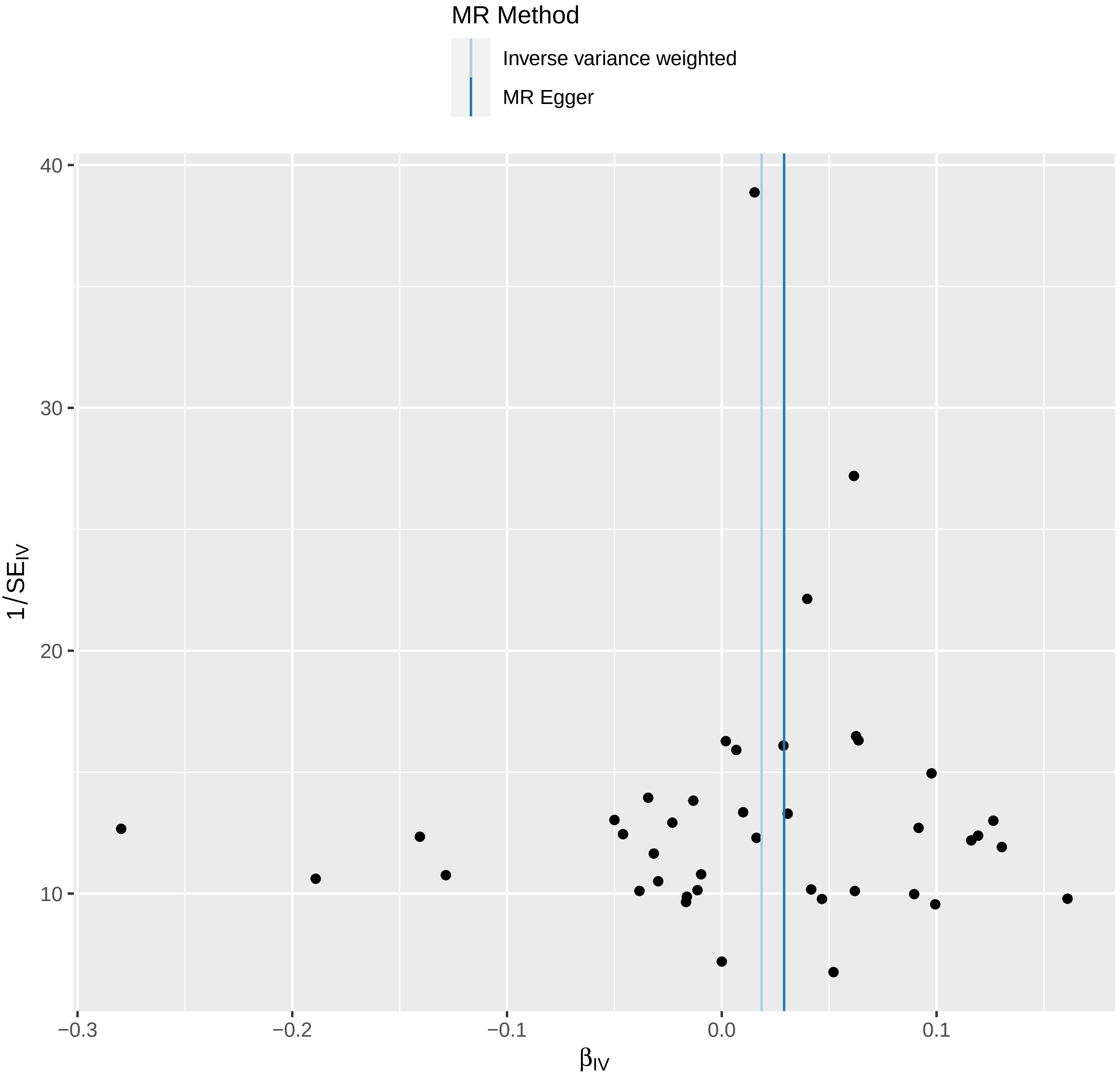

Batch 116 : Ulcerative colitis on IDP T1 FAST ROIs L cerebellum VIIb

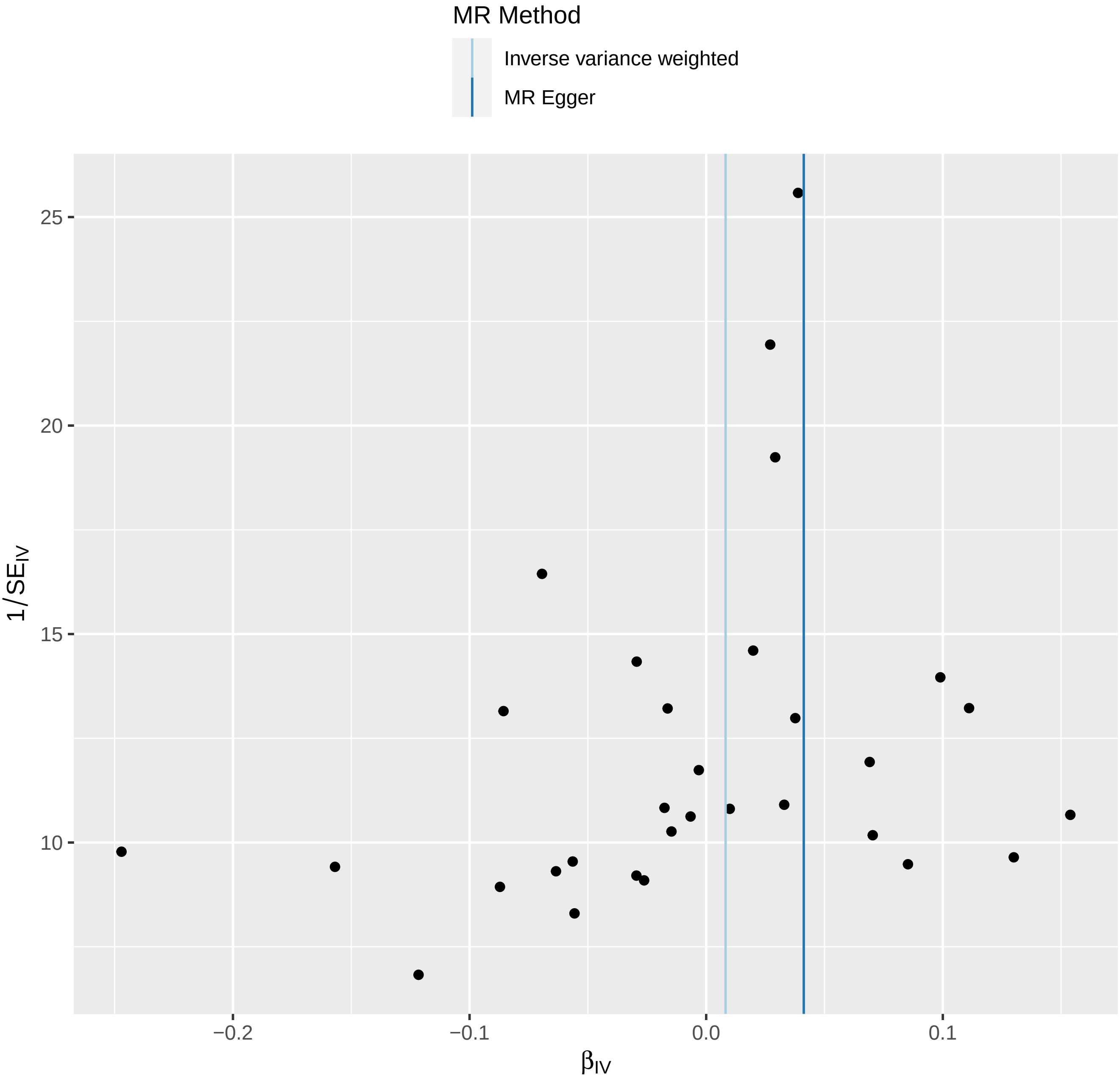

Batch 117 : Crohn's disease on IDP T1 FAST ROIs V cerebellum VIIb

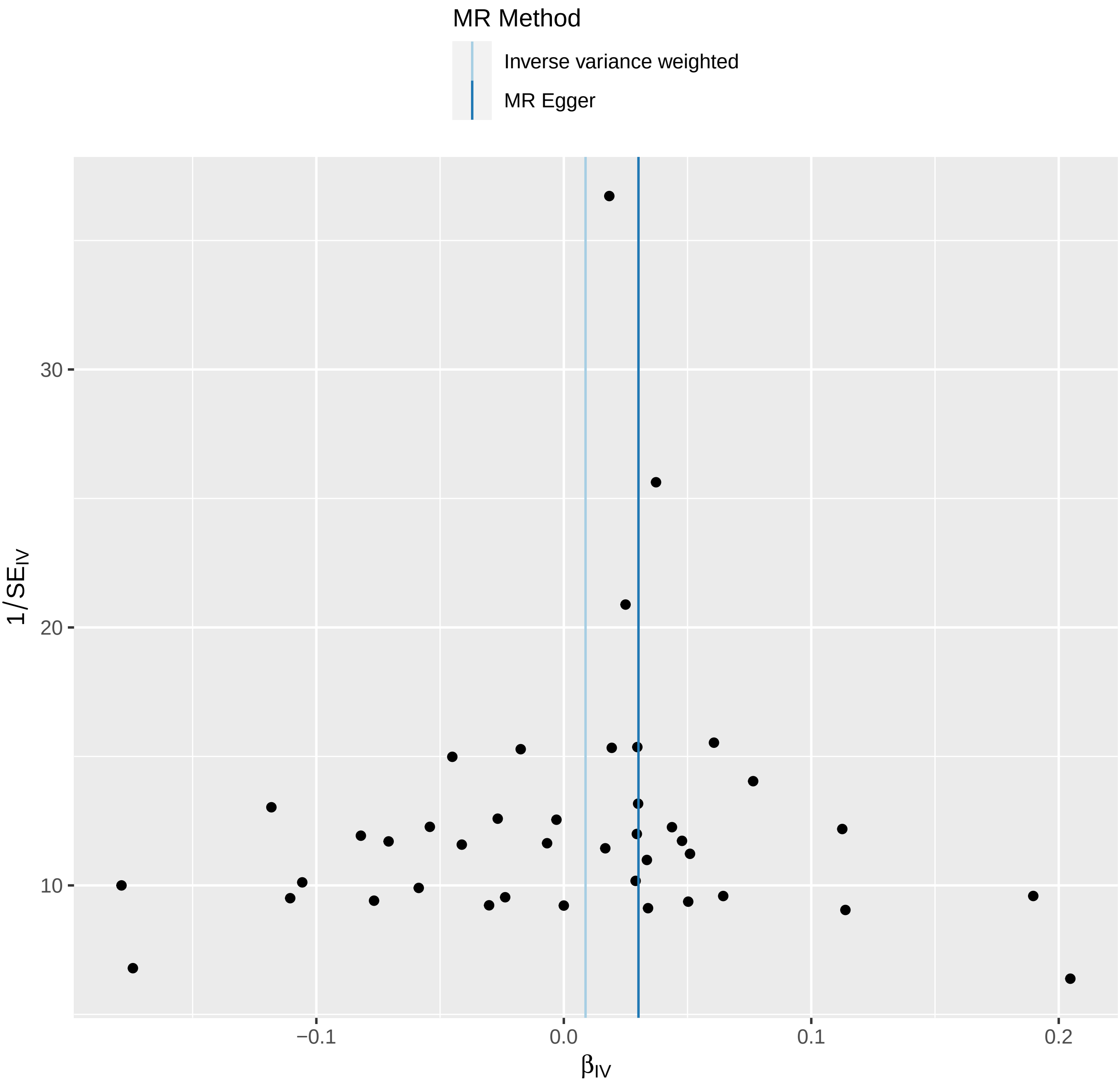

Batch 118 : Ulcerative colitis on IDP T1 FAST ROIs V cerebellum VIIb

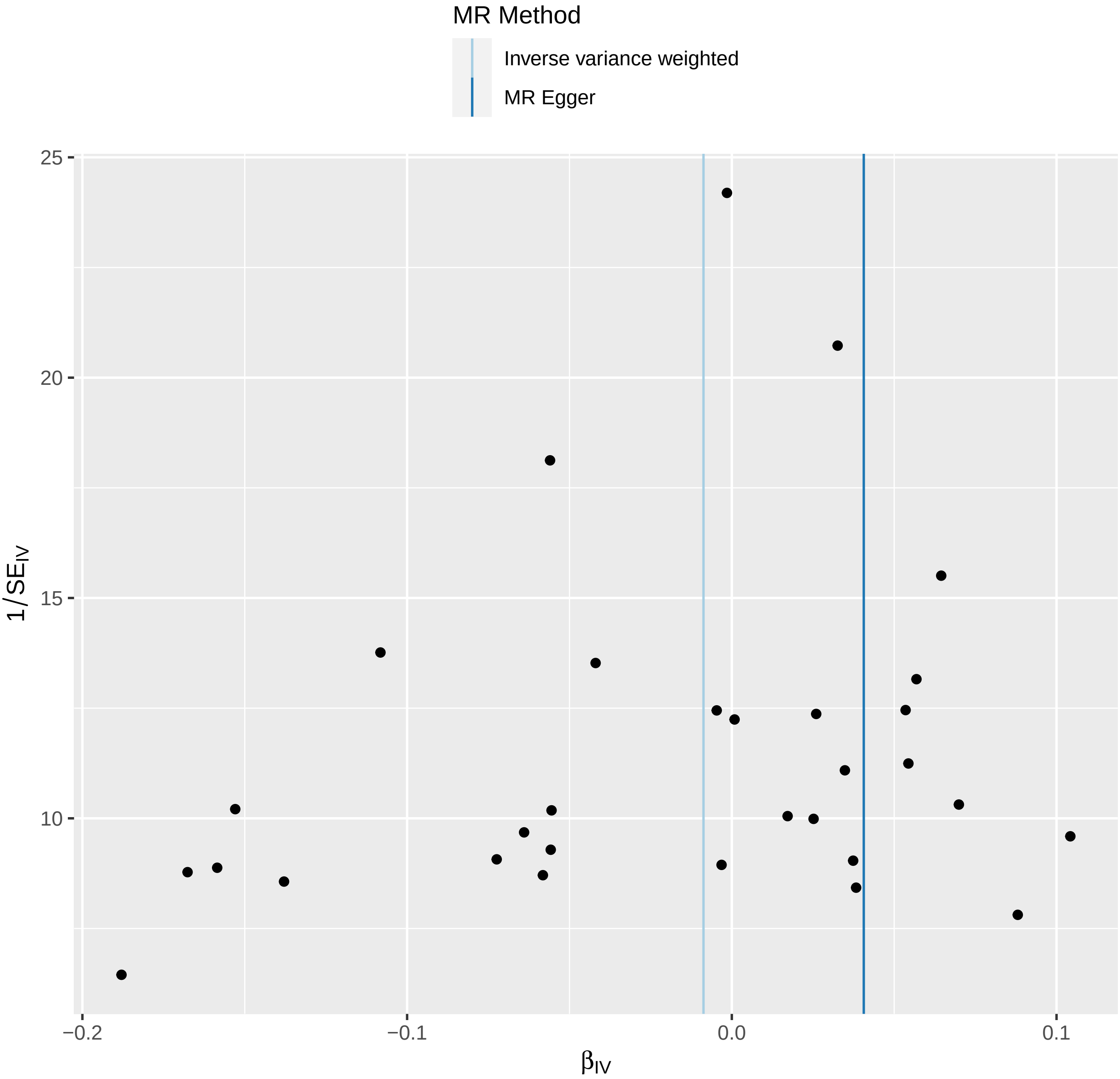

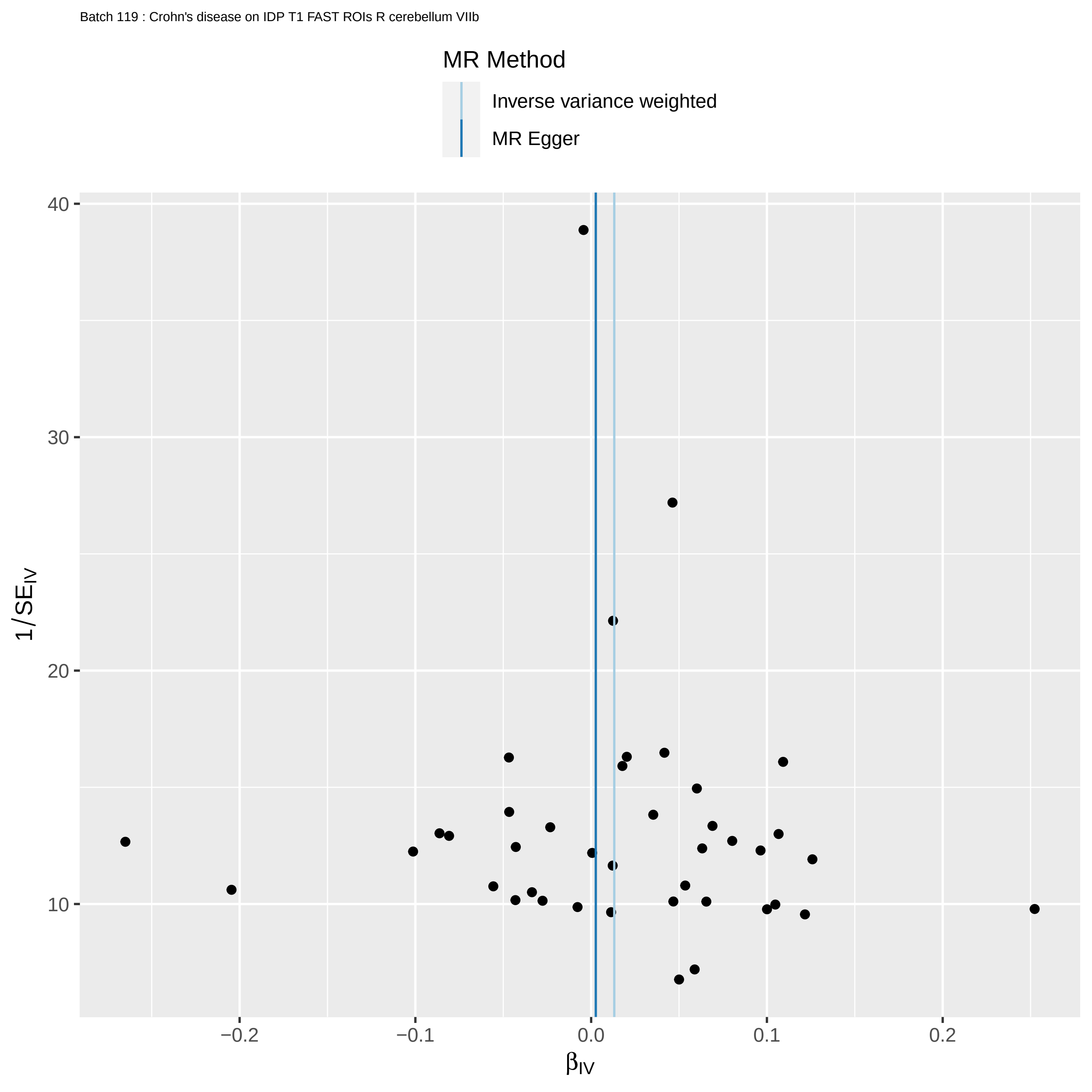

Batch 119 : Crohn's disease on IDP T1 FAST ROIs R cerebellum VIIb
MR Method
Inverse variance weighted
MR Egger
40
30
20
10
1/SE_IV
−0.2
−0.1
0.0
0.1
0.2
β_IV

Batch 120 : Ulcerative colitis on IDP T1 FAST ROIs R cerebellum VIIb

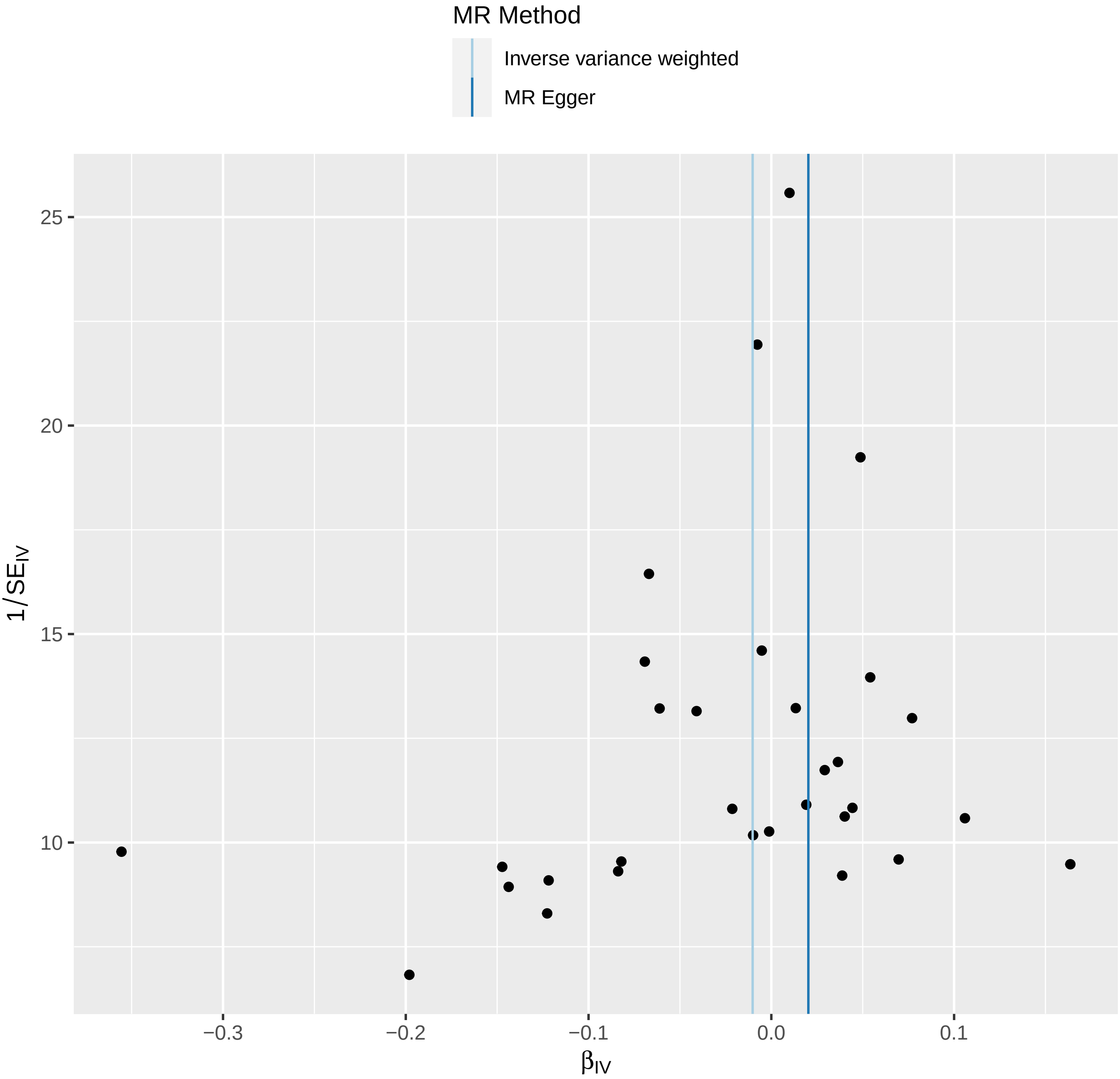

Batch 121 : Crohn's disease on IDP T1 FAST ROIs L cerebellum VIIIa

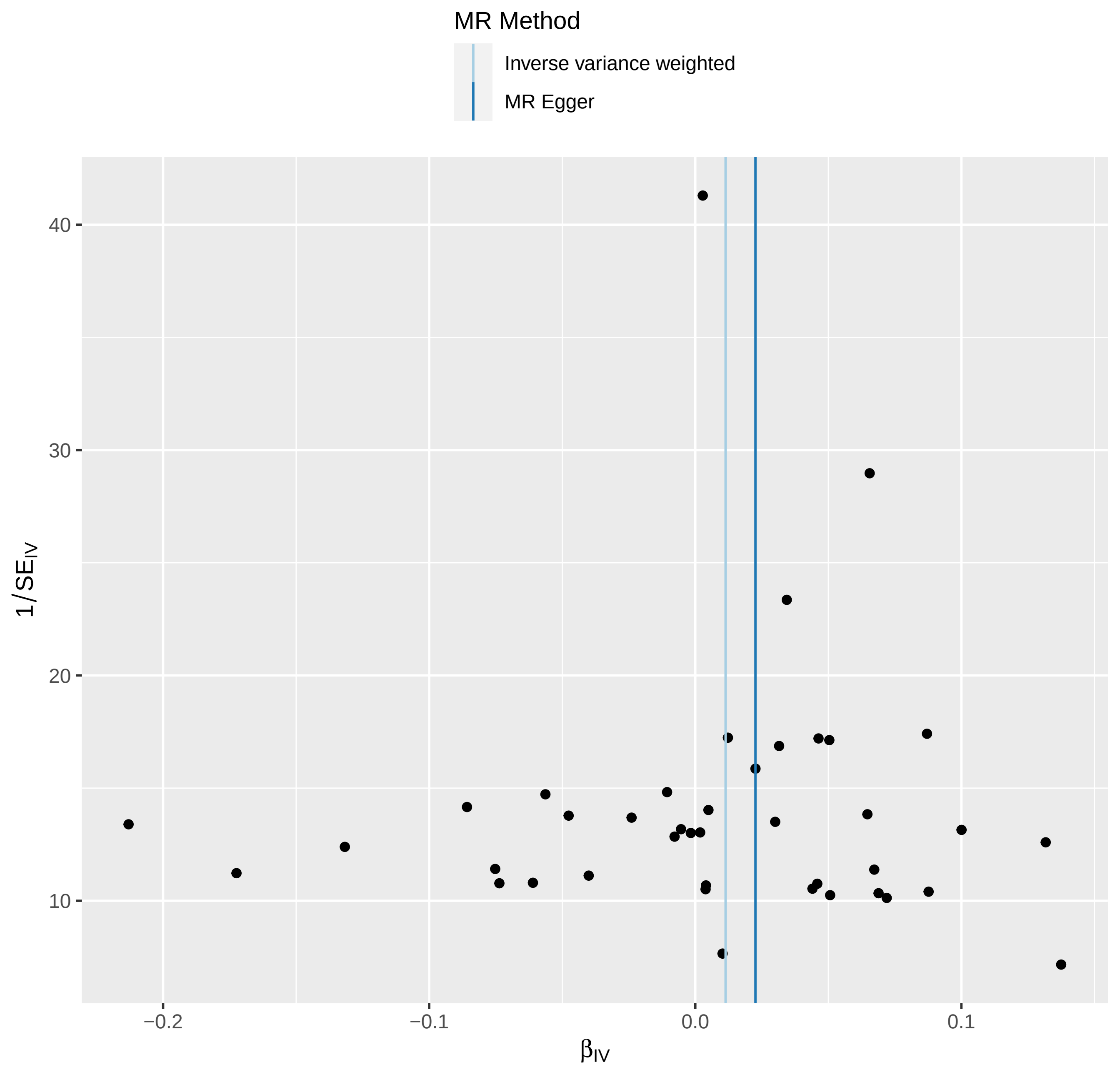

Batch 122 : Ulcerative colitis on IDP T1 FAST ROIs L cerebellum VIIIa

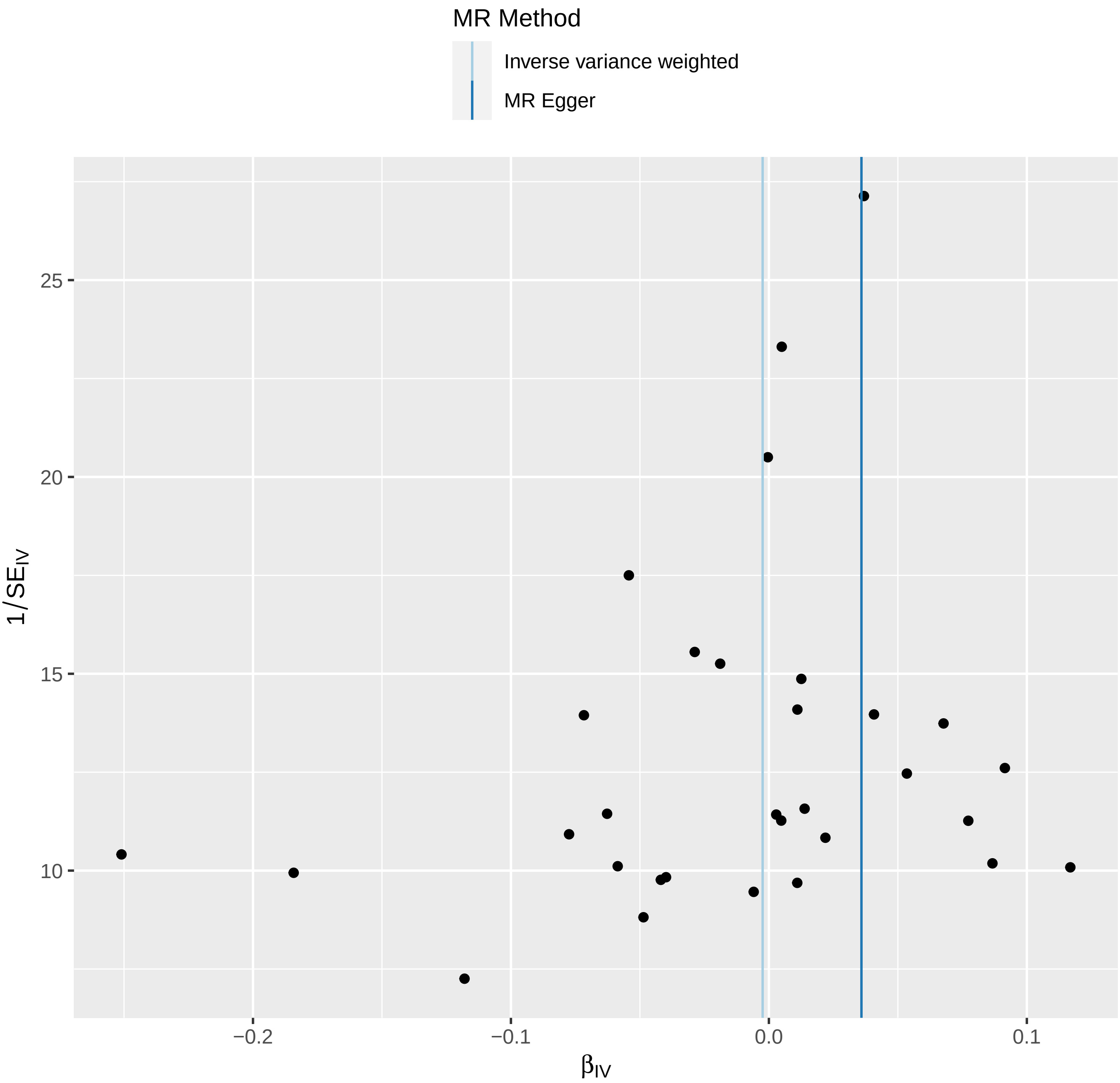

Batch 123 : Crohn's disease on IDP T1 FAST ROIs V cerebellum VIIIa

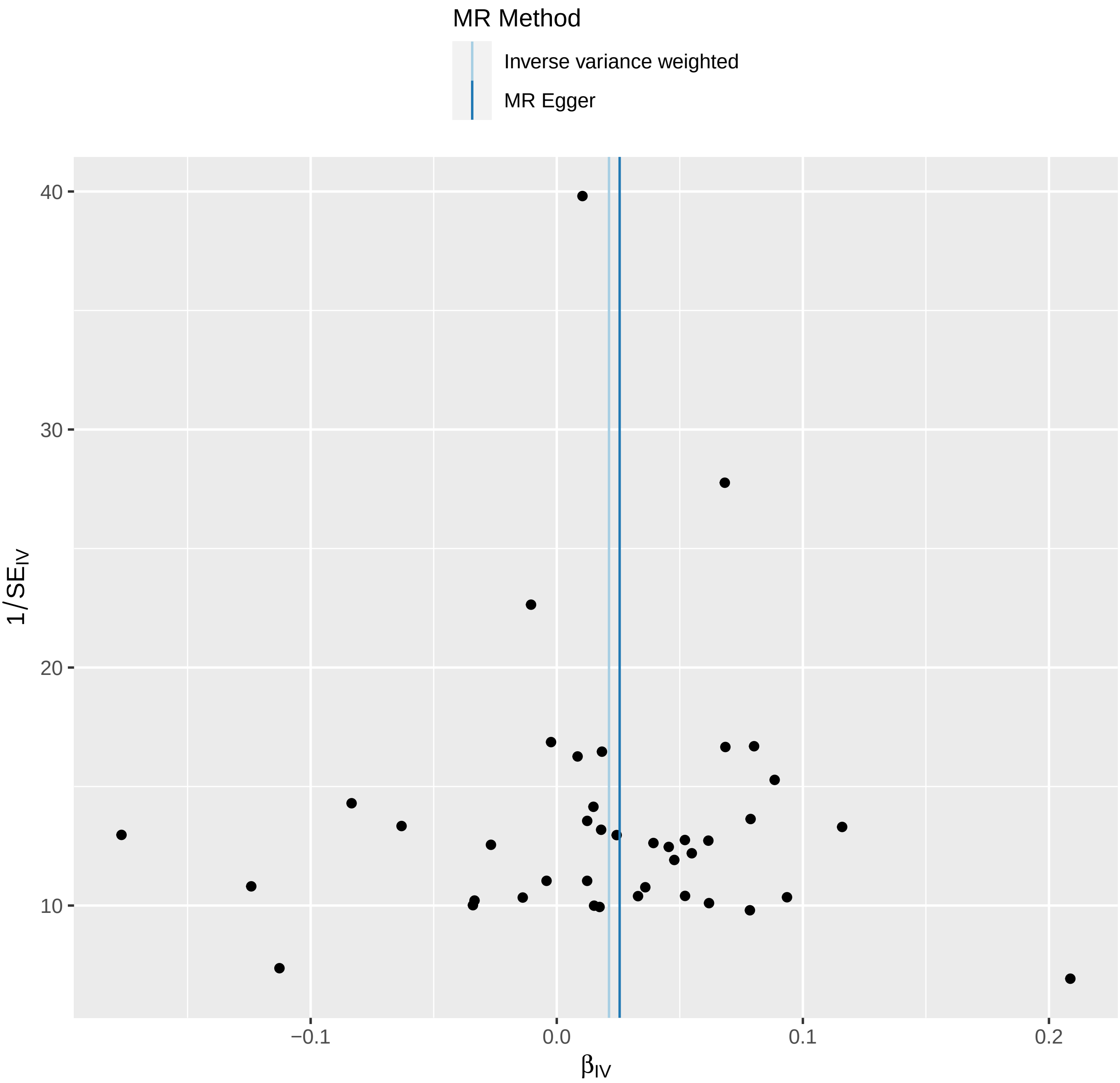

Batch 124 : Ulcerative colitis on IDP T1 FAST ROIs V cerebellum VIIIa

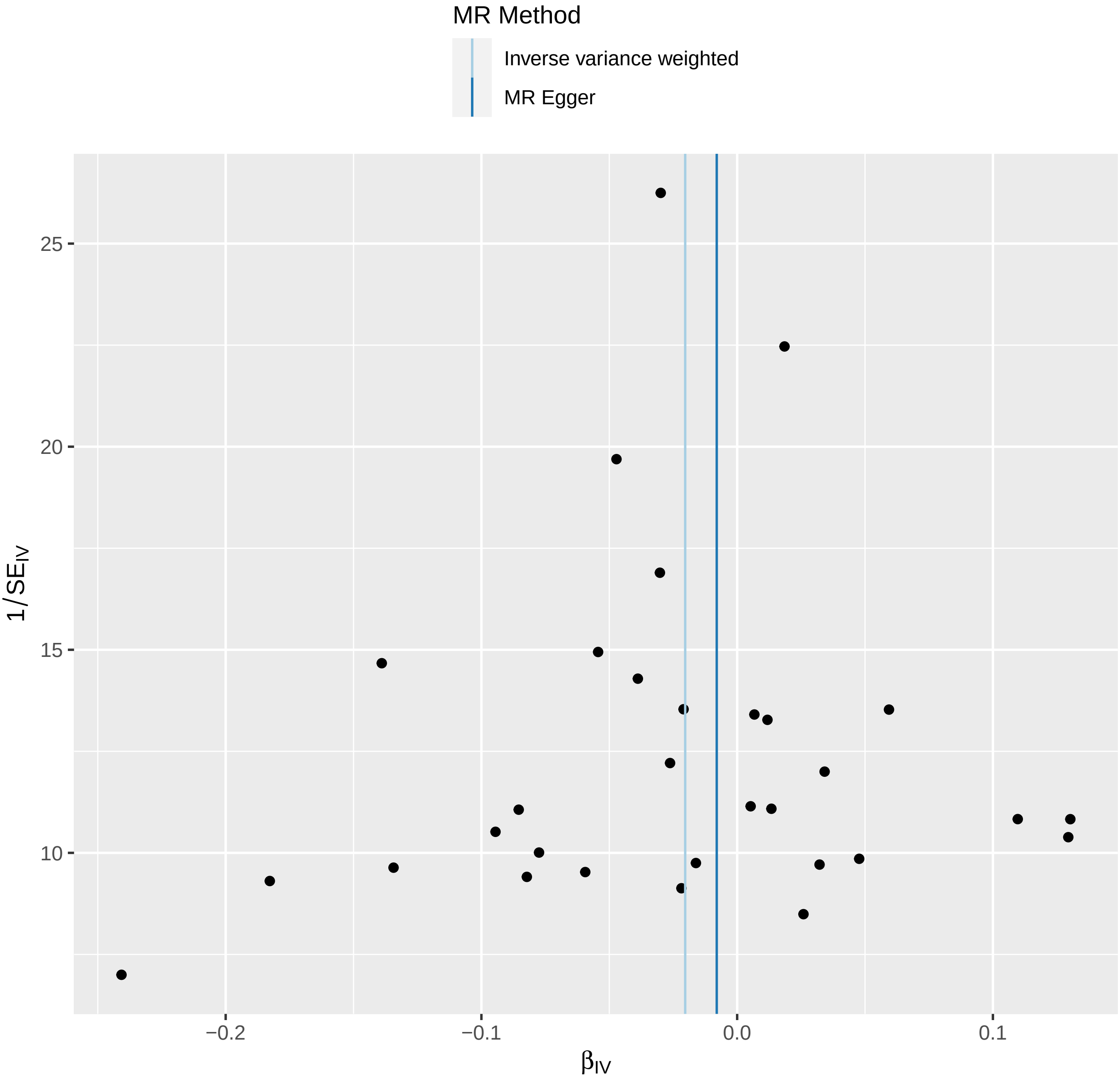

Batch 125 : Crohn's disease on IDP T1 FAST ROIs R cerebellum VIIIa

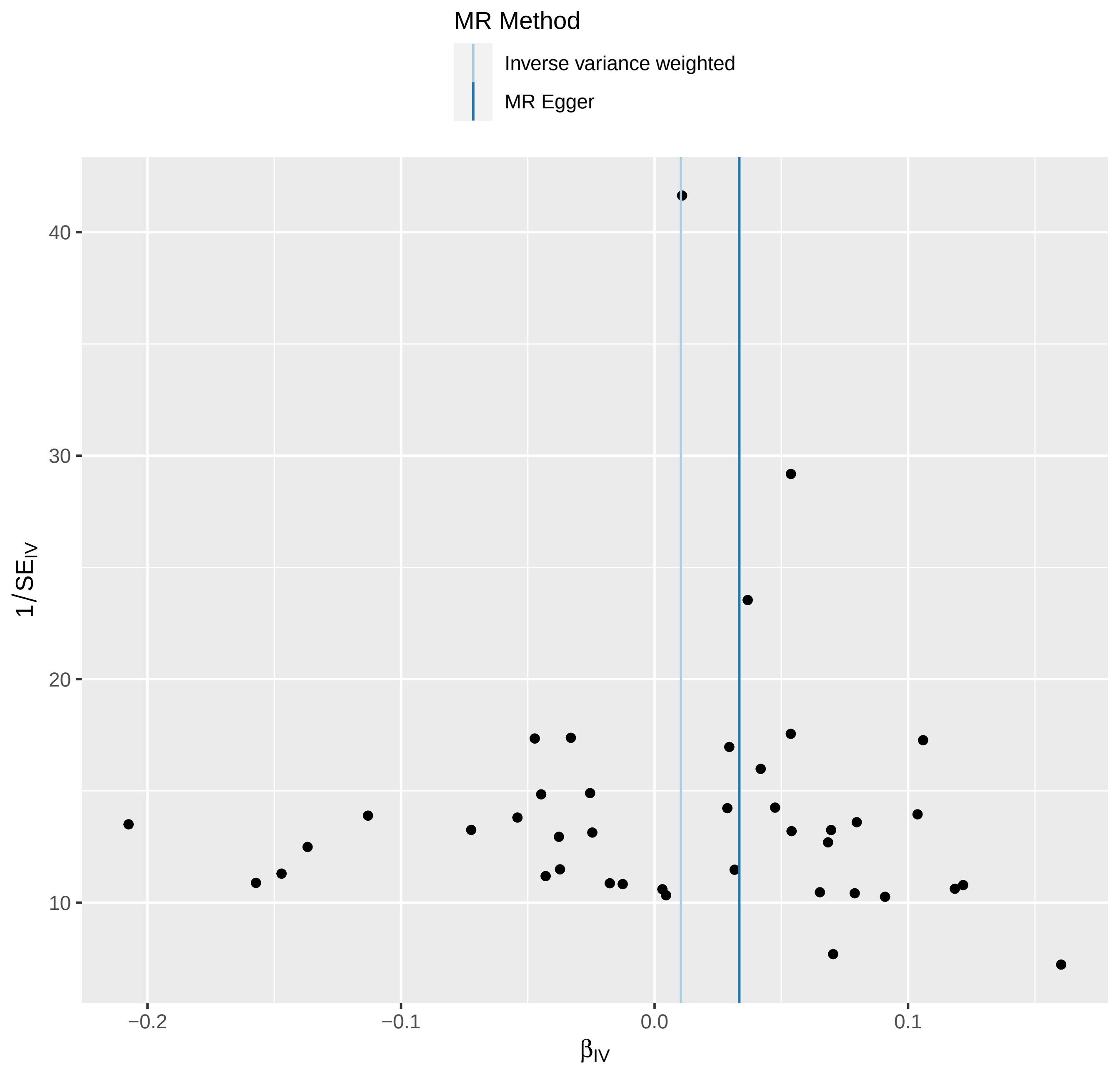

Batch 126 : Ulcerative colitis on IDP T1 FAST ROIs R cerebellum VIIIa

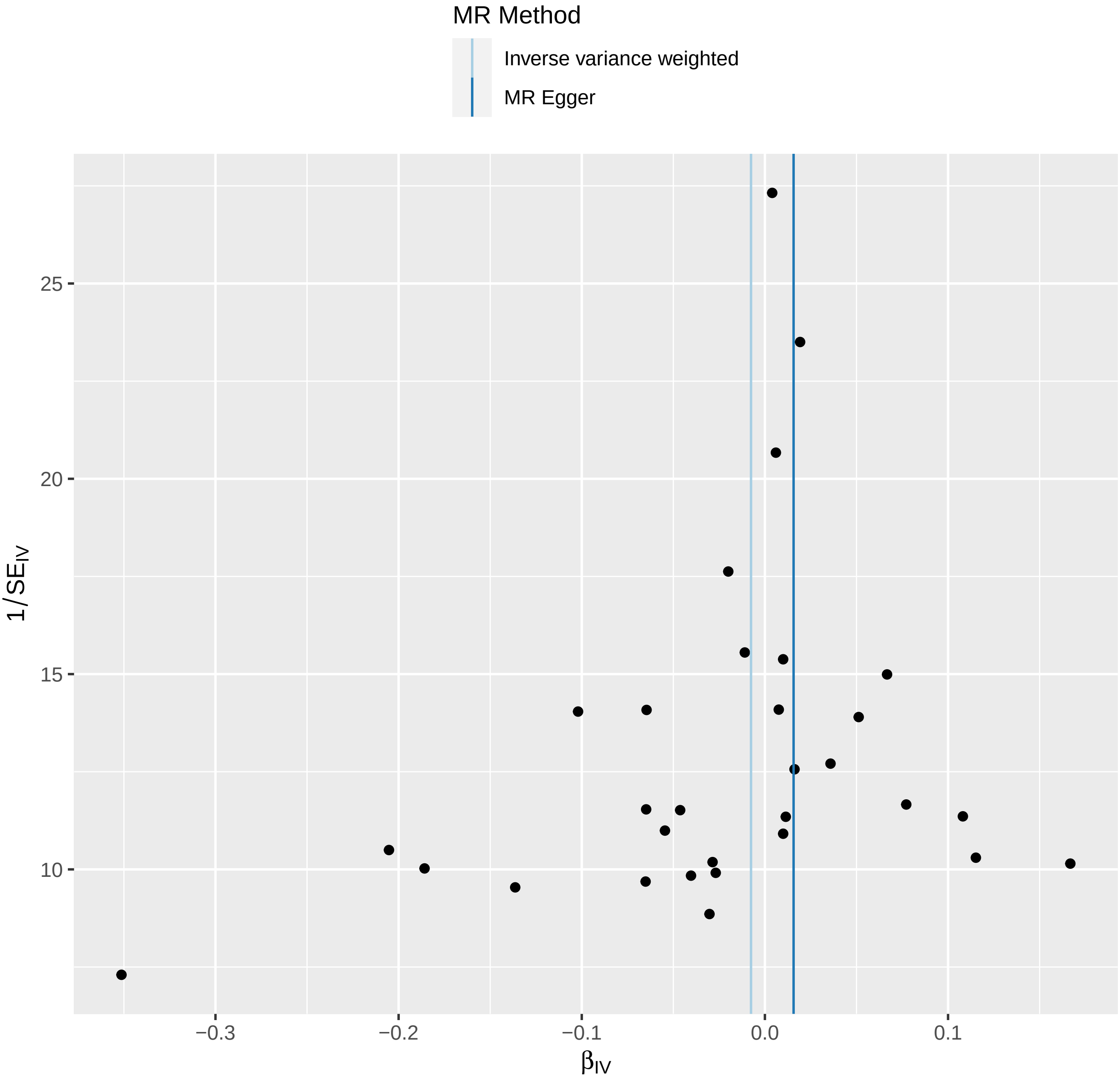

Batch 127 : Crohn's disease on IDP T1 FAST ROIs L cerebellum VIIIb

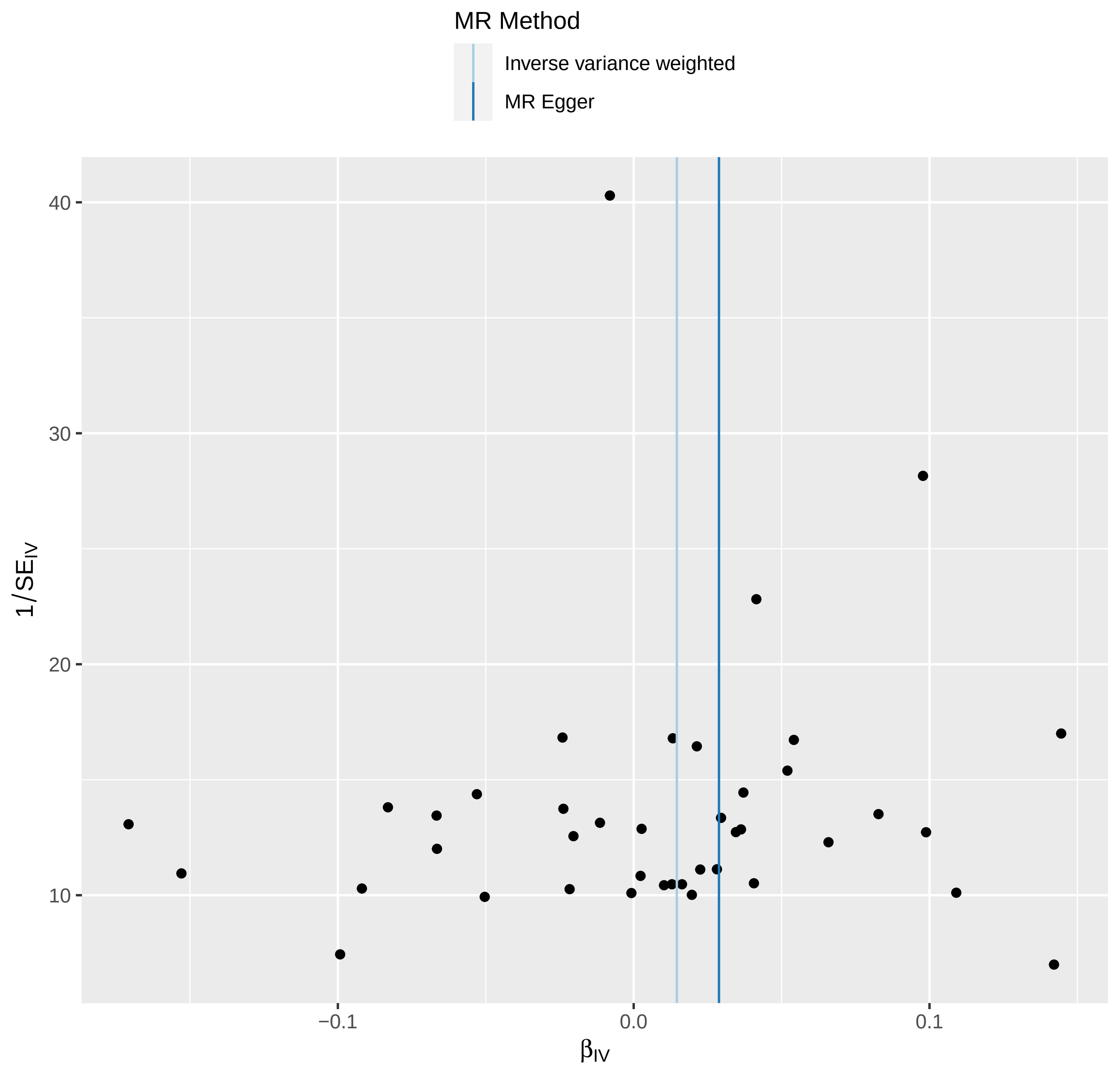

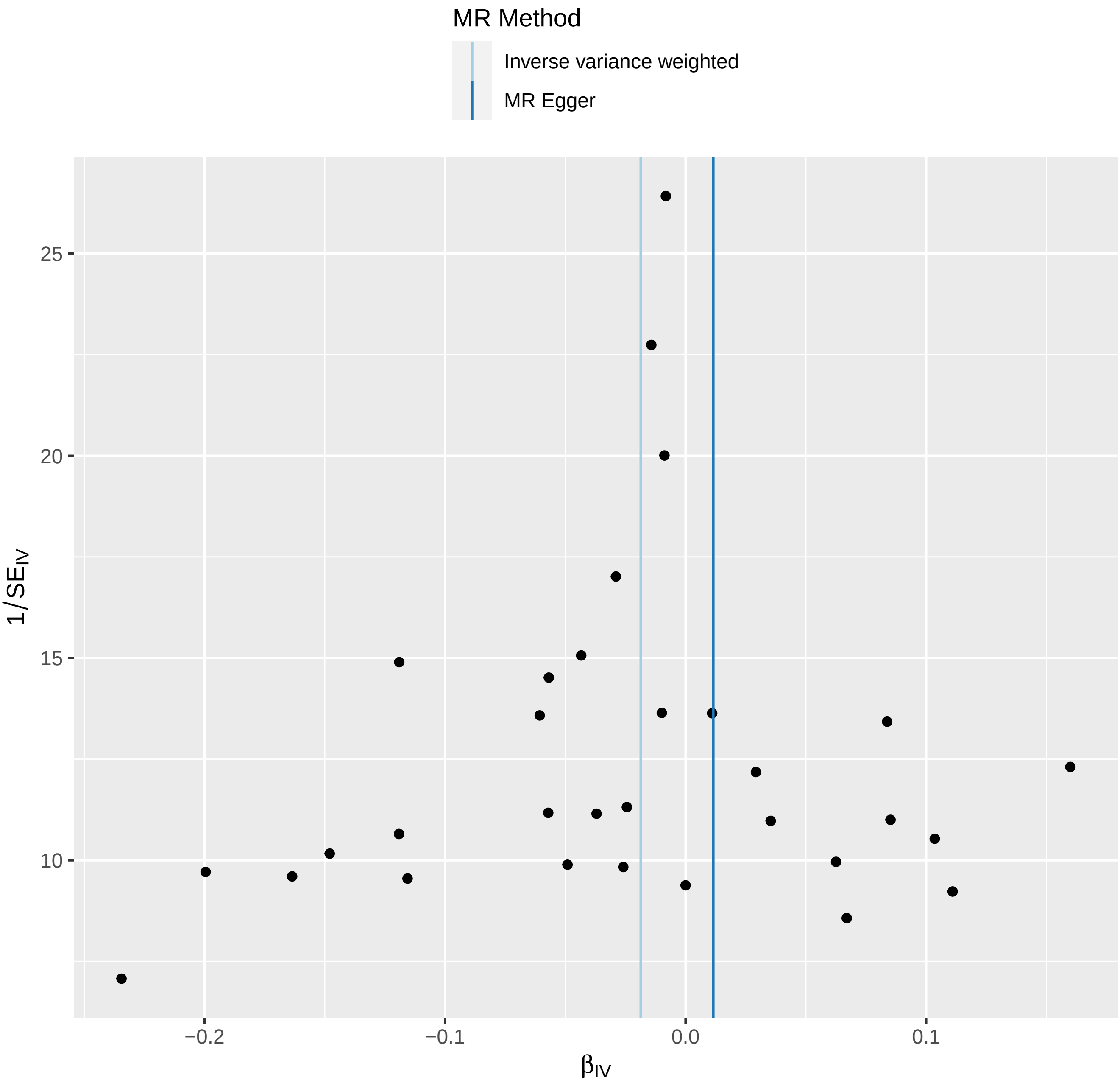
MR Method
Inverse variance weighted
MR Egger
25
20
15
10
1/SE$_{IV}$
−0.2
−0.1
0.0
0.1
β$_{IV}$

Batch 129 : Crohn's disease on IDP T1 FAST ROIs V cerebellum VIIIb

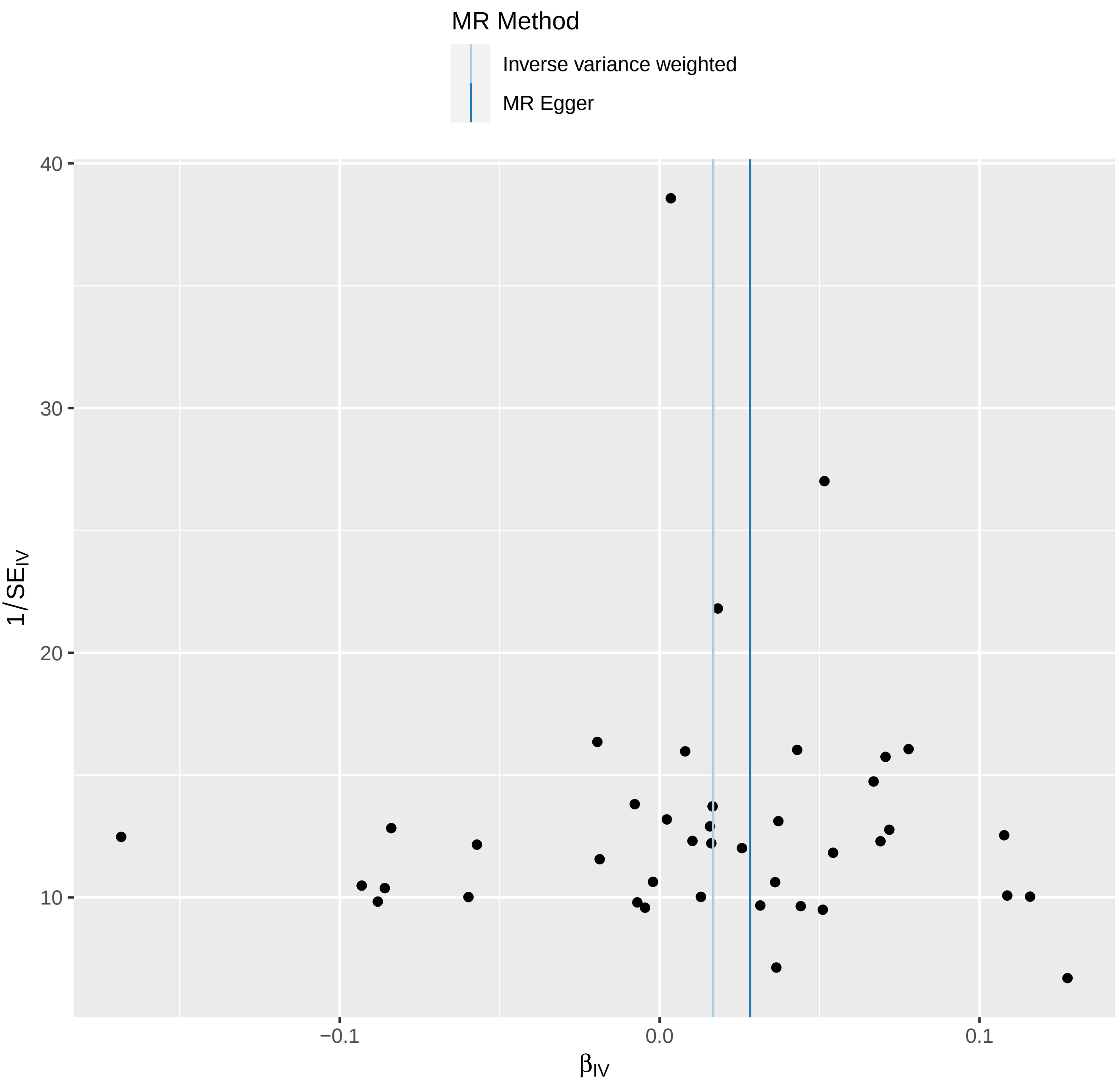

Batch 130 : Ulcerative colitis on IDP T1 FAST ROIs V cerebellum VIIIb

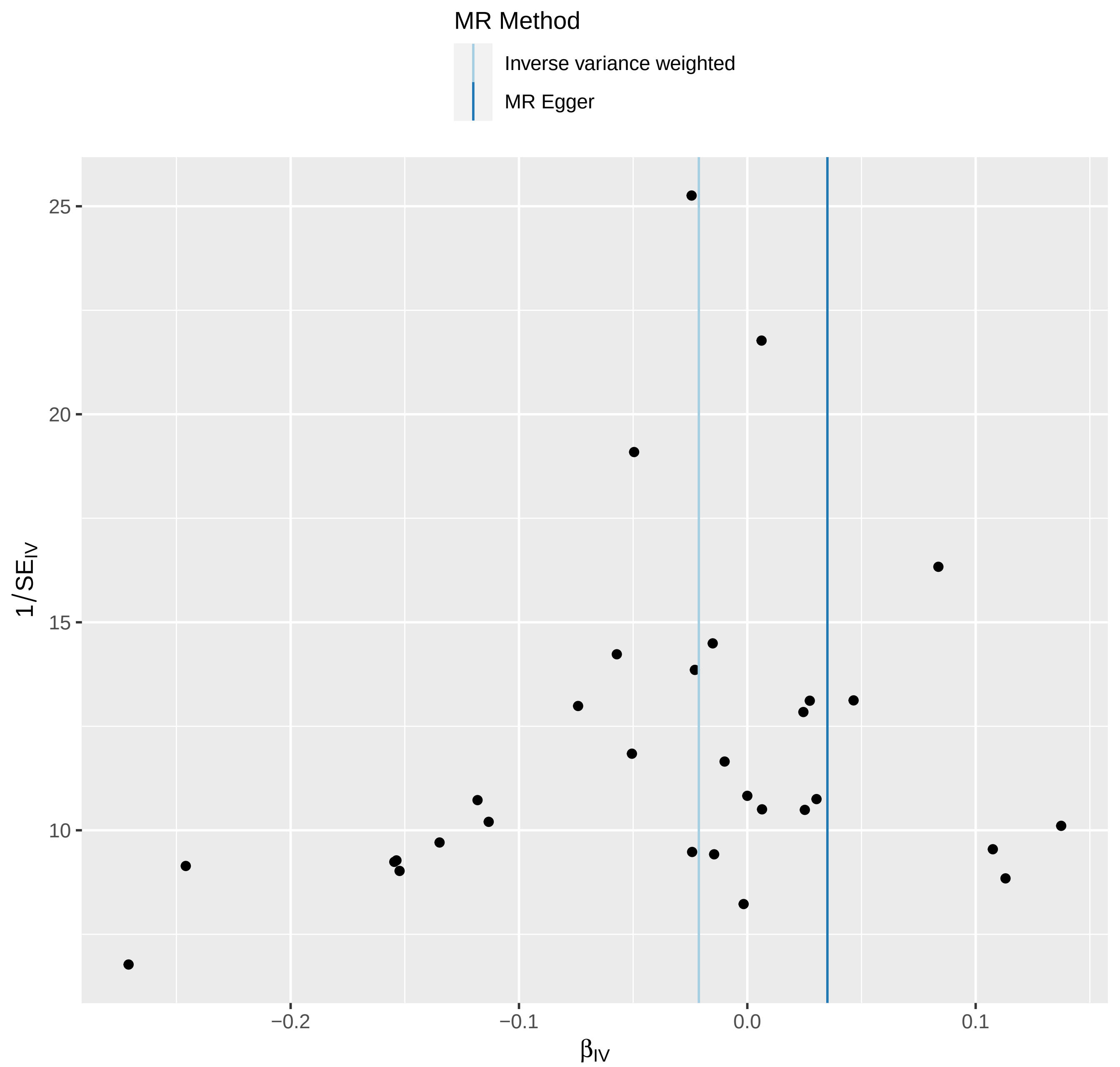

Batch 131 : Crohn's disease on IDP T1 FAST ROIs R cerebellum VIIIb

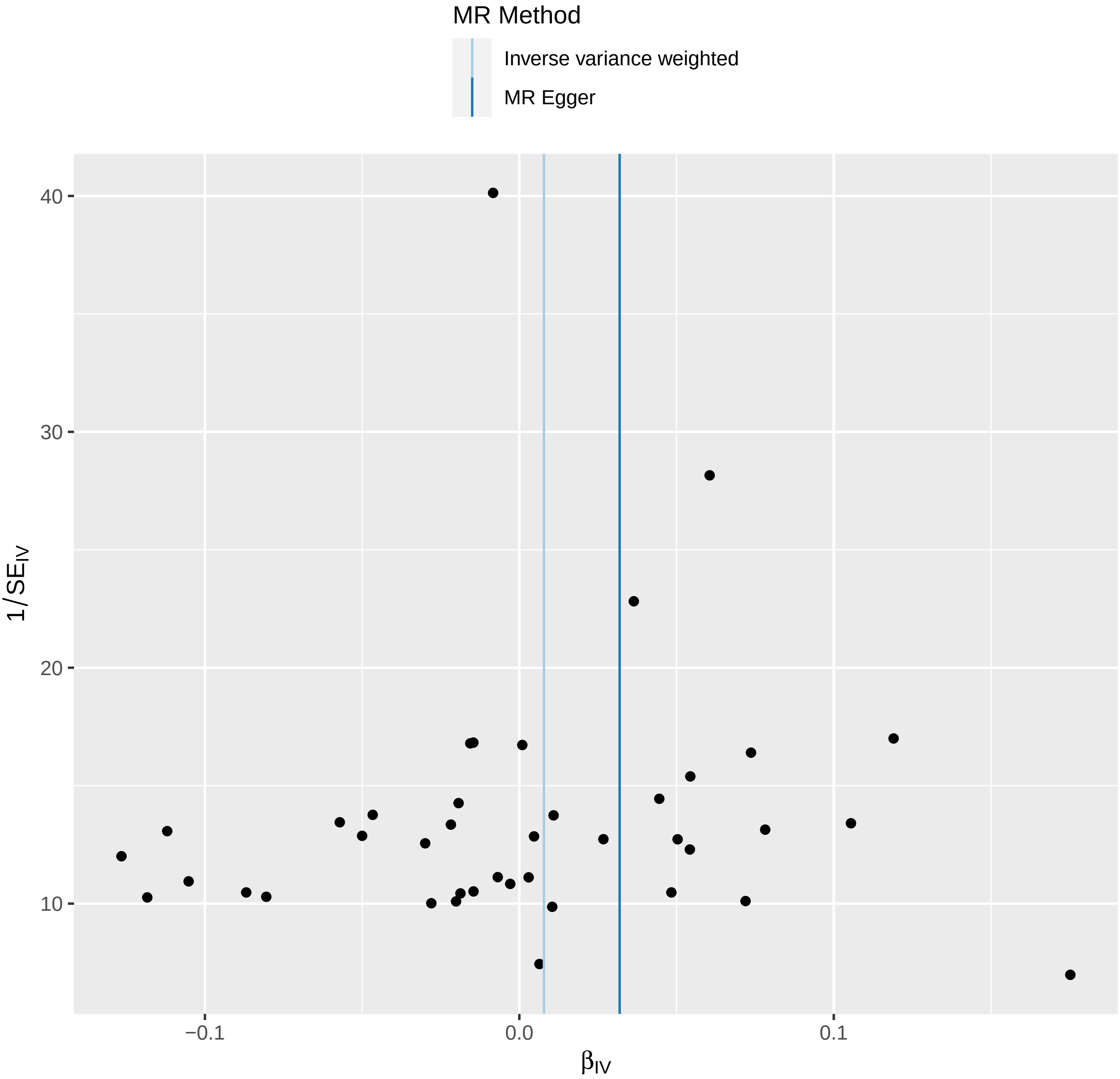

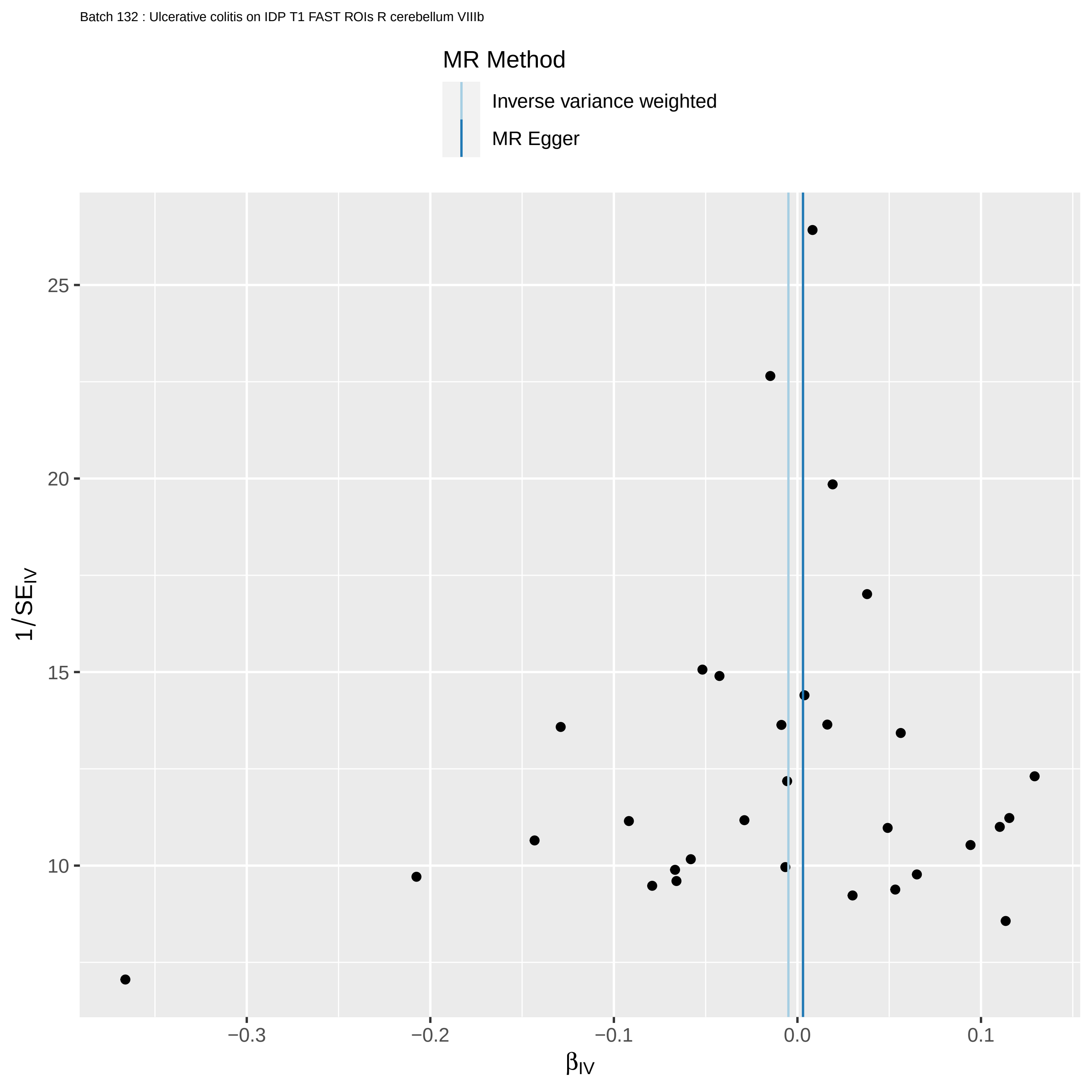

Batch 132 : Ulcerative colitis on IDP T1 FAST ROIs R cerebellum VIIIb
MR Method
Inverse variance weighted
MR Egger
25
20
15
10
1/SE_IV
−0.3
−0.2
−0.1
0.0
0.1
β_IV

Batch 133 : Crohn's disease on IDP T1 FAST ROIs L cerebellum IX

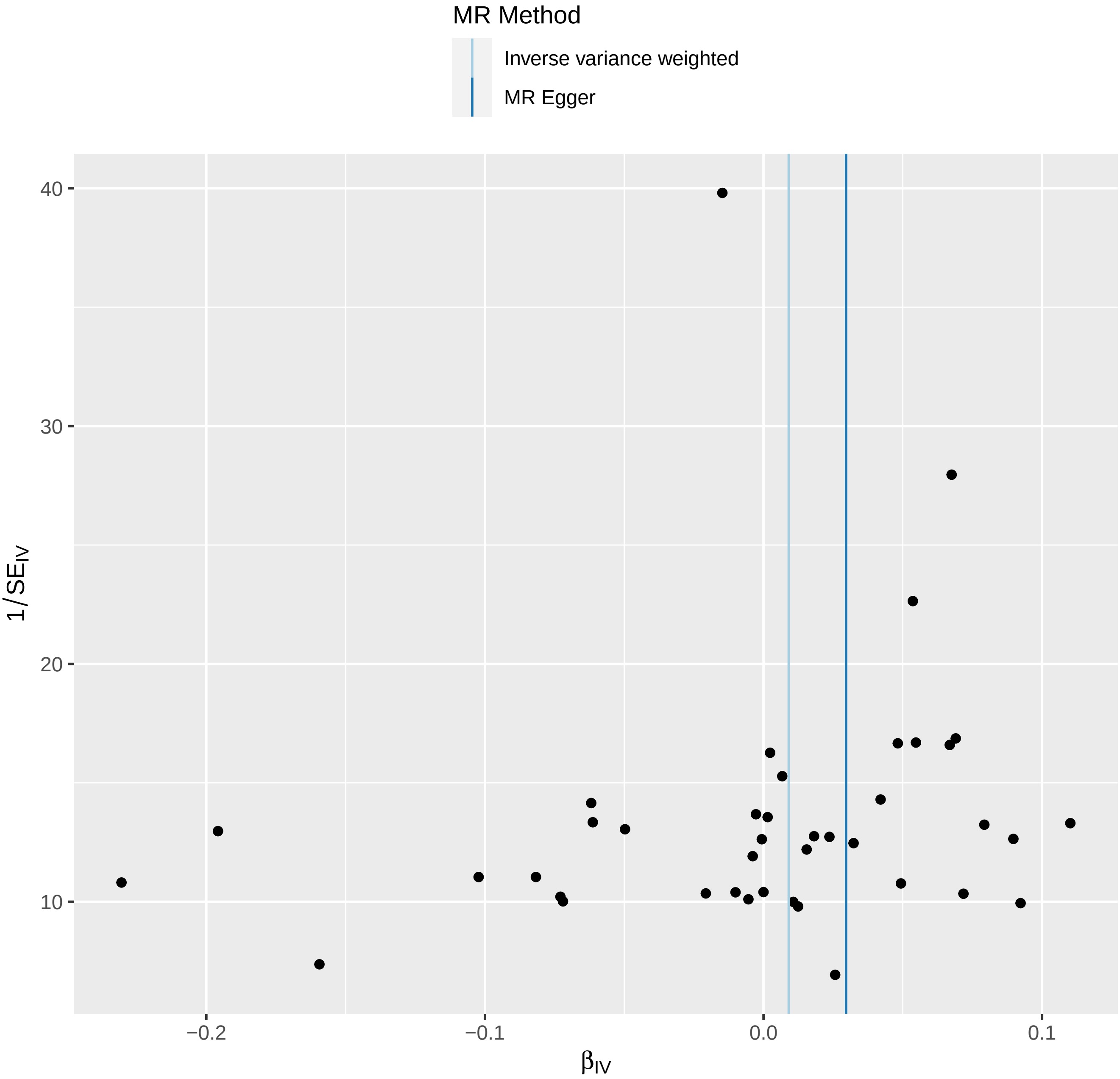

Batch 134 : Ulcerative colitis on IDP T1 FAST ROIs L cerebellum IX

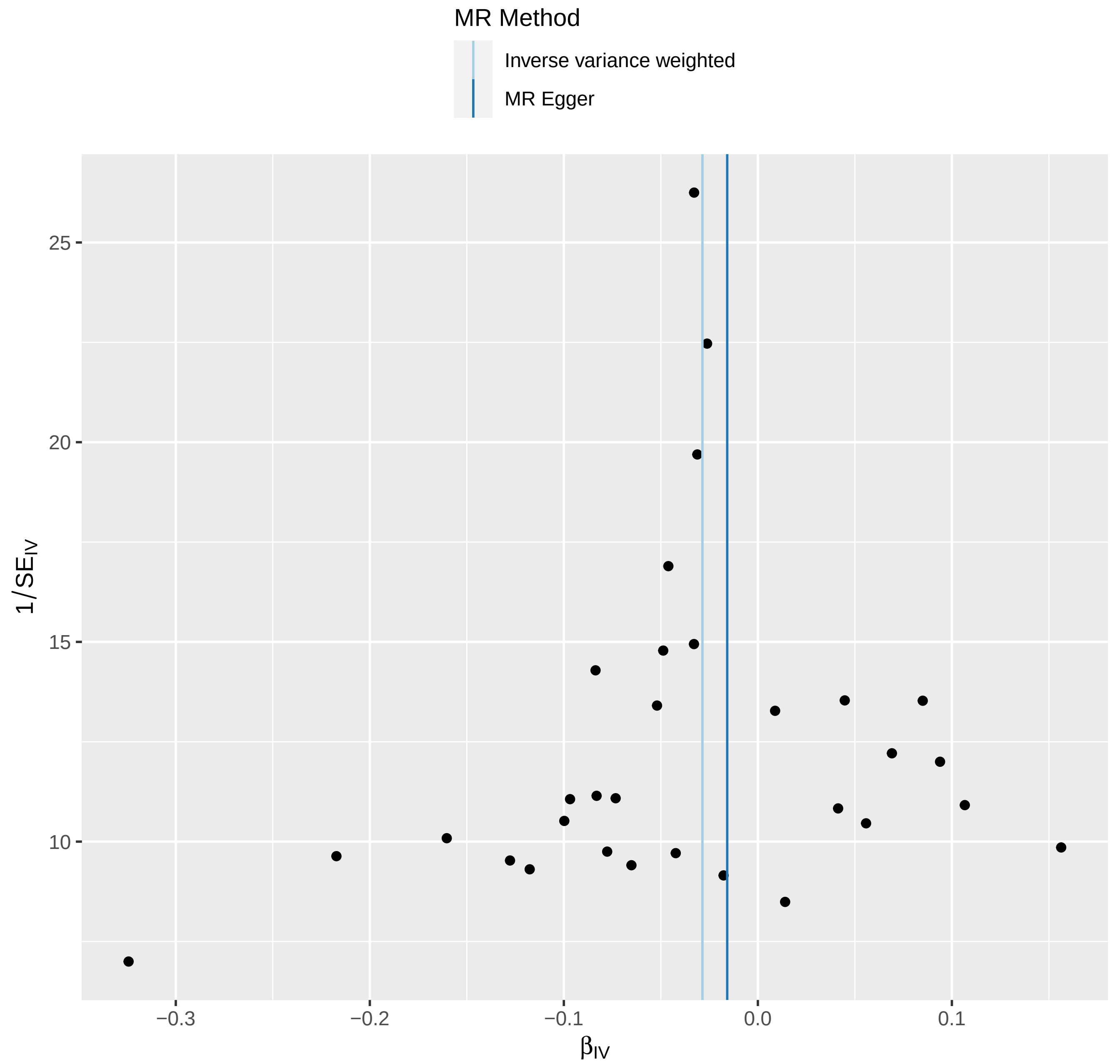

Batch 135 : Crohn's disease on IDP T1 FIRST right putamen volume

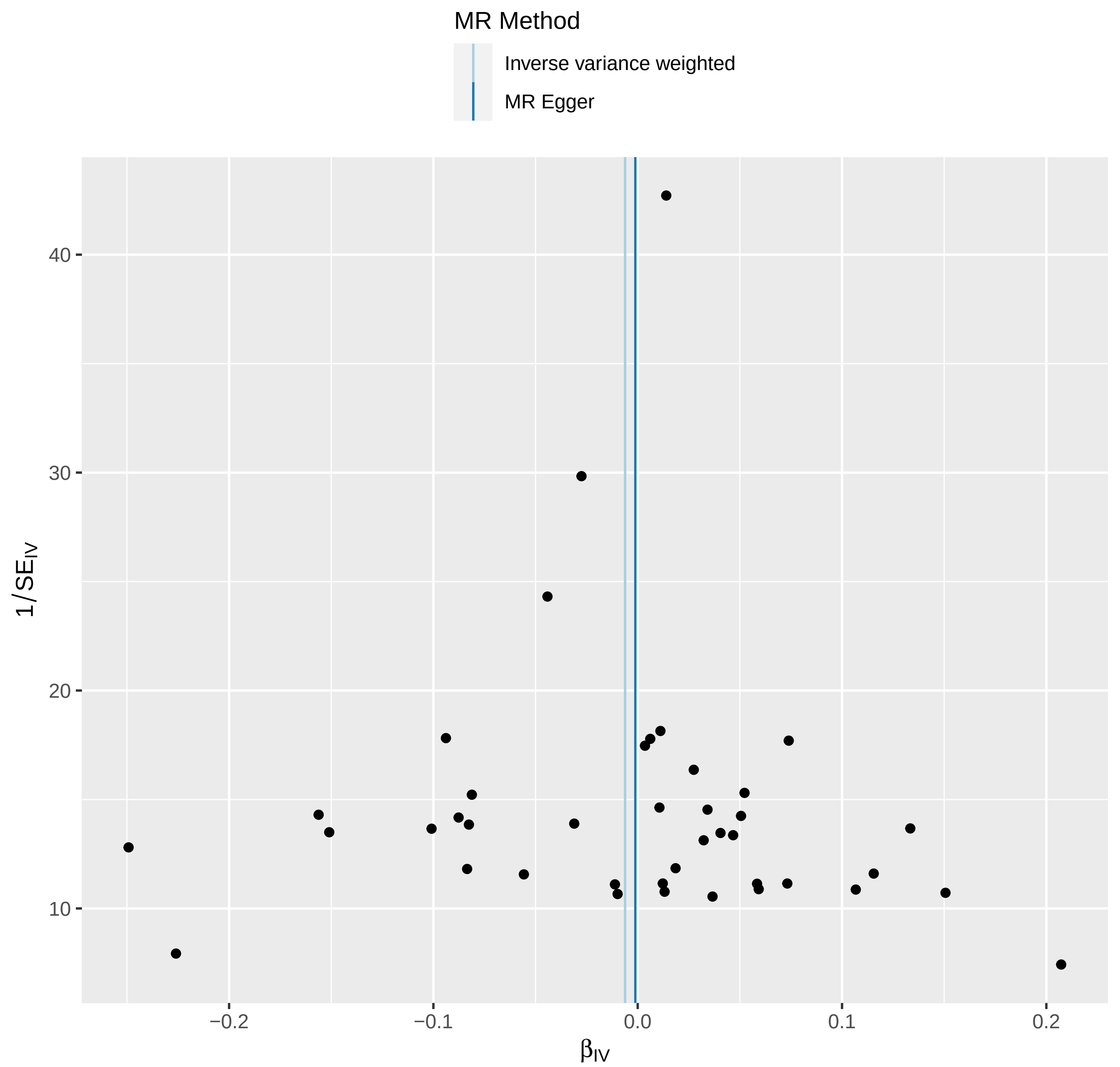

Batch 136 : Ulcerative colitis on IDP T1 FIRST right putamen volume

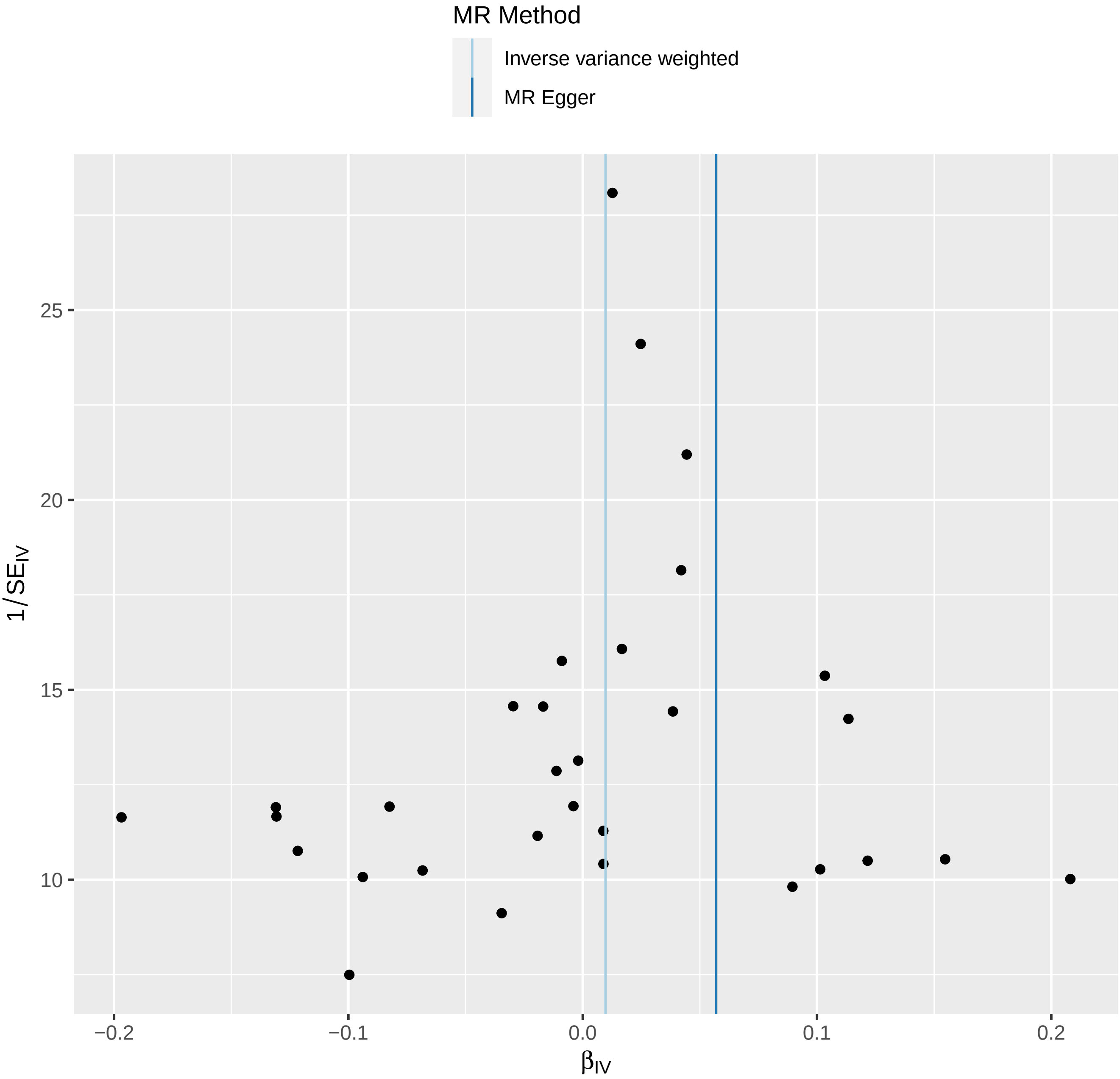

Batch 137 : Crohn's disease on IDP T1 FAST ROIs V cerebellum IX

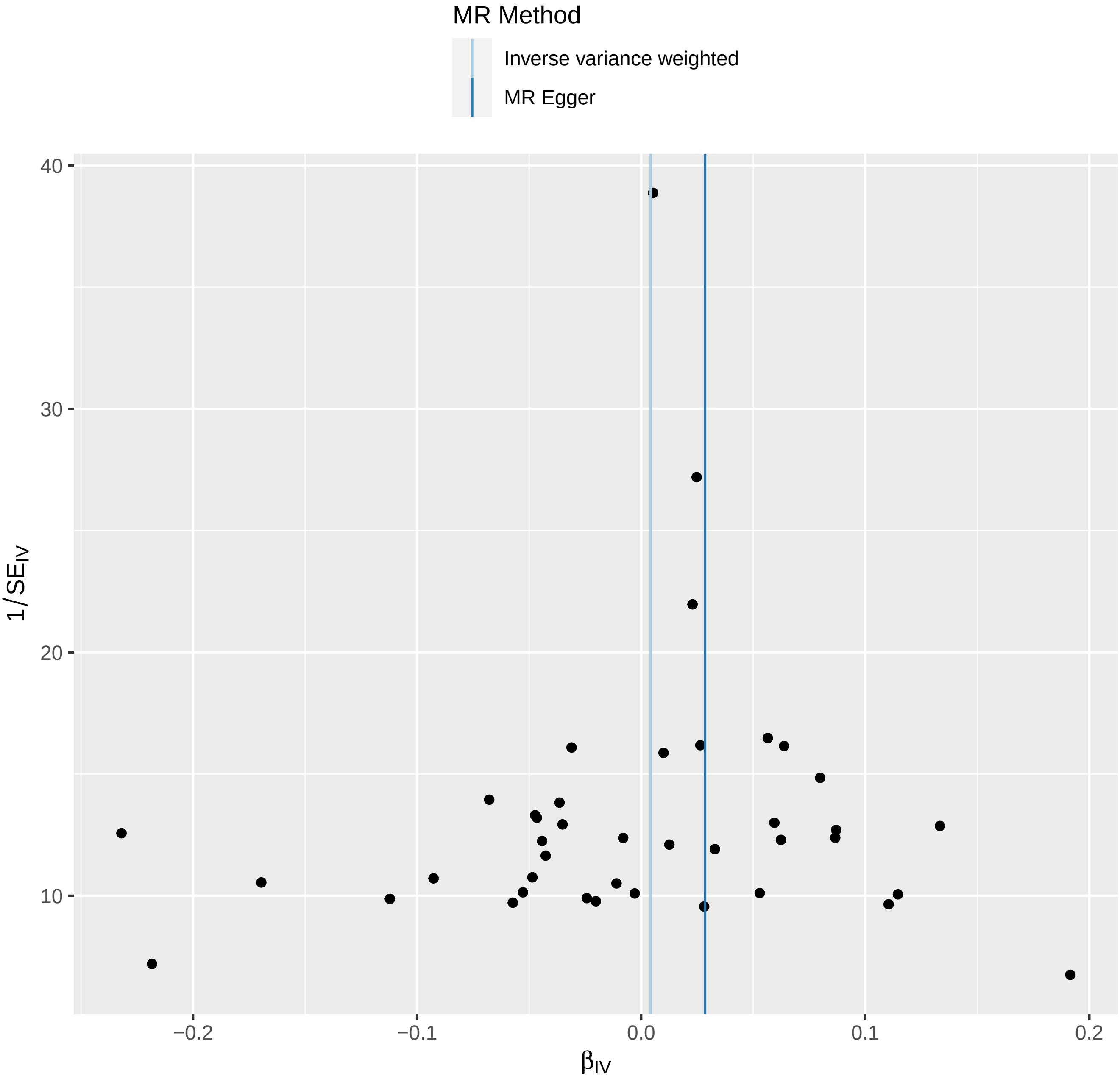

Batch 138 : Ulcerative colitis on IDP T1 FAST ROIs V cerebellum IX

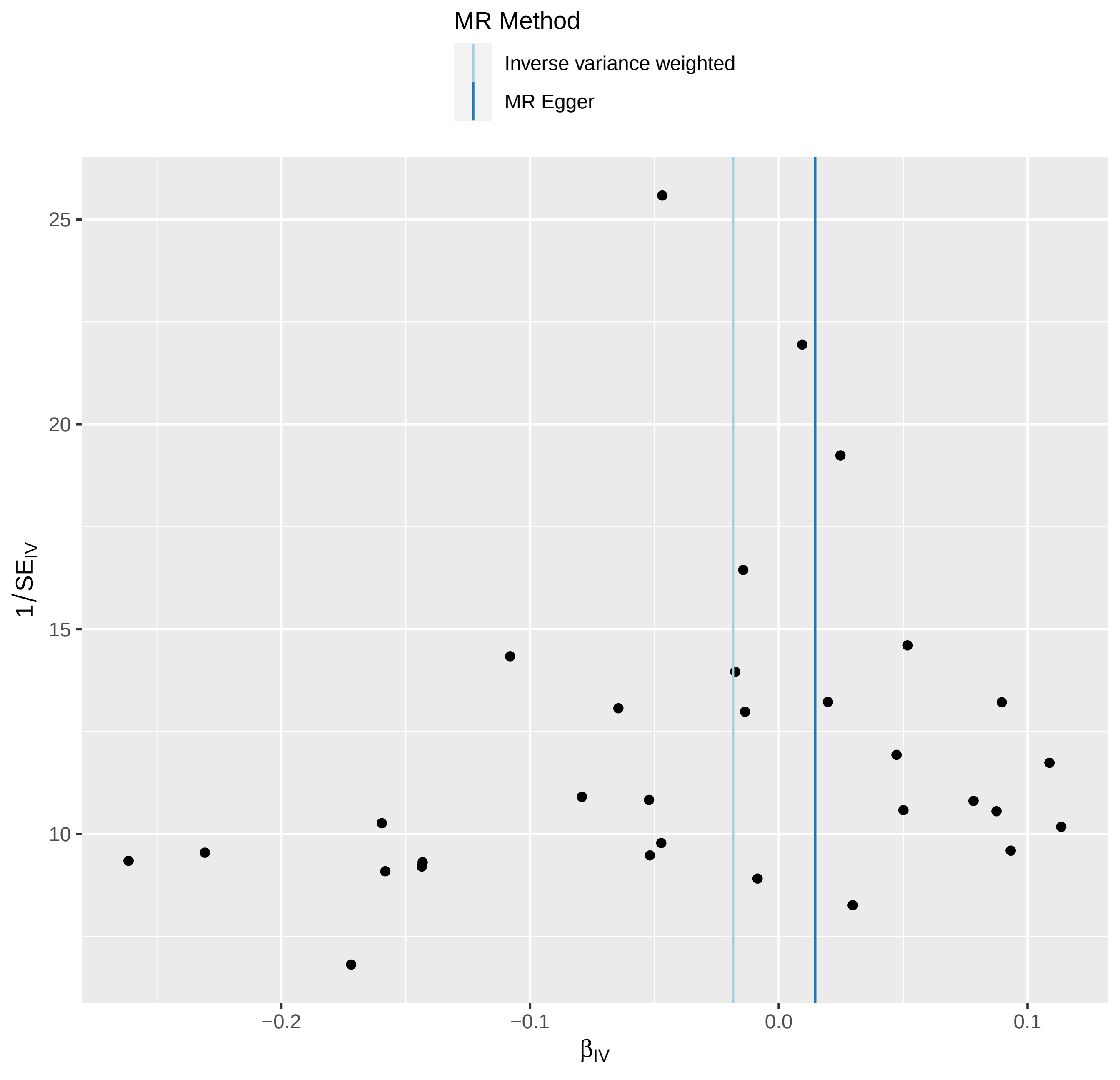

Batch 139 : Crohn's disease on IDP T1 FAST ROIs R cerebellum IX

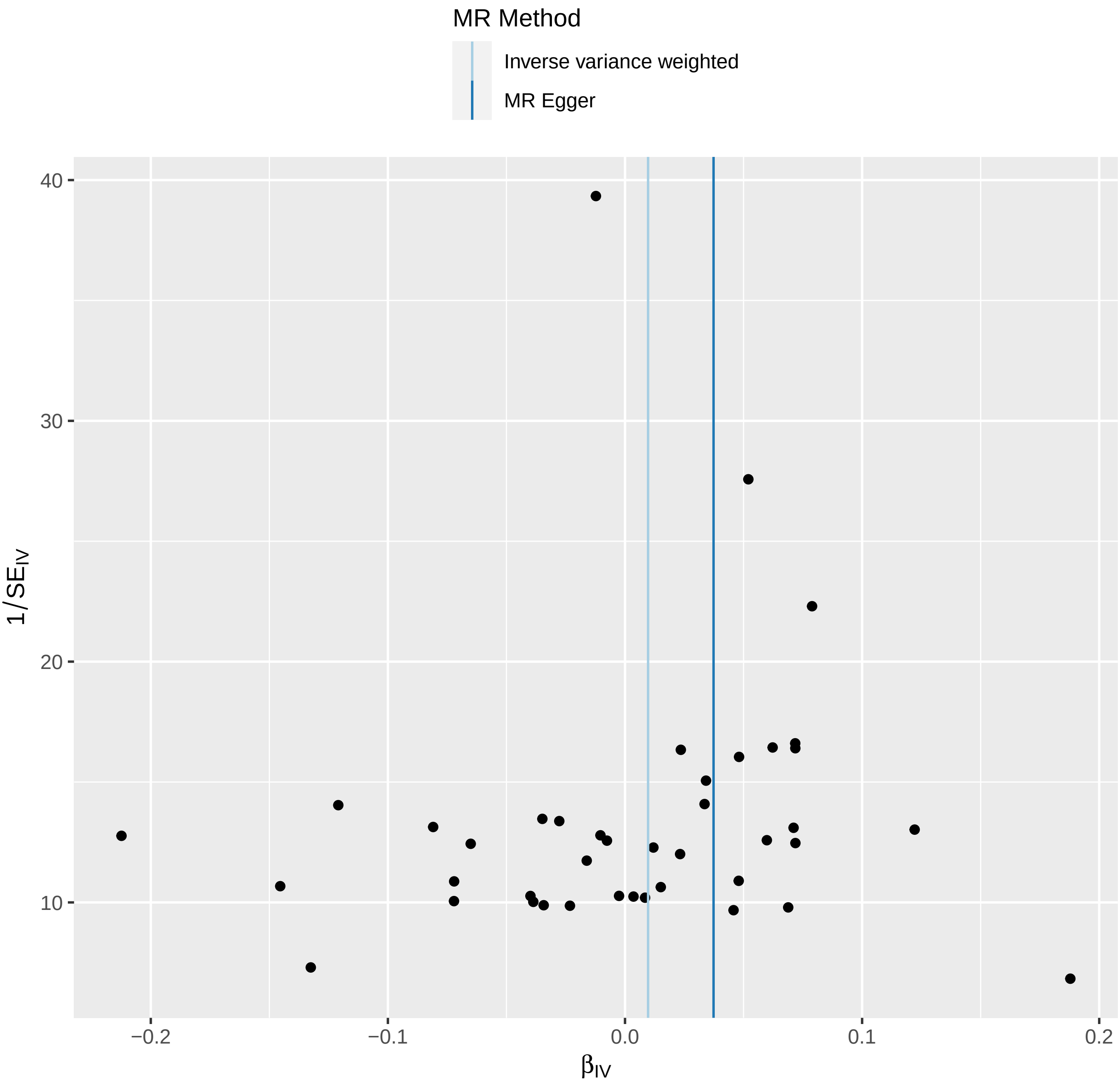

Batch 140 : Ulcerative colitis on IDP T1 FAST ROIs R cerebellum IX

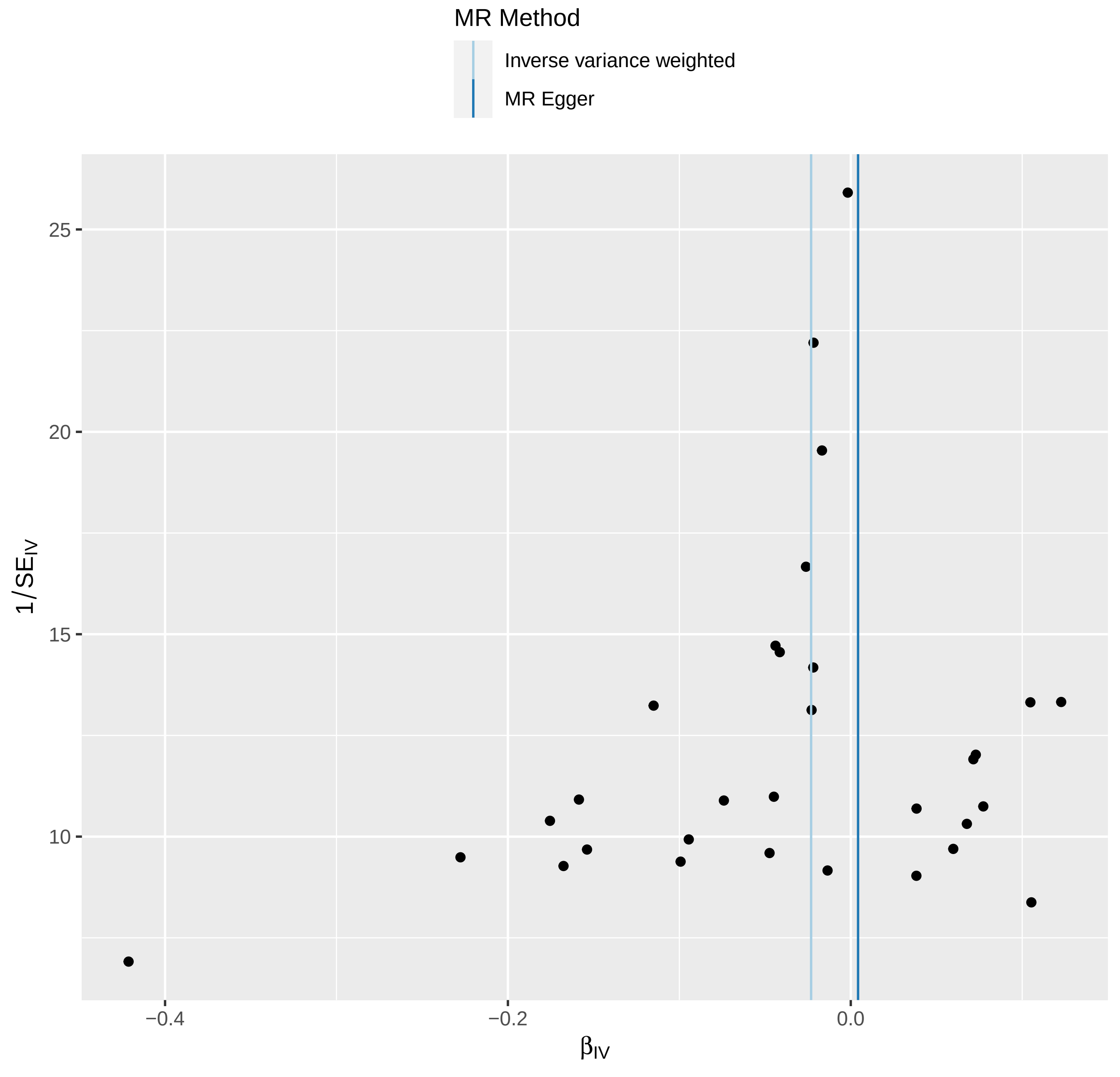

Batch 141 : Crohn's disease on IDP T1 FAST ROIs L cerebellum X

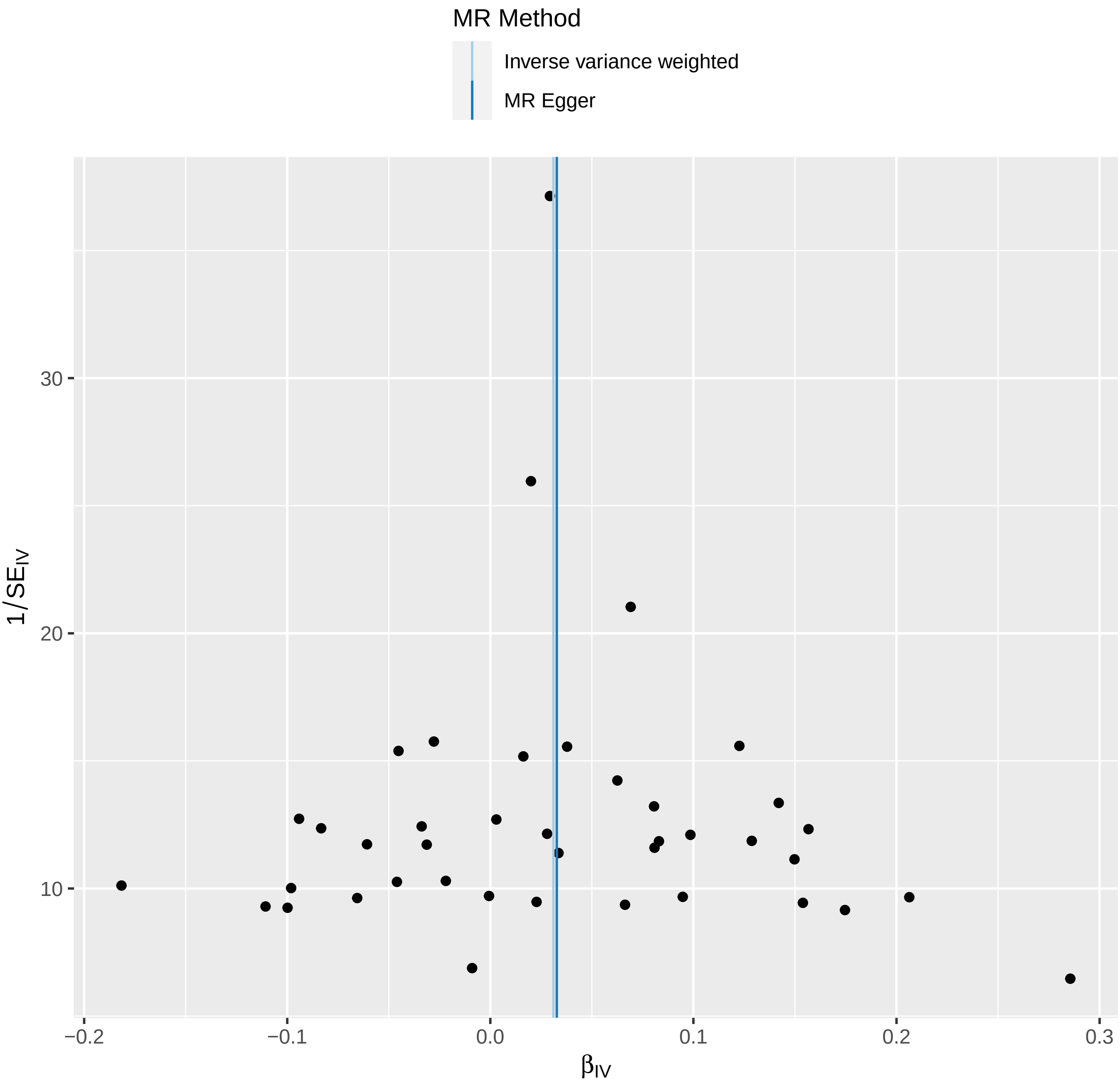

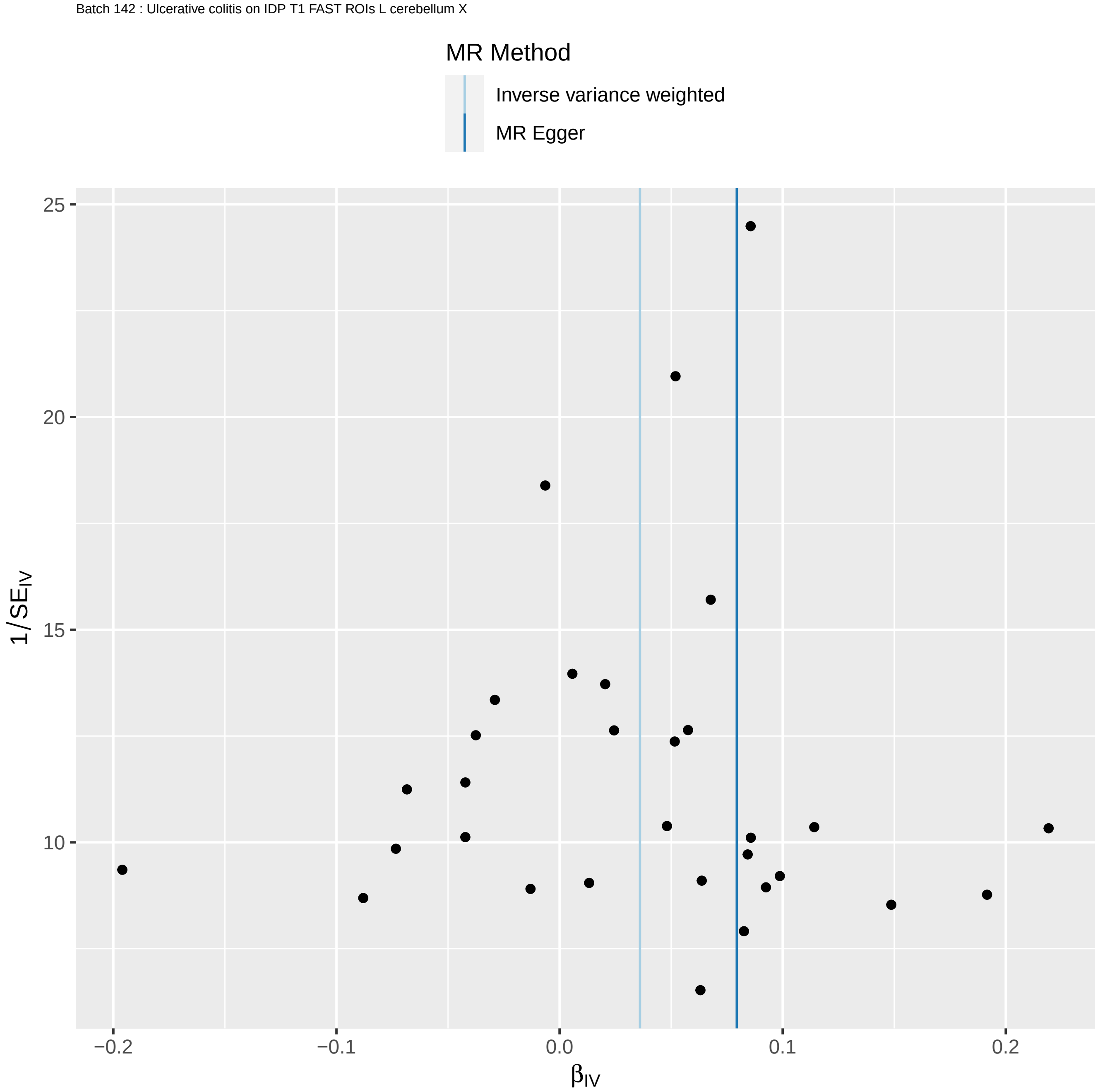

Batch 142 : Ulcerative colitis on IDP T1 FAST ROIs L cerebellum X
MR Method
Inverse variance weighted
MR Egger
25
20
15
10
1/SE$_{IV}$
−0.2
−0.1
0.0
0.1
0.2
β$_{IV}$

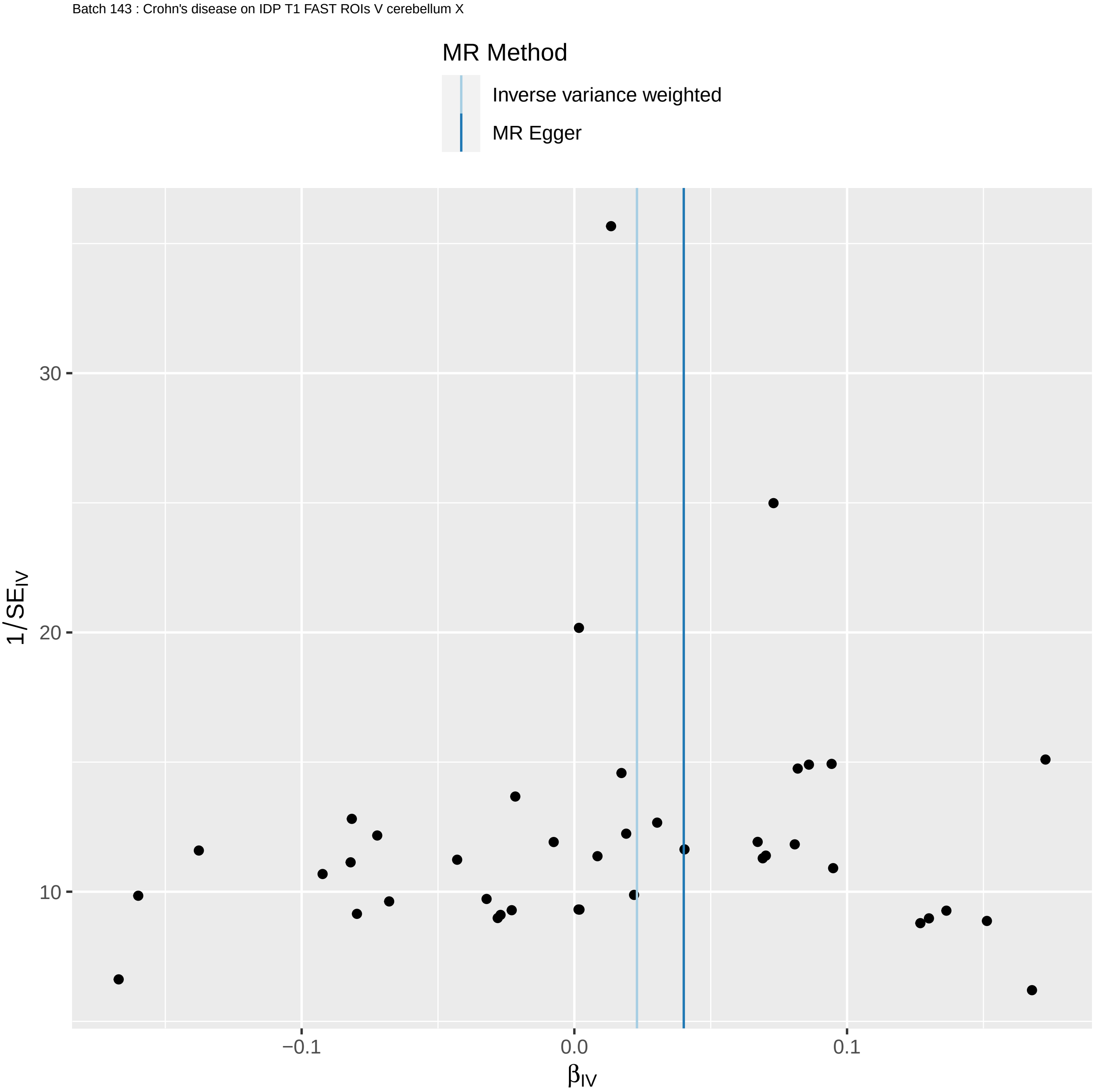

Batch 143 : Crohn's disease on IDP T1 FAST ROIs V cerebellum X
MR Method
Inverse variance weighted
MR Egger
30
20
10
1/SE$_{IV}$
−0.1
0.0
0.1
β$_{IV}$

Batch 144 : Ulcerative colitis on IDP T1 FAST ROIs V cerebellum X

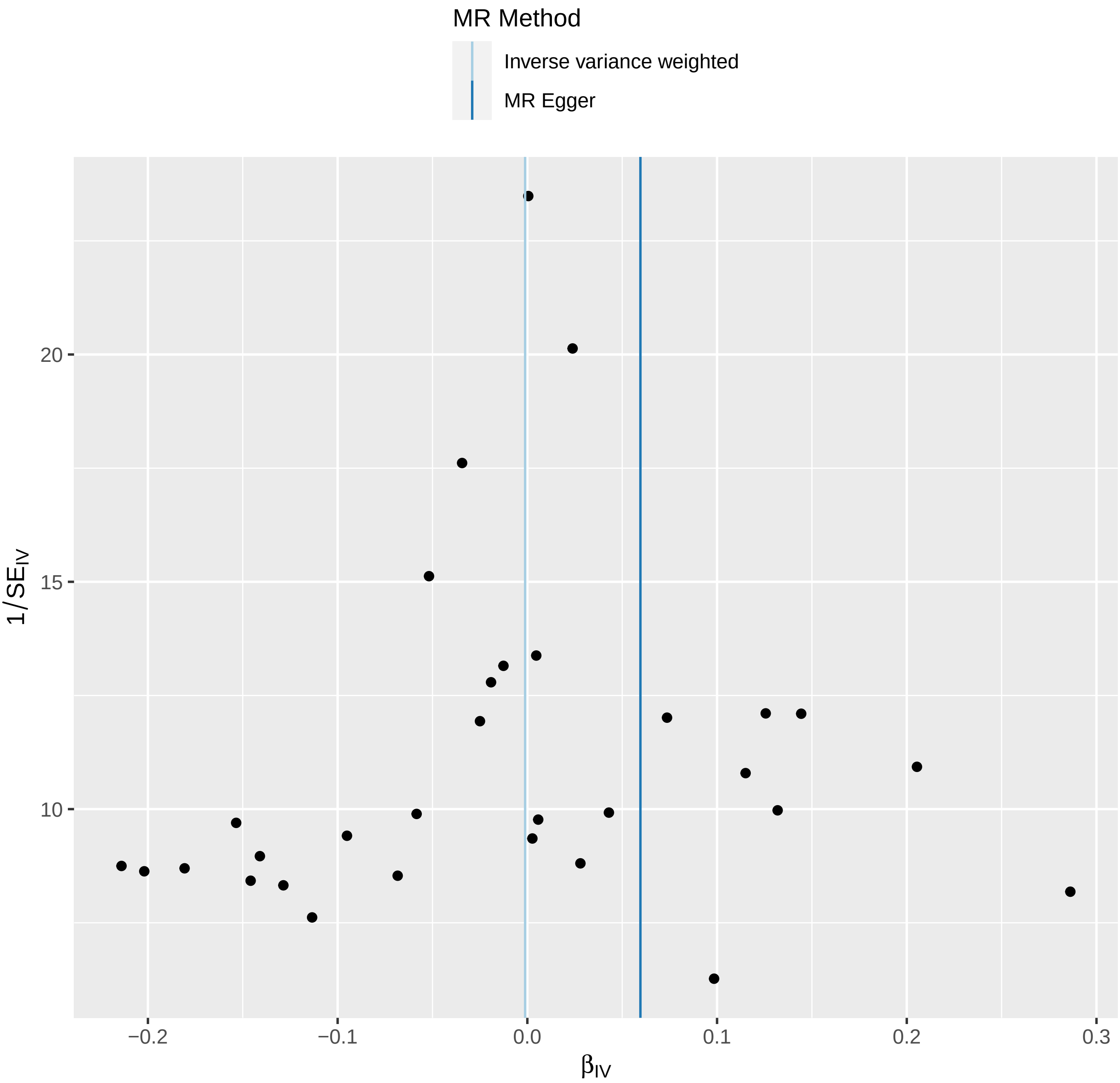

Batch 145 : Crohn's disease on IDP T1 FAST ROIs R cerebellum X

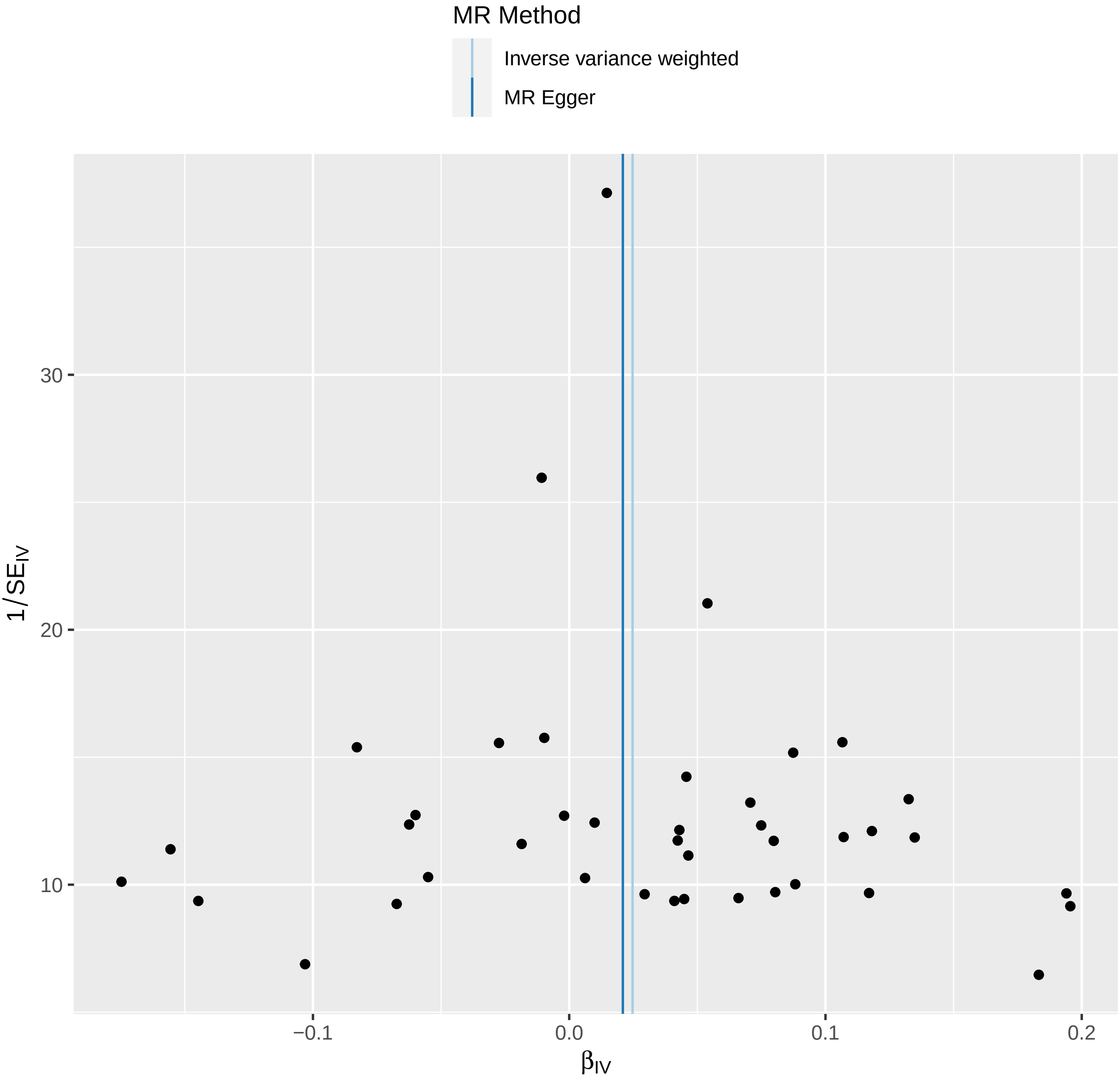

Batch 146 : Ulcerative colitis on IDP T1 FAST ROIs R cerebellum X

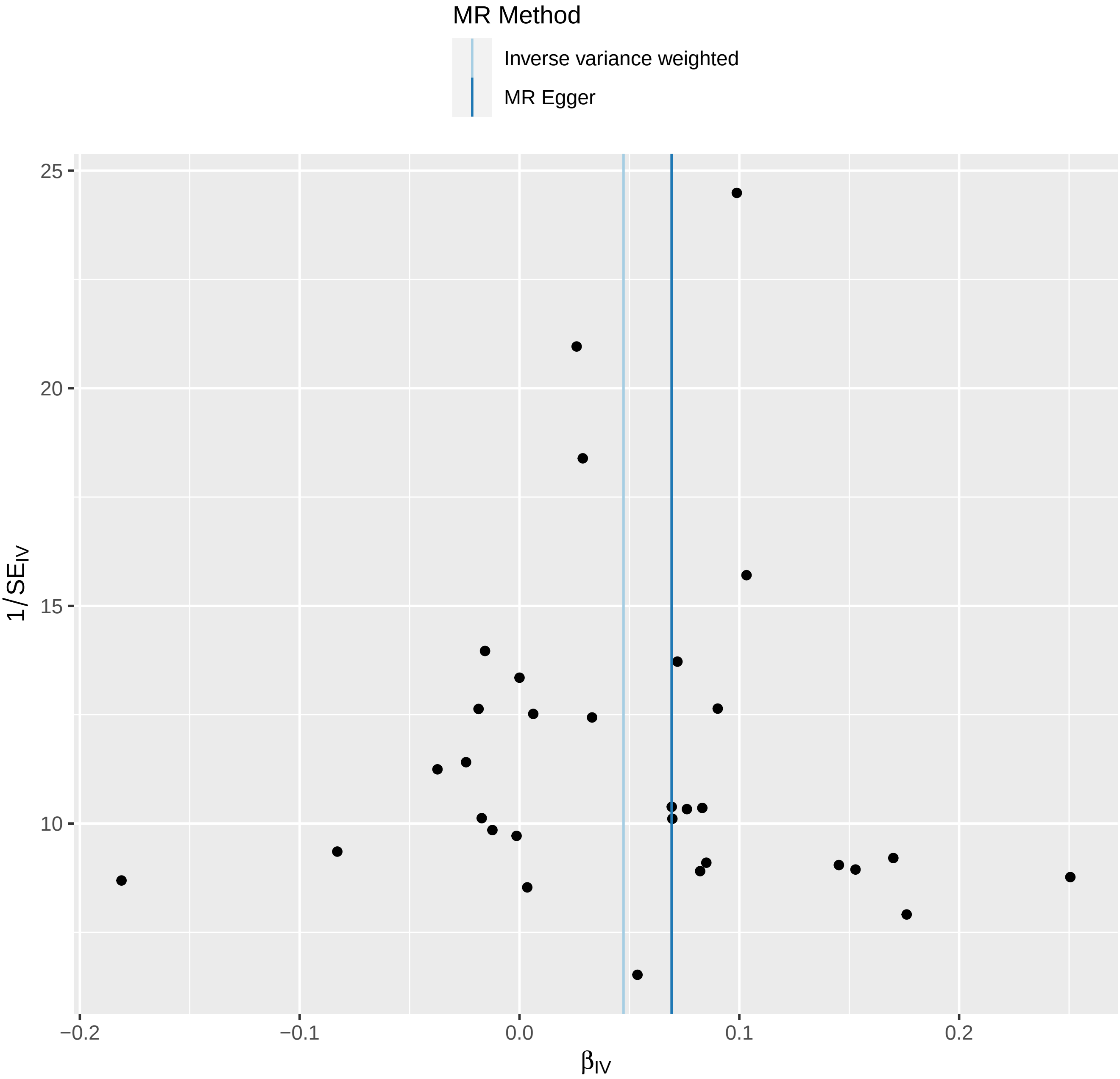

Batch 147 : Crohn's disease on IDP T2 FLAIR BIANCA WMH volume

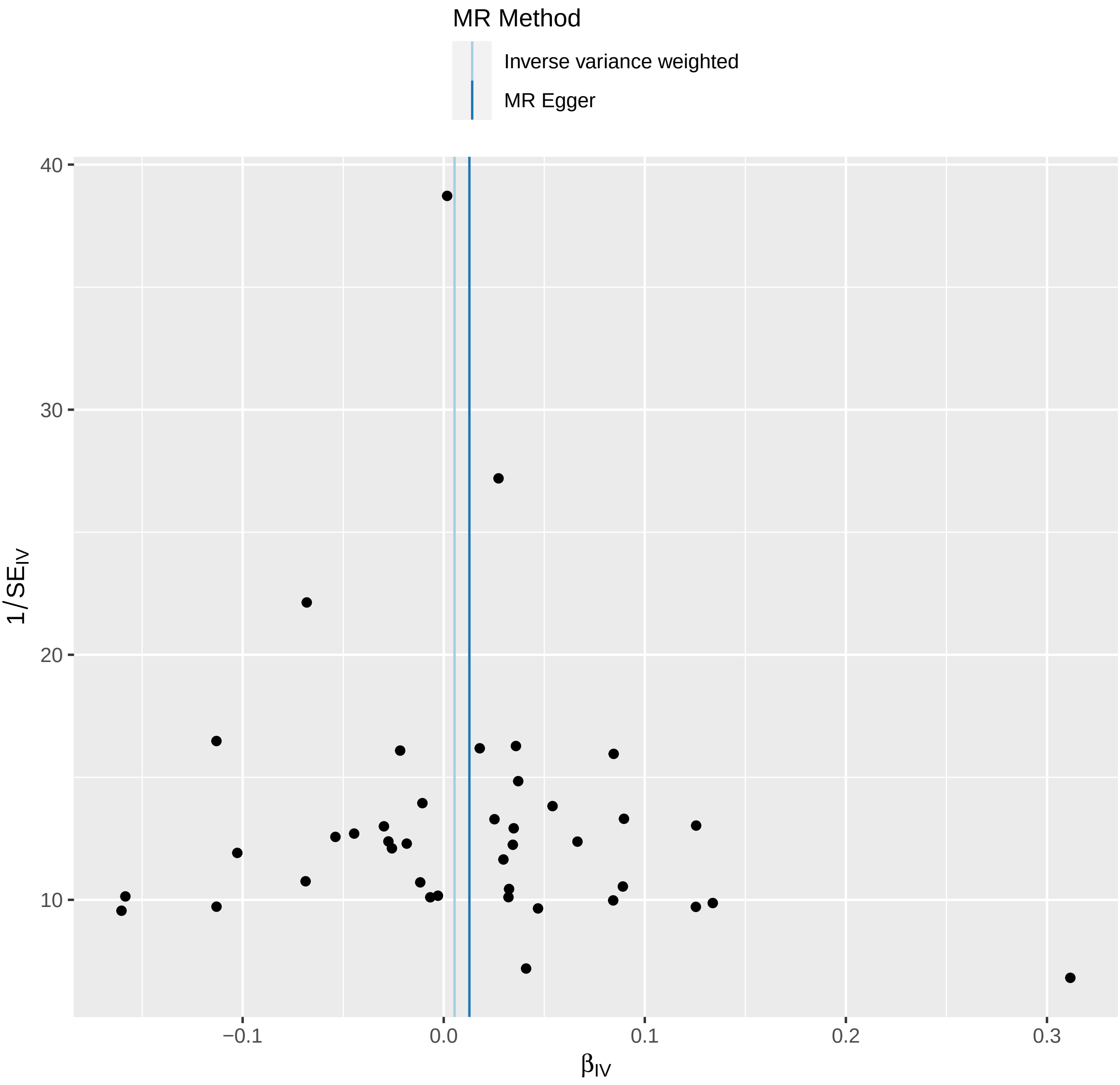

Batch 148 : Ulcerative colitis on IDP T2 FLAIR BIANCA WMH volume

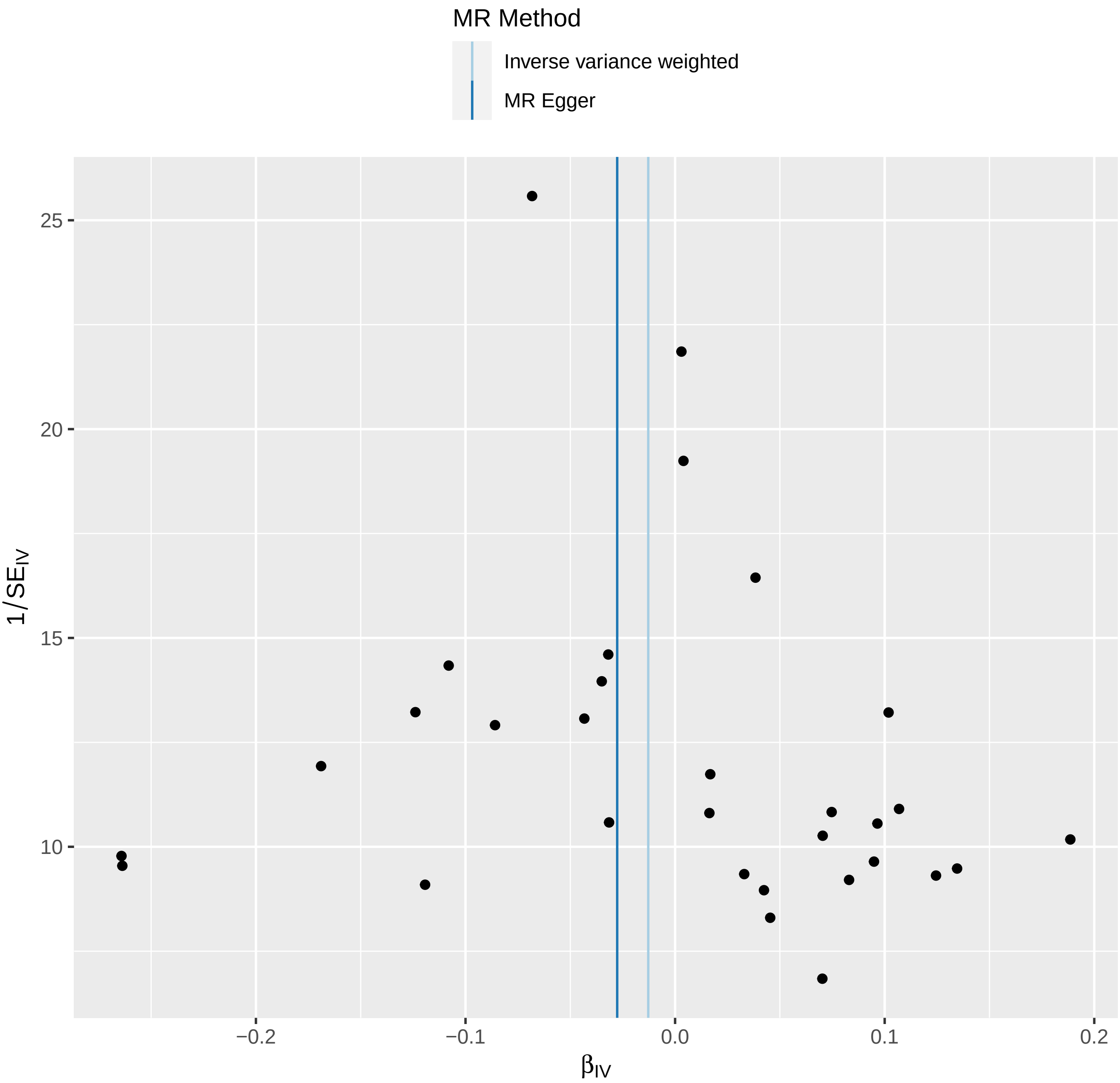

Batch 157 : Crohn's disease on IDP T1 FIRST left pallidum volume

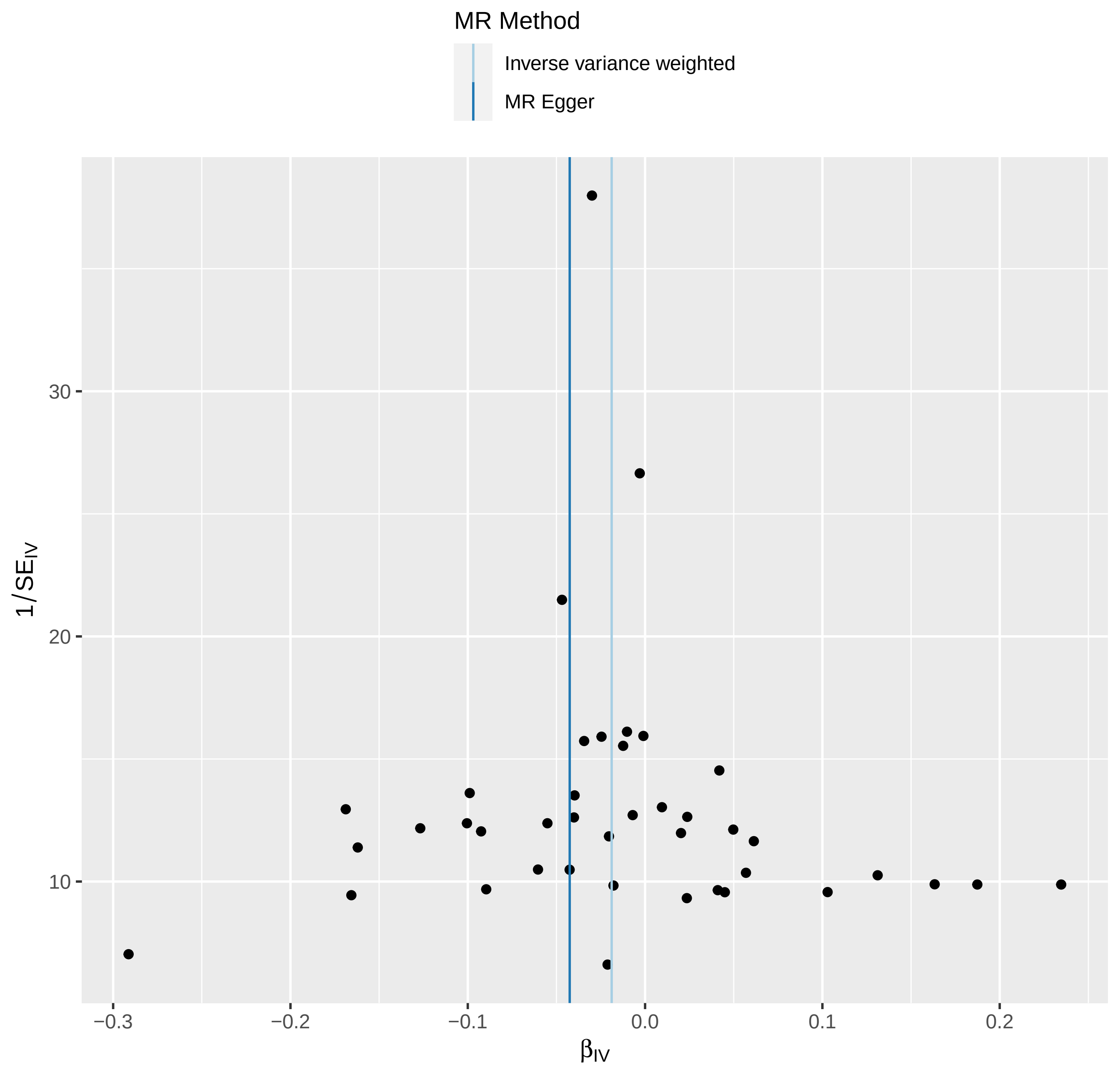

Batch 158 : Ulcerative colitis on IDP T1 FIRST left pallidum volume

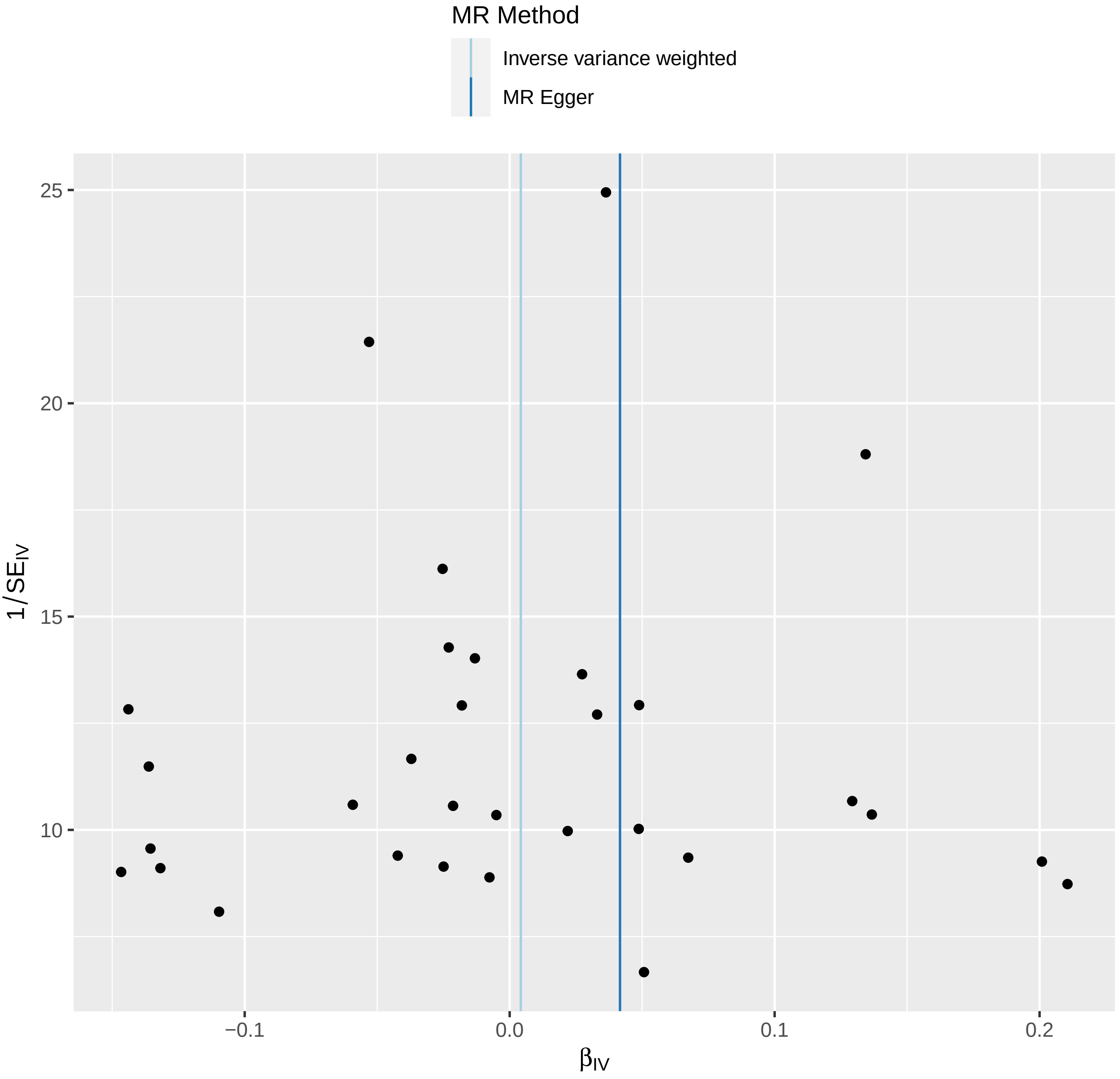

Batch 179 : Crohn's disease on IDP T1 FIRST right pallidum volume

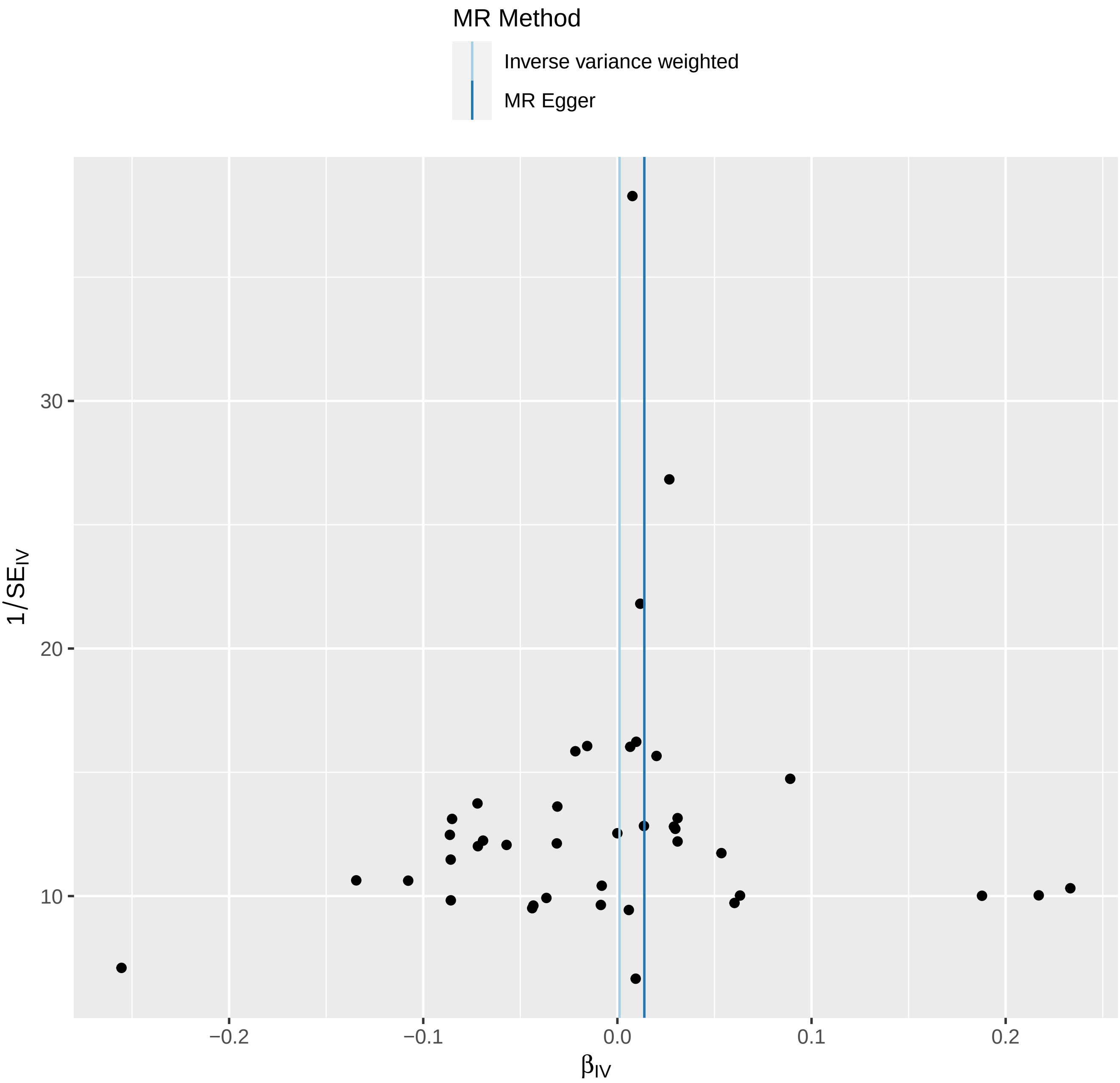

Batch 180 : Ulcerative colitis on IDP T1 FIRST right pallidum volume

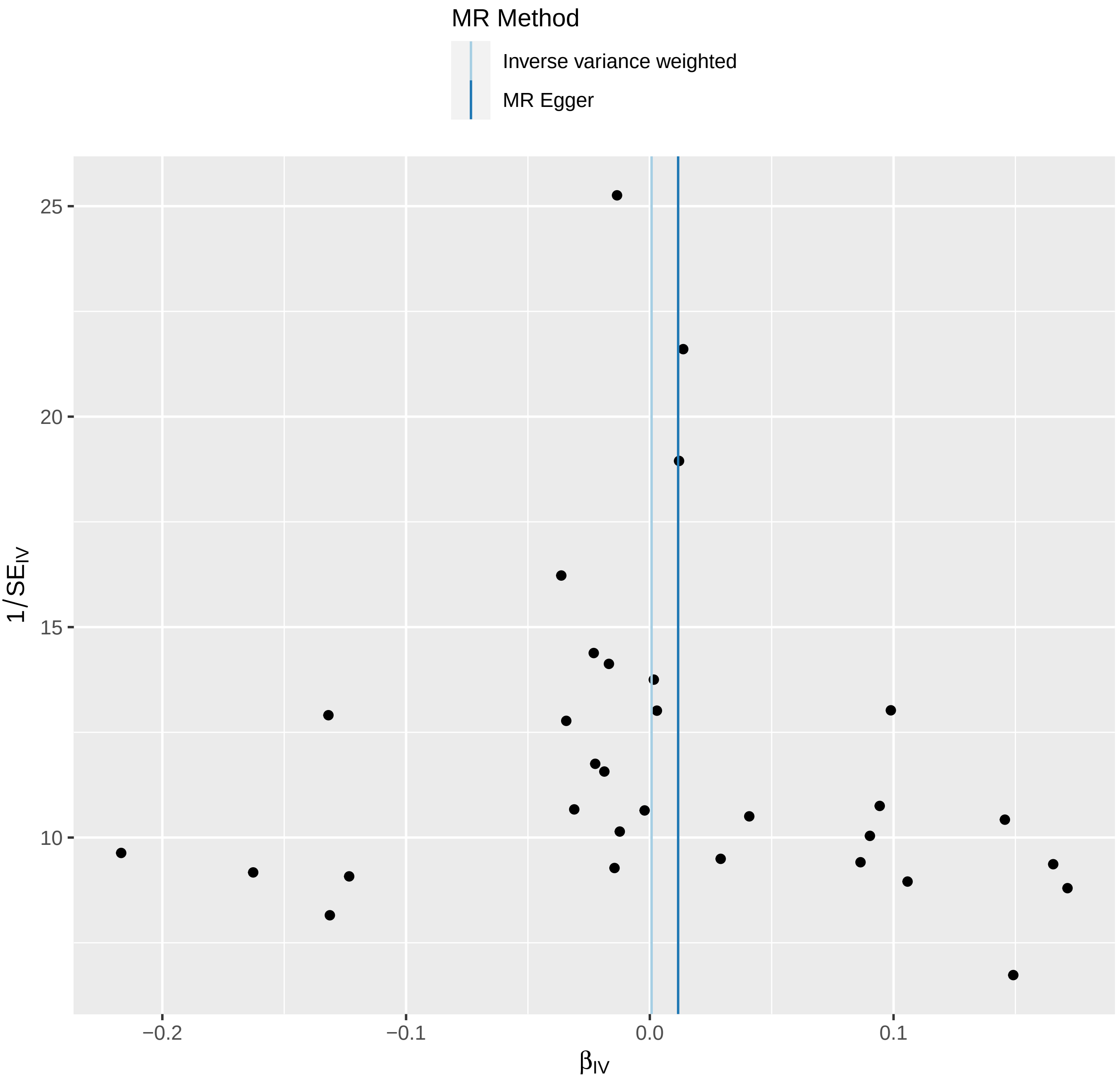

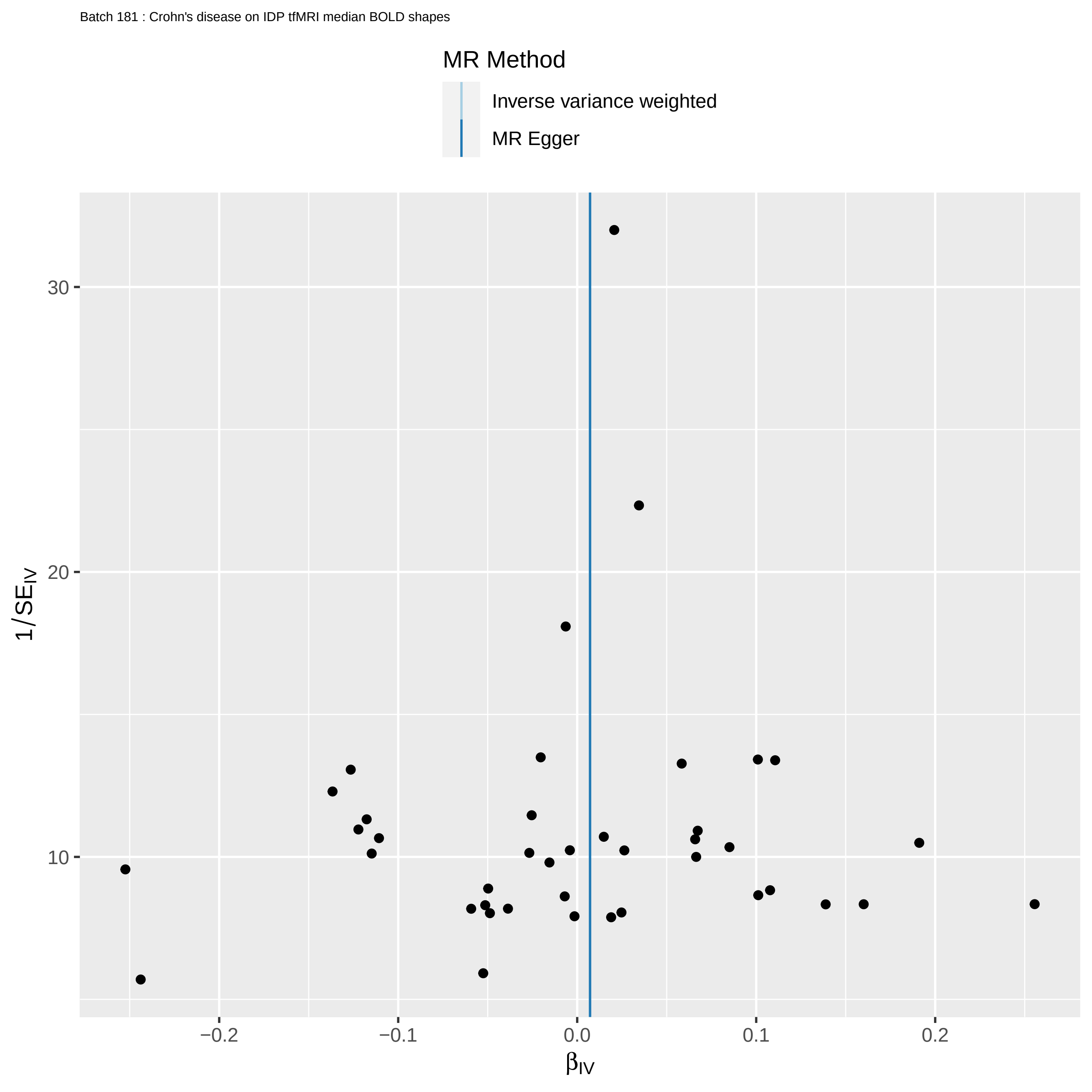

Batch 181 : Crohn's disease on IDP tfMRI median BOLD shapes
MR Method
Inverse variance weighted
MR Egger
30
20
10
1/SE_IV
−0.2
−0.1
0.0
0.1
0.2
β_IV

Batch 182 : Ulcerative colitis on IDP tfMRI median BOLD shapes

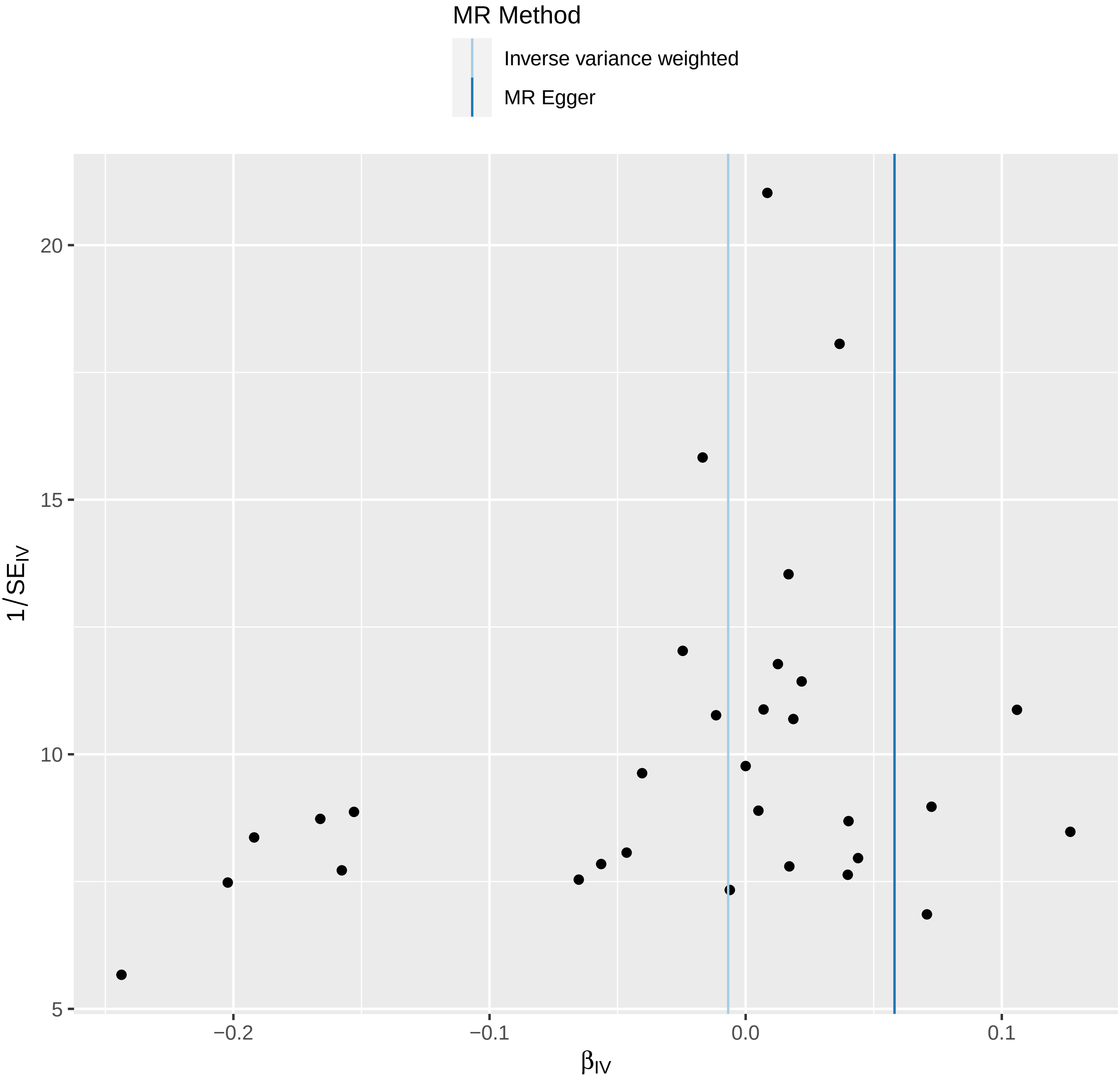

Batch 183 : Crohn's disease on IDP tfMRI 90th−percentile BOLD shapes

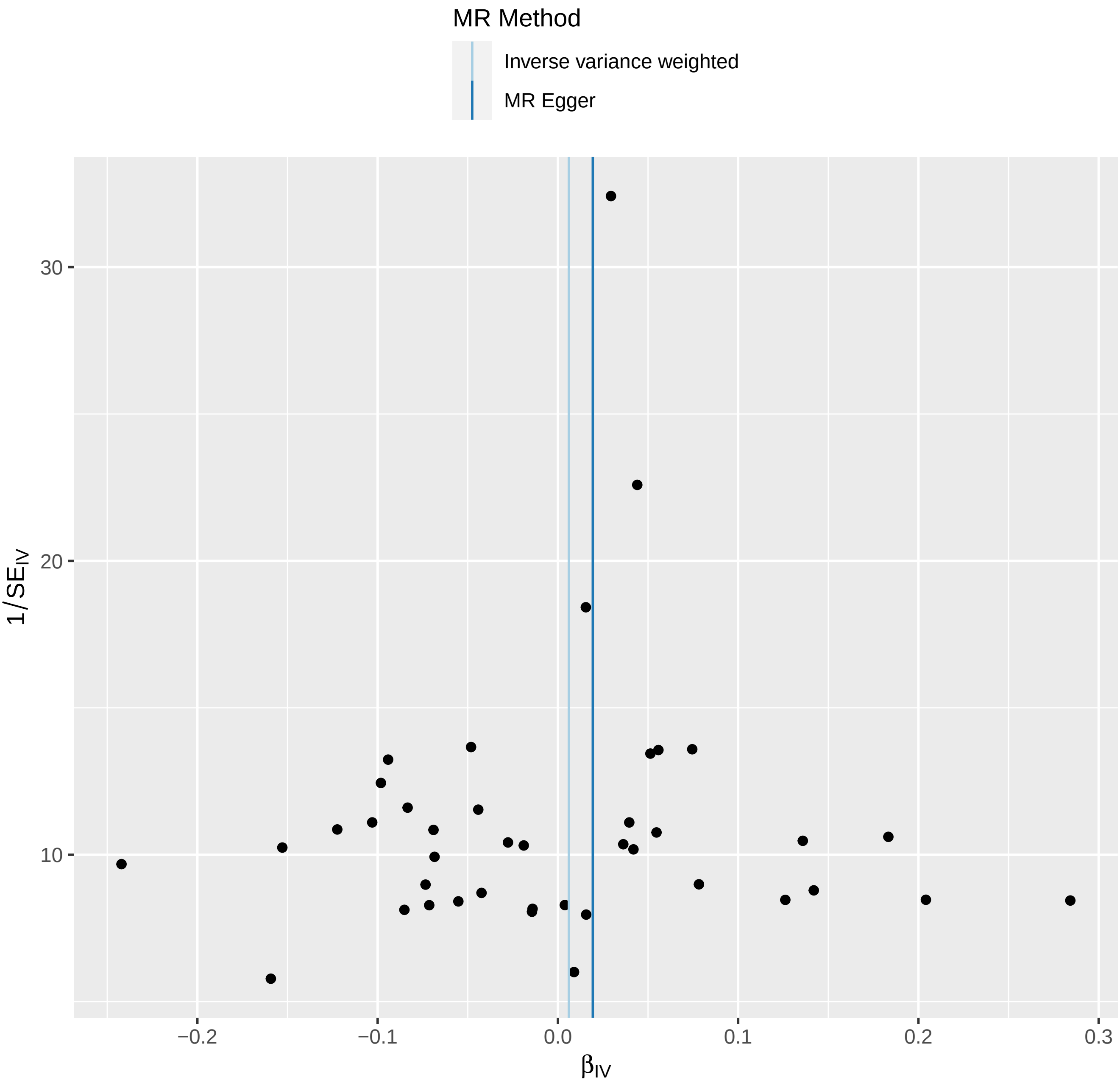

Batch 184 : Ulcerative colitis on IDP tfMRI 90th−percentile BOLD shapes

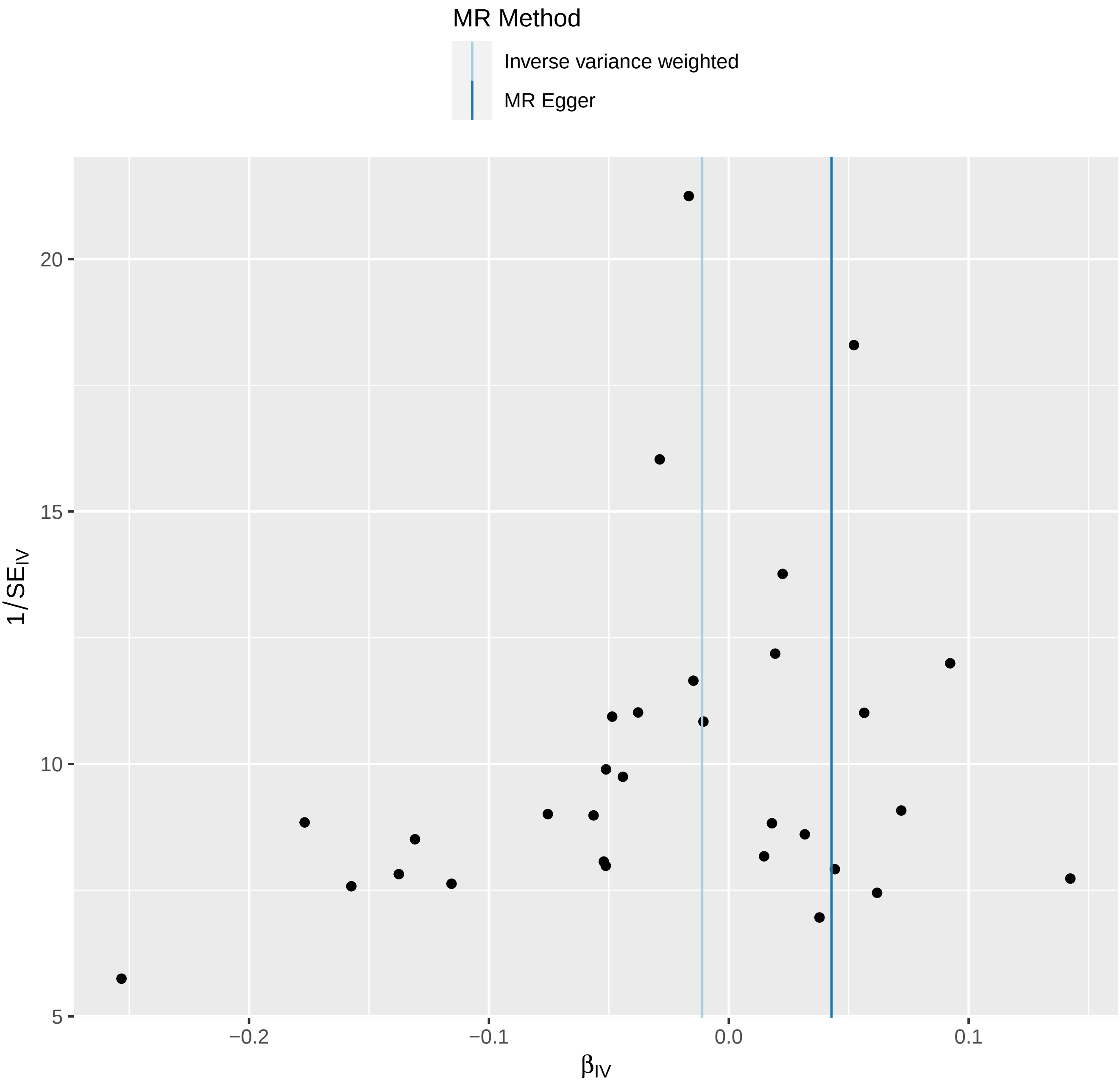

Batch 185 : Crohn's disease on IDP tfMRI median zstat shapes

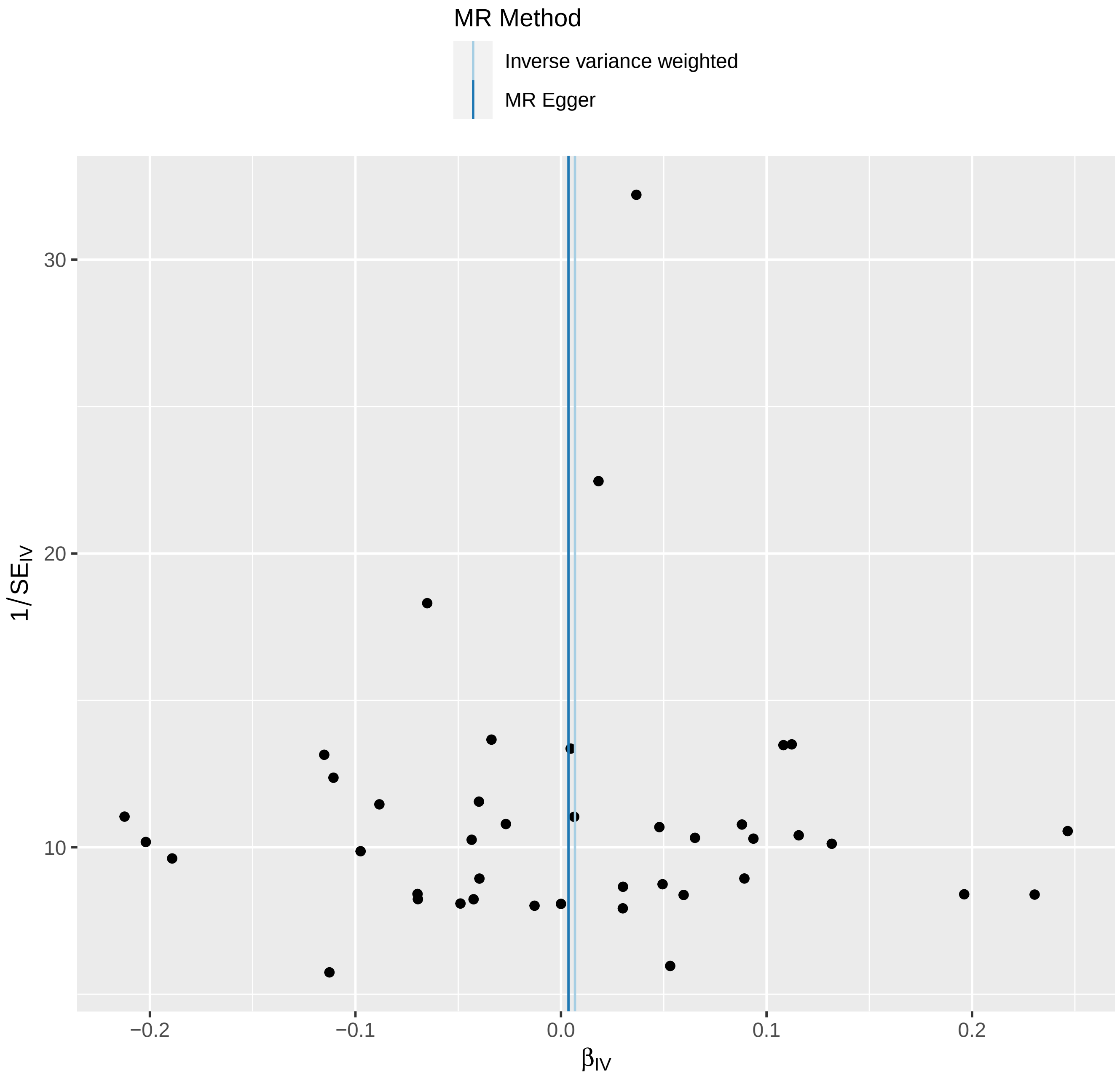

Batch 186 : Ulcerative colitis on IDP tfMRI median zstat shapes

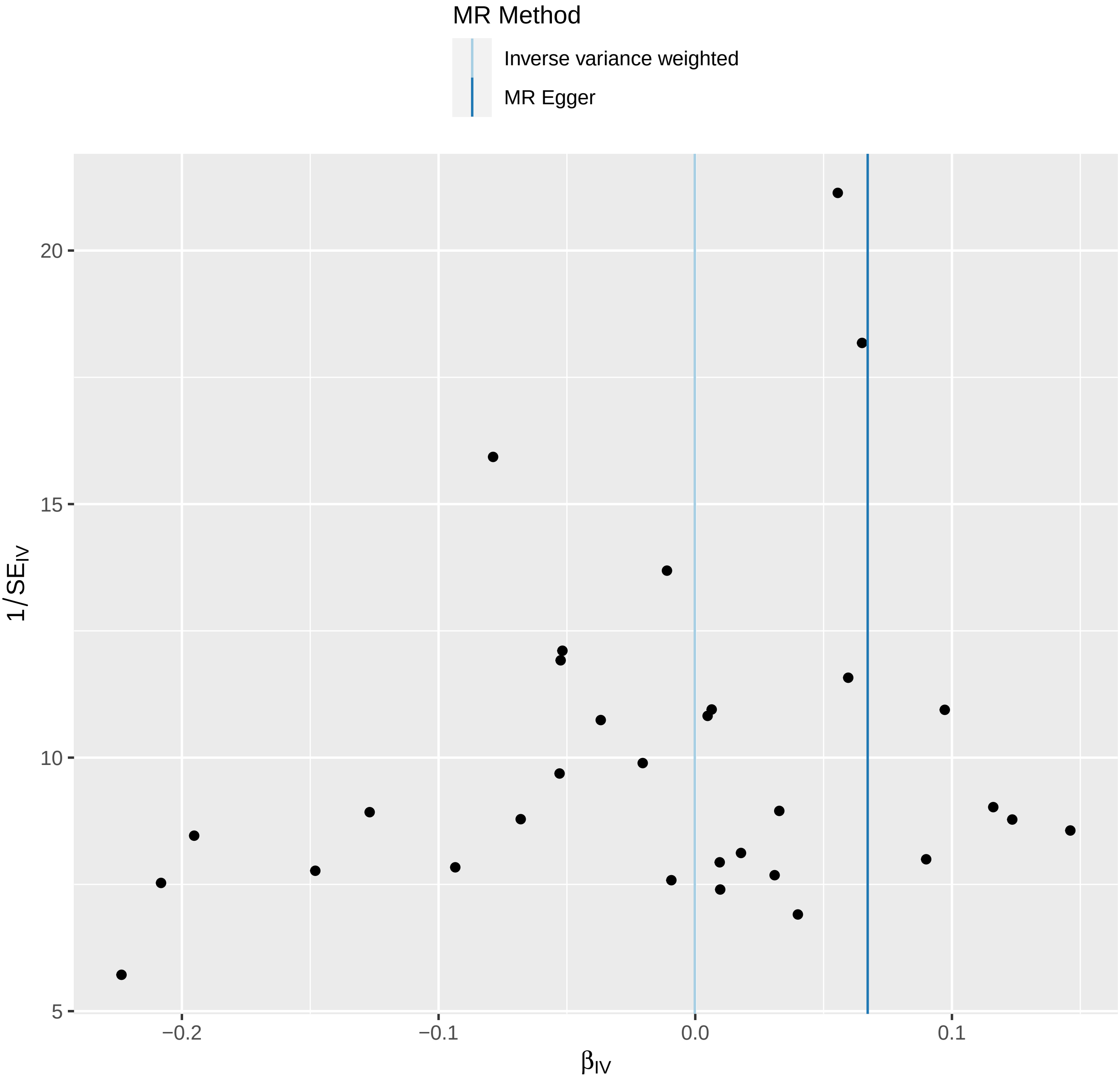

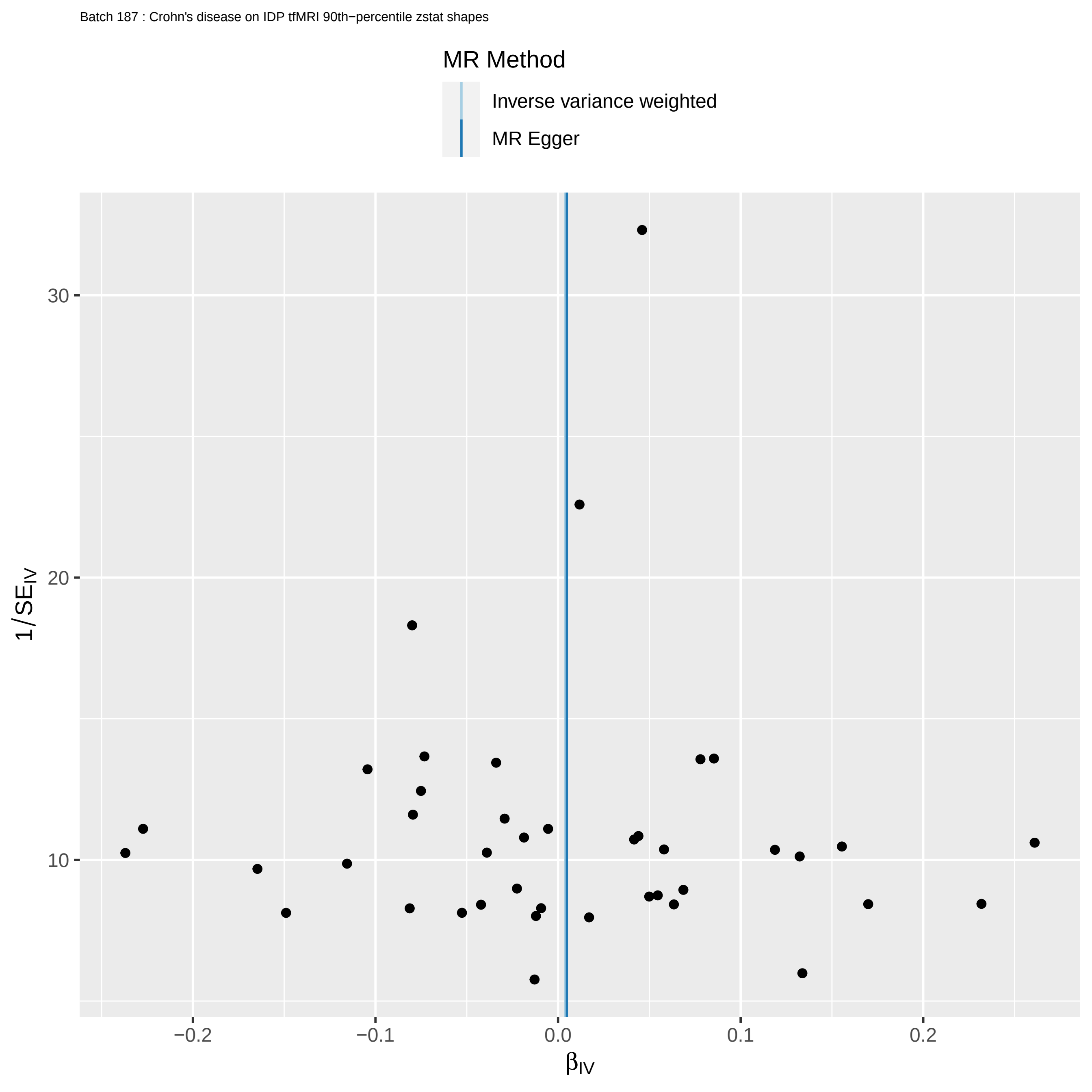
Batch 187 : Crohn's disease on IDP tfMRI 90th−percentile zstat shapes
MR Method
Inverse variance weighted
MR Egger
30
20
10
1/SE$_{IV}$
−0.2
−0.1
0.0
0.1
0.2
β$_{IV}$

Batch 188 : Ulcerative colitis on IDP tfMRI 90th−percentile zstat shapes

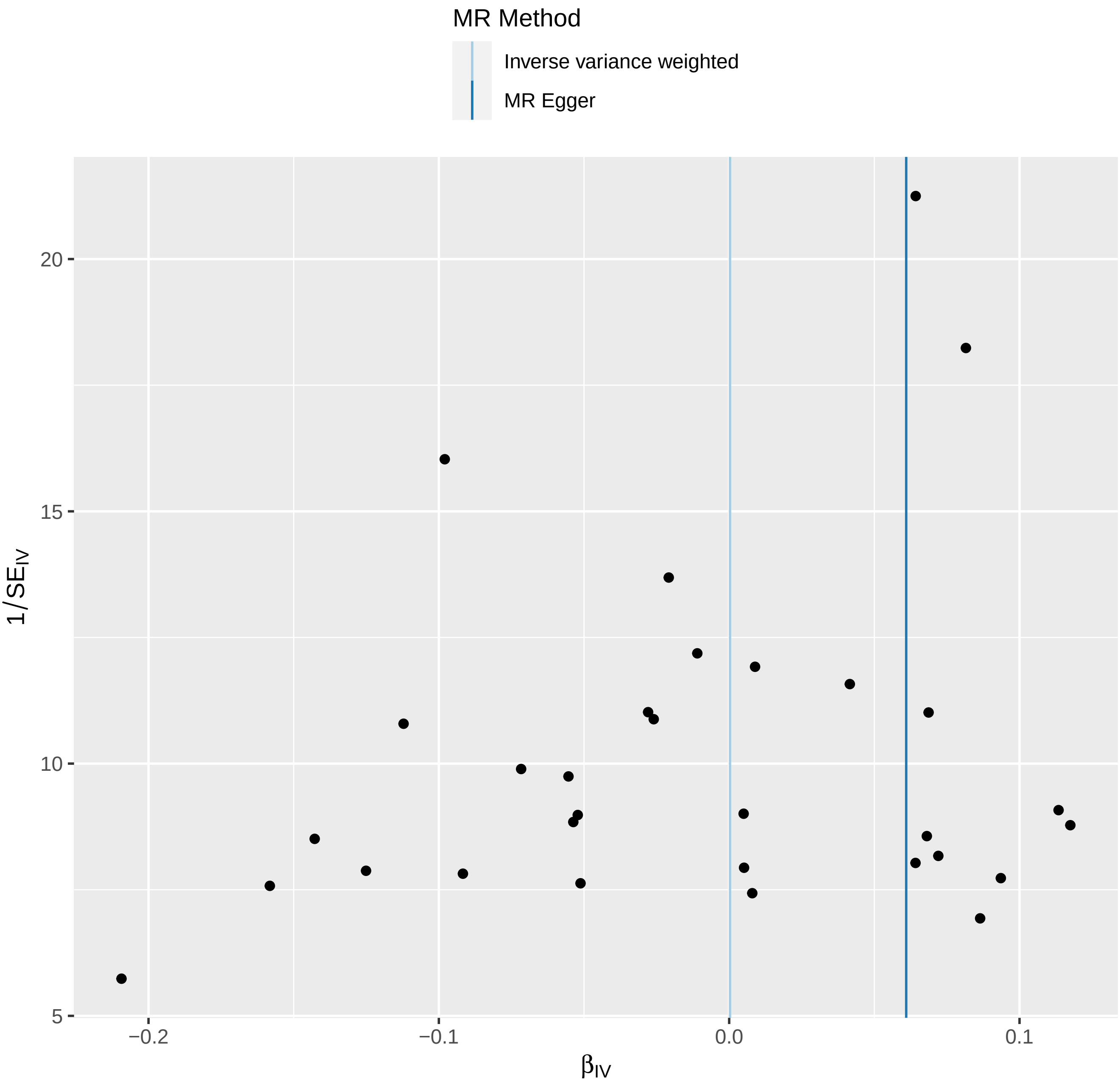

Batch 189 : Crohn's disease on IDP tfMRI median BOLD faces

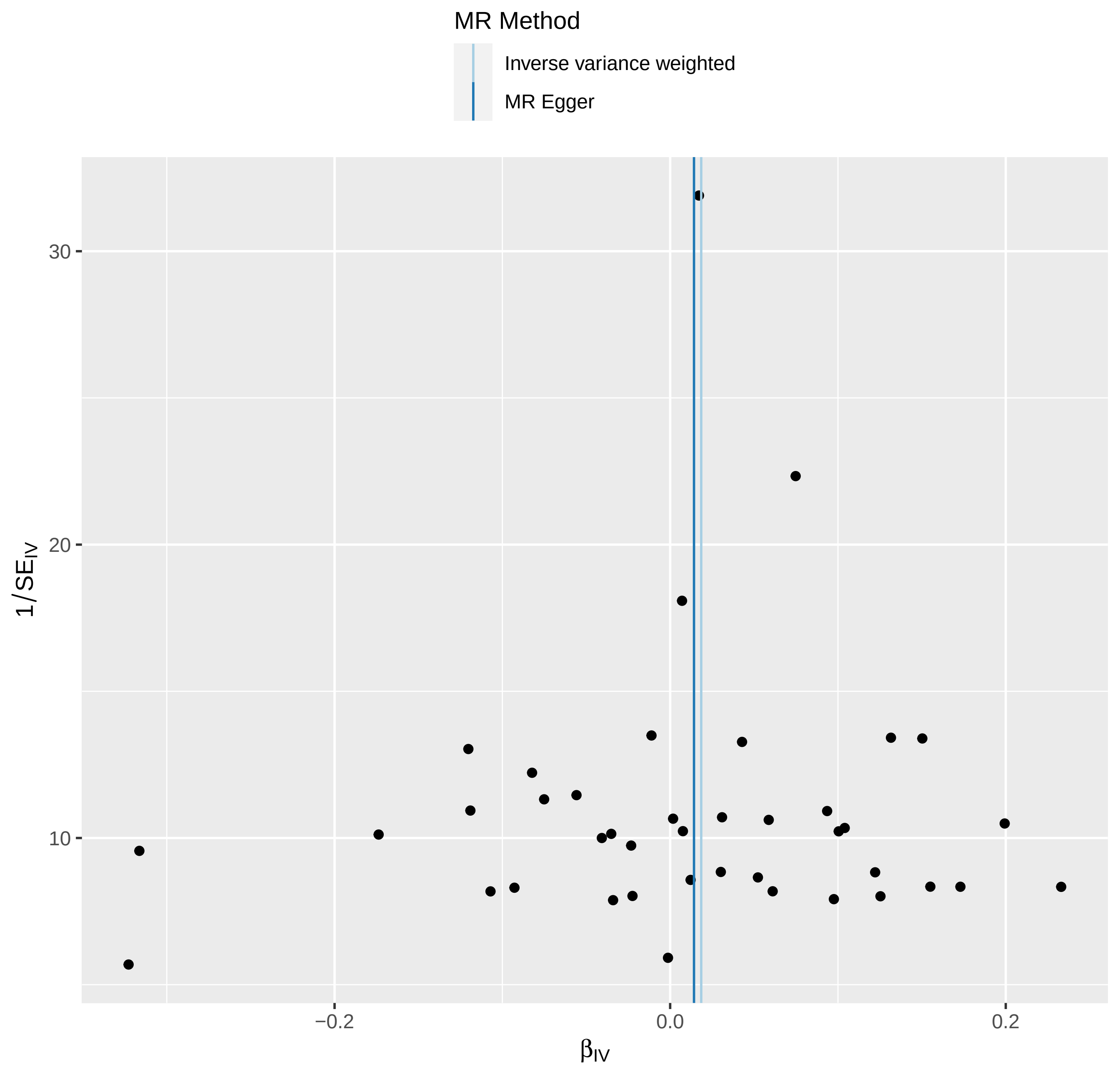

Batch 190 : Ulcerative colitis on IDP tfMRI median BOLD faces

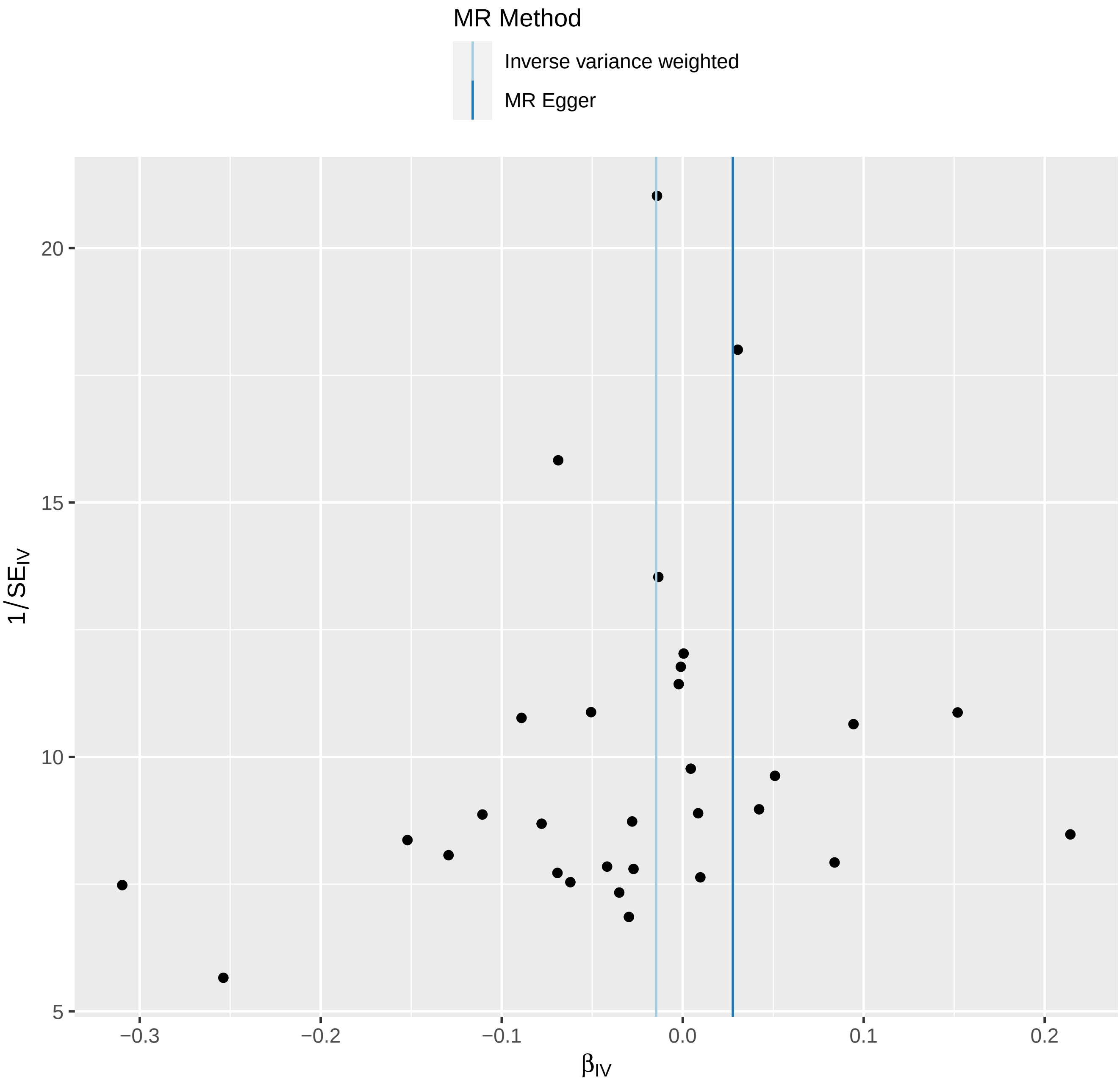

Batch 191 : Crohn's disease on IDP tfMRI 90th−percentile BOLD faces

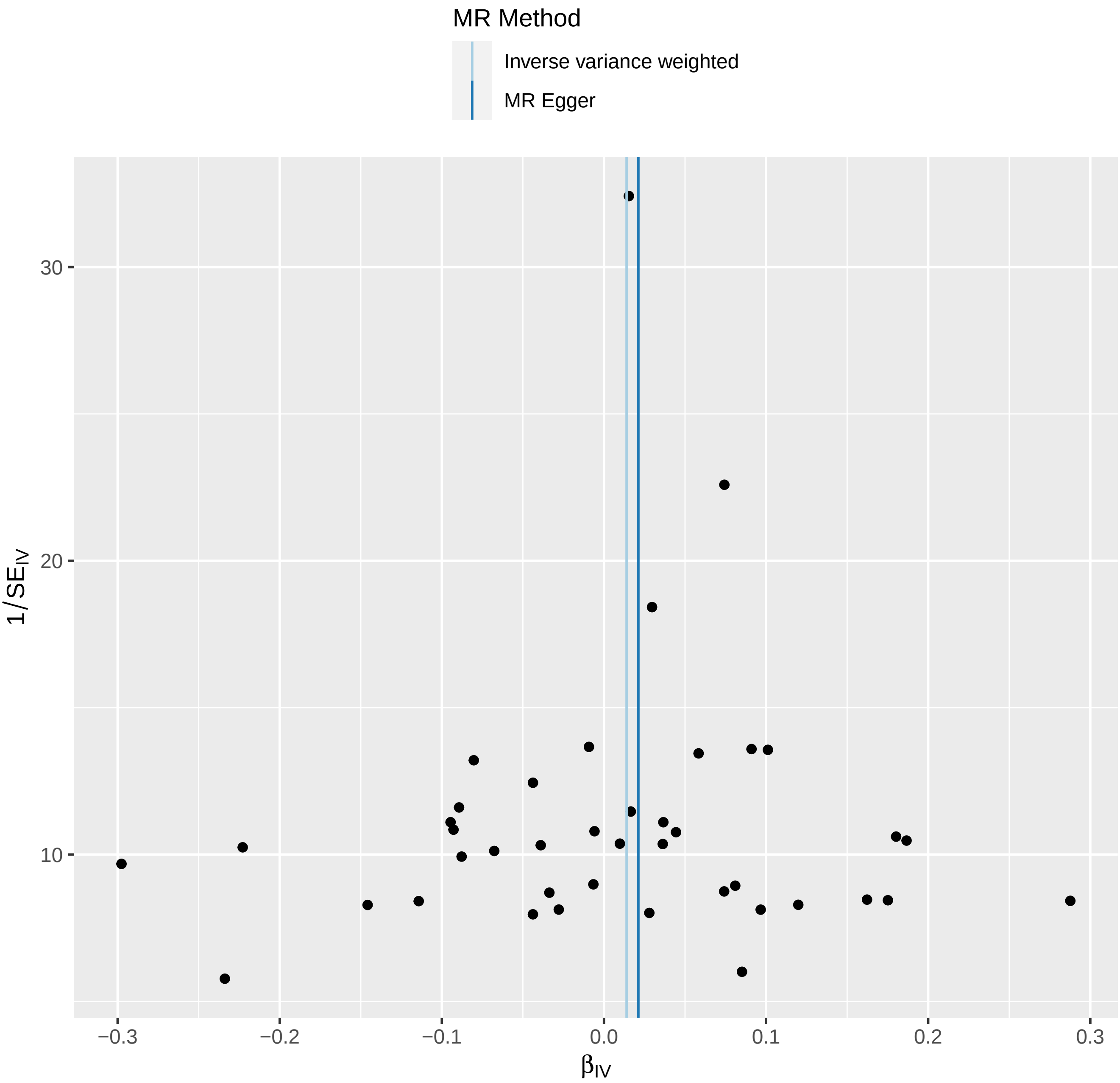

Batch 192 : Ulcerative colitis on IDP tfMRI 90th−percentile BOLD faces

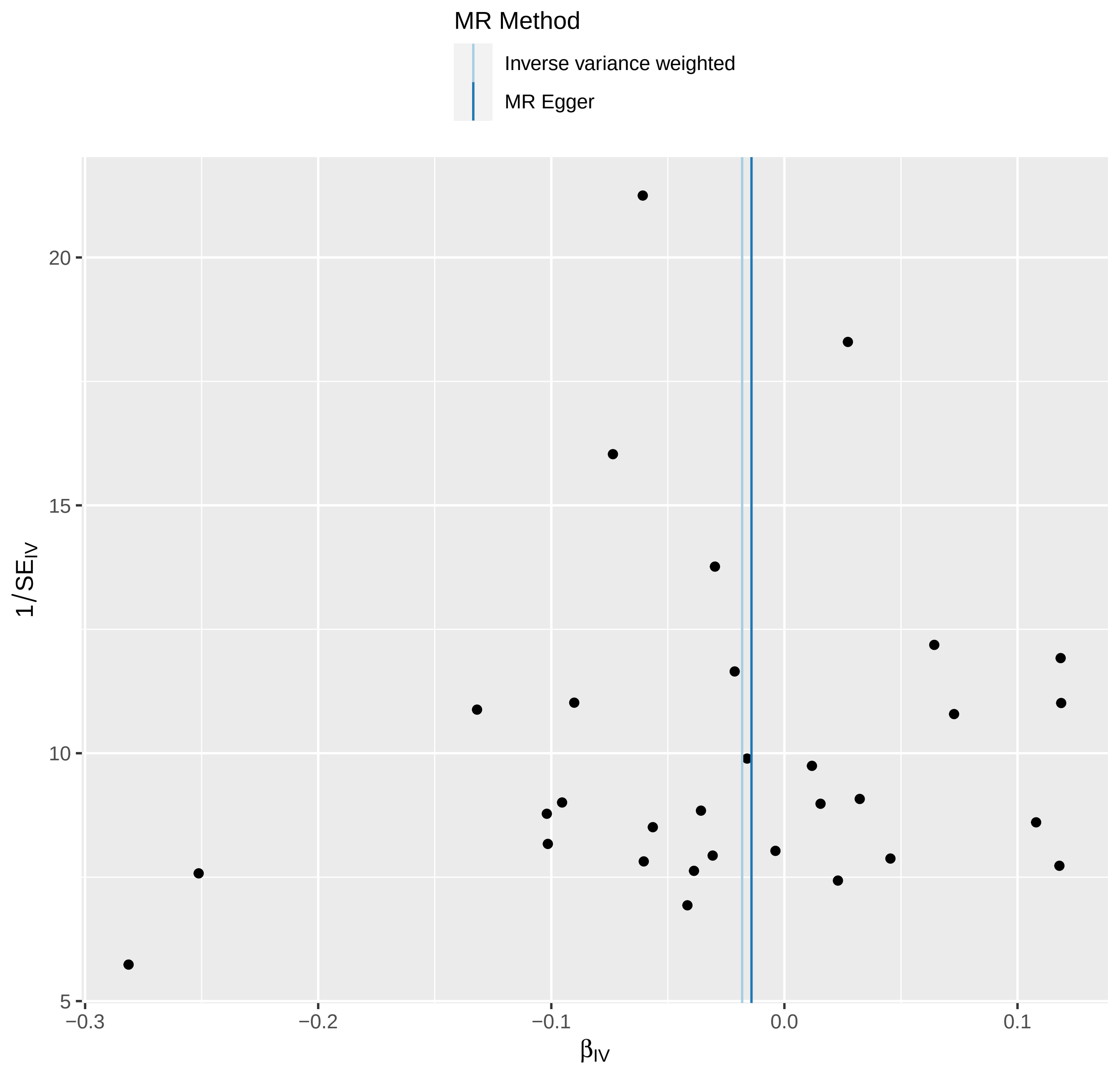

Batch 193 : Crohn's disease on IDP tfMRI median zstat faces

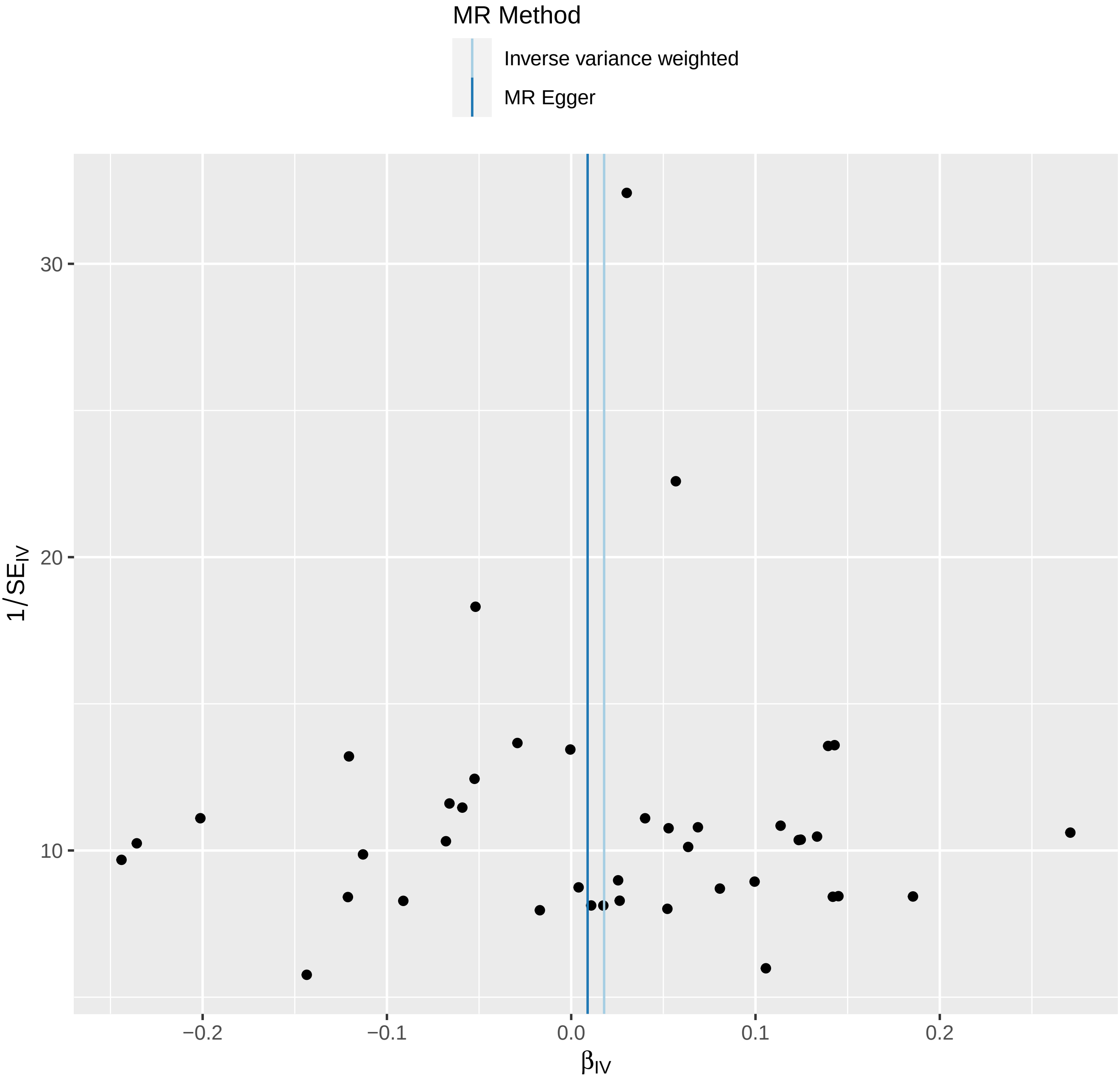

Batch 194 : Ulcerative colitis on IDP tfMRI median zstat faces

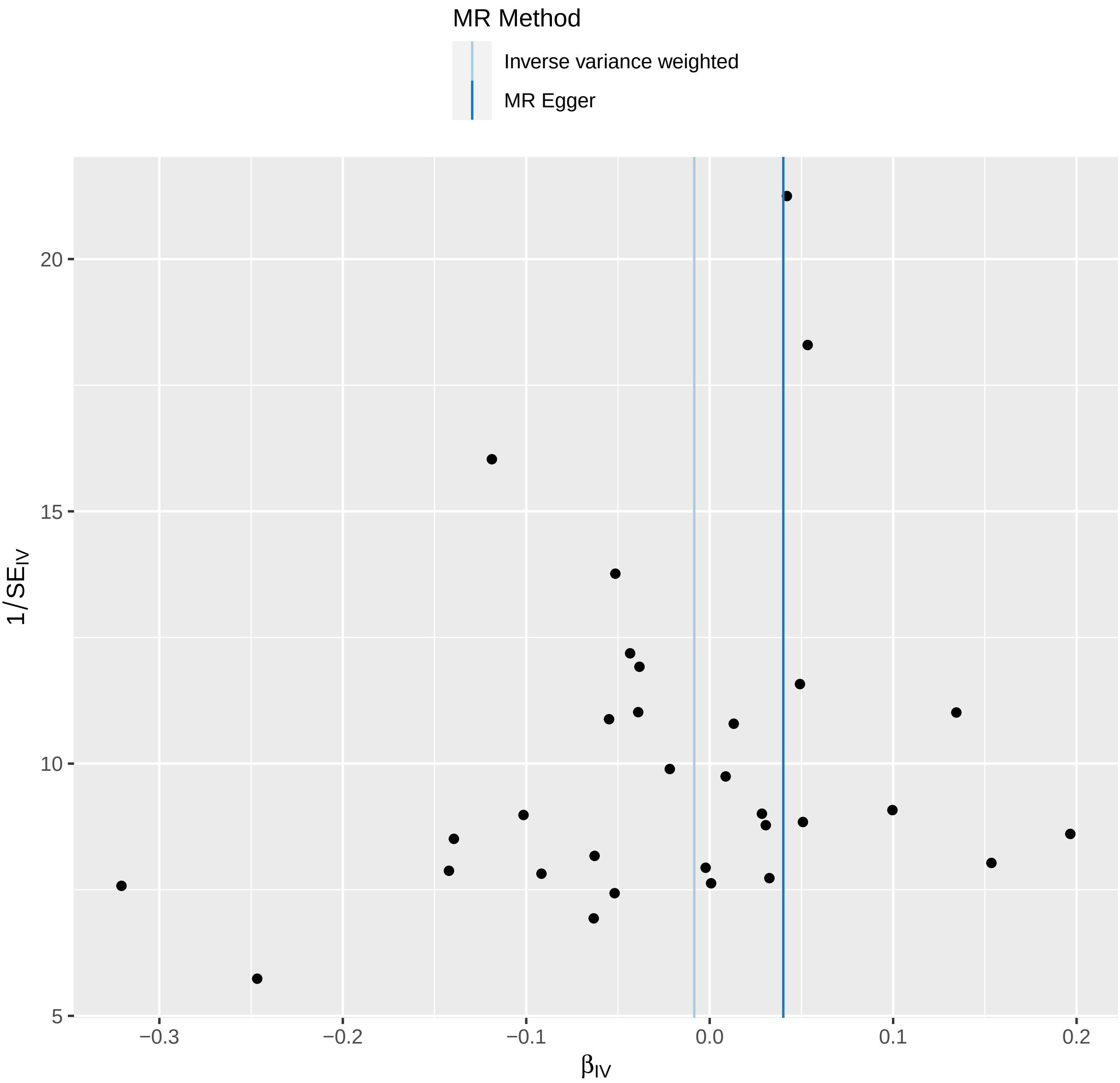

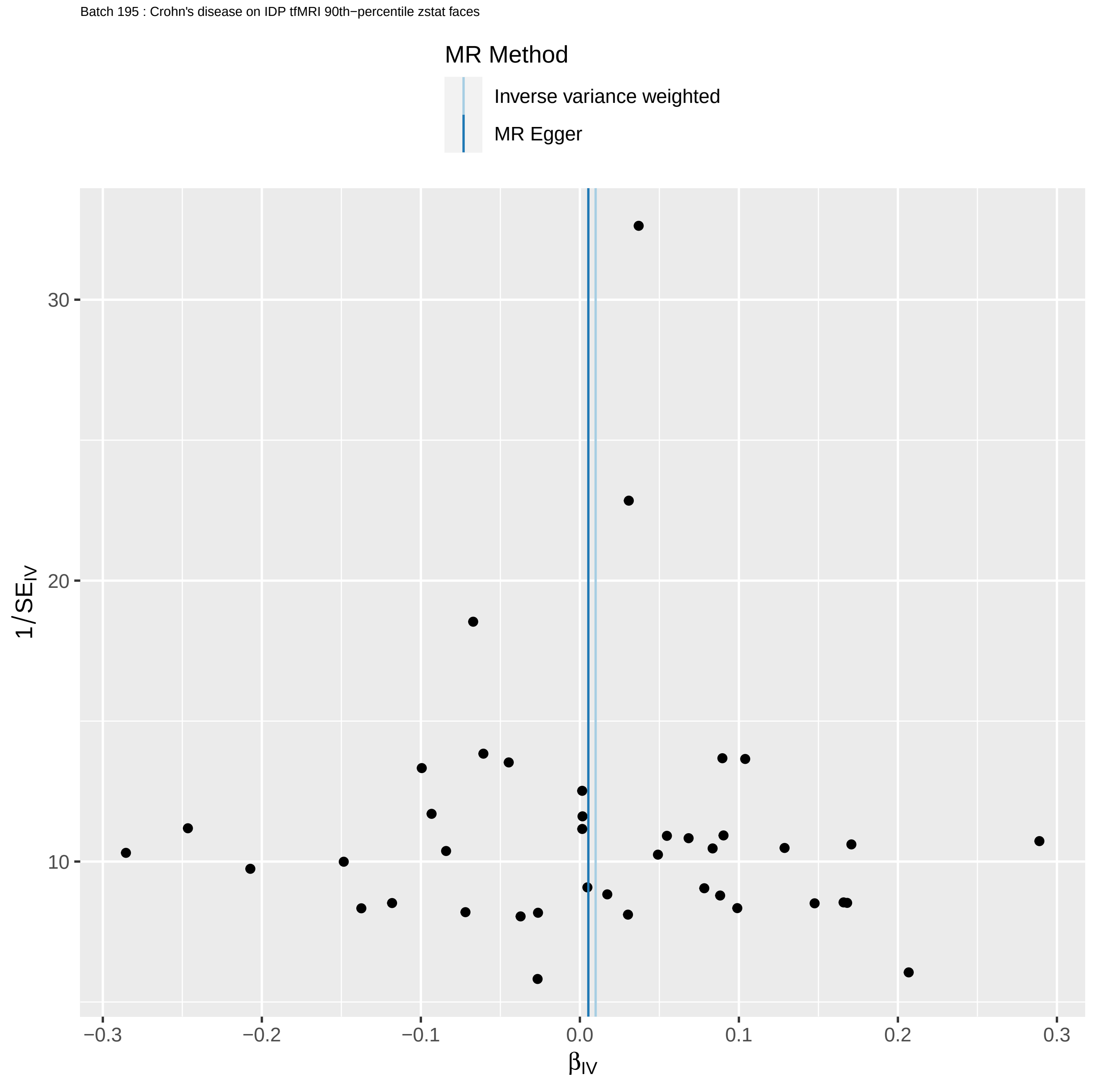

Batch 195 : Crohn's disease on IDP tfMRI 90th−percentile zstat faces
MR Method
Inverse variance weighted
MR Egger
30
20
10
$1/SE_{IV}$
−0.3
−0.2
−0.1
0.0
0.1
0.2
0.3
β_{IV}

Batch 196 : Ulcerative colitis on IDP tfMRI 90th−percentile zstat faces

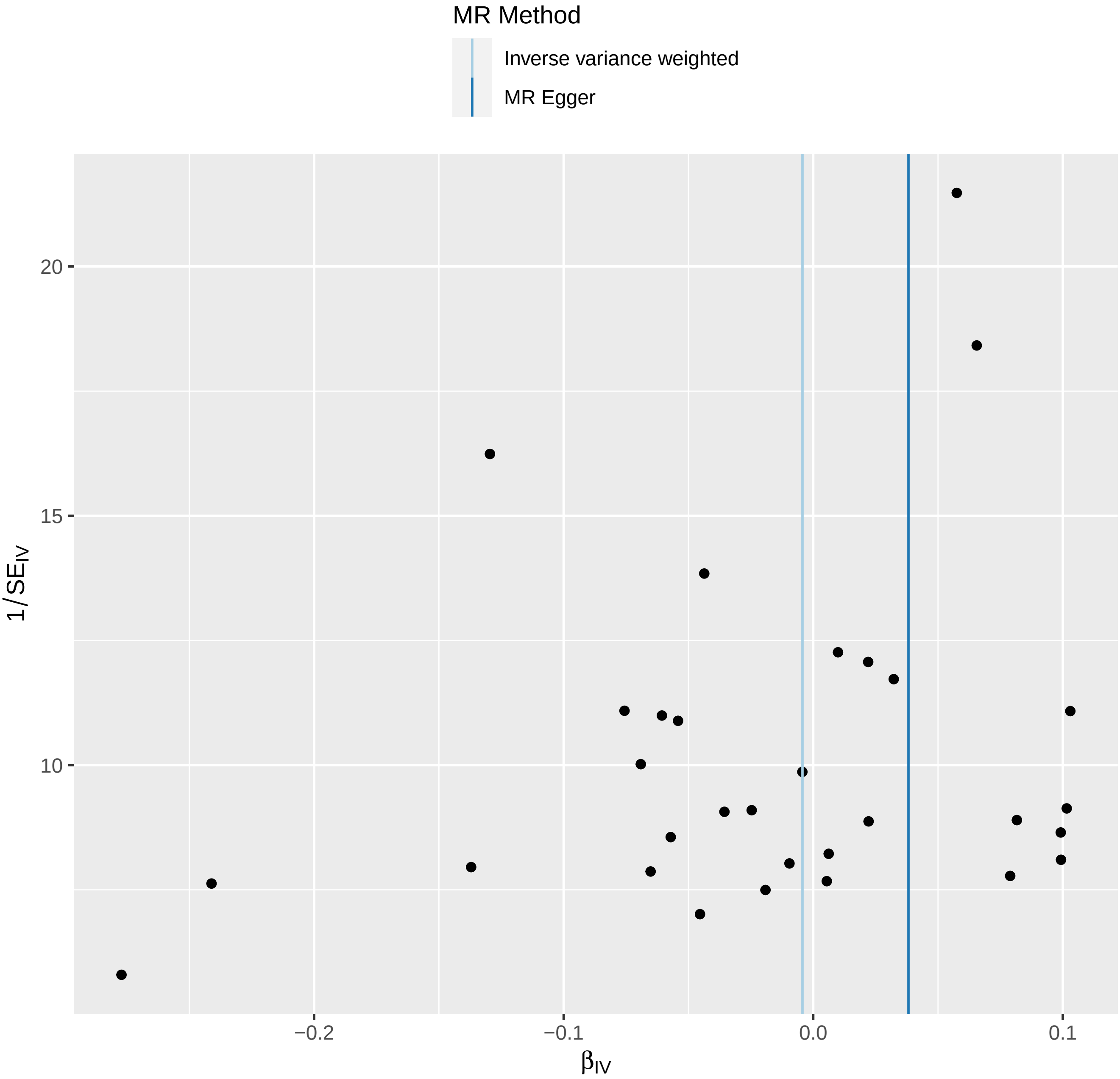

Batch 197 : Crohn's disease on IDP tfMRI median BOLD faces−shapes

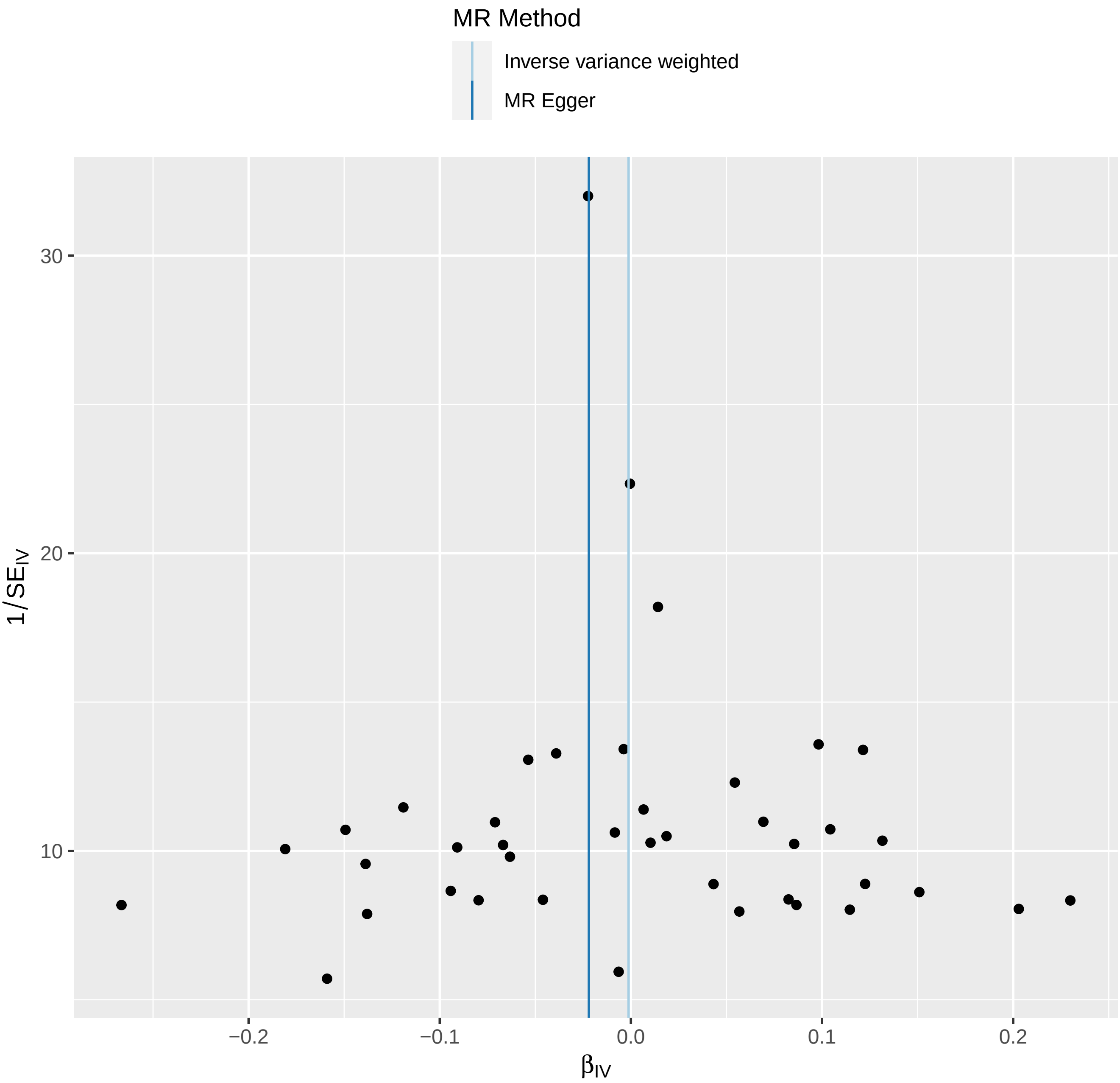

Batch 198 : Ulcerative colitis on IDP tfMRI median BOLD faces−shapes

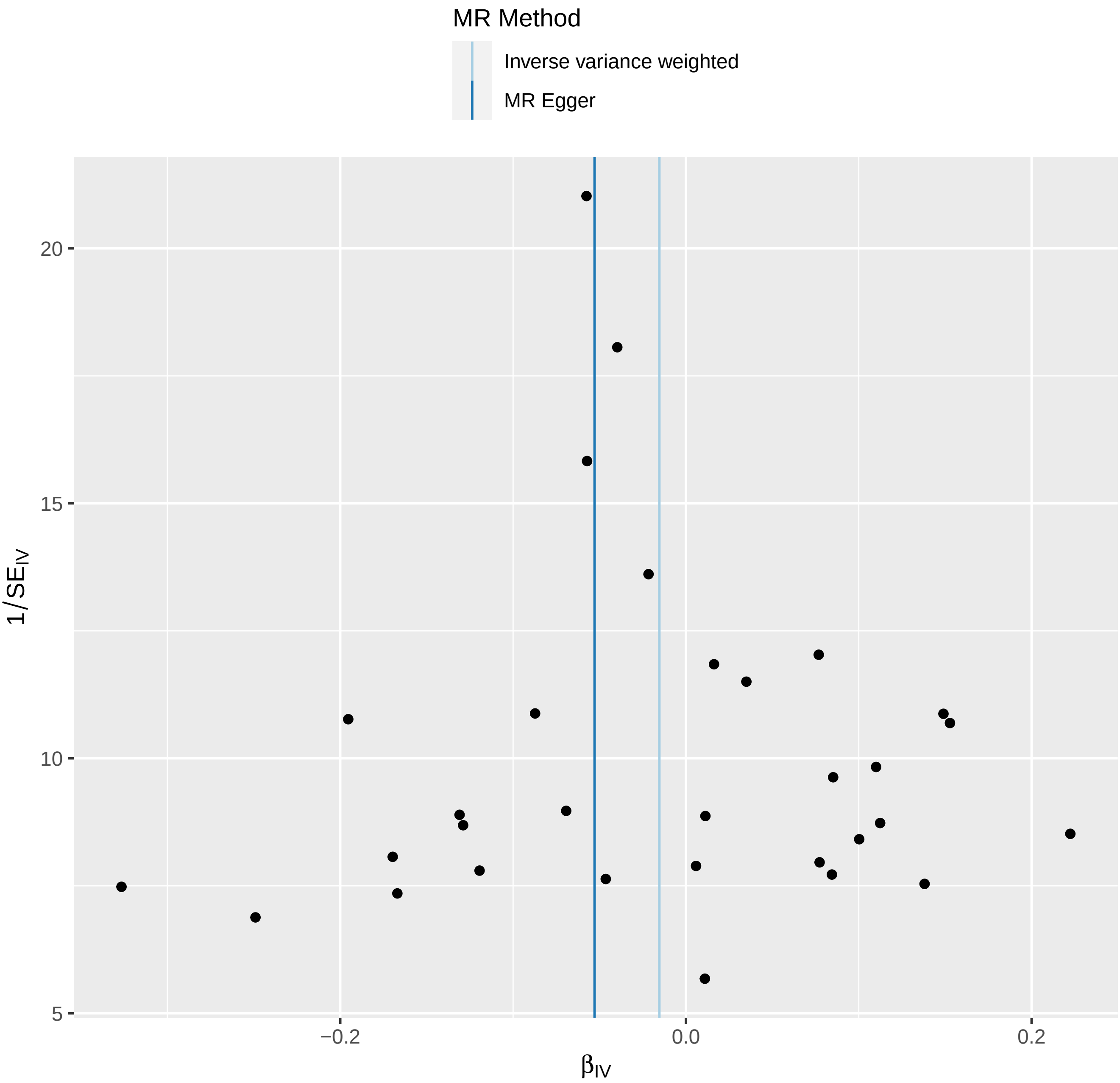

Batch 199 : Crohn's disease on IDP tfMRI 90th−percentile BOLD faces−shapes

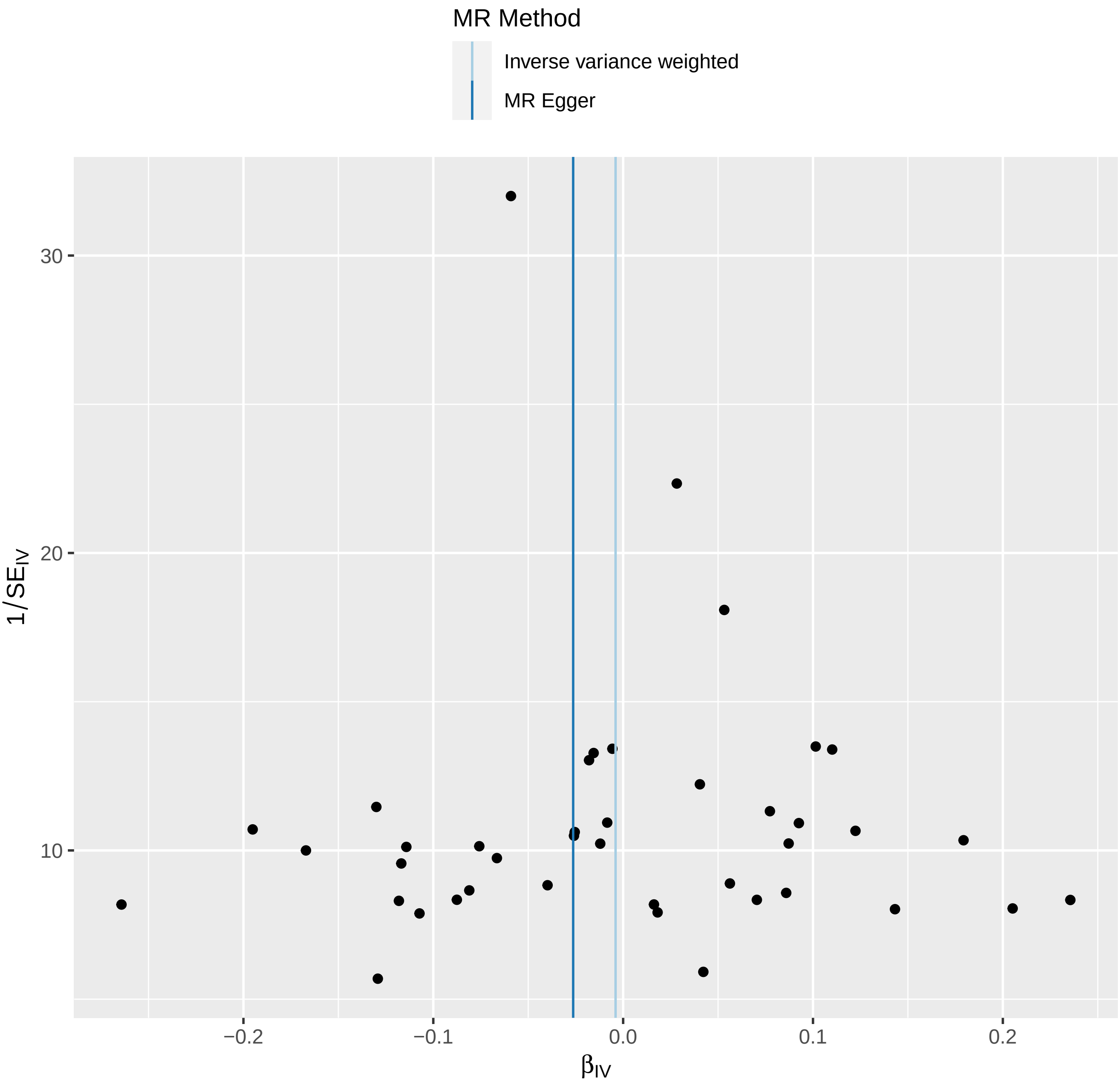

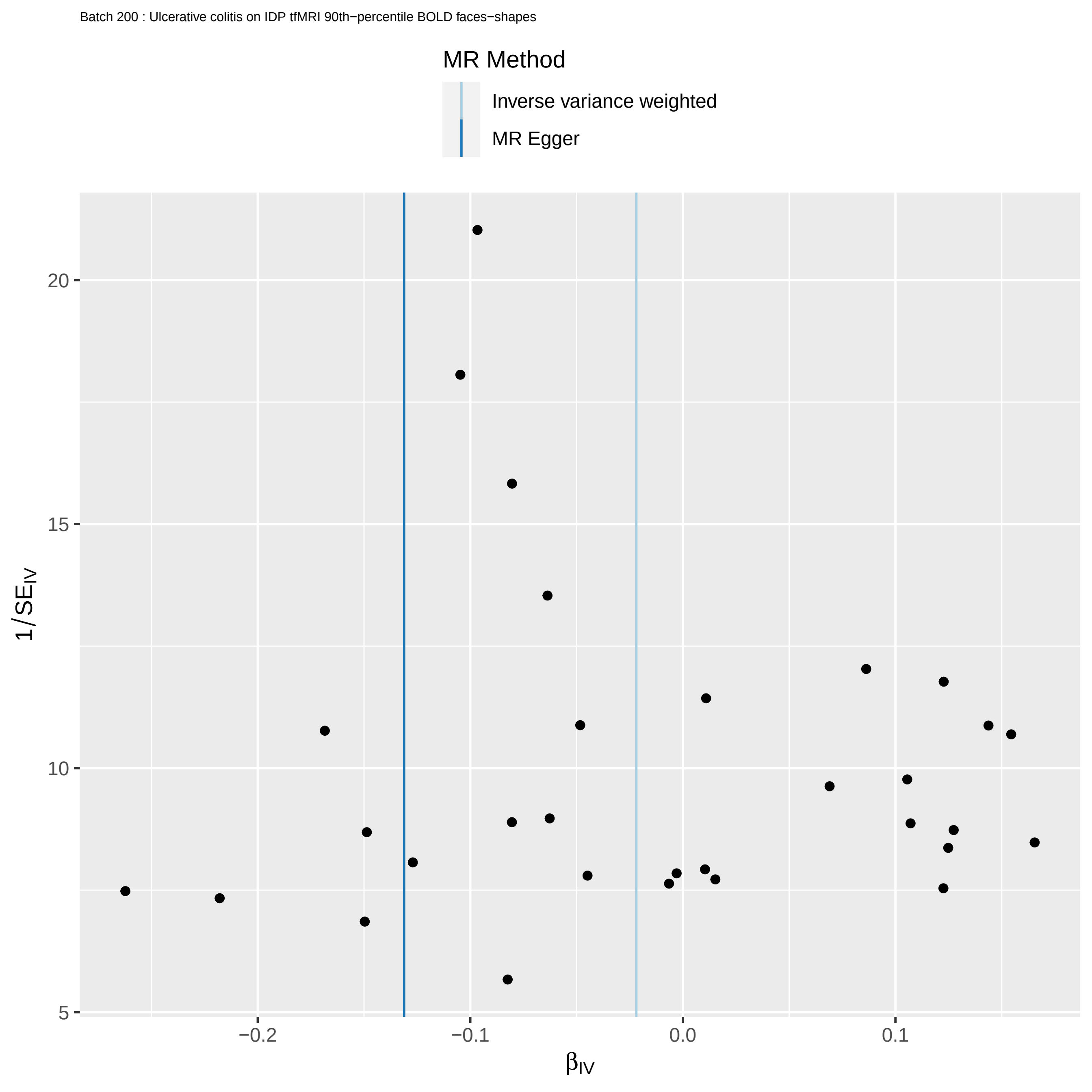

Batch 200 : Ulcerative colitis on IDP tfMRI 90th−percentile BOLD faces−shapes
MR Method
Inverse variance weighted
MR Egger
1/SE$_{IV}$
20
15
10
5
−0.2
−0.1
0.0
0.1
β$_{IV}$

Batch 201 : Crohn's disease on IDP T1 FIRST left hippocampus volume

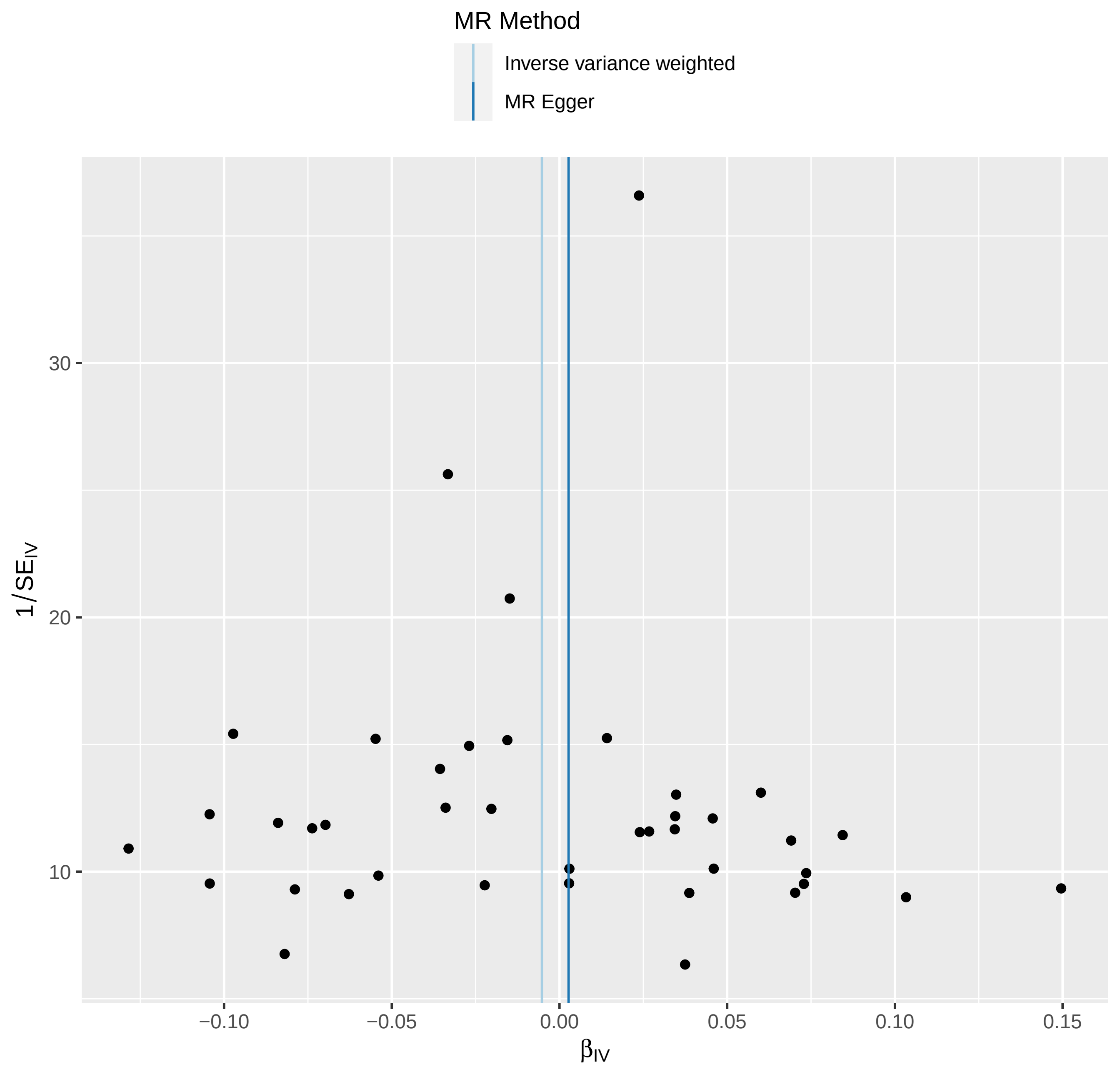

Batch 202 : Ulcerative colitis on IDP T1 FIRST left hippocampus volume

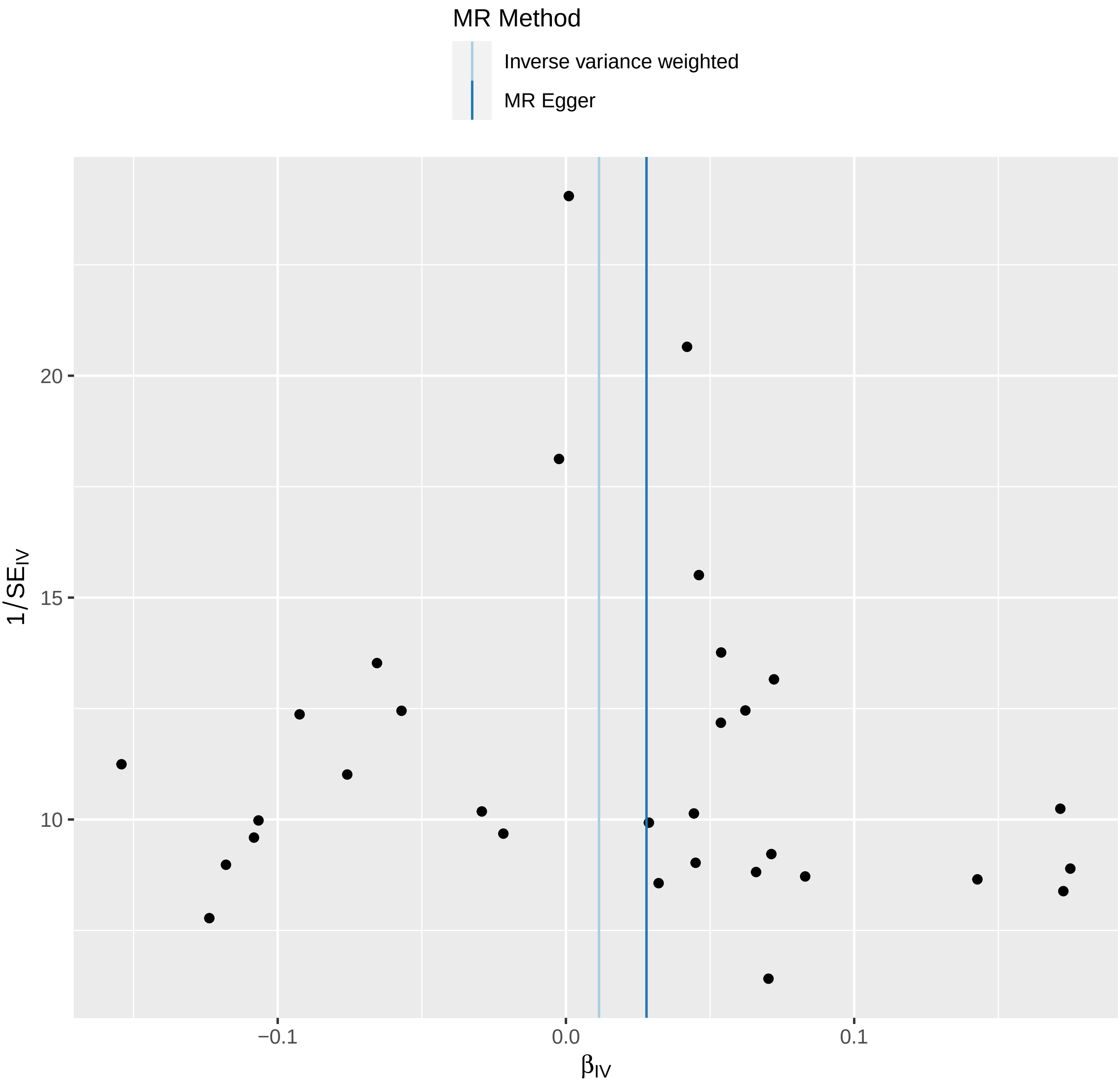

Batch 203 : Crohn's disease on IDP tfMRI median zstat faces−shapes

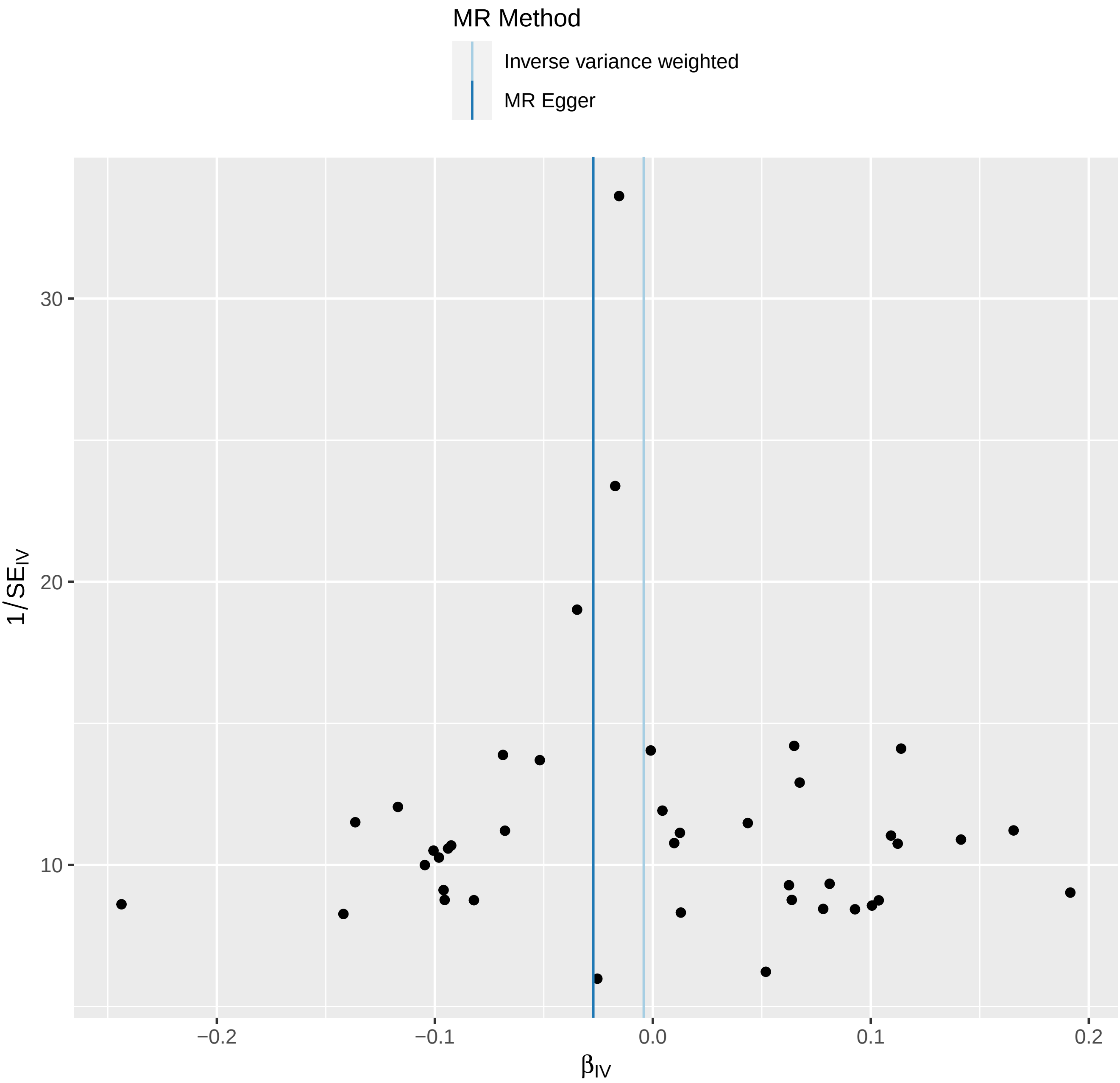

Batch 204 : Ulcerative colitis on IDP tfMRI median zstat faces−shapes

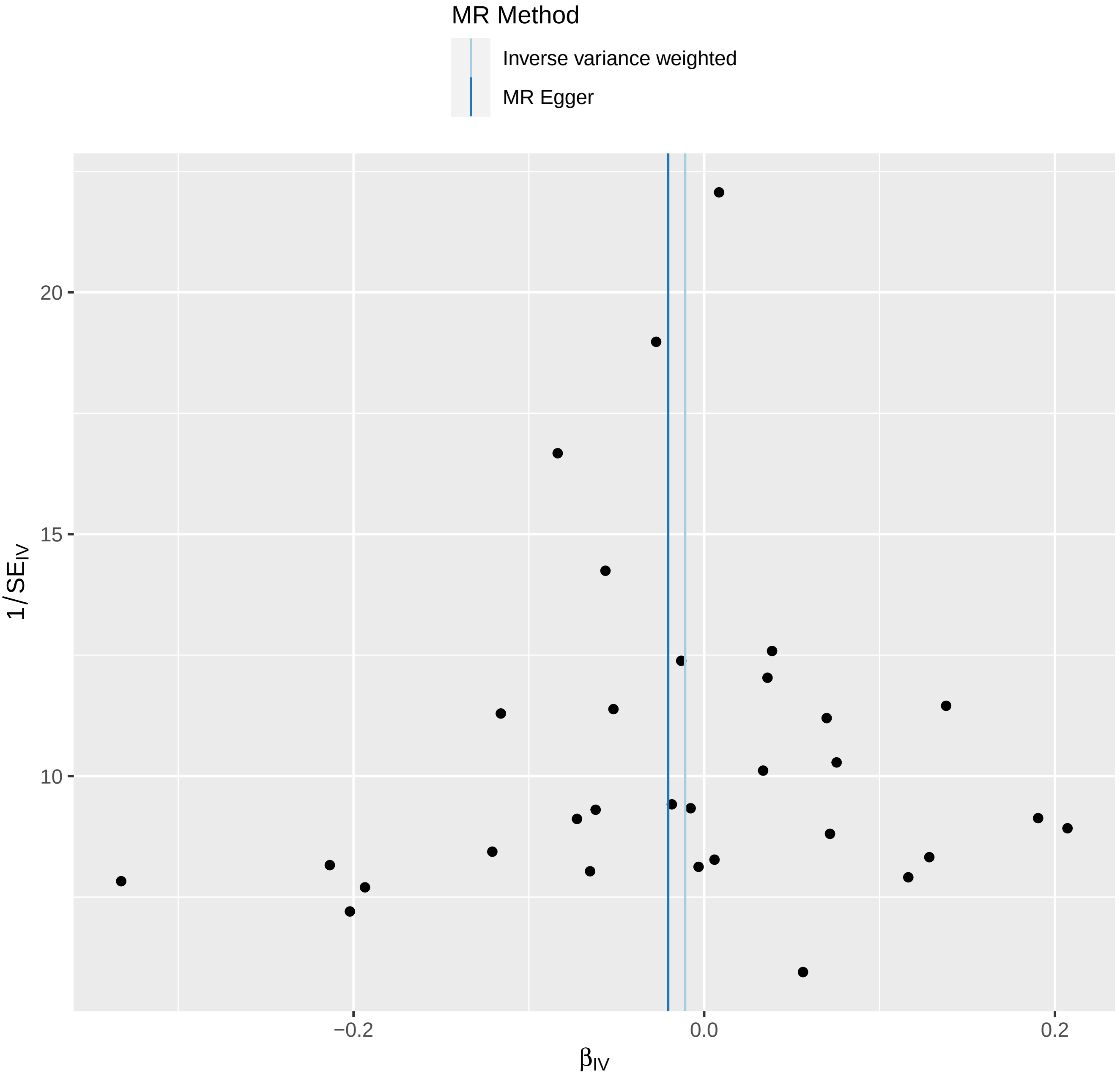

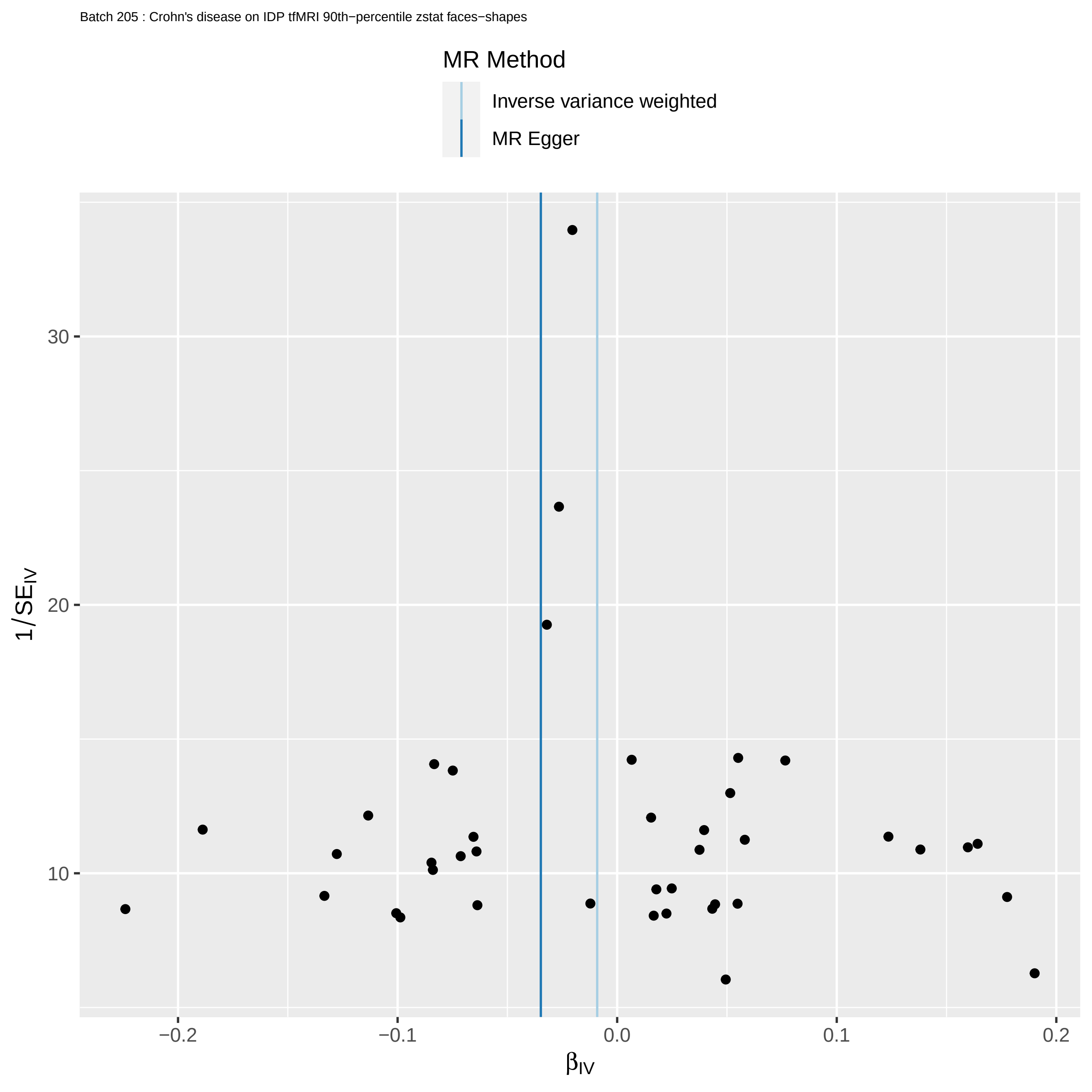

Batch 205 : Crohn's disease on IDP tfMRI 90th−percentile zstat faces−shapes
MR Method
Inverse variance weighted
MR Egger
30
20
10
1/SE$_{IV}$
−0.2
−0.1
0.0
0.1
0.2
β$_{IV}$

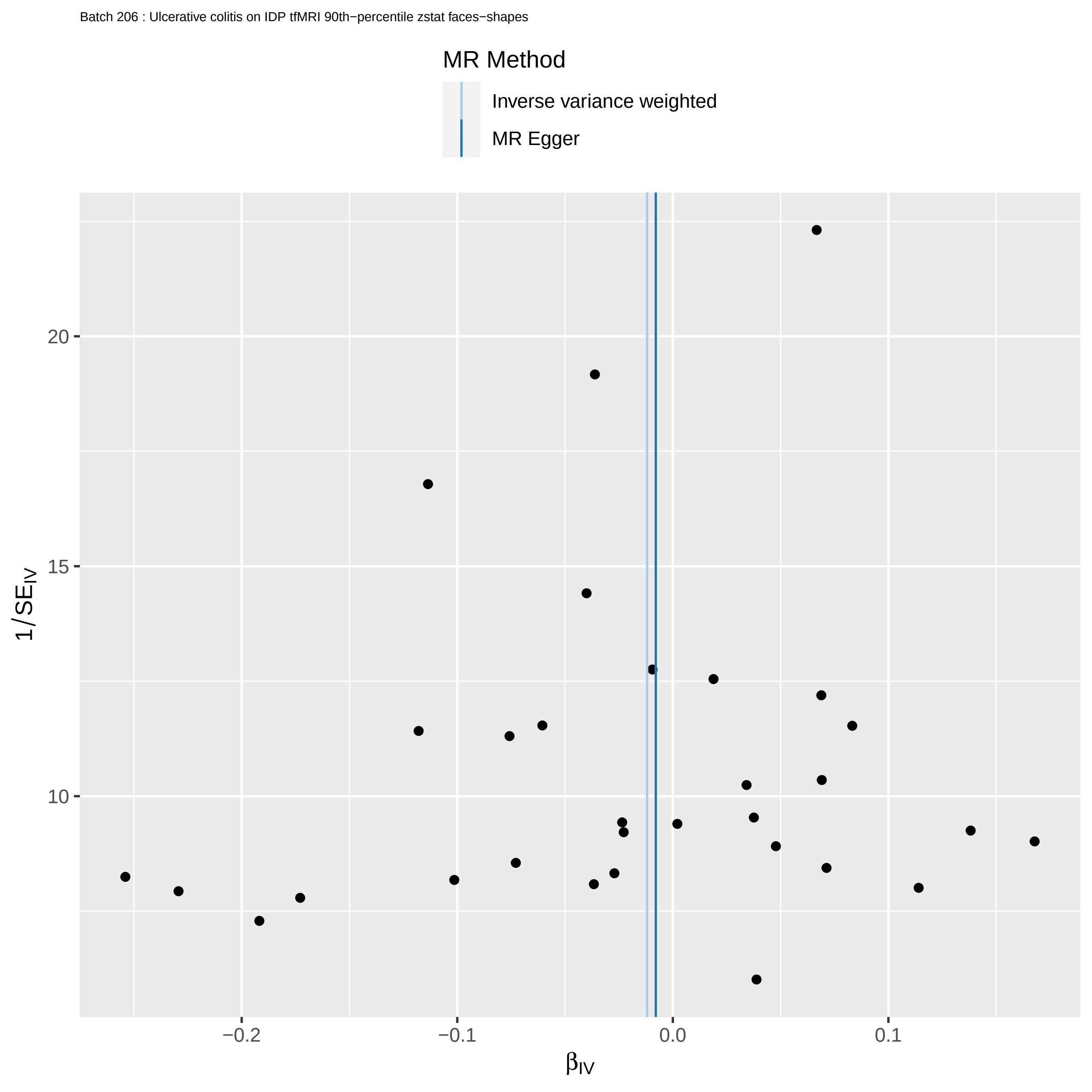
Batch 206 : Ulcerative colitis on IDP tfMRI 90th−percentile zstat faces−shapes
MR Method
Inverse variance weighted
MR Egger
20
15
10
1/SE$_{IV}$
−0.2
−0.1
0.0
0.1
β$_{IV}$

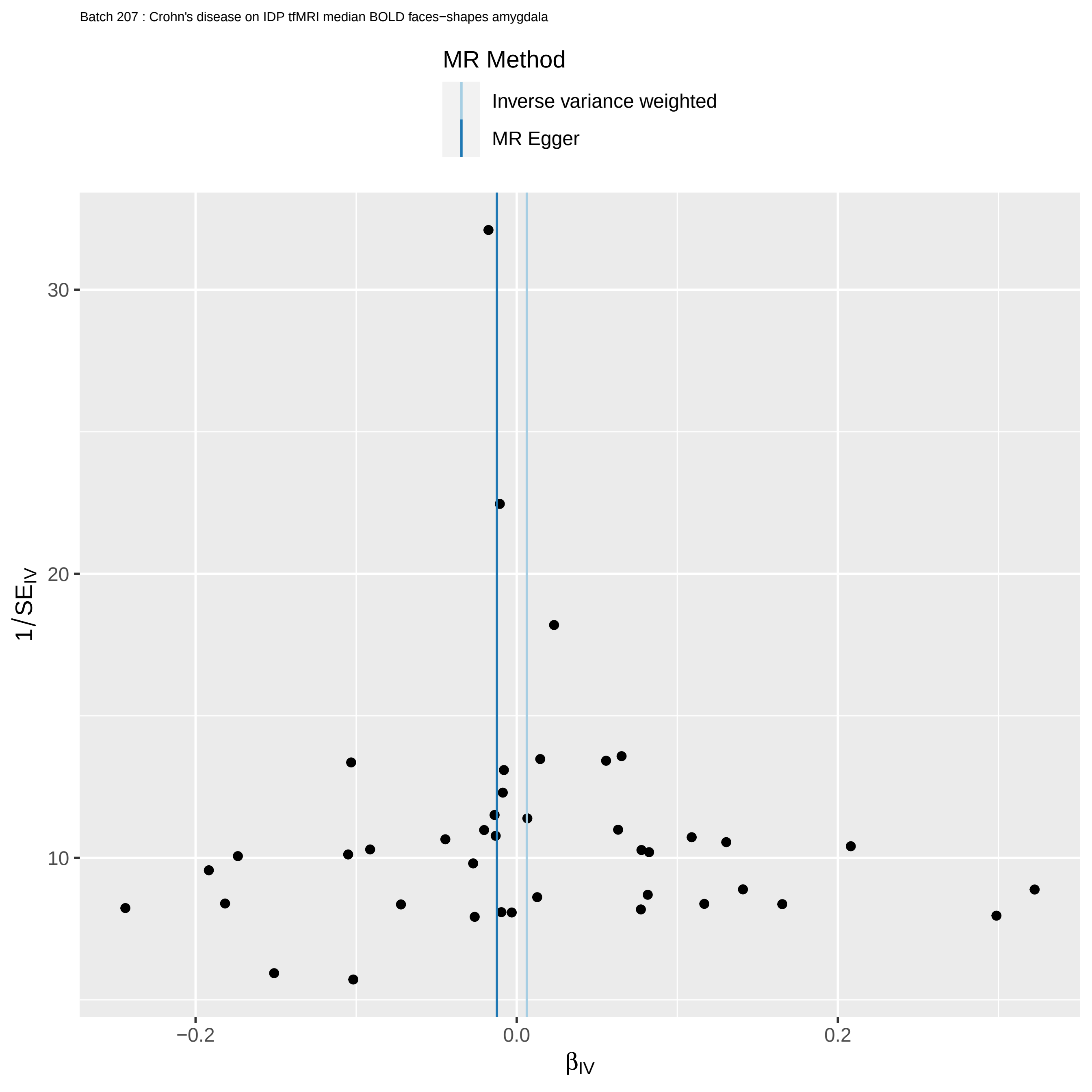

Batch 207 : Crohn's disease on IDP tfMRI median BOLD faces−shapes amygdala
MR Method
Inverse variance weighted
MR Egger
30
20
10
1/SE$_{IV}$
−0.2
0.0
0.2
β$_{IV}$

Batch 208 : Ulcerative colitis on IDP tfMRI median BOLD faces−shapes amygdala

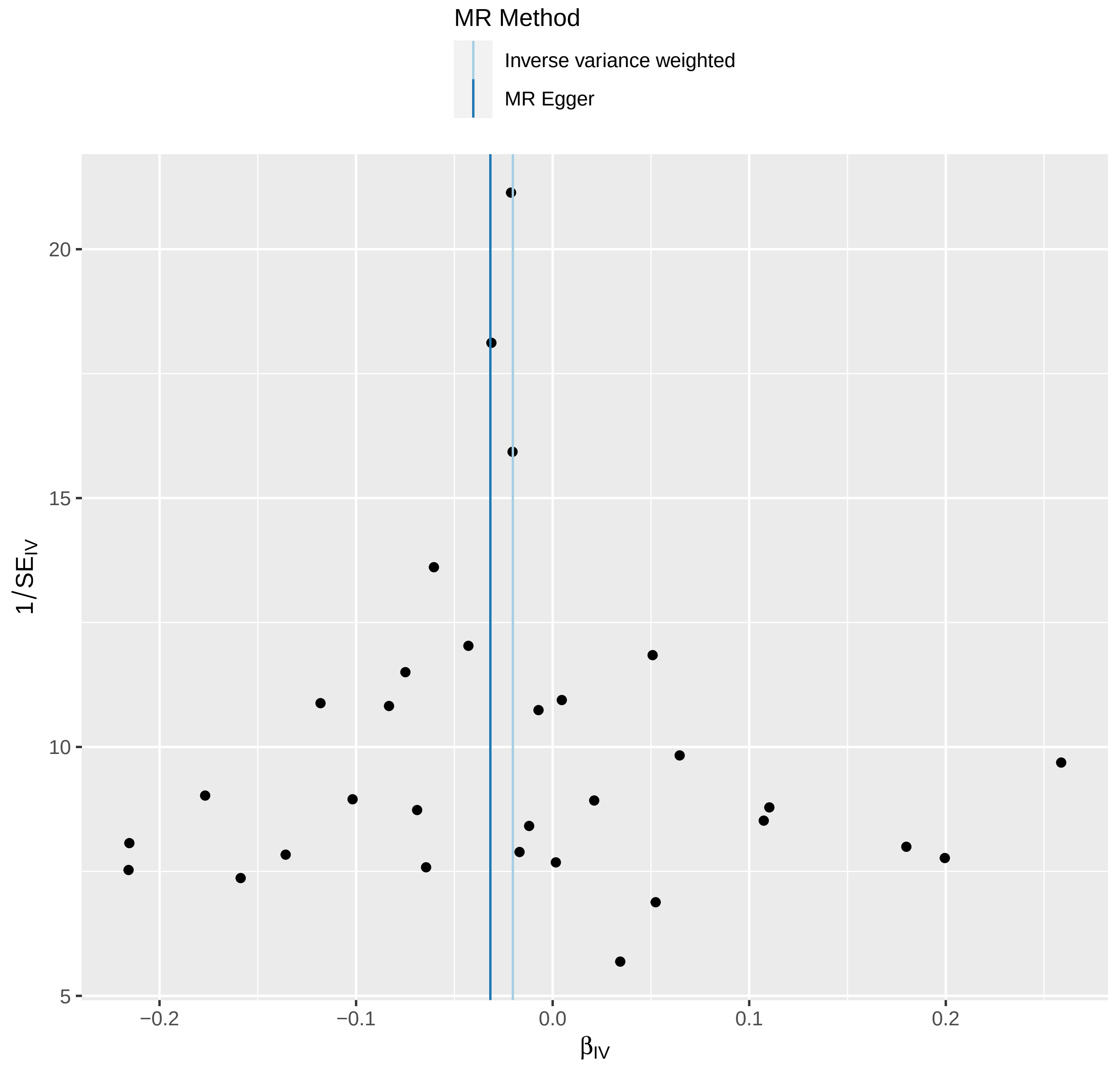

Batch 209 : Crohn's disease on IDP tfMRI 90th−percentile BOLD faces−shapes amygdala

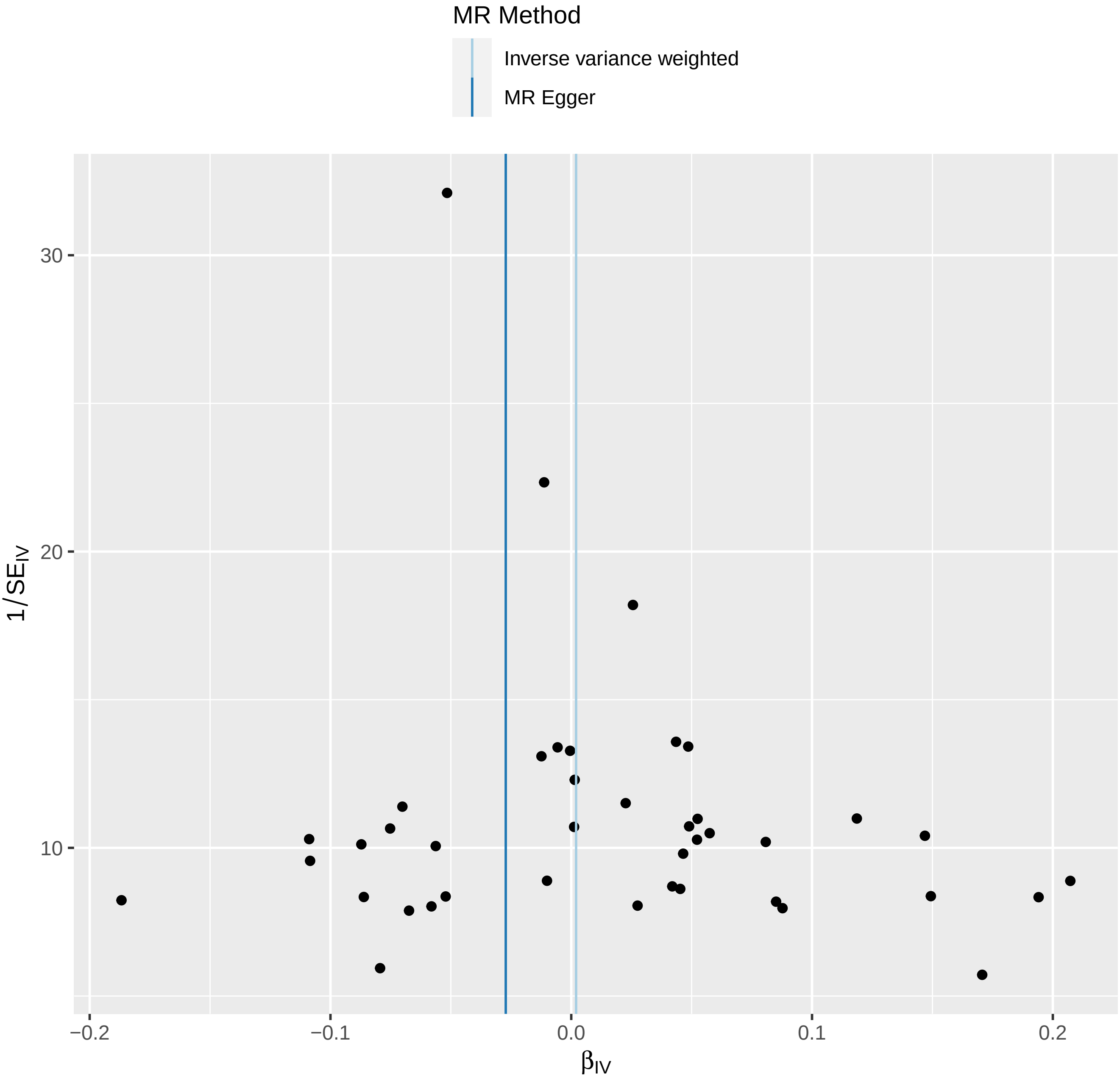

Batch 210 : Ulcerative colitis on IDP tfMRI 90th−percentile BOLD faces−shapes amygdala

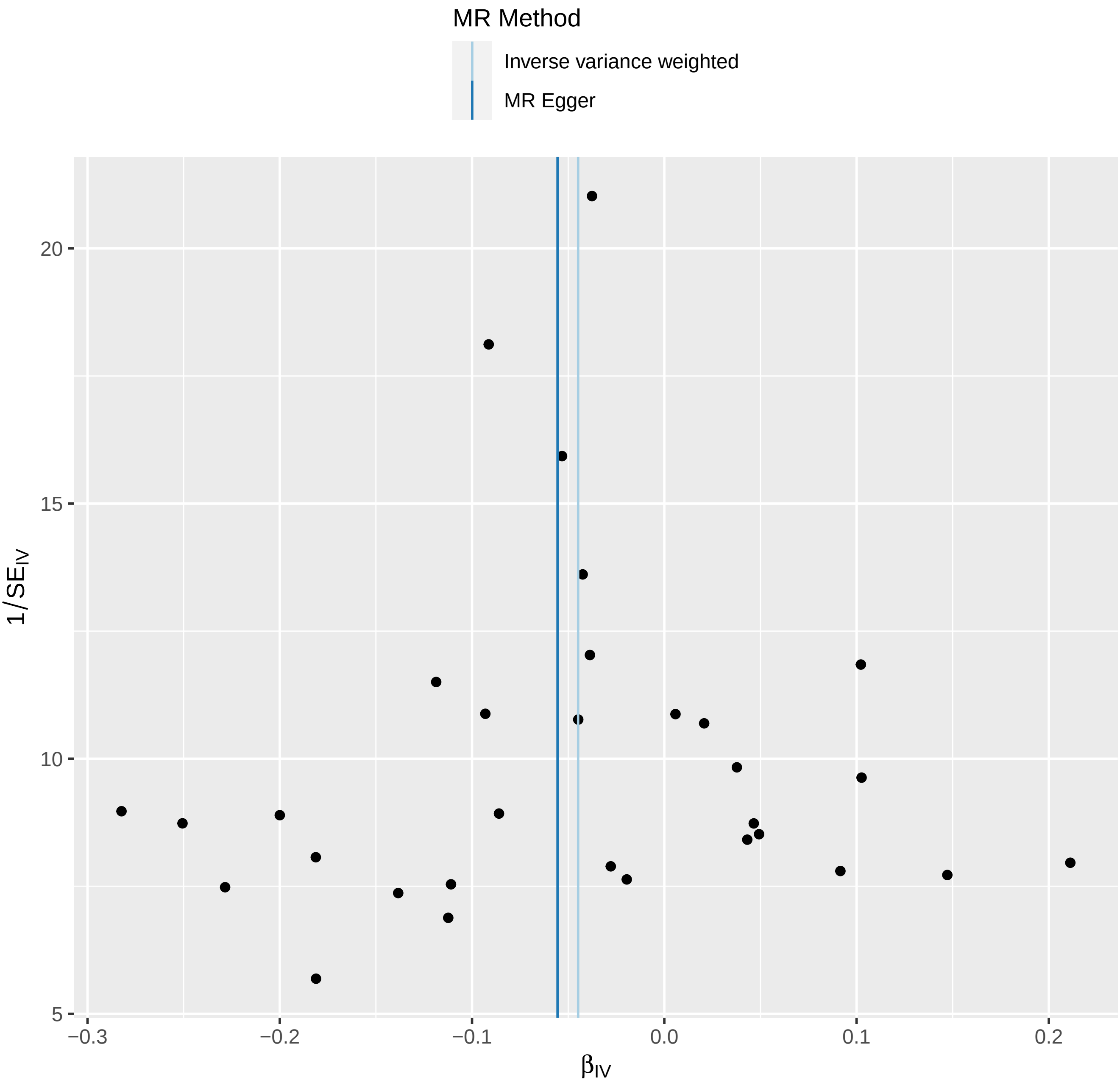

Batch 211 : Crohn's disease on IDP tfMRI median zstat faces−shapes amygdala

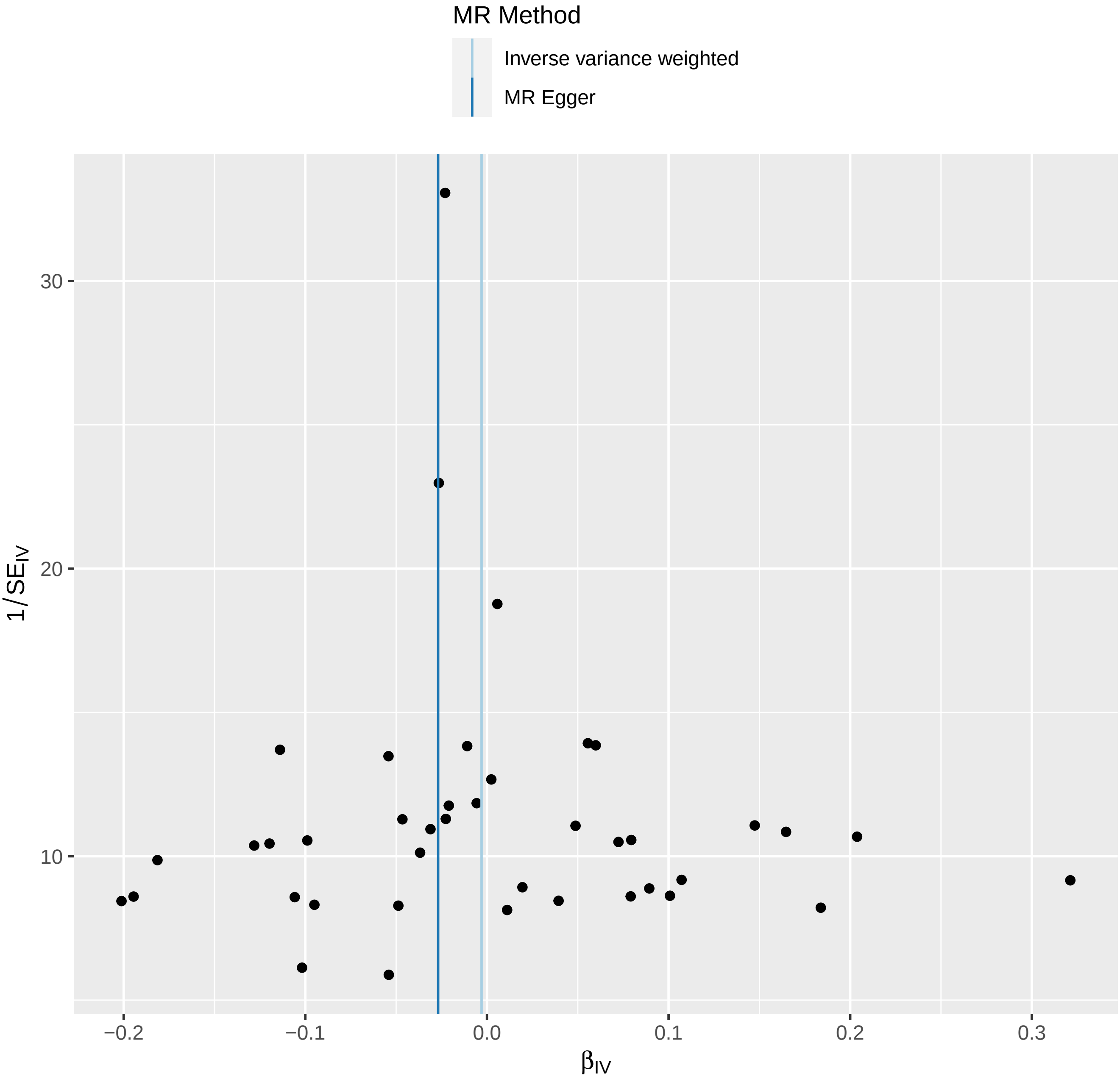

Batch 212 : Ulcerative colitis on IDP tfMRI median zstat faces−shapes amygdala

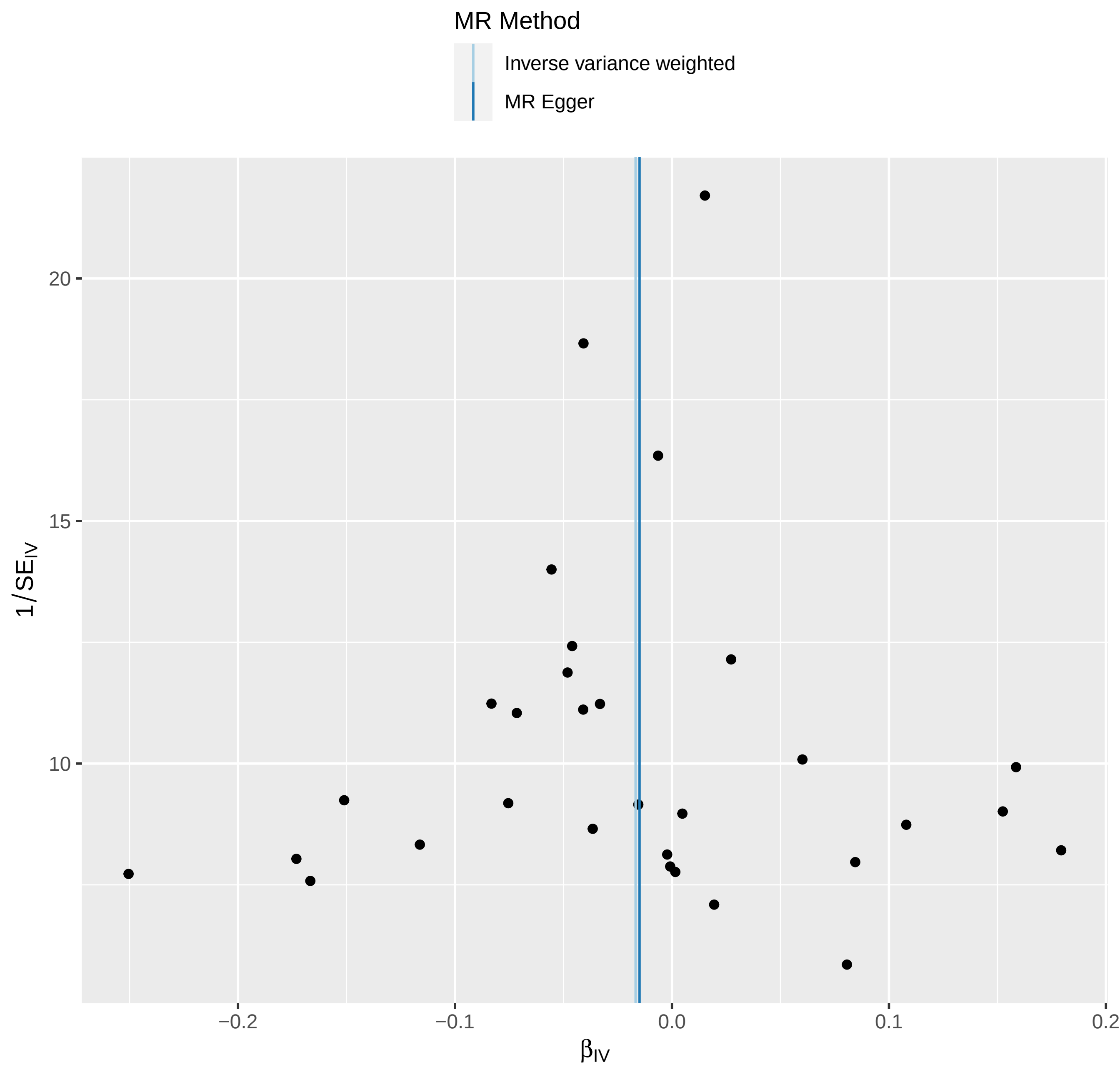

Batch 213 : Crohn's disease on IDP tfMRI 90th−percentile zstat faces−shapes amygdala

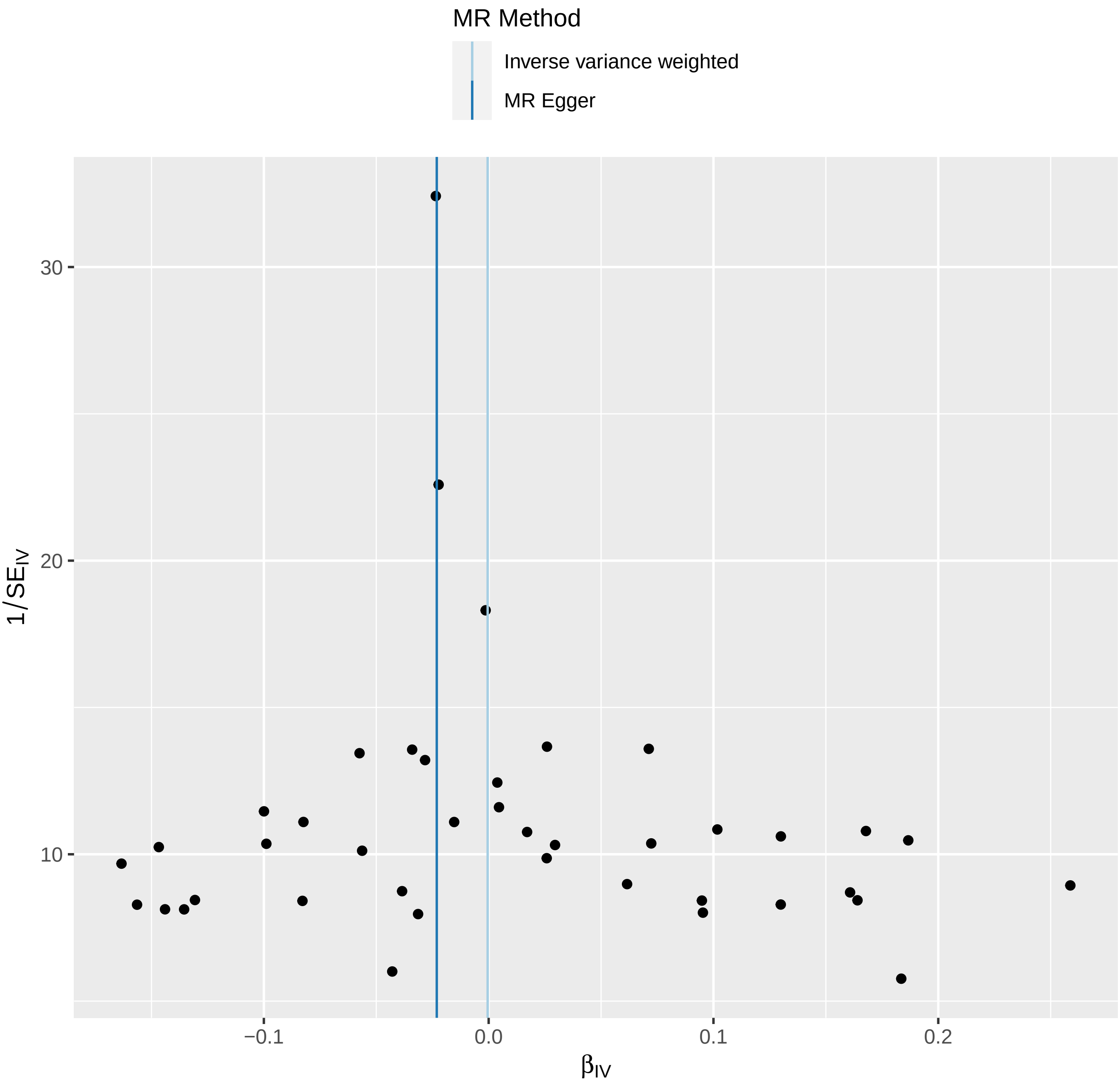

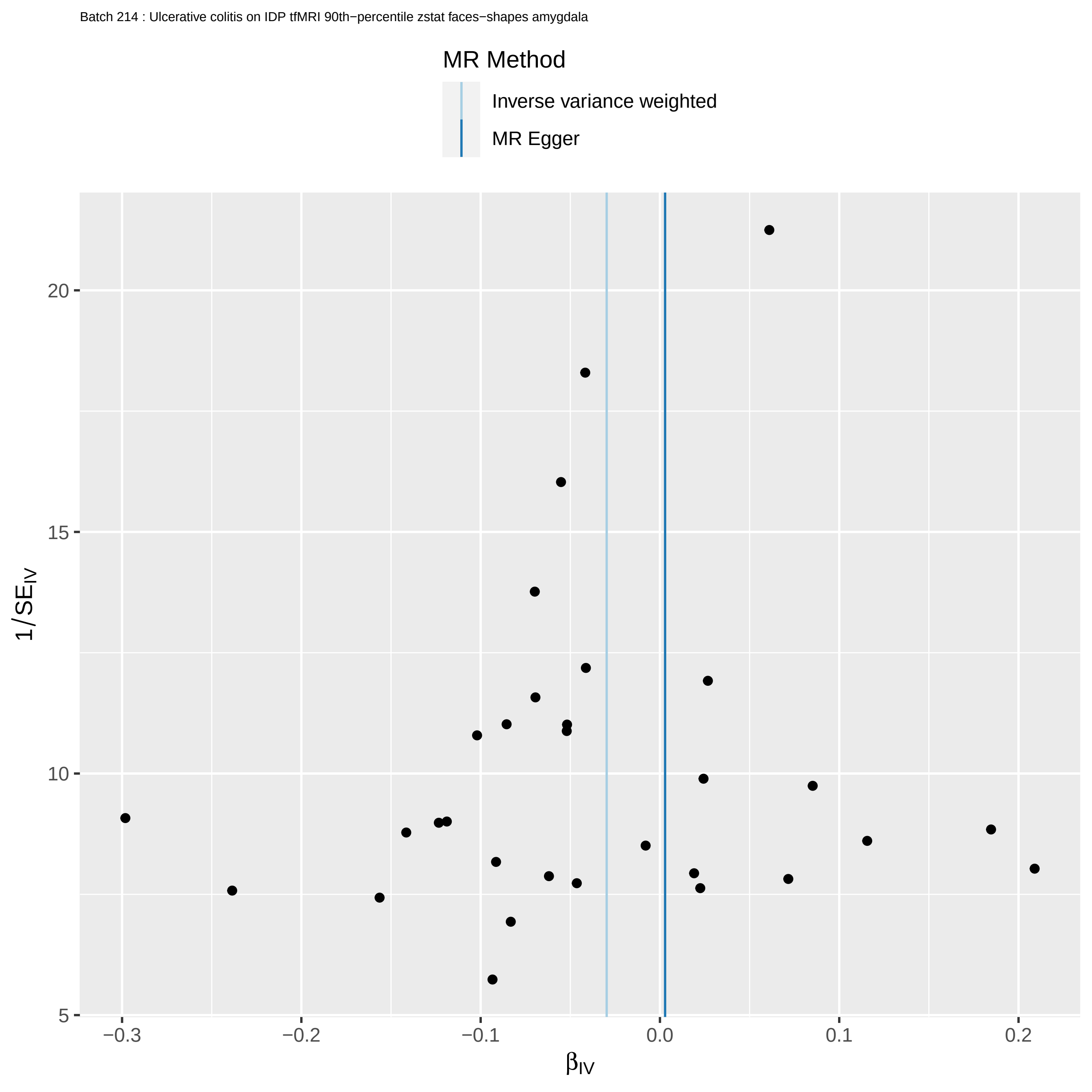

Batch 214 : Ulcerative colitis on IDP tfMRI 90th−percentile zstat faces−shapes amygdala
MR Method
Inverse variance weighted
MR Egger
20
15
10
5
$1/SE_{IV}$
−0.3
−0.2
−0.1
0.0
0.1
0.2
β_{IV}

Batch 223 : Crohn's disease on IDP T1 SIENAX peripheral grey unnormalised volume

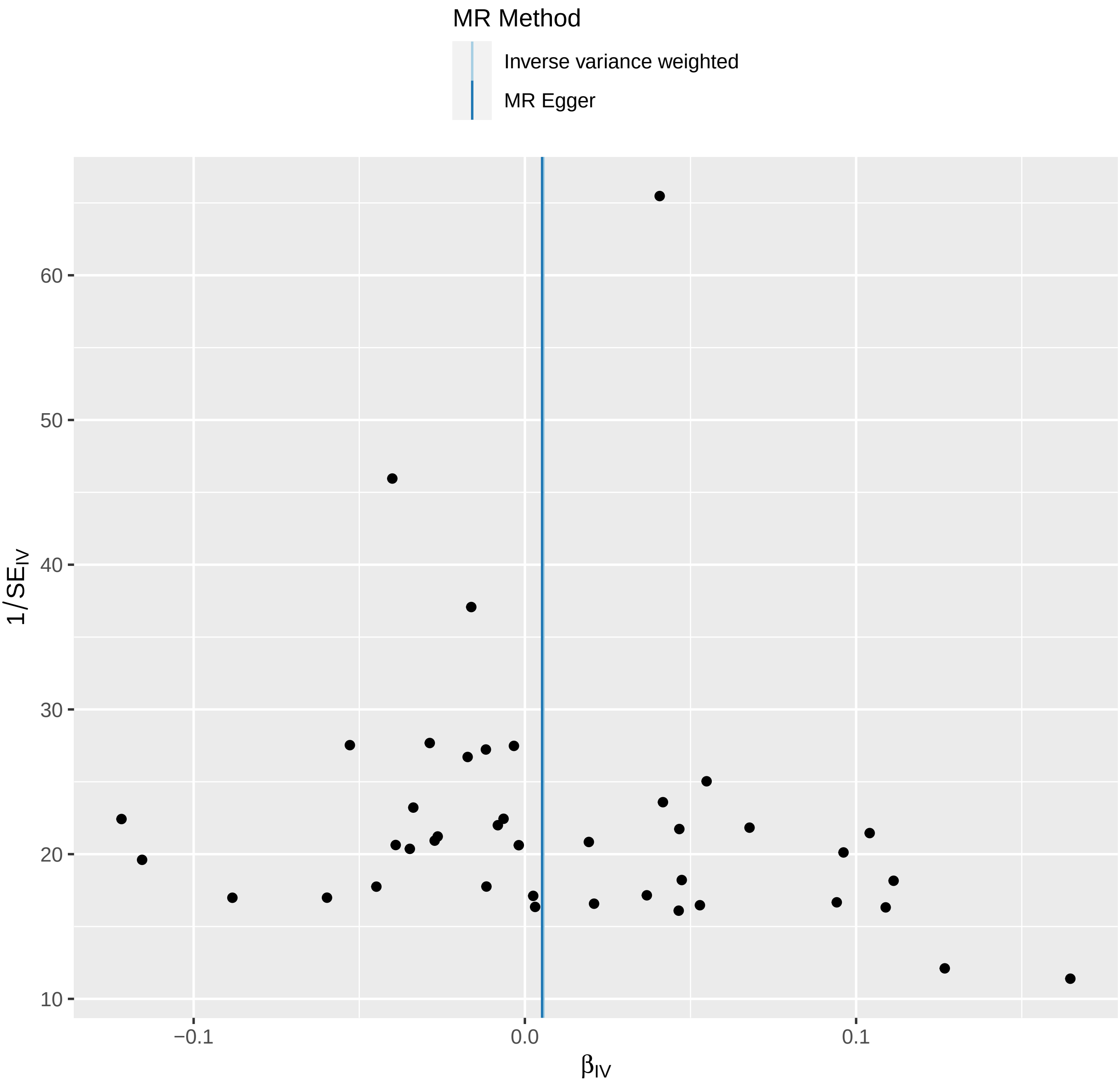

Batch 224 : Ulcerative colitis on IDP T1 SIENAX peripheral grey unnormalised volume

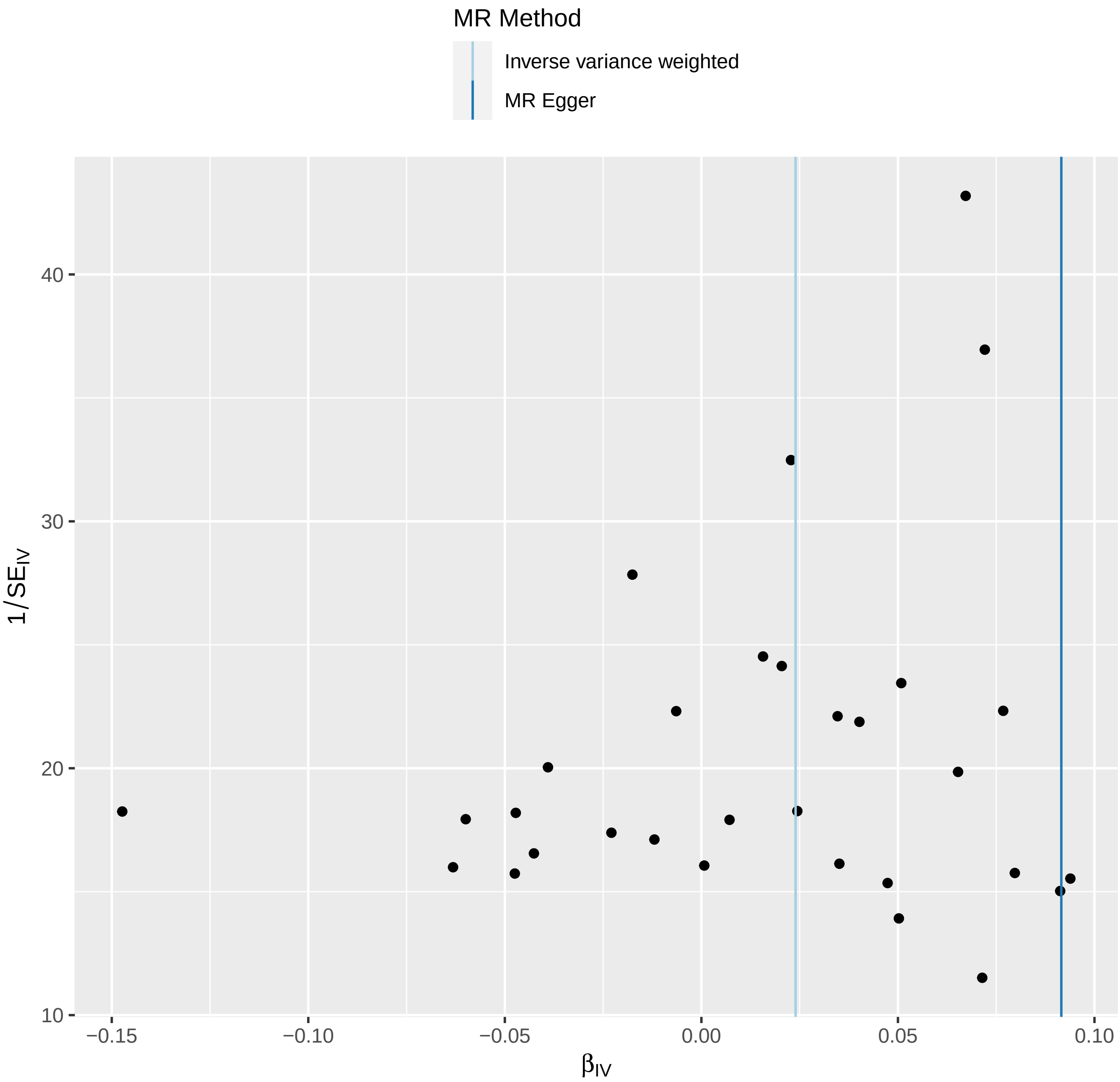

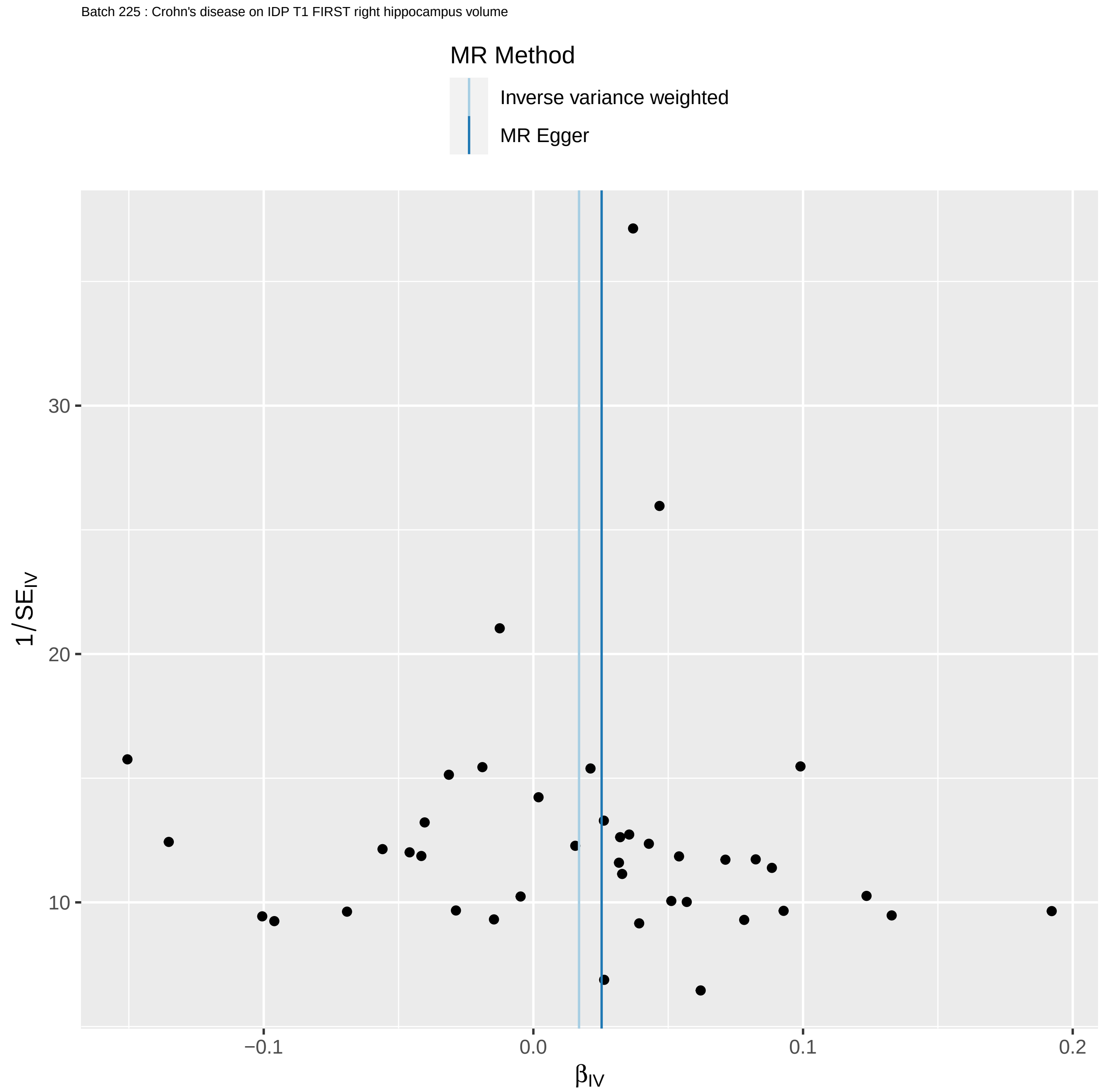
Batch 225 : Crohn's disease on IDP T1 FIRST right hippocampus volume
MR Method
Inverse variance weighted
MR Egger
30
20
10
1/SE$_{IV}$
−0.1
0.0
0.1
0.2
β$_{IV}$

Batch 226 : Ulcerative colitis on IDP T1 FIRST right hippocampus volume

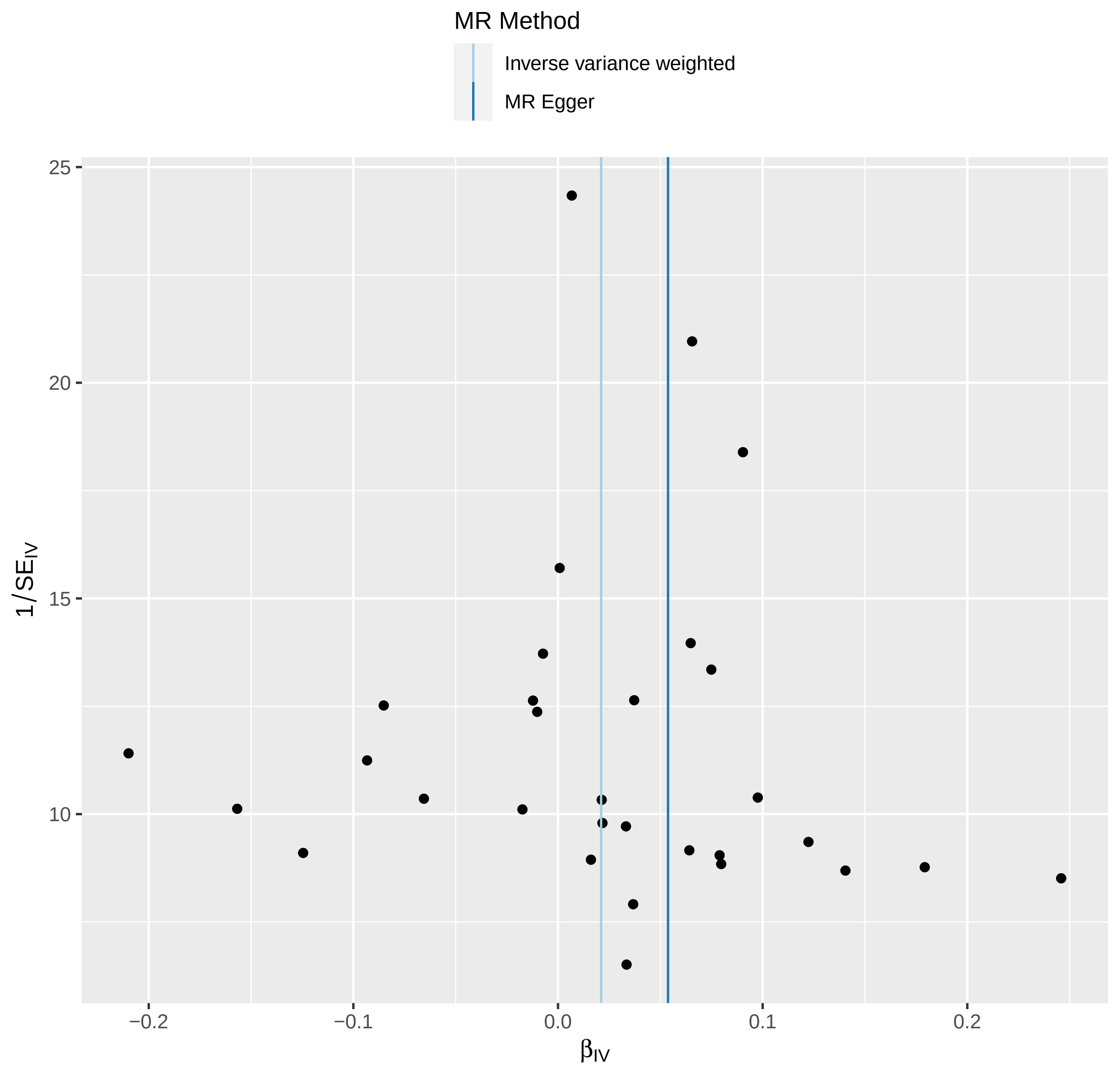

Batch 247 : Crohn's disease on IDP T1 FIRST left amygdala volume

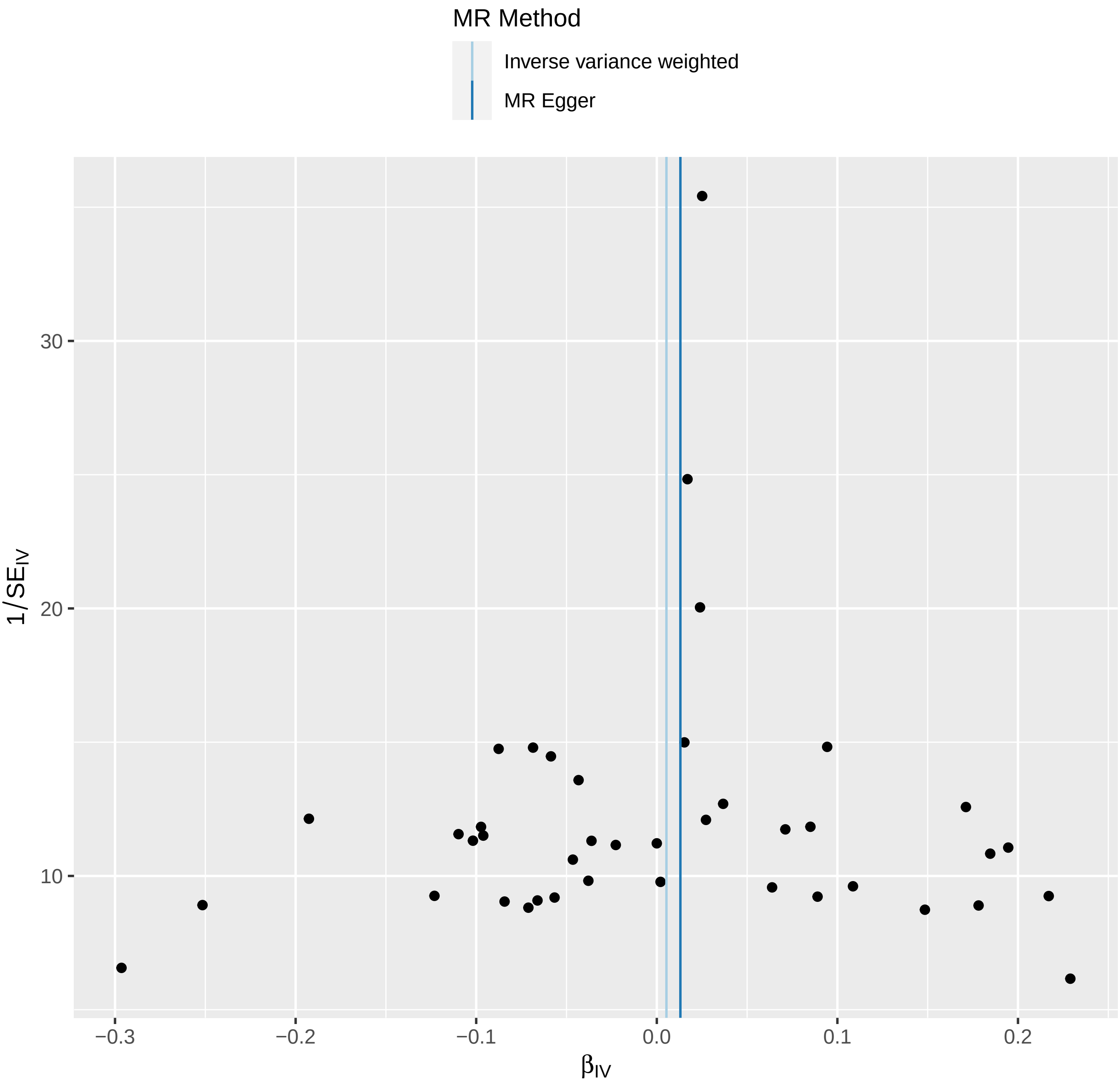

Batch 248 : Ulcerative colitis on IDP T1 FIRST left amygdala volume

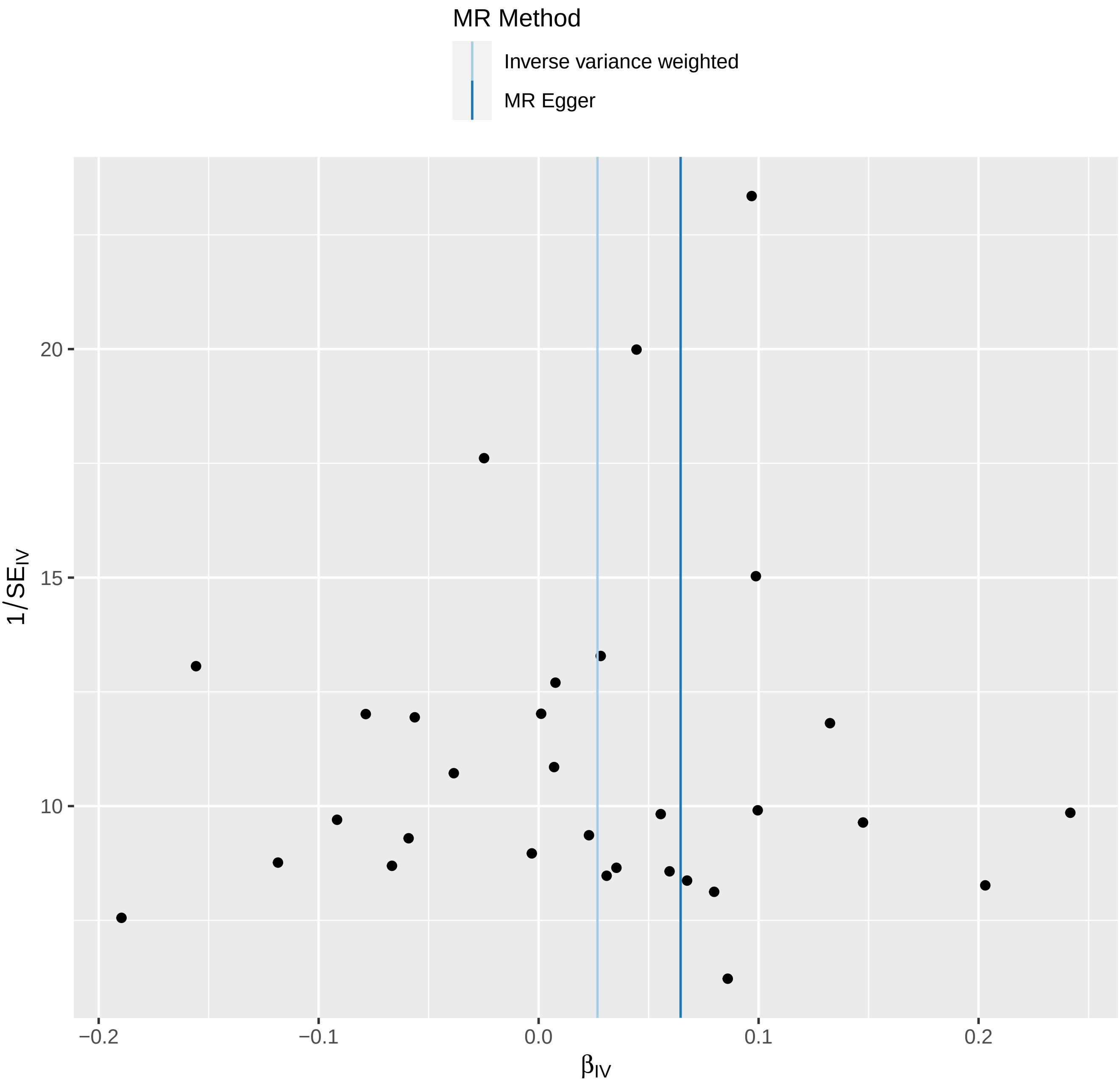

Batch 269 : Crohn's disease on IDP T1 FIRST right amygdala volume

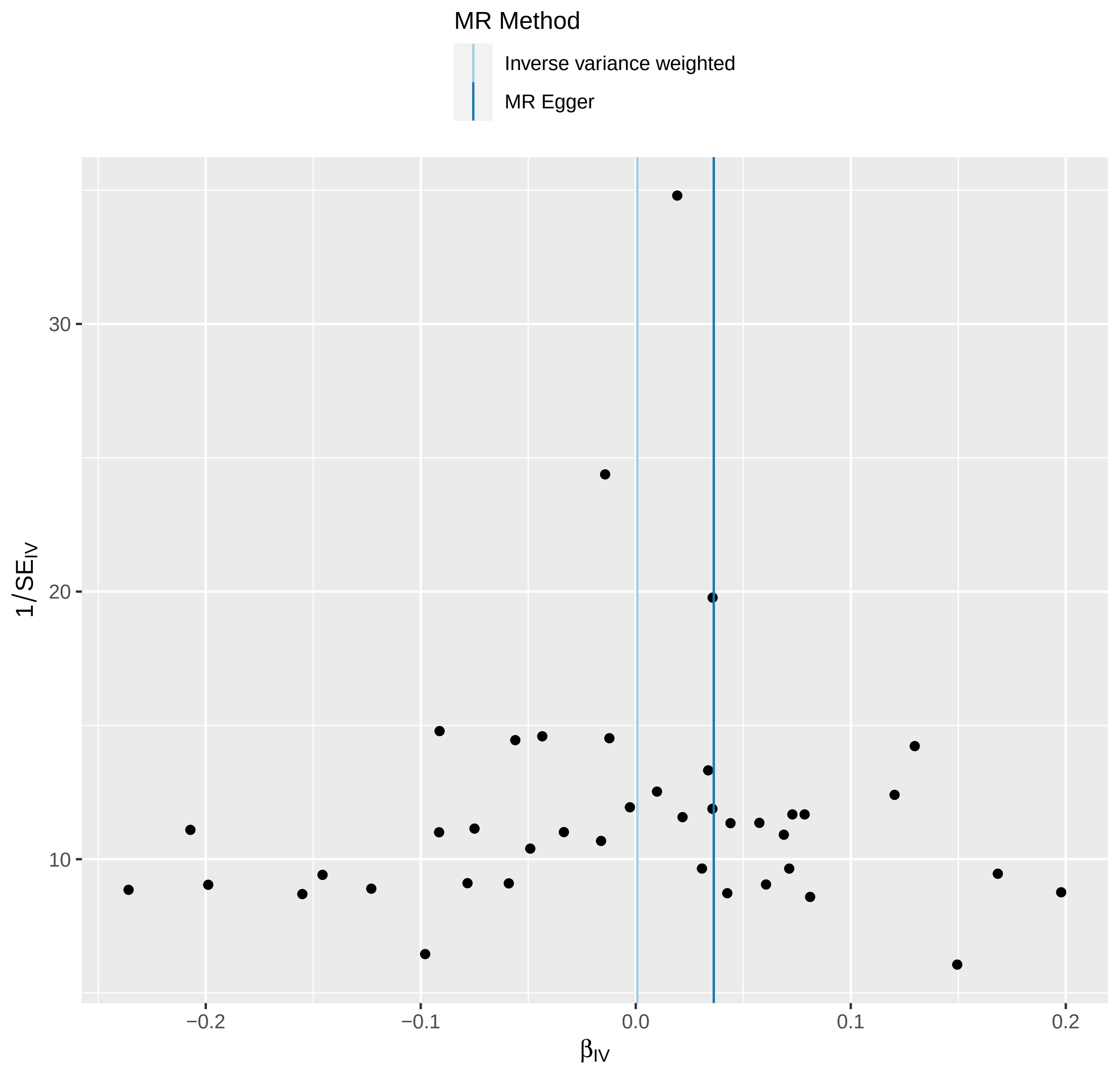

Batch 270 : Ulcerative colitis on IDP T1 FIRST right amygdala volume

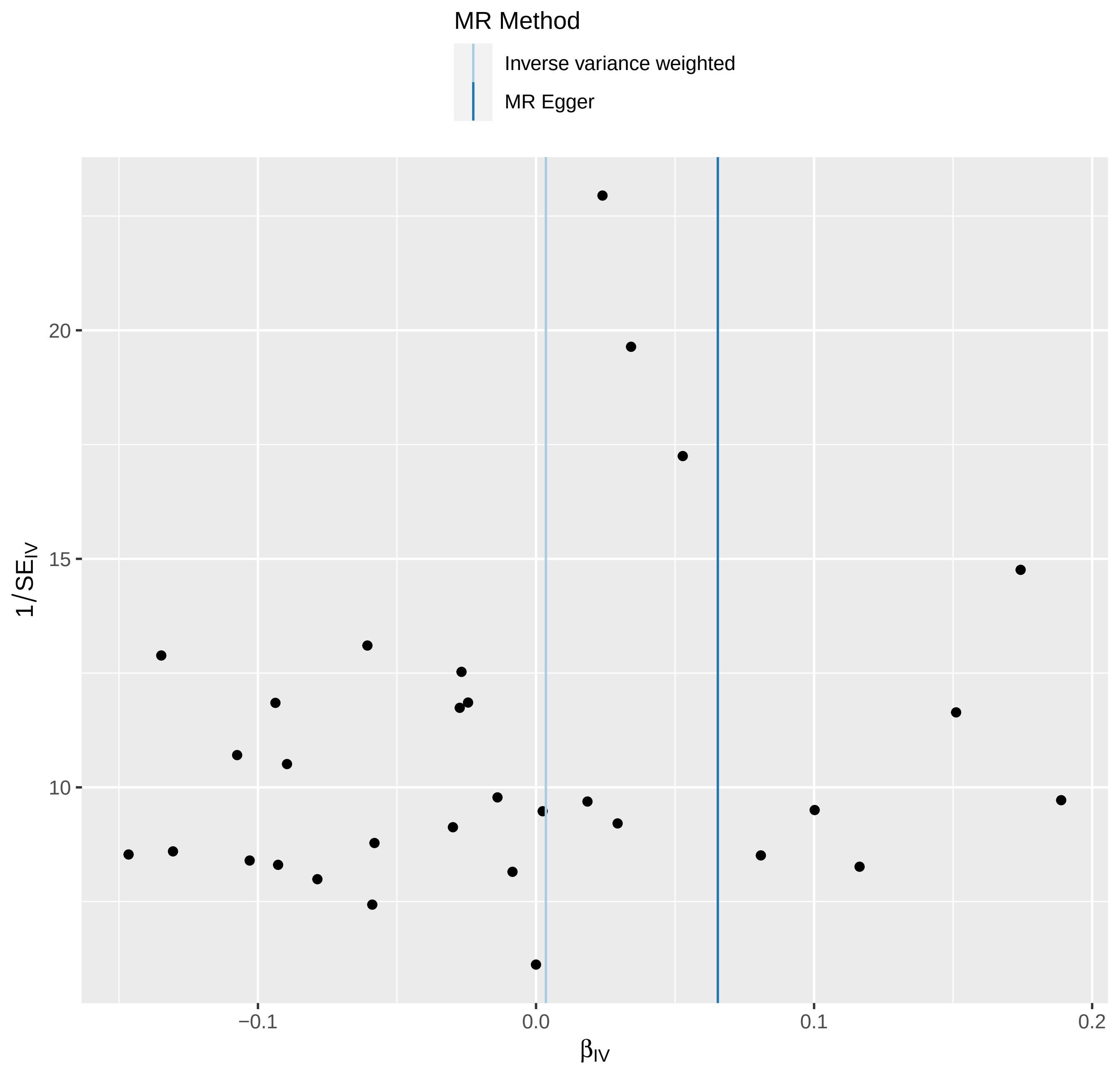

Batch 291 : Crohn's disease on IDP T1 FIRST left accumbens volume

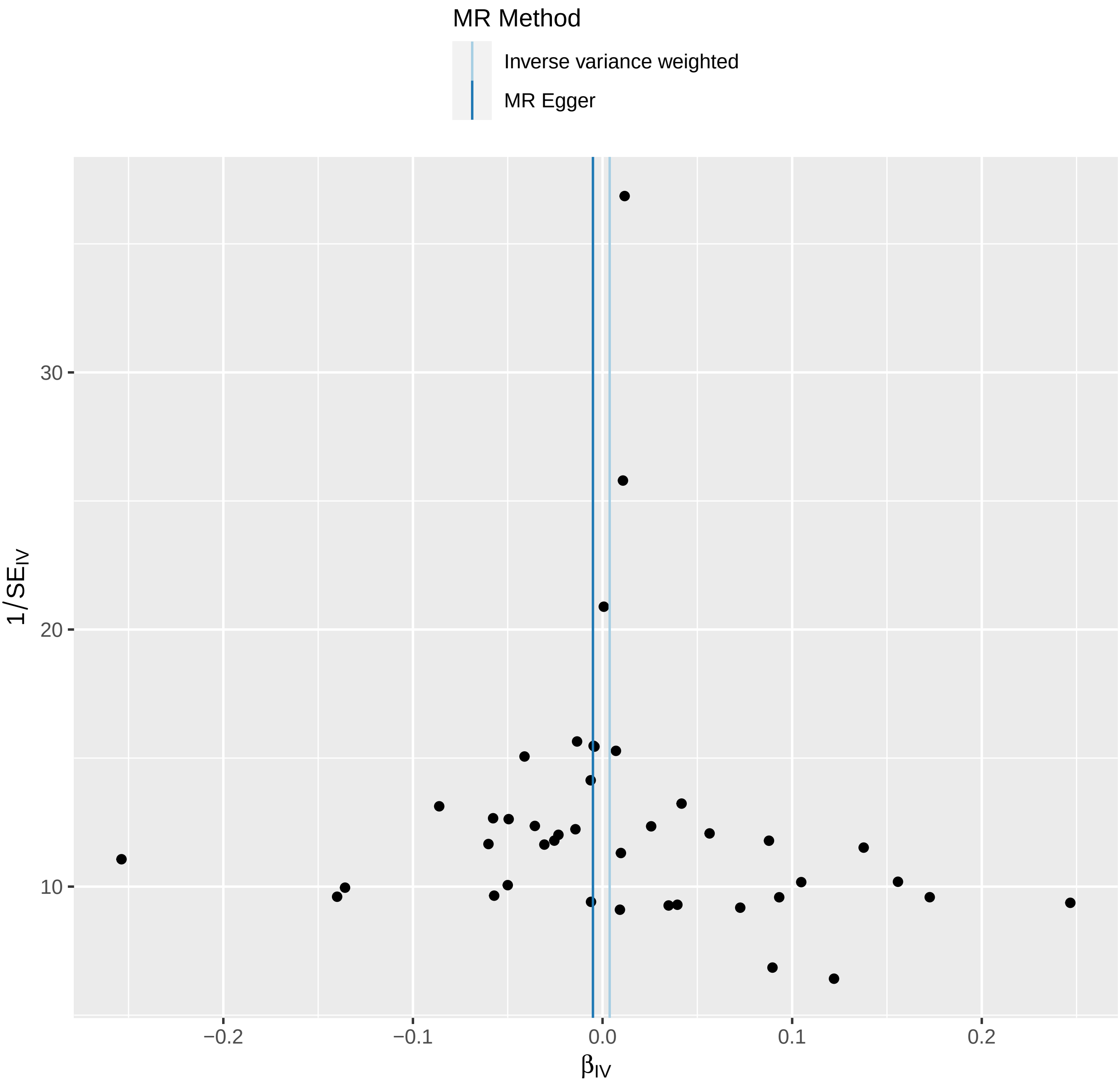

Batch 292 : Ulcerative colitis on IDP T1 FIRST left accumbens volume

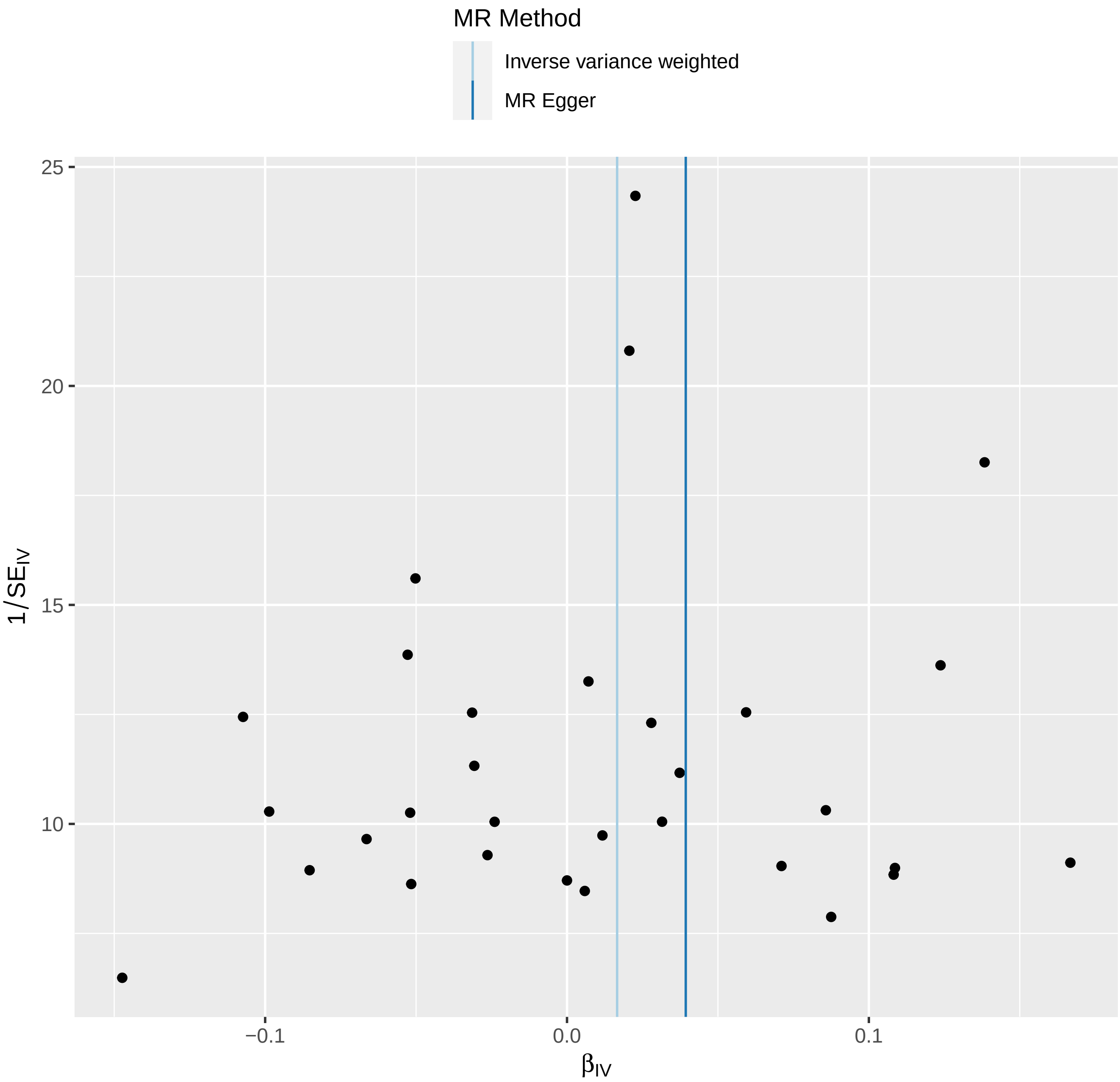

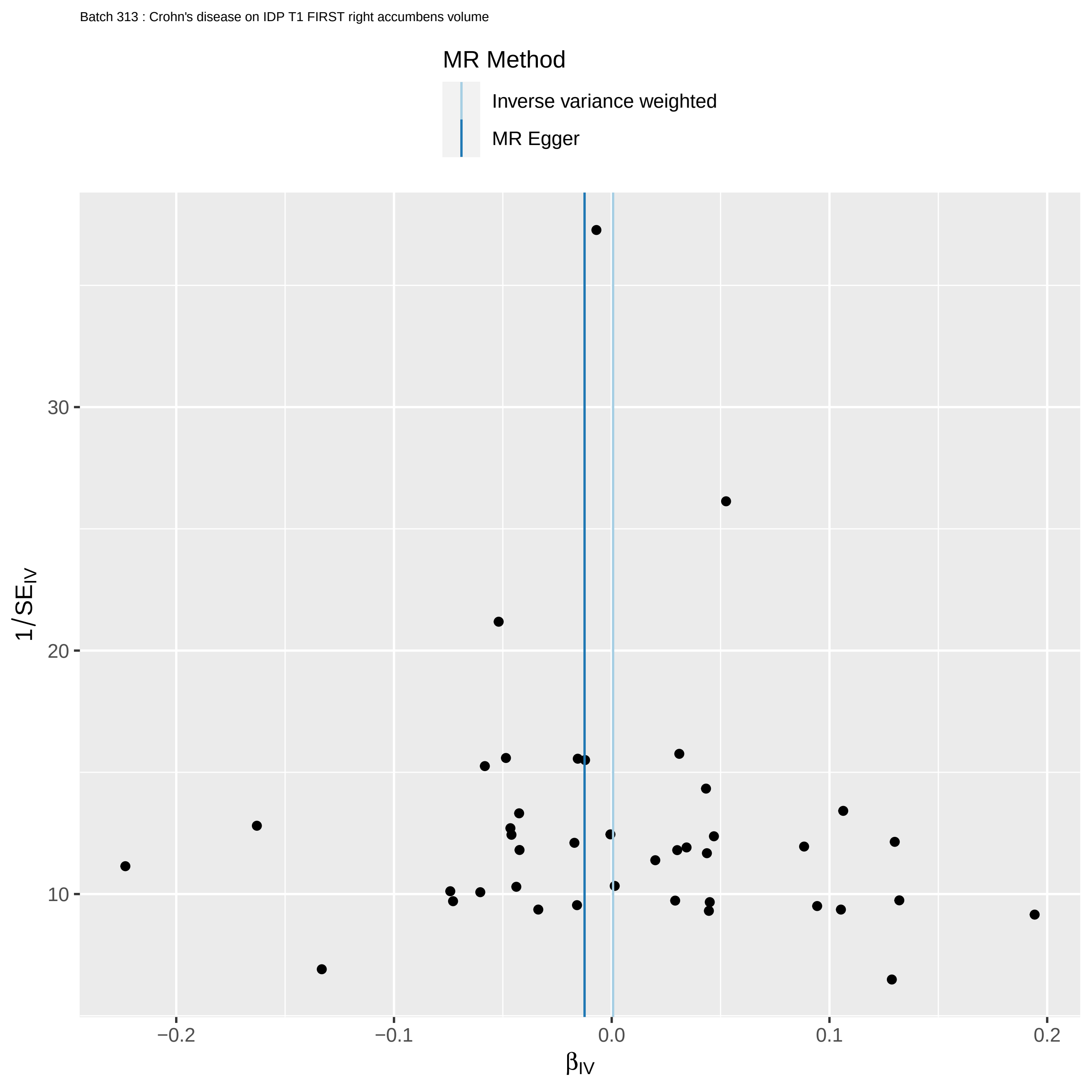

Batch 313 : Crohn's disease on IDP T1 FIRST right accumbens volume
MR Method
Inverse variance weighted
MR Egger
30
20
10
1/SE$_{IV}$
−0.2
−0.1
0.0
0.1
0.2
β$_{IV}$

Batch 314 : Ulcerative colitis on IDP T1 FIRST right accumbens volume

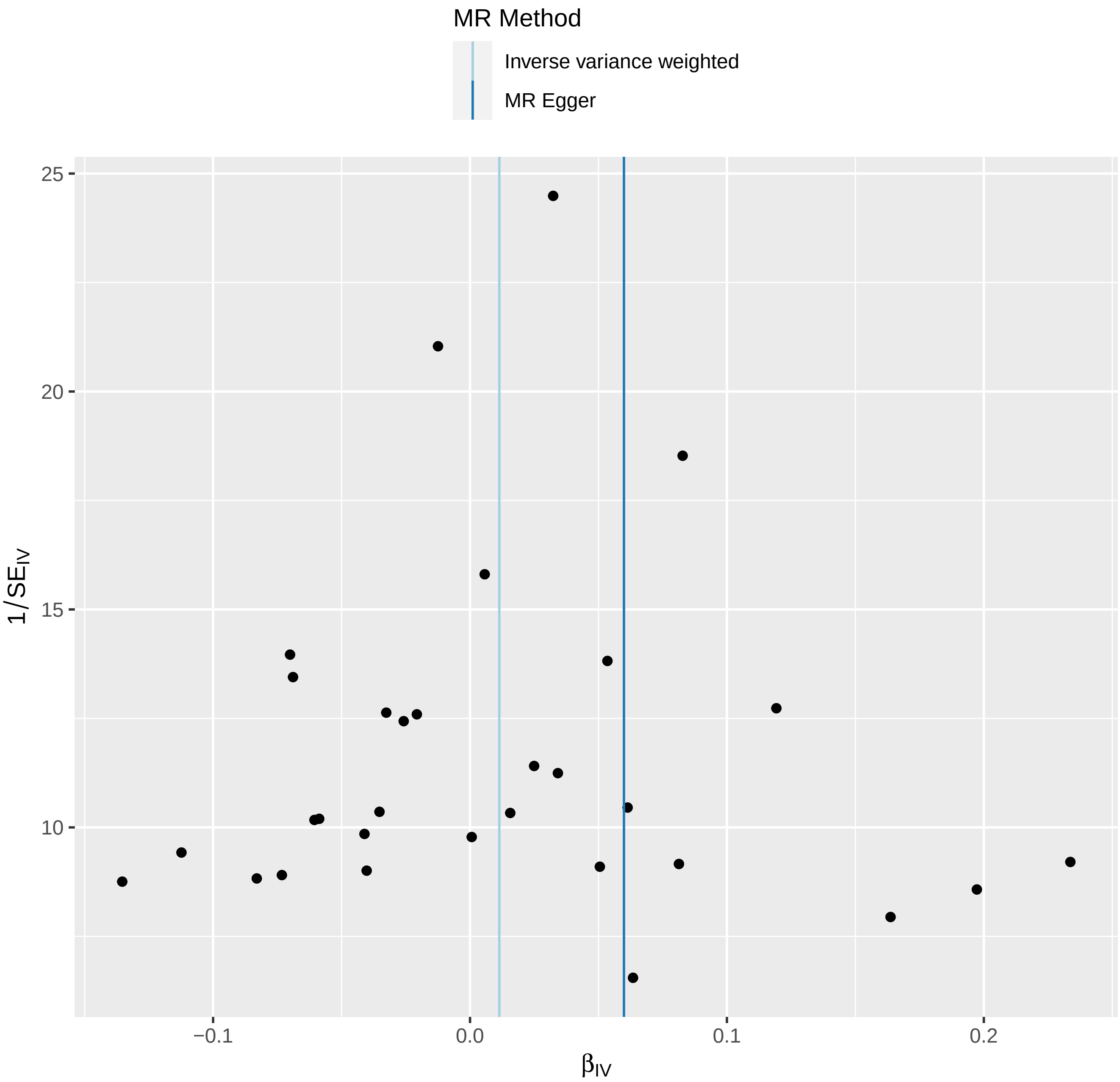

Batch 335 : Crohn's disease on IDP T1 FIRST brain stem+4th ventricle volume

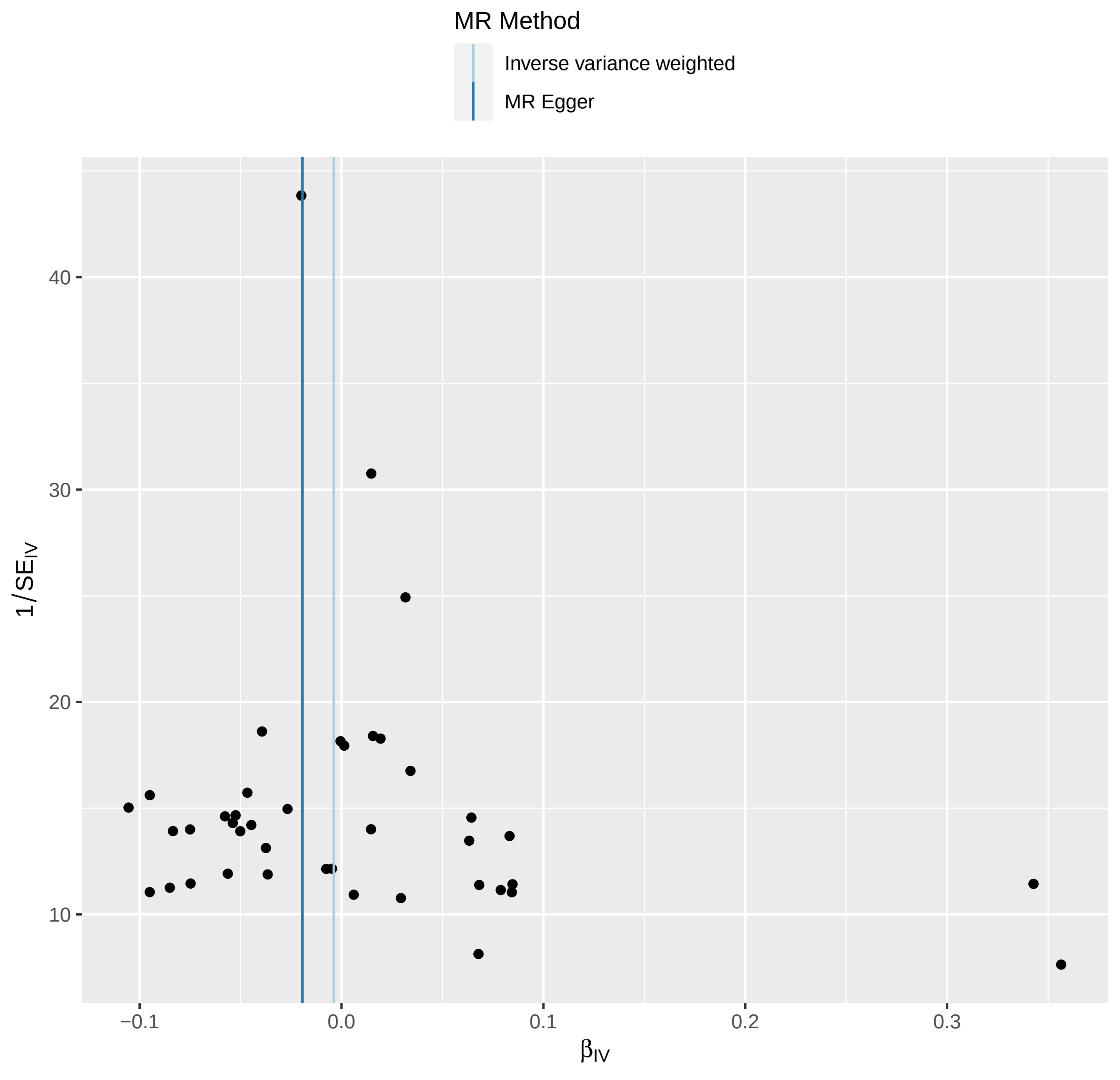

Batch 336 : Ulcerative colitis on IDP T1 FIRST brain stem+4th ventricle volume

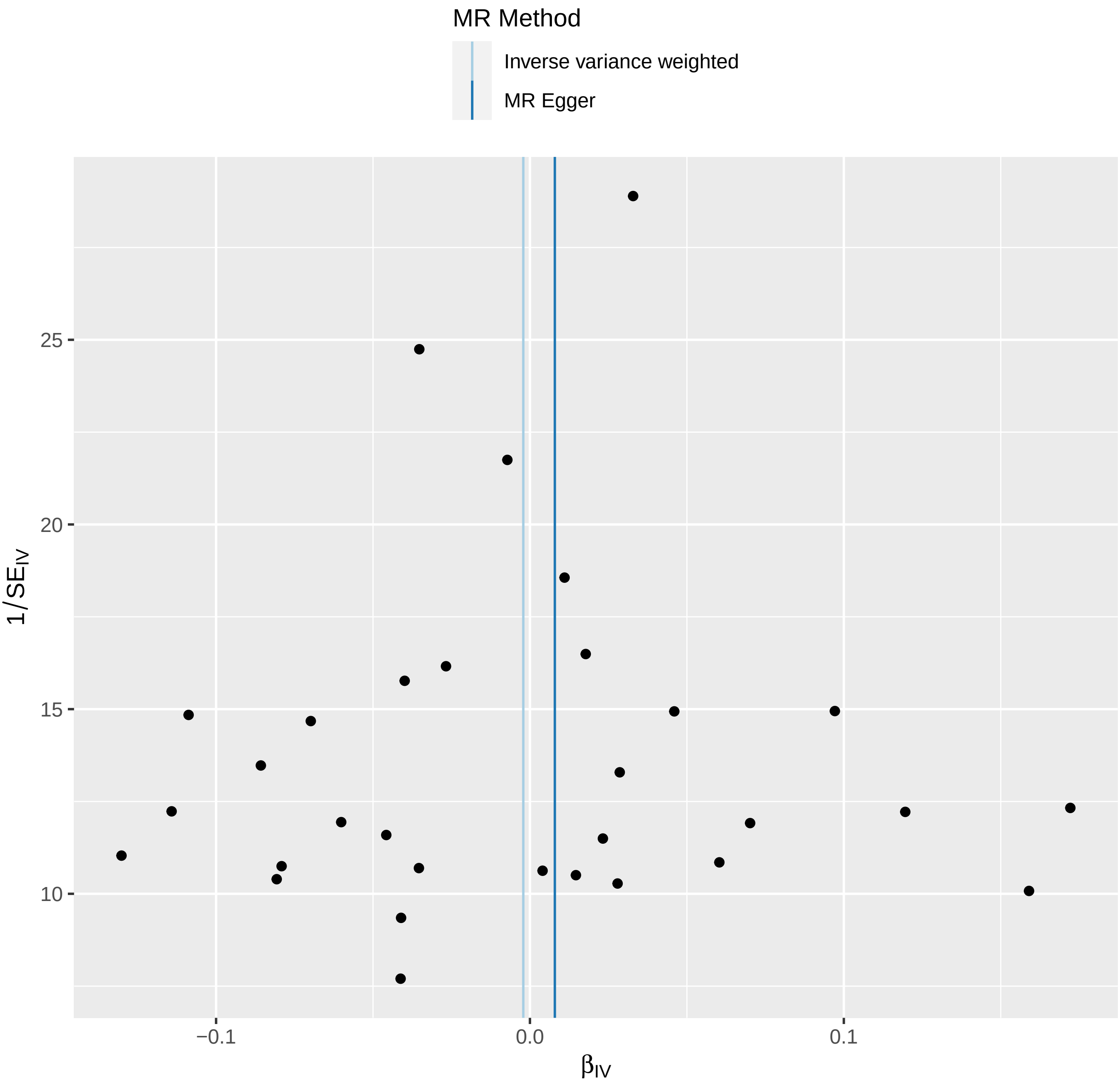

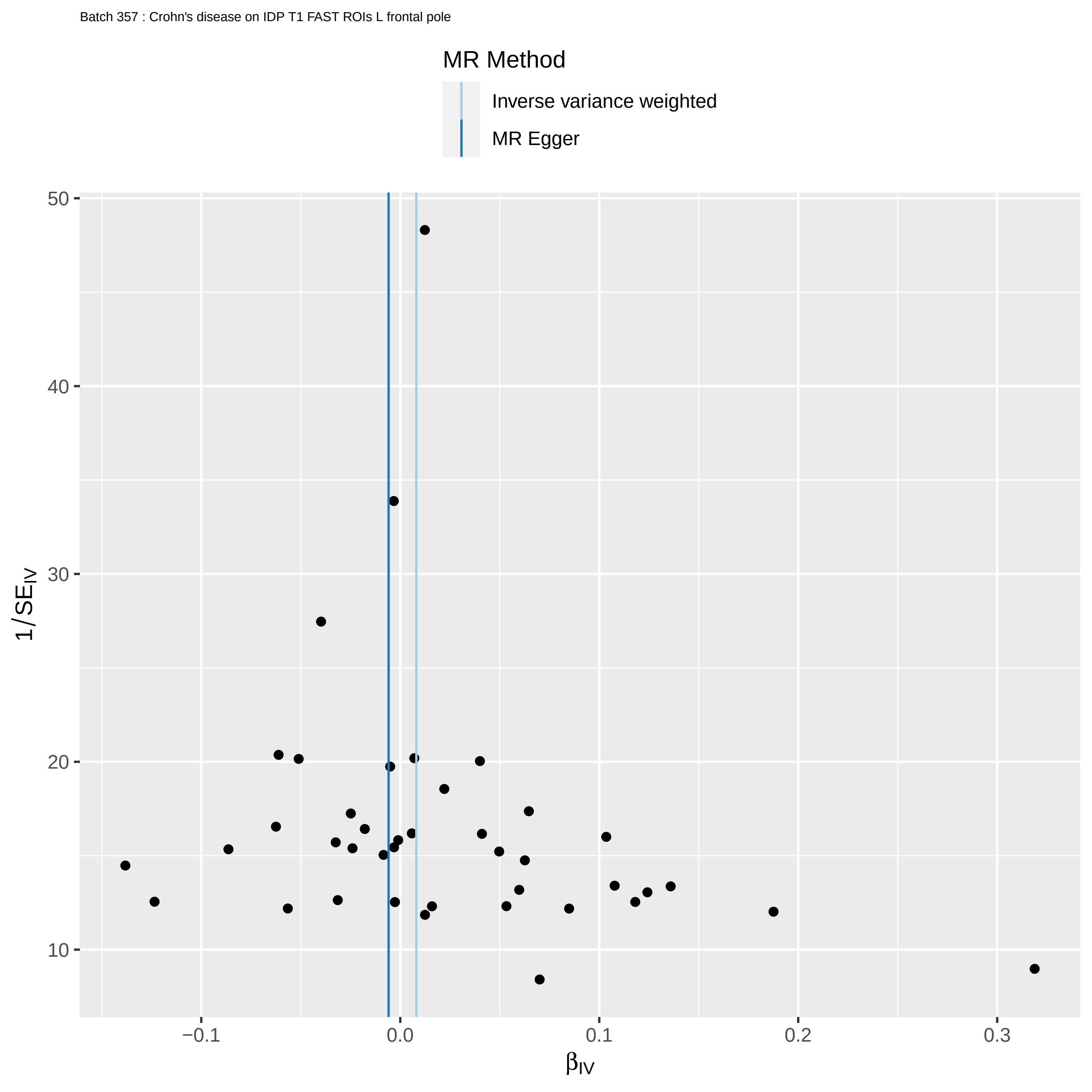

Batch 357 : Crohn's disease on IDP T1 FAST ROIs L frontal pole
MR Method
Inverse variance weighted
MR Egger
50
40
30
20
10
1/SE$_{IV}$
−0.1
0.0
0.1
0.2
0.3
β$_{IV}$

Batch 358 : Ulcerative colitis on IDP T1 FAST ROIs L frontal pole

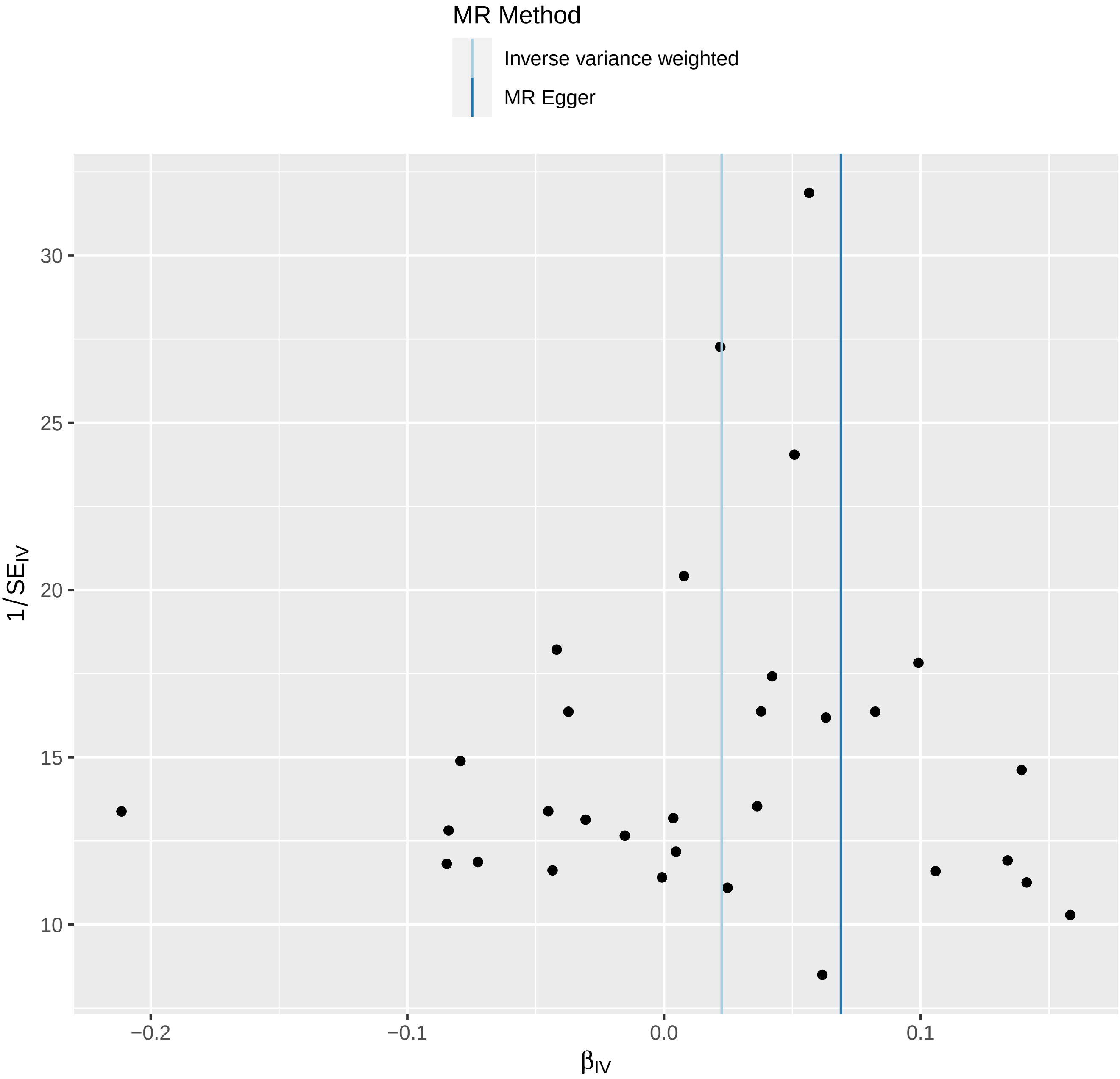

Batch 369 : Crohn's disease on IDP T1 FIRST left thalamus volume plus IDP T1 FIRST right thalamus volume

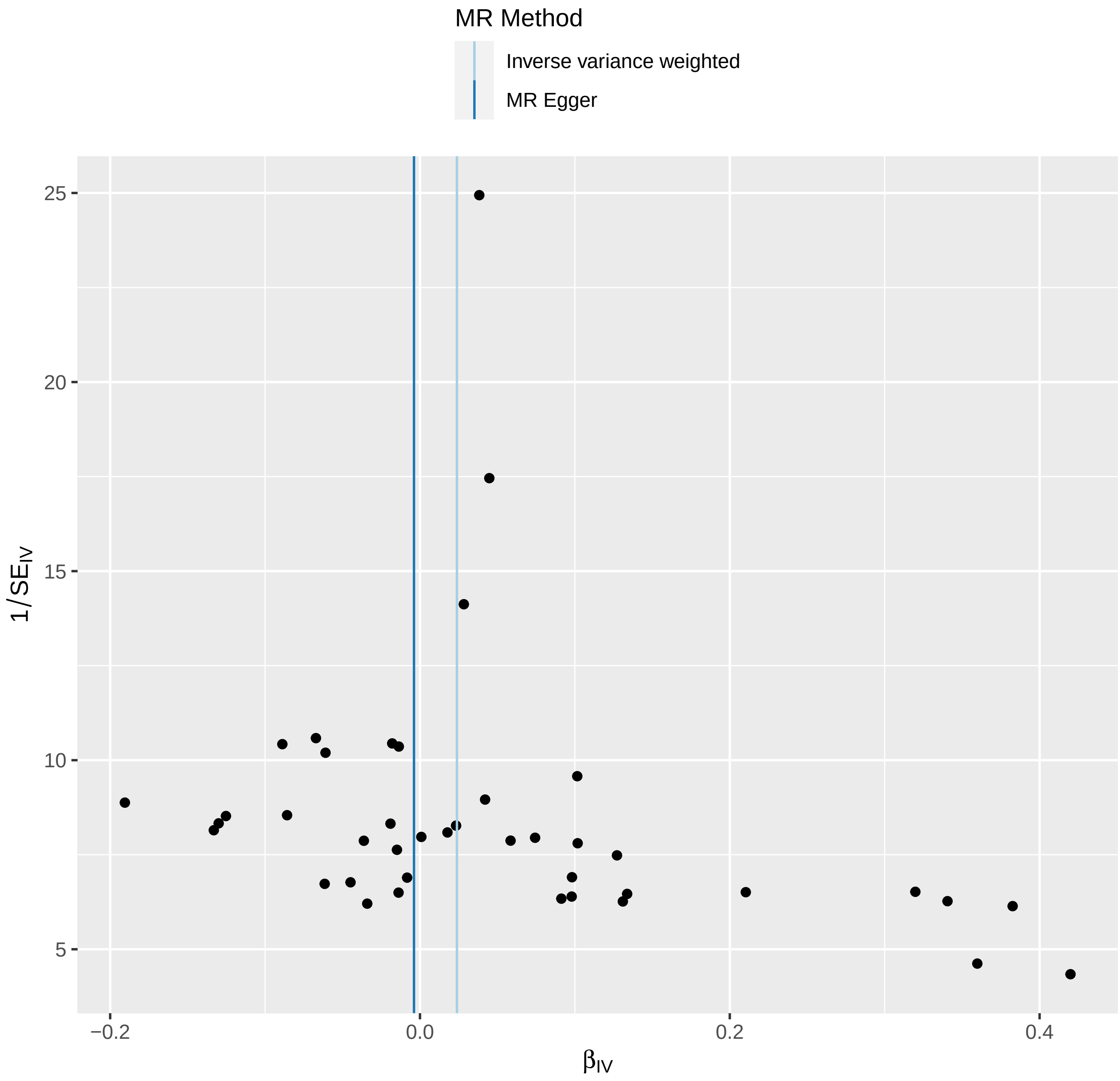

Batch 370 : Ulcerative colitis on IDP T1 FIRST left thalamus volume plus IDP T1 FIRST right thalamus volume

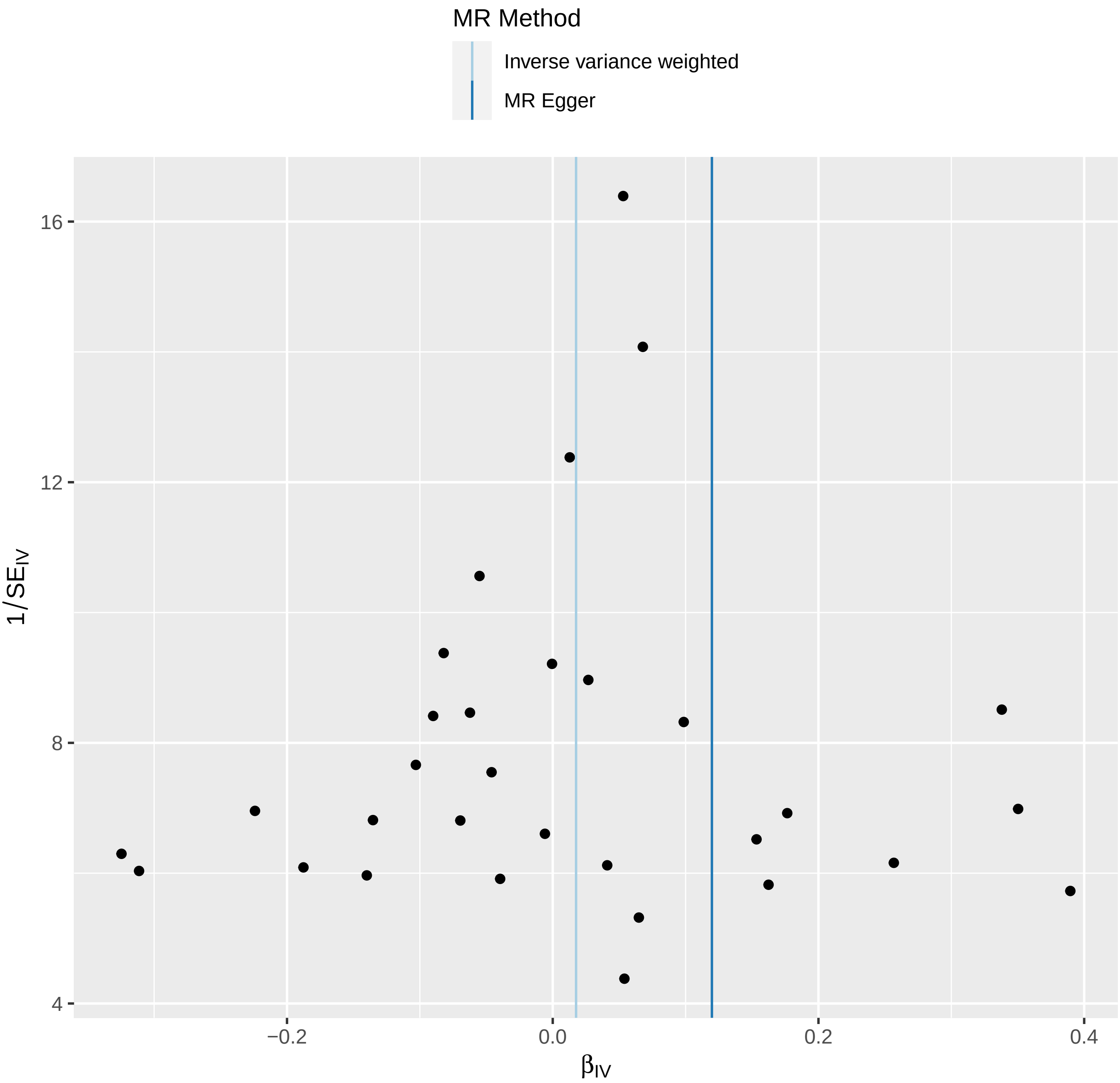

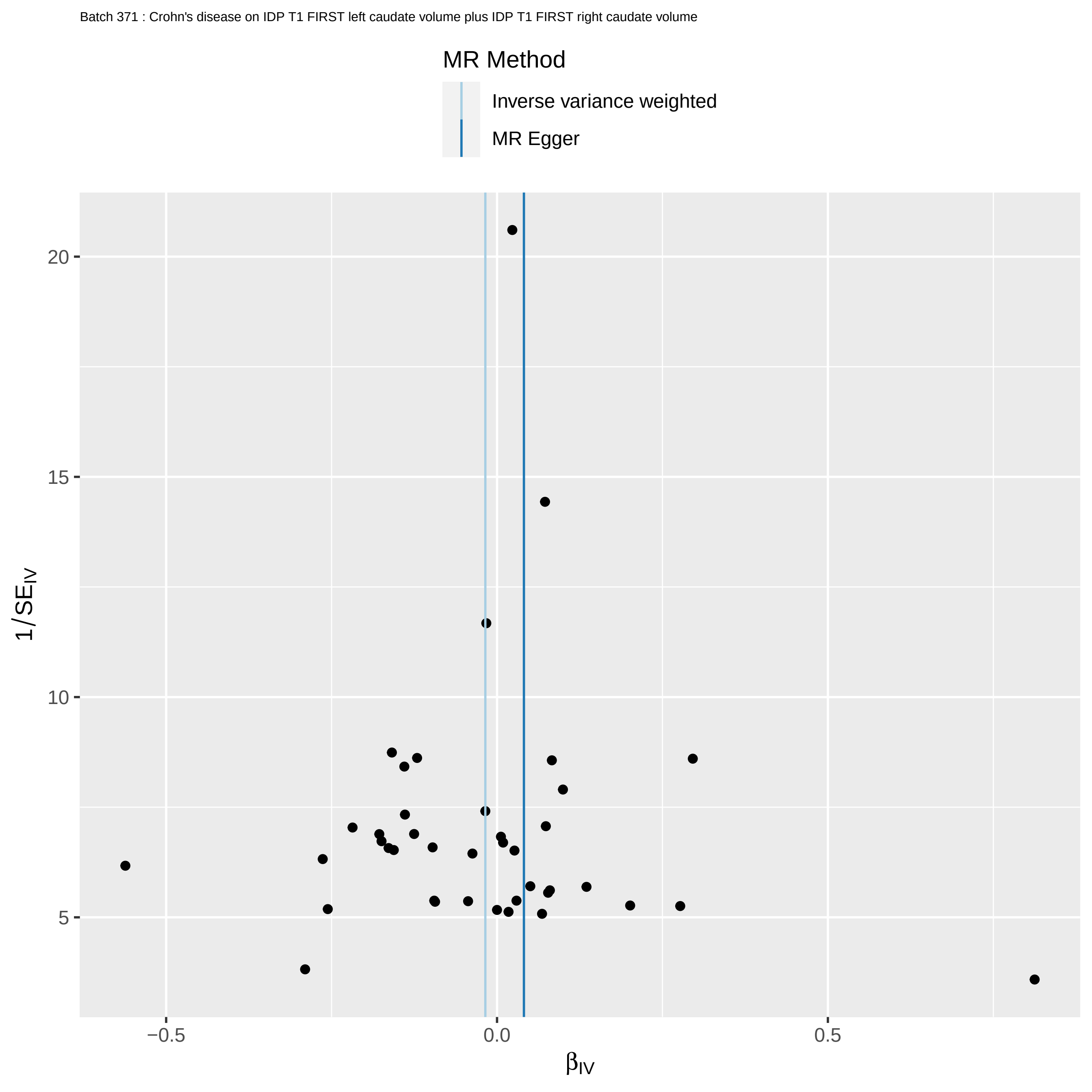

Batch 371 : Crohn's disease on IDP T1 FIRST left caudate volume plus IDP T1 FIRST right caudate volume
MR Method
Inverse variance weighted
MR Egger
20
15
10
5
1/SE$_{IV}$
−0.5
0.0
0.5
β$_{IV}$

Batch 372 : Ulcerative colitis on IDP T1 FIRST left caudate volume plus IDP T1 FIRST right caudate volume

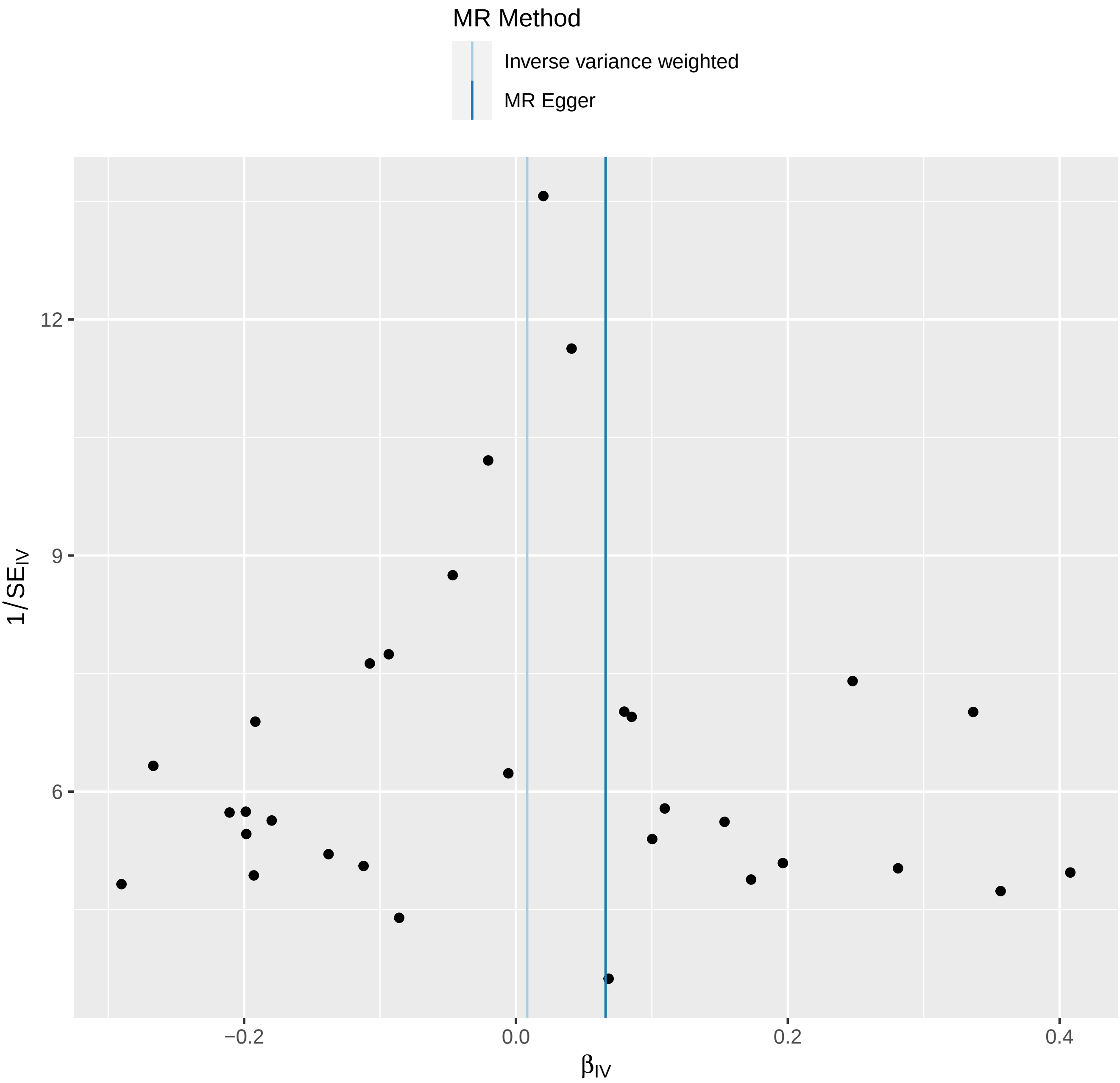

Batch 373 : Crohn's disease on IDP T1 FIRST left putamen volume plus IDP T1 FIRST right putamen volume

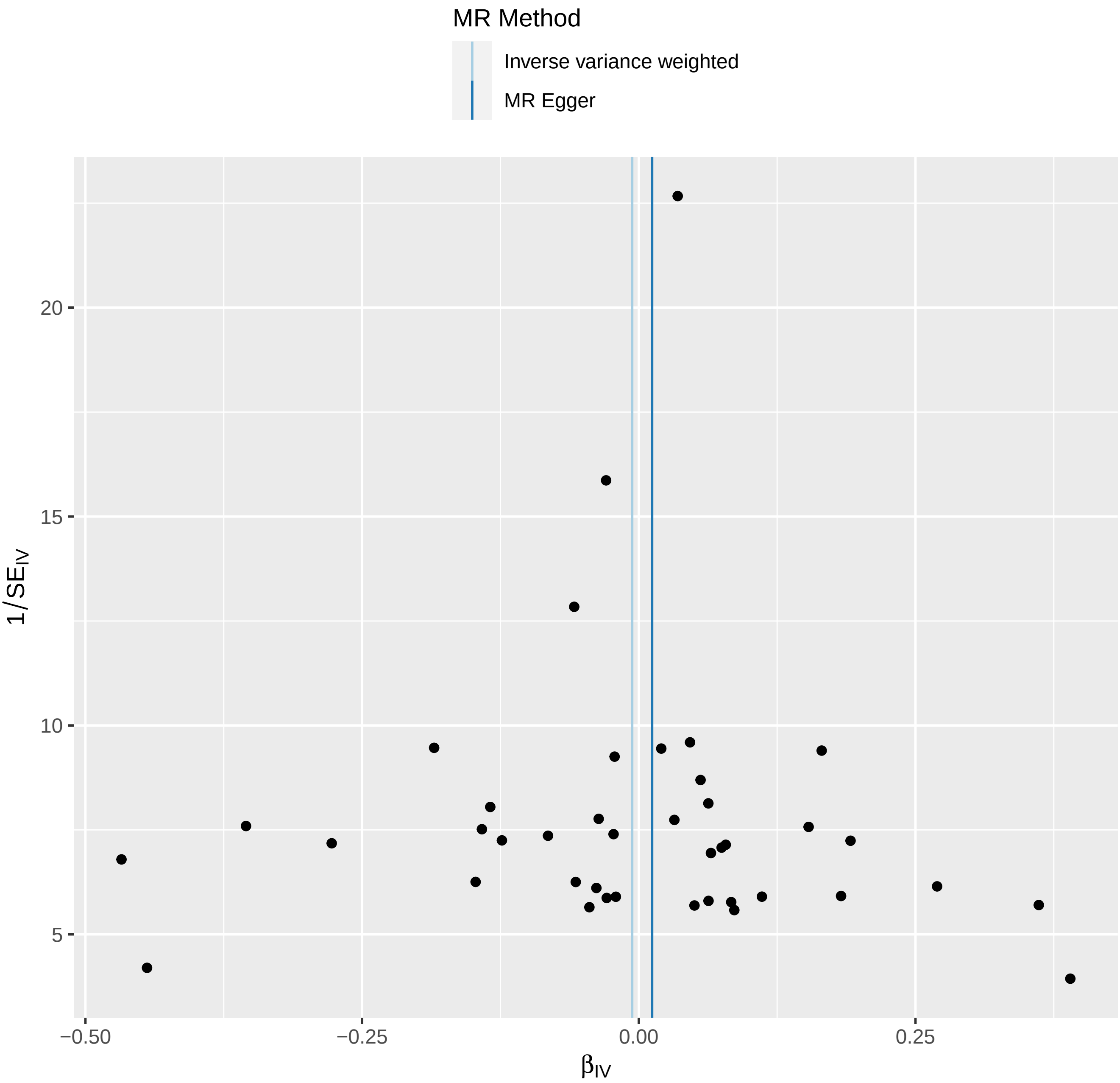

Batch 374 : Ulcerative colitis on IDP T1 FIRST left putamen volume plus IDP T1 FIRST right putamen volume

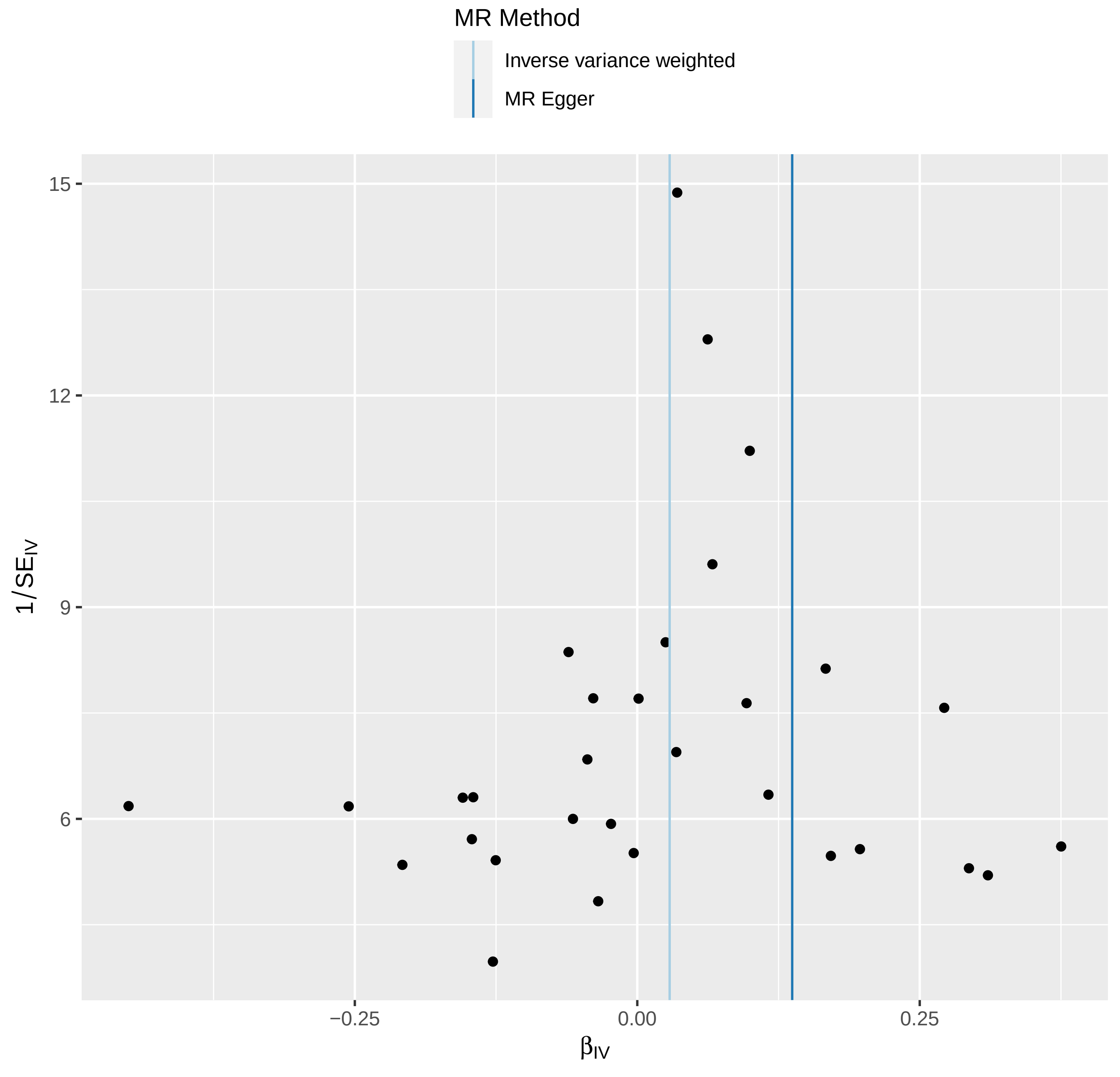

Batch 375 : Crohn's disease on IDP T1 FIRST left pallidum volume plus IDP T1 FIRST right pallidum volume

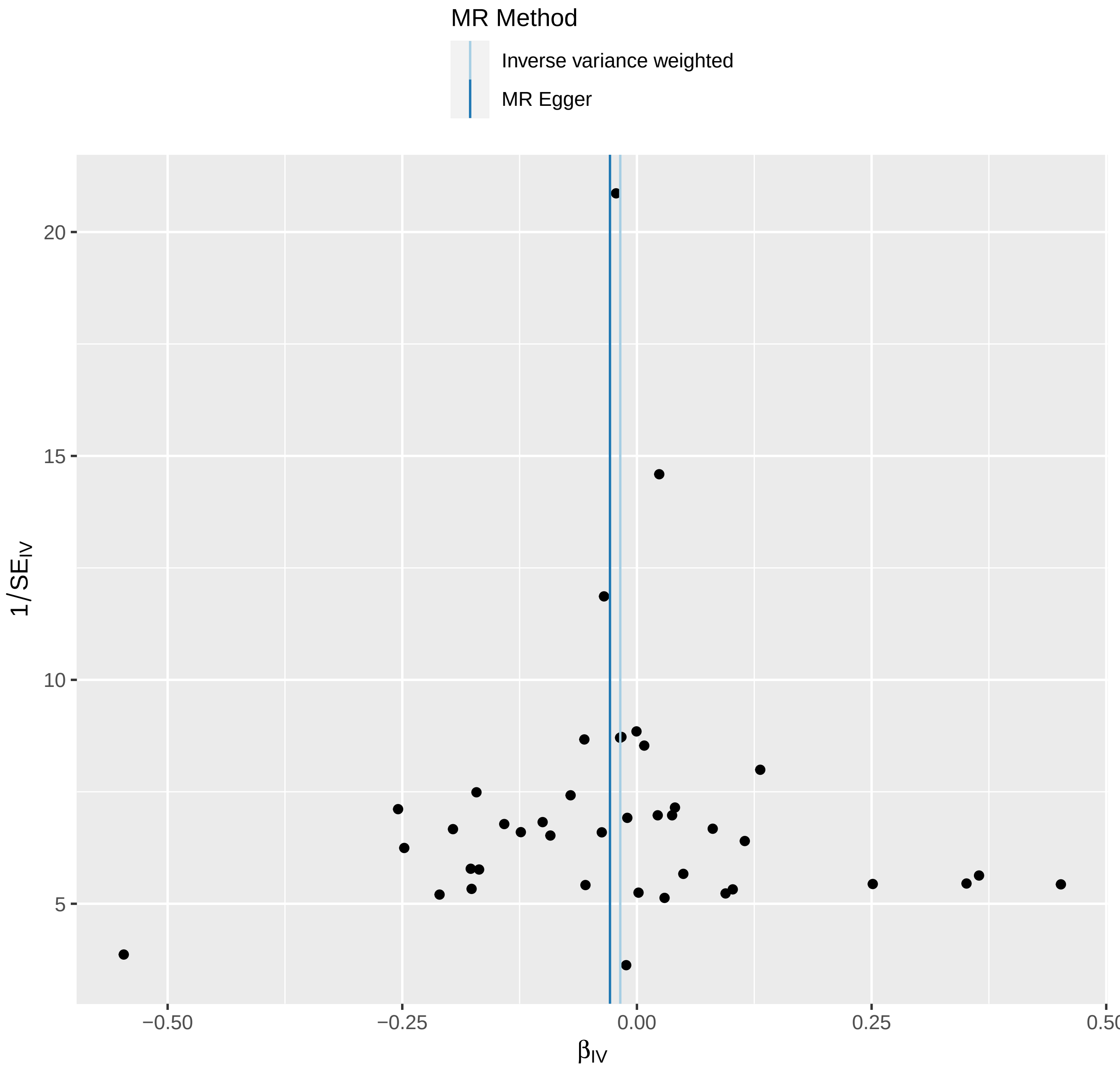

Batch 376 : Ulcerative colitis on IDP T1 FIRST left pallidum volume plus IDP T1 FIRST right pallidum volume

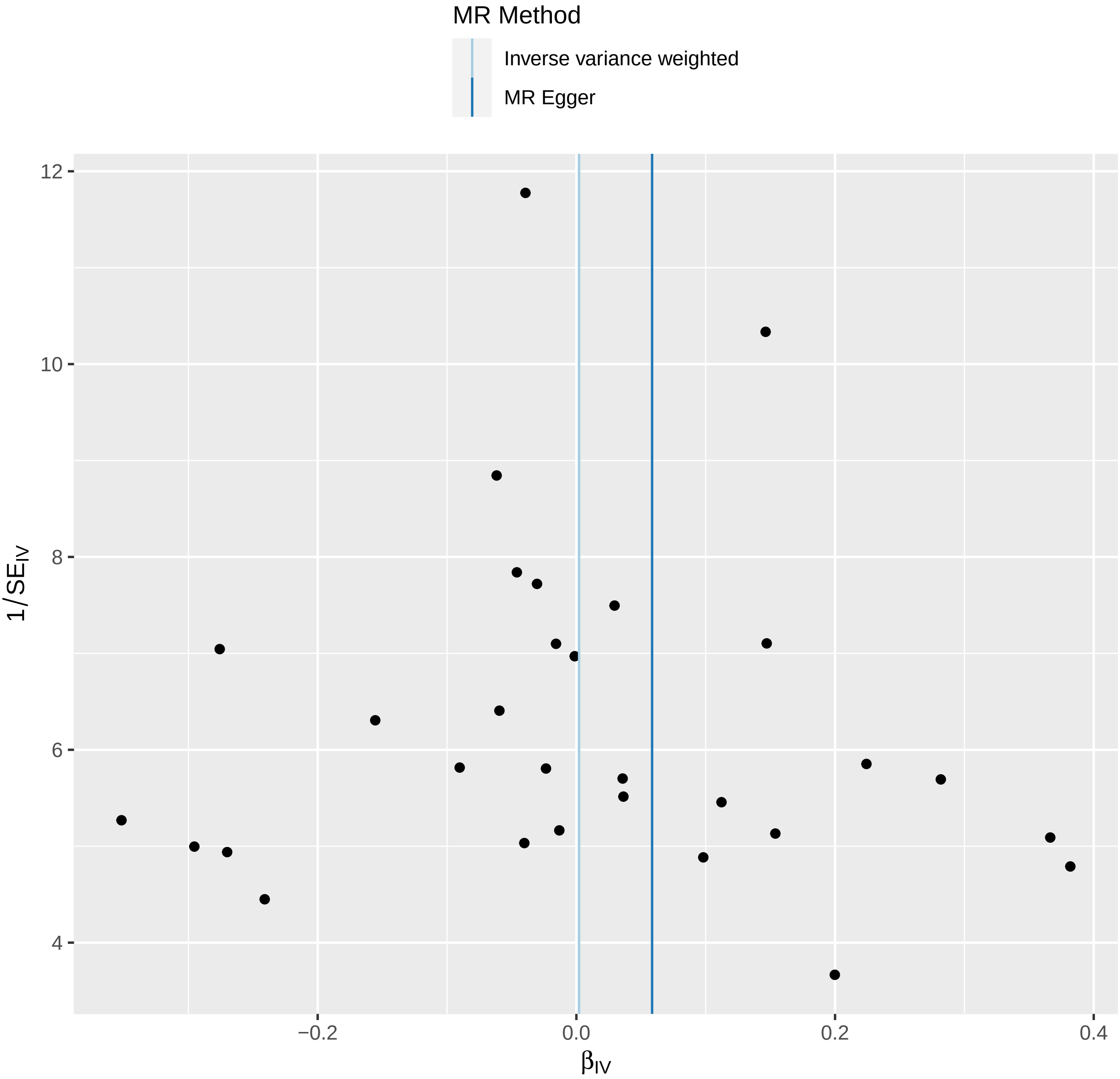

Batch 377 : Crohn's disease on IDP T1 FIRST left hippocampus volume plus IDP T1 FIRST right hippocampus volume

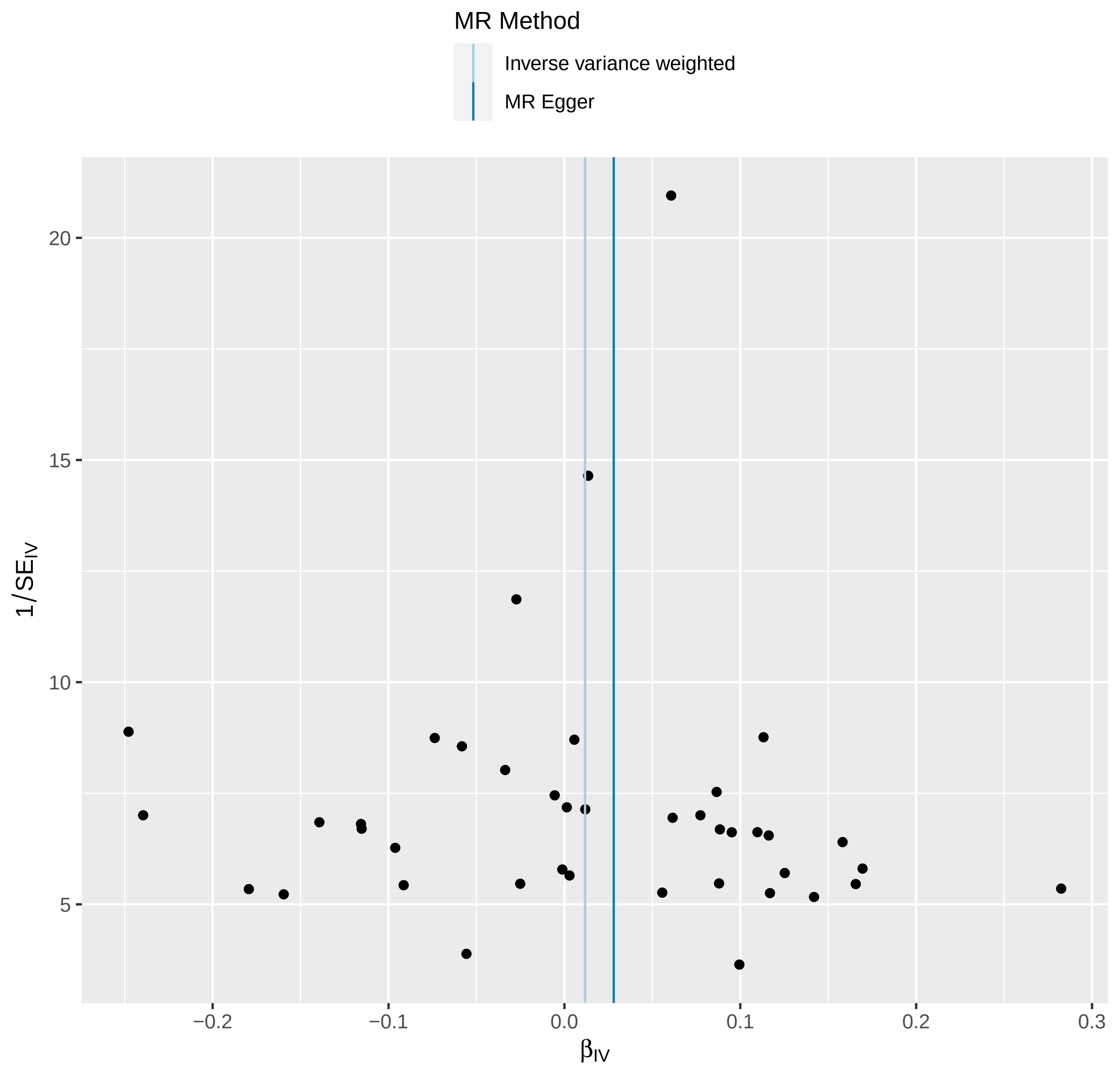

Batch 378 : Ulcerative colitis on IDP T1 FIRST left hippocampus volume plus IDP T1 FIRST right hippocampus volume

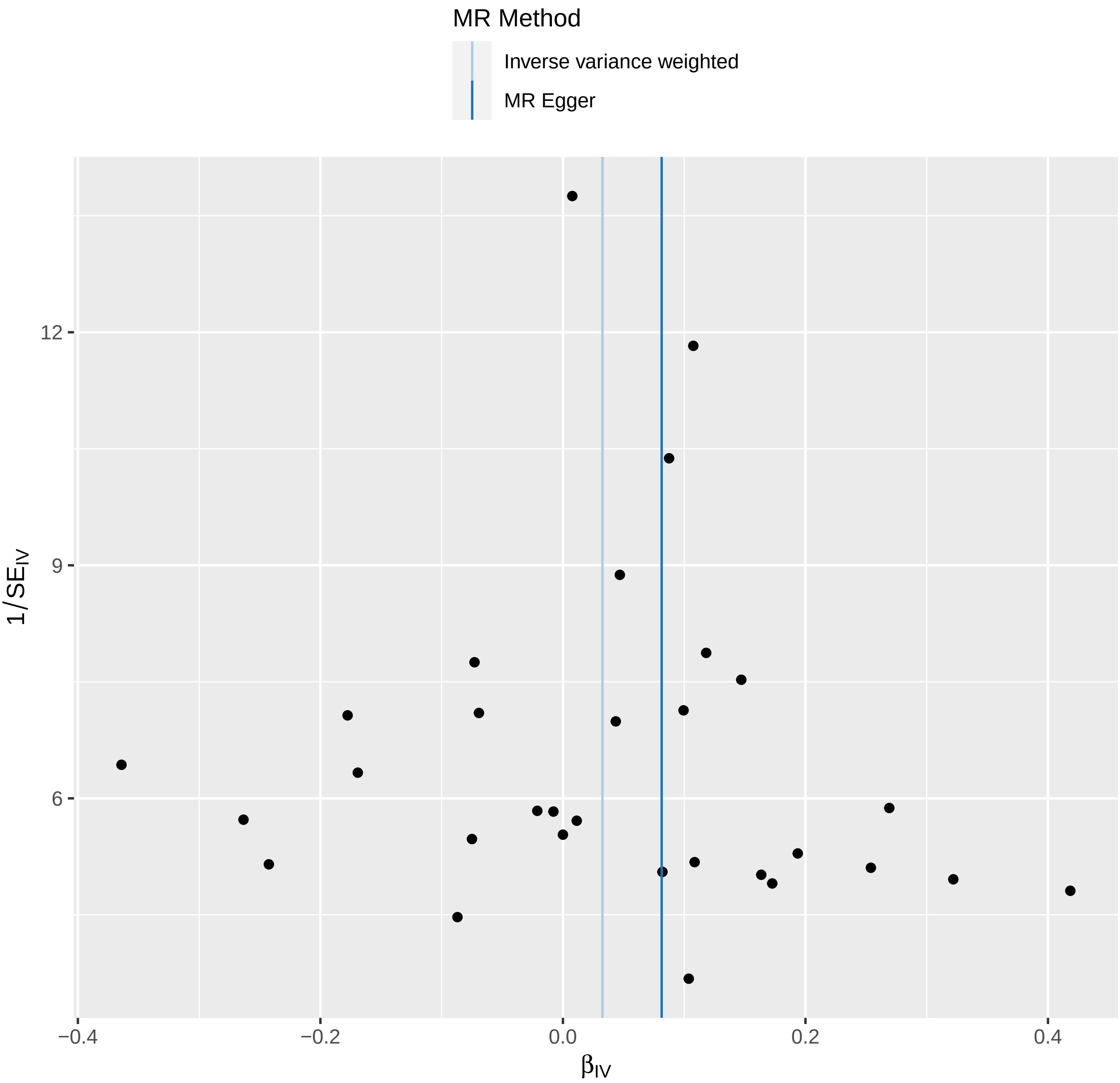

Batch 379 : Crohn's disease on IDP T1 FIRST left amygdala volume plus IDP T1 FIRST right amygdala volume

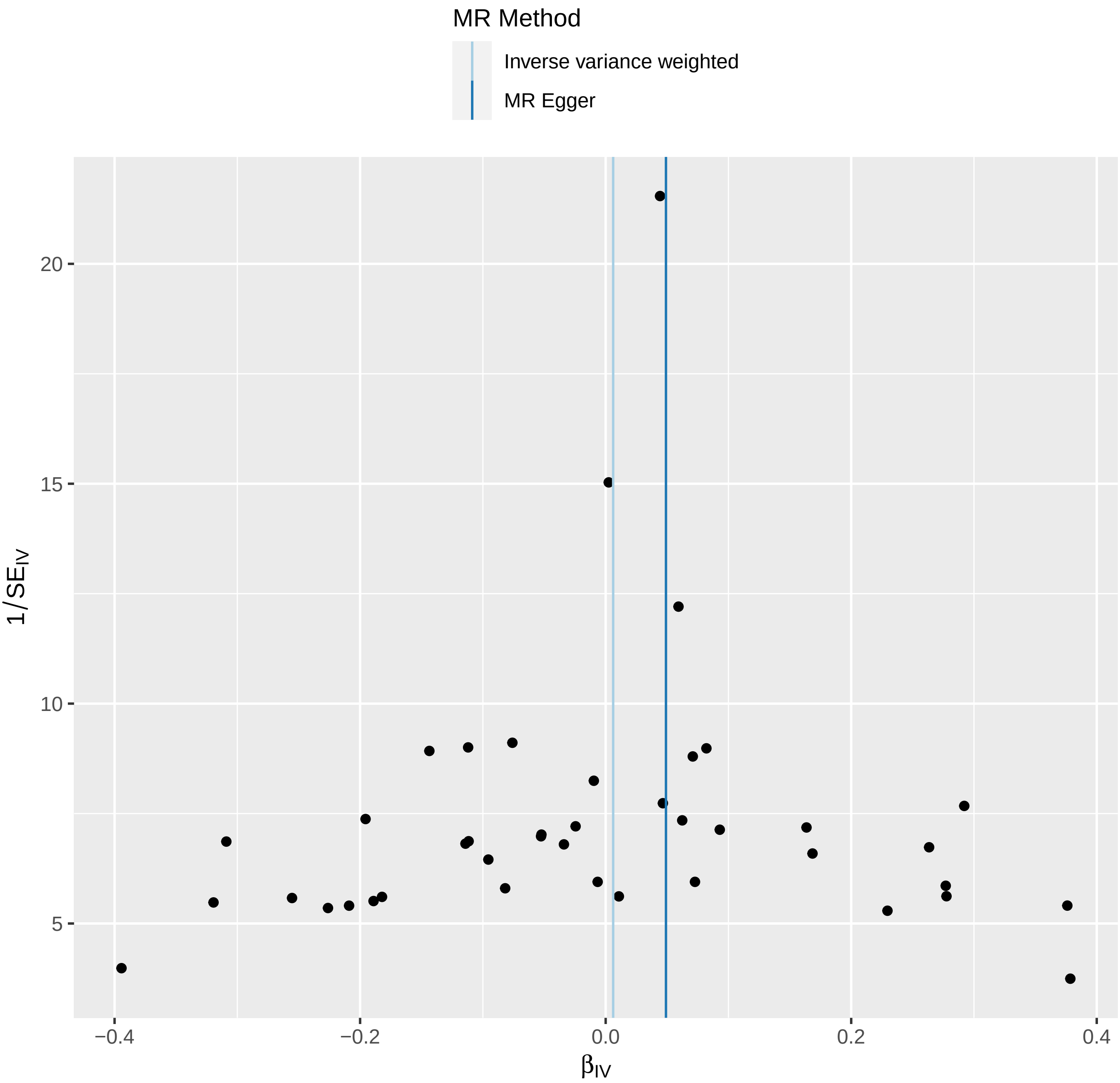

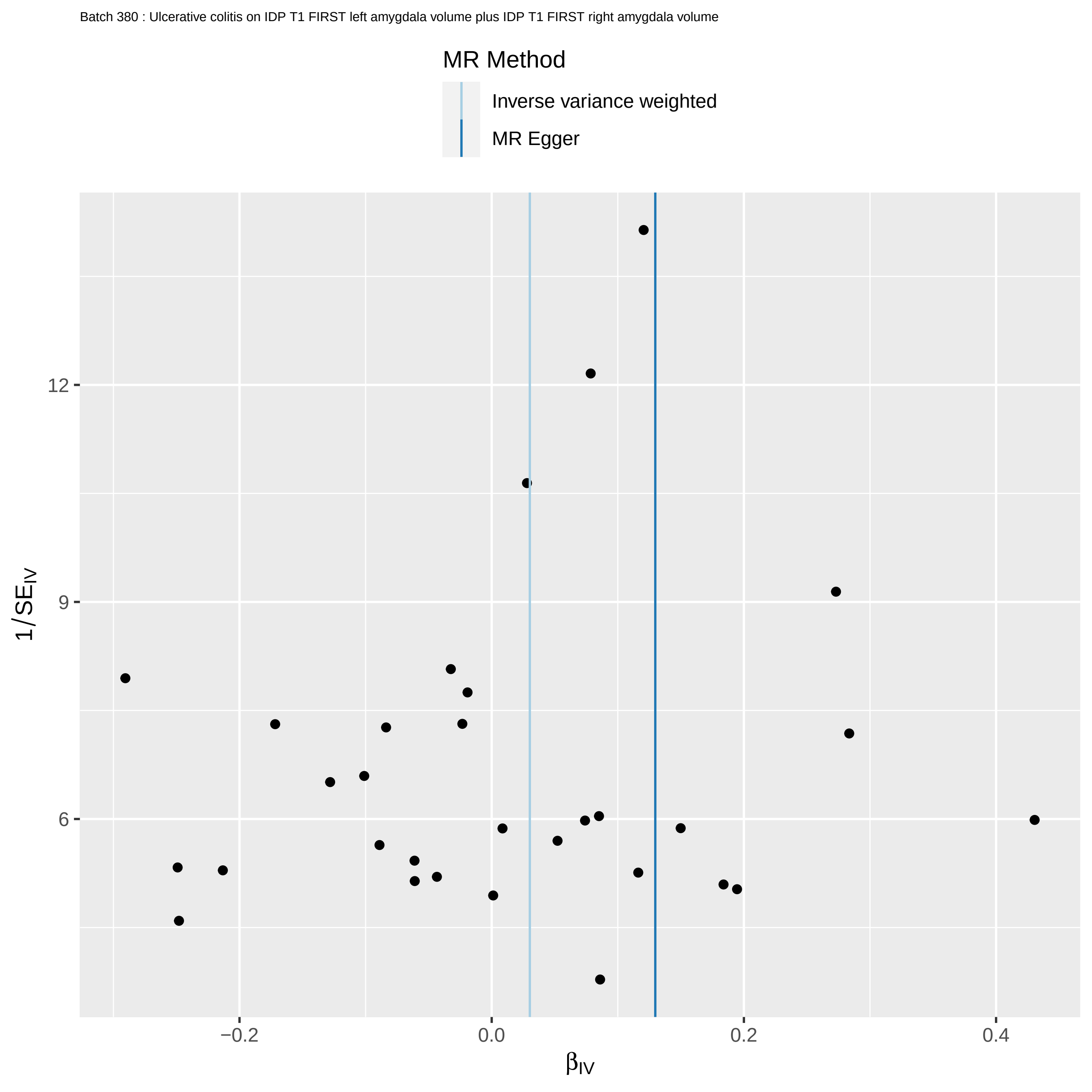

Batch 380 : Ulcerative colitis on IDP T1 FIRST left amygdala volume plus IDP T1 FIRST right amygdala volume
MR Method
Inverse variance weighted
MR Egger
1/SE$_{IV}$
12
9
6
−0.2
0.0
0.2
0.4
β_{IV}

Batch 381 : Crohn's disease on IDP T1 FIRST left accumbens volume plus IDP T1 FIRST right accumbens volume

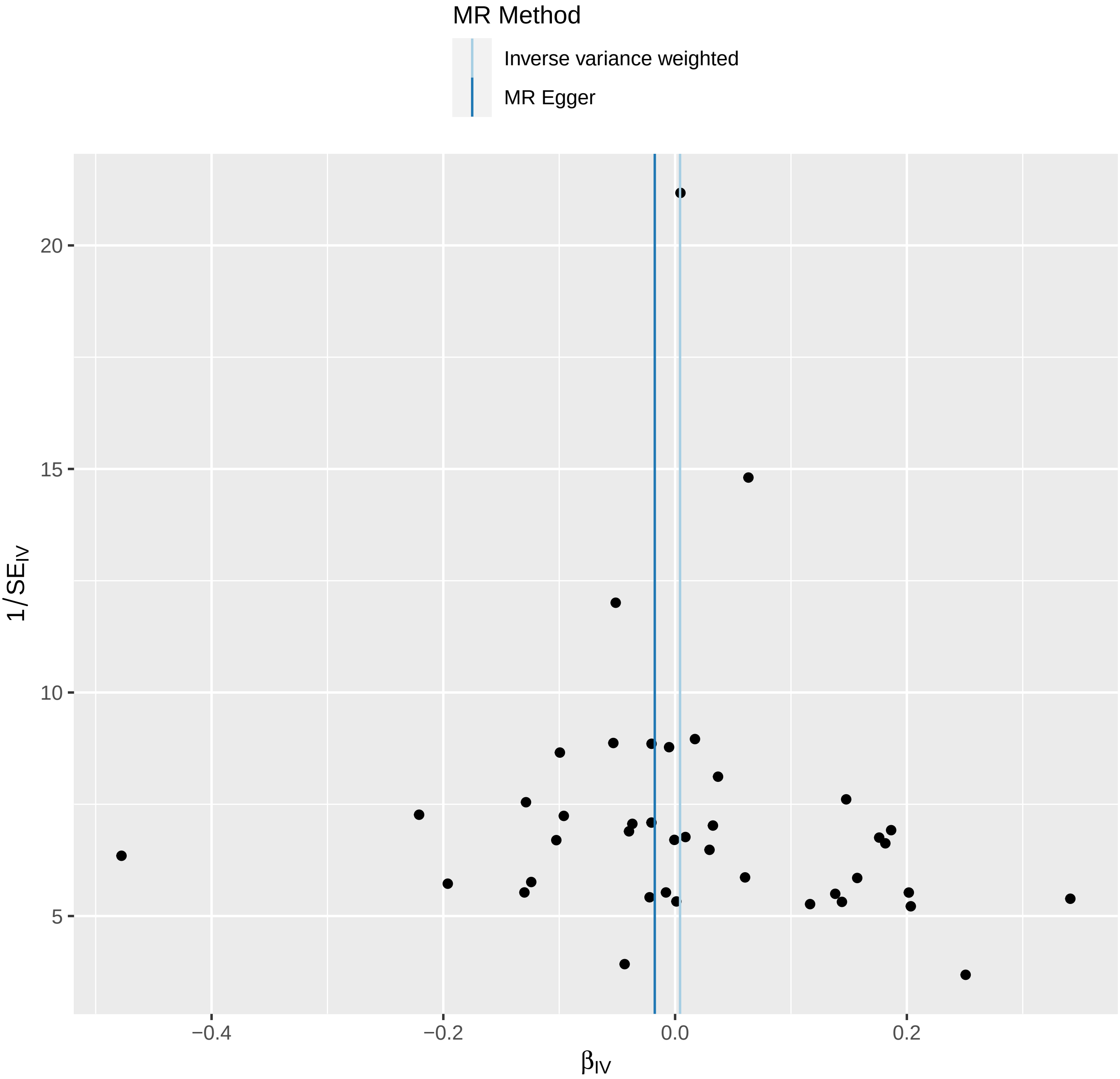

Batch 382 : Ulcerative colitis on IDP T1 FIRST left accumbens volume plus IDP T1 FIRST right accumbens volume

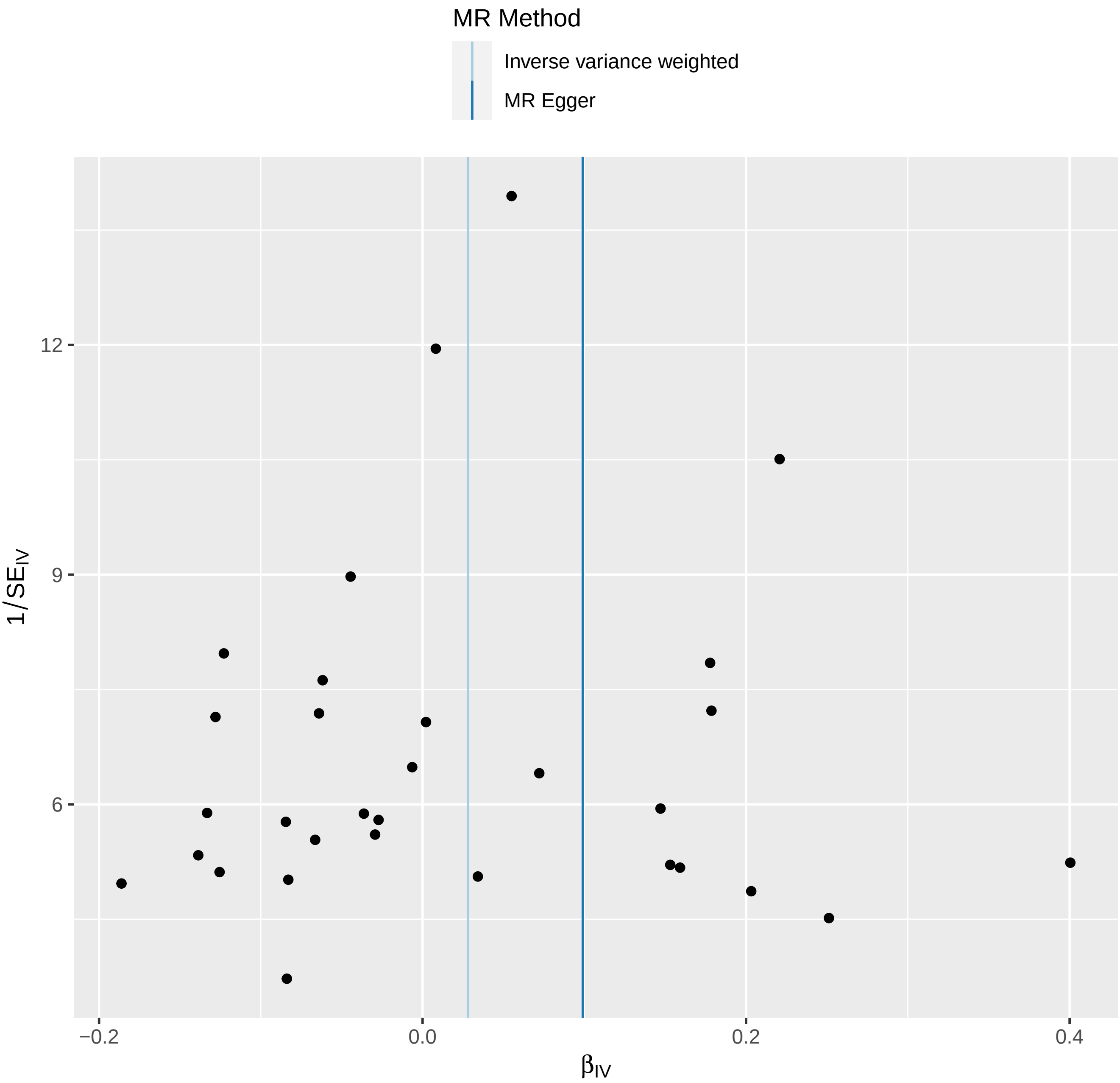

Batch 495 : Crohn's disease on IDP T1 FAST ROIs R frontal pole

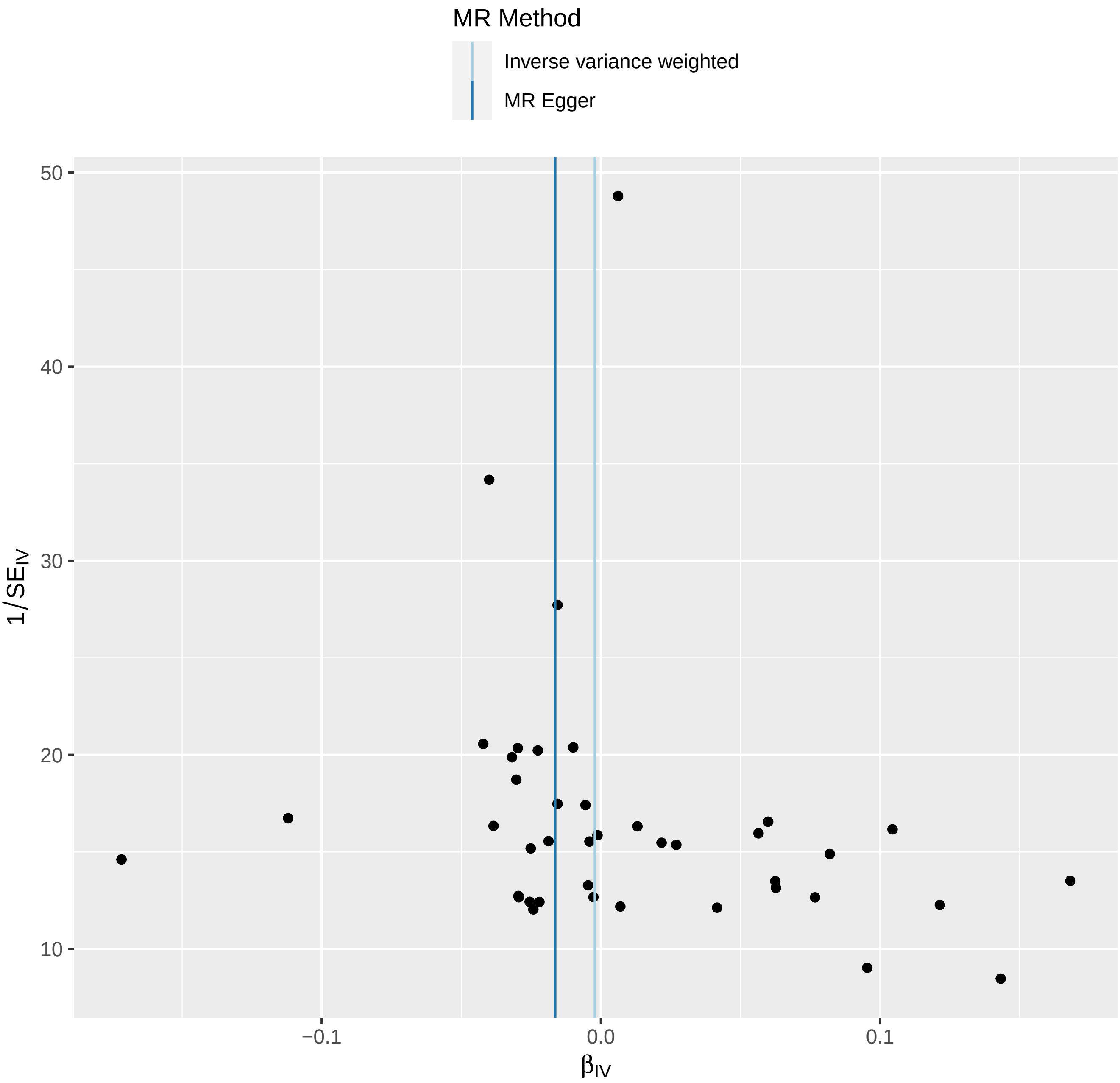

Batch 496 : Ulcerative colitis on IDP T1 FAST ROIs R frontal pole

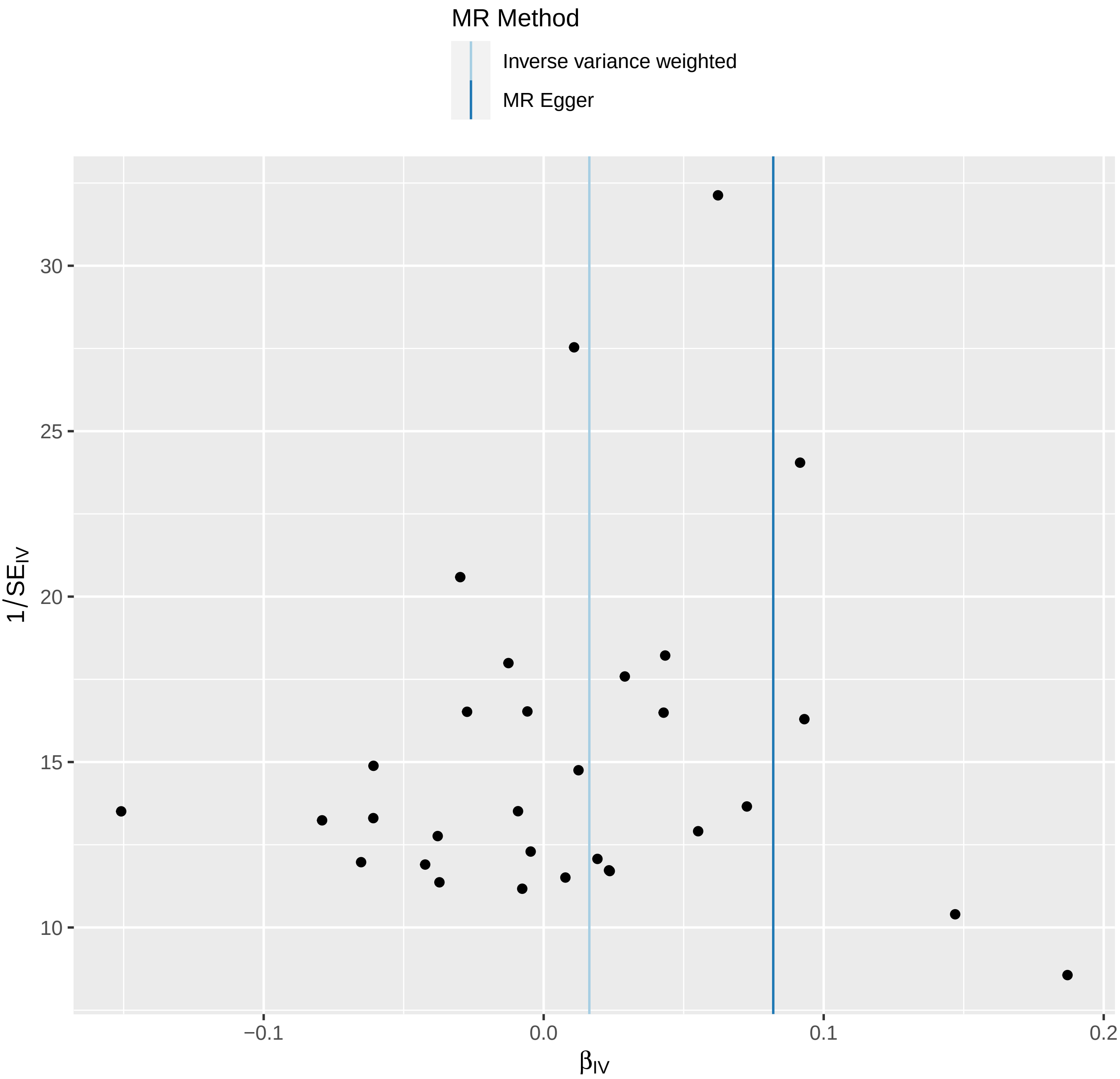

Batch 529 : Crohn's disease on DKTatlas lh caudalanteriorcingulate area

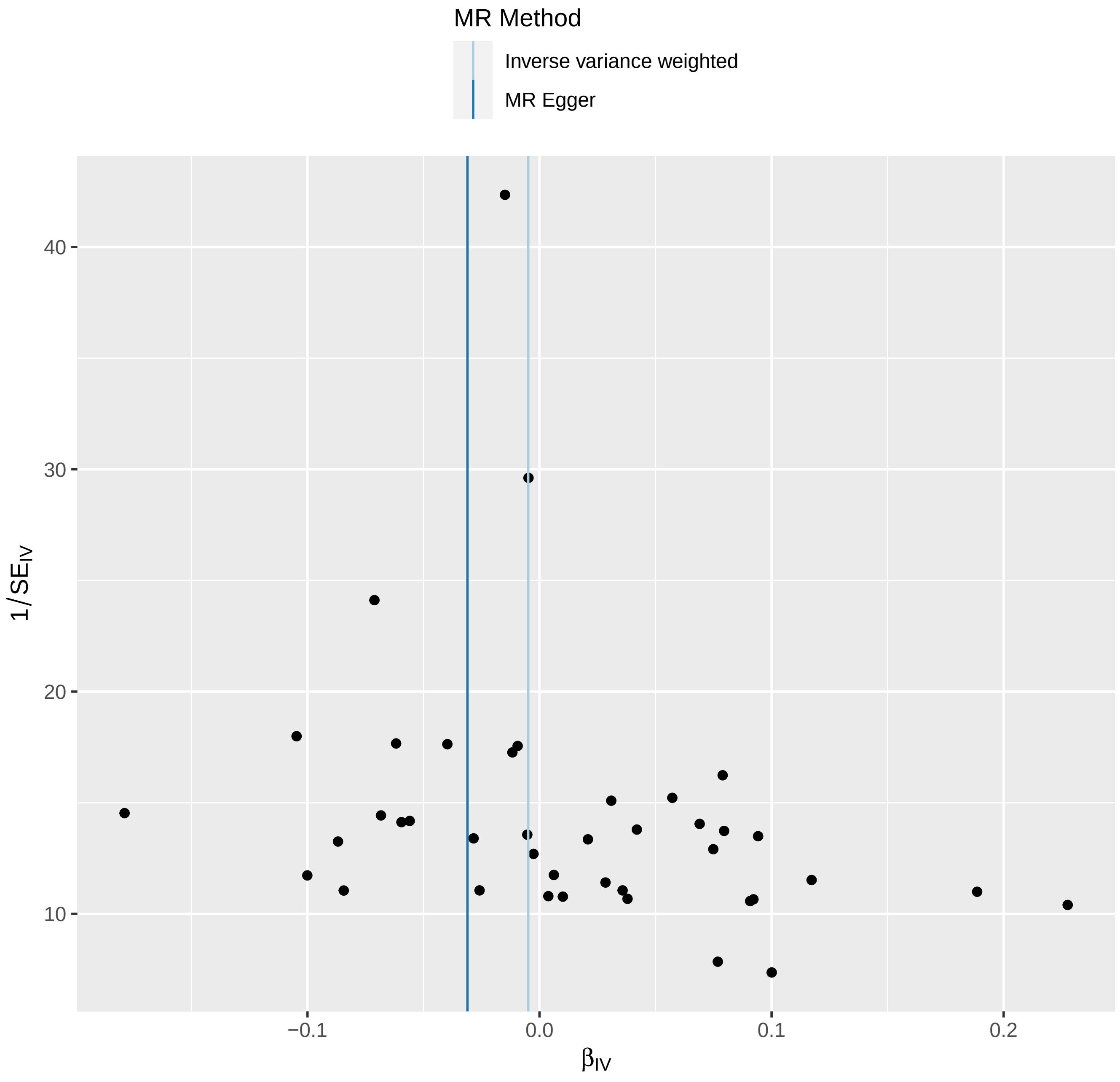

Batch 530 : Ulcerative colitis on DKTatlas lh caudalanteriorcingulate area

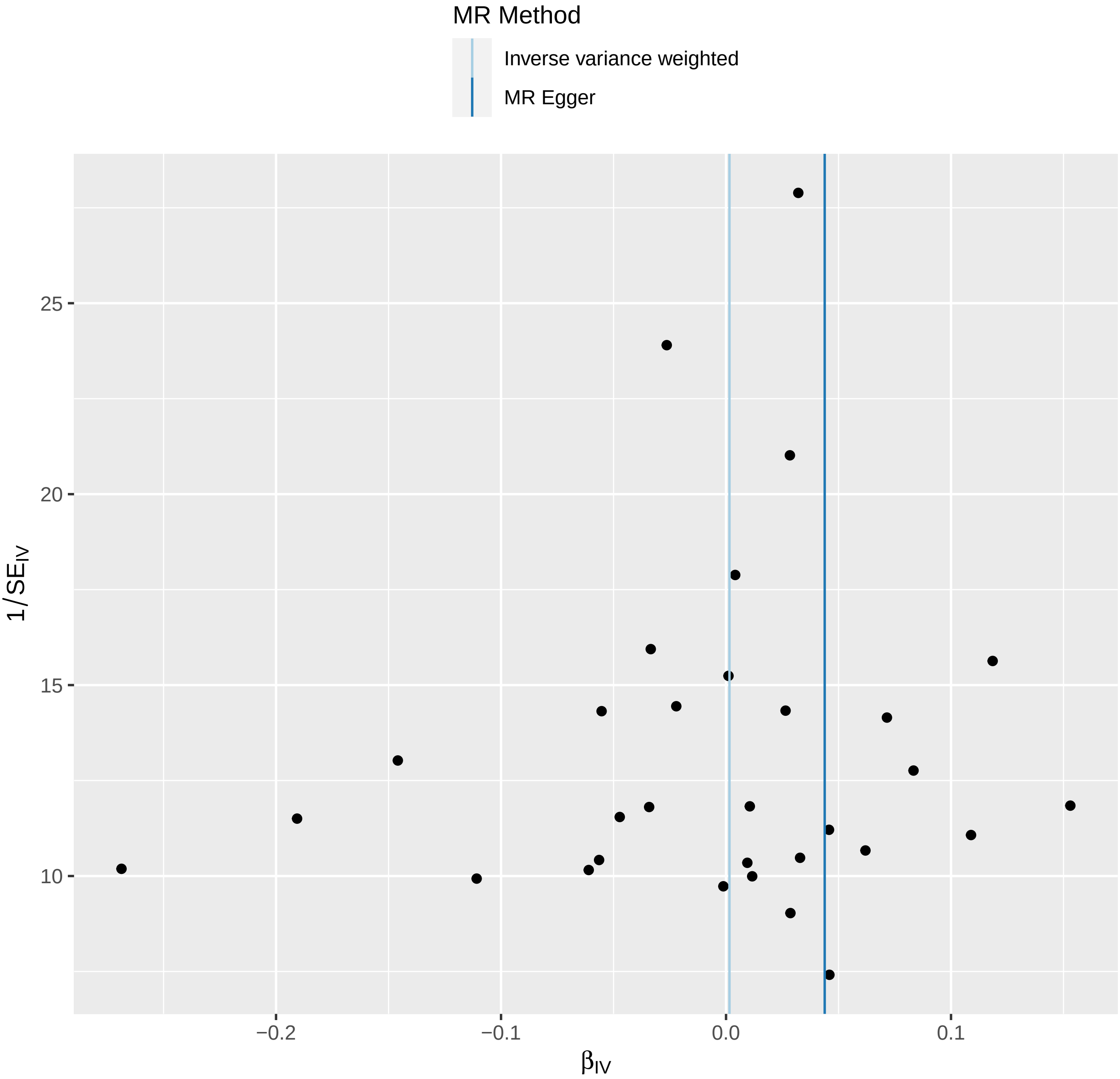

Batch 531 : Crohn's disease on DKTatlas lh caudalmiddlefrontal area

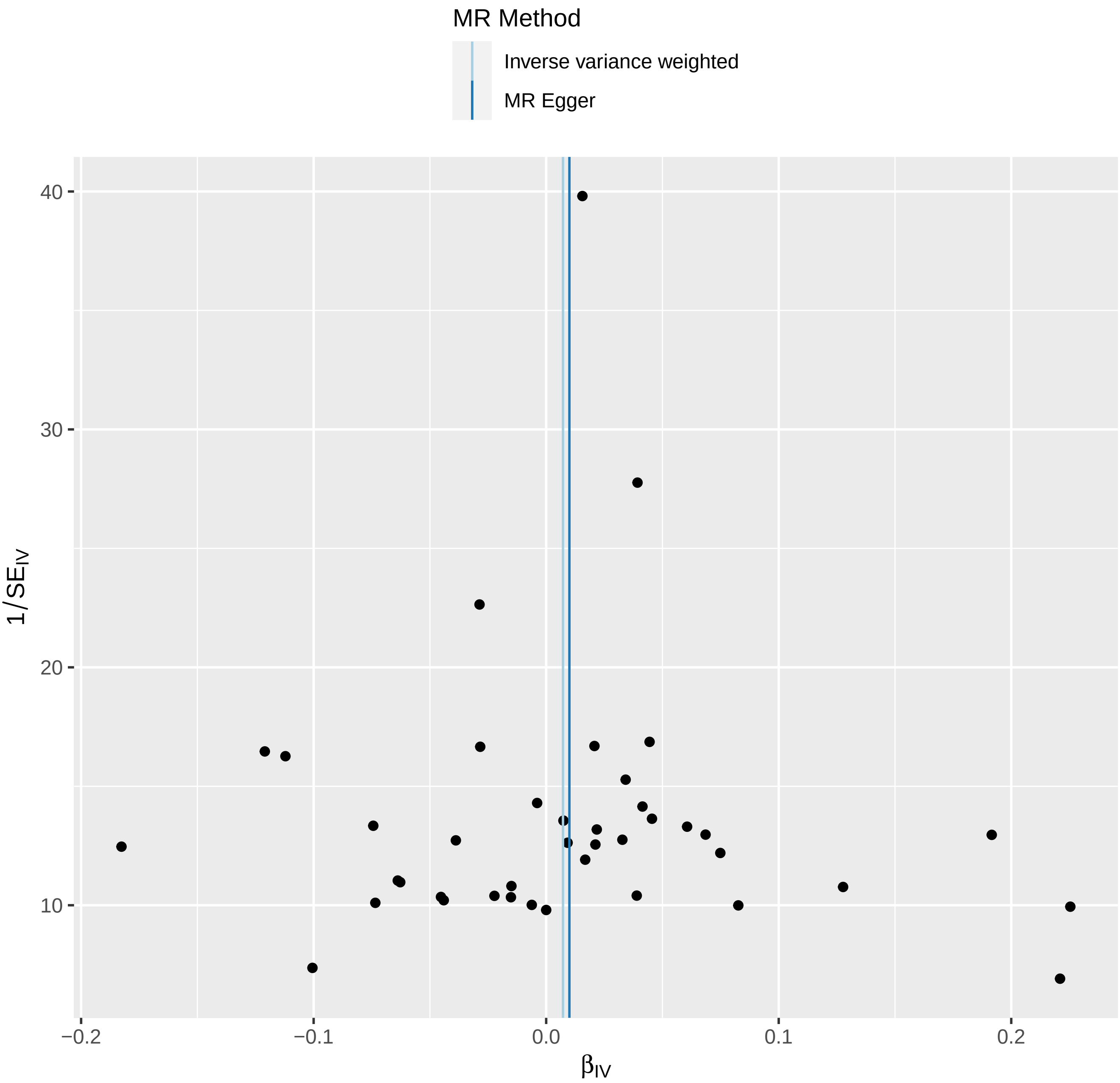

Batch 532 : Ulcerative colitis on DKTatlas lh caudalmiddlefrontal area

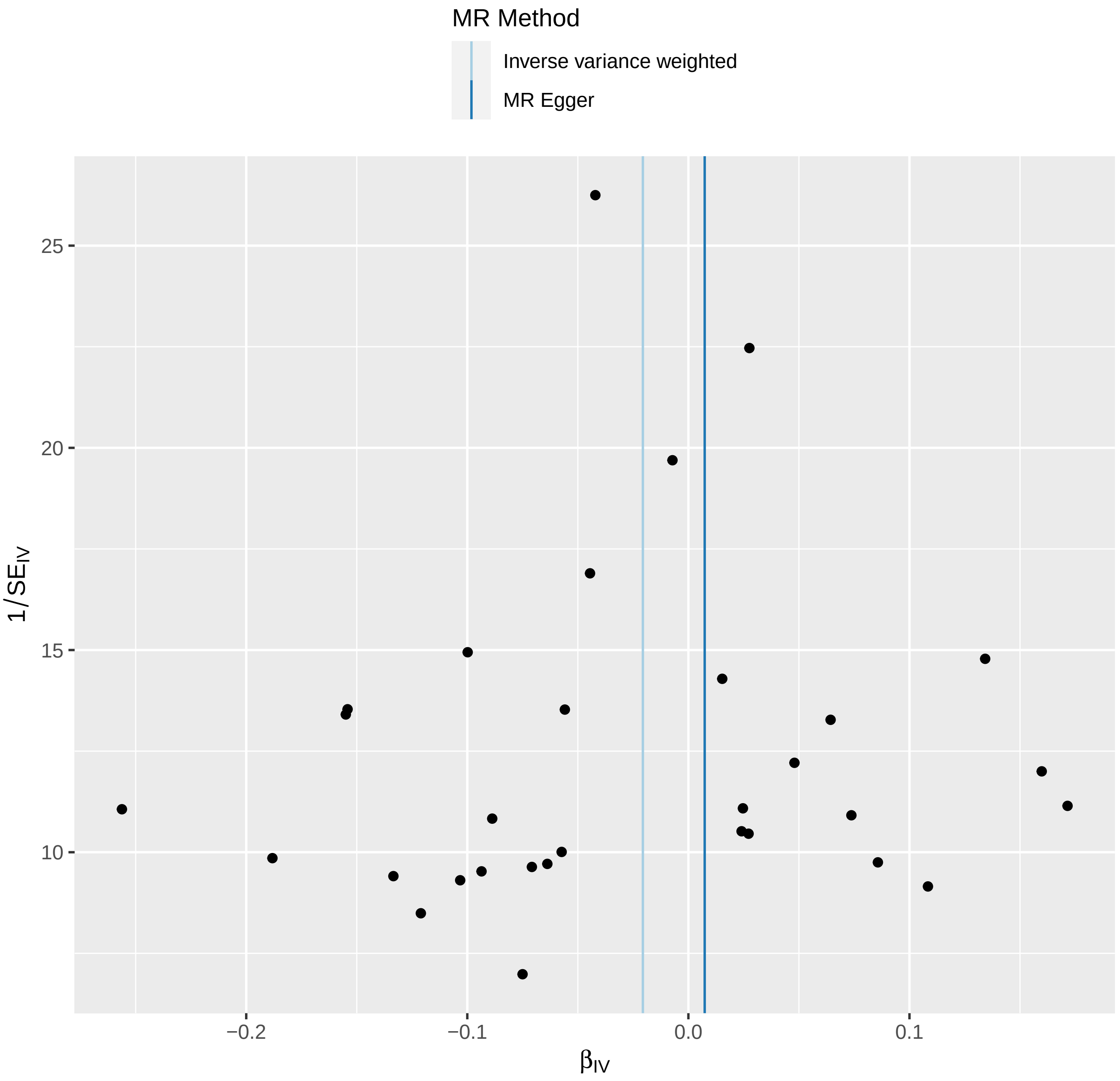

Batch 533 : Crohn's disease on DKTatlas lh cuneus area

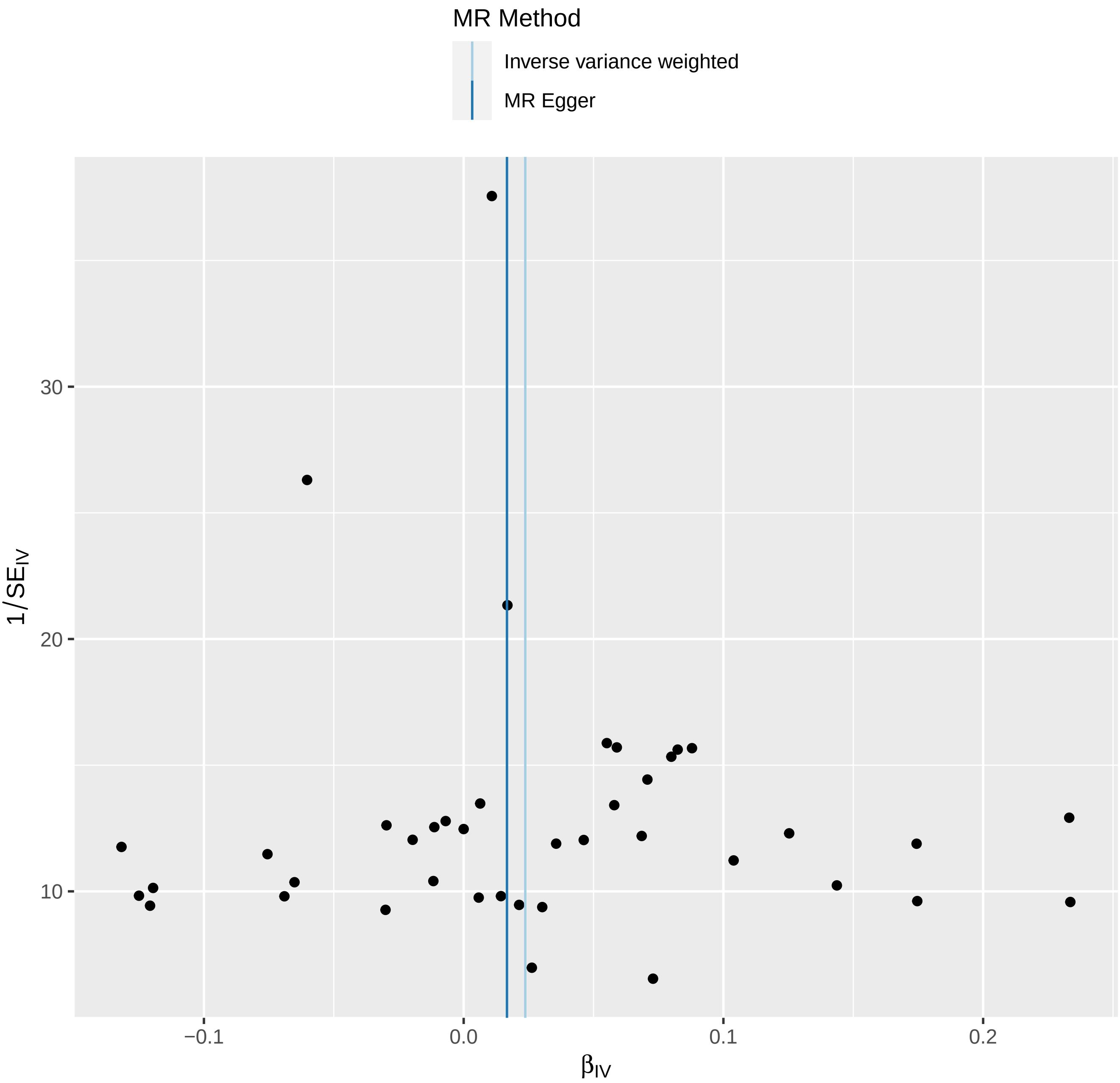

Batch 534 : Ulcerative colitis on DKTatlas lh cuneus area

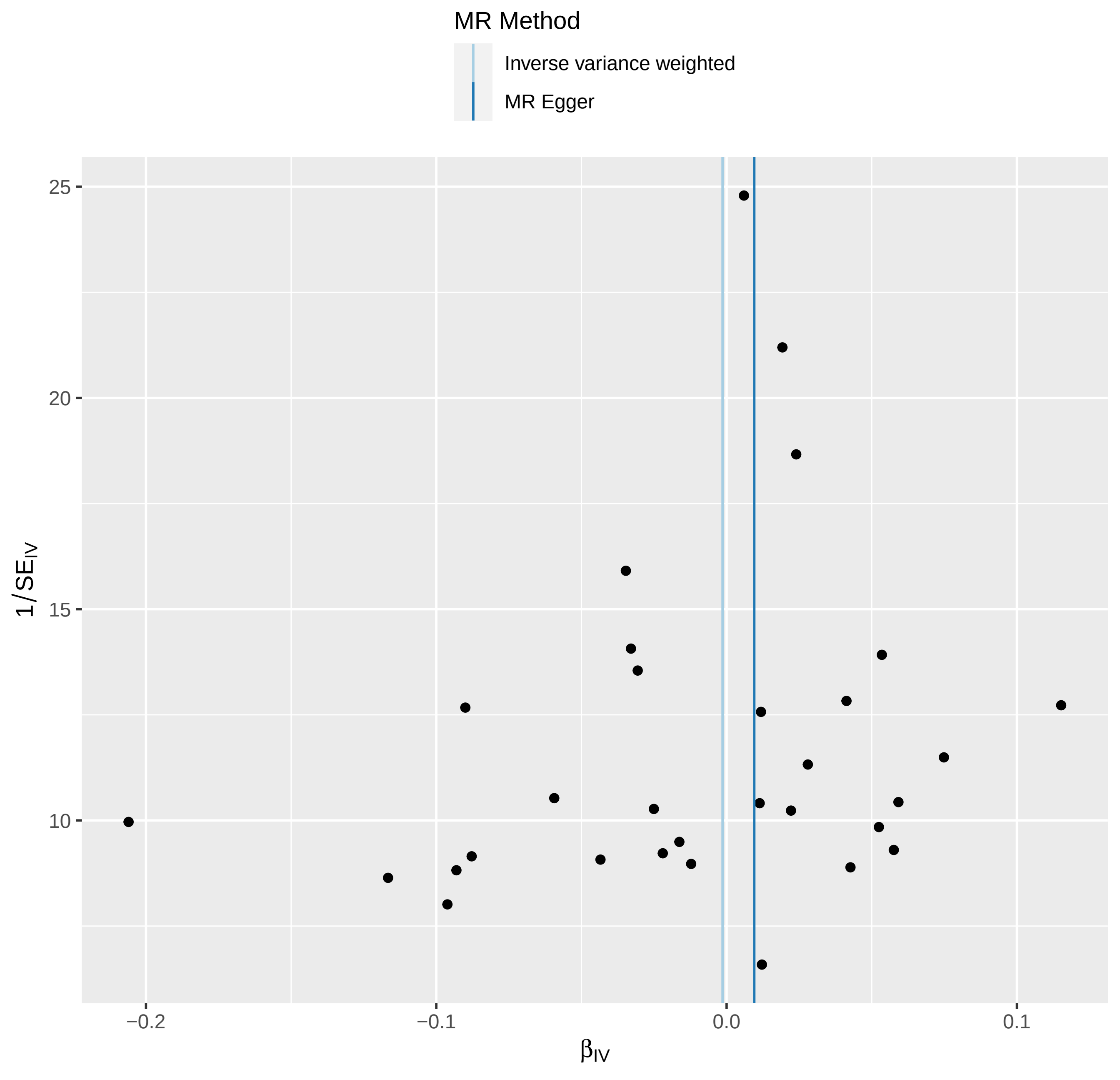

Batch 535 : Crohn's disease on DKTatlas lh entorhinal area

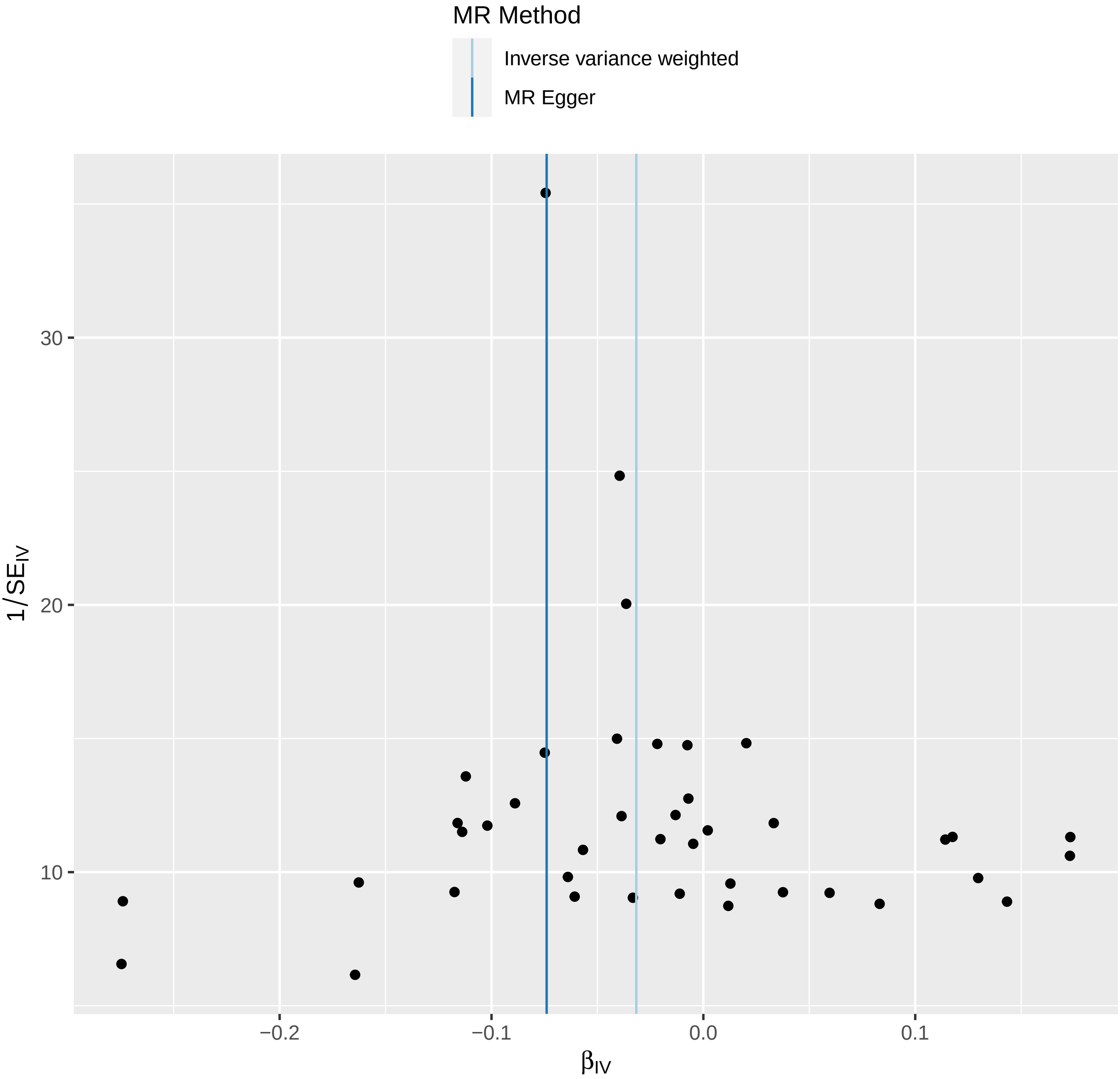

Batch 536 : Ulcerative colitis on DKTatlas lh entorhinal area

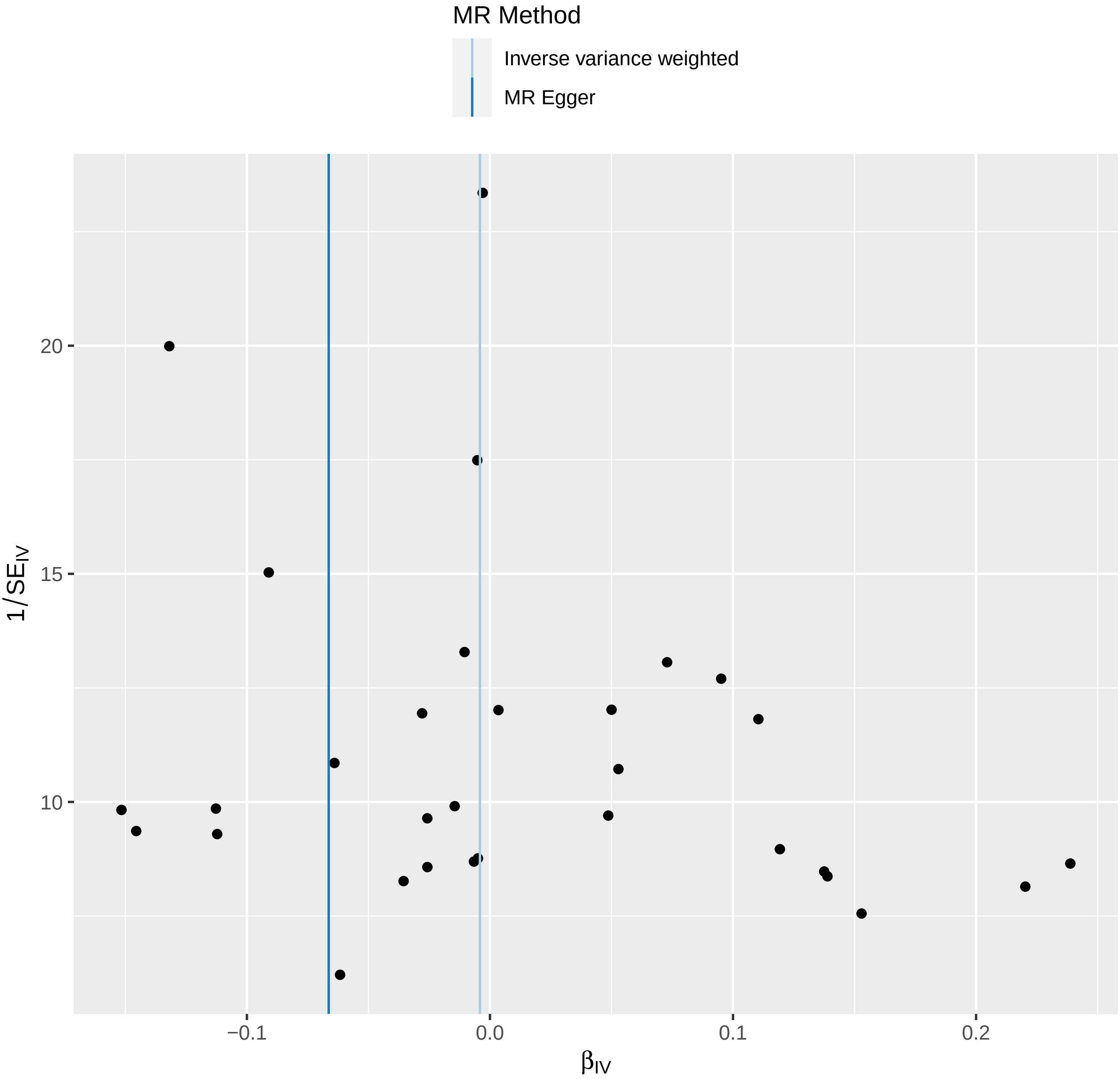

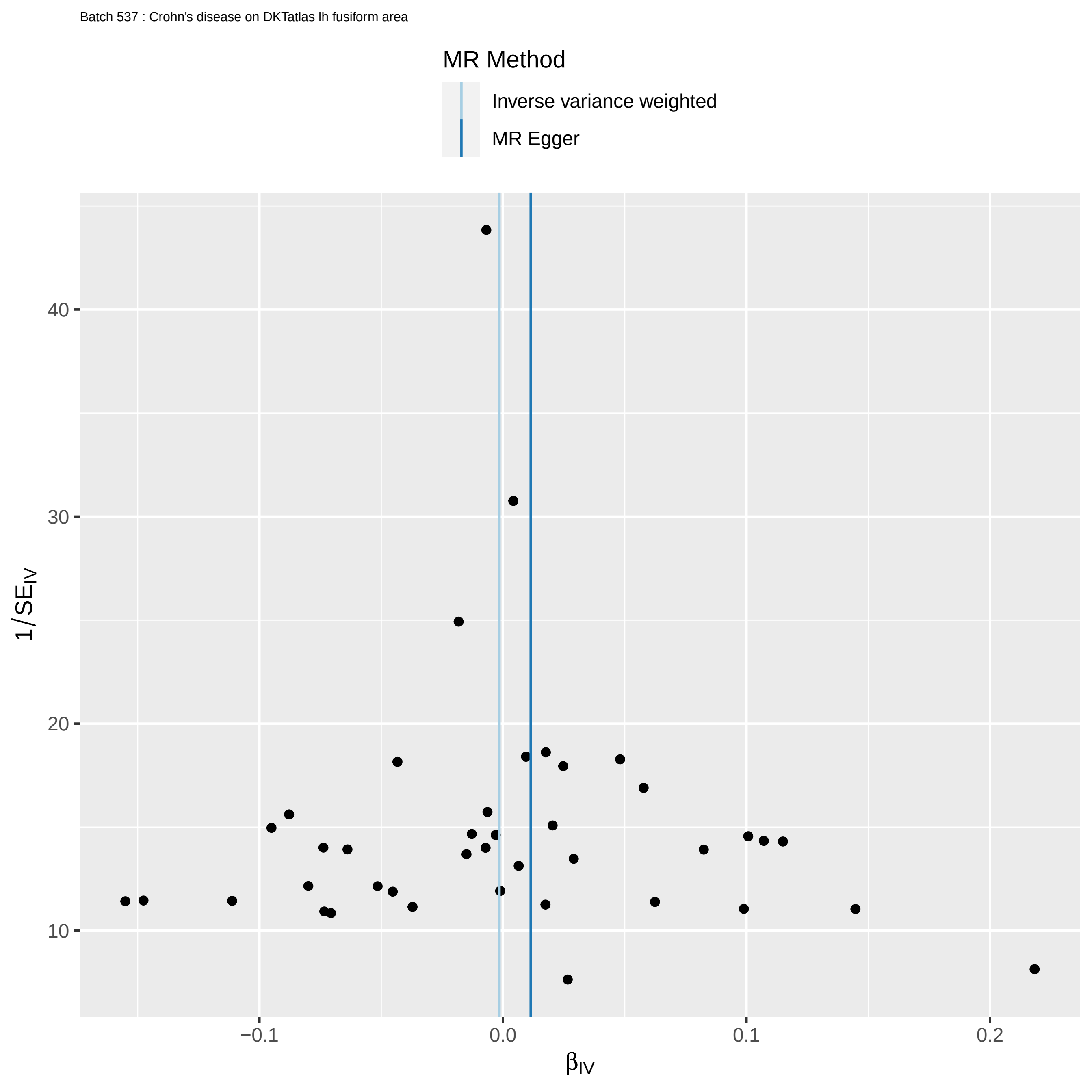
Batch 537 : Crohn's disease on DKTatlas lh fusiform area
MR Method
Inverse variance weighted
MR Egger
40
30
20
10
1/SE$_{IV}$
−0.1
0.0
0.1
0.2
β$_{IV}$

Batch 538 : Ulcerative colitis on DKTatlas lh fusiform area

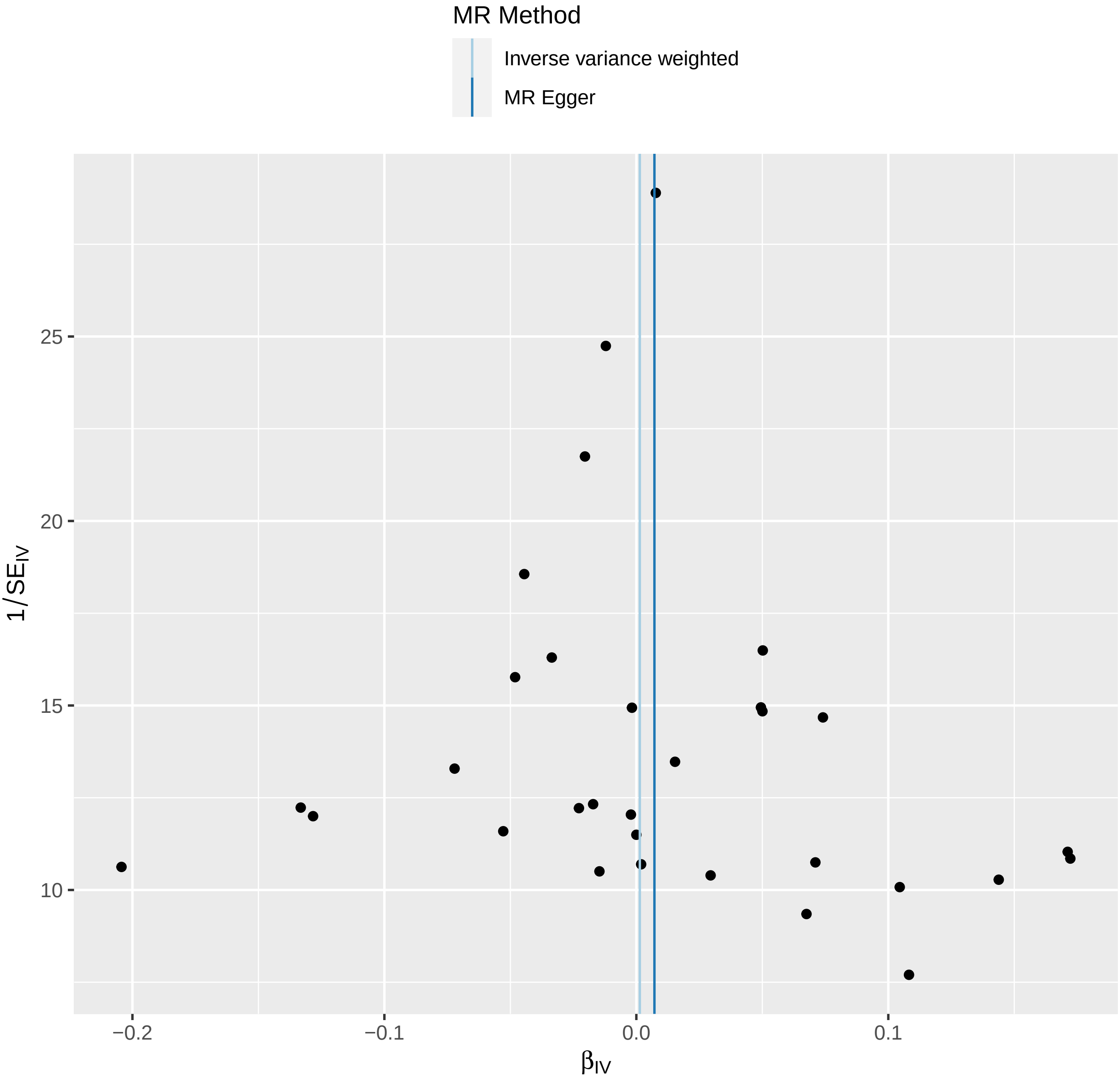

Batch 541 : Crohn's disease on DKTatlas lh inferiorparietal area

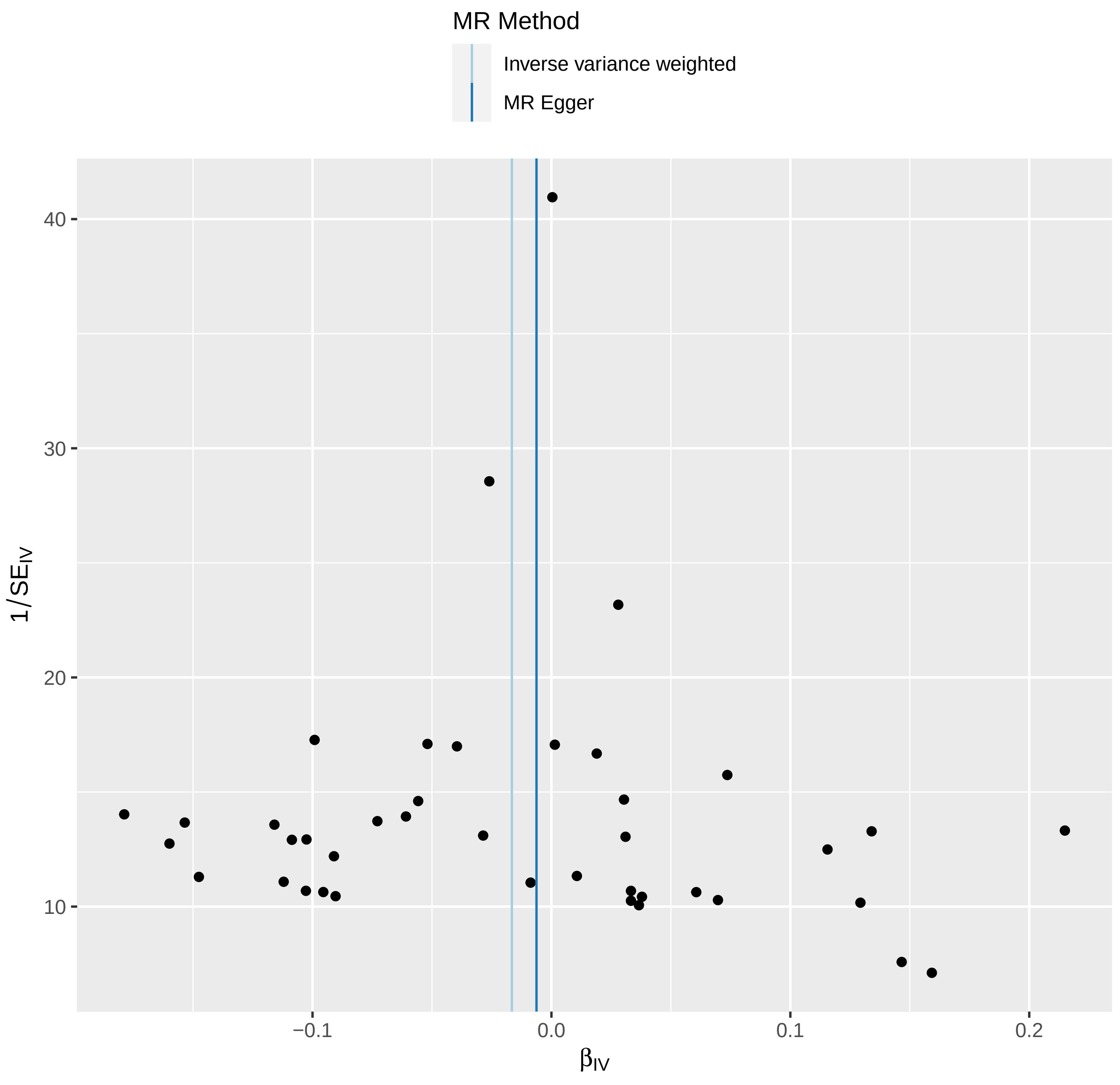

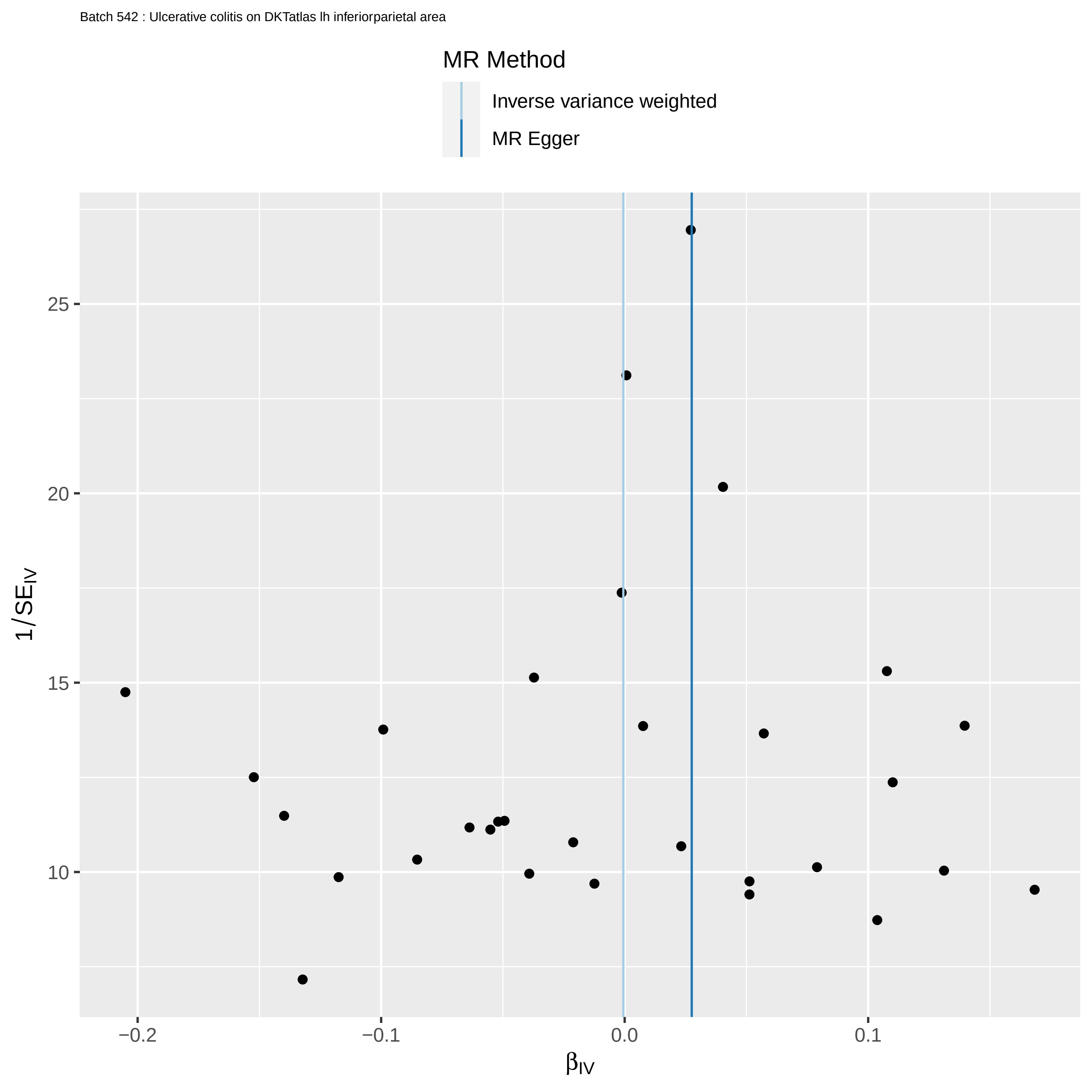
Batch 542 : Ulcerative colitis on DKTatlas lh inferiorparietal area
MR Method
Inverse variance weighted
MR Egger
25
20
15
10
−0.2
−0.1
0.0
0.1
1/SE_IV
β_IV

Batch 543 : Crohn's disease on DKTatlas lh inferiortemporal area

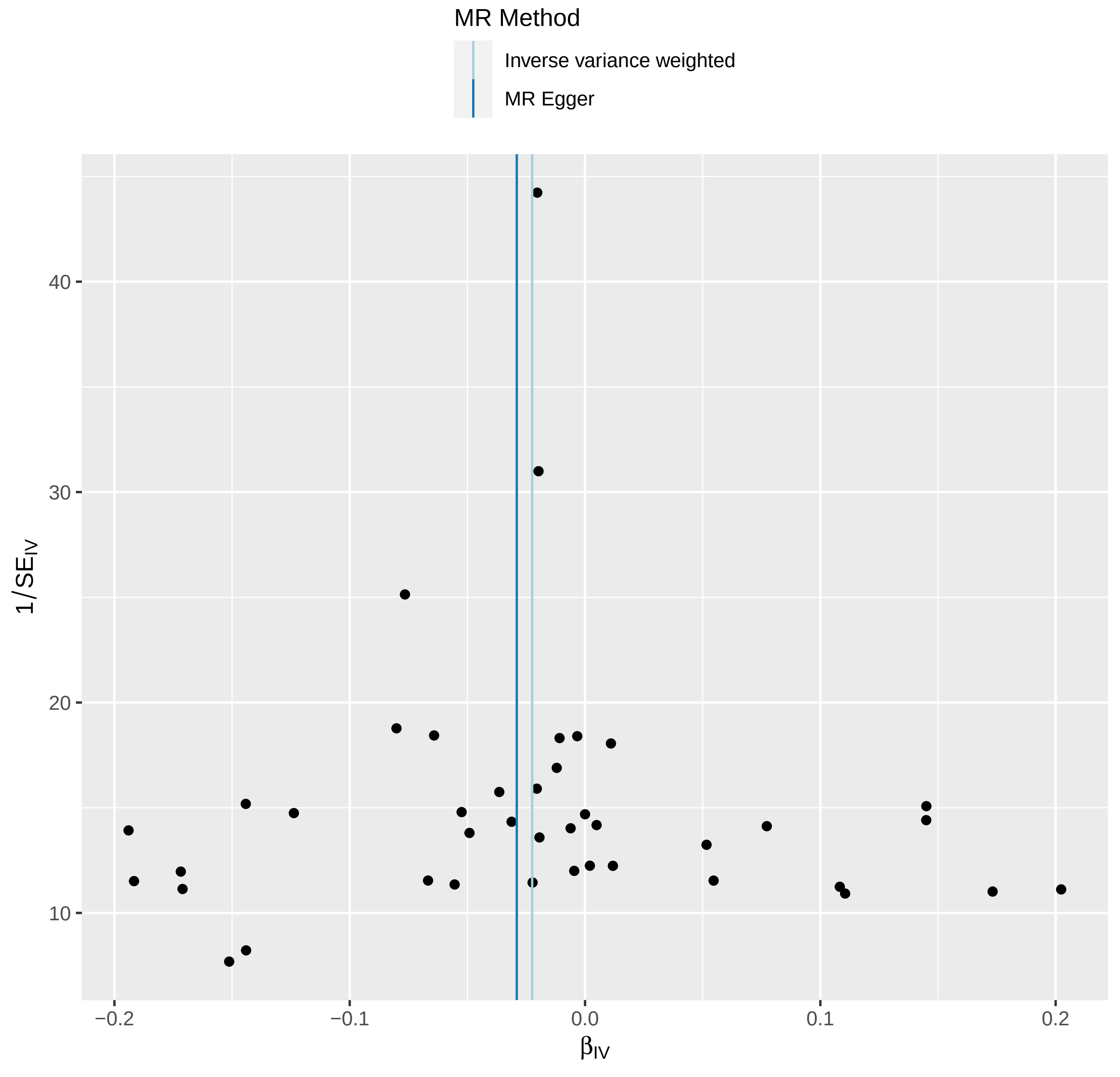

Batch 544 : Ulcerative colitis on DKTatlas lh inferiortemporal area

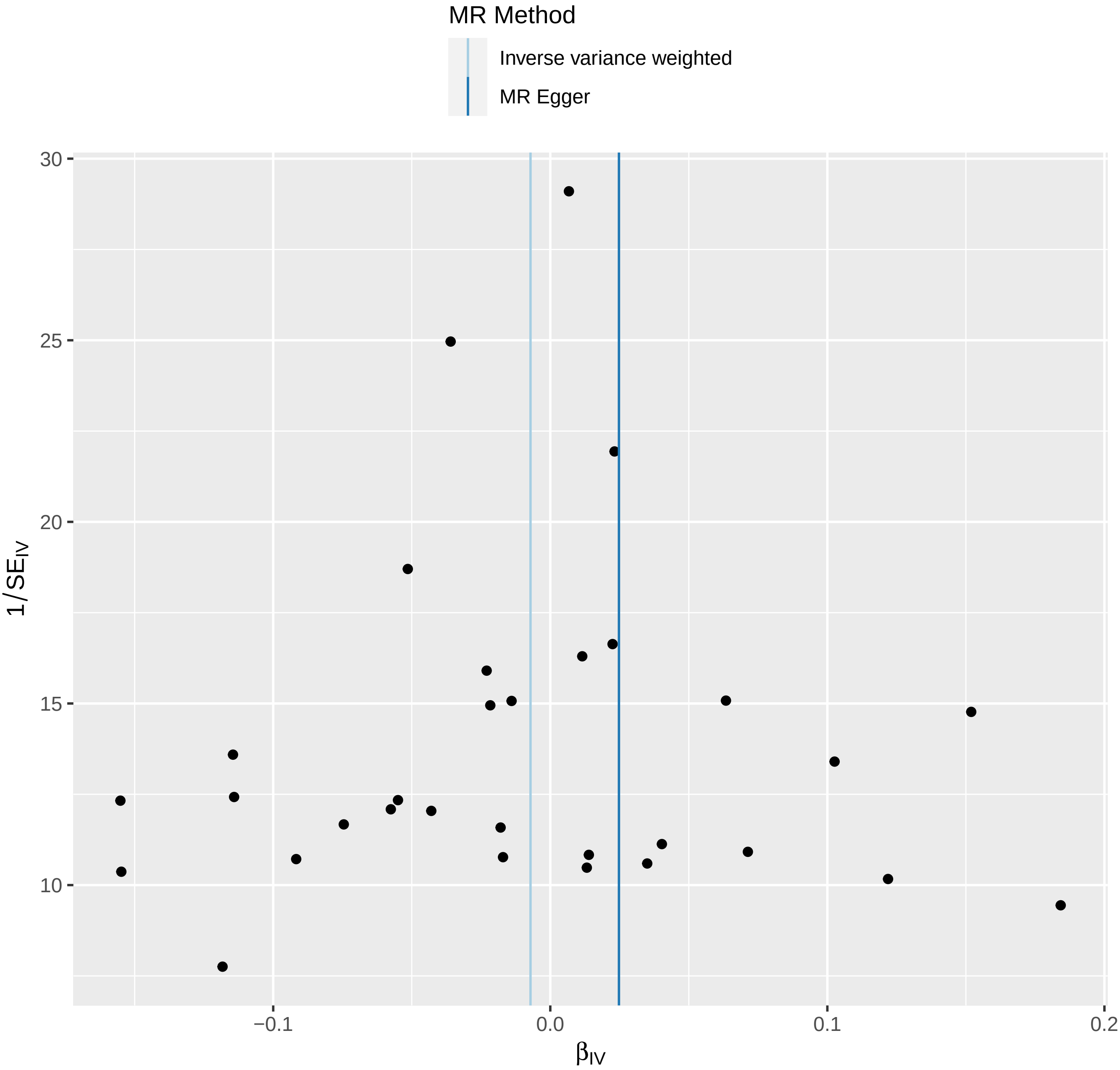

Batch 546 : Ulcerative colitis on DKTatlas lh isthmuscingulate area

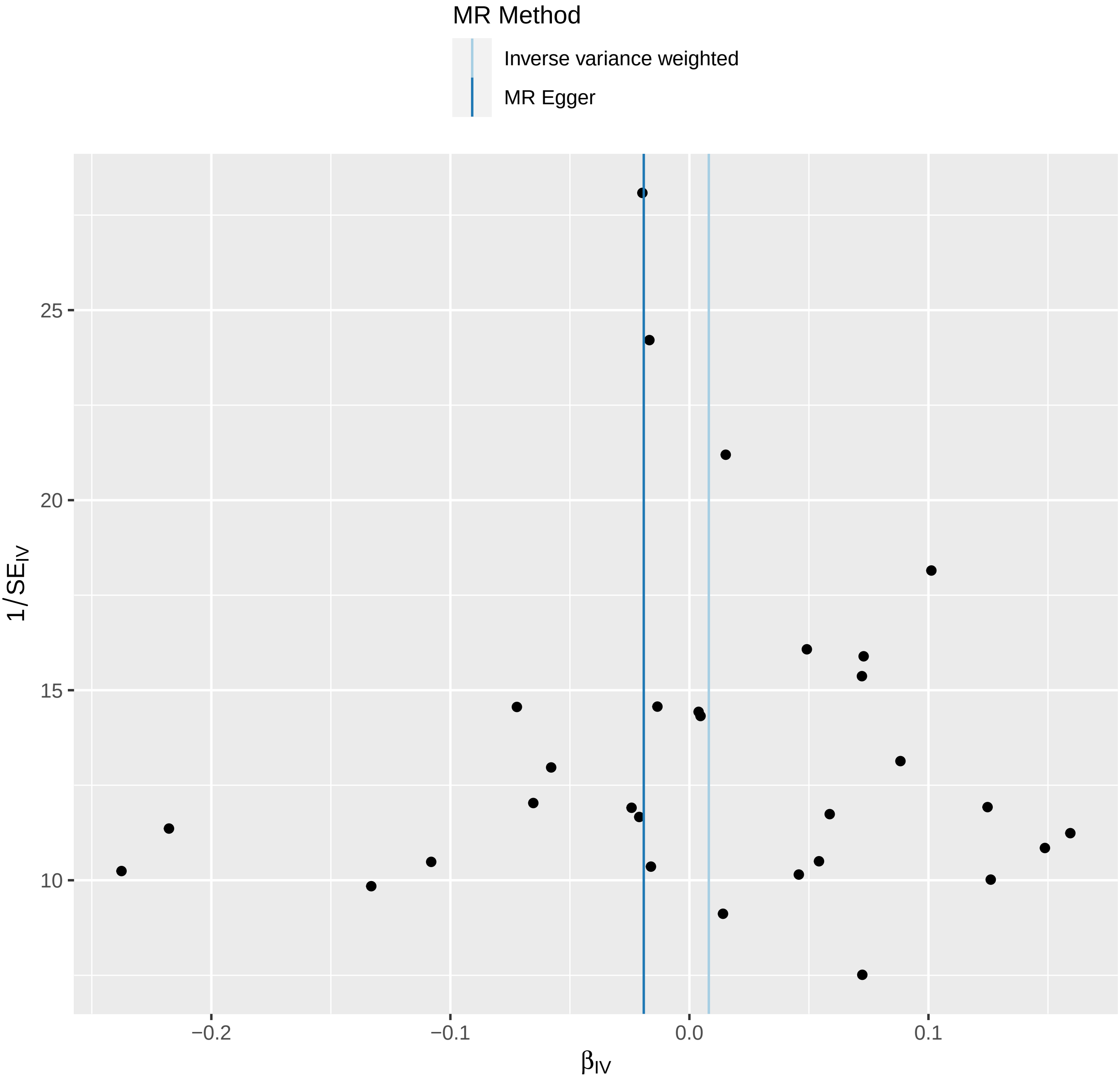

Batch 547 : Crohn's disease on DKTatlas lh lateraloccipital area

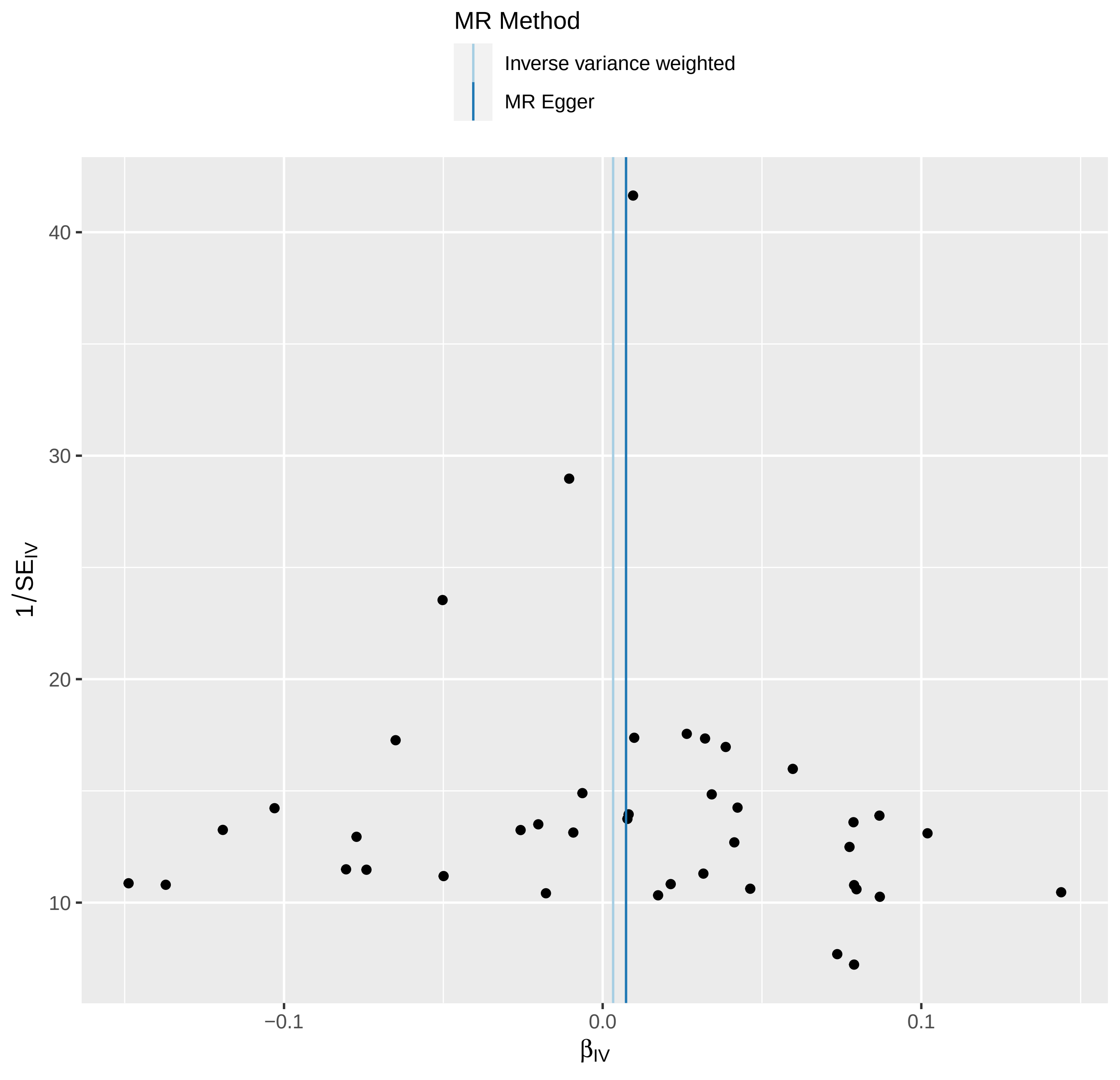

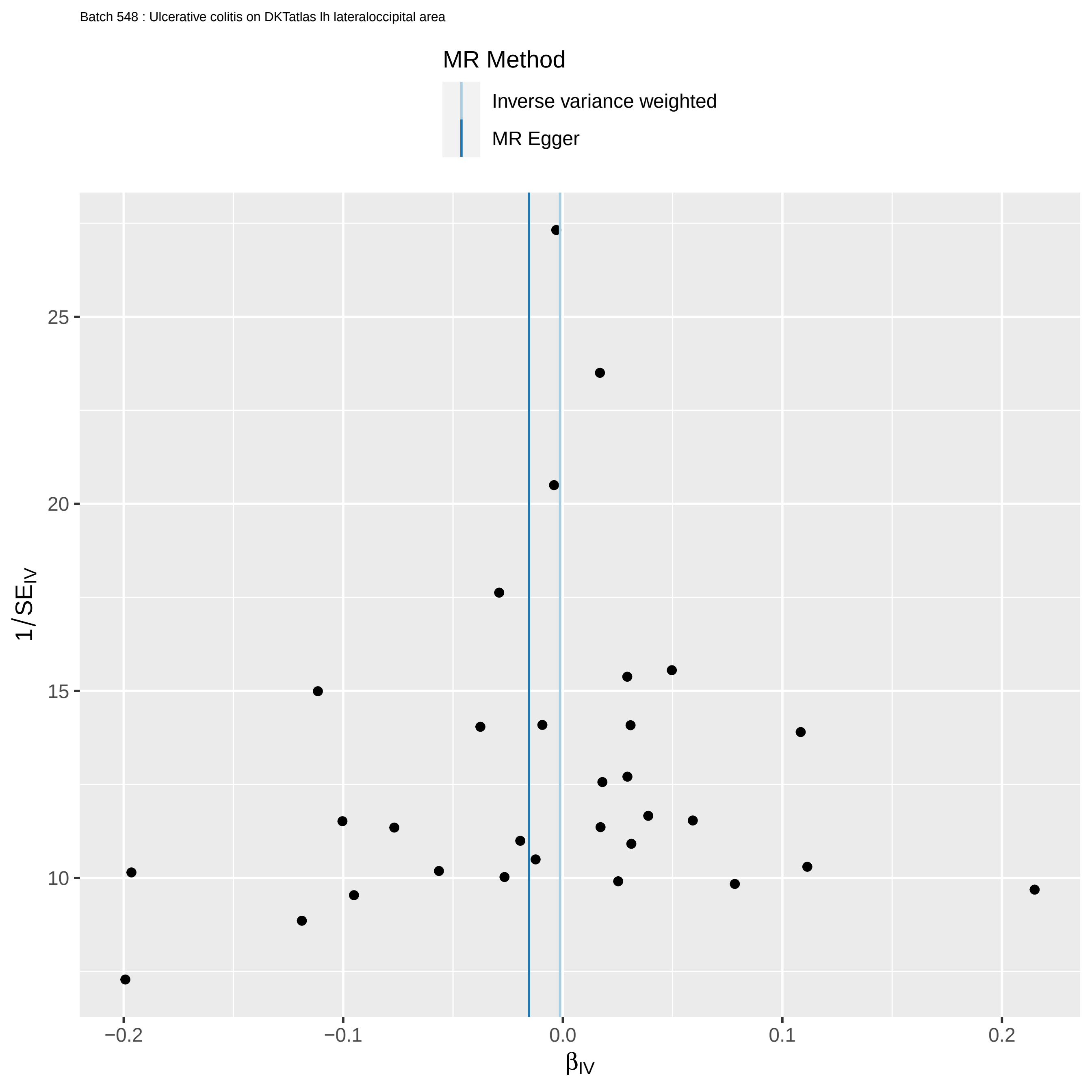

Batch 548 : Ulcerative colitis on DKTatlas lh lateraloccipital area
MR Method
Inverse variance weighted
MR Egger
1/SE_IV
β_IV
25
20
15
10
−0.2
−0.1
0.0
0.1
0.2

Batch 549 : Crohn's disease on DKTatlas lh lateralorbitofrontal area

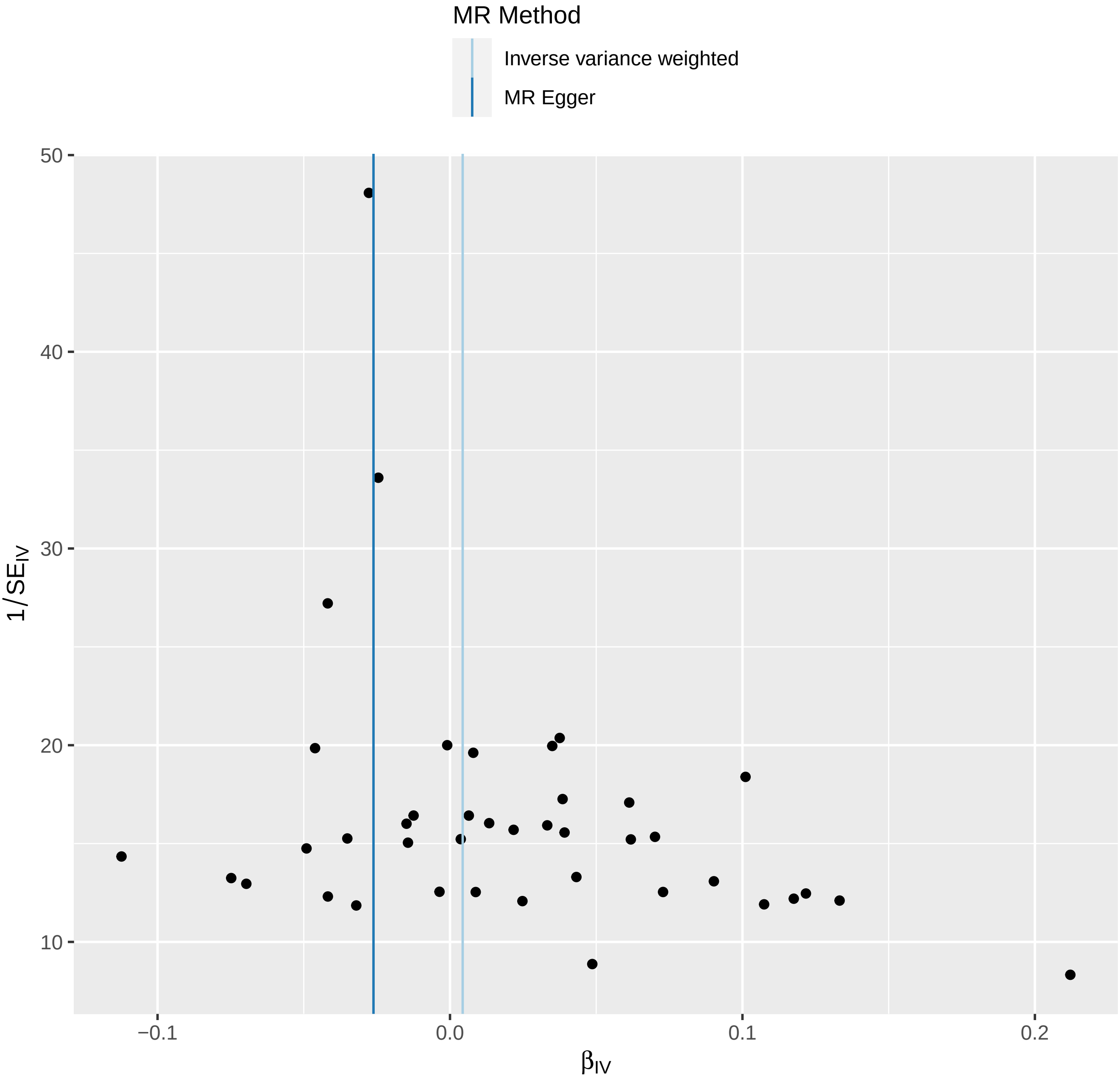

Batch 550 : Ulcerative colitis on DKTatlas lh lateralorbitofrontal area

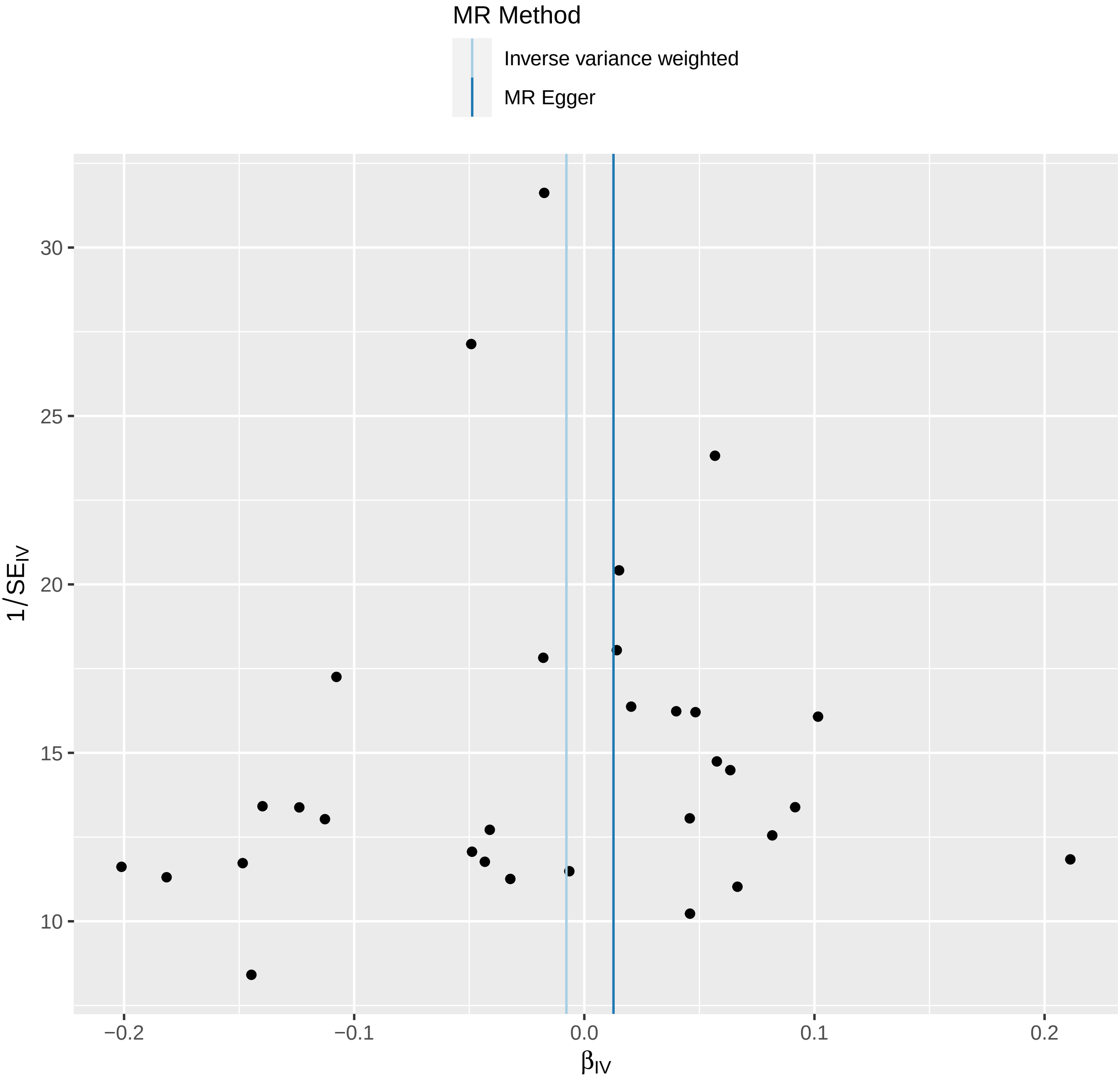

Batch 551 : Crohn's disease on DKTatlas lh lingual area

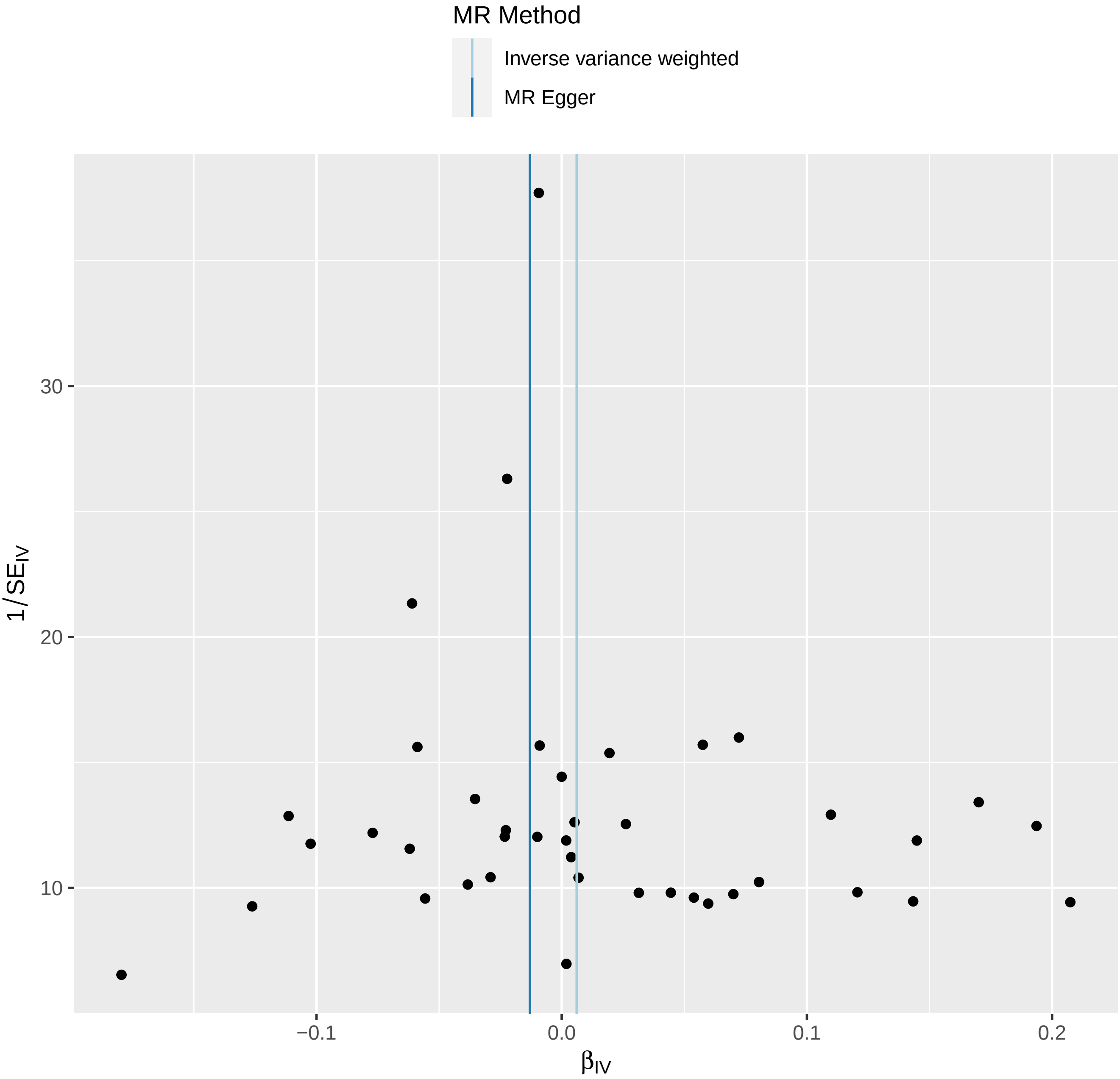

Batch 552 : Ulcerative colitis on DKTatlas lh lingual area

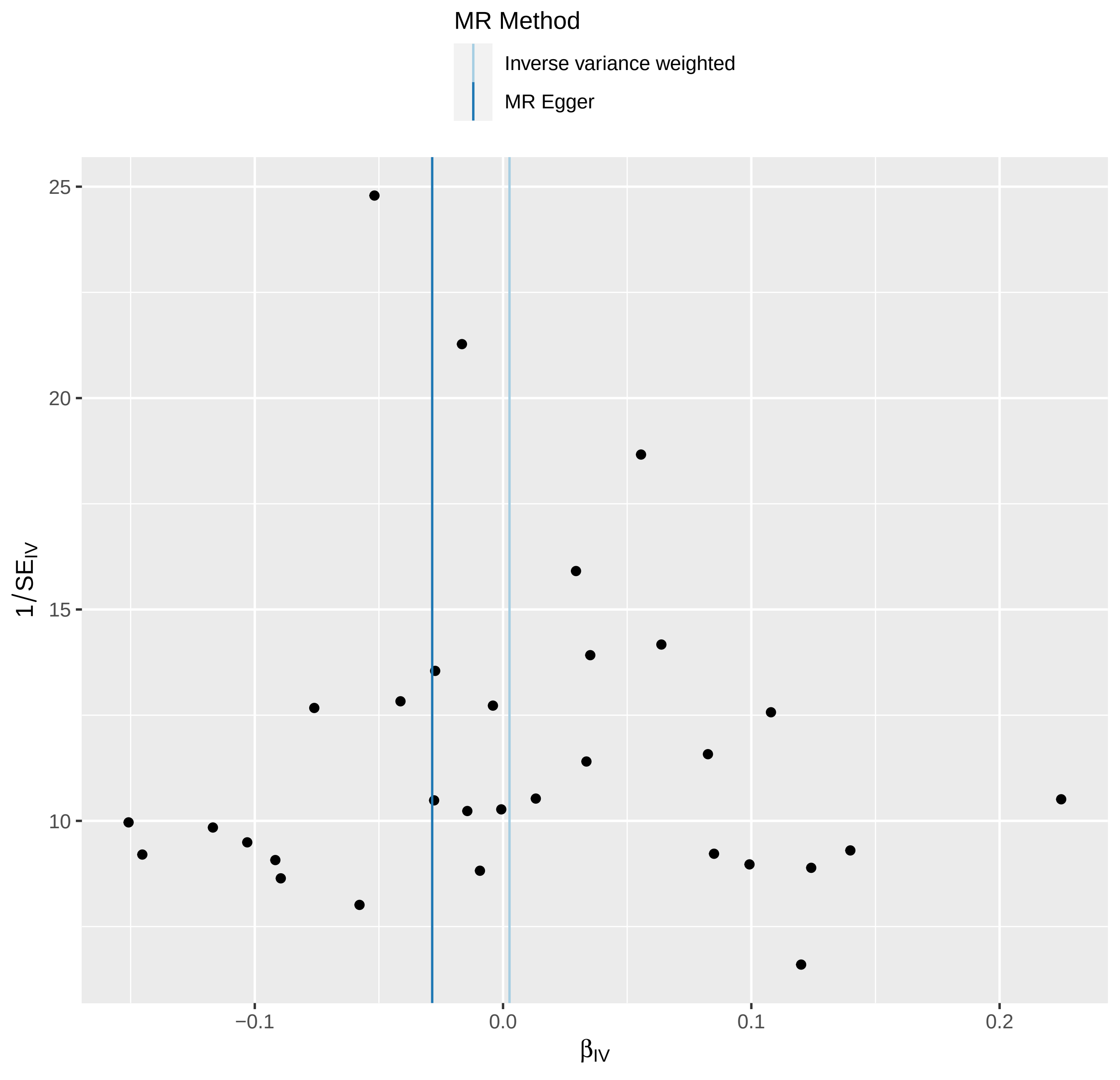

Batch 553 : Crohn's disease on DKTatlas lh medialorbitofrontal area

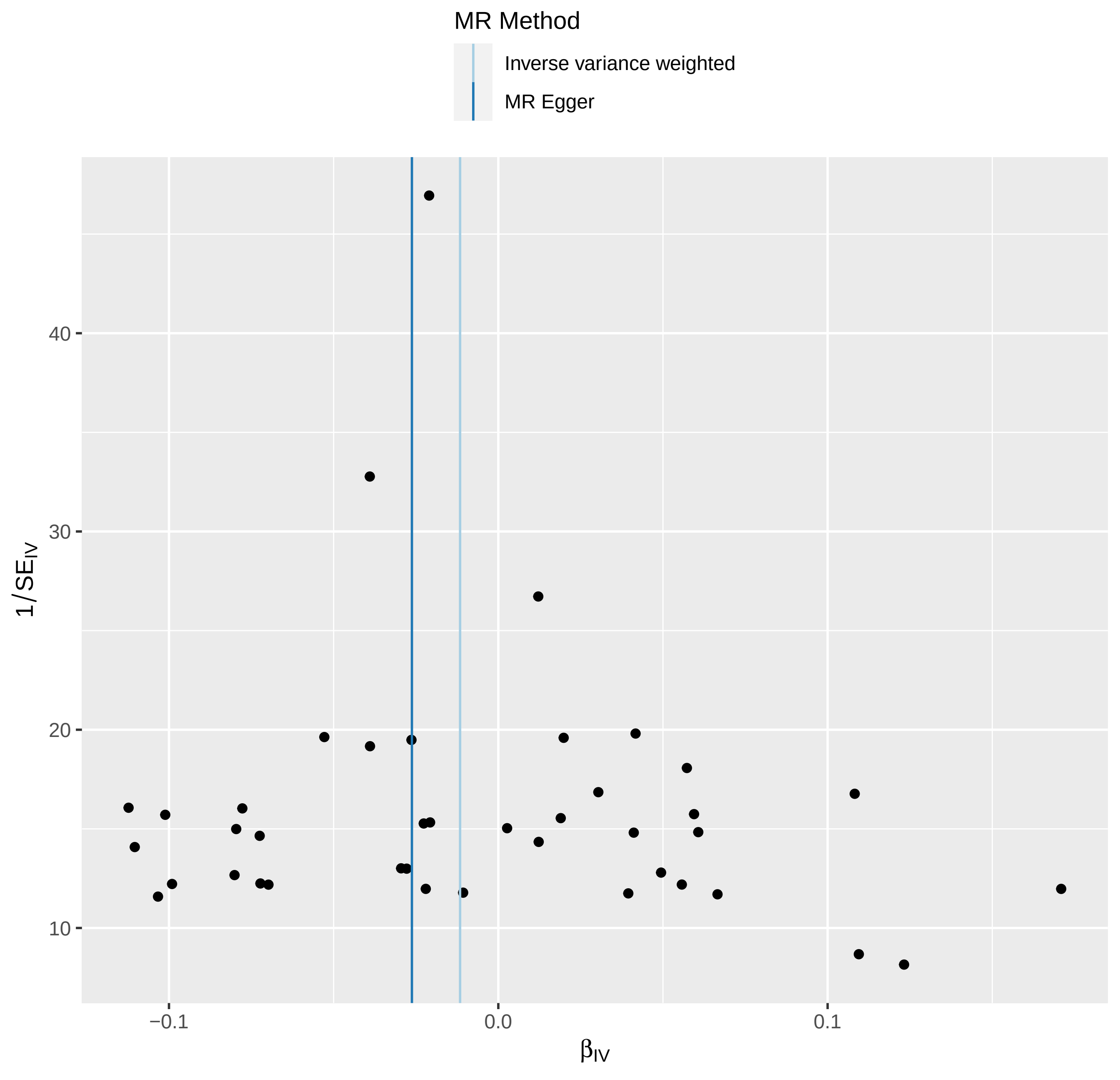

Batch 554 : Ulcerative colitis on DKTatlas lh medialorbitofrontal area

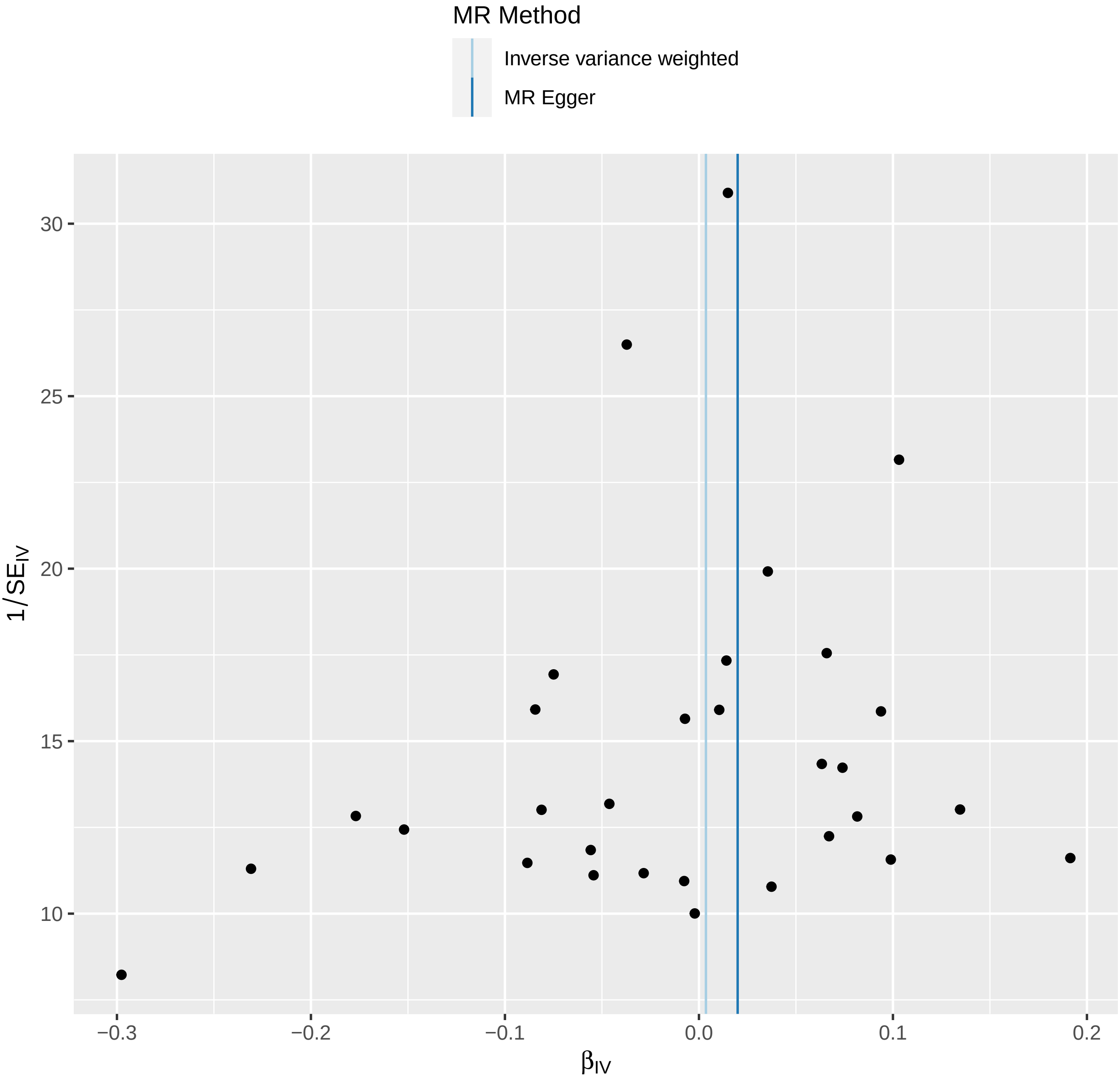

Batch 555 : Crohn's disease on DKTatlas lh middletemporal area

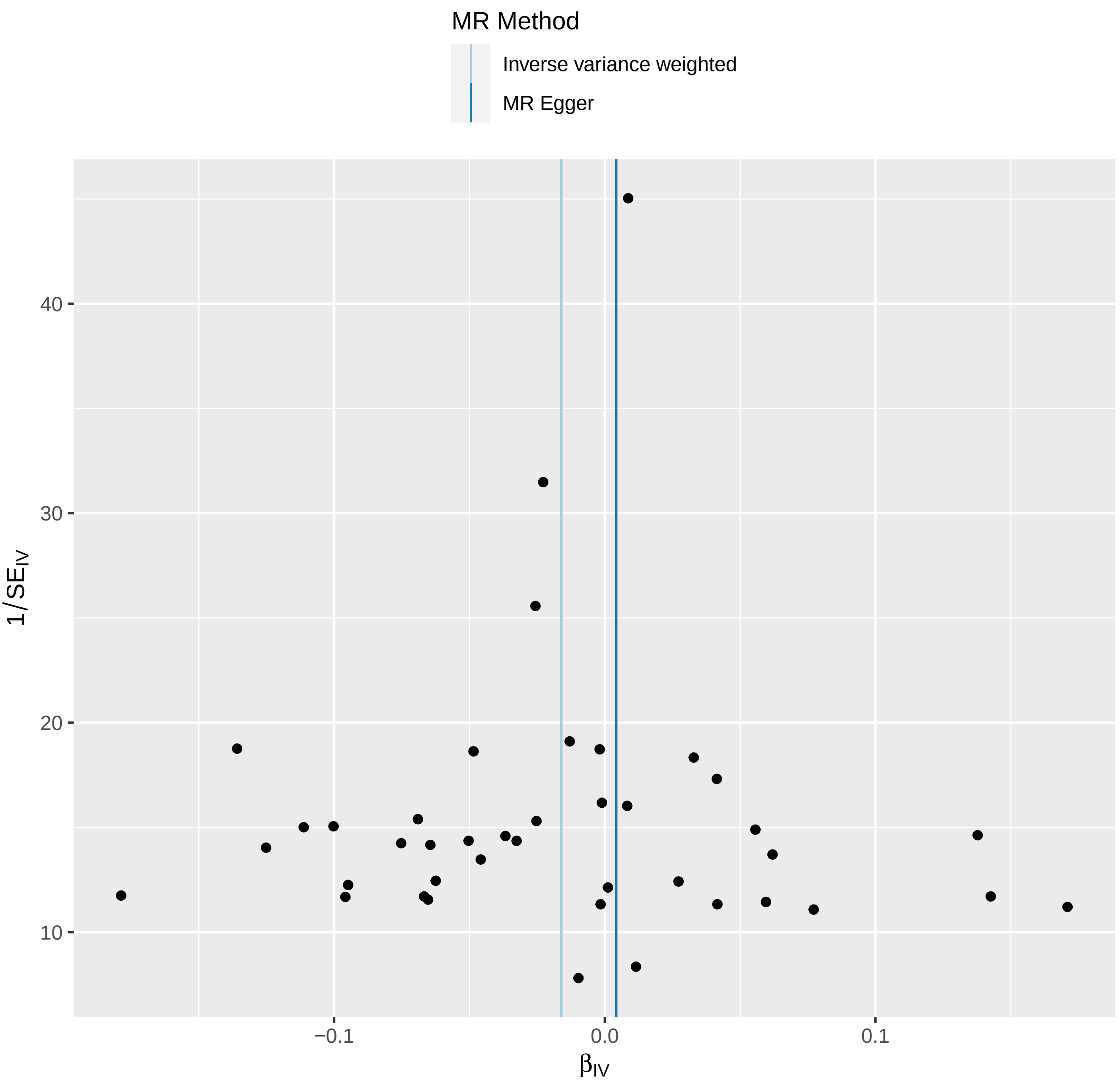

Batch 556 : Ulcerative colitis on DKTatlas lh middletemporal area

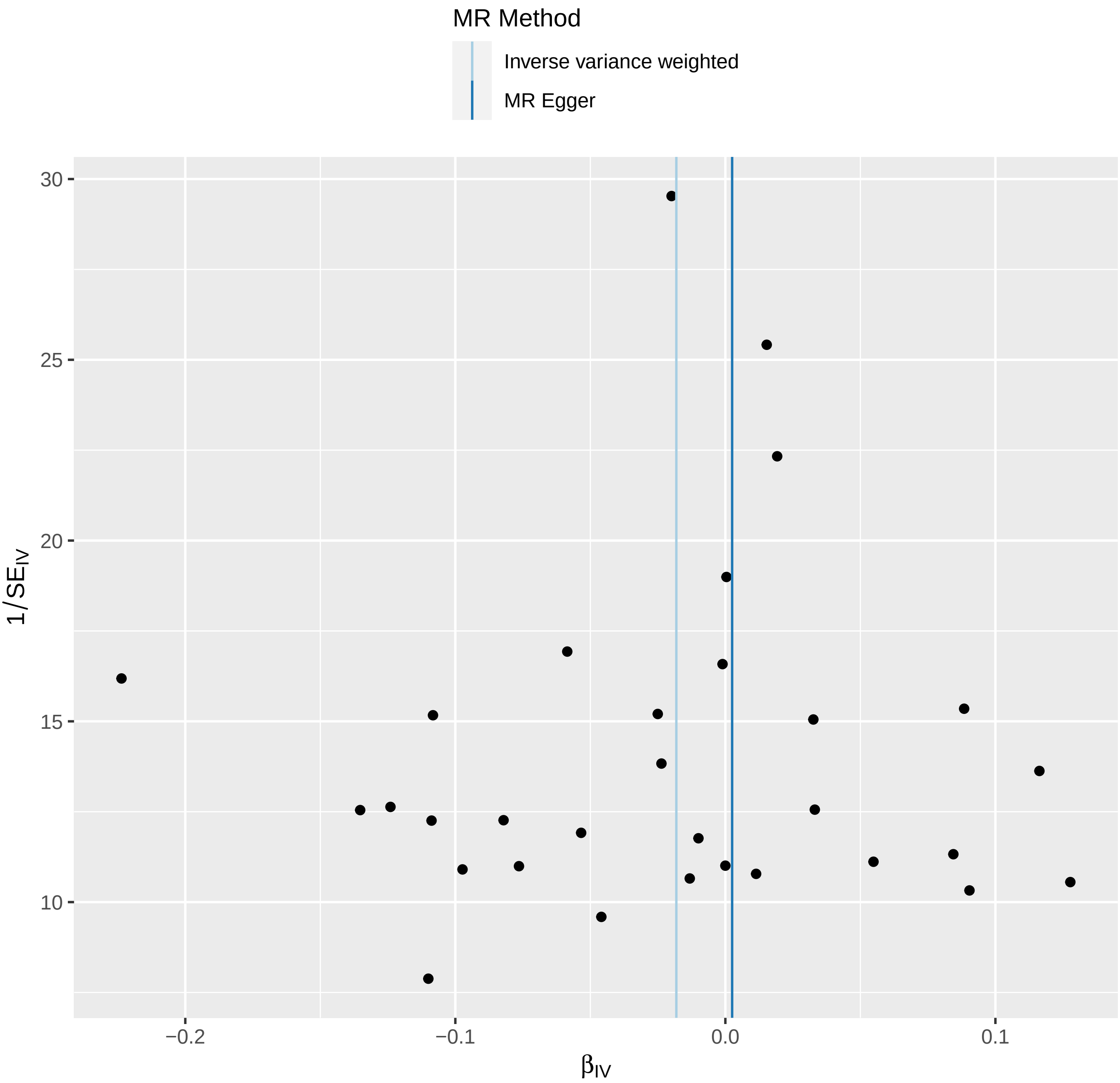

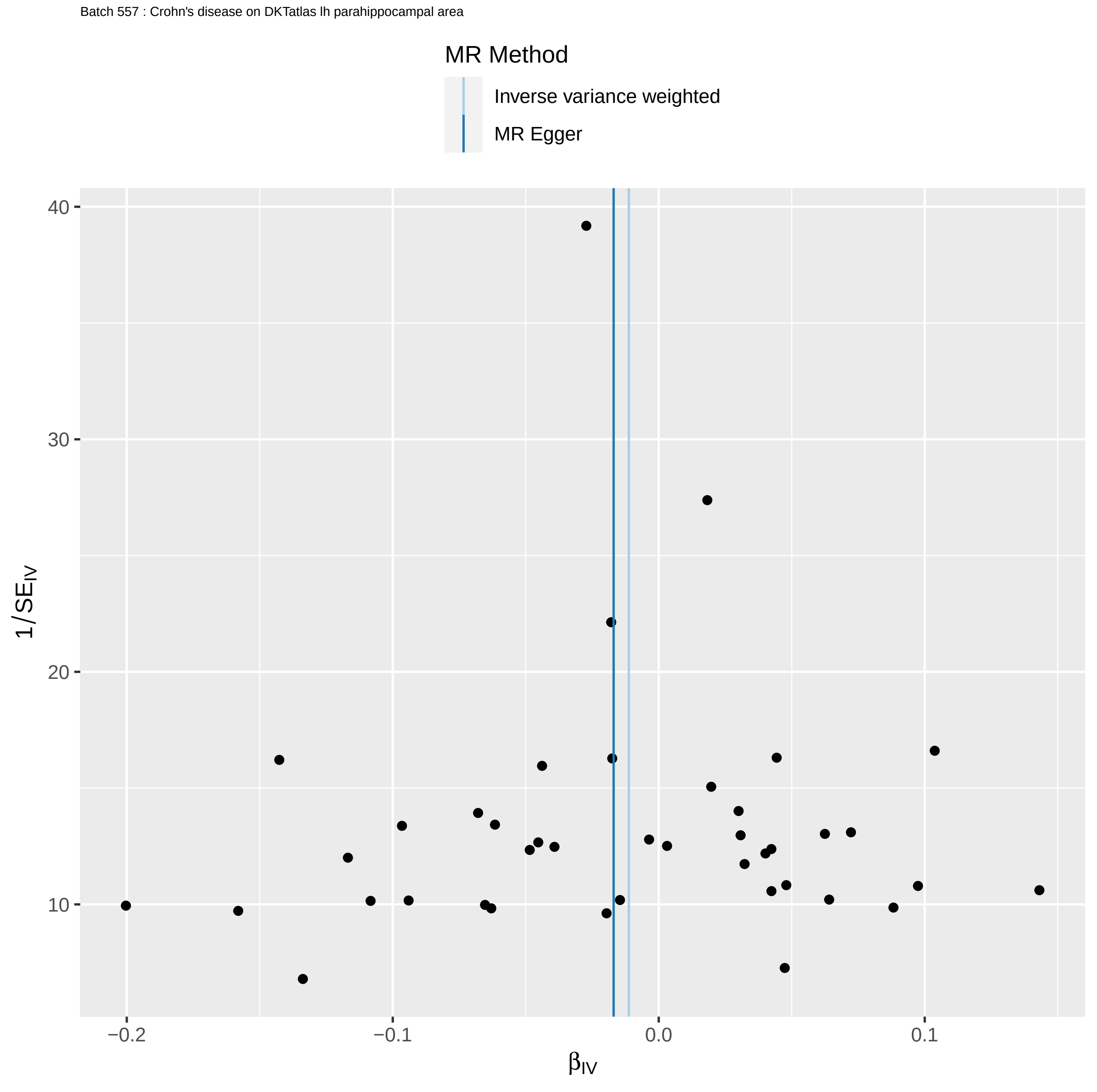

Batch 557 : Crohn's disease on DKTatlas lh parahippocampal area
MR Method
Inverse variance weighted
MR Egger
40
30
20
10
1/SE_IV
−0.2
−0.1
0.0
0.1
β_IV

Batch 558 : Ulcerative colitis on DKTatlas lh parahippocampal area

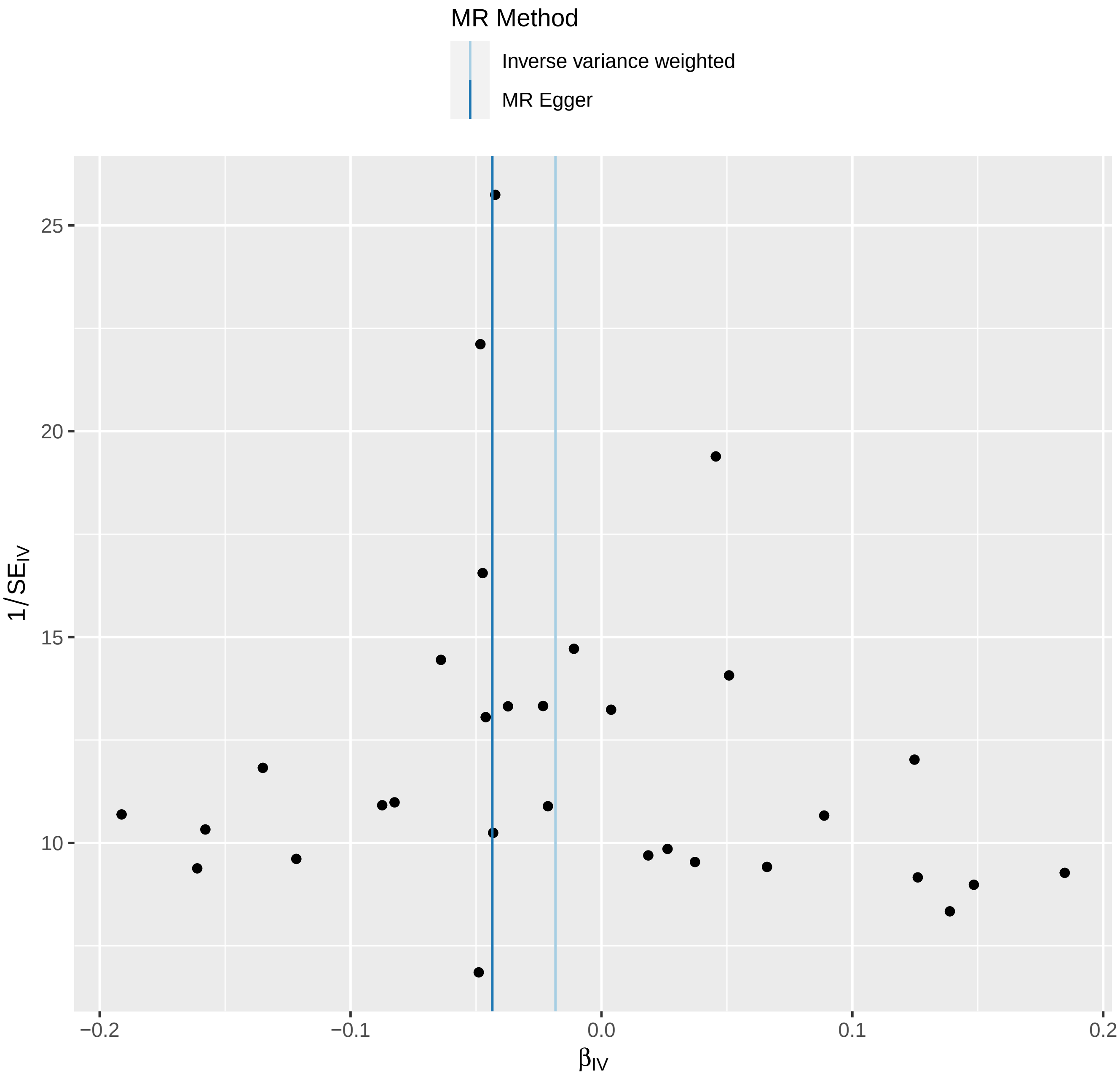

Batch 559 : Crohn's disease on DKTatlas lh paracentral area

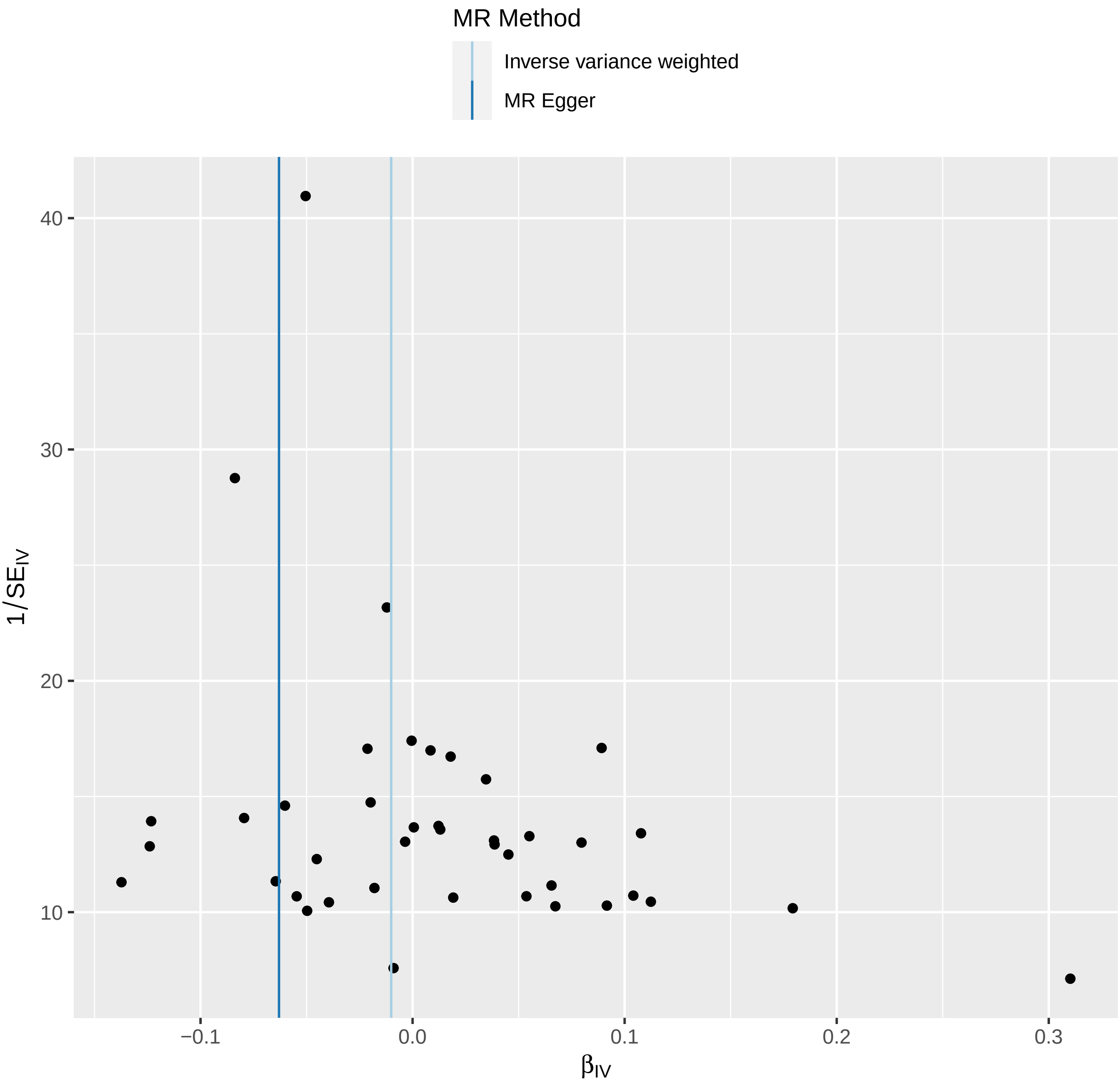

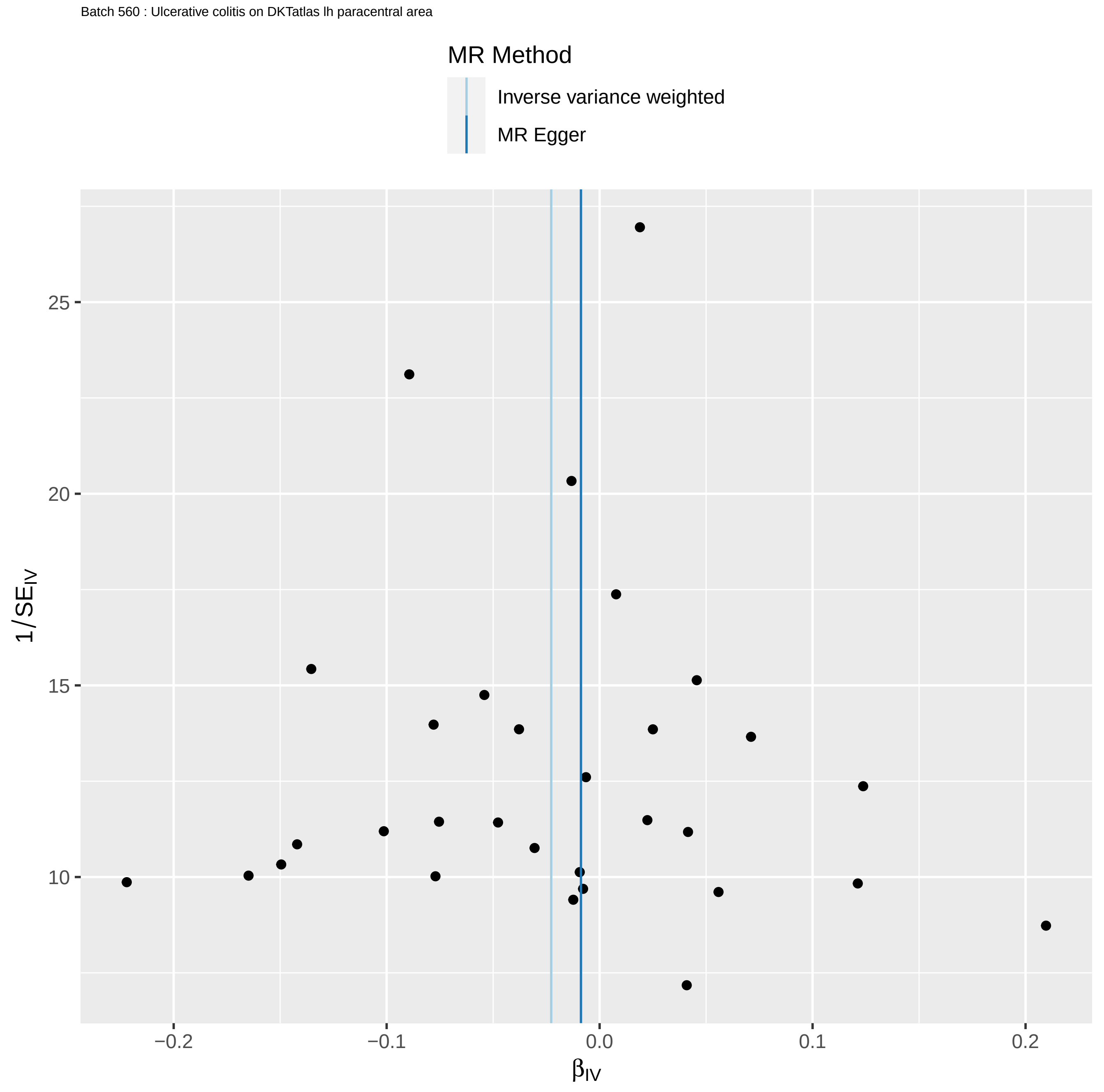

Batch 560 : Ulcerative colitis on DKTatlas lh paracentral area
MR Method
Inverse variance weighted
MR Egger
25
20
15
10
1/SE_IV
−0.2
−0.1
0.0
0.1
0.2
β_IV

Batch 563 : Crohn's disease on DKTatlas lh parsopercularis area

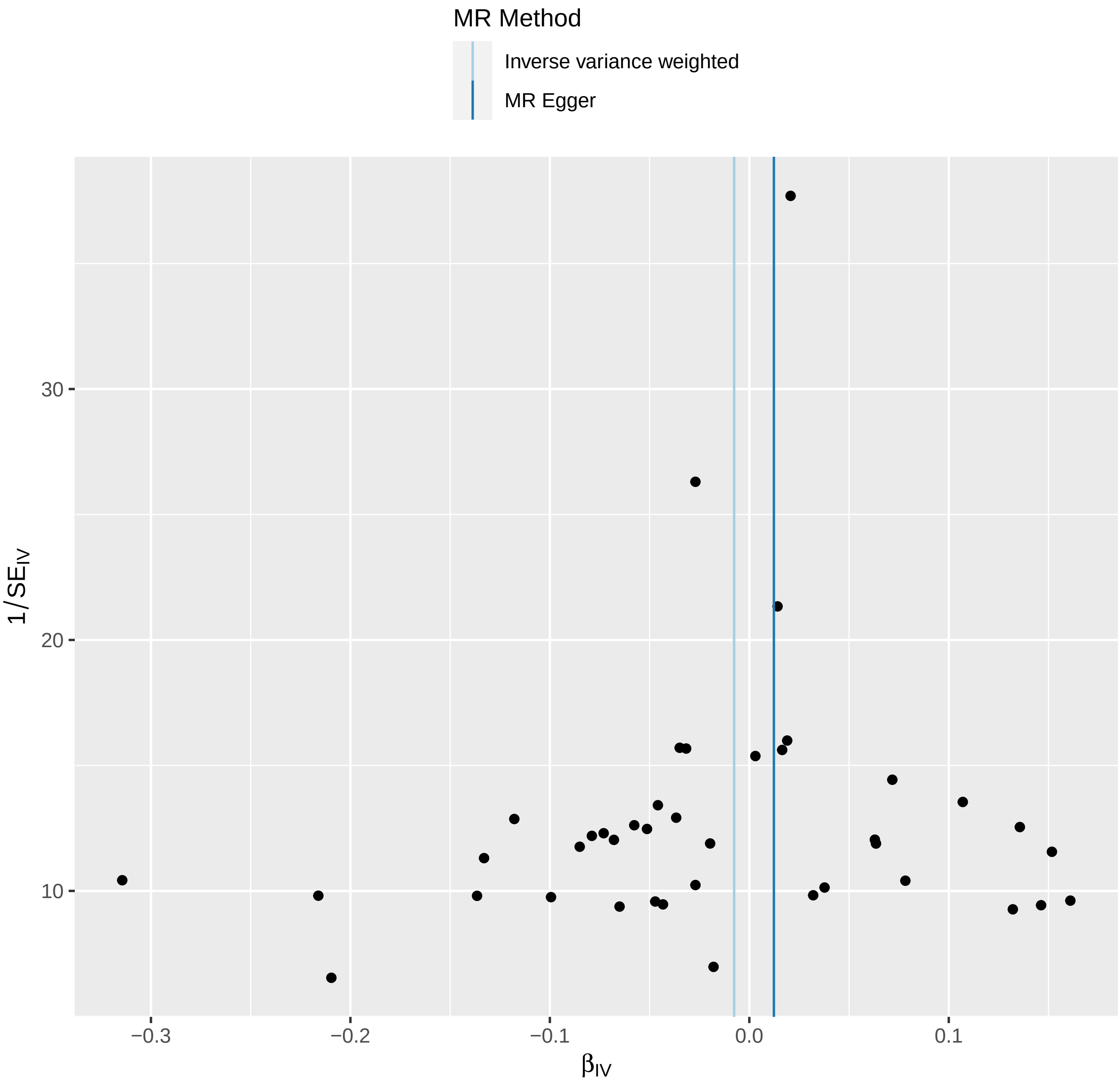

Batch 564 : Ulcerative colitis on DKTatlas lh parsopercularis area

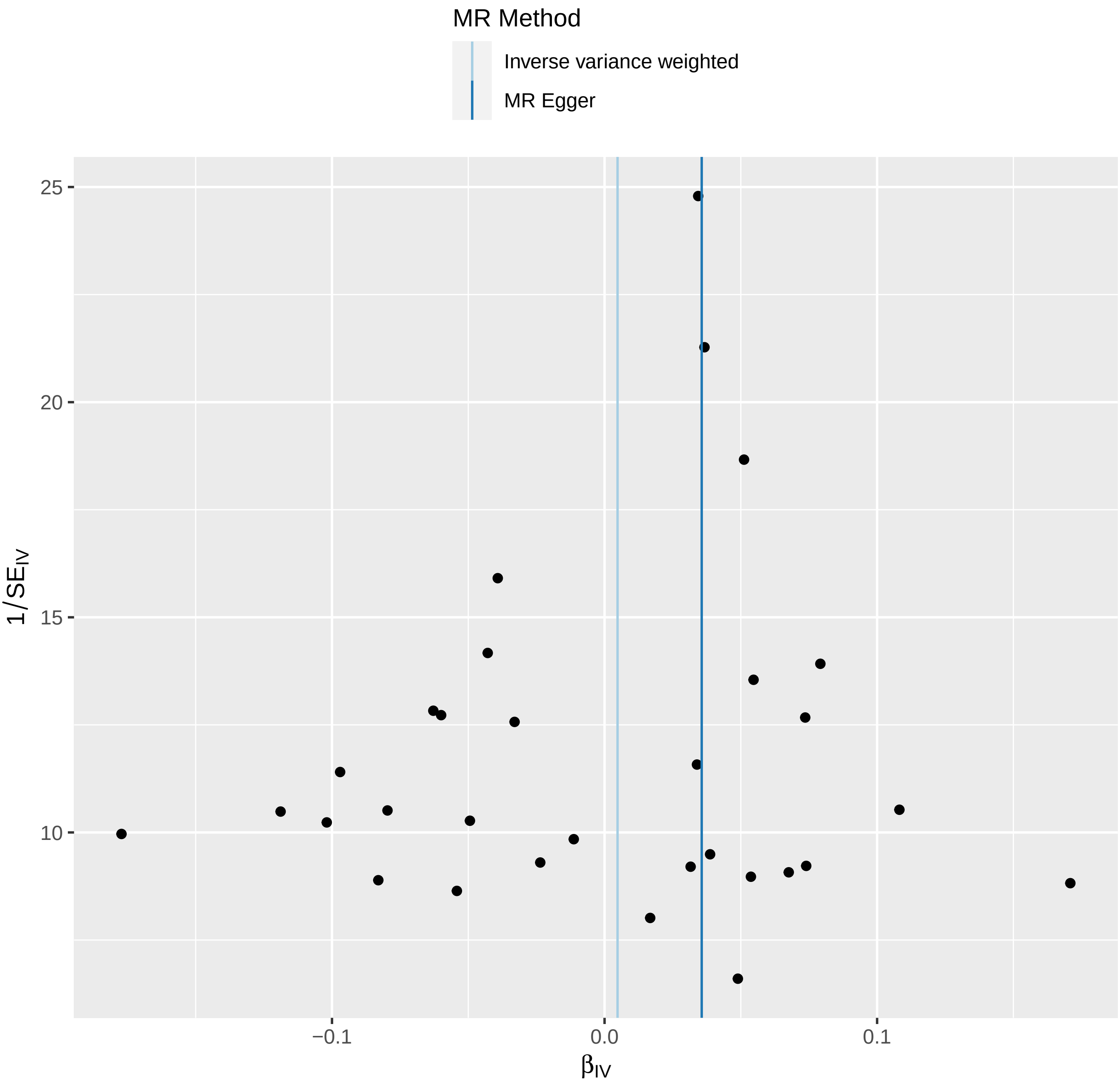

Batch 565 : Crohn's disease on DKTatlas lh parsorbitalis area

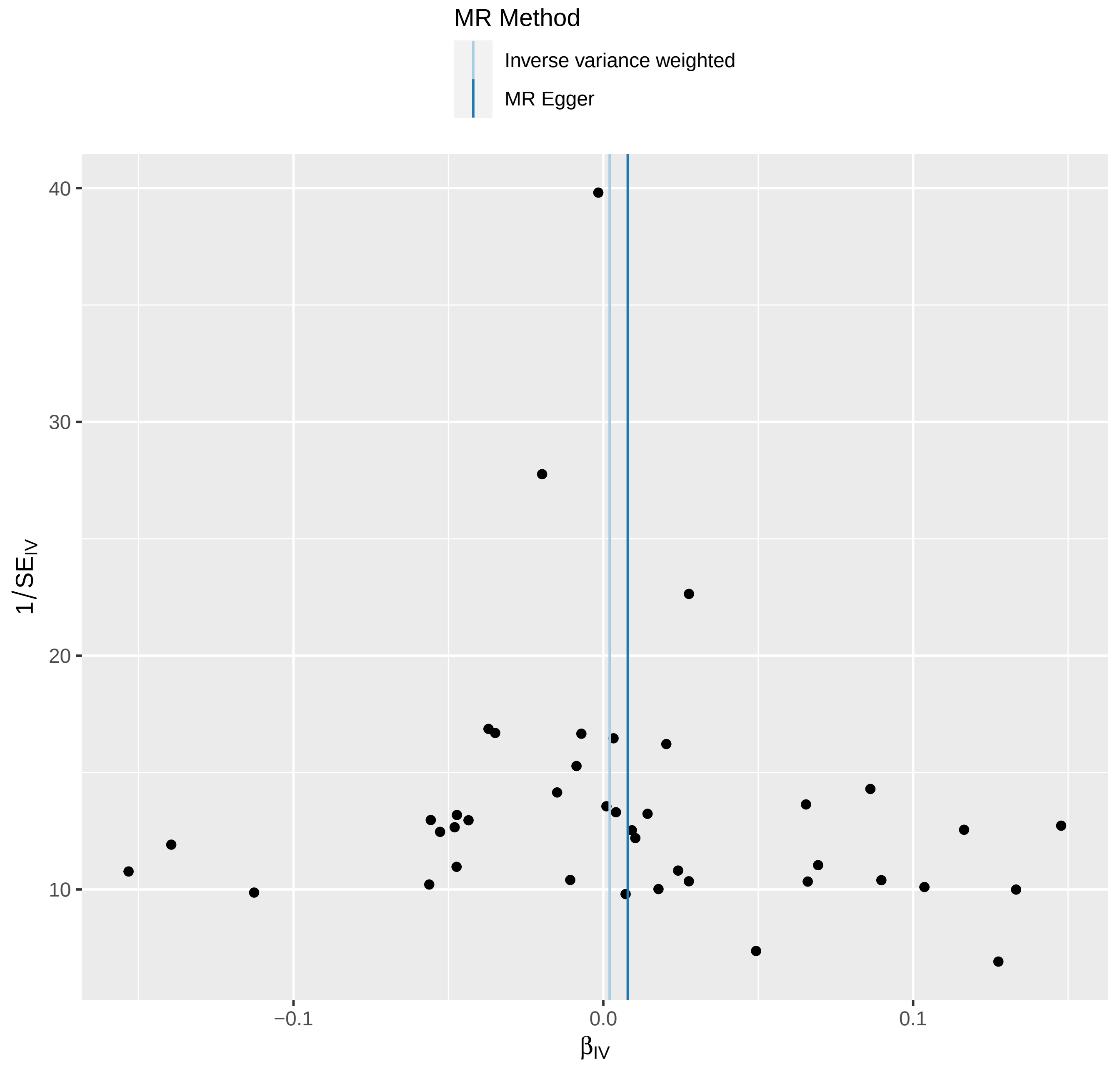

Batch 566 : Ulcerative colitis on DKTatlas lh parsorbitalis area

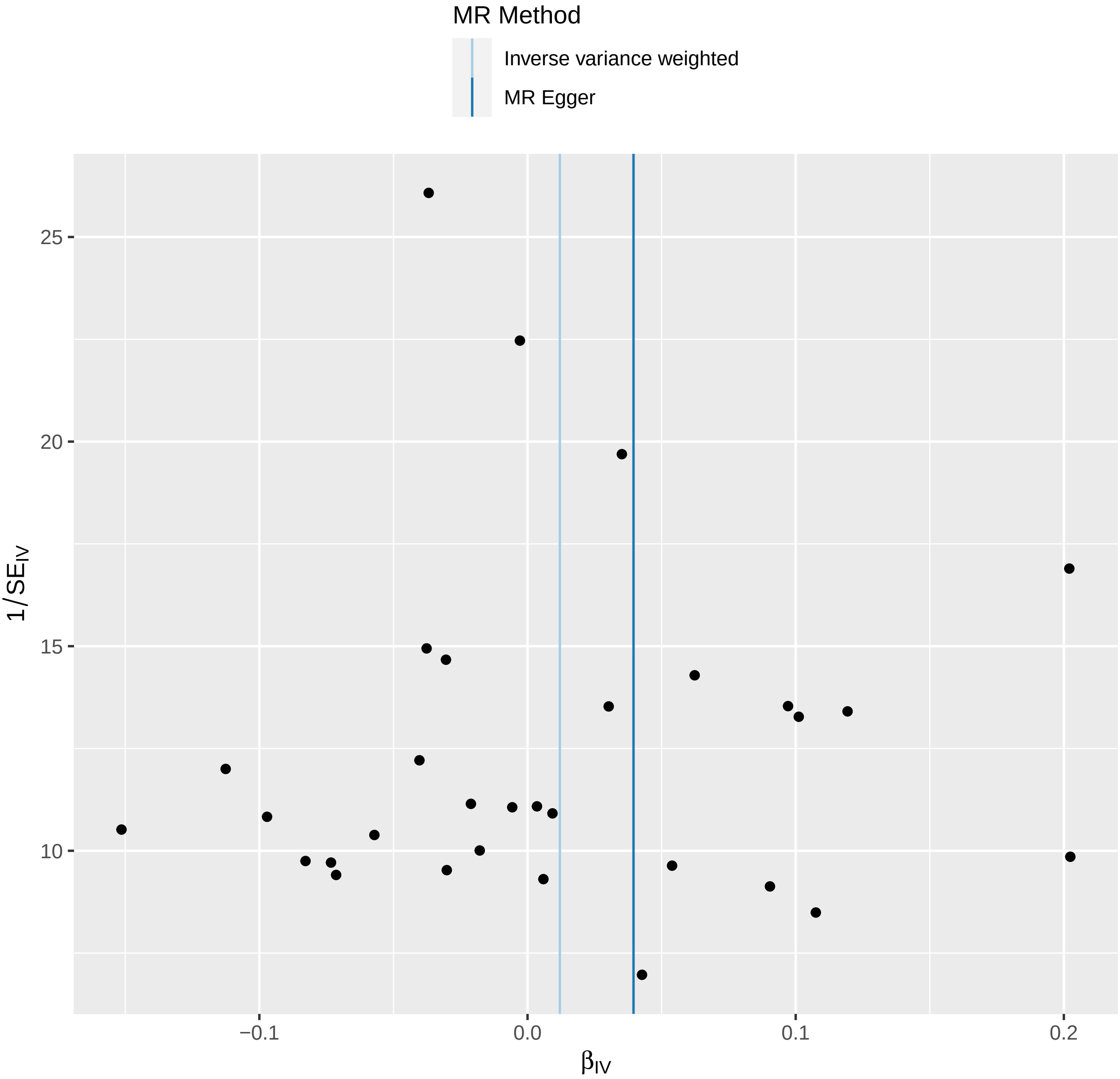

Batch 567 : Crohn's disease on DKTatlas lh parstriangularis area

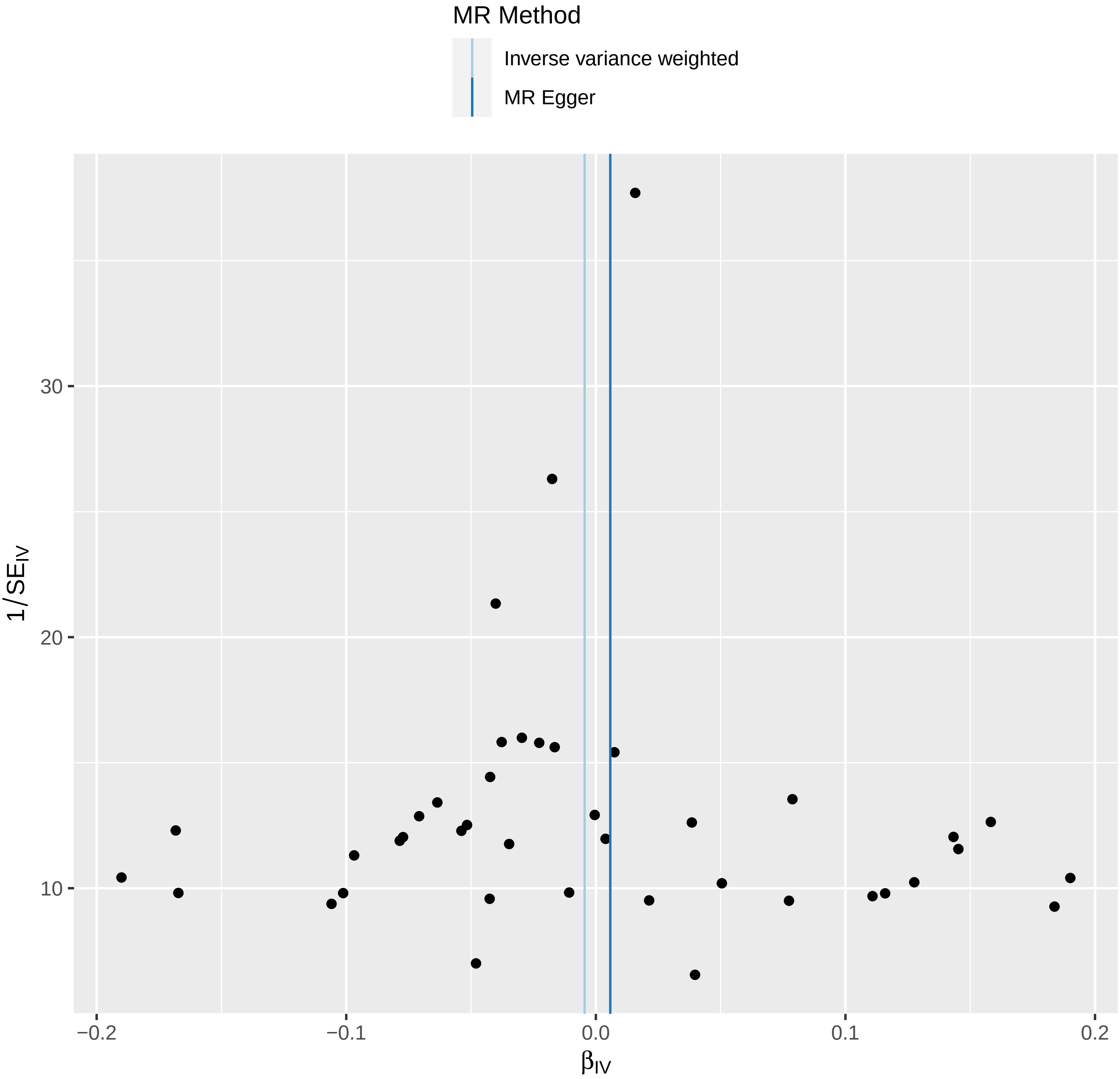

Batch 568 : Ulcerative colitis on DKTatlas lh parstriangularis area

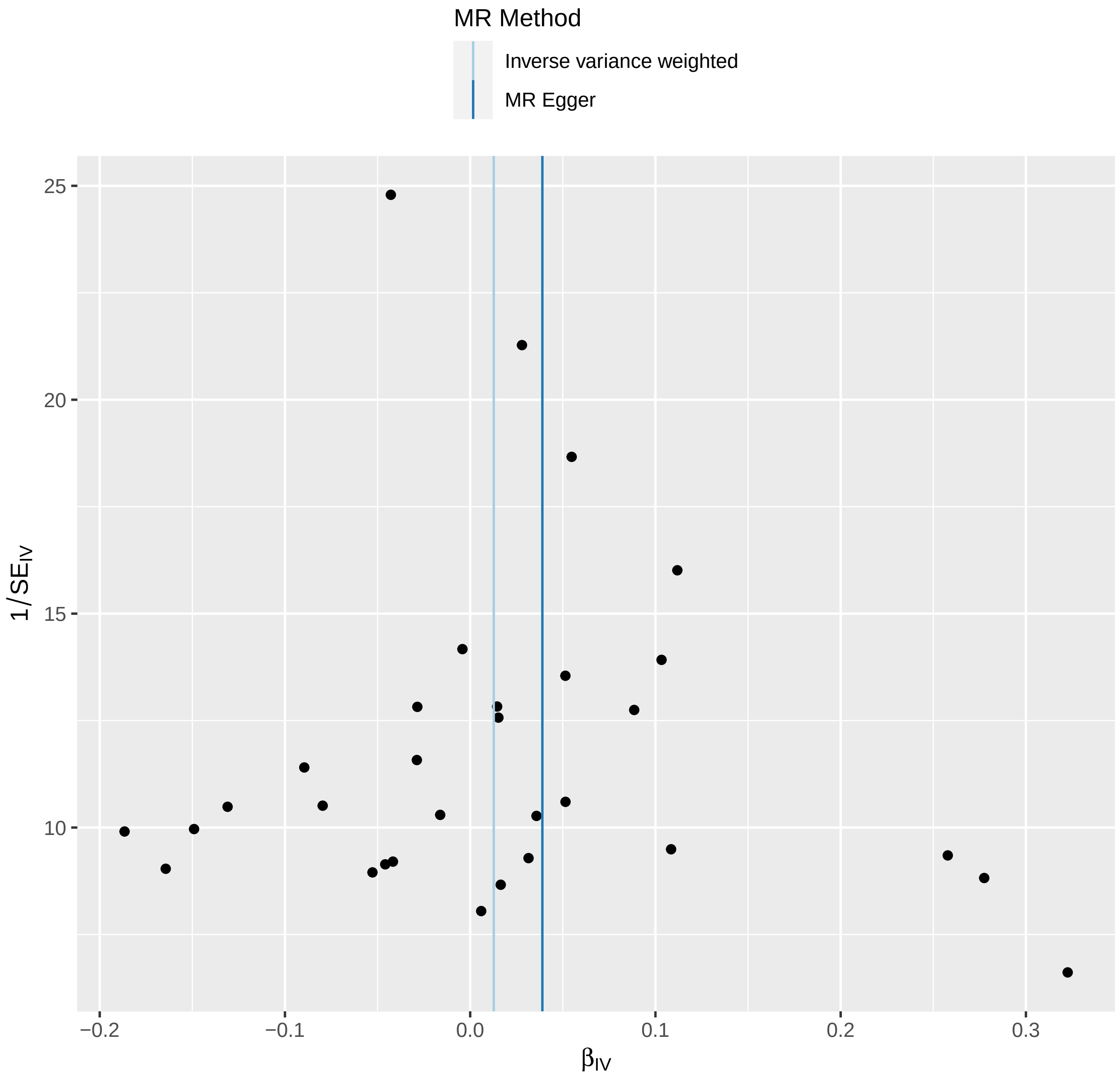

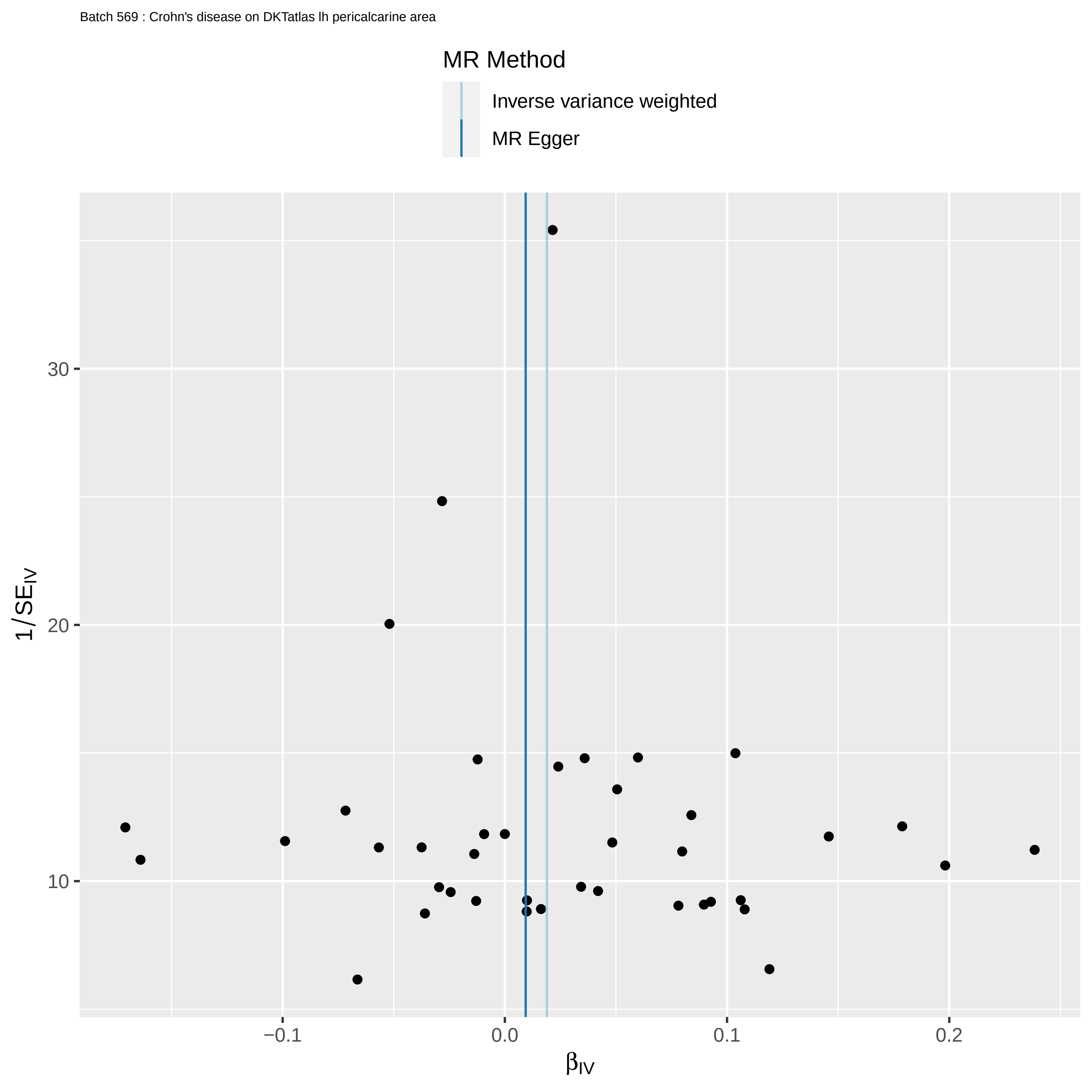
Batch 569 : Crohn's disease on DKTatlas lh pericalcarine area
MR Method
Inverse variance weighted
MR Egger
1/SE_IV
30
20
10
β_IV
−0.1
0.0
0.1
0.2

Batch 570 : Ulcerative colitis on DKTatlas lh pericalcarine area

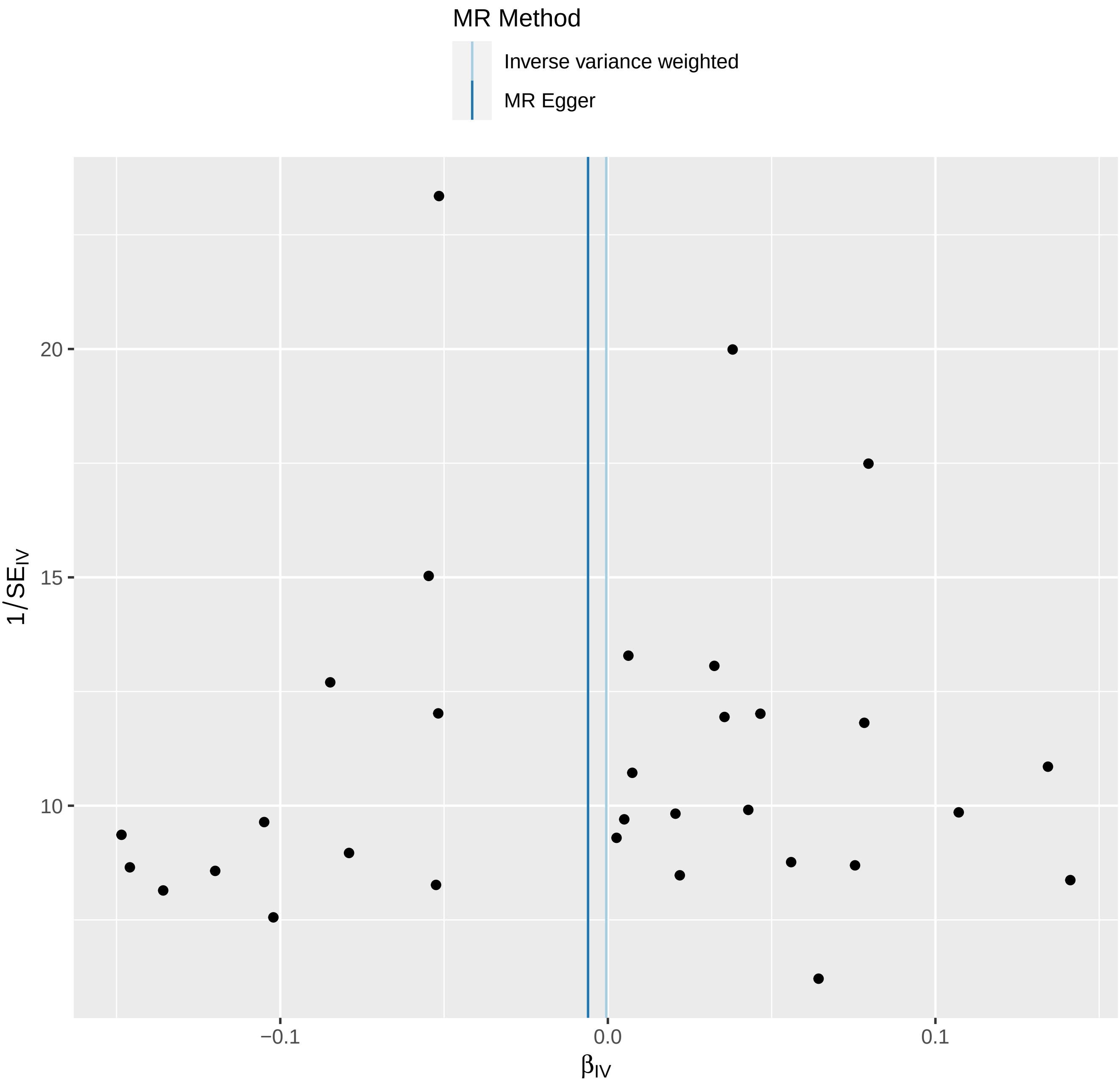

Batch 571 : Crohn's disease on DKTatlas lh postcentral area

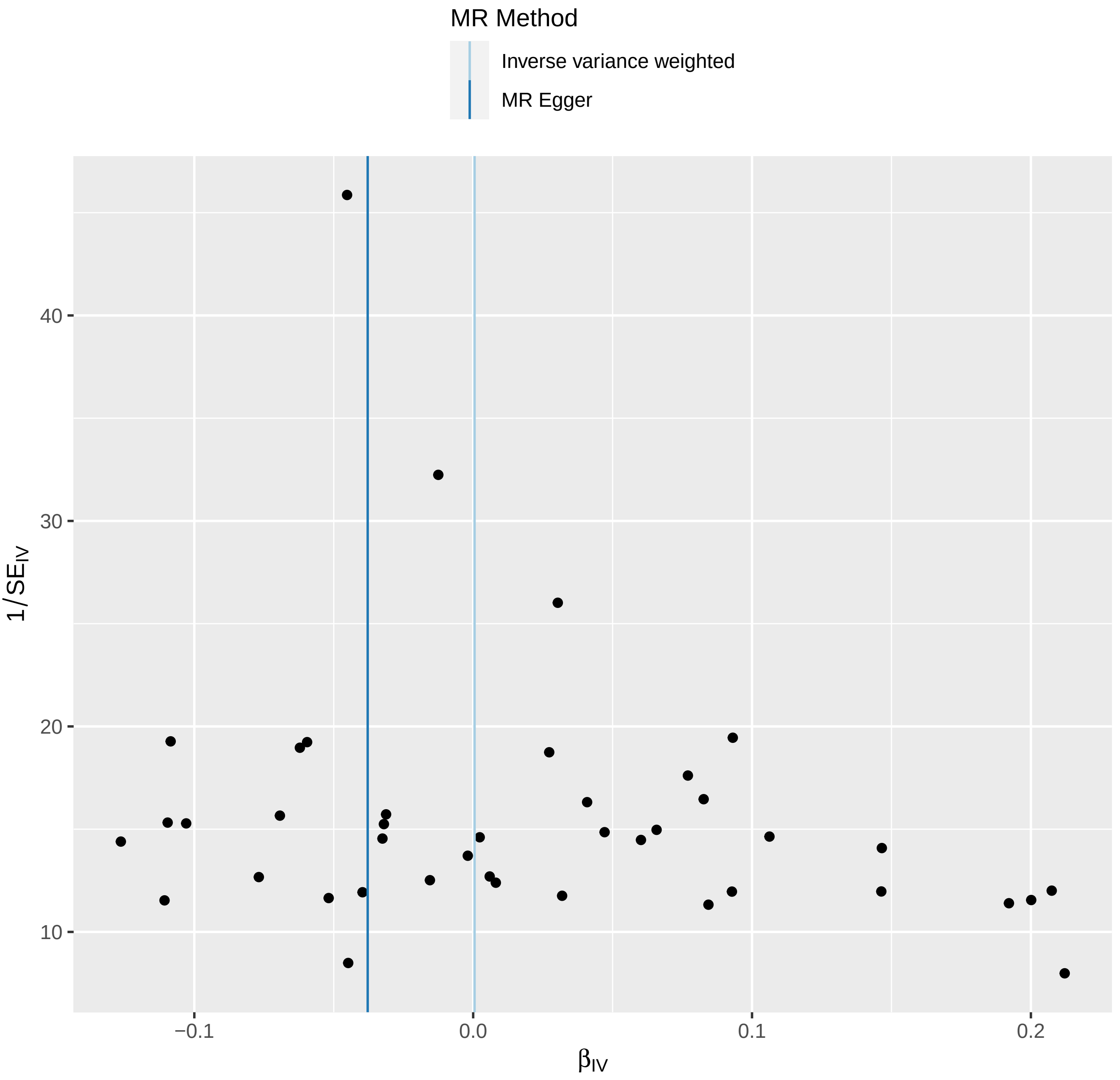

Batch 572 : Ulcerative colitis on DKTatlas lh postcentral area

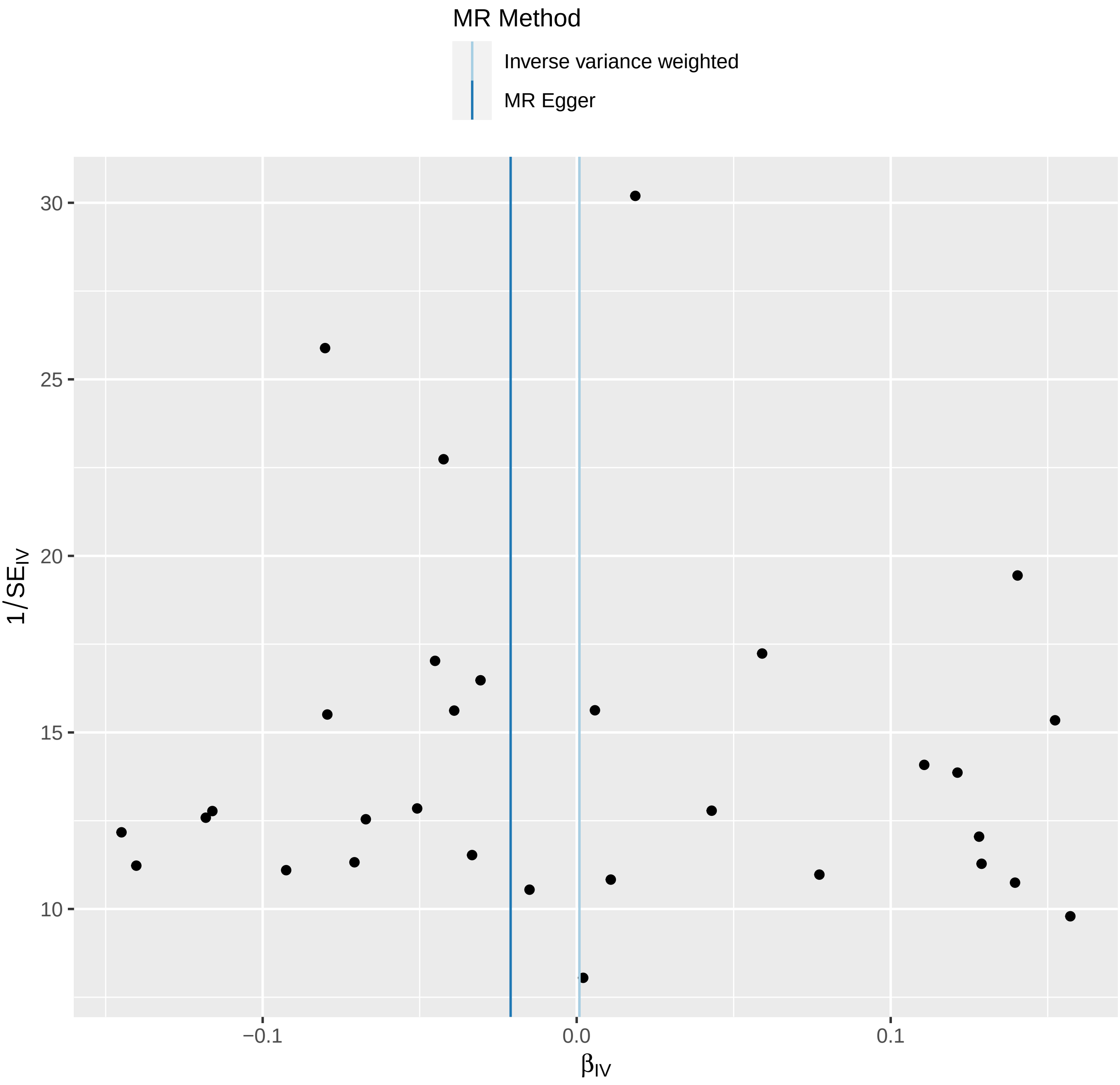

Batch 573 : Crohn's disease on DKTatlas lh posteriorcingulate area

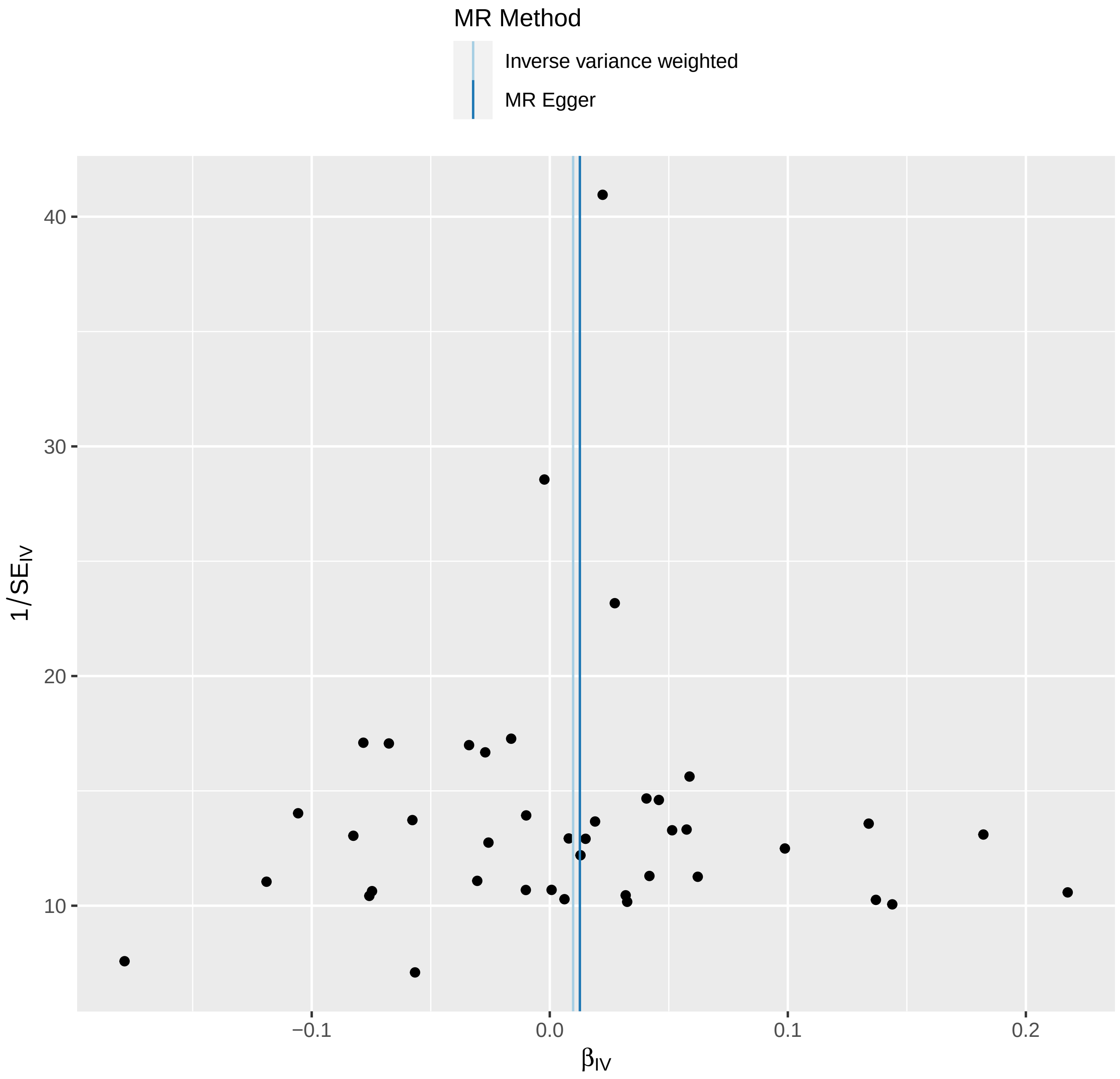

Batch 574 : Ulcerative colitis on DKTatlas lh posteriorcingulate area

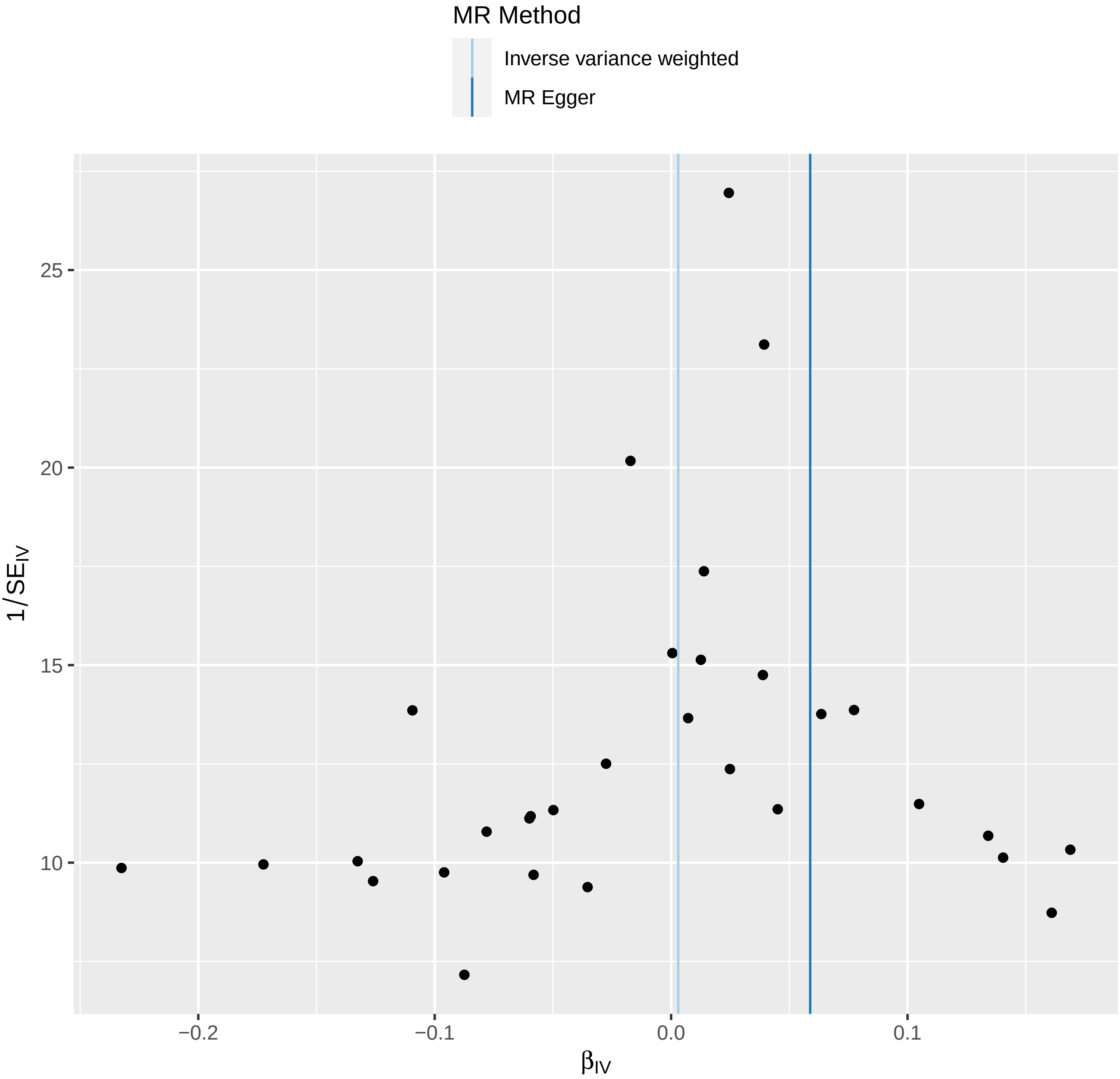

Batch 575 : Crohn's disease on DKTatlas lh precentral area

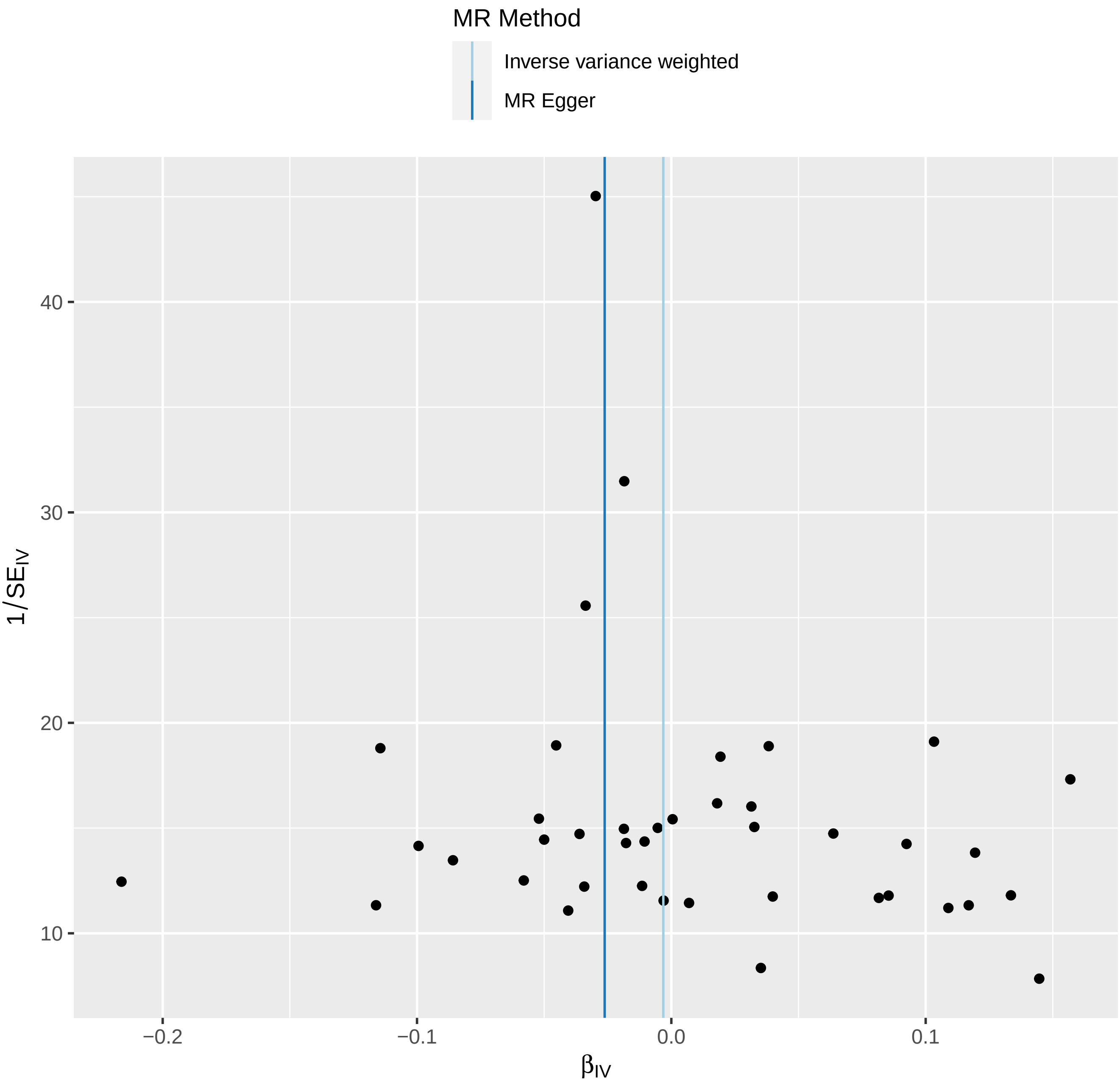

Batch 576 : Ulcerative colitis on DKTatlas lh precentral area

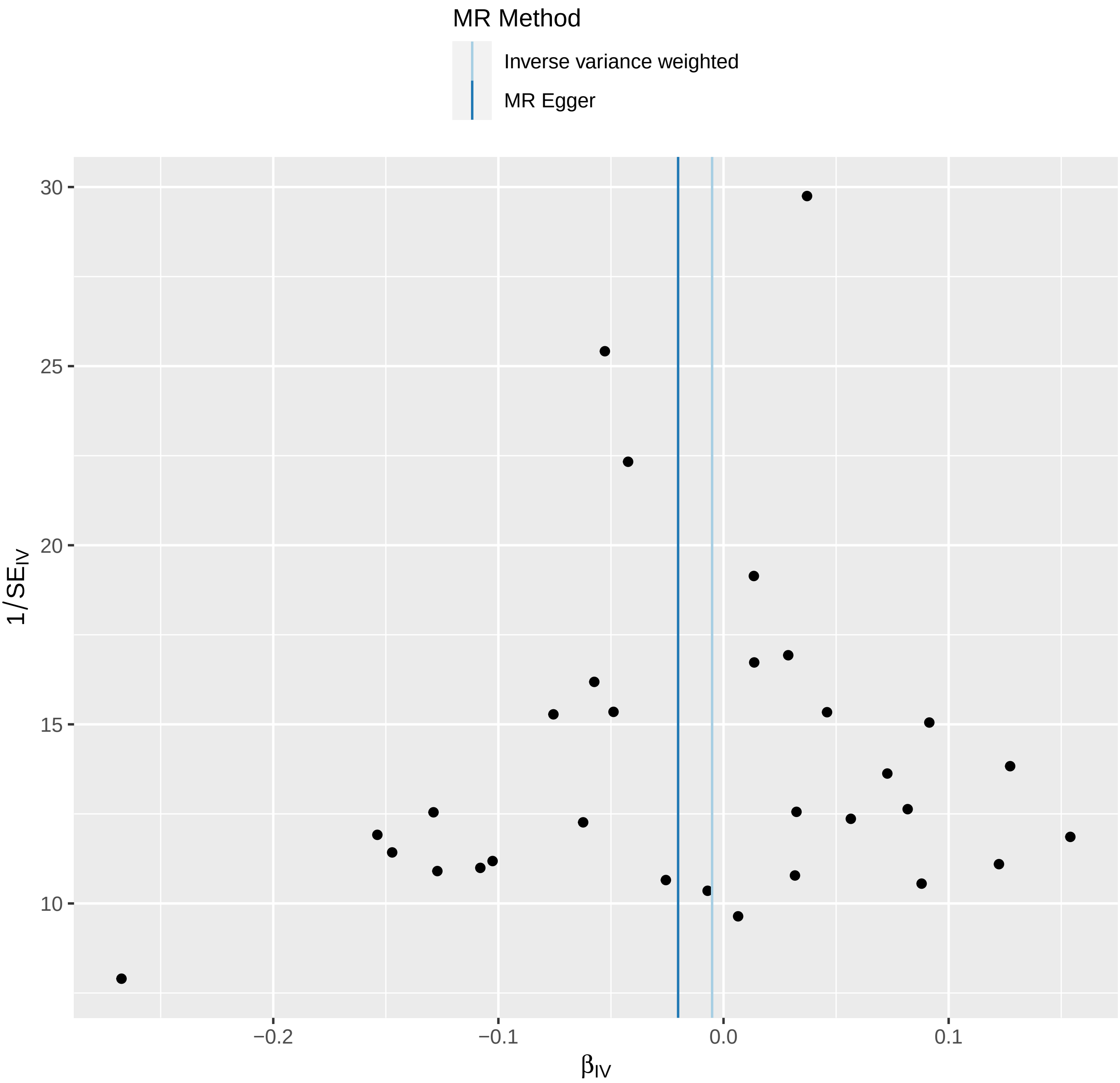

Batch 577 : Crohn's disease on DKTatlas lh precuneus area

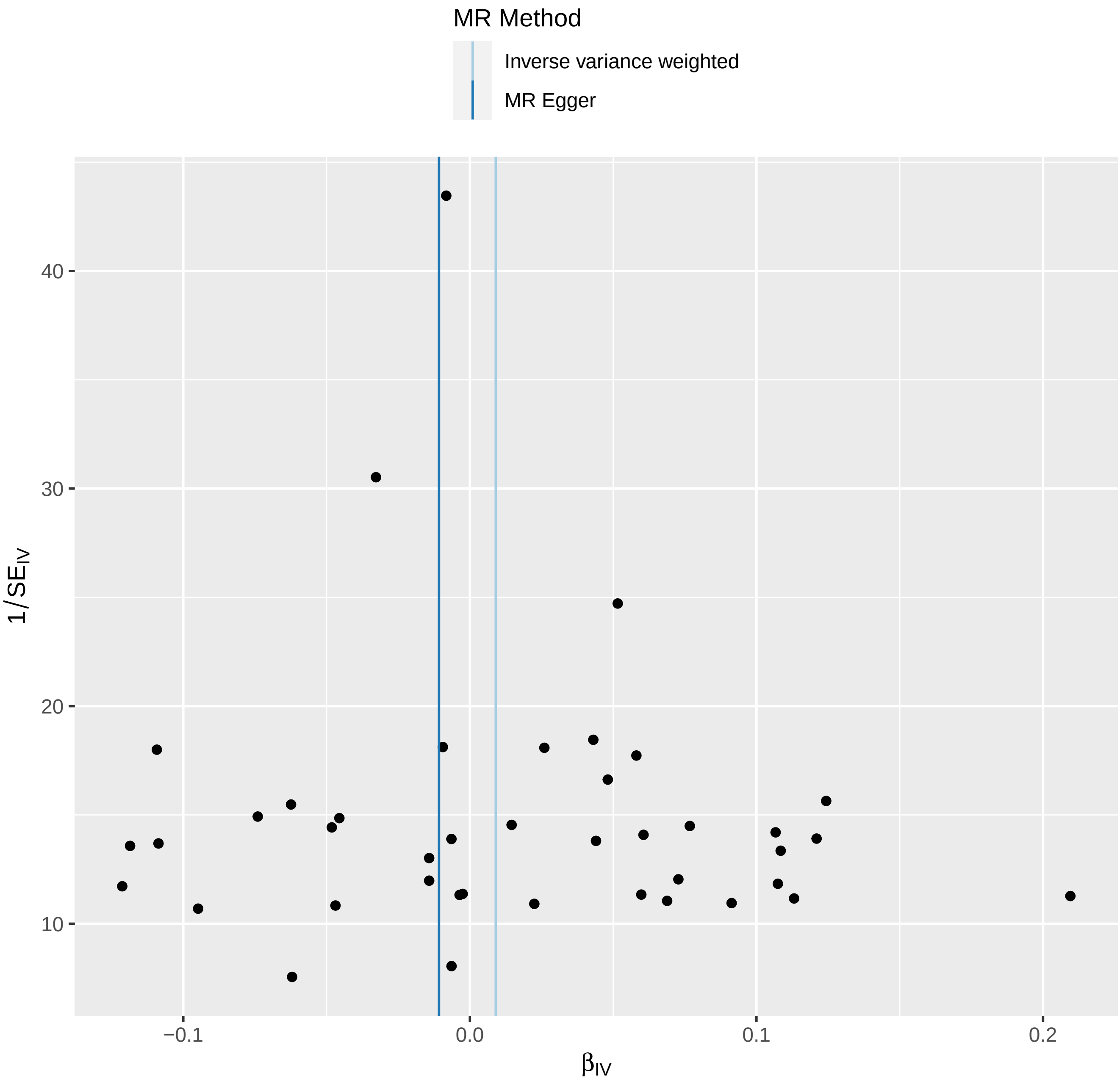

Batch 578 : Ulcerative colitis on DKTatlas lh precuneus area

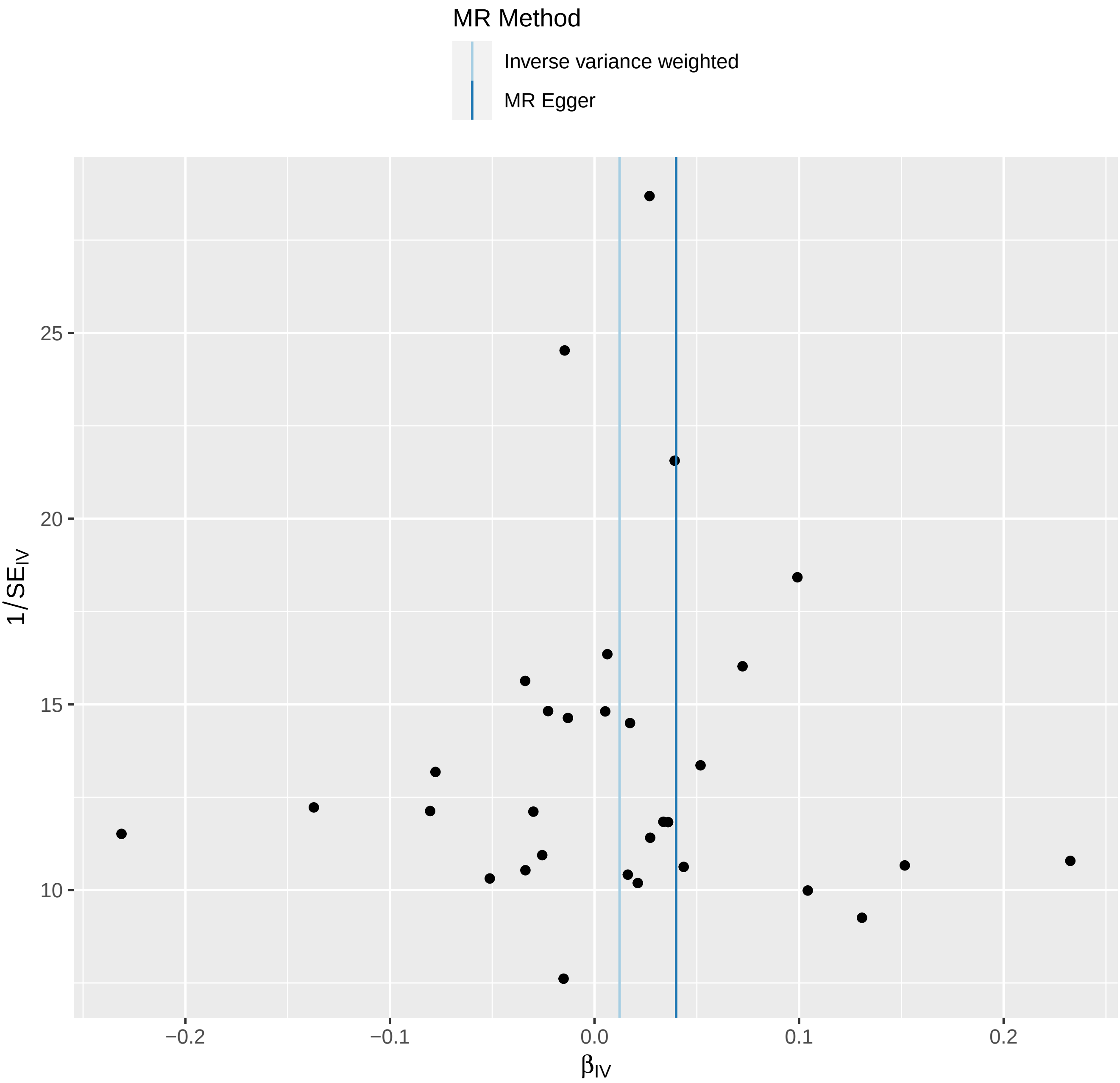

Batch 579 : Crohn's disease on DKTatlas lh rostralanteriorcingulate area

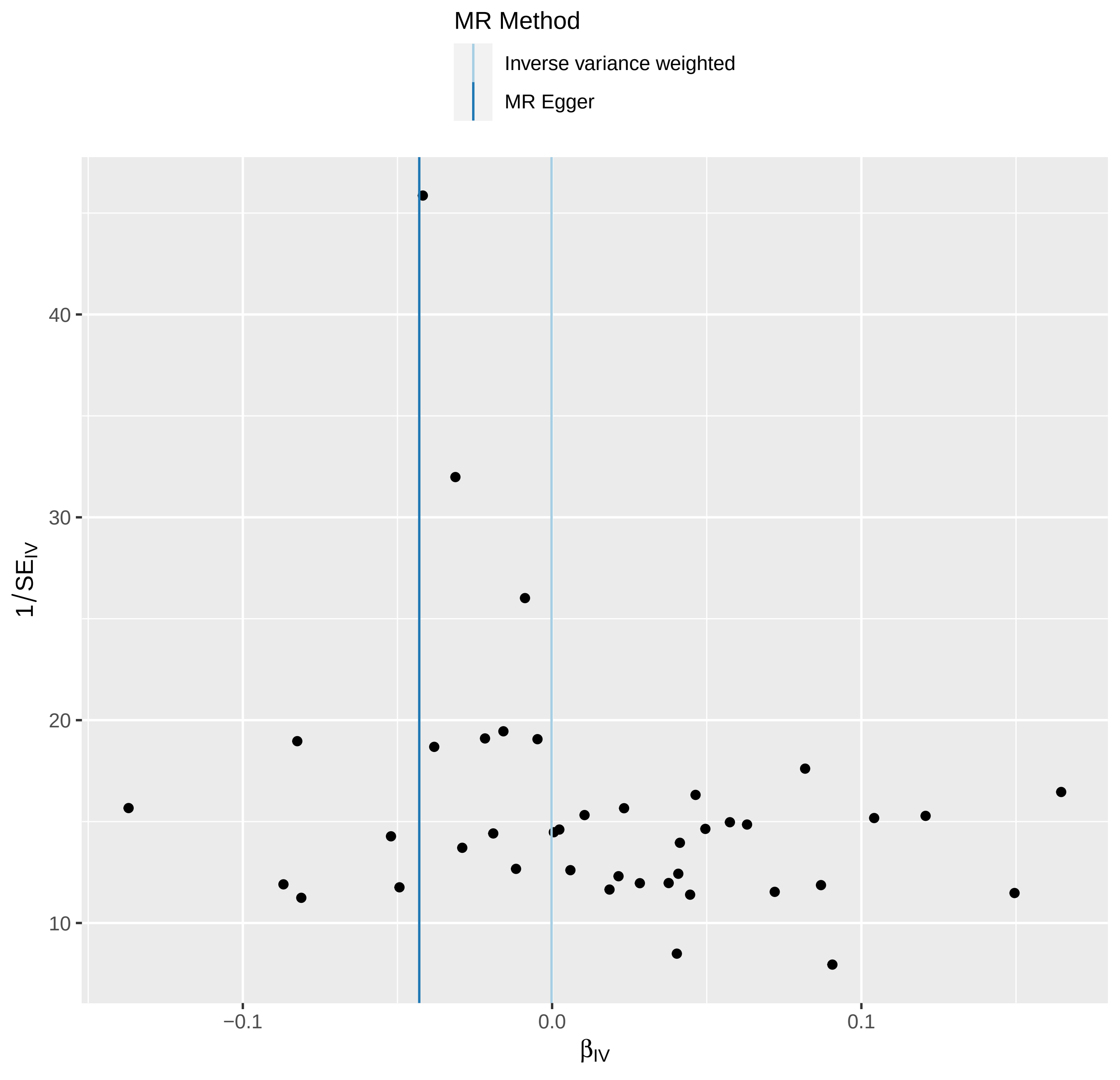

Batch 580 : Ulcerative colitis on DKTatlas lh rostralanteriorcingulate area

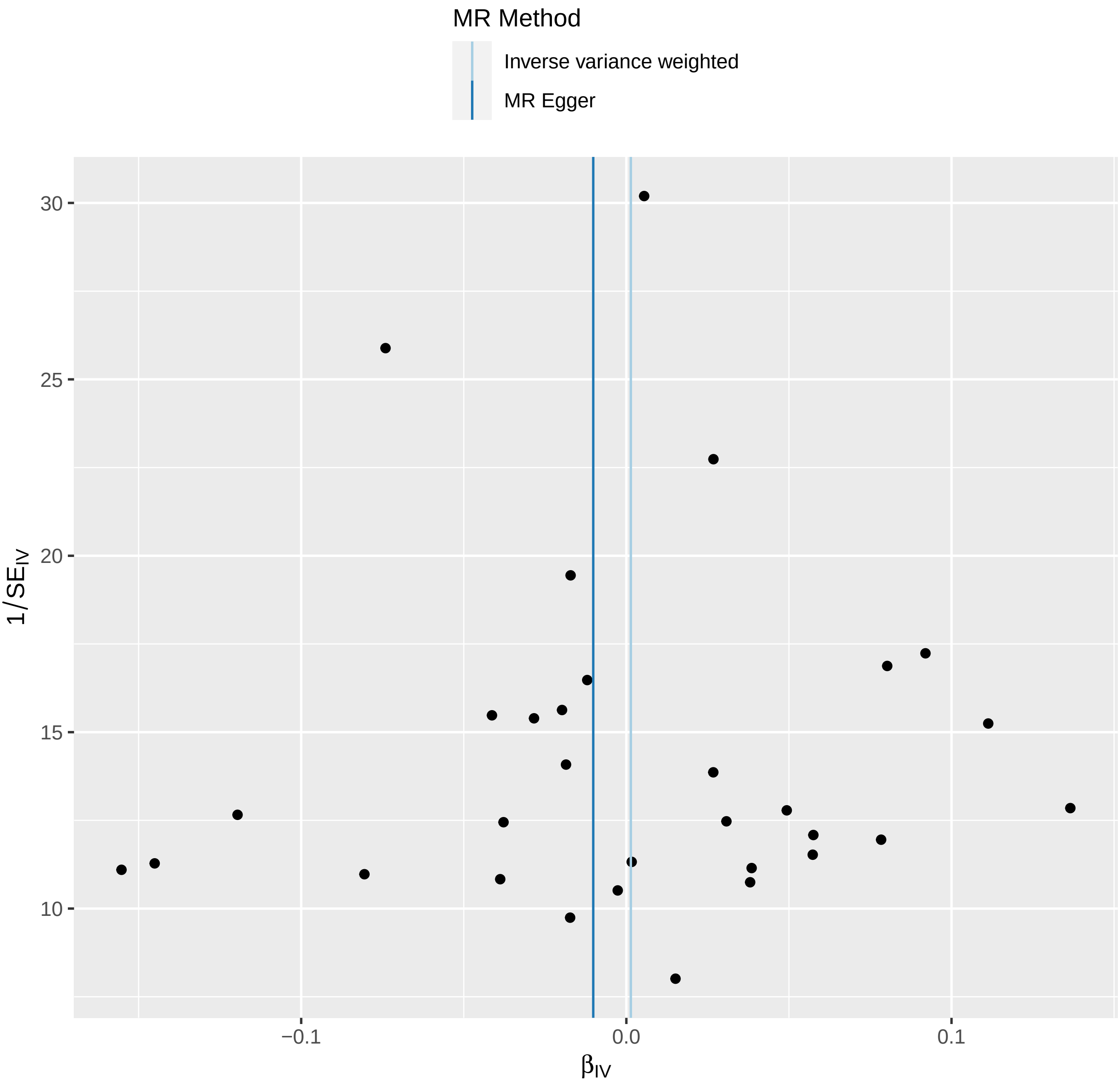

Batch 581 : Crohn's disease on DKTatlas lh rostralmiddlefrontal area

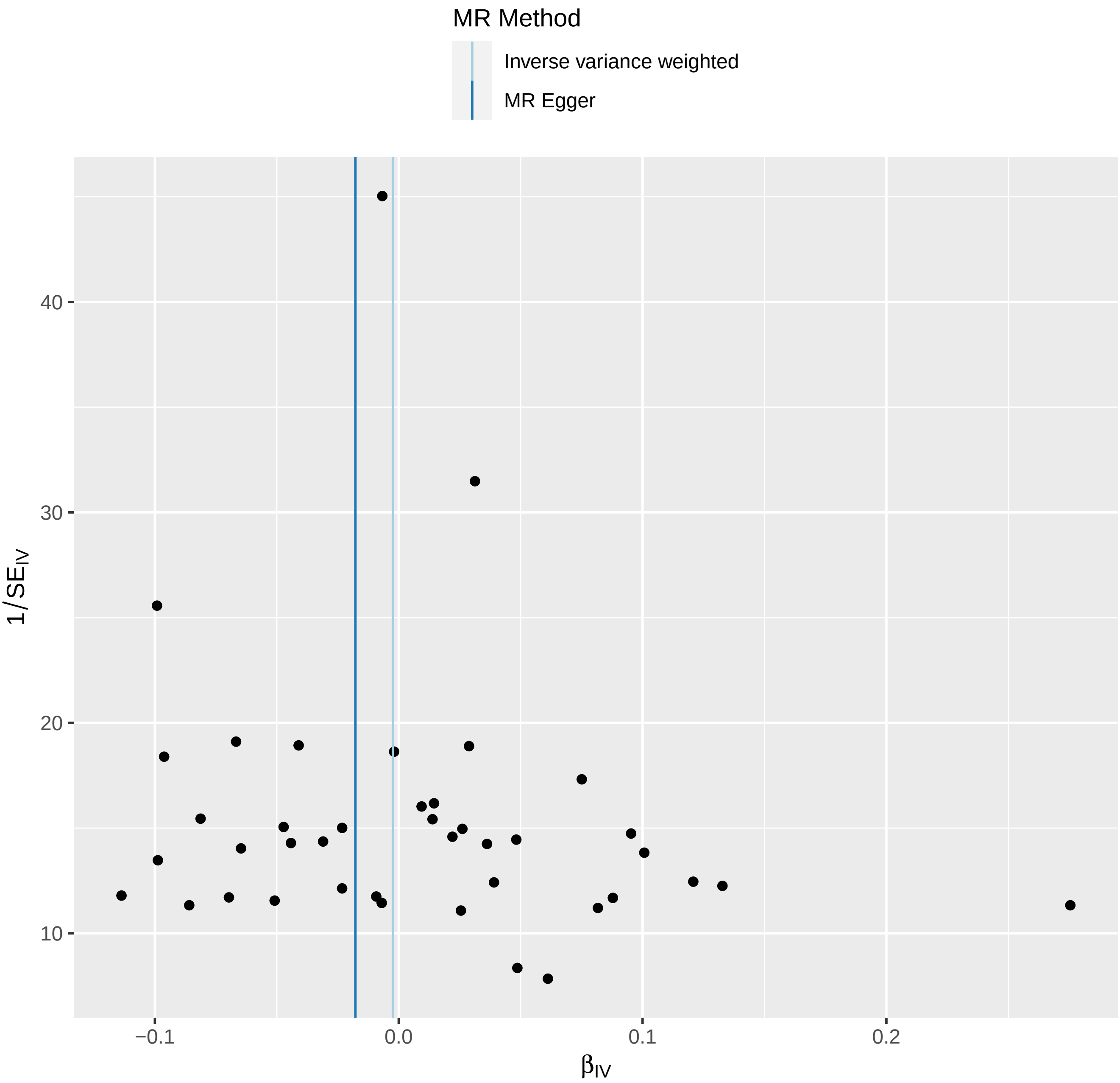

Batch 582 : Ulcerative colitis on DKTatlas lh rostralmiddlefrontal area

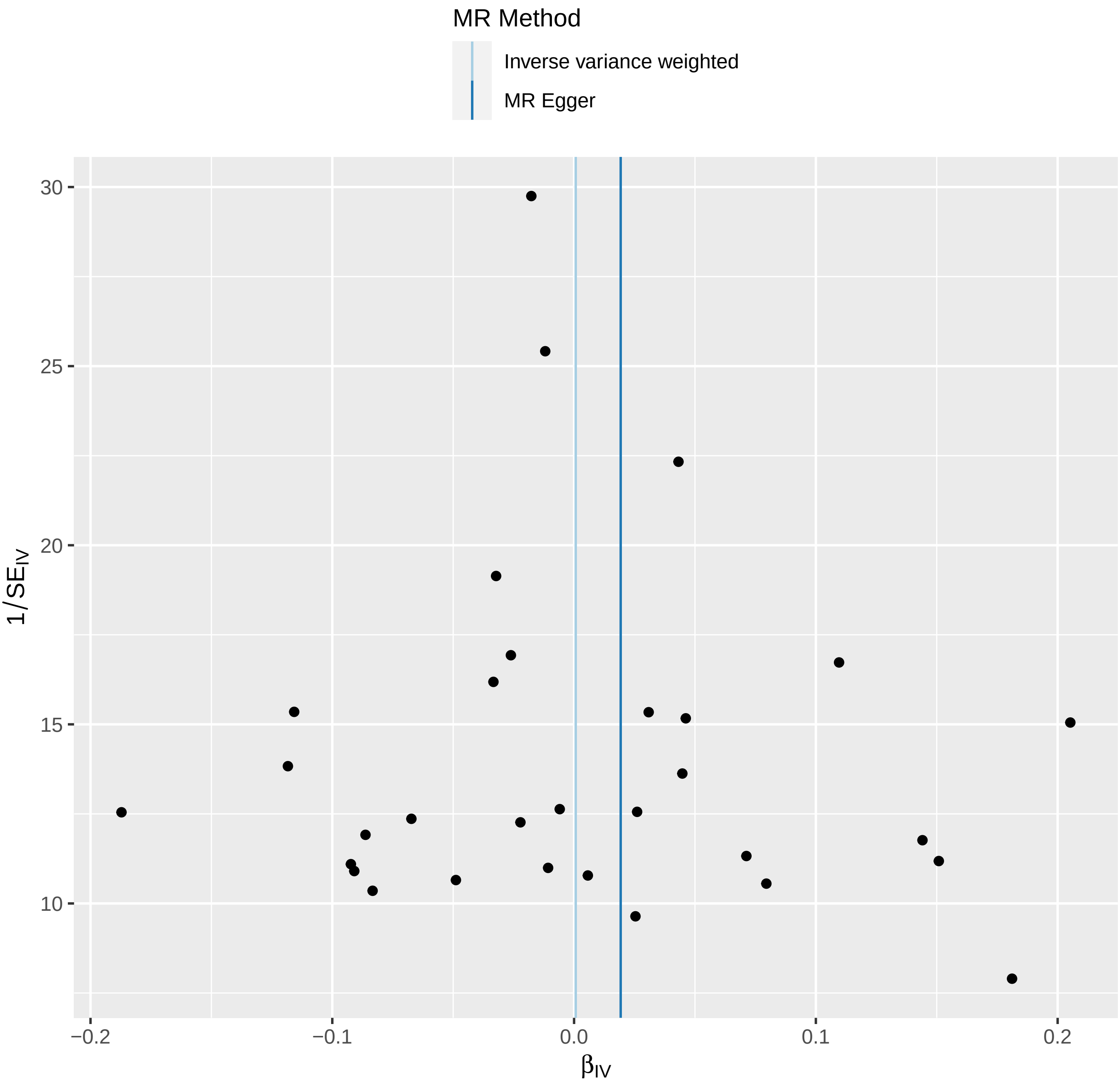

Batch 585 : Crohn's disease on DKTatlas lh superiorfrontal area

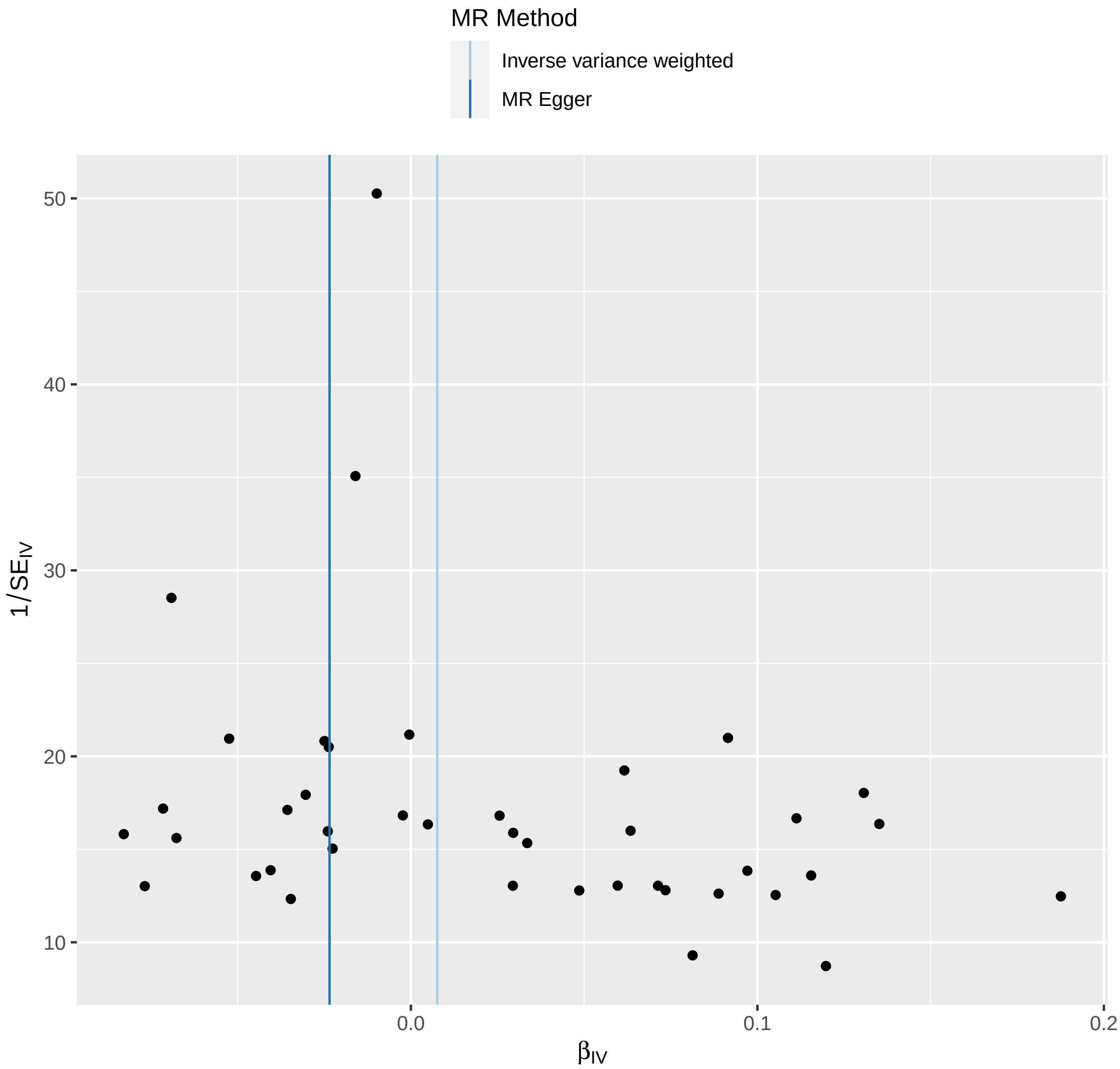

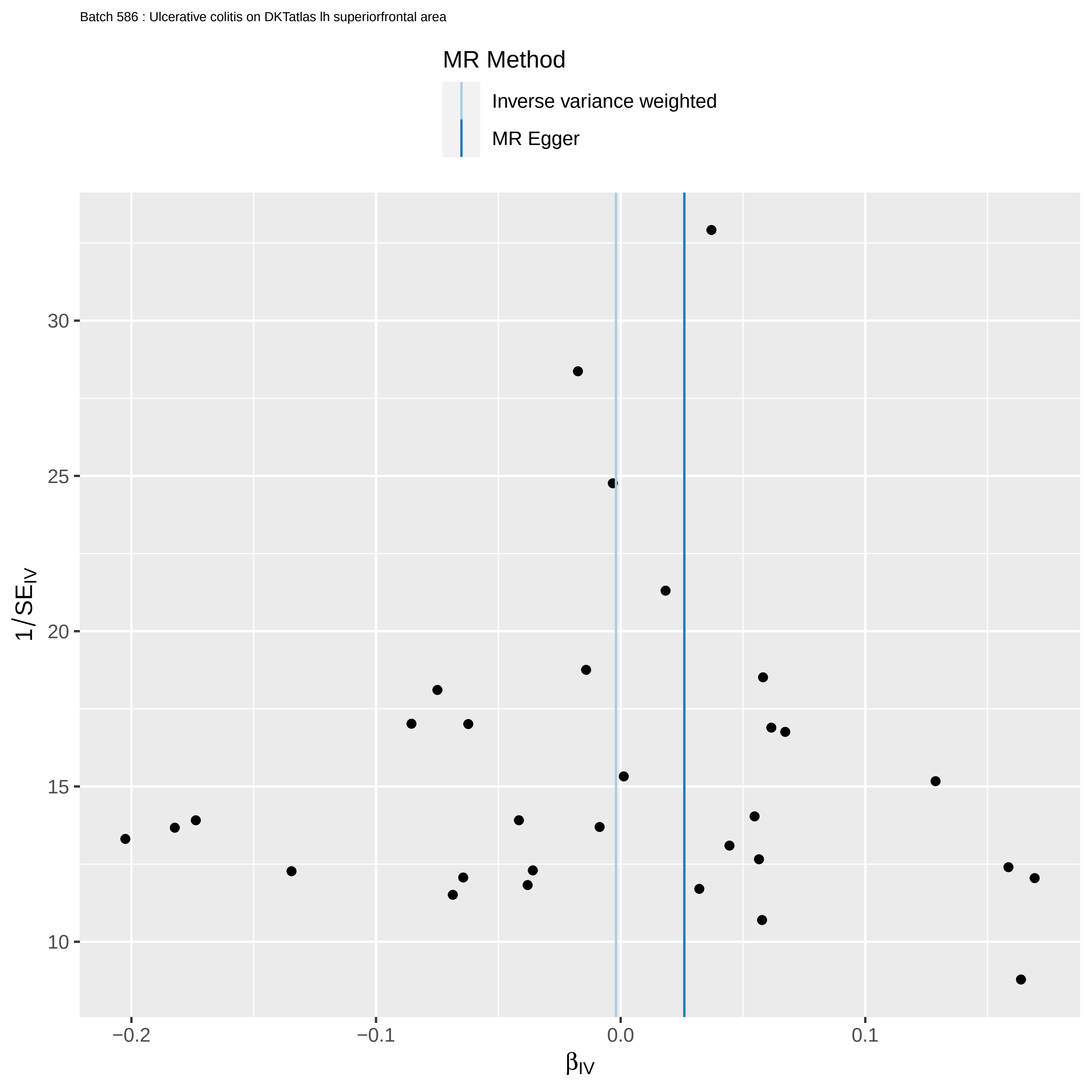

Batch 586 : Ulcerative colitis on DKTatlas lh superiorfrontal area
MR Method
Inverse variance weighted
MR Egger
30
25
20
15
10
$1/SE_{IV}$
−0.2
−0.1
0.0
0.1
β_{IV}

Batch 587 : Crohn's disease on DKTatlas lh superiorparietal area

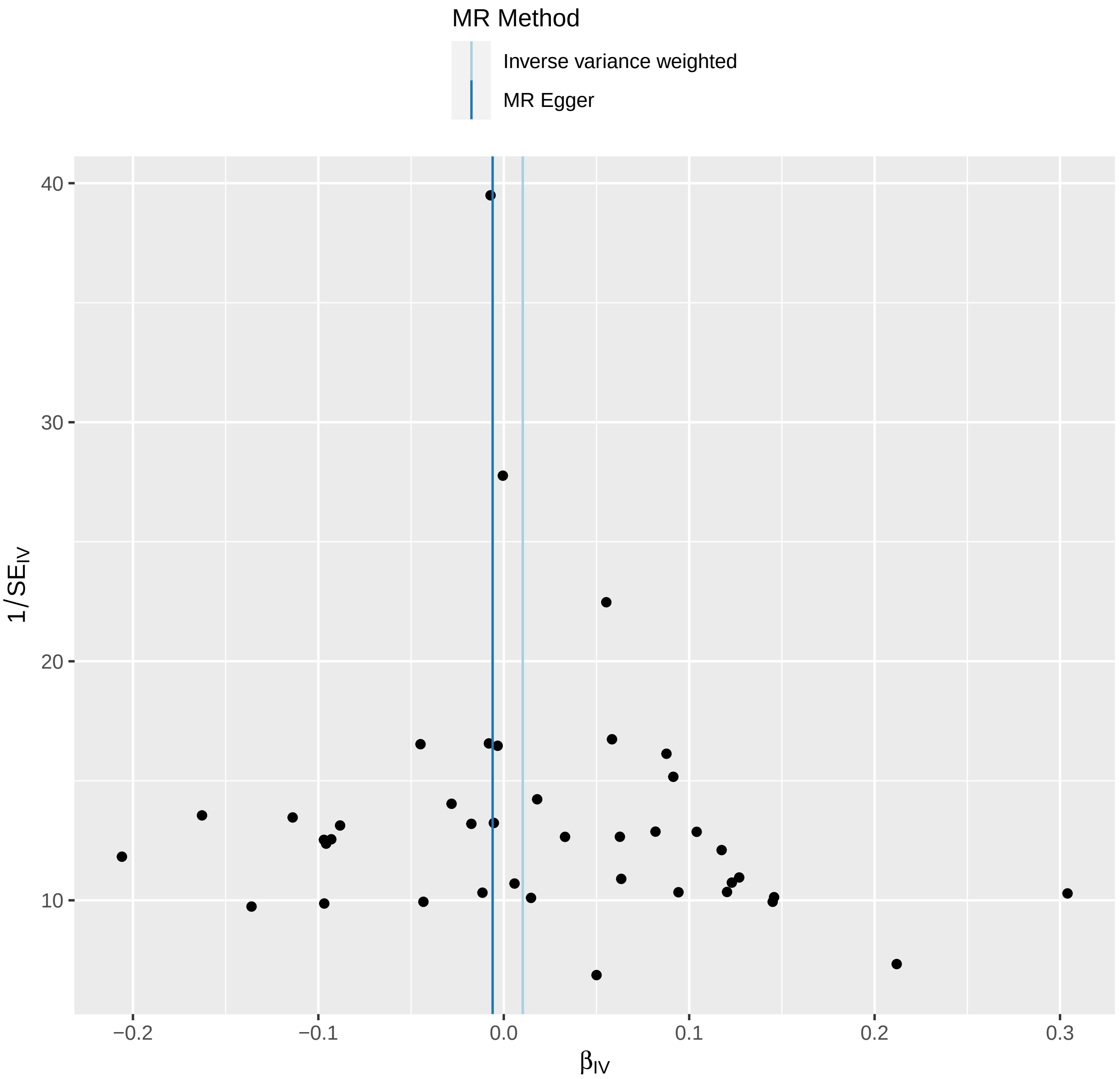

Batch 588 : Ulcerative colitis on DKTatlas lh superiorparietal area

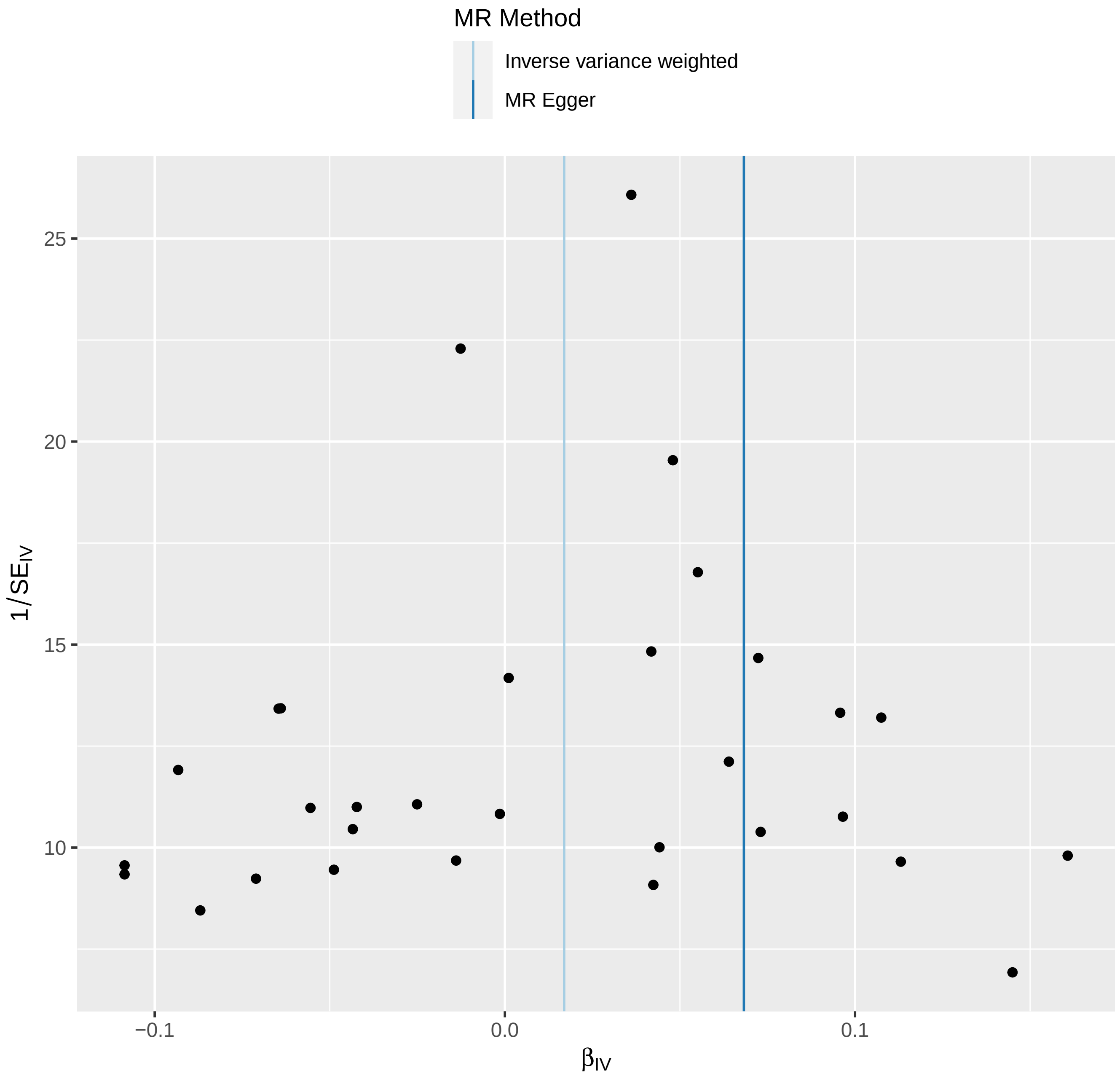

Batch 589 : Crohn's disease on DKTatlas lh superiortemporal area

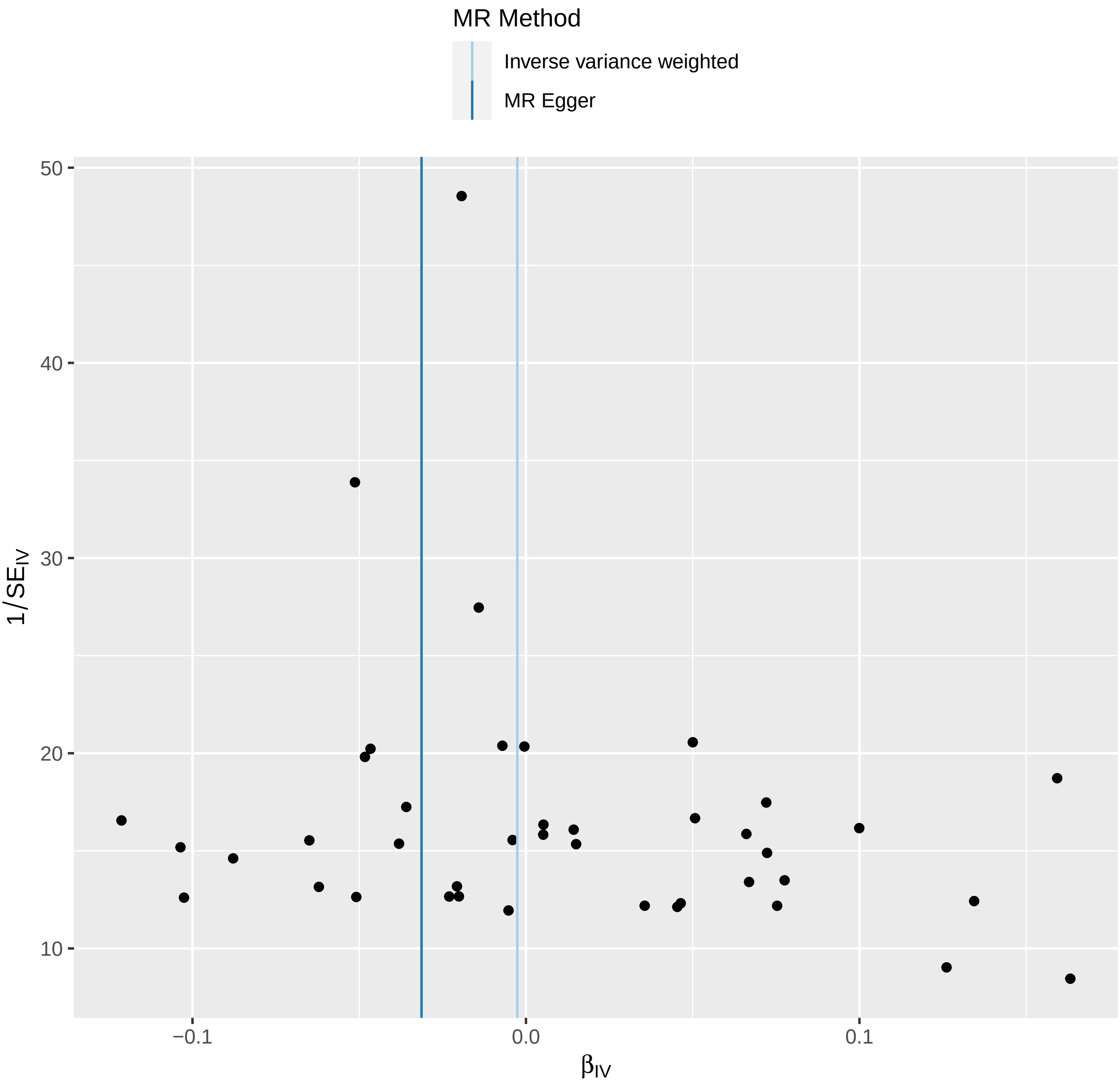

Batch 590 : Ulcerative colitis on DKTatlas lh superiortemporal area

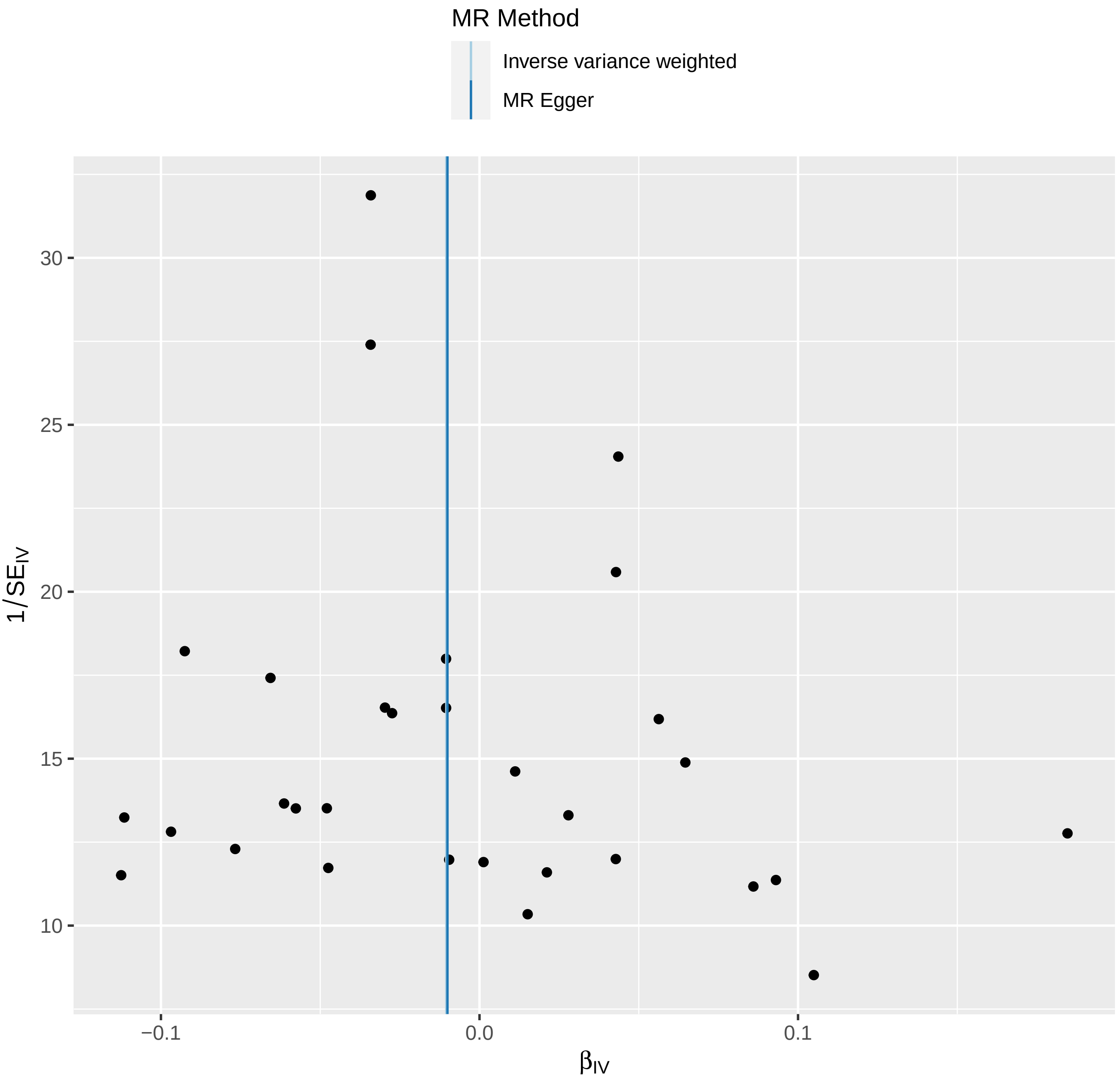

Batch 591 : Crohn's disease on DKTatlas lh supramarginal area

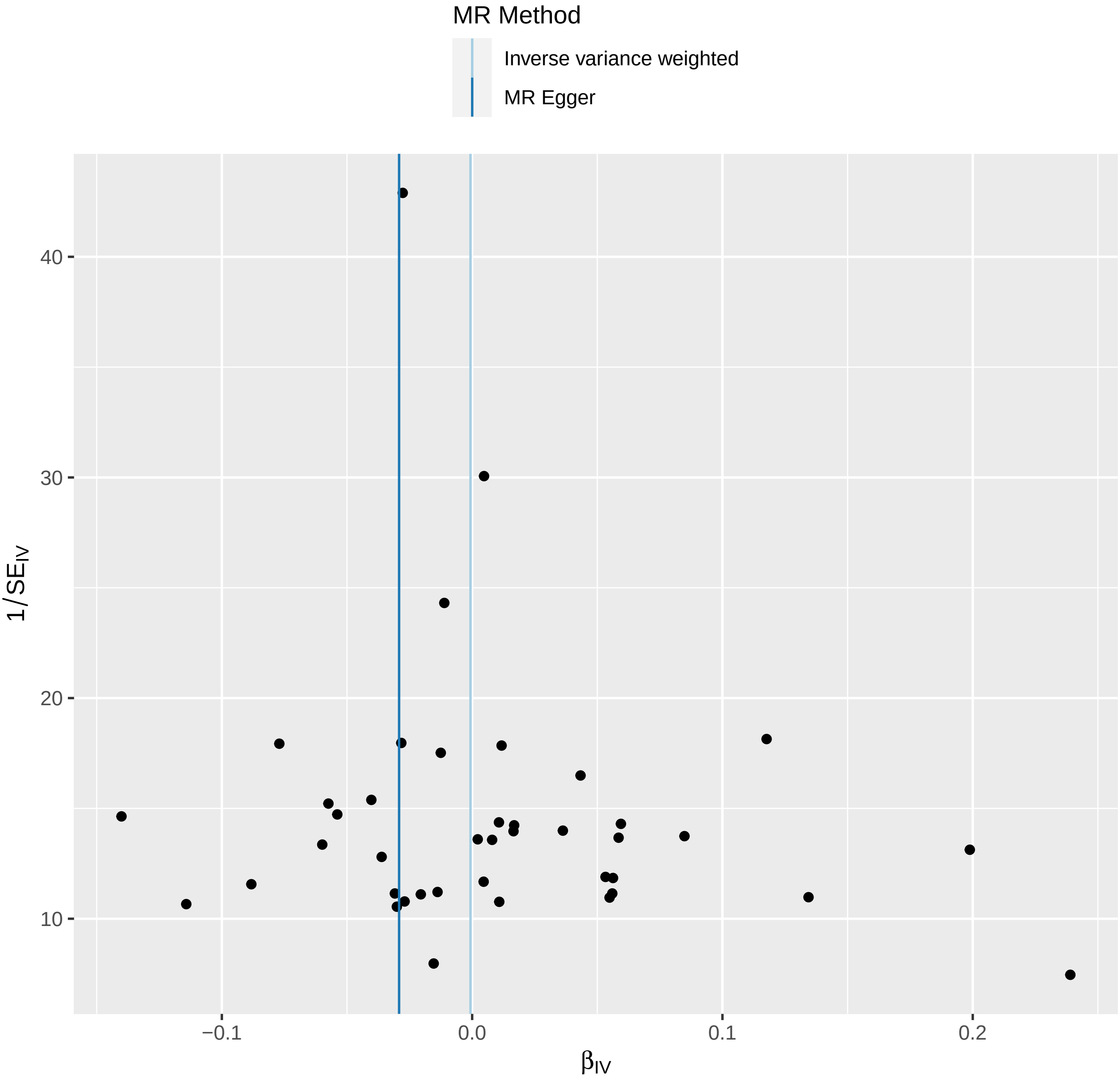

Batch 592 : Ulcerative colitis on DKTatlas lh supramarginal area

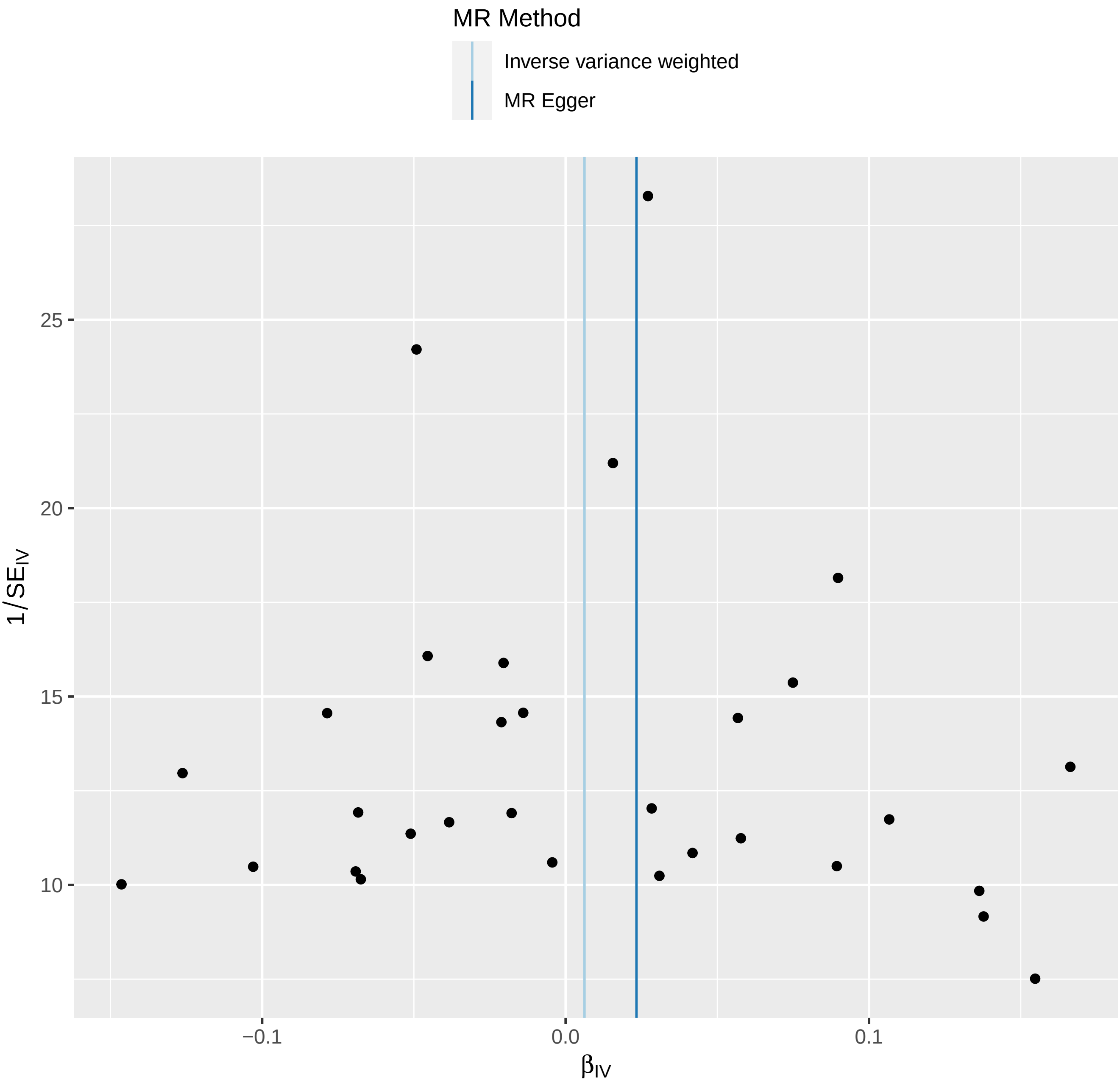

Batch 593 : Crohn's disease on DKTatlas lh transversetemporal area

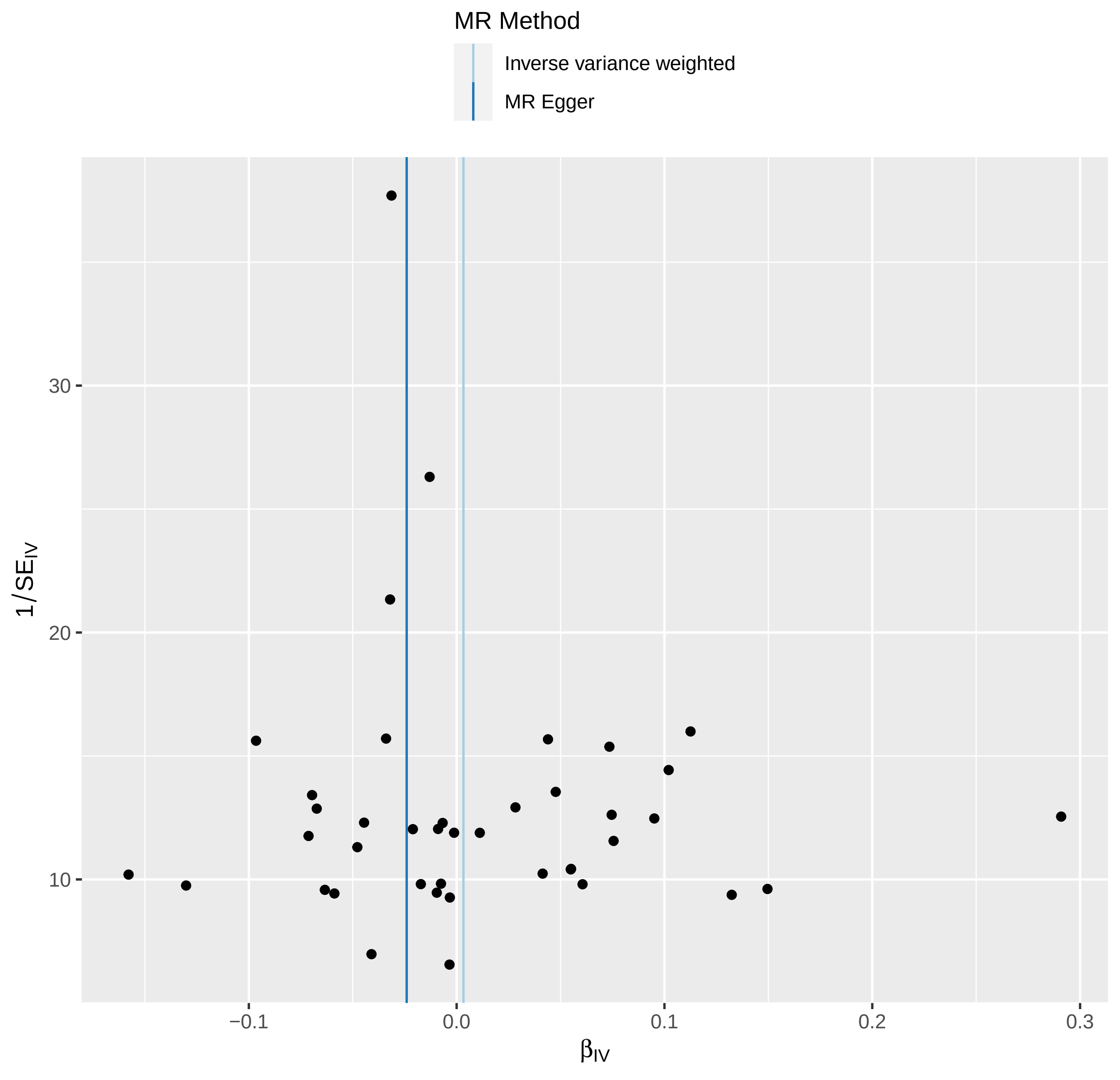

Batch 594 : Ulcerative colitis on DKTatlas lh transversetemporal area

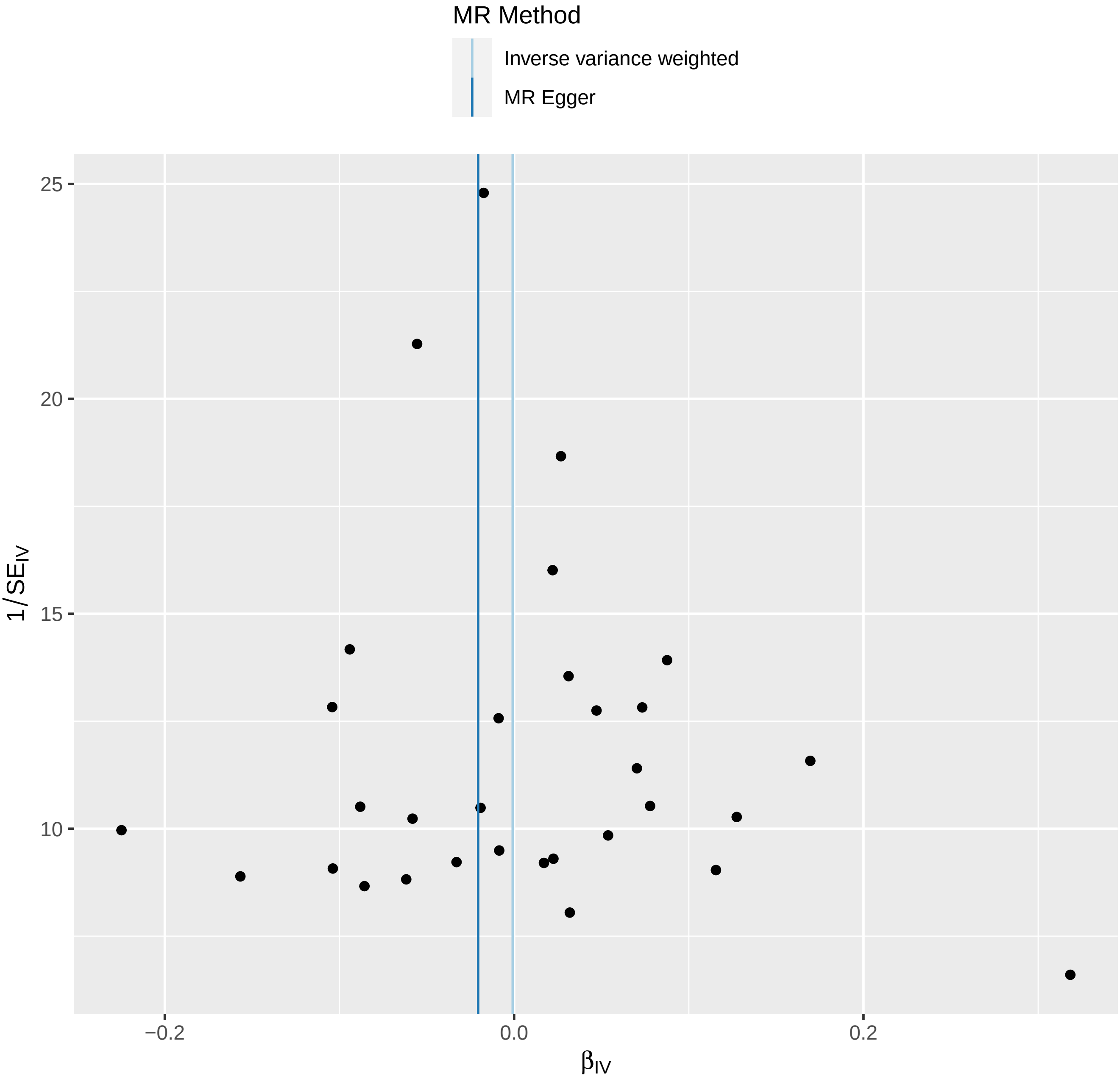

Batch 595 : Crohn's disease on DKTatlas lh insula area

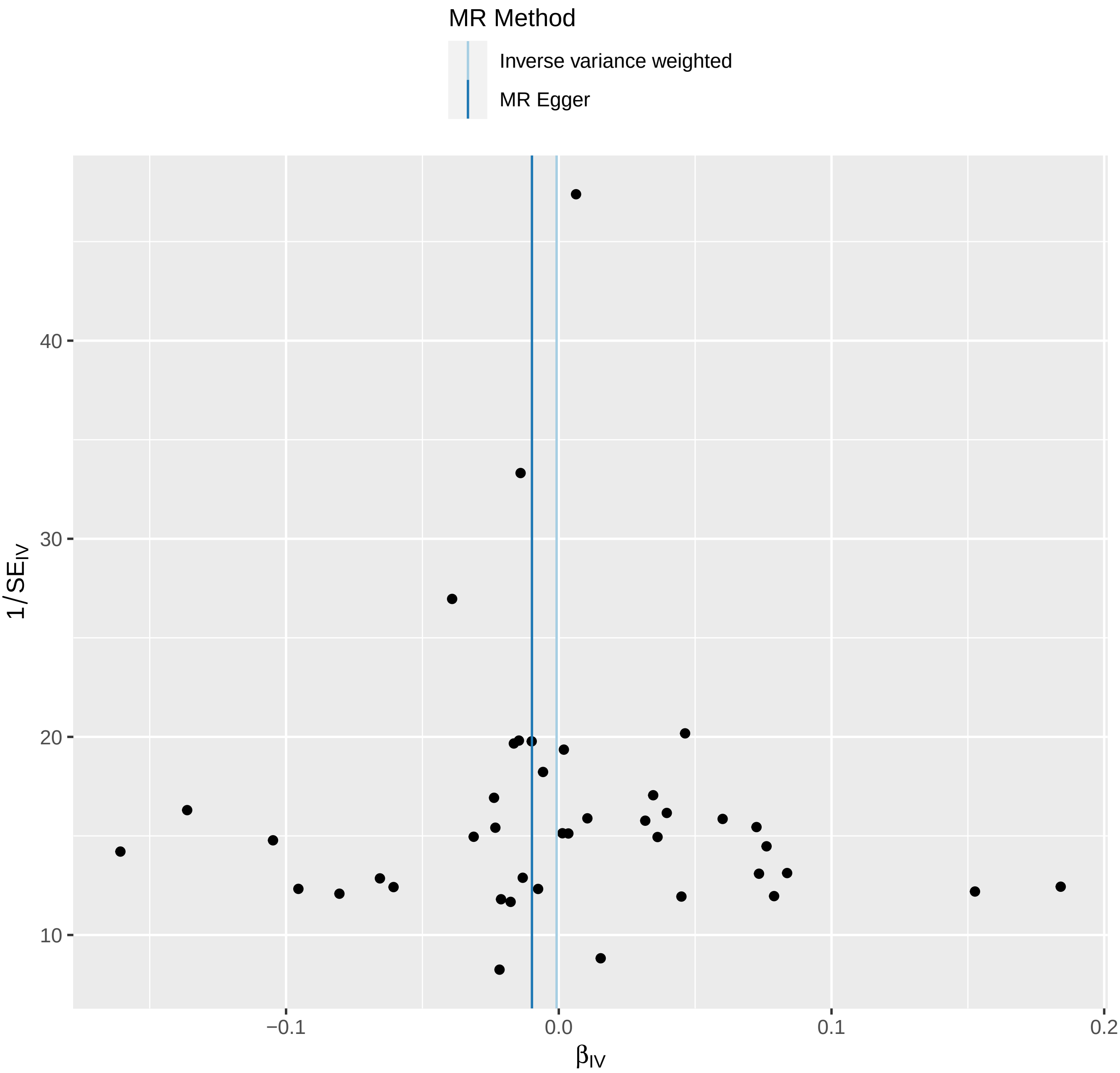

Batch 596 : Ulcerative colitis on DKTatlas lh insula area

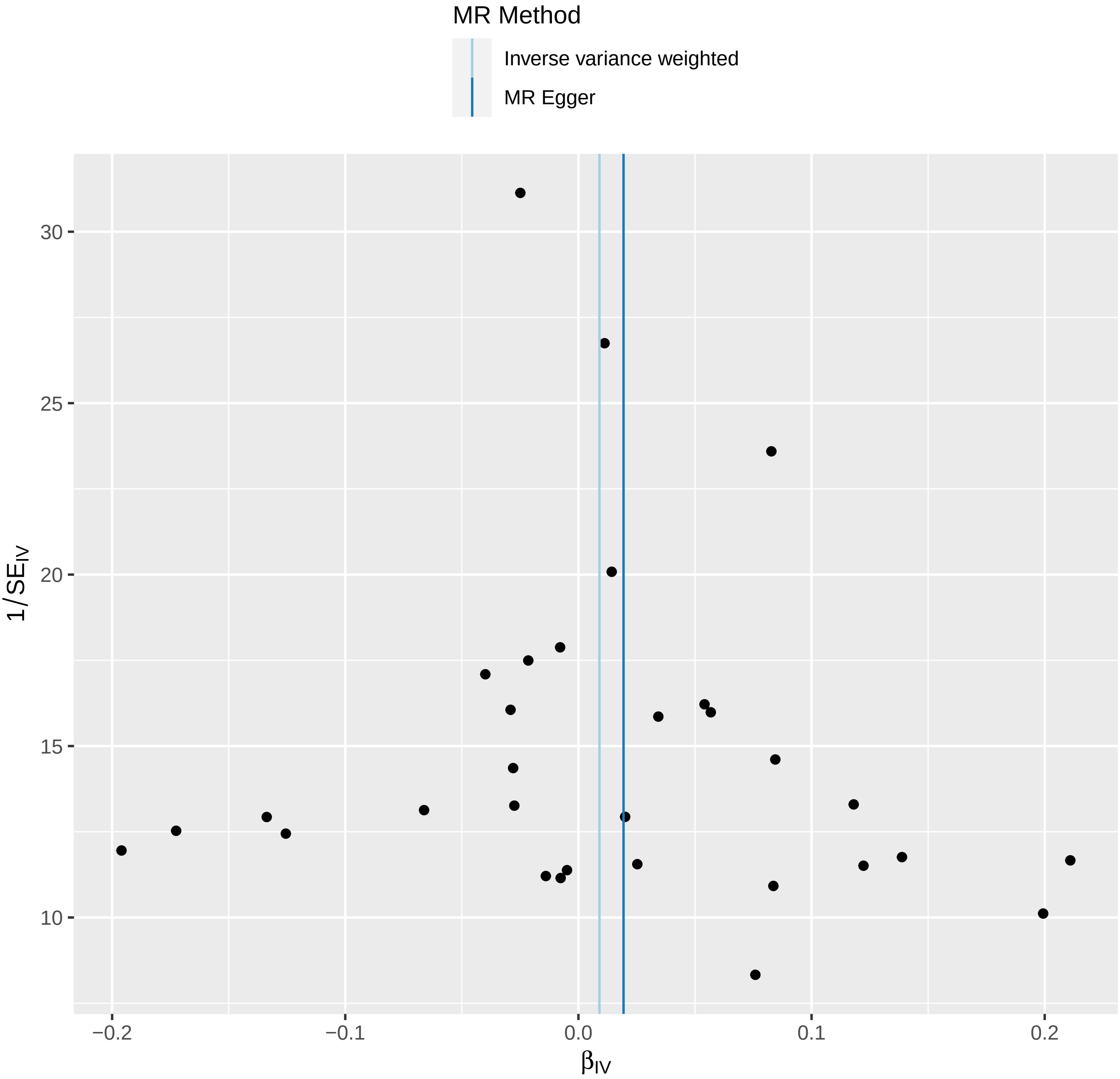

Batch 597 : Crohn's disease on DKTatlas lh WhiteSurfArea area

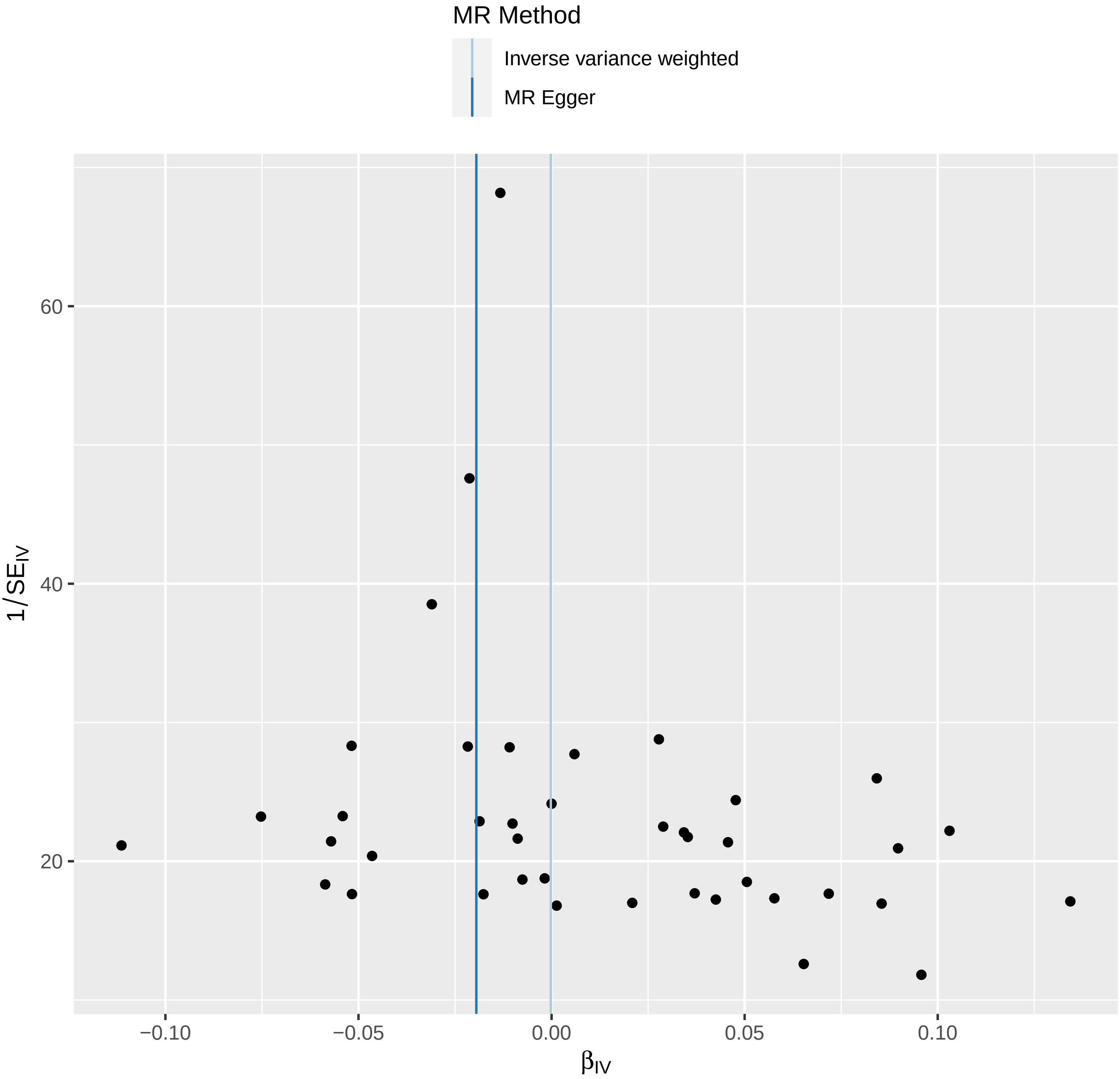

Batch 598 : Ulcerative colitis on DKTatlas lh WhiteSurfArea area

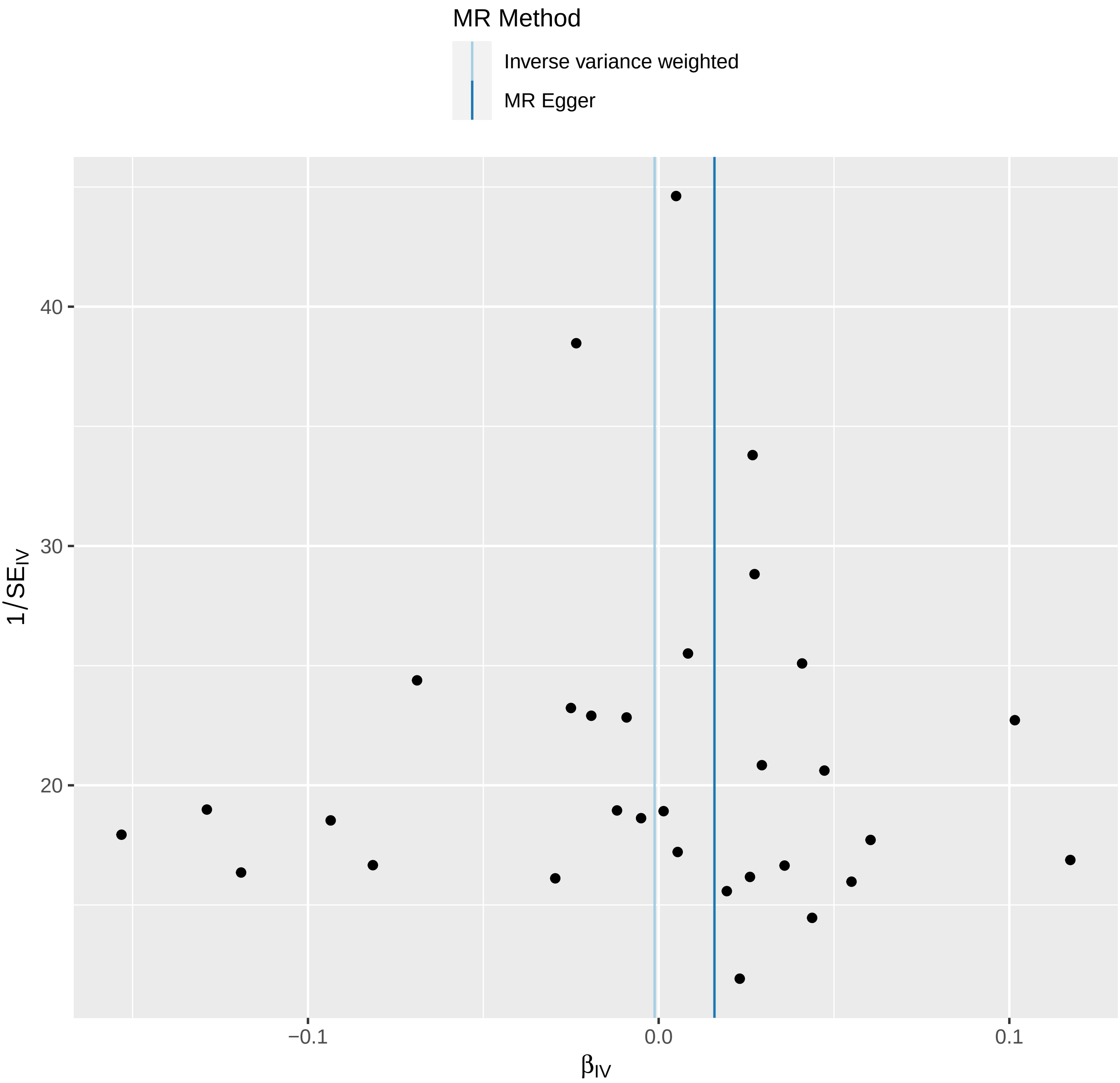

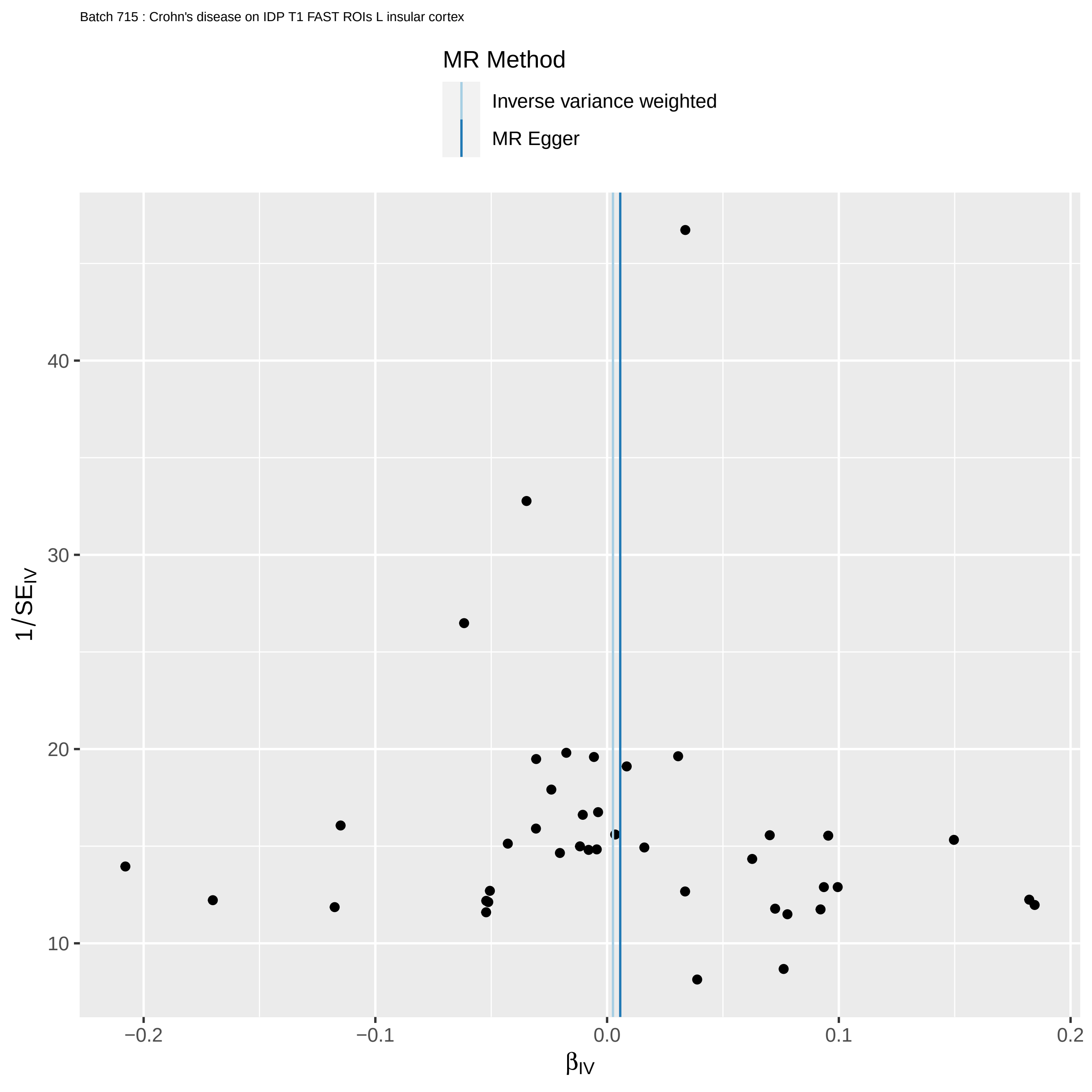

Batch 715 : Crohn's disease on IDP T1 FAST ROIs L insular cortex
MR Method
Inverse variance weighted
MR Egger
40
30
20
10
−0.2
−0.1
0.0
0.1
0.2
1/SE_IV
β_IV

Batch 716 : Ulcerative colitis on IDP T1 FAST ROIs L insular cortex

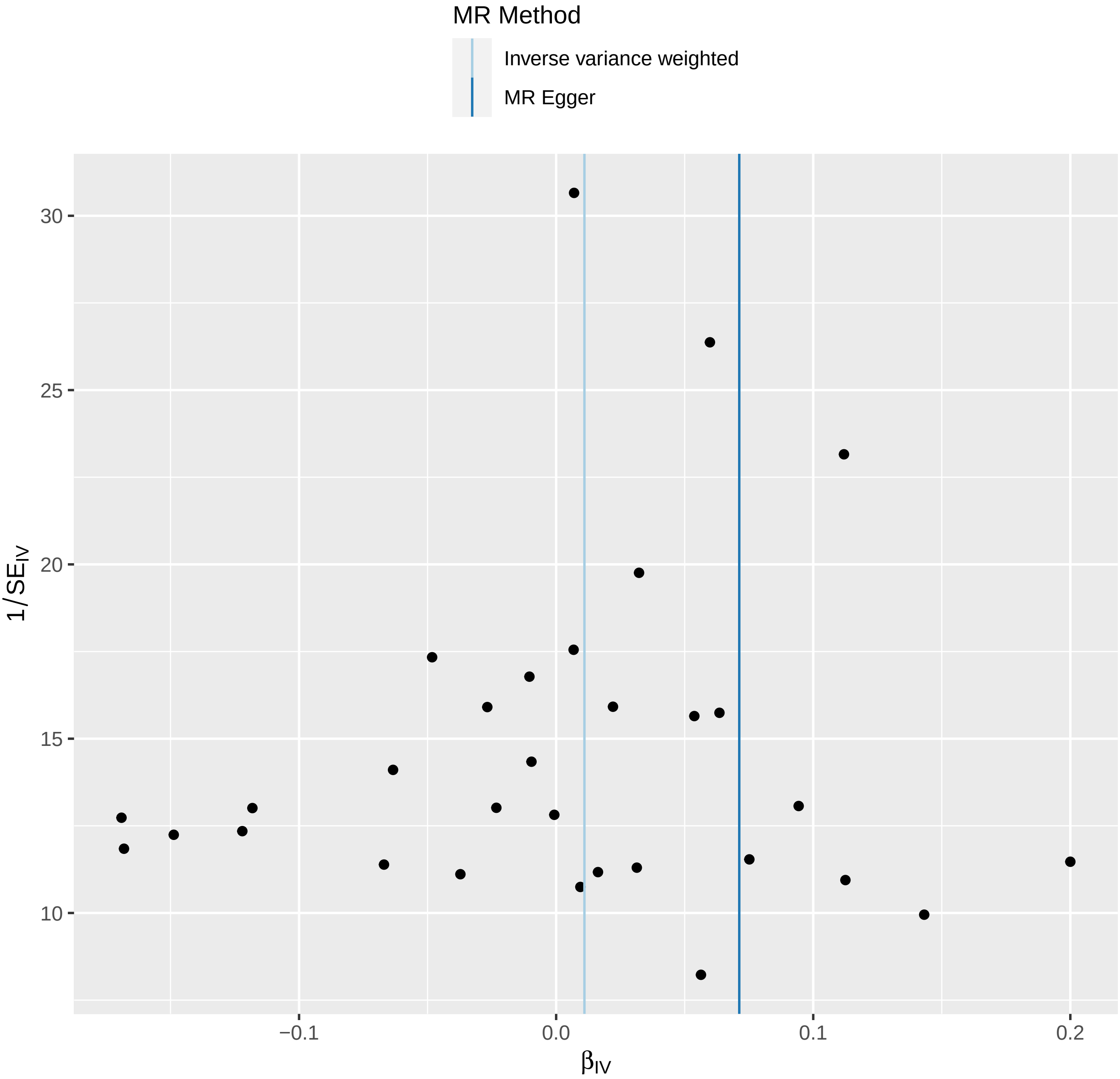

Batch 765 : Crohn's disease on DKTatlas rh caudalanteriorcingulate area

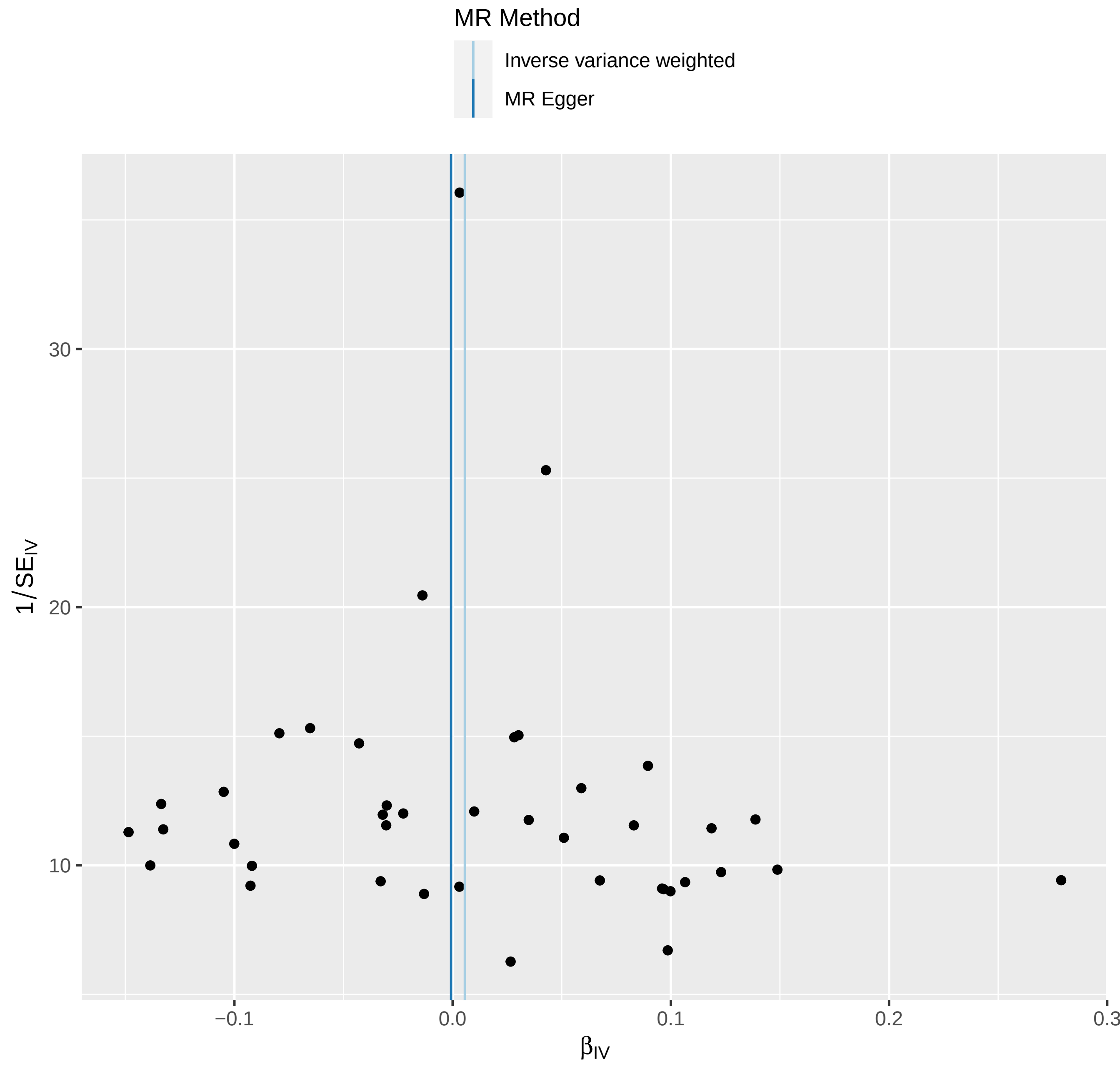

Batch 766 : Ulcerative colitis on DKTatlas rh caudalanteriorcingulate area

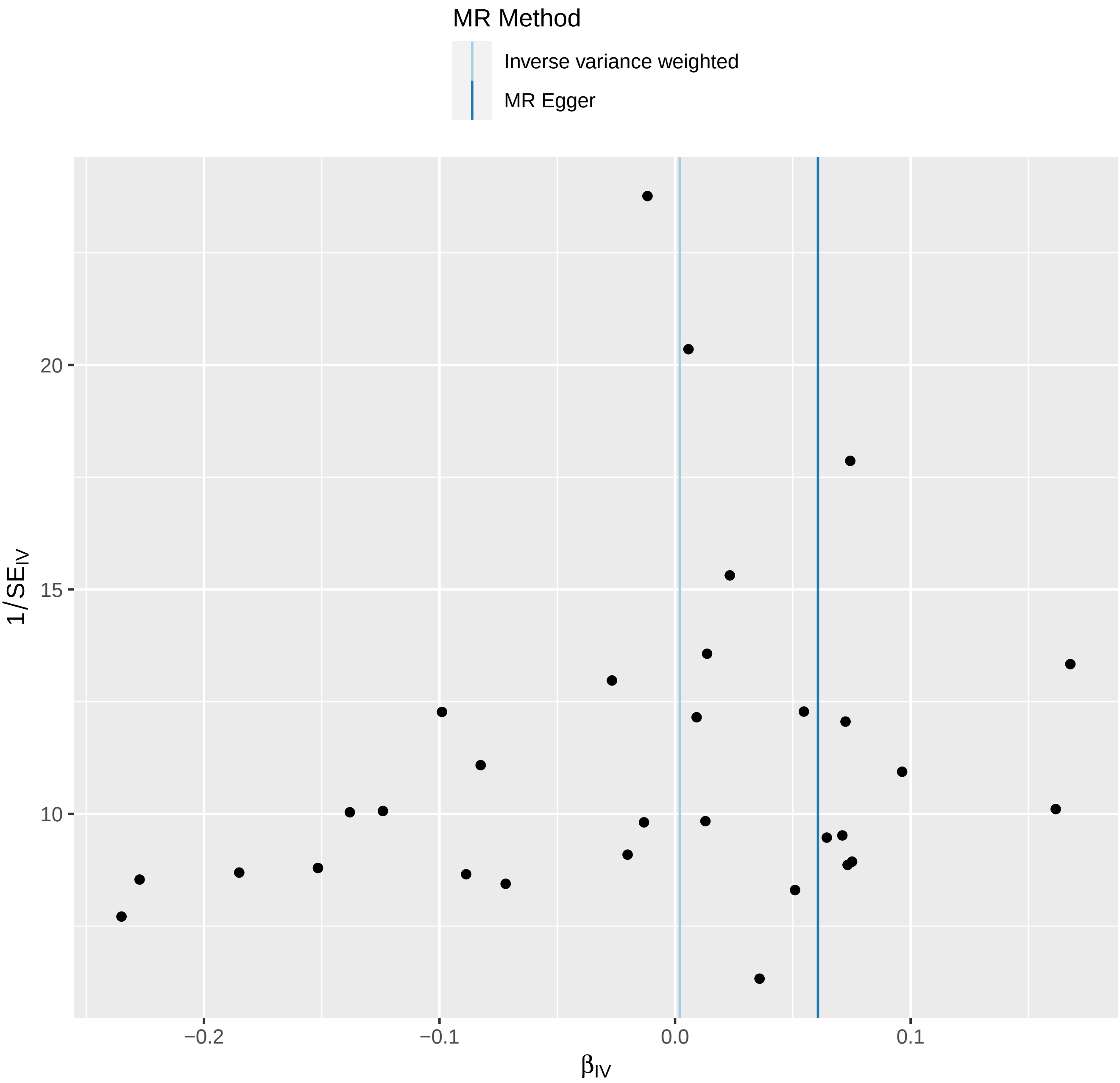

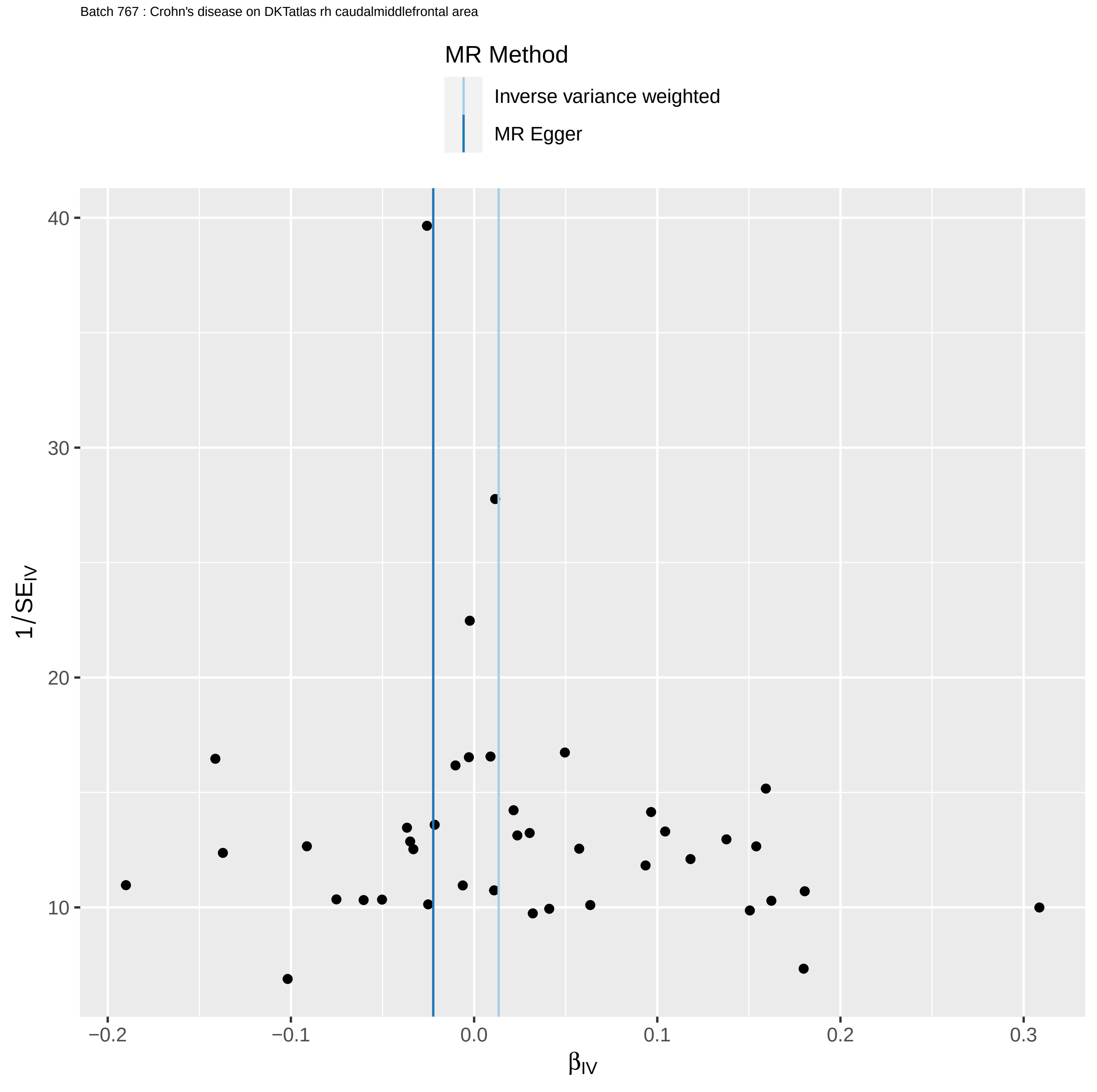

Batch 767 : Crohn's disease on DKTatlas rh caudalmiddlefrontal area
MR Method
Inverse variance weighted
MR Egger
40
30
20
10
1/SE$_{IV}$
−0.2
−0.1
0.0
0.1
0.2
0.3
β$_{IV}$

Batch 768 : Ulcerative colitis on DKTatlas rh caudalmiddlefrontal area

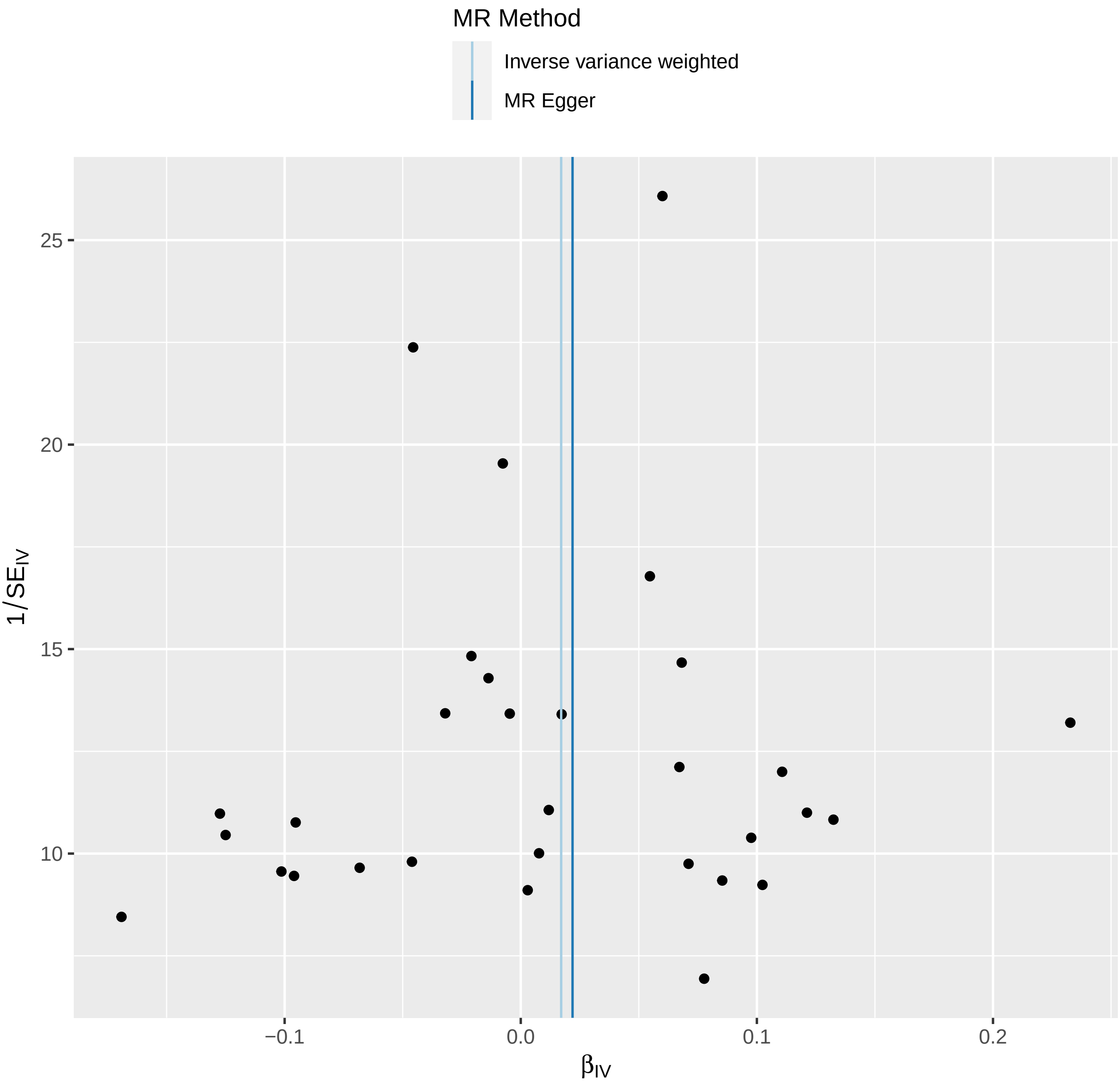

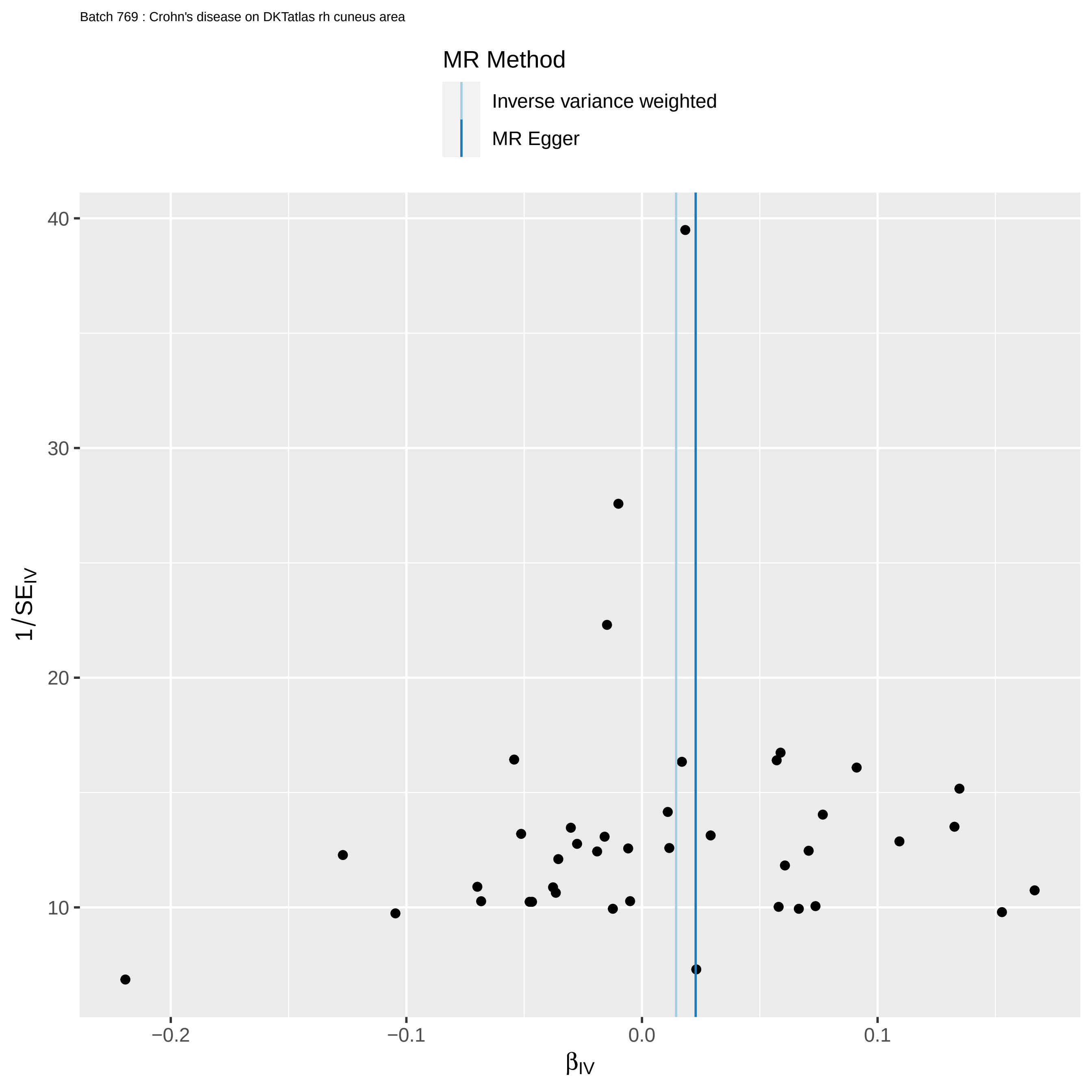
Batch 769 : Crohn's disease on DKTatlas rh cuneus area
MR Method
Inverse variance weighted
MR Egger
40
30
20
10
1/SE$_{IV}$
−0.2
−0.1
0.0
0.1
β_{IV}

Batch 770 : Ulcerative colitis on DKTatlas rh cuneus area

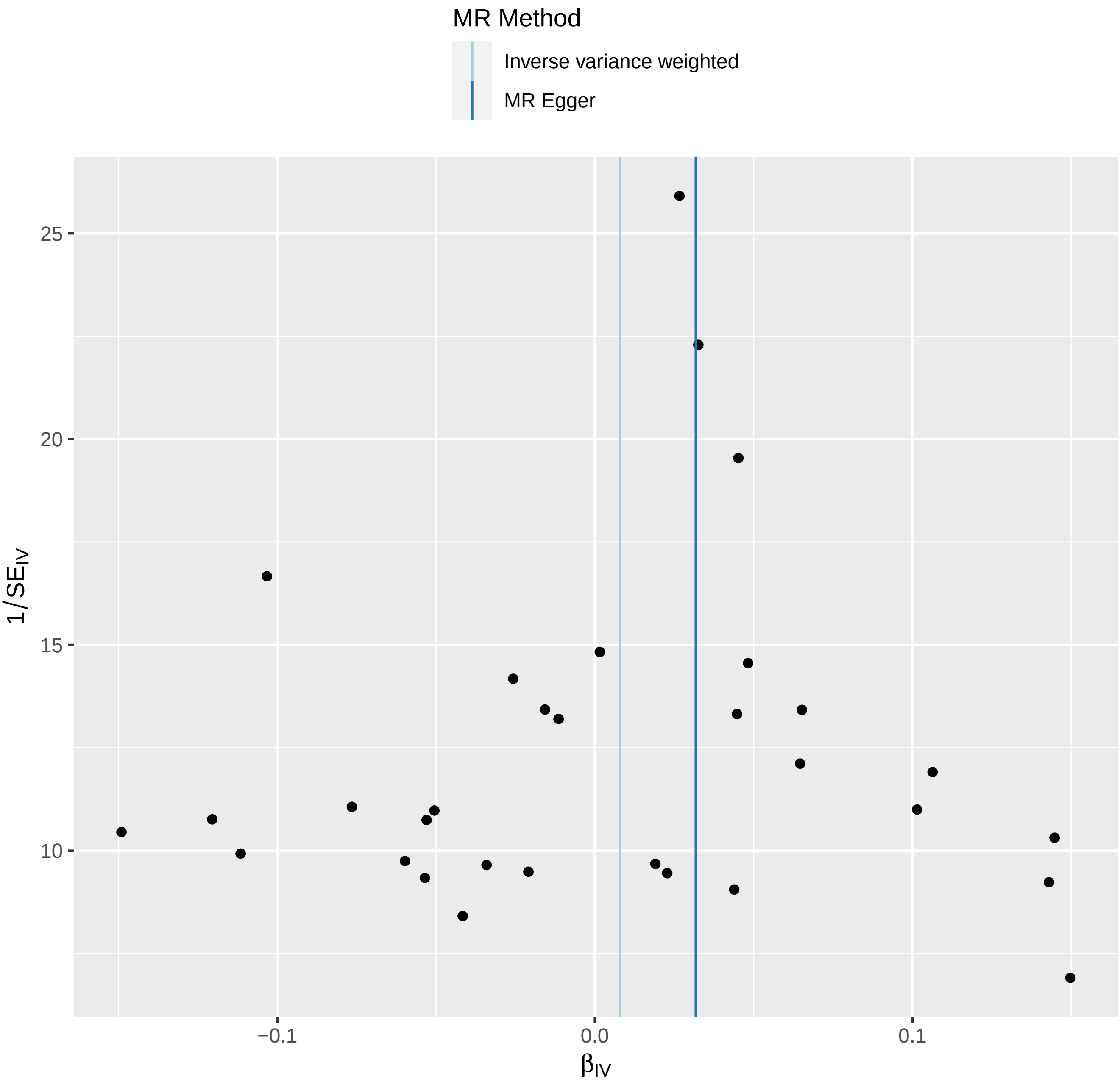

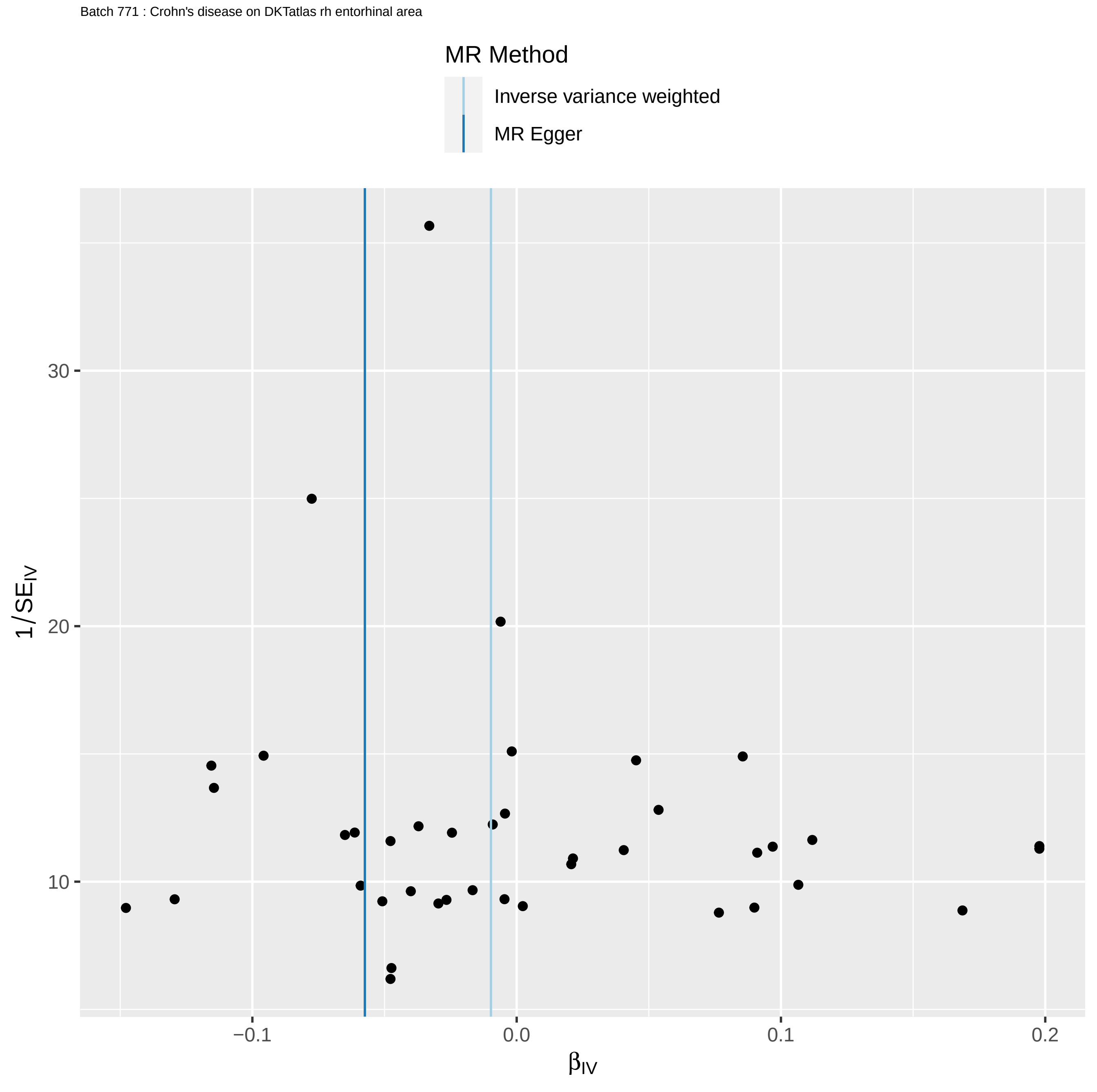
Batch 771 : Crohn's disease on DKTatlas rh entorhinal area
MR Method
Inverse variance weighted
MR Egger
30
20
10
1/SE_IV
−0.1
0.0
0.1
0.2
β_IV

Batch 772 : Ulcerative colitis on DKTatlas rh entorhinal area

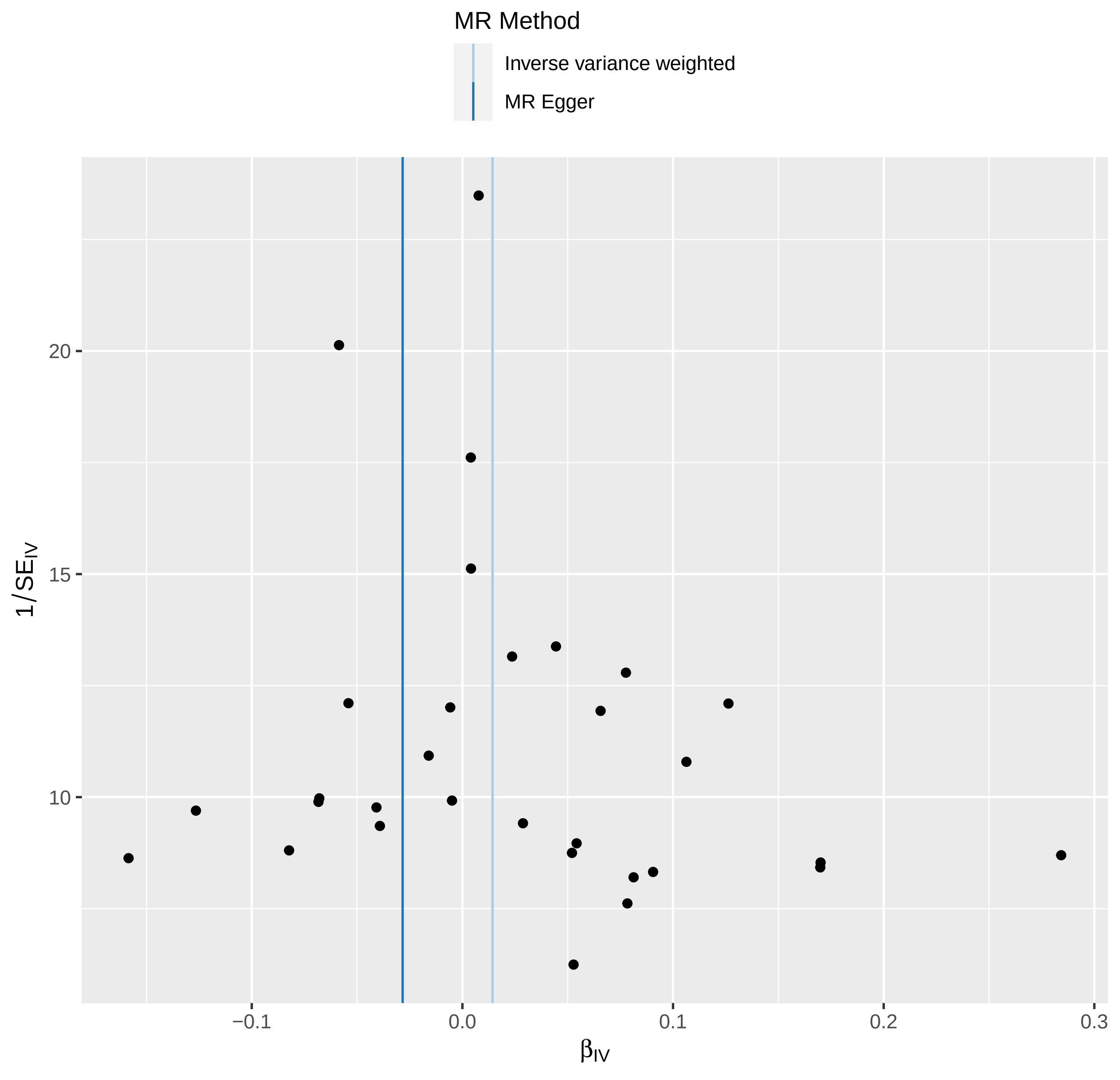

Batch 773 : Crohn's disease on DKTatlas rh fusiform area

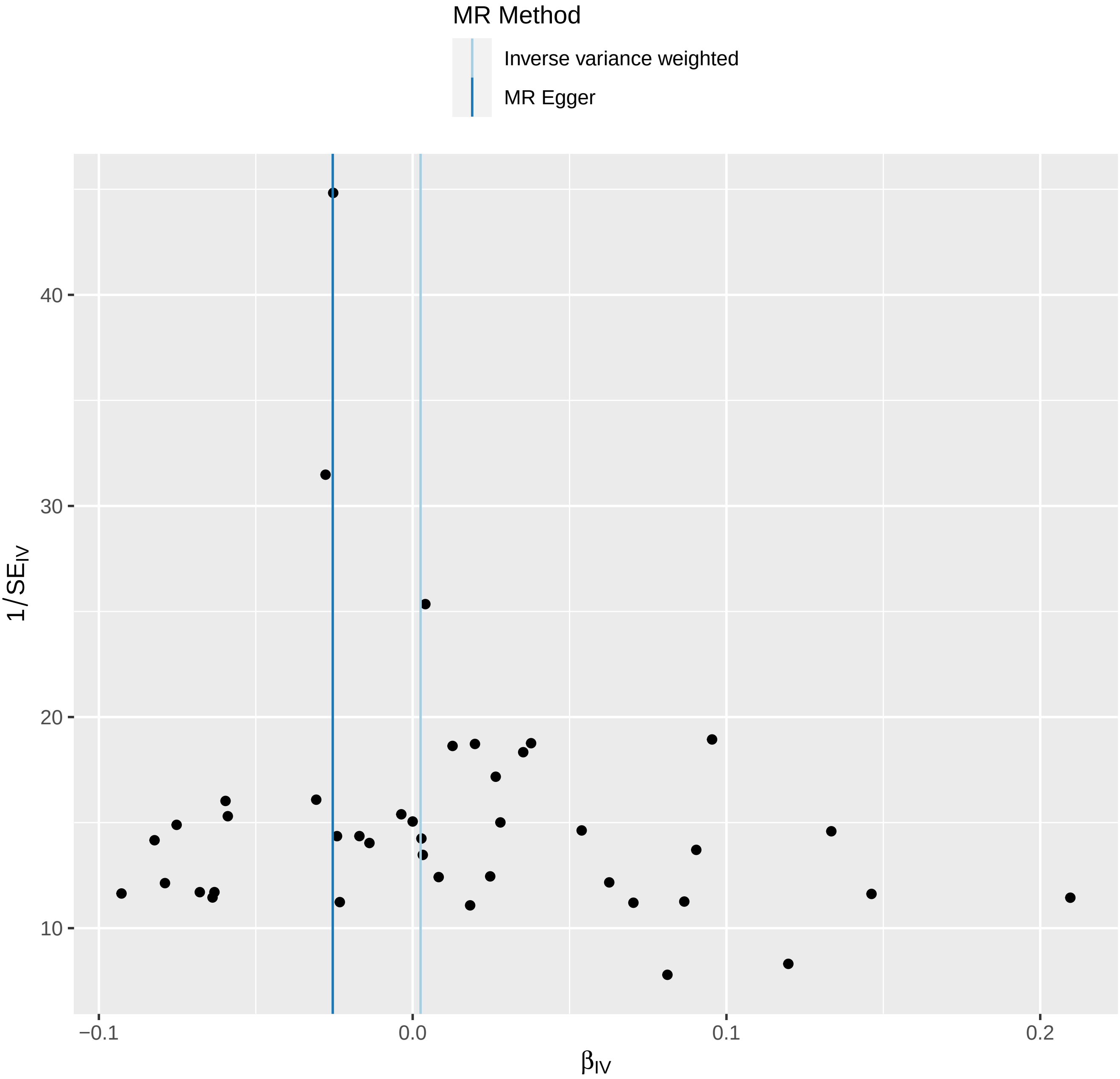

Batch 774 : Ulcerative colitis on DKTatlas rh fusiform area

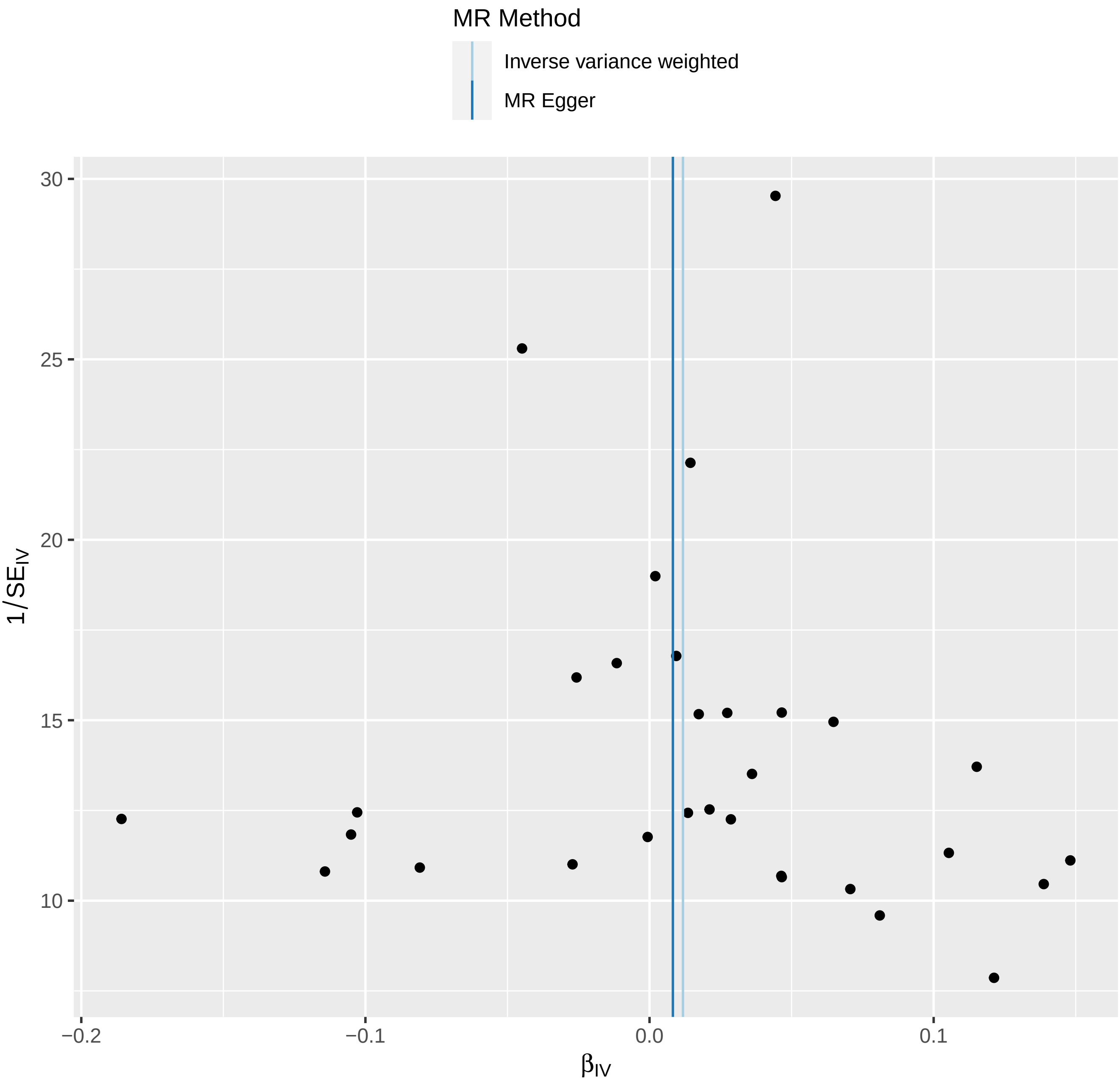

Batch 775 : Crohn's disease on DKTatlas rh inferiorparietal area

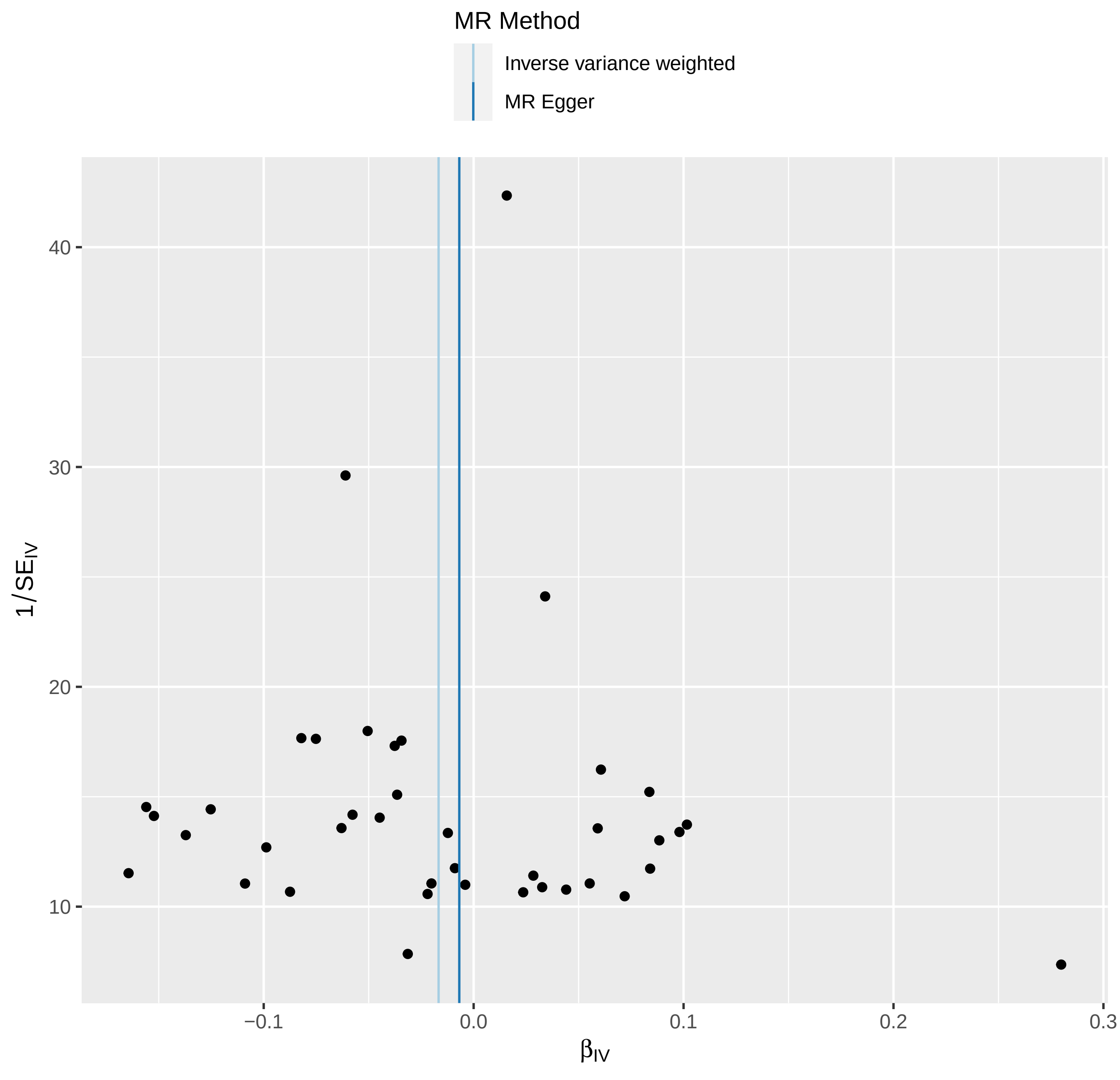

Batch 776 : Ulcerative colitis on DKTatlas rh inferiorparietal area

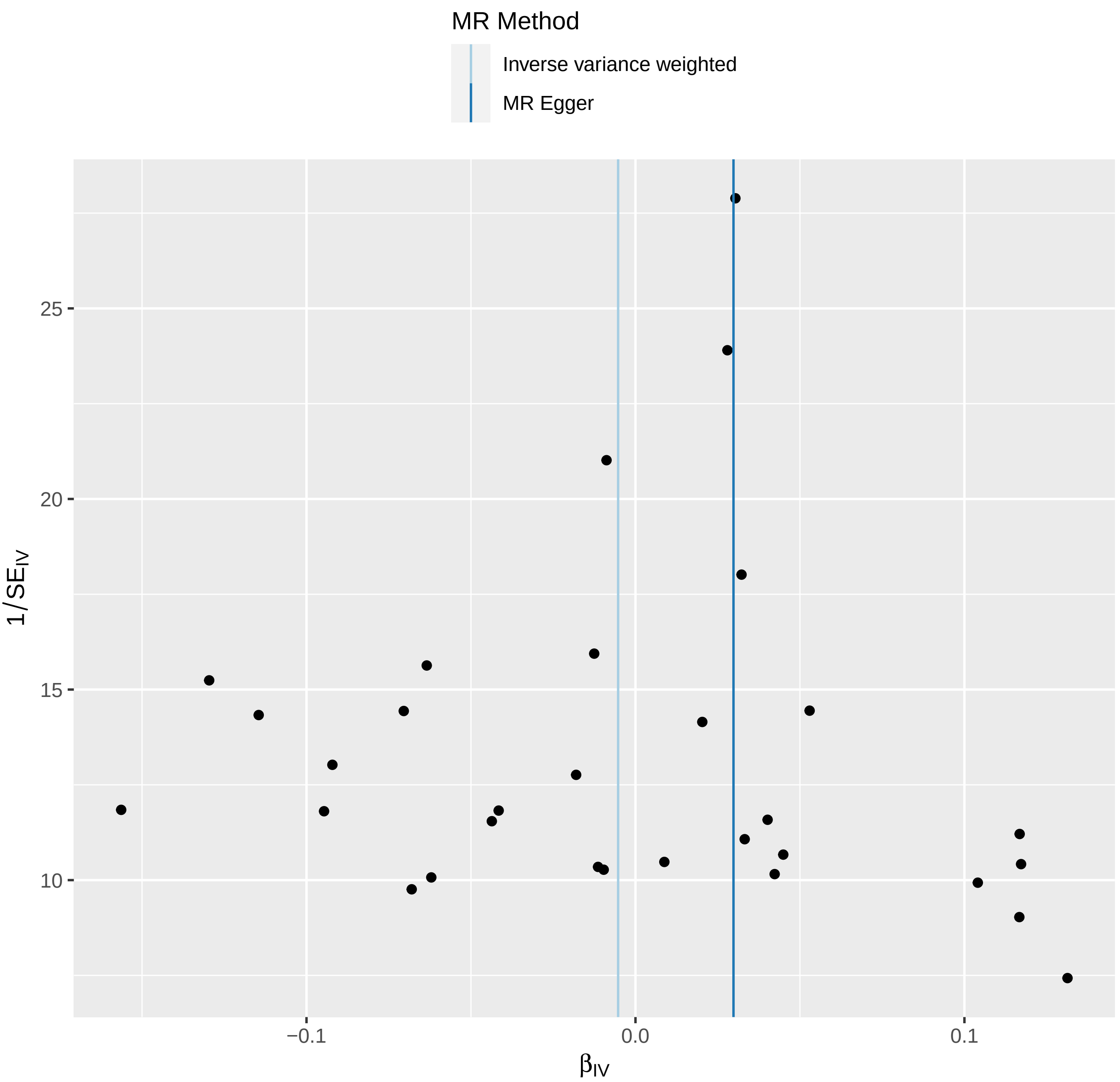

Batch 777 : Crohn's disease on DKTatlas rh inferiortemporal area

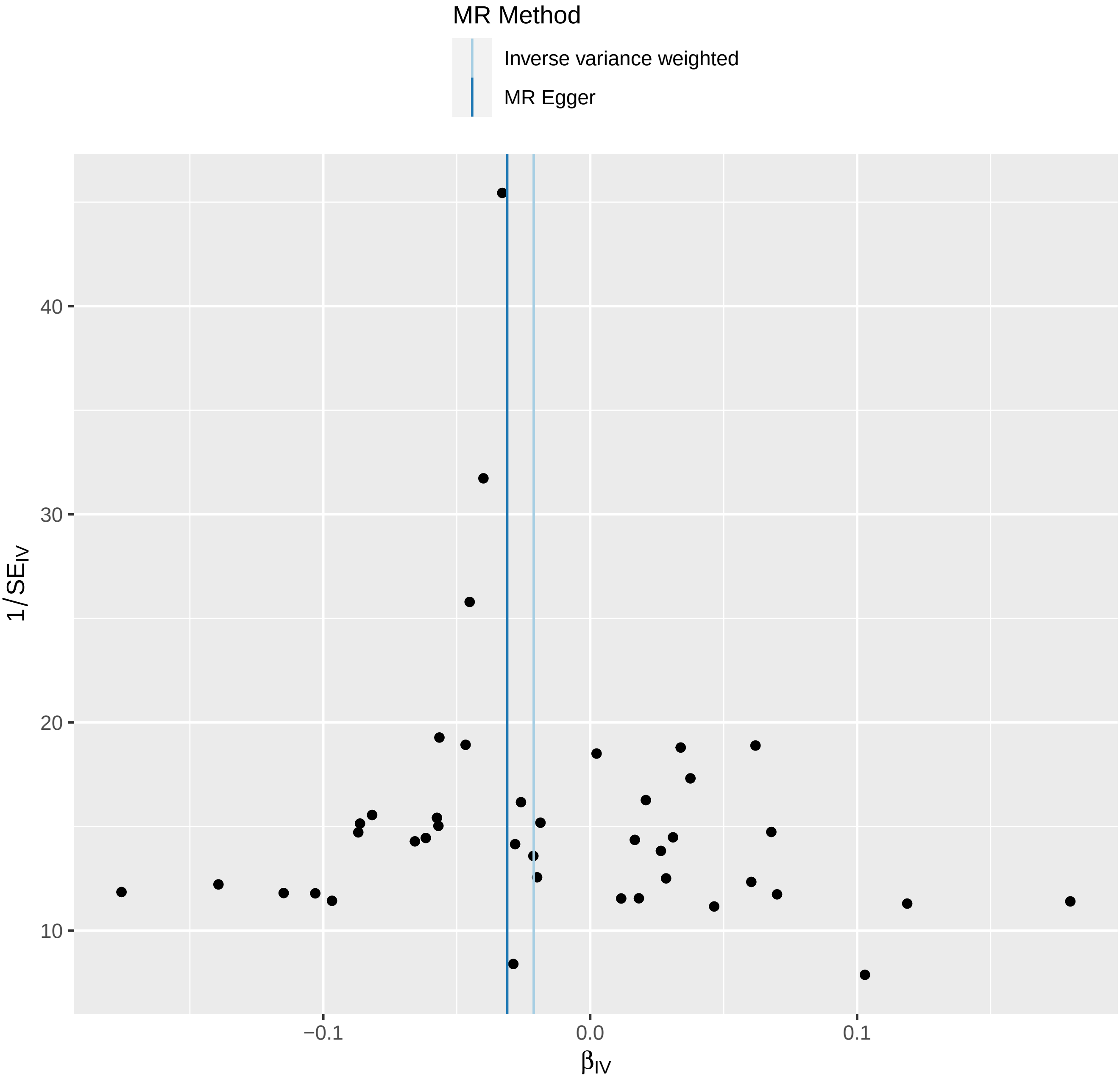

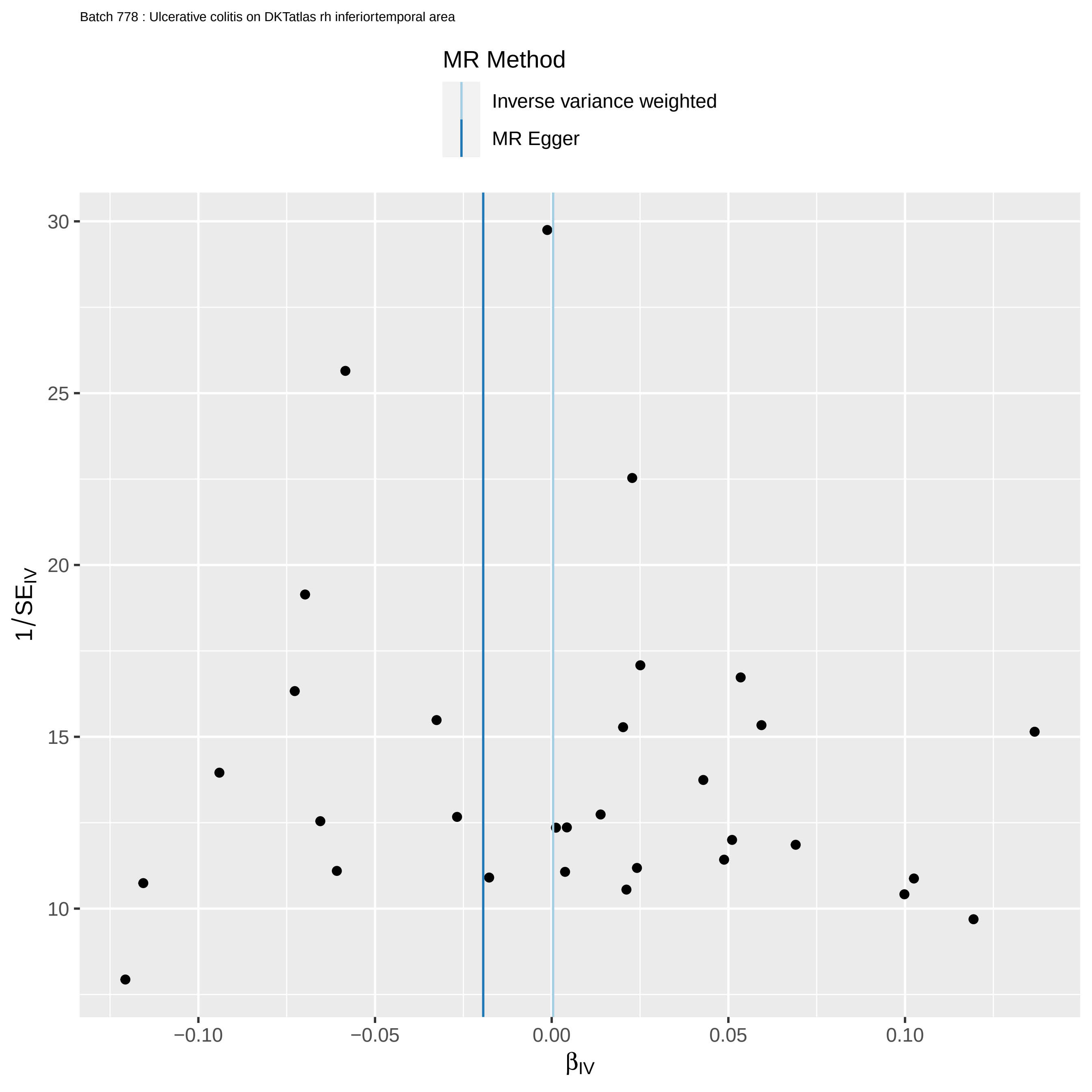
Batch 778 : Ulcerative colitis on DKTatlas rh inferiortemporal area
MR Method
Inverse variance weighted
MR Egger
30
25
20
15
10
1/SE$_{IV}$
−0.10
−0.05
0.00
0.05
0.10
β$_{IV}$

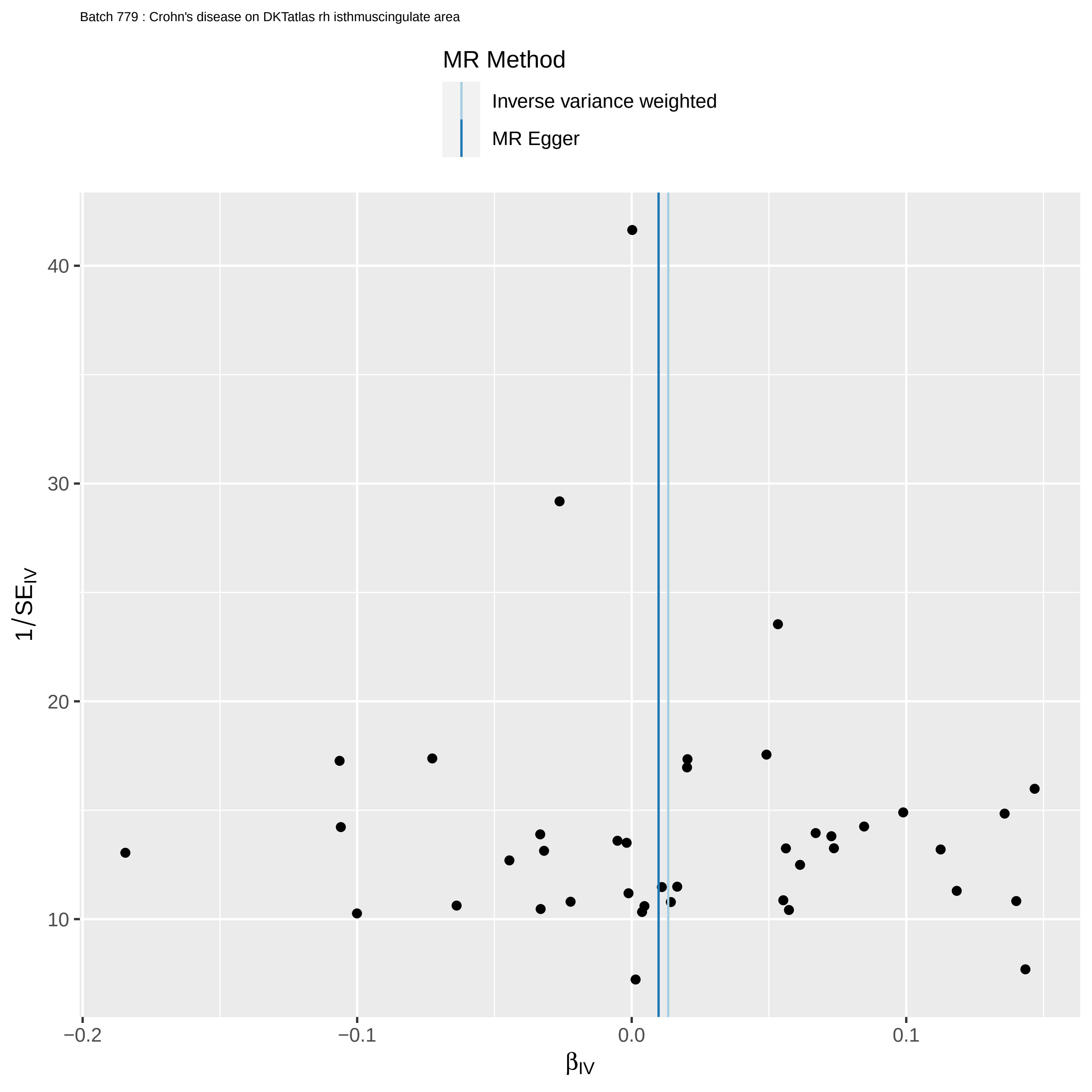
Batch 779 : Crohn's disease on DKTatlas rh isthmuscingulate area
MR Method
Inverse variance weighted
MR Egger
1/SE_IV
40
30
20
10
−0.2
−0.1
0.0
0.1
β_IV

Batch 780 : Ulcerative colitis on DKTatlas rh isthmuscingulate area

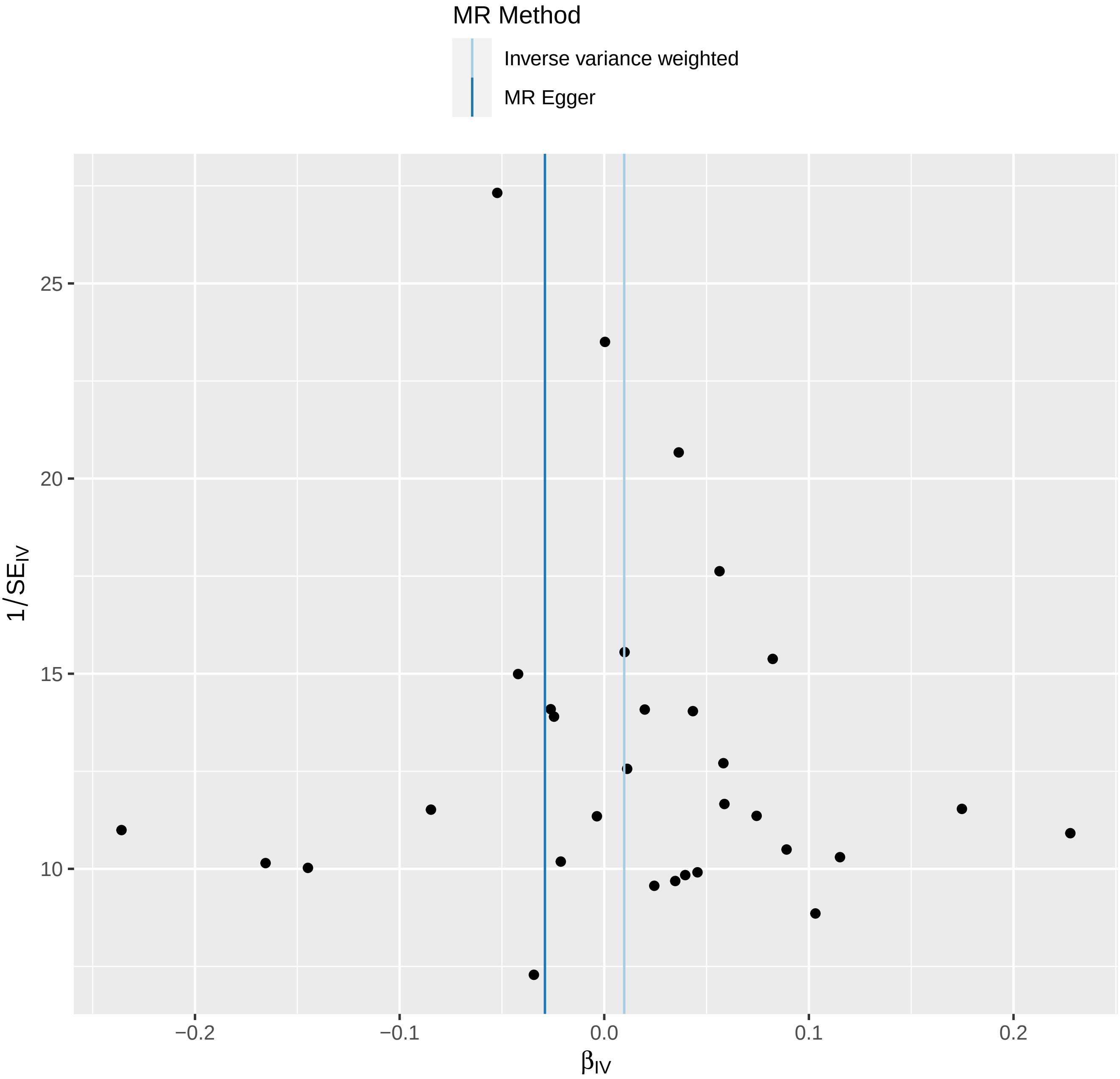

Batch 781 : Crohn's disease on DKTatlas rh lateraloccipital area

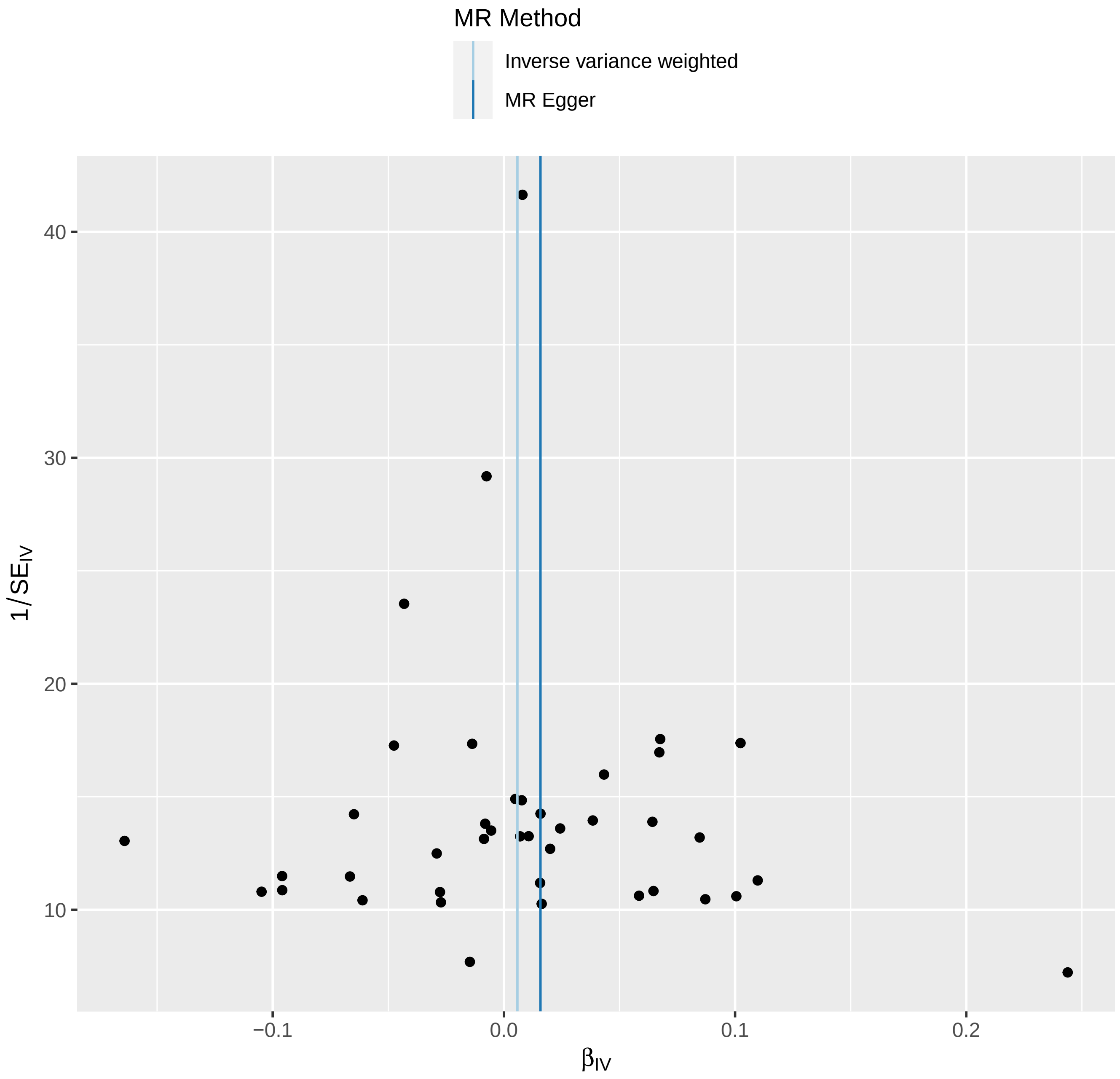

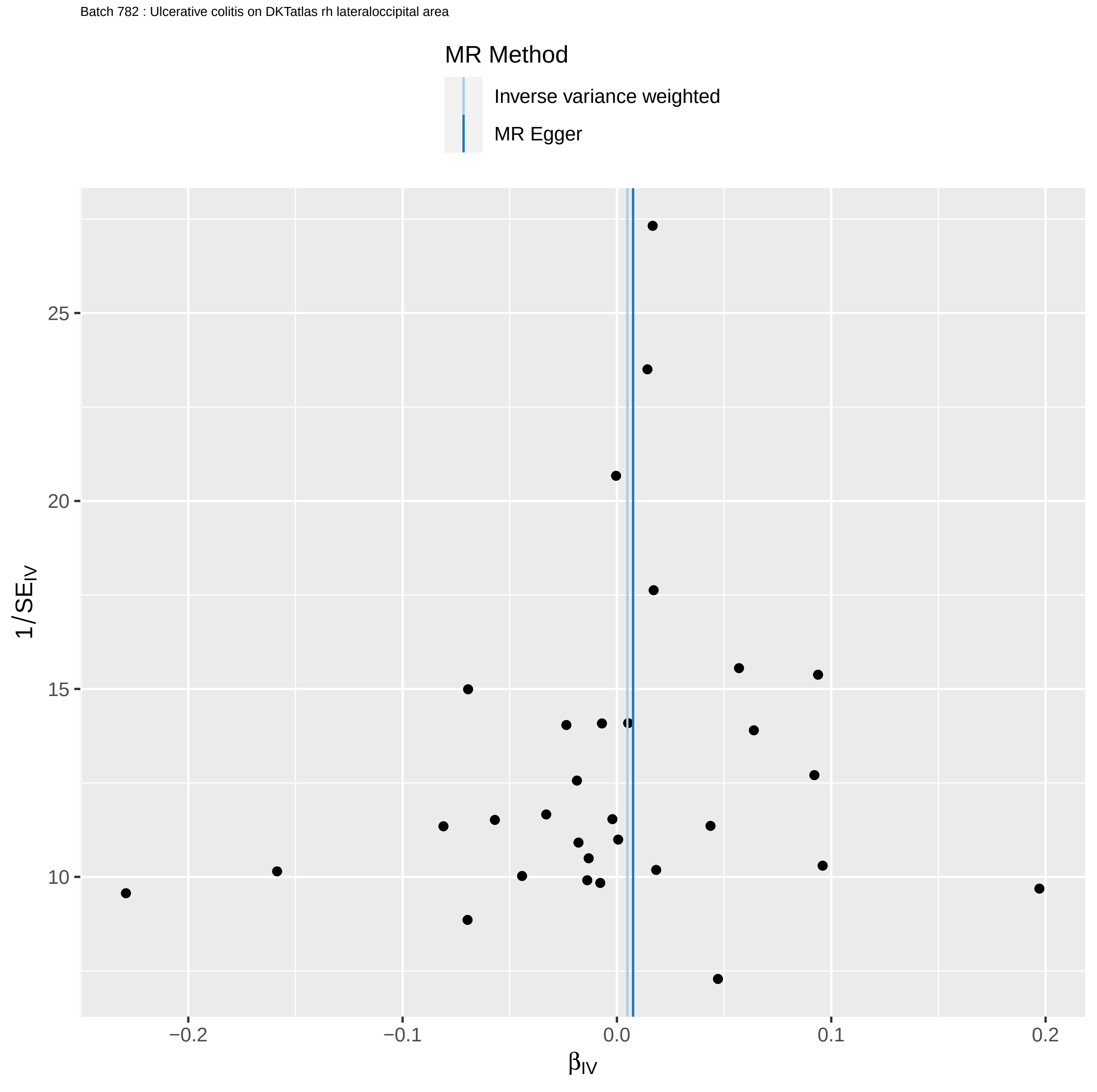

Batch 782 : Ulcerative colitis on DKTatlas rh lateraloccipital area
MR Method
Inverse variance weighted
MR Egger
25
20
15
10
1/SE$_{IV}$
−0.2
−0.1
0.0
0.1
0.2
β$_{IV}$

Batch 785 : Crohn's disease on DKTatlas rh lateralorbitofrontal area

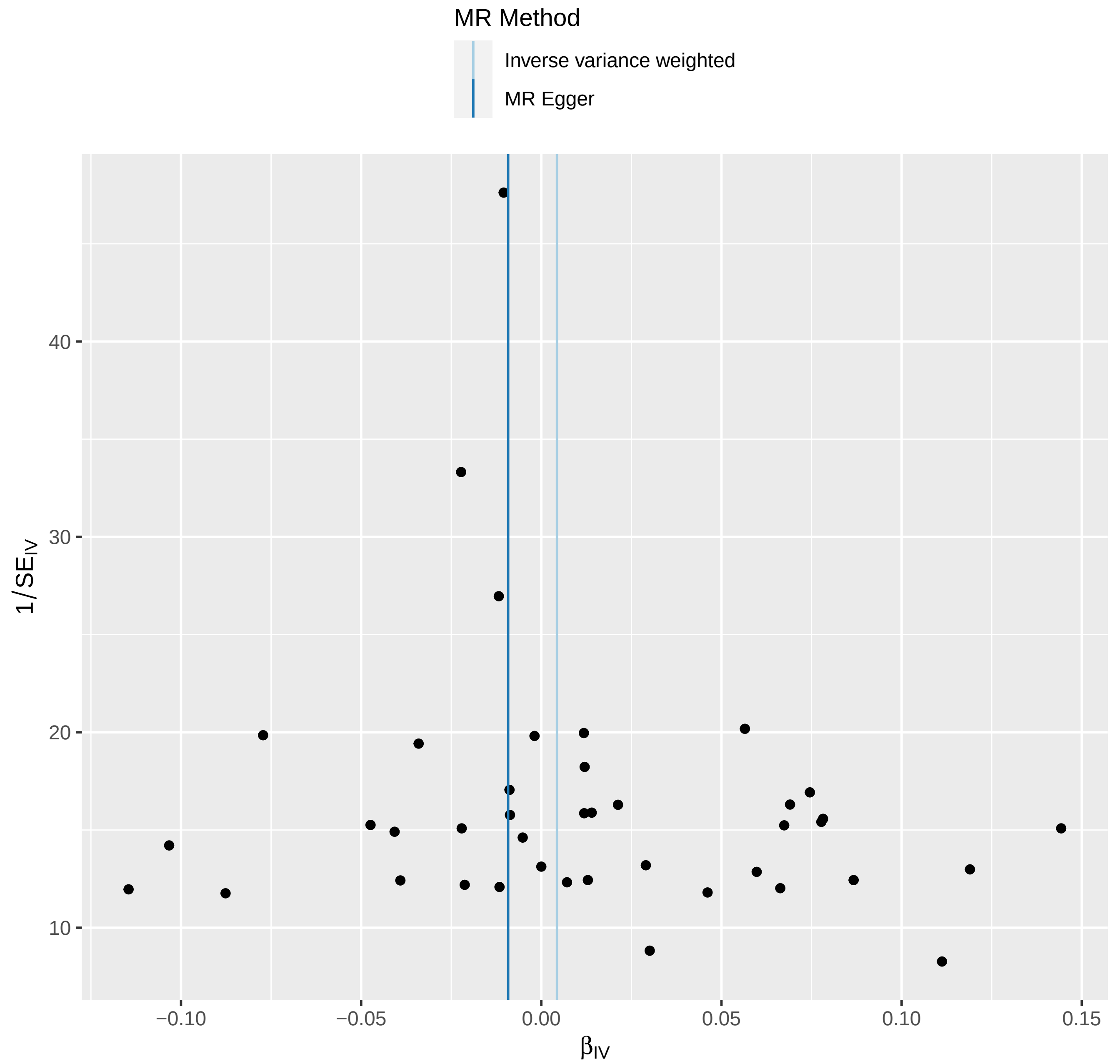

Batch 786 : Ulcerative colitis on DKTatlas rh lateralorbitofrontal area

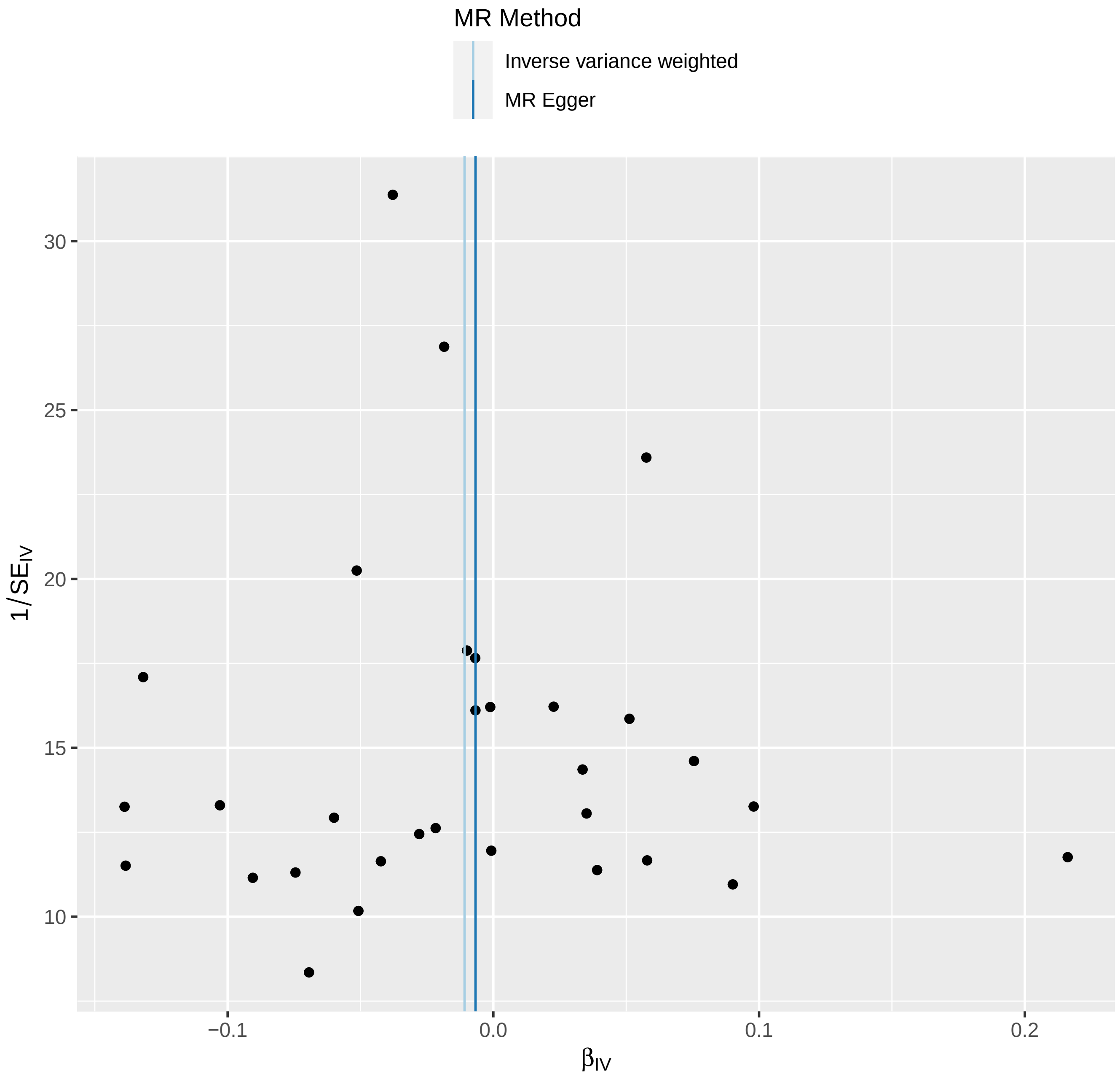

Batch 787 : Crohn's disease on DKTatlas rh lingual area

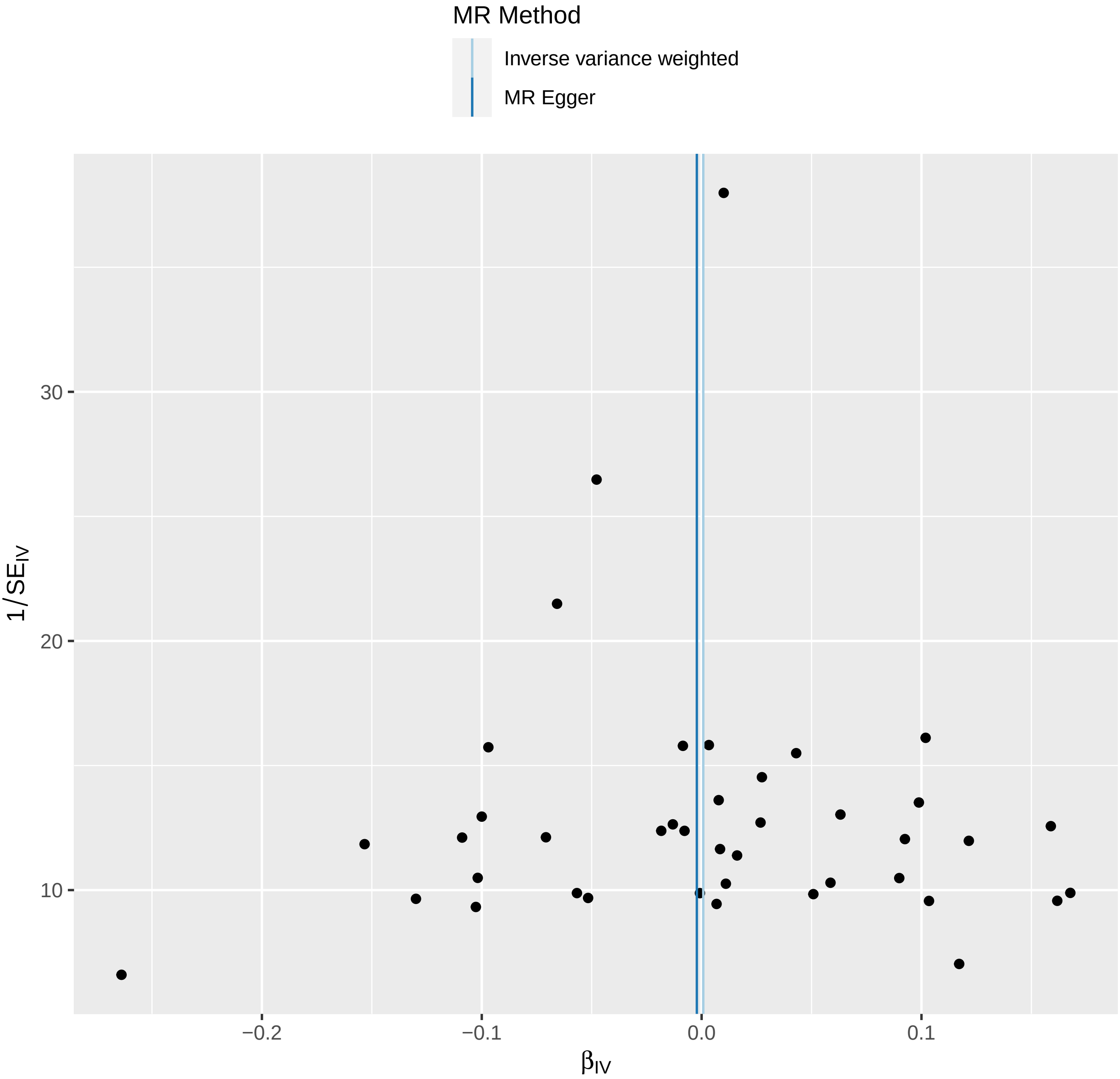

Batch 788 : Ulcerative colitis on DKTatlas rh lingual area

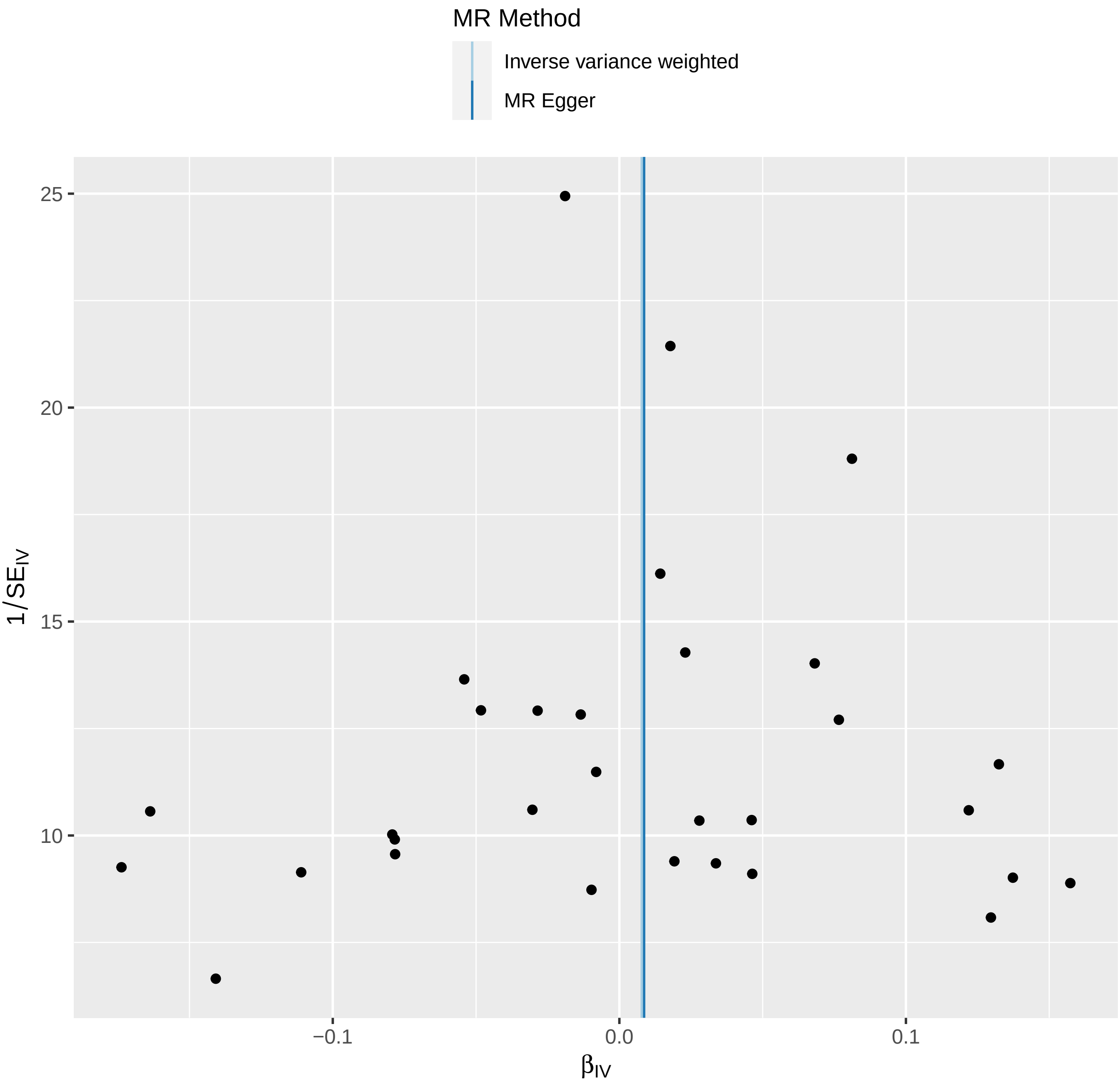

Batch 789 : Crohn's disease on DKTatlas rh medialorbitofrontal area

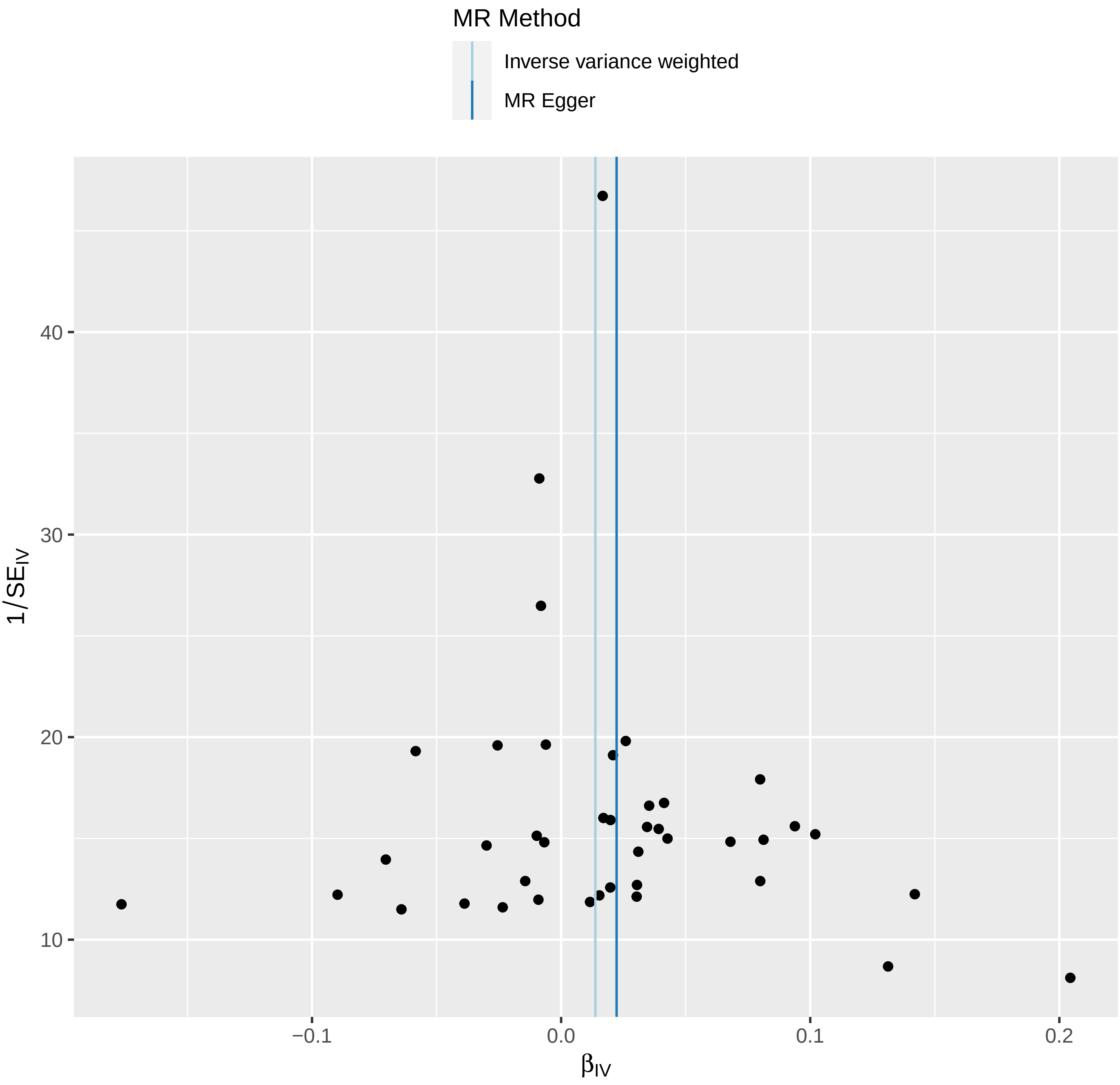

Batch 790 : Ulcerative colitis on DKTatlas rh medialorbitofrontal area

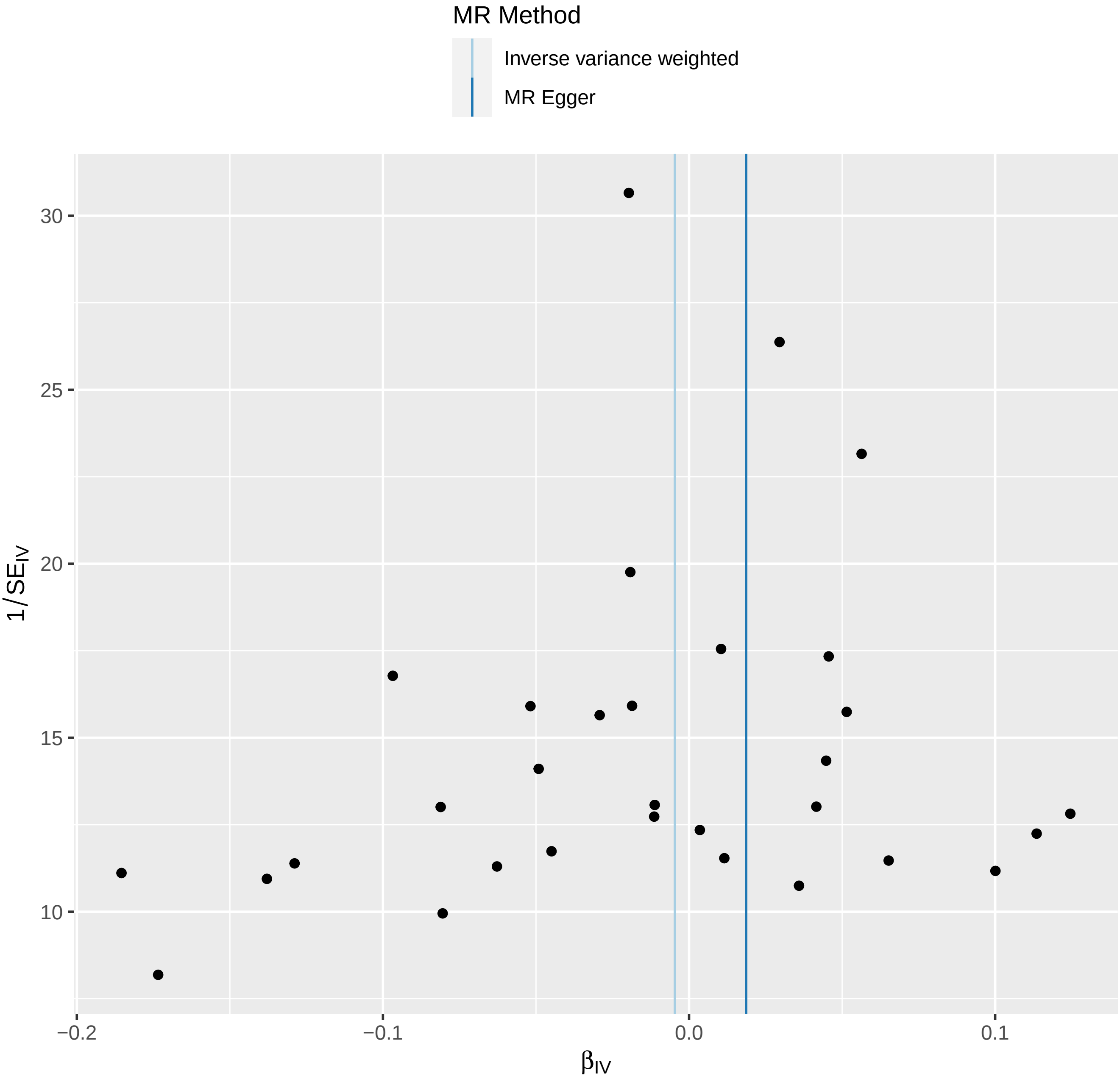

Batch 791 : Crohn's disease on DKTatlas rh middletemporal area

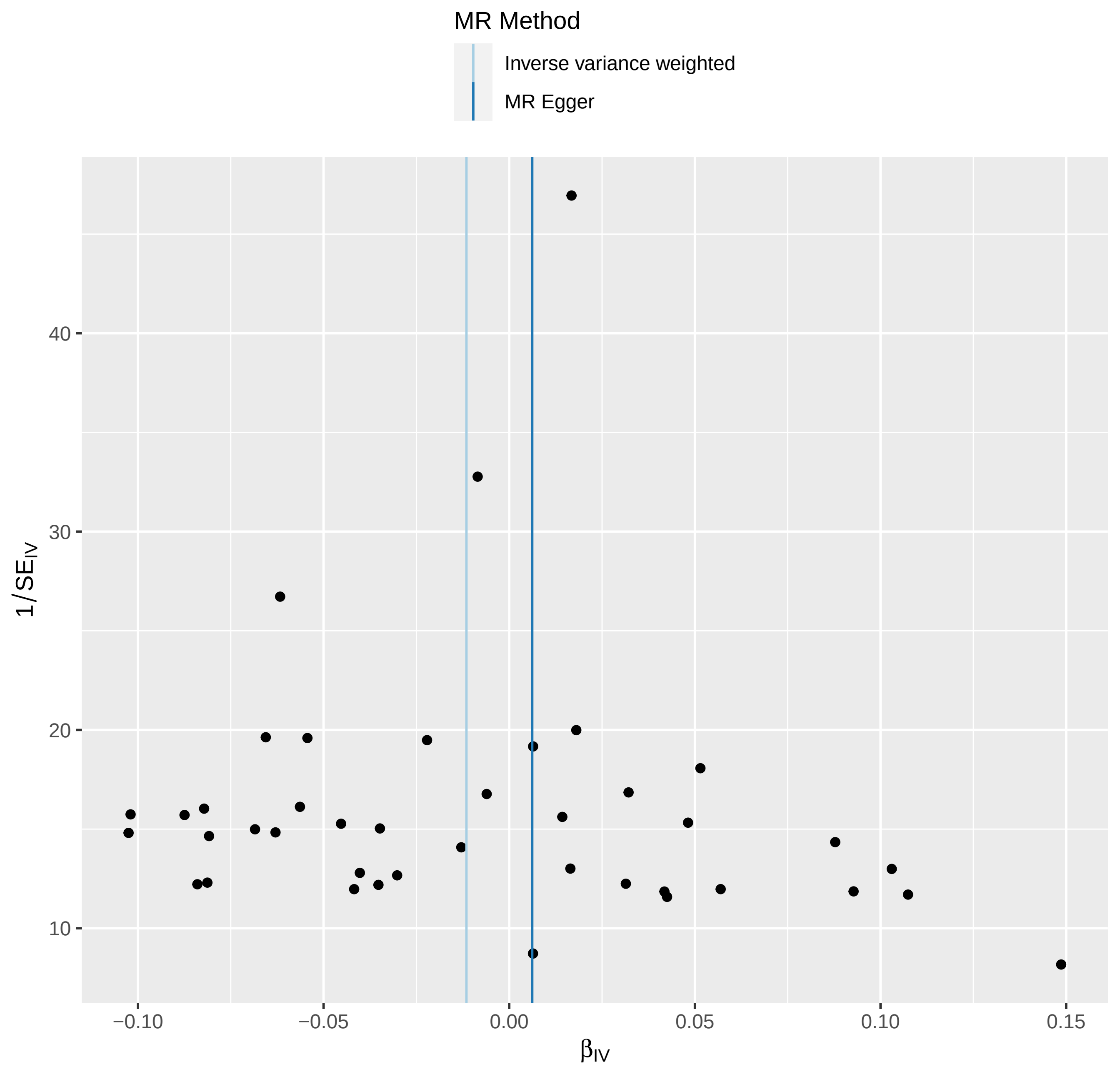

Batch 792 : Ulcerative colitis on DKTatlas rh middletemporal area

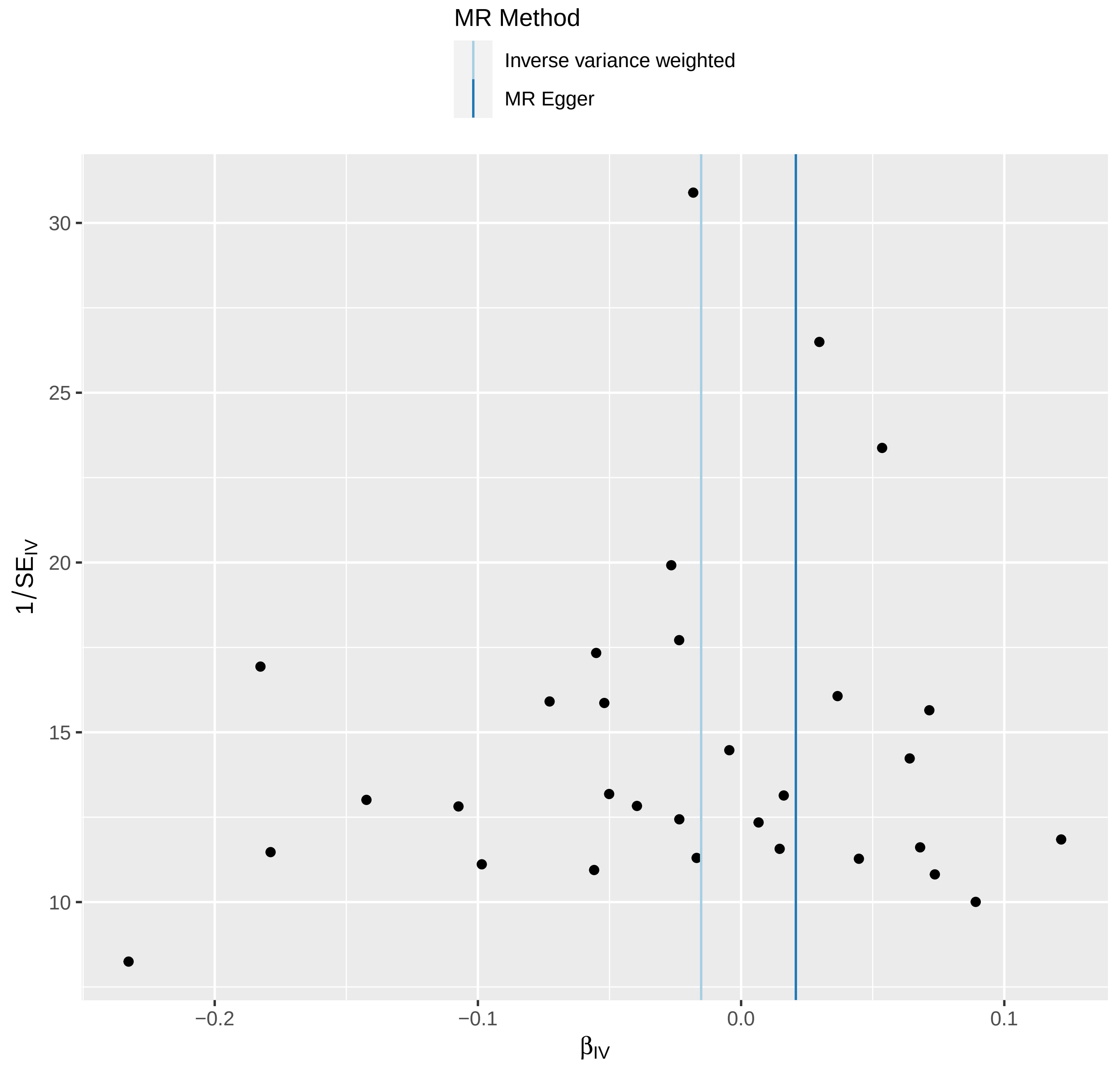

Batch 793 : Crohn's disease on DKTatlas rh parahippocampal area

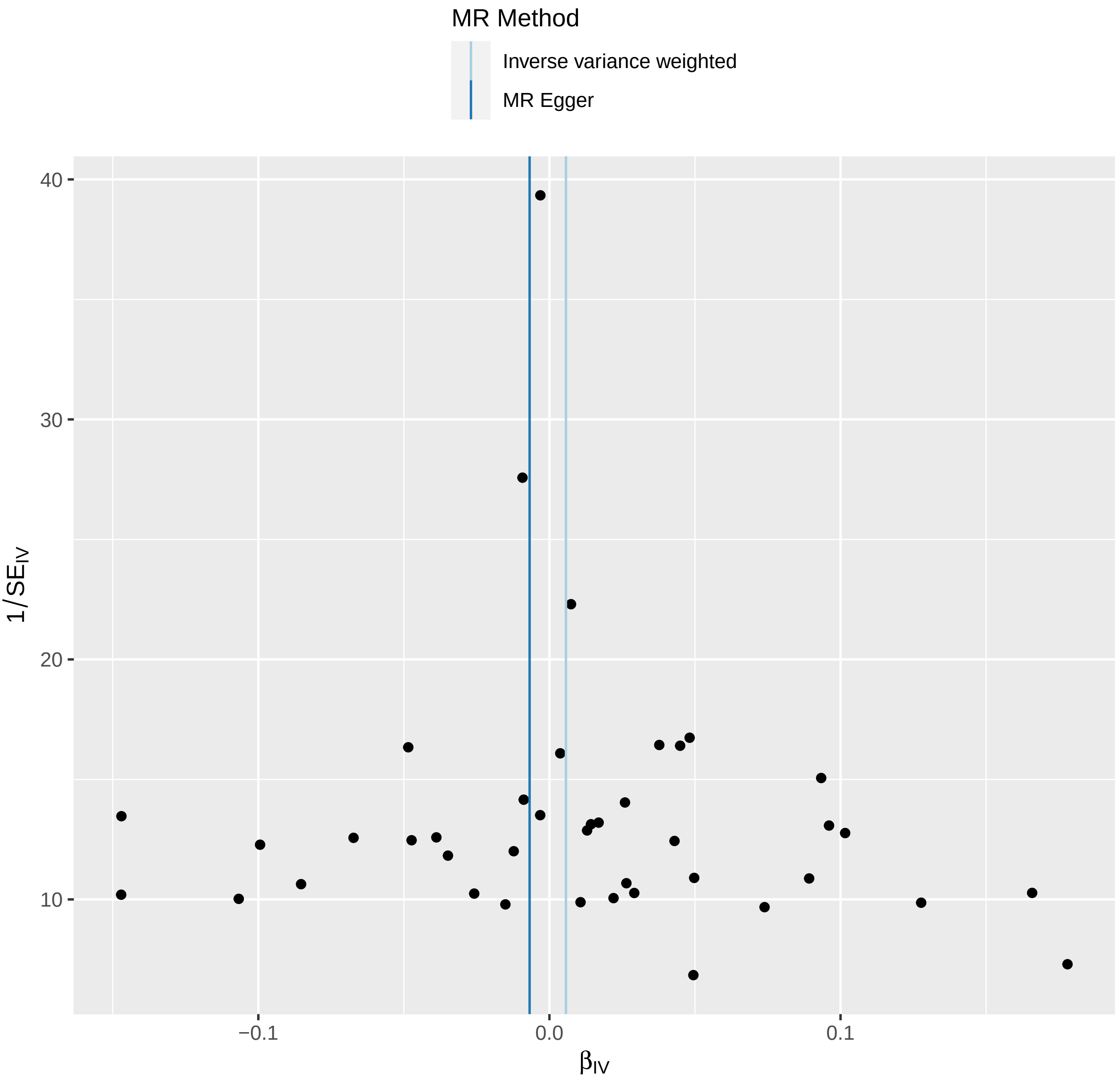

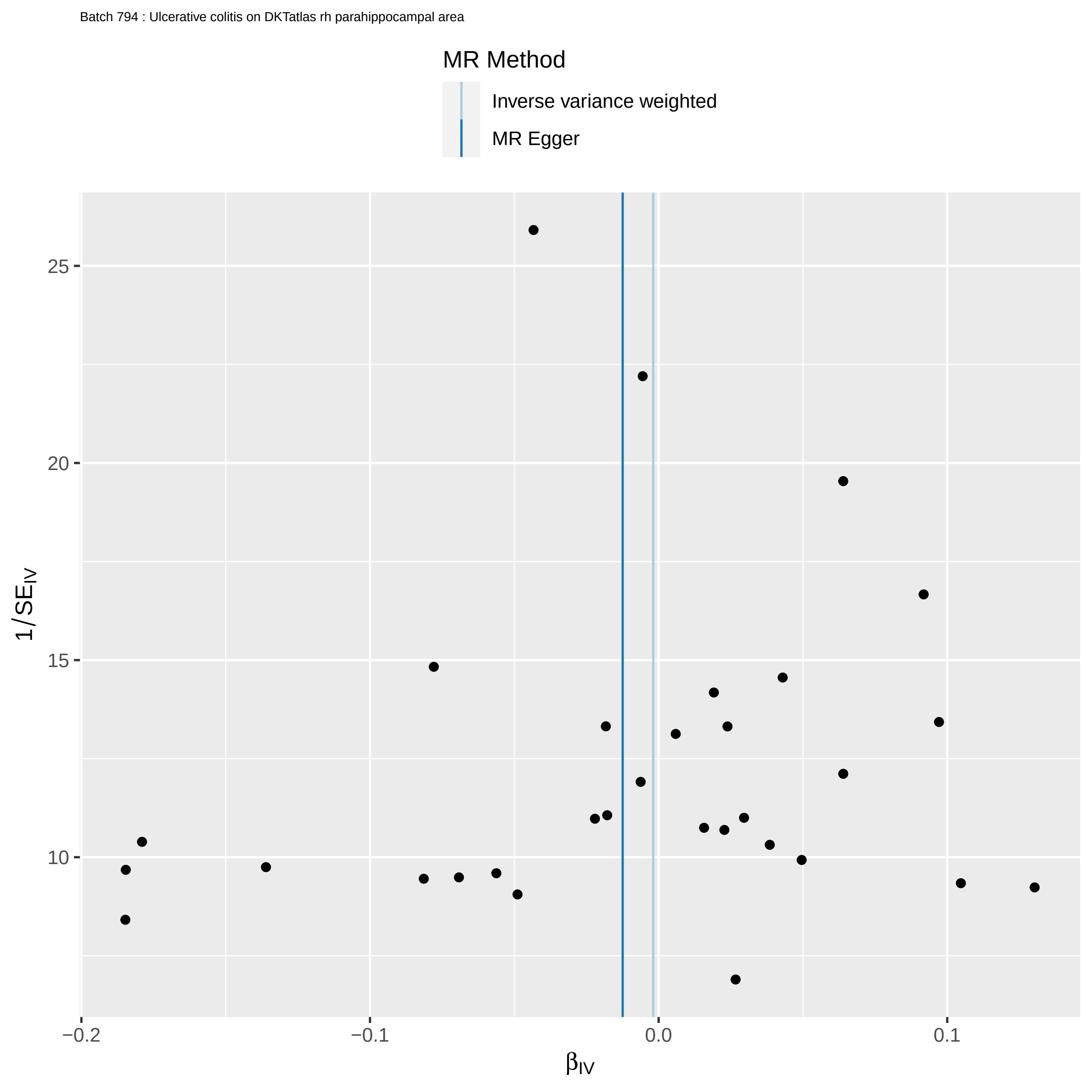
Batch 794 : Ulcerative colitis on DKTatlas rh parahippocampal area
MR Method
Inverse variance weighted
MR Egger
25
20
15
10
−0.2
−0.1
0.0
0.1
$1/SE_{IV}$
β_{IV}

Batch 795 : Crohn's disease on DKTatlas rh paracentral area

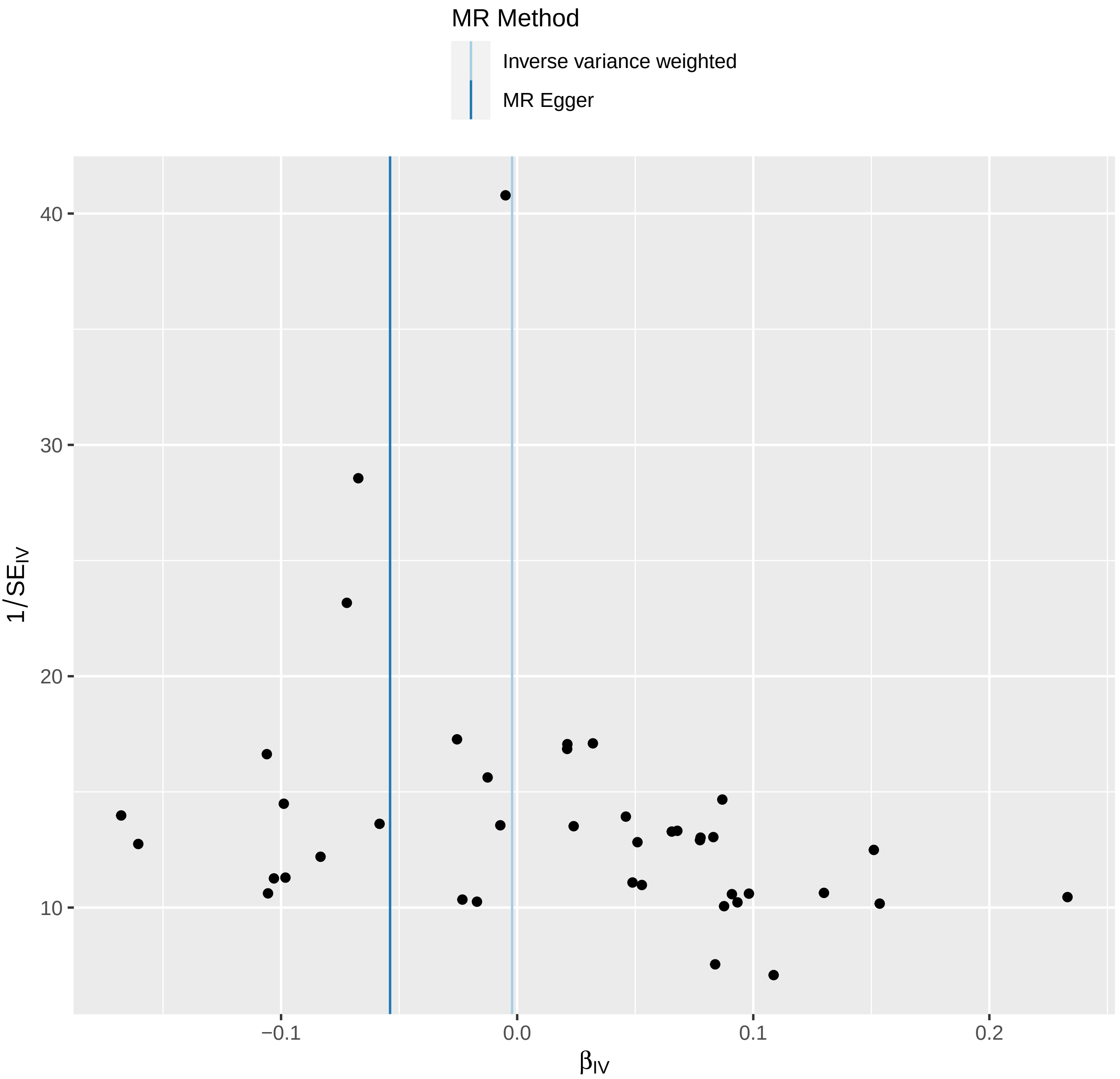

Batch 796 : Ulcerative colitis on DKTatlas rh paracentral area

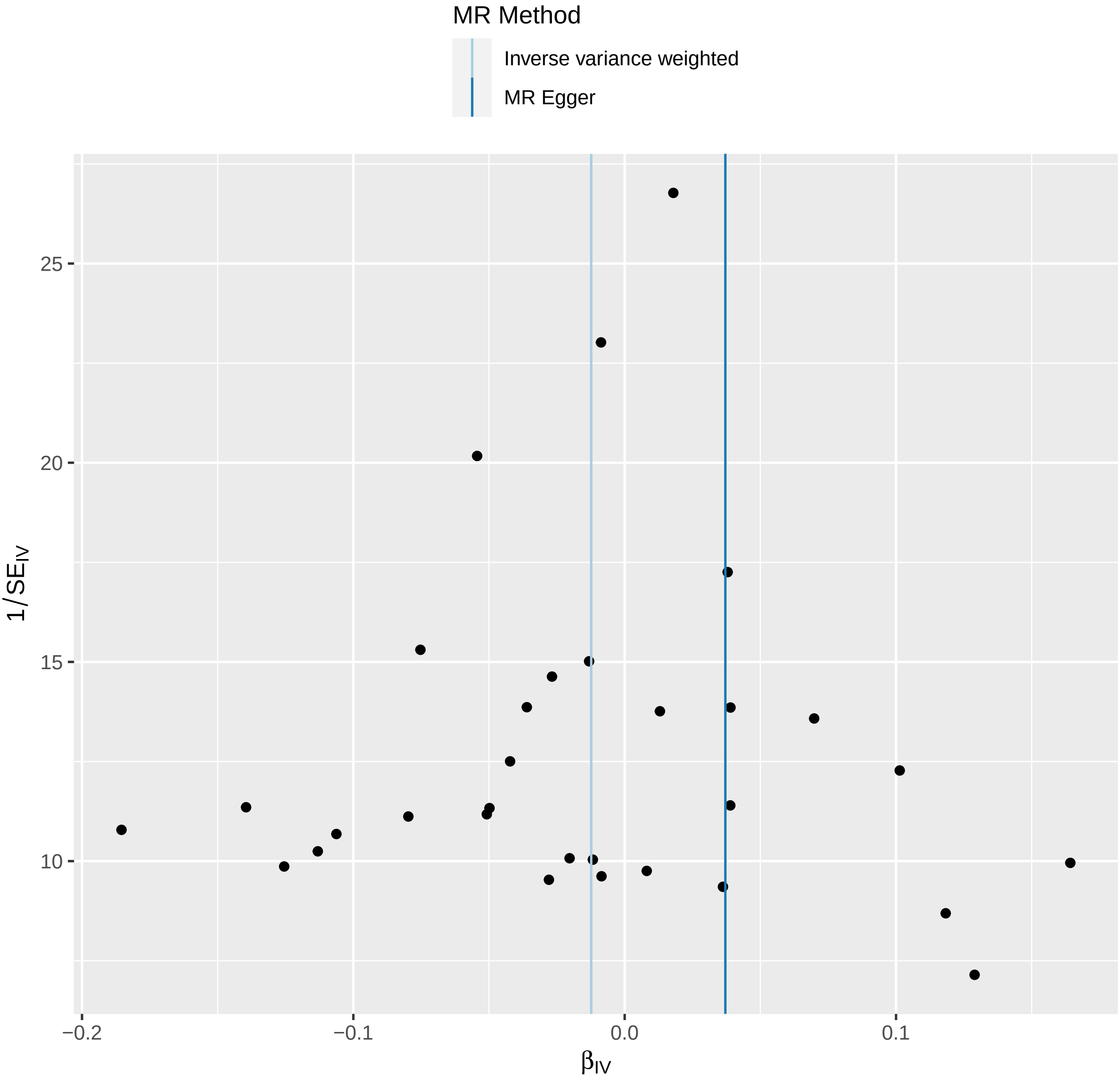

Batch 797 : Crohn's disease on DKTatlas rh parsopercularis area

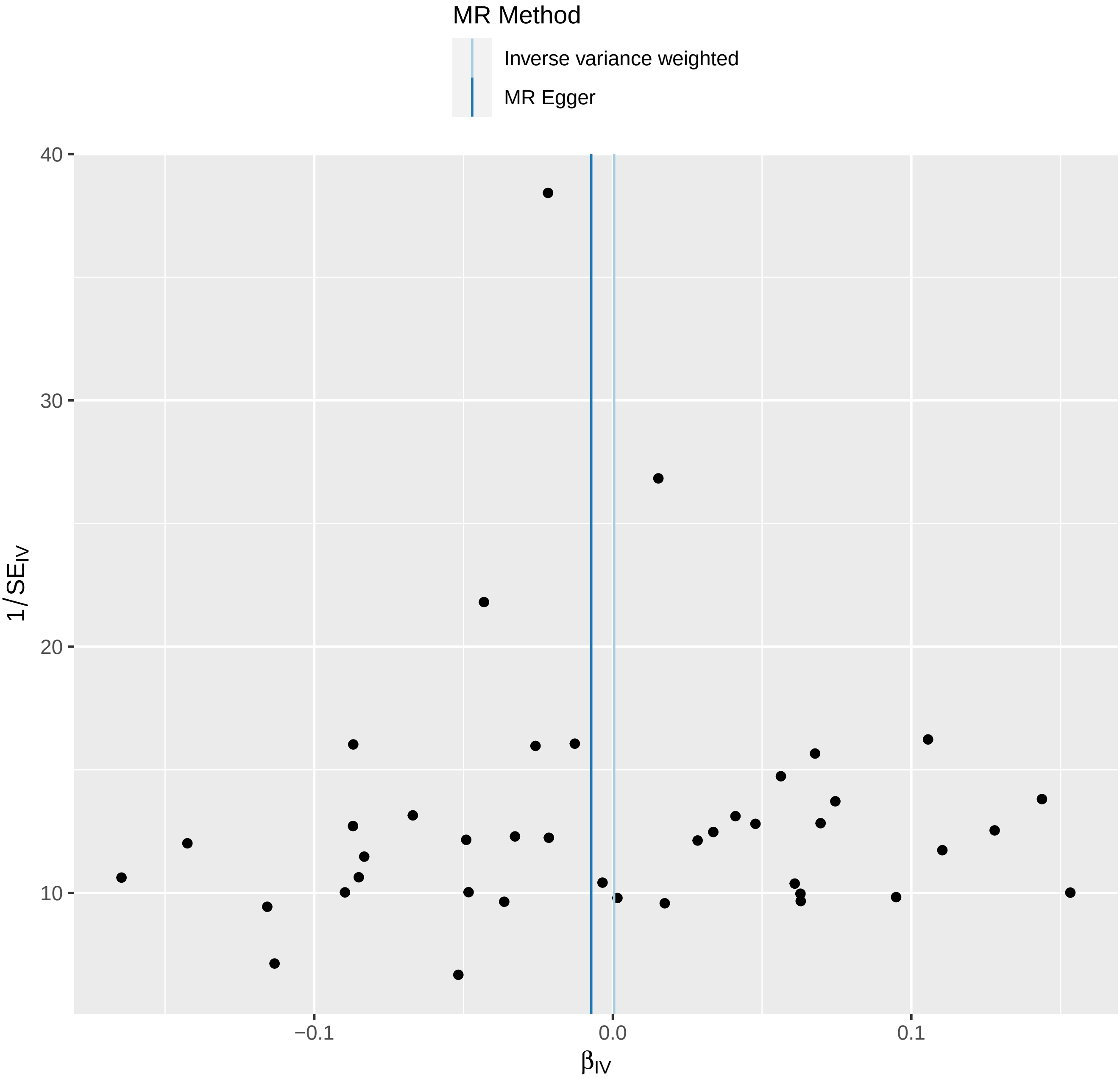

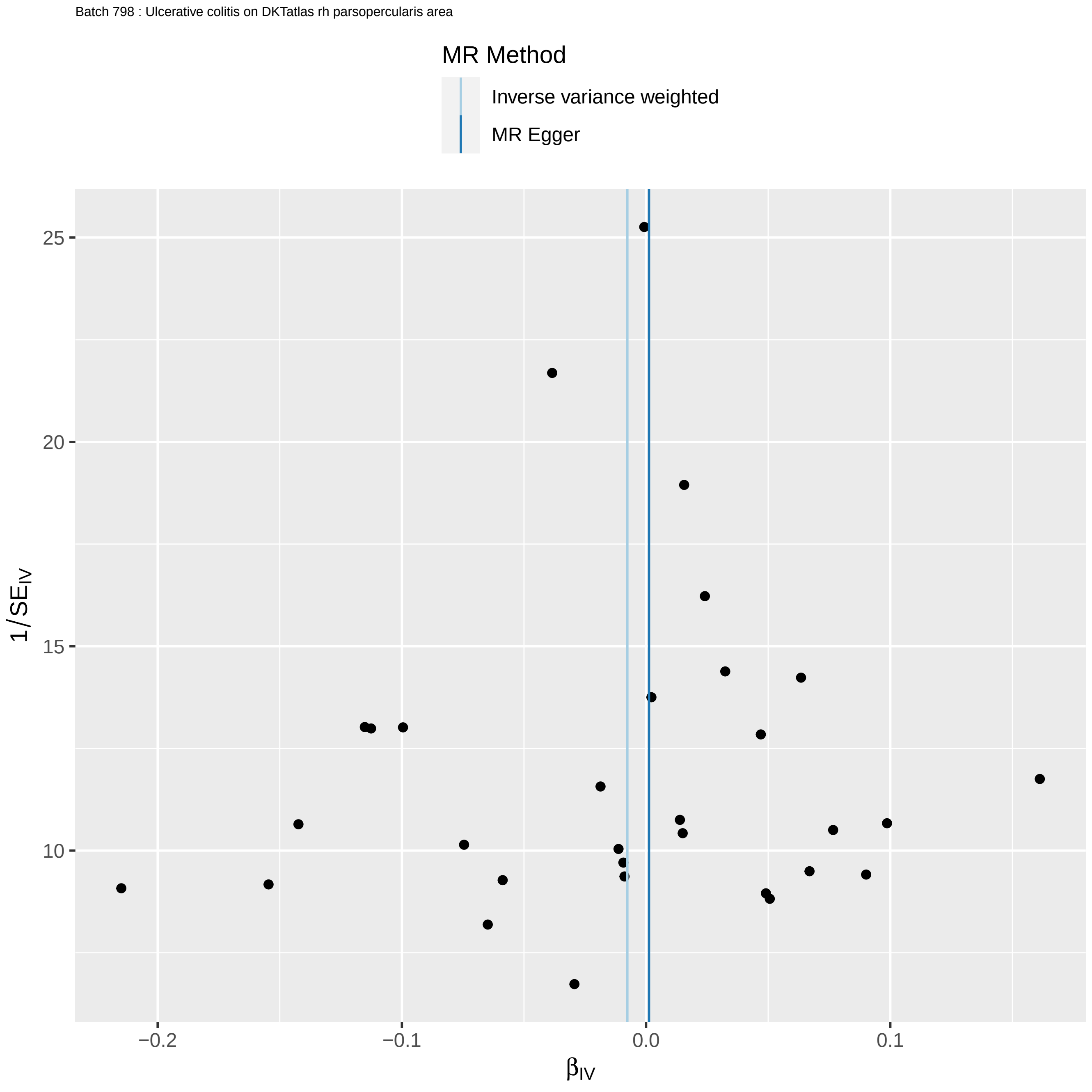

Batch 798 : Ulcerative colitis on DKTatlas rh parsopercularis area
MR Method
Inverse variance weighted
MR Egger
25
20
15
10
1/SE$_{IV}$
−0.2
−0.1
0.0
0.1
β_{IV}

Batch 799 : Crohn's disease on DKTatlas rh parsorbitalis area

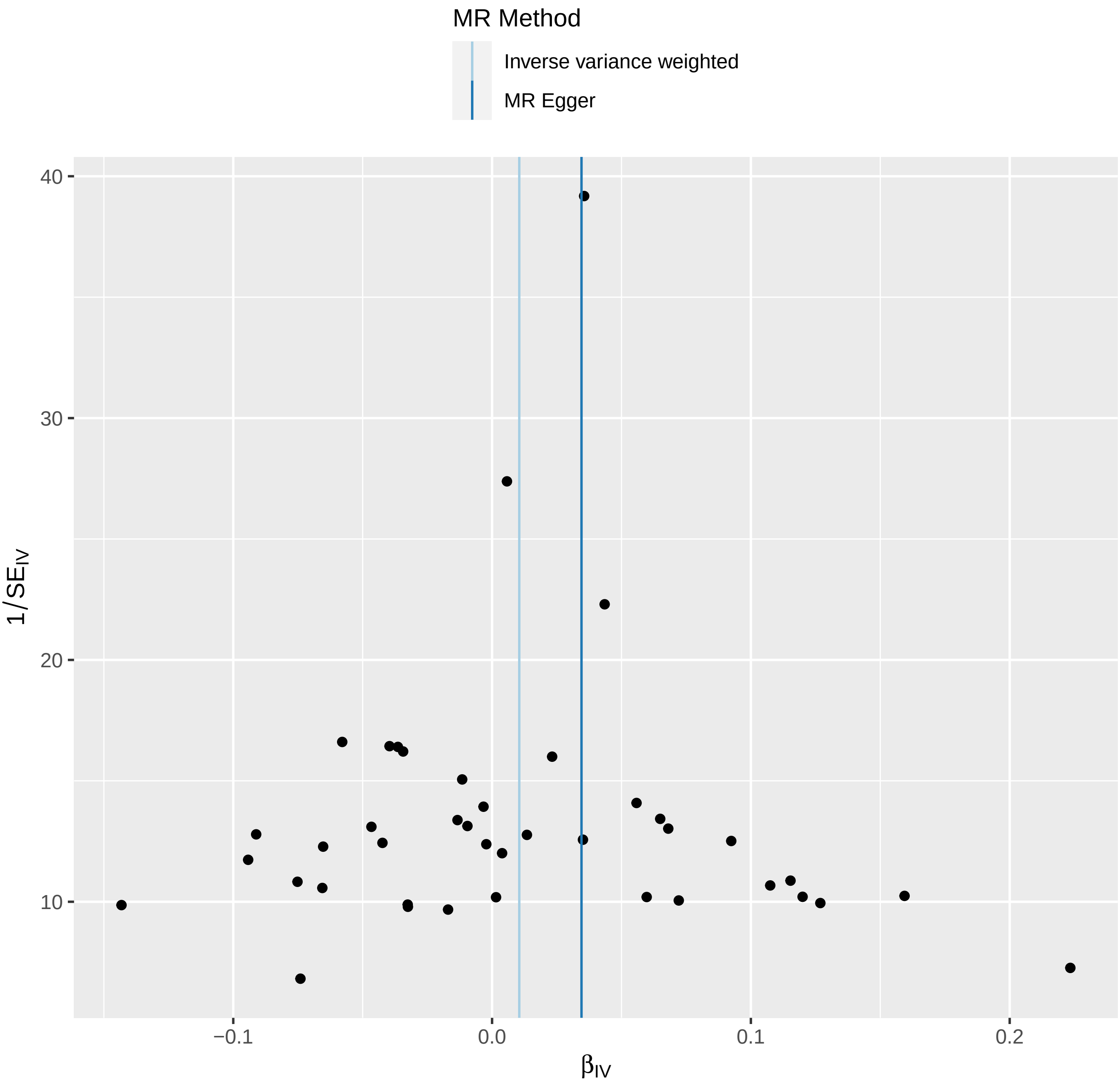

Batch 800 : Ulcerative colitis on DKTatlas rh parsorbitalis area

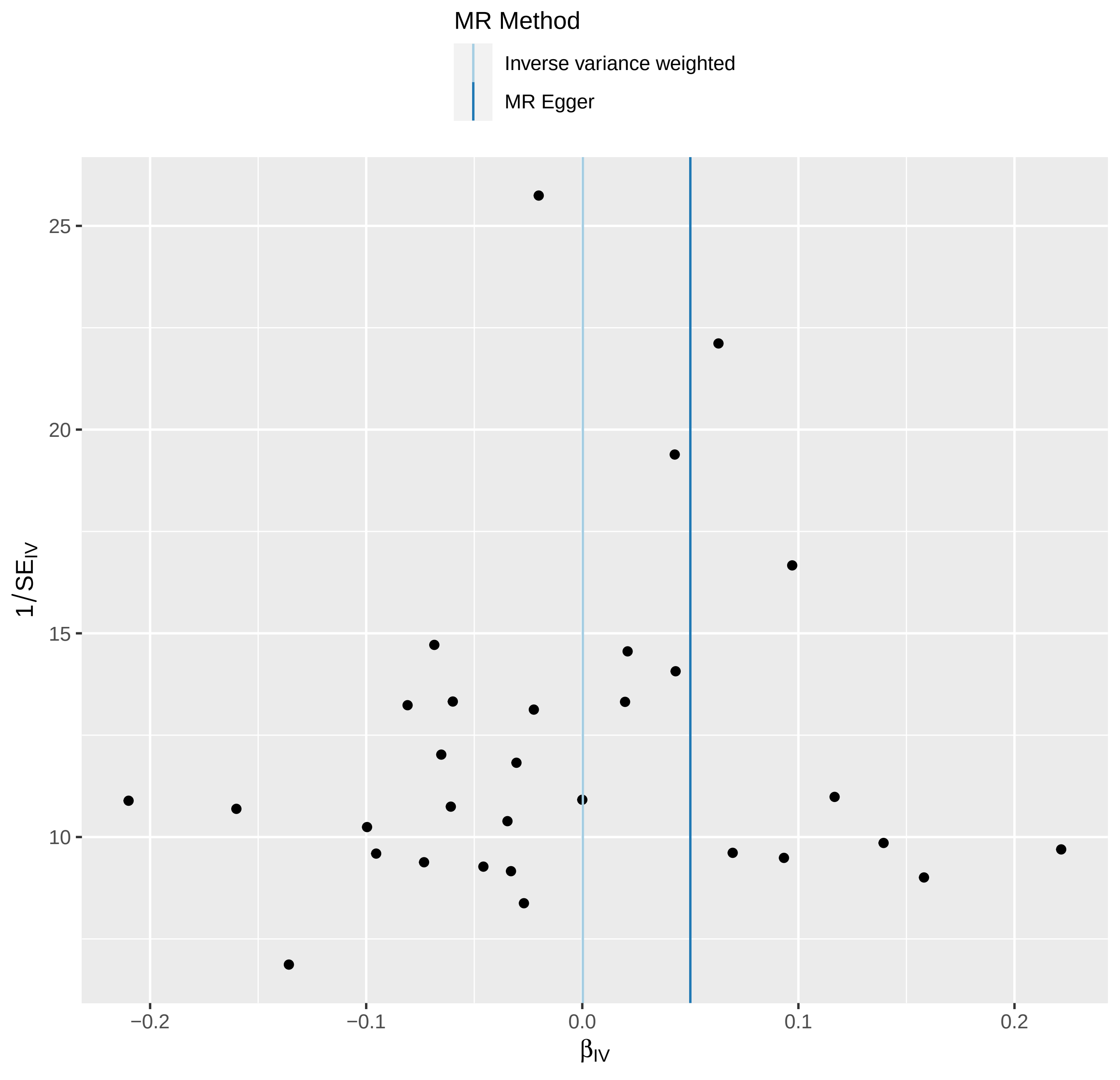

Batch 801 : Crohn's disease on DKTatlas rh parstriangularis area

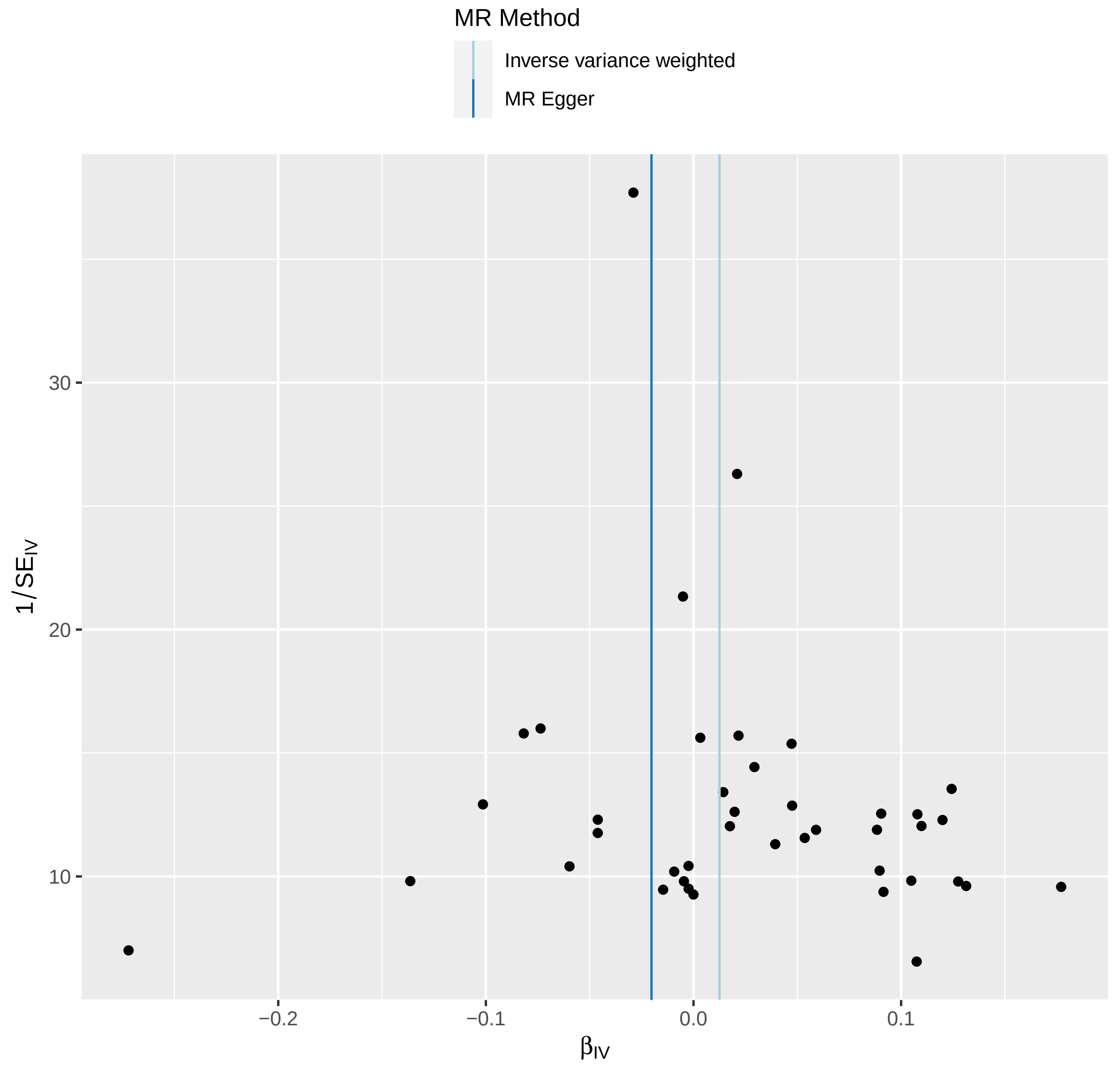

Batch 802 : Ulcerative colitis on DKTatlas rh parstriangularis area

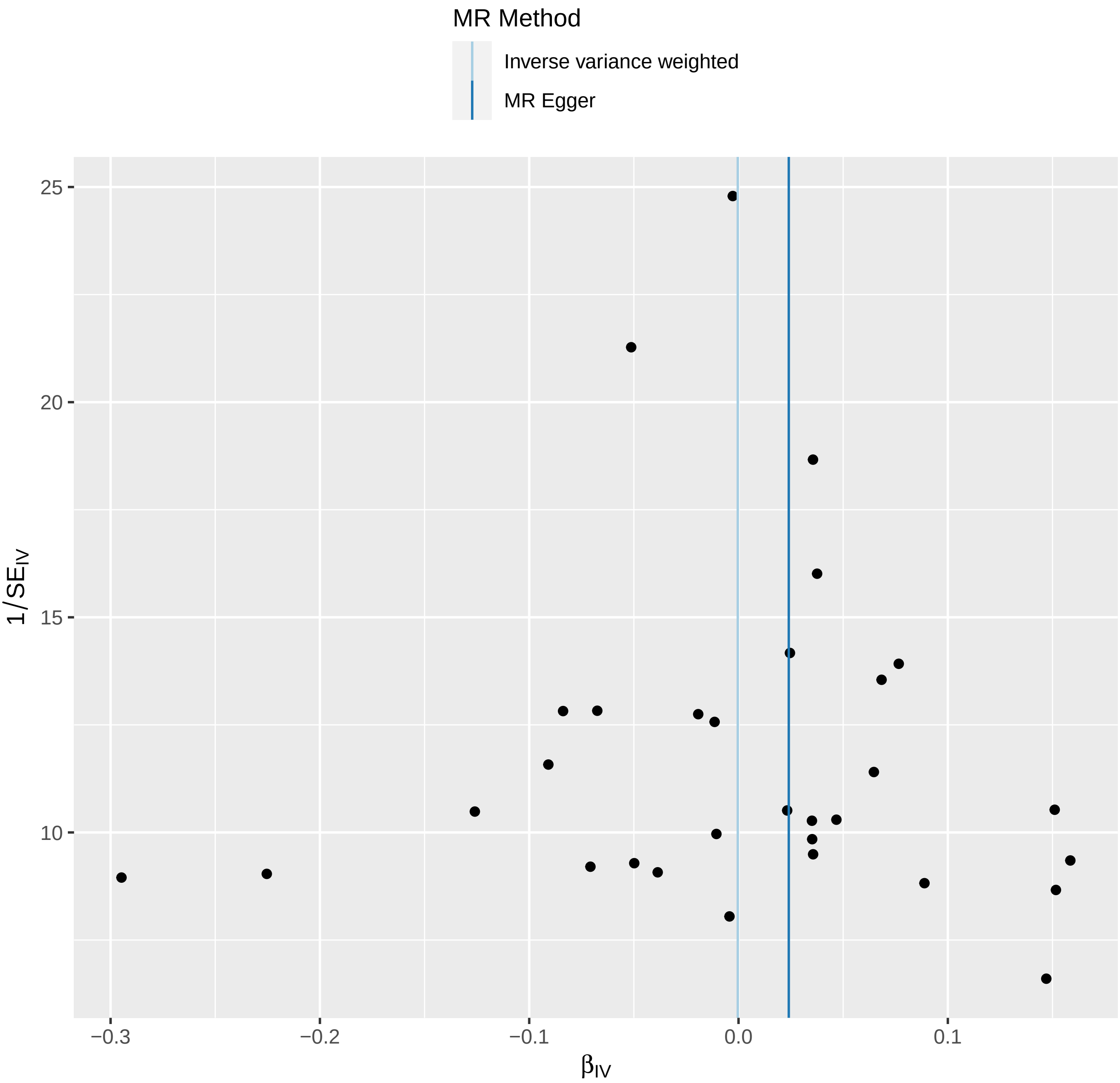

Batch 803 : Crohn's disease on DKTatlas rh pericalcarine area

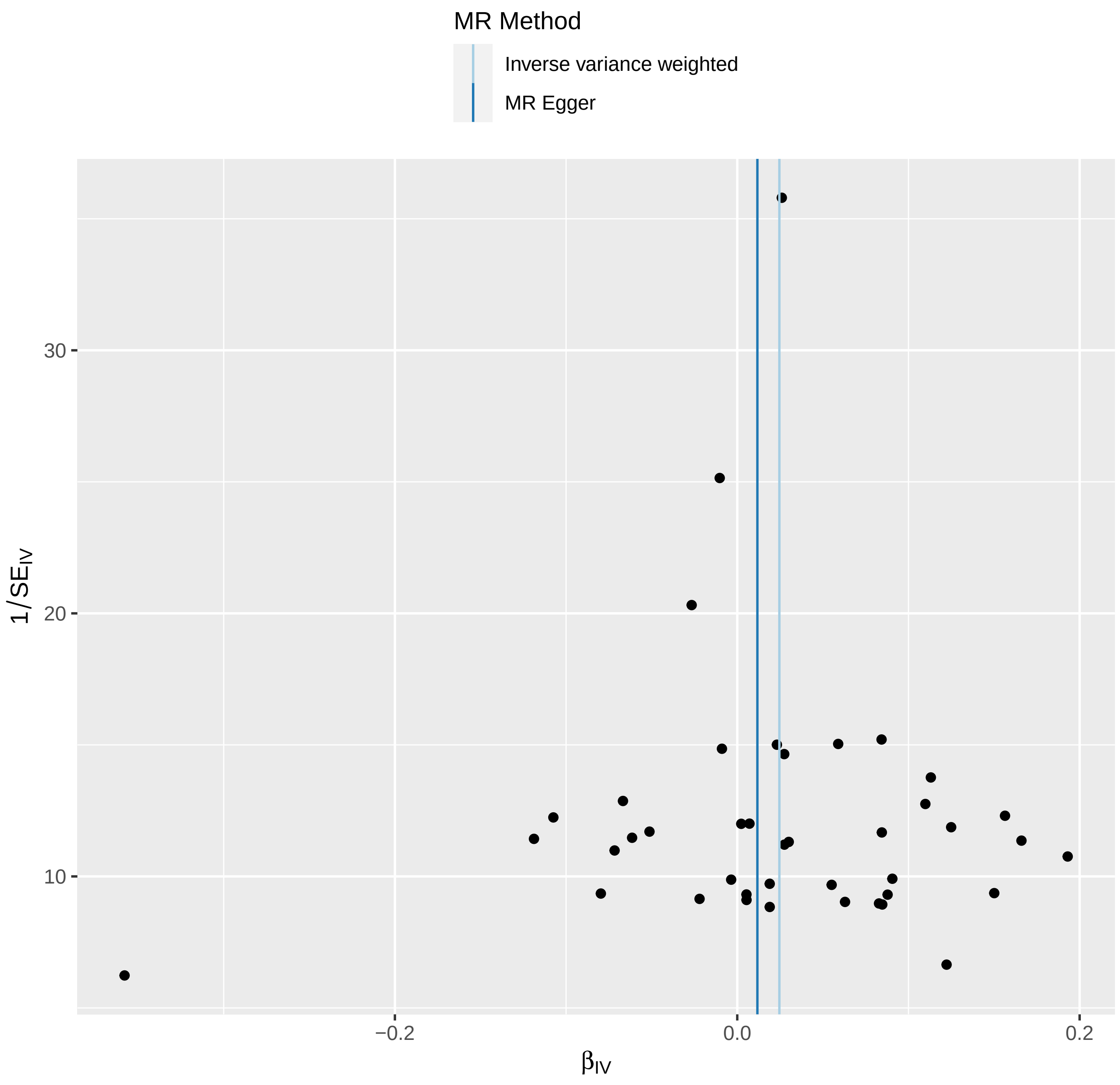

Batch 804 : Ulcerative colitis on DKTatlas rh pericalcarine area

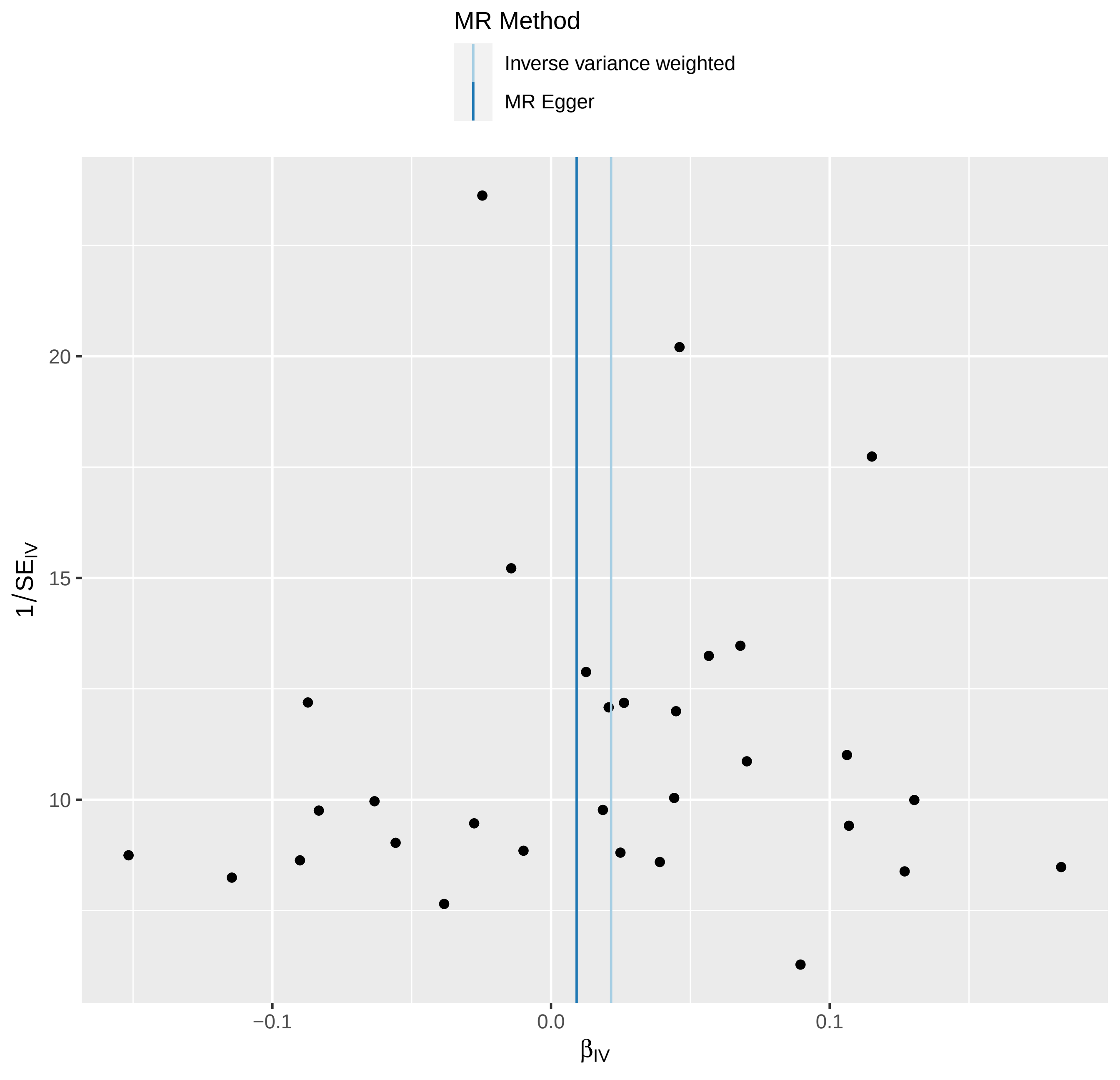

Batch 807 : Crohn's disease on DKTatlas rh postcentral area

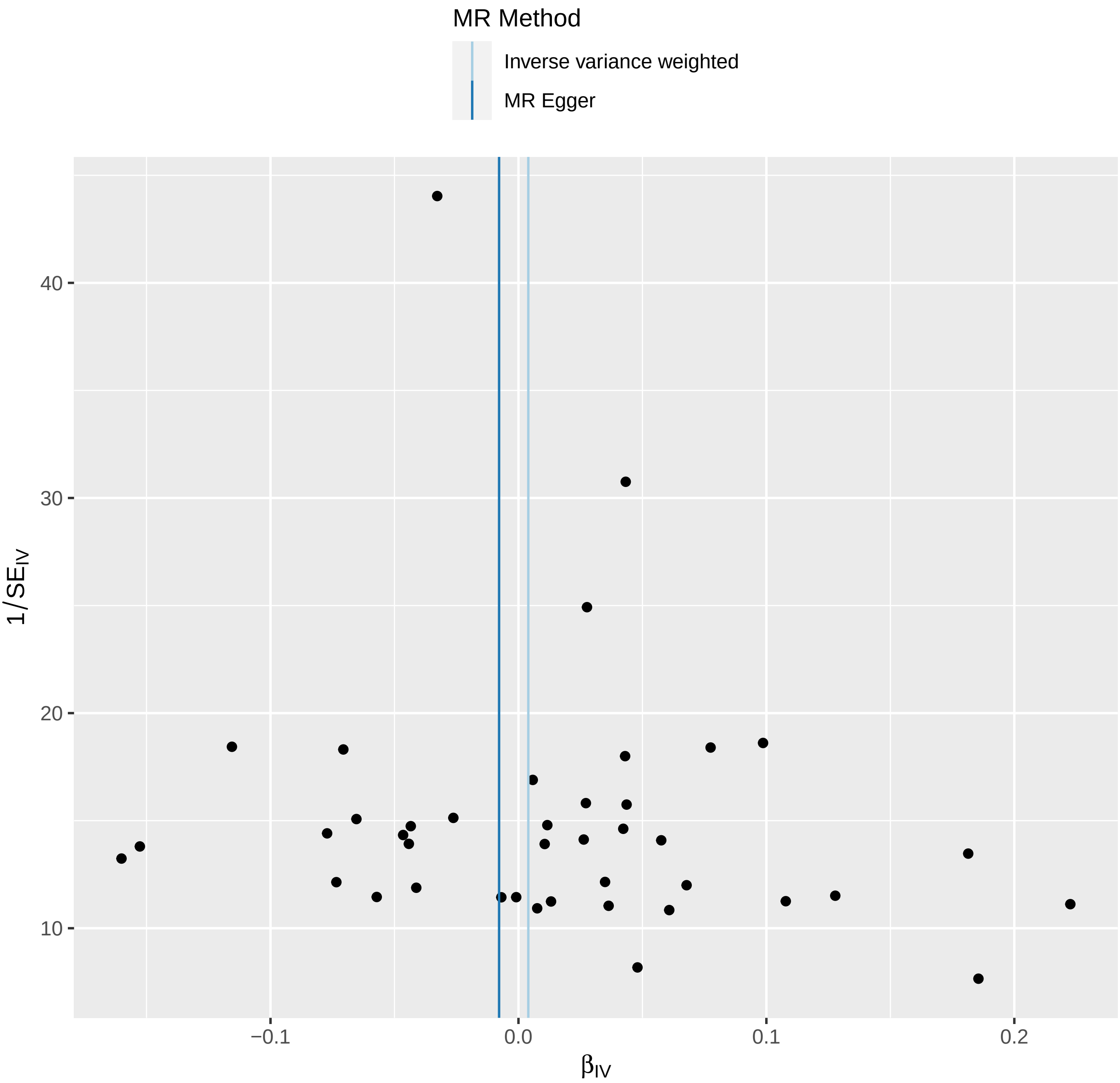

Batch 808 : Ulcerative colitis on DKTatlas rh postcentral area

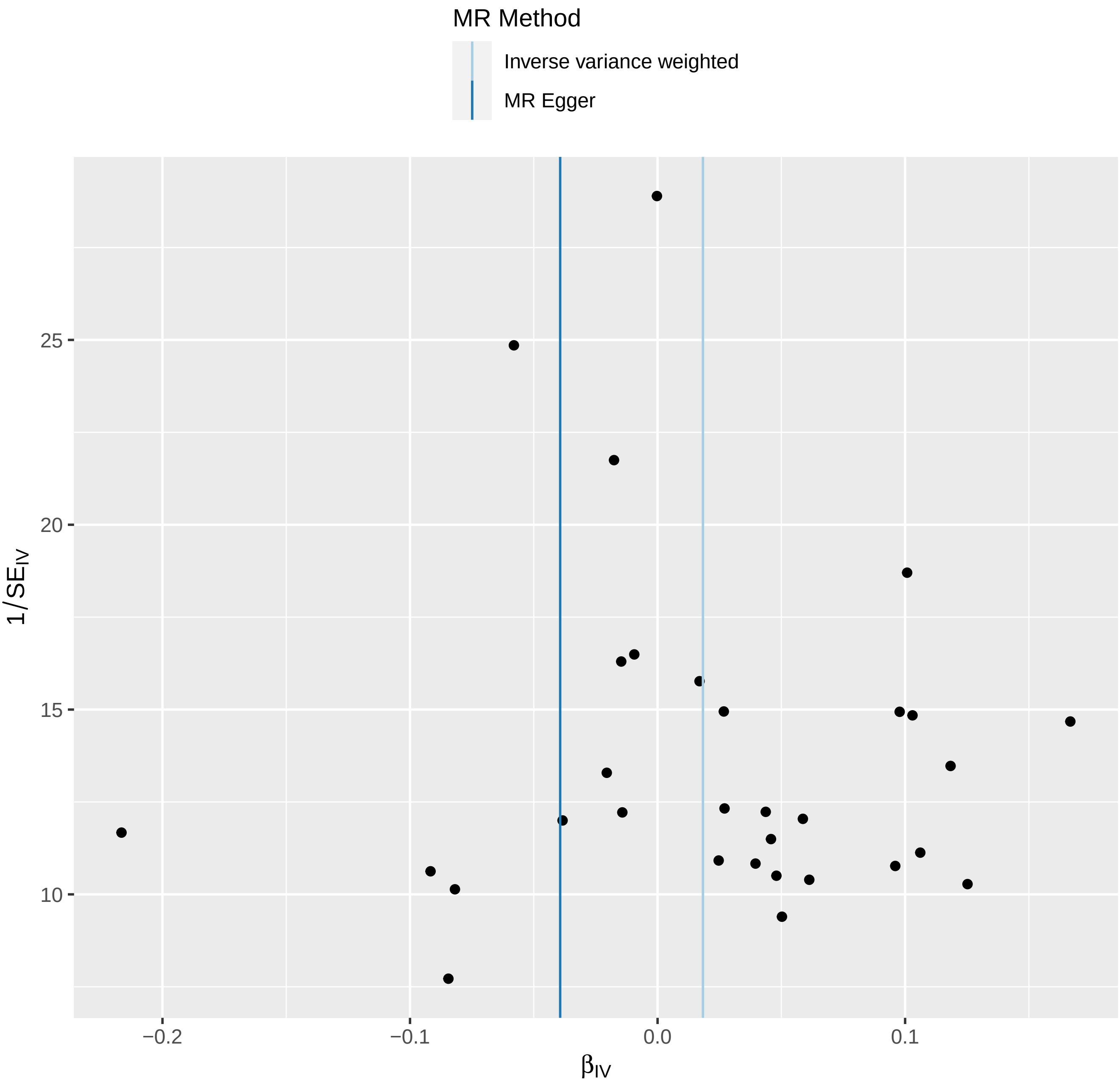

Batch 809 : Crohn's disease on DKTatlas rh posteriorcingulate area

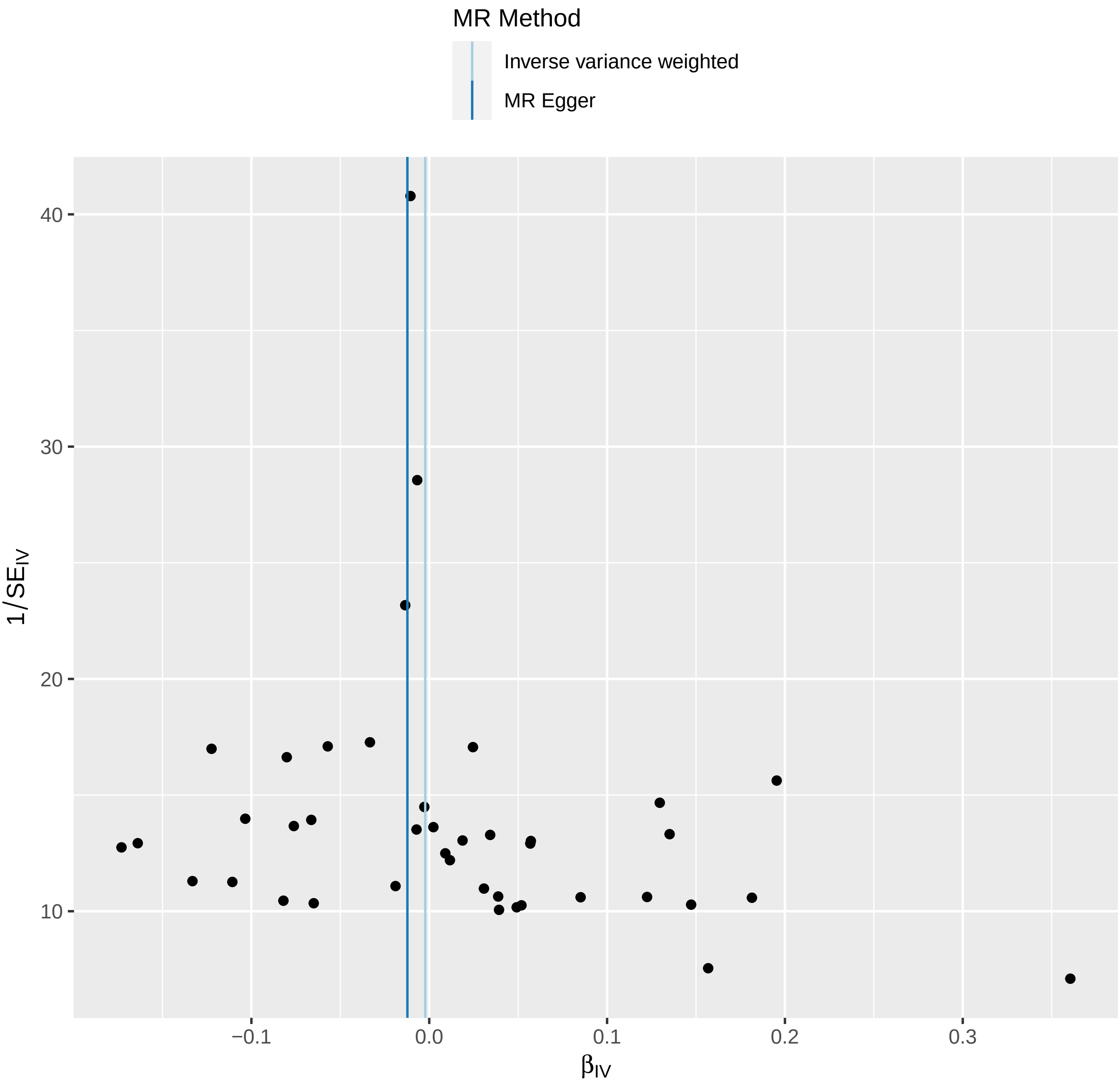

Batch 810 : Ulcerative colitis on DKTatlas rh posteriorcingulate area

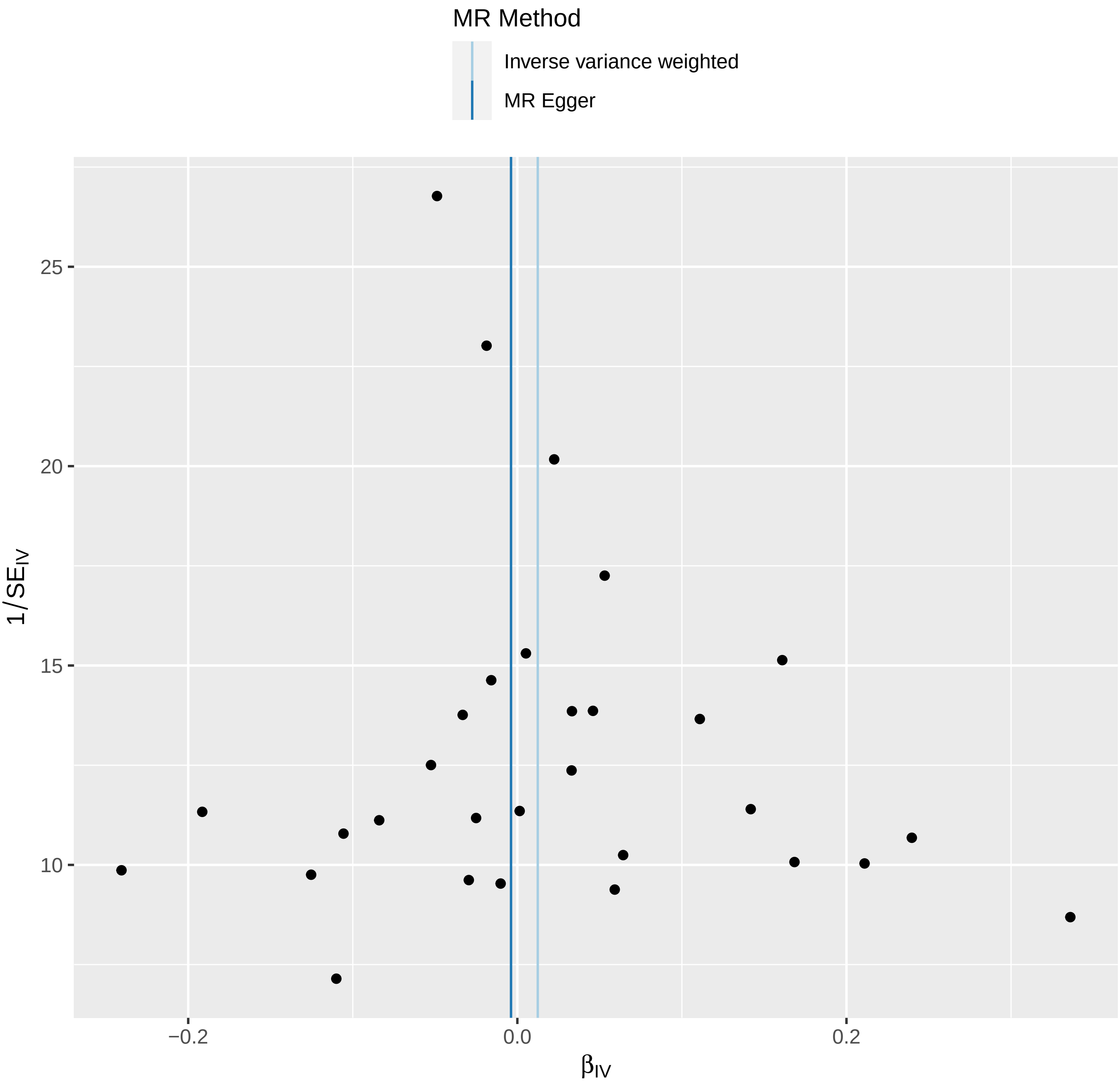

Batch 811 : Crohn's disease on DKTatlas rh precentral area

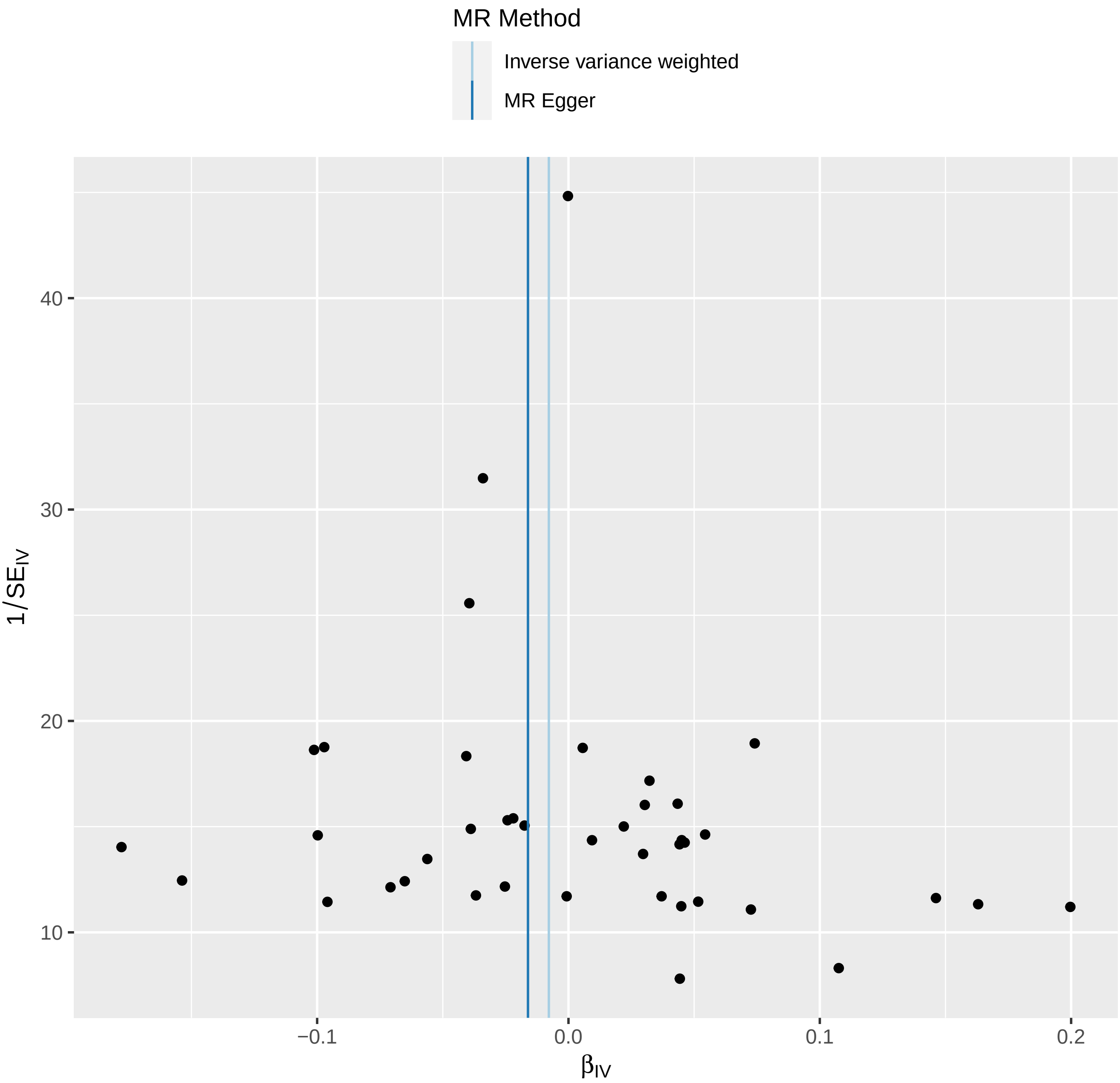

Batch 812 : Ulcerative colitis on DKTatlas rh precentral area

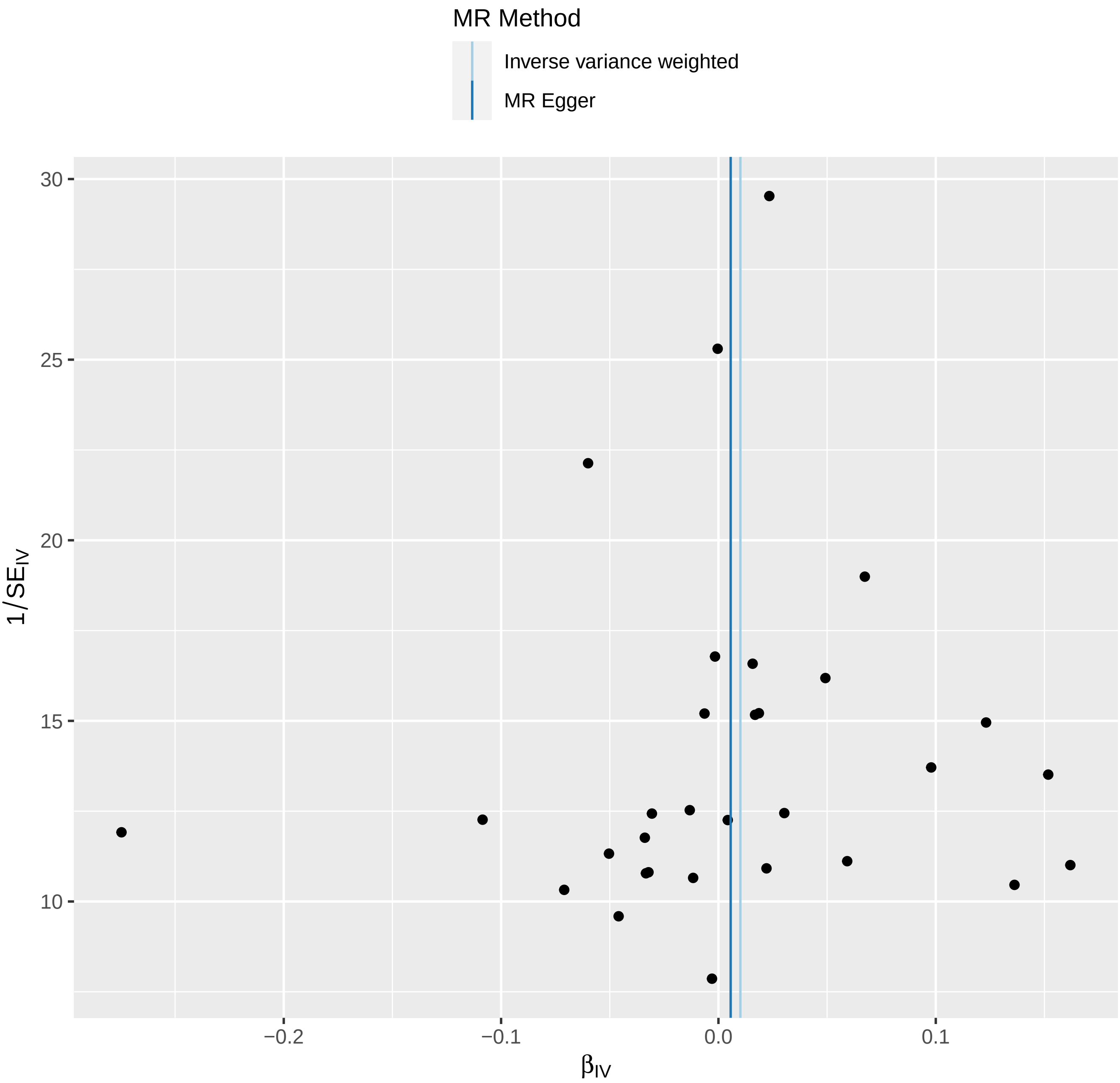

Batch 813 : Crohn's disease on DKTatlas rh precuneus area

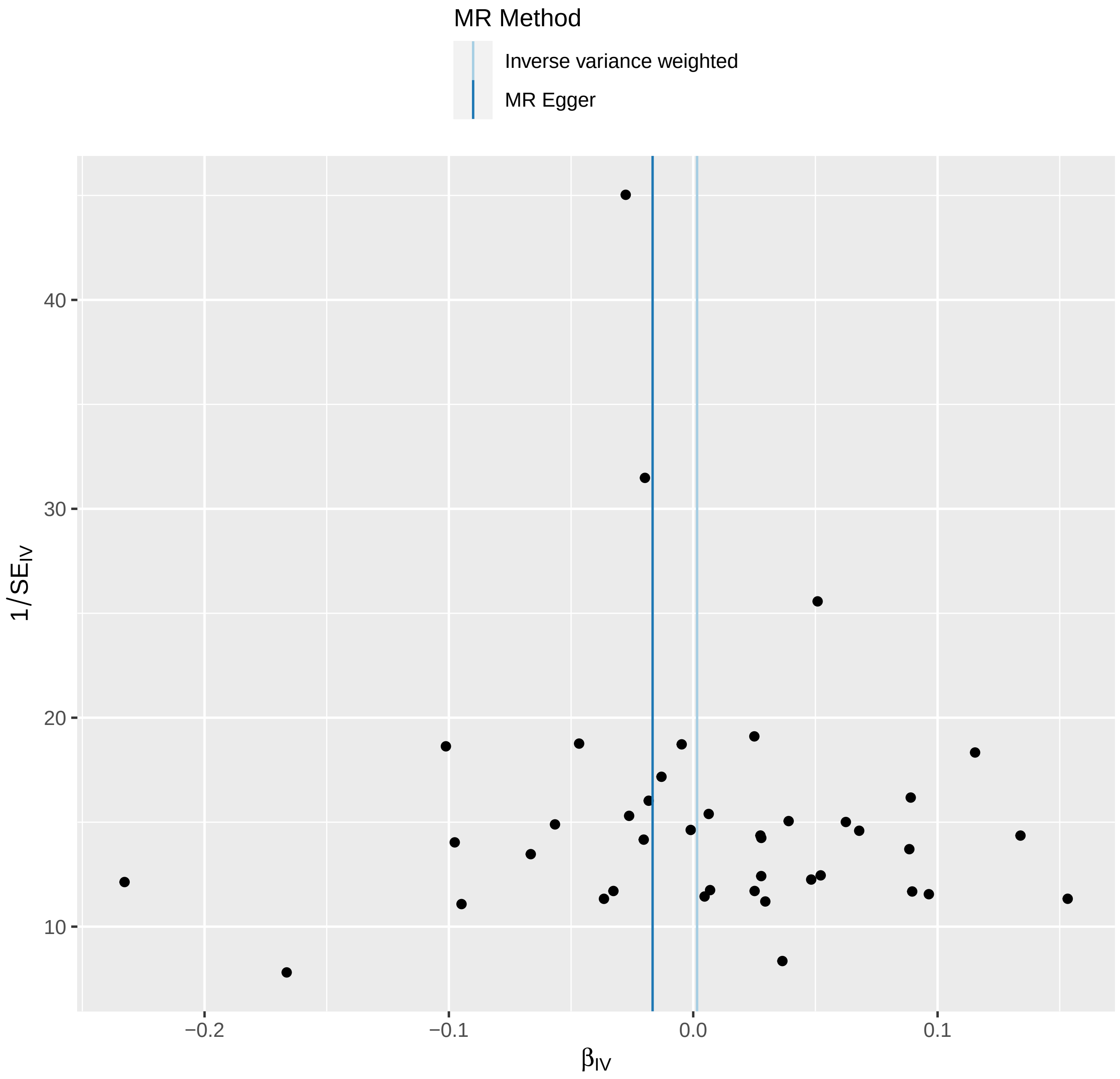

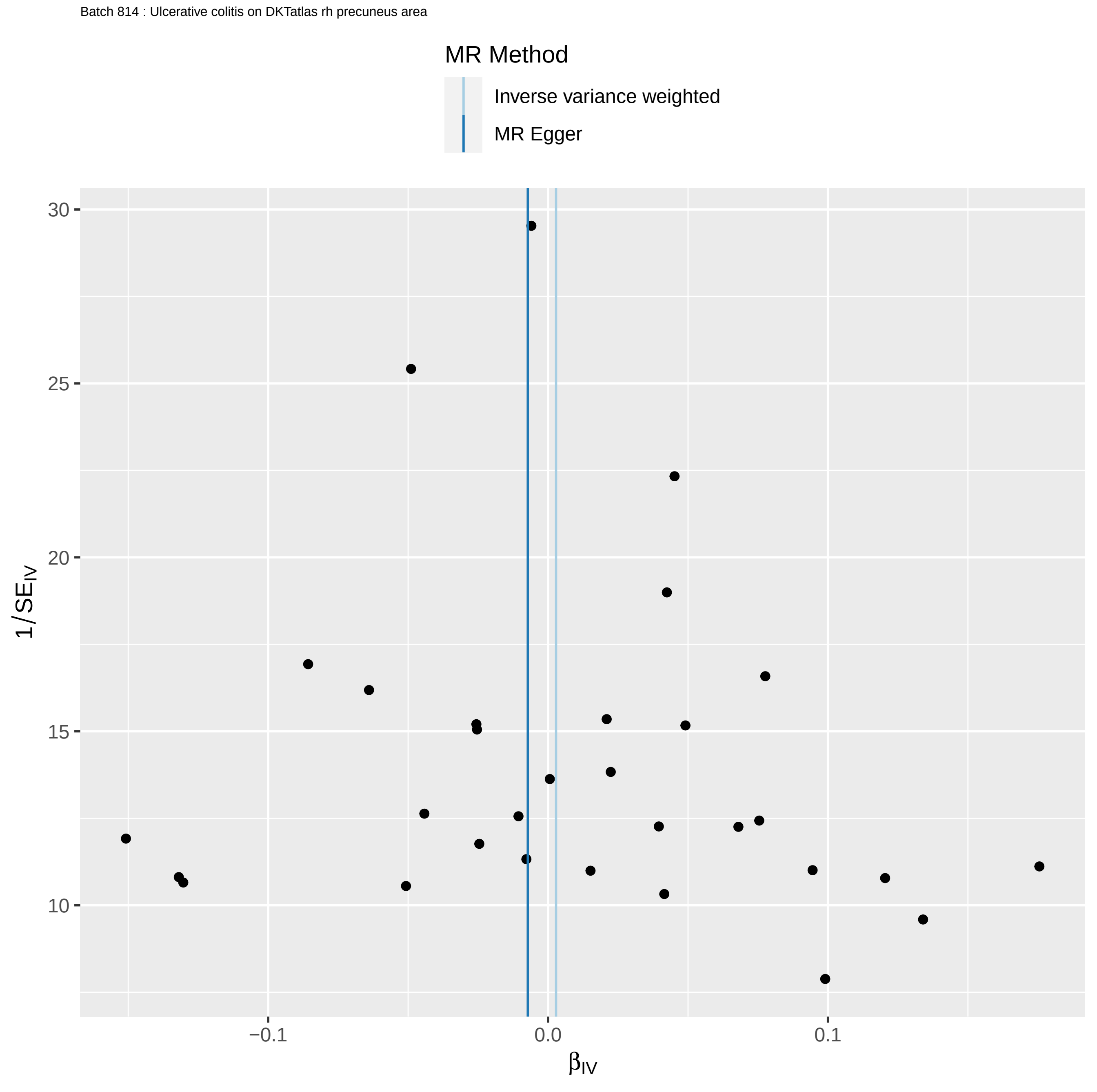

Batch 814 : Ulcerative colitis on DKTatlas rh precuneus area
MR Method
Inverse variance weighted
MR Egger
30
25
20
15
10
1/SE$_{IV}$
−0.1
0.0
0.1
β$_{IV}$

Batch 815 : Crohn's disease on DKTatlas rh rostralanteriorcingulate area

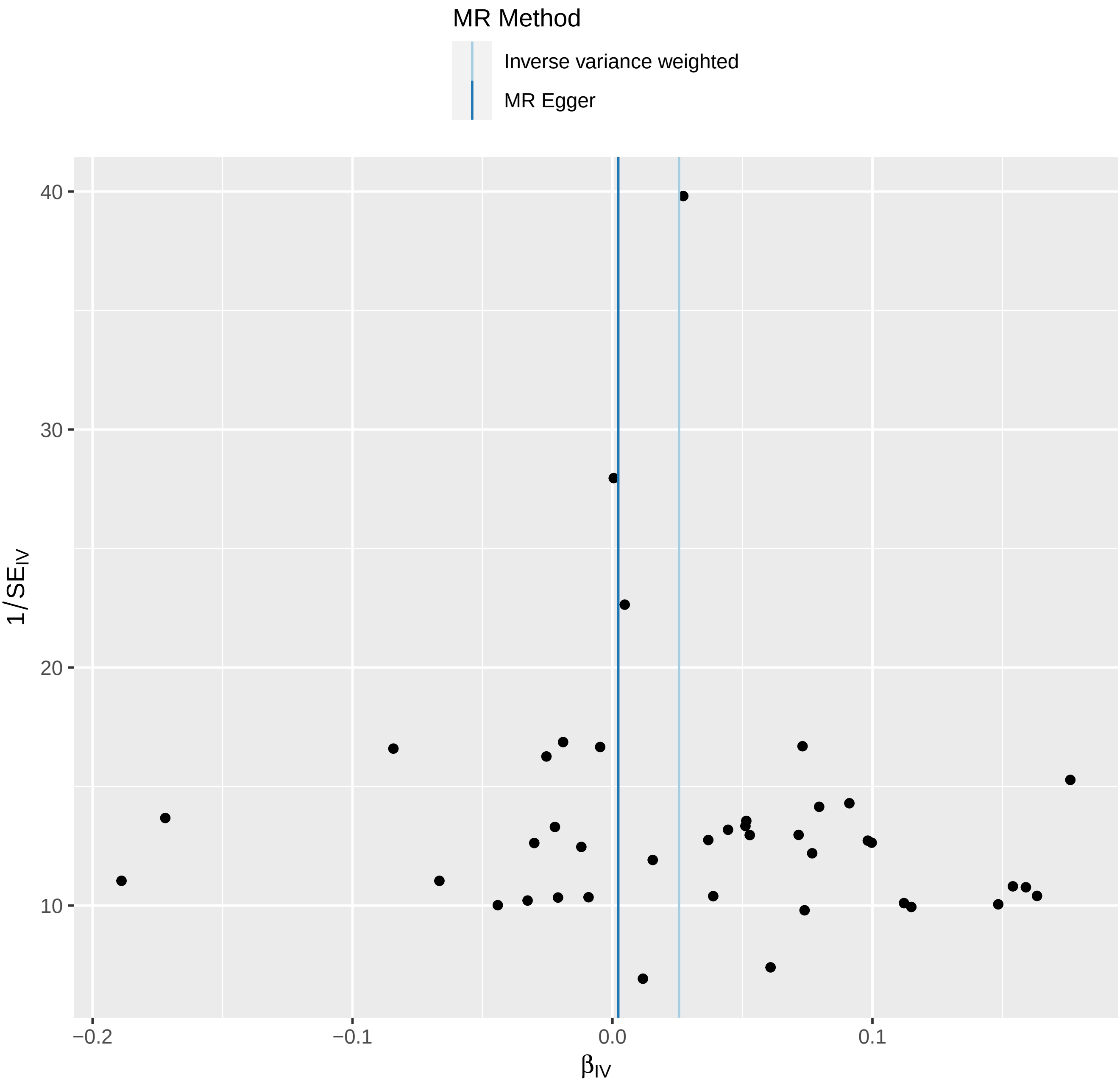

Batch 816 : Ulcerative colitis on DKTatlas rh rostralanteriorcingulate area

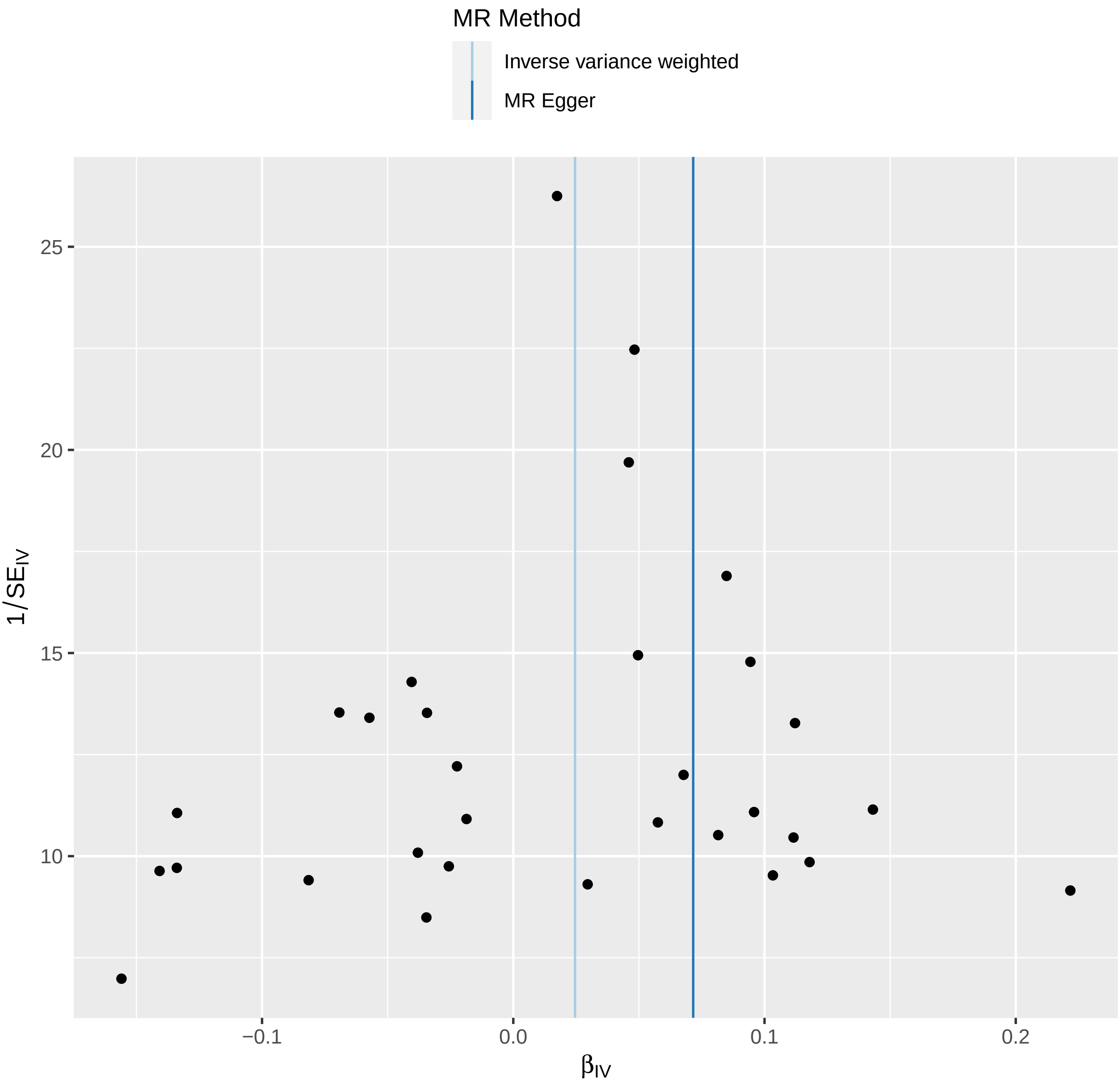

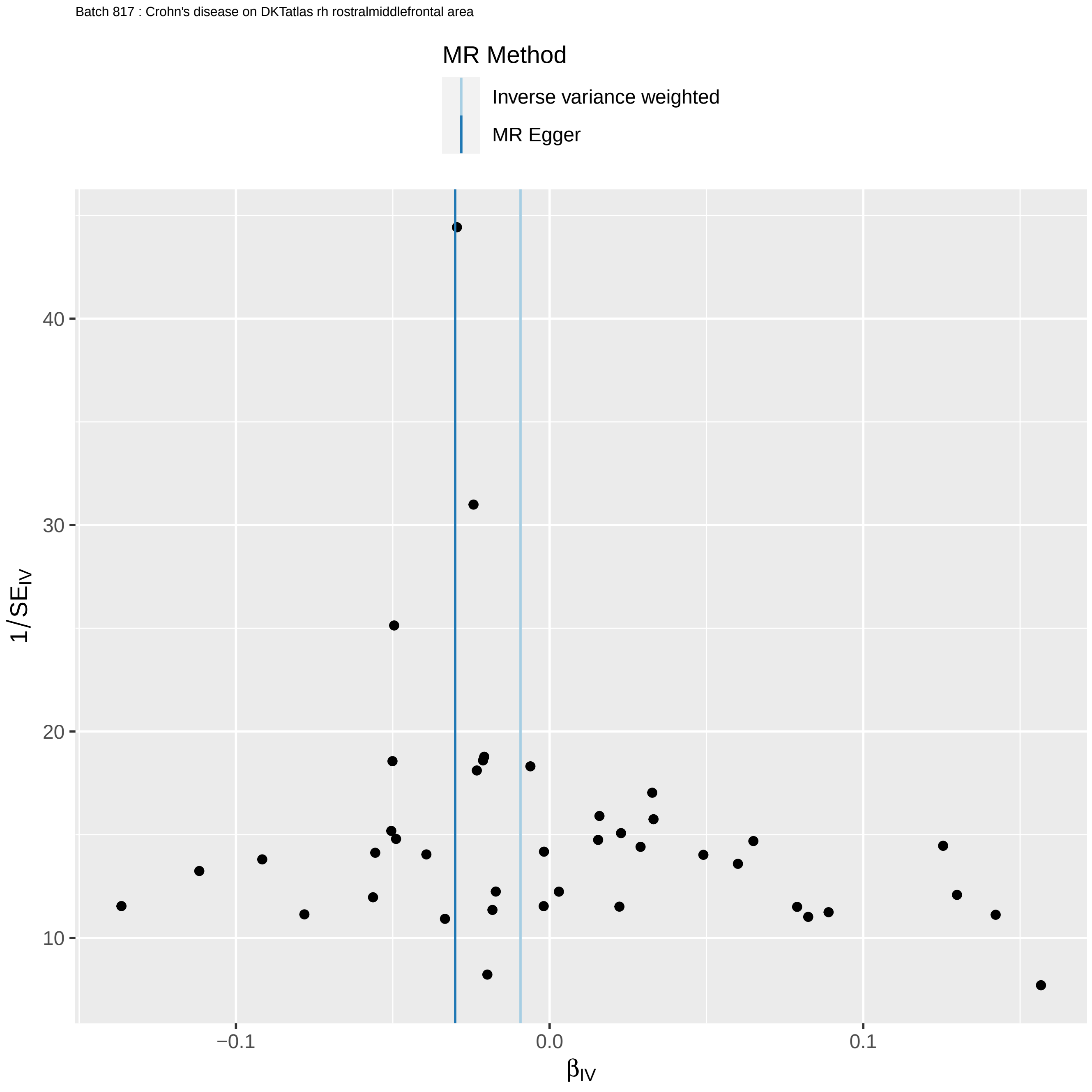

Batch 817 : Crohn's disease on DKTatlas rh rostralmiddlefrontal area
MR Method
Inverse variance weighted
MR Egger
40
30
20
10
1/SE_IV
−0.1
0.0
0.1
β_IV

Batch 818 : Ulcerative colitis on DKTatlas rh rostralmiddlefrontal area

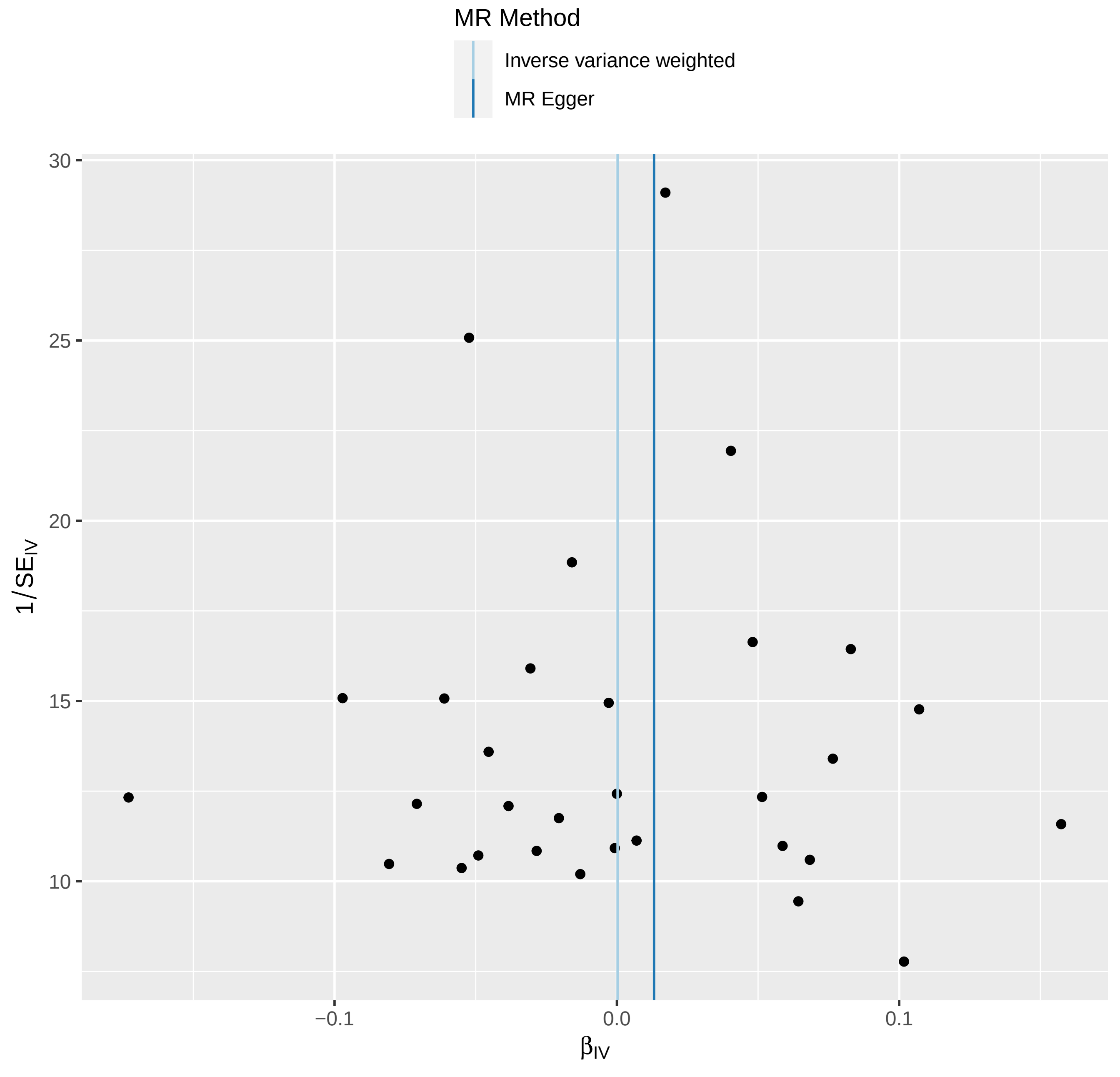

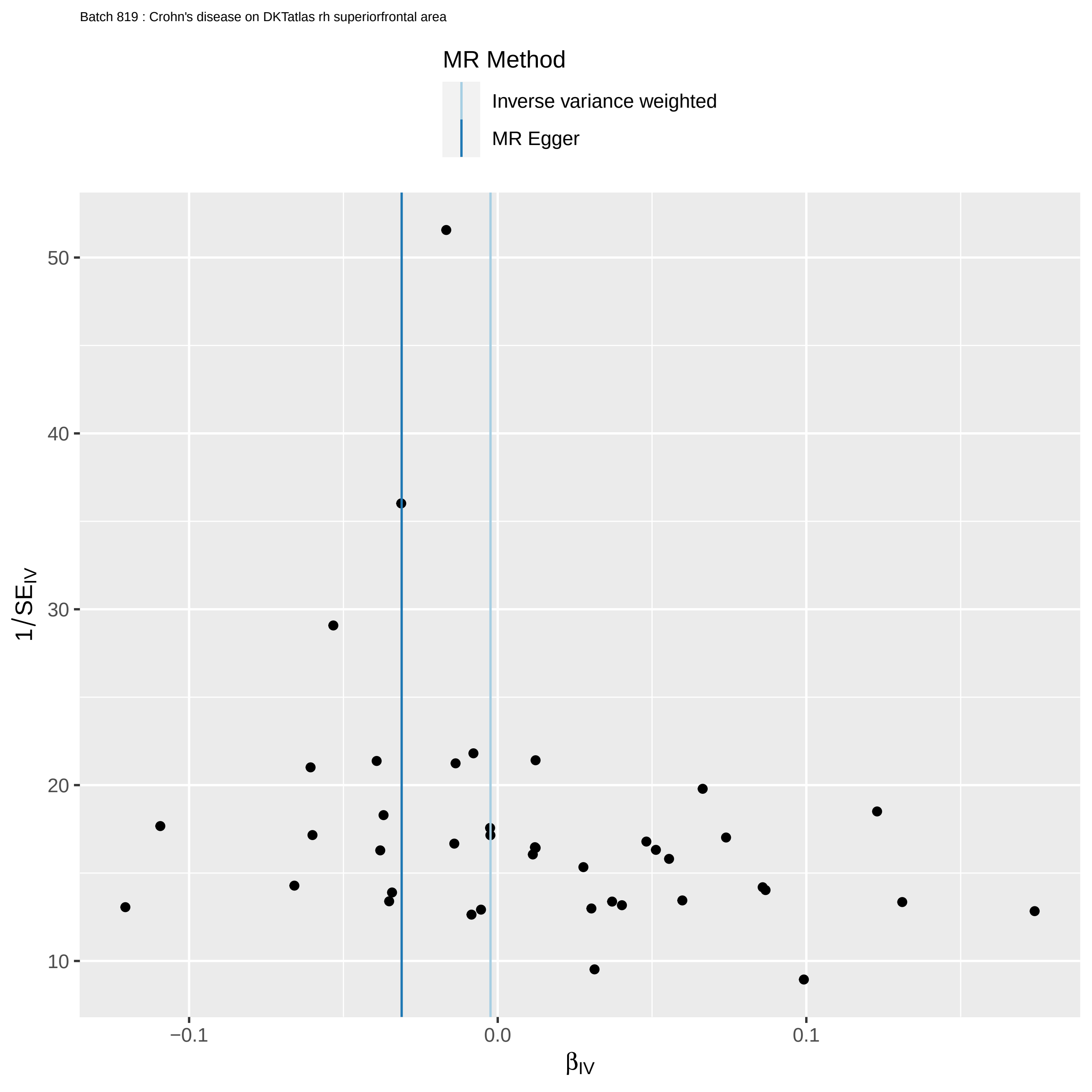

Batch 819 : Crohn's disease on DKTatlas rh superiorfrontal area
MR Method
Inverse variance weighted
MR Egger
50
40
30
20
10
1/SE_IV
−0.1
0.0
0.1
β_IV

Batch 820 : Ulcerative colitis on DKTatlas rh superiorfrontal area

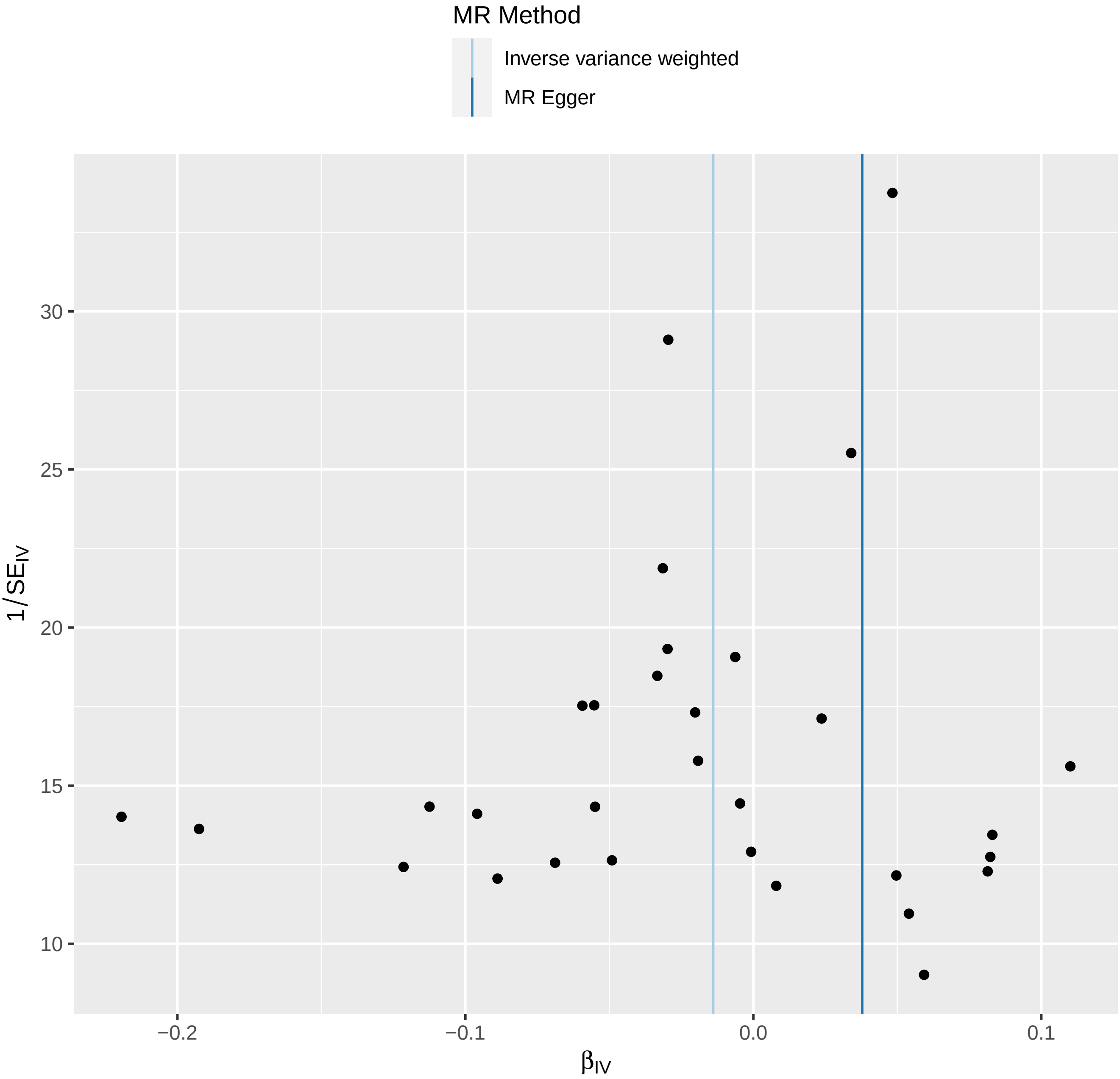

Batch 821 : Crohn's disease on DKTatlas rh superiorparietal area

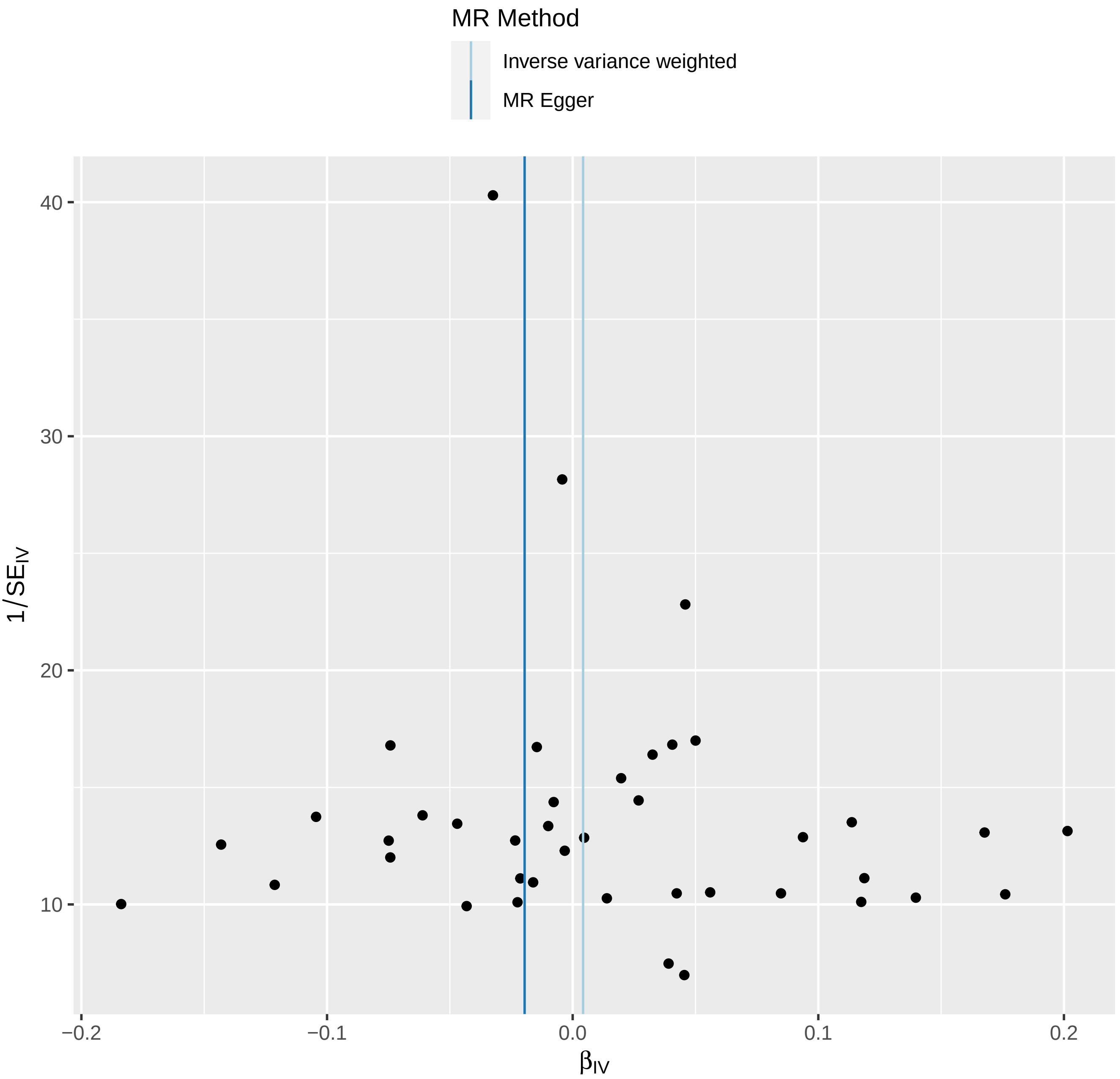

Batch 822 : Ulcerative colitis on DKTatlas rh superiorparietal area

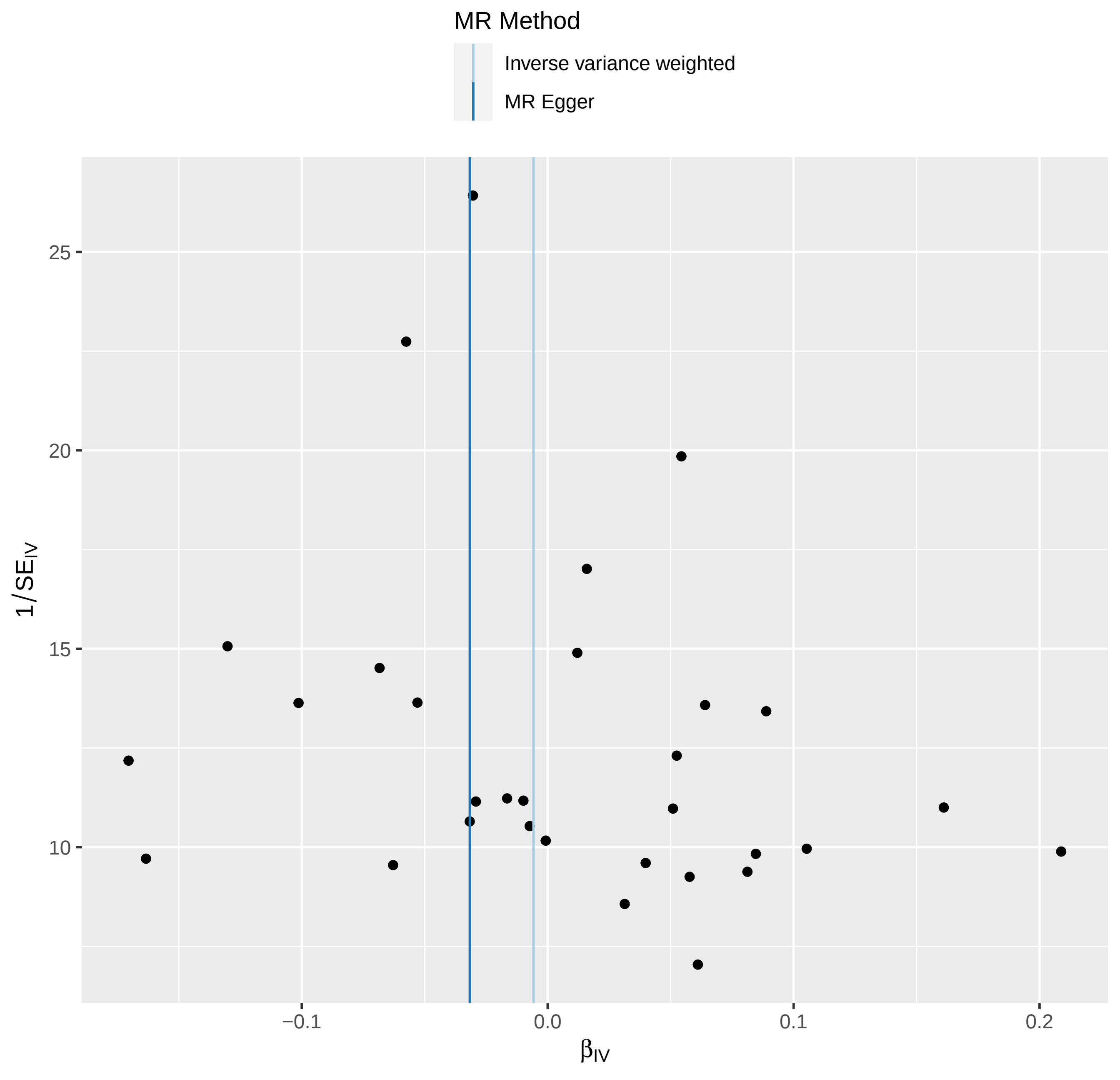

Batch 823 : Crohn's disease on DKTatlas rh superiortemporal area

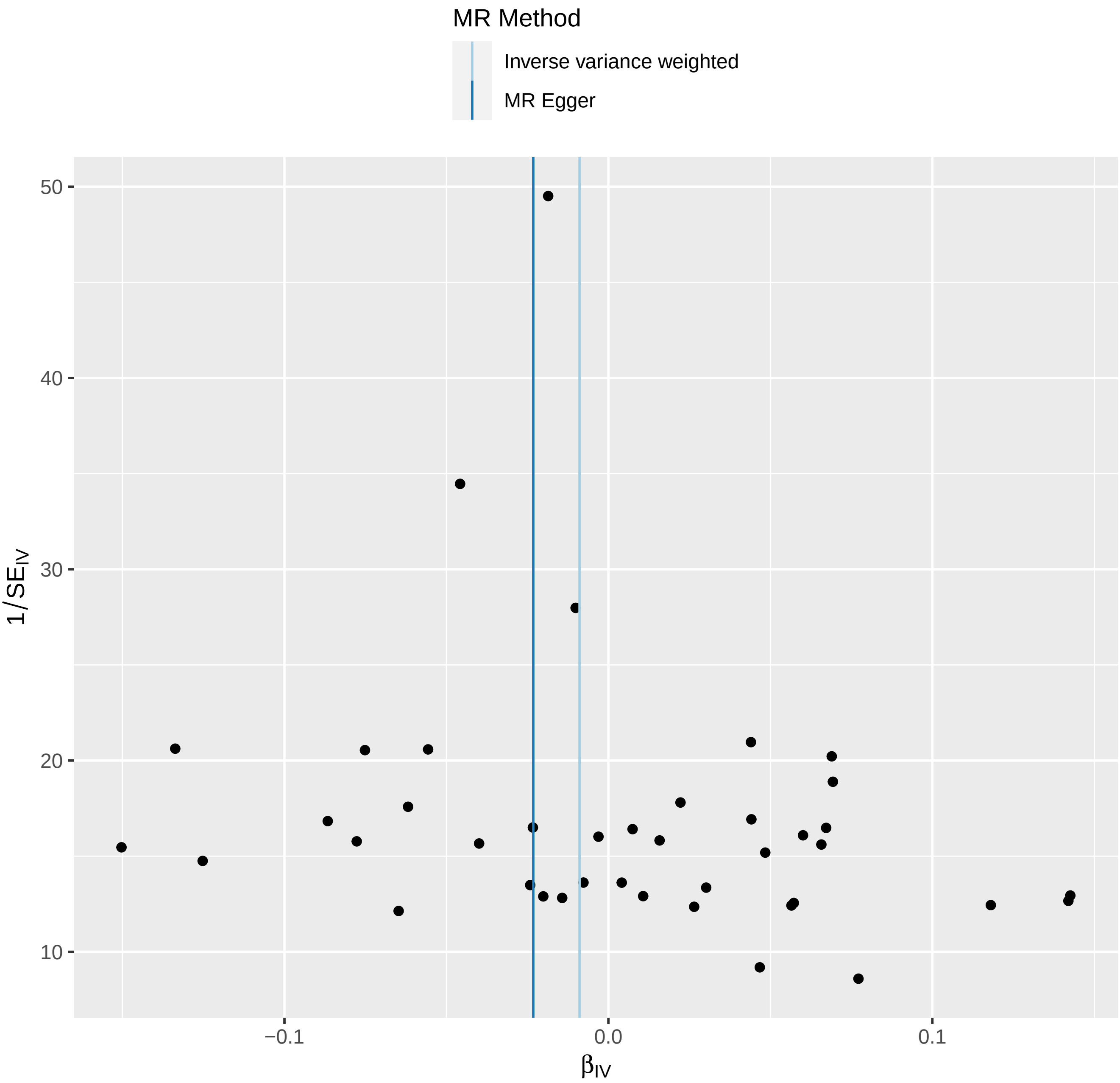

Batch 824 : Ulcerative colitis on DKTatlas rh superiortemporal area

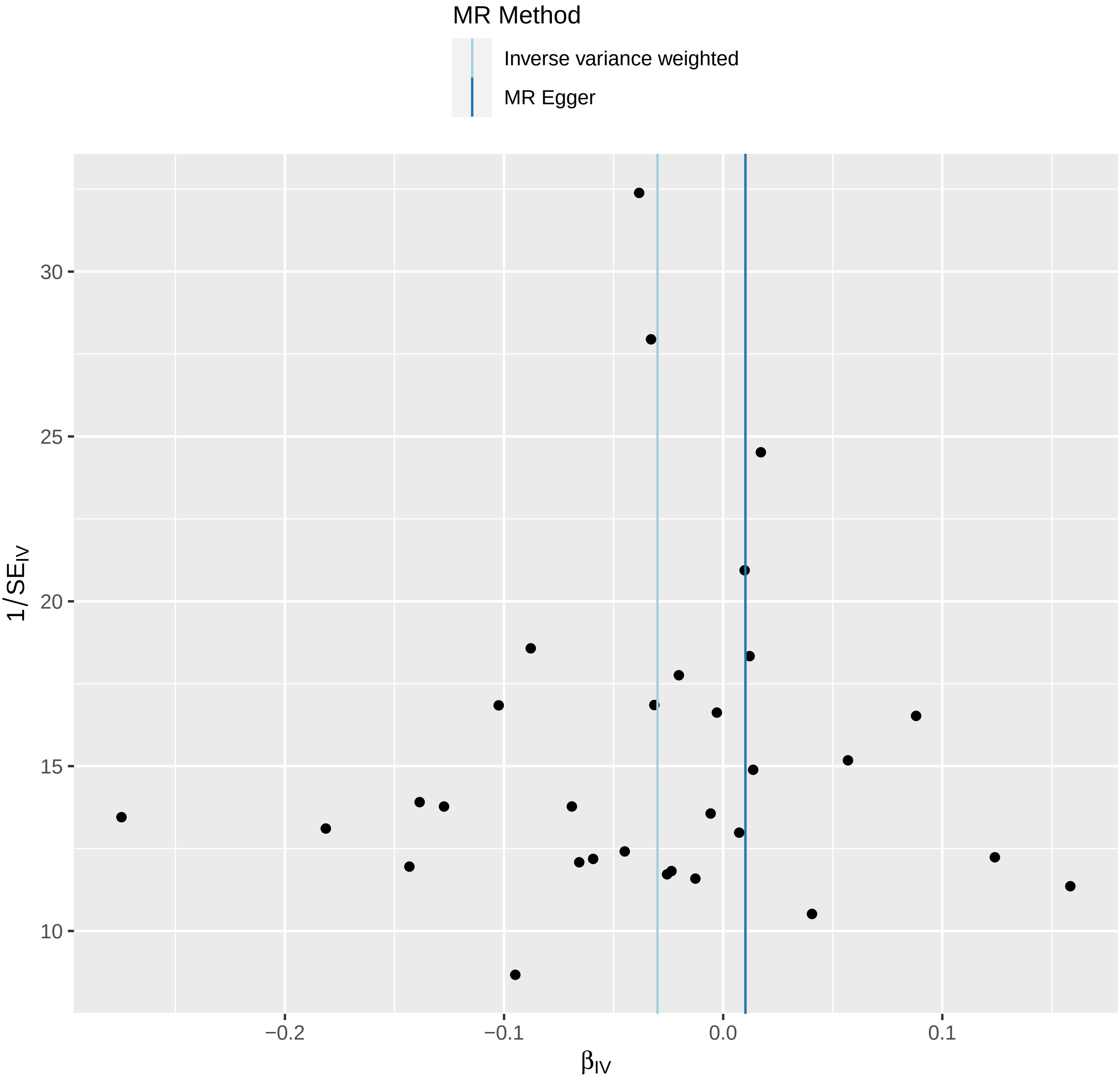

Batch 825 : Crohn's disease on DKTatlas rh supramarginal area

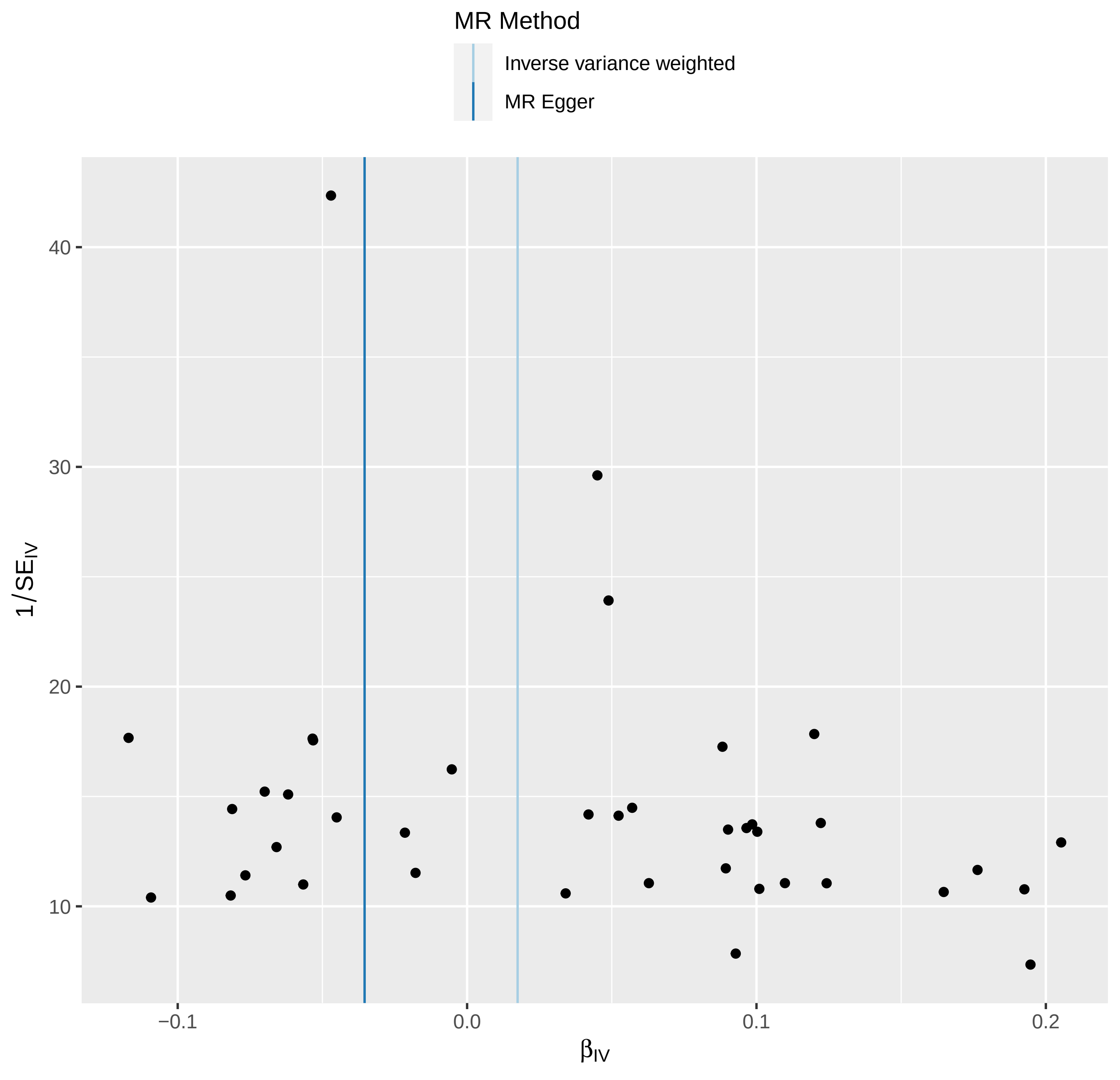

Batch 826 : Ulcerative colitis on DKTatlas rh supramarginal area

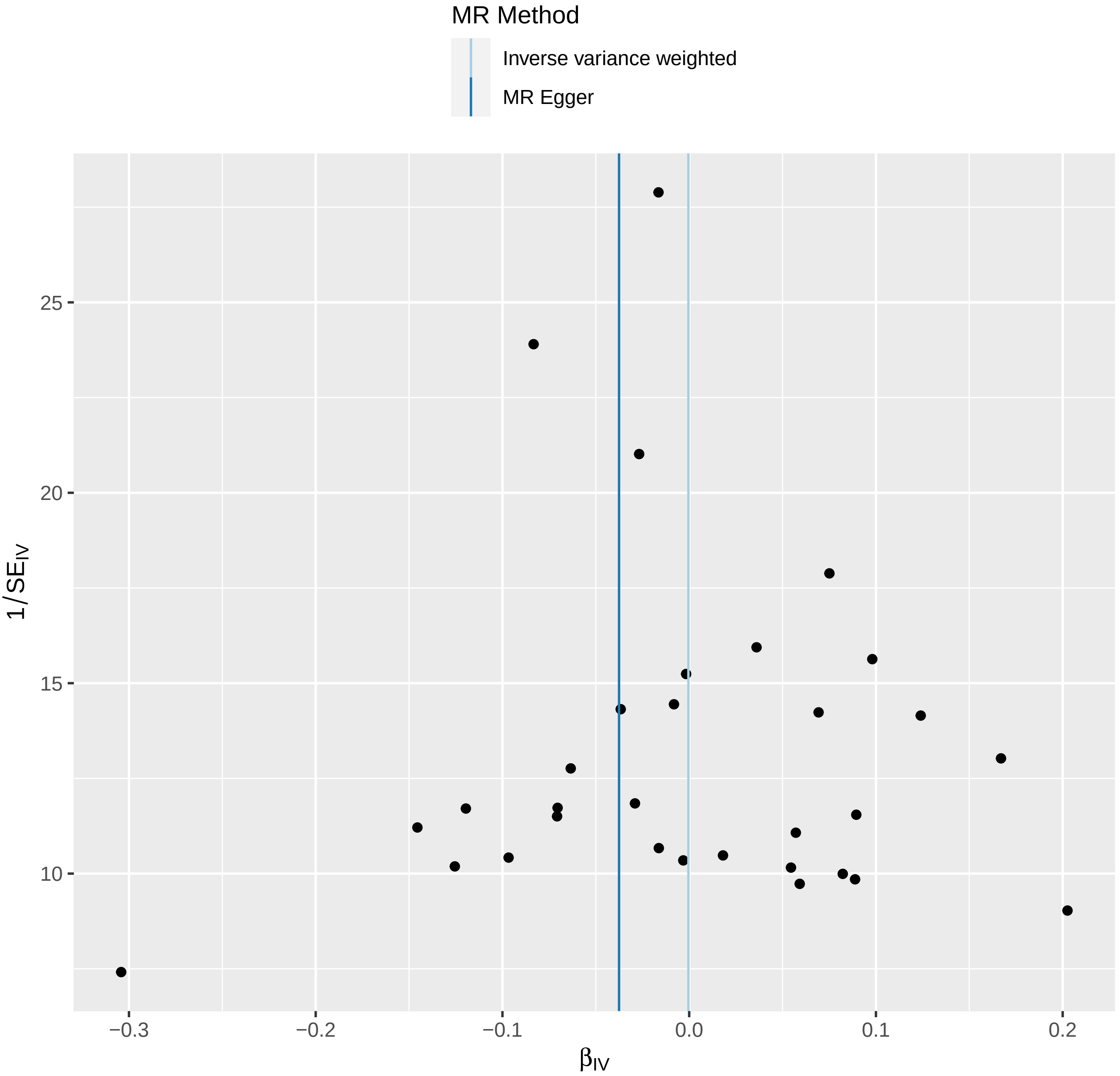

Batch 829 : Crohn's disease on DKTatlas rh transversetemporal area

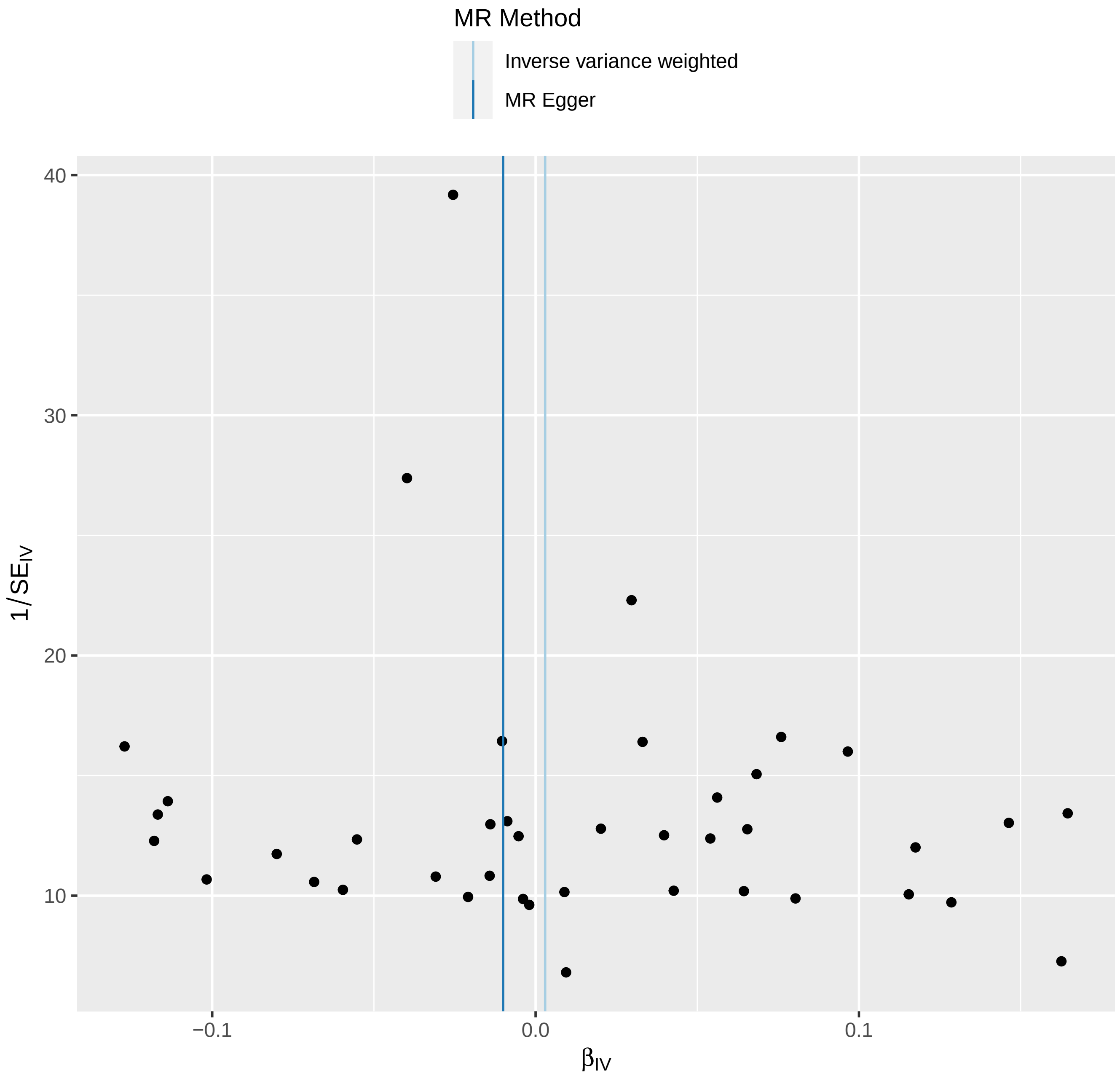

Batch 830 : Ulcerative colitis on DKTatlas rh transversetemporal area

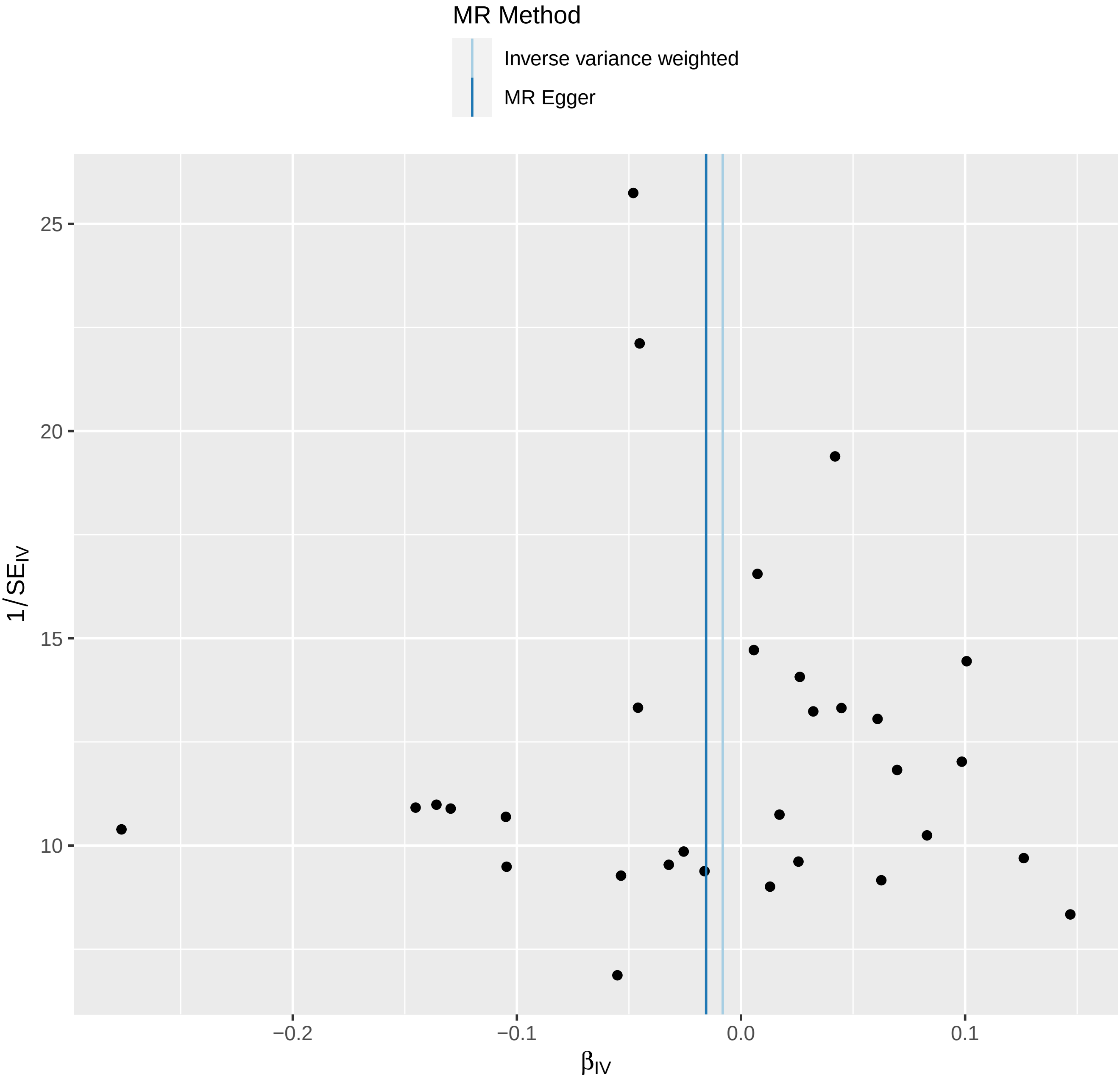

Batch 831 : Crohn's disease on DKTatlas rh insula area

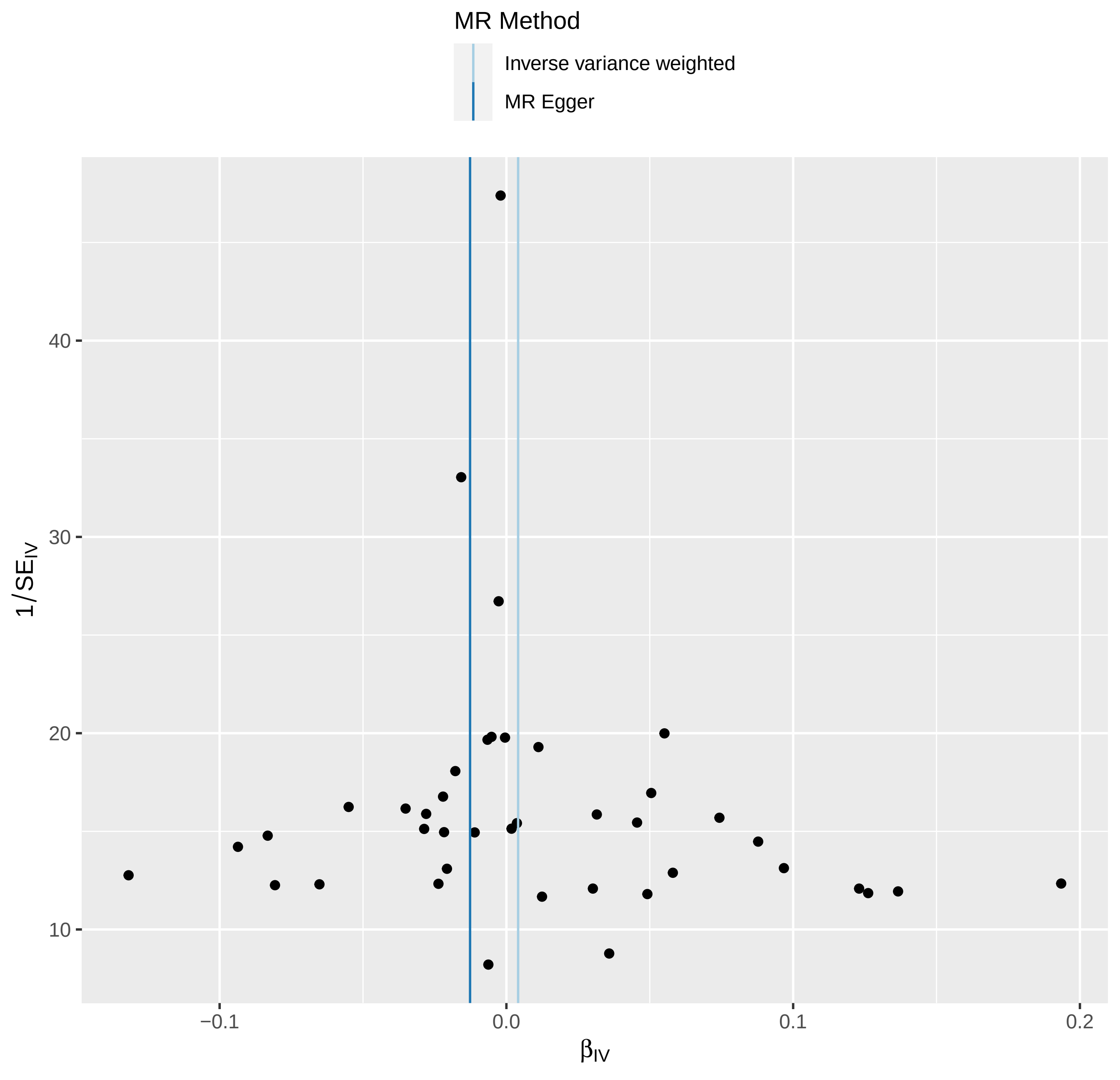

Batch 832 : Ulcerative colitis on DKTatlas rh insula area

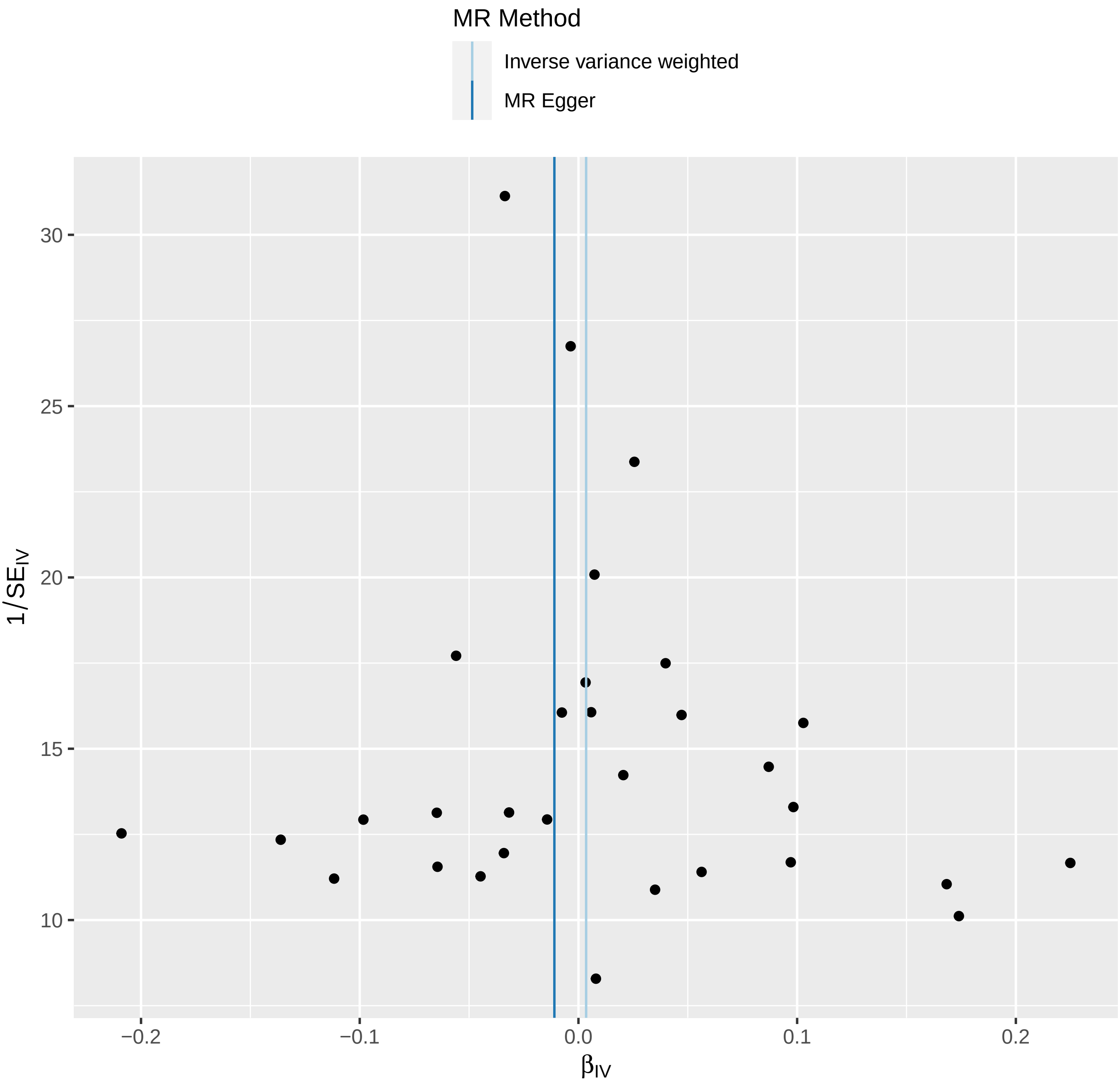

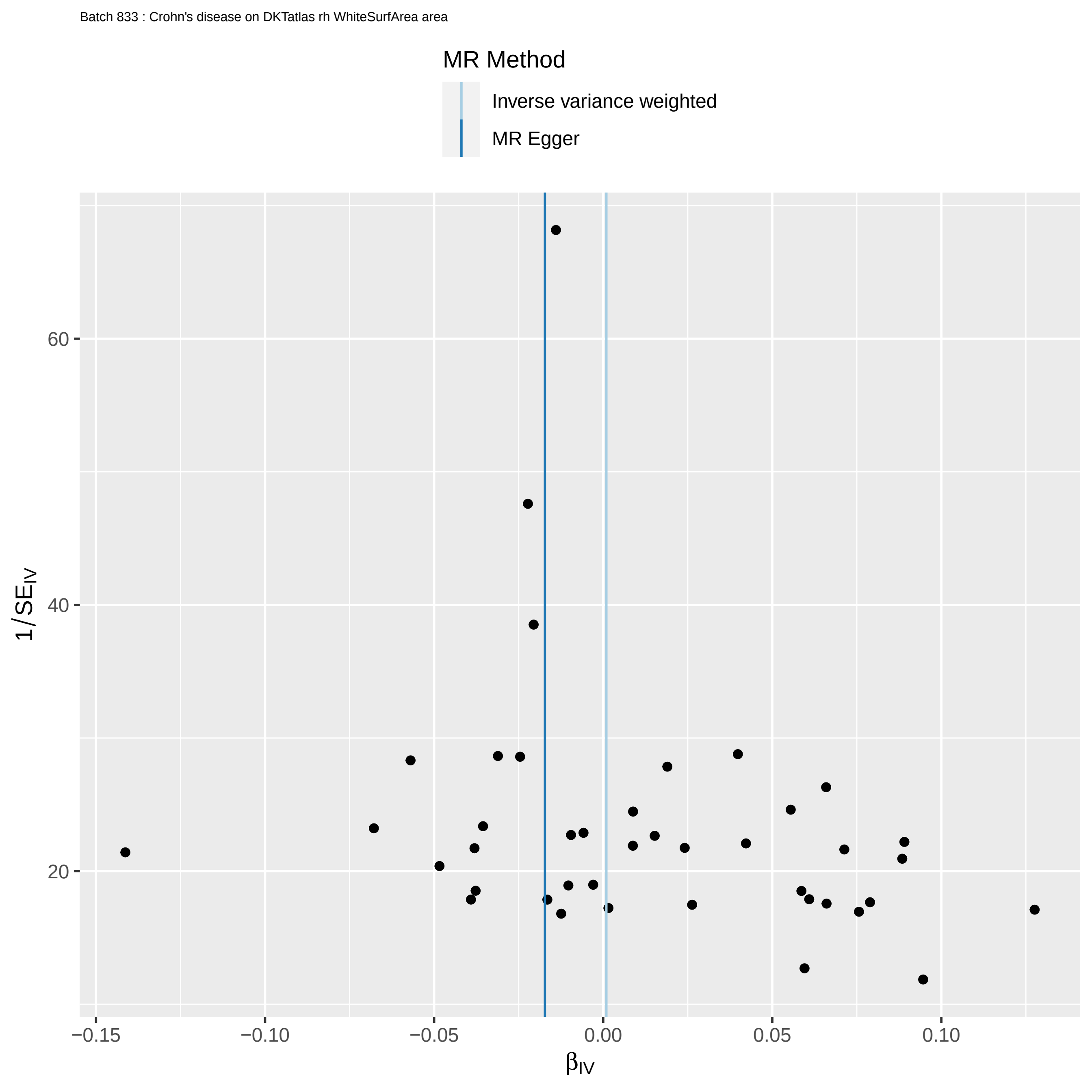
Batch 833 : Crohn's disease on DKTatlas rh WhiteSurfArea area
MR Method
Inverse variance weighted
MR Egger
60
40
20
1/SE$_{IV}$
−0.15
−0.10
−0.05
0.00
0.05
0.10
β$_{IV}$

Batch 834 : Ulcerative colitis on DKTatlas rh WhiteSurfArea area

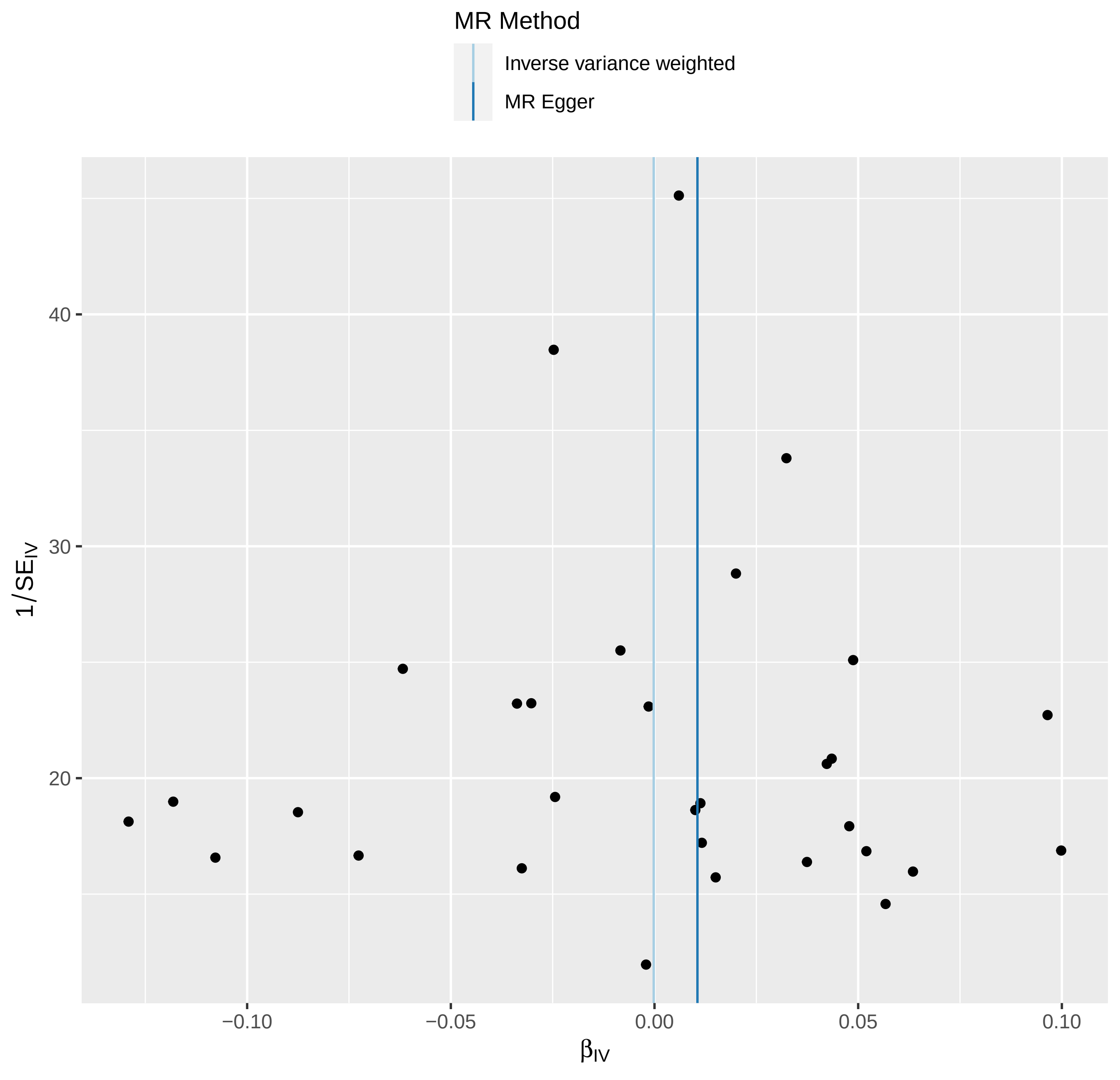

Batch 937 : Crohn's disease on IDP T1 FAST ROIs R insular cortex

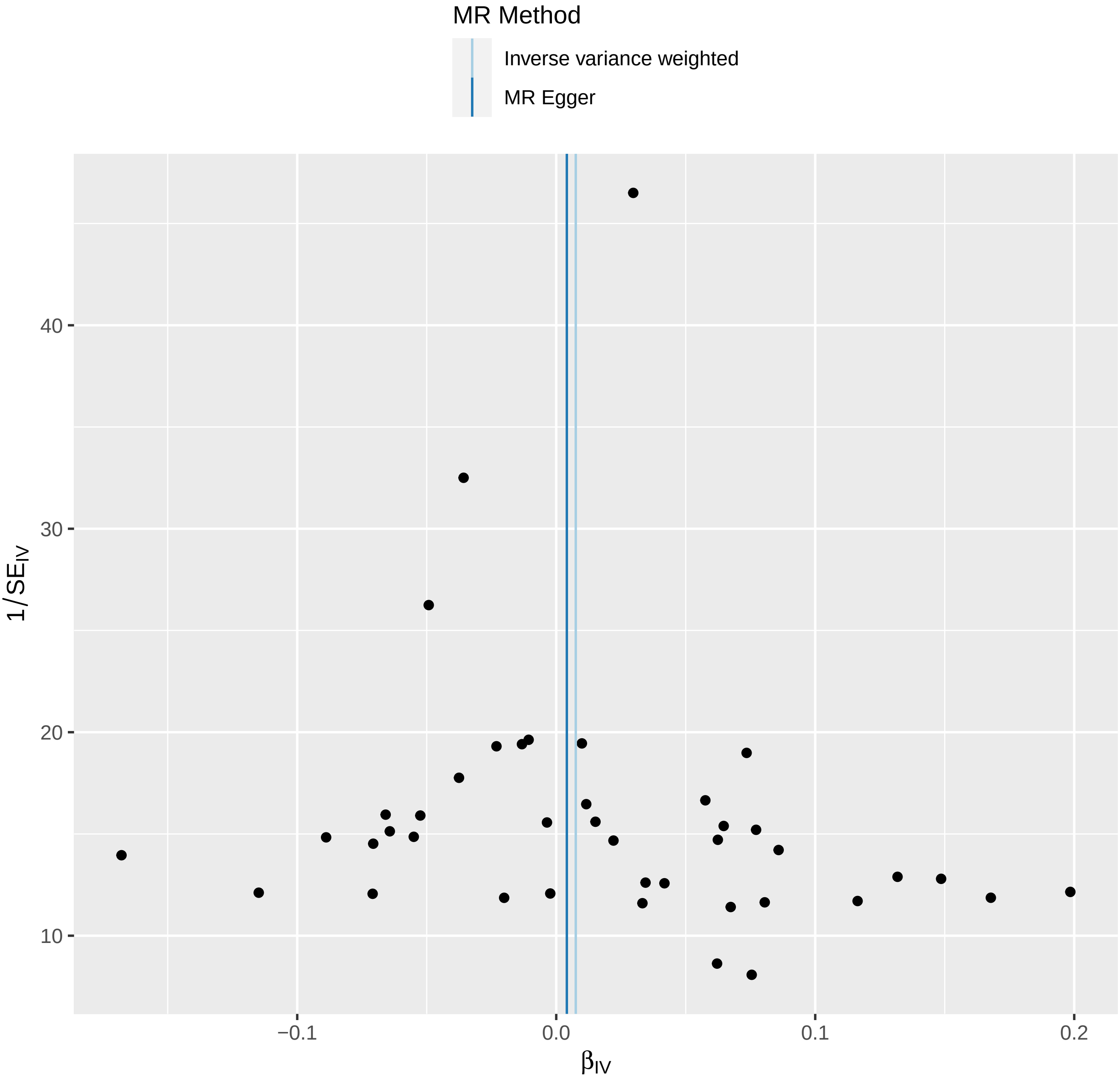

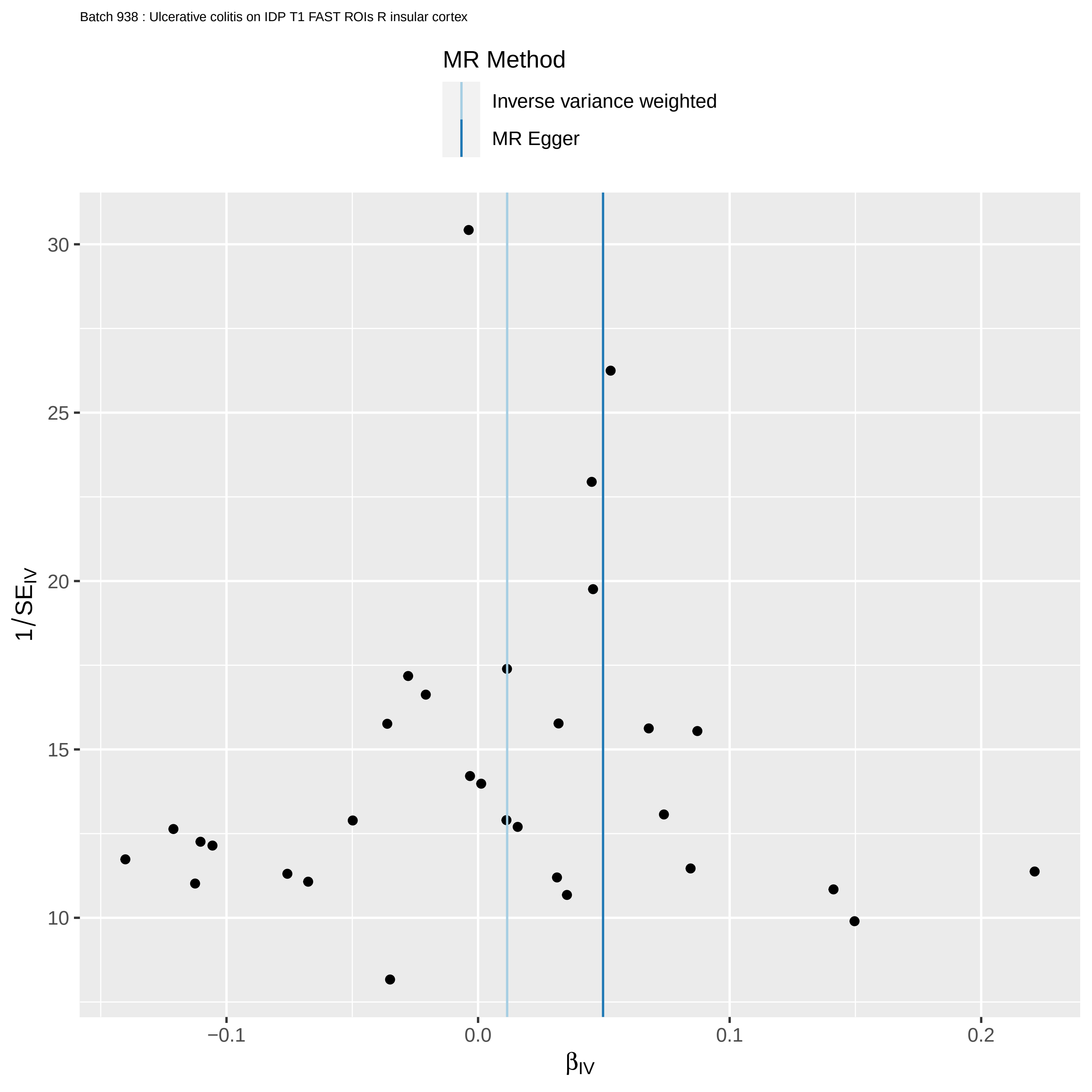
Batch 938 : Ulcerative colitis on IDP T1 FAST ROIs R insular cortex
MR Method
Inverse variance weighted
MR Egger
30
25
20
15
10
1/SE$_{IV}$
−0.1
0.0
0.1
0.2
β$_{IV}$

Batch 1159 : Crohn's disease on IDP T1 SIENAX CSF normalised volume

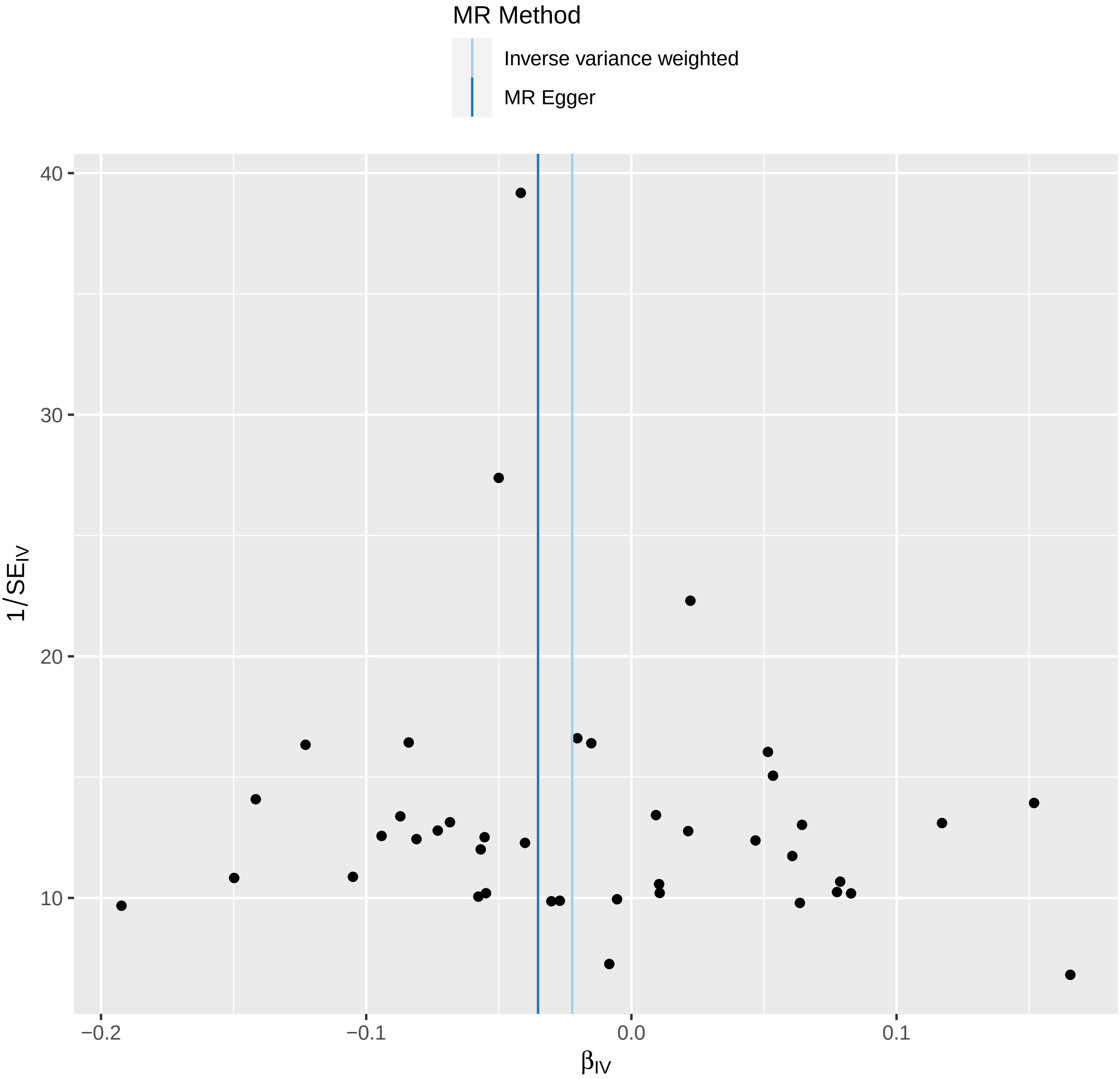

Batch 1160 : Ulcerative colitis on IDP T1 SIENAX CSF normalised volume

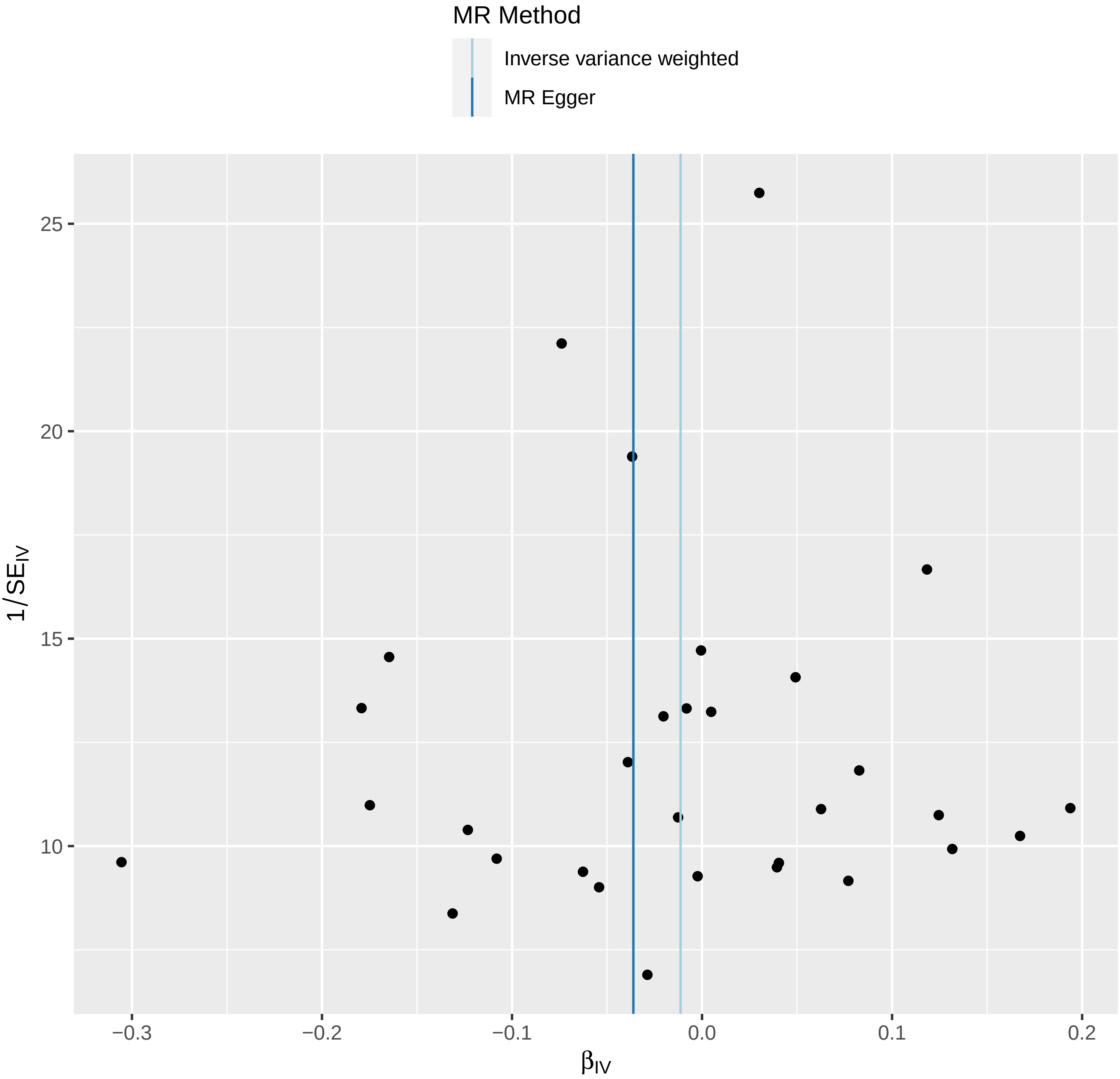

Batch 1161 : Crohn's disease on IDP T1 FAST ROIs L sup front gyrus

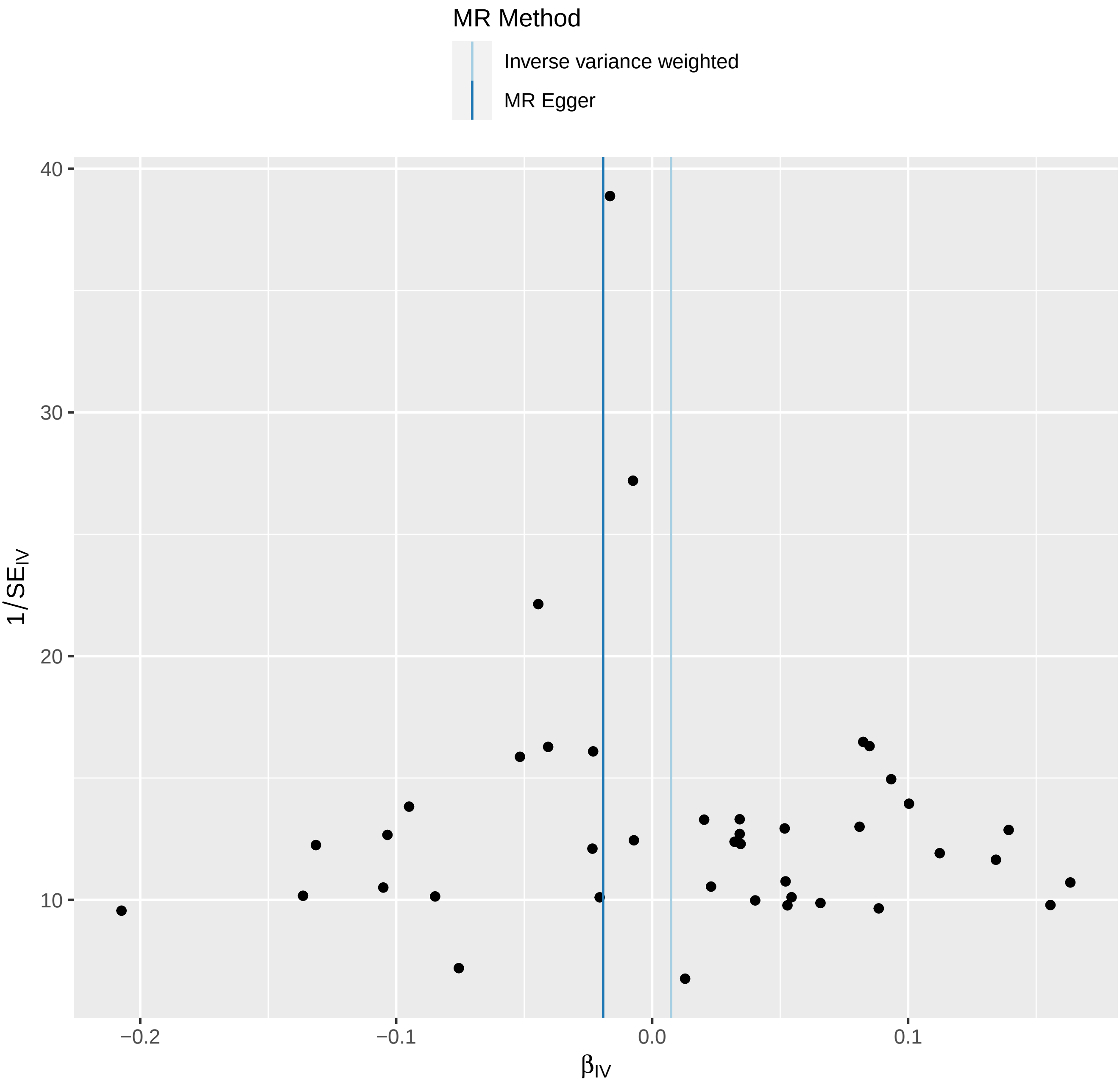

Batch 1162 : Ulcerative colitis on IDP T1 FAST ROIs L sup front gyrus

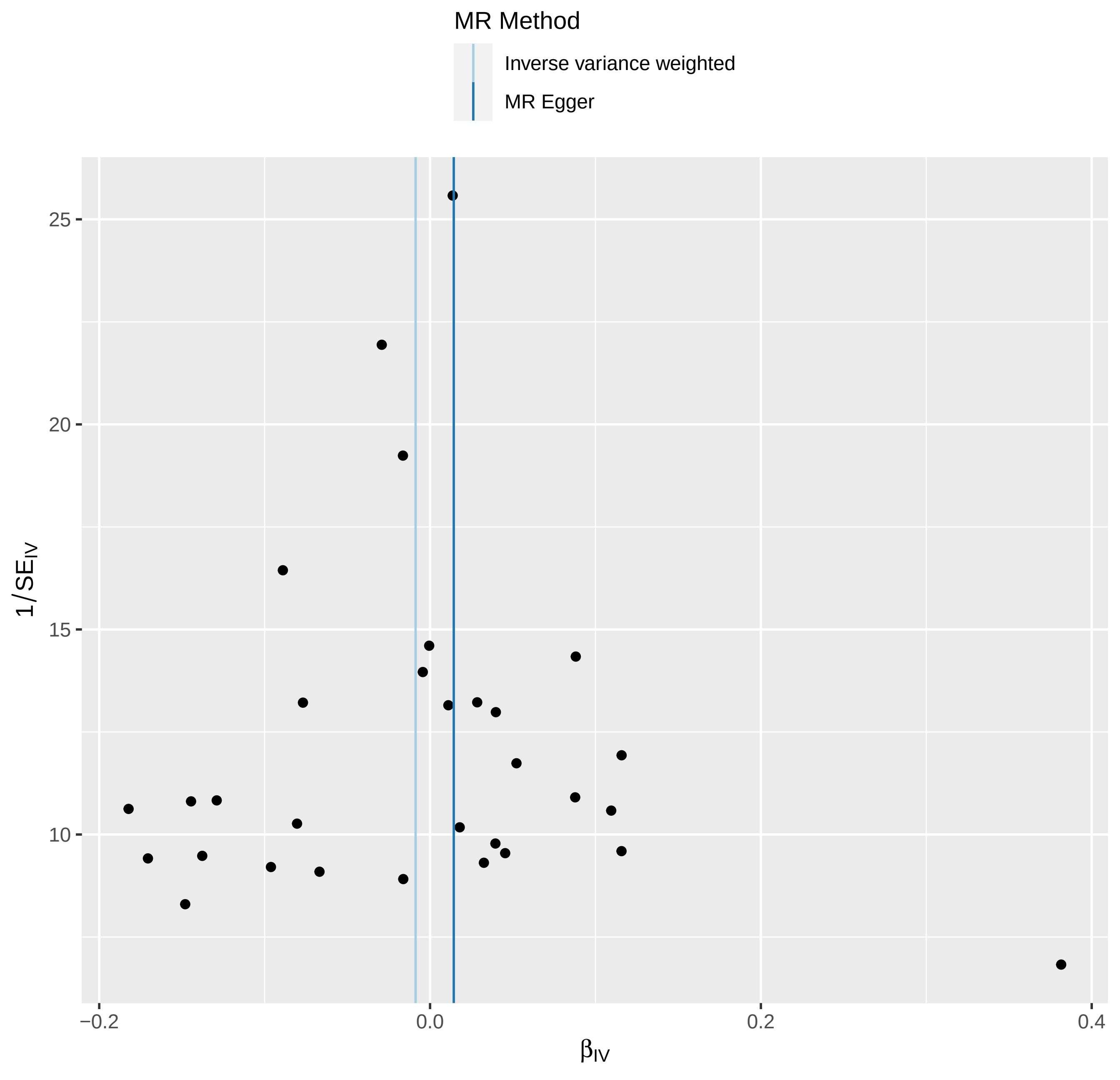

Batch 1383 : Crohn's disease on IDP T1 FAST ROIs R sup front gyrus

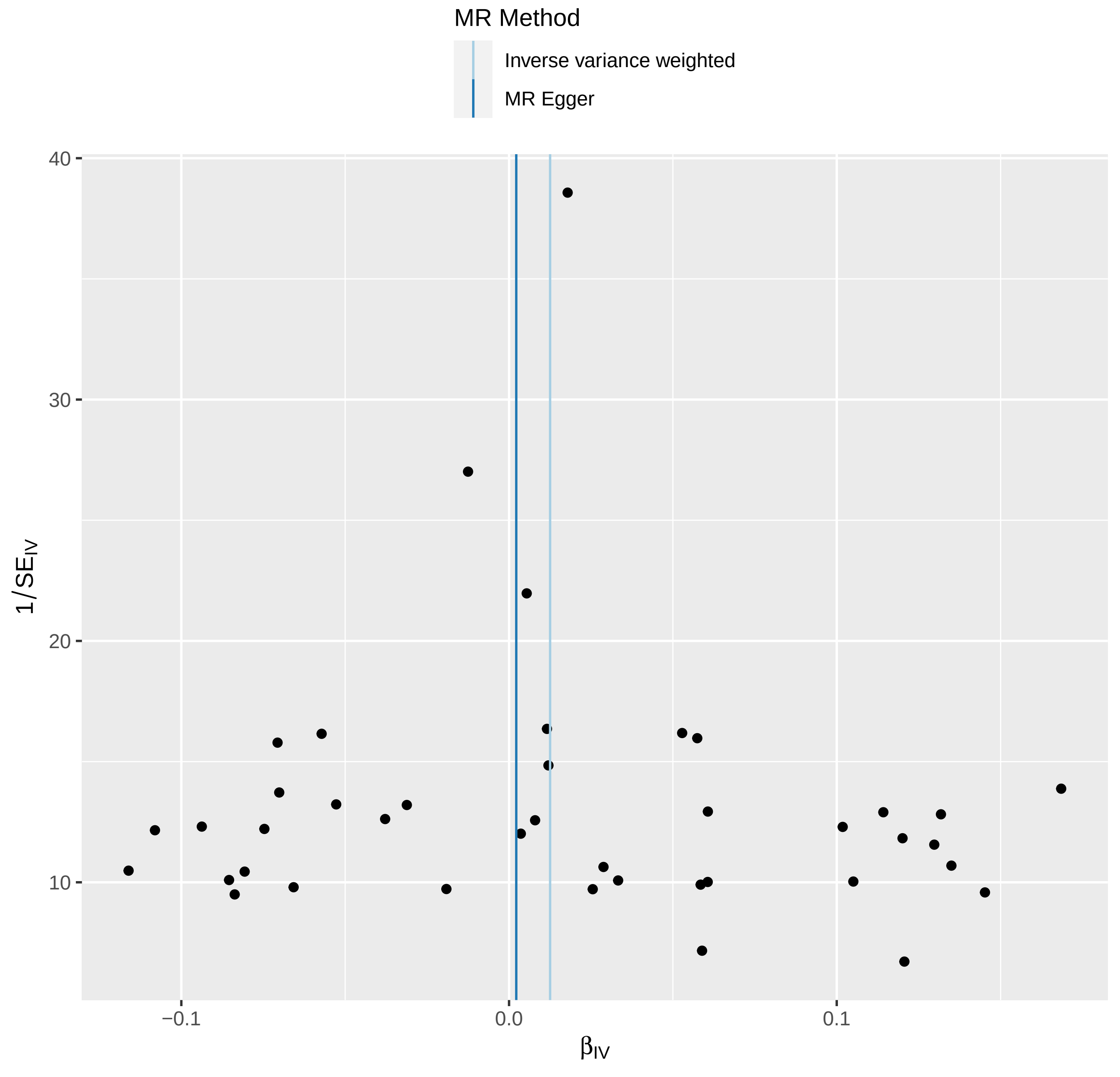

Batch 1384 : Ulcerative colitis on IDP T1 FAST ROIs R sup front gyrus

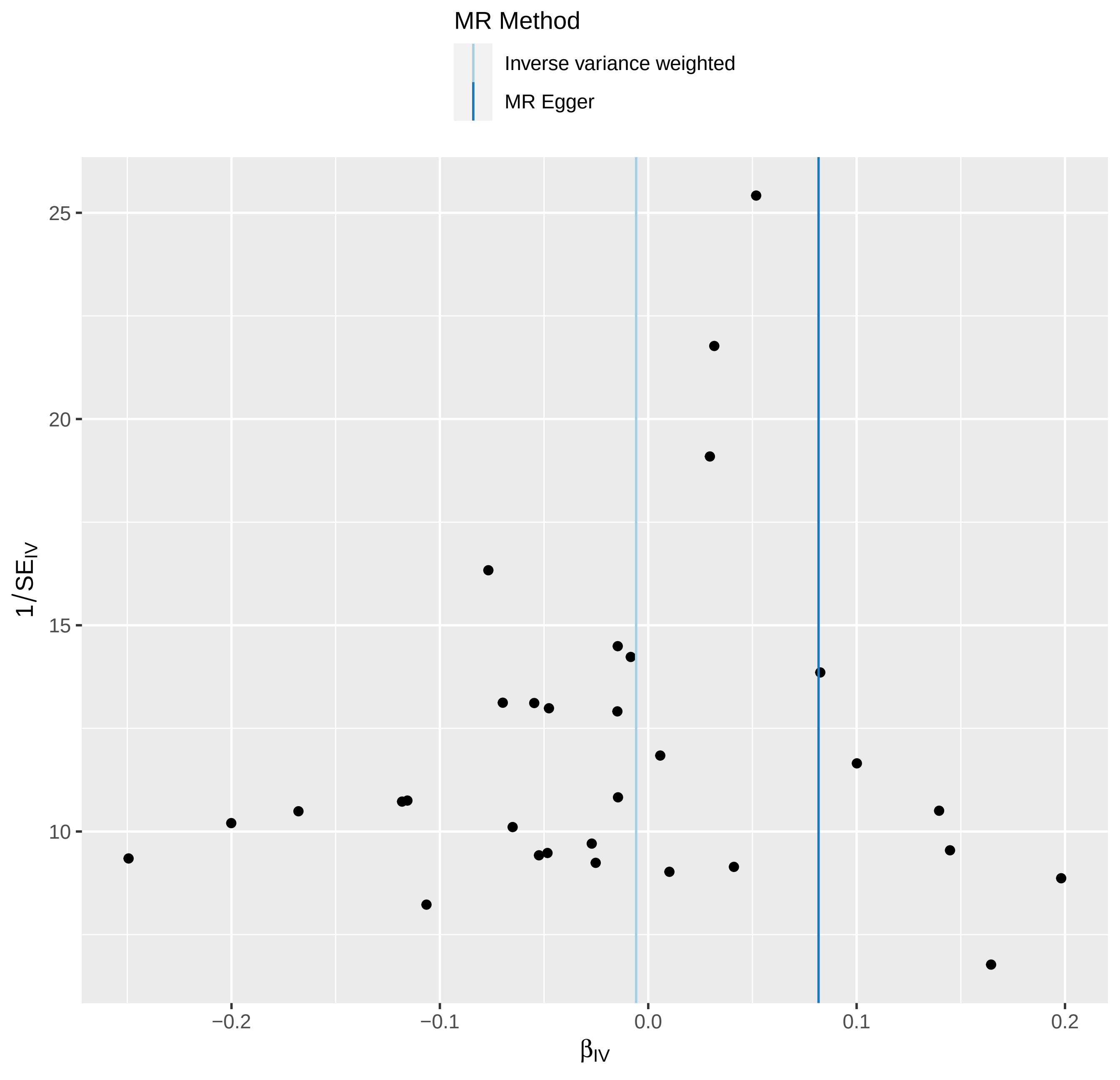

Batch 1483 : Crohn's disease on IDP T1 FAST ROIs L mid front gyrus

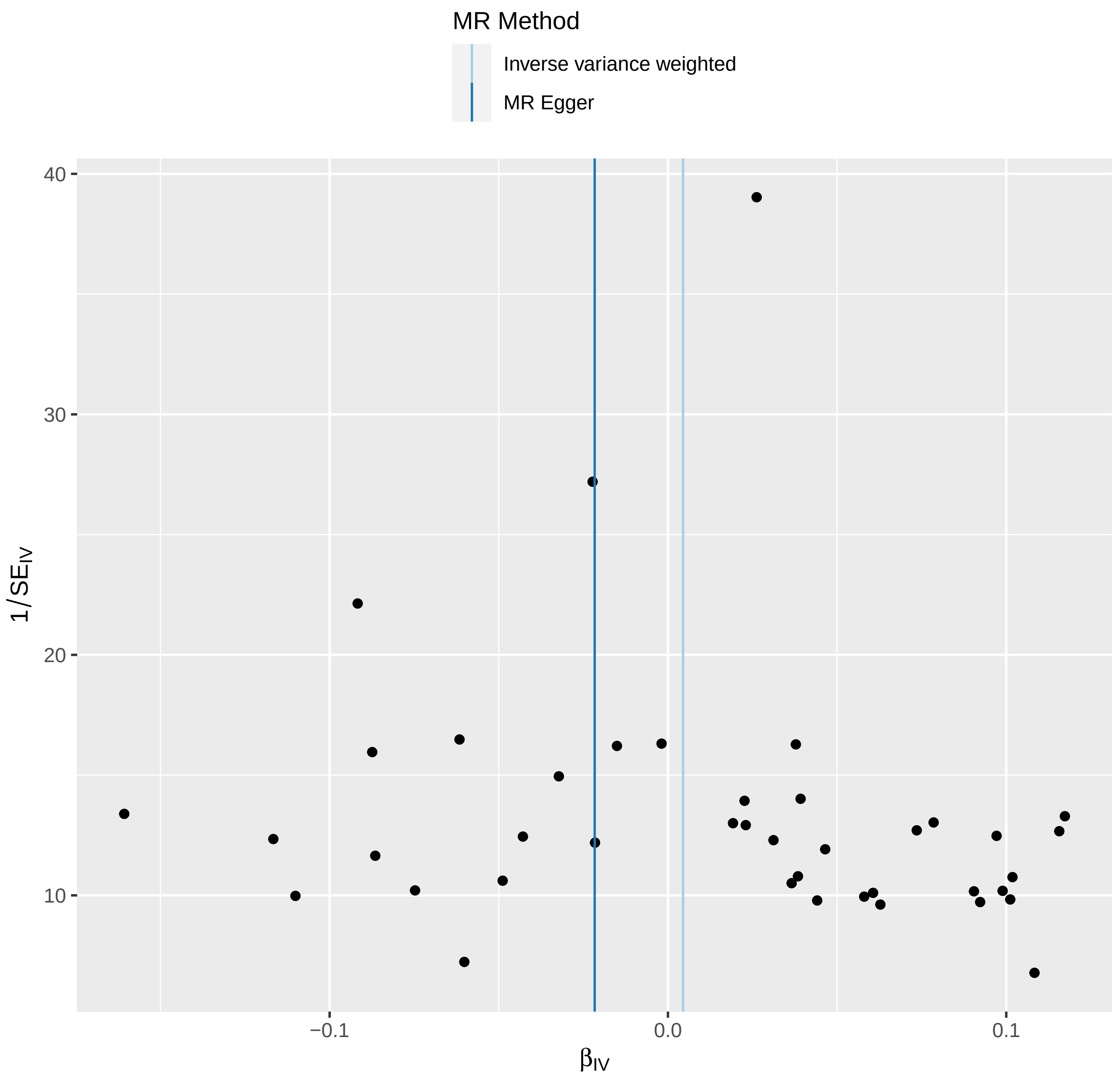

Batch 1484 : Ulcerative colitis on IDP T1 FAST ROIs L mid front gyrus

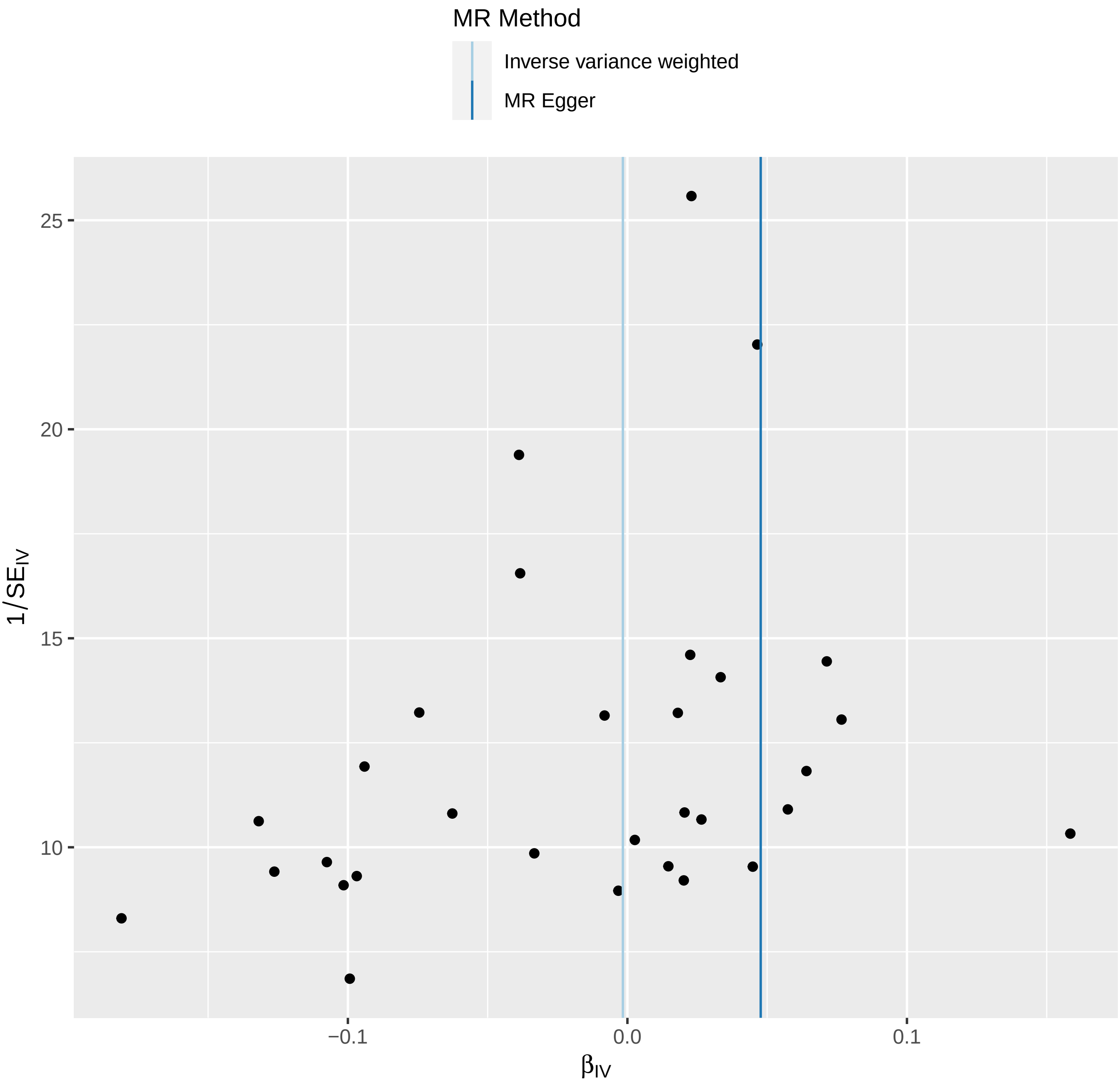

Batch 1505 : Crohn's disease on IDP T1 FAST ROIs R mid front gyrus

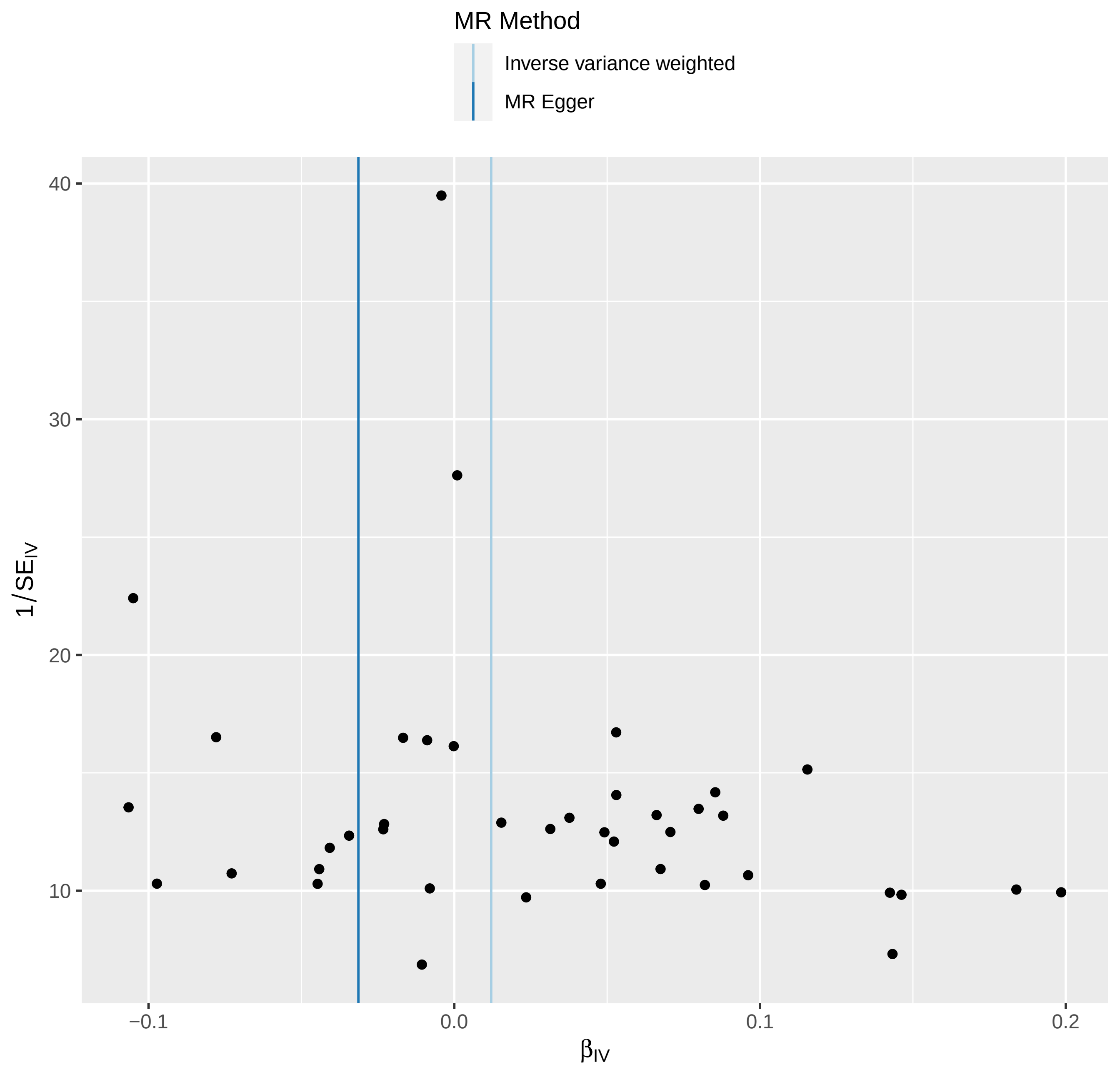

Batch 1506 : Ulcerative colitis on IDP T1 FAST ROIs R mid front gyrus

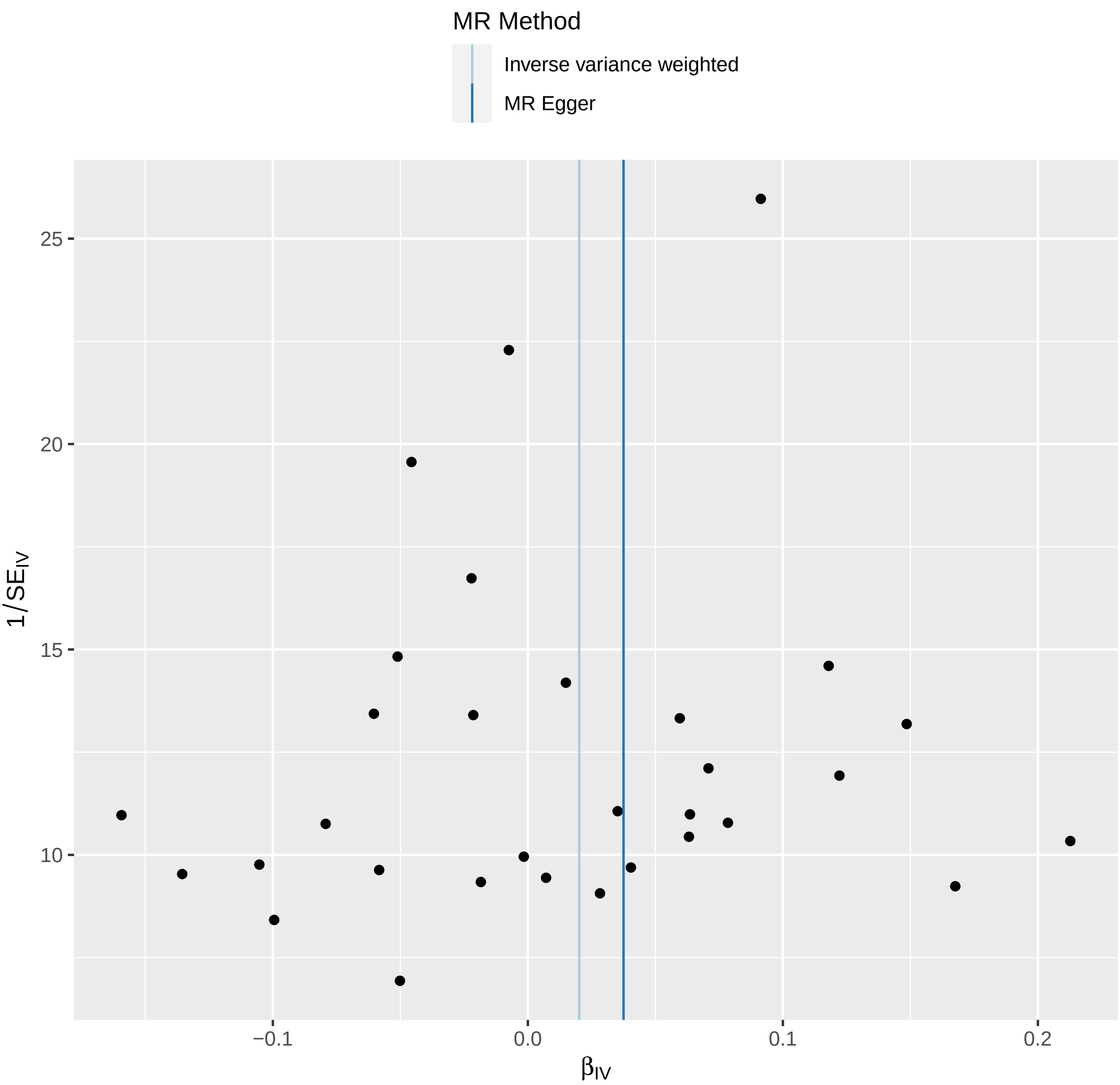

Batch 1527 : Crohn's disease on IDP T1 FAST ROIs L inf front gyrus parstri

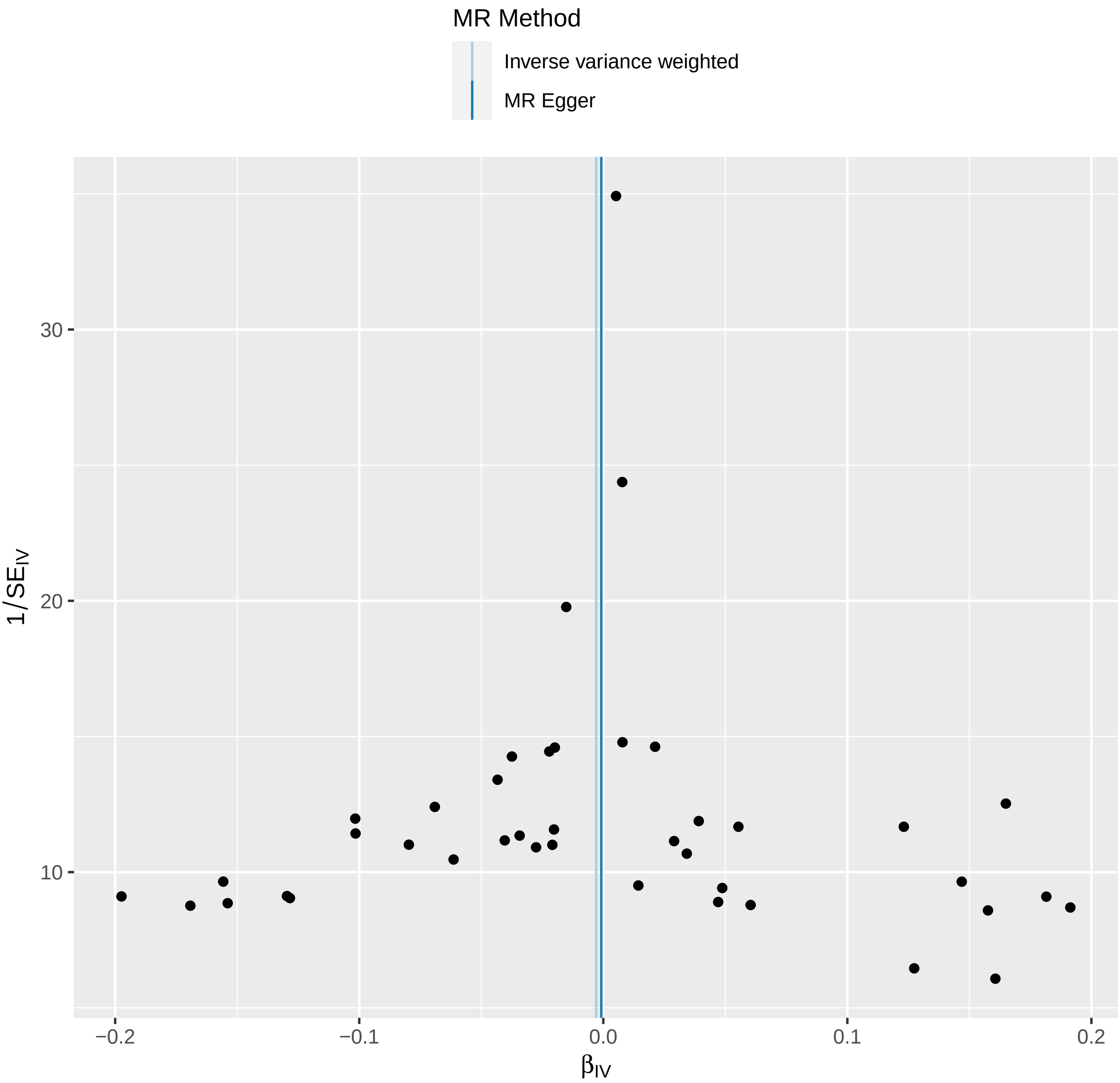

Batch 1528 : Ulcerative colitis on IDP T1 FAST ROIs L inf front gyrus parstri

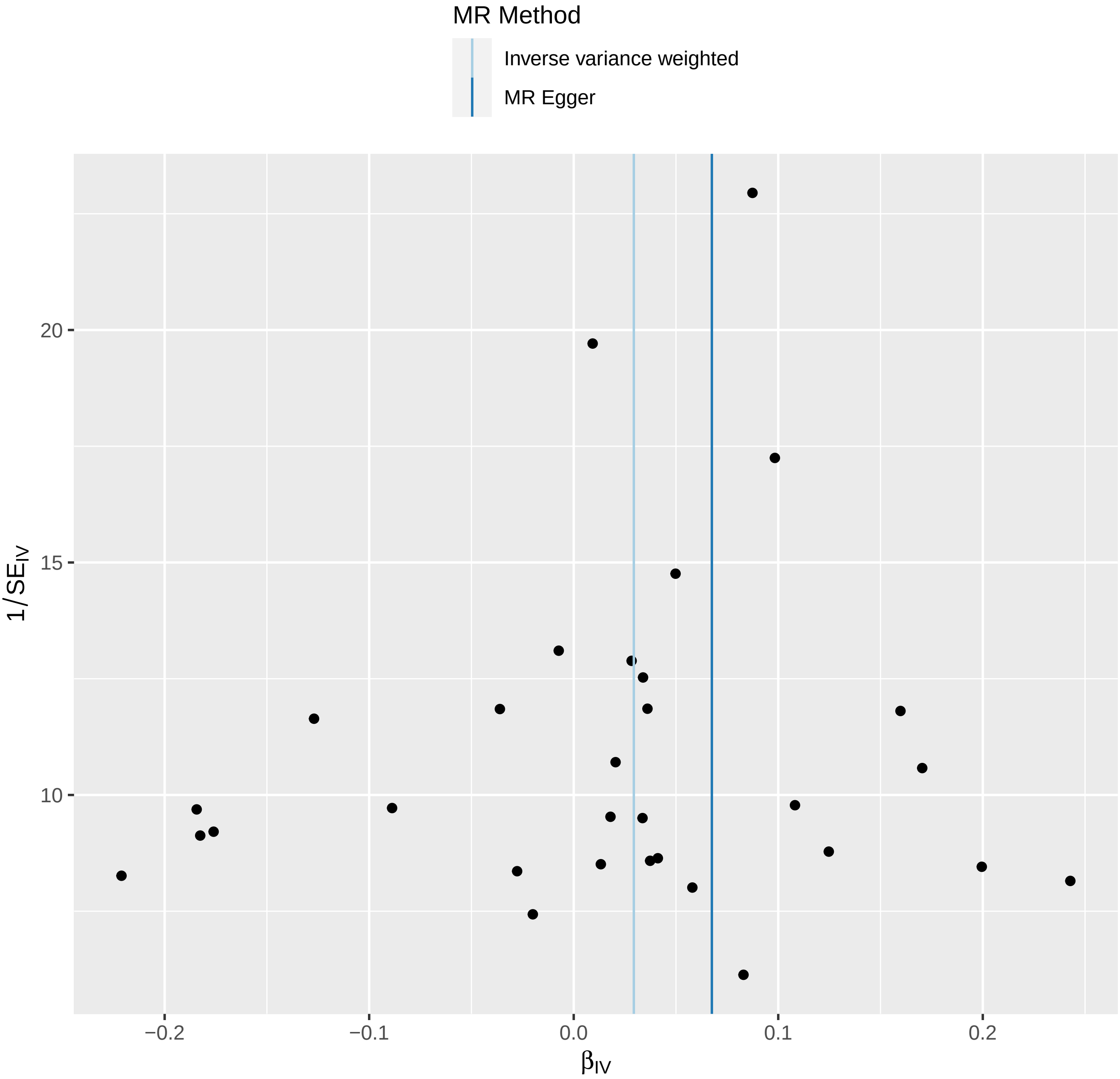

Batch 1549 : Crohn's disease on IDP T1 FAST ROIs R inf front gyrus parstri

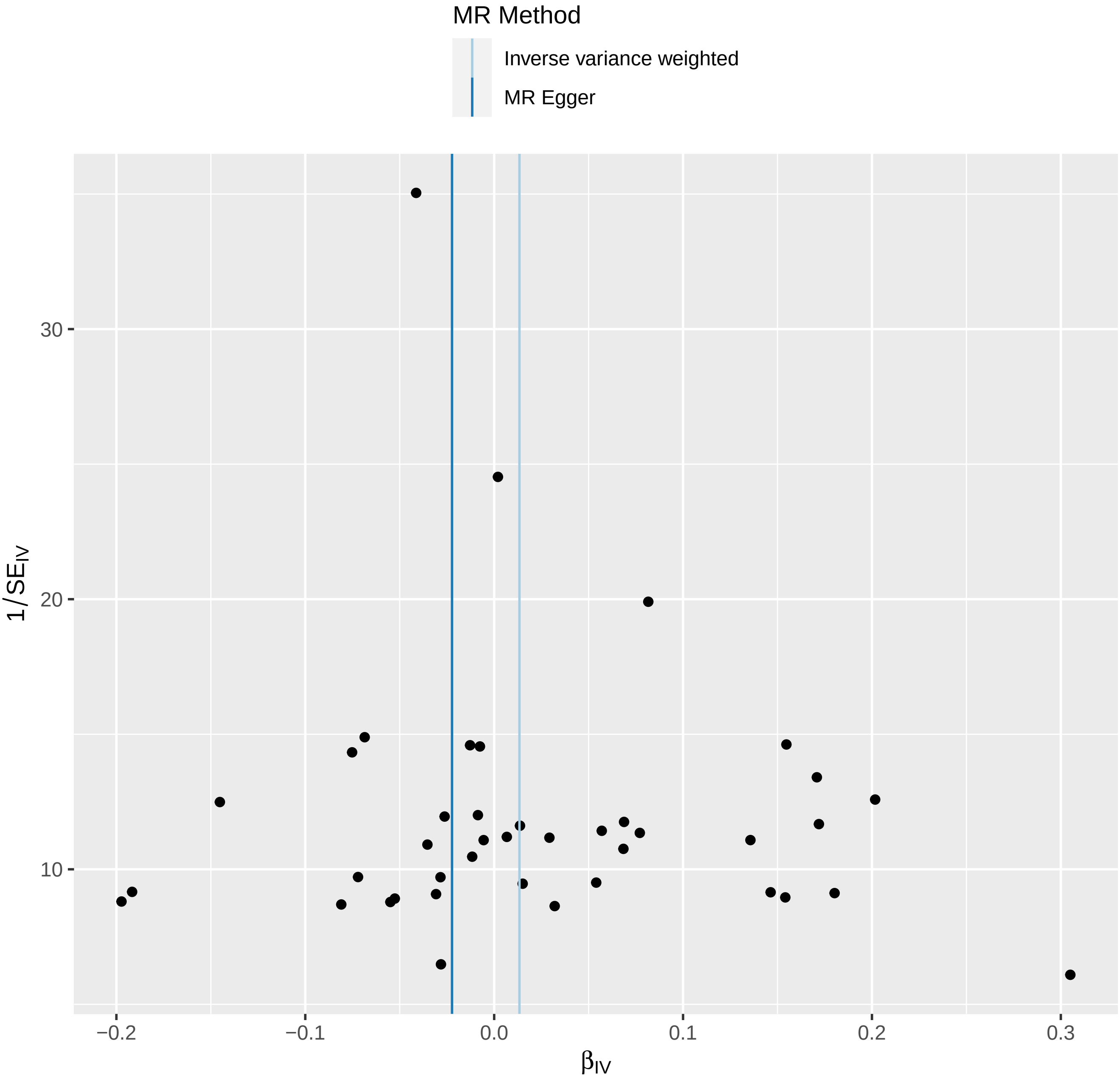

Batch 1550 : Ulcerative colitis on IDP T1 FAST ROIs R inf front gyrus parstri

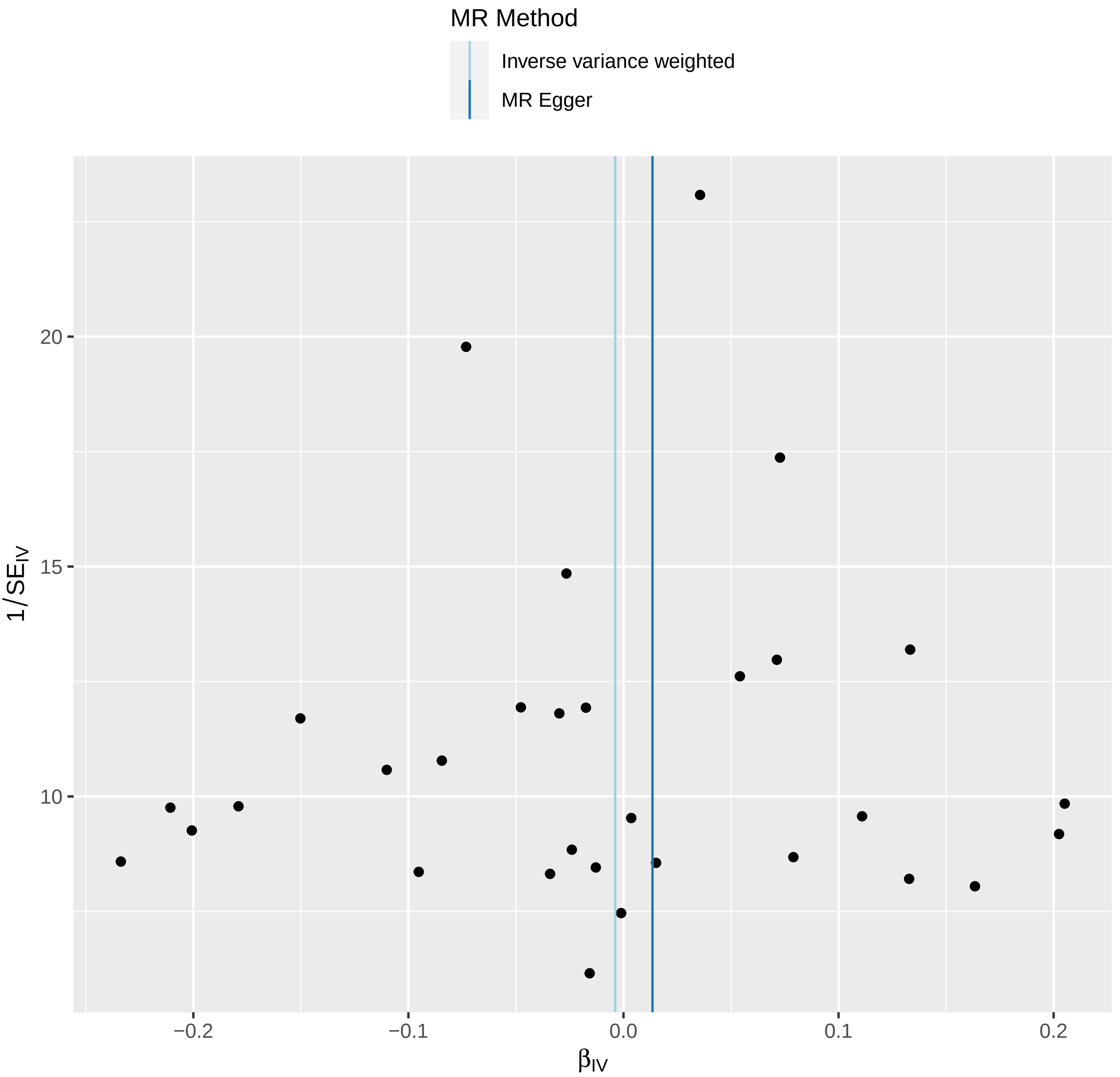

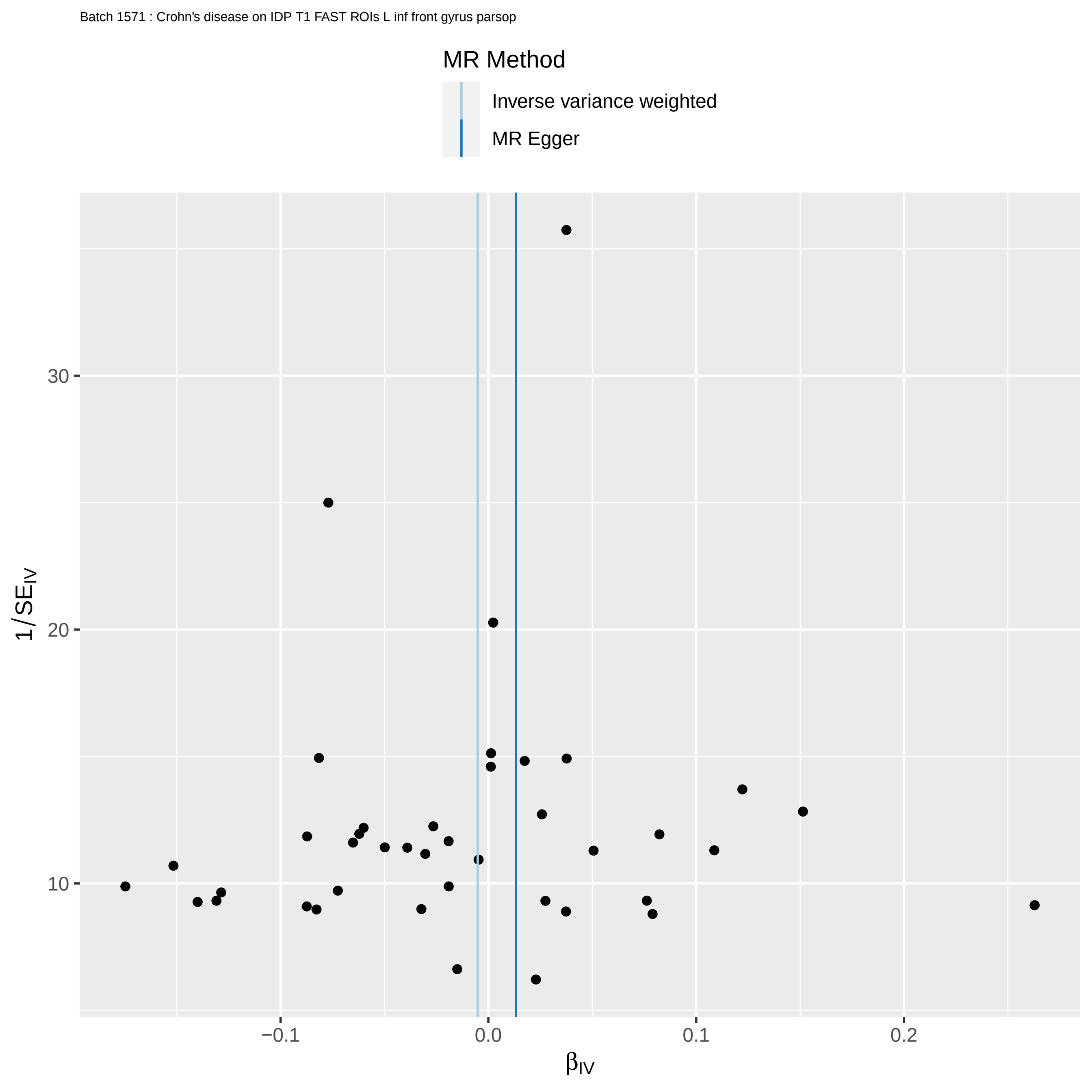
Batch 1571 : Crohn's disease on IDP T1 FAST ROIs L inf front gyrus parsop
MR Method
Inverse variance weighted
MR Egger
30
20
10
1/SE_IV
−0.1
0.0
0.1
0.2
β_IV

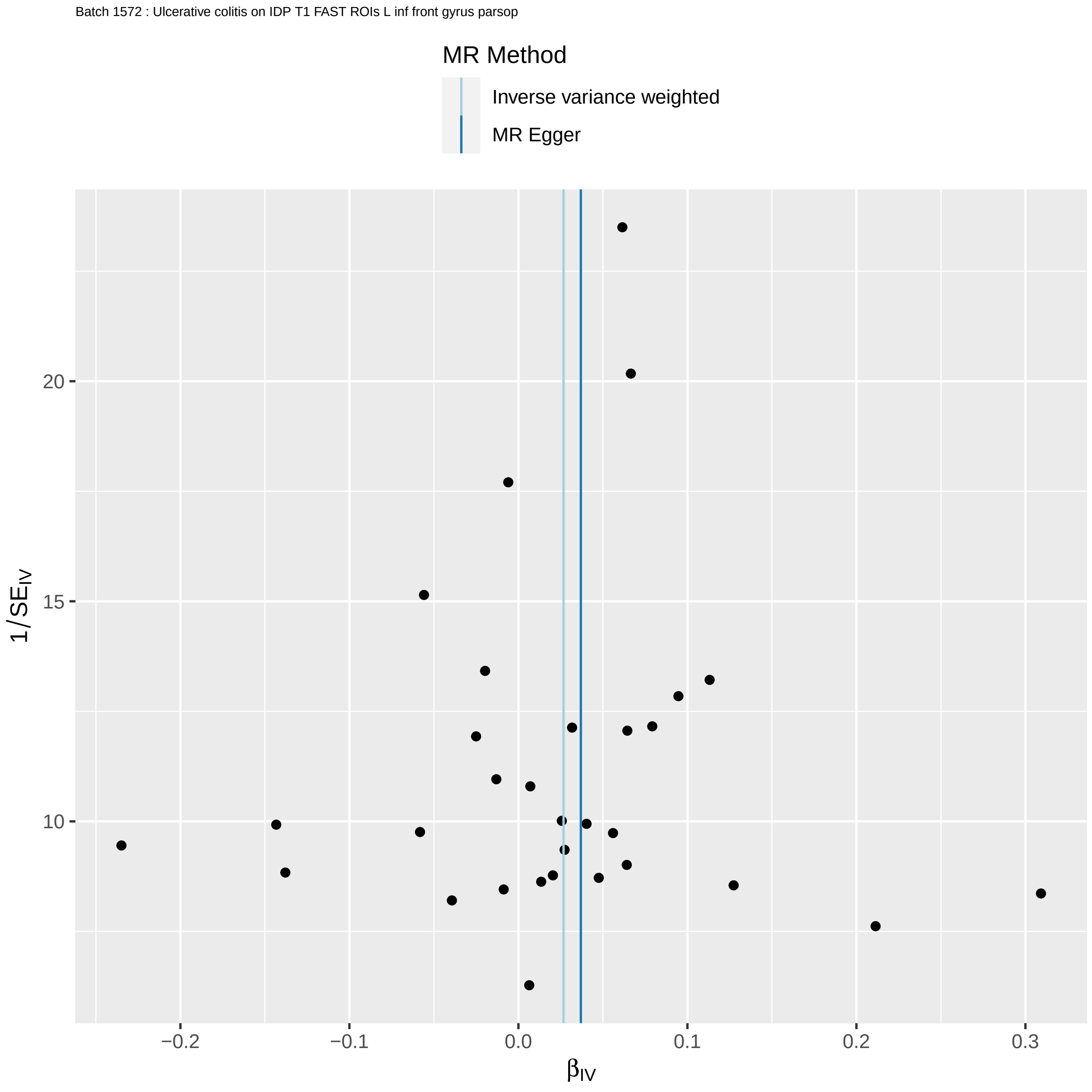

Batch 1572 : Ulcerative colitis on IDP T1 FAST ROIs L inf front gyrus parsop
MR Method
Inverse variance weighted
MR Egger
1/SE_IV
20
15
10
−0.2
−0.1
0.0
0.1
0.2
0.3
β_IV

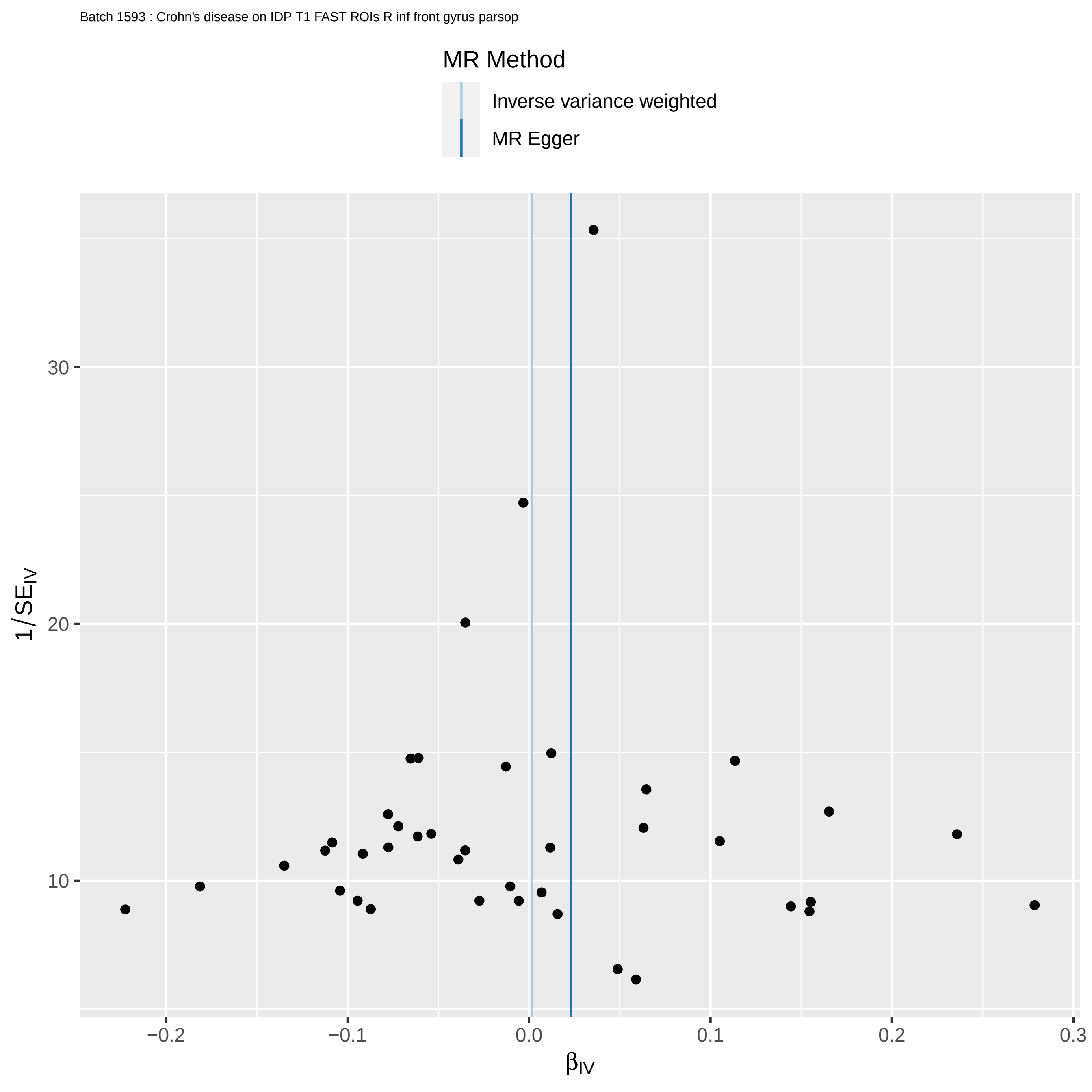

Batch 1593 : Crohn's disease on IDP T1 FAST ROIs R inf front gyrus parsop
MR Method
Inverse variance weighted
MR Egger
30
20
10
$1/SE_{IV}$
−0.2
−0.1
0.0
0.1
0.2
0.3
β_{IV}

Batch 1594 : Ulcerative colitis on IDP T1 FAST ROIs R inf front gyrus parsop

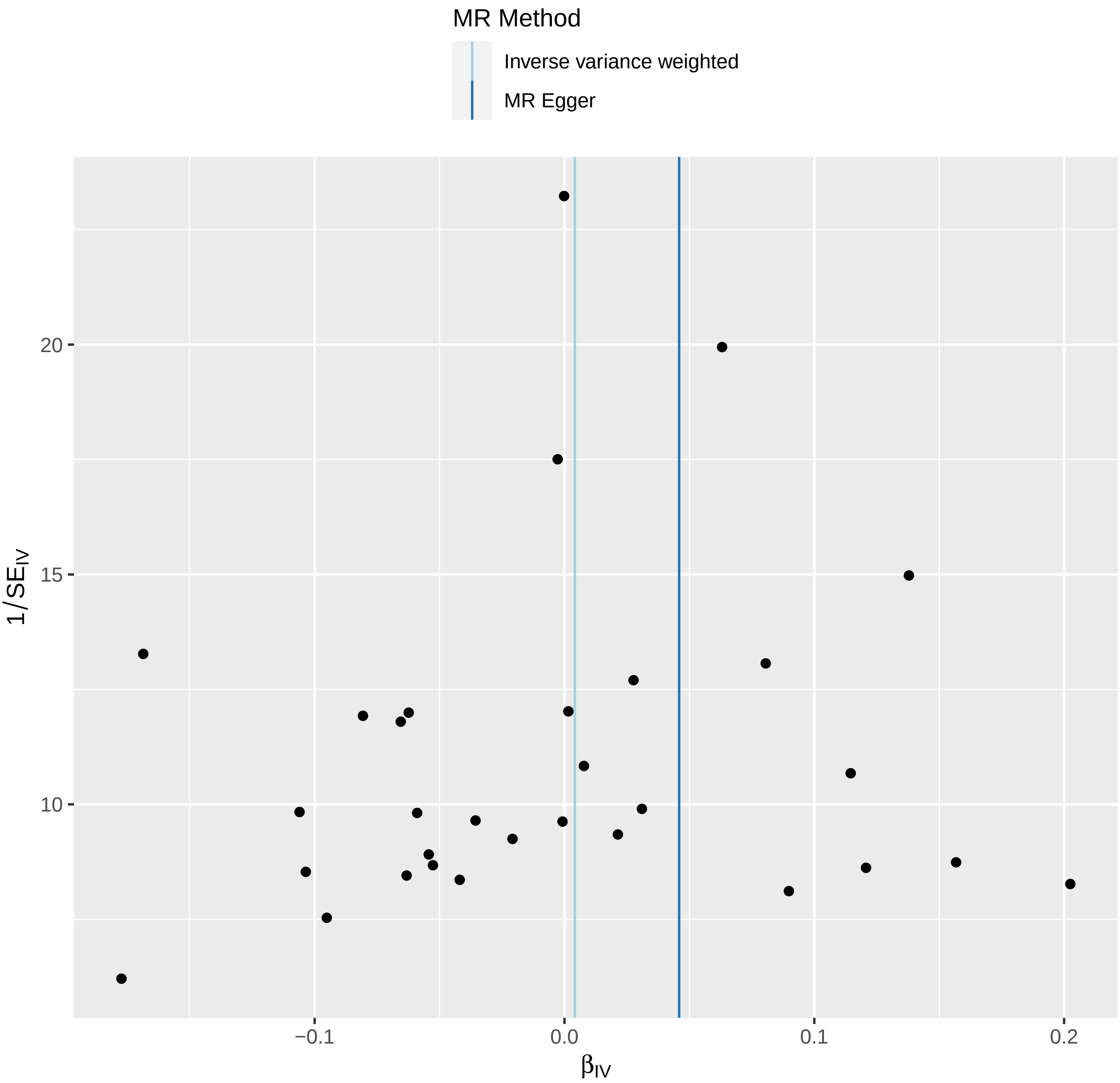

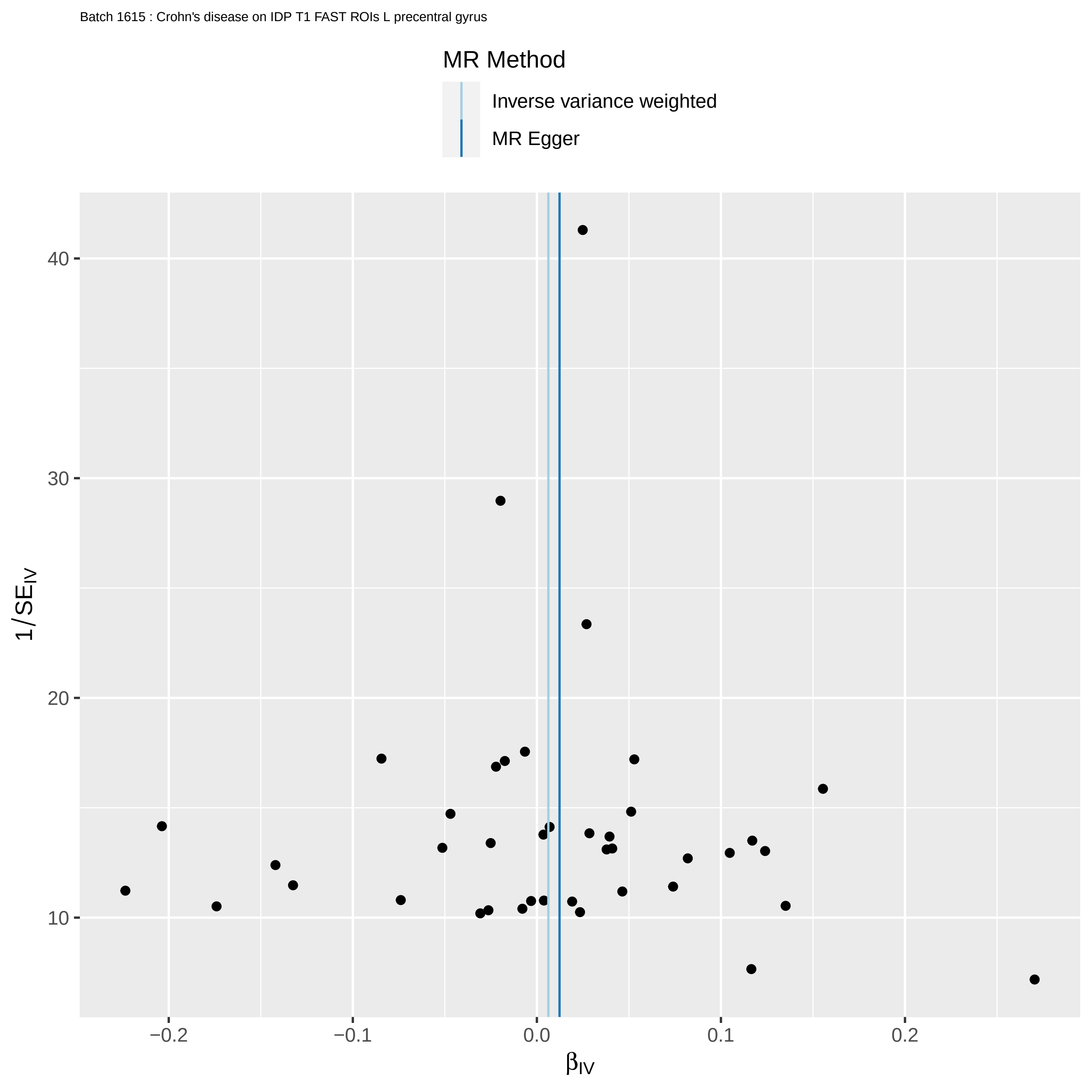

Batch 1615 : Crohn's disease on IDP T1 FAST ROIs L precentral gyrus
MR Method
Inverse variance weighted
MR Egger
40
30
20
10
1/SE$_{IV}$
−0.2
−0.1
0.0
0.1
0.2
β$_{IV}$

Batch 1616 : Ulcerative colitis on IDP T1 FAST ROIs L precentral gyrus

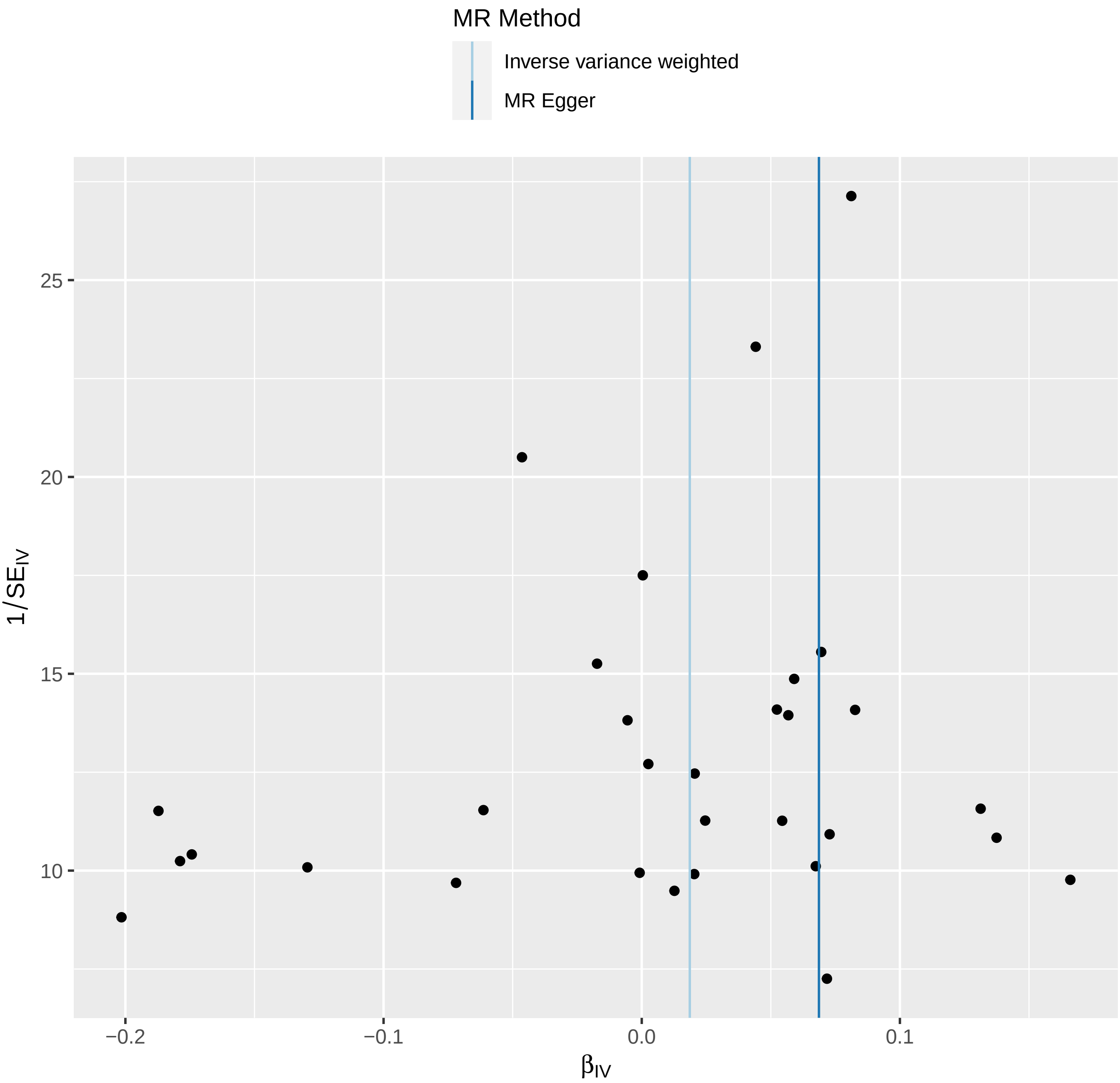

Batch 1637 : Crohn's disease on IDP T1 FAST ROIs R precentral gyrus

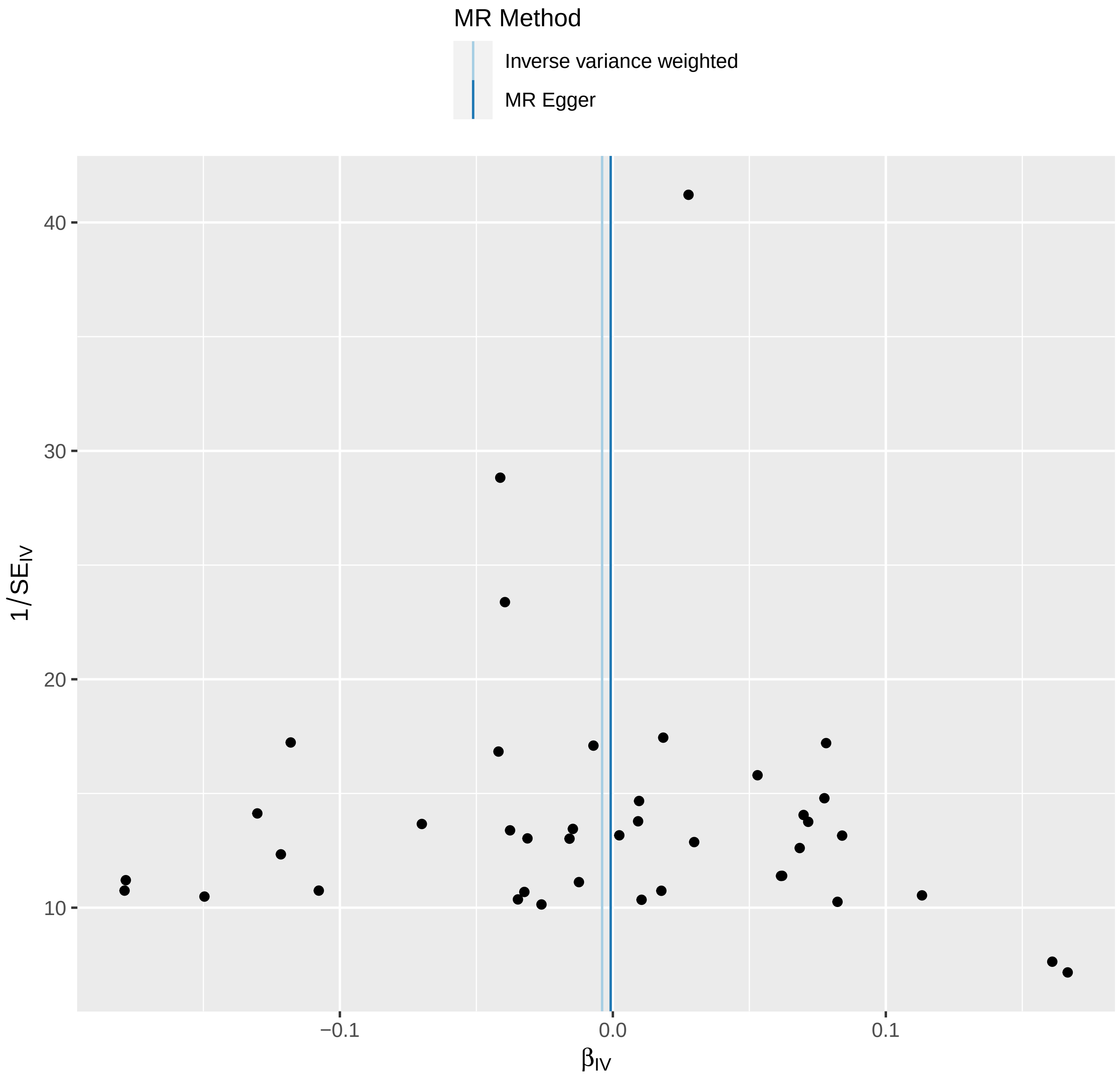

Batch 1638 : Ulcerative colitis on IDP T1 FAST ROIs R precentral gyrus

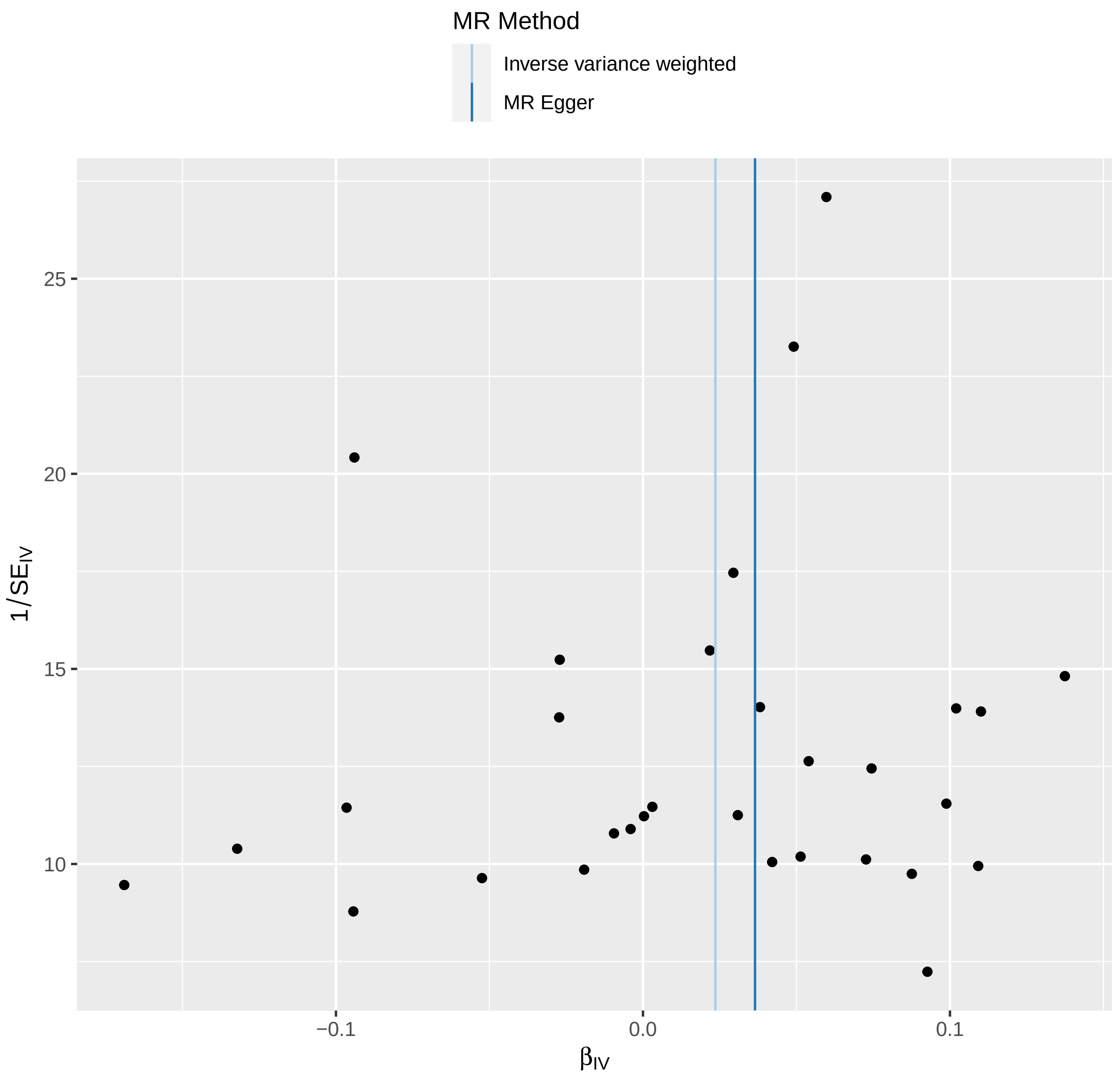

Batch 1659 : Crohn's disease on IDP T1 SIENAX CSF unnormalised volume

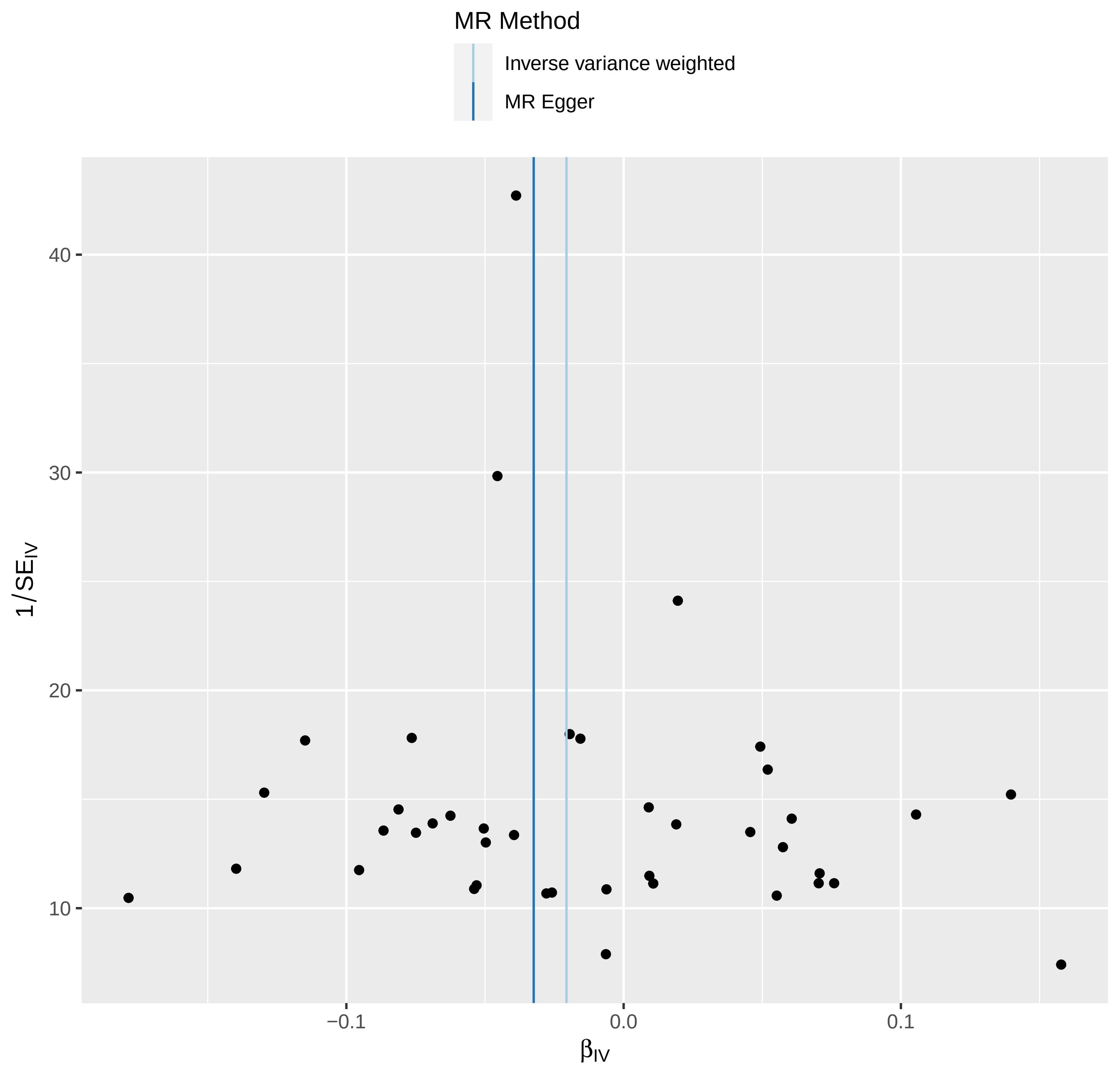

Batch 1660 : Ulcerative colitis on IDP T1 SIENAX CSF unnormalised volume

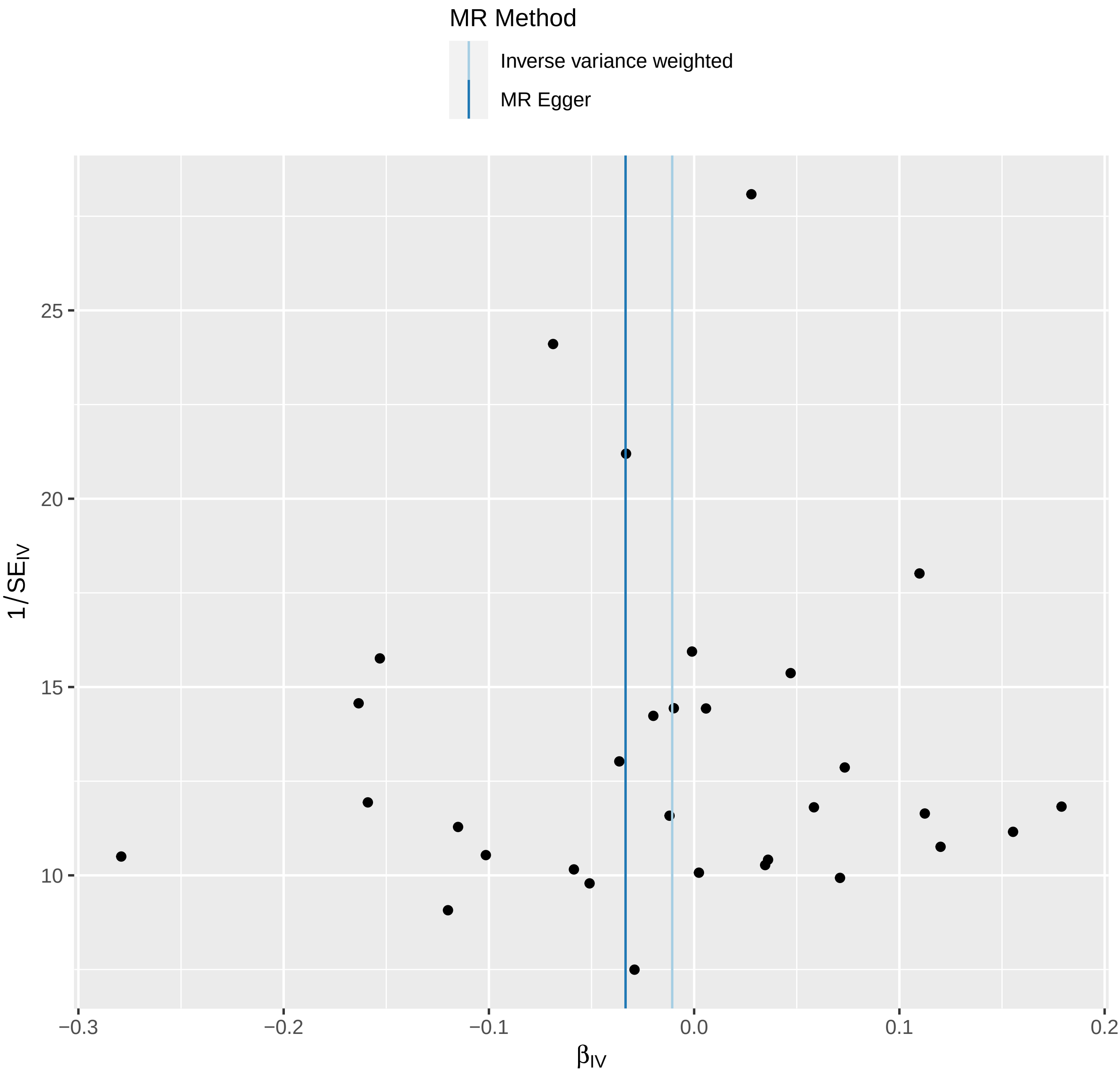

Batch 1661 : Crohn's disease on IDP T1 FAST ROIs L temporal pole

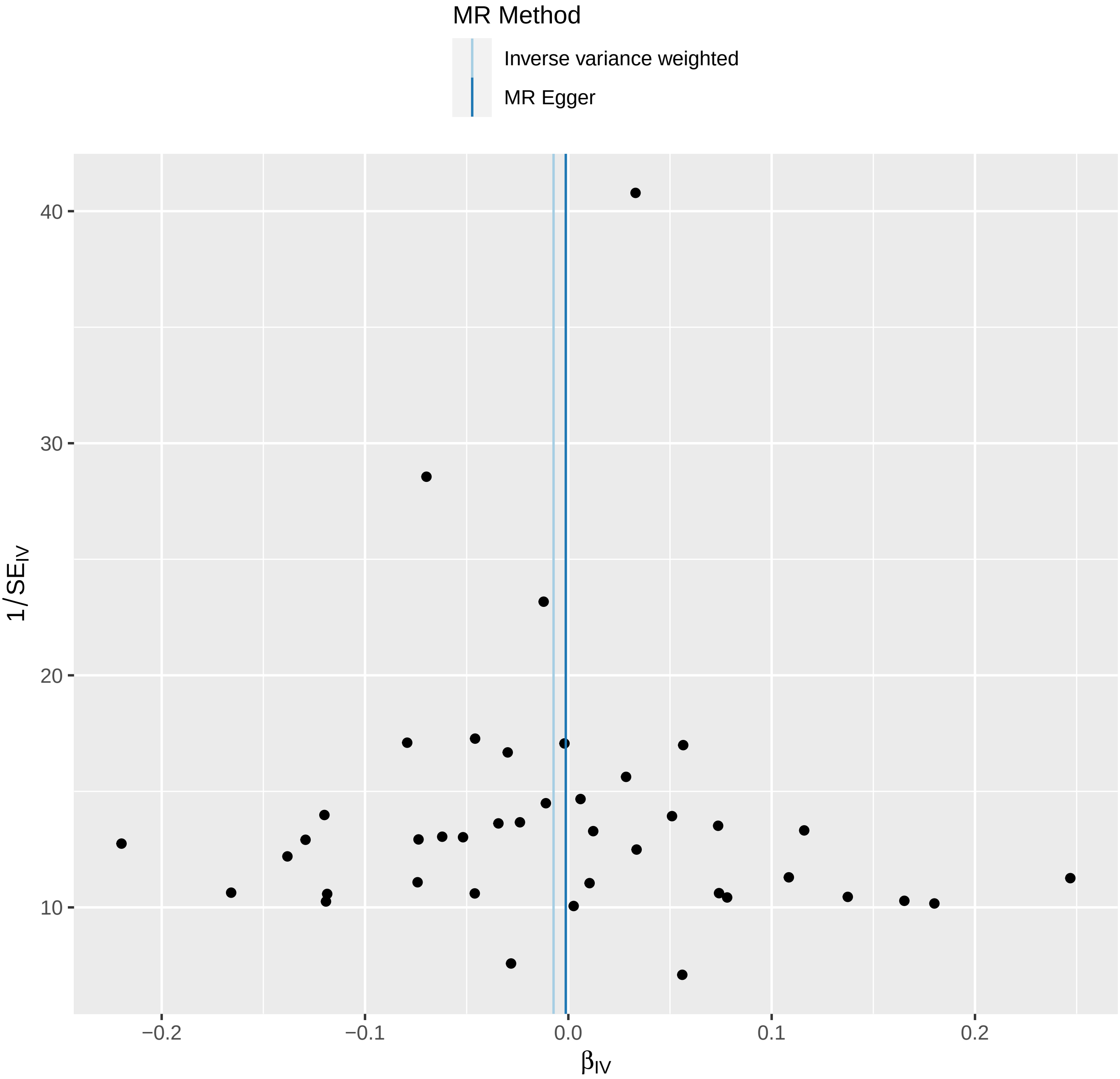

Batch 1662 : Ulcerative colitis on IDP T1 FAST ROIs L temporal pole

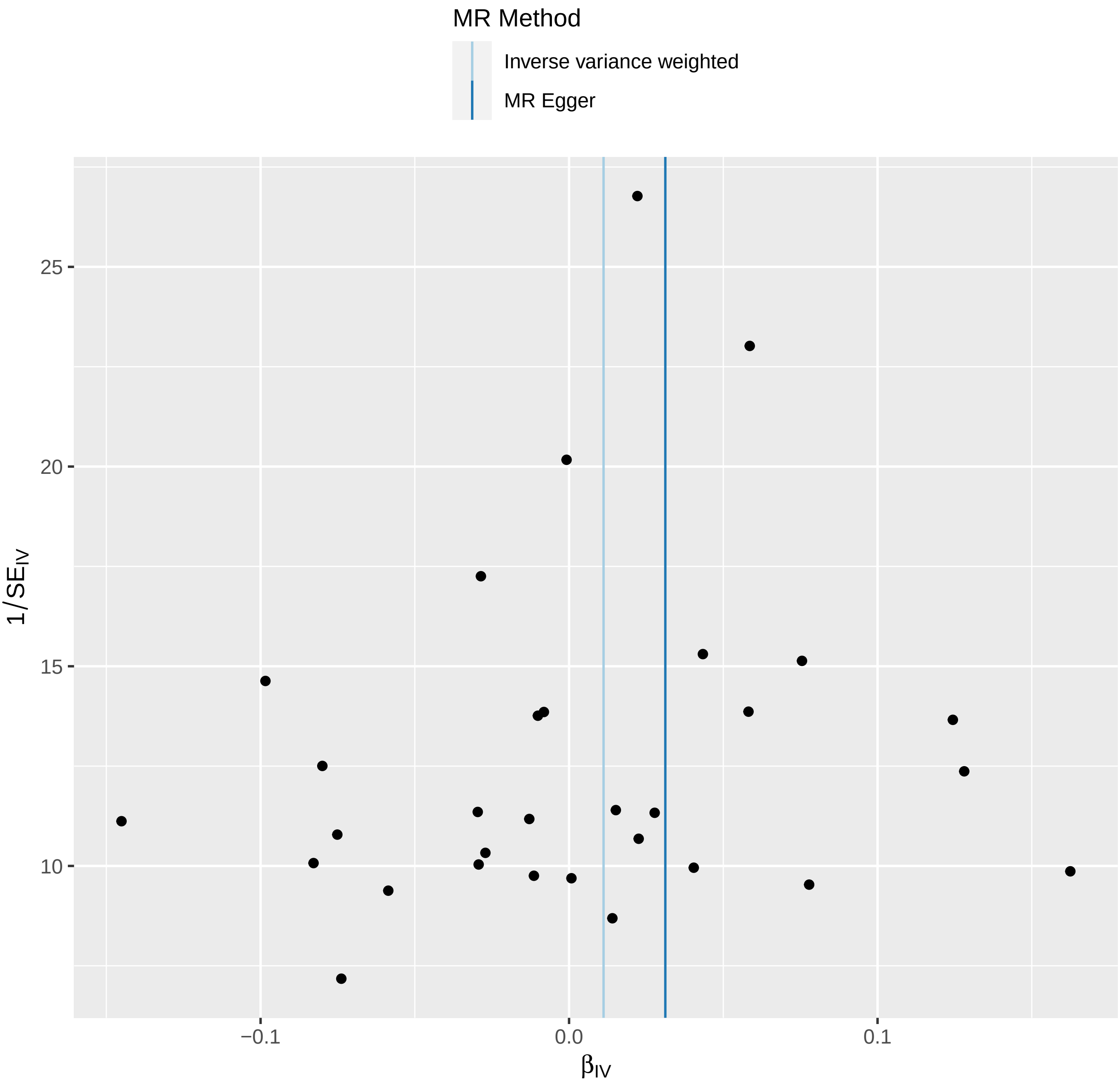

Batch 1683 : Crohn's disease on IDP T1 FAST ROIs R temporal pole

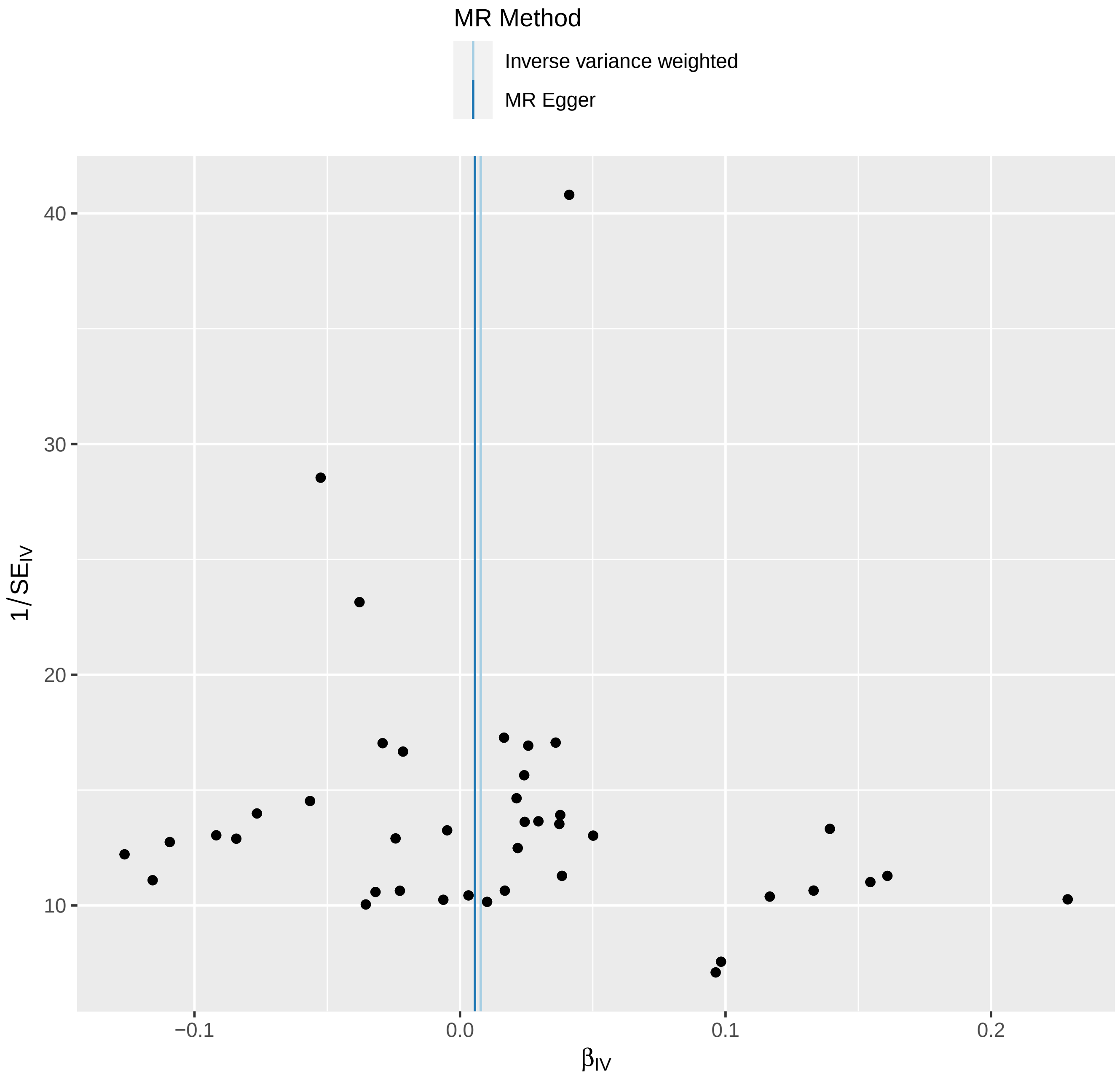

Batch 1684 : Ulcerative colitis on IDP T1 FAST ROIs R temporal pole

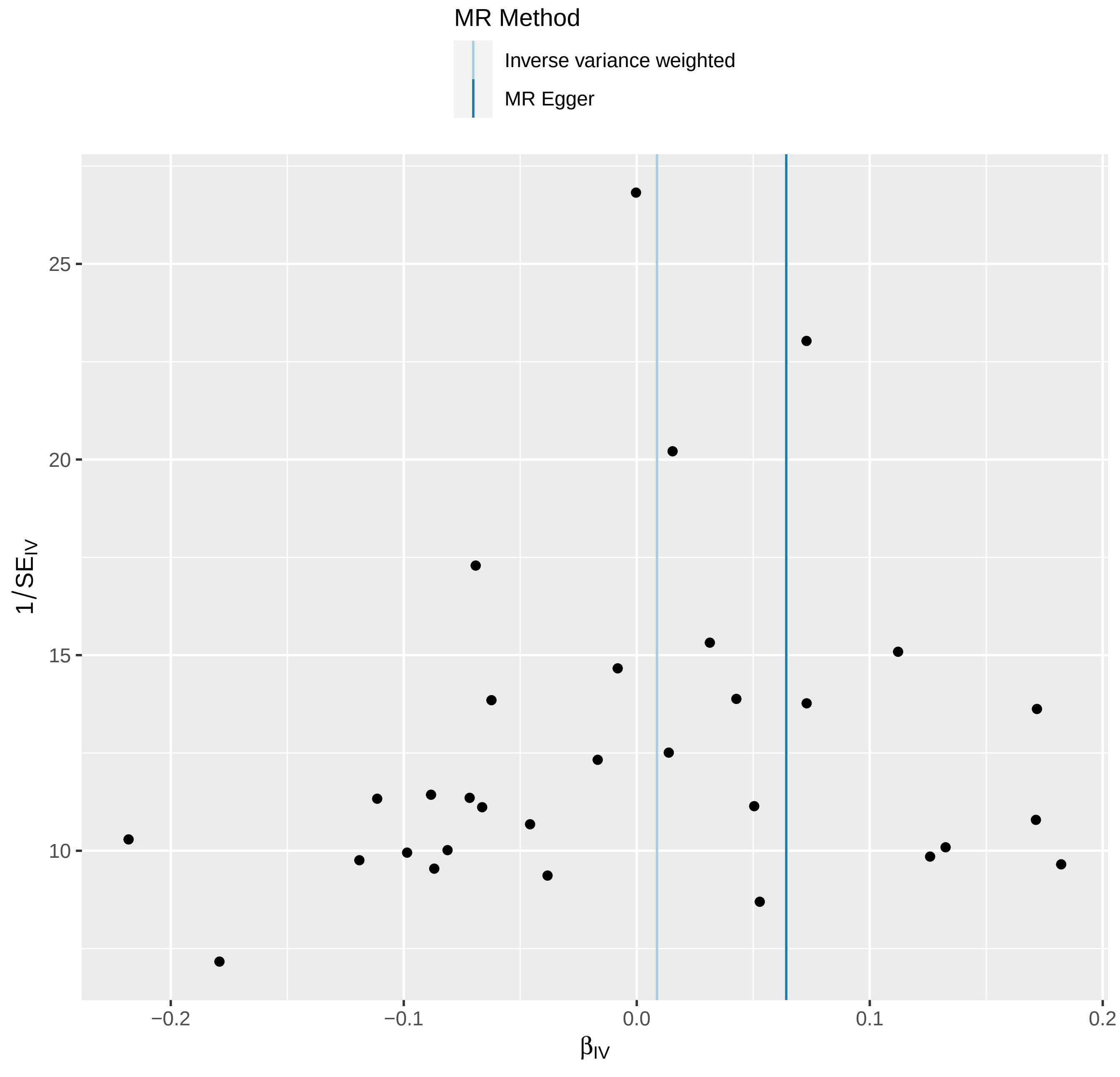

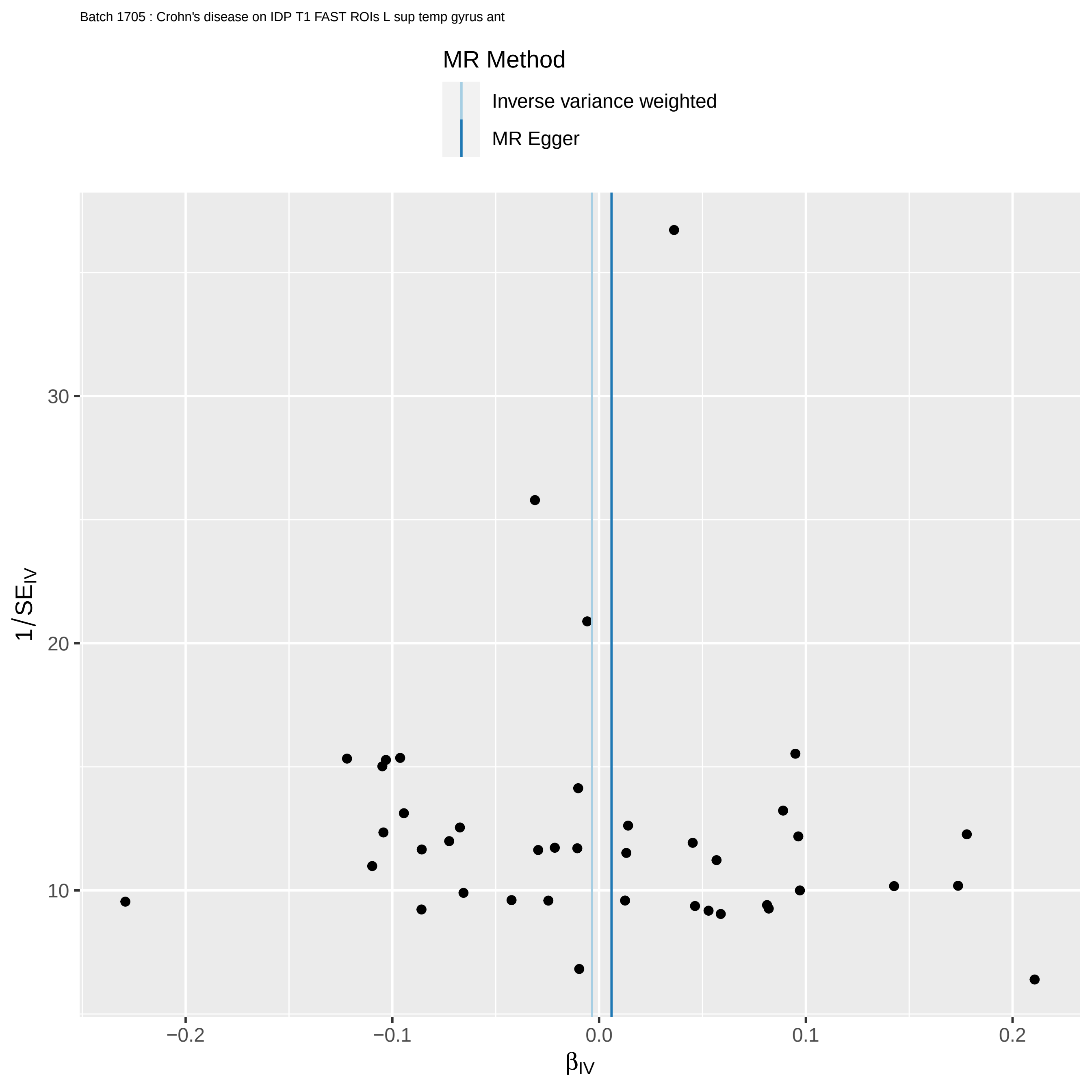

Batch 1705 : Crohn's disease on IDP T1 FAST ROIs L sup temp gyrus ant
MR Method
Inverse variance weighted
MR Egger
30
20
10
1/SE$_{IV}$
−0.2
−0.1
0.0
0.1
0.2
β_{IV}

Batch 1706 : Ulcerative colitis on IDP T1 FAST ROIs L sup temp gyrus ant

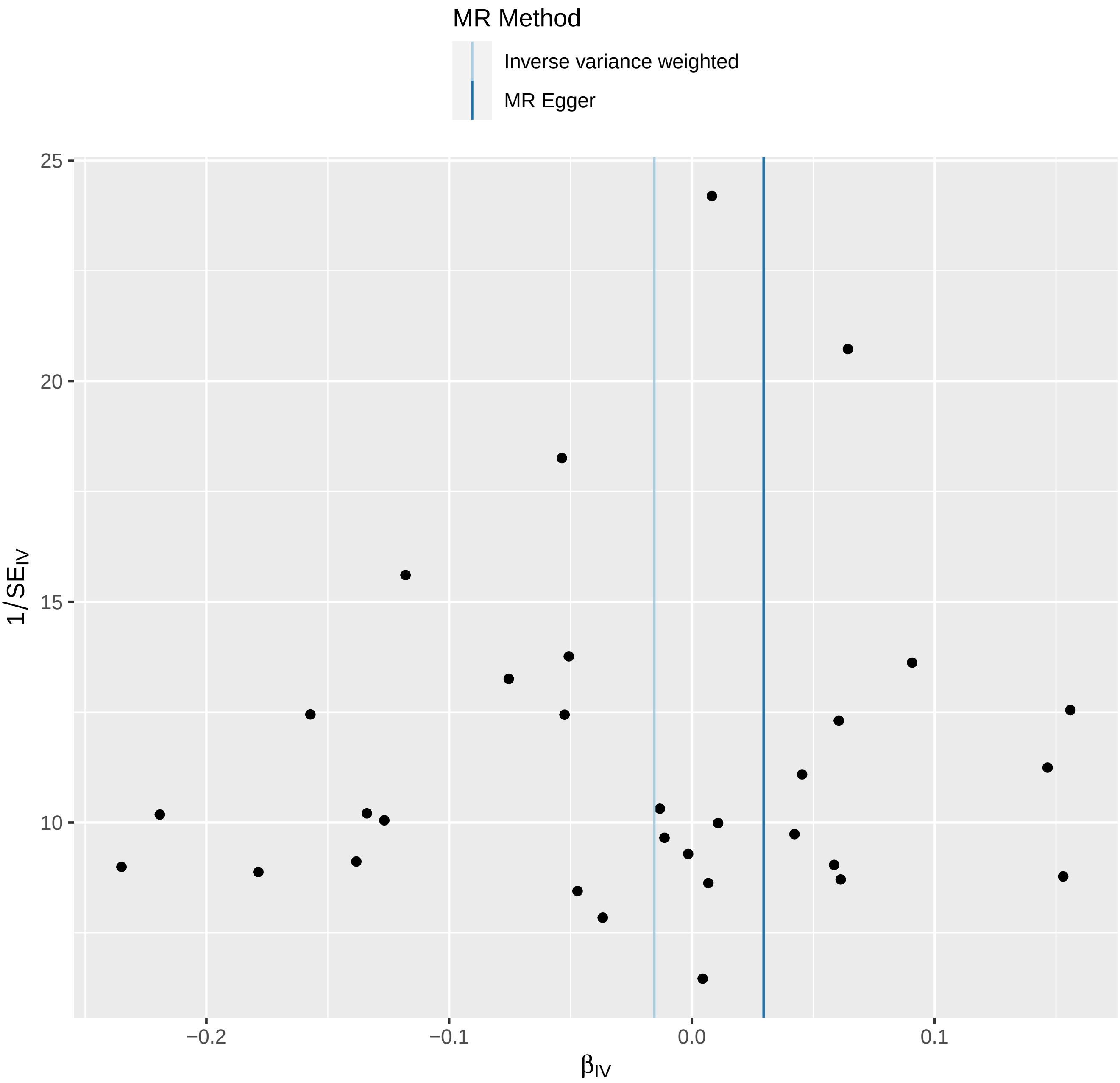

Batch 1727 : Crohn's disease on IDP T1 FAST ROIs R sup temp gyrus ant

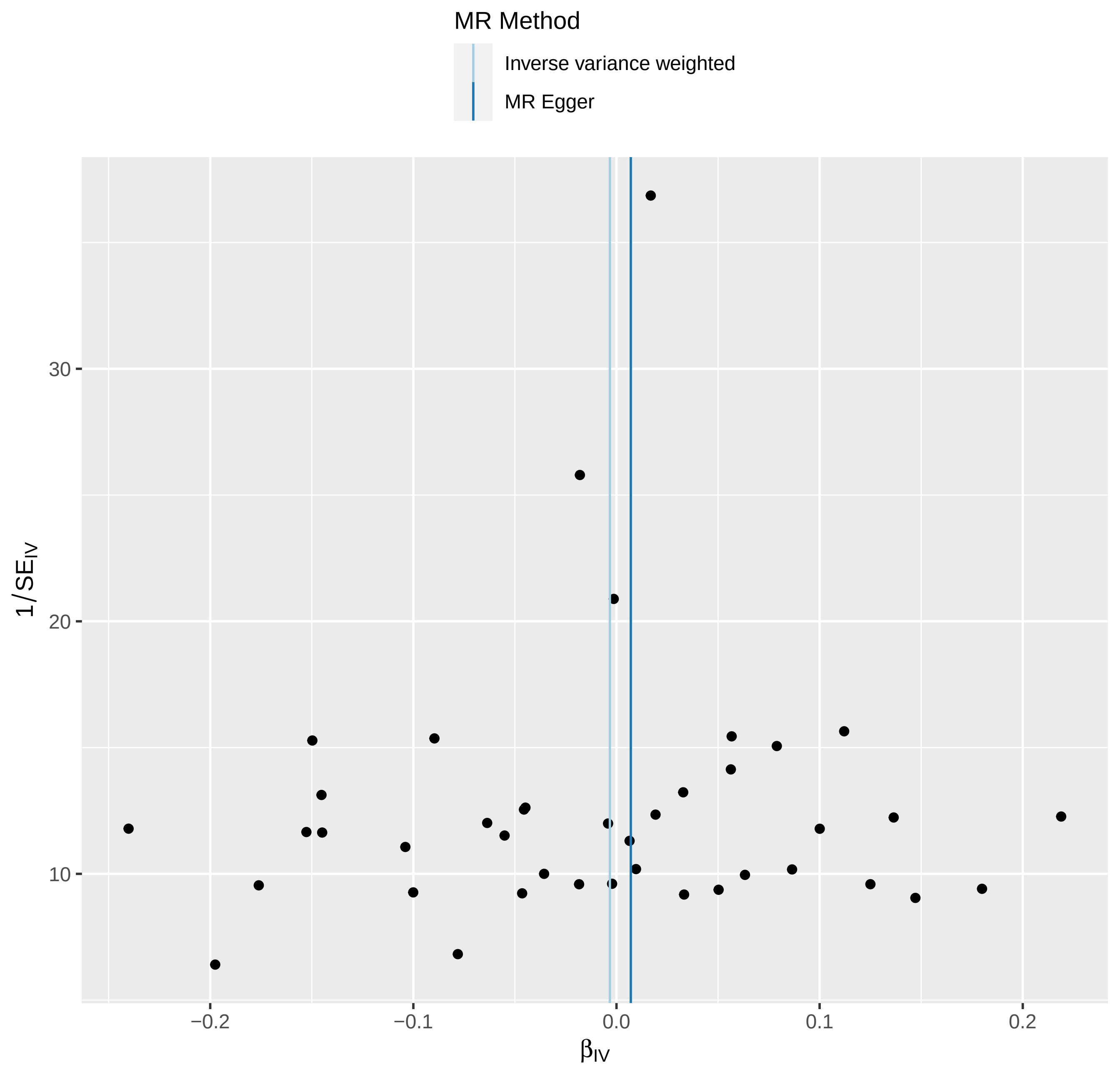

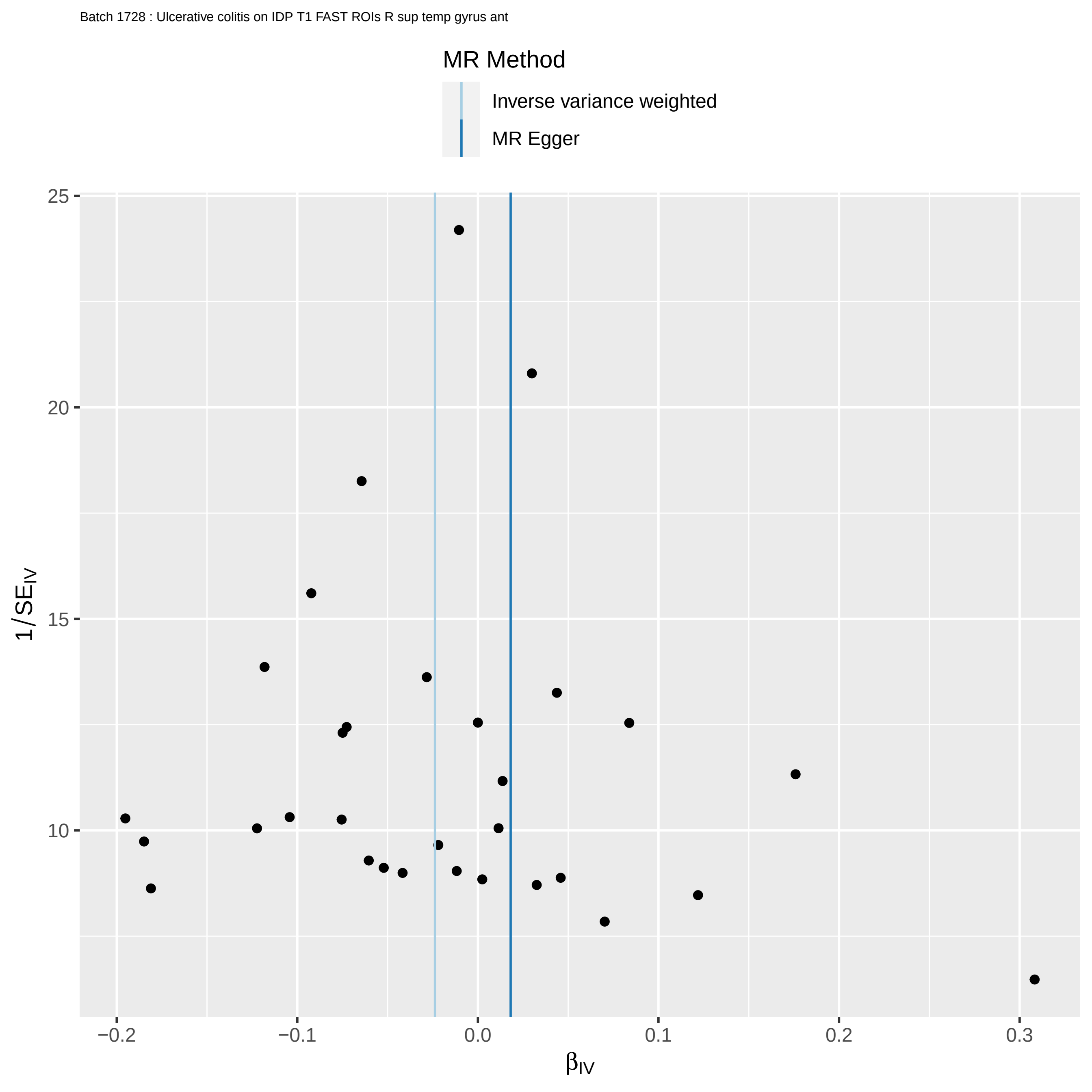

Batch 1728 : Ulcerative colitis on IDP T1 FAST ROIs R sup temp gyrus ant
MR Method
Inverse variance weighted
MR Egger
25
20
15
10
1/SE$_{IV}$
−0.2
−0.1
0.0
0.1
0.2
0.3
β_{IV}

Batch 1749 : Crohn's disease on IDP T1 FAST ROIs L sup temp gyrus post

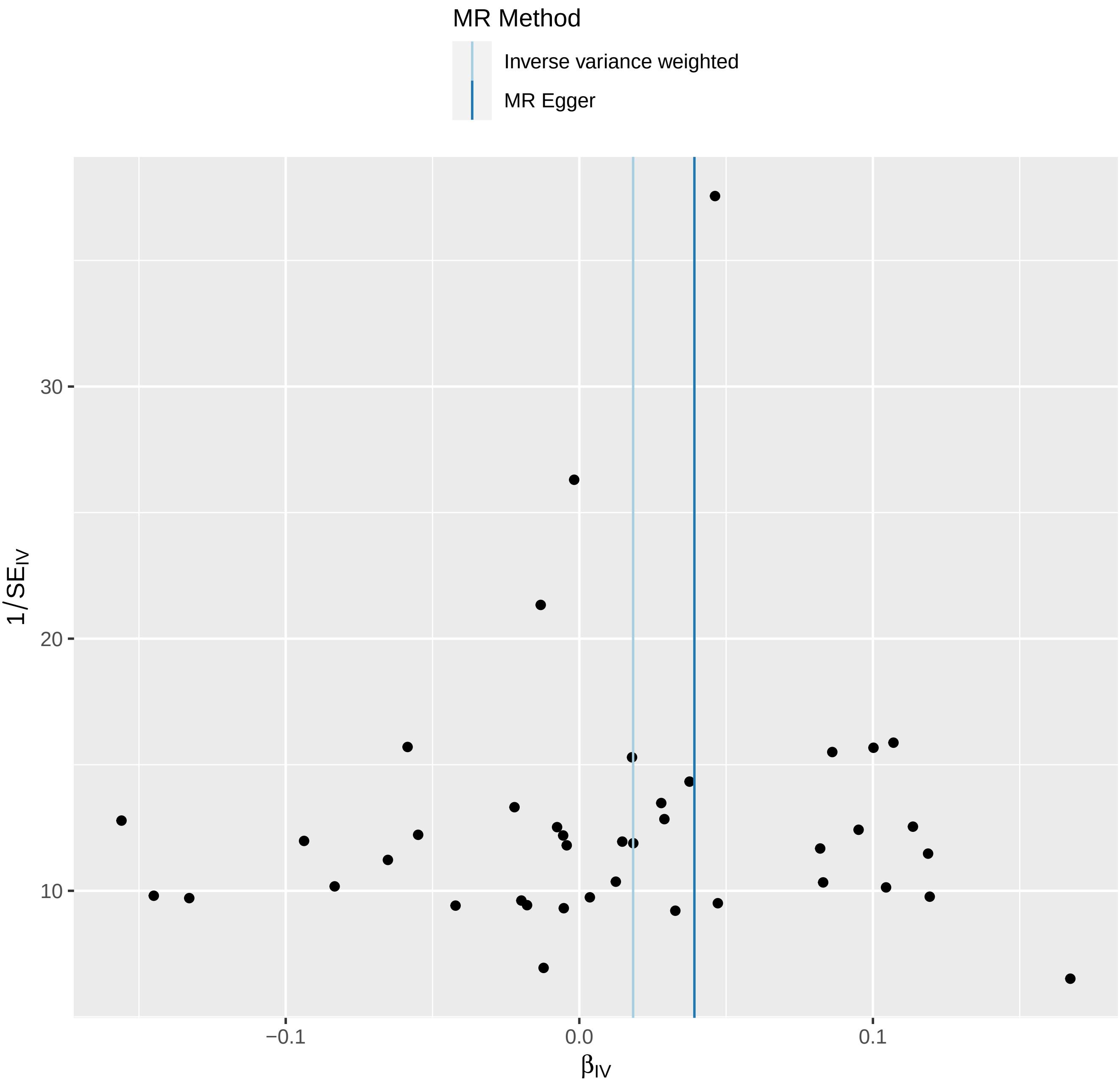

Batch 1750 : Ulcerative colitis on IDP T1 FAST ROIs L sup temp gyrus post

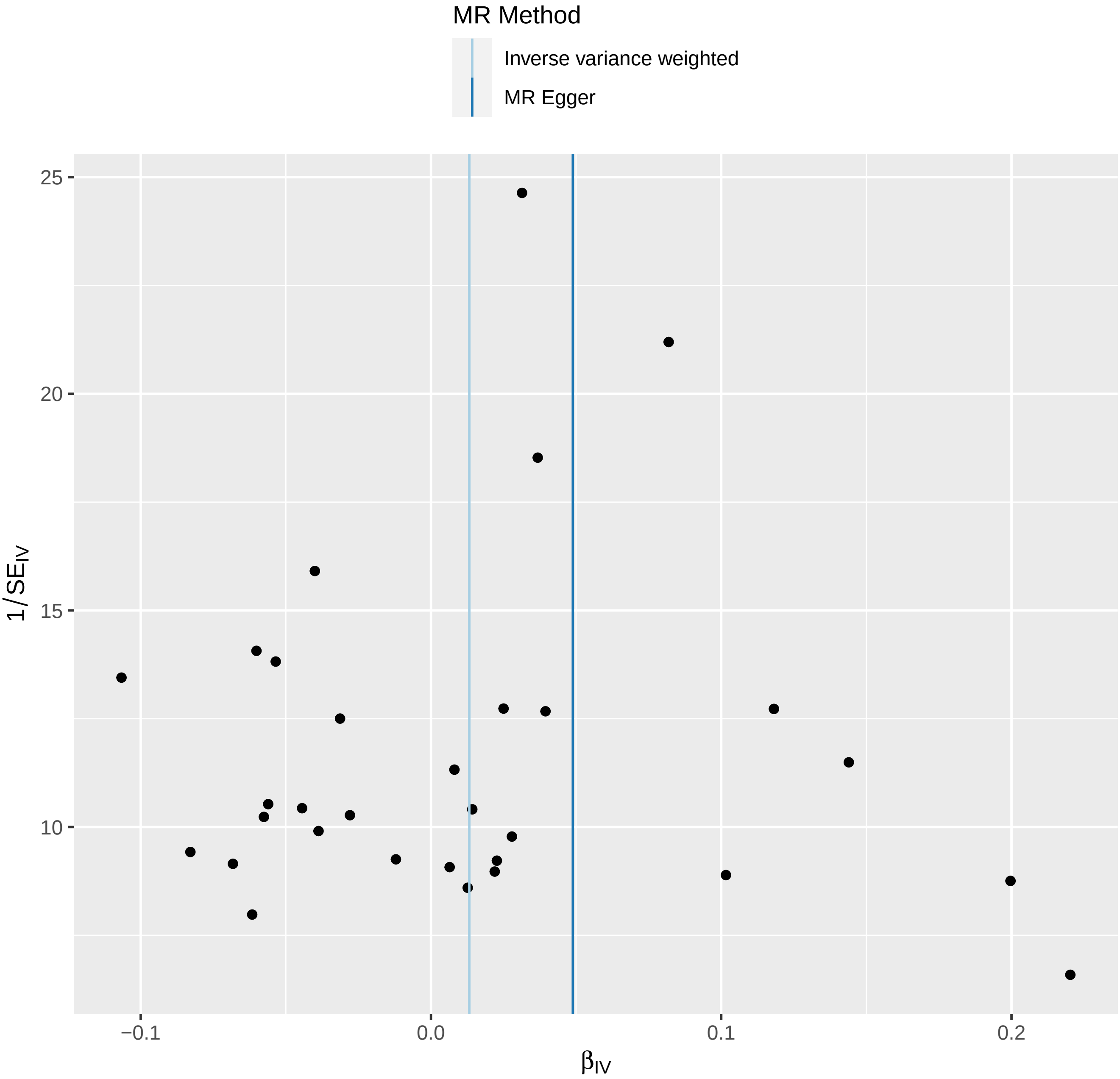

Batch 1771 : Crohn's disease on IDP T1 FAST ROIs R sup temp gyrus post

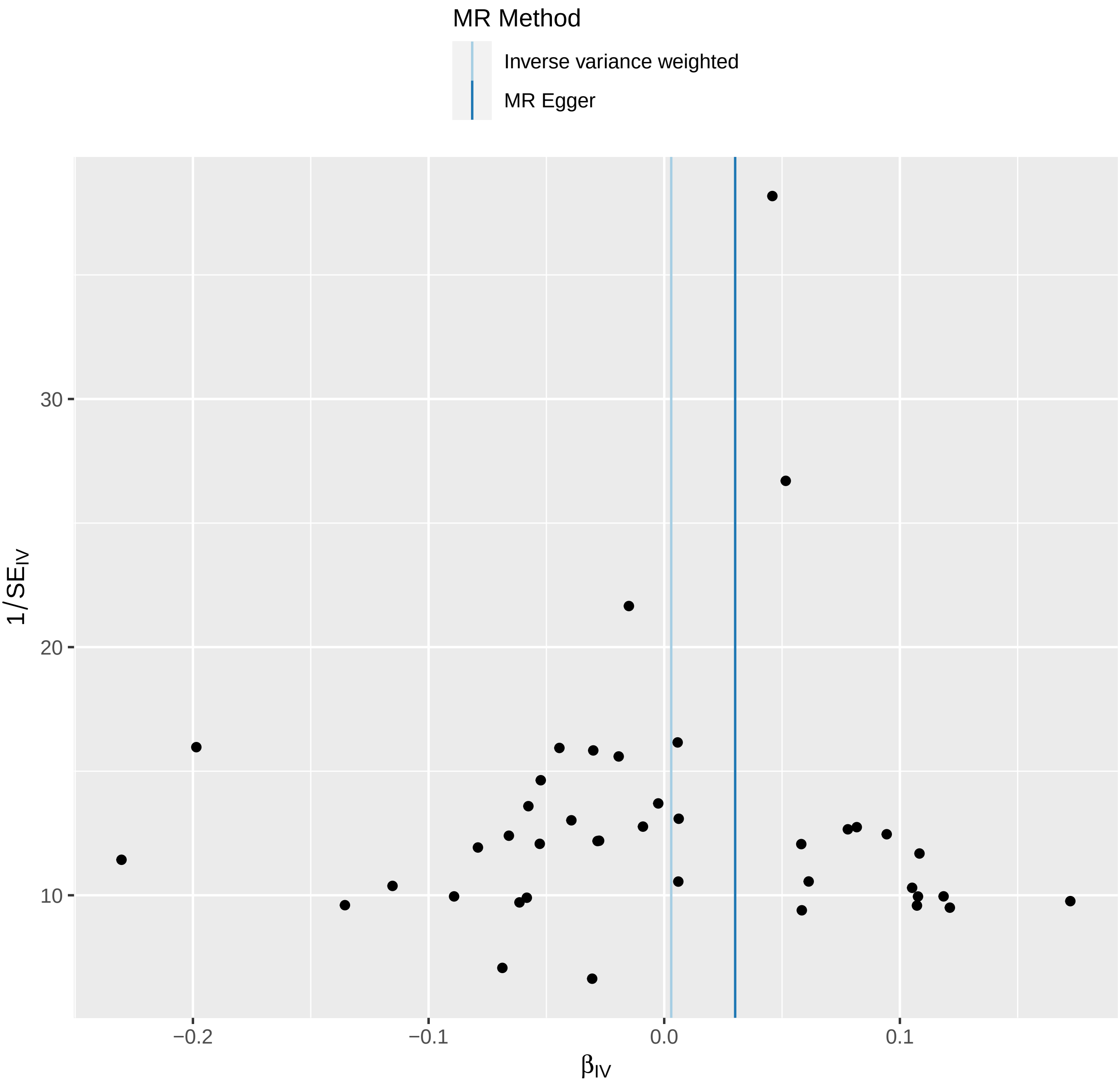

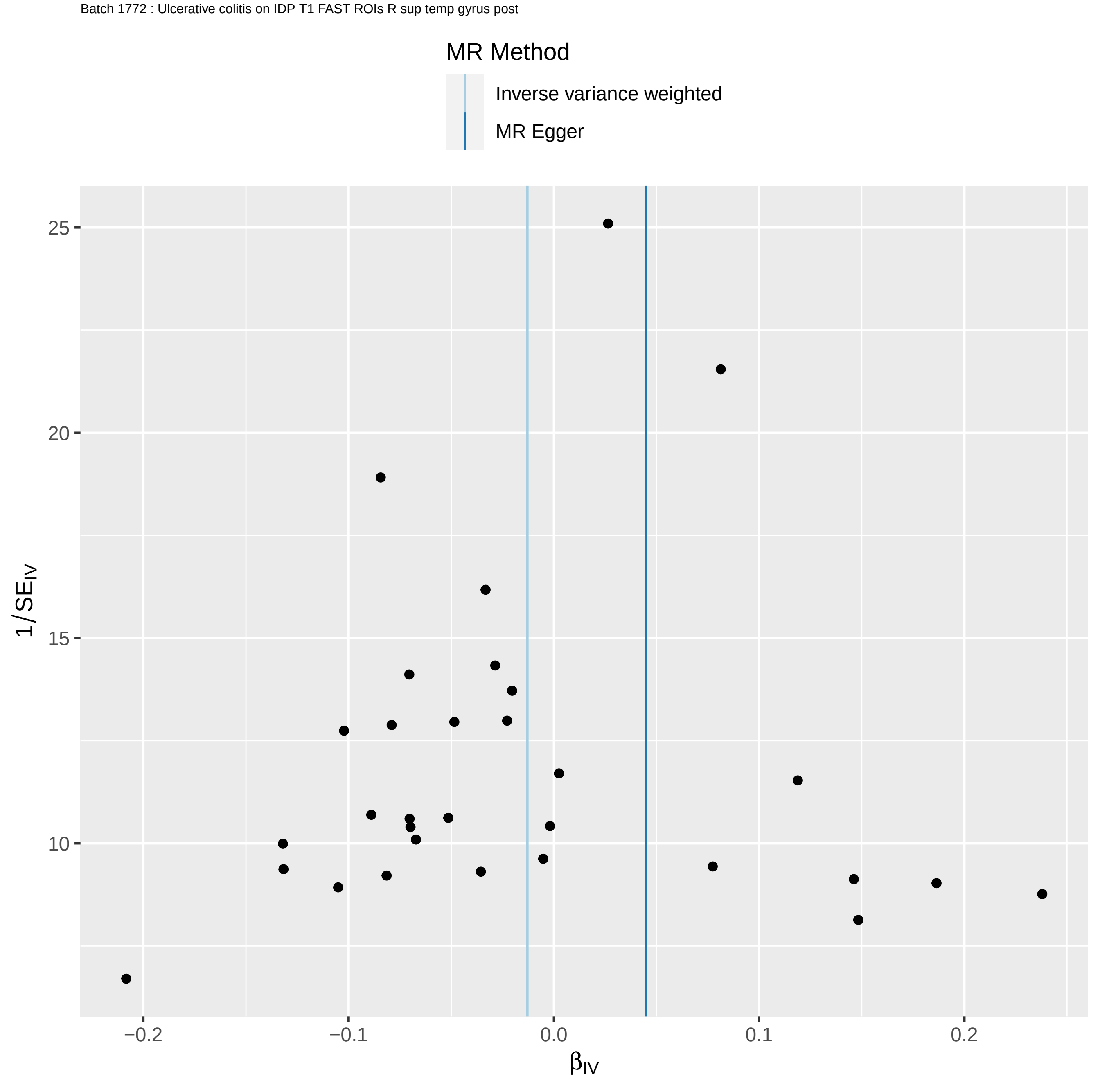

Batch 1772 : Ulcerative colitis on IDP T1 FAST ROIs R sup temp gyrus post
MR Method
Inverse variance weighted
MR Egger
25
20
15
10
$1/SE_{IV}$
−0.2
−0.1
0.0
0.1
0.2
β_{IV}

Batch 1793 : Crohn's disease on IDP T1 FAST ROIs L mid temp gyrus ant

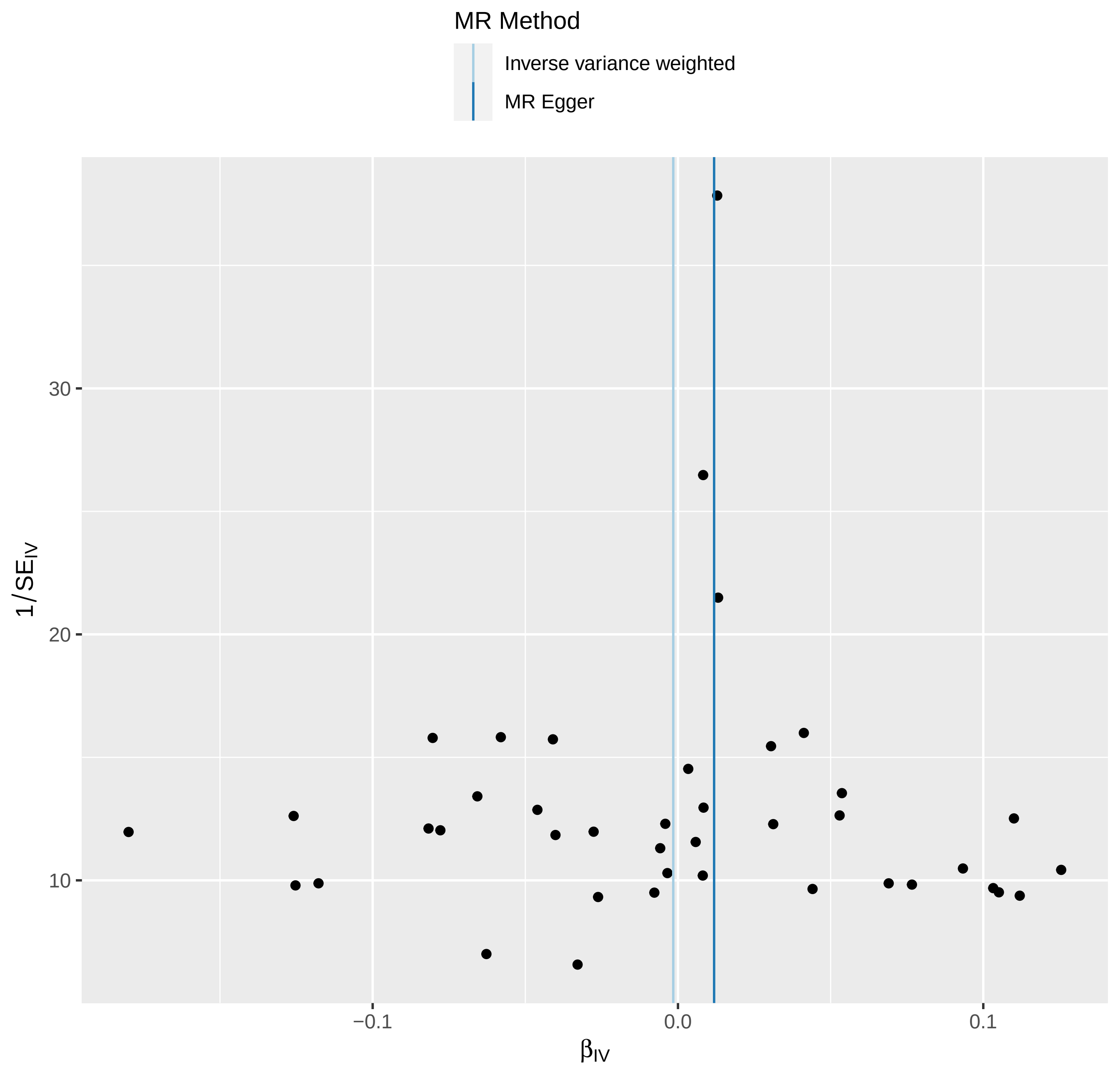

Batch 1794 : Ulcerative colitis on IDP T1 FAST ROIs L mid temp gyrus ant

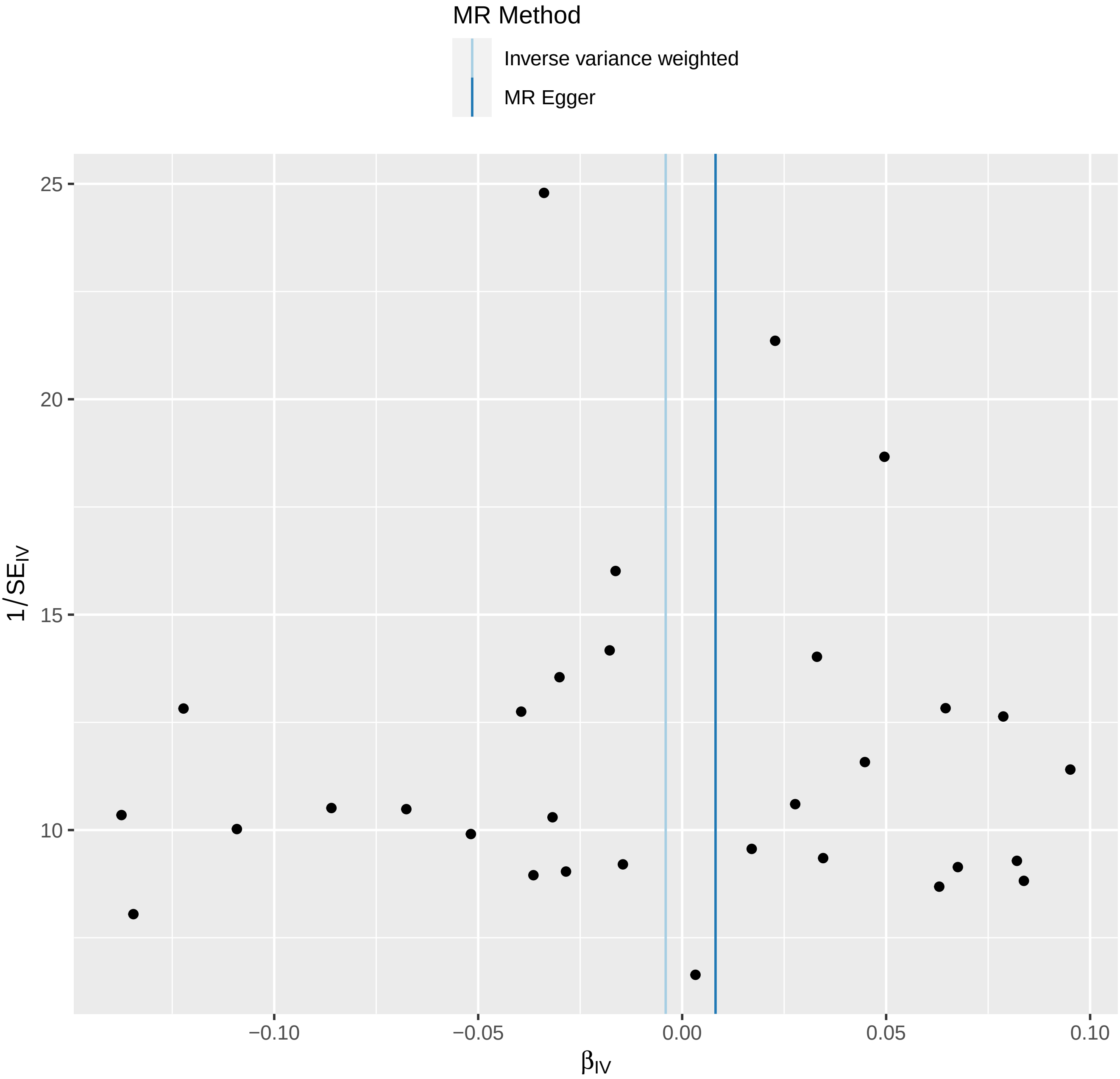

Batch 1815 : Crohn's disease on IDP T1 FAST ROIs R mid temp gyrus ant

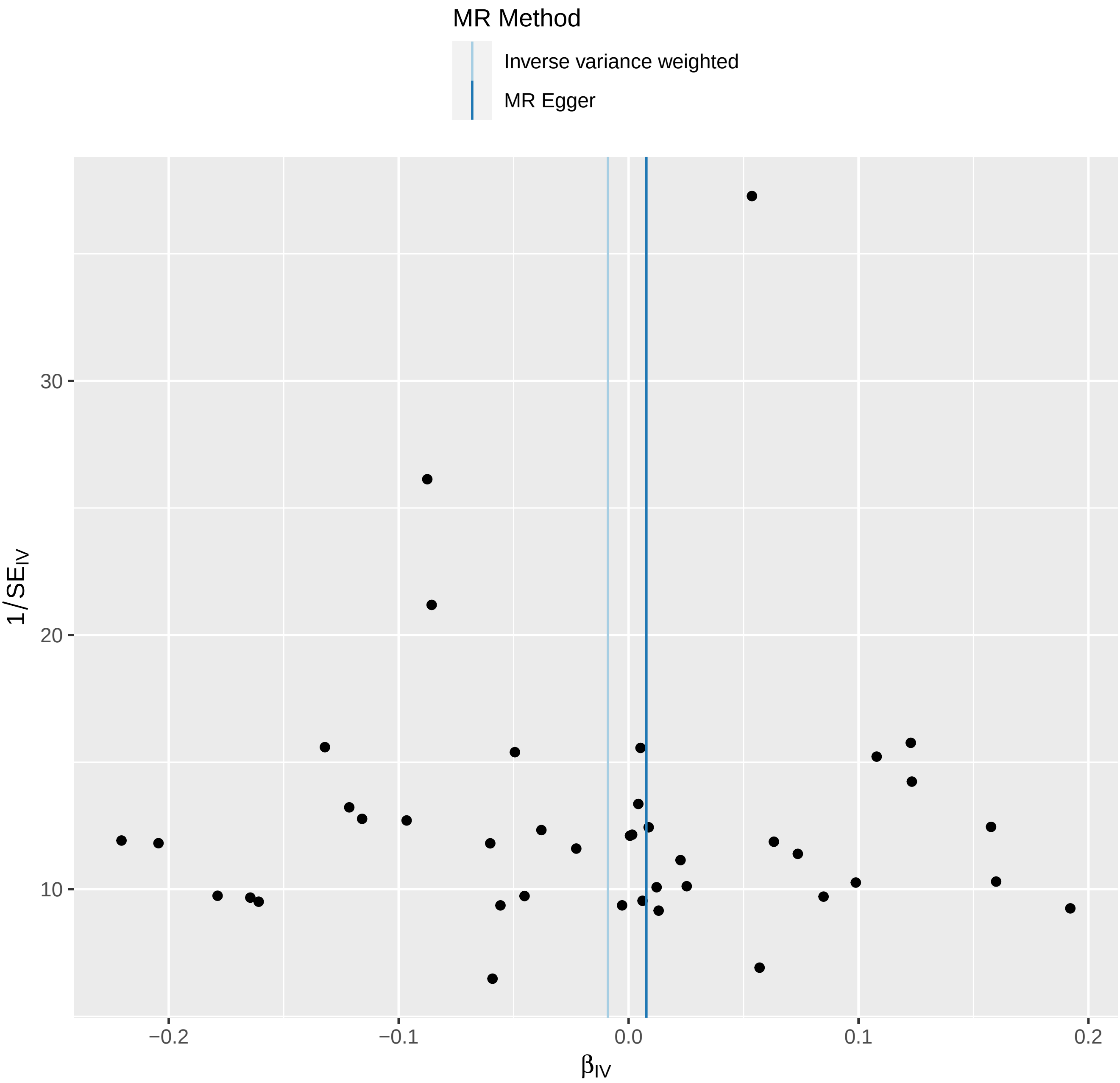

Batch 1816 : Ulcerative colitis on IDP T1 FAST ROIs R mid temp gyrus ant

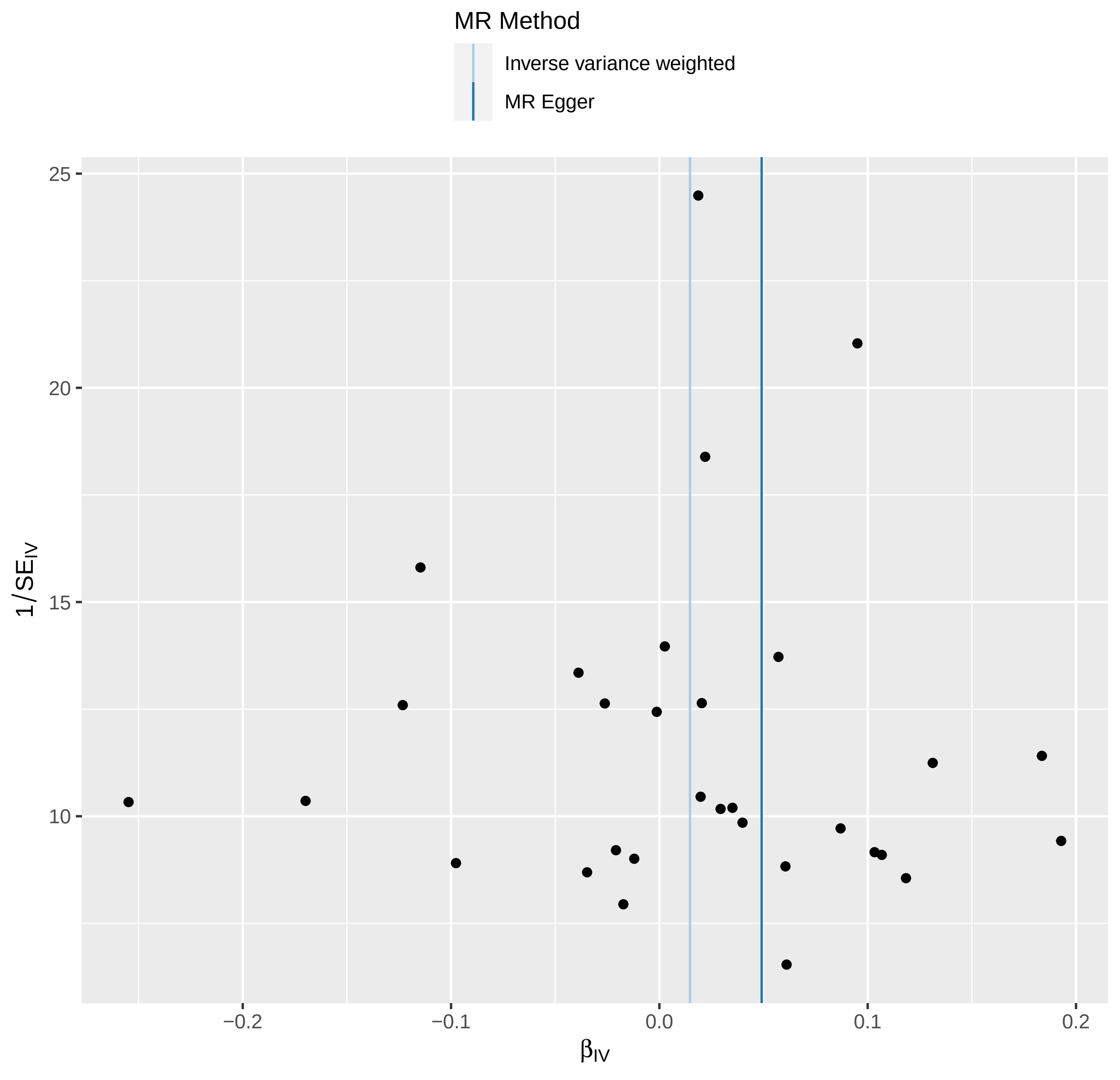

Batch 1837 : Crohn's disease on IDP T1 FAST ROIs L mid temp gyrus post

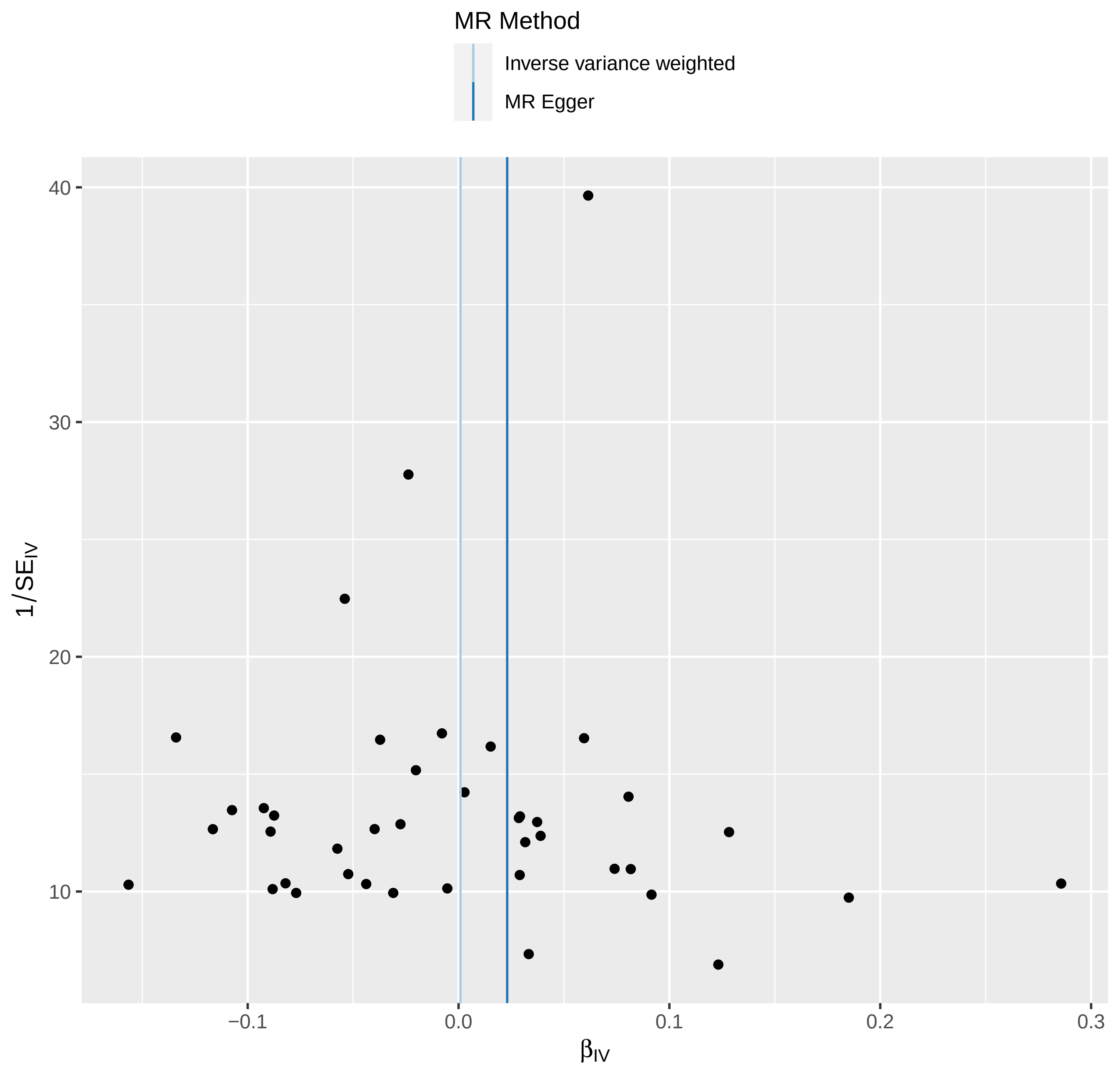

Batch 1838 : Ulcerative colitis on IDP T1 FAST ROIs L mid temp gyrus post

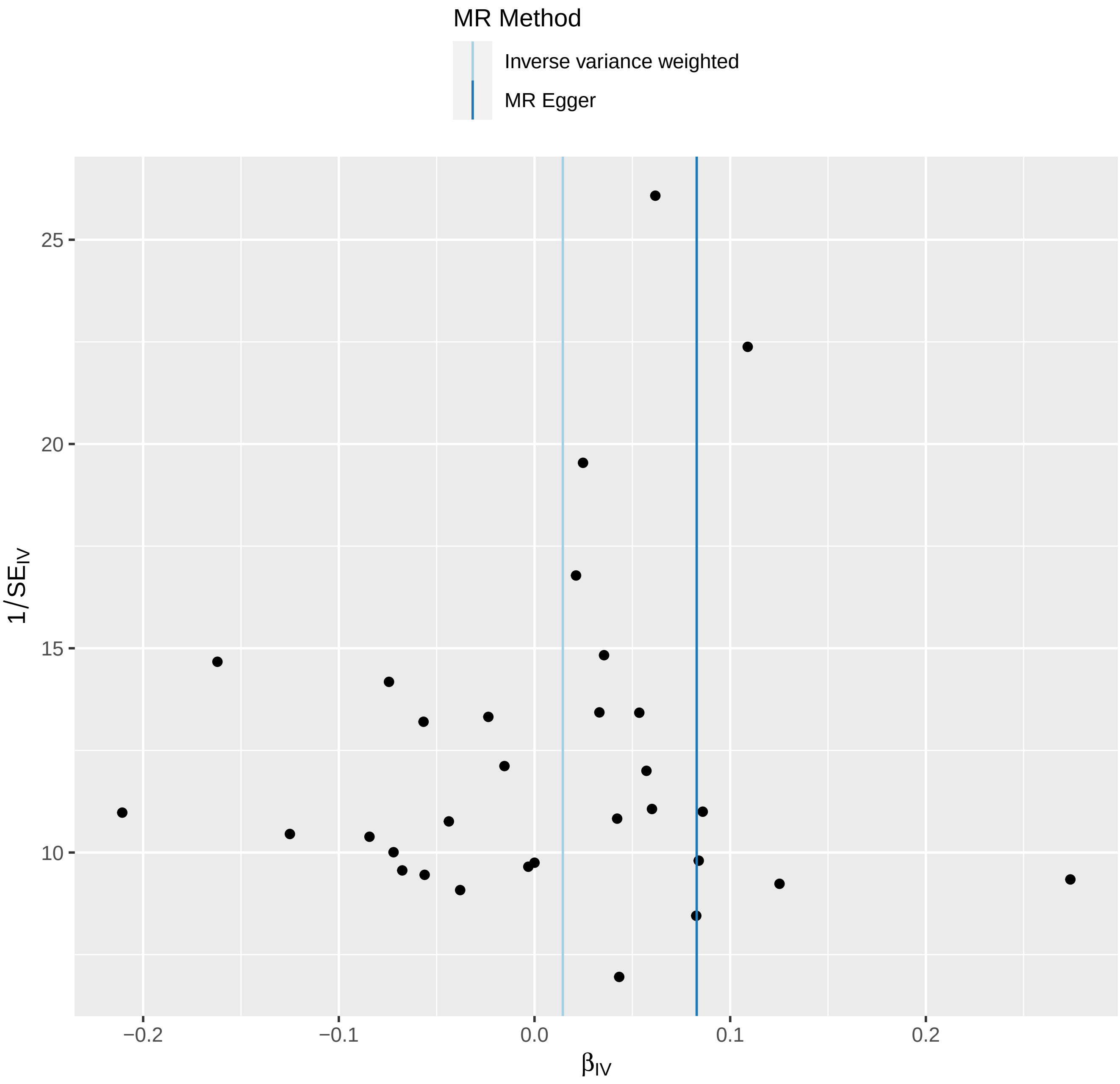

Batch 1859 : Crohn's disease on IDP T1 FAST ROIs R mid temp gyrus post

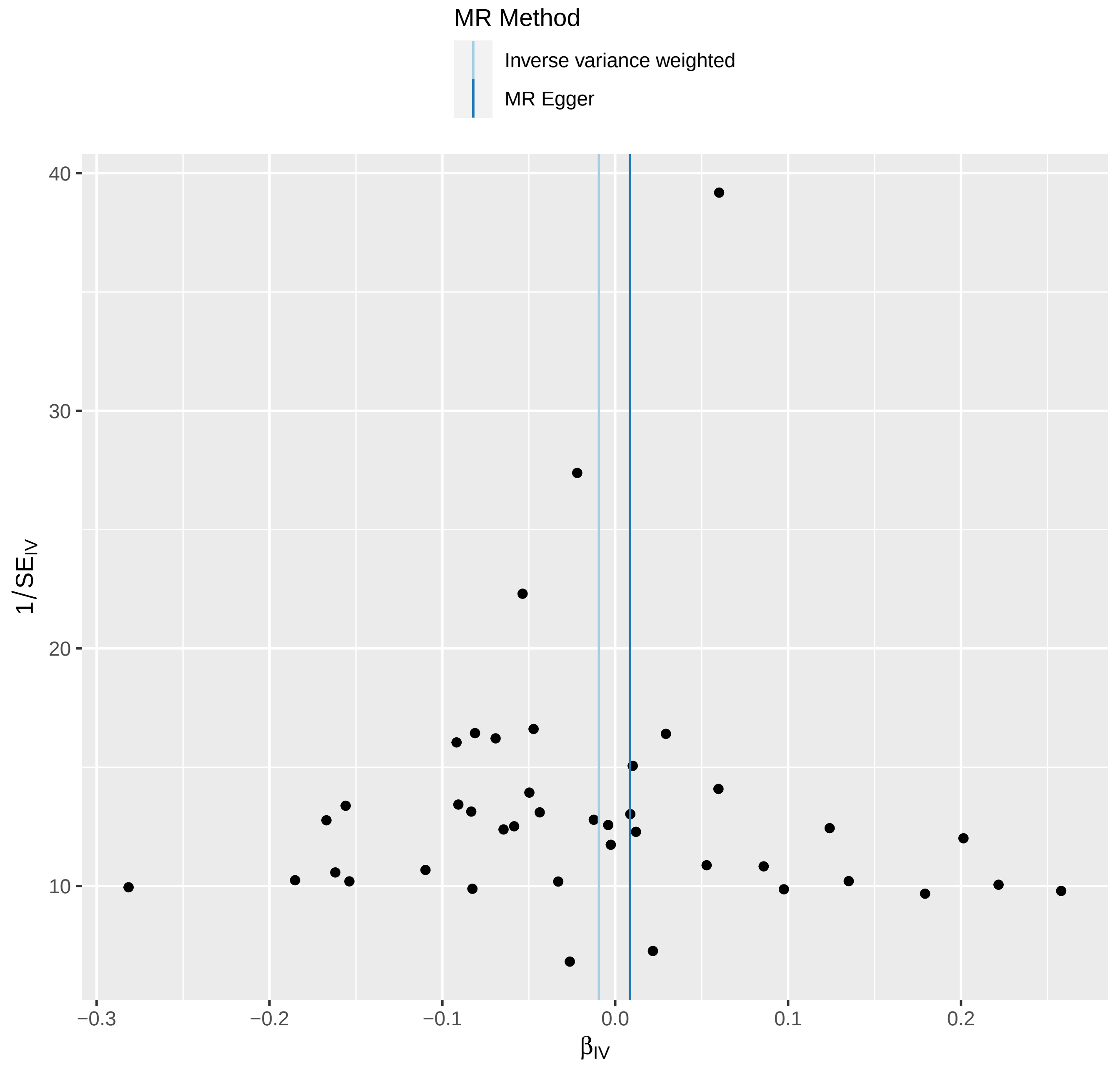

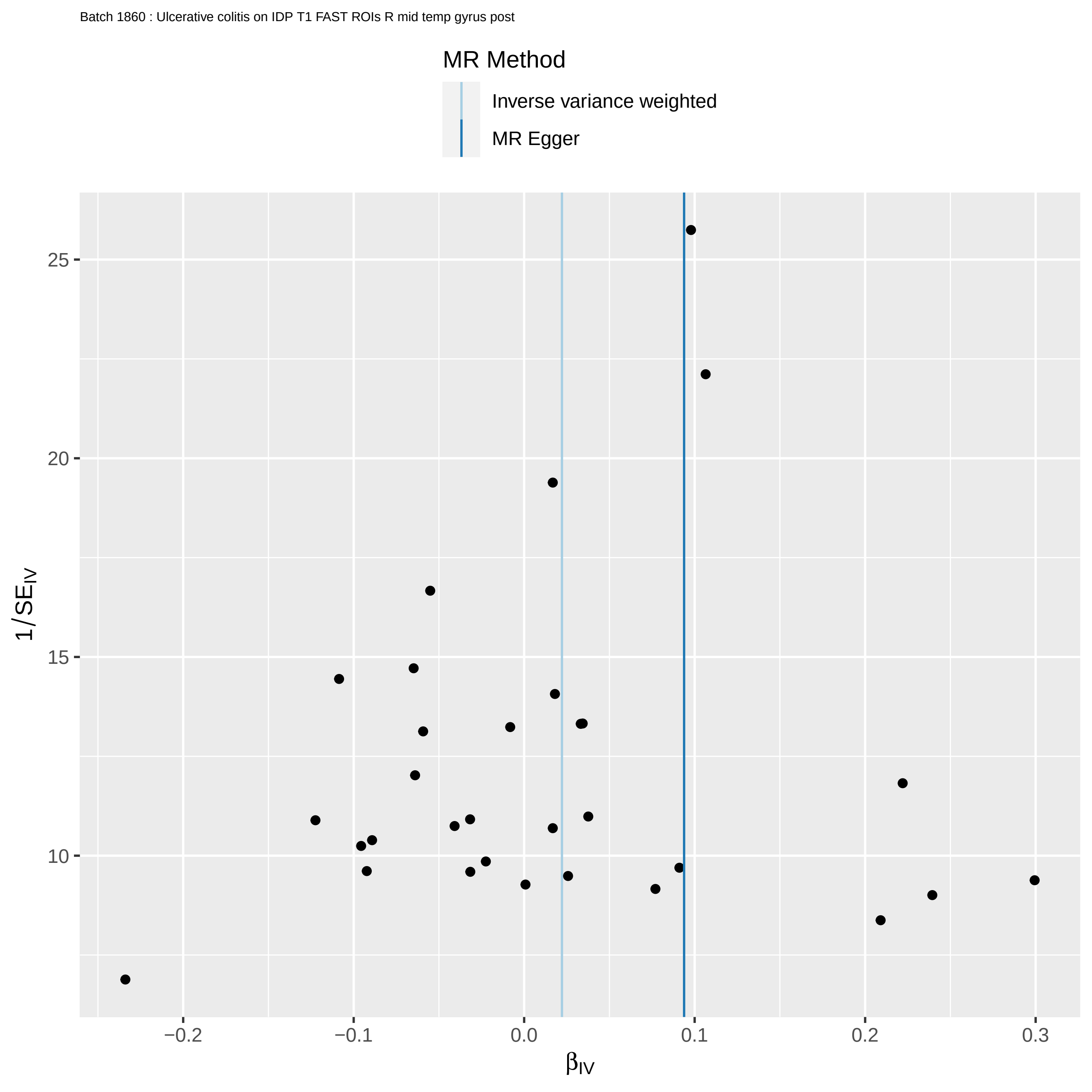
Batch 1860 : Ulcerative colitis on IDP T1 FAST ROIs R mid temp gyrus post
MR Method
Inverse variance weighted
MR Egger
1/SE_IV
β_IV
25
20
15
10
−0.2
−0.1
0.0
0.1
0.2
0.3

Batch 1881 : Crohn's disease on IDP T1 SIENAX grey normalised volume

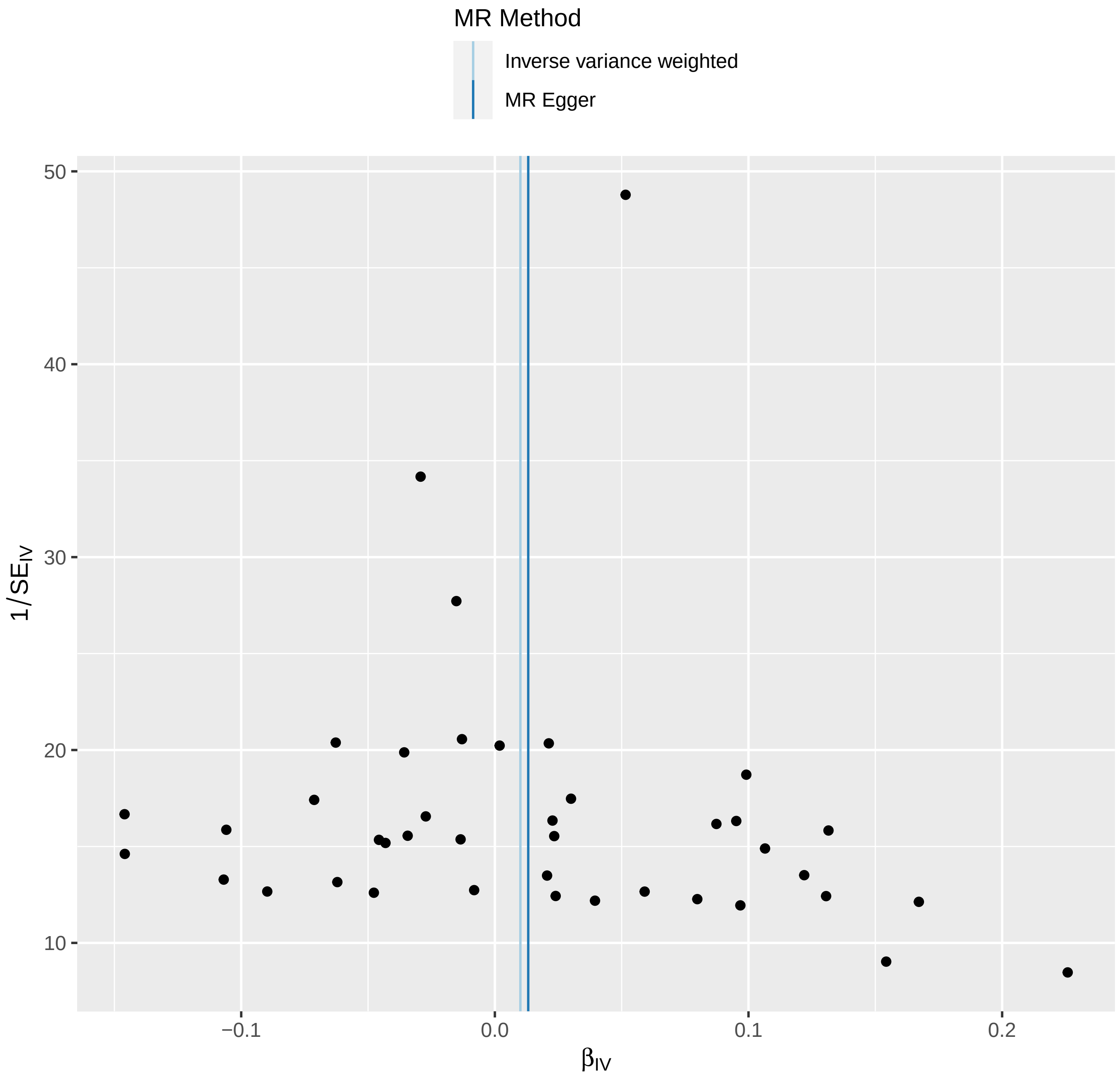

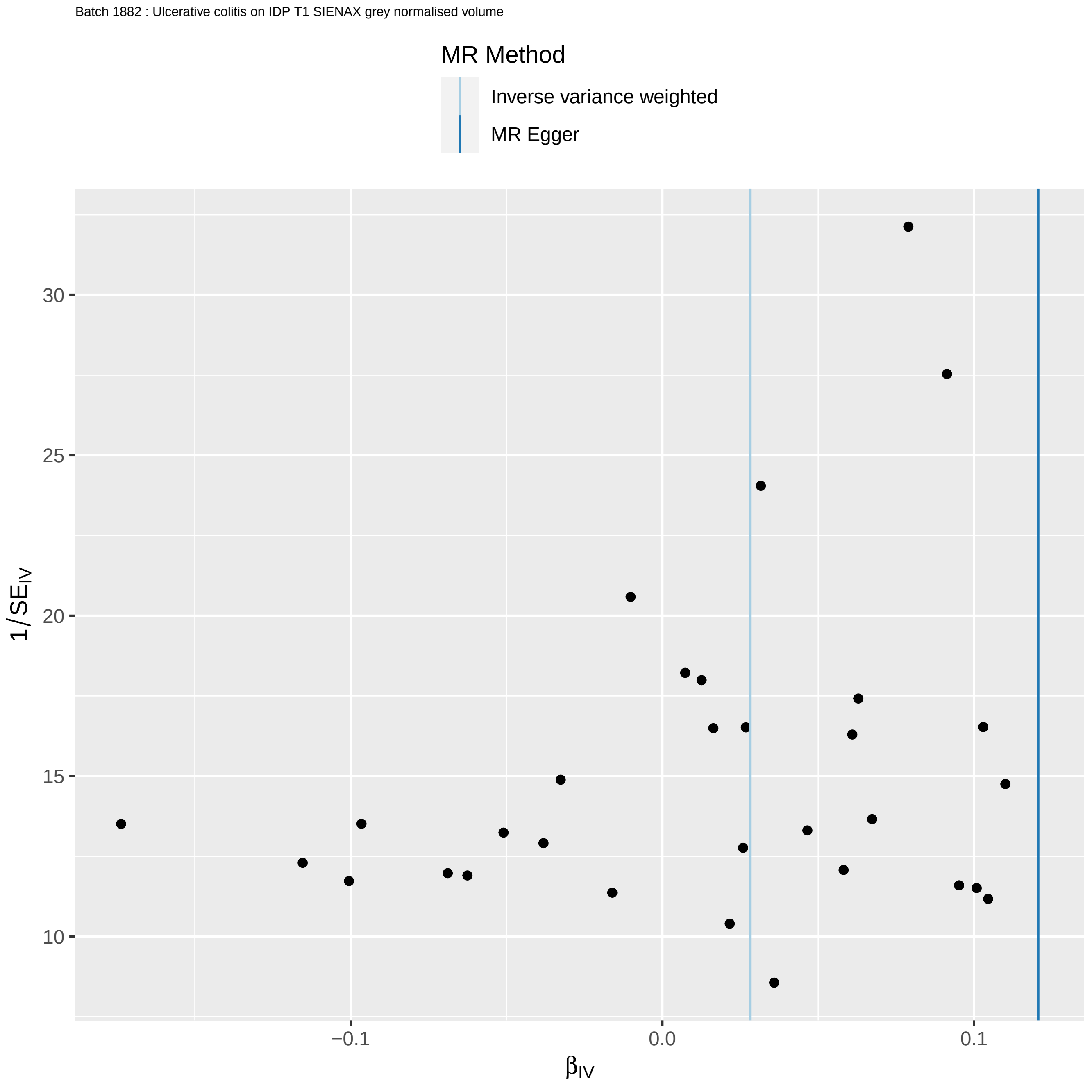
Batch 1882 : Ulcerative colitis on IDP T1 SIENAX grey normalised volume
MR Method
Inverse variance weighted
MR Egger
30
25
20
15
10
1/SE_IV
−0.1
0.0
0.1
β_IV

Batch 1883 : Crohn's disease on IDP T1 FAST ROIs L mid temp gyrus tempocc

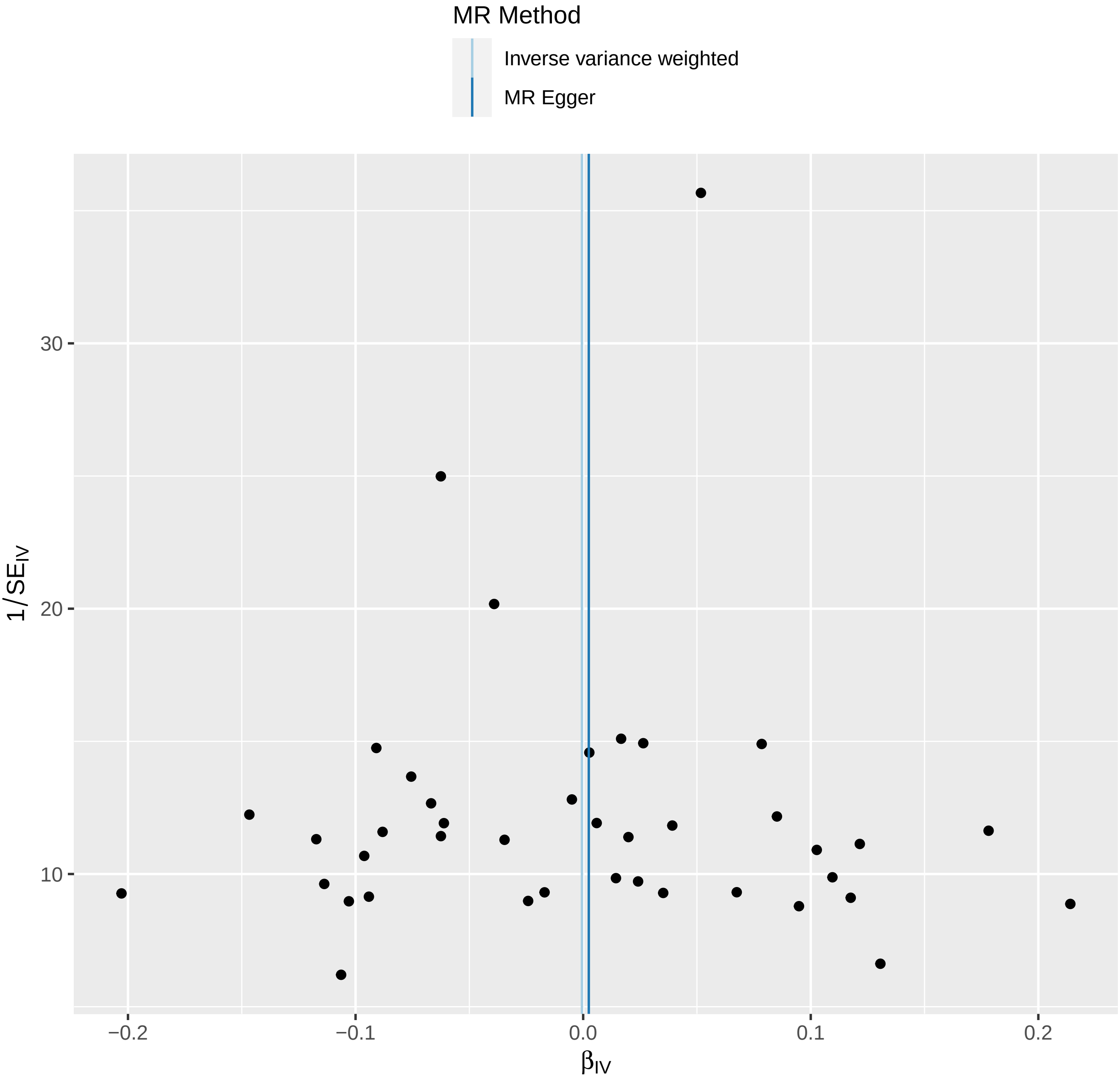

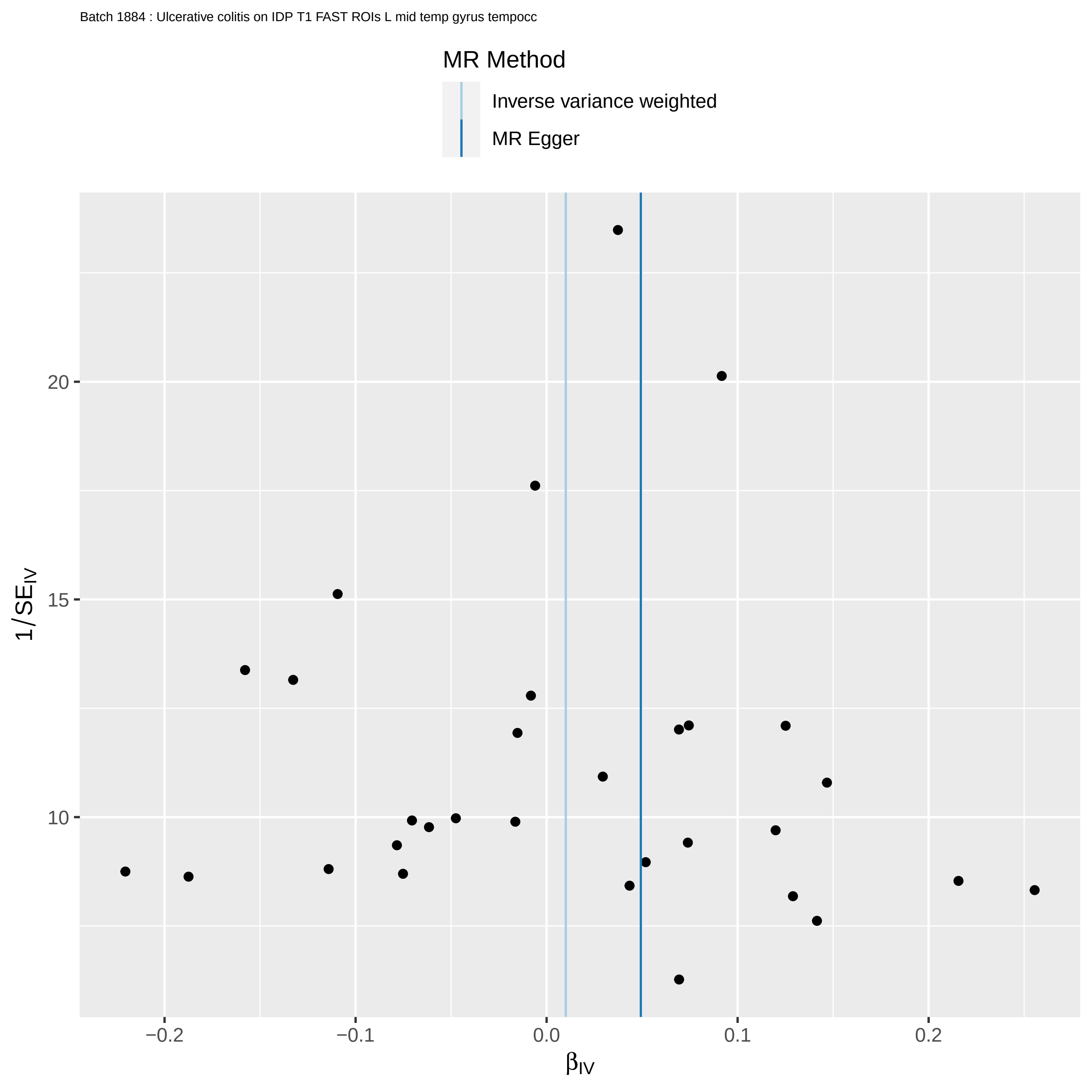

Batch 1884 : Ulcerative colitis on IDP T1 FAST ROIs L mid temp gyrus tempocc
MR Method
Inverse variance weighted
MR Egger
20
15
10
1/SE_IV
−0.2
−0.1
0.0
0.1
0.2
β_IV

Batch 1905 : Crohn's disease on IDP T1 FAST ROIs R mid temp gyrus tempocc

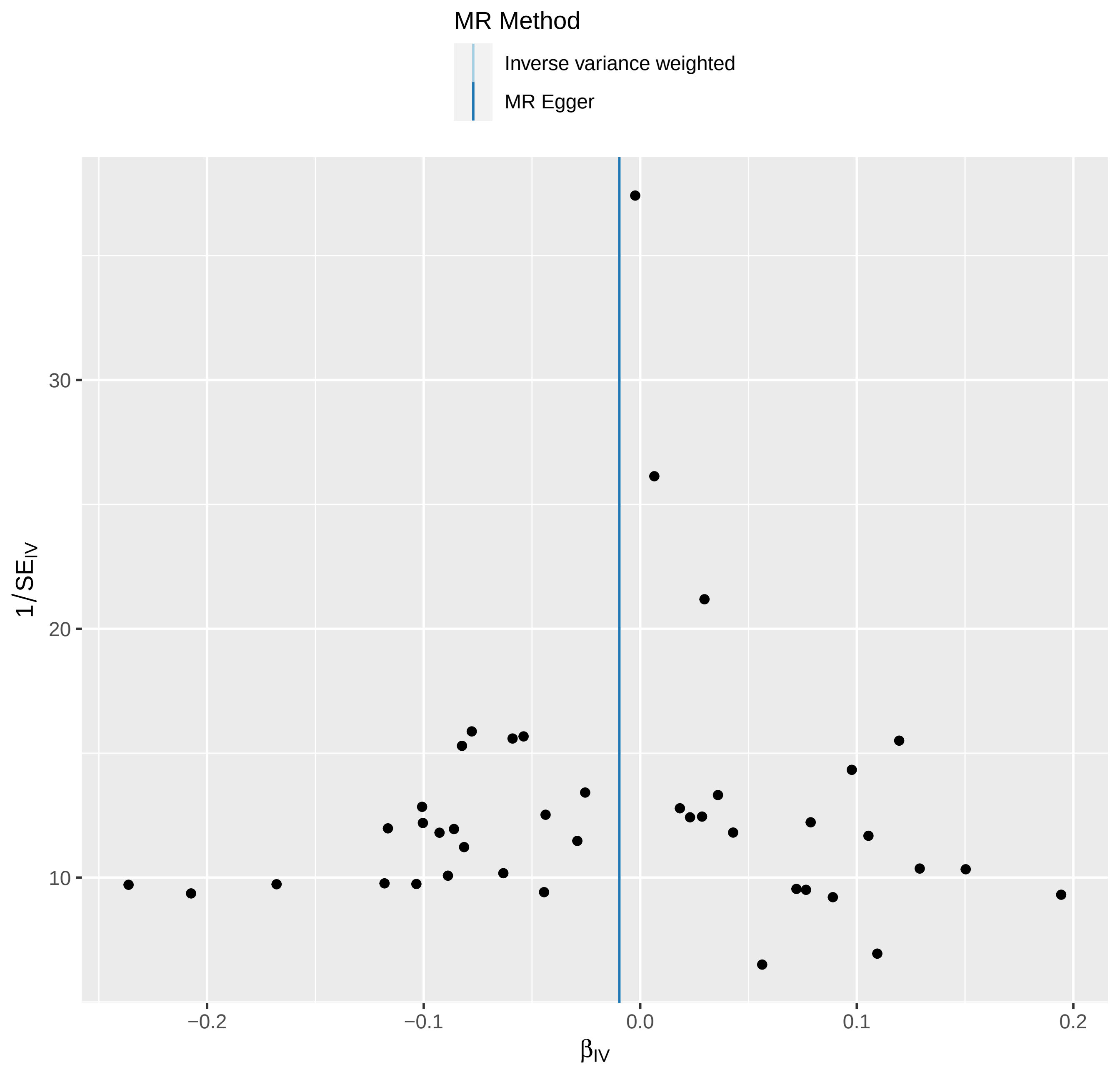

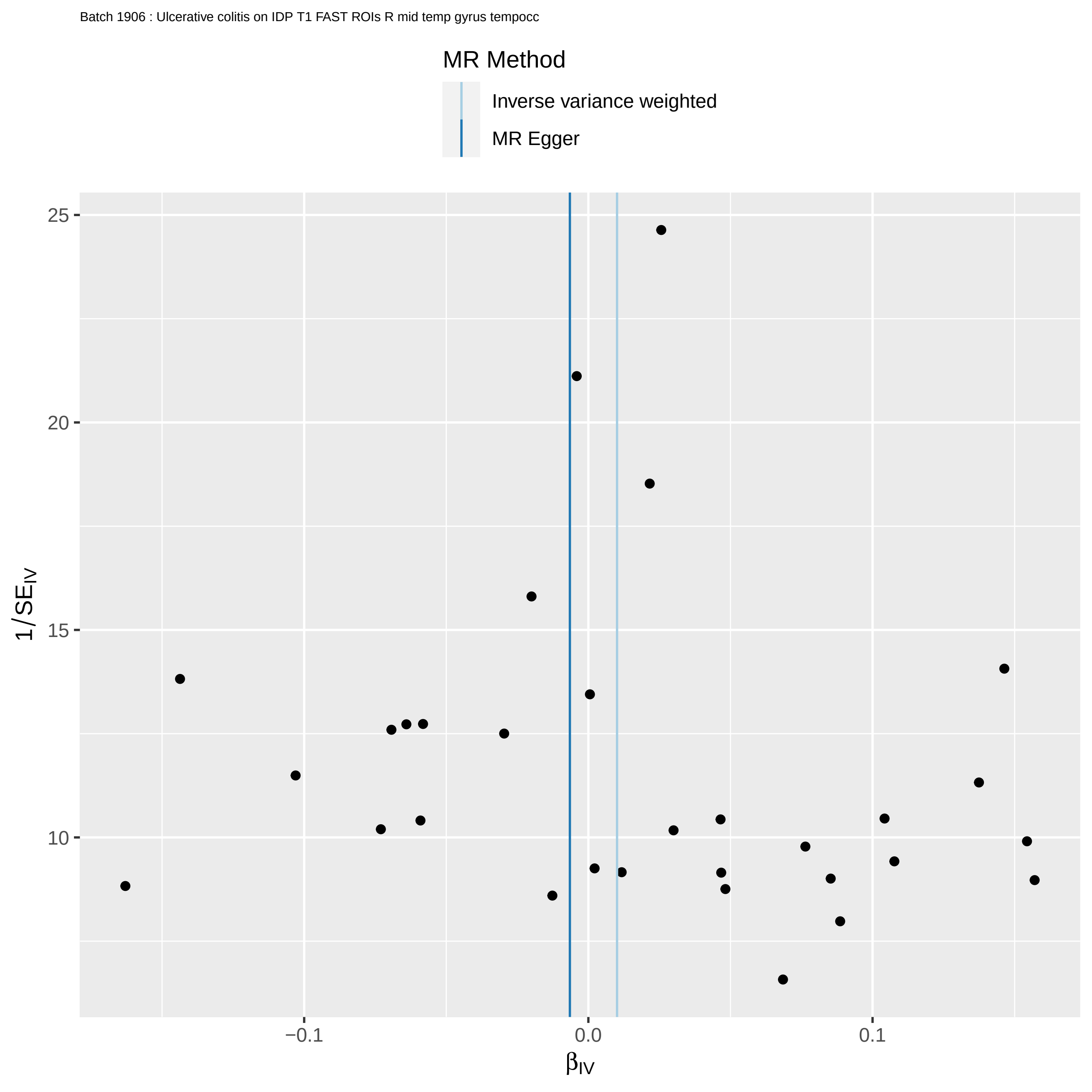

Batch 1906 : Ulcerative colitis on IDP T1 FAST ROIs R mid temp gyrus tempocc
MR Method
Inverse variance weighted
MR Egger
25
20
15
10
1/SE$_{IV}$
−0.1
0.0
0.1
β$_{IV}$

Batch 1927 : Crohn's disease on IDP T1 FAST ROIs L inf temp gyrus ant

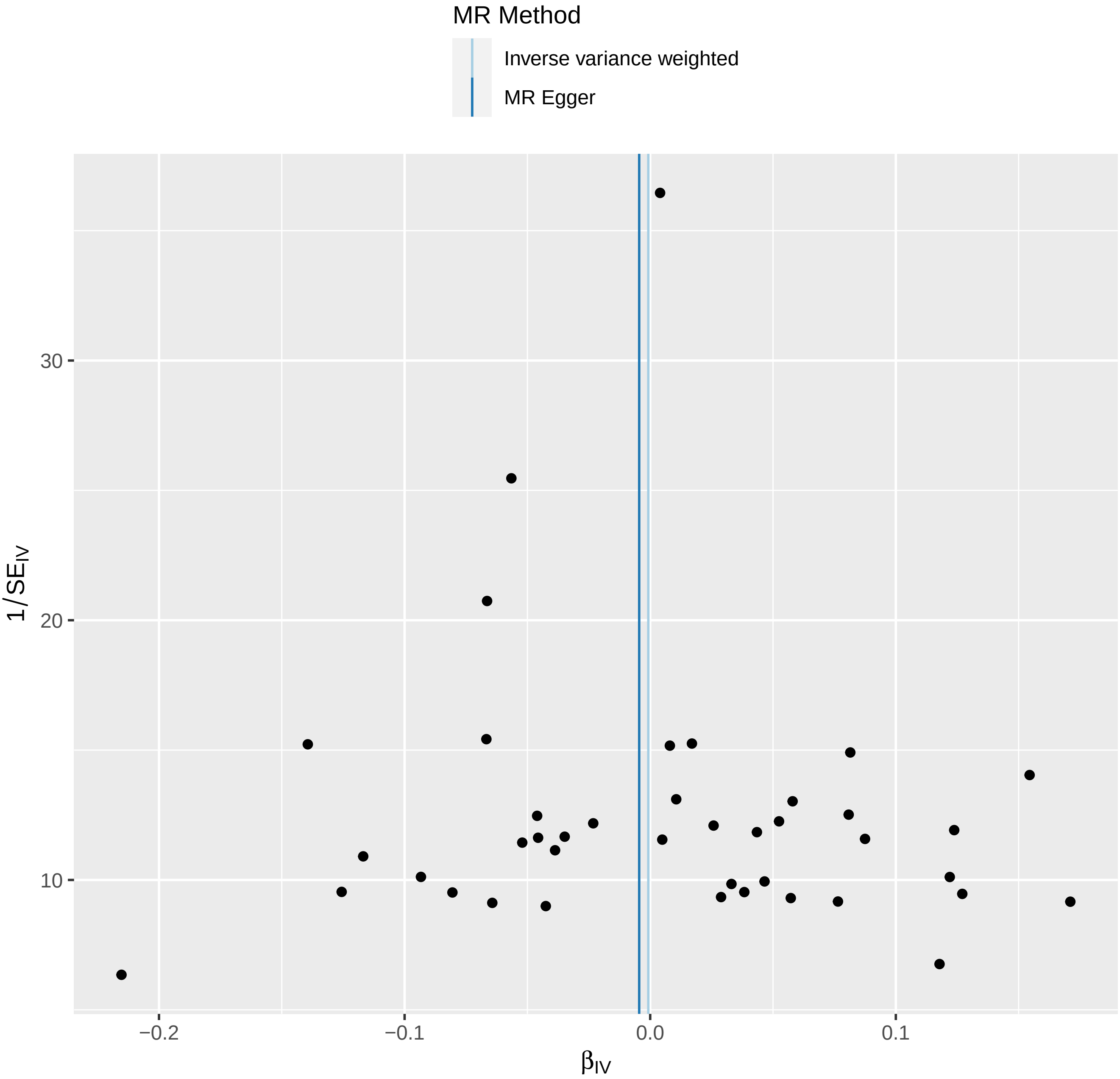

Batch 1928 : Ulcerative colitis on IDP T1 FAST ROIs L inf temp gyrus ant

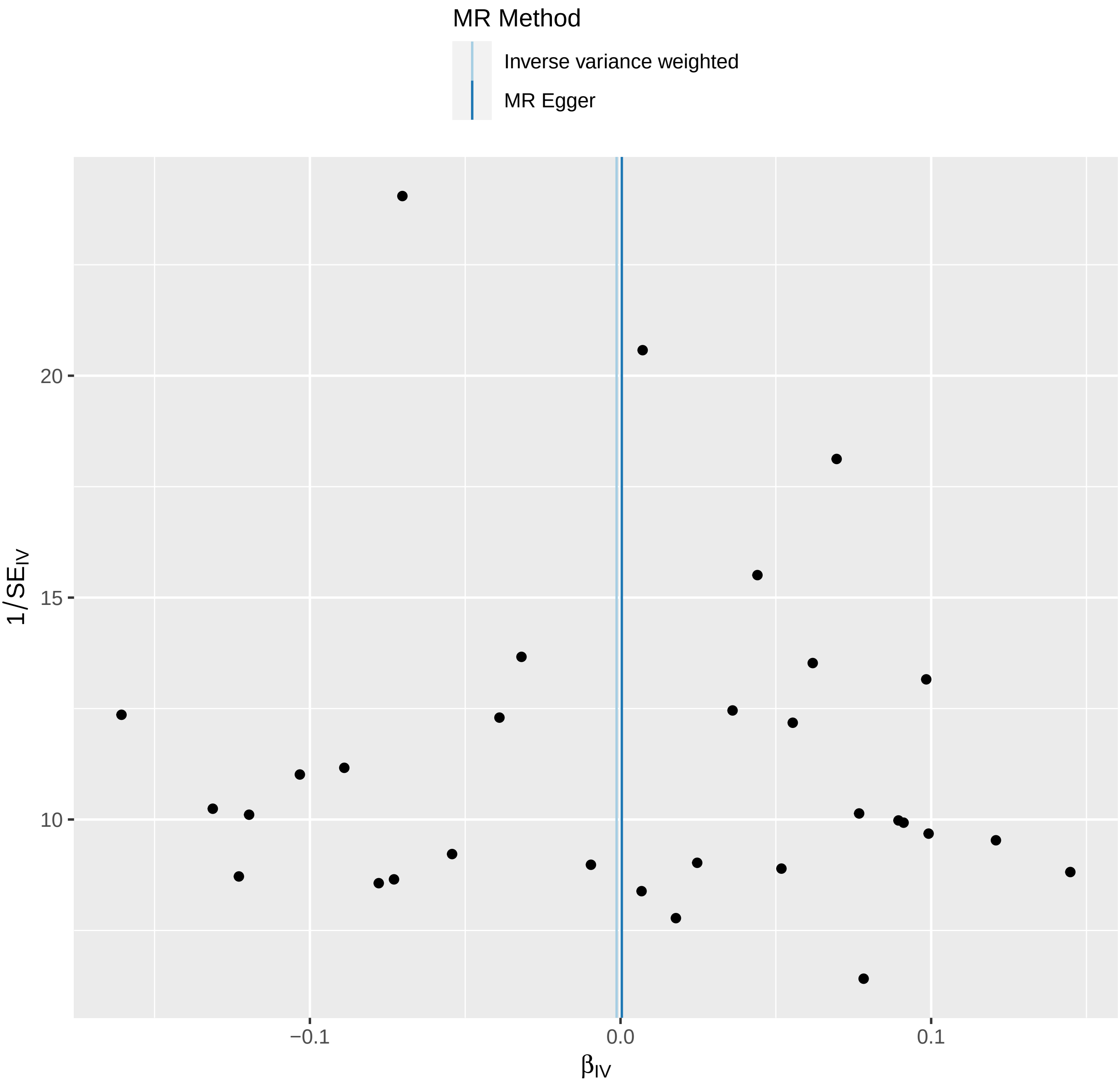

Batch 1949 : Crohn's disease on IDP T1 FAST ROIs R inf temp gyrus ant

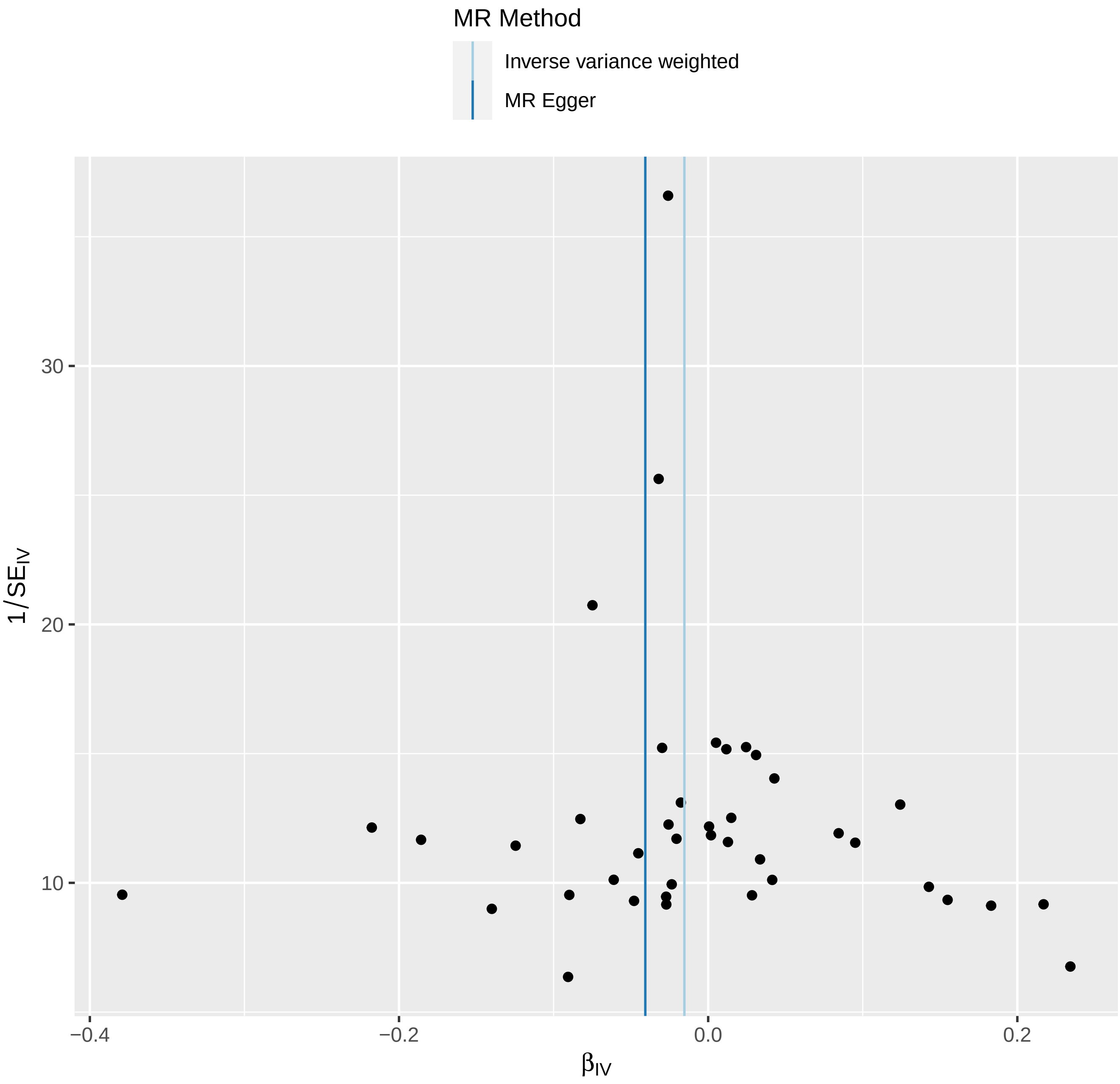

Batch 1950 : Ulcerative colitis on IDP T1 FAST ROIs R inf temp gyrus ant

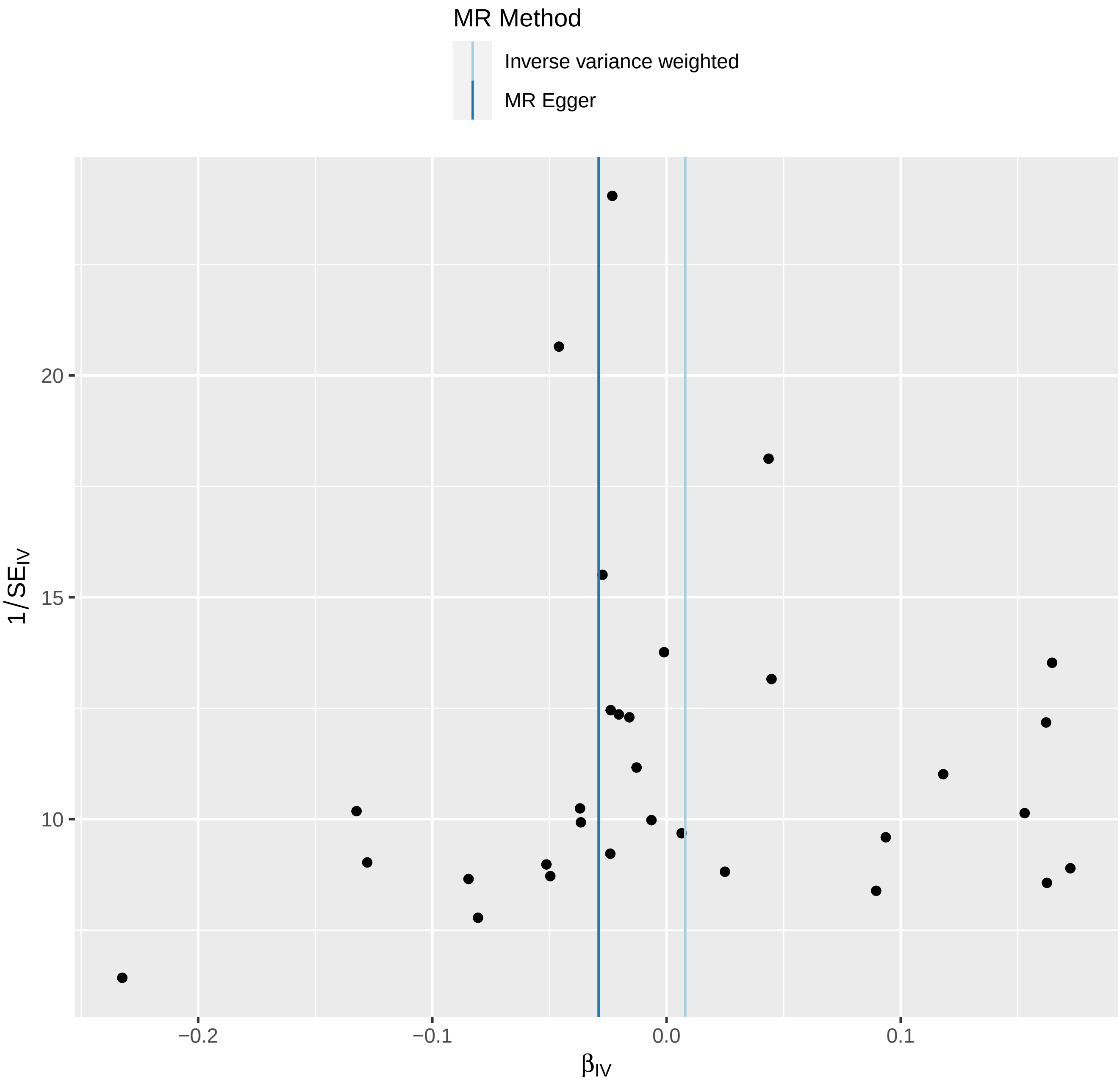

Batch 1971 : Crohn's disease on IDP T1 FAST ROIs L inf temp gyrus post

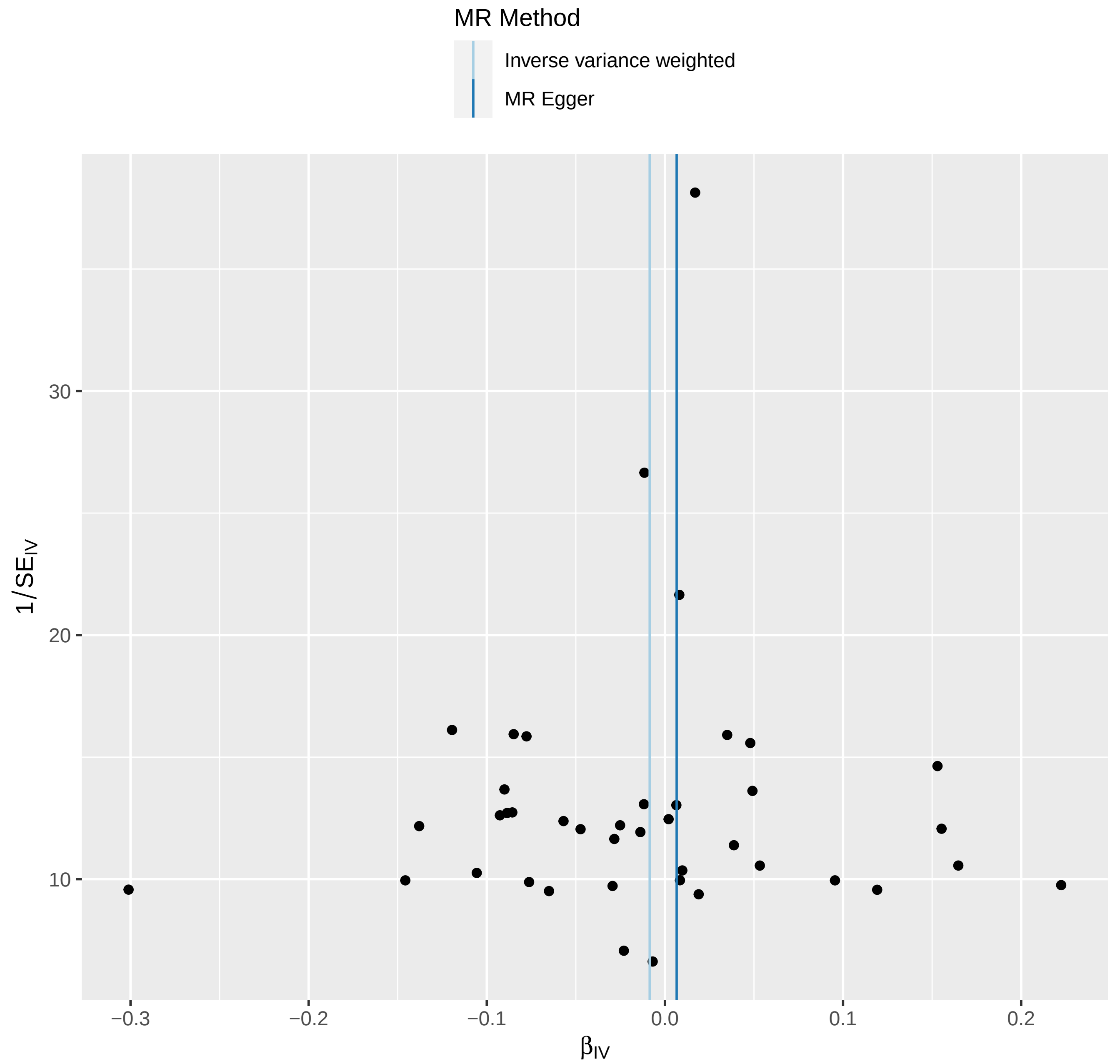

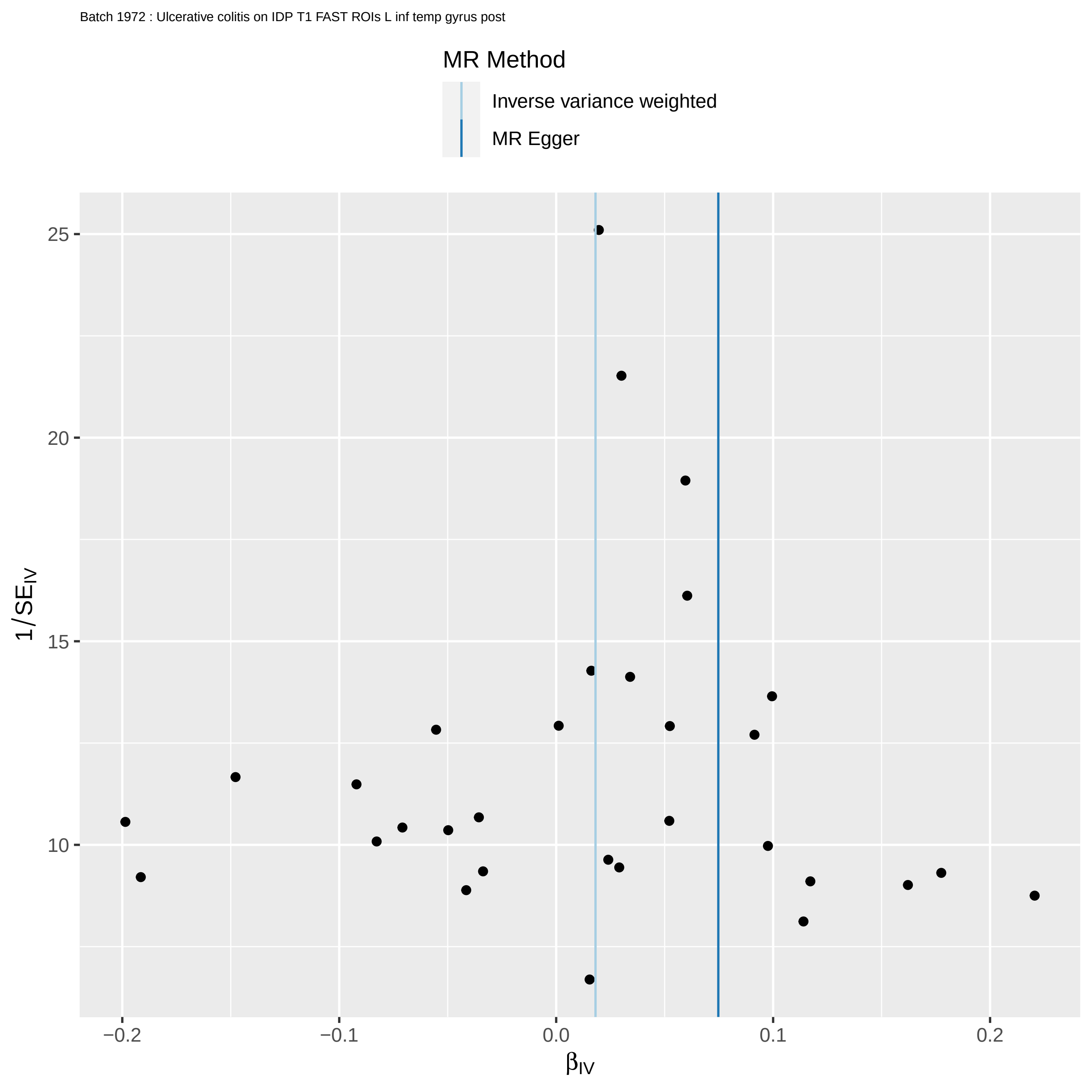

Batch 1972 : Ulcerative colitis on IDP T1 FAST ROIs L inf temp gyrus post
MR Method
Inverse variance weighted
MR Egger
25
20
15
10
1/SE$_{IV}$
−0.2
−0.1
0.0
0.1
0.2
β$_{IV}$

Batch 1993 : Crohn's disease on IDP T1 FAST ROIs R inf temp gyrus post

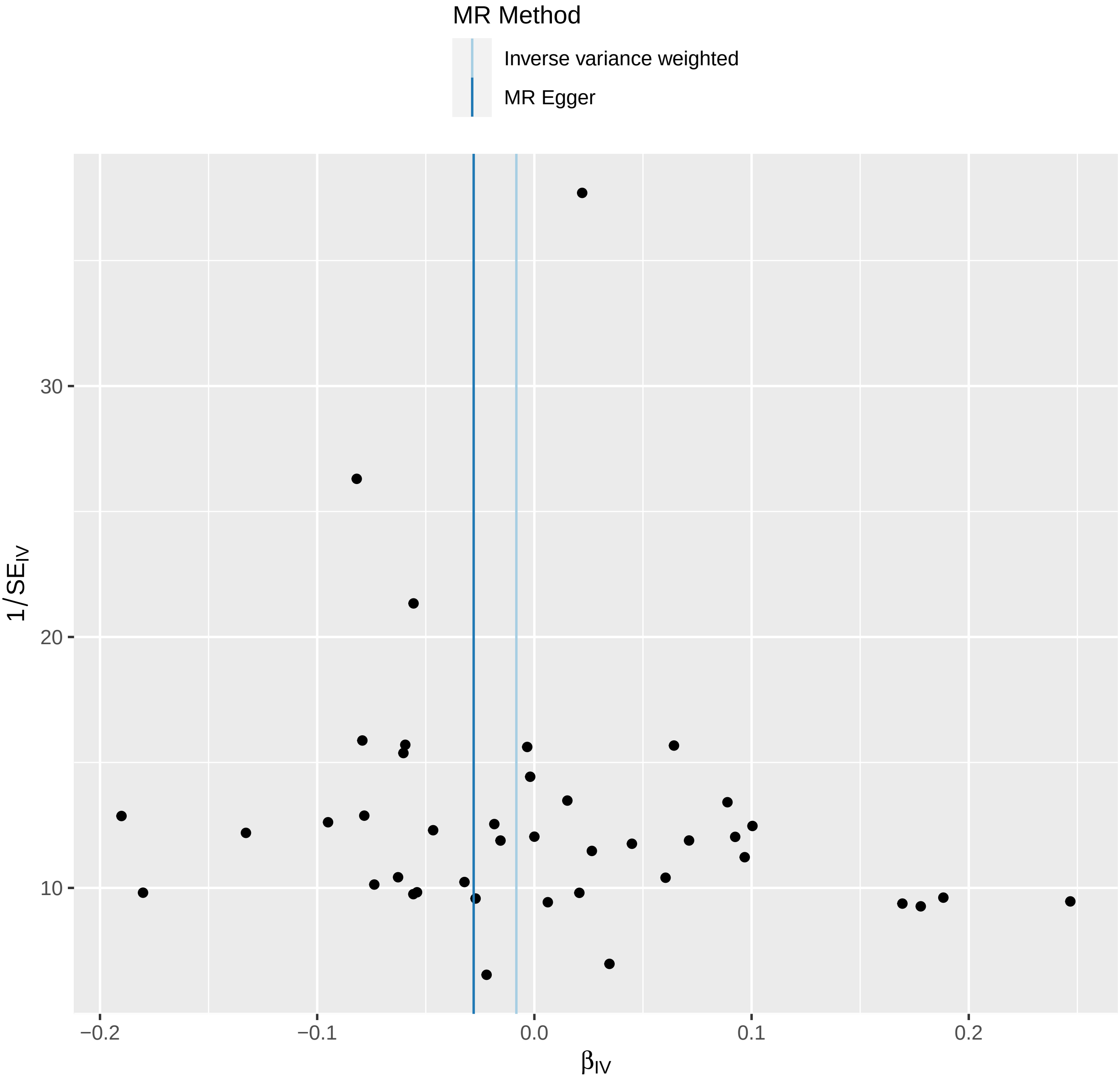

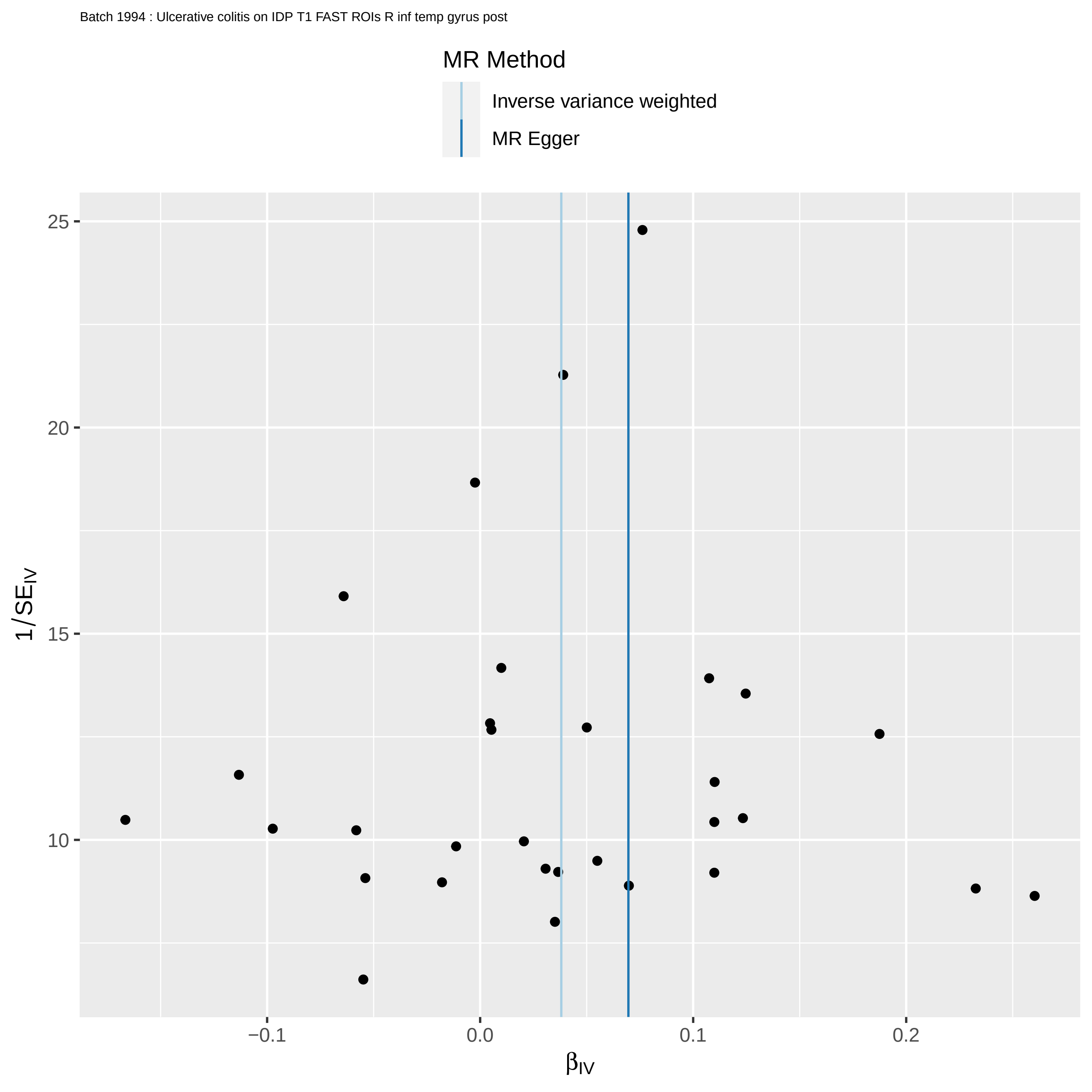

Batch 1994 : Ulcerative colitis on IDP T1 FAST ROIs R inf temp gyrus post
MR Method
Inverse variance weighted
MR Egger
25
20
15
10
1/SE$_{IV}$
−0.1
0.0
0.1
0.2
β$_{IV}$

Batch 2015 : Crohn's disease on IDP T1 FAST ROIs L inf temp gyrus tempocc

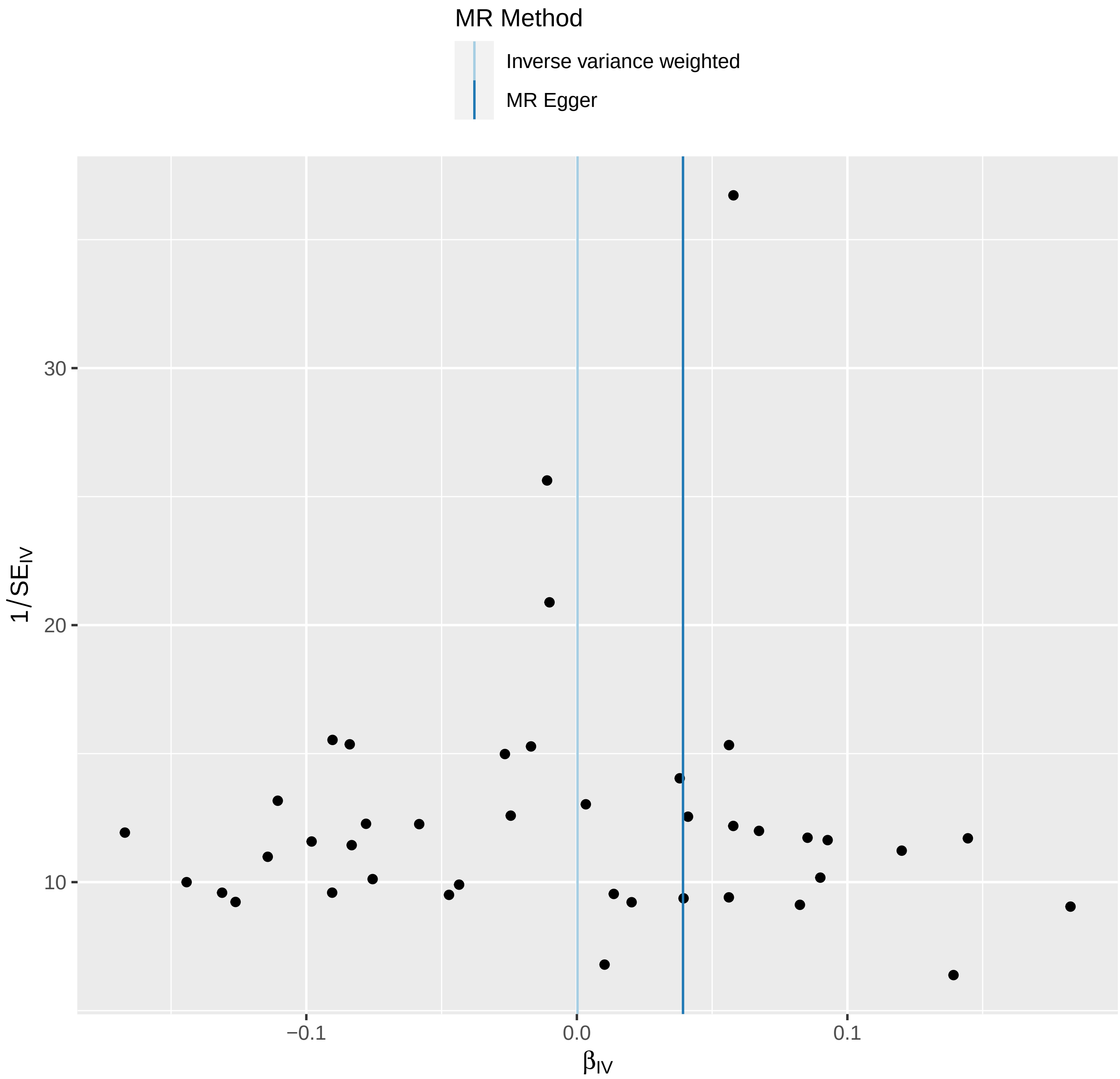

Batch 2016 : Ulcerative colitis on IDP T1 FAST ROIs L inf temp gyrus tempocc

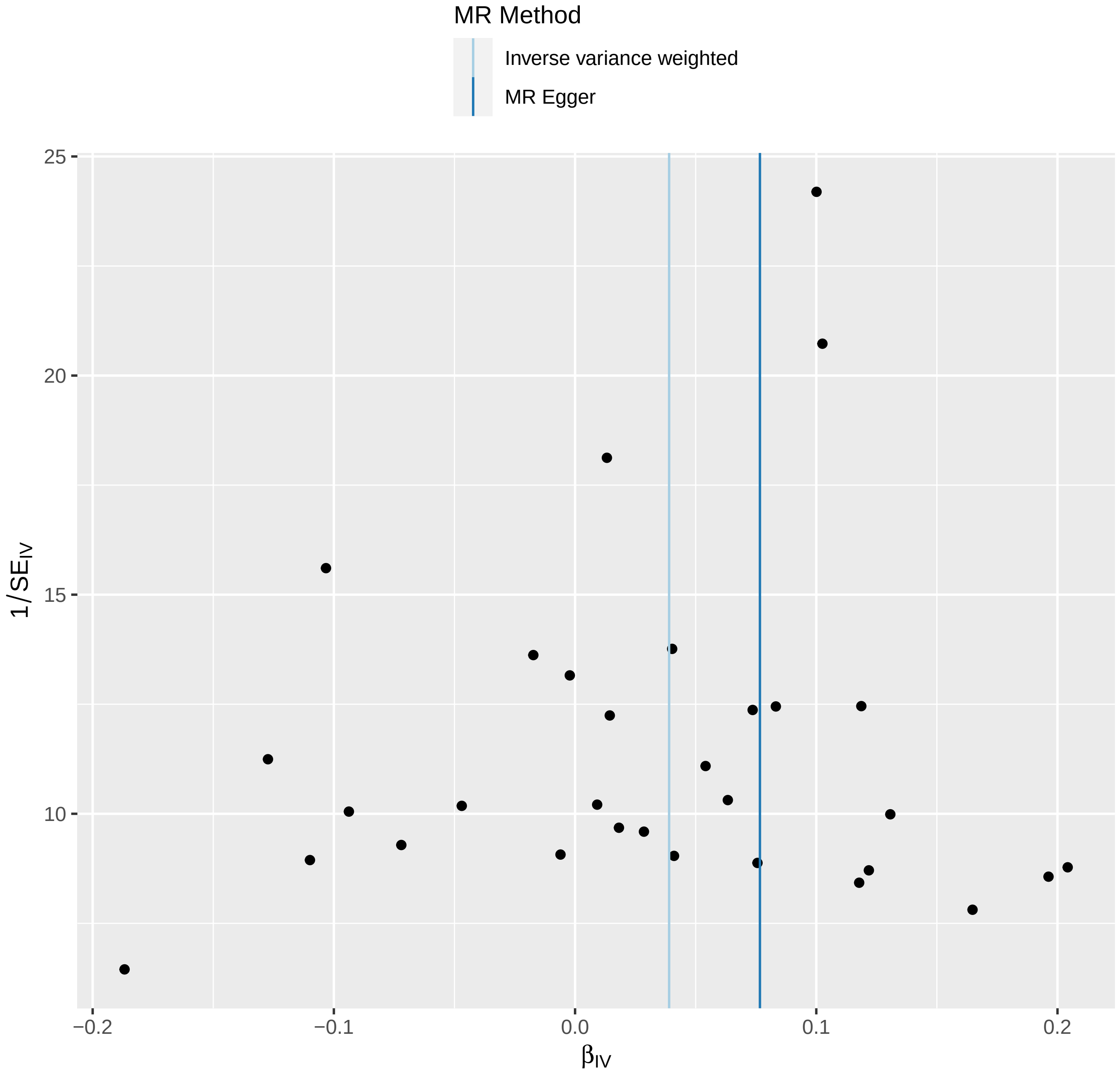

Batch 2037 : Crohn's disease on IDP T1 FAST ROIs R inf temp gyrus tempocc

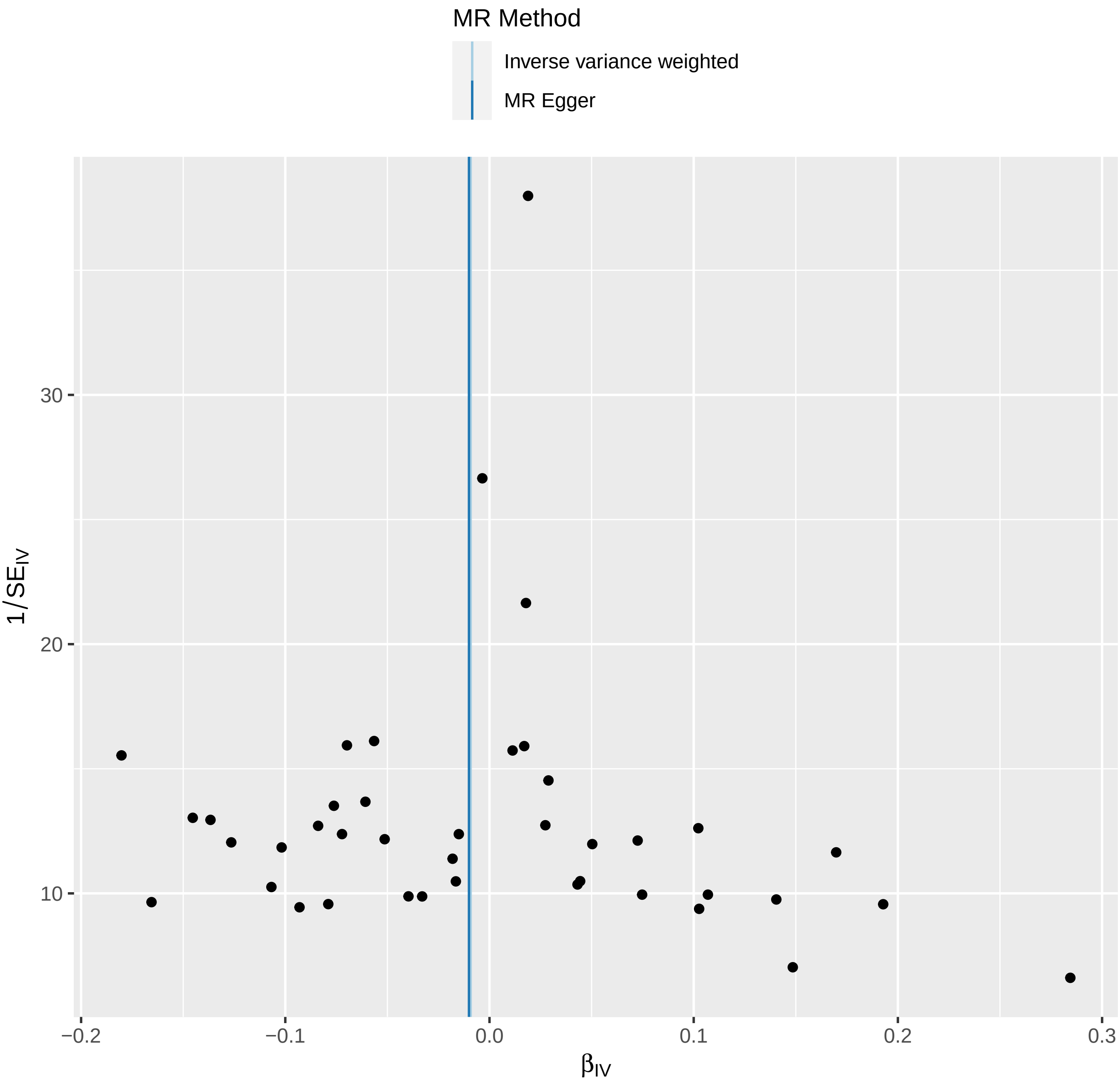

Batch 2038 : Ulcerative colitis on IDP T1 FAST ROIs R inf temp gyrus tempocc

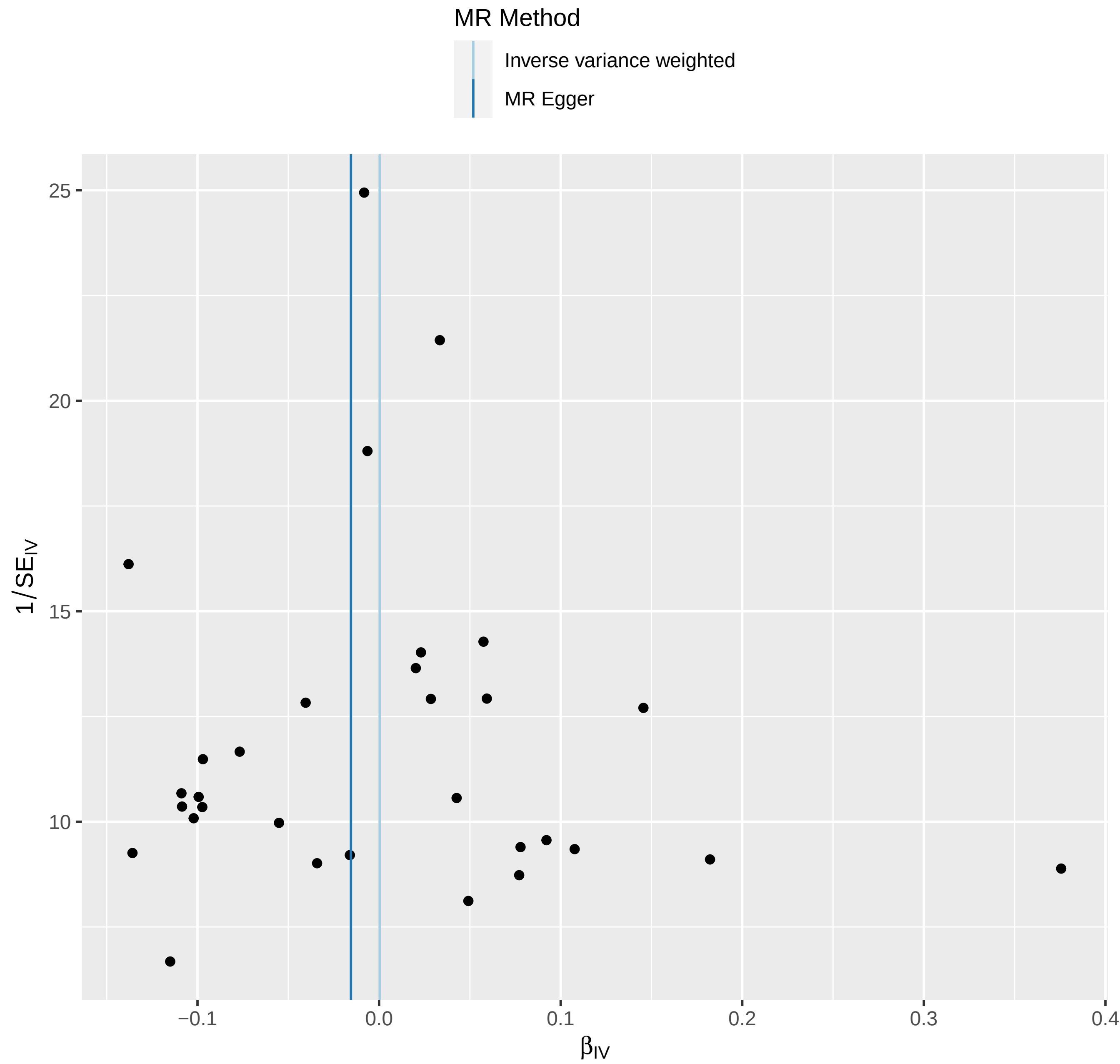

Batch 2059 : Crohn's disease on IDP T1 FAST ROIs L postcent gyrus

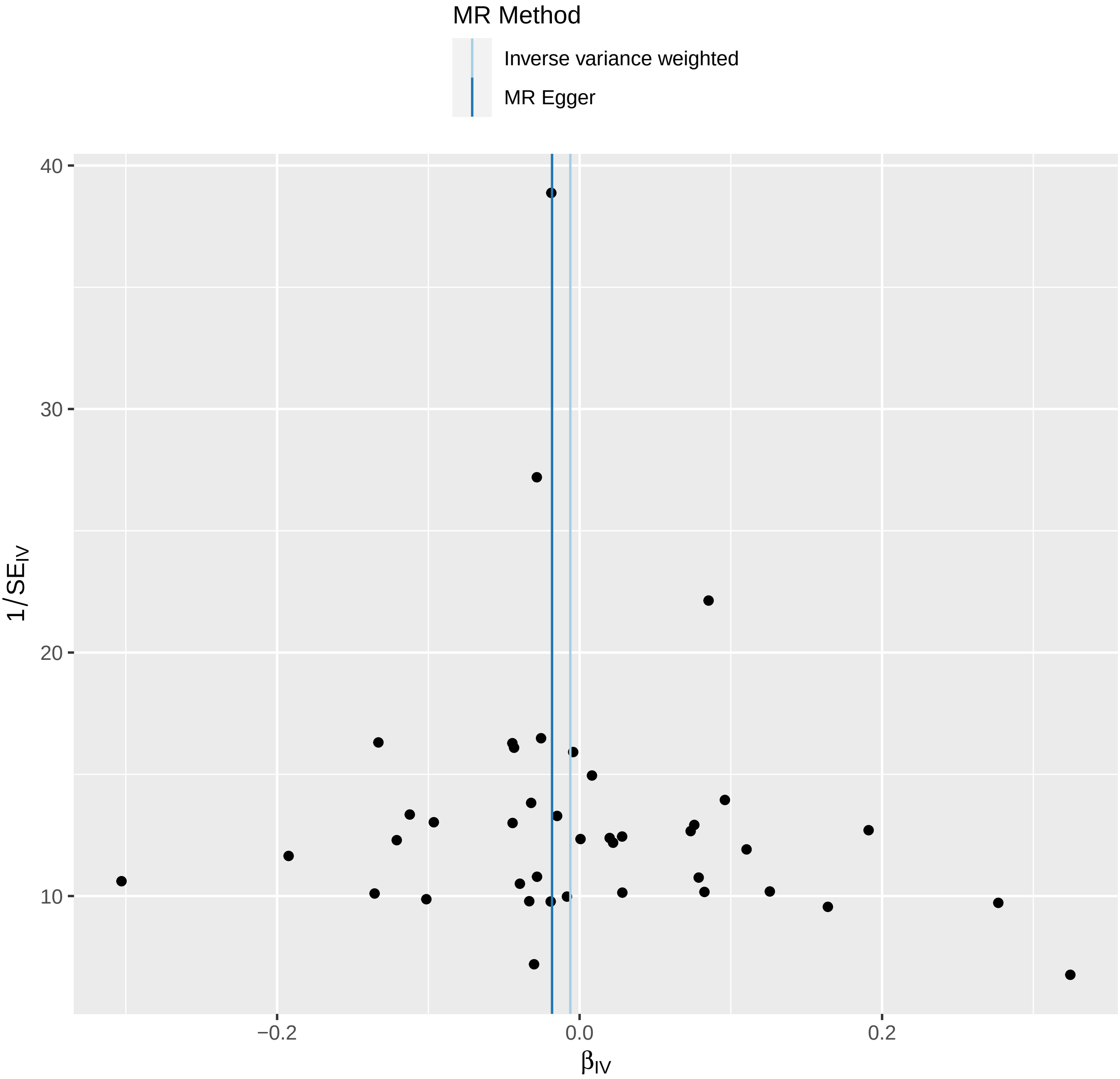

Batch 2060 : Ulcerative colitis on IDP T1 FAST ROIs L postcent gyrus

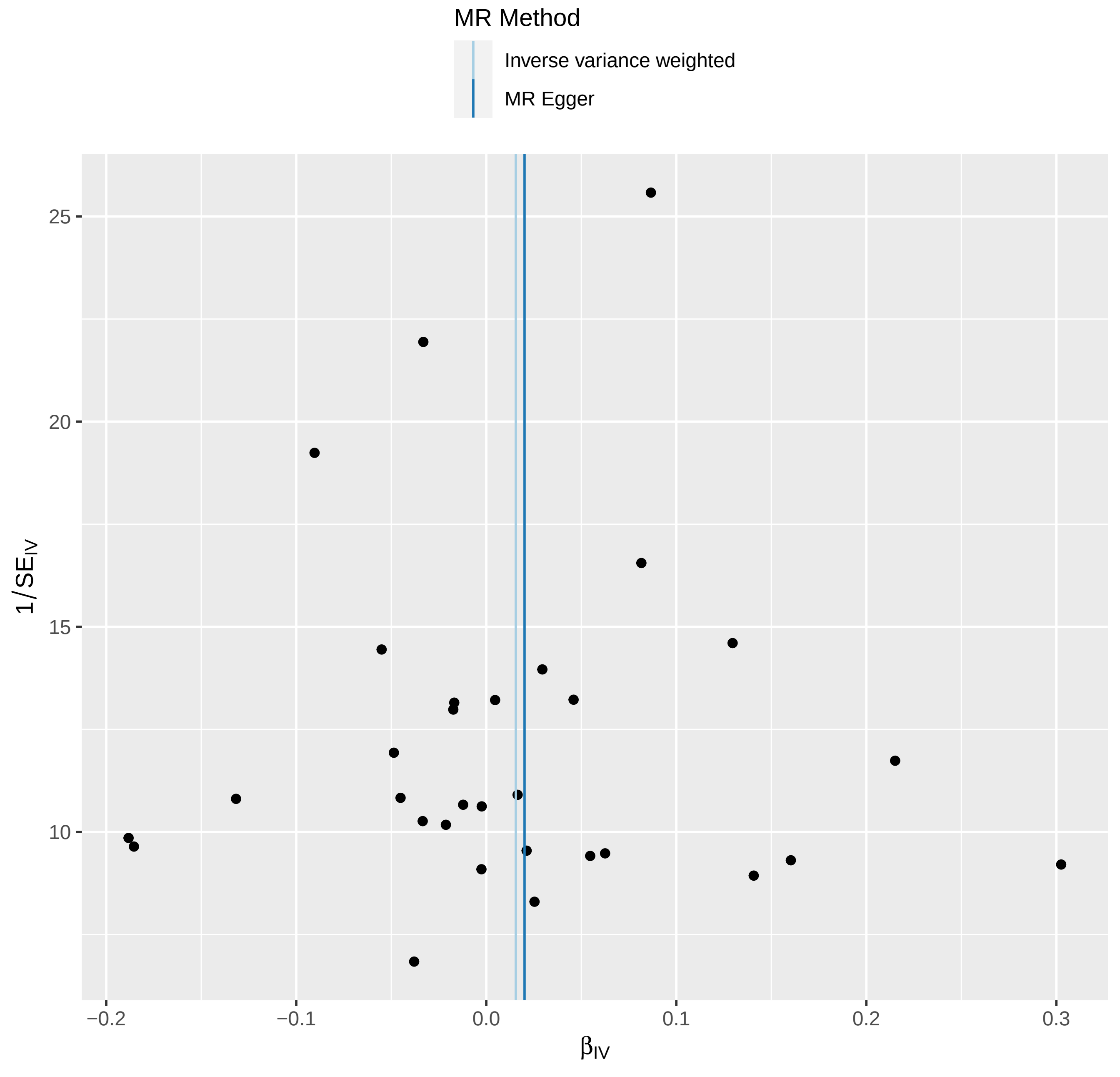

Batch 2081 : Crohn's disease on IDP T1 FAST ROIs R postcent gyrus

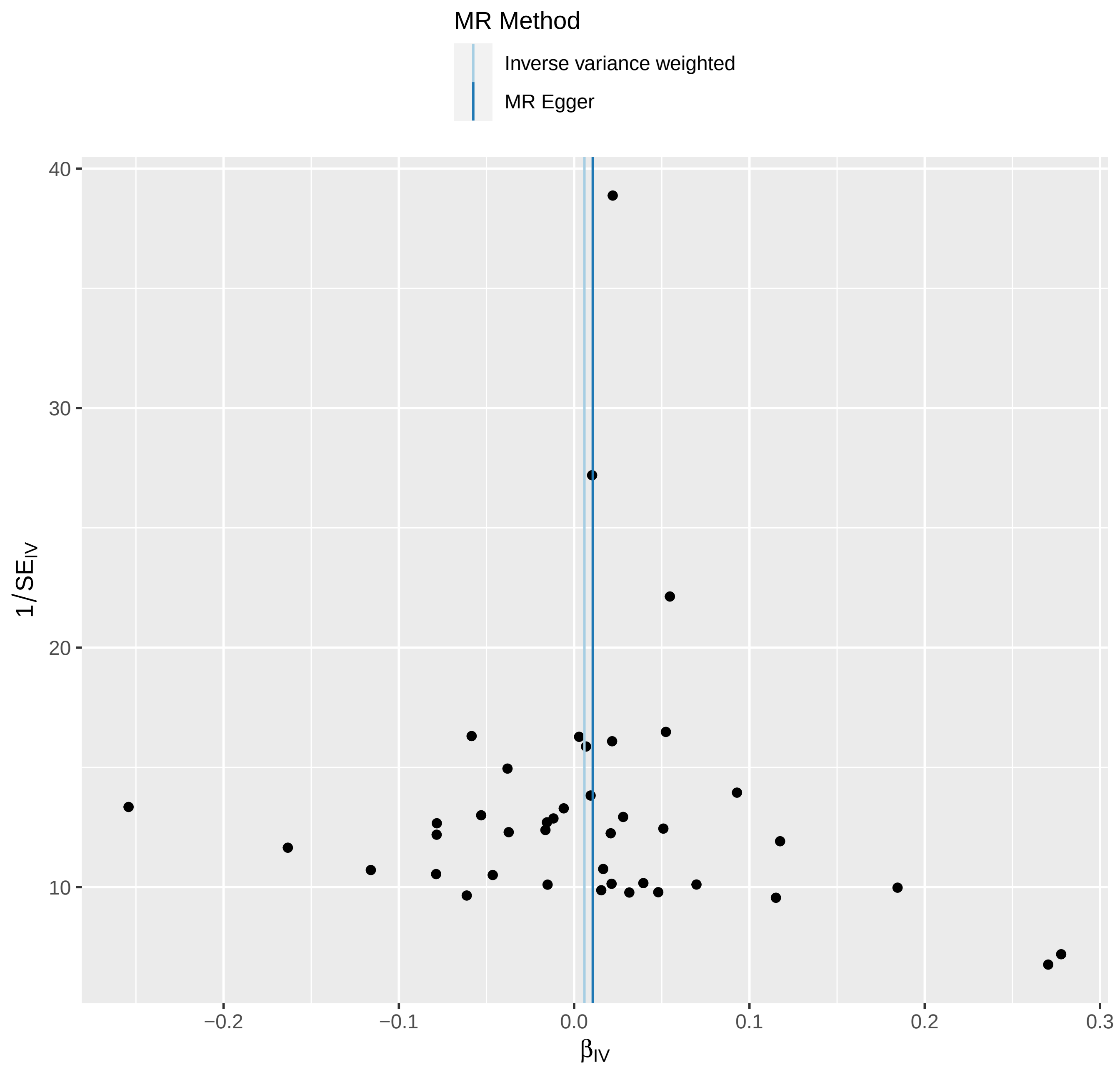

Batch 2082 : Ulcerative colitis on IDP T1 FAST ROIs R postcent gyrus

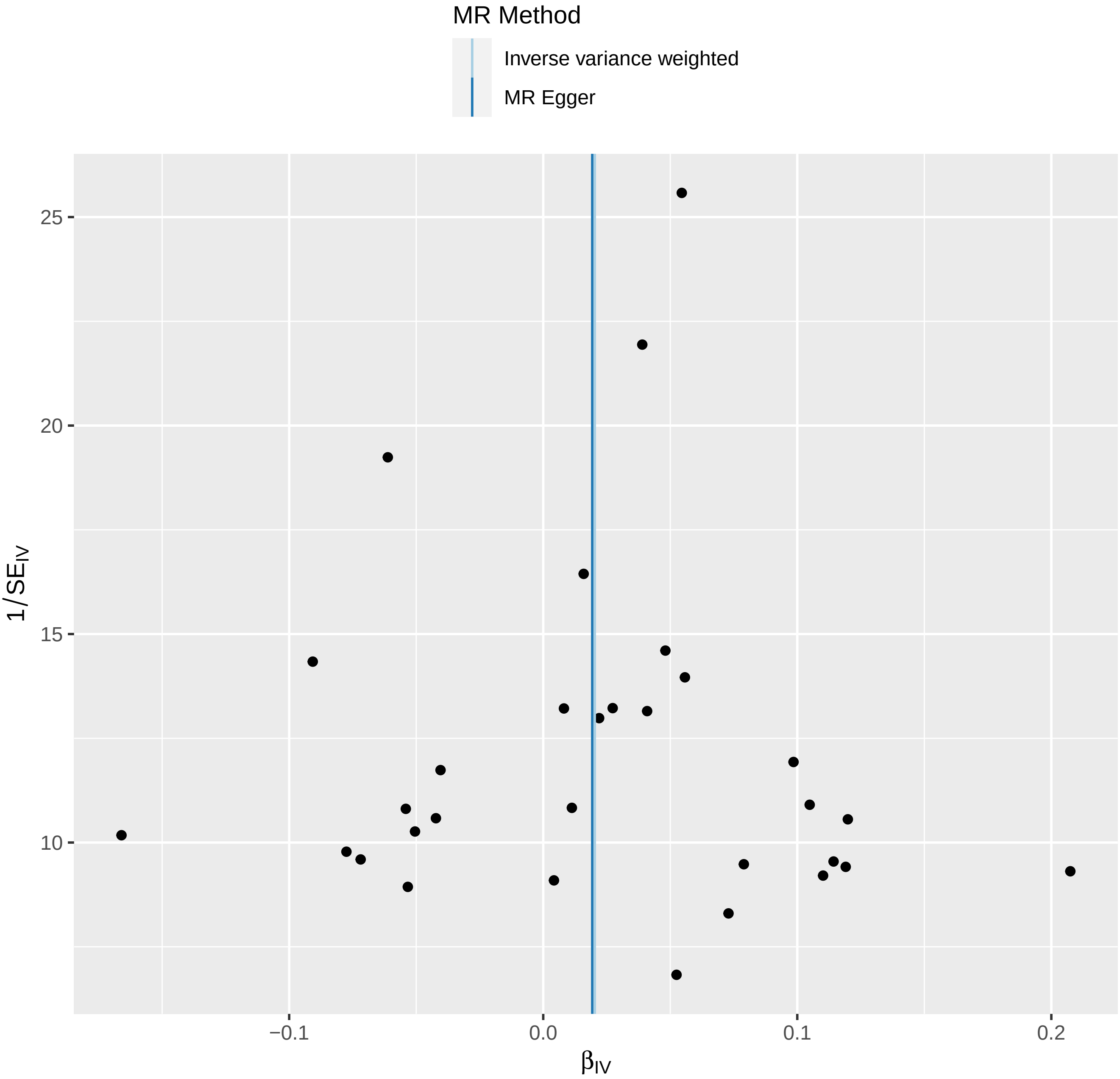

Batch 2103 : Crohn's disease on IDP T1 SIENAX grey unnormalised volume

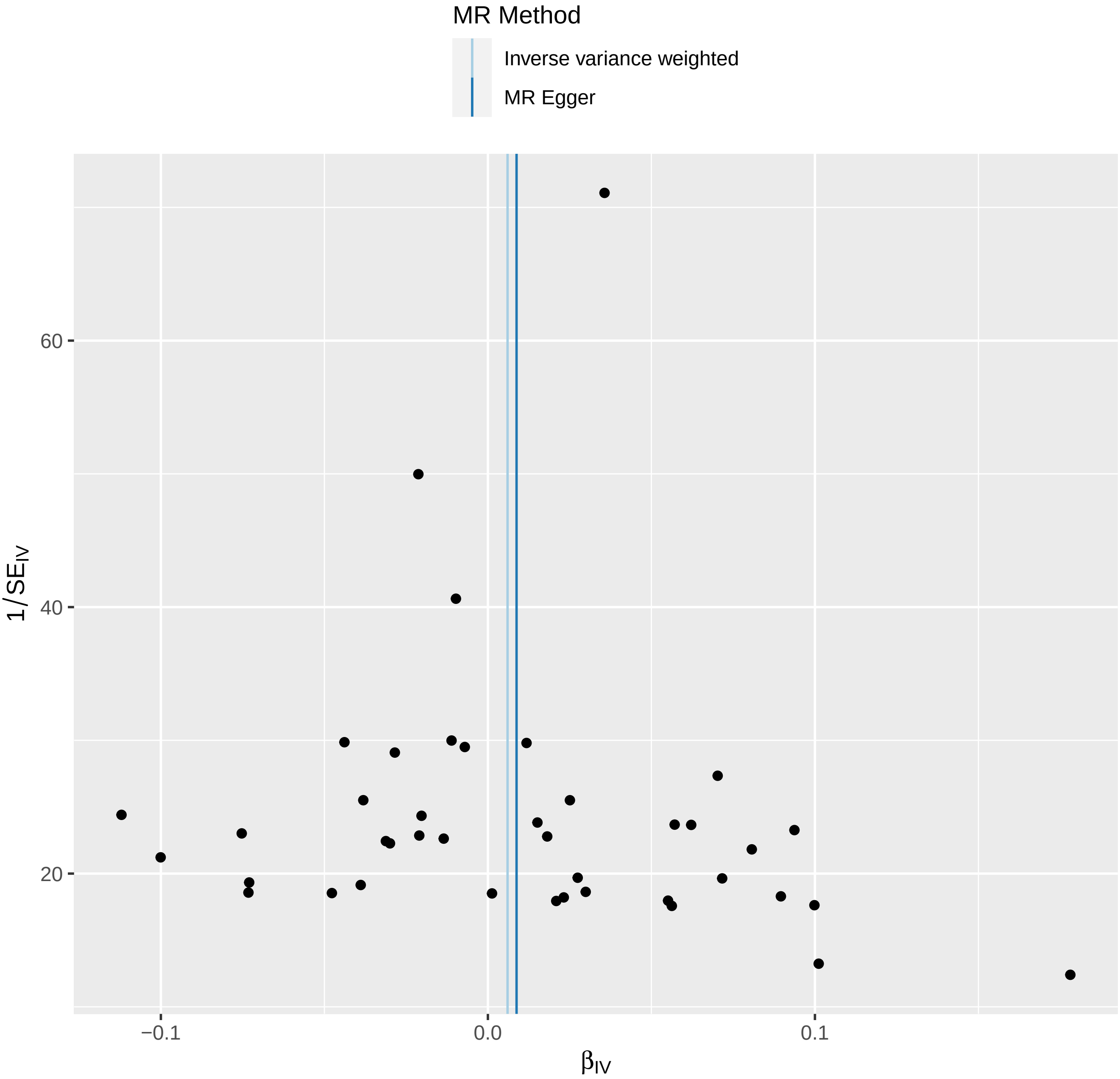

Batch 2104 : Ulcerative colitis on IDP T1 SIENAX grey unnormalised volume

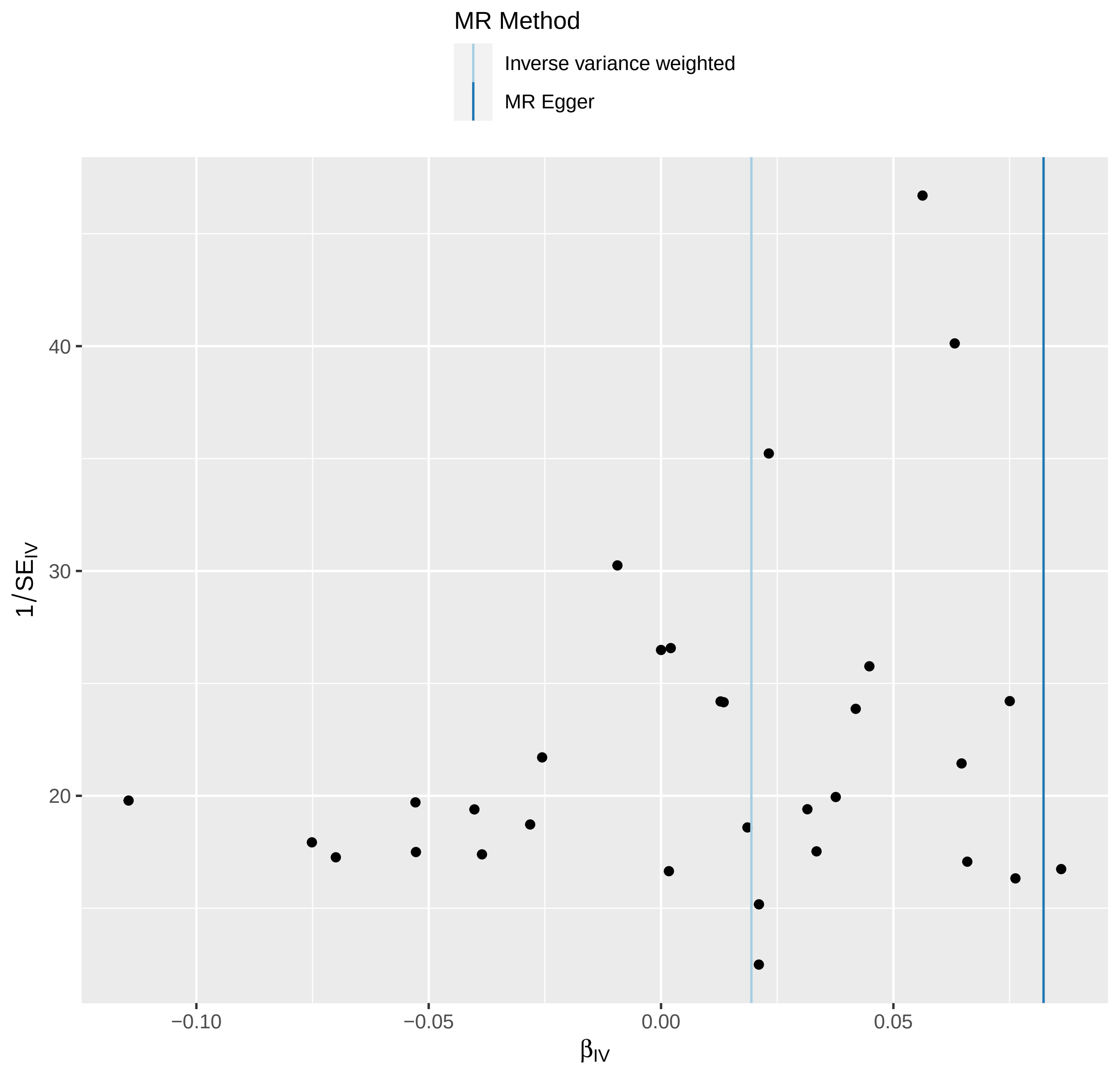

Batch 2105 : Crohn's disease on IDP T1 FAST ROIs L sup parietal lobule

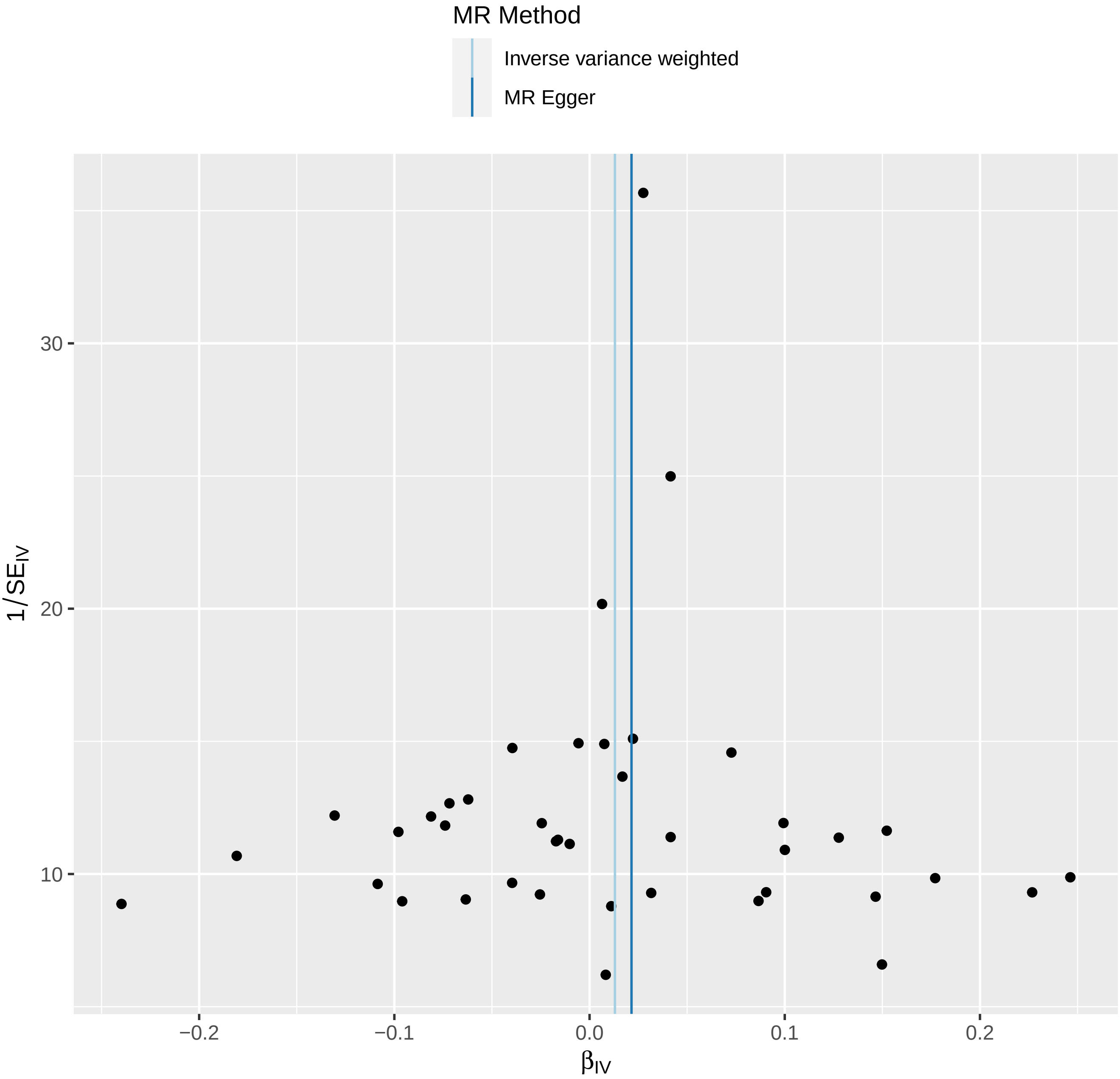

Batch 2106 : Ulcerative colitis on IDP T1 FAST ROIs L sup parietal lobule

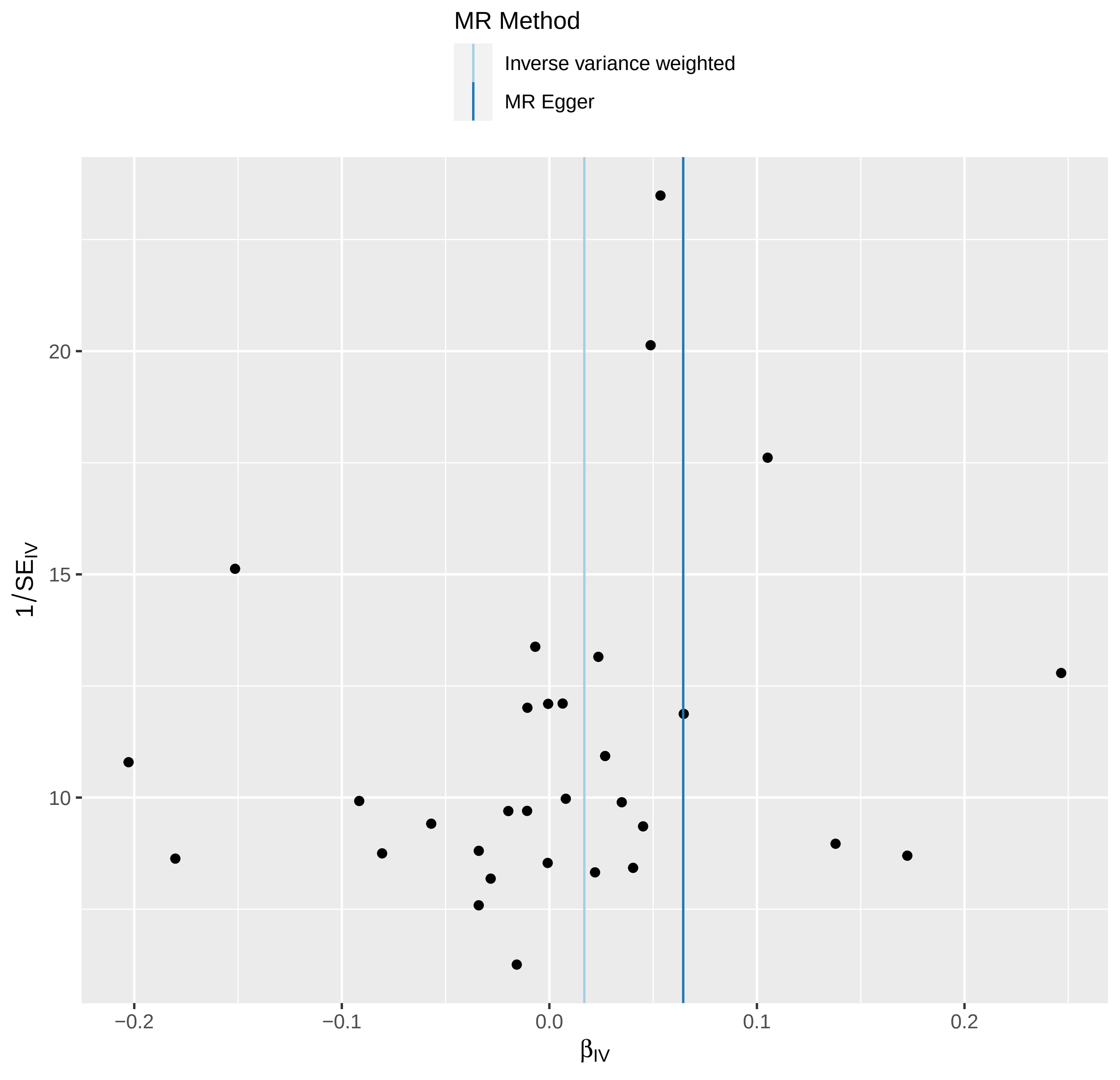

Batch 2127 : Crohn's disease on IDP T1 FAST ROIs R sup parietal lobule

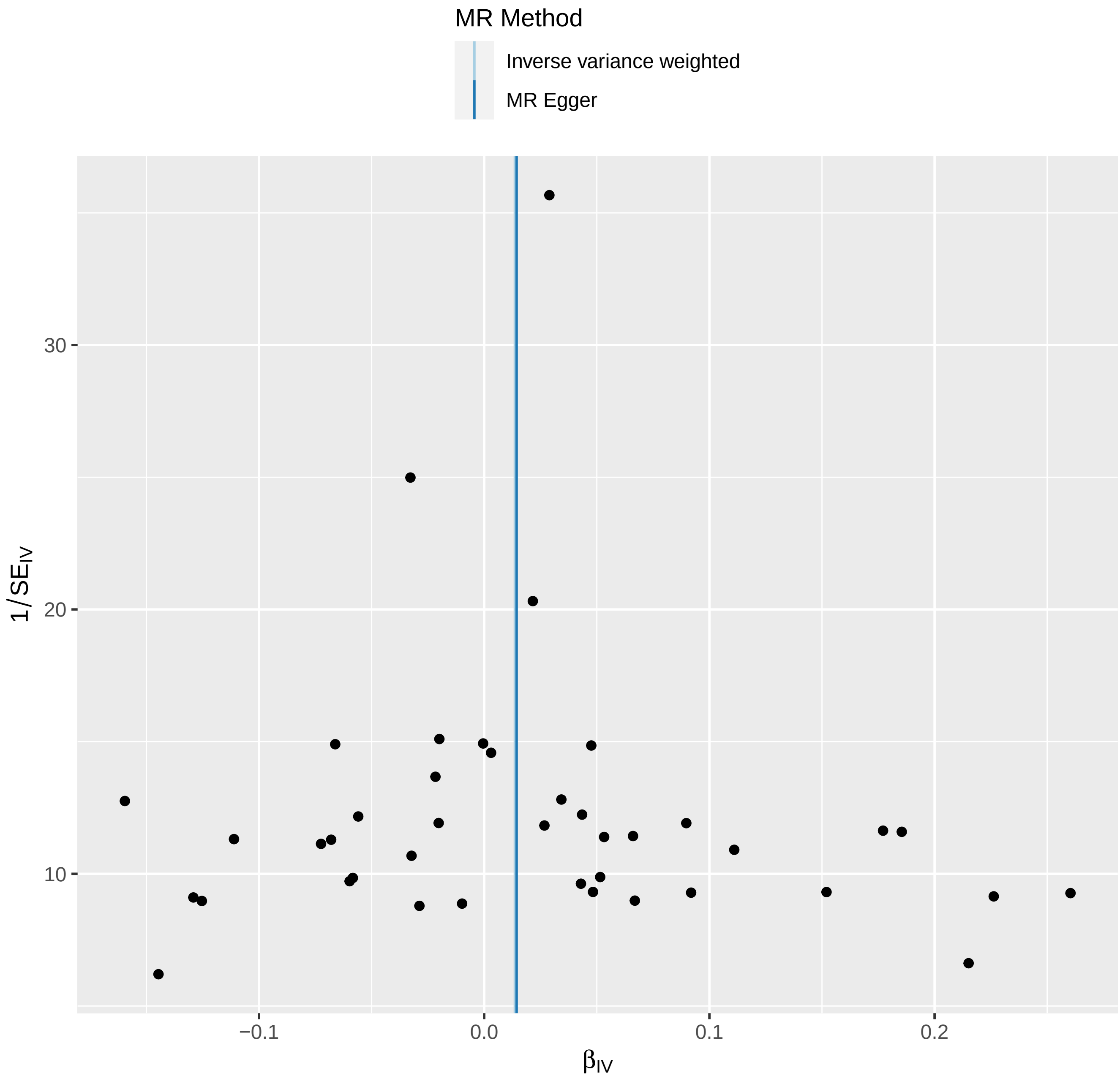

Batch 2128 : Ulcerative colitis on IDP T1 FAST ROIs R sup parietal lobule

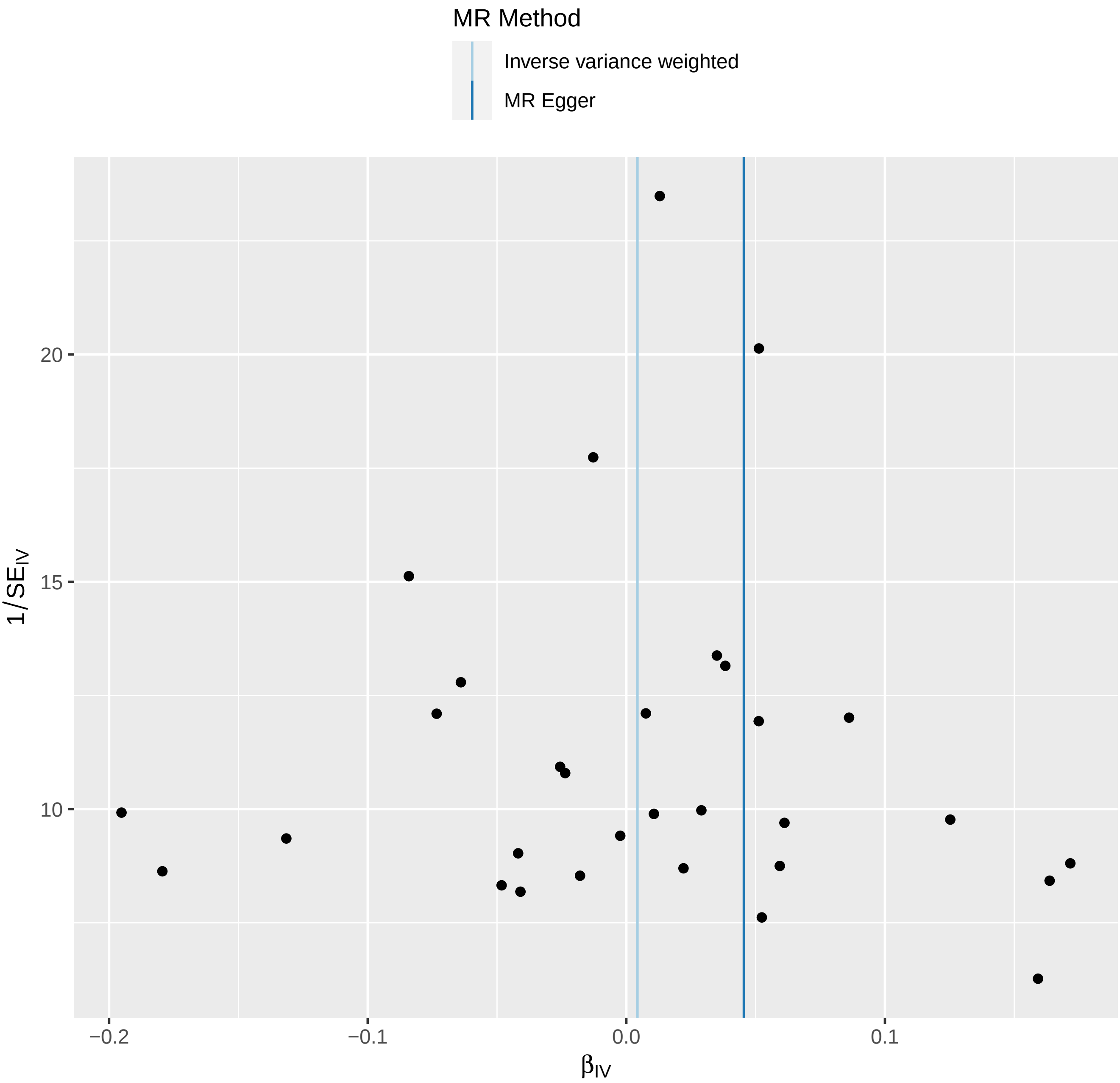

Batch 2149 : Crohn's disease on IDP T1 FAST ROIs L supramarg gyrus ant

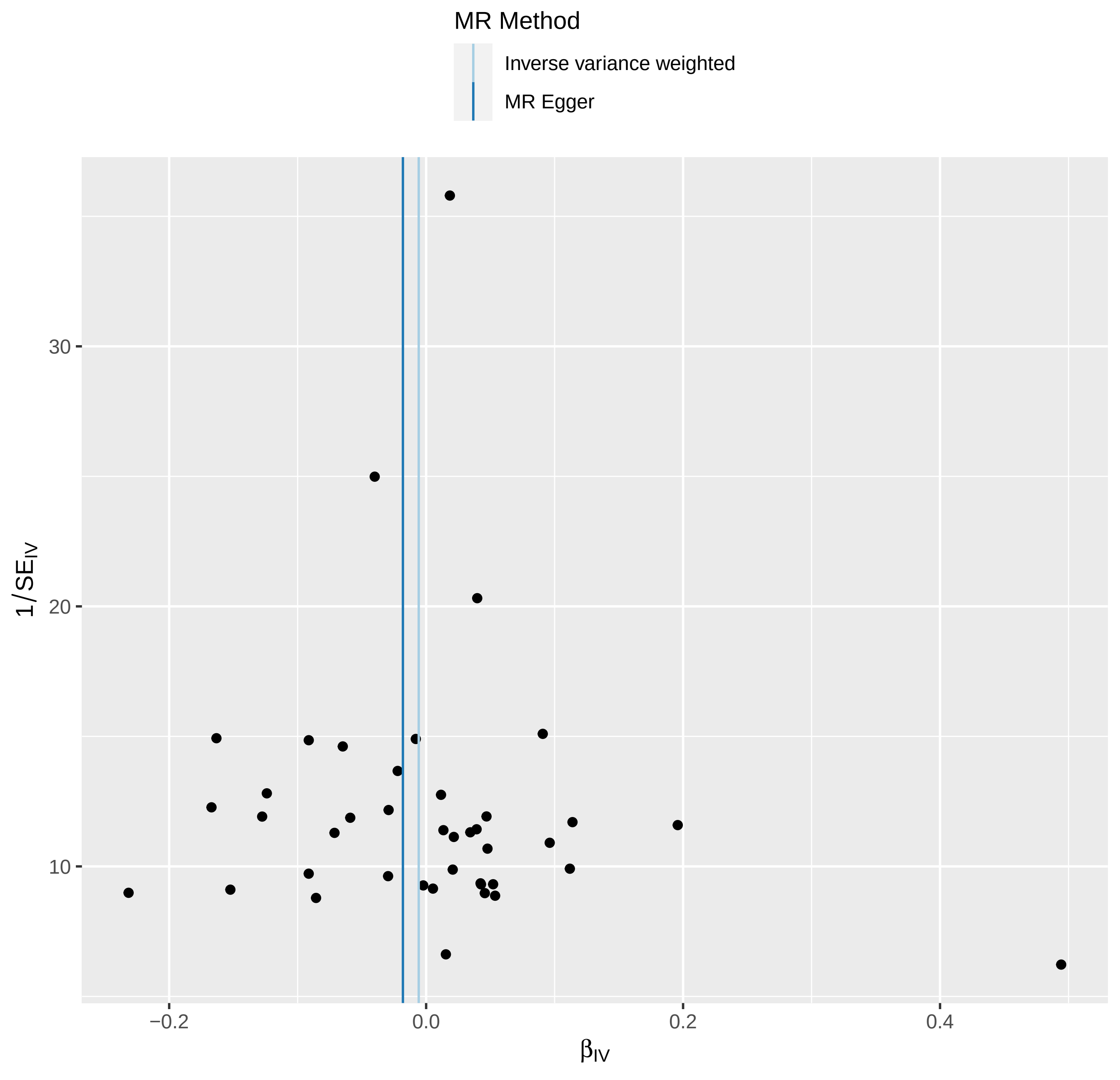

Batch 2150 : Ulcerative colitis on IDP T1 FAST ROIs L supramarg gyrus ant

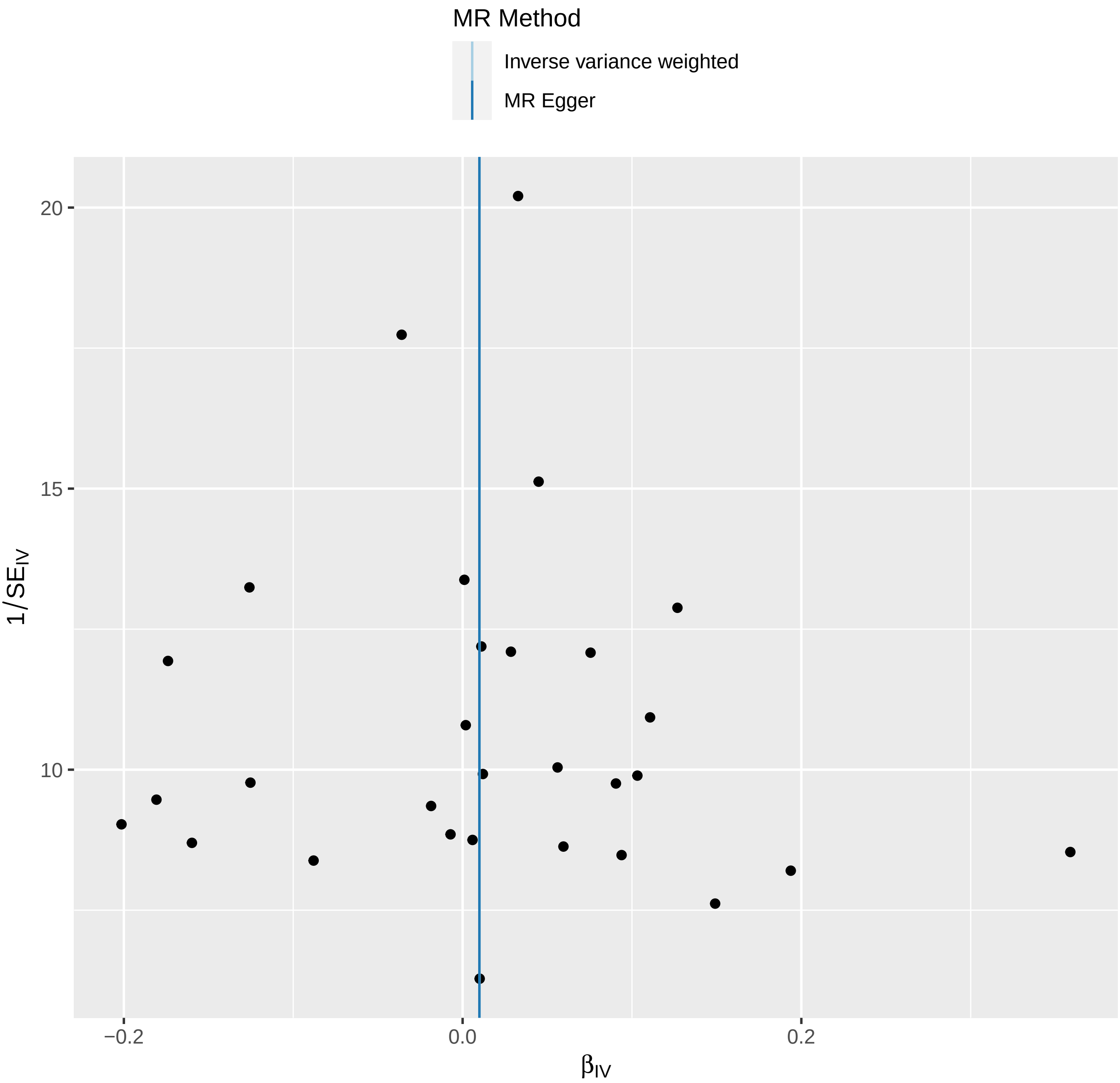

Batch 2171 : Crohn's disease on IDP T1 FAST ROIs R supramarg gyrus ant

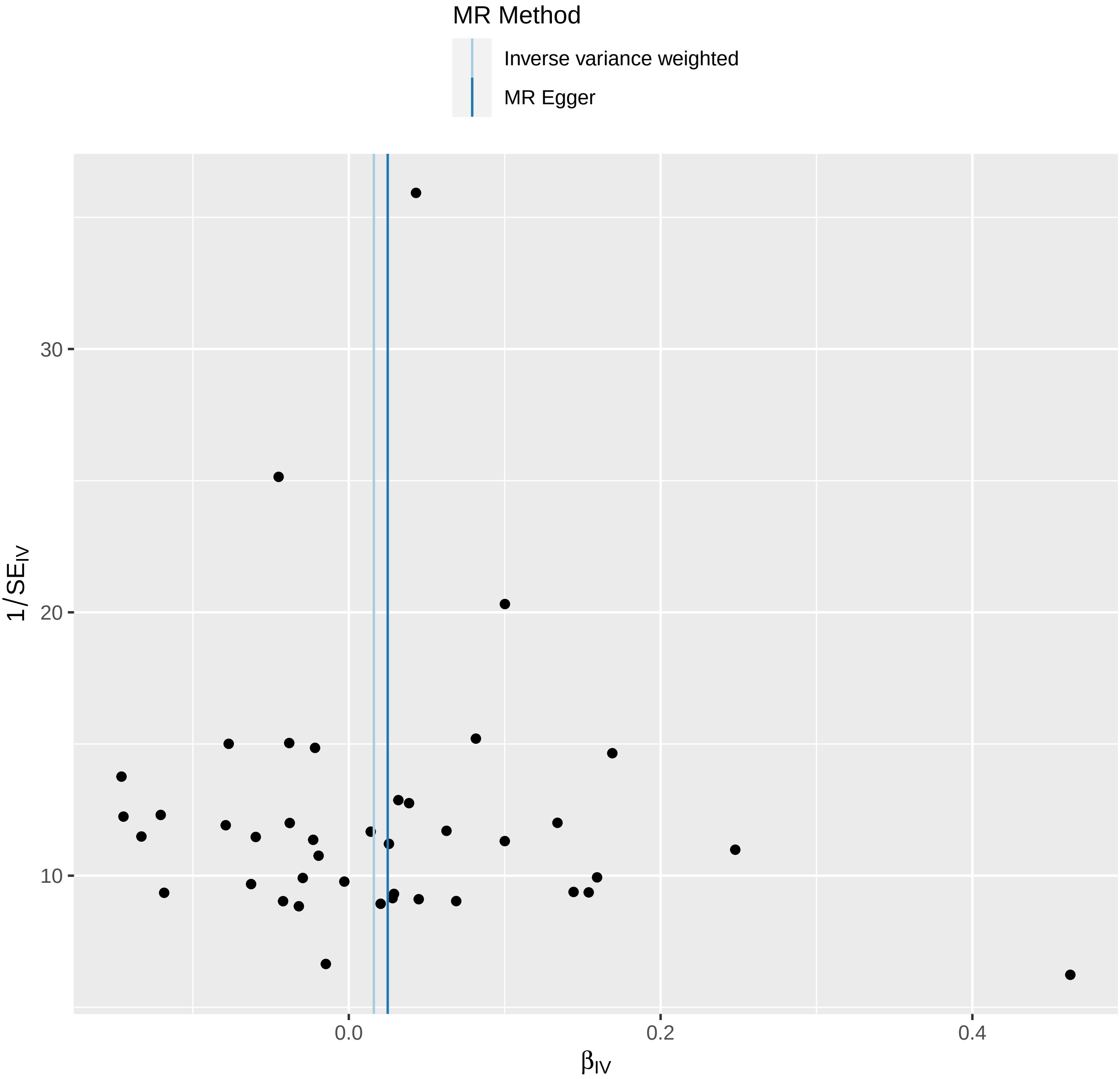

Batch 2172 : Ulcerative colitis on IDP T1 FAST ROIs R supramarg gyrus ant

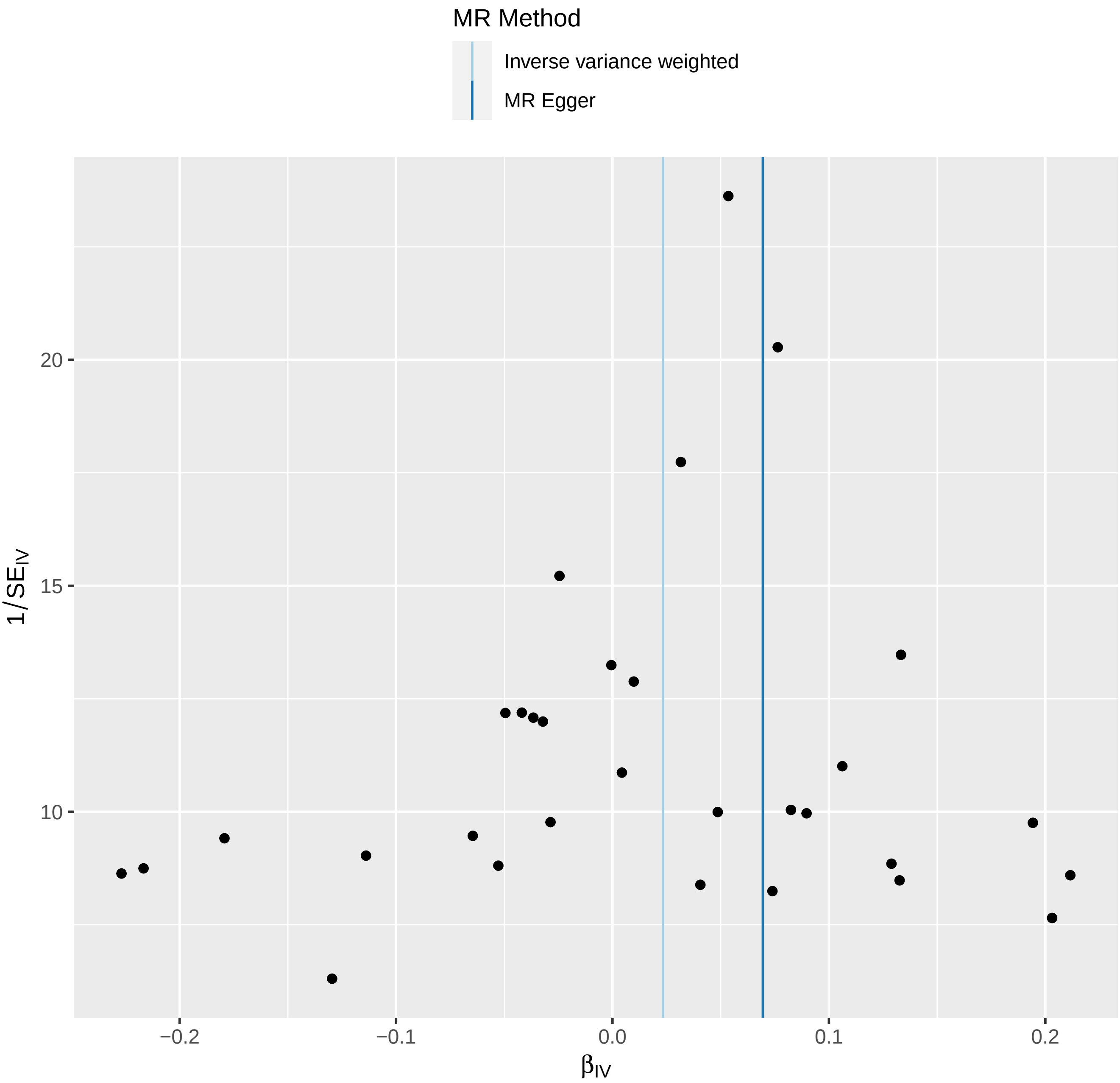

Batch 2193 : Crohn's disease on IDP T1 FAST ROIs L supramarg gyrus post

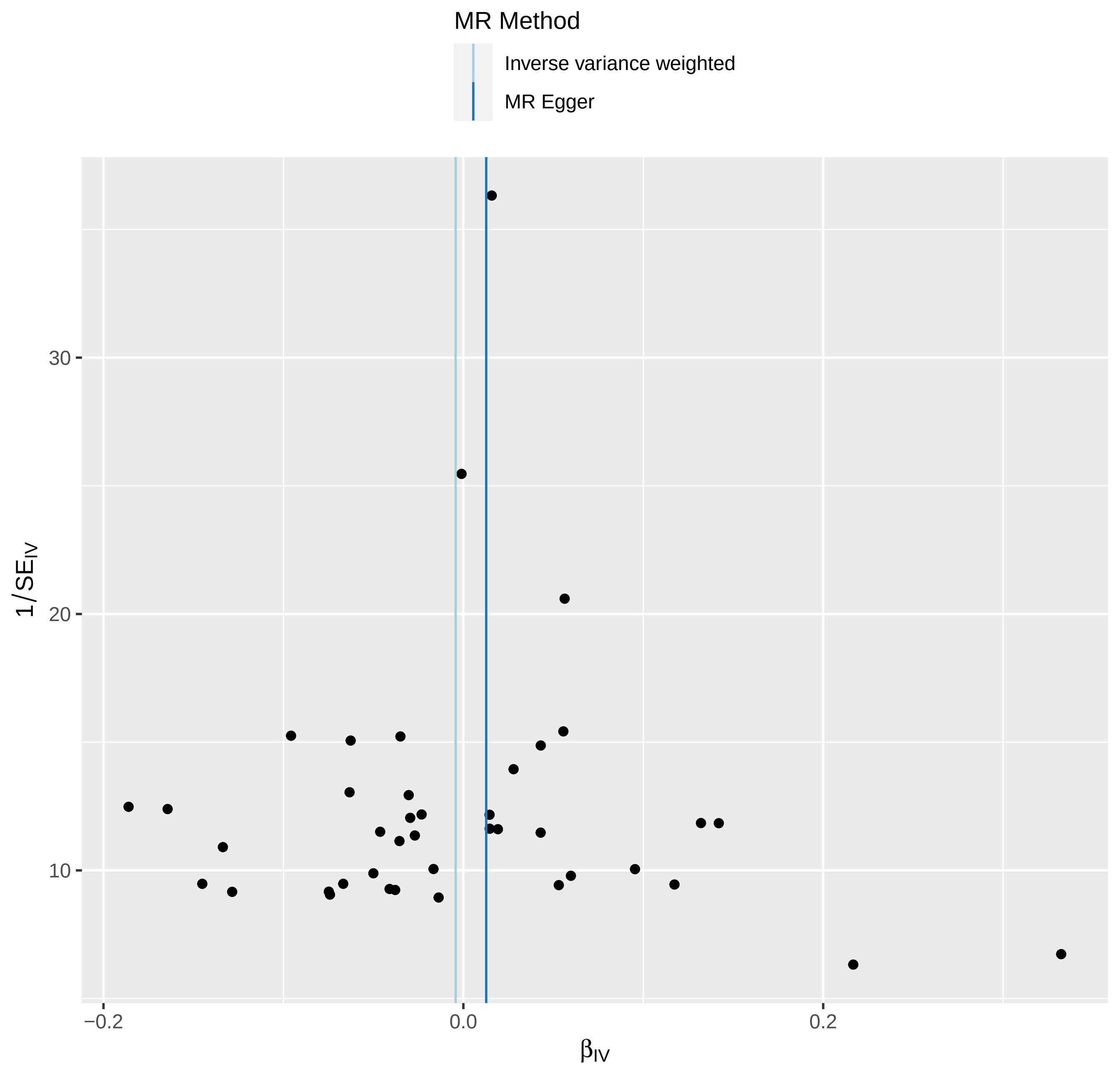

Batch 2194 : Ulcerative colitis on IDP T1 FAST ROIs L supramarg gyrus post

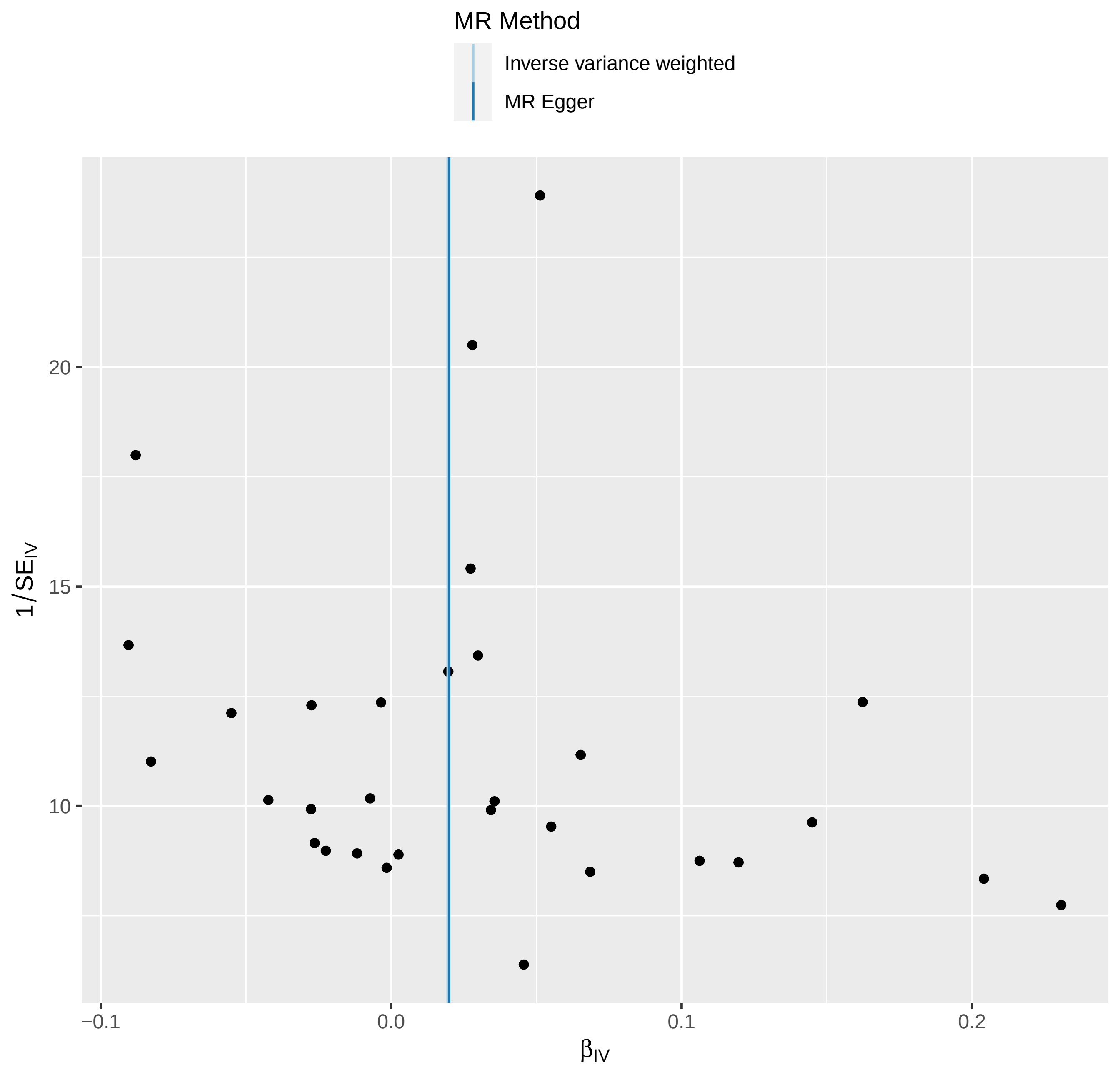

Batch 2215 : Crohn's disease on IDP T1 FAST ROIs R supramarg gyrus post

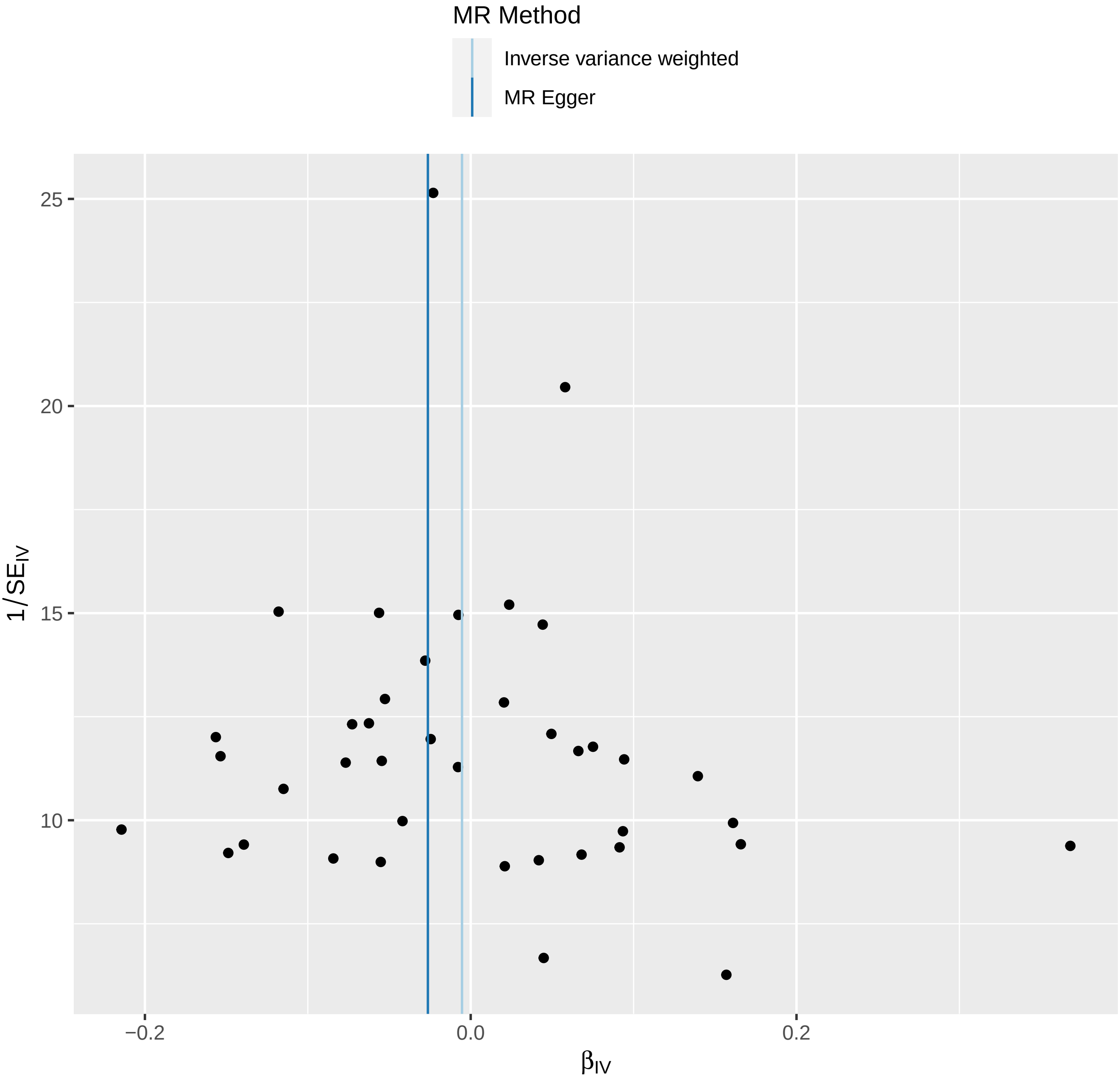

Batch 2216 : Ulcerative colitis on IDP T1 FAST ROIs R supramarg gyrus post

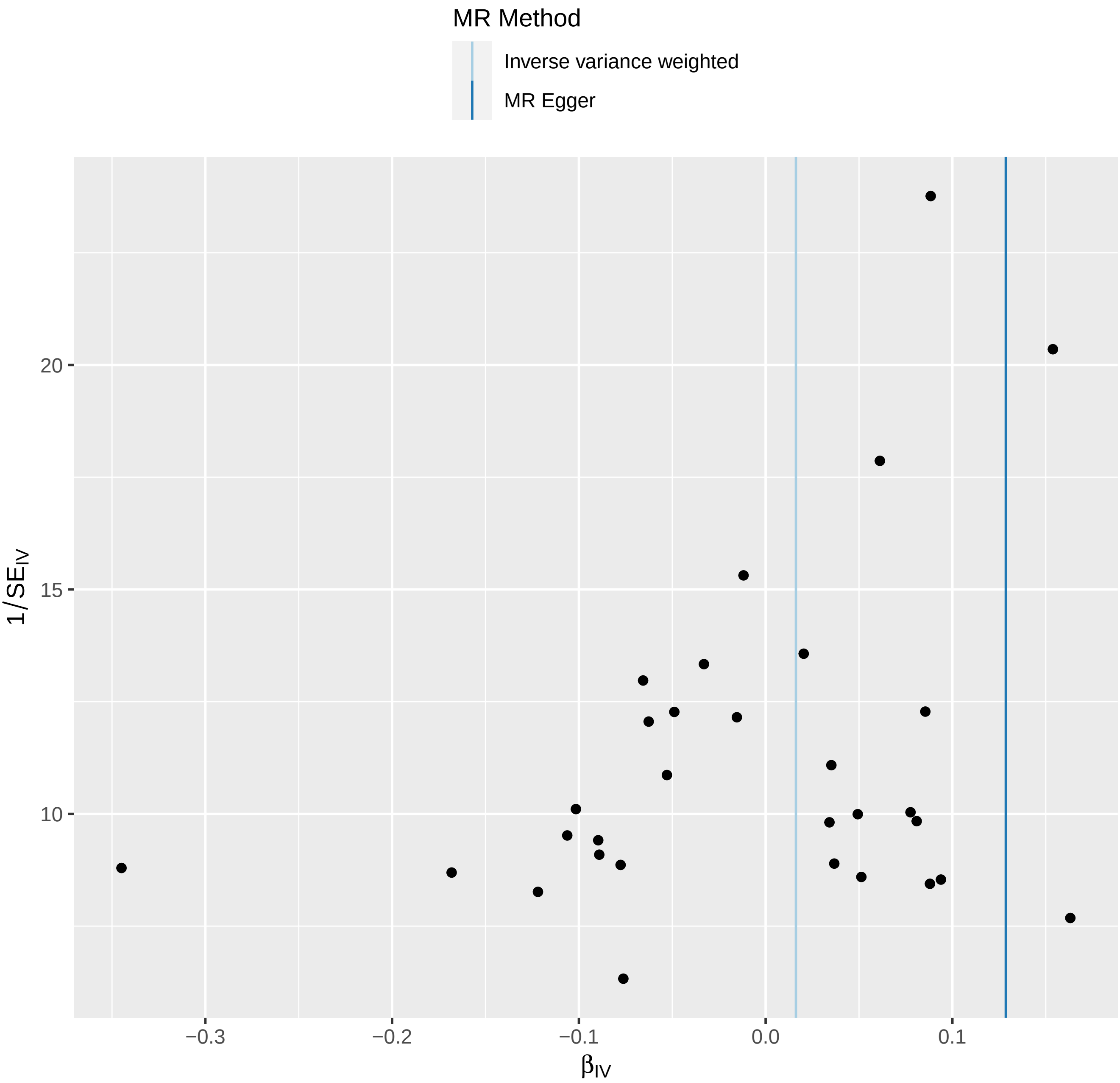

Batch 2237 : Crohn's disease on IDP T1 FAST ROIs L angular gyrus

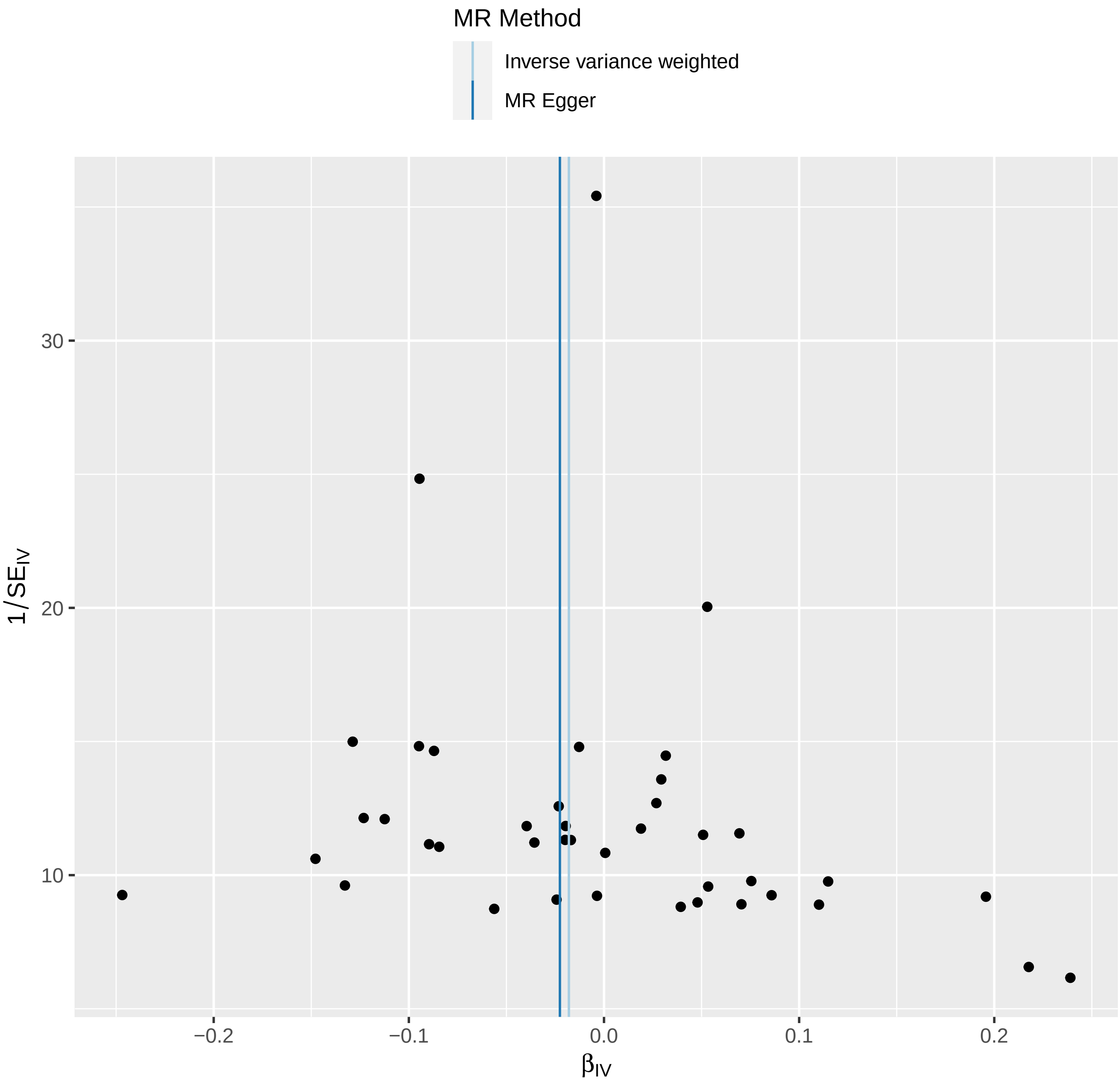

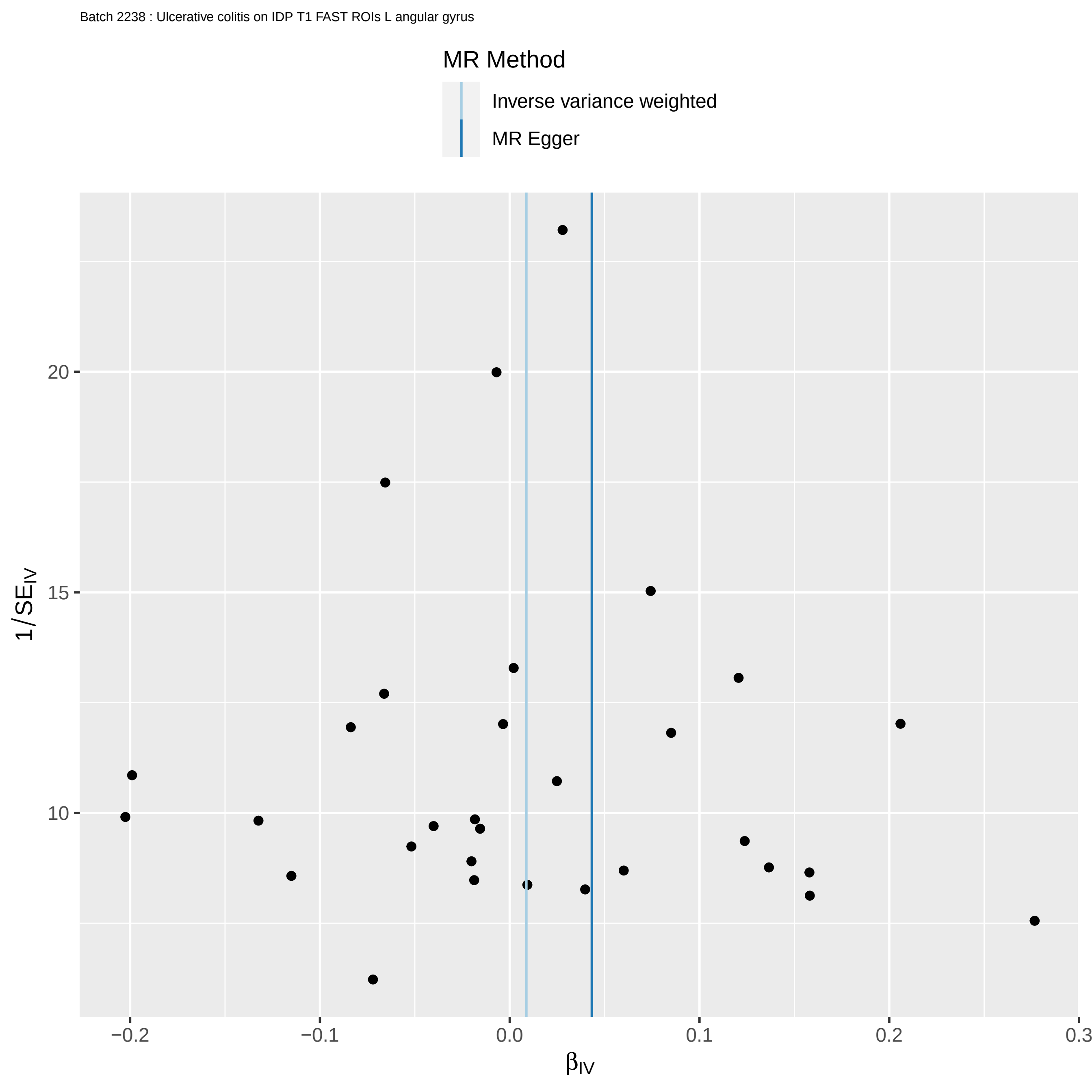
Batch 2238 : Ulcerative colitis on IDP T1 FAST ROIs L angular gyrus
MR Method
Inverse variance weighted
MR Egger
20
15
10
1/SE$_{IV}$
−0.2
−0.1
0.0
0.1
0.2
0.3
β$_{IV}$

Batch 2259 : Crohn's disease on IDP T1 FAST ROIs R angular gyrus

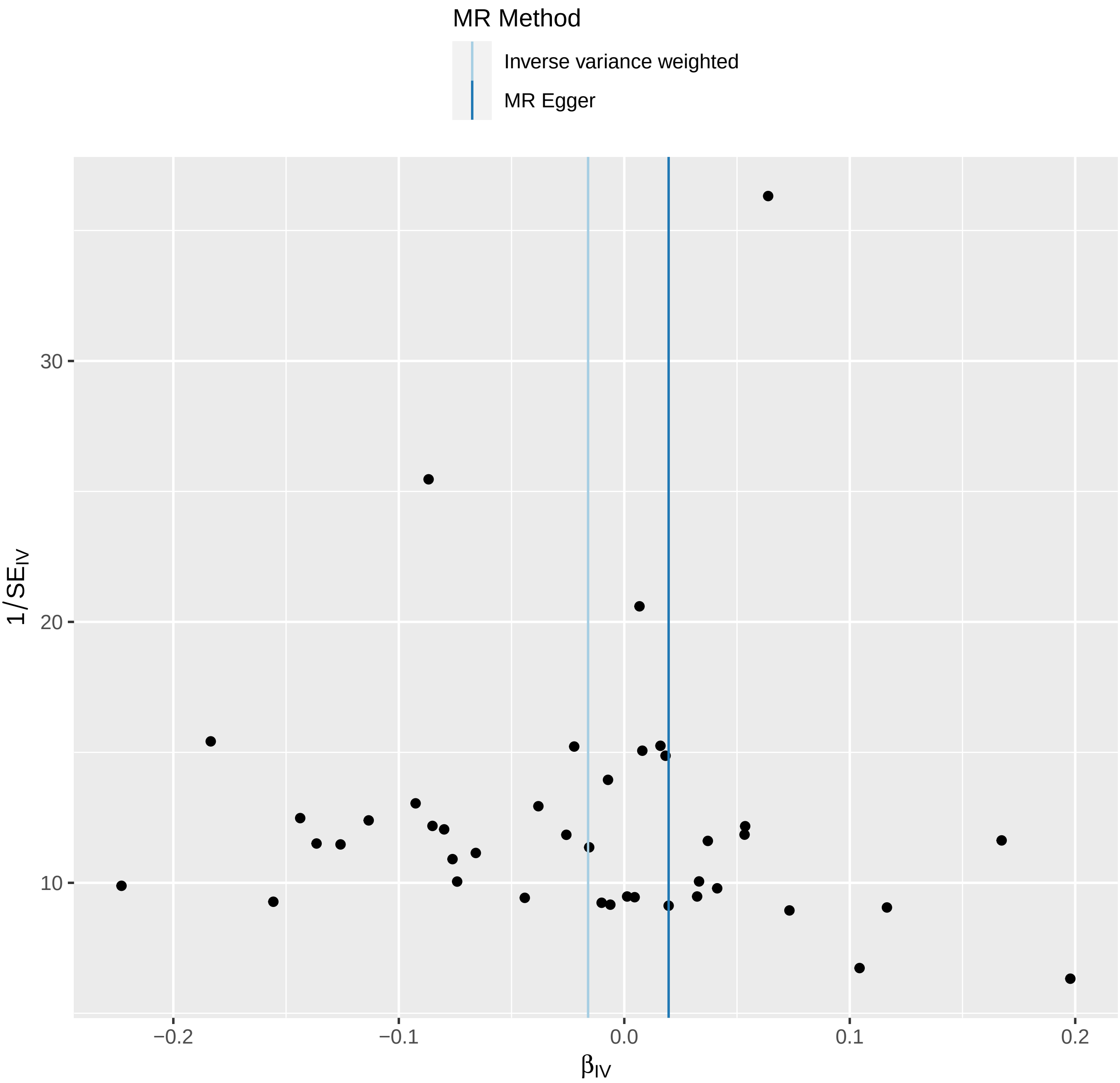

Batch 2260 : Ulcerative colitis on IDP T1 FAST ROIs R angular gyrus

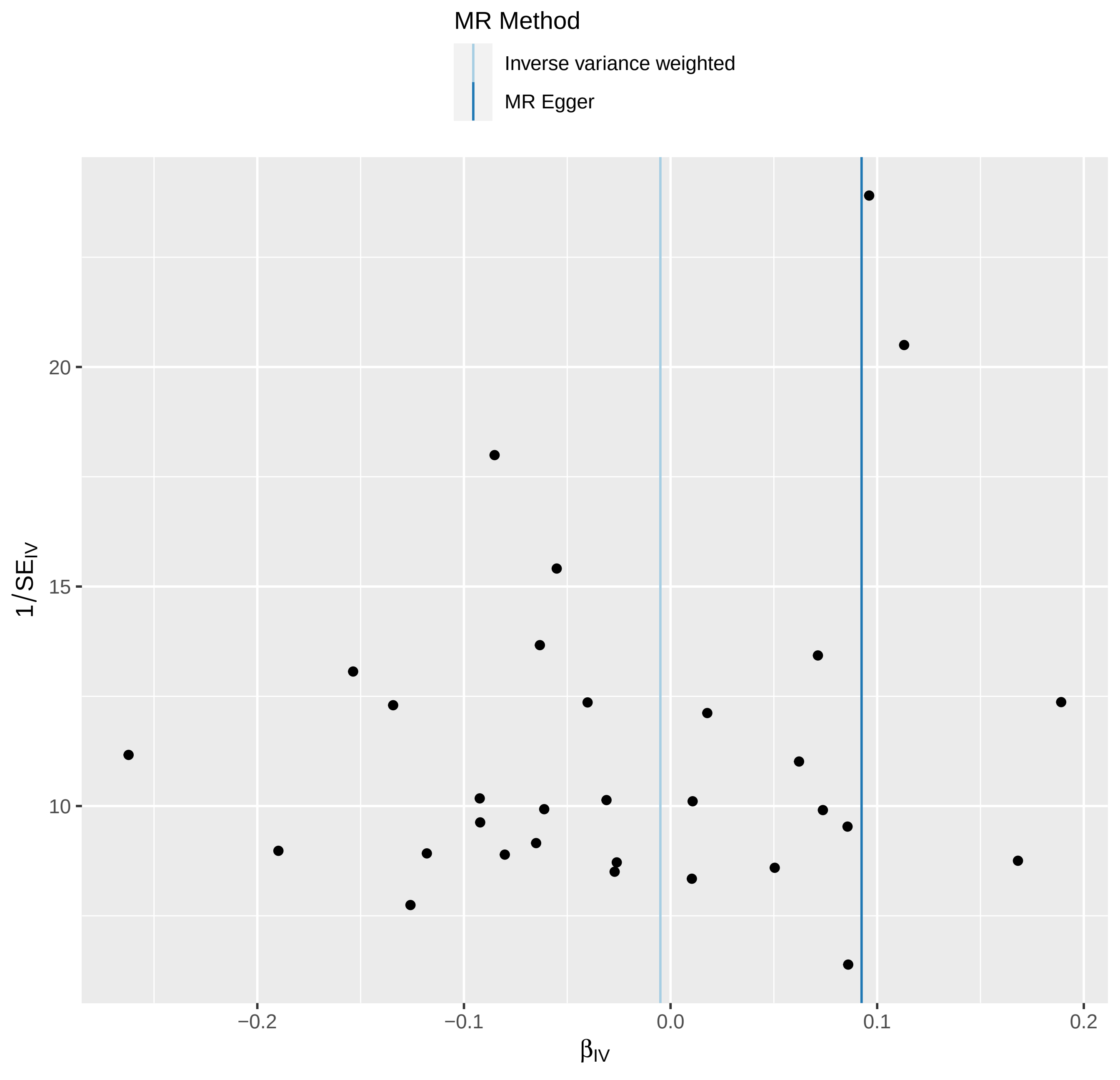

Batch 2281 : Crohn's disease on IDP T1 FAST ROIs L latocc cortex sup

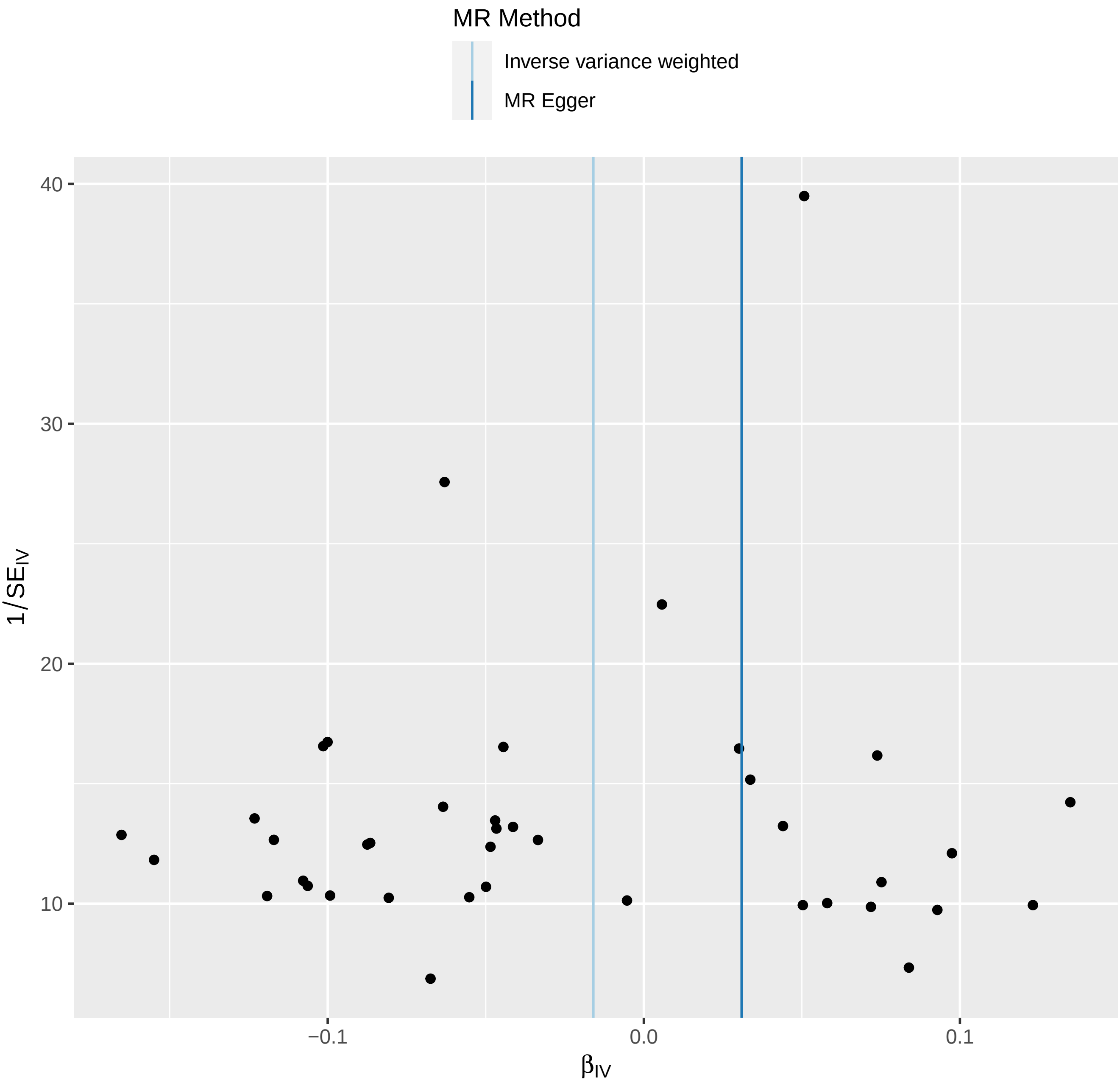

Batch 2282 : Ulcerative colitis on IDP T1 FAST ROIs L latocc cortex sup

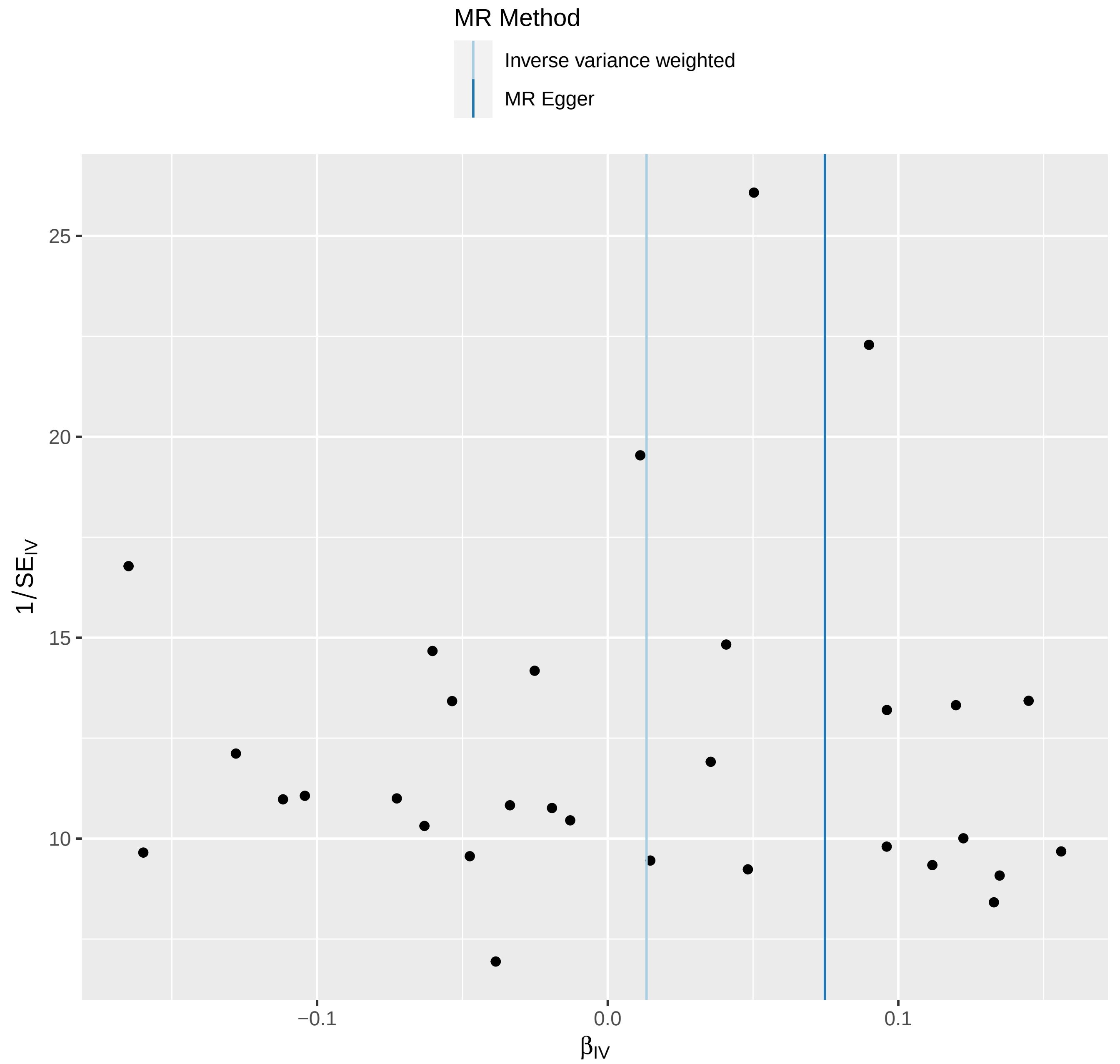

Batch 2303 : Crohn's disease on IDP T1 FAST ROIs R latocc cortex sup

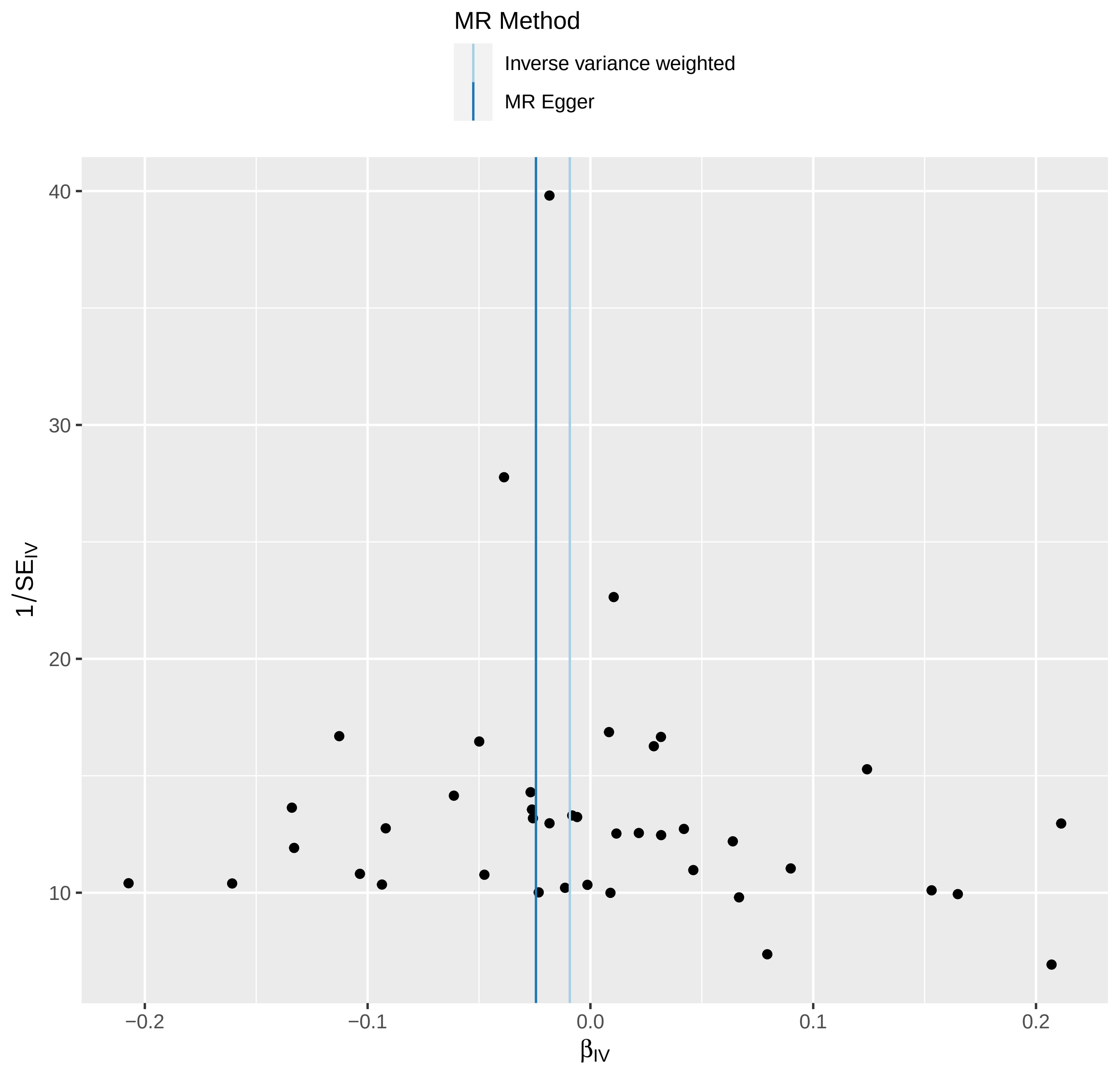

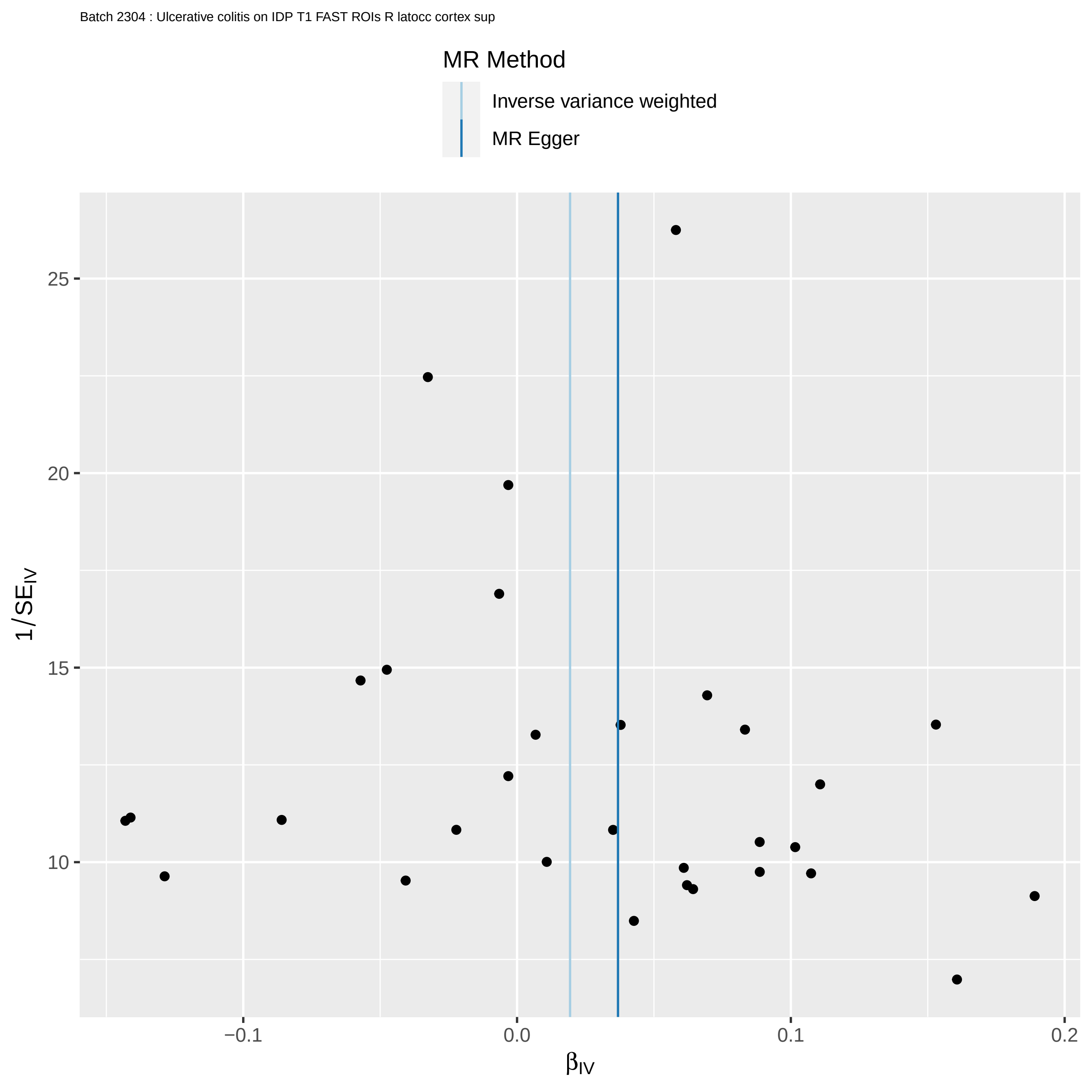

Batch 2304 : Ulcerative colitis on IDP T1 FAST ROIs R latocc cortex sup
MR Method
Inverse variance weighted
MR Egger
25
20
15
10
1/SE$_{IV}$
−0.1
0.0
0.1
0.2
β$_{IV}$

Batch 2325 : Crohn's disease on IDP T1 SIENAX white normalised volume

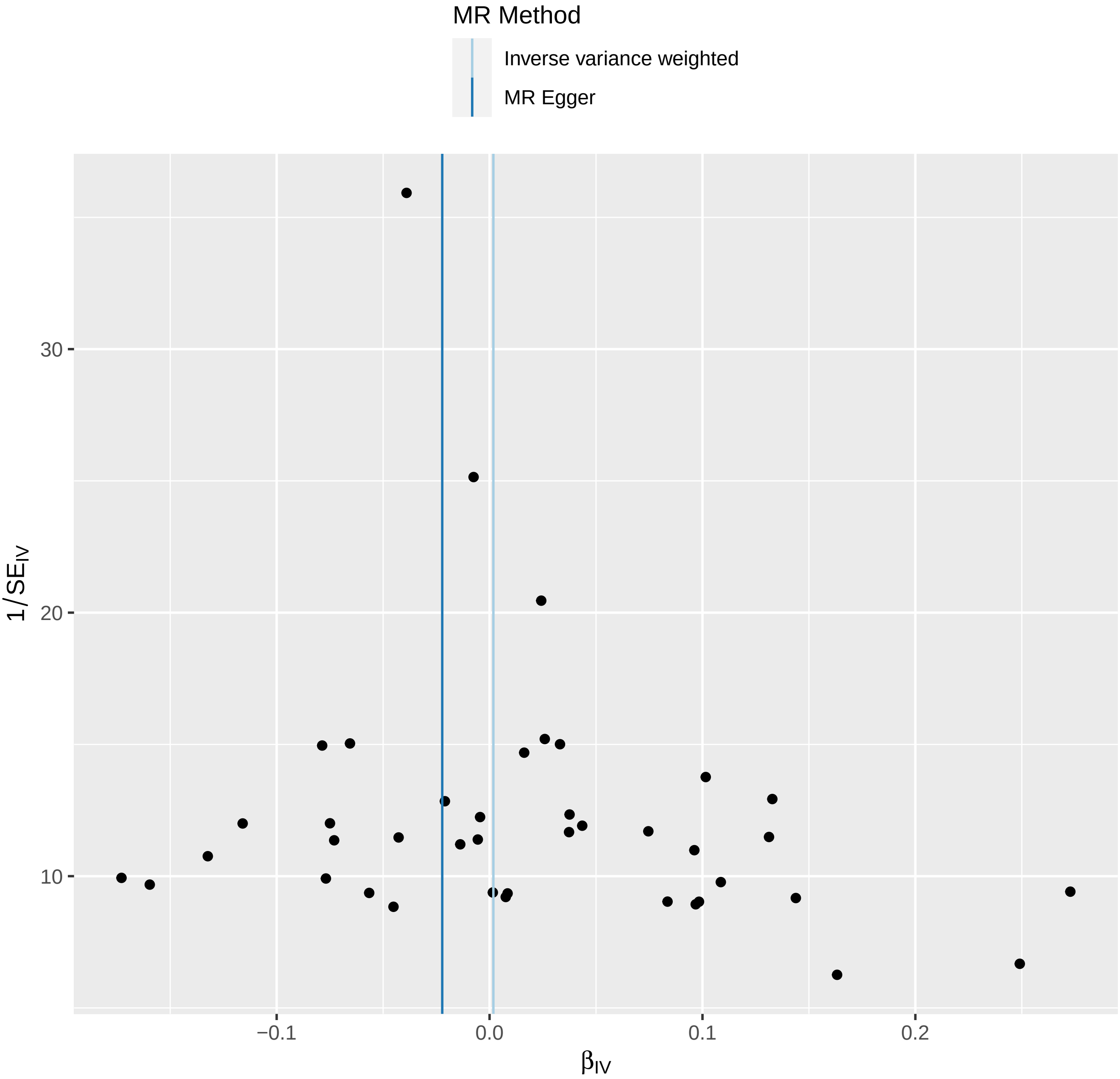

Batch 2326 : Ulcerative colitis on IDP T1 SIENAX white normalised volume

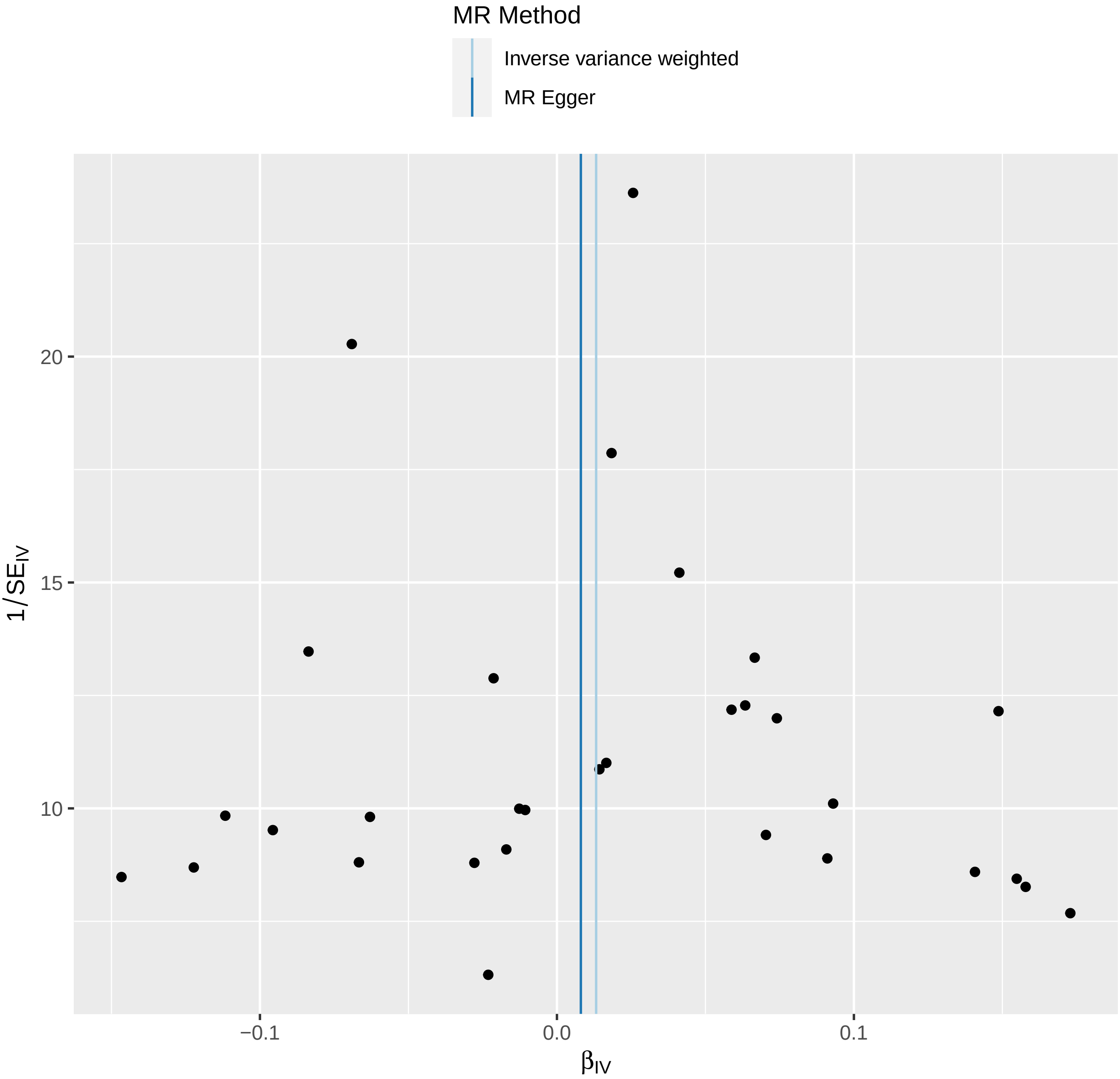

Batch 2327 : Crohn's disease on IDP T1 FAST ROIs L latocc cortex inf

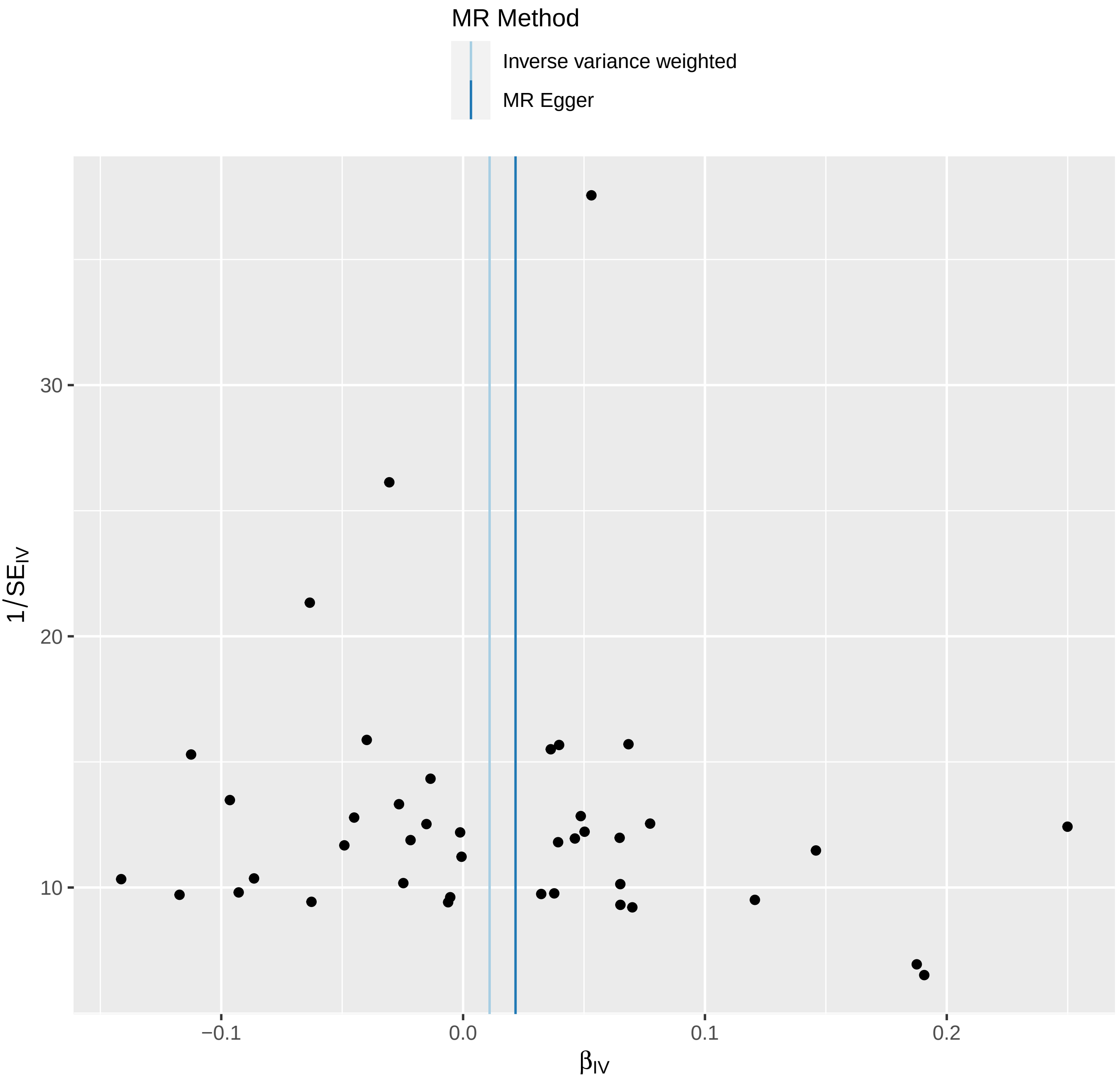

Batch 2328 : Ulcerative colitis on IDP T1 FAST ROIs L latocc cortex inf

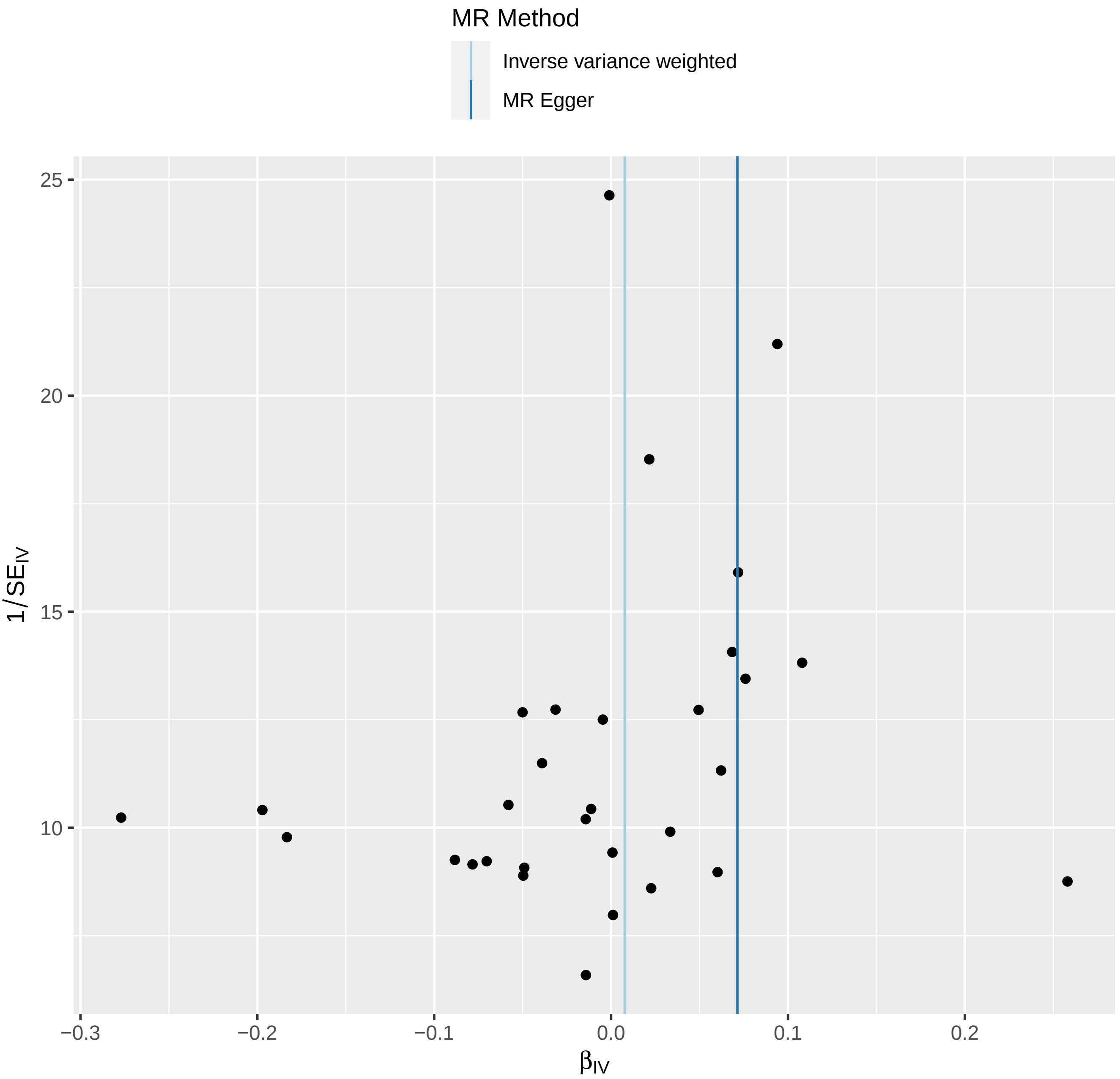

Batch 2349 : Crohn's disease on IDP T1 FAST ROIs R latocc cortex inf

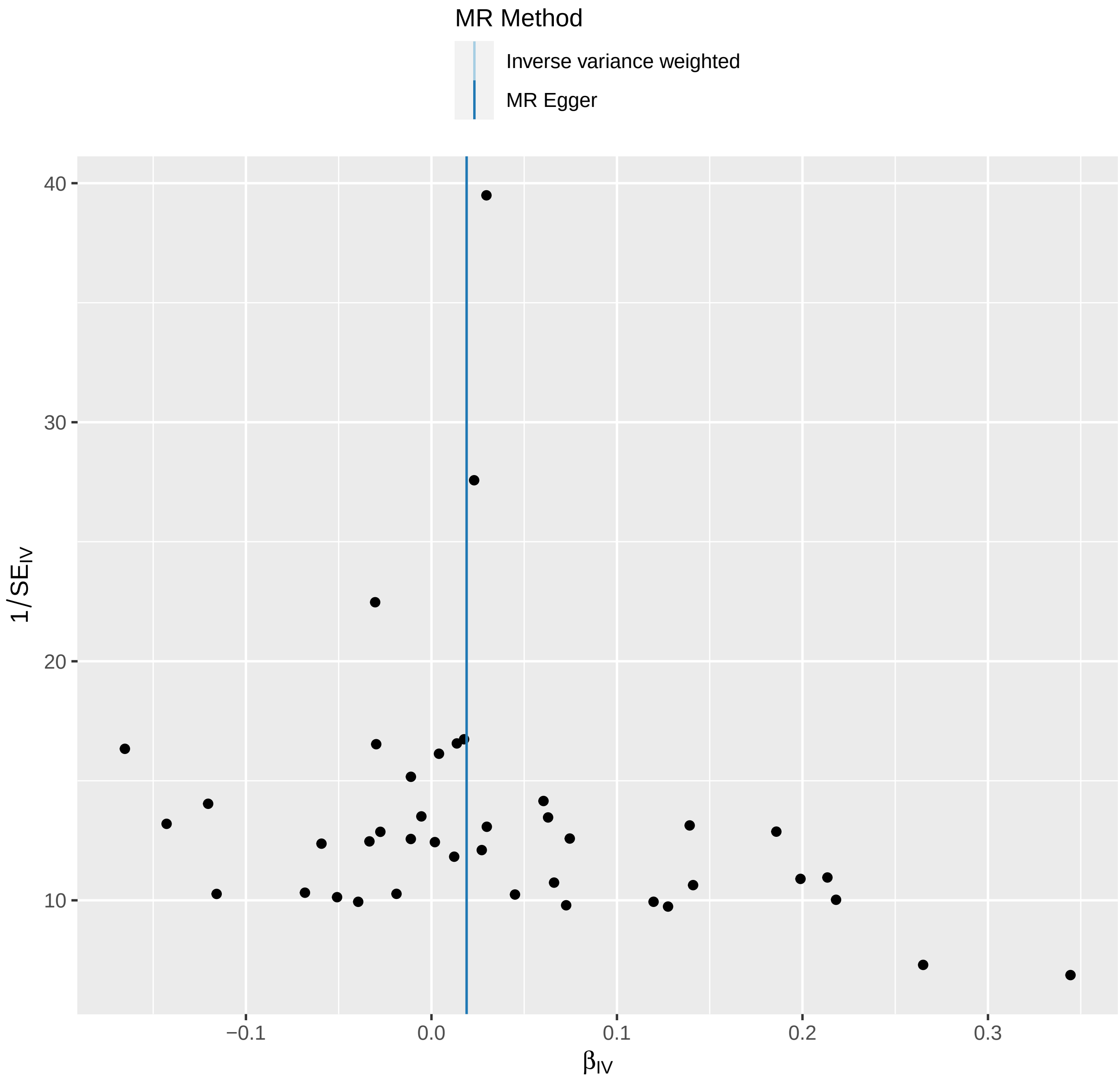

Batch 2350 : Ulcerative colitis on IDP T1 FAST ROIs R latocc cortex inf

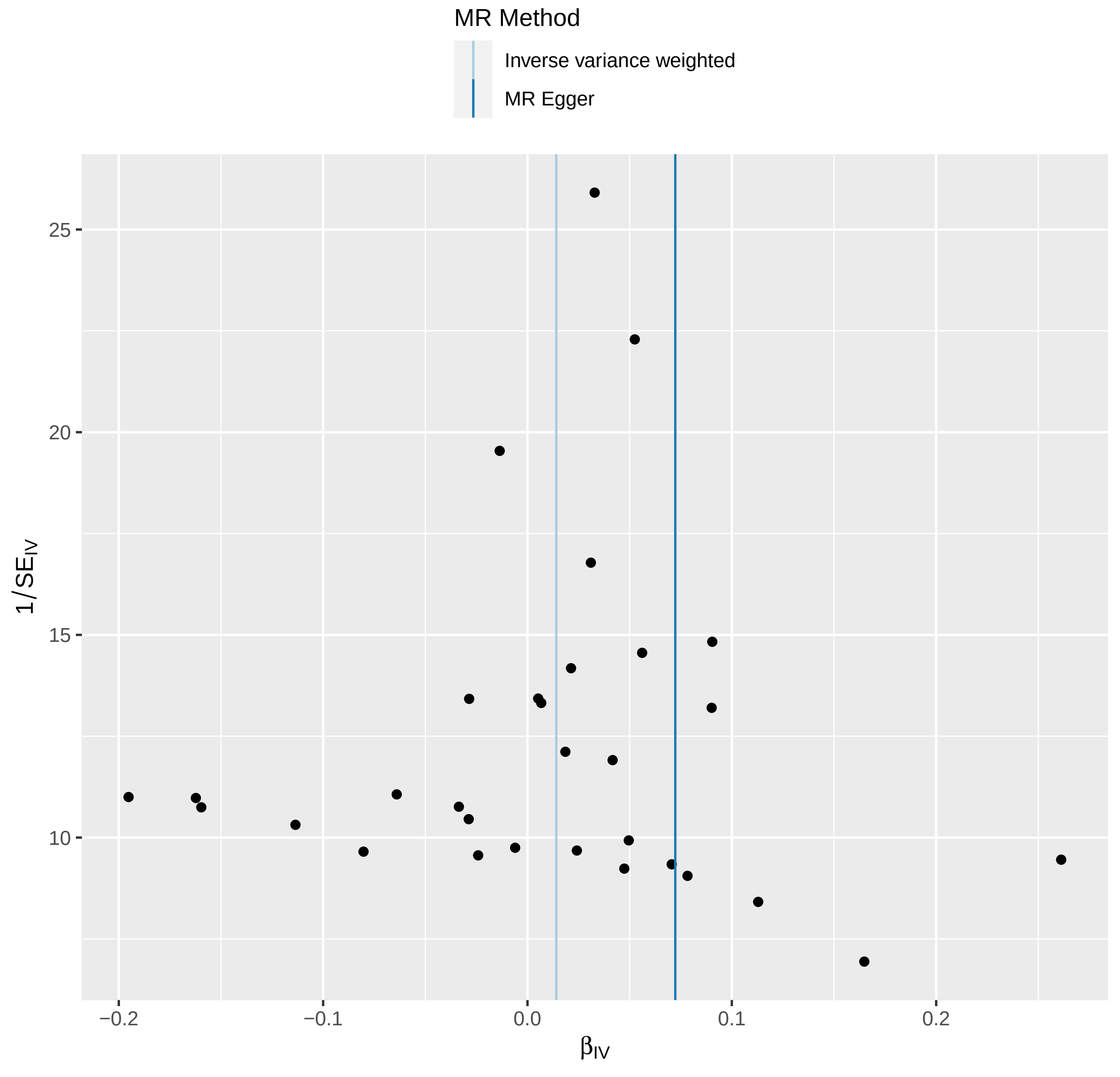

Batch 2371 : Crohn's disease on IDP T1 FAST ROIs L intracalc cortex

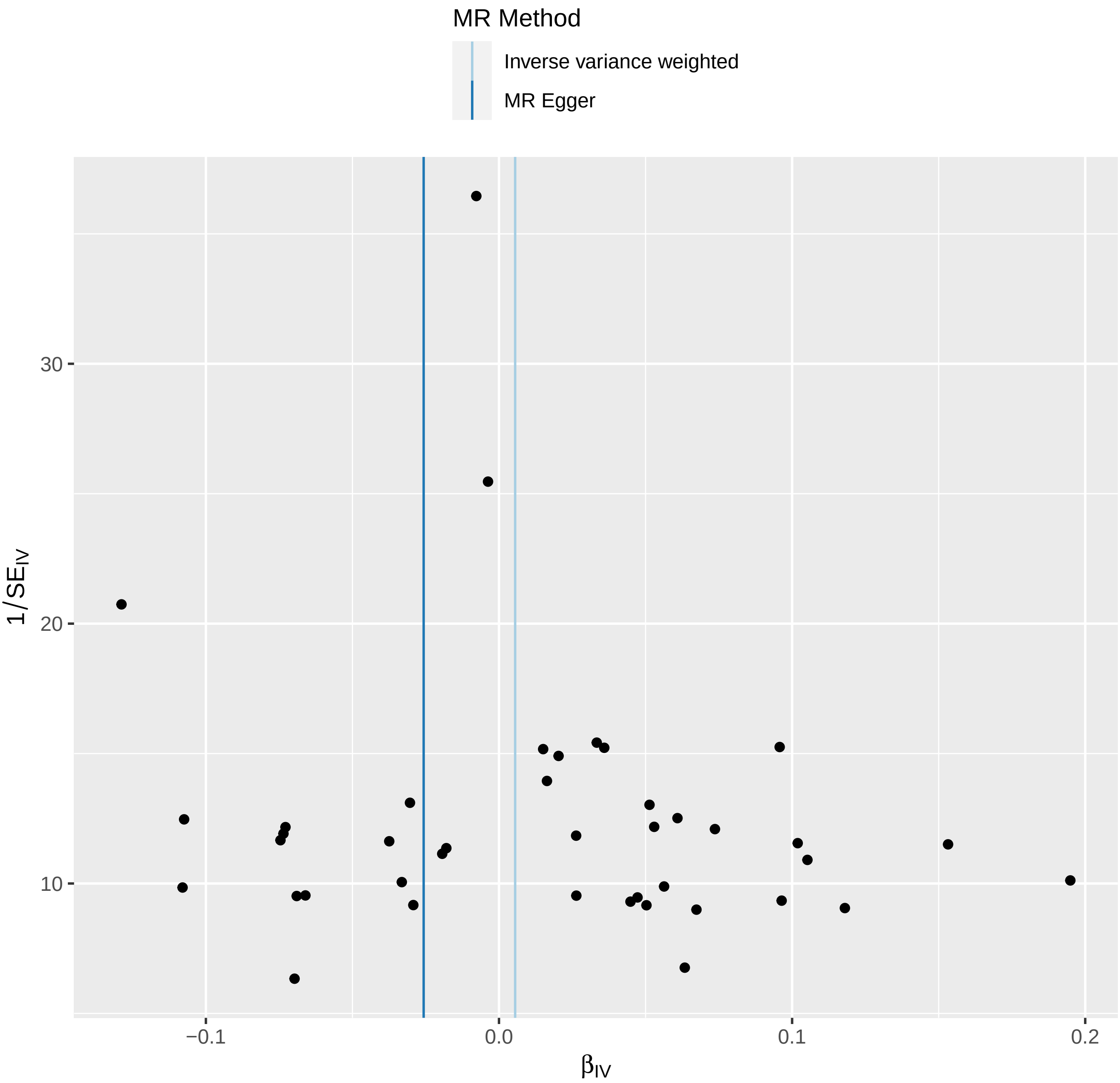

Batch 2372 : Ulcerative colitis on IDP T1 FAST ROIs L intracalc cortex

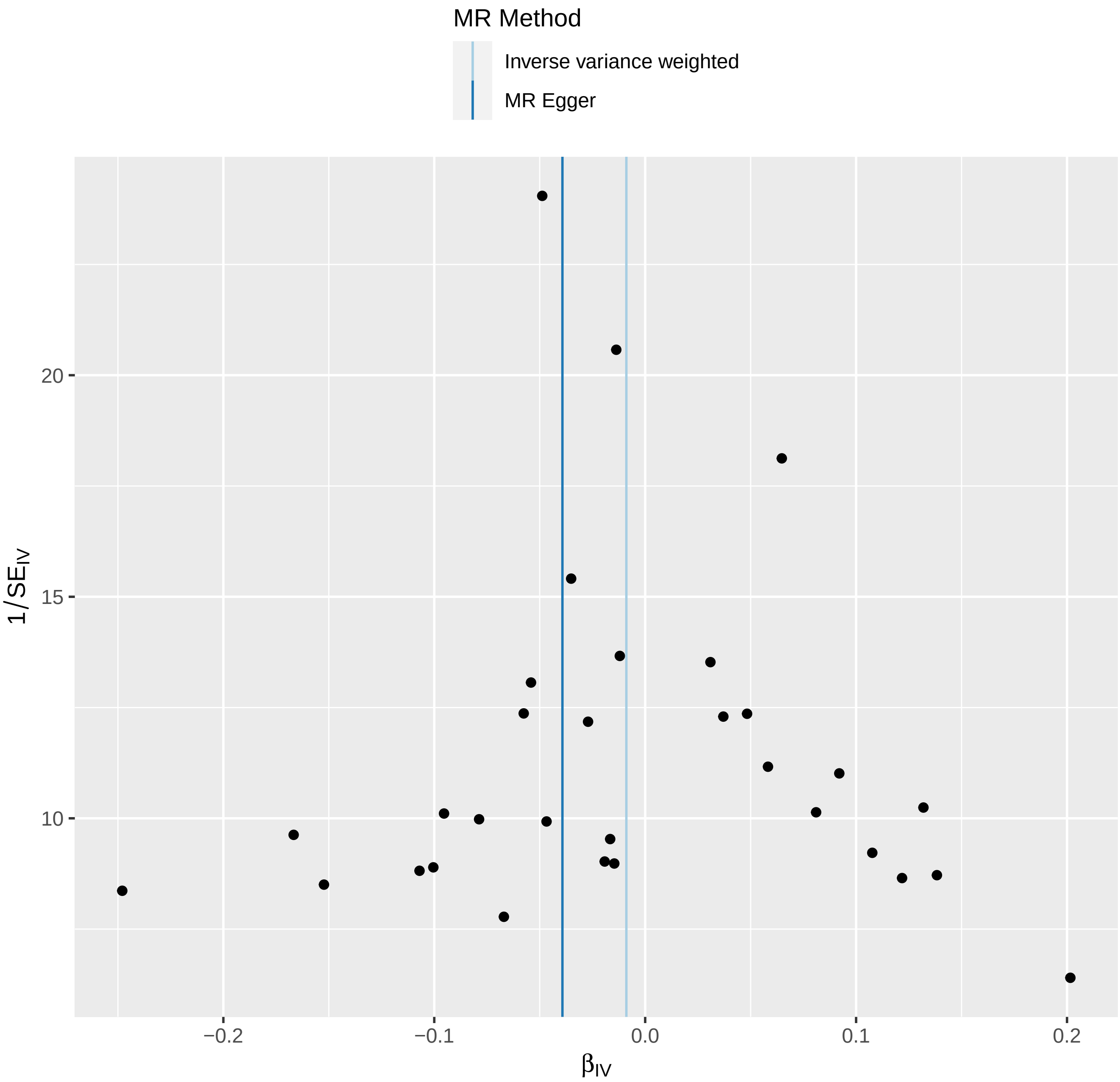

Batch 2393 : Crohn's disease on IDP T1 FAST ROIs R intracalc cortex

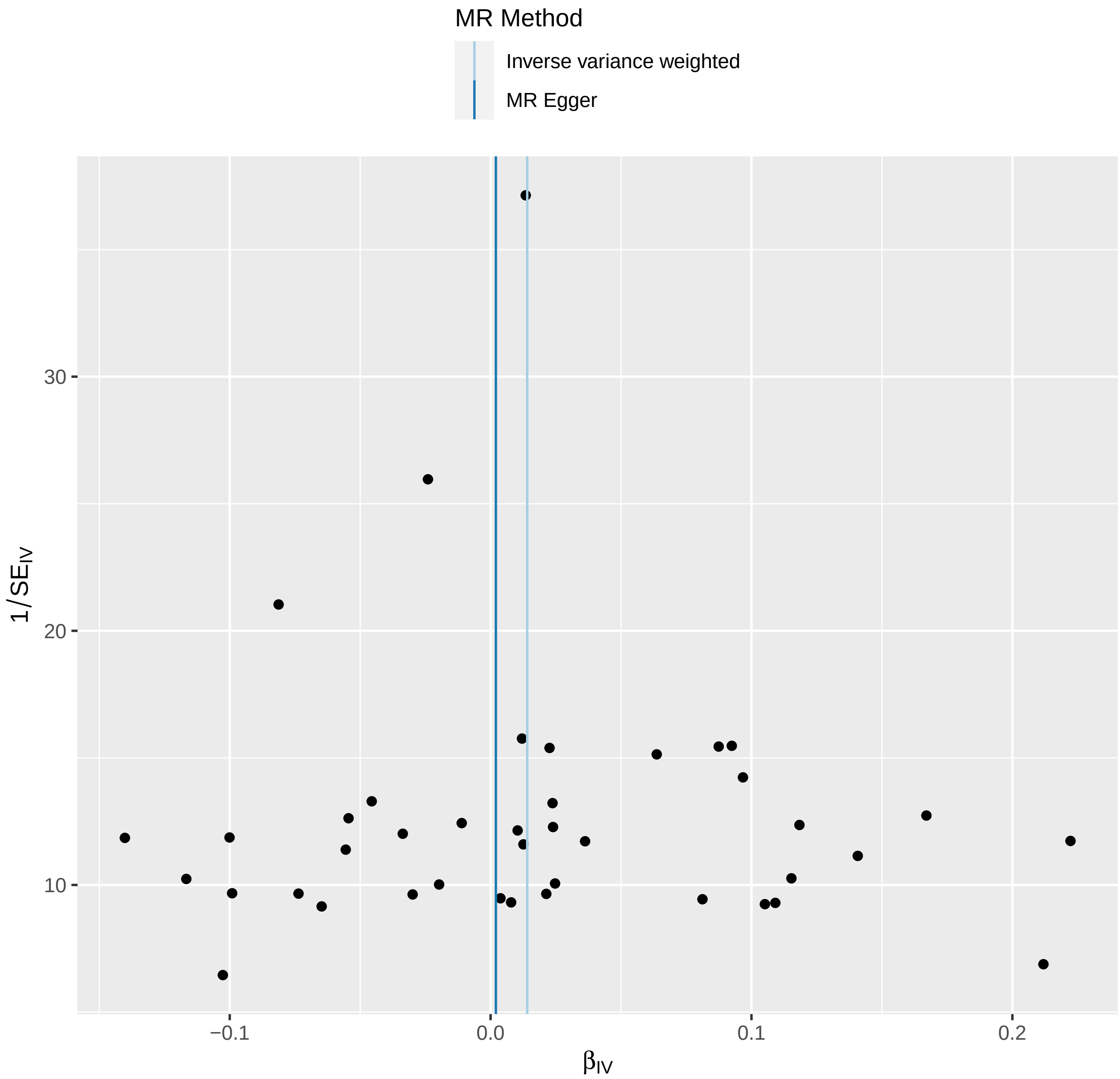

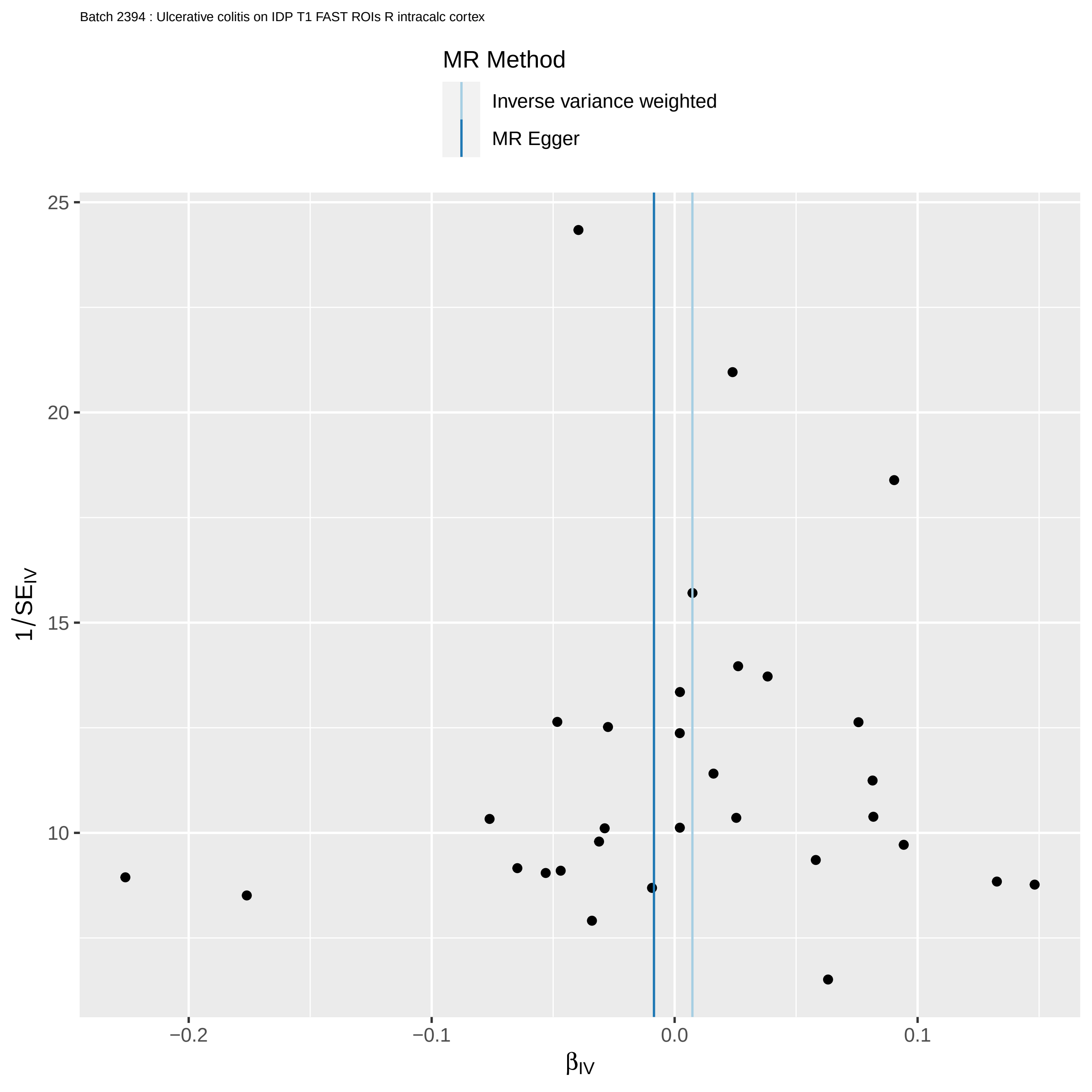

Batch 2394 : Ulcerative colitis on IDP T1 FAST ROIs R intracalc cortex
MR Method
Inverse variance weighted
MR Egger
25
20
15
10
1/SE$_{IV}$
−0.2
−0.1
0.0
0.1
β$_{IV}$

Batch 2415 : Crohn's disease on IDP T1 FAST ROIs L front med cortex

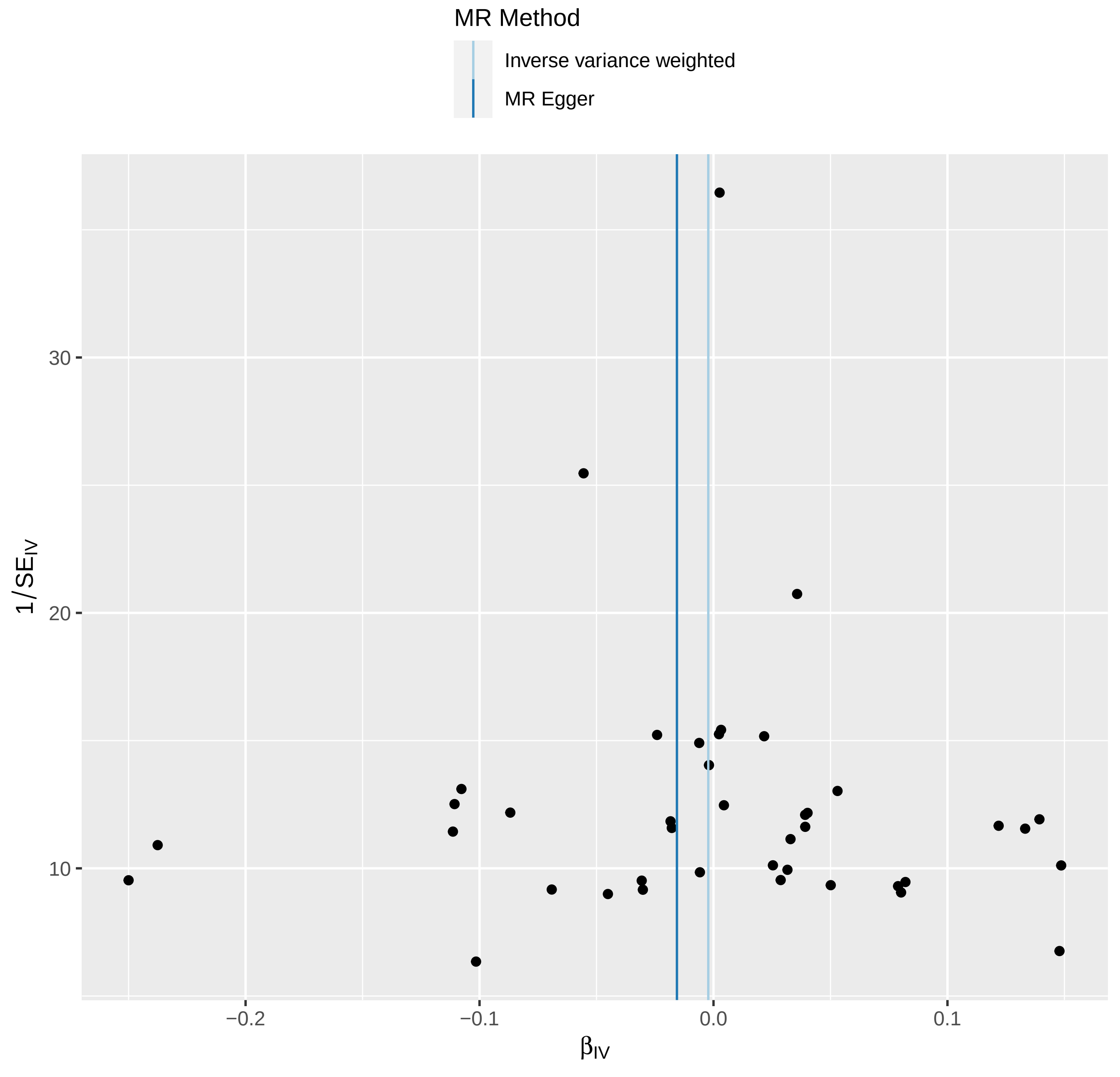

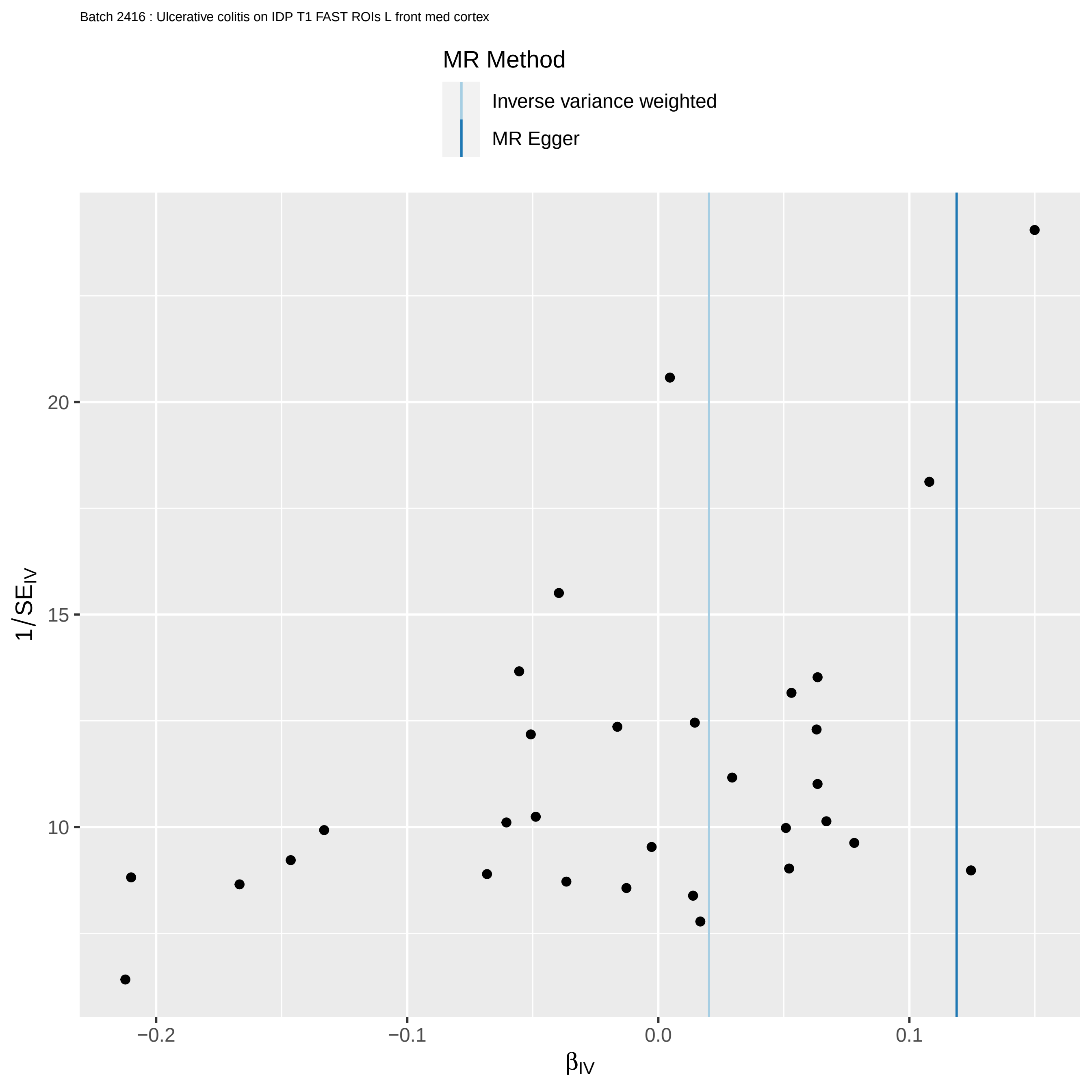

Batch 2416 : Ulcerative colitis on IDP T1 FAST ROIs L front med cortex
MR Method
Inverse variance weighted
MR Egger
20
15
10
1/SE_IV
−0.2
−0.1
0.0
0.1
β_IV

Batch 2437 : Crohn's disease on IDP T1 FAST ROIs R front med cortex

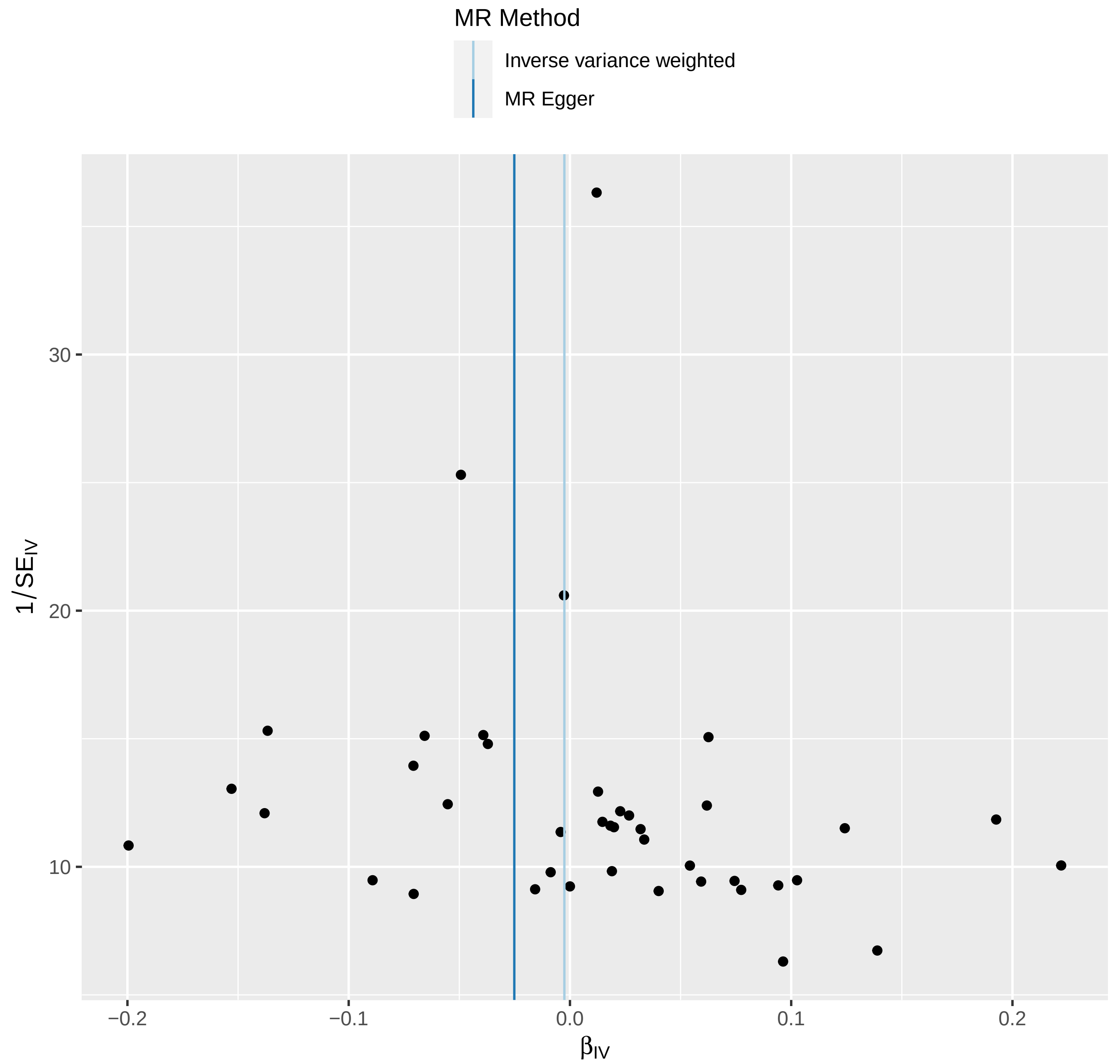

Batch 2438 : Ulcerative colitis on IDP T1 FAST ROIs R front med cortex

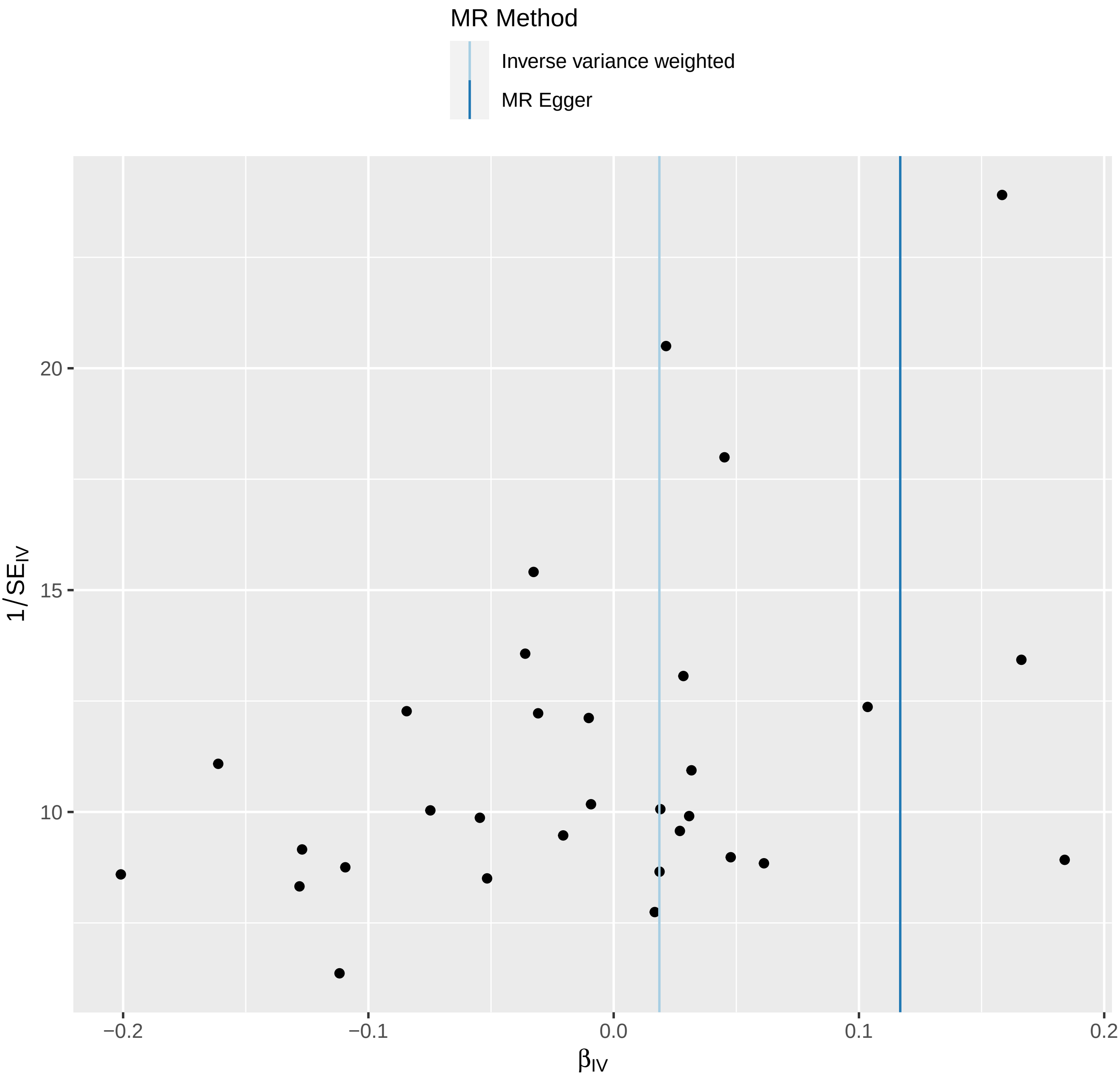

Batch 2459 : Crohn's disease on IDP T1 FAST ROIs L juxtapos lobule cortex

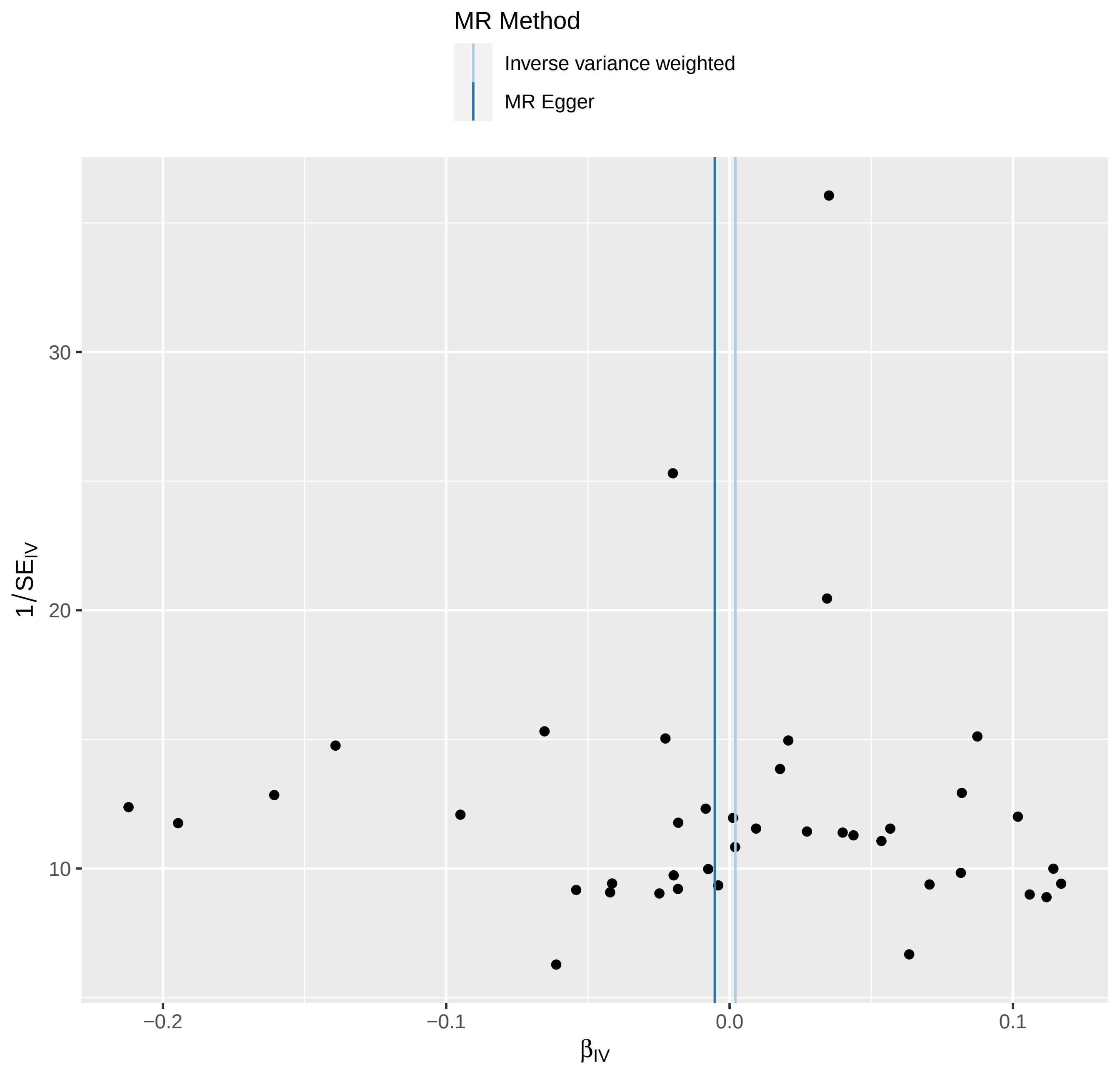

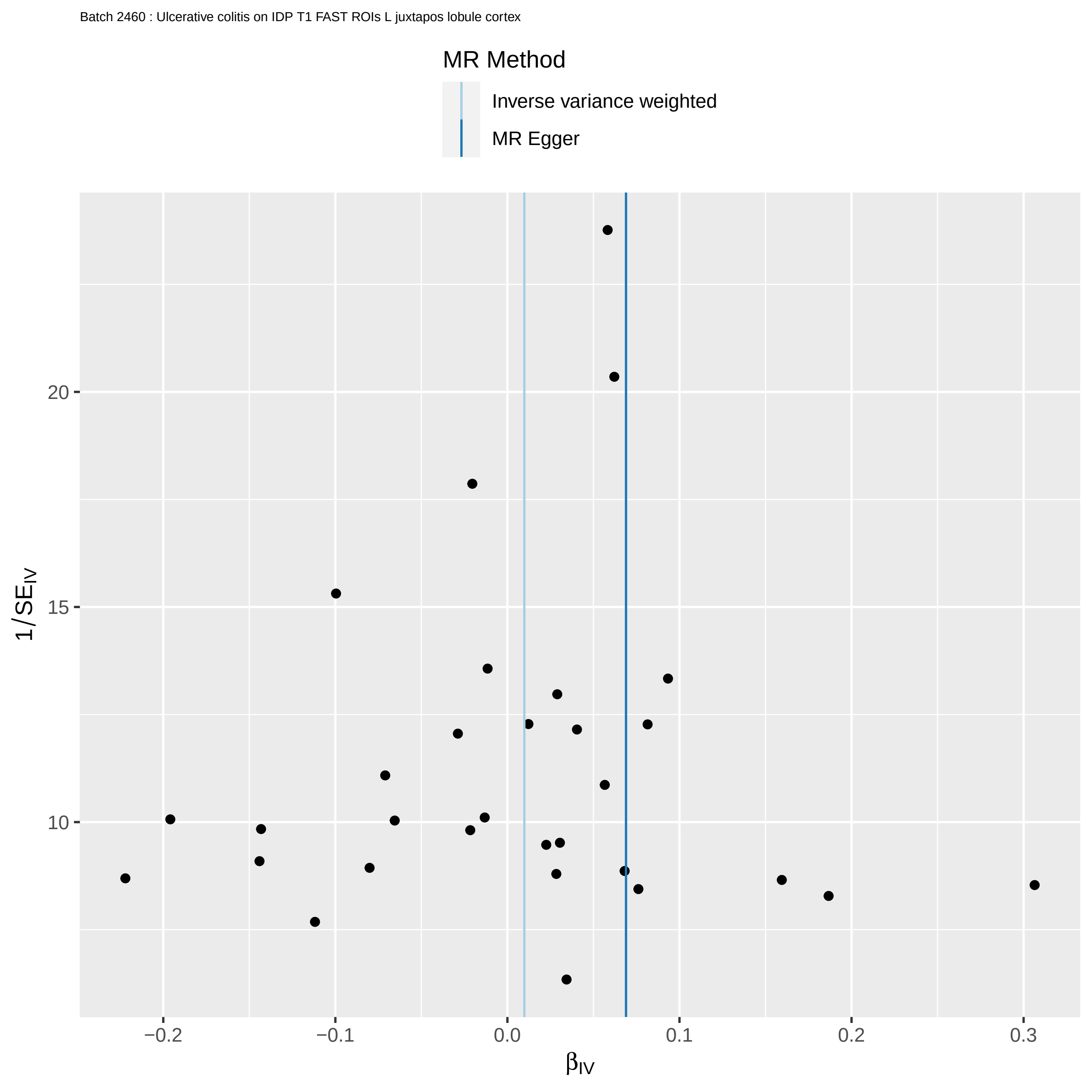

Batch 2460 : Ulcerative colitis on IDP T1 FAST ROIs L juxtapos lobule cortex
MR Method
Inverse variance weighted
MR Egger
20
15
10
1/SE_IV
−0.2
−0.1
0.0
0.1
0.2
0.3
β_IV

Batch 2481 : Crohn's disease on IDP T1 FAST ROIs R juxtapos lobule cortex

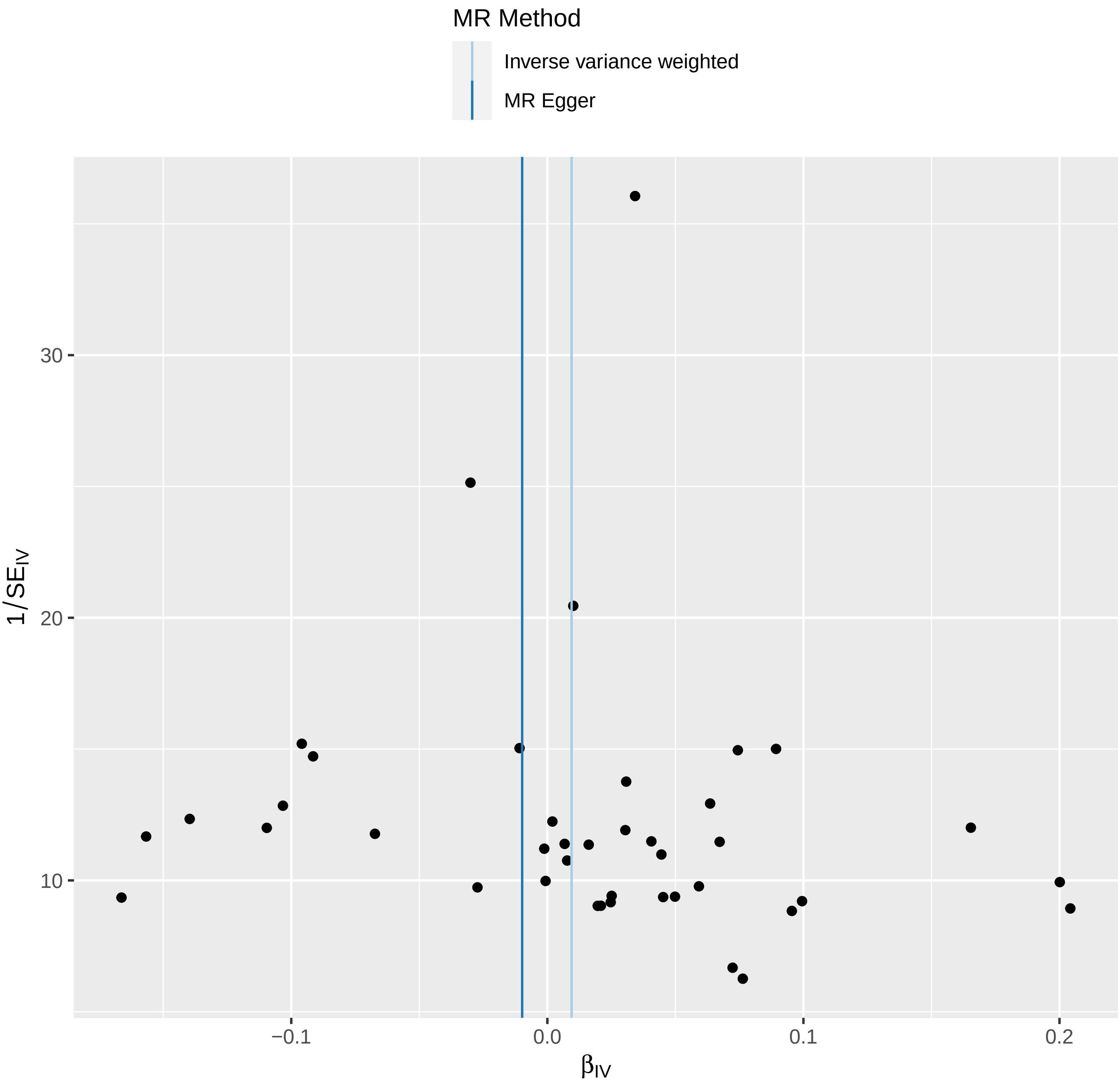

Batch 2482 : Ulcerative colitis on IDP T1 FAST ROIs R juxtapos lobule cortex

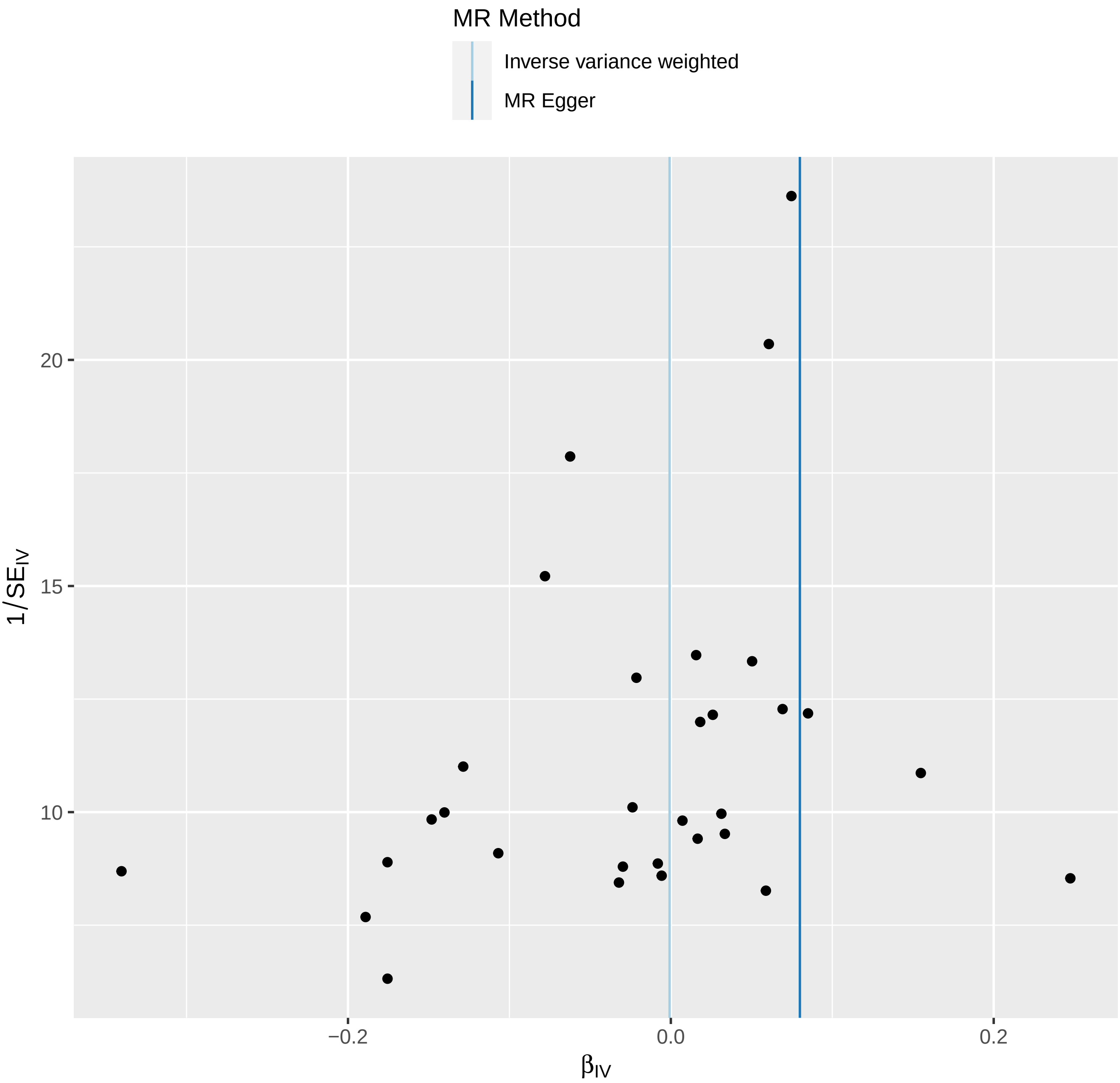

Batch 2503 : Crohn's disease on IDP T1 FAST ROIs L subcallosal cortex

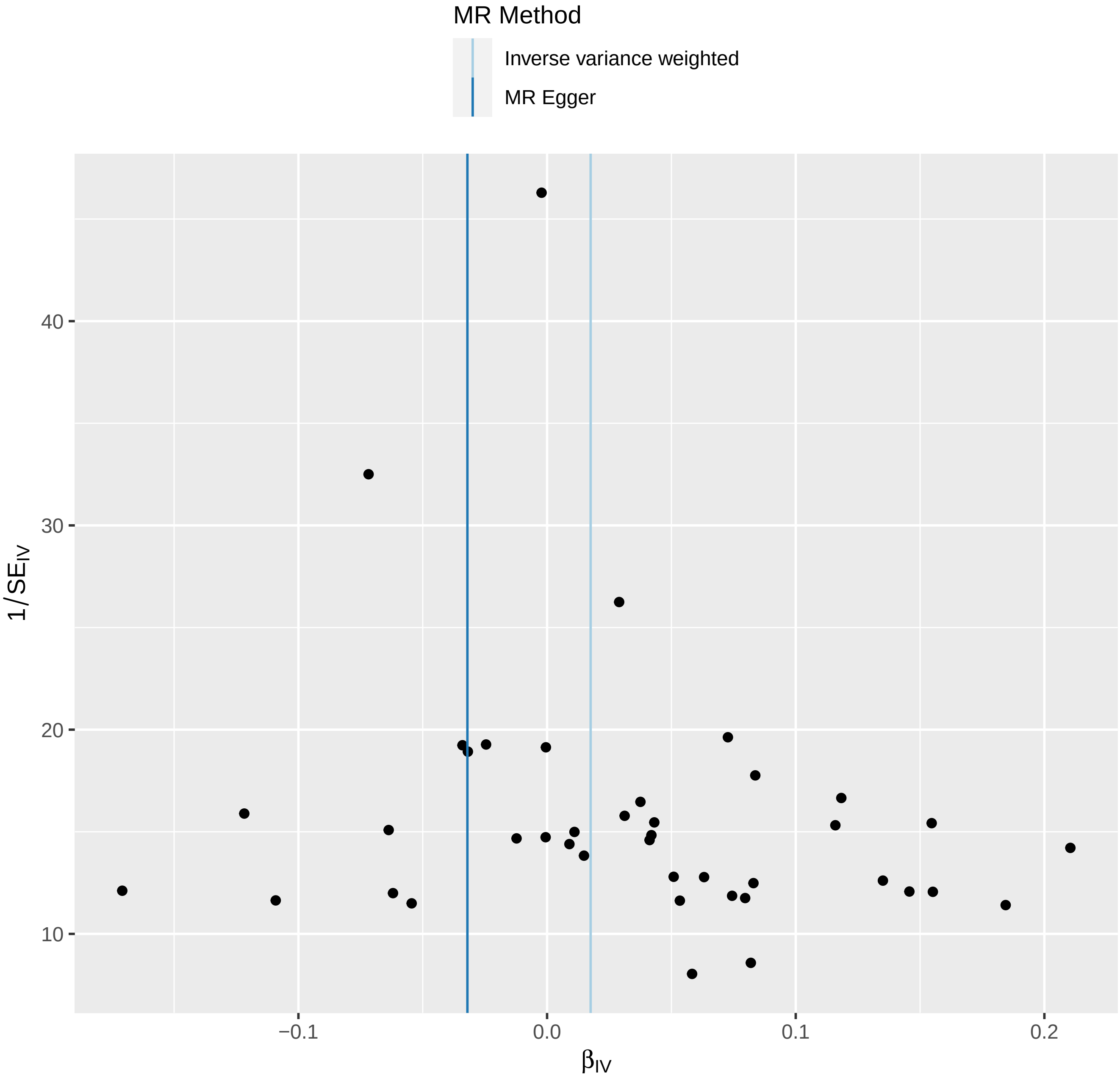

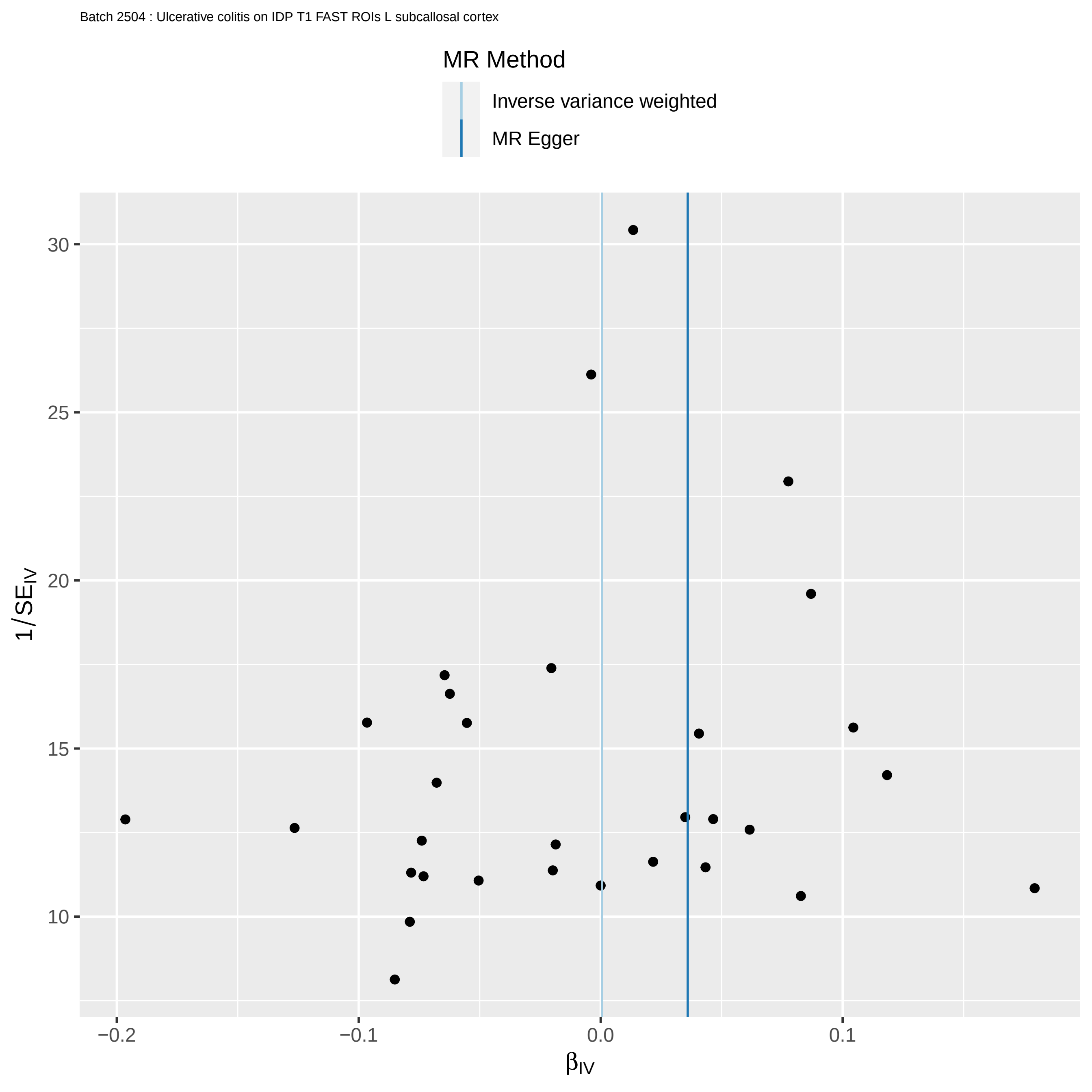

Batch 2504 : Ulcerative colitis on IDP T1 FAST ROIs L subcallosal cortex
MR Method
Inverse variance weighted
MR Egger
30
25
20
15
10
1/SE$_{IV}$
−0.2
−0.1
0.0
0.1
β$_{IV}$

Batch 2525 : Crohn's disease on IDP T1 FAST ROIs R subcallosal cortex

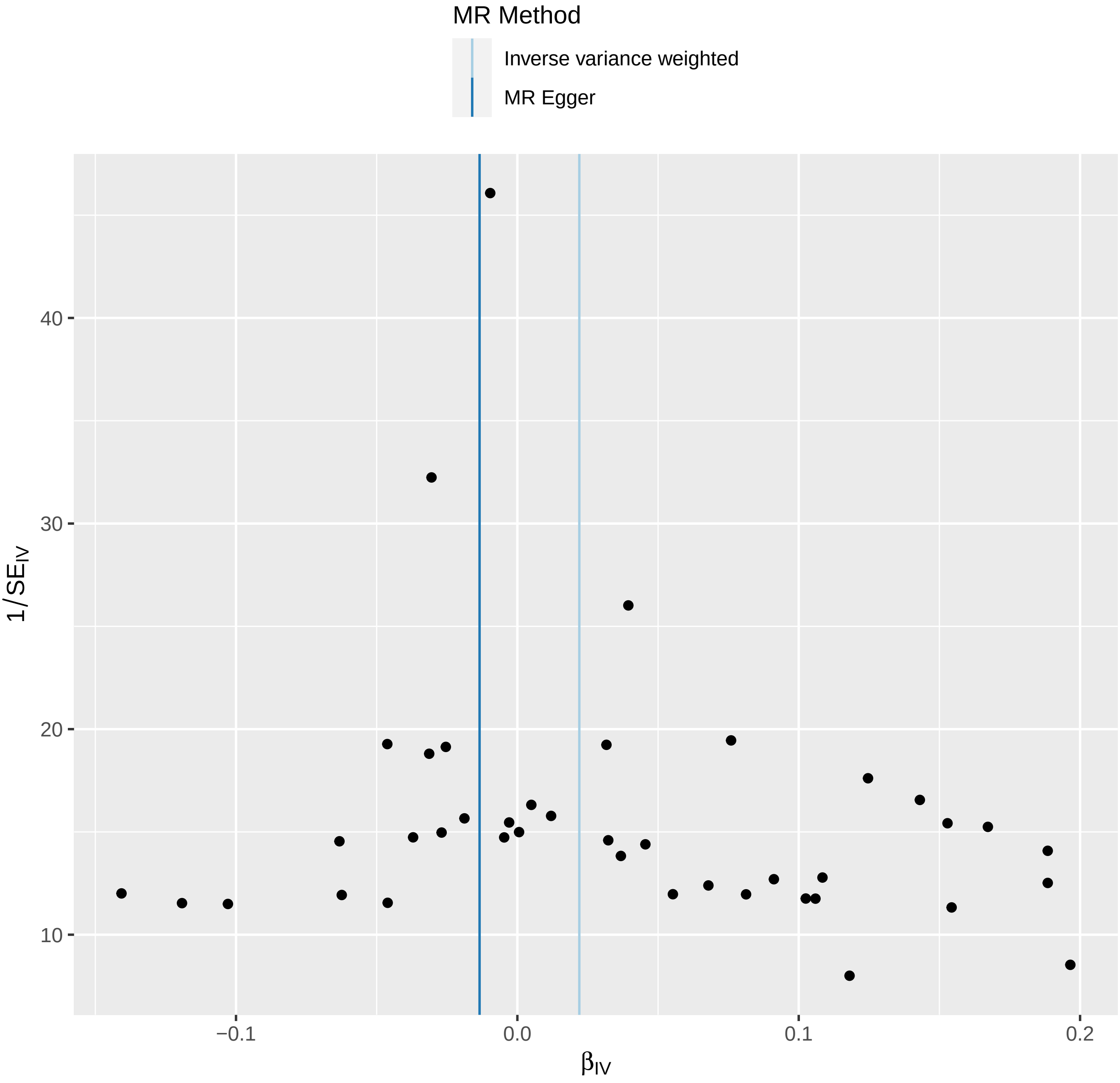

Batch 2526 : Ulcerative colitis on IDP T1 FAST ROIs R subcallosal cortex

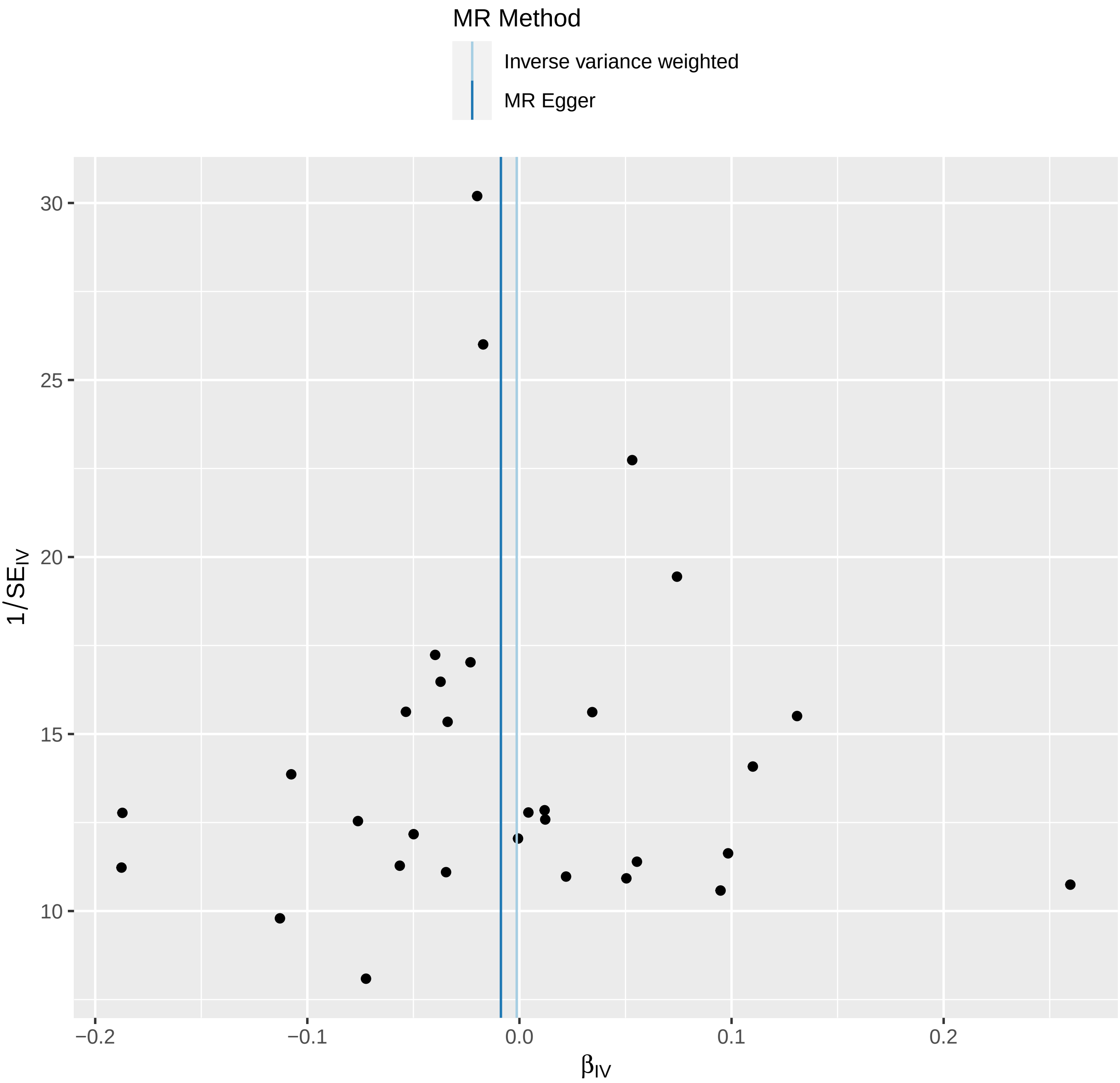

Batch 2547 : Crohn's disease on IDP T1 SIENAX white unnormalised volume

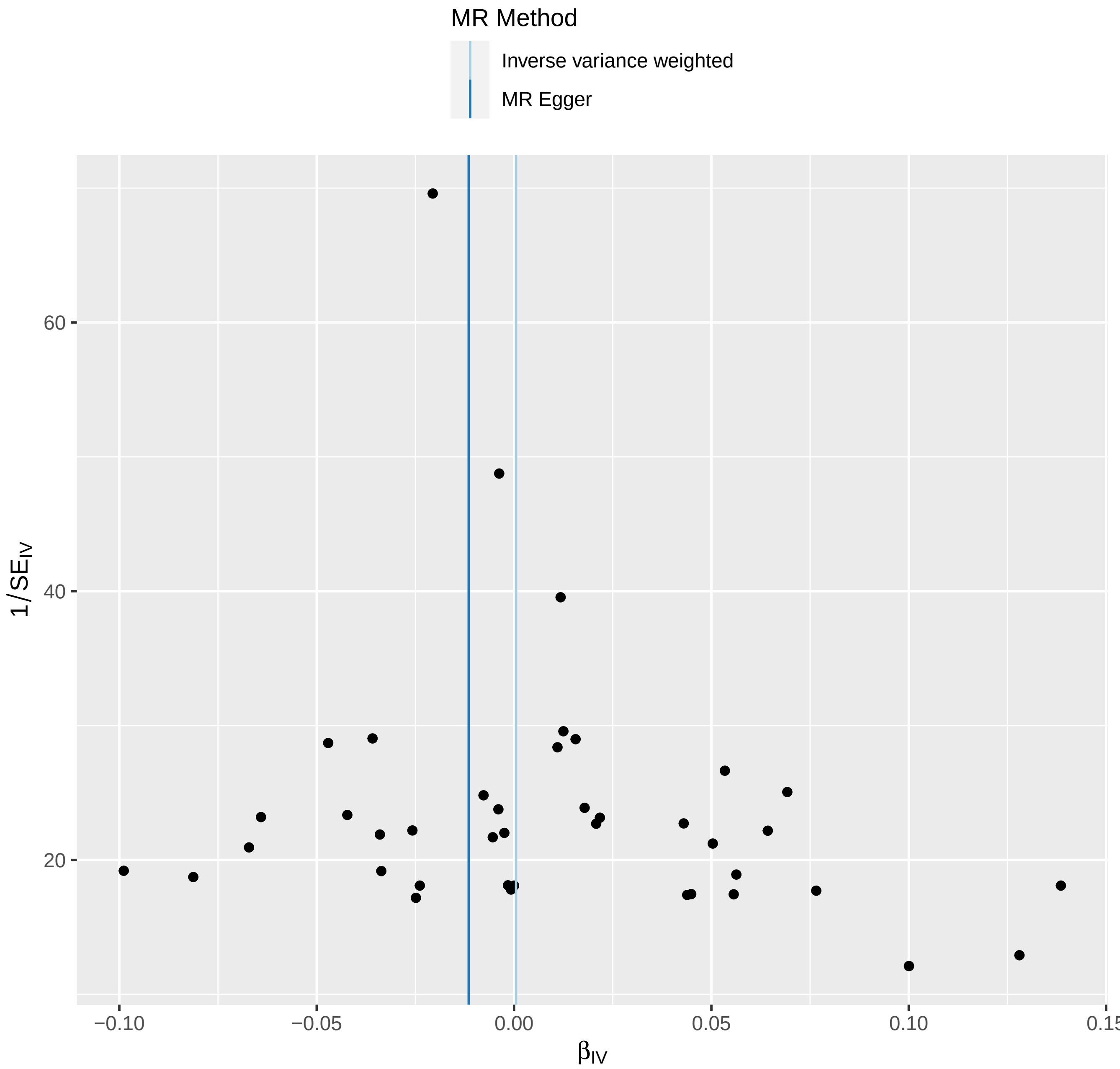

Batch 2548 : Ulcerative colitis on IDP T1 SIENAX white unnormalised volume

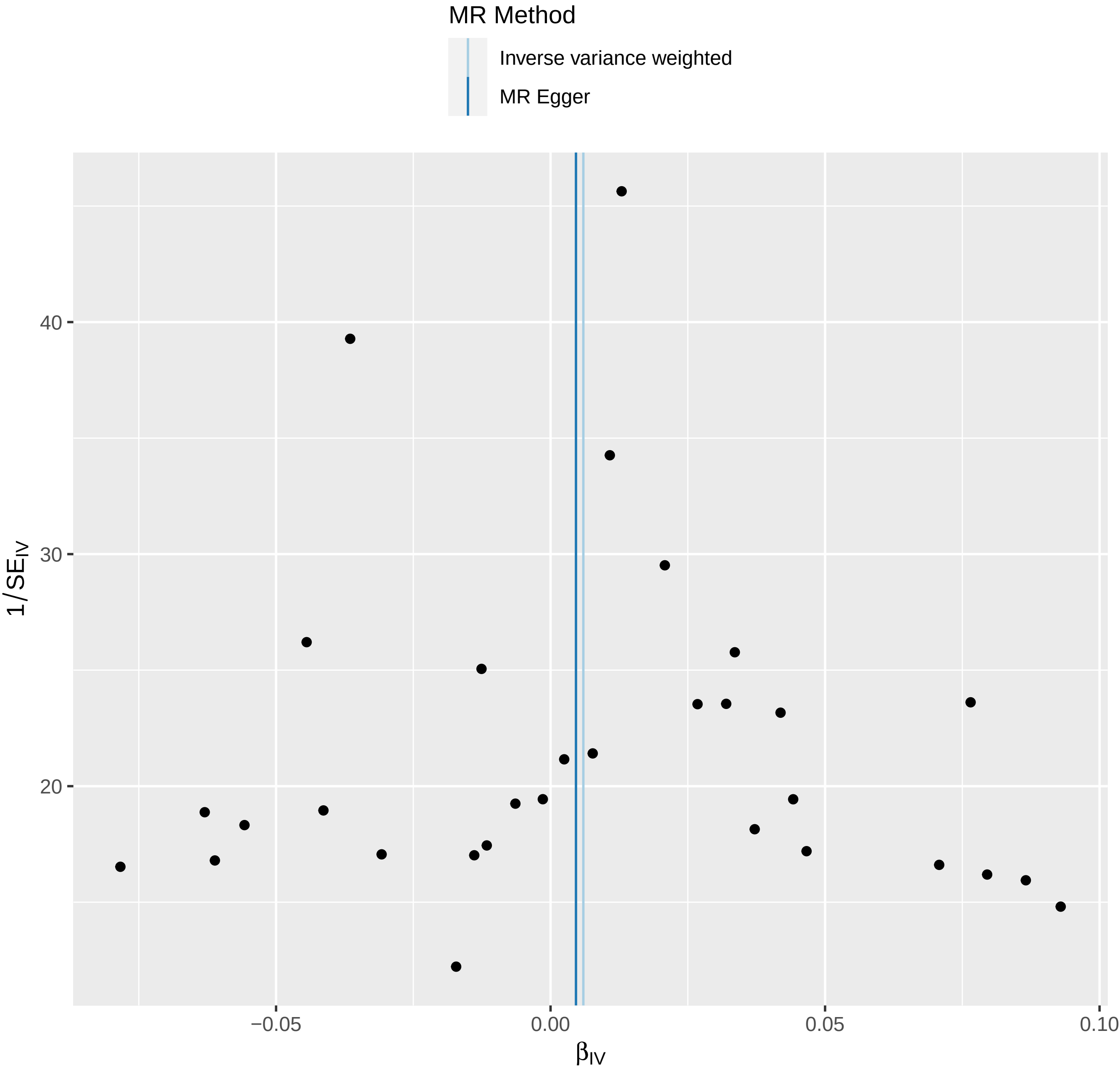

Batch 2549 : Crohn's disease on IDP T1 FAST ROIs L paracing gyrus

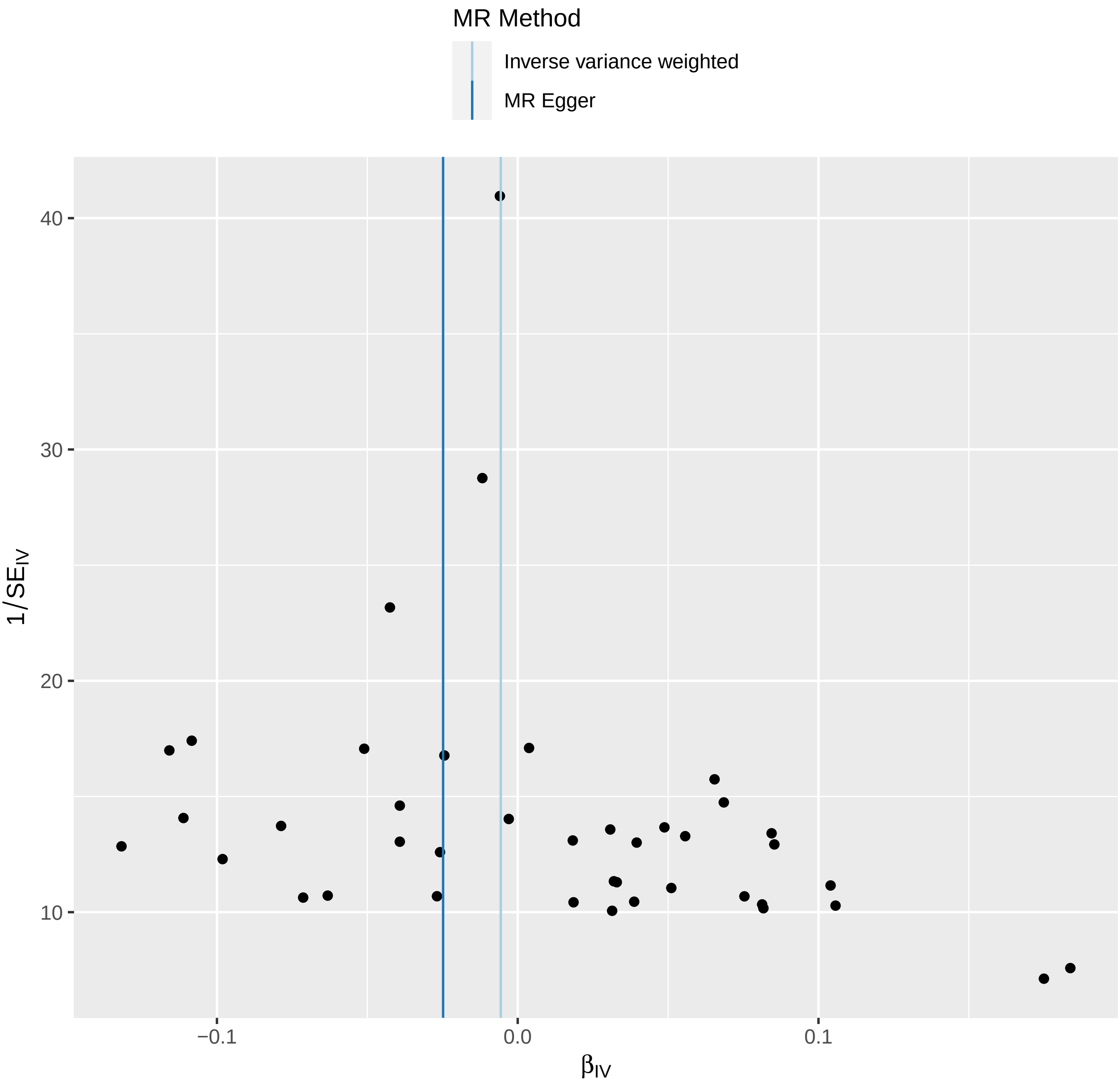

Batch 2550 : Ulcerative colitis on IDP T1 FAST ROIs L paracing gyrus

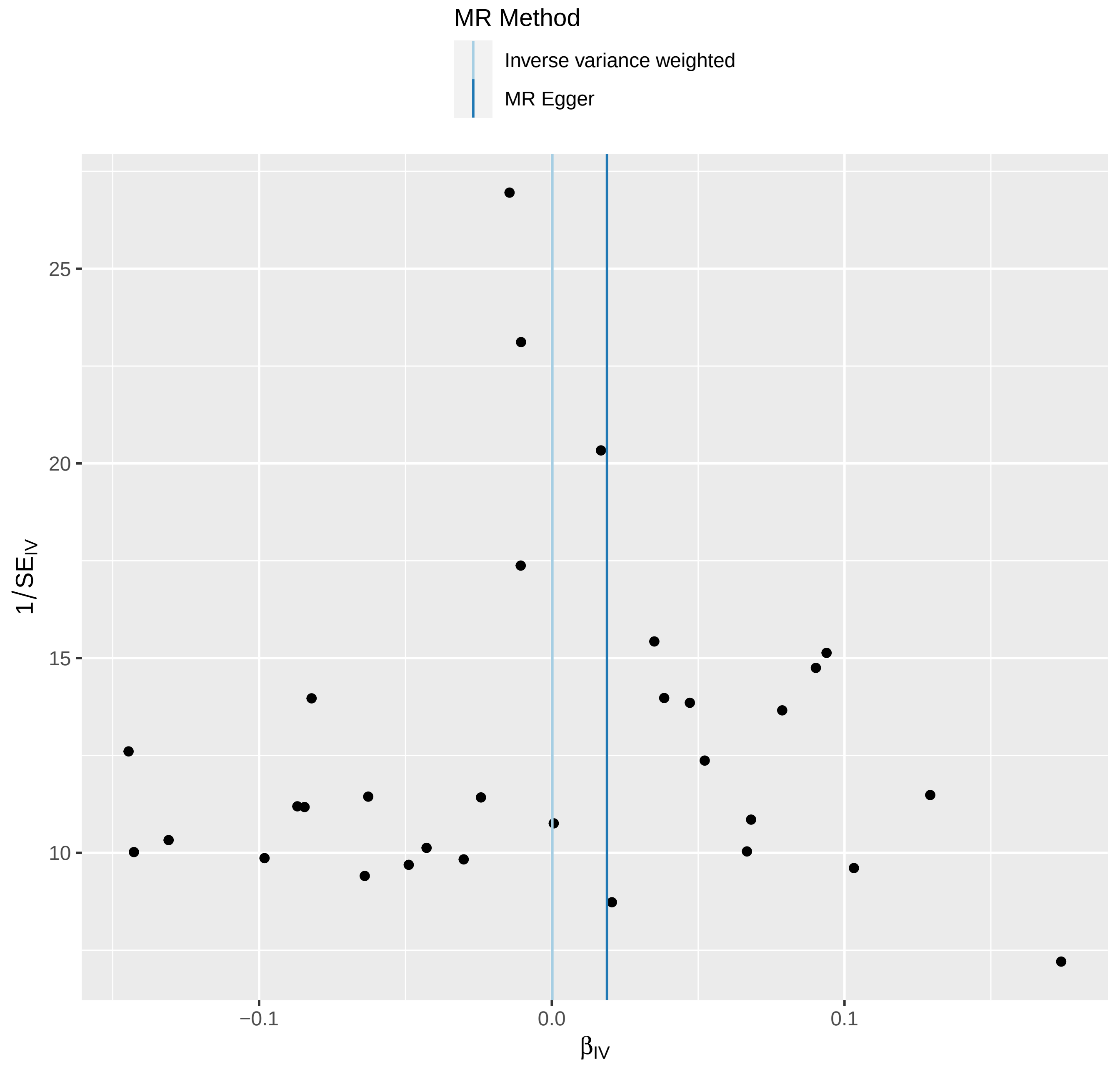

Batch 2571 : Crohn's disease on IDP T1 FAST ROIs R paracing gyrus

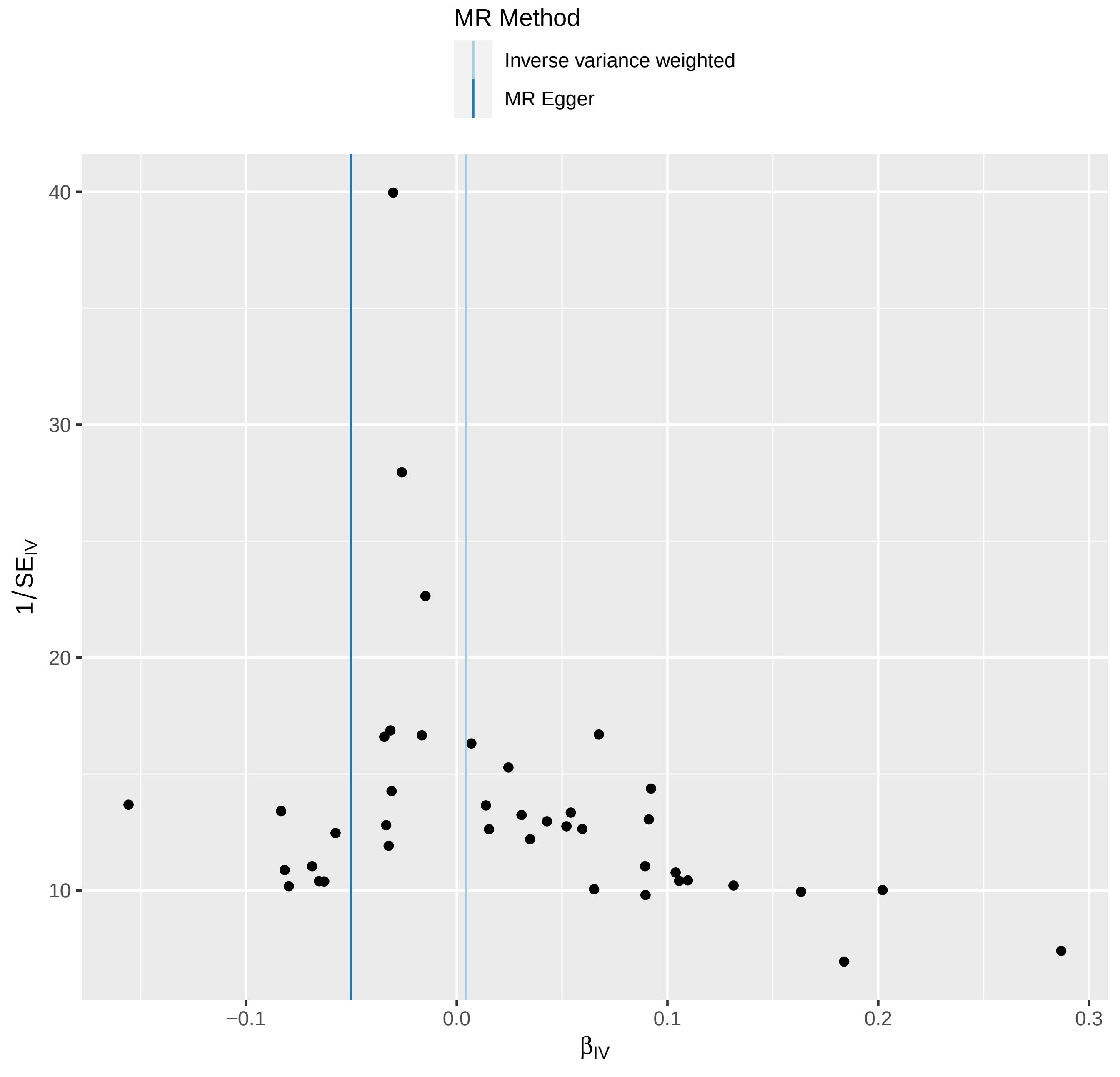

Batch 2572 : Ulcerative colitis on IDP T1 FAST ROIs R paracing gyrus

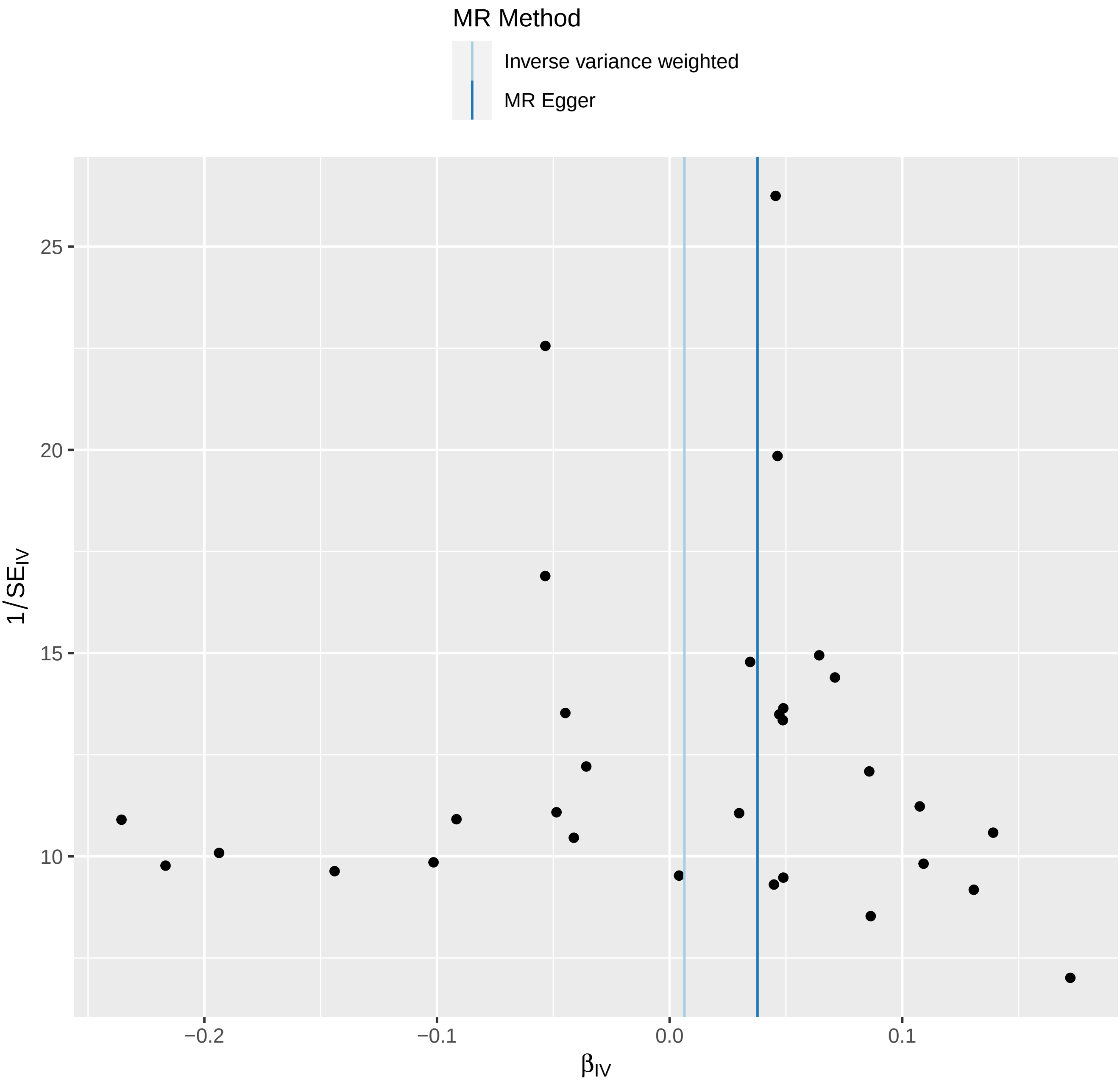

Batch 2593 : Crohn's disease on IDP T1 FAST ROIs L cing gyrus ant

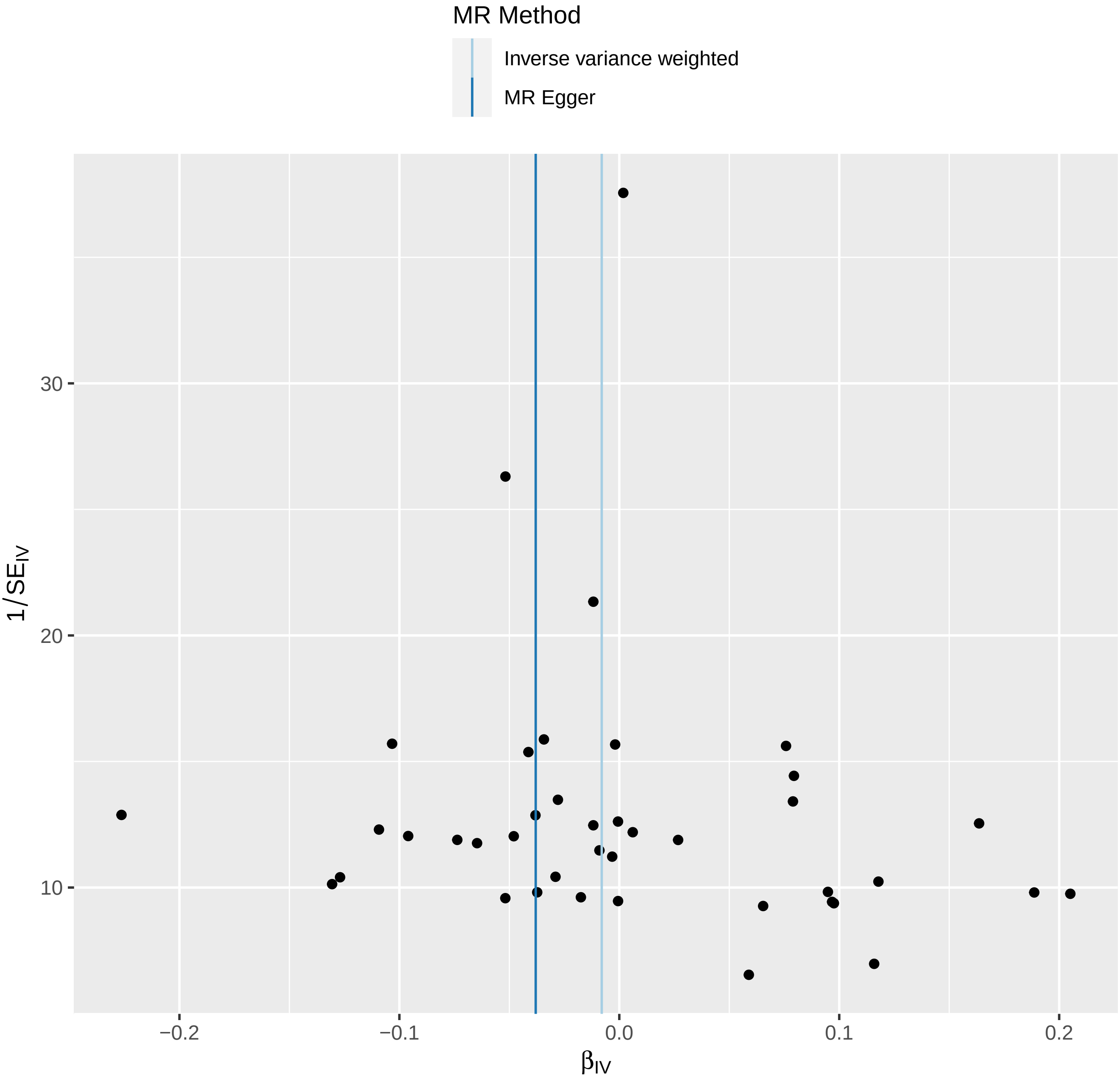

Batch 2594 : Ulcerative colitis on IDP T1 FAST ROIs L cing gyrus ant

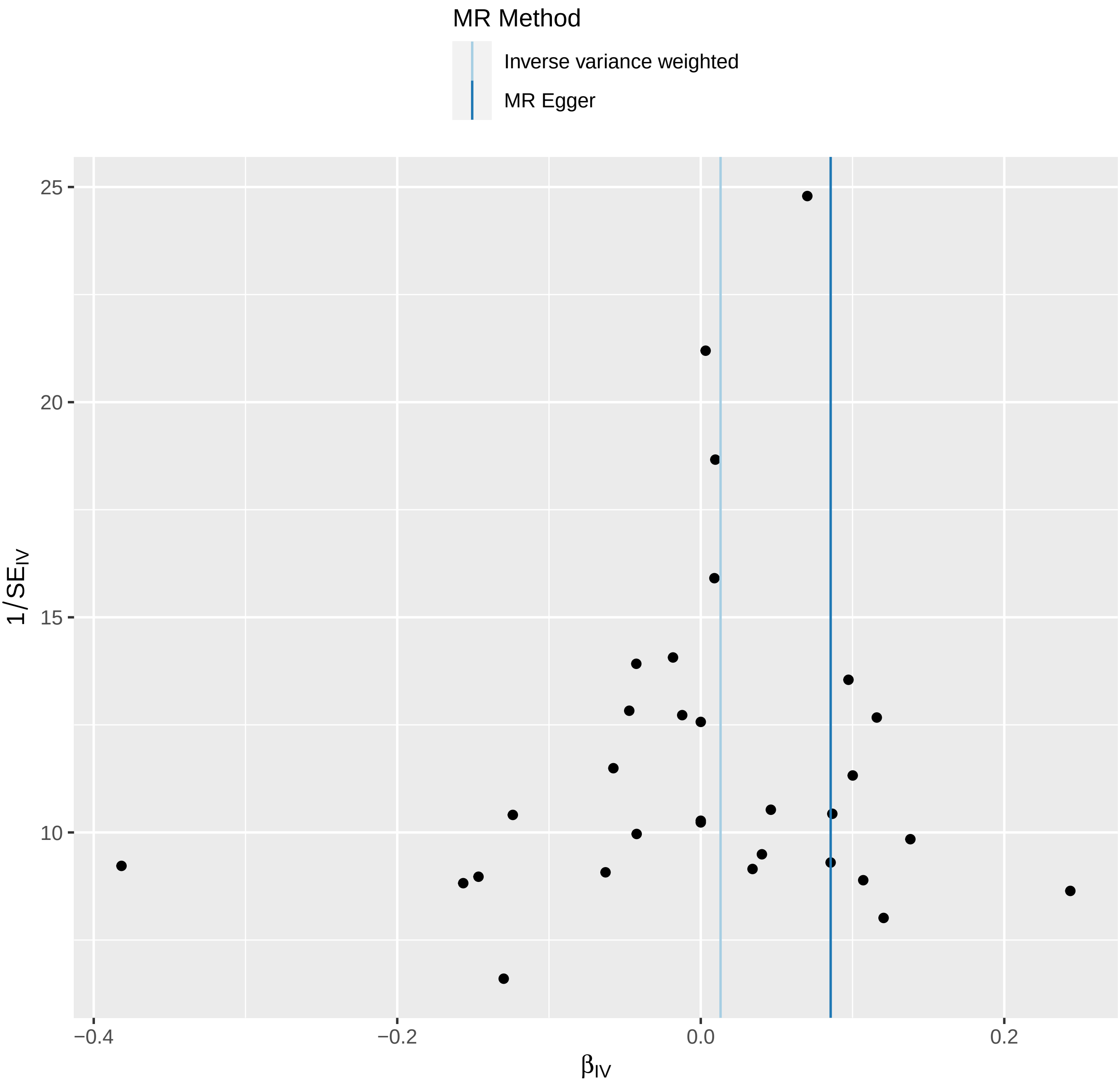

Batch 2615 : Crohn's disease on IDP T1 FAST ROIs R cing gyrus ant

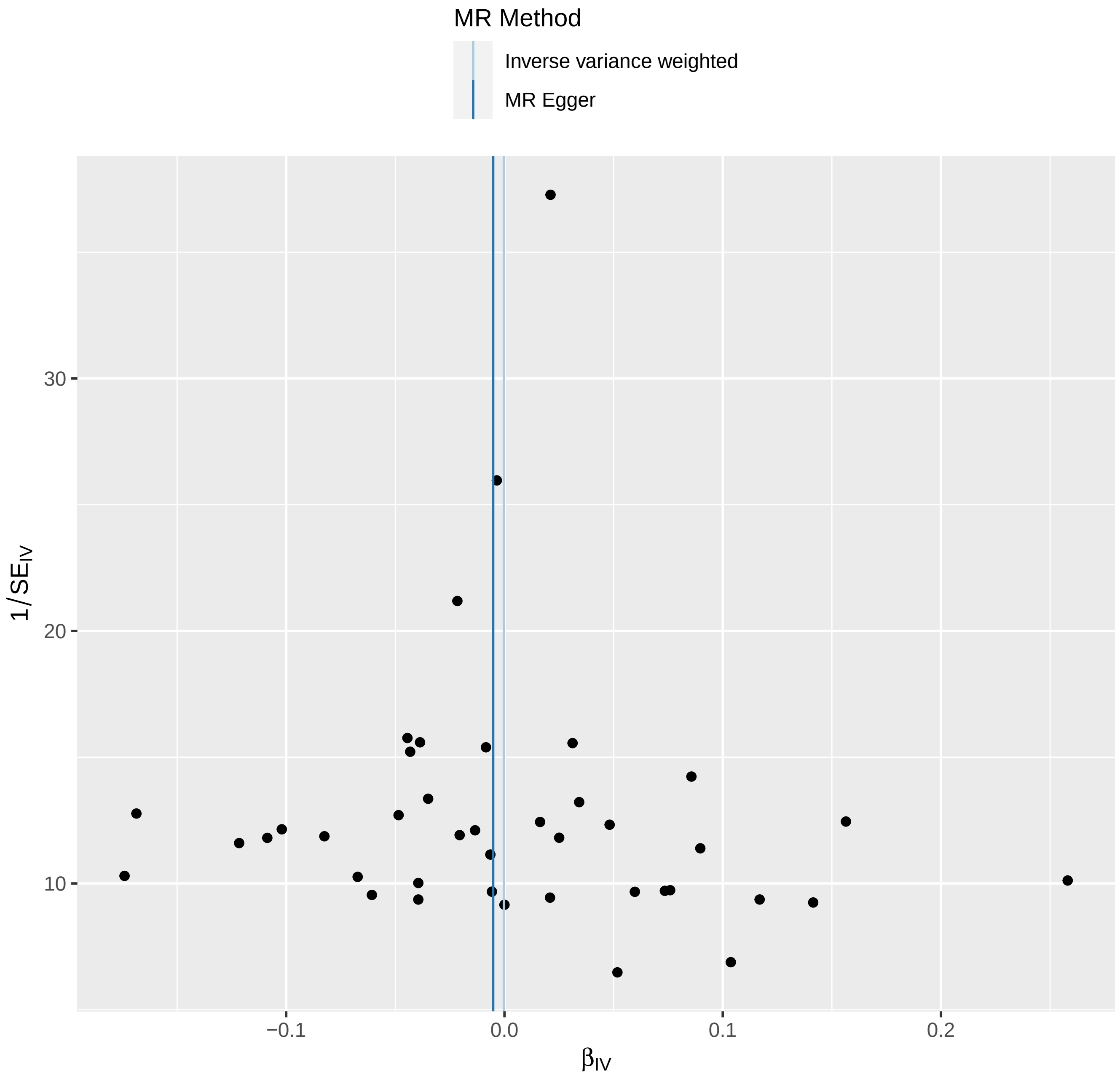

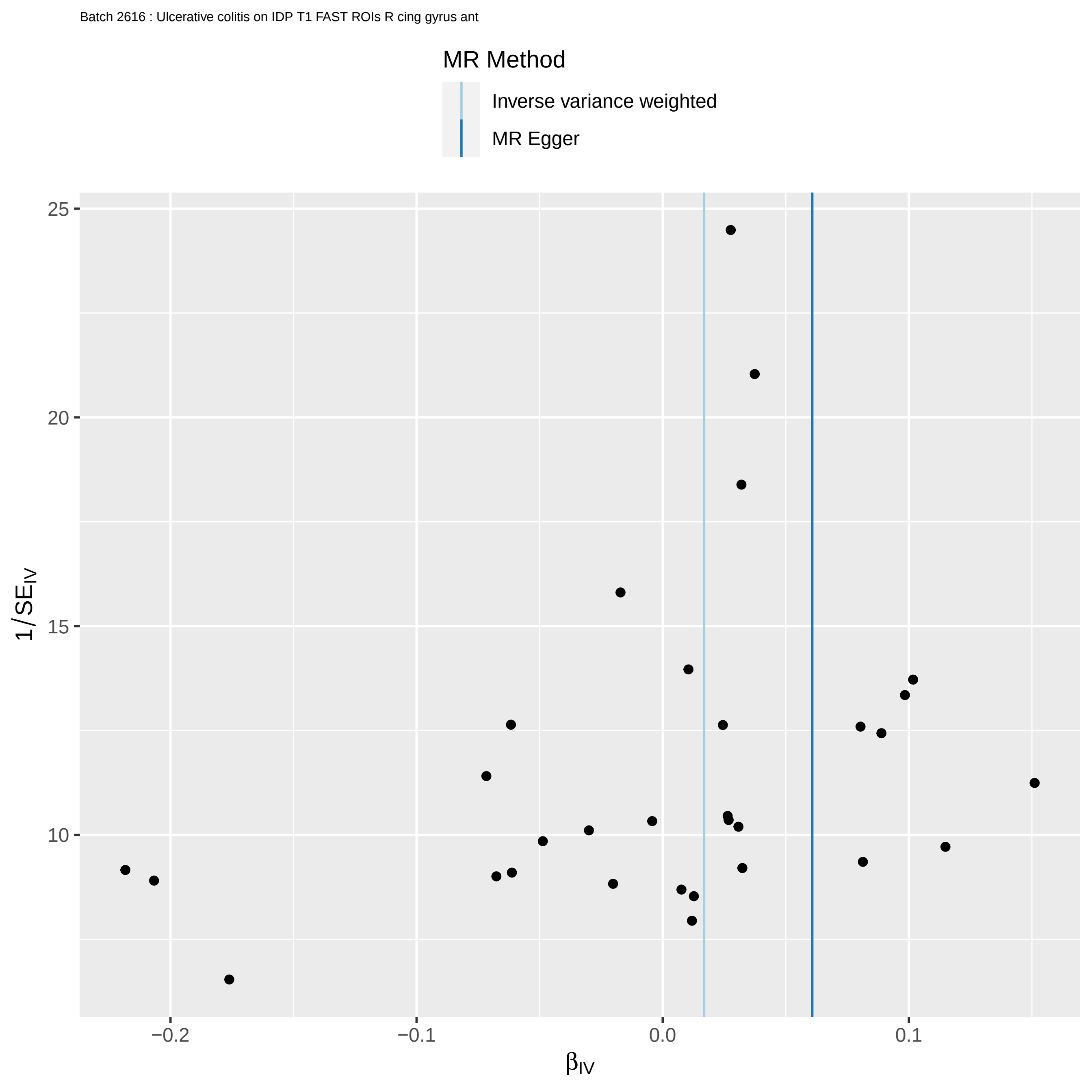
Batch 2616 : Ulcerative colitis on IDP T1 FAST ROIs R cing gyrus ant
MR Method
Inverse variance weighted
MR Egger
25
20
15
10
1/SE$_{IV}$
−0.2
−0.1
0.0
0.1
β$_{IV}$

Batch 2637 : Crohn's disease on IDP T1 FAST ROIs L cing gyrus post

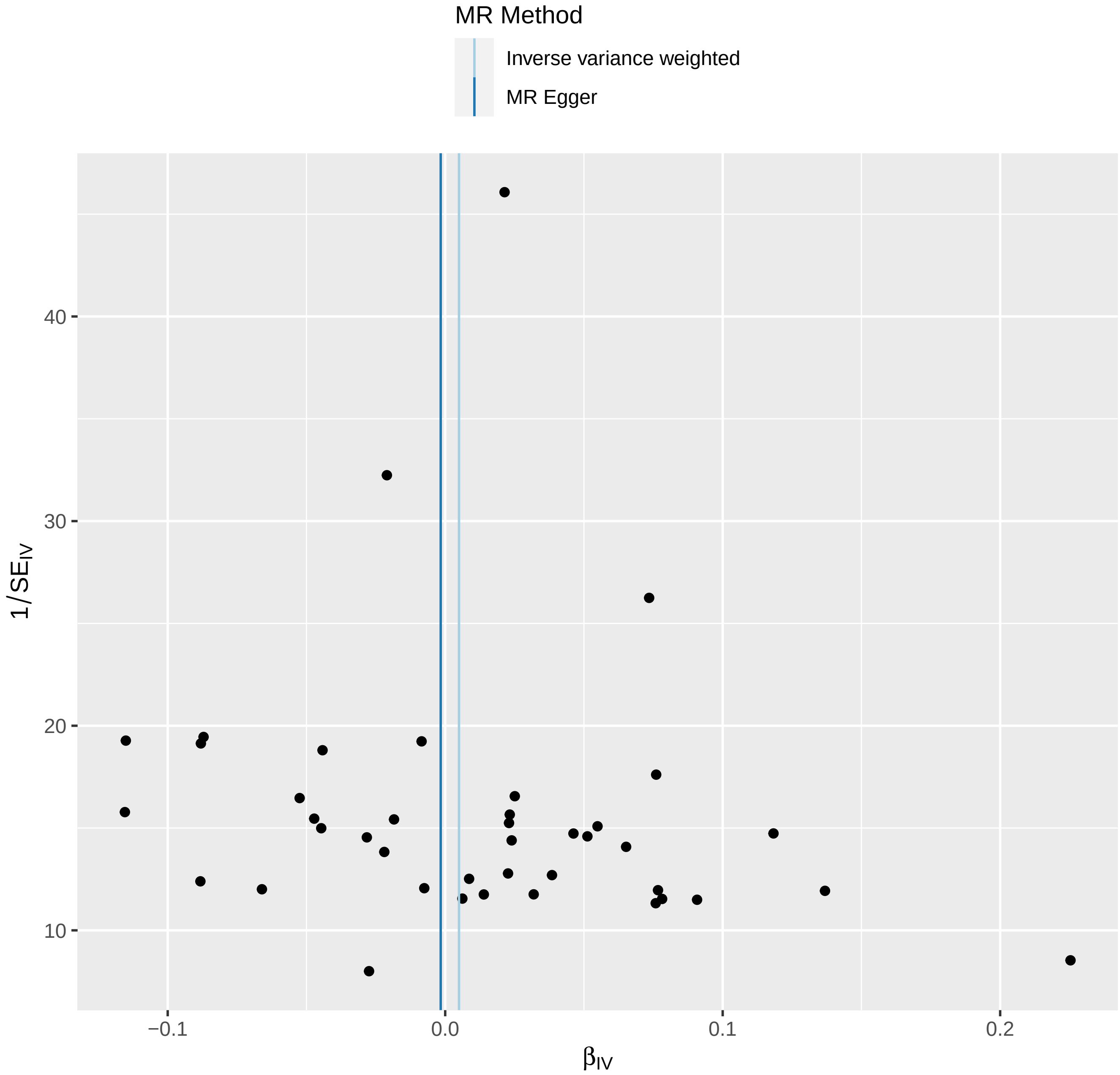

Batch 2638 : Ulcerative colitis on IDP T1 FAST ROIs L cing gyrus post

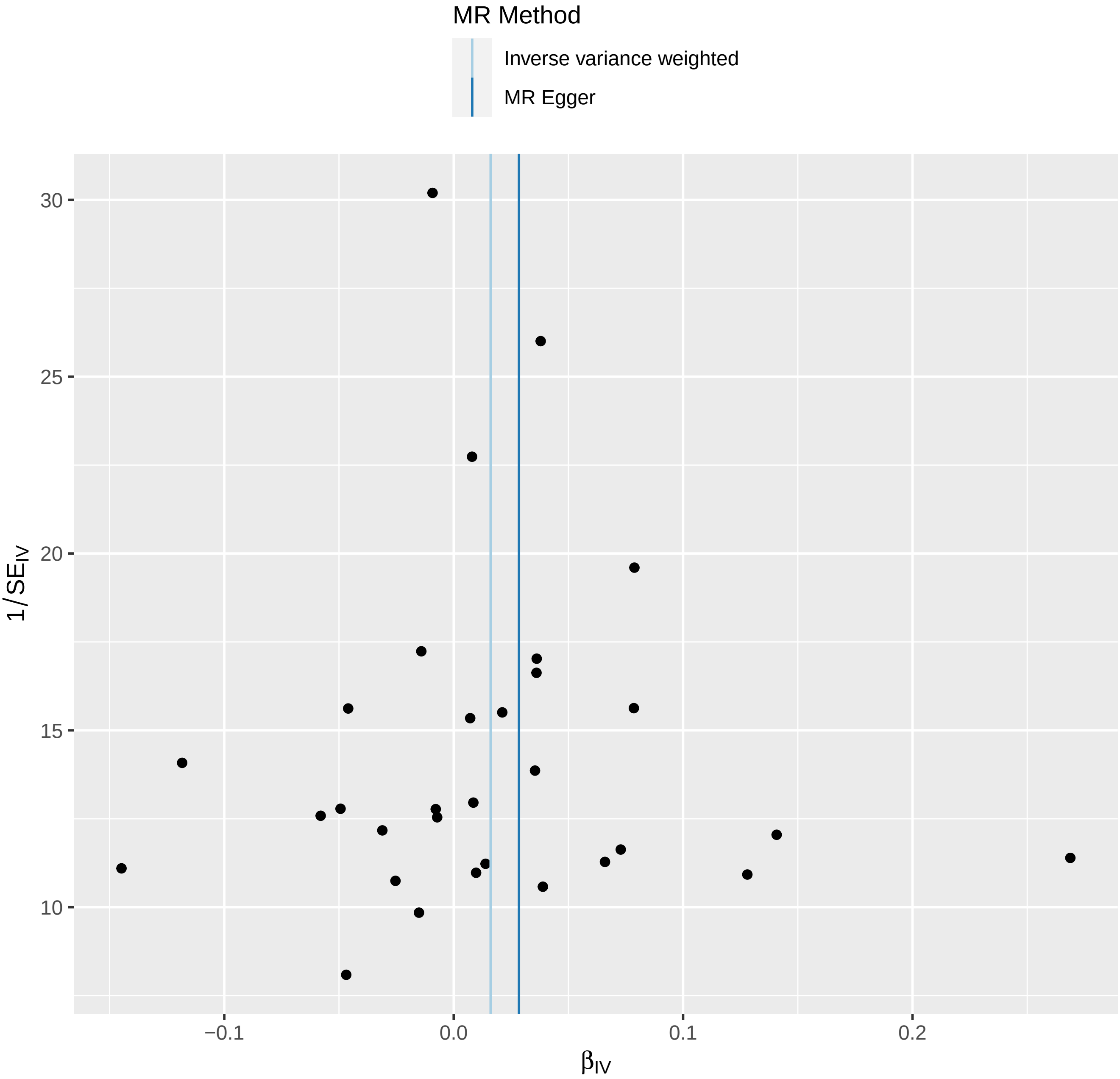

Batch 2659 : Crohn's disease on IDP T1 FAST ROIs R cing gyrus post

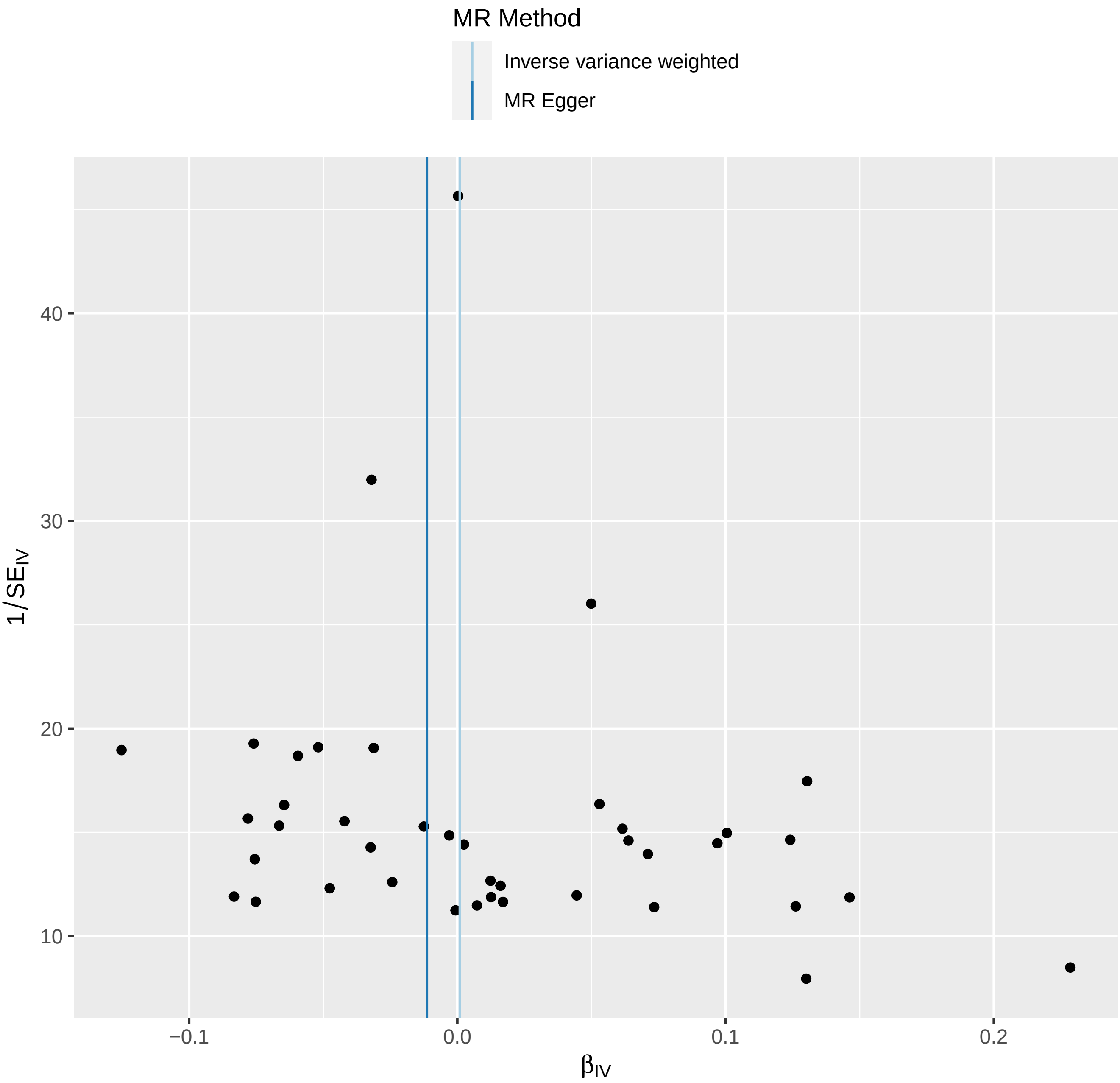

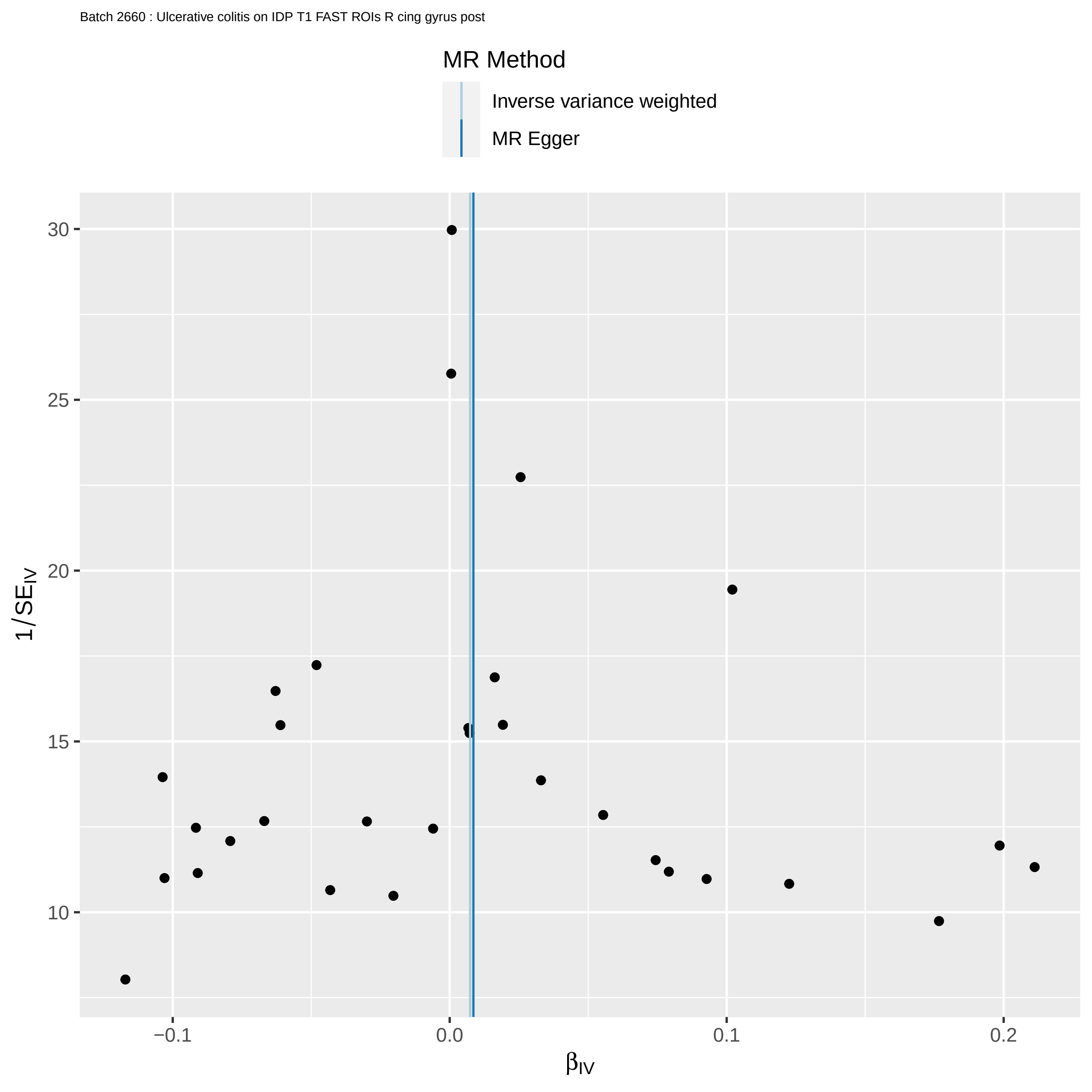
Batch 2660 : Ulcerative colitis on IDP T1 FAST ROIs R cing gyrus post
MR Method
Inverse variance weighted
MR Egger
30
25
20
15
10
1/SE$_{IV}$
−0.1
0.0
0.1
0.2
β$_{IV}$

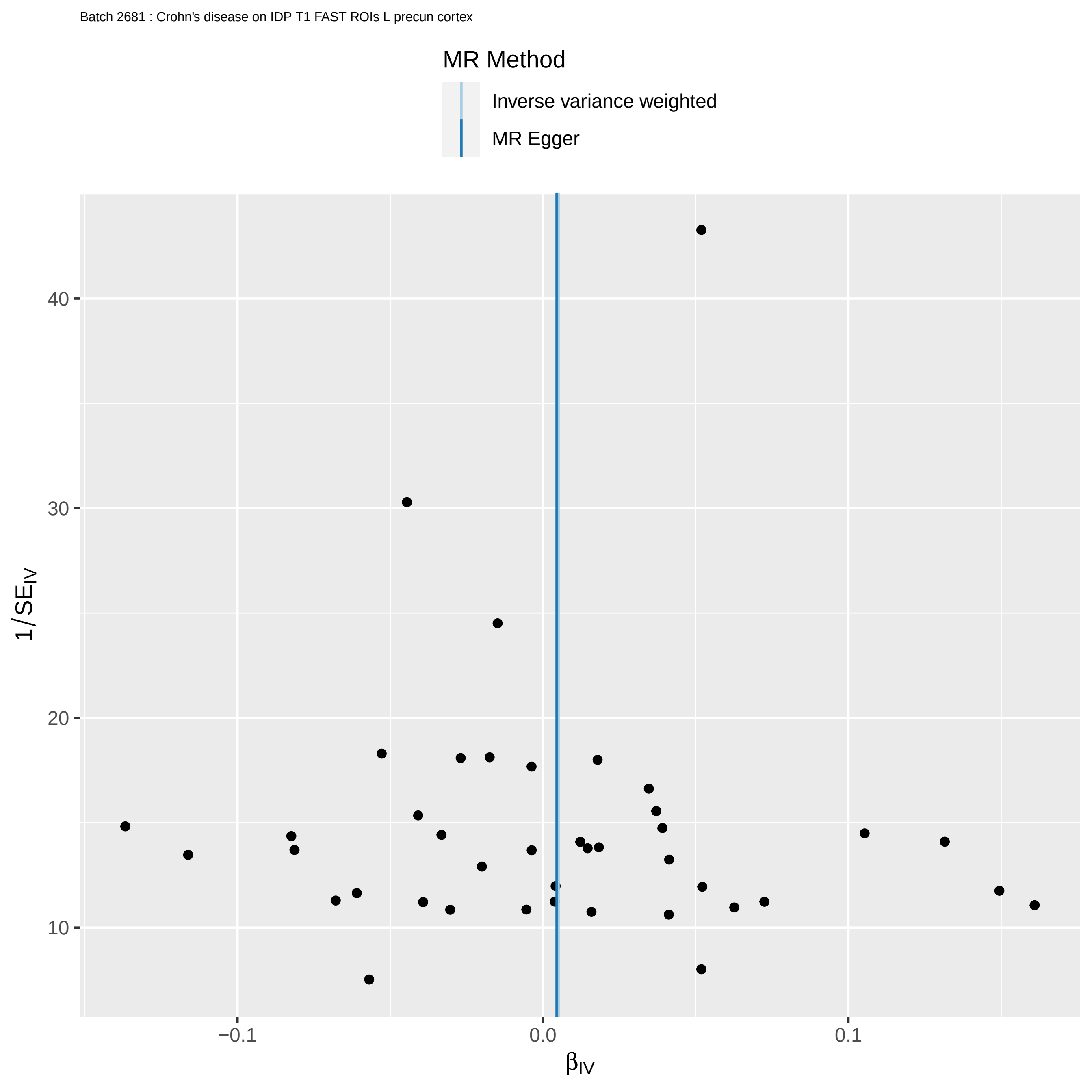

Batch 2681 : Crohn's disease on IDP T1 FAST ROIs L precun cortex
MR Method
Inverse variance weighted
MR Egger
40
30
20
10
$1/SE_{IV}$
−0.1
0.0
0.1
β_{IV}

Batch 2682 : Ulcerative colitis on IDP T1 FAST ROIs L precun cortex

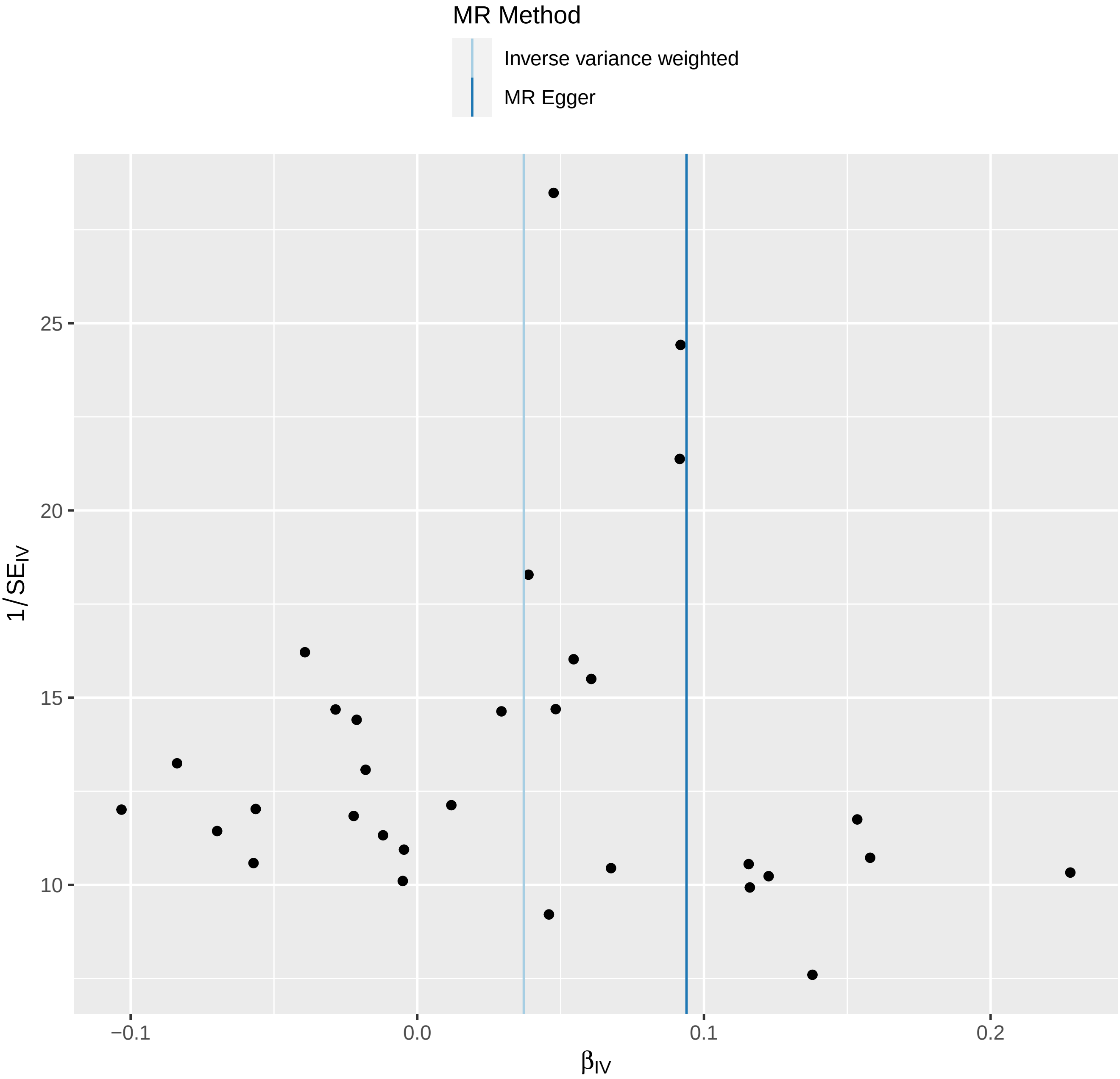

Batch 2703 : Crohn's disease on IDP T1 FAST ROIs R precun cortex

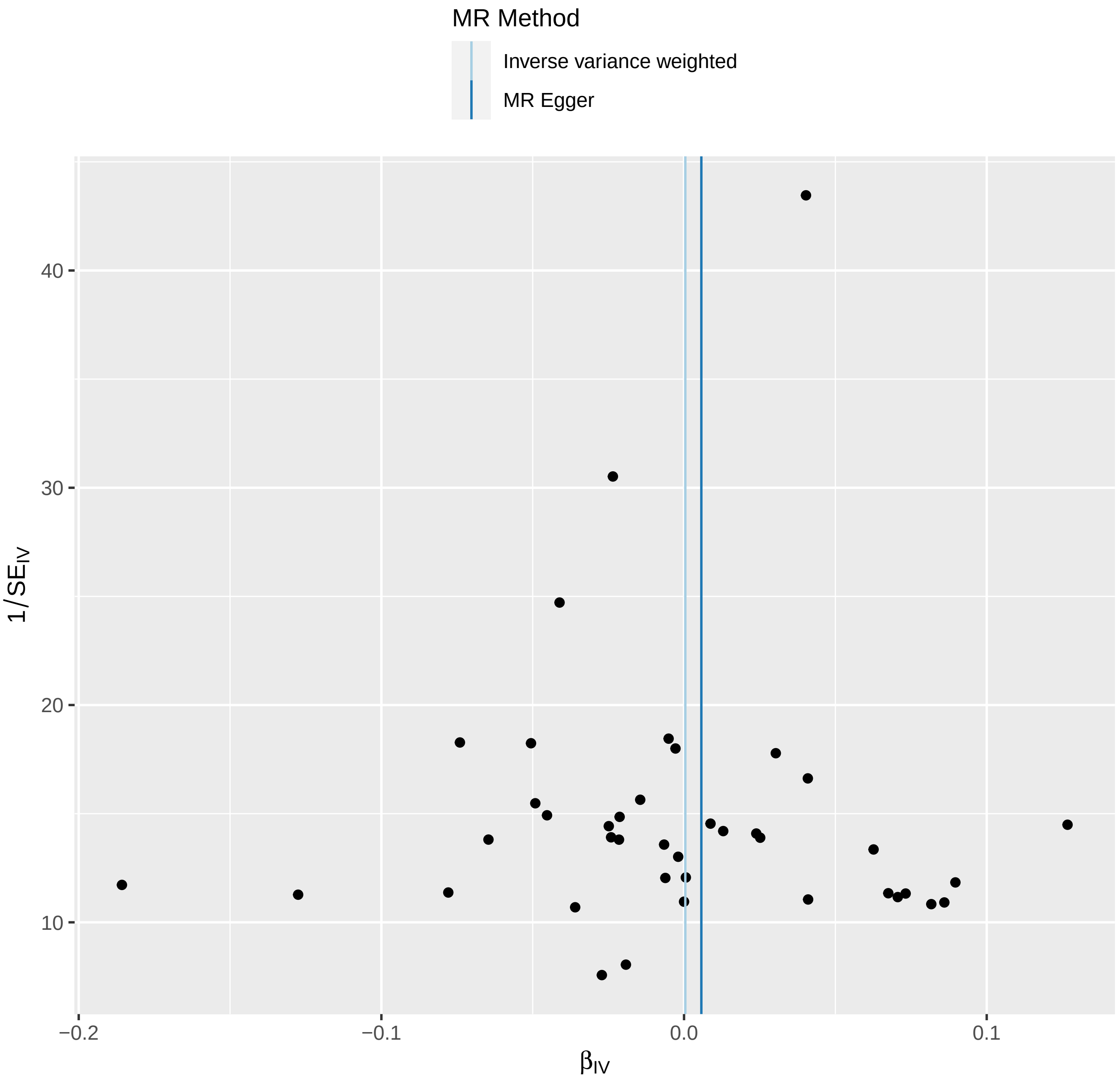

Batch 2704 : Ulcerative colitis on IDP T1 FAST ROIs R precun cortex

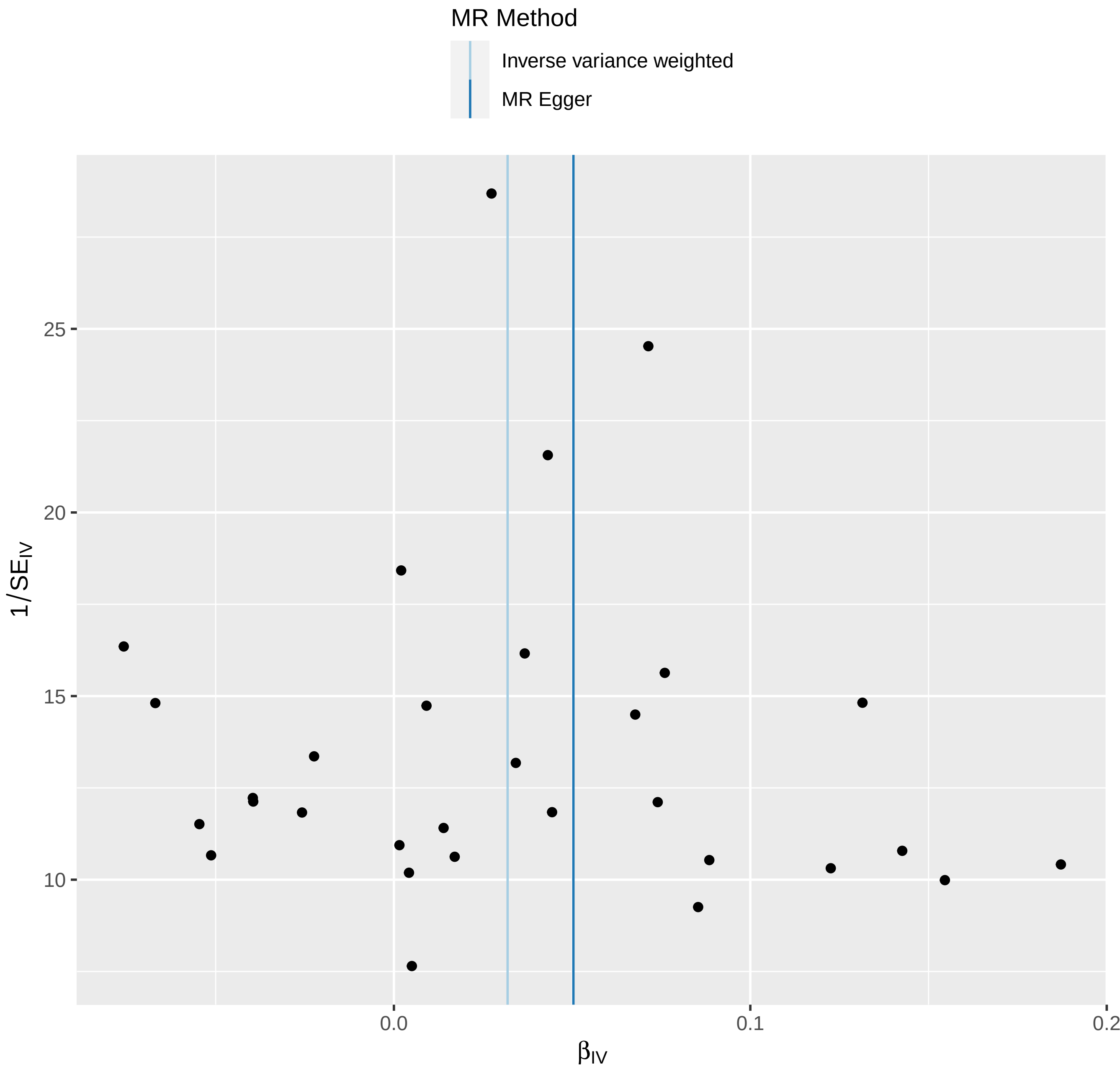

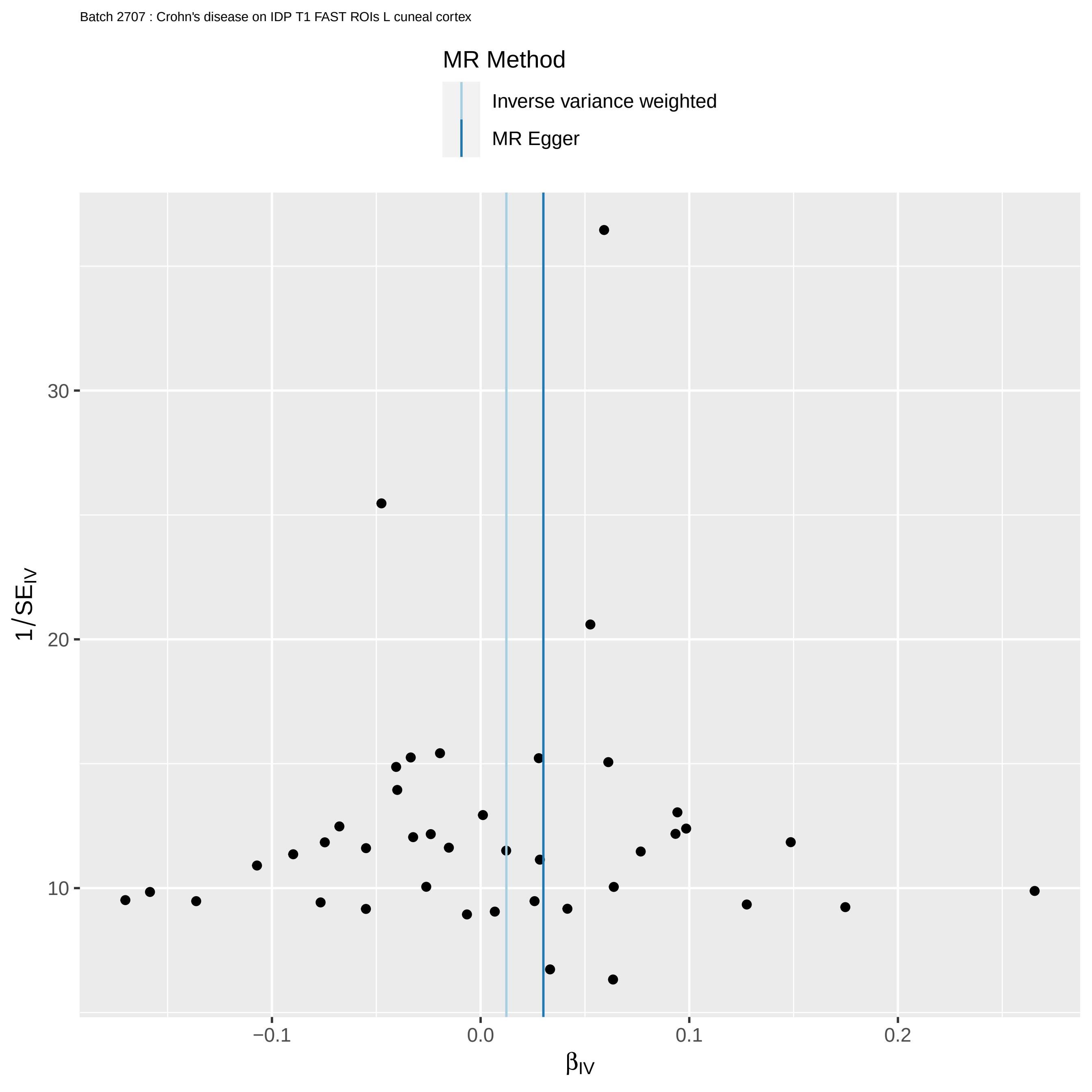

Batch 2707 : Crohn's disease on IDP T1 FAST ROIs L cuneal cortex
MR Method
Inverse variance weighted
MR Egger
1/SE_IV
30
20
10
−0.1
0.0
0.1
0.2
β_IV

Batch 2708 : Ulcerative colitis on IDP T1 FAST ROIs L cuneal cortex

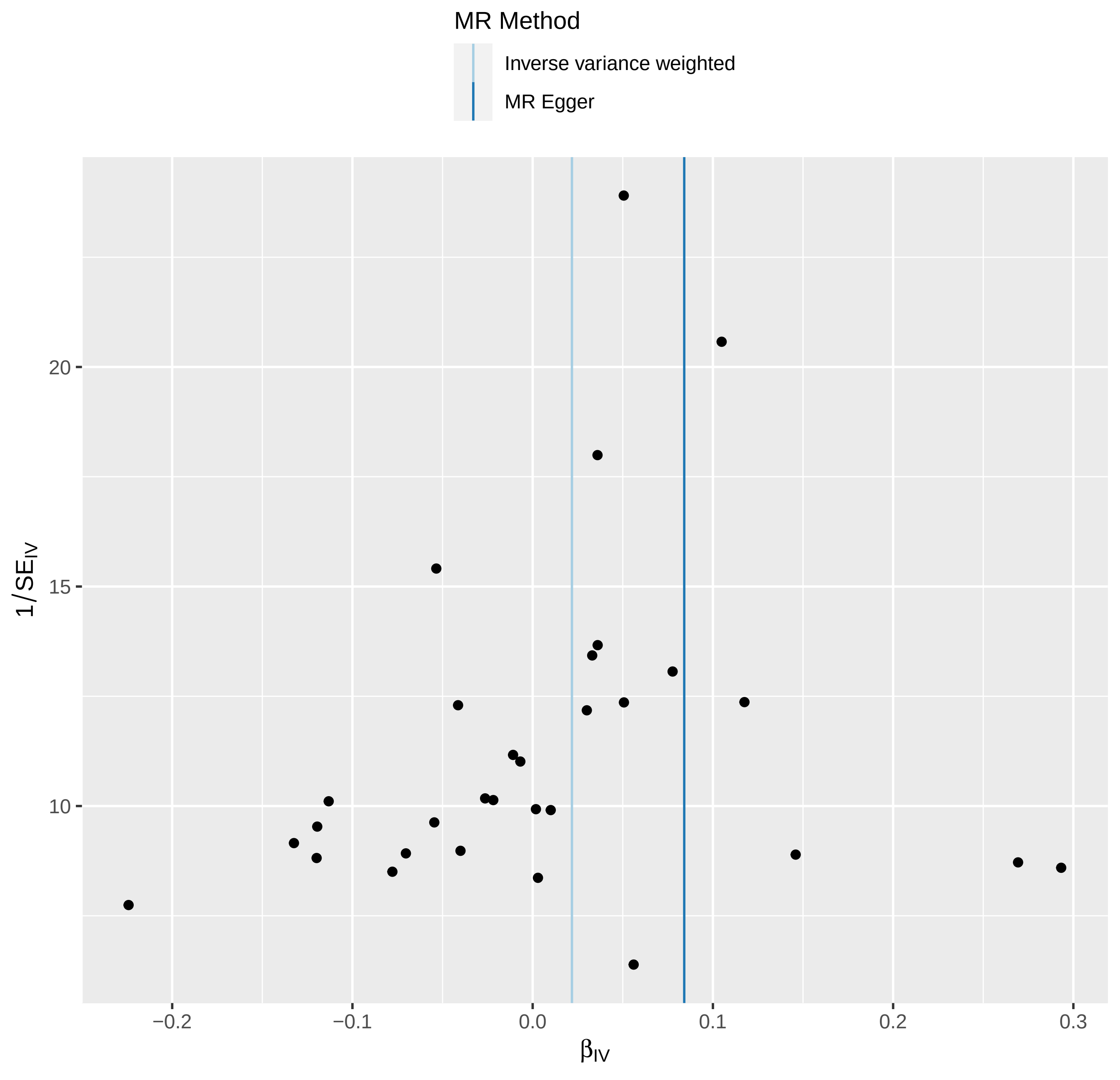

Batch 2709 : Crohn's disease on IDP T1 FAST ROIs R cuneal cortex

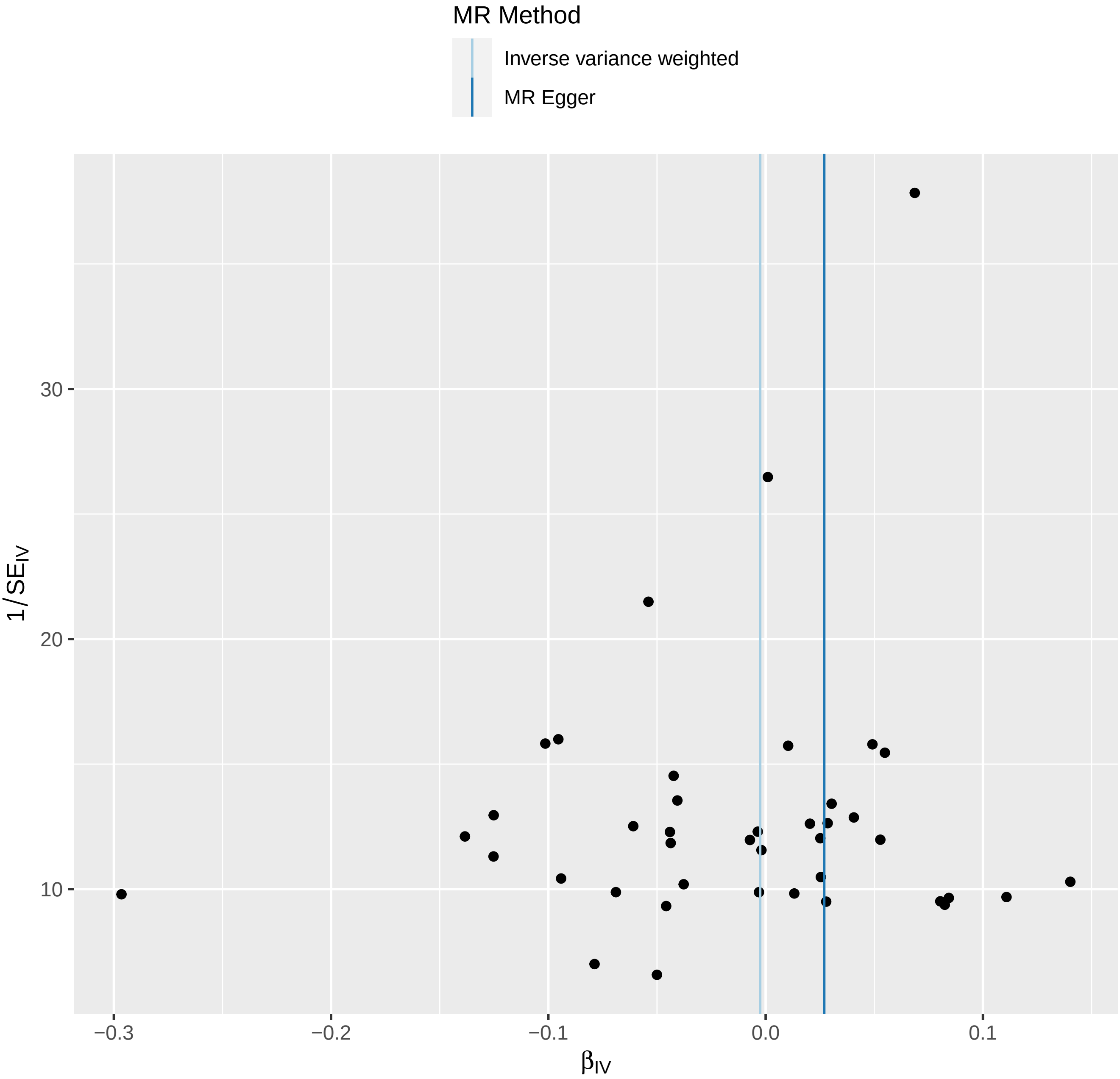

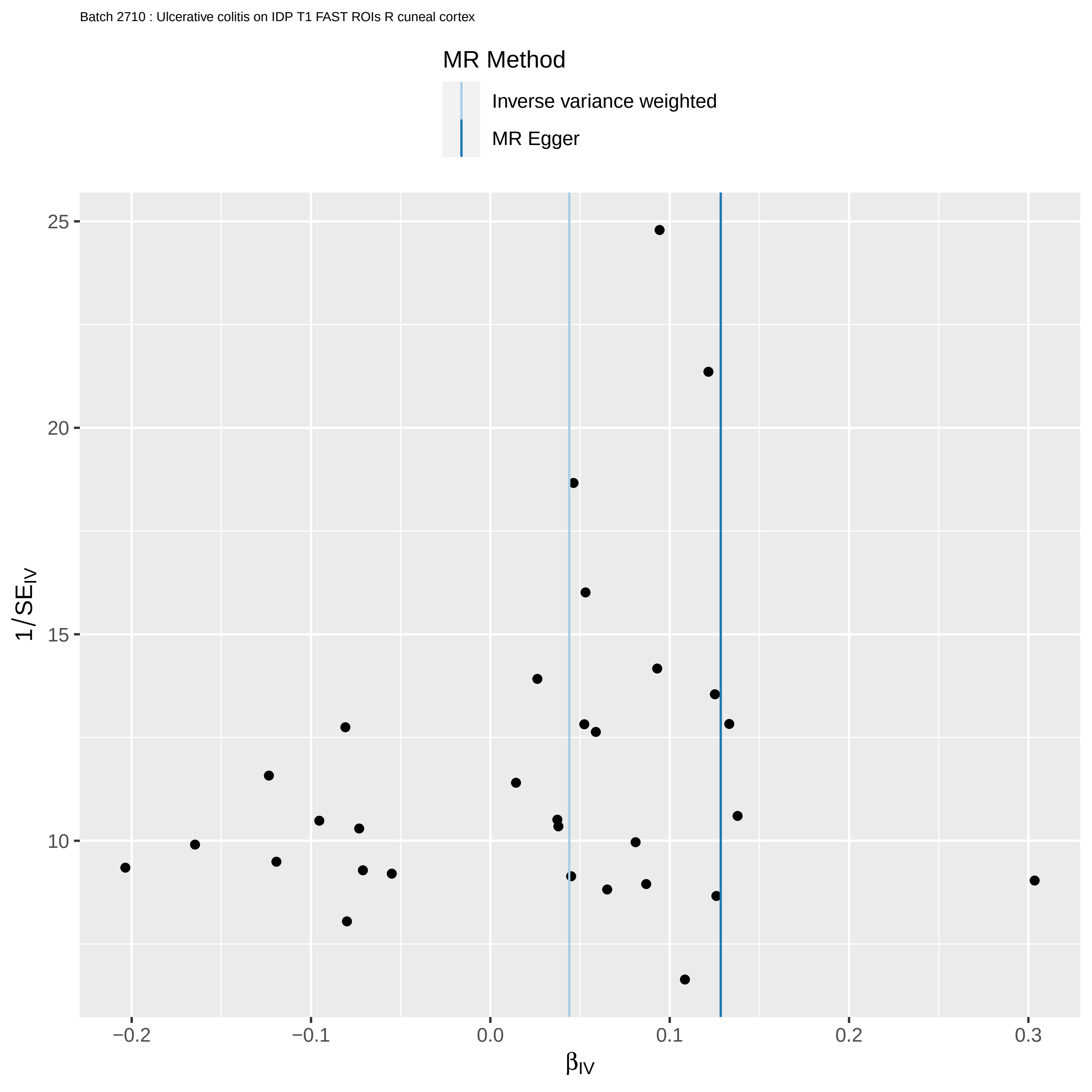

Batch 2710 : Ulcerative colitis on IDP T1 FAST ROIs R cuneal cortex
MR Method
Inverse variance weighted
MR Egger
25
20
15
10
1/SE_IV
−0.2
−0.1
0.0
0.1
0.2
0.3
β_IV

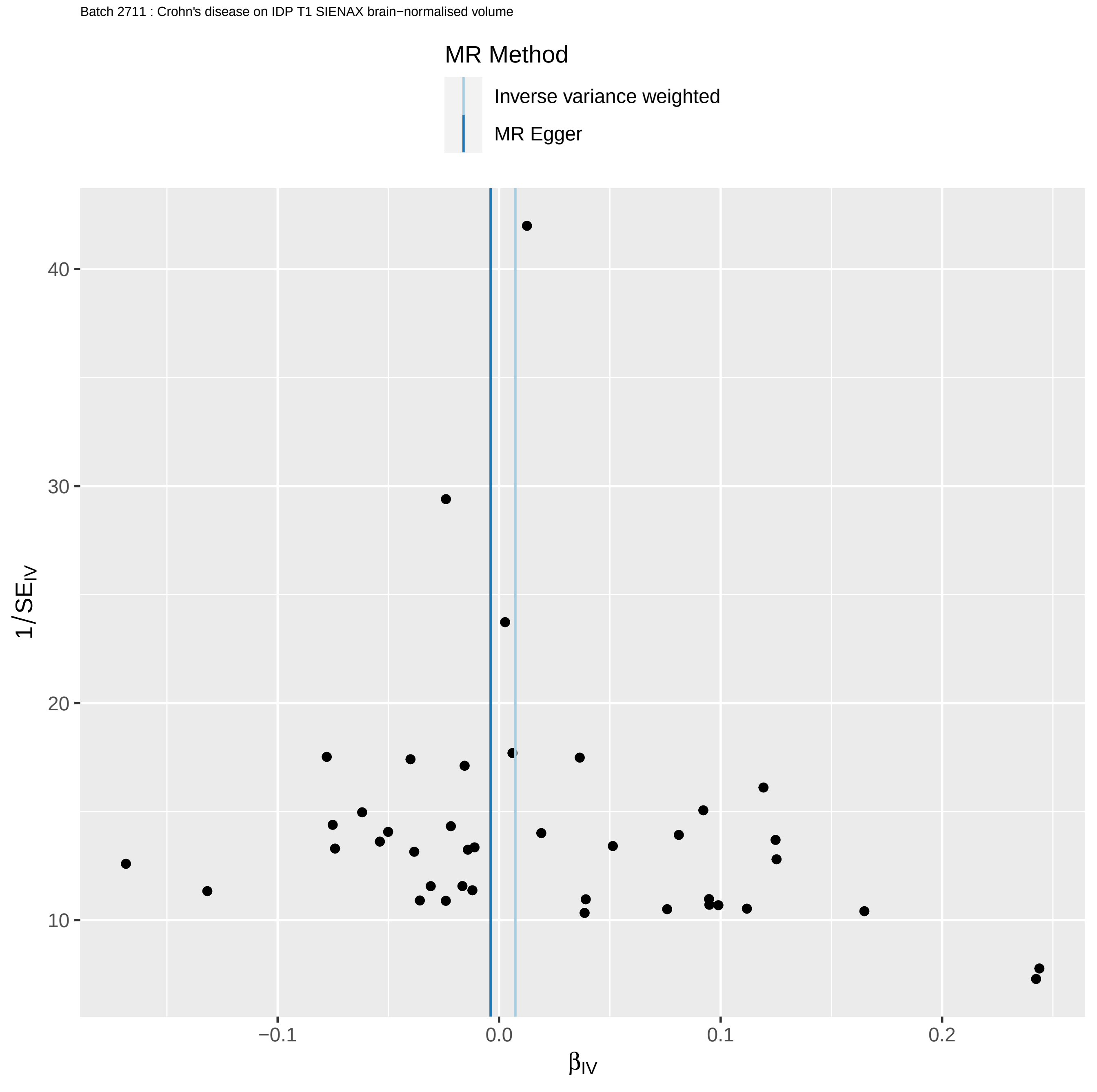
Batch 2711 : Crohn's disease on IDP T1 SIENAX brain−normalised volume
MR Method
Inverse variance weighted
MR Egger
40
30
20
10
$1/SE_{IV}$
−0.1
0.0
0.1
0.2
β_{IV}

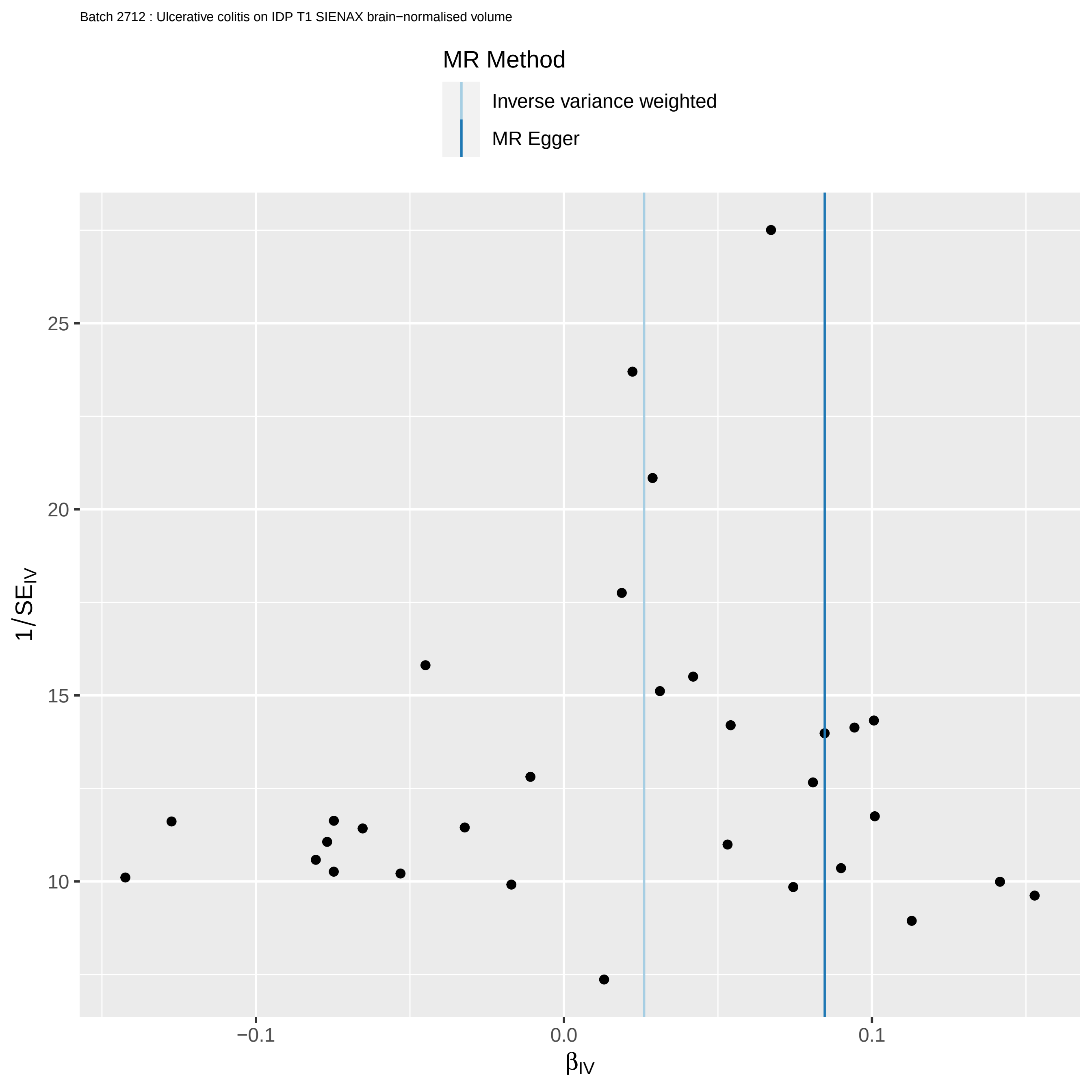

Batch 2712 : Ulcerative colitis on IDP T1 SIENAX brain−normalised volume
MR Method
Inverse variance weighted
MR Egger
25
20
15
10
1/SE_IV
−0.1
0.0
0.1
β_IV

Batch 2713 : Crohn's disease on IDP T1 FAST ROIs L front orb cortex

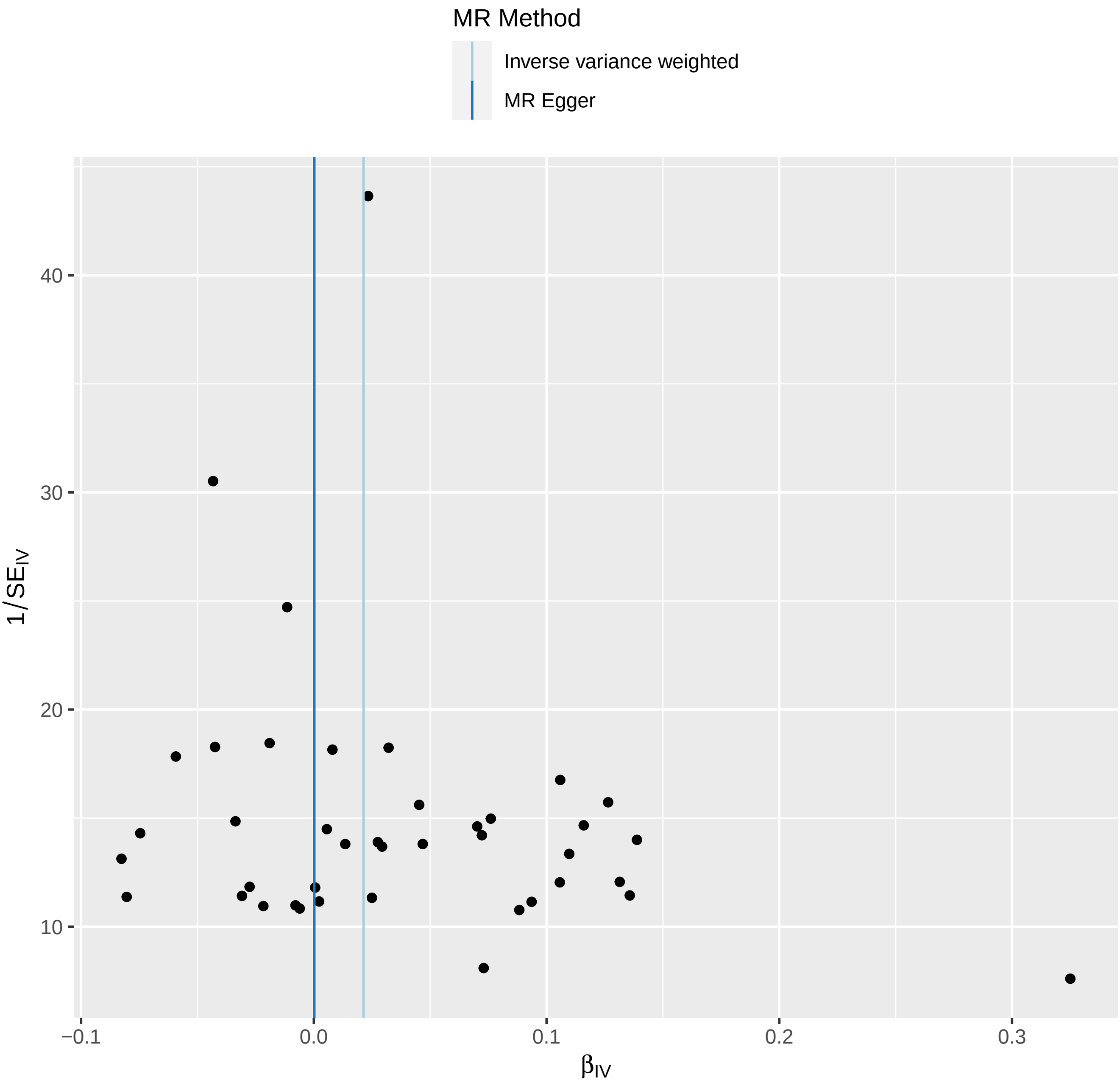

Batch 2714 : Ulcerative colitis on IDP T1 FAST ROIs L front orb cortex

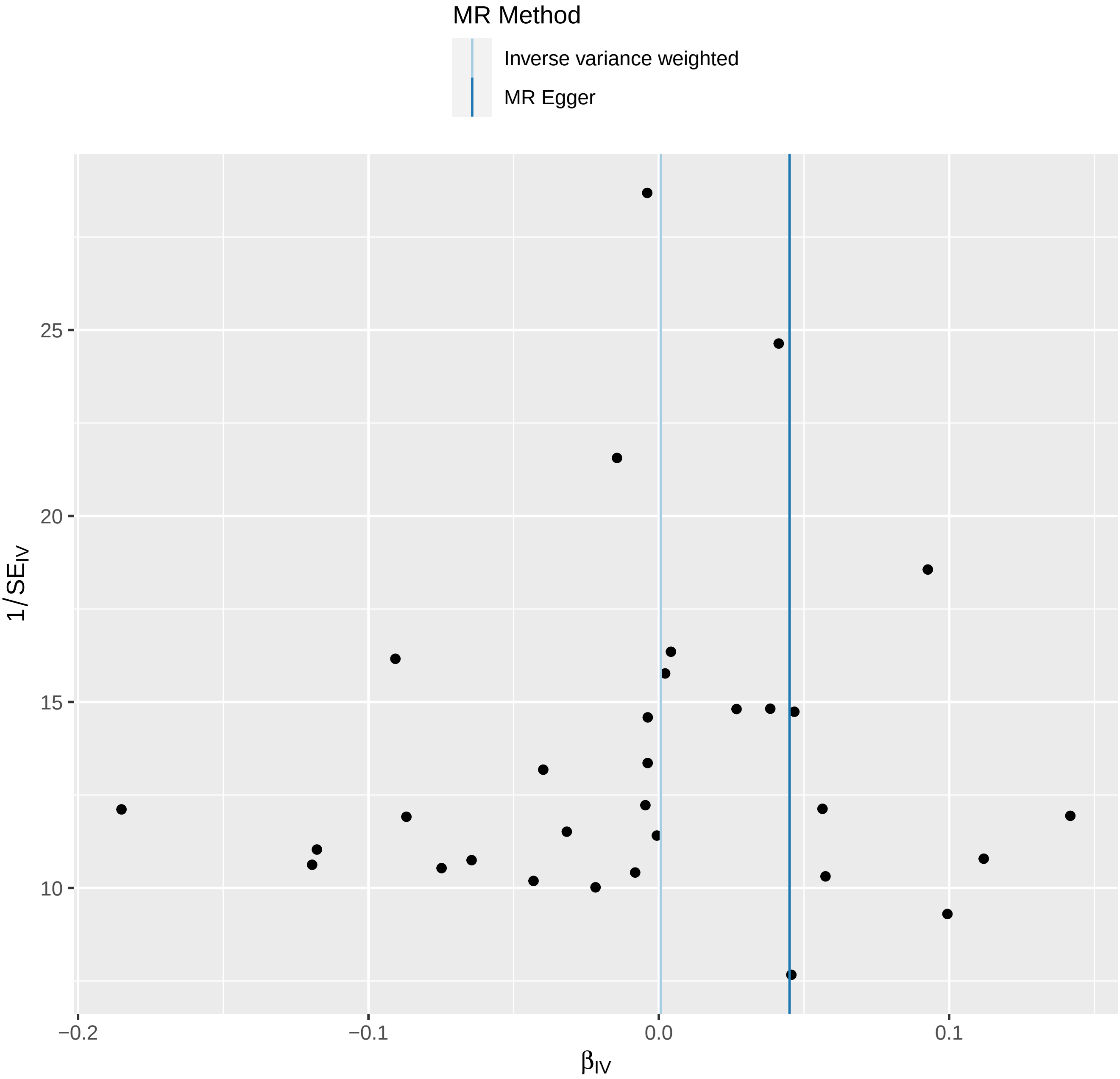

Batch 2715 : Crohn's disease on IDP T1 FAST ROIs R front orb cortex

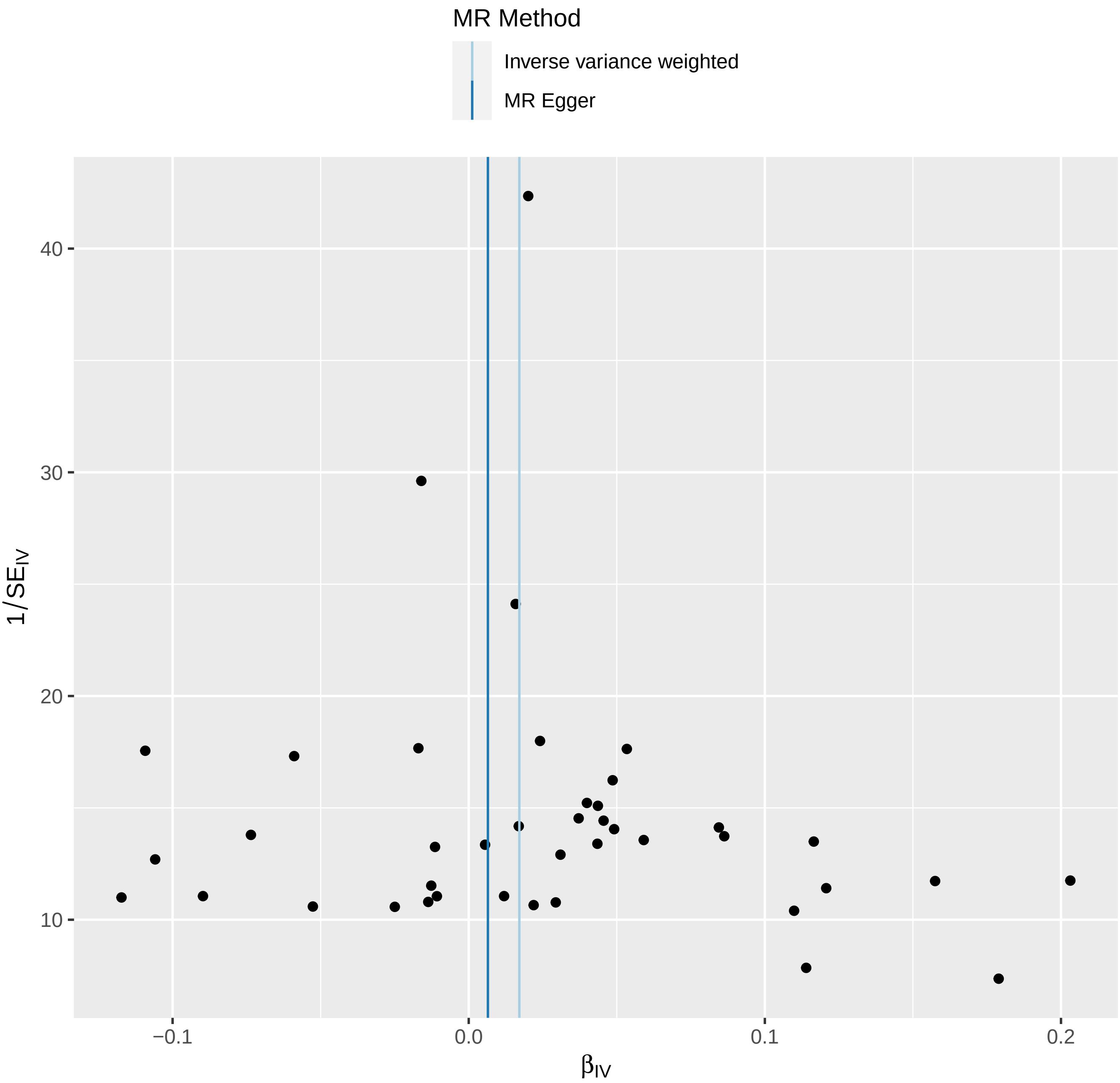

Batch 2716 : Ulcerative colitis on IDP T1 FAST ROIs R front orb cortex

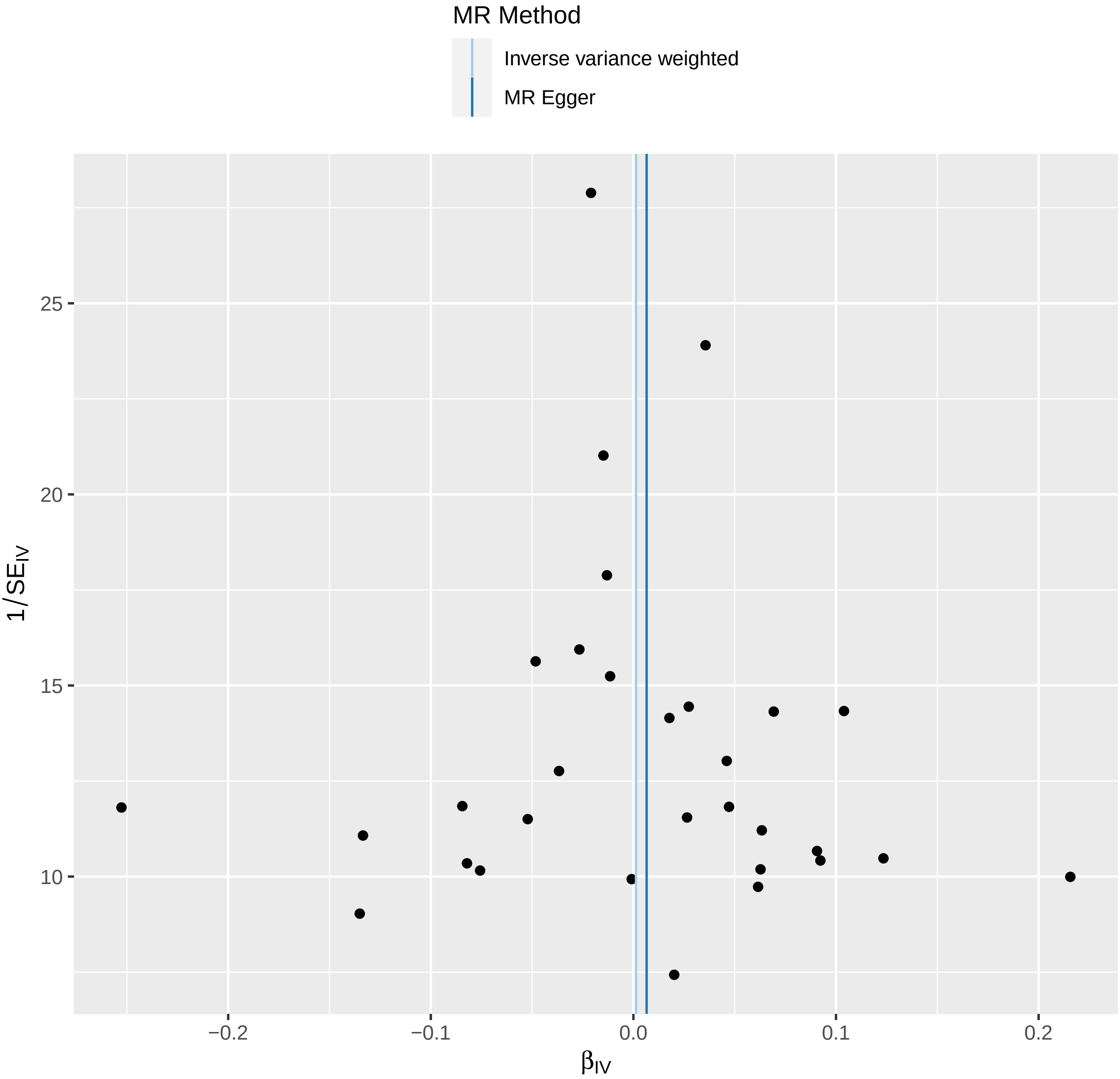

Batch 2717 : Crohn's disease on IDP T1 FAST ROIs L parahipp gyrus ant

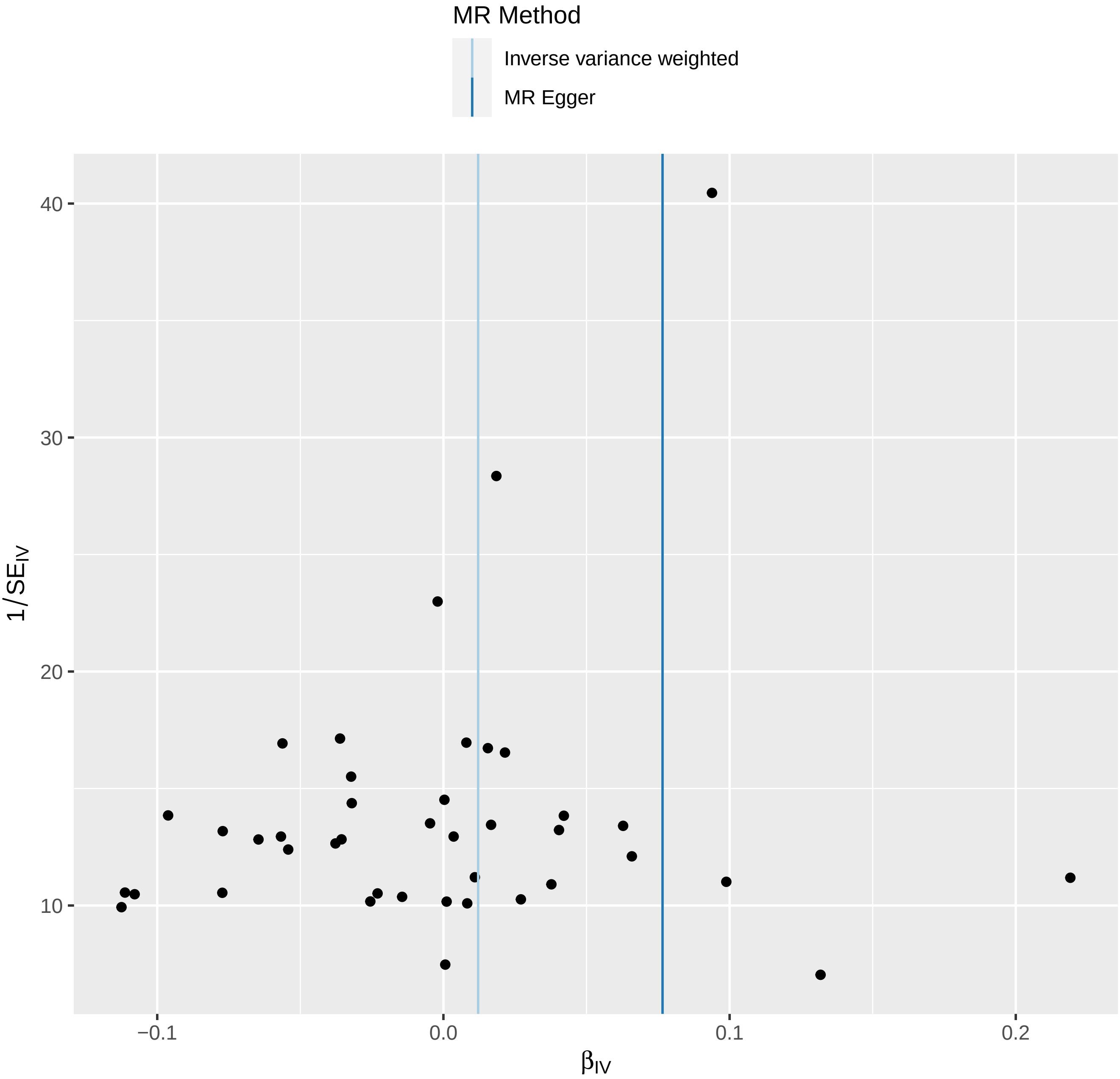

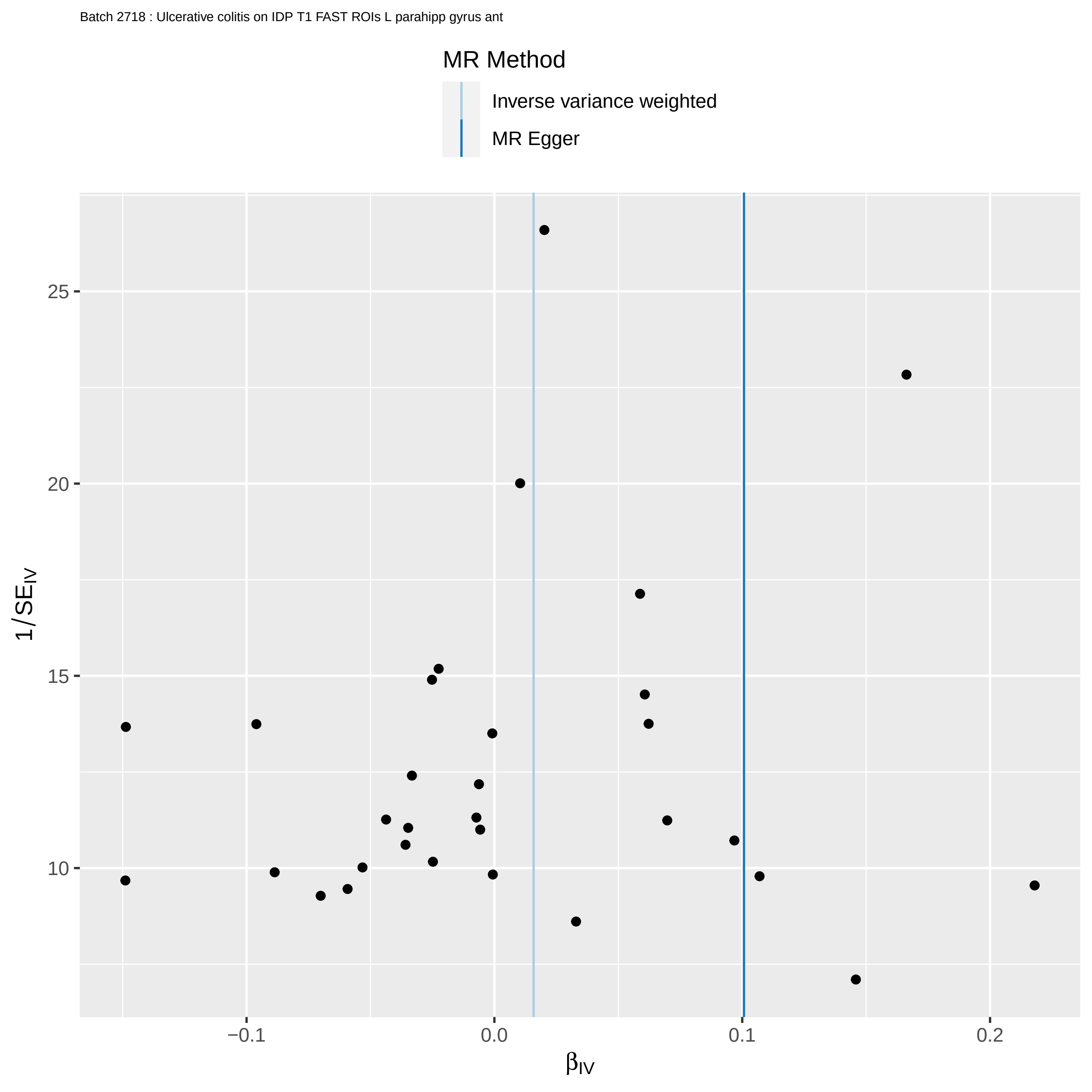

Batch 2718 : Ulcerative colitis on IDP T1 FAST ROIs L parahipp gyrus ant
MR Method
Inverse variance weighted
MR Egger
25
20
15
10
1/SE_IV
−0.1
0.0
0.1
0.2
β_IV

Batch 2719 : Crohn's disease on IDP T1 FAST ROIs R parahipp gyrus ant

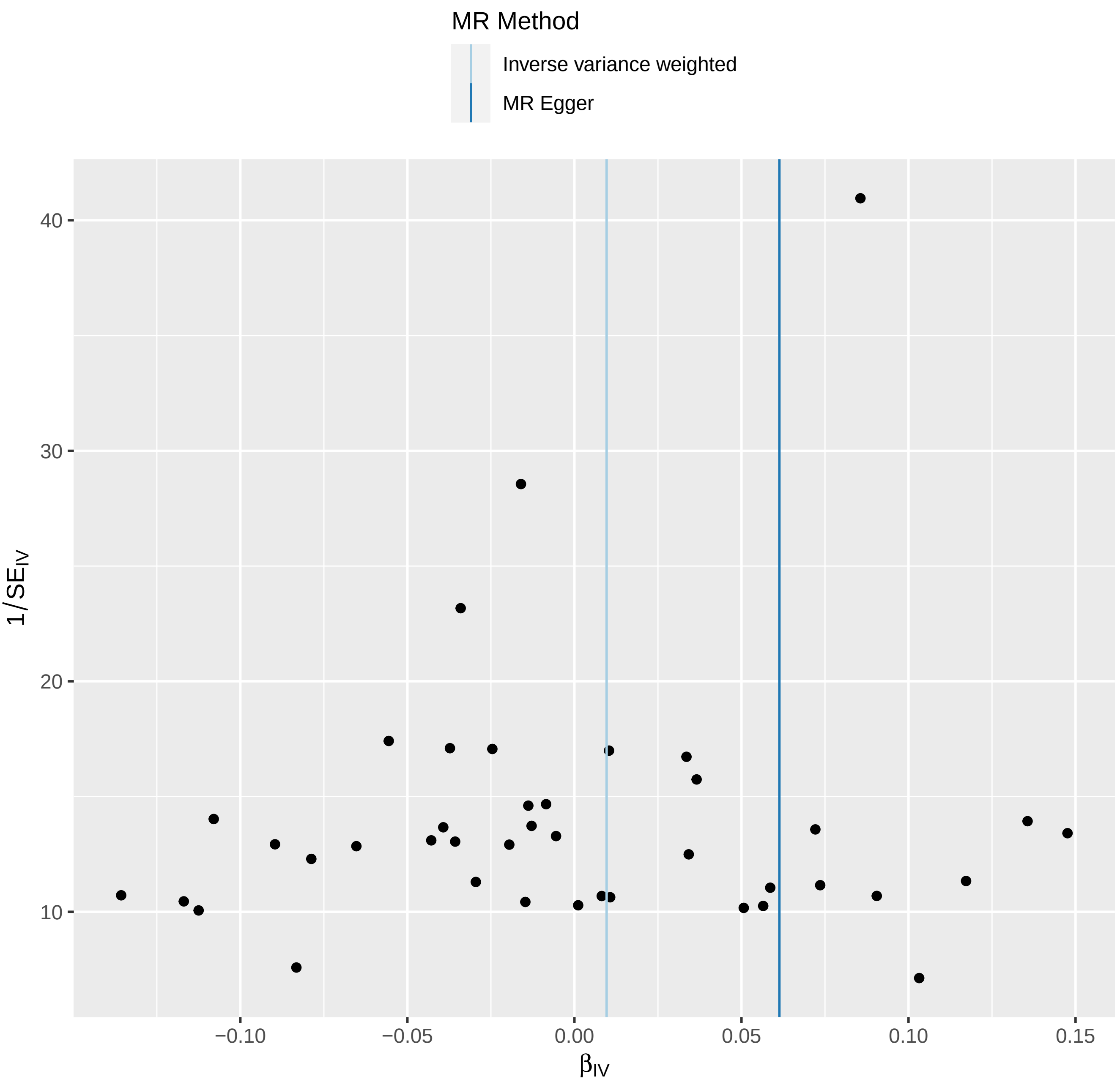

Batch 2720 : Ulcerative colitis on IDP T1 FAST ROIs R parahipp gyrus ant

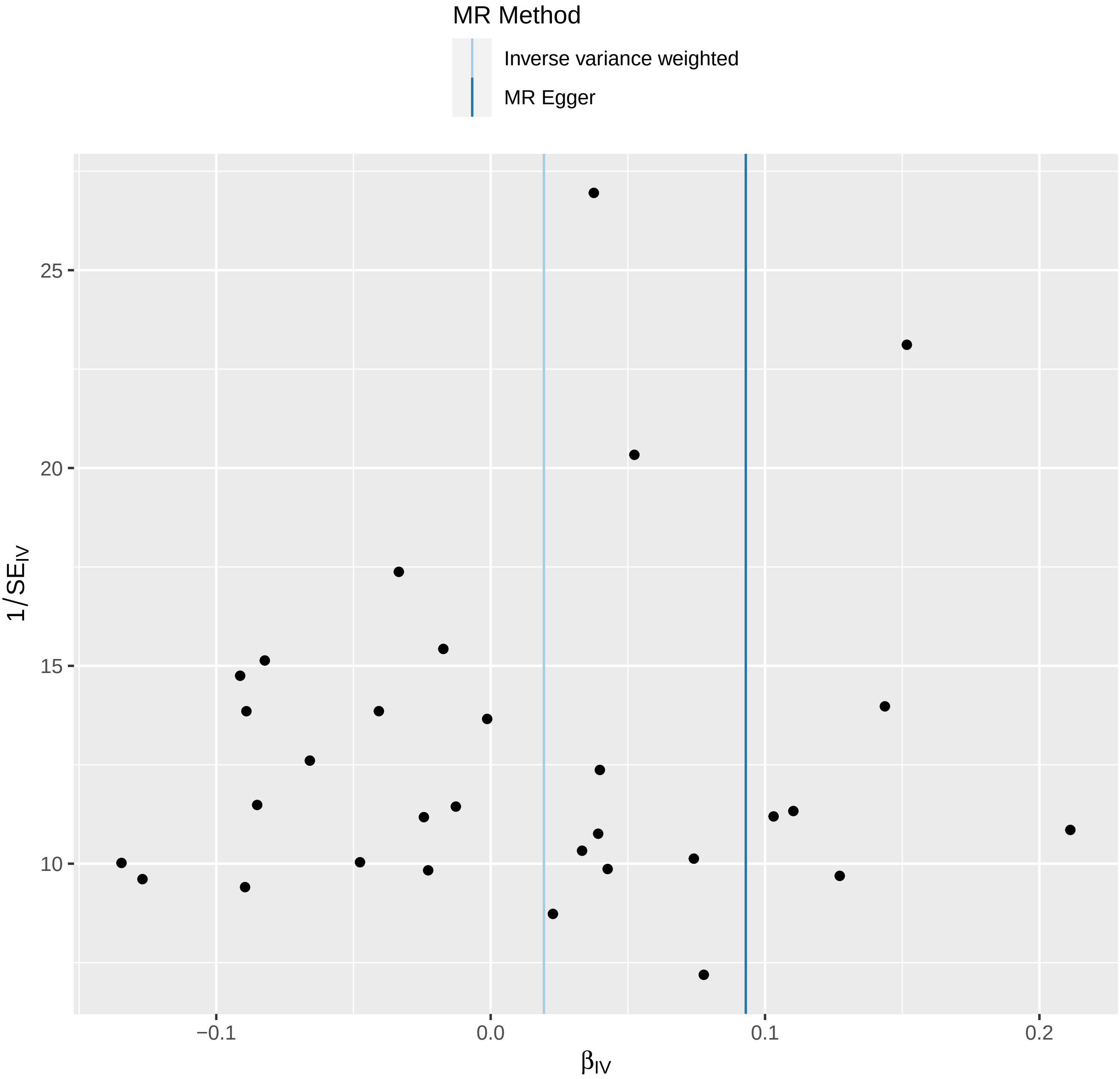

Batch 2721 : Crohn's disease on IDP T1 FAST ROIs L parahipp gyrus post

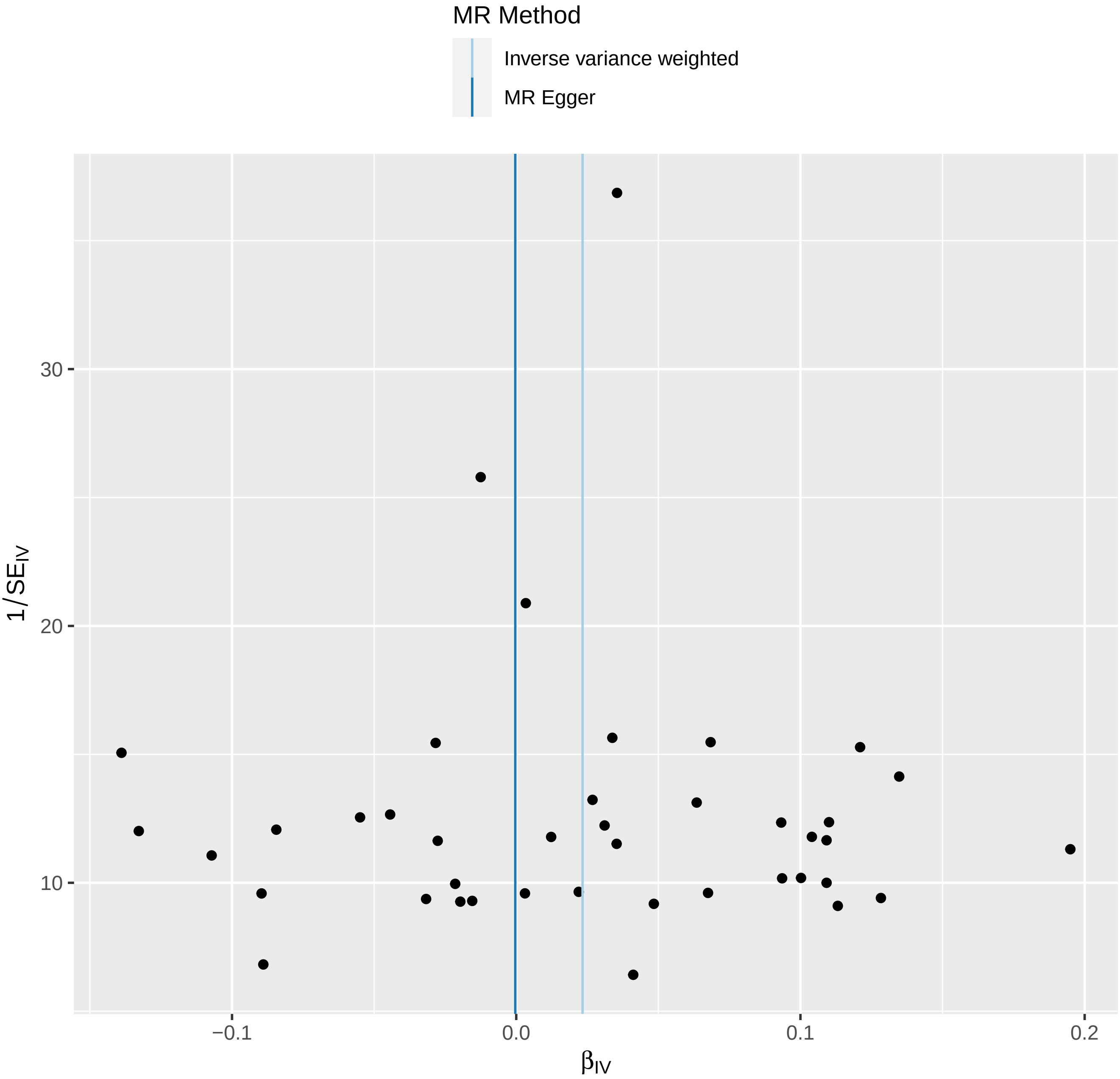

Batch 2722 : Ulcerative colitis on IDP T1 FAST ROIs L parahipp gyrus post

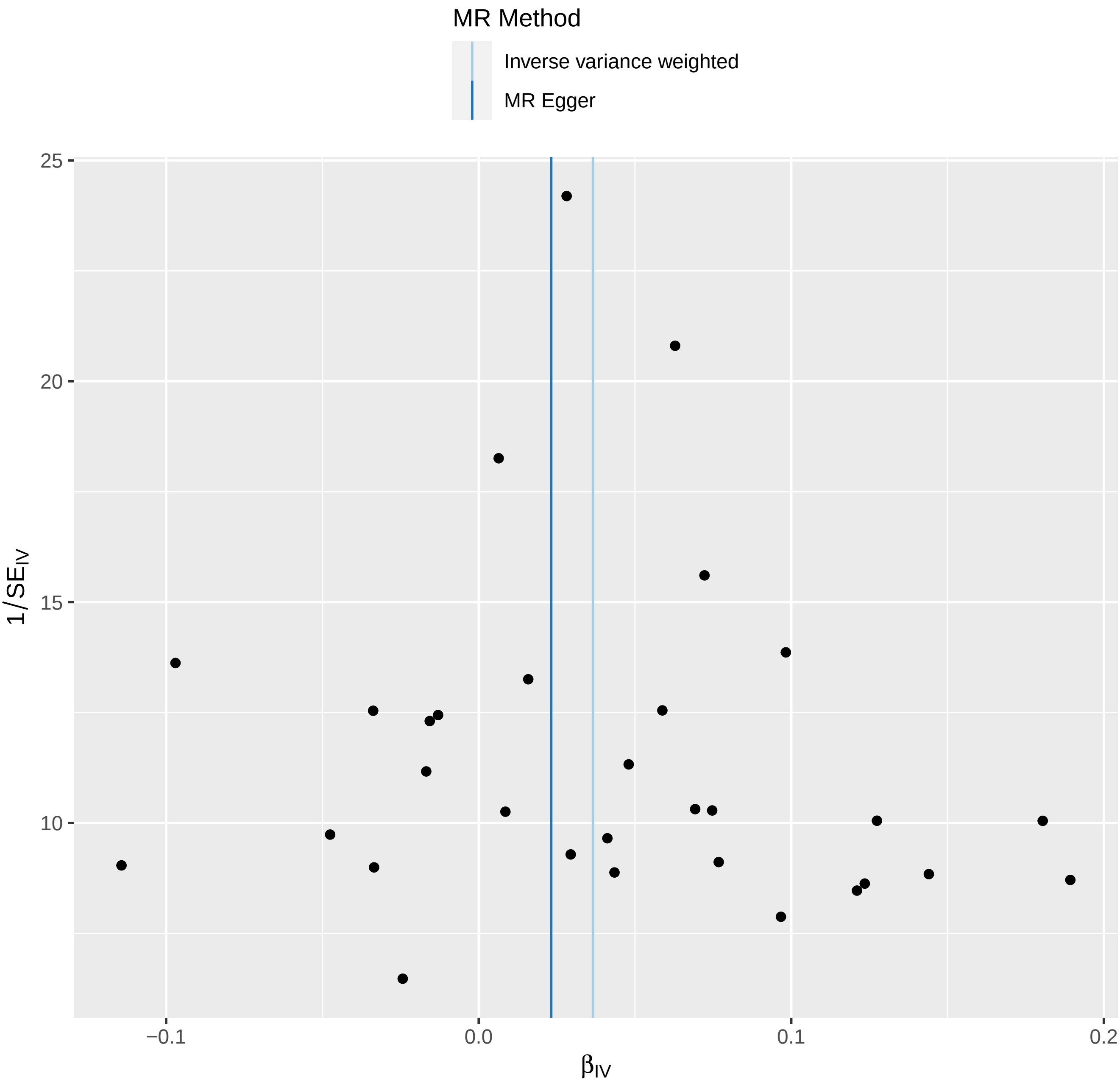

Batch 2723 : Crohn's disease on IDP T1 FAST ROIs R parahipp gyrus post

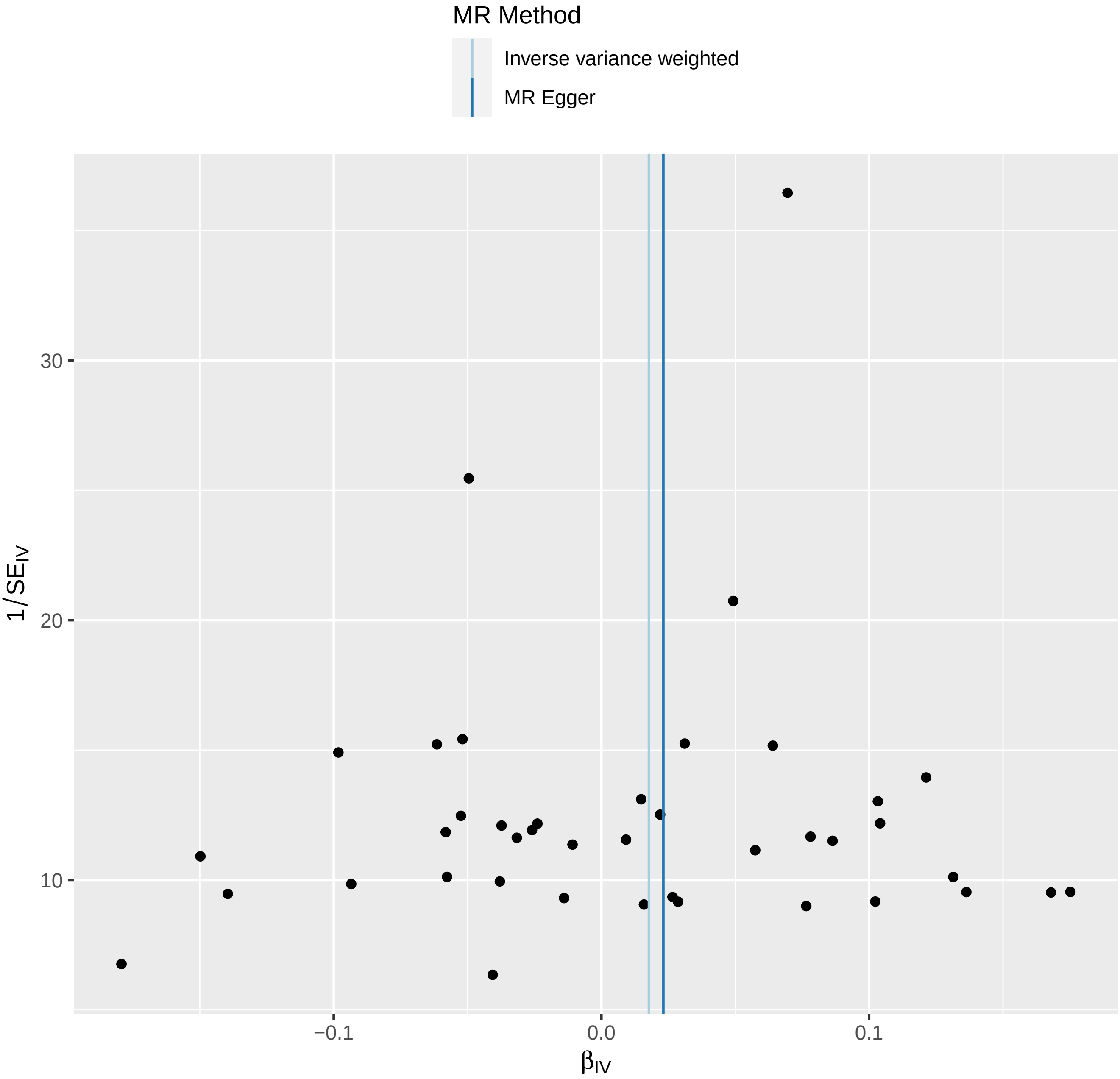

Batch 2724 : Ulcerative colitis on IDP T1 FAST ROIs R parahipp gyrus post

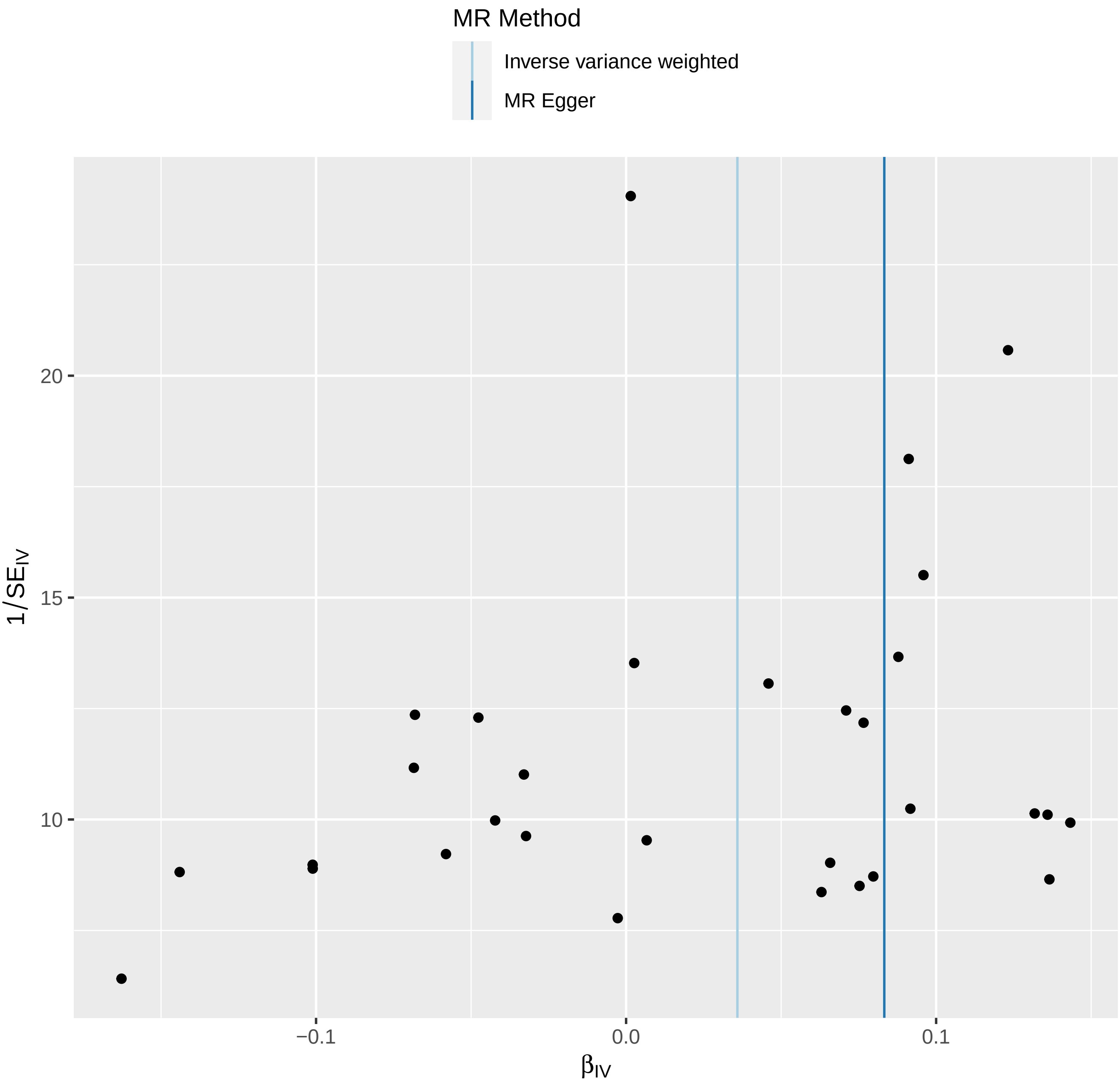

Batch 2725 : Crohn's disease on IDP T1 FAST ROIs L lingual gyrus

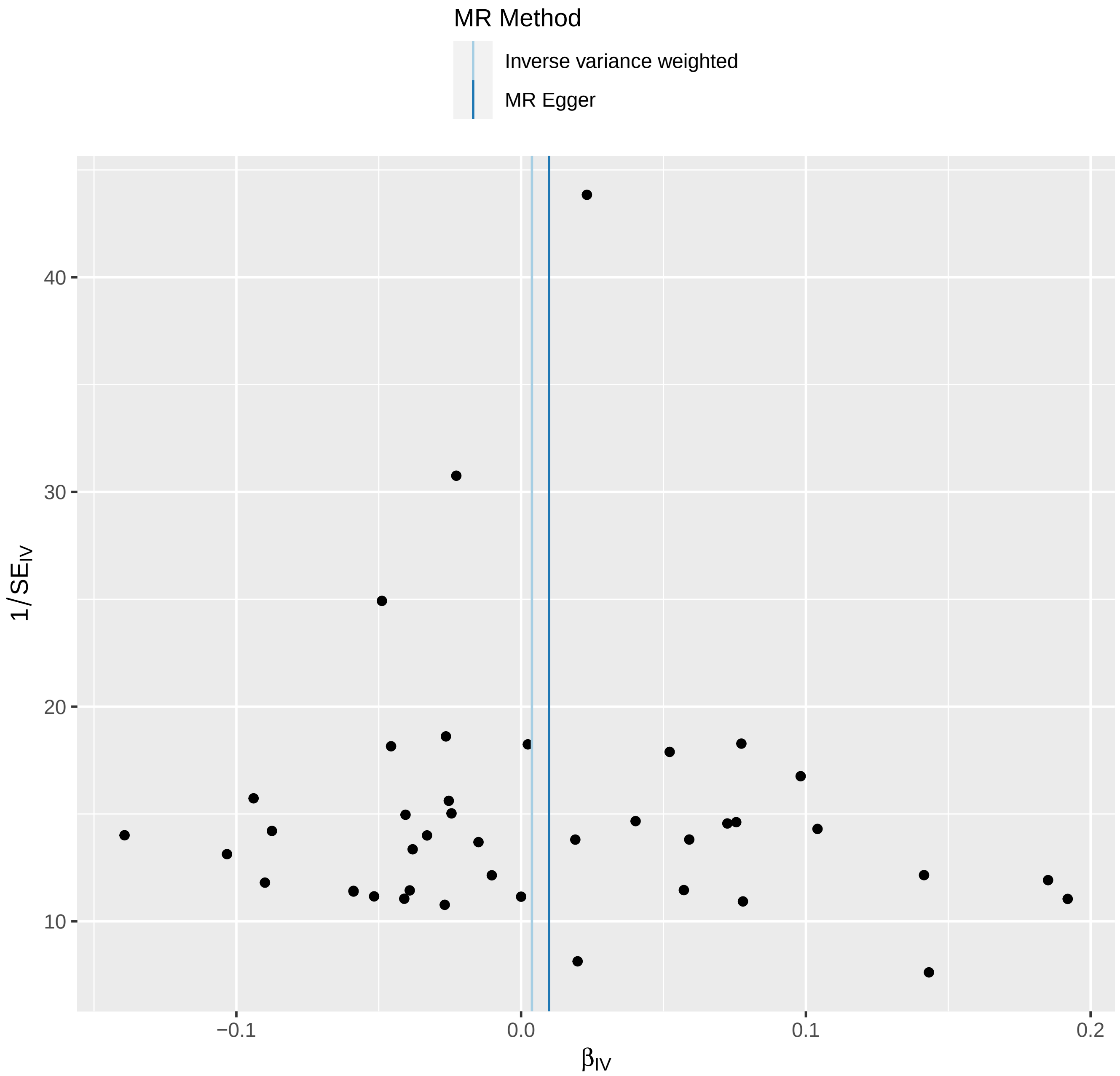

Batch 2726 : Ulcerative colitis on IDP T1 FAST ROIs L lingual gyrus

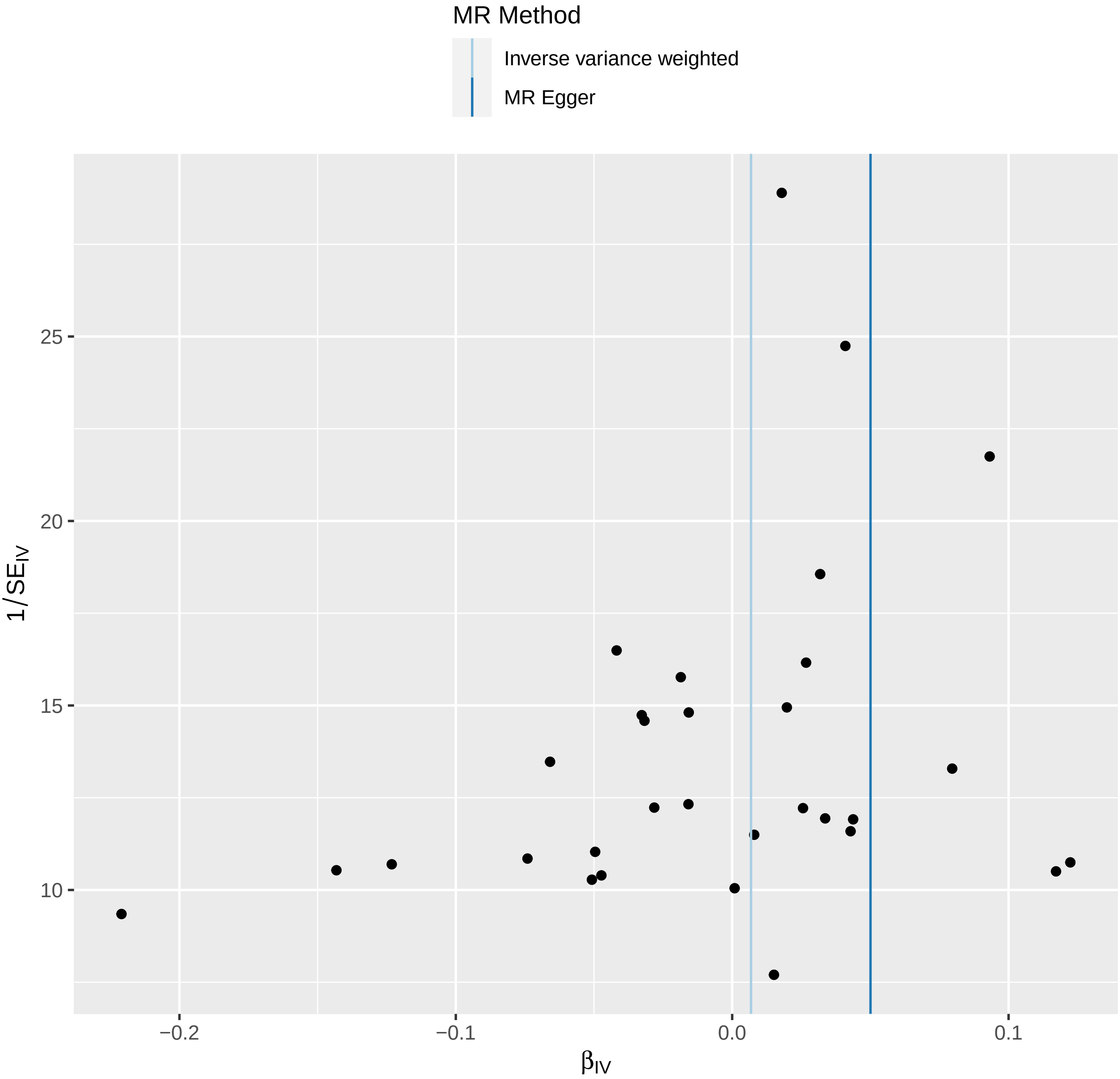

Batch 2727 : Crohn's disease on IDP T1 FAST ROIs R lingual gyrus

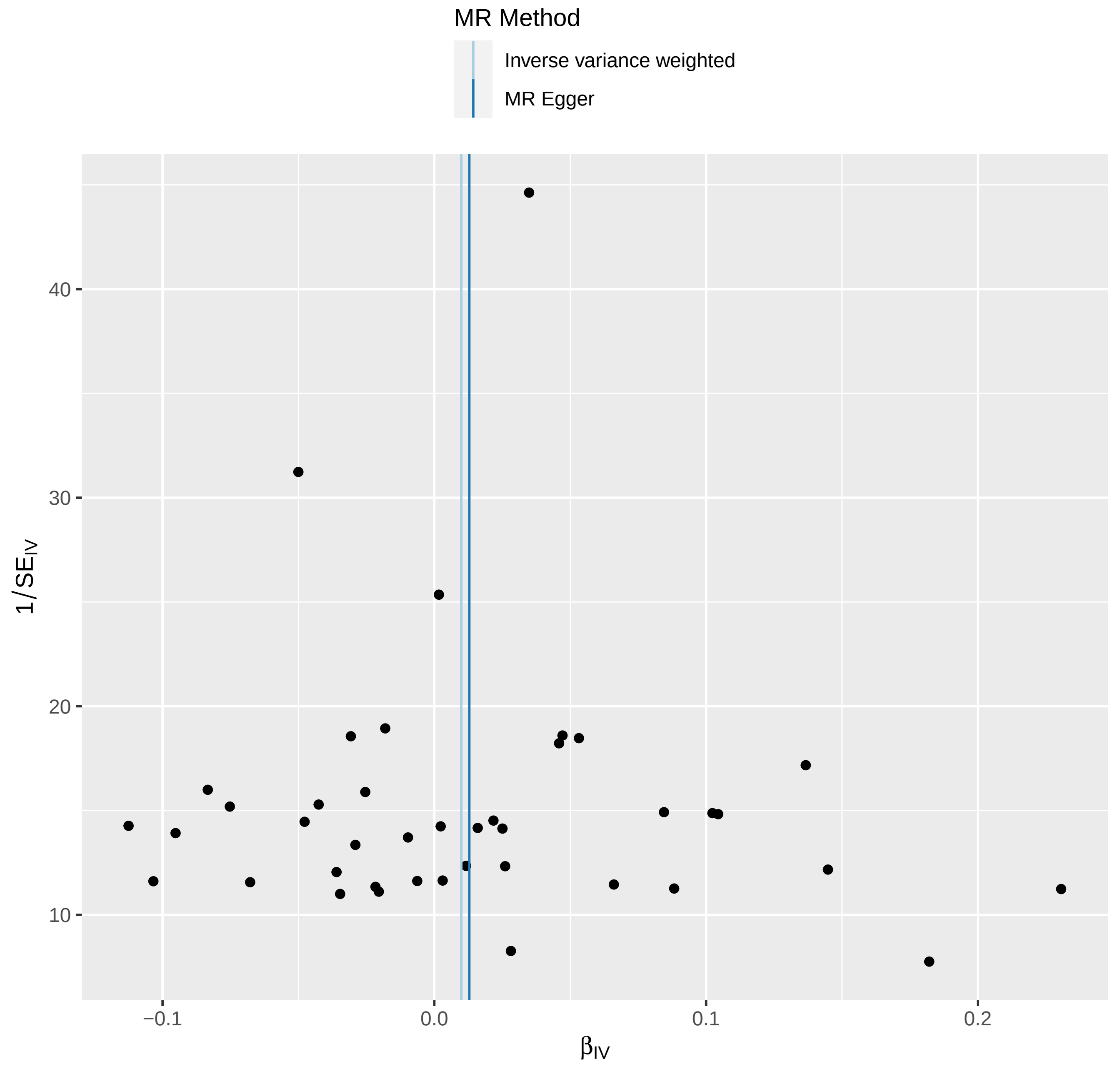

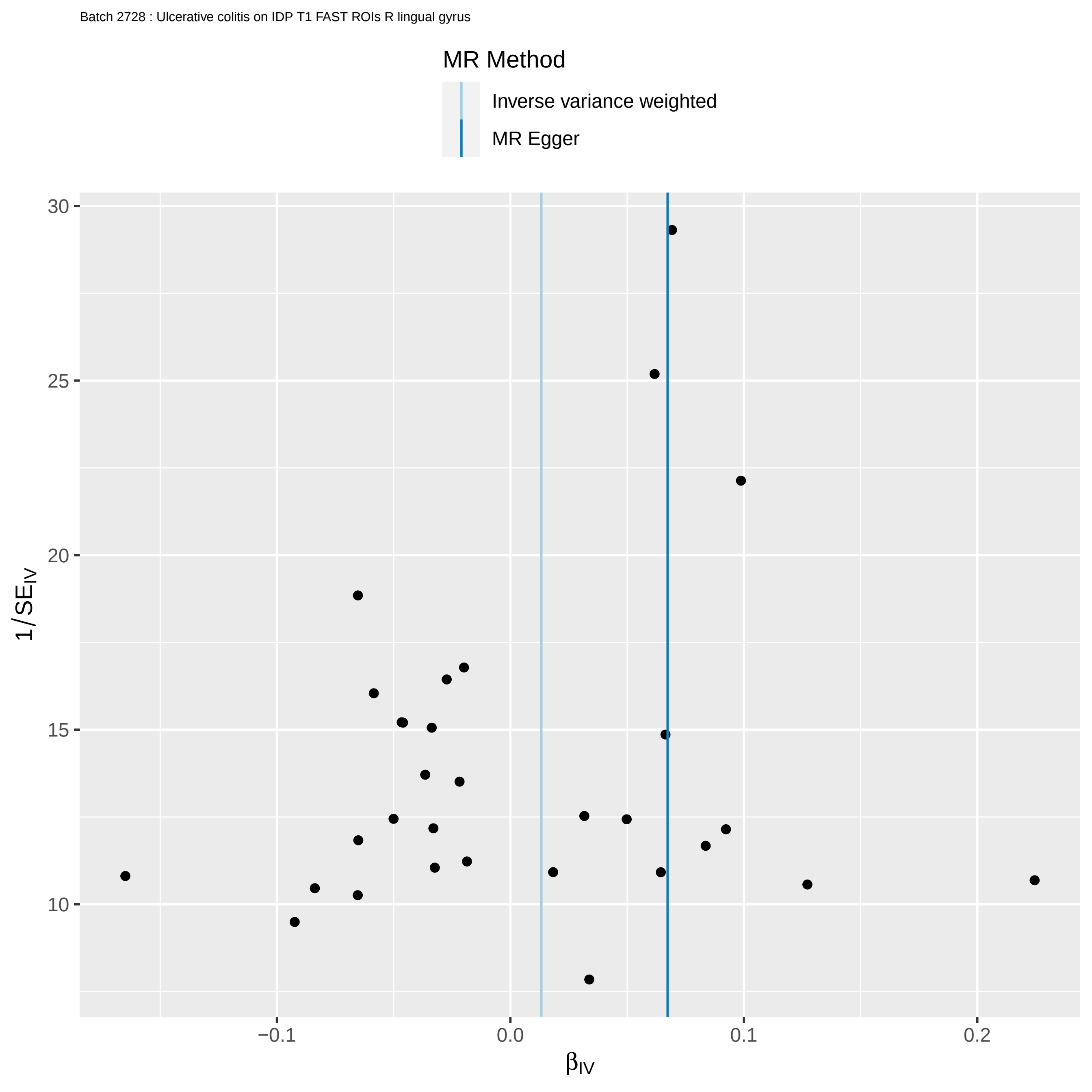

Batch 2728 : Ulcerative colitis on IDP T1 FAST ROIs R lingual gyrus
MR Method
Inverse variance weighted
MR Egger
30
25
20
15
10
1/SE$_{IV}$
−0.1
0.0
0.1
0.2
β$_{IV}$

Batch 2729 : Crohn's disease on IDP T1 FAST ROIs L temp fusif cortex ant

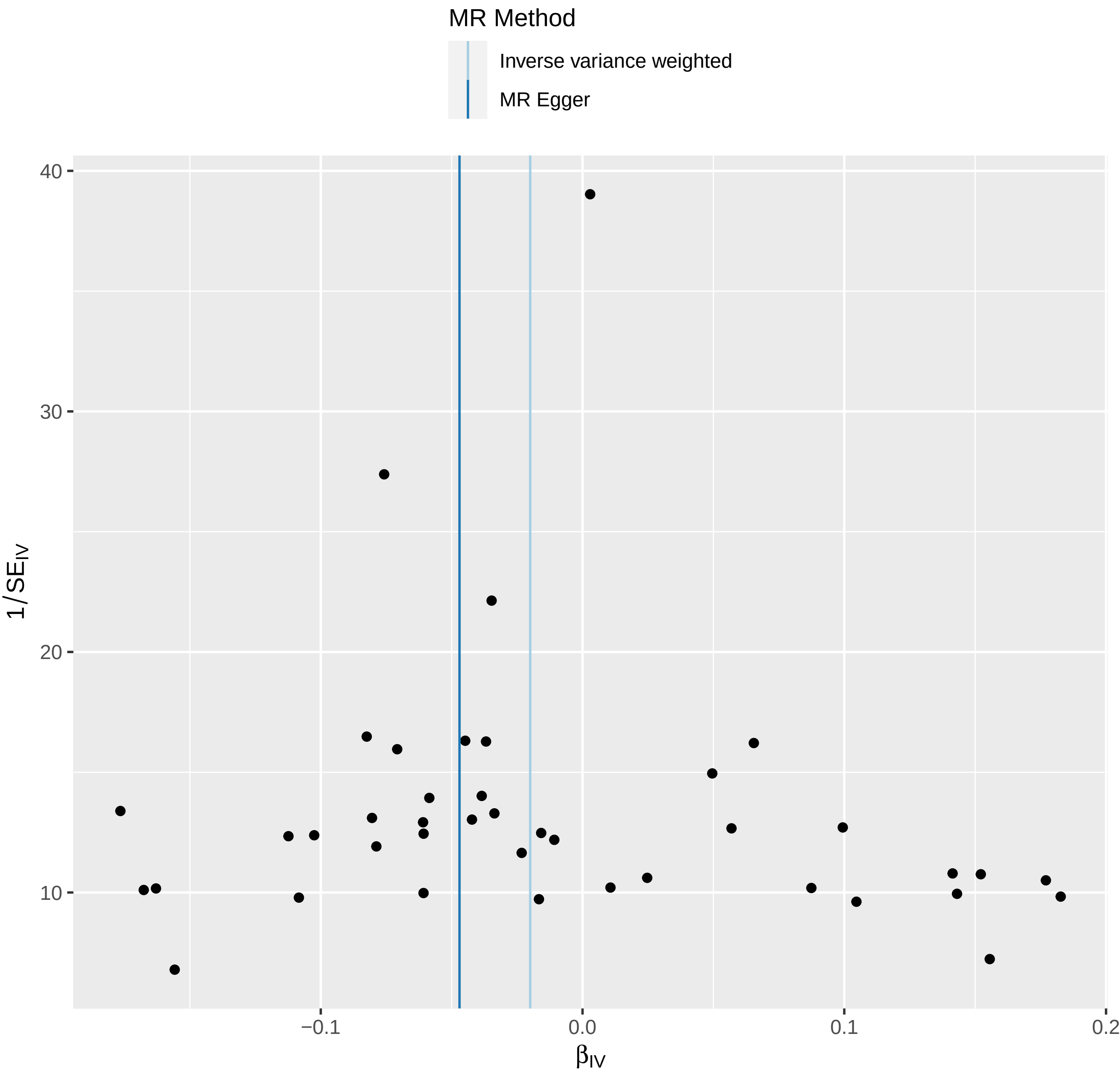

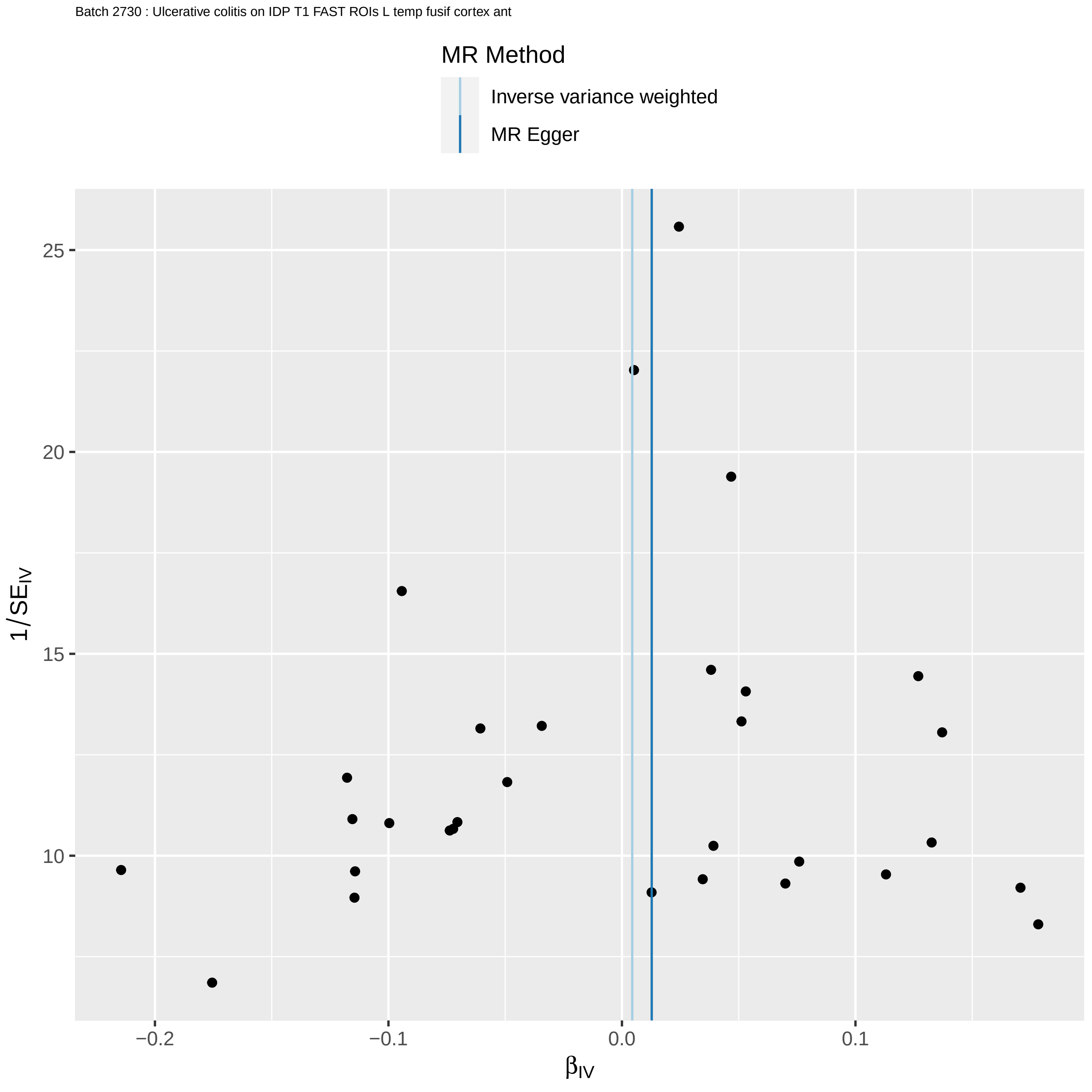

Batch 2730 : Ulcerative colitis on IDP T1 FAST ROIs L temp fusif cortex ant
MR Method
Inverse variance weighted
MR Egger
25
20
15
10
1/SE_IV
−0.2
−0.1
0.0
0.1
β_IV

Batch 2731 : Crohn's disease on IDP T1 FAST ROIs R temp fusif cortex ant

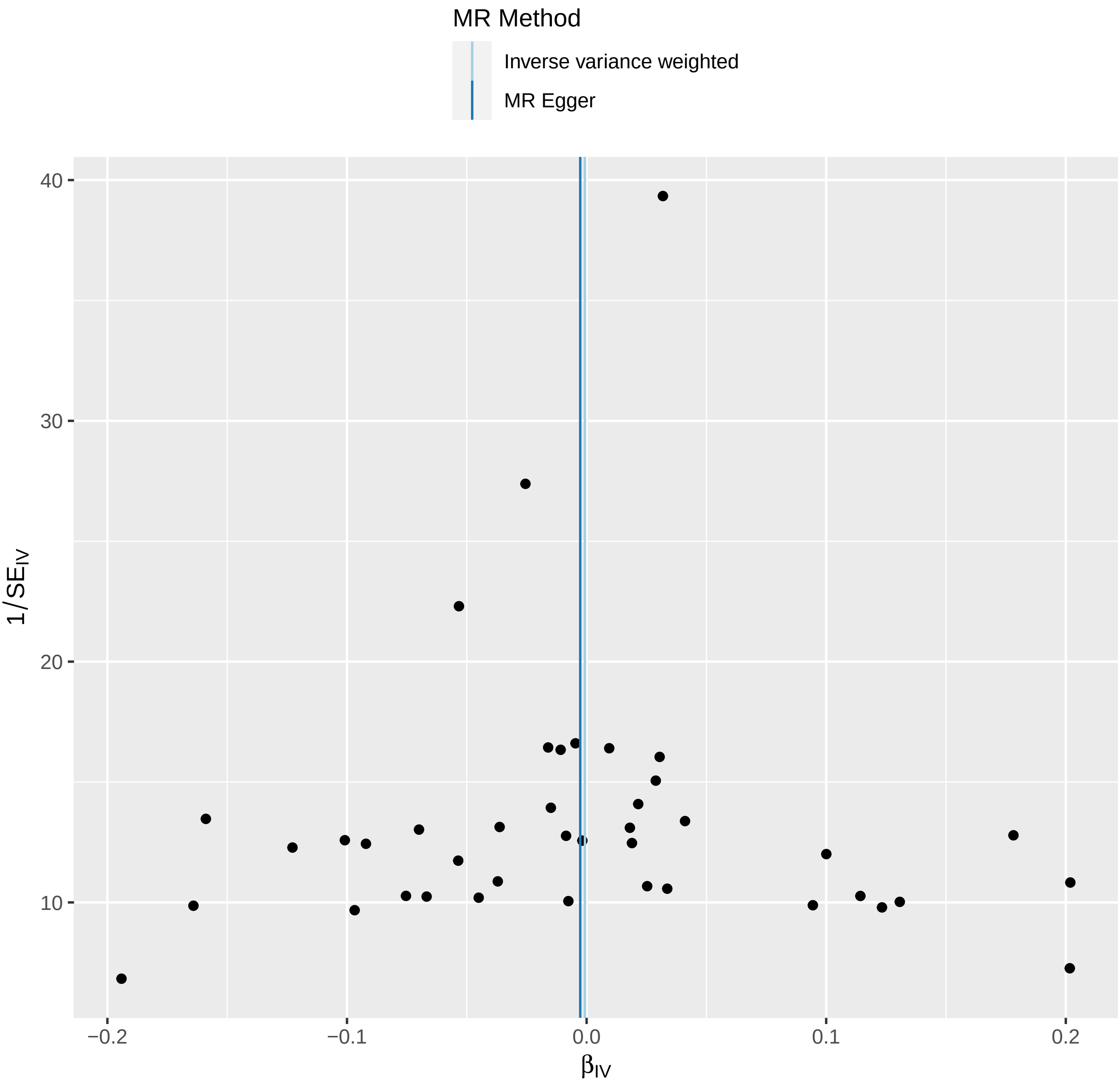

Batch 2732 : Ulcerative colitis on IDP T1 FAST ROIs R temp fusif cortex ant

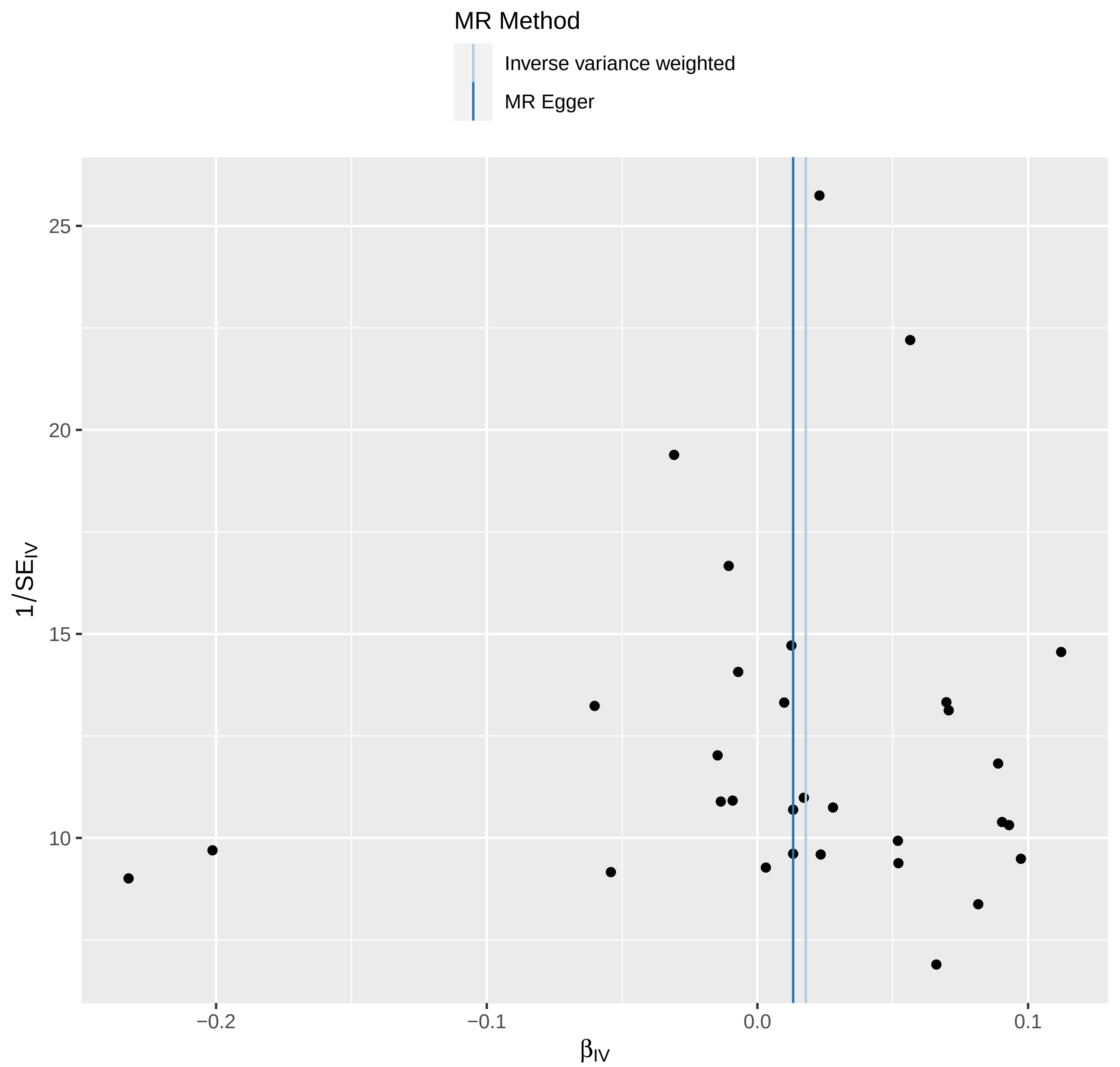

Batch 2734 : IDP T1 SIENAX brain−unnormalised volume on Crohn's disease

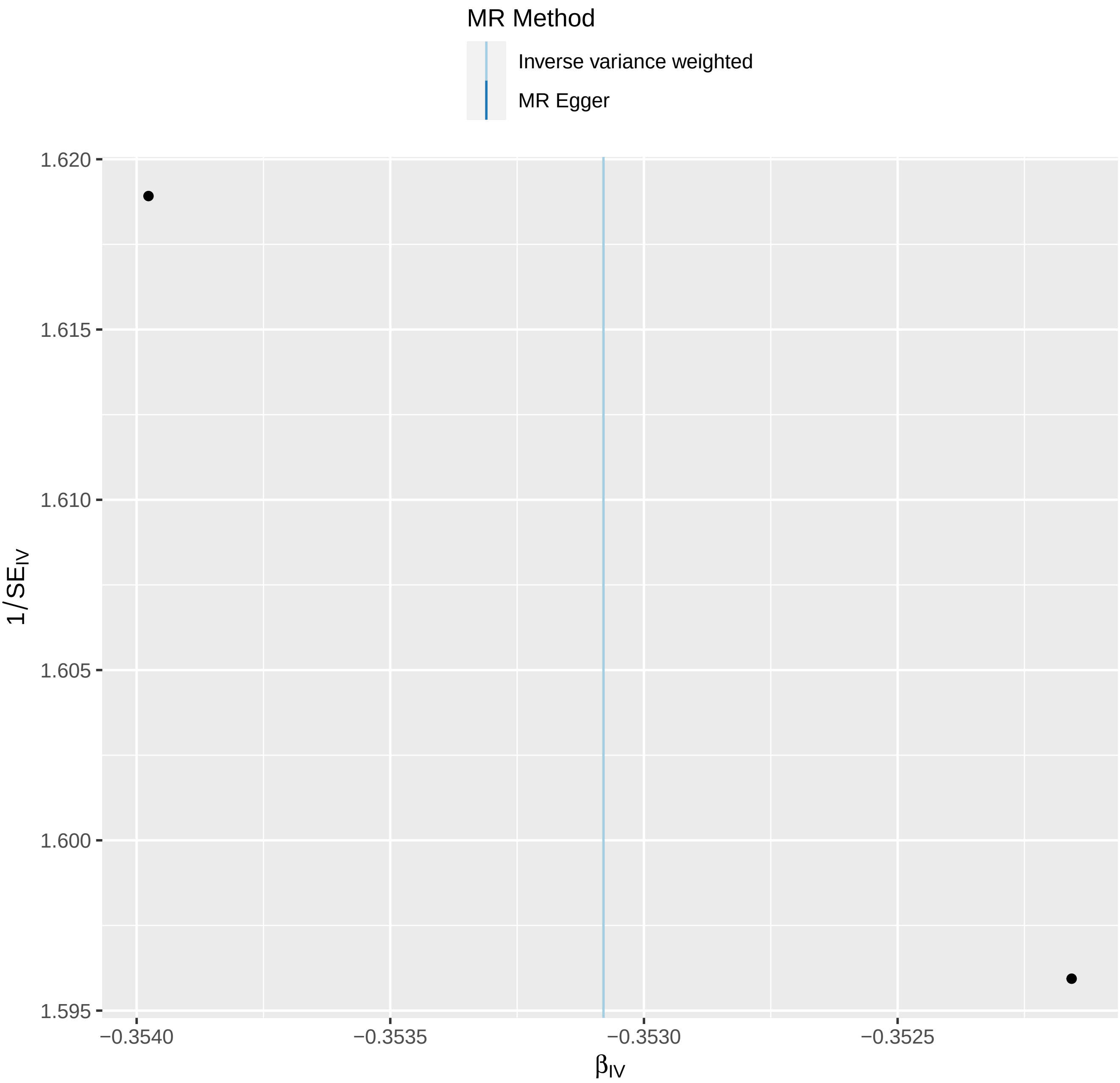

Batch 2736 : IDP T1 FAST ROIs R temp fusif cortex post on Crohn's disease

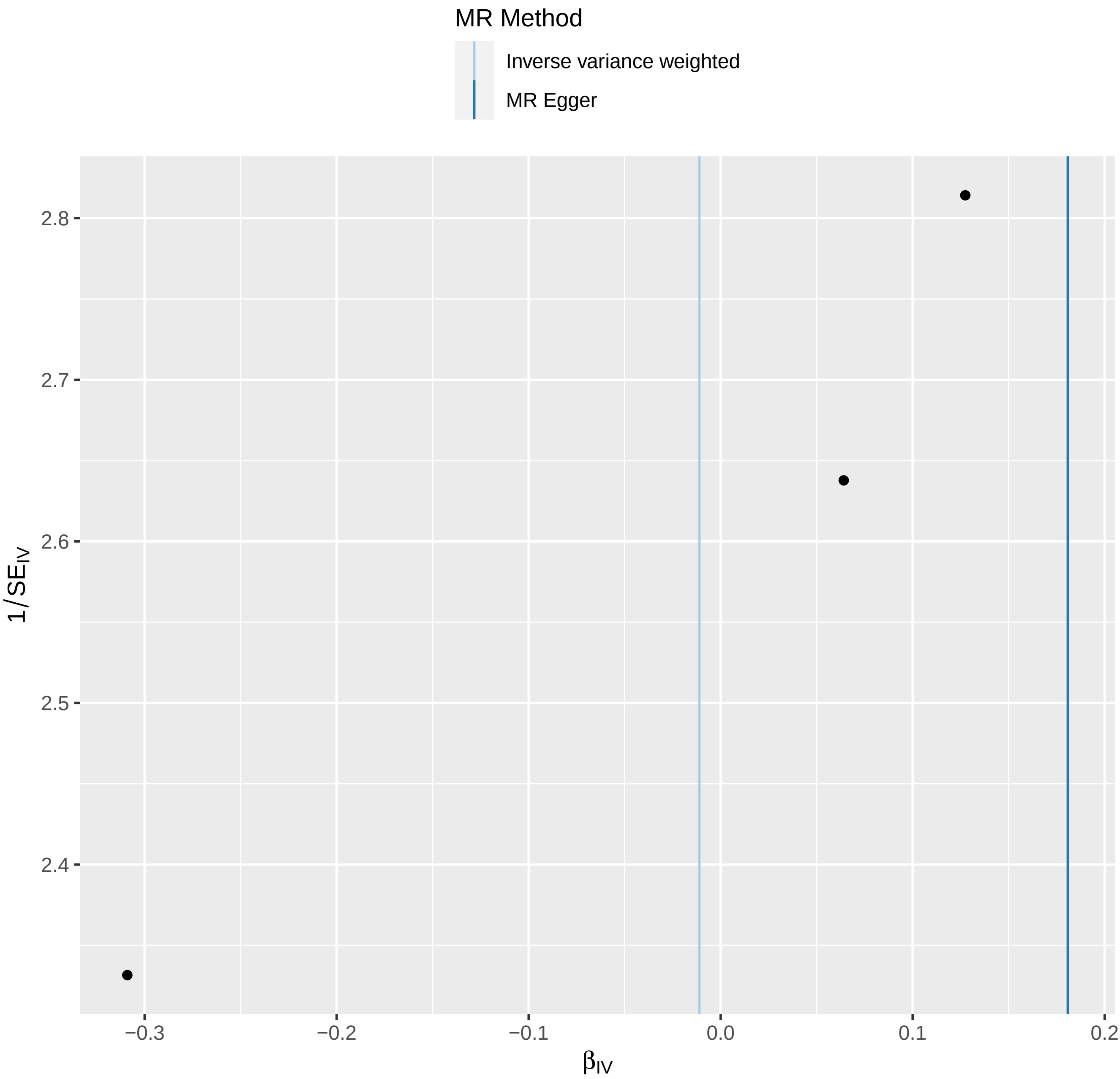

Batch 2742 : IDP T1 FAST ROIs R front operc cortex on Crohn's disease

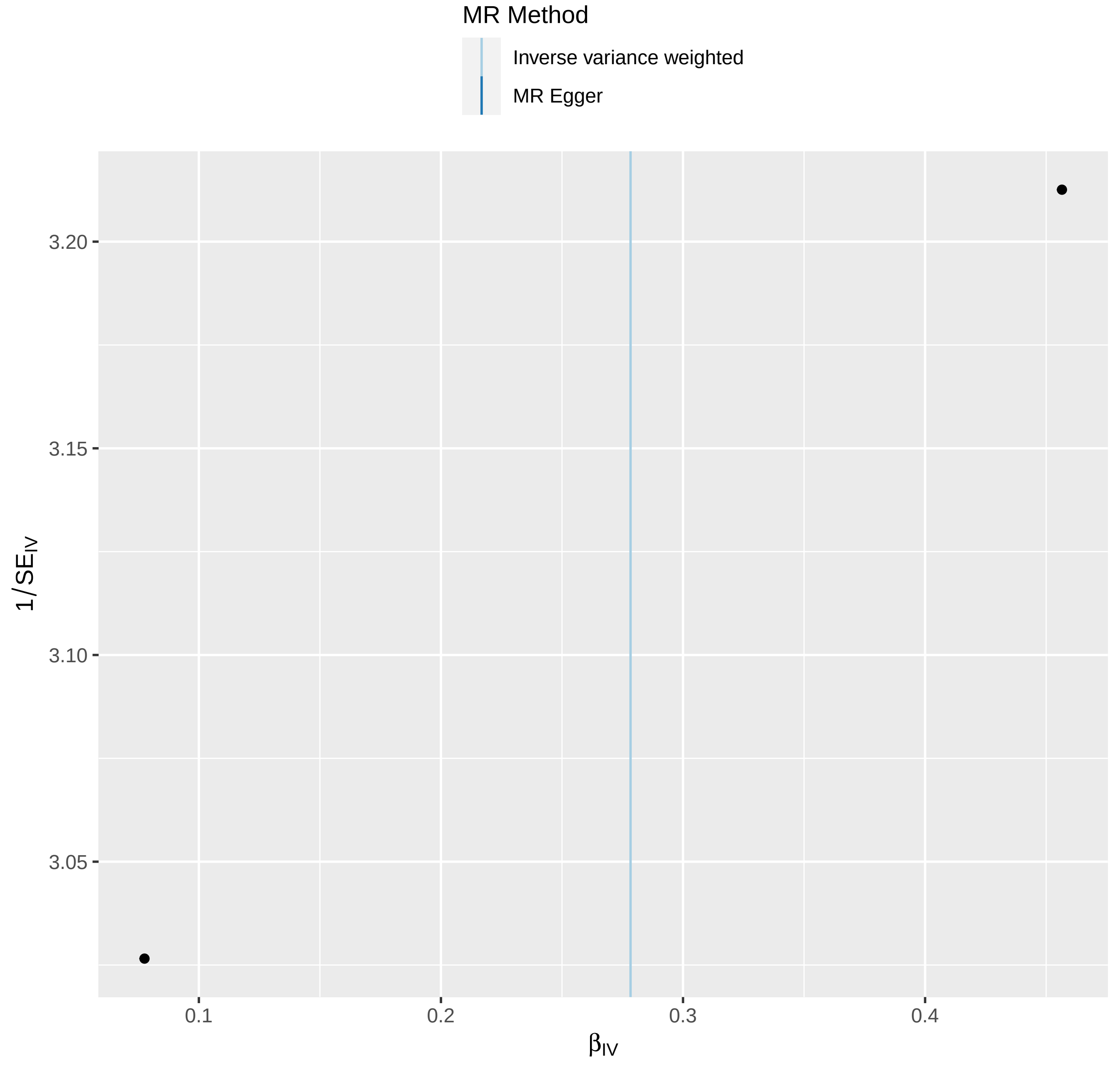

Batch 2745 : IDP T1 FIRST left thalamus volume on Crohn's disease

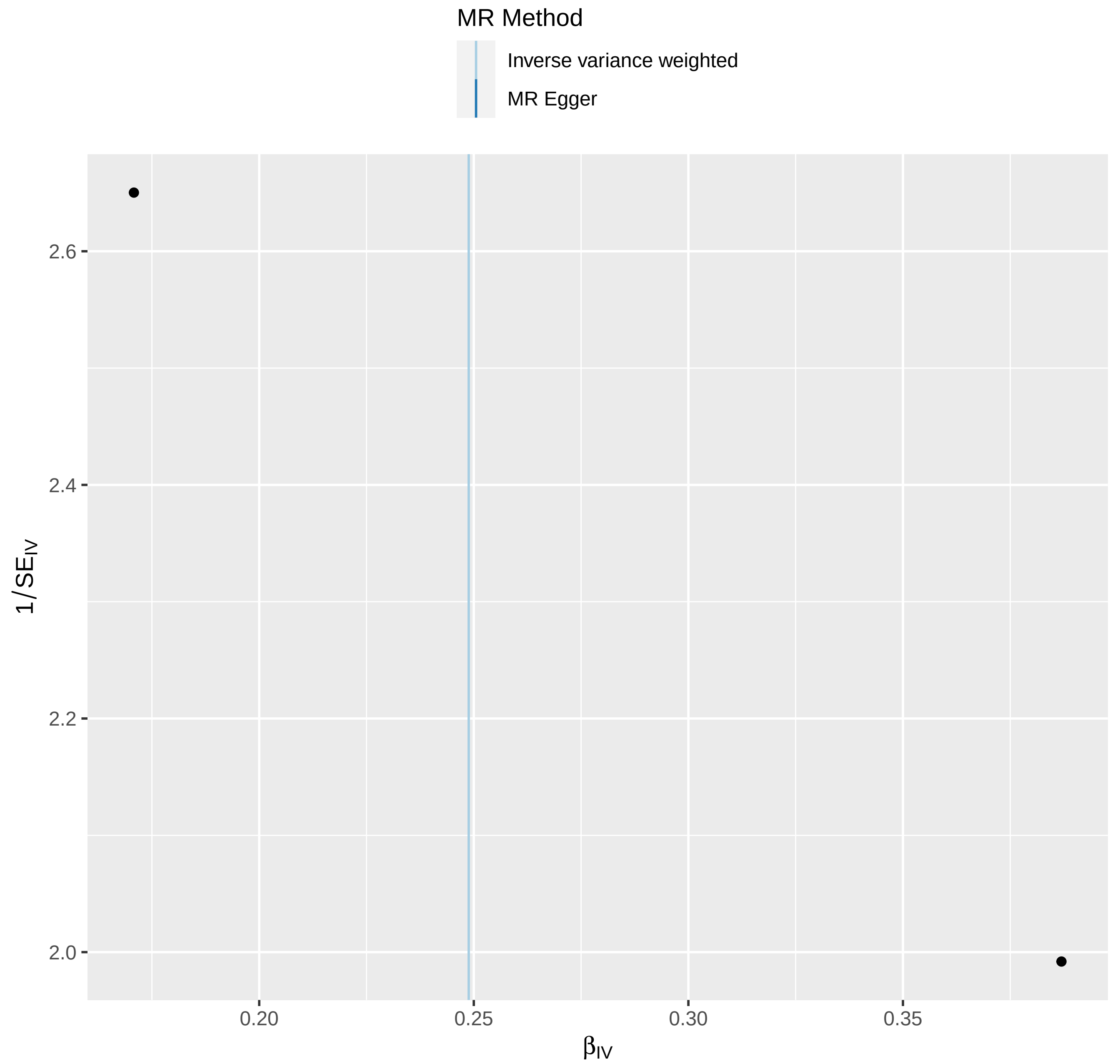

Batch 2746 : IDP T1 FAST ROIs L parietal operc cortex on Crohn's disease

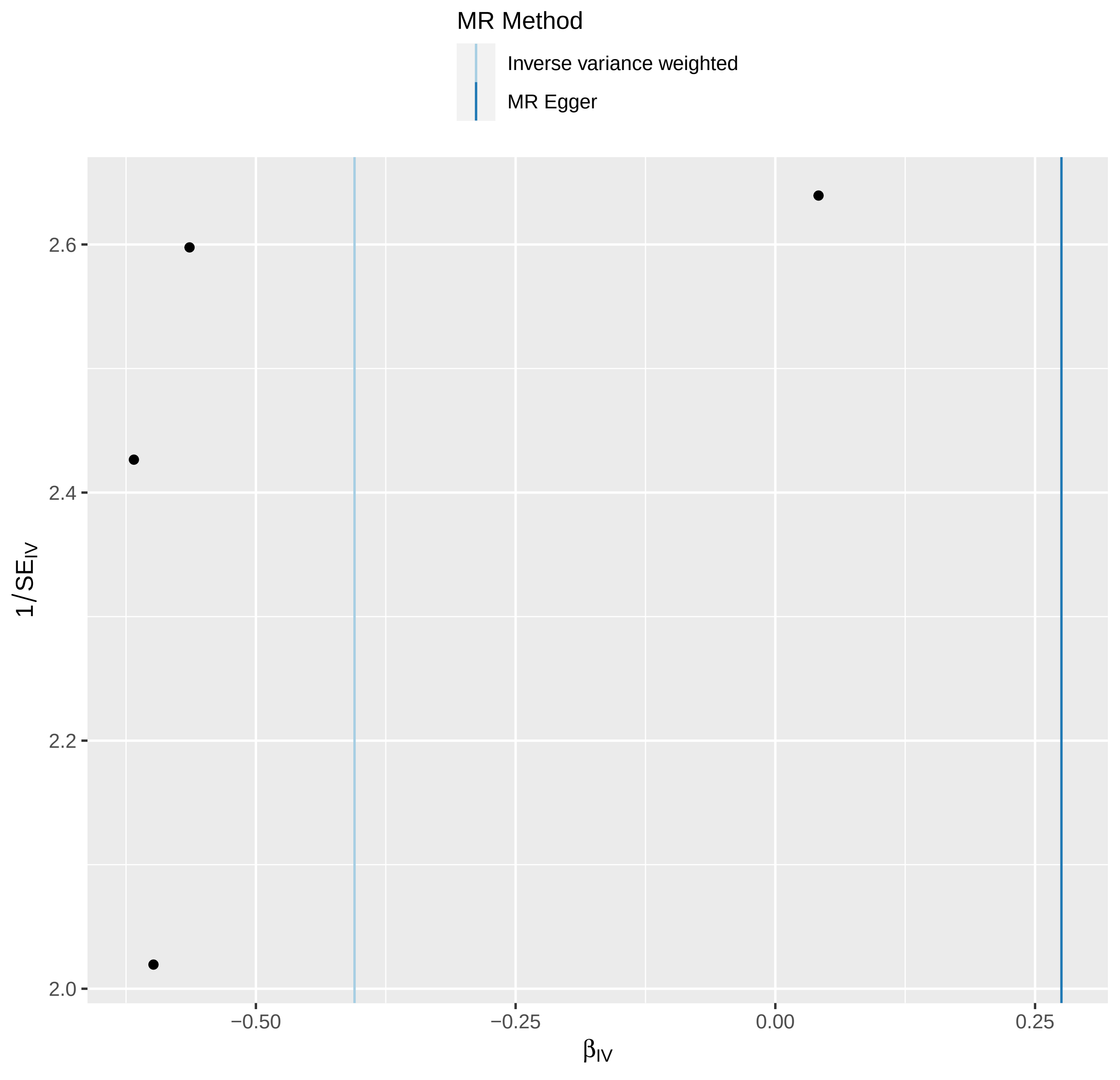

Batch 2747 : IDP T1 FAST ROIs R parietal operc cortex on Crohn's disease

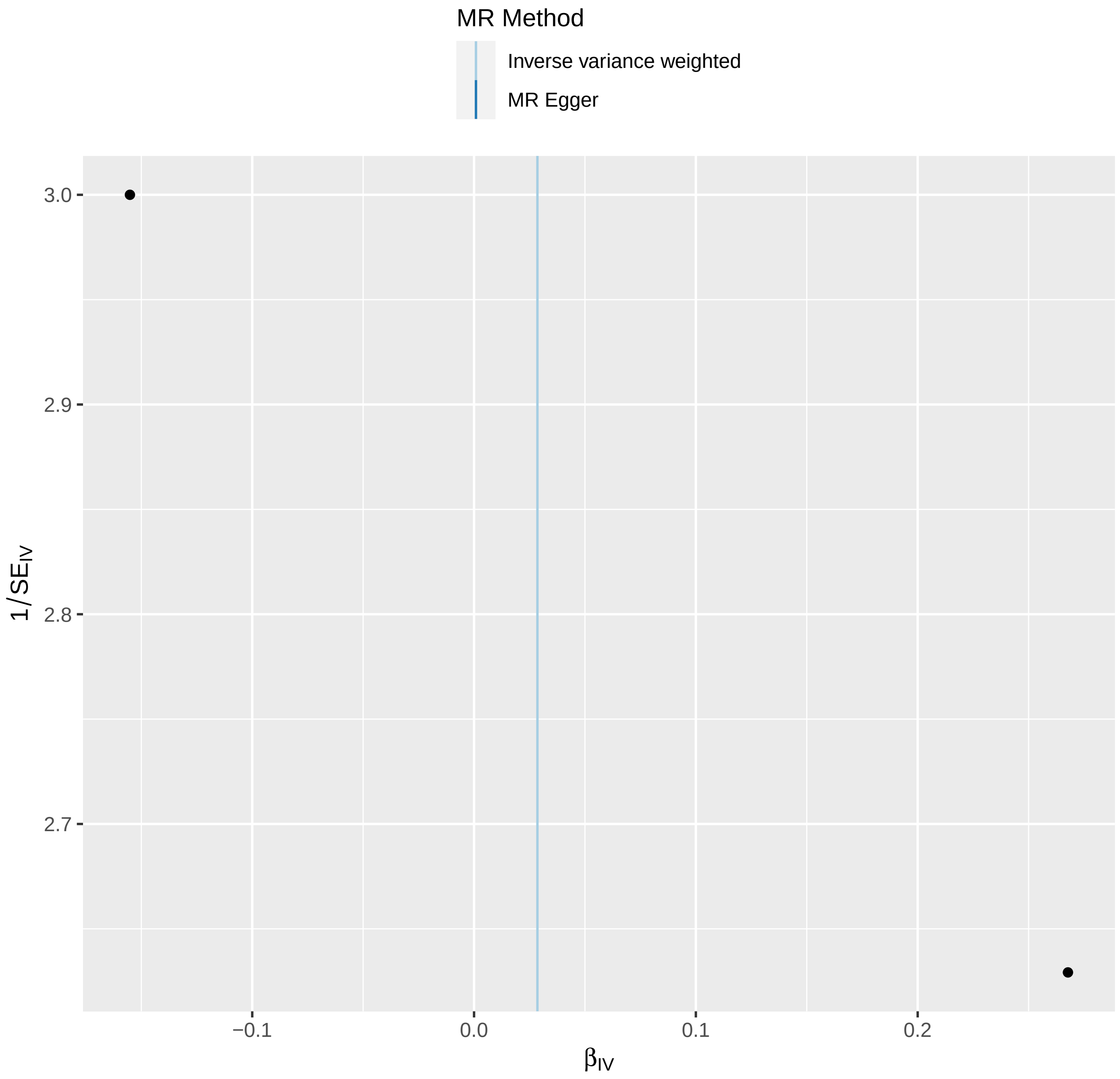

Batch 2748 : IDP T1 FAST ROIs L planum polare on Crohn's disease

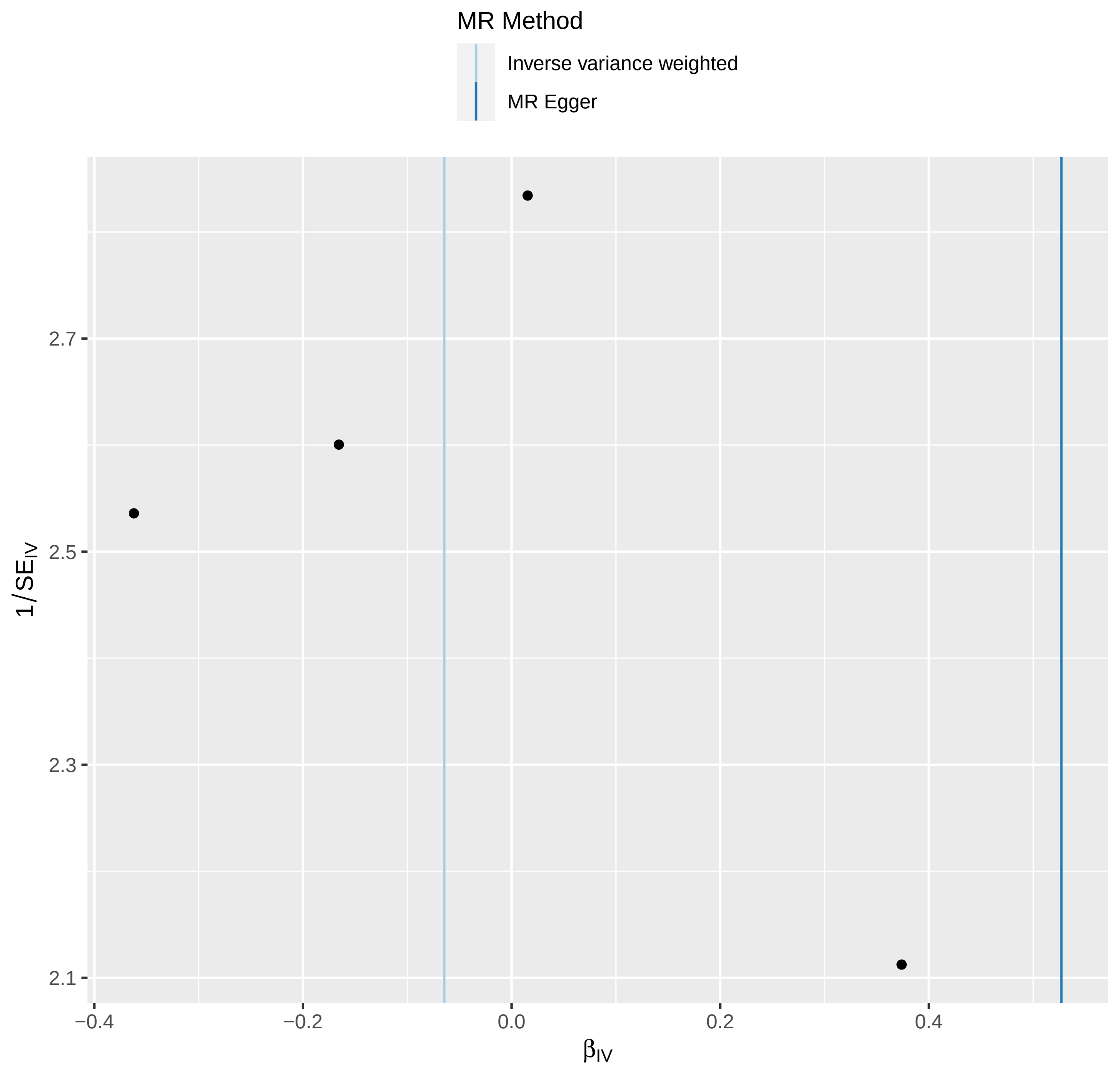

Batch 2750 : IDP T1 FAST ROIs L heschl gyrus on Crohn's disease

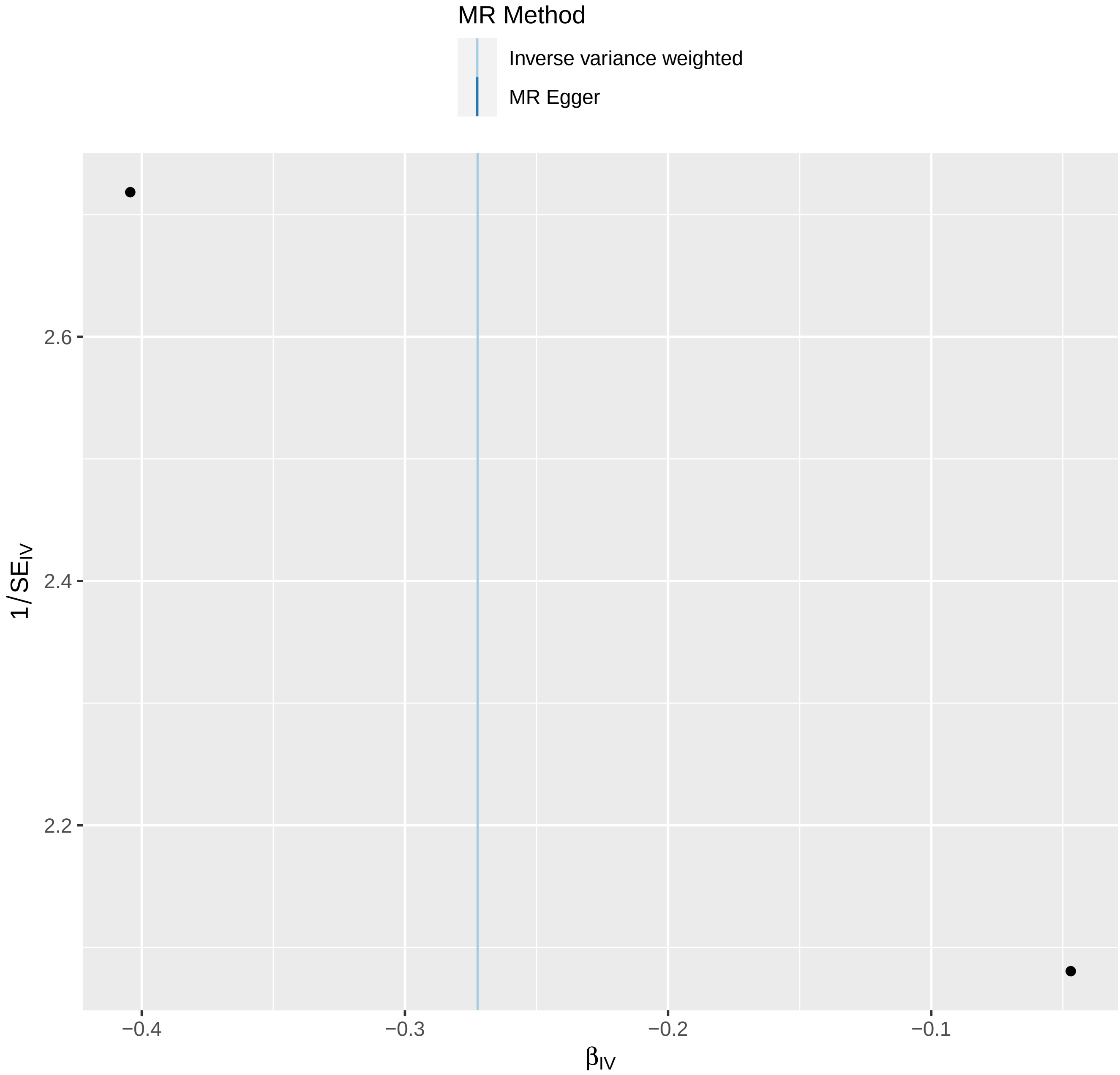

Batch 2751 : IDP T1 FAST ROIs R heschl gyrus on Crohn's disease

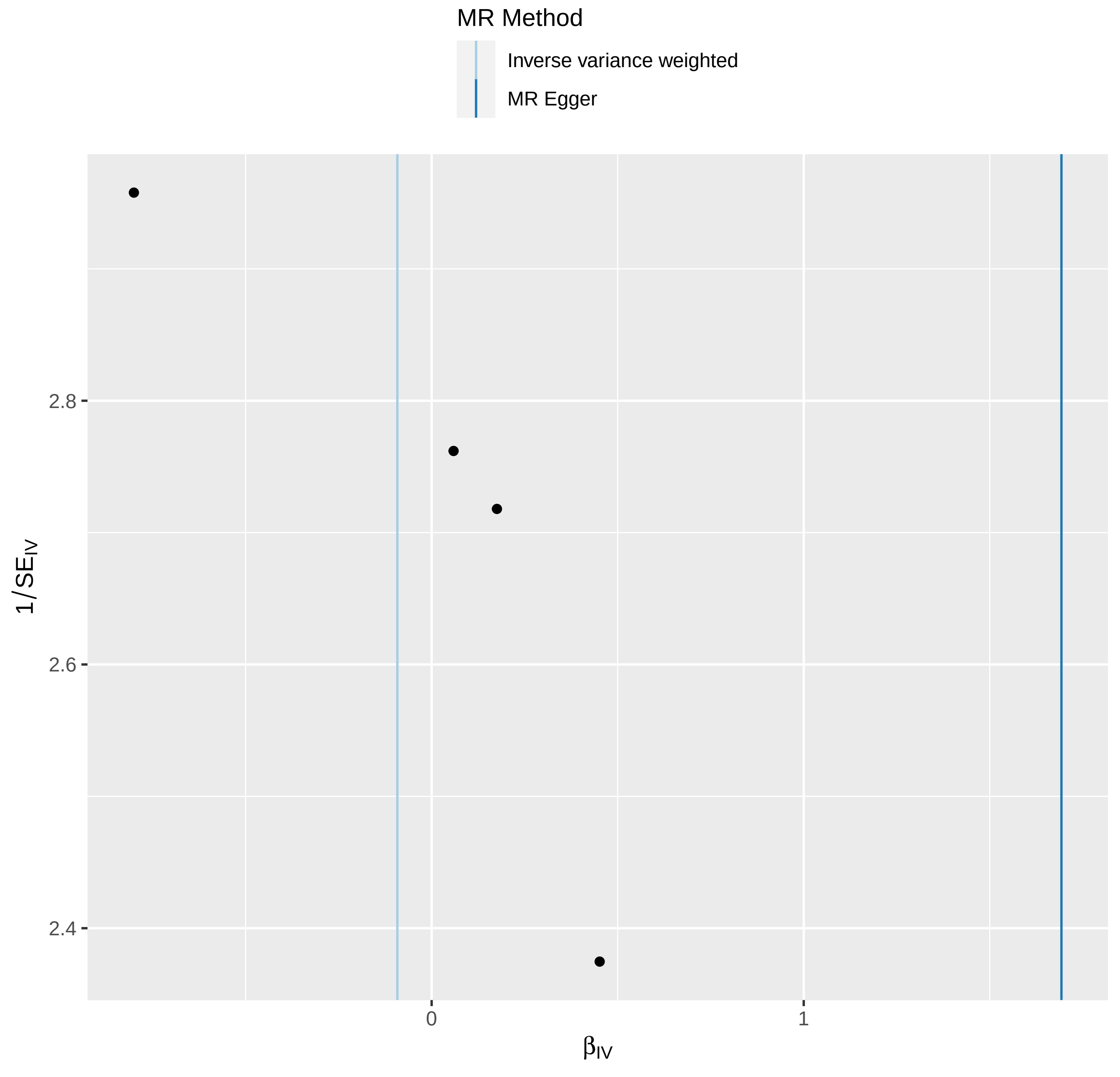

Batch 2753 : IDP T1 FAST ROIs R planum temporale on Crohn's disease

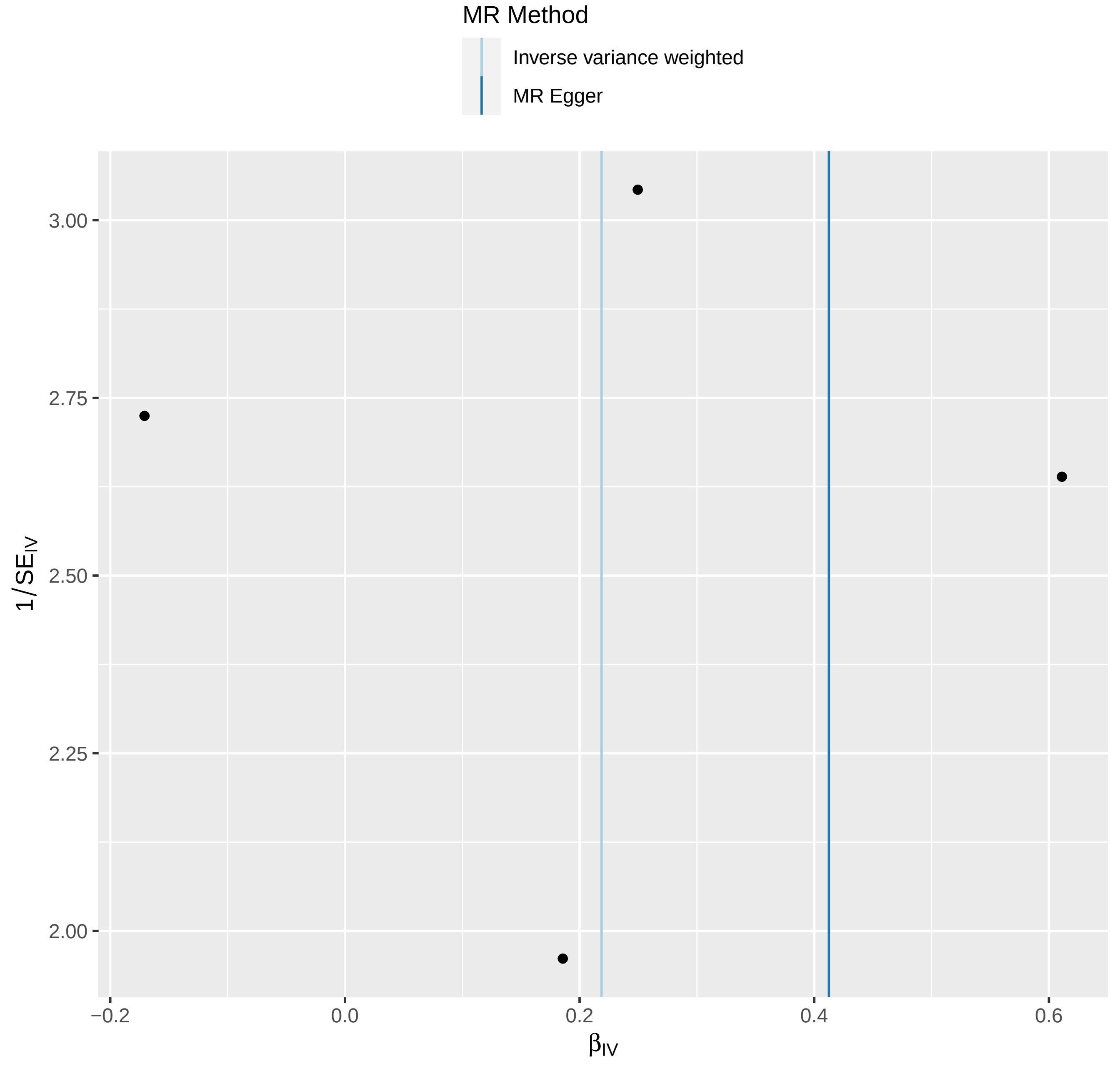

Batch 2754 : IDP T1 FAST ROIs L supracalc cortex on Crohn's disease

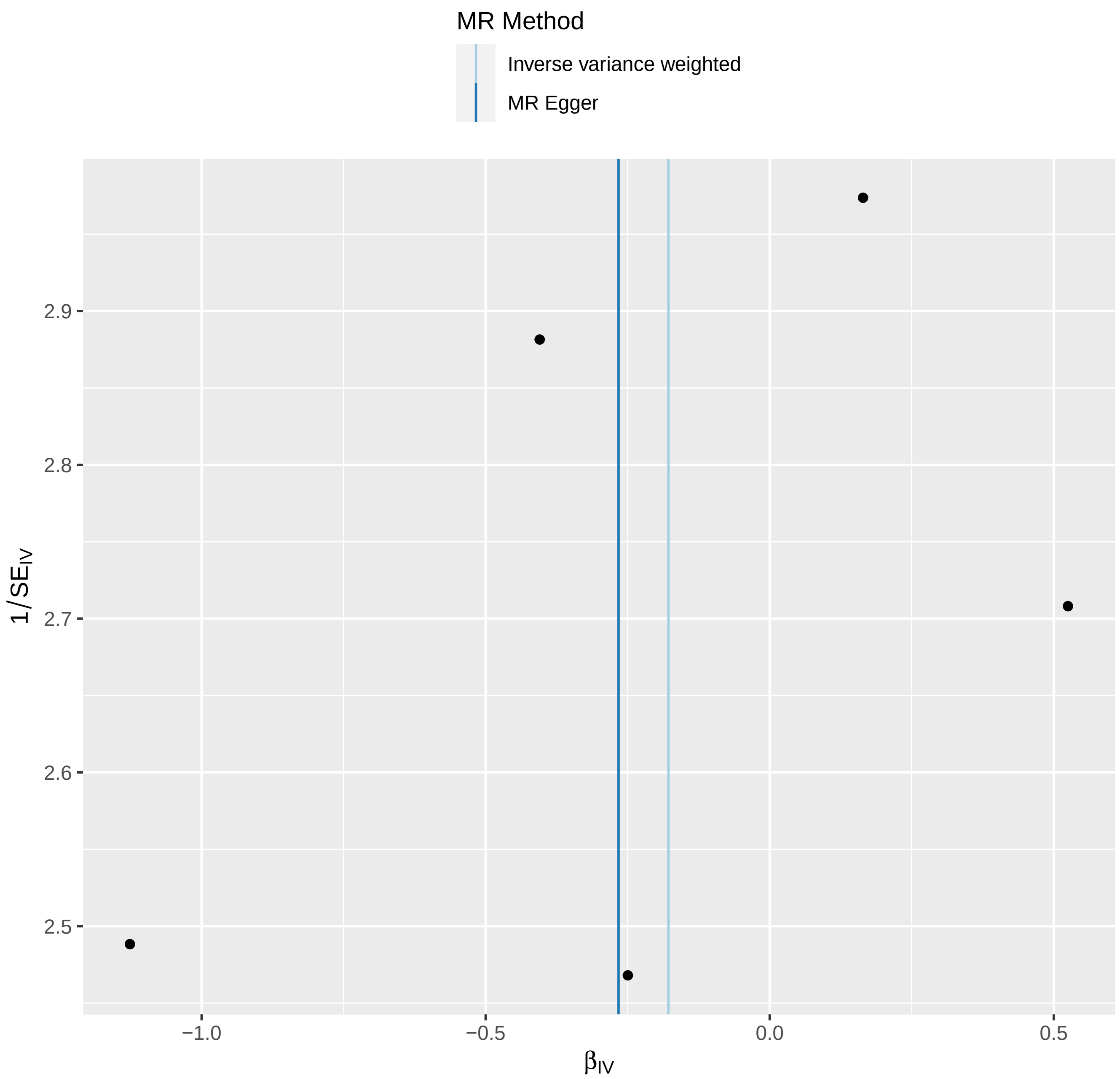

Batch 2755 : IDP T1 FAST ROIs R supracalc cortex on Crohn's disease

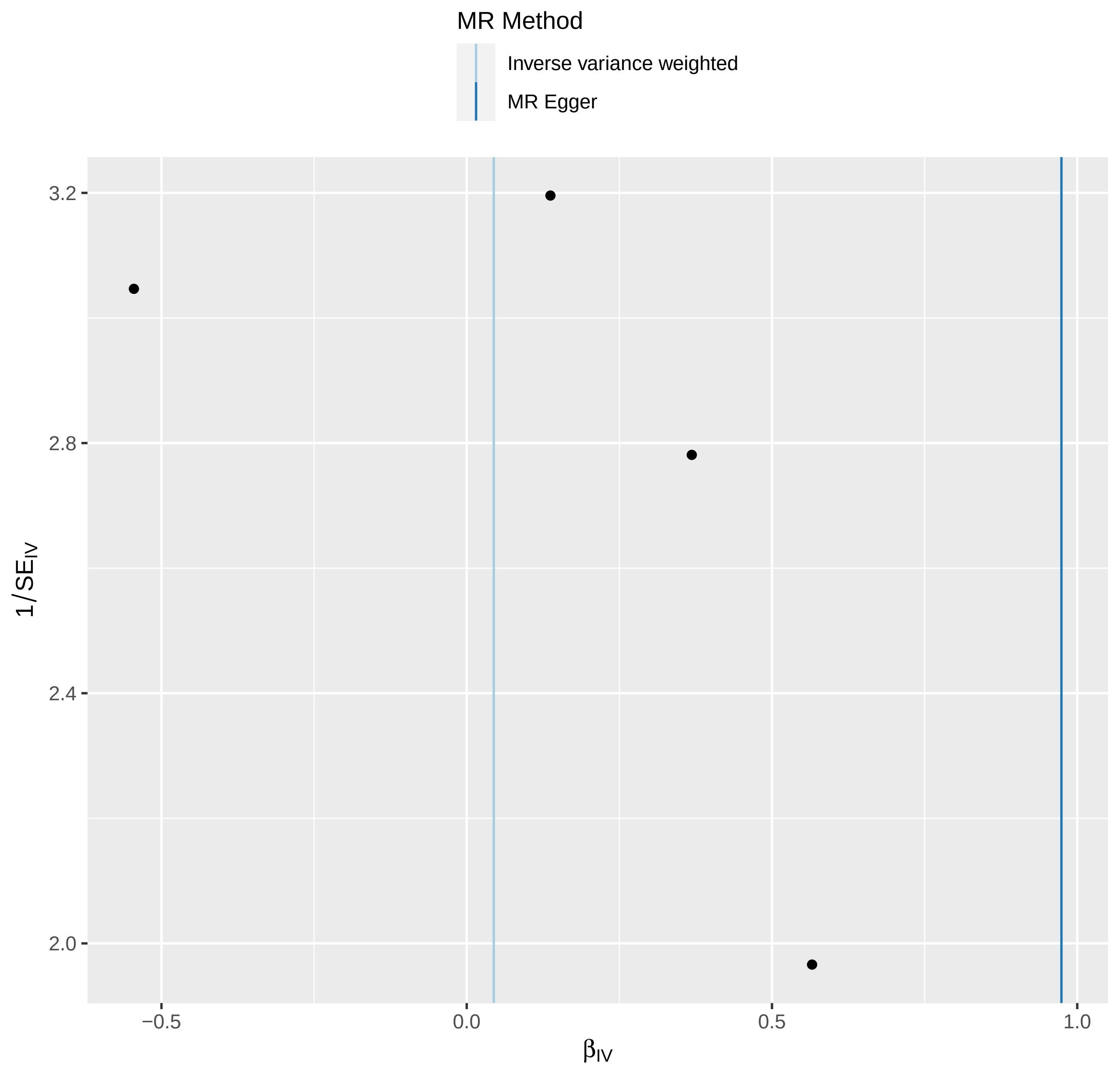

Batch 2757 : IDP T1 FAST ROIs L occ pole on Crohn's disease

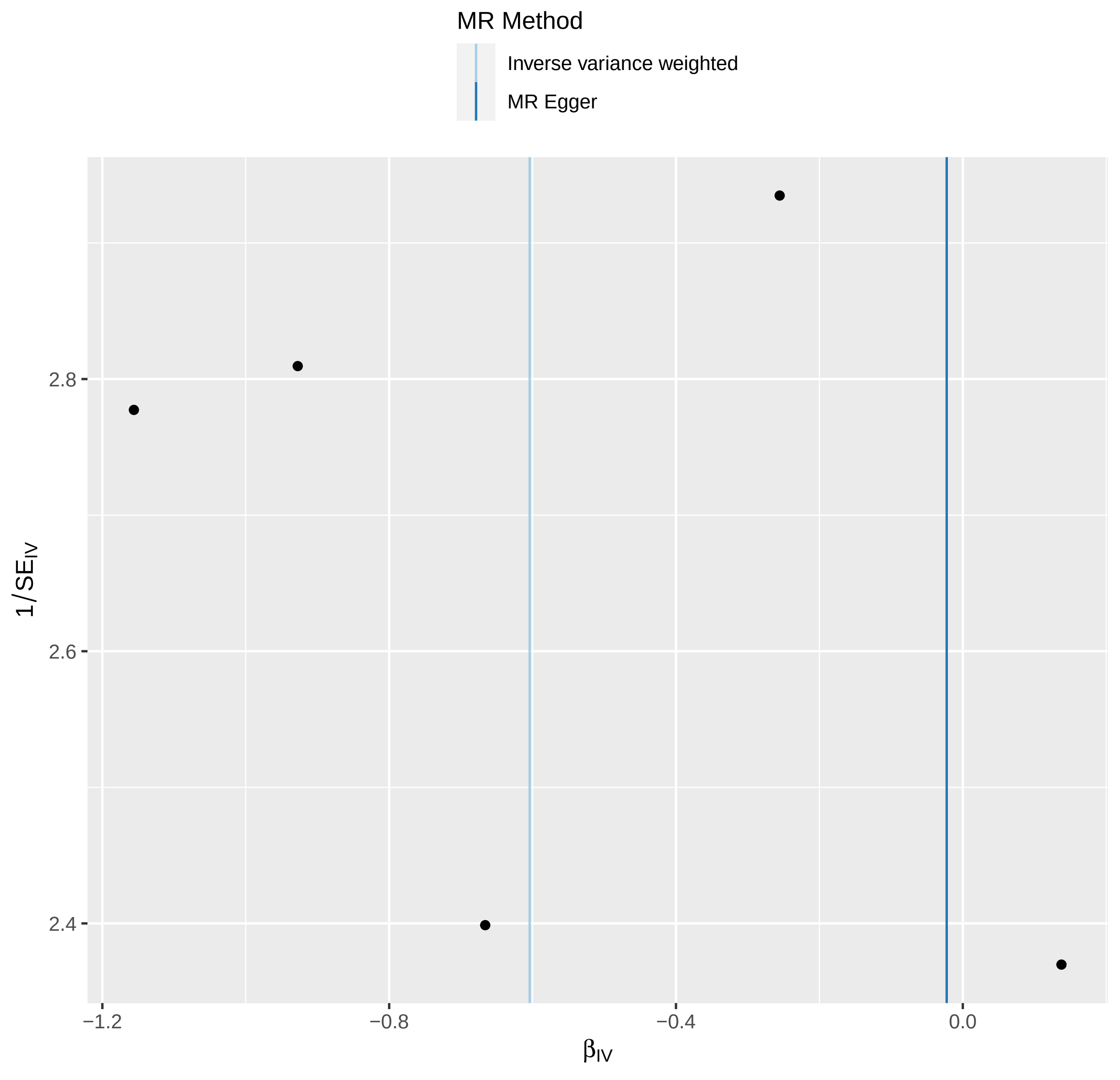

Batch 2759 : IDP T1 FAST ROIs L thalamus on Crohn's disease

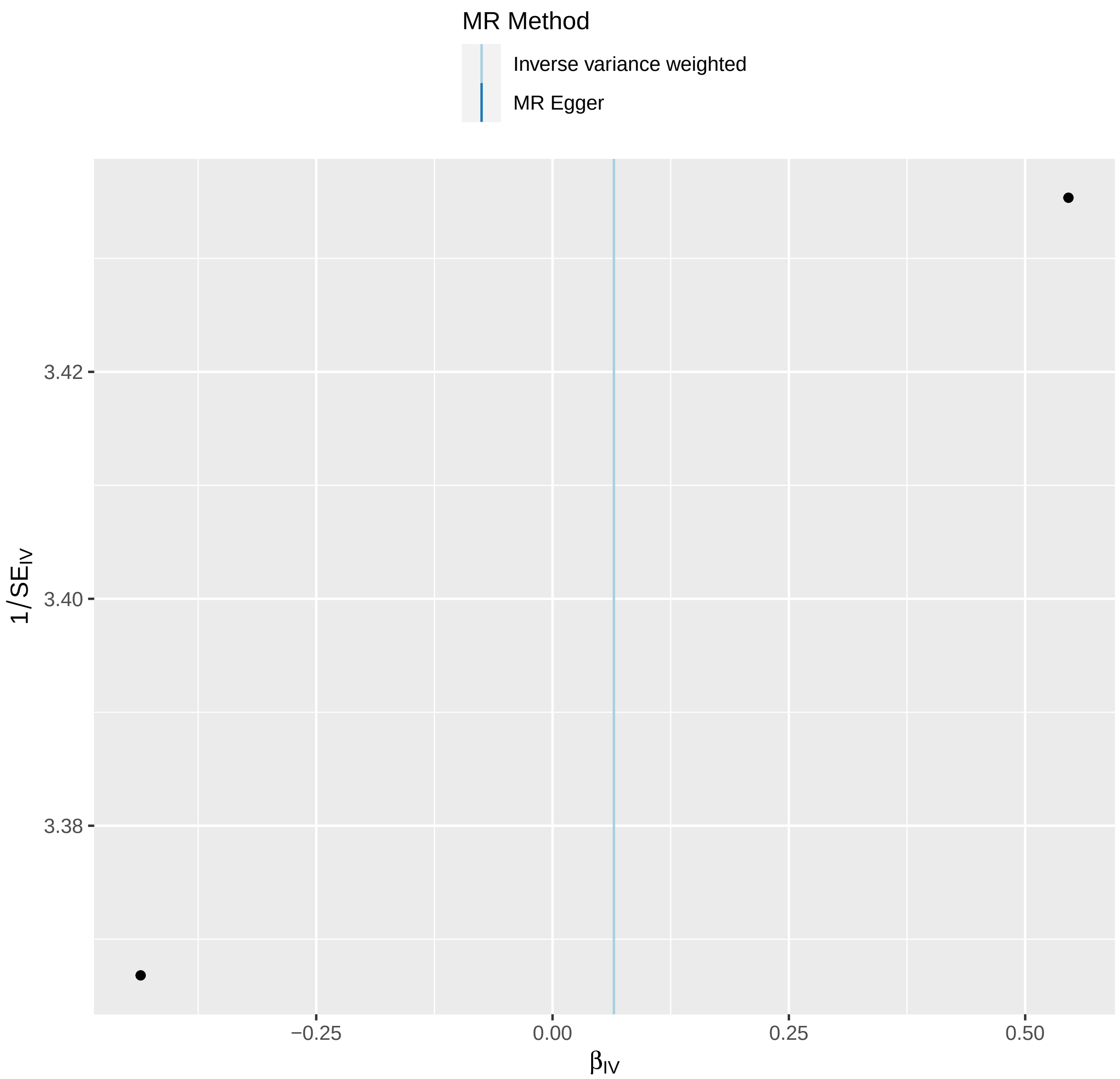

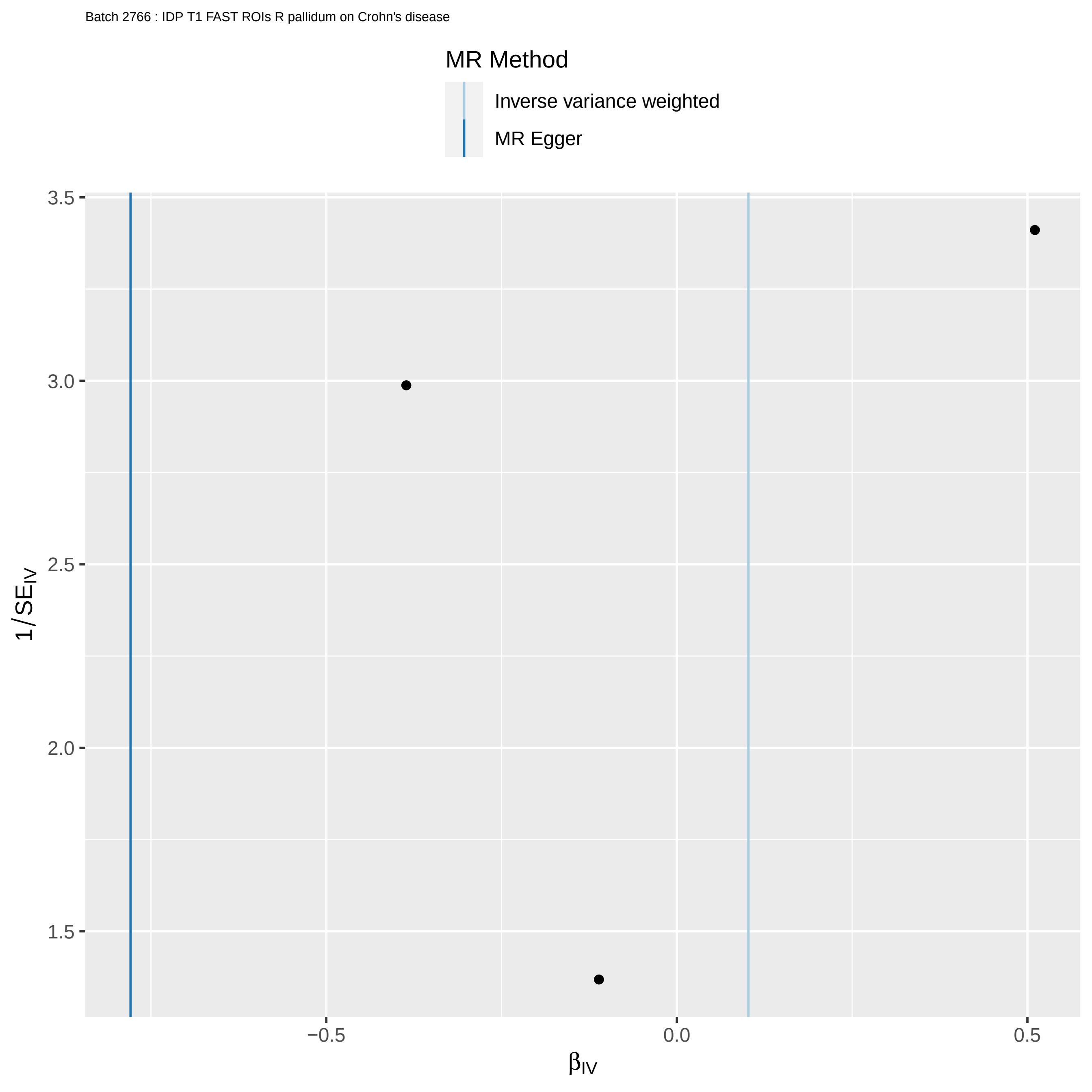
Batch 2766 : IDP T1 FAST ROIs R pallidum on Crohn's disease
MR Method
Inverse variance weighted
MR Egger
3.5
3.0
2.5
2.0
1.5
1/SE_IV
−0.5
0.0
0.5
β_IV

Batch 2768 : IDP T1 FAST ROIs L hippocampus on Crohn's disease

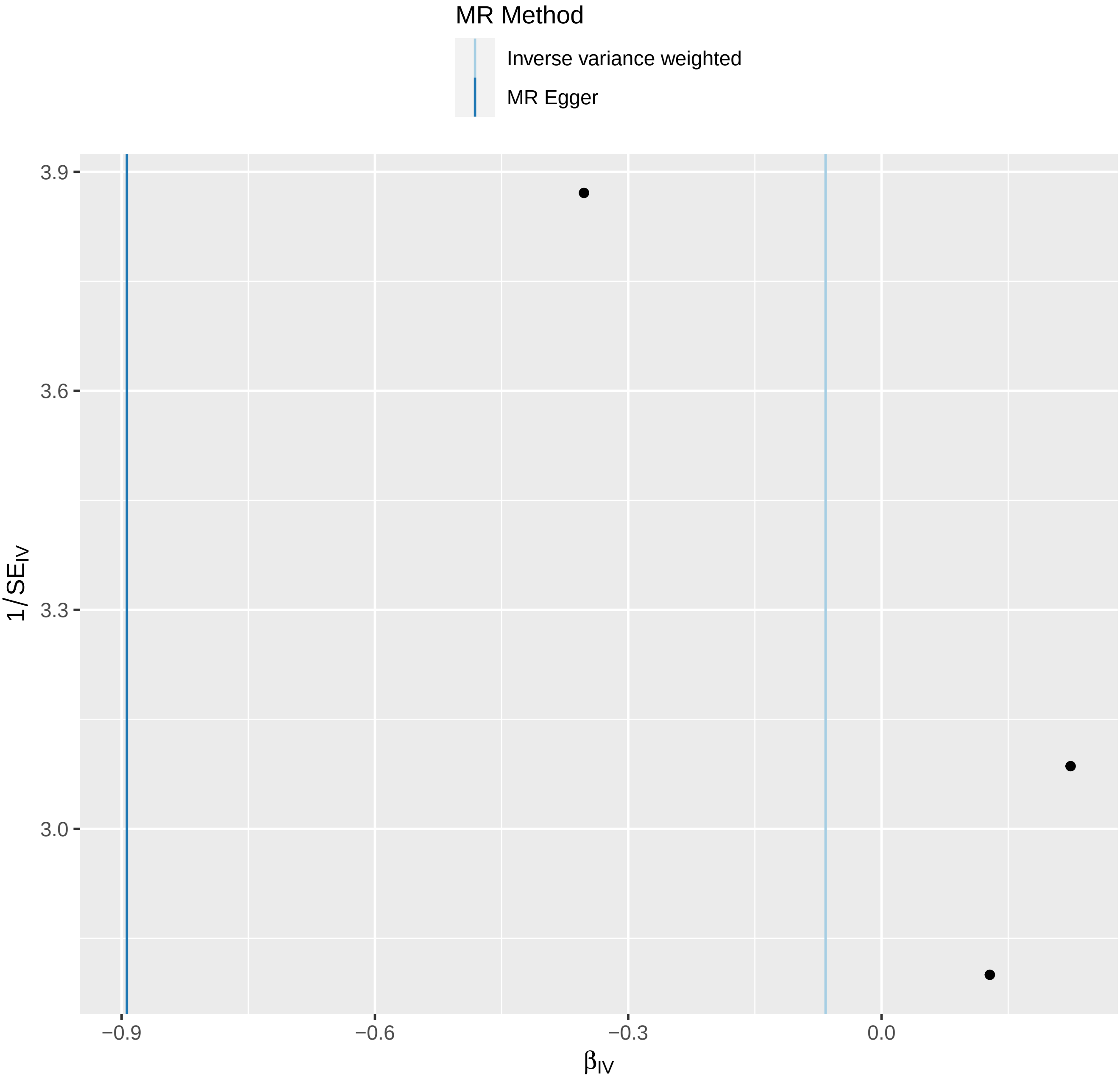

Batch 2769 : IDP T1 FAST ROIs R hippocampus on Crohn's disease

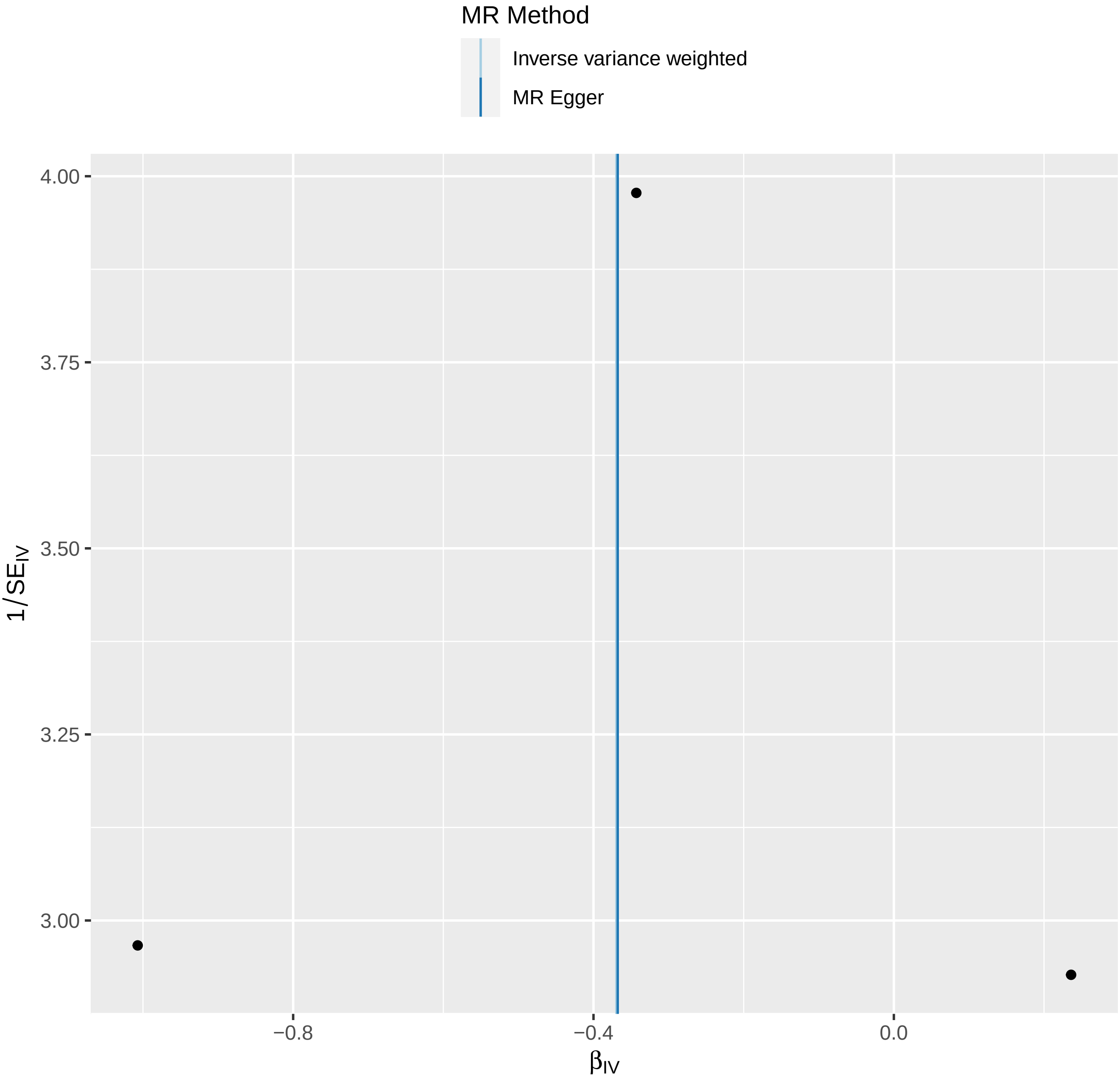

Batch 2773 : IDP T1 FAST ROIs R ventral striatum on Crohn's disease

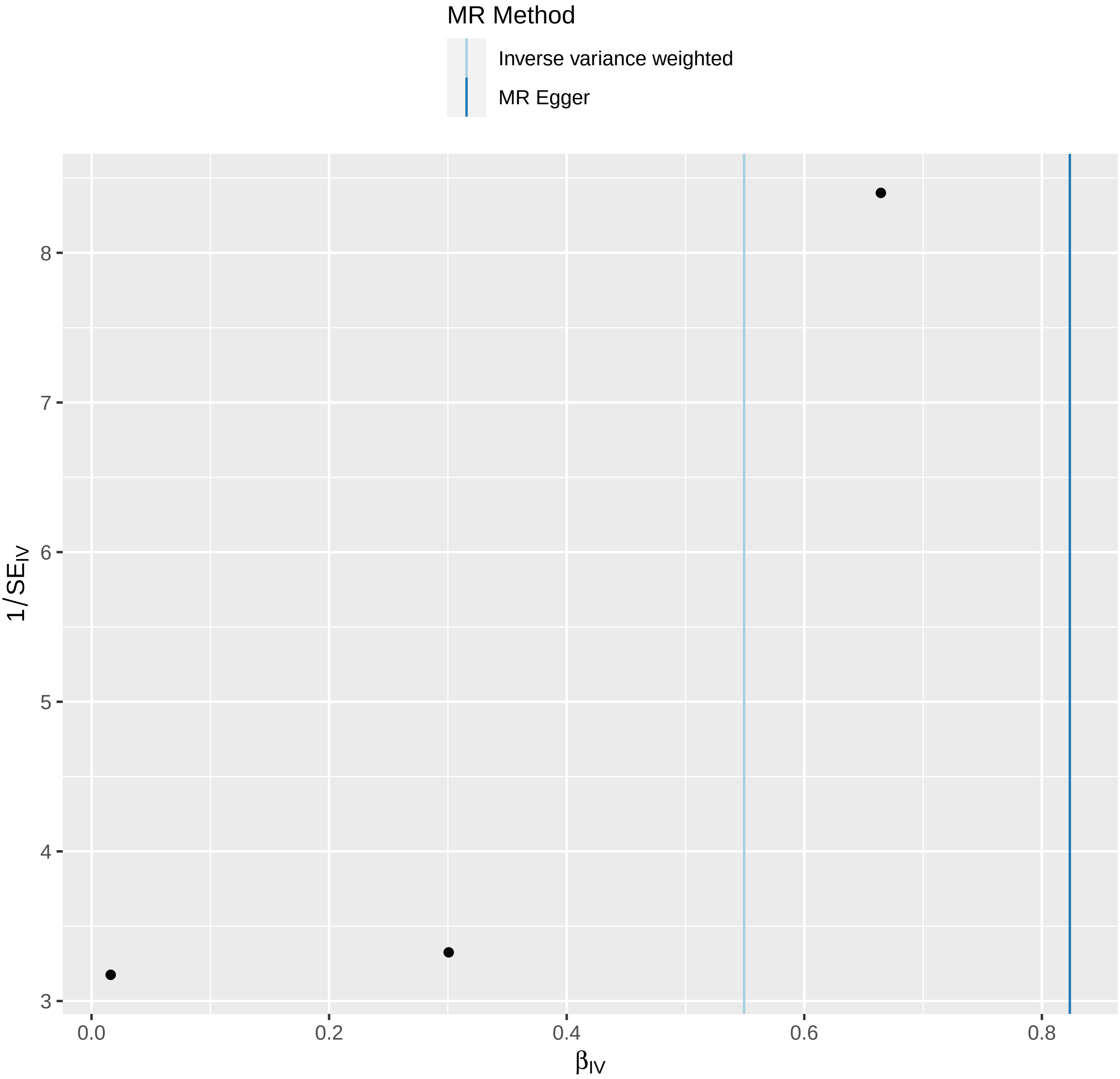

Batch 2775 : IDP T1 FAST ROIs L cerebellum I−IV on Crohn's disease

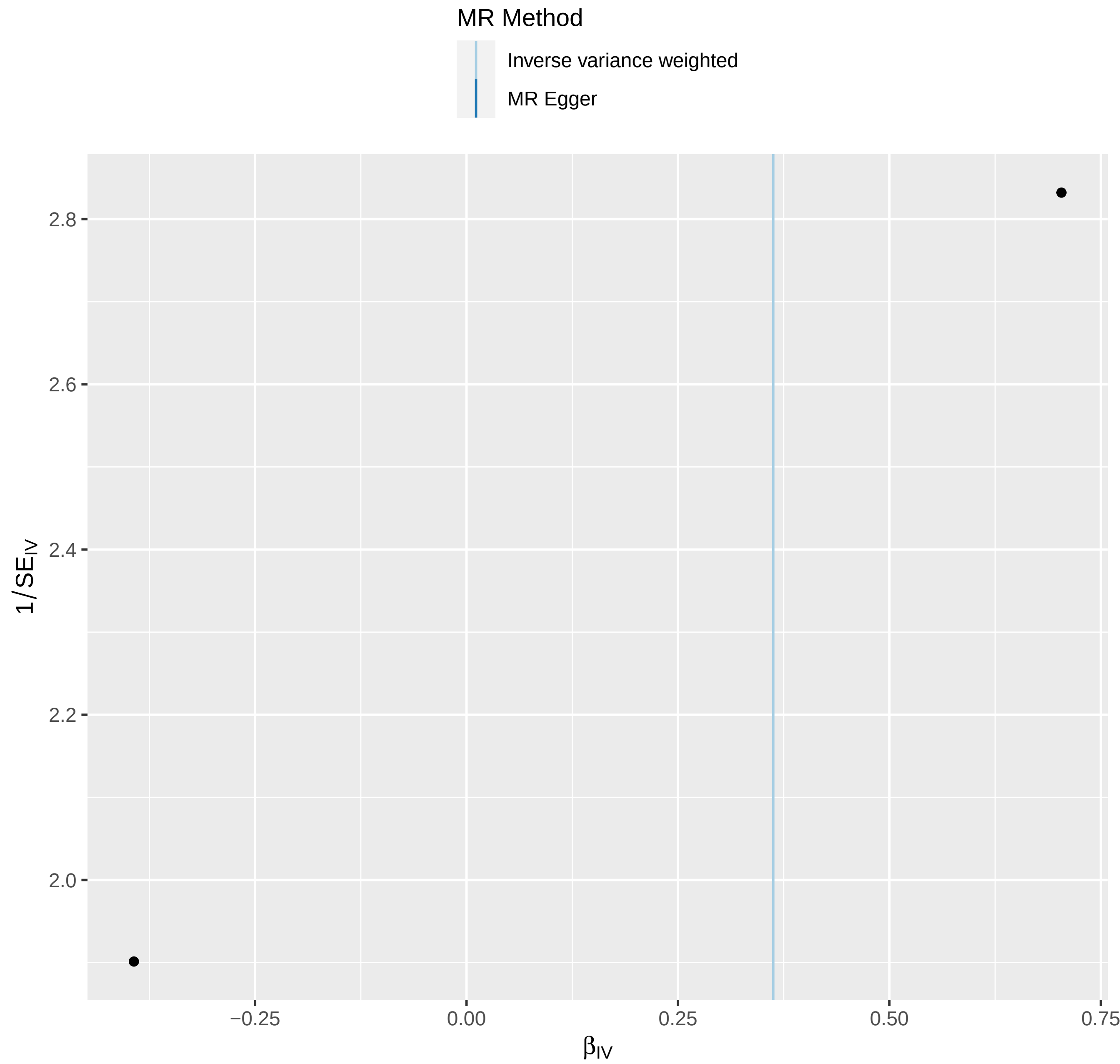

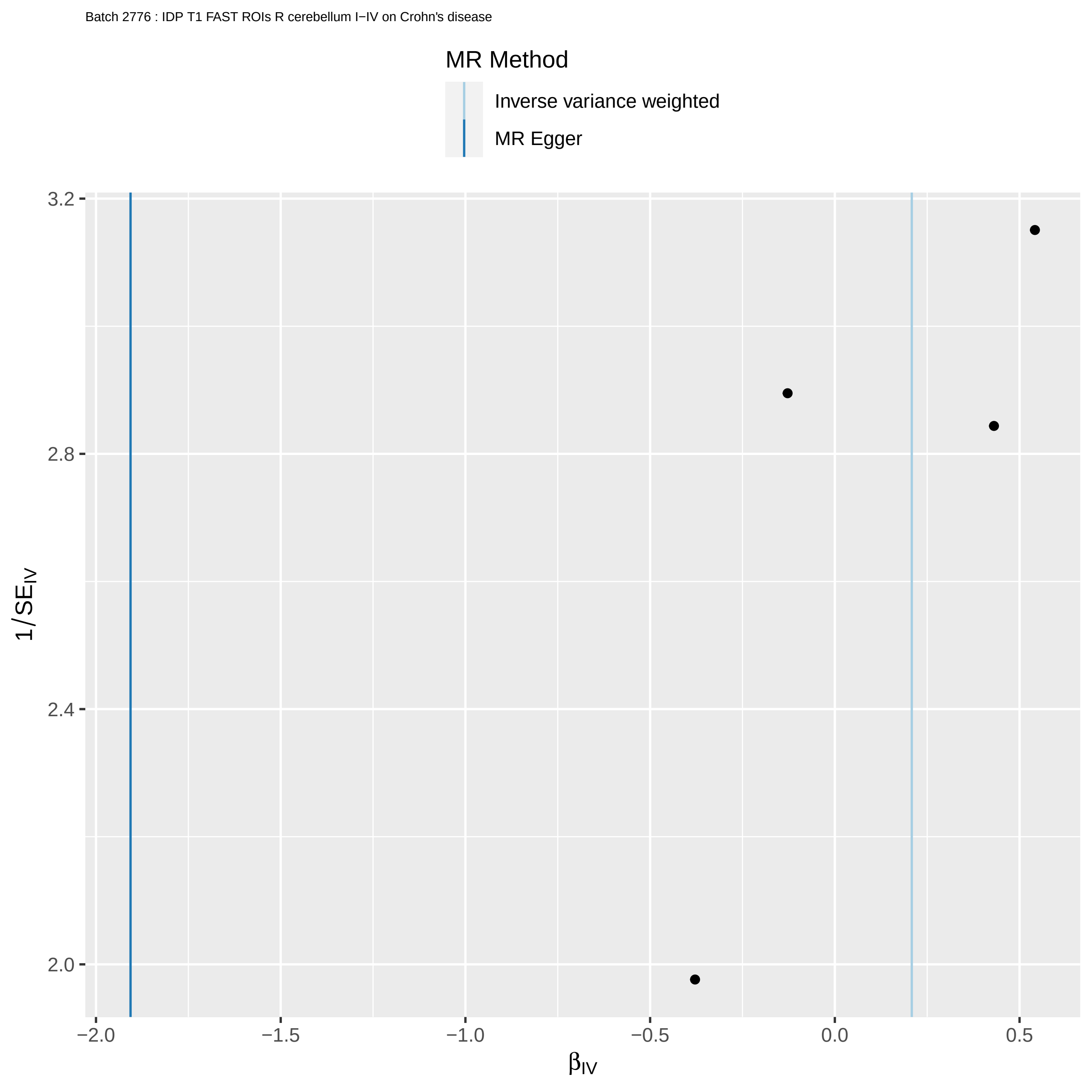

Batch 2776 : IDP T1 FAST ROIs R cerebellum I−IV on Crohn's disease
MR Method
Inverse variance weighted
MR Egger
3.2
2.8
2.4
2.0
1/SE_IV
−2.0
−1.5
−1.0
−0.5
0.0
0.5
β_IV

Batch 2777 : IDP T1 FAST ROIs L cerebellum V on Crohn's disease

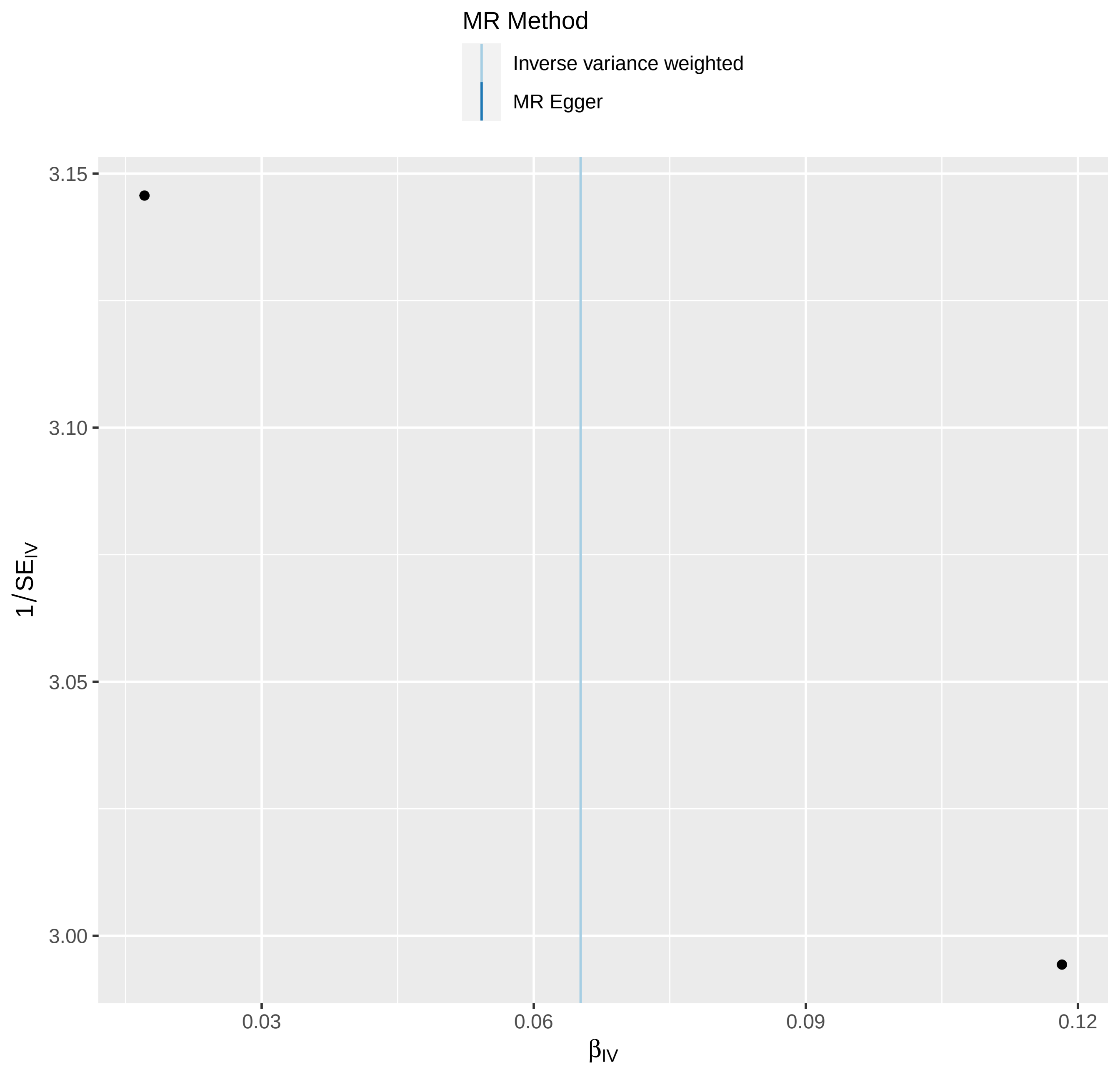

Batch 2779 : IDP T1 FAST ROIs R cerebellum V on Crohn's disease

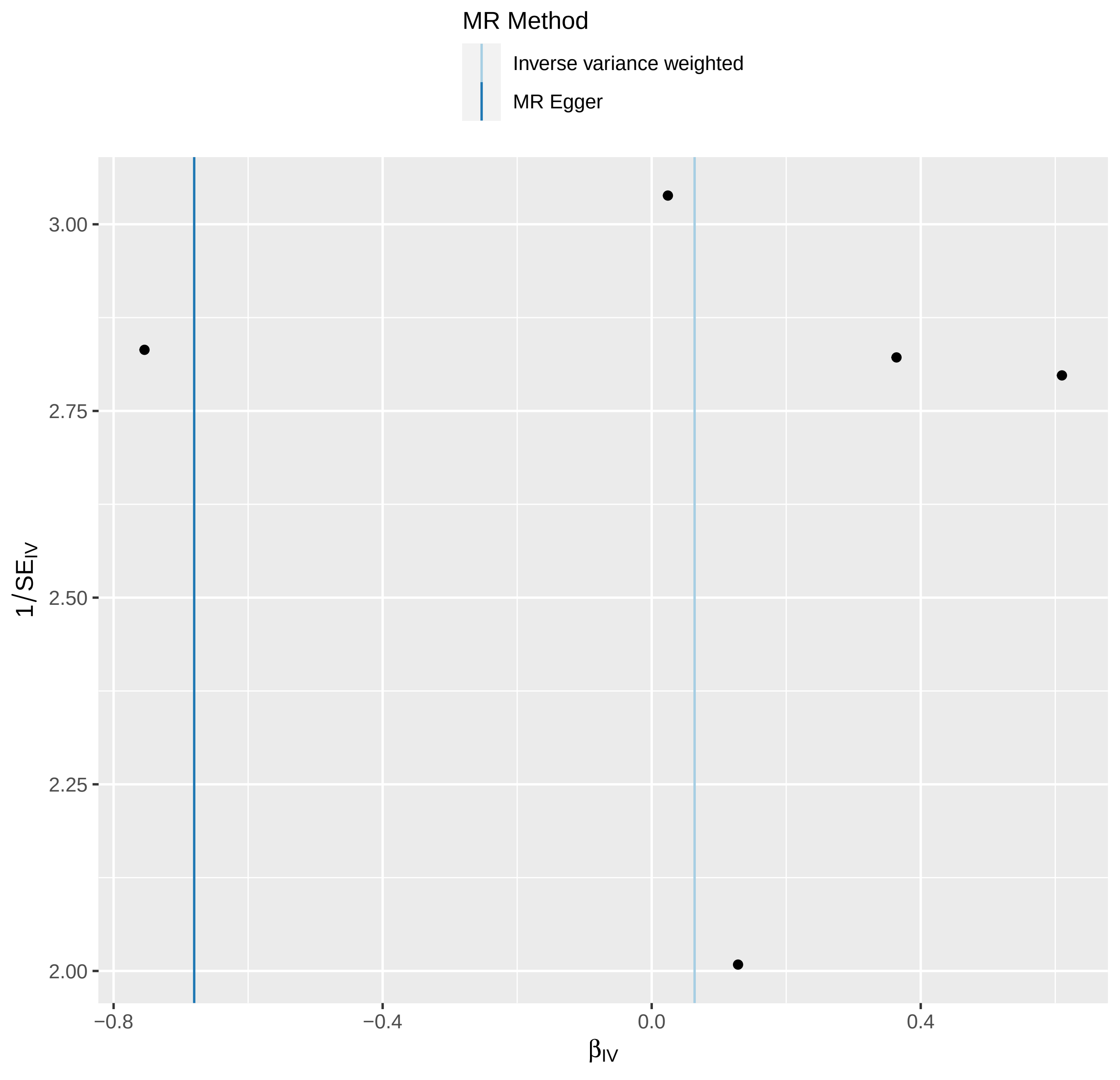

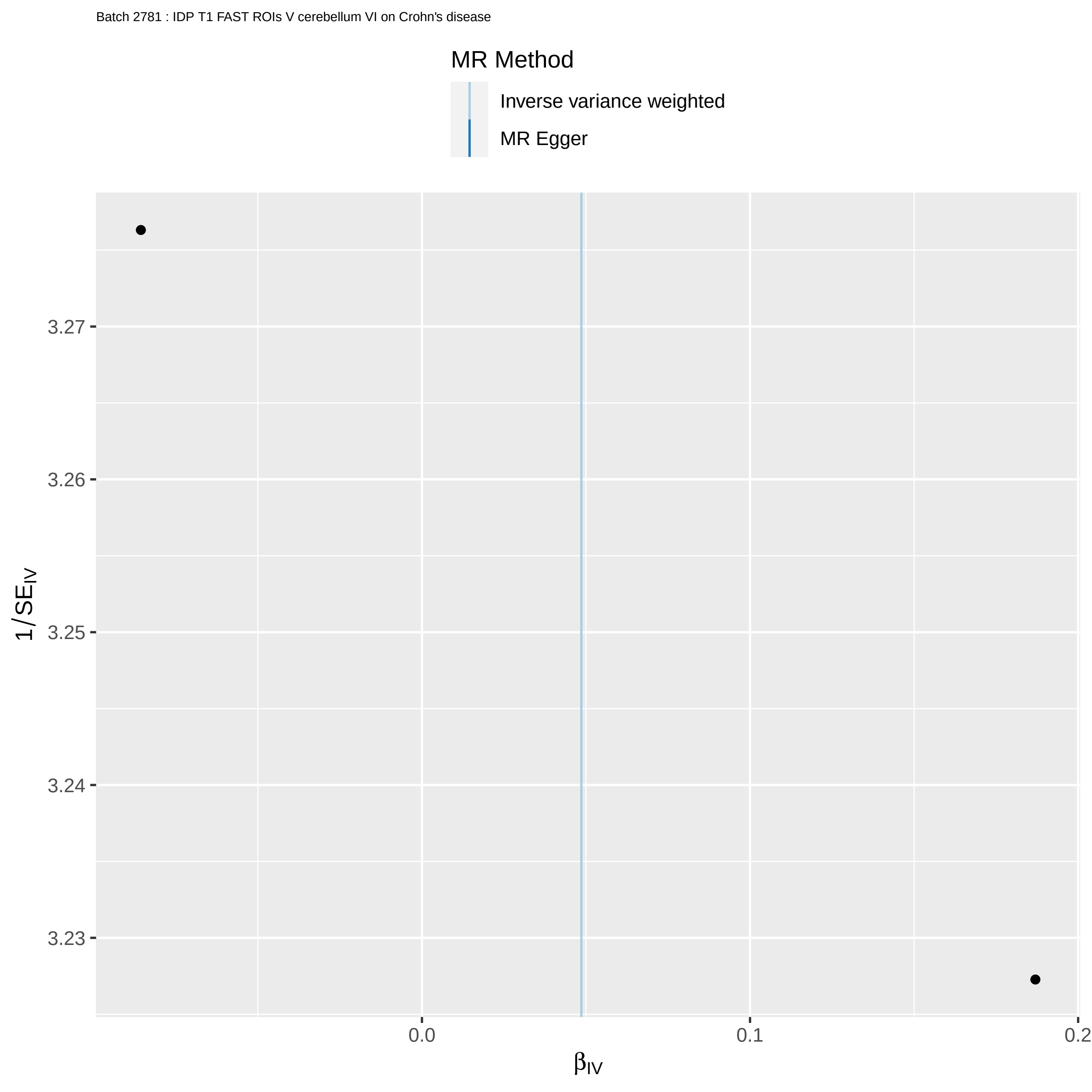

Batch 2781 : IDP T1 FAST ROIs V cerebellum VI on Crohn's disease
MR Method
Inverse variance weighted
MR Egger
3.27
3.26
3.25
3.24
3.23
$1/SE_{IV}$
0.0
0.1
0.2
β_{IV}

Batch 2783 : IDP T1 FAST ROIs L cerebellum crus I on Crohn's disease

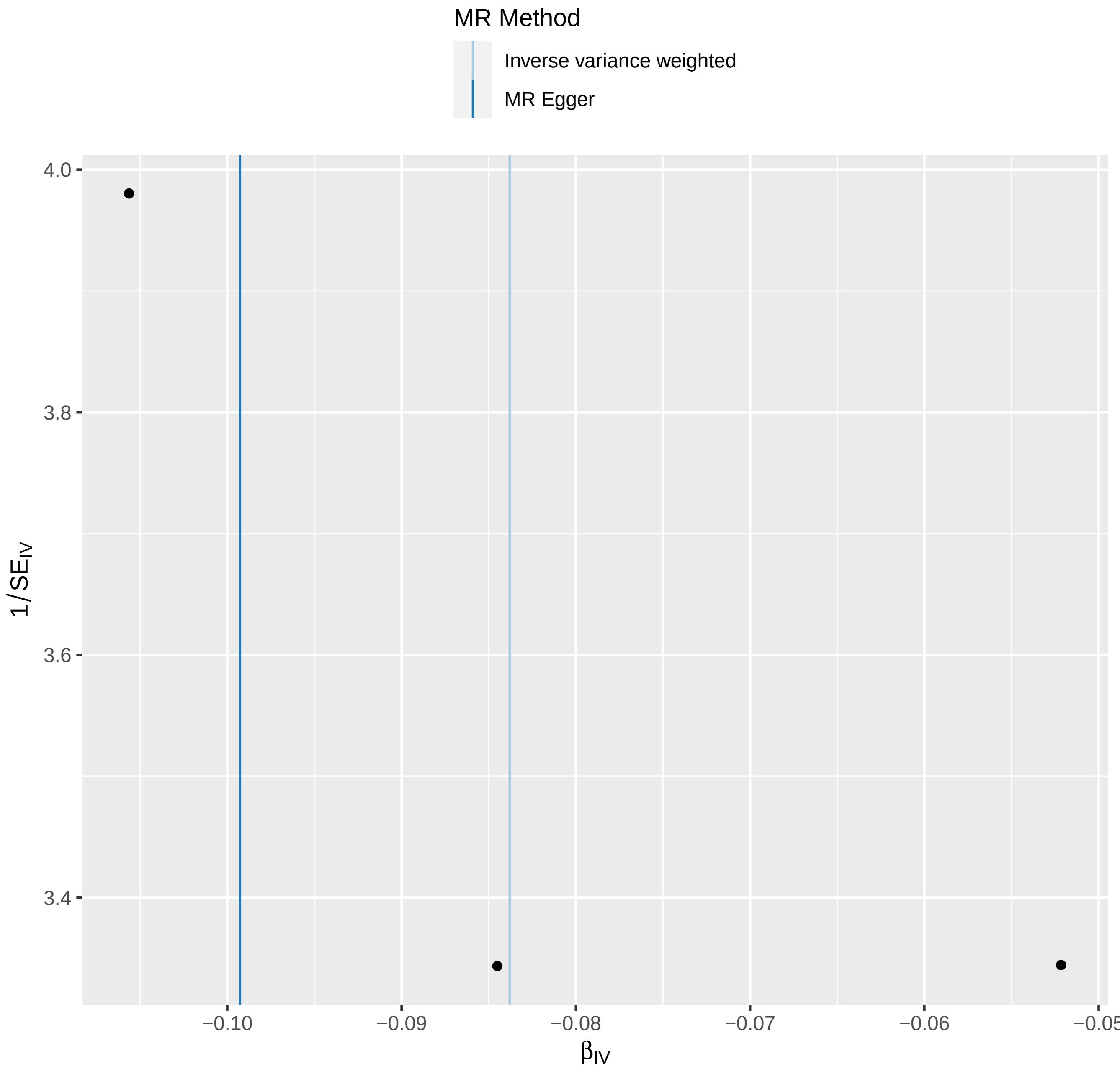

Batch 2785 : IDP T1 FAST ROIs R cerebellum crus I on Crohn's disease

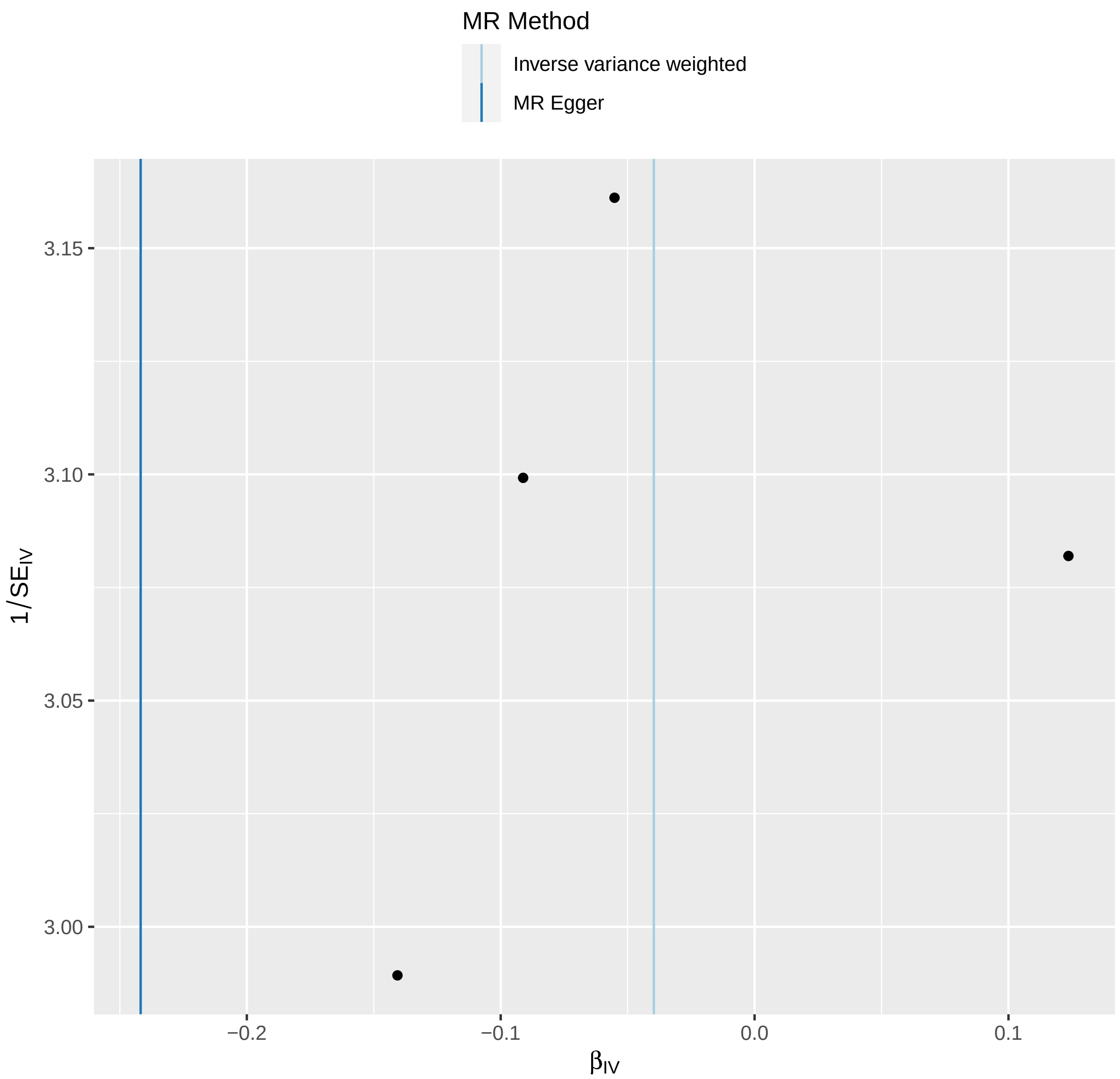

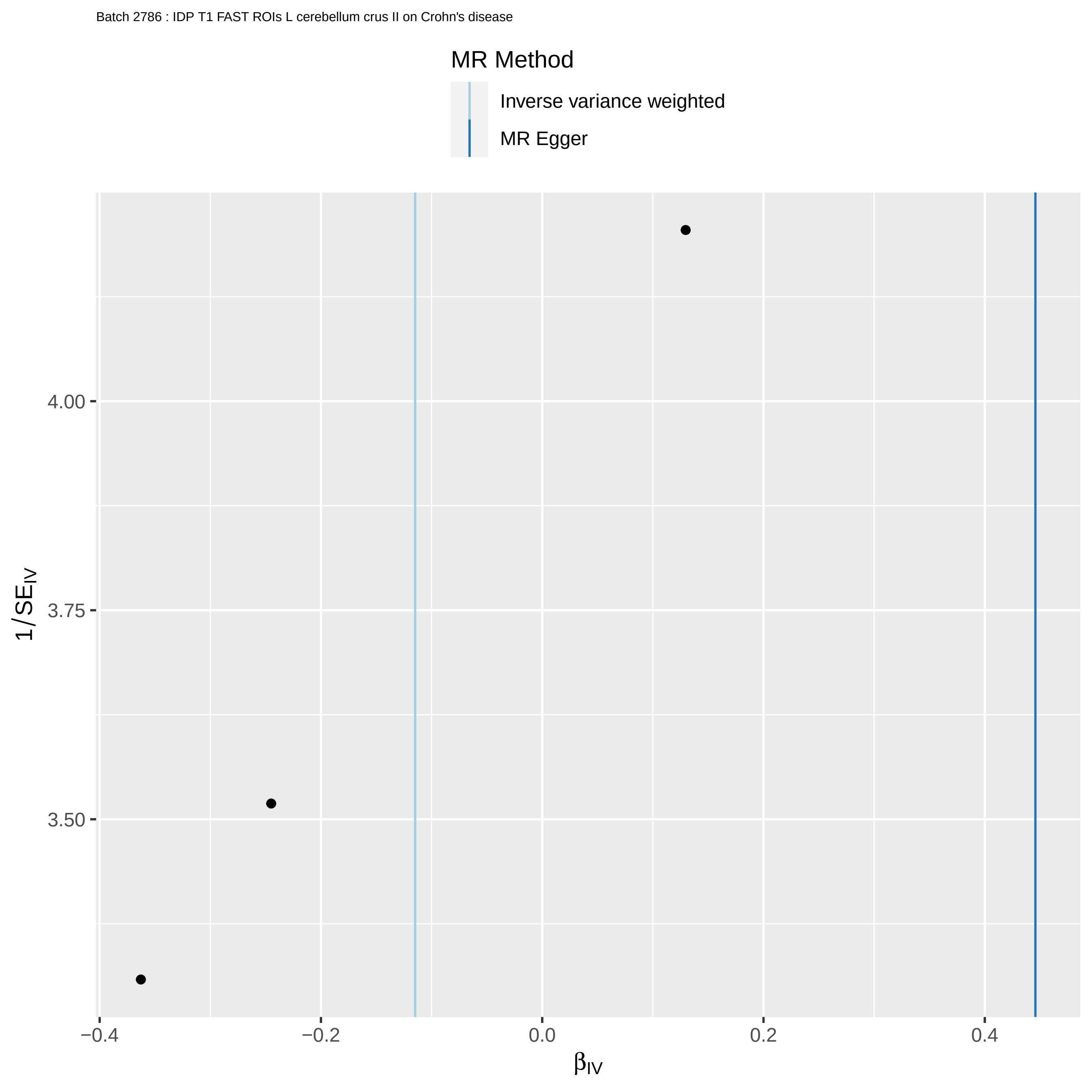

Batch 2786 : IDP T1 FAST ROIs L cerebellum crus II on Crohn's disease
MR Method
Inverse variance weighted
MR Egger
4.00
3.75
3.50
1/SE_IV
−0.4
−0.2
0.0
0.2
0.4
β_IV

Batch 2787 : IDP T1 FAST ROIs V cerebellum crus II on Crohn's disease

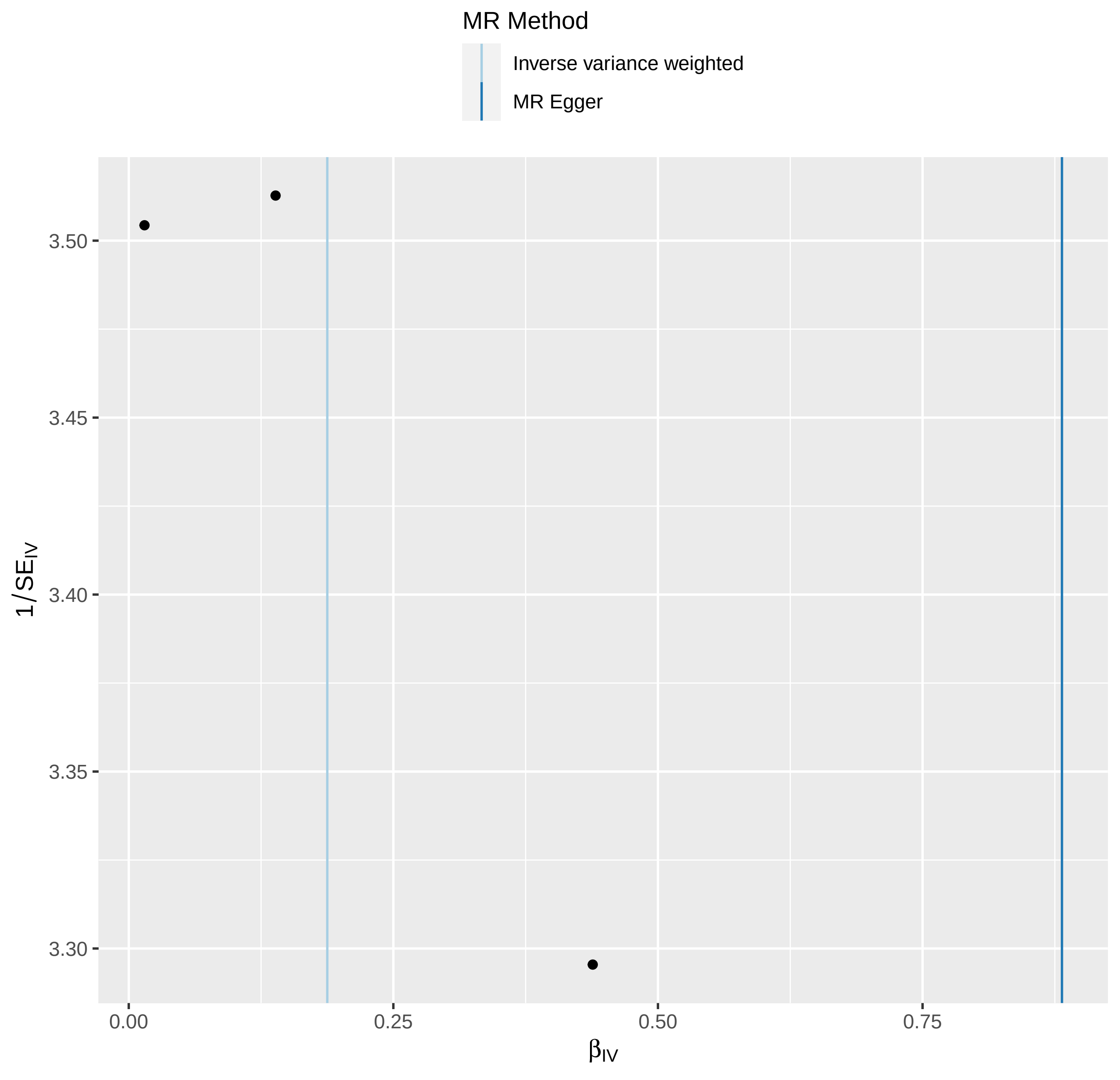

Batch 2788 : IDP T1 FAST ROIs R cerebellum crus II on Crohn's disease

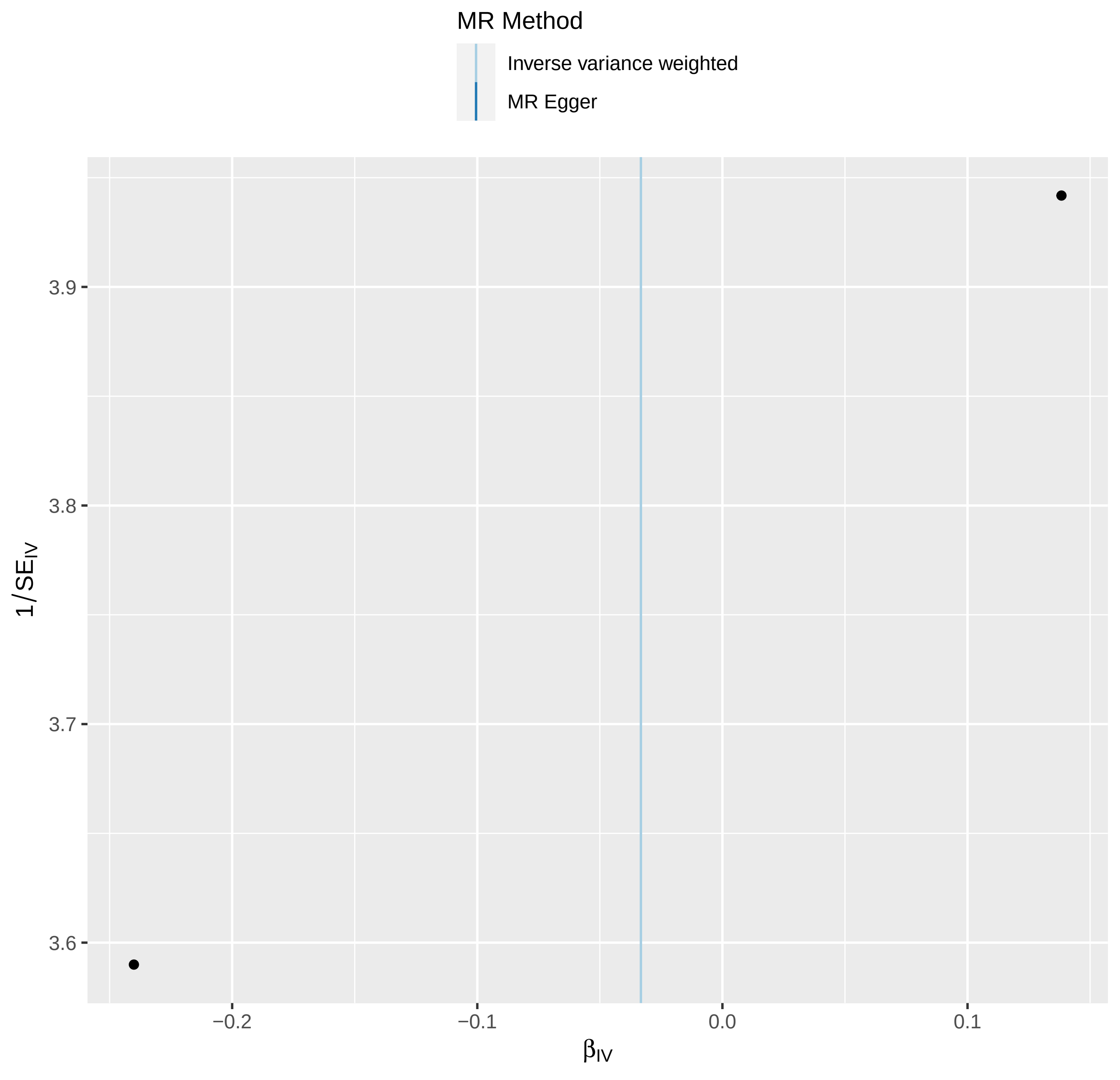

Batch 2791 : IDP T1 FAST ROIs V cerebellum VIIb on Crohn's disease

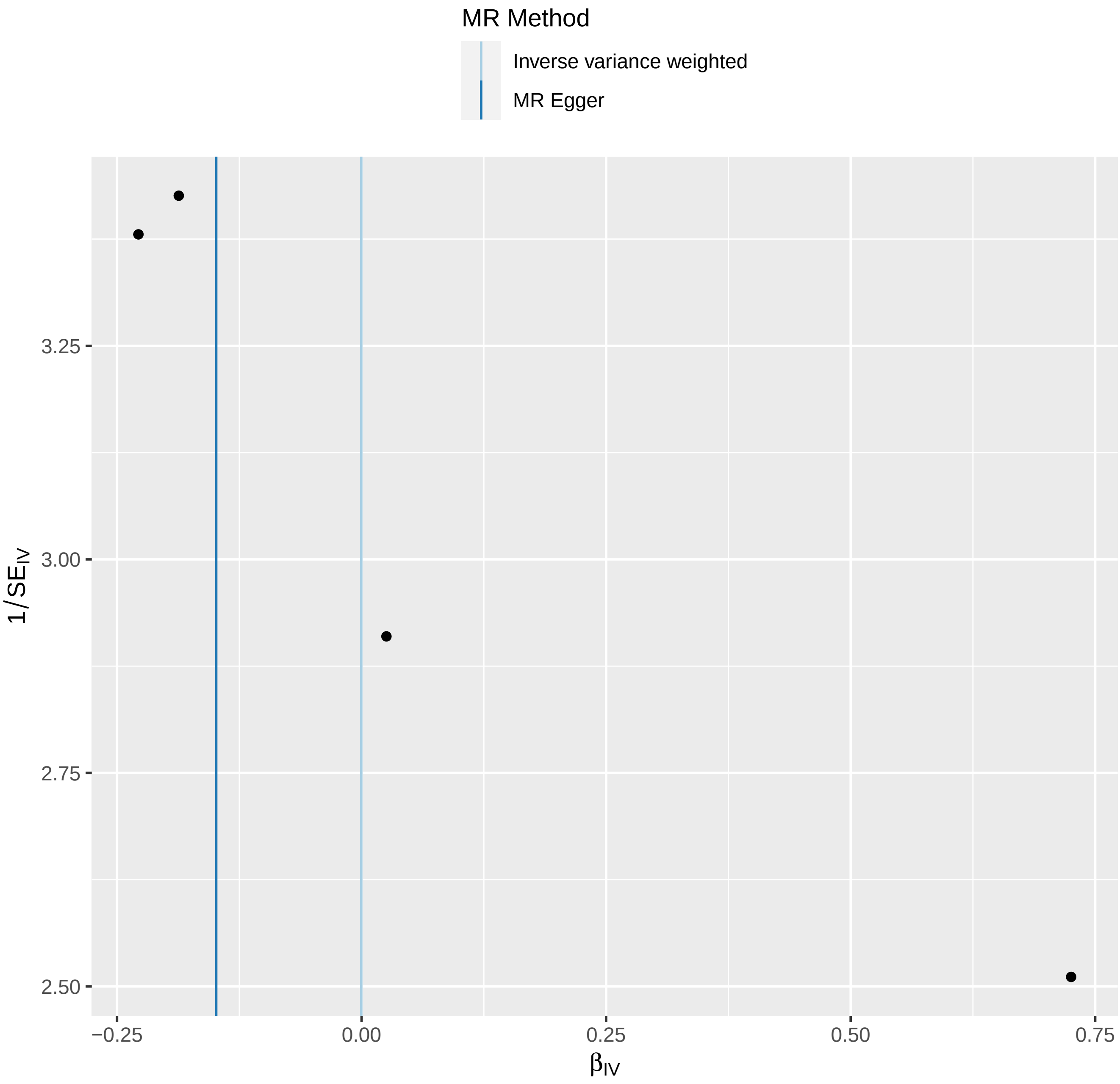

Batch 2794 : IDP T1 FAST ROIs V cerebellum VIIIa on Crohn's disease

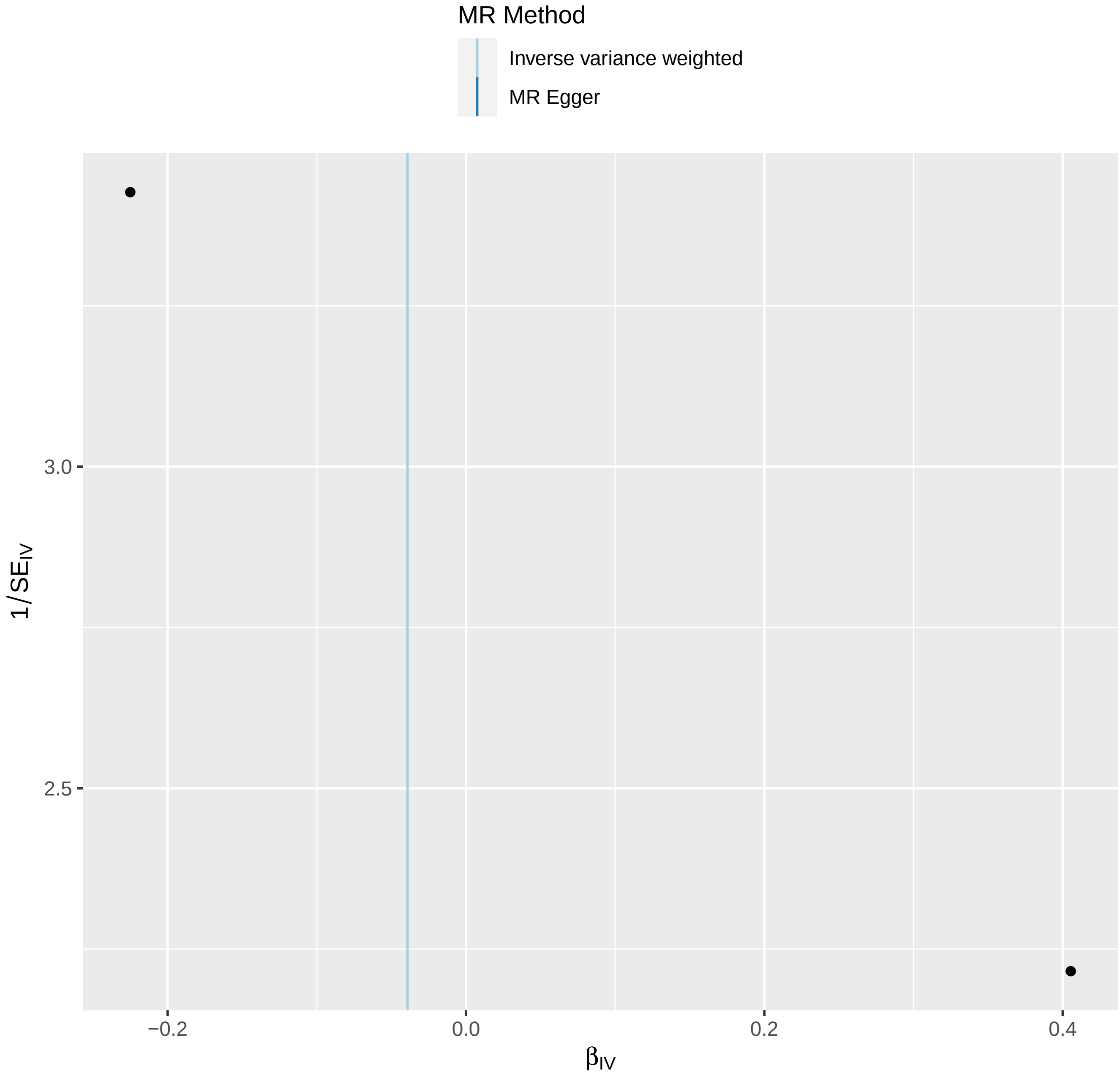

Batch 2803 : IDP T1 FAST ROIs L cerebellum X on Crohn's disease

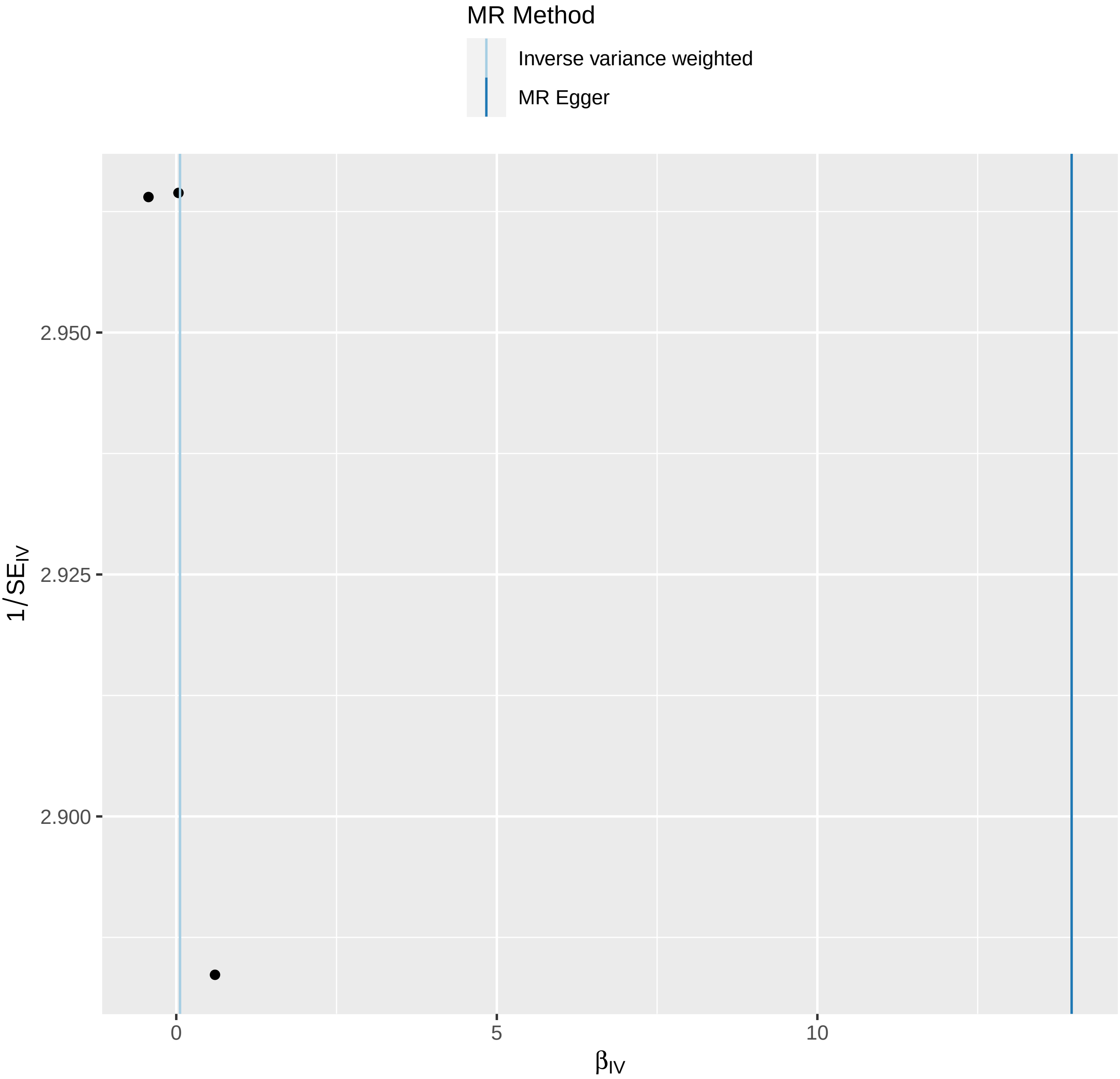

Batch 2805 : IDP T1 FAST ROIs R cerebellum X on Crohn's disease

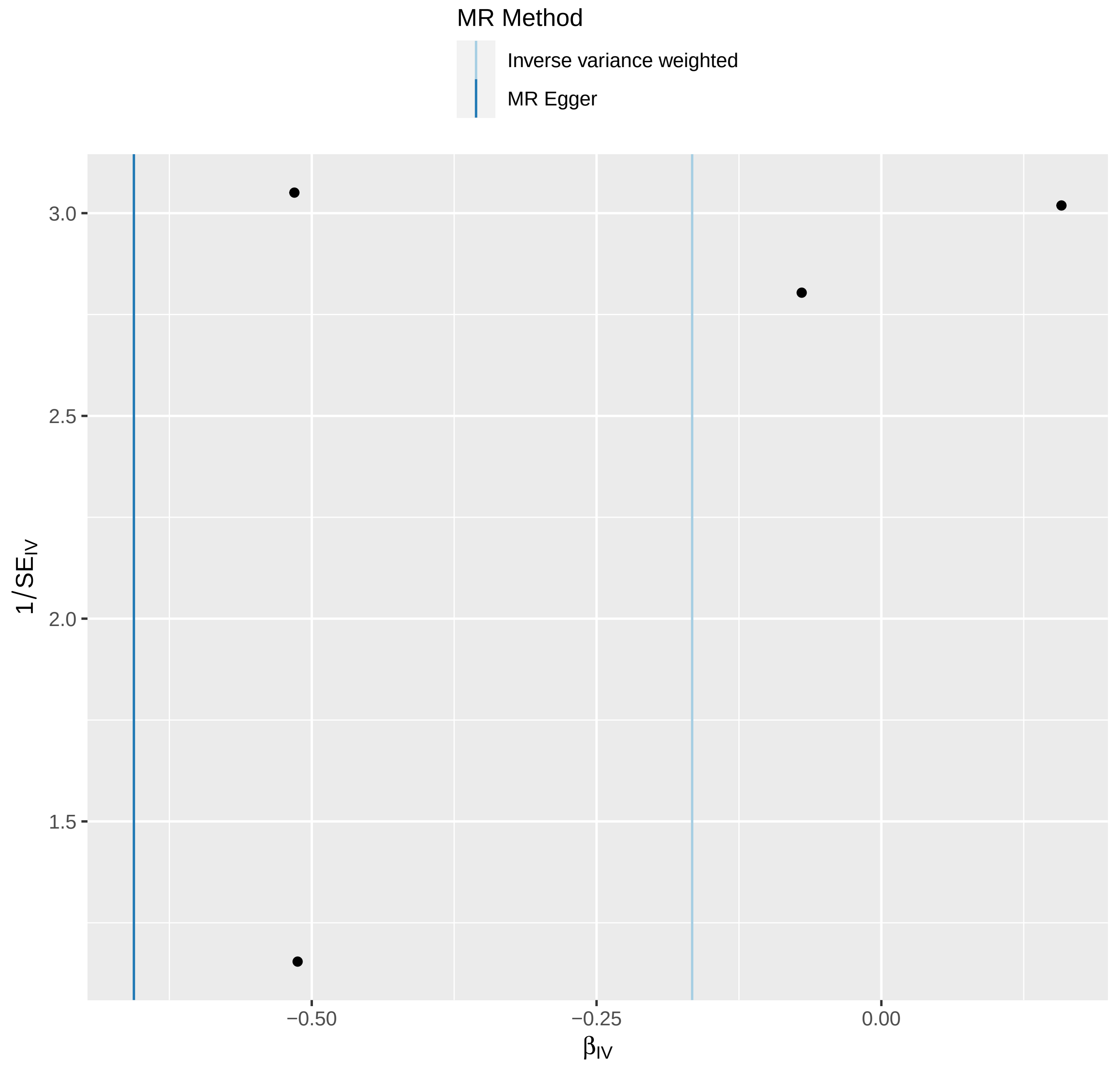

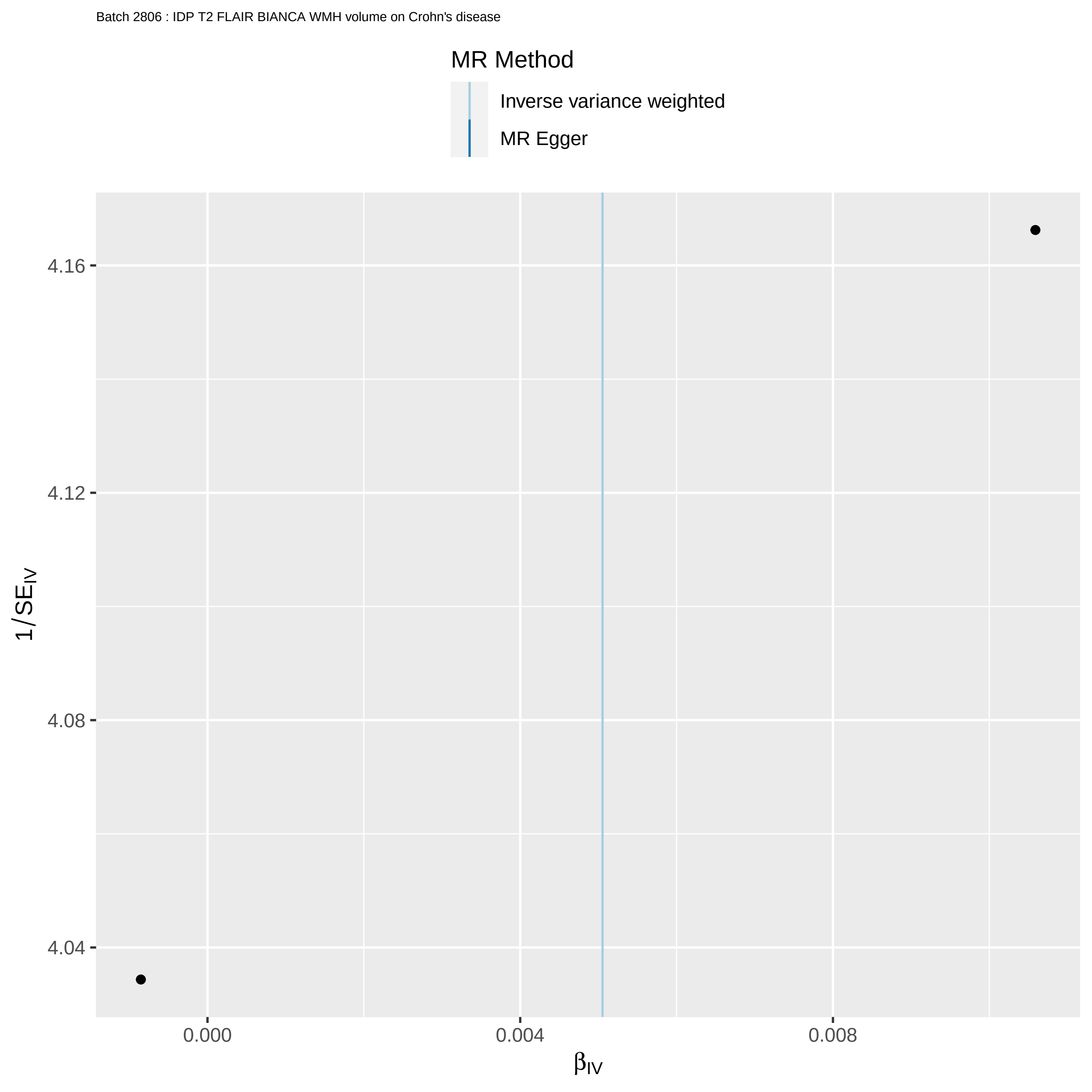

Batch 2806 : IDP T2 FLAIR BIANCA WMH volume on Crohn's disease
MR Method
Inverse variance weighted
MR Egger
4.16
4.12
4.08
4.04
$1/SE_{IV}$
0.000
0.004
0.008
β_{IV}

Batch 2824 : IDP tfMRI 90th−percentile BOLD shapes on Crohn's disease

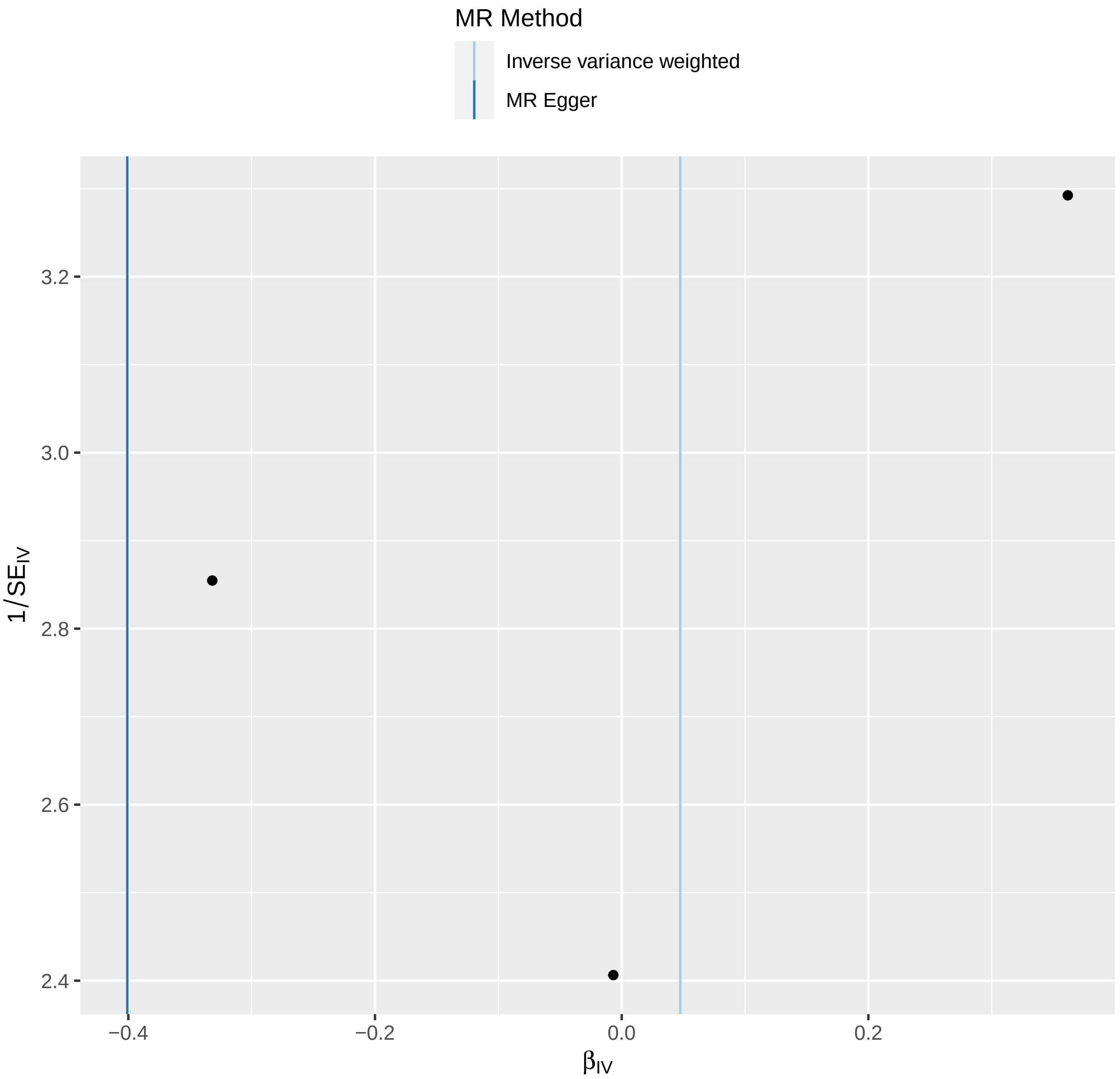

Batch 2825 : IDP tfMRI median zstat shapes on Crohn's disease

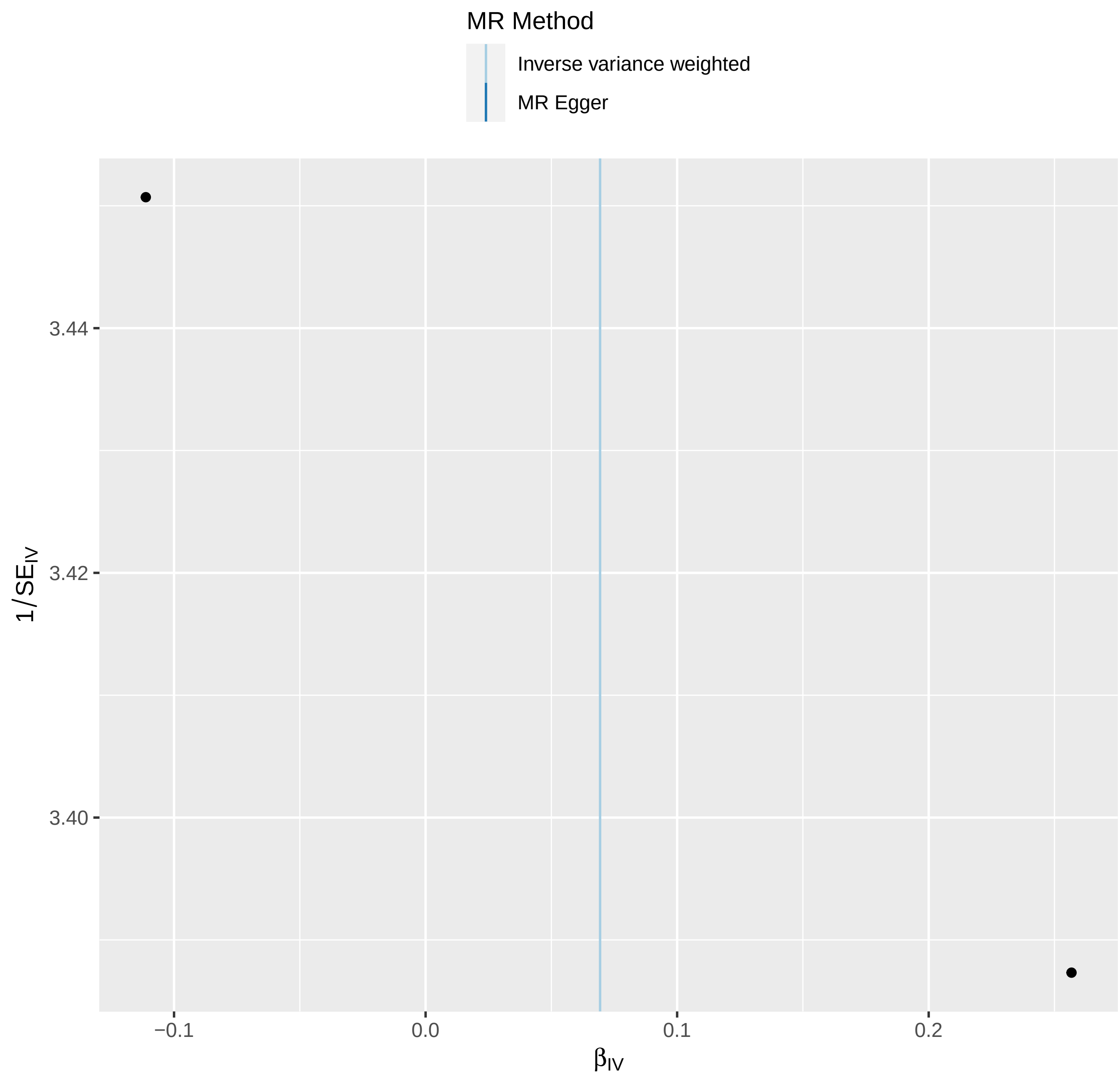

Batch 2826 : IDP tfMRI 90th−percentile zstat shapes on Crohn's disease

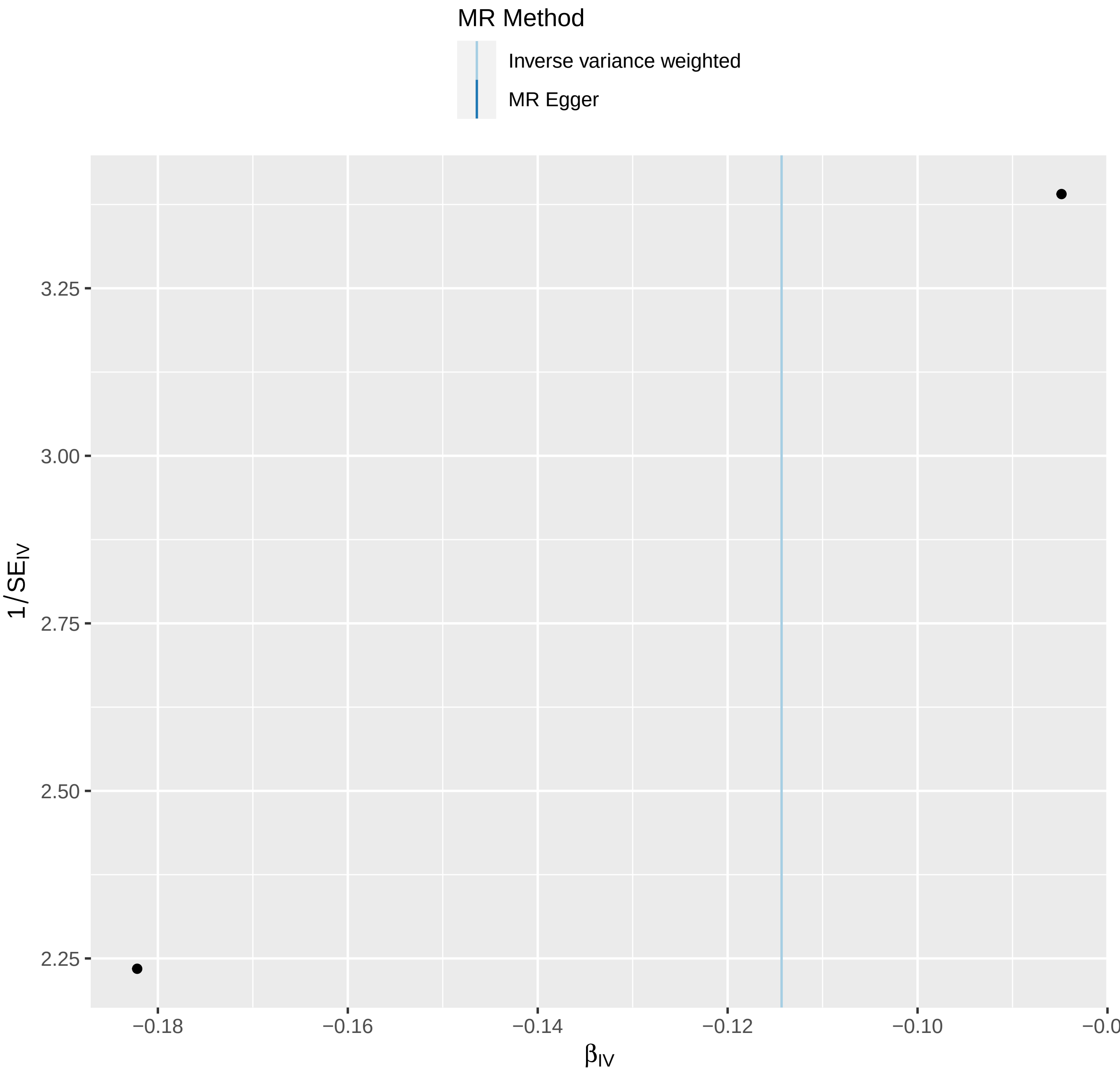

Batch 2827 : IDP tfMRI median BOLD faces on Crohn's disease

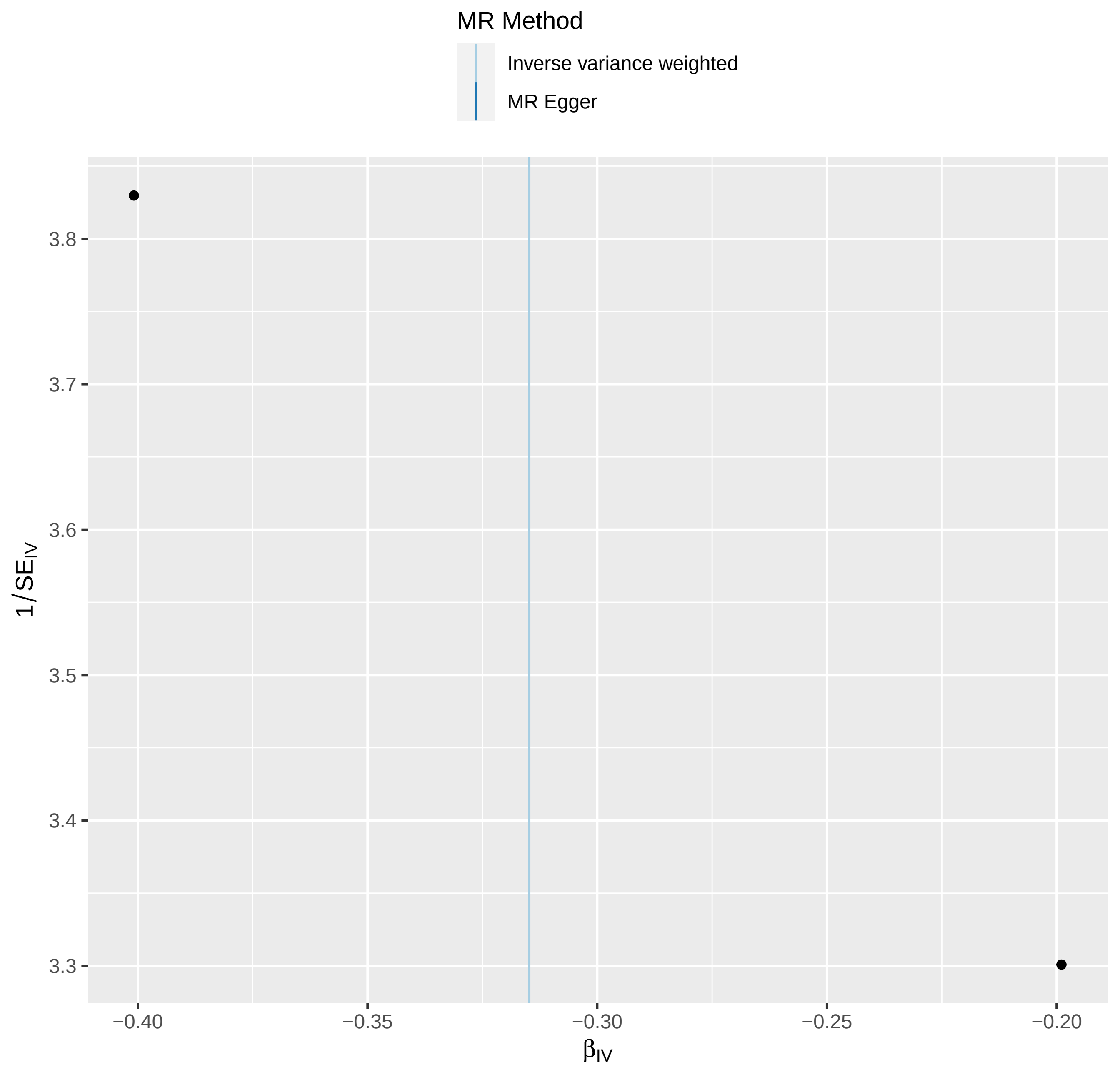

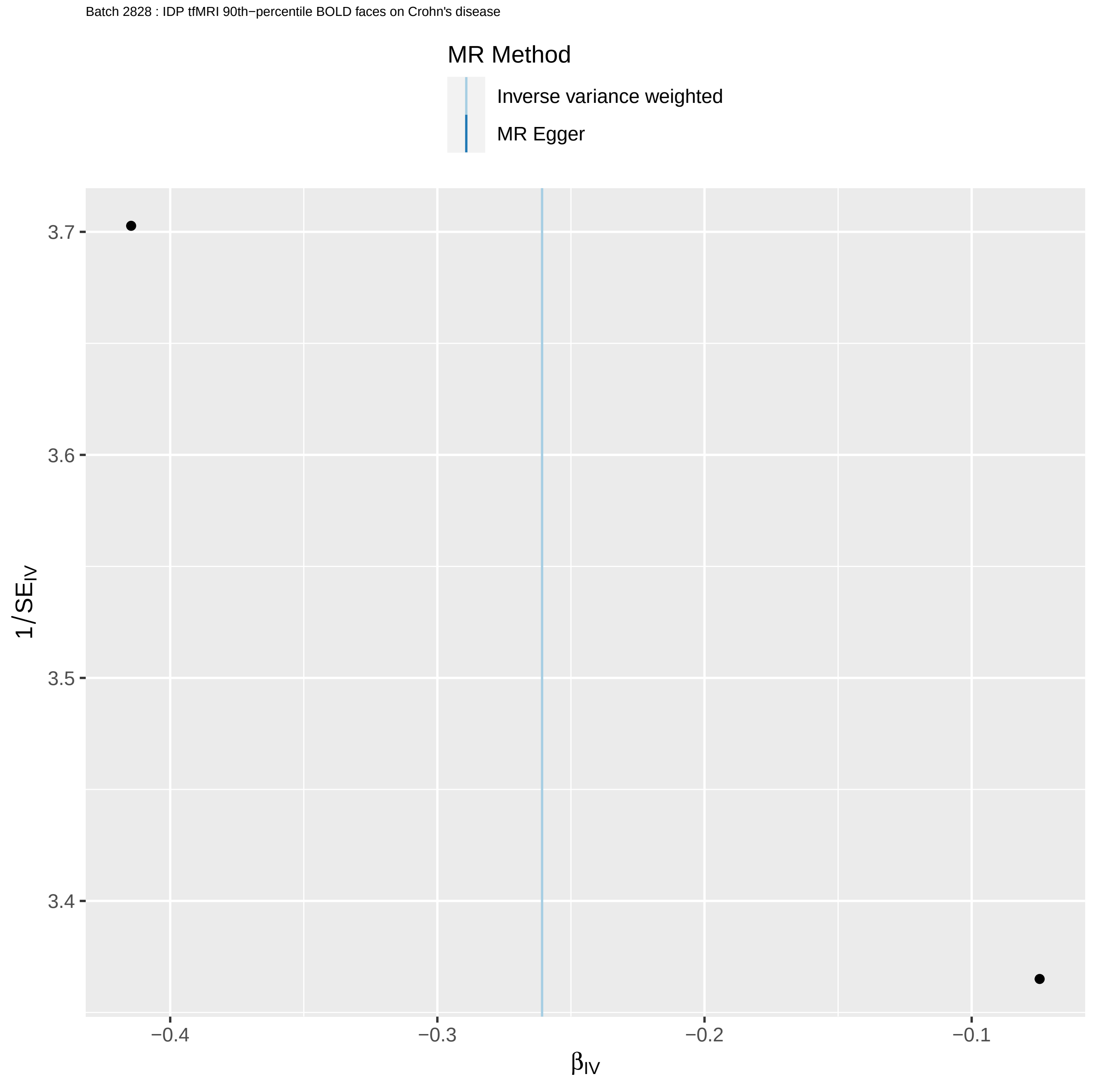
Batch 2828 : IDP tfMRI 90th−percentile BOLD faces on Crohn's disease
MR Method
Inverse variance weighted
MR Egger
3.7
3.6
3.5
3.4
1/SE_IV
−0.4
−0.3
−0.2
−0.1
β_IV

Batch 2829 : IDP tfMRI median zstat faces on Crohn's disease

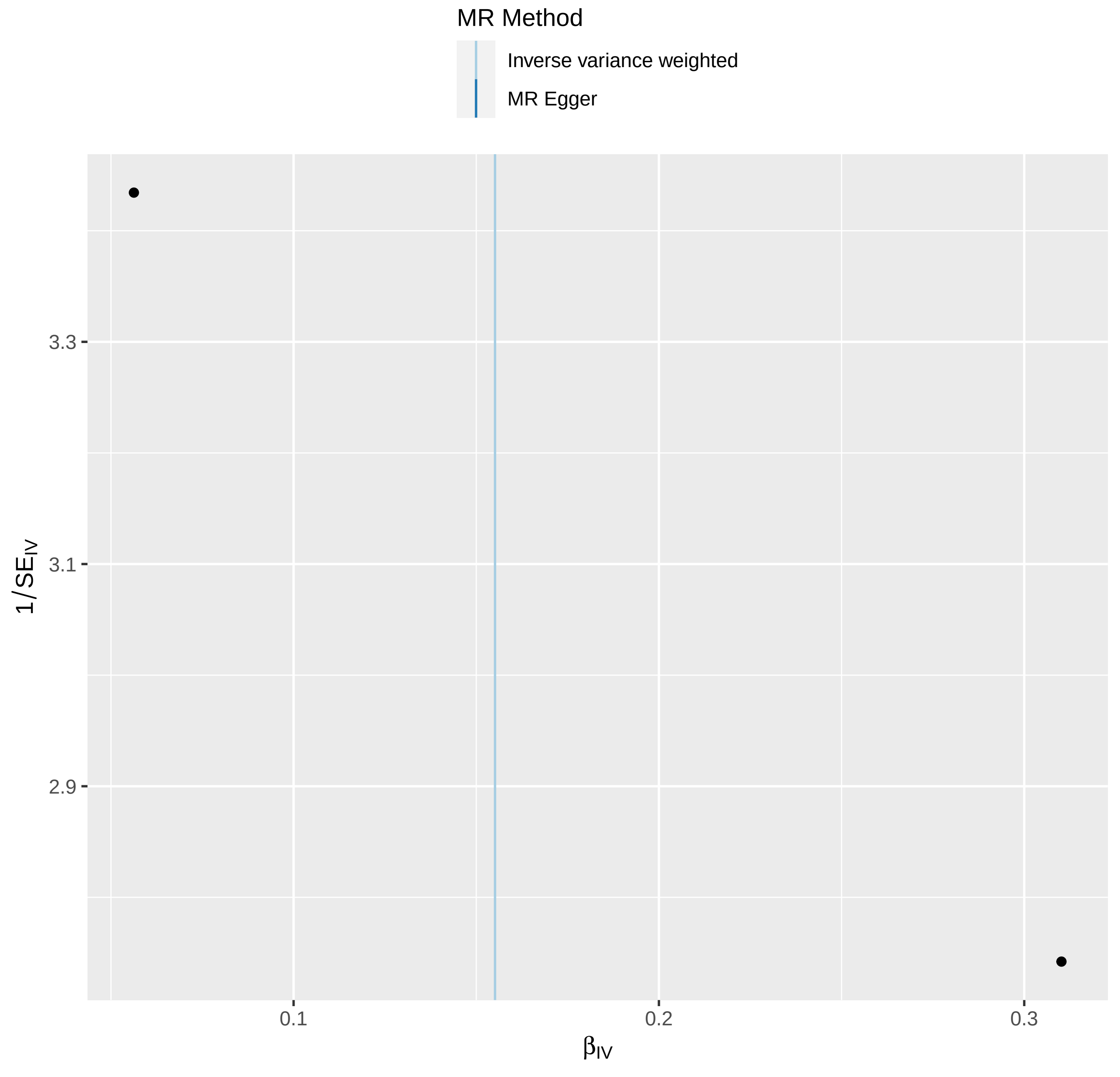

Batch 2832 : IDP tfMRI 90th−percentile BOLD faces−shapes on Crohn's disease

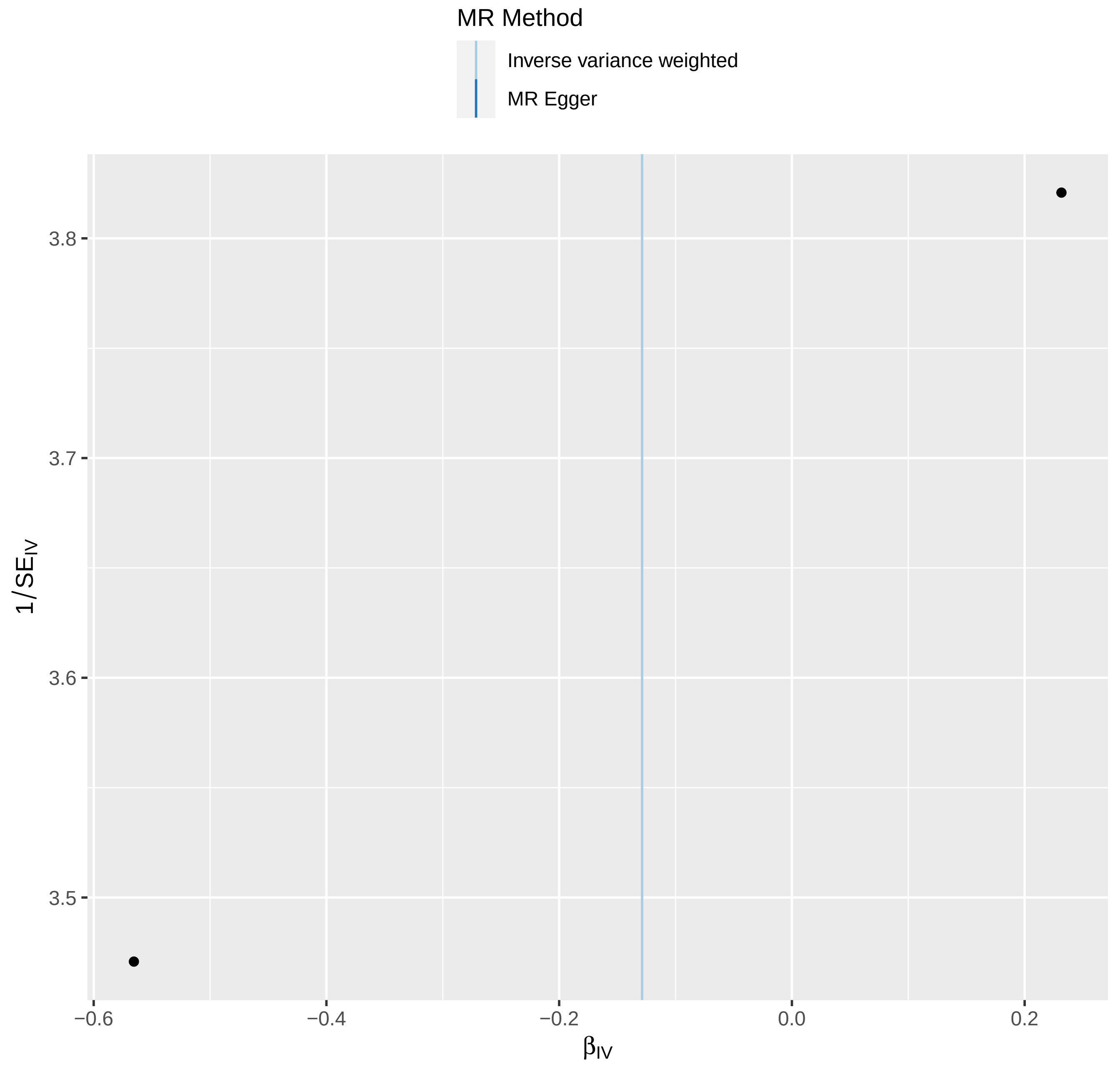

Batch 2833 : IDP T1 FIRST left hippocampus volume on Crohn's disease

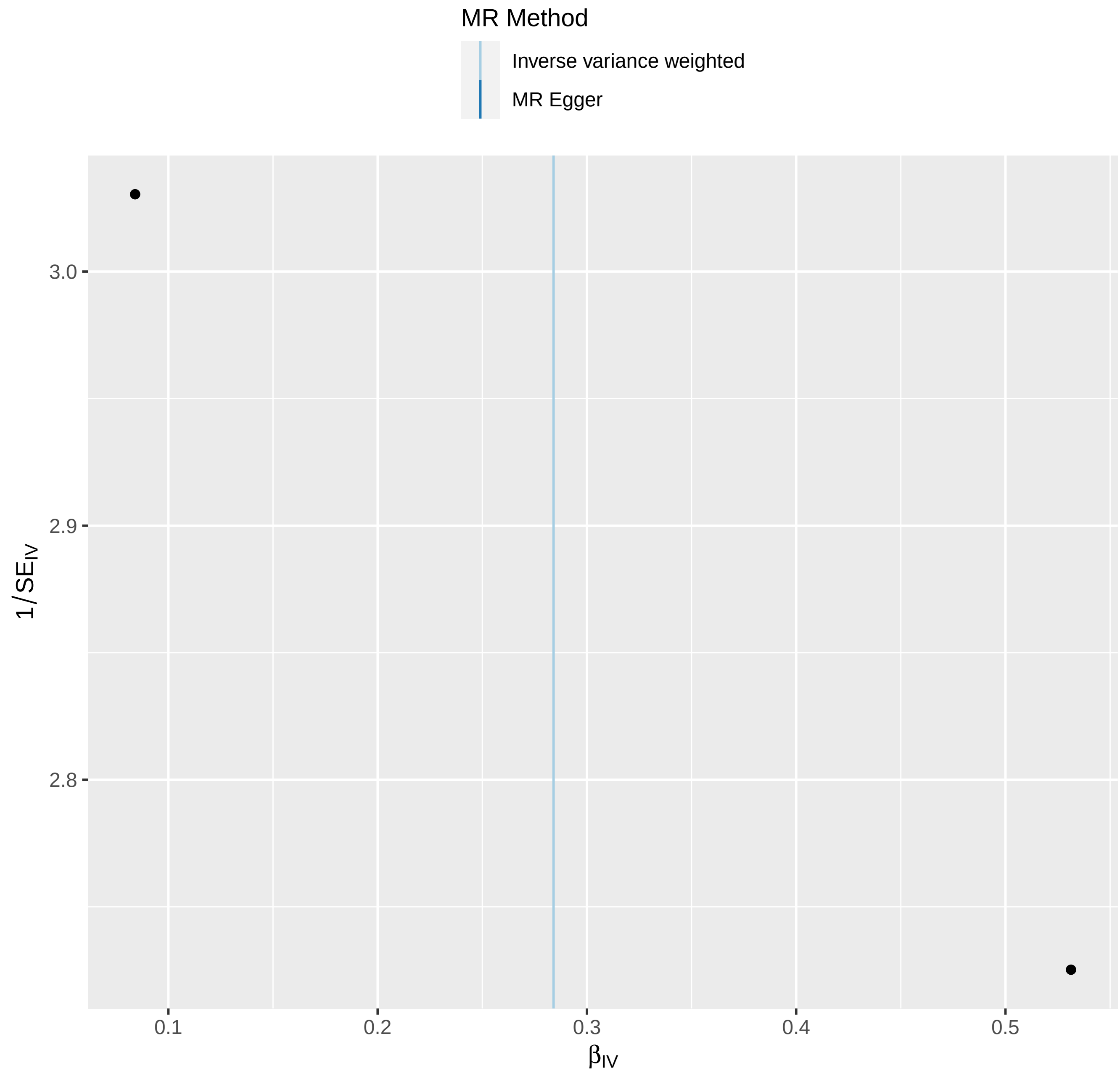

Batch 2856 : IDP T1 FIRST left amygdala volume on Crohn's disease

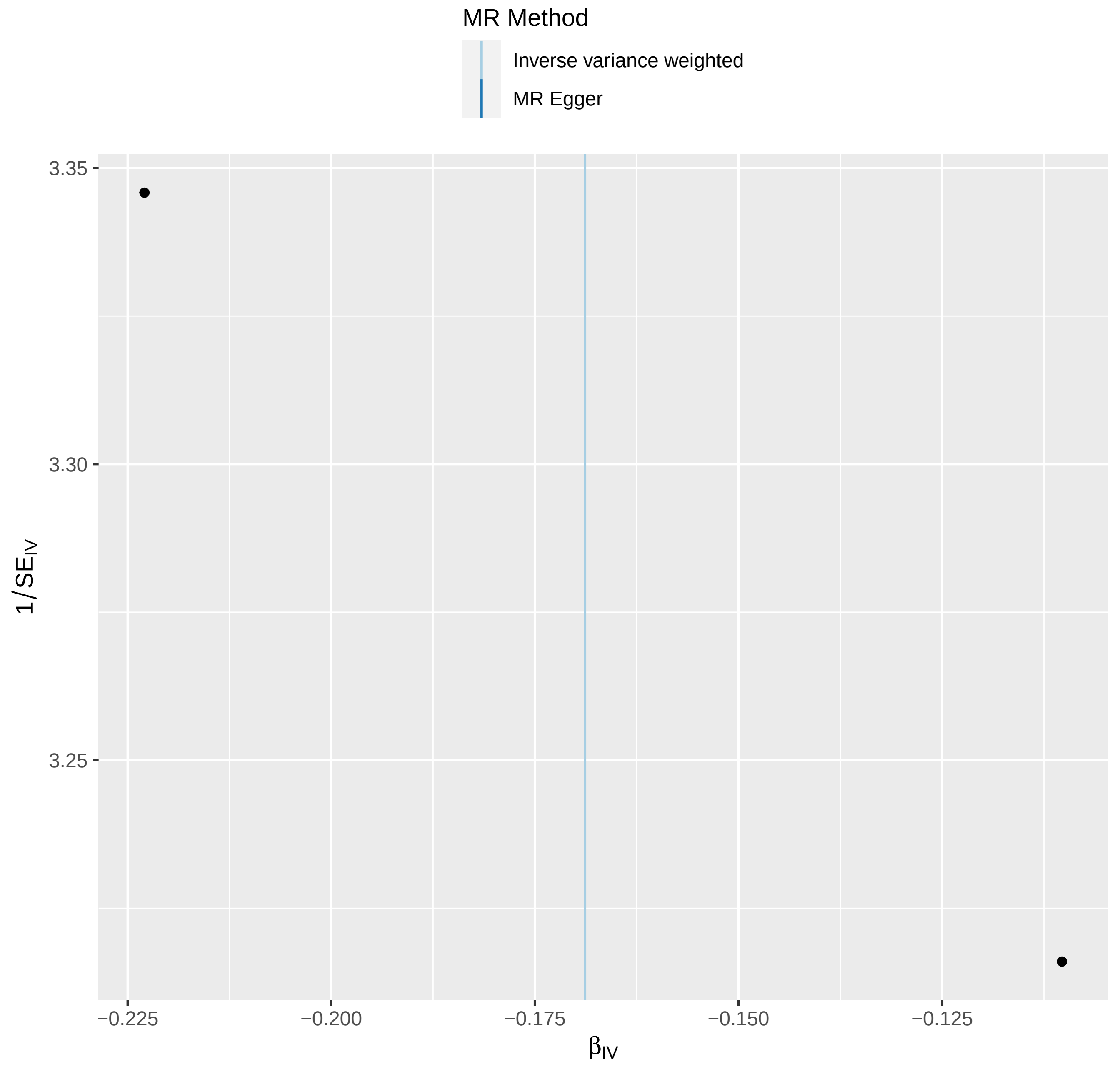

Batch 2889 : IDP T1 FIRST right accumbens volume on Crohn's disease

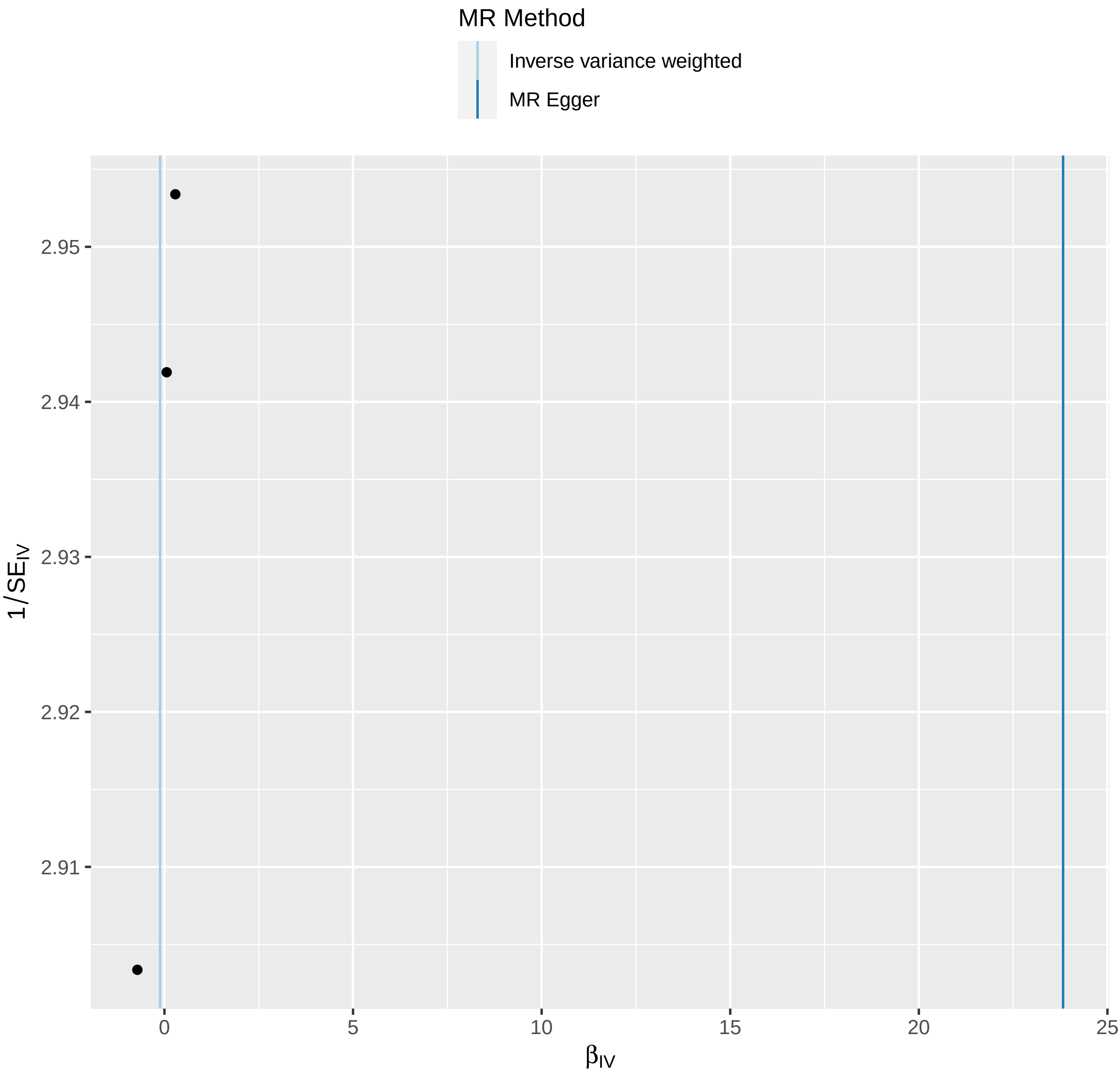

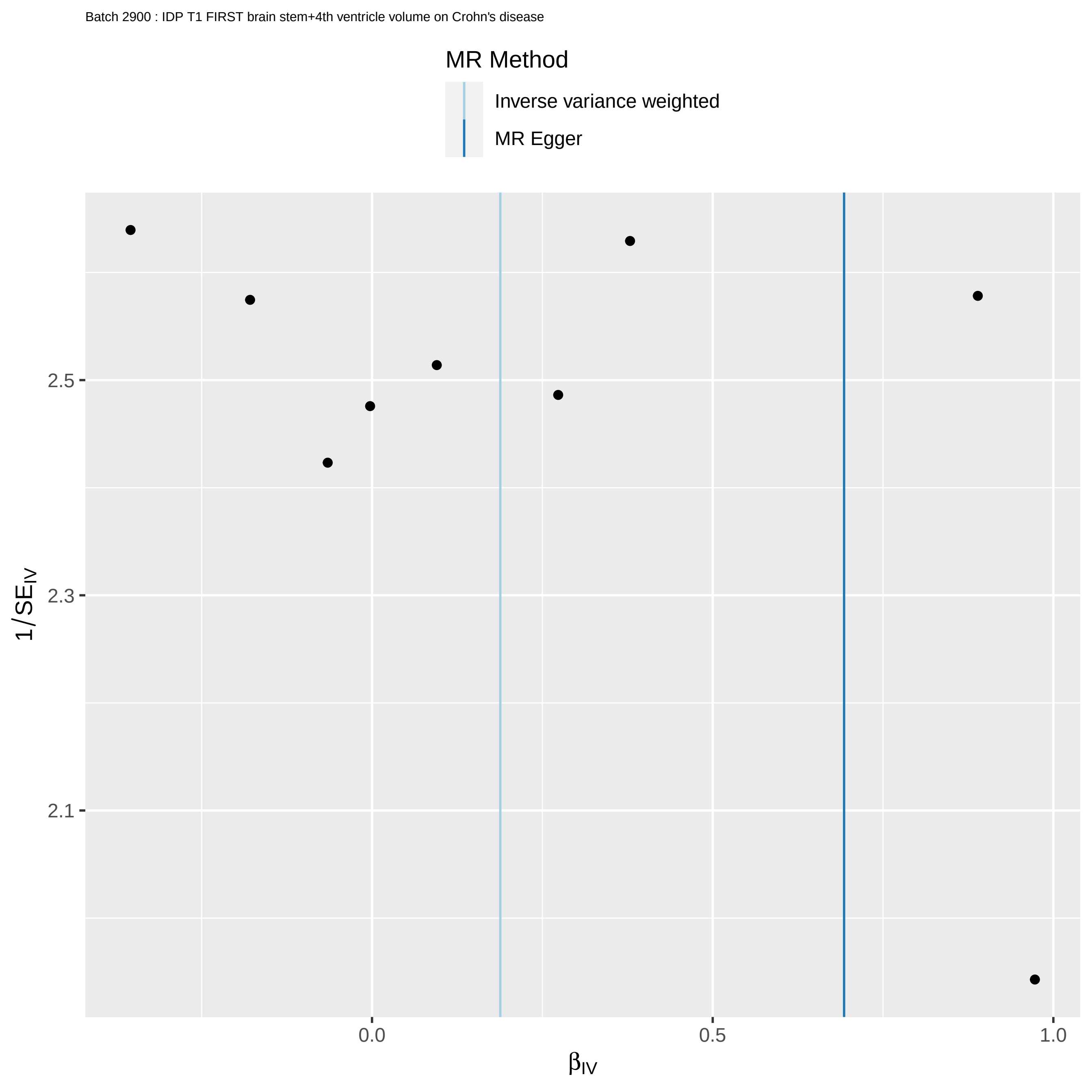

Batch 2900 : IDP T1 FIRST brain stem+4th ventricle volume on Crohn's disease
MR Method
Inverse variance weighted
MR Egger
2.5
2.3
2.1
1/SE$_{IV}$
0.0
0.5
1.0
β$_{IV}$

Batch 2919 : IDP T1 FIRST left putamen volume plus IDP T1 FIRST right putamen volume on Crohn's disease

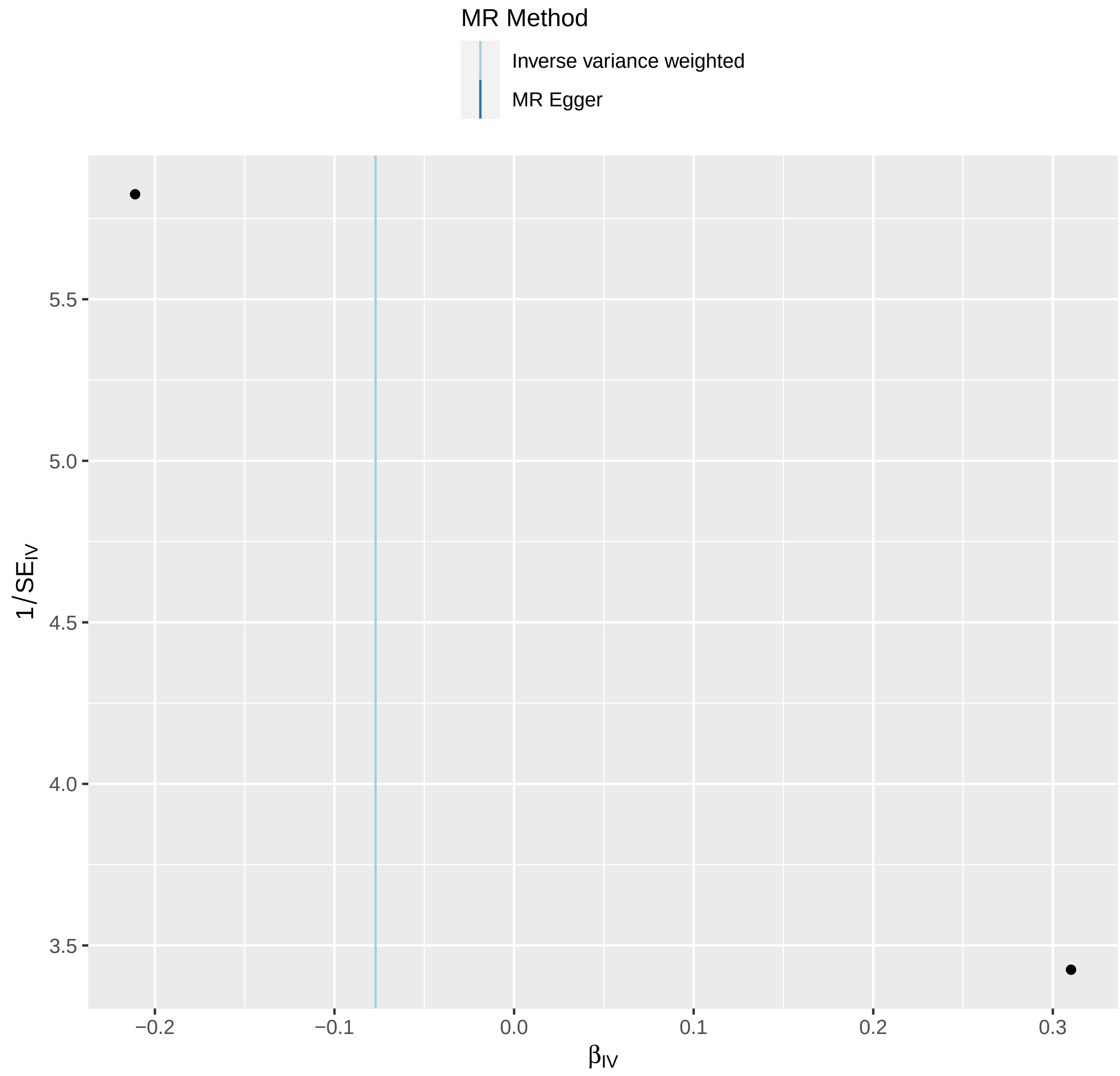

Batch 2921 : IDP T1 FIRST left hippocampus volume plus IDP T1 FIRST right hippocampus volume on Crohn's disease

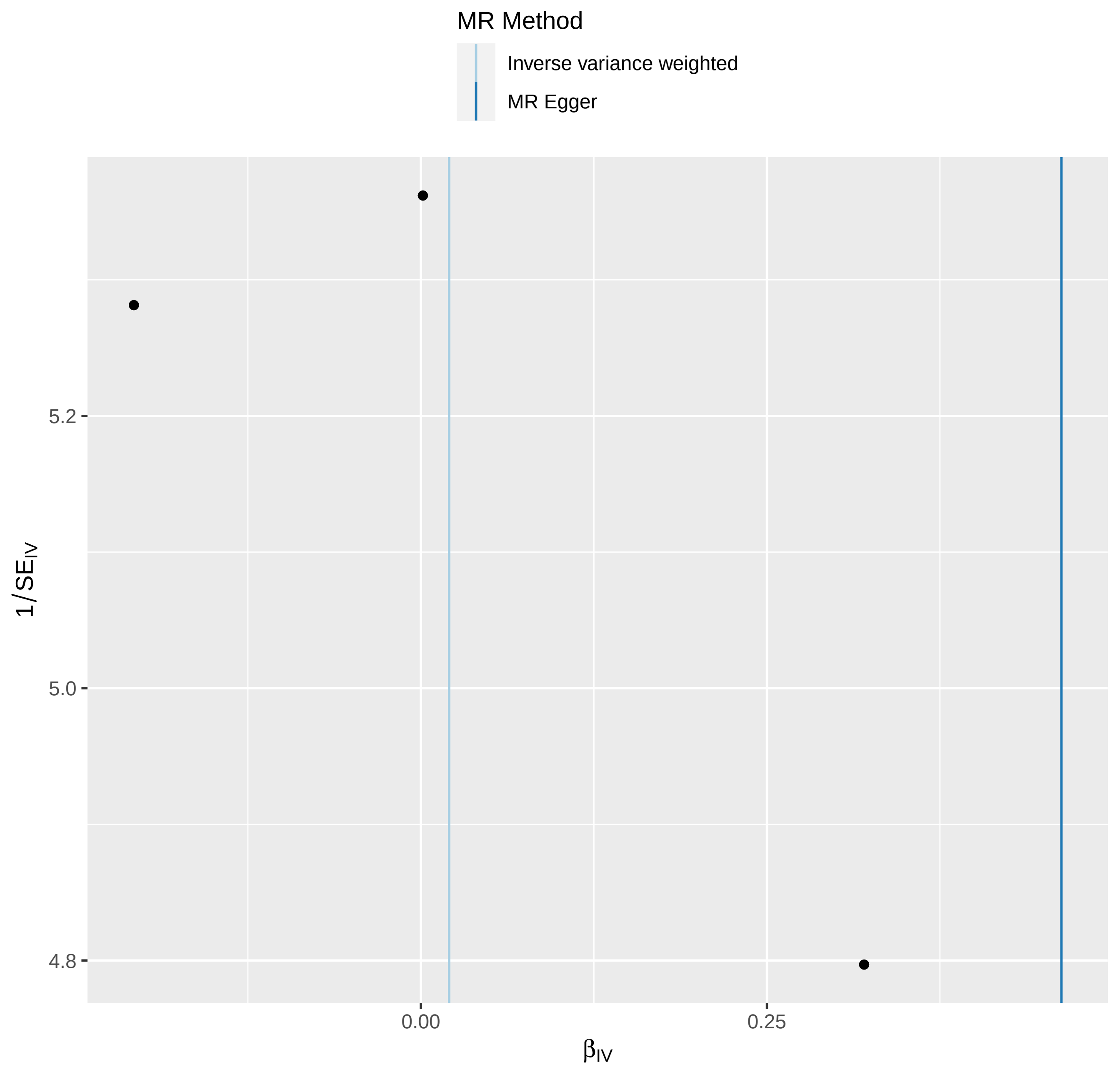

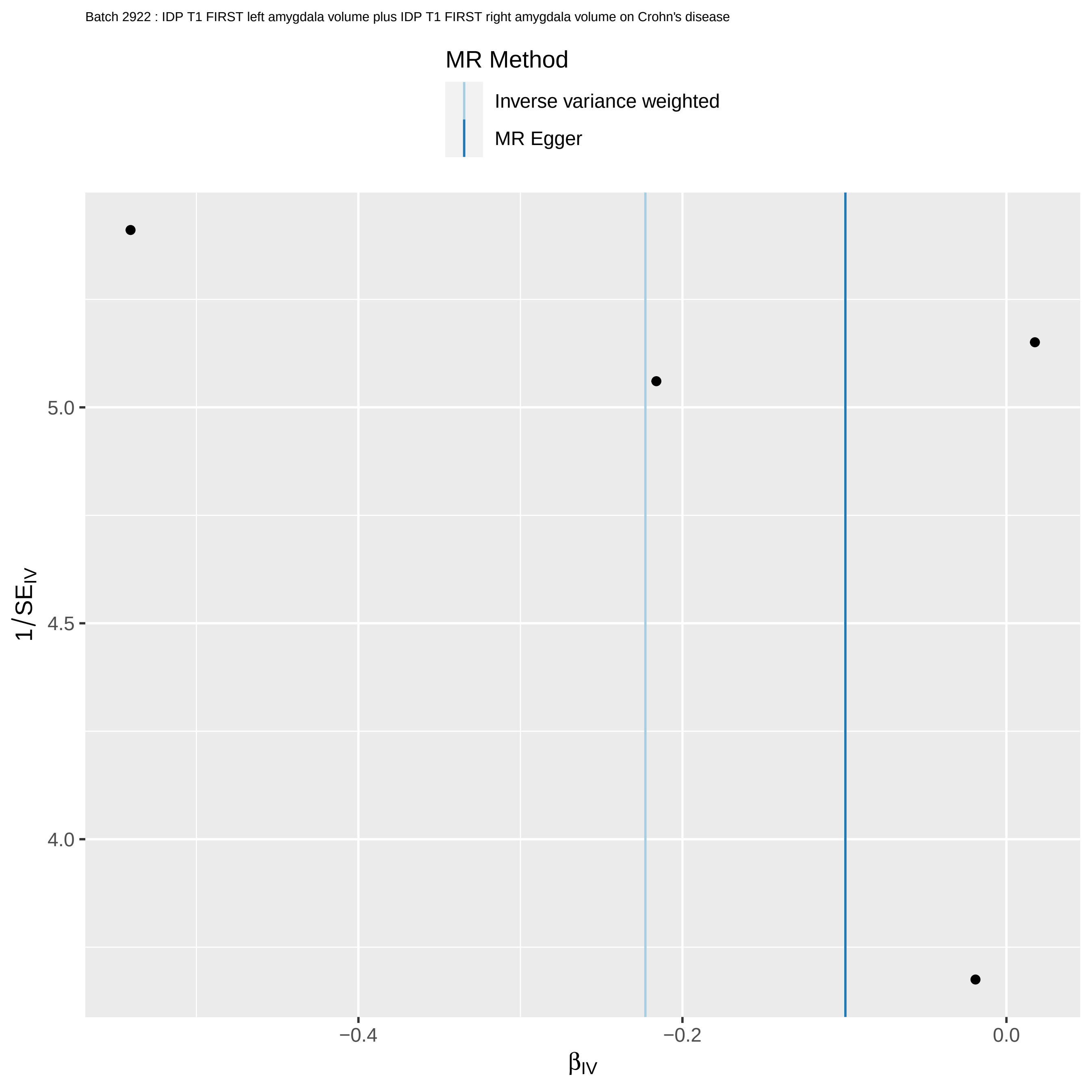
Batch 2922 : IDP T1 FIRST left amygdala volume plus IDP T1 FIRST right amygdala volume on Crohn's disease
MR Method
Inverse variance weighted
MR Egger
5.0
4.5
4.0
1/SE$_{IV}$
−0.4
−0.2
0.0
β_{IV}

Batch 2997 : DKTatlas lh caudalanteriorcingulate area on Crohn's disease

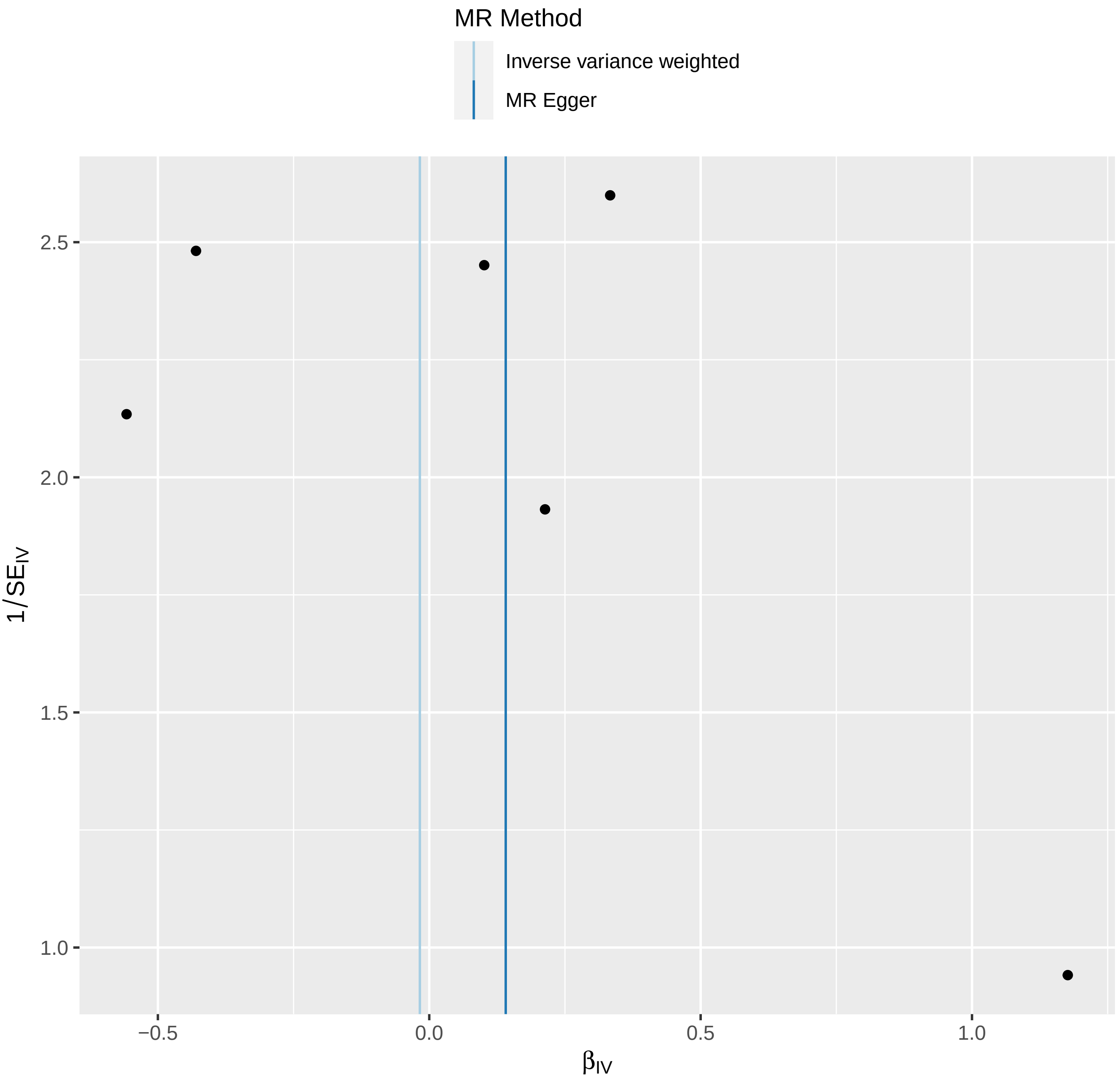

Batch 3006 : DKTatlas lh lateraloccipital area on Crohn's disease

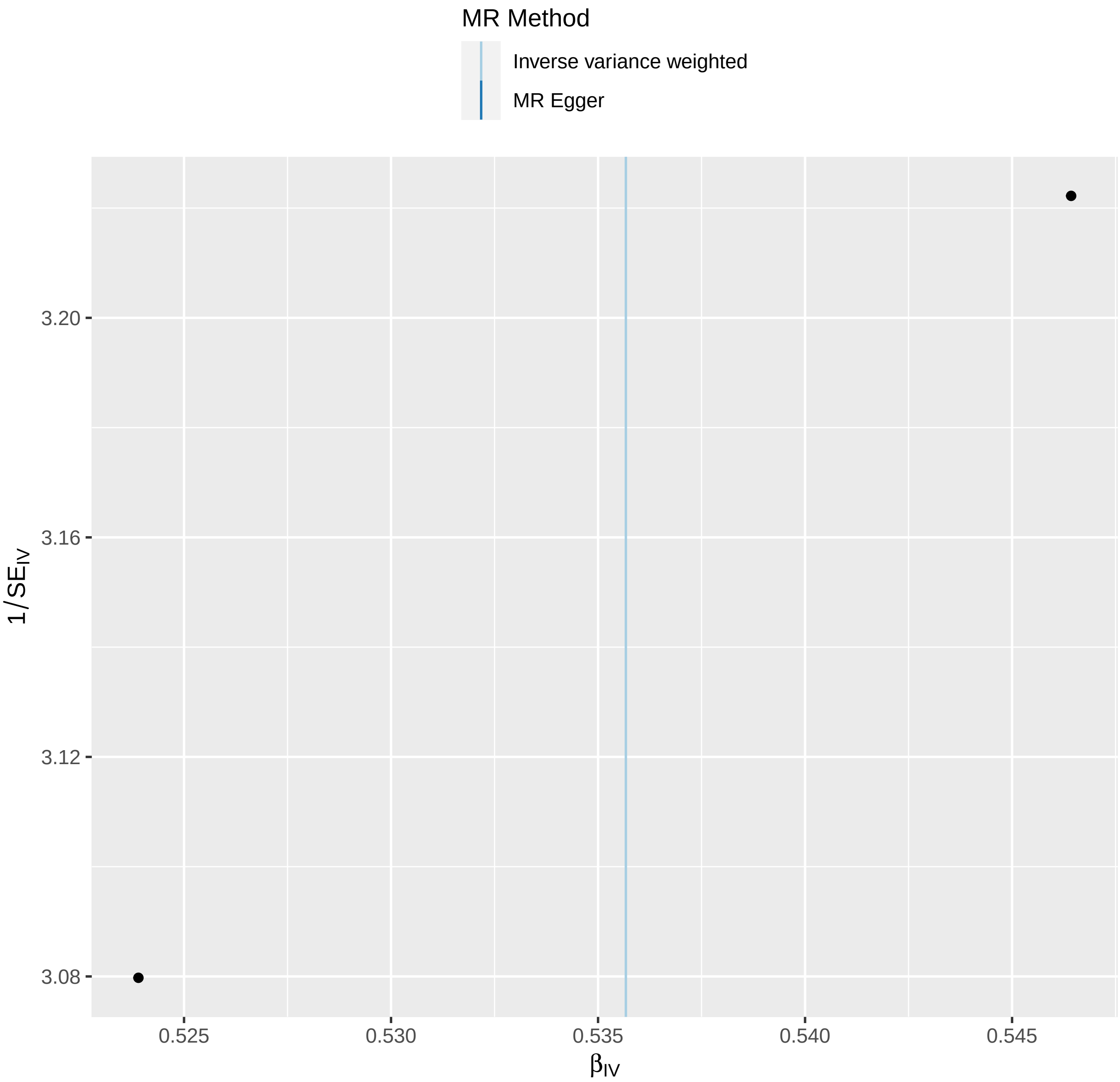

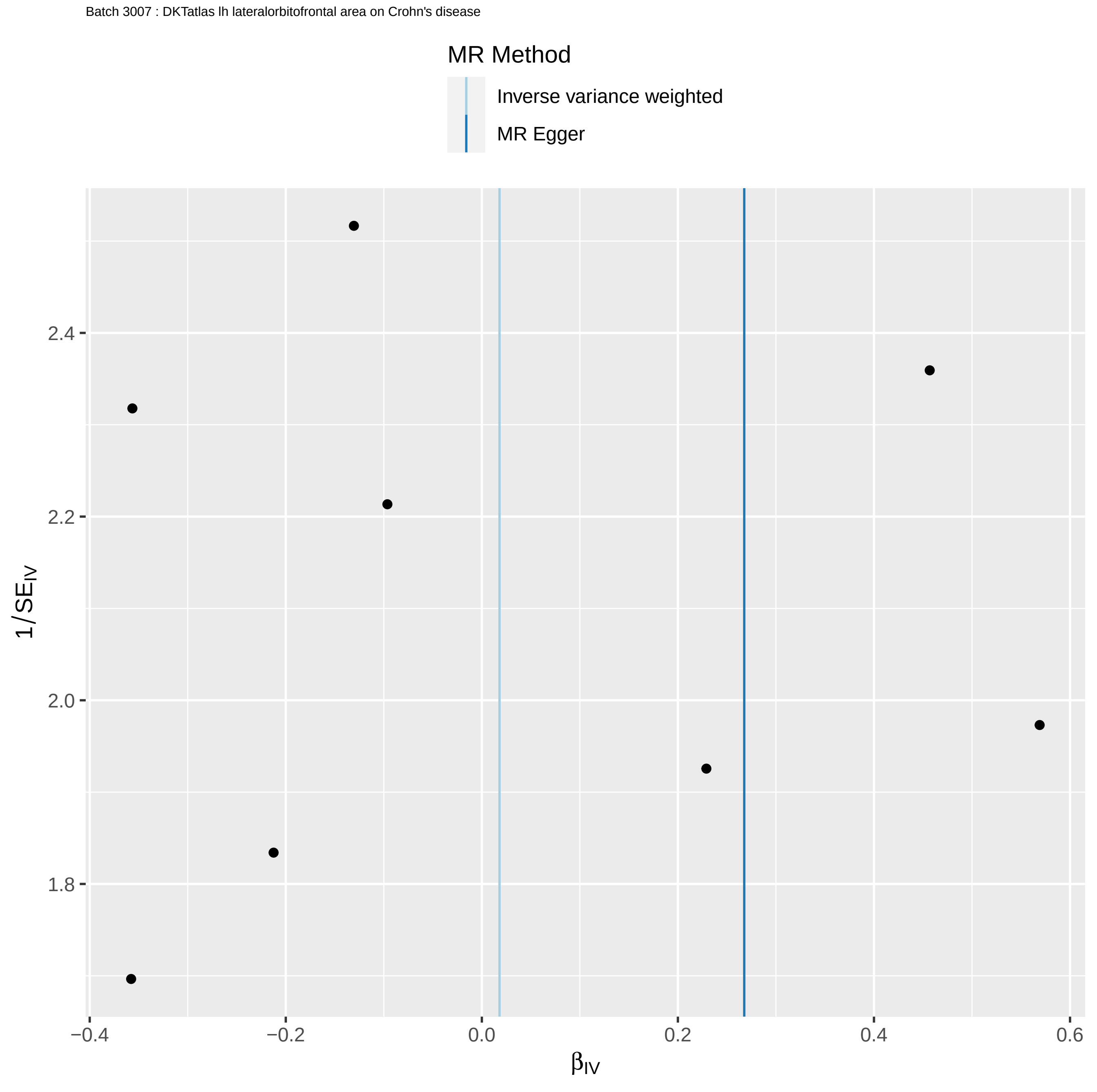
Batch 3007 : DKTatlas lh lateralorbitofrontal area on Crohn's disease
MR Method
Inverse variance weighted
MR Egger
2.4
2.2
2.0
1.8
1/SE$_{IV}$
−0.4
−0.2
0.0
0.2
0.4
0.6
β$_{IV}$

Batch 3009 : DKTatlas lh medialorbitofrontal area on Crohn's disease

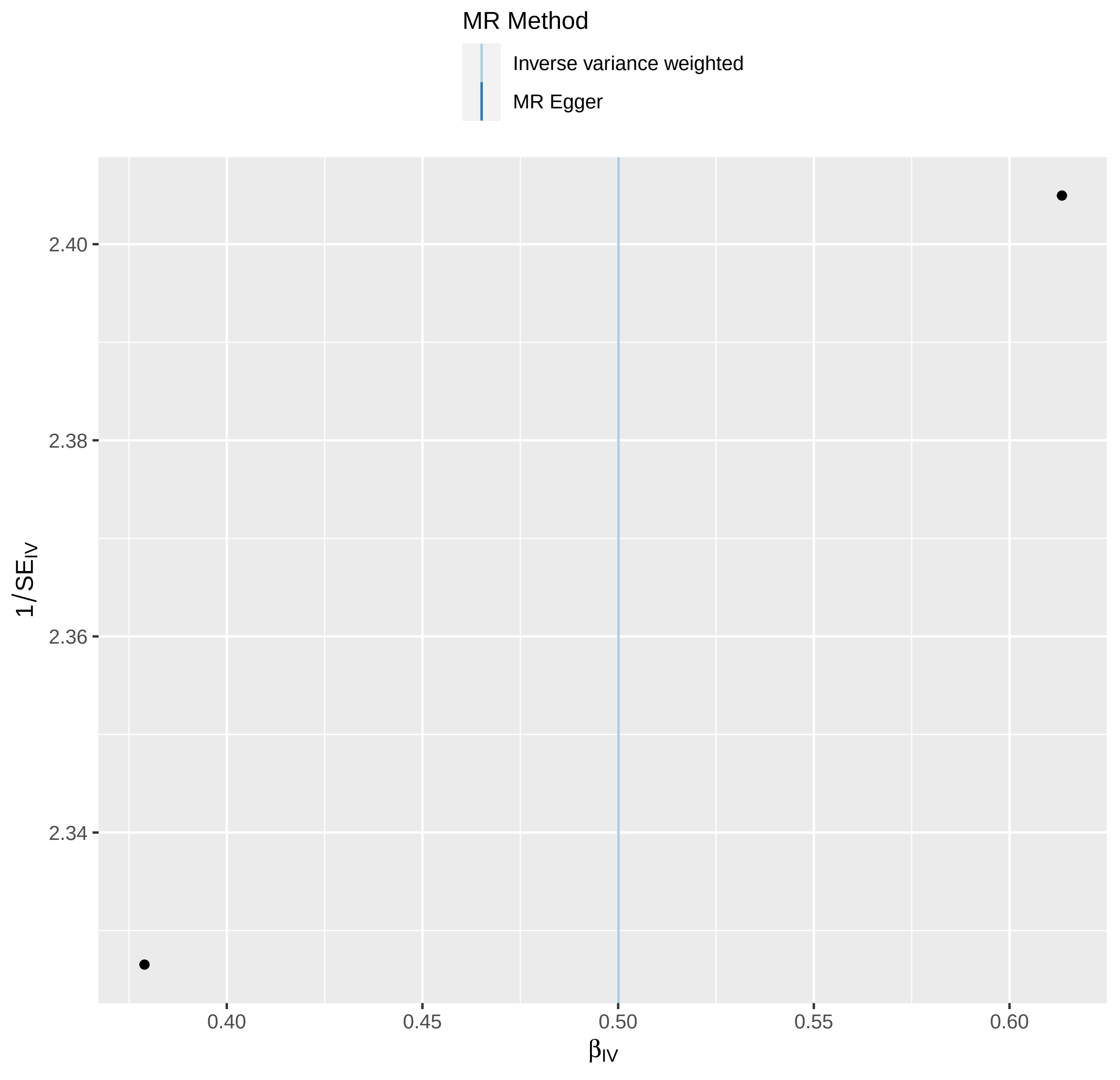

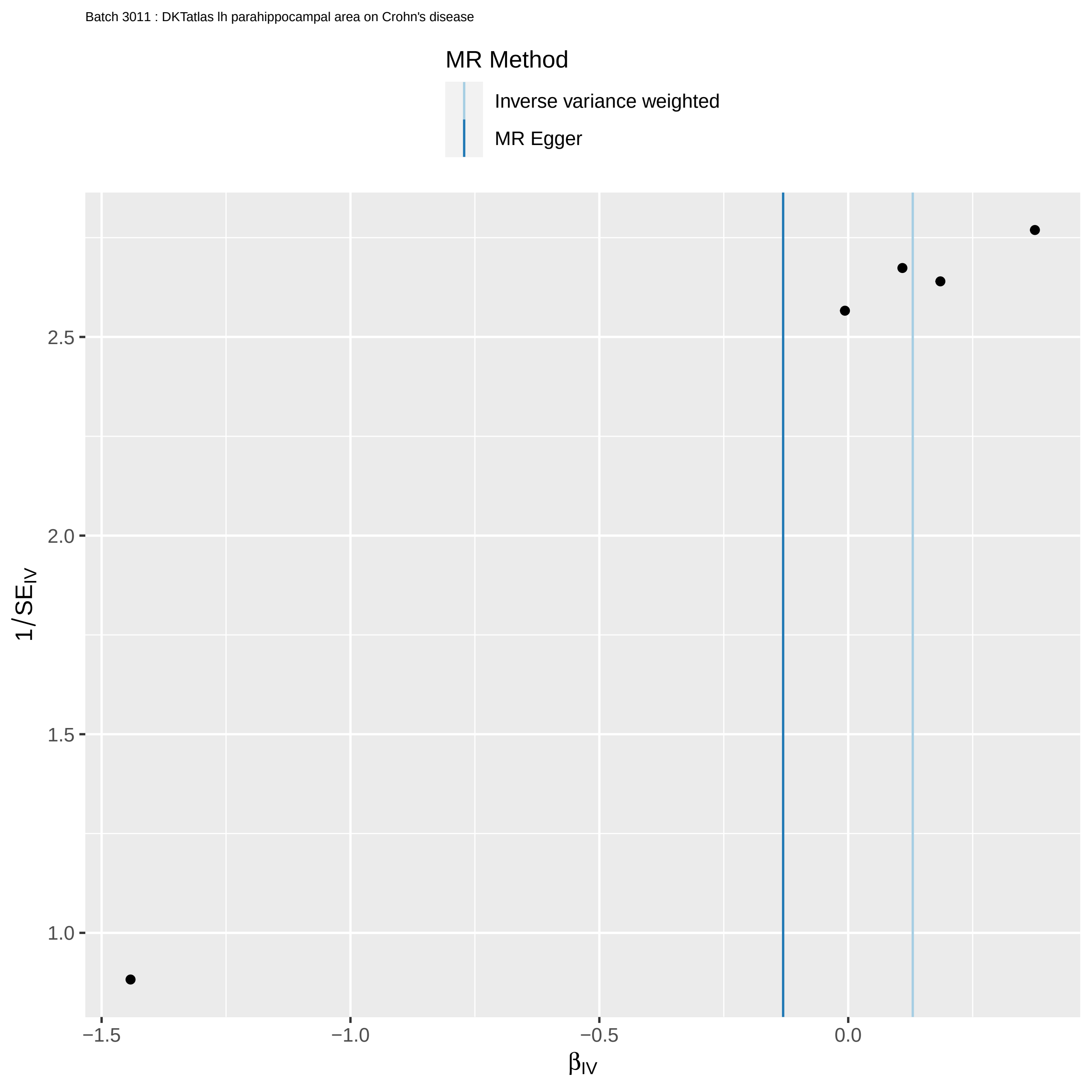
Batch 3011 : DKTatlas lh parahippocampal area on Crohn's disease
MR Method
Inverse variance weighted
MR Egger
2.5
2.0
1.5
1.0
1/SE$_{IV}$
−1.5
−1.0
−0.5
0.0
β$_{IV}$

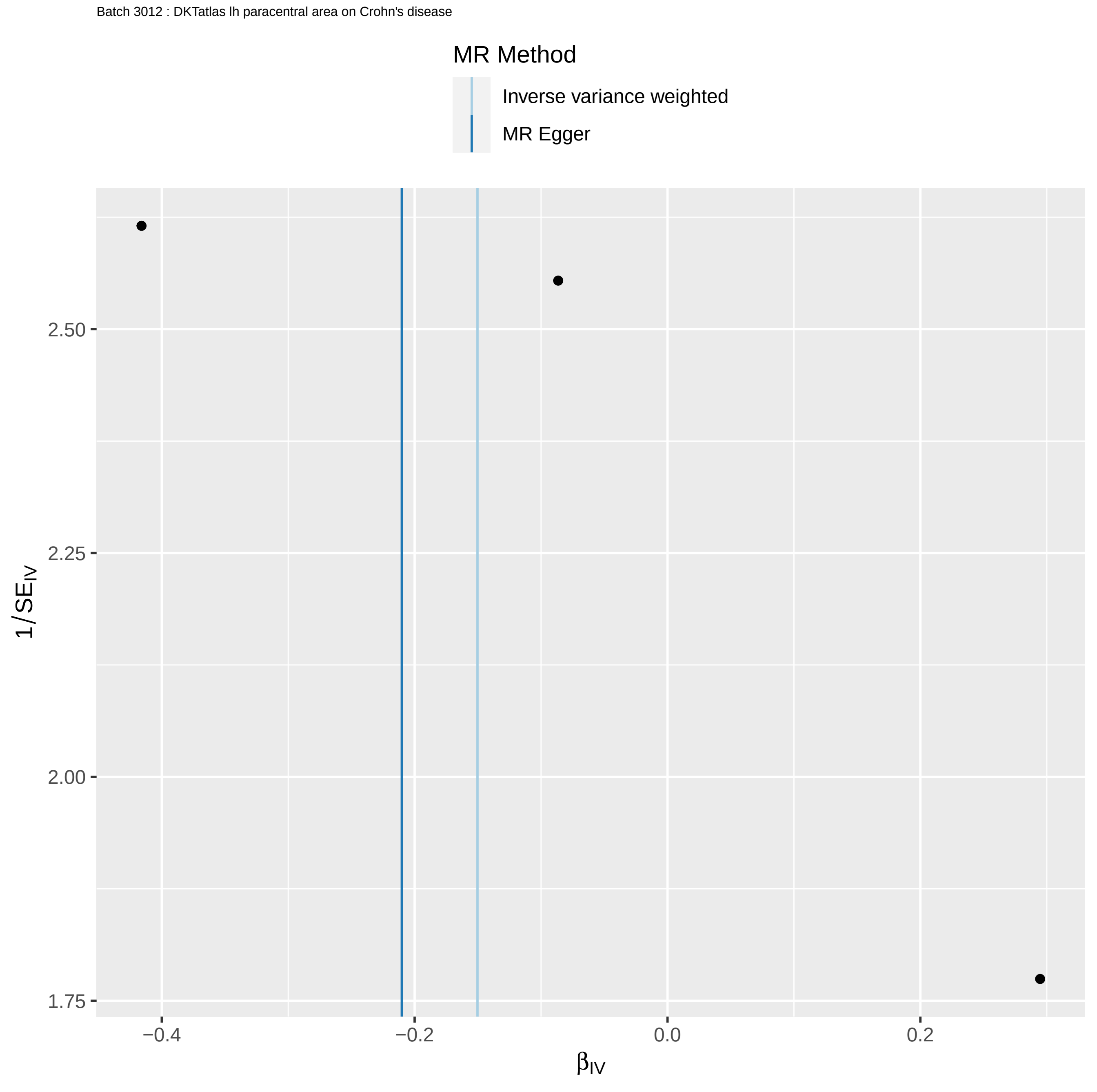
Batch 3012 : DKTatlas lh paracentral area on Crohn's disease
MR Method
Inverse variance weighted
MR Egger
2.50
2.25
2.00
1.75
1/SE_IV
−0.4
−0.2
0.0
0.2
β_IV

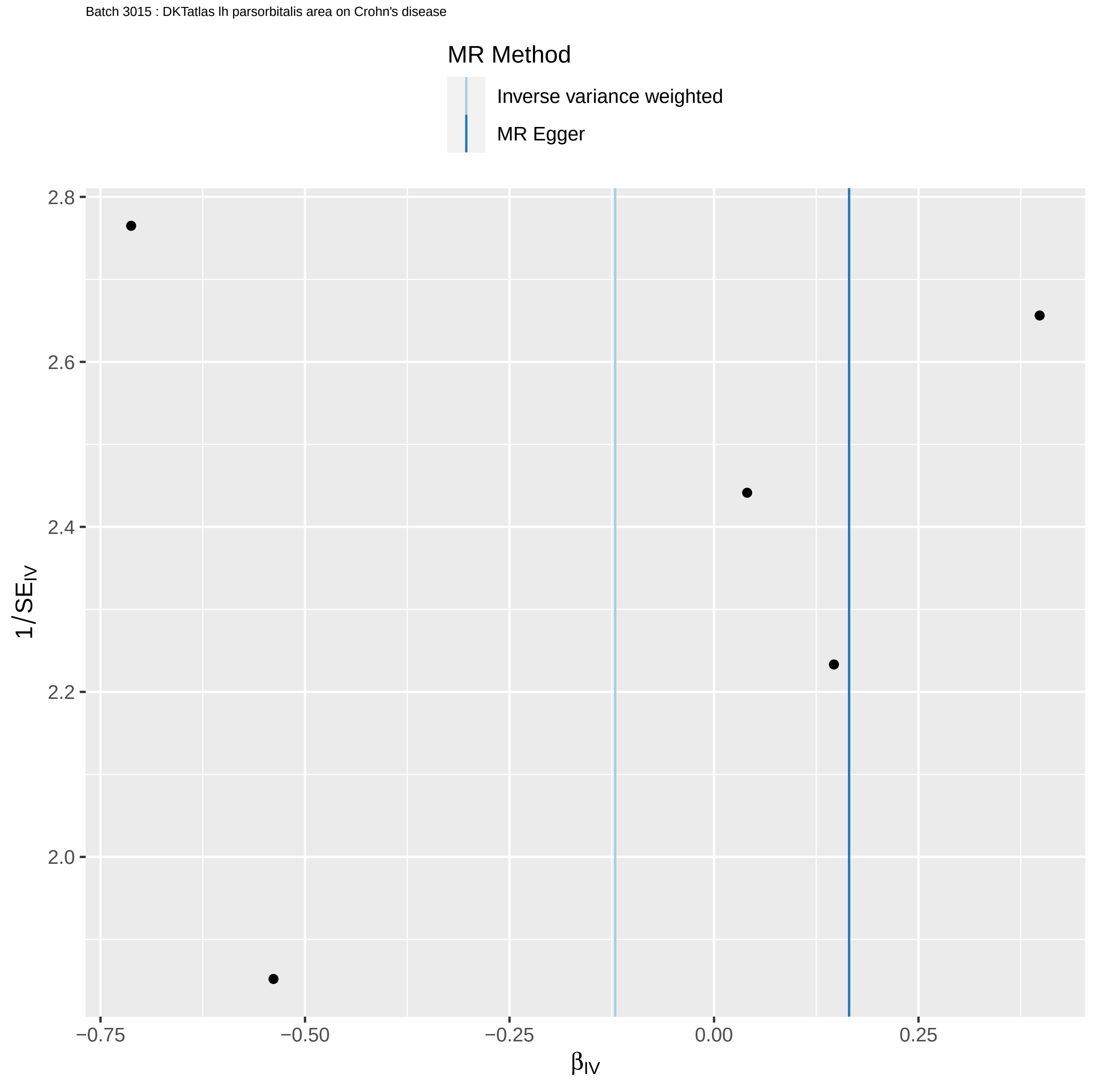
Batch 3015 : DKTatlas lh parsorbitalis area on Crohn's disease
MR Method
Inverse variance weighted
MR Egger
2.8
2.6
2.4
2.2
2.0
1/SE_IV
−0.75
−0.50
−0.25
0.00
0.25
β_IV

Batch 3018 : DKTatlas lh postcentral area on Crohn's disease

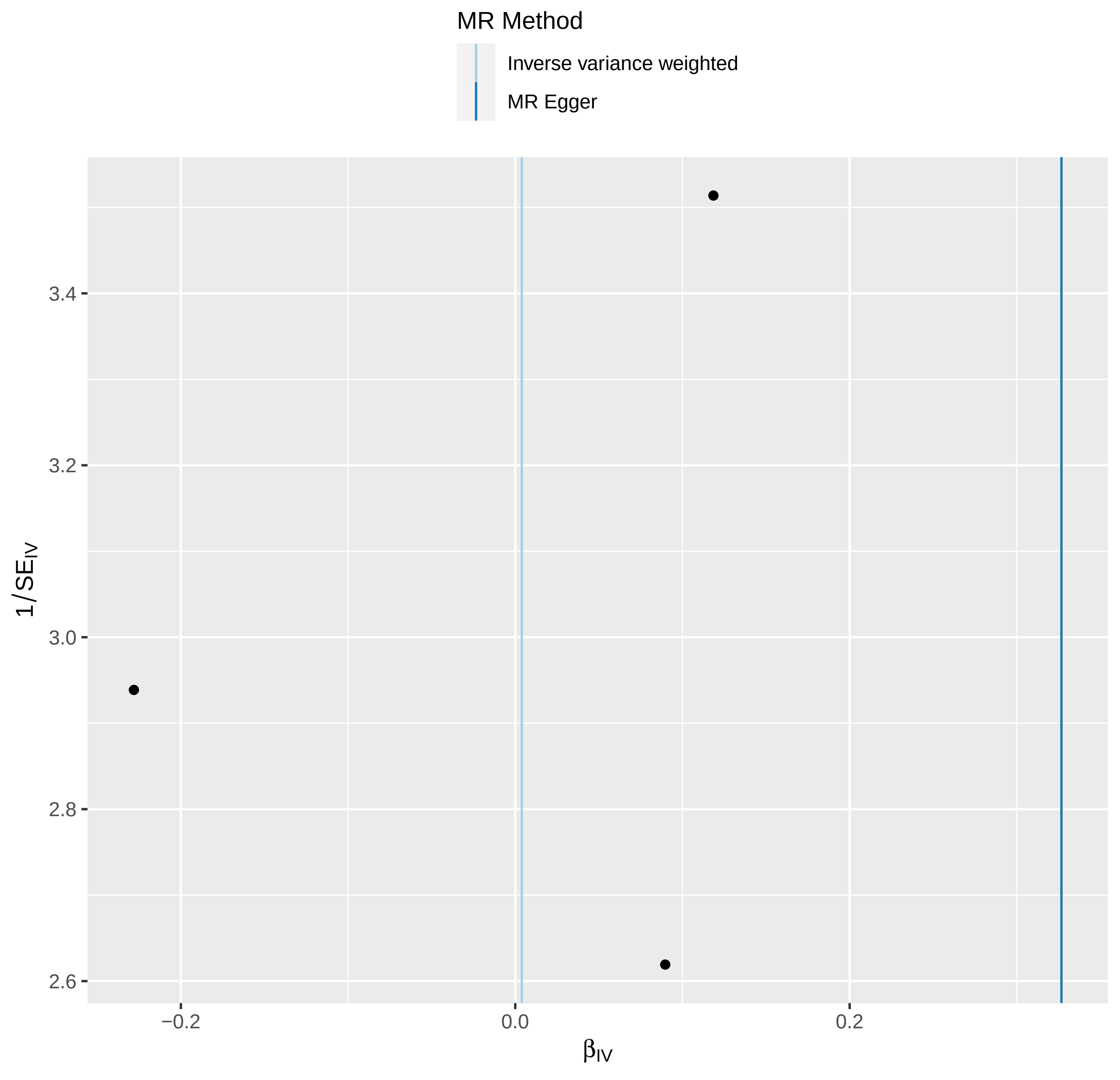

Batch 3021 : DKTatlas lh precuneus area on Crohn's disease

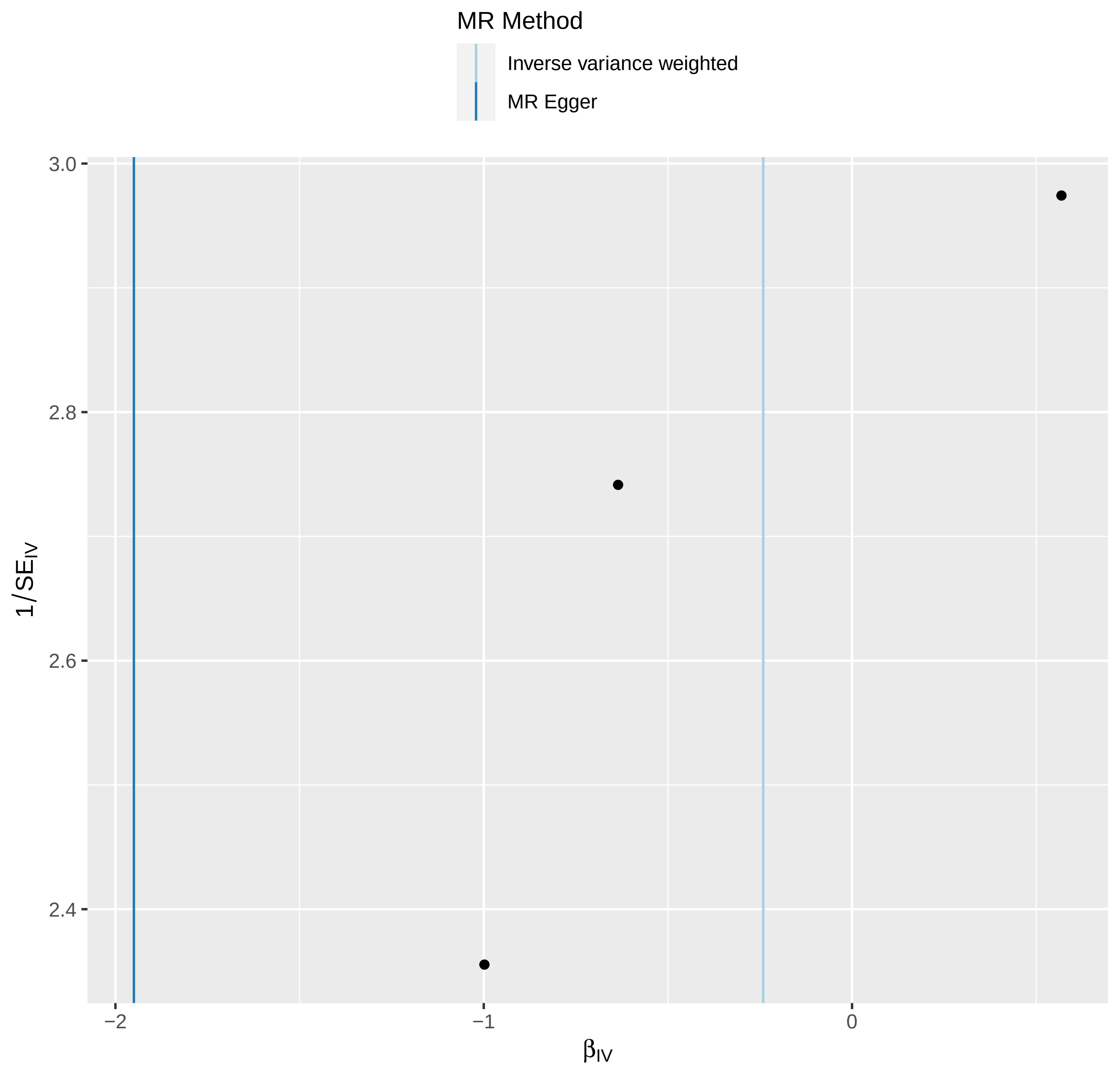

Batch 3022 : DKTatlas lh rostralanteriorcingulate area on Crohn's disease

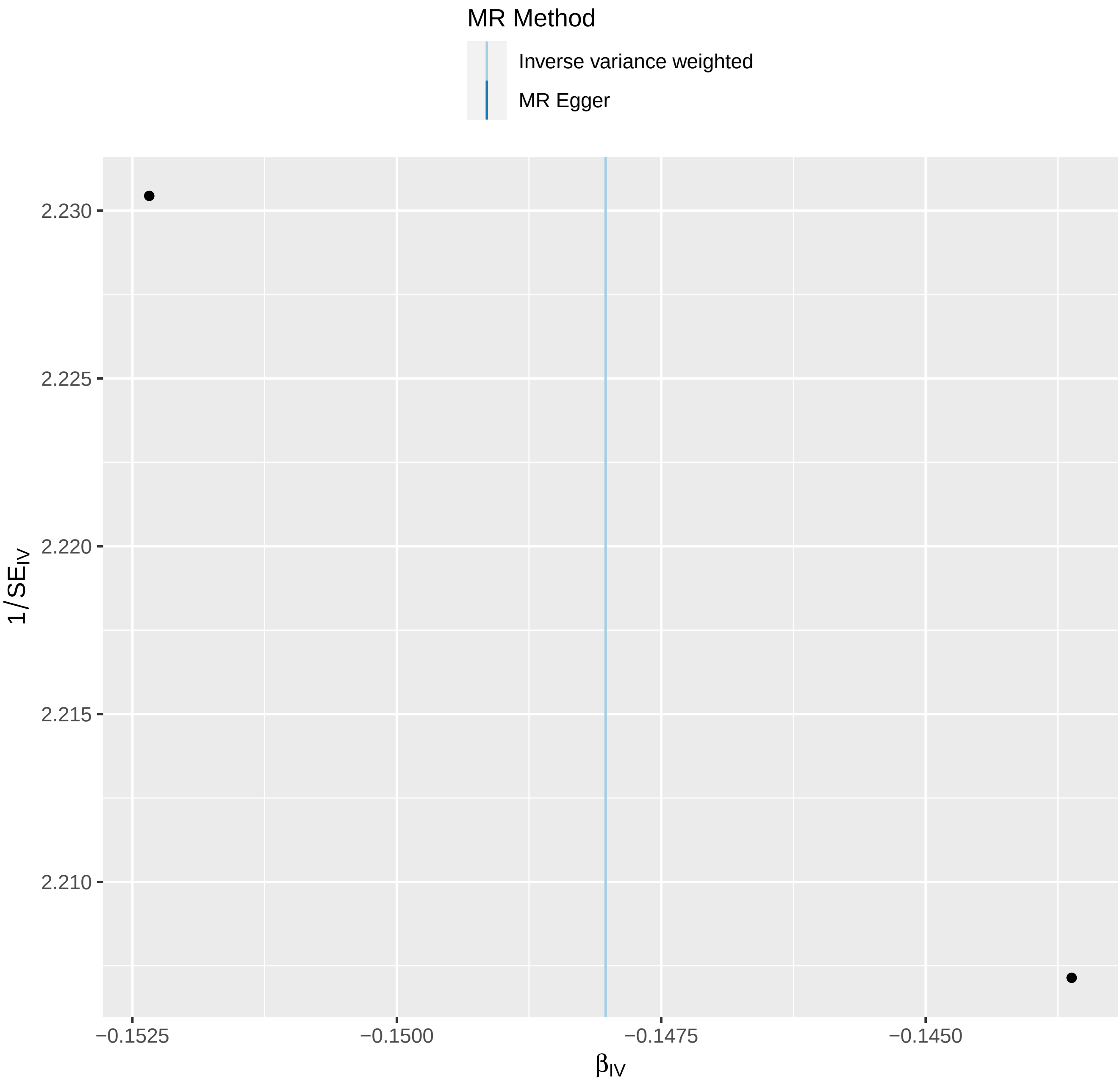

Batch 3023 : DKTatlas lh rostralmiddlefrontal area on Crohn's disease

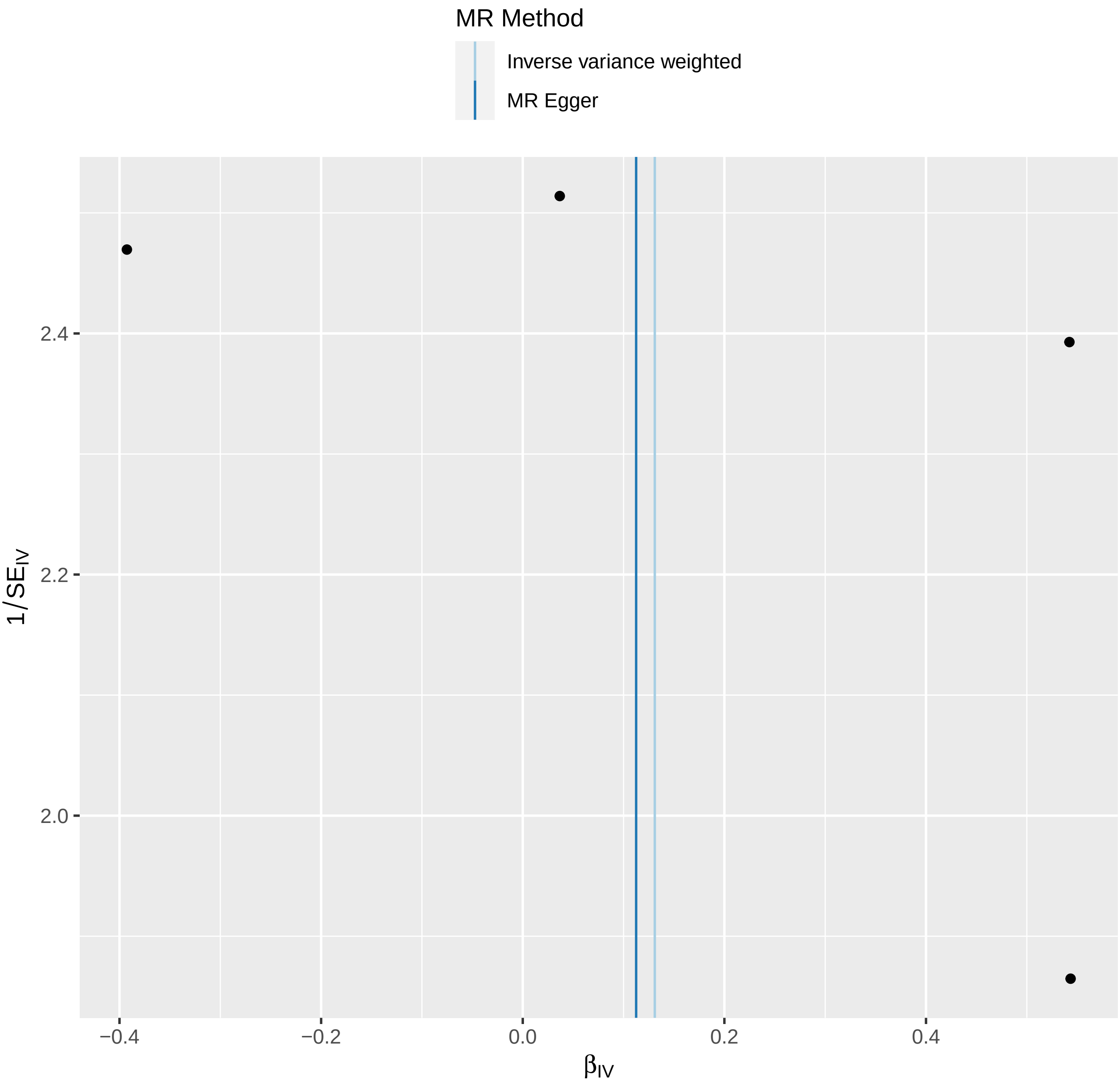

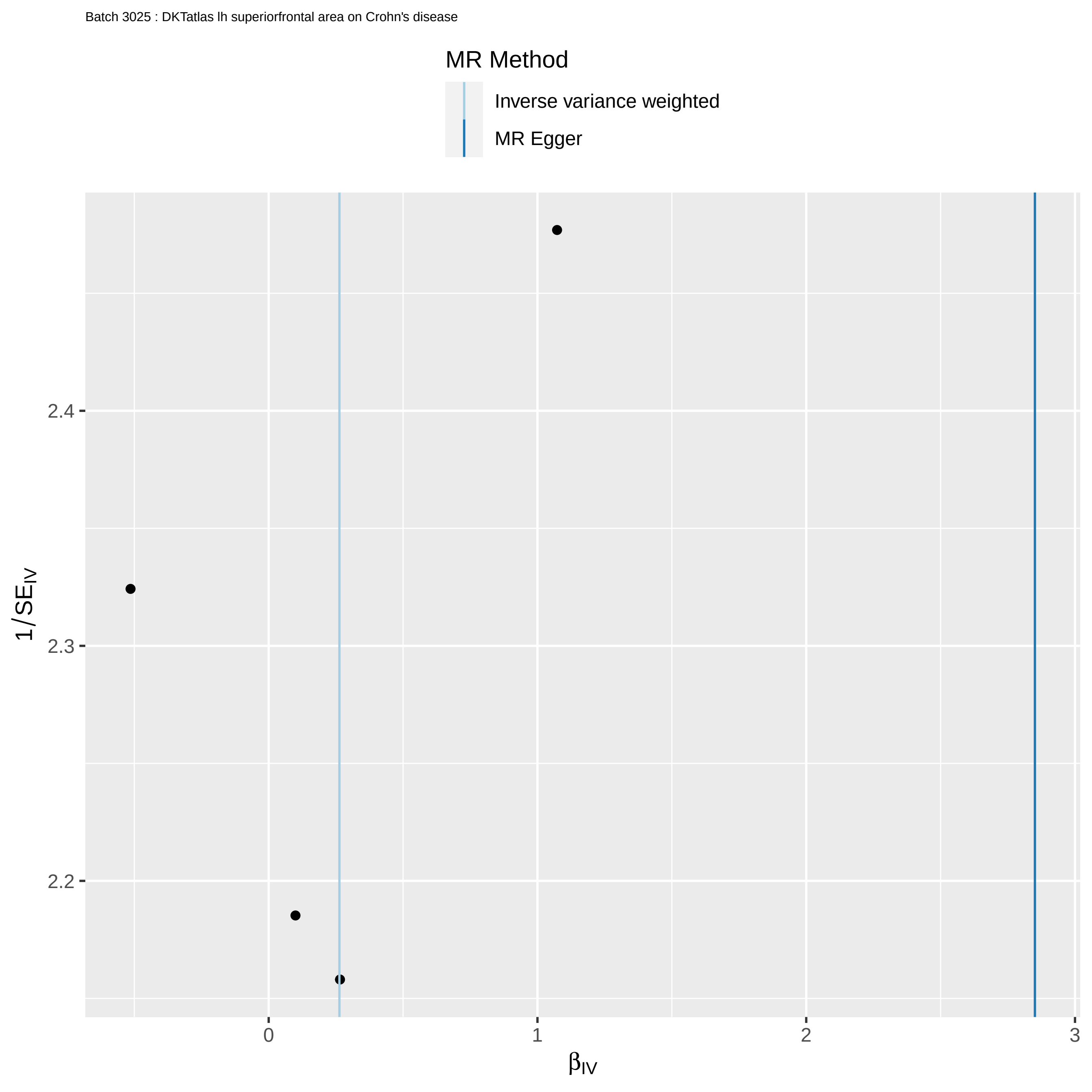
Batch 3025 : DKTatlas lh superiorfrontal area on Crohn's disease
MR Method
Inverse variance weighted
MR Egger
2.4
2.3
2.2
1/SE$_{IV}$
0
1
2
3
β_{IV}

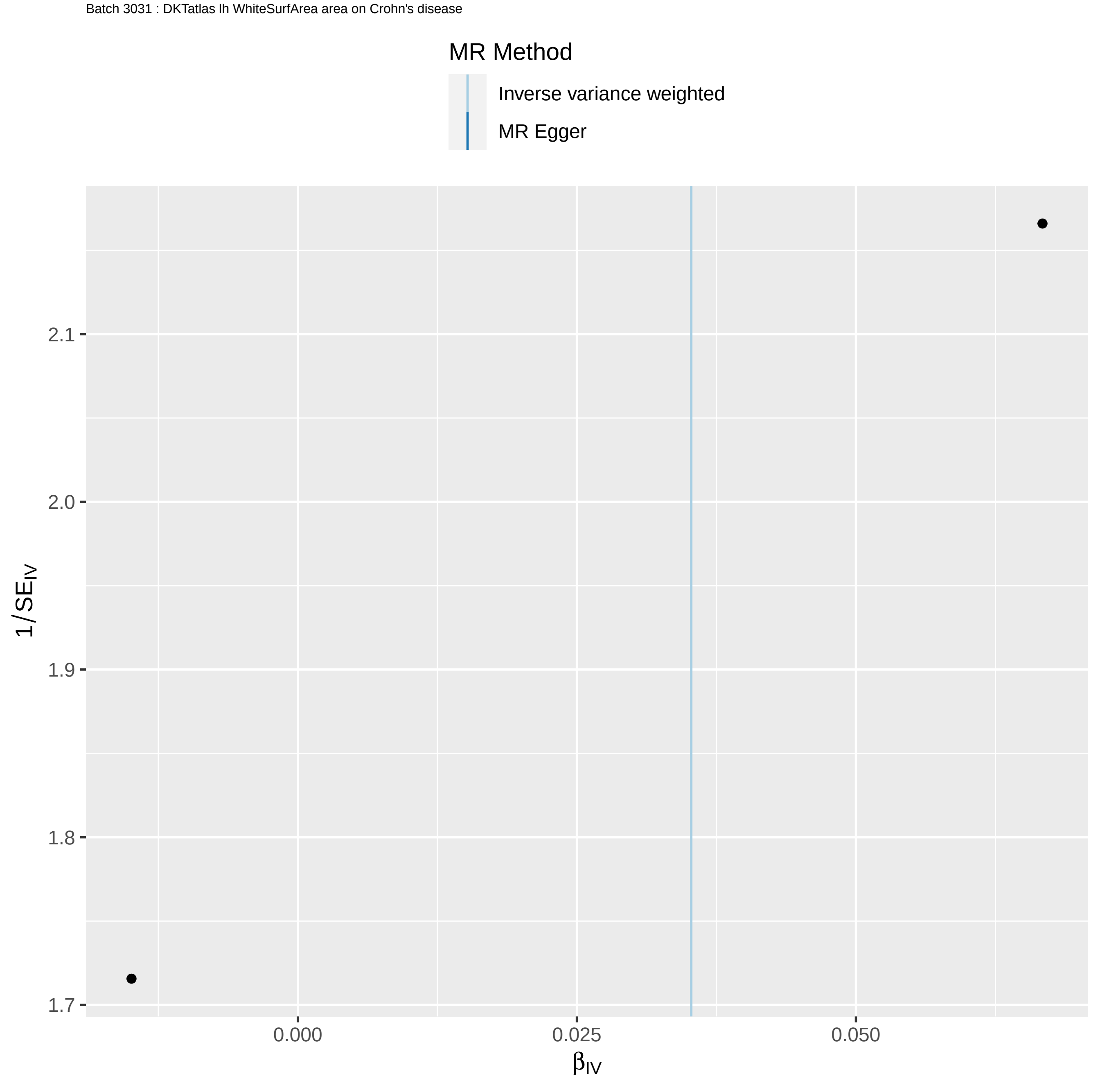

Batch 3031 : DKTatlas lh WhiteSurfArea area on Crohn's disease
MR Method
Inverse variance weighted
MR Egger
2.1
2.0
1.9
1.8
1.7
$1/SE_{IV}$
0.000
0.025
0.050
β_{IV}

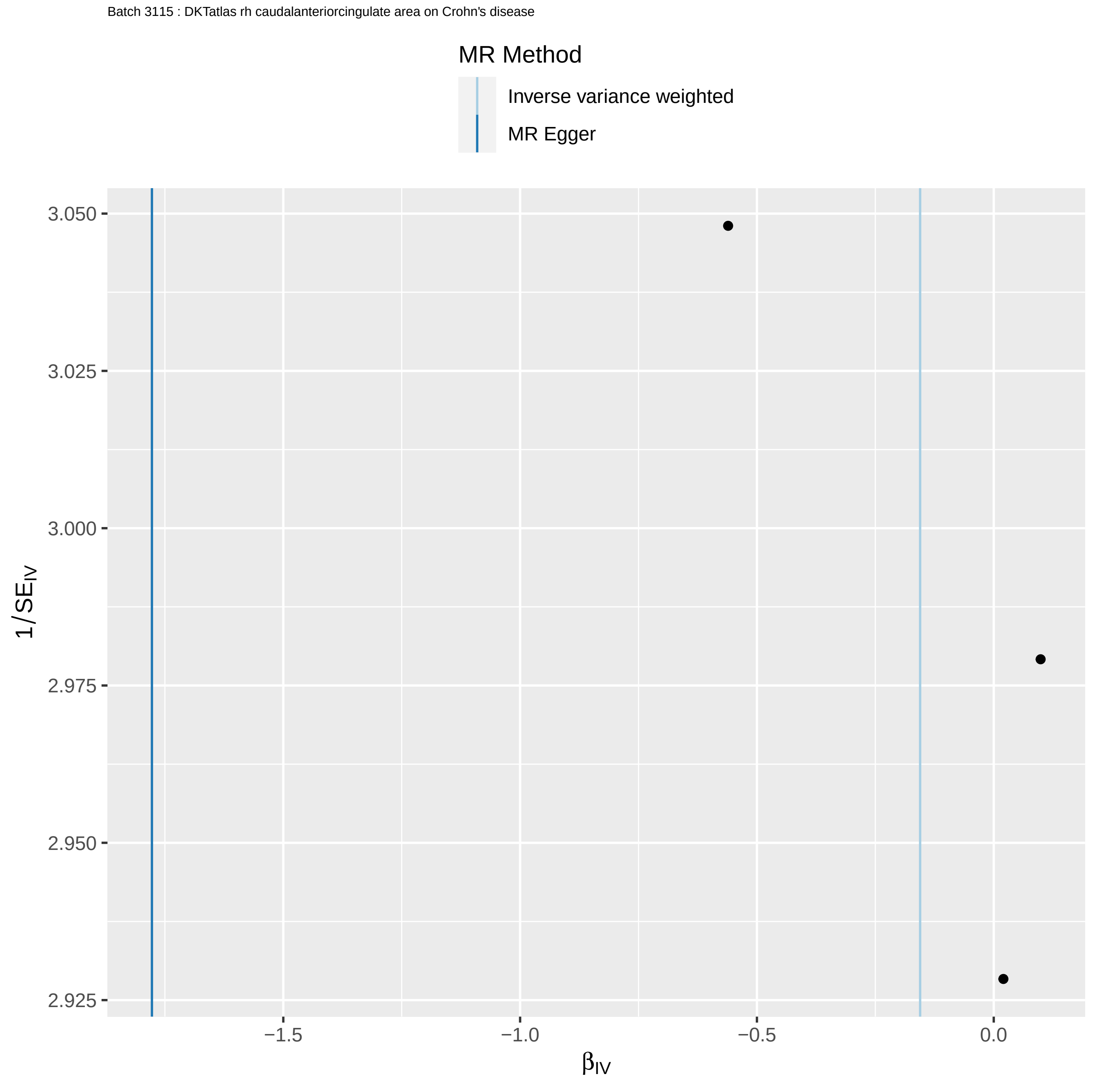
Batch 3115 : DKTatlas rh caudalanteriorcingulate area on Crohn's disease
MR Method
Inverse variance weighted
MR Egger
3.050
3.025
3.000
2.975
2.950
2.925
1/SE_IV
−1.5
−1.0
−0.5
0.0
β_IV

Batch 3118 : DKTatlas rh entorhinal area on Crohn's disease

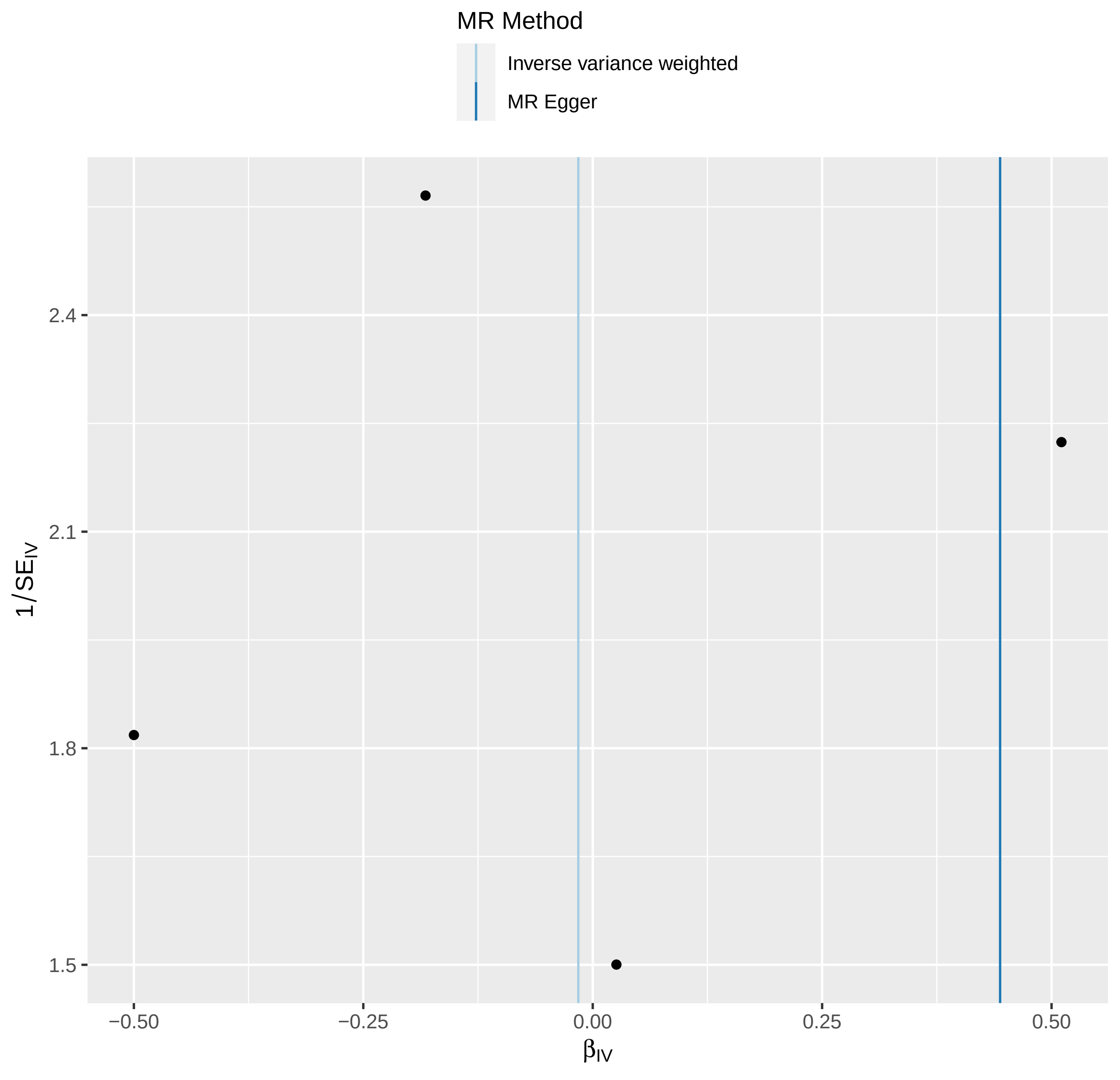

Batch 3122 : DKTatlas rh isthmuscingulate area on Crohn's disease

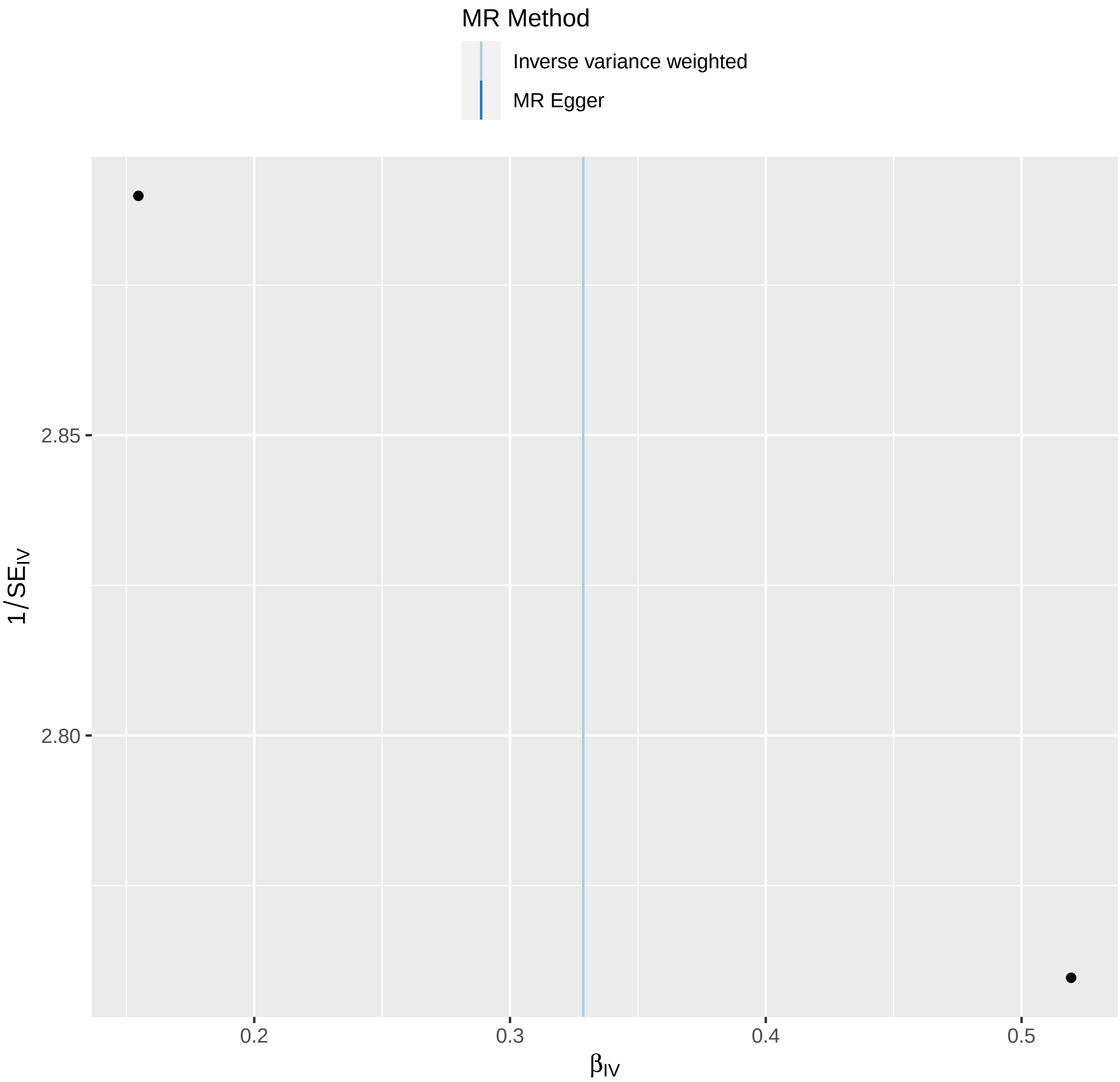

Batch 3128 : DKTatlas rh middletemporal area on Crohn's disease

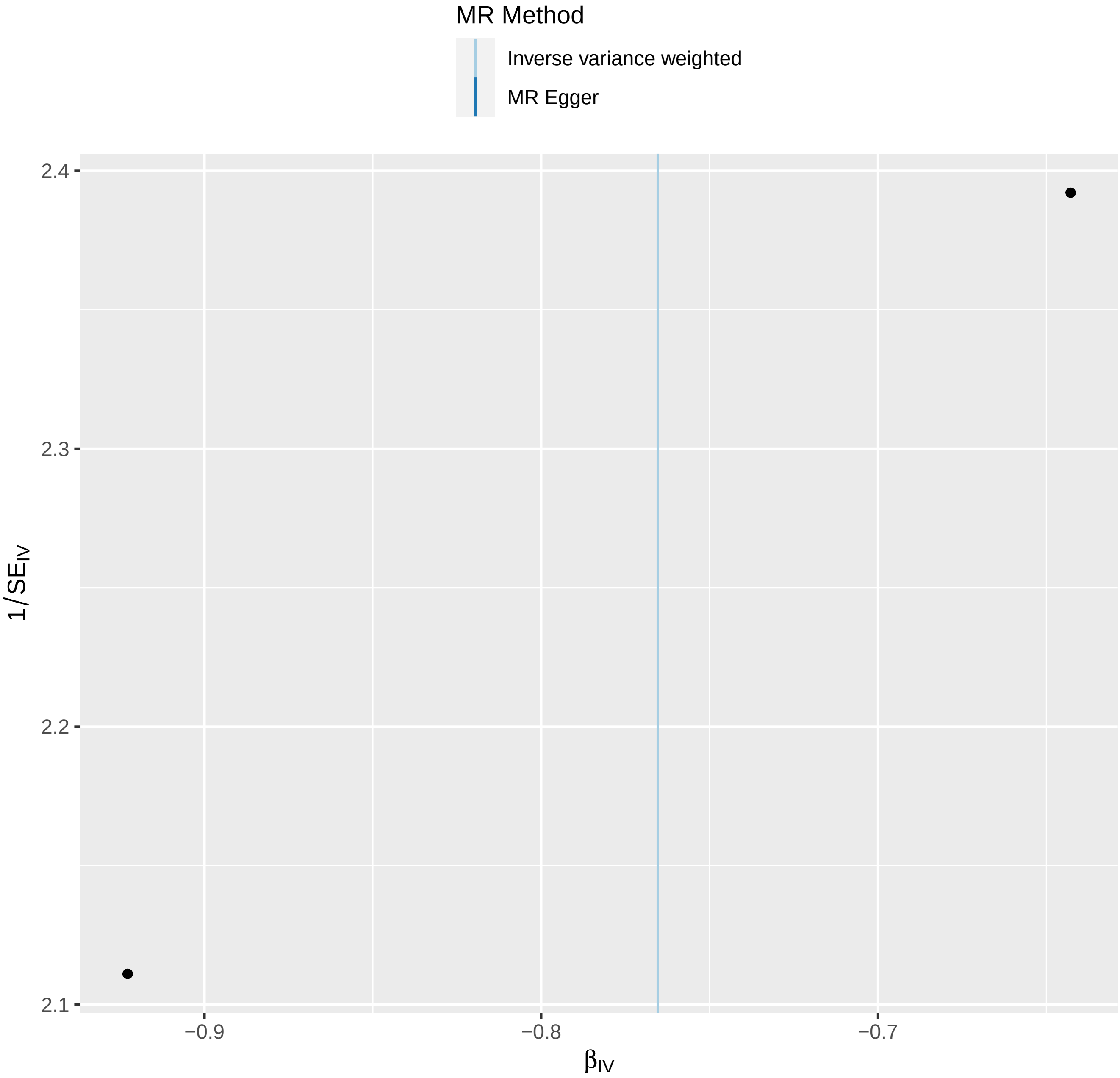

Batch 3131 : DKTatlas rh parsopercularis area on Crohn's disease

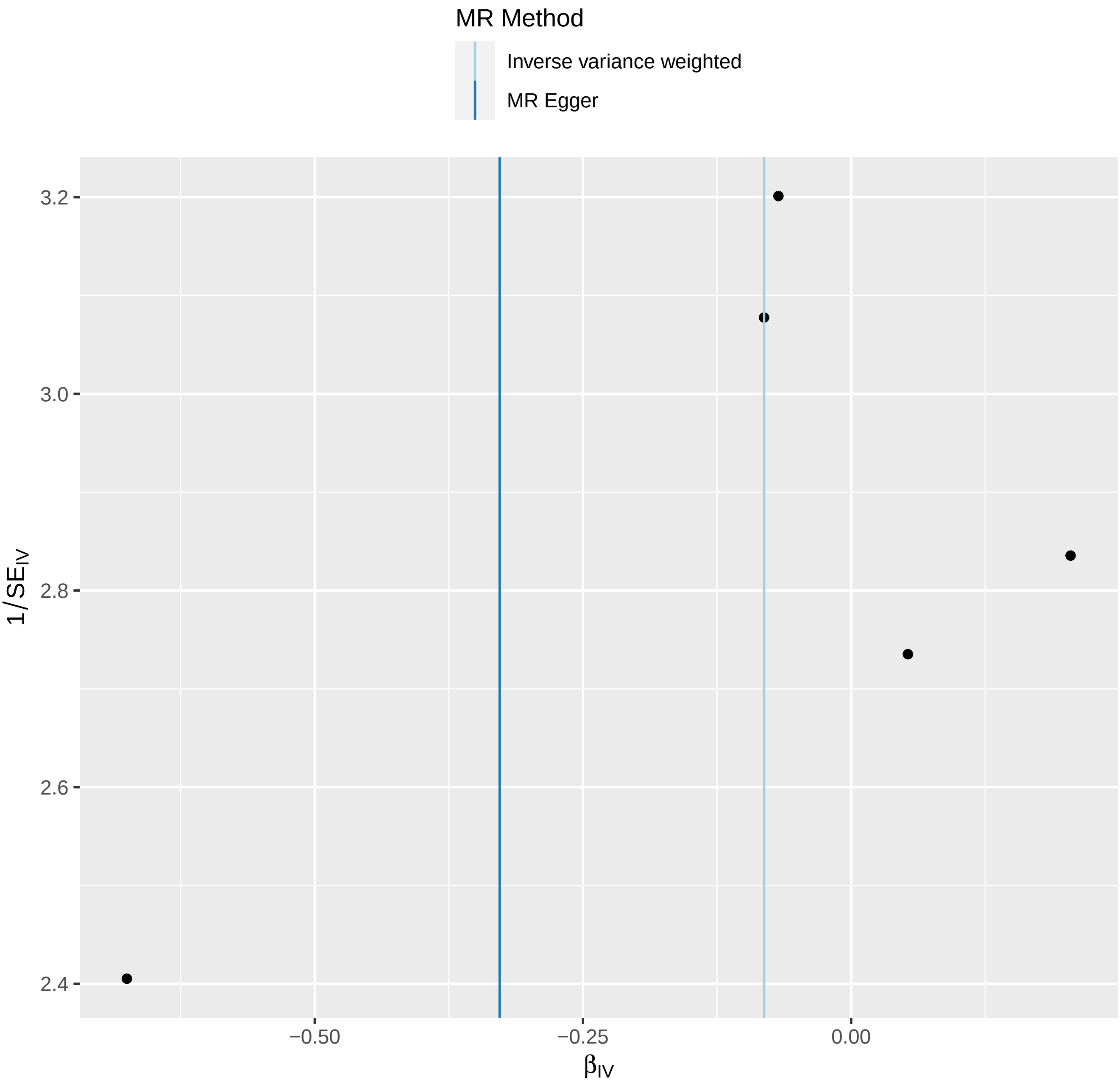

Batch 3134 : DKTatlas rh pericalcarine area on Crohn's disease

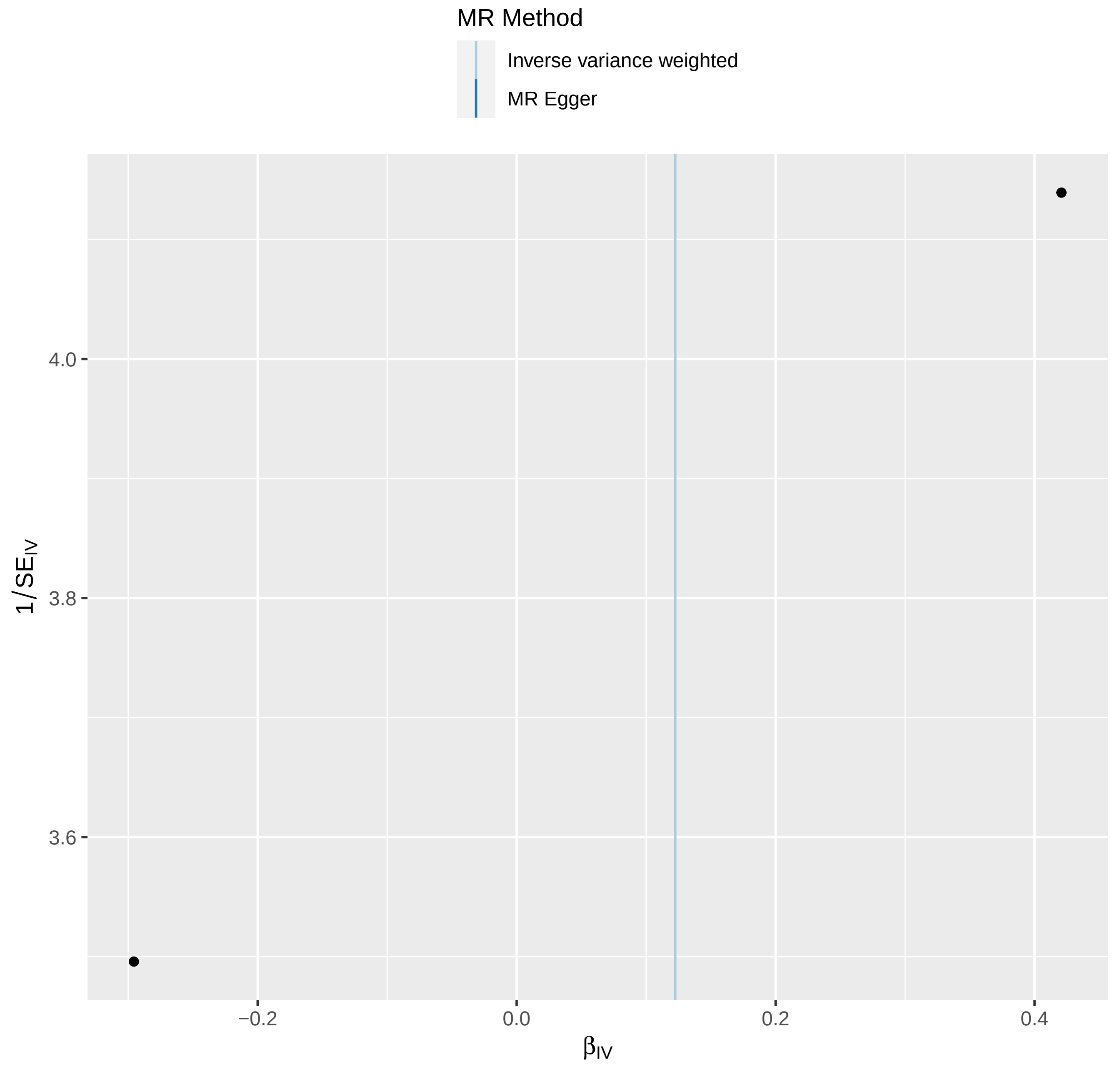

Batch 3136 : DKTatlas rh postcentral area on Crohn's disease

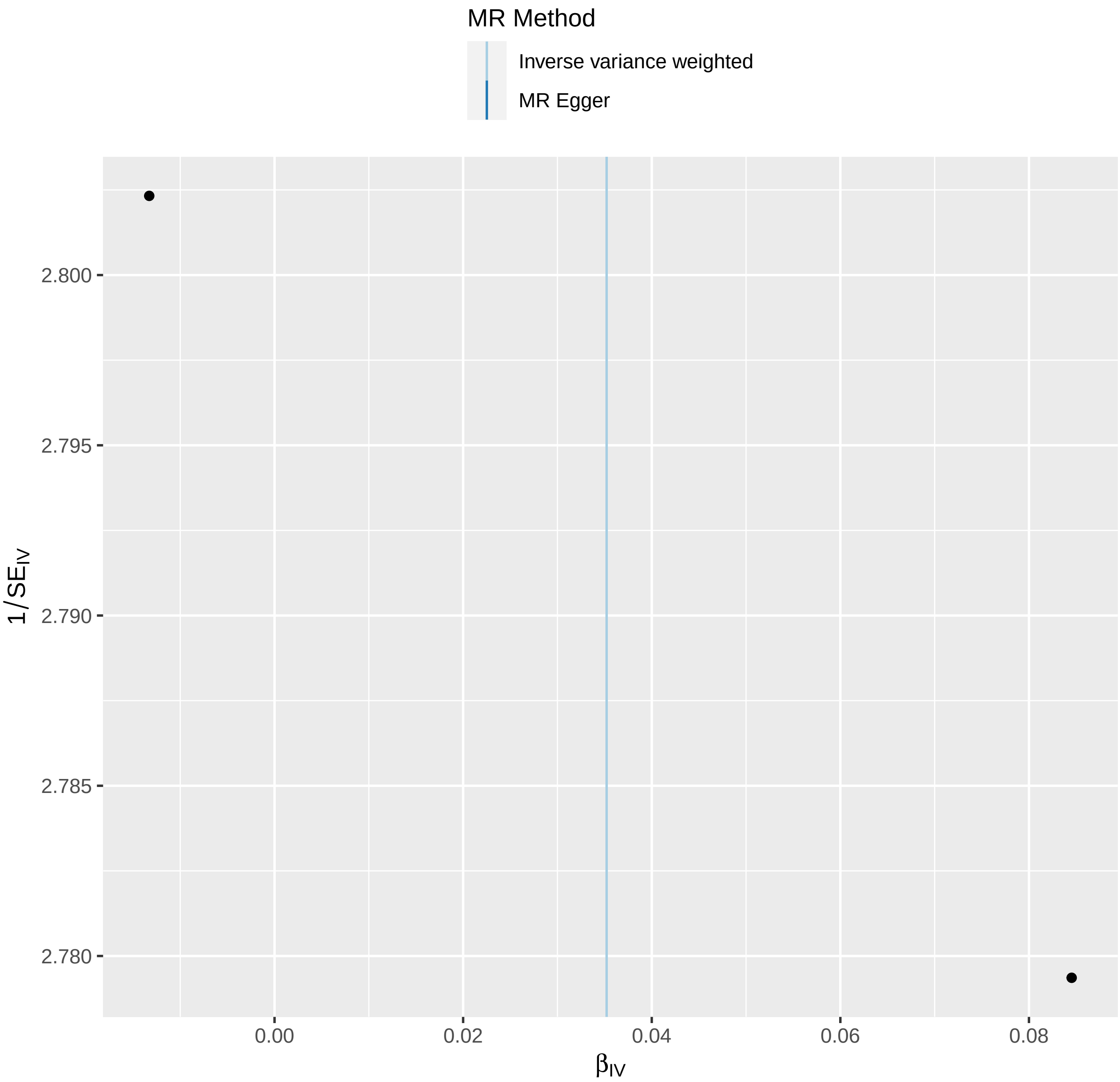

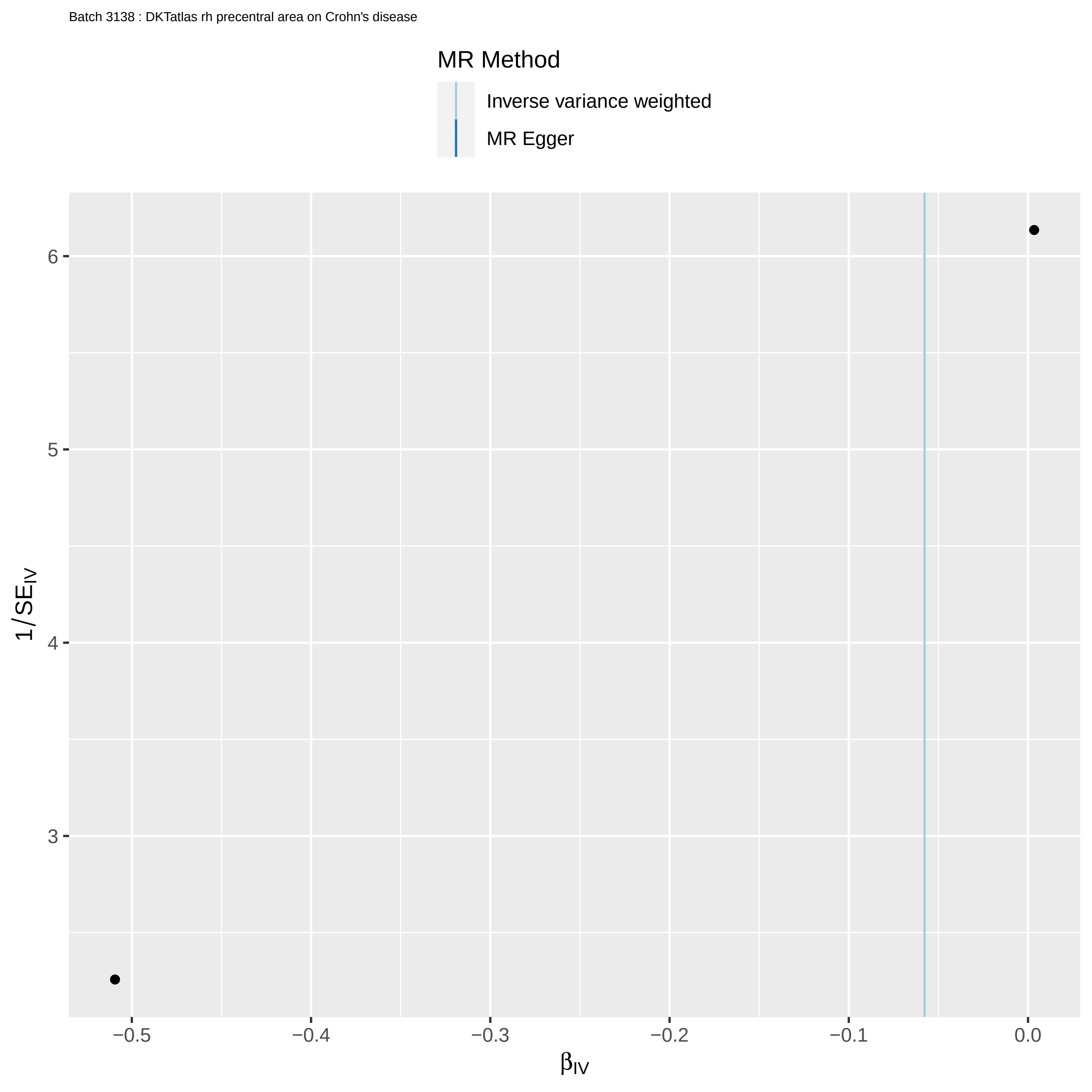

Batch 3138 : DKTatlas rh precentral area on Crohn's disease
MR Method
Inverse variance weighted
MR Egger
1/SE_IV
6
5
4
3
−0.5
−0.4
−0.3
−0.2
−0.1
0.0
β_IV

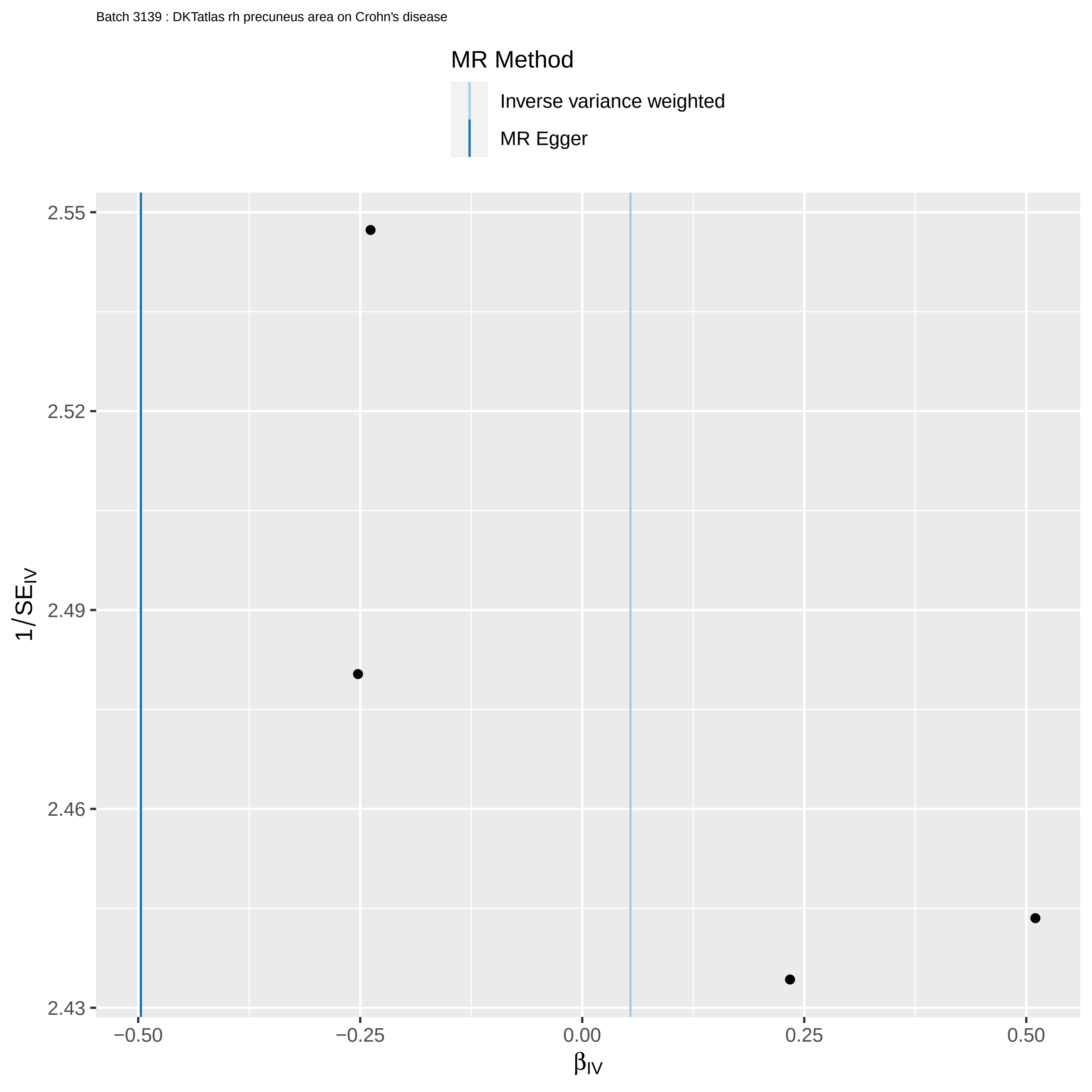
Batch 3139 : DKTatlas rh precuneus area on Crohn's disease
MR Method
Inverse variance weighted
MR Egger
2.55
2.52
2.49
2.46
2.43
1/SE_IV
−0.50
−0.25
0.00
0.25
0.50
β_IV

Batch 3142 : DKTatlas rh superiorfrontal area on Crohn's disease

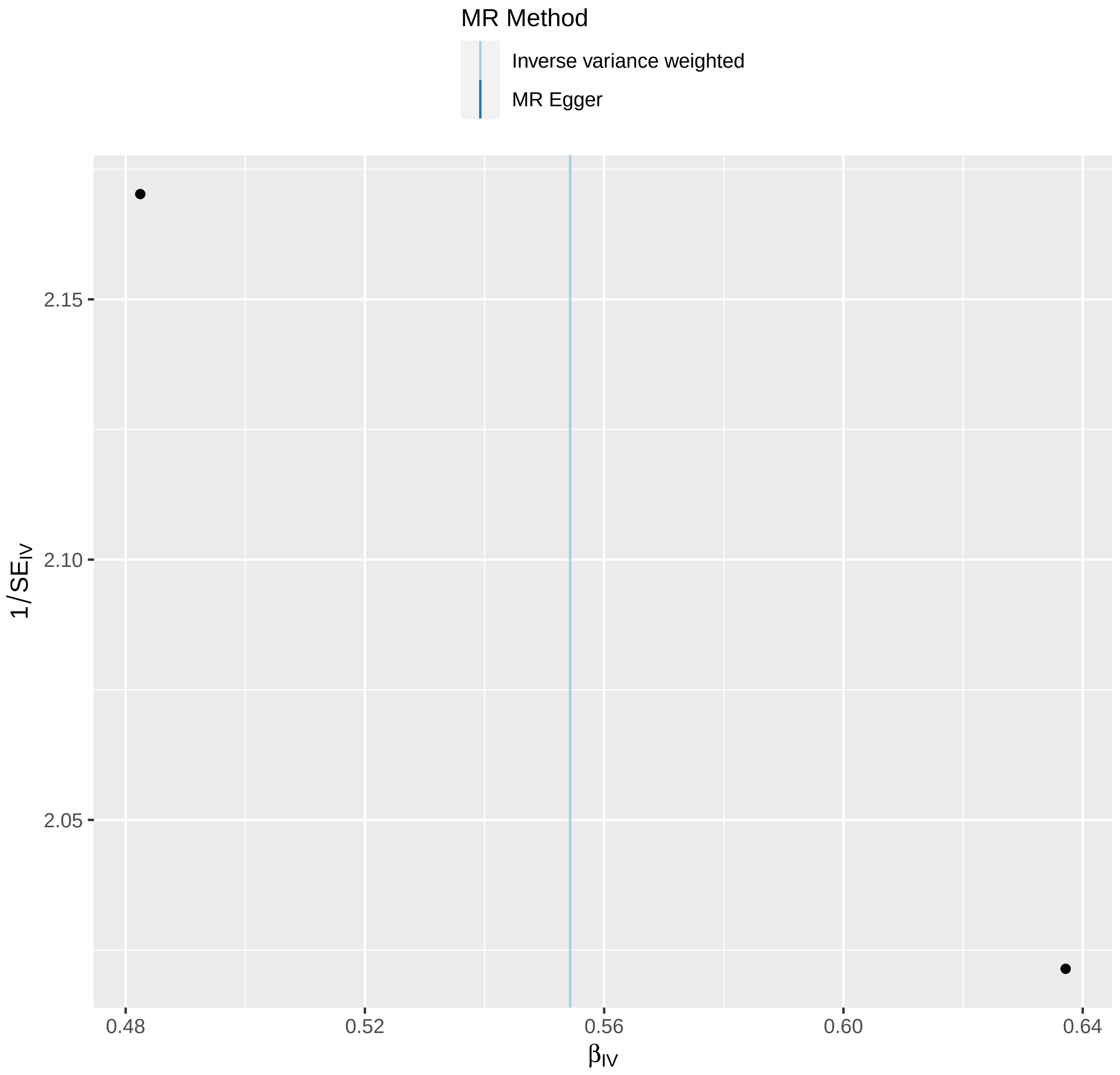

Batch 3144 : DKTatlas rh superiortemporal area on Crohn's disease

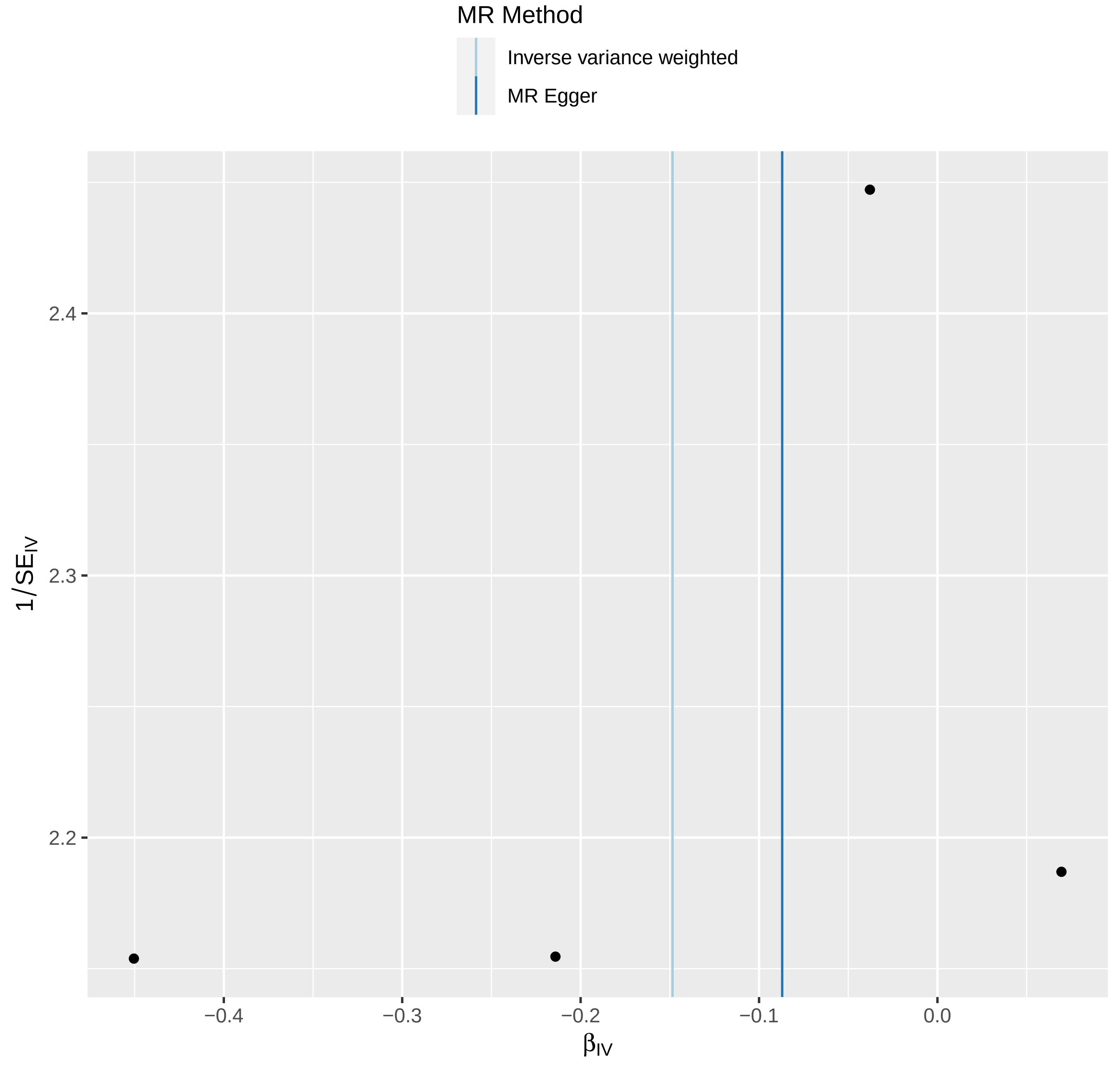

Batch 3147 : DKTatlas rh transversetemporal area on Crohn's disease

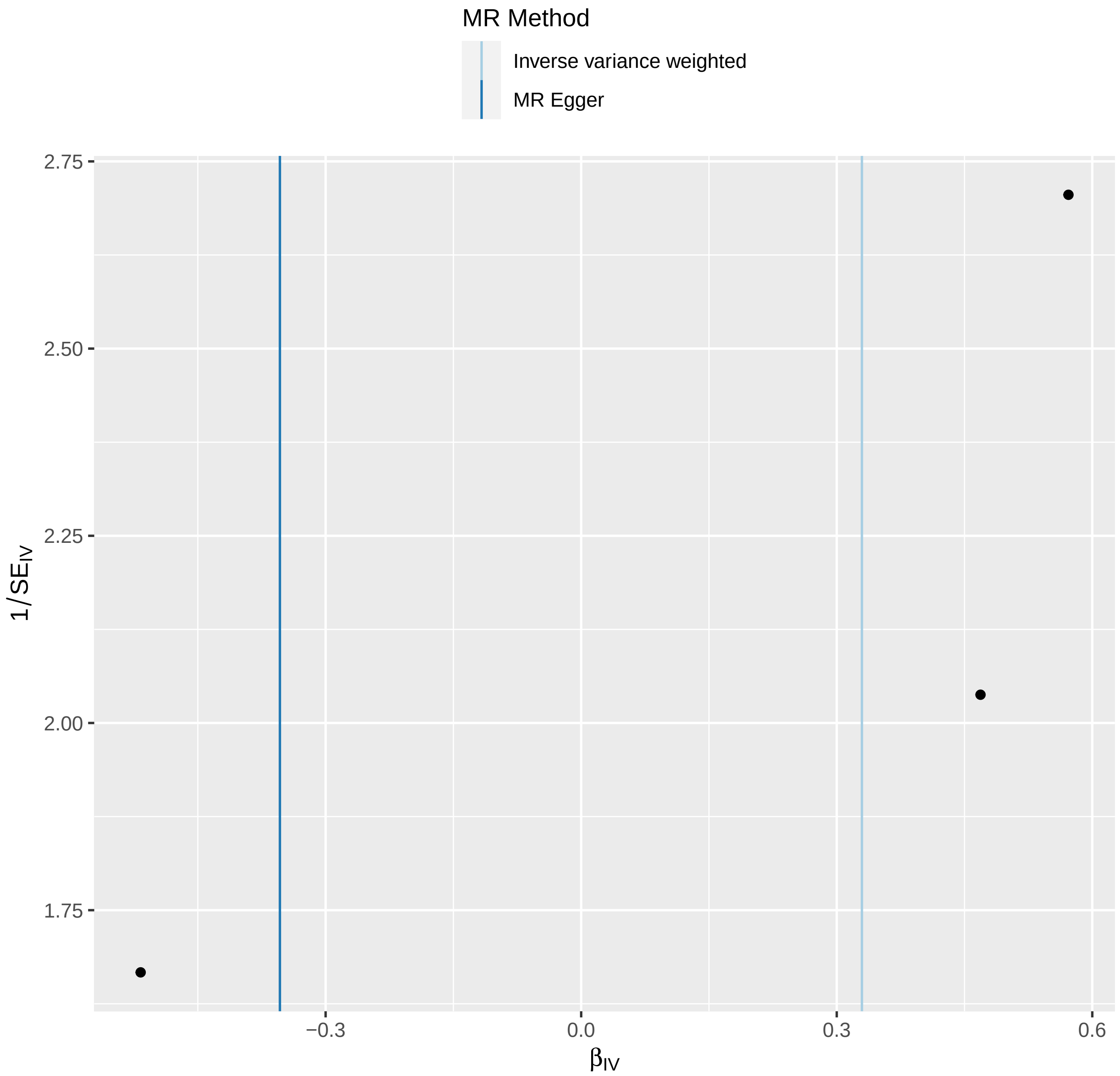

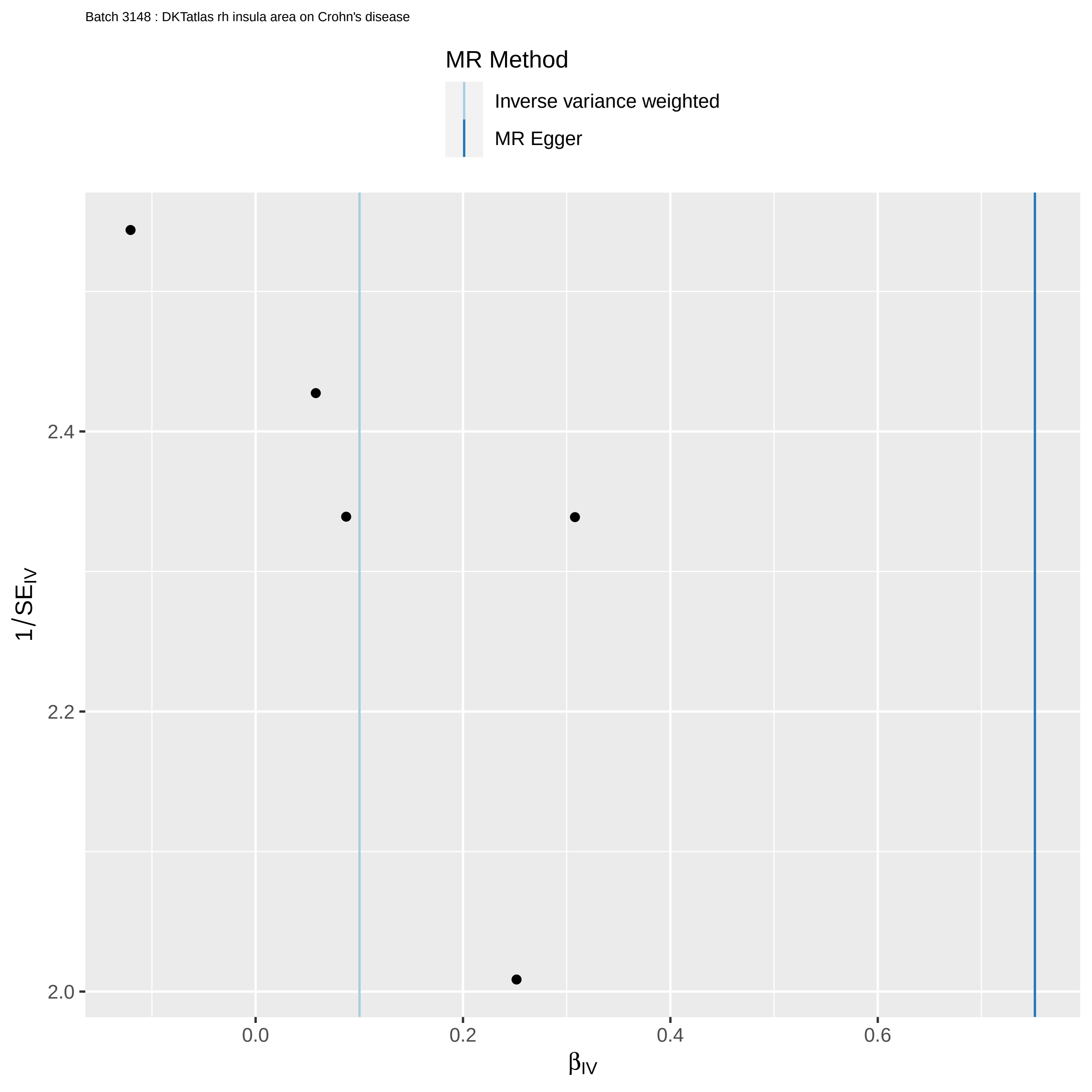

Batch 3148 : DKTatlas rh insula area on Crohn's disease
MR Method
Inverse variance weighted
MR Egger
2.4
2.2
2.0
1/SE_IV
0.0
0.2
0.4
0.6
β_IV

Batch 3201 : IDP T1 FAST ROIs R insular cortex on Crohn's disease

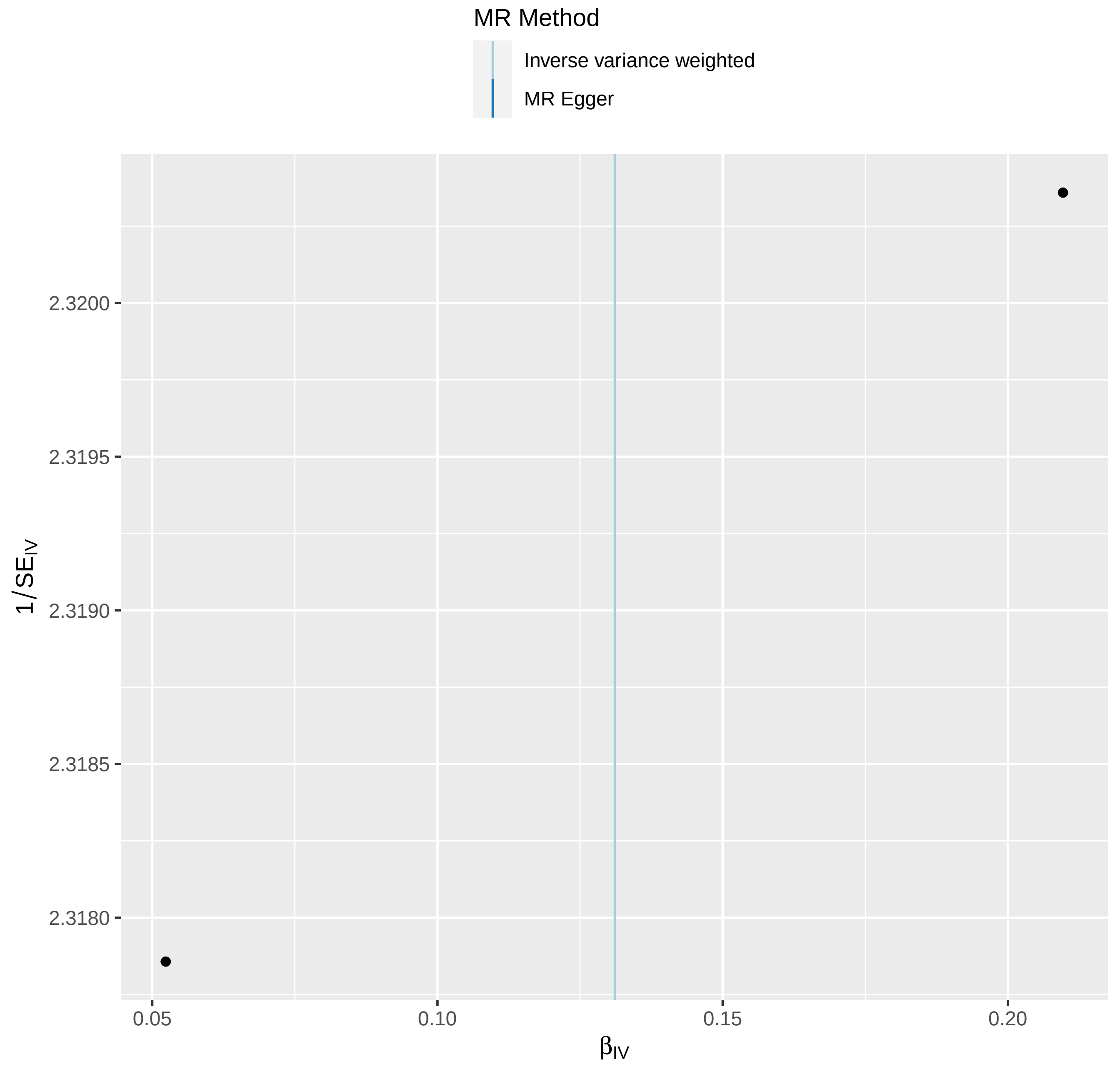

Batch 3312 : IDP T1 SIENAX CSF normalised volume on Crohn's disease

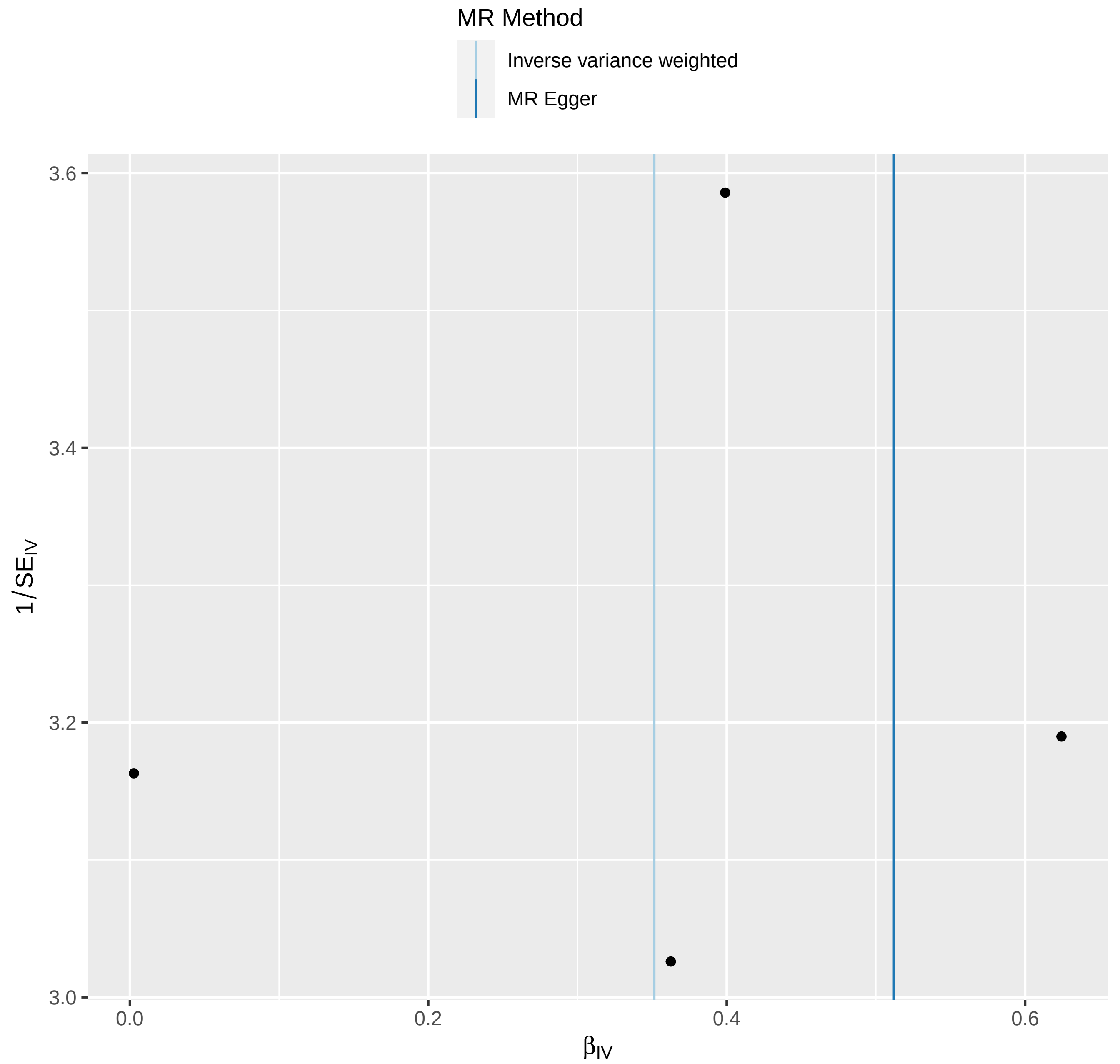

Batch 3424 : IDP T1 FAST ROIs R sup front gyrus on Crohn's disease

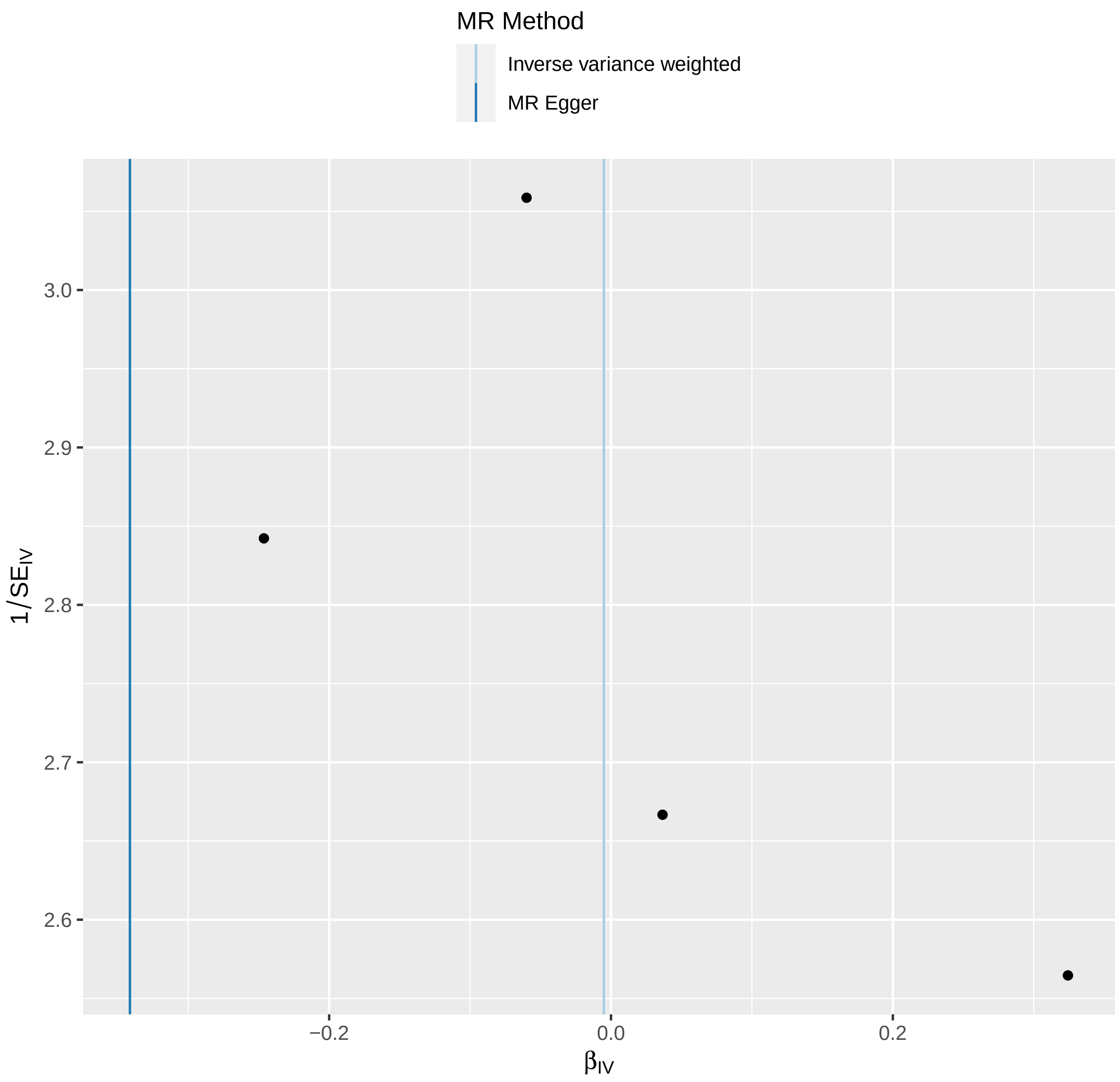

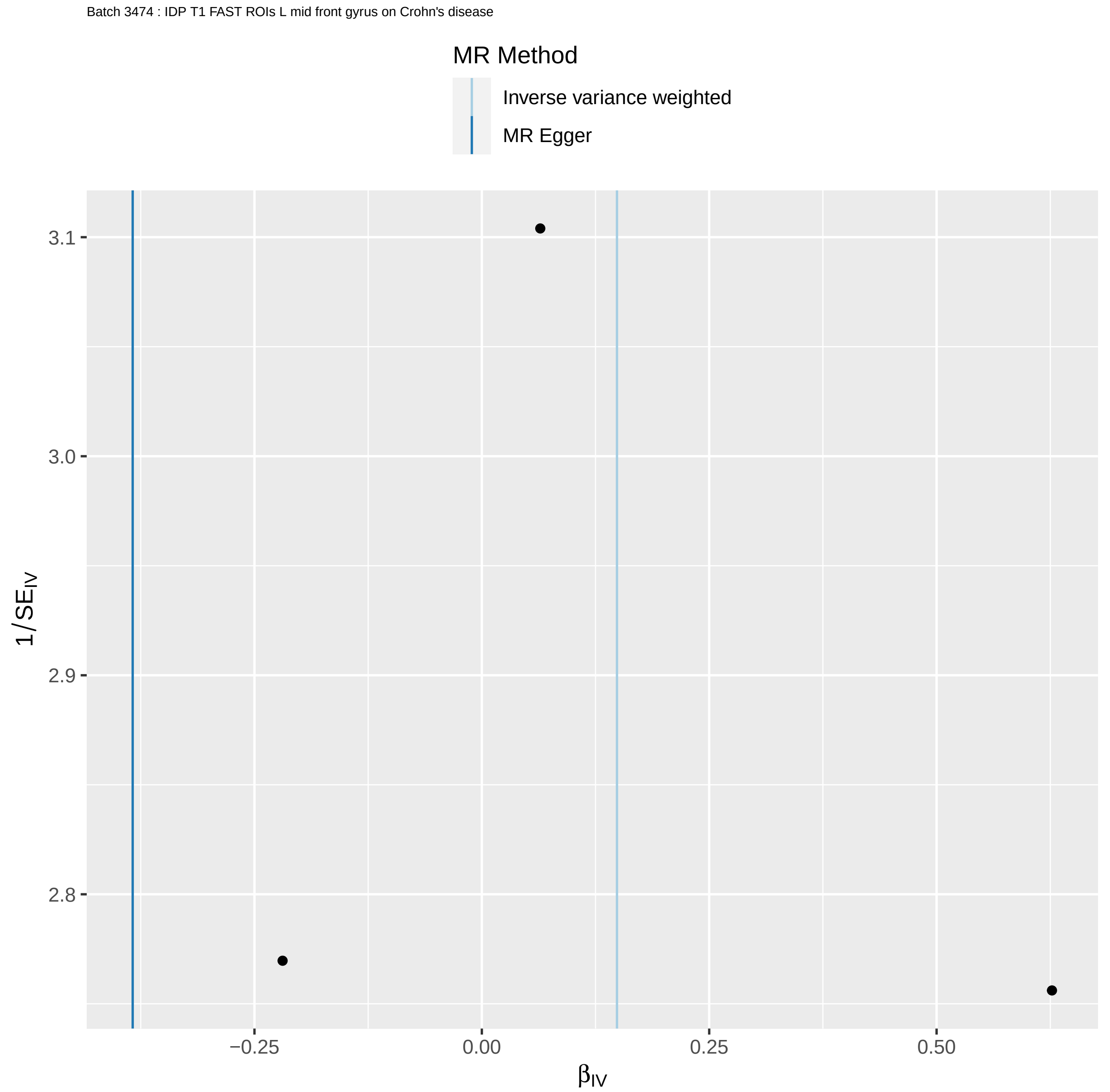

Batch 3474 : IDP T1 FAST ROIs L mid front gyrus on Crohn's disease
MR Method
Inverse variance weighted
MR Egger
3.1
3.0
2.9
2.8
$1/SE_{IV}$
−0.25
0.00
0.25
0.50
β_{IV}

Batch 3496 : IDP T1 FAST ROIs L inf front gyrus parstri on Crohn's disease

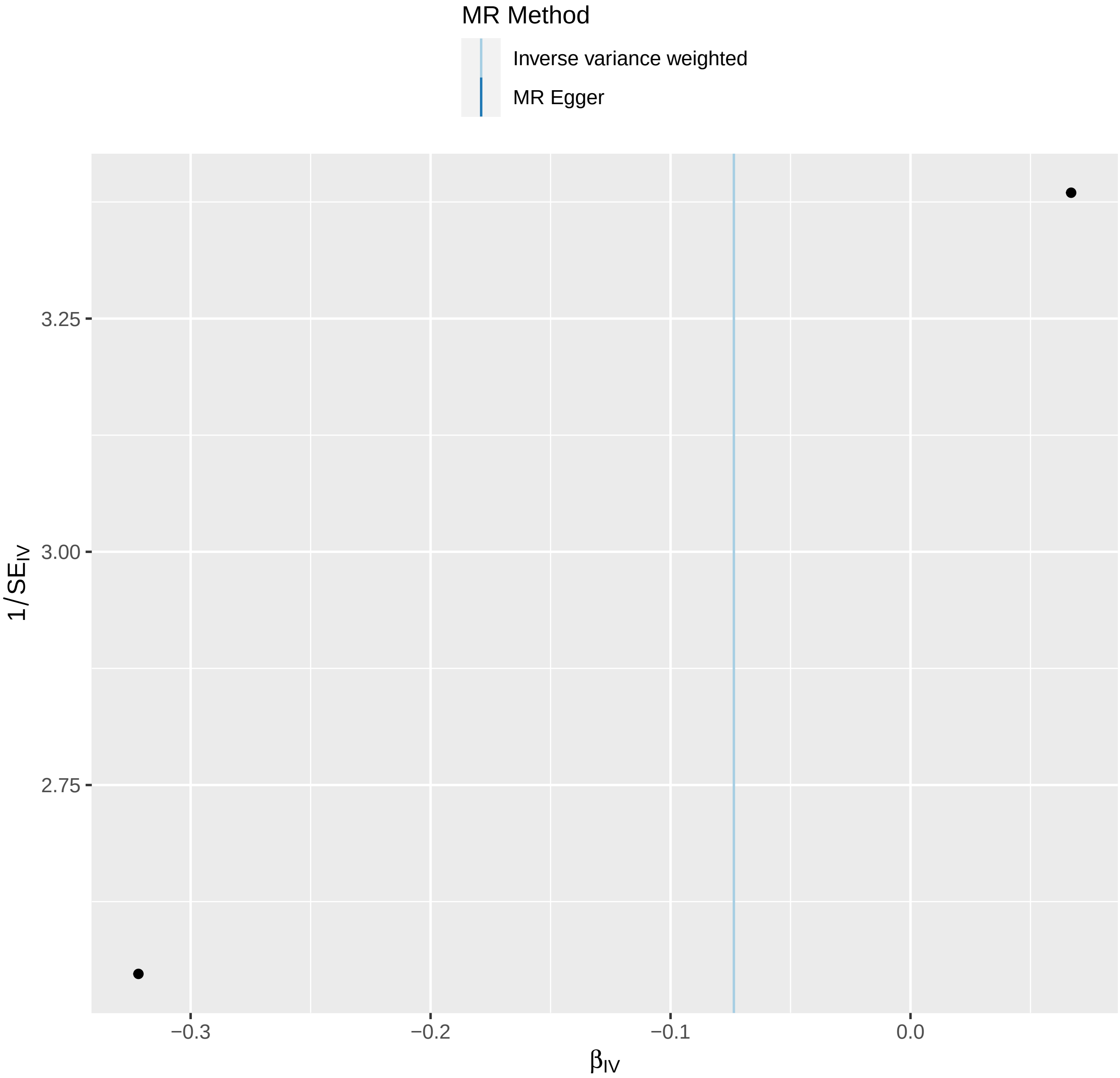

Batch 3561 : IDP T1 SIENAX CSF unnormalised volume on Crohn's disease

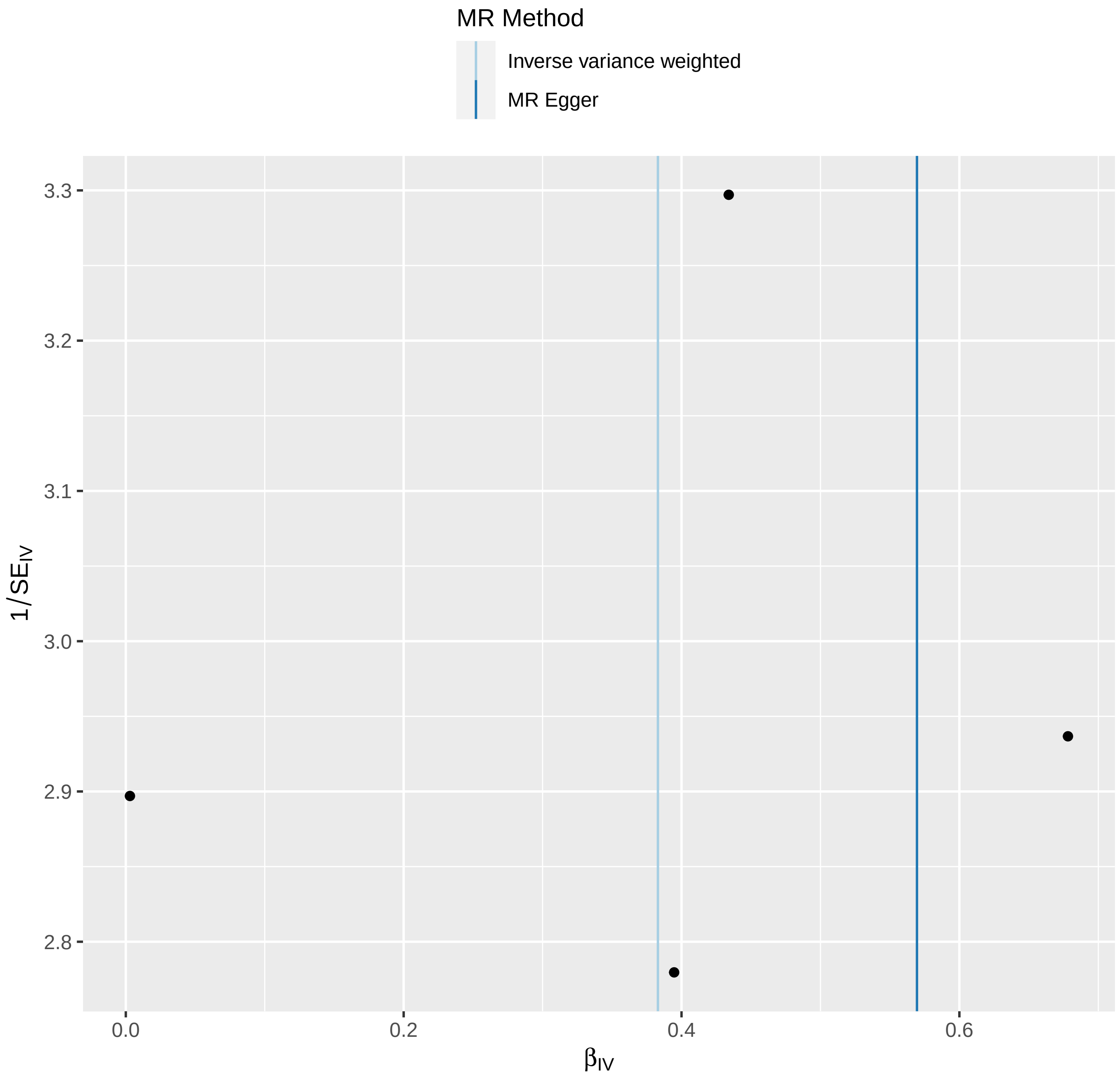

Batch 3583 : IDP T1 FAST ROIs L sup temp gyrus ant on Crohn's disease

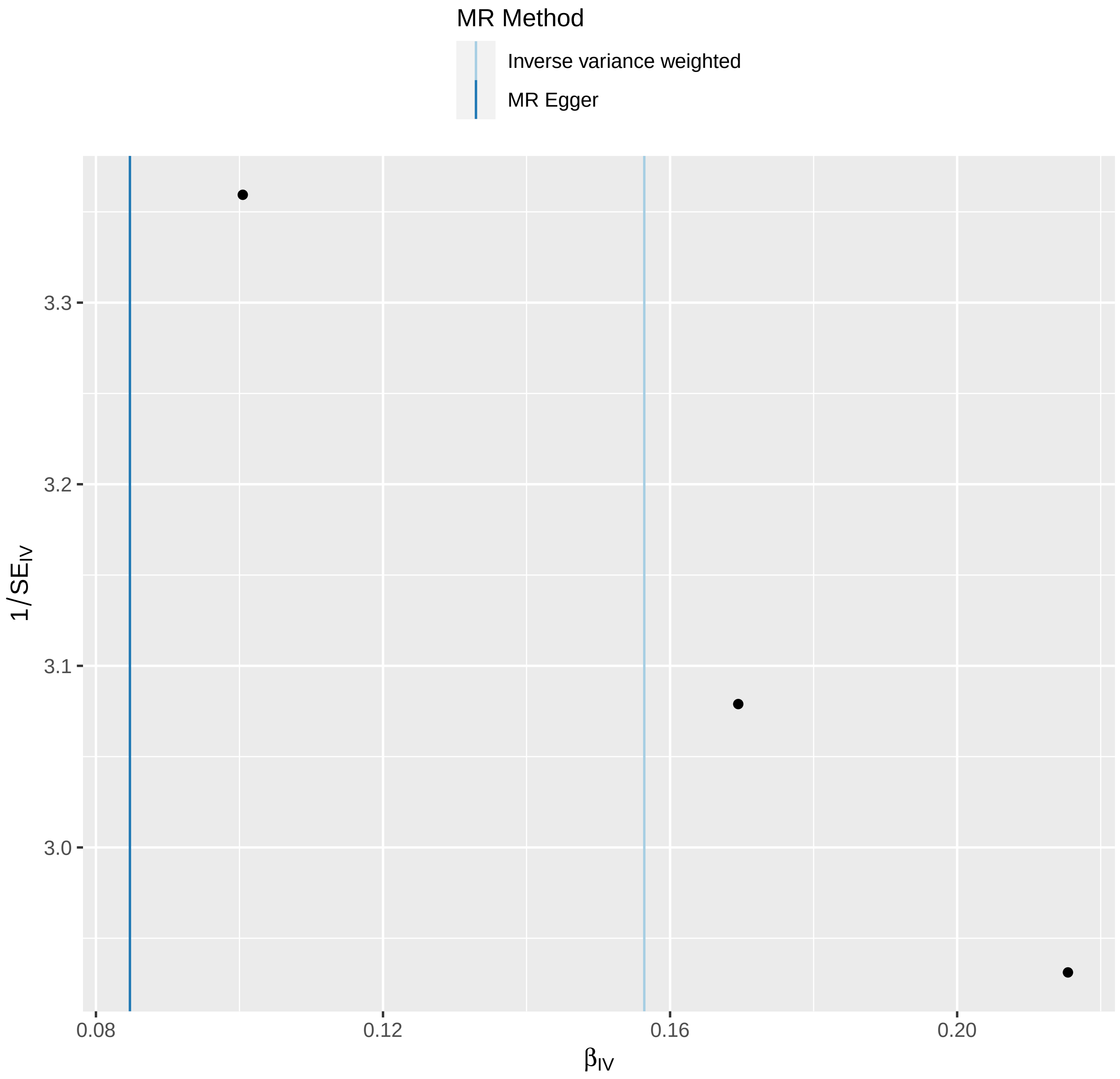

Batch 3594 : IDP T1 FAST ROIs R sup temp gyrus ant on Crohn's disease

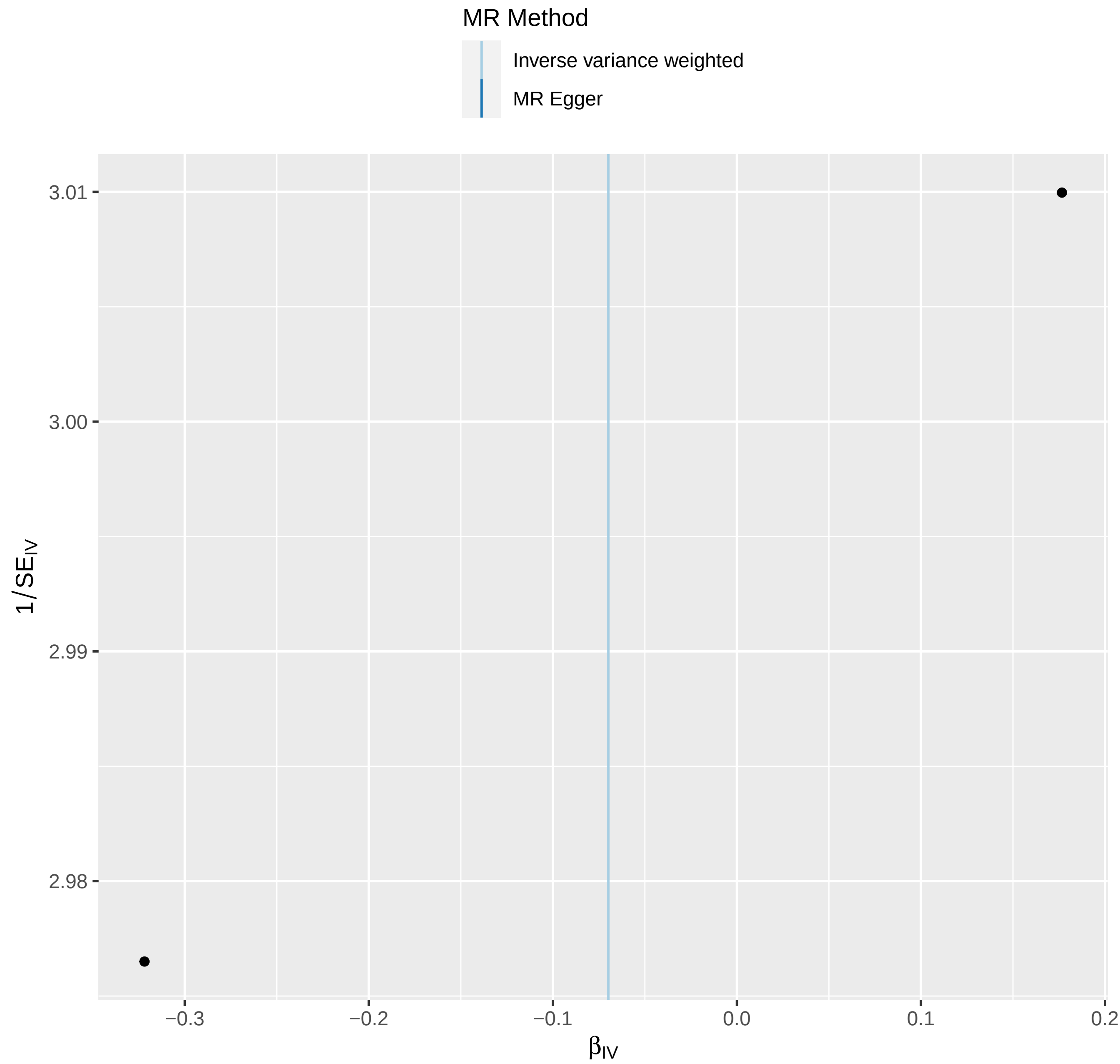

Batch 3625 : IDP T1 FAST ROIs L mid temp gyrus ant on Crohn's disease

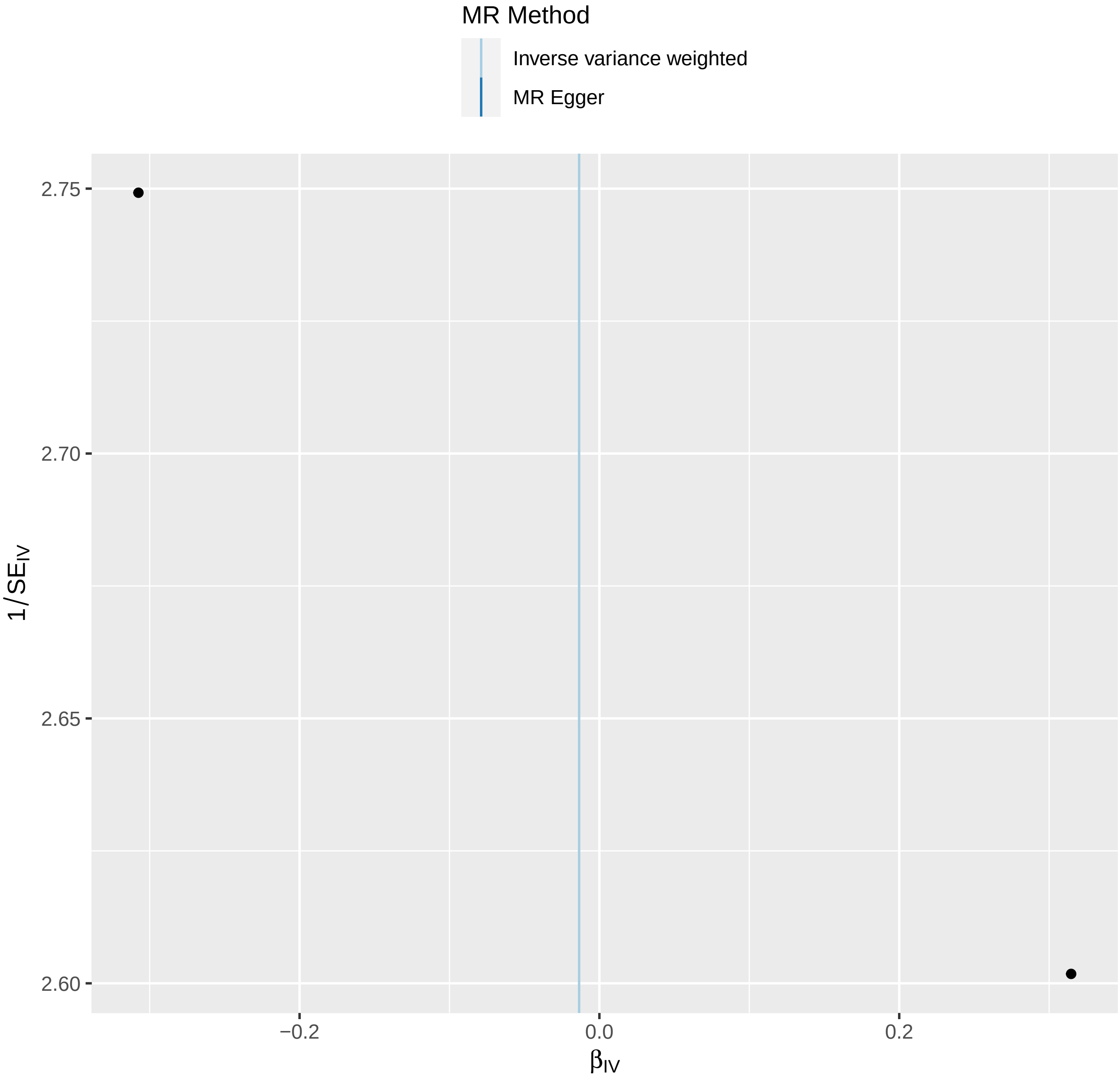

Batch 3669 : IDP T1 SIENAX grey normalised volume on Crohn's disease

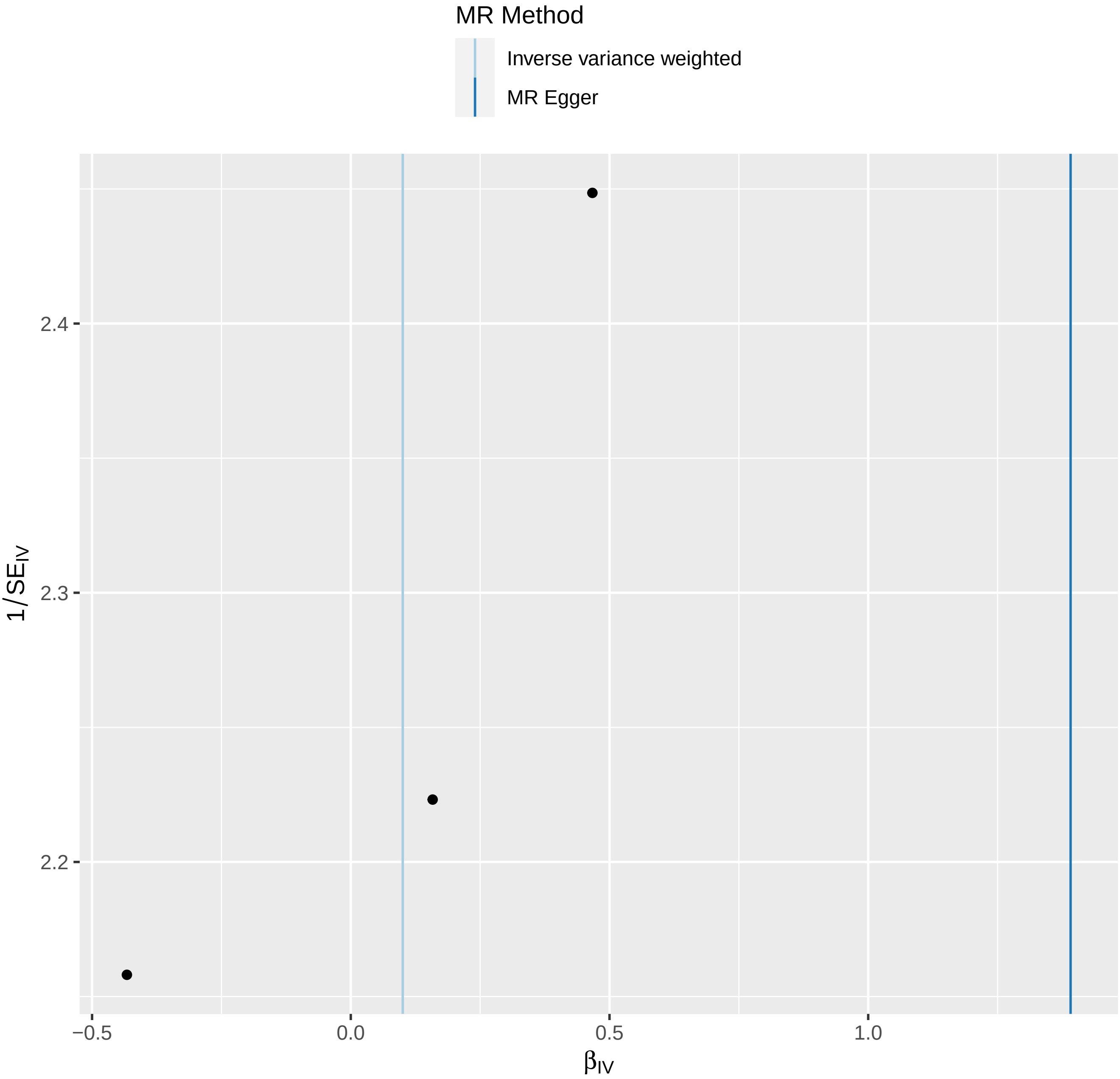

Batch 3681 : IDP T1 FAST ROIs R mid temp gyrus tempocc on Crohn's disease

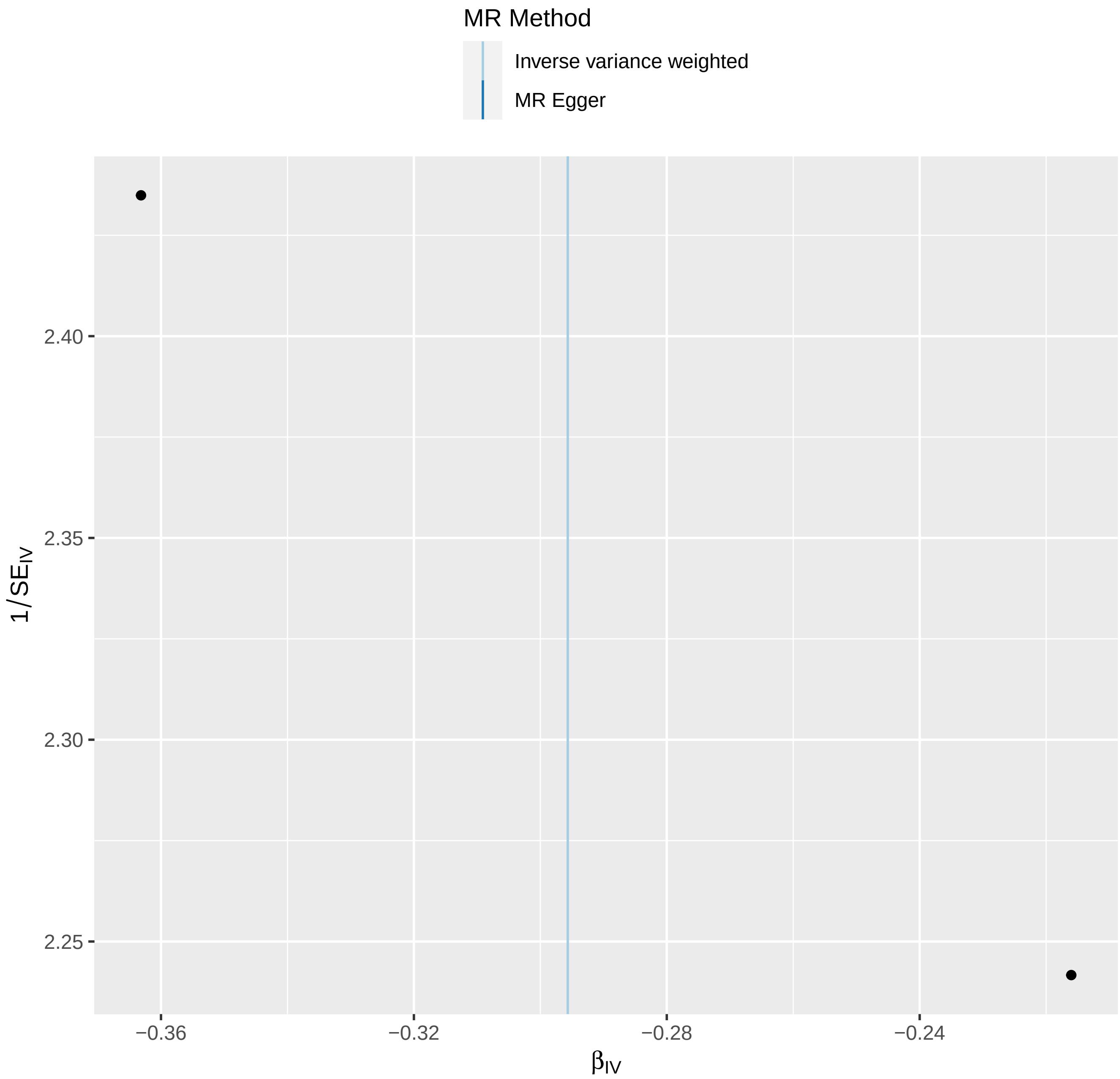

Batch 3692 : IDP T1 FAST ROIs L inf temp gyrus ant on Crohn's disease

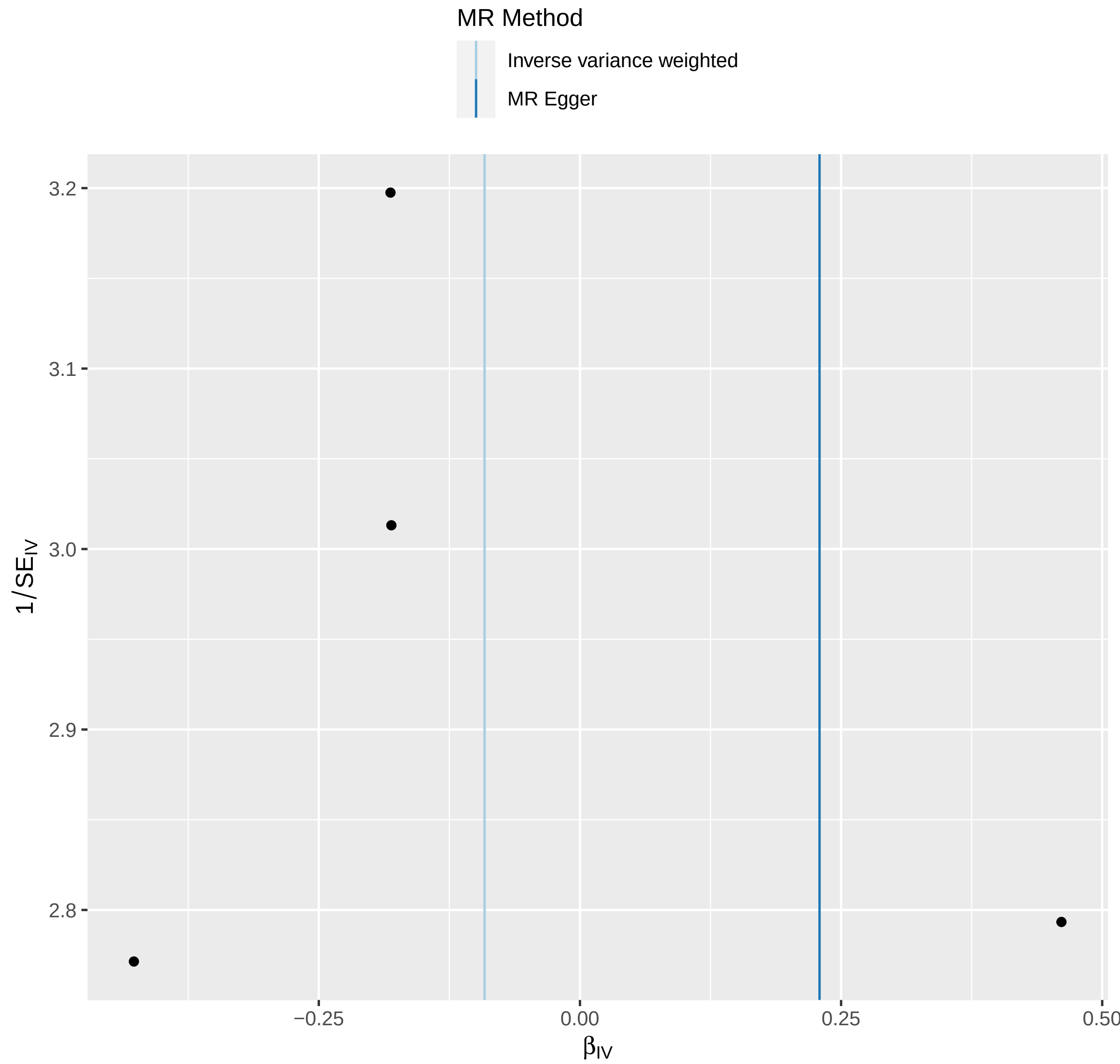

Batch 3703 : IDP T1 FAST ROIs R inf temp gyrus ant on Crohn's disease

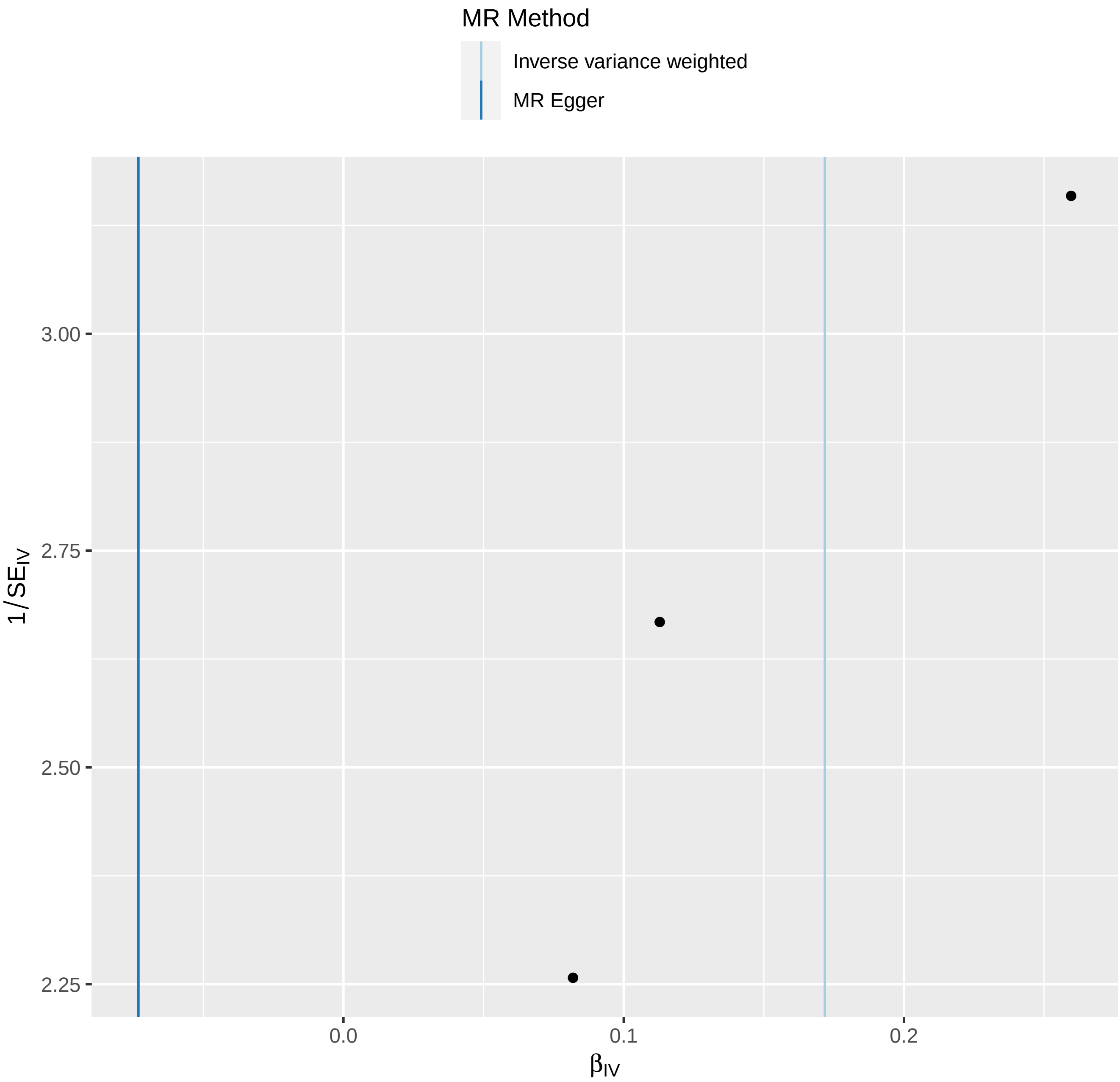

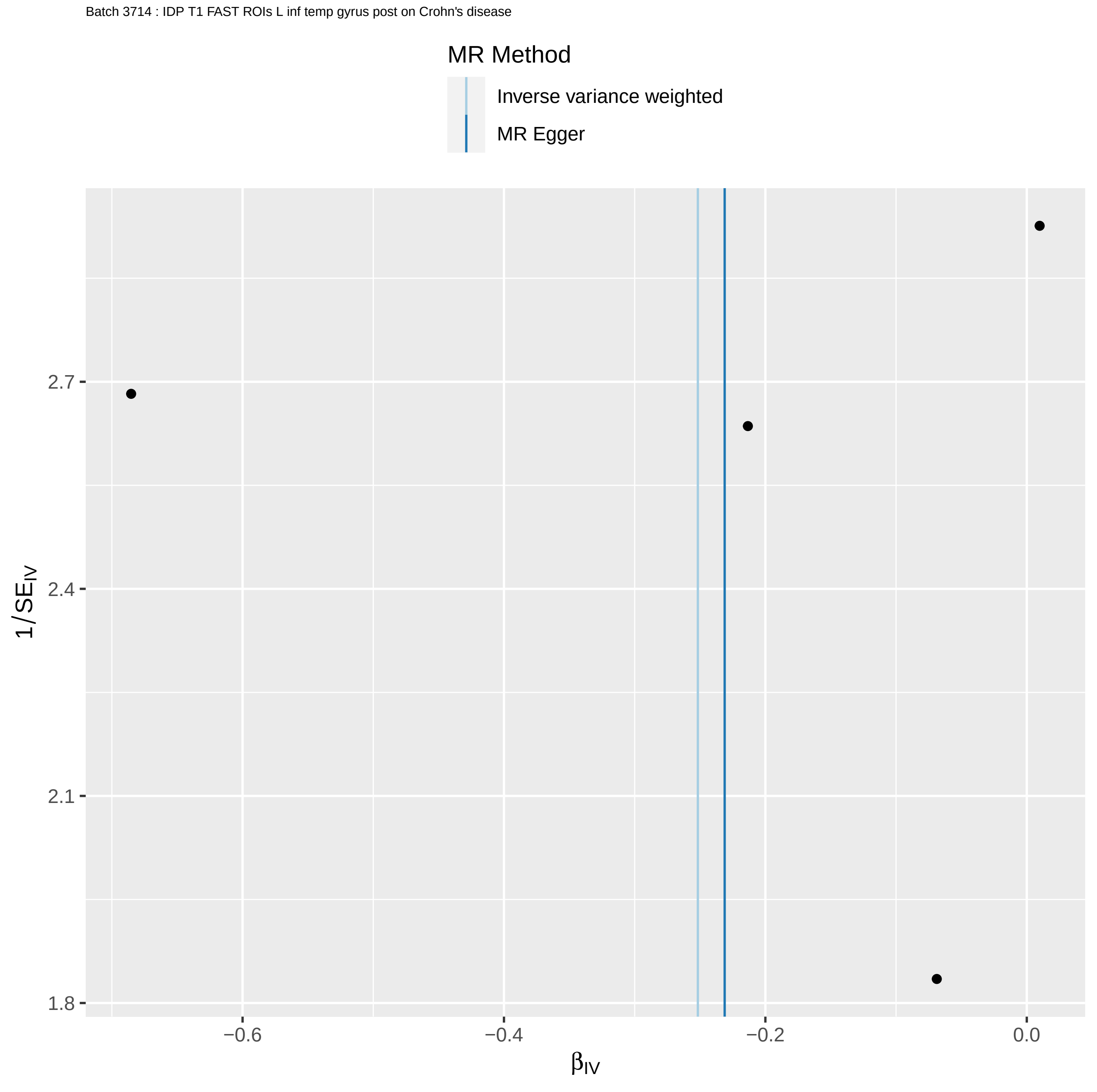

Batch 3714 : IDP T1 FAST ROIs L inf temp gyrus post on Crohn's disease
MR Method
Inverse variance weighted
MR Egger
2.7
2.4
2.1
1.8
1/SE_IV
−0.6
−0.4
−0.2
0.0
β_IV

Batch 3736 : IDP T1 FAST ROIs L inf temp gyrus tempocc on Crohn's disease

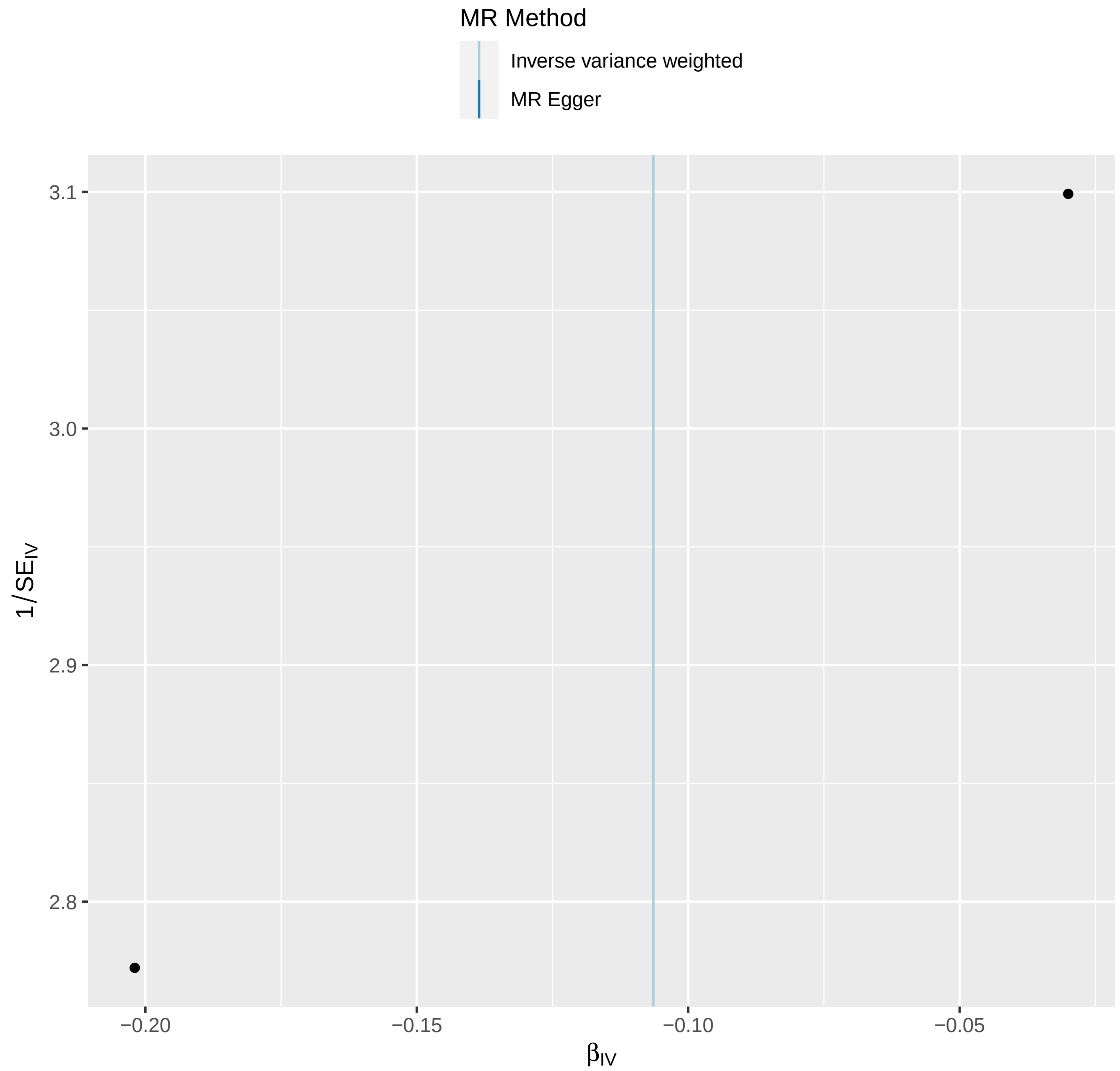

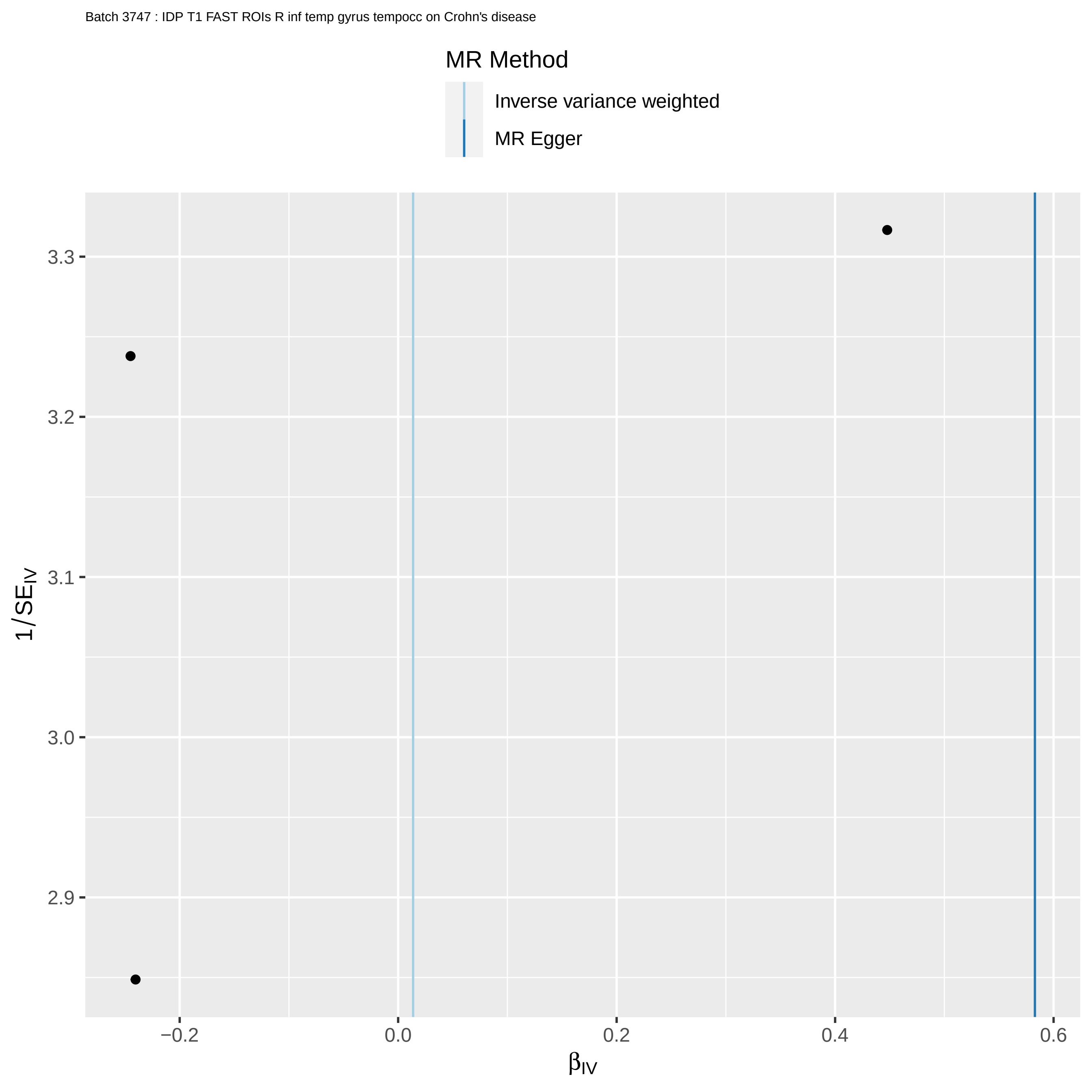

Batch 3747 : IDP T1 FAST ROIs R inf temp gyrus tempocc on Crohn's disease
MR Method
Inverse variance weighted
MR Egger
3.3
3.2
3.1
3.0
2.9
1/SE_IV
−0.2
0.0
0.2
0.4
0.6
β_IV

Batch 3758 : IDP T1 FAST ROIs L postcent gyrus on Crohn's disease

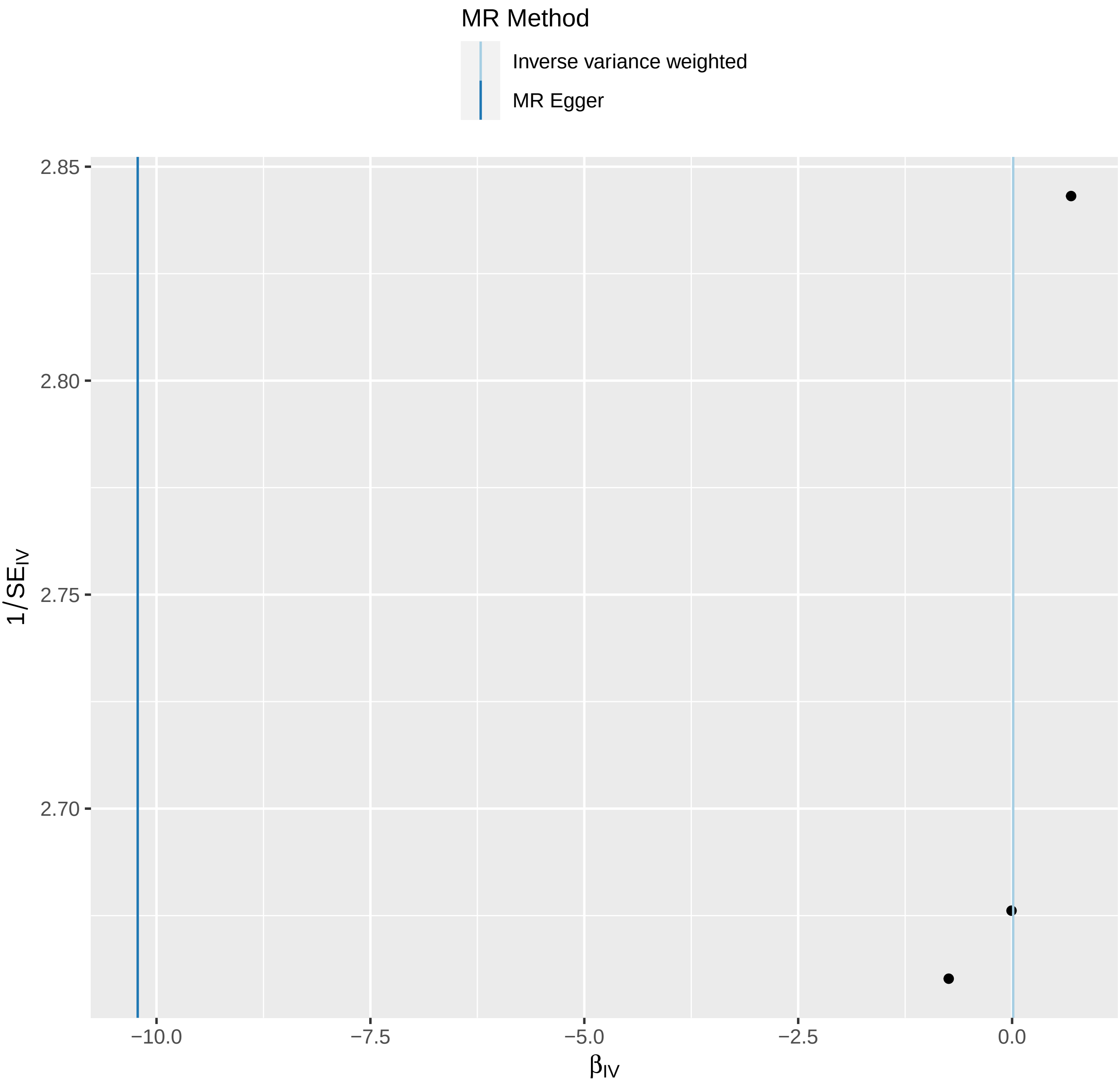

Batch 3780 : IDP T1 SIENAX grey unnormalised volume on Crohn's disease

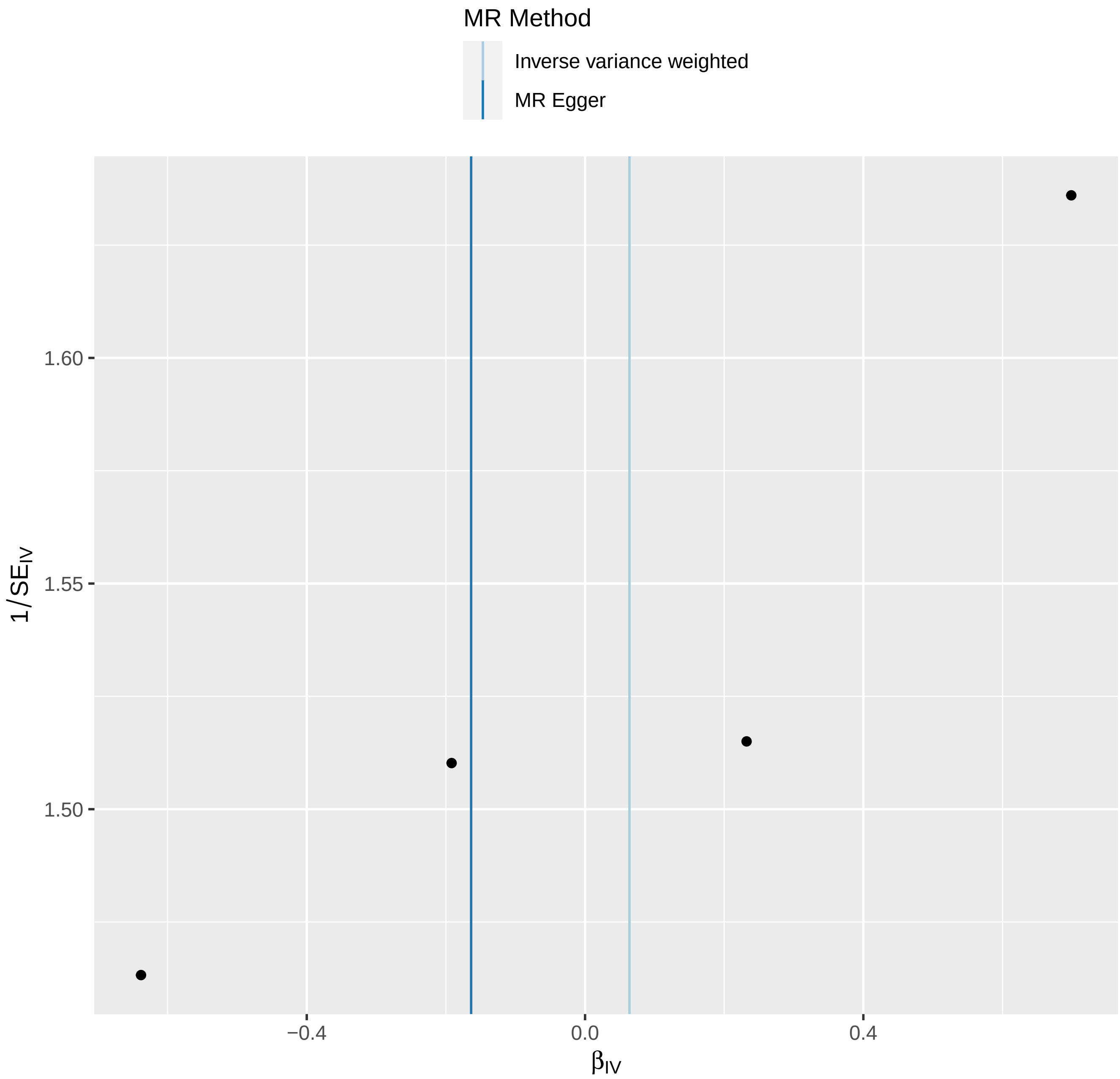

Batch 3781 : IDP T1 FAST ROIs L sup parietal lobule on Crohn's disease

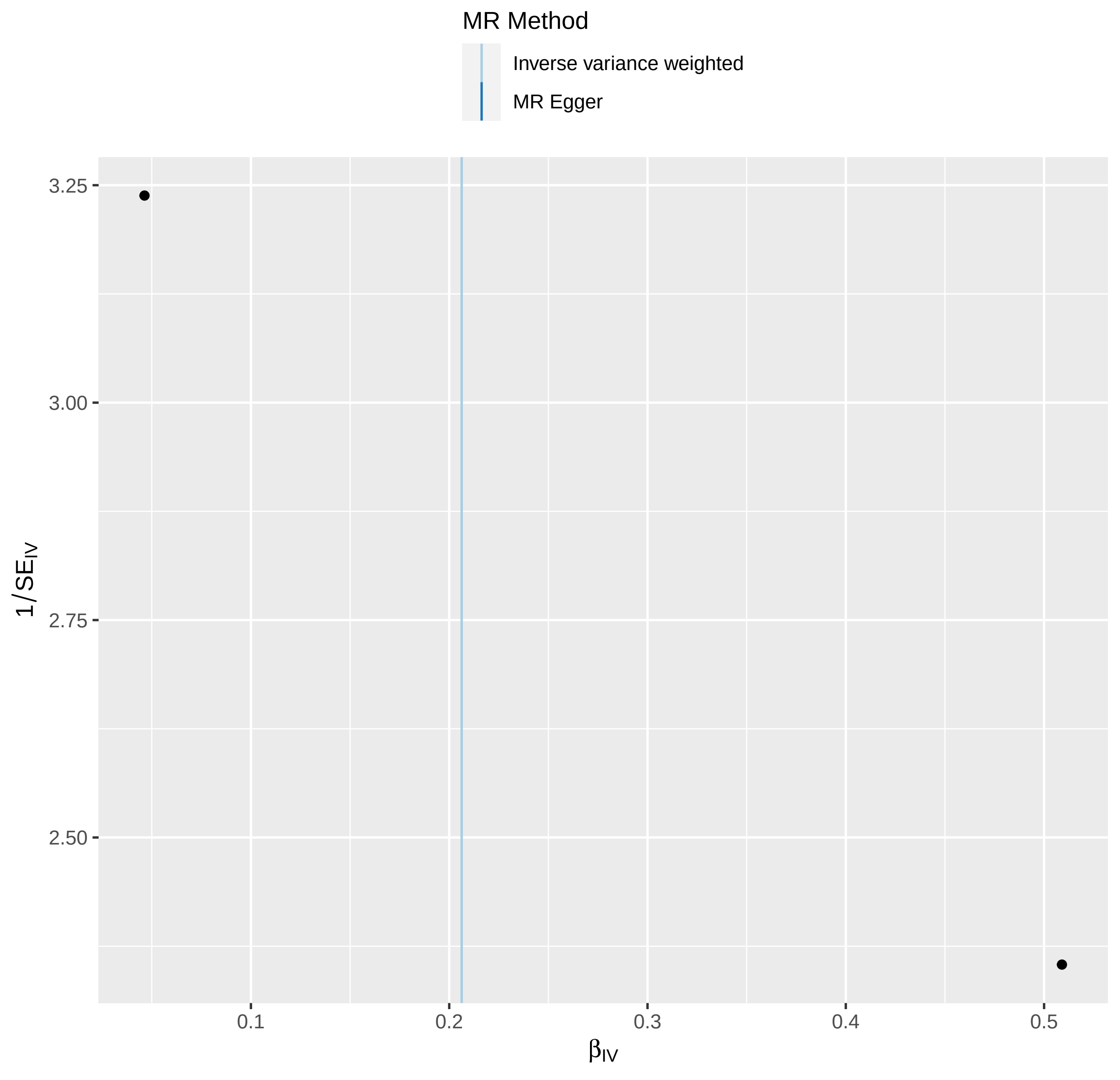

Batch 3803 : IDP T1 FAST ROIs L supramarg gyrus ant on Crohn's disease

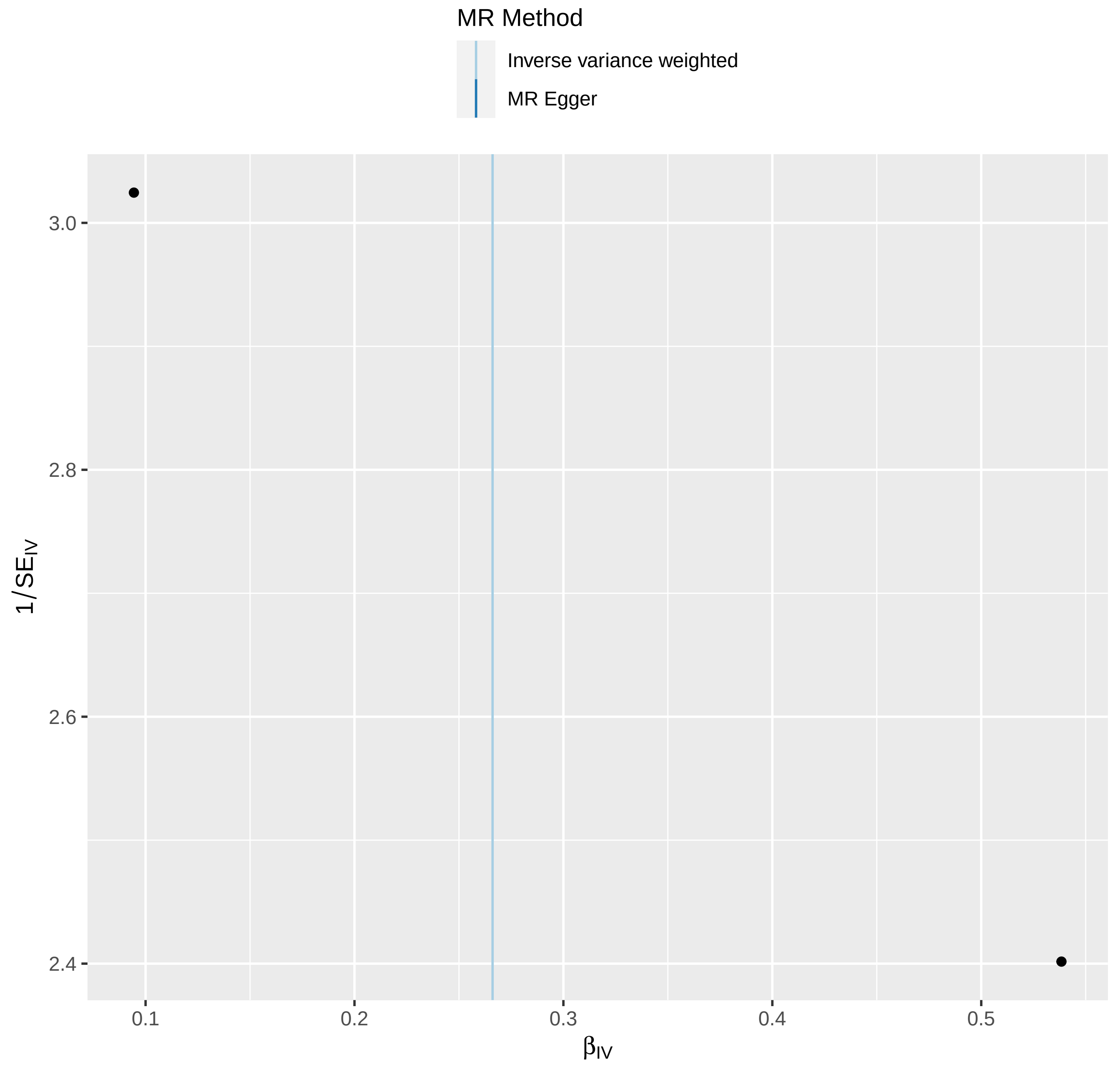

Batch 3847 : IDP T1 FAST ROIs L angular gyrus on Crohn's disease

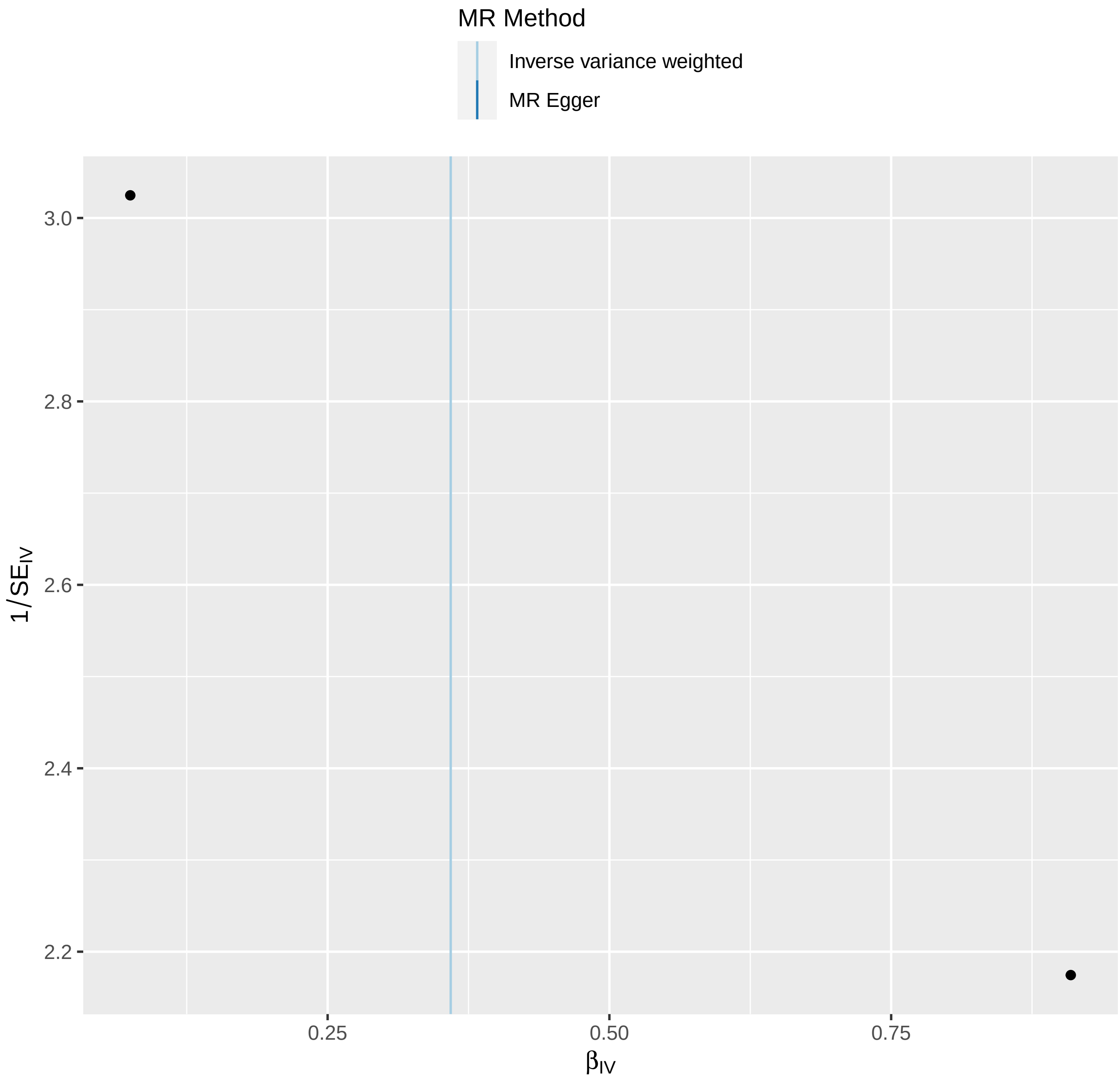

Batch 3869 : IDP T1 FAST ROIs L latocc cortex sup on Crohn's disease

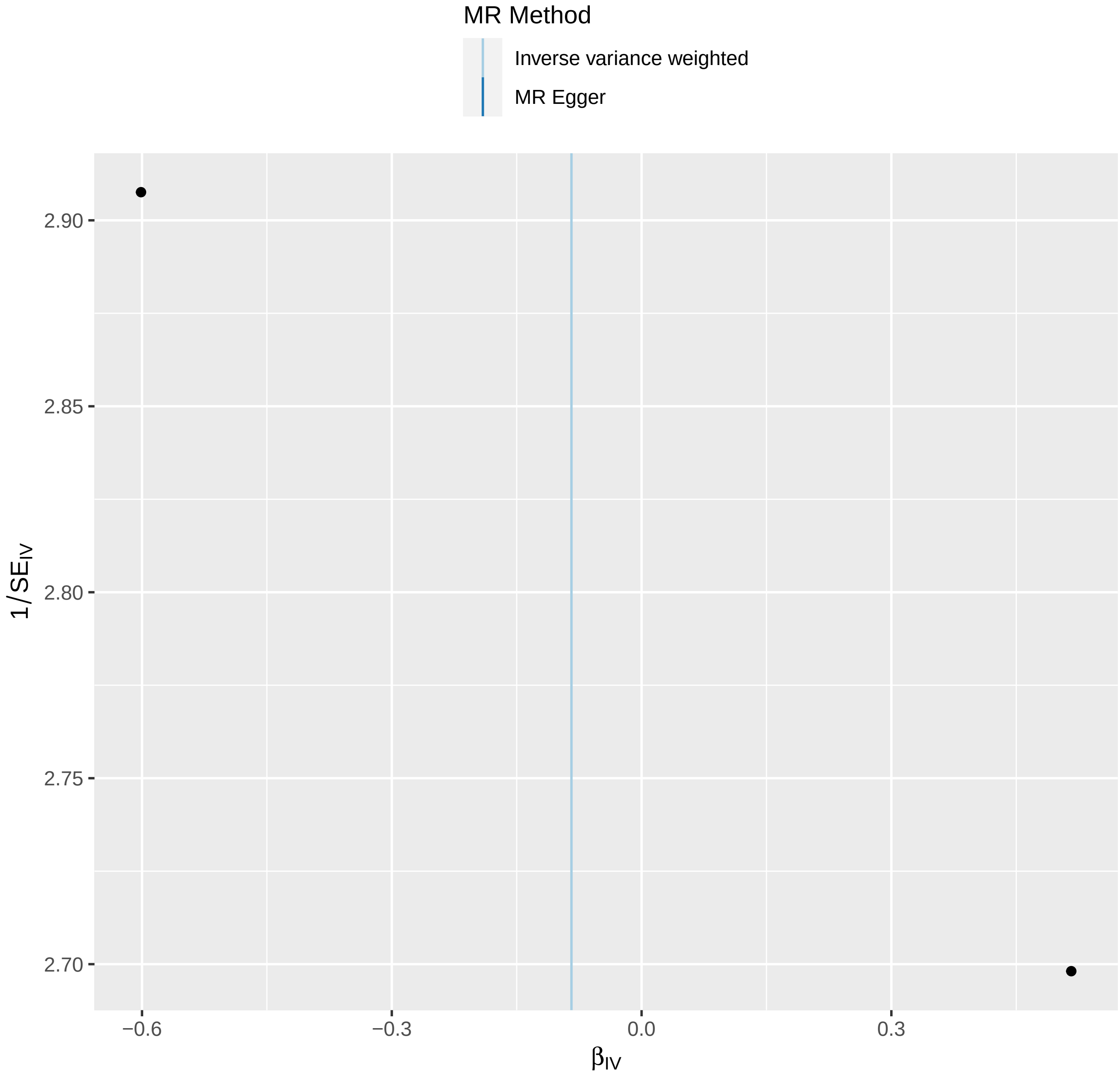

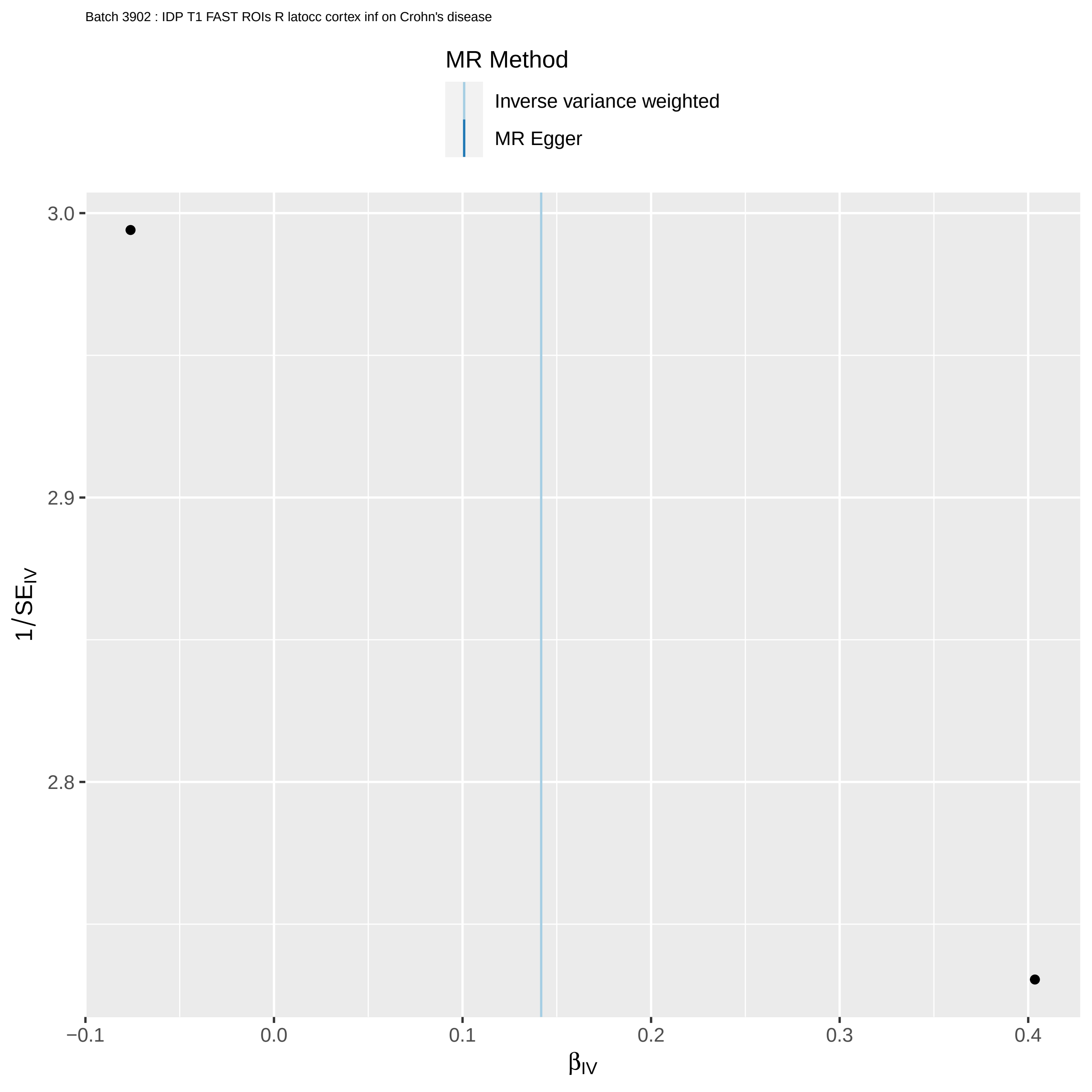
Batch 3902 : IDP T1 FAST ROIs R latocc cortex inf on Crohn's disease
MR Method
Inverse variance weighted
MR Egger
3.0
2.9
2.8
1/SE_IV
−0.1
0.0
0.1
0.2
0.3
0.4
β_IV

Batch 3968 : IDP T1 FAST ROIs R juxtapos lobule cortex on Crohn's disease

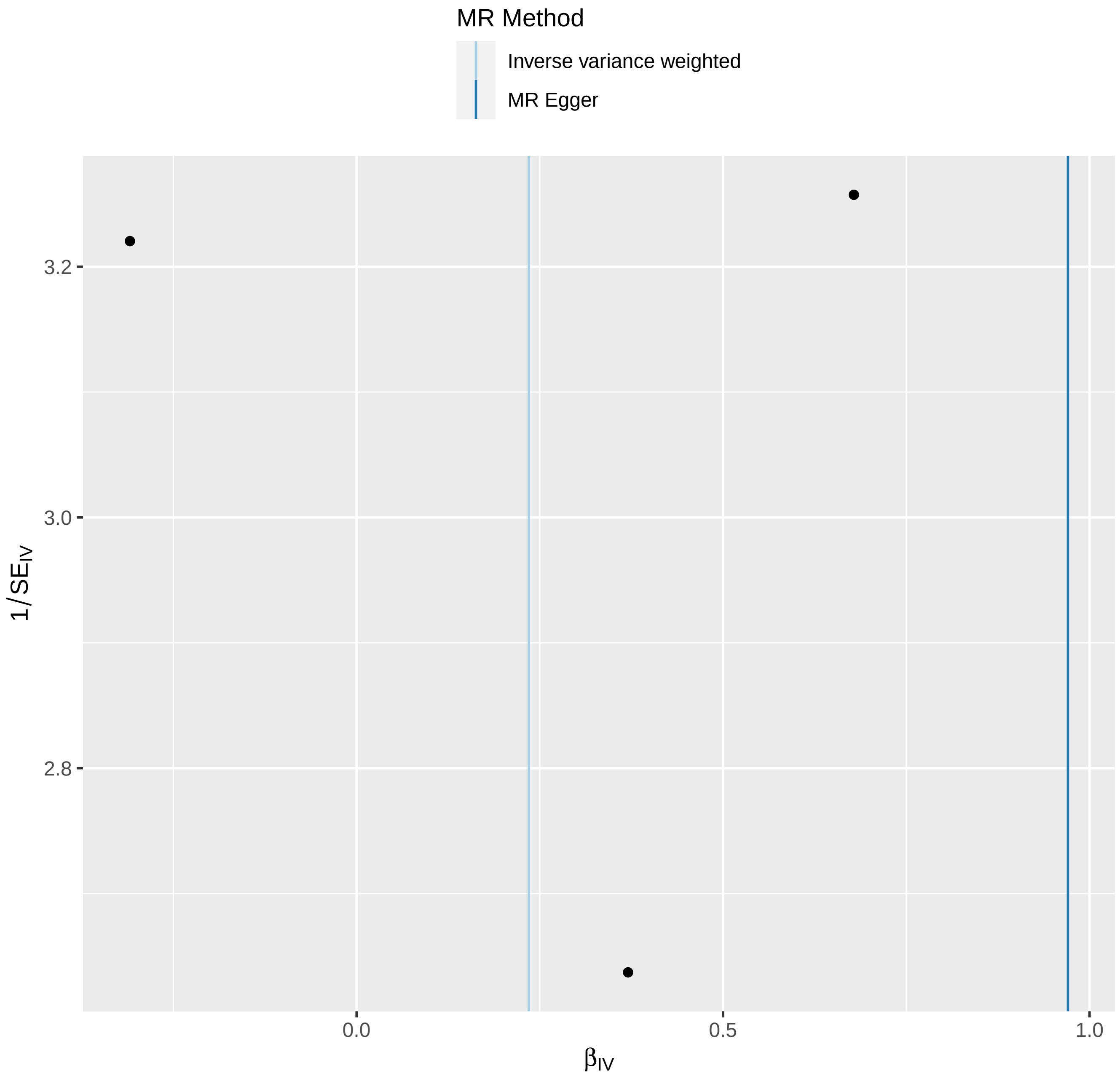

Batch 3979 : IDP T1 FAST ROIs L subcallosal cortex on Crohn's disease

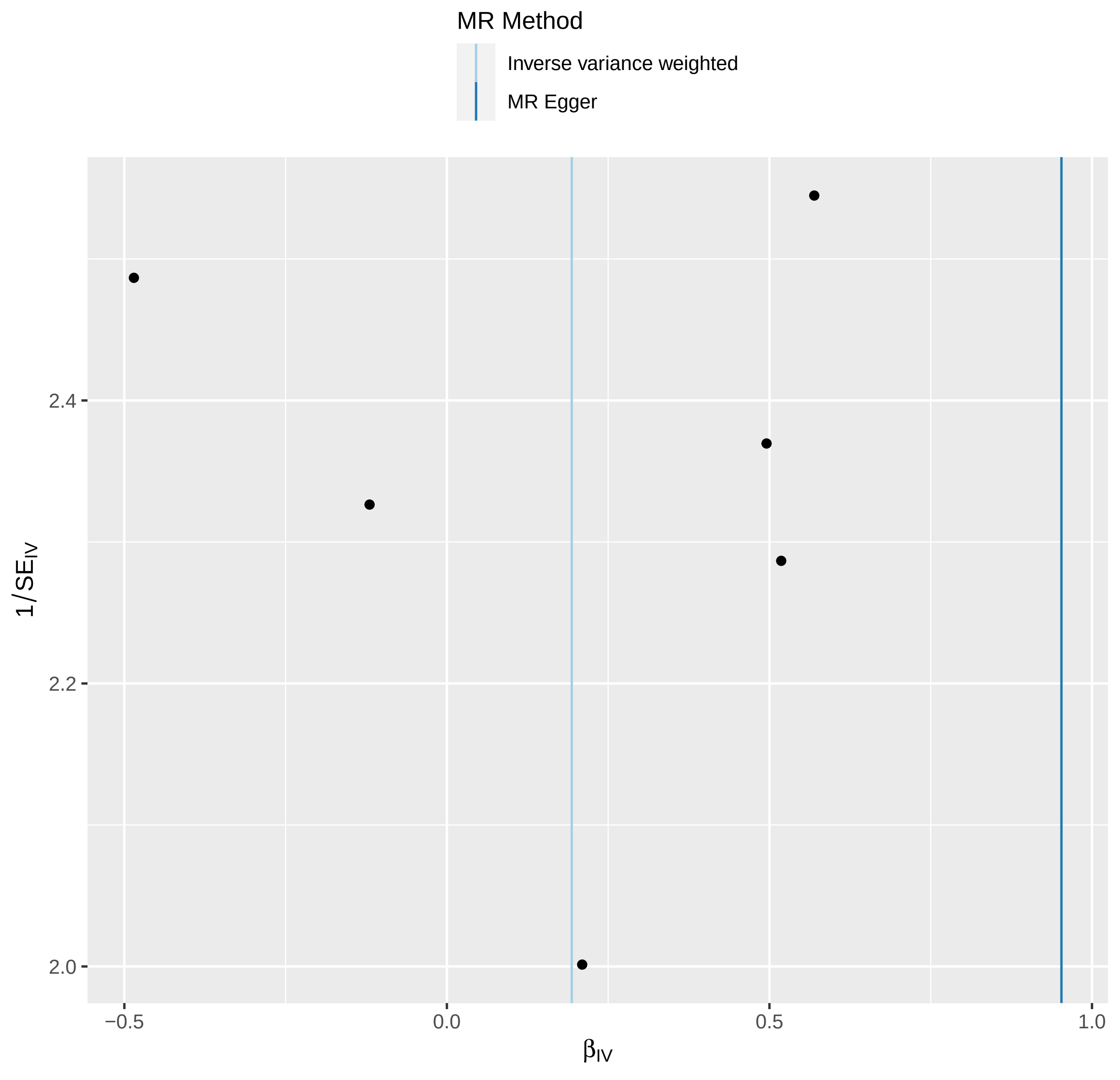

Batch 3990 : IDP T1 FAST ROIs R subcallosal cortex on Crohn's disease

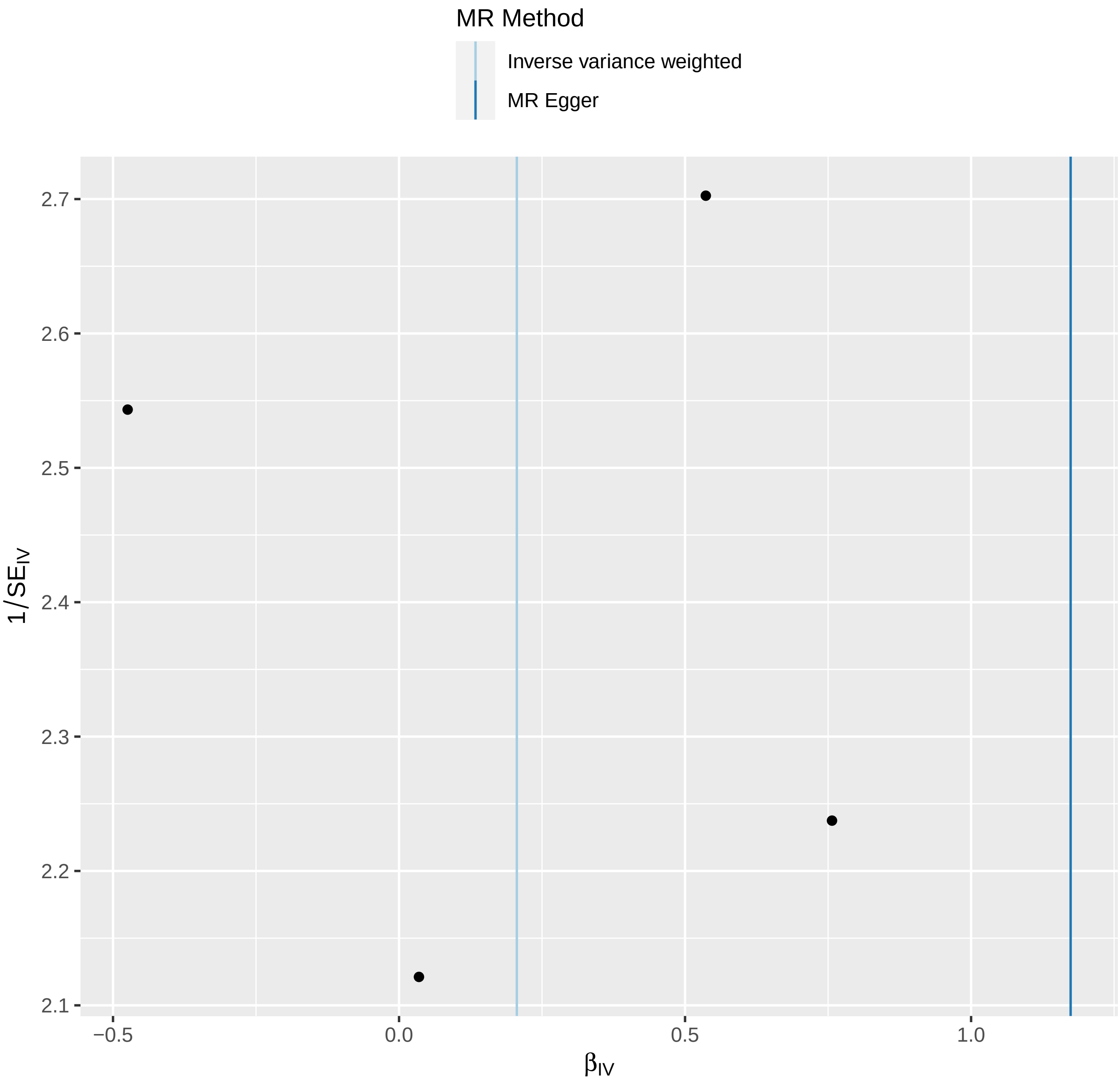

Batch 4035 : IDP T1 FAST ROIs R cing gyrus ant on Crohn's disease

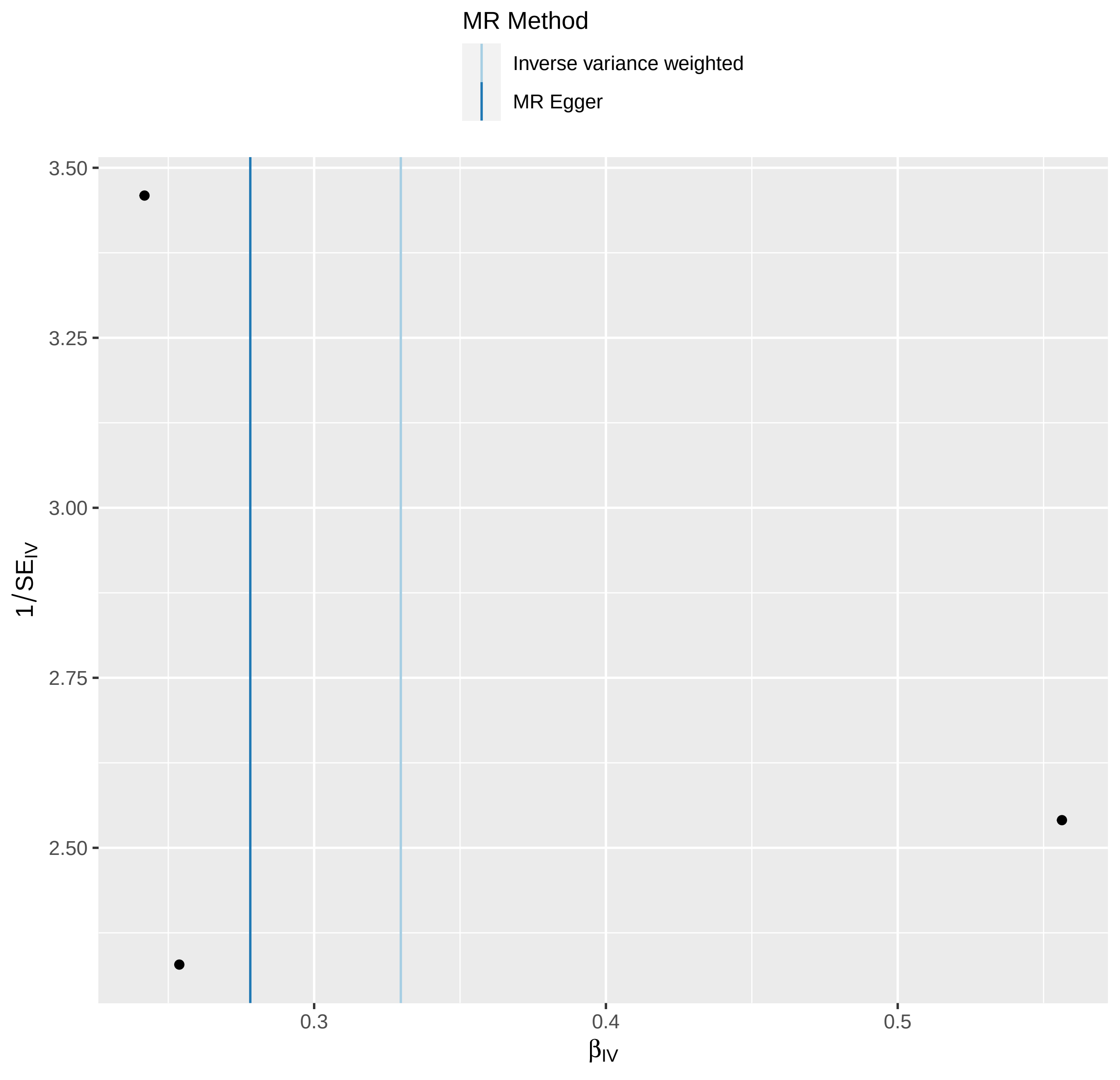

Batch 4057 : IDP T1 FAST ROIs R cing gyrus post on Crohn's disease

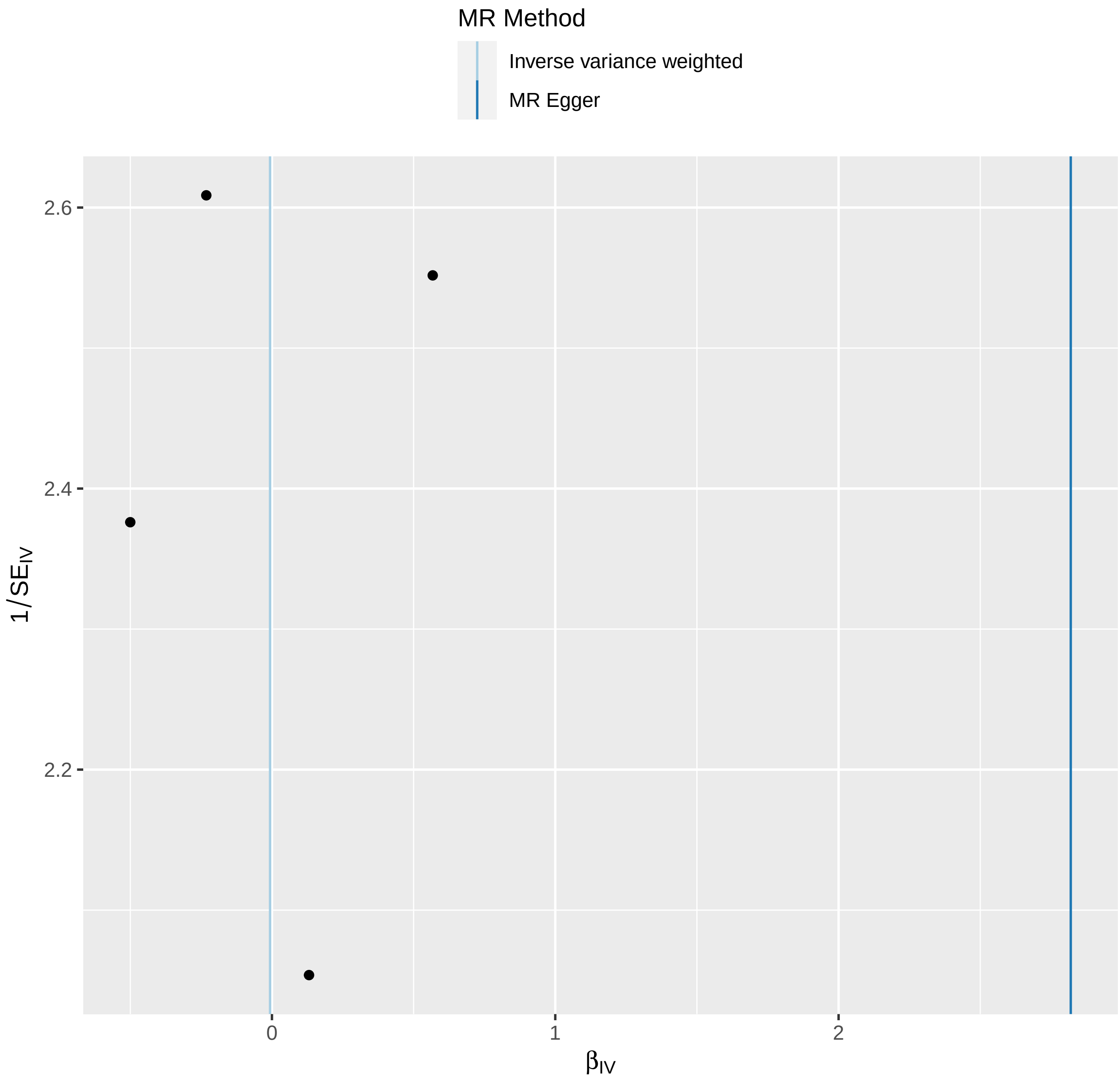

Batch 4068 : IDP T1 FAST ROIs L precun cortex on Crohn's disease

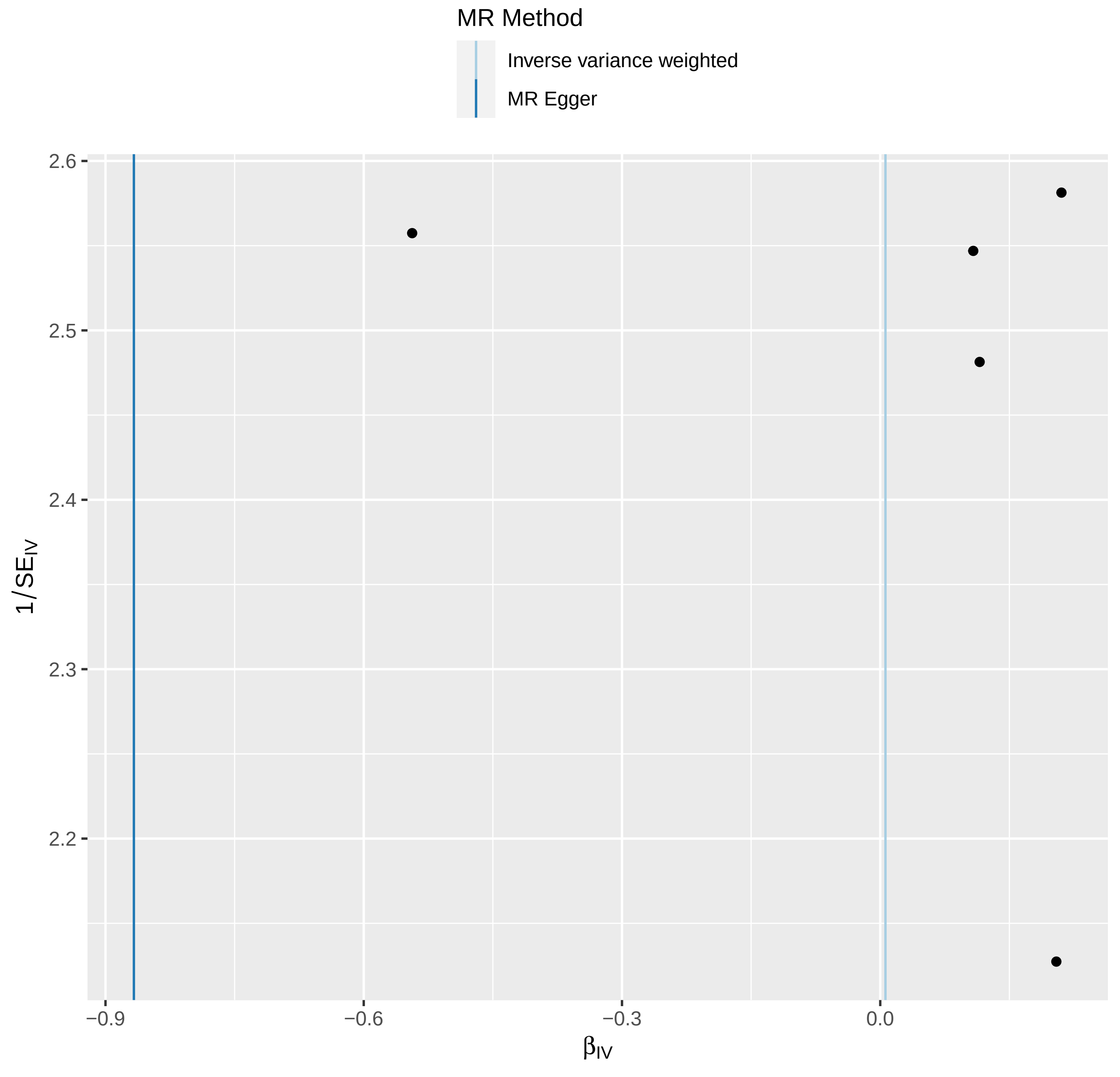

Batch 4081 : IDP T1 FAST ROIs L cuneal cortex on Crohn's disease

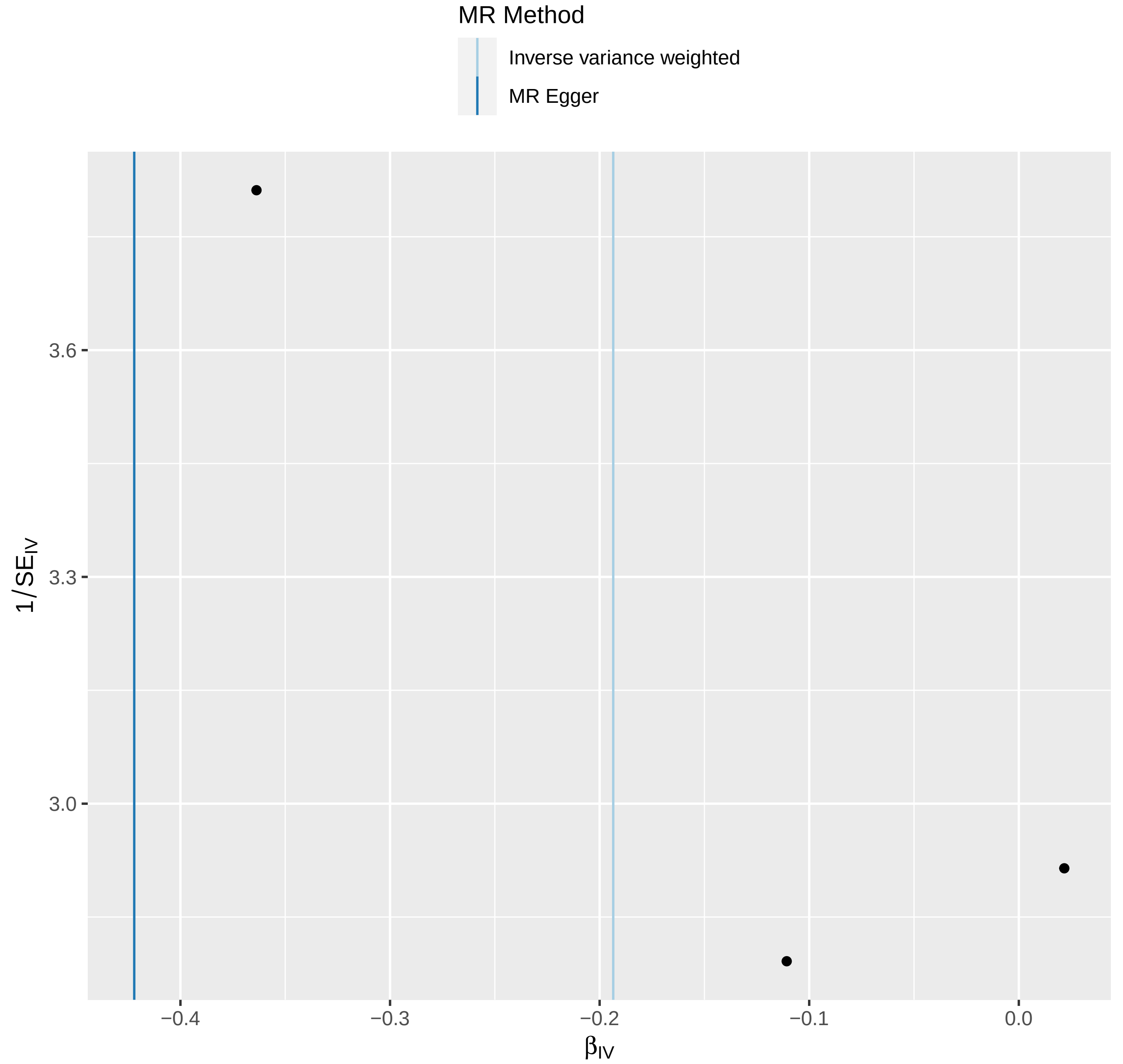

Batch 4083 : IDP T1 SIENAX brain−normalised volume on Crohn's disease

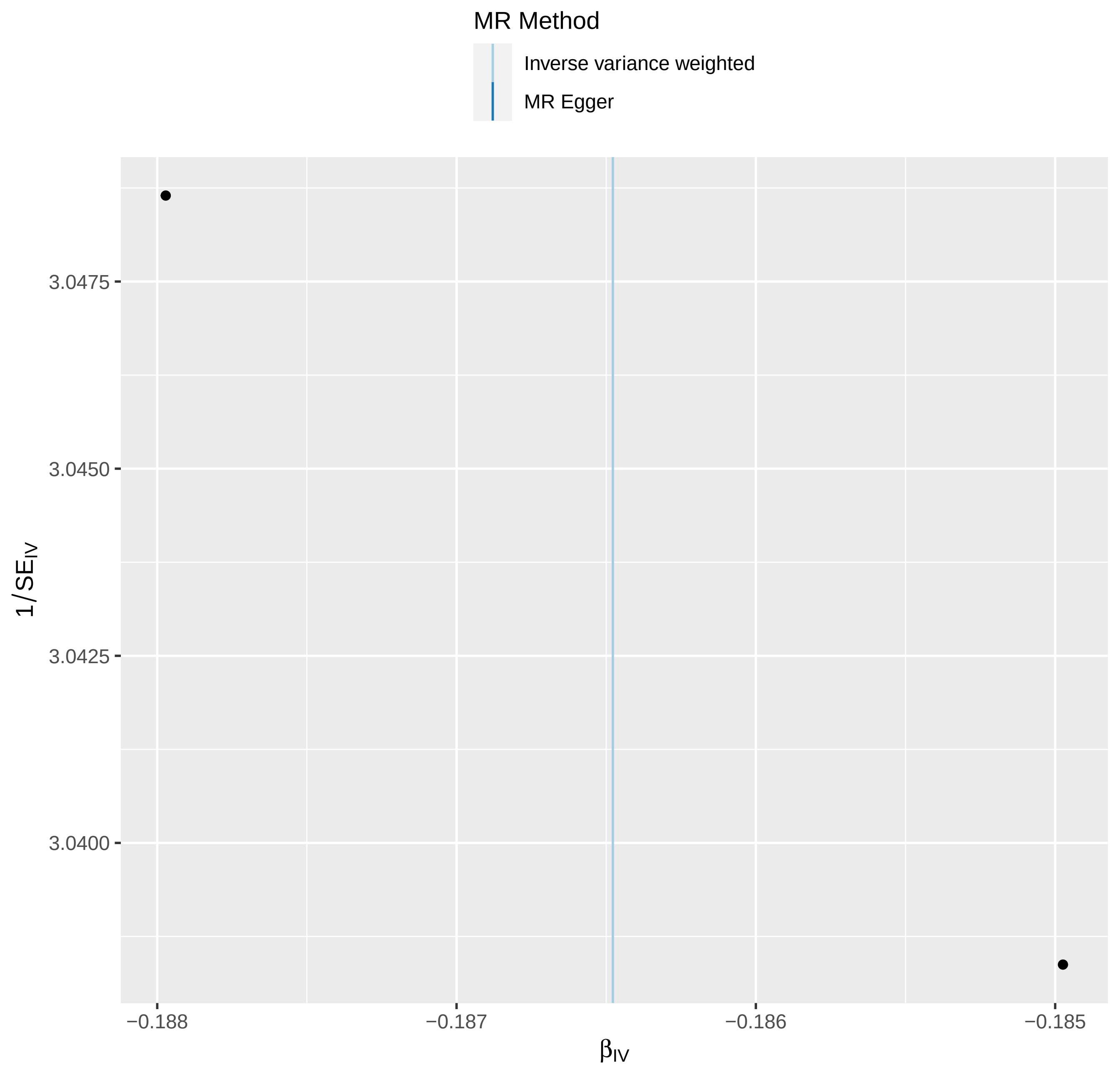

Batch 4084 : IDP T1 FAST ROIs L front orb cortex on Crohn's disease

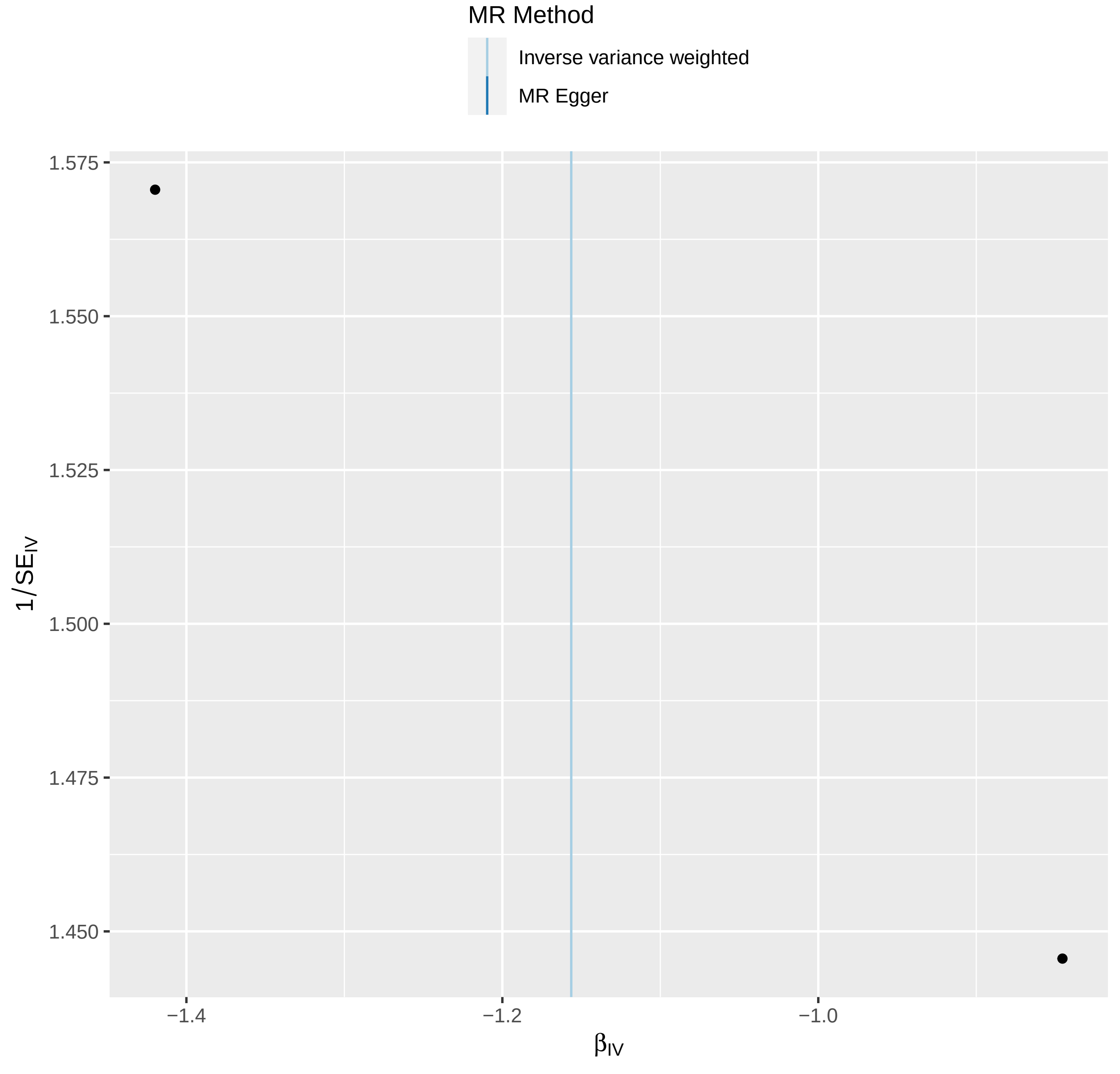

Batch 4086 : IDP T1 FAST ROIs L parahipp gyrus ant on Crohn's disease

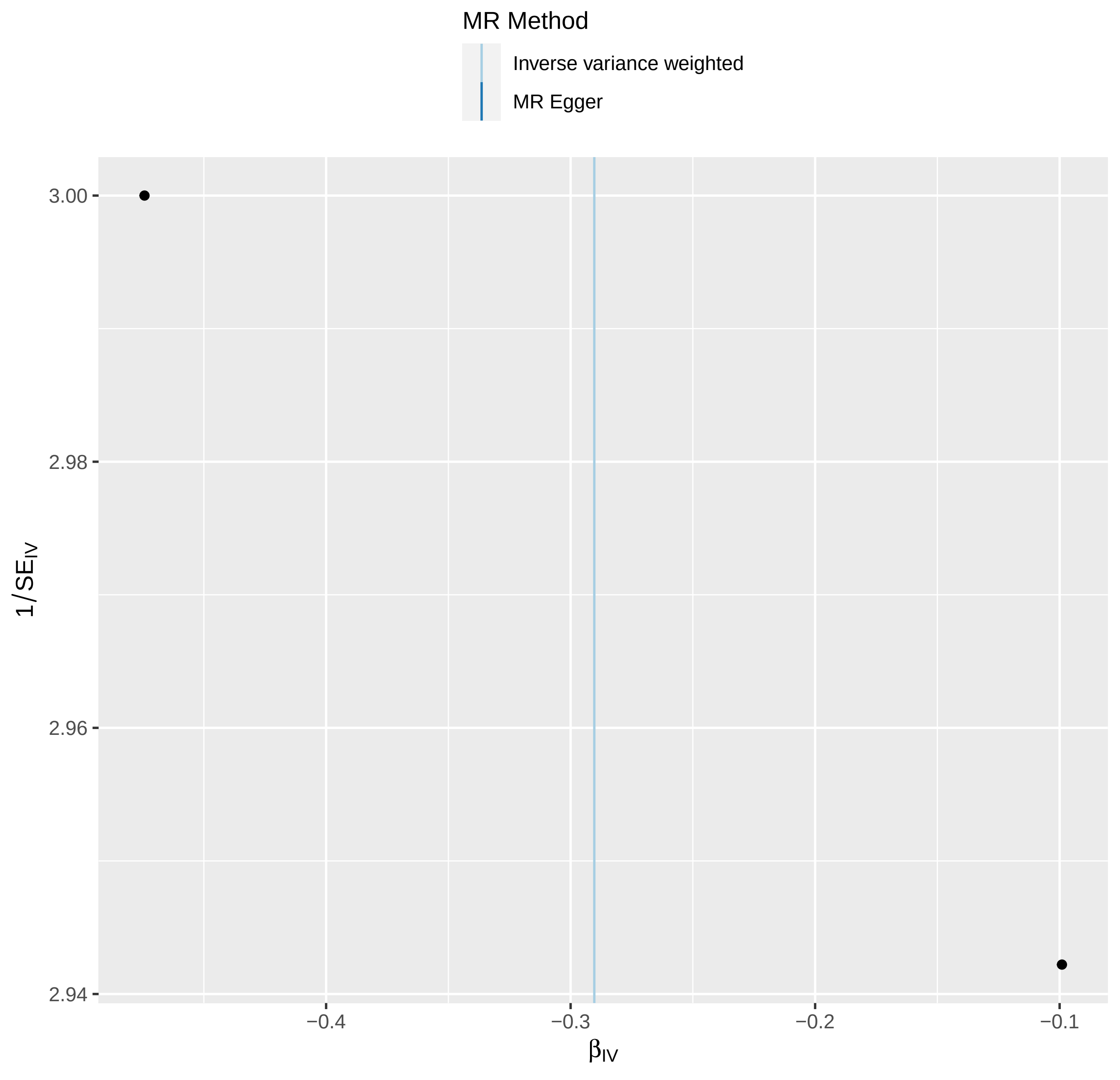

Batch 4087 : IDP T1 FAST ROIs R parahipp gyrus ant on Crohn's disease

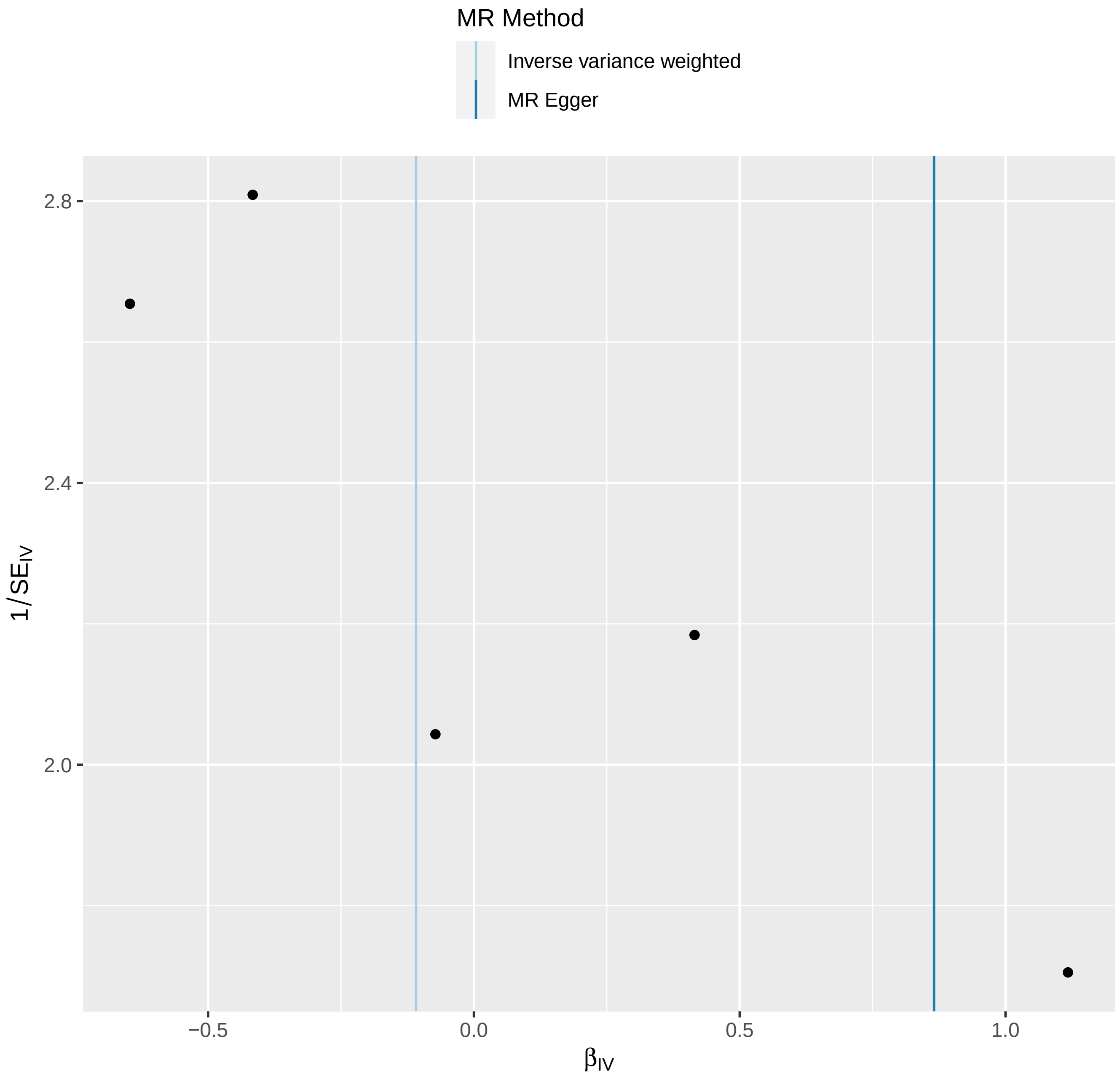

Batch 4089 : IDP T1 FAST ROIs R parahipp gyrus post on Crohn's disease

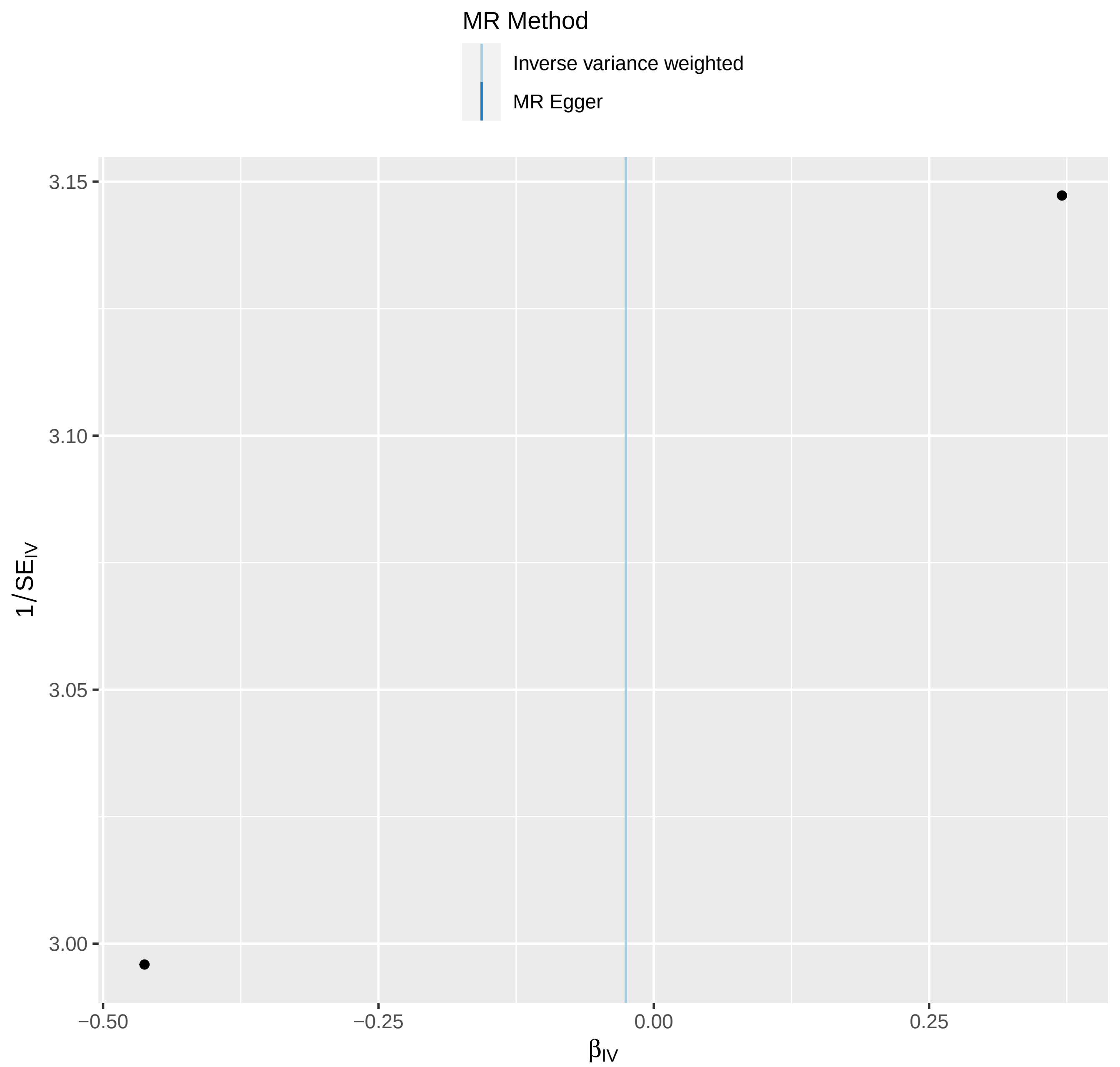

Batch 4091 : IDP T1 FAST ROIs R lingual gyrus on Crohn's disease

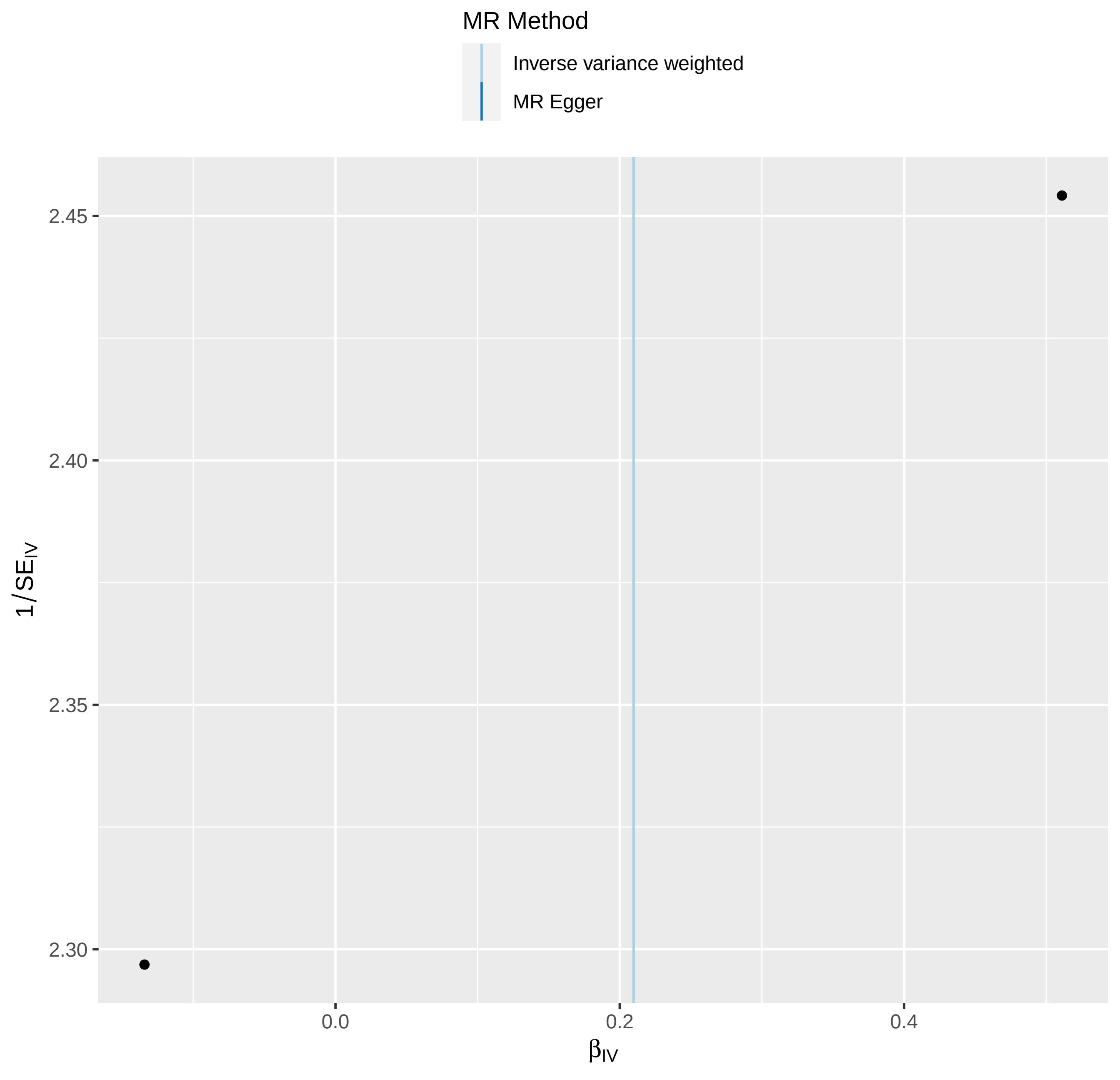

Batch 4095 : IDP T1 SIENAX brain−unnormalised volume on Ulcerative colitis

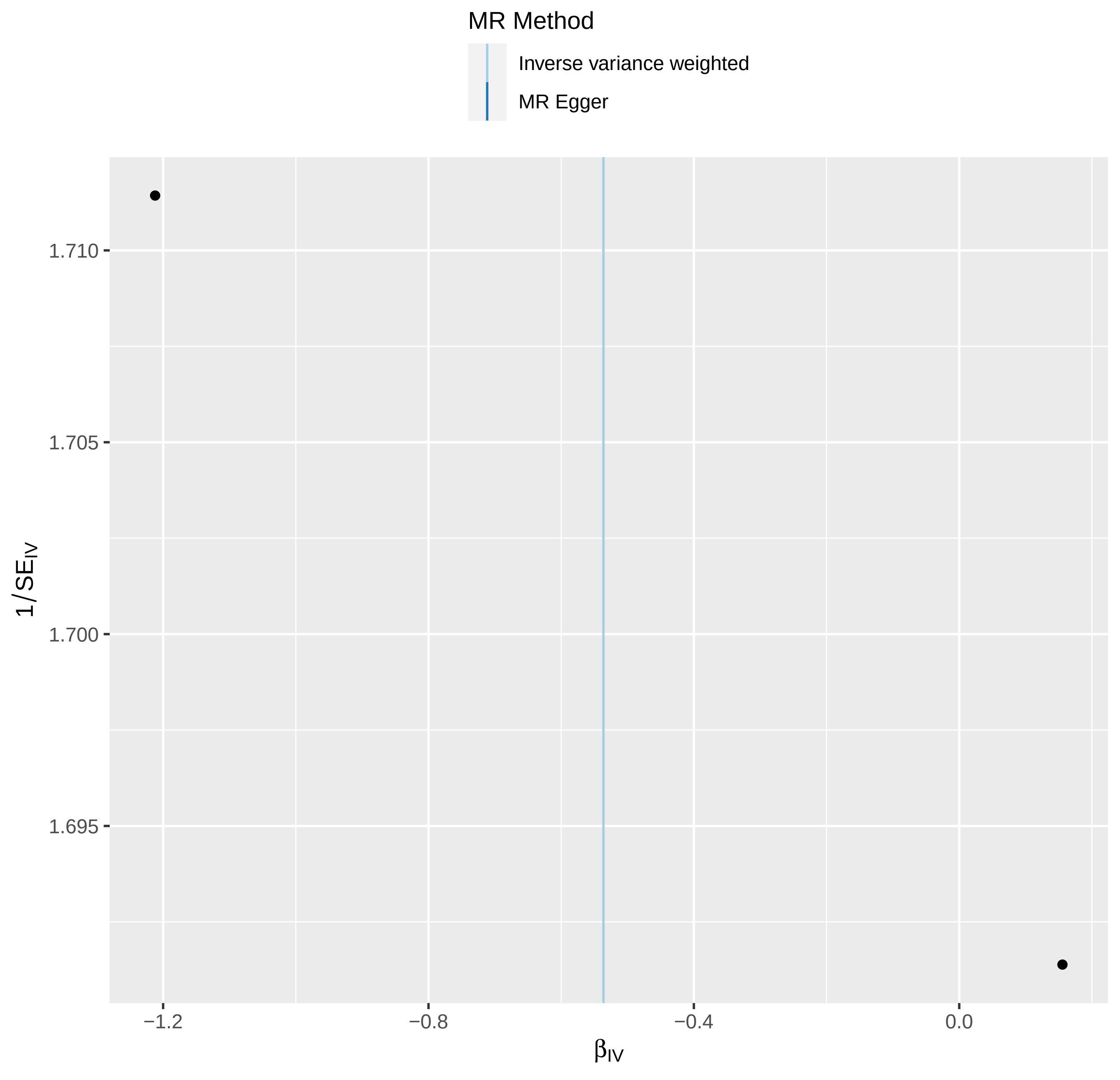

Batch 4097 : IDP T1 FAST ROIs R temp fusif cortex post on Ulcerative colitis

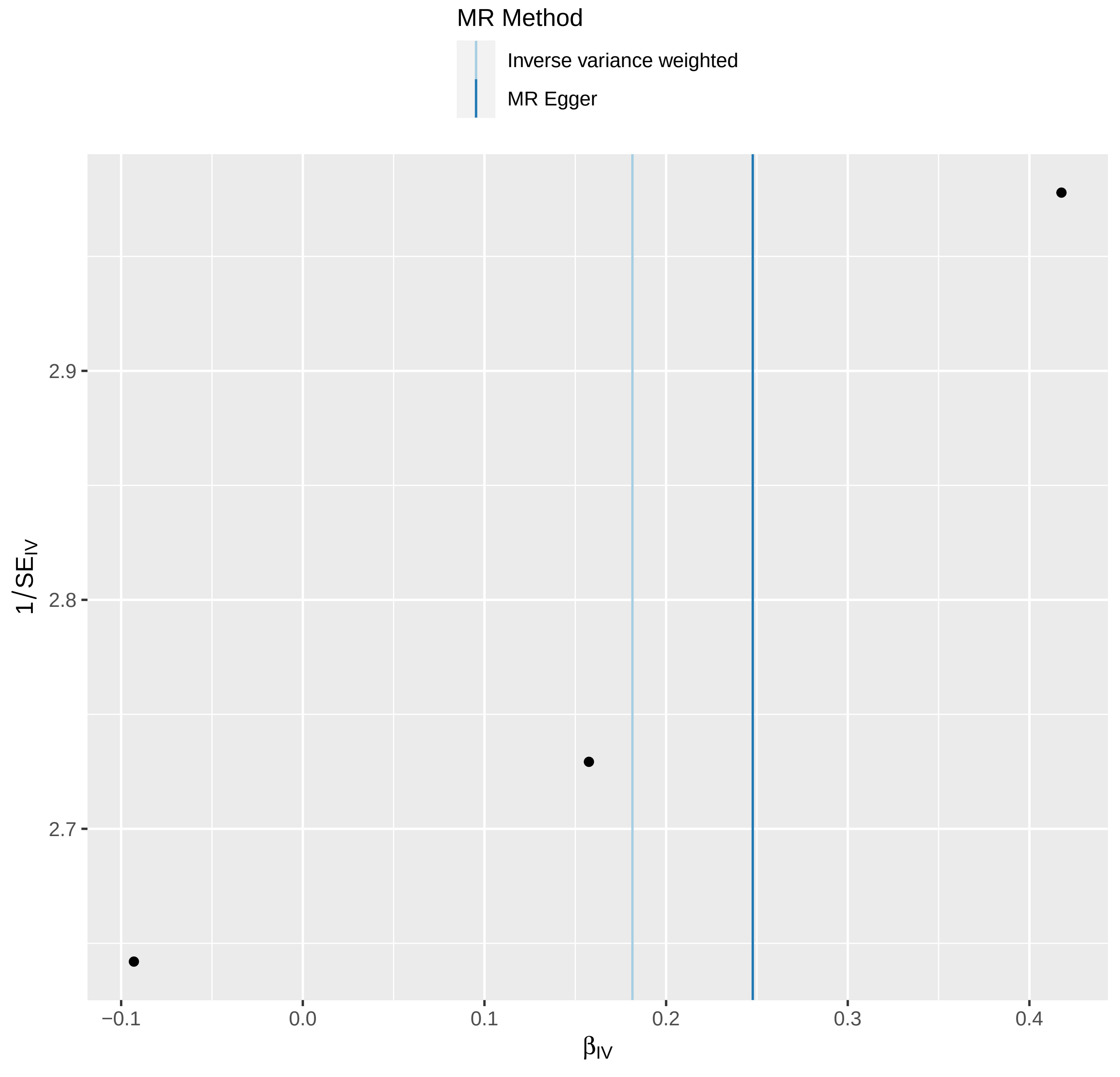

Batch 4103 : IDP T1 FAST ROIs R front operc cortex on Ulcerative colitis

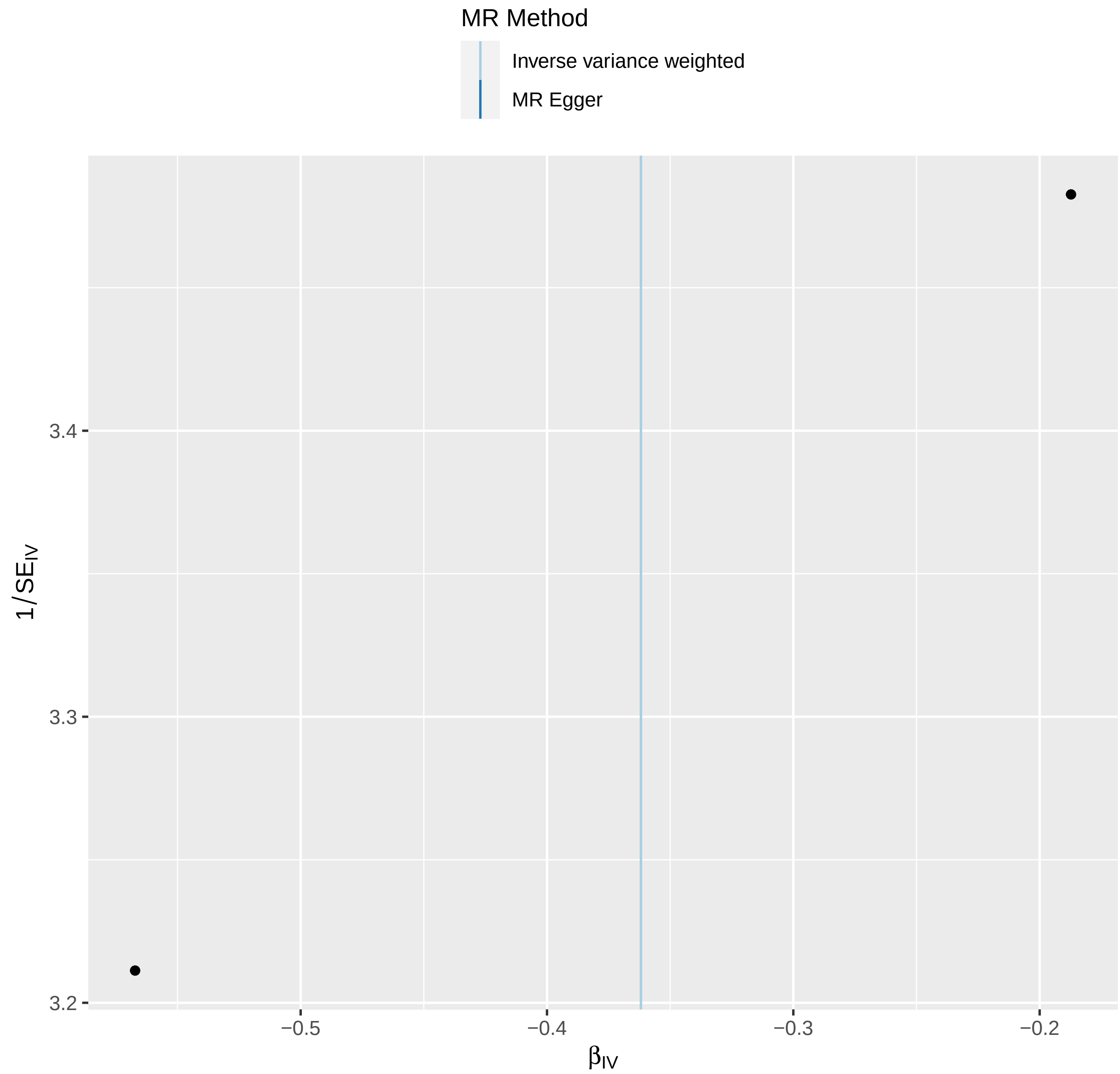

Batch 4106 : IDP T1 FIRST left thalamus volume on Ulcerative colitis

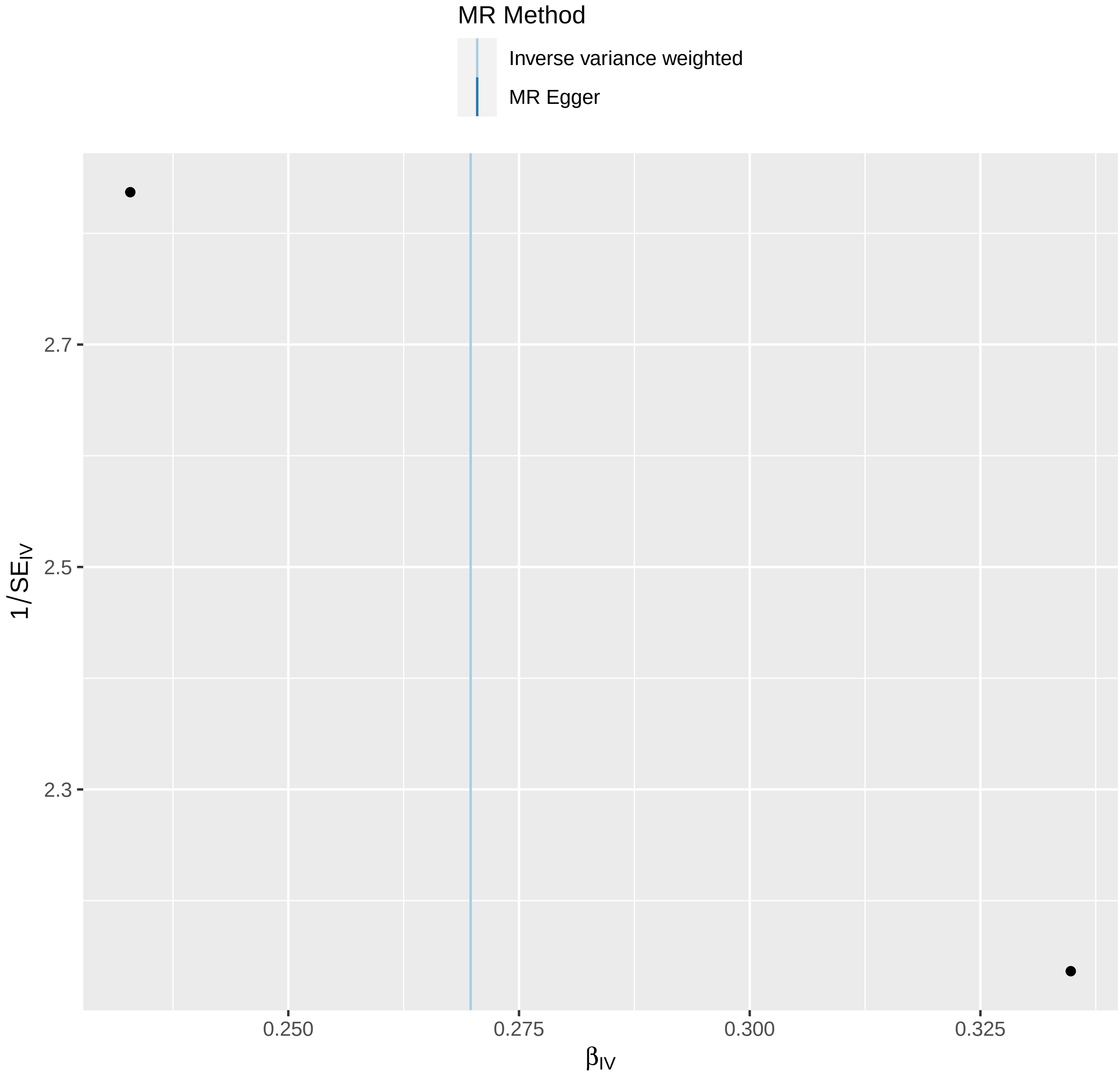

Batch 4107 : IDP T1 FAST ROIs L parietal operc cortex on Ulcerative colitis

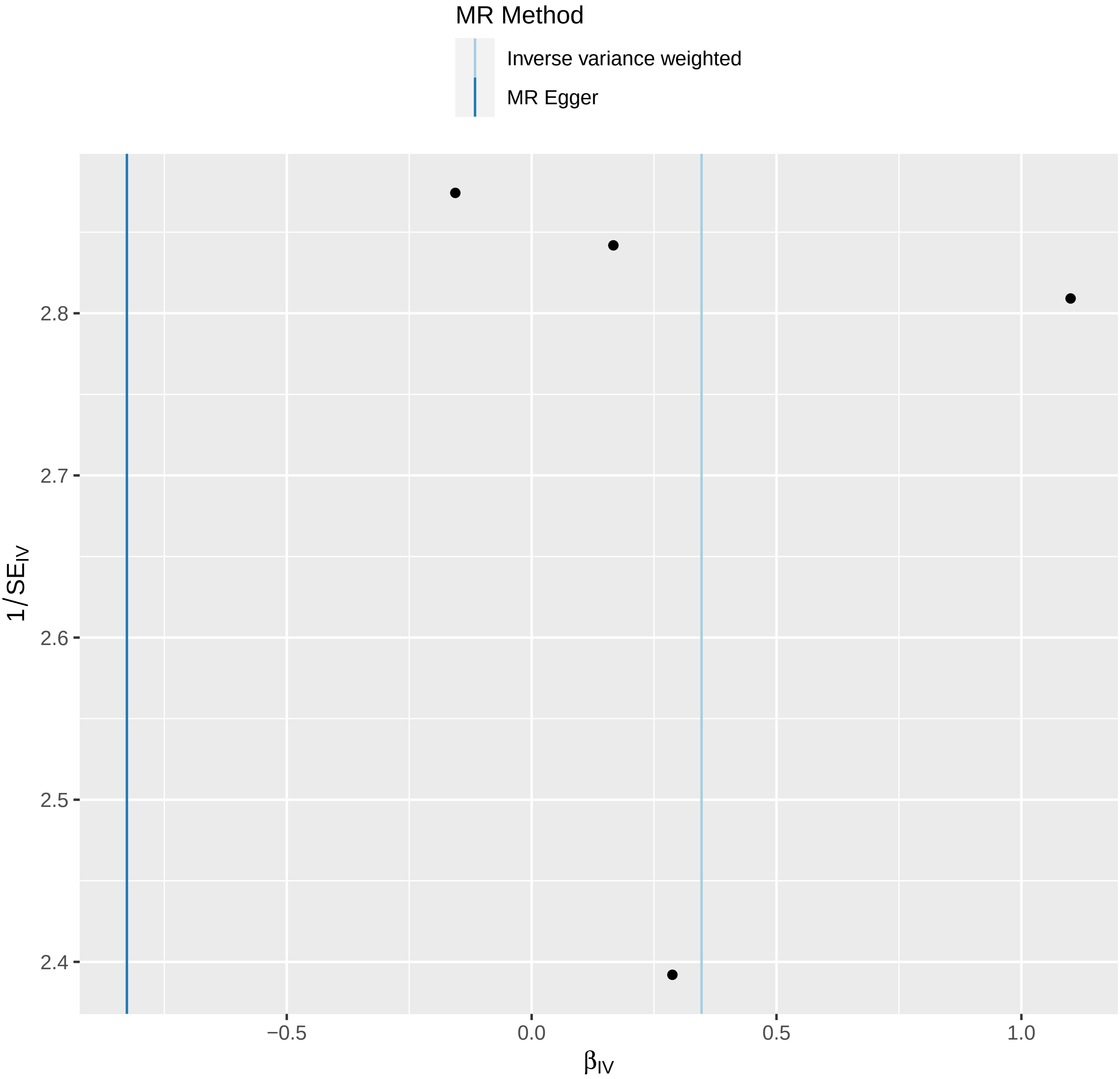

Batch 4108 : IDP T1 FAST ROIs R parietal operc cortex on Ulcerative colitis

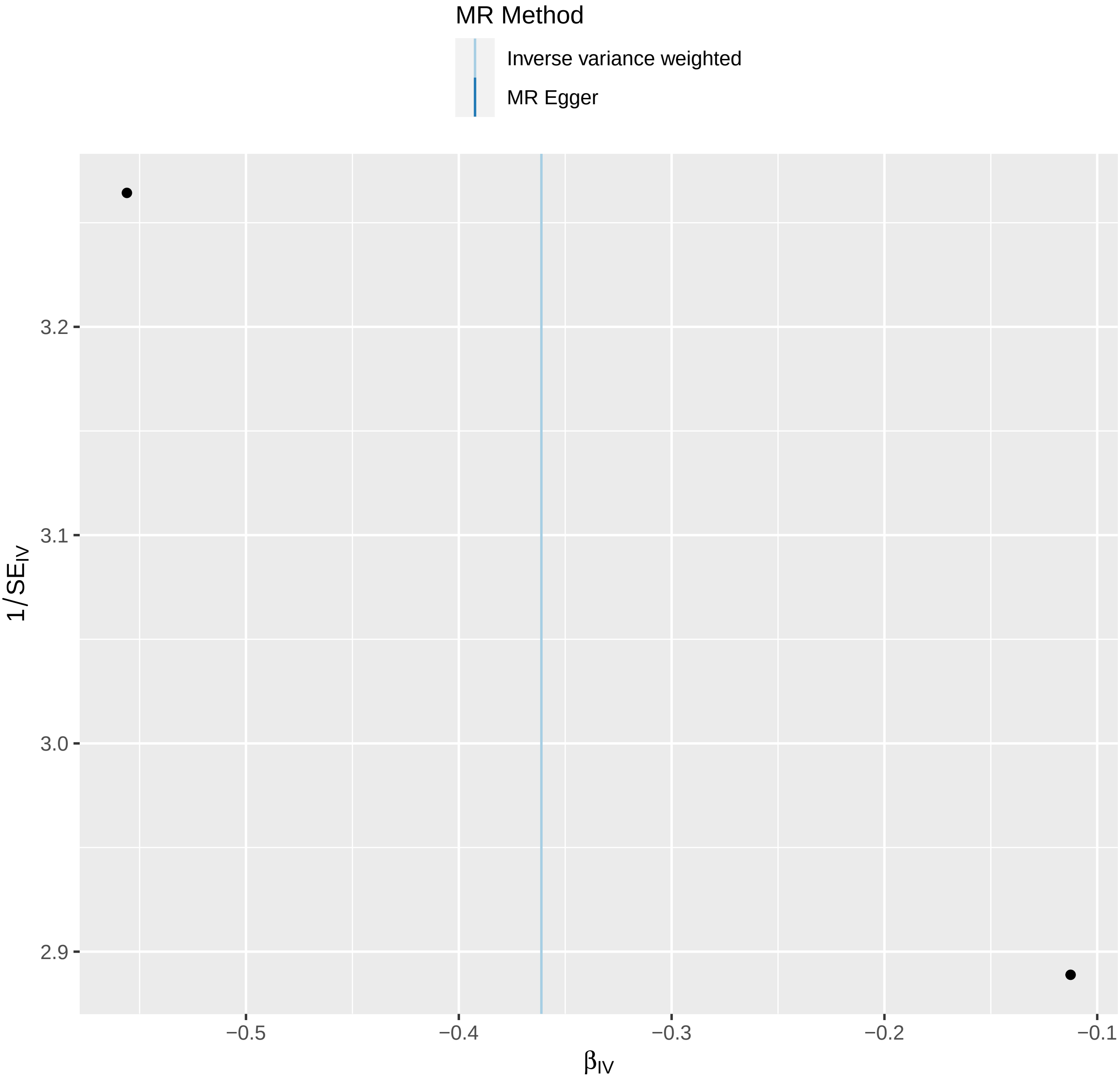

Batch 4109 : IDP T1 FAST ROIs L planum polare on Ulcerative colitis

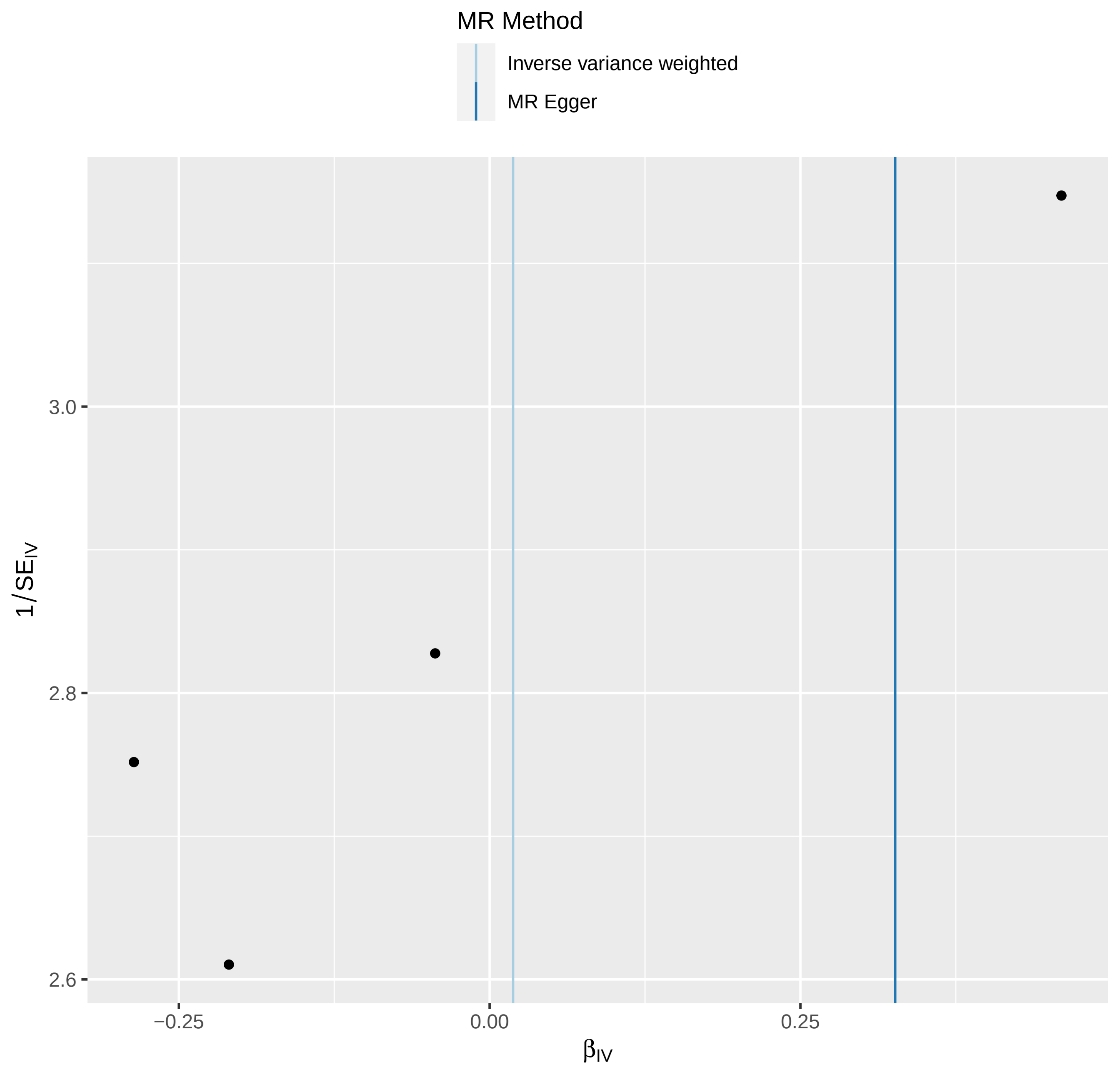

Batch 4111 : IDP T1 FAST ROIs L heschl gyrus on Ulcerative colitis

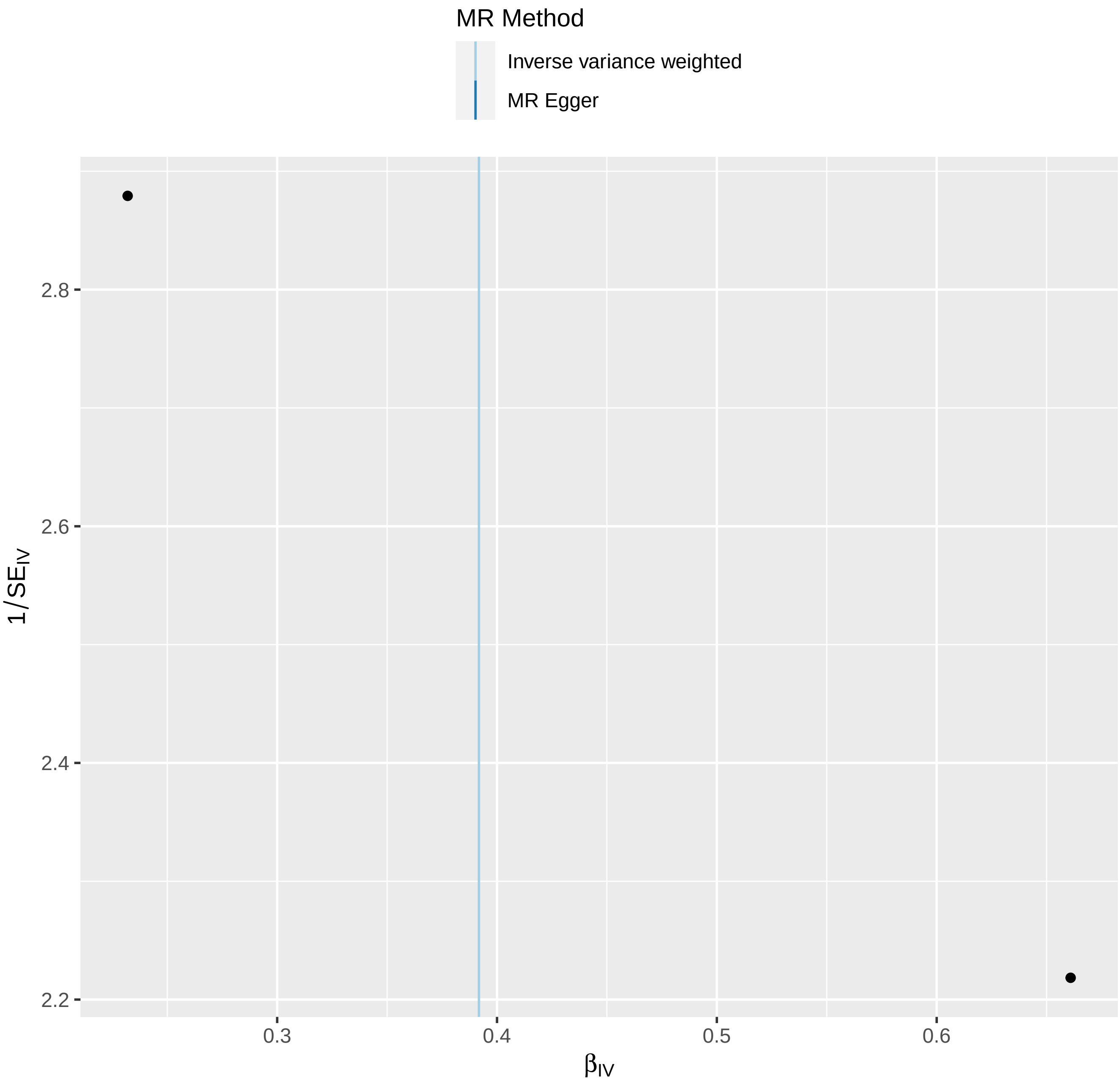

Batch 4112 : IDP T1 FAST ROIs R heschl gyrus on Ulcerative colitis

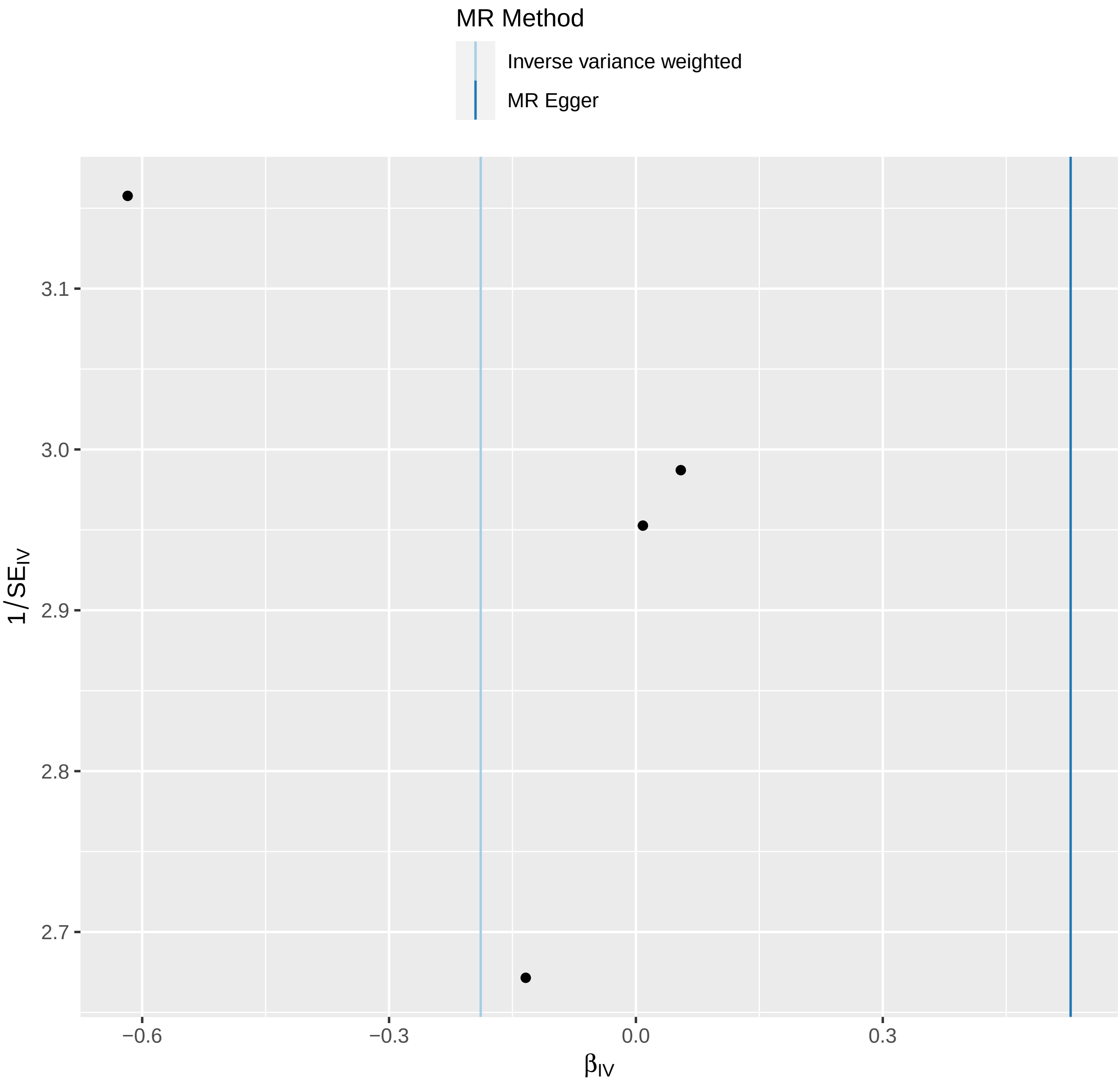

Batch 4114 : IDP T1 FAST ROIs R planum temporale on Ulcerative colitis

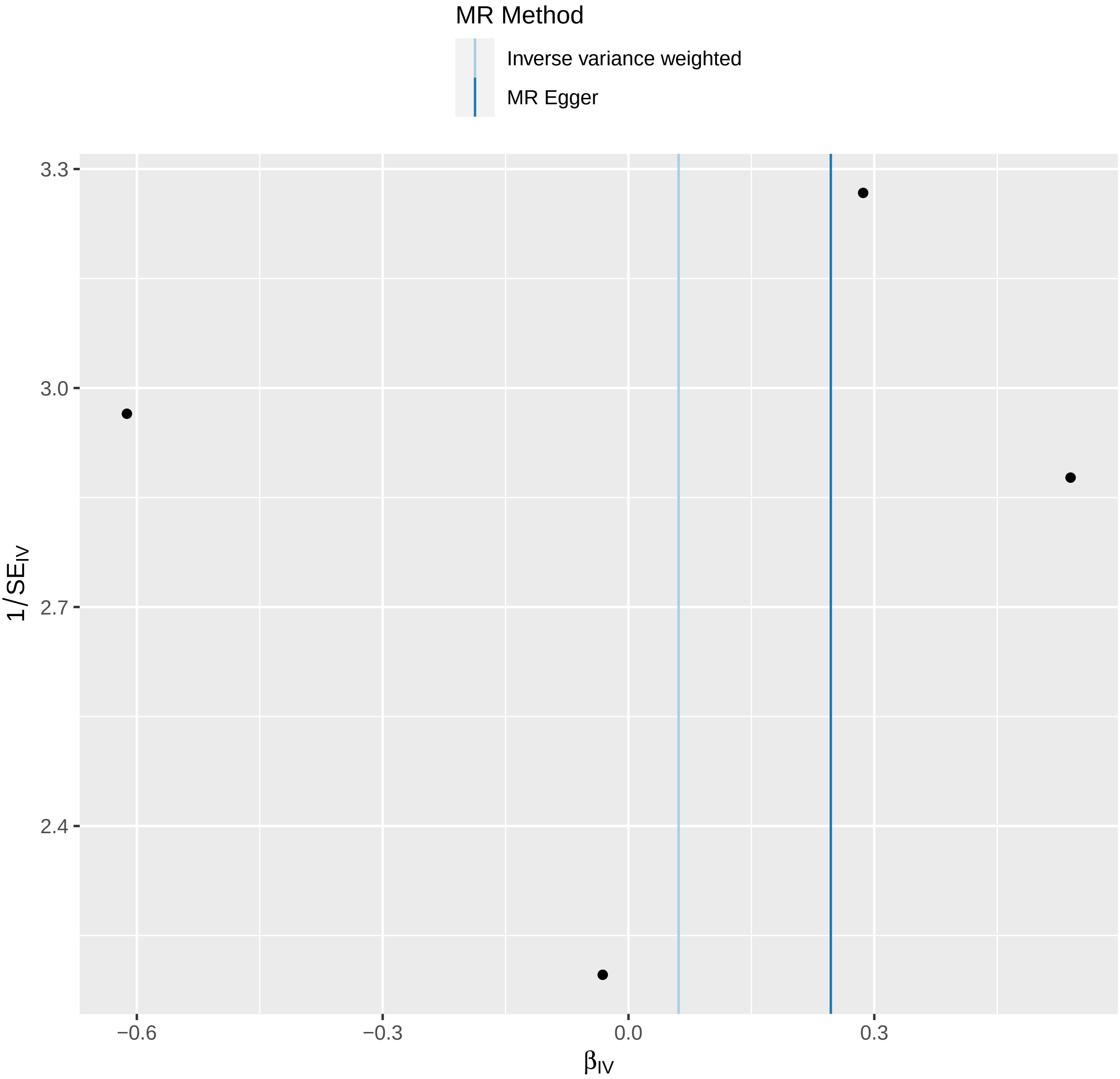

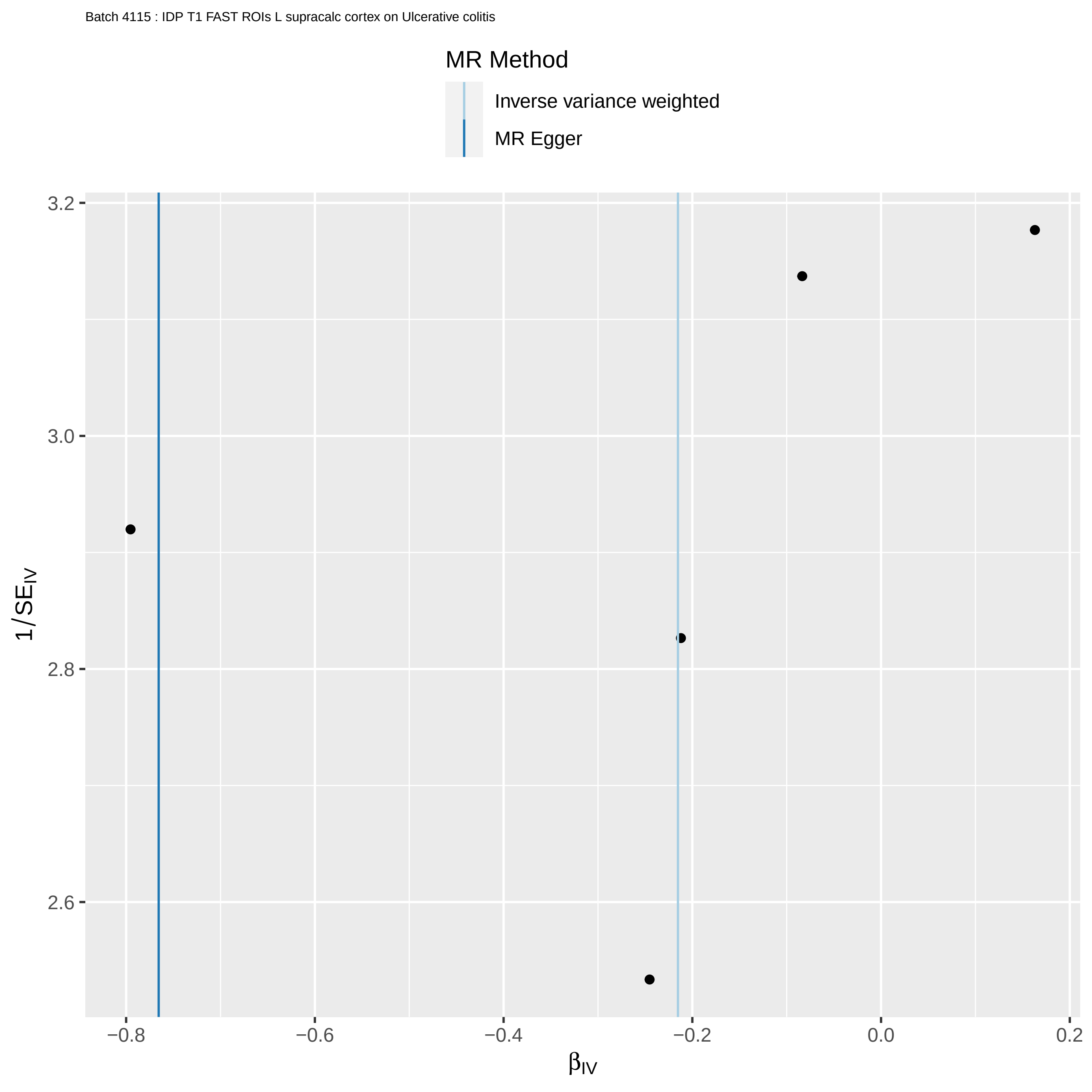

Batch 4115 : IDP T1 FAST ROIs L supracalc cortex on Ulcerative colitis
MR Method
Inverse variance weighted
MR Egger
3.2
3.0
2.8
2.6
1/SE_IV
−0.8
−0.6
−0.4
−0.2
0.0
0.2
β_IV

Batch 4116 : IDP T1 FAST ROIs R supracalc cortex on Ulcerative colitis

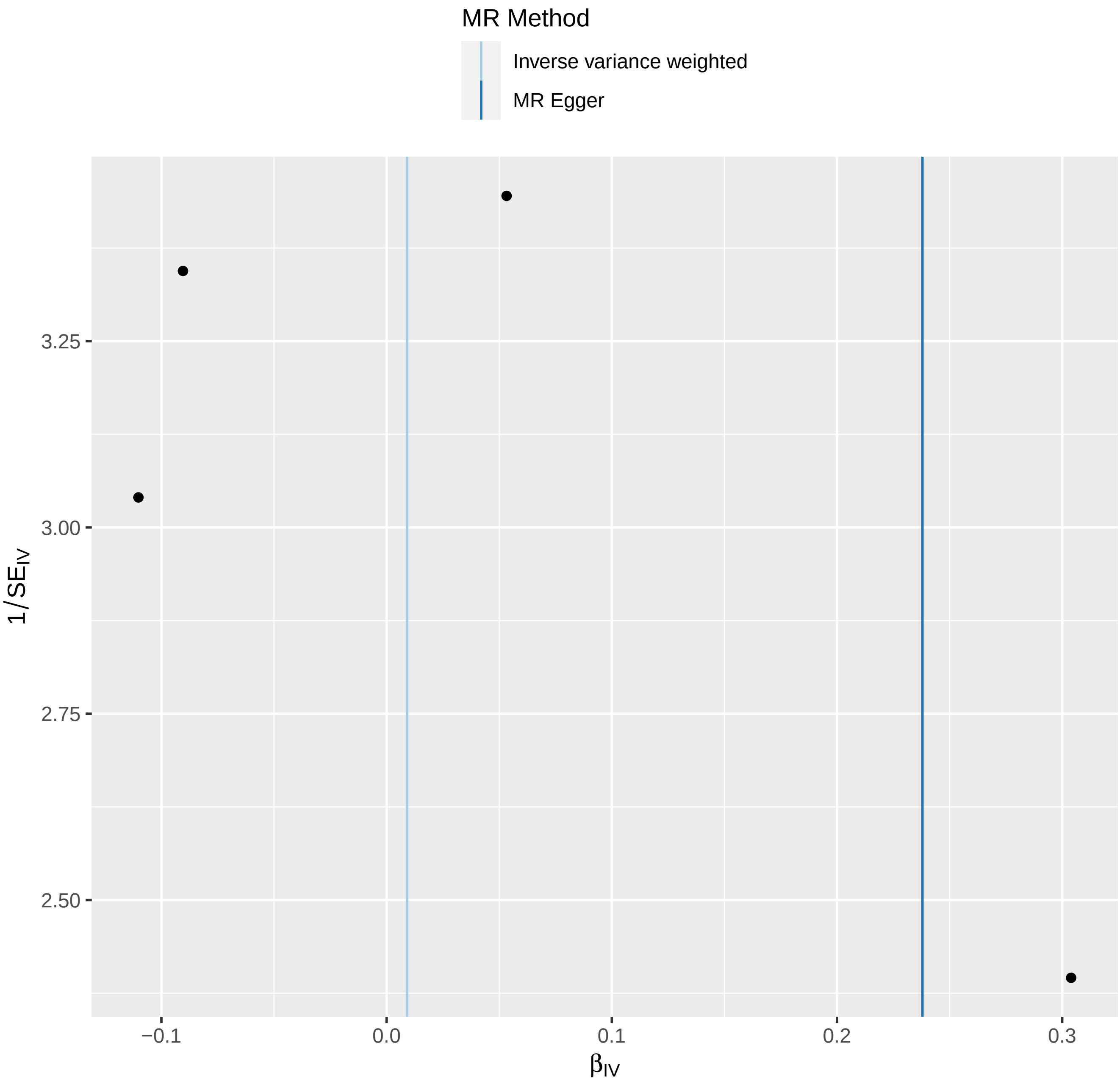

Batch 4118 : IDP T1 FAST ROIs L occ pole on Ulcerative colitis

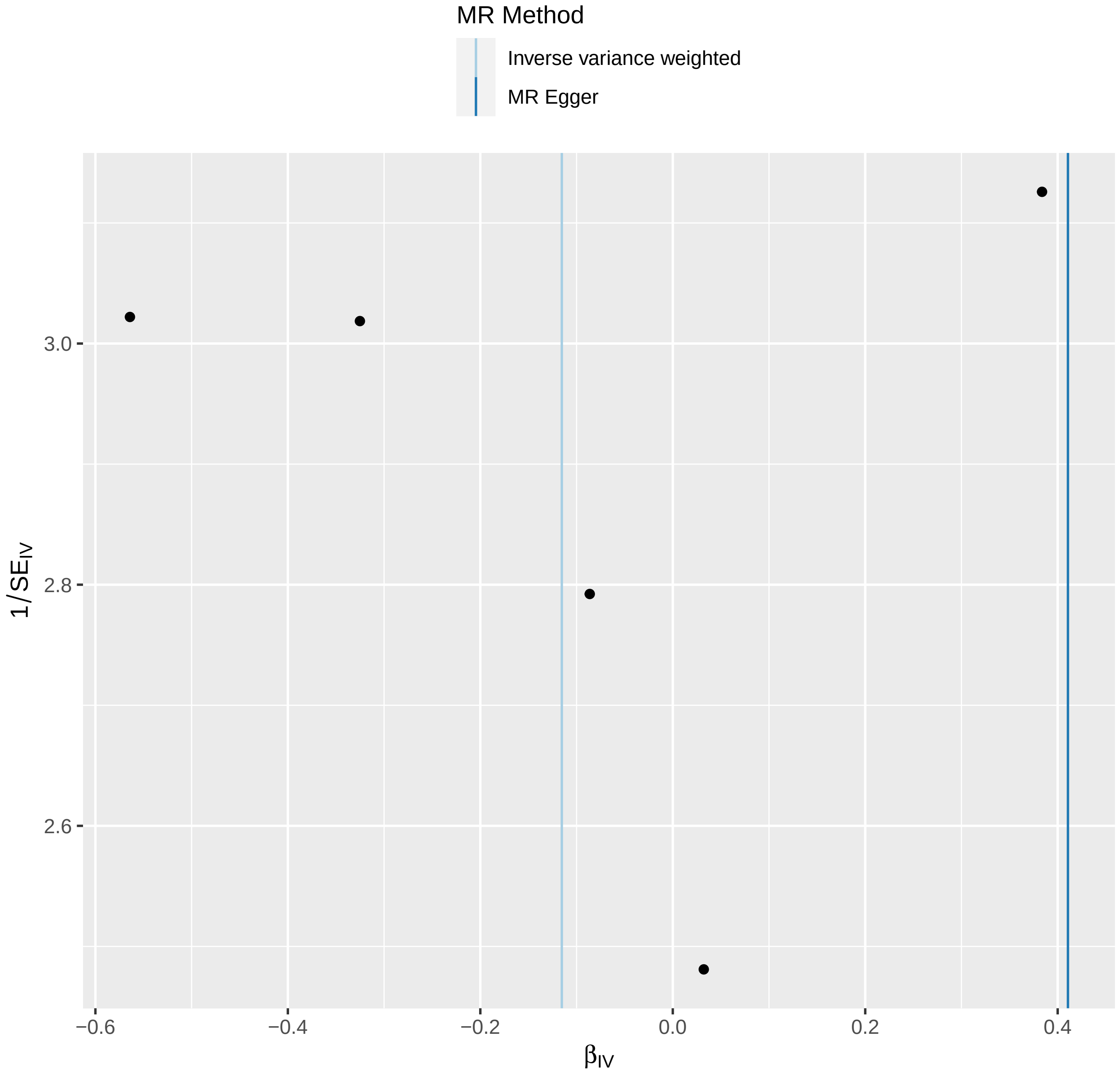

Batch 4120 : IDP T1 FAST ROIs L thalamus on Ulcerative colitis

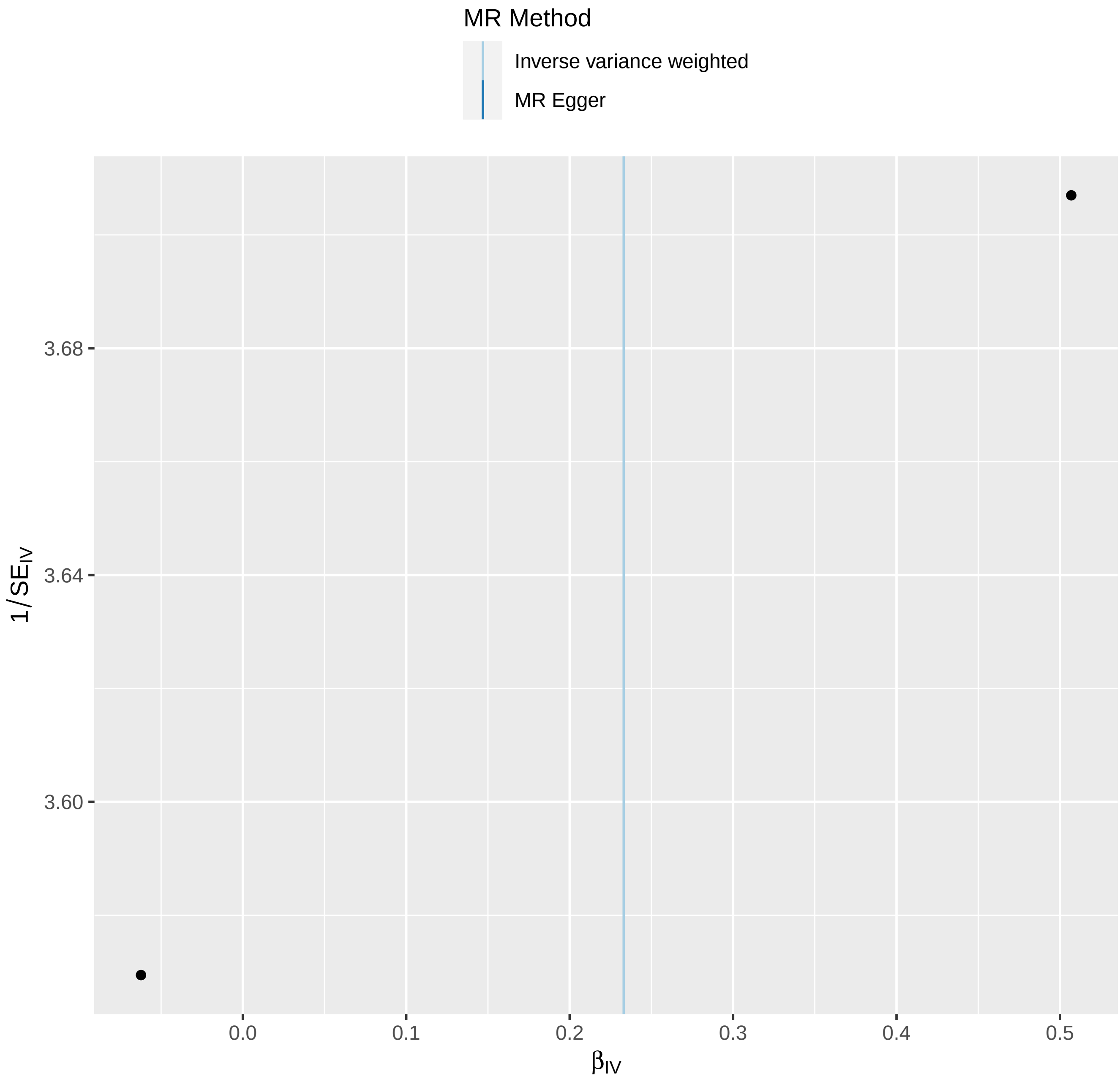

Batch 4124 : IDP T1 FAST ROIs L putamen on Ulcerative colitis

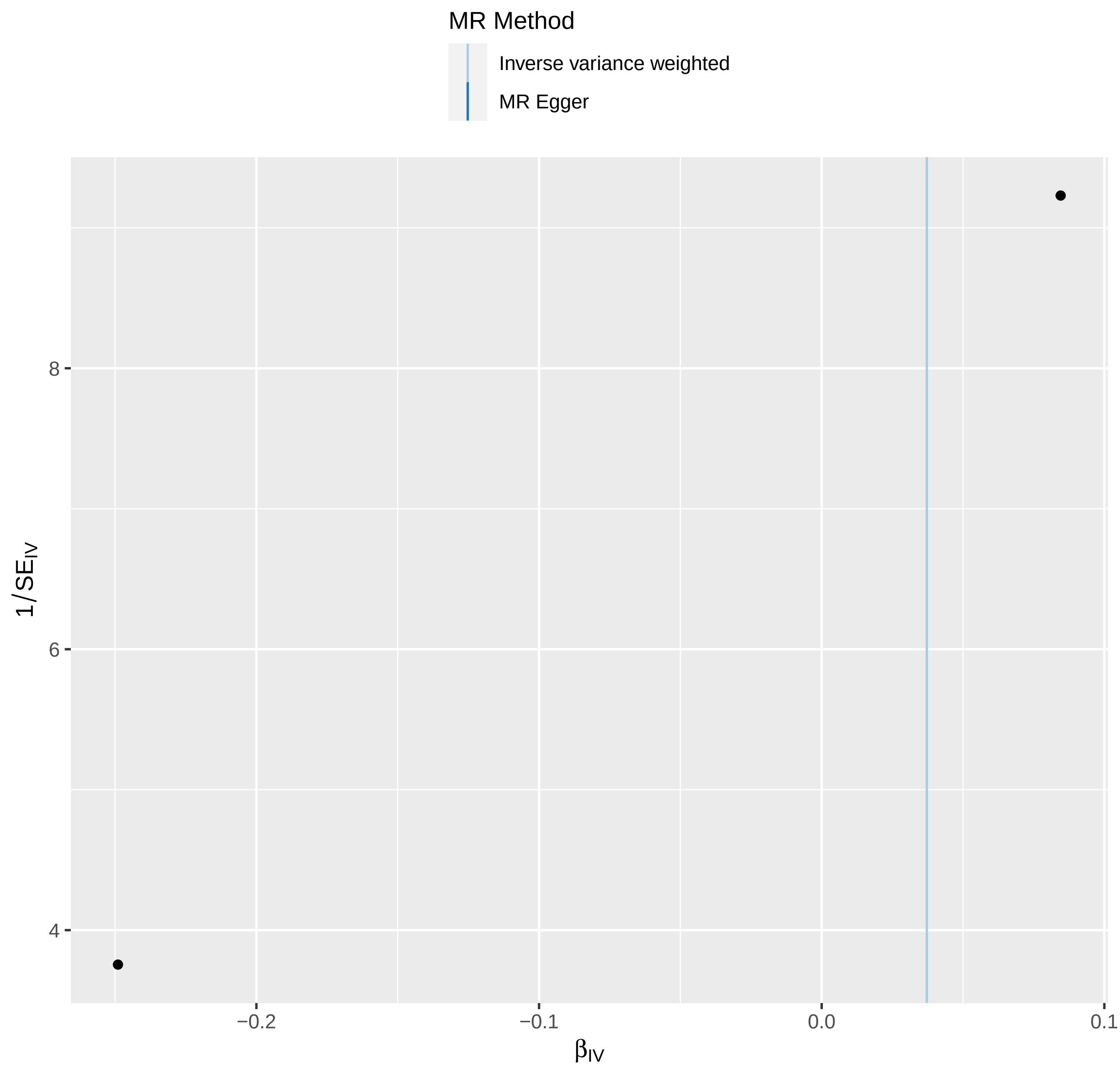

Batch 4125 : IDP T1 FAST ROIs R putamen on Ulcerative colitis

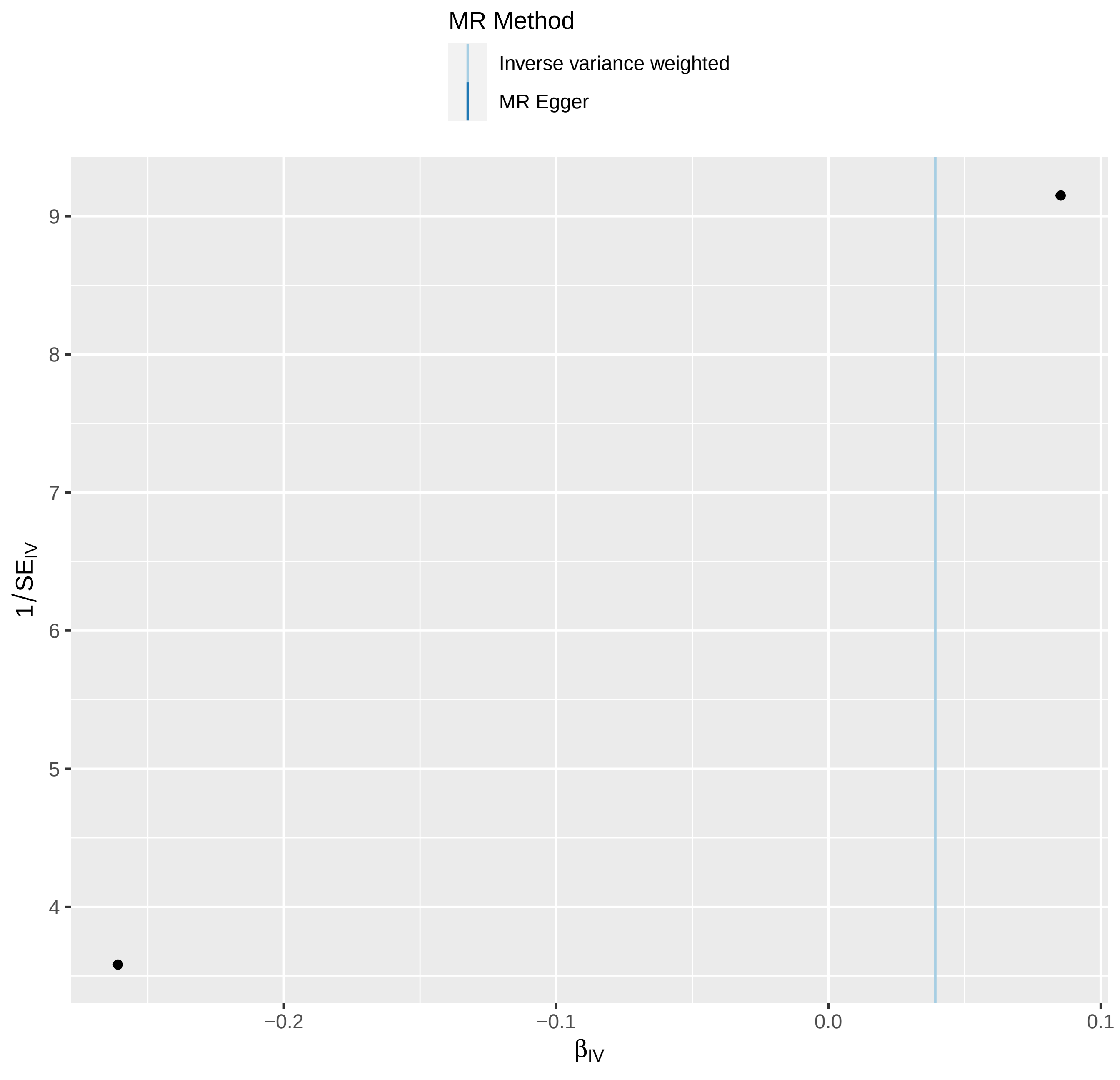

Batch 4127 : IDP T1 FAST ROIs R pallidum on Ulcerative colitis

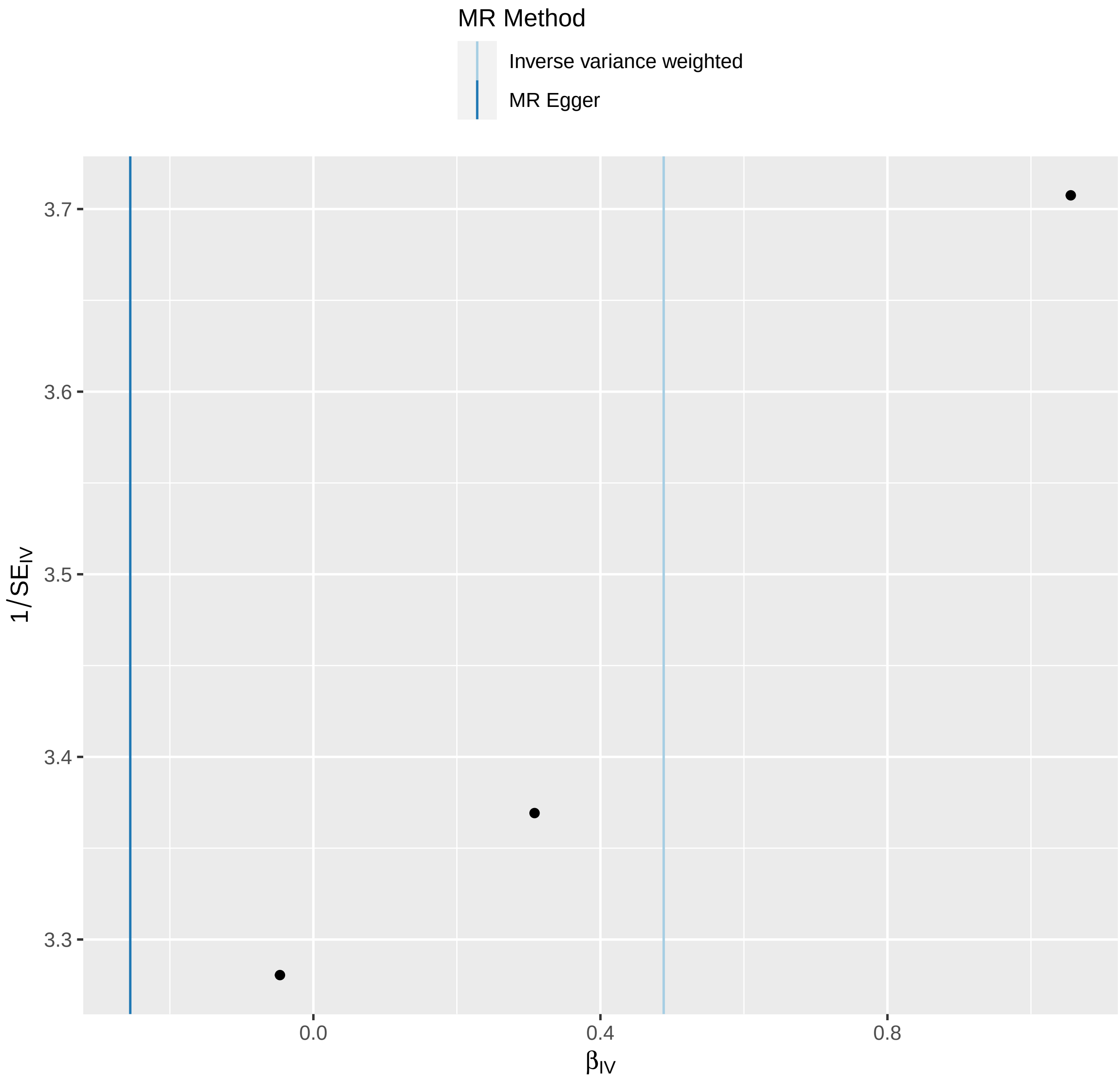

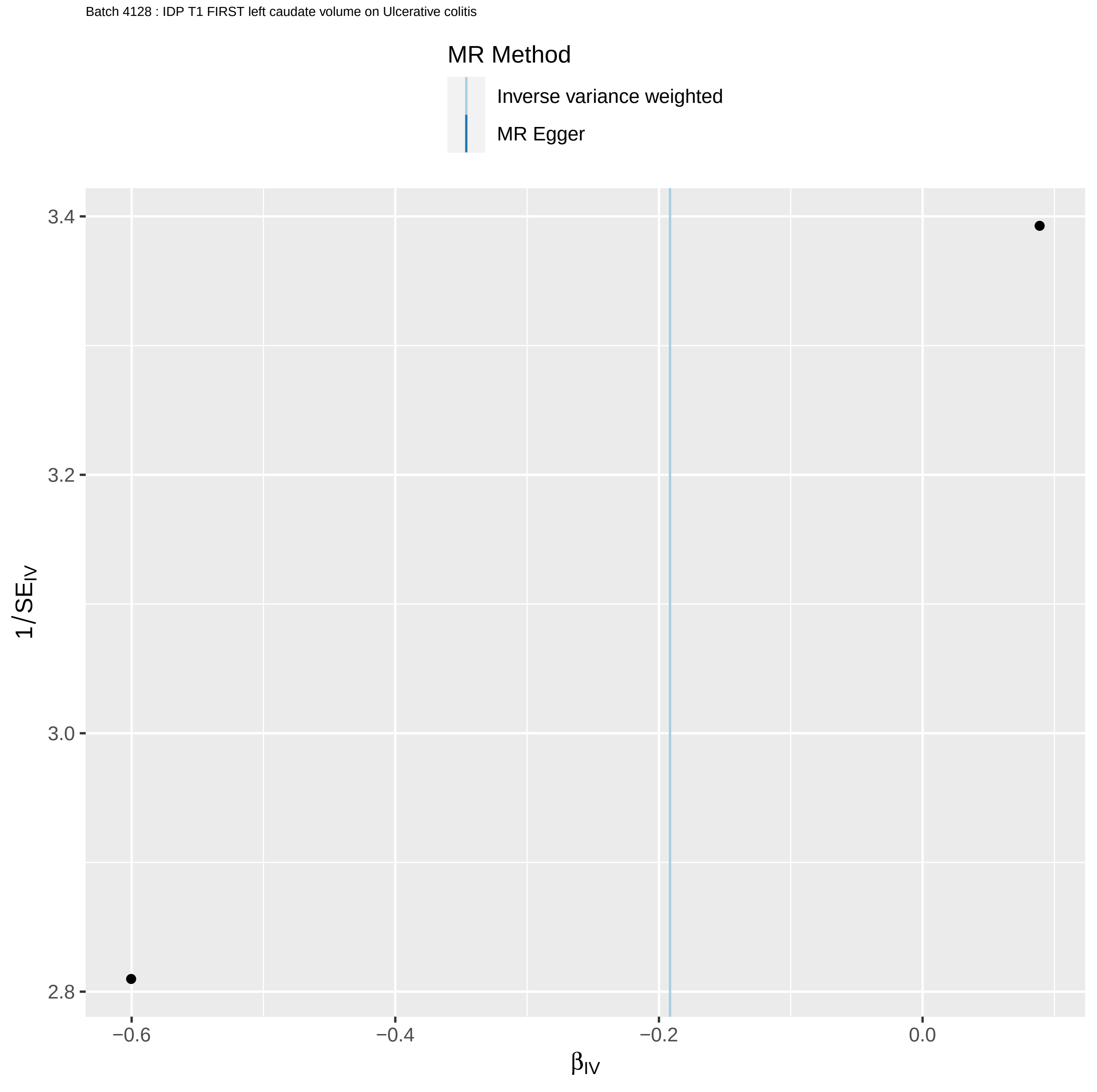
Batch 4128 : IDP T1 FIRST left caudate volume on Ulcerative colitis
MR Method
Inverse variance weighted
MR Egger
3.4
3.2
3.0
2.8
1/SE_IV
−0.6
−0.4
−0.2
0.0
β_IV

Batch 4129 : IDP T1 FAST ROIs L hippocampus on Ulcerative colitis

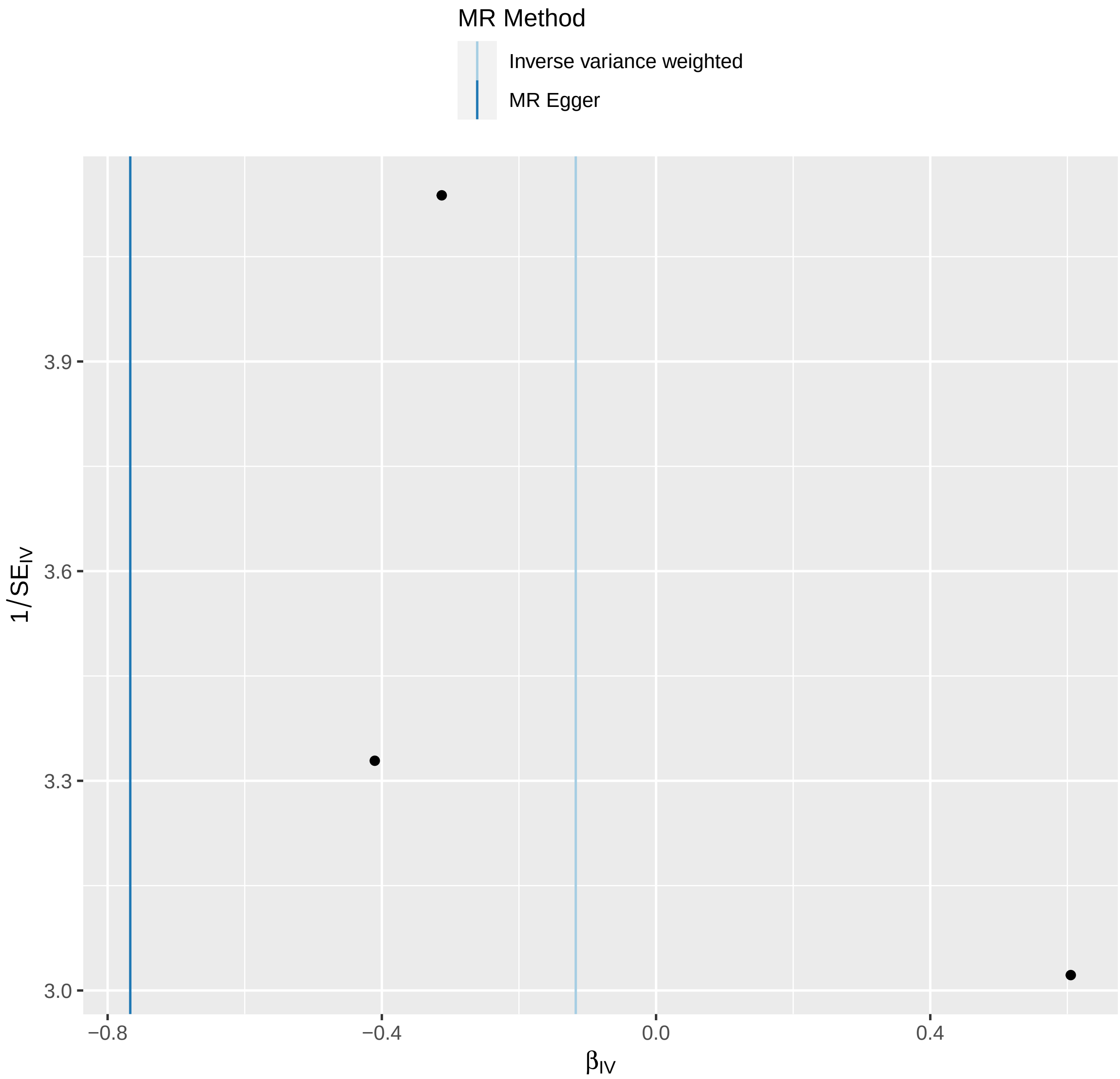

Batch 4130 : IDP T1 FAST ROIs R hippocampus on Ulcerative colitis

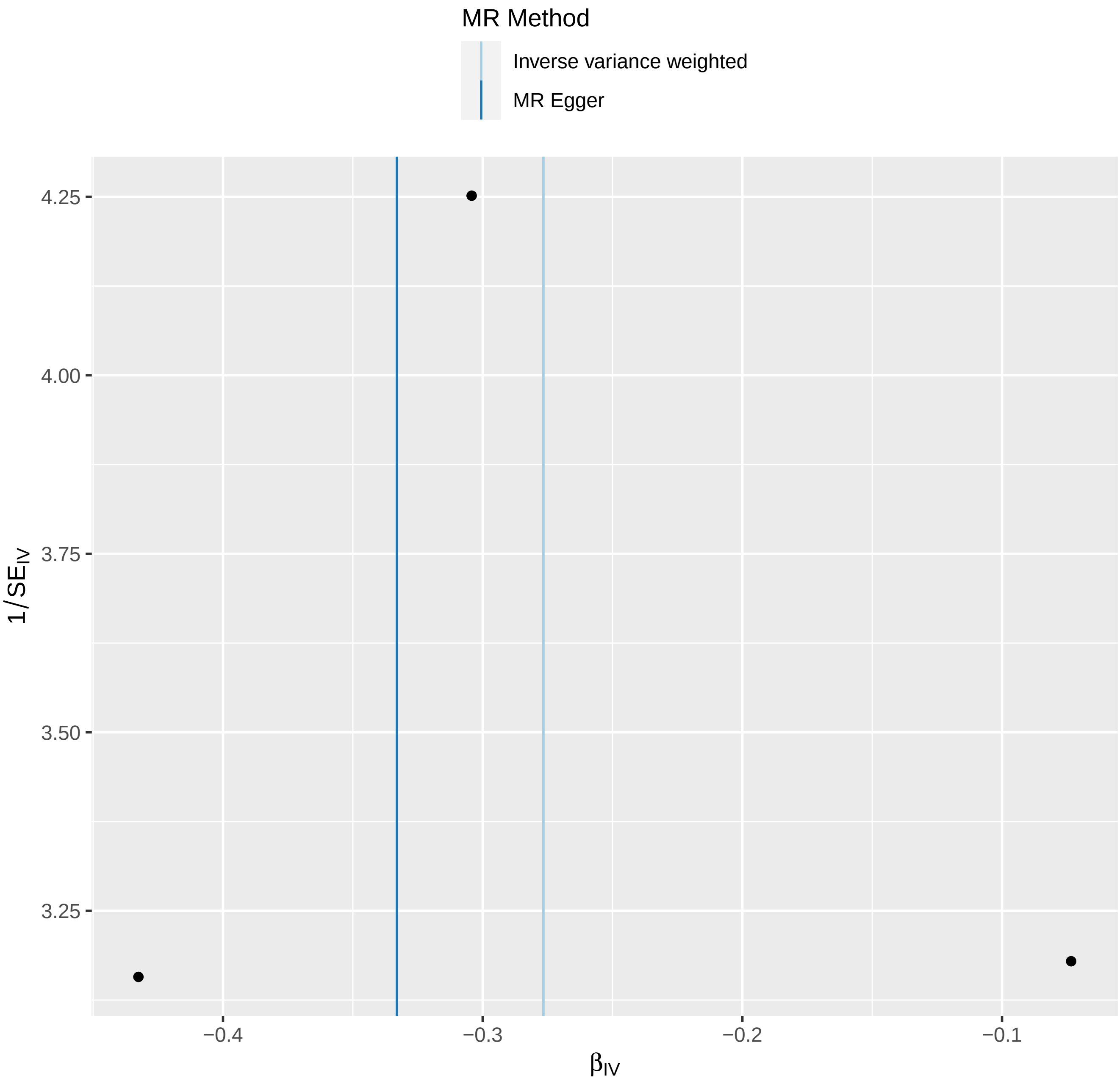

Batch 4133 : IDP T1 FAST ROIs L ventral striatum on Ulcerative colitis

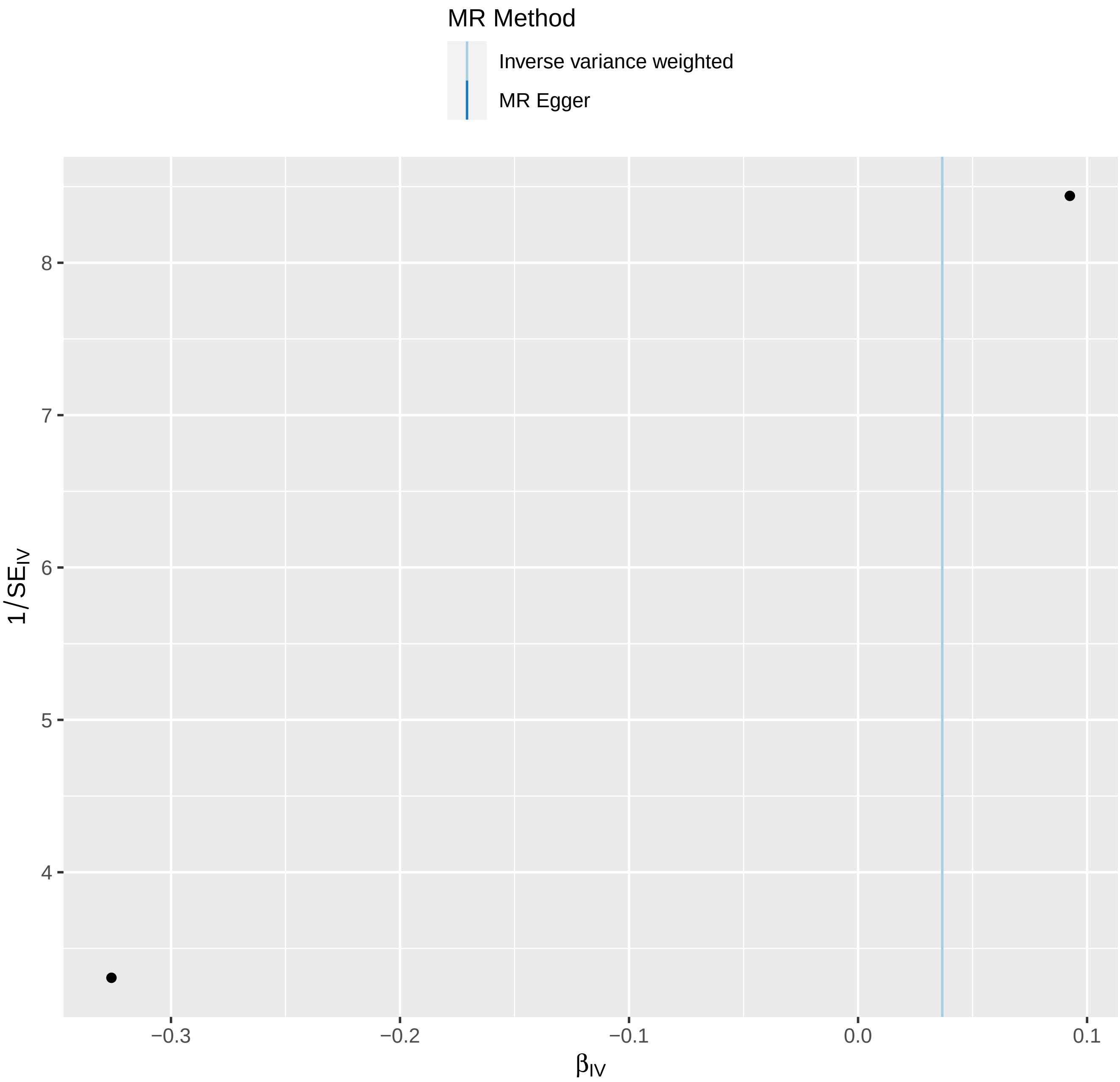

Batch 4134 : IDP T1 FAST ROIs R ventral striatum on Ulcerative colitis

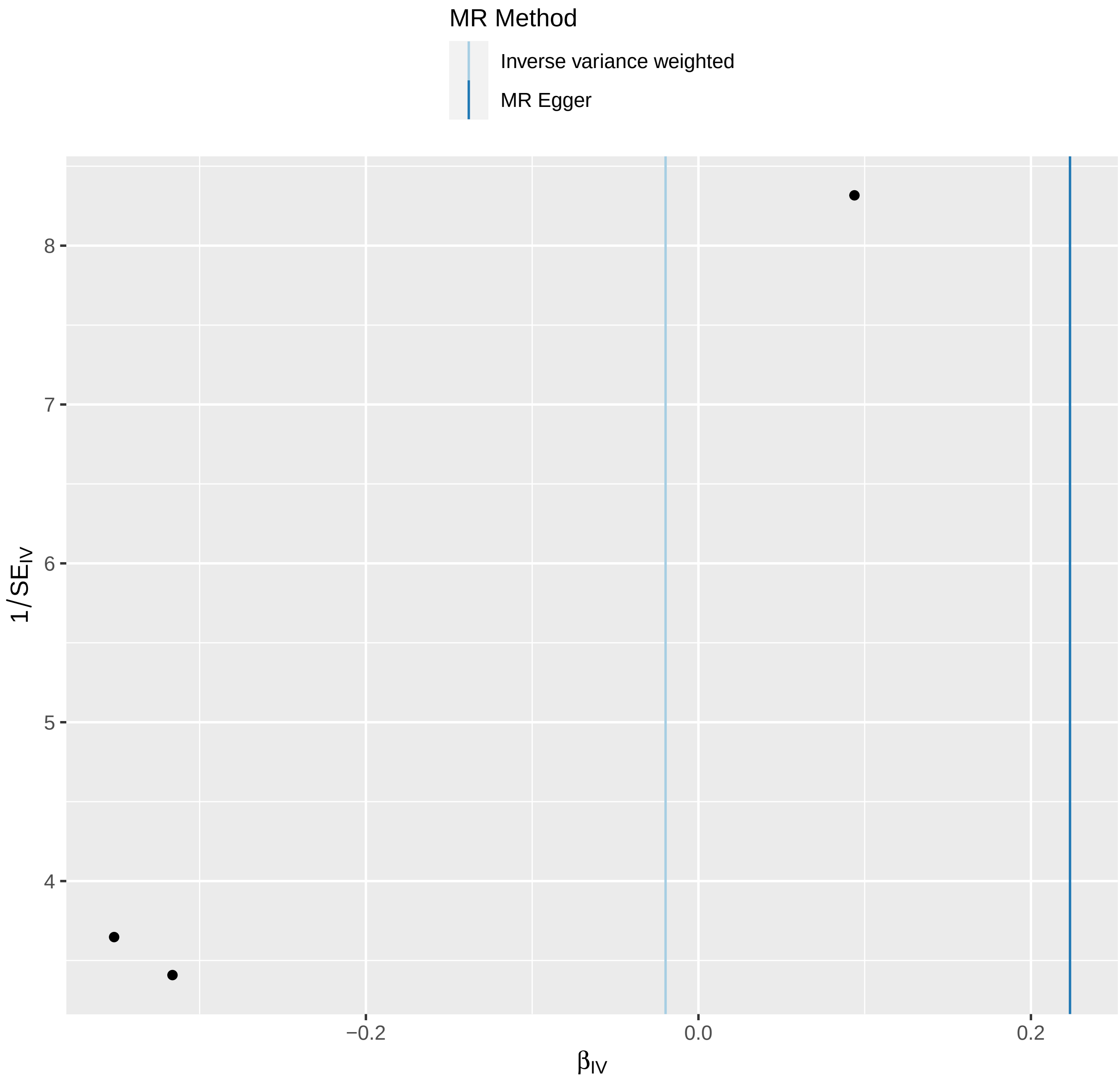

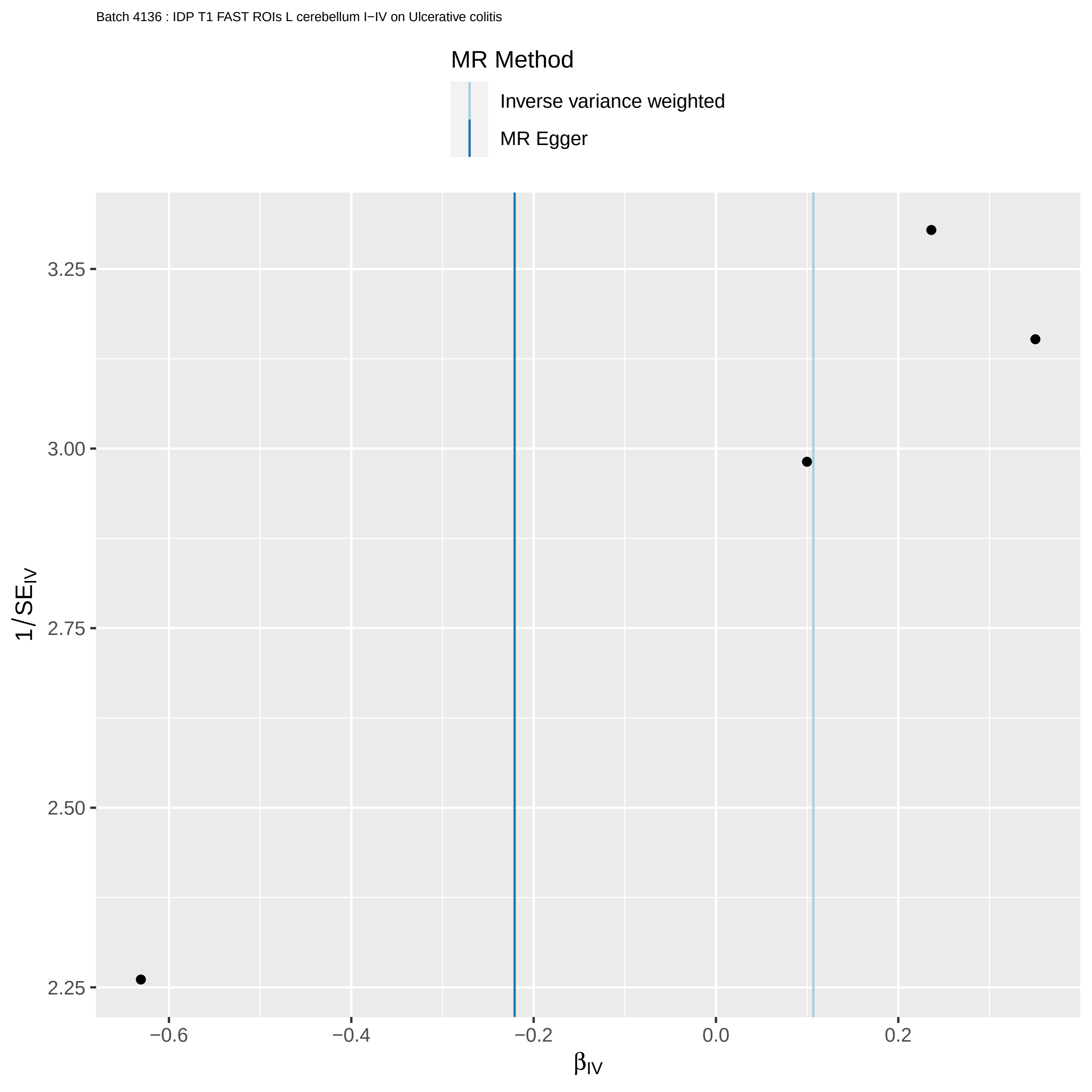
Batch 4136 : IDP T1 FAST ROIs L cerebellum I−IV on Ulcerative colitis
MR Method
Inverse variance weighted
MR Egger
3.25
3.00
2.75
2.50
2.25
1/SE_IV
−0.6
−0.4
−0.2
0.0
0.2
β_IV

Batch 4137 : IDP T1 FAST ROIs R cerebellum I−IV on Ulcerative colitis

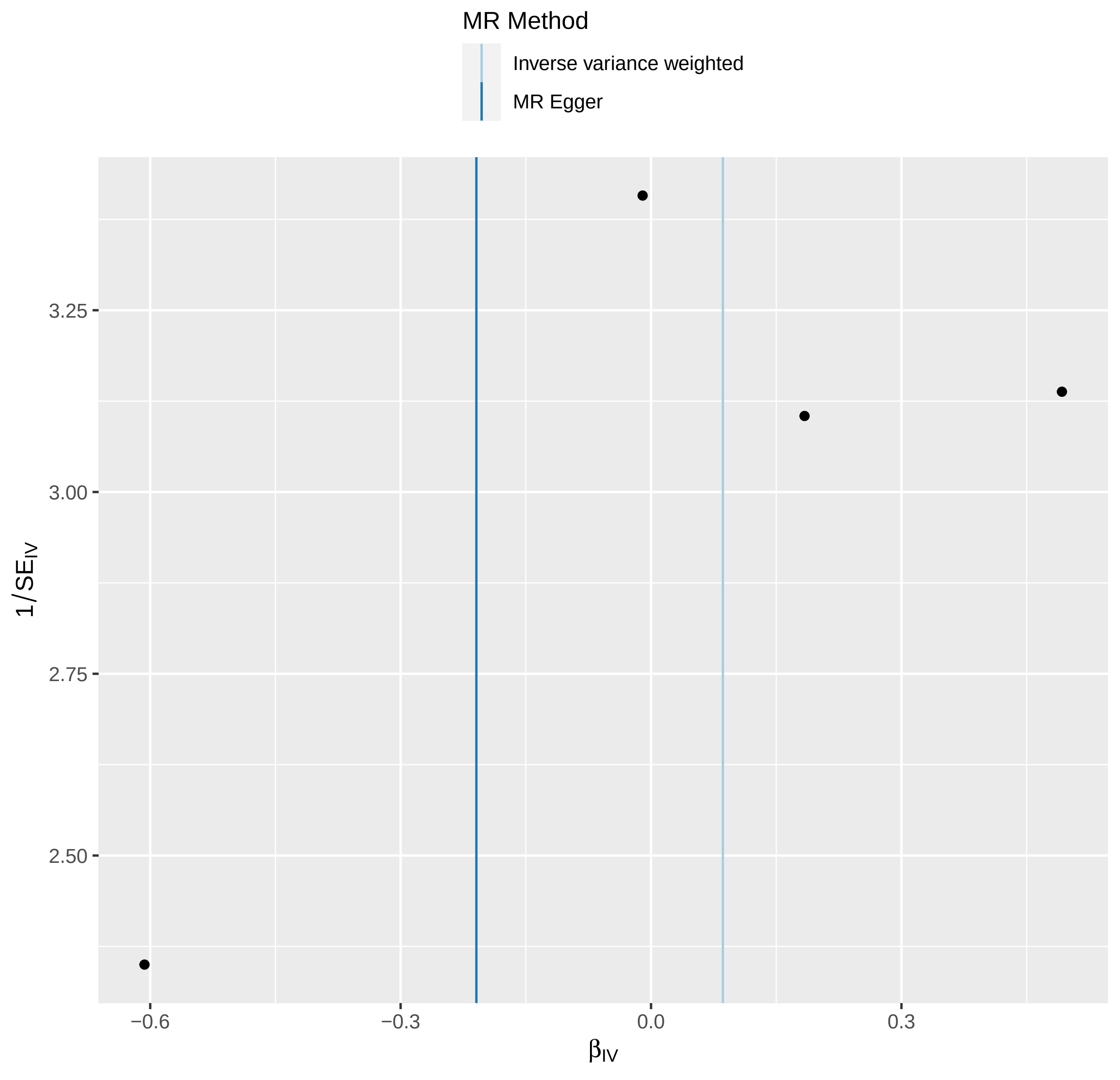

Batch 4138 : IDP T1 FAST ROIs L cerebellum V on Ulcerative colitis

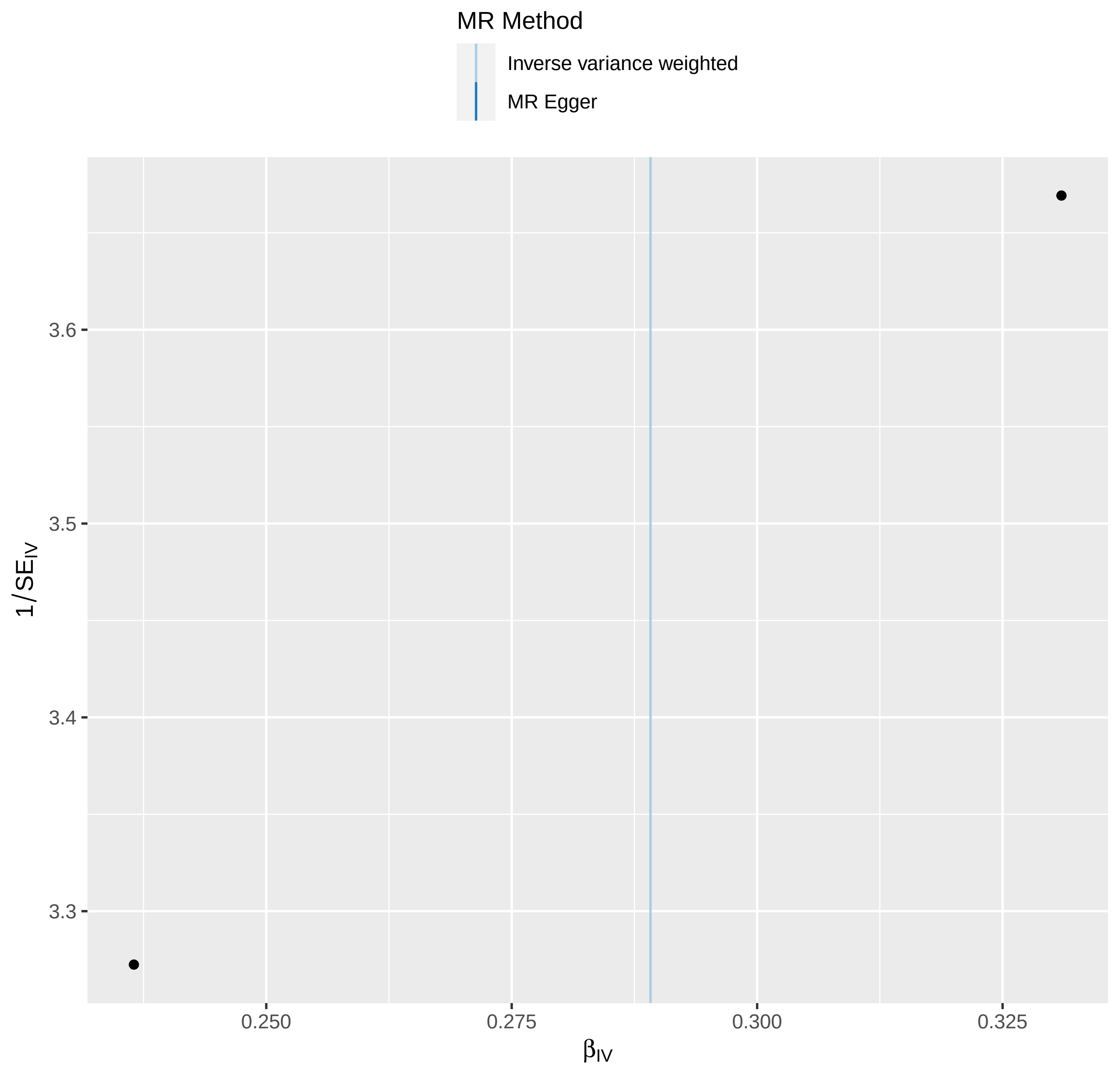

Batch 4140 : IDP T1 FAST ROIs R cerebellum V on Ulcerative colitis

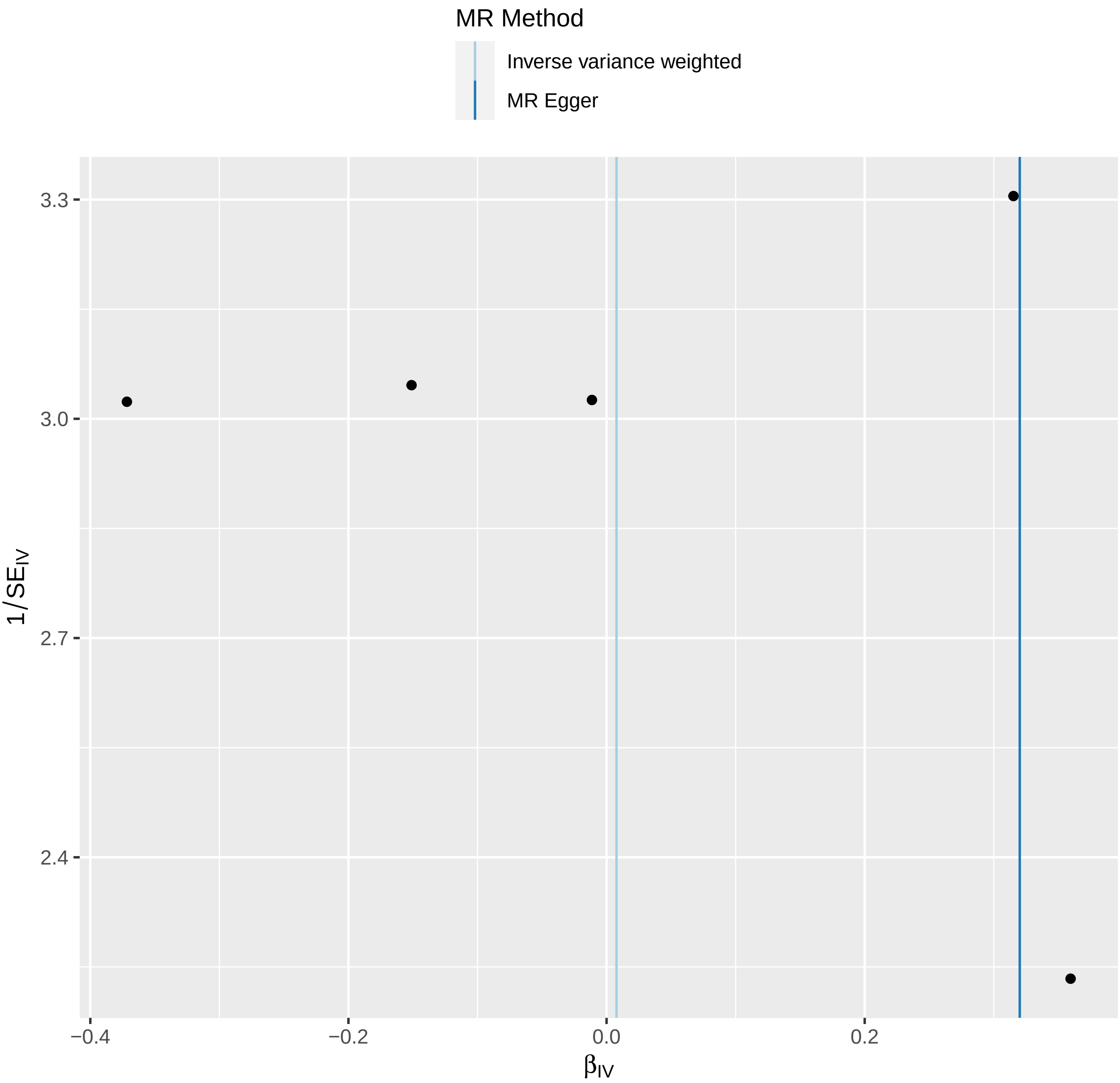

Batch 4142 : IDP T1 FAST ROIs V cerebellum VI on Ulcerative colitis

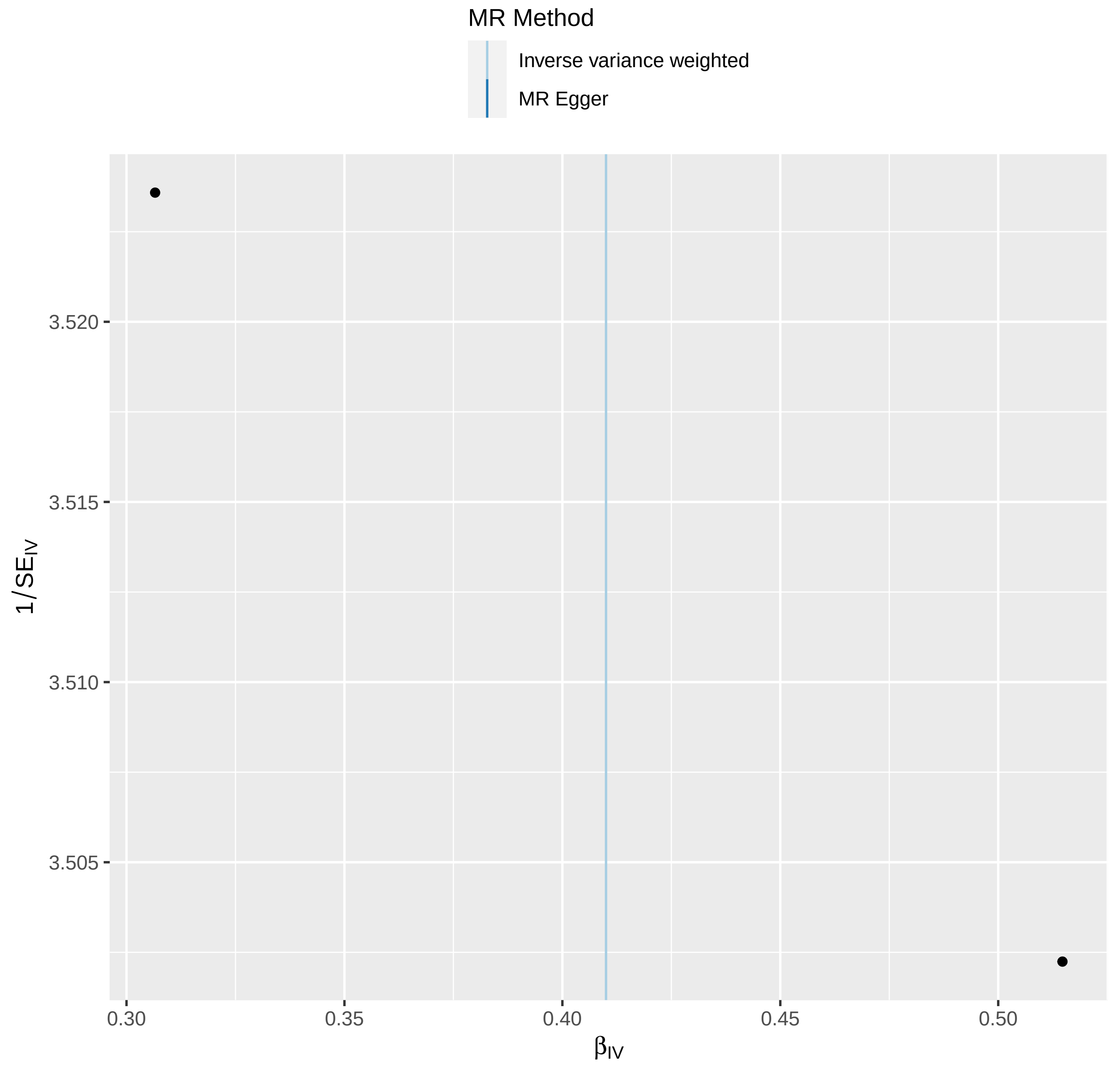

Batch 4144 : IDP T1 FAST ROIs L cerebellum crus I on Ulcerative colitis

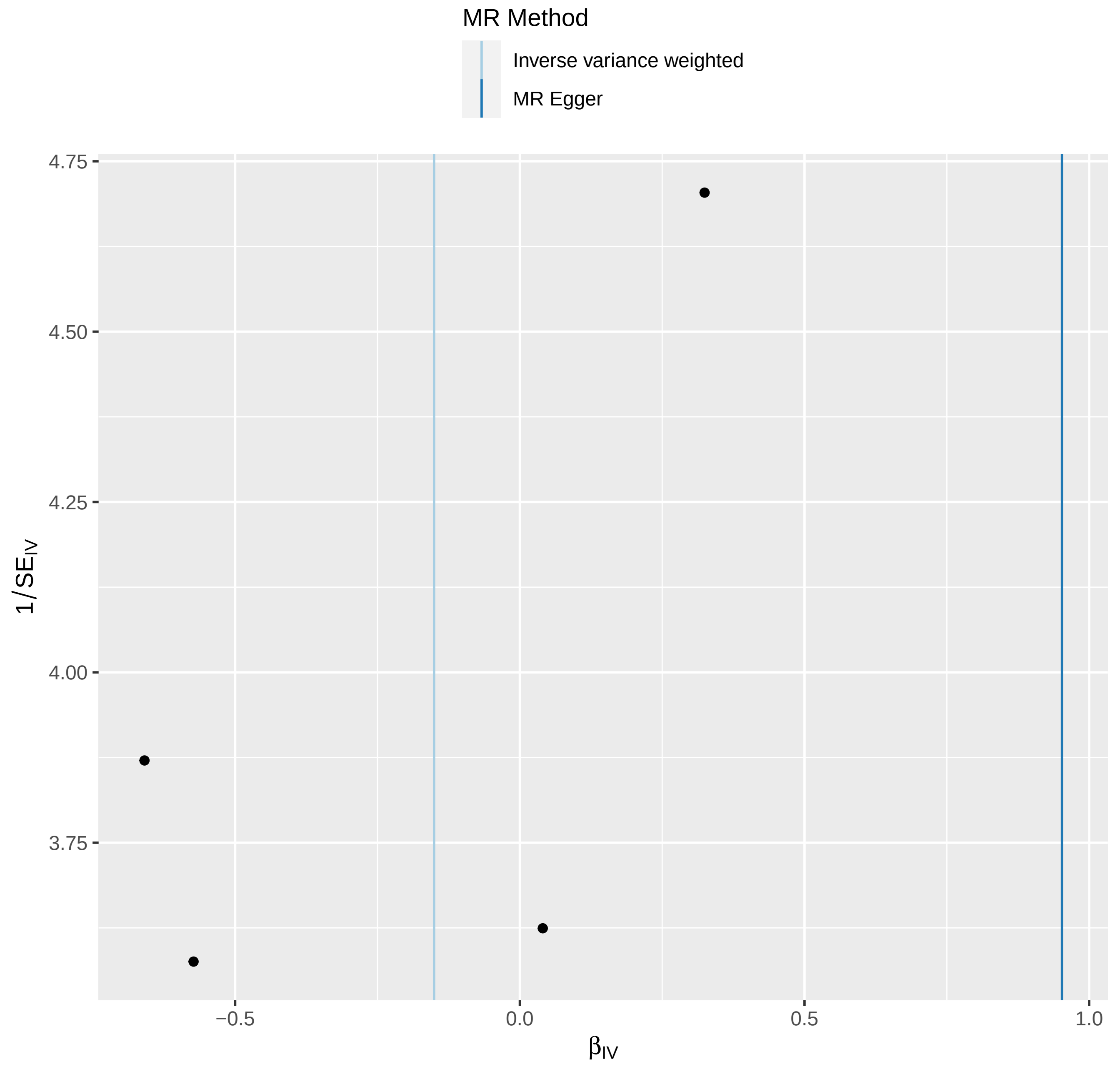

Batch 4146 : IDP T1 FAST ROIs R cerebellum crus I on Ulcerative colitis

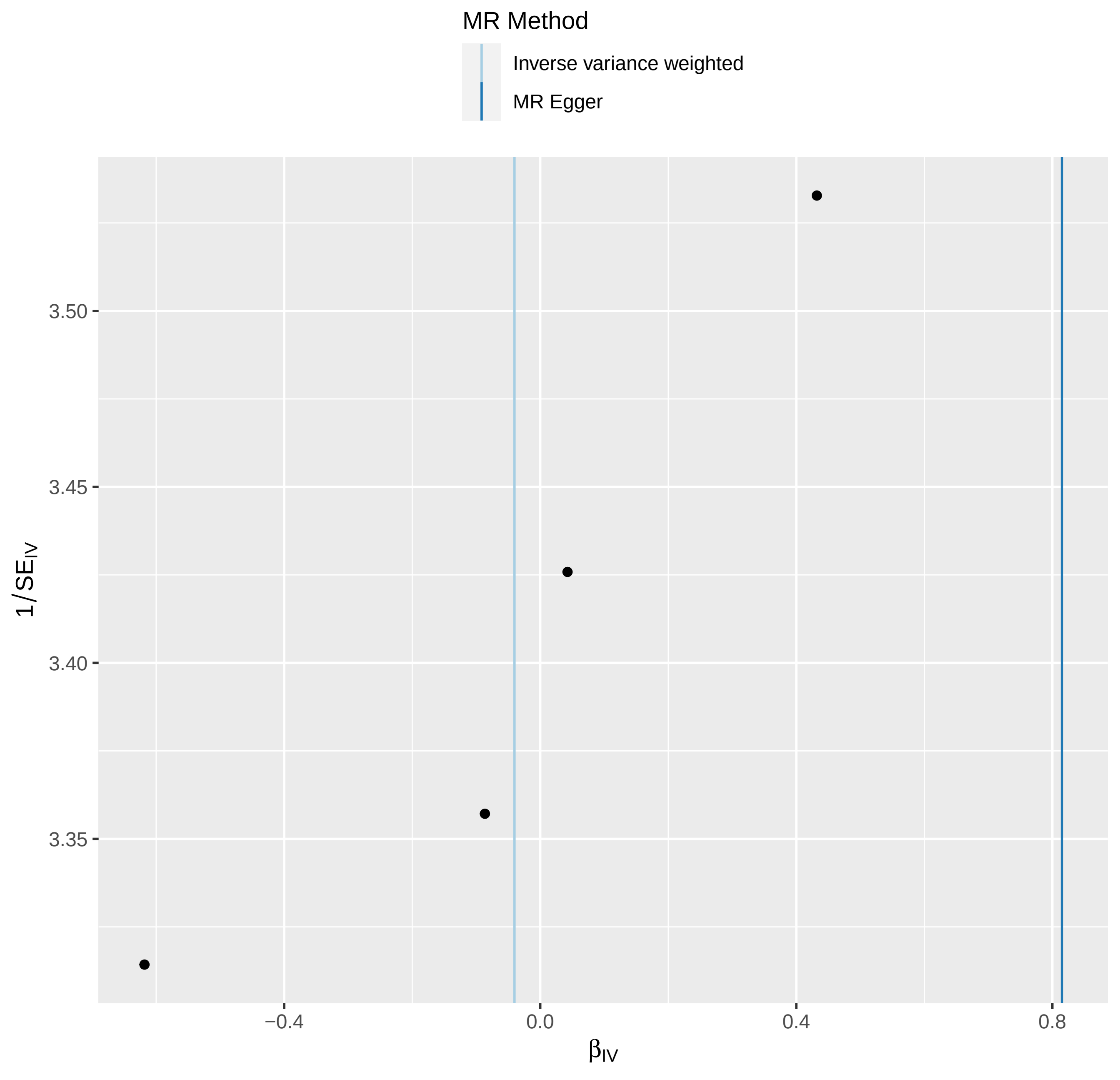

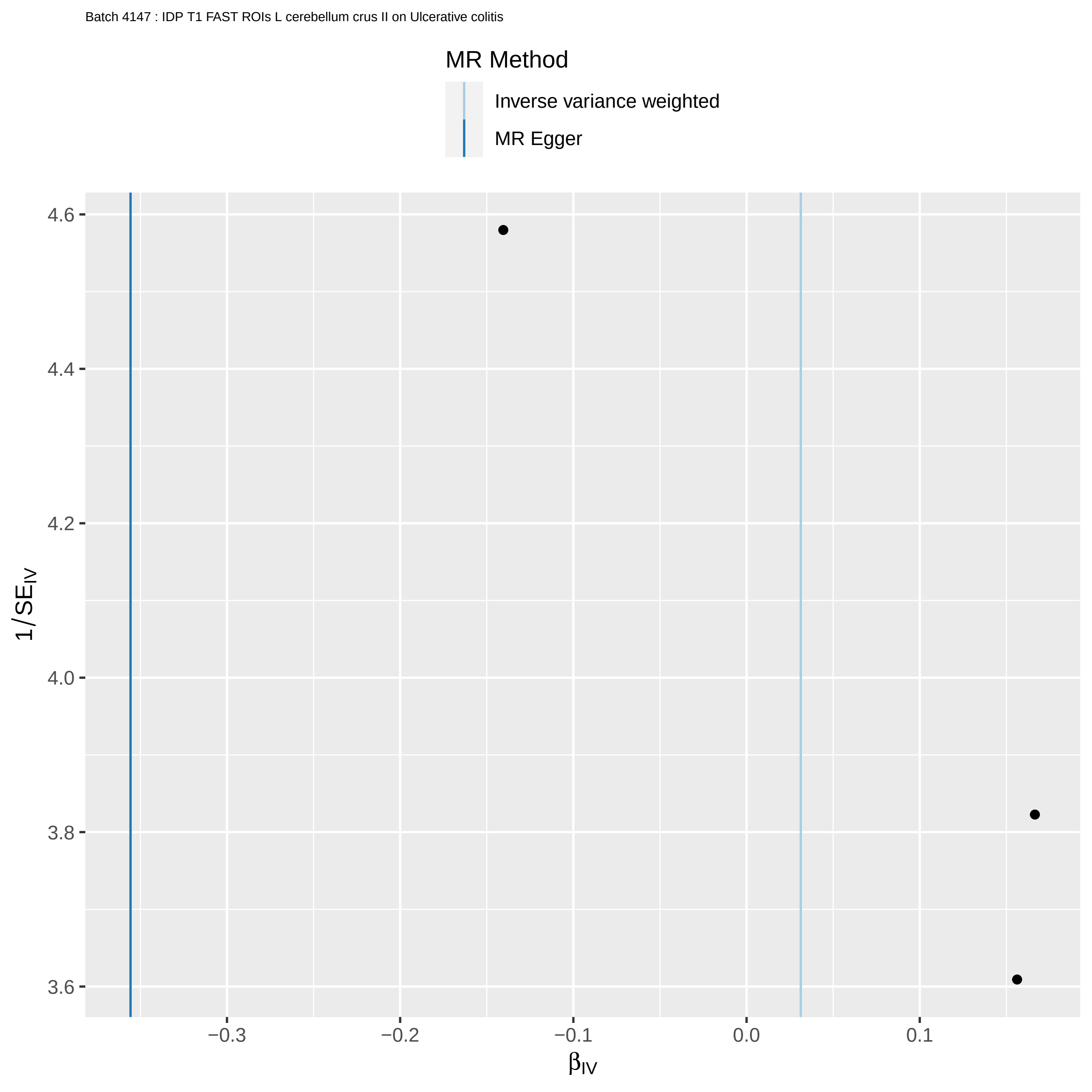
Batch 4147 : IDP T1 FAST ROIs L cerebellum crus II on Ulcerative colitis
MR Method
Inverse variance weighted
MR Egger
4.6
4.4
4.2
4.0
3.8
3.6
1/SE_IV
−0.3
−0.2
−0.1
0.0
0.1
β_IV

Batch 4148 : IDP T1 FAST ROIs V cerebellum crus II on Ulcerative colitis

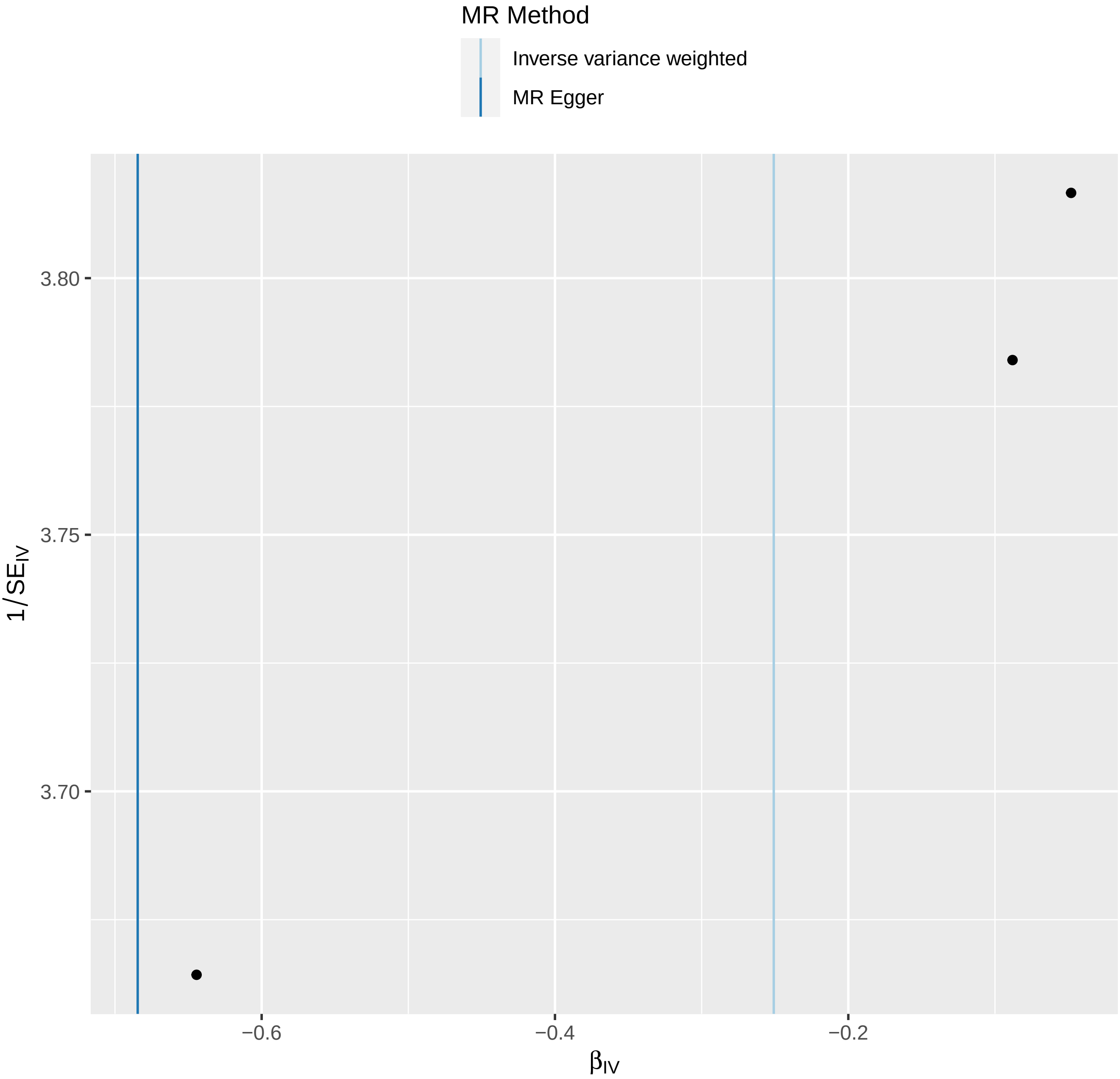

Batch 4149 : IDP T1 FAST ROIs R cerebellum crus II on Ulcerative colitis

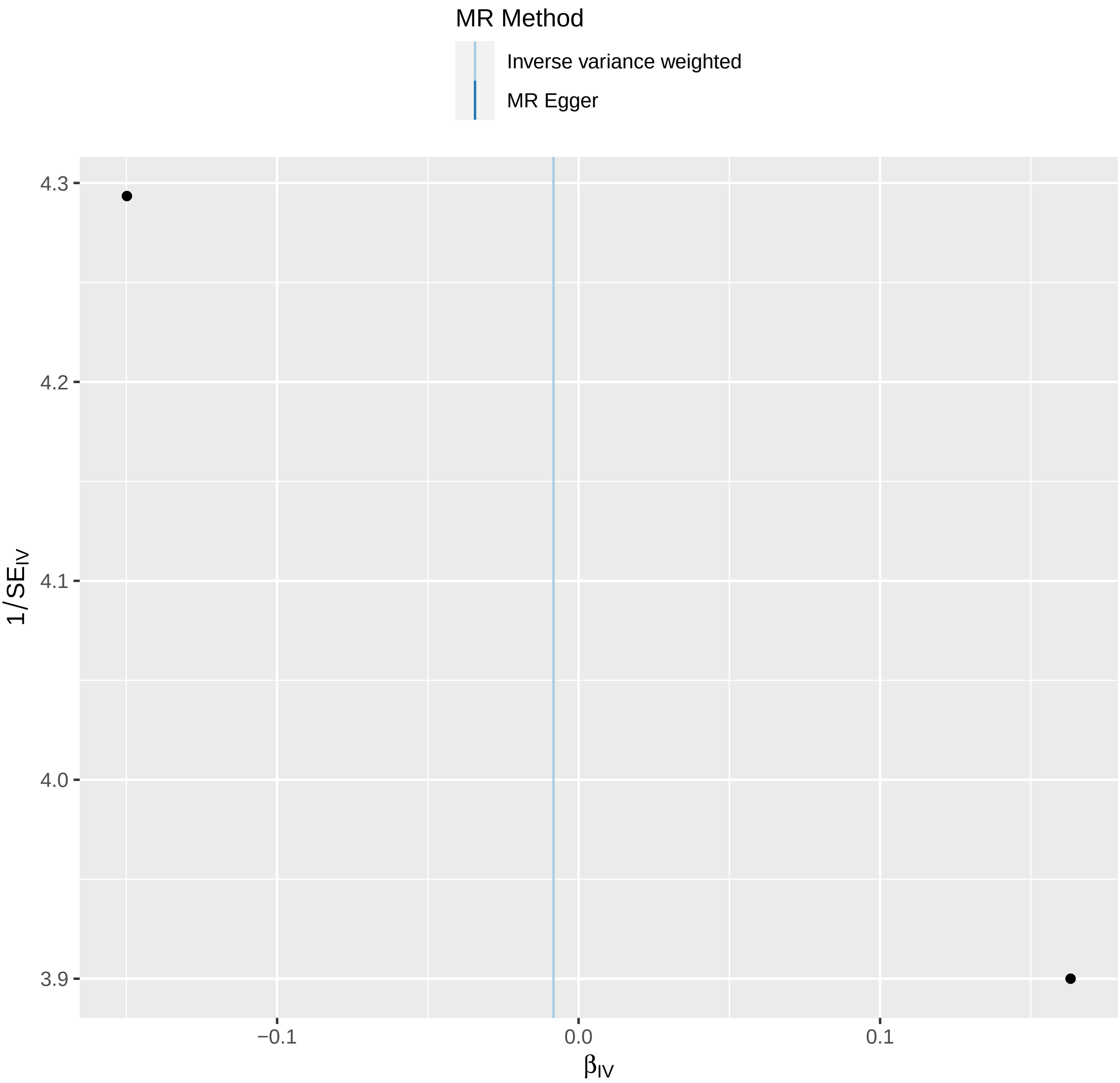

Batch 4152 : IDP T1 FAST ROIs V cerebellum VIIb on Ulcerative colitis

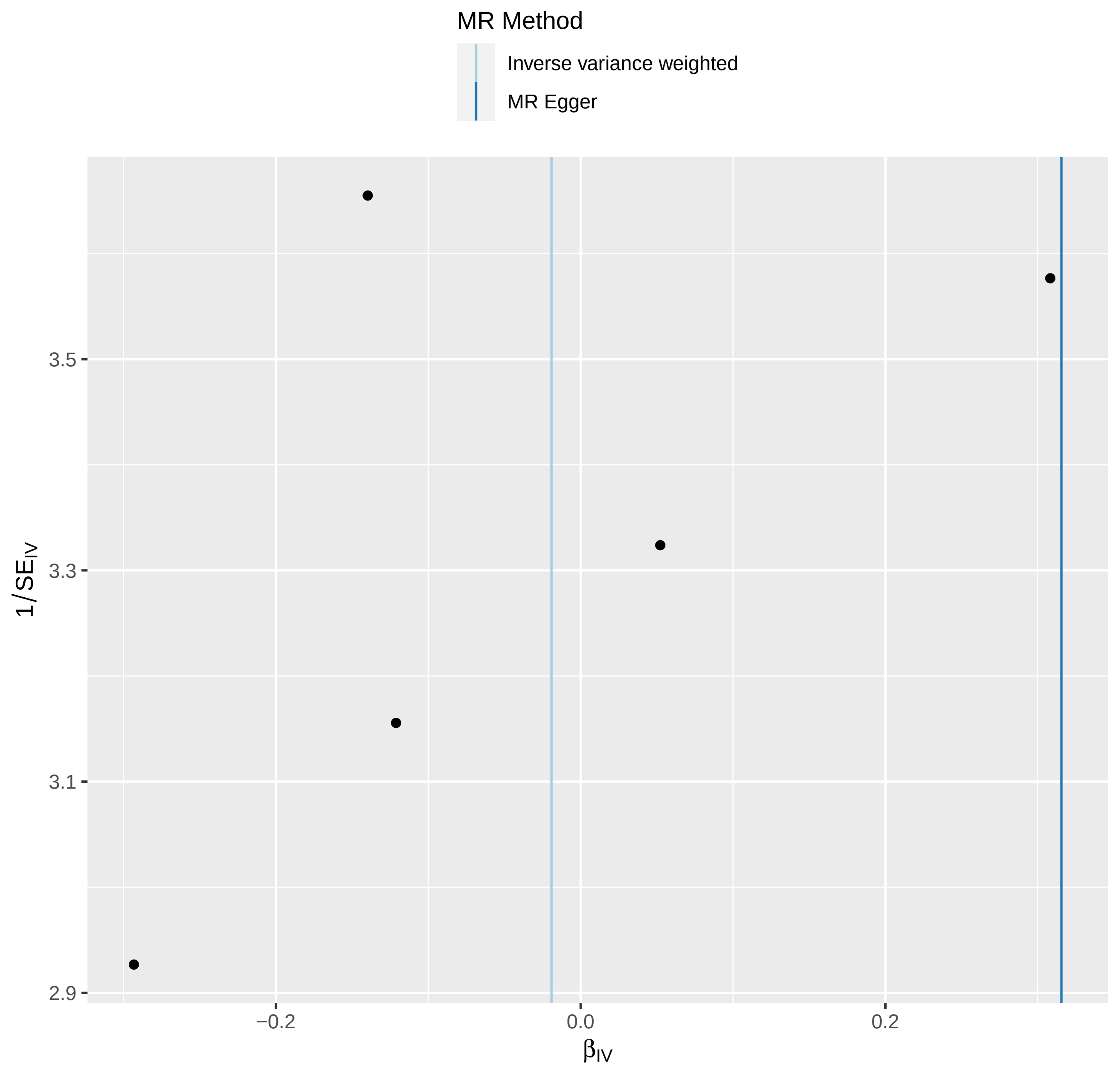

Batch 4155 : IDP T1 FAST ROIs V cerebellum VIIIa on Ulcerative colitis

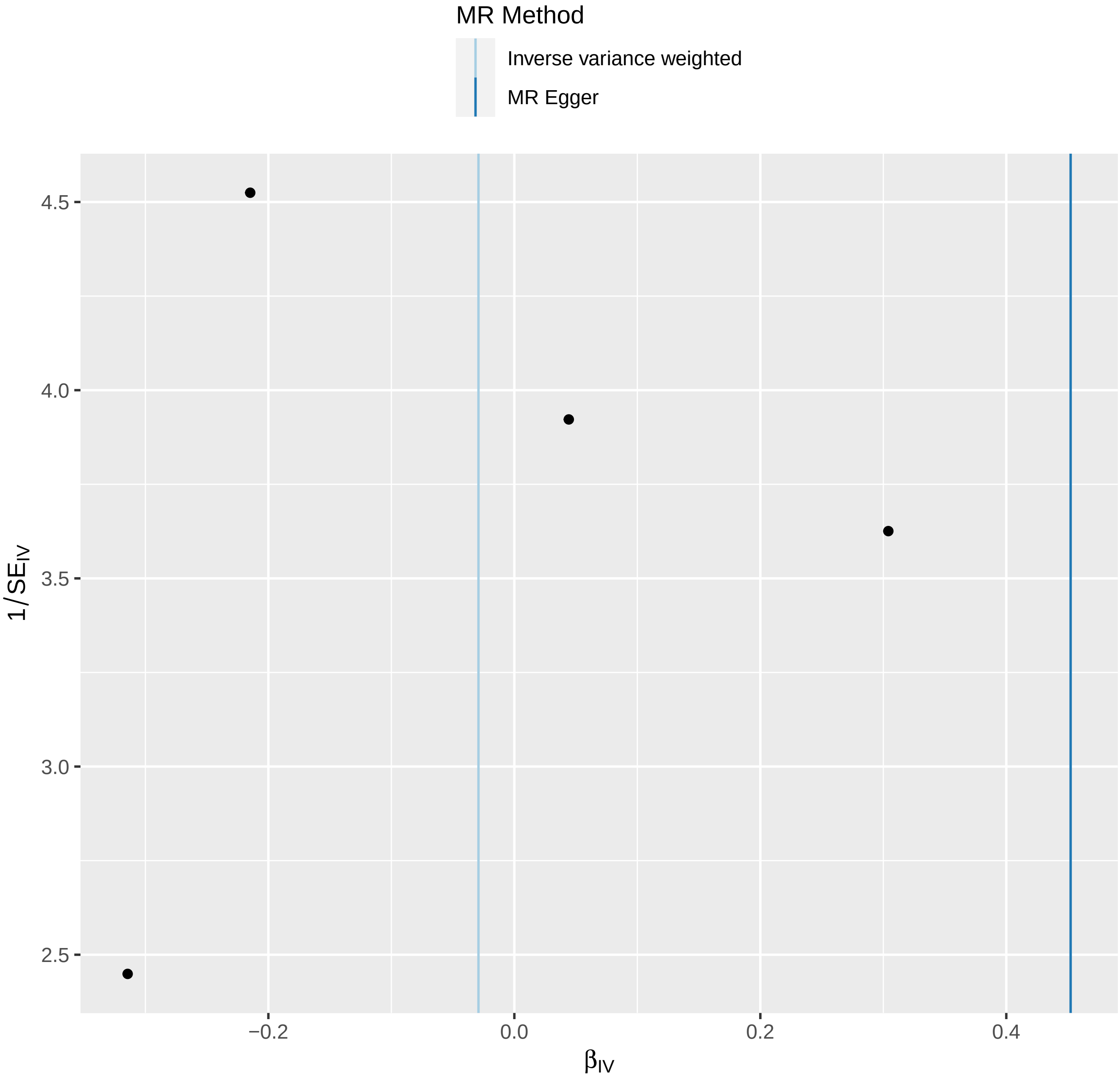

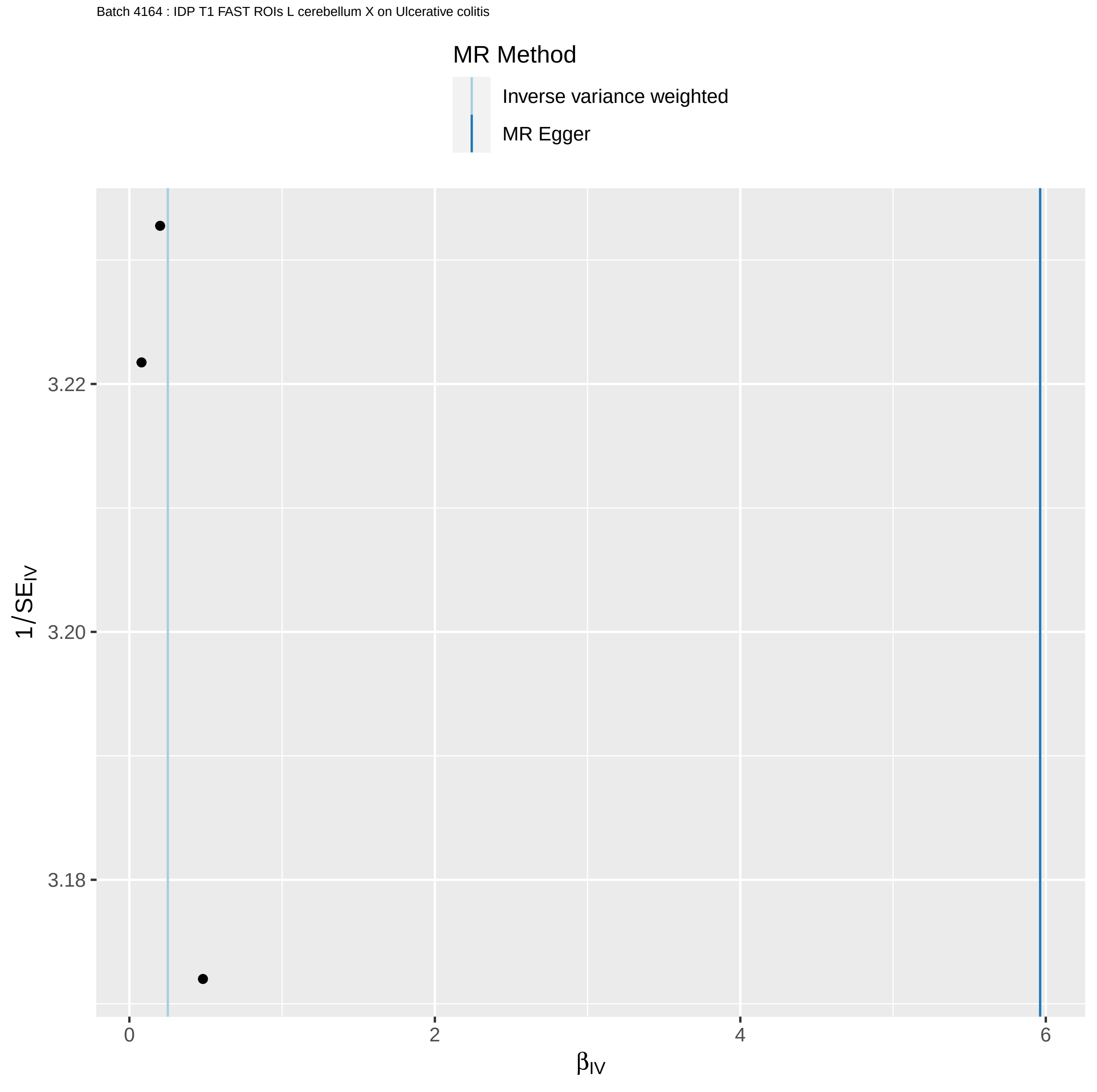
Batch 4164 : IDP T1 FAST ROIs L cerebellum X on Ulcerative colitis
MR Method
Inverse variance weighted
MR Egger
3.22
3.20
3.18
$1/SE_{IV}$
0
2
4
6
β_{IV}

Batch 4166 : IDP T1 FAST ROIs R cerebellum X on Ulcerative colitis

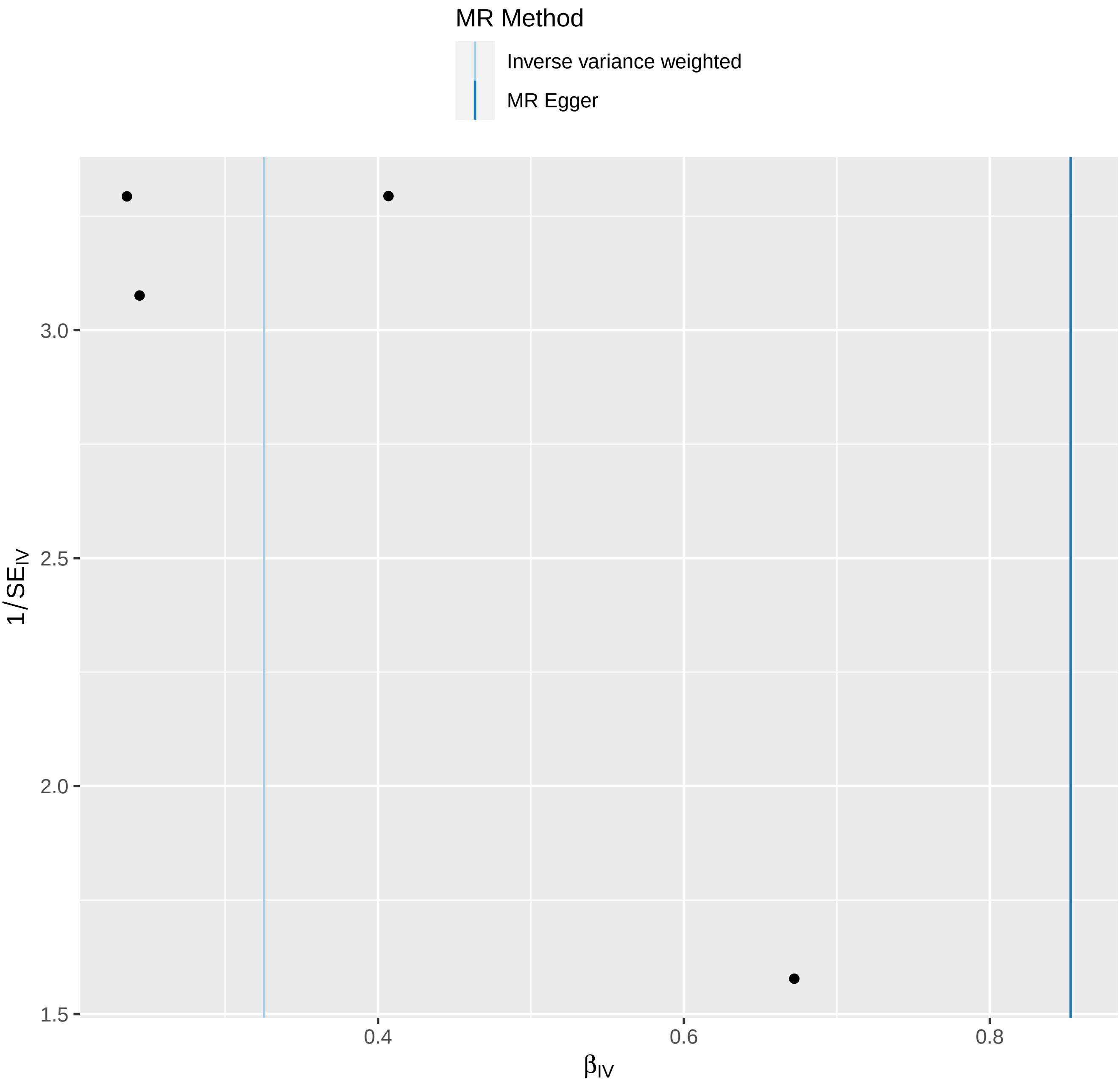

Batch 4167 : IDP T2 FLAIR BIANCA WMH volume on Ulcerative colitis

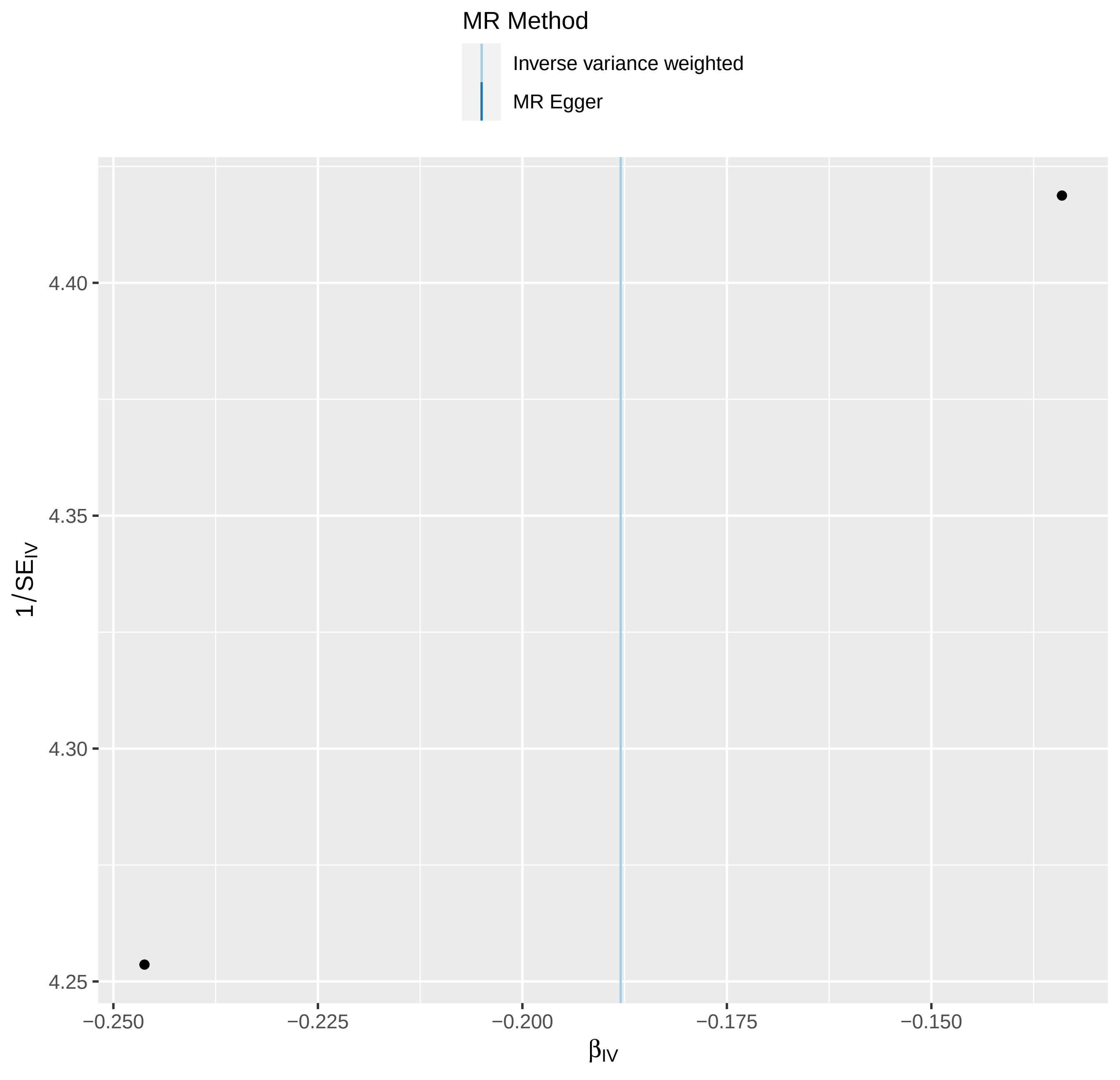

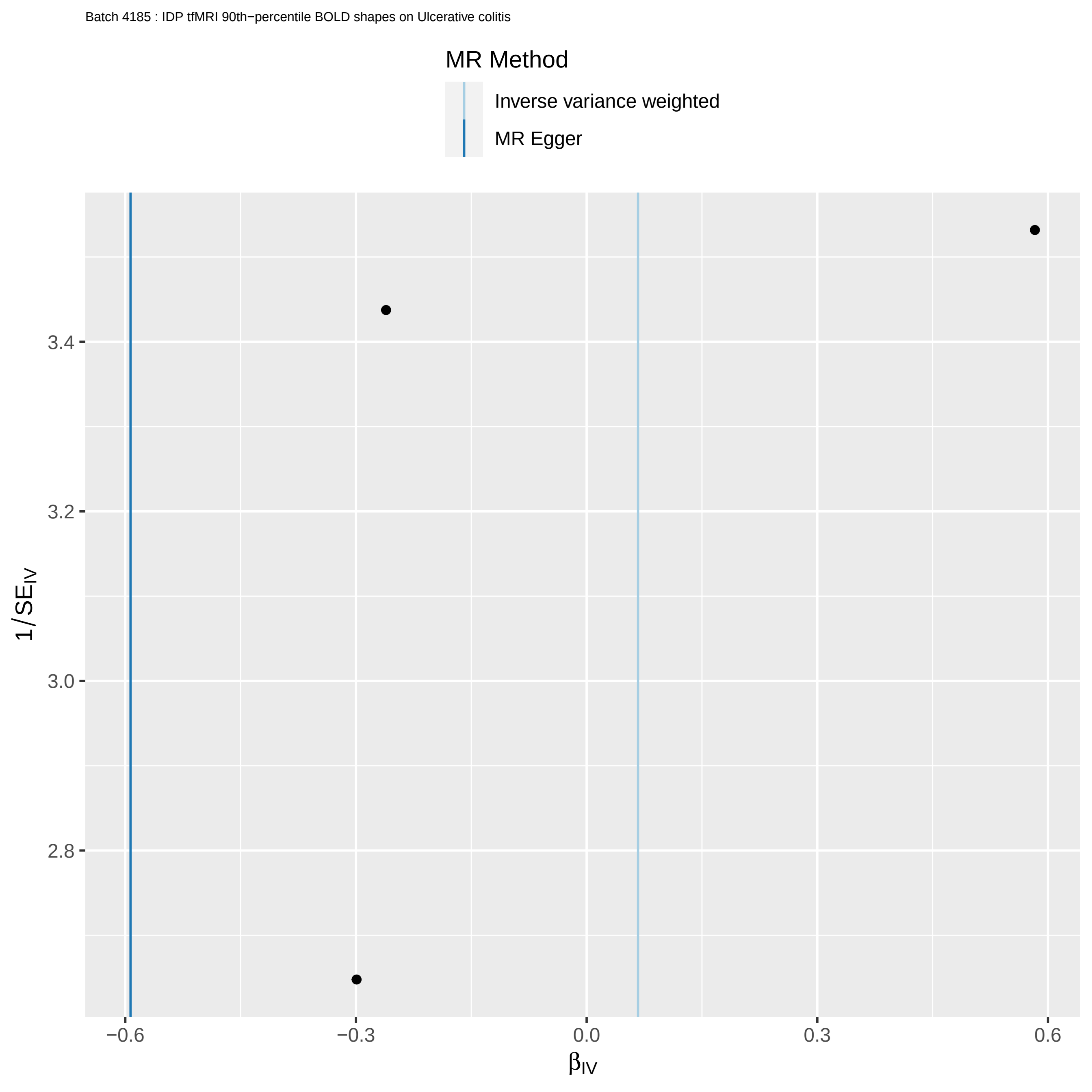

Batch 4185 : IDP tfMRI 90th−percentile BOLD shapes on Ulcerative colitis
MR Method
Inverse variance weighted
MR Egger
3.4
3.2
3.0
2.8
1/SE_IV
−0.6
−0.3
0.0
0.3
0.6
β_IV

Batch 4186 : IDP tfMRI median zstat shapes on Ulcerative colitis

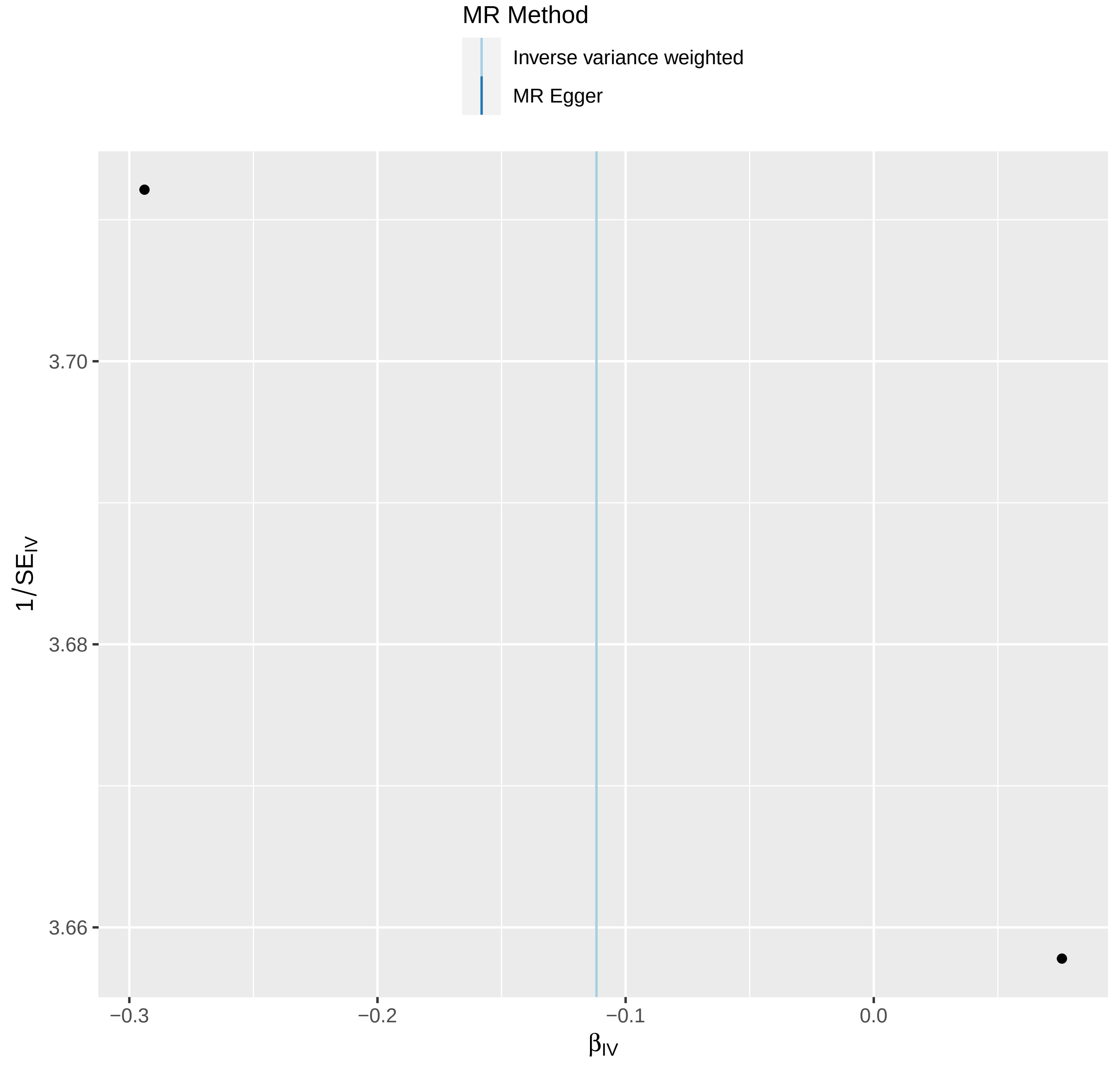

Batch 4187 : IDP tfMRI 90th−percentile zstat shapes on Ulcerative colitis

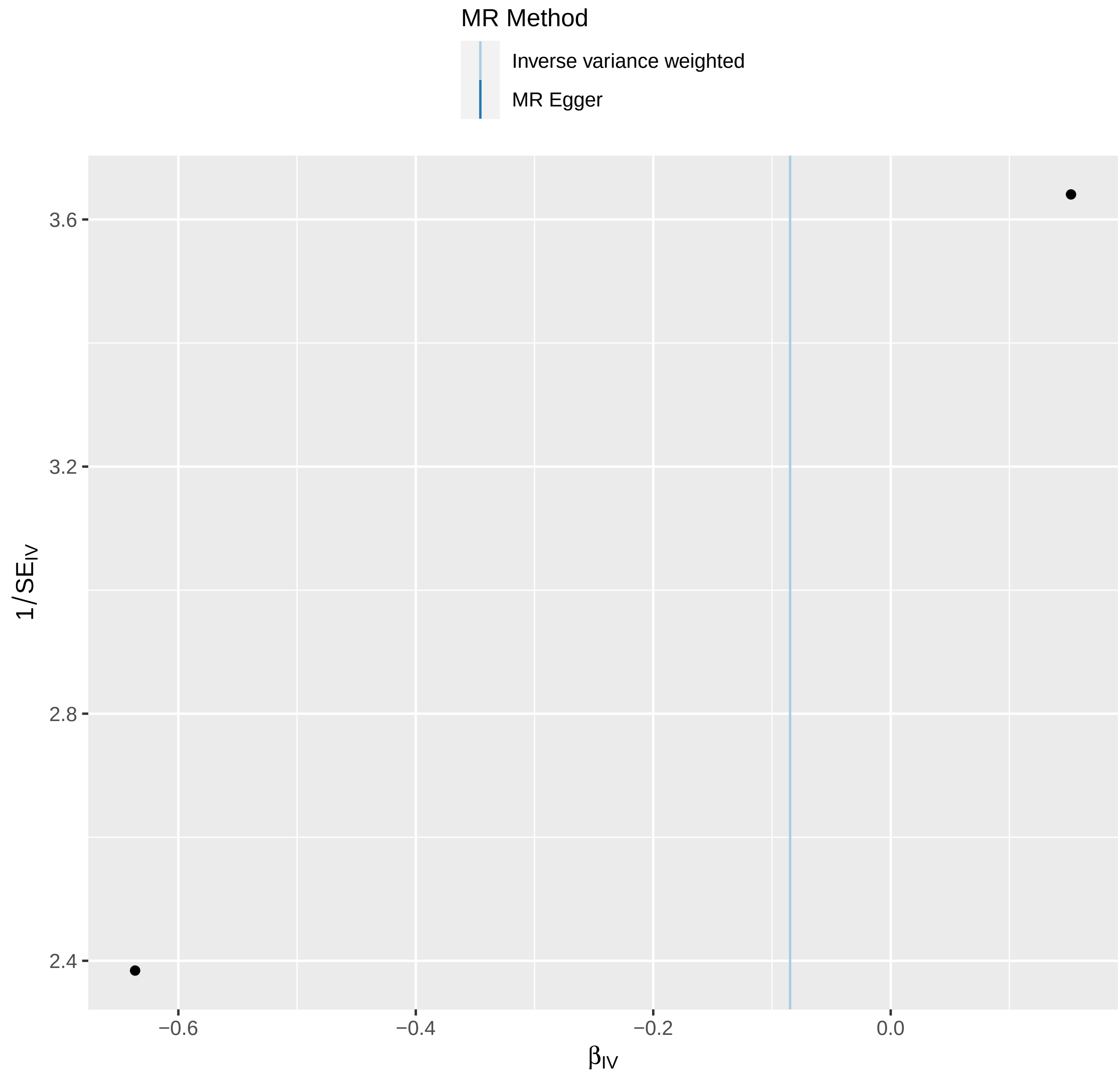

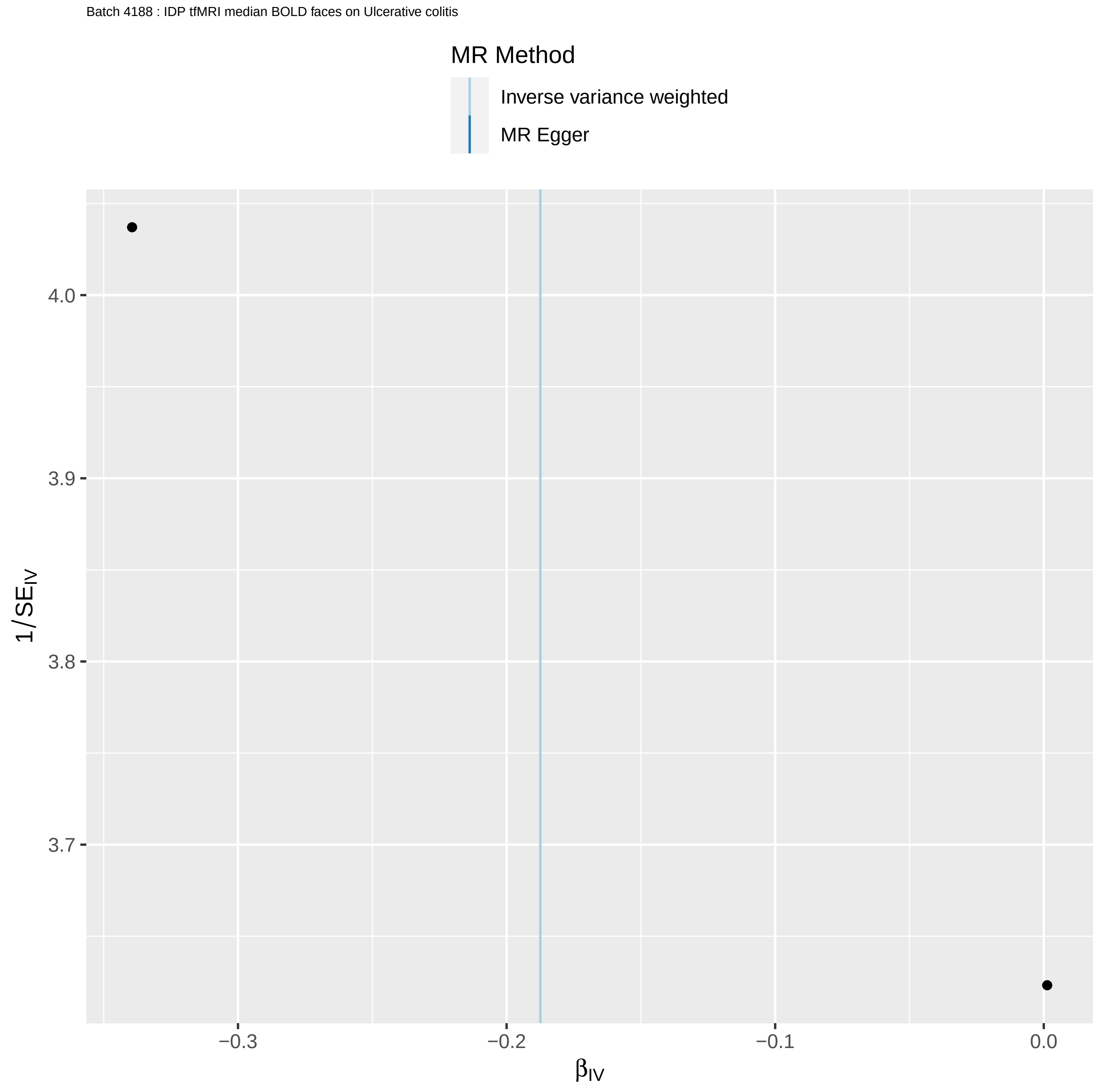
Batch 4188 : IDP tfMRI median BOLD faces on Ulcerative colitis
MR Method
Inverse variance weighted
MR Egger
4.0
3.9
3.8
3.7
1/SE$_{IV}$
−0.3
−0.2
−0.1
0.0
β_{IV}

Batch 4189 : IDP tfMRI 90th−percentile BOLD faces on Ulcerative colitis

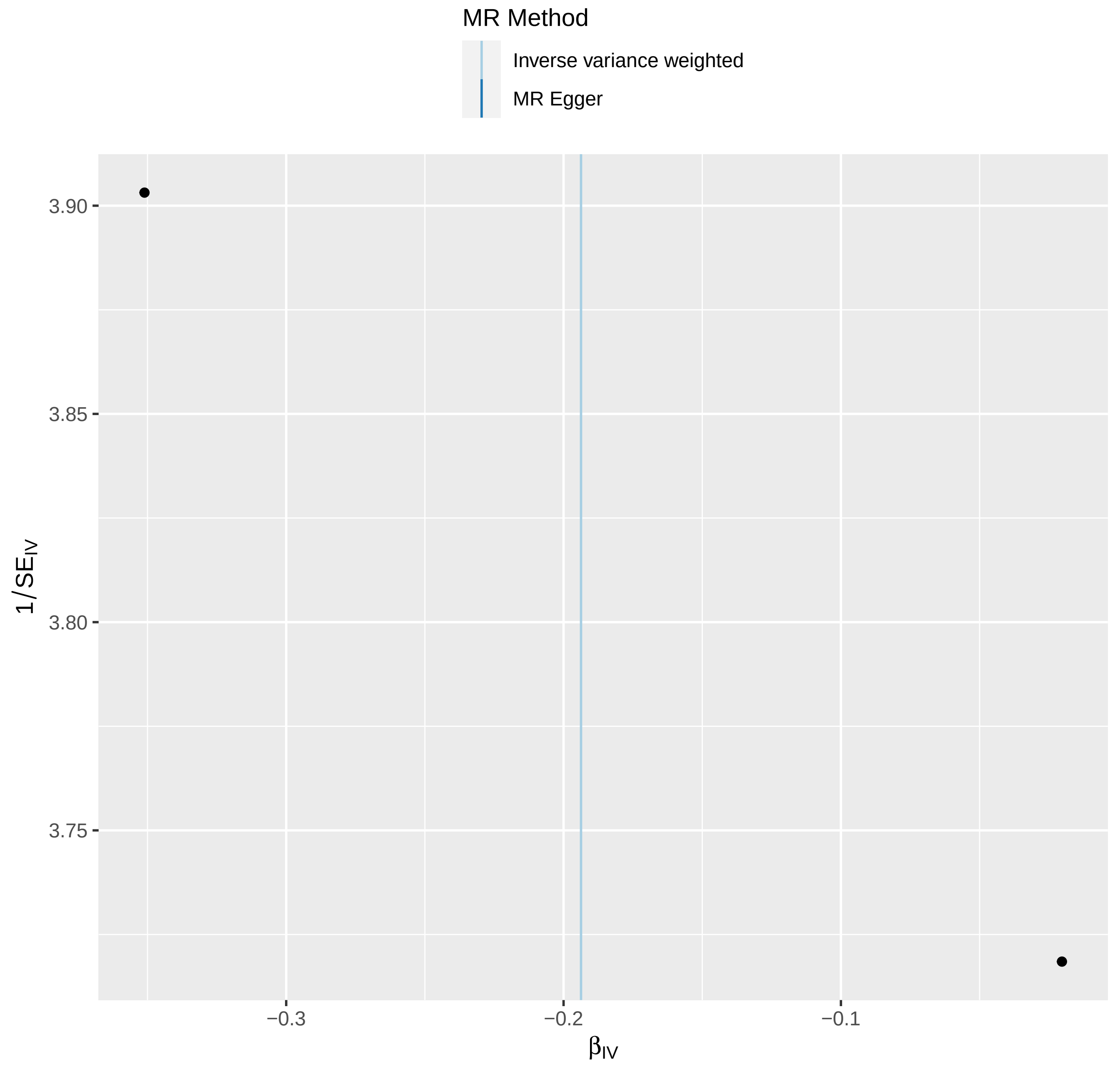

Batch 4190 : IDP tfMRI median zstat faces on Ulcerative colitis

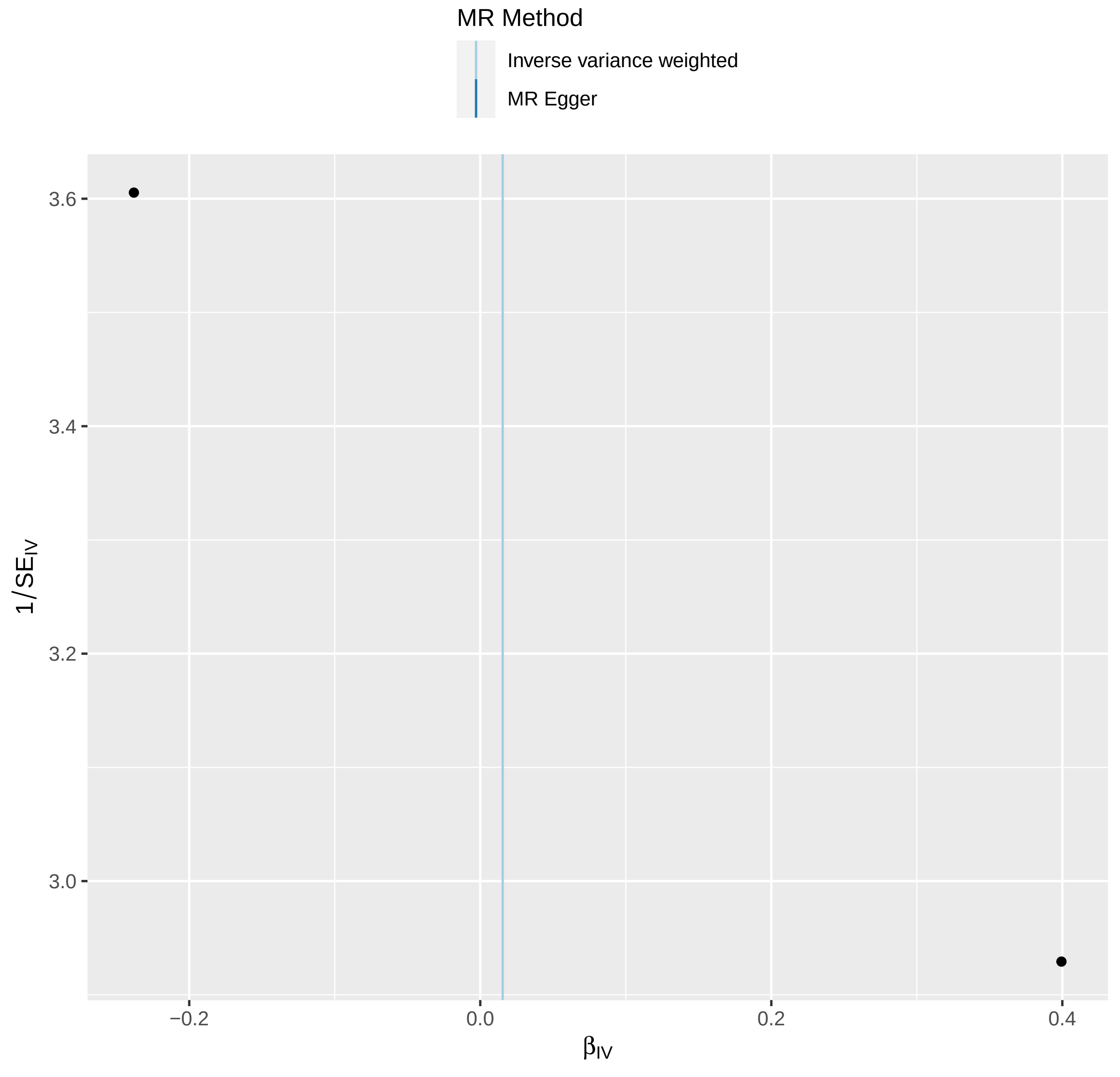

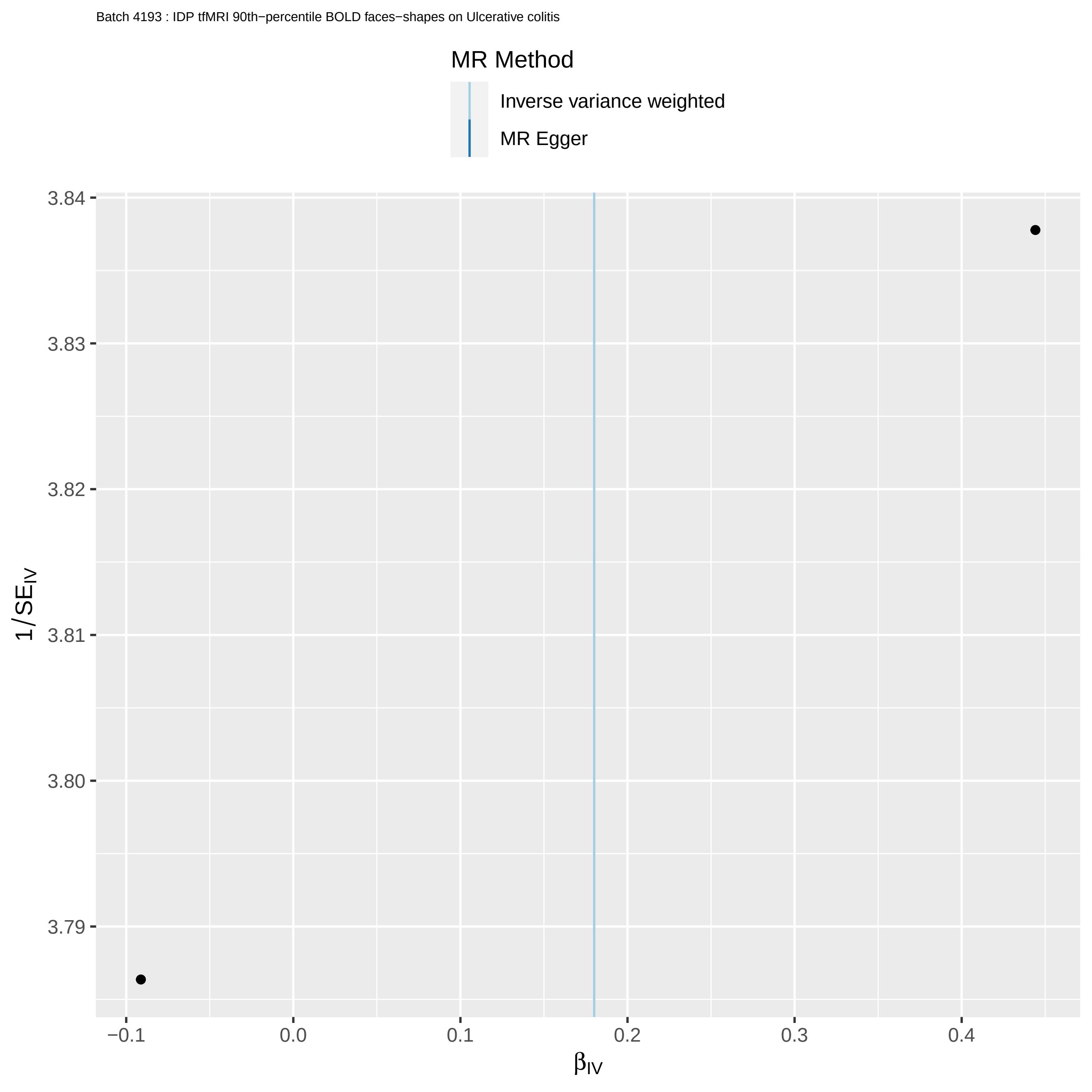
Batch 4193 : IDP tfMRI 90th−percentile BOLD faces−shapes on Ulcerative colitis
MR Method
Inverse variance weighted
MR Egger
3.84
3.83
3.82
3.81
3.80
3.79
1/SE_IV
−0.1
0.0
0.1
0.2
0.3
0.4
β_IV

Batch 4194 : IDP T1 FIRST left hippocampus volume on Ulcerative colitis

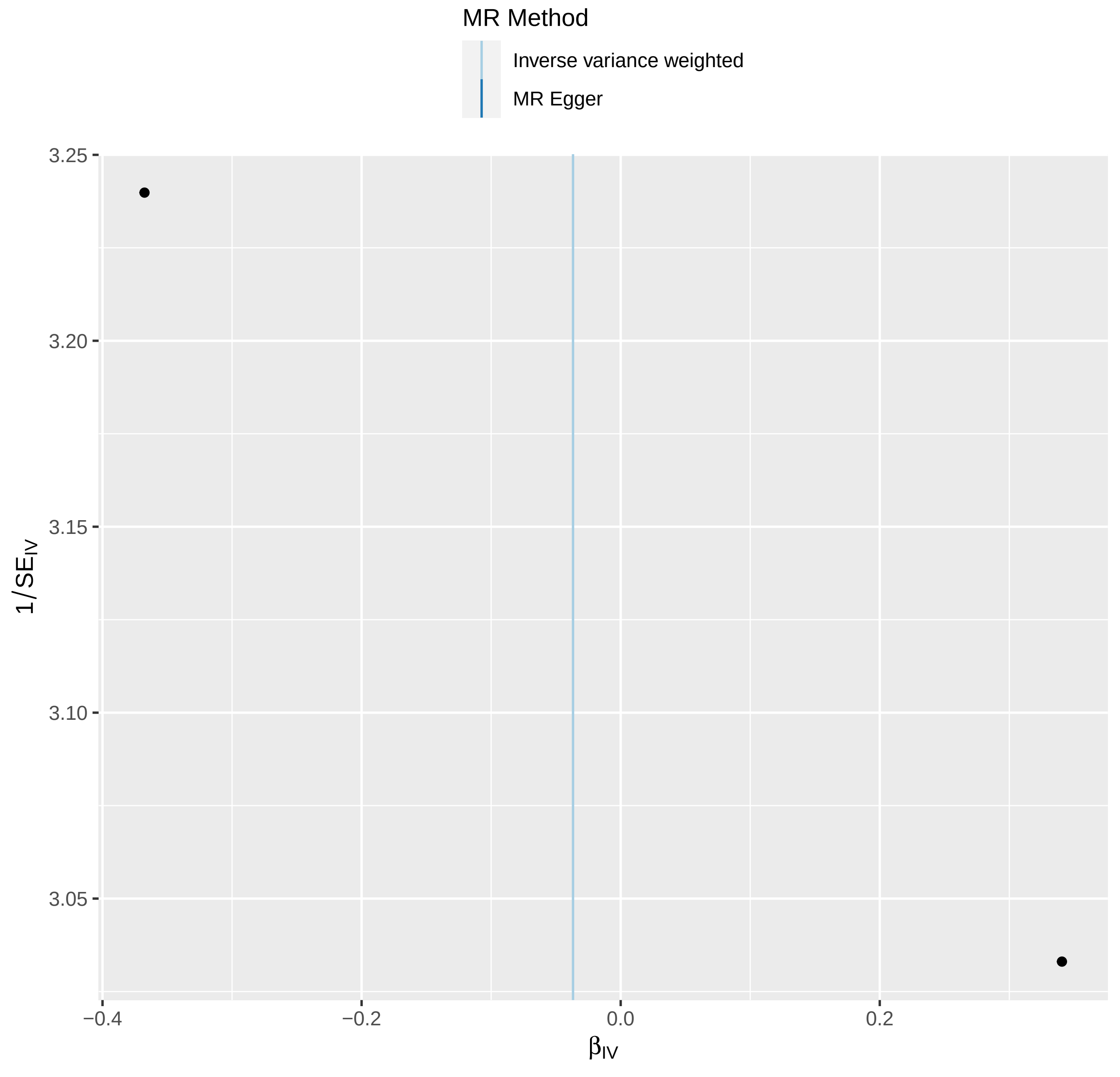

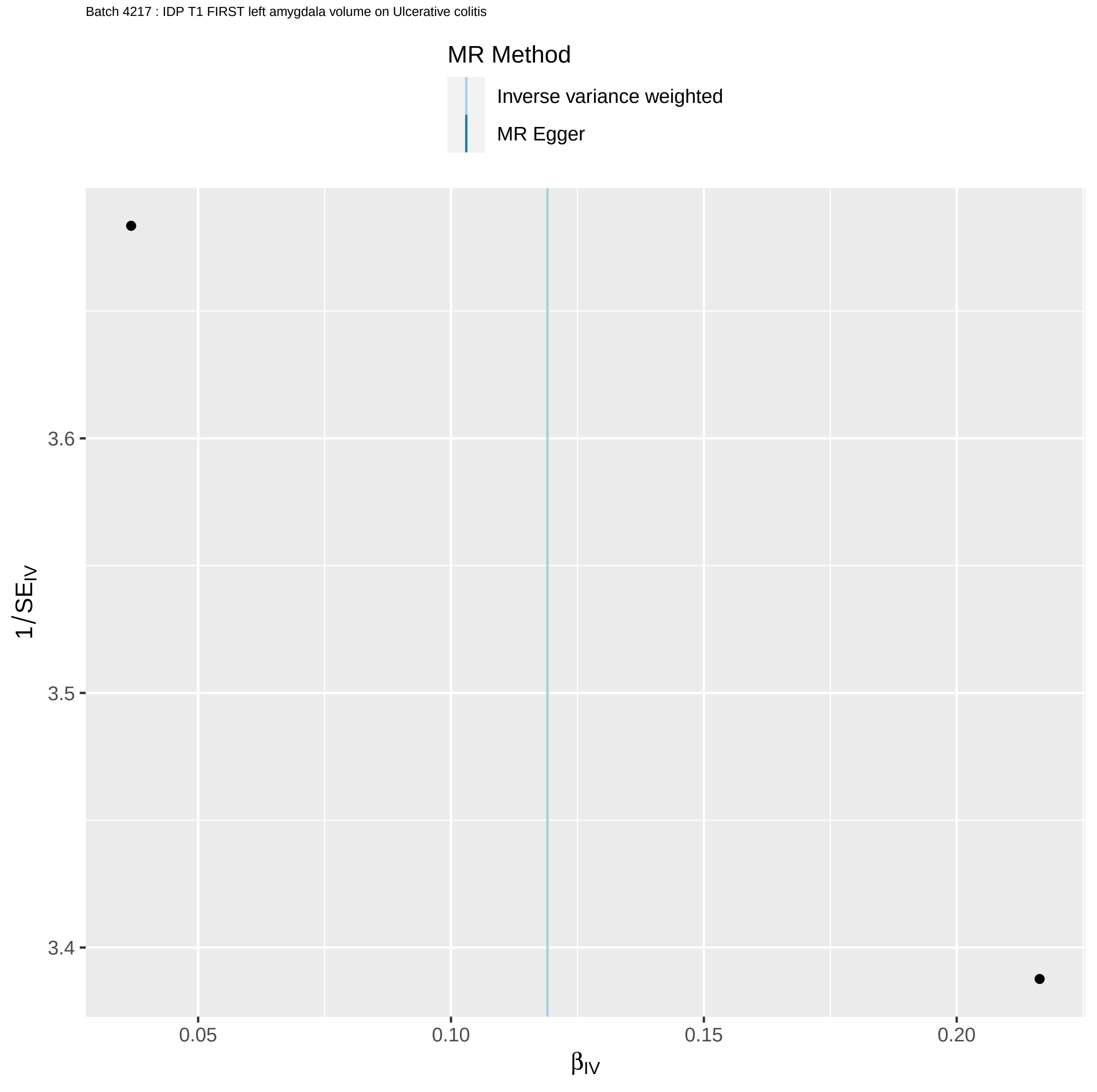

Batch 4217 : IDP T1 FIRST left amygdala volume on Ulcerative colitis
MR Method
Inverse variance weighted
MR Egger
3.6
3.5
3.4
1/SE_IV
0.05
0.10
0.15
0.20
β_IV

Batch 4250 : IDP T1 FIRST right accumbens volume on Ulcerative colitis

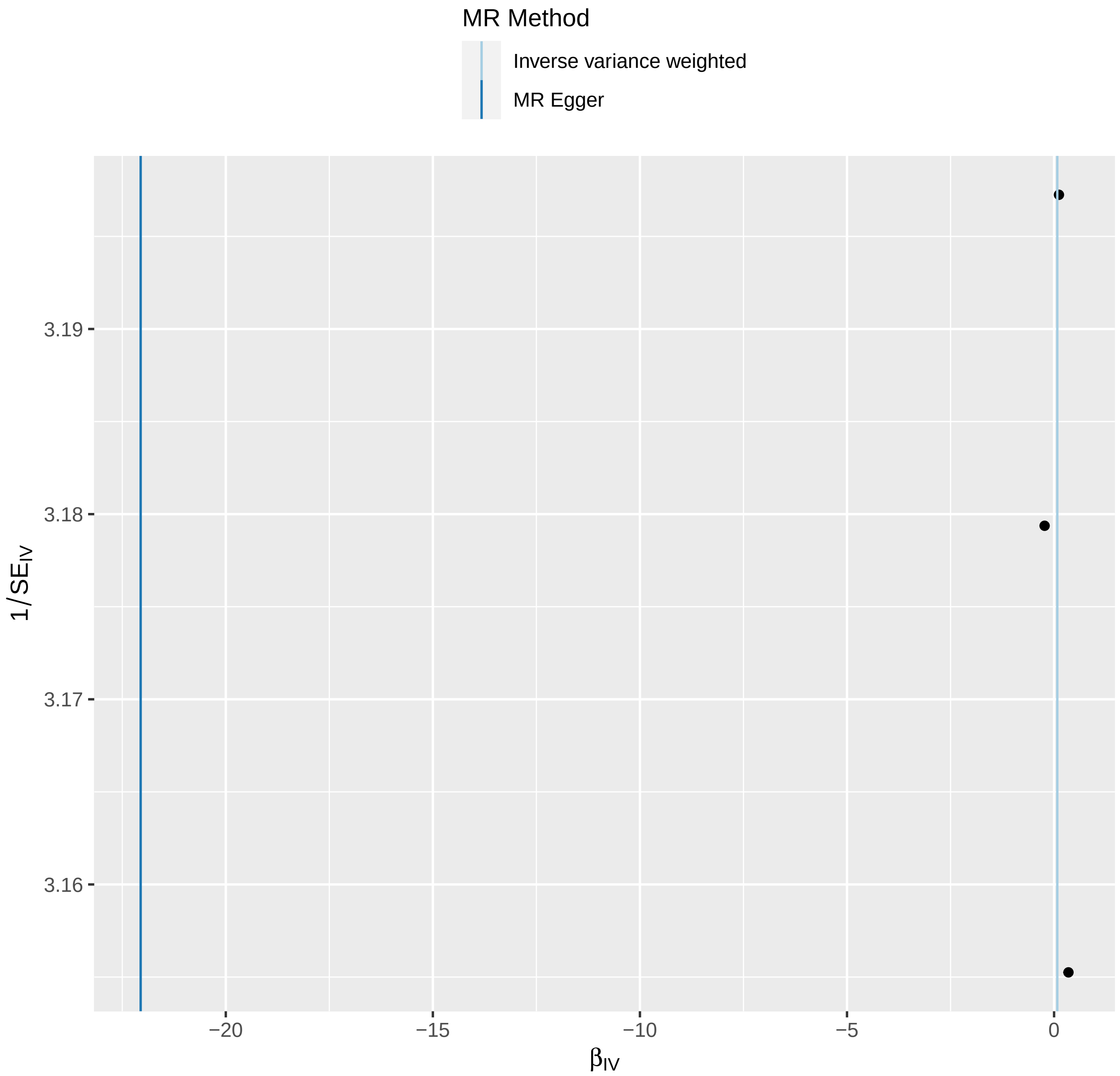

Batch 4261 : IDP T1 FIRST brain stem+4th ventricle volume on Ulcerative colitis

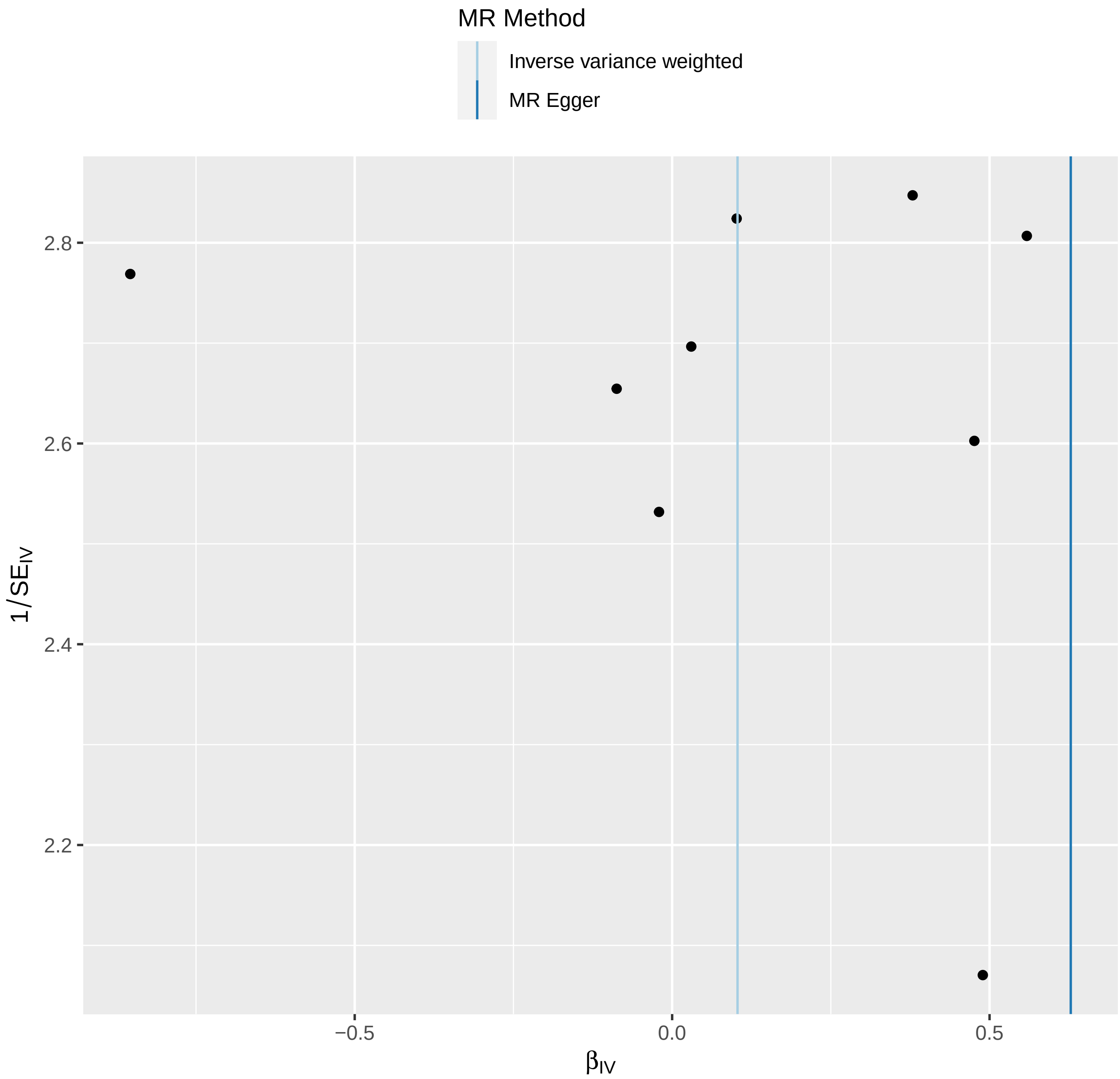

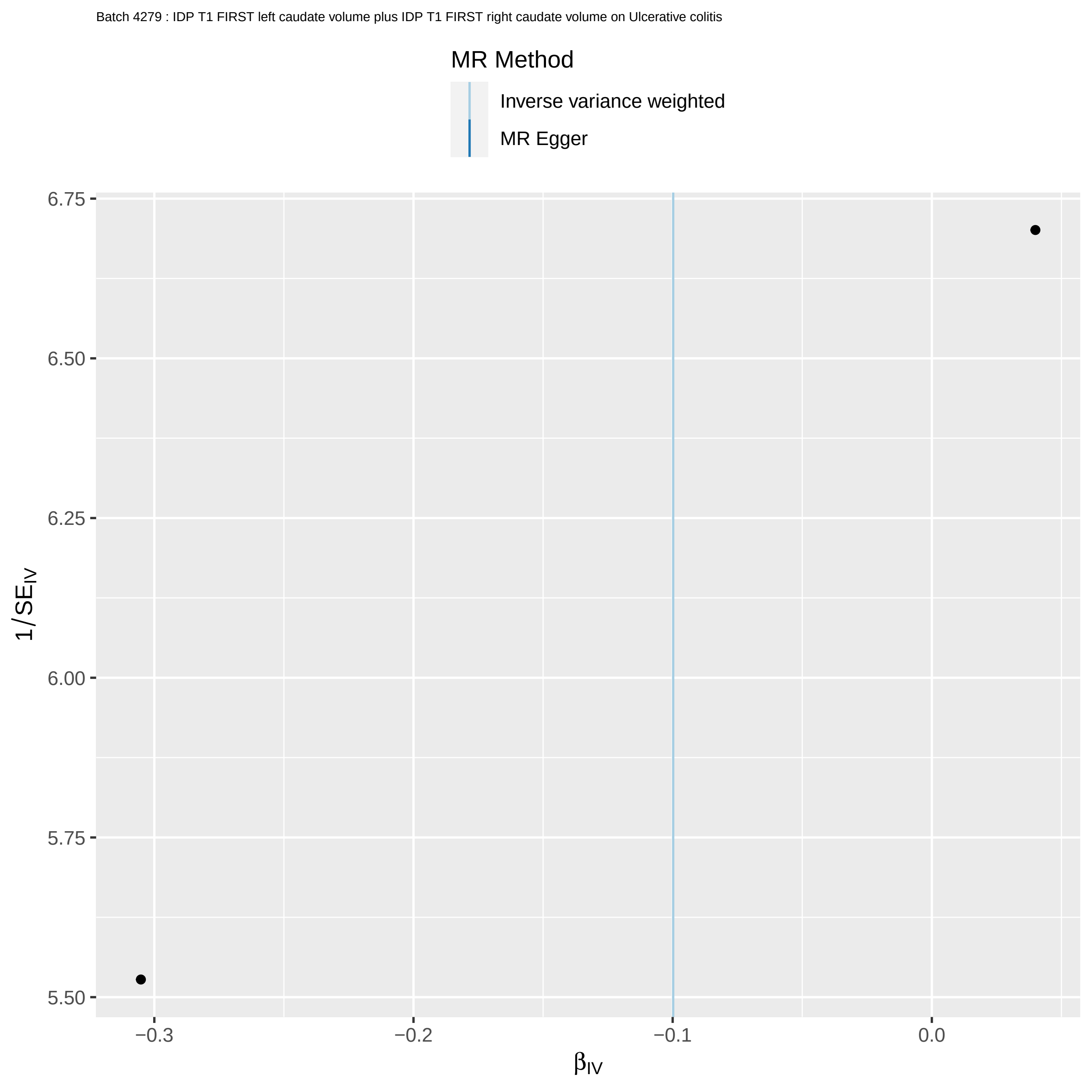

Batch 4279 : IDP T1 FIRST left caudate volume plus IDP T1 FIRST right caudate volume on Ulcerative colitis
MR Method
Inverse variance weighted
MR Egger
6.75
6.50
6.25
6.00
5.75
5.50
1/SE$_{IV}$
−0.3
−0.2
−0.1
0.0
β_{IV}

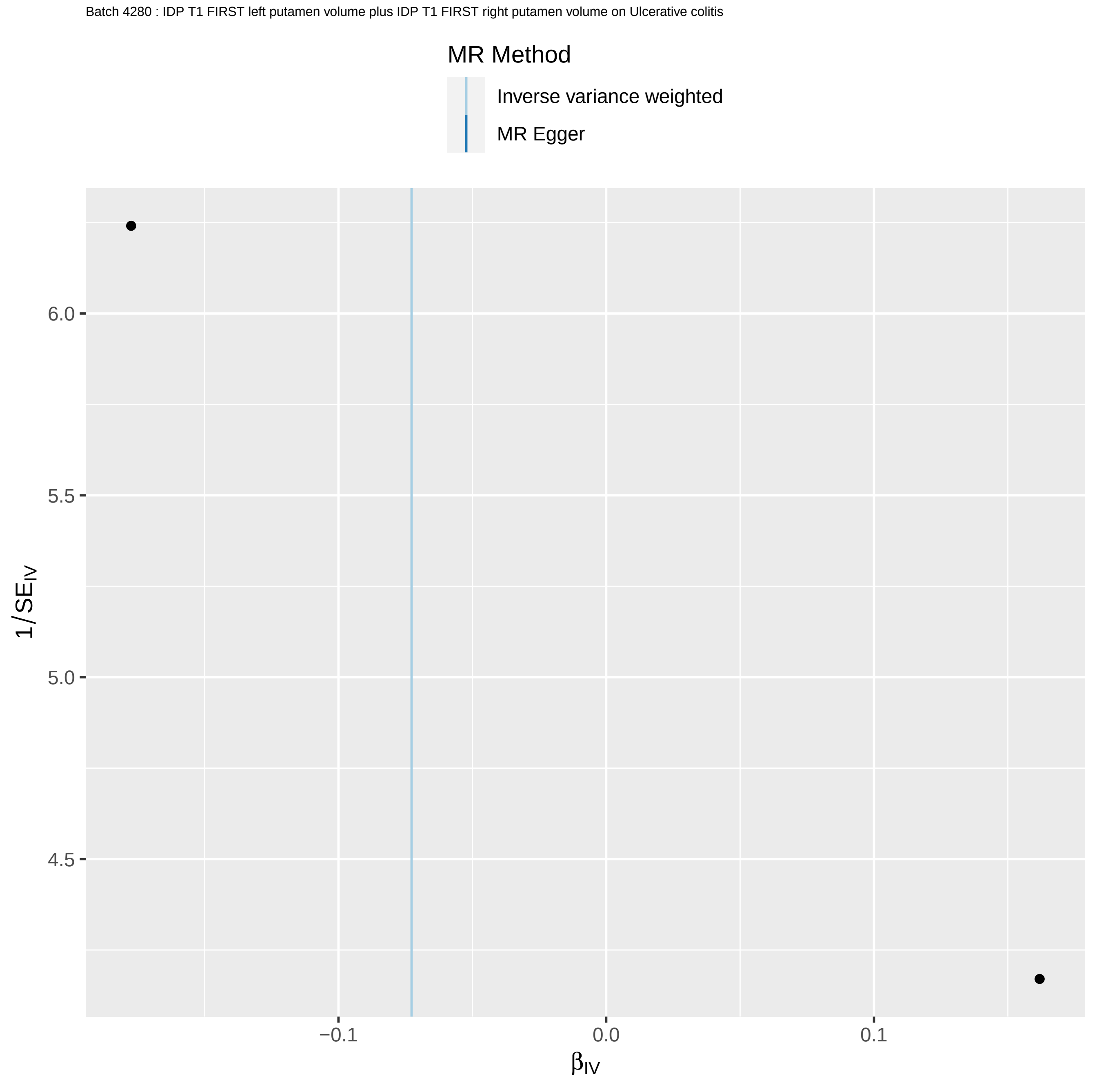

Batch 4280 : IDP T1 FIRST left putamen volume plus IDP T1 FIRST right putamen volume on Ulcerative colitis
MR Method
Inverse variance weighted
MR Egger
6.0
5.5
5.0
4.5
1/SE$_{IV}$
−0.1
0.0
0.1
β_{IV}

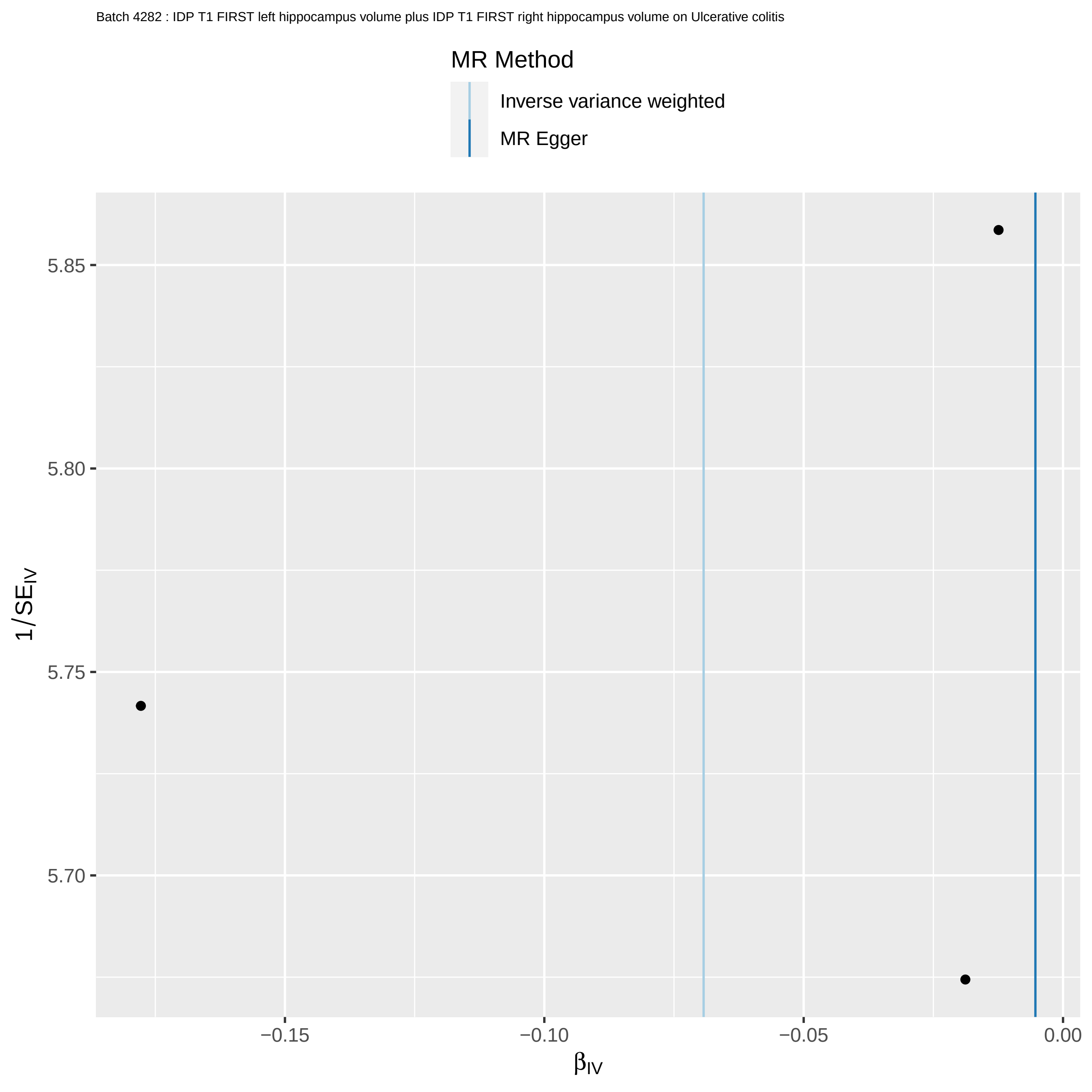
Batch 4282 : IDP T1 FIRST left hippocampus volume plus IDP T1 FIRST right hippocampus volume on Ulcerative colitis
MR Method
Inverse variance weighted
MR Egger
5.85
5.80
5.75
5.70
1/SE$_{IV}$
−0.15
−0.10
−0.05
0.00
β$_{IV}$

Batch 4283 : IDP T1 FIRST left amygdala volume plus IDP T1 FIRST right amygdala volume on Ulcerative colitis

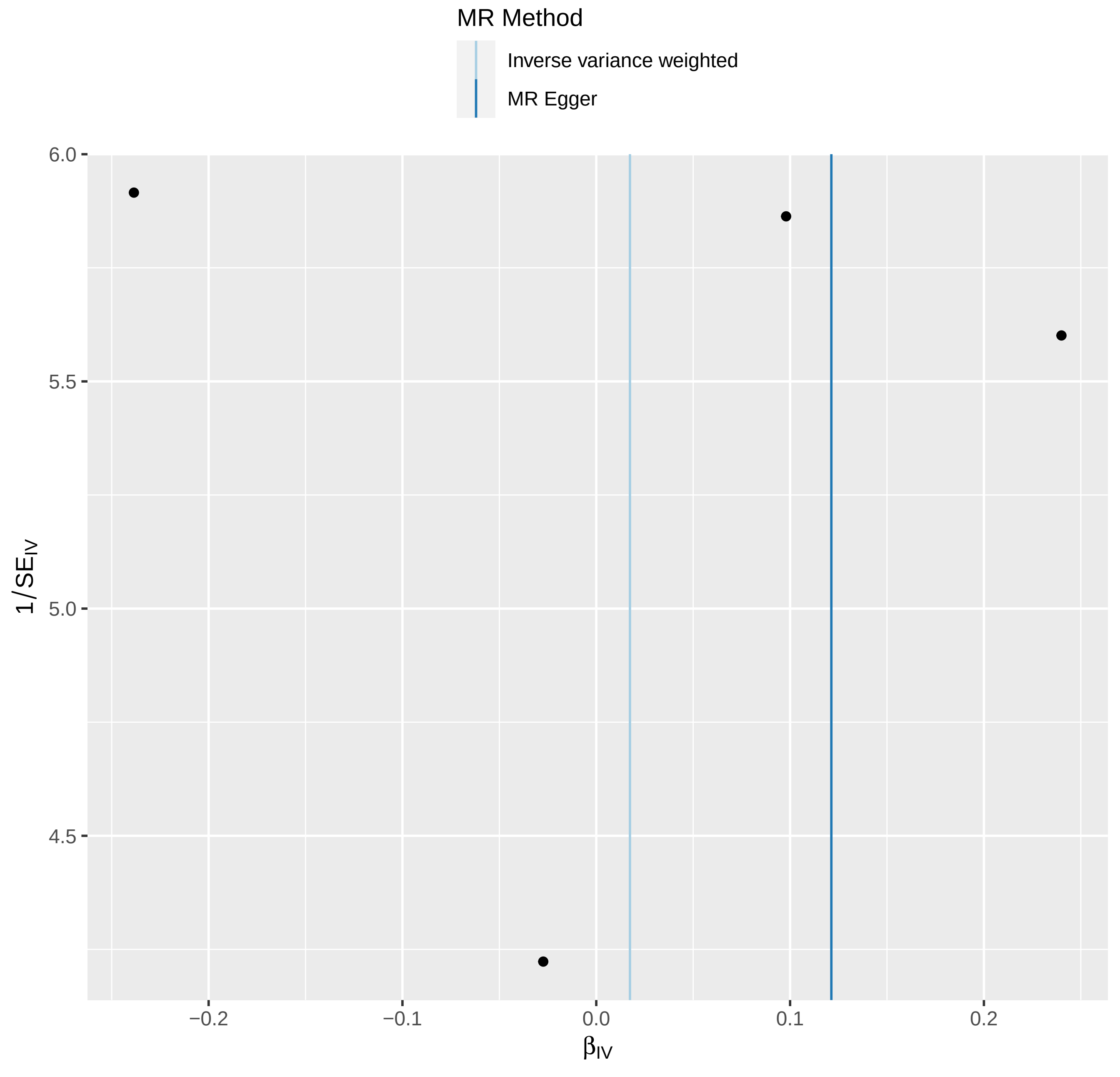

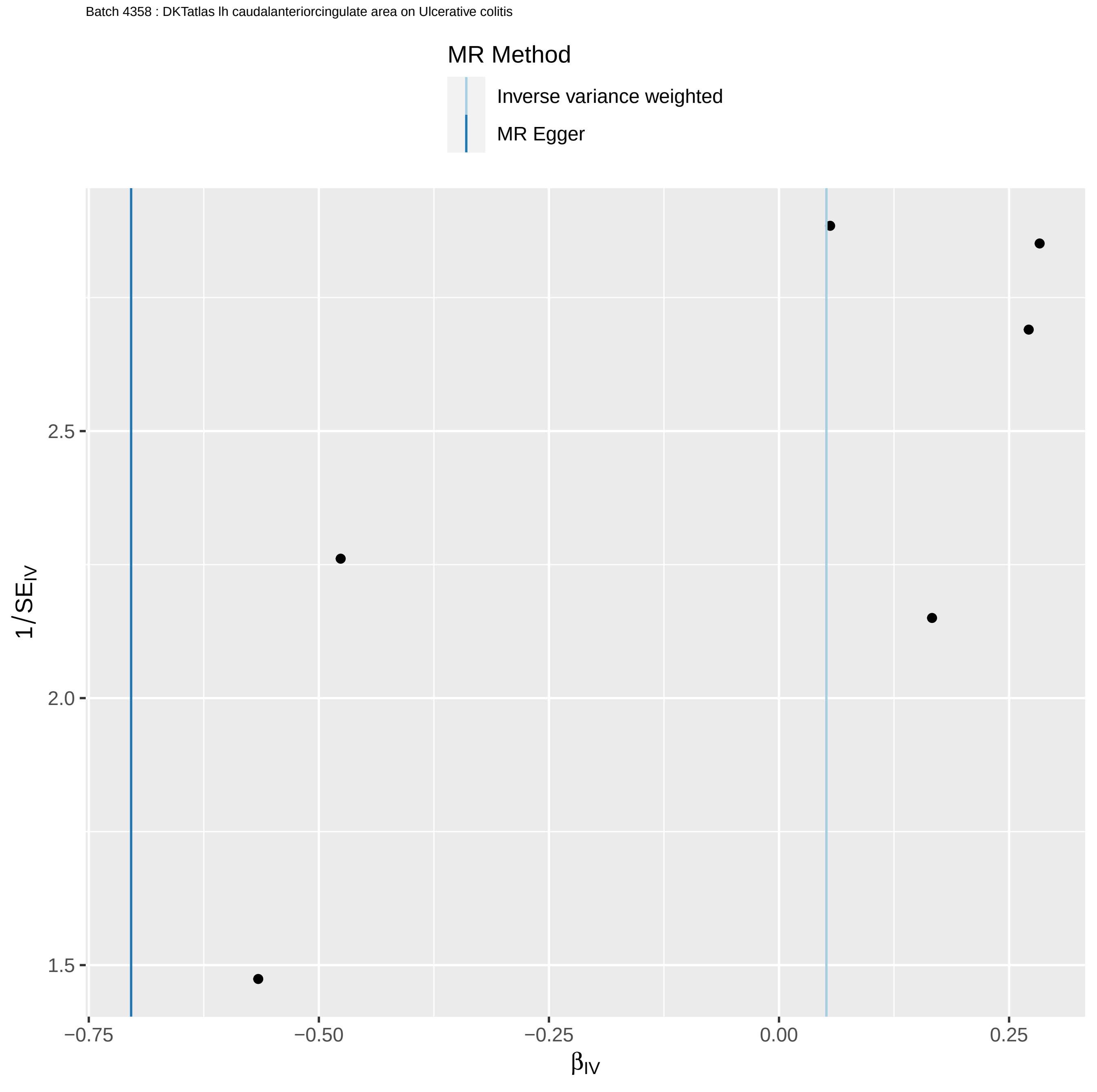

Batch 4358 : DKTatlas lh caudalanteriorcingulate area on Ulcerative colitis
MR Method
Inverse variance weighted
MR Egger
2.5
2.0
1.5
1/SE_IV
−0.75
−0.50
−0.25
0.00
0.25
β_IV

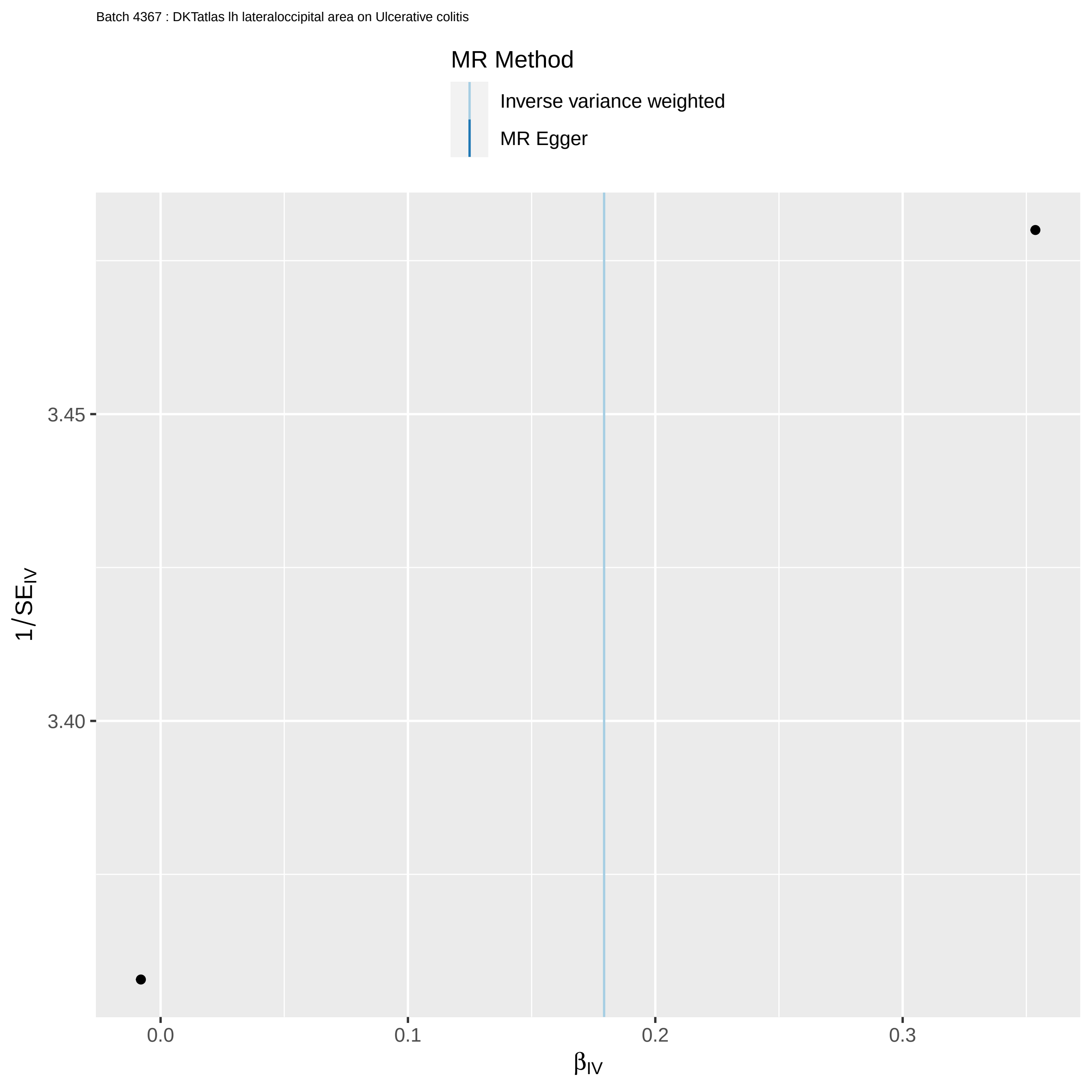

Batch 4367 : DKTatlas lh lateraloccipital area on Ulcerative colitis
MR Method
Inverse variance weighted
MR Egger
3.45
3.40
$1/SE_{IV}$
0.0
0.1
0.2
0.3
β_{IV}

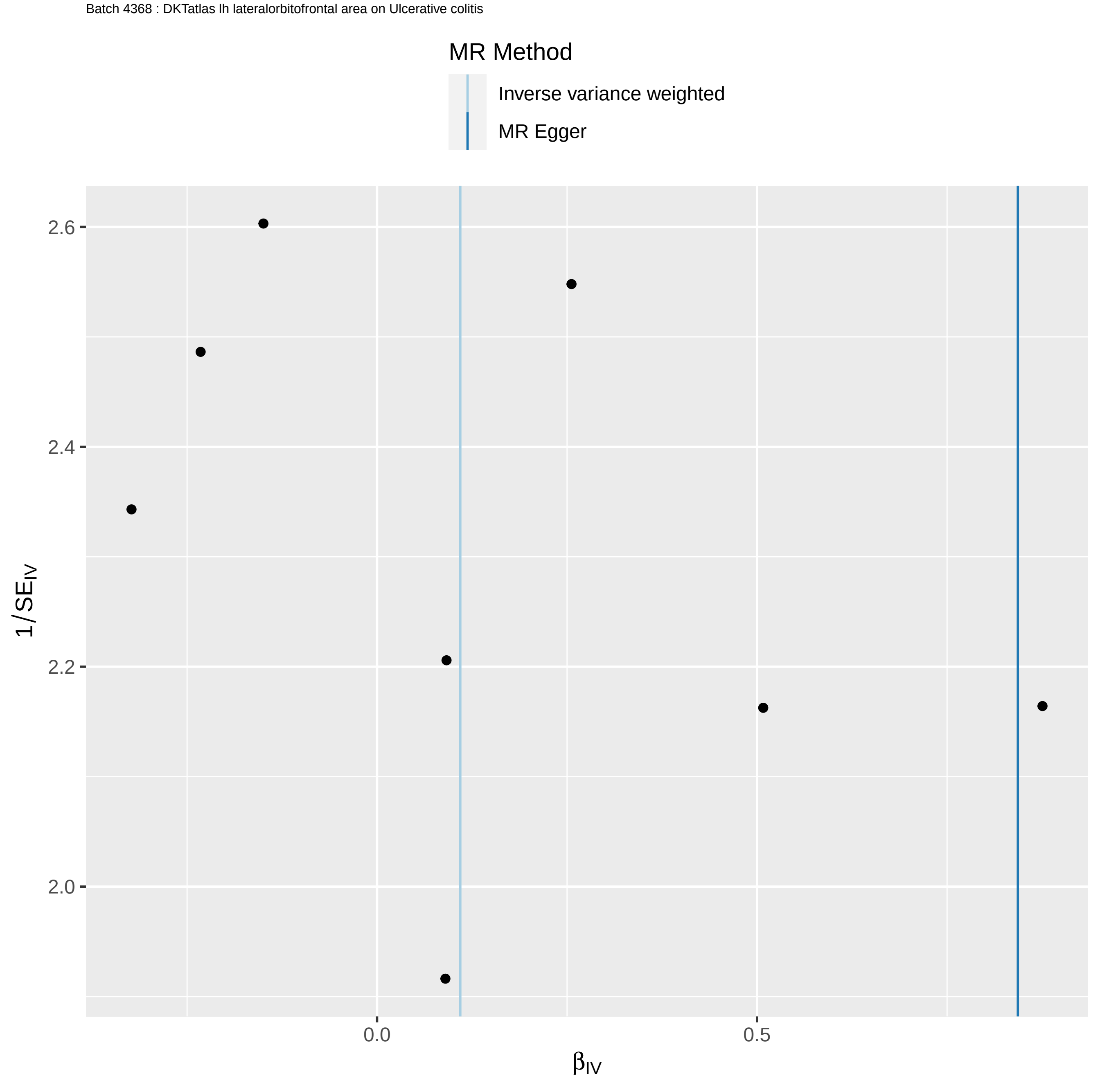
Batch 4368 : DKTatlas lh lateralorbitofrontal area on Ulcerative colitis
MR Method
Inverse variance weighted
MR Egger
2.6
2.4
2.2
2.0
1/SE_IV
0.0
0.5
β_IV

Batch 4370 : DKTatlas lh medialorbitofrontal area on Ulcerative colitis

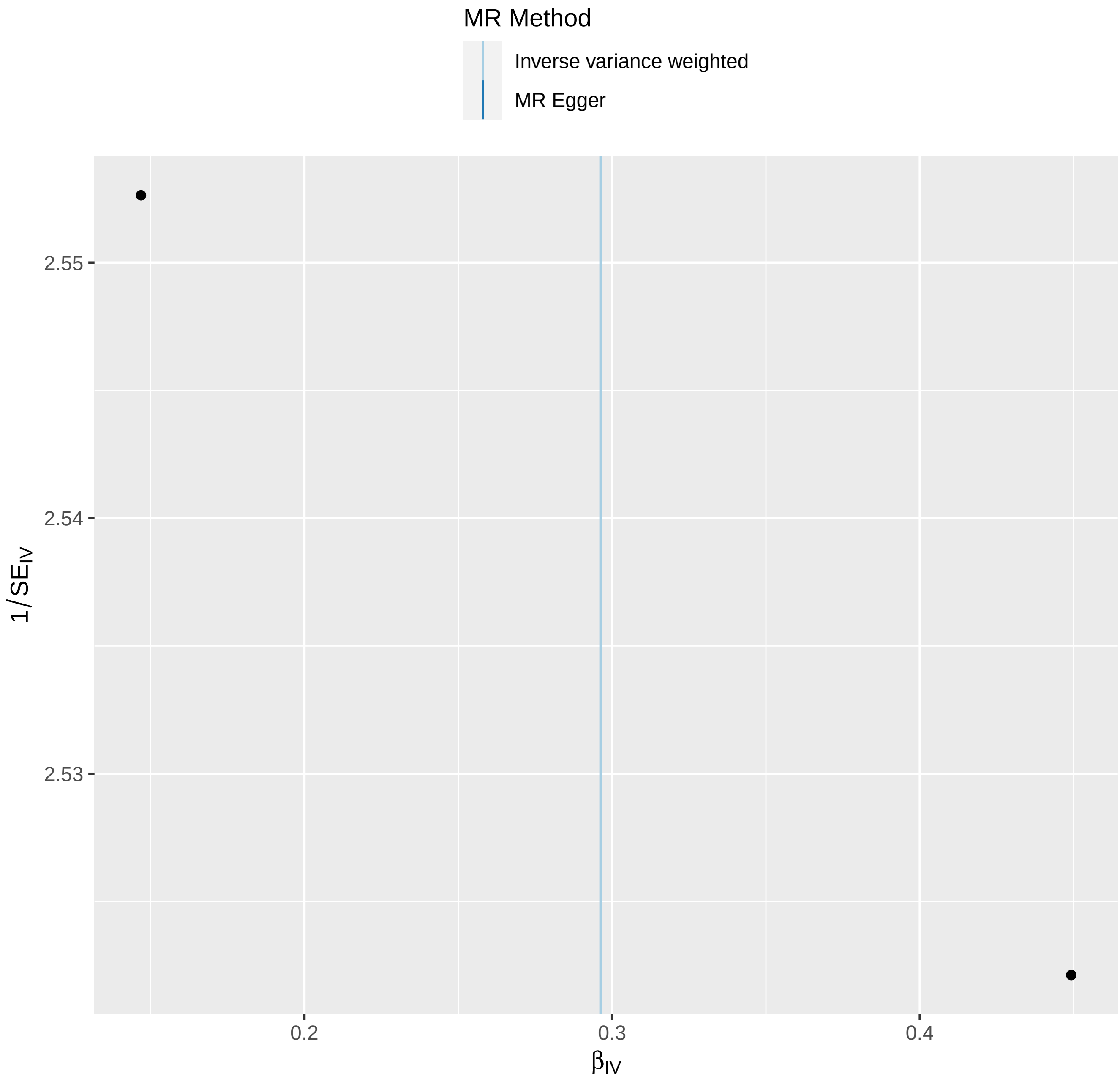

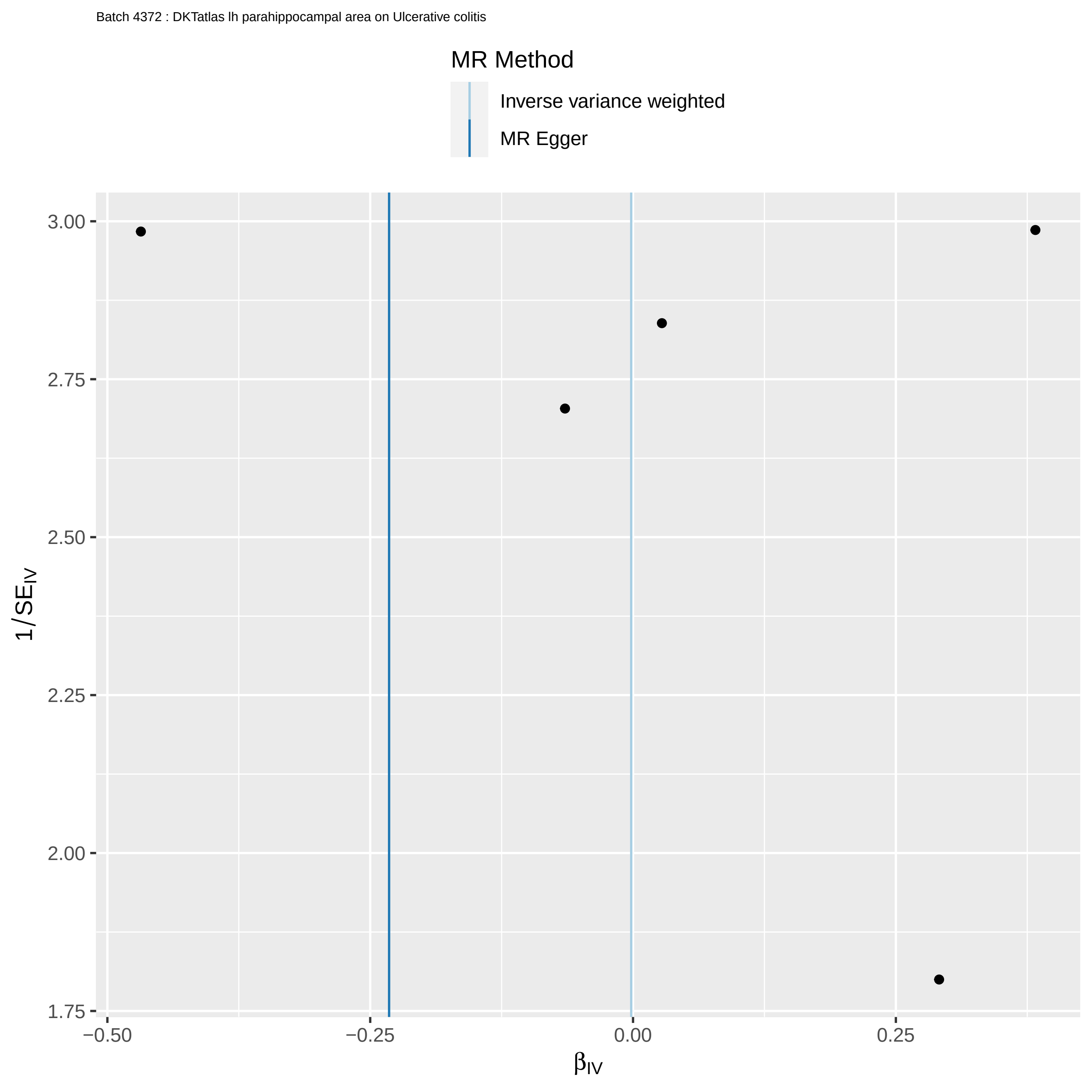
Batch 4372 : DKTatlas lh parahippocampal area on Ulcerative colitis
MR Method
Inverse variance weighted
MR Egger
3.00
2.75
2.50
2.25
2.00
1.75
1/SE_IV
−0.50
−0.25
0.00
0.25
β_IV

Batch 4373 : DKTatlas lh paracentral area on Ulcerative colitis

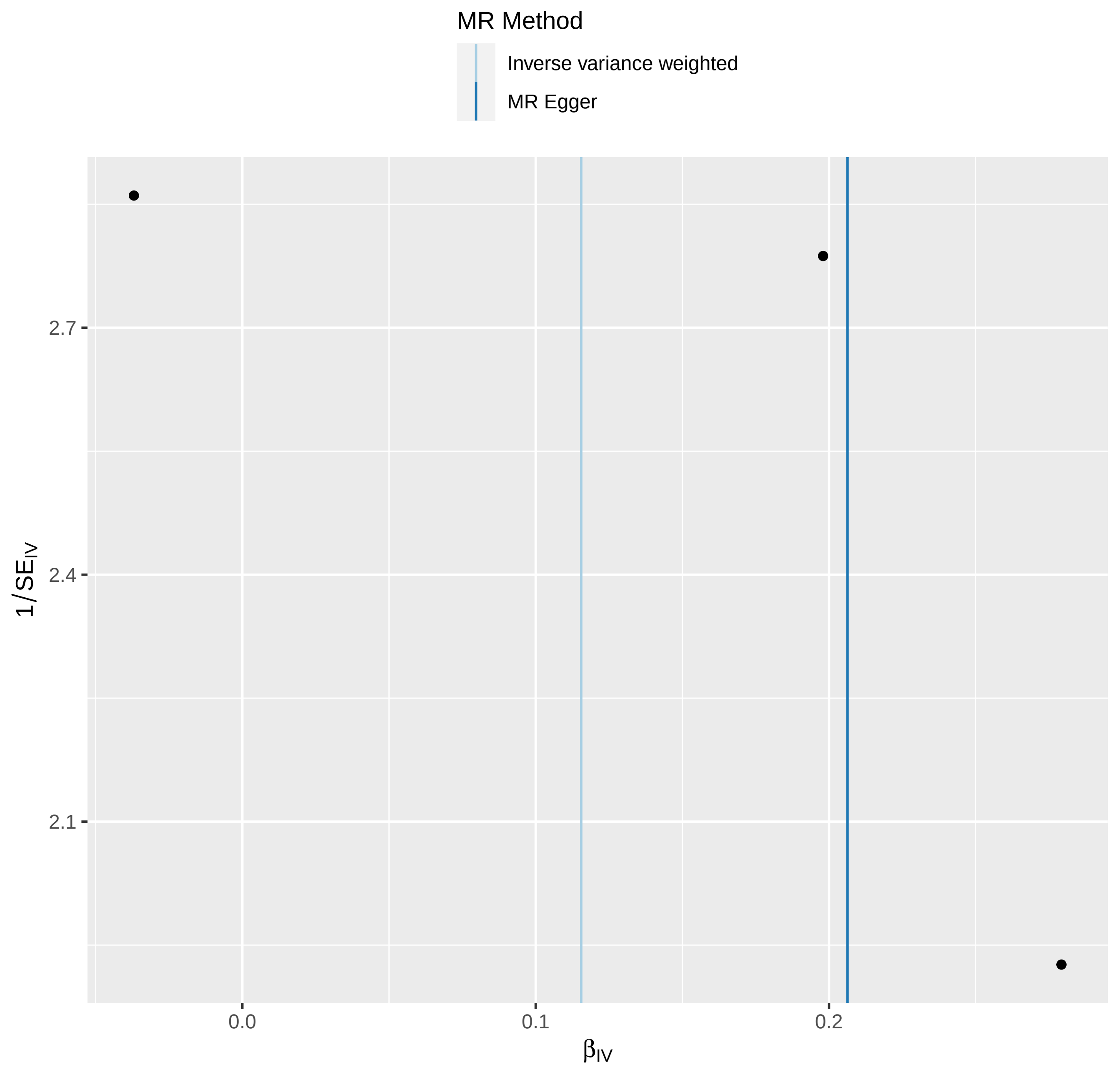

Batch 4376 : DKTatlas lh parsorbitalis area on Ulcerative colitis

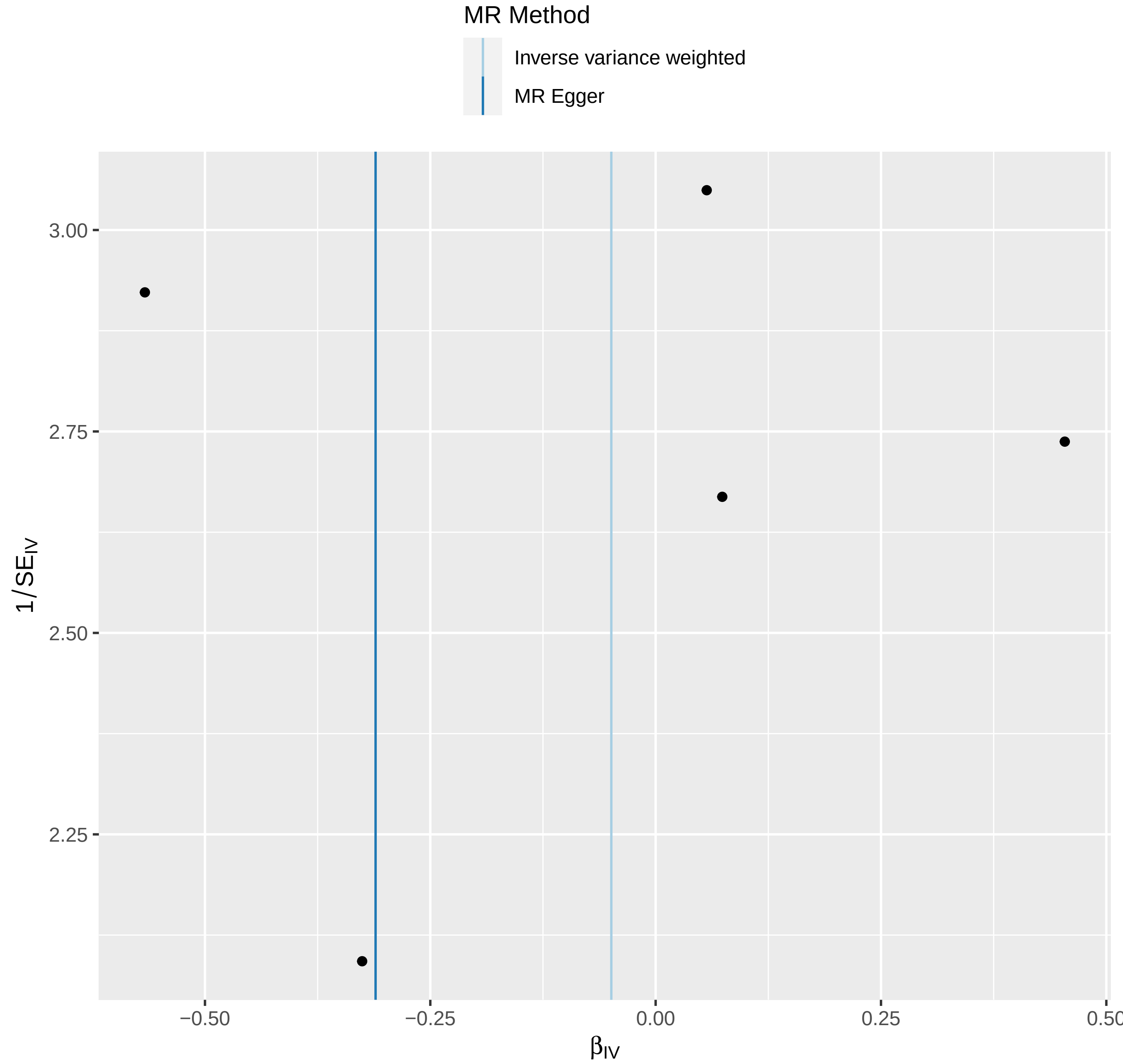

Batch 4379 : DKTatlas lh postcentral area on Ulcerative colitis

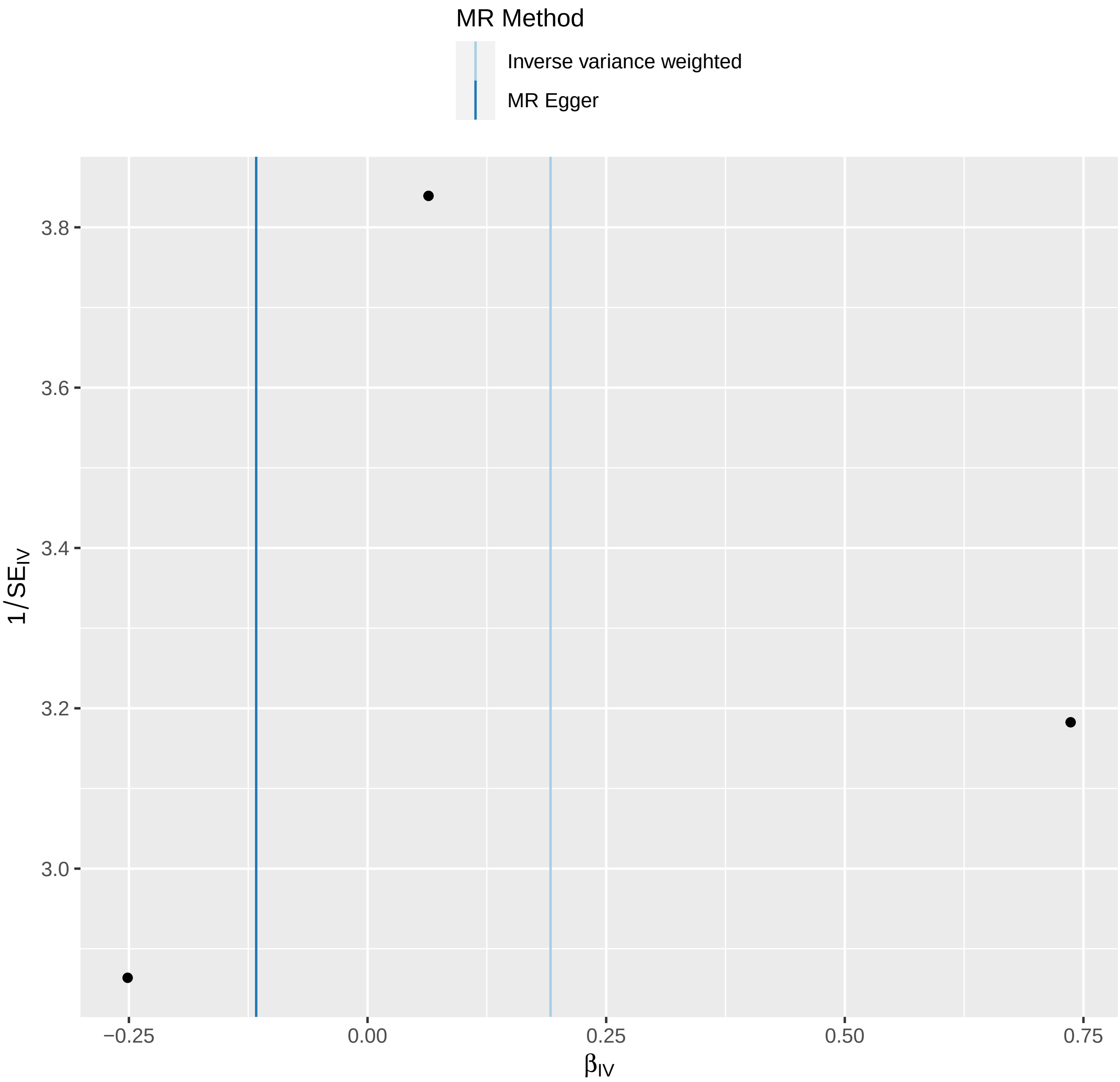

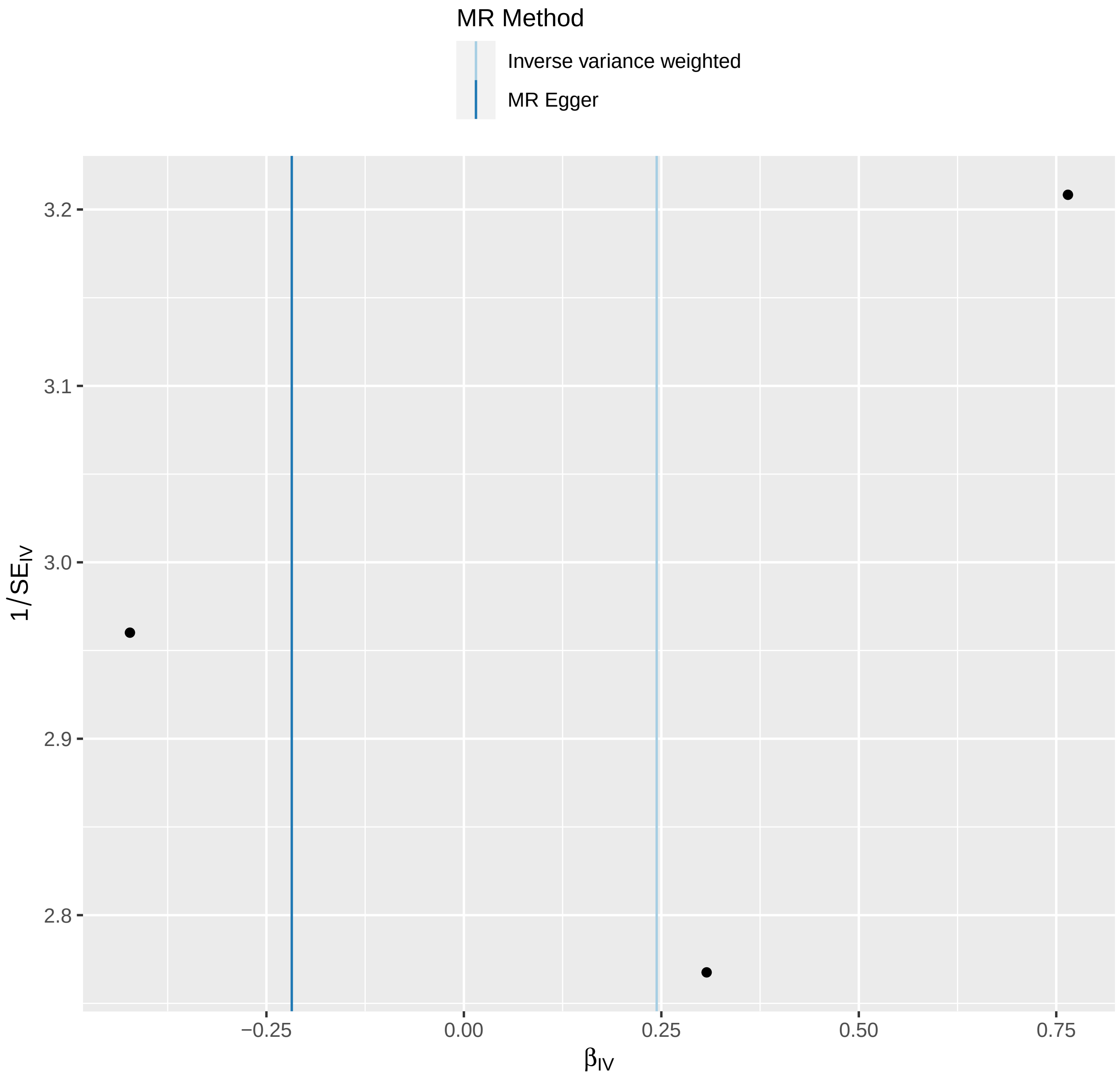
MR Method
Inverse variance weighted
MR Egger
3.2
3.1
3.0
2.9
2.8
1/SE$_{IV}$
−0.25
0.00
0.25
0.50
0.75
β$_{IV}$

Batch 4383 : DKTatlas lh rostralanteriorcingulate area on Ulcerative colitis

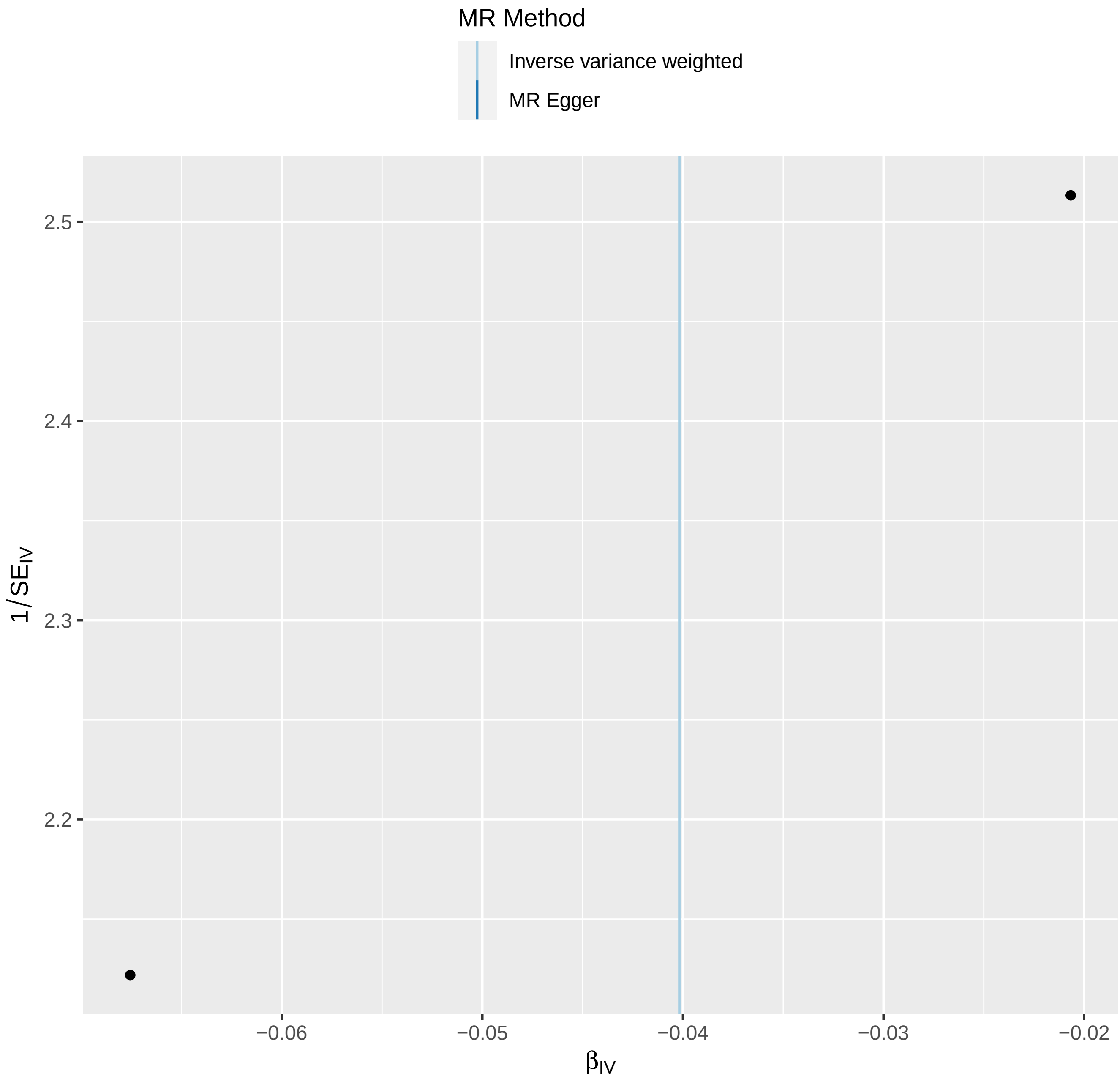

Batch 4384 : DKTatlas lh rostralmiddlefrontal area on Ulcerative colitis

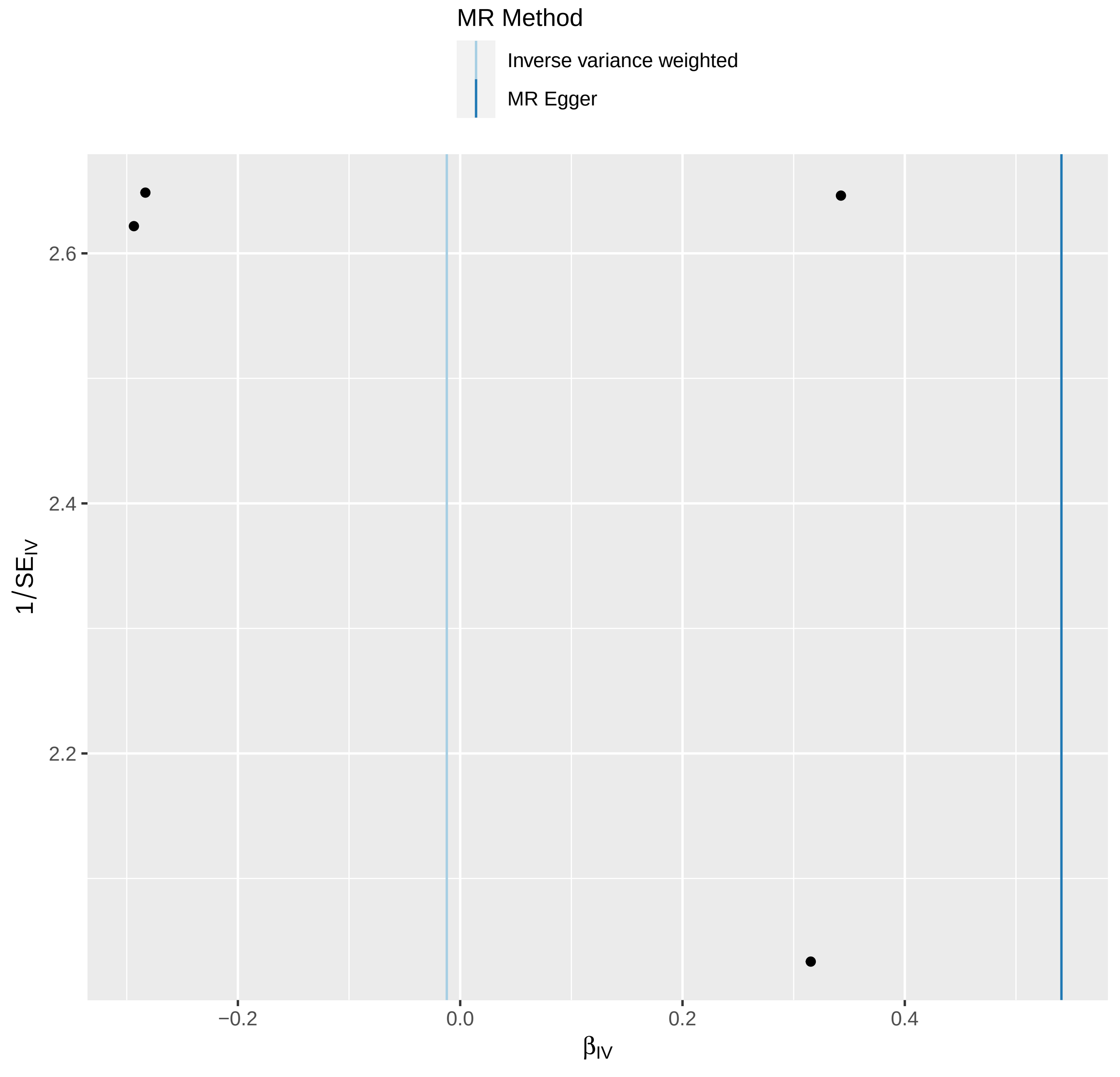

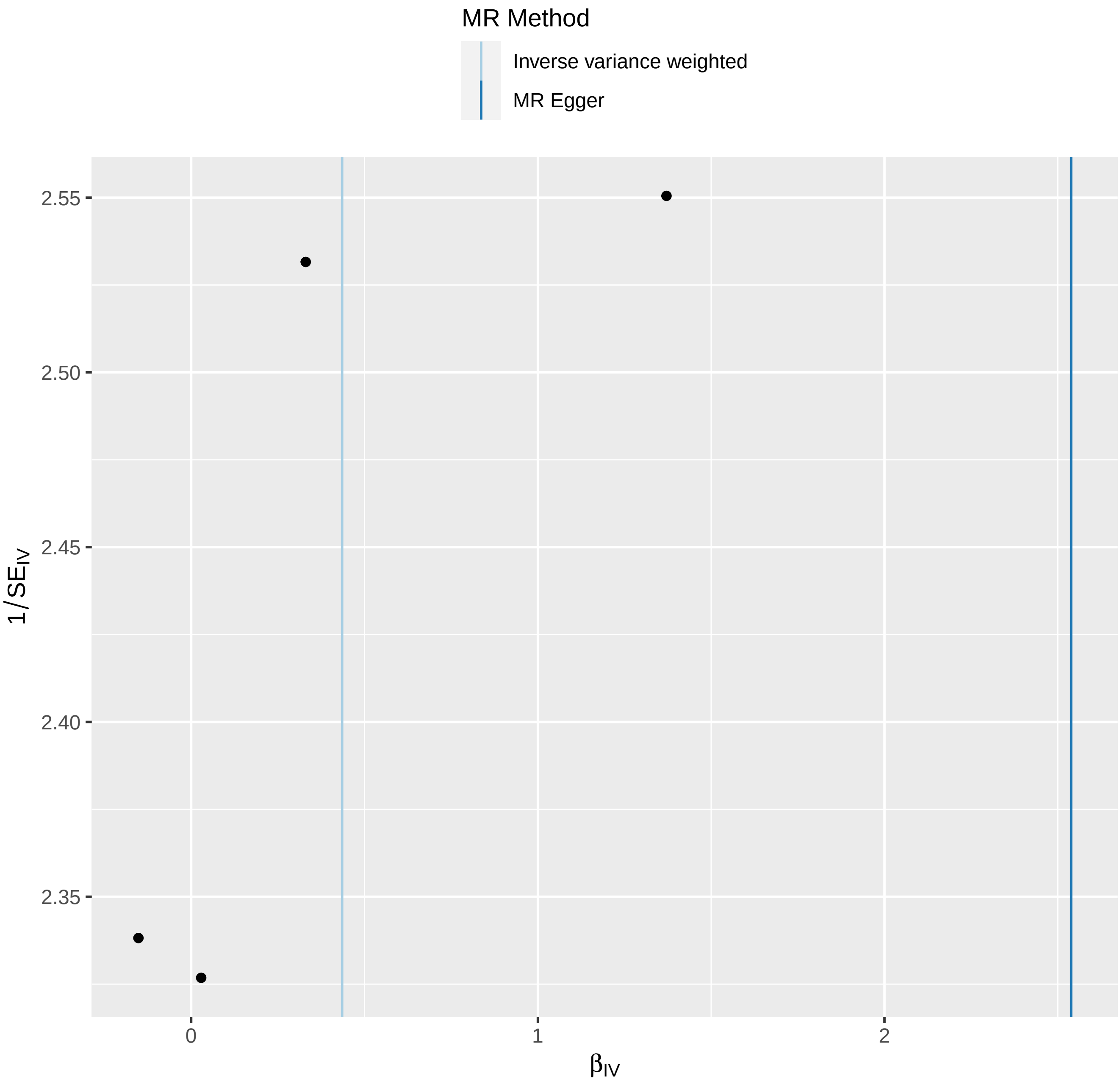
MR Method
Inverse variance weighted
MR Egger
2.55
2.50
2.45
2.40
2.35
$1/SE_{IV}$
0
1
2
β_{IV}

Batch 4392 : DKTatlas lh WhiteSurfArea area on Ulcerative colitis

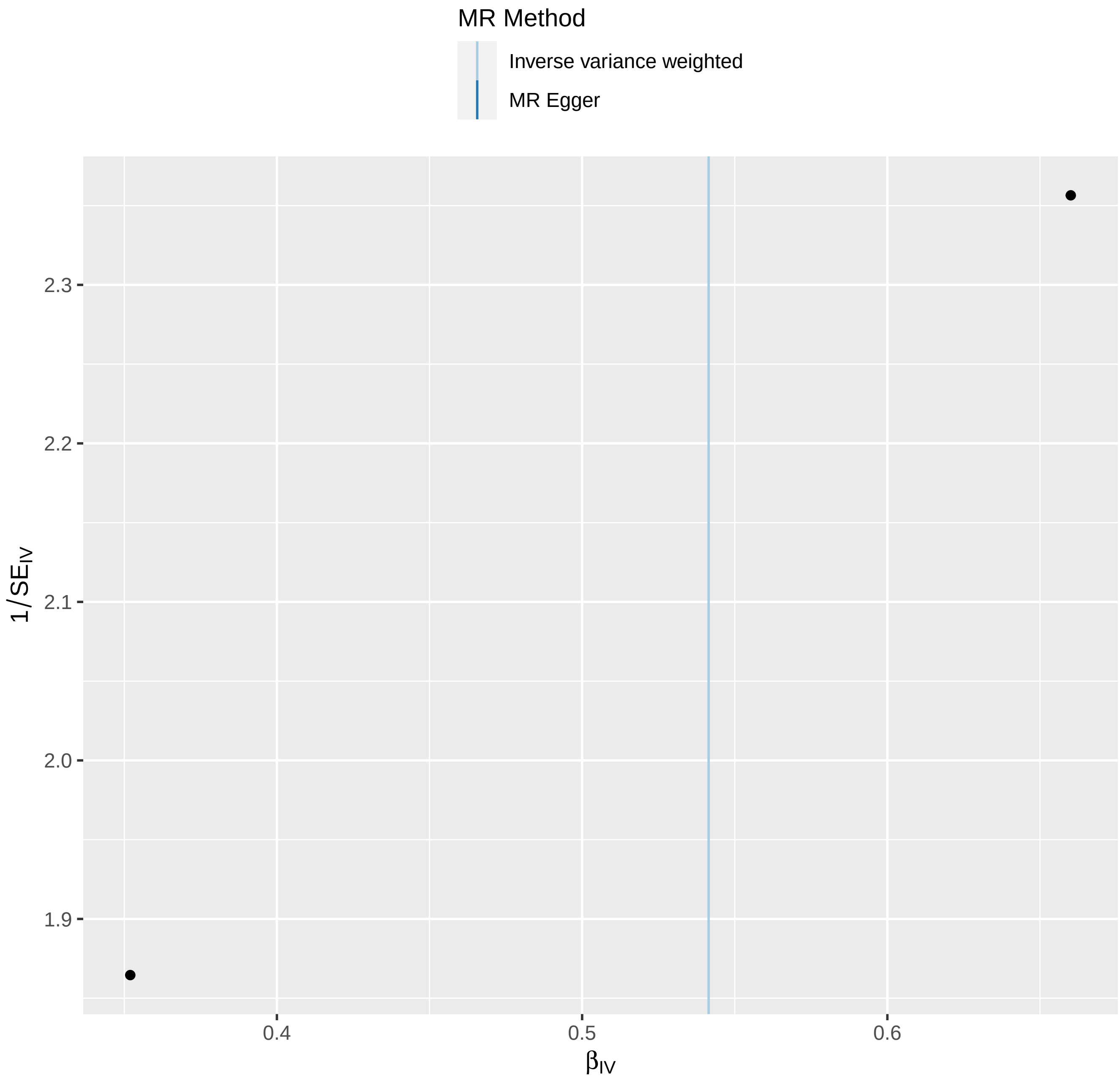

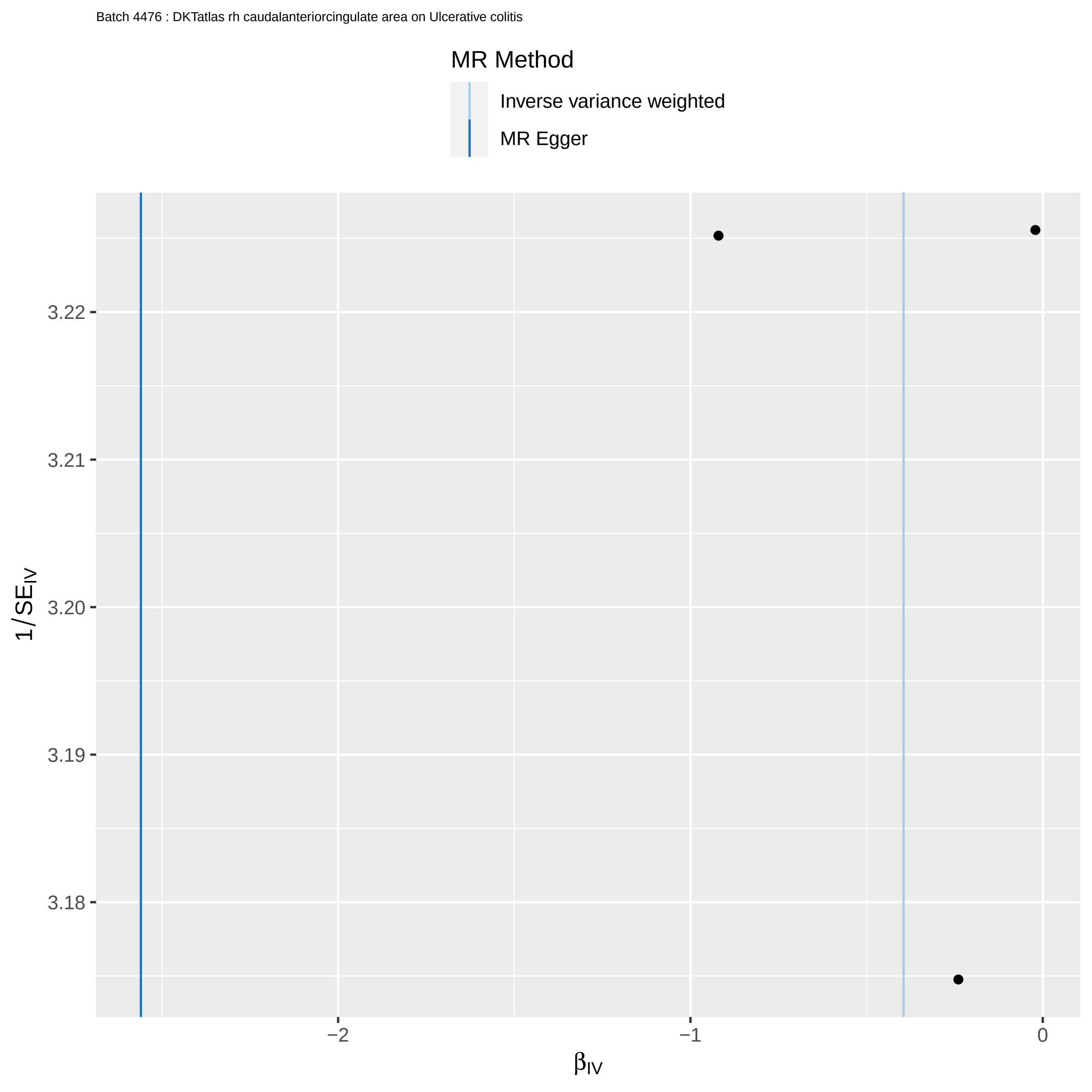

Batch 4476 : DKTatlas rh caudalanteriorcingulate area on Ulcerative colitis
MR Method
Inverse variance weighted
MR Egger
3.22
3.21
3.20
3.19
3.18
1/SE_IV
−2
−1
0
β_IV

Batch 4479 : DKTatlas rh entorhinal area on Ulcerative colitis

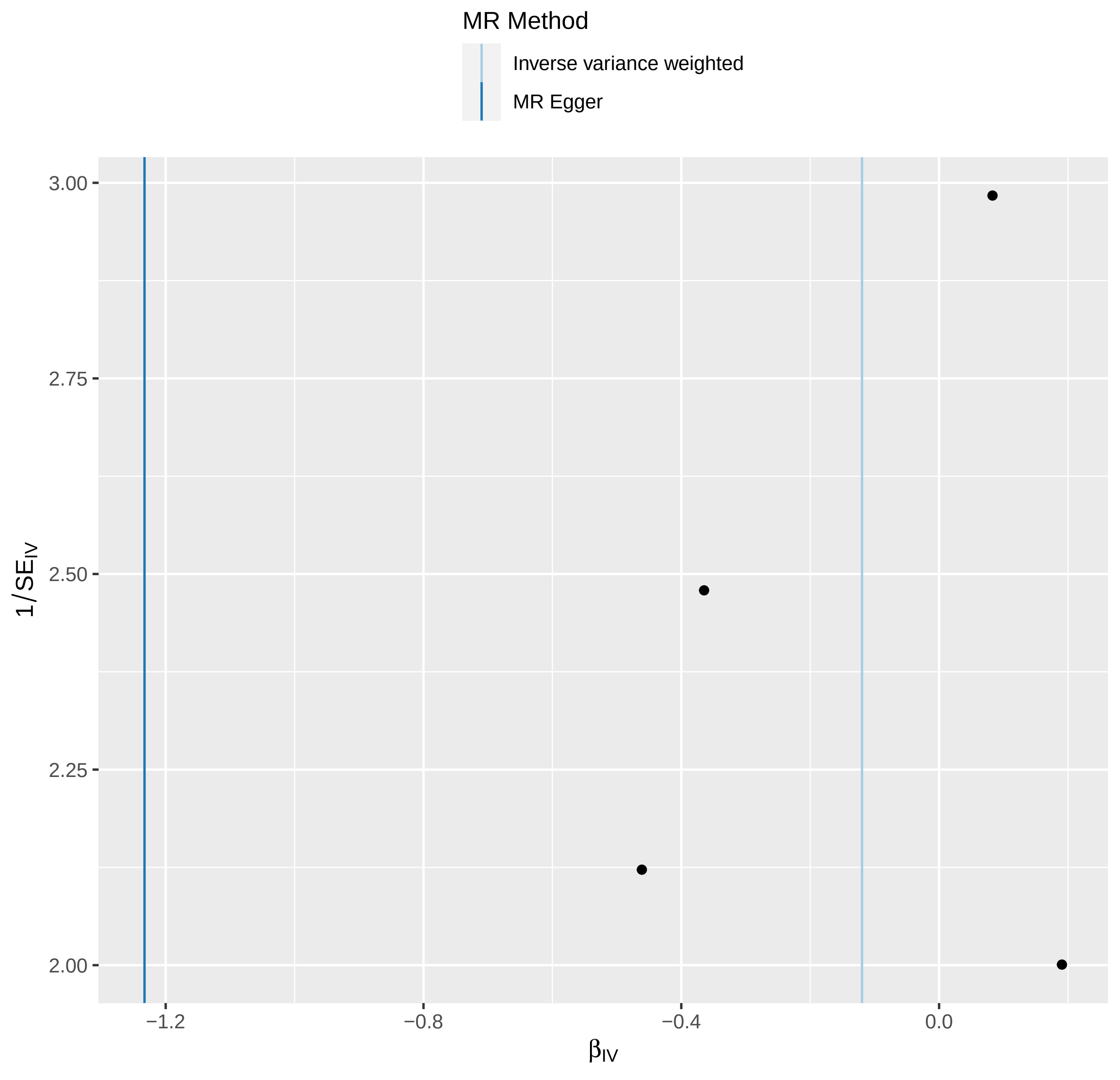

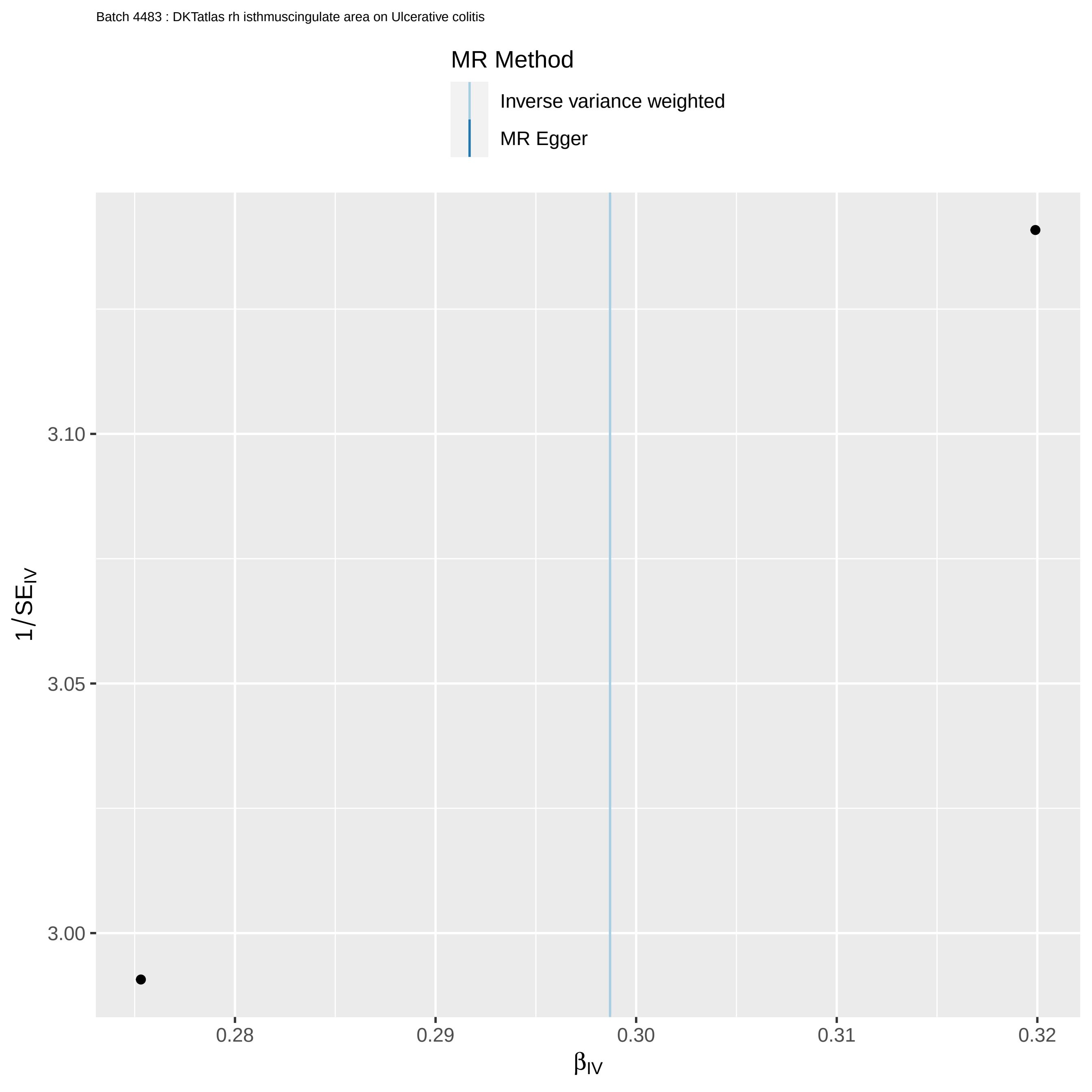

Batch 4483 : DKTatlas rh isthmuscingulate area on Ulcerative colitis
MR Method
Inverse variance weighted
MR Egger
3.10
3.05
3.00
1/SE$_{IV}$
0.28
0.29
0.30
0.31
0.32
β$_{IV}$

Batch 4484 : DKTatlas rh lateraloccipital area on Ulcerative colitis

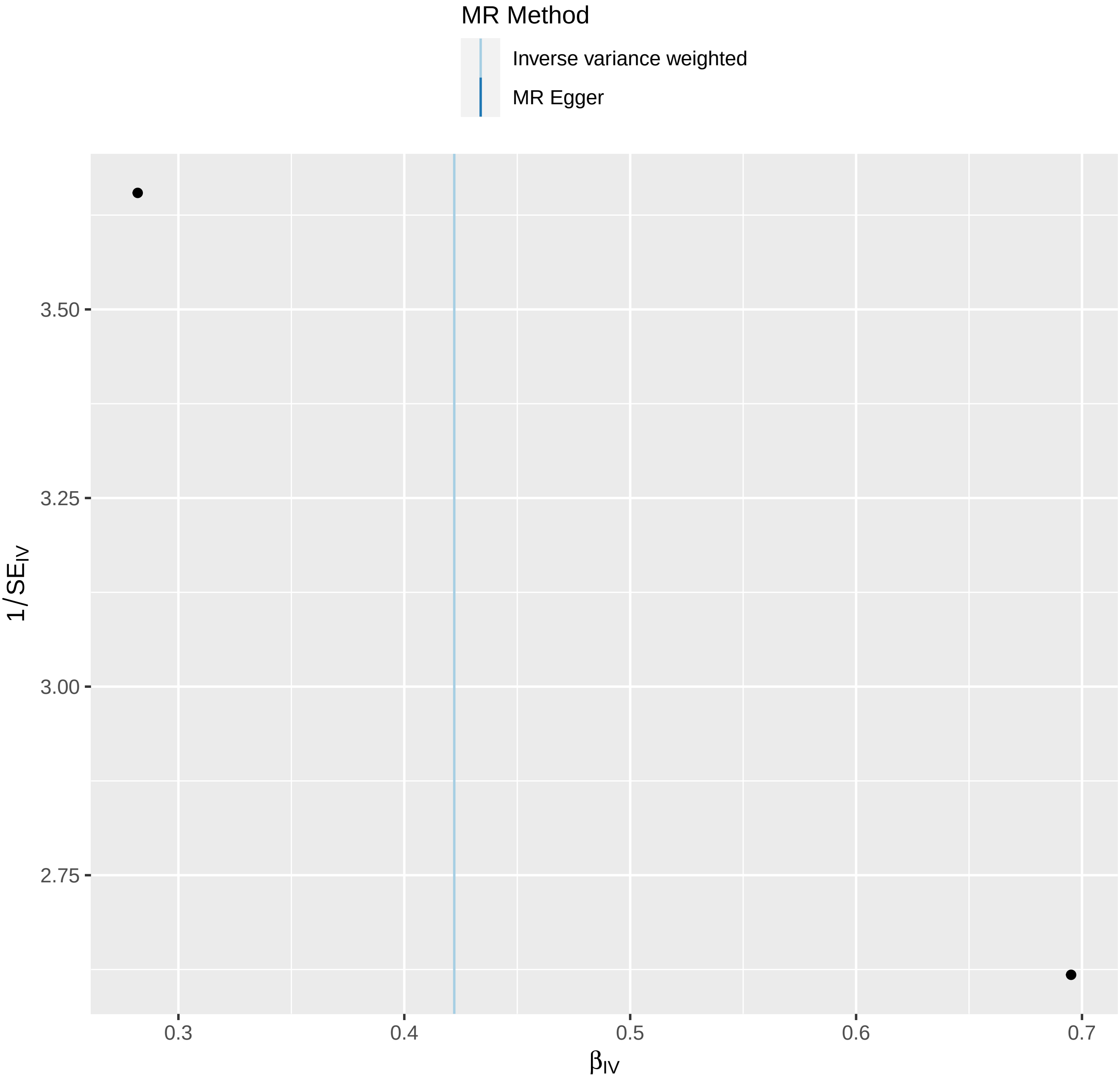

Batch 4489 : DKTatlas rh middletemporal area on Ulcerative colitis

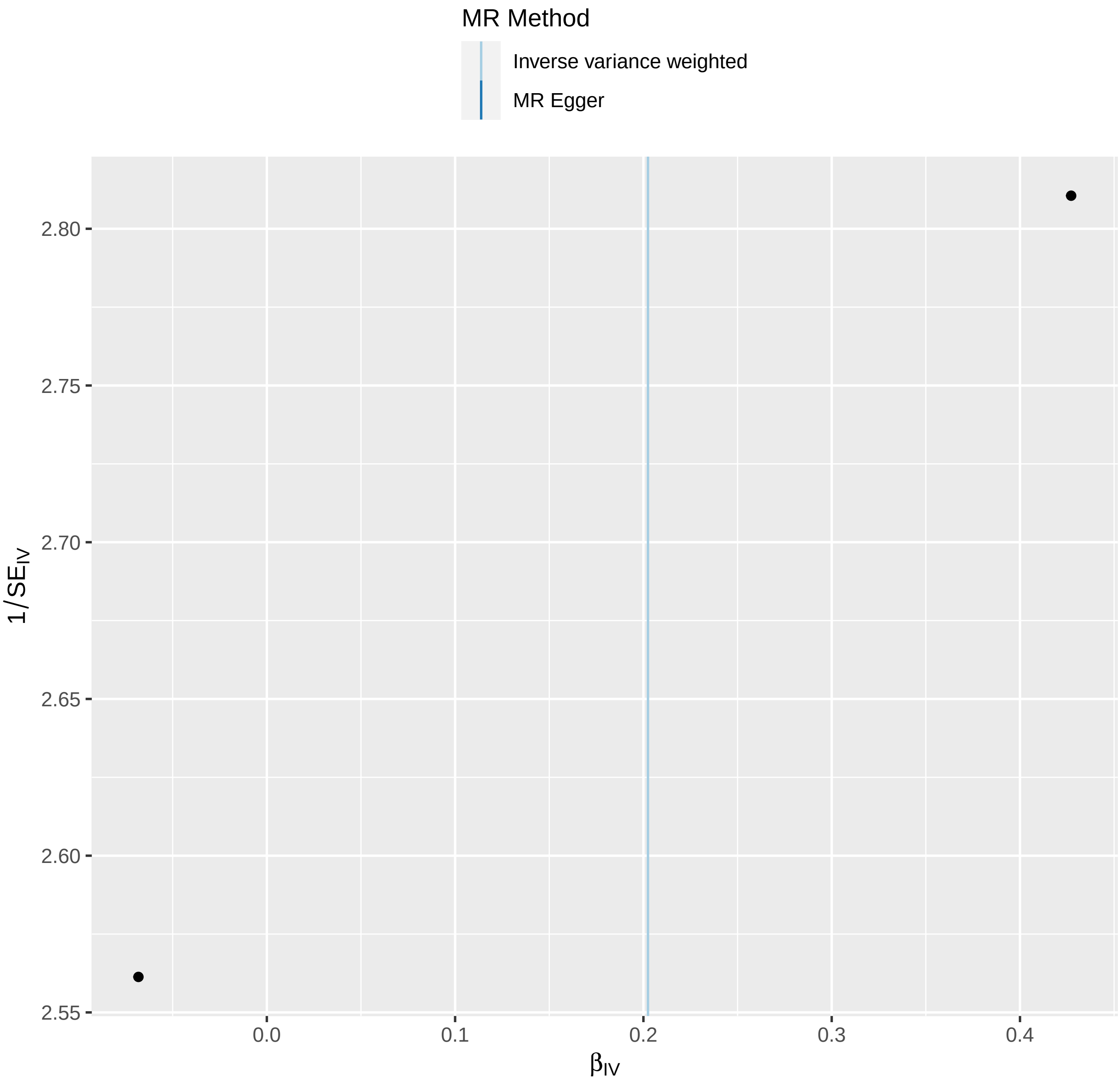

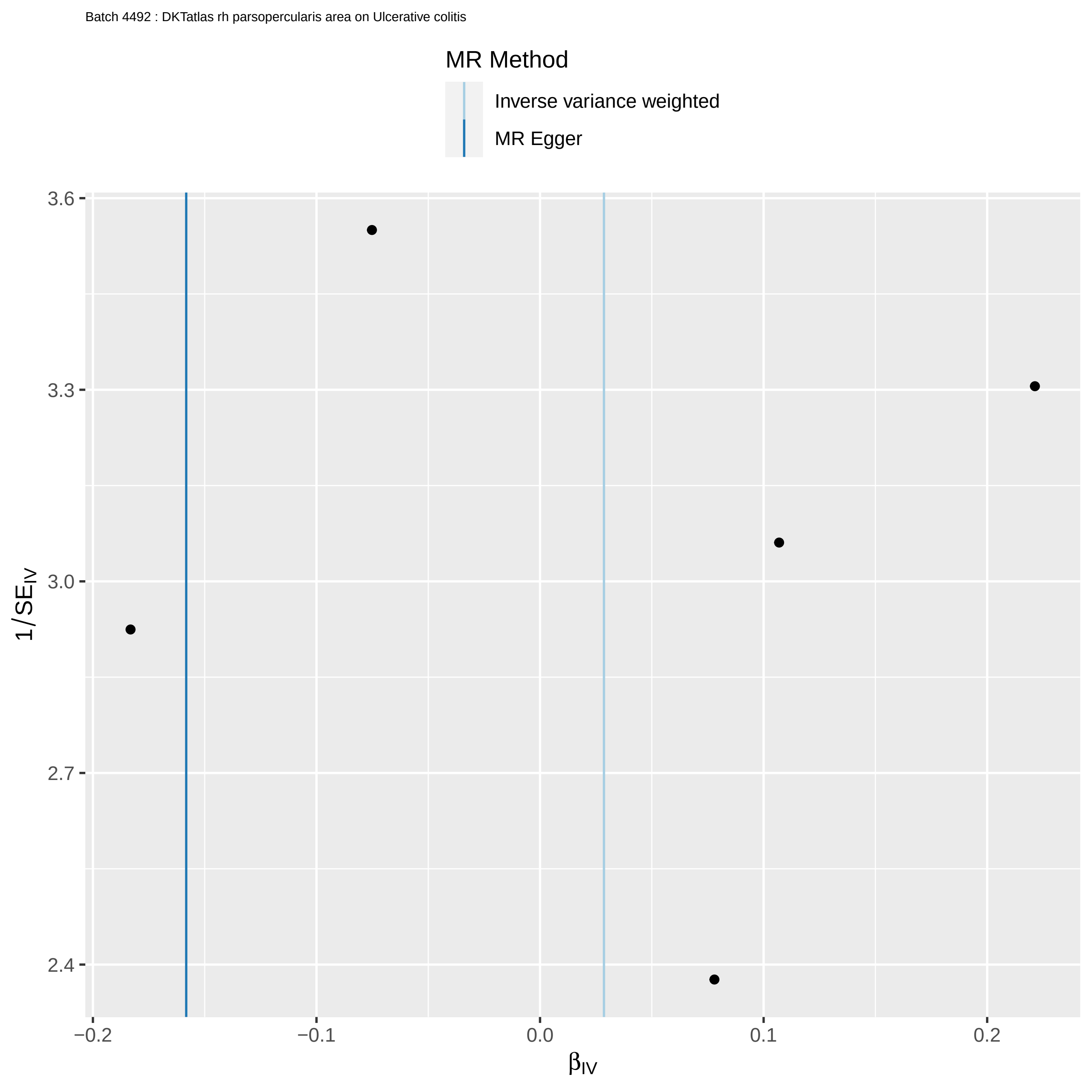
Batch 4492 : DKTatlas rh parsopercularis area on Ulcerative colitis
MR Method
Inverse variance weighted
MR Egger
3.6
3.3
3.0
2.7
2.4
1/SE_IV
−0.2
−0.1
0.0
0.1
0.2
β_IV

Batch 4495 : DKTatlas rh pericalcarine area on Ulcerative colitis

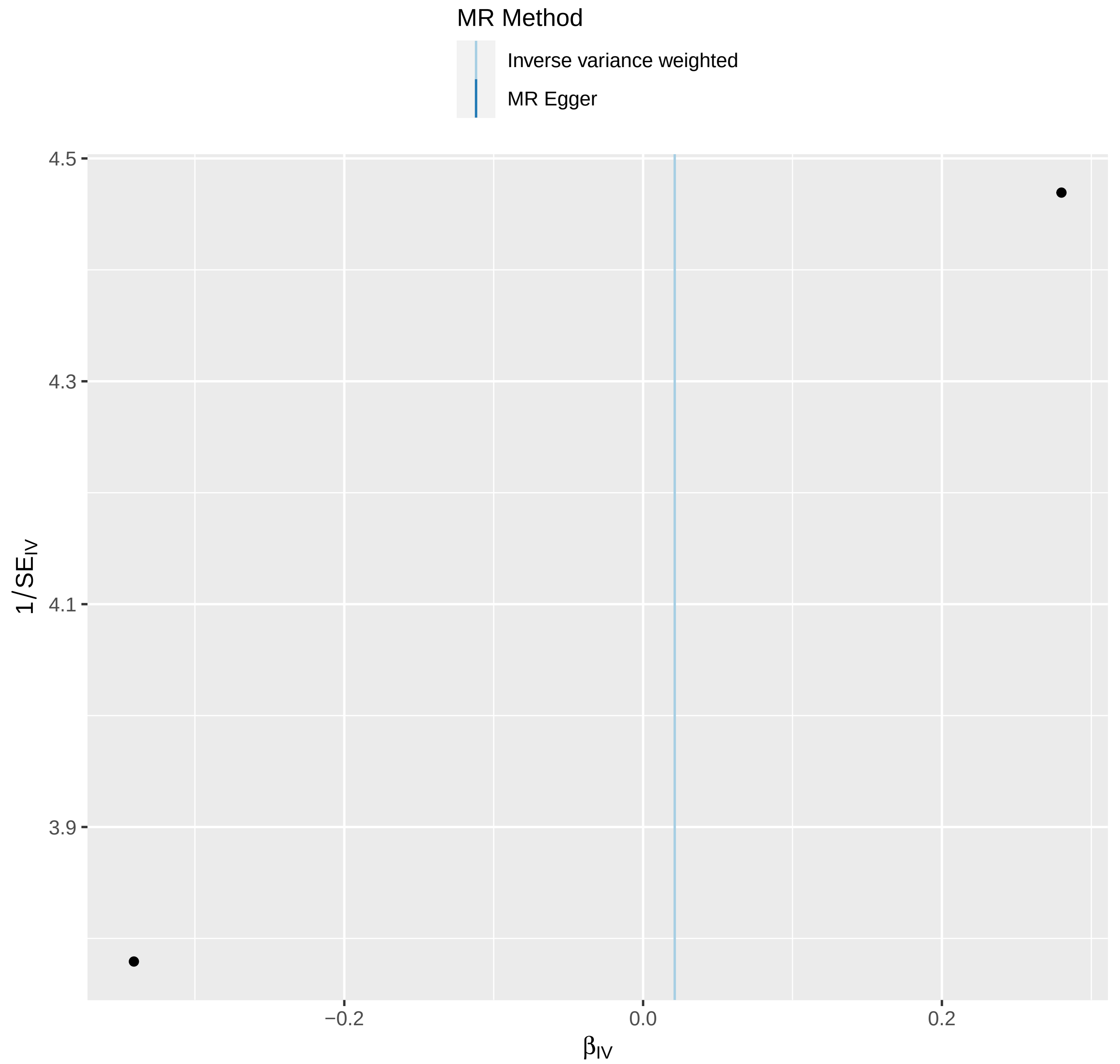

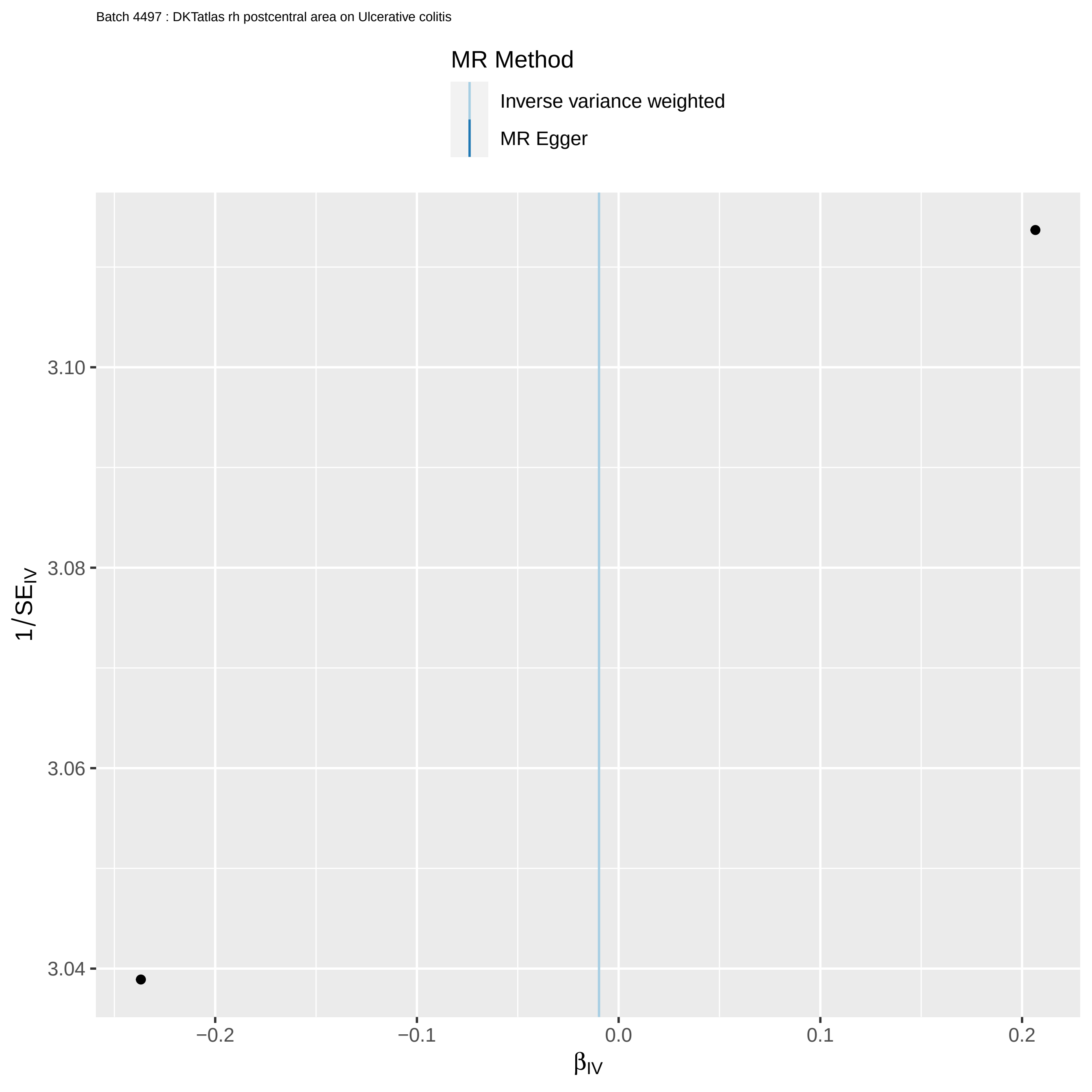
Batch 4497 : DKTatlas rh postcentral area on Ulcerative colitis
MR Method
Inverse variance weighted
MR Egger
3.10
3.08
3.06
3.04
$1/SE_{IV}$
−0.2
−0.1
0.0
0.1
0.2
β_{IV}

Batch 4499 : DKTatlas rh precentral area on Ulcerative colitis

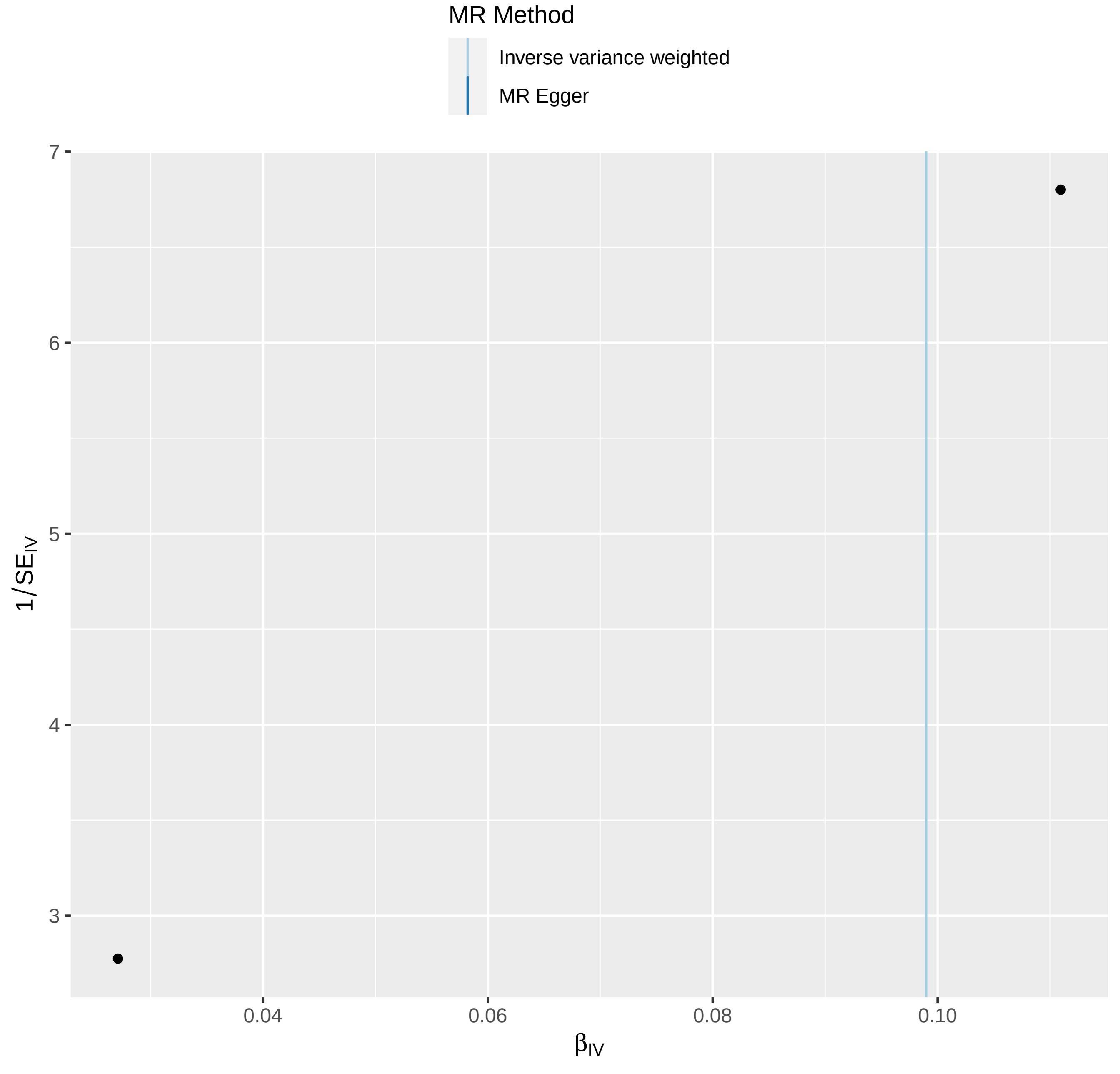

Batch 4500 : DKTatlas rh precuneus area on Ulcerative colitis

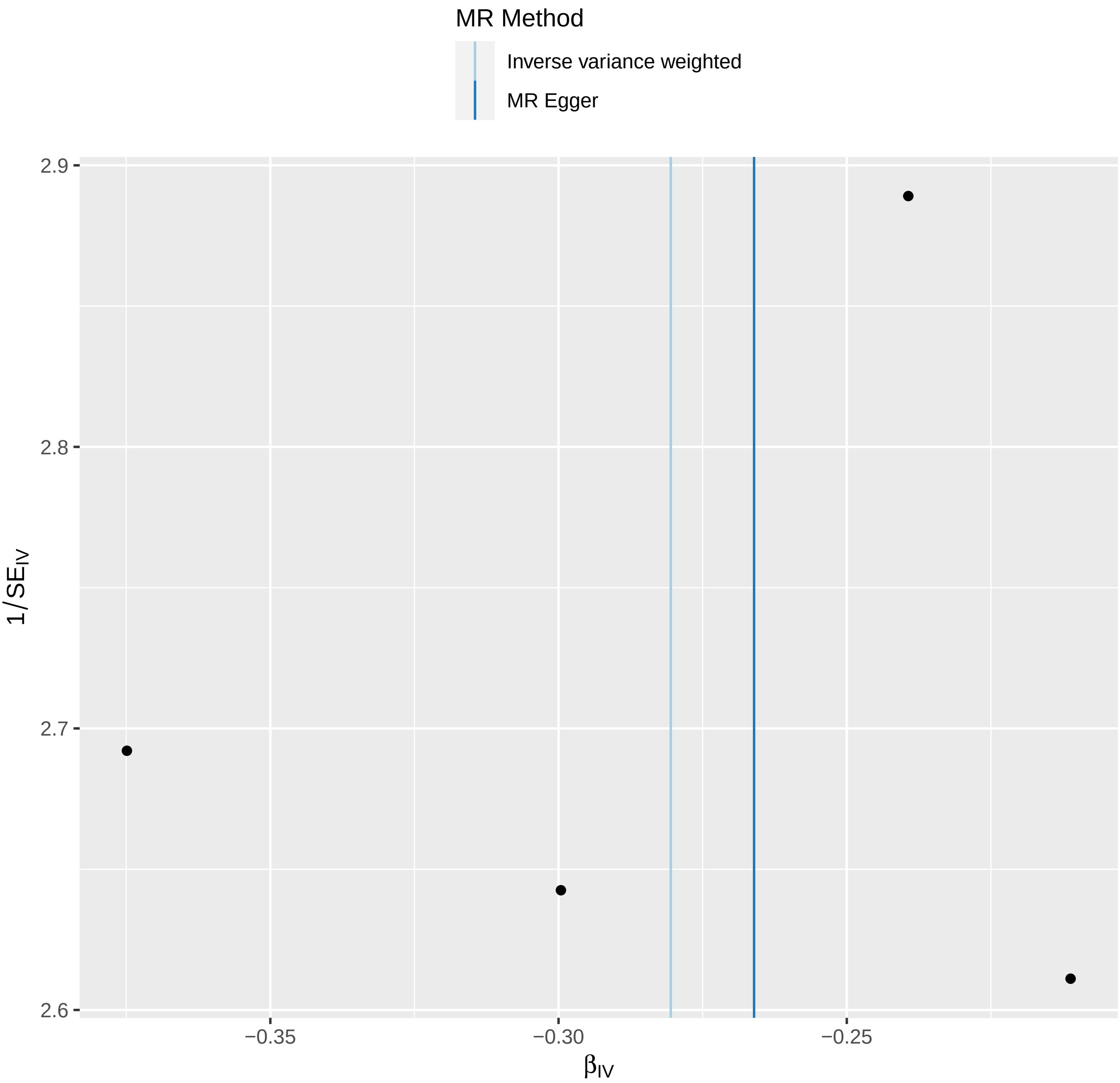

Batch 4503 : DKTatlas rh superiorfrontal area on Ulcerative colitis

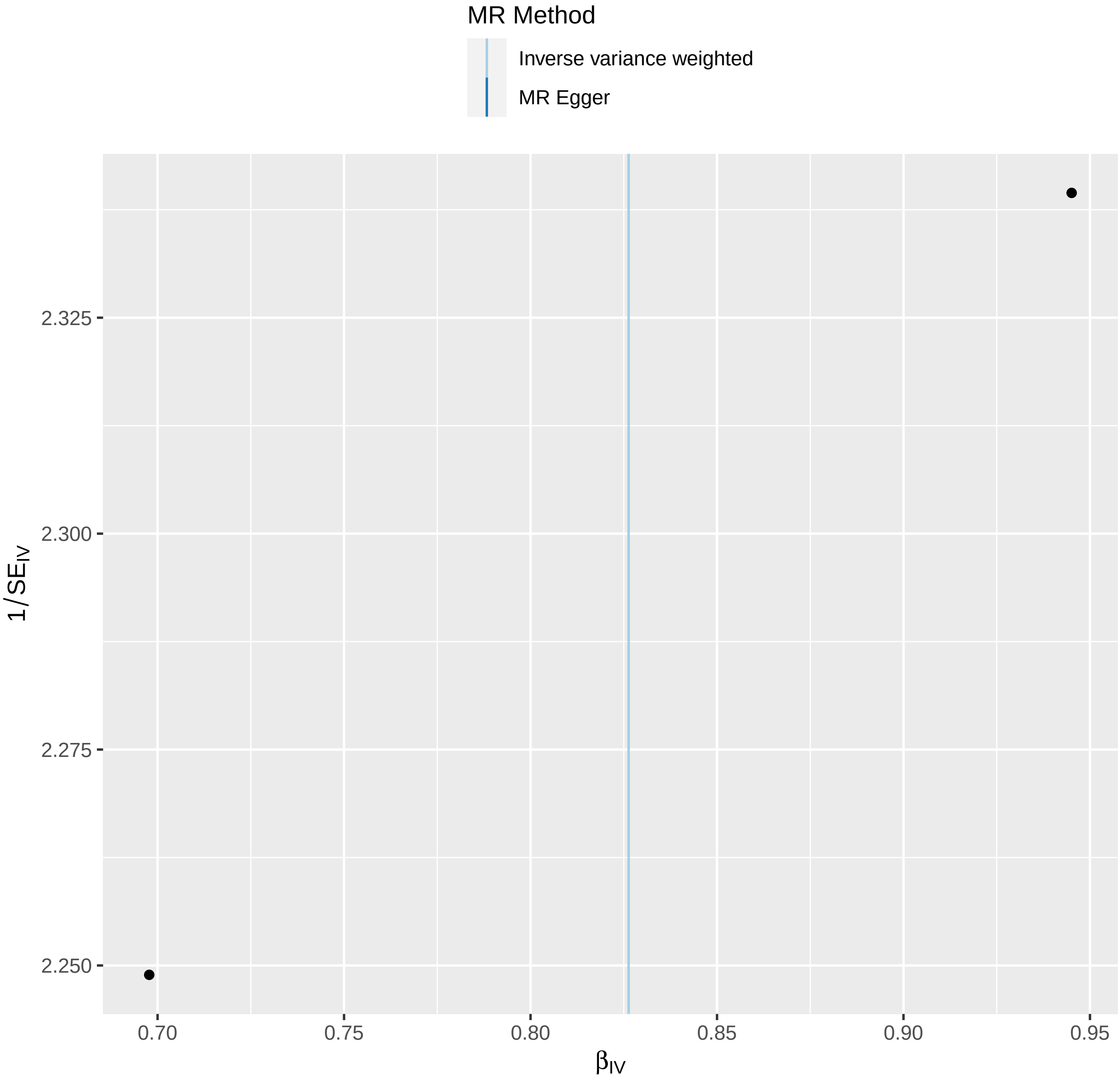

Batch 4505 : DKTatlas rh superiortemporal area on Ulcerative colitis

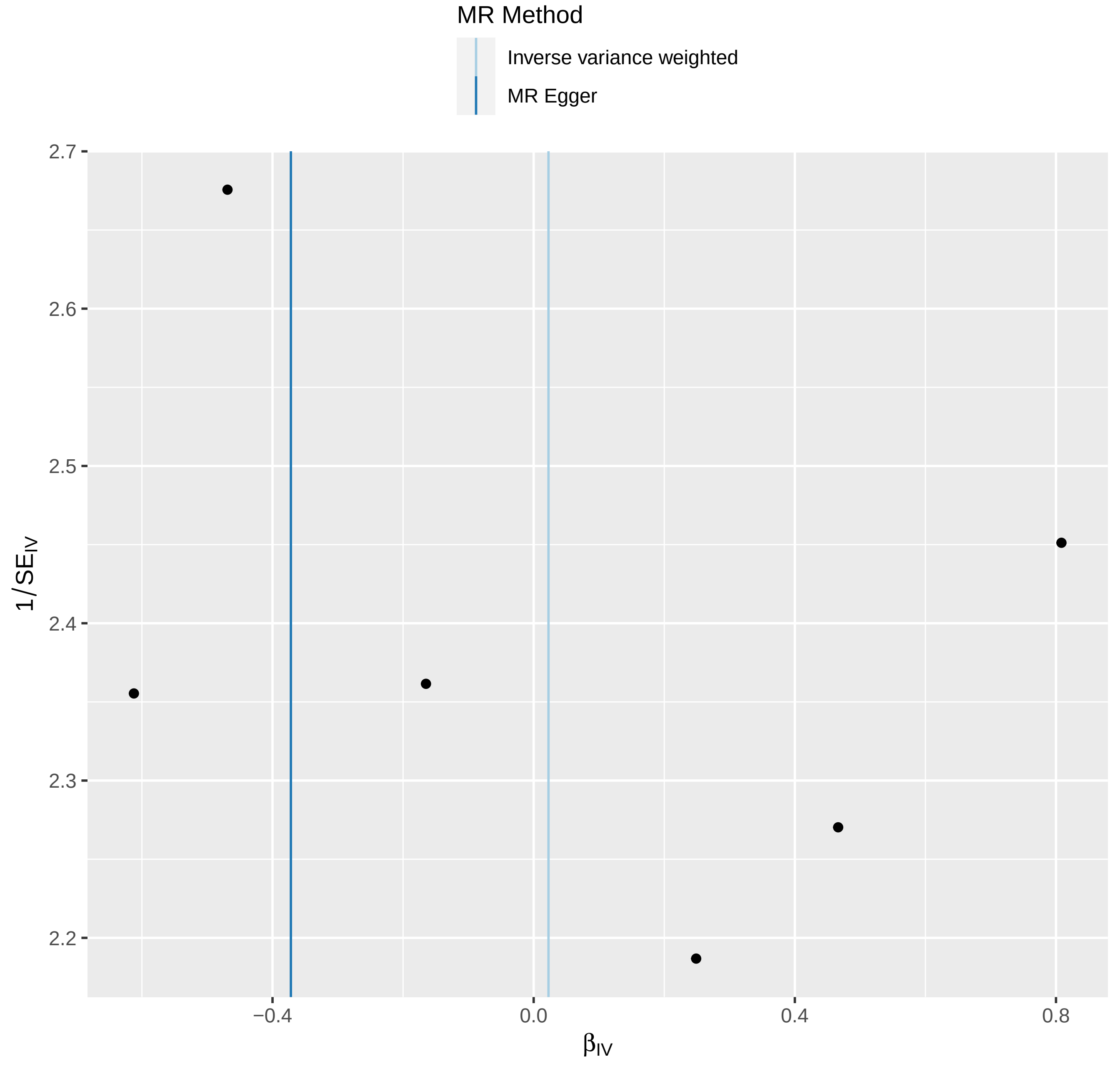

Batch 4508 : DKTatlas rh transversetemporal area on Ulcerative colitis

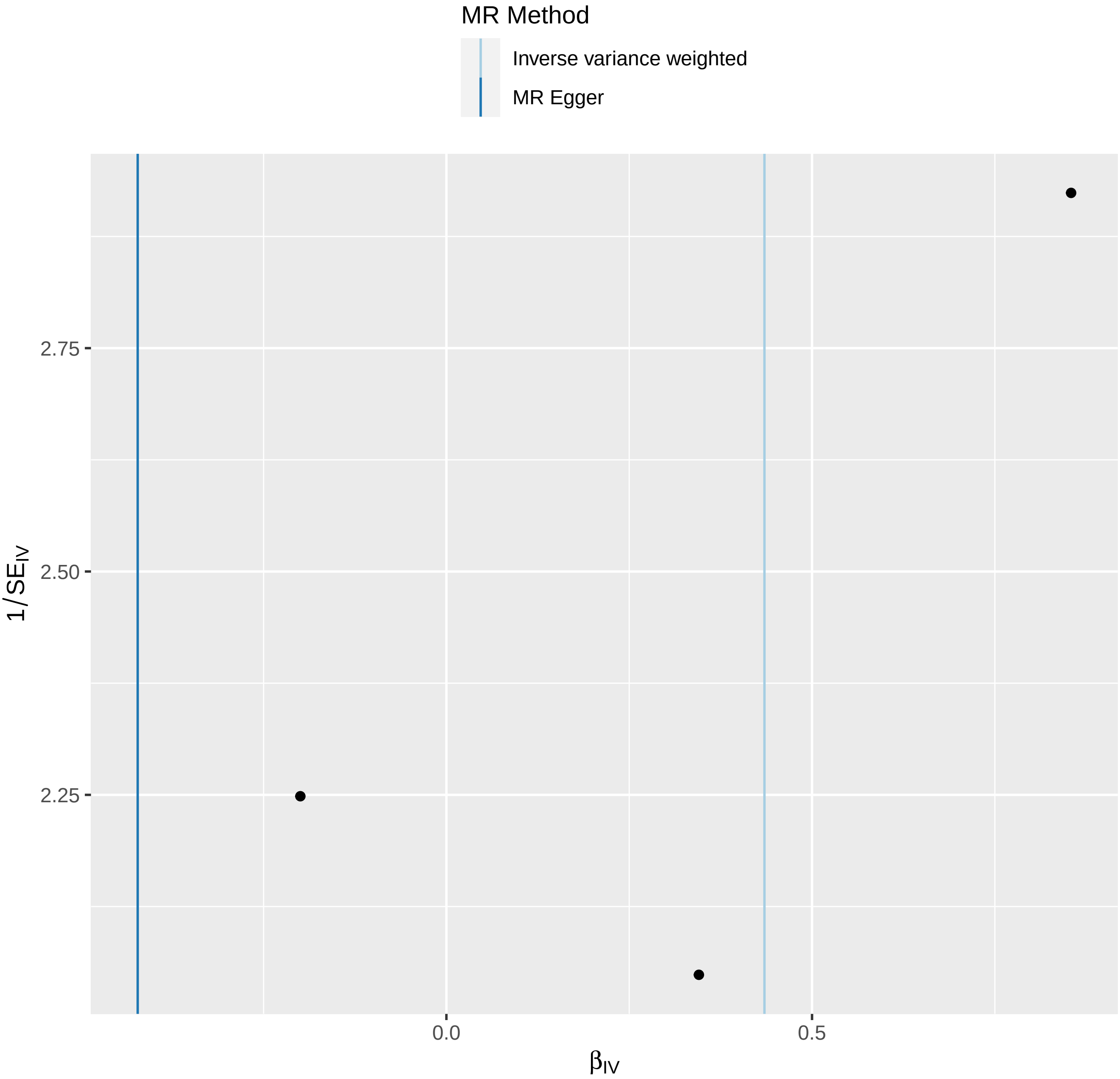

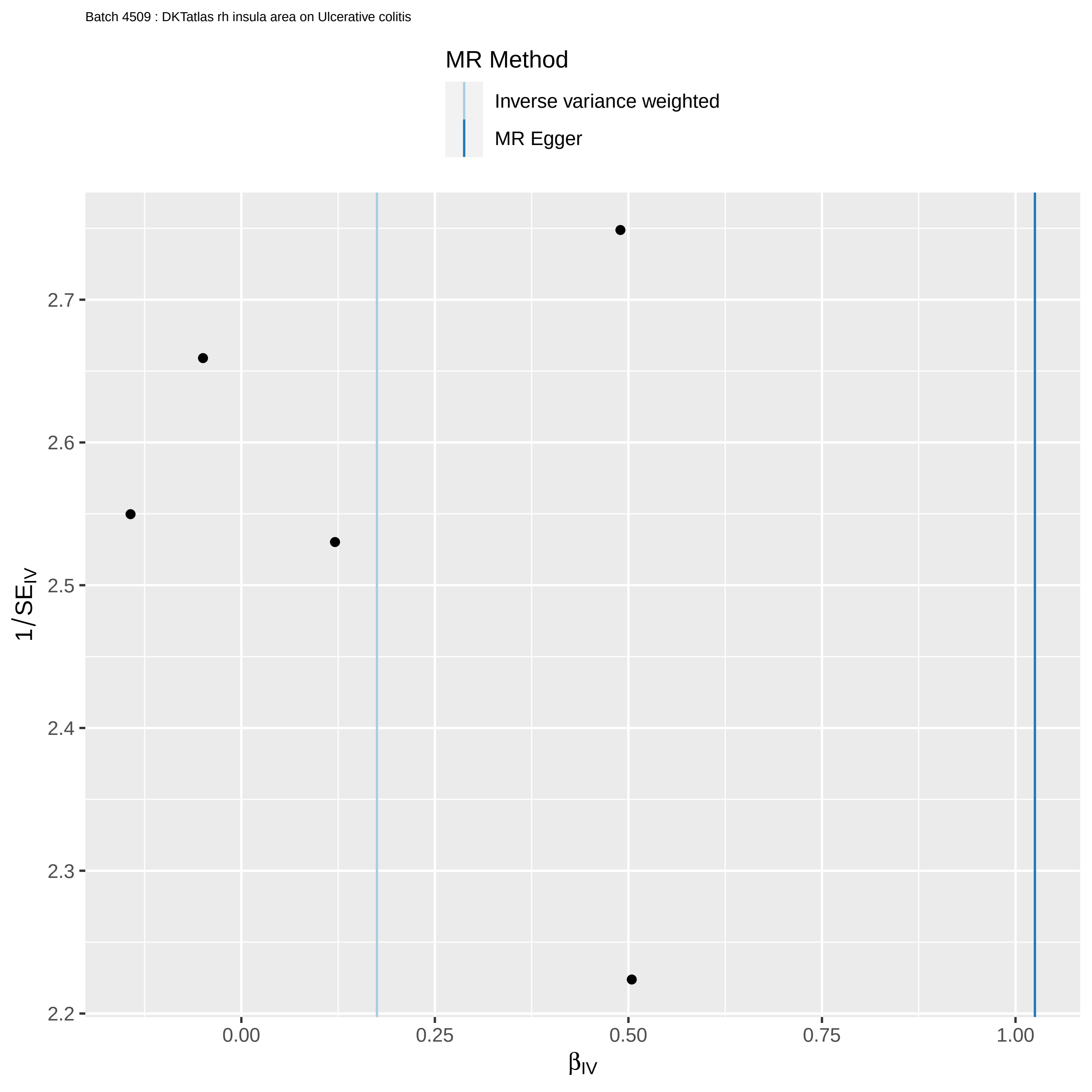

Batch 4509 : DKTatlas rh insula area on Ulcerative colitis
MR Method
Inverse variance weighted
MR Egger
2.7
2.6
2.5
2.4
2.3
2.2
$1/SE_{IV}$
0.00
0.25
0.50
0.75
1.00
β_{IV}

Batch 4510 : DKTatlas rh WhiteSurfArea area on Ulcerative colitis

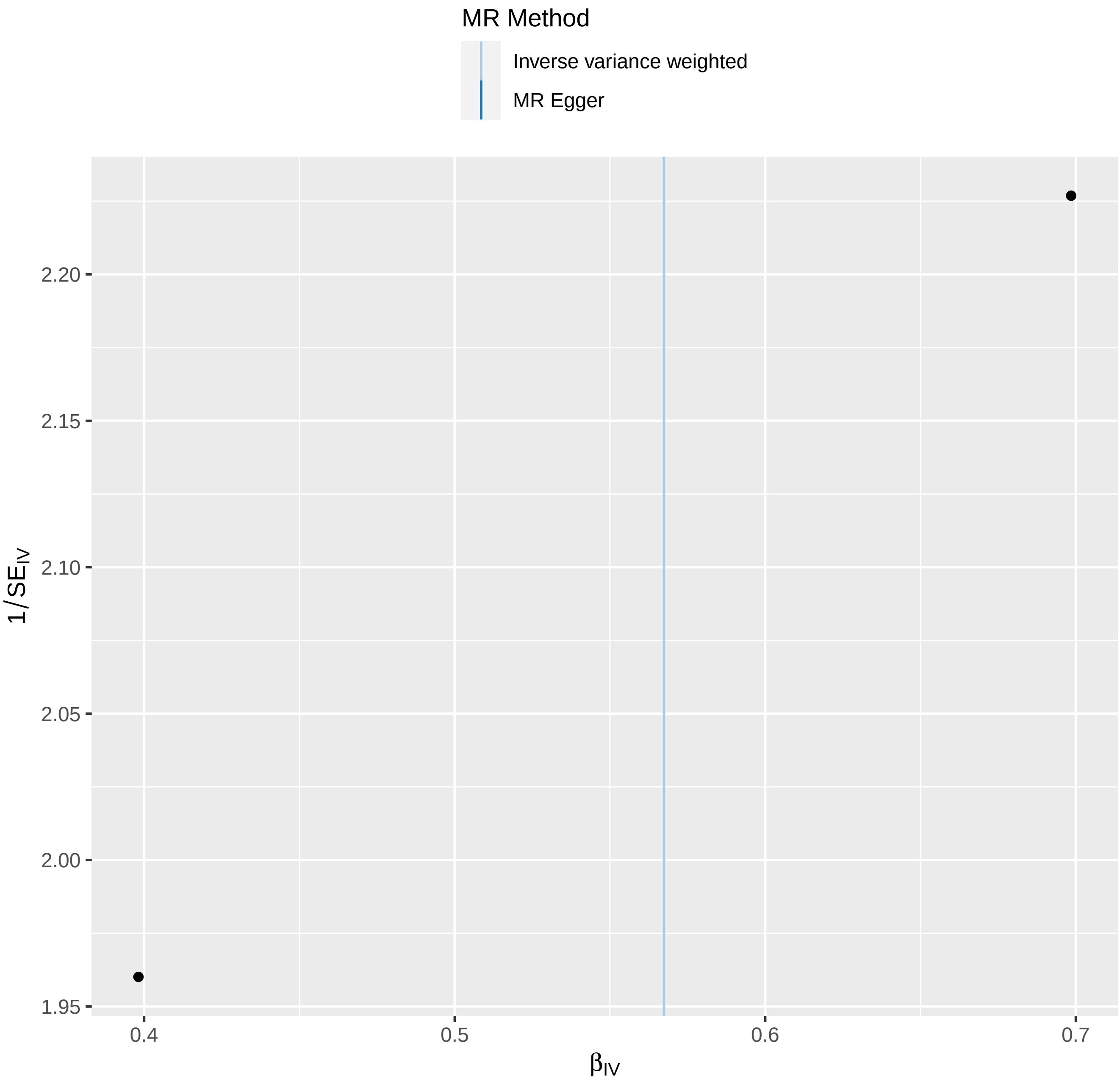

Batch 4562 : IDP T1 FAST ROIs R insular cortex on Ulcerative colitis

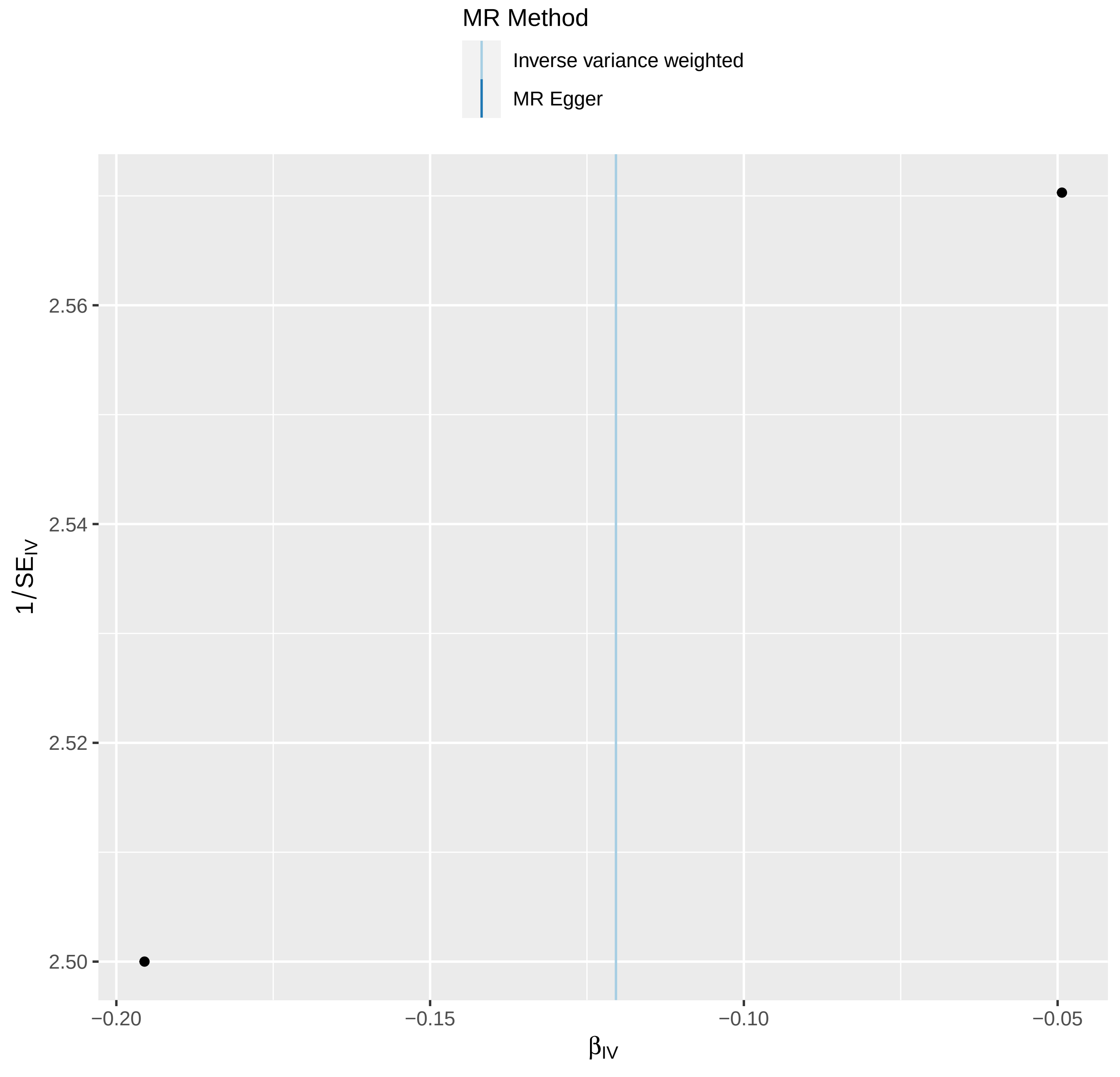

Batch 4673 : IDP T1 SIENAX CSF normalised volume on Ulcerative colitis

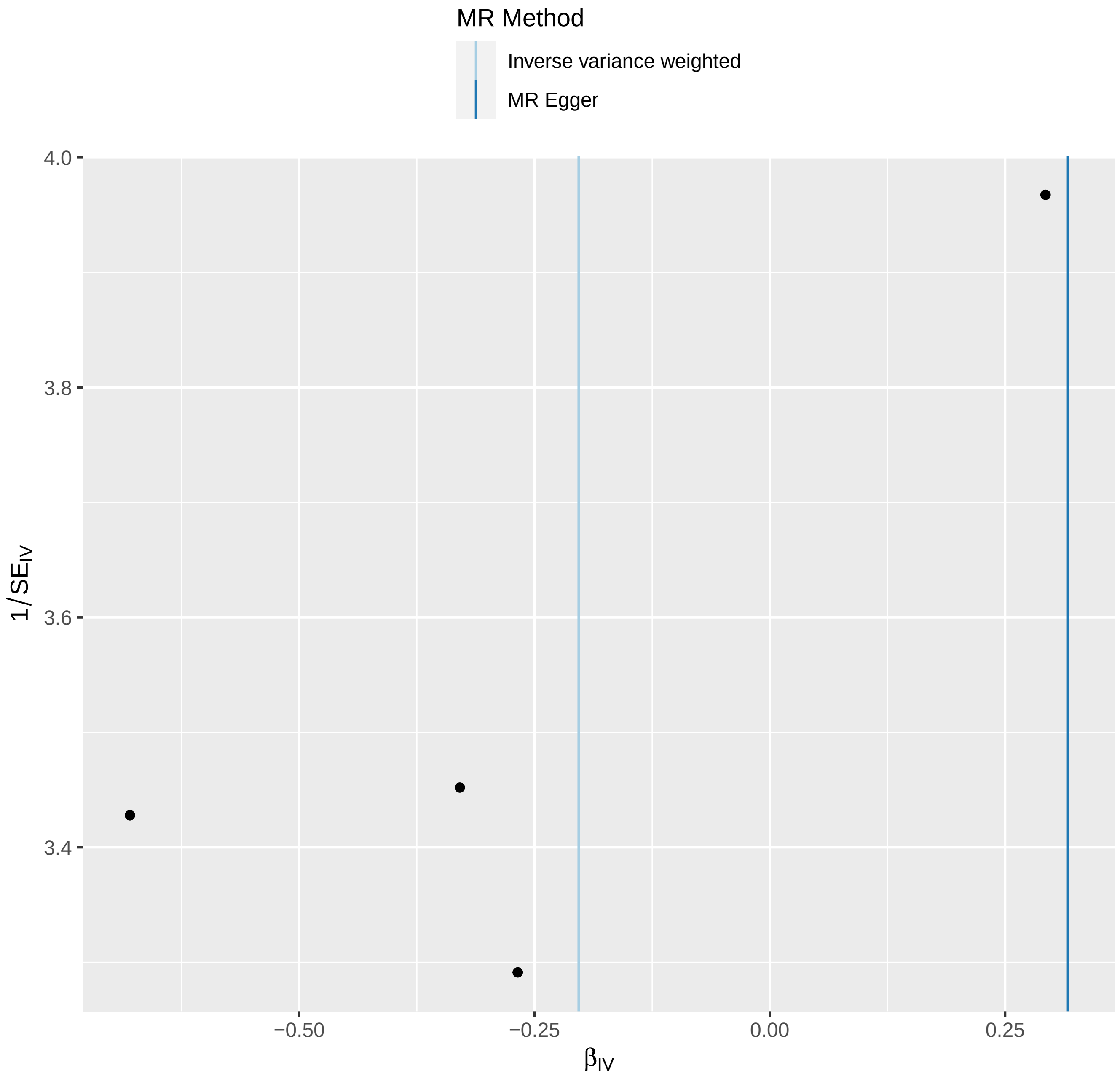

Batch 4785 : IDP T1 FAST ROIs R sup front gyrus on Ulcerative colitis

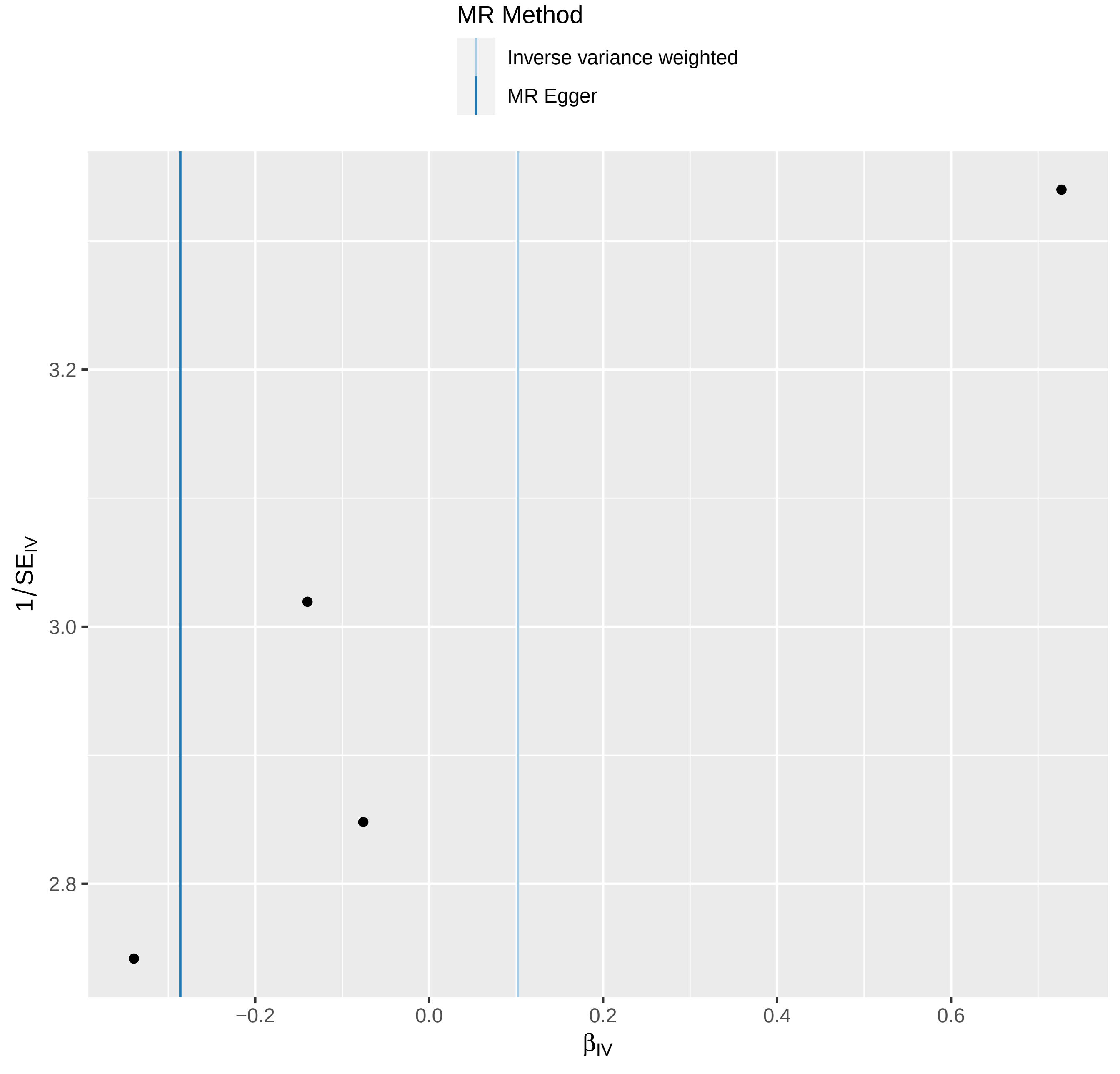

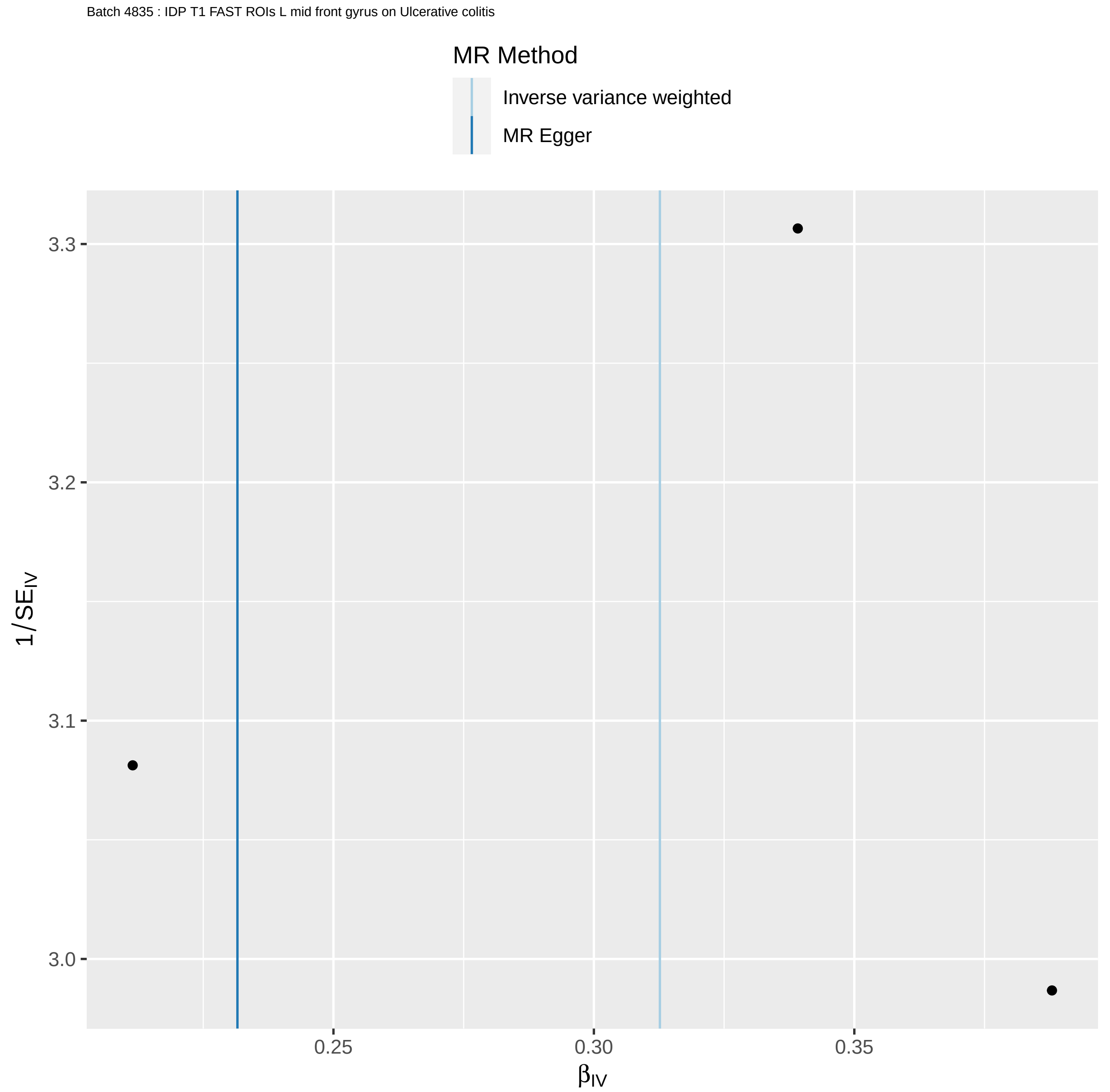
Batch 4835 : IDP T1 FAST ROIs L mid front gyrus on Ulcerative colitis
MR Method
Inverse variance weighted
MR Egger
3.3
3.2
3.1
3.0
0.25
0.30
0.35
β_{IV}
$1/SE_{IV}$

Batch 4857 : IDP T1 FAST ROIs L inf front gyrus parstri on Ulcerative colitis

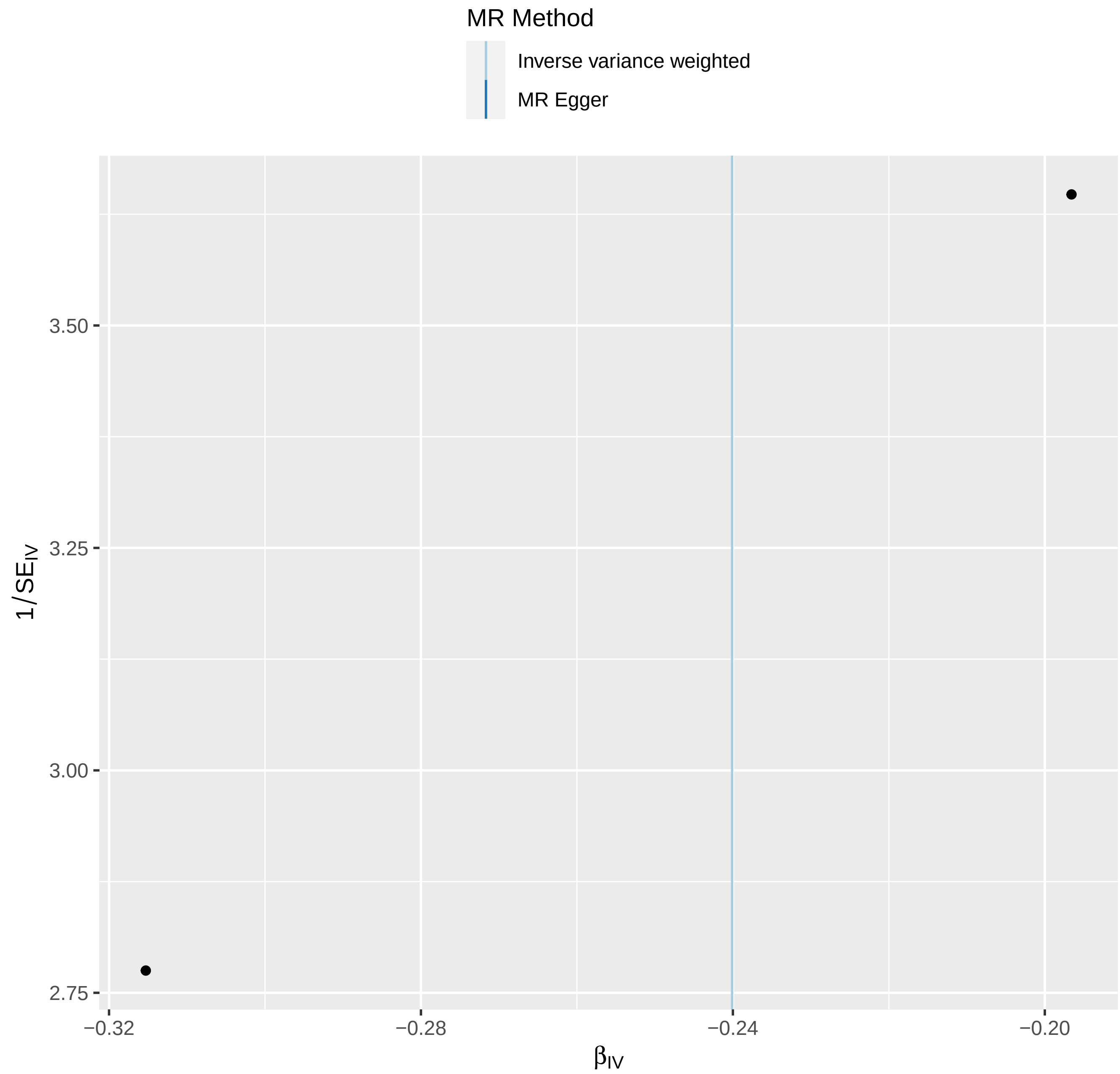

Batch 4922 : IDP T1 SIENAX CSF unnormalised volume on Ulcerative colitis

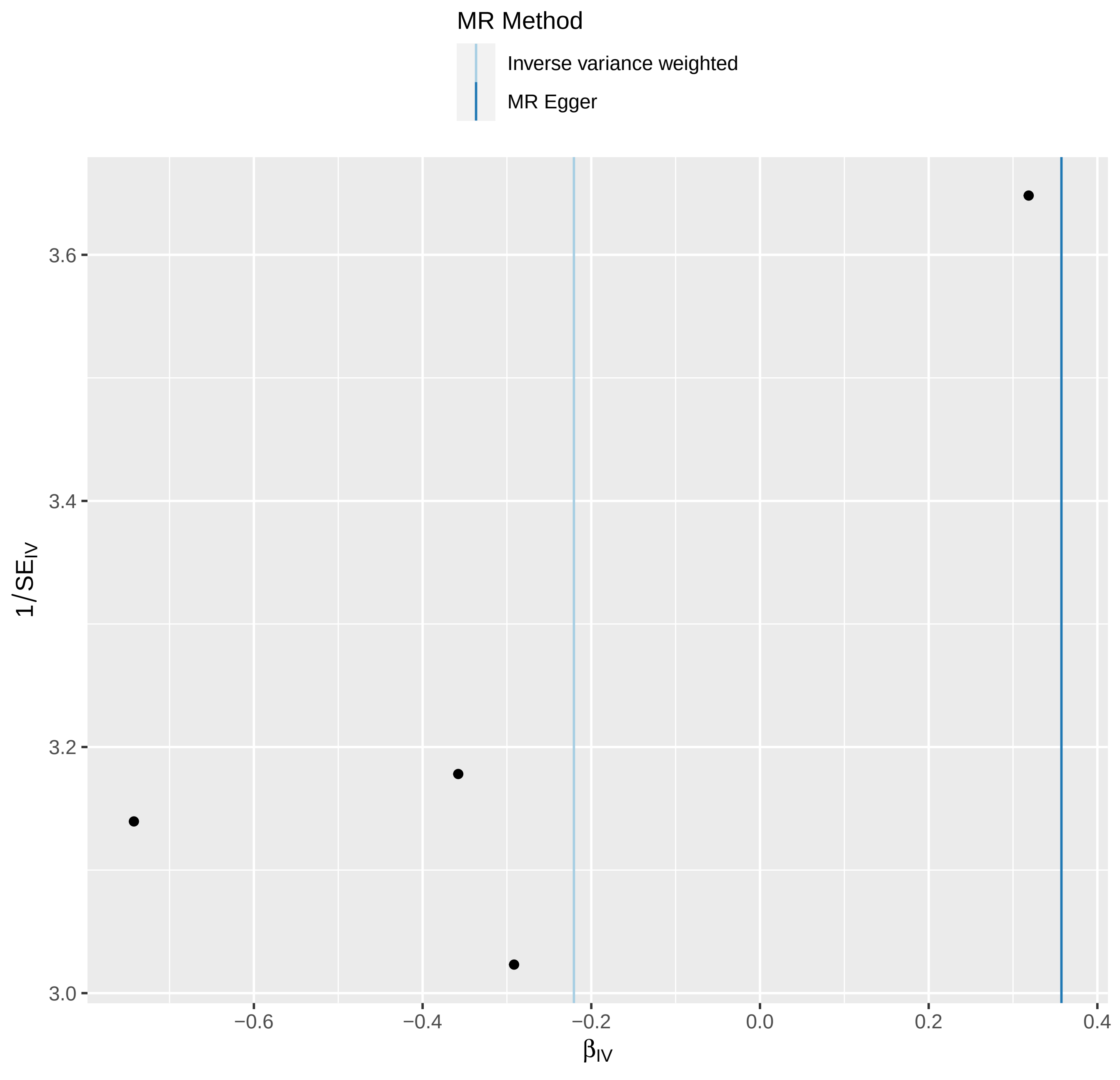

Batch 4944 : IDP T1 FAST ROIs L sup temp gyrus ant on Ulcerative colitis

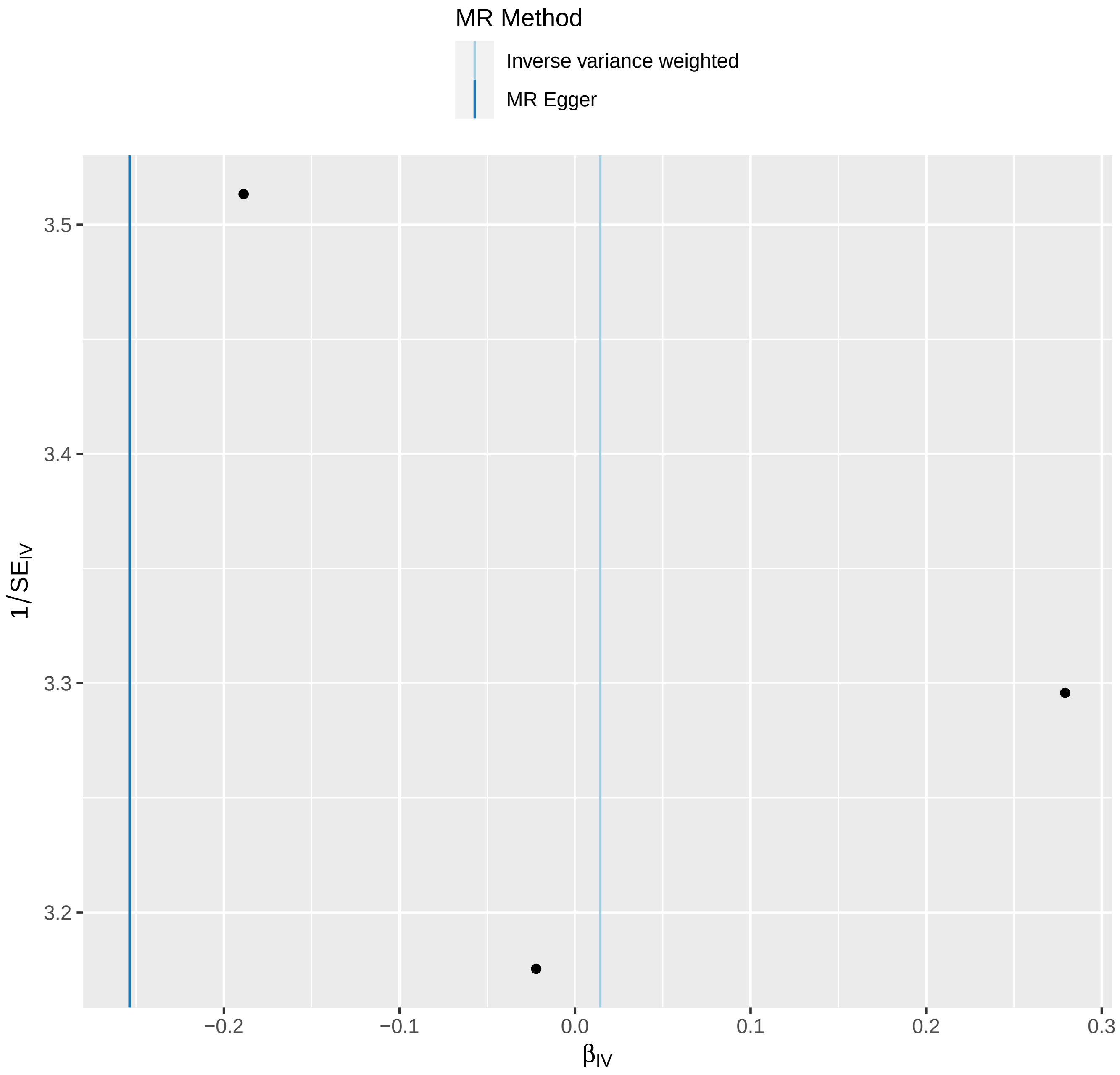

Batch 4955 : IDP T1 FAST ROIs R sup temp gyrus ant on Ulcerative colitis

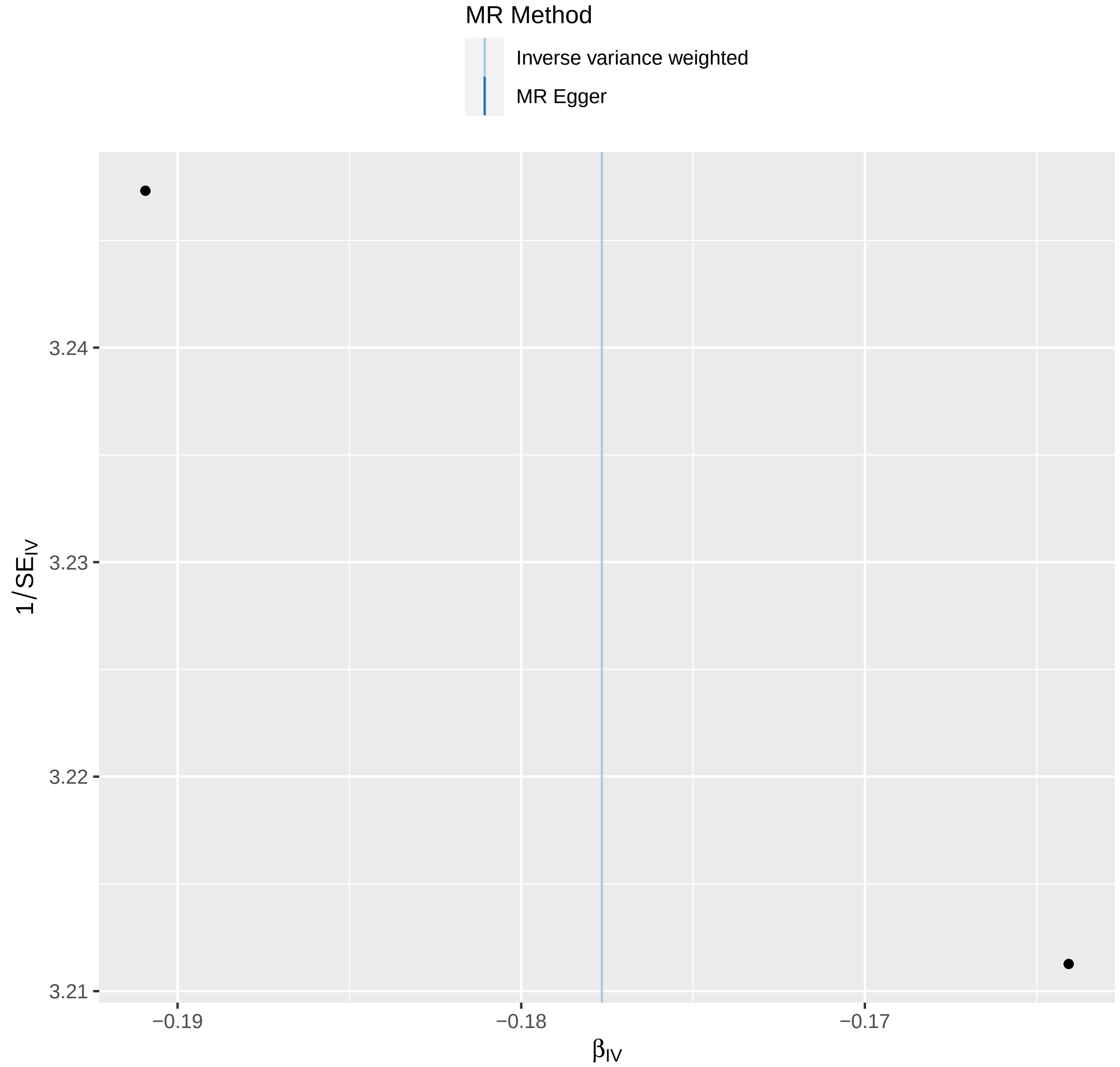

Batch 4966 : IDP T1 FAST ROIs L sup temp gyrus post on Ulcerative colitis

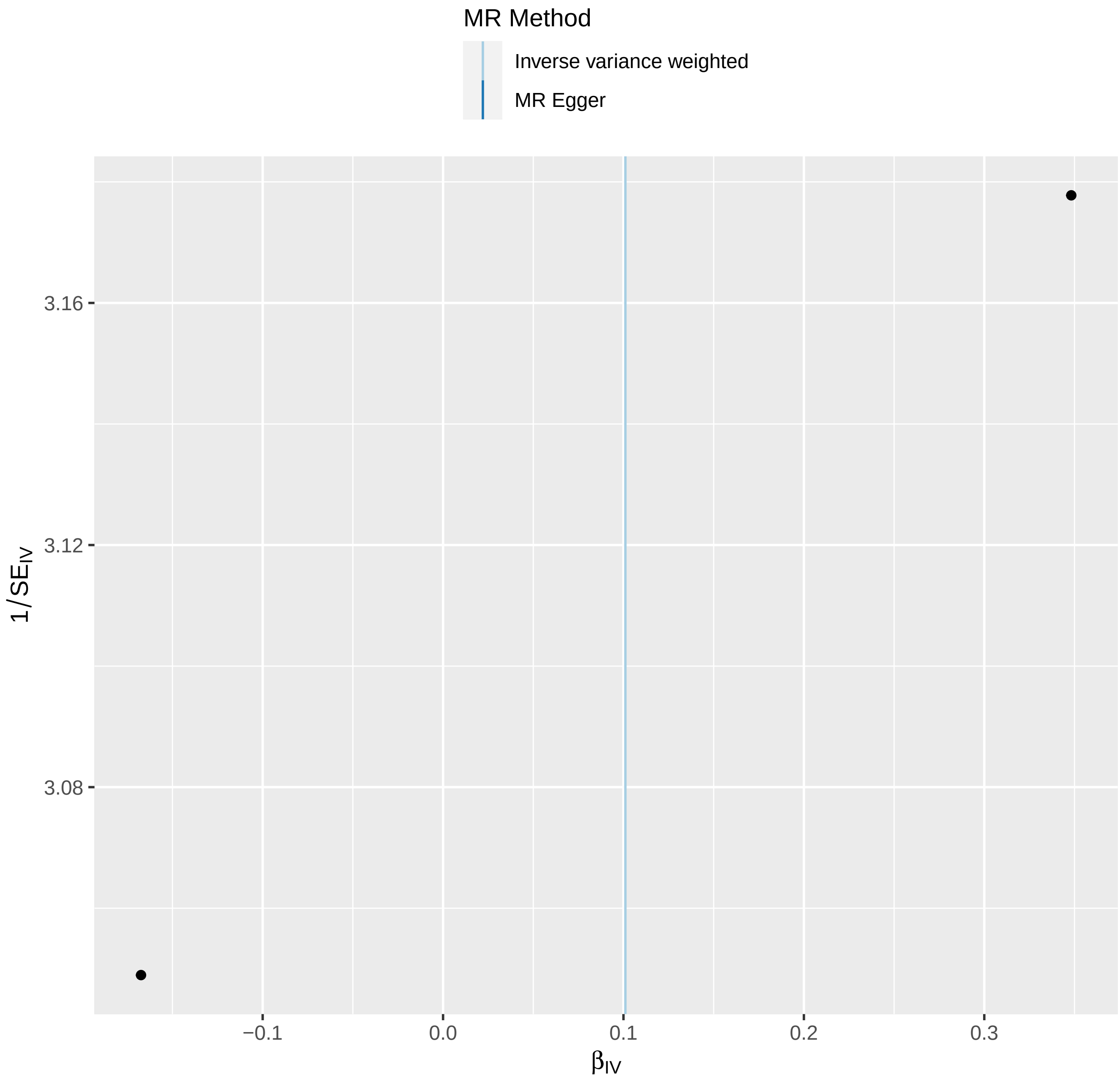

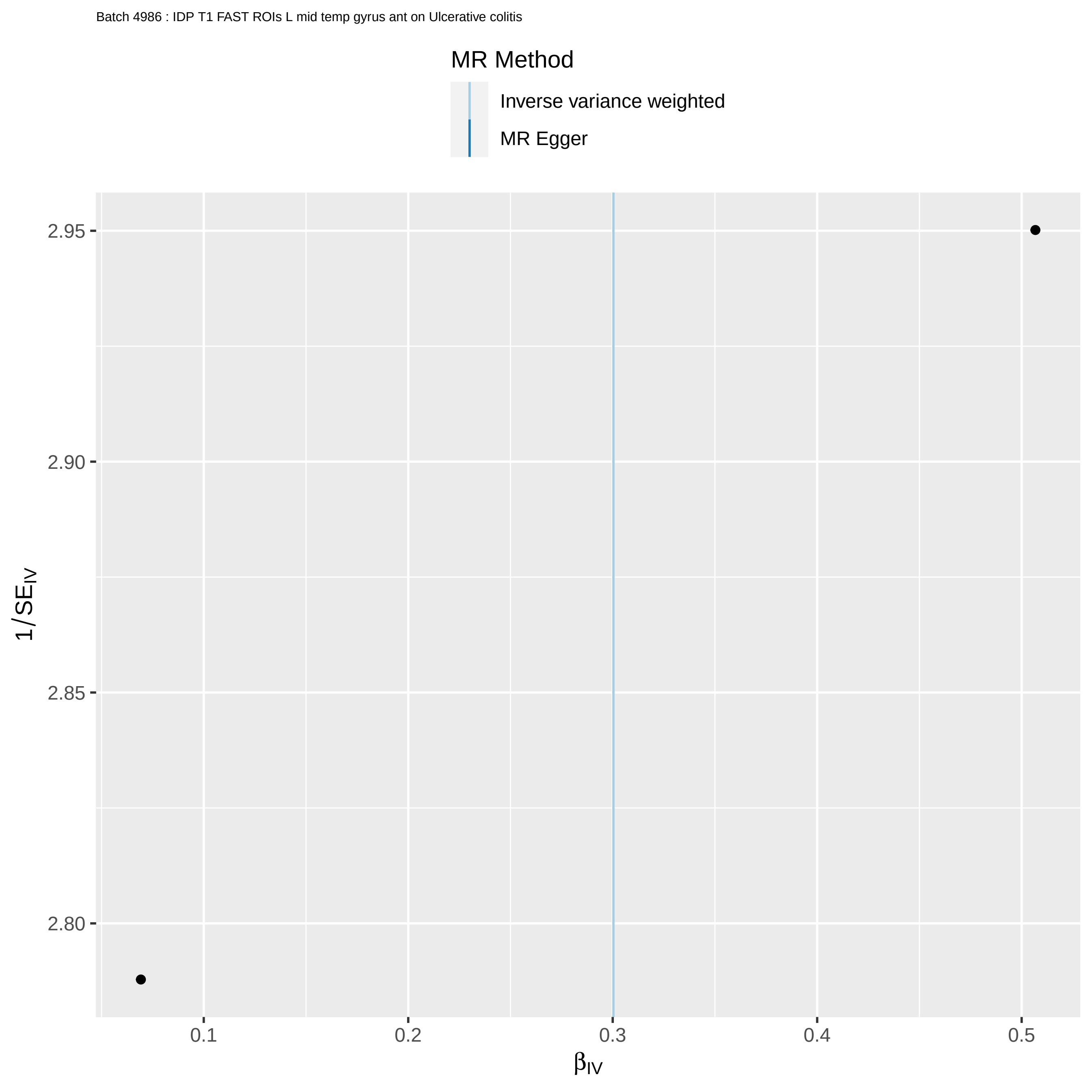

Batch 4986 : IDP T1 FAST ROIs L mid temp gyrus ant on Ulcerative colitis
MR Method
Inverse variance weighted
MR Egger
2.95
2.90
2.85
2.80
1/SE_IV
0.1
0.2
0.3
0.4
0.5
β_IV

Batch 5030 : IDP T1 SIENAX grey normalised volume on Ulcerative colitis

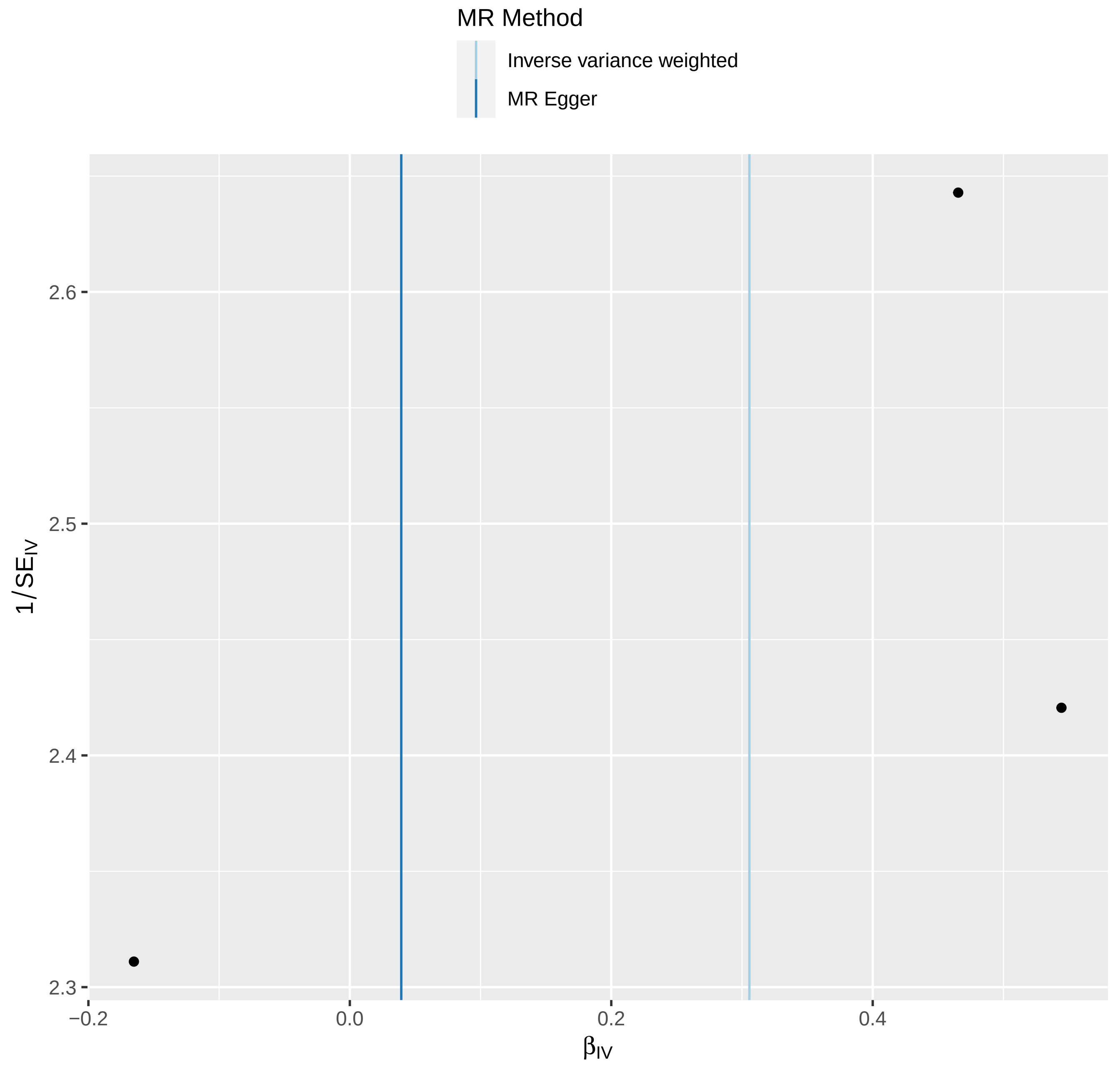

Batch 5042 : IDP T1 FAST ROIs R mid temp gyrus tempocc on Ulcerative colitis

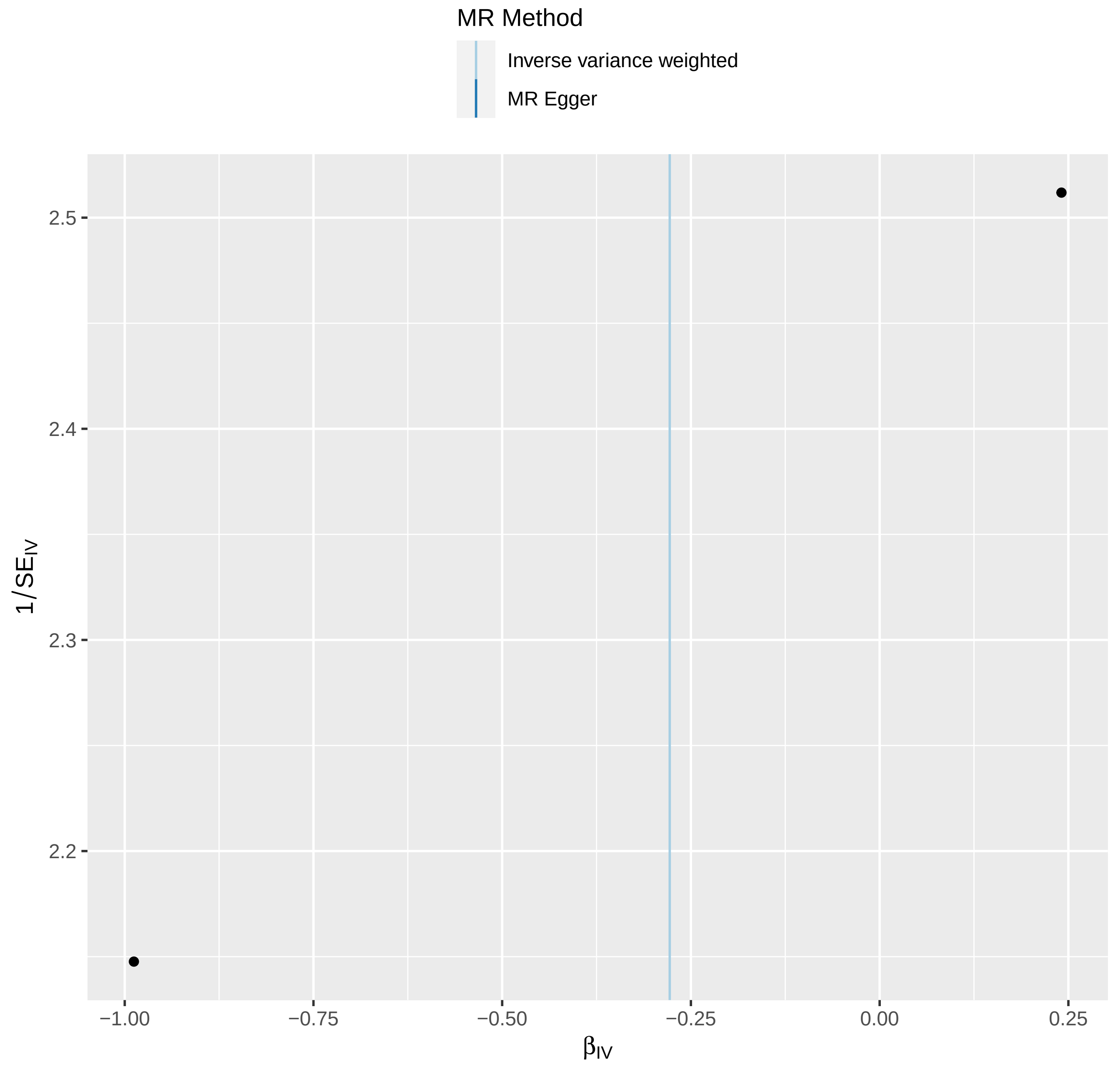

Batch 5053 : IDP T1 FAST ROIs L inf temp gyrus ant on Ulcerative colitis

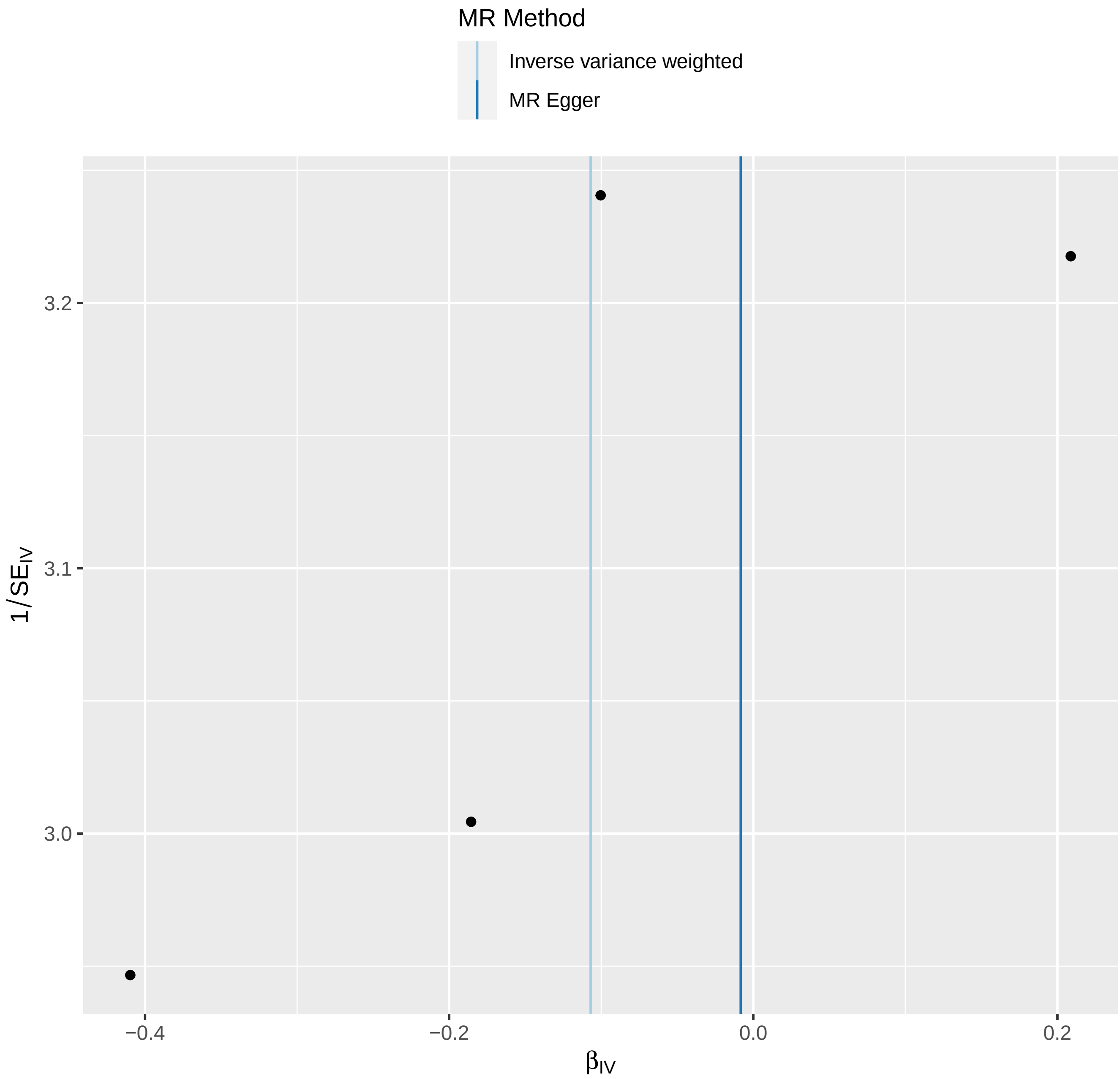

Batch 5064 : IDP T1 FAST ROIs R inf temp gyrus ant on Ulcerative colitis

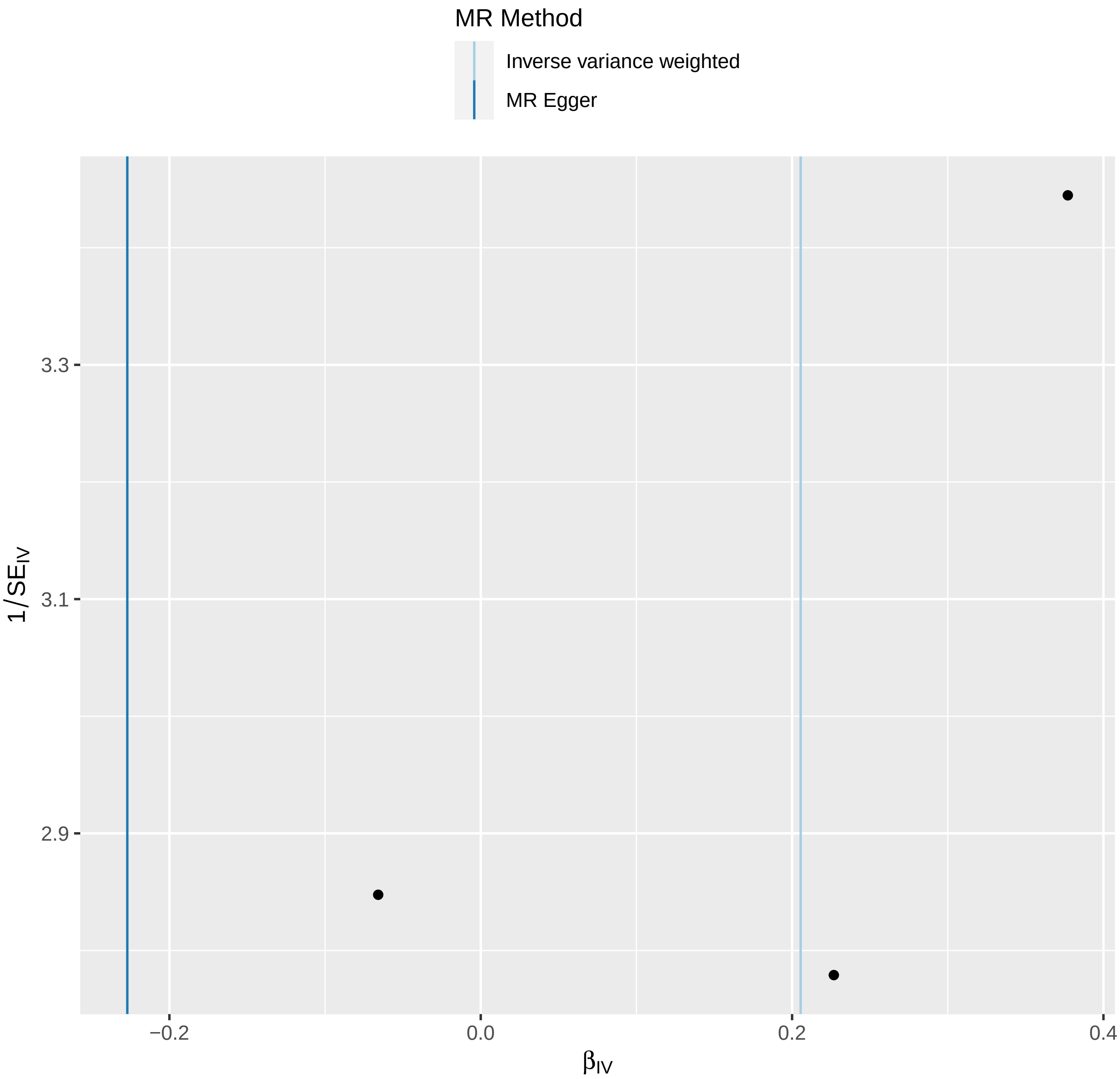

Batch 5075 : IDP T1 FAST ROIs L inf temp gyrus post on Ulcerative colitis

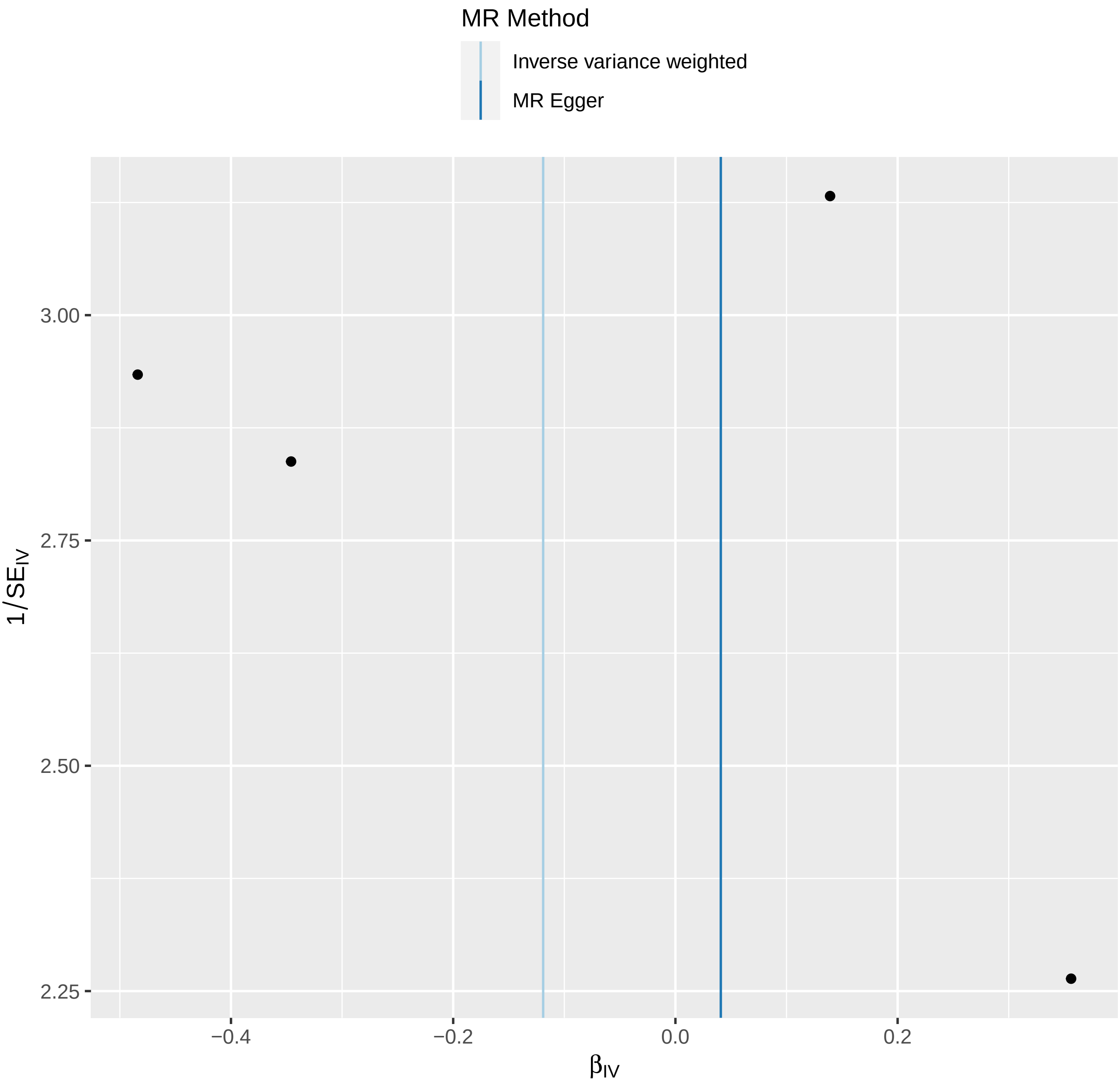

Batch 5097 : IDP T1 FAST ROIs L inf temp gyrus tempocc on Ulcerative colitis

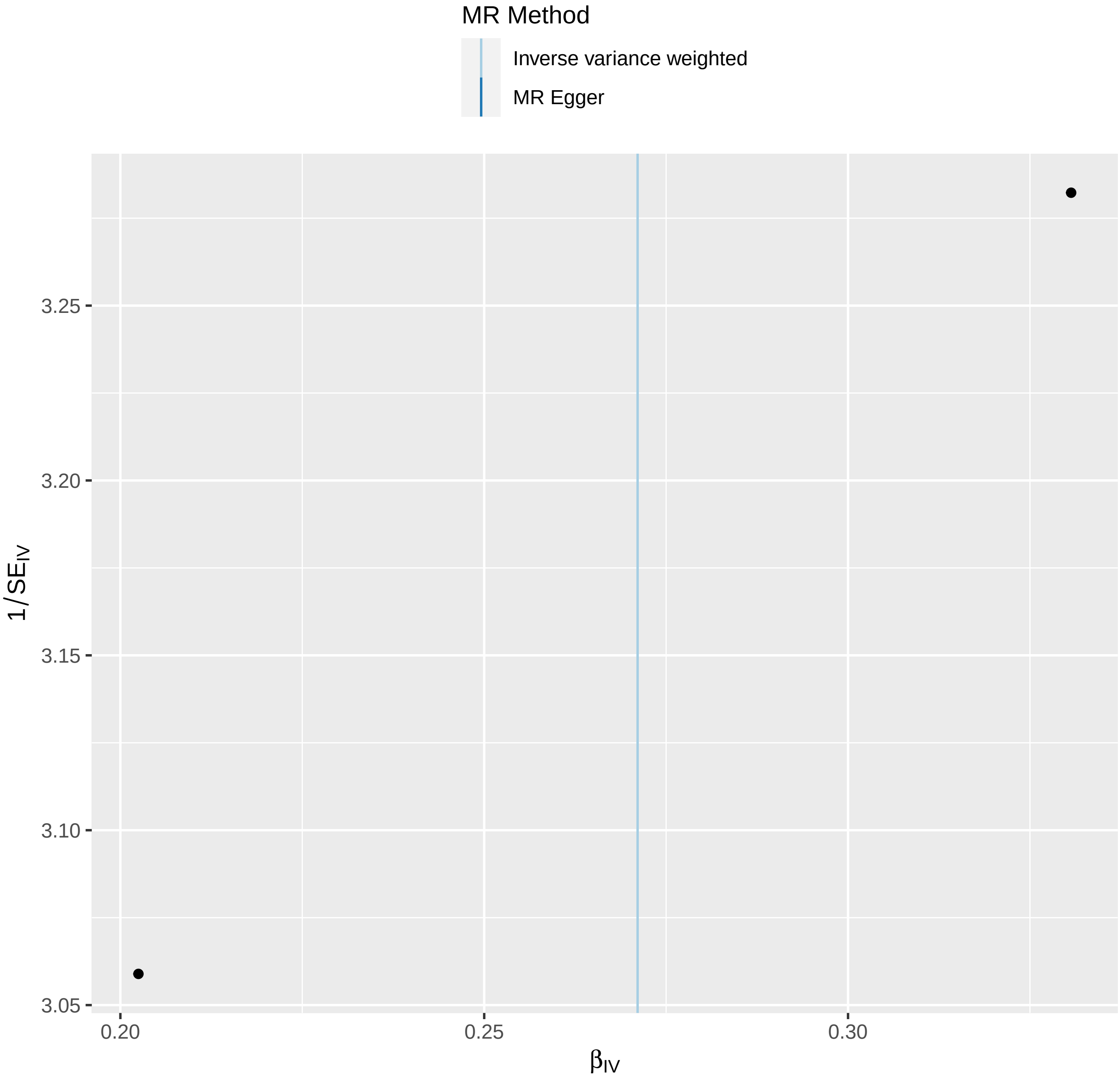

Batch 5108 : IDP T1 FAST ROIs R inf temp gyrus tempocc on Ulcerative colitis

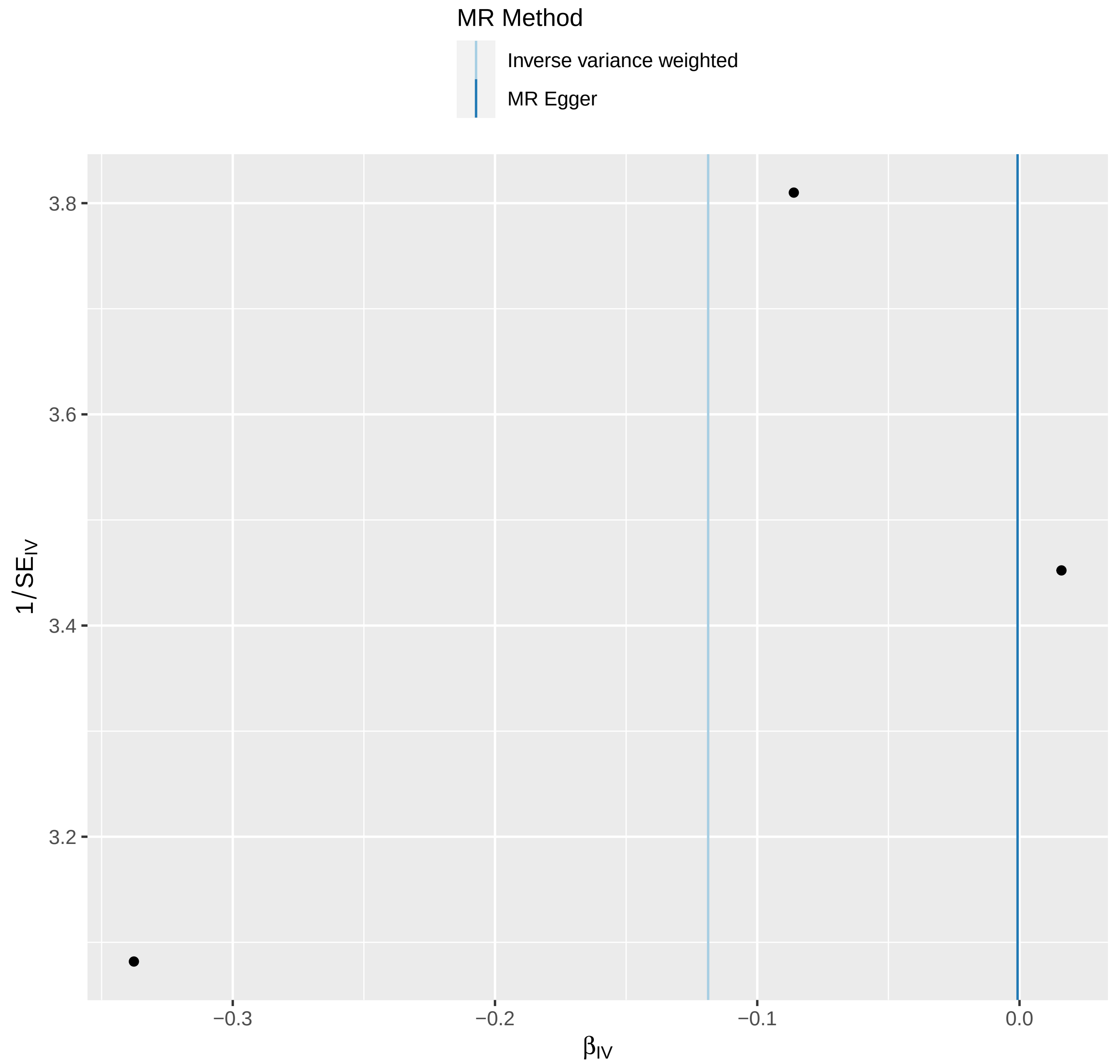

Batch 5119 : IDP T1 FAST ROIs L postcent gyrus on Ulcerative colitis

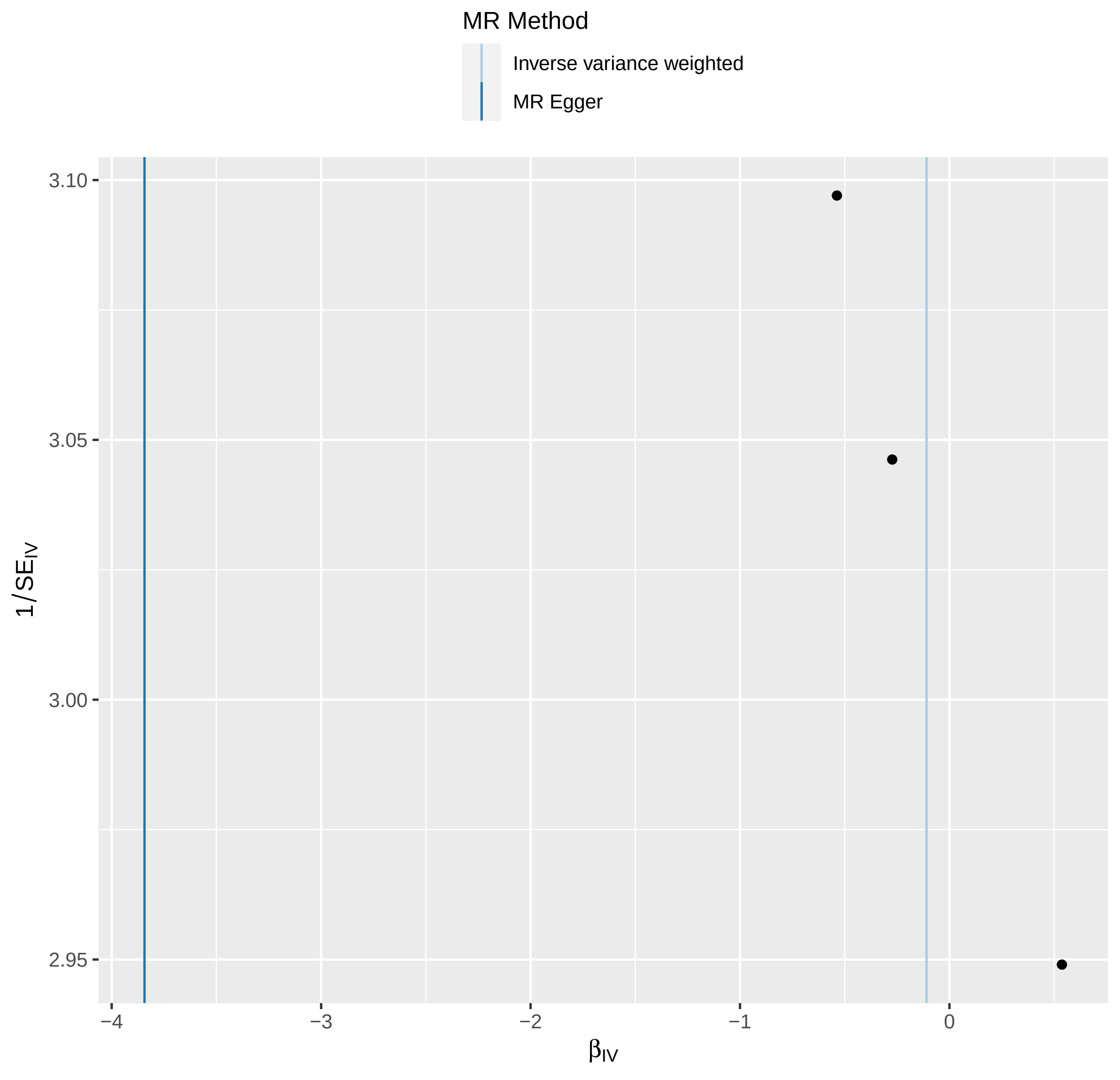

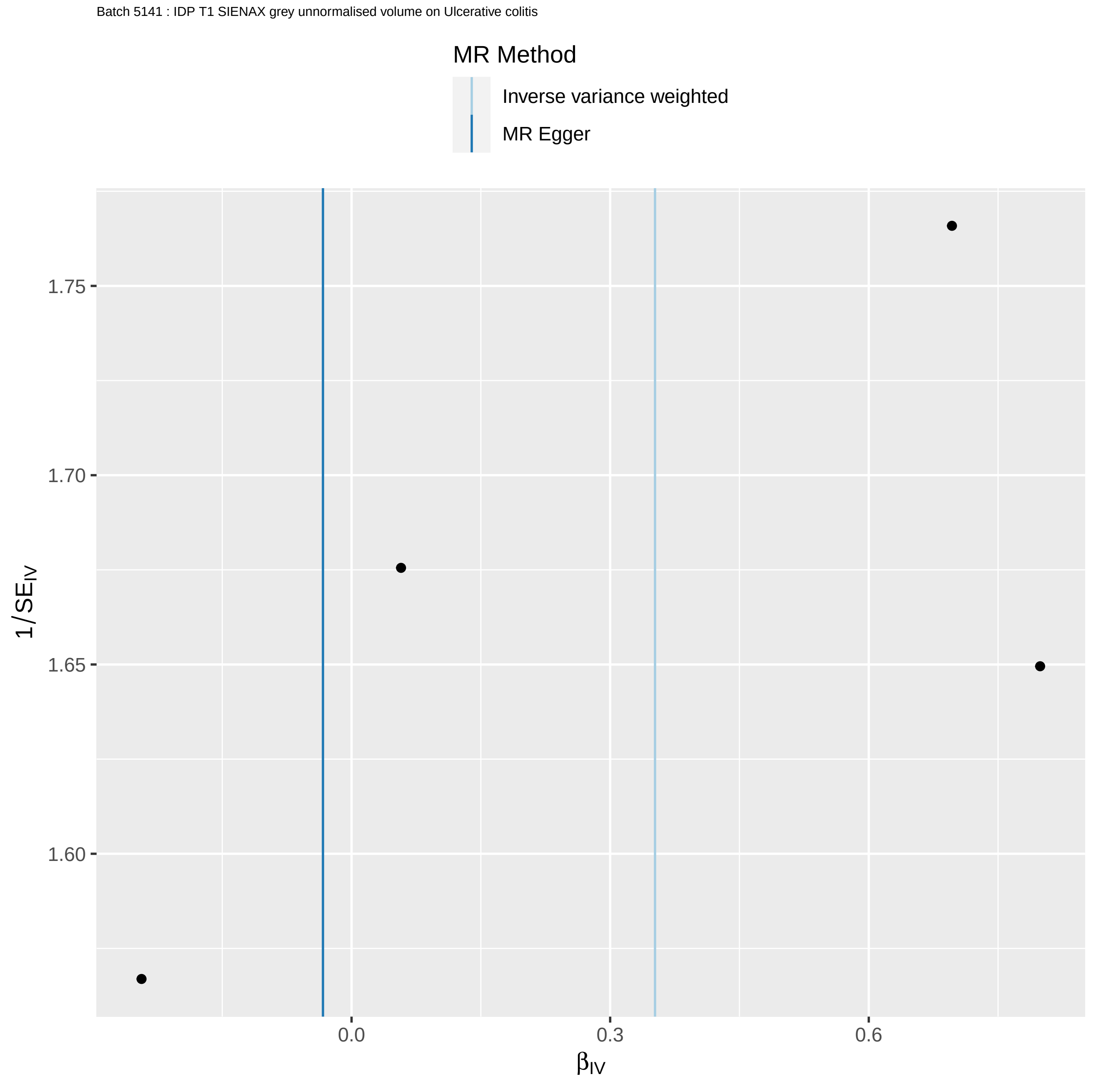

Batch 5141 : IDP T1 SIENAX grey unnormalised volume on Ulcerative colitis
MR Method
Inverse variance weighted
MR Egger
1.75
1.70
1.65
1.60
1/SE_IV
0.0
0.3
0.6
β_IV

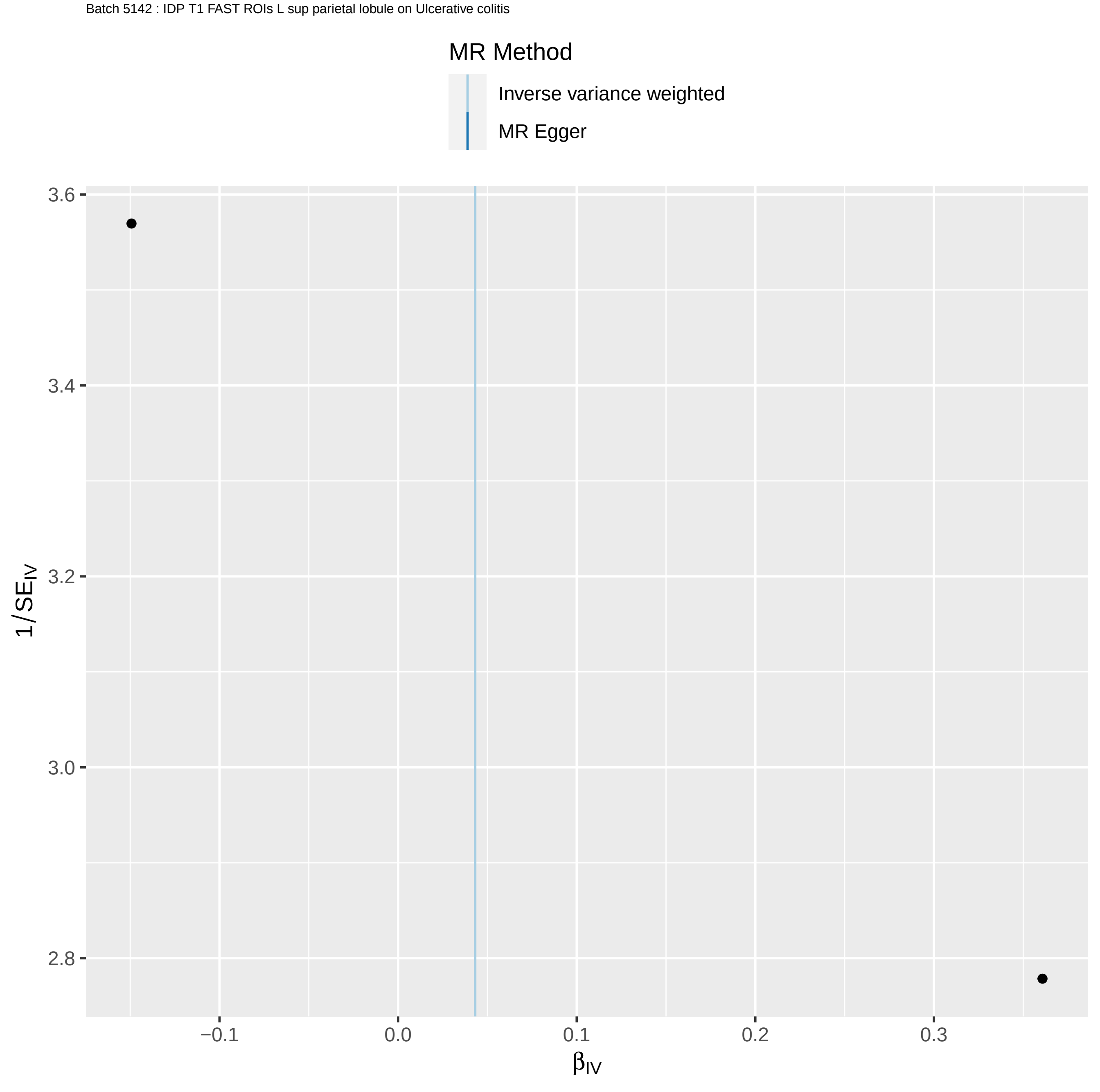

Batch 5142 : IDP T1 FAST ROIs L sup parietal lobule on Ulcerative colitis
MR Method
Inverse variance weighted
MR Egger
3.6
3.4
3.2
3.0
2.8
1/SE$_{IV}$
−0.1
0.0
0.1
0.2
0.3
β$_{IV}$

Batch 5164 : IDP T1 FAST ROIs L supramarg gyrus ant on Ulcerative colitis

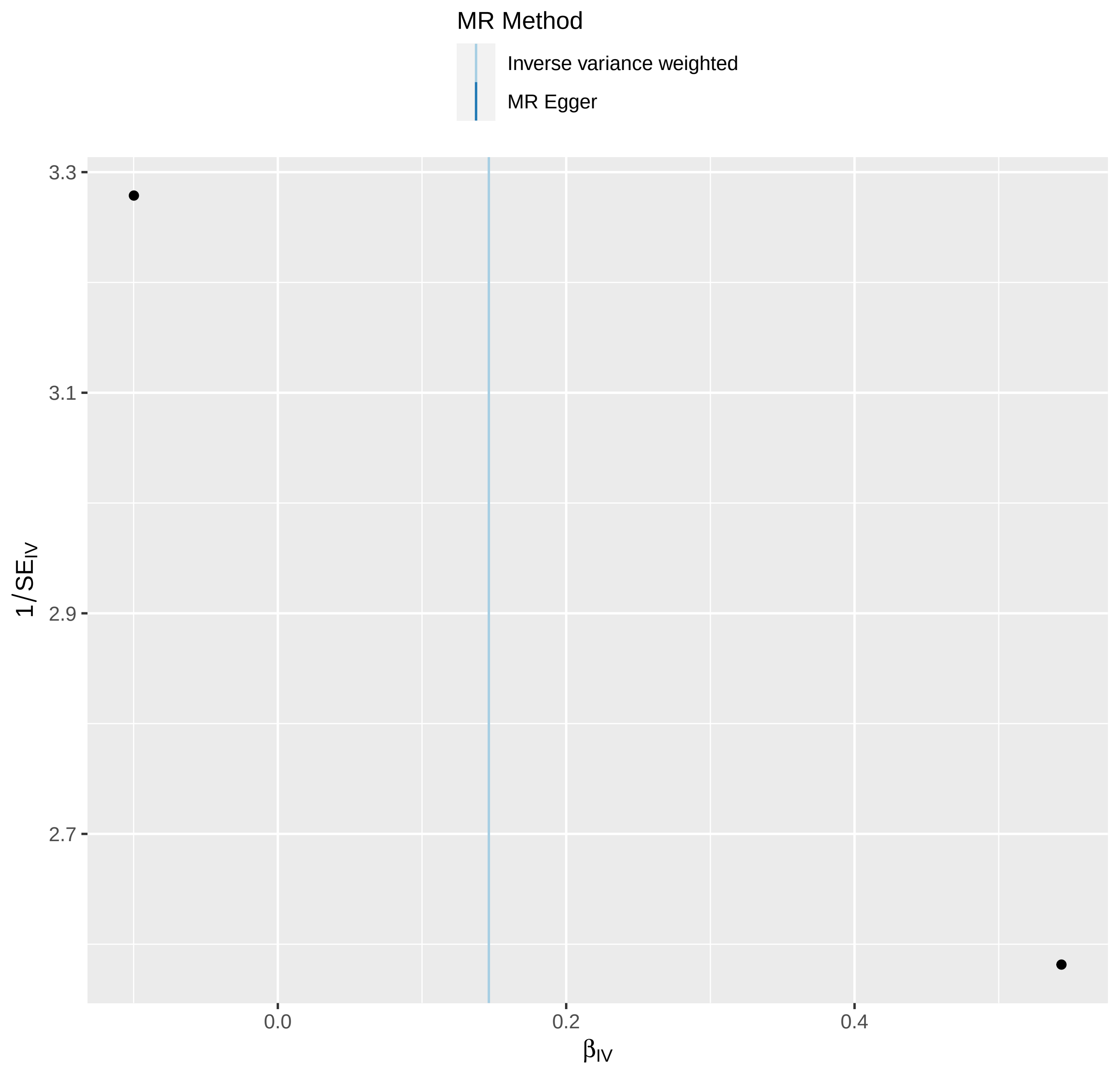

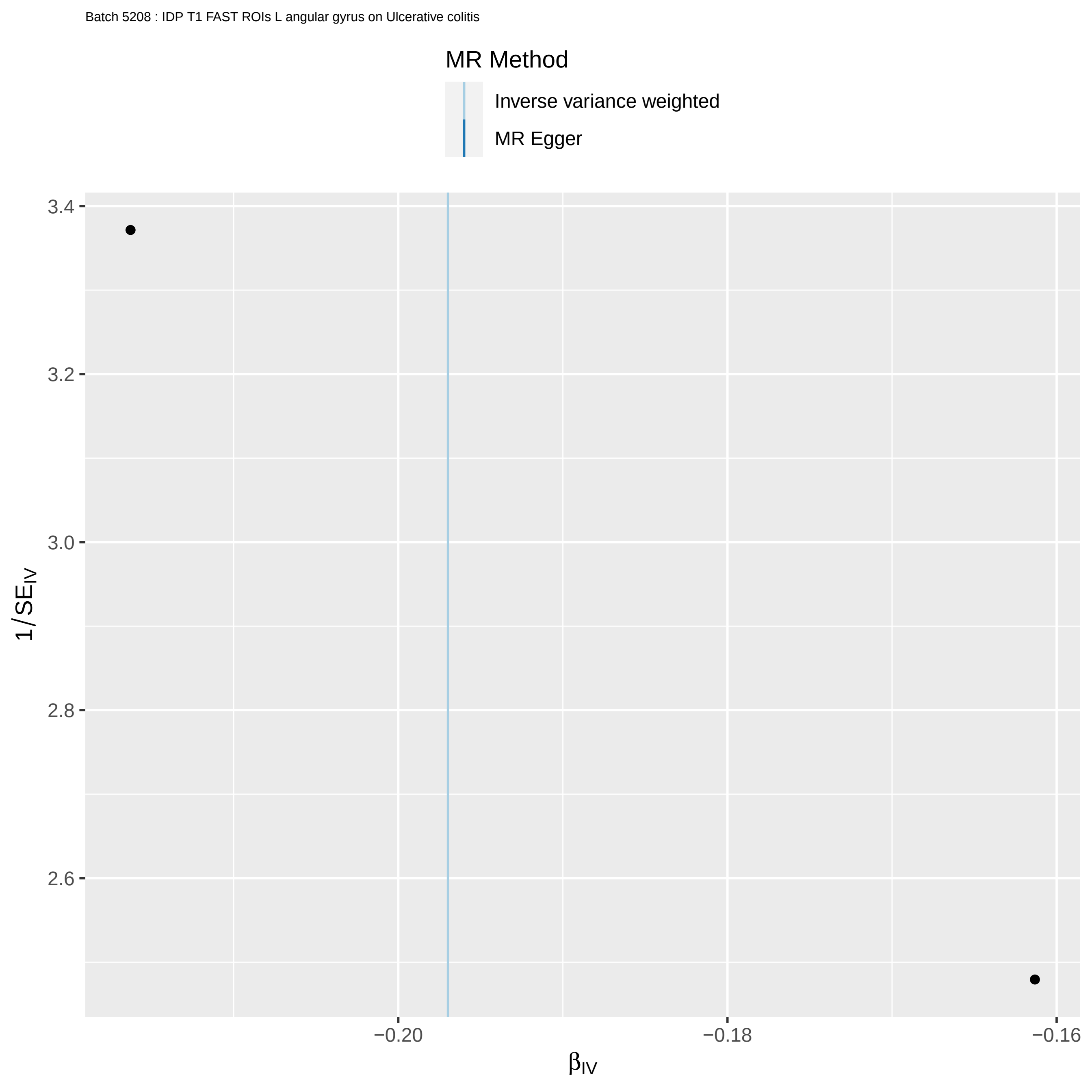

Batch 5208 : IDP T1 FAST ROIs L angular gyrus on Ulcerative colitis
MR Method
Inverse variance weighted
MR Egger
3.4
3.2
3.0
2.8
2.6
1/SE$_{IV}$
−0.20
−0.18
−0.16
β$_{IV}$

Batch 5230 : IDP T1 FAST ROIs L latocc cortex sup on Ulcerative colitis

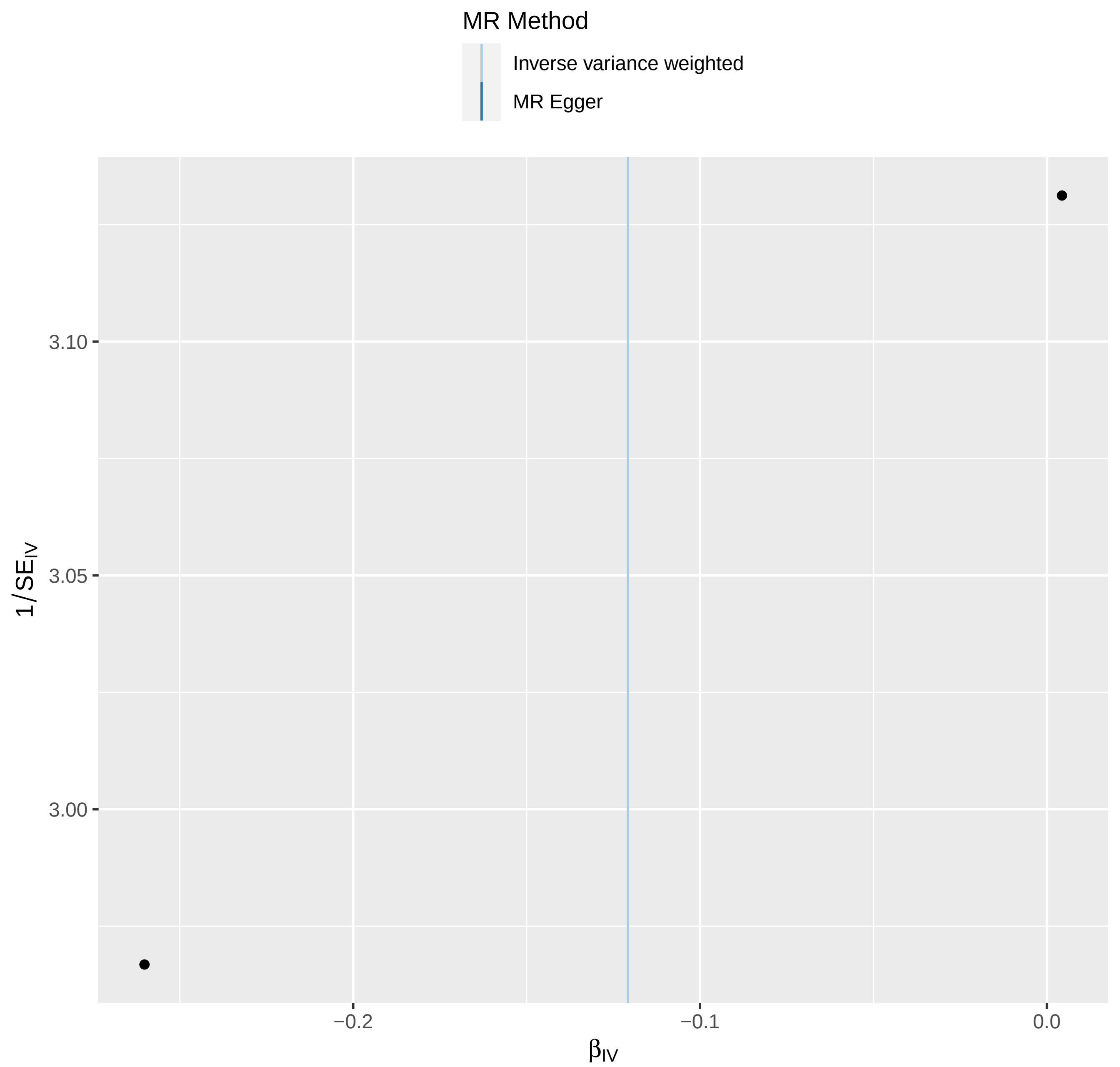

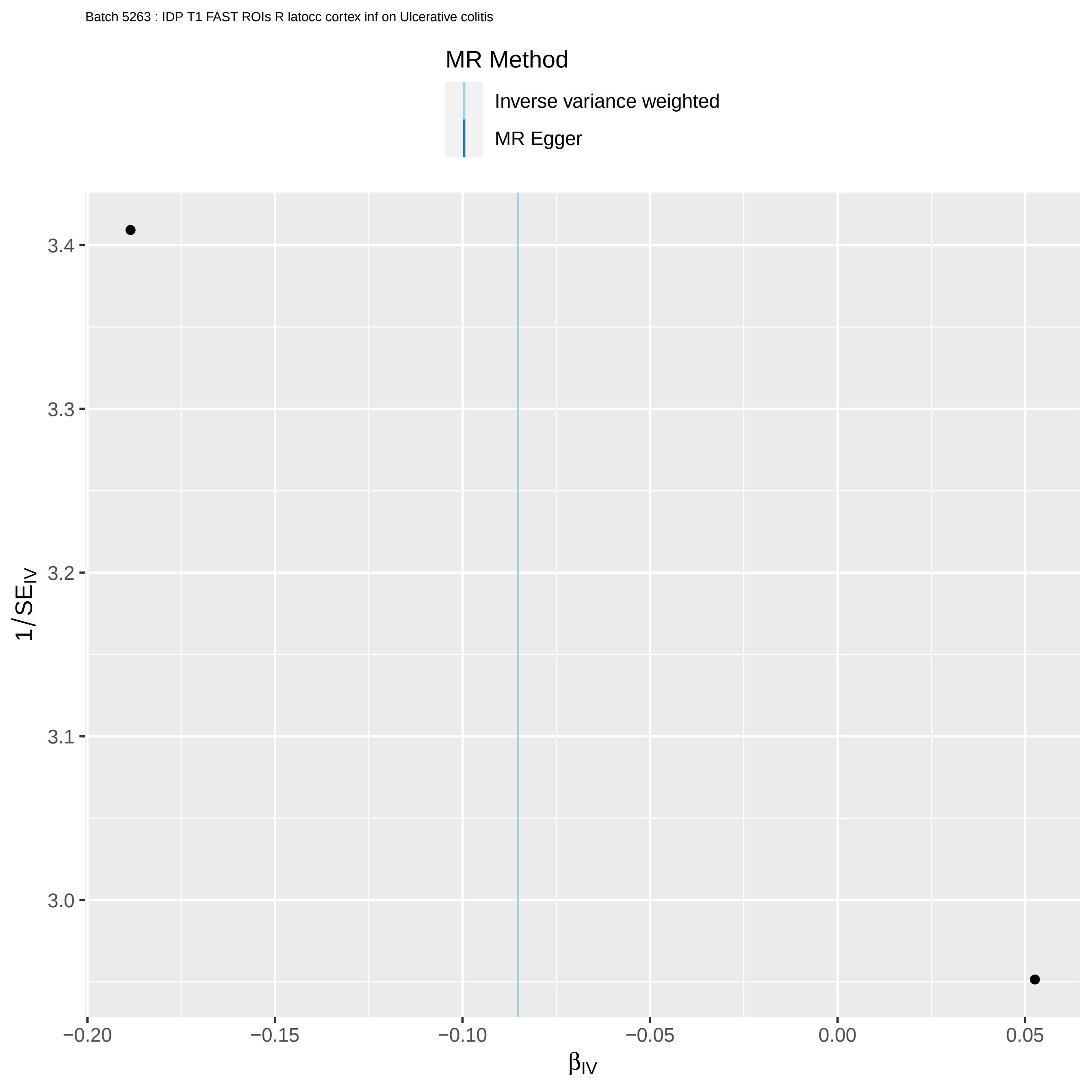

Batch 5263 : IDP T1 FAST ROIs R latocc cortex inf on Ulcerative colitis
MR Method
Inverse variance weighted
MR Egger
3.4
3.3
3.2
3.1
3.0
1/SE_IV
−0.20
−0.15
−0.10
−0.05
0.00
0.05
β_IV

Batch 5274 : IDP T1 FAST ROIs L intracalc cortex on Ulcerative colitis

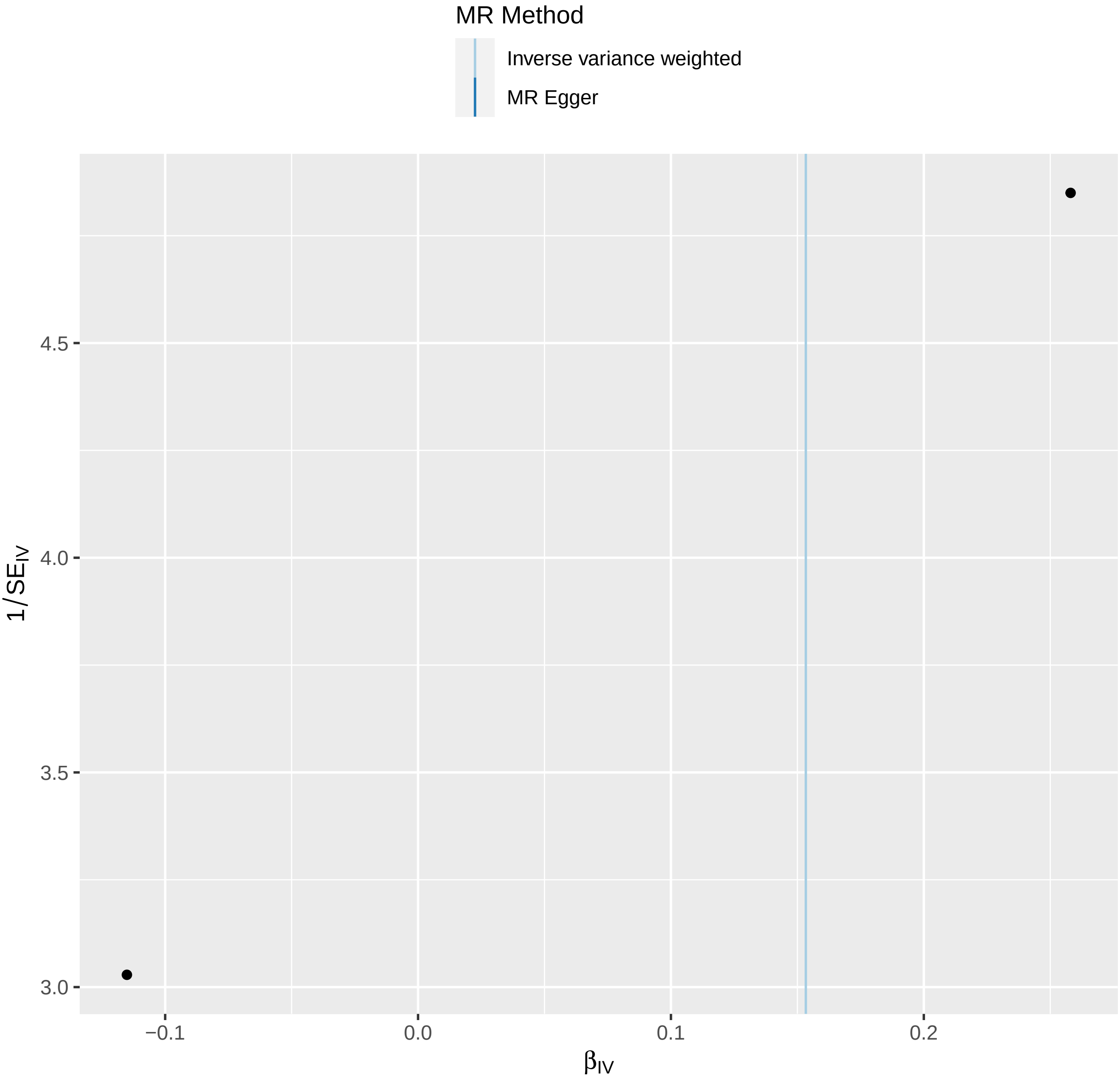

Batch 5329 : IDP T1 FAST ROIs R juxtapos lobule cortex on Ulcerative colitis

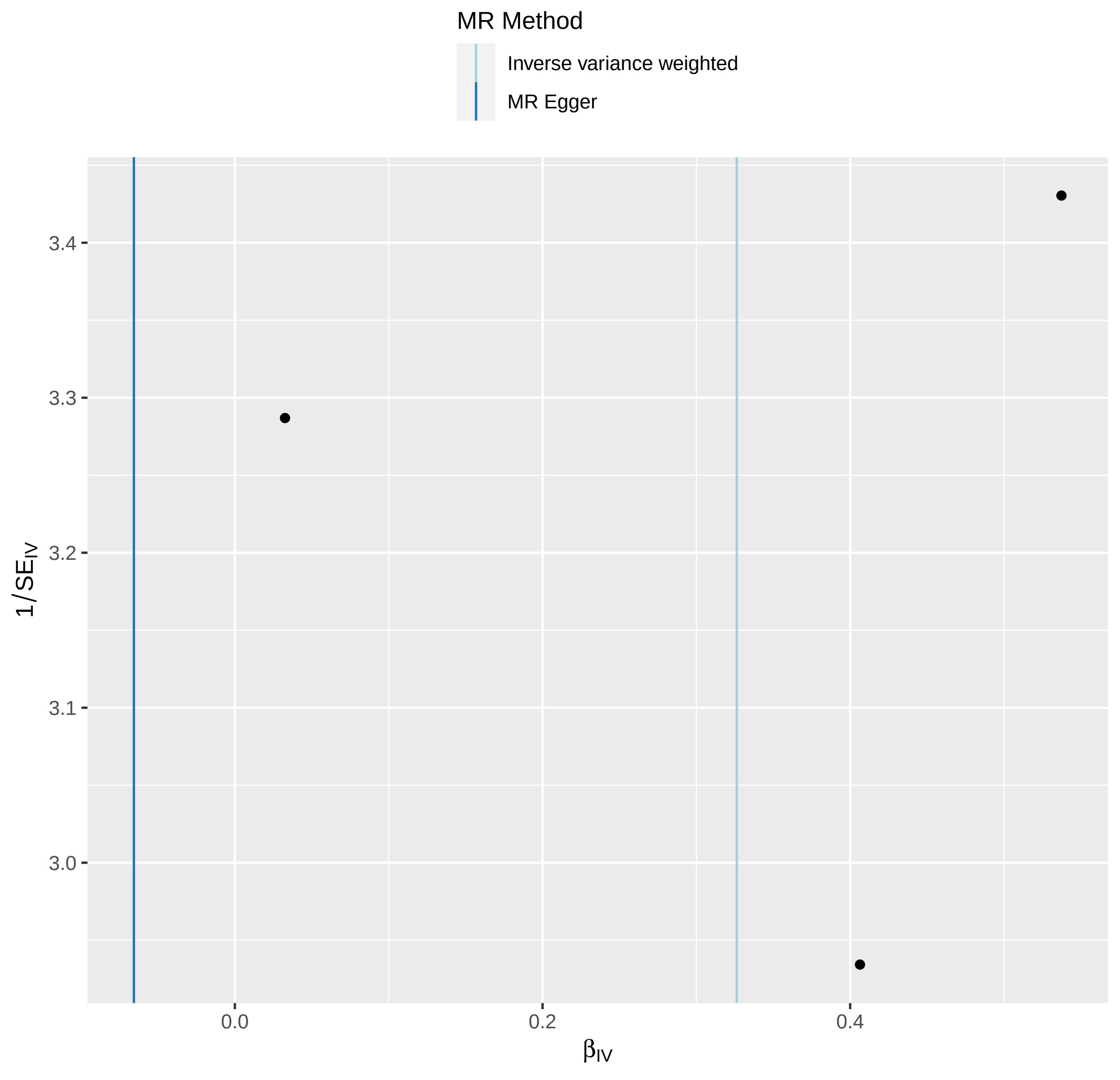

Batch 5340 : IDP T1 FAST ROIs L subcallosal cortex on Ulcerative colitis

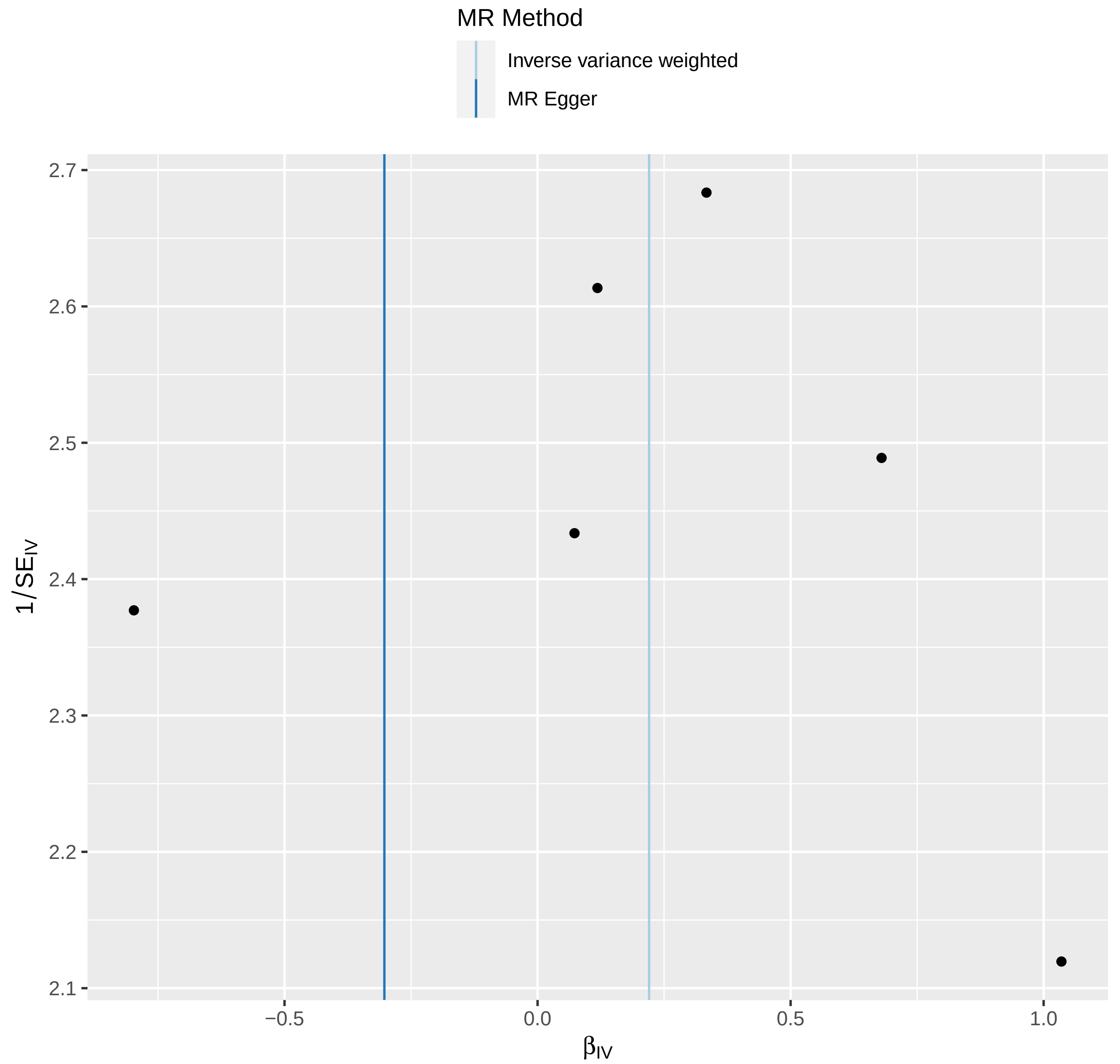

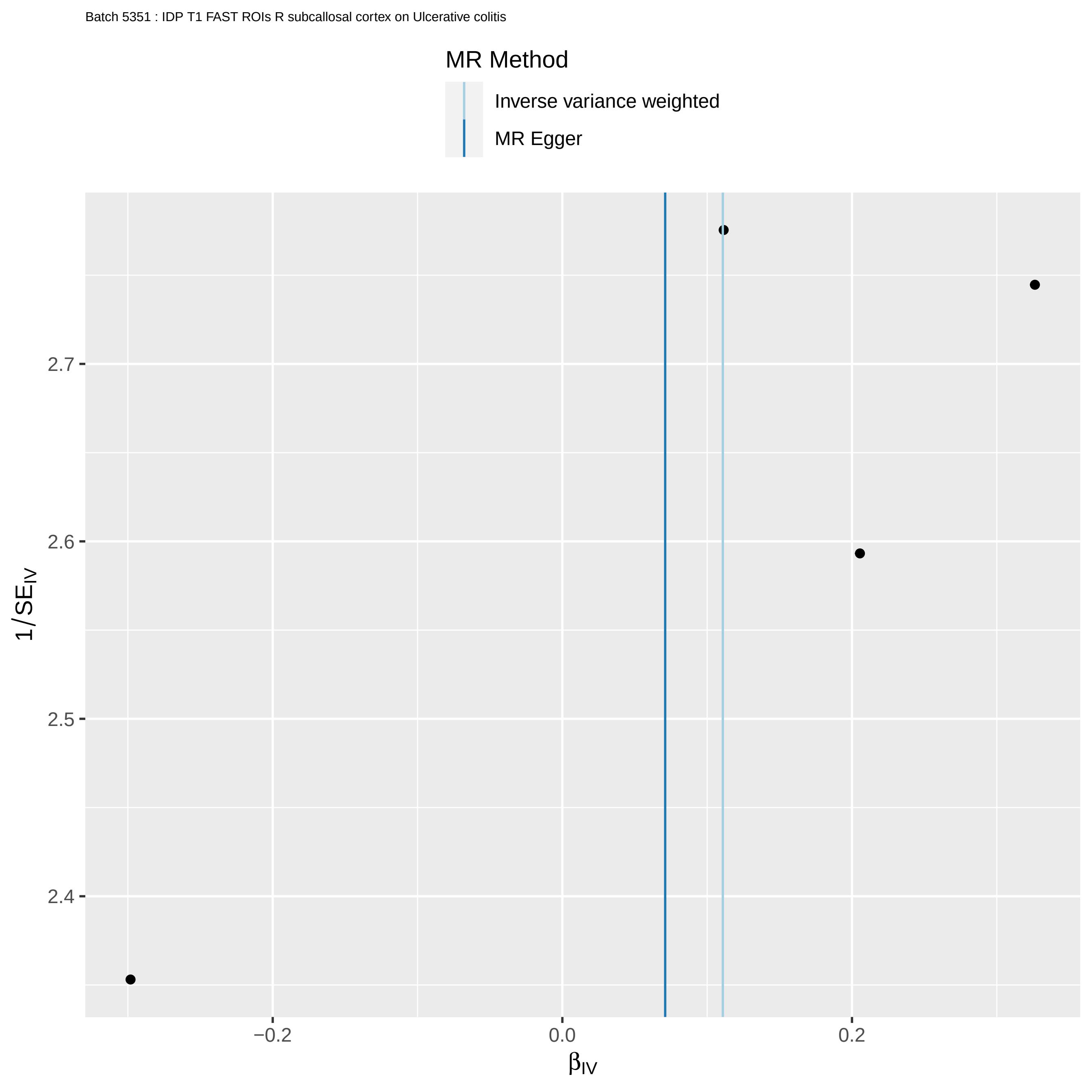
Batch 5351 : IDP T1 FAST ROIs R subcallosal cortex on Ulcerative colitis
MR Method
Inverse variance weighted
MR Egger
2.7
2.6
2.5
2.4
1/SE_IV
−0.2
0.0
0.2
β_IV

Batch 5396 : IDP T1 FAST ROIs R cing gyrus ant on Ulcerative colitis

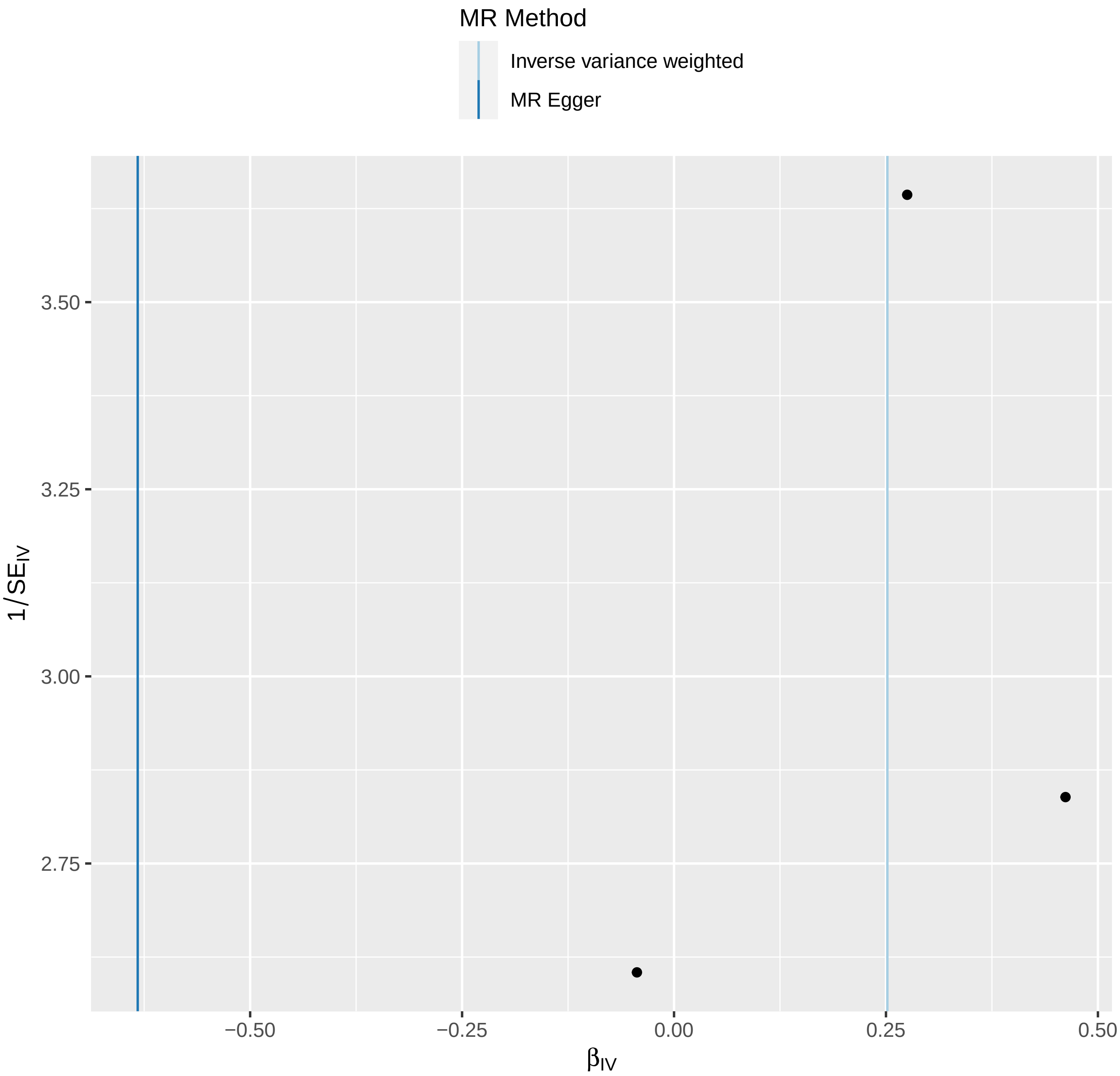

Batch 5418 : IDP T1 FAST ROIs R cing gyrus post on Ulcerative colitis

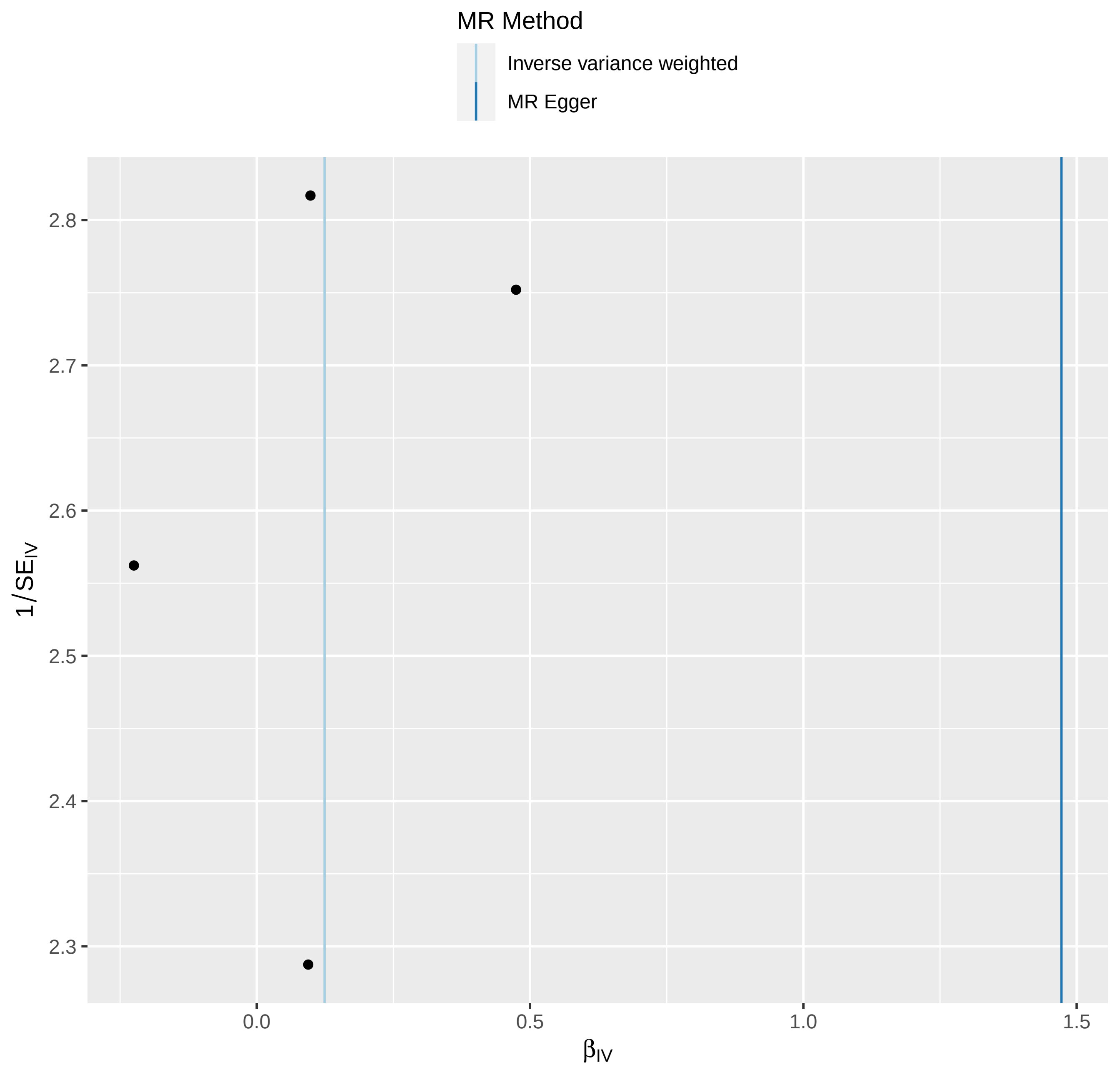

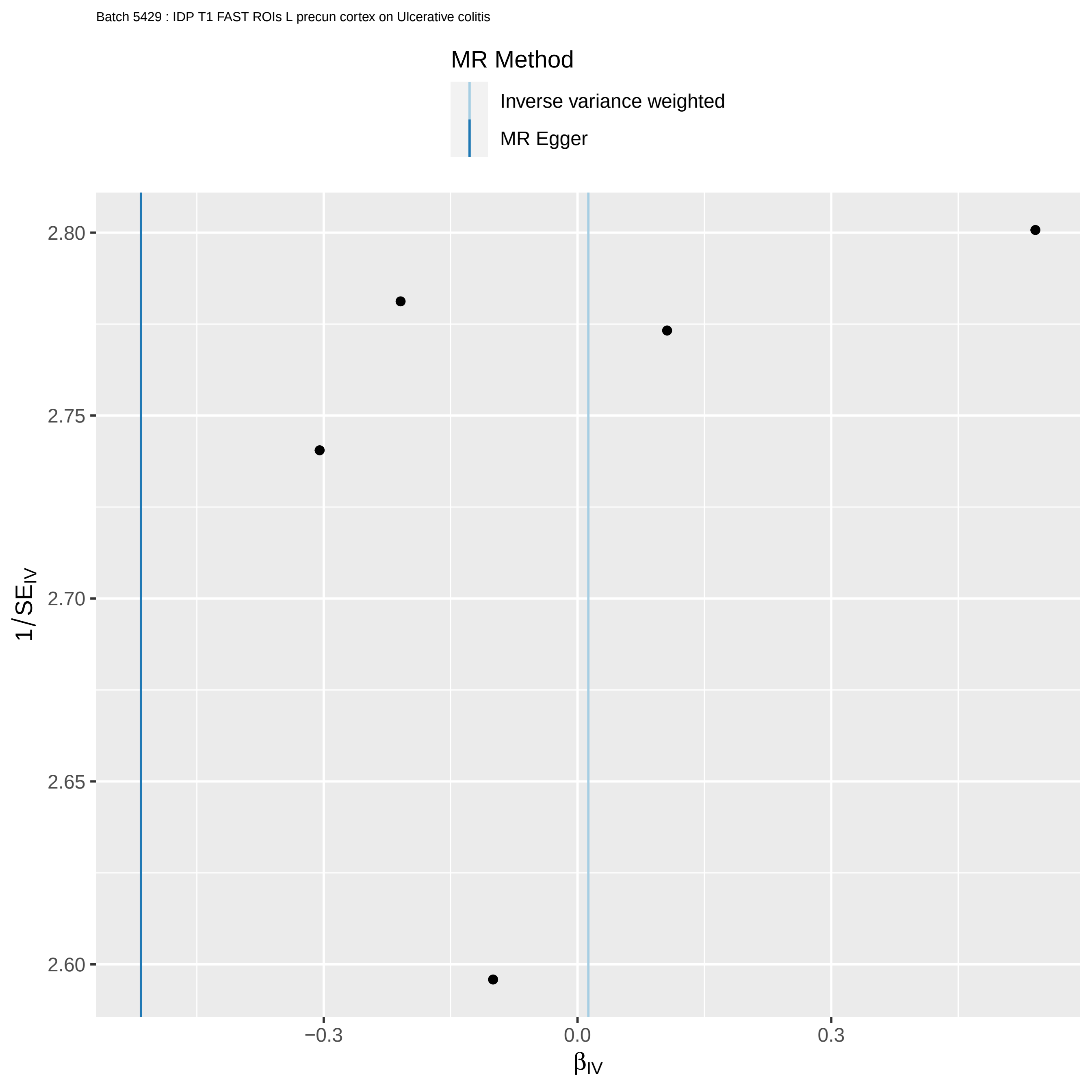
Batch 5429 : IDP T1 FAST ROIs L precun cortex on Ulcerative colitis
MR Method
Inverse variance weighted
MR Egger
2.80
2.75
2.70
2.65
2.60
1/SE_IV
−0.3
0.0
0.3
β_IV

Batch 5442 : IDP T1 FAST ROIs L cuneal cortex on Ulcerative colitis

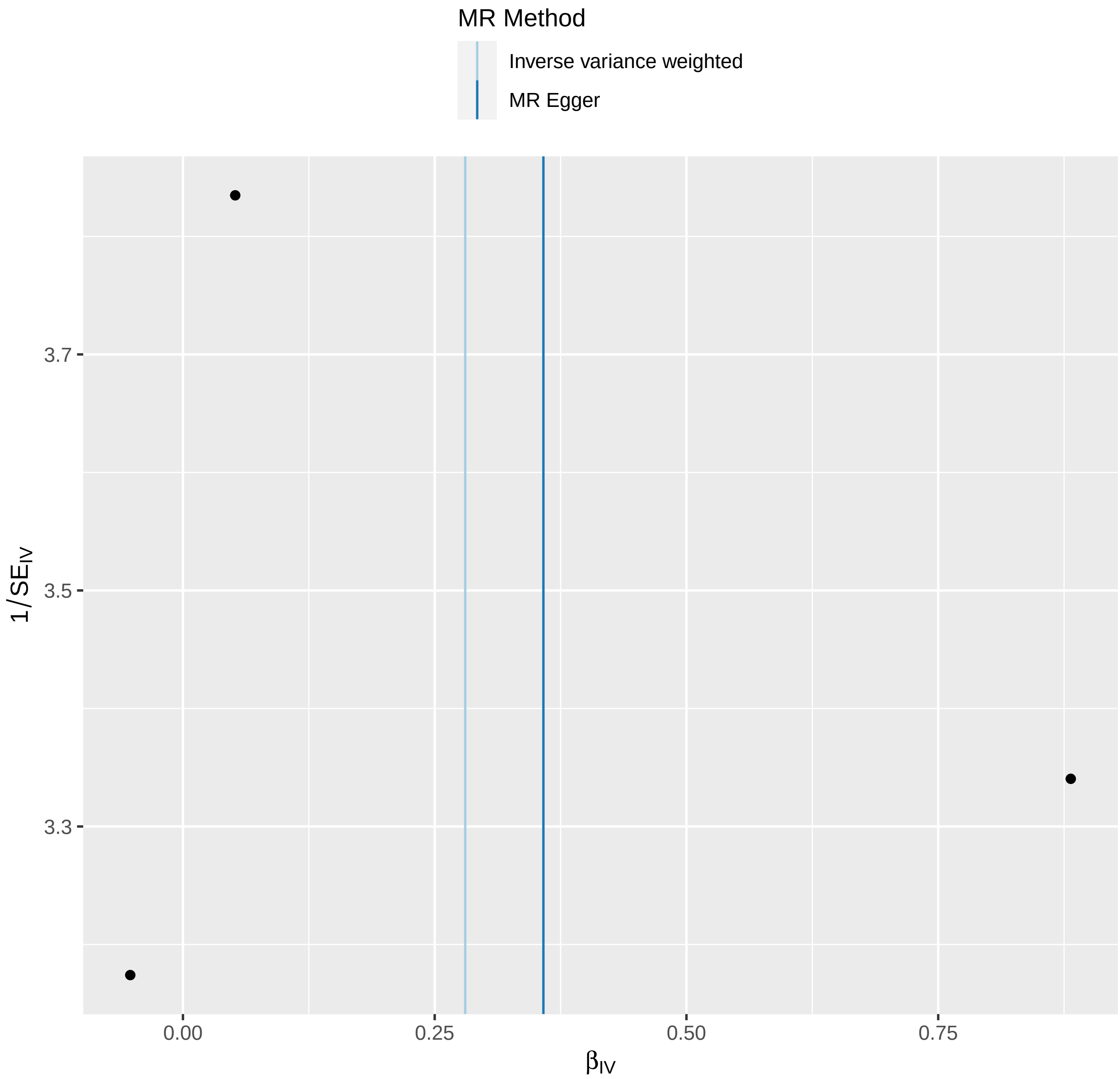

Batch 5444 : IDP T1 SIENAX brain−normalised volume on Ulcerative colitis

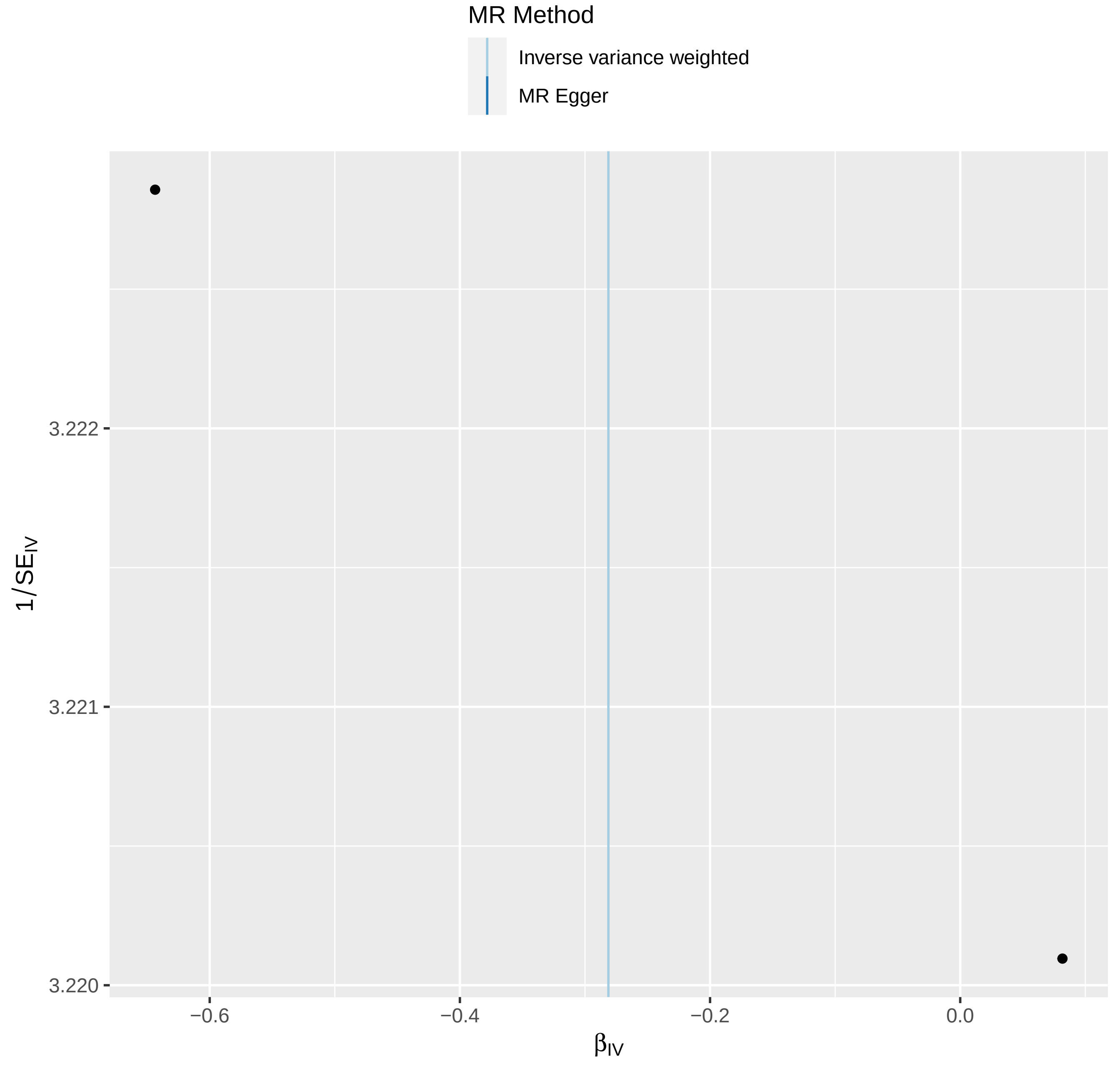

Batch 5445 : IDP T1 FAST ROIs L front orb cortex on Ulcerative colitis

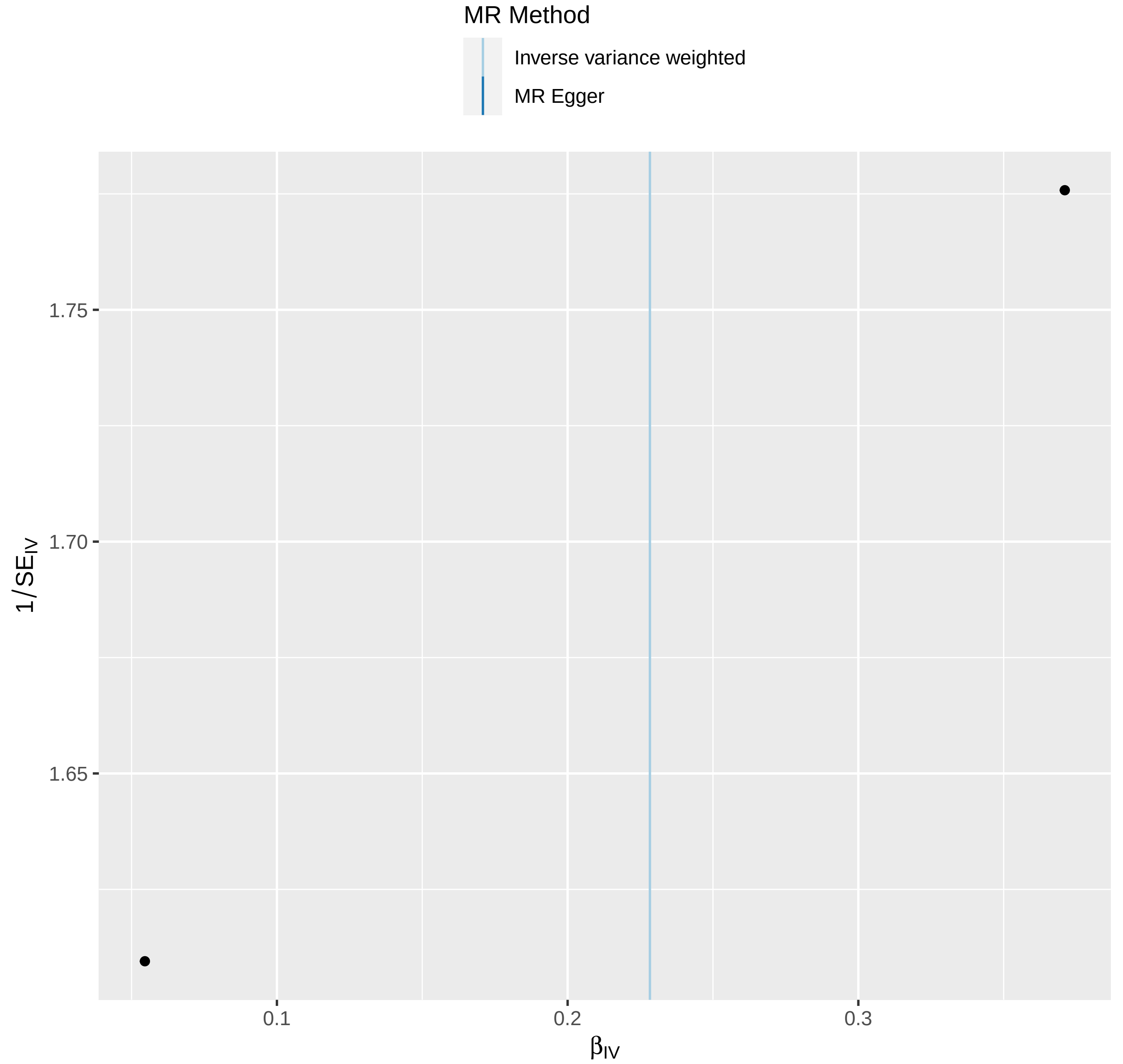

Batch 5447 : IDP T1 FAST ROIs L parahipp gyrus ant on Ulcerative colitis

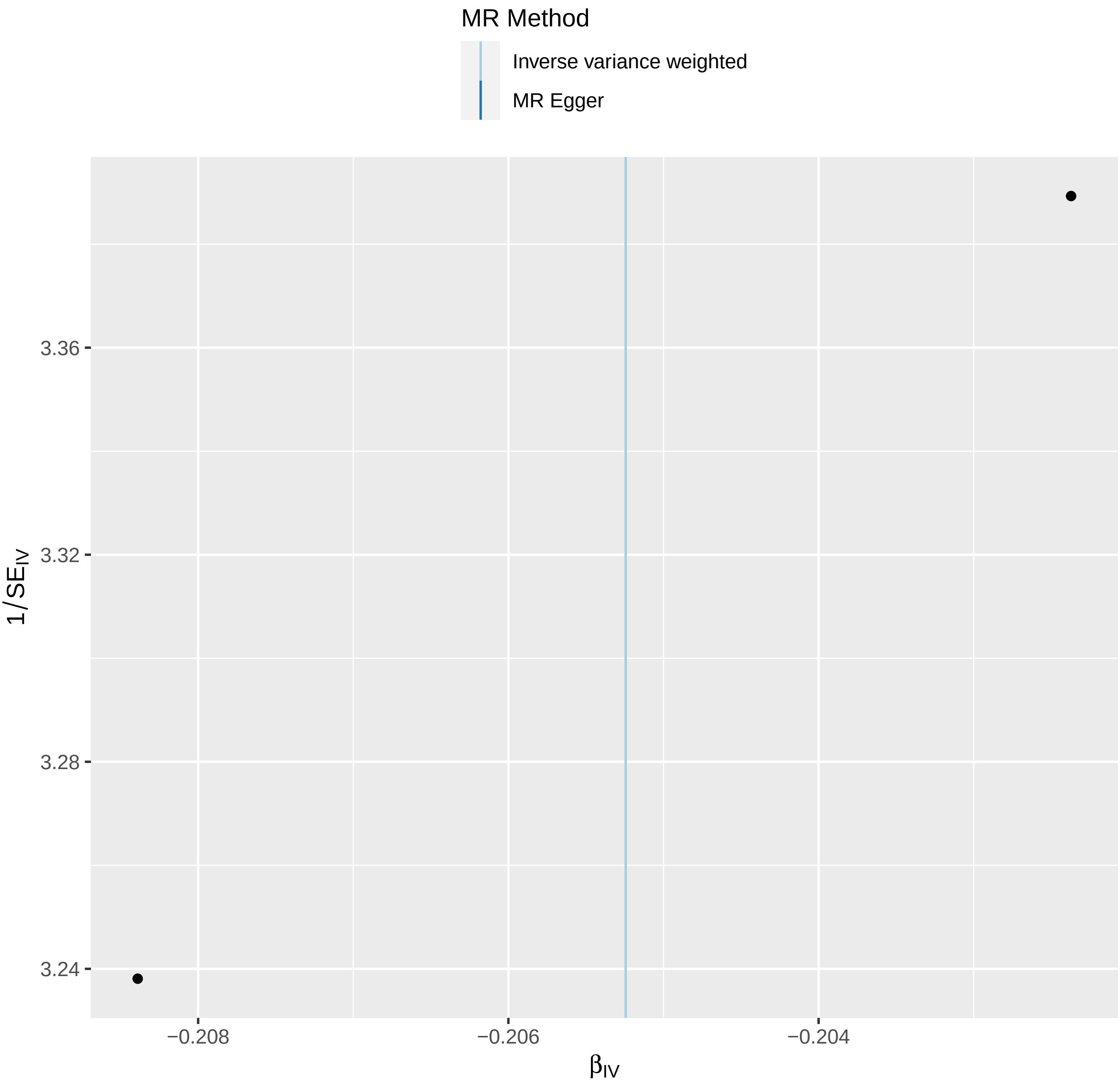

Batch 5448 : IDP T1 FAST ROIs R parahipp gyrus ant on Ulcerative colitis

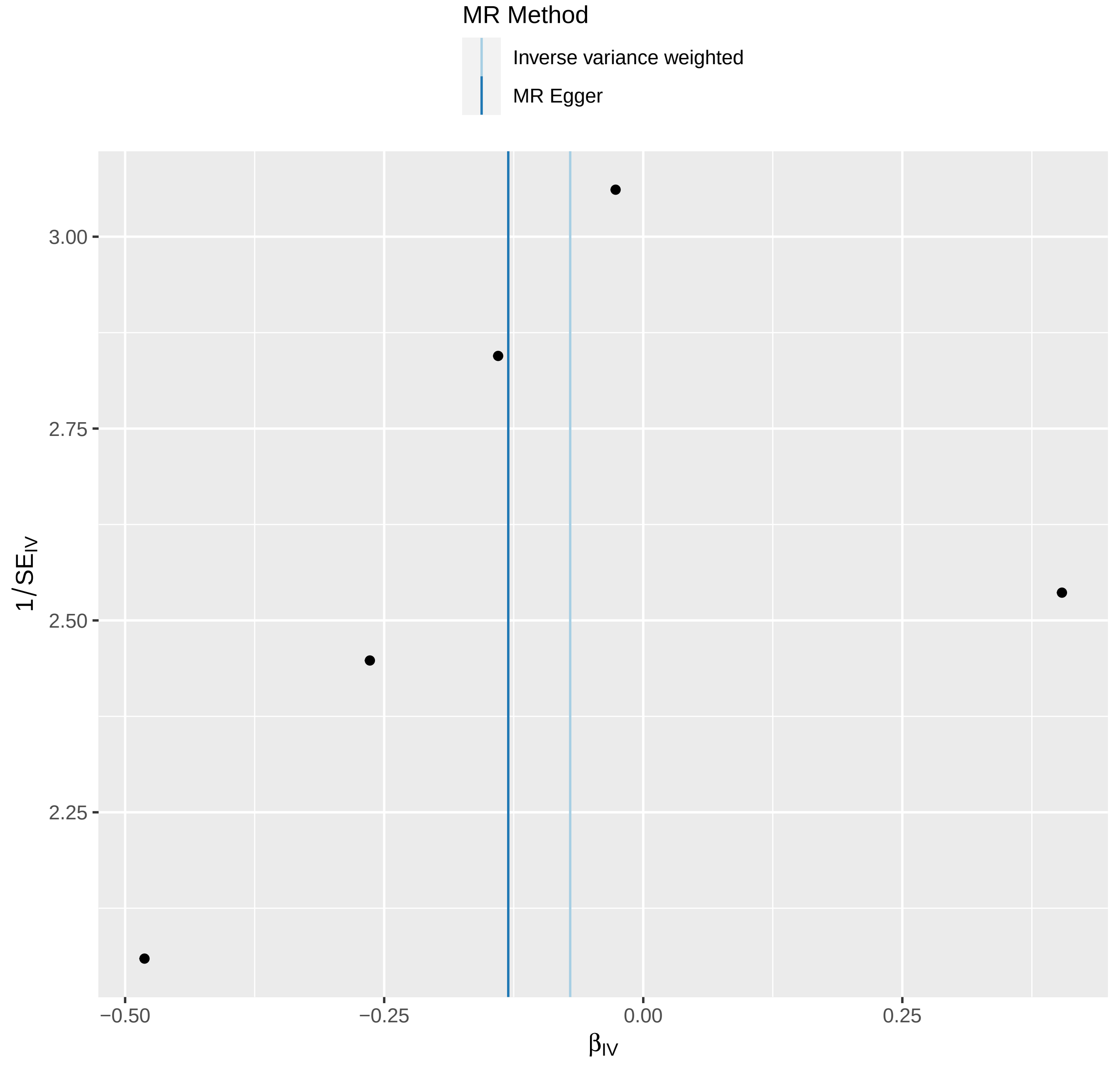

Batch 5450 : IDP T1 FAST ROIs R parahipp gyrus post on Ulcerative colitis

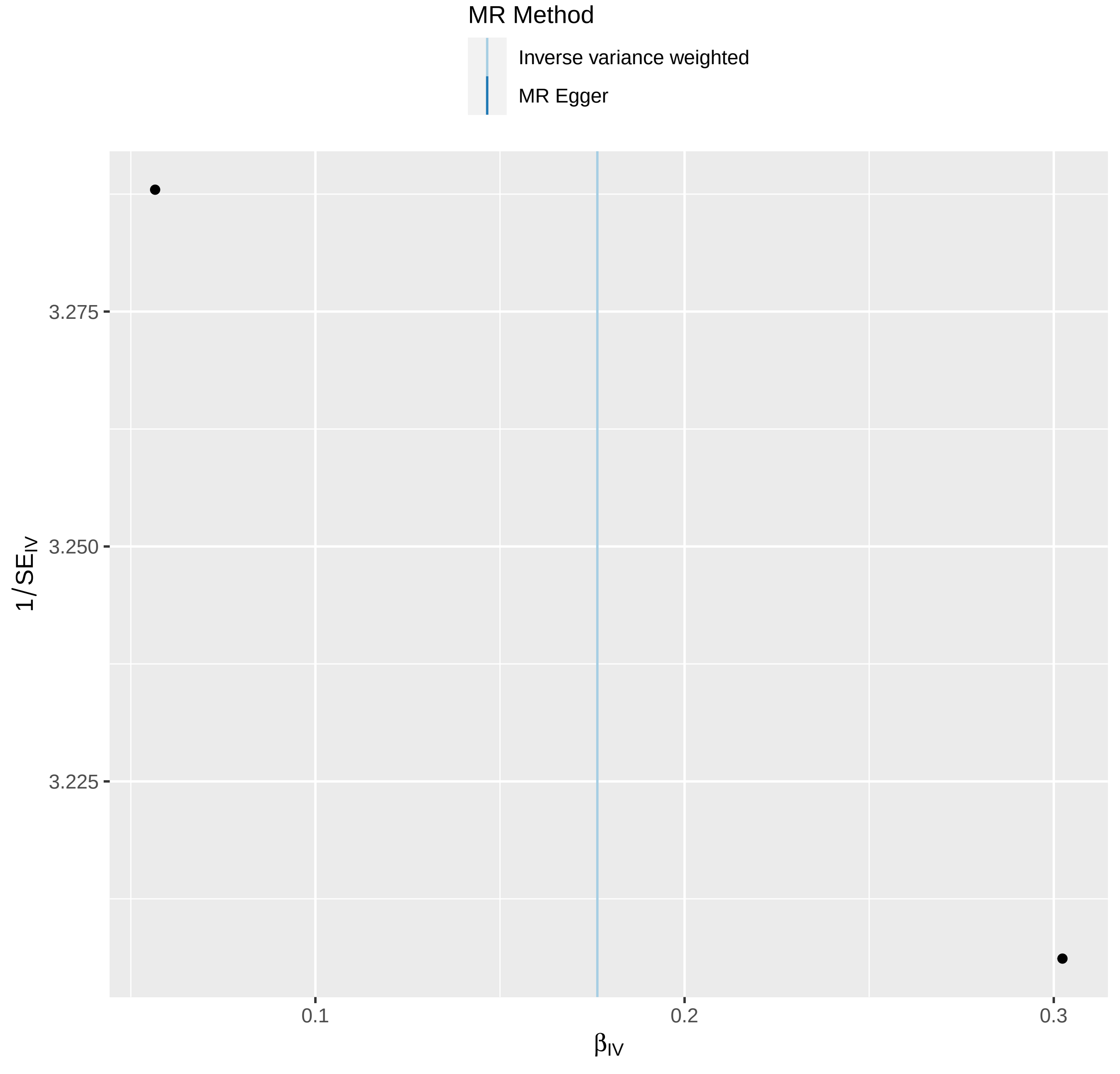

Batch 5452 : IDP T1 FAST ROIs R lingual gyrus on Ulcerative colitis

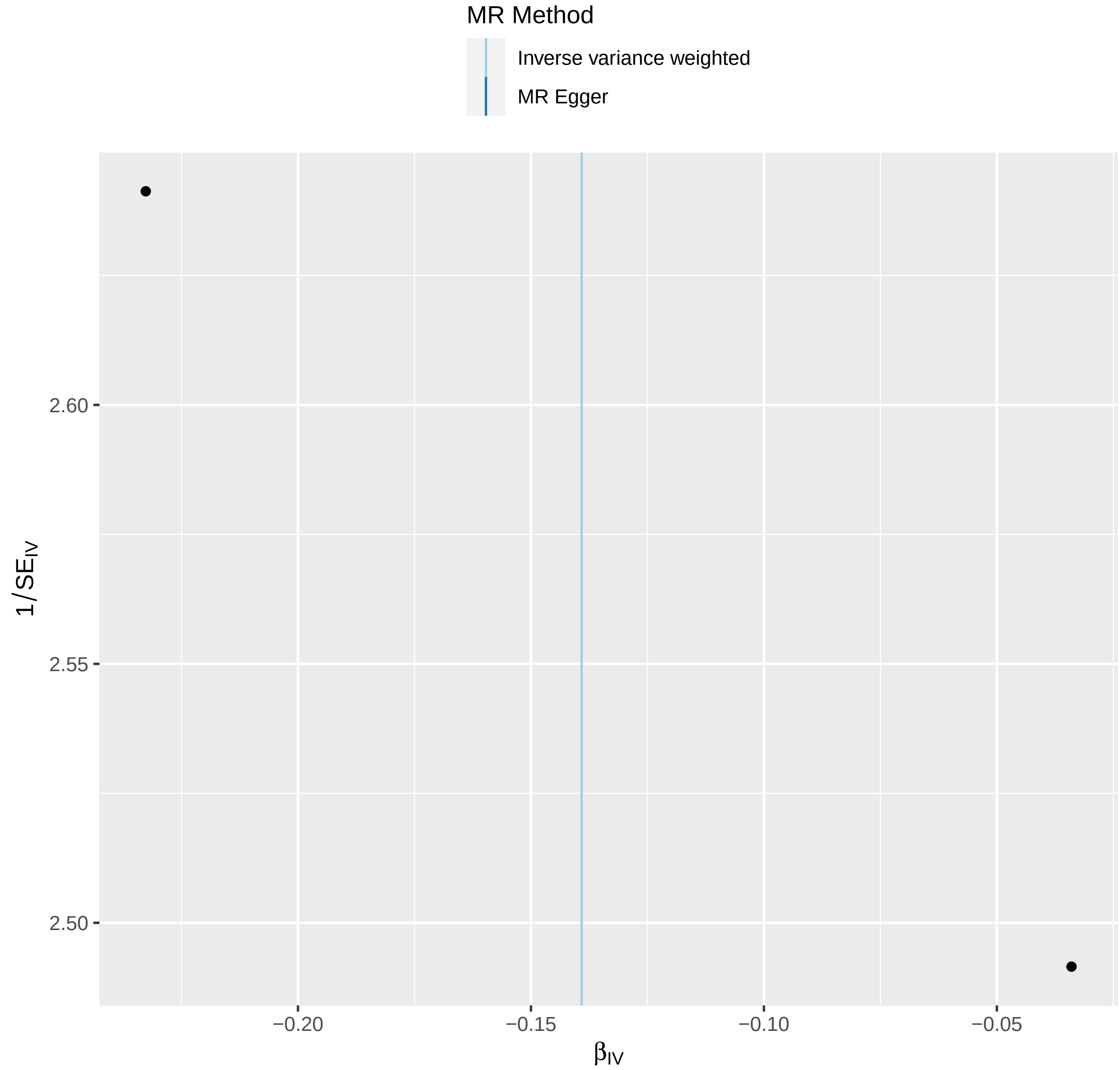

Supplement: Supplementary file 6 [file DataSheet_4.pdf]
