# Supplementary material for: Brain imaging derived phenotypes: a biomarker for the onset of inflammatory bowel disease and a potential mediator of mental complications
Source: Front Immunol. 2024 Feb 26;15:1359540. doi: 10.3389/fimmu.2024.1359540 (PMC10925669; doi:10.3389/fimmu.2024.1359540)
Supplement: Supplementary file 5 [file DataSheet_3.pdf]

Batch 1 : Crohn's disease on IDP T1 SIENAX peripheral grey normalised volume

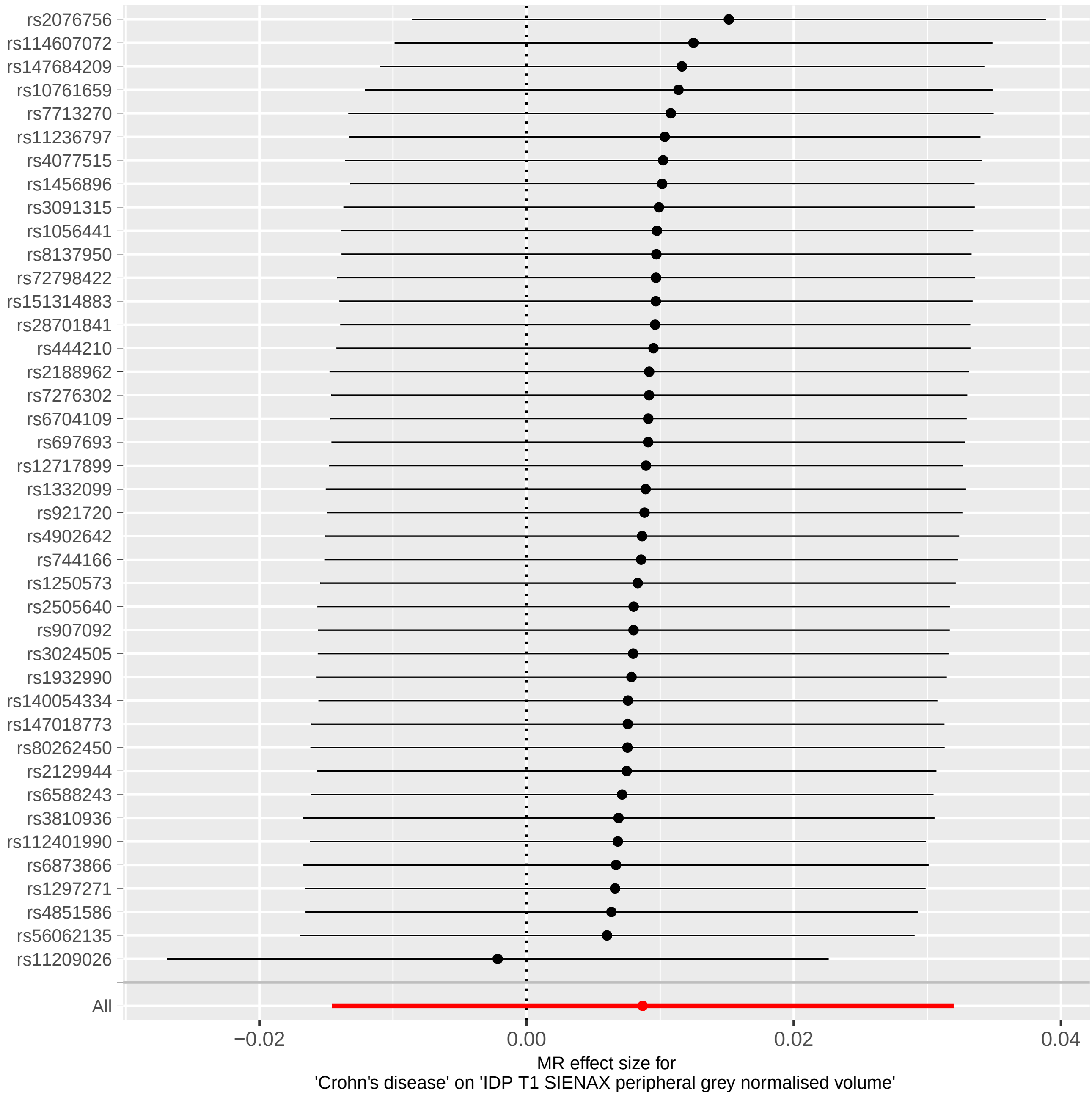

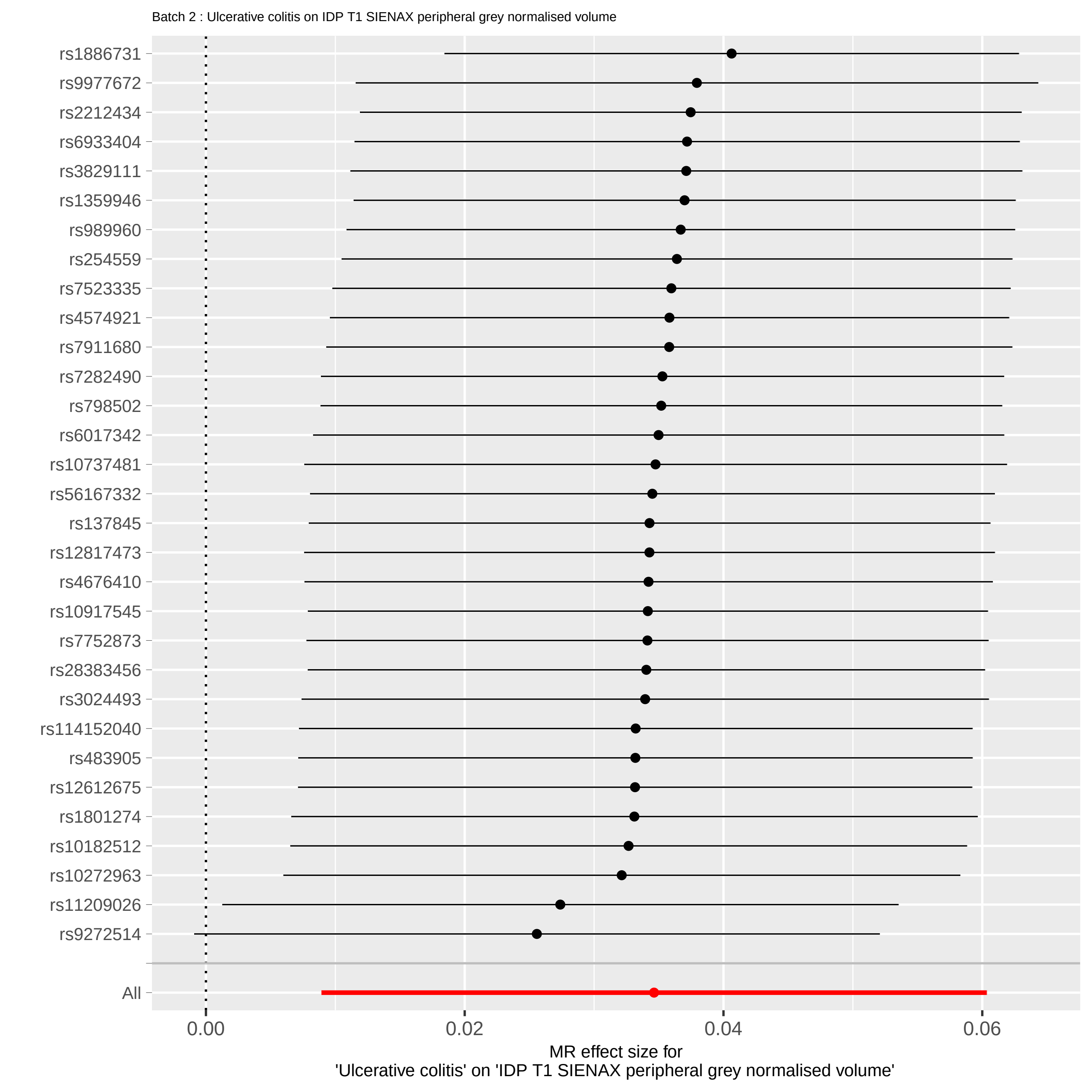
Batch 2 : Ulcerative colitis on IDP T1 SIENAX peripheral grey normalised volume
rs1886731
rs9977672
rs2212434
rs6933404
rs3829111
rs1359946
rs989960
rs254559
rs7523335
rs4574921
rs7911680
rs7282490
rs798502
rs6017342
rs10737481
rs56167332
rs137845
rs12817473
rs4676410
rs10917545
rs7752873
rs28383456
rs3024493
rs114152040
rs483905
rs12612675
rs1801274
rs10182512
rs10272963
rs11209026
rs9272514
All
0.00
0.02
0.04
0.06
MR effect size for
'Ulcerative colitis' on 'IDP T1 SIENAX peripheral grey normalised volume'

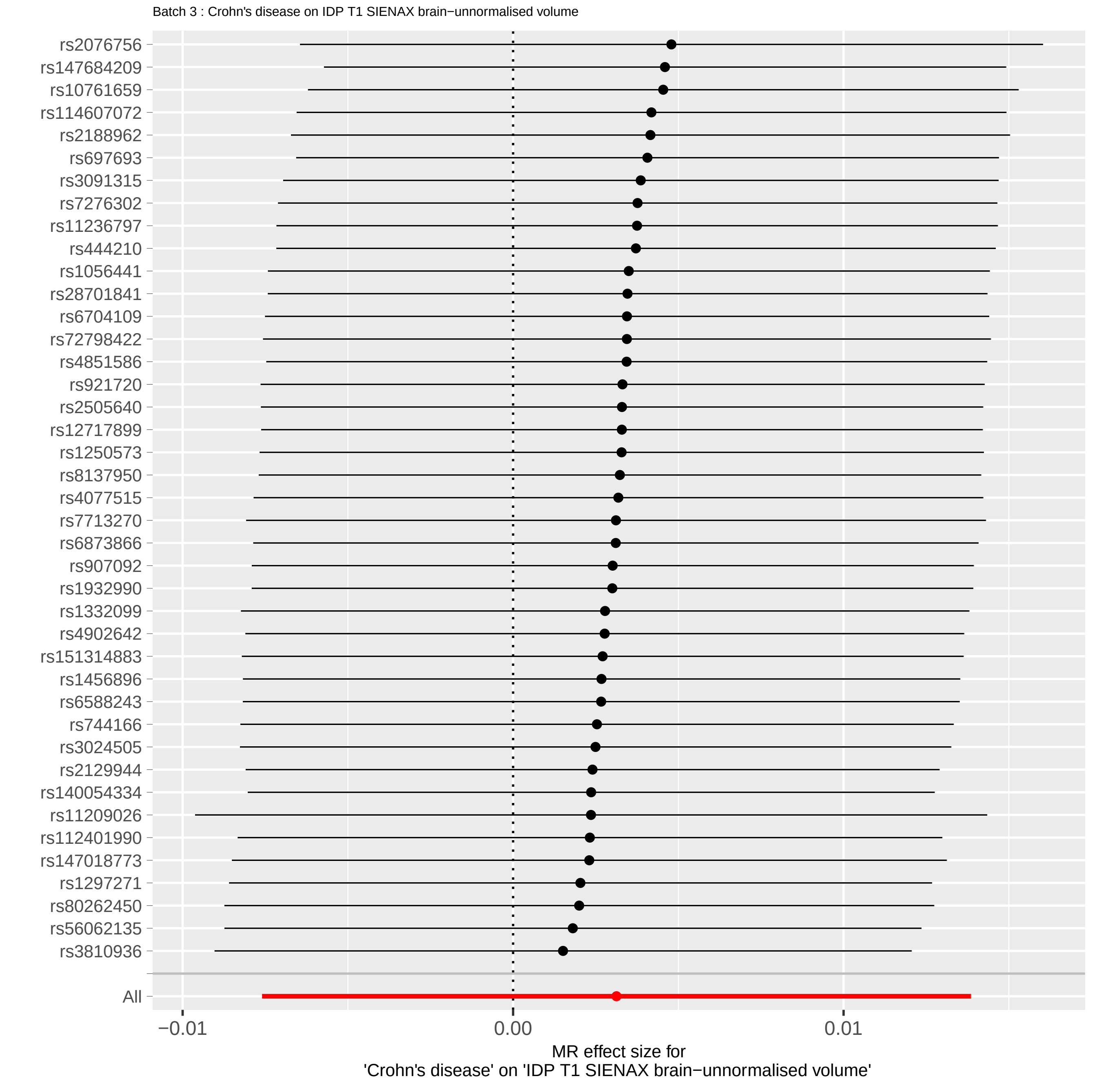

Batch 3 : Crohn's disease on IDP T1 SIENAX brain−unnormalised volume
rs2076756
rs147684209
rs10761659
rs114607072
rs2188962
rs697693
rs3091315
rs7276302
rs11236797
rs444210
rs1056441
rs28701841
rs6704109
rs72798422
rs4851586
rs921720
rs2505640
rs12717899
rs1250573
rs8137950
rs4077515
rs7713270
rs6873866
rs907092
rs1932990
rs1332099
rs4902642
rs151314883
rs1456896
rs6588243
rs744166
rs3024505
rs2129944
rs140054334
rs11209026
rs112401990
rs147018773
rs1297271
rs80262450
rs56062135
rs3810936
All
−0.01
0.00
0.01
MR effect size for
'Crohn's disease' on 'IDP T1 SIENAX brain−unnormalised volume'

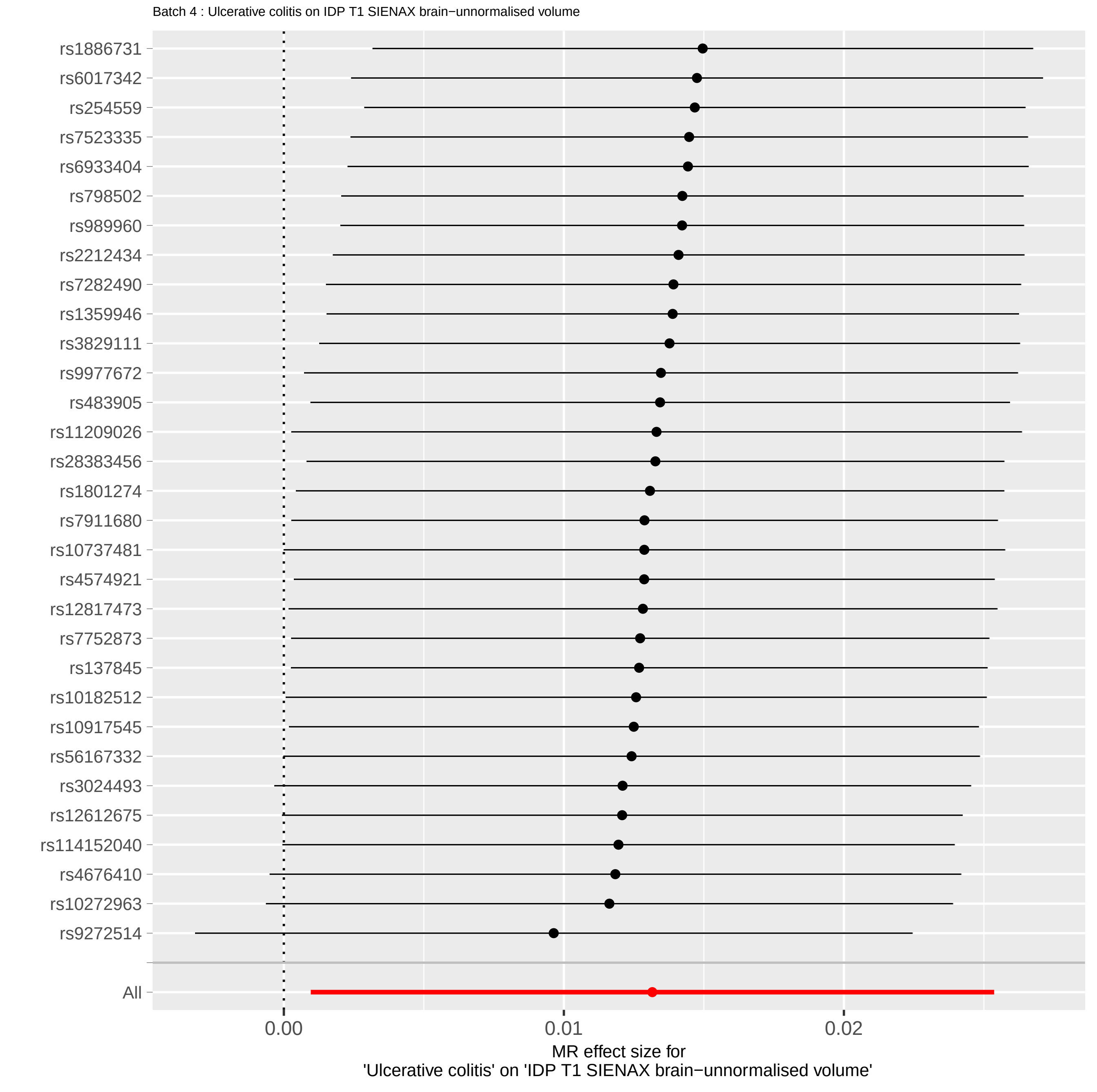
Batch 4 : Ulcerative colitis on IDP T1 SIENAX brain−unnormalised volume
rs1886731
rs6017342
rs254559
rs7523335
rs6933404
rs798502
rs989960
rs2212434
rs7282490
rs1359946
rs3829111
rs9977672
rs483905
rs11209026
rs28383456
rs1801274
rs7911680
rs10737481
rs4574921
rs12817473
rs7752873
rs137845
rs10182512
rs10917545
rs56167332
rs3024493
rs12612675
rs114152040
rs4676410
rs10272963
rs9272514
All
0.00
0.01
0.02
MR effect size for
'Ulcerative colitis' on 'IDP T1 SIENAX brain−unnormalised volume'

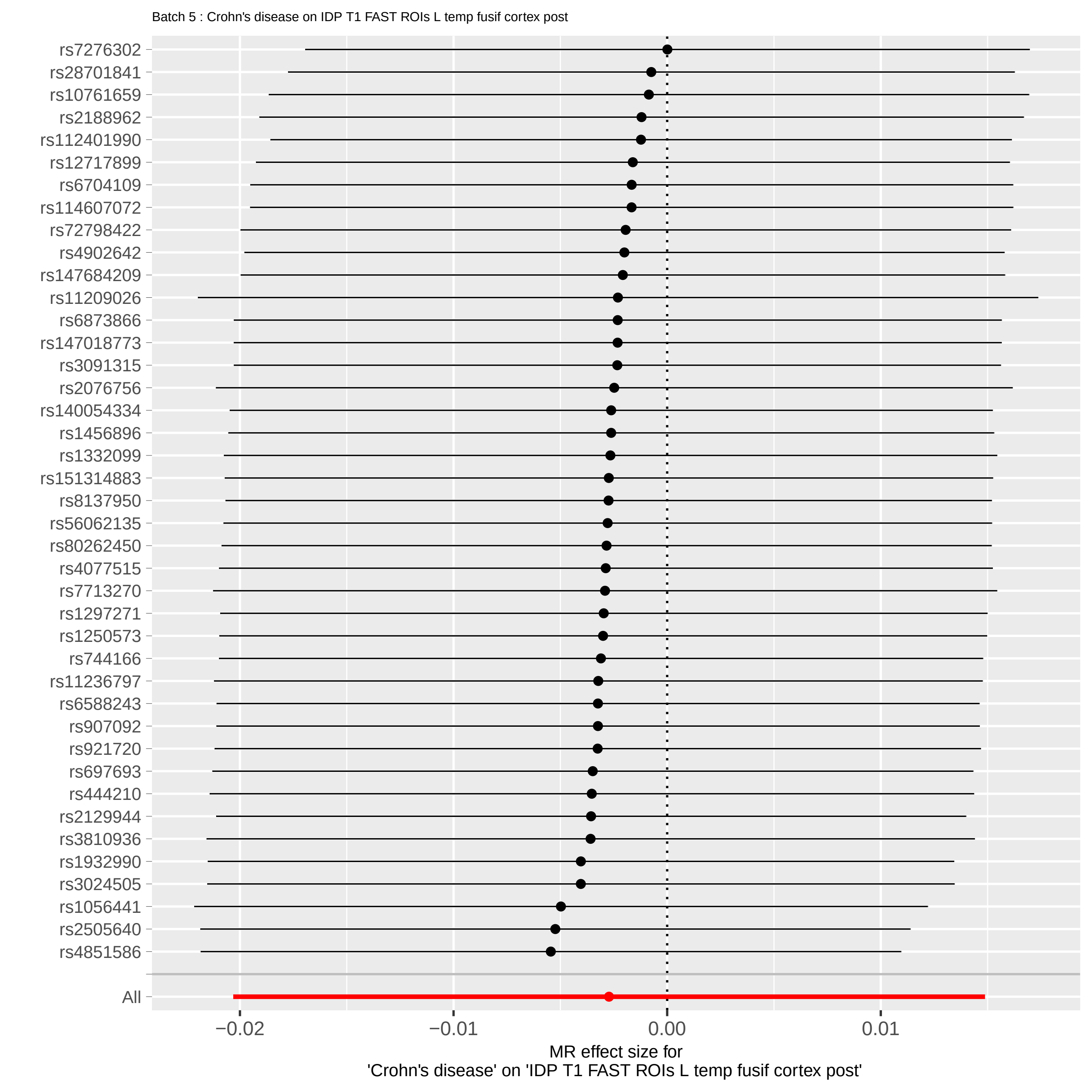

Batch 5 : Crohn's disease on IDP T1 FAST ROIs L temp fusif cortex post
rs7276302
rs28701841
rs10761659
rs2188962
rs112401990
rs12717899
rs6704109
rs114607072
rs72798422
rs4902642
rs147684209
rs11209026
rs6873866
rs147018773
rs3091315
rs2076756
rs140054334
rs1456896
rs1332099
rs151314883
rs8137950
rs56062135
rs80262450
rs4077515
rs7713270
rs1297271
rs1250573
rs744166
rs11236797
rs6588243
rs907092
rs921720
rs697693
rs444210
rs2129944
rs3810936
rs1932990
rs3024505
rs1056441
rs2505640
rs4851586
All
−0.02
−0.01
0.00
0.01
MR effect size for
'Crohn's disease' on 'IDP T1 FAST ROIs L temp fusif cortex post'

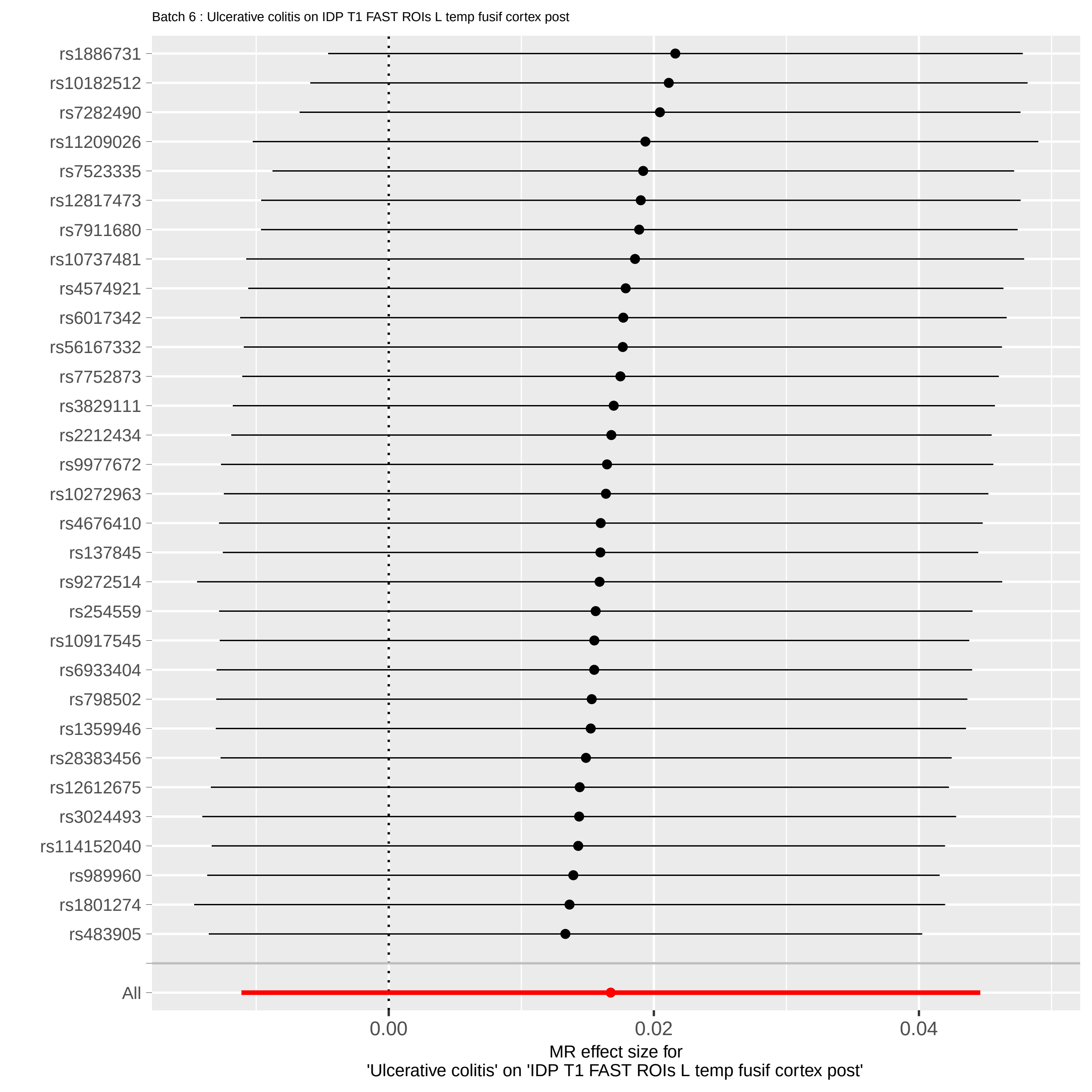
Batch 6 : Ulcerative colitis on IDP T1 FAST ROIs L temp fusif cortex post
rs1886731
rs10182512
rs7282490
rs11209026
rs7523335
rs12817473
rs7911680
rs10737481
rs4574921
rs6017342
rs56167332
rs7752873
rs3829111
rs2212434
rs9977672
rs10272963
rs4676410
rs137845
rs9272514
rs254559
rs10917545
rs6933404
rs798502
rs1359946
rs28383456
rs12612675
rs3024493
rs114152040
rs989960
rs1801274
rs483905
All
0.00
0.02
0.04
MR effect size for
'Ulcerative colitis' on 'IDP T1 FAST ROIs L temp fusif cortex post'

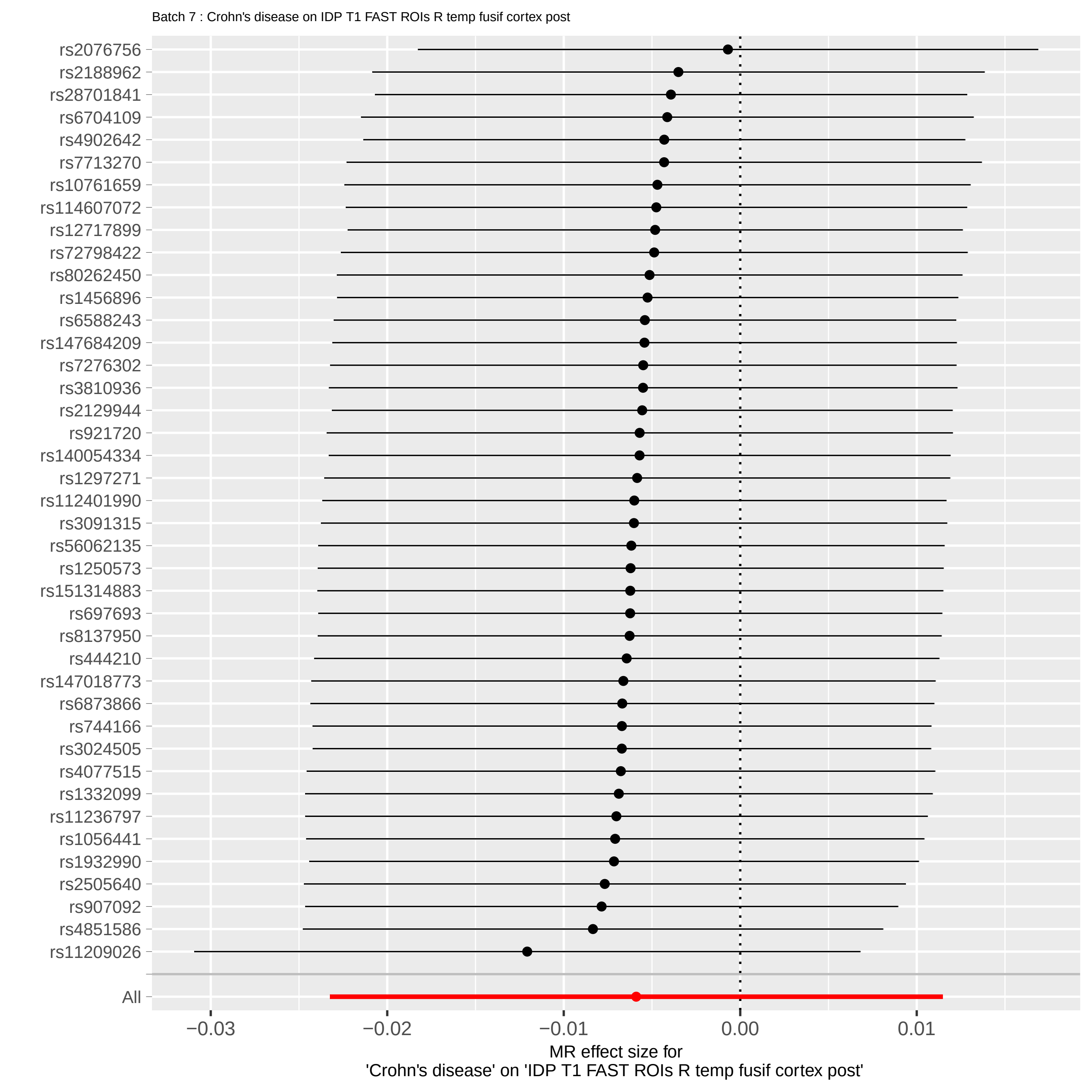

Batch 7 : Crohn's disease on IDP T1 FAST ROIs R temp fusif cortex post
rs2076756
rs2188962
rs28701841
rs6704109
rs4902642
rs7713270
rs10761659
rs114607072
rs12717899
rs72798422
rs80262450
rs1456896
rs6588243
rs147684209
rs7276302
rs3810936
rs2129944
rs921720
rs140054334
rs1297271
rs112401990
rs3091315
rs56062135
rs1250573
rs151314883
rs697693
rs8137950
rs444210
rs147018773
rs6873866
rs744166
rs3024505
rs4077515
rs1332099
rs11236797
rs1056441
rs1932990
rs2505640
rs907092
rs4851586
rs11209026
All
−0.03
−0.02
−0.01
0.00
0.01
MR effect size for
'Crohn's disease' on 'IDP T1 FAST ROIs R temp fusif cortex post'

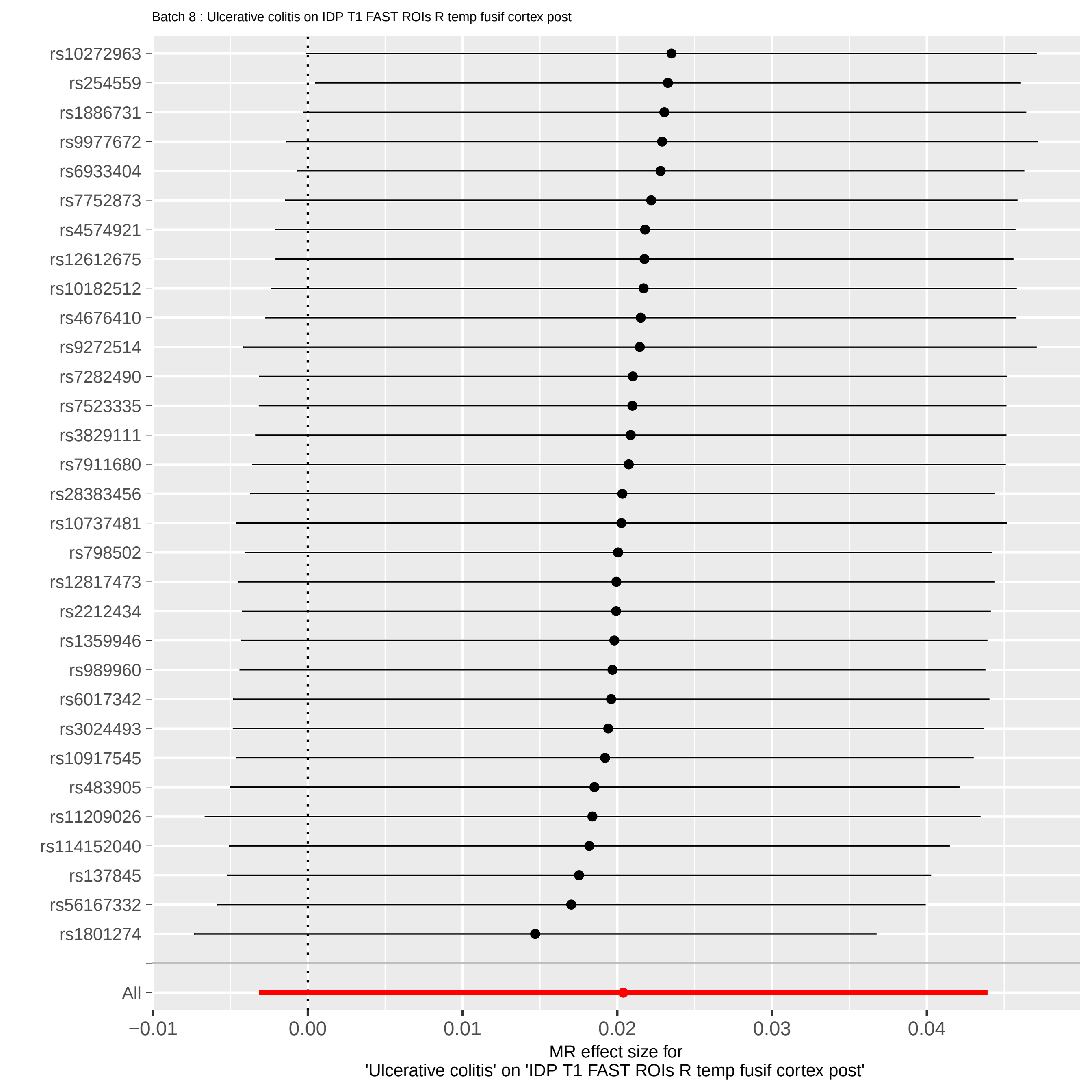

Batch 8 : Ulcerative colitis on IDP T1 FAST ROIs R temp fusif cortex post
rs10272963
rs254559
rs1886731
rs9977672
rs6933404
rs7752873
rs4574921
rs12612675
rs10182512
rs4676410
rs9272514
rs7282490
rs7523335
rs3829111
rs7911680
rs28383456
rs10737481
rs798502
rs12817473
rs2212434
rs1359946
rs989960
rs6017342
rs3024493
rs10917545
rs483905
rs11209026
rs114152040
rs137845
rs56167332
rs1801274
All
−0.01
0.00
0.01
0.02
0.03
0.04
MR effect size for
'Ulcerative colitis' on 'IDP T1 FAST ROIs R temp fusif cortex post'

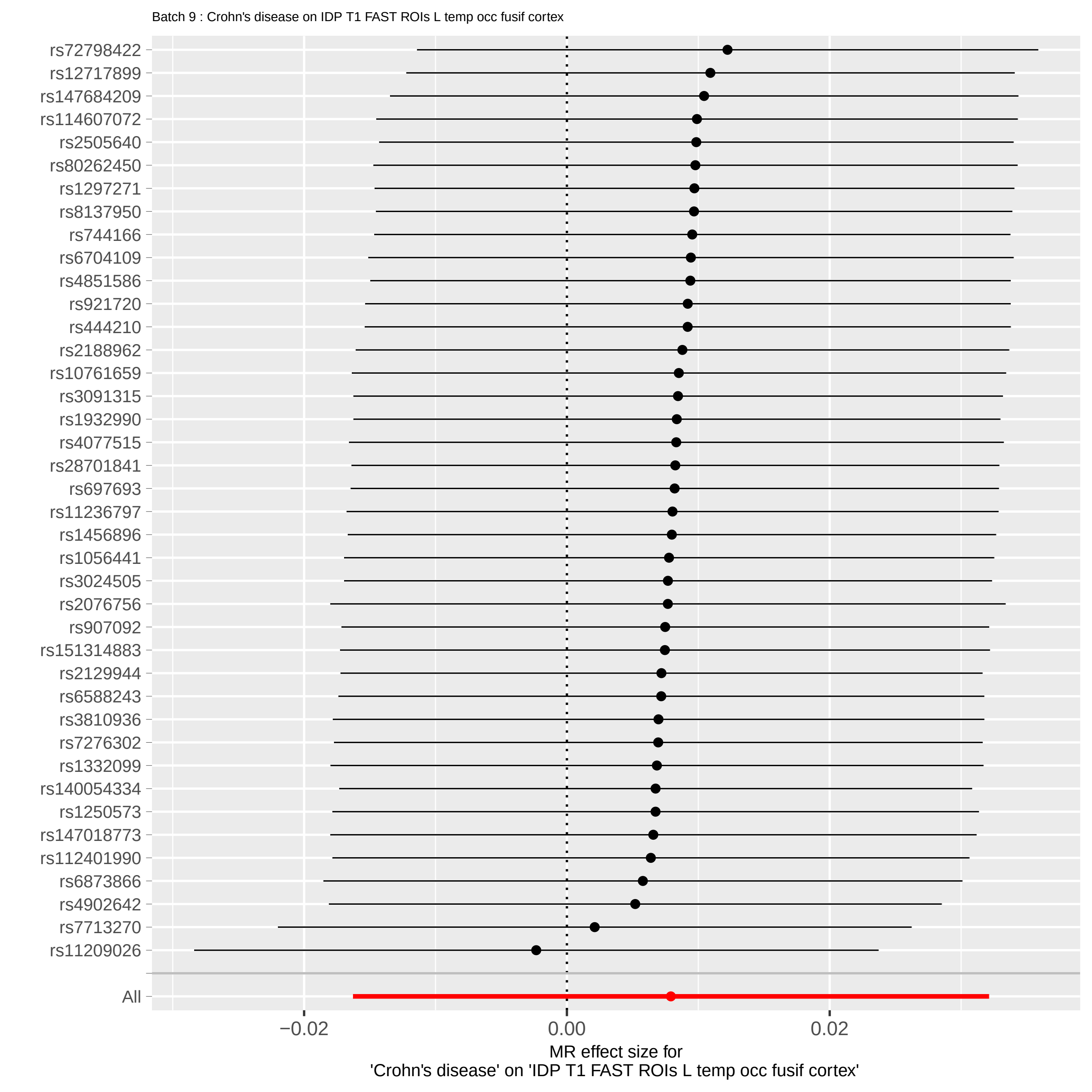

Batch 9 : Crohn's disease on IDP T1 FAST ROIs L temp occ fusif cortex
rs72798422
rs12717899
rs147684209
rs114607072
rs2505640
rs80262450
rs1297271
rs8137950
rs744166
rs6704109
rs4851586
rs921720
rs444210
rs2188962
rs10761659
rs3091315
rs1932990
rs4077515
rs28701841
rs697693
rs11236797
rs1456896
rs1056441
rs3024505
rs2076756
rs907092
rs151314883
rs2129944
rs6588243
rs3810936
rs7276302
rs1332099
rs140054334
rs1250573
rs147018773
rs112401990
rs6873866
rs4902642
rs7713270
rs11209026
All
−0.02
0.00
0.02
MR effect size for
'Crohn's disease' on 'IDP T1 FAST ROIs L temp occ fusif cortex'

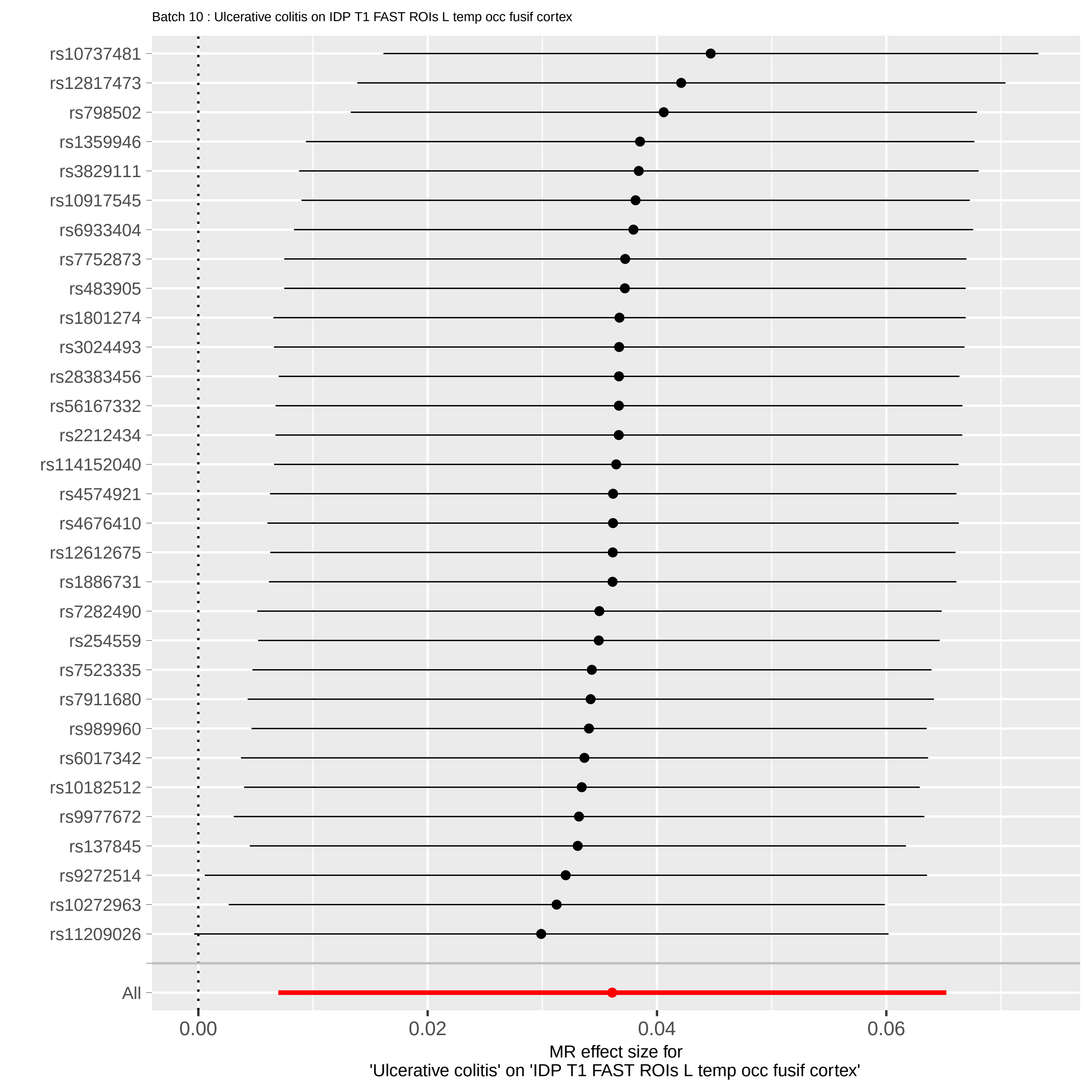

Batch 10 : Ulcerative colitis on IDP T1 FAST ROIs L temp occ fusif cortex
rs10737481
rs12817473
rs798502
rs1359946
rs3829111
rs10917545
rs6933404
rs7752873
rs483905
rs1801274
rs3024493
rs28383456
rs56167332
rs2212434
rs114152040
rs4574921
rs4676410
rs12612675
rs1886731
rs7282490
rs254559
rs7523335
rs7911680
rs989960
rs6017342
rs10182512
rs9977672
rs137845
rs9272514
rs10272963
rs11209026
All
0.00
0.02
0.04
0.06
MR effect size for
'Ulcerative colitis' on 'IDP T1 FAST ROIs L temp occ fusif cortex'

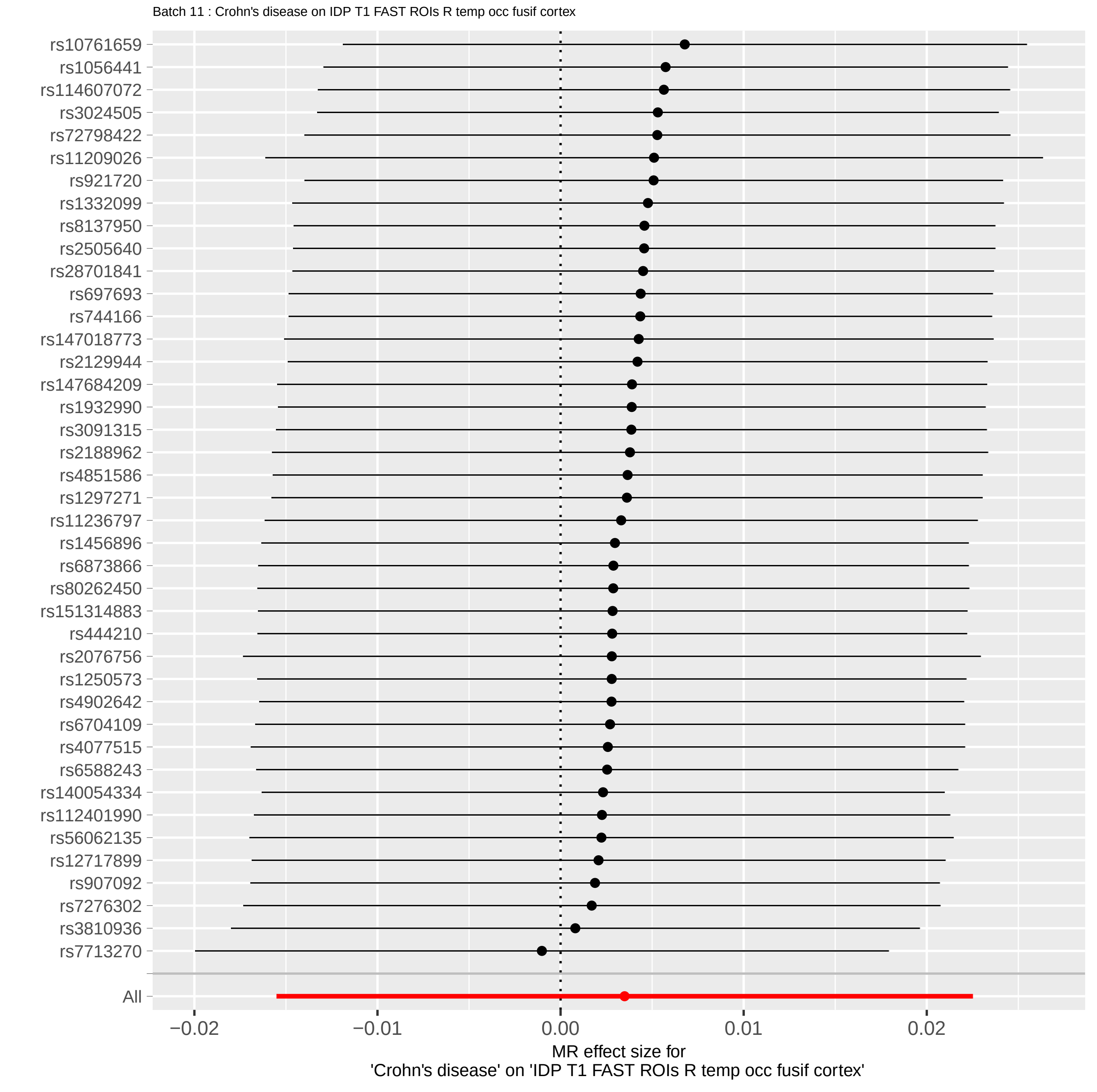

Batch 11 : Crohn's disease on IDP T1 FAST ROIs R temp occ fusif cortex
rs10761659
rs1056441
rs114607072
rs3024505
rs72798422
rs11209026
rs921720
rs1332099
rs8137950
rs2505640
rs28701841
rs697693
rs744166
rs147018773
rs2129944
rs147684209
rs1932990
rs3091315
rs2188962
rs4851586
rs1297271
rs11236797
rs1456896
rs6873866
rs80262450
rs151314883
rs444210
rs2076756
rs1250573
rs4902642
rs6704109
rs4077515
rs6588243
rs140054334
rs112401990
rs56062135
rs12717899
rs907092
rs7276302
rs3810936
rs7713270
All
−0.02
−0.01
0.00
0.01
0.02
MR effect size for
'Crohn's disease' on 'IDP T1 FAST ROIs R temp occ fusif cortex'

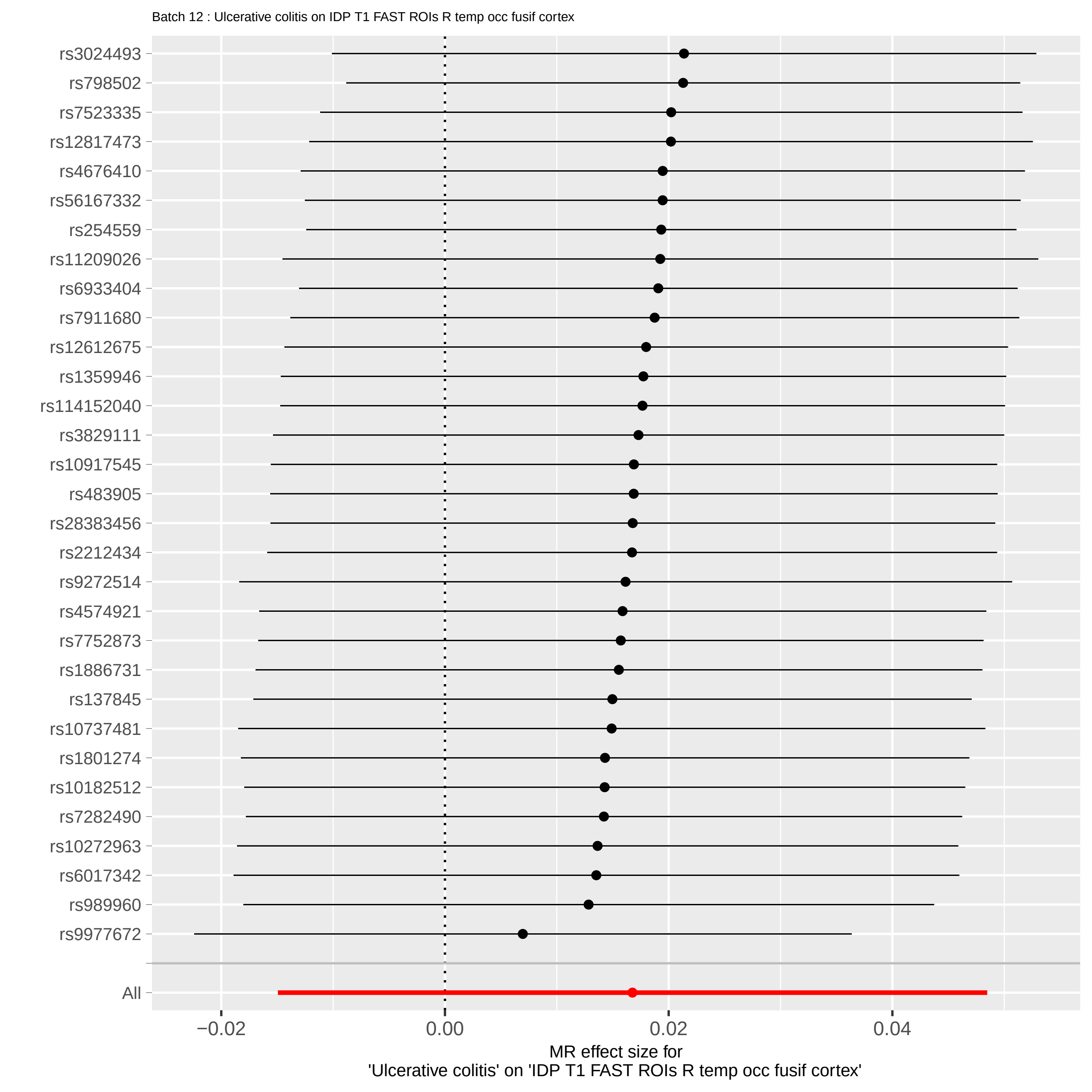

Batch 12 : Ulcerative colitis on IDP T1 FAST ROIs R temp occ fusif cortex
rs3024493
rs798502
rs7523335
rs12817473
rs4676410
rs56167332
rs254559
rs11209026
rs6933404
rs7911680
rs12612675
rs1359946
rs114152040
rs3829111
rs10917545
rs483905
rs28383456
rs2212434
rs9272514
rs4574921
rs7752873
rs1886731
rs137845
rs10737481
rs1801274
rs10182512
rs7282490
rs10272963
rs6017342
rs989960
rs9977672
All
−0.02
0.00
0.02
0.04
MR effect size for
'Ulcerative colitis' on 'IDP T1 FAST ROIs R temp occ fusif cortex'

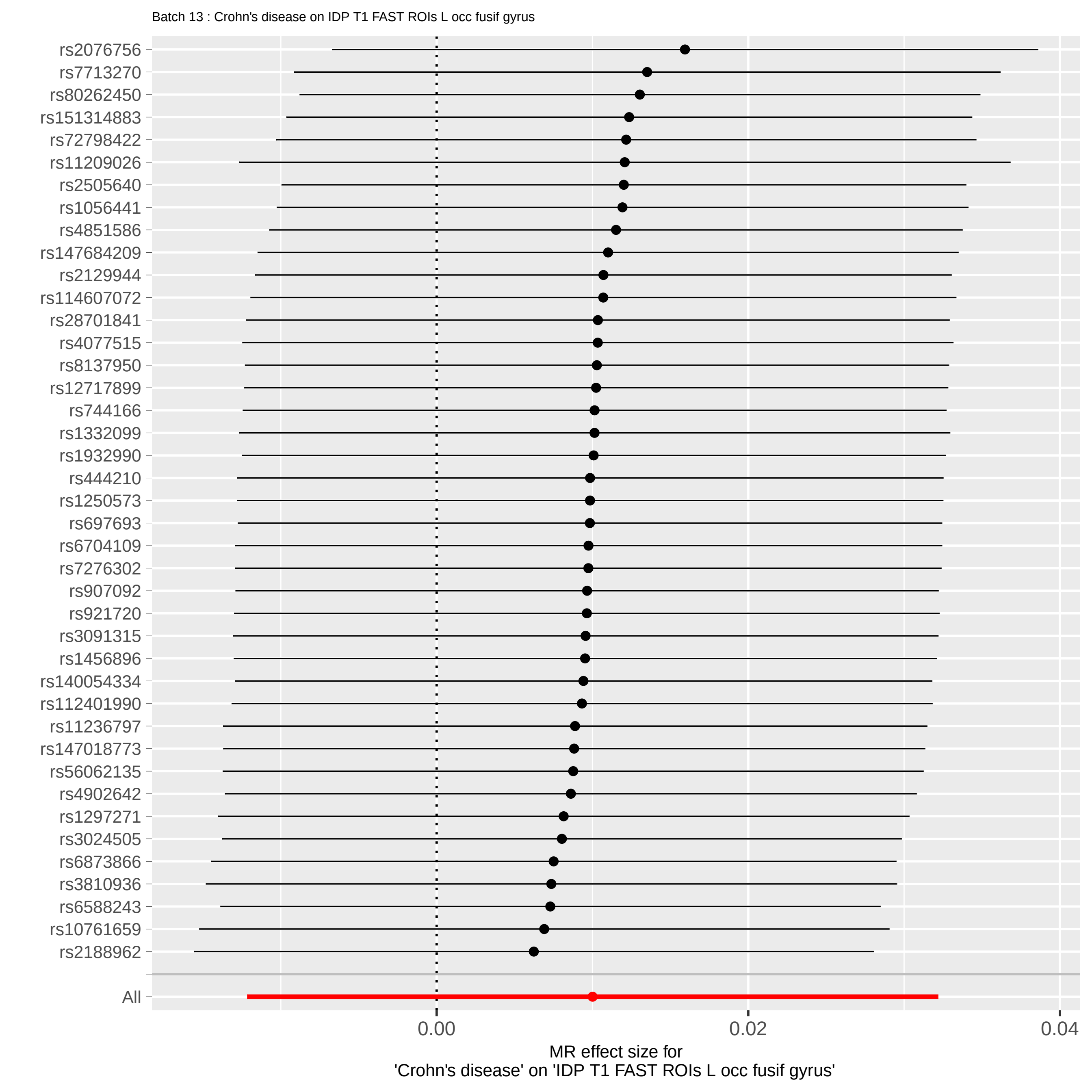

Batch 13 : Crohn's disease on IDP T1 FAST ROIs L occ fusif gyrus
rs2076756
rs7713270
rs80262450
rs151314883
rs72798422
rs11209026
rs2505640
rs1056441
rs4851586
rs147684209
rs2129944
rs114607072
rs28701841
rs4077515
rs8137950
rs12717899
rs744166
rs1332099
rs1932990
rs444210
rs1250573
rs697693
rs6704109
rs7276302
rs907092
rs921720
rs3091315
rs1456896
rs140054334
rs112401990
rs11236797
rs147018773
rs56062135
rs4902642
rs1297271
rs3024505
rs6873866
rs3810936
rs6588243
rs10761659
rs2188962
All
0.00
0.02
0.04
MR effect size for
'Crohn's disease' on 'IDP T1 FAST ROIs L occ fusif gyrus'

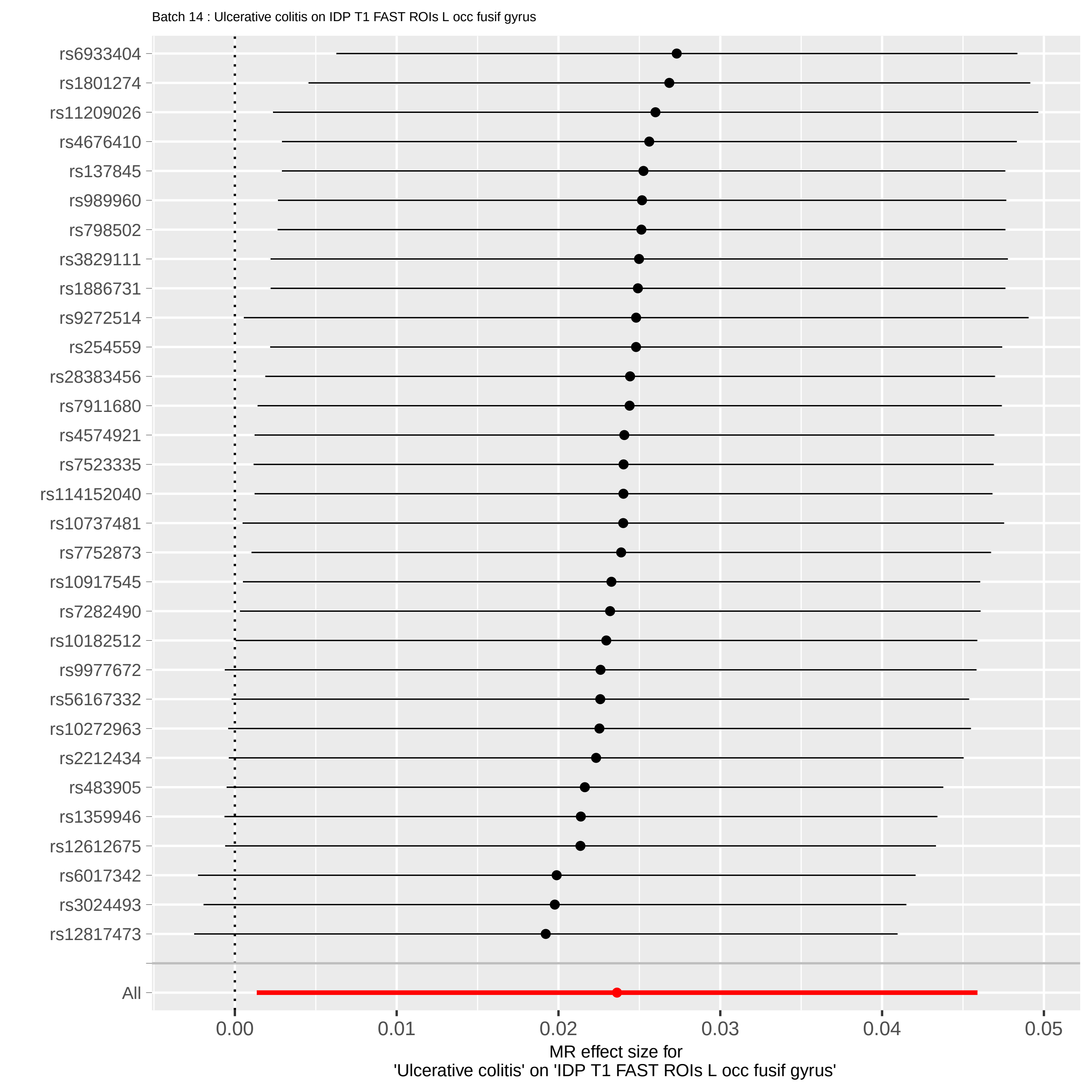

Batch 14 : Ulcerative colitis on IDP T1 FAST ROIs L occ fusif gyrus
rs6933404
rs1801274
rs11209026
rs4676410
rs137845
rs989960
rs798502
rs3829111
rs1886731
rs9272514
rs254559
rs28383456
rs7911680
rs4574921
rs7523335
rs114152040
rs10737481
rs7752873
rs10917545
rs7282490
rs10182512
rs9977672
rs56167332
rs10272963
rs2212434
rs483905
rs1359946
rs12612675
rs6017342
rs3024493
rs12817473
All
0.00
0.01
0.02
0.03
0.04
0.05
MR effect size for
'Ulcerative colitis' on 'IDP T1 FAST ROIs L occ fusif gyrus'

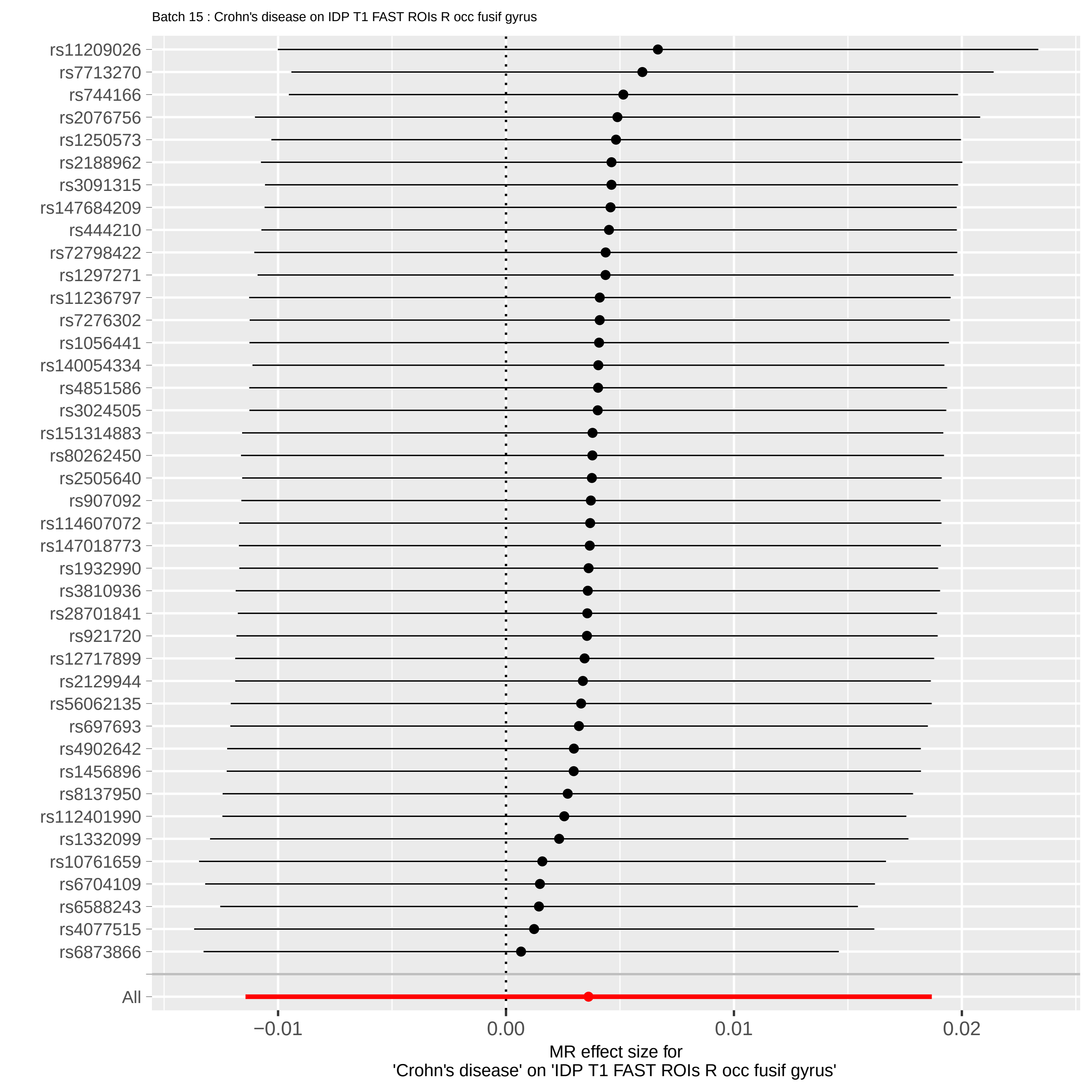

Batch 15 : Crohn's disease on IDP T1 FAST ROIs R occ fusif gyrus
rs11209026
rs7713270
rs744166
rs2076756
rs1250573
rs2188962
rs3091315
rs147684209
rs444210
rs72798422
rs1297271
rs11236797
rs7276302
rs1056441
rs140054334
rs4851586
rs3024505
rs151314883
rs80262450
rs2505640
rs907092
rs114607072
rs147018773
rs1932990
rs3810936
rs28701841
rs921720
rs12717899
rs2129944
rs56062135
rs697693
rs4902642
rs1456896
rs8137950
rs112401990
rs1332099
rs10761659
rs6704109
rs6588243
rs4077515
rs6873866
All
−0.01
0.00
0.01
0.02
MR effect size for
'Crohn's disease' on 'IDP T1 FAST ROIs R occ fusif gyrus'

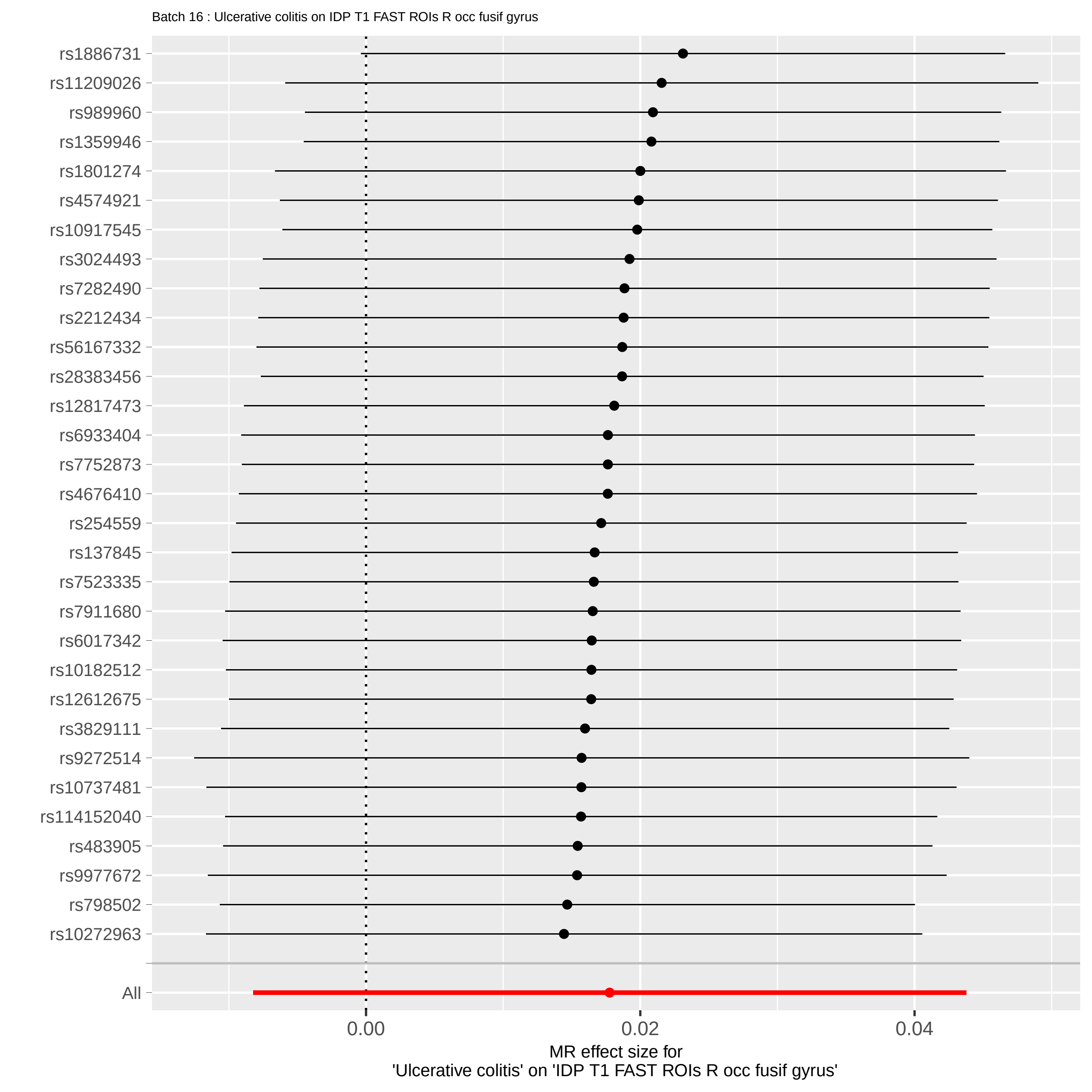
Batch 16 : Ulcerative colitis on IDP T1 FAST ROIs R occ fusif gyrus
rs1886731
rs11209026
rs989960
rs1359946
rs1801274
rs4574921
rs10917545
rs3024493
rs7282490
rs2212434
rs56167332
rs28383456
rs12817473
rs6933404
rs7752873
rs4676410
rs254559
rs137845
rs7523335
rs7911680
rs6017342
rs10182512
rs12612675
rs3829111
rs9272514
rs10737481
rs114152040
rs483905
rs9977672
rs798502
rs10272963
All
0.00
0.02
0.04
MR effect size for
'Ulcerative colitis' on 'IDP T1 FAST ROIs R occ fusif gyrus'

Batch 17 : Crohn's disease on IDP T1 FAST ROIs L front operc cortex

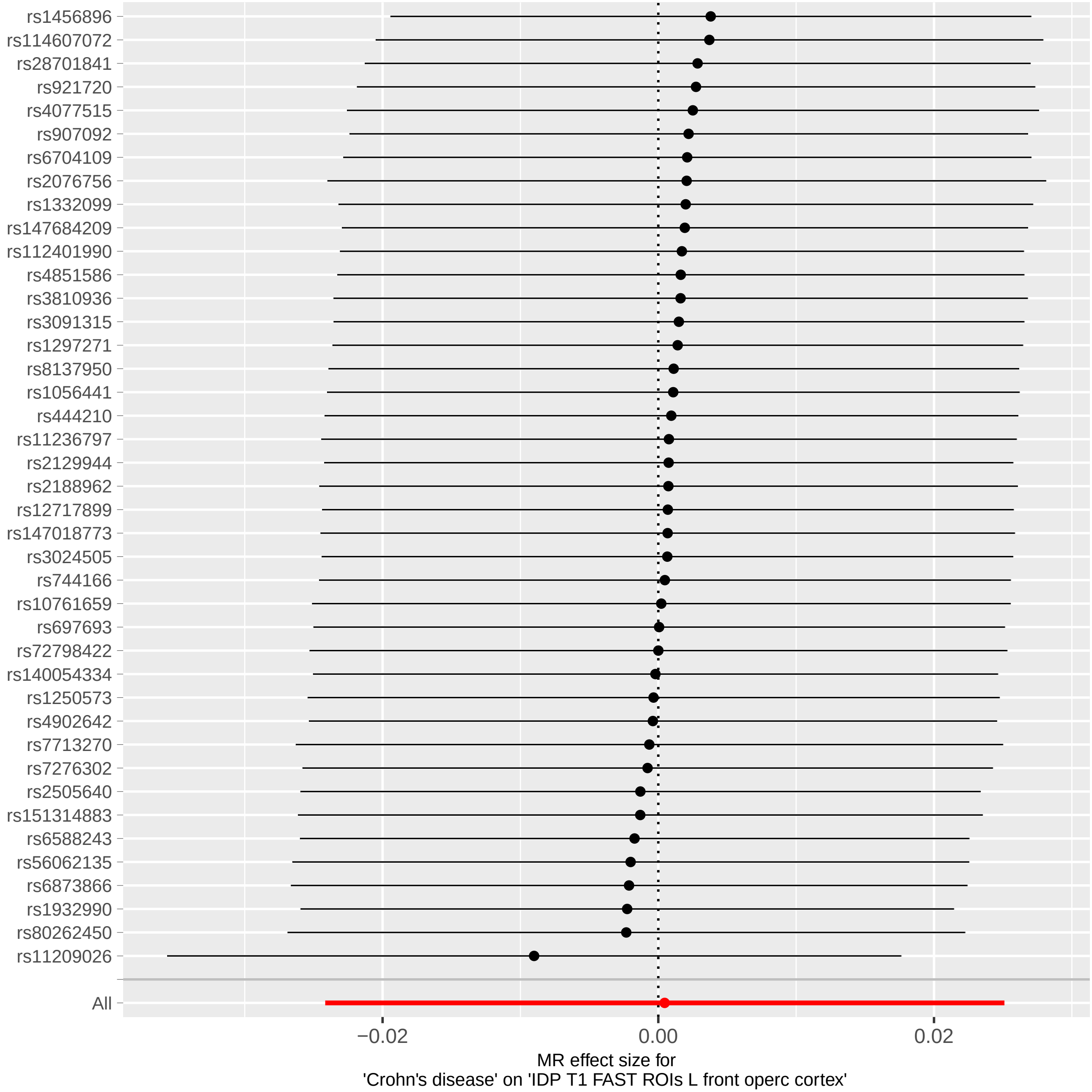

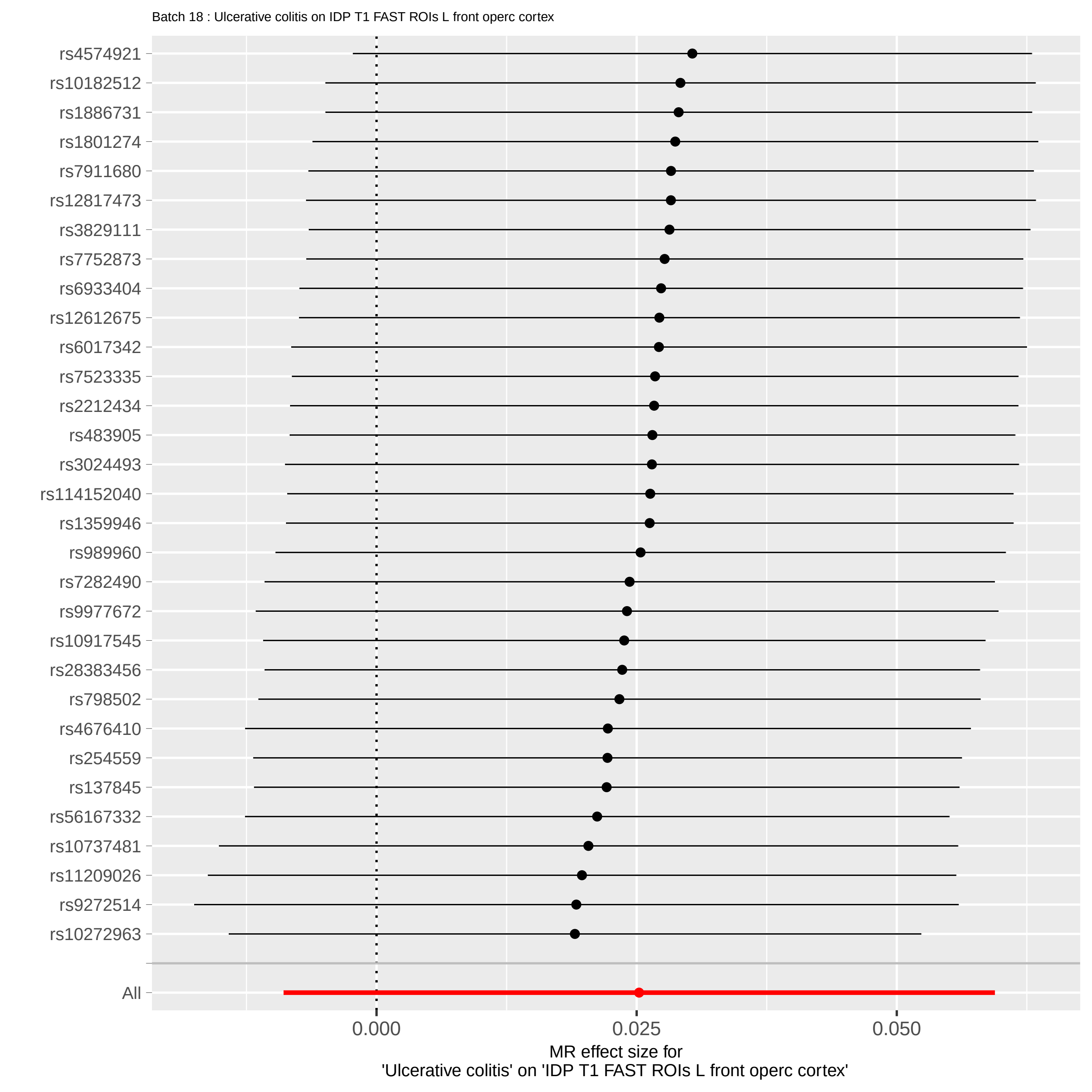
Batch 18 : Ulcerative colitis on IDP T1 FAST ROIs L front operc cortex
rs4574921
rs10182512
rs1886731
rs1801274
rs7911680
rs12817473
rs3829111
rs7752873
rs6933404
rs12612675
rs6017342
rs7523335
rs2212434
rs483905
rs3024493
rs114152040
rs1359946
rs989960
rs7282490
rs9977672
rs10917545
rs28383456
rs798502
rs4676410
rs254559
rs137845
rs56167332
rs10737481
rs11209026
rs9272514
rs10272963
All
0.000
0.025
0.050
MR effect size for
'Ulcerative colitis' on 'IDP T1 FAST ROIs L front operc cortex'

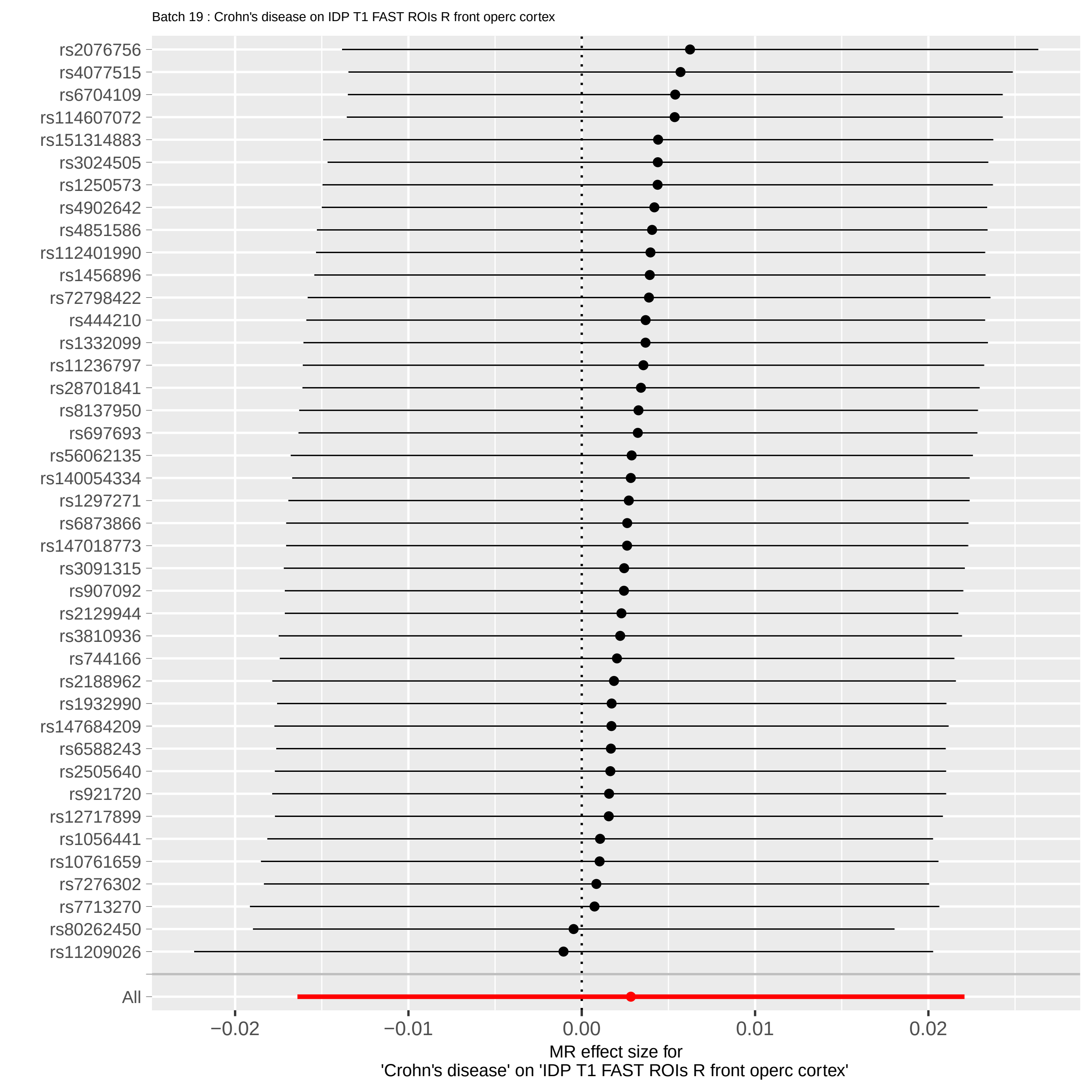

Batch 19 : Crohn's disease on IDP T1 FAST ROIs R front operc cortex
rs2076756
rs4077515
rs6704109
rs114607072
rs151314883
rs3024505
rs1250573
rs4902642
rs4851586
rs112401990
rs1456896
rs72798422
rs444210
rs1332099
rs11236797
rs28701841
rs8137950
rs697693
rs56062135
rs140054334
rs1297271
rs6873866
rs147018773
rs3091315
rs907092
rs2129944
rs3810936
rs744166
rs2188962
rs1932990
rs147684209
rs6588243
rs2505640
rs921720
rs12717899
rs1056441
rs10761659
rs7276302
rs7713270
rs80262450
rs11209026
All
−0.02
−0.01
0.00
0.01
0.02
MR effect size for
'Crohn's disease' on 'IDP T1 FAST ROIs R front operc cortex'

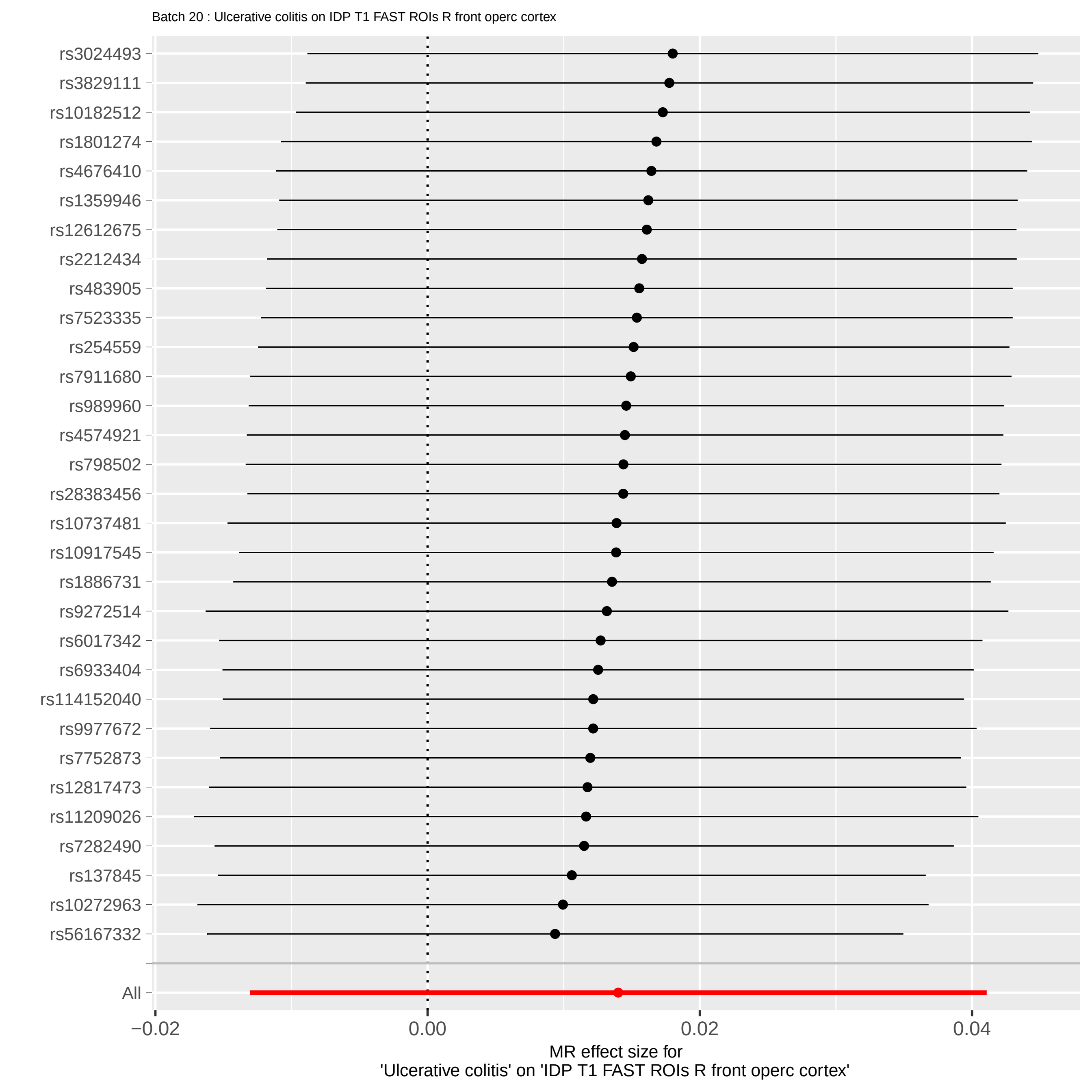

Batch 20 : Ulcerative colitis on IDP T1 FAST ROIs R front operc cortex
rs3024493
rs3829111
rs10182512
rs1801274
rs4676410
rs1359946
rs12612675
rs2212434
rs483905
rs7523335
rs254559
rs7911680
rs989960
rs4574921
rs798502
rs28383456
rs10737481
rs10917545
rs1886731
rs9272514
rs6017342
rs6933404
rs114152040
rs9977672
rs7752873
rs12817473
rs11209026
rs7282490
rs137845
rs10272963
rs56167332
All
−0.02
0.00
0.02
0.04
MR effect size for
'Ulcerative colitis' on 'IDP T1 FAST ROIs R front operc cortex'

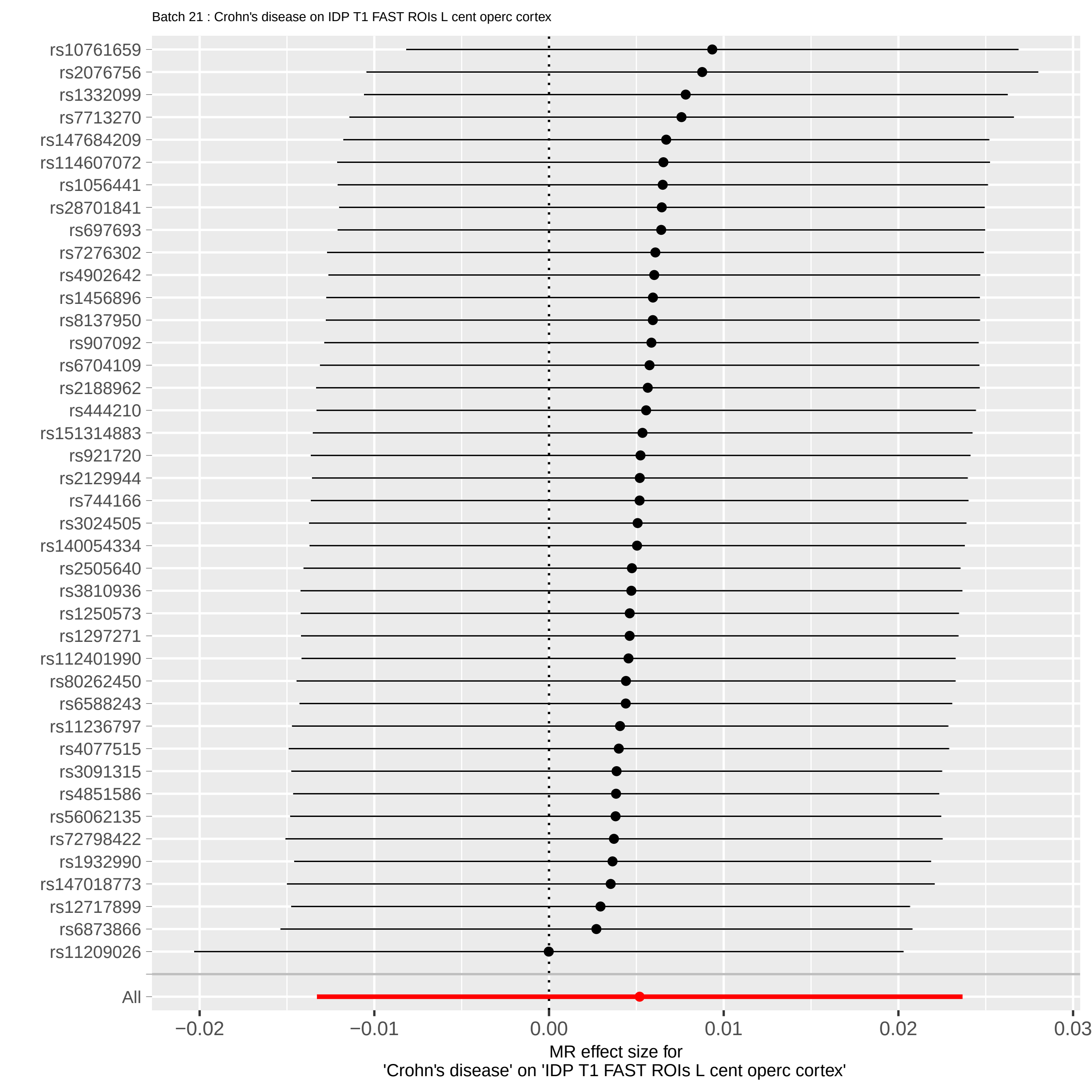

Batch 21 : Crohn's disease on IDP T1 FAST ROIs L cent operc cortex
rs10761659
rs2076756
rs1332099
rs7713270
rs147684209
rs114607072
rs1056441
rs28701841
rs697693
rs7276302
rs4902642
rs1456896
rs8137950
rs907092
rs6704109
rs2188962
rs444210
rs151314883
rs921720
rs2129944
rs744166
rs3024505
rs140054334
rs2505640
rs3810936
rs1250573
rs1297271
rs112401990
rs80262450
rs6588243
rs11236797
rs4077515
rs3091315
rs4851586
rs56062135
rs72798422
rs1932990
rs147018773
rs12717899
rs6873866
rs11209026
All
−0.02
−0.01
0.00
0.01
0.02
0.03
MR effect size for
'Crohn's disease' on 'IDP T1 FAST ROIs L cent operc cortex'

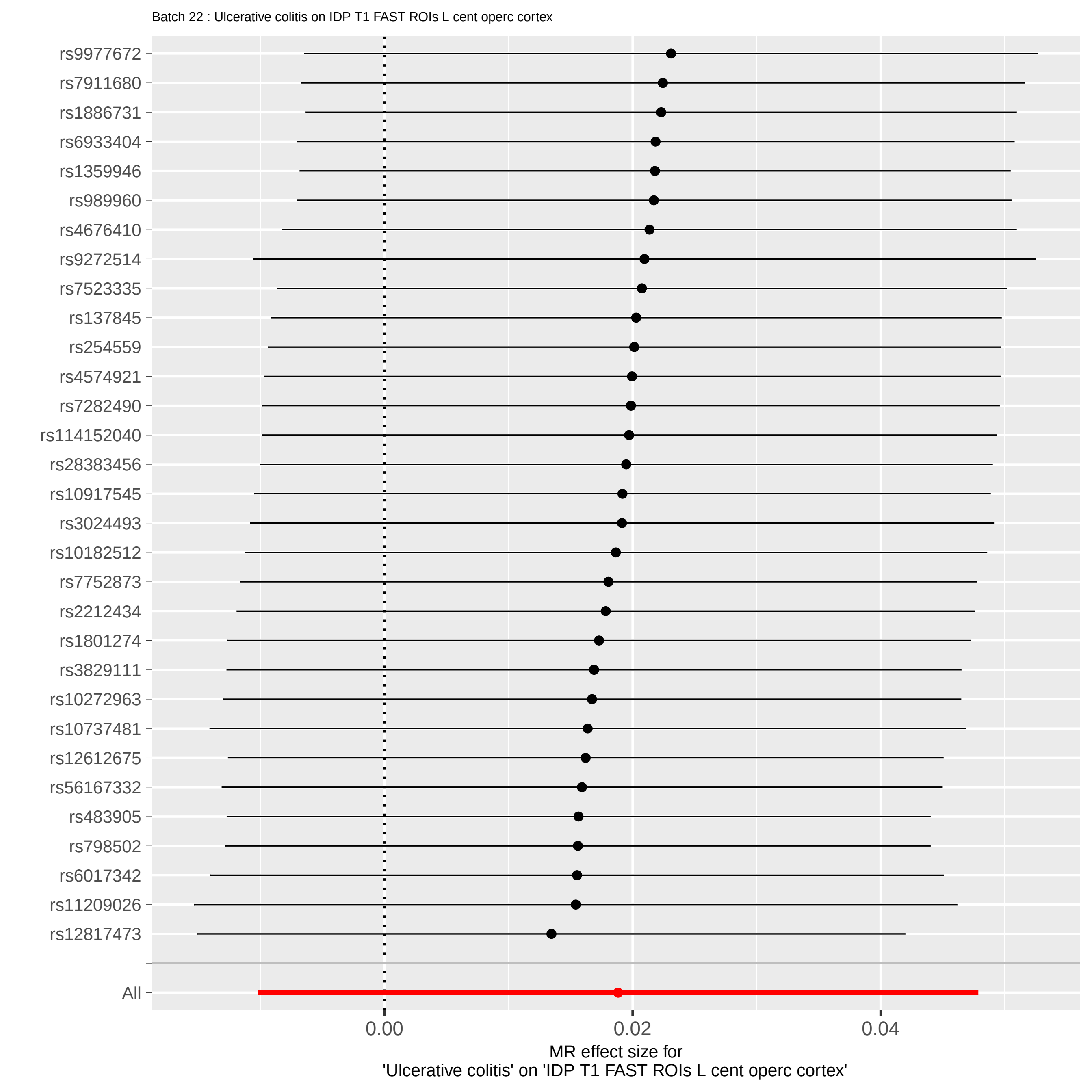

Batch 22 : Ulcerative colitis on IDP T1 FAST ROIs L cent operc cortex
rs9977672
rs7911680
rs1886731
rs6933404
rs1359946
rs989960
rs4676410
rs9272514
rs7523335
rs137845
rs254559
rs4574921
rs7282490
rs114152040
rs28383456
rs10917545
rs3024493
rs10182512
rs7752873
rs2212434
rs1801274
rs3829111
rs10272963
rs10737481
rs12612675
rs56167332
rs483905
rs798502
rs6017342
rs11209026
rs12817473
All
0.00
0.02
0.04
MR effect size for
'Ulcerative colitis' on 'IDP T1 FAST ROIs L cent operc cortex'

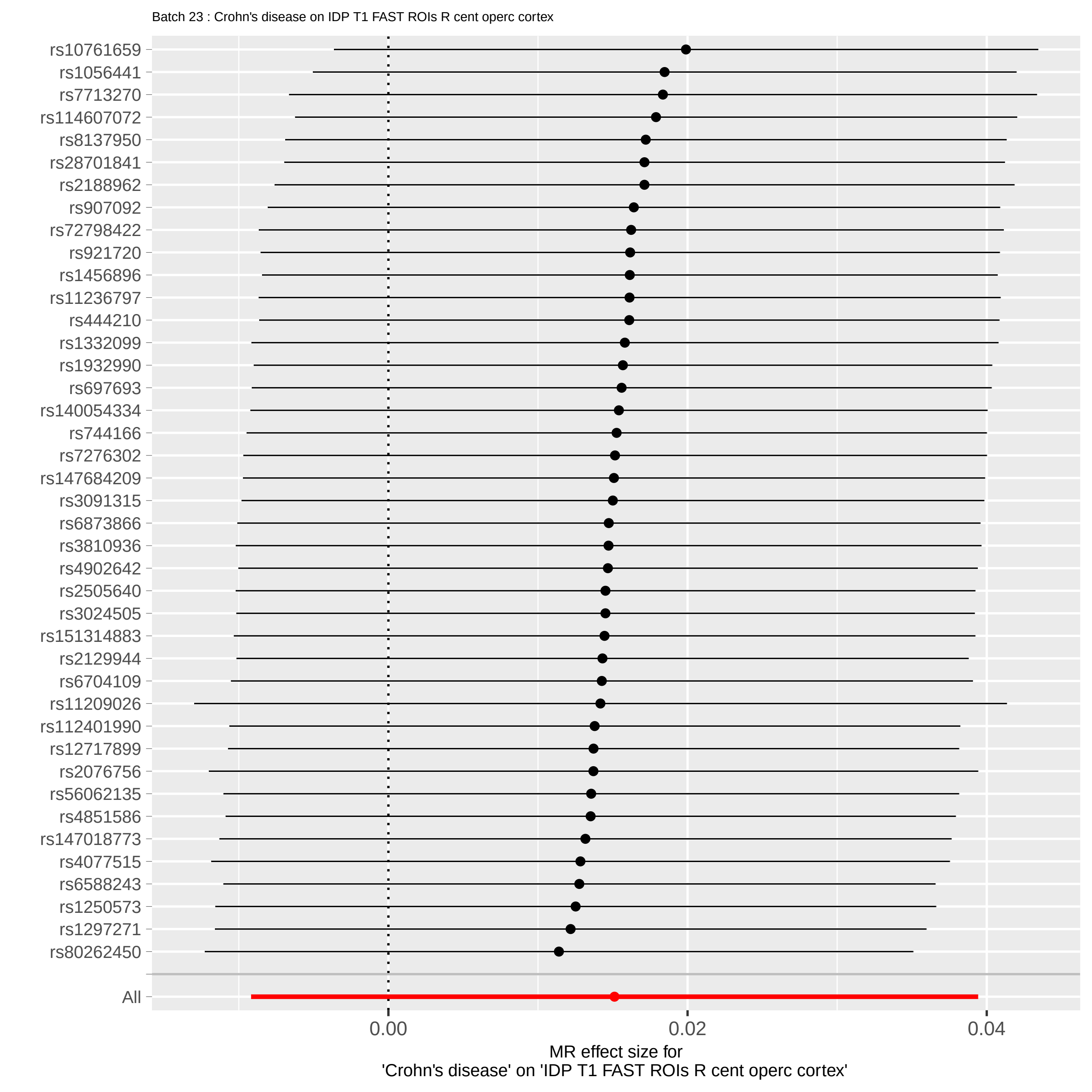
Batch 23 : Crohn's disease on IDP T1 FAST ROIs R cent operc cortex
rs10761659
rs1056441
rs7713270
rs114607072
rs8137950
rs28701841
rs2188962
rs907092
rs72798422
rs921720
rs1456896
rs11236797
rs444210
rs1332099
rs1932990
rs697693
rs140054334
rs744166
rs7276302
rs147684209
rs3091315
rs6873866
rs3810936
rs4902642
rs2505640
rs3024505
rs151314883
rs2129944
rs6704109
rs11209026
rs112401990
rs12717899
rs2076756
rs56062135
rs4851586
rs147018773
rs4077515
rs6588243
rs1250573
rs1297271
rs80262450
All
0.00
0.02
0.04
MR effect size for
'Crohn's disease' on 'IDP T1 FAST ROIs R cent operc cortex'

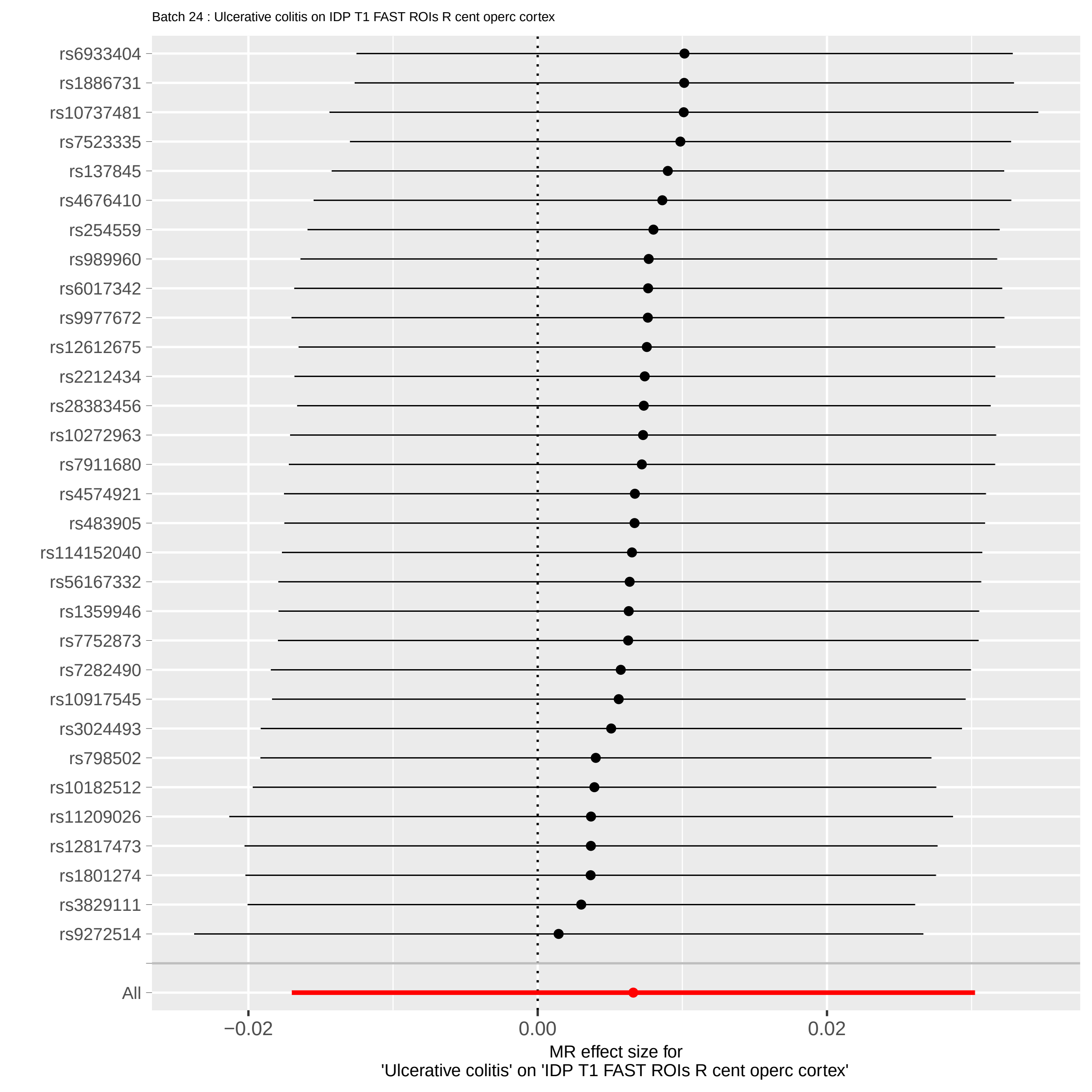

Batch 24 : Ulcerative colitis on IDP T1 FAST ROIs R cent operc cortex
rs6933404
rs1886731
rs10737481
rs7523335
rs137845
rs4676410
rs254559
rs989960
rs6017342
rs9977672
rs12612675
rs2212434
rs28383456
rs10272963
rs7911680
rs4574921
rs483905
rs114152040
rs56167332
rs1359946
rs7752873
rs7282490
rs10917545
rs3024493
rs798502
rs10182512
rs11209026
rs12817473
rs1801274
rs3829111
rs9272514
All
−0.02
0.00
0.02
MR effect size for
'Ulcerative colitis' on 'IDP T1 FAST ROIs R cent operc cortex'

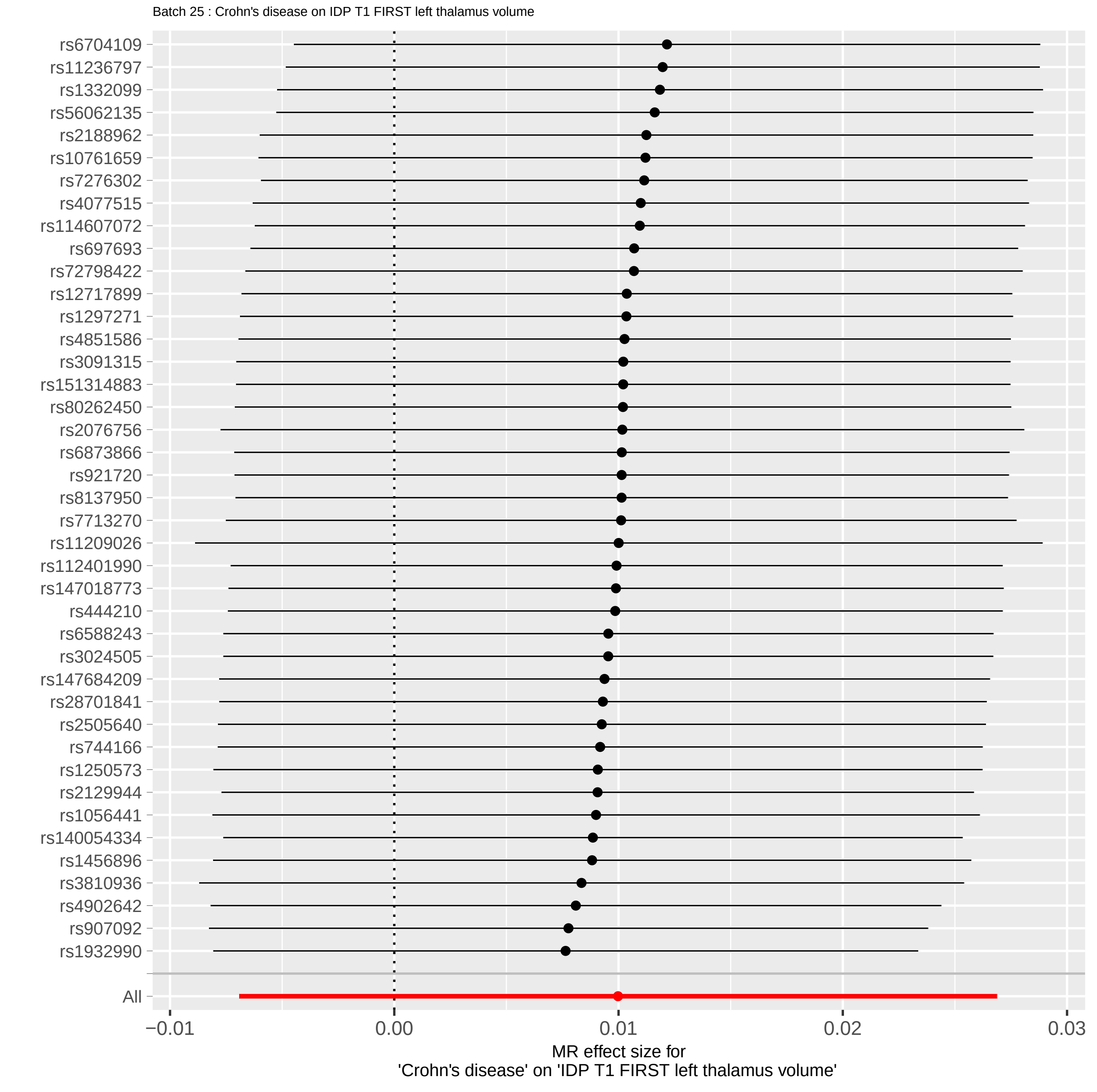
Batch 25 : Crohn's disease on IDP T1 FIRST left thalamus volume
rs6704109
rs11236797
rs1332099
rs56062135
rs2188962
rs10761659
rs7276302
rs4077515
rs114607072
rs697693
rs72798422
rs12717899
rs1297271
rs4851586
rs3091315
rs151314883
rs80262450
rs2076756
rs6873866
rs921720
rs8137950
rs7713270
rs11209026
rs112401990
rs147018773
rs444210
rs6588243
rs3024505
rs147684209
rs28701841
rs2505640
rs744166
rs1250573
rs2129944
rs1056441
rs140054334
rs1456896
rs3810936
rs4902642
rs907092
rs1932990
All
−0.01
0.00
0.01
0.02
0.03
MR effect size for
'Crohn's disease' on 'IDP T1 FIRST left thalamus volume'

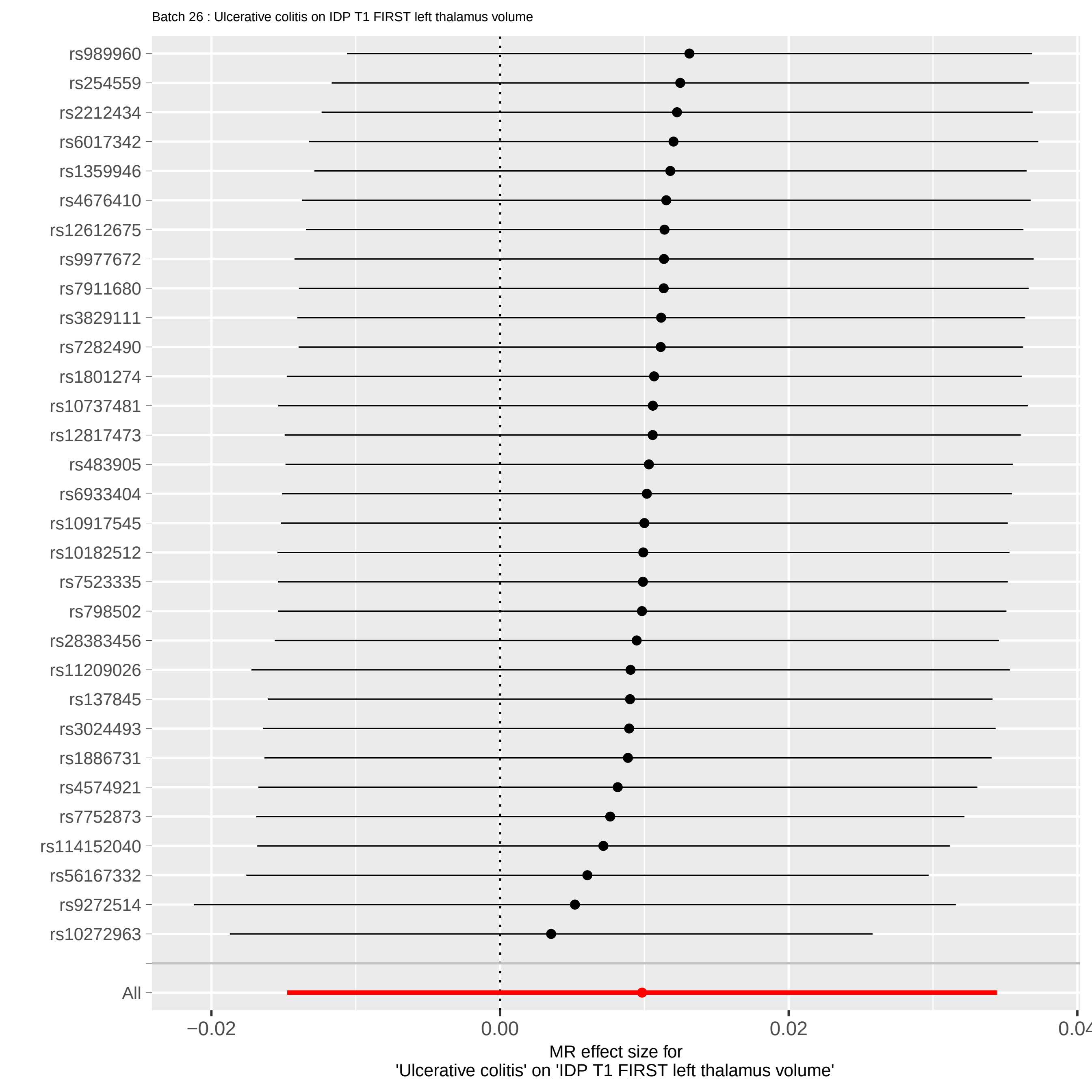

Batch 26 : Ulcerative colitis on IDP T1 FIRST left thalamus volume
rs989960
rs254559
rs2212434
rs6017342
rs1359946
rs4676410
rs12612675
rs9977672
rs7911680
rs3829111
rs7282490
rs1801274
rs10737481
rs12817473
rs483905
rs6933404
rs10917545
rs10182512
rs7523335
rs798502
rs28383456
rs11209026
rs137845
rs3024493
rs1886731
rs4574921
rs7752873
rs114152040
rs56167332
rs9272514
rs10272963
All
−0.02
0.00
0.02
0.04
MR effect size for
'Ulcerative colitis' on 'IDP T1 FIRST left thalamus volume'

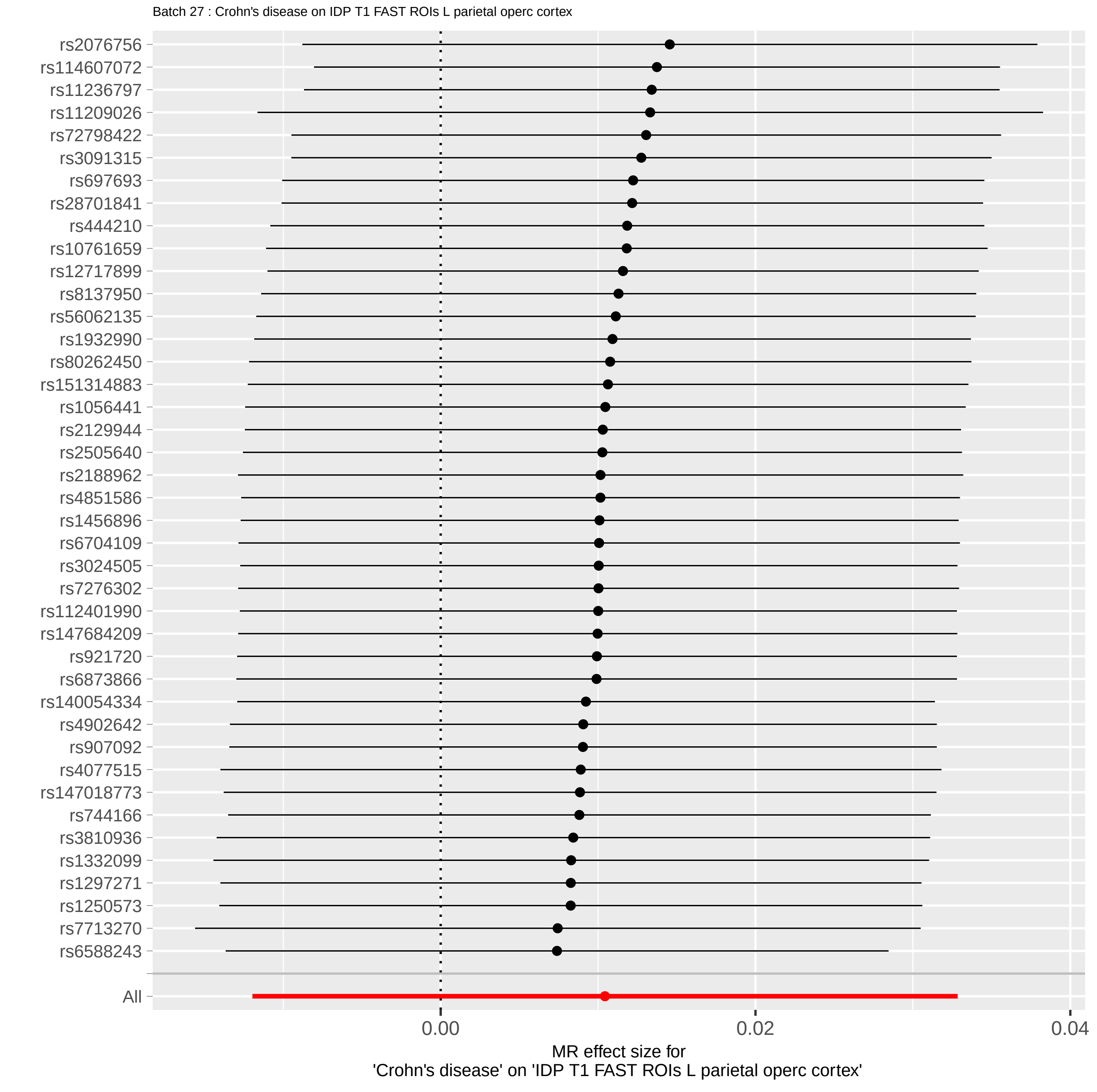

Batch 27 : Crohn's disease on IDP T1 FAST ROIs L parietal operc cortex
rs2076756
rs114607072
rs11236797
rs11209026
rs72798422
rs3091315
rs697693
rs28701841
rs444210
rs10761659
rs12717899
rs8137950
rs56062135
rs1932990
rs80262450
rs151314883
rs1056441
rs2129944
rs2505640
rs2188962
rs4851586
rs1456896
rs6704109
rs3024505
rs7276302
rs112401990
rs147684209
rs921720
rs6873866
rs140054334
rs4902642
rs907092
rs4077515
rs147018773
rs744166
rs3810936
rs1332099
rs1297271
rs1250573
rs7713270
rs6588243
All
0.00
0.02
0.04
MR effect size for
'Crohn's disease' on 'IDP T1 FAST ROIs L parietal operc cortex'

Batch 28 : Ulcerative colitis on IDP T1 FAST ROIs L parietal operc cortex

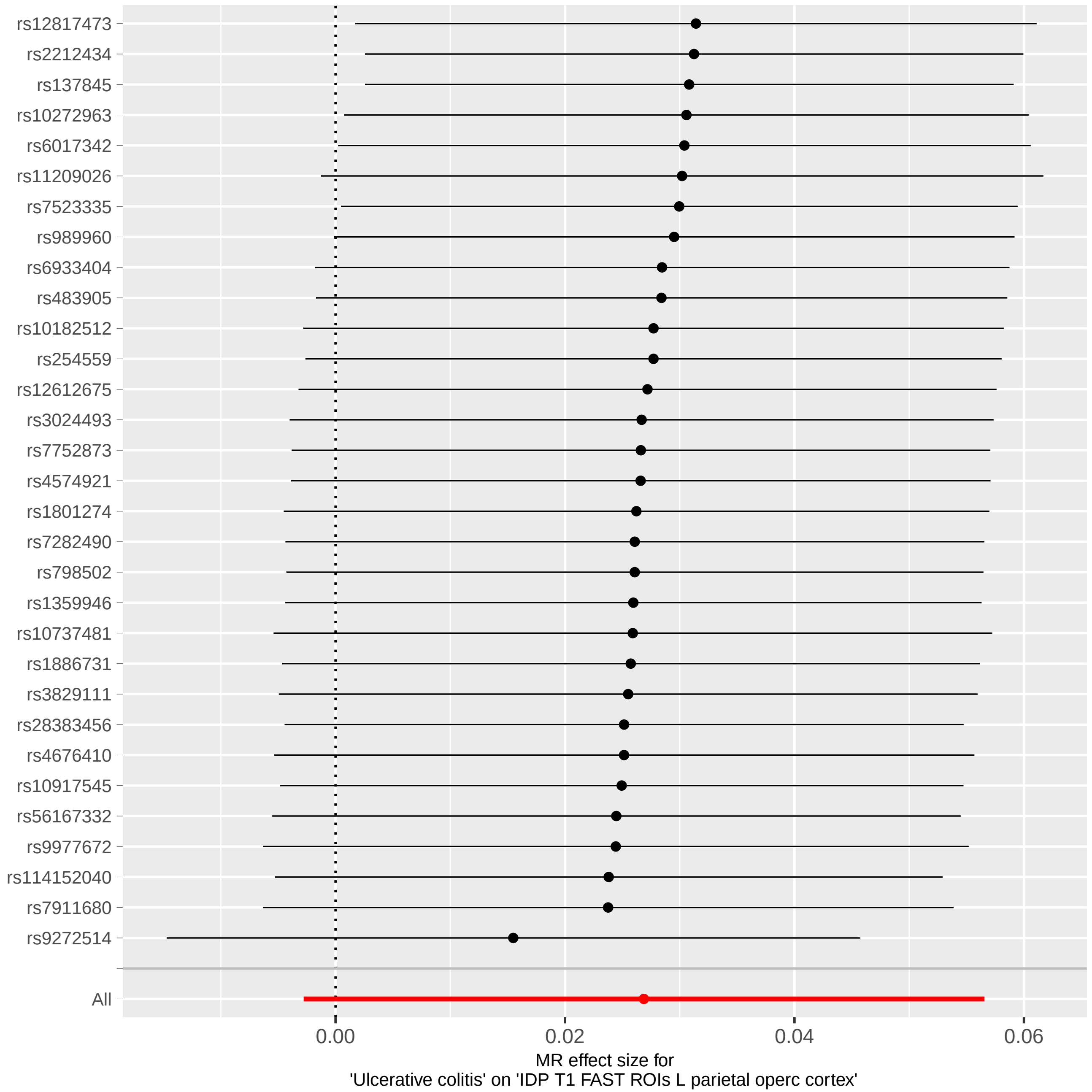

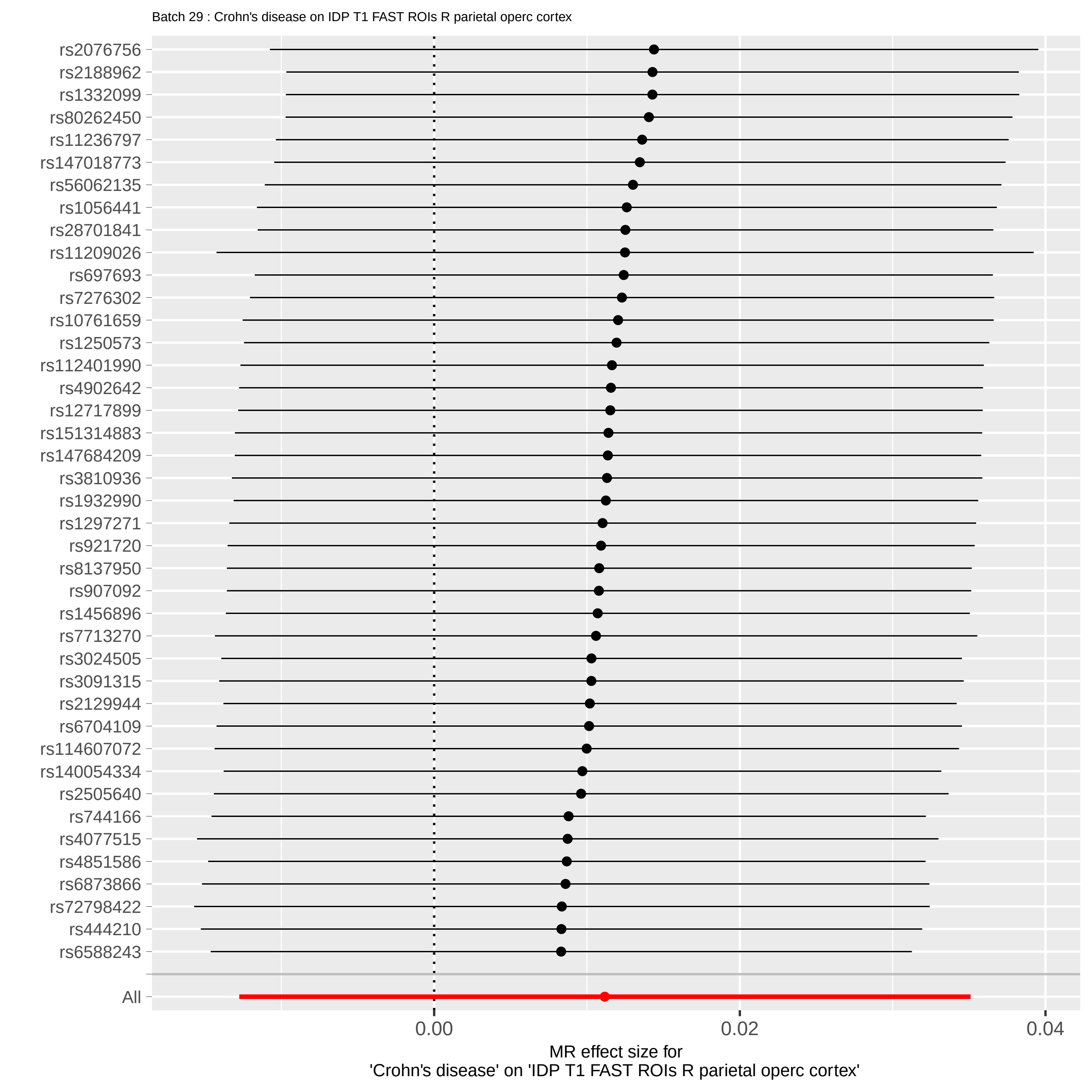
Batch 29 : Crohn's disease on IDP T1 FAST ROIs R parietal operc cortex
rs2076756
rs2188962
rs1332099
rs80262450
rs11236797
rs147018773
rs56062135
rs1056441
rs28701841
rs11209026
rs697693
rs7276302
rs10761659
rs1250573
rs112401990
rs4902642
rs12717899
rs151314883
rs147684209
rs3810936
rs1932990
rs1297271
rs921720
rs8137950
rs907092
rs1456896
rs7713270
rs3024505
rs3091315
rs2129944
rs6704109
rs114607072
rs140054334
rs2505640
rs744166
rs4077515
rs4851586
rs6873866
rs72798422
rs444210
rs6588243
All
0.00
0.02
0.04
MR effect size for
'Crohn's disease' on 'IDP T1 FAST ROIs R parietal operc cortex'

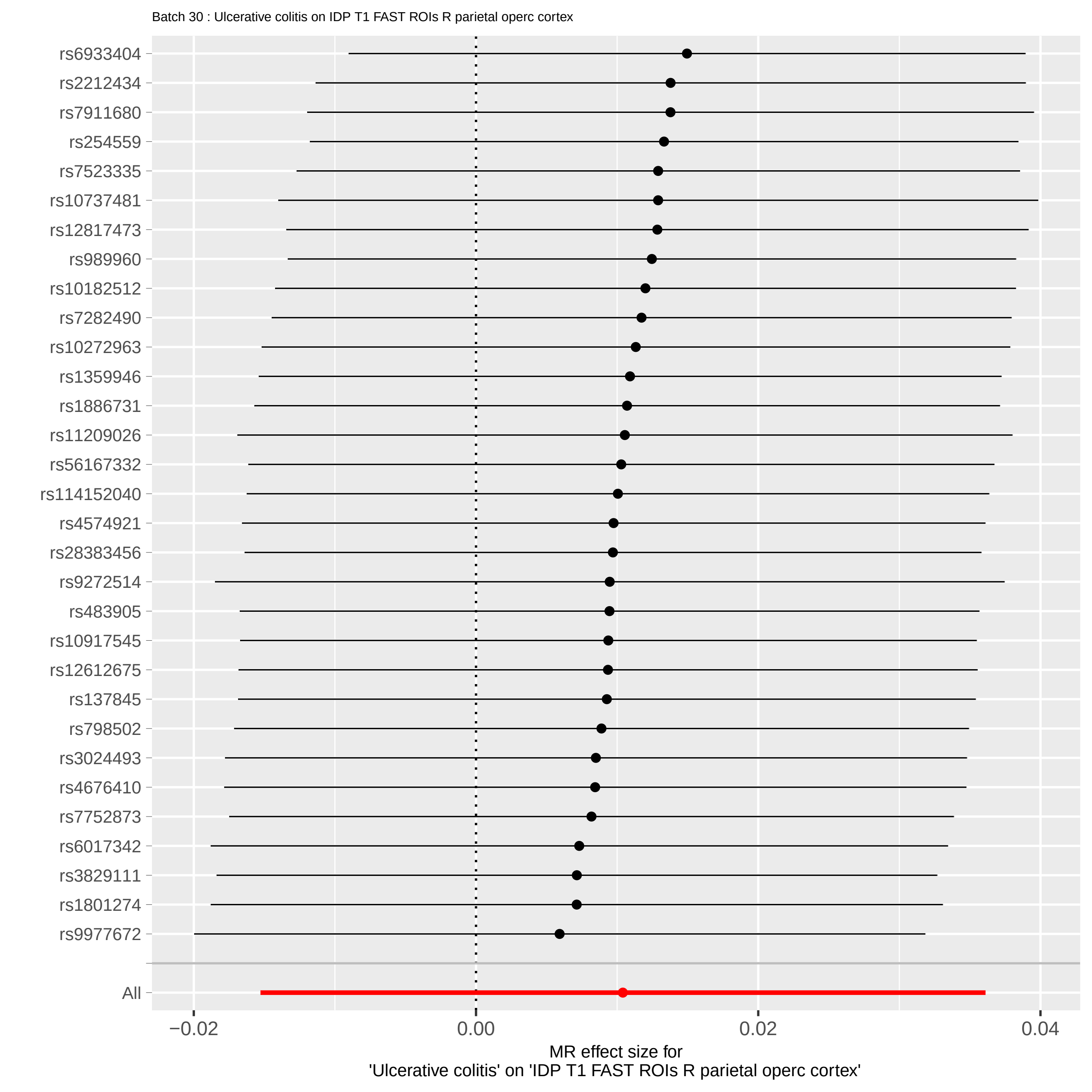

Batch 30 : Ulcerative colitis on IDP T1 FAST ROIs R parietal operc cortex
rs6933404
rs2212434
rs7911680
rs254559
rs7523335
rs10737481
rs12817473
rs989960
rs10182512
rs7282490
rs10272963
rs1359946
rs1886731
rs11209026
rs56167332
rs114152040
rs4574921
rs28383456
rs9272514
rs483905
rs10917545
rs12612675
rs137845
rs798502
rs3024493
rs4676410
rs7752873
rs6017342
rs3829111
rs1801274
rs9977672
All
−0.02
0.00
0.02
0.04
MR effect size for
'Ulcerative colitis' on 'IDP T1 FAST ROIs R parietal operc cortex'

Batch 31 : Crohn's disease on IDP T1 FAST ROIs L planum polare

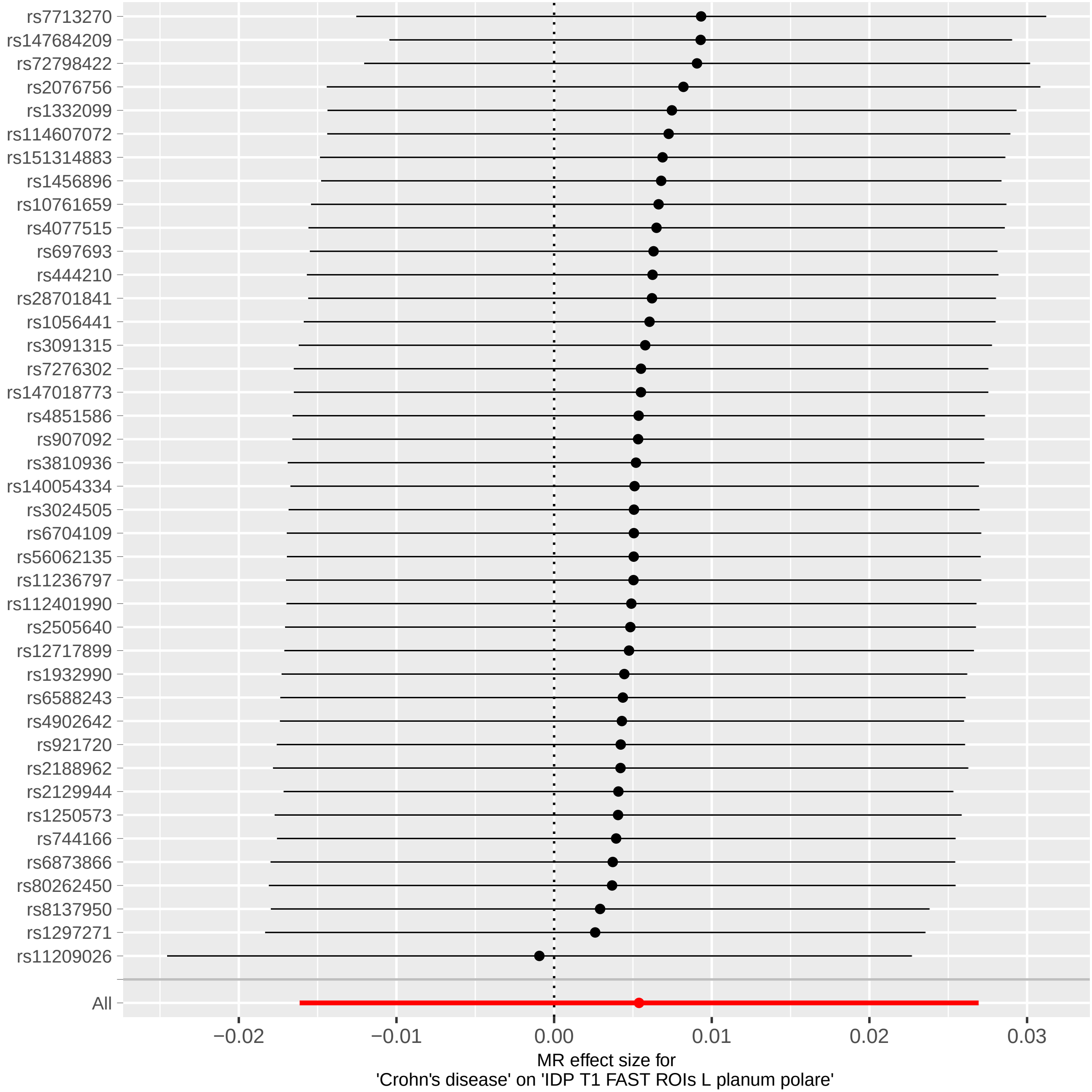

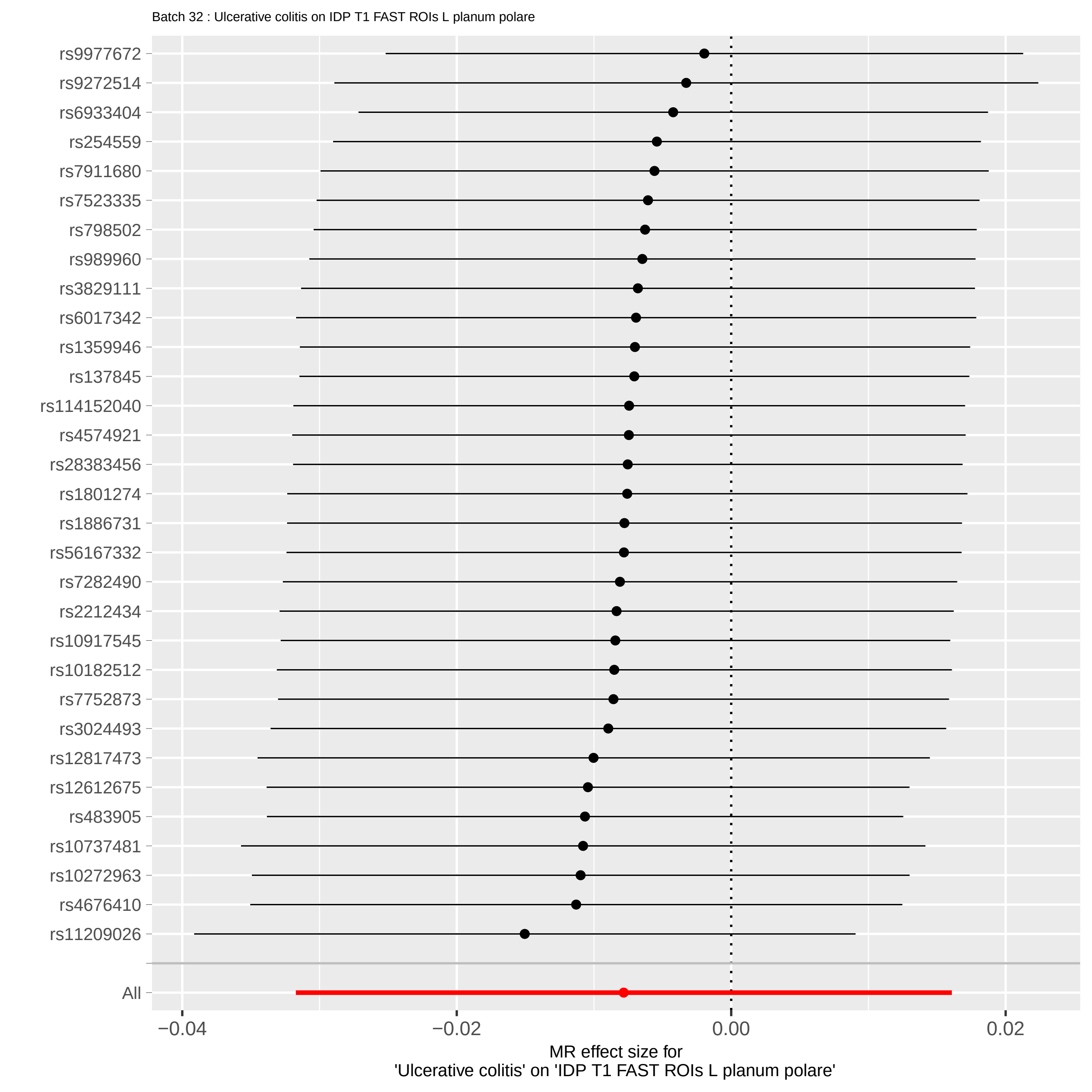
Batch 32 : Ulcerative colitis on IDP T1 FAST ROIs L planum polare
rs9977672
rs9272514
rs6933404
rs254559
rs7911680
rs7523335
rs798502
rs989960
rs3829111
rs6017342
rs1359946
rs137845
rs114152040
rs4574921
rs28383456
rs1801274
rs1886731
rs56167332
rs7282490
rs2212434
rs10917545
rs10182512
rs7752873
rs3024493
rs12817473
rs12612675
rs483905
rs10737481
rs10272963
rs4676410
rs11209026
All
−0.04
−0.02
0.00
0.02
MR effect size for
'Ulcerative colitis' on 'IDP T1 FAST ROIs L planum polare'

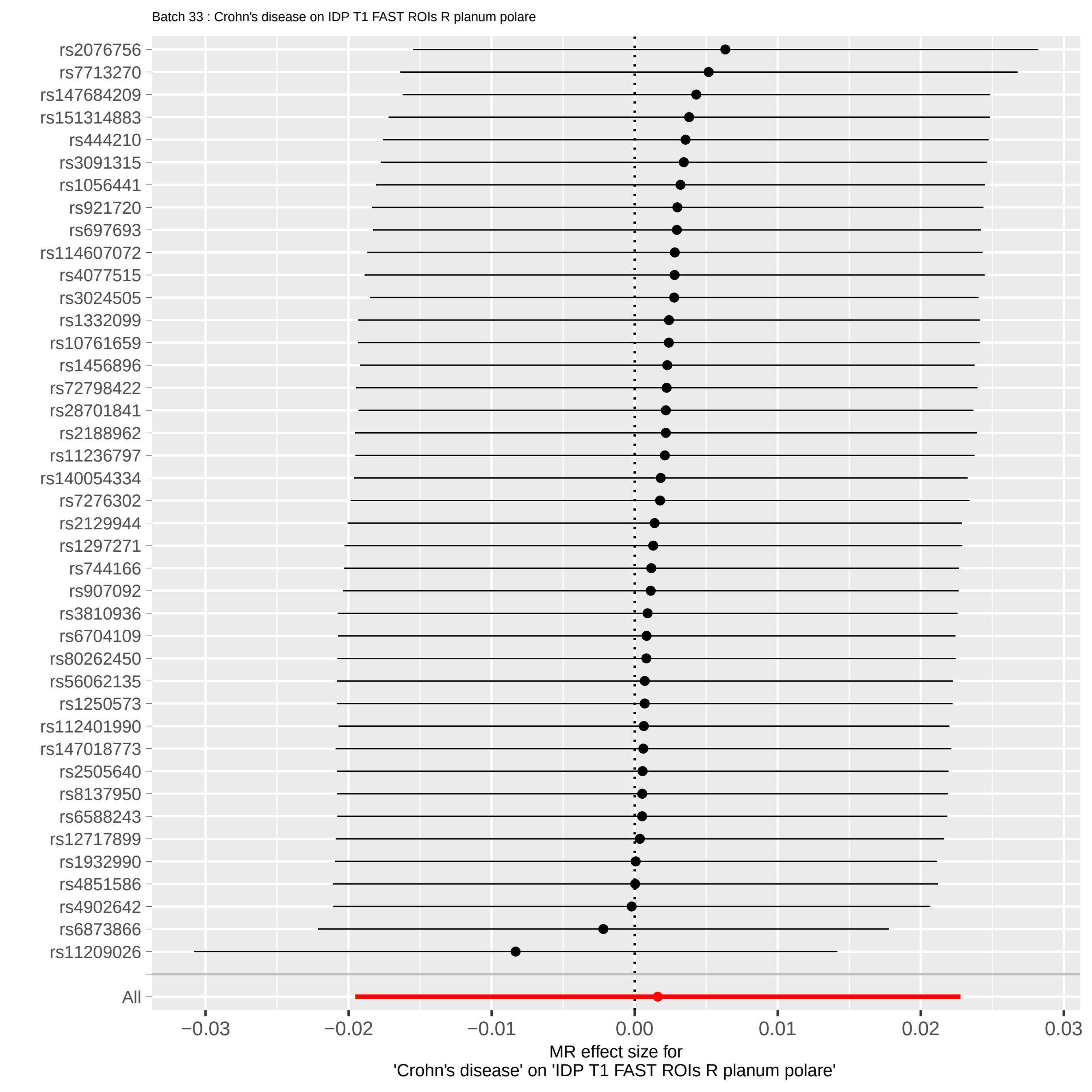

Batch 33 : Crohn's disease on IDP T1 FAST ROIs R planum polare
rs2076756
rs7713270
rs147684209
rs151314883
rs444210
rs3091315
rs1056441
rs921720
rs697693
rs114607072
rs4077515
rs3024505
rs1332099
rs10761659
rs1456896
rs72798422
rs28701841
rs2188962
rs11236797
rs140054334
rs7276302
rs2129944
rs1297271
rs744166
rs907092
rs3810936
rs6704109
rs80262450
rs56062135
rs1250573
rs112401990
rs147018773
rs2505640
rs8137950
rs6588243
rs12717899
rs1932990
rs4851586
rs4902642
rs6873866
rs11209026
All
−0.03
−0.02
−0.01
0.00
0.01
0.02
0.03
MR effect size for
'Crohn's disease' on 'IDP T1 FAST ROIs R planum polare'

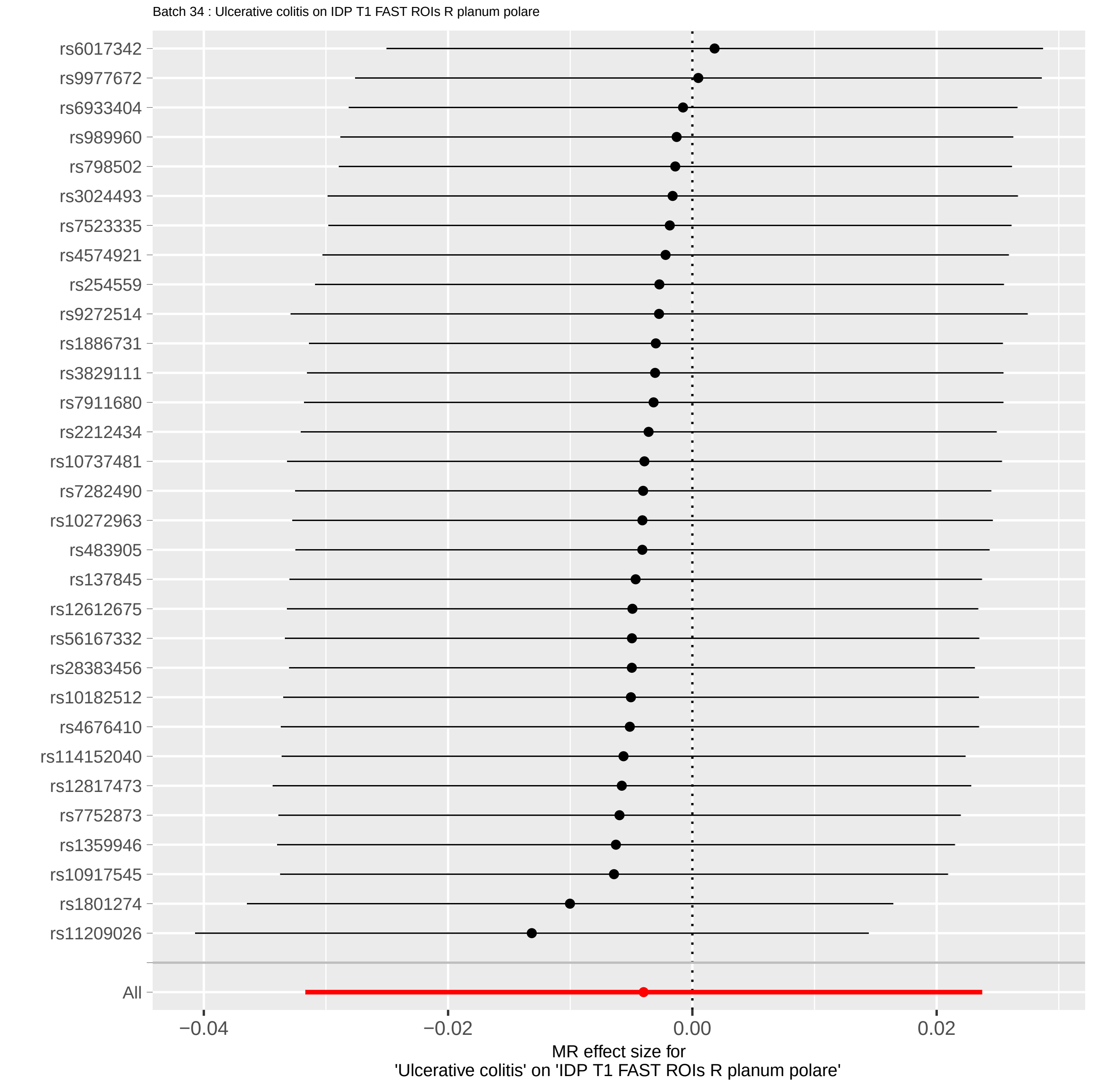

Batch 34 : Ulcerative colitis on IDP T1 FAST ROIs R planum polare
rs6017342
rs9977672
rs6933404
rs989960
rs798502
rs3024493
rs7523335
rs4574921
rs254559
rs9272514
rs1886731
rs3829111
rs7911680
rs2212434
rs10737481
rs7282490
rs10272963
rs483905
rs137845
rs12612675
rs56167332
rs28383456
rs10182512
rs4676410
rs114152040
rs12817473
rs7752873
rs1359946
rs10917545
rs1801274
rs11209026
All
−0.04
−0.02
0.00
0.02
MR effect size for
'Ulcerative colitis' on 'IDP T1 FAST ROIs R planum polare'

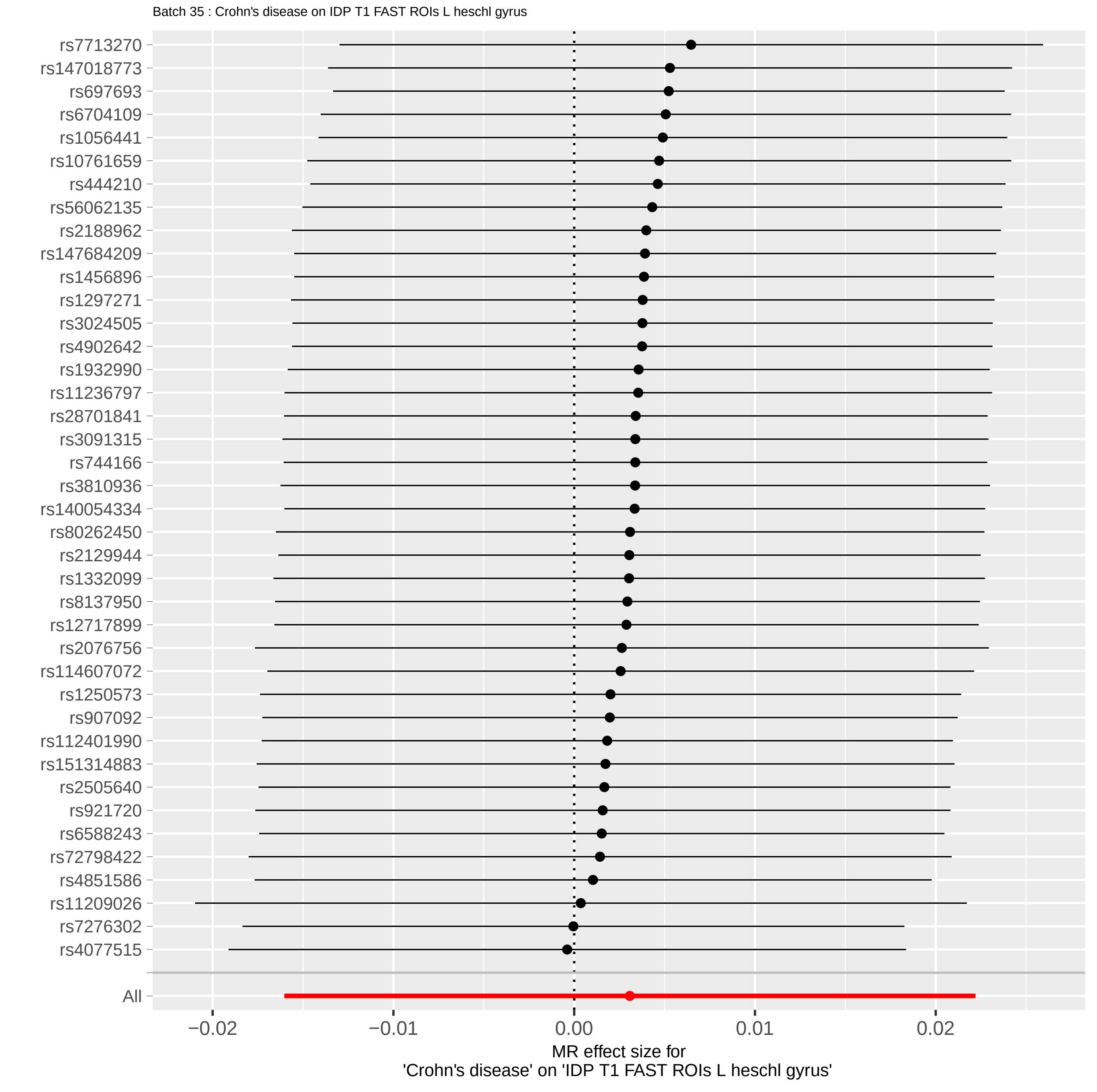

Batch 35 : Crohn's disease on IDP T1 FAST ROIs L heschl gyrus
rs7713270
rs147018773
rs697693
rs6704109
rs1056441
rs10761659
rs444210
rs56062135
rs2188962
rs147684209
rs1456896
rs1297271
rs3024505
rs4902642
rs1932990
rs11236797
rs28701841
rs3091315
rs744166
rs3810936
rs140054334
rs80262450
rs2129944
rs1332099
rs8137950
rs12717899
rs2076756
rs114607072
rs1250573
rs907092
rs112401990
rs151314883
rs2505640
rs921720
rs6588243
rs72798422
rs4851586
rs11209026
rs7276302
rs4077515
All
−0.02
−0.01
0.00
0.01
0.02
MR effect size for
'Crohn's disease' on 'IDP T1 FAST ROIs L heschl gyrus'

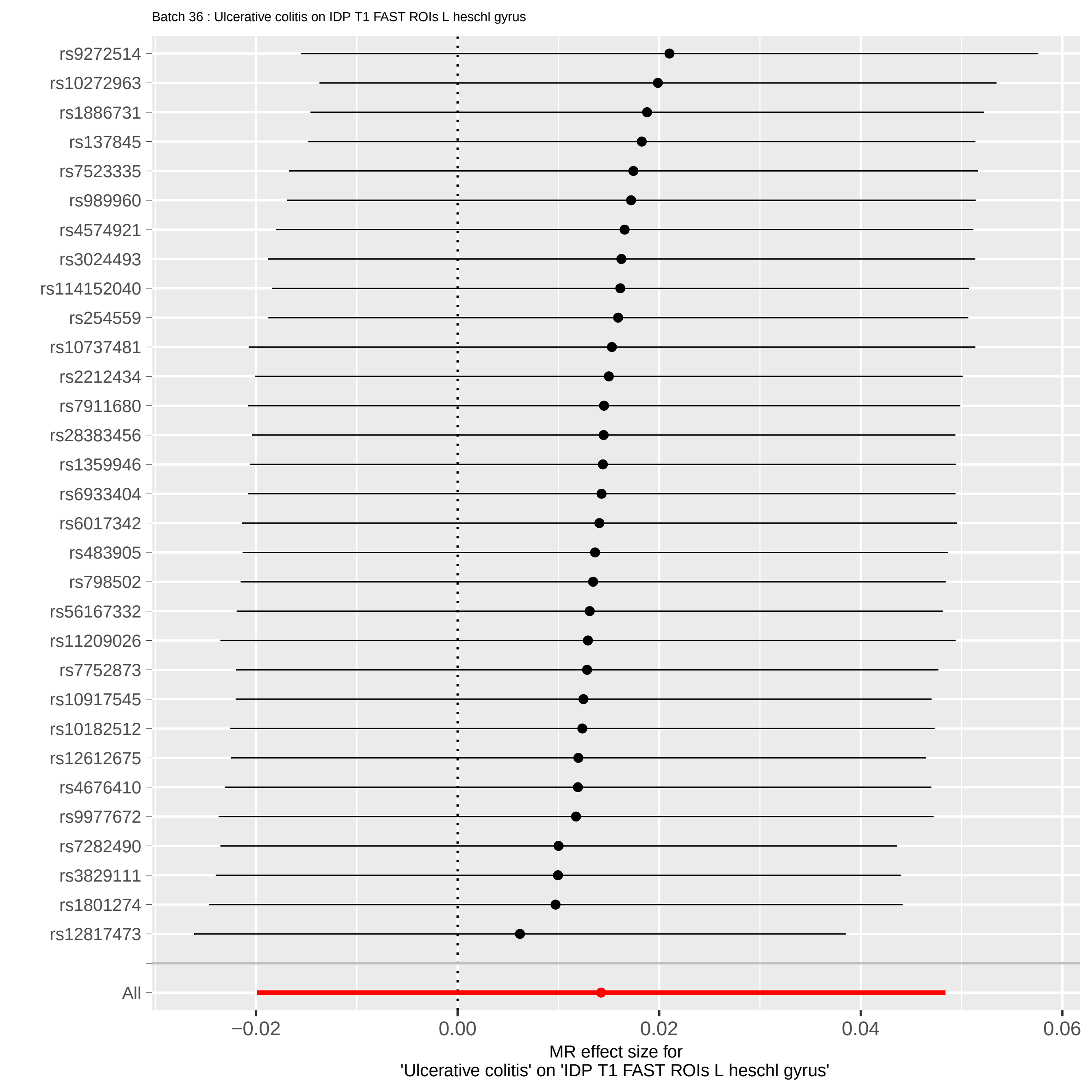

Batch 36 : Ulcerative colitis on IDP T1 FAST ROIs L heschl gyrus
rs9272514
rs10272963
rs1886731
rs137845
rs7523335
rs989960
rs4574921
rs3024493
rs114152040
rs254559
rs10737481
rs2212434
rs7911680
rs28383456
rs1359946
rs6933404
rs6017342
rs483905
rs798502
rs56167332
rs11209026
rs7752873
rs10917545
rs10182512
rs12612675
rs4676410
rs9977672
rs7282490
rs3829111
rs1801274
rs12817473
All
−0.02
0.00
0.02
0.04
0.06
MR effect size for
'Ulcerative colitis' on 'IDP T1 FAST ROIs L heschl gyrus'

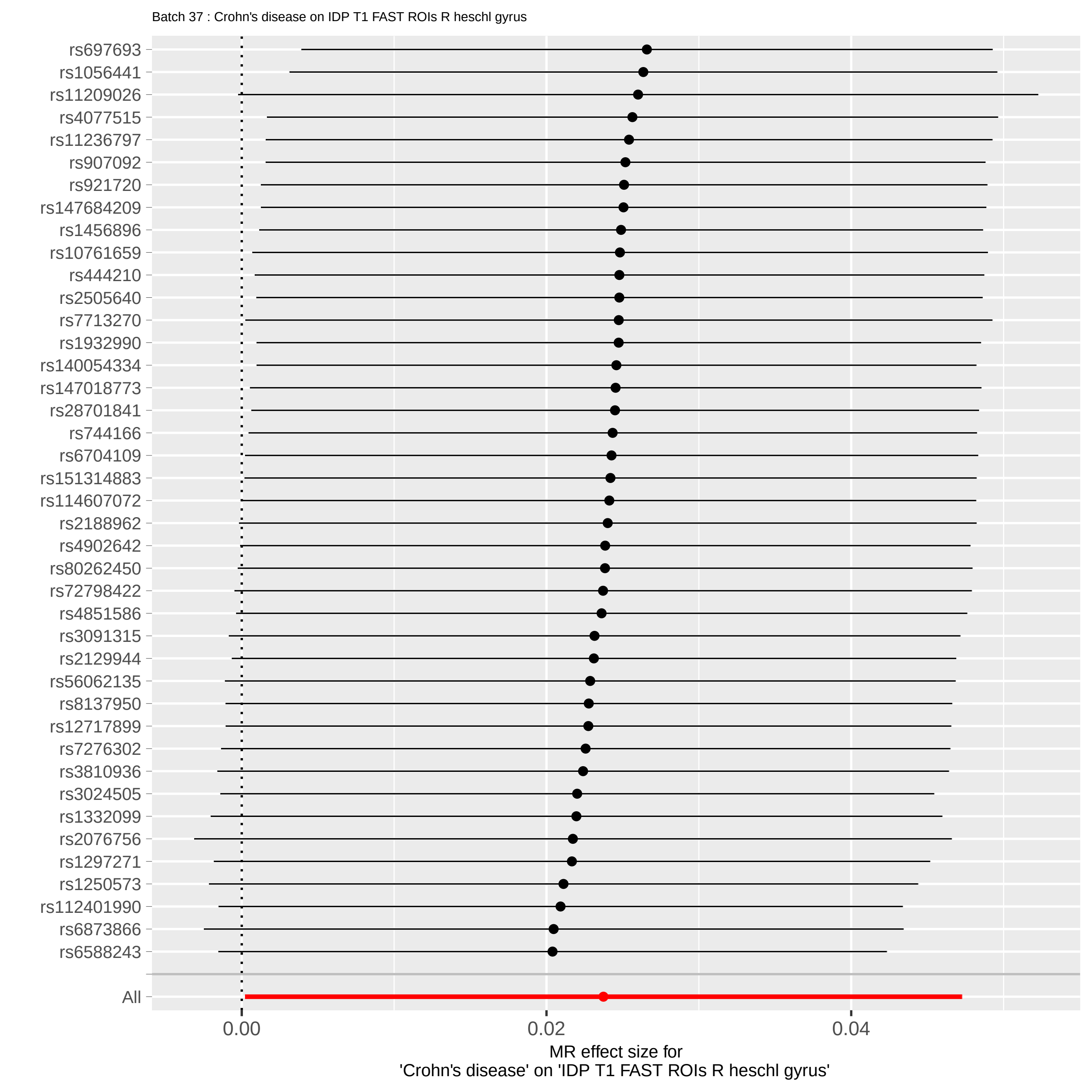

Batch 37 : Crohn's disease on IDP T1 FAST ROIs R heschl gyrus
rs697693
rs1056441
rs11209026
rs4077515
rs11236797
rs907092
rs921720
rs147684209
rs1456896
rs10761659
rs444210
rs2505640
rs7713270
rs1932990
rs140054334
rs147018773
rs28701841
rs744166
rs6704109
rs151314883
rs114607072
rs2188962
rs4902642
rs80262450
rs72798422
rs4851586
rs3091315
rs2129944
rs56062135
rs8137950
rs12717899
rs7276302
rs3810936
rs3024505
rs1332099
rs2076756
rs1297271
rs1250573
rs112401990
rs6873866
rs6588243
All
0.00
0.02
0.04
MR effect size for
'Crohn's disease' on 'IDP T1 FAST ROIs R heschl gyrus'

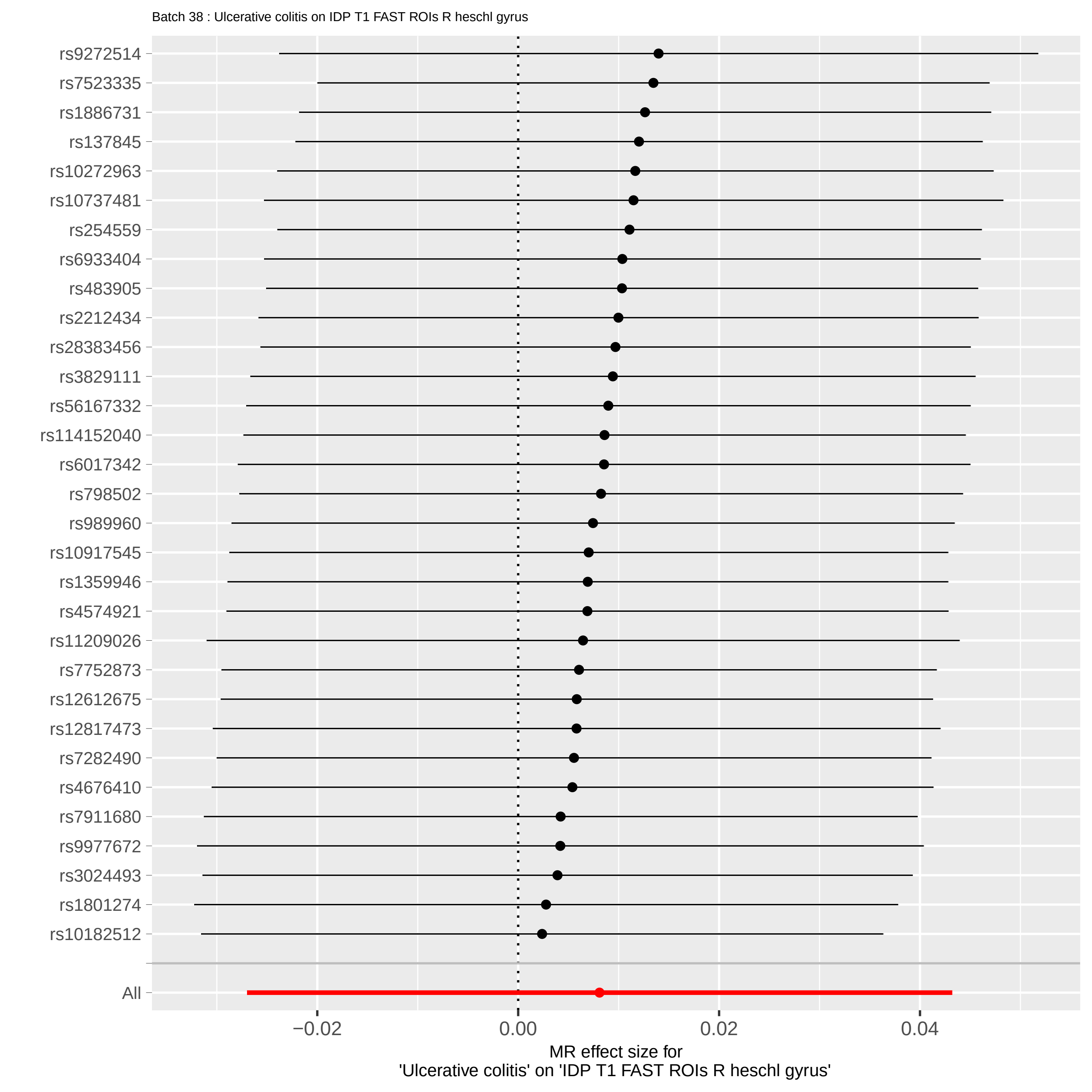
Batch 38 : Ulcerative colitis on IDP T1 FAST ROIs R heschl gyrus
rs9272514
rs7523335
rs1886731
rs137845
rs10272963
rs10737481
rs254559
rs6933404
rs483905
rs2212434
rs28383456
rs3829111
rs56167332
rs114152040
rs6017342
rs798502
rs989960
rs10917545
rs1359946
rs4574921
rs11209026
rs7752873
rs12612675
rs12817473
rs7282490
rs4676410
rs7911680
rs9977672
rs3024493
rs1801274
rs10182512
All
−0.02
0.00
0.02
0.04
MR effect size for
'Ulcerative colitis' on 'IDP T1 FAST ROIs R heschl gyrus'

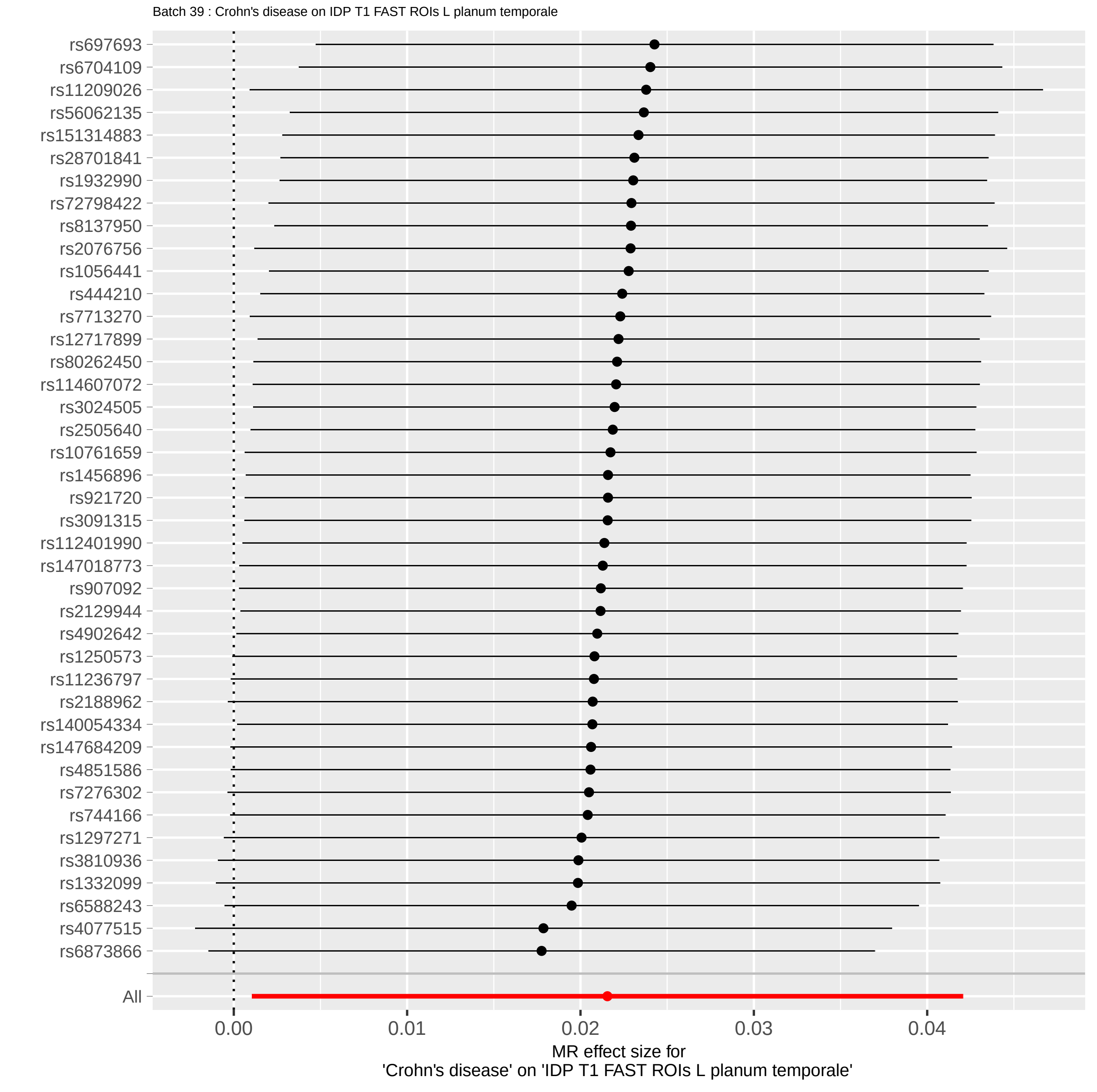

Batch 39 : Crohn's disease on IDP T1 FAST ROIs L planum temporale
rs697693
rs6704109
rs11209026
rs56062135
rs151314883
rs28701841
rs1932990
rs72798422
rs8137950
rs2076756
rs1056441
rs444210
rs7713270
rs12717899
rs80262450
rs114607072
rs3024505
rs2505640
rs10761659
rs1456896
rs921720
rs3091315
rs112401990
rs147018773
rs907092
rs2129944
rs4902642
rs1250573
rs11236797
rs2188962
rs140054334
rs147684209
rs4851586
rs7276302
rs744166
rs1297271
rs3810936
rs1332099
rs6588243
rs4077515
rs6873866
All
0.00
0.01
0.02
0.03
0.04
MR effect size for
'Crohn's disease' on 'IDP T1 FAST ROIs L planum temporale'

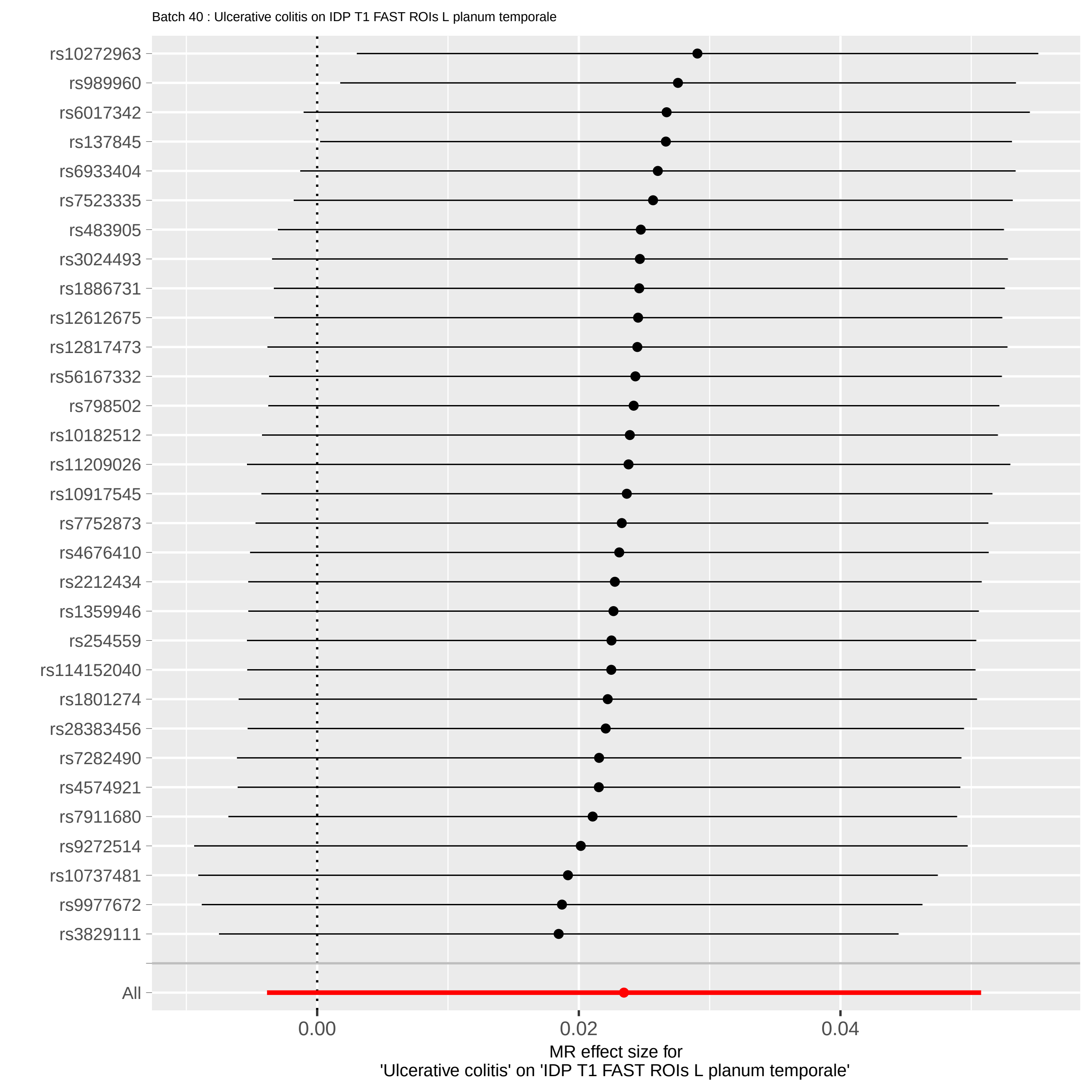
Batch 40 : Ulcerative colitis on IDP T1 FAST ROIs L planum temporale
rs10272963
rs989960
rs6017342
rs137845
rs6933404
rs7523335
rs483905
rs3024493
rs1886731
rs12612675
rs12817473
rs56167332
rs798502
rs10182512
rs11209026
rs10917545
rs7752873
rs4676410
rs2212434
rs1359946
rs254559
rs114152040
rs1801274
rs28383456
rs7282490
rs4574921
rs7911680
rs9272514
rs10737481
rs9977672
rs3829111
All
0.00
0.02
0.04
MR effect size for
'Ulcerative colitis' on 'IDP T1 FAST ROIs L planum temporale'

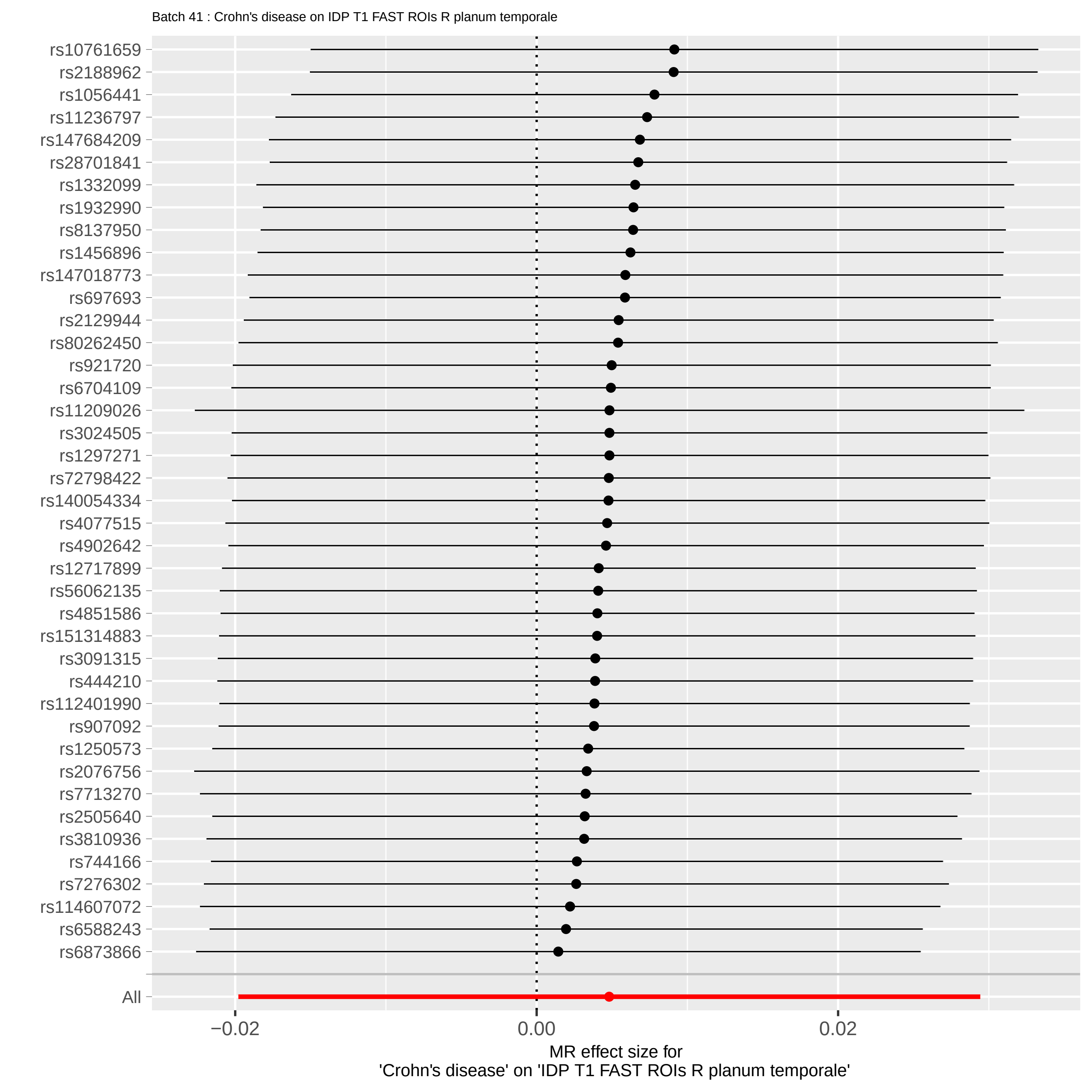
Batch 41 : Crohn's disease on IDP T1 FAST ROIs R planum temporale
rs10761659
rs2188962
rs1056441
rs11236797
rs147684209
rs28701841
rs1332099
rs1932990
rs8137950
rs1456896
rs147018773
rs697693
rs2129944
rs80262450
rs921720
rs6704109
rs11209026
rs3024505
rs1297271
rs72798422
rs140054334
rs4077515
rs4902642
rs12717899
rs56062135
rs4851586
rs151314883
rs3091315
rs444210
rs112401990
rs907092
rs1250573
rs2076756
rs7713270
rs2505640
rs3810936
rs744166
rs7276302
rs114607072
rs6588243
rs6873866
All
−0.02
0.00
0.02
MR effect size for
'Crohn's disease' on 'IDP T1 FAST ROIs R planum temporale'

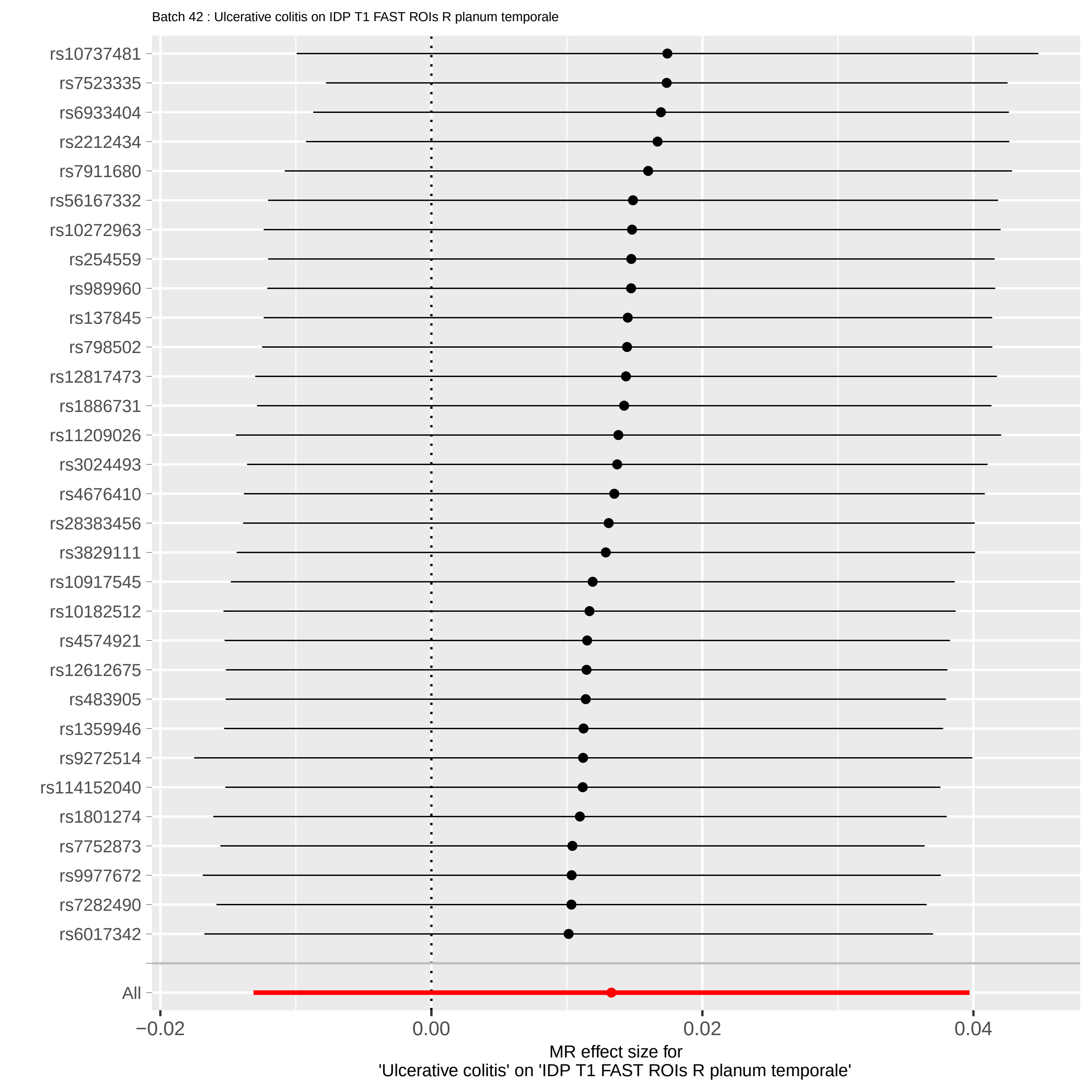

Batch 42 : Ulcerative colitis on IDP T1 FAST ROIs R planum temporale
rs10737481
rs7523335
rs6933404
rs2212434
rs7911680
rs56167332
rs10272963
rs254559
rs989960
rs137845
rs798502
rs12817473
rs1886731
rs11209026
rs3024493
rs4676410
rs28383456
rs3829111
rs10917545
rs10182512
rs4574921
rs12612675
rs483905
rs1359946
rs9272514
rs114152040
rs1801274
rs7752873
rs9977672
rs7282490
rs6017342
All
−0.02
0.00
0.02
0.04
MR effect size for
'Ulcerative colitis' on 'IDP T1 FAST ROIs R planum temporale'

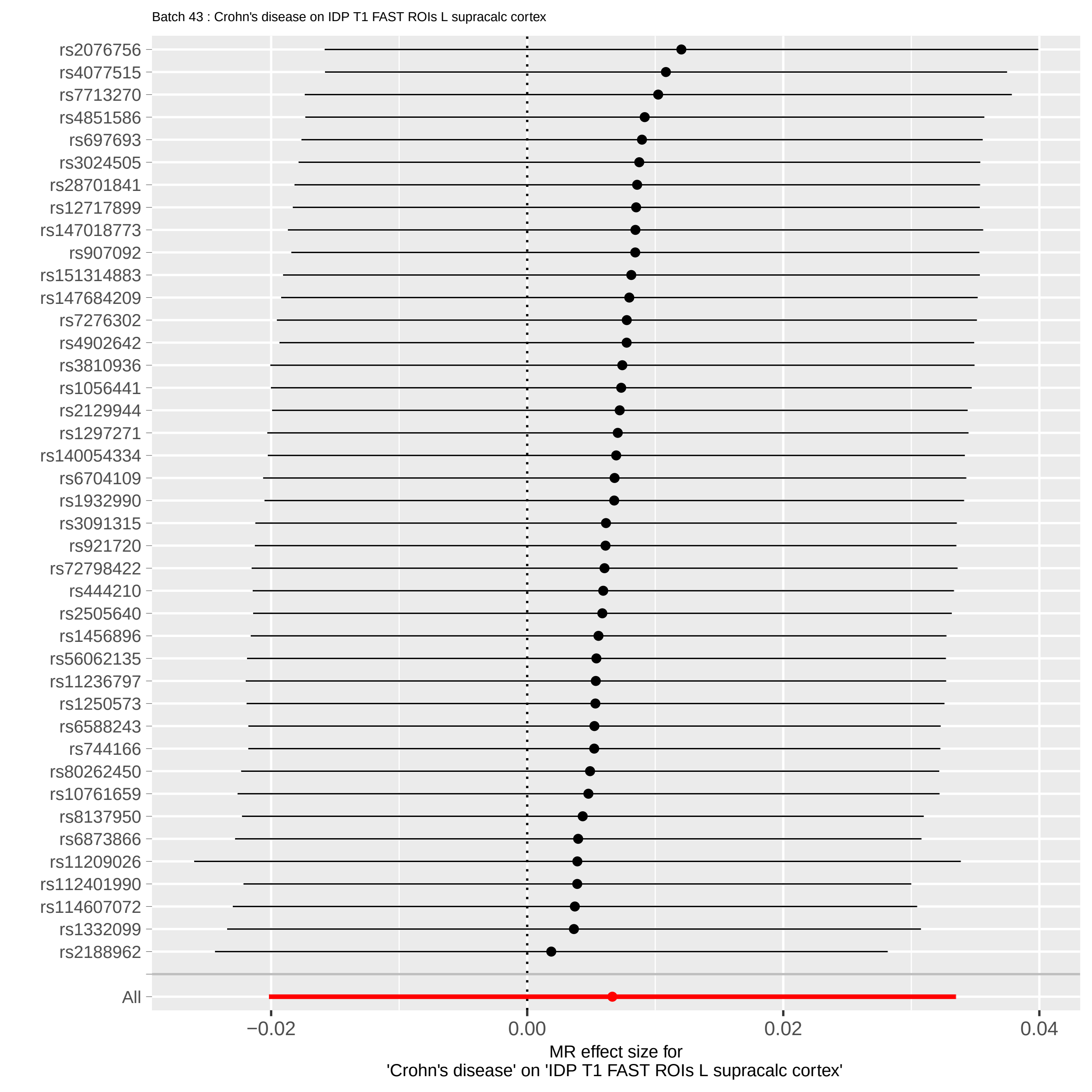

Batch 43 : Crohn's disease on IDP T1 FAST ROIs L supracalc cortex
rs2076756
rs4077515
rs7713270
rs4851586
rs697693
rs3024505
rs28701841
rs12717899
rs147018773
rs907092
rs151314883
rs147684209
rs7276302
rs4902642
rs3810936
rs1056441
rs2129944
rs1297271
rs140054334
rs6704109
rs1932990
rs3091315
rs921720
rs72798422
rs444210
rs2505640
rs1456896
rs56062135
rs11236797
rs1250573
rs6588243
rs744166
rs80262450
rs10761659
rs8137950
rs6873866
rs11209026
rs112401990
rs114607072
rs1332099
rs2188962
All
−0.02
0.00
0.02
0.04
MR effect size for
'Crohn's disease' on 'IDP T1 FAST ROIs L supracalc cortex'

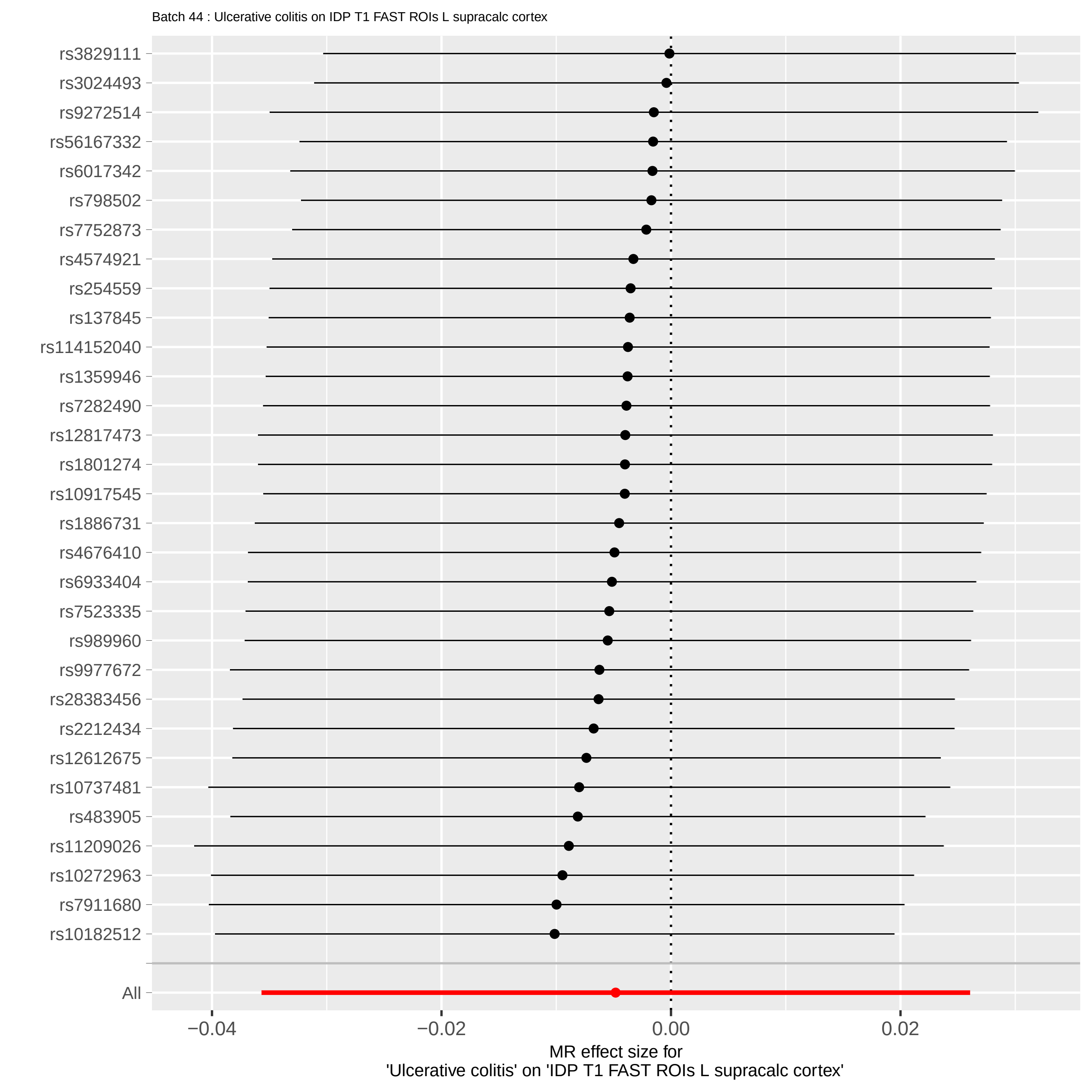
Batch 44 : Ulcerative colitis on IDP T1 FAST ROIs L supracalc cortex
rs3829111
rs3024493
rs9272514
rs56167332
rs6017342
rs798502
rs7752873
rs4574921
rs254559
rs137845
rs114152040
rs1359946
rs7282490
rs12817473
rs1801274
rs10917545
rs1886731
rs4676410
rs6933404
rs7523335
rs989960
rs9977672
rs28383456
rs2212434
rs12612675
rs10737481
rs483905
rs11209026
rs10272963
rs7911680
rs10182512
All
−0.04
−0.02
0.00
0.02
MR effect size for
'Ulcerative colitis' on 'IDP T1 FAST ROIs L supracalc cortex'

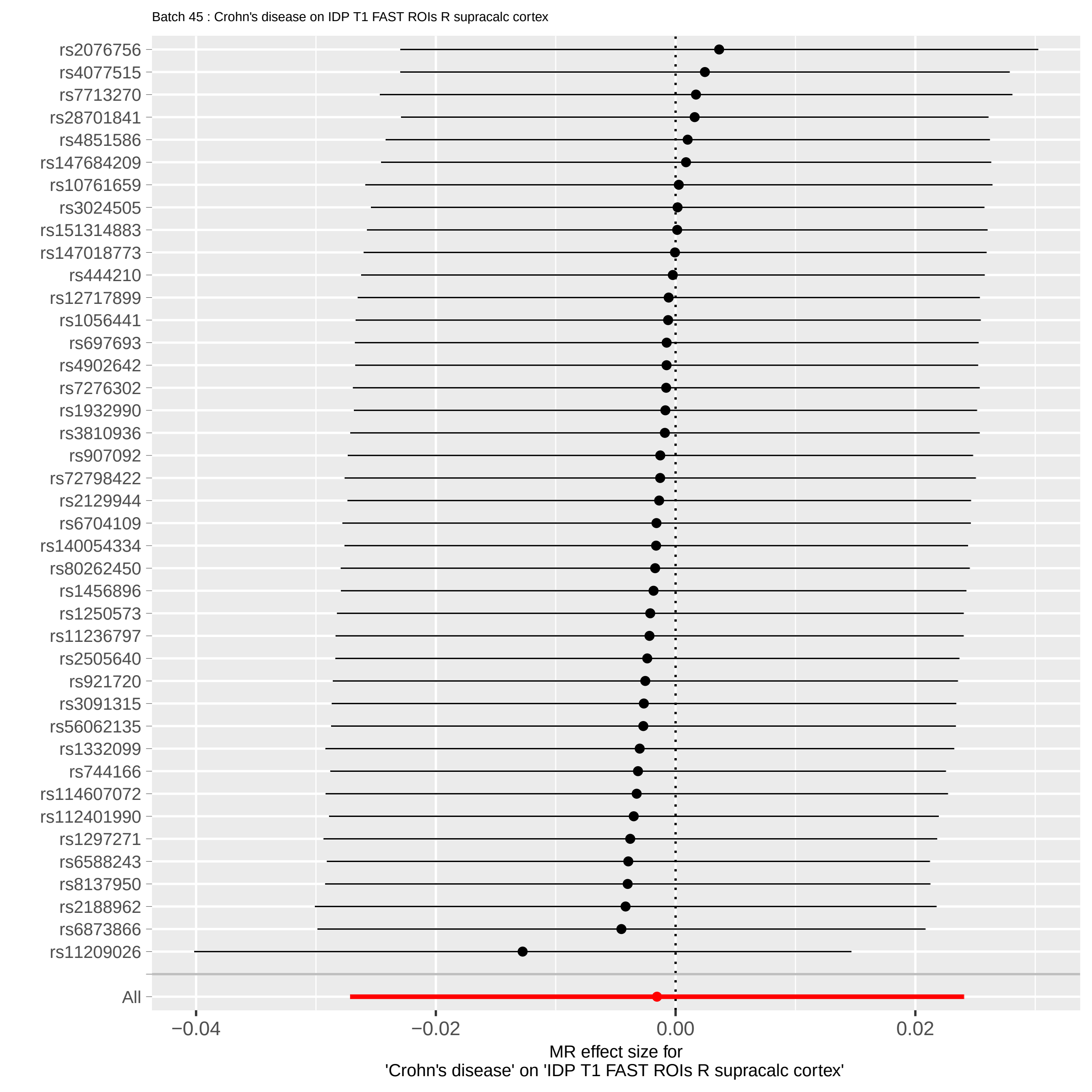
Batch 45 : Crohn's disease on IDP T1 FAST ROIs R supracalc cortex
rs2076756
rs4077515
rs7713270
rs28701841
rs4851586
rs147684209
rs10761659
rs3024505
rs151314883
rs147018773
rs444210
rs12717899
rs1056441
rs697693
rs4902642
rs7276302
rs1932990
rs3810936
rs907092
rs72798422
rs2129944
rs6704109
rs140054334
rs80262450
rs1456896
rs1250573
rs11236797
rs2505640
rs921720
rs3091315
rs56062135
rs1332099
rs744166
rs114607072
rs112401990
rs1297271
rs6588243
rs8137950
rs2188962
rs6873866
rs11209026
All
−0.04
−0.02
0.00
0.02
MR effect size for
'Crohn's disease' on 'IDP T1 FAST ROIs R supracalc cortex'

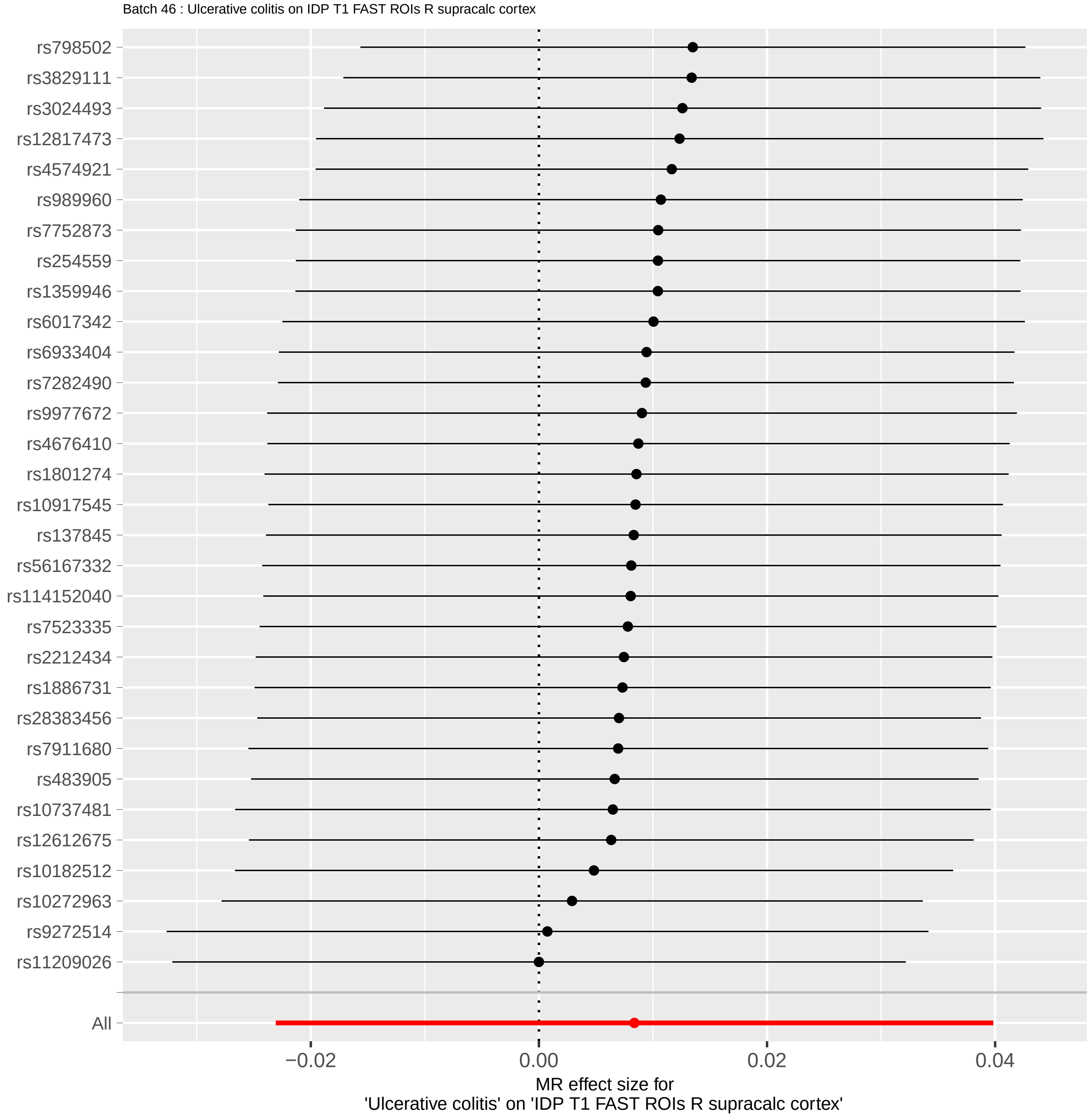

Batch 46 : Ulcerative colitis on IDP T1 FAST ROIs R supracalc cortex
rs798502
rs3829111
rs3024493
rs12817473
rs4574921
rs989960
rs7752873
rs254559
rs1359946
rs6017342
rs6933404
rs7282490
rs9977672
rs4676410
rs1801274
rs10917545
rs137845
rs56167332
rs114152040
rs7523335
rs2212434
rs1886731
rs28383456
rs7911680
rs483905
rs10737481
rs12612675
rs10182512
rs10272963
rs9272514
rs11209026
All
−0.02
0.00
0.02
0.04
MR effect size for
'Ulcerative colitis' on 'IDP T1 FAST ROIs R supracalc cortex'

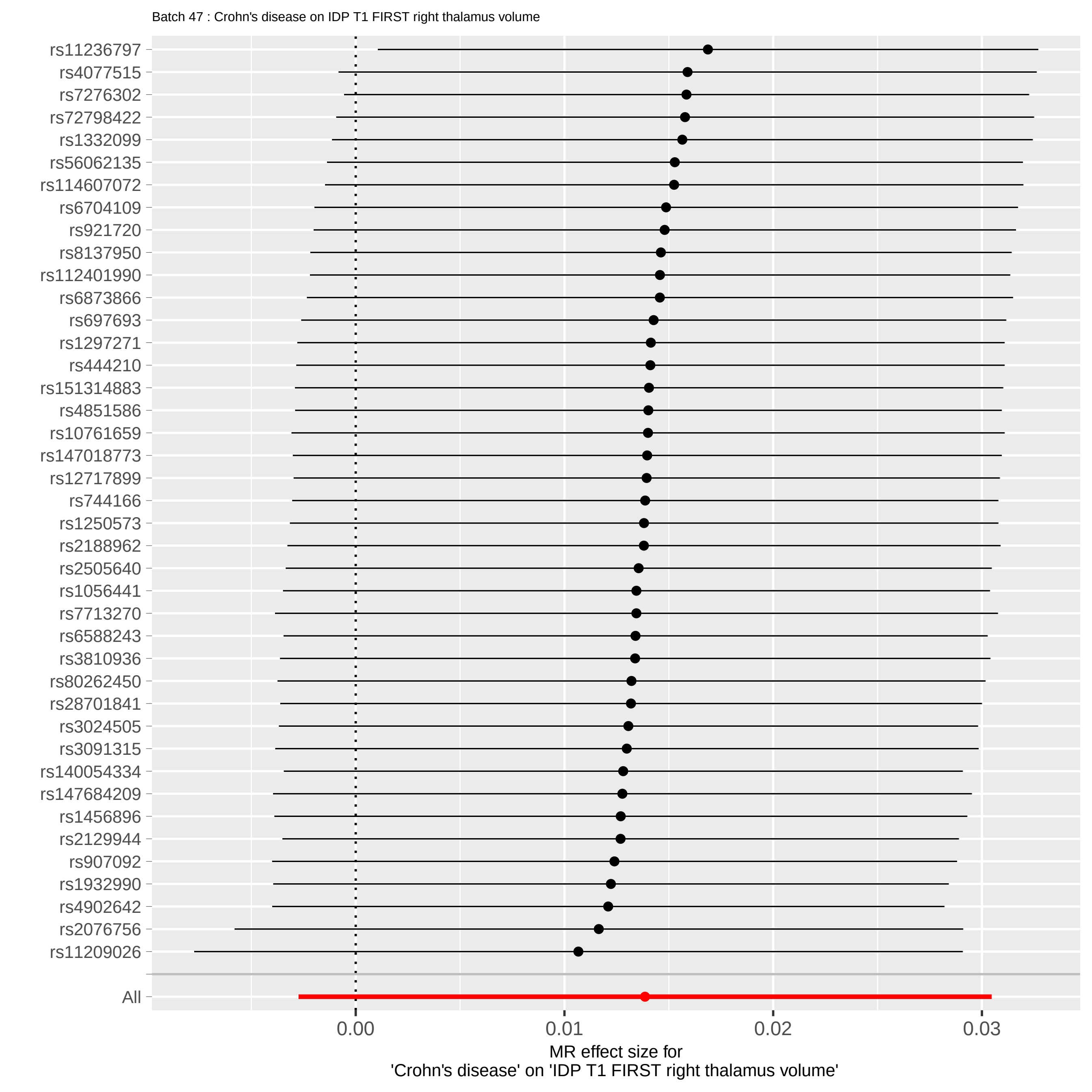

Batch 47 : Crohn's disease on IDP T1 FIRST right thalamus volume
rs11236797
rs4077515
rs7276302
rs72798422
rs1332099
rs56062135
rs114607072
rs6704109
rs921720
rs8137950
rs112401990
rs6873866
rs697693
rs1297271
rs444210
rs151314883
rs4851586
rs10761659
rs147018773
rs12717899
rs744166
rs1250573
rs2188962
rs2505640
rs1056441
rs7713270
rs6588243
rs3810936
rs80262450
rs28701841
rs3024505
rs3091315
rs140054334
rs147684209
rs1456896
rs2129944
rs907092
rs1932990
rs4902642
rs2076756
rs11209026
All
0.00
0.01
0.02
0.03
MR effect size for
'Crohn's disease' on 'IDP T1 FIRST right thalamus volume'

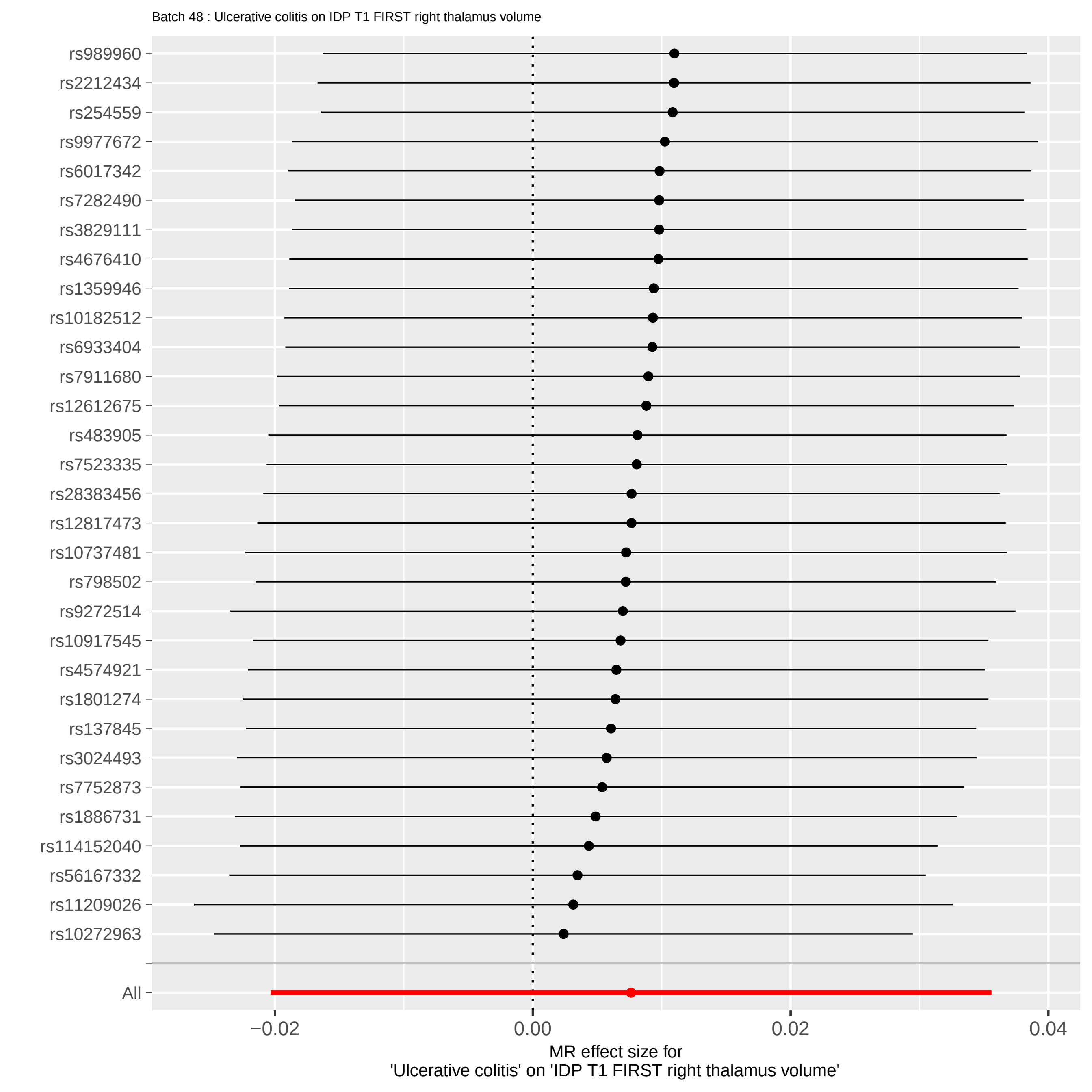

Batch 48 : Ulcerative colitis on IDP T1 FIRST right thalamus volume
rs989960
rs2212434
rs254559
rs9977672
rs6017342
rs7282490
rs3829111
rs4676410
rs1359946
rs10182512
rs6933404
rs7911680
rs12612675
rs483905
rs7523335
rs28383456
rs12817473
rs10737481
rs798502
rs9272514
rs10917545
rs4574921
rs1801274
rs137845
rs3024493
rs7752873
rs1886731
rs114152040
rs56167332
rs11209026
rs10272963
All
−0.02
0.00
0.02
0.04
MR effect size for
'Ulcerative colitis' on 'IDP T1 FIRST right thalamus volume'

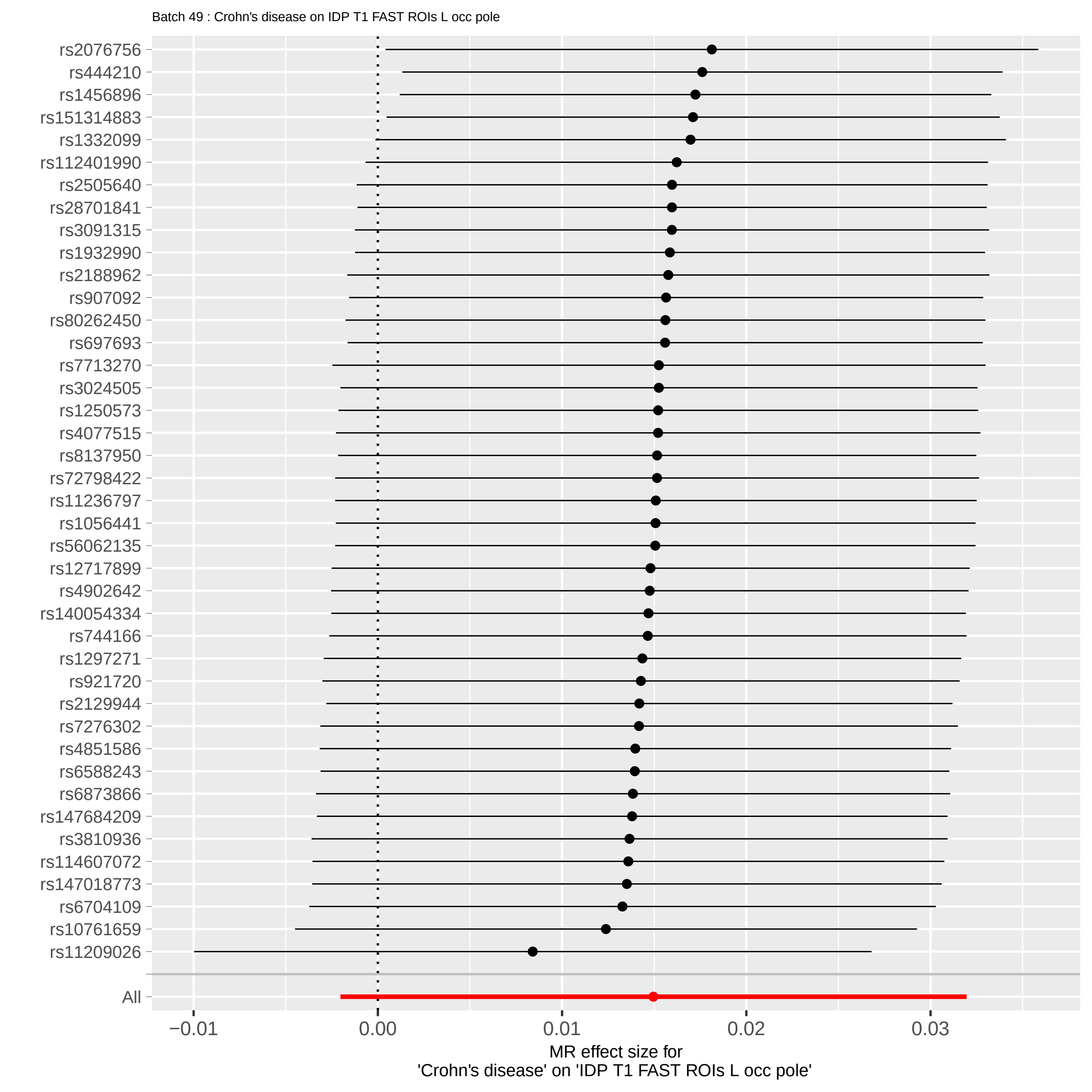
Batch 49 : Crohn's disease on IDP T1 FAST ROIs L occ pole
rs2076756
rs444210
rs1456896
rs151314883
rs1332099
rs112401990
rs2505640
rs28701841
rs3091315
rs1932990
rs2188962
rs907092
rs80262450
rs697693
rs7713270
rs3024505
rs1250573
rs4077515
rs8137950
rs72798422
rs11236797
rs1056441
rs56062135
rs12717899
rs4902642
rs140054334
rs744166
rs1297271
rs921720
rs2129944
rs7276302
rs4851586
rs6588243
rs6873866
rs147684209
rs3810936
rs114607072
rs147018773
rs6704109
rs10761659
rs11209026
All
−0.01
0.00
0.01
0.02
0.03
MR effect size for
'Crohn's disease' on 'IDP T1 FAST ROIs L occ pole'

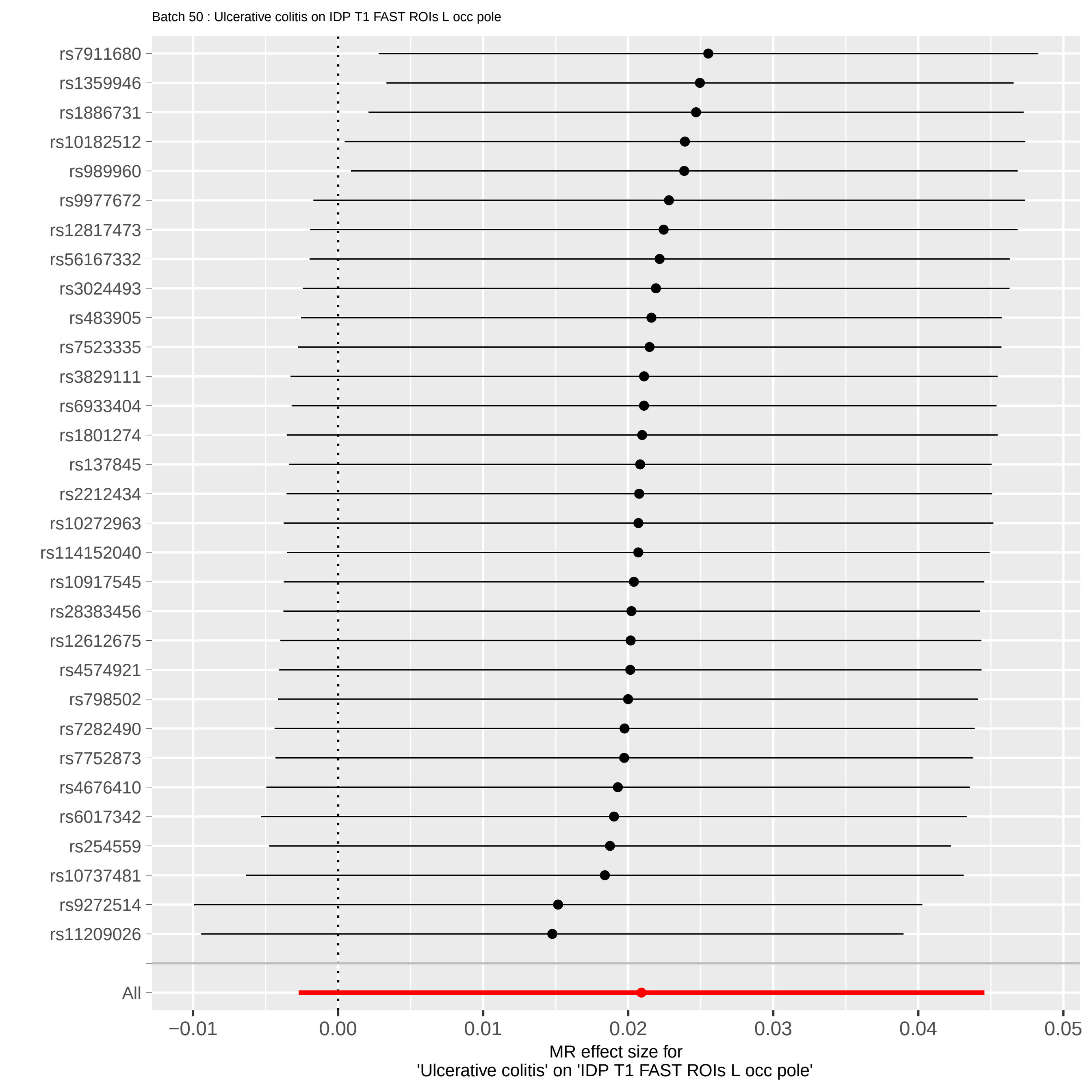

Batch 50 : Ulcerative colitis on IDP T1 FAST ROIs L occ pole
rs7911680
rs1359946
rs1886731
rs10182512
rs989960
rs9977672
rs12817473
rs56167332
rs3024493
rs483905
rs7523335
rs3829111
rs6933404
rs1801274
rs137845
rs2212434
rs10272963
rs114152040
rs10917545
rs28383456
rs12612675
rs4574921
rs798502
rs7282490
rs7752873
rs4676410
rs6017342
rs254559
rs10737481
rs9272514
rs11209026
All
−0.01
0.00
0.01
0.02
0.03
0.04
0.05
MR effect size for
'Ulcerative colitis' on 'IDP T1 FAST ROIs L occ pole'

Batch 51 : Crohn's disease on IDP T1 FAST ROIs R occ pole

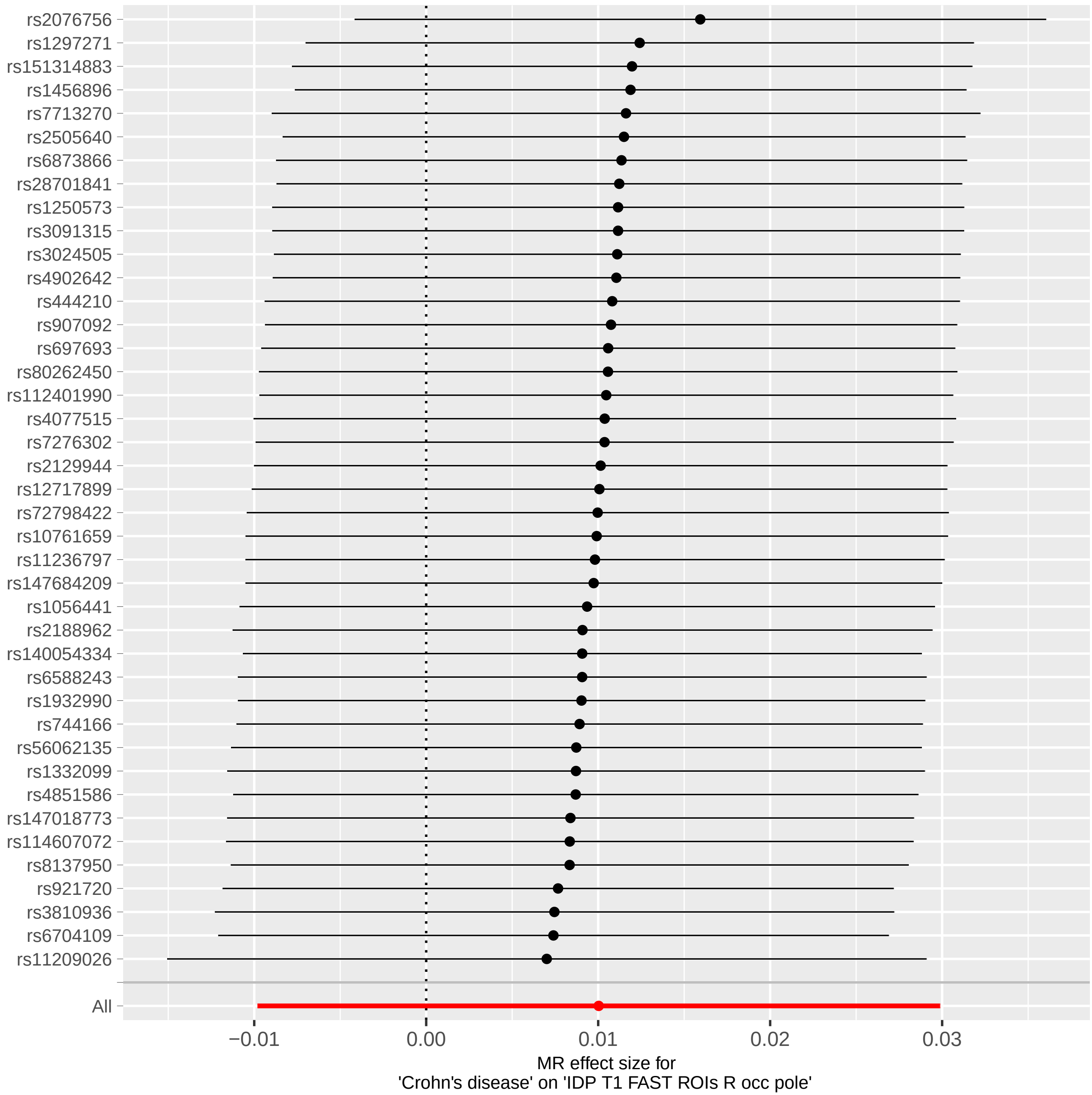

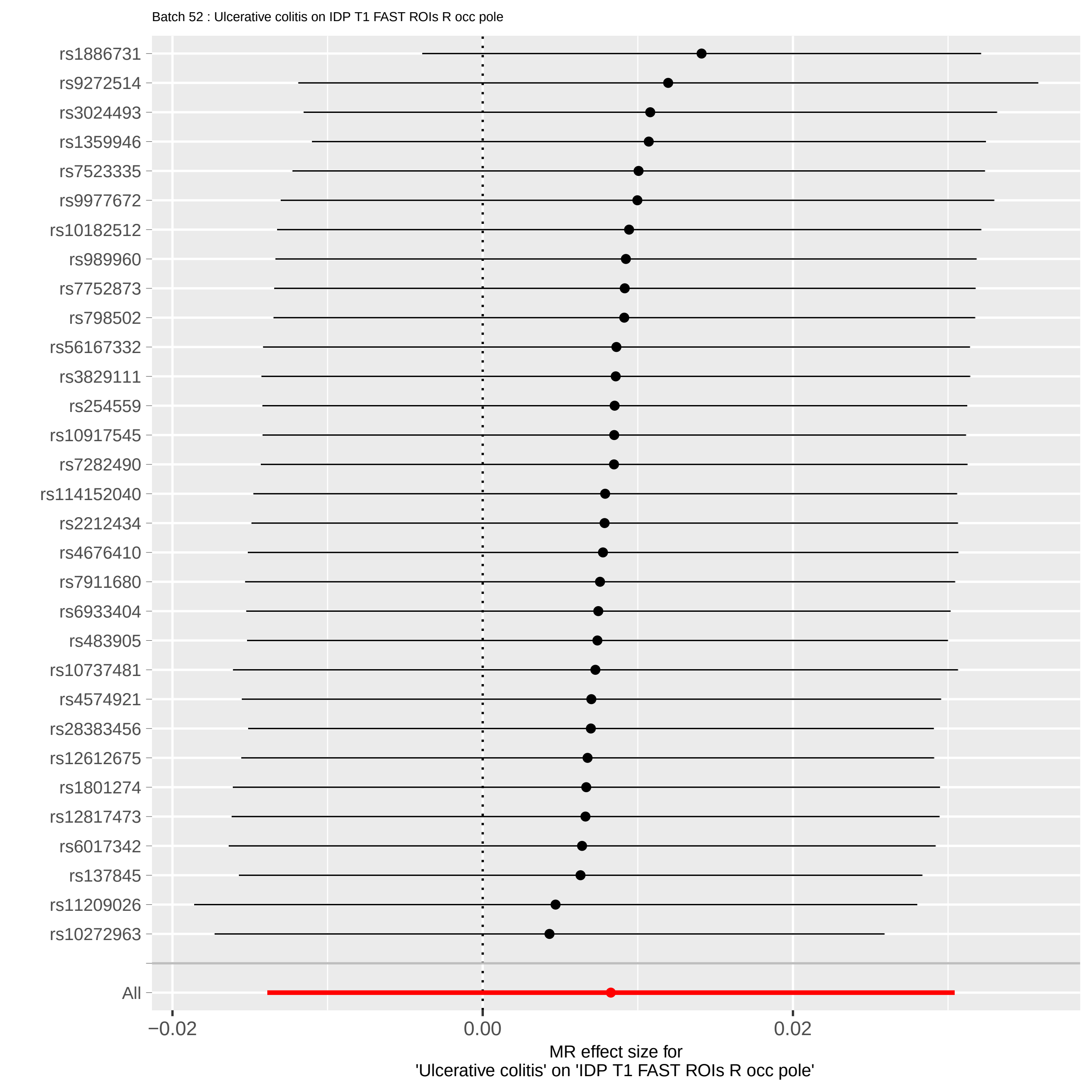

Batch 52 : Ulcerative colitis on IDP T1 FAST ROIs R occ pole
rs1886731
rs9272514
rs3024493
rs1359946
rs7523335
rs9977672
rs10182512
rs989960
rs7752873
rs798502
rs56167332
rs3829111
rs254559
rs10917545
rs7282490
rs114152040
rs2212434
rs4676410
rs7911680
rs6933404
rs483905
rs10737481
rs4574921
rs28383456
rs12612675
rs1801274
rs12817473
rs6017342
rs137845
rs11209026
rs10272963
All
−0.02
0.00
0.02
MR effect size for
'Ulcerative colitis' on 'IDP T1 FAST ROIs R occ pole'

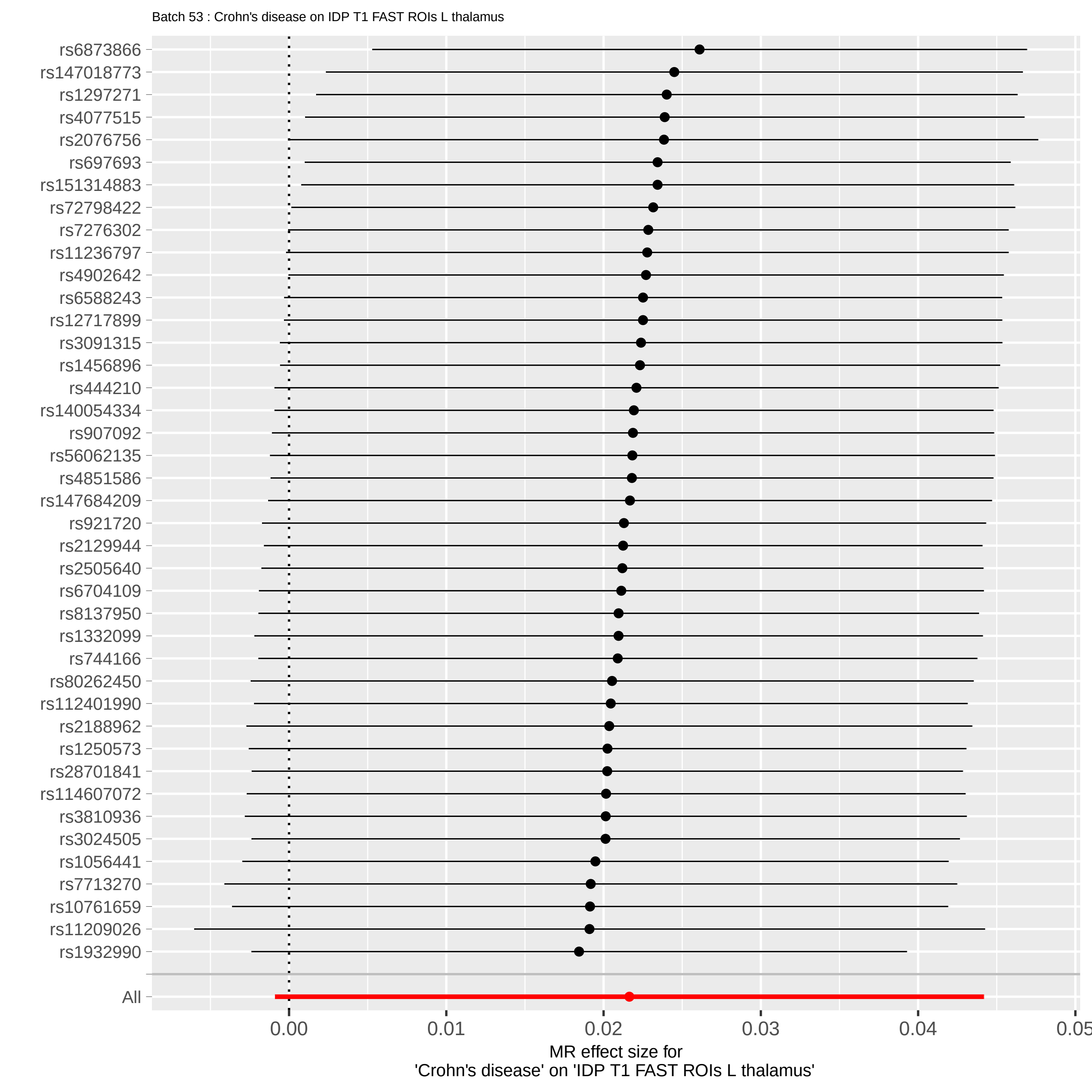
Batch 53 : Crohn's disease on IDP T1 FAST ROIs L thalamus
rs6873866
rs147018773
rs1297271
rs4077515
rs2076756
rs697693
rs151314883
rs72798422
rs7276302
rs11236797
rs4902642
rs6588243
rs12717899
rs3091315
rs1456896
rs444210
rs140054334
rs907092
rs56062135
rs4851586
rs147684209
rs921720
rs2129944
rs2505640
rs6704109
rs8137950
rs1332099
rs744166
rs80262450
rs112401990
rs2188962
rs1250573
rs28701841
rs114607072
rs3810936
rs3024505
rs1056441
rs7713270
rs10761659
rs11209026
rs1932990
All
0.00
0.01
0.02
0.03
0.04
0.05
MR effect size for
'Crohn's disease' on 'IDP T1 FAST ROIs L thalamus'

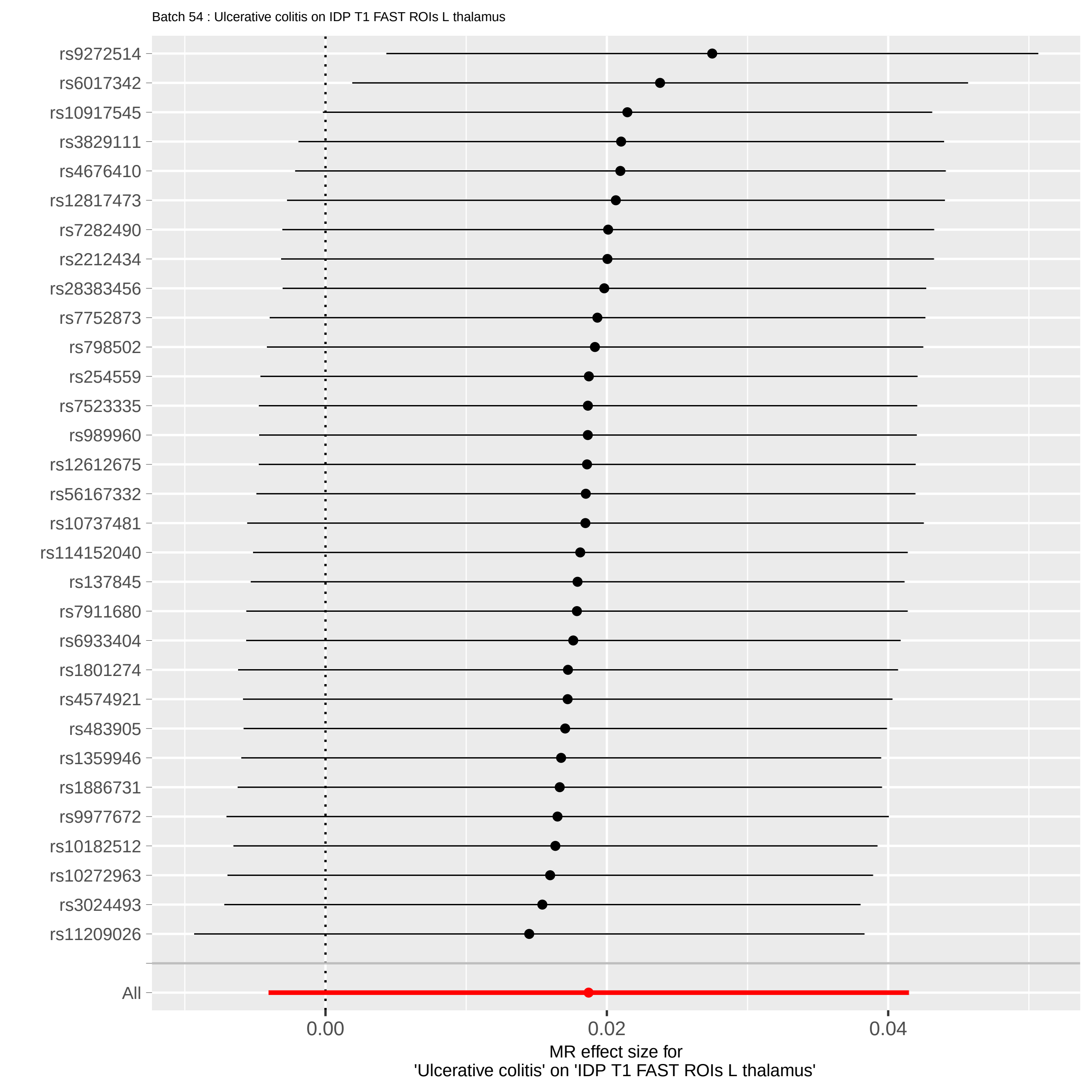

Batch 54 : Ulcerative colitis on IDP T1 FAST ROIs L thalamus
rs9272514
rs6017342
rs10917545
rs3829111
rs4676410
rs12817473
rs7282490
rs2212434
rs28383456
rs7752873
rs798502
rs254559
rs7523335
rs989960
rs12612675
rs56167332
rs10737481
rs114152040
rs137845
rs7911680
rs6933404
rs1801274
rs4574921
rs483905
rs1359946
rs1886731
rs9977672
rs10182512
rs10272963
rs3024493
rs11209026
All
0.00
0.02
0.04
MR effect size for
'Ulcerative colitis' on 'IDP T1 FAST ROIs L thalamus'

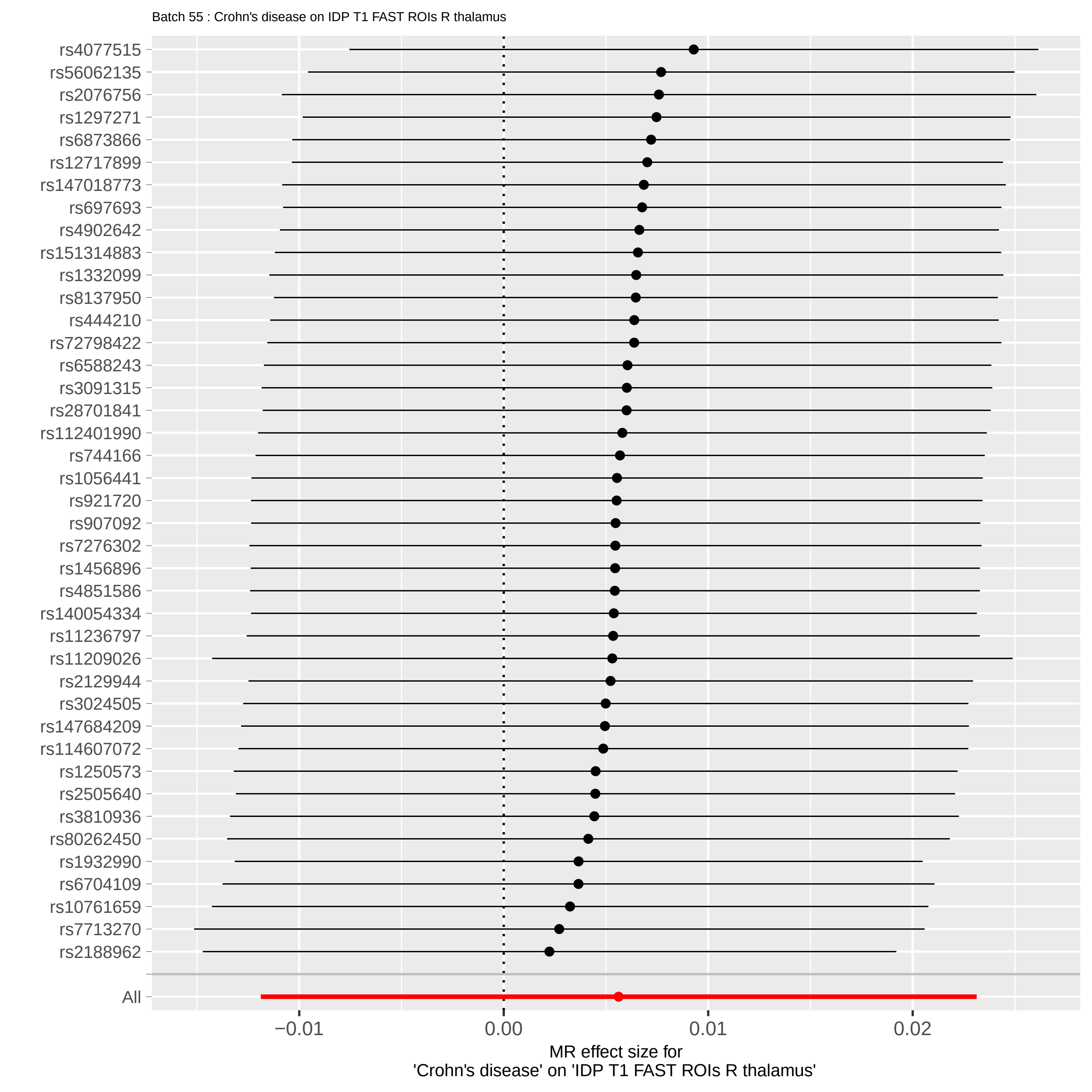

Batch 55 : Crohn's disease on IDP T1 FAST ROIs R thalamus
rs4077515
rs56062135
rs2076756
rs1297271
rs6873866
rs12717899
rs147018773
rs697693
rs4902642
rs151314883
rs1332099
rs8137950
rs444210
rs72798422
rs6588243
rs3091315
rs28701841
rs112401990
rs744166
rs1056441
rs921720
rs907092
rs7276302
rs1456896
rs4851586
rs140054334
rs11236797
rs11209026
rs2129944
rs3024505
rs147684209
rs114607072
rs1250573
rs2505640
rs3810936
rs80262450
rs1932990
rs6704109
rs10761659
rs7713270
rs2188962
All
−0.01
0.00
0.01
0.02
MR effect size for
'Crohn's disease' on 'IDP T1 FAST ROIs R thalamus'

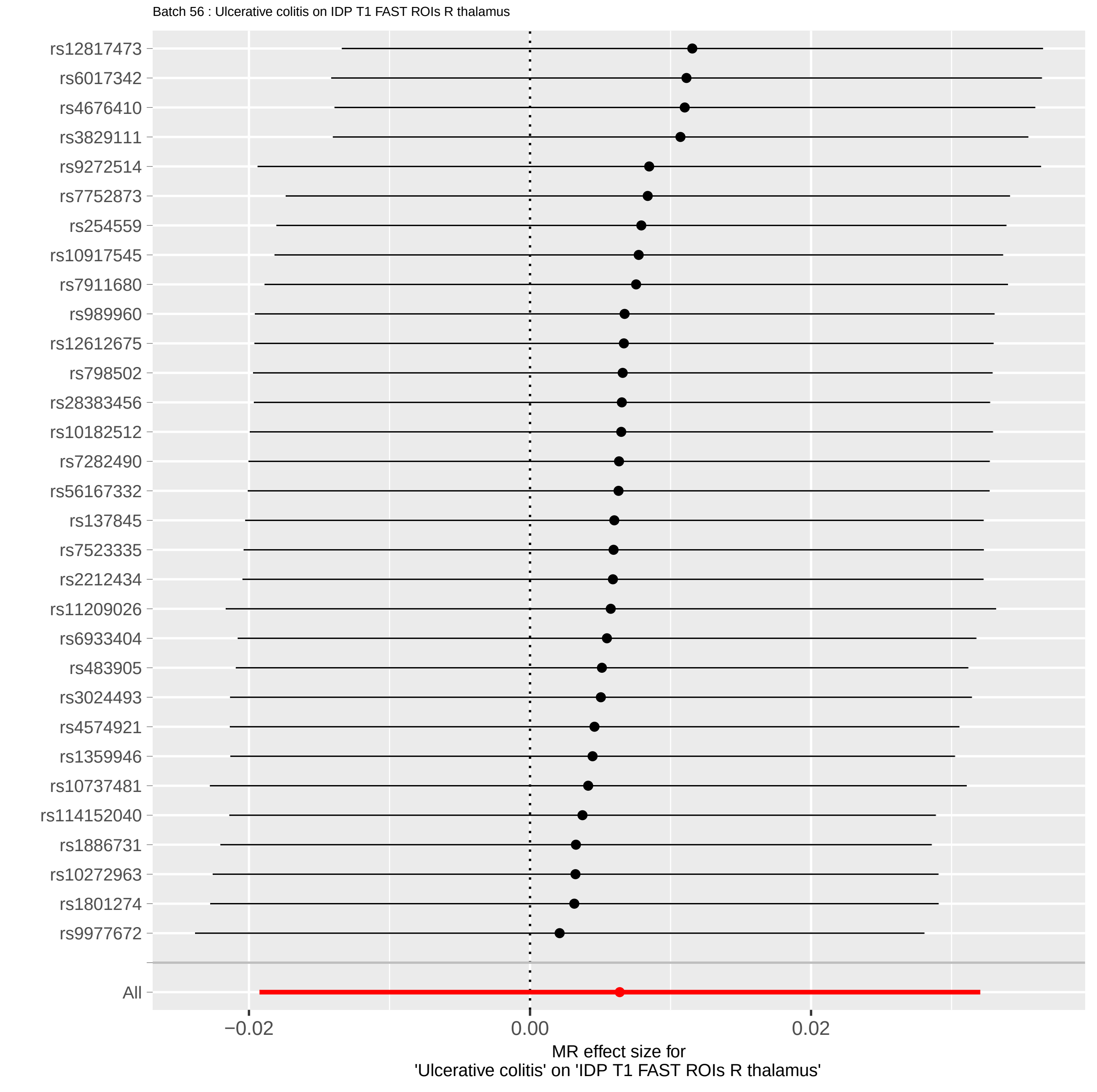
Batch 56 : Ulcerative colitis on IDP T1 FAST ROIs R thalamus
rs12817473
rs6017342
rs4676410
rs3829111
rs9272514
rs7752873
rs254559
rs10917545
rs7911680
rs989960
rs12612675
rs798502
rs28383456
rs10182512
rs7282490
rs56167332
rs137845
rs7523335
rs2212434
rs11209026
rs6933404
rs483905
rs3024493
rs4574921
rs1359946
rs10737481
rs114152040
rs1886731
rs10272963
rs1801274
rs9977672
All
−0.02
0.00
0.02
MR effect size for
'Ulcerative colitis' on 'IDP T1 FAST ROIs R thalamus'

Batch 57 : Crohn's disease on IDP T1 FAST ROIs L caudate

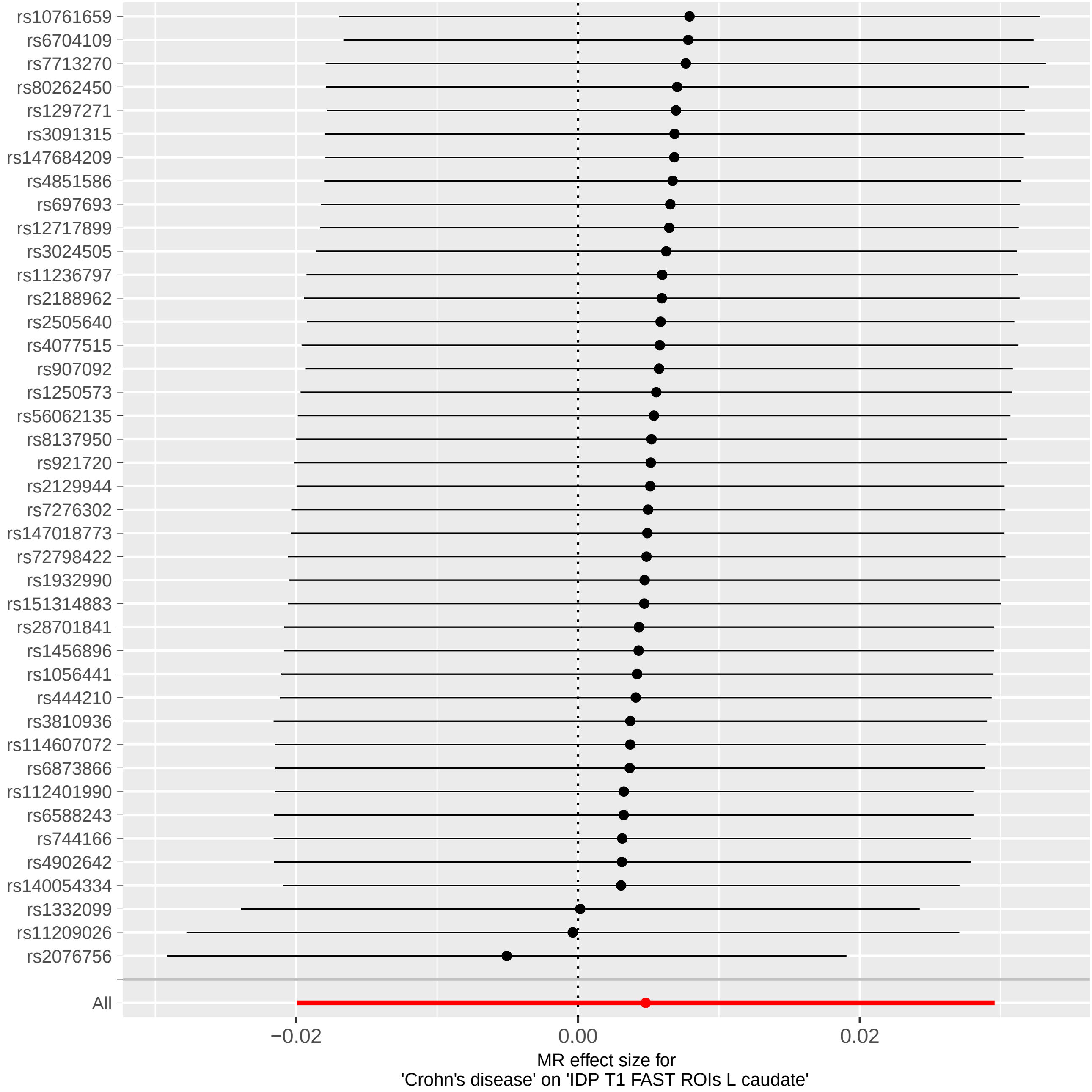

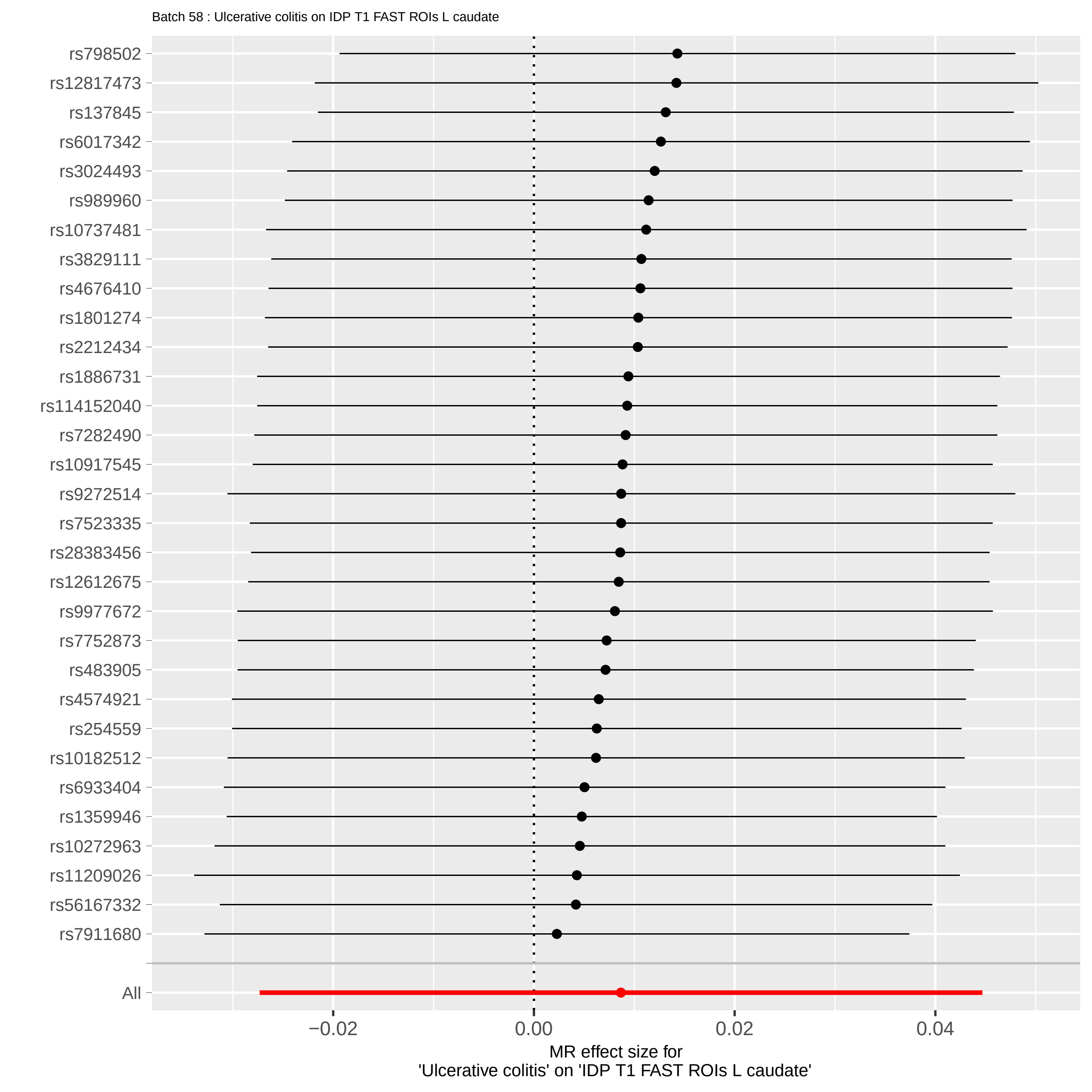

Batch 58 : Ulcerative colitis on IDP T1 FAST ROIs L caudate
rs798502
rs12817473
rs137845
rs6017342
rs3024493
rs989960
rs10737481
rs3829111
rs4676410
rs1801274
rs2212434
rs1886731
rs114152040
rs7282490
rs10917545
rs9272514
rs7523335
rs28383456
rs12612675
rs9977672
rs7752873
rs483905
rs4574921
rs254559
rs10182512
rs6933404
rs1359946
rs10272963
rs11209026
rs56167332
rs7911680
All
−0.02
0.00
0.02
0.04
MR effect size for
'Ulcerative colitis' on 'IDP T1 FAST ROIs L caudate'

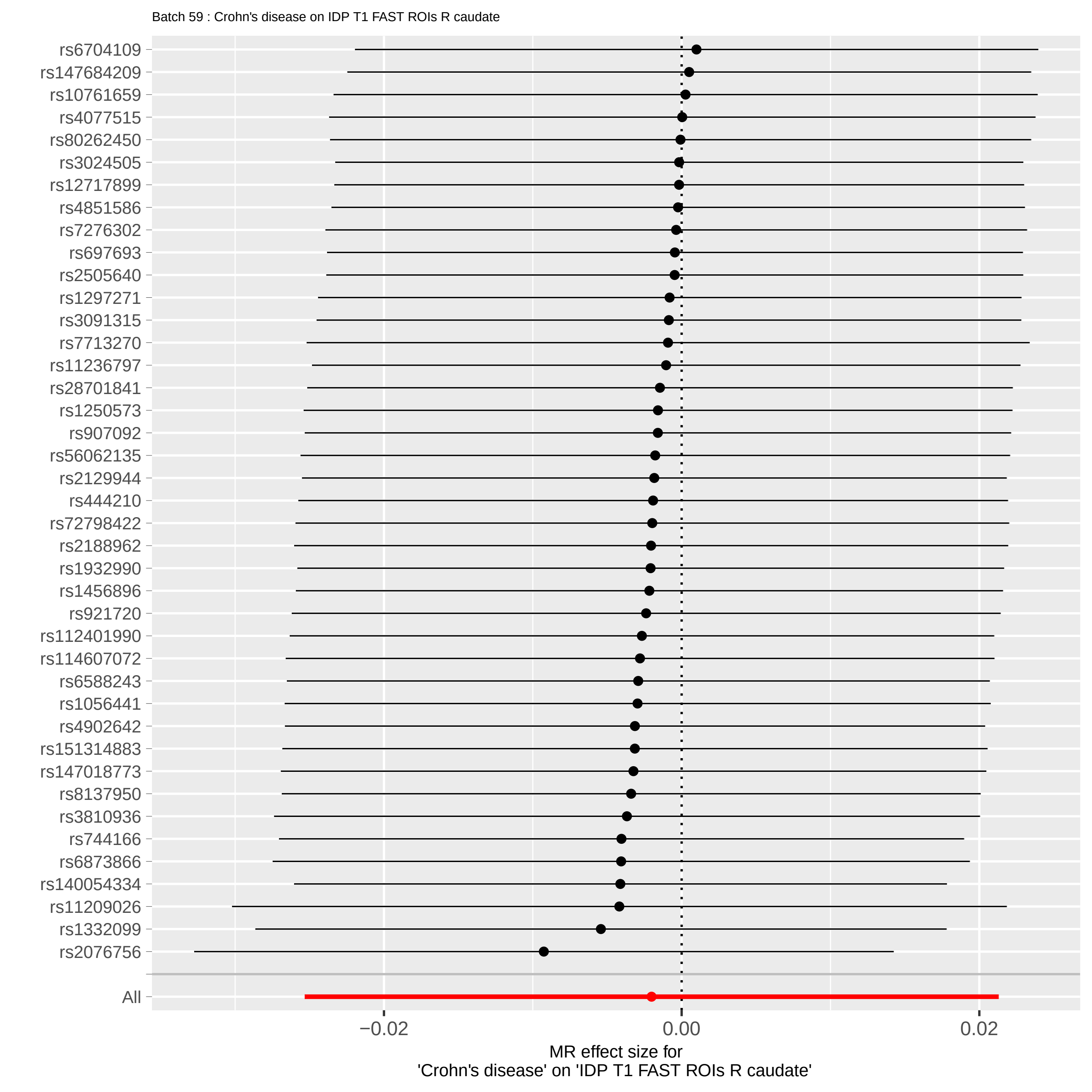
Batch 59 : Crohn's disease on IDP T1 FAST ROIs R caudate
rs6704109
rs147684209
rs10761659
rs4077515
rs80262450
rs3024505
rs12717899
rs4851586
rs7276302
rs697693
rs2505640
rs1297271
rs3091315
rs7713270
rs11236797
rs28701841
rs1250573
rs907092
rs56062135
rs2129944
rs444210
rs72798422
rs2188962
rs1932990
rs1456896
rs921720
rs112401990
rs114607072
rs6588243
rs1056441
rs4902642
rs151314883
rs147018773
rs8137950
rs3810936
rs744166
rs6873866
rs140054334
rs11209026
rs1332099
rs2076756
All
−0.02
0.00
0.02
MR effect size for
'Crohn's disease' on 'IDP T1 FAST ROIs R caudate'

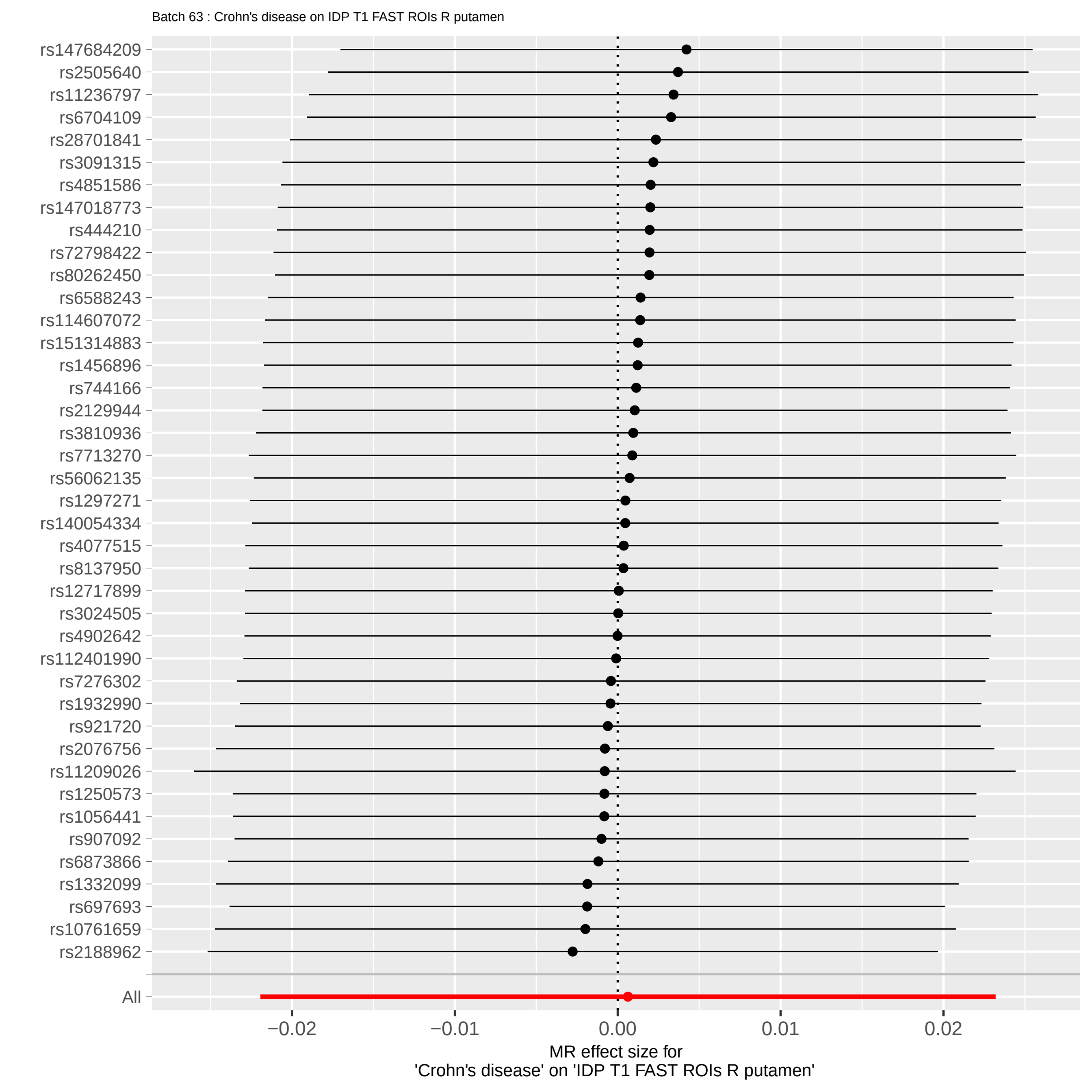

Batch 63 : Crohn's disease on IDP T1 FAST ROIs R putamen
rs147684209
rs2505640
rs11236797
rs6704109
rs28701841
rs3091315
rs4851586
rs147018773
rs444210
rs72798422
rs80262450
rs6588243
rs114607072
rs151314883
rs1456896
rs744166
rs2129944
rs3810936
rs7713270
rs56062135
rs1297271
rs140054334
rs4077515
rs8137950
rs12717899
rs3024505
rs4902642
rs112401990
rs7276302
rs1932990
rs921720
rs2076756
rs11209026
rs1250573
rs1056441
rs907092
rs6873866
rs1332099
rs697693
rs10761659
rs2188962
All
−0.02
−0.01
0.00
0.01
0.02
MR effect size for
'Crohn's disease' on 'IDP T1 FAST ROIs R putamen'

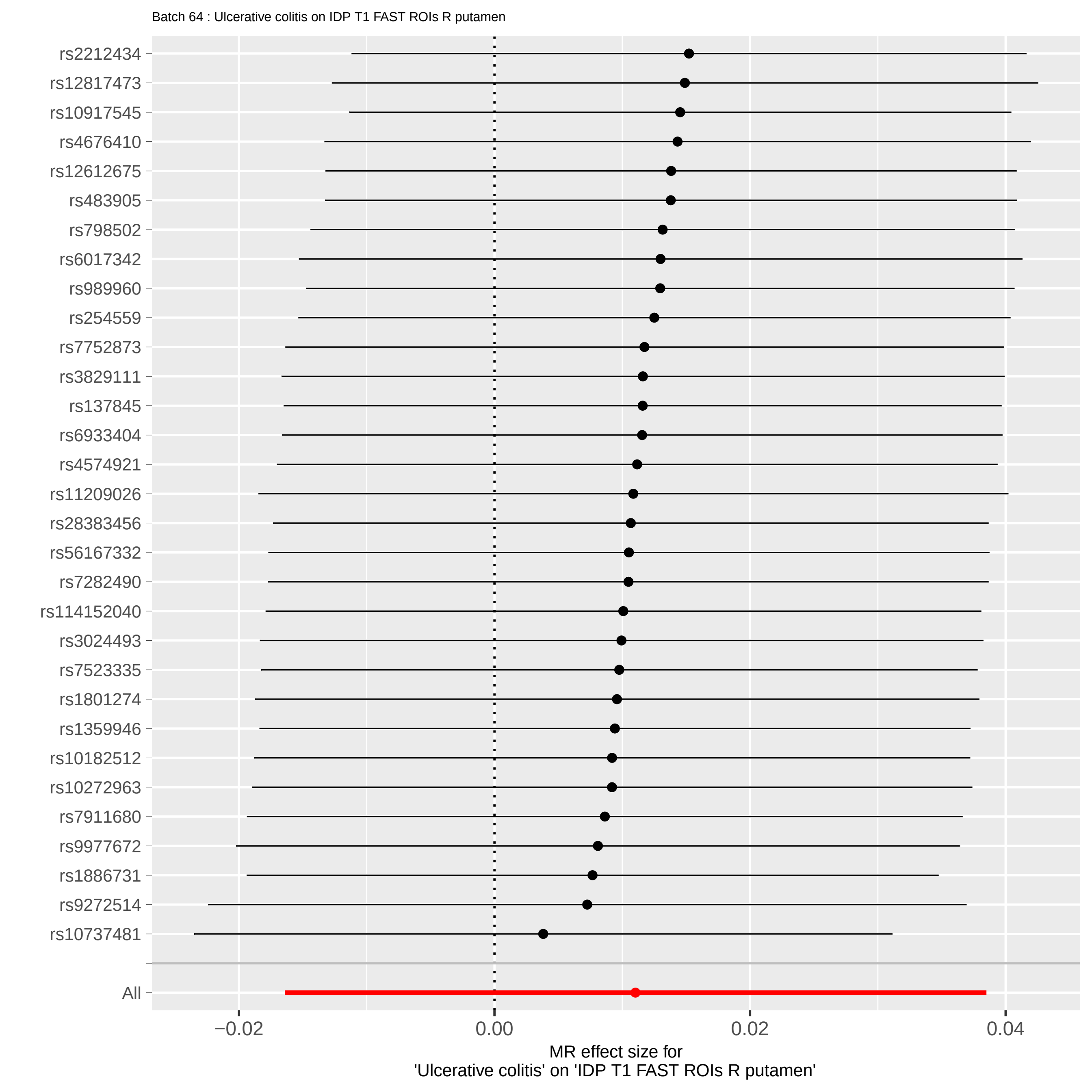
Batch 64 : Ulcerative colitis on IDP T1 FAST ROIs R putamen
rs2212434
rs12817473
rs10917545
rs4676410
rs12612675
rs483905
rs798502
rs6017342
rs989960
rs254559
rs7752873
rs3829111
rs137845
rs6933404
rs4574921
rs11209026
rs28383456
rs56167332
rs7282490
rs114152040
rs3024493
rs7523335
rs1801274
rs1359946
rs10182512
rs10272963
rs7911680
rs9977672
rs1886731
rs9272514
rs10737481
All
−0.02
0.00
0.02
0.04
MR effect size for
'Ulcerative colitis' on 'IDP T1 FAST ROIs R putamen'

Batch 65 : Crohn's disease on IDP T1 FAST ROIs L pallidum

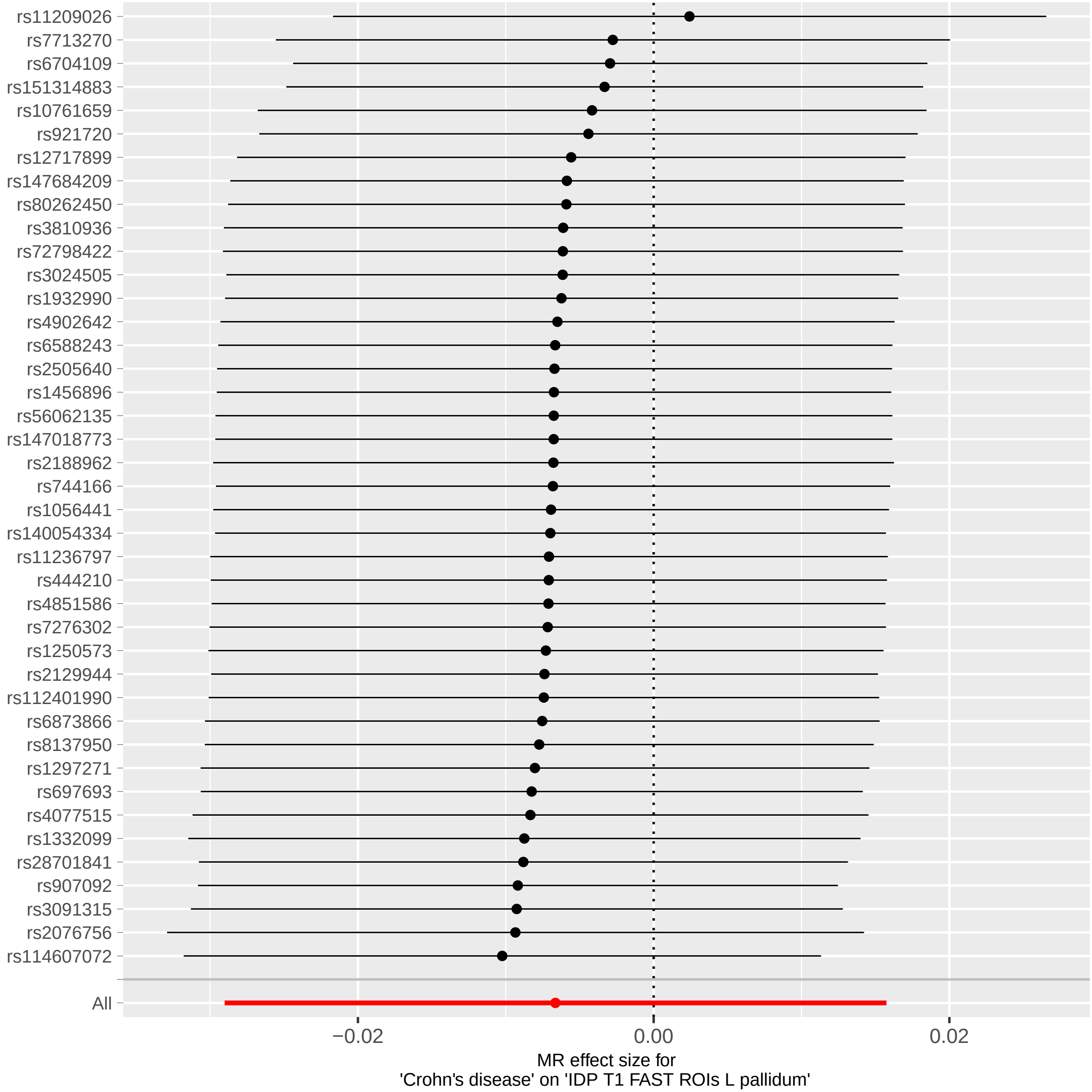

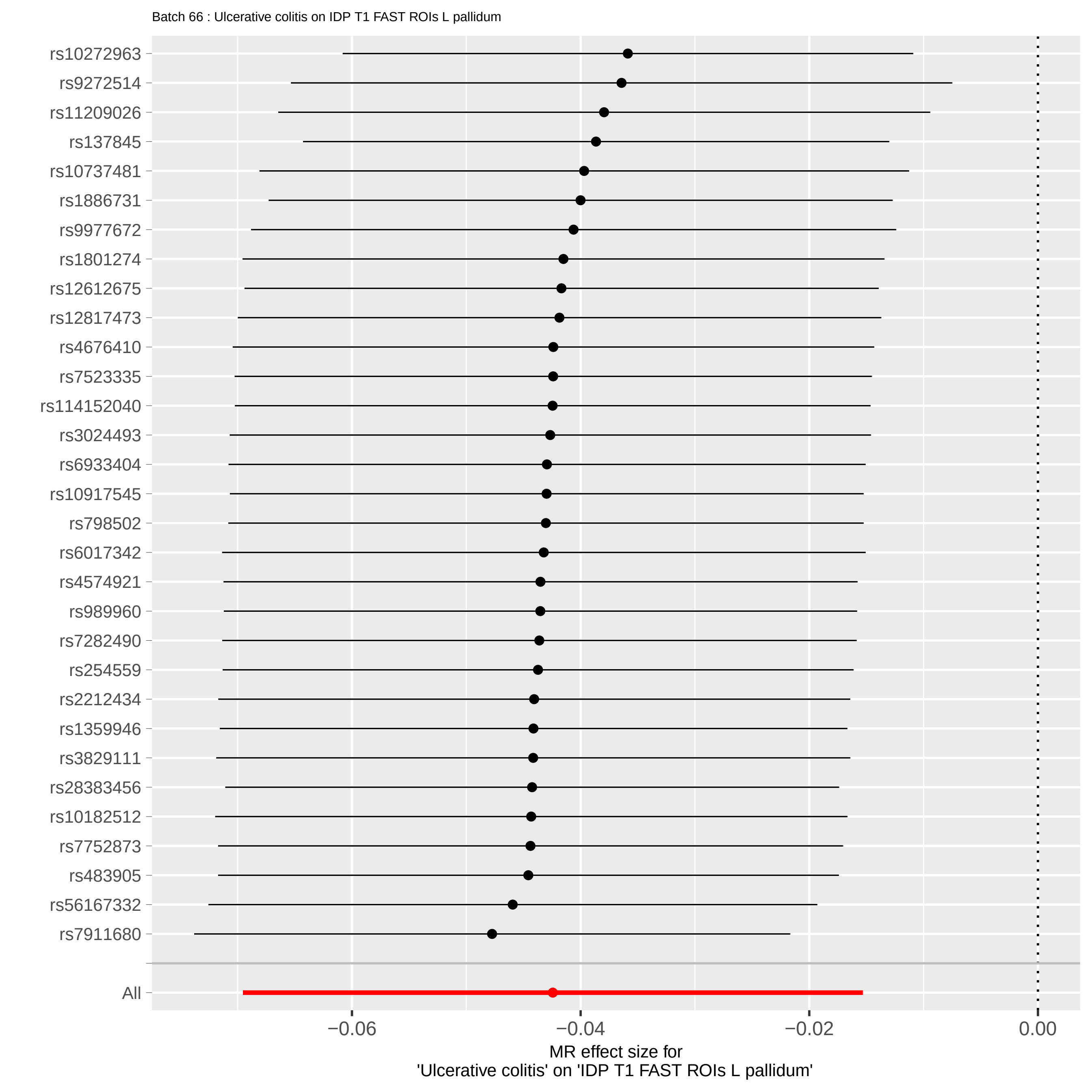

Batch 66 : Ulcerative colitis on IDP T1 FAST ROIs L pallidum
rs10272963
rs9272514
rs11209026
rs137845
rs10737481
rs1886731
rs9977672
rs1801274
rs12612675
rs12817473
rs4676410
rs7523335
rs114152040
rs3024493
rs6933404
rs10917545
rs798502
rs6017342
rs4574921
rs989960
rs7282490
rs254559
rs2212434
rs1359946
rs3829111
rs28383456
rs10182512
rs7752873
rs483905
rs56167332
rs7911680
All
−0.06
−0.04
−0.02
0.00
MR effect size for
'Ulcerative colitis' on 'IDP T1 FAST ROIs L pallidum'

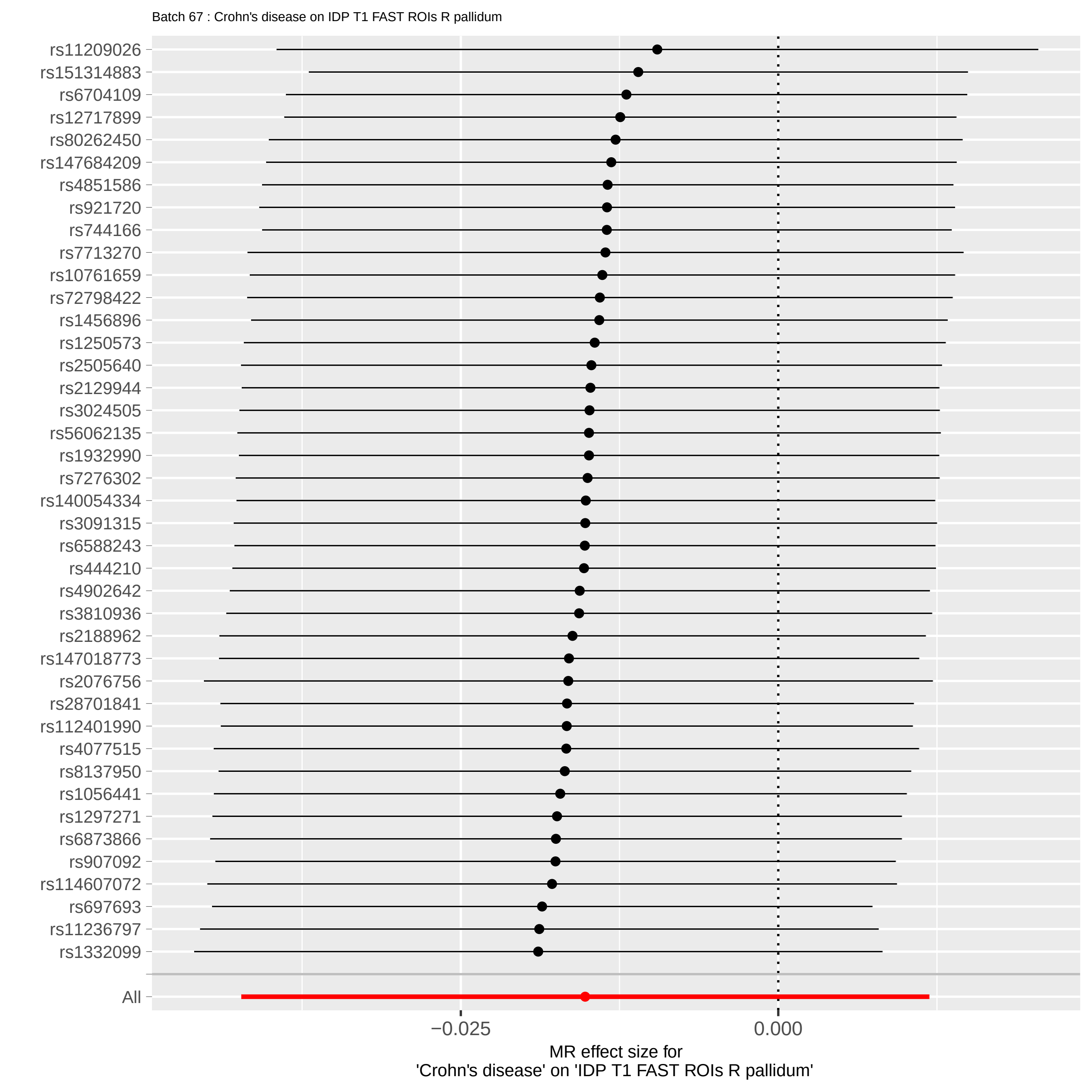

Batch 67 : Crohn's disease on IDP T1 FAST ROIs R pallidum
rs11209026
rs151314883
rs6704109
rs12717899
rs80262450
rs147684209
rs4851586
rs921720
rs744166
rs7713270
rs10761659
rs72798422
rs1456896
rs1250573
rs2505640
rs2129944
rs3024505
rs56062135
rs1932990
rs7276302
rs140054334
rs3091315
rs6588243
rs444210
rs4902642
rs3810936
rs2188962
rs147018773
rs2076756
rs28701841
rs112401990
rs4077515
rs8137950
rs1056441
rs1297271
rs6873866
rs907092
rs114607072
rs697693
rs11236797
rs1332099
All
−0.025
0.000
MR effect size for
'Crohn's disease' on 'IDP T1 FAST ROIs R pallidum'

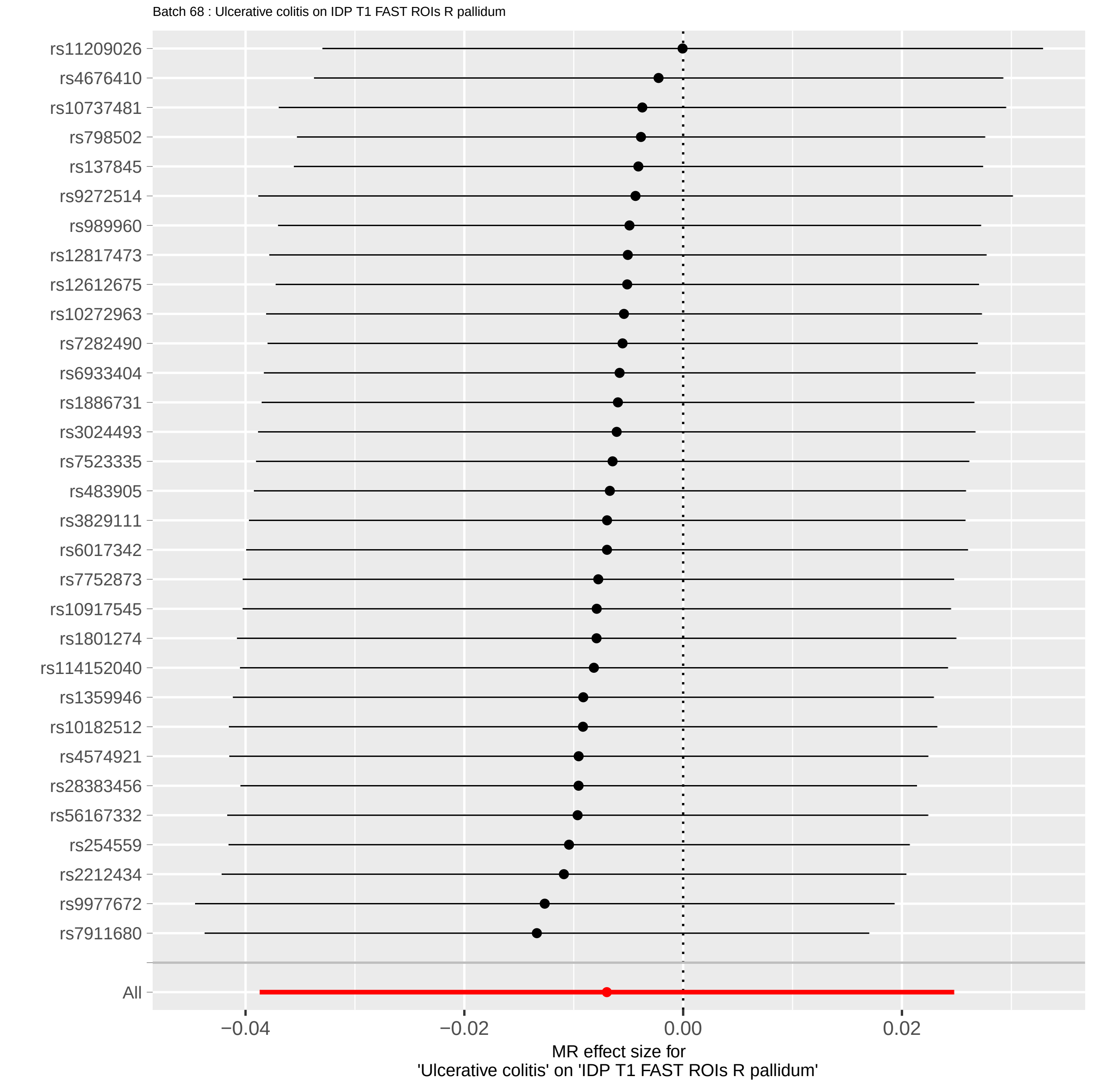
Batch 68 : Ulcerative colitis on IDP T1 FAST ROIs R pallidum
rs11209026
rs4676410
rs10737481
rs798502
rs137845
rs9272514
rs989960
rs12817473
rs12612675
rs10272963
rs7282490
rs6933404
rs1886731
rs3024493
rs7523335
rs483905
rs3829111
rs6017342
rs7752873
rs10917545
rs1801274
rs114152040
rs1359946
rs10182512
rs4574921
rs28383456
rs56167332
rs254559
rs2212434
rs9977672
rs7911680
All
−0.04
−0.02
0.00
0.02
MR effect size for
'Ulcerative colitis' on 'IDP T1 FAST ROIs R pallidum'

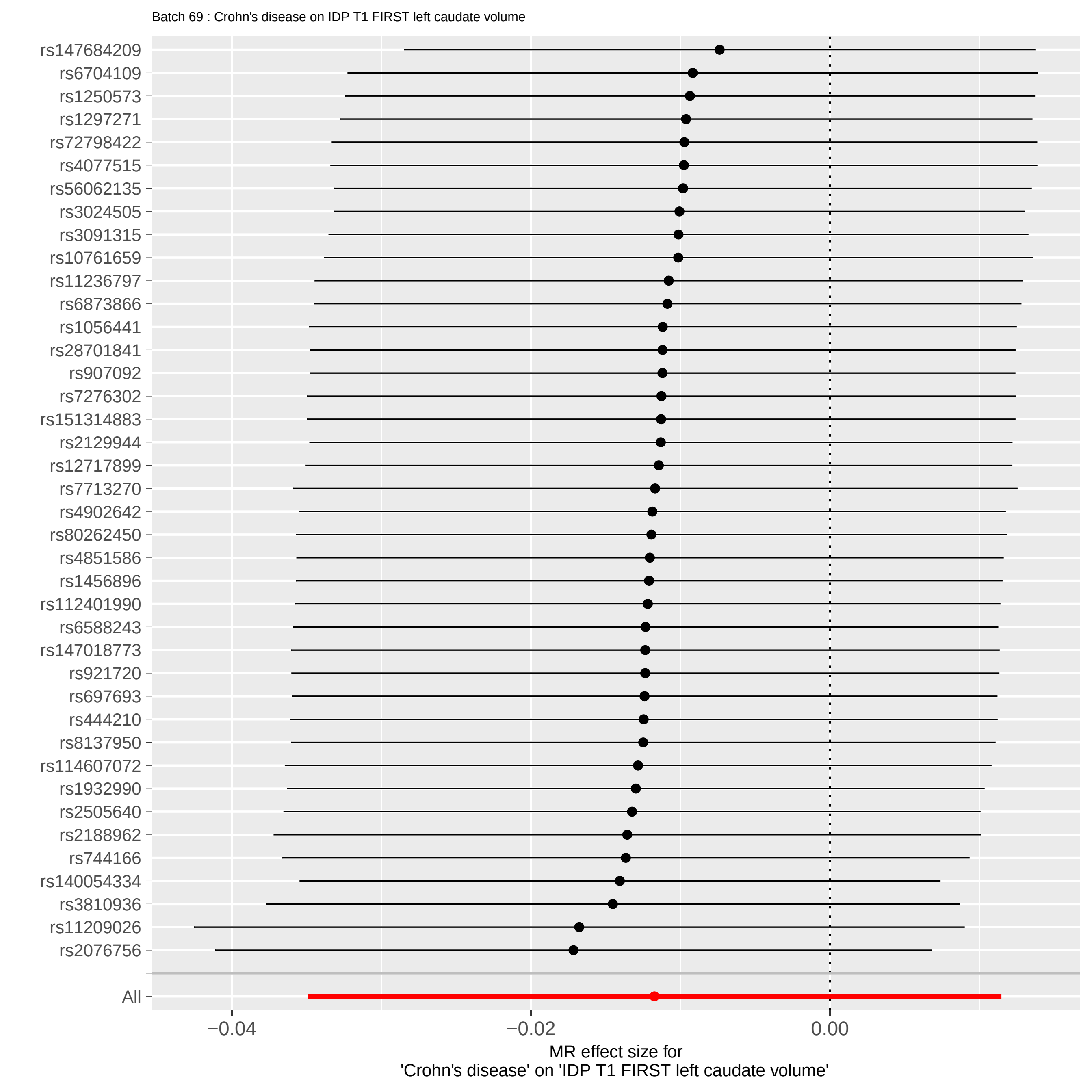
Batch 69 : Crohn's disease on IDP T1 FIRST left caudate volume
rs147684209
rs6704109
rs1250573
rs1297271
rs72798422
rs4077515
rs56062135
rs3024505
rs3091315
rs10761659
rs11236797
rs6873866
rs1056441
rs28701841
rs907092
rs7276302
rs151314883
rs2129944
rs12717899
rs7713270
rs4902642
rs80262450
rs4851586
rs1456896
rs112401990
rs6588243
rs147018773
rs921720
rs697693
rs444210
rs8137950
rs114607072
rs1932990
rs2505640
rs2188962
rs744166
rs140054334
rs3810936
rs11209026
rs2076756
All
−0.04
−0.02
0.00
MR effect size for
'Crohn's disease' on 'IDP T1 FIRST left caudate volume'

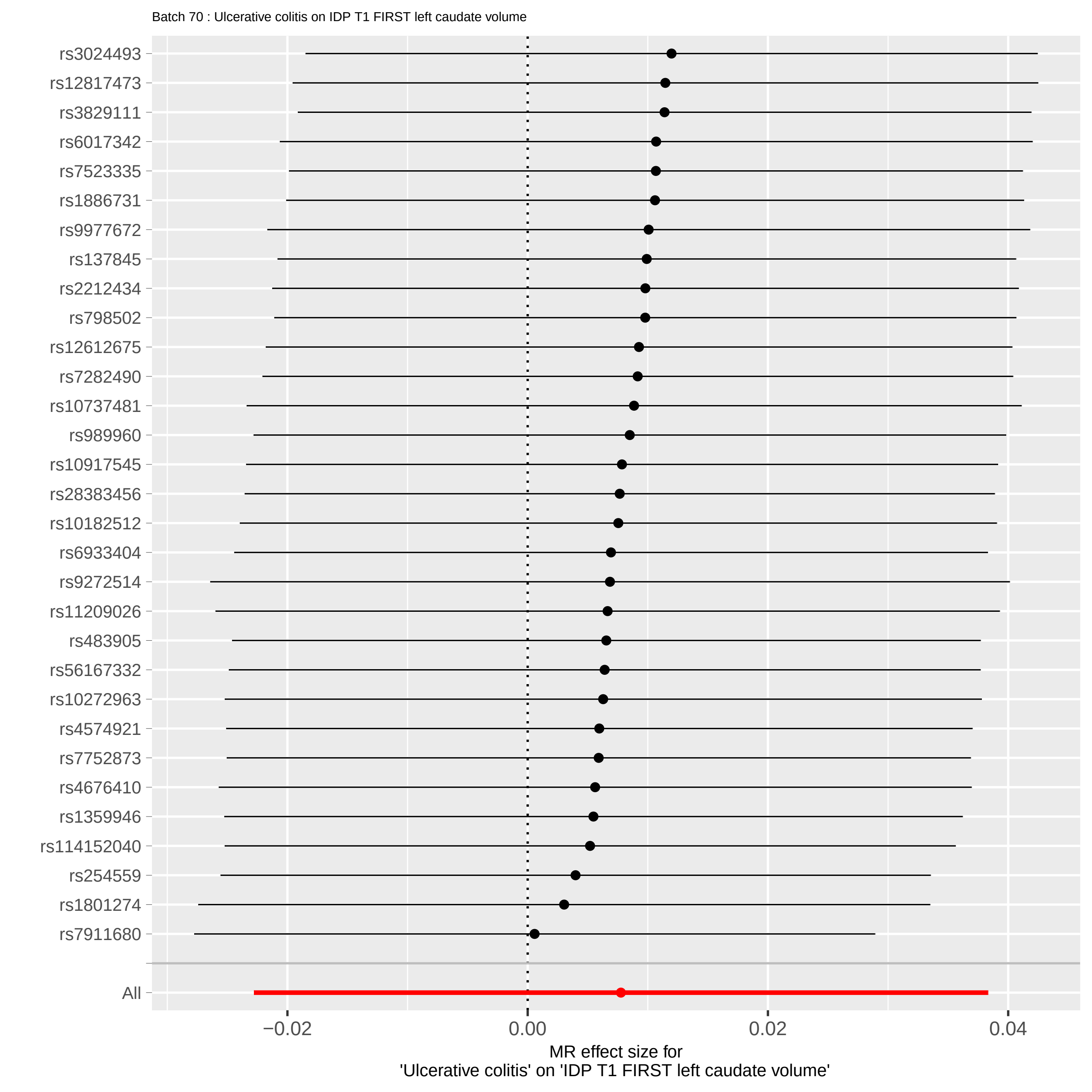

Batch 70 : Ulcerative colitis on IDP T1 FIRST left caudate volume
rs3024493
rs12817473
rs3829111
rs6017342
rs7523335
rs1886731
rs9977672
rs137845
rs2212434
rs798502
rs12612675
rs7282490
rs10737481
rs989960
rs10917545
rs28383456
rs10182512
rs6933404
rs9272514
rs11209026
rs483905
rs56167332
rs10272963
rs4574921
rs7752873
rs4676410
rs1359946
rs114152040
rs254559
rs1801274
rs7911680
All
−0.02
0.00
0.02
0.04
MR effect size for
'Ulcerative colitis' on 'IDP T1 FIRST left caudate volume'

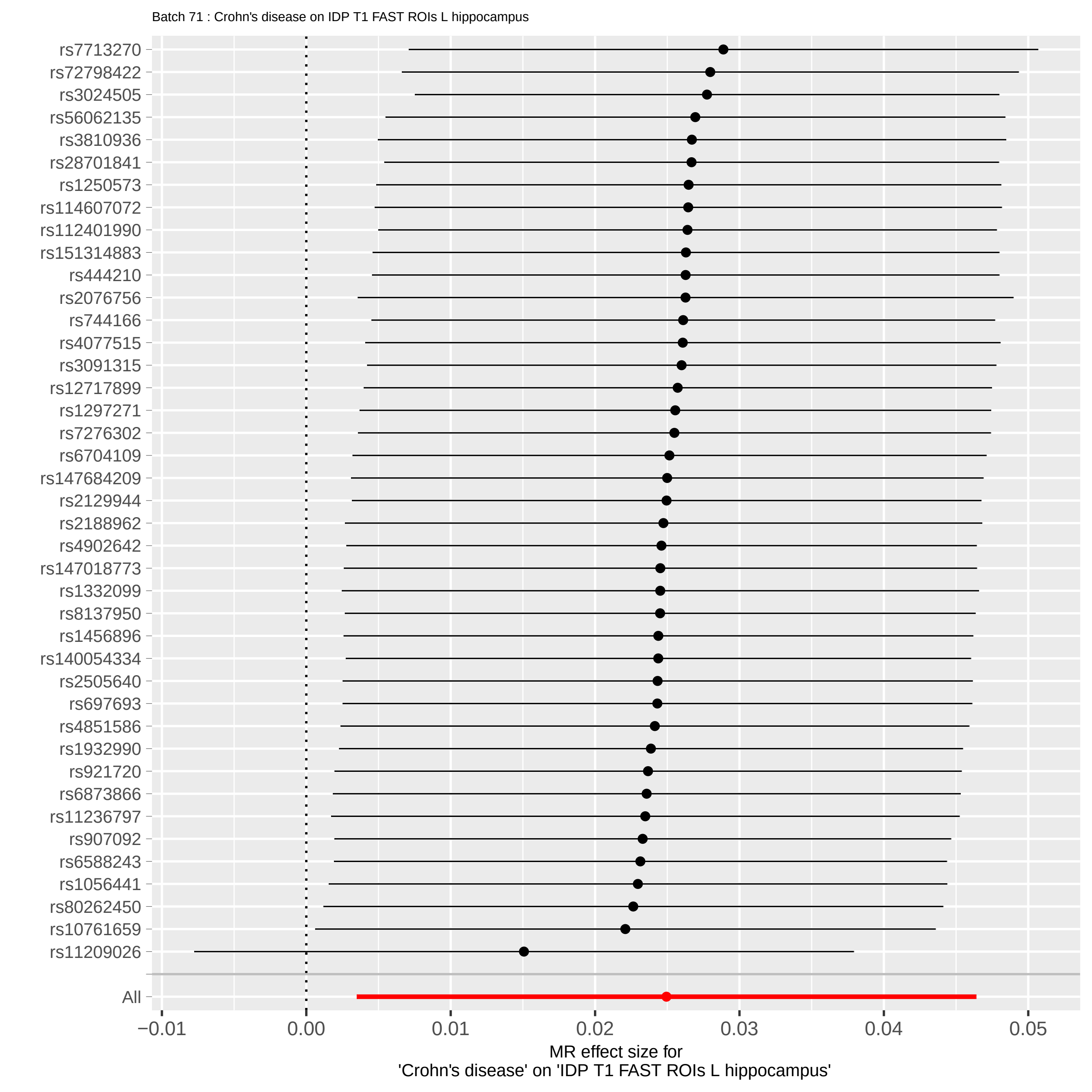

Batch 71 : Crohn's disease on IDP T1 FAST ROIs L hippocampus
rs7713270
rs72798422
rs3024505
rs56062135
rs3810936
rs28701841
rs1250573
rs114607072
rs112401990
rs151314883
rs444210
rs2076756
rs744166
rs4077515
rs3091315
rs12717899
rs1297271
rs7276302
rs6704109
rs147684209
rs2129944
rs2188962
rs4902642
rs147018773
rs1332099
rs8137950
rs1456896
rs140054334
rs2505640
rs697693
rs4851586
rs1932990
rs921720
rs6873866
rs11236797
rs907092
rs6588243
rs1056441
rs80262450
rs10761659
rs11209026
All
−0.01
0.00
0.01
0.02
0.03
0.04
0.05
MR effect size for
'Crohn's disease' on 'IDP T1 FAST ROIs L hippocampus'

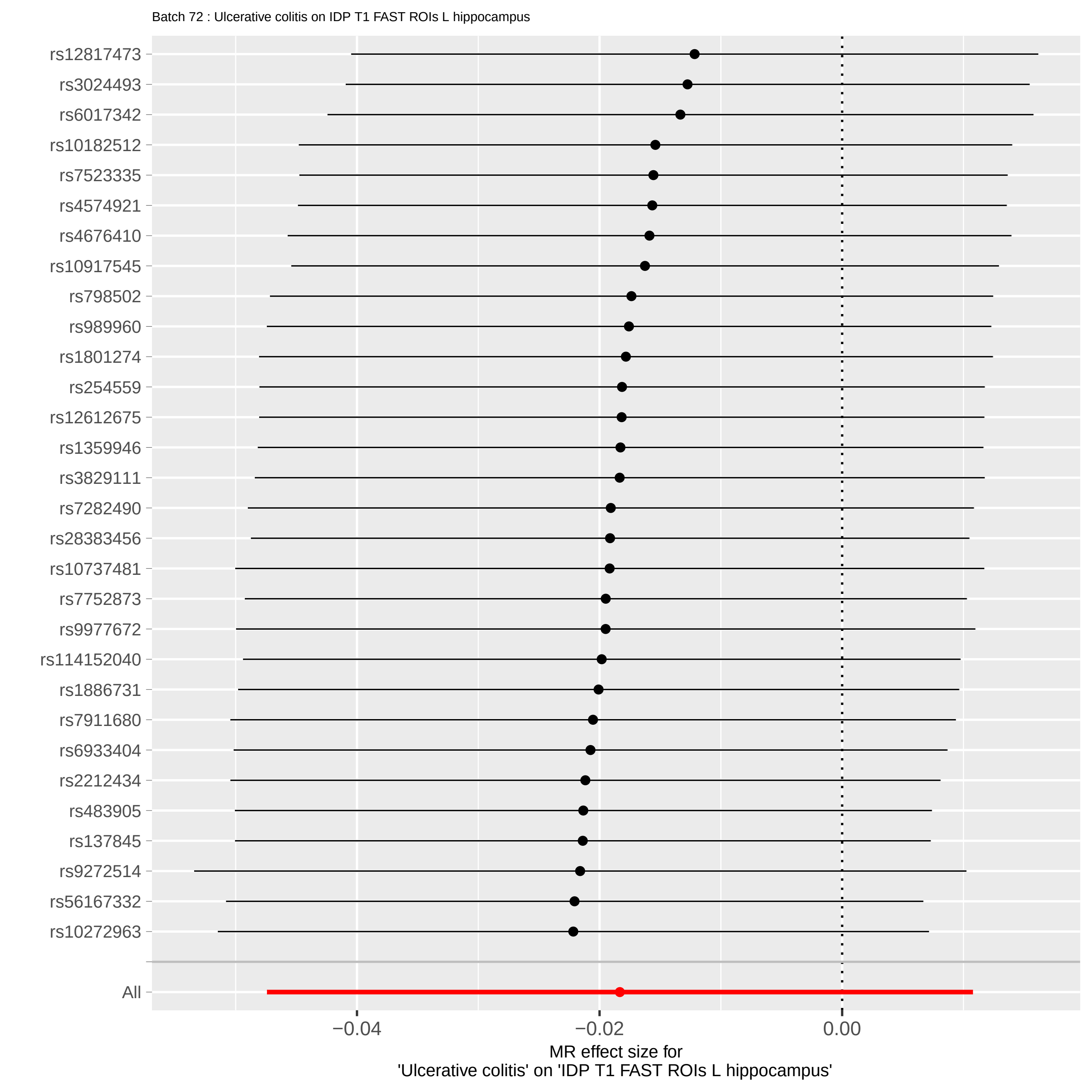

Batch 72 : Ulcerative colitis on IDP T1 FAST ROIs L hippocampus
rs12817473
rs3024493
rs6017342
rs10182512
rs7523335
rs4574921
rs4676410
rs10917545
rs798502
rs989960
rs1801274
rs254559
rs12612675
rs1359946
rs3829111
rs7282490
rs28383456
rs10737481
rs7752873
rs9977672
rs114152040
rs1886731
rs7911680
rs6933404
rs2212434
rs483905
rs137845
rs9272514
rs56167332
rs10272963
All
−0.04
−0.02
0.00
MR effect size for
'Ulcerative colitis' on 'IDP T1 FAST ROIs L hippocampus'

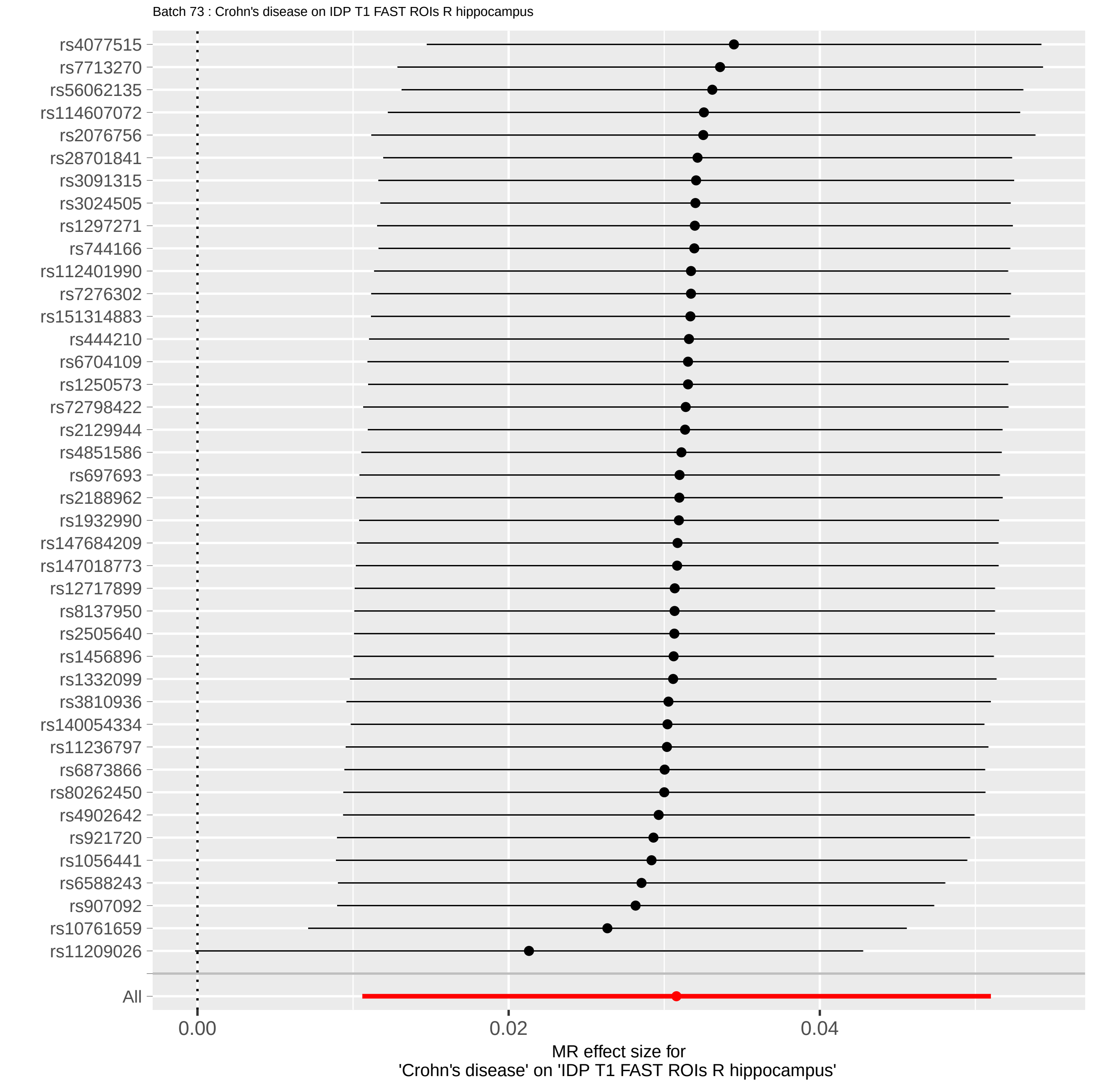

Batch 73 : Crohn's disease on IDP T1 FAST ROIs R hippocampus
rs4077515
rs7713270
rs56062135
rs114607072
rs2076756
rs28701841
rs3091315
rs3024505
rs1297271
rs744166
rs112401990
rs7276302
rs151314883
rs444210
rs6704109
rs1250573
rs72798422
rs2129944
rs4851586
rs697693
rs2188962
rs1932990
rs147684209
rs147018773
rs12717899
rs8137950
rs2505640
rs1456896
rs1332099
rs3810936
rs140054334
rs11236797
rs6873866
rs80262450
rs4902642
rs921720
rs1056441
rs6588243
rs907092
rs10761659
rs11209026
All
0.00
0.02
0.04
MR effect size for
'Crohn's disease' on 'IDP T1 FAST ROIs R hippocampus'

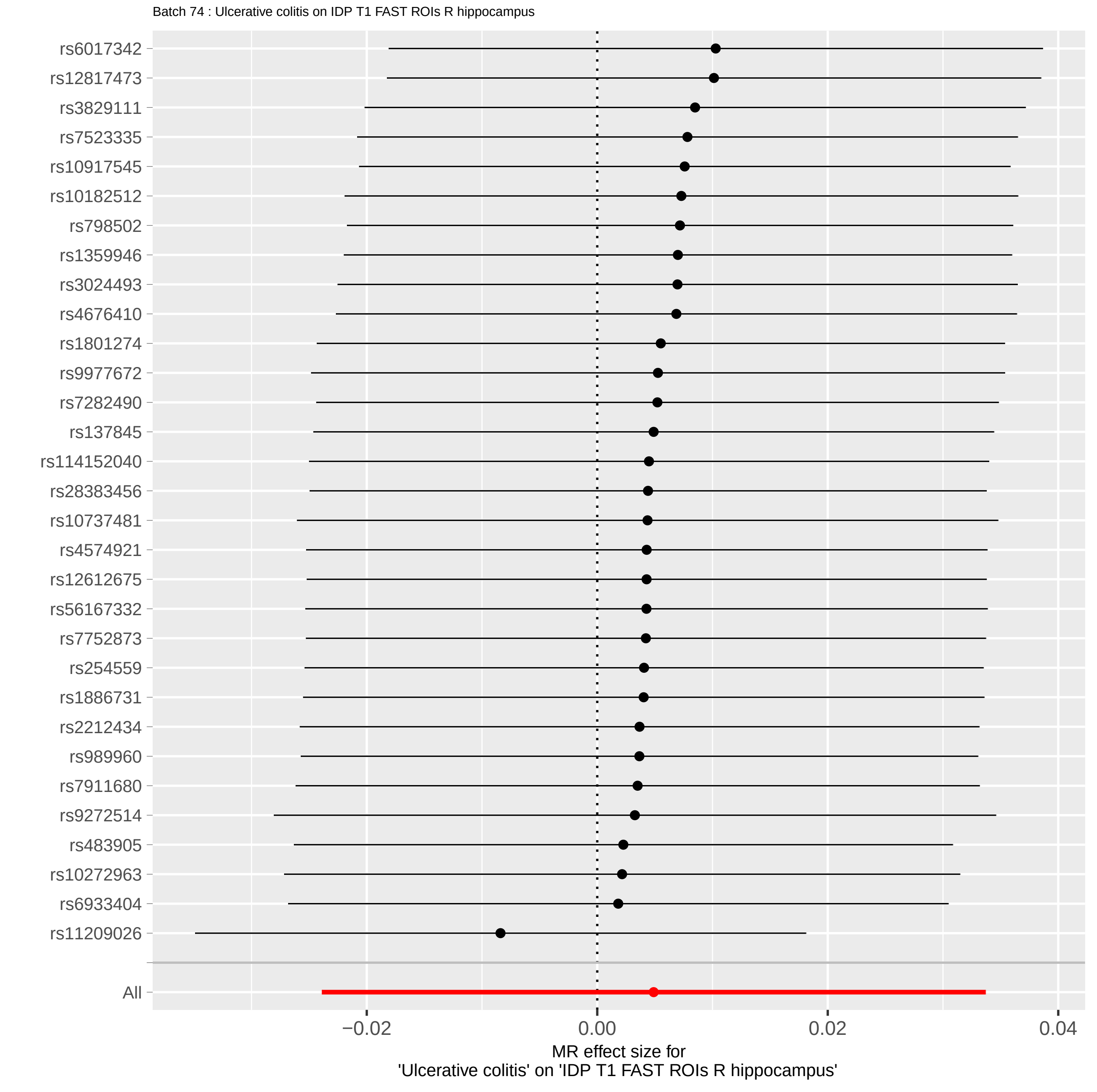
Batch 74 : Ulcerative colitis on IDP T1 FAST ROIs R hippocampus
rs6017342
rs12817473
rs3829111
rs7523335
rs10917545
rs10182512
rs798502
rs1359946
rs3024493
rs4676410
rs1801274
rs9977672
rs7282490
rs137845
rs114152040
rs28383456
rs10737481
rs4574921
rs12612675
rs56167332
rs7752873
rs254559
rs1886731
rs2212434
rs989960
rs7911680
rs9272514
rs483905
rs10272963
rs6933404
rs11209026
All
−0.02
0.00
0.02
0.04
MR effect size for
'Ulcerative colitis' on 'IDP T1 FAST ROIs R hippocampus'

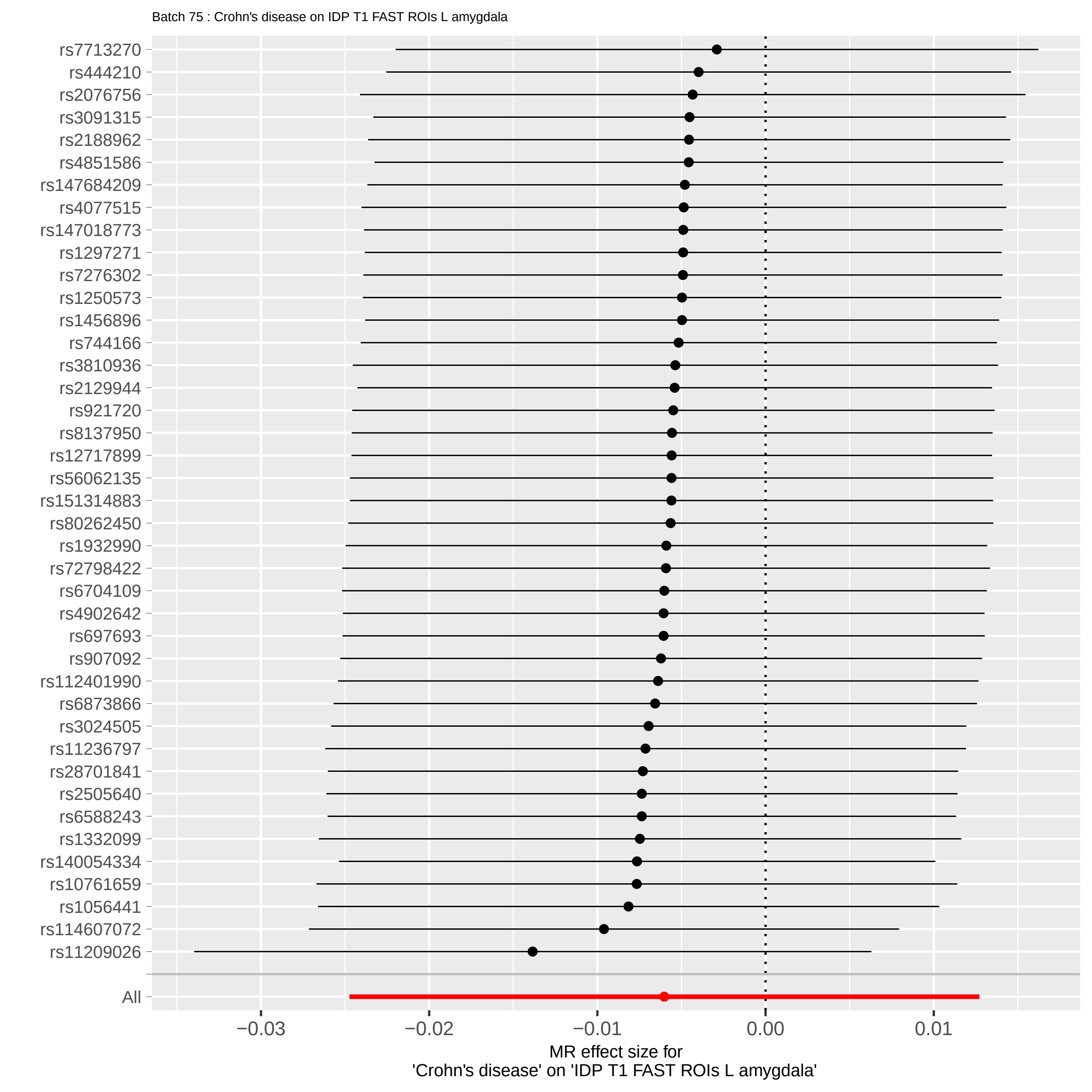

Batch 75 : Crohn's disease on IDP T1 FAST ROIs L amygdala
rs7713270
rs444210
rs2076756
rs3091315
rs2188962
rs4851586
rs147684209
rs4077515
rs147018773
rs1297271
rs7276302
rs1250573
rs1456896
rs744166
rs3810936
rs2129944
rs921720
rs8137950
rs12717899
rs56062135
rs151314883
rs80262450
rs1932990
rs72798422
rs6704109
rs4902642
rs697693
rs907092
rs112401990
rs6873866
rs3024505
rs11236797
rs28701841
rs2505640
rs6588243
rs1332099
rs140054334
rs10761659
rs1056441
rs114607072
rs11209026
All
−0.03
−0.02
−0.01
0.00
0.01
MR effect size for
'Crohn's disease' on 'IDP T1 FAST ROIs L amygdala'

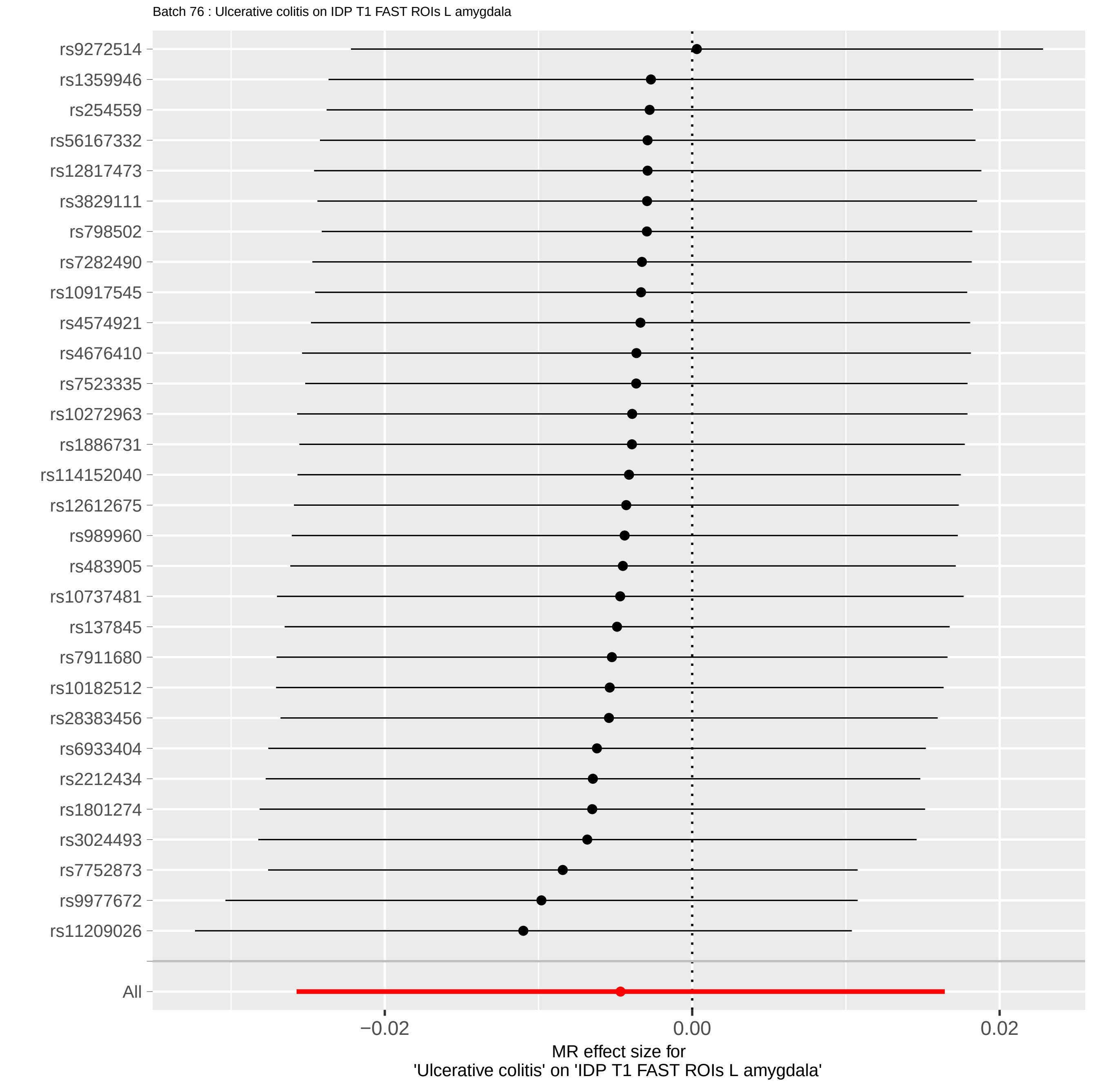
Batch 76 : Ulcerative colitis on IDP T1 FAST ROIs L amygdala
rs9272514
rs1359946
rs254559
rs56167332
rs12817473
rs3829111
rs798502
rs7282490
rs10917545
rs4574921
rs4676410
rs7523335
rs10272963
rs1886731
rs114152040
rs12612675
rs989960
rs483905
rs10737481
rs137845
rs7911680
rs10182512
rs28383456
rs6933404
rs2212434
rs1801274
rs3024493
rs7752873
rs9977672
rs11209026
All
−0.02
0.00
0.02
MR effect size for
'Ulcerative colitis' on 'IDP T1 FAST ROIs L amygdala'

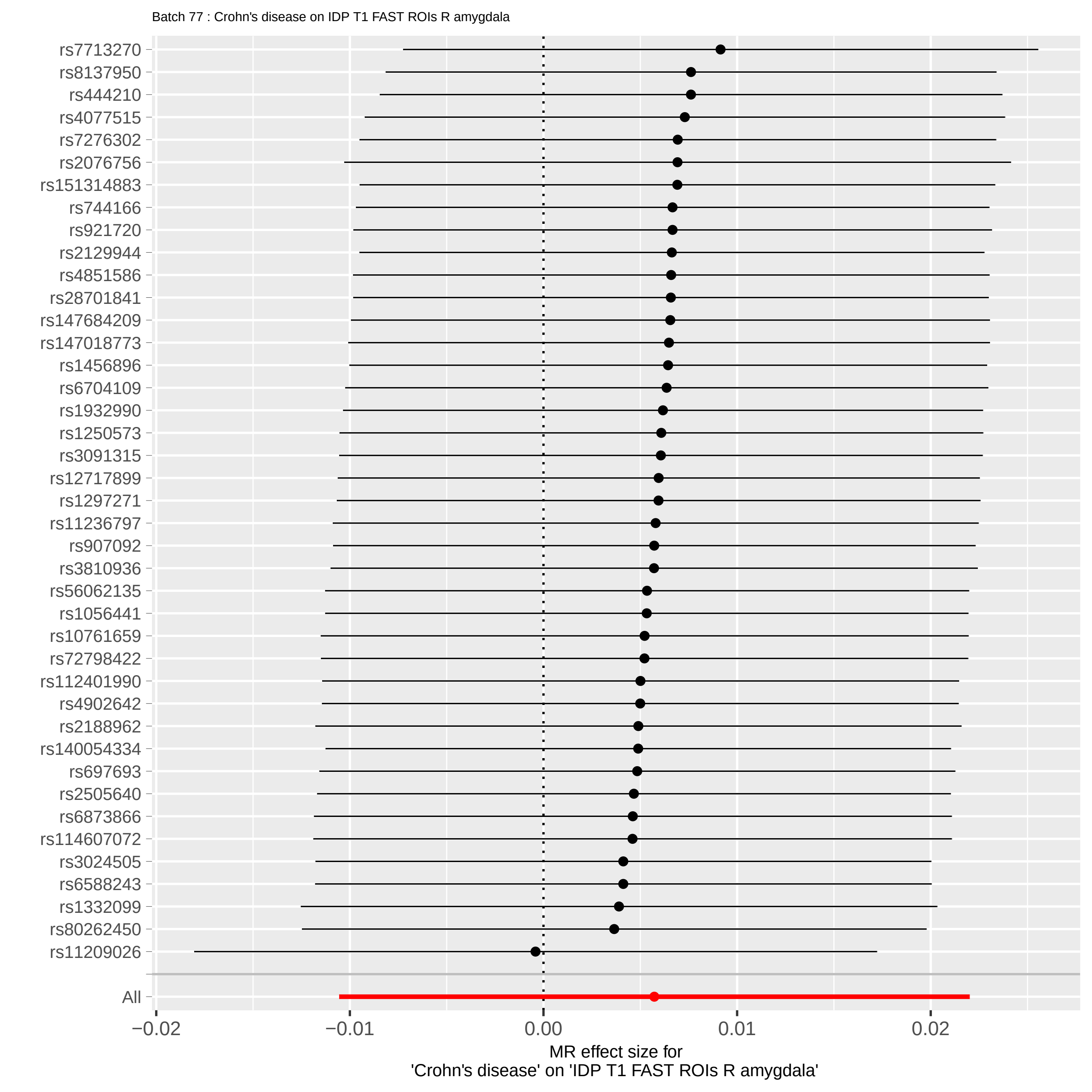

Batch 77 : Crohn's disease on IDP T1 FAST ROIs R amygdala
rs7713270
rs8137950
rs444210
rs4077515
rs7276302
rs2076756
rs151314883
rs744166
rs921720
rs2129944
rs4851586
rs28701841
rs147684209
rs147018773
rs1456896
rs6704109
rs1932990
rs1250573
rs3091315
rs12717899
rs1297271
rs11236797
rs907092
rs3810936
rs56062135
rs1056441
rs10761659
rs72798422
rs112401990
rs4902642
rs2188962
rs140054334
rs697693
rs2505640
rs6873866
rs114607072
rs3024505
rs6588243
rs1332099
rs80262450
rs11209026
All
−0.02
−0.01
0.00
0.01
0.02
MR effect size for
'Crohn's disease' on 'IDP T1 FAST ROIs R amygdala'

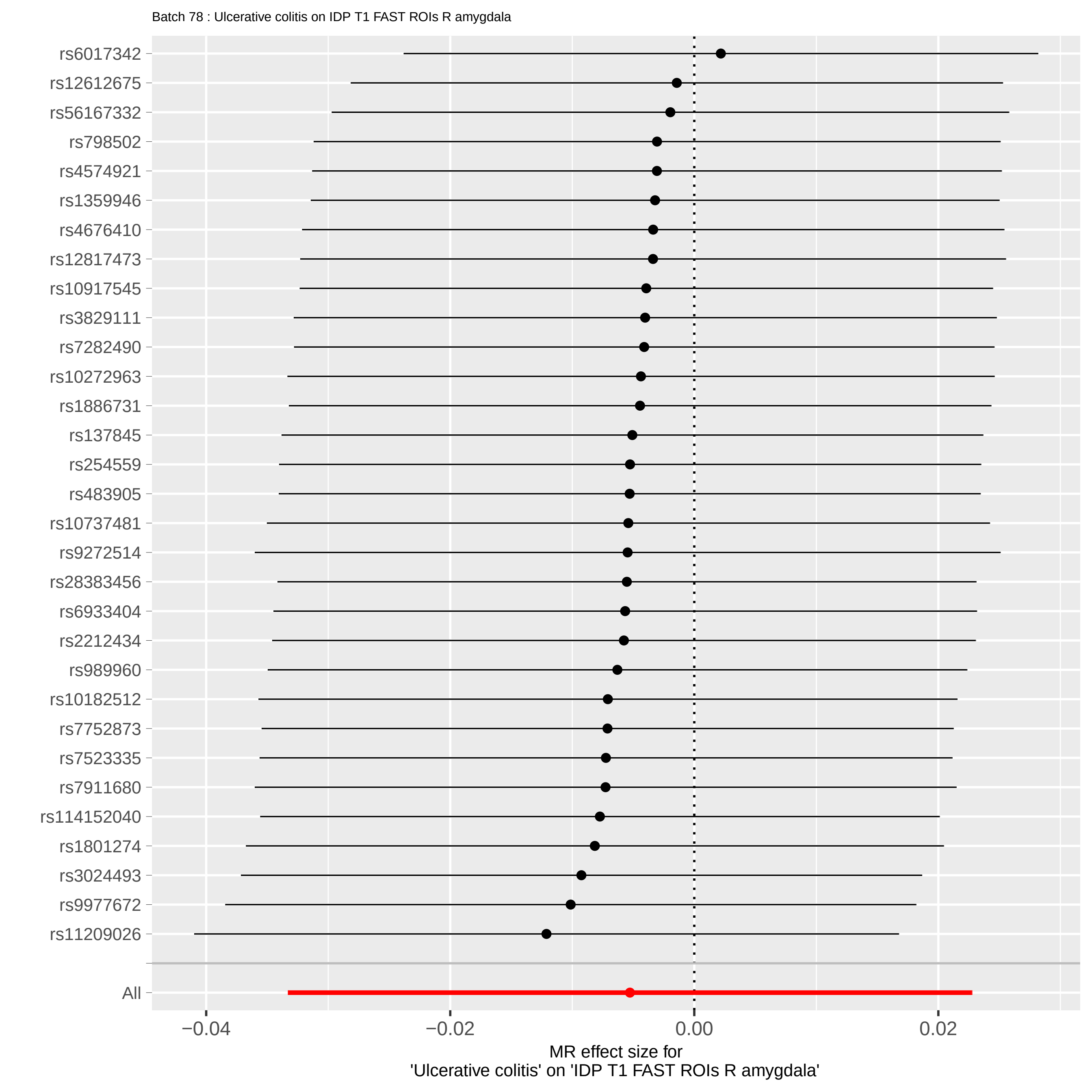
Batch 78 : Ulcerative colitis on IDP T1 FAST ROIs R amygdala
rs6017342
rs12612675
rs56167332
rs798502
rs4574921
rs1359946
rs4676410
rs12817473
rs10917545
rs3829111
rs7282490
rs10272963
rs1886731
rs137845
rs254559
rs483905
rs10737481
rs9272514
rs28383456
rs6933404
rs2212434
rs989960
rs10182512
rs7752873
rs7523335
rs7911680
rs114152040
rs1801274
rs3024493
rs9977672
rs11209026
All
−0.04
−0.02
0.00
0.02
MR effect size for
'Ulcerative colitis' on 'IDP T1 FAST ROIs R amygdala'

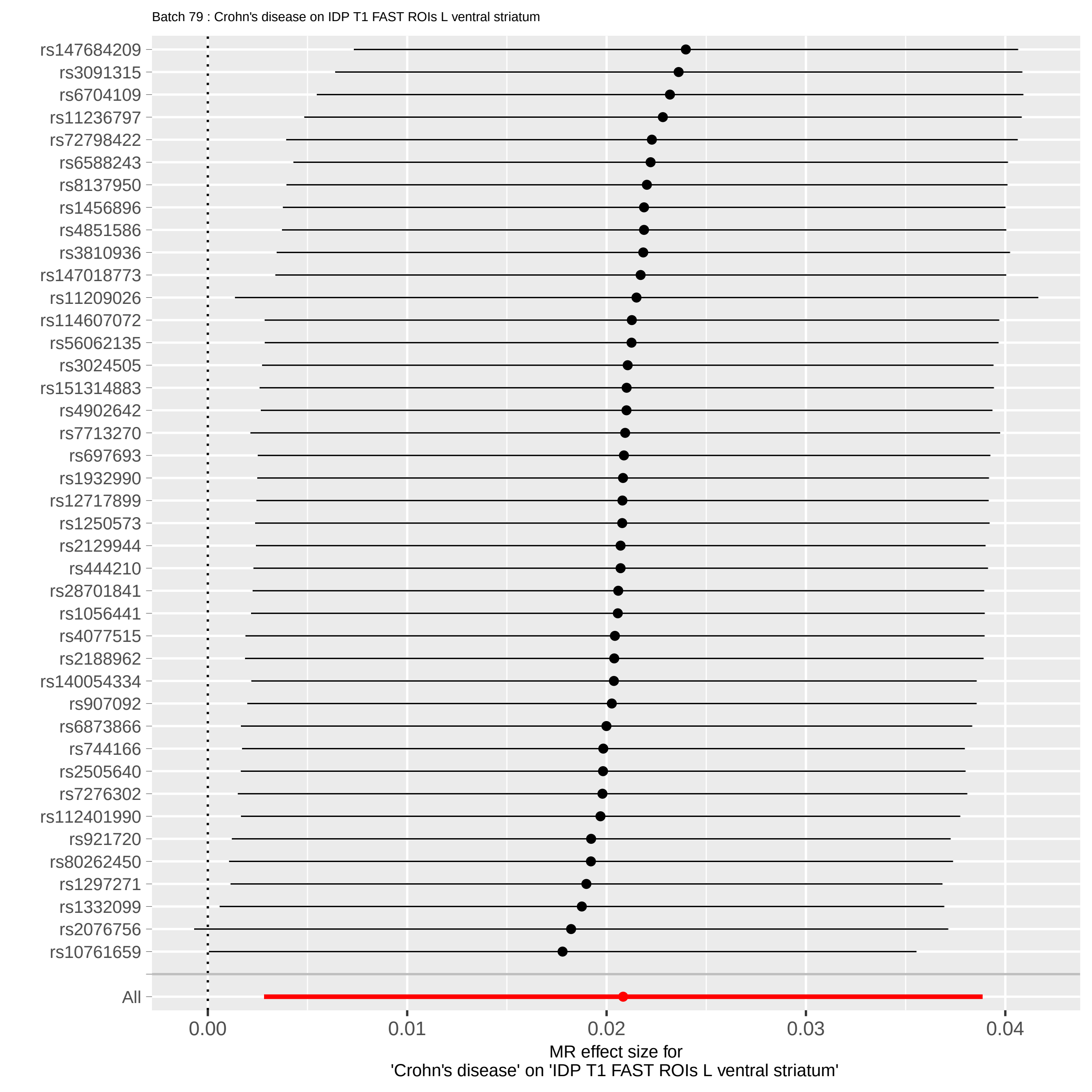

Batch 79 : Crohn's disease on IDP T1 FAST ROIs L ventral striatum
rs147684209
rs3091315
rs6704109
rs11236797
rs72798422
rs6588243
rs8137950
rs1456896
rs4851586
rs3810936
rs147018773
rs11209026
rs114607072
rs56062135
rs3024505
rs151314883
rs4902642
rs7713270
rs697693
rs1932990
rs12717899
rs1250573
rs2129944
rs444210
rs28701841
rs1056441
rs4077515
rs2188962
rs140054334
rs907092
rs6873866
rs744166
rs2505640
rs7276302
rs112401990
rs921720
rs80262450
rs1297271
rs1332099
rs2076756
rs10761659
All
0.00
0.01
0.02
0.03
0.04
MR effect size for
'Crohn's disease' on 'IDP T1 FAST ROIs L ventral striatum'

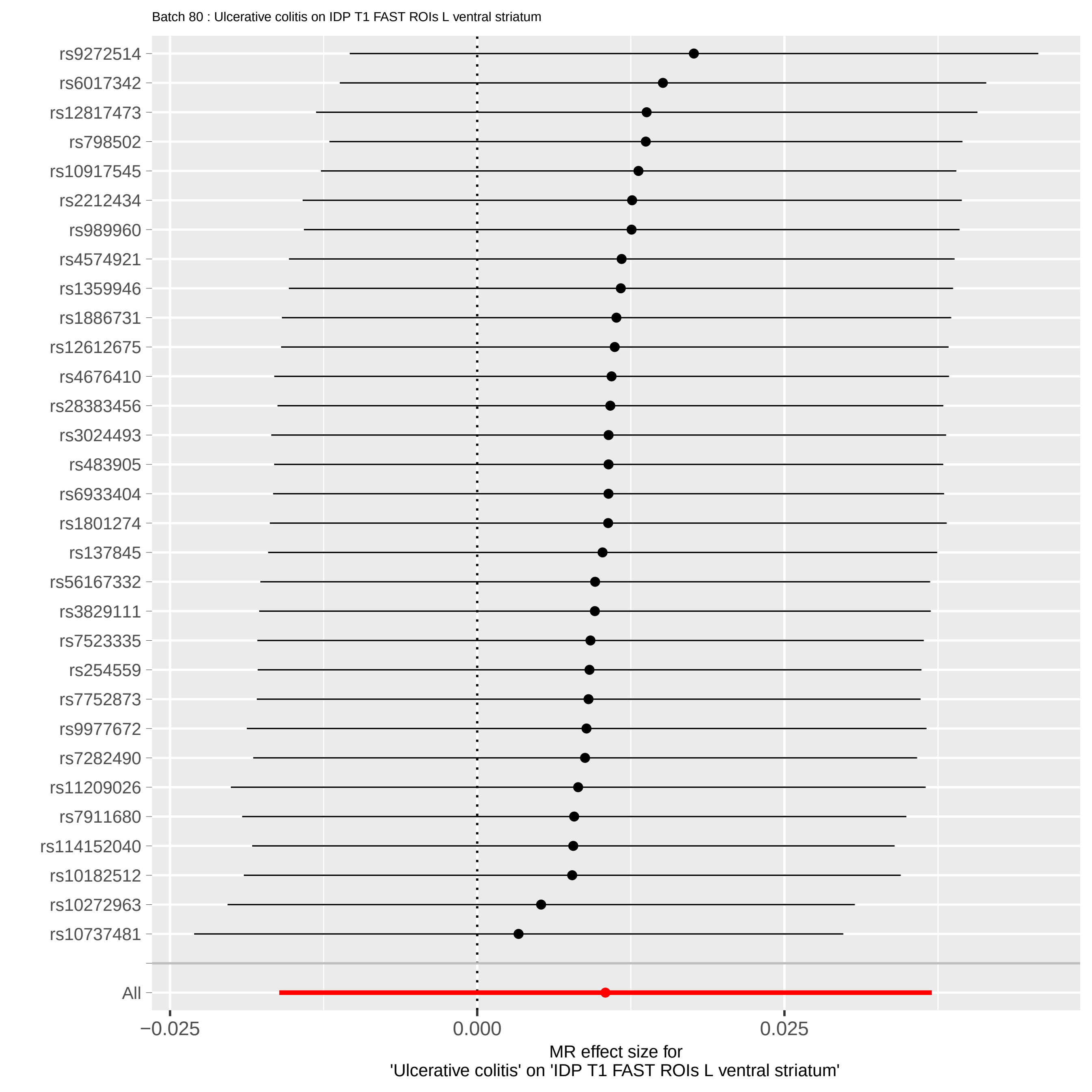
Batch 80 : Ulcerative colitis on IDP T1 FAST ROIs L ventral striatum
rs9272514
rs6017342
rs12817473
rs798502
rs10917545
rs2212434
rs989960
rs4574921
rs1359946
rs1886731
rs12612675
rs4676410
rs28383456
rs3024493
rs483905
rs6933404
rs1801274
rs137845
rs56167332
rs3829111
rs7523335
rs254559
rs7752873
rs9977672
rs7282490
rs11209026
rs7911680
rs114152040
rs10182512
rs10272963
rs10737481
All
−0.025
0.000
0.025
MR effect size for
'Ulcerative colitis' on 'IDP T1 FAST ROIs L ventral striatum'

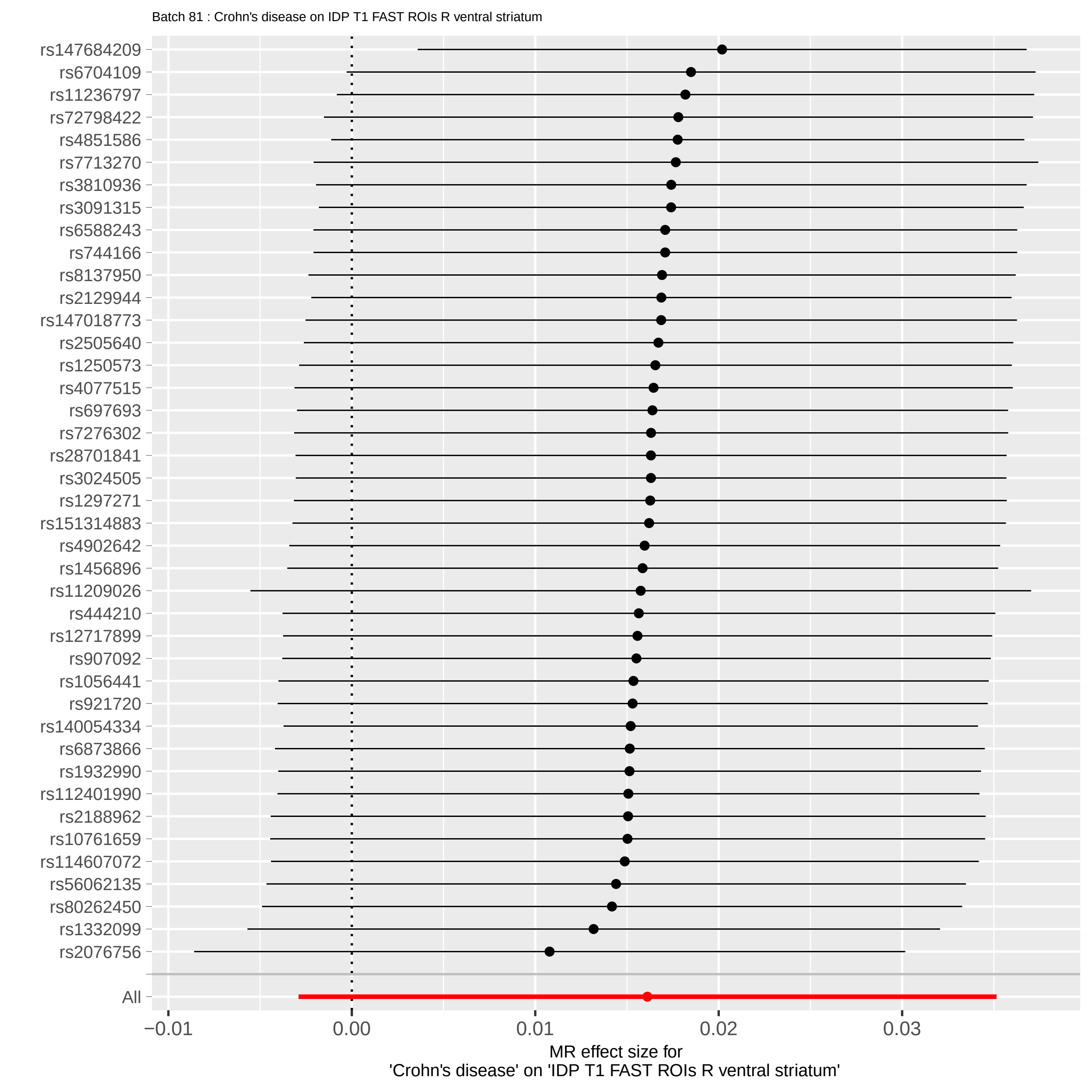

Batch 81 : Crohn's disease on IDP T1 FAST ROIs R ventral striatum
rs147684209
rs6704109
rs11236797
rs72798422
rs4851586
rs7713270
rs3810936
rs3091315
rs6588243
rs744166
rs8137950
rs2129944
rs147018773
rs2505640
rs1250573
rs4077515
rs697693
rs7276302
rs28701841
rs3024505
rs1297271
rs151314883
rs4902642
rs1456896
rs11209026
rs444210
rs12717899
rs907092
rs1056441
rs921720
rs140054334
rs6873866
rs1932990
rs112401990
rs2188962
rs10761659
rs114607072
rs56062135
rs80262450
rs1332099
rs2076756
All
−0.01
0.00
0.01
0.02
0.03
MR effect size for
'Crohn's disease' on 'IDP T1 FAST ROIs R ventral striatum'

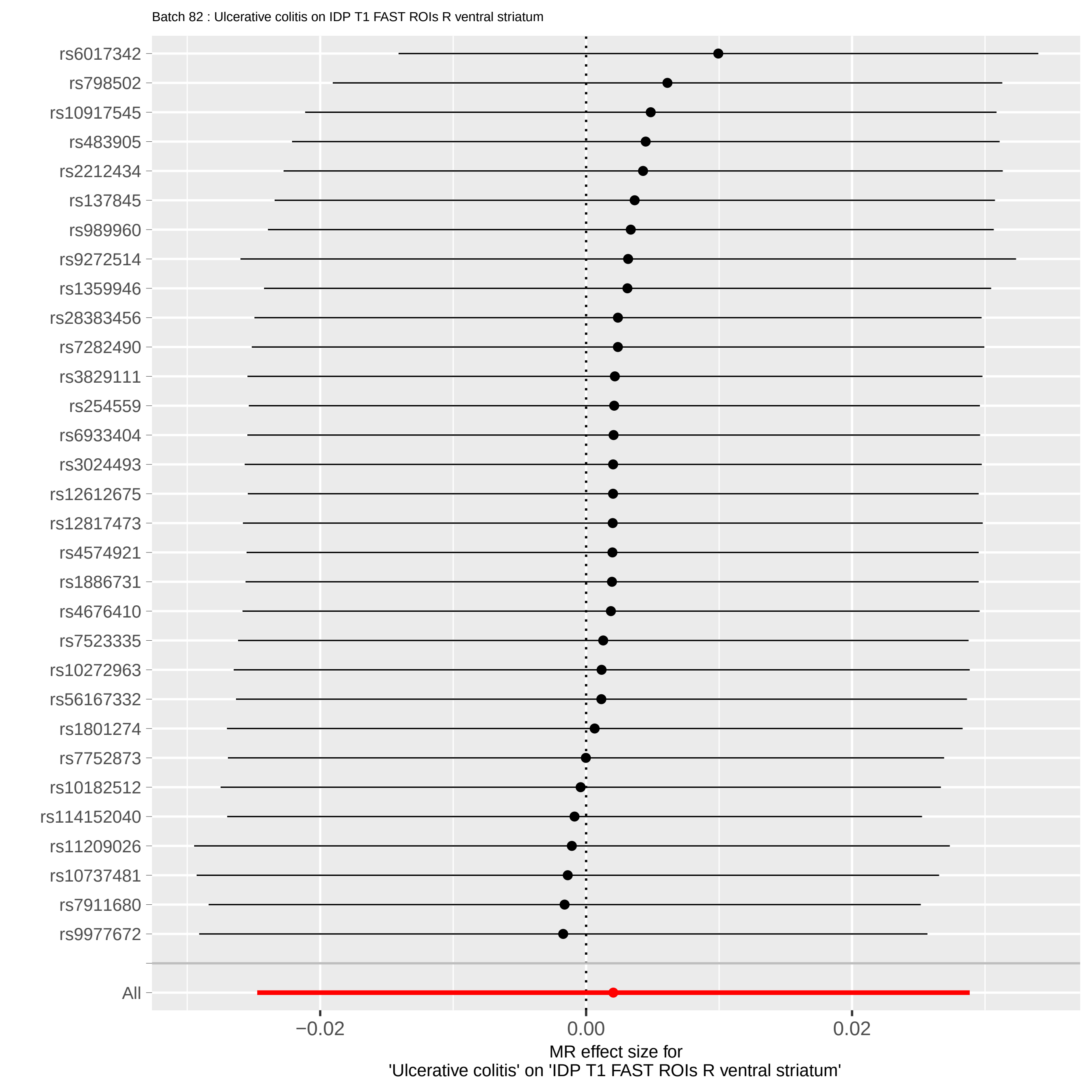
Batch 82 : Ulcerative colitis on IDP T1 FAST ROIs R ventral striatum
rs6017342
rs798502
rs10917545
rs483905
rs2212434
rs137845
rs989960
rs9272514
rs1359946
rs28383456
rs7282490
rs3829111
rs254559
rs6933404
rs3024493
rs12612675
rs12817473
rs4574921
rs1886731
rs4676410
rs7523335
rs10272963
rs56167332
rs1801274
rs7752873
rs10182512
rs114152040
rs11209026
rs10737481
rs7911680
rs9977672
All
−0.02
0.00
0.02
MR effect size for
'Ulcerative colitis' on 'IDP T1 FAST ROIs R ventral striatum'

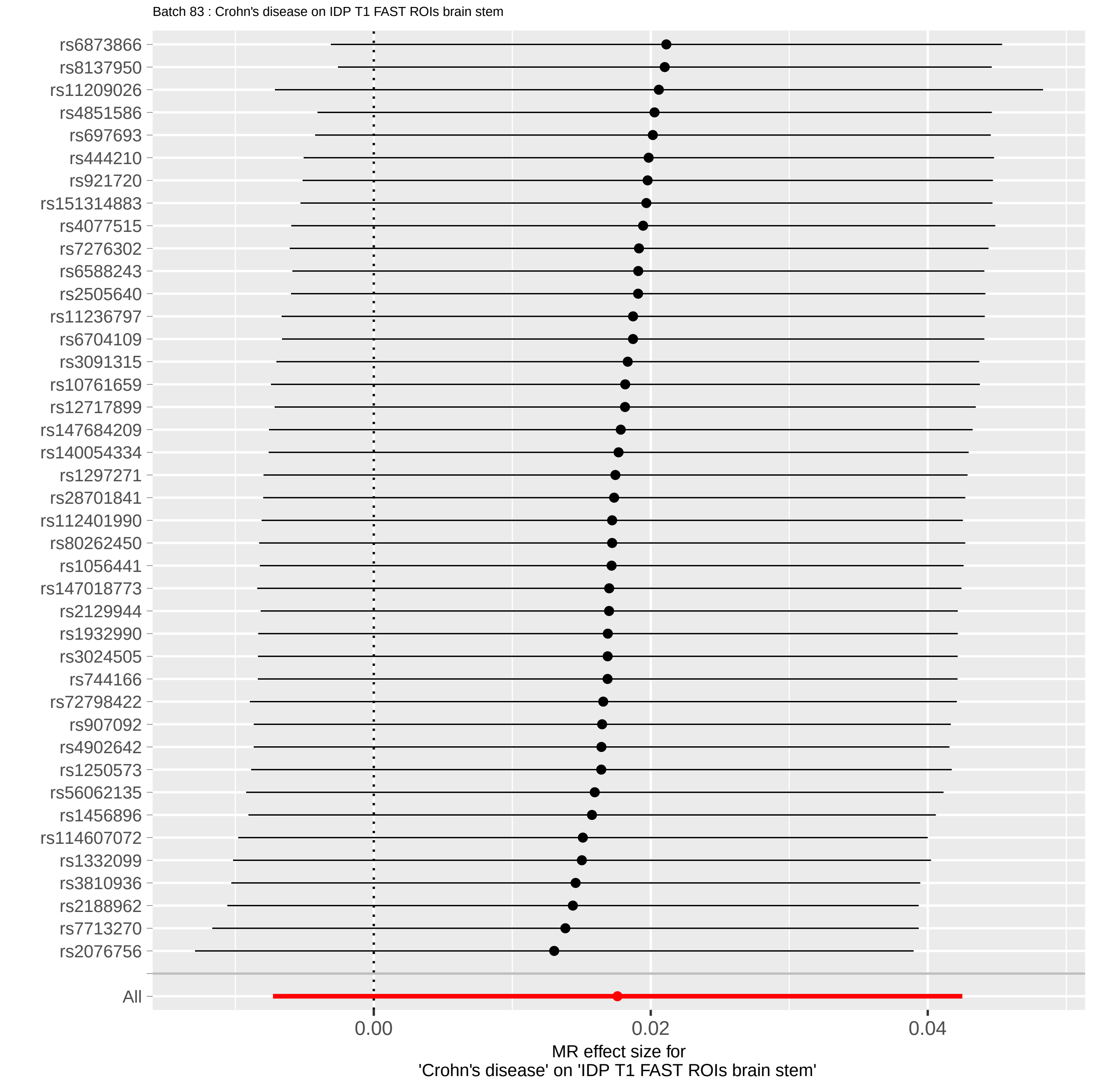

Batch 83 : Crohn's disease on IDP T1 FAST ROIs brain stem
rs6873866
rs8137950
rs11209026
rs4851586
rs697693
rs444210
rs921720
rs151314883
rs4077515
rs7276302
rs6588243
rs2505640
rs11236797
rs6704109
rs3091315
rs10761659
rs12717899
rs147684209
rs140054334
rs1297271
rs28701841
rs112401990
rs80262450
rs1056441
rs147018773
rs2129944
rs1932990
rs3024505
rs744166
rs72798422
rs907092
rs4902642
rs1250573
rs56062135
rs1456896
rs114607072
rs1332099
rs3810936
rs2188962
rs7713270
rs2076756
All
0.00
0.02
0.04
MR effect size for
'Crohn's disease' on 'IDP T1 FAST ROIs brain stem'

Batch 84 : Ulcerative colitis on IDP T1 FAST ROIs brain stem

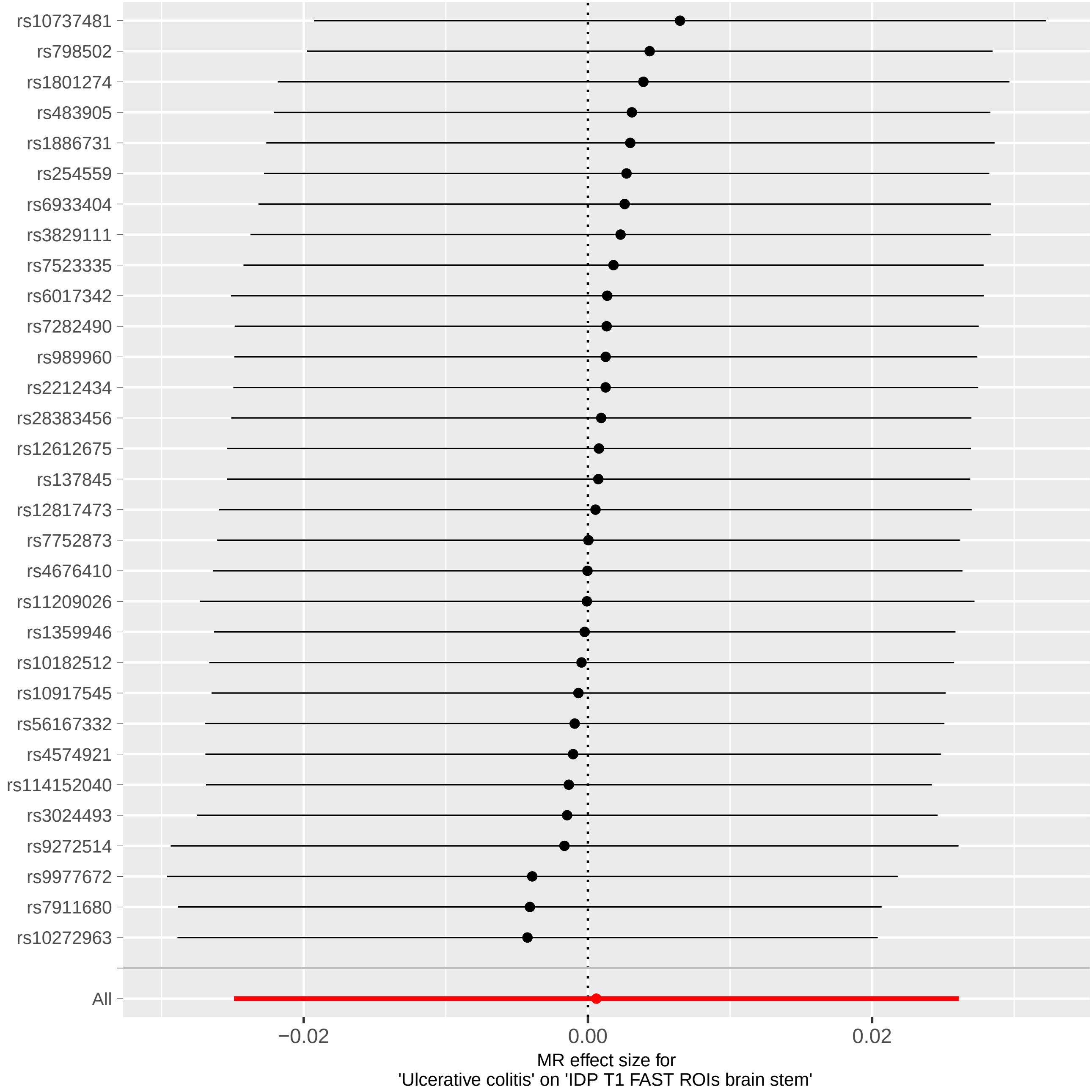

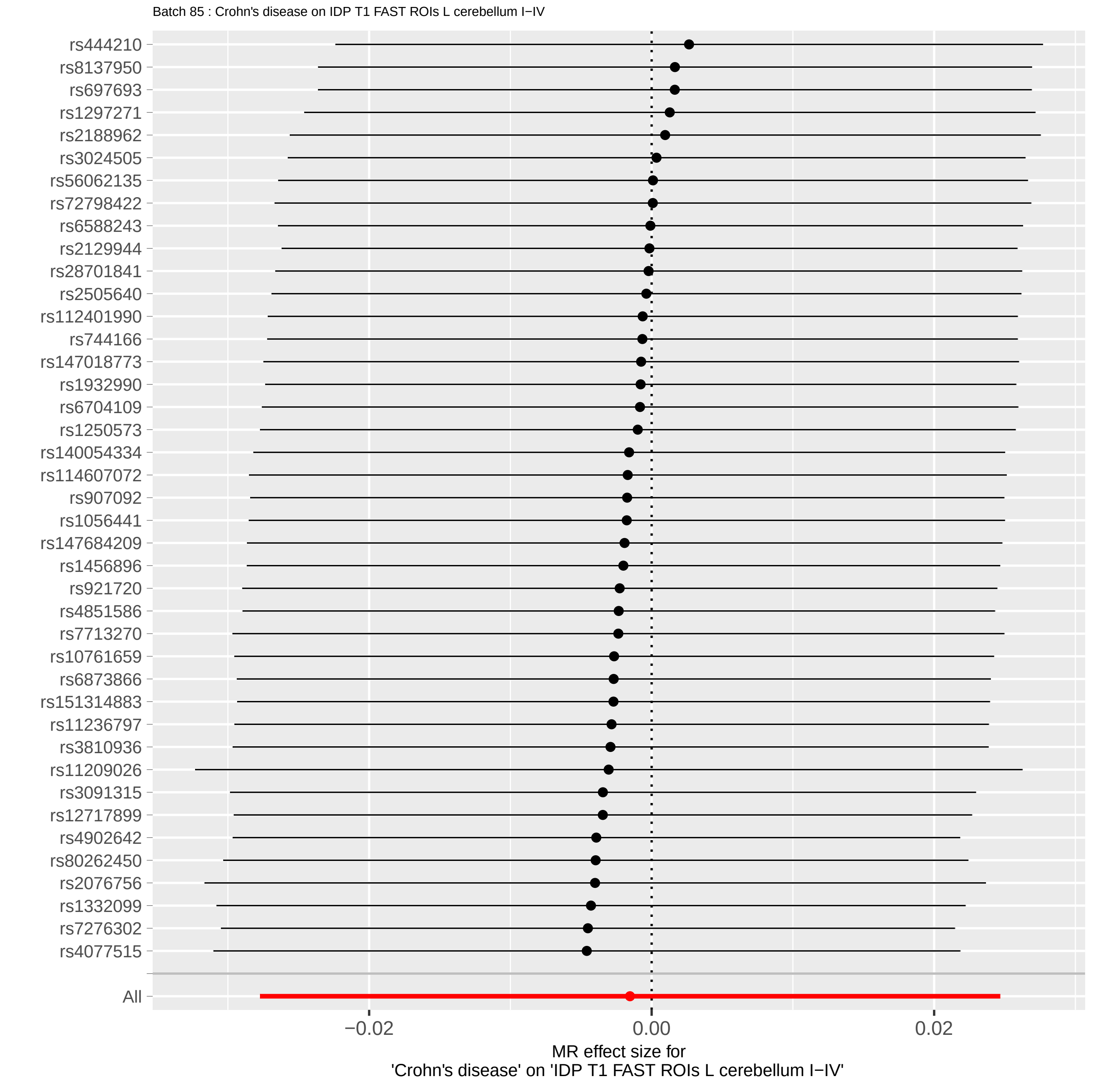

Batch 85 : Crohn's disease on IDP T1 FAST ROIs L cerebellum I−IV
rs444210
rs8137950
rs697693
rs1297271
rs2188962
rs3024505
rs56062135
rs72798422
rs6588243
rs2129944
rs28701841
rs2505640
rs112401990
rs744166
rs147018773
rs1932990
rs6704109
rs1250573
rs140054334
rs114607072
rs907092
rs1056441
rs147684209
rs1456896
rs921720
rs4851586
rs7713270
rs10761659
rs6873866
rs151314883
rs11236797
rs3810936
rs11209026
rs3091315
rs12717899
rs4902642
rs80262450
rs2076756
rs1332099
rs7276302
rs4077515
All
−0.02
0.00
0.02
MR effect size for
'Crohn's disease' on 'IDP T1 FAST ROIs L cerebellum I−IV'

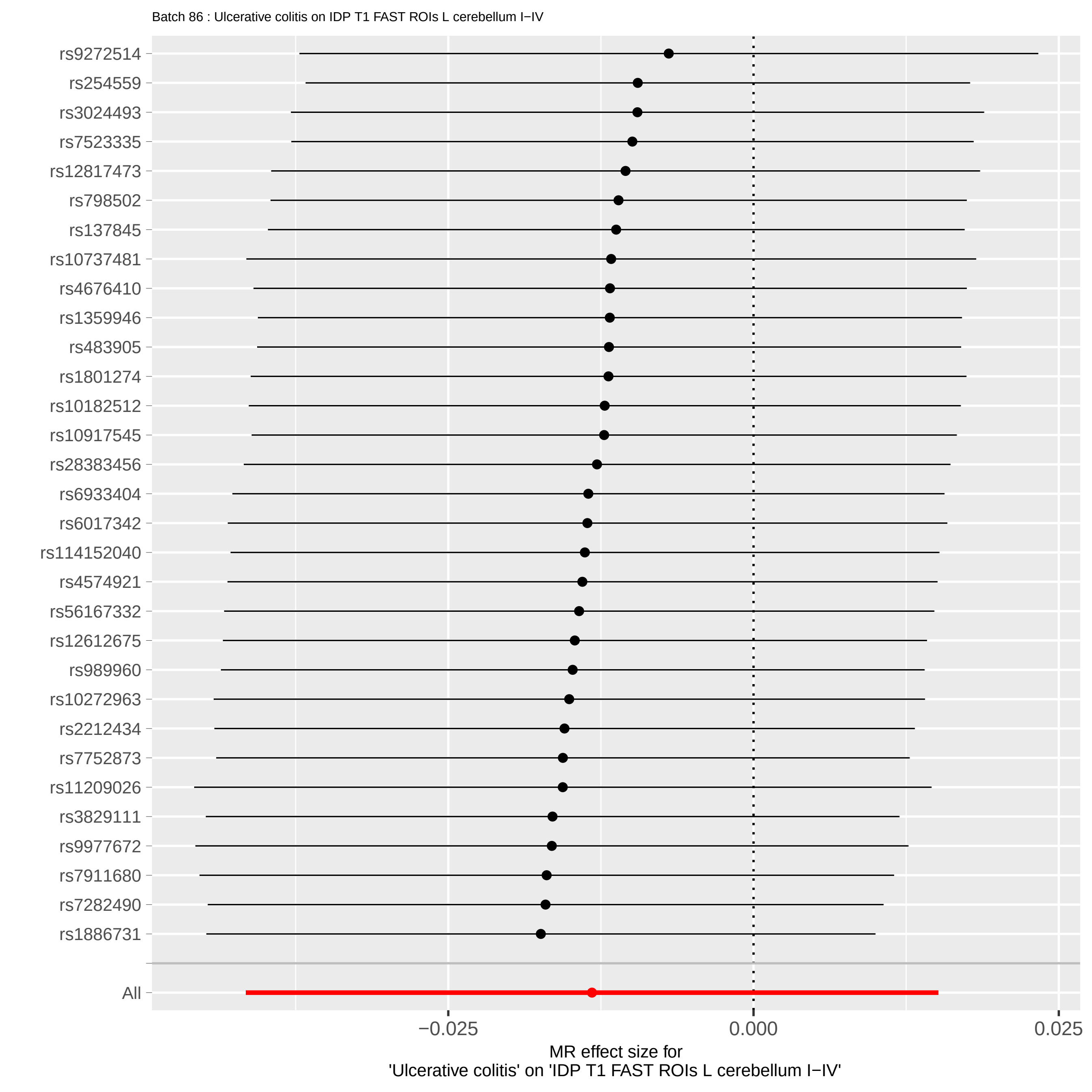

Batch 86 : Ulcerative colitis on IDP T1 FAST ROIs L cerebellum I−IV
rs9272514
rs254559
rs3024493
rs7523335
rs12817473
rs798502
rs137845
rs10737481
rs4676410
rs1359946
rs483905
rs1801274
rs10182512
rs10917545
rs28383456
rs6933404
rs6017342
rs114152040
rs4574921
rs56167332
rs12612675
rs989960
rs10272963
rs2212434
rs7752873
rs11209026
rs3829111
rs9977672
rs7911680
rs7282490
rs1886731
All
−0.025
0.000
0.025
MR effect size for
'Ulcerative colitis' on 'IDP T1 FAST ROIs L cerebellum I−IV'

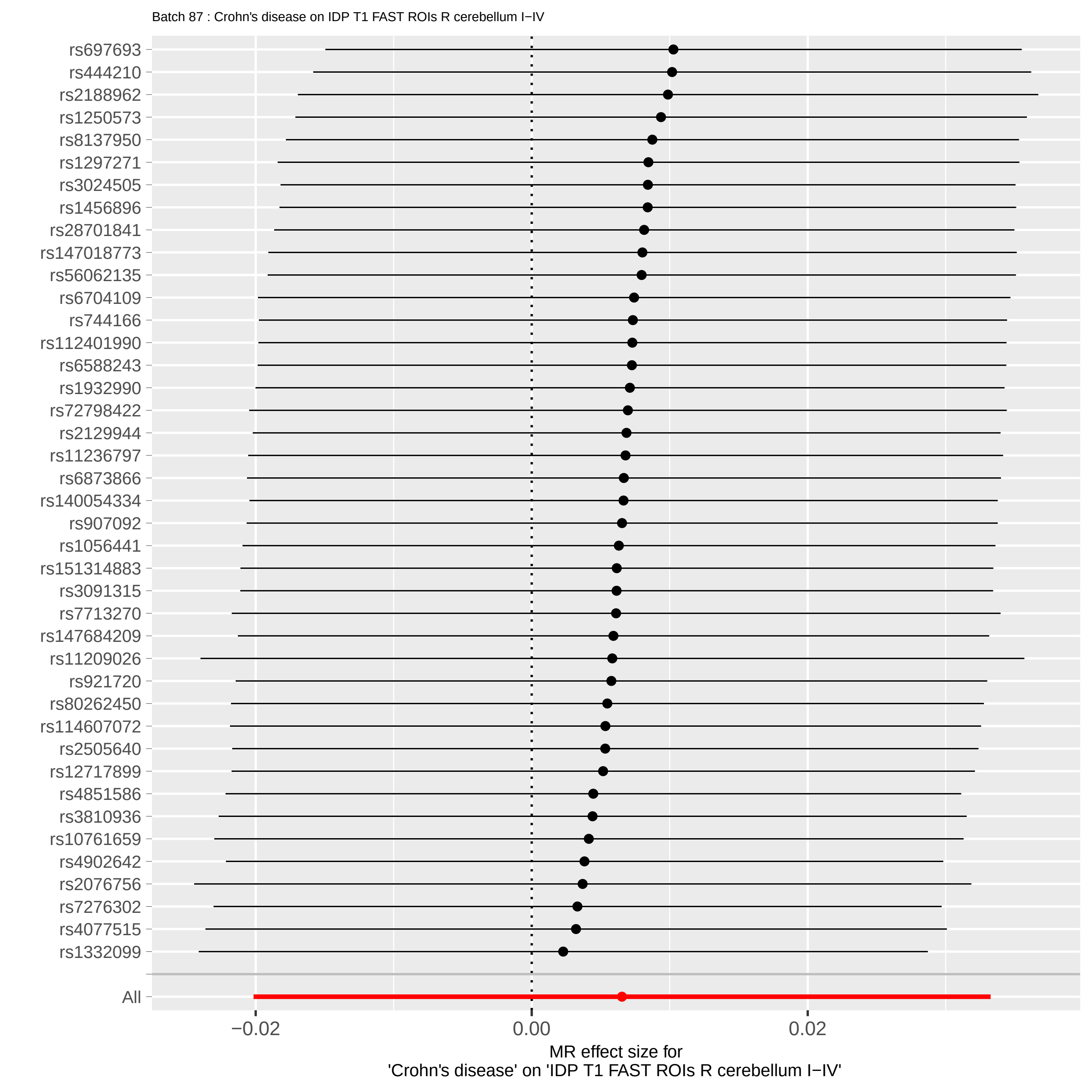
Batch 87 : Crohn's disease on IDP T1 FAST ROIs R cerebellum I−IV
rs697693
rs444210
rs2188962
rs1250573
rs8137950
rs1297271
rs3024505
rs1456896
rs28701841
rs147018773
rs56062135
rs6704109
rs744166
rs112401990
rs6588243
rs1932990
rs72798422
rs2129944
rs11236797
rs6873866
rs140054334
rs907092
rs1056441
rs151314883
rs3091315
rs7713270
rs147684209
rs11209026
rs921720
rs80262450
rs114607072
rs2505640
rs12717899
rs4851586
rs3810936
rs10761659
rs4902642
rs2076756
rs7276302
rs4077515
rs1332099
All
−0.02
0.00
0.02
MR effect size for
'Crohn's disease' on 'IDP T1 FAST ROIs R cerebellum I−IV'

Batch 88 : Ulcerative colitis on IDP T1 FAST ROIs R cerebellum I−IV

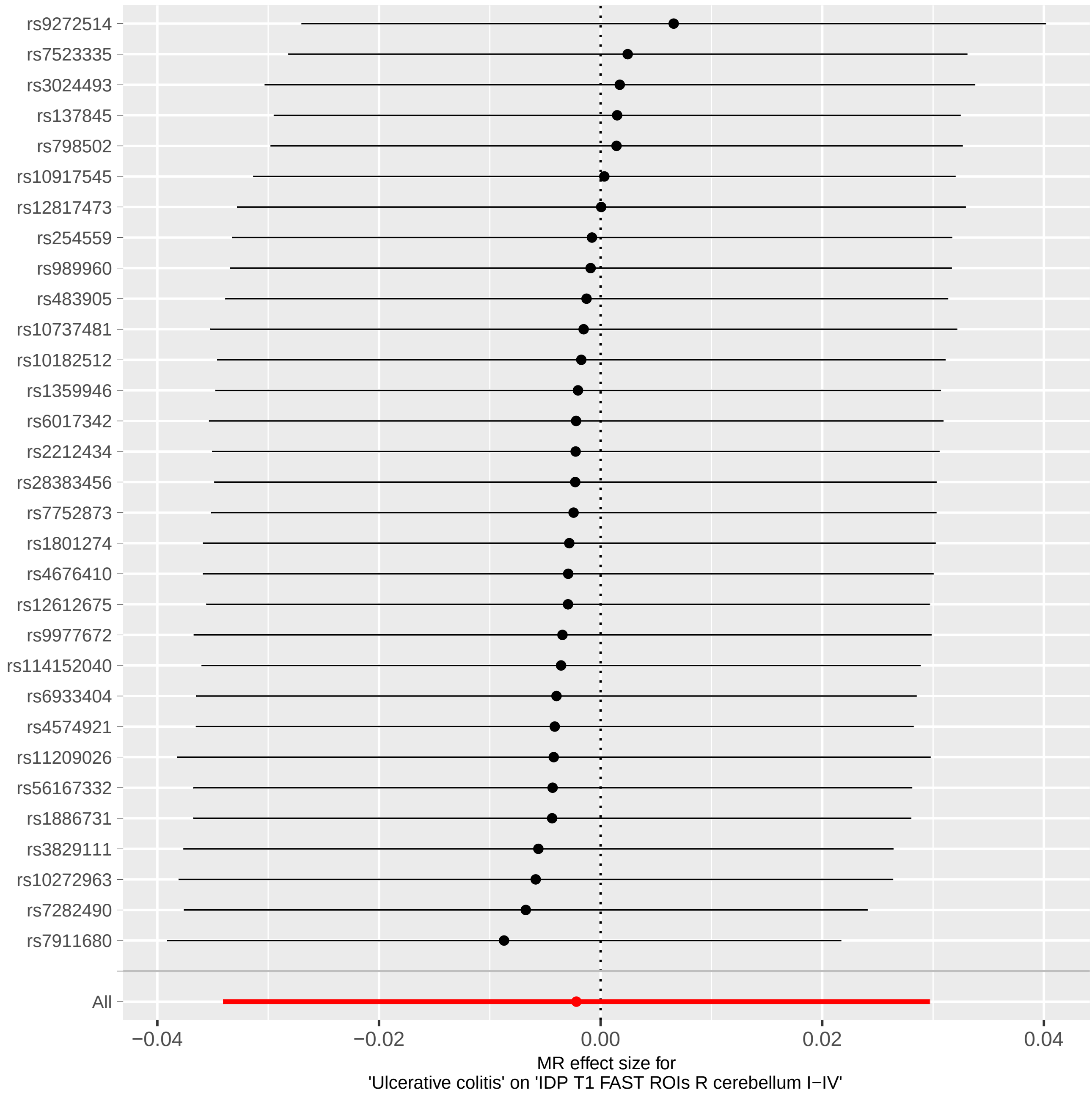

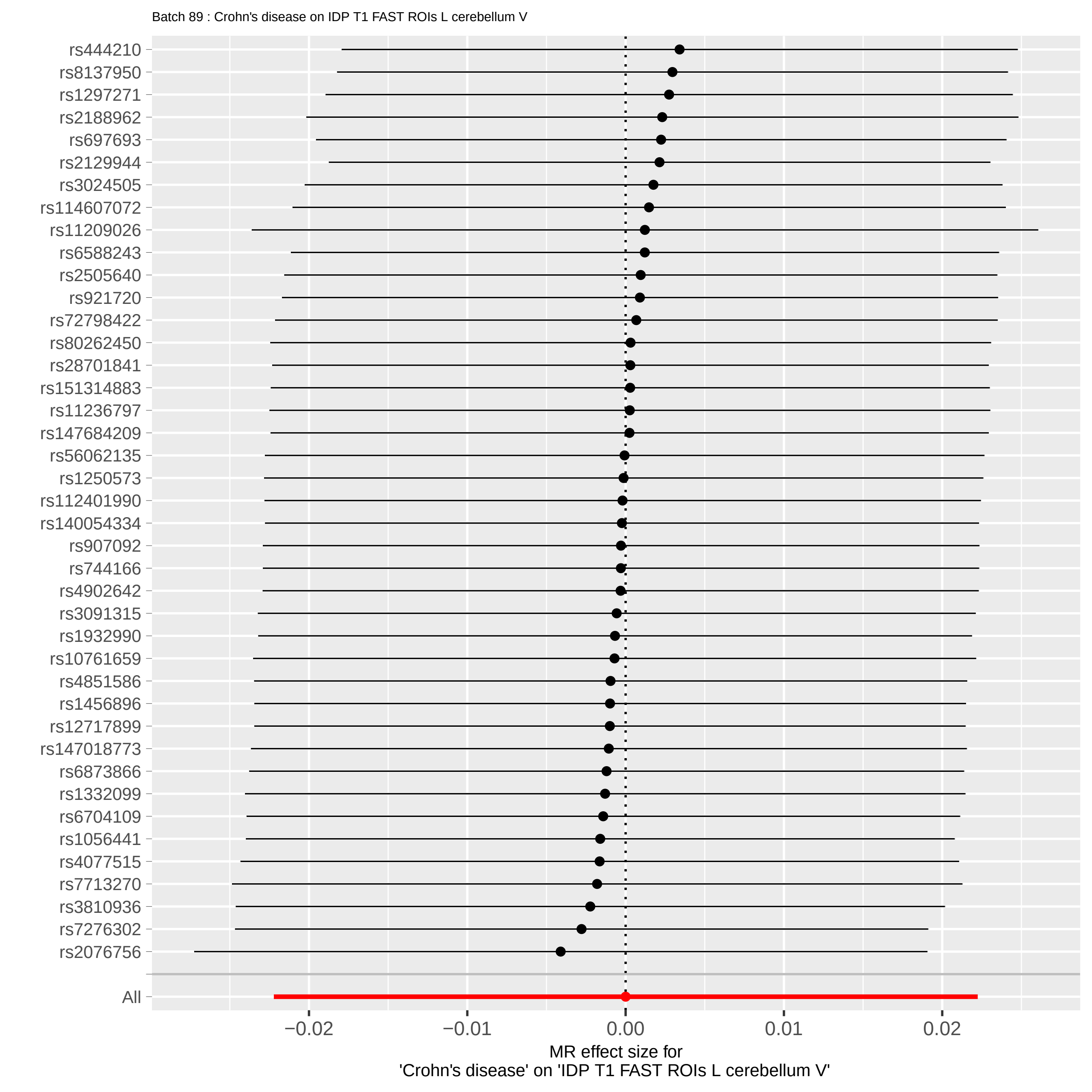

Batch 89 : Crohn's disease on IDP T1 FAST ROIs L cerebellum V
rs444210
rs8137950
rs1297271
rs2188962
rs697693
rs2129944
rs3024505
rs114607072
rs11209026
rs6588243
rs2505640
rs921720
rs72798422
rs80262450
rs28701841
rs151314883
rs11236797
rs147684209
rs56062135
rs1250573
rs112401990
rs140054334
rs907092
rs744166
rs4902642
rs3091315
rs1932990
rs10761659
rs4851586
rs1456896
rs12717899
rs147018773
rs6873866
rs1332099
rs6704109
rs1056441
rs4077515
rs7713270
rs3810936
rs7276302
rs2076756
All
−0.02
−0.01
0.00
0.01
0.02
MR effect size for
'Crohn's disease' on 'IDP T1 FAST ROIs L cerebellum V'

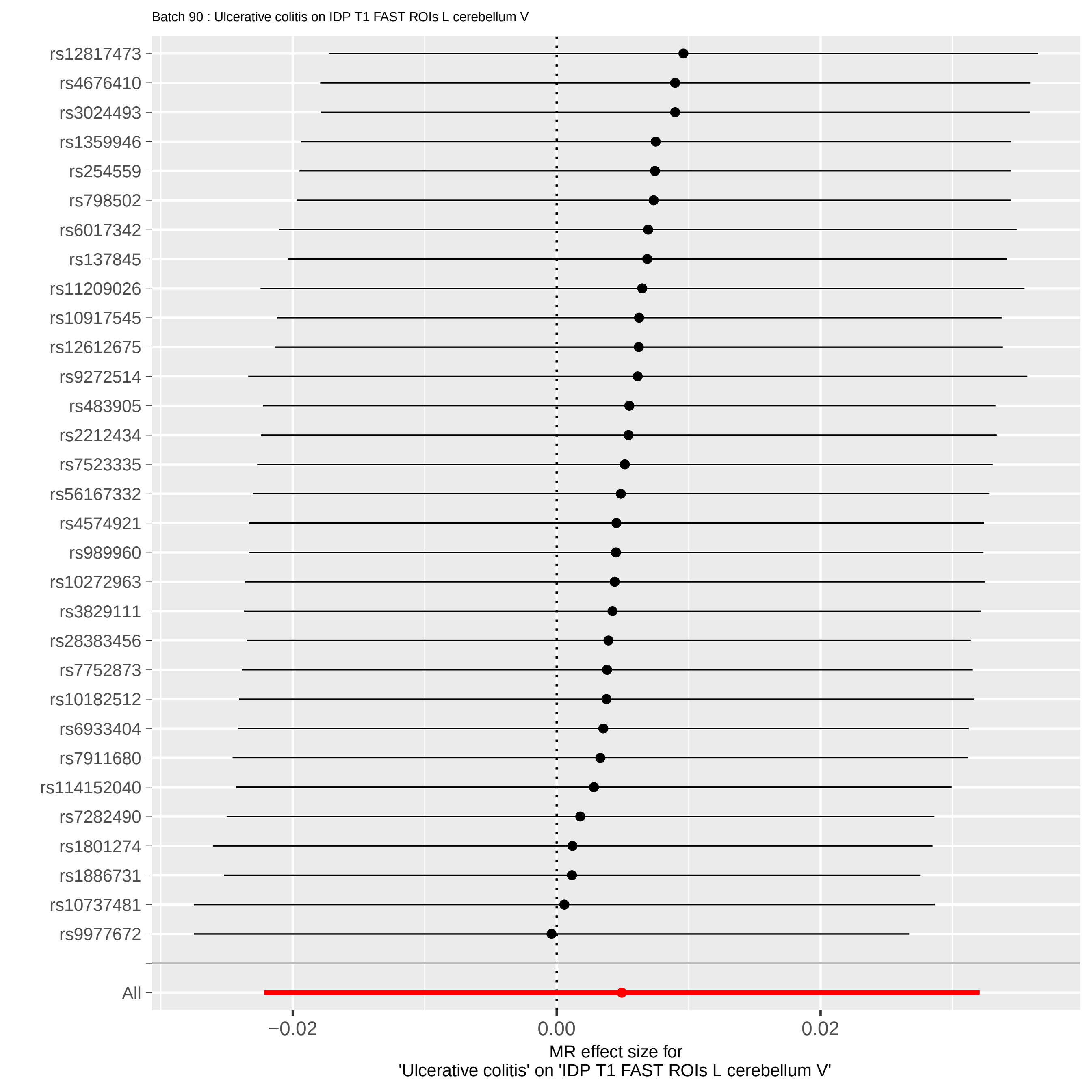

Batch 90 : Ulcerative colitis on IDP T1 FAST ROIs L cerebellum V
rs12817473
rs4676410
rs3024493
rs1359946
rs254559
rs798502
rs6017342
rs137845
rs11209026
rs10917545
rs12612675
rs9272514
rs483905
rs2212434
rs7523335
rs56167332
rs4574921
rs989960
rs10272963
rs3829111
rs28383456
rs7752873
rs10182512
rs6933404
rs7911680
rs114152040
rs7282490
rs1801274
rs1886731
rs10737481
rs9977672
All
−0.02
0.00
0.02
MR effect size for
'Ulcerative colitis' on 'IDP T1 FAST ROIs L cerebellum V'

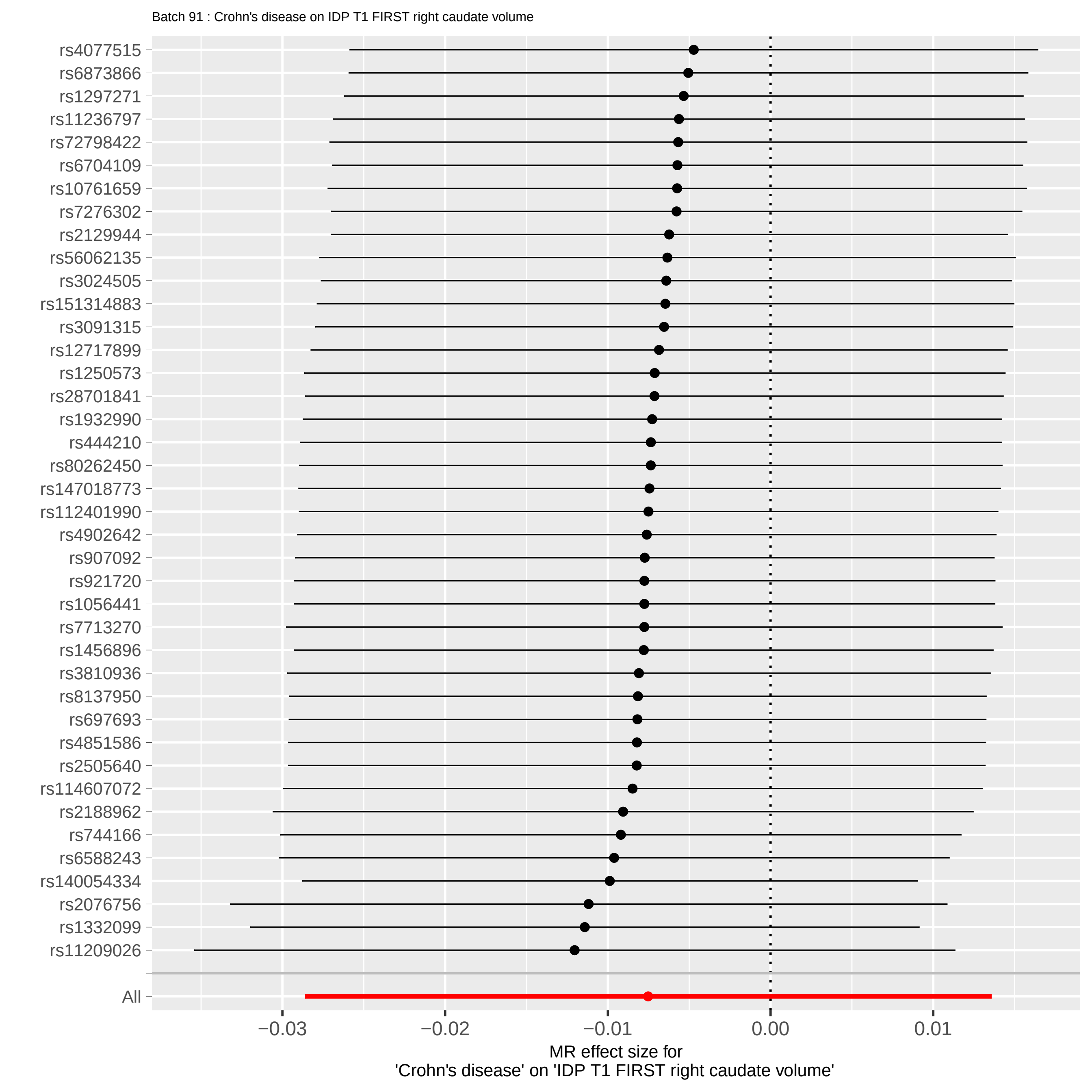
Batch 91 : Crohn's disease on IDP T1 FIRST right caudate volume
rs4077515
rs6873866
rs1297271
rs11236797
rs72798422
rs6704109
rs10761659
rs7276302
rs2129944
rs56062135
rs3024505
rs151314883
rs3091315
rs12717899
rs1250573
rs28701841
rs1932990
rs444210
rs80262450
rs147018773
rs112401990
rs4902642
rs907092
rs921720
rs1056441
rs7713270
rs1456896
rs3810936
rs8137950
rs697693
rs4851586
rs2505640
rs114607072
rs2188962
rs744166
rs6588243
rs140054334
rs2076756
rs1332099
rs11209026
All
−0.03
−0.02
−0.01
0.00
0.01
MR effect size for
'Crohn's disease' on 'IDP T1 FIRST right caudate volume'

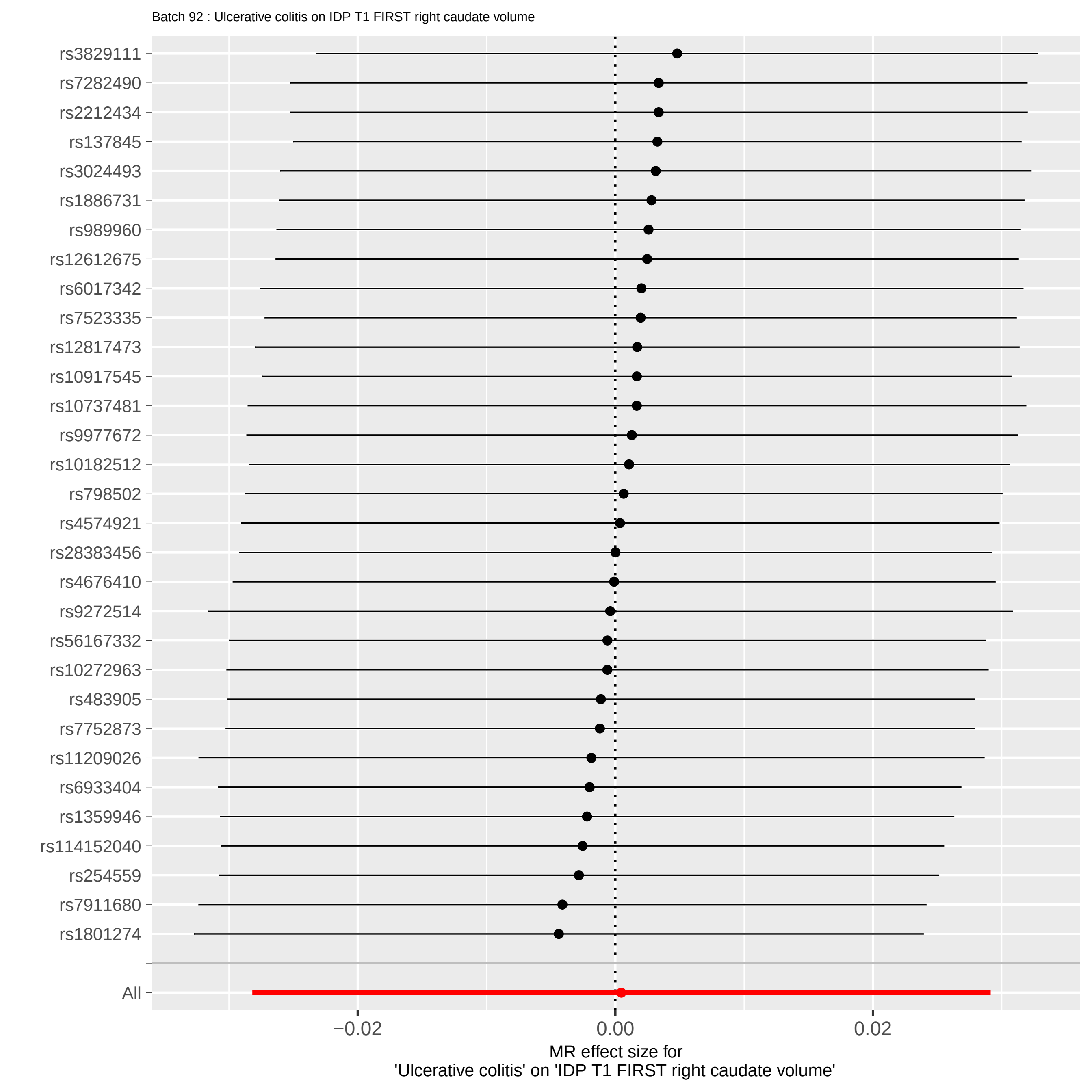
Batch 92 : Ulcerative colitis on IDP T1 FIRST right caudate volume
rs3829111
rs7282490
rs2212434
rs137845
rs3024493
rs1886731
rs989960
rs12612675
rs6017342
rs7523335
rs12817473
rs10917545
rs10737481
rs9977672
rs10182512
rs798502
rs4574921
rs28383456
rs4676410
rs9272514
rs56167332
rs10272963
rs483905
rs7752873
rs11209026
rs6933404
rs1359946
rs114152040
rs254559
rs7911680
rs1801274
All
−0.02
0.00
0.02
MR effect size for
'Ulcerative colitis' on 'IDP T1 FIRST right caudate volume'

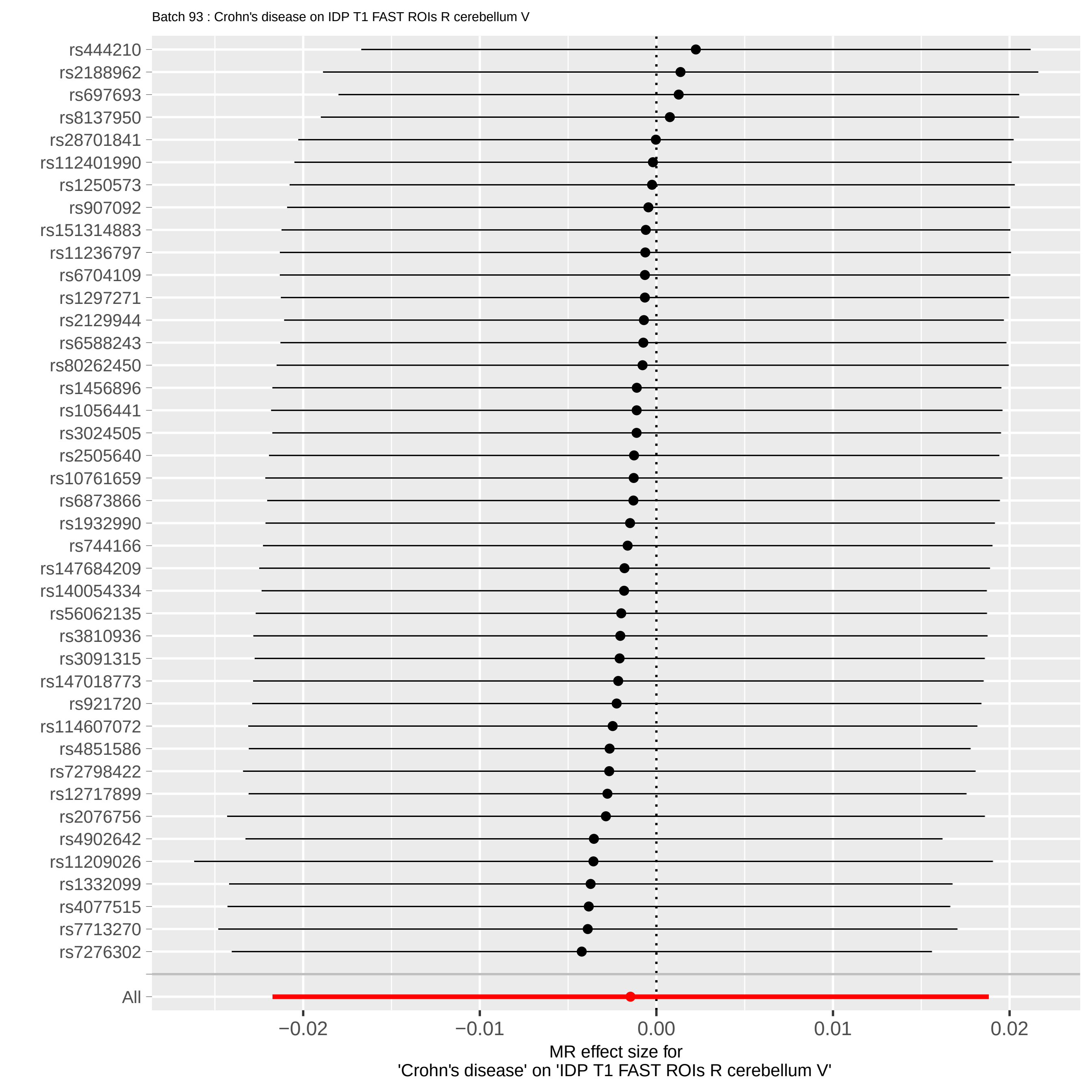
Batch 93 : Crohn's disease on IDP T1 FAST ROIs R cerebellum V
rs444210
rs2188962
rs697693
rs8137950
rs28701841
rs112401990
rs1250573
rs907092
rs151314883
rs11236797
rs6704109
rs1297271
rs2129944
rs6588243
rs80262450
rs1456896
rs1056441
rs3024505
rs2505640
rs10761659
rs6873866
rs1932990
rs744166
rs147684209
rs140054334
rs56062135
rs3810936
rs3091315
rs147018773
rs921720
rs114607072
rs4851586
rs72798422
rs12717899
rs2076756
rs4902642
rs11209026
rs1332099
rs4077515
rs7713270
rs7276302
All
−0.02
−0.01
0.00
0.01
0.02
MR effect size for
'Crohn's disease' on 'IDP T1 FAST ROIs R cerebellum V'

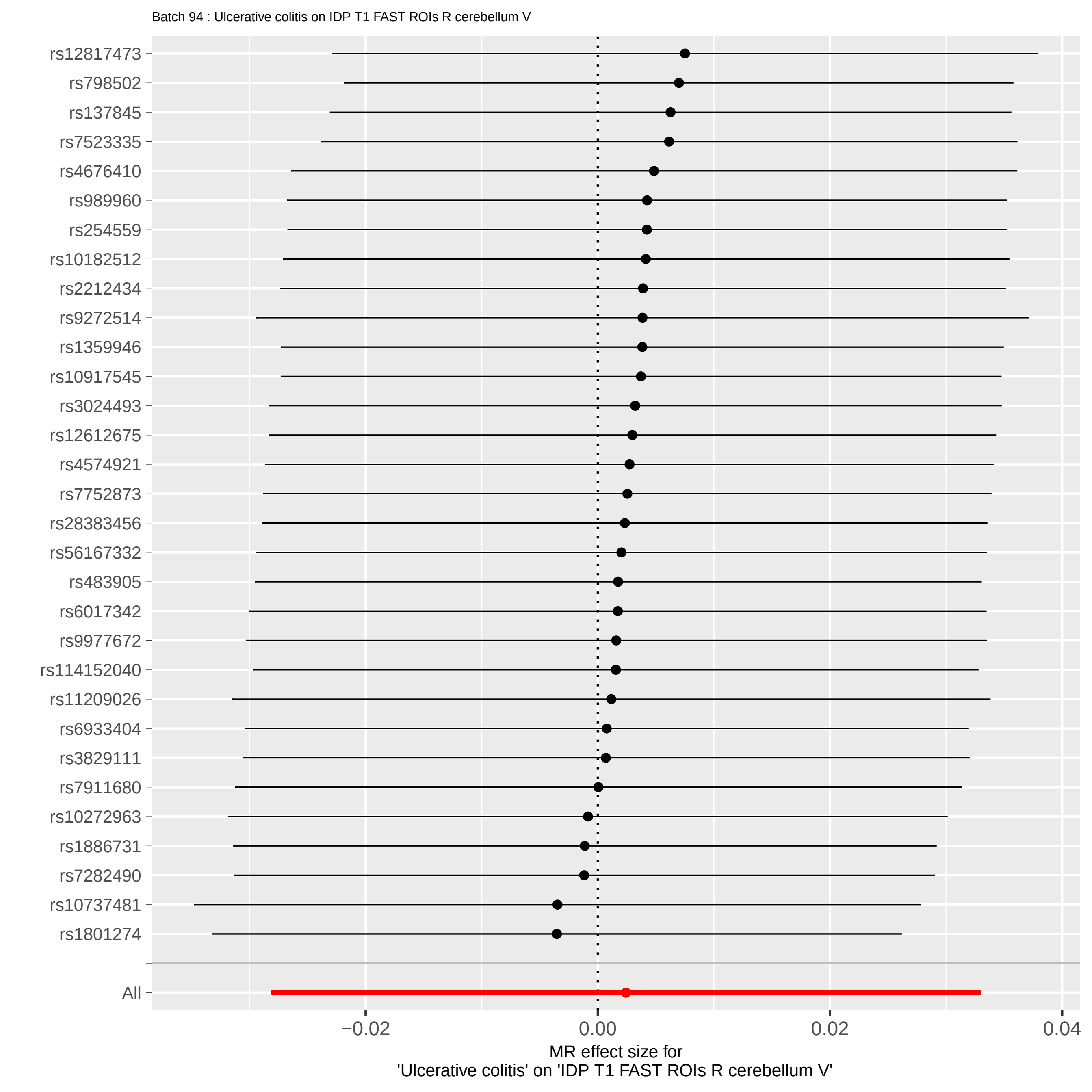

Batch 94 : Ulcerative colitis on IDP T1 FAST ROIs R cerebellum V
rs12817473
rs798502
rs137845
rs7523335
rs4676410
rs989960
rs254559
rs10182512
rs2212434
rs9272514
rs1359946
rs10917545
rs3024493
rs12612675
rs4574921
rs7752873
rs28383456
rs56167332
rs483905
rs6017342
rs9977672
rs114152040
rs11209026
rs6933404
rs3829111
rs7911680
rs10272963
rs1886731
rs7282490
rs10737481
rs1801274
All
−0.02
0.00
0.02
0.04
MR effect size for
'Ulcerative colitis' on 'IDP T1 FAST ROIs R cerebellum V'

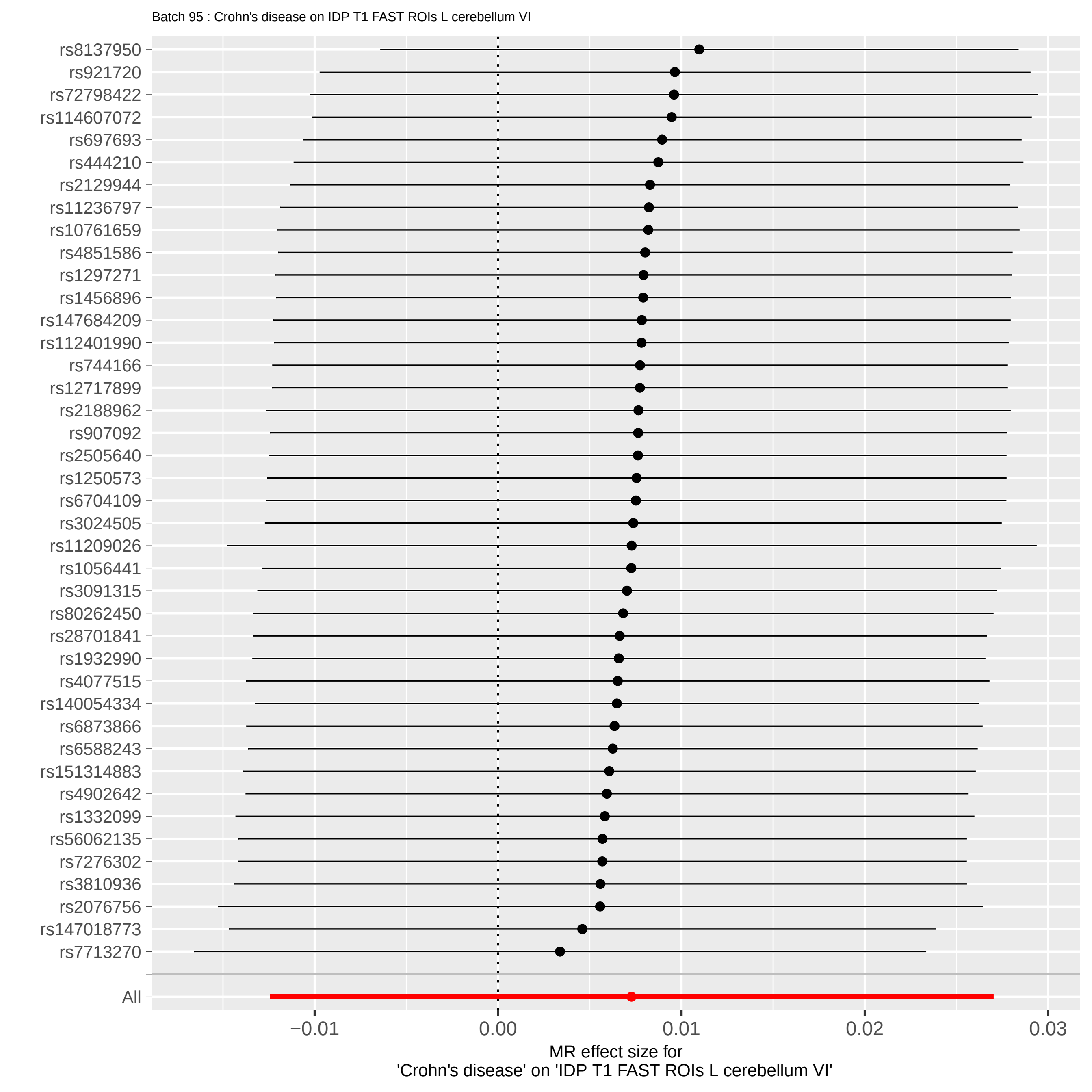

Batch 95 : Crohn's disease on IDP T1 FAST ROIs L cerebellum VI
rs8137950
rs921720
rs72798422
rs114607072
rs697693
rs444210
rs2129944
rs11236797
rs10761659
rs4851586
rs1297271
rs1456896
rs147684209
rs112401990
rs744166
rs12717899
rs2188962
rs907092
rs2505640
rs1250573
rs6704109
rs3024505
rs11209026
rs1056441
rs3091315
rs80262450
rs28701841
rs1932990
rs4077515
rs140054334
rs6873866
rs6588243
rs151314883
rs4902642
rs1332099
rs56062135
rs7276302
rs3810936
rs2076756
rs147018773
rs7713270
All
−0.01
0.00
0.01
0.02
0.03
MR effect size for
'Crohn's disease' on 'IDP T1 FAST ROIs L cerebellum VI'

Batch 96 : Ulcerative colitis on IDP T1 FAST ROIs L cerebellum VI

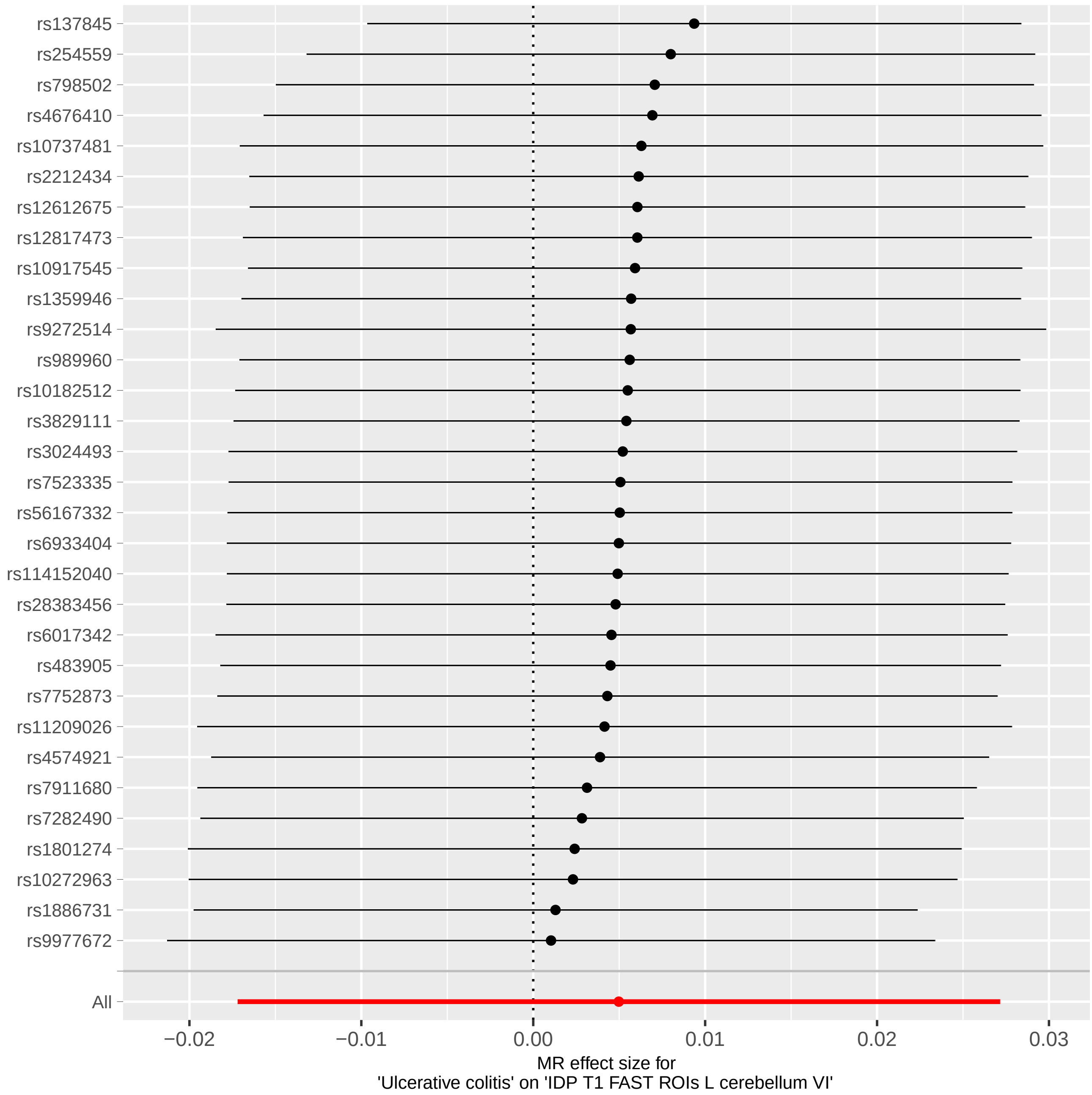

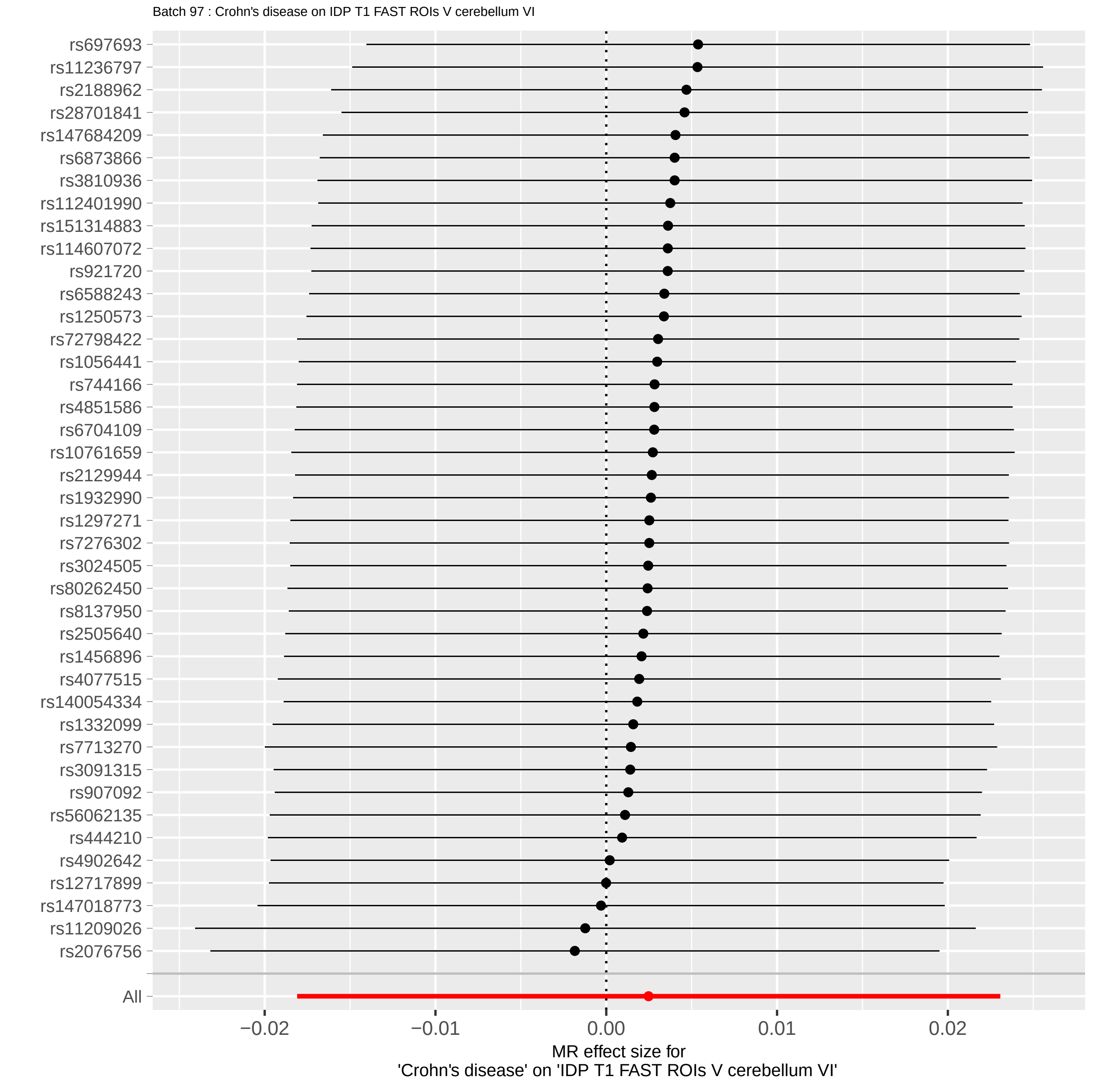
Batch 97 : Crohn's disease on IDP T1 FAST ROIs V cerebellum VI
rs697693
rs11236797
rs2188962
rs28701841
rs147684209
rs6873866
rs3810936
rs112401990
rs151314883
rs114607072
rs921720
rs6588243
rs1250573
rs72798422
rs1056441
rs744166
rs4851586
rs6704109
rs10761659
rs2129944
rs1932990
rs1297271
rs7276302
rs3024505
rs80262450
rs8137950
rs2505640
rs1456896
rs4077515
rs140054334
rs1332099
rs7713270
rs3091315
rs907092
rs56062135
rs444210
rs4902642
rs12717899
rs147018773
rs11209026
rs2076756
All
−0.02
−0.01
0.00
0.01
0.02
MR effect size for
'Crohn's disease' on 'IDP T1 FAST ROIs V cerebellum VI'

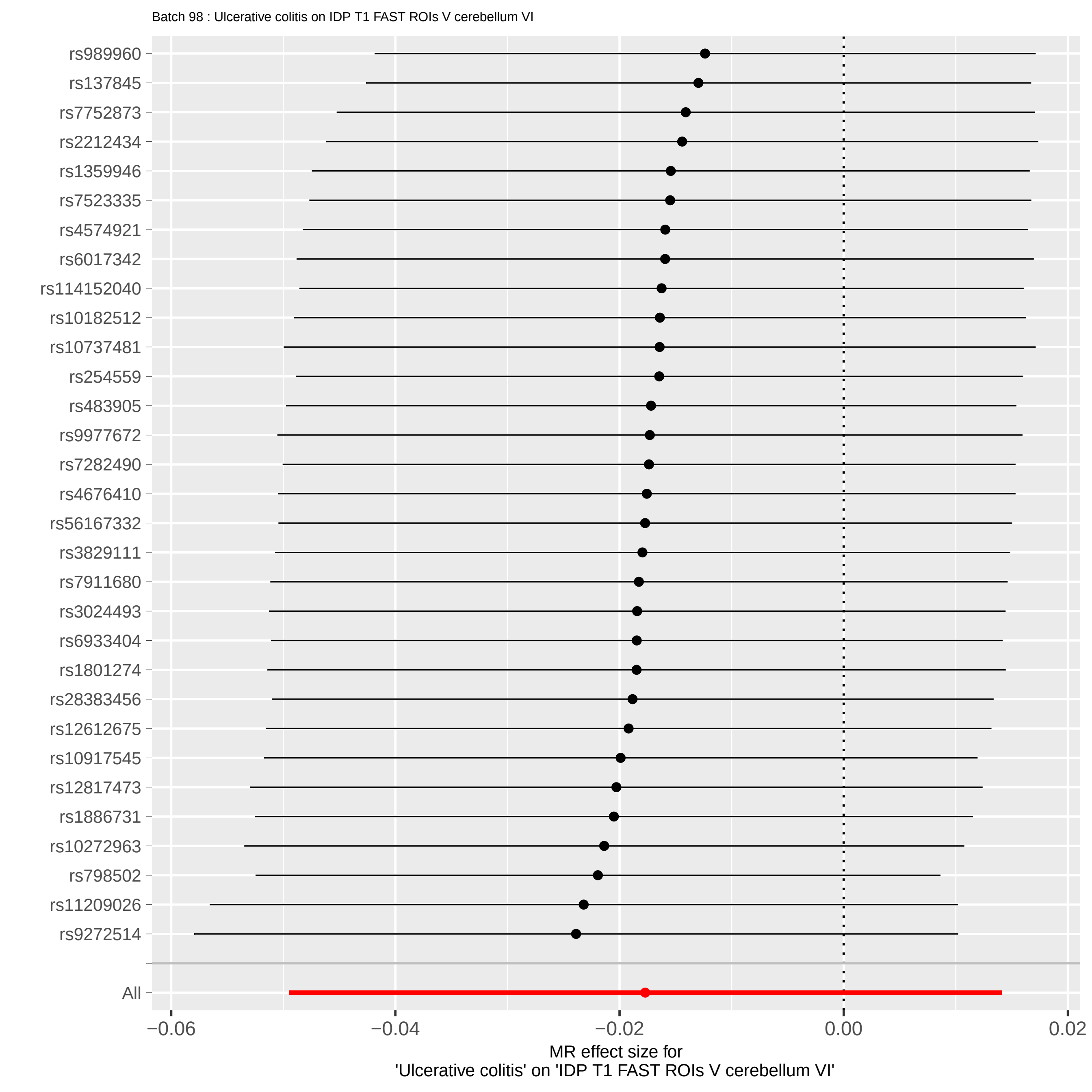
Batch 98 : Ulcerative colitis on IDP T1 FAST ROIs V cerebellum VI
rs989960
rs137845
rs7752873
rs2212434
rs1359946
rs7523335
rs4574921
rs6017342
rs114152040
rs10182512
rs10737481
rs254559
rs483905
rs9977672
rs7282490
rs4676410
rs56167332
rs3829111
rs7911680
rs3024493
rs6933404
rs1801274
rs28383456
rs12612675
rs10917545
rs12817473
rs1886731
rs10272963
rs798502
rs11209026
rs9272514
All
−0.06
−0.04
−0.02
0.00
0.02
MR effect size for
'Ulcerative colitis' on 'IDP T1 FAST ROIs V cerebellum VI'

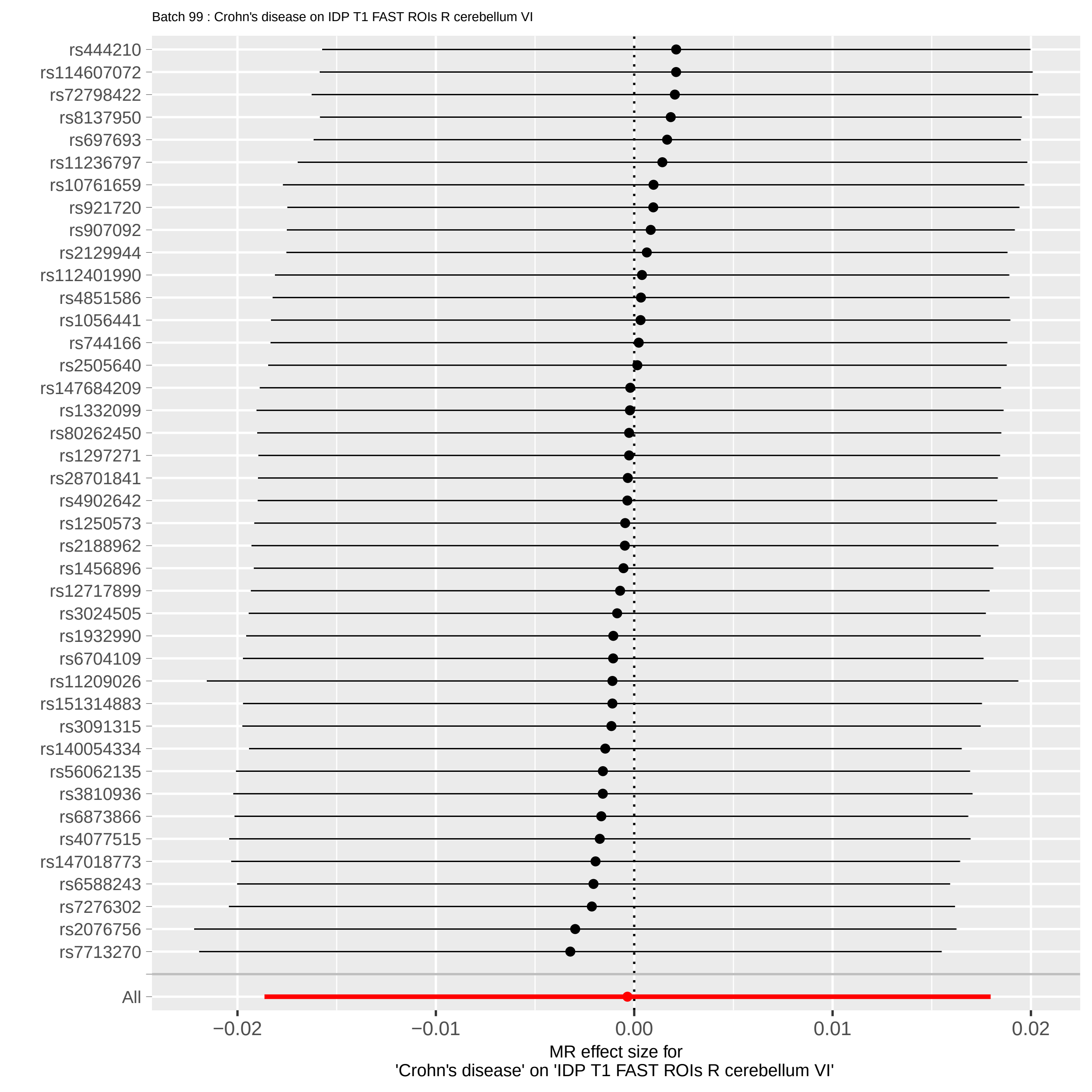
Batch 99 : Crohn's disease on IDP T1 FAST ROIs R cerebellum VI
rs444210
rs114607072
rs72798422
rs8137950
rs697693
rs11236797
rs10761659
rs921720
rs907092
rs2129944
rs112401990
rs4851586
rs1056441
rs744166
rs2505640
rs147684209
rs1332099
rs80262450
rs1297271
rs28701841
rs4902642
rs1250573
rs2188962
rs1456896
rs12717899
rs3024505
rs1932990
rs6704109
rs11209026
rs151314883
rs3091315
rs140054334
rs56062135
rs3810936
rs6873866
rs4077515
rs147018773
rs6588243
rs7276302
rs2076756
rs7713270
All
−0.02
−0.01
0.00
0.01
0.02
MR effect size for
'Crohn's disease' on 'IDP T1 FAST ROIs R cerebellum VI'

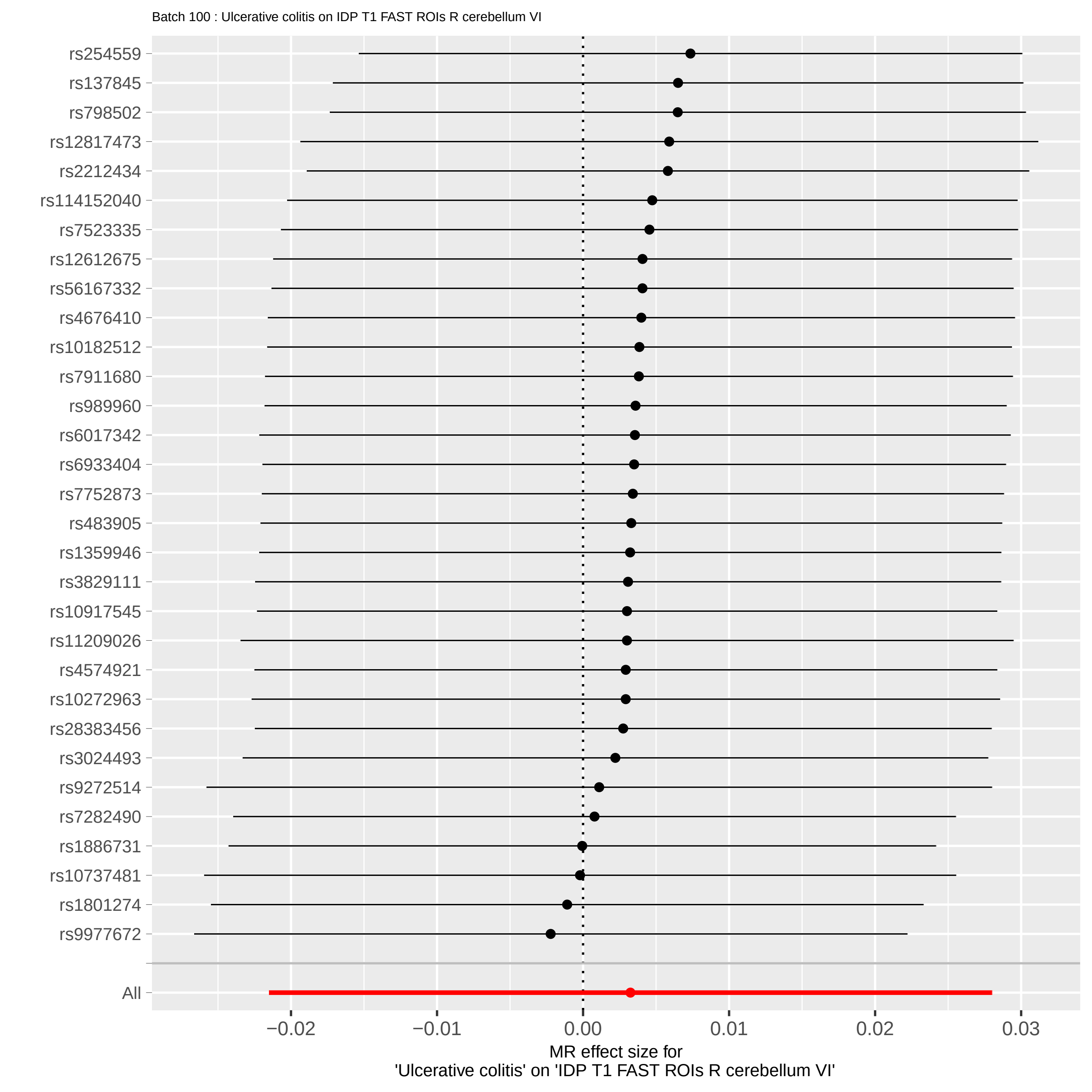

Batch 100 : Ulcerative colitis on IDP T1 FAST ROIs R cerebellum VI
rs254559
rs137845
rs798502
rs12817473
rs2212434
rs114152040
rs7523335
rs12612675
rs56167332
rs4676410
rs10182512
rs7911680
rs989960
rs6017342
rs6933404
rs7752873
rs483905
rs1359946
rs3829111
rs10917545
rs11209026
rs4574921
rs10272963
rs28383456
rs3024493
rs9272514
rs7282490
rs1886731
rs10737481
rs1801274
rs9977672
All
−0.02
−0.01
0.00
0.01
0.02
0.03
MR effect size for
'Ulcerative colitis' on 'IDP T1 FAST ROIs R cerebellum VI'

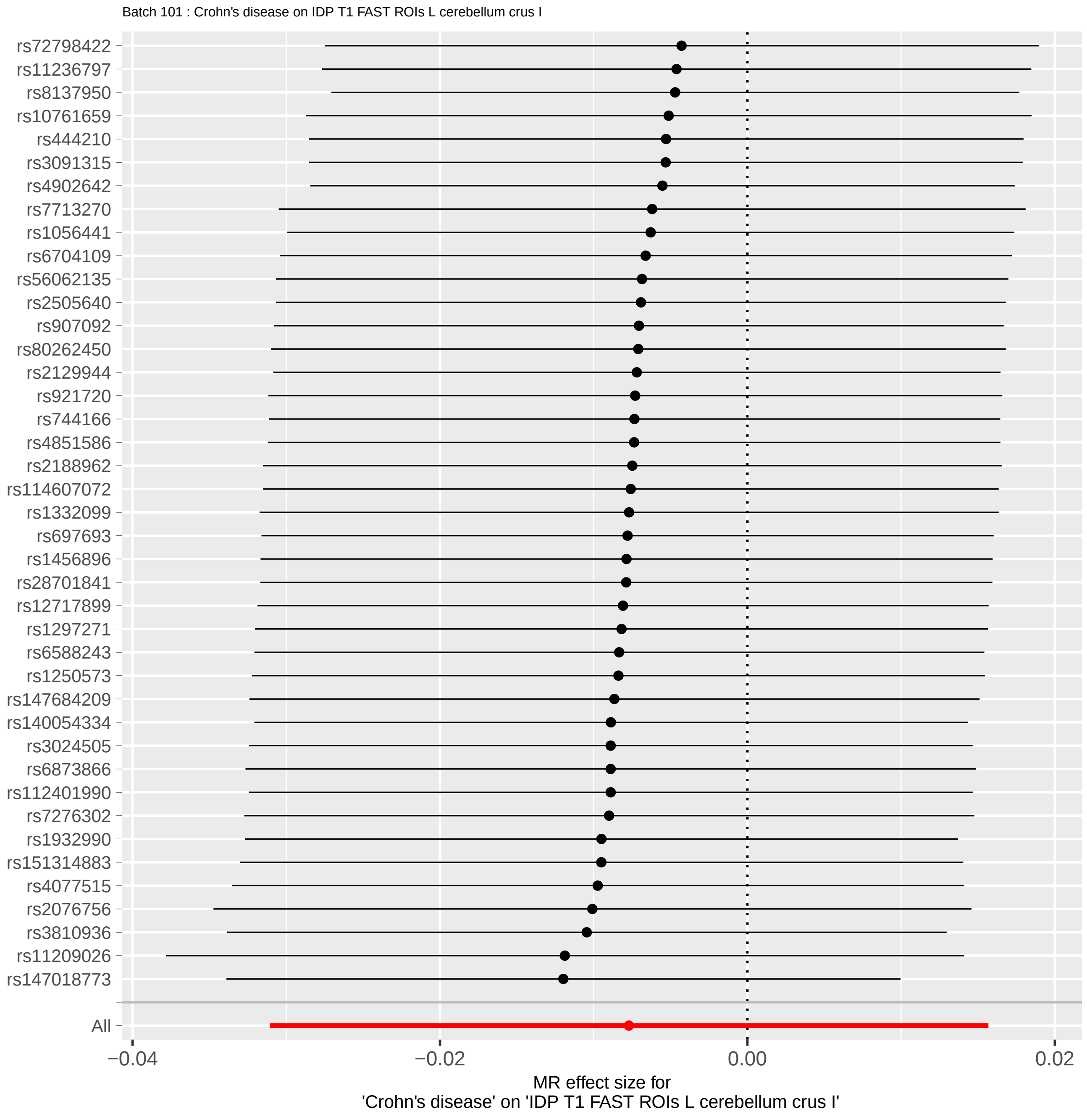
Batch 101 : Crohn's disease on IDP T1 FAST ROIs L cerebellum crus I
rs72798422
rs11236797
rs8137950
rs10761659
rs444210
rs3091315
rs4902642
rs7713270
rs1056441
rs6704109
rs56062135
rs2505640
rs907092
rs80262450
rs2129944
rs921720
rs744166
rs4851586
rs2188962
rs114607072
rs1332099
rs697693
rs1456896
rs28701841
rs12717899
rs1297271
rs6588243
rs1250573
rs147684209
rs140054334
rs3024505
rs6873866
rs112401990
rs7276302
rs1932990
rs151314883
rs4077515
rs2076756
rs3810936
rs11209026
rs147018773
All
−0.04
−0.02
0.00
0.02
MR effect size for
'Crohn's disease' on 'IDP T1 FAST ROIs L cerebellum crus I'

Batch 102 : Ulcerative colitis on IDP T1 FAST ROIs L cerebellum crus I

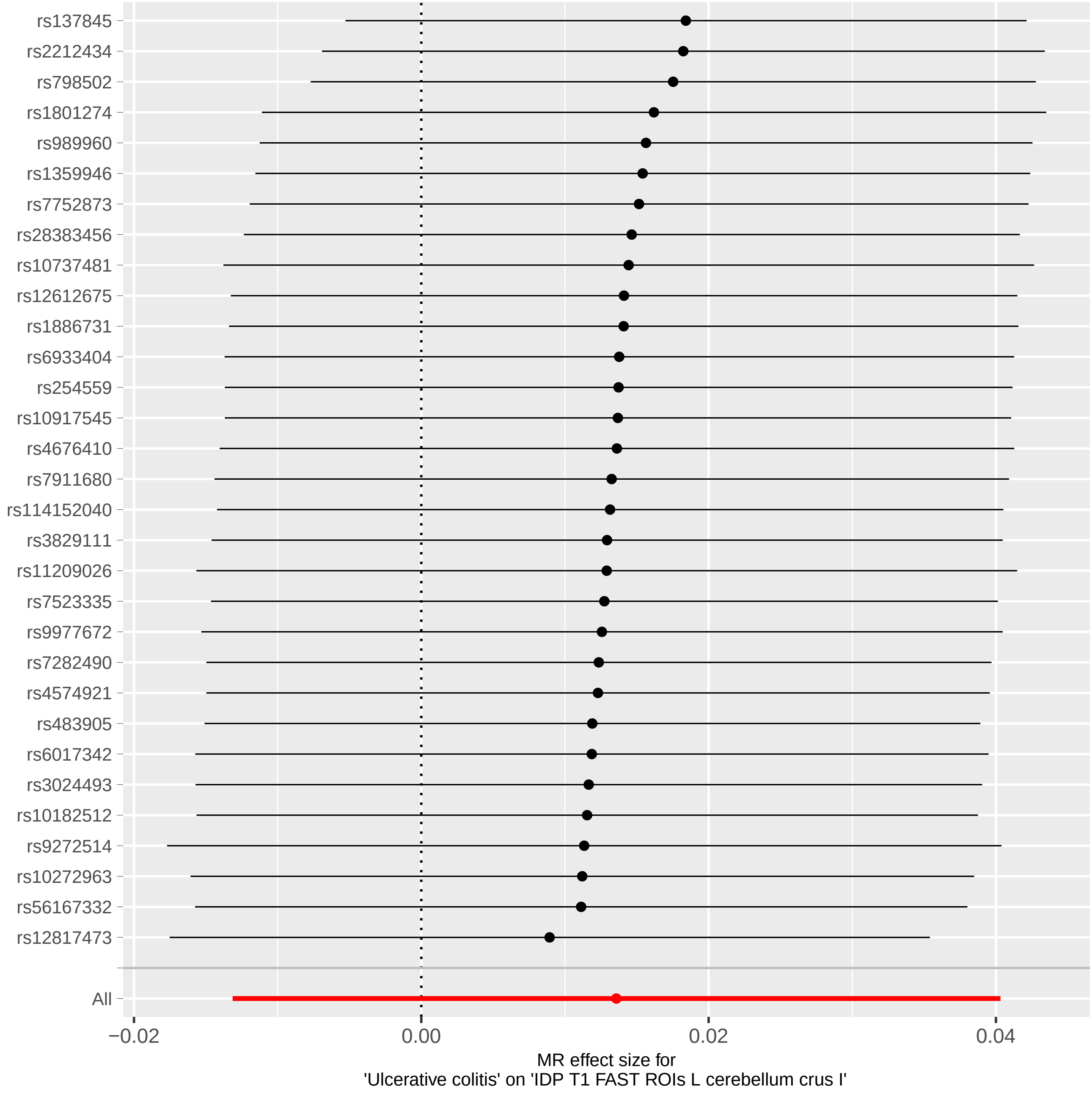

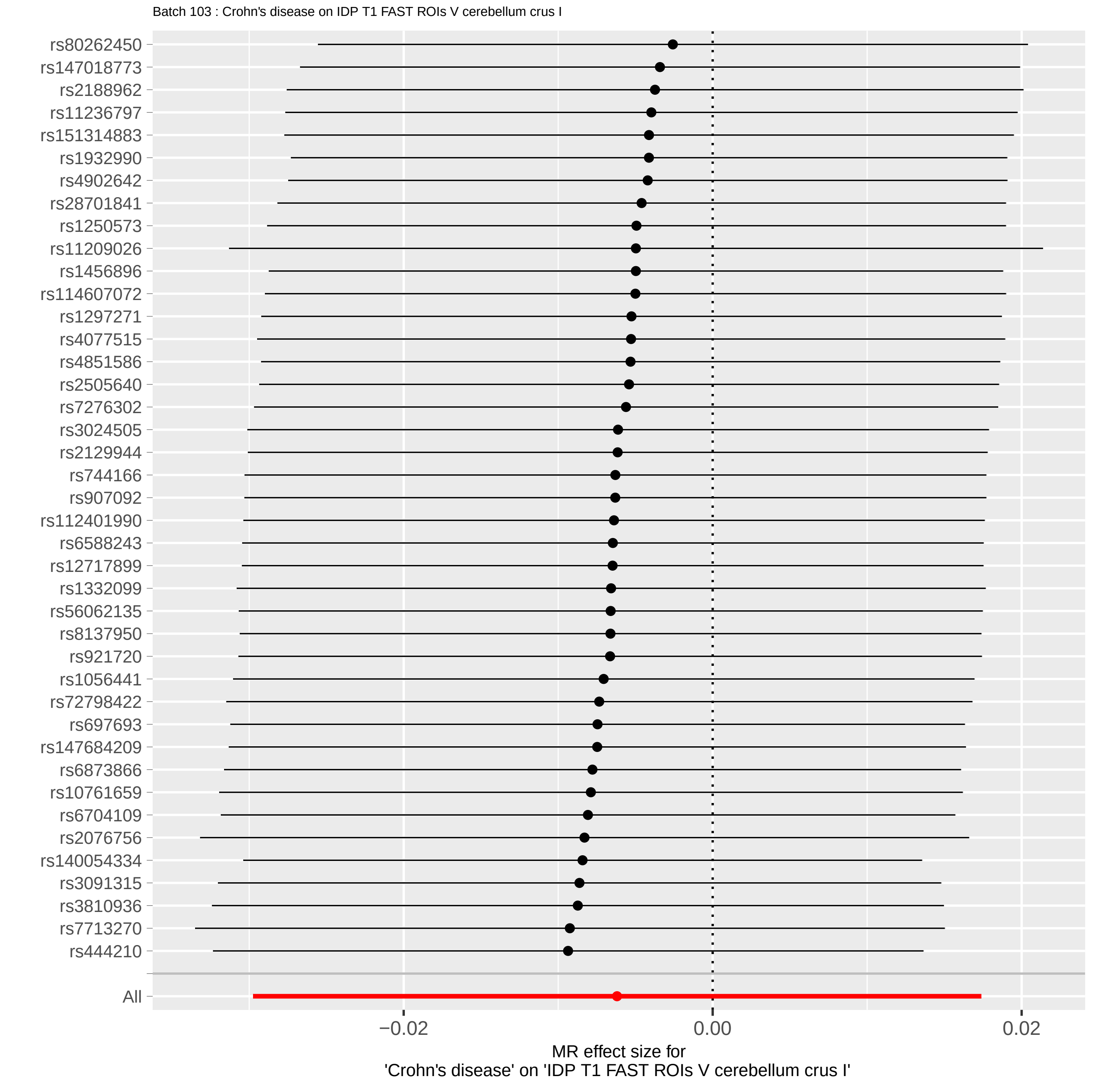
Batch 103 : Crohn's disease on IDP T1 FAST ROIs V cerebellum crus I
rs80262450
rs147018773
rs2188962
rs11236797
rs151314883
rs1932990
rs4902642
rs28701841
rs1250573
rs11209026
rs1456896
rs114607072
rs1297271
rs4077515
rs4851586
rs2505640
rs7276302
rs3024505
rs2129944
rs744166
rs907092
rs112401990
rs6588243
rs12717899
rs1332099
rs56062135
rs8137950
rs921720
rs1056441
rs72798422
rs697693
rs147684209
rs6873866
rs10761659
rs6704109
rs2076756
rs140054334
rs3091315
rs3810936
rs7713270
rs444210
All
−0.02
0.00
0.02
MR effect size for
'Crohn's disease' on 'IDP T1 FAST ROIs V cerebellum crus I'

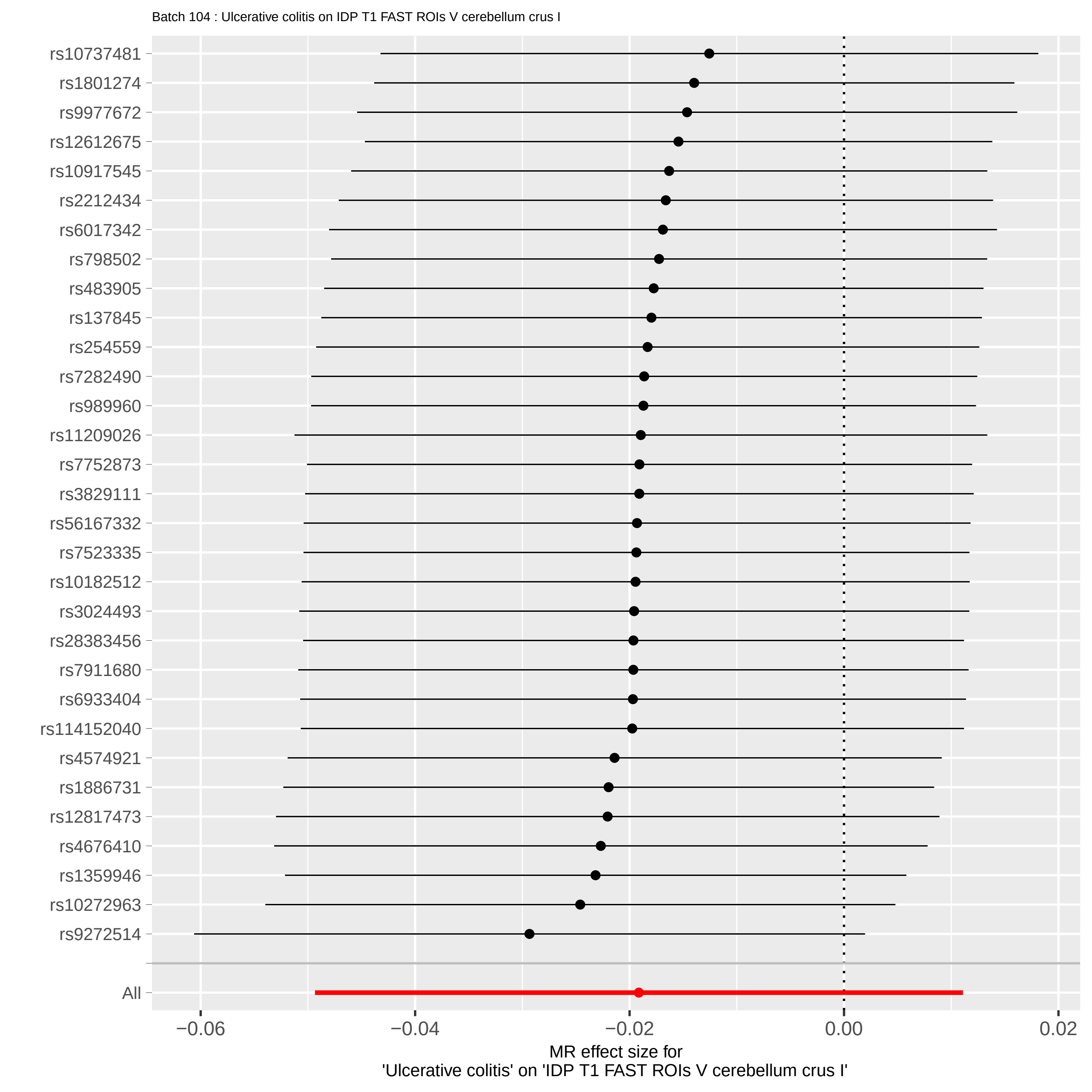
Batch 104 : Ulcerative colitis on IDP T1 FAST ROIs V cerebellum crus I
rs10737481
rs1801274
rs9977672
rs12612675
rs10917545
rs2212434
rs6017342
rs798502
rs483905
rs137845
rs254559
rs7282490
rs989960
rs11209026
rs7752873
rs3829111
rs56167332
rs7523335
rs10182512
rs3024493
rs28383456
rs7911680
rs6933404
rs114152040
rs4574921
rs1886731
rs12817473
rs4676410
rs1359946
rs10272963
rs9272514
All
−0.06
−0.04
−0.02
0.00
0.02
MR effect size for
'Ulcerative colitis' on 'IDP T1 FAST ROIs V cerebellum crus I'

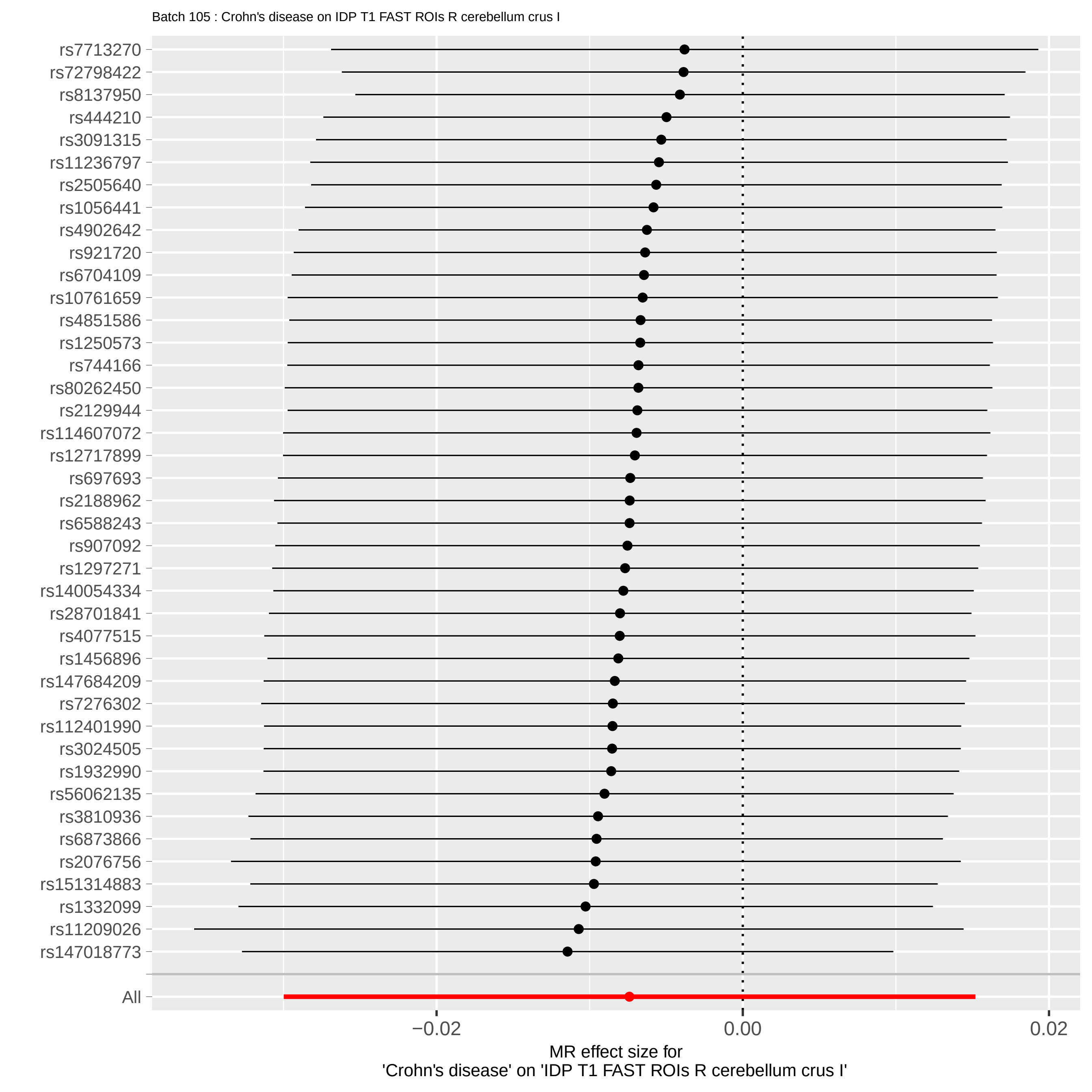

Batch 105 : Crohn's disease on IDP T1 FAST ROIs R cerebellum crus I
rs7713270
rs72798422
rs8137950
rs444210
rs3091315
rs11236797
rs2505640
rs1056441
rs4902642
rs921720
rs6704109
rs10761659
rs4851586
rs1250573
rs744166
rs80262450
rs2129944
rs114607072
rs12717899
rs697693
rs2188962
rs6588243
rs907092
rs1297271
rs140054334
rs28701841
rs4077515
rs1456896
rs147684209
rs7276302
rs112401990
rs3024505
rs1932990
rs56062135
rs3810936
rs6873866
rs2076756
rs151314883
rs1332099
rs11209026
rs147018773
All
−0.02
0.00
0.02
MR effect size for
'Crohn's disease' on 'IDP T1 FAST ROIs R cerebellum crus I'

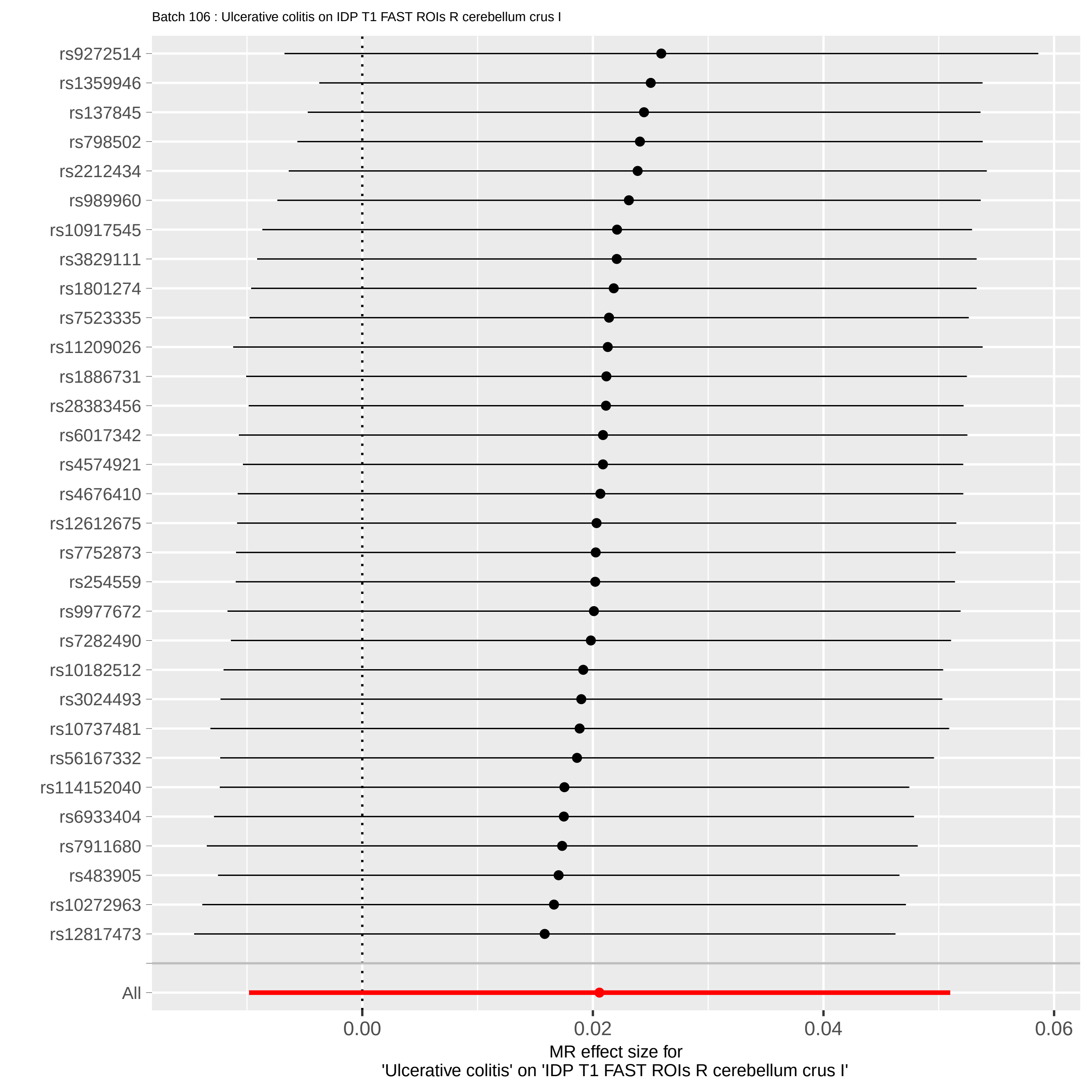

Batch 106 : Ulcerative colitis on IDP T1 FAST ROIs R cerebellum crus I
rs9272514
rs1359946
rs137845
rs798502
rs2212434
rs989960
rs10917545
rs3829111
rs1801274
rs7523335
rs11209026
rs1886731
rs28383456
rs6017342
rs4574921
rs4676410
rs12612675
rs7752873
rs254559
rs9977672
rs7282490
rs10182512
rs3024493
rs10737481
rs56167332
rs114152040
rs6933404
rs7911680
rs483905
rs10272963
rs12817473
All
0.00
0.02
0.04
0.06
MR effect size for
'Ulcerative colitis' on 'IDP T1 FAST ROIs R cerebellum crus I'

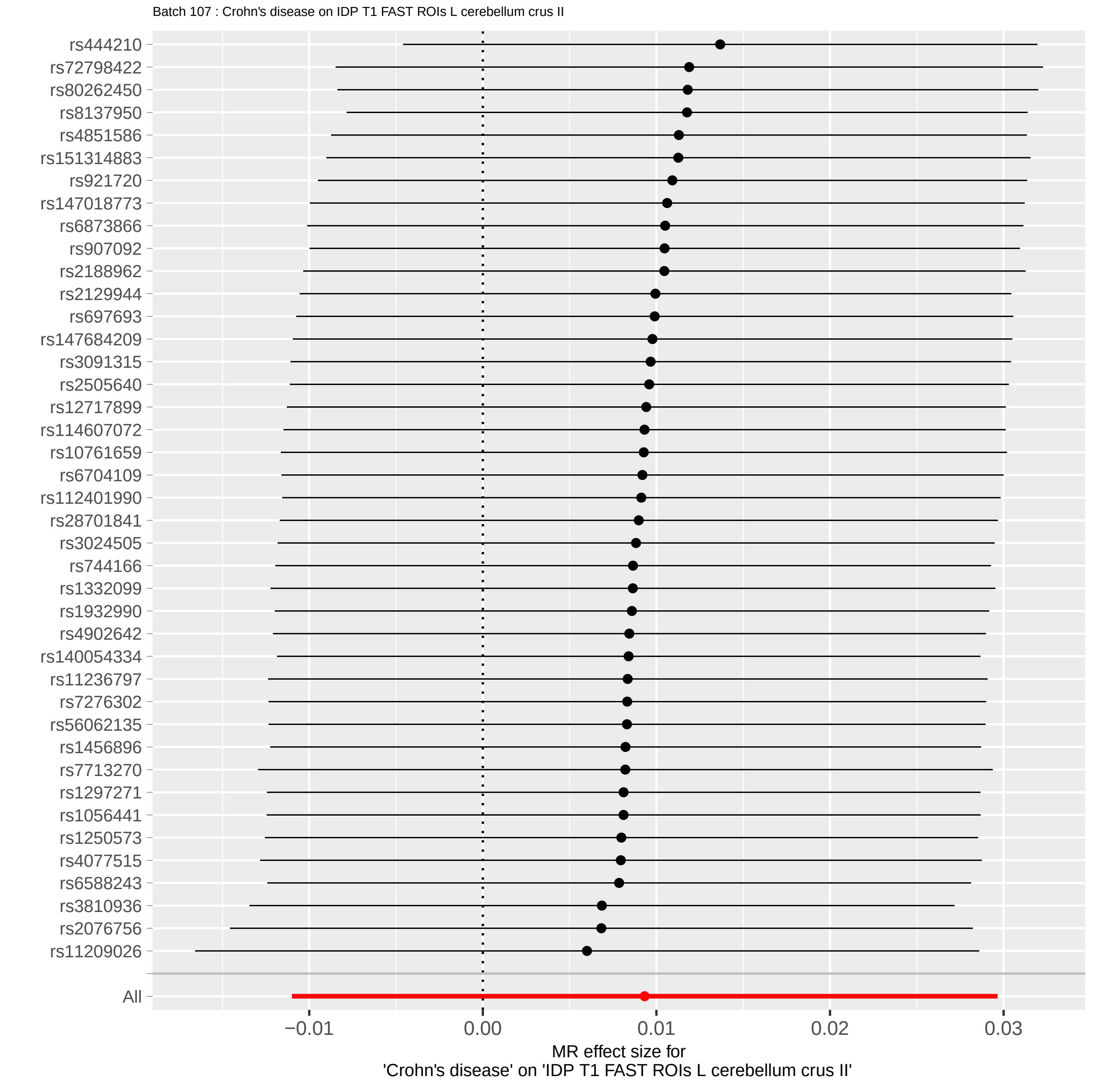
Batch 107 : Crohn's disease on IDP T1 FAST ROIs L cerebellum crus II
rs444210
rs72798422
rs80262450
rs8137950
rs4851586
rs151314883
rs921720
rs147018773
rs6873866
rs907092
rs2188962
rs2129944
rs697693
rs147684209
rs3091315
rs2505640
rs12717899
rs114607072
rs10761659
rs6704109
rs112401990
rs28701841
rs3024505
rs744166
rs1332099
rs1932990
rs4902642
rs140054334
rs11236797
rs7276302
rs56062135
rs1456896
rs7713270
rs1297271
rs1056441
rs1250573
rs4077515
rs6588243
rs3810936
rs2076756
rs11209026
All
−0.01
0.00
0.01
0.02
0.03
MR effect size for
'Crohn's disease' on 'IDP T1 FAST ROIs L cerebellum crus II'

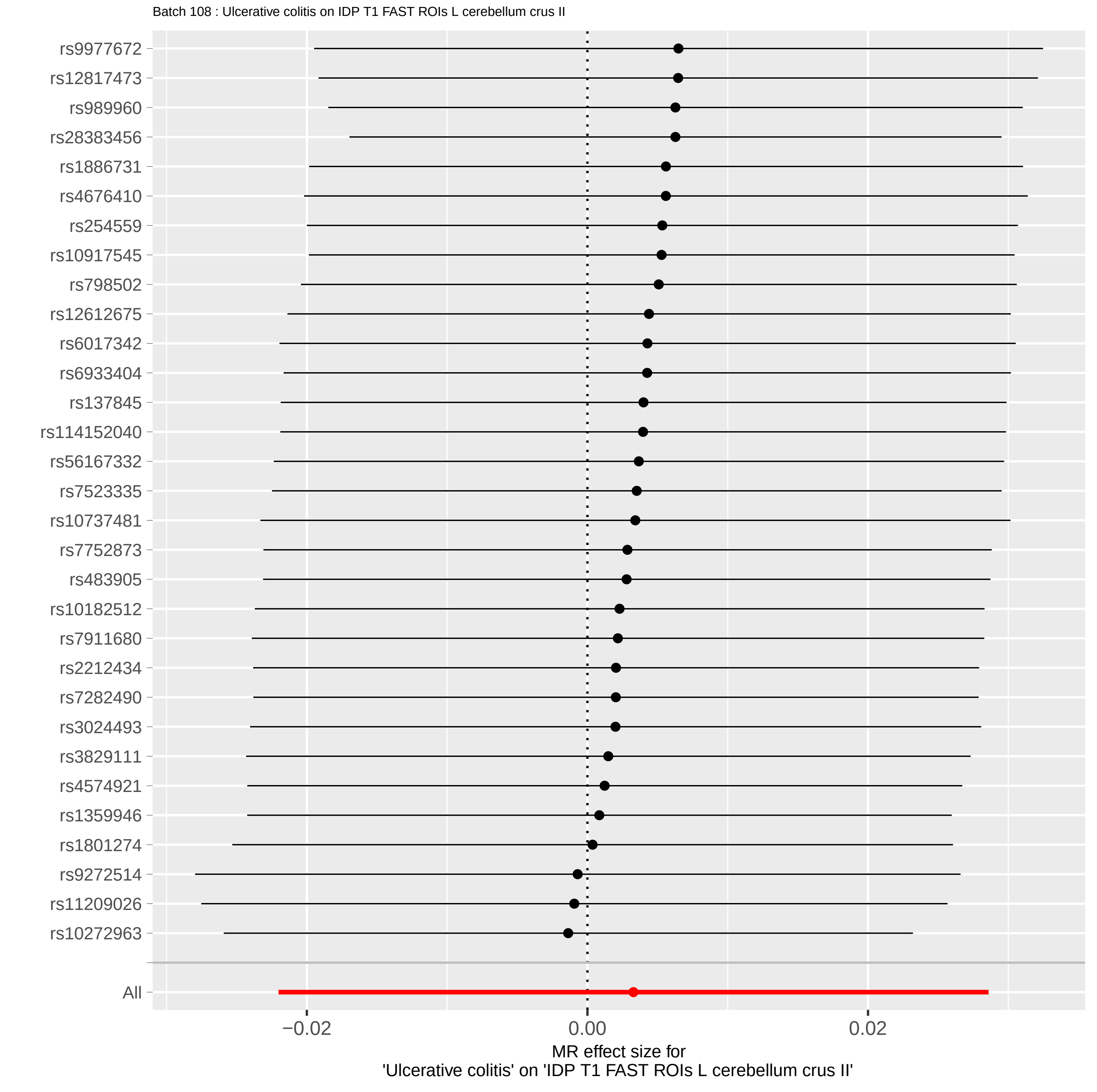

Batch 108 : Ulcerative colitis on IDP T1 FAST ROIs L cerebellum crus II
rs9977672
rs12817473
rs989960
rs28383456
rs1886731
rs4676410
rs254559
rs10917545
rs798502
rs12612675
rs6017342
rs6933404
rs137845
rs114152040
rs56167332
rs7523335
rs10737481
rs7752873
rs483905
rs10182512
rs7911680
rs2212434
rs7282490
rs3024493
rs3829111
rs4574921
rs1359946
rs1801274
rs9272514
rs11209026
rs10272963
All
−0.02
0.00
0.02
MR effect size for
'Ulcerative colitis' on 'IDP T1 FAST ROIs L cerebellum crus II'

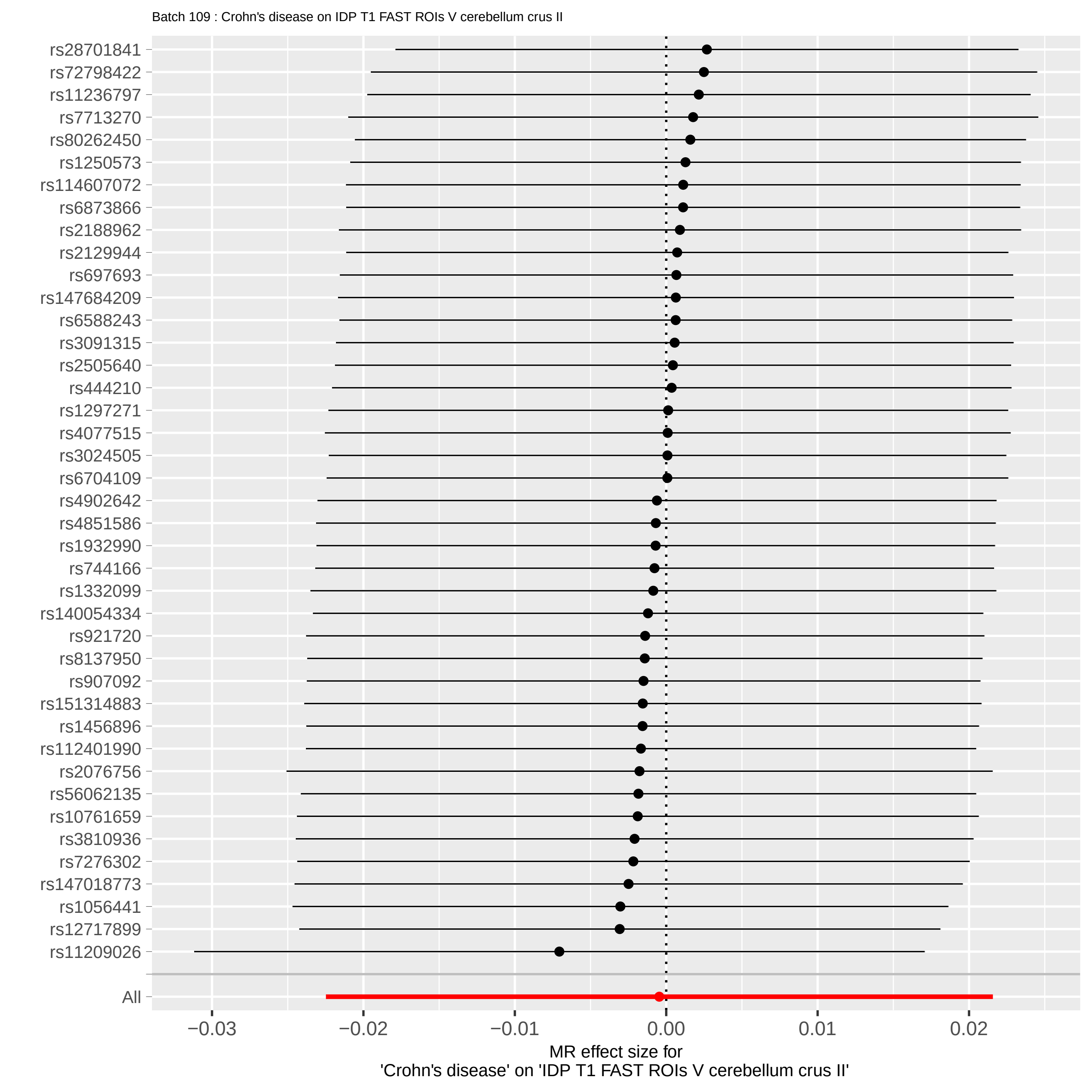

Batch 109 : Crohn's disease on IDP T1 FAST ROIs V cerebellum crus II
rs28701841
rs72798422
rs11236797
rs7713270
rs80262450
rs1250573
rs114607072
rs6873866
rs2188962
rs2129944
rs697693
rs147684209
rs6588243
rs3091315
rs2505640
rs444210
rs1297271
rs4077515
rs3024505
rs6704109
rs4902642
rs4851586
rs1932990
rs744166
rs1332099
rs140054334
rs921720
rs8137950
rs907092
rs151314883
rs1456896
rs112401990
rs2076756
rs56062135
rs10761659
rs3810936
rs7276302
rs147018773
rs1056441
rs12717899
rs11209026
All
−0.03
−0.02
−0.01
0.00
0.01
0.02
MR effect size for
'Crohn's disease' on 'IDP T1 FAST ROIs V cerebellum crus II'

Batch 110 : Ulcerative colitis on IDP T1 FAST ROIs V cerebellum crus II

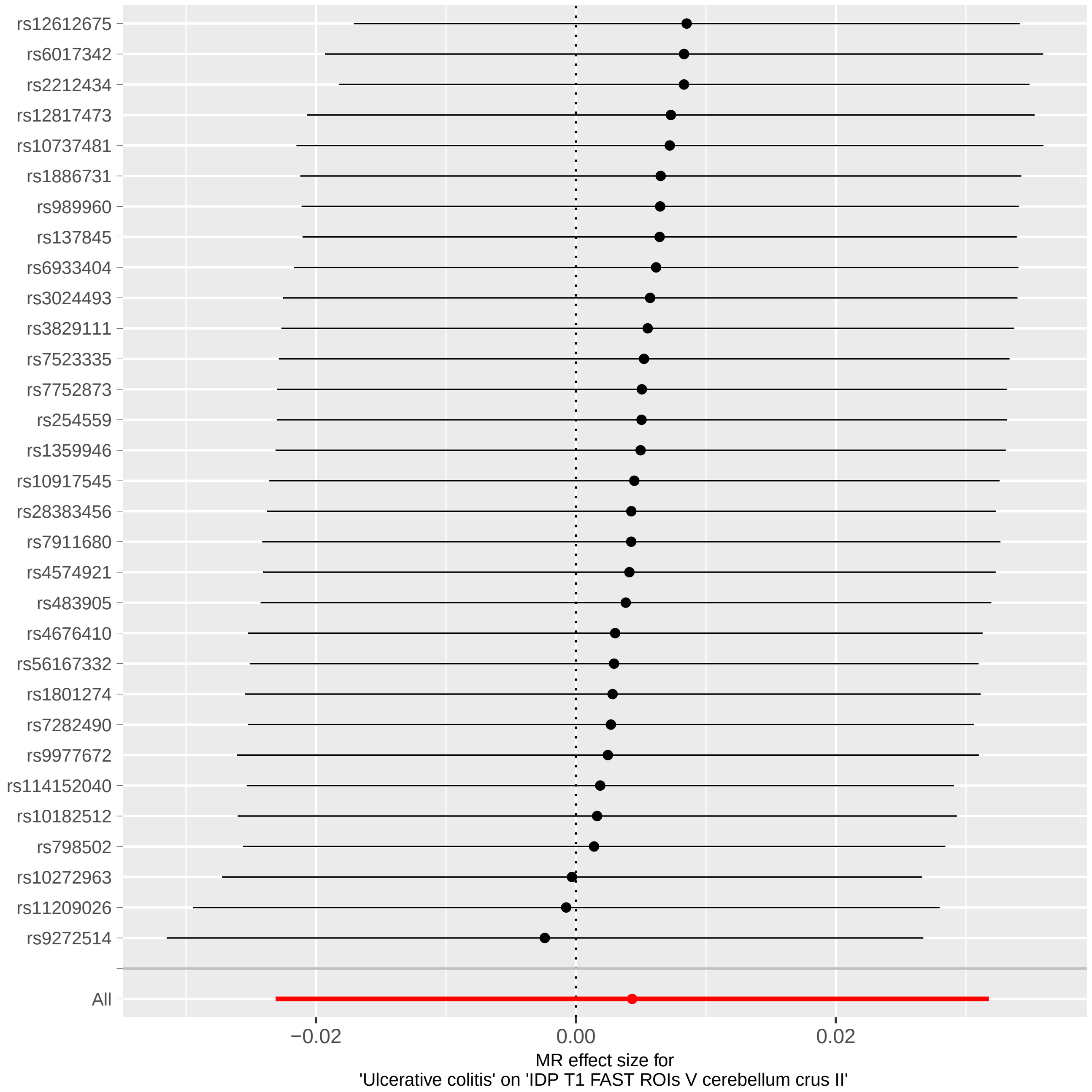

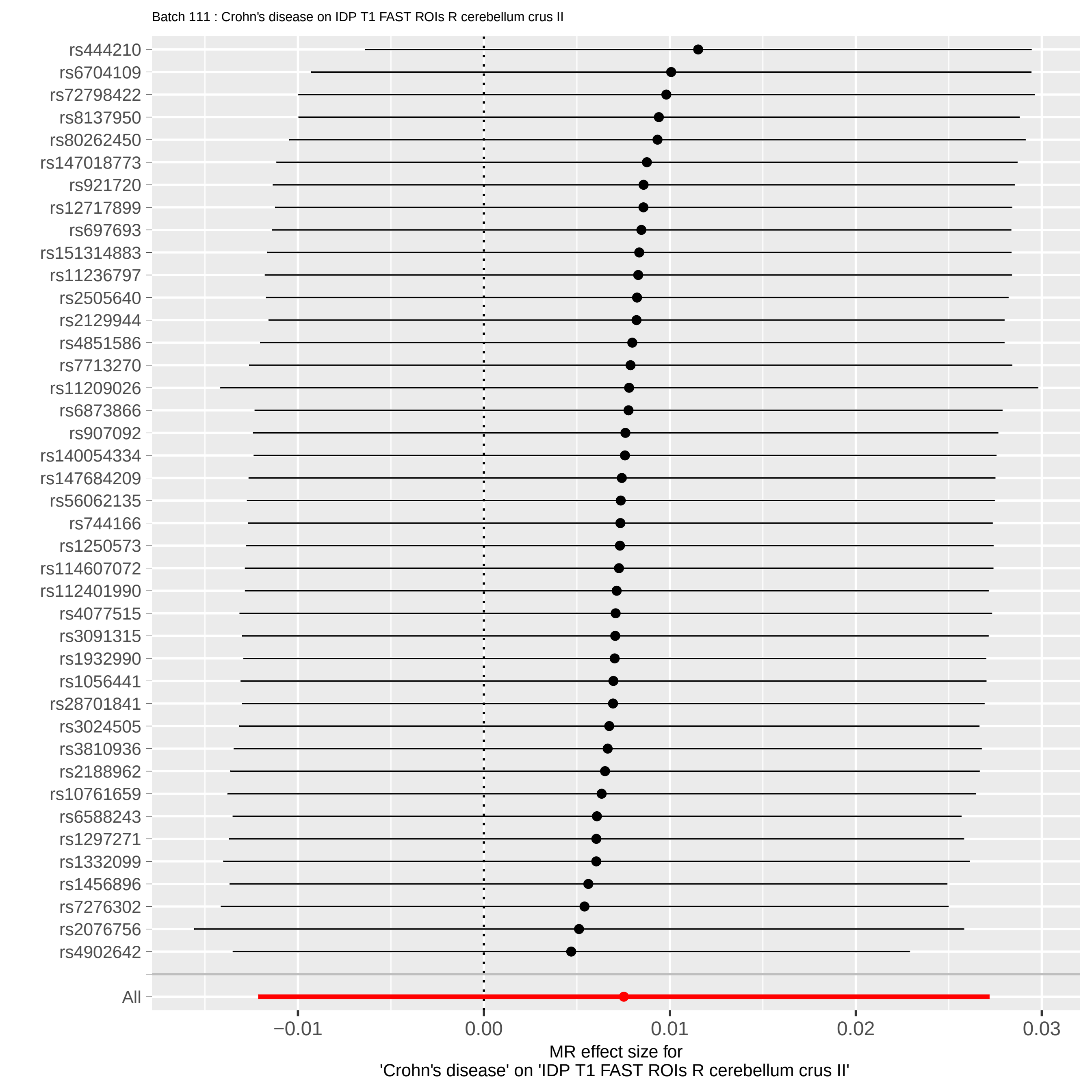

Batch 111 : Crohn's disease on IDP T1 FAST ROIs R cerebellum crus II
rs444210
rs6704109
rs72798422
rs8137950
rs80262450
rs147018773
rs921720
rs12717899
rs697693
rs151314883
rs11236797
rs2505640
rs2129944
rs4851586
rs7713270
rs11209026
rs6873866
rs907092
rs140054334
rs147684209
rs56062135
rs744166
rs1250573
rs114607072
rs112401990
rs4077515
rs3091315
rs1932990
rs1056441
rs28701841
rs3024505
rs3810936
rs2188962
rs10761659
rs6588243
rs1297271
rs1332099
rs1456896
rs7276302
rs2076756
rs4902642
All
−0.01
0.00
0.01
0.02
0.03
MR effect size for
'Crohn's disease' on 'IDP T1 FAST ROIs R cerebellum crus II'

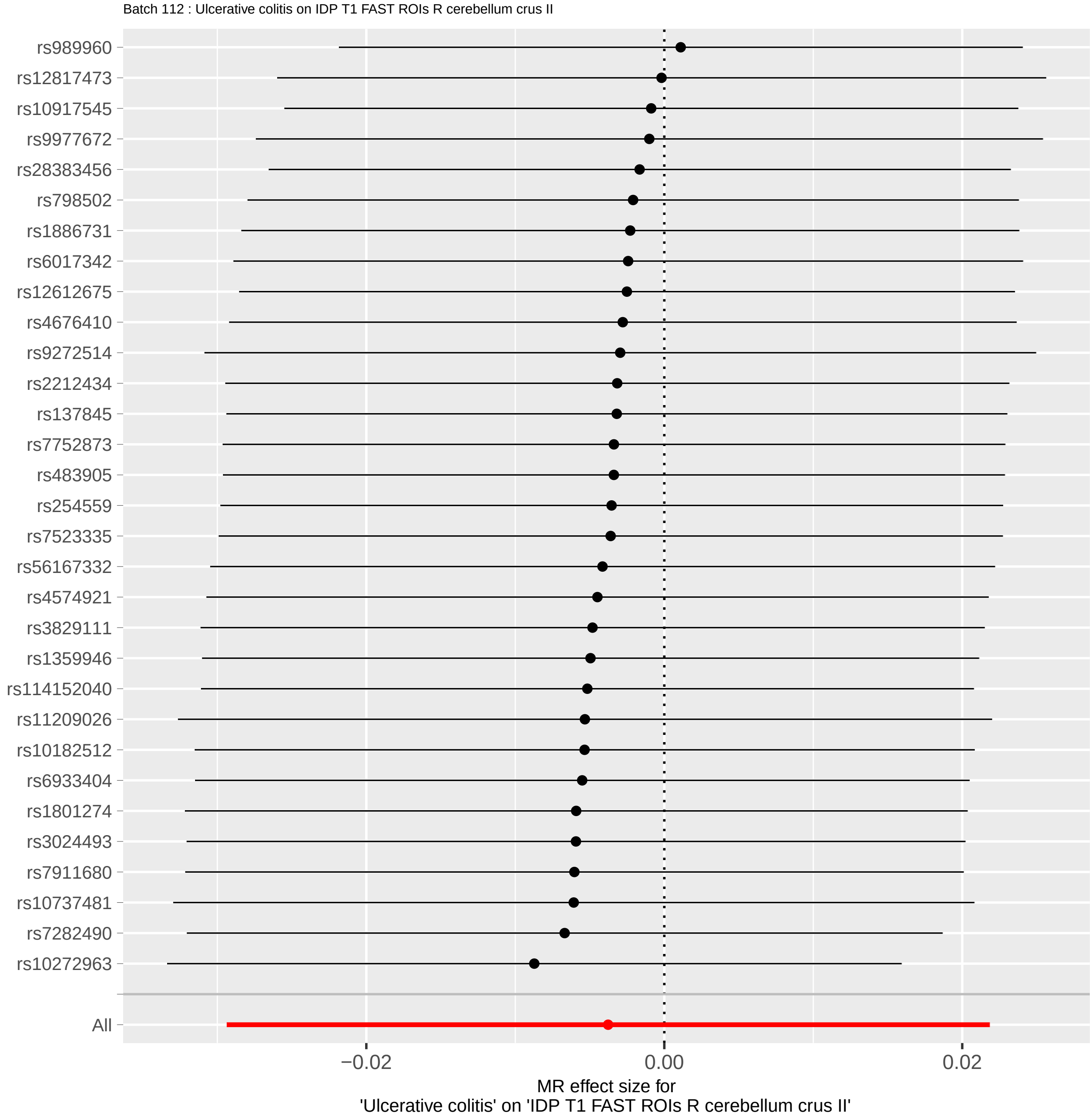
Batch 112 : Ulcerative colitis on IDP T1 FAST ROIs R cerebellum crus II
rs989960
rs12817473
rs10917545
rs9977672
rs28383456
rs798502
rs1886731
rs6017342
rs12612675
rs4676410
rs9272514
rs2212434
rs137845
rs7752873
rs483905
rs254559
rs7523335
rs56167332
rs4574921
rs3829111
rs1359946
rs114152040
rs11209026
rs10182512
rs6933404
rs1801274
rs3024493
rs7911680
rs10737481
rs7282490
rs10272963
All
−0.02
0.00
0.02
MR effect size for
'Ulcerative colitis' on 'IDP T1 FAST ROIs R cerebellum crus II'

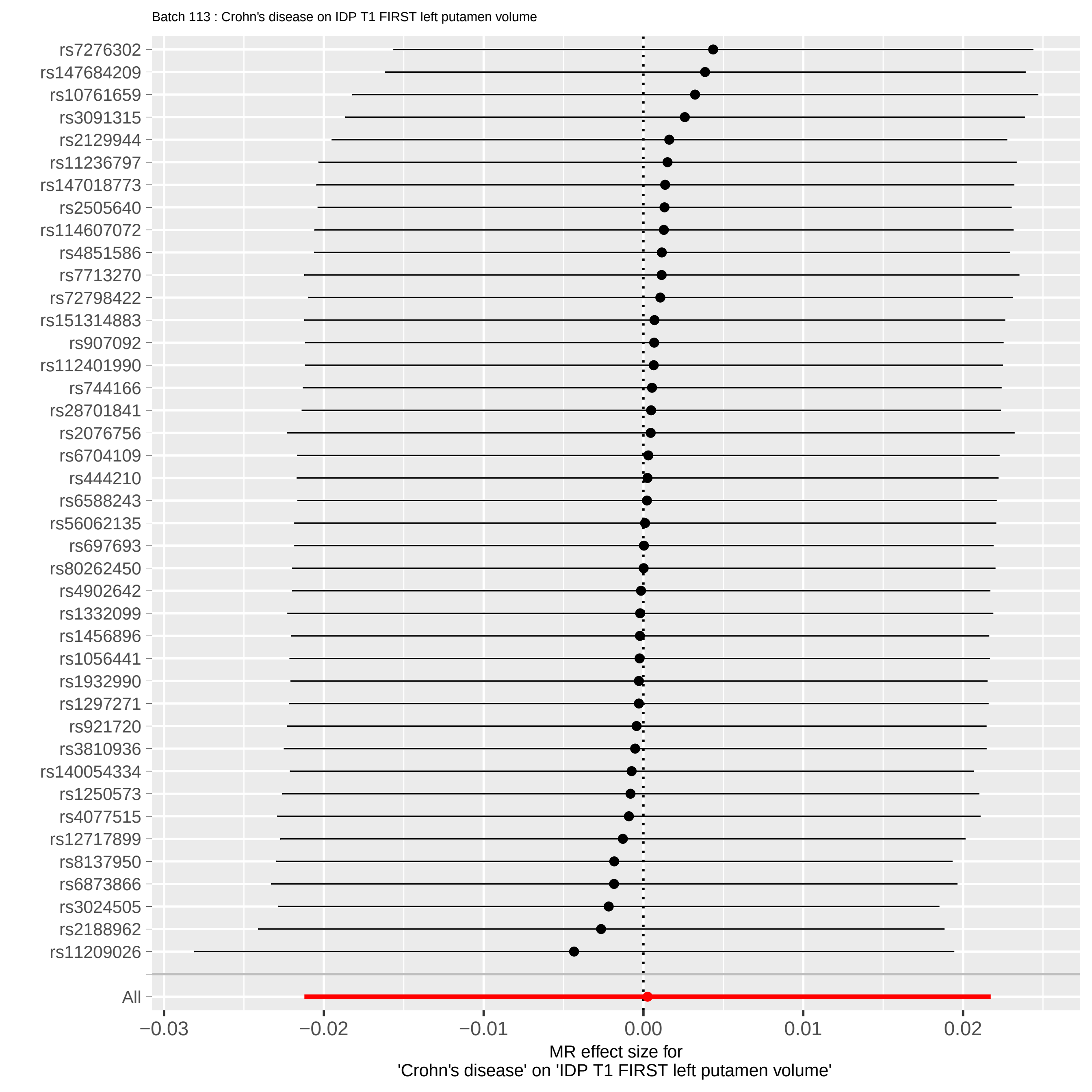

Batch 113 : Crohn's disease on IDP T1 FIRST left putamen volume
rs7276302
rs147684209
rs10761659
rs3091315
rs2129944
rs11236797
rs147018773
rs2505640
rs114607072
rs4851586
rs7713270
rs72798422
rs151314883
rs907092
rs112401990
rs744166
rs28701841
rs2076756
rs6704109
rs444210
rs6588243
rs56062135
rs697693
rs80262450
rs4902642
rs1332099
rs1456896
rs1056441
rs1932990
rs1297271
rs921720
rs3810936
rs140054334
rs1250573
rs4077515
rs12717899
rs8137950
rs6873866
rs3024505
rs2188962
rs11209026
All
−0.03
−0.02
−0.01
0.00
0.01
0.02
MR effect size for
'Crohn's disease' on 'IDP T1 FIRST left putamen volume'

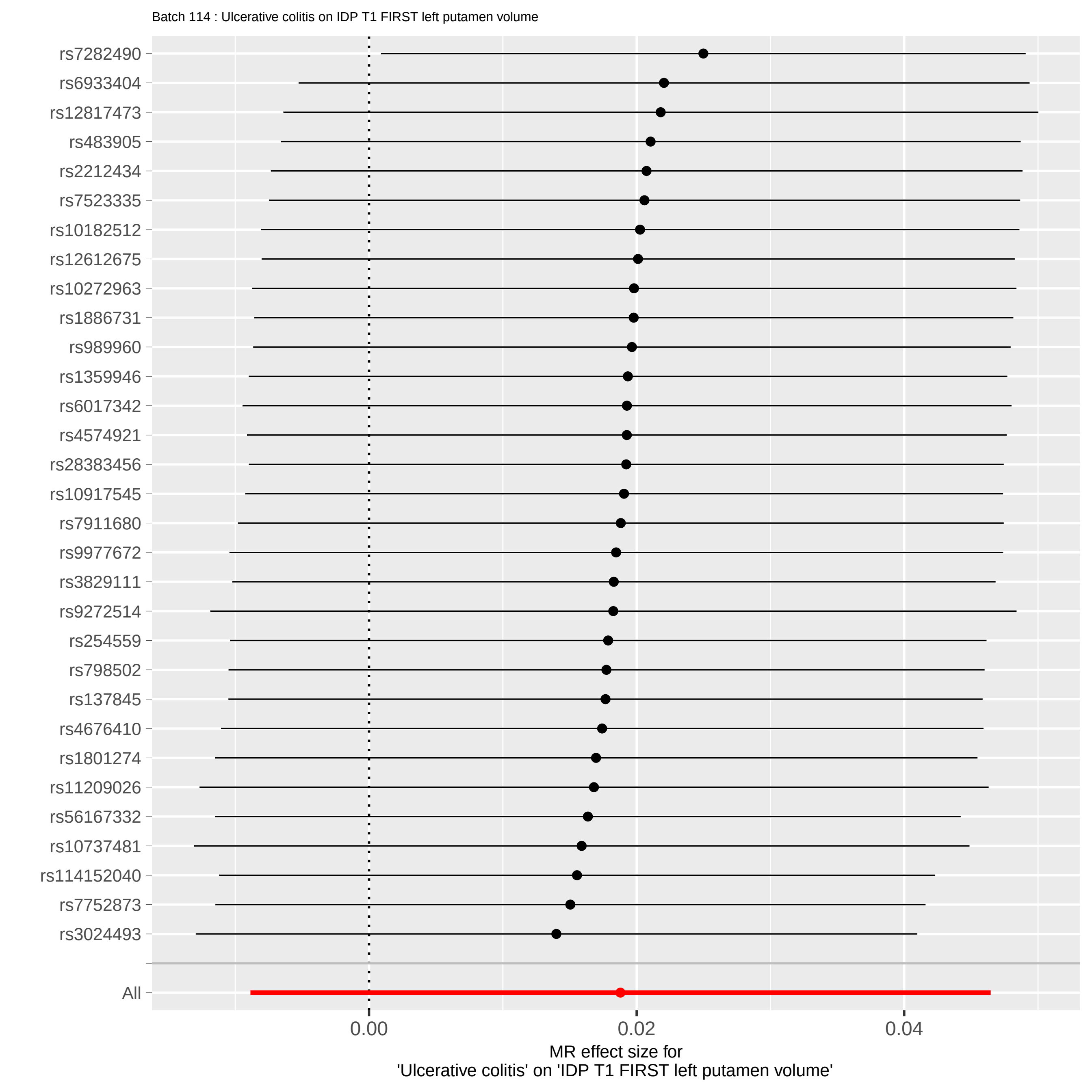

Batch 114 : Ulcerative colitis on IDP T1 FIRST left putamen volume
rs7282490
rs6933404
rs12817473
rs483905
rs2212434
rs7523335
rs10182512
rs12612675
rs10272963
rs1886731
rs989960
rs1359946
rs6017342
rs4574921
rs28383456
rs10917545
rs7911680
rs9977672
rs3829111
rs9272514
rs254559
rs798502
rs137845
rs4676410
rs1801274
rs11209026
rs56167332
rs10737481
rs114152040
rs7752873
rs3024493
All
0.00
0.02
0.04
MR effect size for
'Ulcerative colitis' on 'IDP T1 FIRST left putamen volume'

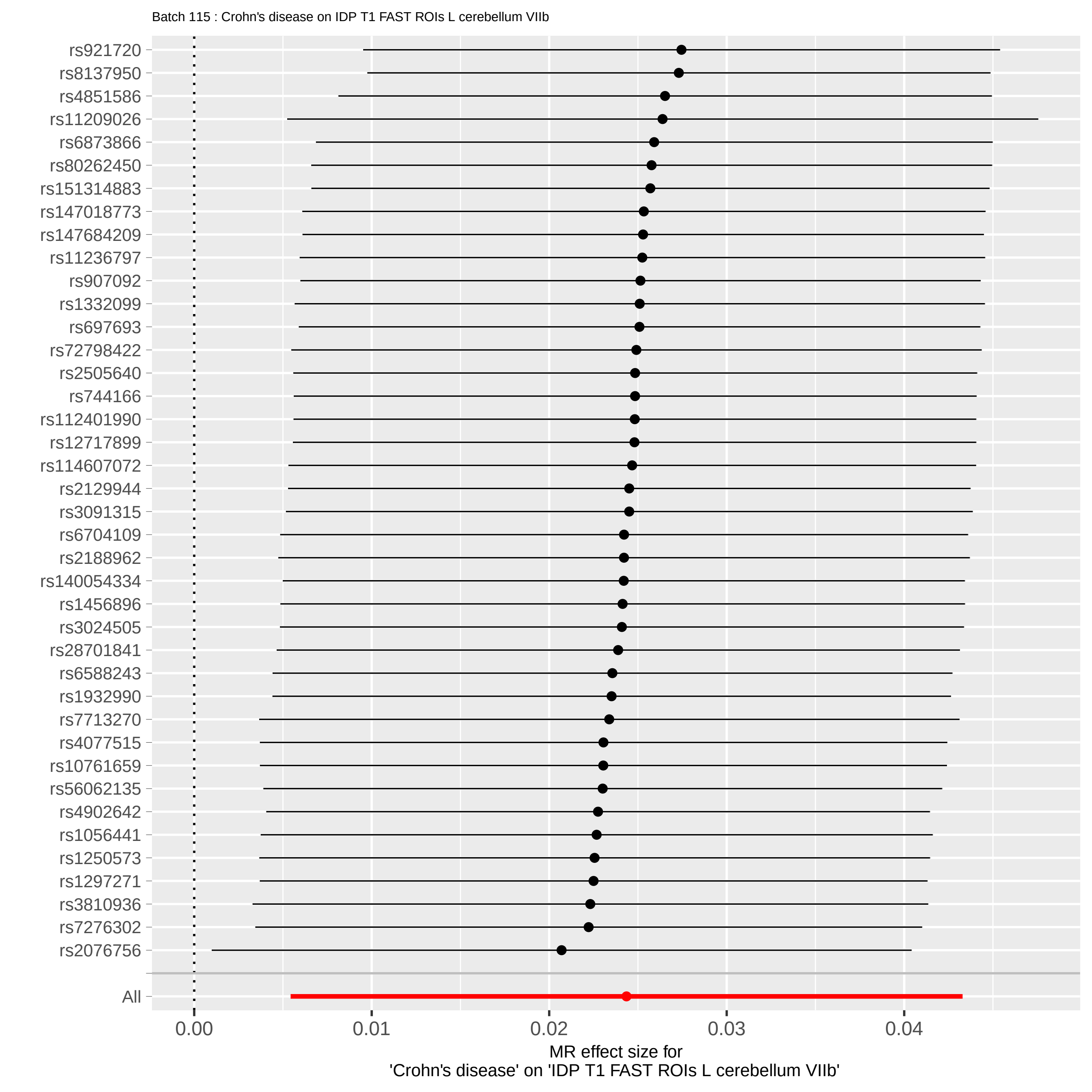

Batch 115 : Crohn's disease on IDP T1 FAST ROIs L cerebellum VIIb
rs921720
rs8137950
rs4851586
rs11209026
rs6873866
rs80262450
rs151314883
rs147018773
rs147684209
rs11236797
rs907092
rs1332099
rs697693
rs72798422
rs2505640
rs744166
rs112401990
rs12717899
rs114607072
rs2129944
rs3091315
rs6704109
rs2188962
rs140054334
rs1456896
rs3024505
rs28701841
rs6588243
rs1932990
rs7713270
rs4077515
rs10761659
rs56062135
rs4902642
rs1056441
rs1250573
rs1297271
rs3810936
rs7276302
rs2076756
All
0.00
0.01
0.02
0.03
0.04
MR effect size for
'Crohn's disease' on 'IDP T1 FAST ROIs L cerebellum VIIb'

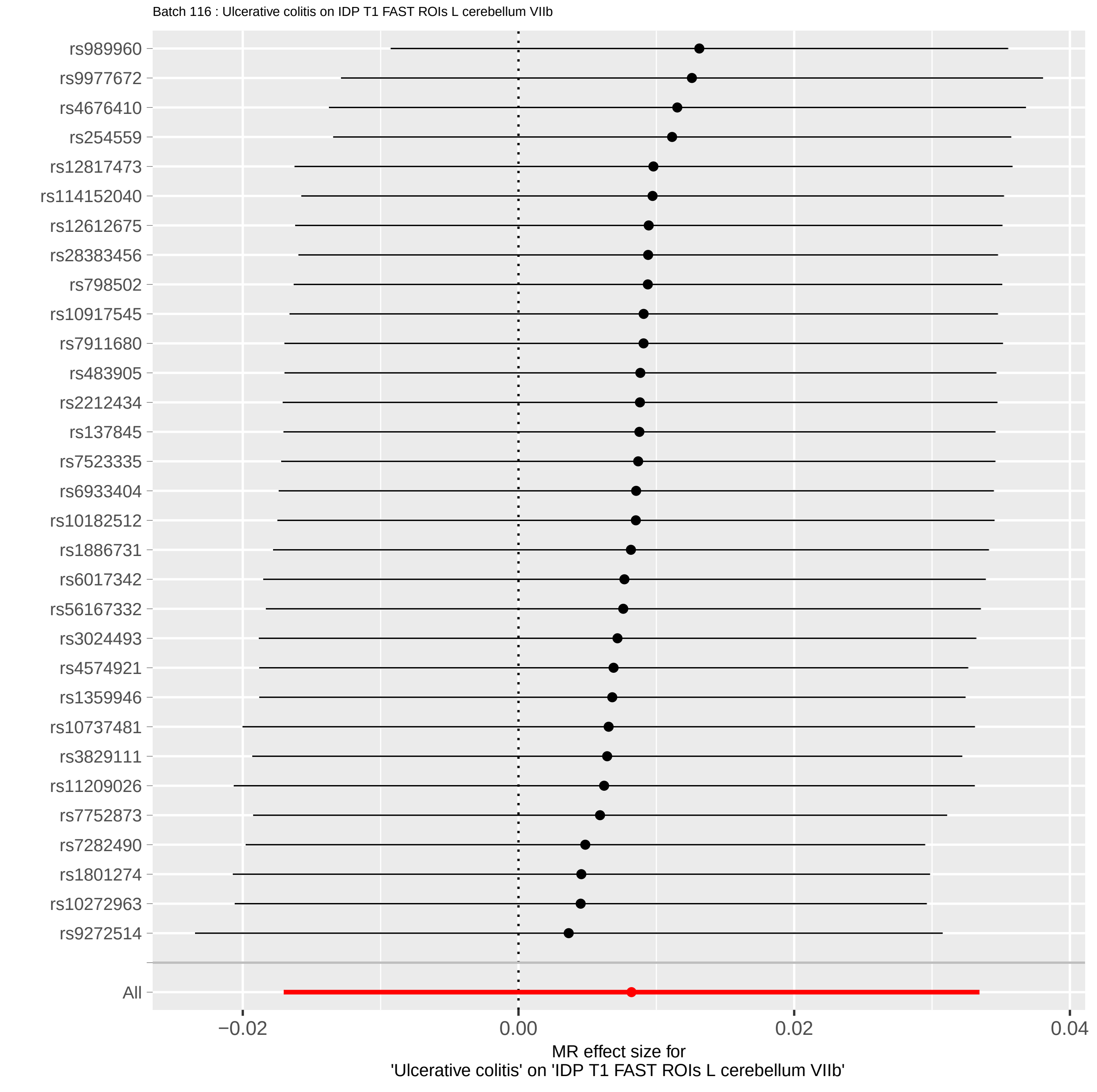

Batch 116 : Ulcerative colitis on IDP T1 FAST ROIs L cerebellum VIIb
rs989960
rs9977672
rs4676410
rs254559
rs12817473
rs114152040
rs12612675
rs28383456
rs798502
rs10917545
rs7911680
rs483905
rs2212434
rs137845
rs7523335
rs6933404
rs10182512
rs1886731
rs6017342
rs56167332
rs3024493
rs4574921
rs1359946
rs10737481
rs3829111
rs11209026
rs7752873
rs7282490
rs1801274
rs10272963
rs9272514
All
−0.02
0.00
0.02
0.04
MR effect size for
'Ulcerative colitis' on 'IDP T1 FAST ROIs L cerebellum VIIb'

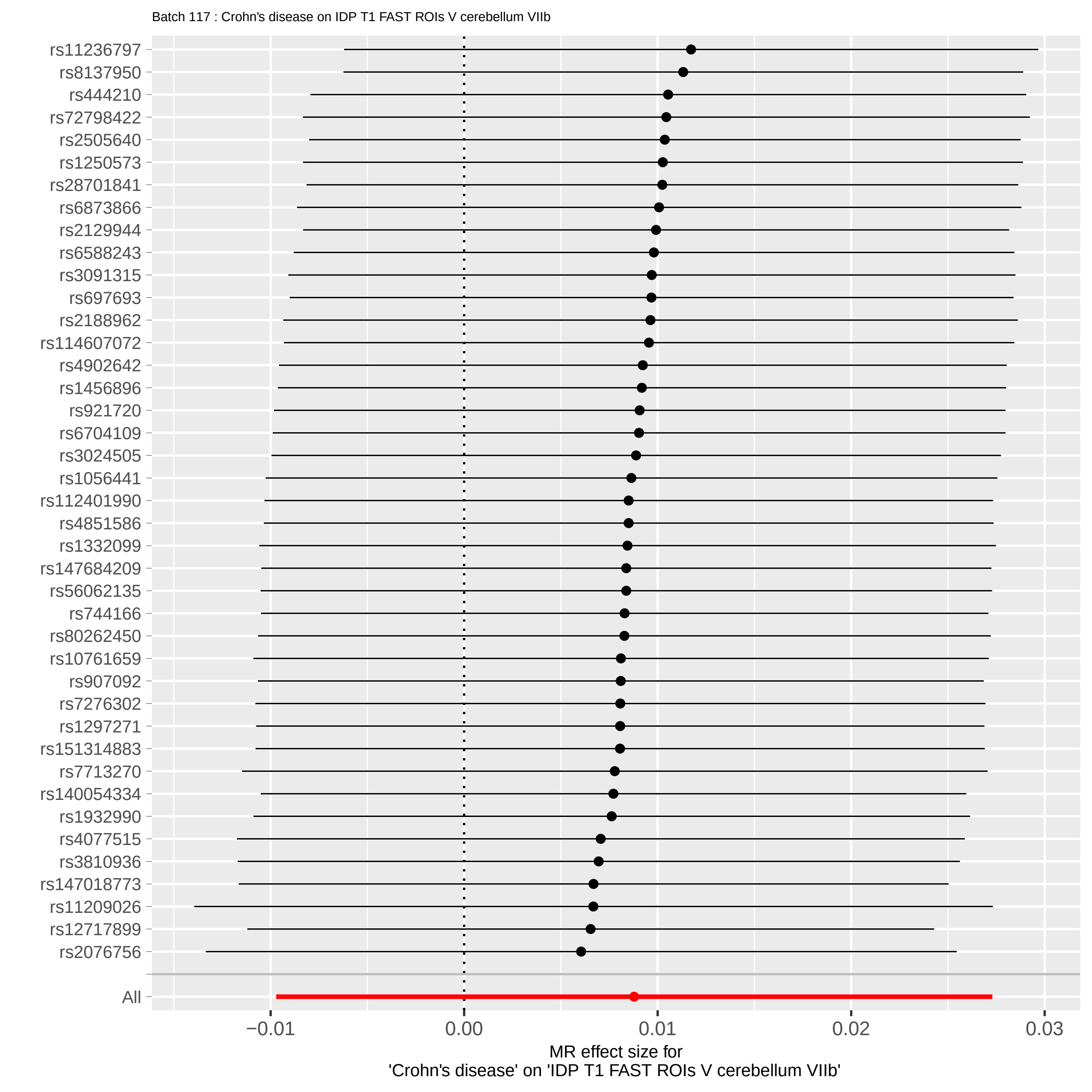

Batch 117 : Crohn's disease on IDP T1 FAST ROIs V cerebellum VIIb
rs11236797
rs8137950
rs444210
rs72798422
rs2505640
rs1250573
rs28701841
rs6873866
rs2129944
rs6588243
rs3091315
rs697693
rs2188962
rs114607072
rs4902642
rs1456896
rs921720
rs6704109
rs3024505
rs1056441
rs112401990
rs4851586
rs1332099
rs147684209
rs56062135
rs744166
rs80262450
rs10761659
rs907092
rs7276302
rs1297271
rs151314883
rs7713270
rs140054334
rs1932990
rs4077515
rs3810936
rs147018773
rs11209026
rs12717899
rs2076756
All
−0.01
0.00
0.01
0.02
0.03
MR effect size for
'Crohn's disease' on 'IDP T1 FAST ROIs V cerebellum VIIb'

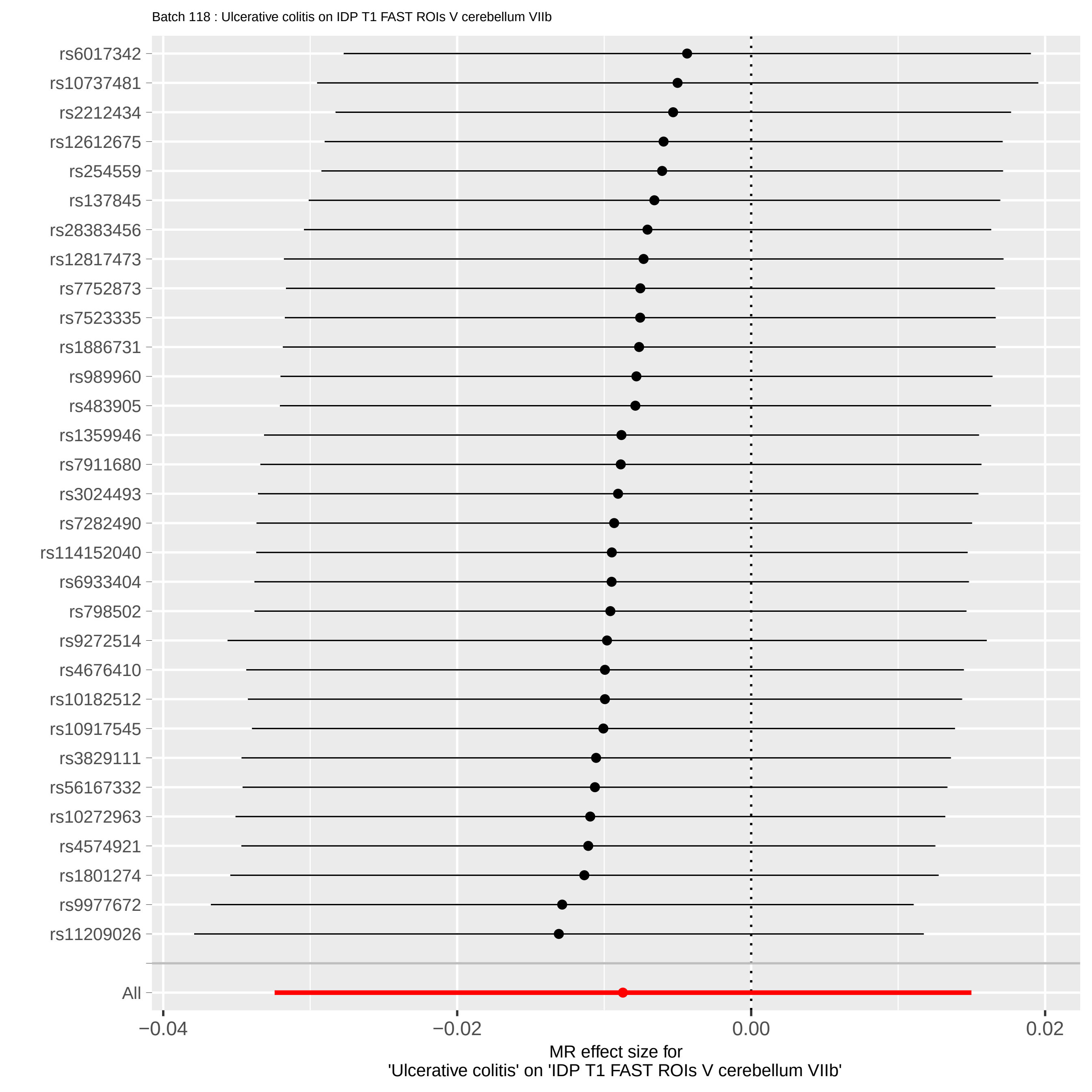

Batch 118 : Ulcerative colitis on IDP T1 FAST ROIs V cerebellum VIIb
rs6017342
rs10737481
rs2212434
rs12612675
rs254559
rs137845
rs28383456
rs12817473
rs7752873
rs7523335
rs1886731
rs989960
rs483905
rs1359946
rs7911680
rs3024493
rs7282490
rs114152040
rs6933404
rs798502
rs9272514
rs4676410
rs10182512
rs10917545
rs3829111
rs56167332
rs10272963
rs4574921
rs1801274
rs9977672
rs11209026
All
−0.04
−0.02
0.00
0.02
MR effect size for
'Ulcerative colitis' on 'IDP T1 FAST ROIs V cerebellum VIIb'

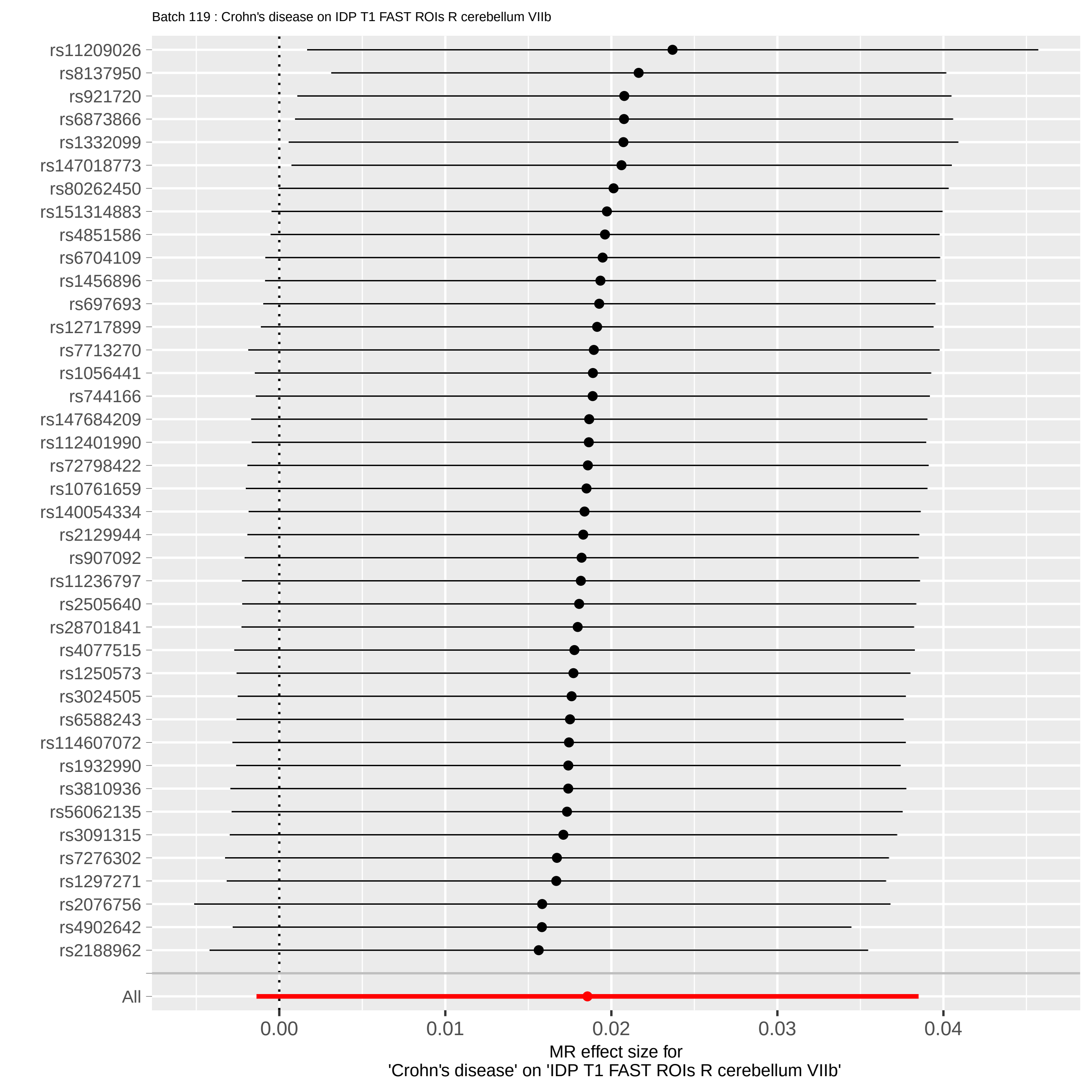

Batch 119 : Crohn's disease on IDP T1 FAST ROIs R cerebellum VIIb
rs11209026
rs8137950
rs921720
rs6873866
rs1332099
rs147018773
rs80262450
rs151314883
rs4851586
rs6704109
rs1456896
rs697693
rs12717899
rs7713270
rs1056441
rs744166
rs147684209
rs112401990
rs72798422
rs10761659
rs140054334
rs2129944
rs907092
rs11236797
rs2505640
rs28701841
rs4077515
rs1250573
rs3024505
rs6588243
rs114607072
rs1932990
rs3810936
rs56062135
rs3091315
rs7276302
rs1297271
rs2076756
rs4902642
rs2188962
All
0.00
0.01
0.02
0.03
0.04
MR effect size for
'Crohn's disease' on 'IDP T1 FAST ROIs R cerebellum VIIb'

Batch 120 : Ulcerative colitis on IDP T1 FAST ROIs R cerebellum VIIb

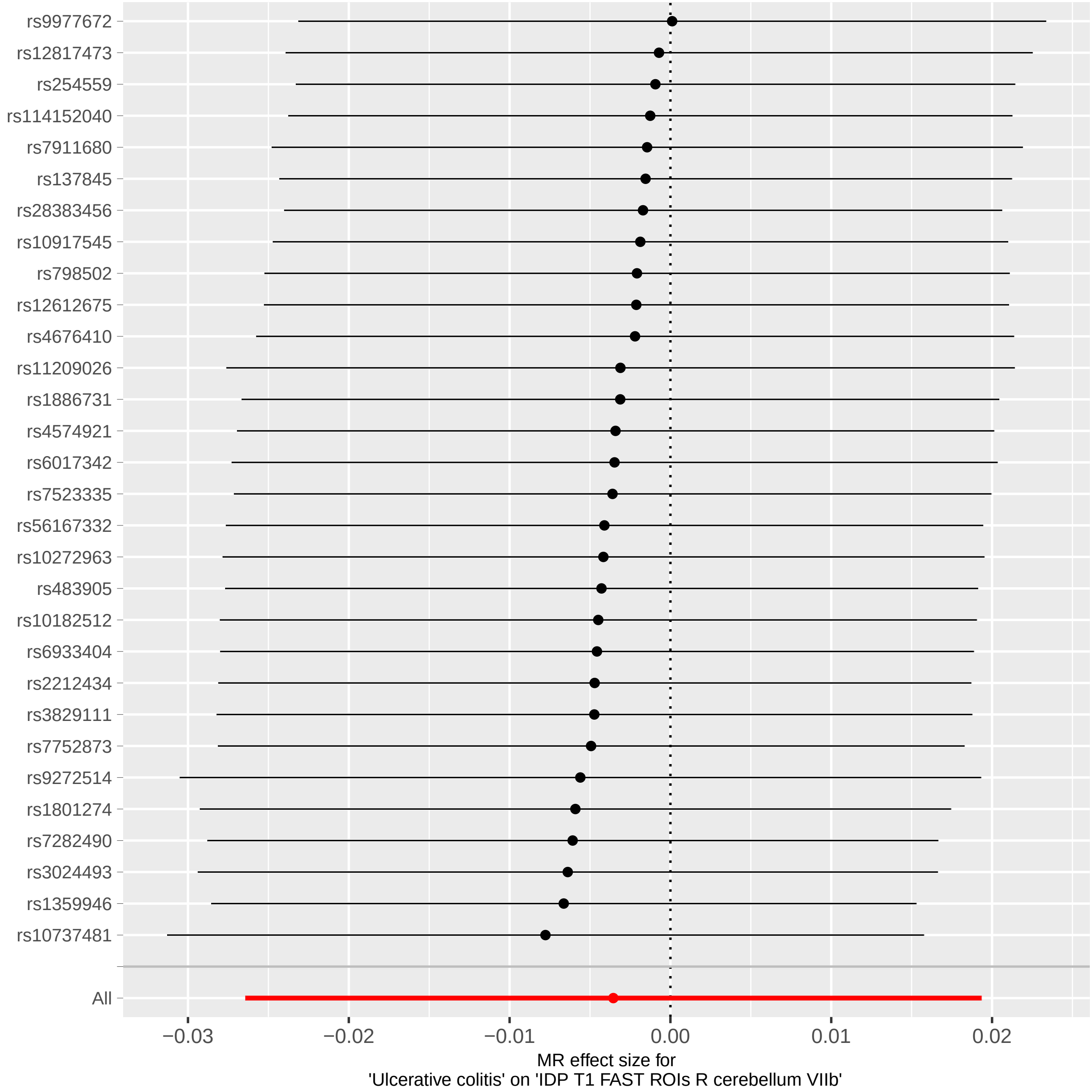

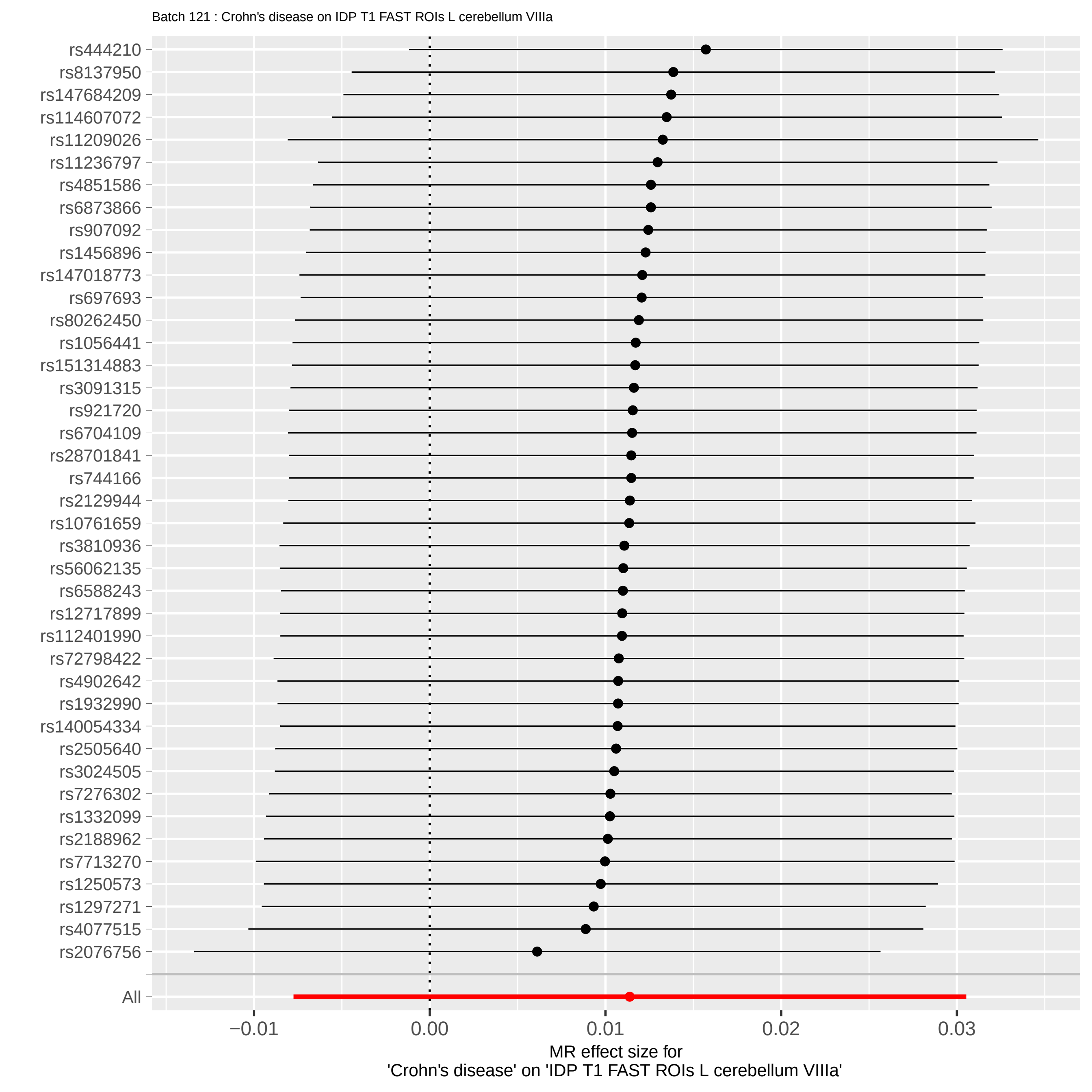

Batch 121 : Crohn's disease on IDP T1 FAST ROIs L cerebellum VIIIa
rs444210
rs8137950
rs147684209
rs114607072
rs11209026
rs11236797
rs4851586
rs6873866
rs907092
rs1456896
rs147018773
rs697693
rs80262450
rs1056441
rs151314883
rs3091315
rs921720
rs6704109
rs28701841
rs744166
rs2129944
rs10761659
rs3810936
rs56062135
rs6588243
rs12717899
rs112401990
rs72798422
rs4902642
rs1932990
rs140054334
rs2505640
rs3024505
rs7276302
rs1332099
rs2188962
rs7713270
rs1250573
rs1297271
rs4077515
rs2076756
All
−0.01
0.00
0.01
0.02
0.03
MR effect size for
'Crohn's disease' on 'IDP T1 FAST ROIs L cerebellum VIIIa'

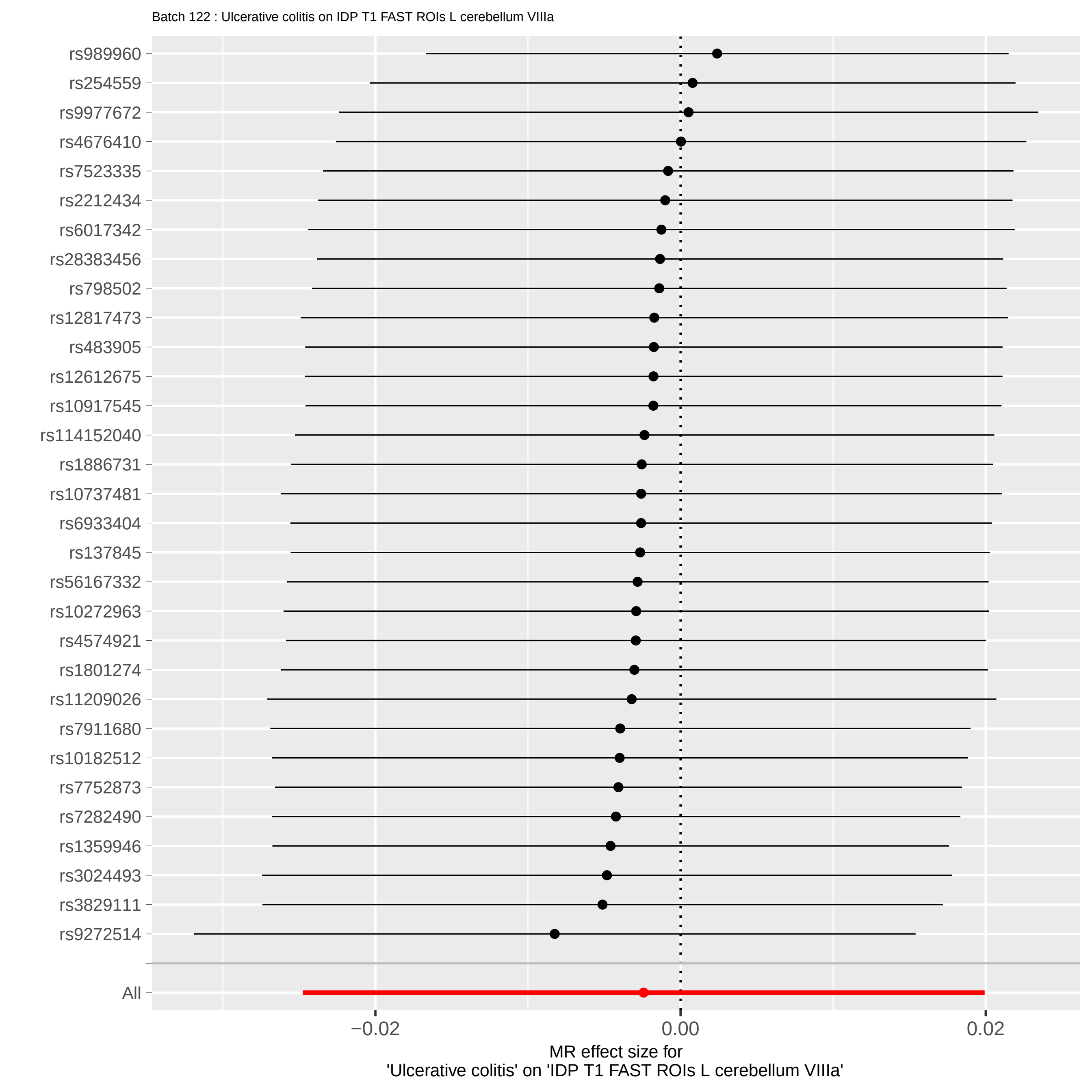

Batch 122 : Ulcerative colitis on IDP T1 FAST ROIs L cerebellum VIIIa
rs989960
rs254559
rs9977672
rs4676410
rs7523335
rs2212434
rs6017342
rs28383456
rs798502
rs12817473
rs483905
rs12612675
rs10917545
rs114152040
rs1886731
rs10737481
rs6933404
rs137845
rs56167332
rs10272963
rs4574921
rs1801274
rs11209026
rs7911680
rs10182512
rs7752873
rs7282490
rs1359946
rs3024493
rs3829111
rs9272514
All
−0.02
0.00
0.02
MR effect size for
'Ulcerative colitis' on 'IDP T1 FAST ROIs L cerebellum VIIIa'

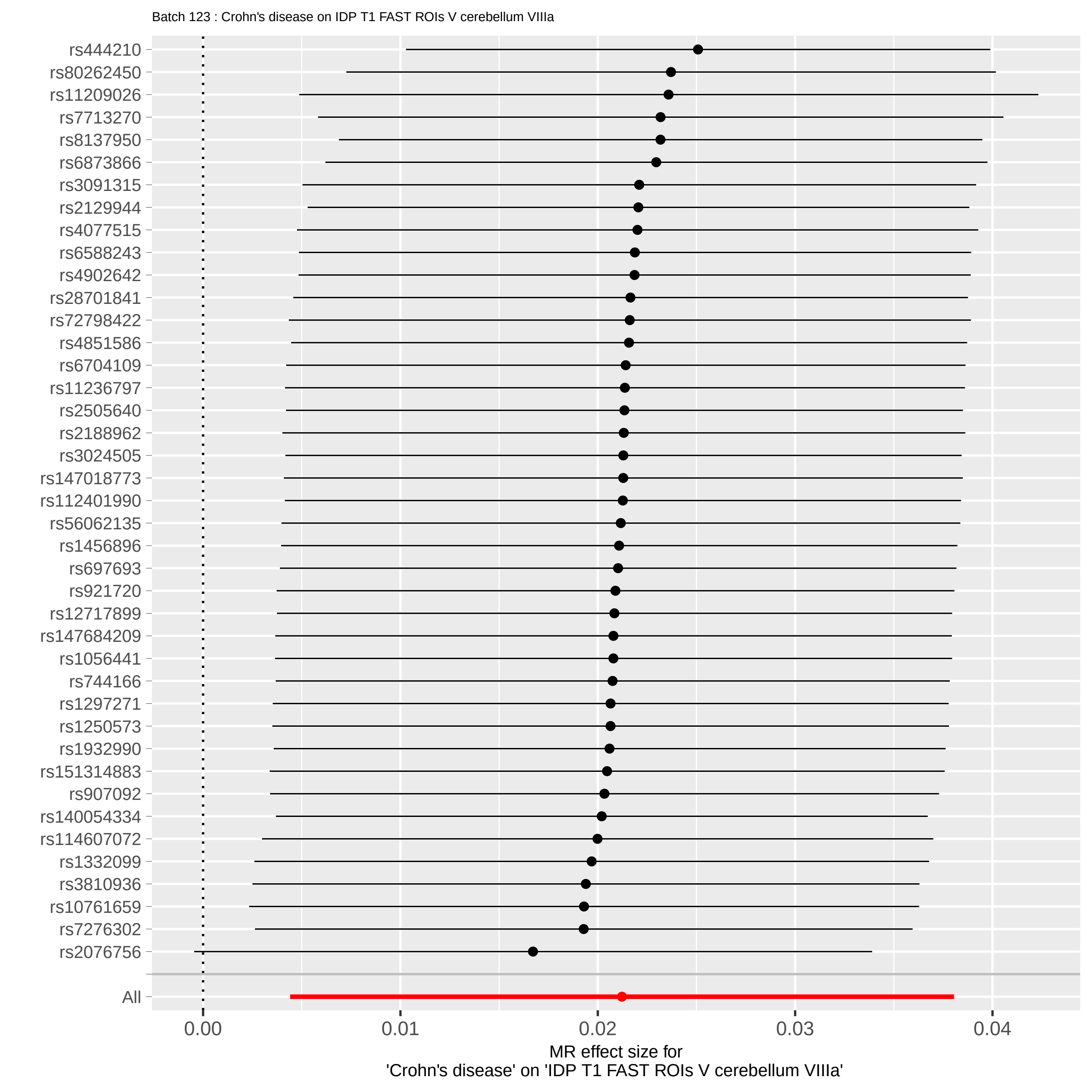

Batch 123 : Crohn's disease on IDP T1 FAST ROIs V cerebellum VIIIa
rs444210
rs80262450
rs11209026
rs7713270
rs8137950
rs6873866
rs3091315
rs2129944
rs4077515
rs6588243
rs4902642
rs28701841
rs72798422
rs4851586
rs6704109
rs11236797
rs2505640
rs2188962
rs3024505
rs147018773
rs112401990
rs56062135
rs1456896
rs697693
rs921720
rs12717899
rs147684209
rs1056441
rs744166
rs1297271
rs1250573
rs1932990
rs151314883
rs907092
rs140054334
rs114607072
rs1332099
rs3810936
rs10761659
rs7276302
rs2076756
All
0.00
0.01
0.02
0.03
0.04
MR effect size for
'Crohn's disease' on 'IDP T1 FAST ROIs V cerebellum VIIIa'

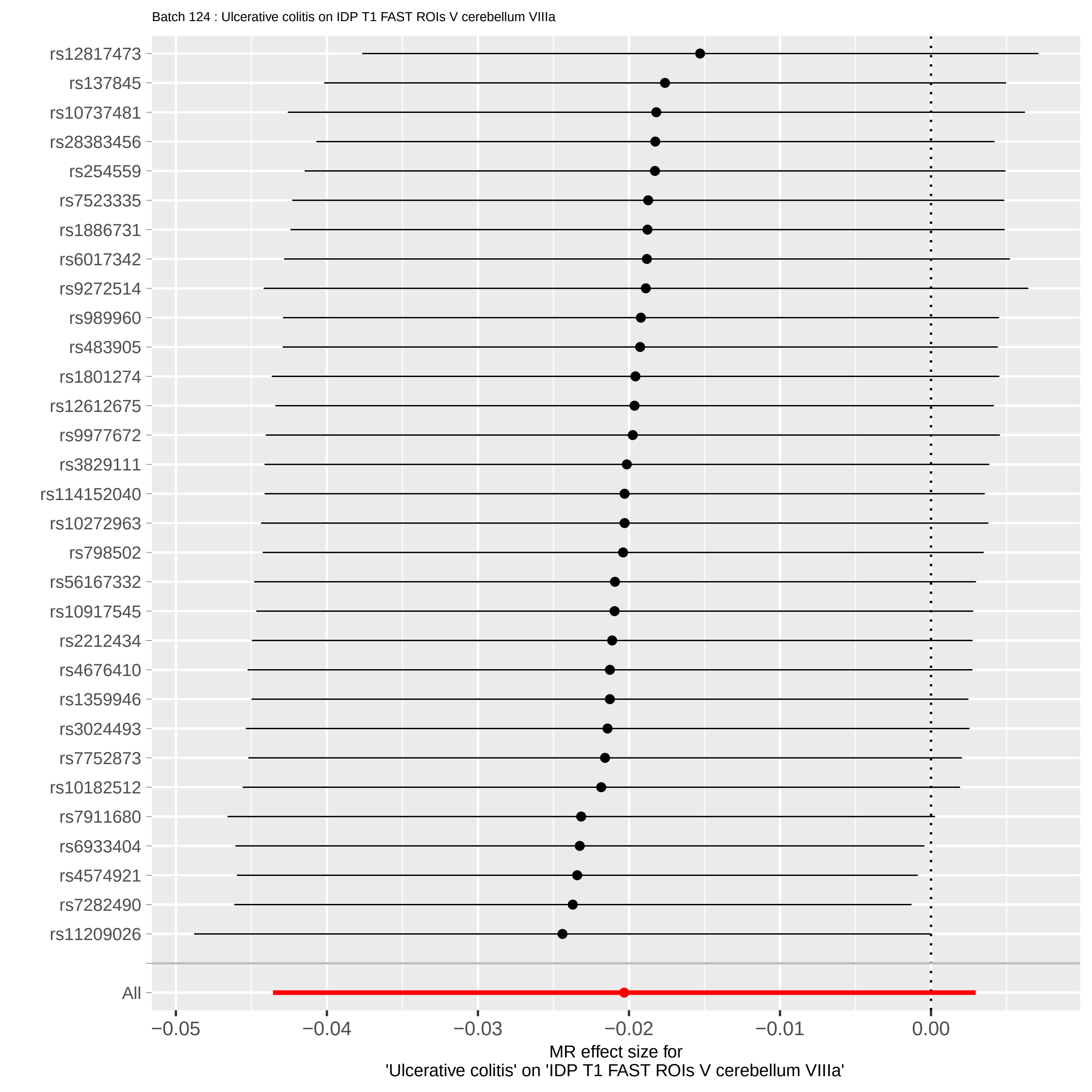

Batch 124 : Ulcerative colitis on IDP T1 FAST ROIs V cerebellum VIIIa
rs12817473
rs137845
rs10737481
rs28383456
rs254559
rs7523335
rs1886731
rs6017342
rs9272514
rs989960
rs483905
rs1801274
rs12612675
rs9977672
rs3829111
rs114152040
rs10272963
rs798502
rs56167332
rs10917545
rs2212434
rs4676410
rs1359946
rs3024493
rs7752873
rs10182512
rs7911680
rs6933404
rs4574921
rs7282490
rs11209026
All
−0.05
−0.04
−0.03
−0.02
−0.01
0.00
MR effect size for
'Ulcerative colitis' on 'IDP T1 FAST ROIs V cerebellum VIIIa'

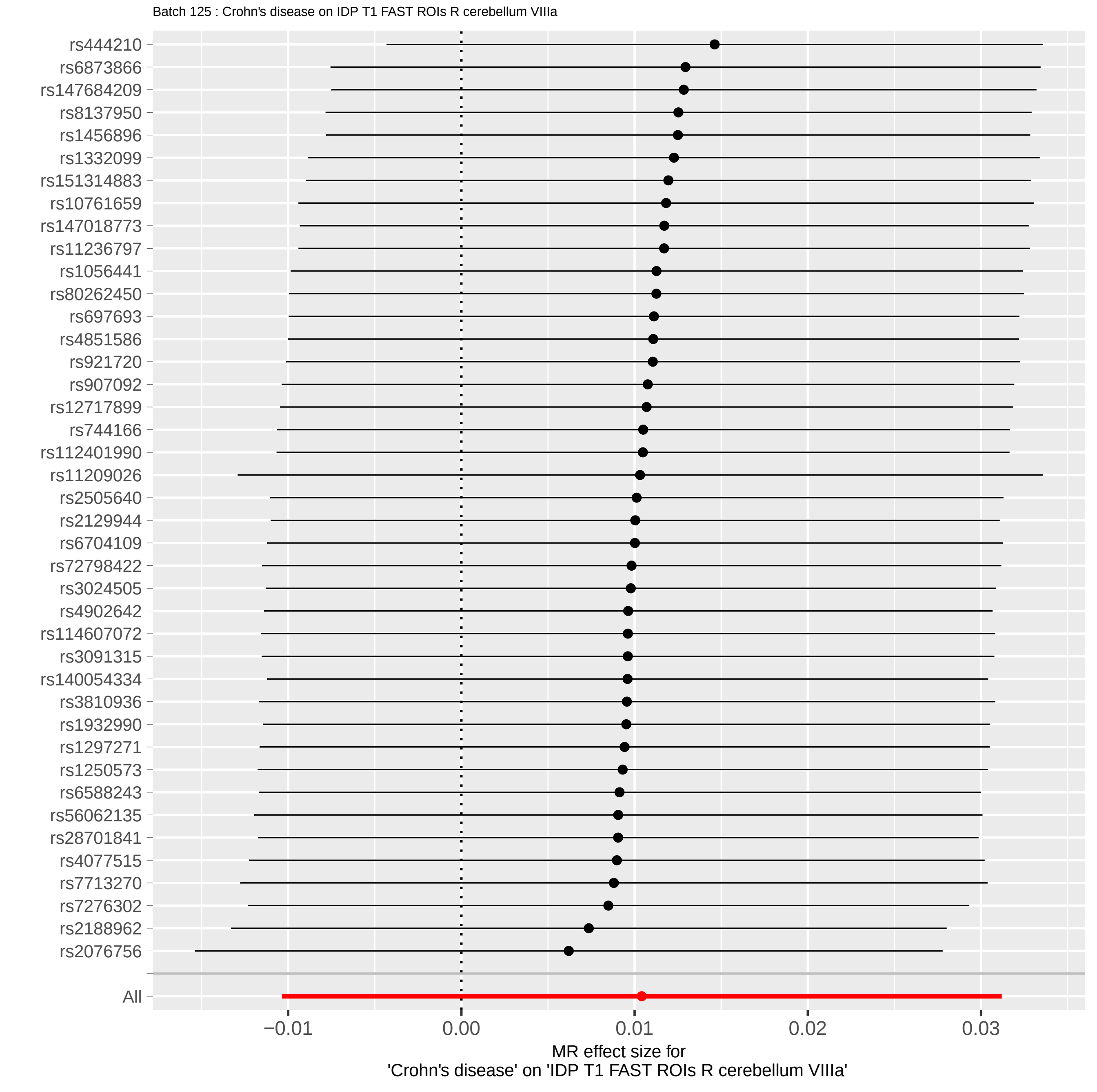

Batch 125 : Crohn's disease on IDP T1 FAST ROIs R cerebellum VIIIa
rs444210
rs6873866
rs147684209
rs8137950
rs1456896
rs1332099
rs151314883
rs10761659
rs147018773
rs11236797
rs1056441
rs80262450
rs697693
rs4851586
rs921720
rs907092
rs12717899
rs744166
rs112401990
rs11209026
rs2505640
rs2129944
rs6704109
rs72798422
rs3024505
rs4902642
rs114607072
rs3091315
rs140054334
rs3810936
rs1932990
rs1297271
rs1250573
rs6588243
rs56062135
rs28701841
rs4077515
rs7713270
rs7276302
rs2188962
rs2076756
All
−0.01
0.00
0.01
0.02
0.03
MR effect size for
'Crohn's disease' on 'IDP T1 FAST ROIs R cerebellum VIIIa'

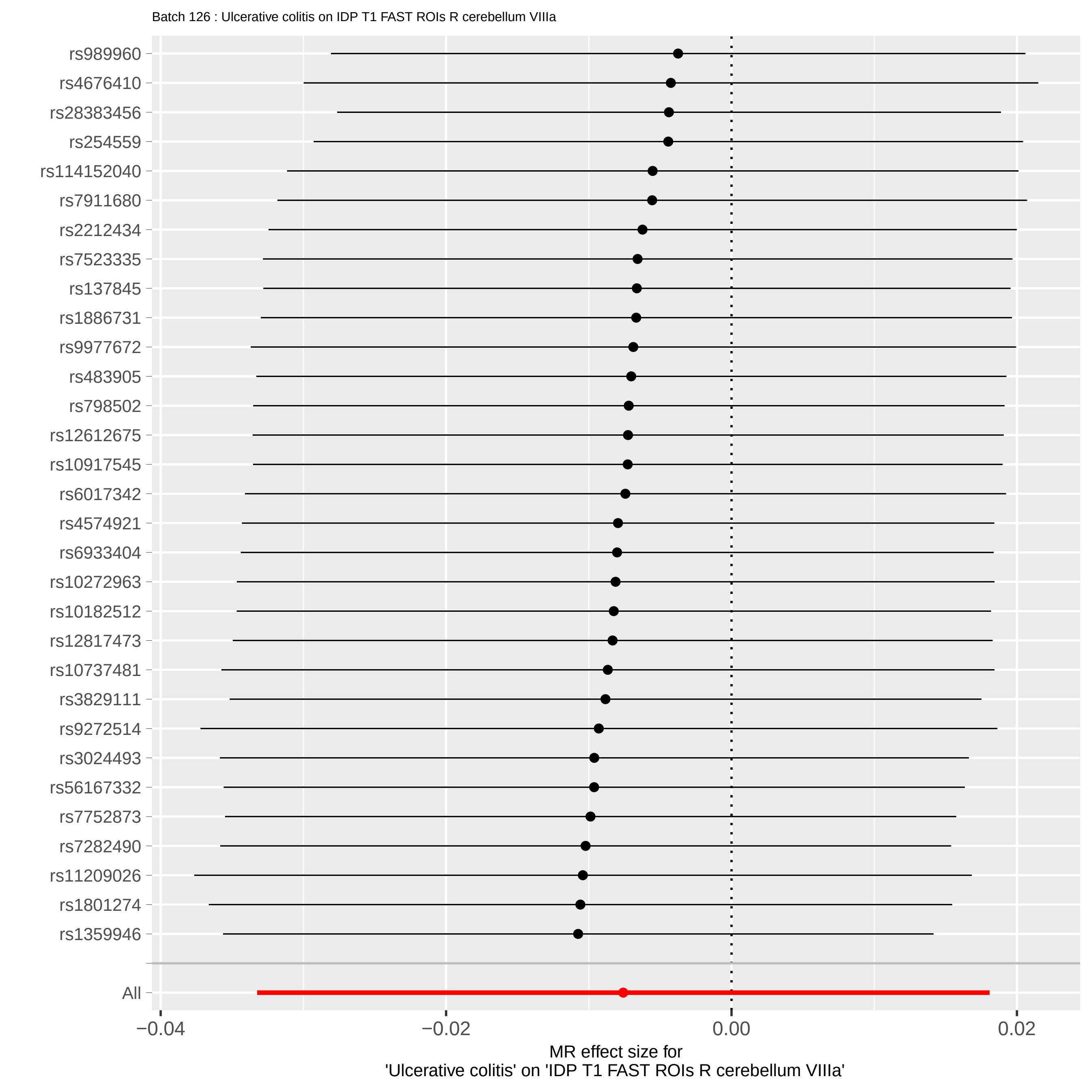

Batch 126 : Ulcerative colitis on IDP T1 FAST ROIs R cerebellum VIIIa
rs989960
rs4676410
rs28383456
rs254559
rs114152040
rs7911680
rs2212434
rs7523335
rs137845
rs1886731
rs9977672
rs483905
rs798502
rs12612675
rs10917545
rs6017342
rs4574921
rs6933404
rs10272963
rs10182512
rs12817473
rs10737481
rs3829111
rs9272514
rs3024493
rs56167332
rs7752873
rs7282490
rs11209026
rs1801274
rs1359946
All
−0.04
−0.02
0.00
0.02
MR effect size for
'Ulcerative colitis' on 'IDP T1 FAST ROIs R cerebellum VIIIa'

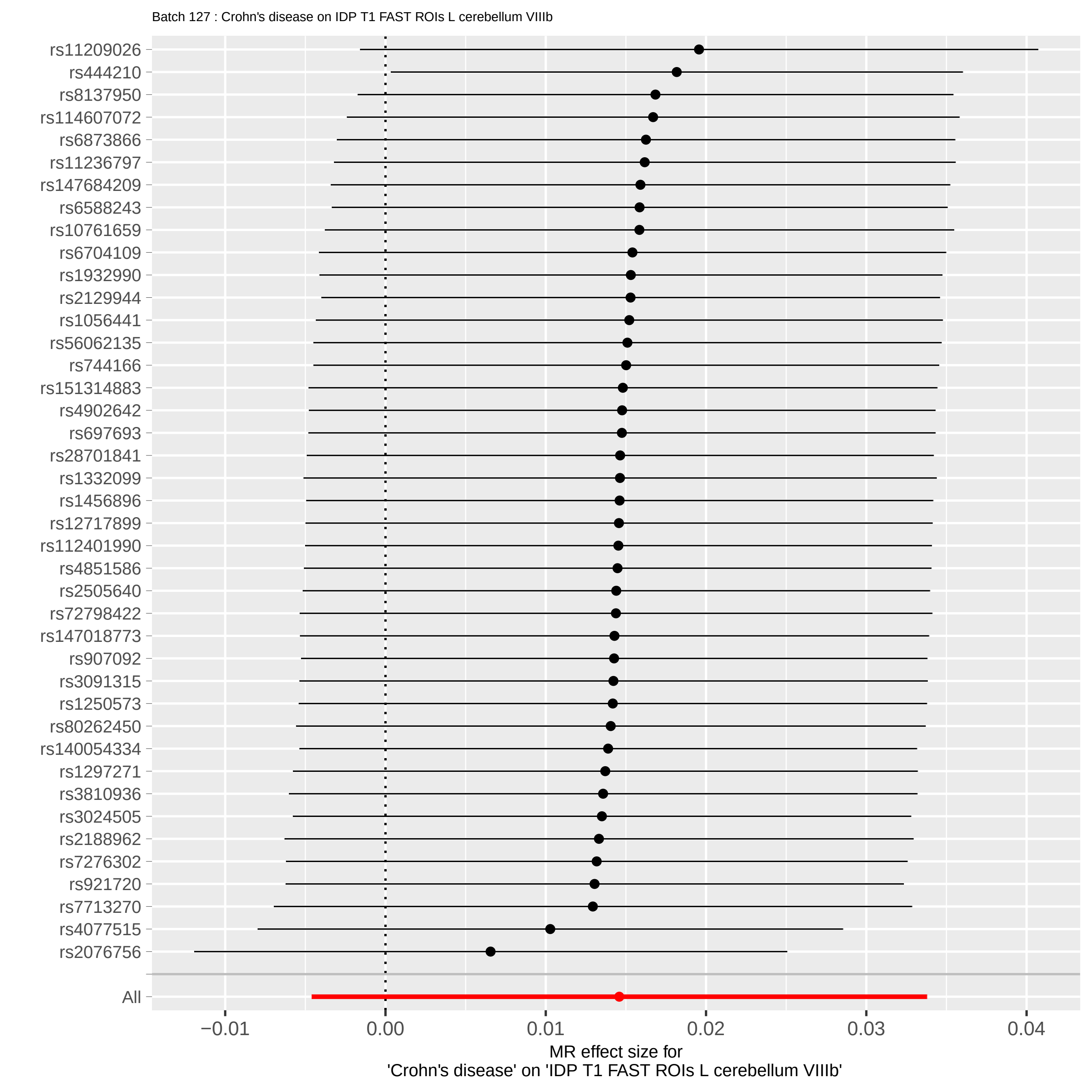
Batch 127 : Crohn's disease on IDP T1 FAST ROIs L cerebellum VIIIb
rs11209026
rs444210
rs8137950
rs114607072
rs6873866
rs11236797
rs147684209
rs6588243
rs10761659
rs6704109
rs1932990
rs2129944
rs1056441
rs56062135
rs744166
rs151314883
rs4902642
rs697693
rs28701841
rs1332099
rs1456896
rs12717899
rs112401990
rs4851586
rs2505640
rs72798422
rs147018773
rs907092
rs3091315
rs1250573
rs80262450
rs140054334
rs1297271
rs3810936
rs3024505
rs2188962
rs7276302
rs921720
rs7713270
rs4077515
rs2076756
All
−0.01
0.00
0.01
0.02
0.03
0.04
MR effect size for
'Crohn's disease' on 'IDP T1 FAST ROIs L cerebellum VIIIb'

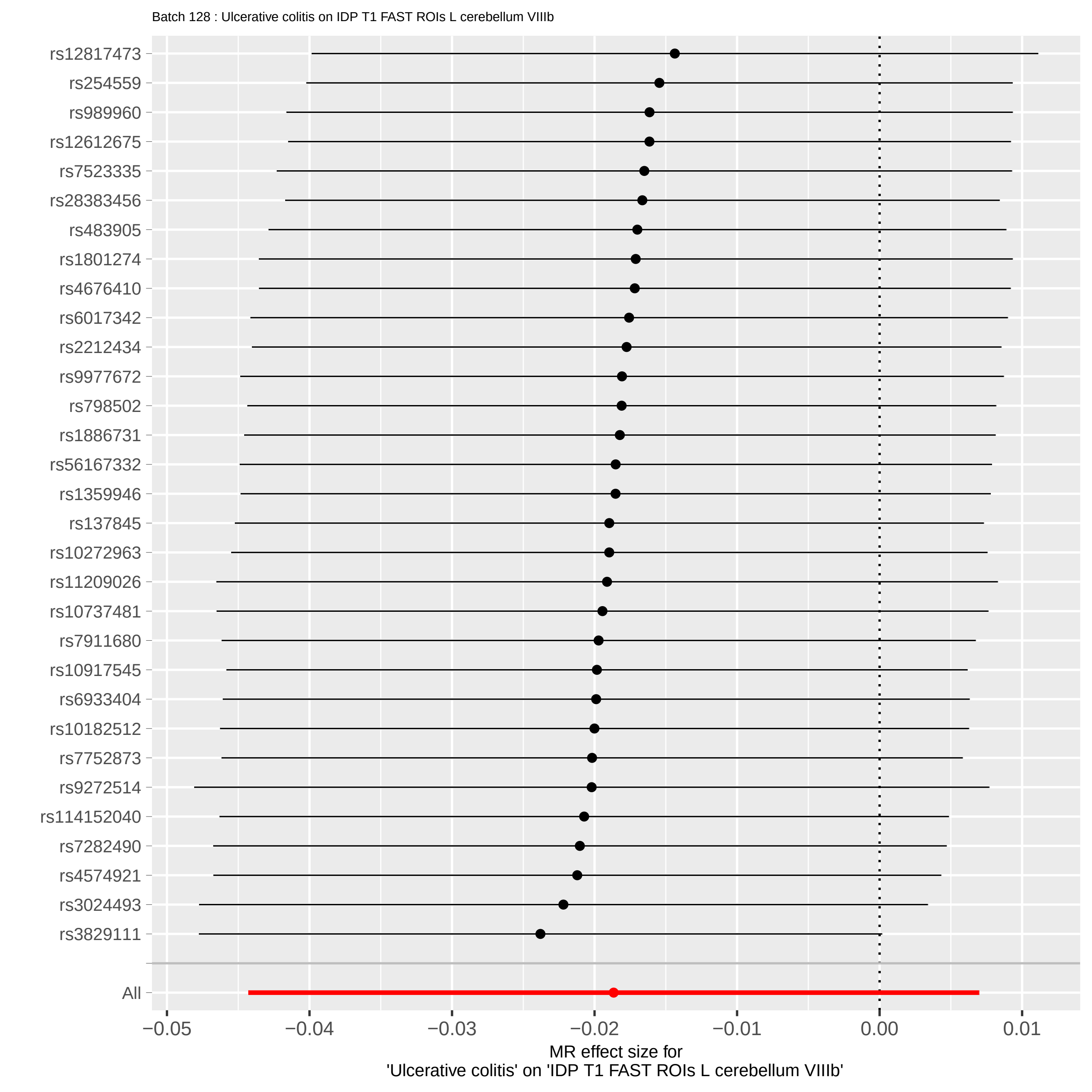
Batch 128 : Ulcerative colitis on IDP T1 FAST ROIs L cerebellum VIIIb
rs12817473
rs254559
rs989960
rs12612675
rs7523335
rs28383456
rs483905
rs1801274
rs4676410
rs6017342
rs2212434
rs9977672
rs798502
rs1886731
rs56167332
rs1359946
rs137845
rs10272963
rs11209026
rs10737481
rs7911680
rs10917545
rs6933404
rs10182512
rs7752873
rs9272514
rs114152040
rs7282490
rs4574921
rs3024493
rs3829111
All
−0.05
−0.04
−0.03
−0.02
−0.01
0.00
0.01
MR effect size for
'Ulcerative colitis' on 'IDP T1 FAST ROIs L cerebellum VIIIb'

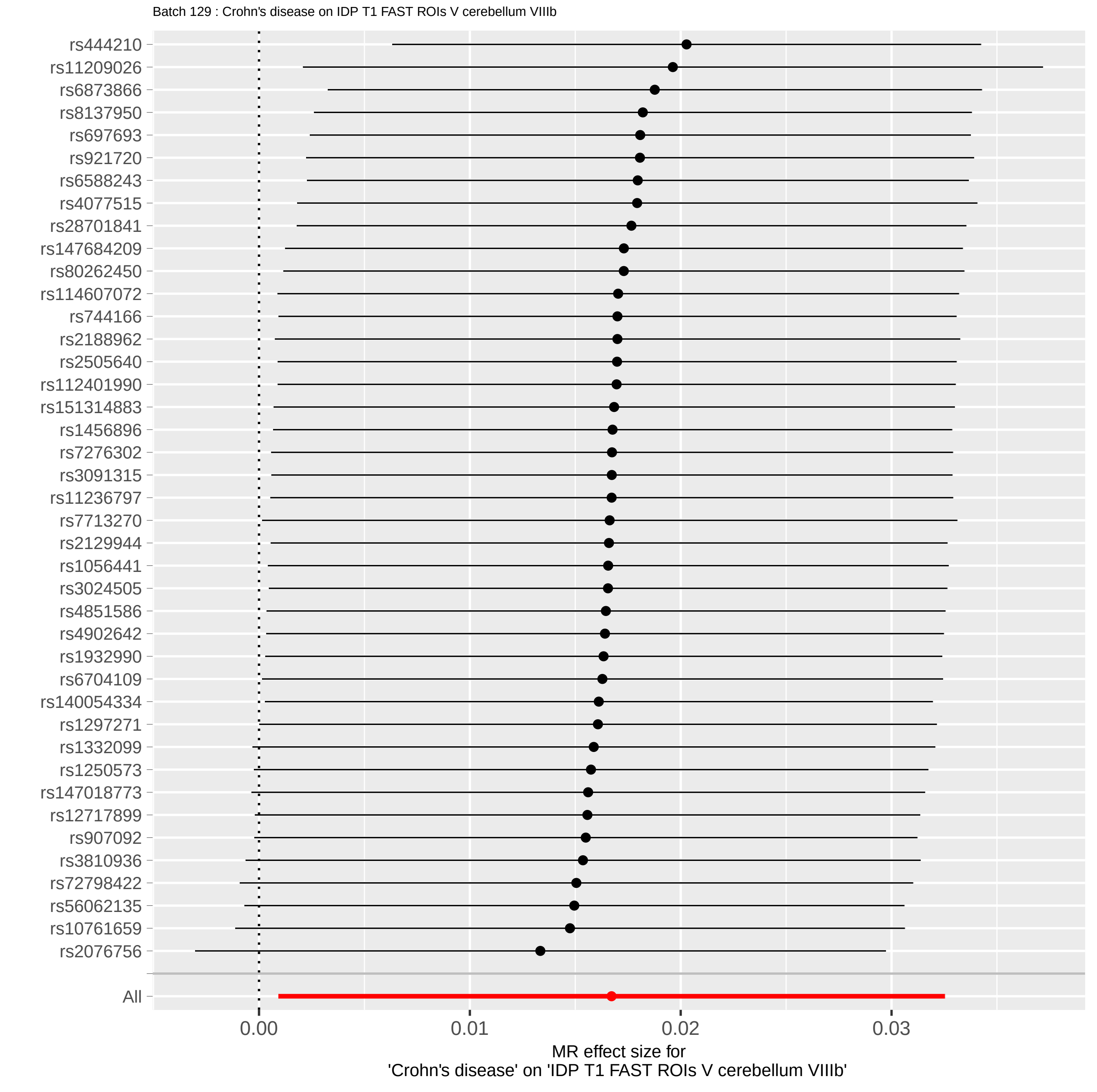
Batch 129 : Crohn's disease on IDP T1 FAST ROIs V cerebellum VIIIb
rs444210
rs11209026
rs6873866
rs8137950
rs697693
rs921720
rs6588243
rs4077515
rs28701841
rs147684209
rs80262450
rs114607072
rs744166
rs2188962
rs2505640
rs112401990
rs151314883
rs1456896
rs7276302
rs3091315
rs11236797
rs7713270
rs2129944
rs1056441
rs3024505
rs4851586
rs4902642
rs1932990
rs6704109
rs140054334
rs1297271
rs1332099
rs1250573
rs147018773
rs12717899
rs907092
rs3810936
rs72798422
rs56062135
rs10761659
rs2076756
All
0.00
0.01
0.02
0.03
MR effect size for
'Crohn's disease' on 'IDP T1 FAST ROIs V cerebellum VIIIb'

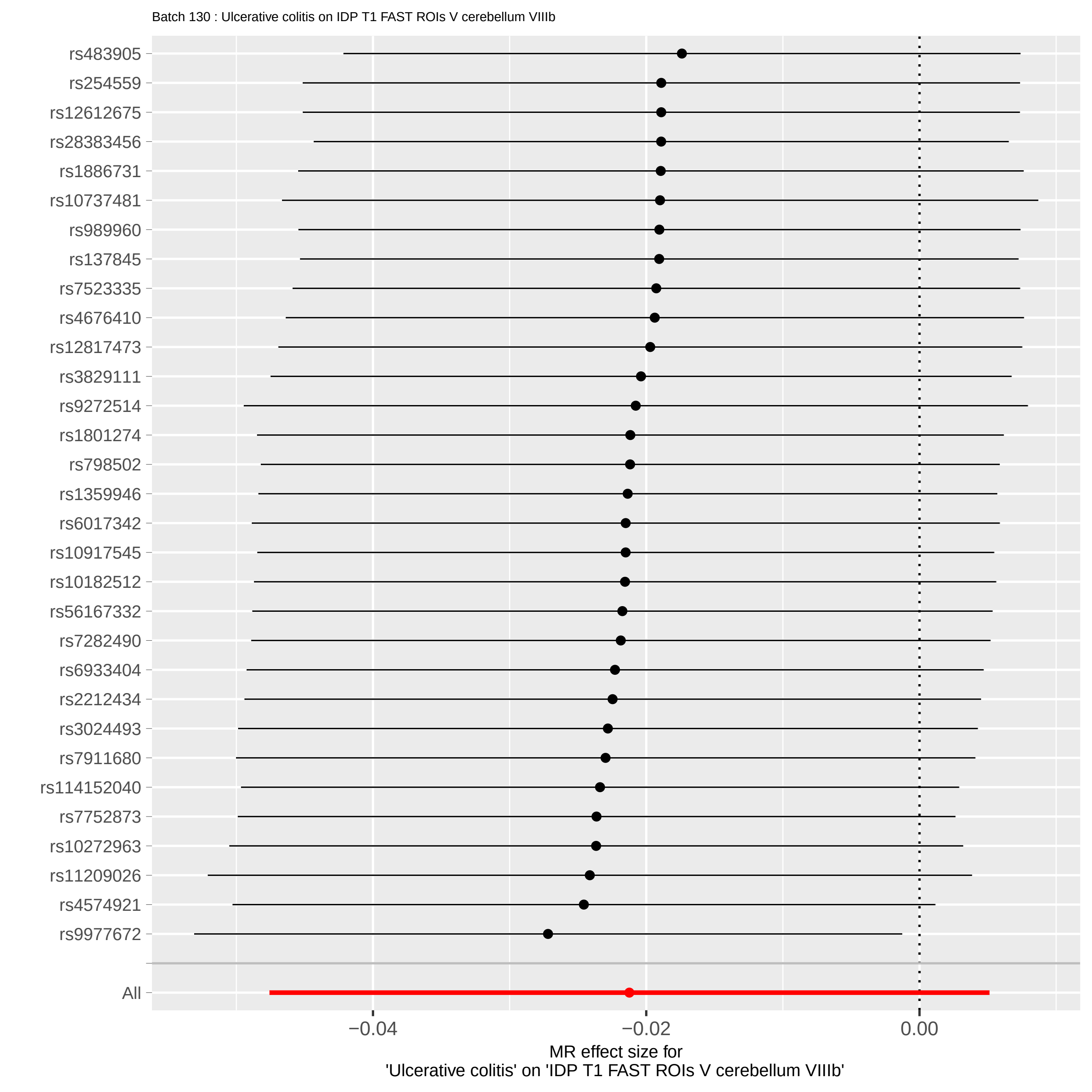
Batch 130 : Ulcerative colitis on IDP T1 FAST ROIs V cerebellum VIIIb
rs483905
rs254559
rs12612675
rs28383456
rs1886731
rs10737481
rs989960
rs137845
rs7523335
rs4676410
rs12817473
rs3829111
rs9272514
rs1801274
rs798502
rs1359946
rs6017342
rs10917545
rs10182512
rs56167332
rs7282490
rs6933404
rs2212434
rs3024493
rs7911680
rs114152040
rs7752873
rs10272963
rs11209026
rs4574921
rs9977672
All
−0.04
−0.02
0.00
MR effect size for
'Ulcerative colitis' on 'IDP T1 FAST ROIs V cerebellum VIIIb'

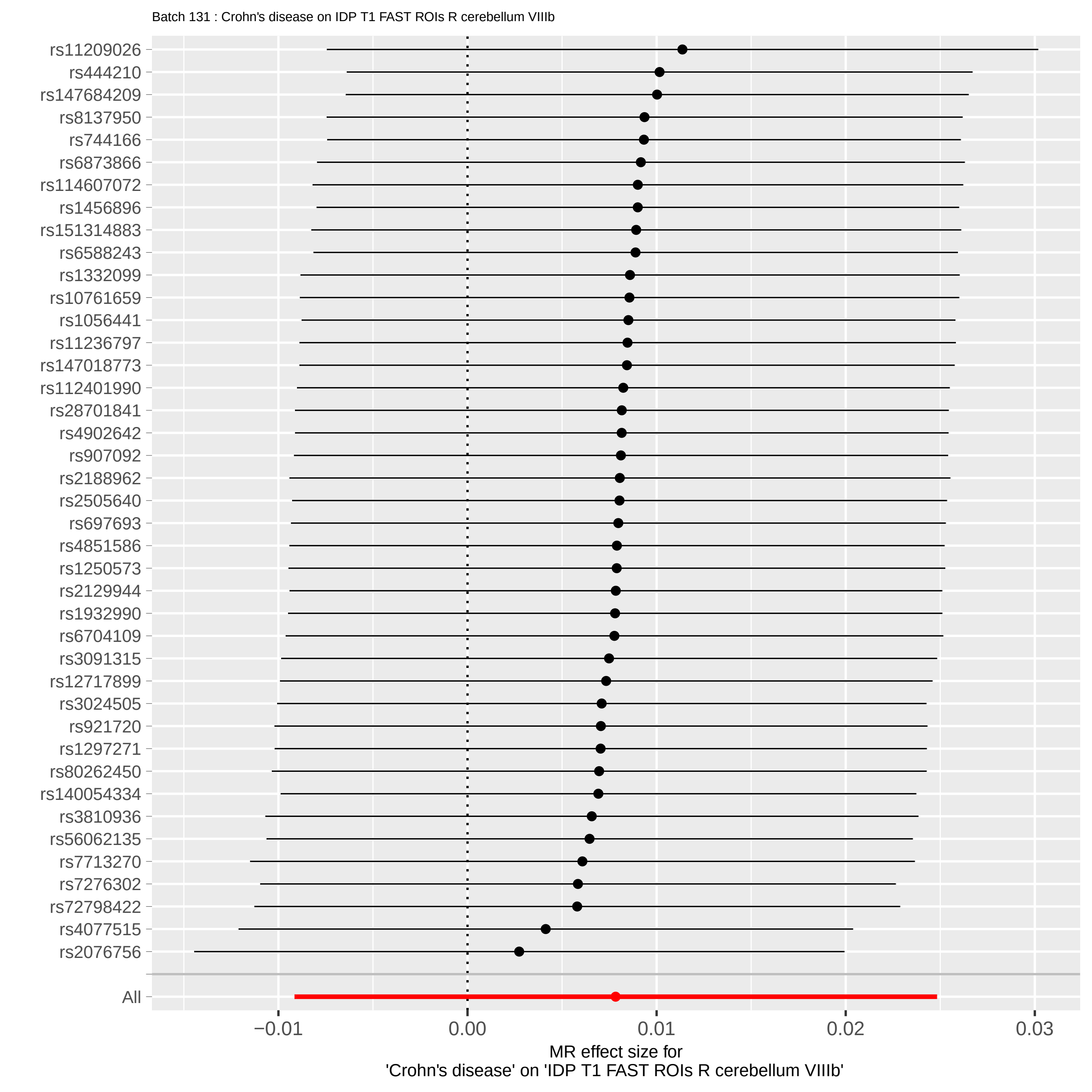
Batch 131 : Crohn's disease on IDP T1 FAST ROIs R cerebellum VIIIb
rs11209026
rs444210
rs147684209
rs8137950
rs744166
rs6873866
rs114607072
rs1456896
rs151314883
rs6588243
rs1332099
rs10761659
rs1056441
rs11236797
rs147018773
rs112401990
rs28701841
rs4902642
rs907092
rs2188962
rs2505640
rs697693
rs4851586
rs1250573
rs2129944
rs1932990
rs6704109
rs3091315
rs12717899
rs3024505
rs921720
rs1297271
rs80262450
rs140054334
rs3810936
rs56062135
rs7713270
rs7276302
rs72798422
rs4077515
rs2076756
All
−0.01
0.00
0.01
0.02
0.03
MR effect size for
'Crohn's disease' on 'IDP T1 FAST ROIs R cerebellum VIIIb'

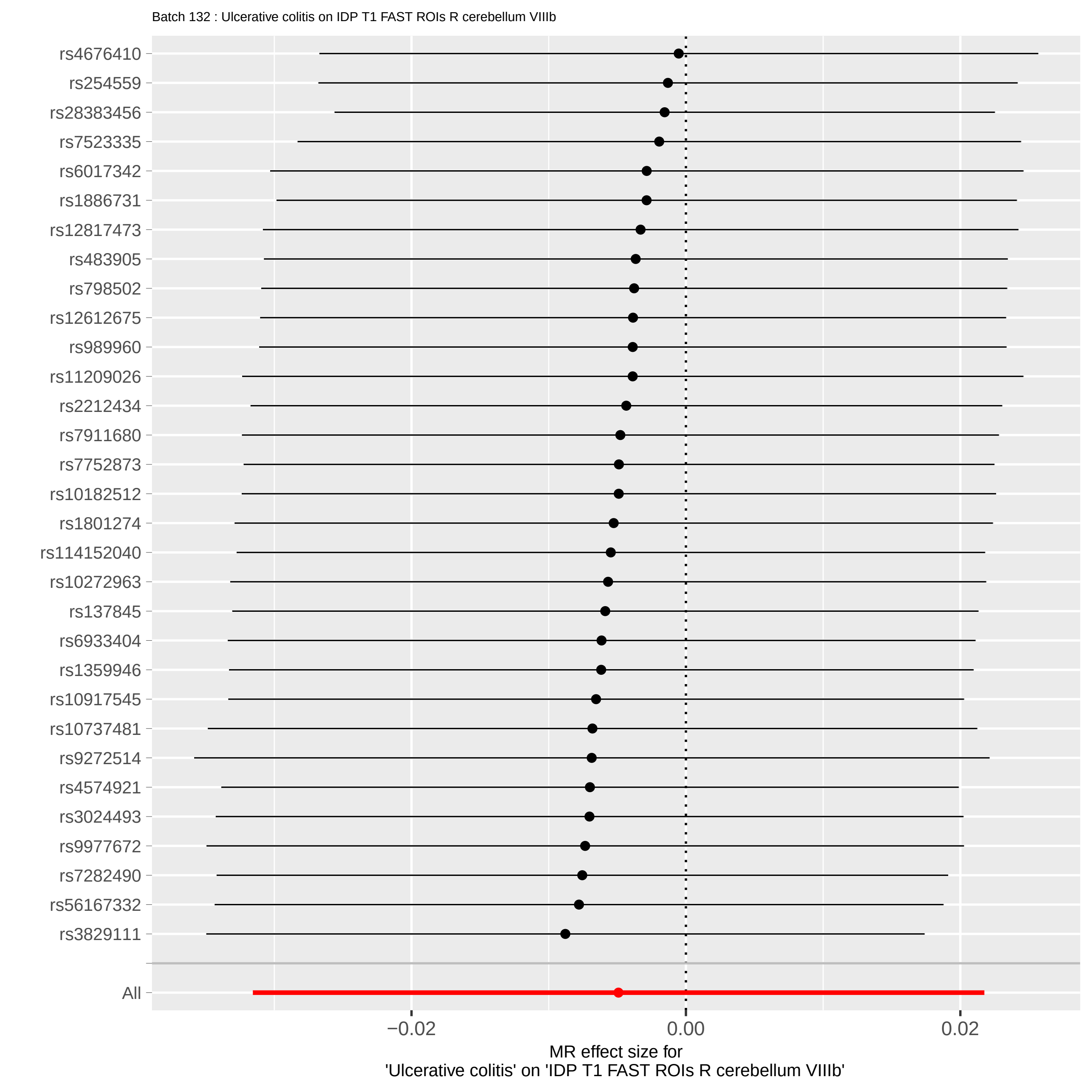
Batch 132 : Ulcerative colitis on IDP T1 FAST ROIs R cerebellum VIIIb
rs4676410
rs254559
rs28383456
rs7523335
rs6017342
rs1886731
rs12817473
rs483905
rs798502
rs12612675
rs989960
rs11209026
rs2212434
rs7911680
rs7752873
rs10182512
rs1801274
rs114152040
rs10272963
rs137845
rs6933404
rs1359946
rs10917545
rs10737481
rs9272514
rs4574921
rs3024493
rs9977672
rs7282490
rs56167332
rs3829111
All
−0.02
0.00
0.02
MR effect size for
'Ulcerative colitis' on 'IDP T1 FAST ROIs R cerebellum VIIIb'

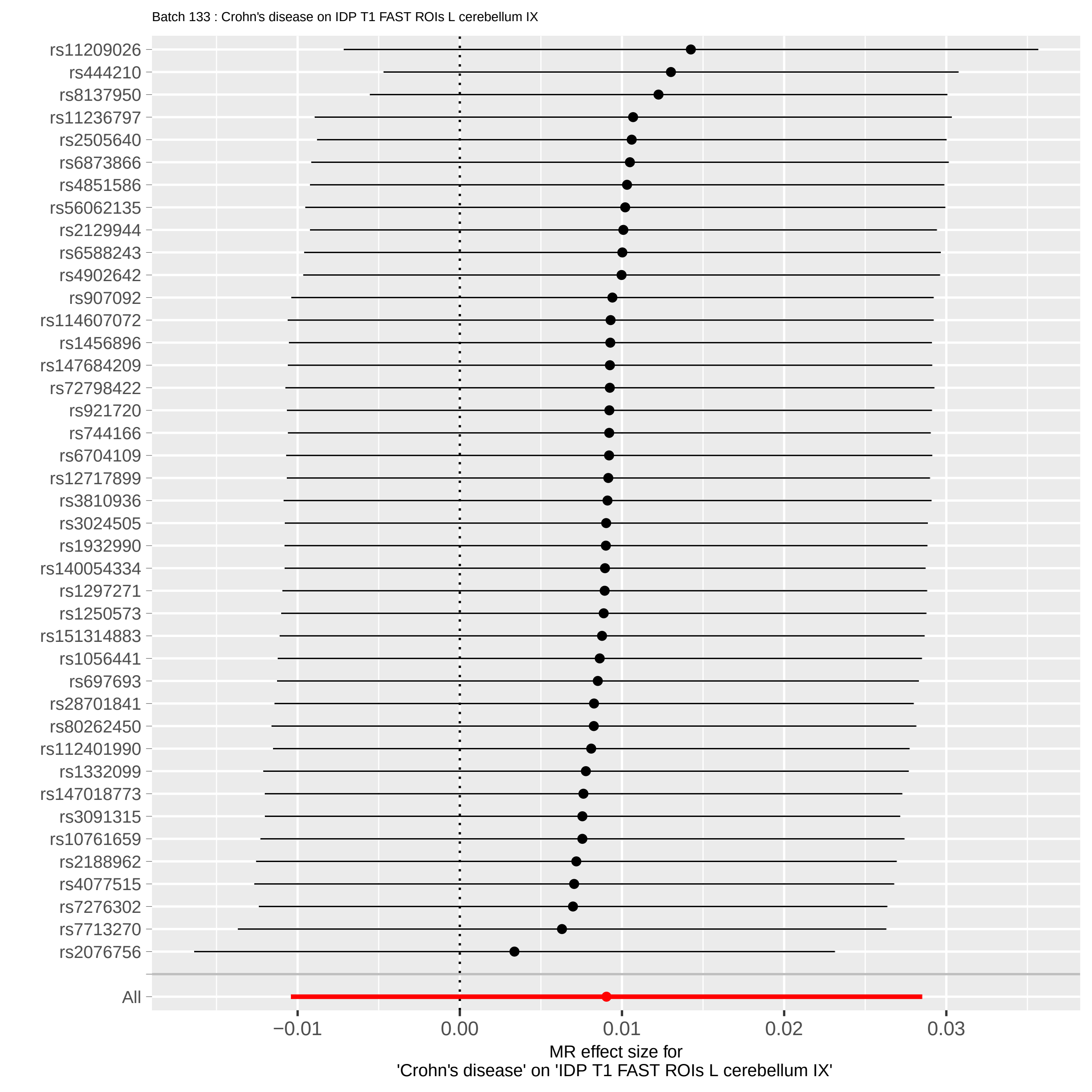
Batch 133 : Crohn's disease on IDP T1 FAST ROIs L cerebellum IX
rs11209026
rs444210
rs8137950
rs11236797
rs2505640
rs6873866
rs4851586
rs56062135
rs2129944
rs6588243
rs4902642
rs907092
rs114607072
rs1456896
rs147684209
rs72798422
rs921720
rs744166
rs6704109
rs12717899
rs3810936
rs3024505
rs1932990
rs140054334
rs1297271
rs1250573
rs151314883
rs1056441
rs697693
rs28701841
rs80262450
rs112401990
rs1332099
rs147018773
rs3091315
rs10761659
rs2188962
rs4077515
rs7276302
rs7713270
rs2076756
All
−0.01
0.00
0.01
0.02
0.03
MR effect size for
'Crohn's disease' on 'IDP T1 FAST ROIs L cerebellum IX'

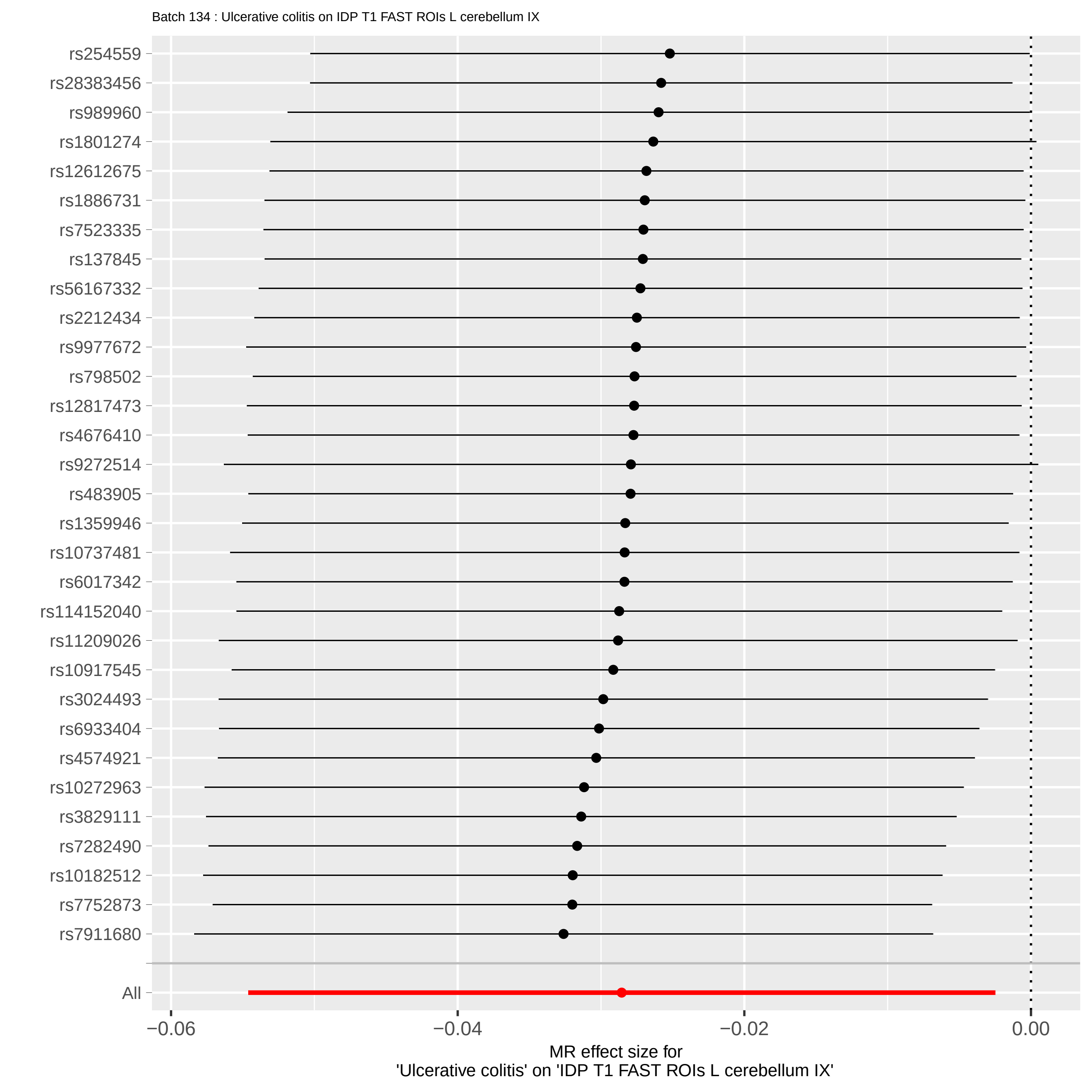

Batch 134 : Ulcerative colitis on IDP T1 FAST ROIs L cerebellum IX
rs254559
rs28383456
rs989960
rs1801274
rs12612675
rs1886731
rs7523335
rs137845
rs56167332
rs2212434
rs9977672
rs798502
rs12817473
rs4676410
rs9272514
rs483905
rs1359946
rs10737481
rs6017342
rs114152040
rs11209026
rs10917545
rs3024493
rs6933404
rs4574921
rs10272963
rs3829111
rs7282490
rs10182512
rs7752873
rs7911680
All
−0.06
−0.04
−0.02
0.00
MR effect size for
'Ulcerative colitis' on 'IDP T1 FAST ROIs L cerebellum IX'

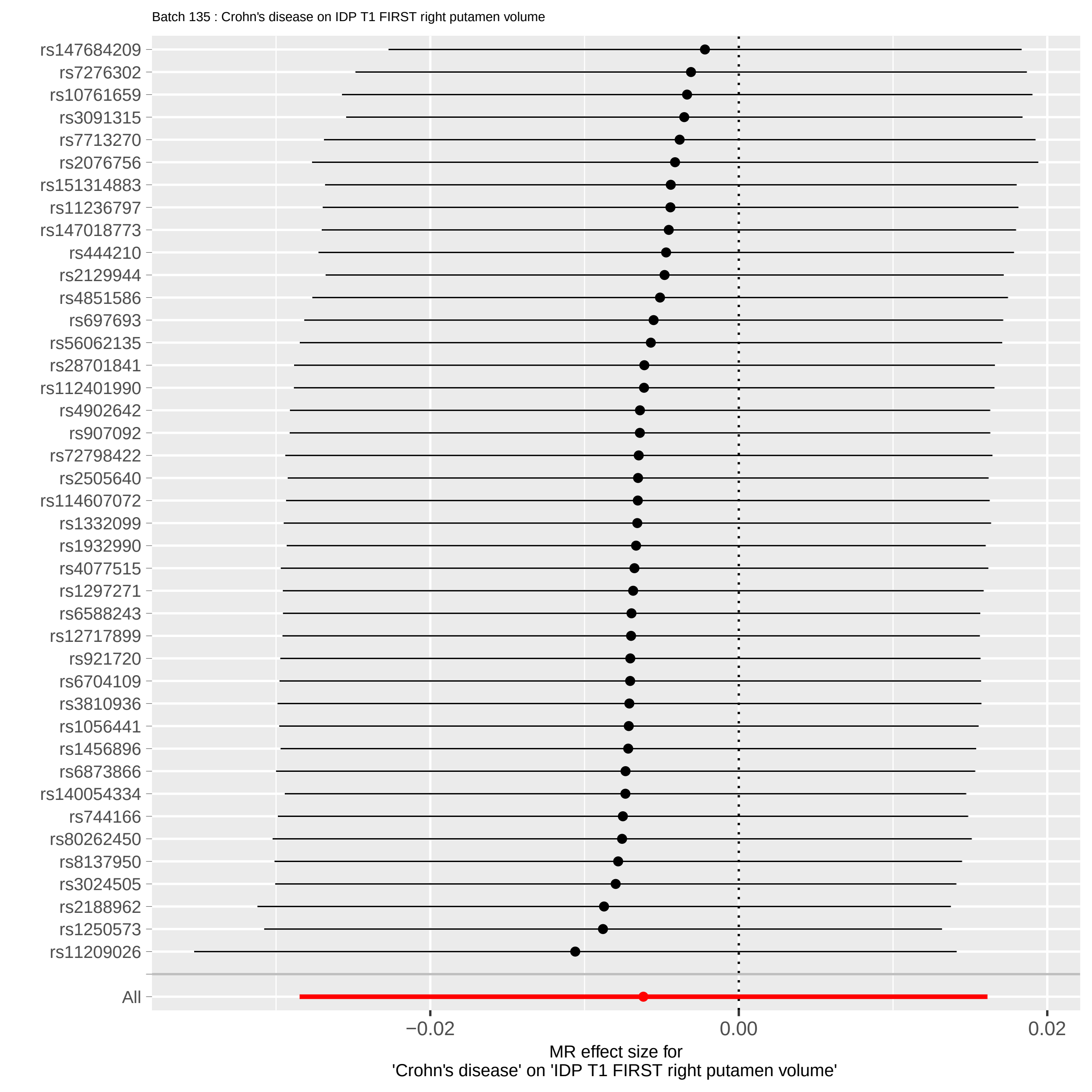

Batch 135 : Crohn's disease on IDP T1 FIRST right putamen volume
rs147684209
rs7276302
rs10761659
rs3091315
rs7713270
rs2076756
rs151314883
rs11236797
rs147018773
rs444210
rs2129944
rs4851586
rs697693
rs56062135
rs28701841
rs112401990
rs4902642
rs907092
rs72798422
rs2505640
rs114607072
rs1332099
rs1932990
rs4077515
rs1297271
rs6588243
rs12717899
rs921720
rs6704109
rs3810936
rs1056441
rs1456896
rs6873866
rs140054334
rs744166
rs80262450
rs8137950
rs3024505
rs2188962
rs1250573
rs11209026
All
−0.02
0.00
0.02
MR effect size for
'Crohn's disease' on 'IDP T1 FIRST right putamen volume'

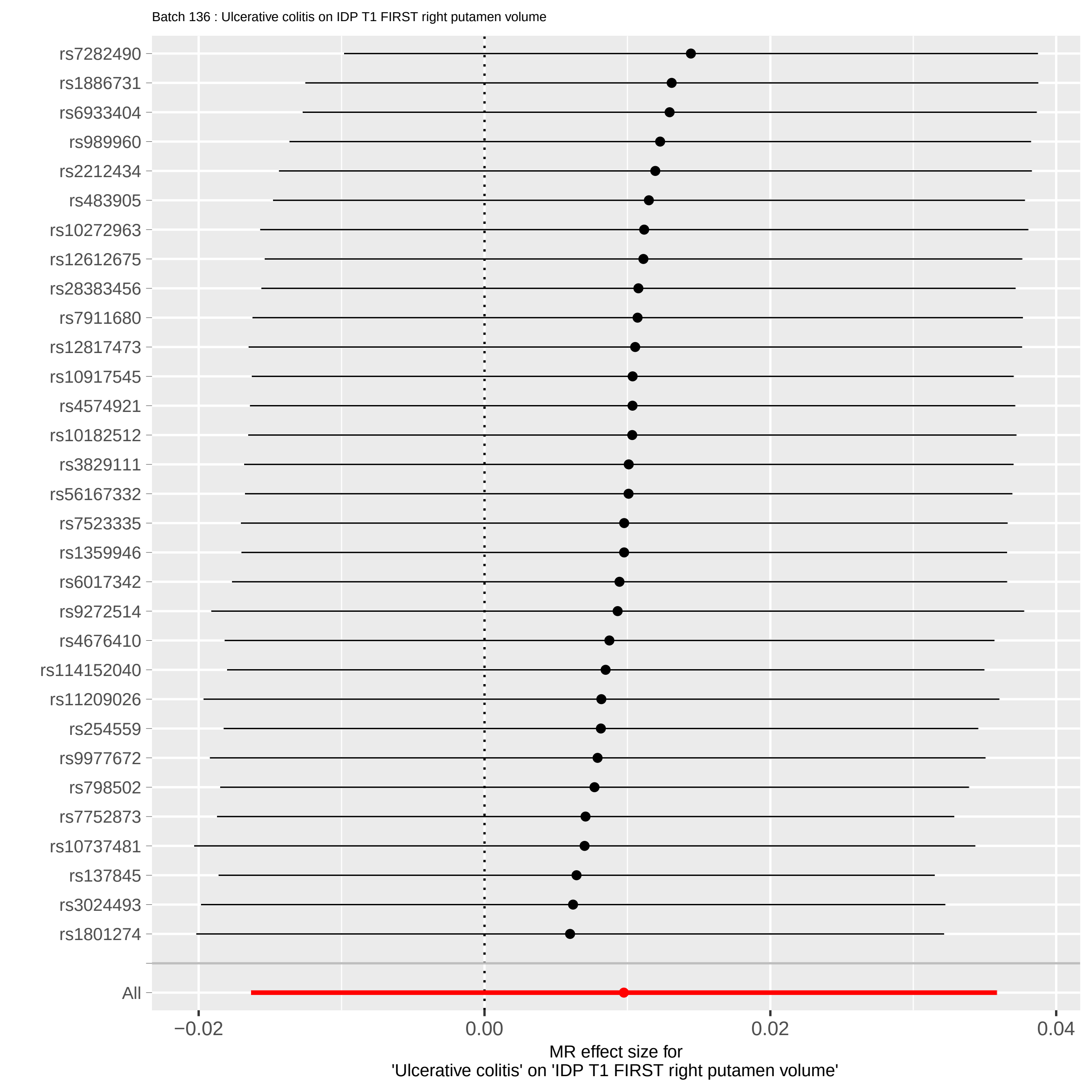

Batch 136 : Ulcerative colitis on IDP T1 FIRST right putamen volume
rs7282490
rs1886731
rs6933404
rs989960
rs2212434
rs483905
rs10272963
rs12612675
rs28383456
rs7911680
rs12817473
rs10917545
rs4574921
rs10182512
rs3829111
rs56167332
rs7523335
rs1359946
rs6017342
rs9272514
rs4676410
rs114152040
rs11209026
rs254559
rs9977672
rs798502
rs7752873
rs10737481
rs137845
rs3024493
rs1801274
All
−0.02
0.00
0.02
0.04
MR effect size for
'Ulcerative colitis' on 'IDP T1 FIRST right putamen volume'

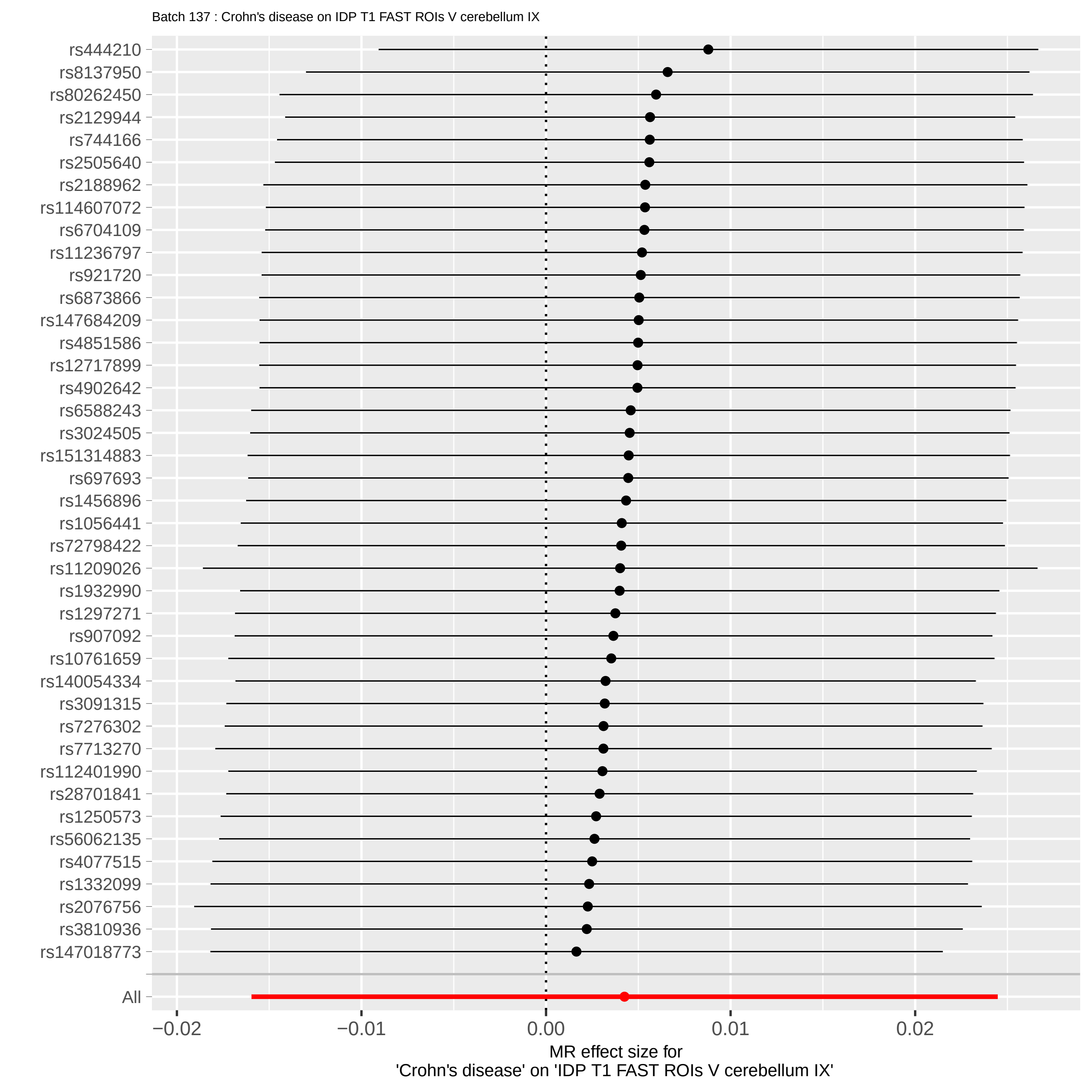

Batch 137 : Crohn's disease on IDP T1 FAST ROIs V cerebellum IX
rs444210
rs8137950
rs80262450
rs2129944
rs744166
rs2505640
rs2188962
rs114607072
rs6704109
rs11236797
rs921720
rs6873866
rs147684209
rs4851586
rs12717899
rs4902642
rs6588243
rs3024505
rs151314883
rs697693
rs1456896
rs1056441
rs72798422
rs11209026
rs1932990
rs1297271
rs907092
rs10761659
rs140054334
rs3091315
rs7276302
rs7713270
rs112401990
rs28701841
rs1250573
rs56062135
rs4077515
rs1332099
rs2076756
rs3810936
rs147018773
All
−0.02
−0.01
0.00
0.01
0.02
MR effect size for
'Crohn's disease' on 'IDP T1 FAST ROIs V cerebellum IX'

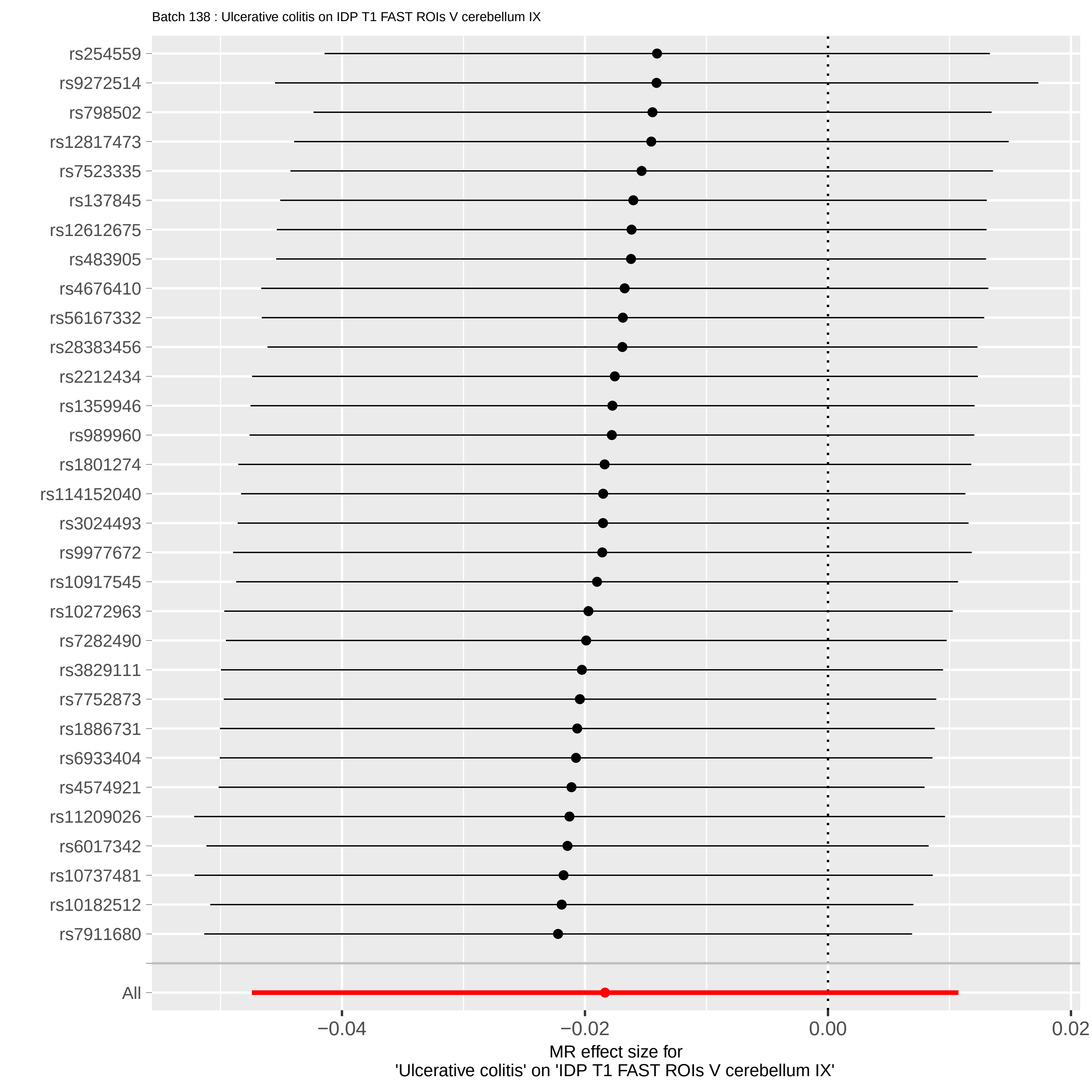

Batch 138 : Ulcerative colitis on IDP T1 FAST ROIs V cerebellum IX
rs254559
rs9272514
rs798502
rs12817473
rs7523335
rs137845
rs12612675
rs483905
rs4676410
rs56167332
rs28383456
rs2212434
rs1359946
rs989960
rs1801274
rs114152040
rs3024493
rs9977672
rs10917545
rs10272963
rs7282490
rs3829111
rs7752873
rs1886731
rs6933404
rs4574921
rs11209026
rs6017342
rs10737481
rs10182512
rs7911680
All
−0.04
−0.02
0.00
0.02
MR effect size for
'Ulcerative colitis' on 'IDP T1 FAST ROIs V cerebellum IX'

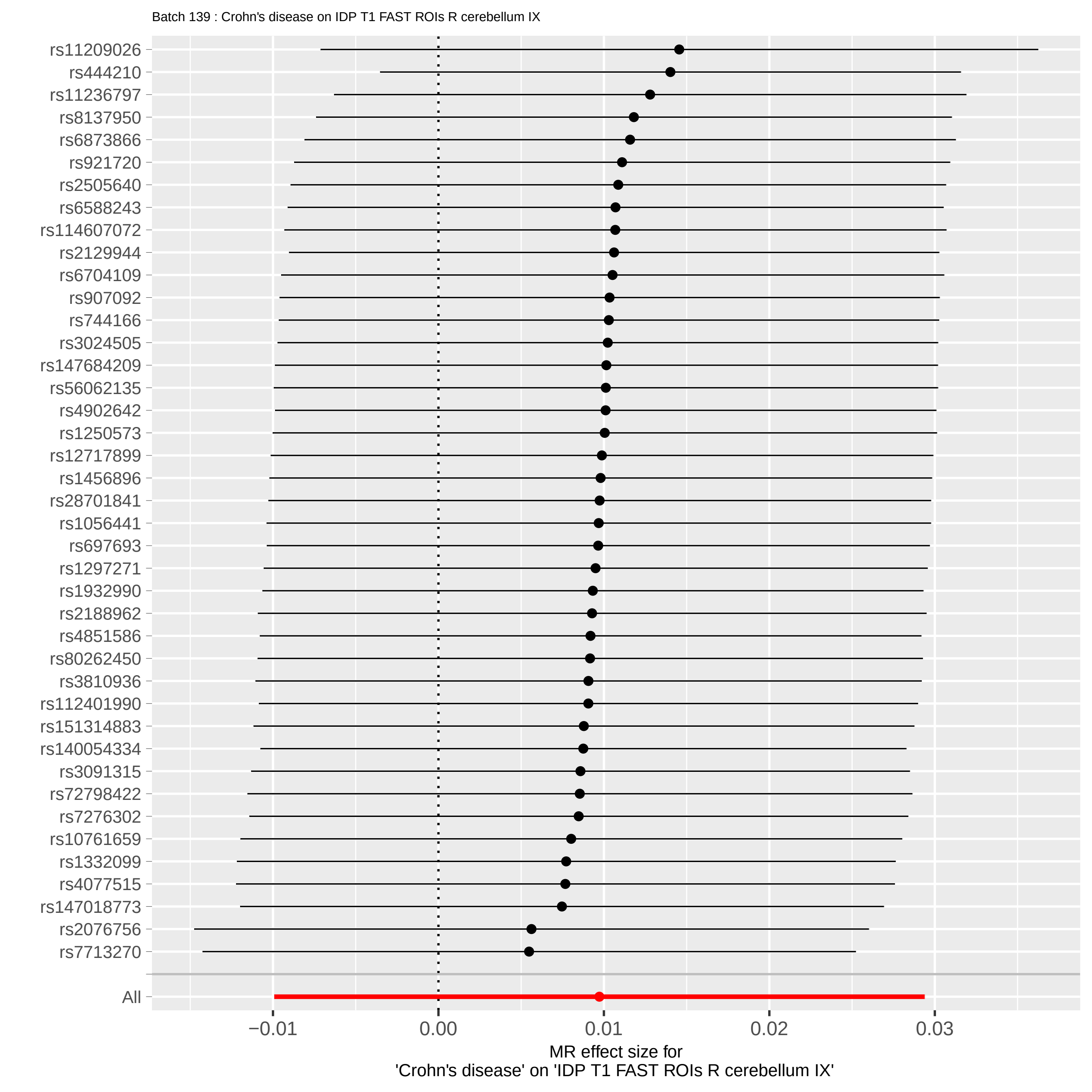
Batch 139 : Crohn's disease on IDP T1 FAST ROIs R cerebellum IX
rs11209026
rs444210
rs11236797
rs8137950
rs6873866
rs921720
rs2505640
rs6588243
rs114607072
rs2129944
rs6704109
rs907092
rs744166
rs3024505
rs147684209
rs56062135
rs4902642
rs1250573
rs12717899
rs1456896
rs28701841
rs1056441
rs697693
rs1297271
rs1932990
rs2188962
rs4851586
rs80262450
rs3810936
rs112401990
rs151314883
rs140054334
rs3091315
rs72798422
rs7276302
rs10761659
rs1332099
rs4077515
rs147018773
rs2076756
rs7713270
All
−0.01
0.00
0.01
0.02
0.03
MR effect size for
'Crohn's disease' on 'IDP T1 FAST ROIs R cerebellum IX'

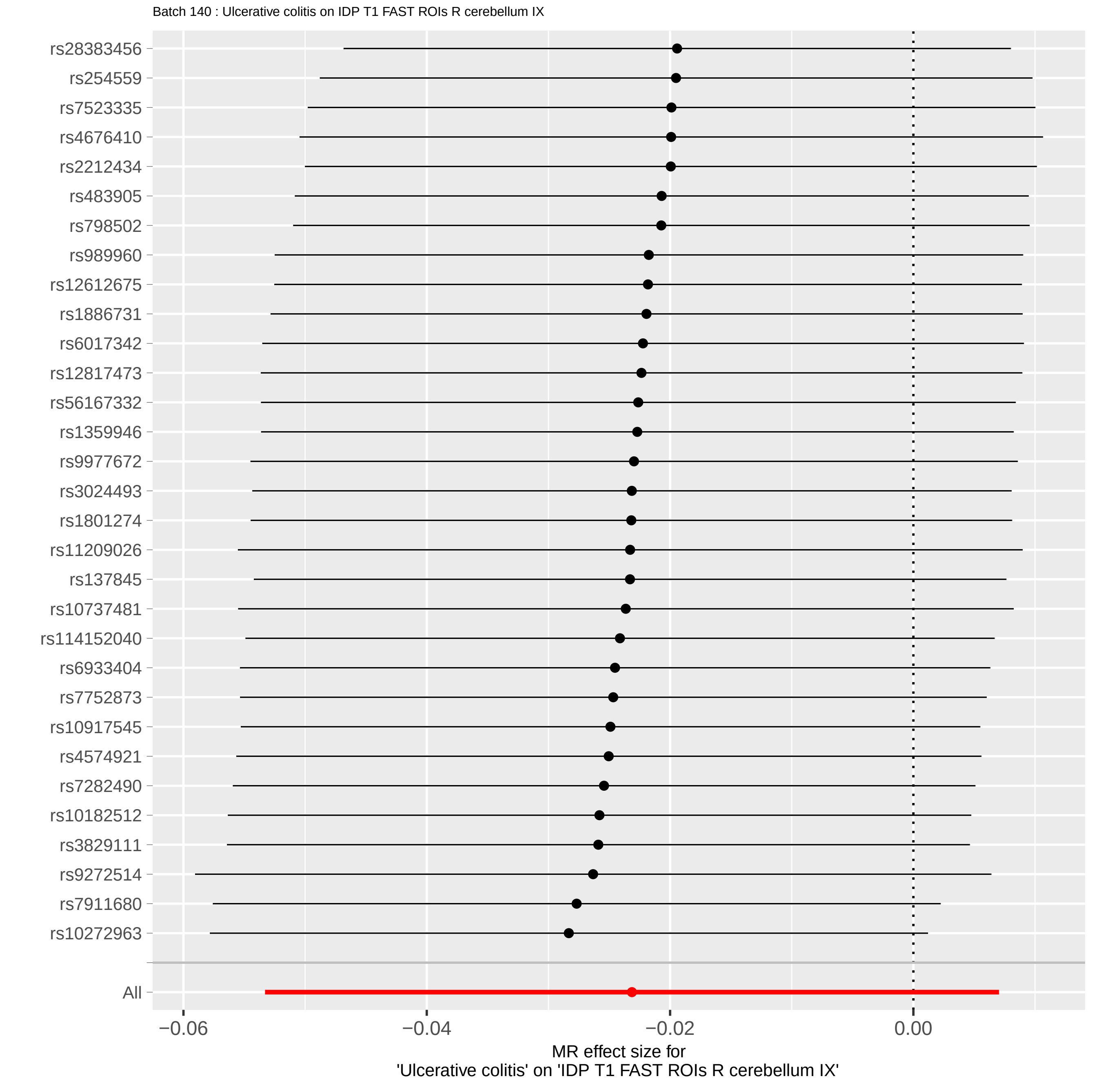
Batch 140 : Ulcerative colitis on IDP T1 FAST ROIs R cerebellum IX
rs28383456
rs254559
rs7523335
rs4676410
rs2212434
rs483905
rs798502
rs989960
rs12612675
rs1886731
rs6017342
rs12817473
rs56167332
rs1359946
rs9977672
rs3024493
rs1801274
rs11209026
rs137845
rs10737481
rs114152040
rs6933404
rs7752873
rs10917545
rs4574921
rs7282490
rs10182512
rs3829111
rs9272514
rs7911680
rs10272963
All
−0.06
−0.04
−0.02
0.00
MR effect size for
'Ulcerative colitis' on 'IDP T1 FAST ROIs R cerebellum IX'

Batch 141 : Crohn's disease on IDP T1 FAST ROIs L cerebellum X

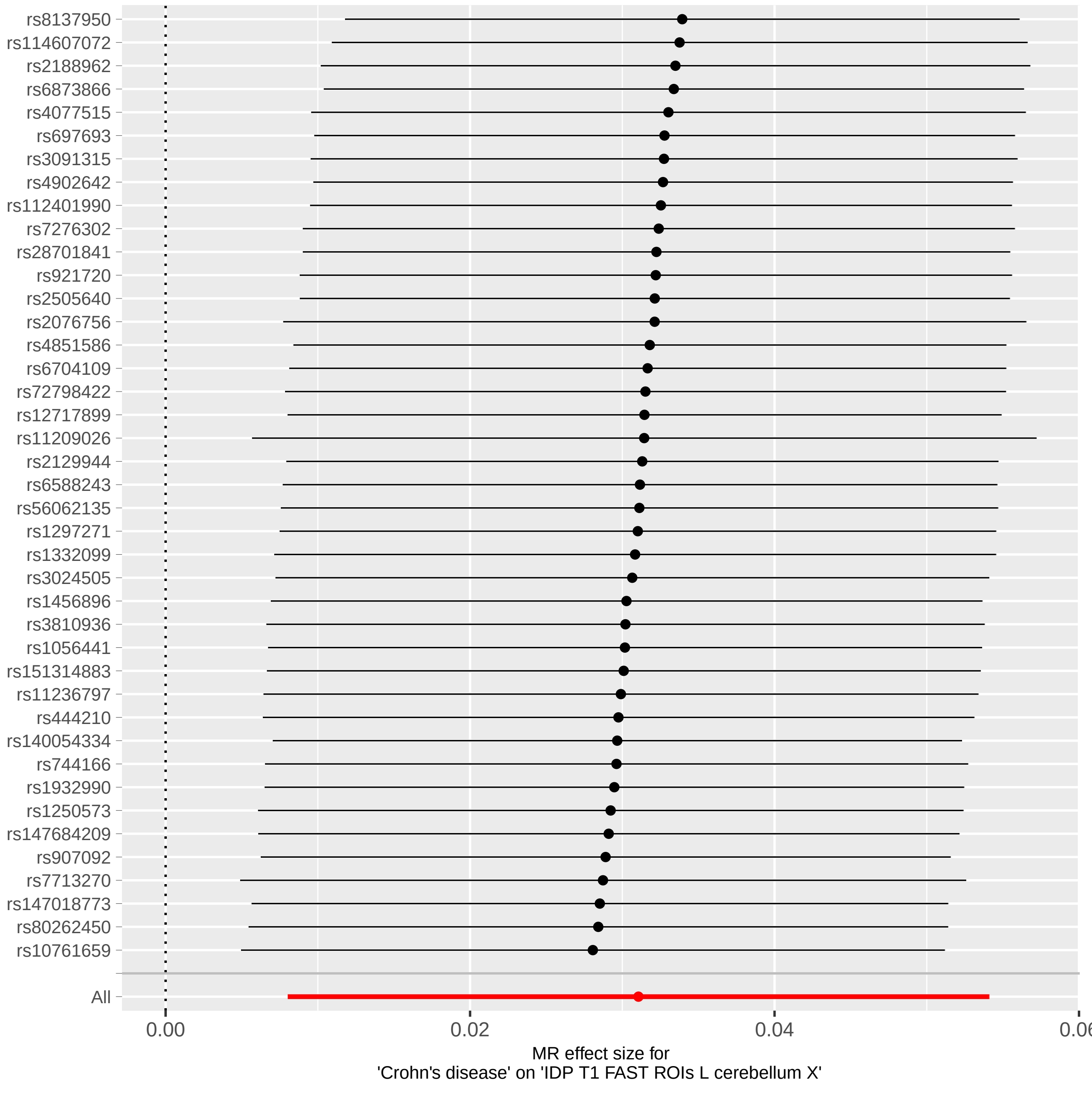

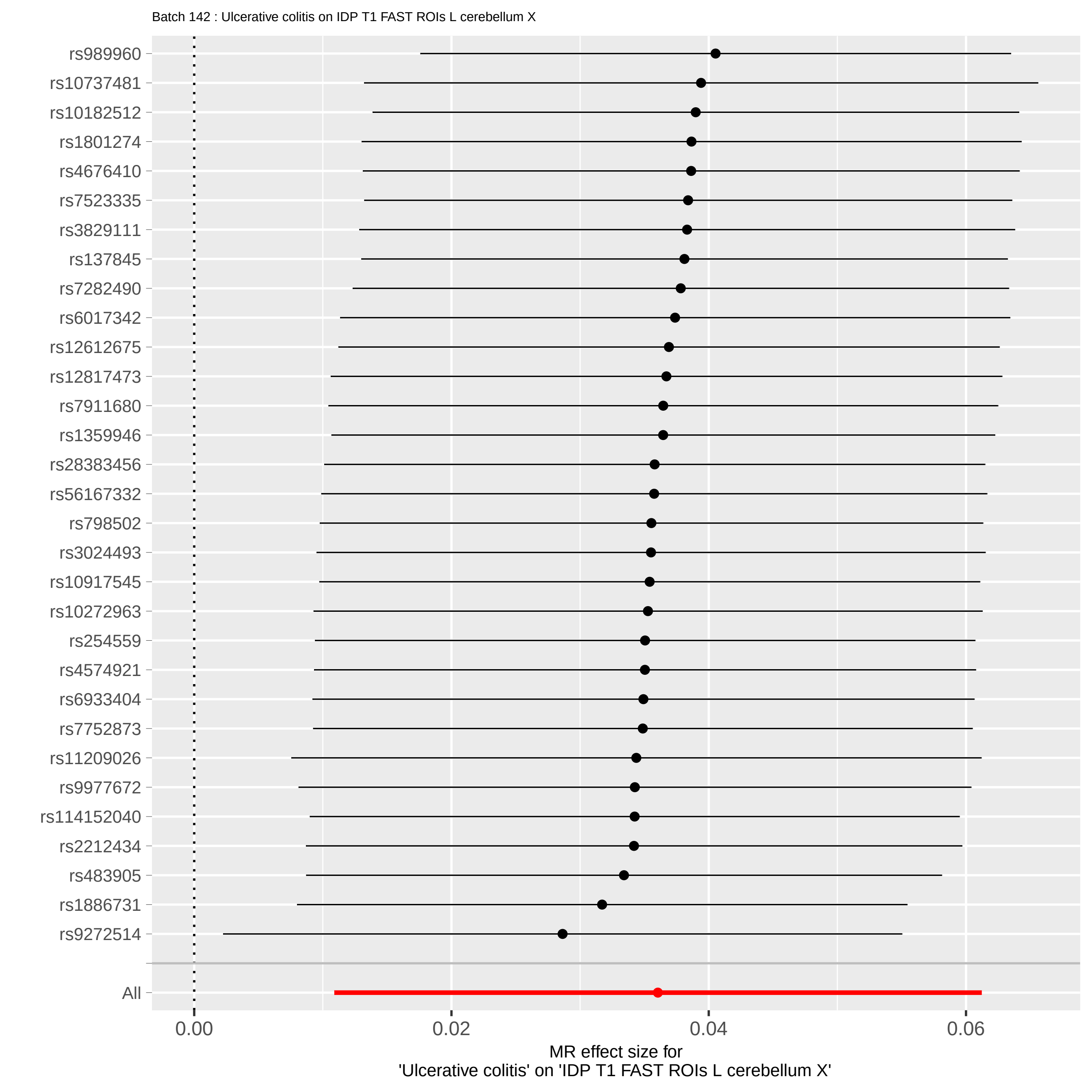

Batch 142 : Ulcerative colitis on IDP T1 FAST ROIs L cerebellum X
rs989960
rs10737481
rs10182512
rs1801274
rs4676410
rs7523335
rs3829111
rs137845
rs7282490
rs6017342
rs12612675
rs12817473
rs7911680
rs1359946
rs28383456
rs56167332
rs798502
rs3024493
rs10917545
rs10272963
rs254559
rs4574921
rs6933404
rs7752873
rs11209026
rs9977672
rs114152040
rs2212434
rs483905
rs1886731
rs9272514
All
0.00
0.02
0.04
0.06
MR effect size for
'Ulcerative colitis' on 'IDP T1 FAST ROIs L cerebellum X'

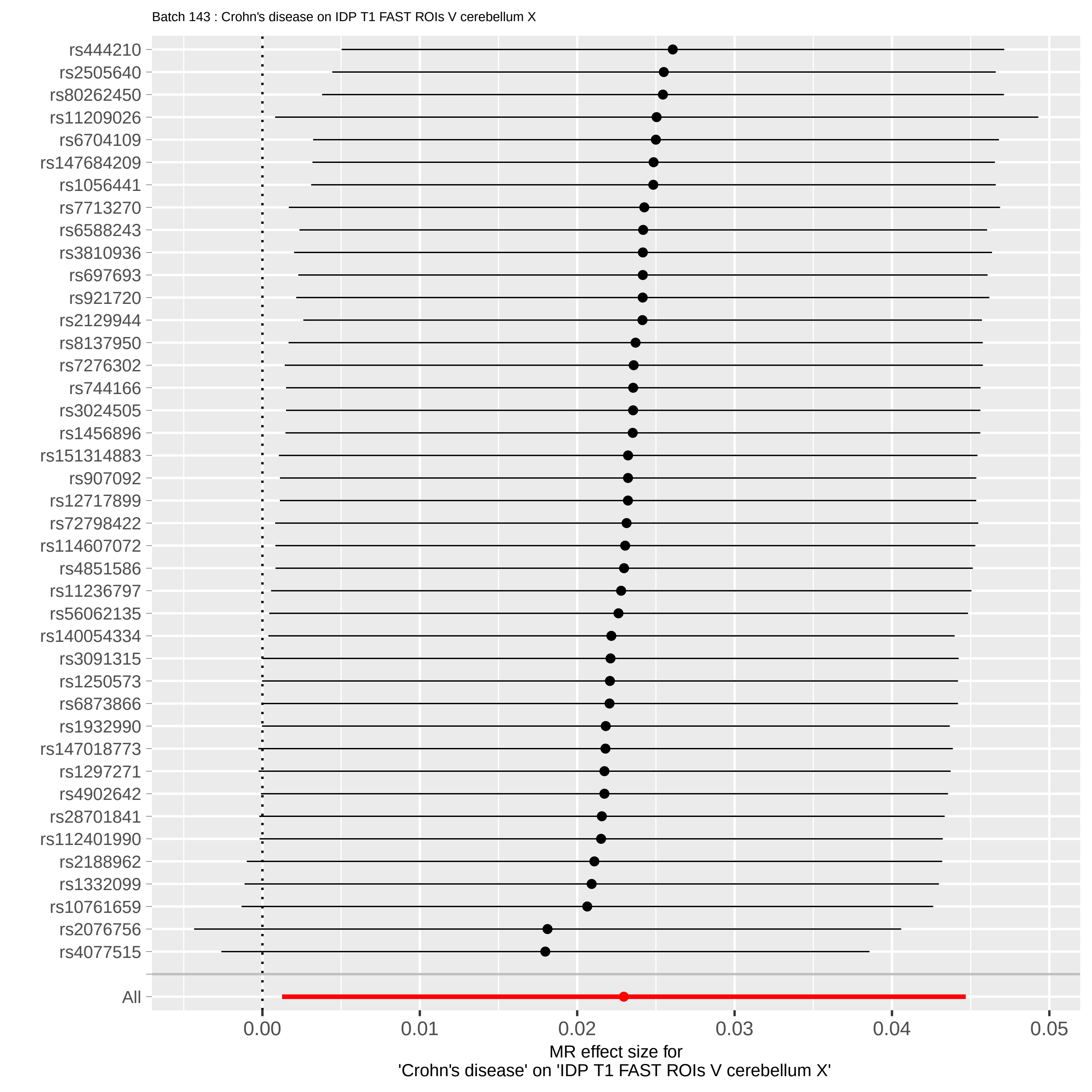

Batch 143 : Crohn's disease on IDP T1 FAST ROIs V cerebellum X
rs444210
rs2505640
rs80262450
rs11209026
rs6704109
rs147684209
rs1056441
rs7713270
rs6588243
rs3810936
rs697693
rs921720
rs2129944
rs8137950
rs7276302
rs744166
rs3024505
rs1456896
rs151314883
rs907092
rs12717899
rs72798422
rs114607072
rs4851586
rs11236797
rs56062135
rs140054334
rs3091315
rs1250573
rs6873866
rs1932990
rs147018773
rs1297271
rs4902642
rs28701841
rs112401990
rs2188962
rs1332099
rs10761659
rs2076756
rs4077515
All
0.00
0.01
0.02
0.03
0.04
0.05
MR effect size for
'Crohn's disease' on 'IDP T1 FAST ROIs V cerebellum X'

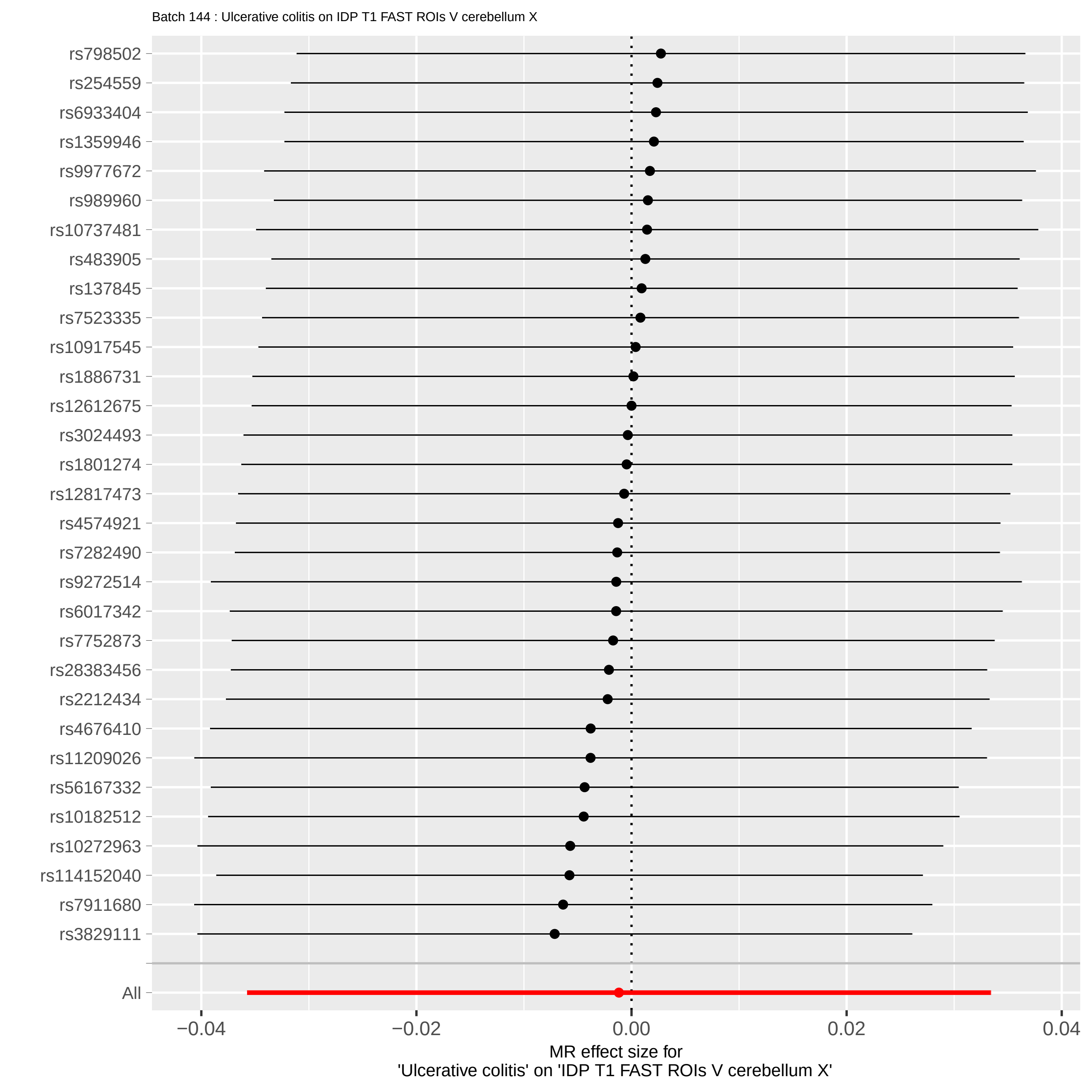
Batch 144 : Ulcerative colitis on IDP T1 FAST ROIs V cerebellum X
rs798502
rs254559
rs6933404
rs1359946
rs9977672
rs989960
rs10737481
rs483905
rs137845
rs7523335
rs10917545
rs1886731
rs12612675
rs3024493
rs1801274
rs12817473
rs4574921
rs7282490
rs9272514
rs6017342
rs7752873
rs28383456
rs2212434
rs4676410
rs11209026
rs56167332
rs10182512
rs10272963
rs114152040
rs7911680
rs3829111
All
−0.04
−0.02
0.00
0.02
0.04
MR effect size for
'Ulcerative colitis' on 'IDP T1 FAST ROIs V cerebellum X'

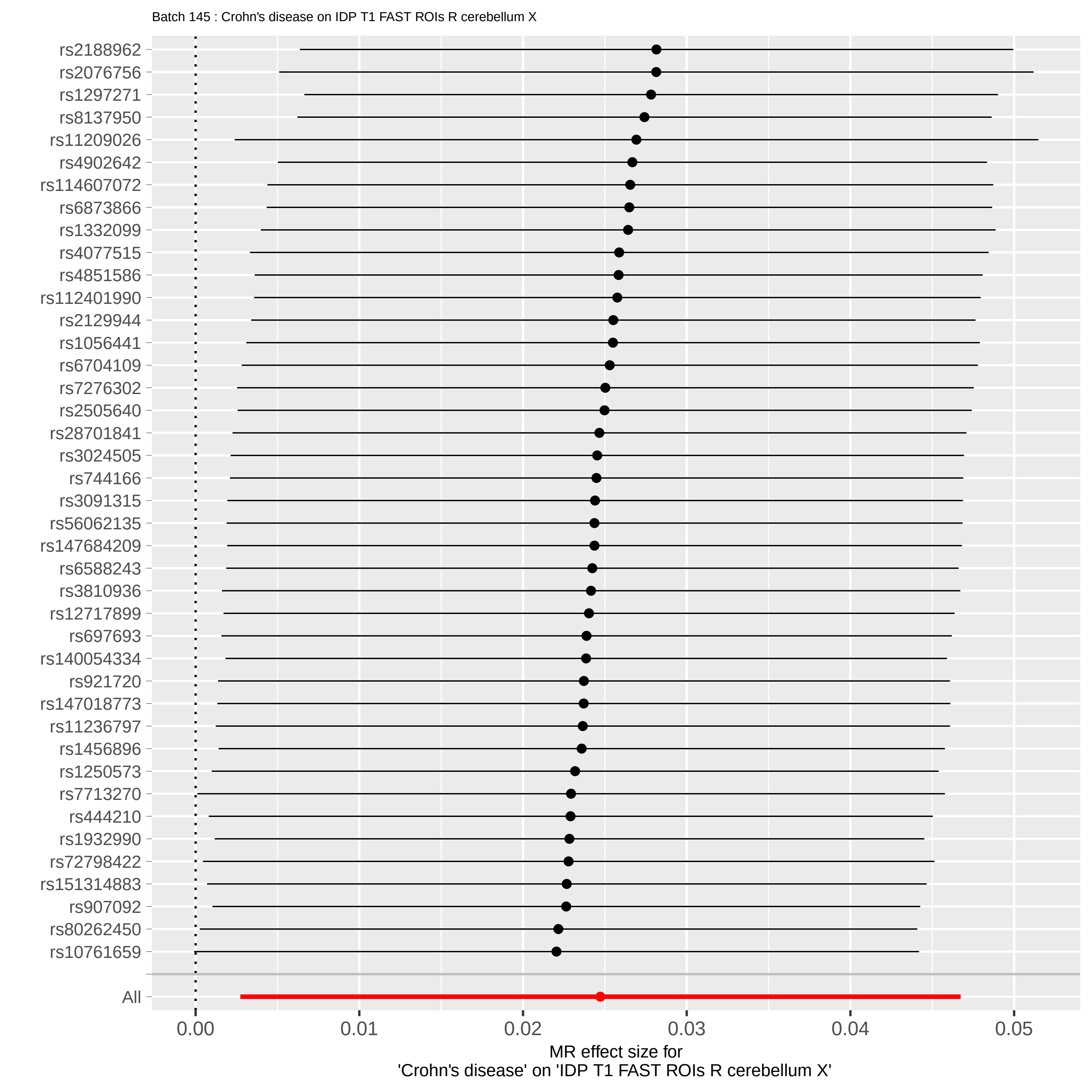
Batch 145 : Crohn's disease on IDP T1 FAST ROIs R cerebellum X
rs2188962
rs2076756
rs1297271
rs8137950
rs11209026
rs4902642
rs114607072
rs6873866
rs1332099
rs4077515
rs4851586
rs112401990
rs2129944
rs1056441
rs6704109
rs7276302
rs2505640
rs28701841
rs3024505
rs744166
rs3091315
rs56062135
rs147684209
rs6588243
rs3810936
rs12717899
rs697693
rs140054334
rs921720
rs147018773
rs11236797
rs1456896
rs1250573
rs7713270
rs444210
rs1932990
rs72798422
rs151314883
rs907092
rs80262450
rs10761659
All
0.00
0.01
0.02
0.03
0.04
0.05
MR effect size for
'Crohn's disease' on 'IDP T1 FAST ROIs R cerebellum X'

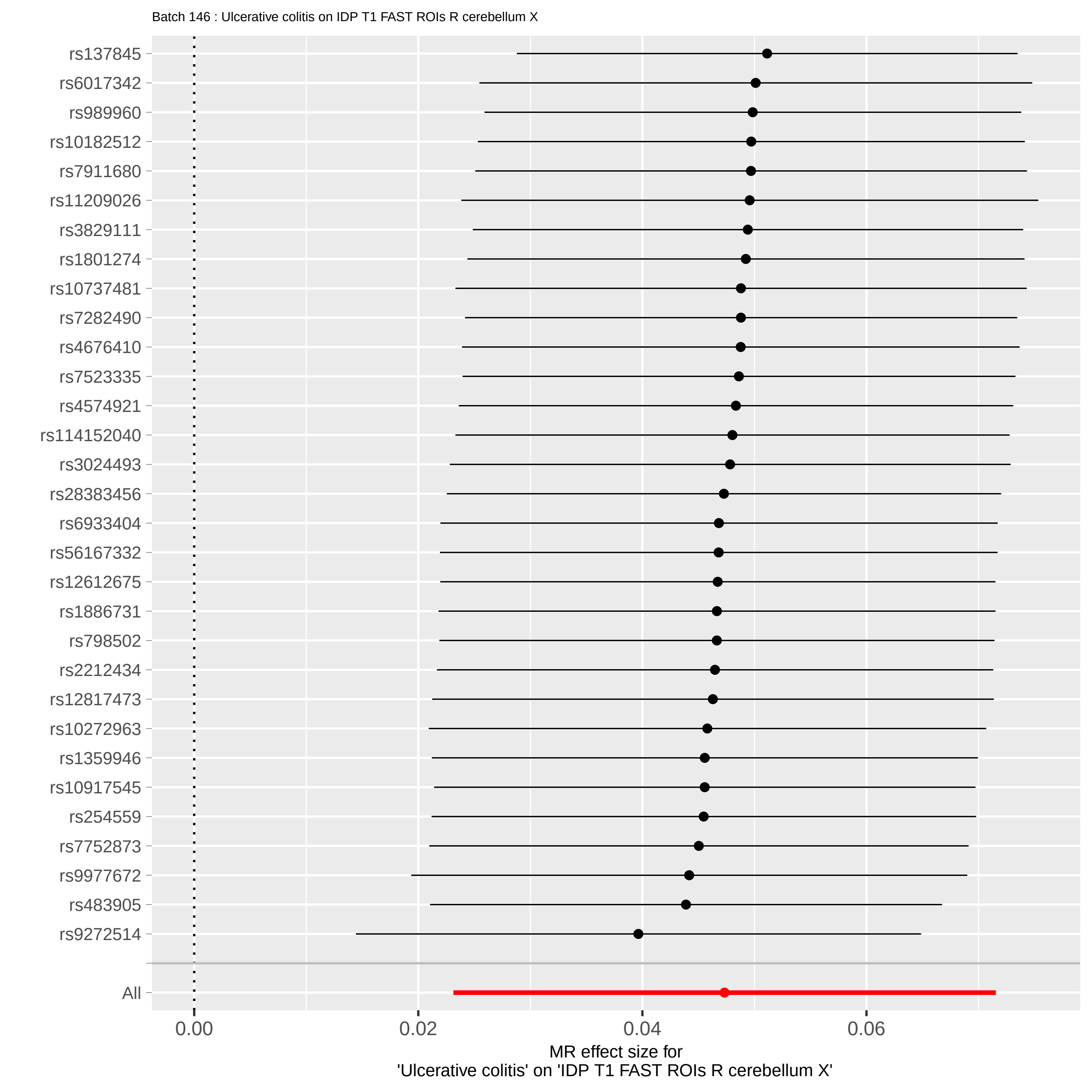

Batch 146 : Ulcerative colitis on IDP T1 FAST ROIs R cerebellum X
rs137845
rs6017342
rs989960
rs10182512
rs7911680
rs11209026
rs3829111
rs1801274
rs10737481
rs7282490
rs4676410
rs7523335
rs4574921
rs114152040
rs3024493
rs28383456
rs6933404
rs56167332
rs12612675
rs1886731
rs798502
rs2212434
rs12817473
rs10272963
rs1359946
rs10917545
rs254559
rs7752873
rs9977672
rs483905
rs9272514
All
0.00
0.02
0.04
0.06
MR effect size for
'Ulcerative colitis' on 'IDP T1 FAST ROIs R cerebellum X'

Batch 147 : Crohn's disease on IDP T2 FLAIR BIANCA WMH volume

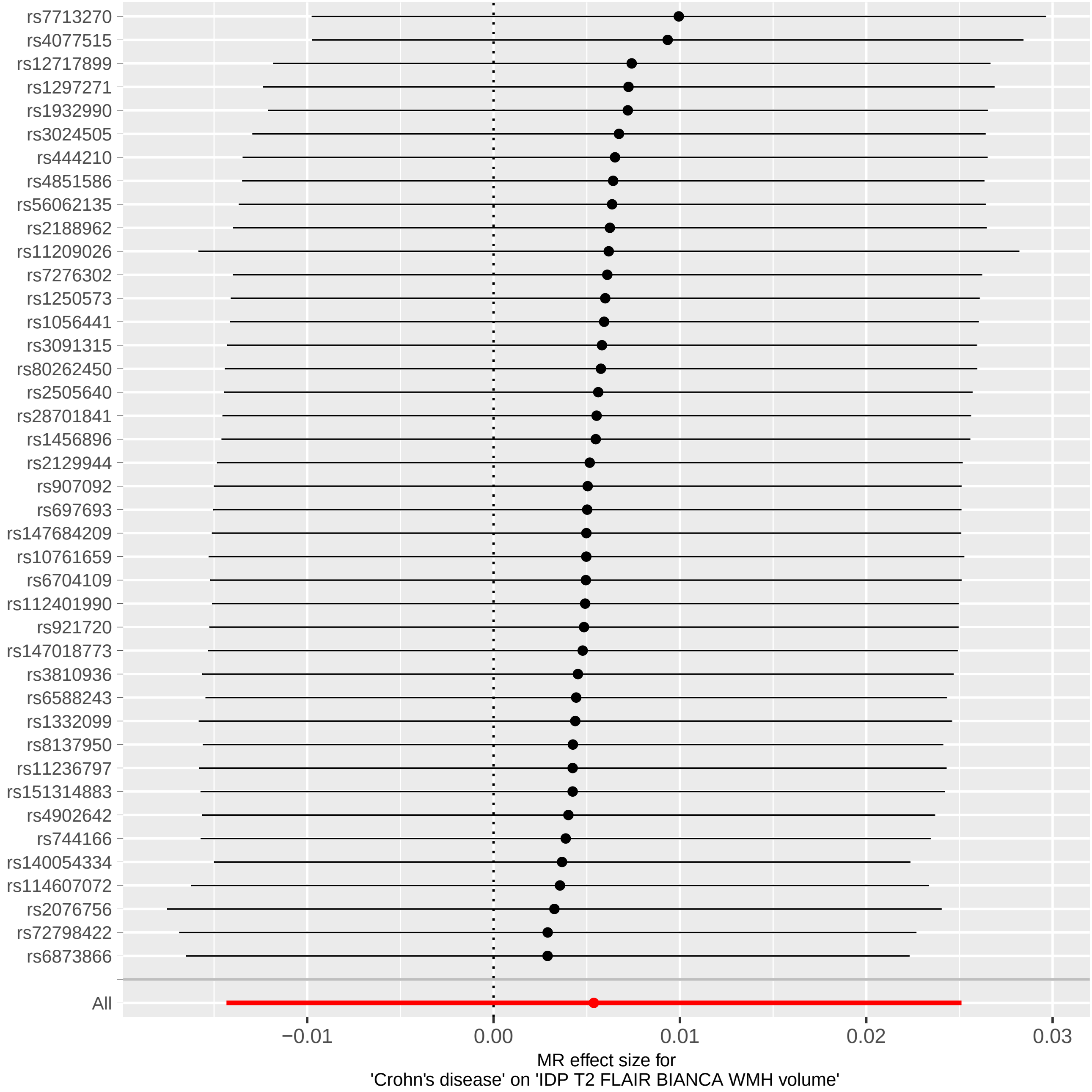

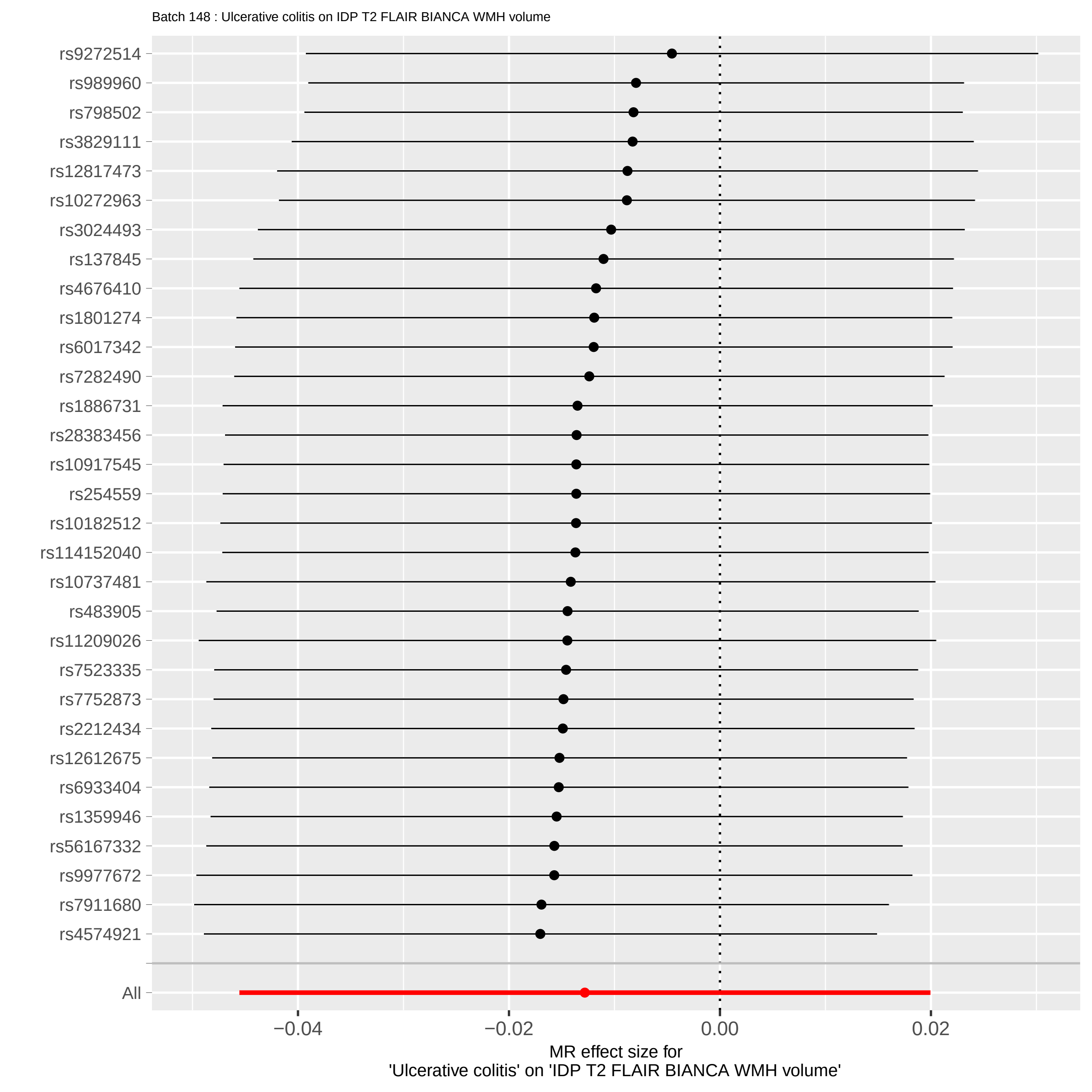

Batch 148 : Ulcerative colitis on IDP T2 FLAIR BIANCA WMH volume
rs9272514
rs989960
rs798502
rs3829111
rs12817473
rs10272963
rs3024493
rs137845
rs4676410
rs1801274
rs6017342
rs7282490
rs1886731
rs28383456
rs10917545
rs254559
rs10182512
rs114152040
rs10737481
rs483905
rs11209026
rs7523335
rs7752873
rs2212434
rs12612675
rs6933404
rs1359946
rs56167332
rs9977672
rs7911680
rs4574921
All
−0.04
−0.02
0.00
0.02
MR effect size for
'Ulcerative colitis' on 'IDP T2 FLAIR BIANCA WMH volume'

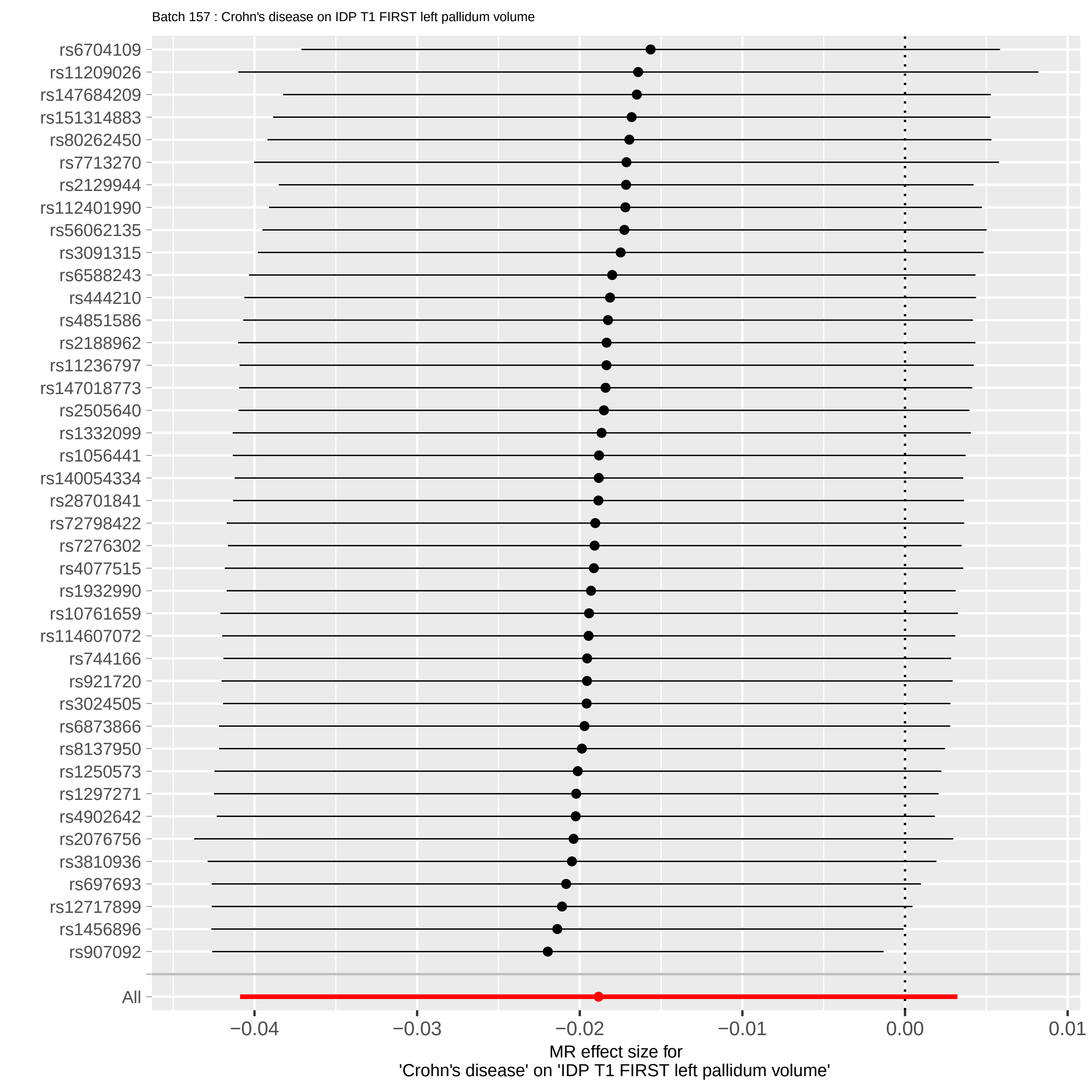
Batch 157 : Crohn's disease on IDP T1 FIRST left pallidum volume
rs6704109
rs11209026
rs147684209
rs151314883
rs80262450
rs7713270
rs2129944
rs112401990
rs56062135
rs3091315
rs6588243
rs444210
rs4851586
rs2188962
rs11236797
rs147018773
rs2505640
rs1332099
rs1056441
rs140054334
rs28701841
rs72798422
rs7276302
rs4077515
rs1932990
rs10761659
rs114607072
rs744166
rs921720
rs3024505
rs6873866
rs8137950
rs1250573
rs1297271
rs4902642
rs2076756
rs3810936
rs697693
rs12717899
rs1456896
rs907092
All
−0.04
−0.03
−0.02
−0.01
0.00
0.01
MR effect size for
'Crohn's disease' on 'IDP T1 FIRST left pallidum volume'

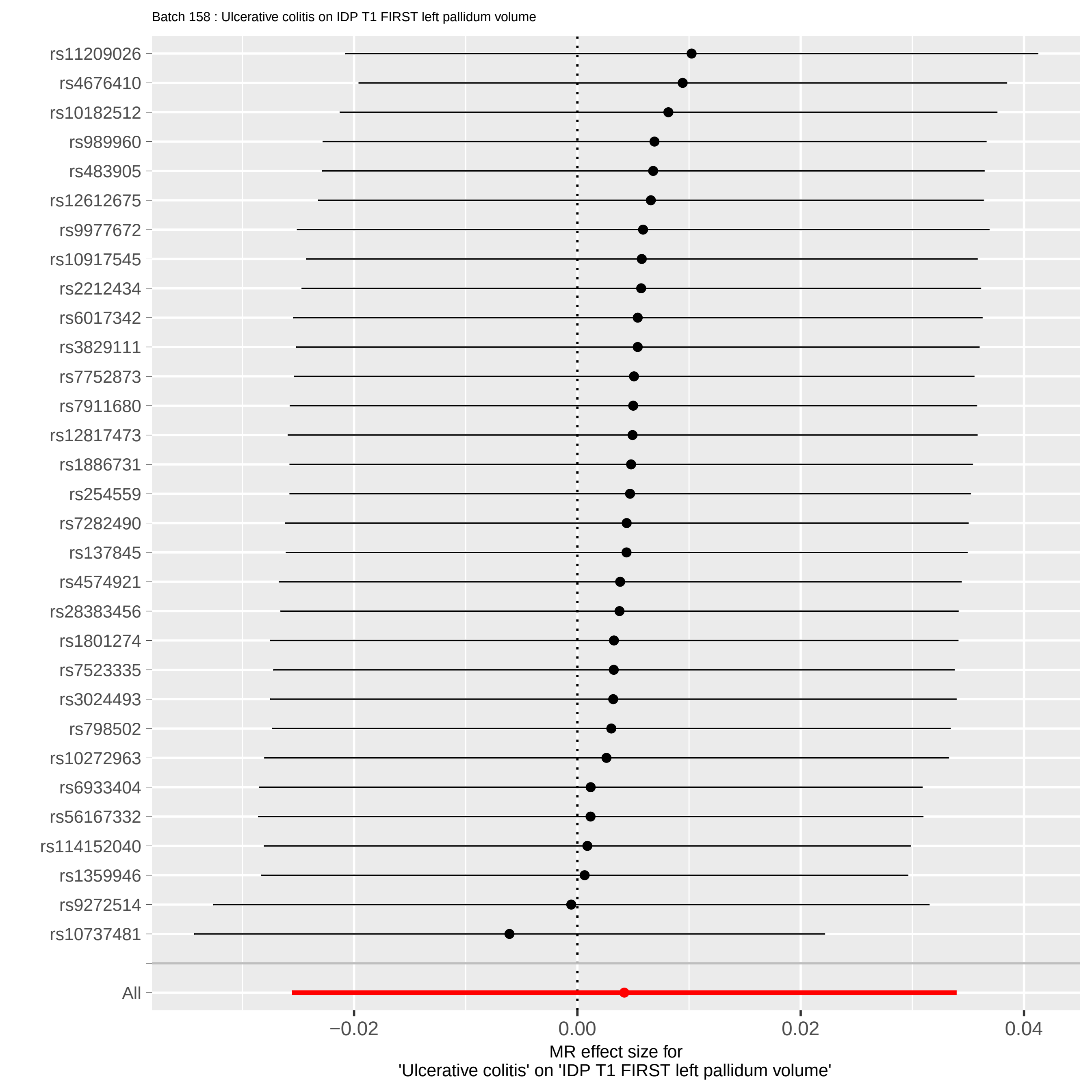
Batch 158 : Ulcerative colitis on IDP T1 FIRST left pallidum volume
rs11209026
rs4676410
rs10182512
rs989960
rs483905
rs12612675
rs9977672
rs10917545
rs2212434
rs6017342
rs3829111
rs7752873
rs7911680
rs12817473
rs1886731
rs254559
rs7282490
rs137845
rs4574921
rs28383456
rs1801274
rs7523335
rs3024493
rs798502
rs10272963
rs6933404
rs56167332
rs114152040
rs1359946
rs9272514
rs10737481
All
−0.02
0.00
0.02
0.04
MR effect size for
'Ulcerative colitis' on 'IDP T1 FIRST left pallidum volume'

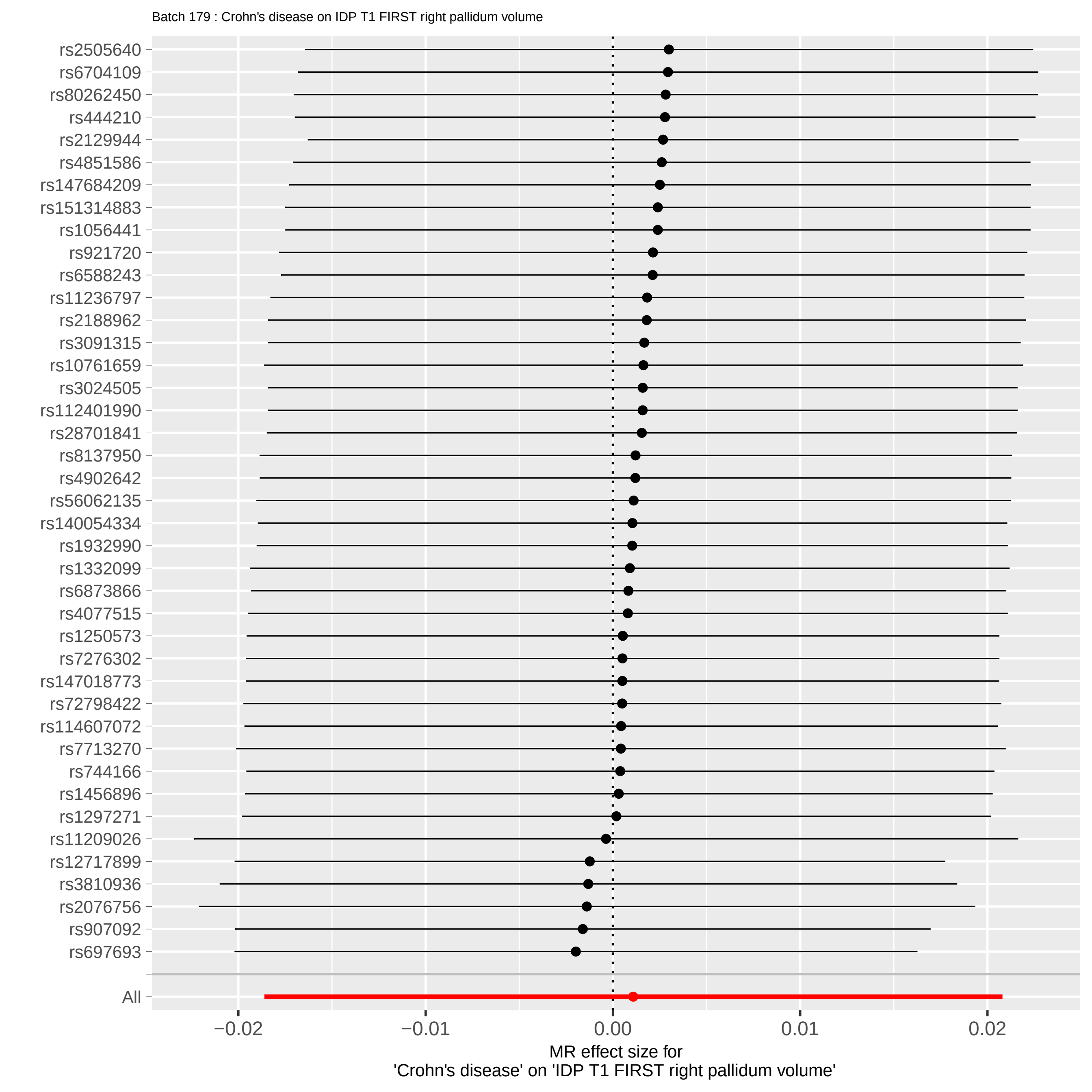

Batch 179 : Crohn's disease on IDP T1 FIRST right pallidum volume
rs2505640
rs6704109
rs80262450
rs444210
rs2129944
rs4851586
rs147684209
rs151314883
rs1056441
rs921720
rs6588243
rs11236797
rs2188962
rs3091315
rs10761659
rs3024505
rs112401990
rs28701841
rs8137950
rs4902642
rs56062135
rs140054334
rs1932990
rs1332099
rs6873866
rs4077515
rs1250573
rs7276302
rs147018773
rs72798422
rs114607072
rs7713270
rs744166
rs1456896
rs1297271
rs11209026
rs12717899
rs3810936
rs2076756
rs907092
rs697693
All
−0.02
−0.01
0.00
0.01
0.02
MR effect size for
'Crohn's disease' on 'IDP T1 FIRST right pallidum volume'

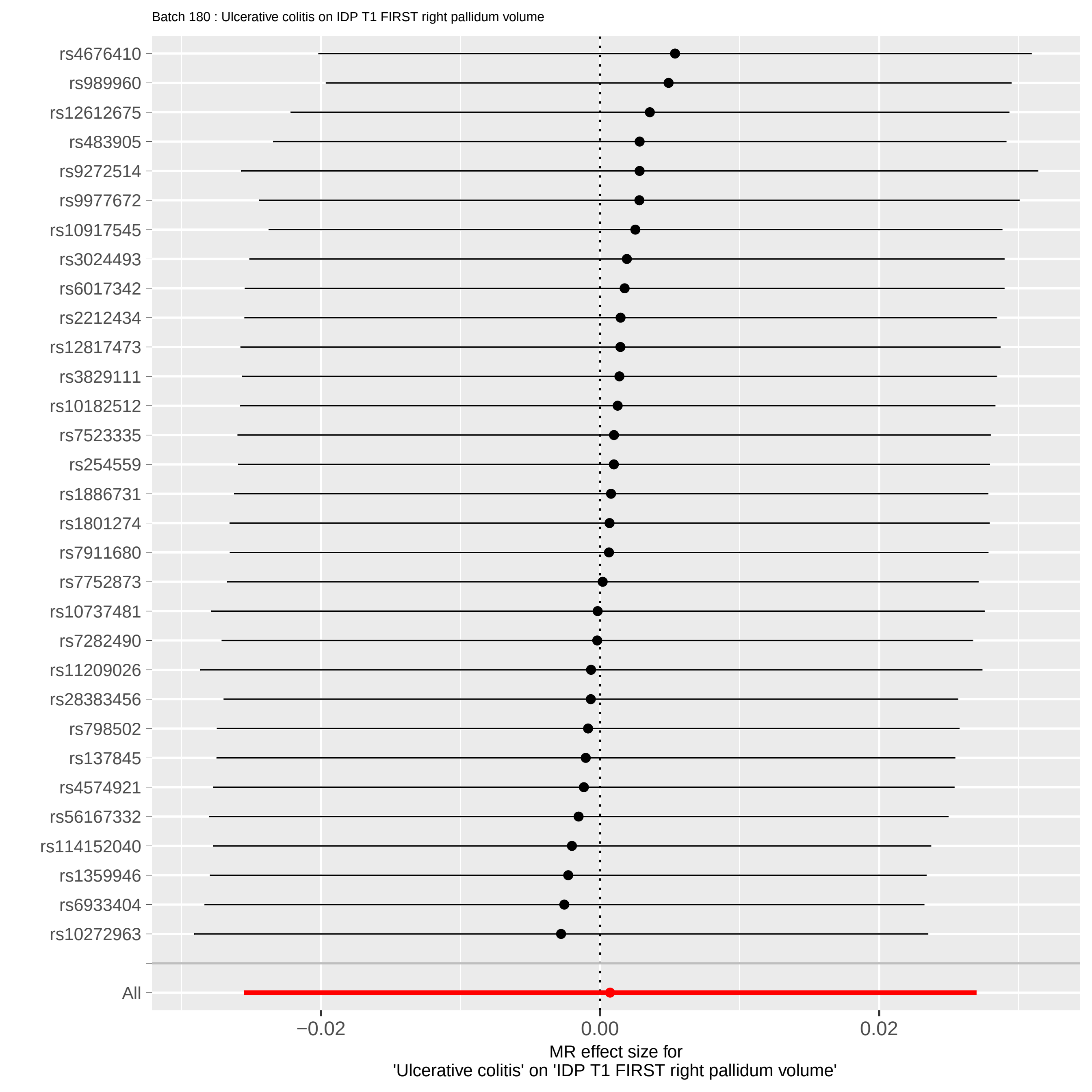
Batch 180 : Ulcerative colitis on IDP T1 FIRST right pallidum volume
rs4676410
rs989960
rs12612675
rs483905
rs9272514
rs9977672
rs10917545
rs3024493
rs6017342
rs2212434
rs12817473
rs3829111
rs10182512
rs7523335
rs254559
rs1886731
rs1801274
rs7911680
rs7752873
rs10737481
rs7282490
rs11209026
rs28383456
rs798502
rs137845
rs4574921
rs56167332
rs114152040
rs1359946
rs6933404
rs10272963
All
−0.02
0.00
0.02
MR effect size for
'Ulcerative colitis' on 'IDP T1 FIRST right pallidum volume'

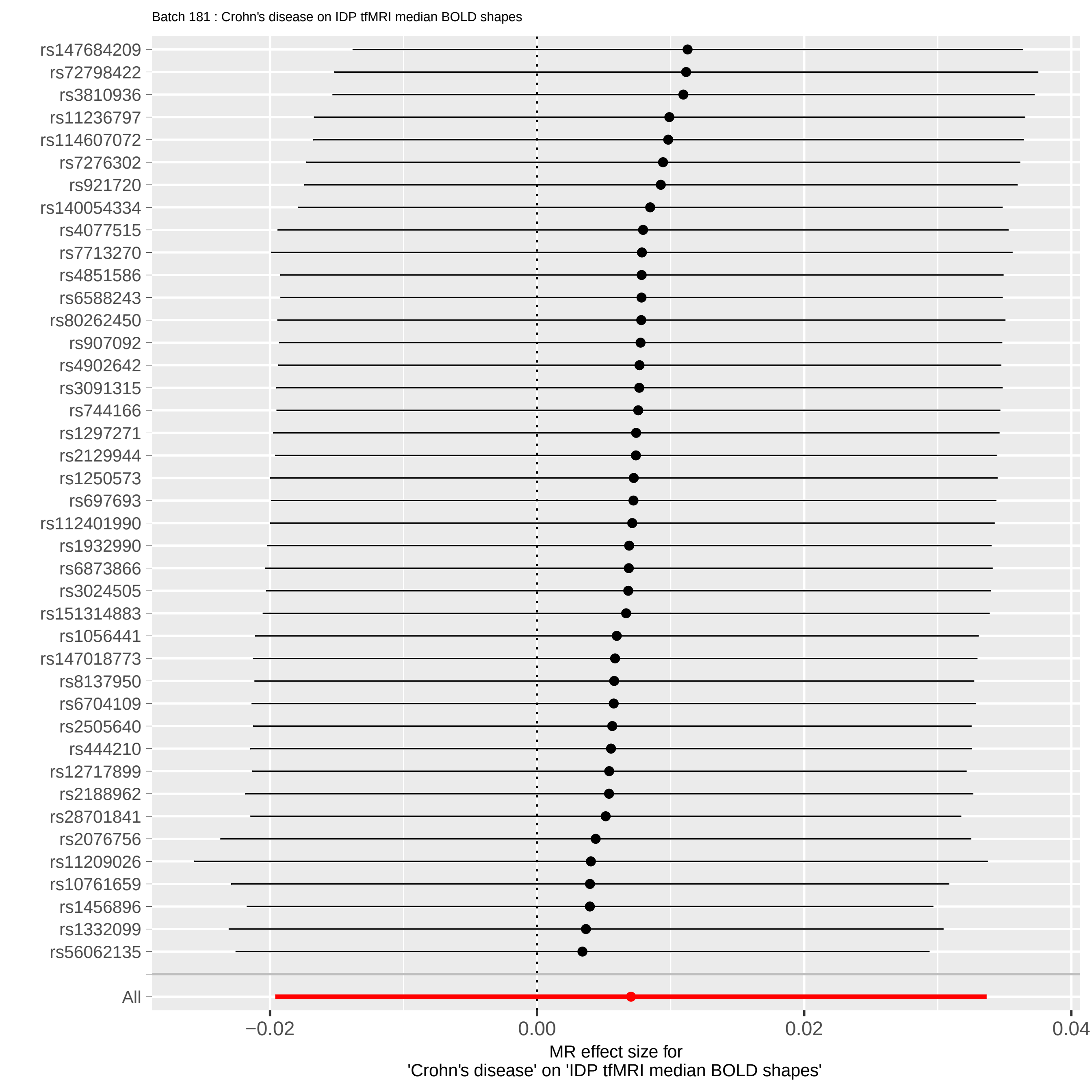
Batch 181 : Crohn's disease on IDP tfMRI median BOLD shapes
rs147684209
rs72798422
rs3810936
rs11236797
rs114607072
rs7276302
rs921720
rs140054334
rs4077515
rs7713270
rs4851586
rs6588243
rs80262450
rs907092
rs4902642
rs3091315
rs744166
rs1297271
rs2129944
rs1250573
rs697693
rs112401990
rs1932990
rs6873866
rs3024505
rs151314883
rs1056441
rs147018773
rs8137950
rs6704109
rs2505640
rs444210
rs12717899
rs2188962
rs28701841
rs2076756
rs11209026
rs10761659
rs1456896
rs1332099
rs56062135
All
−0.02
0.00
0.02
0.04
MR effect size for
'Crohn's disease' on 'IDP tfMRI median BOLD shapes'

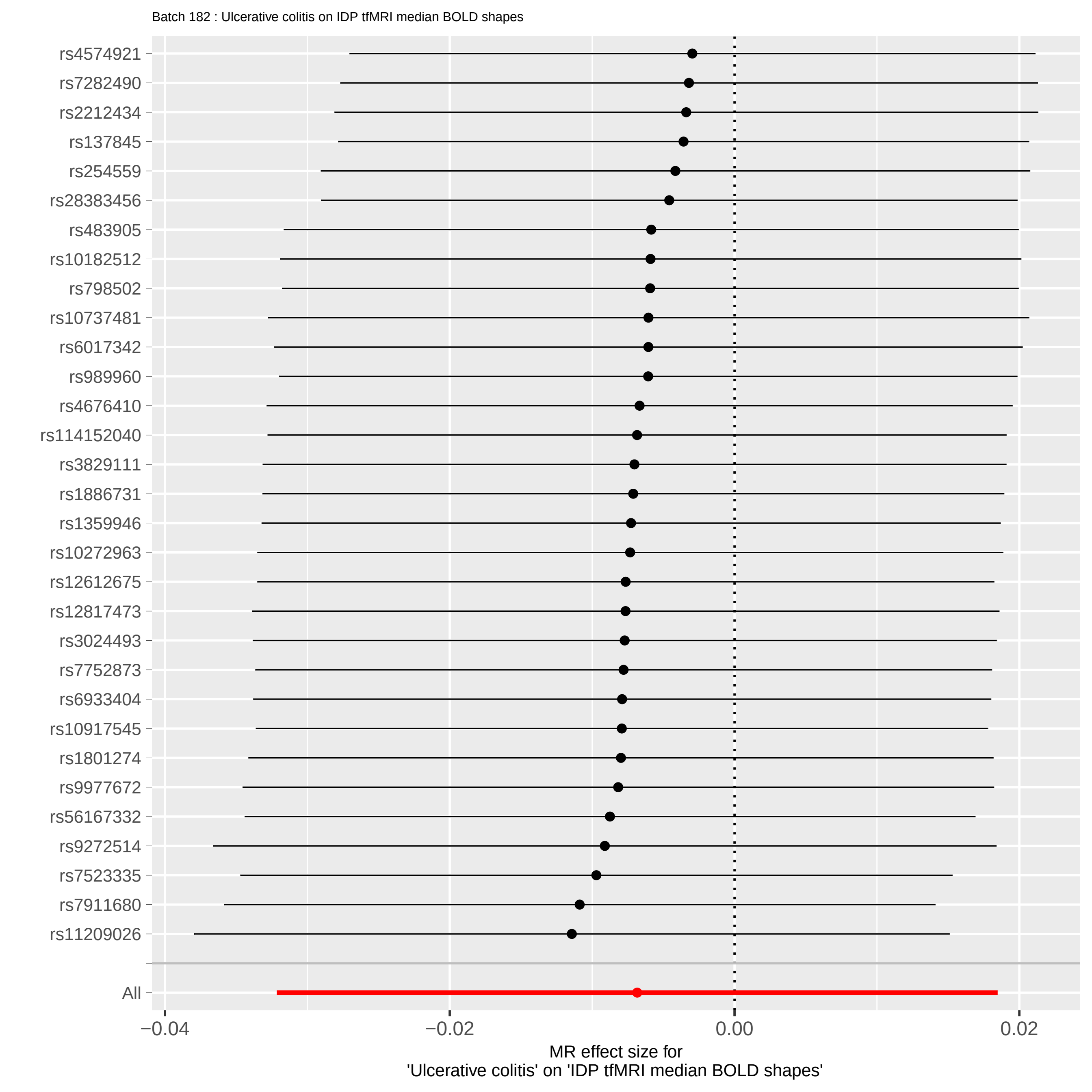

Batch 182 : Ulcerative colitis on IDP tfMRI median BOLD shapes
rs4574921
rs7282490
rs2212434
rs137845
rs254559
rs28383456
rs483905
rs10182512
rs798502
rs10737481
rs6017342
rs989960
rs4676410
rs114152040
rs3829111
rs1886731
rs1359946
rs10272963
rs12612675
rs12817473
rs3024493
rs7752873
rs6933404
rs10917545
rs1801274
rs9977672
rs56167332
rs9272514
rs7523335
rs7911680
rs11209026
All
−0.04
−0.02
0.00
0.02
MR effect size for
'Ulcerative colitis' on 'IDP tfMRI median BOLD shapes'

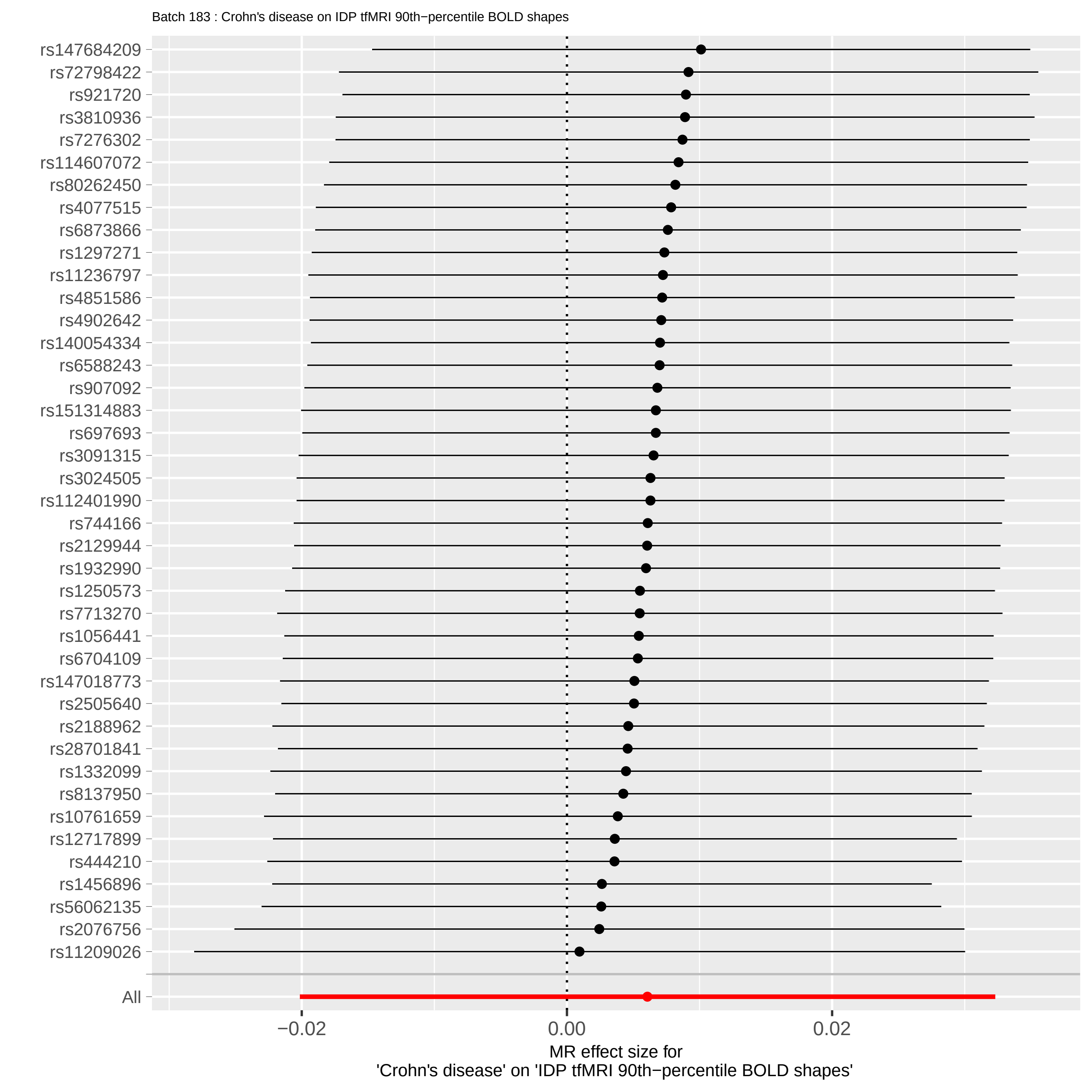

Batch 183 : Crohn's disease on IDP tfMRI 90th−percentile BOLD shapes
rs147684209
rs72798422
rs921720
rs3810936
rs7276302
rs114607072
rs80262450
rs4077515
rs6873866
rs1297271
rs11236797
rs4851586
rs4902642
rs140054334
rs6588243
rs907092
rs151314883
rs697693
rs3091315
rs3024505
rs112401990
rs744166
rs2129944
rs1932990
rs1250573
rs7713270
rs1056441
rs6704109
rs147018773
rs2505640
rs2188962
rs28701841
rs1332099
rs8137950
rs10761659
rs12717899
rs444210
rs1456896
rs56062135
rs2076756
rs11209026
All
−0.02
0.00
0.02
MR effect size for
'Crohn's disease' on 'IDP tfMRI 90th−percentile BOLD shapes'

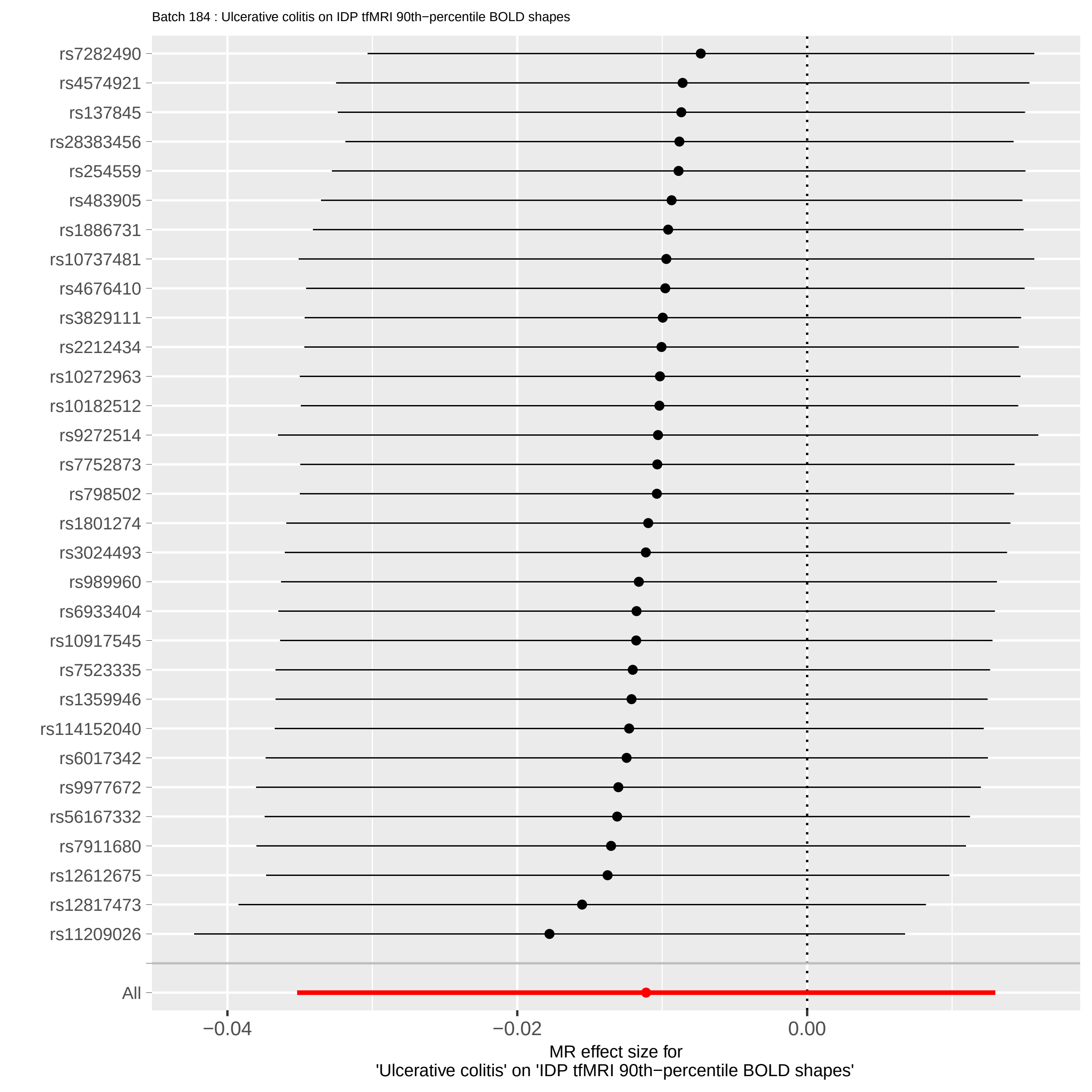

Batch 184 : Ulcerative colitis on IDP tfMRI 90th−percentile BOLD shapes
rs7282490
rs4574921
rs137845
rs28383456
rs254559
rs483905
rs1886731
rs10737481
rs4676410
rs3829111
rs2212434
rs10272963
rs10182512
rs9272514
rs7752873
rs798502
rs1801274
rs3024493
rs989960
rs6933404
rs10917545
rs7523335
rs1359946
rs114152040
rs6017342
rs9977672
rs56167332
rs7911680
rs12612675
rs12817473
rs11209026
All
−0.04
−0.02
0.00
MR effect size for
'Ulcerative colitis' on 'IDP tfMRI 90th−percentile BOLD shapes'

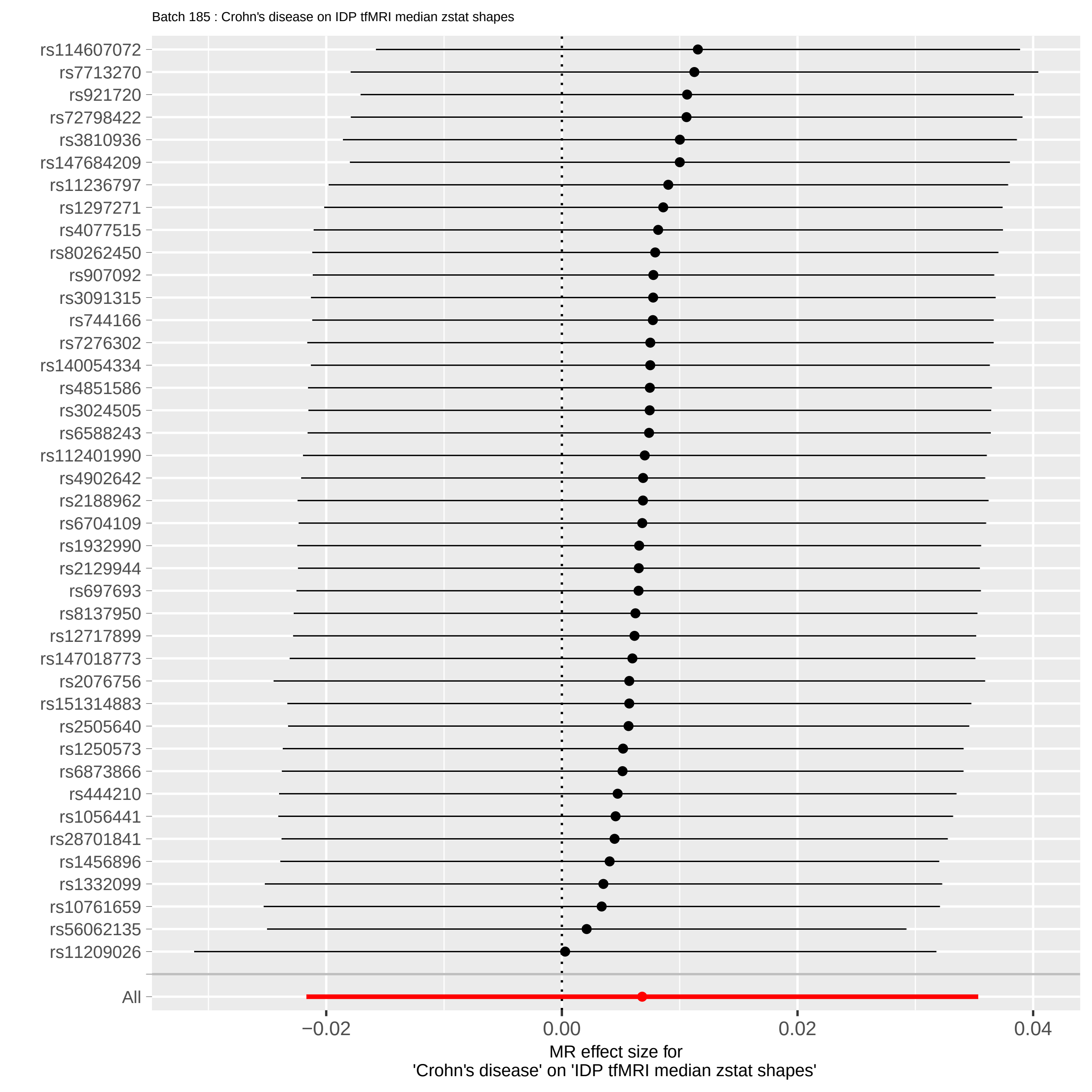
Batch 185 : Crohn's disease on IDP tfMRI median zstat shapes
rs114607072
rs7713270
rs921720
rs72798422
rs3810936
rs147684209
rs11236797
rs1297271
rs4077515
rs80262450
rs907092
rs3091315
rs744166
rs7276302
rs140054334
rs4851586
rs3024505
rs6588243
rs112401990
rs4902642
rs2188962
rs6704109
rs1932990
rs2129944
rs697693
rs8137950
rs12717899
rs147018773
rs2076756
rs151314883
rs2505640
rs1250573
rs6873866
rs444210
rs1056441
rs28701841
rs1456896
rs1332099
rs10761659
rs56062135
rs11209026
All
−0.02
0.00
0.02
0.04
MR effect size for
'Crohn's disease' on 'IDP tfMRI median zstat shapes'

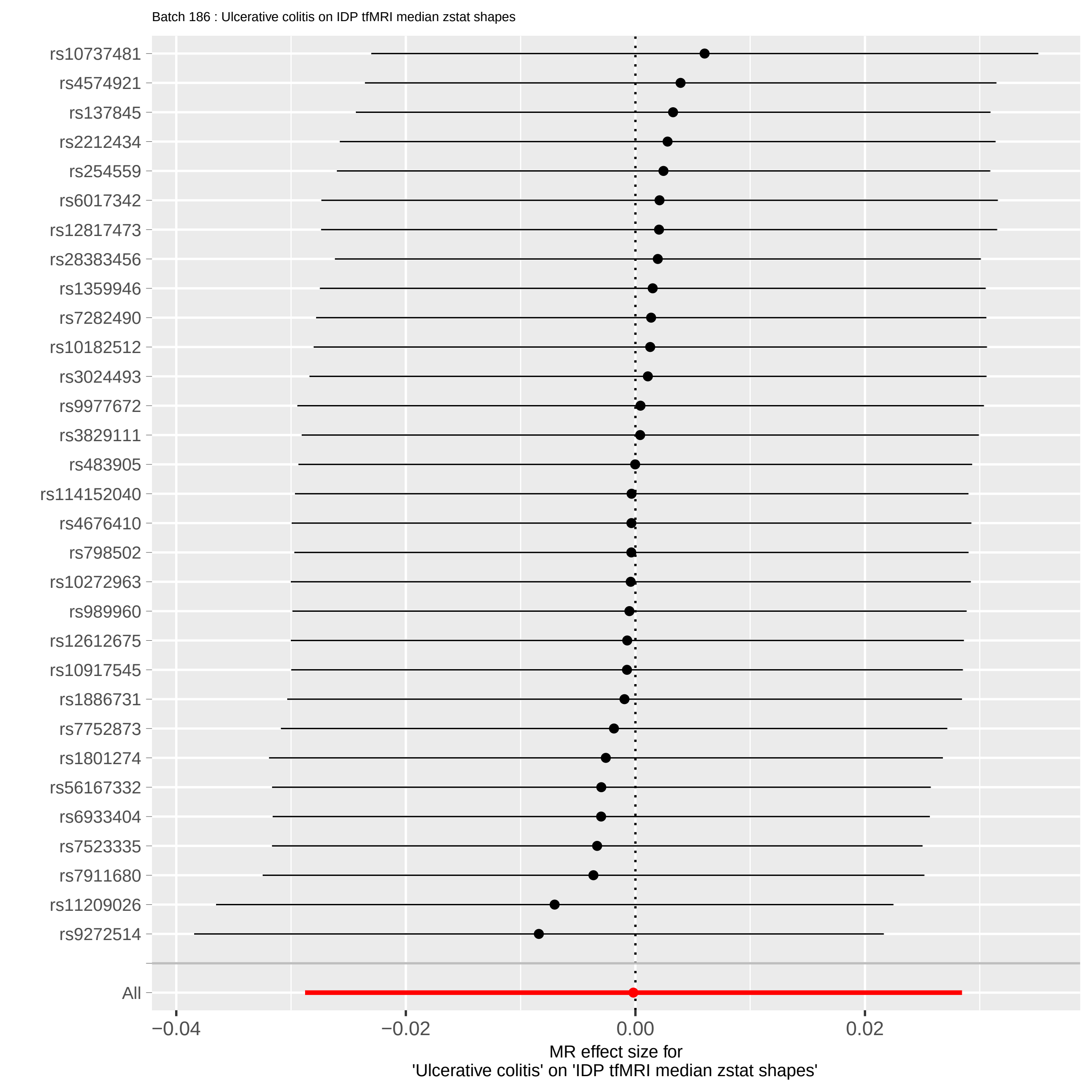

Batch 186 : Ulcerative colitis on IDP tfMRI median zstat shapes
rs10737481
rs4574921
rs137845
rs2212434
rs254559
rs6017342
rs12817473
rs28383456
rs1359946
rs7282490
rs10182512
rs3024493
rs9977672
rs3829111
rs483905
rs114152040
rs4676410
rs798502
rs10272963
rs989960
rs12612675
rs10917545
rs1886731
rs7752873
rs1801274
rs56167332
rs6933404
rs7523335
rs7911680
rs11209026
rs9272514
All
−0.04
−0.02
0.00
0.02
MR effect size for
'Ulcerative colitis' on 'IDP tfMRI median zstat shapes'

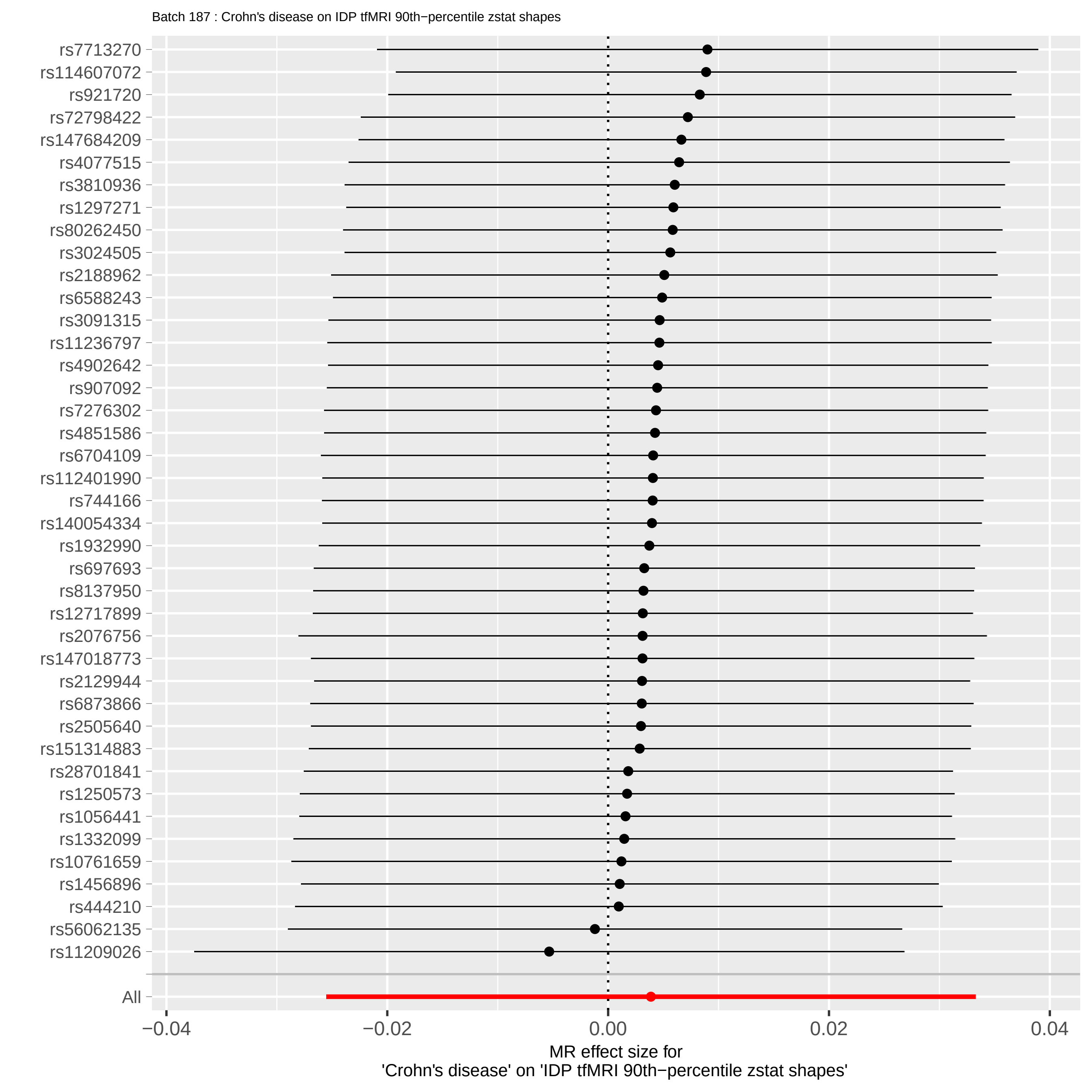

Batch 187 : Crohn's disease on IDP tfMRI 90th−percentile zstat shapes
rs7713270
rs114607072
rs921720
rs72798422
rs147684209
rs4077515
rs3810936
rs1297271
rs80262450
rs3024505
rs2188962
rs6588243
rs3091315
rs11236797
rs4902642
rs907092
rs7276302
rs4851586
rs6704109
rs112401990
rs744166
rs140054334
rs1932990
rs697693
rs8137950
rs12717899
rs2076756
rs147018773
rs2129944
rs6873866
rs2505640
rs151314883
rs28701841
rs1250573
rs1056441
rs1332099
rs10761659
rs1456896
rs444210
rs56062135
rs11209026
All
−0.04
−0.02
0.00
0.02
0.04
MR effect size for
'Crohn's disease' on 'IDP tfMRI 90th−percentile zstat shapes'

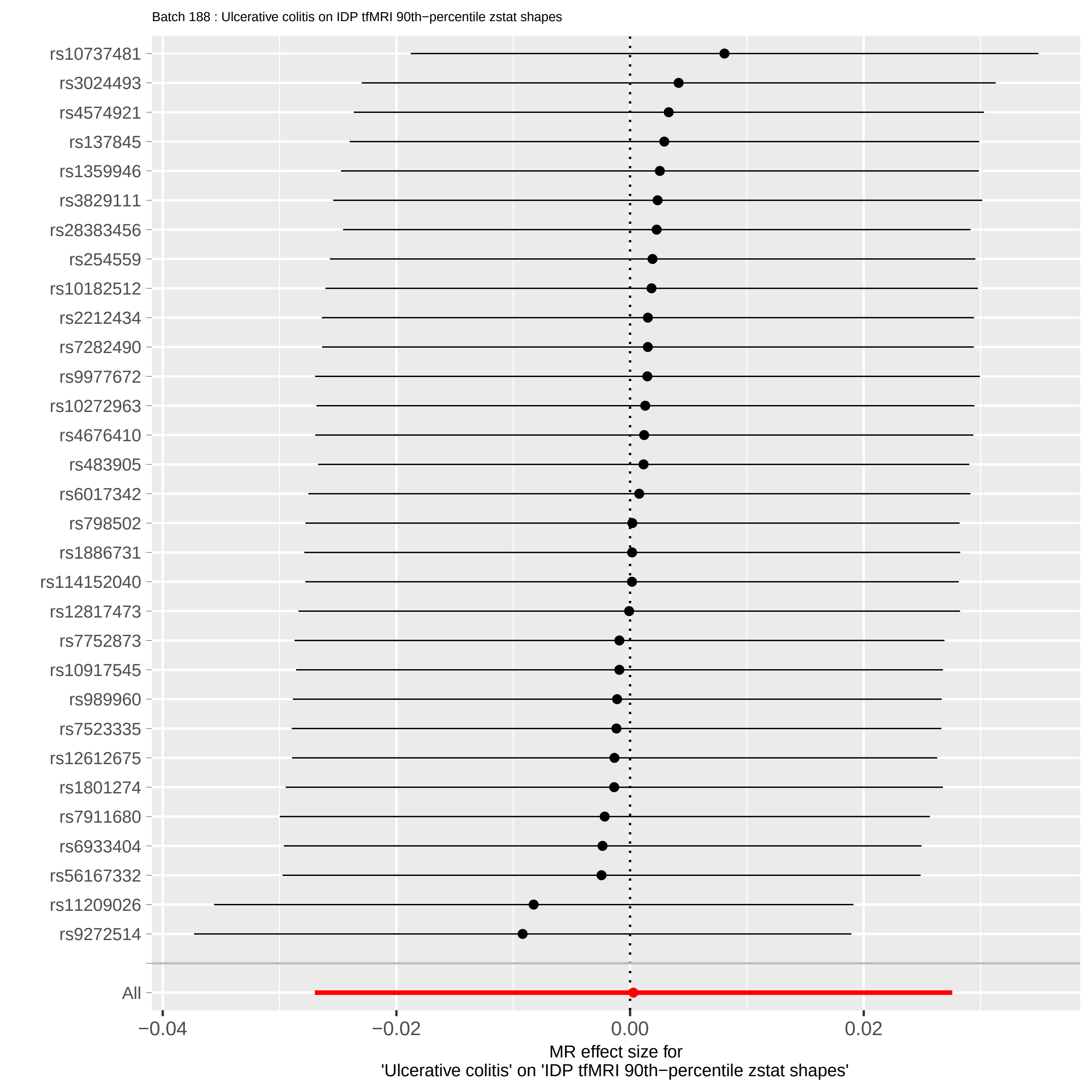

Batch 188 : Ulcerative colitis on IDP tfMRI 90th−percentile zstat shapes
rs10737481
rs3024493
rs4574921
rs137845
rs1359946
rs3829111
rs28383456
rs254559
rs10182512
rs2212434
rs7282490
rs9977672
rs10272963
rs4676410
rs483905
rs6017342
rs798502
rs1886731
rs114152040
rs12817473
rs7752873
rs10917545
rs989960
rs7523335
rs12612675
rs1801274
rs7911680
rs6933404
rs56167332
rs11209026
rs9272514
All
−0.04
−0.02
0.00
0.02
MR effect size for
'Ulcerative colitis' on 'IDP tfMRI 90th−percentile zstat shapes'

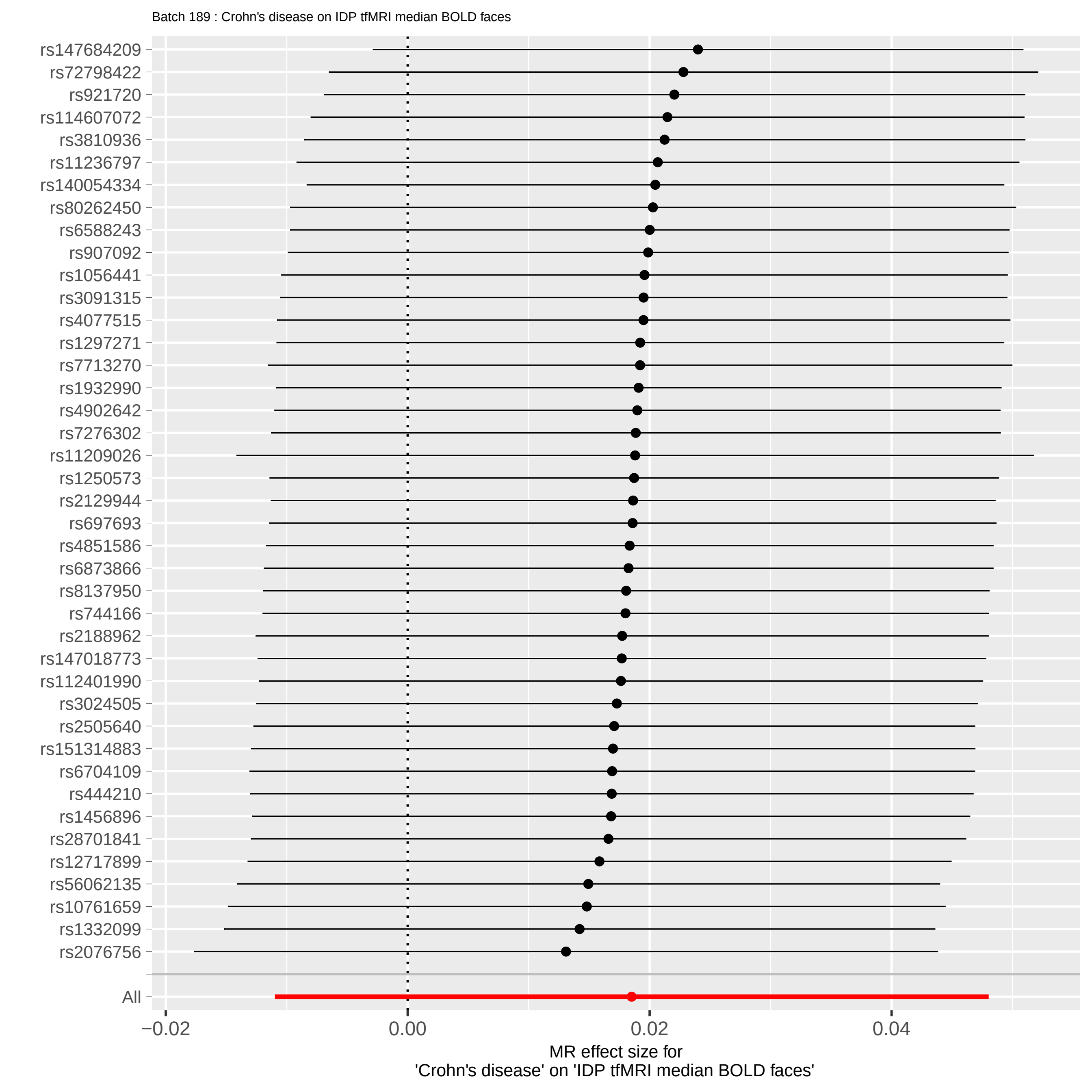

Batch 189 : Crohn's disease on IDP tfMRI median BOLD faces
rs147684209
rs72798422
rs921720
rs114607072
rs3810936
rs11236797
rs140054334
rs80262450
rs6588243
rs907092
rs1056441
rs3091315
rs4077515
rs1297271
rs7713270
rs1932990
rs4902642
rs7276302
rs11209026
rs1250573
rs2129944
rs697693
rs4851586
rs6873866
rs8137950
rs744166
rs2188962
rs147018773
rs112401990
rs3024505
rs2505640
rs151314883
rs6704109
rs444210
rs1456896
rs28701841
rs12717899
rs56062135
rs10761659
rs1332099
rs2076756
All
−0.02
0.00
0.02
0.04
MR effect size for
'Crohn's disease' on 'IDP tfMRI median BOLD faces'

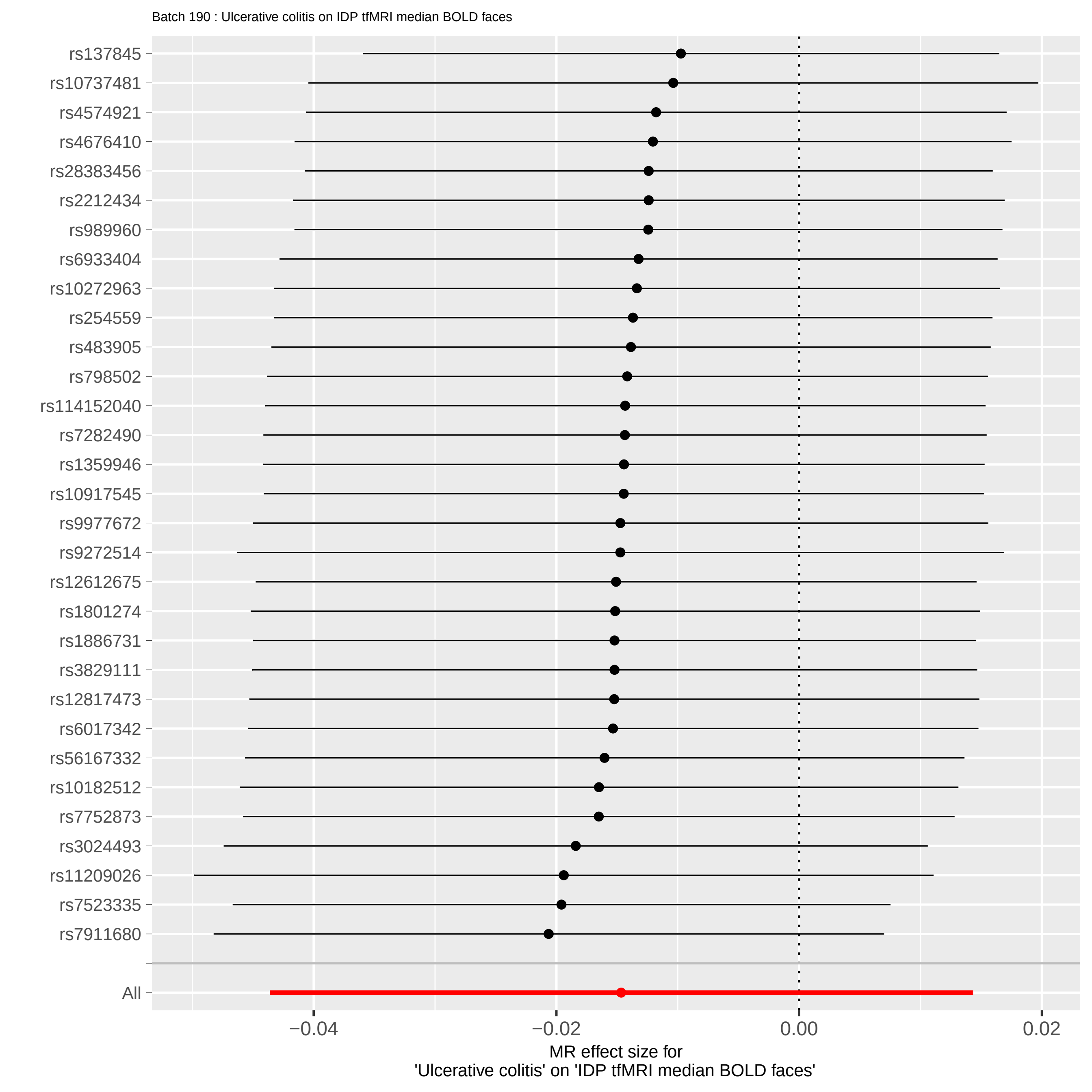
Batch 190 : Ulcerative colitis on IDP tfMRI median BOLD faces
rs137845
rs10737481
rs4574921
rs4676410
rs28383456
rs2212434
rs989960
rs6933404
rs10272963
rs254559
rs483905
rs798502
rs114152040
rs7282490
rs1359946
rs10917545
rs9977672
rs9272514
rs12612675
rs1801274
rs1886731
rs3829111
rs12817473
rs6017342
rs56167332
rs10182512
rs7752873
rs3024493
rs11209026
rs7523335
rs7911680
All
−0.04
−0.02
0.00
0.02
MR effect size for
'Ulcerative colitis' on 'IDP tfMRI median BOLD faces'

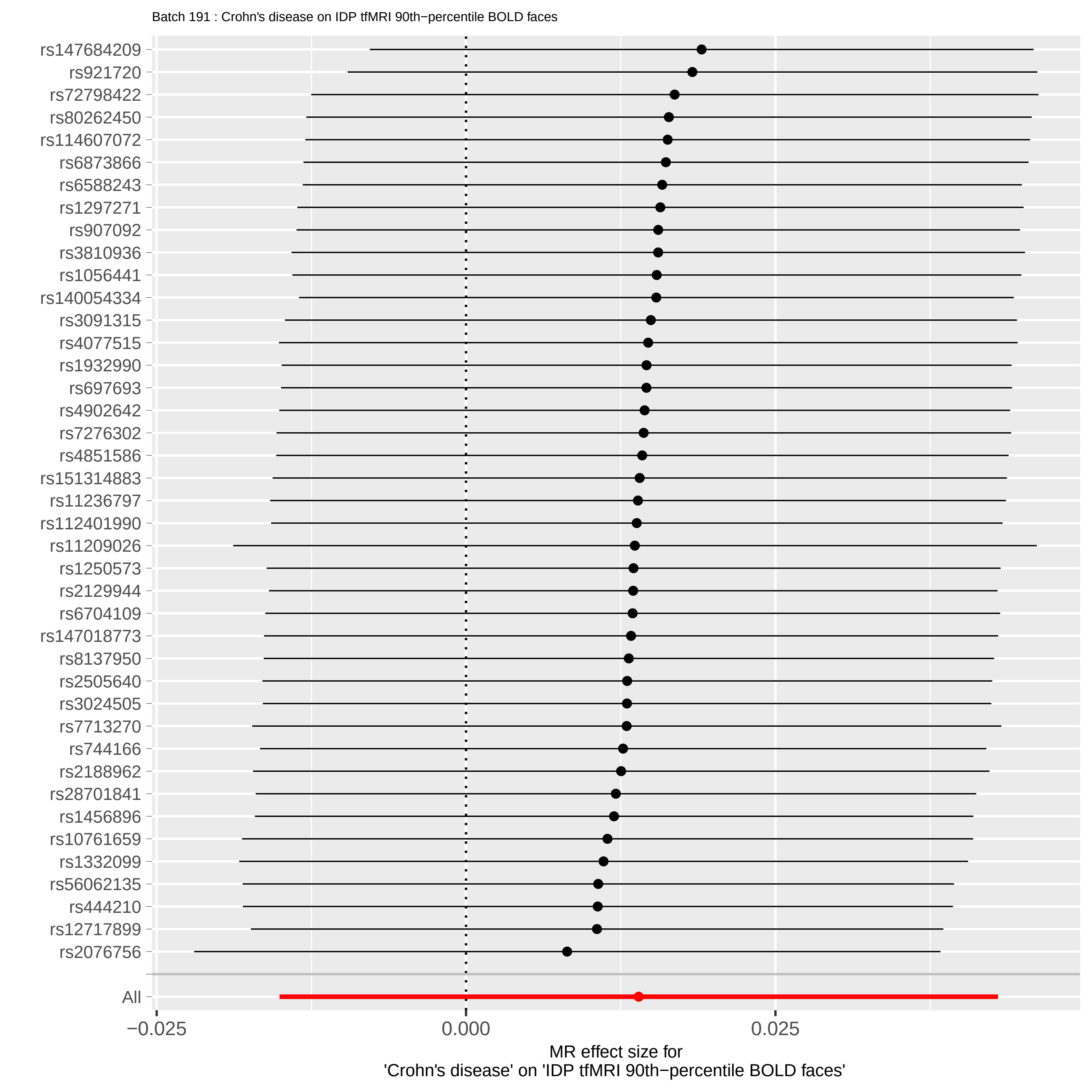

Batch 191 : Crohn's disease on IDP tfMRI 90th−percentile BOLD faces
rs147684209
rs921720
rs72798422
rs80262450
rs114607072
rs6873866
rs6588243
rs1297271
rs907092
rs3810936
rs1056441
rs140054334
rs3091315
rs4077515
rs1932990
rs697693
rs4902642
rs7276302
rs4851586
rs151314883
rs11236797
rs112401990
rs11209026
rs1250573
rs2129944
rs6704109
rs147018773
rs8137950
rs2505640
rs3024505
rs7713270
rs744166
rs2188962
rs28701841
rs1456896
rs10761659
rs1332099
rs56062135
rs444210
rs12717899
rs2076756
All
−0.025
0.000
0.025
MR effect size for
'Crohn's disease' on 'IDP tfMRI 90th−percentile BOLD faces'

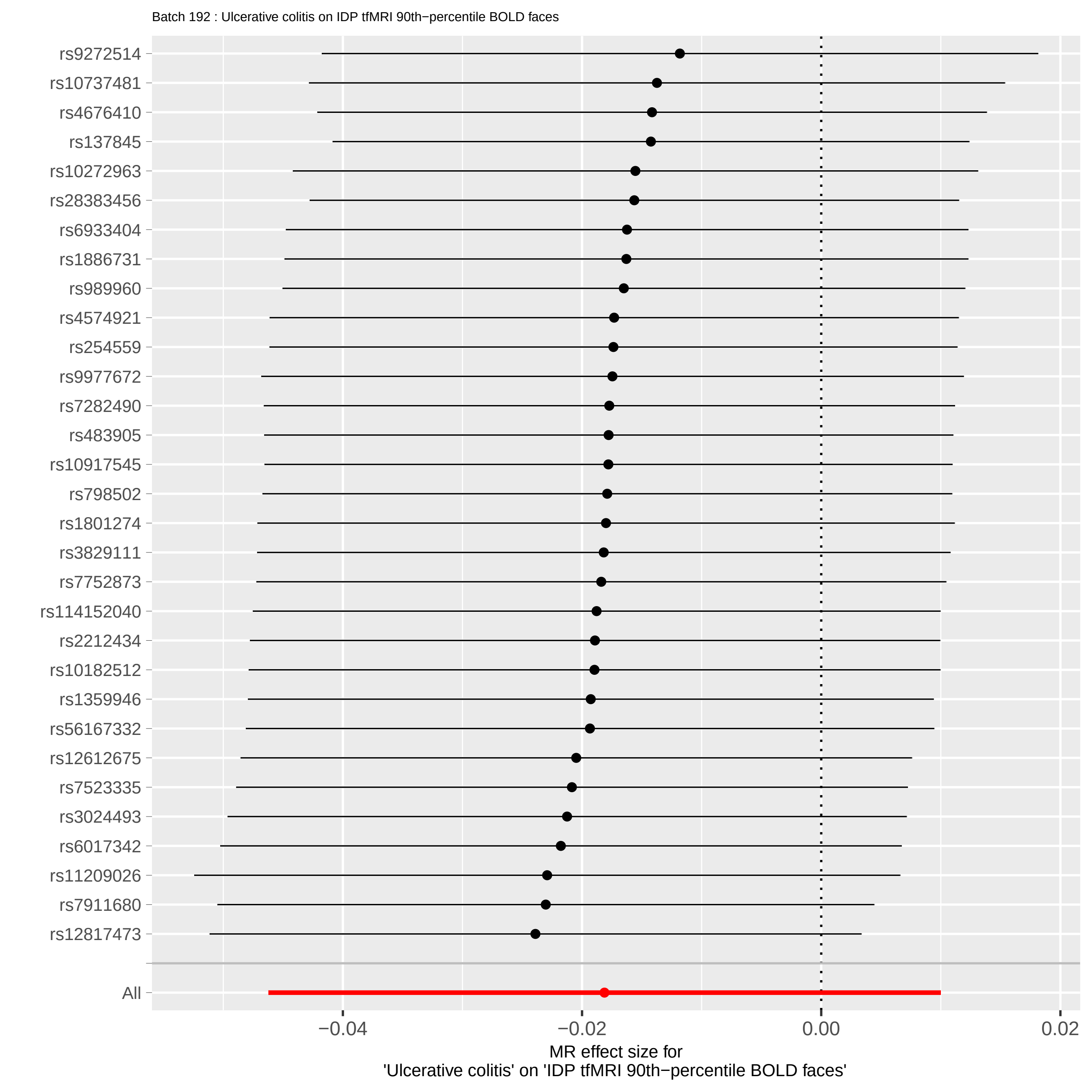

Batch 192 : Ulcerative colitis on IDP tfMRI 90th−percentile BOLD faces
rs9272514
rs10737481
rs4676410
rs137845
rs10272963
rs28383456
rs6933404
rs1886731
rs989960
rs4574921
rs254559
rs9977672
rs7282490
rs483905
rs10917545
rs798502
rs1801274
rs3829111
rs7752873
rs114152040
rs2212434
rs10182512
rs1359946
rs56167332
rs12612675
rs7523335
rs3024493
rs6017342
rs11209026
rs7911680
rs12817473
All
−0.04
−0.02
0.00
0.02
MR effect size for
'Ulcerative colitis' on 'IDP tfMRI 90th−percentile BOLD faces'

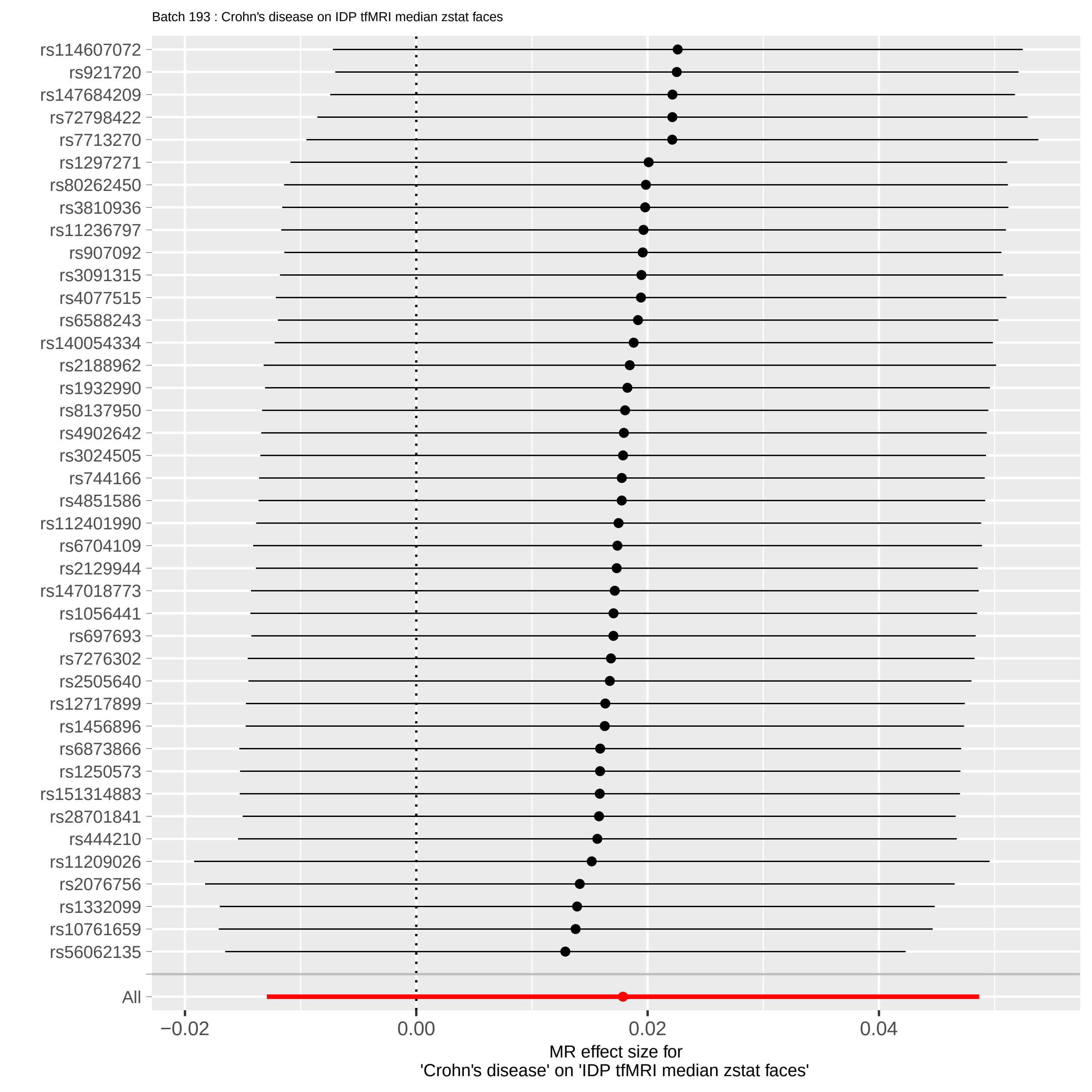

Batch 193 : Crohn's disease on IDP tfMRI median zstat faces
rs114607072
rs921720
rs147684209
rs72798422
rs7713270
rs1297271
rs80262450
rs3810936
rs11236797
rs907092
rs3091315
rs4077515
rs6588243
rs140054334
rs2188962
rs1932990
rs8137950
rs4902642
rs3024505
rs744166
rs4851586
rs112401990
rs6704109
rs2129944
rs147018773
rs1056441
rs697693
rs7276302
rs2505640
rs12717899
rs1456896
rs6873866
rs1250573
rs151314883
rs28701841
rs444210
rs11209026
rs2076756
rs1332099
rs10761659
rs56062135
All
−0.02
0.00
0.02
0.04
MR effect size for
'Crohn's disease' on 'IDP tfMRI median zstat faces'

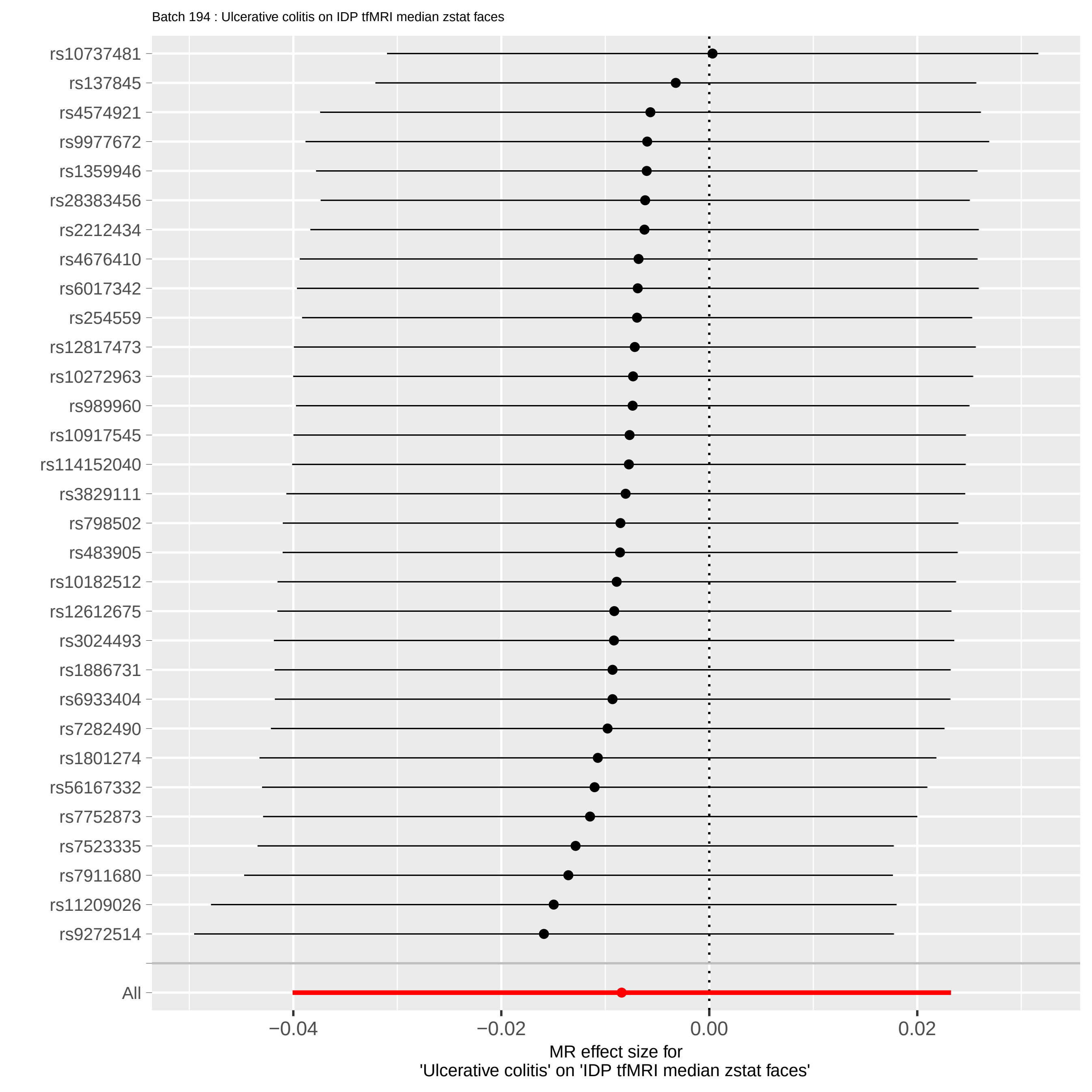
Batch 194 : Ulcerative colitis on IDP tfMRI median zstat faces
rs10737481
rs137845
rs4574921
rs9977672
rs1359946
rs28383456
rs2212434
rs4676410
rs6017342
rs254559
rs12817473
rs10272963
rs989960
rs10917545
rs114152040
rs3829111
rs798502
rs483905
rs10182512
rs12612675
rs3024493
rs1886731
rs6933404
rs7282490
rs1801274
rs56167332
rs7752873
rs7523335
rs7911680
rs11209026
rs9272514
All
−0.04
−0.02
0.00
0.02
MR effect size for
'Ulcerative colitis' on 'IDP tfMRI median zstat faces'

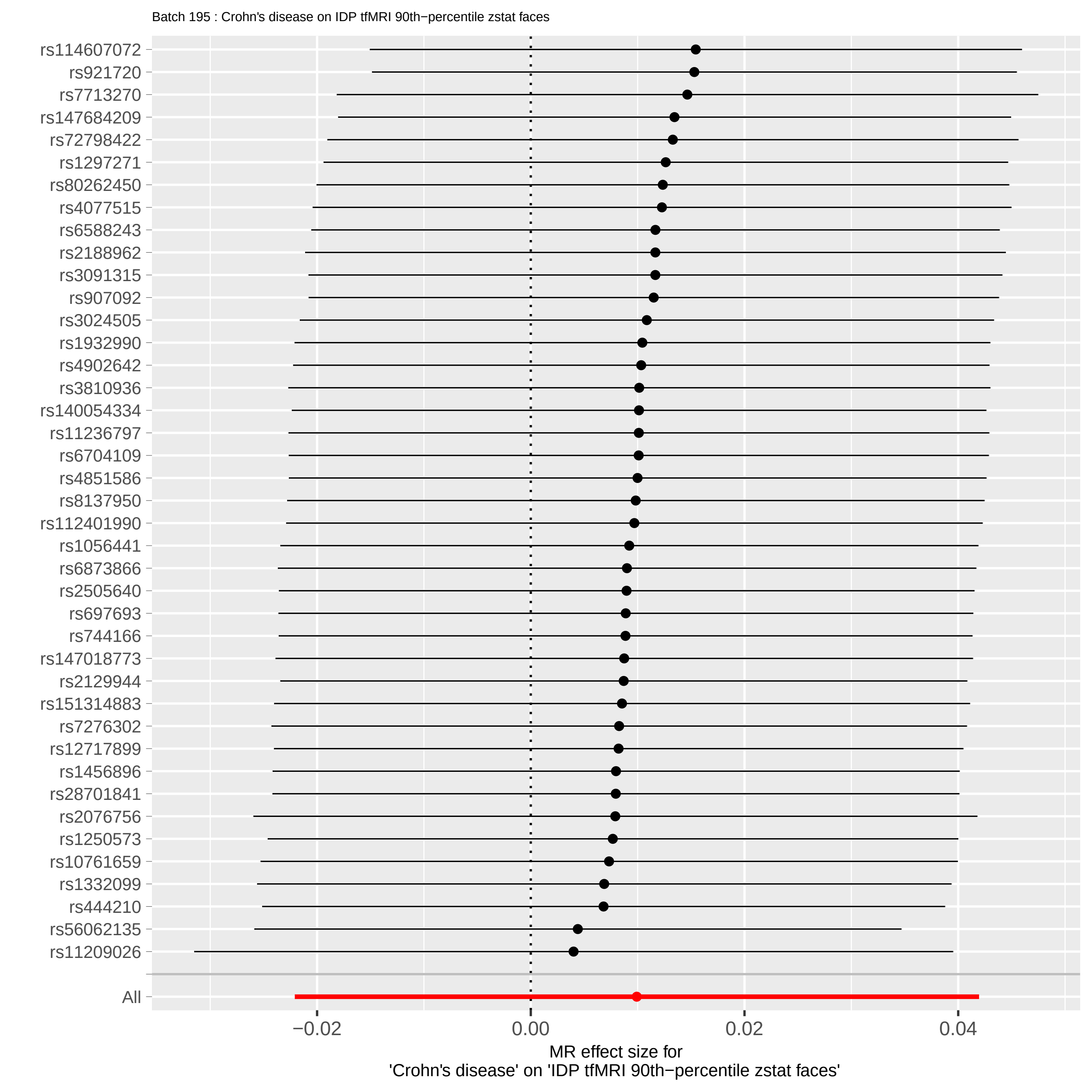
Batch 195 : Crohn's disease on IDP tfMRI 90th−percentile zstat faces
rs114607072
rs921720
rs7713270
rs147684209
rs72798422
rs1297271
rs80262450
rs4077515
rs6588243
rs2188962
rs3091315
rs907092
rs3024505
rs1932990
rs4902642
rs3810936
rs140054334
rs11236797
rs6704109
rs4851586
rs8137950
rs112401990
rs1056441
rs6873866
rs2505640
rs697693
rs744166
rs147018773
rs2129944
rs151314883
rs7276302
rs12717899
rs1456896
rs28701841
rs2076756
rs1250573
rs10761659
rs1332099
rs444210
rs56062135
rs11209026
All
−0.02
0.00
0.02
0.04
MR effect size for
'Crohn's disease' on 'IDP tfMRI 90th−percentile zstat faces'

Batch 196 : Ulcerative colitis on IDP tfMRI 90th−percentile zstat faces

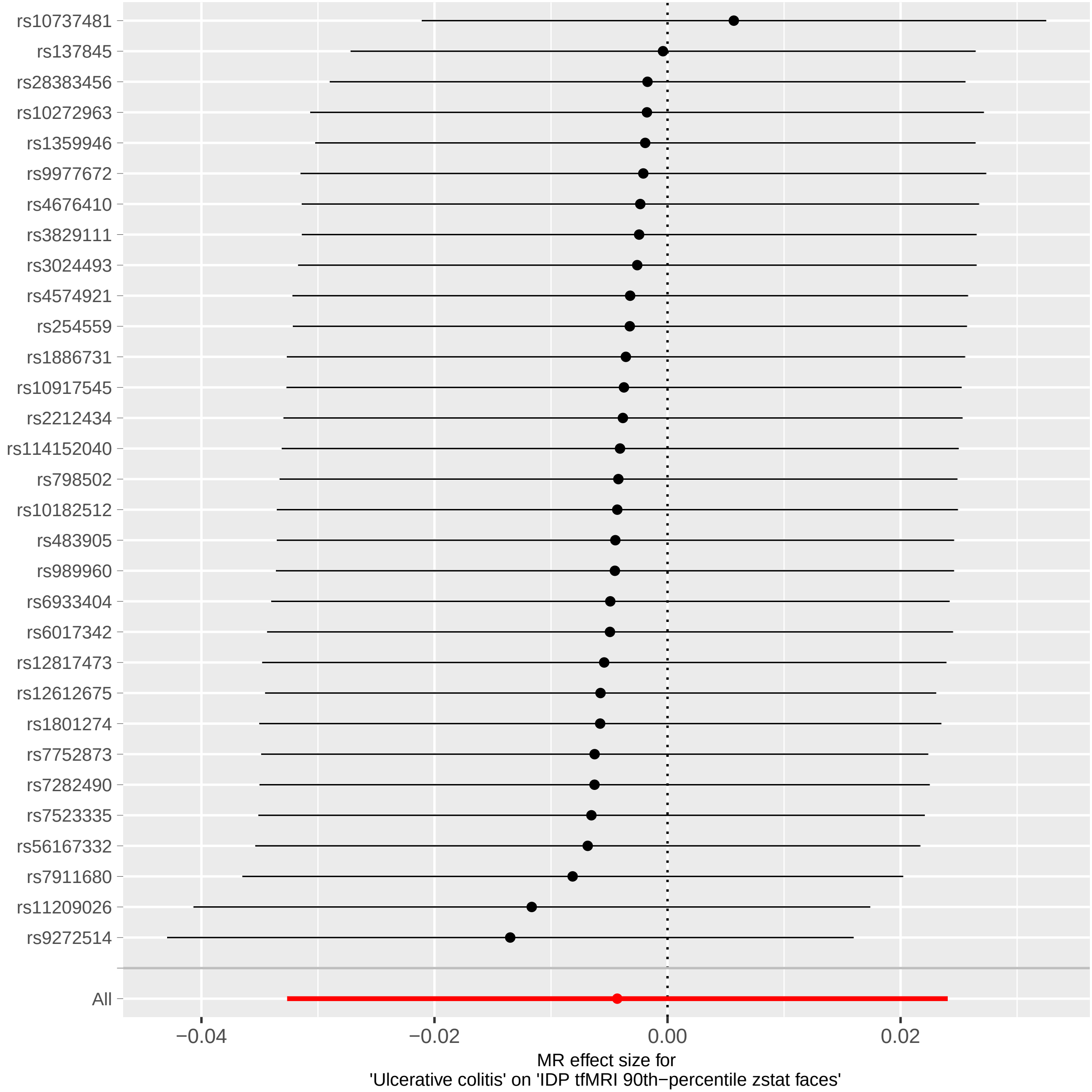

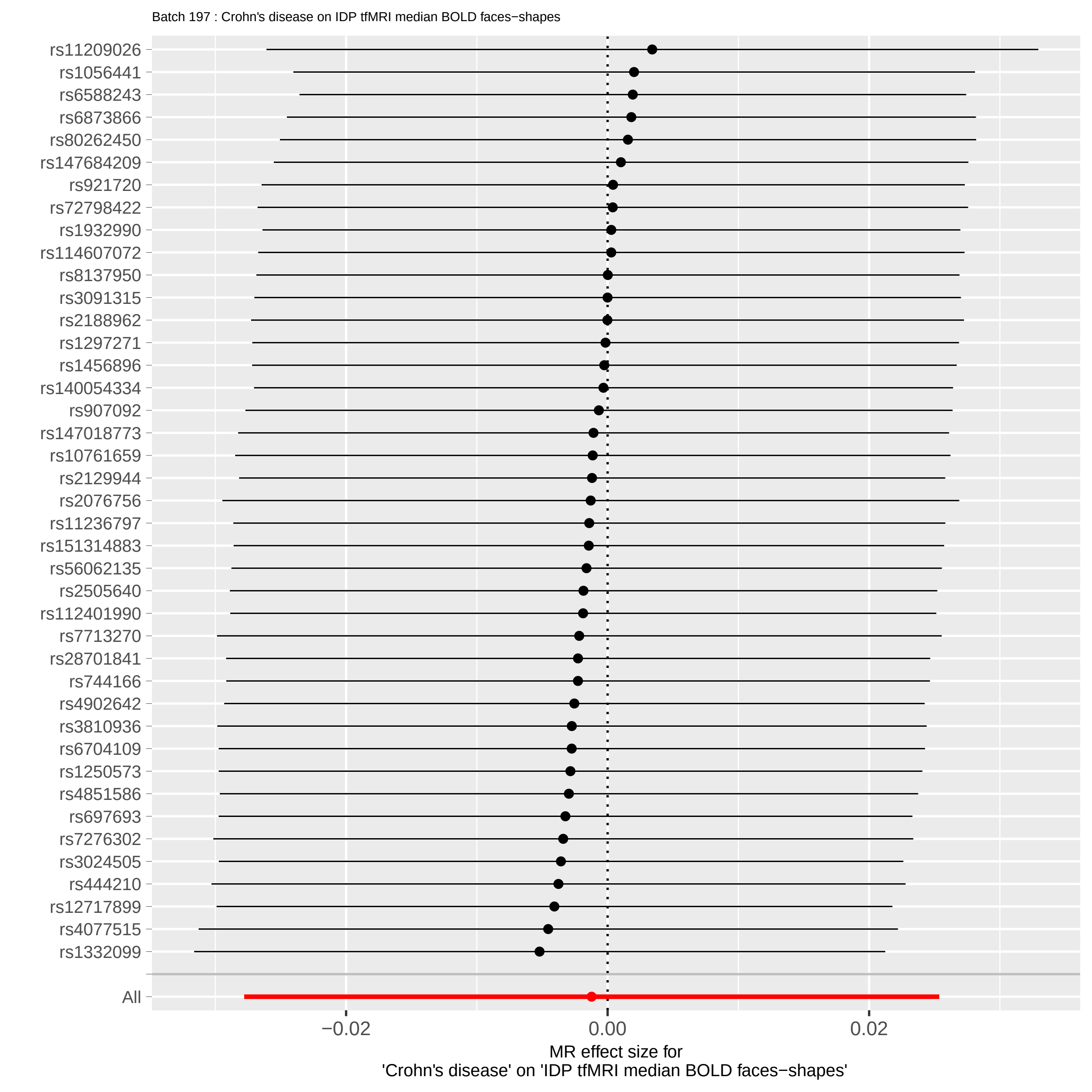

Batch 197 : Crohn's disease on IDP tfMRI median BOLD faces−shapes
rs11209026
rs1056441
rs6588243
rs6873866
rs80262450
rs147684209
rs921720
rs72798422
rs1932990
rs114607072
rs8137950
rs3091315
rs2188962
rs1297271
rs1456896
rs140054334
rs907092
rs147018773
rs10761659
rs2129944
rs2076756
rs11236797
rs151314883
rs56062135
rs2505640
rs112401990
rs7713270
rs28701841
rs744166
rs4902642
rs3810936
rs6704109
rs1250573
rs4851586
rs697693
rs7276302
rs3024505
rs444210
rs12717899
rs4077515
rs1332099
All
−0.02
0.00
0.02
MR effect size for
'Crohn's disease' on 'IDP tfMRI median BOLD faces−shapes'

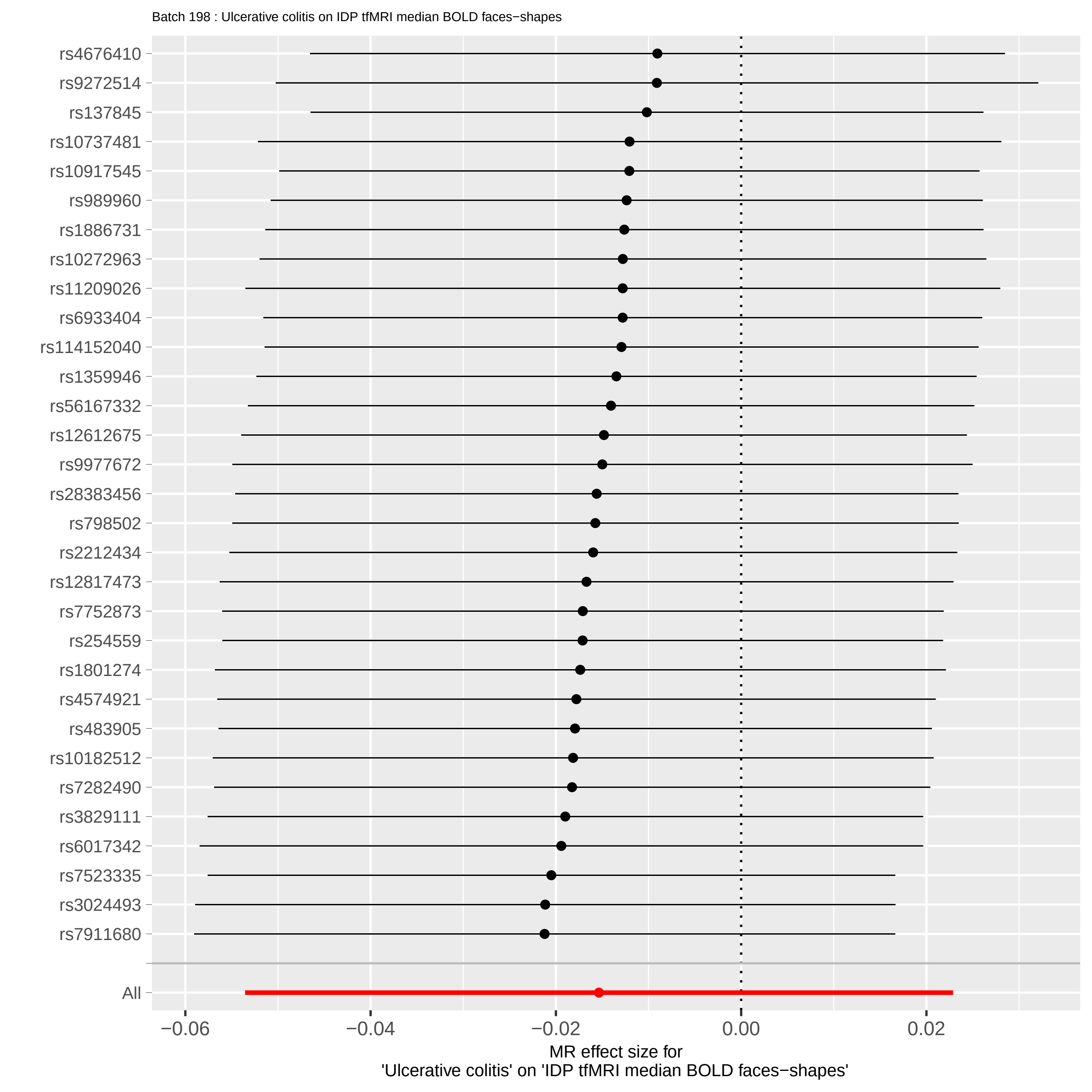

Batch 198 : Ulcerative colitis on IDP tfMRI median BOLD faces−shapes
rs4676410
rs9272514
rs137845
rs10737481
rs10917545
rs989960
rs1886731
rs10272963
rs11209026
rs6933404
rs114152040
rs1359946
rs56167332
rs12612675
rs9977672
rs28383456
rs798502
rs2212434
rs12817473
rs7752873
rs254559
rs1801274
rs4574921
rs483905
rs10182512
rs7282490
rs3829111
rs6017342
rs7523335
rs3024493
rs7911680
All
−0.06
−0.04
−0.02
0.00
0.02
MR effect size for
'Ulcerative colitis' on 'IDP tfMRI median BOLD faces−shapes'

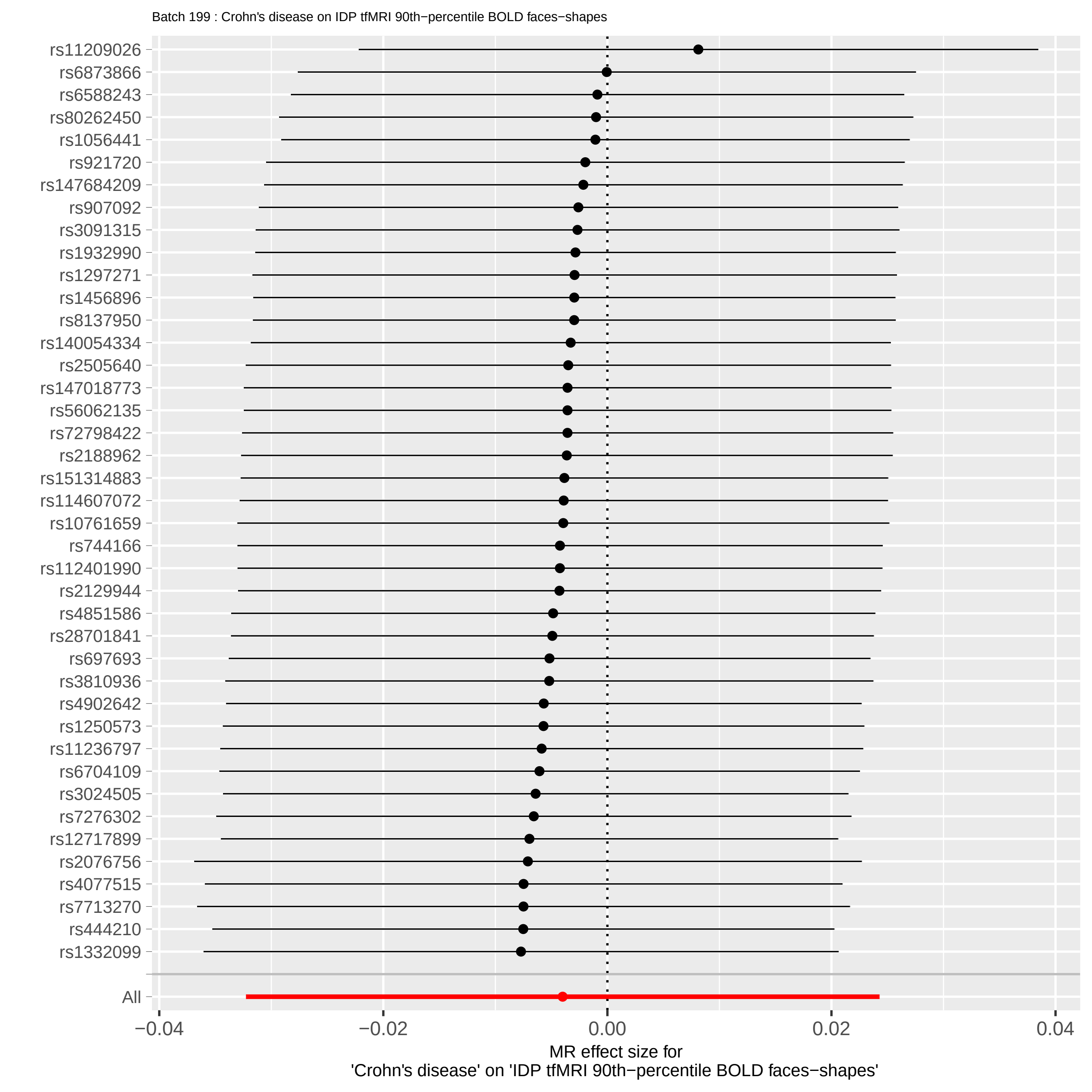

Batch 199 : Crohn's disease on IDP tfMRI 90th−percentile BOLD faces−shapes
rs11209026
rs6873866
rs6588243
rs80262450
rs1056441
rs921720
rs147684209
rs907092
rs3091315
rs1932990
rs1297271
rs1456896
rs8137950
rs140054334
rs2505640
rs147018773
rs56062135
rs72798422
rs2188962
rs151314883
rs114607072
rs10761659
rs744166
rs112401990
rs2129944
rs4851586
rs28701841
rs697693
rs3810936
rs4902642
rs1250573
rs11236797
rs6704109
rs3024505
rs7276302
rs12717899
rs2076756
rs4077515
rs7713270
rs444210
rs1332099
All
−0.04
−0.02
0.00
0.02
0.04
MR effect size for
'Crohn's disease' on 'IDP tfMRI 90th−percentile BOLD faces−shapes'

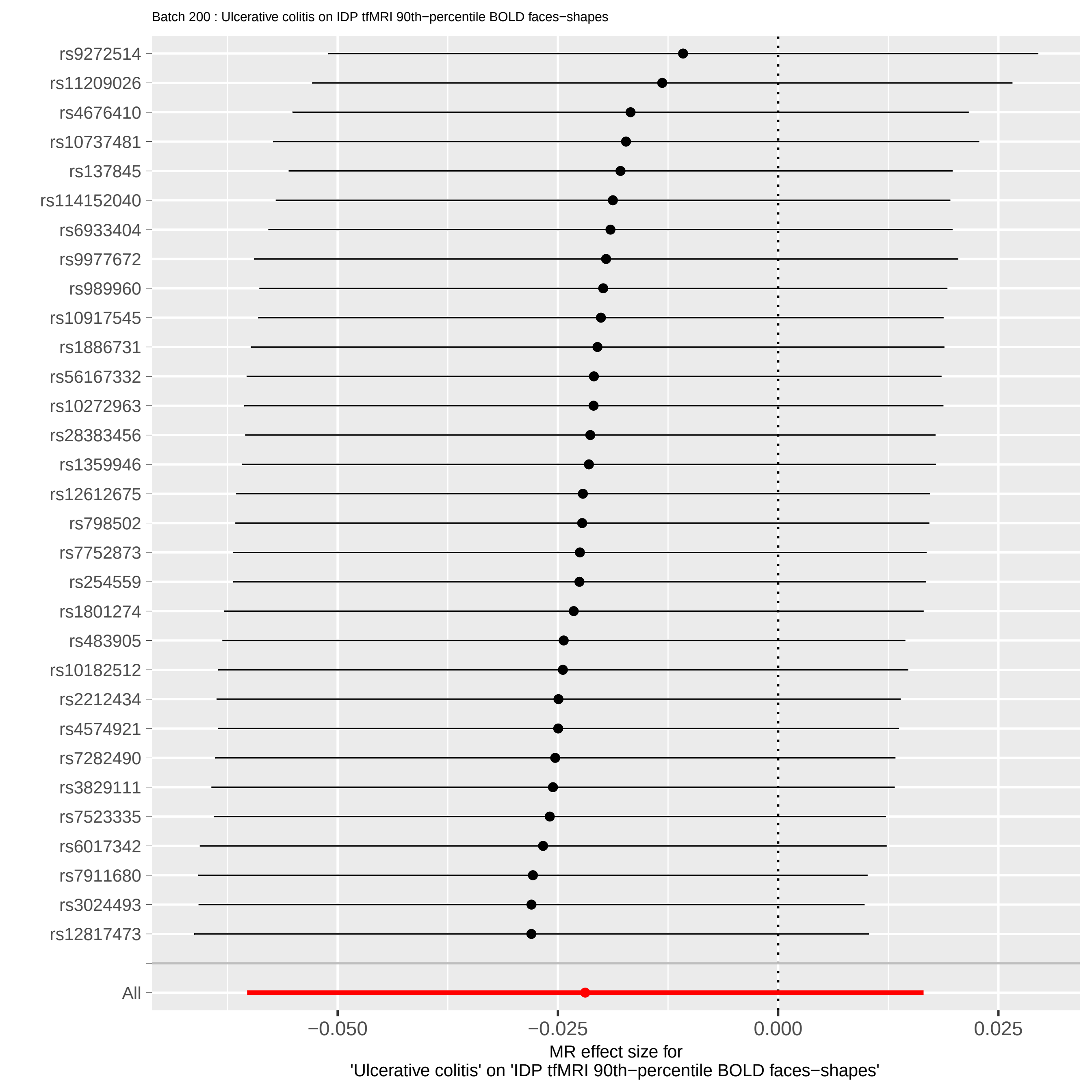

Batch 200 : Ulcerative colitis on IDP tfMRI 90th−percentile BOLD faces−shapes
rs9272514
rs11209026
rs4676410
rs10737481
rs137845
rs114152040
rs6933404
rs9977672
rs989960
rs10917545
rs1886731
rs56167332
rs10272963
rs28383456
rs1359946
rs12612675
rs798502
rs7752873
rs254559
rs1801274
rs483905
rs10182512
rs2212434
rs4574921
rs7282490
rs3829111
rs7523335
rs6017342
rs7911680
rs3024493
rs12817473
All
−0.050
−0.025
0.000
0.025
MR effect size for
'Ulcerative colitis' on 'IDP tfMRI 90th−percentile BOLD faces−shapes'

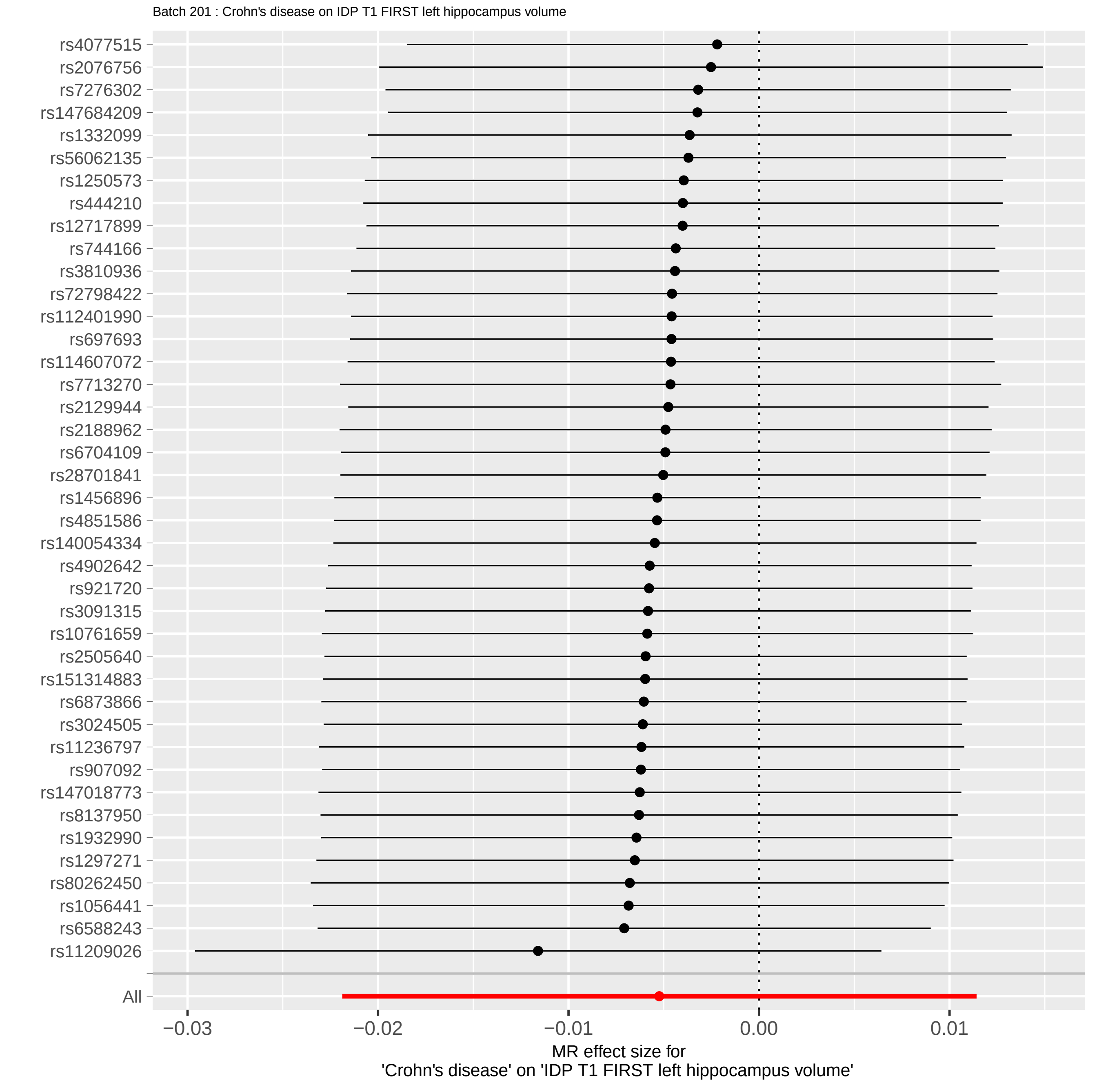

Batch 201 : Crohn's disease on IDP T1 FIRST left hippocampus volume
rs4077515
rs2076756
rs7276302
rs147684209
rs1332099
rs56062135
rs1250573
rs444210
rs12717899
rs744166
rs3810936
rs72798422
rs112401990
rs697693
rs114607072
rs7713270
rs2129944
rs2188962
rs6704109
rs28701841
rs1456896
rs4851586
rs140054334
rs4902642
rs921720
rs3091315
rs10761659
rs2505640
rs151314883
rs6873866
rs3024505
rs11236797
rs907092
rs147018773
rs8137950
rs1932990
rs1297271
rs80262450
rs1056441
rs6588243
rs11209026
All
−0.03
−0.02
−0.01
0.00
0.01
MR effect size for
'Crohn's disease' on 'IDP T1 FIRST left hippocampus volume'

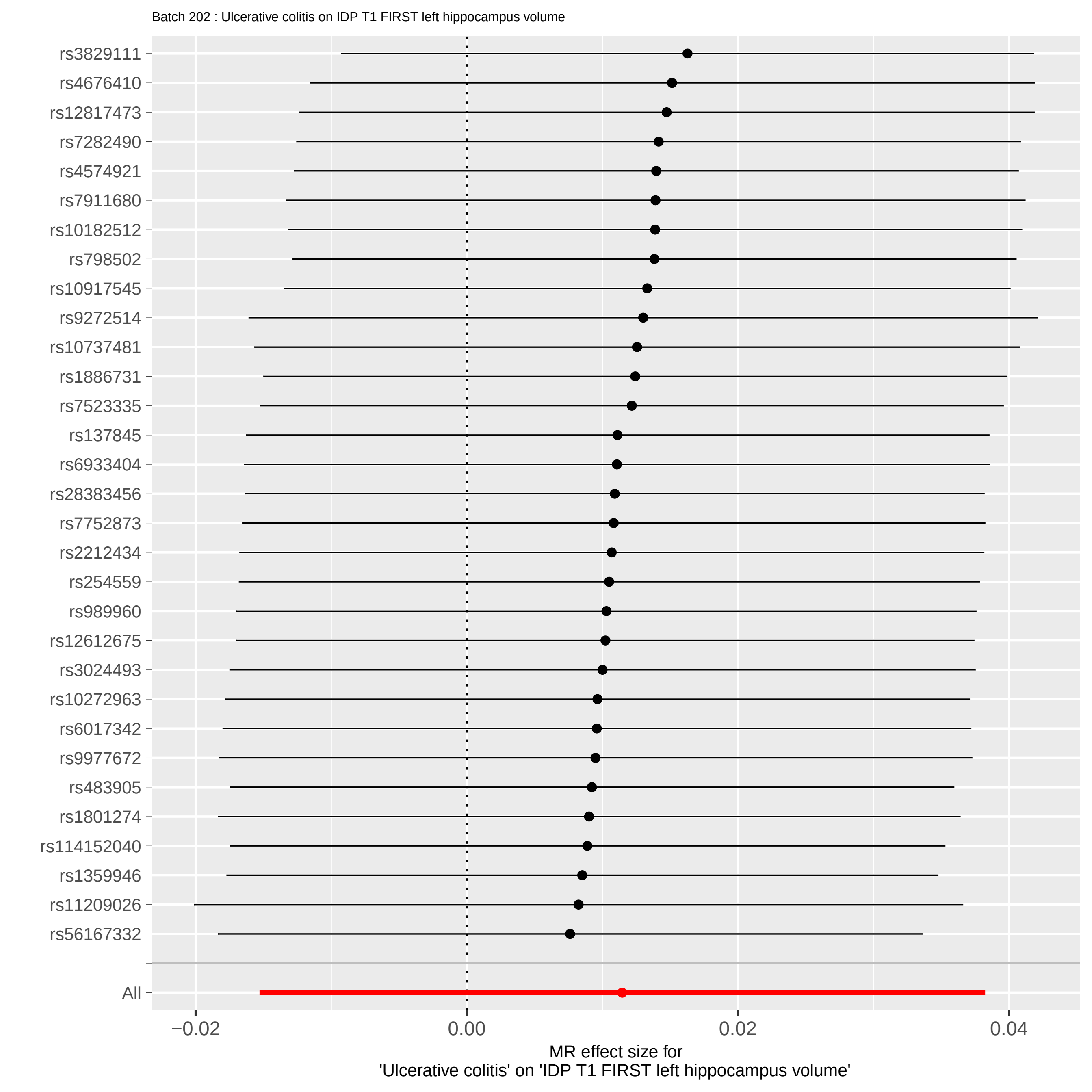

Batch 202 : Ulcerative colitis on IDP T1 FIRST left hippocampus volume
rs3829111
rs4676410
rs12817473
rs7282490
rs4574921
rs7911680
rs10182512
rs798502
rs10917545
rs9272514
rs10737481
rs1886731
rs7523335
rs137845
rs6933404
rs28383456
rs7752873
rs2212434
rs254559
rs989960
rs12612675
rs3024493
rs10272963
rs6017342
rs9977672
rs483905
rs1801274
rs114152040
rs1359946
rs11209026
rs56167332
All
−0.02
0.00
0.02
0.04
MR effect size for
'Ulcerative colitis' on 'IDP T1 FIRST left hippocampus volume'

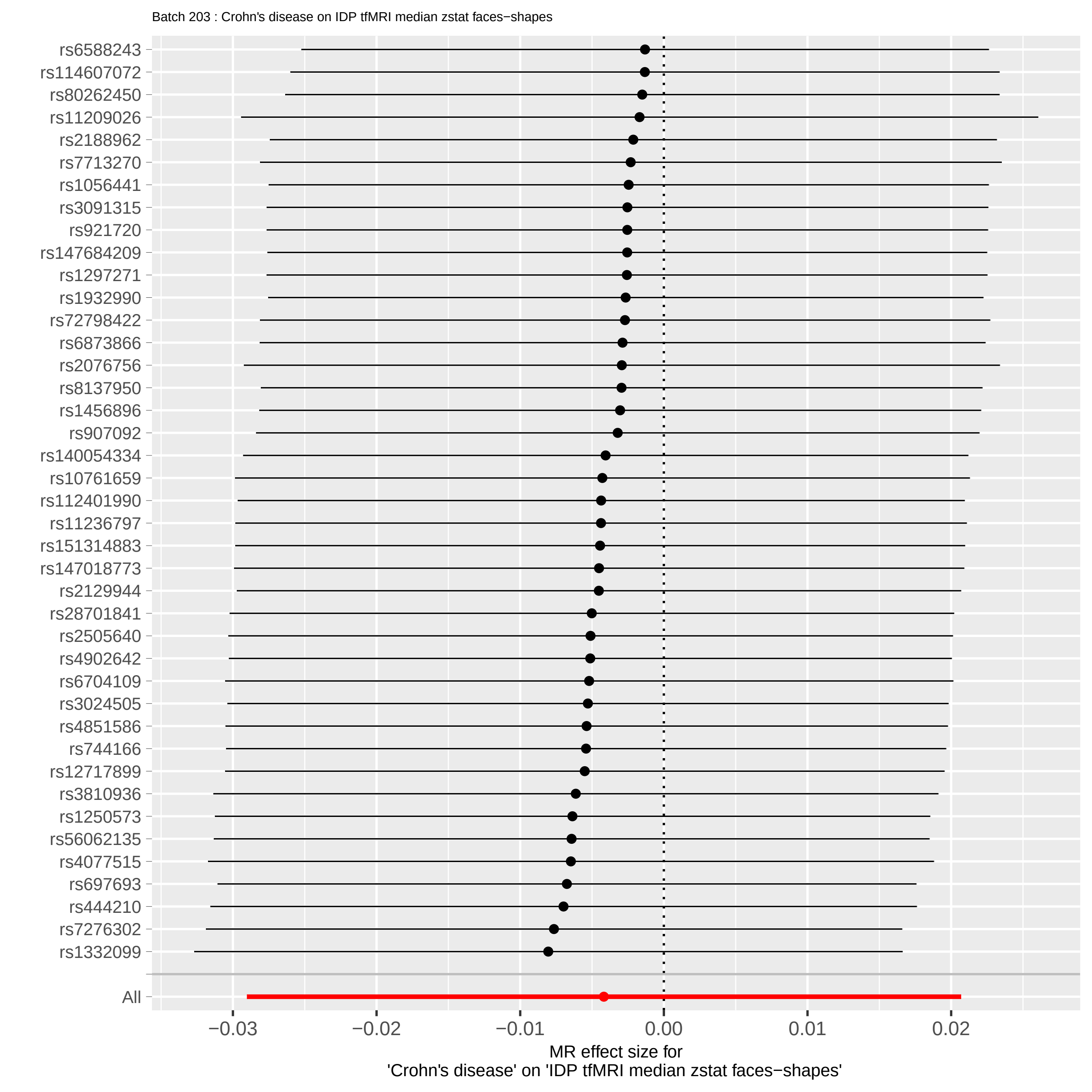
Batch 203 : Crohn's disease on IDP tfMRI median zstat faces−shapes
rs6588243
rs114607072
rs80262450
rs11209026
rs2188962
rs7713270
rs1056441
rs3091315
rs921720
rs147684209
rs1297271
rs1932990
rs72798422
rs6873866
rs2076756
rs8137950
rs1456896
rs907092
rs140054334
rs10761659
rs112401990
rs11236797
rs151314883
rs147018773
rs2129944
rs28701841
rs2505640
rs4902642
rs6704109
rs3024505
rs4851586
rs744166
rs12717899
rs3810936
rs1250573
rs56062135
rs4077515
rs697693
rs444210
rs7276302
rs1332099
All
−0.03
−0.02
−0.01
0.00
0.01
0.02
MR effect size for
'Crohn's disease' on 'IDP tfMRI median zstat faces−shapes'

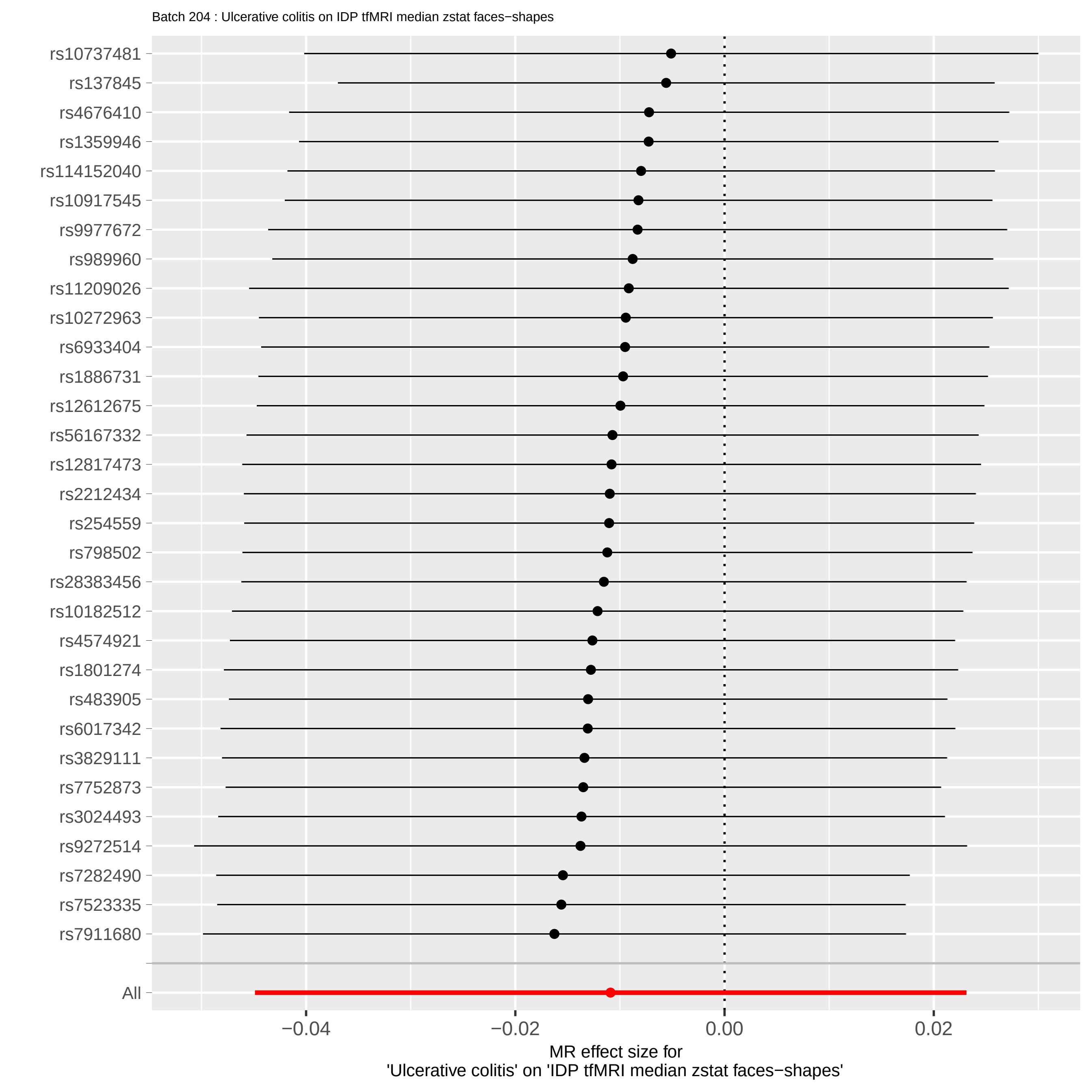

Batch 204 : Ulcerative colitis on IDP tfMRI median zstat faces−shapes
rs10737481
rs137845
rs4676410
rs1359946
rs114152040
rs10917545
rs9977672
rs989960
rs11209026
rs10272963
rs6933404
rs1886731
rs12612675
rs56167332
rs12817473
rs2212434
rs254559
rs798502
rs28383456
rs10182512
rs4574921
rs1801274
rs483905
rs6017342
rs3829111
rs7752873
rs3024493
rs9272514
rs7282490
rs7523335
rs7911680
All
−0.04
−0.02
0.00
0.02
MR effect size for
'Ulcerative colitis' on 'IDP tfMRI median zstat faces−shapes'

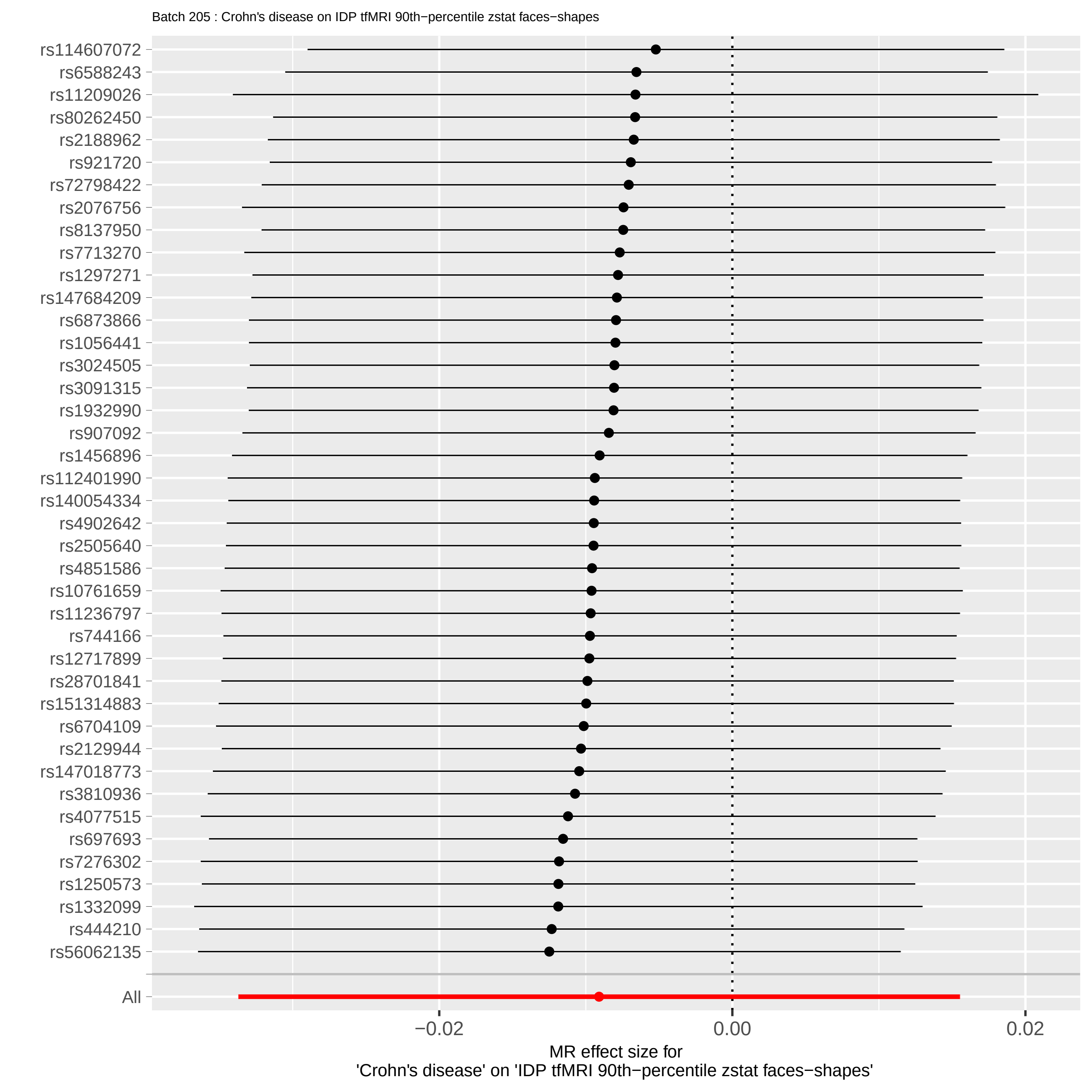

Batch 205 : Crohn's disease on IDP tfMRI 90th−percentile zstat faces−shapes
rs114607072
rs6588243
rs11209026
rs80262450
rs2188962
rs921720
rs72798422
rs2076756
rs8137950
rs7713270
rs1297271
rs147684209
rs6873866
rs1056441
rs3024505
rs3091315
rs1932990
rs907092
rs1456896
rs112401990
rs140054334
rs4902642
rs2505640
rs4851586
rs10761659
rs11236797
rs744166
rs12717899
rs28701841
rs151314883
rs6704109
rs2129944
rs147018773
rs3810936
rs4077515
rs697693
rs7276302
rs1250573
rs1332099
rs444210
rs56062135
All
−0.02
0.00
0.02
MR effect size for
'Crohn's disease' on 'IDP tfMRI 90th−percentile zstat faces−shapes'

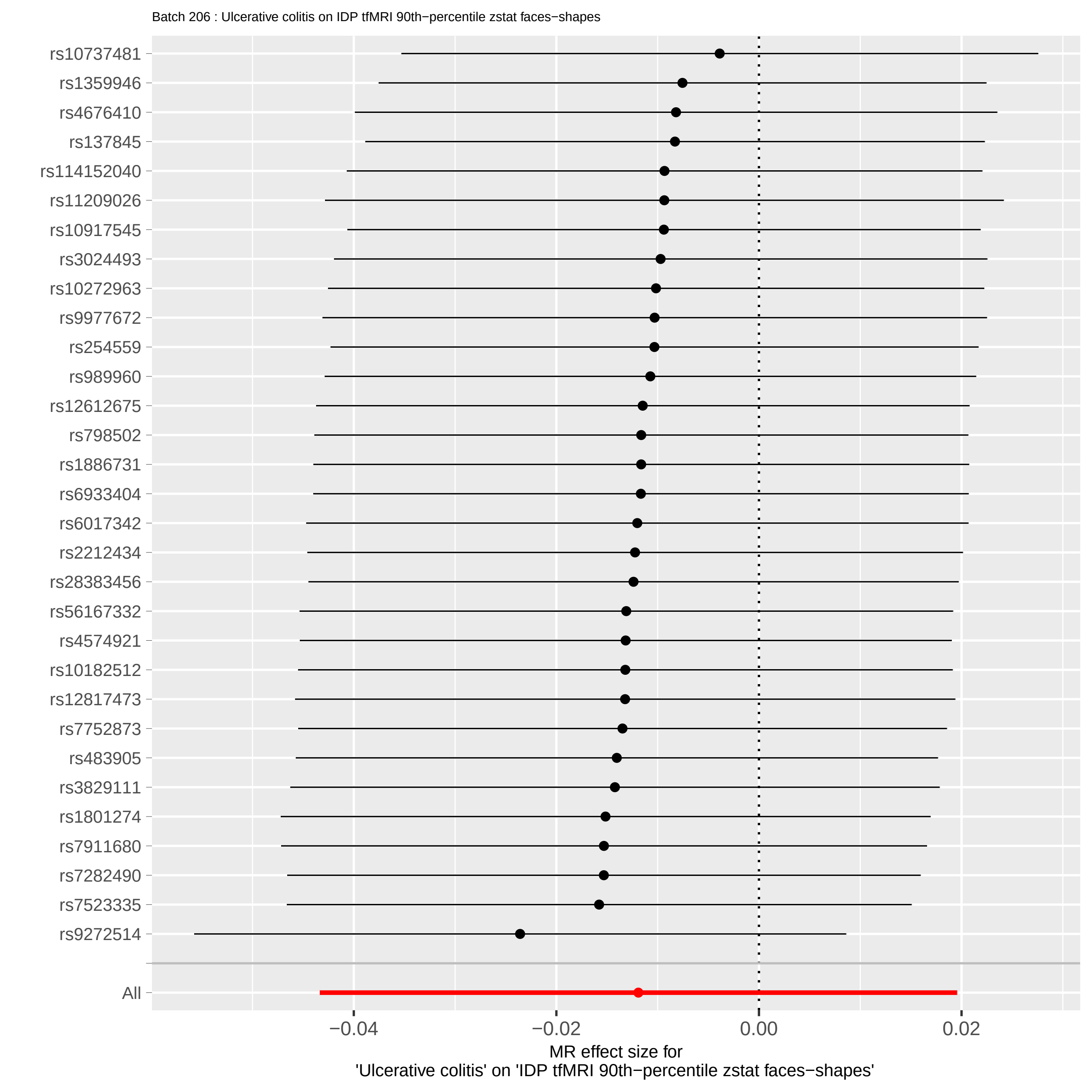

Batch 206 : Ulcerative colitis on IDP tfMRI 90th−percentile zstat faces−shapes
rs10737481
rs1359946
rs4676410
rs137845
rs114152040
rs11209026
rs10917545
rs3024493
rs10272963
rs9977672
rs254559
rs989960
rs12612675
rs798502
rs1886731
rs6933404
rs6017342
rs2212434
rs28383456
rs56167332
rs4574921
rs10182512
rs12817473
rs7752873
rs483905
rs3829111
rs1801274
rs7911680
rs7282490
rs7523335
rs9272514
All
−0.04
−0.02
0.00
0.02
MR effect size for
'Ulcerative colitis' on 'IDP tfMRI 90th−percentile zstat faces−shapes'

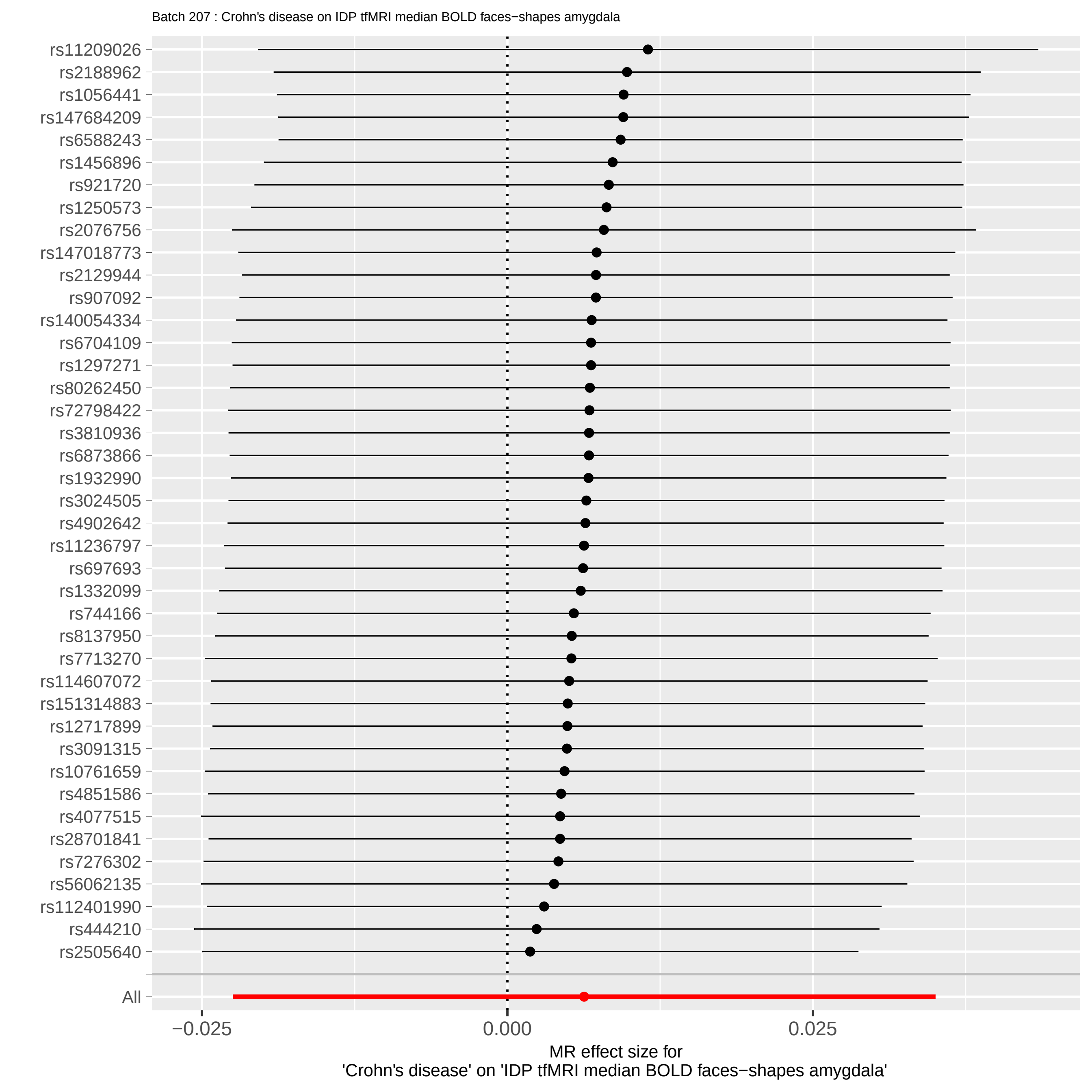
Batch 207 : Crohn's disease on IDP tfMRI median BOLD faces−shapes amygdala
rs11209026
rs2188962
rs1056441
rs147684209
rs6588243
rs1456896
rs921720
rs1250573
rs2076756
rs147018773
rs2129944
rs907092
rs140054334
rs6704109
rs1297271
rs80262450
rs72798422
rs3810936
rs6873866
rs1932990
rs3024505
rs4902642
rs11236797
rs697693
rs1332099
rs744166
rs8137950
rs7713270
rs114607072
rs151314883
rs12717899
rs3091315
rs10761659
rs4851586
rs4077515
rs28701841
rs7276302
rs56062135
rs112401990
rs444210
rs2505640
All
−0.025
0.000
0.025
MR effect size for
'Crohn's disease' on 'IDP tfMRI median BOLD faces−shapes amygdala'

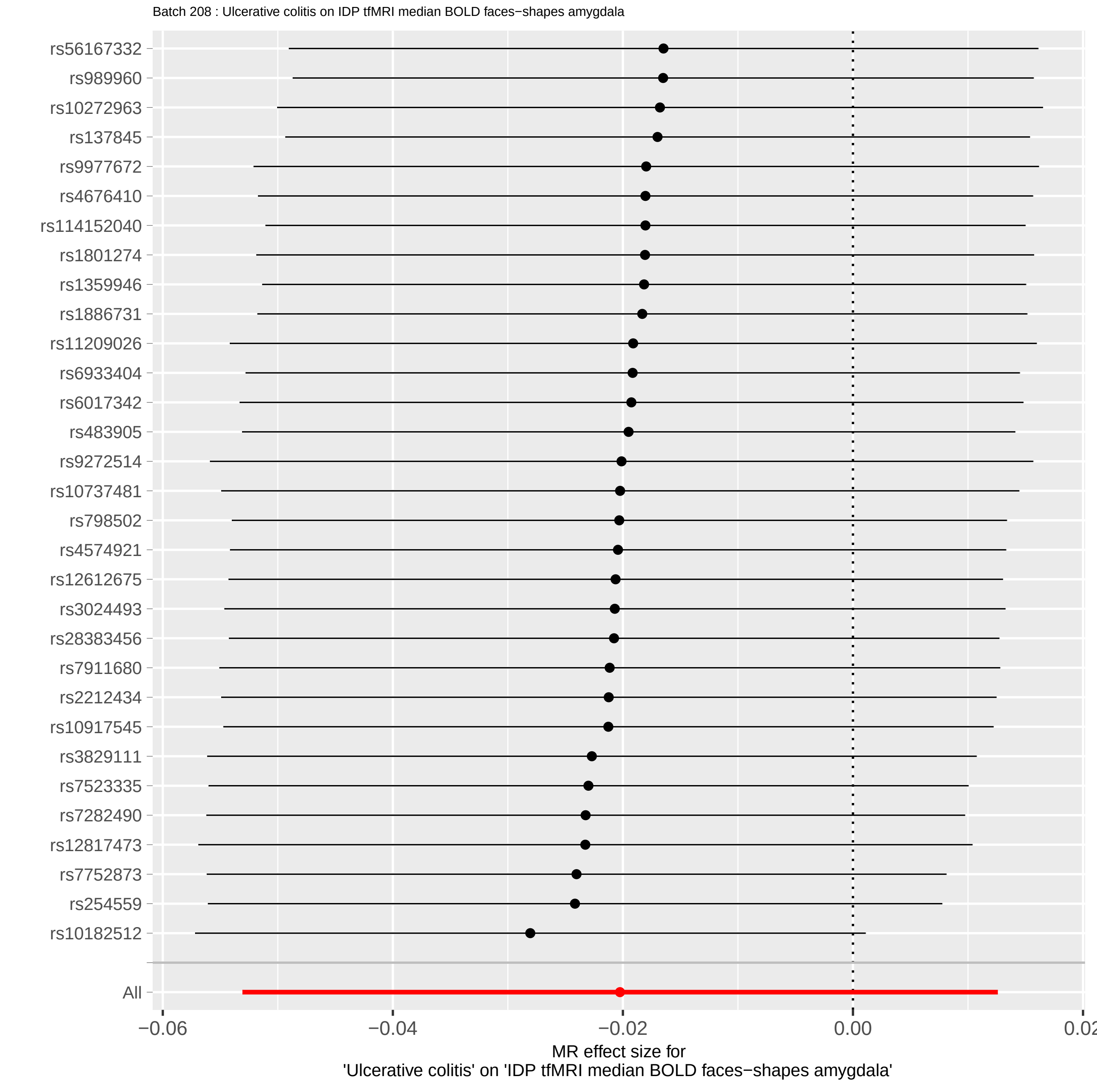

Batch 208 : Ulcerative colitis on IDP tfMRI median BOLD faces−shapes amygdala
rs56167332
rs989960
rs10272963
rs137845
rs9977672
rs4676410
rs114152040
rs1801274
rs1359946
rs1886731
rs11209026
rs6933404
rs6017342
rs483905
rs9272514
rs10737481
rs798502
rs4574921
rs12612675
rs3024493
rs28383456
rs7911680
rs2212434
rs10917545
rs3829111
rs7523335
rs7282490
rs12817473
rs7752873
rs254559
rs10182512
All
−0.06
−0.04
−0.02
0.00
0.02
MR effect size for
'Ulcerative colitis' on 'IDP tfMRI median BOLD faces−shapes amygdala'

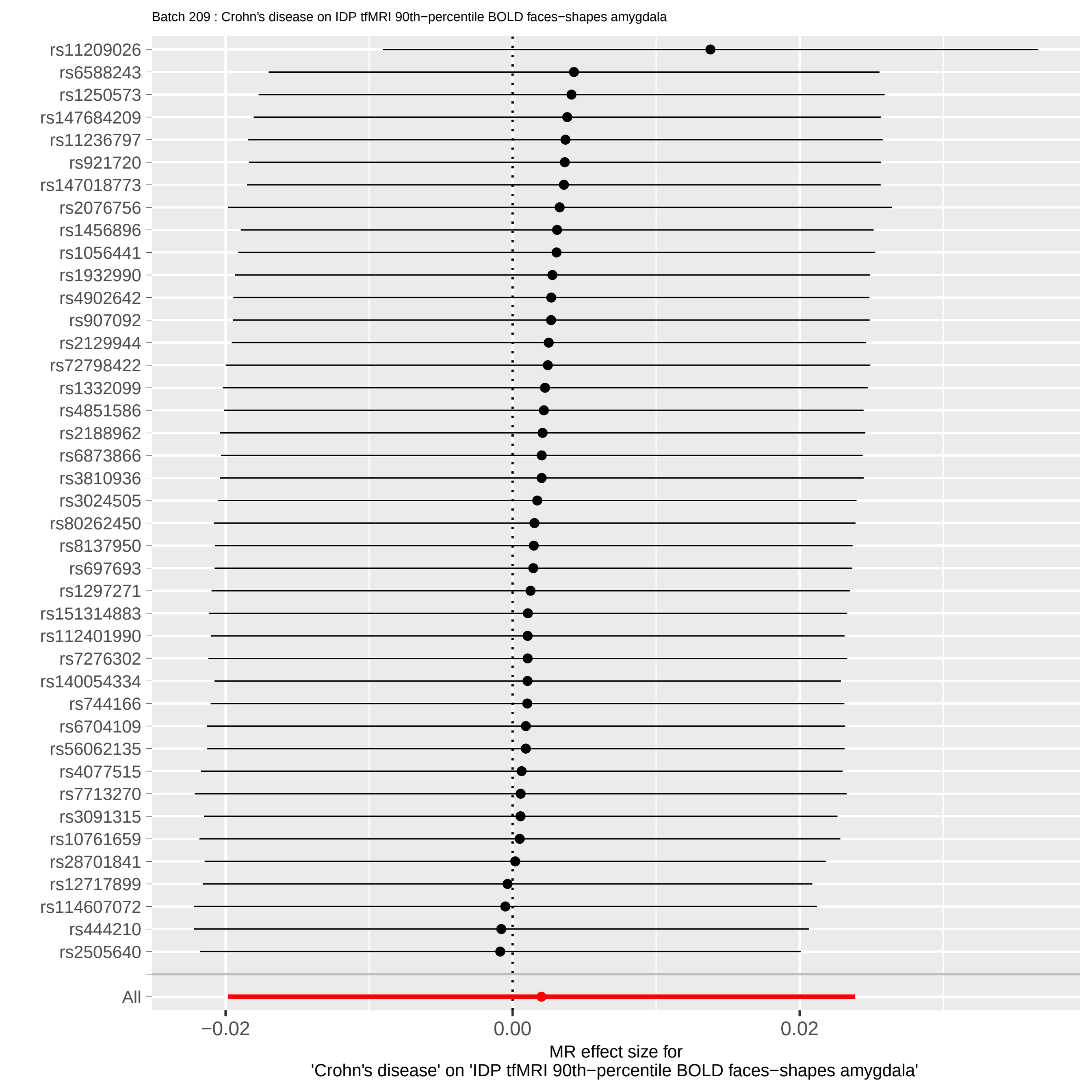

Batch 209 : Crohn's disease on IDP tfMRI 90th−percentile BOLD faces−shapes amygdala
rs11209026
rs6588243
rs1250573
rs147684209
rs11236797
rs921720
rs147018773
rs2076756
rs1456896
rs1056441
rs1932990
rs4902642
rs907092
rs2129944
rs72798422
rs1332099
rs4851586
rs2188962
rs6873866
rs3810936
rs3024505
rs80262450
rs8137950
rs697693
rs1297271
rs151314883
rs112401990
rs7276302
rs140054334
rs744166
rs6704109
rs56062135
rs4077515
rs7713270
rs3091315
rs10761659
rs28701841
rs12717899
rs114607072
rs444210
rs2505640
All
−0.02
0.00
0.02
MR effect size for
'Crohn's disease' on 'IDP tfMRI 90th−percentile BOLD faces−shapes amygdala'

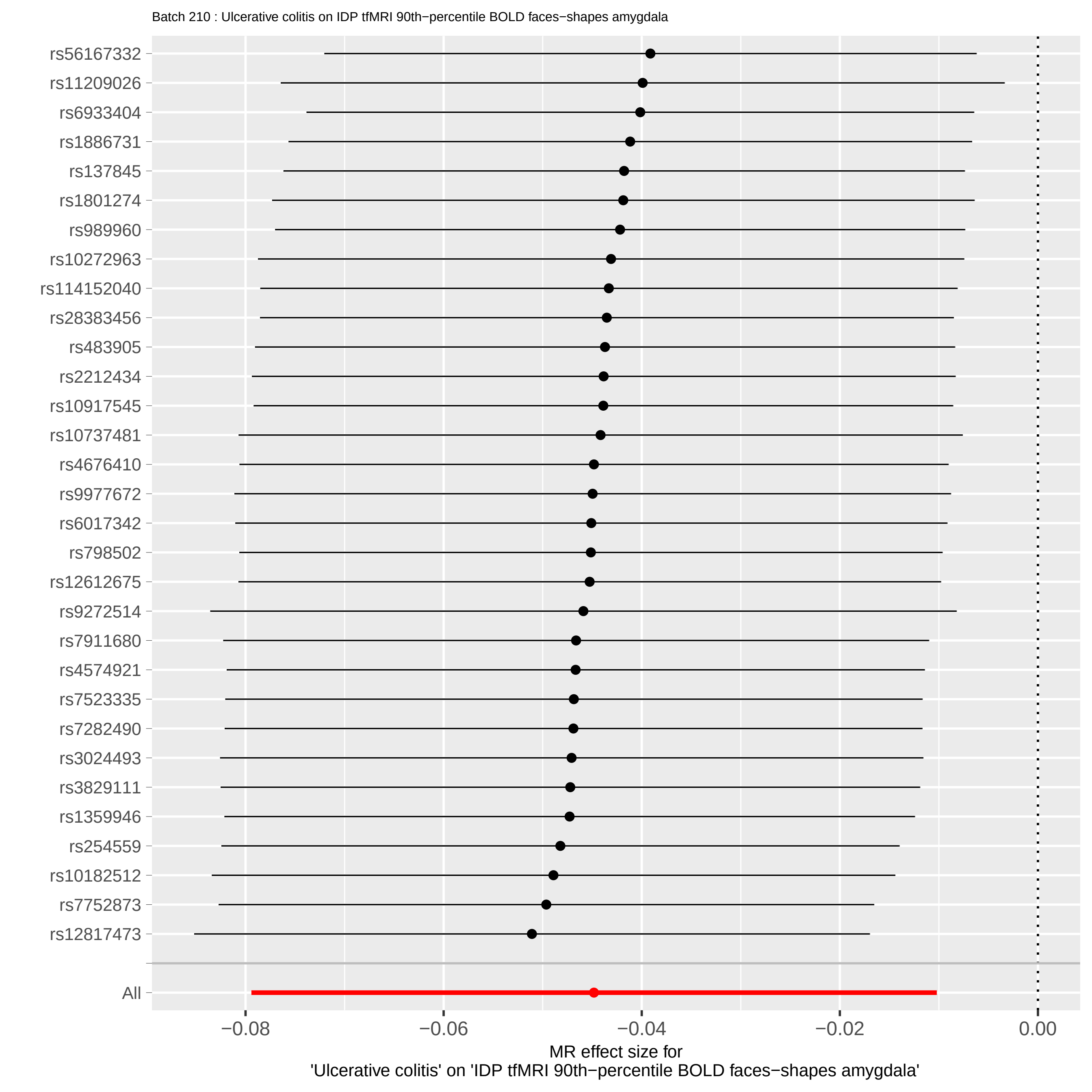

Batch 210 : Ulcerative colitis on IDP tfMRI 90th−percentile BOLD faces−shapes amygdala
rs56167332
rs11209026
rs6933404
rs1886731
rs137845
rs1801274
rs989960
rs10272963
rs114152040
rs28383456
rs483905
rs2212434
rs10917545
rs10737481
rs4676410
rs9977672
rs6017342
rs798502
rs12612675
rs9272514
rs7911680
rs4574921
rs7523335
rs7282490
rs3024493
rs3829111
rs1359946
rs254559
rs10182512
rs7752873
rs12817473
All
−0.08
−0.06
−0.04
−0.02
0.00
MR effect size for
'Ulcerative colitis' on 'IDP tfMRI 90th−percentile BOLD faces−shapes amygdala'

Batch 211 : Crohn's disease on IDP tfMRI median zstat faces−shapes amygdala

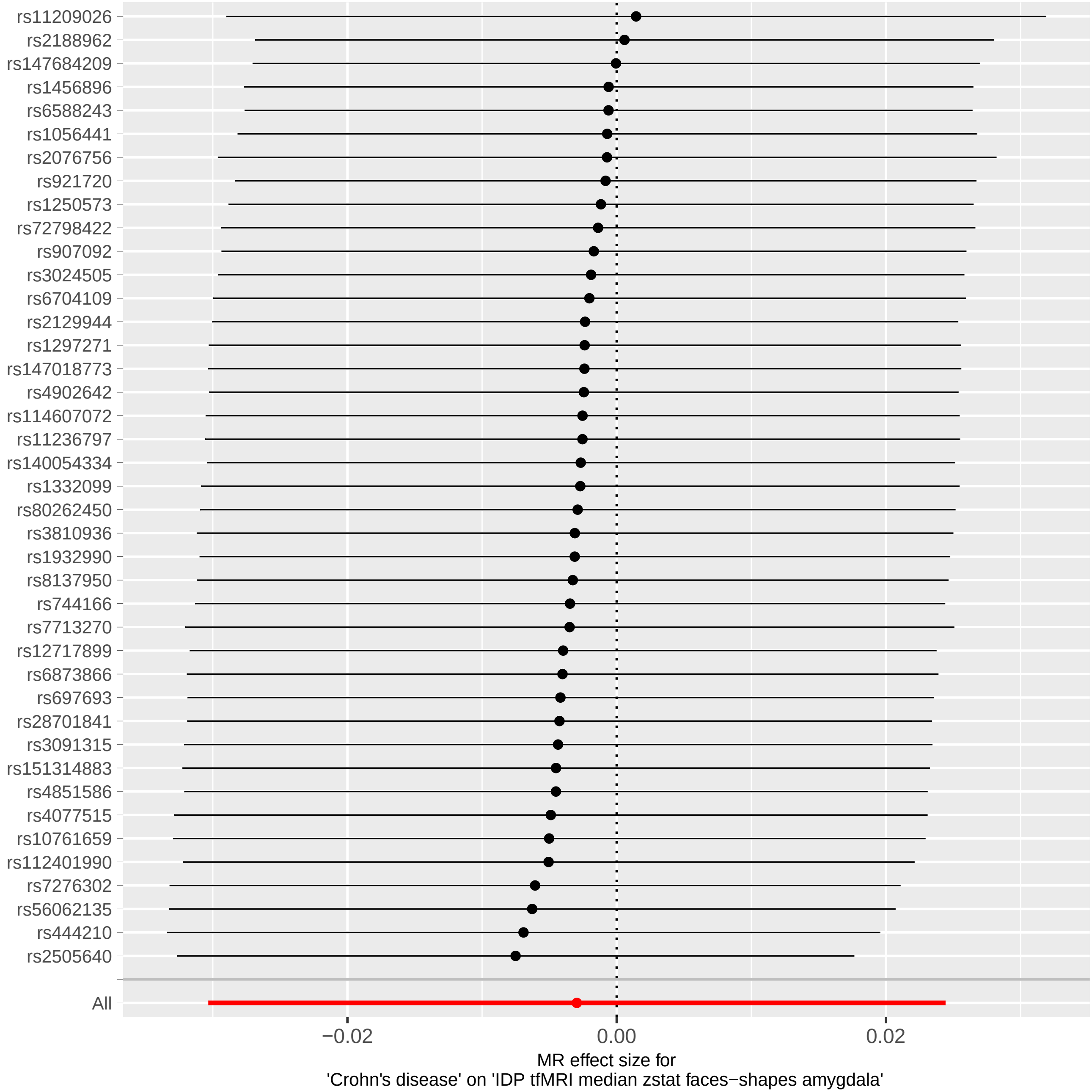

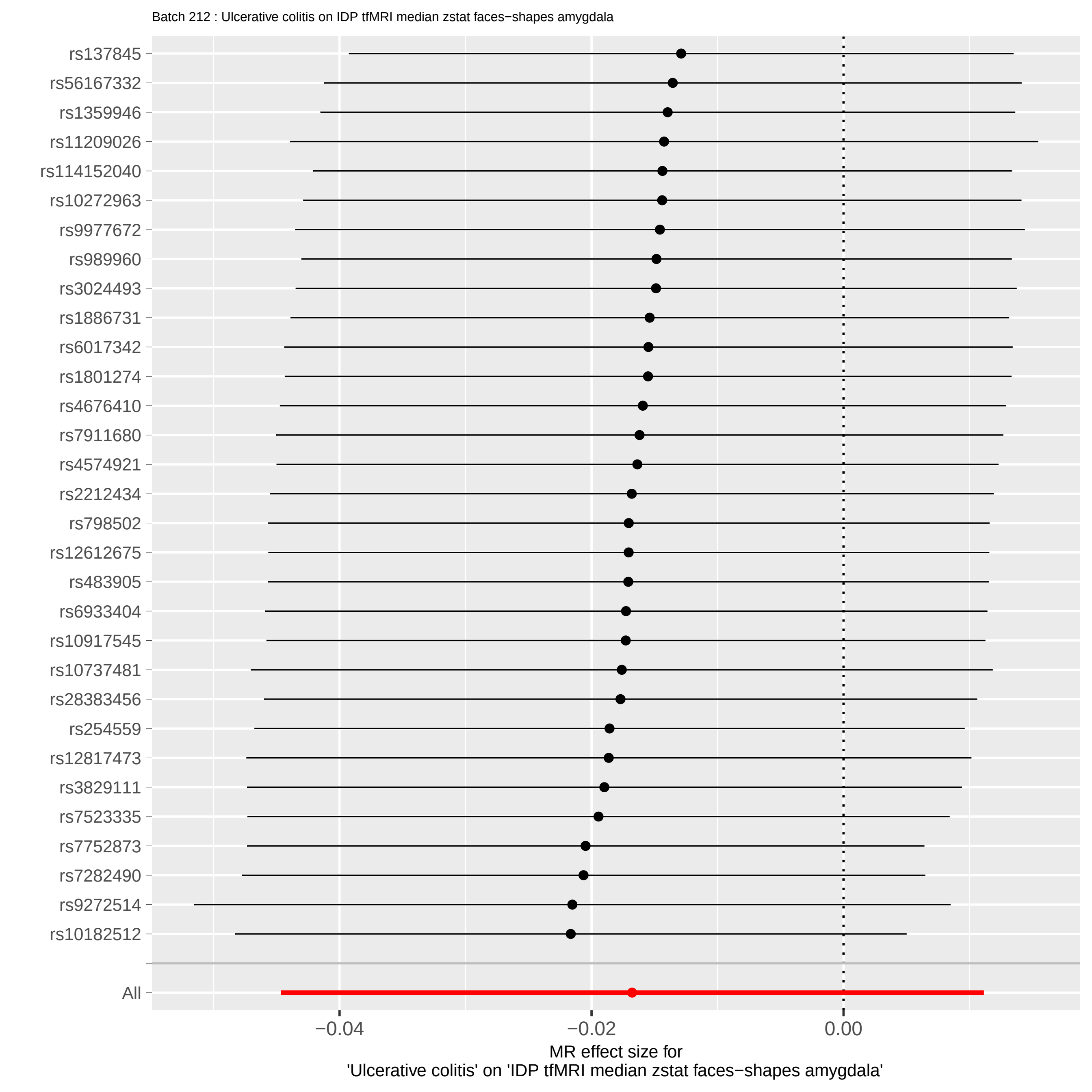

Batch 212 : Ulcerative colitis on IDP tfMRI median zstat faces−shapes amygdala
rs137845
rs56167332
rs1359946
rs11209026
rs114152040
rs10272963
rs9977672
rs989960
rs3024493
rs1886731
rs6017342
rs1801274
rs4676410
rs7911680
rs4574921
rs2212434
rs798502
rs12612675
rs483905
rs6933404
rs10917545
rs10737481
rs28383456
rs254559
rs12817473
rs3829111
rs7523335
rs7752873
rs7282490
rs9272514
rs10182512
All
−0.04
−0.02
0.00
MR effect size for
'Ulcerative colitis' on 'IDP tfMRI median zstat faces−shapes amygdala'

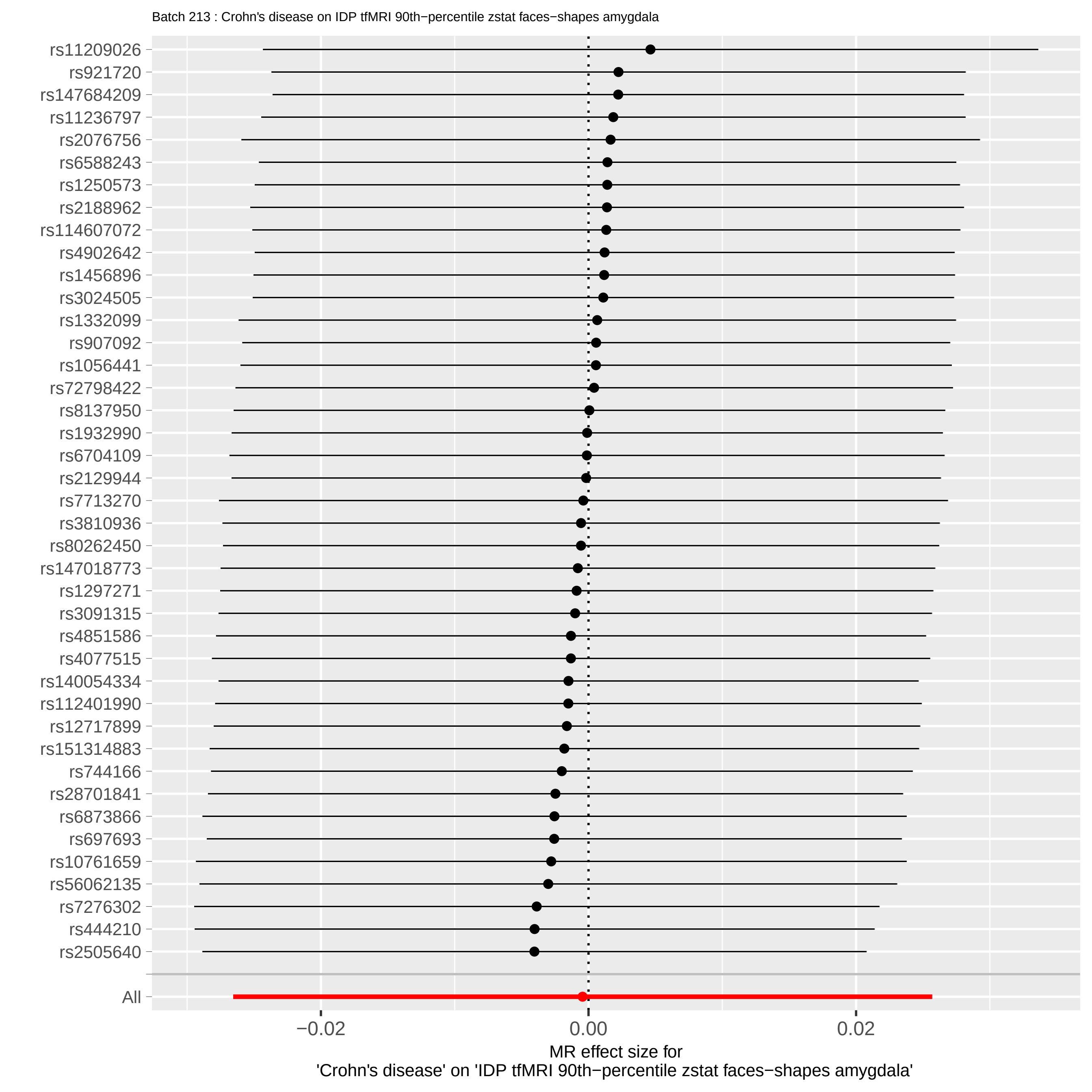

Batch 213 : Crohn's disease on IDP tfMRI 90th−percentile zstat faces−shapes amygdala
rs11209026
rs921720
rs147684209
rs11236797
rs2076756
rs6588243
rs1250573
rs2188962
rs114607072
rs4902642
rs1456896
rs3024505
rs1332099
rs907092
rs1056441
rs72798422
rs8137950
rs1932990
rs6704109
rs2129944
rs7713270
rs3810936
rs80262450
rs147018773
rs1297271
rs3091315
rs4851586
rs4077515
rs140054334
rs112401990
rs12717899
rs151314883
rs744166
rs28701841
rs6873866
rs697693
rs10761659
rs56062135
rs7276302
rs444210
rs2505640
All
−0.02
0.00
0.02
MR effect size for
'Crohn's disease' on 'IDP tfMRI 90th−percentile zstat faces−shapes amygdala'

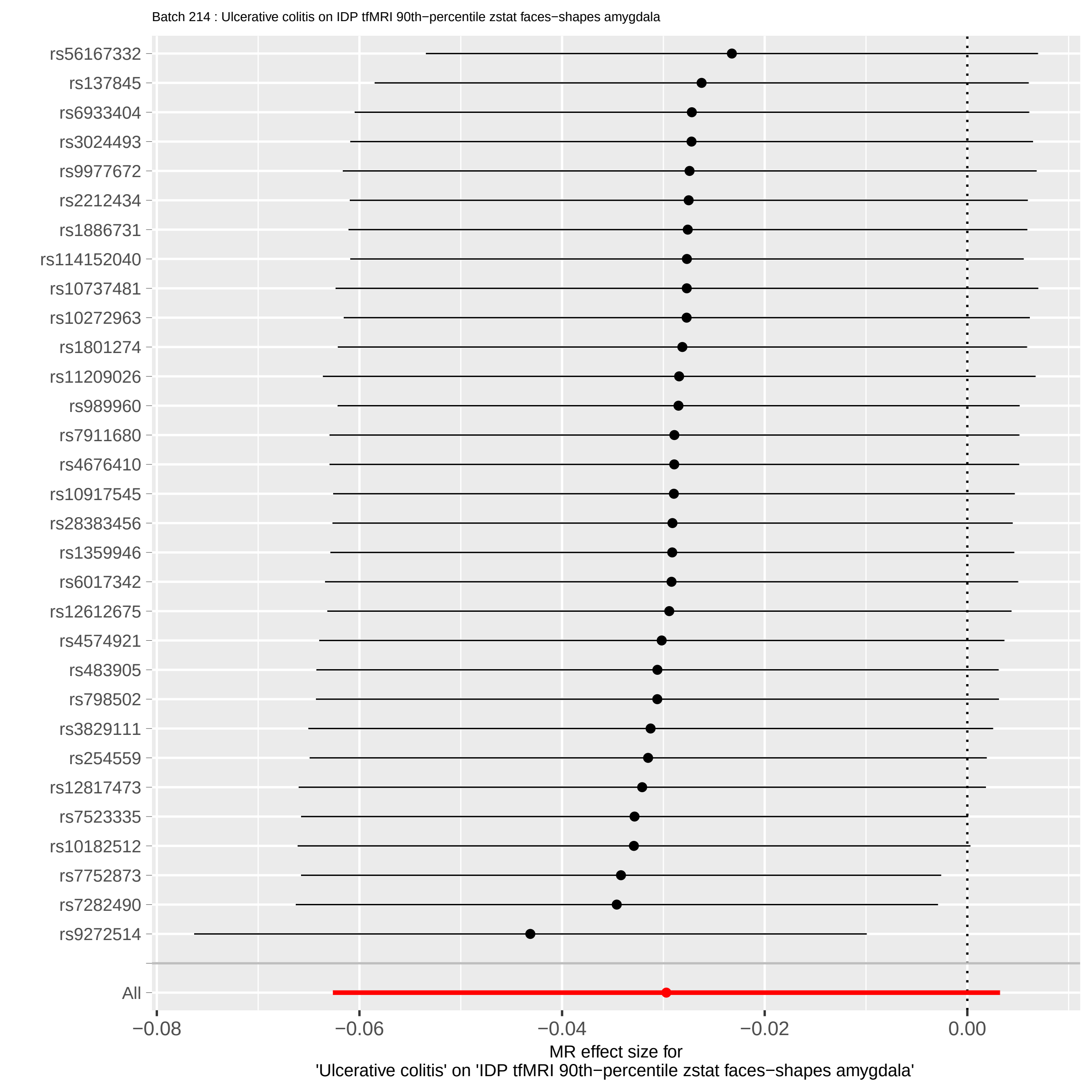

Batch 214 : Ulcerative colitis on IDP tfMRI 90th−percentile zstat faces−shapes amygdala
rs56167332
rs137845
rs6933404
rs3024493
rs9977672
rs2212434
rs1886731
rs114152040
rs10737481
rs10272963
rs1801274
rs11209026
rs989960
rs7911680
rs4676410
rs10917545
rs28383456
rs1359946
rs6017342
rs12612675
rs4574921
rs483905
rs798502
rs3829111
rs254559
rs12817473
rs7523335
rs10182512
rs7752873
rs7282490
rs9272514
All
−0.08
−0.06
−0.04
−0.02
0.00
MR effect size for
'Ulcerative colitis' on 'IDP tfMRI 90th−percentile zstat faces−shapes amygdala'

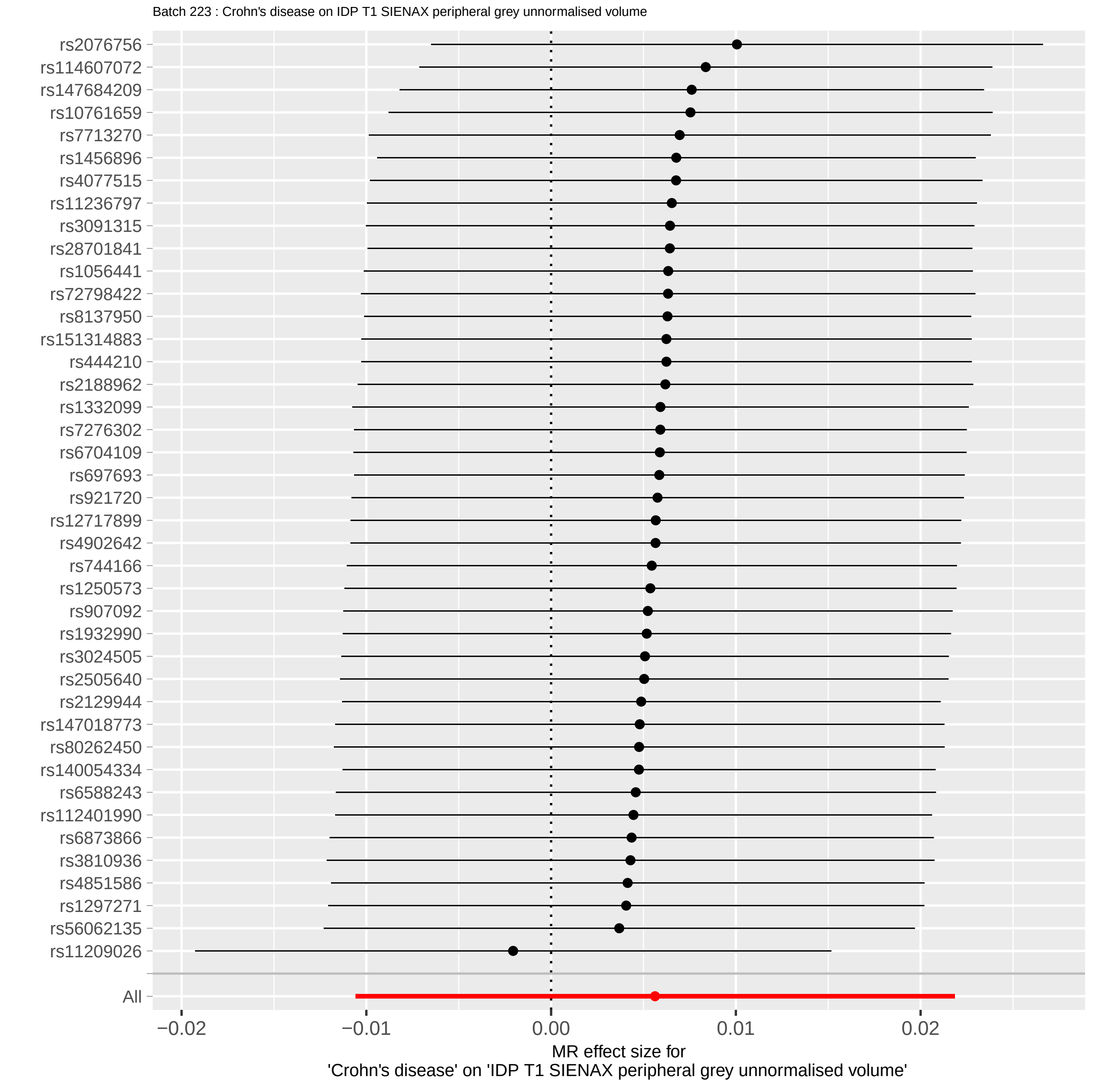

Batch 223 : Crohn's disease on IDP T1 SIENAX peripheral grey unnormalised volume
rs2076756
rs114607072
rs147684209
rs10761659
rs7713270
rs1456896
rs4077515
rs11236797
rs3091315
rs28701841
rs1056441
rs72798422
rs8137950
rs151314883
rs444210
rs2188962
rs1332099
rs7276302
rs6704109
rs697693
rs921720
rs12717899
rs4902642
rs744166
rs1250573
rs907092
rs1932990
rs3024505
rs2505640
rs2129944
rs147018773
rs80262450
rs140054334
rs6588243
rs112401990
rs6873866
rs3810936
rs4851586
rs1297271
rs56062135
rs11209026
All
−0.02
−0.01
0.00
0.01
0.02
MR effect size for
'Crohn's disease' on 'IDP T1 SIENAX peripheral grey unnormalised volume'

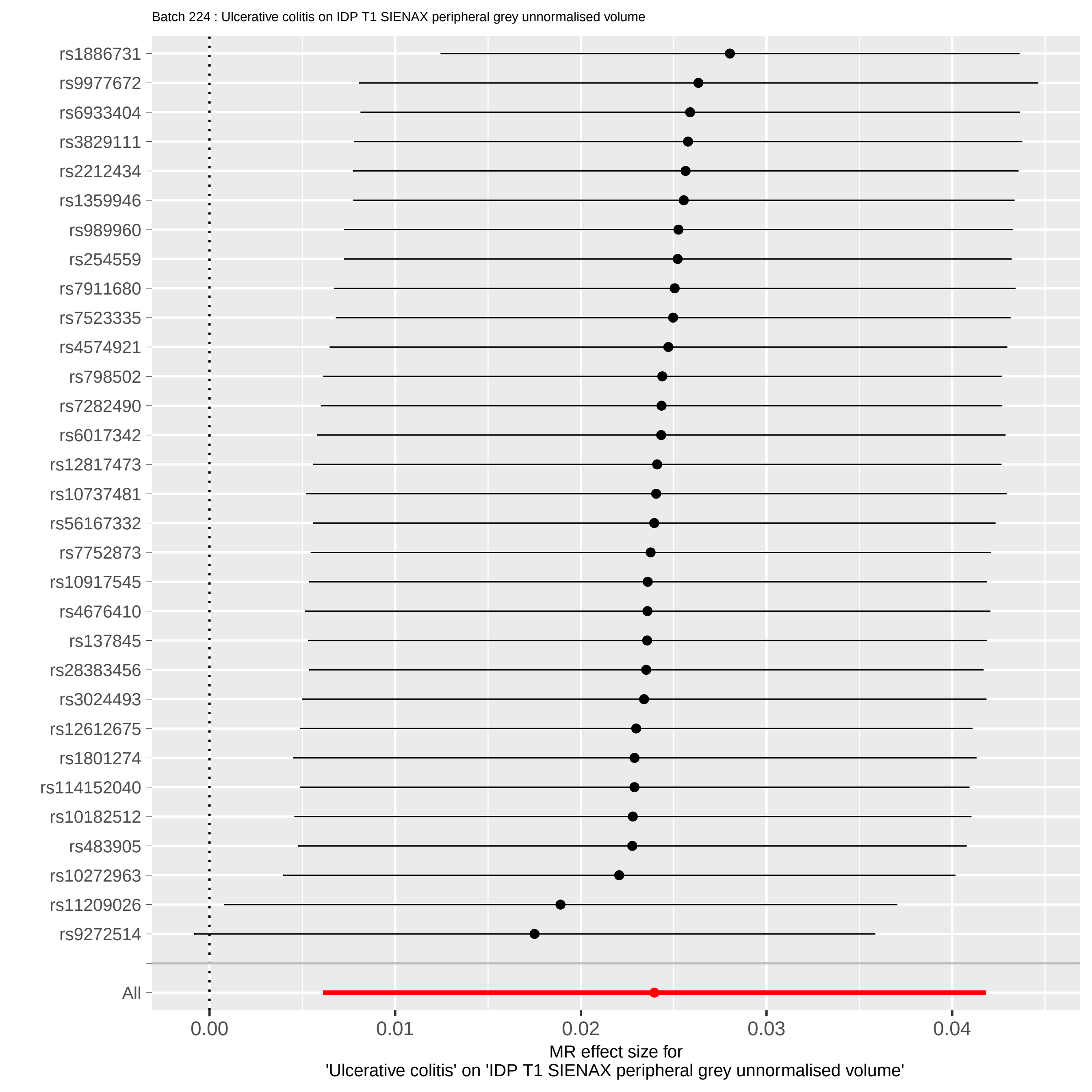

Batch 224 : Ulcerative colitis on IDP T1 SIENAX peripheral grey unnormalised volume
rs1886731
rs9977672
rs6933404
rs3829111
rs2212434
rs1359946
rs989960
rs254559
rs7911680
rs7523335
rs4574921
rs798502
rs7282490
rs6017342
rs12817473
rs10737481
rs56167332
rs7752873
rs10917545
rs4676410
rs137845
rs28383456
rs3024493
rs12612675
rs1801274
rs114152040
rs10182512
rs483905
rs10272963
rs11209026
rs9272514
All
0.00
0.01
0.02
0.03
0.04
MR effect size for
'Ulcerative colitis' on 'IDP T1 SIENAX peripheral grey unnormalised volume'

Batch 225 : Crohn's disease on IDP T1 FIRST right hippocampus volume

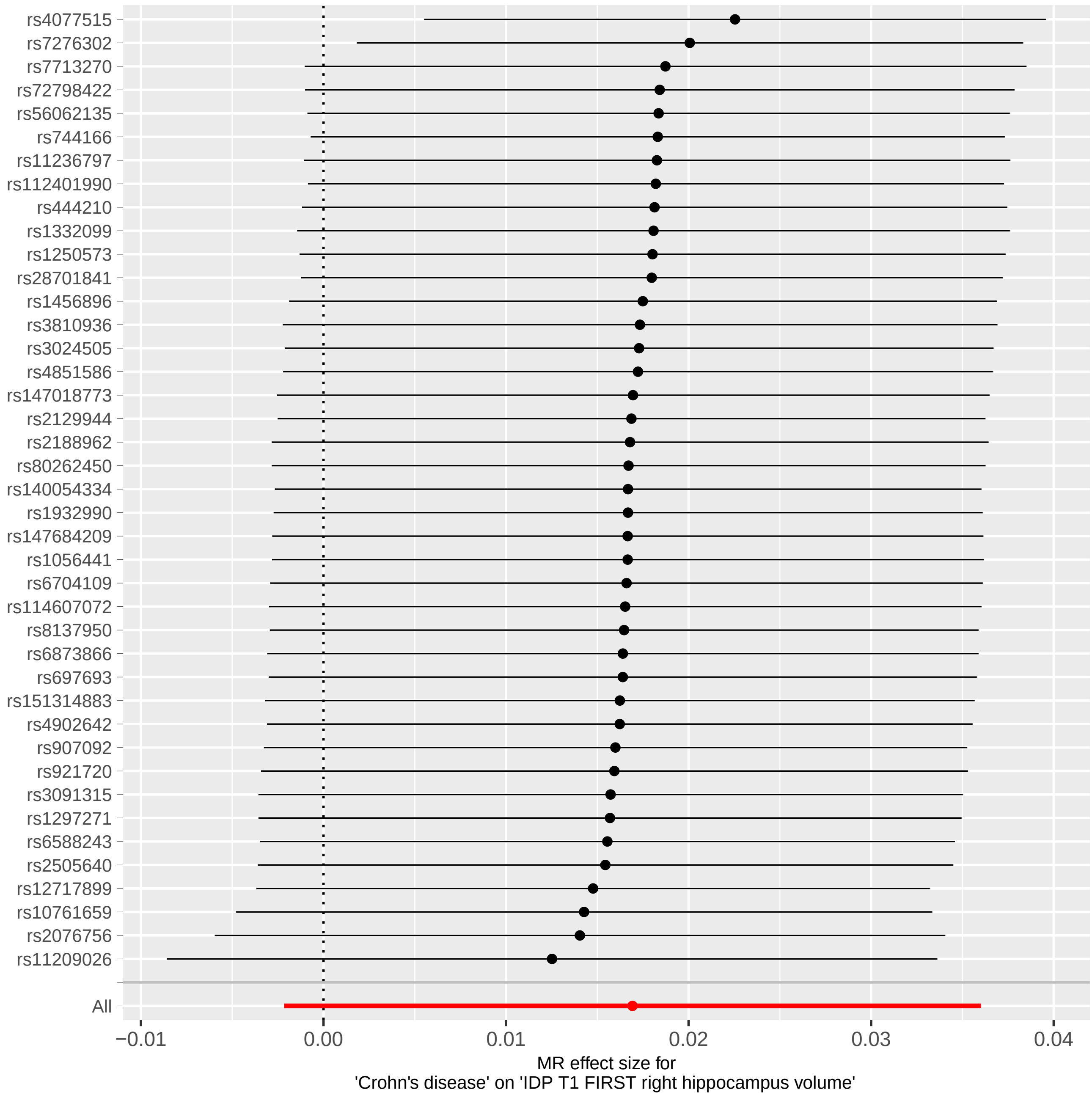

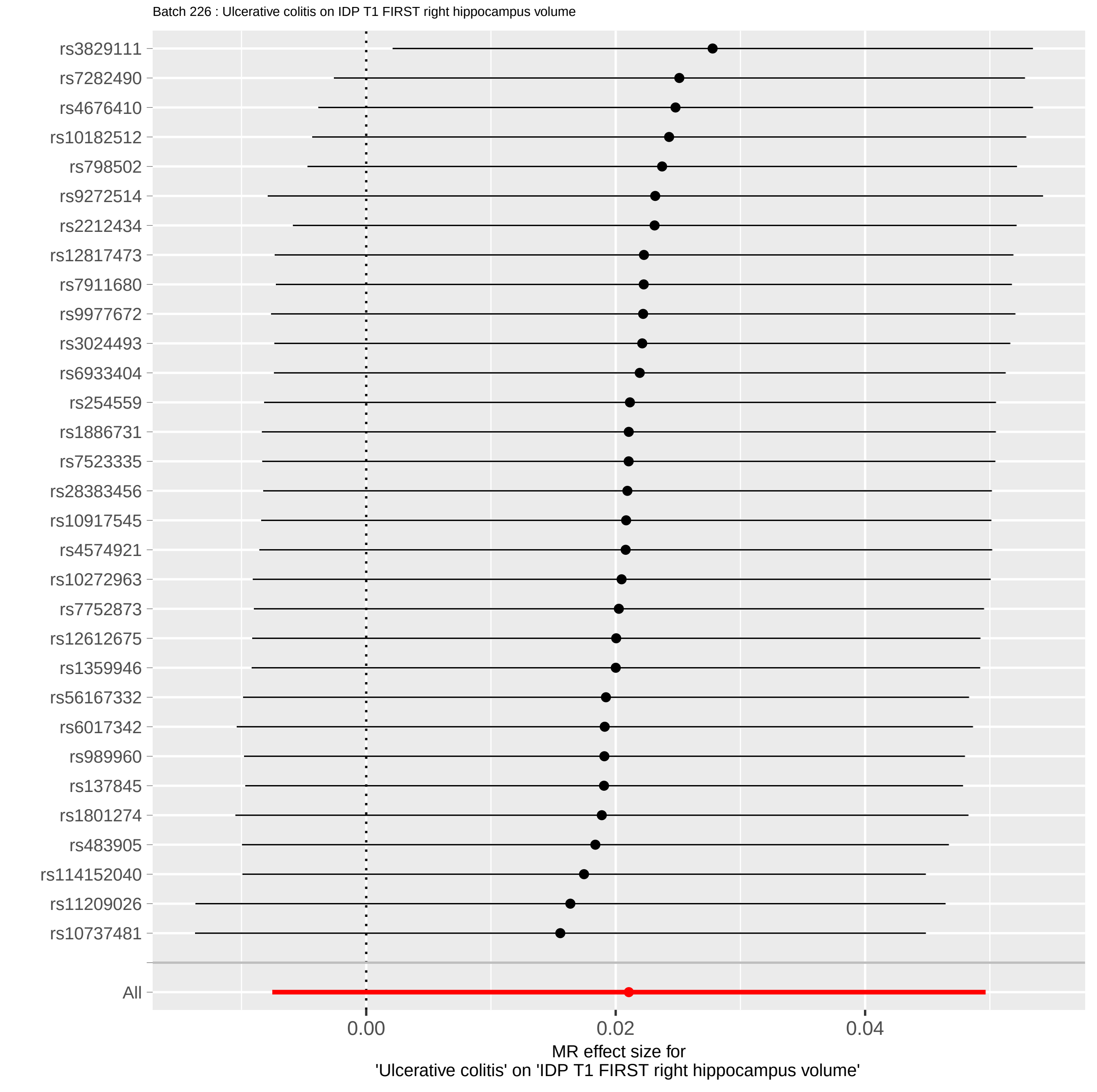

Batch 226 : Ulcerative colitis on IDP T1 FIRST right hippocampus volume
rs3829111
rs7282490
rs4676410
rs10182512
rs798502
rs9272514
rs2212434
rs12817473
rs7911680
rs9977672
rs3024493
rs6933404
rs254559
rs1886731
rs7523335
rs28383456
rs10917545
rs4574921
rs10272963
rs7752873
rs12612675
rs1359946
rs56167332
rs6017342
rs989960
rs137845
rs1801274
rs483905
rs114152040
rs11209026
rs10737481
All
0.00
0.02
0.04
MR effect size for
'Ulcerative colitis' on 'IDP T1 FIRST right hippocampus volume'

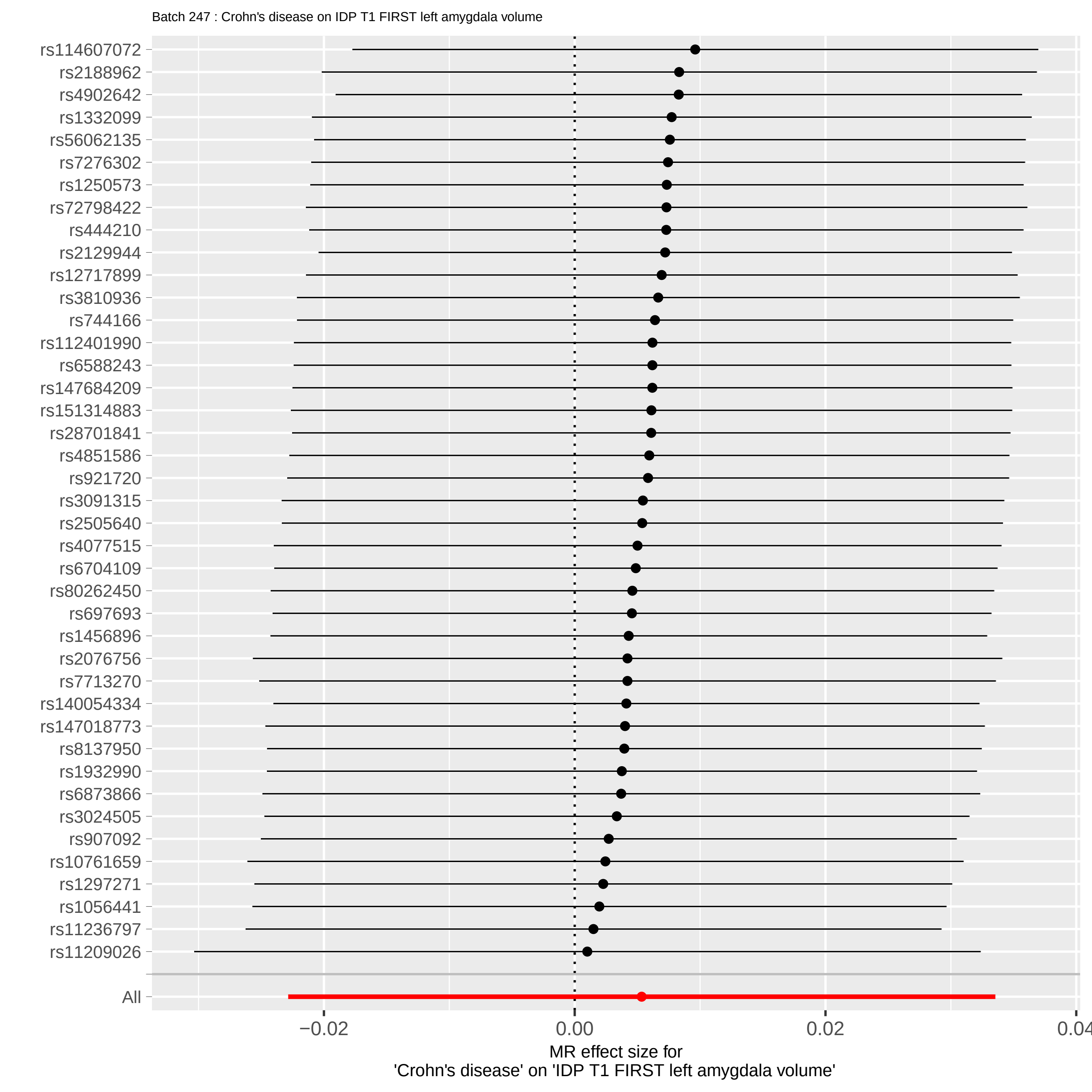

Batch 247 : Crohn's disease on IDP T1 FIRST left amygdala volume
rs114607072
rs2188962
rs4902642
rs1332099
rs56062135
rs7276302
rs1250573
rs72798422
rs444210
rs2129944
rs12717899
rs3810936
rs744166
rs112401990
rs6588243
rs147684209
rs151314883
rs28701841
rs4851586
rs921720
rs3091315
rs2505640
rs4077515
rs6704109
rs80262450
rs697693
rs1456896
rs2076756
rs7713270
rs140054334
rs147018773
rs8137950
rs1932990
rs6873866
rs3024505
rs907092
rs10761659
rs1297271
rs1056441
rs11236797
rs11209026
All
−0.02
0.00
0.02
0.04
MR effect size for
'Crohn's disease' on 'IDP T1 FIRST left amygdala volume'

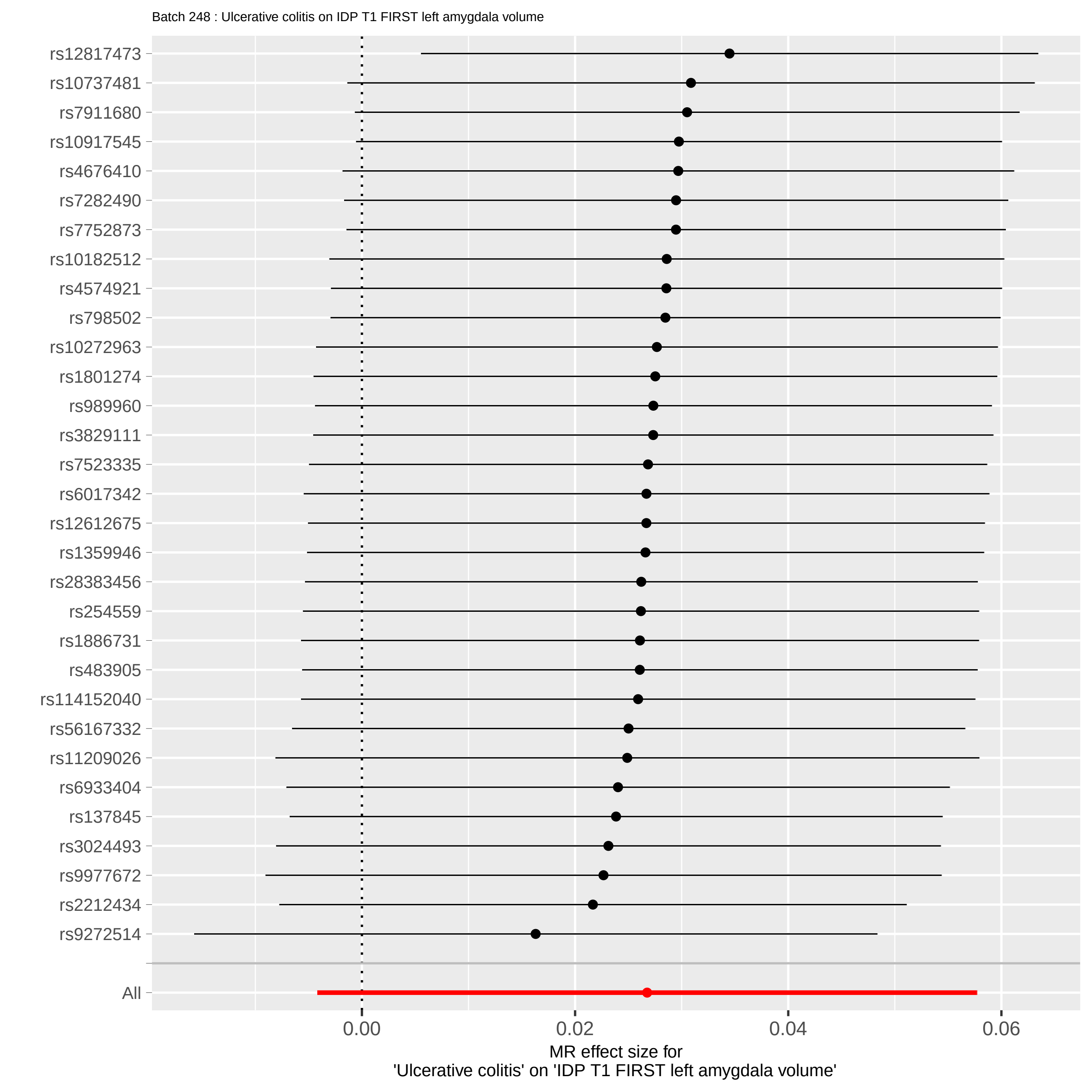

Batch 248 : Ulcerative colitis on IDP T1 FIRST left amygdala volume
rs12817473
rs10737481
rs7911680
rs10917545
rs4676410
rs7282490
rs7752873
rs10182512
rs4574921
rs798502
rs10272963
rs1801274
rs989960
rs3829111
rs7523335
rs6017342
rs12612675
rs1359946
rs28383456
rs254559
rs1886731
rs483905
rs114152040
rs56167332
rs11209026
rs6933404
rs137845
rs3024493
rs9977672
rs2212434
rs9272514
All
0.00
0.02
0.04
0.06
MR effect size for
'Ulcerative colitis' on 'IDP T1 FIRST left amygdala volume'

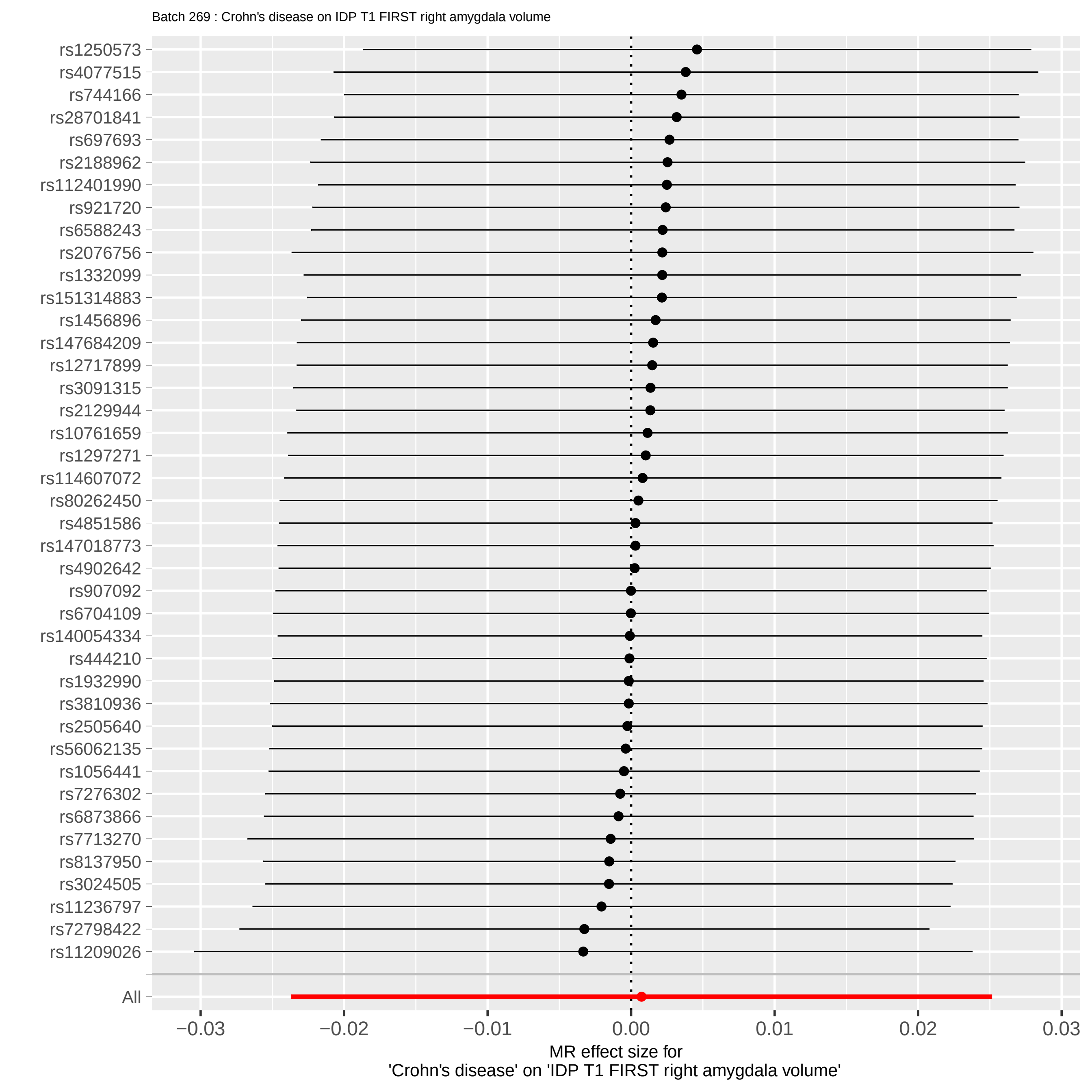

Batch 269 : Crohn's disease on IDP T1 FIRST right amygdala volume
rs1250573
rs4077515
rs744166
rs28701841
rs697693
rs2188962
rs112401990
rs921720
rs6588243
rs2076756
rs1332099
rs151314883
rs1456896
rs147684209
rs12717899
rs3091315
rs2129944
rs10761659
rs1297271
rs114607072
rs80262450
rs4851586
rs147018773
rs4902642
rs907092
rs6704109
rs140054334
rs444210
rs1932990
rs3810936
rs2505640
rs56062135
rs1056441
rs7276302
rs6873866
rs7713270
rs8137950
rs3024505
rs11236797
rs72798422
rs11209026
All
−0.03
−0.02
−0.01
0.00
0.01
0.02
0.03
MR effect size for
'Crohn's disease' on 'IDP T1 FIRST right amygdala volume'

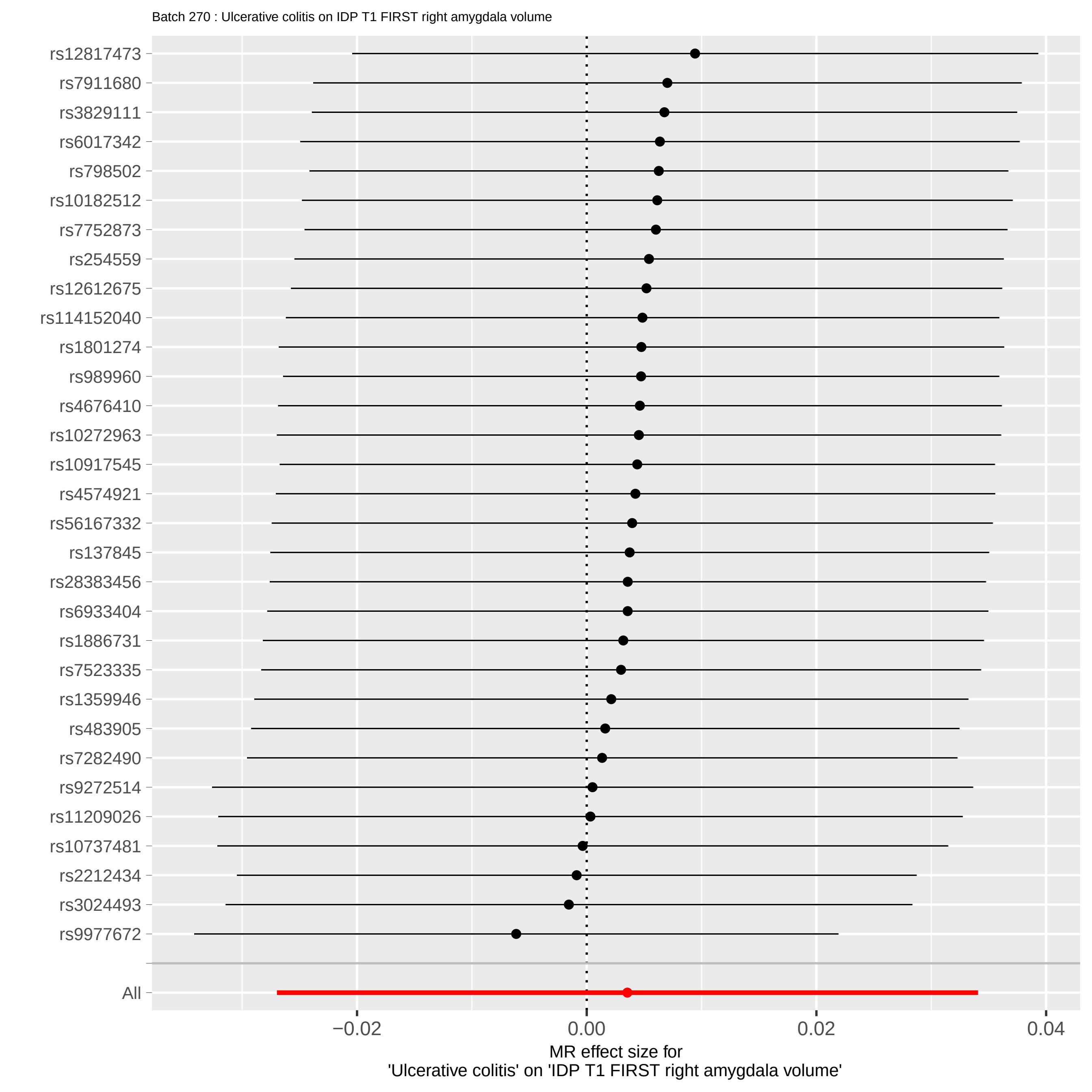

Batch 270 : Ulcerative colitis on IDP T1 FIRST right amygdala volume
rs12817473
rs7911680
rs3829111
rs6017342
rs798502
rs10182512
rs7752873
rs254559
rs12612675
rs114152040
rs1801274
rs989960
rs4676410
rs10272963
rs10917545
rs4574921
rs56167332
rs137845
rs28383456
rs6933404
rs1886731
rs7523335
rs1359946
rs483905
rs7282490
rs9272514
rs11209026
rs10737481
rs2212434
rs3024493
rs9977672
All
−0.02
0.00
0.02
0.04
MR effect size for
'Ulcerative colitis' on 'IDP T1 FIRST right amygdala volume'

Batch 291 : Crohn's disease on IDP T1 FIRST left accumbens volume

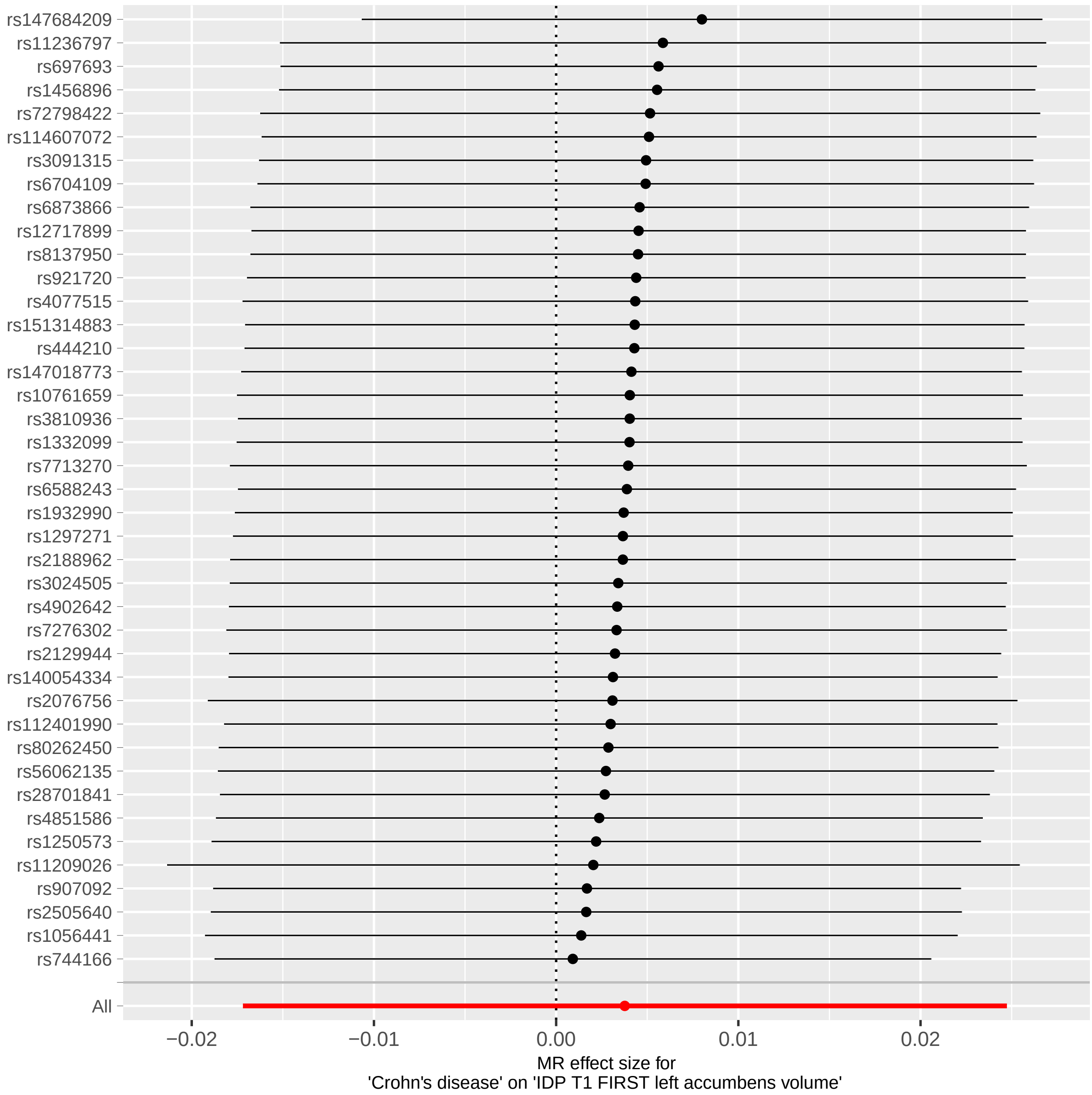

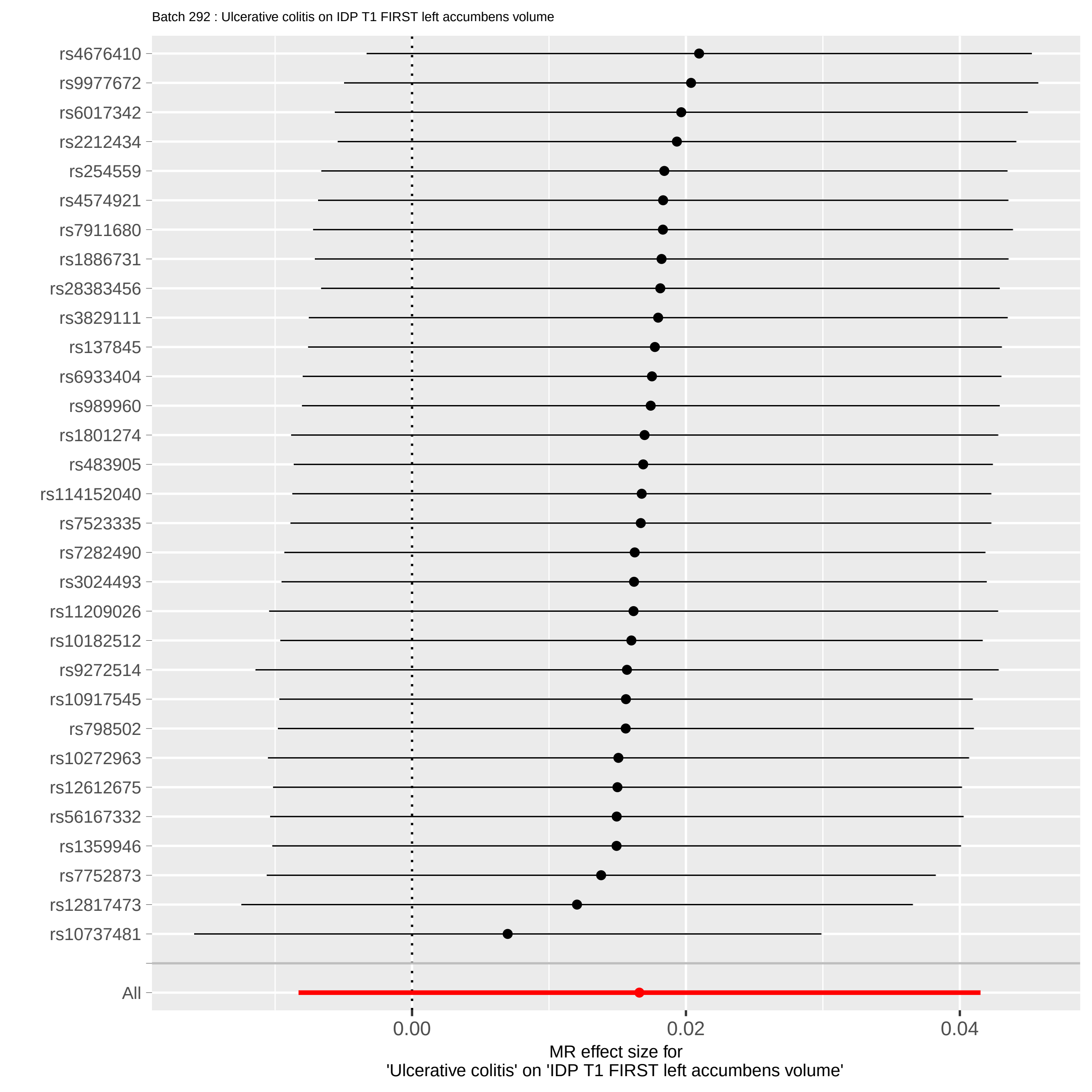

Batch 292 : Ulcerative colitis on IDP T1 FIRST left accumbens volume
rs4676410
rs9977672
rs6017342
rs2212434
rs254559
rs4574921
rs7911680
rs1886731
rs28383456
rs3829111
rs137845
rs6933404
rs989960
rs1801274
rs483905
rs114152040
rs7523335
rs7282490
rs3024493
rs11209026
rs10182512
rs9272514
rs10917545
rs798502
rs10272963
rs12612675
rs56167332
rs1359946
rs7752873
rs12817473
rs10737481
All
0.00
0.02
0.04
MR effect size for
'Ulcerative colitis' on 'IDP T1 FIRST left accumbens volume'

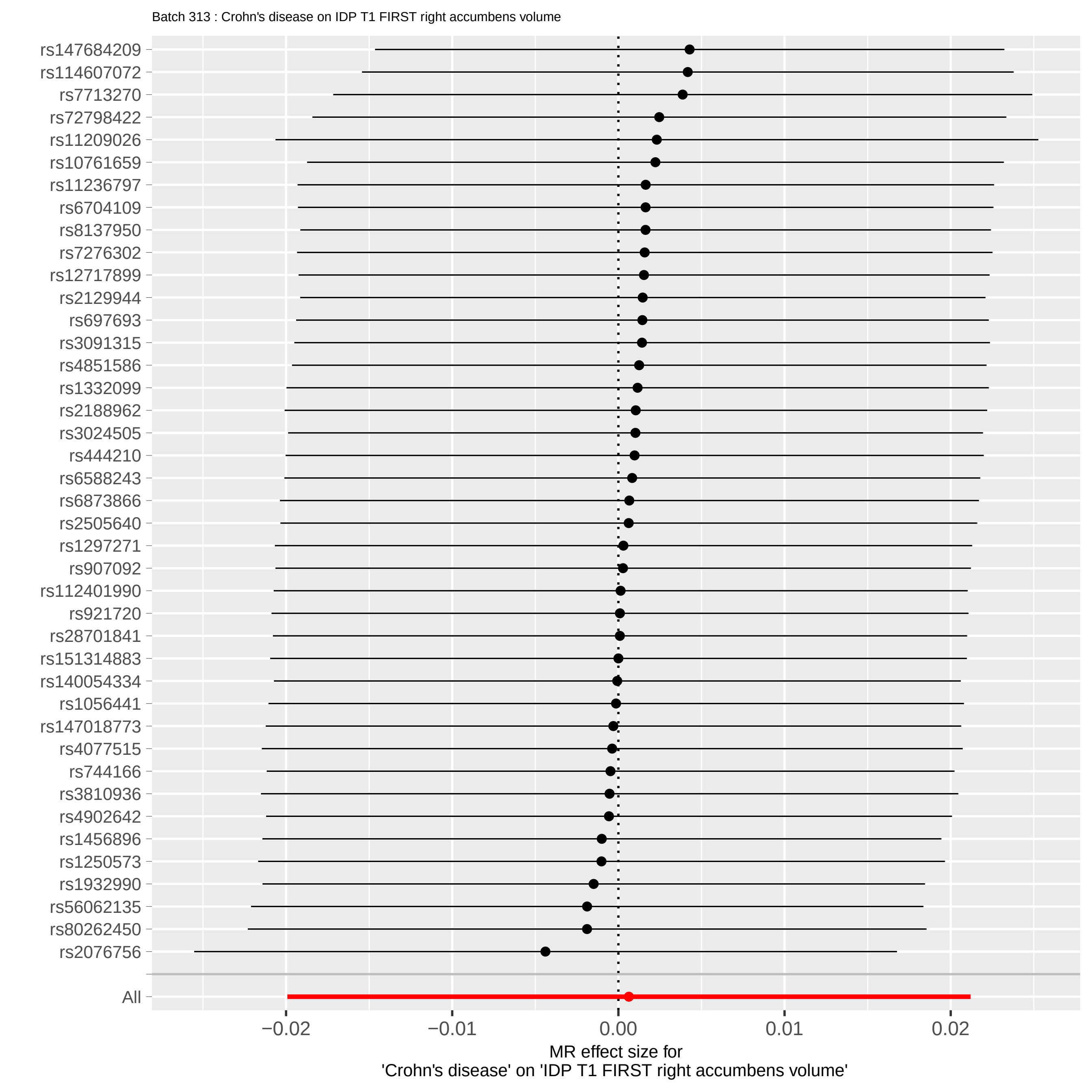

Batch 313 : Crohn's disease on IDP T1 FIRST right accumbens volume
rs147684209
rs114607072
rs7713270
rs72798422
rs11209026
rs10761659
rs11236797
rs6704109
rs8137950
rs7276302
rs12717899
rs2129944
rs697693
rs3091315
rs4851586
rs1332099
rs2188962
rs3024505
rs444210
rs6588243
rs6873866
rs2505640
rs1297271
rs907092
rs112401990
rs921720
rs28701841
rs151314883
rs140054334
rs1056441
rs147018773
rs4077515
rs744166
rs3810936
rs4902642
rs1456896
rs1250573
rs1932990
rs56062135
rs80262450
rs2076756
All
−0.02
−0.01
0.00
0.01
0.02
MR effect size for
'Crohn's disease' on 'IDP T1 FIRST right accumbens volume'

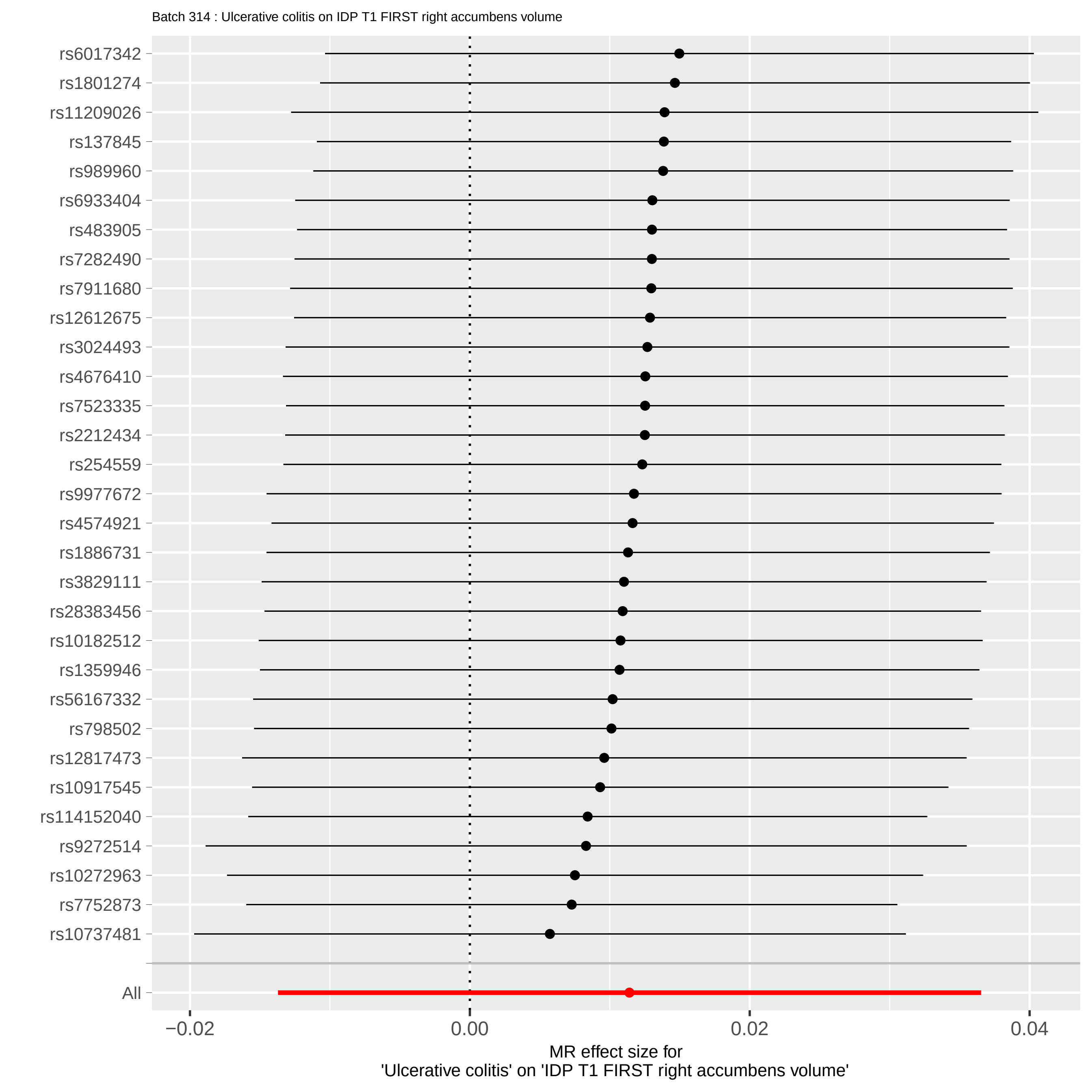

Batch 314 : Ulcerative colitis on IDP T1 FIRST right accumbens volume
rs6017342
rs1801274
rs11209026
rs137845
rs989960
rs6933404
rs483905
rs7282490
rs7911680
rs12612675
rs3024493
rs4676410
rs7523335
rs2212434
rs254559
rs9977672
rs4574921
rs1886731
rs3829111
rs28383456
rs10182512
rs1359946
rs56167332
rs798502
rs12817473
rs10917545
rs114152040
rs9272514
rs10272963
rs7752873
rs10737481
All
−0.02
0.00
0.02
0.04
MR effect size for
'Ulcerative colitis' on 'IDP T1 FIRST right accumbens volume'

Batch 335 : Crohn's disease on IDP T1 FIRST brain stem+4th ventricle volume

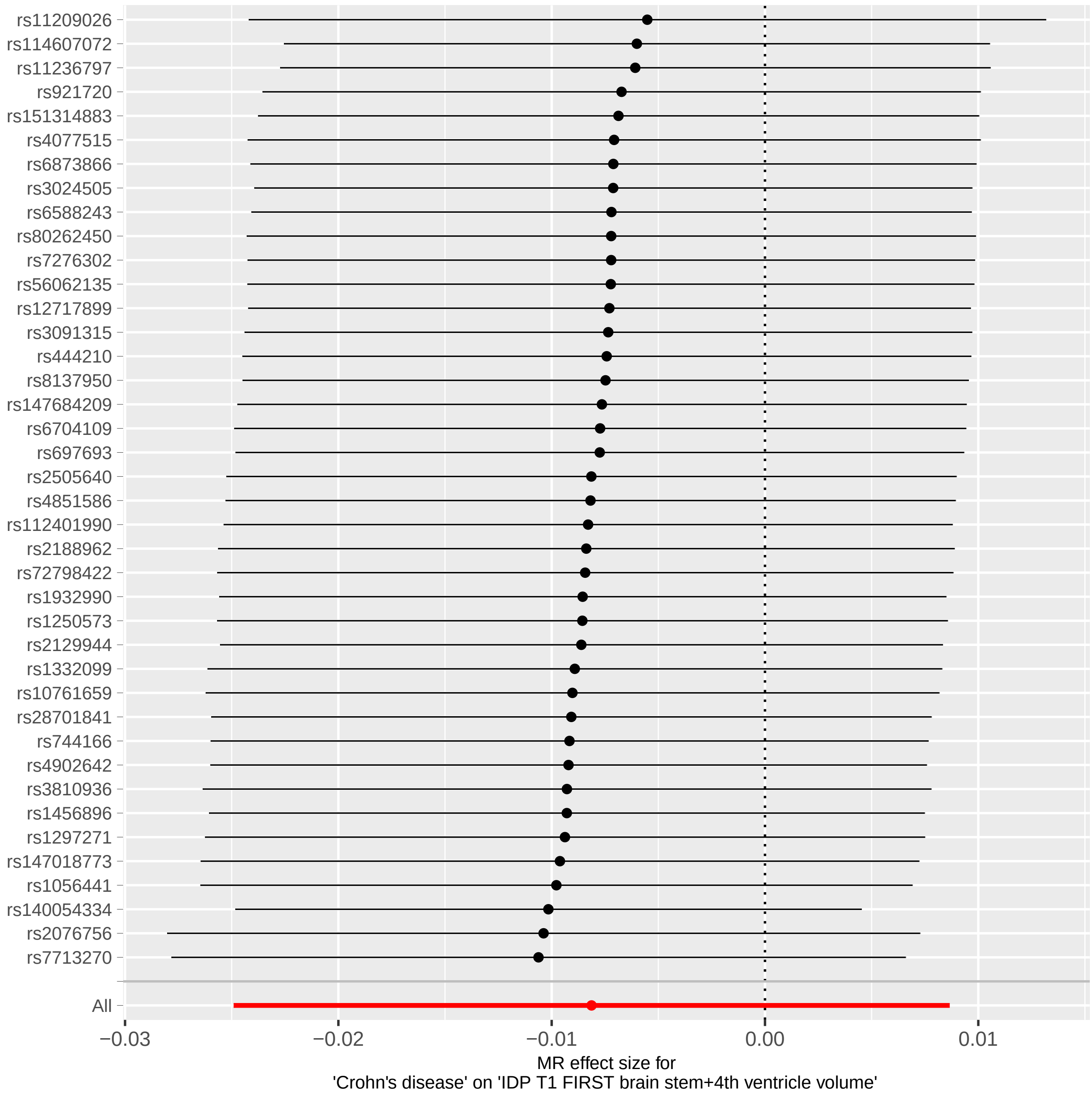

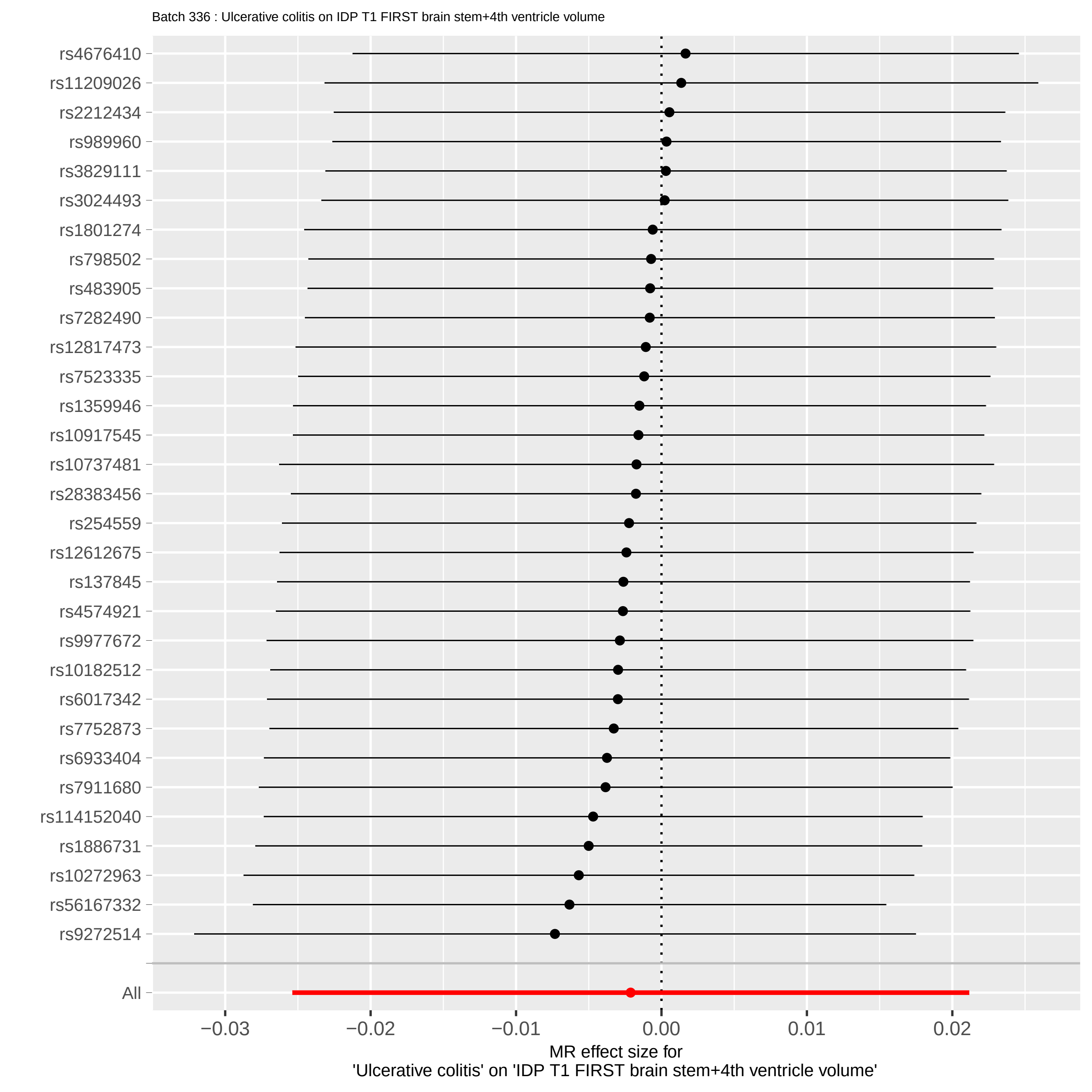
Batch 336 : Ulcerative colitis on IDP T1 FIRST brain stem+4th ventricle volume
rs4676410
rs11209026
rs2212434
rs989960
rs3829111
rs3024493
rs1801274
rs798502
rs483905
rs7282490
rs12817473
rs7523335
rs1359946
rs10917545
rs10737481
rs28383456
rs254559
rs12612675
rs137845
rs4574921
rs9977672
rs10182512
rs6017342
rs7752873
rs6933404
rs7911680
rs114152040
rs1886731
rs10272963
rs56167332
rs9272514
All
−0.03
−0.02
−0.01
0.00
0.01
0.02
MR effect size for
'Ulcerative colitis' on 'IDP T1 FIRST brain stem+4th ventricle volume'

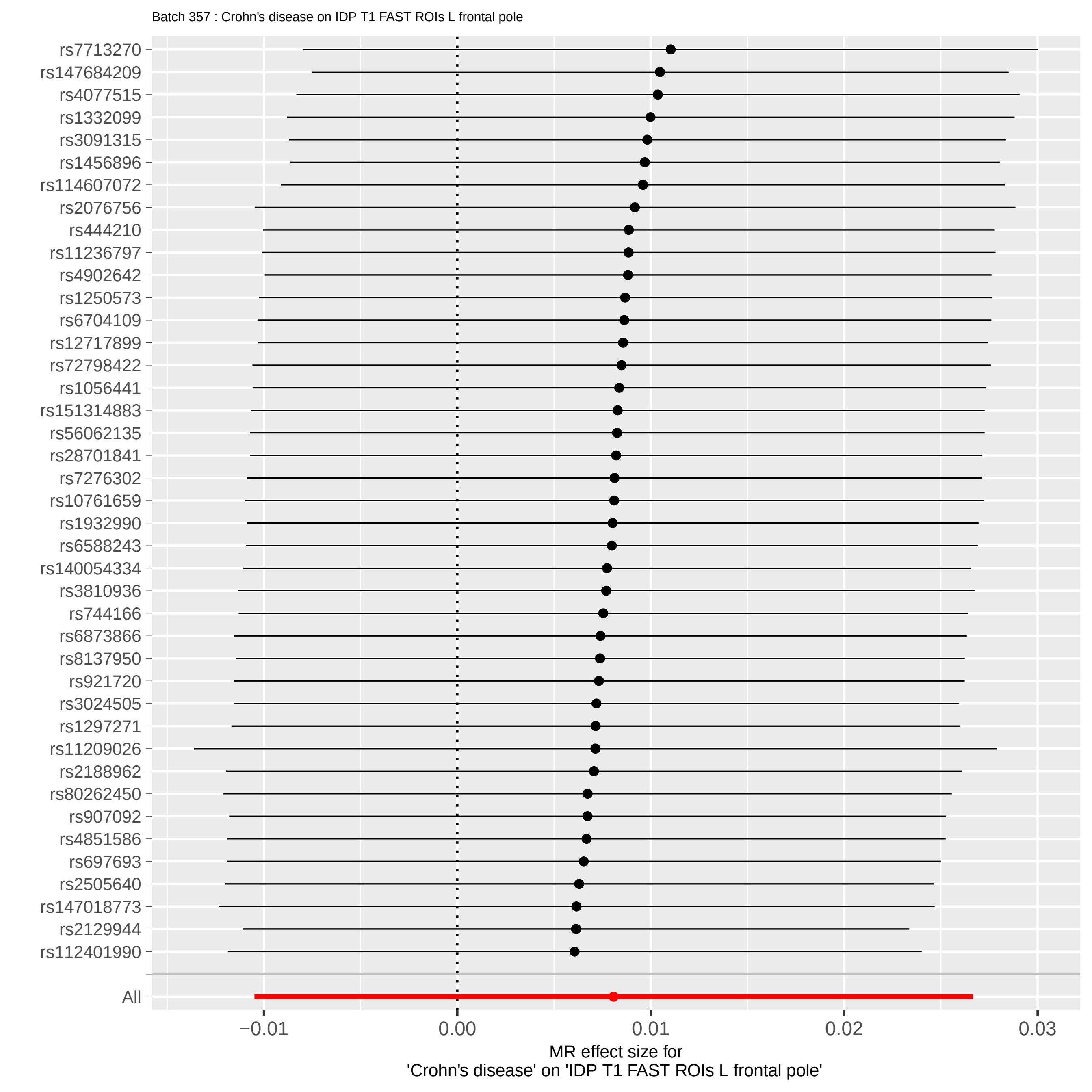

Batch 357 : Crohn's disease on IDP T1 FAST ROIs L frontal pole
rs7713270
rs147684209
rs4077515
rs1332099
rs3091315
rs1456896
rs114607072
rs2076756
rs444210
rs11236797
rs4902642
rs1250573
rs6704109
rs12717899
rs72798422
rs1056441
rs151314883
rs56062135
rs28701841
rs7276302
rs10761659
rs1932990
rs6588243
rs140054334
rs3810936
rs744166
rs6873866
rs8137950
rs921720
rs3024505
rs1297271
rs11209026
rs2188962
rs80262450
rs907092
rs4851586
rs697693
rs2505640
rs147018773
rs2129944
rs112401990
All
−0.01
0.00
0.01
0.02
0.03
MR effect size for
'Crohn's disease' on 'IDP T1 FAST ROIs L frontal pole'

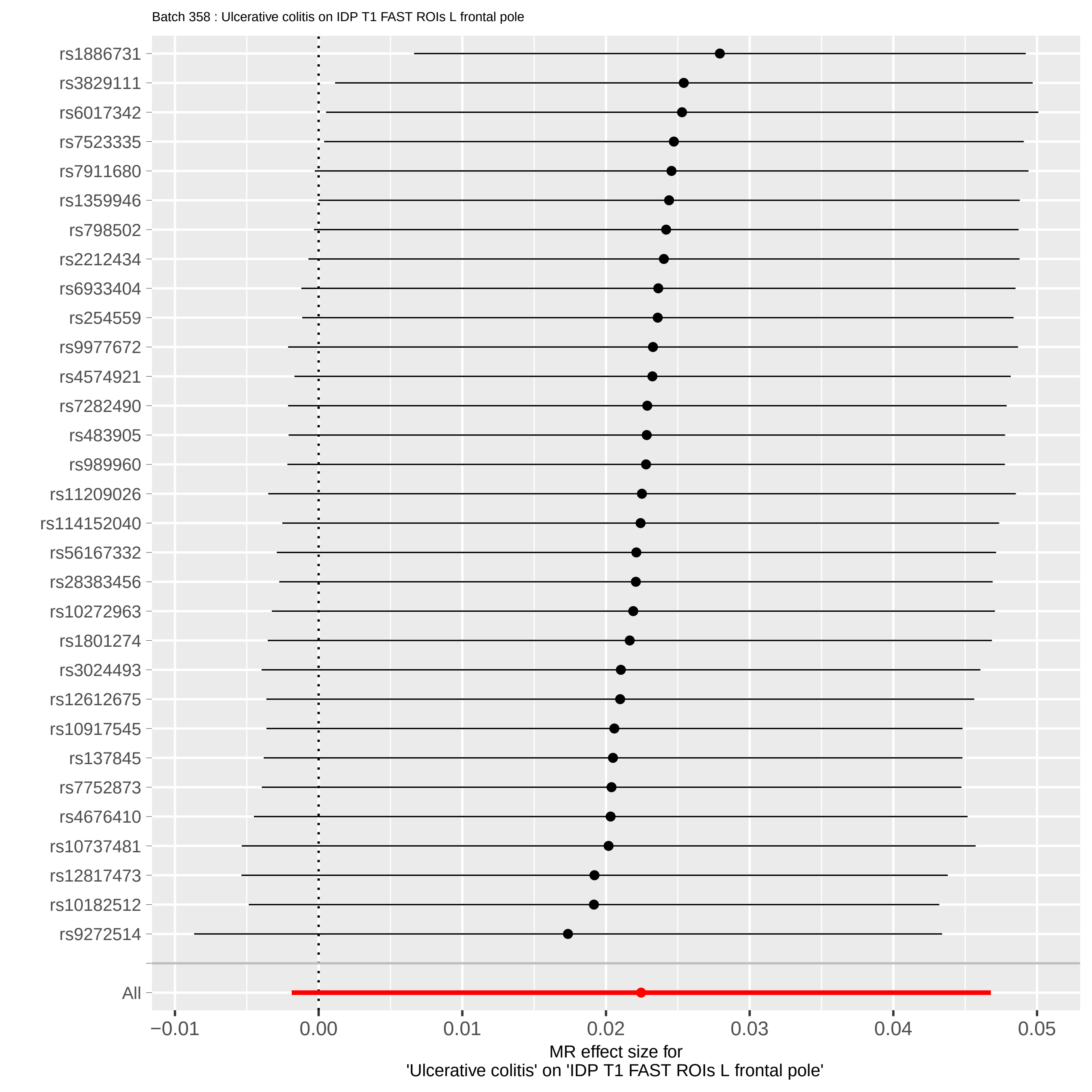
Batch 358 : Ulcerative colitis on IDP T1 FAST ROIs L frontal pole
rs1886731
rs3829111
rs6017342
rs7523335
rs7911680
rs1359946
rs798502
rs2212434
rs6933404
rs254559
rs9977672
rs4574921
rs7282490
rs483905
rs989960
rs11209026
rs114152040
rs56167332
rs28383456
rs10272963
rs1801274
rs3024493
rs12612675
rs10917545
rs137845
rs7752873
rs4676410
rs10737481
rs12817473
rs10182512
rs9272514
All
−0.01
0.00
0.01
0.02
0.03
0.04
0.05
MR effect size for
'Ulcerative colitis' on 'IDP T1 FAST ROIs L frontal pole'

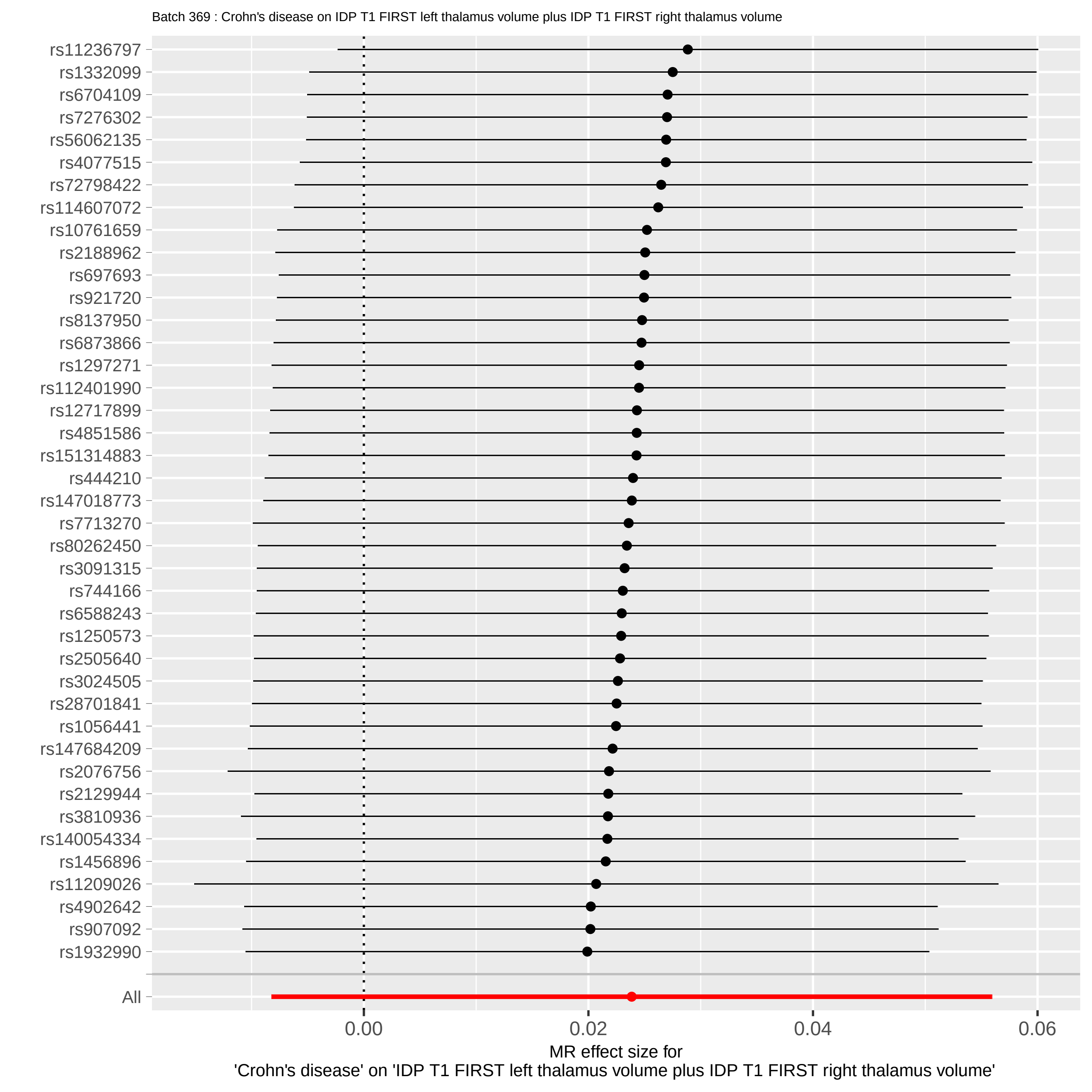

Batch 369 : Crohn's disease on IDP T1 FIRST left thalamus volume plus IDP T1 FIRST right thalamus volume
rs11236797
rs1332099
rs6704109
rs7276302
rs56062135
rs4077515
rs72798422
rs114607072
rs10761659
rs2188962
rs697693
rs921720
rs8137950
rs6873866
rs1297271
rs112401990
rs12717899
rs4851586
rs151314883
rs444210
rs147018773
rs7713270
rs80262450
rs3091315
rs744166
rs6588243
rs1250573
rs2505640
rs3024505
rs28701841
rs1056441
rs147684209
rs2076756
rs2129944
rs3810936
rs140054334
rs1456896
rs11209026
rs4902642
rs907092
rs1932990
All
0.00
0.02
0.04
0.06
MR effect size for
'Crohn's disease' on 'IDP T1 FIRST left thalamus volume plus IDP T1 FIRST right thalamus volume'

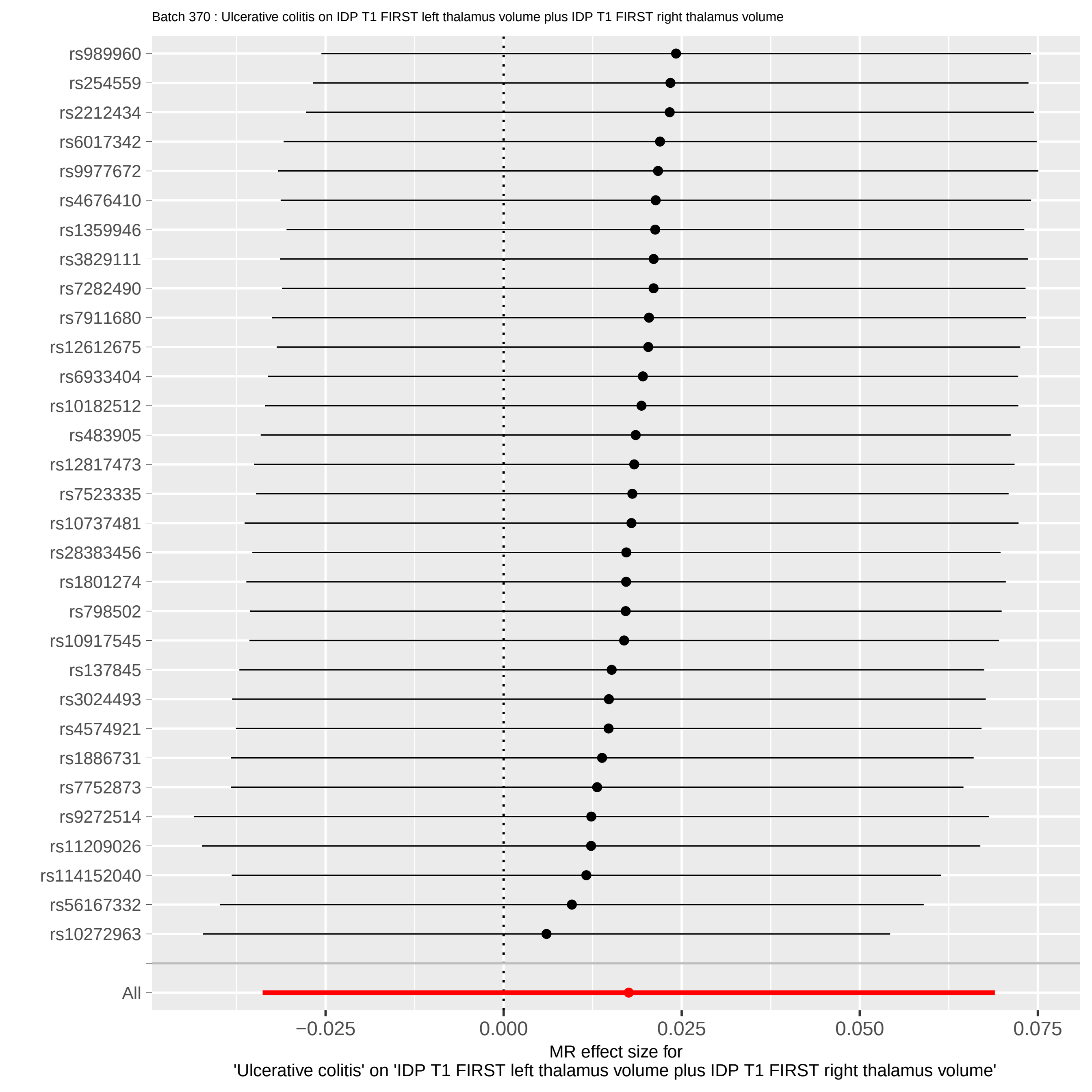

Batch 370 : Ulcerative colitis on IDP T1 FIRST left thalamus volume plus IDP T1 FIRST right thalamus volume
rs989960
rs254559
rs2212434
rs6017342
rs9977672
rs4676410
rs1359946
rs3829111
rs7282490
rs7911680
rs12612675
rs6933404
rs10182512
rs483905
rs12817473
rs7523335
rs10737481
rs28383456
rs1801274
rs798502
rs10917545
rs137845
rs3024493
rs4574921
rs1886731
rs7752873
rs9272514
rs11209026
rs114152040
rs56167332
rs10272963
All
−0.025
0.000
0.025
0.050
0.075
MR effect size for
'Ulcerative colitis' on 'IDP T1 FIRST left thalamus volume plus IDP T1 FIRST right thalamus volume'

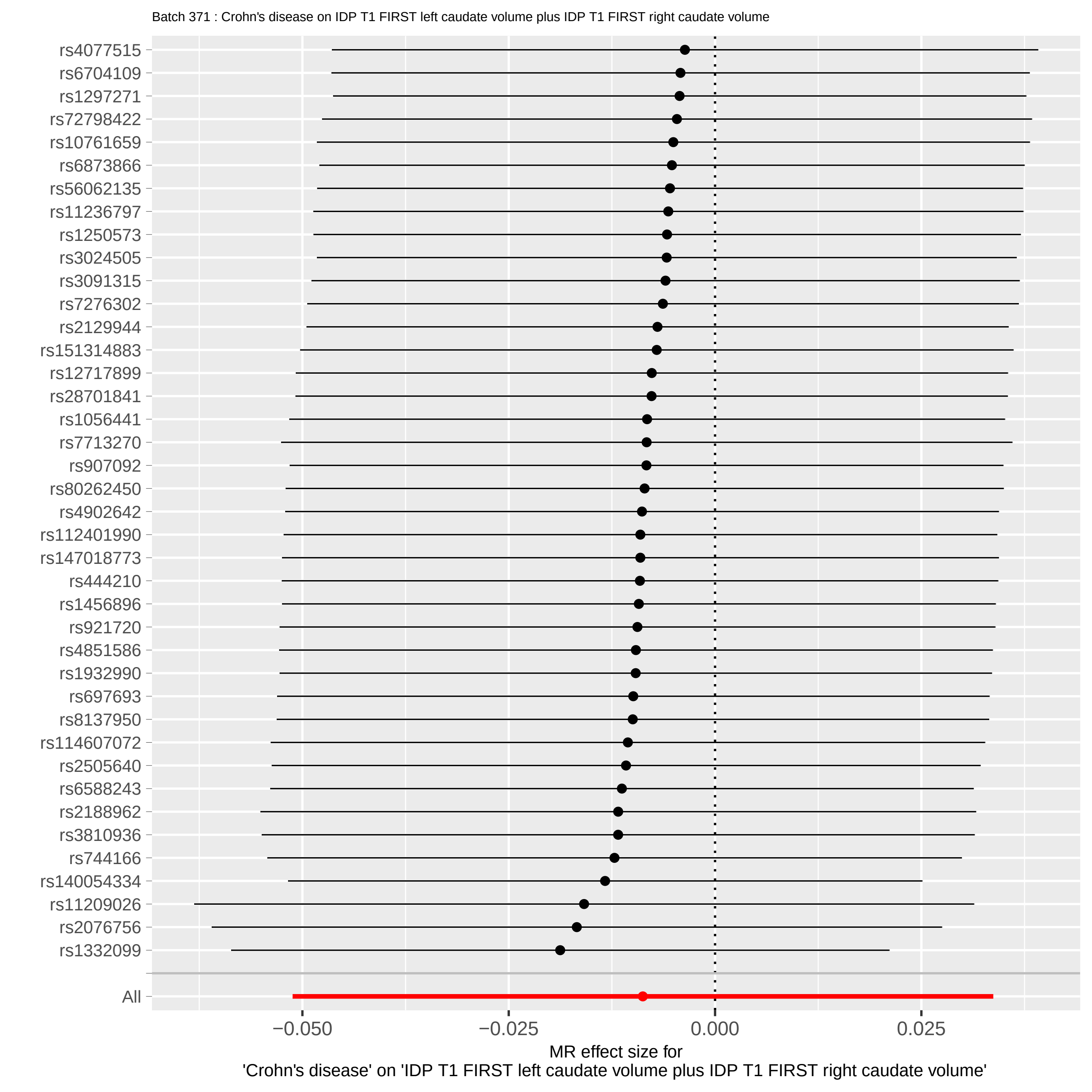
Batch 371 : Crohn's disease on IDP T1 FIRST left caudate volume plus IDP T1 FIRST right caudate volume
rs4077515
rs6704109
rs1297271
rs72798422
rs10761659
rs6873866
rs56062135
rs11236797
rs1250573
rs3024505
rs3091315
rs7276302
rs2129944
rs151314883
rs12717899
rs28701841
rs1056441
rs7713270
rs907092
rs80262450
rs4902642
rs112401990
rs147018773
rs444210
rs1456896
rs921720
rs4851586
rs1932990
rs697693
rs8137950
rs114607072
rs2505640
rs6588243
rs2188962
rs3810936
rs744166
rs140054334
rs11209026
rs2076756
rs1332099
All
−0.050
−0.025
0.000
0.025
MR effect size for
'Crohn's disease' on 'IDP T1 FIRST left caudate volume plus IDP T1 FIRST right caudate volume'

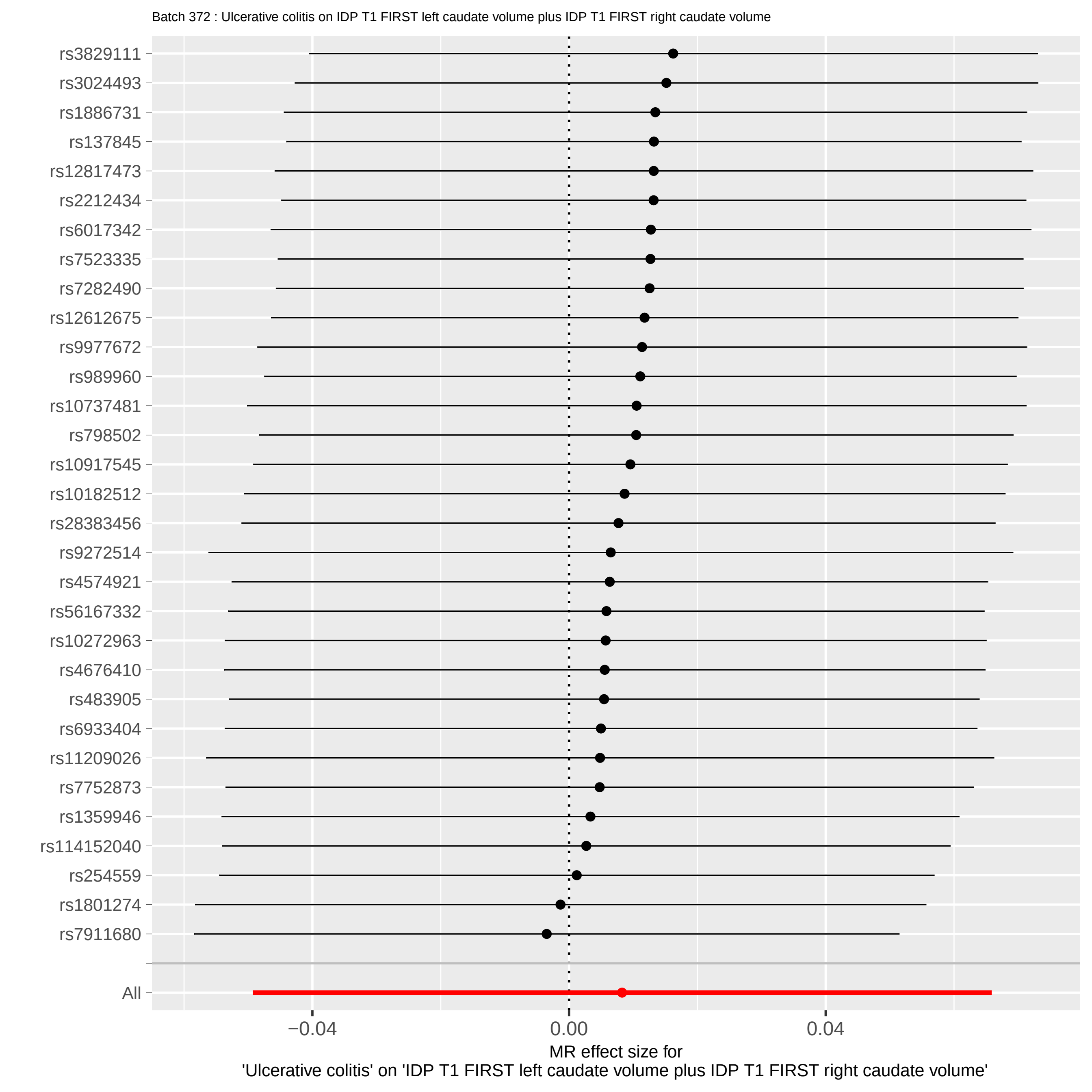

Batch 372 : Ulcerative colitis on IDP T1 FIRST left caudate volume plus IDP T1 FIRST right caudate volume
rs3829111
rs3024493
rs1886731
rs137845
rs12817473
rs2212434
rs6017342
rs7523335
rs7282490
rs12612675
rs9977672
rs989960
rs10737481
rs798502
rs10917545
rs10182512
rs28383456
rs9272514
rs4574921
rs56167332
rs10272963
rs4676410
rs483905
rs6933404
rs11209026
rs7752873
rs1359946
rs114152040
rs254559
rs1801274
rs7911680
All
−0.04
0.00
0.04
MR effect size for
'Ulcerative colitis' on 'IDP T1 FIRST left caudate volume plus IDP T1 FIRST right caudate volume'

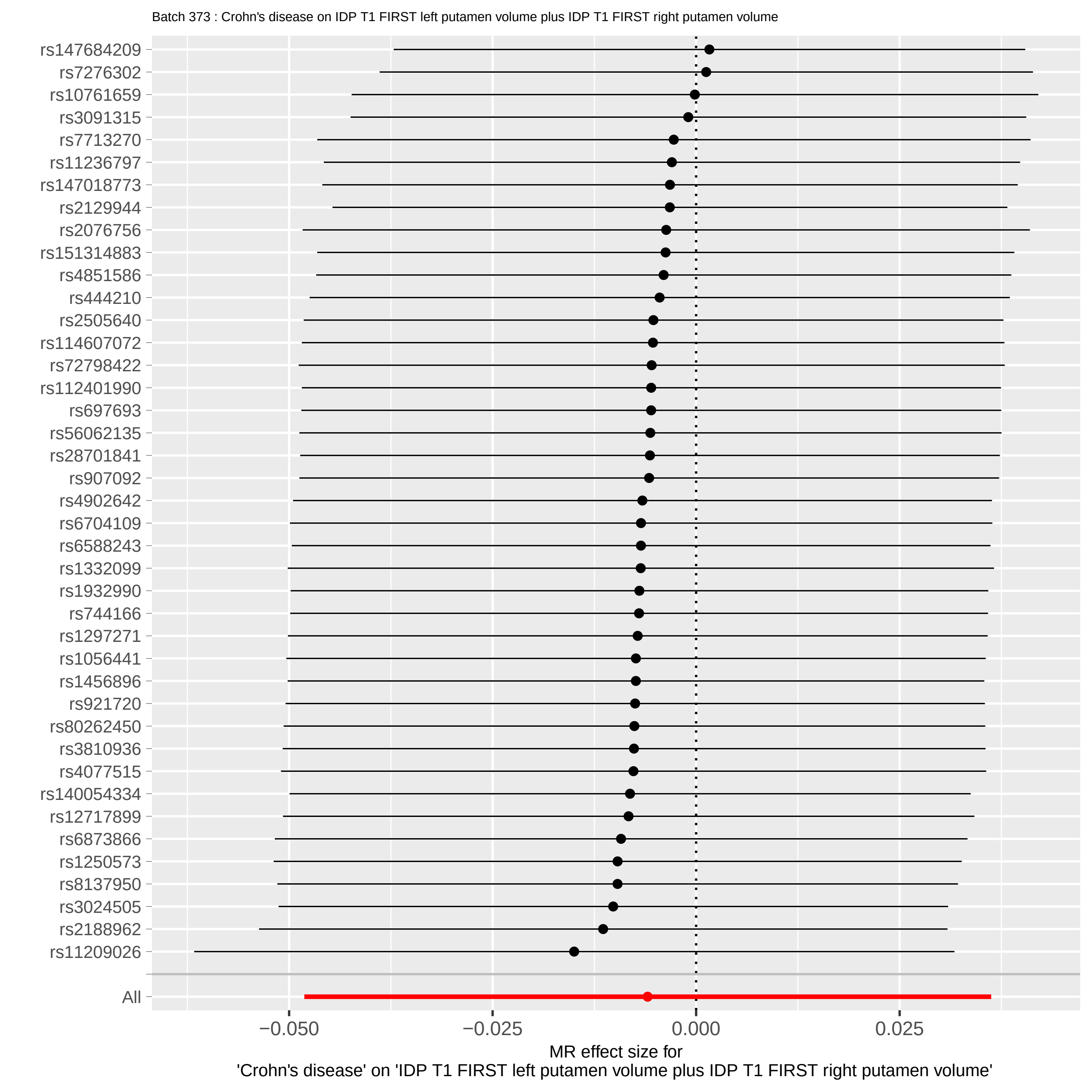

Batch 373 : Crohn's disease on IDP T1 FIRST left putamen volume plus IDP T1 FIRST right putamen volume
rs147684209
rs7276302
rs10761659
rs3091315
rs7713270
rs11236797
rs147018773
rs2129944
rs2076756
rs151314883
rs4851586
rs444210
rs2505640
rs114607072
rs72798422
rs112401990
rs697693
rs56062135
rs28701841
rs907092
rs4902642
rs6704109
rs6588243
rs1332099
rs1932990
rs744166
rs1297271
rs1056441
rs1456896
rs921720
rs80262450
rs3810936
rs4077515
rs140054334
rs12717899
rs6873866
rs1250573
rs8137950
rs3024505
rs2188962
rs11209026
All
−0.050
−0.025
0.000
0.025
MR effect size for
'Crohn's disease' on 'IDP T1 FIRST left putamen volume plus IDP T1 FIRST right putamen volume'

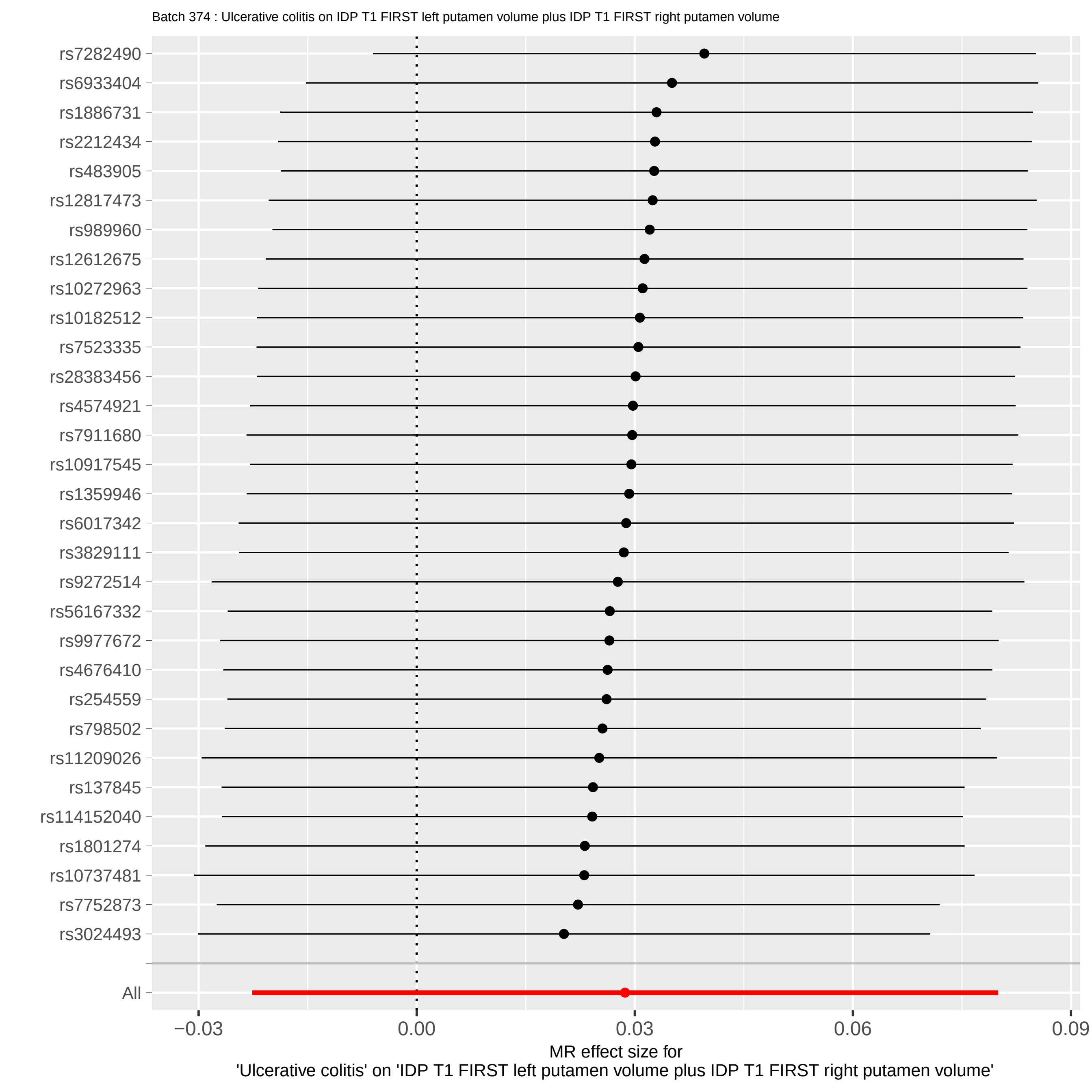
Batch 374 : Ulcerative colitis on IDP T1 FIRST left putamen volume plus IDP T1 FIRST right putamen volume
rs7282490
rs6933404
rs1886731
rs2212434
rs483905
rs12817473
rs989960
rs12612675
rs10272963
rs10182512
rs7523335
rs28383456
rs4574921
rs7911680
rs10917545
rs1359946
rs6017342
rs3829111
rs9272514
rs56167332
rs9977672
rs4676410
rs254559
rs798502
rs11209026
rs137845
rs114152040
rs1801274
rs10737481
rs7752873
rs3024493
All
−0.03
0.00
0.03
0.06
0.09
MR effect size for
'Ulcerative colitis' on 'IDP T1 FIRST left putamen volume plus IDP T1 FIRST right putamen volume'

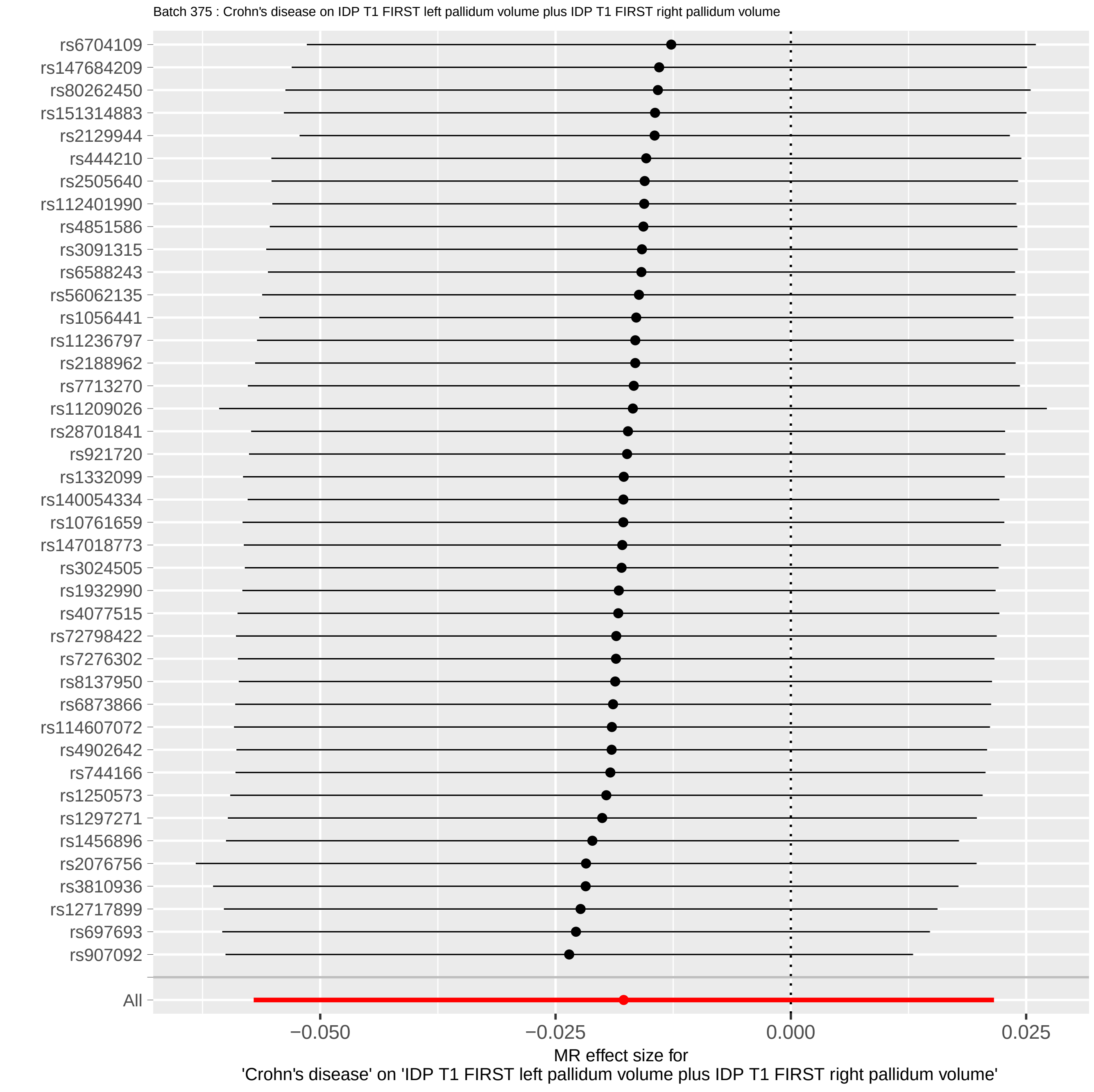

Batch 375 : Crohn's disease on IDP T1 FIRST left pallidum volume plus IDP T1 FIRST right pallidum volume
rs6704109
rs147684209
rs80262450
rs151314883
rs2129944
rs444210
rs2505640
rs112401990
rs4851586
rs3091315
rs6588243
rs56062135
rs1056441
rs11236797
rs2188962
rs7713270
rs11209026
rs28701841
rs921720
rs1332099
rs140054334
rs10761659
rs147018773
rs3024505
rs1932990
rs4077515
rs72798422
rs7276302
rs8137950
rs6873866
rs114607072
rs4902642
rs744166
rs1250573
rs1297271
rs1456896
rs2076756
rs3810936
rs12717899
rs697693
rs907092
All
−0.050
−0.025
0.000
0.025
MR effect size for
'Crohn's disease' on 'IDP T1 FIRST left pallidum volume plus IDP T1 FIRST right pallidum volume'

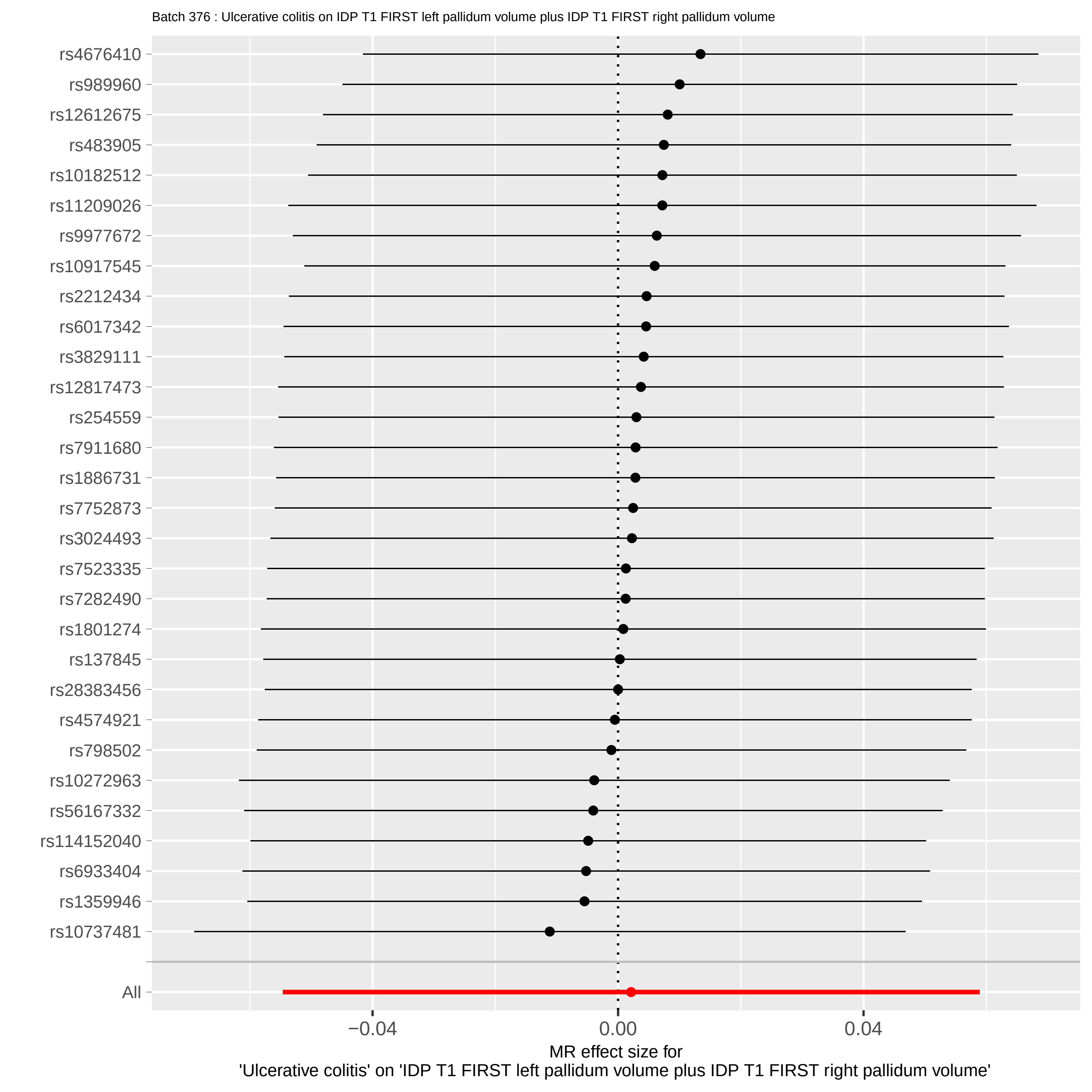

Batch 376 : Ulcerative colitis on IDP T1 FIRST left pallidum volume plus IDP T1 FIRST right pallidum volume
rs4676410
rs989960
rs12612675
rs483905
rs10182512
rs11209026
rs9977672
rs10917545
rs2212434
rs6017342
rs3829111
rs12817473
rs254559
rs7911680
rs1886731
rs7752873
rs3024493
rs7523335
rs7282490
rs1801274
rs137845
rs28383456
rs4574921
rs798502
rs10272963
rs56167332
rs114152040
rs6933404
rs1359946
rs10737481
All
−0.04
0.00
0.04
MR effect size for
'Ulcerative colitis' on 'IDP T1 FIRST left pallidum volume plus IDP T1 FIRST right pallidum volume'

Batch 377 : Crohn's disease on IDP T1 FIRST left hippocampus volume plus IDP T1 FIRST right hippocampus volume

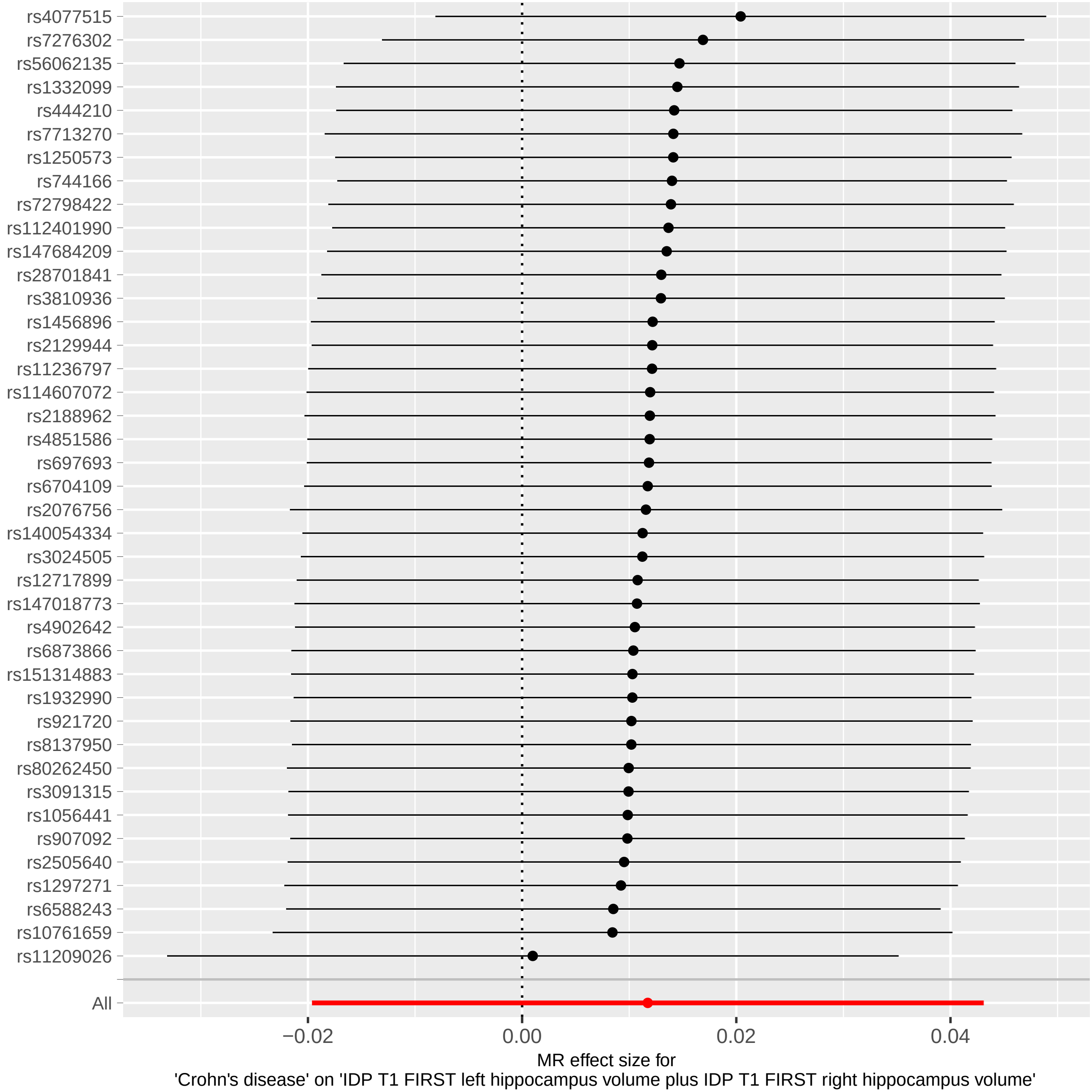

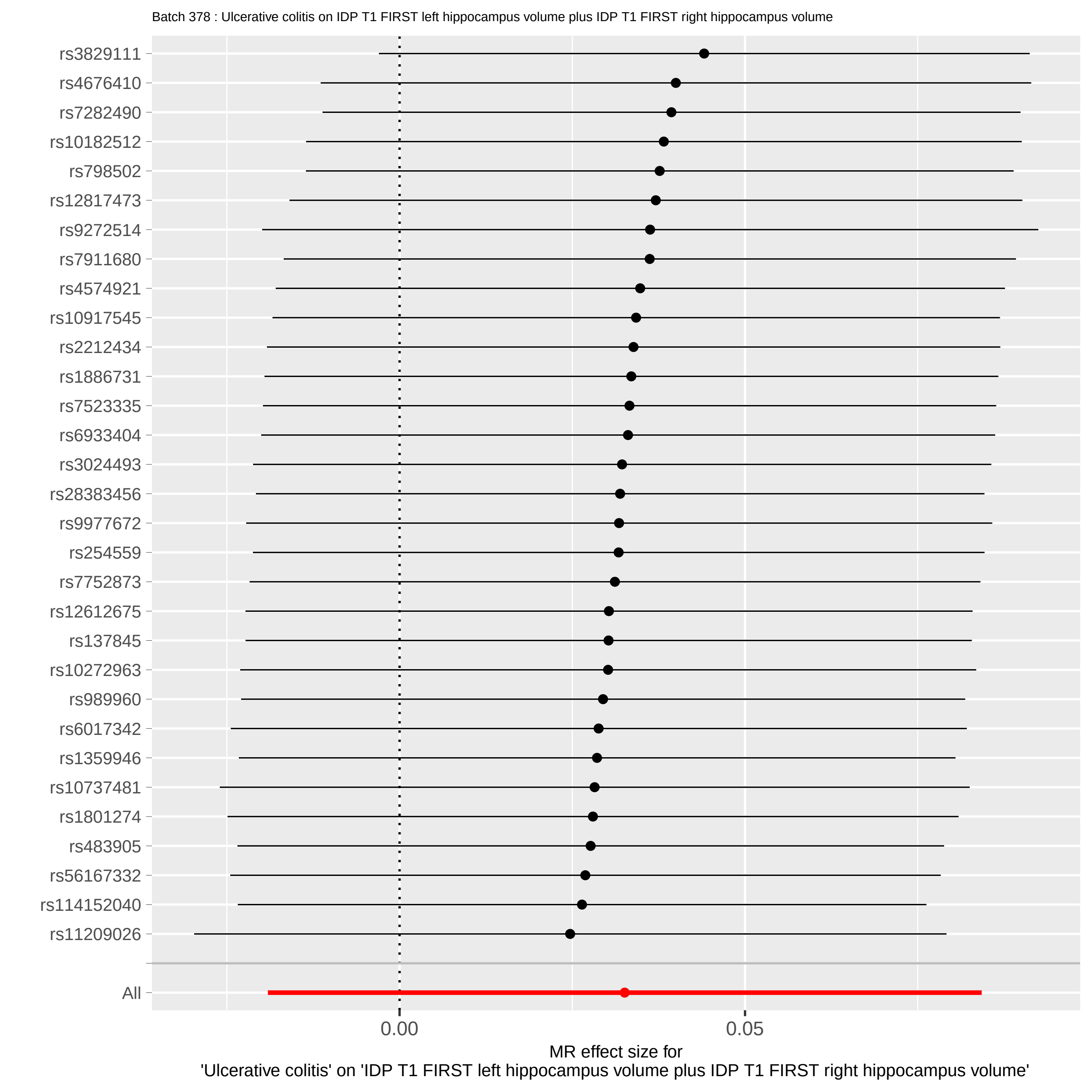

Batch 378 : Ulcerative colitis on IDP T1 FIRST left hippocampus volume plus IDP T1 FIRST right hippocampus volume
rs3829111
rs4676410
rs7282490
rs10182512
rs798502
rs12817473
rs9272514
rs7911680
rs4574921
rs10917545
rs2212434
rs1886731
rs7523335
rs6933404
rs3024493
rs28383456
rs9977672
rs254559
rs7752873
rs12612675
rs137845
rs10272963
rs989960
rs6017342
rs1359946
rs10737481
rs1801274
rs483905
rs56167332
rs114152040
rs11209026
All
0.00
0.05
MR effect size for
'Ulcerative colitis' on 'IDP T1 FIRST left hippocampus volume plus IDP T1 FIRST right hippocampus volume'

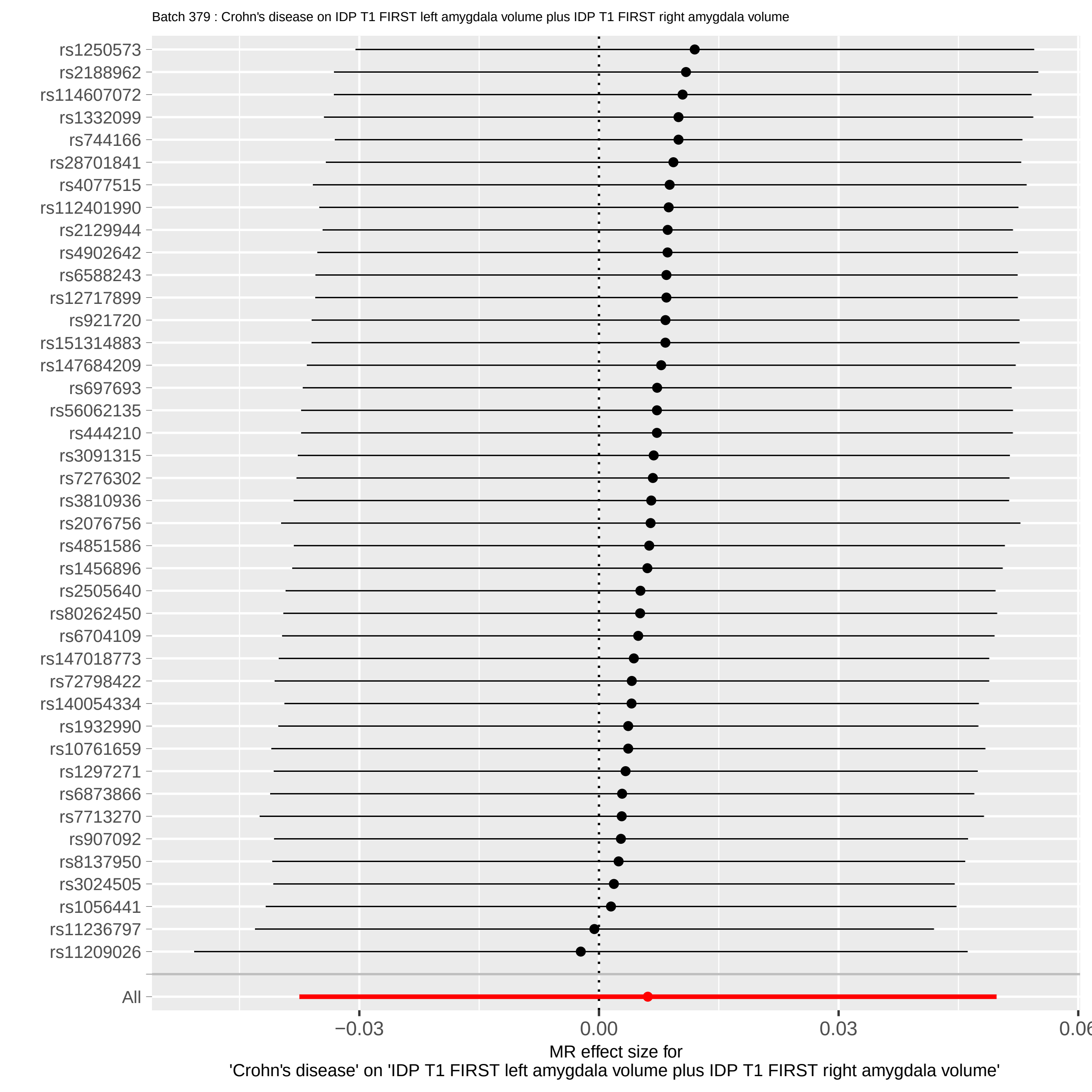

Batch 379 : Crohn's disease on IDP T1 FIRST left amygdala volume plus IDP T1 FIRST right amygdala volume
rs1250573
rs2188962
rs114607072
rs1332099
rs744166
rs28701841
rs4077515
rs112401990
rs2129944
rs4902642
rs6588243
rs12717899
rs921720
rs151314883
rs147684209
rs697693
rs56062135
rs444210
rs3091315
rs7276302
rs3810936
rs2076756
rs4851586
rs1456896
rs2505640
rs80262450
rs6704109
rs147018773
rs72798422
rs140054334
rs1932990
rs10761659
rs1297271
rs6873866
rs7713270
rs907092
rs8137950
rs3024505
rs1056441
rs11236797
rs11209026
All
−0.03
0.00
0.03
0.06
MR effect size for
'Crohn's disease' on 'IDP T1 FIRST left amygdala volume plus IDP T1 FIRST right amygdala volume'

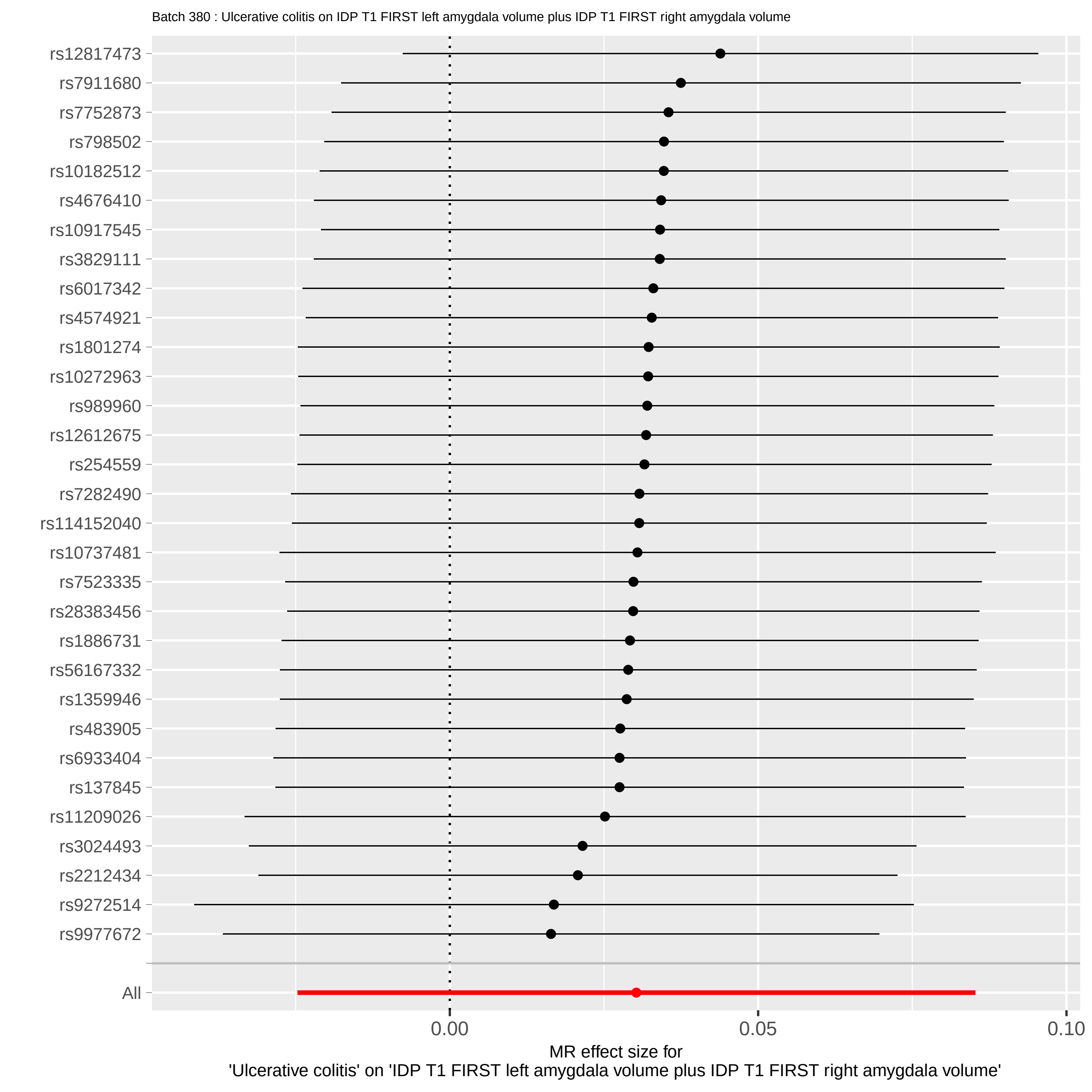

Batch 380 : Ulcerative colitis on IDP T1 FIRST left amygdala volume plus IDP T1 FIRST right amygdala volume
rs12817473
rs7911680
rs7752873
rs798502
rs10182512
rs4676410
rs10917545
rs3829111
rs6017342
rs4574921
rs1801274
rs10272963
rs989960
rs12612675
rs254559
rs7282490
rs114152040
rs10737481
rs7523335
rs28383456
rs1886731
rs56167332
rs1359946
rs483905
rs6933404
rs137845
rs11209026
rs3024493
rs2212434
rs9272514
rs9977672
All
0.00
0.05
0.10
MR effect size for
'Ulcerative colitis' on 'IDP T1 FIRST left amygdala volume plus IDP T1 FIRST right amygdala volume'

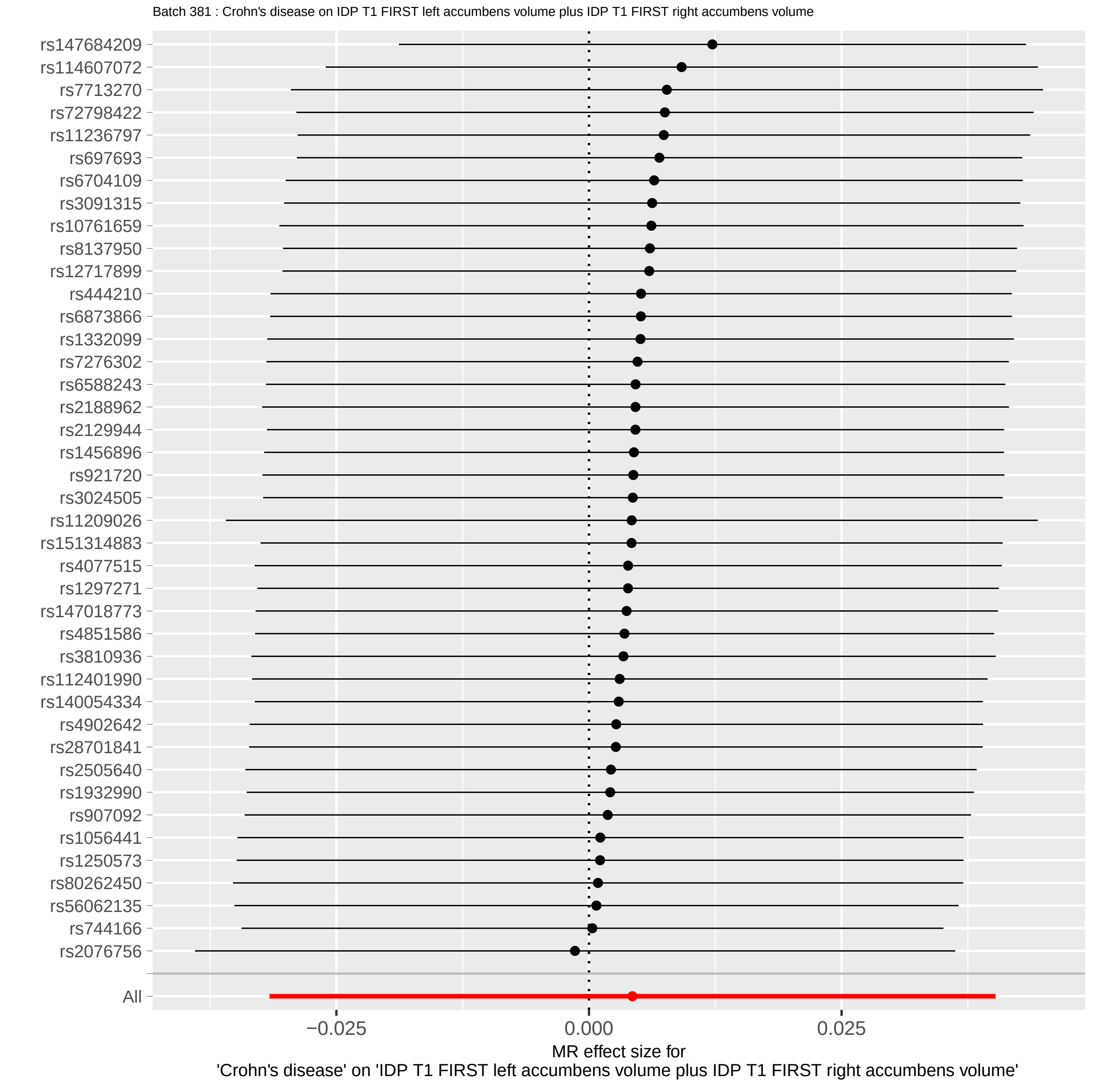

Batch 381 : Crohn's disease on IDP T1 FIRST left accumbens volume plus IDP T1 FIRST right accumbens volume
rs147684209
rs114607072
rs7713270
rs72798422
rs11236797
rs697693
rs6704109
rs3091315
rs10761659
rs8137950
rs12717899
rs444210
rs6873866
rs1332099
rs7276302
rs6588243
rs2188962
rs2129944
rs1456896
rs921720
rs3024505
rs11209026
rs151314883
rs4077515
rs1297271
rs147018773
rs4851586
rs3810936
rs112401990
rs140054334
rs4902642
rs28701841
rs2505640
rs1932990
rs907092
rs1056441
rs1250573
rs80262450
rs56062135
rs744166
rs2076756
All
−0.025
0.000
0.025
MR effect size for
'Crohn's disease' on 'IDP T1 FIRST left accumbens volume plus IDP T1 FIRST right accumbens volume'

Batch 382 : Ulcerative colitis on IDP T1 FIRST left accumbens volume plus IDP T1 FIRST right accumbens volume

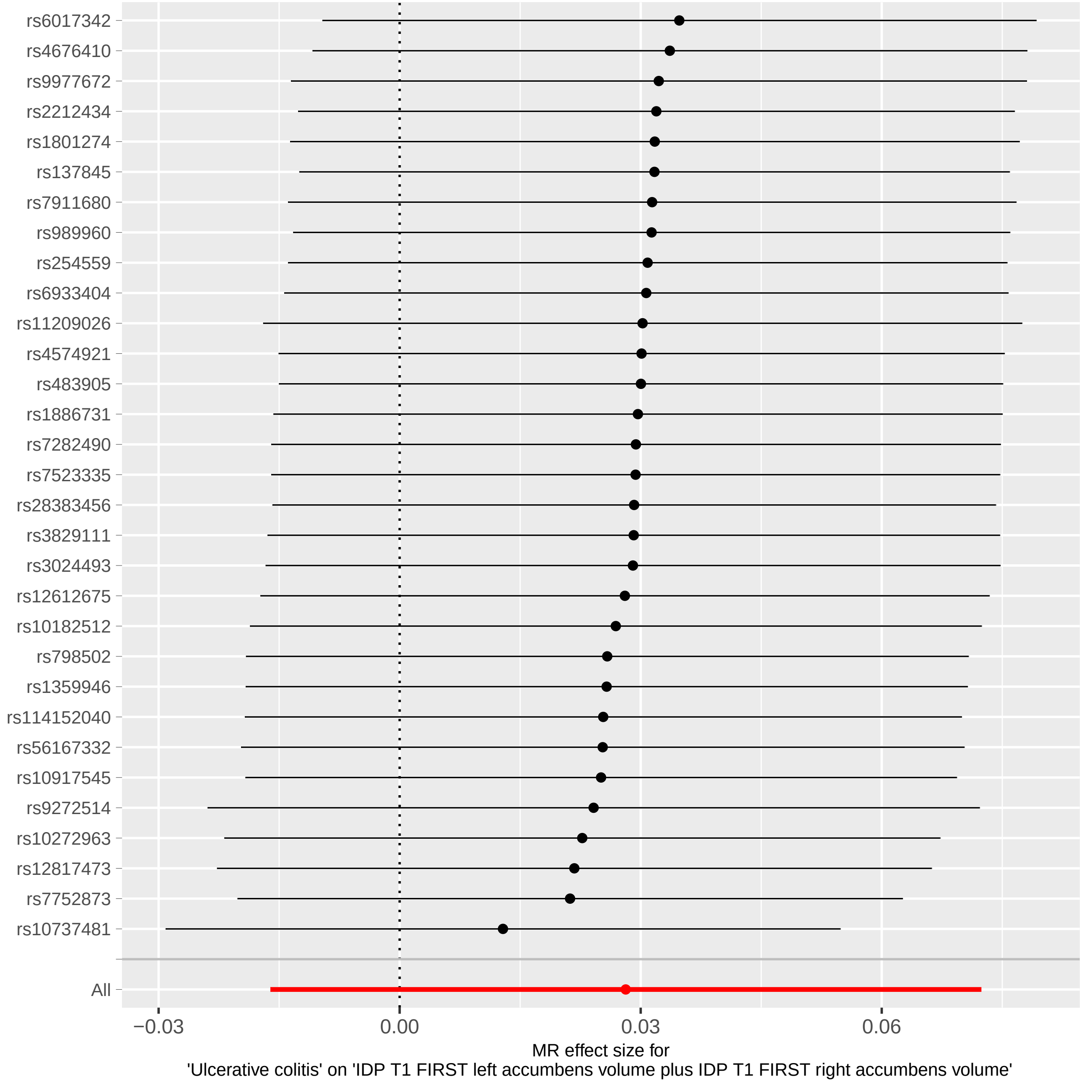

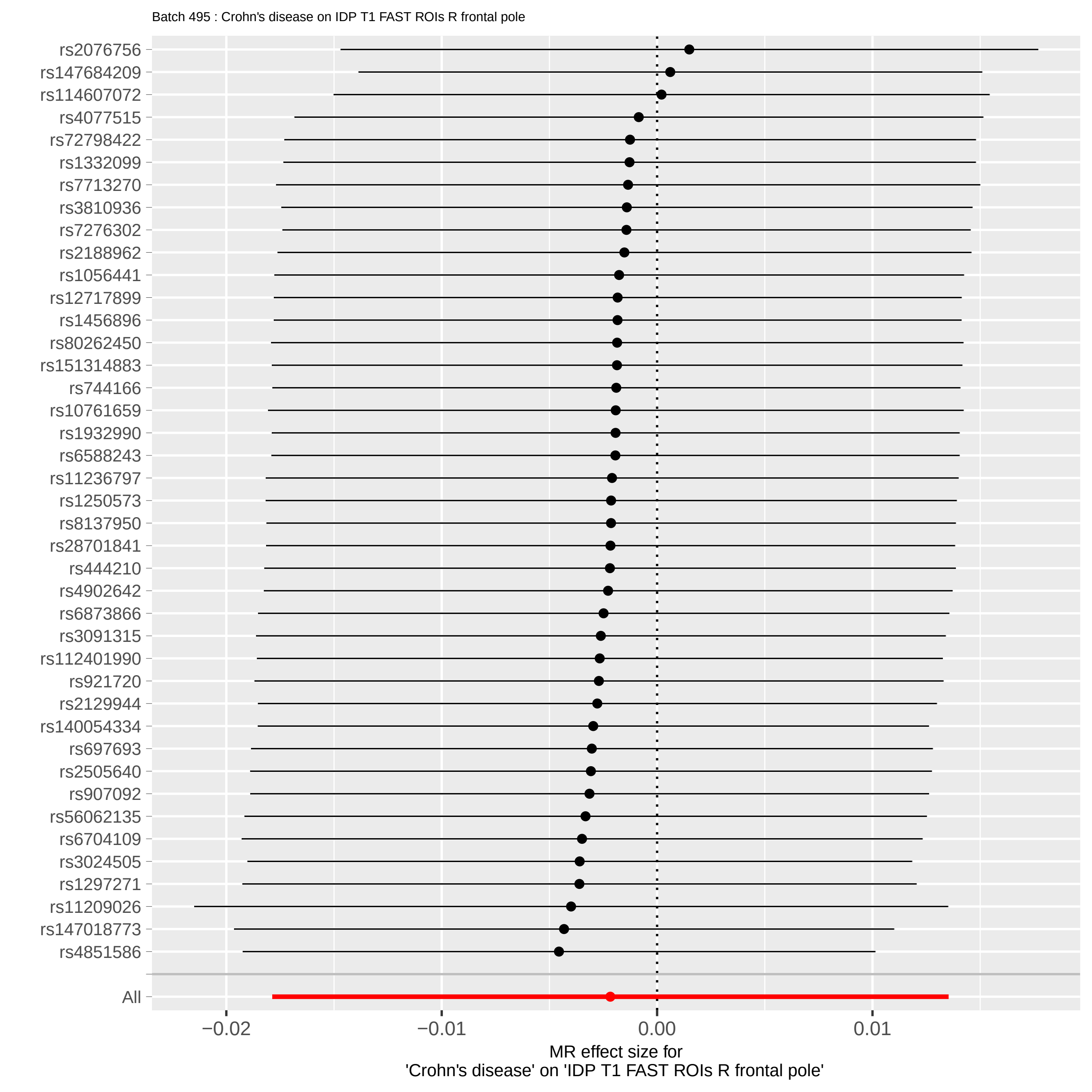

Batch 495 : Crohn's disease on IDP T1 FAST ROIs R frontal pole
rs2076756
rs147684209
rs114607072
rs4077515
rs72798422
rs1332099
rs7713270
rs3810936
rs7276302
rs2188962
rs1056441
rs12717899
rs1456896
rs80262450
rs151314883
rs744166
rs10761659
rs1932990
rs6588243
rs11236797
rs1250573
rs8137950
rs28701841
rs444210
rs4902642
rs6873866
rs3091315
rs112401990
rs921720
rs2129944
rs140054334
rs697693
rs2505640
rs907092
rs56062135
rs6704109
rs3024505
rs1297271
rs11209026
rs147018773
rs4851586
All
−0.02
−0.01
0.00
0.01
MR effect size for
'Crohn's disease' on 'IDP T1 FAST ROIs R frontal pole'

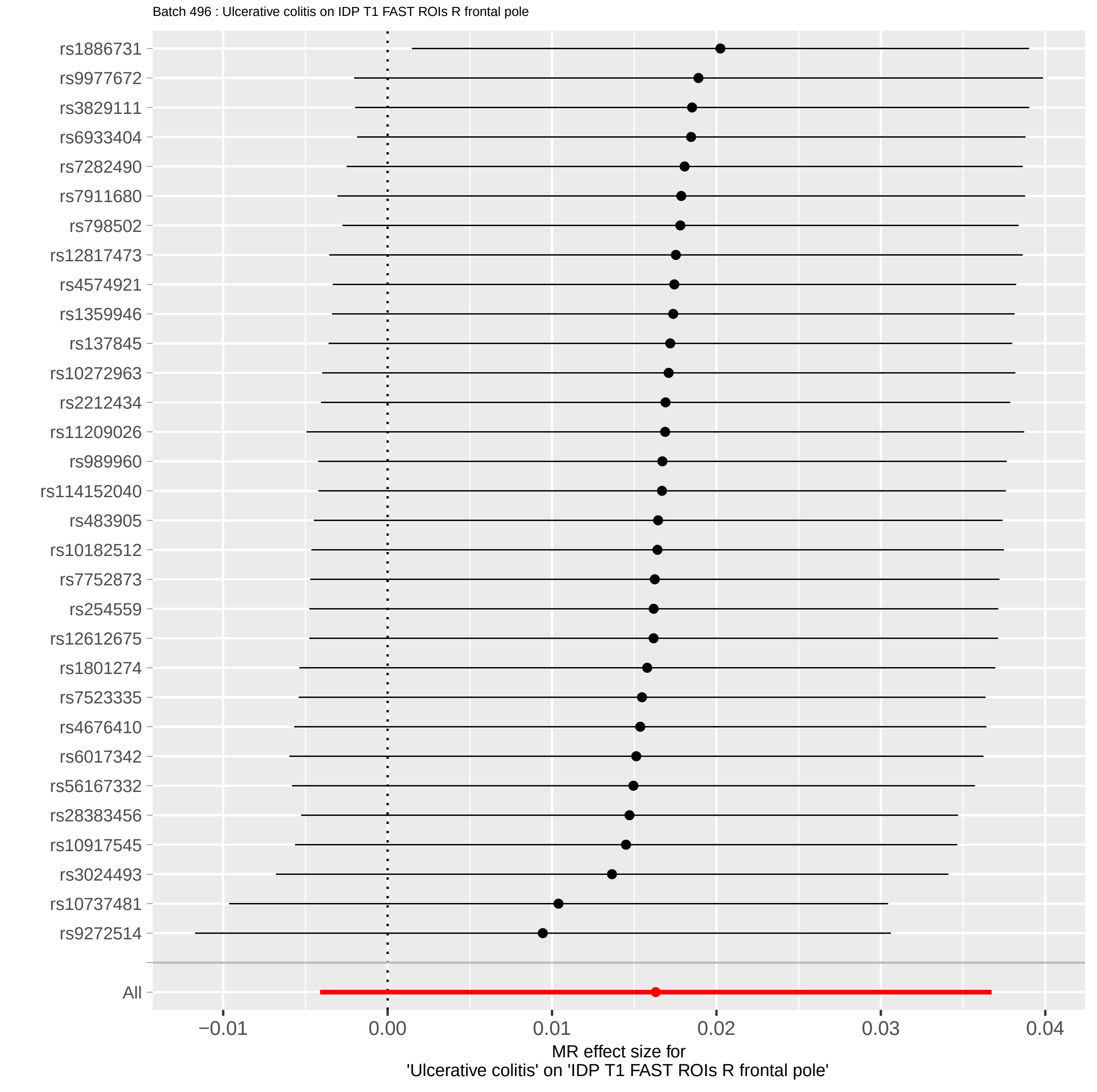

Batch 496 : Ulcerative colitis on IDP T1 FAST ROIs R frontal pole
rs1886731
rs9977672
rs3829111
rs6933404
rs7282490
rs7911680
rs798502
rs12817473
rs4574921
rs1359946
rs137845
rs10272963
rs2212434
rs11209026
rs989960
rs114152040
rs483905
rs10182512
rs7752873
rs254559
rs12612675
rs1801274
rs7523335
rs4676410
rs6017342
rs56167332
rs28383456
rs10917545
rs3024493
rs10737481
rs9272514
All
−0.01
0.00
0.01
0.02
0.03
0.04
MR effect size for
'Ulcerative colitis' on 'IDP T1 FAST ROIs R frontal pole'

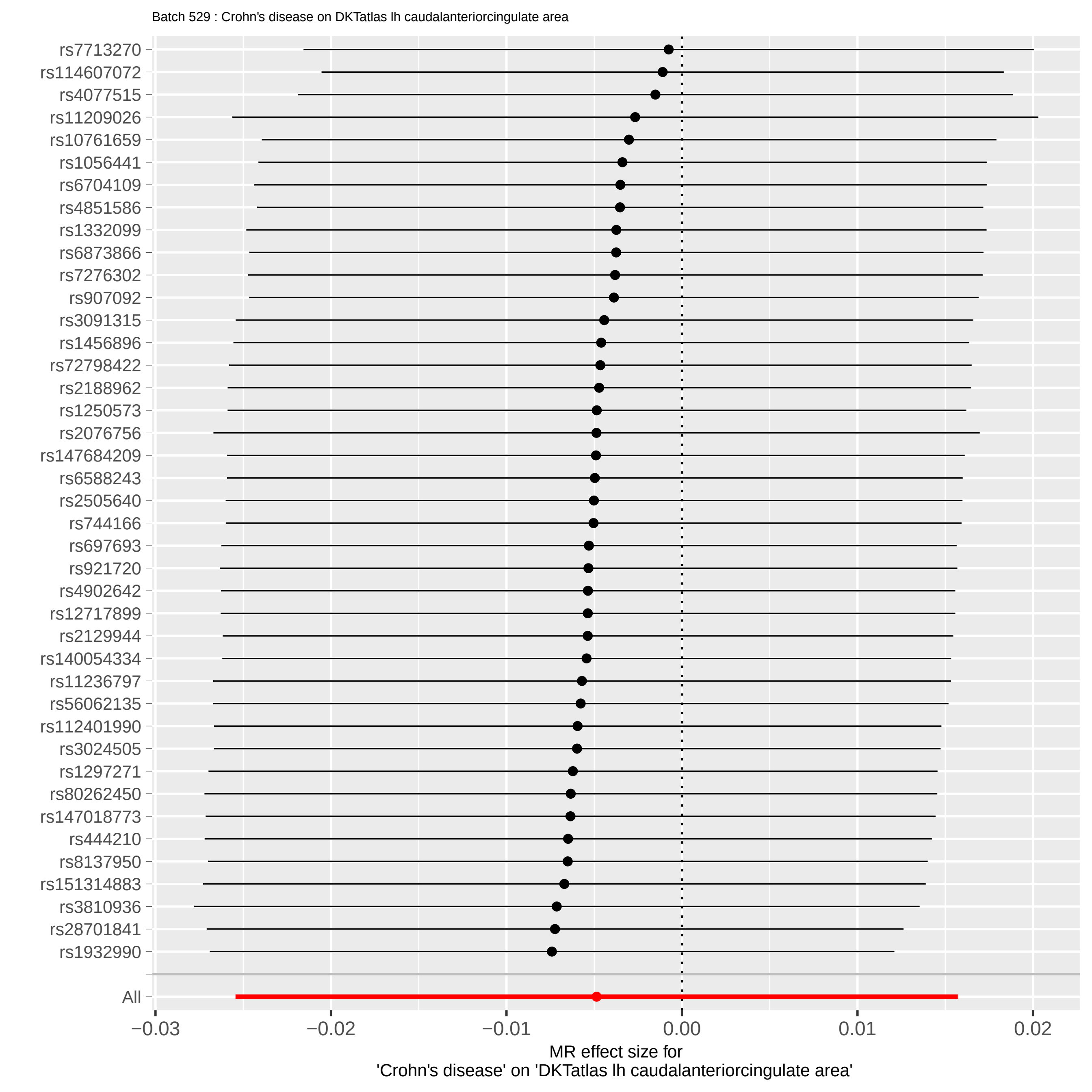

Batch 529 : Crohn's disease on DKTatlas lh caudalanteriorcingulate area
rs7713270
rs114607072
rs4077515
rs11209026
rs10761659
rs1056441
rs6704109
rs4851586
rs1332099
rs6873866
rs7276302
rs907092
rs3091315
rs1456896
rs72798422
rs2188962
rs1250573
rs2076756
rs147684209
rs6588243
rs2505640
rs744166
rs697693
rs921720
rs4902642
rs12717899
rs2129944
rs140054334
rs11236797
rs56062135
rs112401990
rs3024505
rs1297271
rs80262450
rs147018773
rs444210
rs8137950
rs151314883
rs3810936
rs28701841
rs1932990
All
−0.03
−0.02
−0.01
0.00
0.01
0.02
MR effect size for
'Crohn's disease' on 'DKTatlas lh caudalanteriorcingulate area'

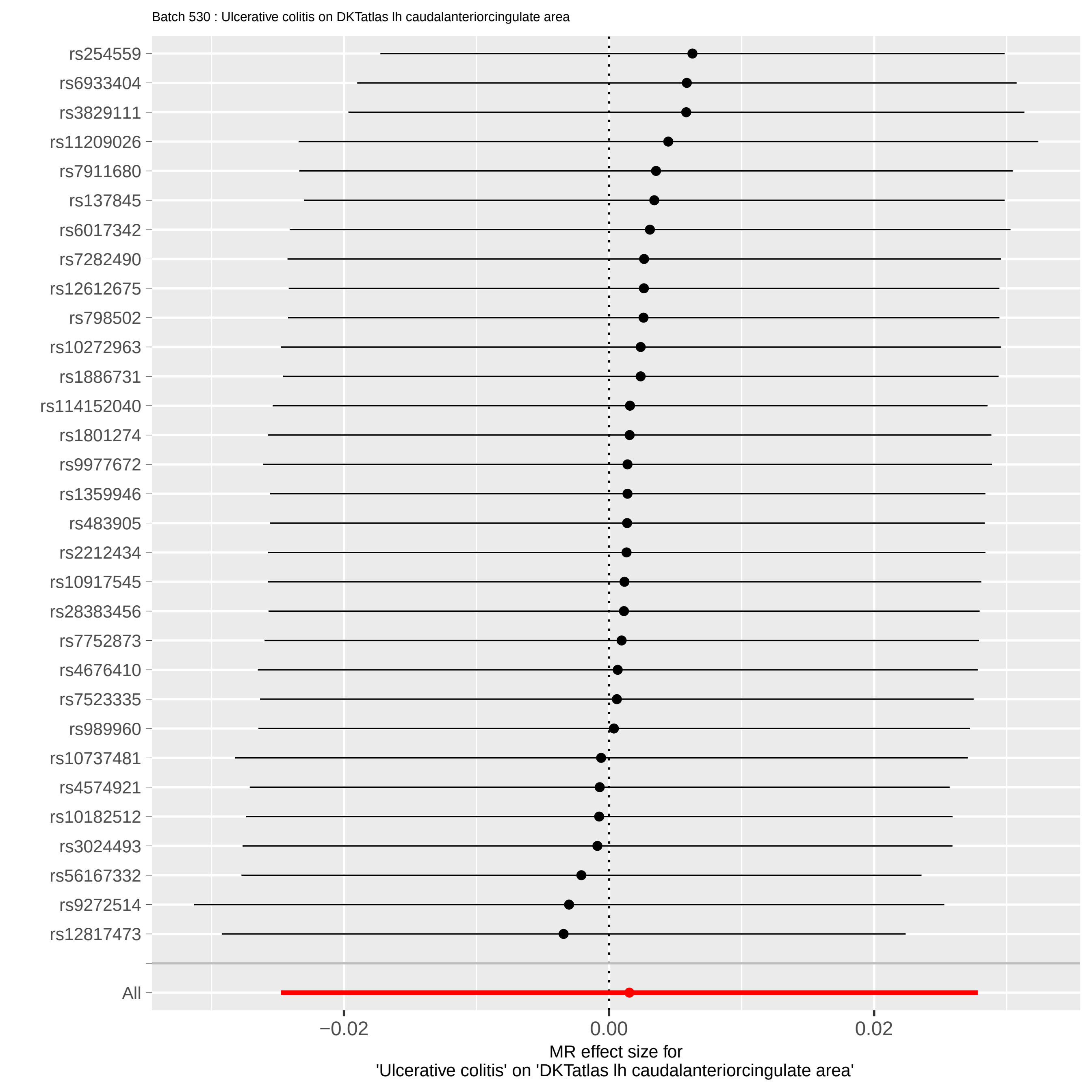
Batch 530 : Ulcerative colitis on DKTatlas lh caudalanteriorcingulate area
rs254559
rs6933404
rs3829111
rs11209026
rs7911680
rs137845
rs6017342
rs7282490
rs12612675
rs798502
rs10272963
rs1886731
rs114152040
rs1801274
rs9977672
rs1359946
rs483905
rs2212434
rs10917545
rs28383456
rs7752873
rs4676410
rs7523335
rs989960
rs10737481
rs4574921
rs10182512
rs3024493
rs56167332
rs9272514
rs12817473
All
−0.02
0.00
0.02
MR effect size for
'Ulcerative colitis' on 'DKTatlas lh caudalanteriorcingulate area'

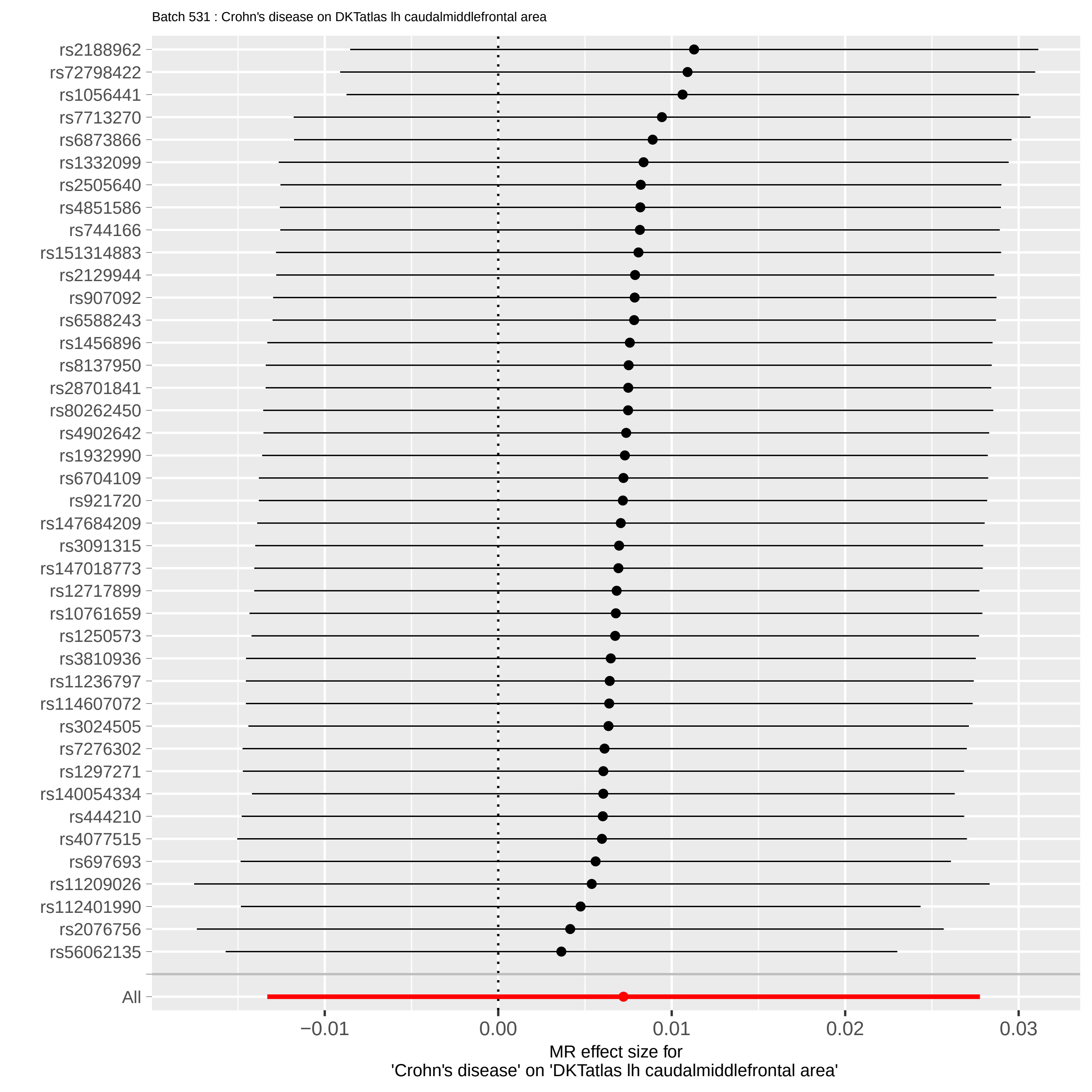
Batch 531 : Crohn's disease on DKTatlas lh caudalmiddlefrontal area
rs2188962
rs72798422
rs1056441
rs7713270
rs6873866
rs1332099
rs2505640
rs4851586
rs744166
rs151314883
rs2129944
rs907092
rs6588243
rs1456896
rs8137950
rs28701841
rs80262450
rs4902642
rs1932990
rs6704109
rs921720
rs147684209
rs3091315
rs147018773
rs12717899
rs10761659
rs1250573
rs3810936
rs11236797
rs114607072
rs3024505
rs7276302
rs1297271
rs140054334
rs444210
rs4077515
rs697693
rs11209026
rs112401990
rs2076756
rs56062135
All
−0.01
0.00
0.01
0.02
0.03
MR effect size for
'Crohn's disease' on 'DKTatlas lh caudalmiddlefrontal area'

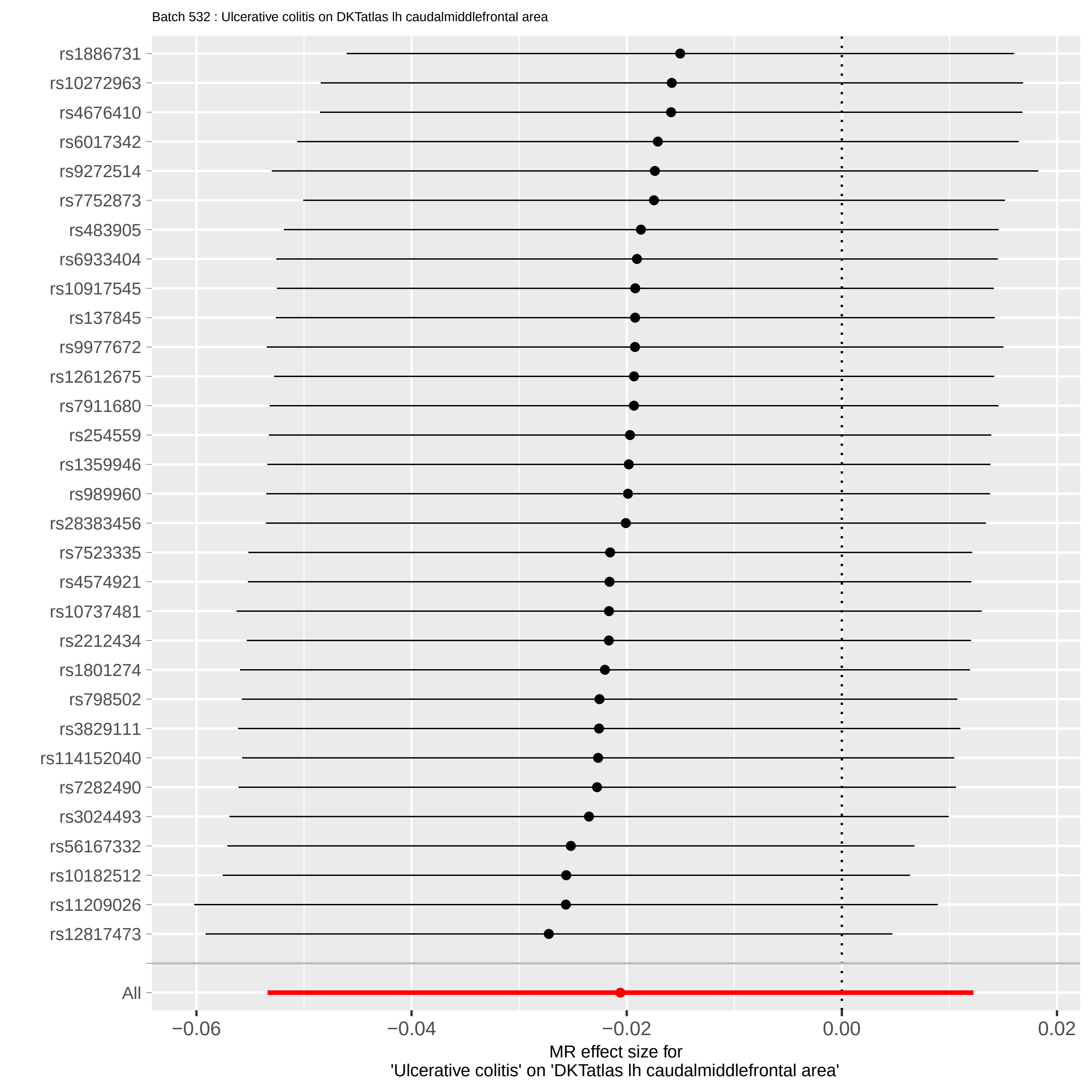

Batch 532 : Ulcerative colitis on DKTatlas lh caudalmiddlefrontal area
rs1886731
rs10272963
rs4676410
rs6017342
rs9272514
rs7752873
rs483905
rs6933404
rs10917545
rs137845
rs9977672
rs12612675
rs7911680
rs254559
rs1359946
rs989960
rs28383456
rs7523335
rs4574921
rs10737481
rs2212434
rs1801274
rs798502
rs3829111
rs114152040
rs7282490
rs3024493
rs56167332
rs10182512
rs11209026
rs12817473
All
−0.06
−0.04
−0.02
0.00
0.02
MR effect size for
'Ulcerative colitis' on 'DKTatlas lh caudalmiddlefrontal area'

Batch 533 : Crohn's disease on DKTatlas lh cuneus area

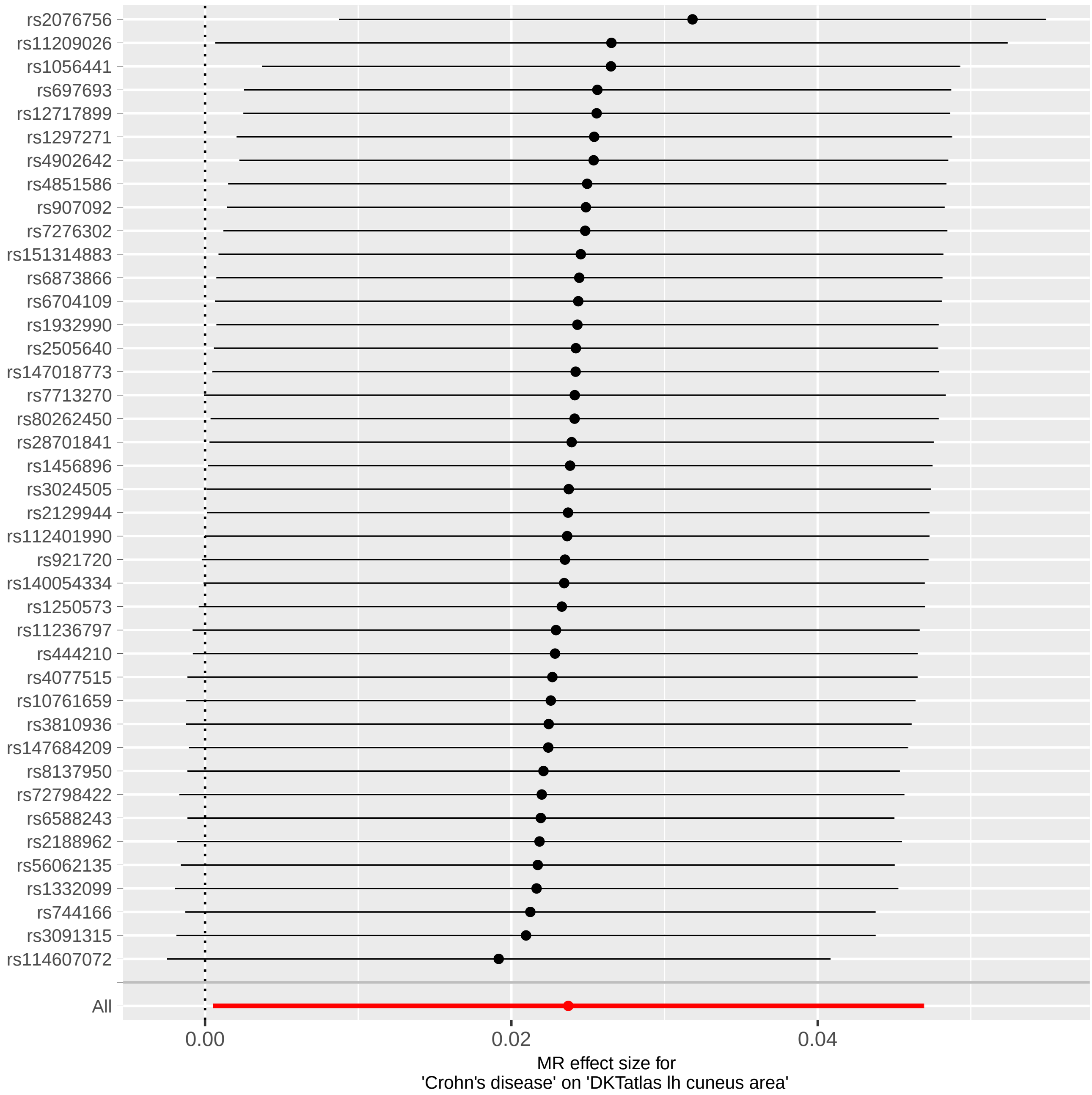

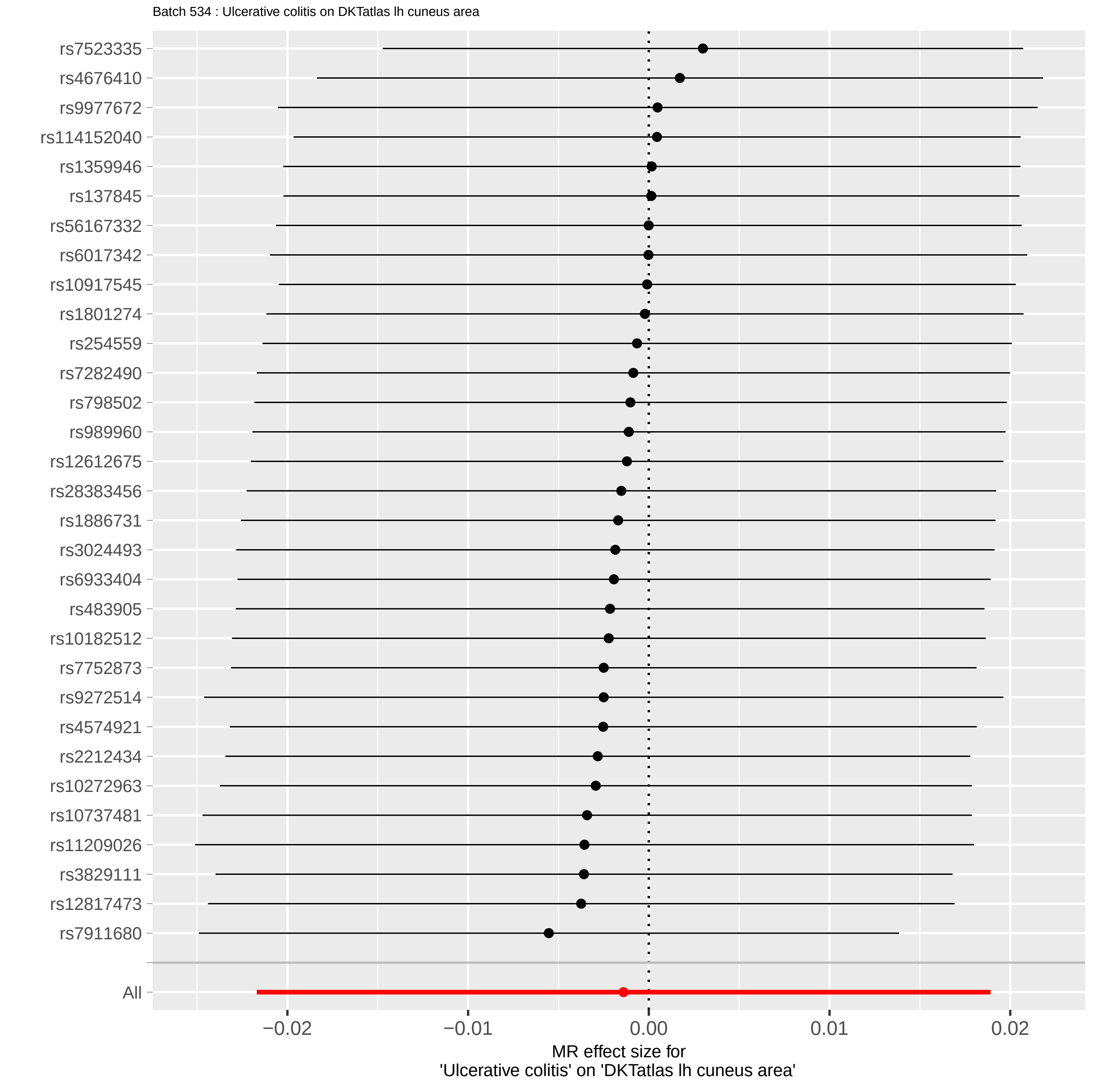
Batch 534 : Ulcerative colitis on DKTatlas lh cuneus area
rs7523335
rs4676410
rs9977672
rs114152040
rs1359946
rs137845
rs56167332
rs6017342
rs10917545
rs1801274
rs254559
rs7282490
rs798502
rs989960
rs12612675
rs28383456
rs1886731
rs3024493
rs6933404
rs483905
rs10182512
rs7752873
rs9272514
rs4574921
rs2212434
rs10272963
rs10737481
rs11209026
rs3829111
rs12817473
rs7911680
All
−0.02
−0.01
0.00
0.01
0.02
MR effect size for
'Ulcerative colitis' on 'DKTatlas lh cuneus area'

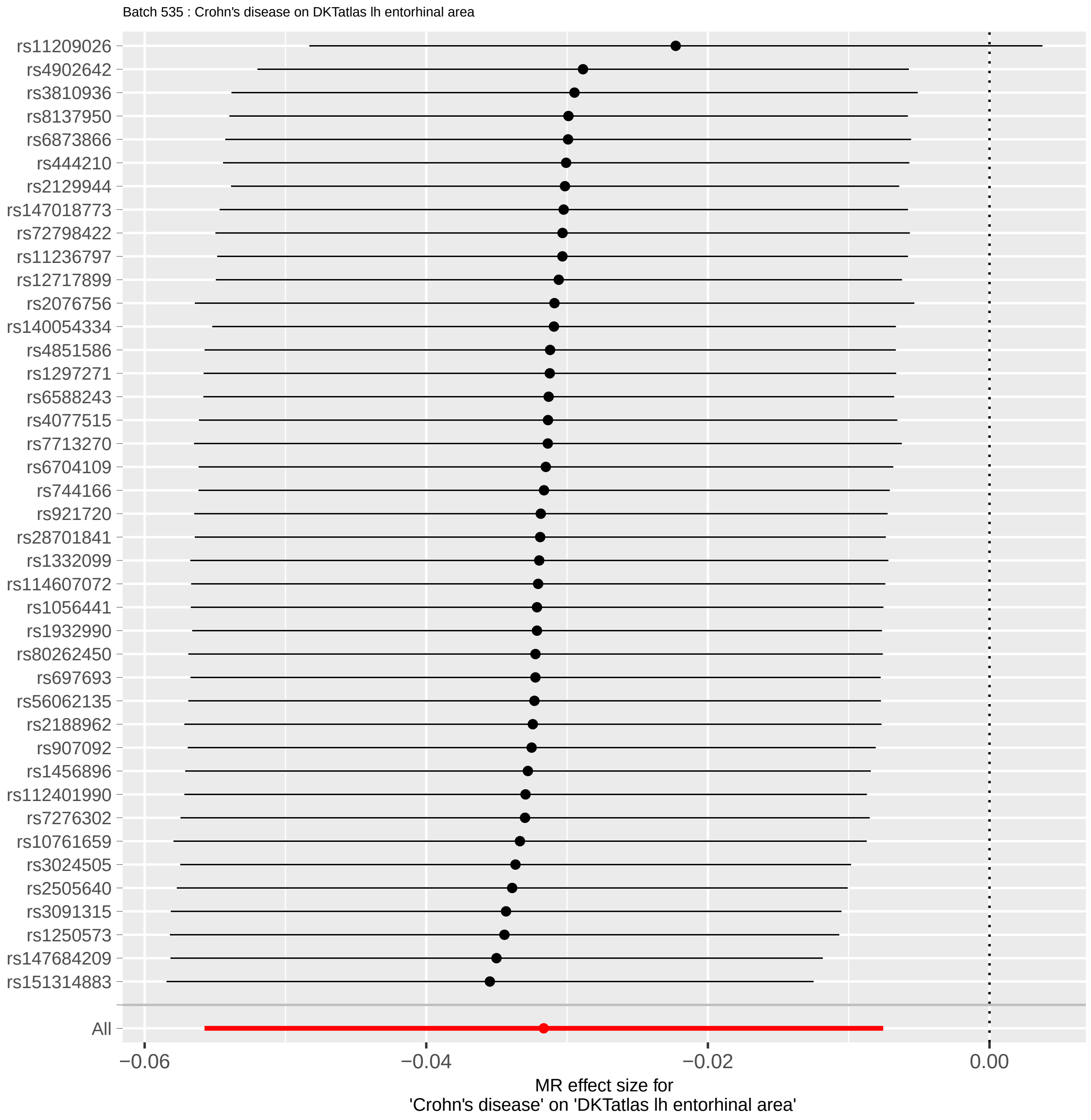

Batch 535 : Crohn's disease on DKTatlas lh entorhinal area
rs11209026
rs4902642
rs3810936
rs8137950
rs6873866
rs444210
rs2129944
rs147018773
rs72798422
rs11236797
rs12717899
rs2076756
rs140054334
rs4851586
rs1297271
rs6588243
rs4077515
rs7713270
rs6704109
rs744166
rs921720
rs28701841
rs1332099
rs114607072
rs1056441
rs1932990
rs80262450
rs697693
rs56062135
rs2188962
rs907092
rs1456896
rs112401990
rs7276302
rs10761659
rs3024505
rs2505640
rs3091315
rs1250573
rs147684209
rs151314883
All
−0.06
−0.04
−0.02
0.00
MR effect size for
'Crohn's disease' on 'DKTatlas lh entorhinal area'

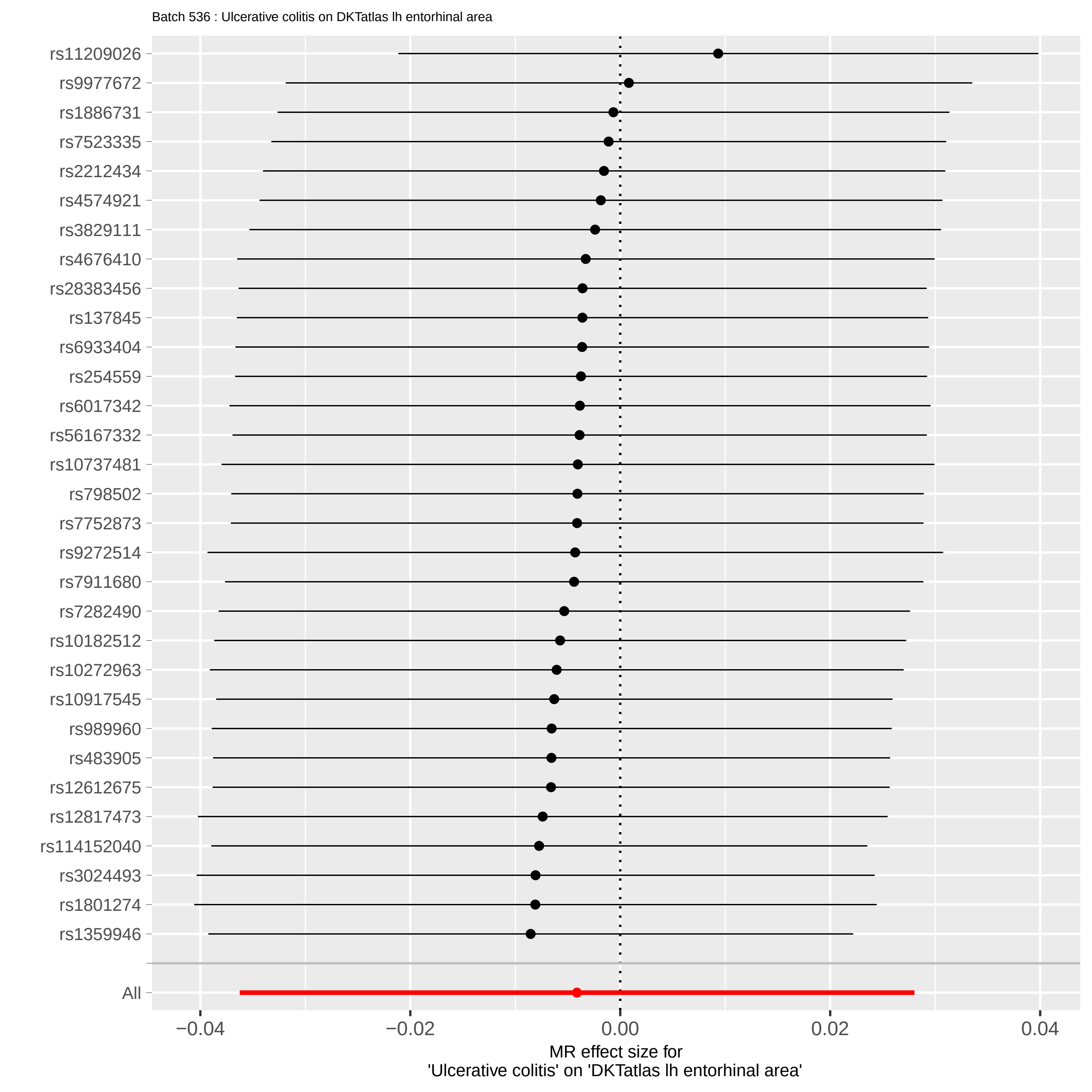

Batch 536 : Ulcerative colitis on DKTatlas lh entorhinal area
rs11209026
rs9977672
rs1886731
rs7523335
rs2212434
rs4574921
rs3829111
rs4676410
rs28383456
rs137845
rs6933404
rs254559
rs6017342
rs56167332
rs10737481
rs798502
rs7752873
rs9272514
rs7911680
rs7282490
rs10182512
rs10272963
rs10917545
rs989960
rs483905
rs12612675
rs12817473
rs114152040
rs3024493
rs1801274
rs1359946
All
−0.04
−0.02
0.00
0.02
0.04
MR effect size for
'Ulcerative colitis' on 'DKTatlas lh entorhinal area'

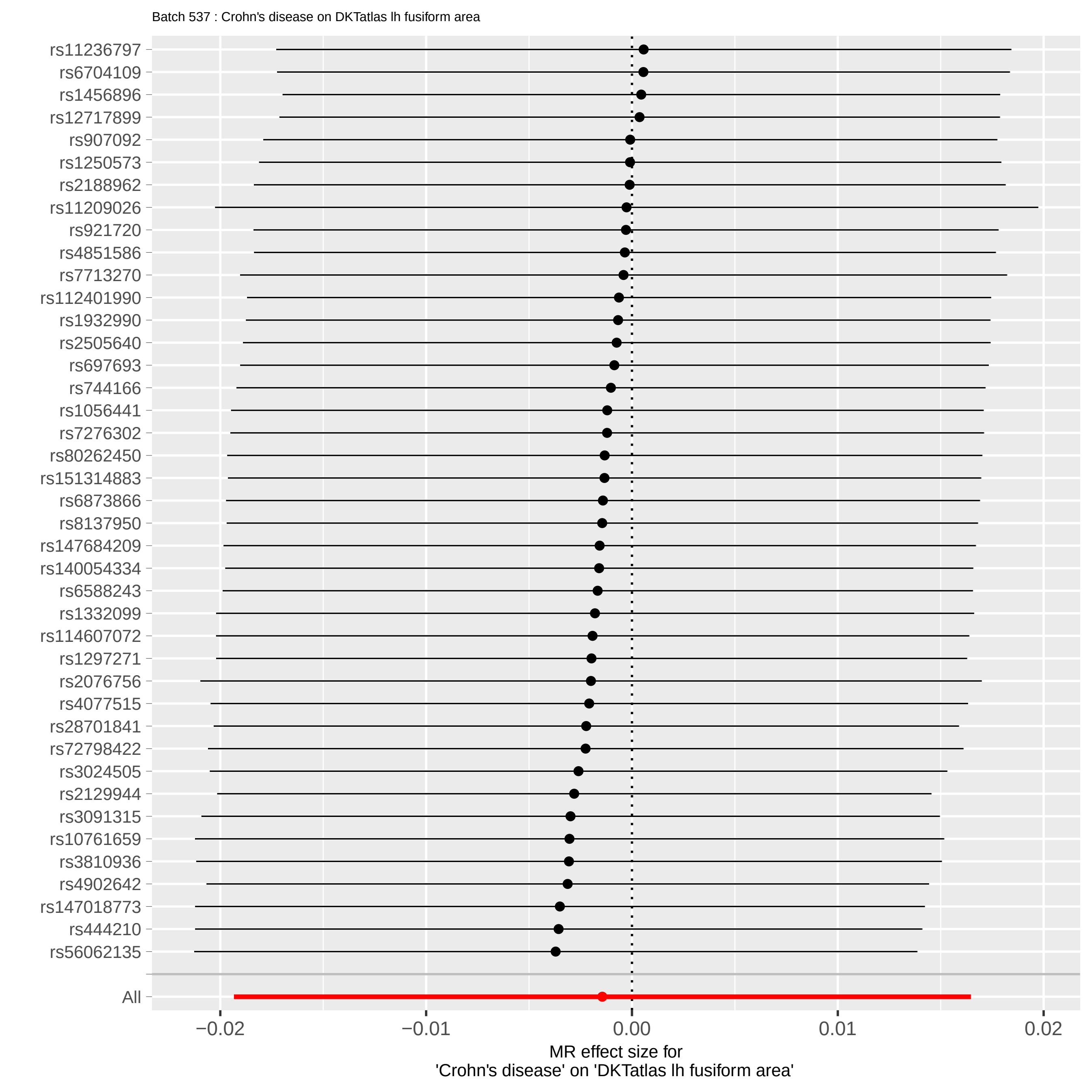
Batch 537 : Crohn's disease on DKTatlas lh fusiform area
rs11236797
rs6704109
rs1456896
rs12717899
rs907092
rs1250573
rs2188962
rs11209026
rs921720
rs4851586
rs7713270
rs112401990
rs1932990
rs2505640
rs697693
rs744166
rs1056441
rs7276302
rs80262450
rs151314883
rs6873866
rs8137950
rs147684209
rs140054334
rs6588243
rs1332099
rs114607072
rs1297271
rs2076756
rs4077515
rs28701841
rs72798422
rs3024505
rs2129944
rs3091315
rs10761659
rs3810936
rs4902642
rs147018773
rs444210
rs56062135
All
−0.02
−0.01
0.00
0.01
0.02
MR effect size for
'Crohn's disease' on 'DKTatlas lh fusiform area'

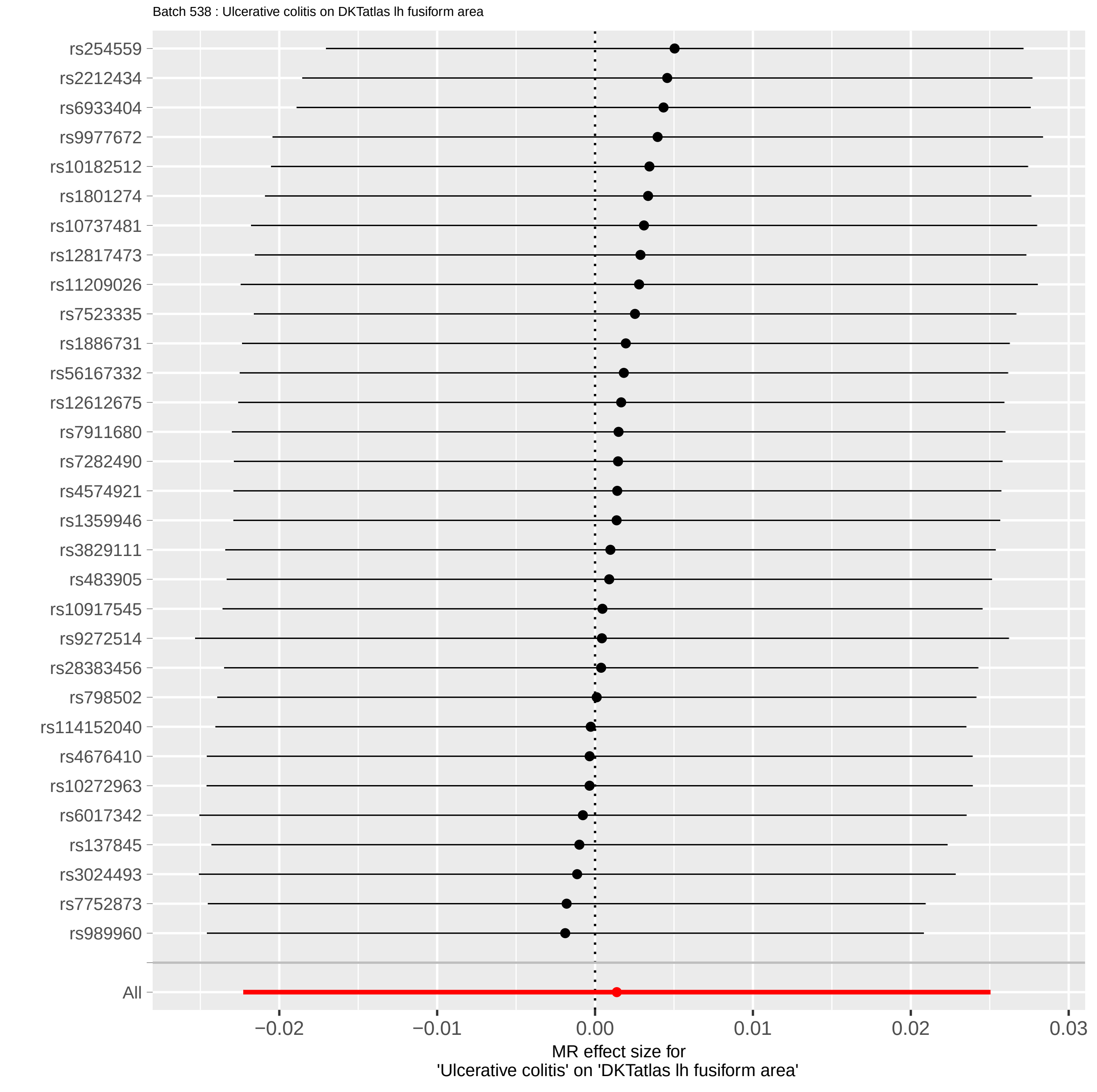

Batch 538 : Ulcerative colitis on DKTatlas lh fusiform area
rs254559
rs2212434
rs6933404
rs9977672
rs10182512
rs1801274
rs10737481
rs12817473
rs11209026
rs7523335
rs1886731
rs56167332
rs12612675
rs7911680
rs7282490
rs4574921
rs1359946
rs3829111
rs483905
rs10917545
rs9272514
rs28383456
rs798502
rs114152040
rs4676410
rs10272963
rs6017342
rs137845
rs3024493
rs7752873
rs989960
All
−0.02
−0.01
0.00
0.01
0.02
0.03
MR effect size for
'Ulcerative colitis' on 'DKTatlas lh fusiform area'

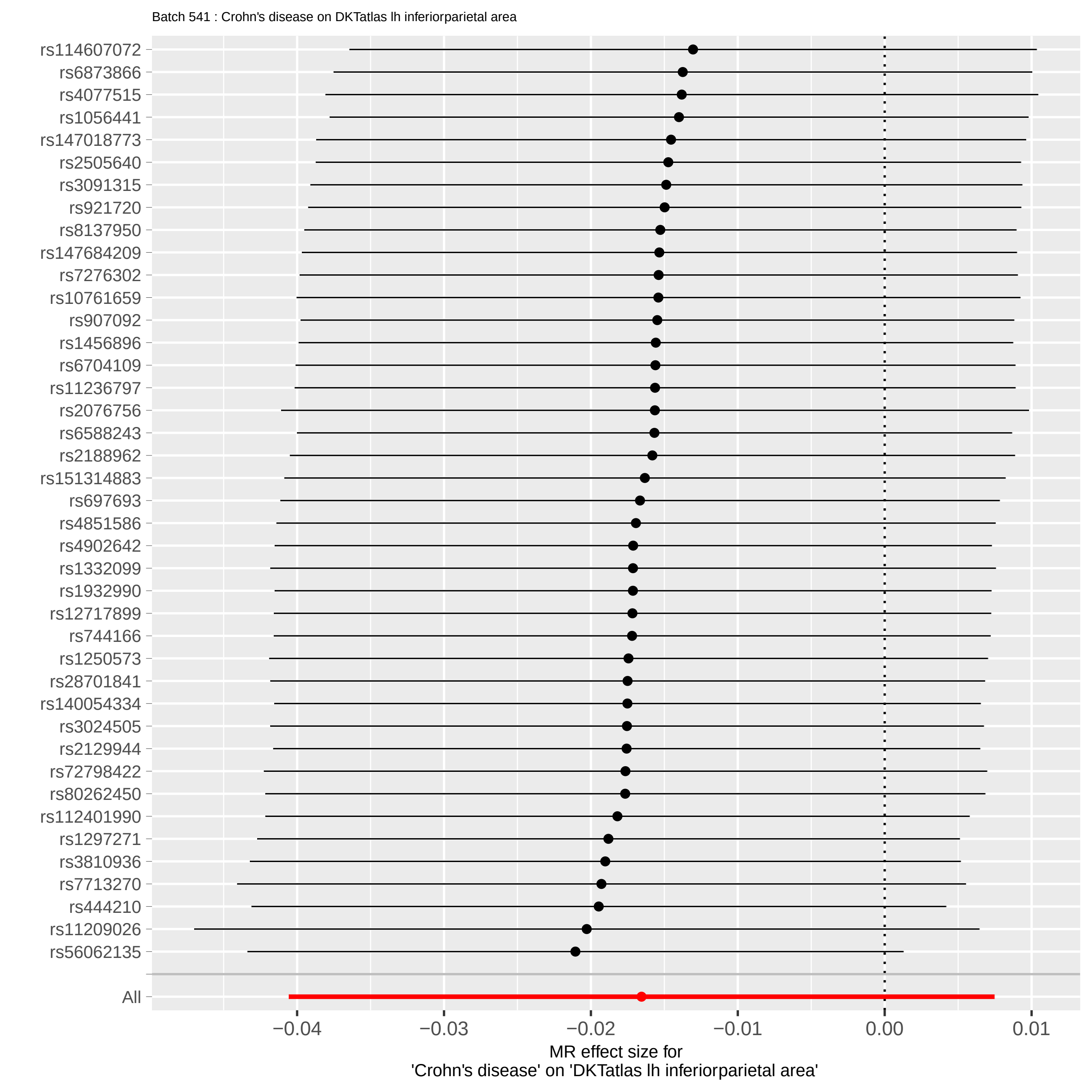

Batch 541 : Crohn's disease on DKTatlas lh inferiorparietal area
rs114607072
rs6873866
rs4077515
rs1056441
rs147018773
rs2505640
rs3091315
rs921720
rs8137950
rs147684209
rs7276302
rs10761659
rs907092
rs1456896
rs6704109
rs11236797
rs2076756
rs6588243
rs2188962
rs151314883
rs697693
rs4851586
rs4902642
rs1332099
rs1932990
rs12717899
rs744166
rs1250573
rs28701841
rs140054334
rs3024505
rs2129944
rs72798422
rs80262450
rs112401990
rs1297271
rs3810936
rs7713270
rs444210
rs11209026
rs56062135
All
−0.04
−0.03
−0.02
−0.01
0.00
0.01
MR effect size for
'Crohn's disease' on 'DKTatlas lh inferiorparietal area'

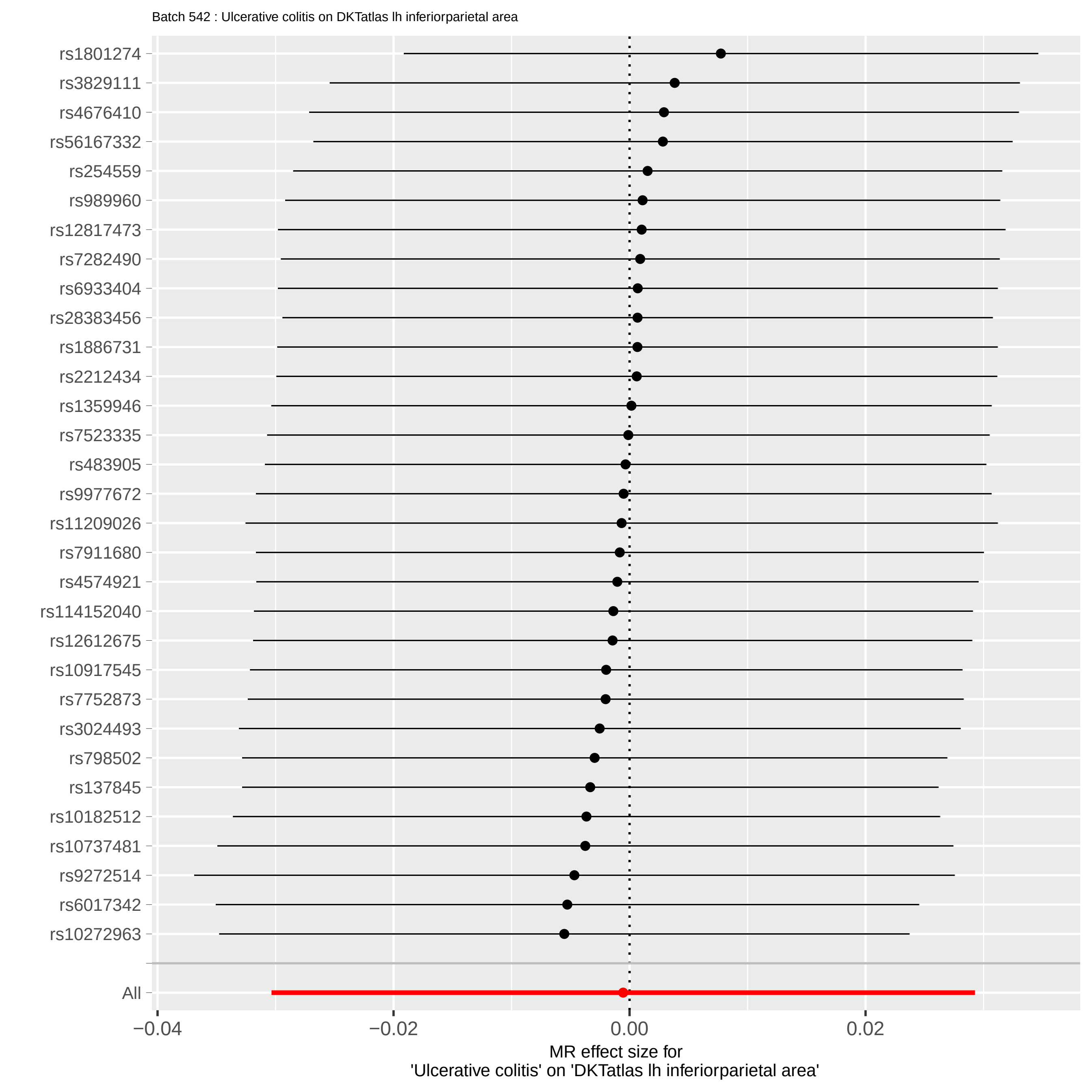

Batch 542 : Ulcerative colitis on DKTatlas lh inferiorparietal area
rs1801274
rs3829111
rs4676410
rs56167332
rs254559
rs989960
rs12817473
rs7282490
rs6933404
rs28383456
rs1886731
rs2212434
rs1359946
rs7523335
rs483905
rs9977672
rs11209026
rs7911680
rs4574921
rs114152040
rs12612675
rs10917545
rs7752873
rs3024493
rs798502
rs137845
rs10182512
rs10737481
rs9272514
rs6017342
rs10272963
All
−0.04
−0.02
0.00
0.02
MR effect size for
'Ulcerative colitis' on 'DKTatlas lh inferiorparietal area'

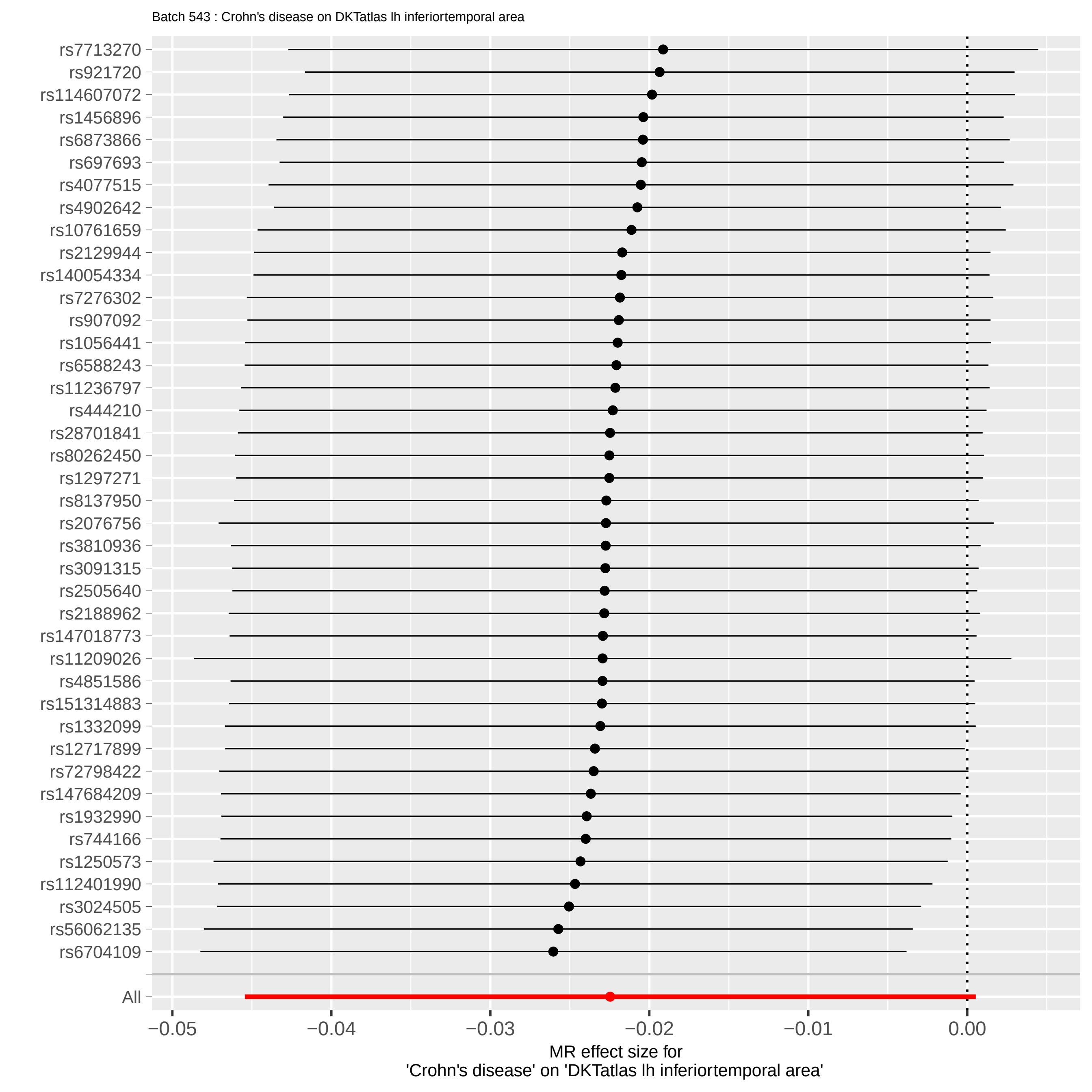

Batch 543 : Crohn's disease on DKTatlas lh inferiortemporal area
rs7713270
rs921720
rs114607072
rs1456896
rs6873866
rs697693
rs4077515
rs4902642
rs10761659
rs2129944
rs140054334
rs7276302
rs907092
rs1056441
rs6588243
rs11236797
rs444210
rs28701841
rs80262450
rs1297271
rs8137950
rs2076756
rs3810936
rs3091315
rs2505640
rs2188962
rs147018773
rs11209026
rs4851586
rs151314883
rs1332099
rs12717899
rs72798422
rs147684209
rs1932990
rs744166
rs1250573
rs112401990
rs3024505
rs56062135
rs6704109
All
−0.05
−0.04
−0.03
−0.02
−0.01
0.00
MR effect size for
'Crohn's disease' on 'DKTatlas lh inferiortemporal area'

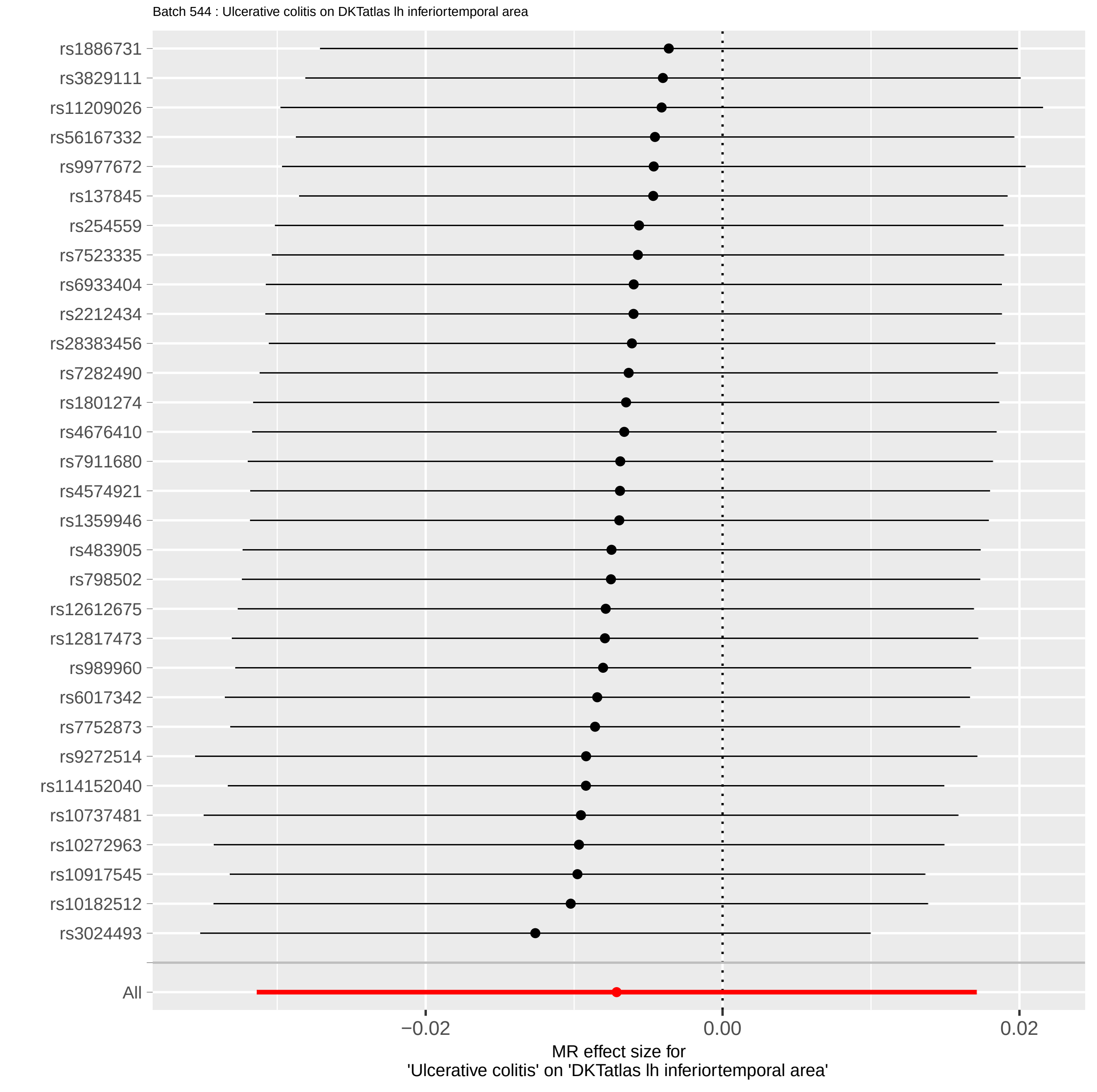

Batch 544 : Ulcerative colitis on DKTatlas lh inferiortemporal area
rs1886731
rs3829111
rs11209026
rs56167332
rs9977672
rs137845
rs254559
rs7523335
rs6933404
rs2212434
rs28383456
rs7282490
rs1801274
rs4676410
rs7911680
rs4574921
rs1359946
rs483905
rs798502
rs12612675
rs12817473
rs989960
rs6017342
rs7752873
rs9272514
rs114152040
rs10737481
rs10272963
rs10917545
rs10182512
rs3024493
All
−0.02
0.00
0.02
MR effect size for
'Ulcerative colitis' on 'DKTatlas lh inferiortemporal area'

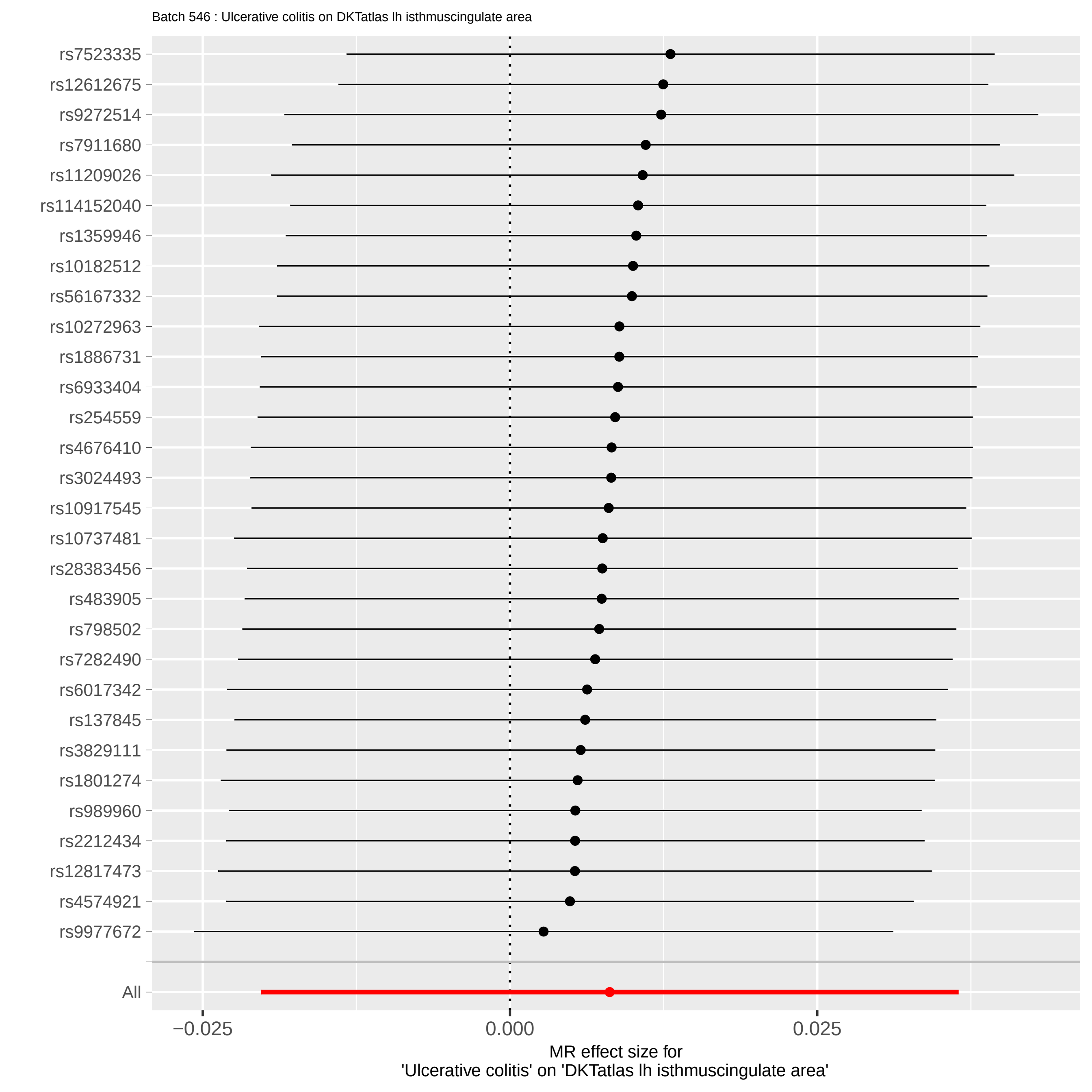

Batch 546 : Ulcerative colitis on DKTatlas lh isthmuscingulate area
rs7523335
rs12612675
rs9272514
rs7911680
rs11209026
rs114152040
rs1359946
rs10182512
rs56167332
rs10272963
rs1886731
rs6933404
rs254559
rs4676410
rs3024493
rs10917545
rs10737481
rs28383456
rs483905
rs798502
rs7282490
rs6017342
rs137845
rs3829111
rs1801274
rs989960
rs2212434
rs12817473
rs4574921
rs9977672
All
−0.025
0.000
0.025
MR effect size for
'Ulcerative colitis' on 'DKTatlas lh isthmuscingulate area'

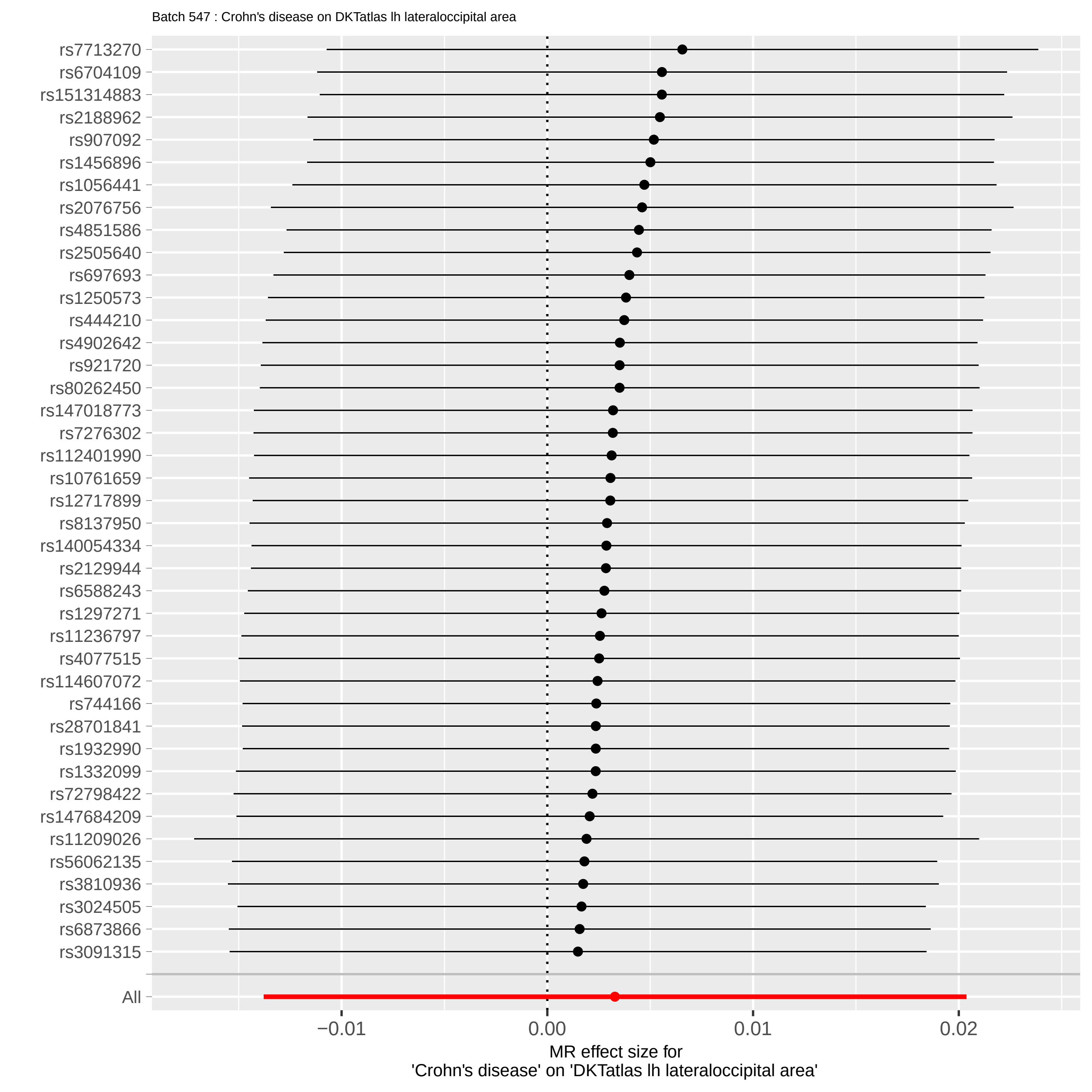

Batch 547 : Crohn's disease on DKTatlas lh lateraloccipital area
rs7713270
rs6704109
rs151314883
rs2188962
rs907092
rs1456896
rs1056441
rs2076756
rs4851586
rs2505640
rs697693
rs1250573
rs444210
rs4902642
rs921720
rs80262450
rs147018773
rs7276302
rs112401990
rs10761659
rs12717899
rs8137950
rs140054334
rs2129944
rs6588243
rs1297271
rs11236797
rs4077515
rs114607072
rs744166
rs28701841
rs1932990
rs1332099
rs72798422
rs147684209
rs11209026
rs56062135
rs3810936
rs3024505
rs6873866
rs3091315
All
−0.01
0.00
0.01
0.02
MR effect size for
'Crohn's disease' on 'DKTatlas lh lateraloccipital area'

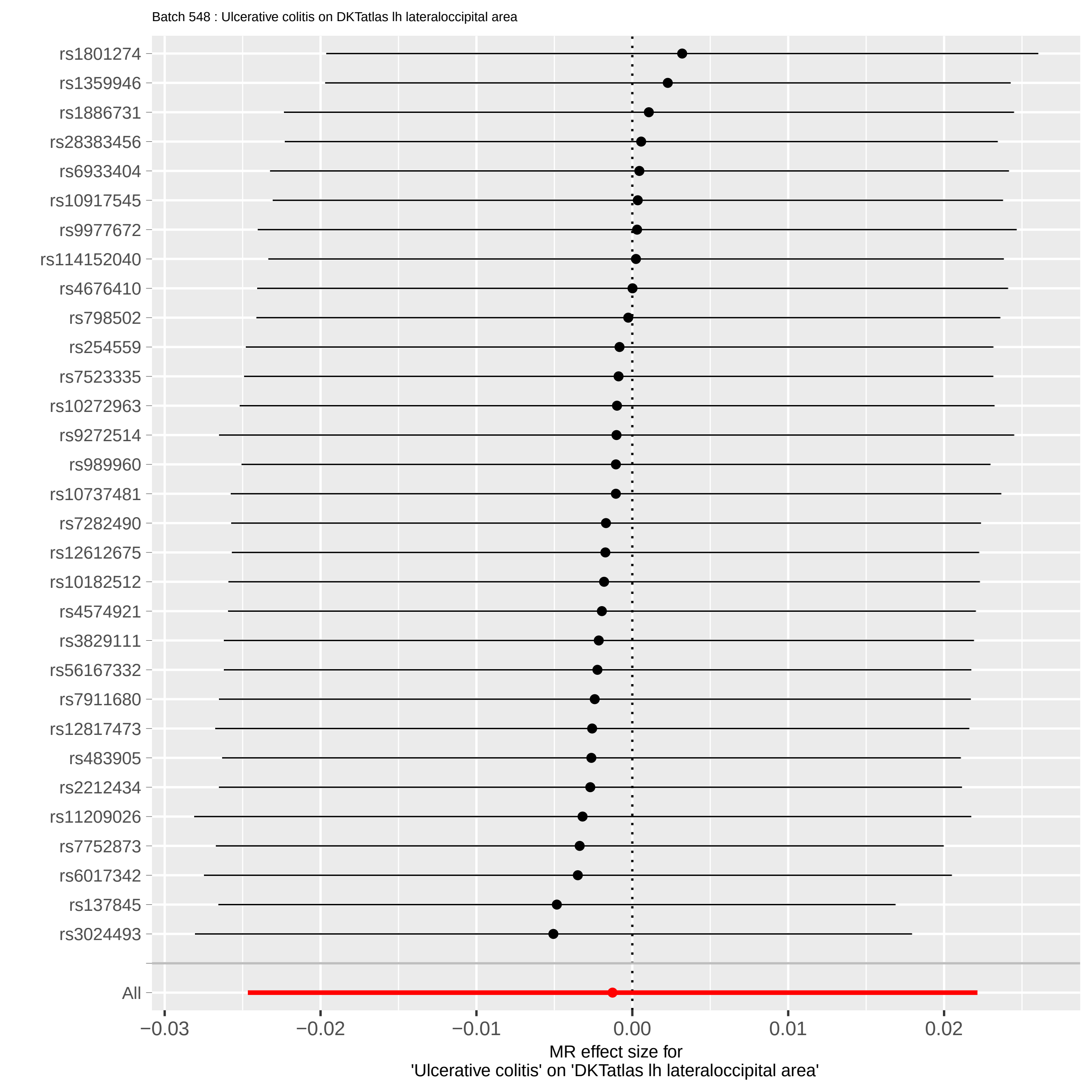

Batch 548 : Ulcerative colitis on DKTatlas lh lateraloccipital area
rs1801274
rs1359946
rs1886731
rs28383456
rs6933404
rs10917545
rs9977672
rs114152040
rs4676410
rs798502
rs254559
rs7523335
rs10272963
rs9272514
rs989960
rs10737481
rs7282490
rs12612675
rs10182512
rs4574921
rs3829111
rs56167332
rs7911680
rs12817473
rs483905
rs2212434
rs11209026
rs7752873
rs6017342
rs137845
rs3024493
All
−0.03
−0.02
−0.01
0.00
0.01
0.02
MR effect size for
'Ulcerative colitis' on 'DKTatlas lh lateraloccipital area'

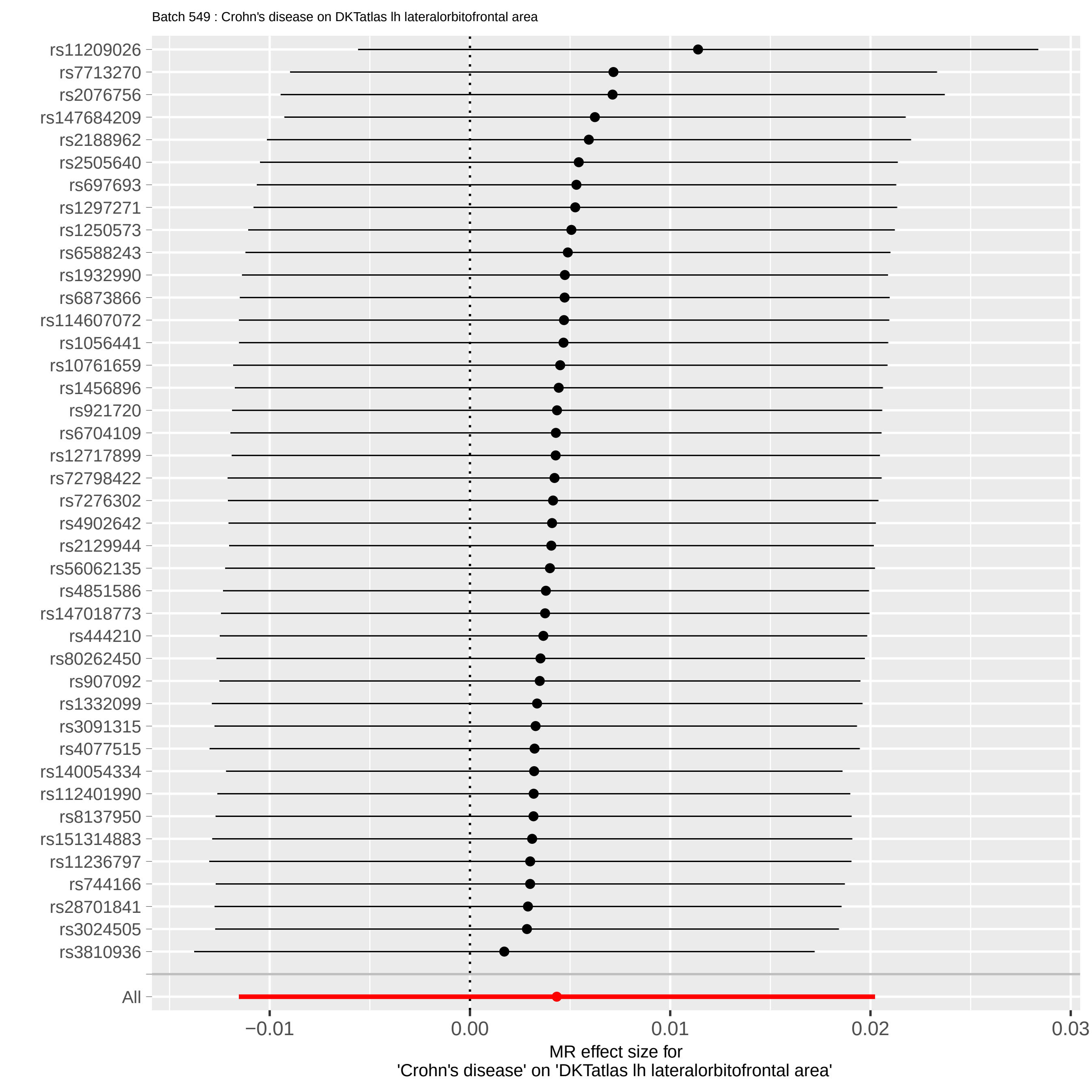

Batch 549 : Crohn's disease on DKTatlas lh lateralorbitofrontal area
rs11209026
rs7713270
rs2076756
rs147684209
rs2188962
rs2505640
rs697693
rs1297271
rs1250573
rs6588243
rs1932990
rs6873866
rs114607072
rs1056441
rs10761659
rs1456896
rs921720
rs6704109
rs12717899
rs72798422
rs7276302
rs4902642
rs2129944
rs56062135
rs4851586
rs147018773
rs444210
rs80262450
rs907092
rs1332099
rs3091315
rs4077515
rs140054334
rs112401990
rs8137950
rs151314883
rs11236797
rs744166
rs28701841
rs3024505
rs3810936
All
−0.01
0.00
0.01
0.02
0.03
MR effect size for
'Crohn's disease' on 'DKTatlas lh lateralorbitofrontal area'

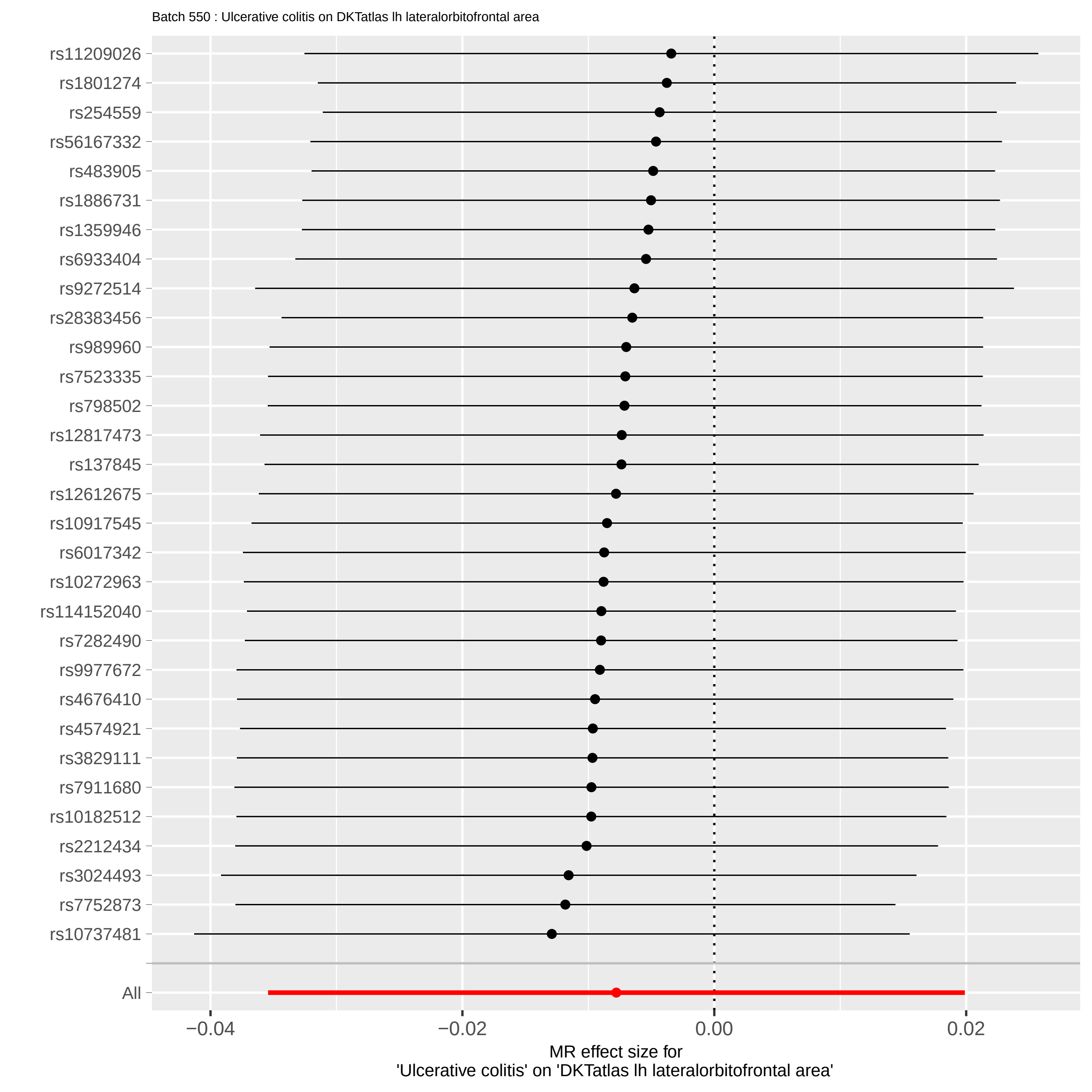
Batch 550 : Ulcerative colitis on DKTatlas lh lateralorbitofrontal area
rs11209026
rs1801274
rs254559
rs56167332
rs483905
rs1886731
rs1359946
rs6933404
rs9272514
rs28383456
rs989960
rs7523335
rs798502
rs12817473
rs137845
rs12612675
rs10917545
rs6017342
rs10272963
rs114152040
rs7282490
rs9977672
rs4676410
rs4574921
rs3829111
rs7911680
rs10182512
rs2212434
rs3024493
rs7752873
rs10737481
All
−0.04
−0.02
0.00
0.02
MR effect size for
'Ulcerative colitis' on 'DKTatlas lh lateralorbitofrontal area'

Batch 551 : Crohn's disease on DKTatlas lh lingual area

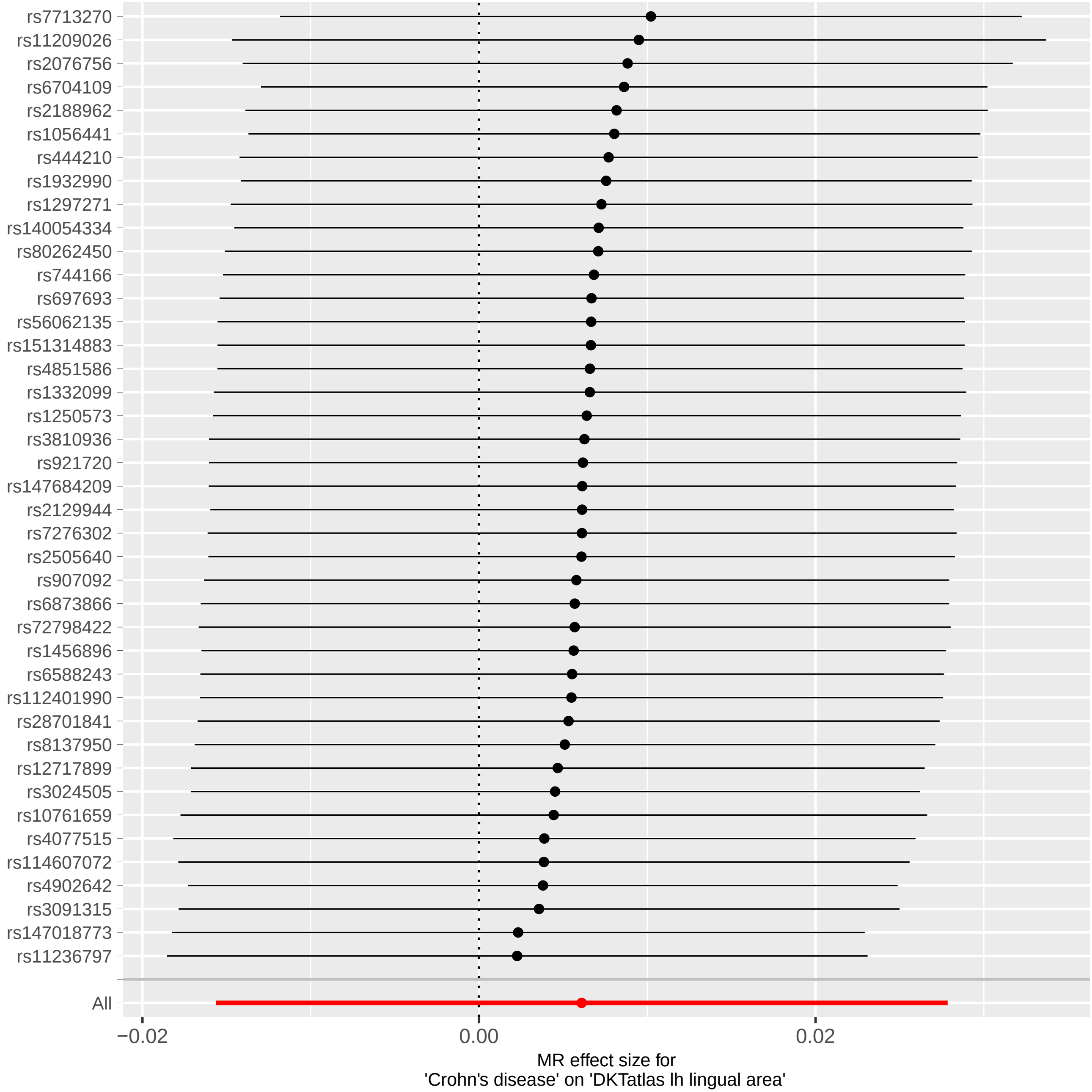

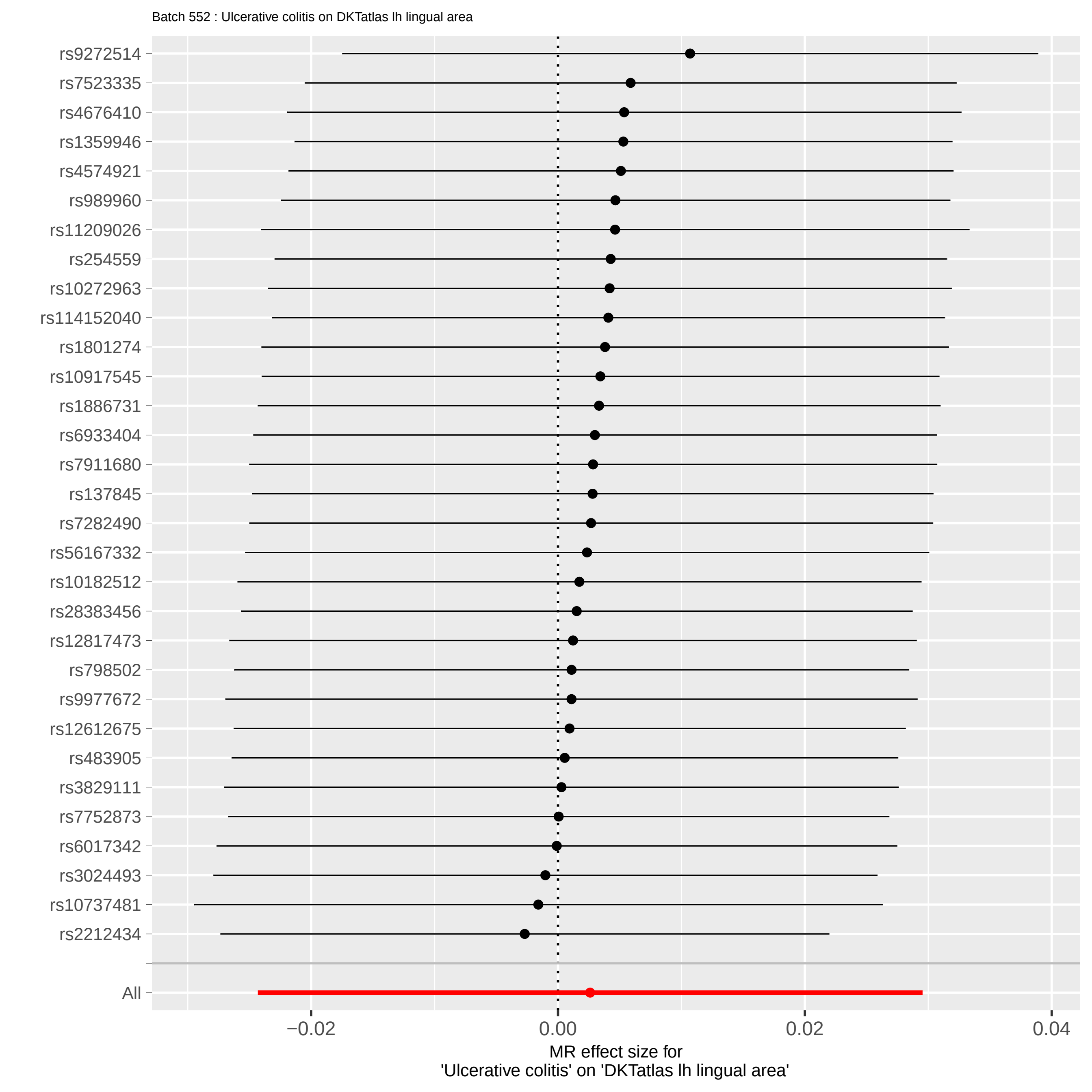
Batch 552 : Ulcerative colitis on DKTatlas lh lingual area
rs9272514
rs7523335
rs4676410
rs1359946
rs4574921
rs989960
rs11209026
rs254559
rs10272963
rs114152040
rs1801274
rs10917545
rs1886731
rs6933404
rs7911680
rs137845
rs7282490
rs56167332
rs10182512
rs28383456
rs12817473
rs798502
rs9977672
rs12612675
rs483905
rs3829111
rs7752873
rs6017342
rs3024493
rs10737481
rs2212434
All
−0.02
0.00
0.02
0.04
MR effect size for
'Ulcerative colitis' on 'DKTatlas lh lingual area'

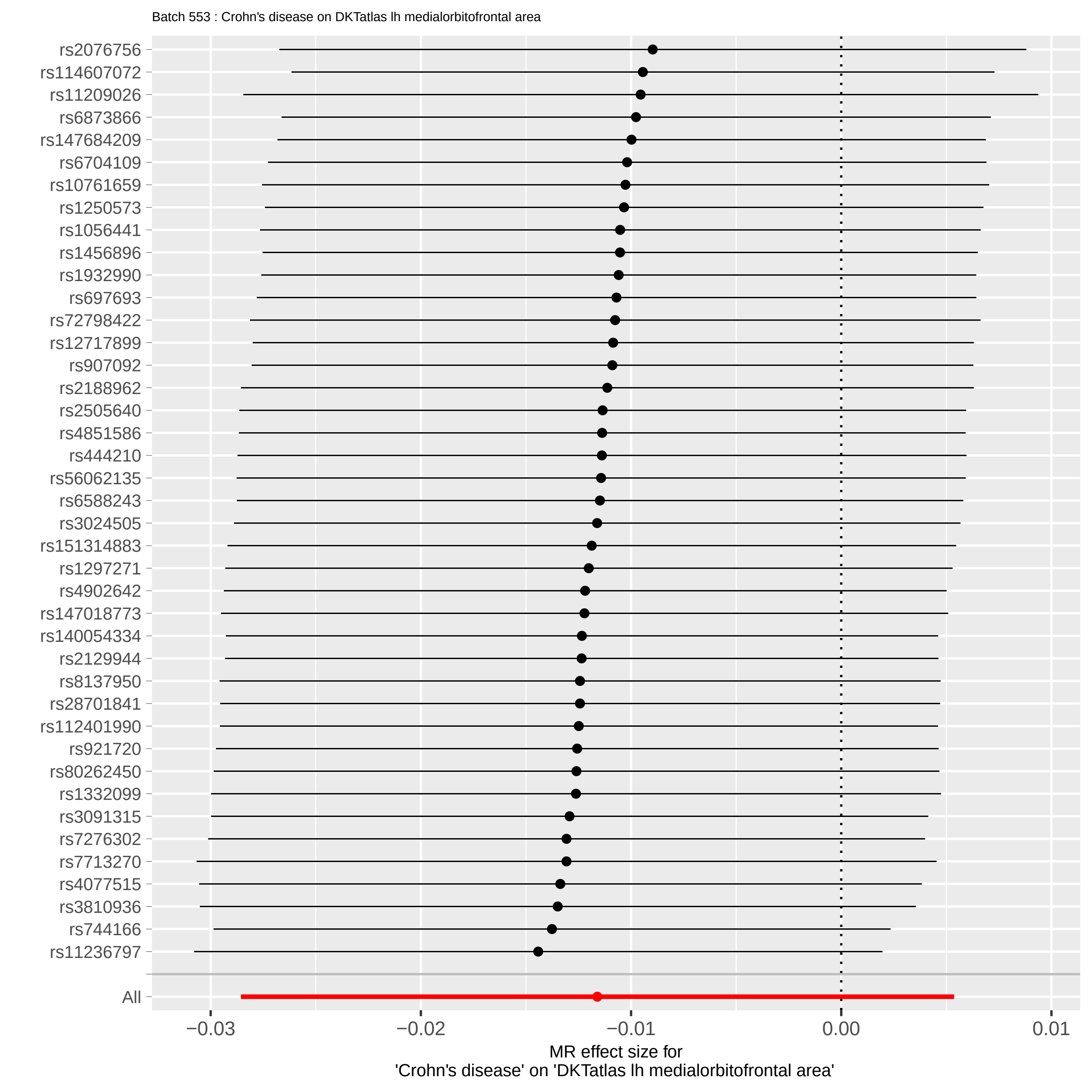

Batch 553 : Crohn's disease on DKTatlas lh medialorbitofrontal area
rs2076756
rs114607072
rs11209026
rs6873866
rs147684209
rs6704109
rs10761659
rs1250573
rs1056441
rs1456896
rs1932990
rs697693
rs72798422
rs12717899
rs907092
rs2188962
rs2505640
rs4851586
rs444210
rs56062135
rs6588243
rs3024505
rs151314883
rs1297271
rs4902642
rs147018773
rs140054334
rs2129944
rs8137950
rs28701841
rs112401990
rs921720
rs80262450
rs1332099
rs3091315
rs7276302
rs7713270
rs4077515
rs3810936
rs744166
rs11236797
All
−0.03
−0.02
−0.01
0.00
0.01
MR effect size for
'Crohn's disease' on 'DKTatlas lh medialorbitofrontal area'

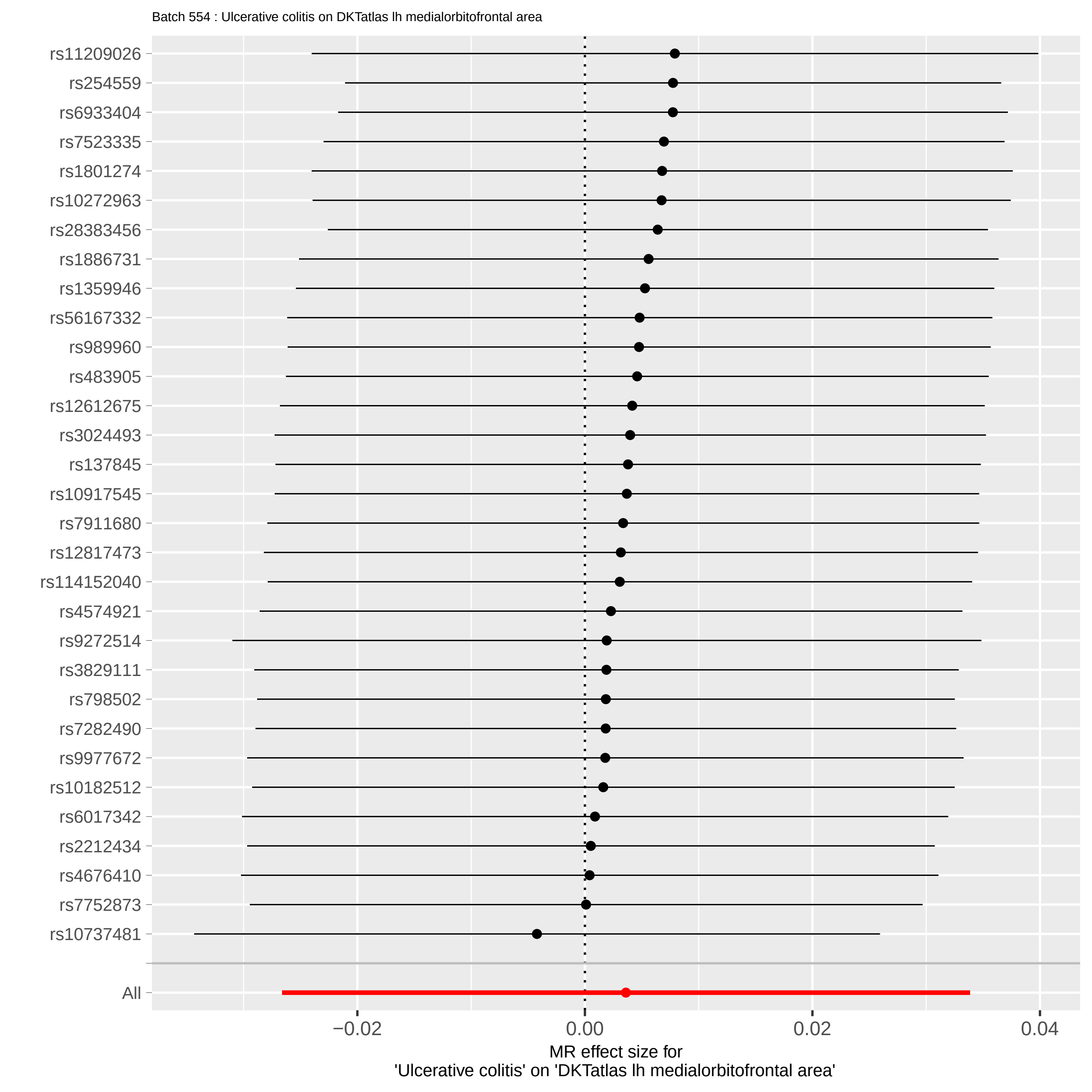

Batch 554 : Ulcerative colitis on DKTatlas lh medialorbitofrontal area
rs11209026
rs254559
rs6933404
rs7523335
rs1801274
rs10272963
rs28383456
rs1886731
rs1359946
rs56167332
rs989960
rs483905
rs12612675
rs3024493
rs137845
rs10917545
rs7911680
rs12817473
rs114152040
rs4574921
rs9272514
rs3829111
rs798502
rs7282490
rs9977672
rs10182512
rs6017342
rs2212434
rs4676410
rs7752873
rs10737481
All
−0.02
0.00
0.02
0.04
MR effect size for
'Ulcerative colitis' on 'DKTatlas lh medialorbitofrontal area'

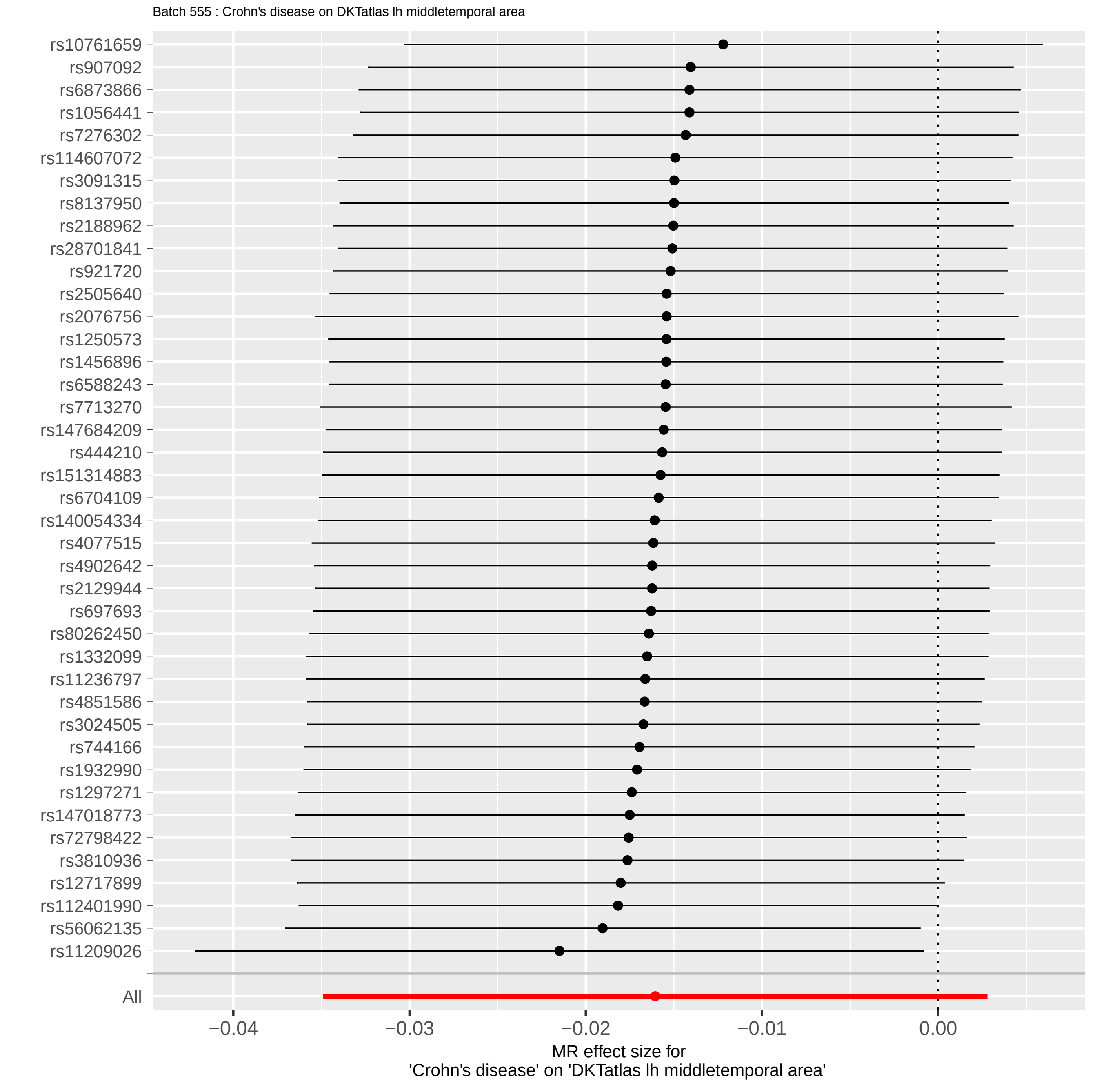

Batch 555 : Crohn's disease on DKTatlas lh middletemporal area
rs10761659
rs907092
rs6873866
rs1056441
rs7276302
rs114607072
rs3091315
rs8137950
rs2188962
rs28701841
rs921720
rs2505640
rs2076756
rs1250573
rs1456896
rs6588243
rs7713270
rs147684209
rs444210
rs151314883
rs6704109
rs140054334
rs4077515
rs4902642
rs2129944
rs697693
rs80262450
rs1332099
rs11236797
rs4851586
rs3024505
rs744166
rs1932990
rs1297271
rs147018773
rs72798422
rs3810936
rs12717899
rs112401990
rs56062135
rs11209026
All
−0.04
−0.03
−0.02
−0.01
0.00
MR effect size for
'Crohn's disease' on 'DKTatlas lh middletemporal area'

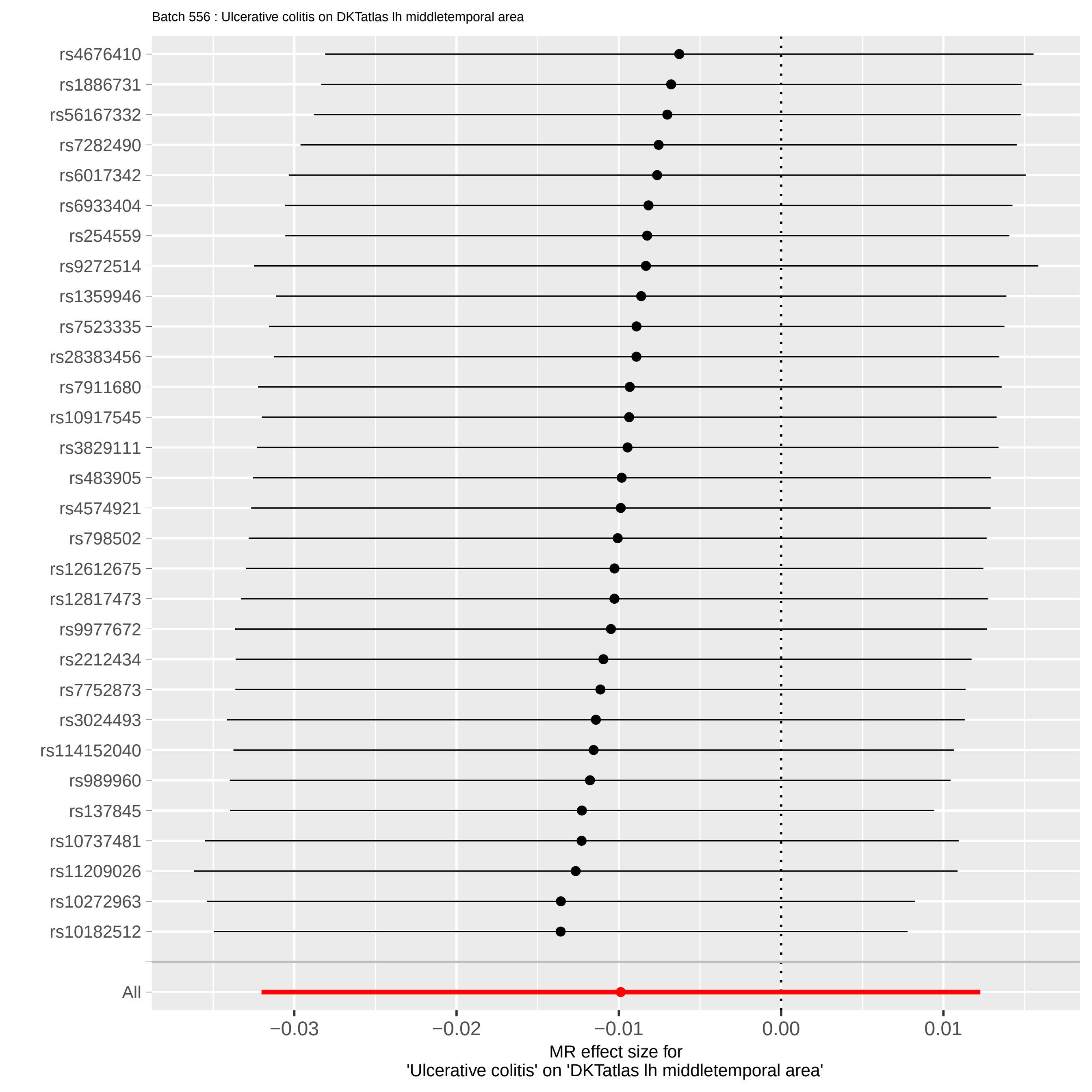

Batch 556 : Ulcerative colitis on DKTatlas lh middletemporal area
rs4676410
rs1886731
rs56167332
rs7282490
rs6017342
rs6933404
rs254559
rs9272514
rs1359946
rs7523335
rs28383456
rs7911680
rs10917545
rs3829111
rs483905
rs4574921
rs798502
rs12612675
rs12817473
rs9977672
rs2212434
rs7752873
rs3024493
rs114152040
rs989960
rs137845
rs10737481
rs11209026
rs10272963
rs10182512
All
−0.03
−0.02
−0.01
0.00
0.01
MR effect size for
'Ulcerative colitis' on 'DKTatlas lh middletemporal area'

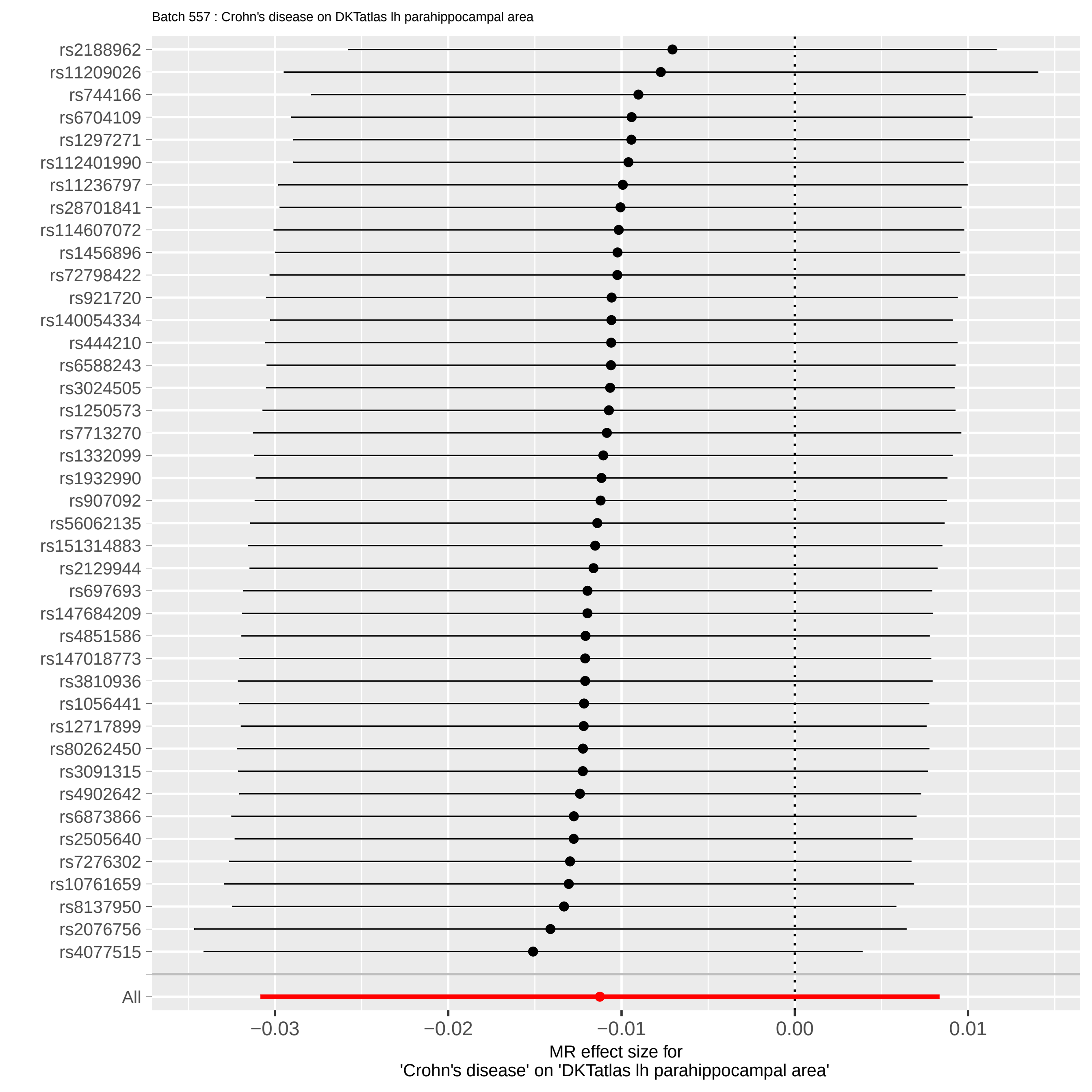

Batch 557 : Crohn's disease on DKTatlas lh parahippocampal area
rs2188962
rs11209026
rs744166
rs6704109
rs1297271
rs112401990
rs11236797
rs28701841
rs114607072
rs1456896
rs72798422
rs921720
rs140054334
rs444210
rs6588243
rs3024505
rs1250573
rs7713270
rs1332099
rs1932990
rs907092
rs56062135
rs151314883
rs2129944
rs697693
rs147684209
rs4851586
rs147018773
rs3810936
rs1056441
rs12717899
rs80262450
rs3091315
rs4902642
rs6873866
rs2505640
rs7276302
rs10761659
rs8137950
rs2076756
rs4077515
All
−0.03
−0.02
−0.01
0.00
0.01
MR effect size for
'Crohn's disease' on 'DKTatlas lh parahippocampal area'

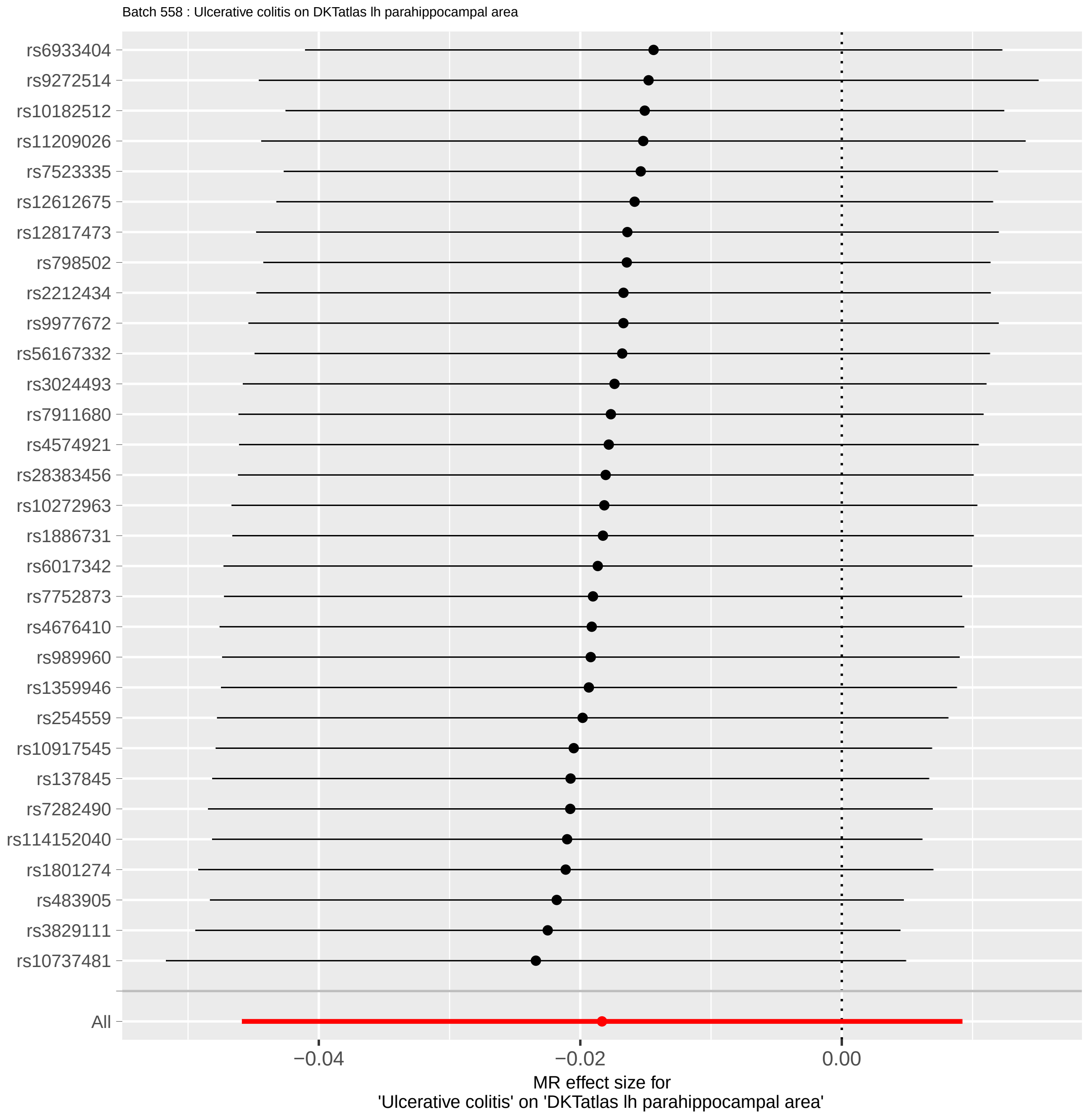
Batch 558 : Ulcerative colitis on DKTatlas lh parahippocampal area
rs6933404
rs9272514
rs10182512
rs11209026
rs7523335
rs12612675
rs12817473
rs798502
rs2212434
rs9977672
rs56167332
rs3024493
rs7911680
rs4574921
rs28383456
rs10272963
rs1886731
rs6017342
rs7752873
rs4676410
rs989960
rs1359946
rs254559
rs10917545
rs137845
rs7282490
rs114152040
rs1801274
rs483905
rs3829111
rs10737481
All
−0.04
−0.02
0.00
MR effect size for
'Ulcerative colitis' on 'DKTatlas lh parahippocampal area'

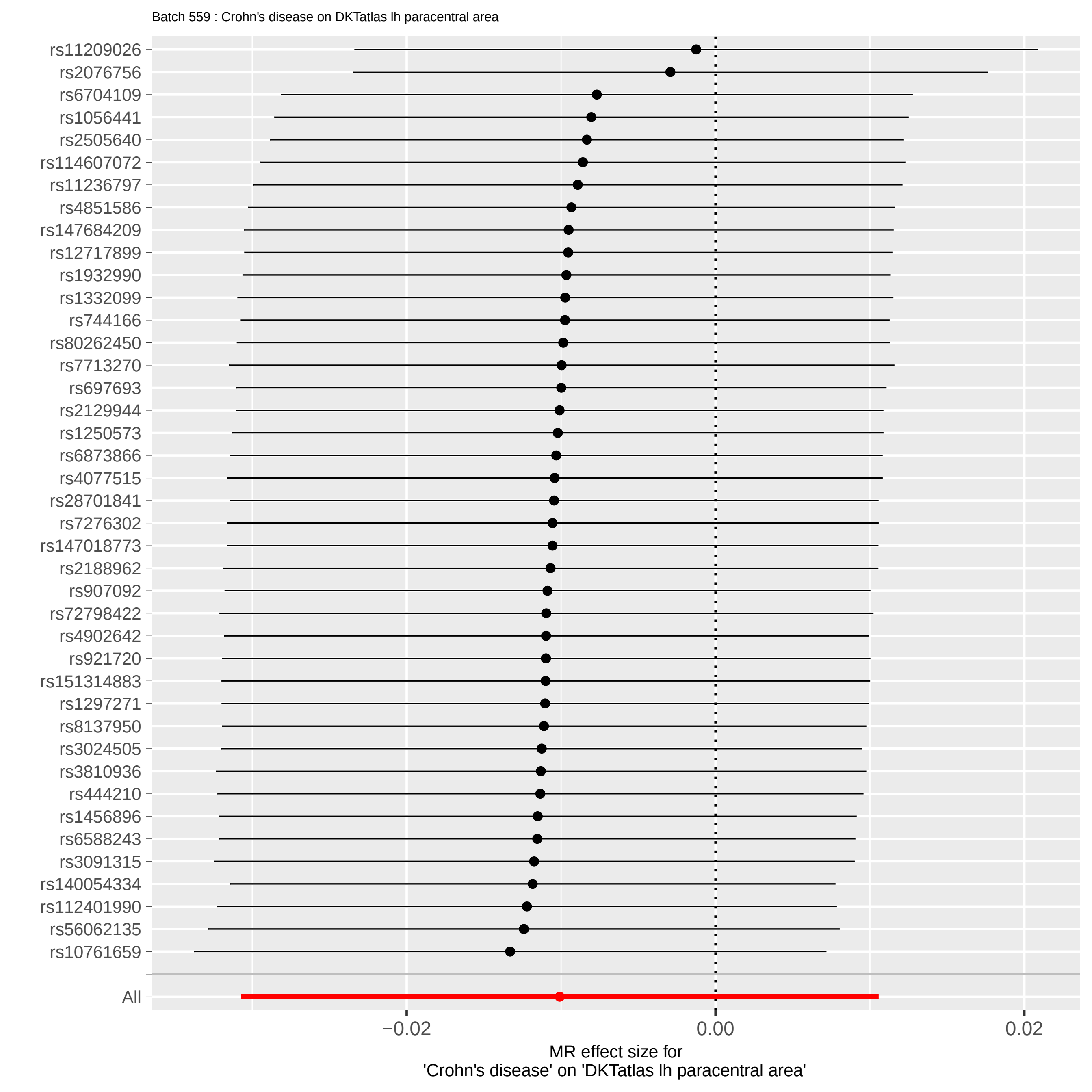

Batch 559 : Crohn's disease on DKTatlas lh paracentral area
rs11209026
rs2076756
rs6704109
rs1056441
rs2505640
rs114607072
rs11236797
rs4851586
rs147684209
rs12717899
rs1932990
rs1332099
rs744166
rs80262450
rs7713270
rs697693
rs2129944
rs1250573
rs6873866
rs4077515
rs28701841
rs7276302
rs147018773
rs2188962
rs907092
rs72798422
rs4902642
rs921720
rs151314883
rs1297271
rs8137950
rs3024505
rs3810936
rs444210
rs1456896
rs6588243
rs3091315
rs140054334
rs112401990
rs56062135
rs10761659
All
−0.02
0.00
0.02
MR effect size for
'Crohn's disease' on 'DKTatlas lh paracentral area'

Batch 560 : Ulcerative colitis on DKTatlas lh paracentral area

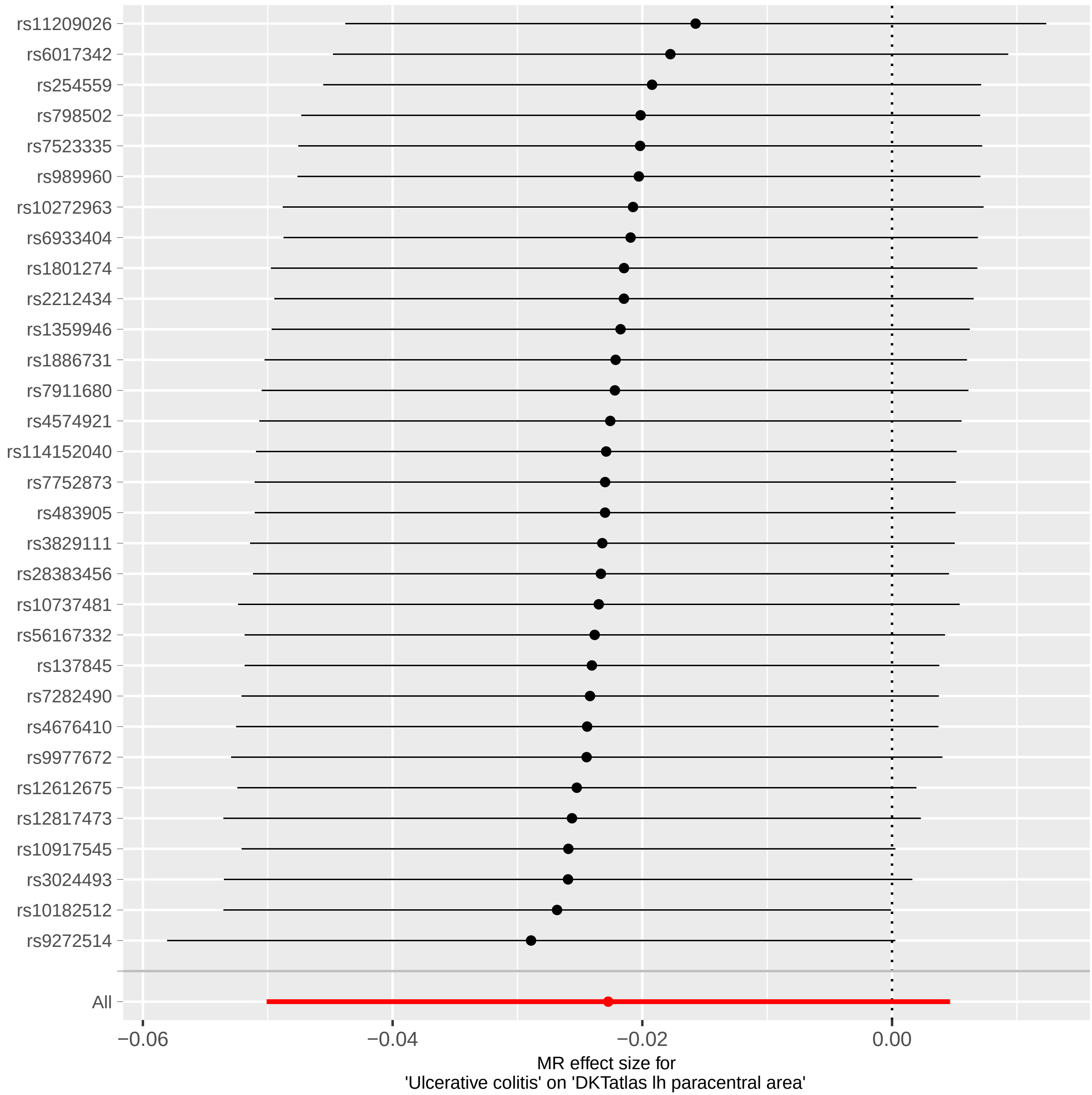

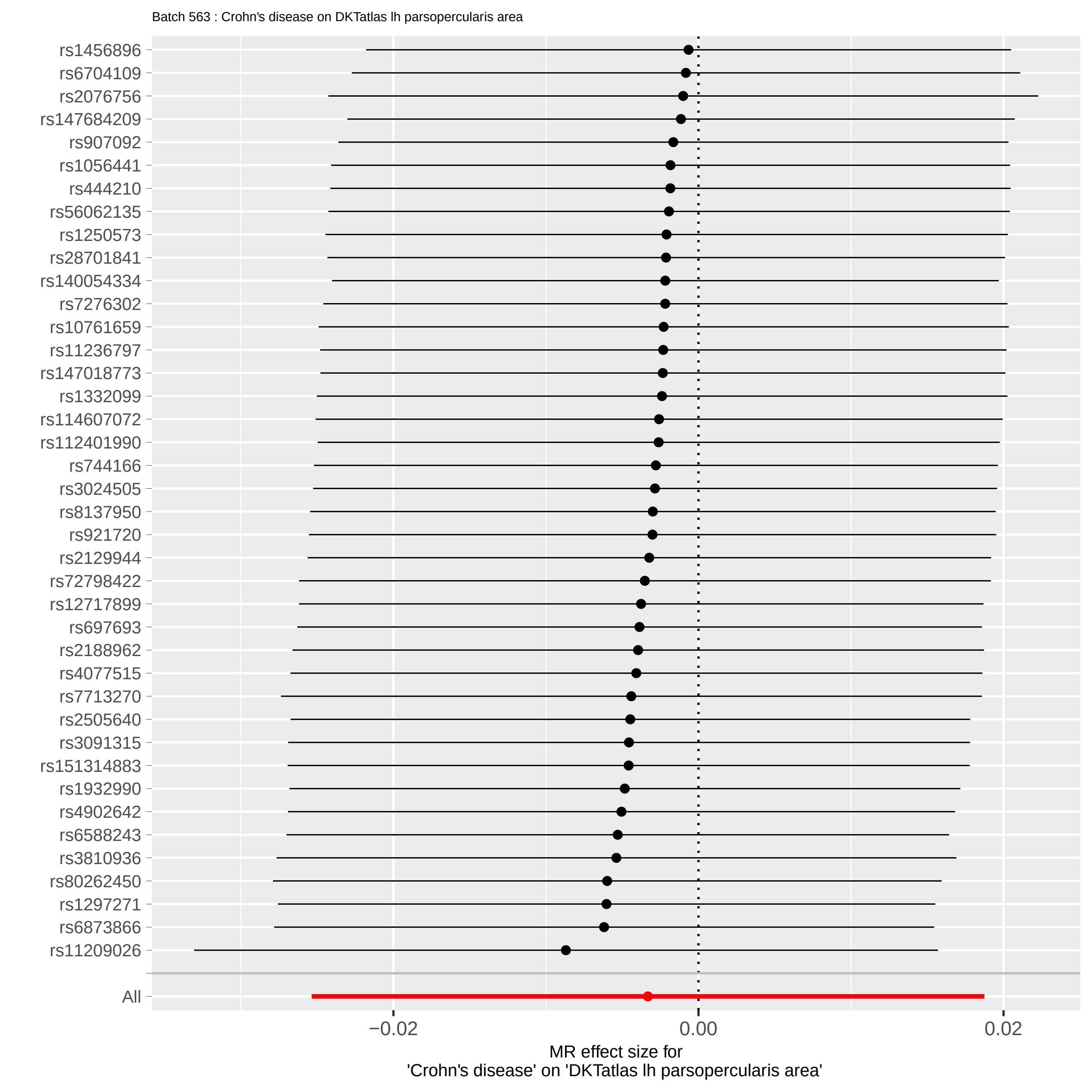
Batch 563 : Crohn's disease on DKTatlas lh parsopercularis area
rs1456896
rs6704109
rs2076756
rs147684209
rs907092
rs1056441
rs444210
rs56062135
rs1250573
rs28701841
rs140054334
rs7276302
rs10761659
rs11236797
rs147018773
rs1332099
rs114607072
rs112401990
rs744166
rs3024505
rs8137950
rs921720
rs2129944
rs72798422
rs12717899
rs697693
rs2188962
rs4077515
rs7713270
rs2505640
rs3091315
rs151314883
rs1932990
rs4902642
rs6588243
rs3810936
rs80262450
rs1297271
rs6873866
rs11209026
All
−0.02
0.00
0.02
MR effect size for
'Crohn's disease' on 'DKTatlas lh parsopercularis area'

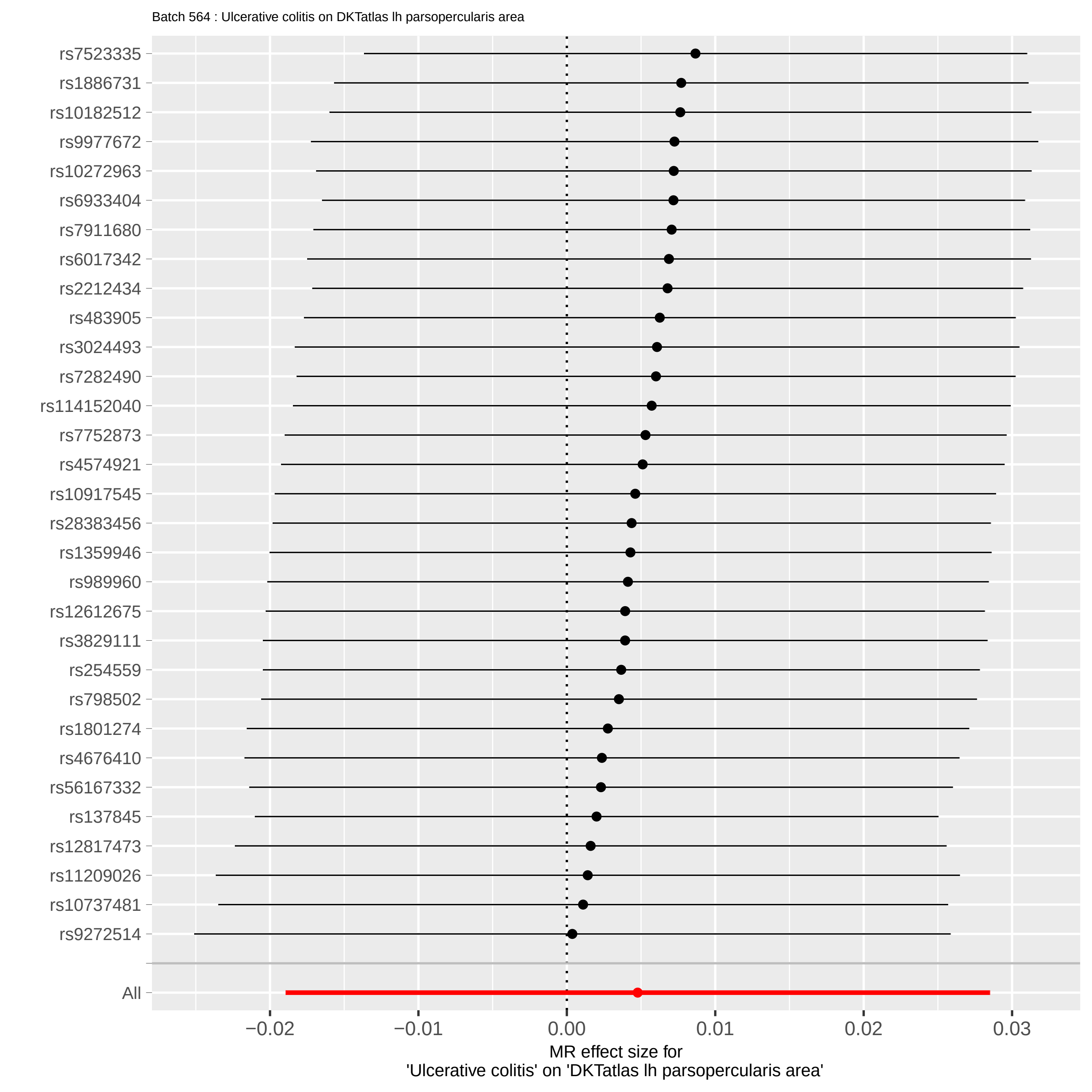

Batch 564 : Ulcerative colitis on DKTatlas lh parsopercularis area
rs7523335
rs1886731
rs10182512
rs9977672
rs10272963
rs6933404
rs7911680
rs6017342
rs2212434
rs483905
rs3024493
rs7282490
rs114152040
rs7752873
rs4574921
rs10917545
rs28383456
rs1359946
rs989960
rs12612675
rs3829111
rs254559
rs798502
rs1801274
rs4676410
rs56167332
rs137845
rs12817473
rs11209026
rs10737481
rs9272514
All
−0.02
−0.01
0.00
0.01
0.02
0.03
MR effect size for
'Ulcerative colitis' on 'DKTatlas lh parsopercularis area'

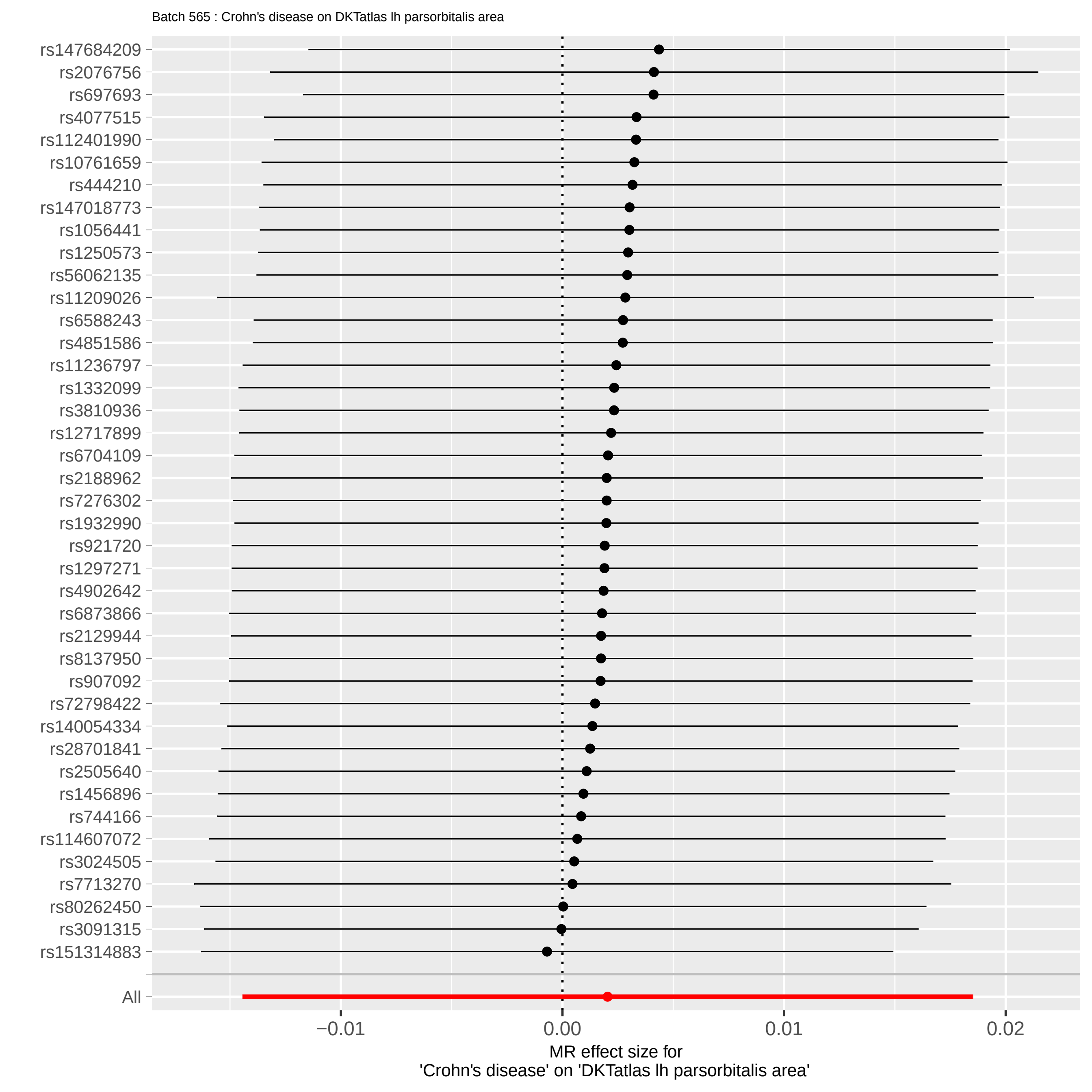

Batch 565 : Crohn's disease on DKTatlas lh parsorbitalis area
rs147684209
rs2076756
rs697693
rs4077515
rs112401990
rs10761659
rs444210
rs147018773
rs1056441
rs1250573
rs56062135
rs11209026
rs6588243
rs4851586
rs11236797
rs1332099
rs3810936
rs12717899
rs6704109
rs2188962
rs7276302
rs1932990
rs921720
rs1297271
rs4902642
rs6873866
rs2129944
rs8137950
rs907092
rs72798422
rs140054334
rs28701841
rs2505640
rs1456896
rs744166
rs114607072
rs3024505
rs7713270
rs80262450
rs3091315
rs151314883
All
−0.01
0.00
0.01
0.02
MR effect size for
'Crohn's disease' on 'DKTatlas lh parsorbitalis area'

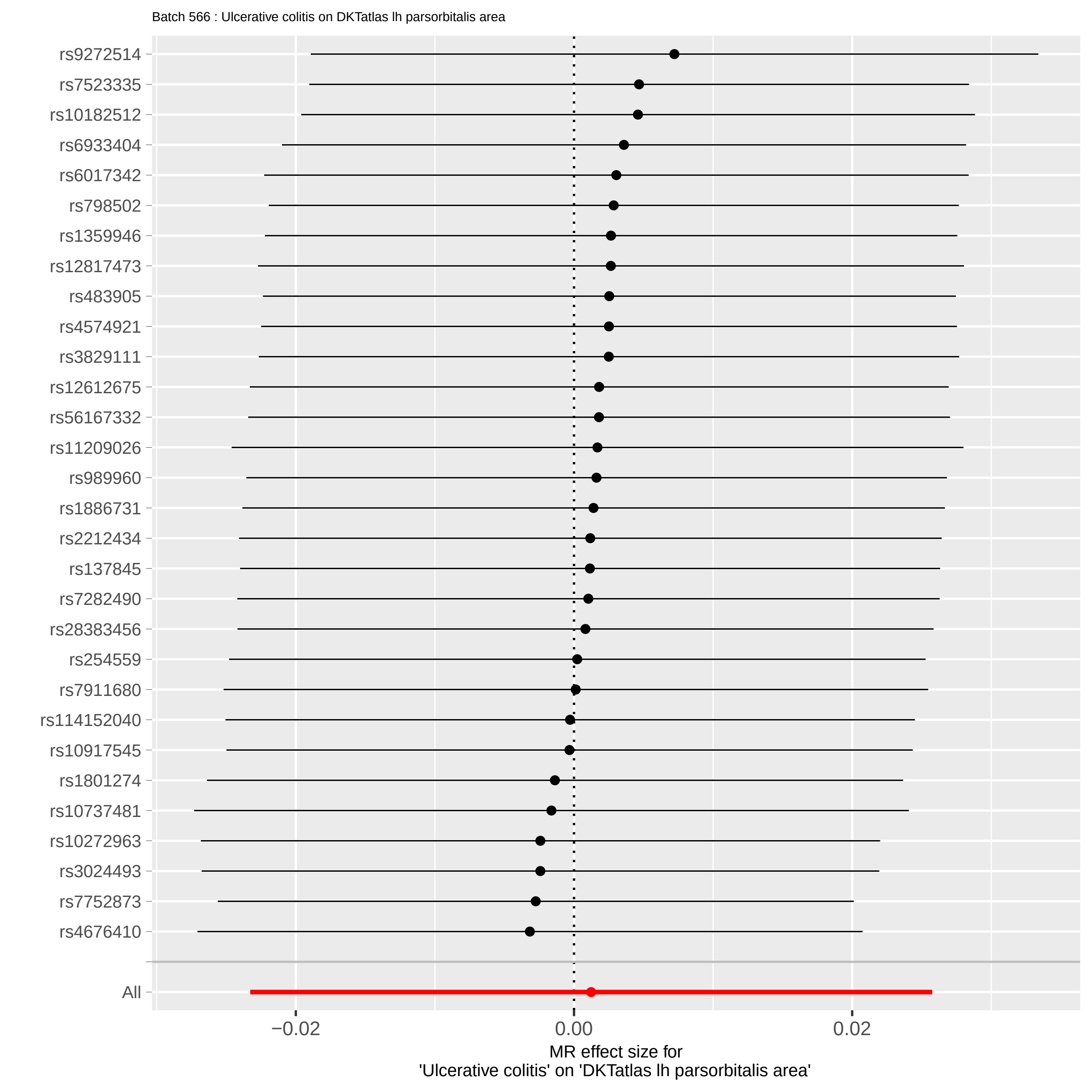

Batch 566 : Ulcerative colitis on DKTatlas lh parsorbitalis area
rs9272514
rs7523335
rs10182512
rs6933404
rs6017342
rs798502
rs1359946
rs12817473
rs483905
rs4574921
rs3829111
rs12612675
rs56167332
rs11209026
rs989960
rs1886731
rs2212434
rs137845
rs7282490
rs28383456
rs254559
rs7911680
rs114152040
rs10917545
rs1801274
rs10737481
rs10272963
rs3024493
rs7752873
rs4676410
All
−0.02
0.00
0.02
MR effect size for
'Ulcerative colitis' on 'DKTatlas lh parsorbitalis area'

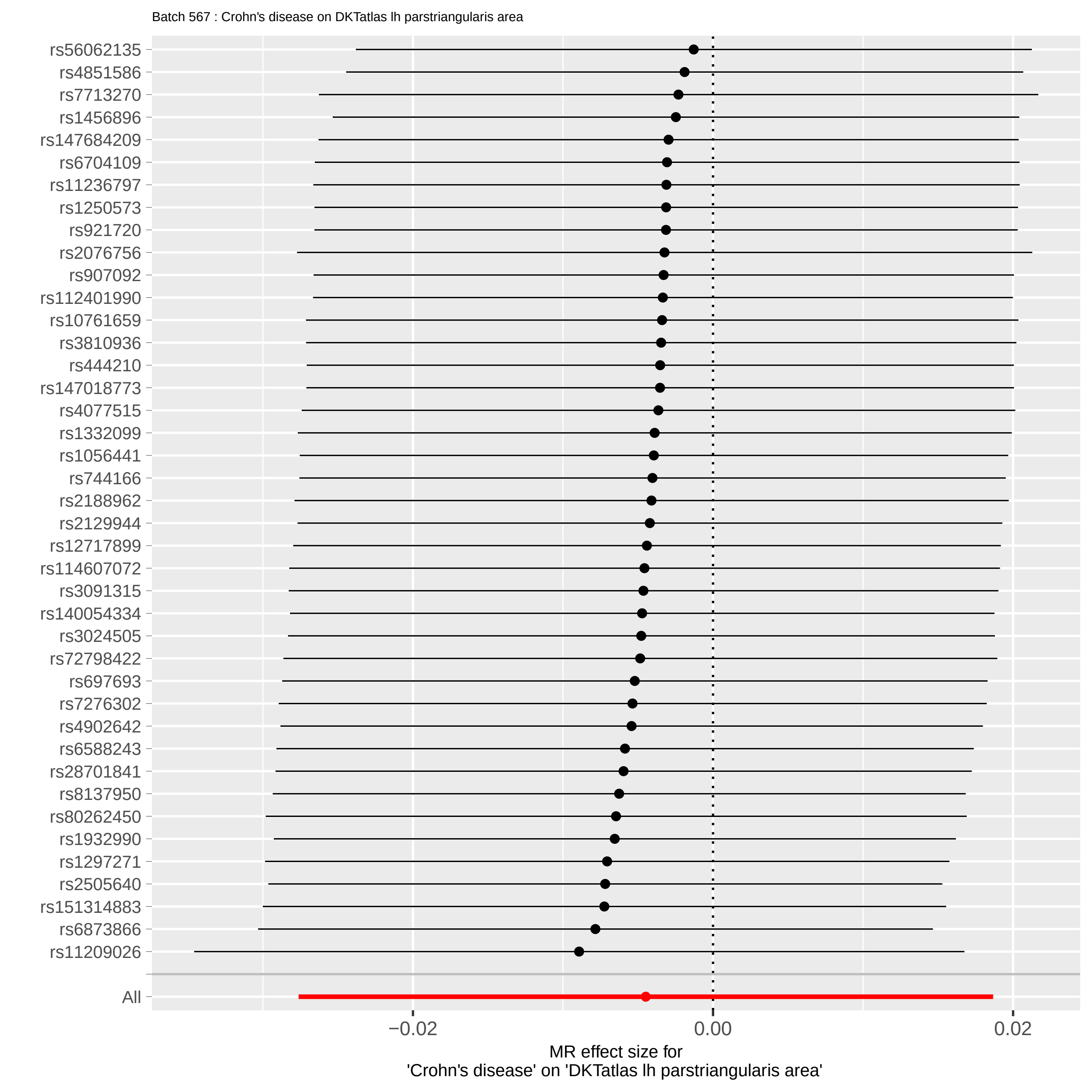
Batch 567 : Crohn's disease on DKTatlas lh parstriangularis area
rs56062135
rs4851586
rs7713270
rs1456896
rs147684209
rs6704109
rs11236797
rs1250573
rs921720
rs2076756
rs907092
rs112401990
rs10761659
rs3810936
rs444210
rs147018773
rs4077515
rs1332099
rs1056441
rs744166
rs2188962
rs2129944
rs12717899
rs114607072
rs3091315
rs140054334
rs3024505
rs72798422
rs697693
rs7276302
rs4902642
rs6588243
rs28701841
rs8137950
rs80262450
rs1932990
rs1297271
rs2505640
rs151314883
rs6873866
rs11209026
All
−0.02
0.00
0.02
MR effect size for
'Crohn's disease' on 'DKTatlas lh parstriangularis area'

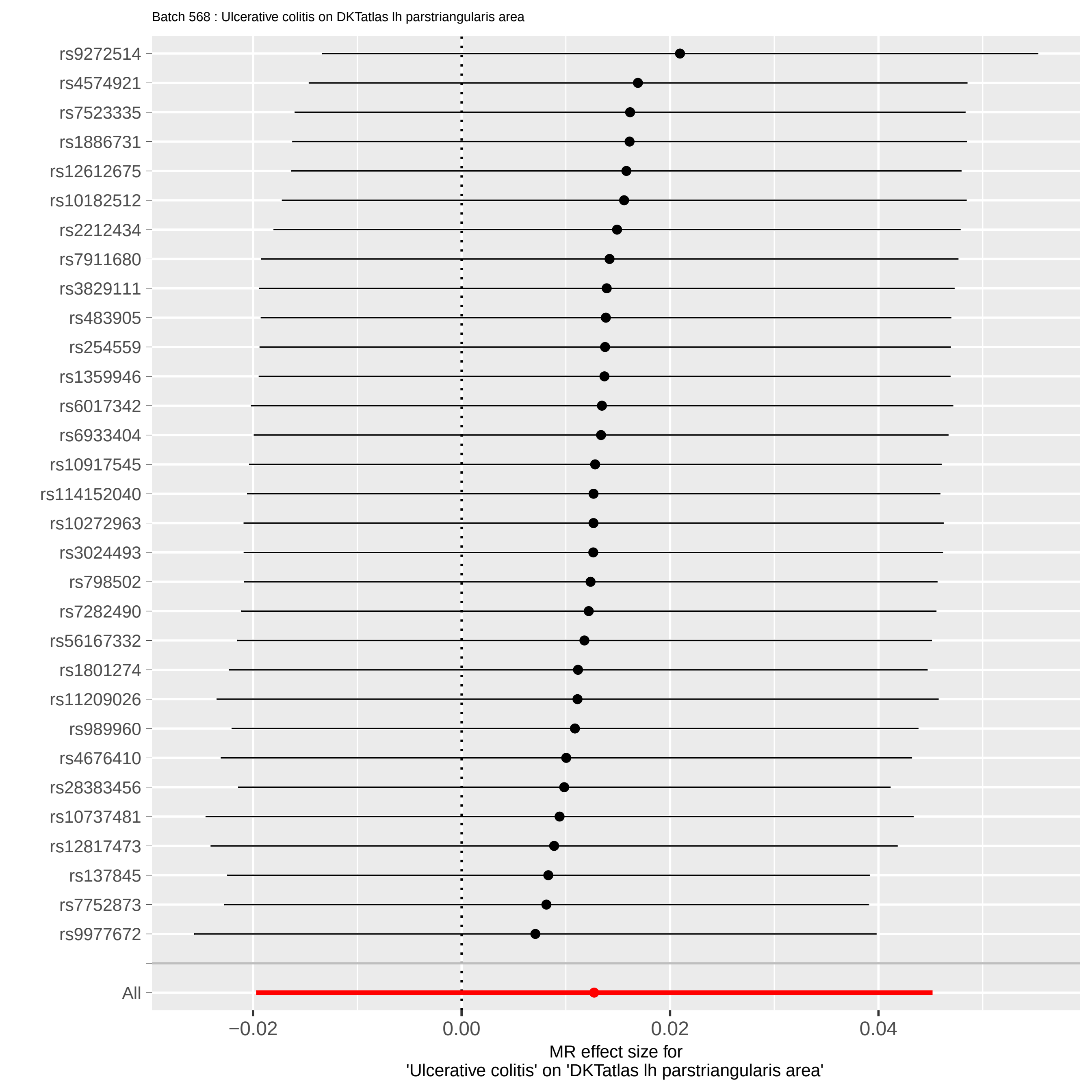

Batch 568 : Ulcerative colitis on DKTatlas lh parstriangularis area
rs9272514
rs4574921
rs7523335
rs1886731
rs12612675
rs10182512
rs2212434
rs7911680
rs3829111
rs483905
rs254559
rs1359946
rs6017342
rs6933404
rs10917545
rs114152040
rs10272963
rs3024493
rs798502
rs7282490
rs56167332
rs1801274
rs11209026
rs989960
rs4676410
rs28383456
rs10737481
rs12817473
rs137845
rs7752873
rs9977672
All
−0.02
0.00
0.02
0.04
MR effect size for
'Ulcerative colitis' on 'DKTatlas lh parstriangularis area'

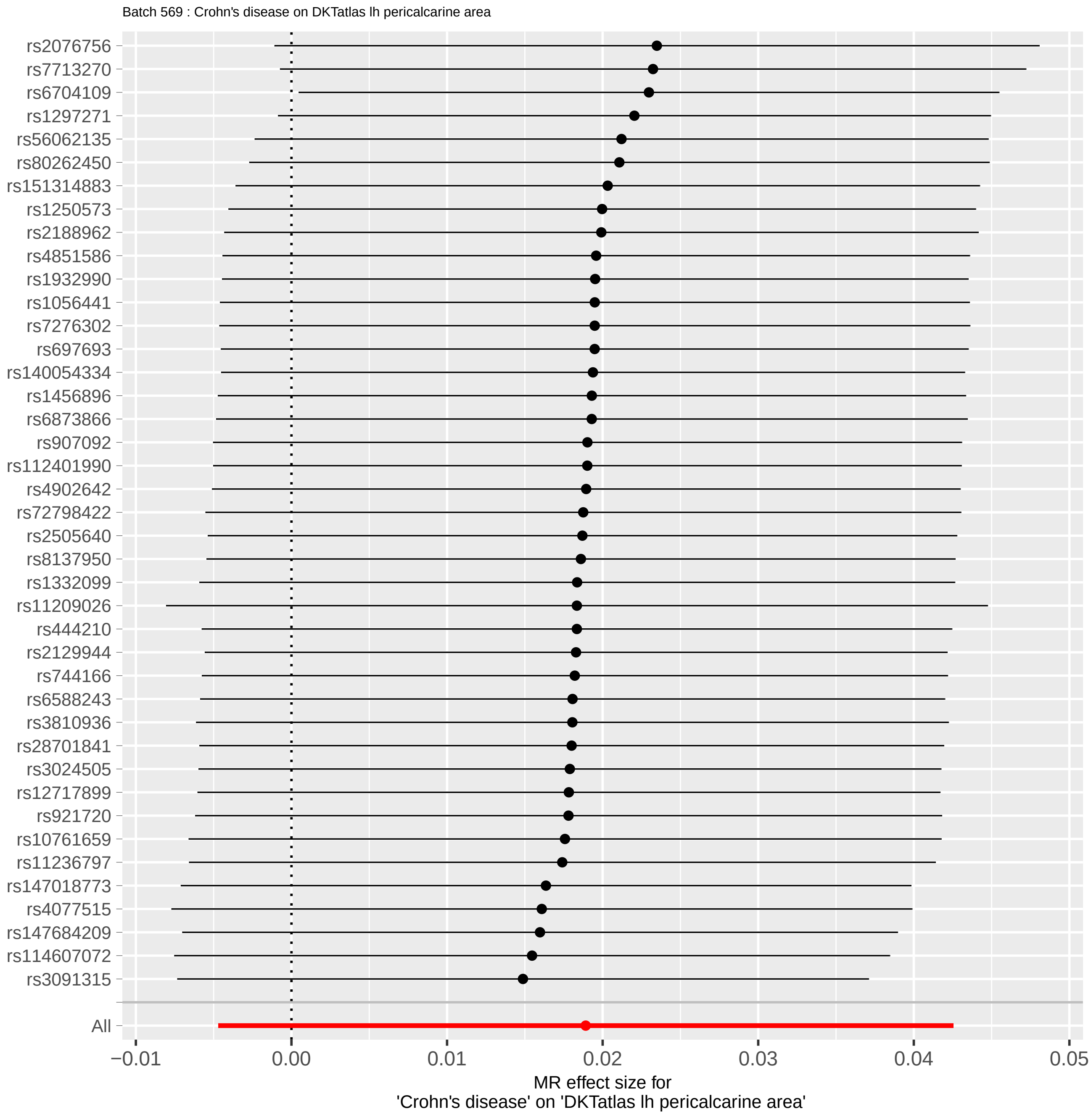

Batch 569 : Crohn's disease on DKTatlas lh pericalcarine area
rs2076756
rs7713270
rs6704109
rs1297271
rs56062135
rs80262450
rs151314883
rs1250573
rs2188962
rs4851586
rs1932990
rs1056441
rs7276302
rs697693
rs140054334
rs1456896
rs6873866
rs907092
rs112401990
rs4902642
rs72798422
rs2505640
rs8137950
rs1332099
rs11209026
rs444210
rs2129944
rs744166
rs6588243
rs3810936
rs28701841
rs3024505
rs12717899
rs921720
rs10761659
rs11236797
rs147018773
rs4077515
rs147684209
rs114607072
rs3091315
All
−0.01
0.00
0.01
0.02
0.03
0.04
0.05
MR effect size for
'Crohn's disease' on 'DKTatlas lh pericalcarine area'

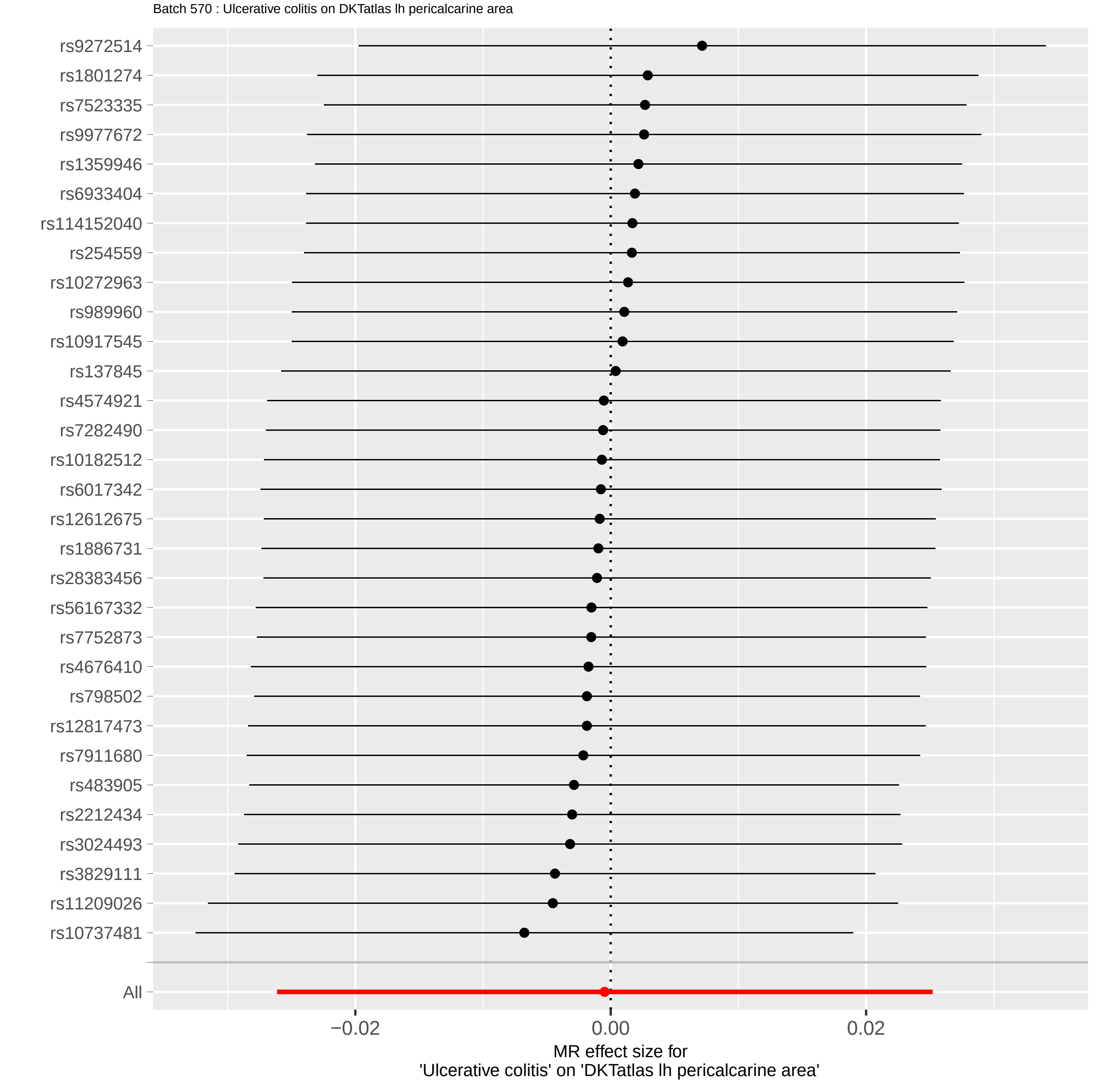

Batch 570 : Ulcerative colitis on DKTatlas lh pericalcarine area
rs9272514
rs1801274
rs7523335
rs9977672
rs1359946
rs6933404
rs114152040
rs254559
rs10272963
rs989960
rs10917545
rs137845
rs4574921
rs7282490
rs10182512
rs6017342
rs12612675
rs1886731
rs28383456
rs56167332
rs7752873
rs4676410
rs798502
rs12817473
rs7911680
rs483905
rs2212434
rs3024493
rs3829111
rs11209026
rs10737481
All
−0.02
0.00
0.02
MR effect size for
'Ulcerative colitis' on 'DKTatlas lh pericalcarine area'

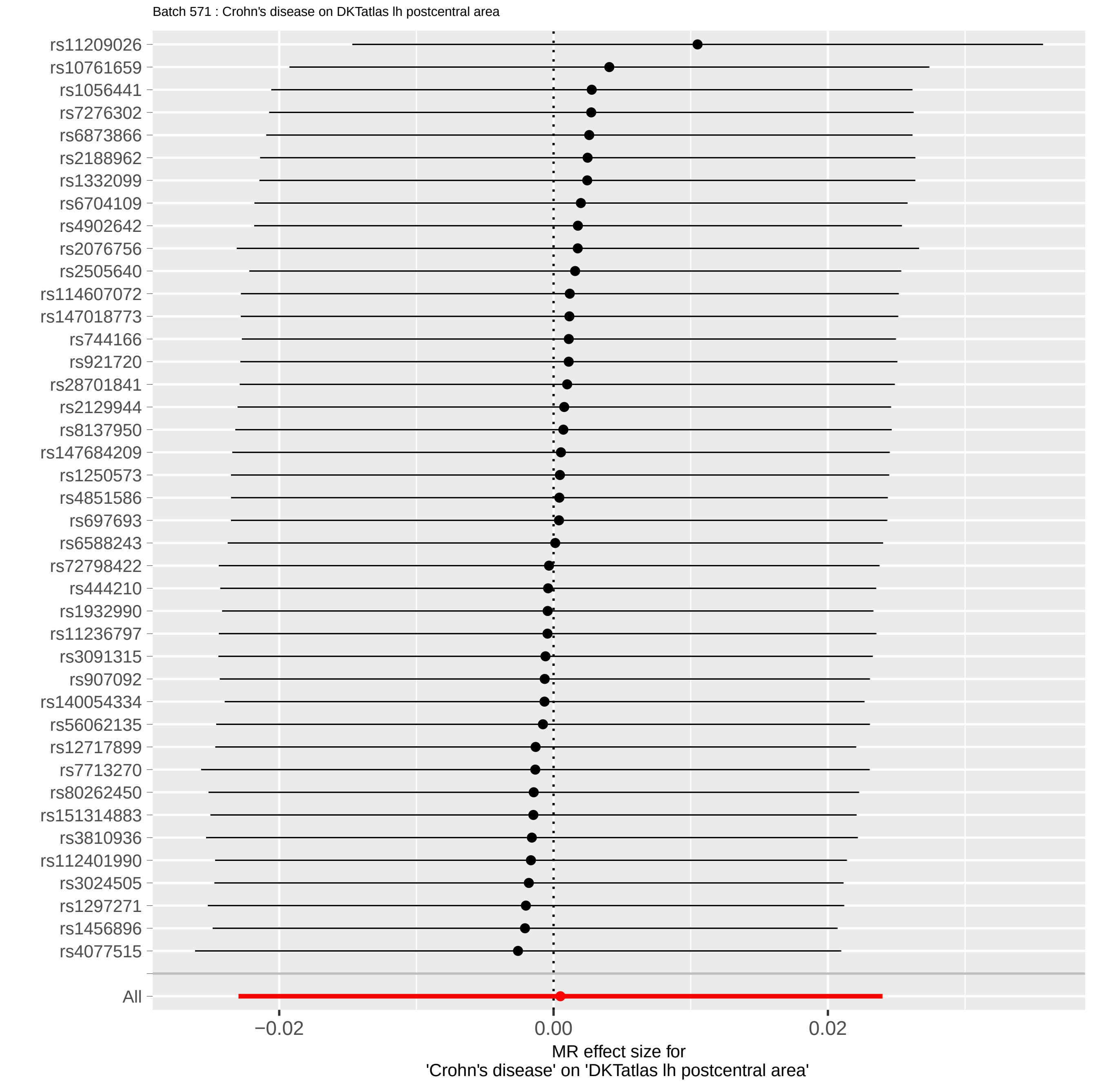
Batch 571 : Crohn's disease on DKTatlas lh postcentral area
rs11209026
rs10761659
rs1056441
rs7276302
rs6873866
rs2188962
rs1332099
rs6704109
rs4902642
rs2076756
rs2505640
rs114607072
rs147018773
rs744166
rs921720
rs28701841
rs2129944
rs8137950
rs147684209
rs1250573
rs4851586
rs697693
rs6588243
rs72798422
rs444210
rs1932990
rs11236797
rs3091315
rs907092
rs140054334
rs56062135
rs12717899
rs7713270
rs80262450
rs151314883
rs3810936
rs112401990
rs3024505
rs1297271
rs1456896
rs4077515
All
−0.02
0.00
0.02
MR effect size for
'Crohn's disease' on 'DKTatlas lh postcentral area'

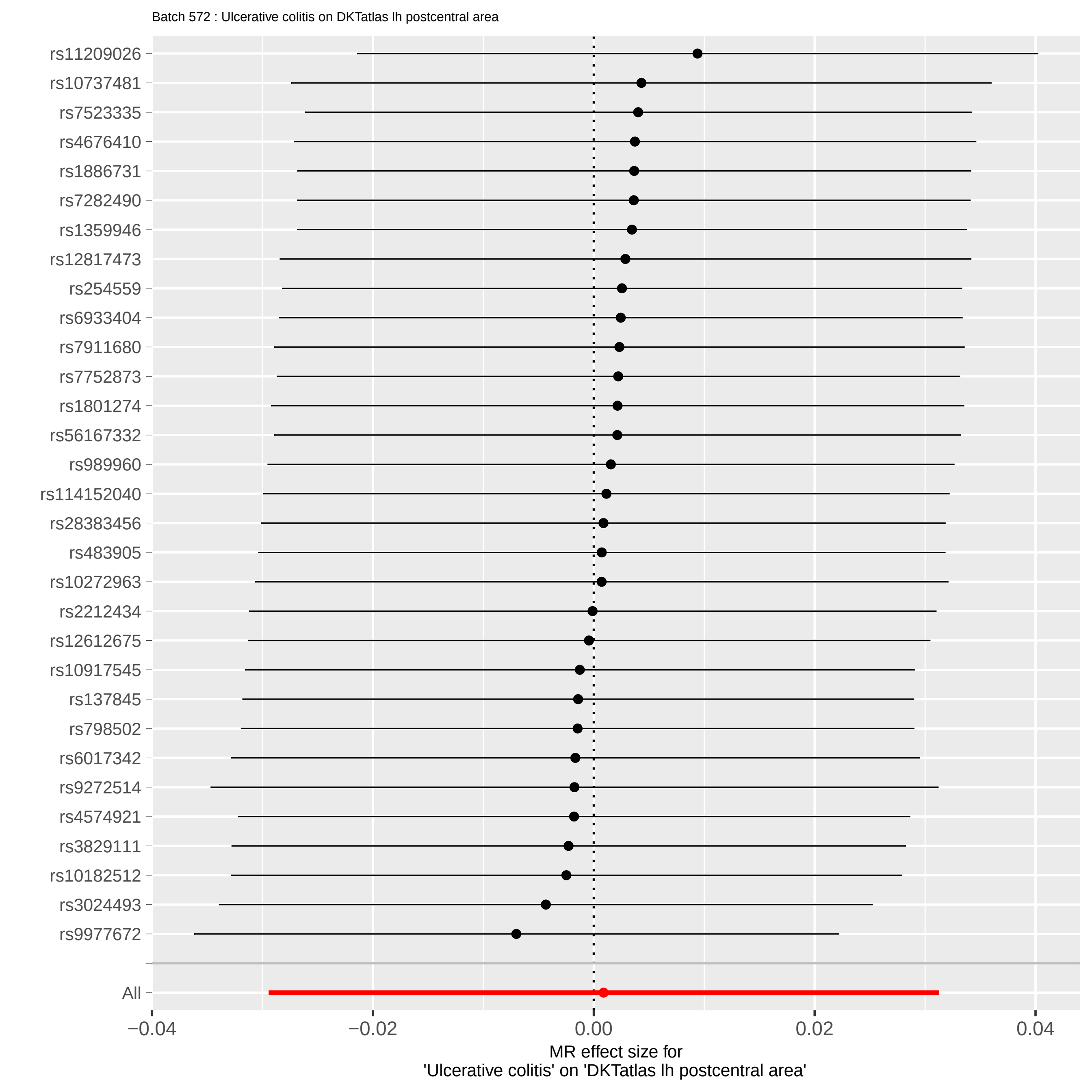

Batch 572 : Ulcerative colitis on DKTatlas lh postcentral area
rs11209026
rs10737481
rs7523335
rs4676410
rs1886731
rs7282490
rs1359946
rs12817473
rs254559
rs6933404
rs7911680
rs7752873
rs1801274
rs56167332
rs989960
rs114152040
rs28383456
rs483905
rs10272963
rs2212434
rs12612675
rs10917545
rs137845
rs798502
rs6017342
rs9272514
rs4574921
rs3829111
rs10182512
rs3024493
rs9977672
All
−0.04
−0.02
0.00
0.02
0.04
MR effect size for
'Ulcerative colitis' on 'DKTatlas lh postcentral area'

Batch 573 : Crohn's disease on DKTatlas lh posteriorcingulate area

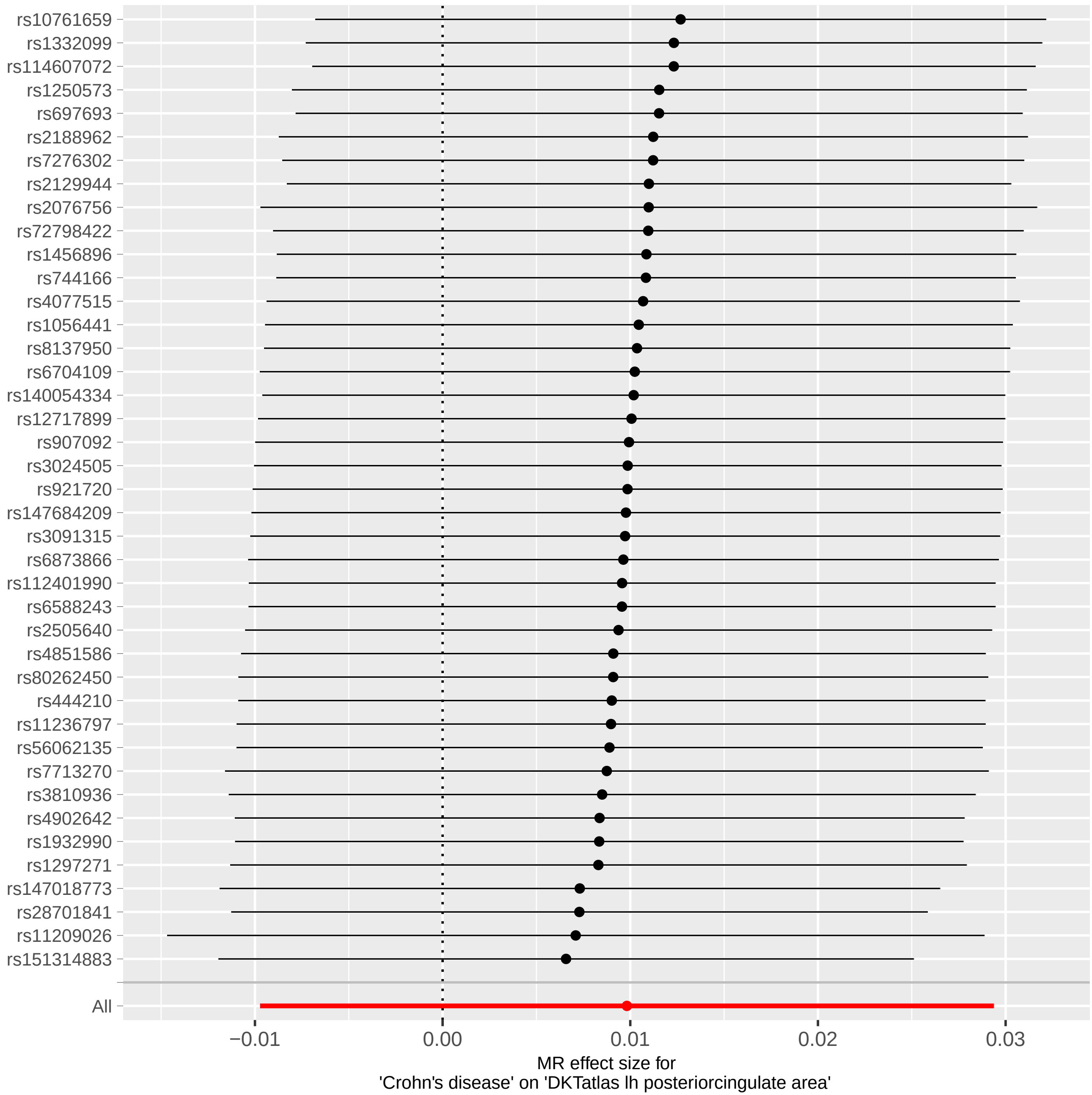

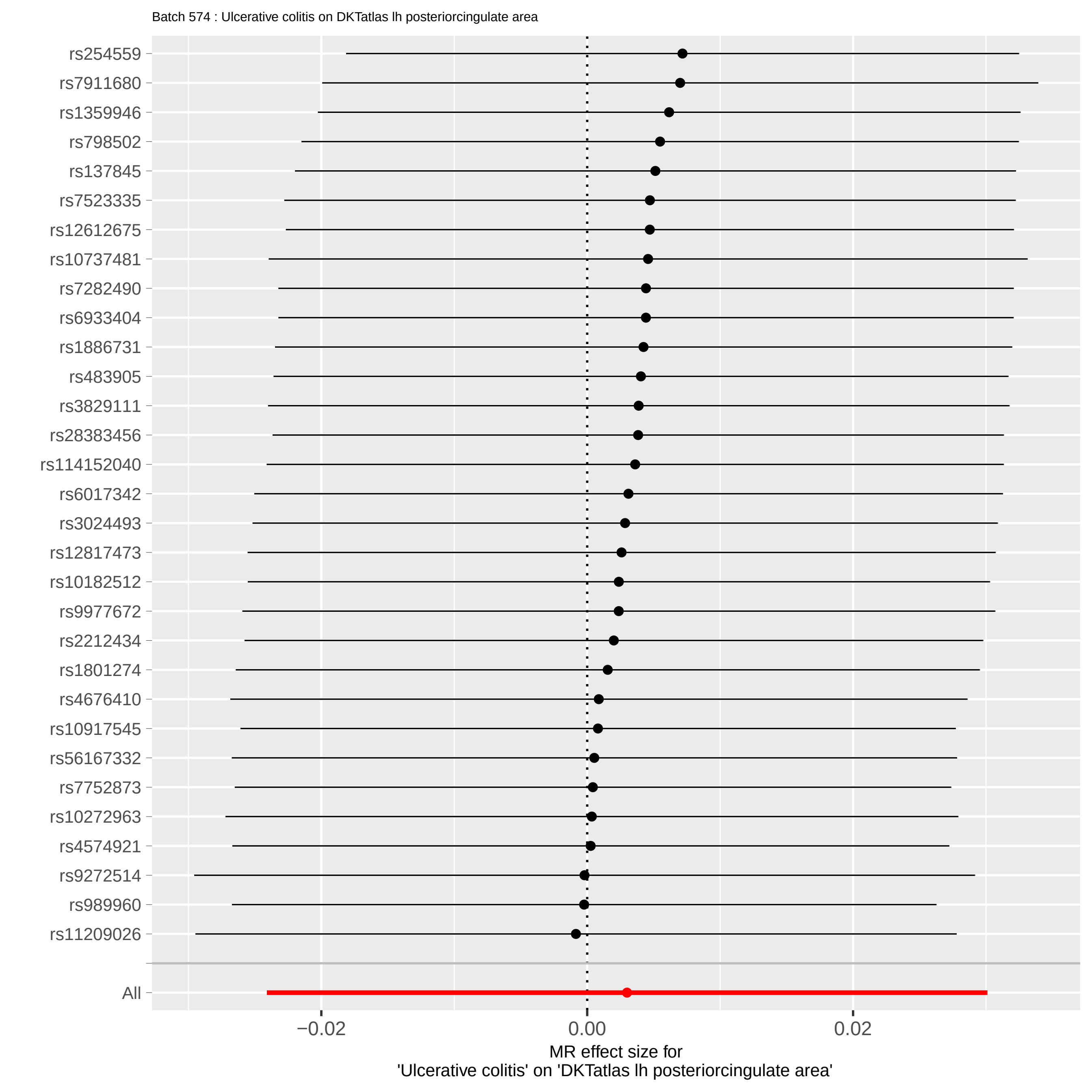

Batch 574 : Ulcerative colitis on DKTatlas lh posteriorcingulate area
rs254559
rs7911680
rs1359946
rs798502
rs137845
rs7523335
rs12612675
rs10737481
rs7282490
rs6933404
rs1886731
rs483905
rs3829111
rs28383456
rs114152040
rs6017342
rs3024493
rs12817473
rs10182512
rs9977672
rs2212434
rs1801274
rs4676410
rs10917545
rs56167332
rs7752873
rs10272963
rs4574921
rs9272514
rs989960
rs11209026
All
−0.02
0.00
0.02
MR effect size for
'Ulcerative colitis' on 'DKTatlas lh posteriorcingulate area'

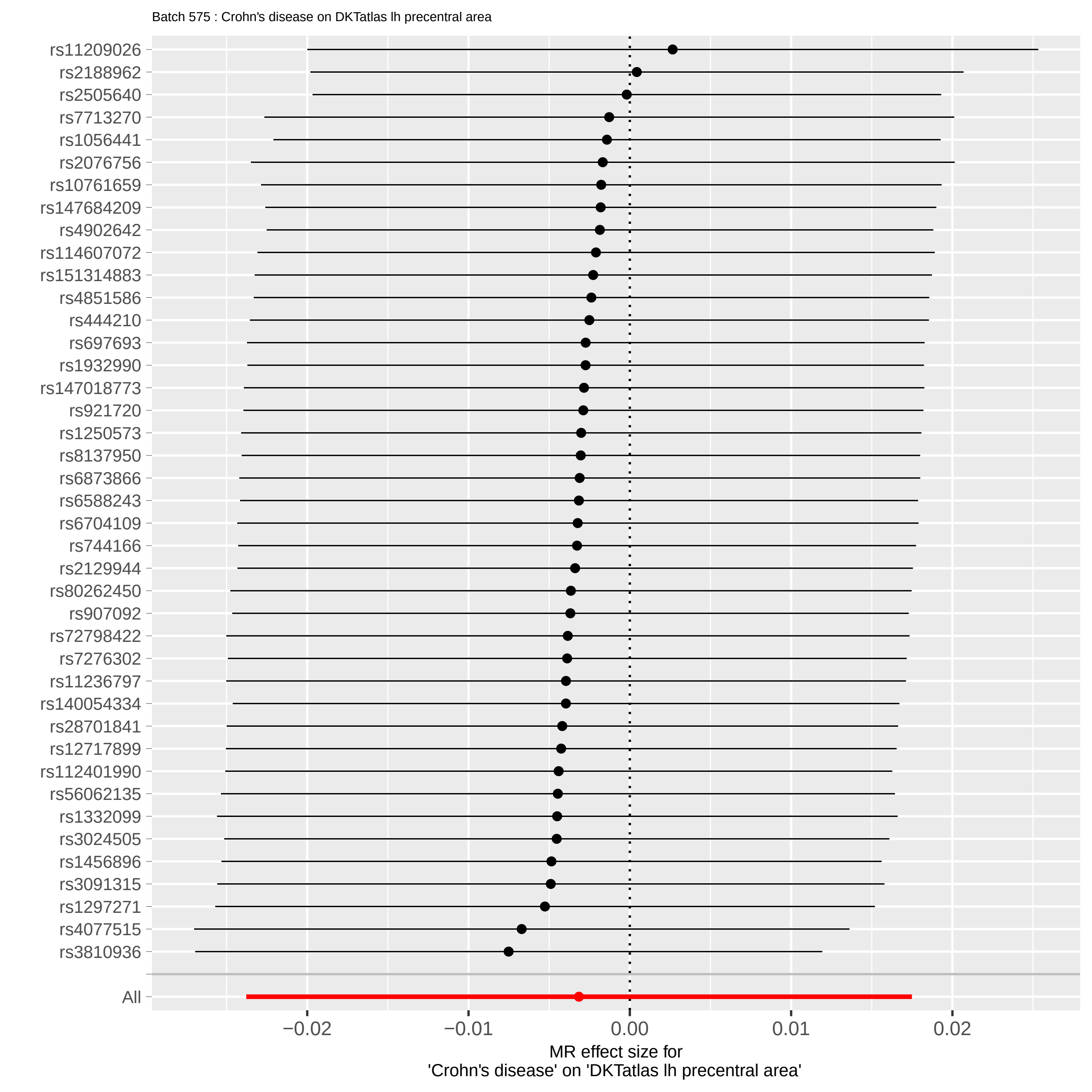

Batch 575 : Crohn's disease on DKTatlas lh precentral area
rs11209026
rs2188962
rs2505640
rs7713270
rs1056441
rs2076756
rs10761659
rs147684209
rs4902642
rs114607072
rs151314883
rs4851586
rs444210
rs697693
rs1932990
rs147018773
rs921720
rs1250573
rs8137950
rs6873866
rs6588243
rs6704109
rs744166
rs2129944
rs80262450
rs907092
rs72798422
rs7276302
rs11236797
rs140054334
rs28701841
rs12717899
rs112401990
rs56062135
rs1332099
rs3024505
rs1456896
rs3091315
rs1297271
rs4077515
rs3810936
All
−0.02
−0.01
0.00
0.01
0.02
MR effect size for
'Crohn's disease' on 'DKTatlas lh precentral area'

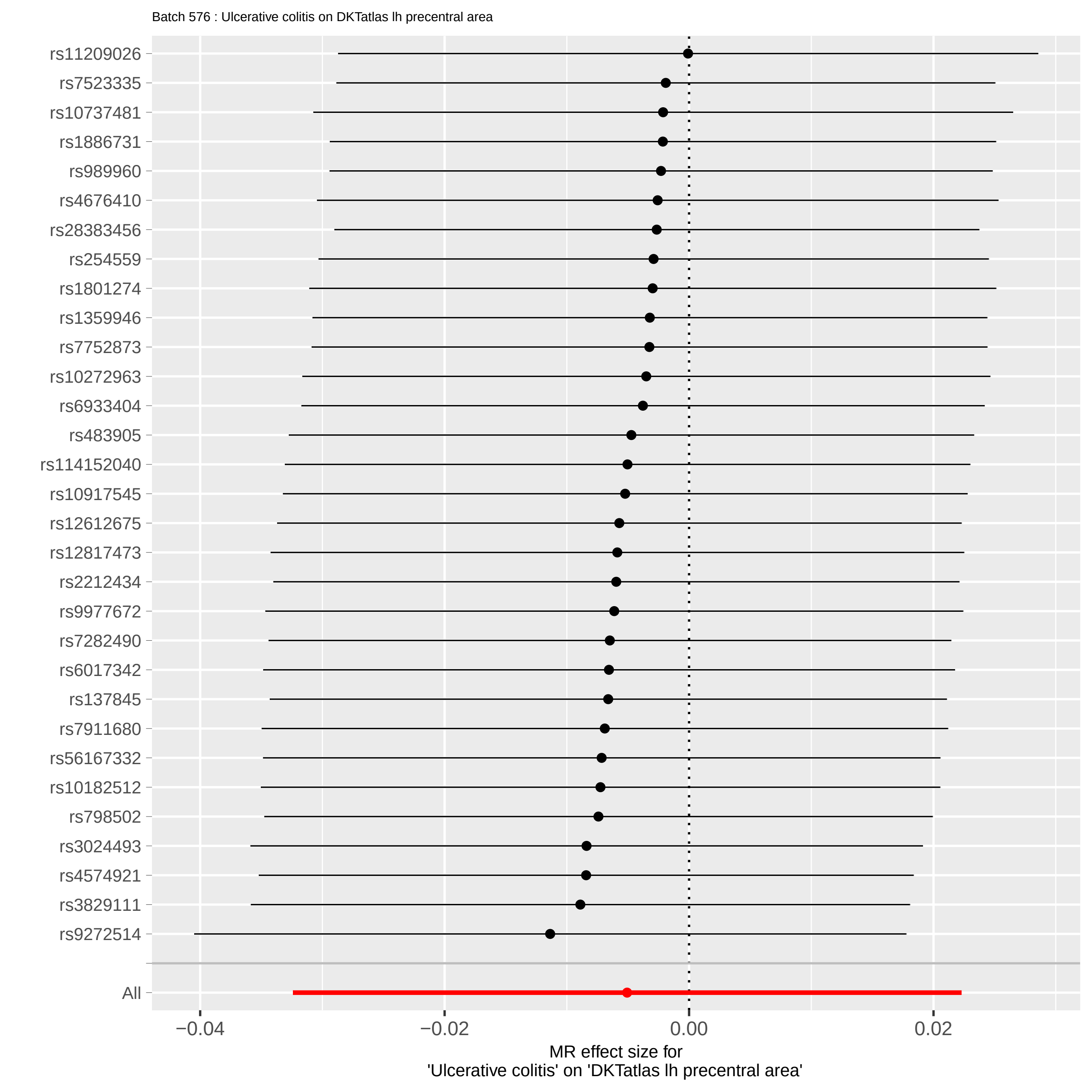

Batch 576 : Ulcerative colitis on DKTatlas lh precentral area
rs11209026
rs7523335
rs10737481
rs1886731
rs989960
rs4676410
rs28383456
rs254559
rs1801274
rs1359946
rs7752873
rs10272963
rs6933404
rs483905
rs114152040
rs10917545
rs12612675
rs12817473
rs2212434
rs9977672
rs7282490
rs6017342
rs137845
rs7911680
rs56167332
rs10182512
rs798502
rs3024493
rs4574921
rs3829111
rs9272514
All
−0.04
−0.02
0.00
0.02
MR effect size for
'Ulcerative colitis' on 'DKTatlas lh precentral area'

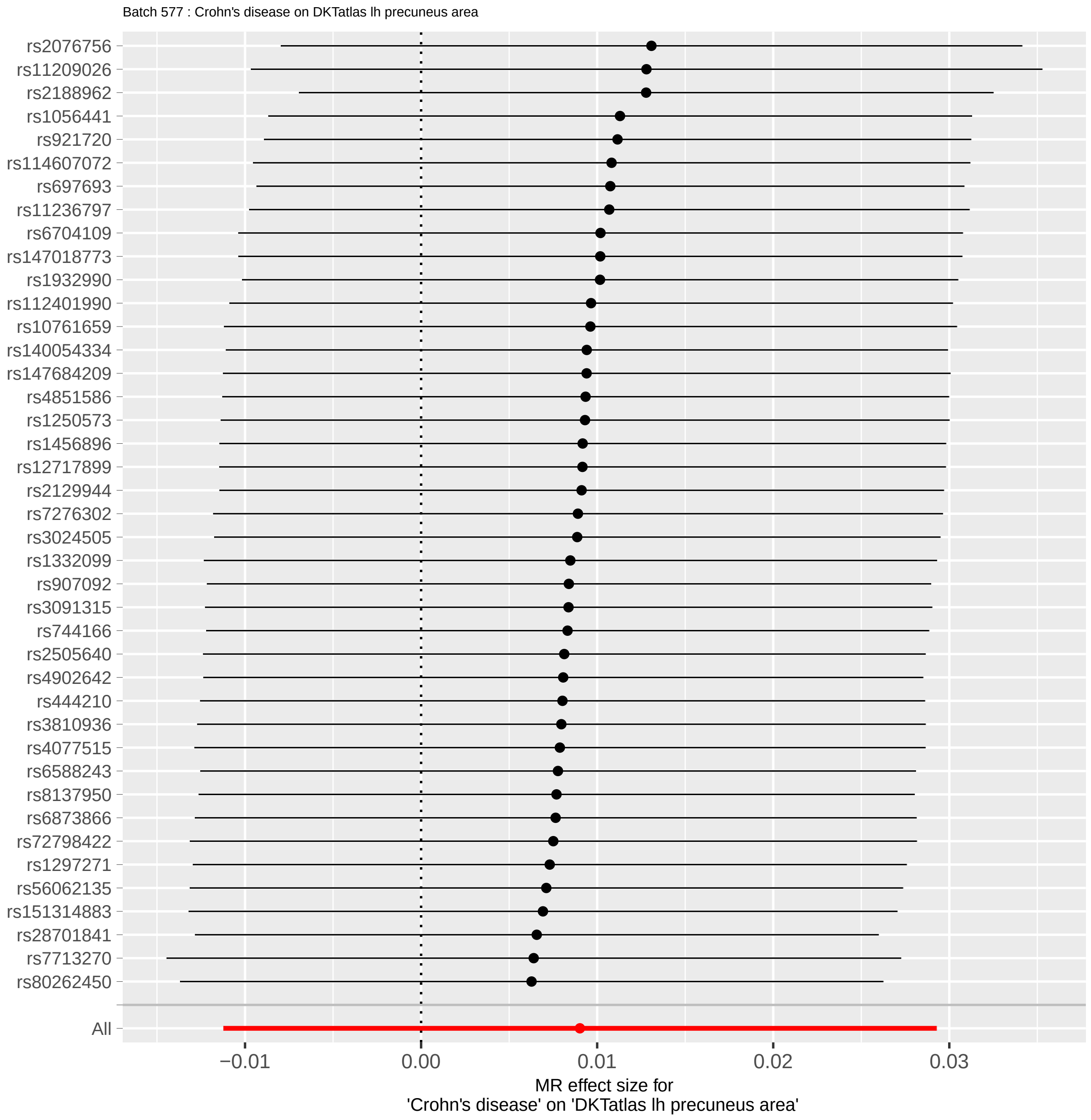
Batch 577 : Crohn's disease on DKTatlas lh precuneus area
rs2076756
rs11209026
rs2188962
rs1056441
rs921720
rs114607072
rs697693
rs11236797
rs6704109
rs147018773
rs1932990
rs112401990
rs10761659
rs140054334
rs147684209
rs4851586
rs1250573
rs1456896
rs12717899
rs2129944
rs7276302
rs3024505
rs1332099
rs907092
rs3091315
rs744166
rs2505640
rs4902642
rs444210
rs3810936
rs4077515
rs6588243
rs8137950
rs6873866
rs72798422
rs1297271
rs56062135
rs151314883
rs28701841
rs7713270
rs80262450
All
−0.01
0.00
0.01
0.02
0.03
MR effect size for
'Crohn's disease' on 'DKTatlas lh precuneus area'

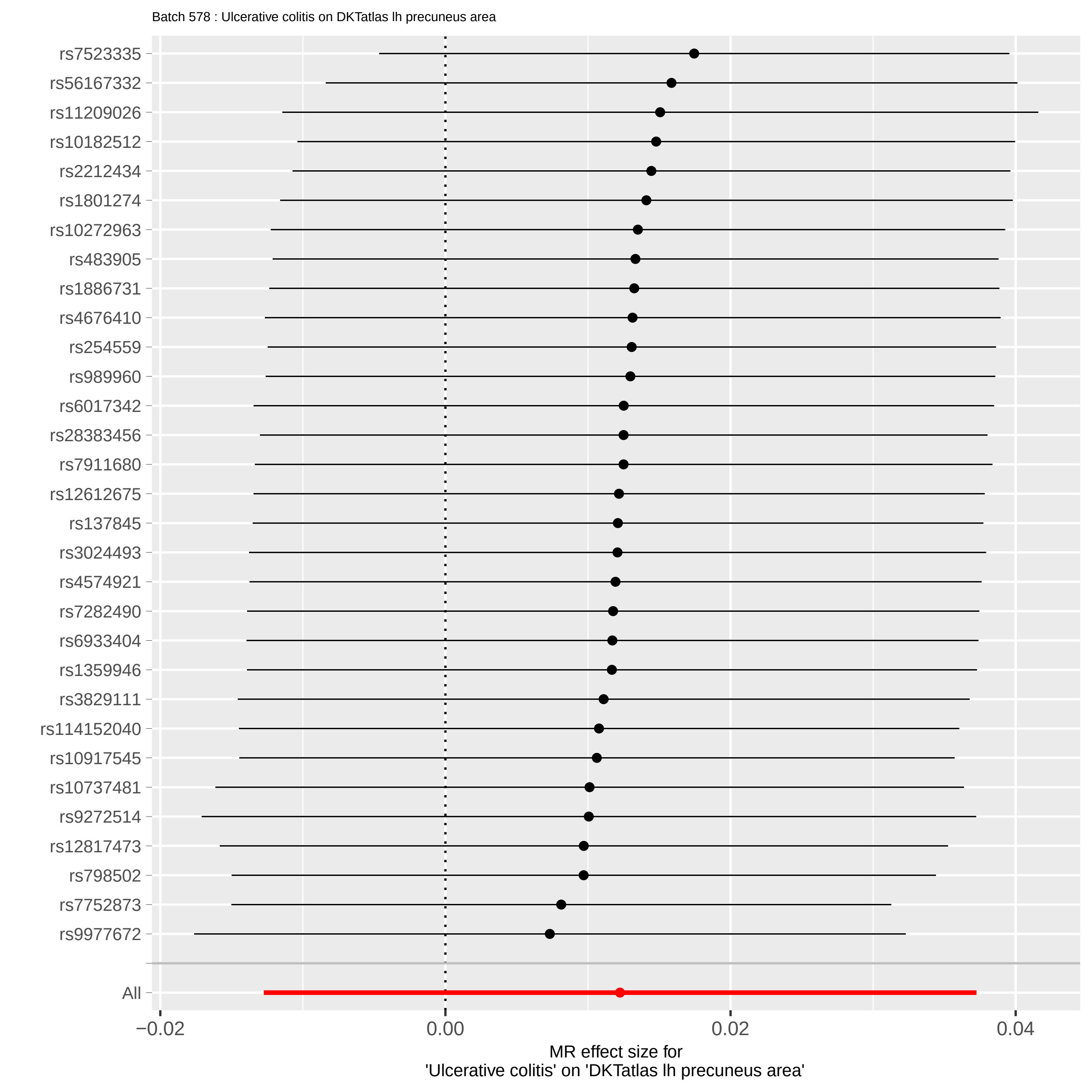
Batch 578 : Ulcerative colitis on DKTatlas lh precuneus area
rs7523335
rs56167332
rs11209026
rs10182512
rs2212434
rs1801274
rs10272963
rs483905
rs1886731
rs4676410
rs254559
rs989960
rs6017342
rs28383456
rs7911680
rs12612675
rs137845
rs3024493
rs4574921
rs7282490
rs6933404
rs1359946
rs3829111
rs114152040
rs10917545
rs10737481
rs9272514
rs12817473
rs798502
rs7752873
rs9977672
All
−0.02
0.00
0.02
0.04
MR effect size for
'Ulcerative colitis' on 'DKTatlas lh precuneus area'

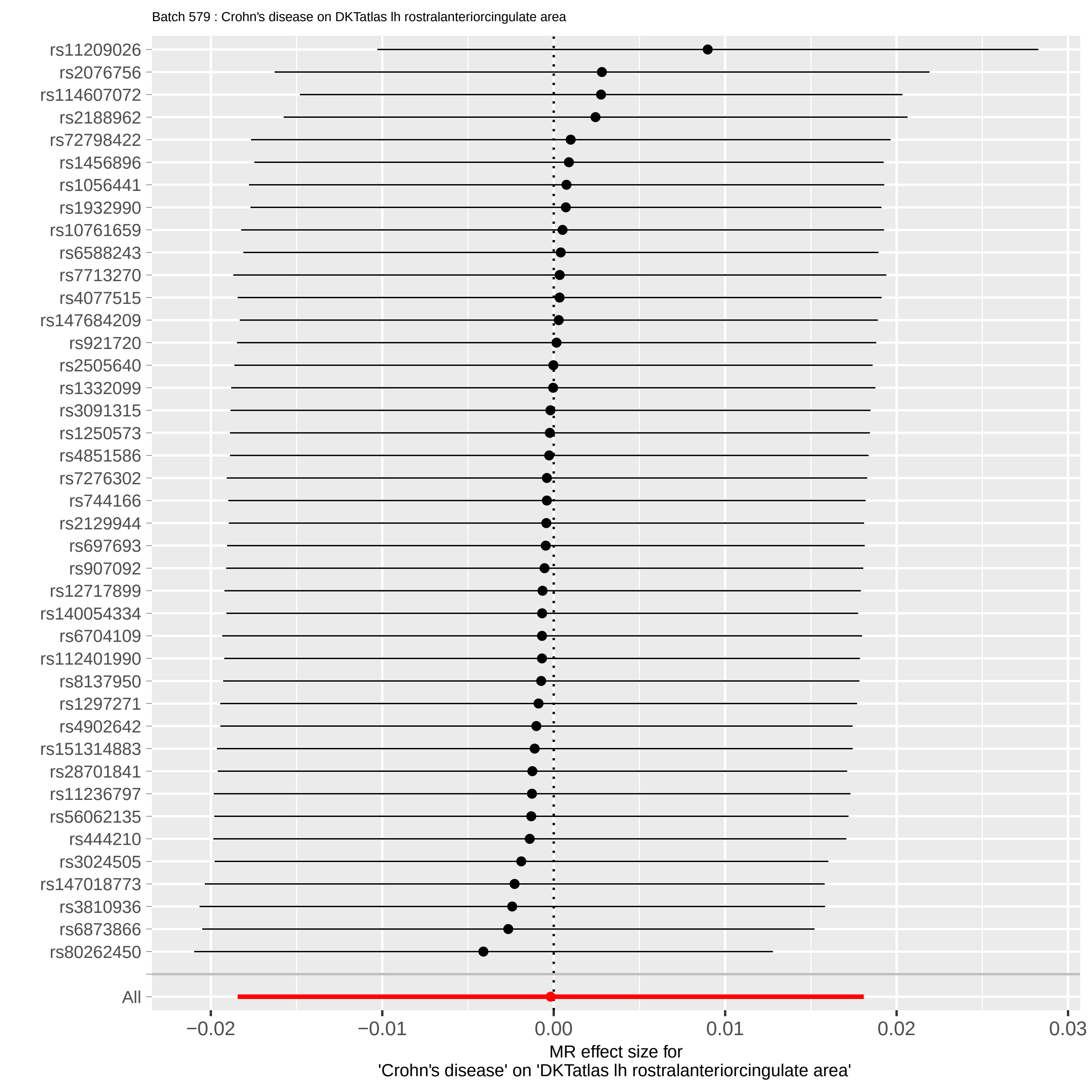

Batch 579 : Crohn's disease on DKTatlas lh rostralanteriorcingulate area
rs11209026
rs2076756
rs114607072
rs2188962
rs72798422
rs1456896
rs1056441
rs1932990
rs10761659
rs6588243
rs7713270
rs4077515
rs147684209
rs921720
rs2505640
rs1332099
rs3091315
rs1250573
rs4851586
rs7276302
rs744166
rs2129944
rs697693
rs907092
rs12717899
rs140054334
rs6704109
rs112401990
rs8137950
rs1297271
rs4902642
rs151314883
rs28701841
rs11236797
rs56062135
rs444210
rs3024505
rs147018773
rs3810936
rs6873866
rs80262450
All
−0.02
−0.01
0.00
0.01
0.02
0.03
MR effect size for
'Crohn's disease' on 'DKTatlas lh rostralanteriorcingulate area'

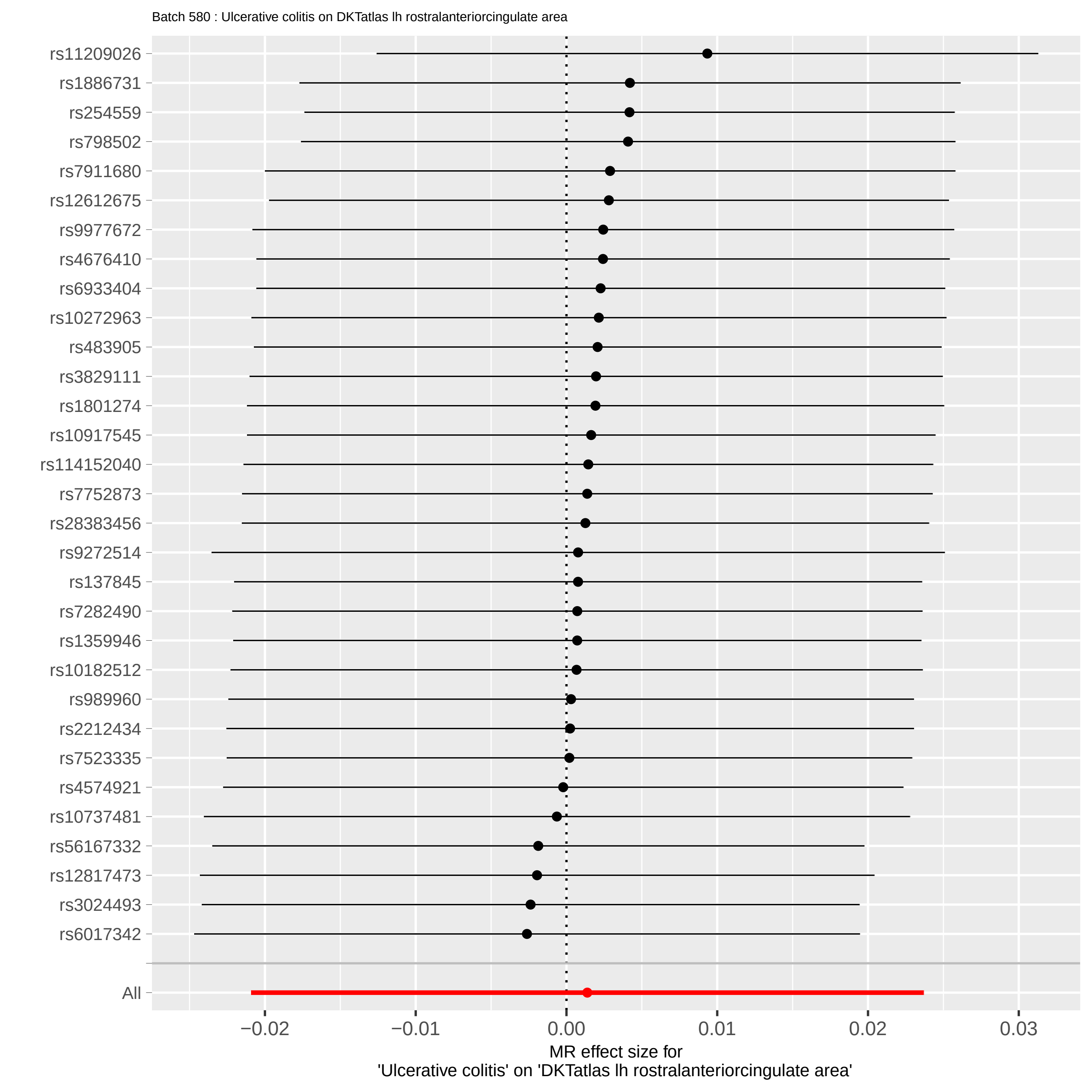

Batch 580 : Ulcerative colitis on DKTatlas lh rostralanteriorcingulate area
rs11209026
rs1886731
rs254559
rs798502
rs7911680
rs12612675
rs9977672
rs4676410
rs6933404
rs10272963
rs483905
rs3829111
rs1801274
rs10917545
rs114152040
rs7752873
rs28383456
rs9272514
rs137845
rs7282490
rs1359946
rs10182512
rs989960
rs2212434
rs7523335
rs4574921
rs10737481
rs56167332
rs12817473
rs3024493
rs6017342
All
−0.02
−0.01
0.00
0.01
0.02
0.03
MR effect size for
'Ulcerative colitis' on 'DKTatlas lh rostralanteriorcingulate area'

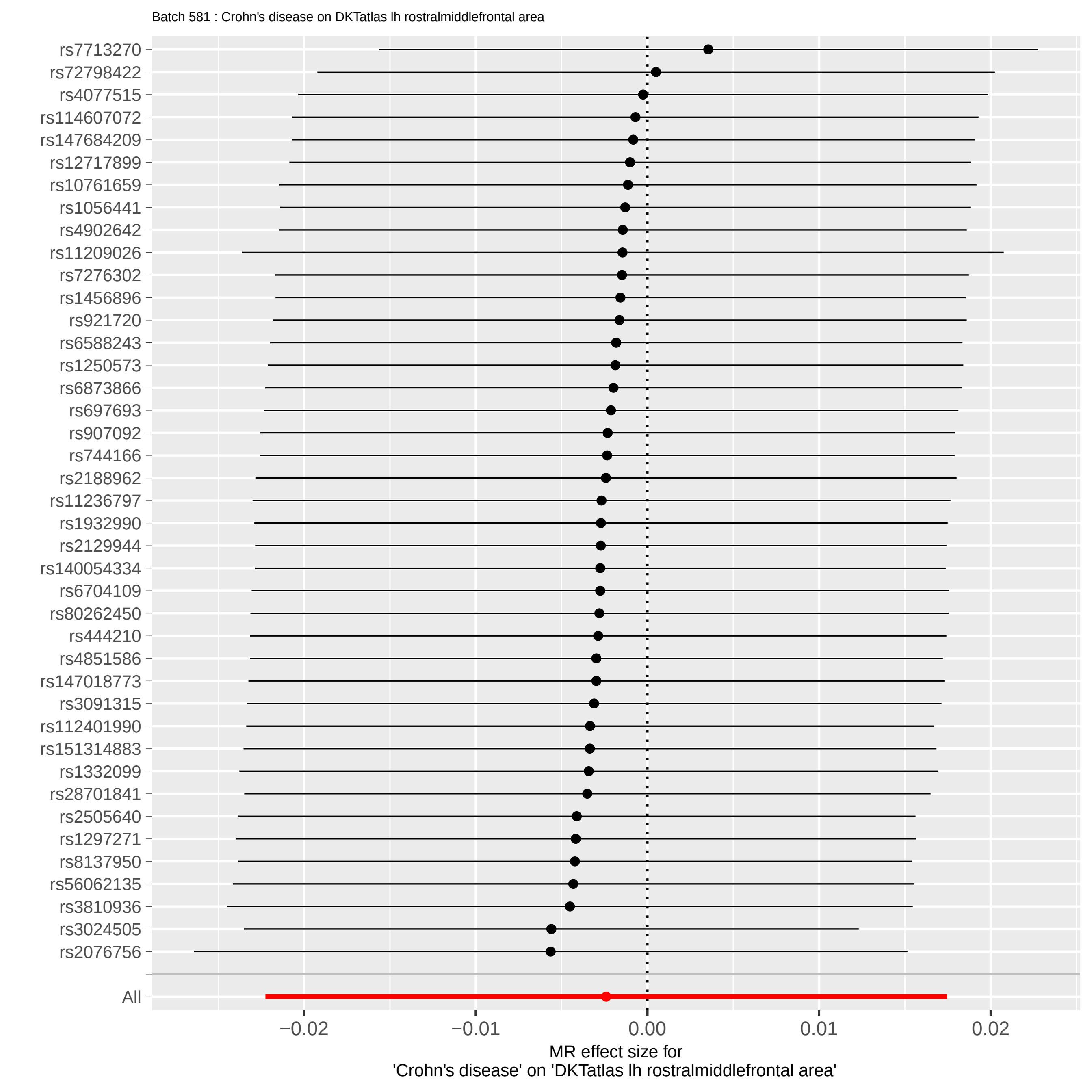

Batch 581 : Crohn's disease on DKTatlas lh rostralmiddlefrontal area
rs7713270
rs72798422
rs4077515
rs114607072
rs147684209
rs12717899
rs10761659
rs1056441
rs4902642
rs11209026
rs7276302
rs1456896
rs921720
rs6588243
rs1250573
rs6873866
rs697693
rs907092
rs744166
rs2188962
rs11236797
rs1932990
rs2129944
rs140054334
rs6704109
rs80262450
rs444210
rs4851586
rs147018773
rs3091315
rs112401990
rs151314883
rs1332099
rs28701841
rs2505640
rs1297271
rs8137950
rs56062135
rs3810936
rs3024505
rs2076756
All
−0.02
−0.01
0.00
0.01
0.02
MR effect size for
'Crohn's disease' on 'DKTatlas lh rostralmiddlefrontal area'

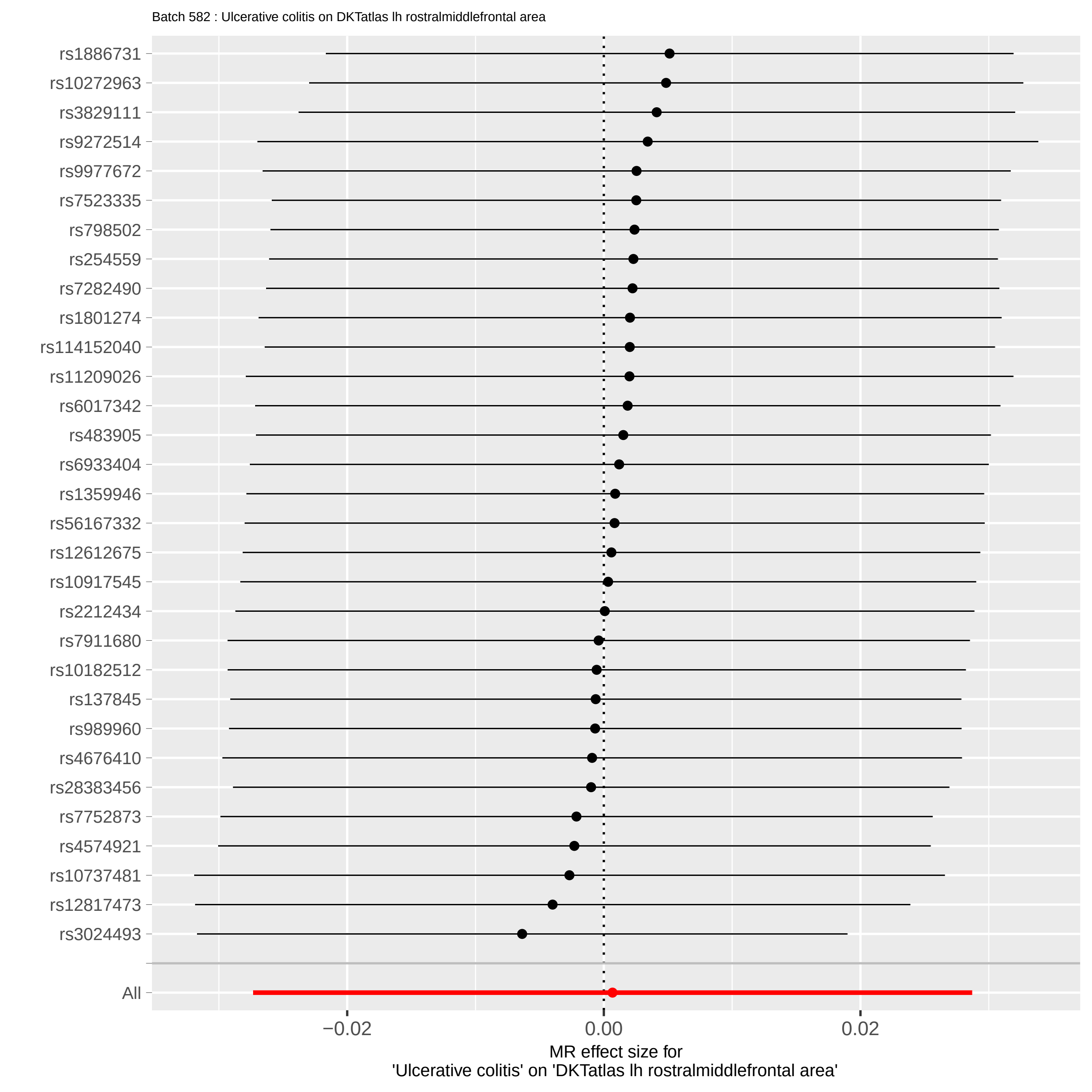

Batch 582 : Ulcerative colitis on DKTatlas lh rostralmiddlefrontal area
rs1886731
rs10272963
rs3829111
rs9272514
rs9977672
rs7523335
rs798502
rs254559
rs7282490
rs1801274
rs114152040
rs11209026
rs6017342
rs483905
rs6933404
rs1359946
rs56167332
rs12612675
rs10917545
rs2212434
rs7911680
rs10182512
rs137845
rs989960
rs4676410
rs28383456
rs7752873
rs4574921
rs10737481
rs12817473
rs3024493
All
−0.02
0.00
0.02
MR effect size for
'Ulcerative colitis' on 'DKTatlas lh rostralmiddlefrontal area'

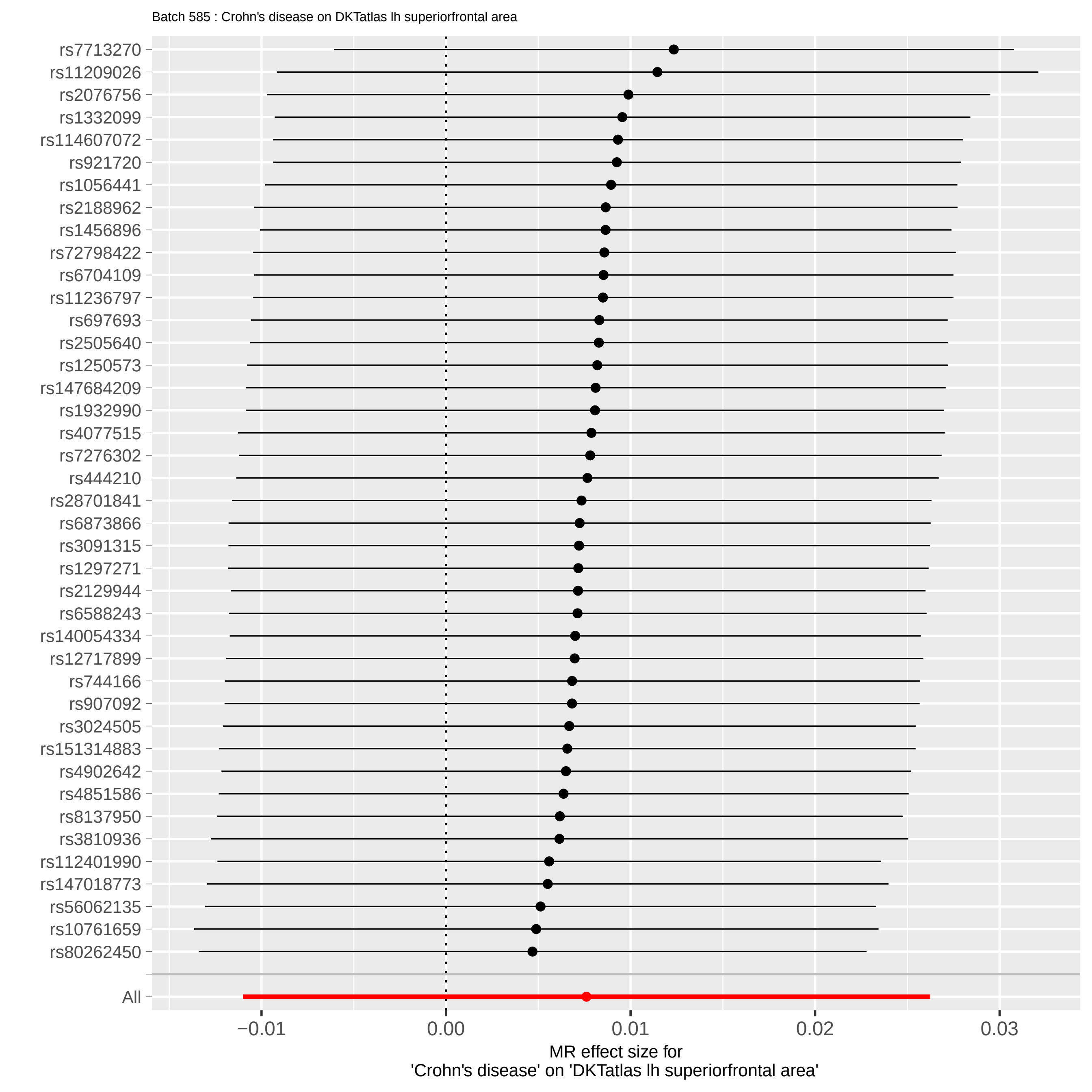

Batch 585 : Crohn's disease on DKTatlas lh superiorfrontal area
rs7713270
rs11209026
rs2076756
rs1332099
rs114607072
rs921720
rs1056441
rs2188962
rs1456896
rs72798422
rs6704109
rs11236797
rs697693
rs2505640
rs1250573
rs147684209
rs1932990
rs4077515
rs7276302
rs444210
rs28701841
rs6873866
rs3091315
rs1297271
rs2129944
rs6588243
rs140054334
rs12717899
rs744166
rs907092
rs3024505
rs151314883
rs4902642
rs4851586
rs8137950
rs3810936
rs112401990
rs147018773
rs56062135
rs10761659
rs80262450
All
−0.01
0.00
0.01
0.02
0.03
MR effect size for
'Crohn's disease' on 'DKTatlas lh superiorfrontal area'

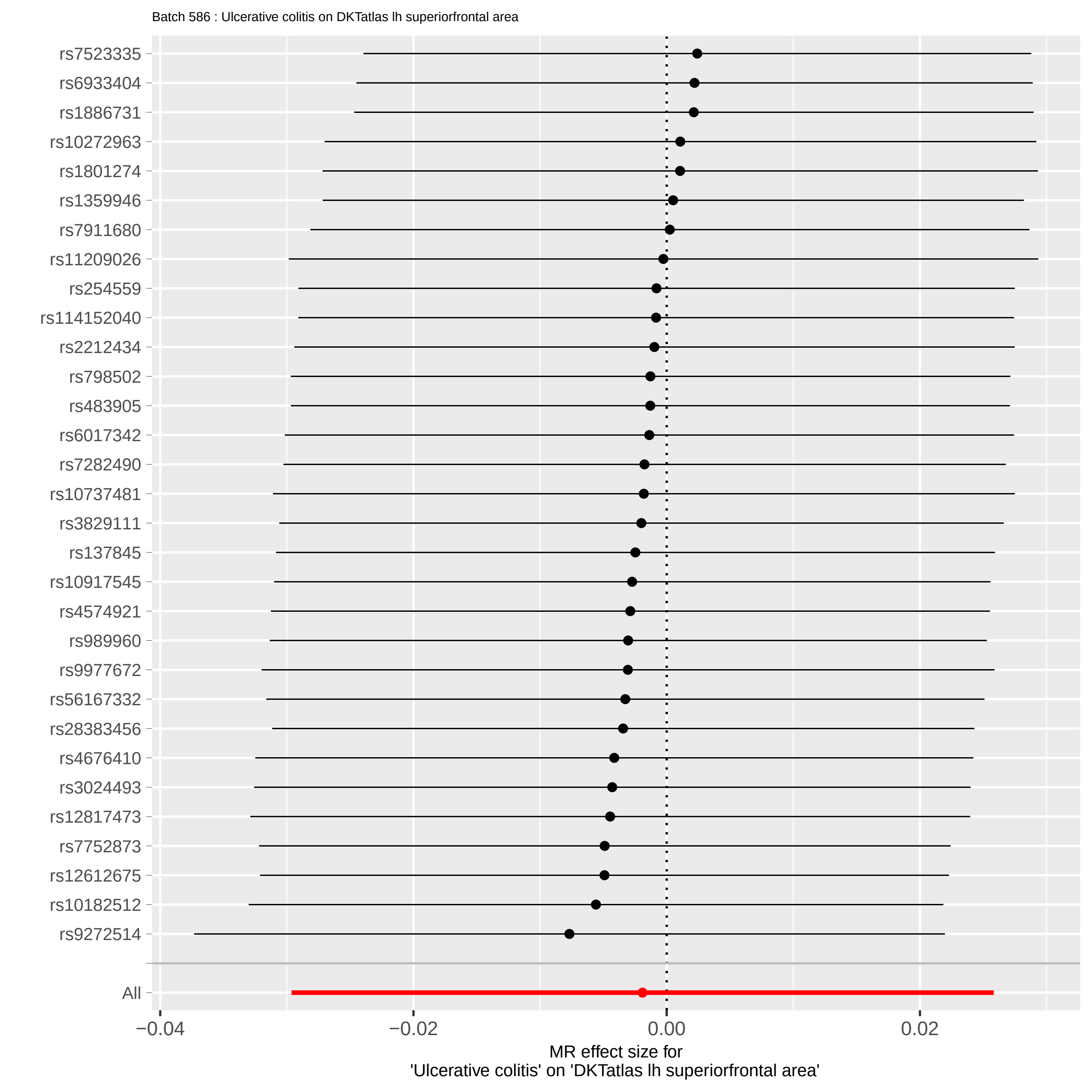

Batch 586 : Ulcerative colitis on DKTatlas lh superiorfrontal area
rs7523335
rs6933404
rs1886731
rs10272963
rs1801274
rs1359946
rs7911680
rs11209026
rs254559
rs114152040
rs2212434
rs798502
rs483905
rs6017342
rs7282490
rs10737481
rs3829111
rs137845
rs10917545
rs4574921
rs989960
rs9977672
rs56167332
rs28383456
rs4676410
rs3024493
rs12817473
rs7752873
rs12612675
rs10182512
rs9272514
All
−0.04
−0.02
0.00
0.02
MR effect size for
'Ulcerative colitis' on 'DKTatlas lh superiorfrontal area'

Batch 587 : Crohn's disease on DKTatlas lh superiorparietal area

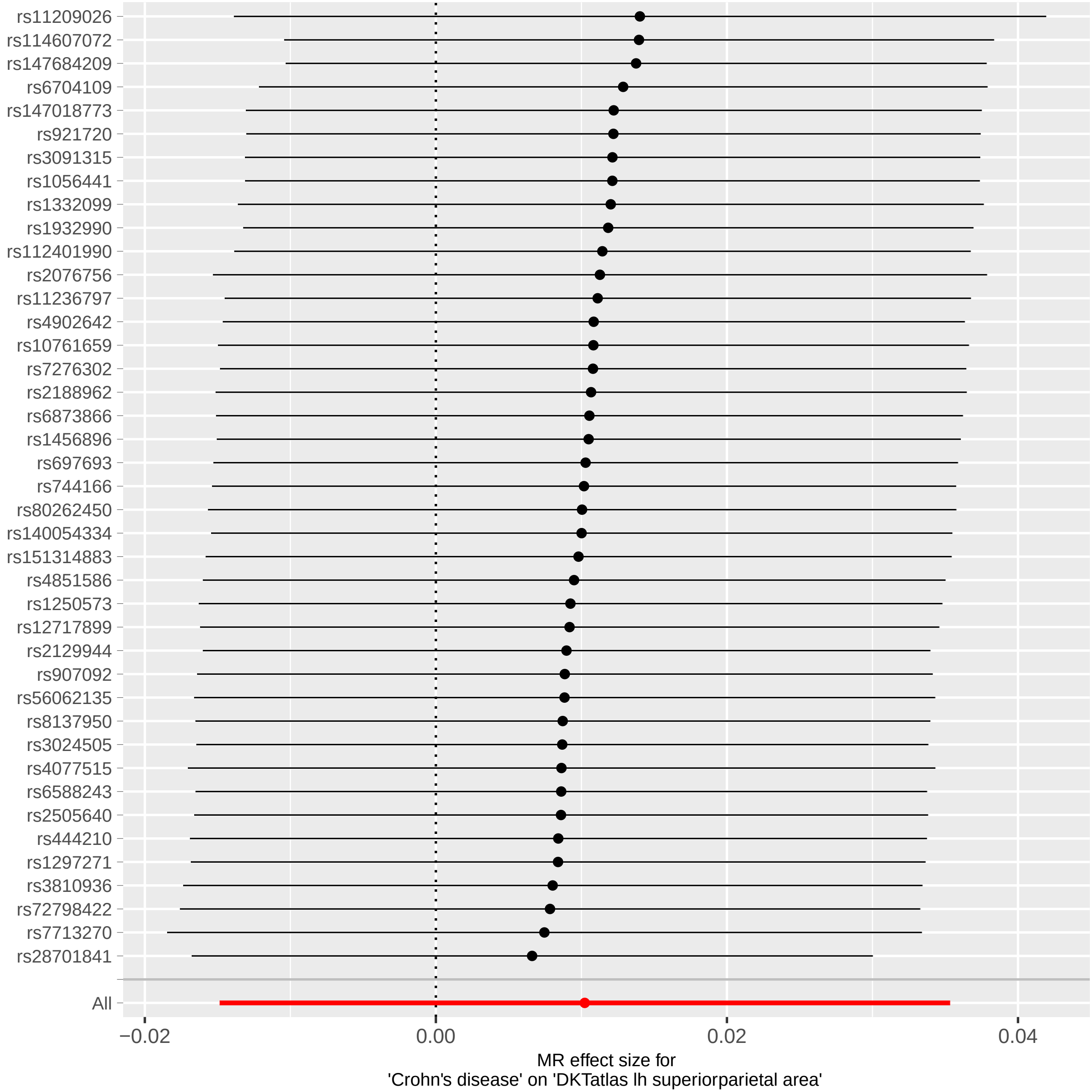

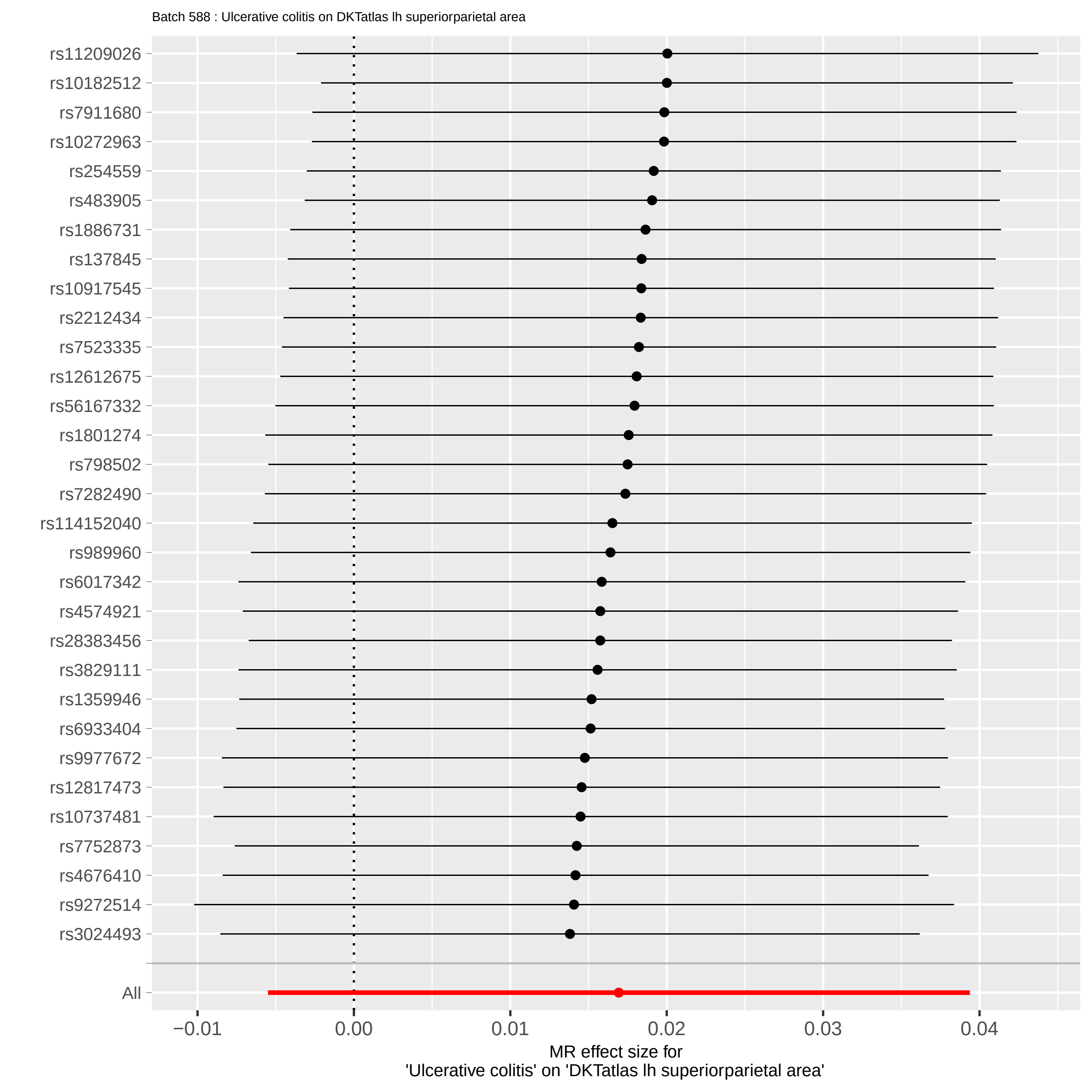

Batch 588 : Ulcerative colitis on DKTatlas lh superiorparietal area
rs11209026
rs10182512
rs7911680
rs10272963
rs254559
rs483905
rs1886731
rs137845
rs10917545
rs2212434
rs7523335
rs12612675
rs56167332
rs1801274
rs798502
rs7282490
rs114152040
rs989960
rs6017342
rs4574921
rs28383456
rs3829111
rs1359946
rs6933404
rs9977672
rs12817473
rs10737481
rs7752873
rs4676410
rs9272514
rs3024493
All
−0.01
0.00
0.01
0.02
0.03
0.04
MR effect size for
'Ulcerative colitis' on 'DKTatlas lh superiorparietal area'

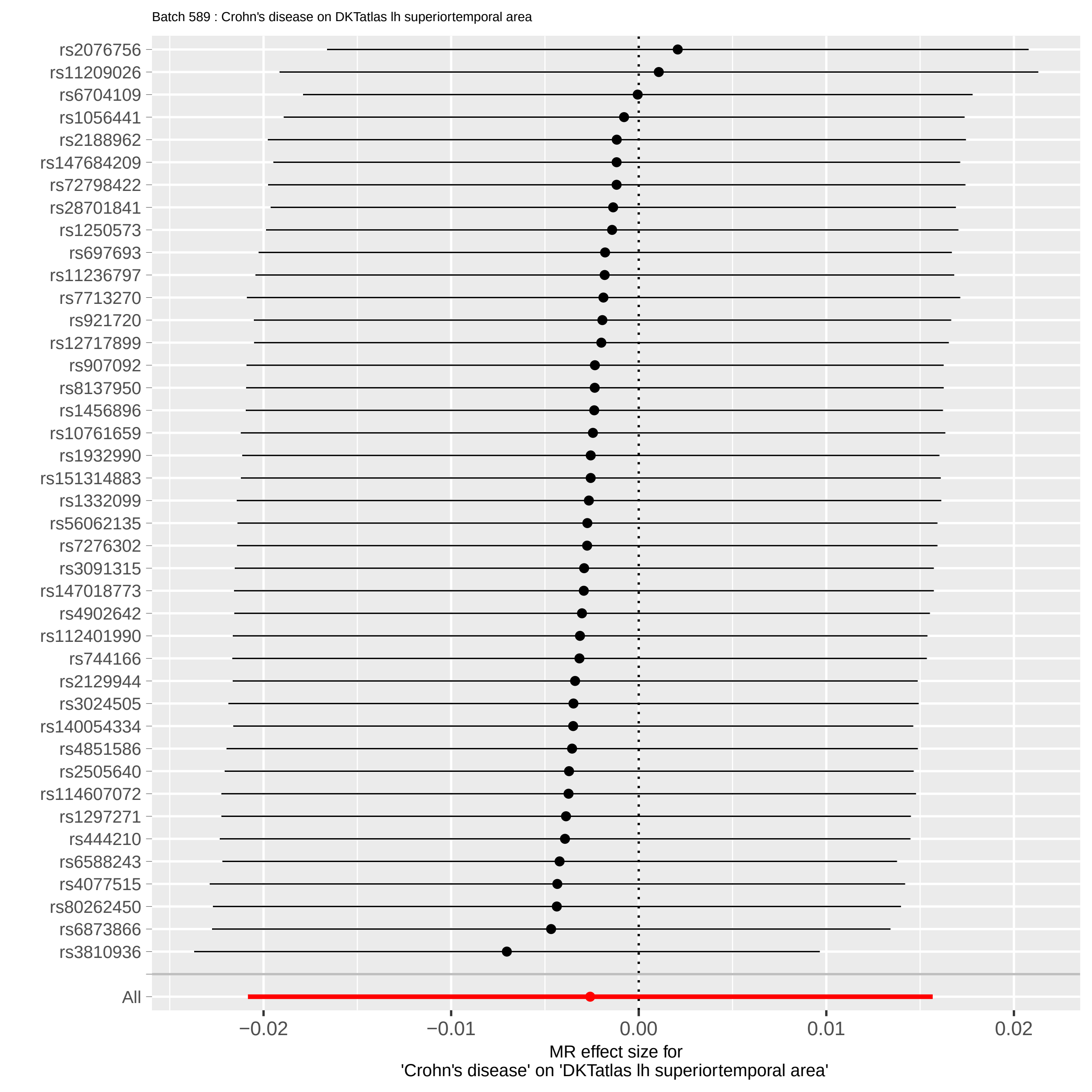

Batch 589 : Crohn's disease on DKTatlas lh superiortemporal area
rs2076756
rs11209026
rs6704109
rs1056441
rs2188962
rs147684209
rs72798422
rs28701841
rs1250573
rs697693
rs11236797
rs7713270
rs921720
rs12717899
rs907092
rs8137950
rs1456896
rs10761659
rs1932990
rs151314883
rs1332099
rs56062135
rs7276302
rs3091315
rs147018773
rs4902642
rs112401990
rs744166
rs2129944
rs3024505
rs140054334
rs4851586
rs2505640
rs114607072
rs1297271
rs444210
rs6588243
rs4077515
rs80262450
rs6873866
rs3810936
All
−0.02
−0.01
0.00
0.01
0.02
MR effect size for
'Crohn's disease' on 'DKTatlas lh superiortemporal area'

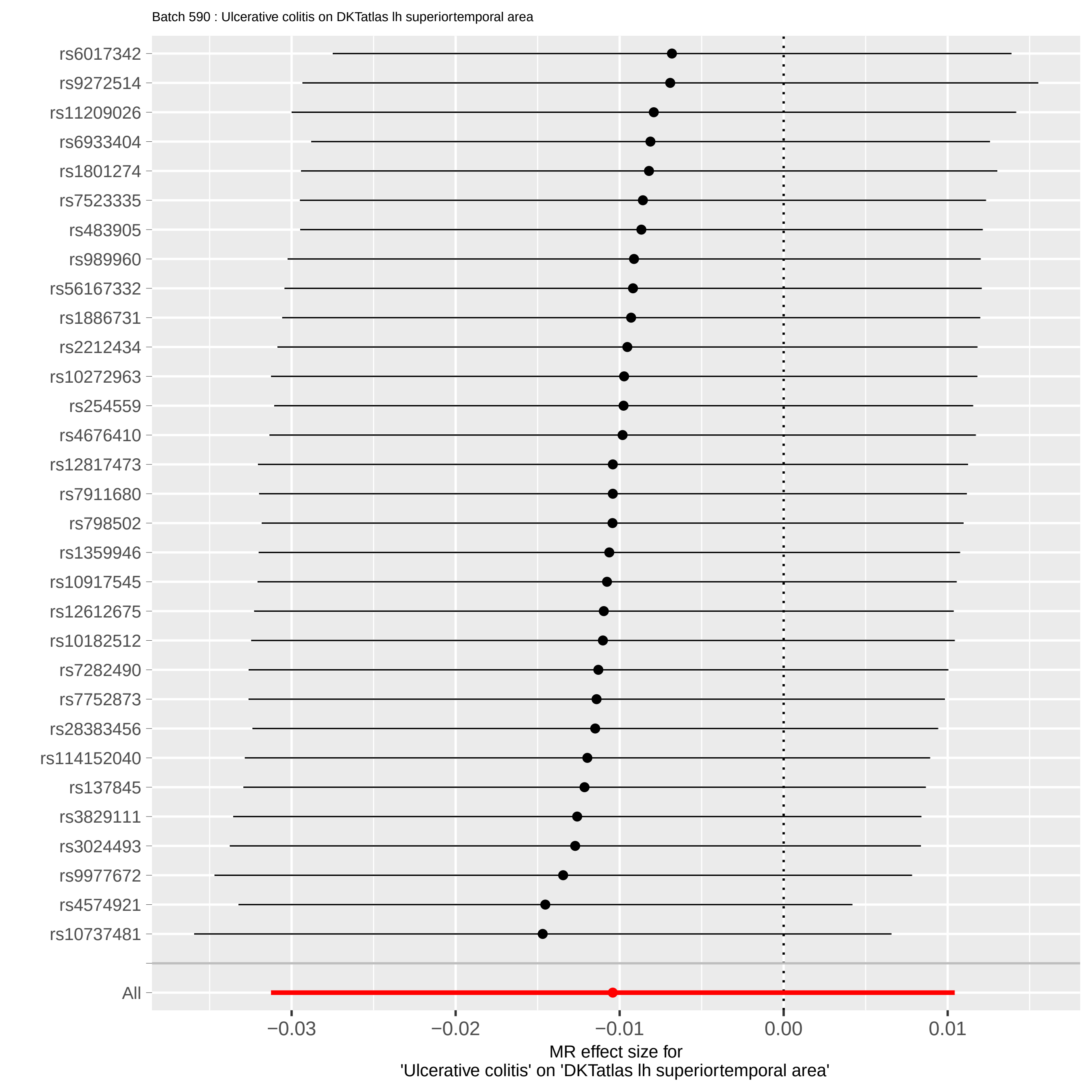

Batch 590 : Ulcerative colitis on DKTatlas lh superiortemporal area
rs6017342
rs9272514
rs11209026
rs6933404
rs1801274
rs7523335
rs483905
rs989960
rs56167332
rs1886731
rs2212434
rs10272963
rs254559
rs4676410
rs12817473
rs7911680
rs798502
rs1359946
rs10917545
rs12612675
rs10182512
rs7282490
rs7752873
rs28383456
rs114152040
rs137845
rs3829111
rs3024493
rs9977672
rs4574921
rs10737481
All
−0.03
−0.02
−0.01
0.00
0.01
MR effect size for
'Ulcerative colitis' on 'DKTatlas lh superiortemporal area'

Batch 591 : Crohn's disease on DKTatlas lh supramarginal area

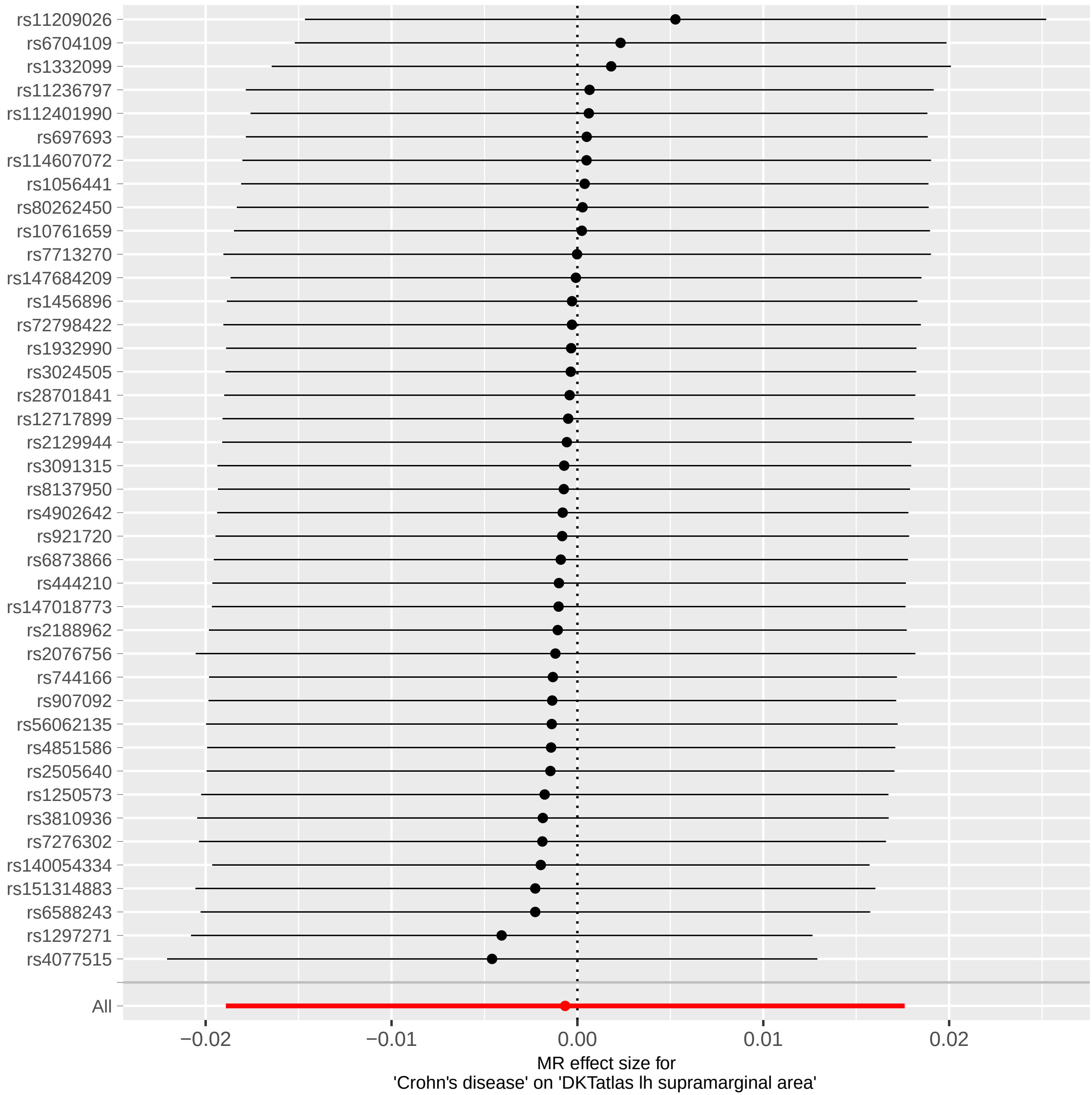

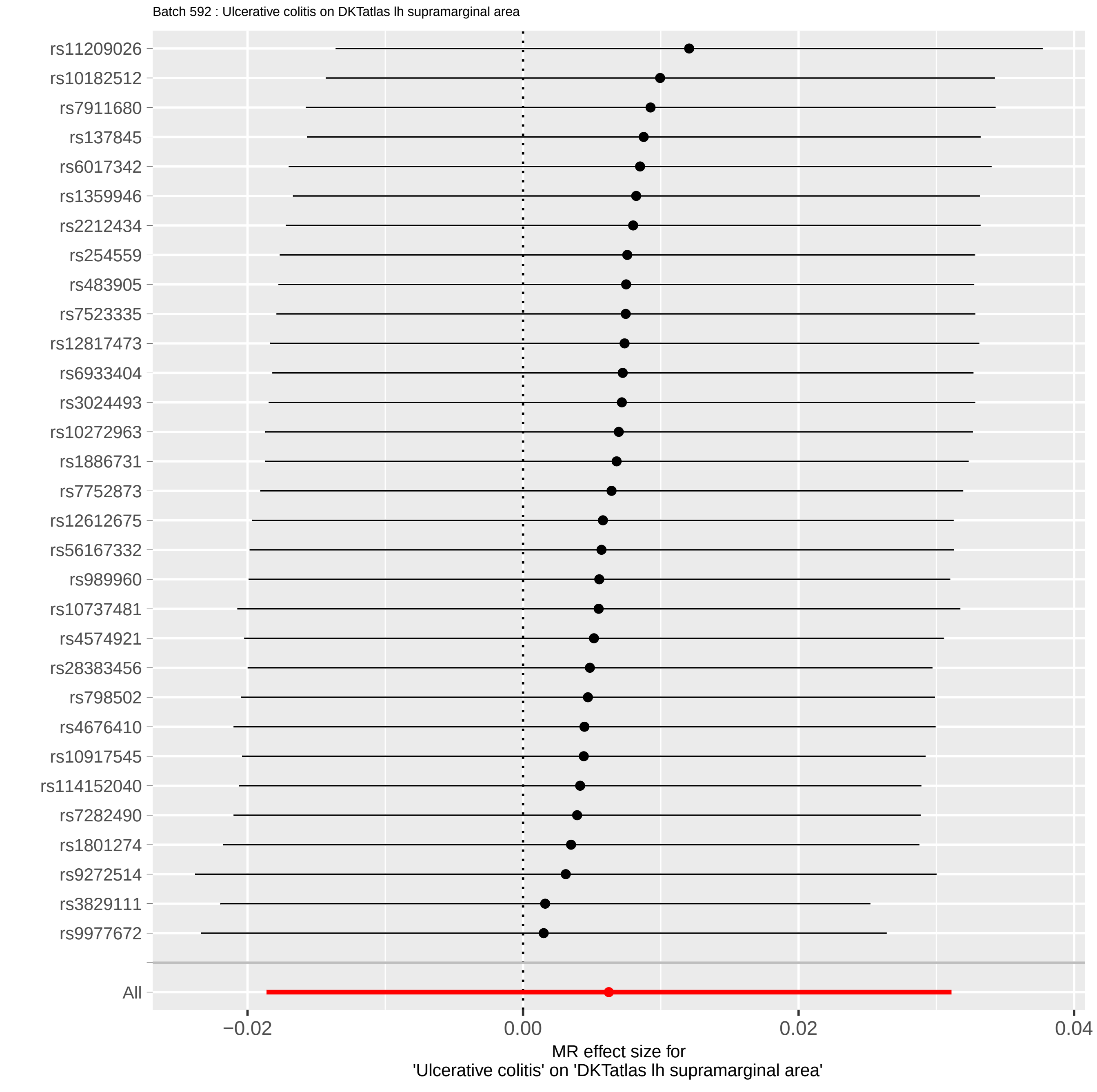

Batch 592 : Ulcerative colitis on DKTatlas lh supramarginal area
rs11209026
rs10182512
rs7911680
rs137845
rs6017342
rs1359946
rs2212434
rs254559
rs483905
rs7523335
rs12817473
rs6933404
rs3024493
rs10272963
rs1886731
rs7752873
rs12612675
rs56167332
rs989960
rs10737481
rs4574921
rs28383456
rs798502
rs4676410
rs10917545
rs114152040
rs7282490
rs1801274
rs9272514
rs3829111
rs9977672
All
−0.02
0.00
0.02
0.04
MR effect size for
'Ulcerative colitis' on 'DKTatlas lh supramarginal area'

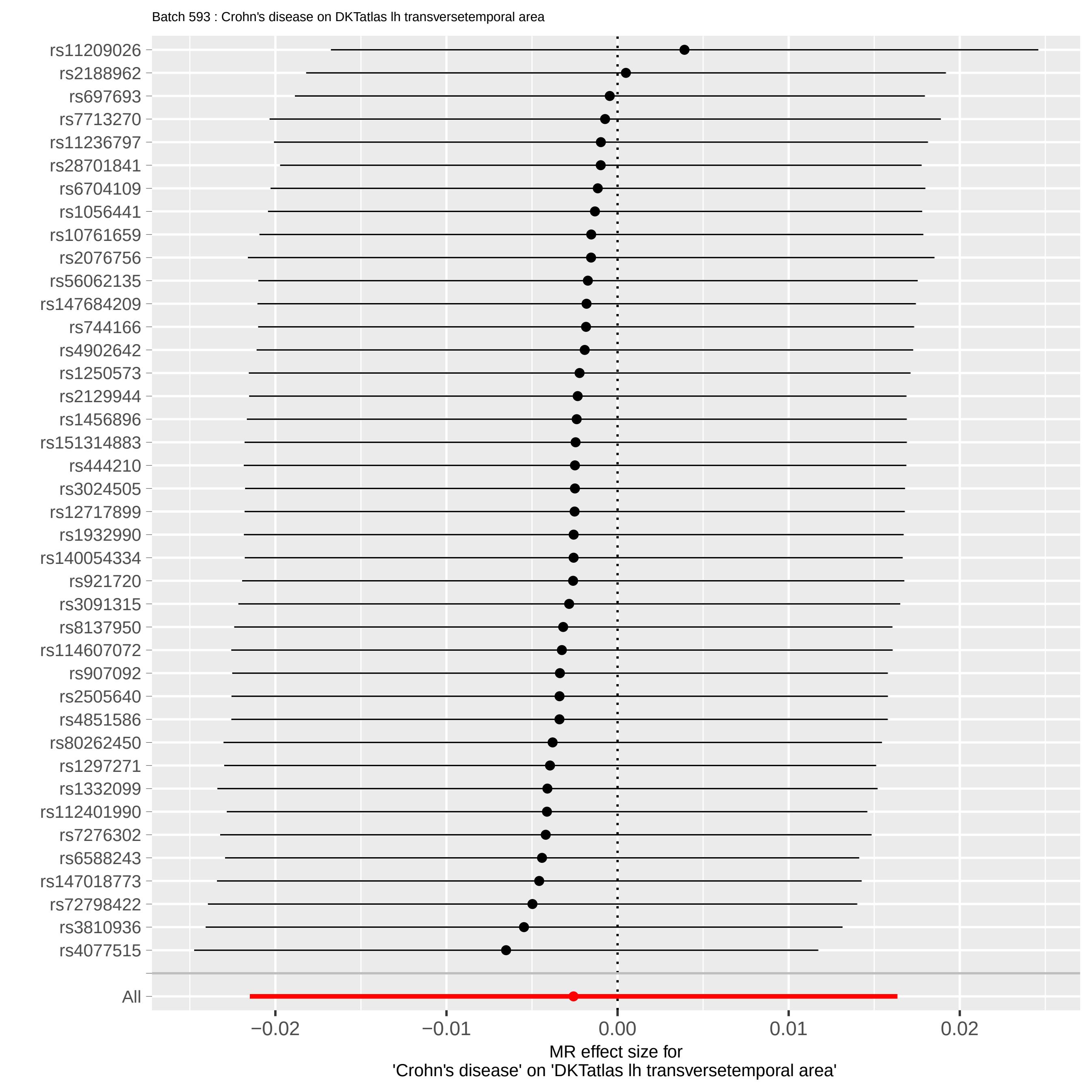

Batch 593 : Crohn's disease on DKTatlas lh transversetemporal area
rs11209026
rs2188962
rs697693
rs7713270
rs11236797
rs28701841
rs6704109
rs1056441
rs10761659
rs2076756
rs56062135
rs147684209
rs744166
rs4902642
rs1250573
rs2129944
rs1456896
rs151314883
rs444210
rs3024505
rs12717899
rs1932990
rs140054334
rs921720
rs3091315
rs8137950
rs114607072
rs907092
rs2505640
rs4851586
rs80262450
rs1297271
rs1332099
rs112401990
rs7276302
rs6588243
rs147018773
rs72798422
rs3810936
rs4077515
All
−0.02
−0.01
0.00
0.01
0.02
MR effect size for
'Crohn's disease' on 'DKTatlas lh transversetemporal area'

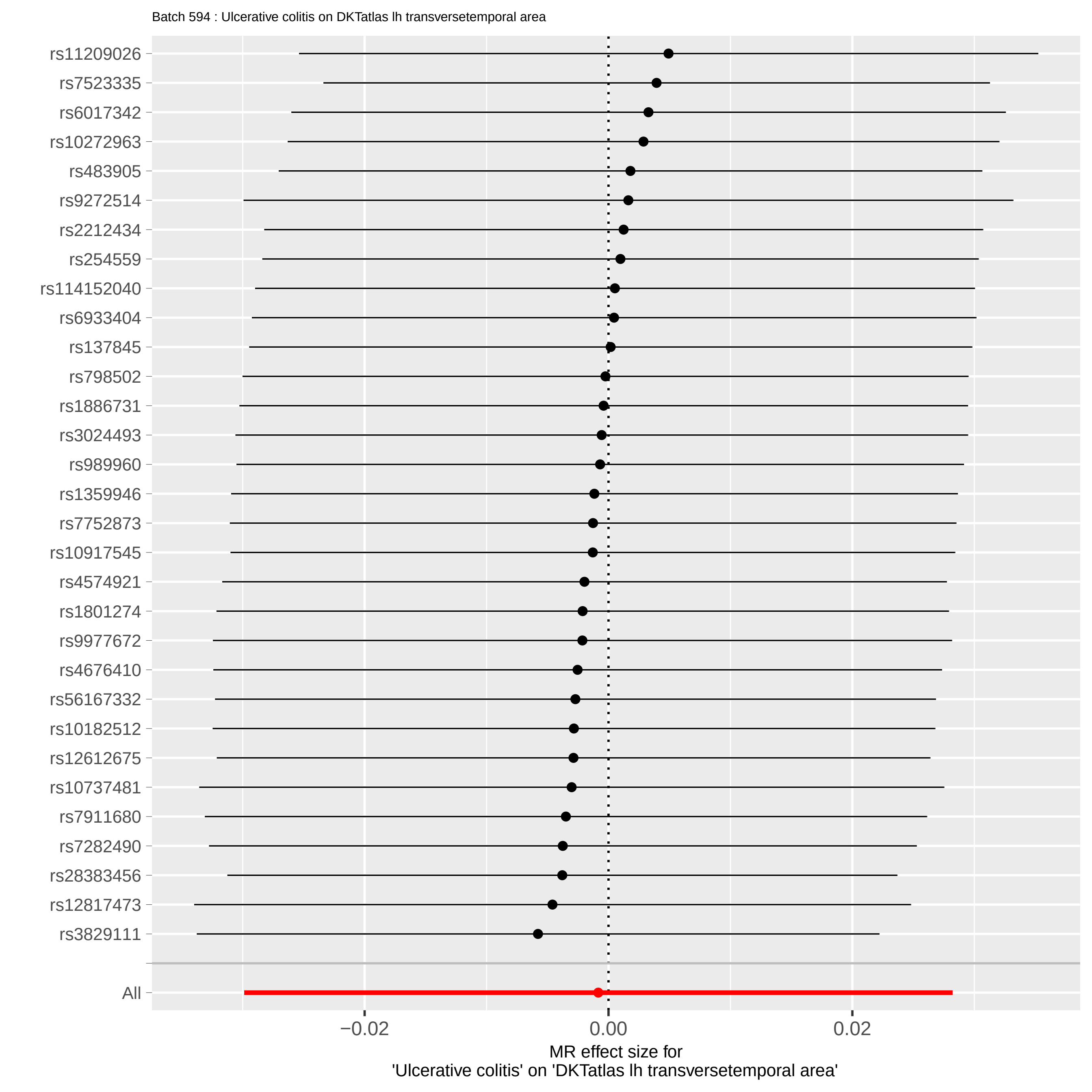

Batch 594 : Ulcerative colitis on DKTatlas lh transversetemporal area
rs11209026
rs7523335
rs6017342
rs10272963
rs483905
rs9272514
rs2212434
rs254559
rs114152040
rs6933404
rs137845
rs798502
rs1886731
rs3024493
rs989960
rs1359946
rs7752873
rs10917545
rs4574921
rs1801274
rs9977672
rs4676410
rs56167332
rs10182512
rs12612675
rs10737481
rs7911680
rs7282490
rs28383456
rs12817473
rs3829111
All
−0.02
0.00
0.02
MR effect size for
'Ulcerative colitis' on 'DKTatlas lh transversetemporal area'

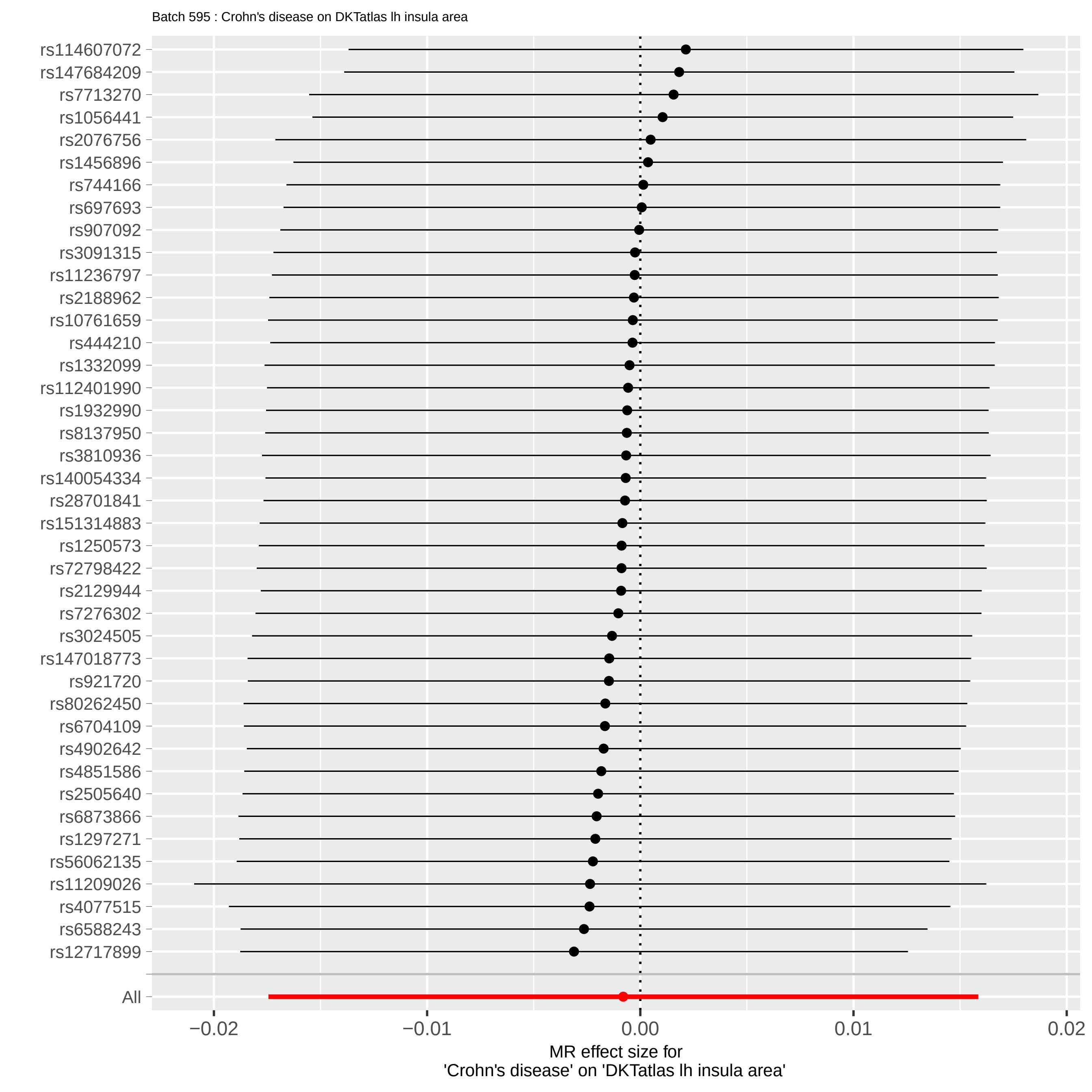

Batch 595 : Crohn's disease on DKTatlas lh insula area
rs114607072
rs147684209
rs7713270
rs1056441
rs2076756
rs1456896
rs744166
rs697693
rs907092
rs3091315
rs11236797
rs2188962
rs10761659
rs444210
rs1332099
rs112401990
rs1932990
rs8137950
rs3810936
rs140054334
rs28701841
rs151314883
rs1250573
rs72798422
rs2129944
rs7276302
rs3024505
rs147018773
rs921720
rs80262450
rs6704109
rs4902642
rs4851586
rs2505640
rs6873866
rs1297271
rs56062135
rs11209026
rs4077515
rs6588243
rs12717899
All
−0.02
−0.01
0.00
0.01
0.02
MR effect size for
'Crohn's disease' on 'DKTatlas lh insula area'

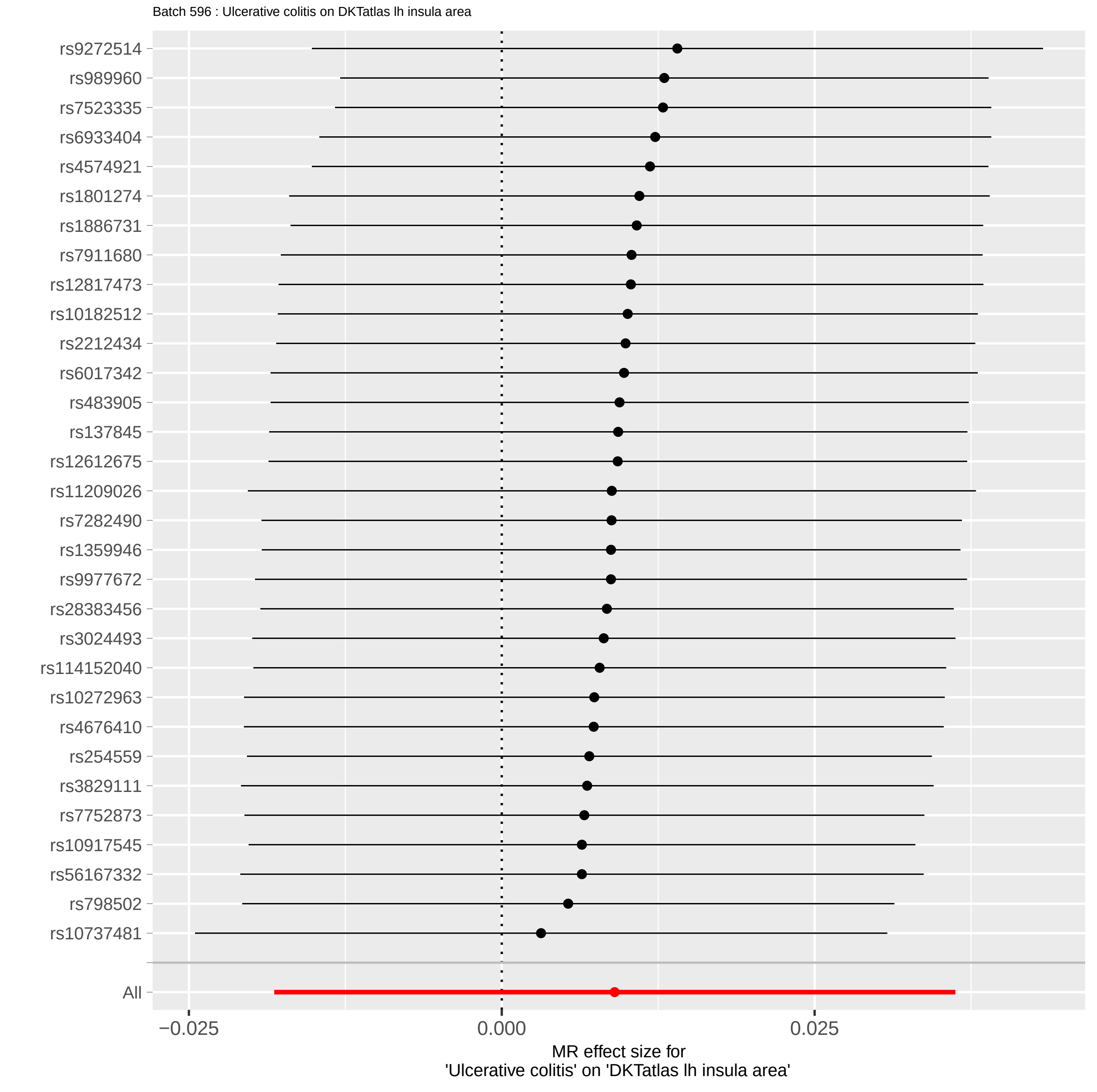

Batch 596 : Ulcerative colitis on DKTatlas lh insula area
rs9272514
rs989960
rs7523335
rs6933404
rs4574921
rs1801274
rs1886731
rs7911680
rs12817473
rs10182512
rs2212434
rs6017342
rs483905
rs137845
rs12612675
rs11209026
rs7282490
rs1359946
rs9977672
rs28383456
rs3024493
rs114152040
rs10272963
rs4676410
rs254559
rs3829111
rs7752873
rs10917545
rs56167332
rs798502
rs10737481
All
−0.025
0.000
0.025
MR effect size for
'Ulcerative colitis' on 'DKTatlas lh insula area'

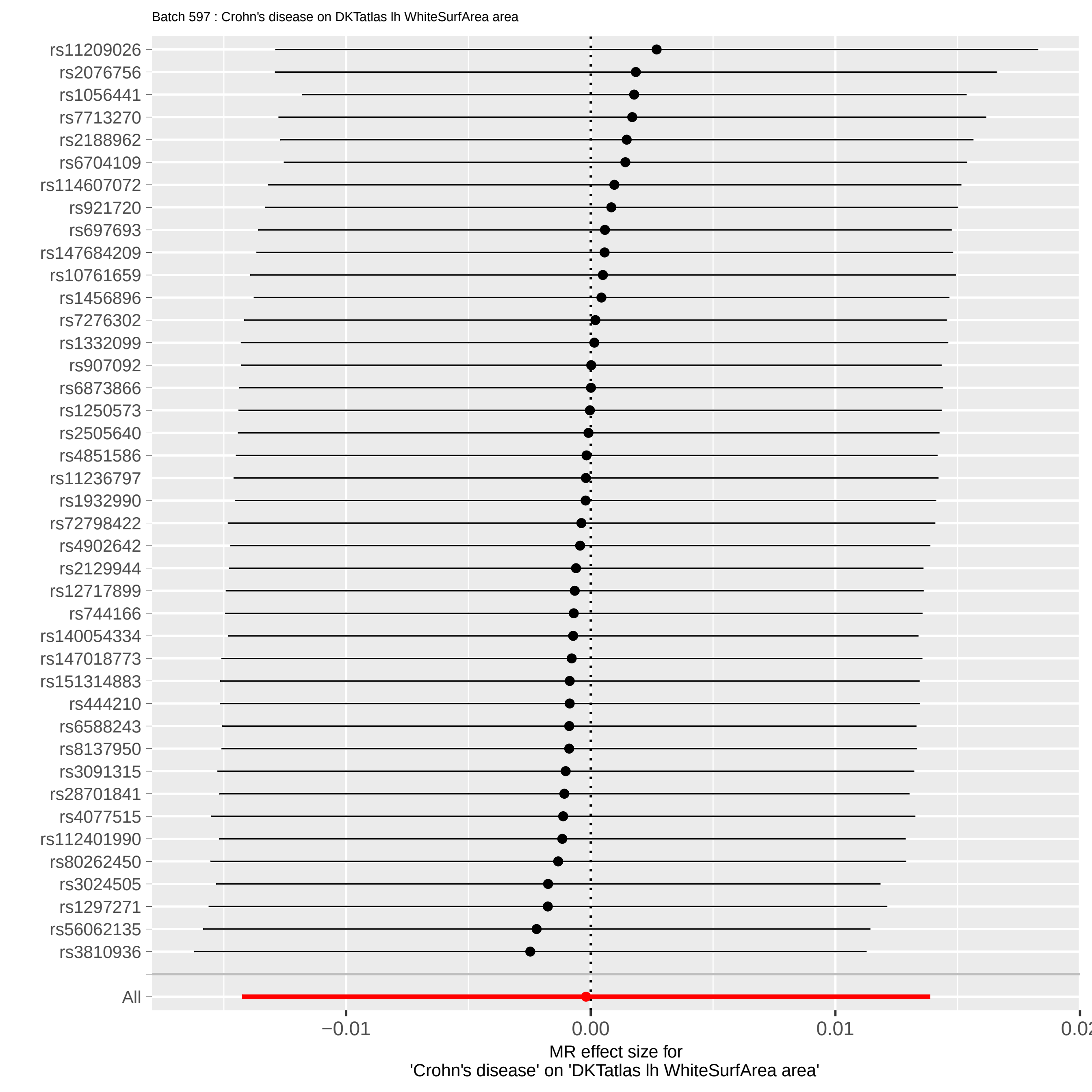
Batch 597 : Crohn's disease on DKTatlas lh WhiteSurfArea area
rs11209026
rs2076756
rs1056441
rs7713270
rs2188962
rs6704109
rs114607072
rs921720
rs697693
rs147684209
rs10761659
rs1456896
rs7276302
rs1332099
rs907092
rs6873866
rs1250573
rs2505640
rs4851586
rs11236797
rs1932990
rs72798422
rs4902642
rs2129944
rs12717899
rs744166
rs140054334
rs147018773
rs151314883
rs444210
rs6588243
rs8137950
rs3091315
rs28701841
rs4077515
rs112401990
rs80262450
rs3024505
rs1297271
rs56062135
rs3810936
All
−0.01
0.00
0.01
MR effect size for
'Crohn's disease' on 'DKTatlas lh WhiteSurfArea area'

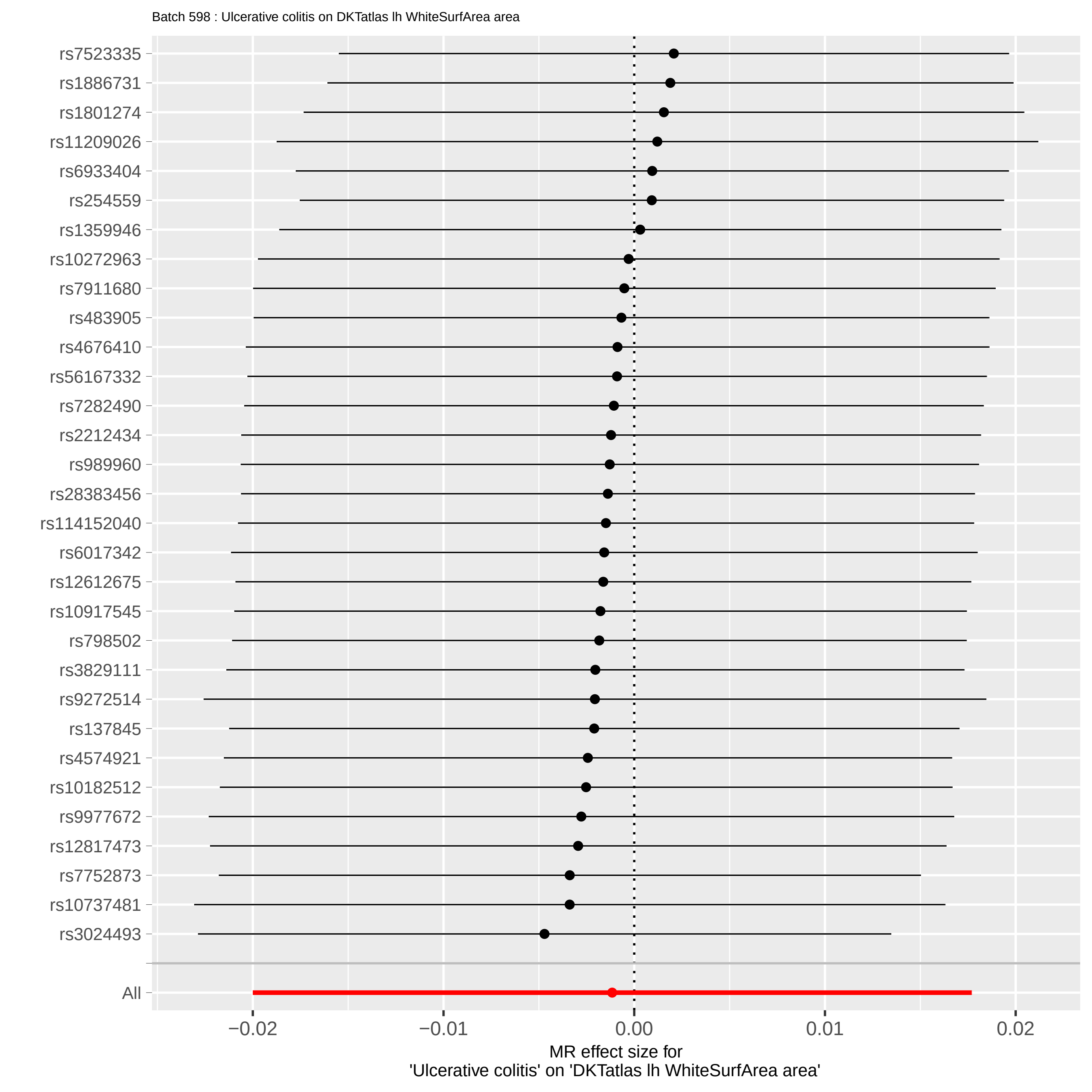
Batch 598 : Ulcerative colitis on DKTatlas lh WhiteSurfArea area
rs7523335
rs1886731
rs1801274
rs11209026
rs6933404
rs254559
rs1359946
rs10272963
rs7911680
rs483905
rs4676410
rs56167332
rs7282490
rs2212434
rs989960
rs28383456
rs114152040
rs6017342
rs12612675
rs10917545
rs798502
rs3829111
rs9272514
rs137845
rs4574921
rs10182512
rs9977672
rs12817473
rs7752873
rs10737481
rs3024493
All
−0.02
−0.01
0.00
0.01
0.02
MR effect size for
'Ulcerative colitis' on 'DKTatlas lh WhiteSurfArea area'

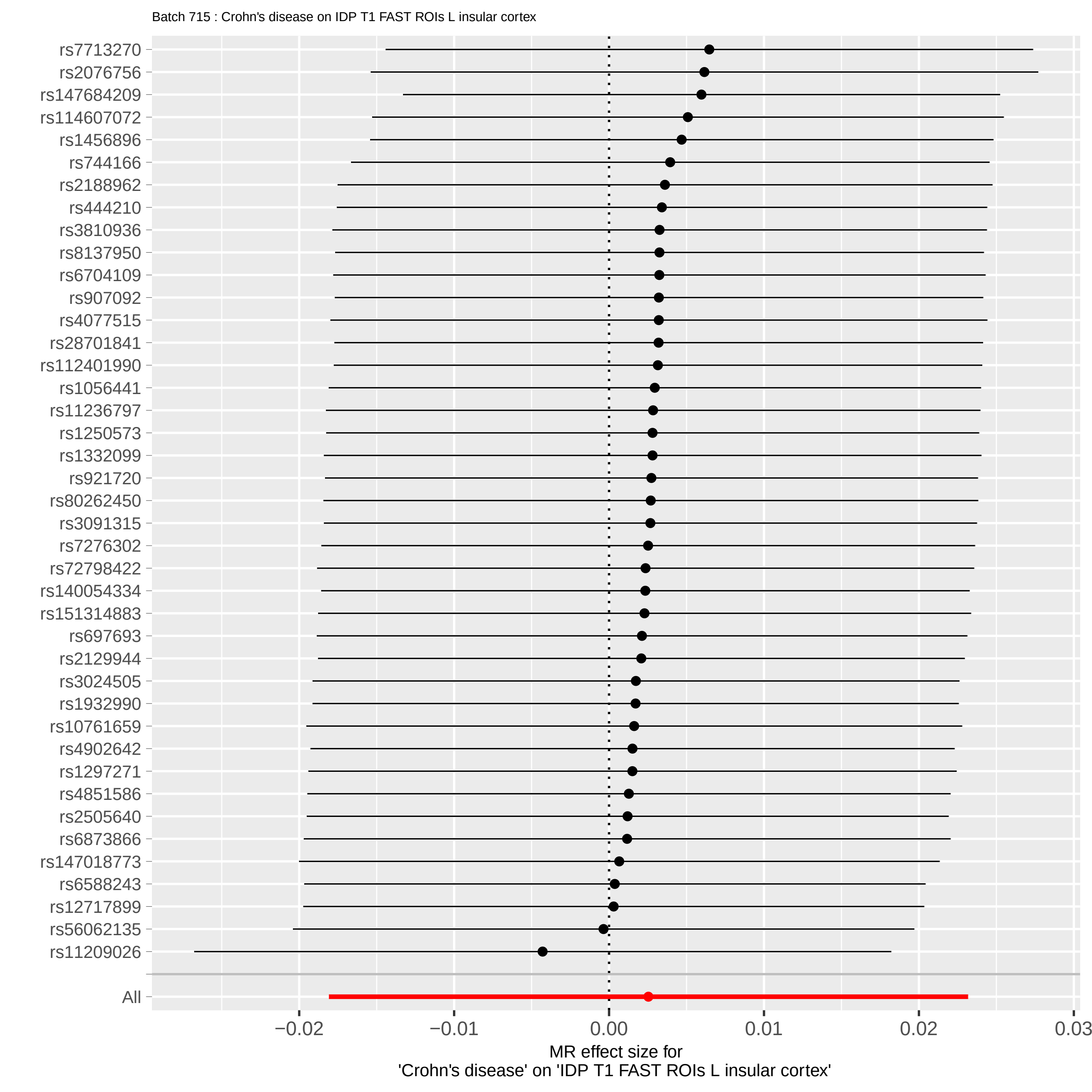

Batch 715 : Crohn's disease on IDP T1 FAST ROIs L insular cortex
rs7713270
rs2076756
rs147684209
rs114607072
rs1456896
rs744166
rs2188962
rs444210
rs3810936
rs8137950
rs6704109
rs907092
rs4077515
rs28701841
rs112401990
rs1056441
rs11236797
rs1250573
rs1332099
rs921720
rs80262450
rs3091315
rs7276302
rs72798422
rs140054334
rs151314883
rs697693
rs2129944
rs3024505
rs1932990
rs10761659
rs4902642
rs1297271
rs4851586
rs2505640
rs6873866
rs147018773
rs6588243
rs12717899
rs56062135
rs11209026
All
−0.02
−0.01
0.00
0.01
0.02
0.03
MR effect size for
'Crohn's disease' on 'IDP T1 FAST ROIs L insular cortex'

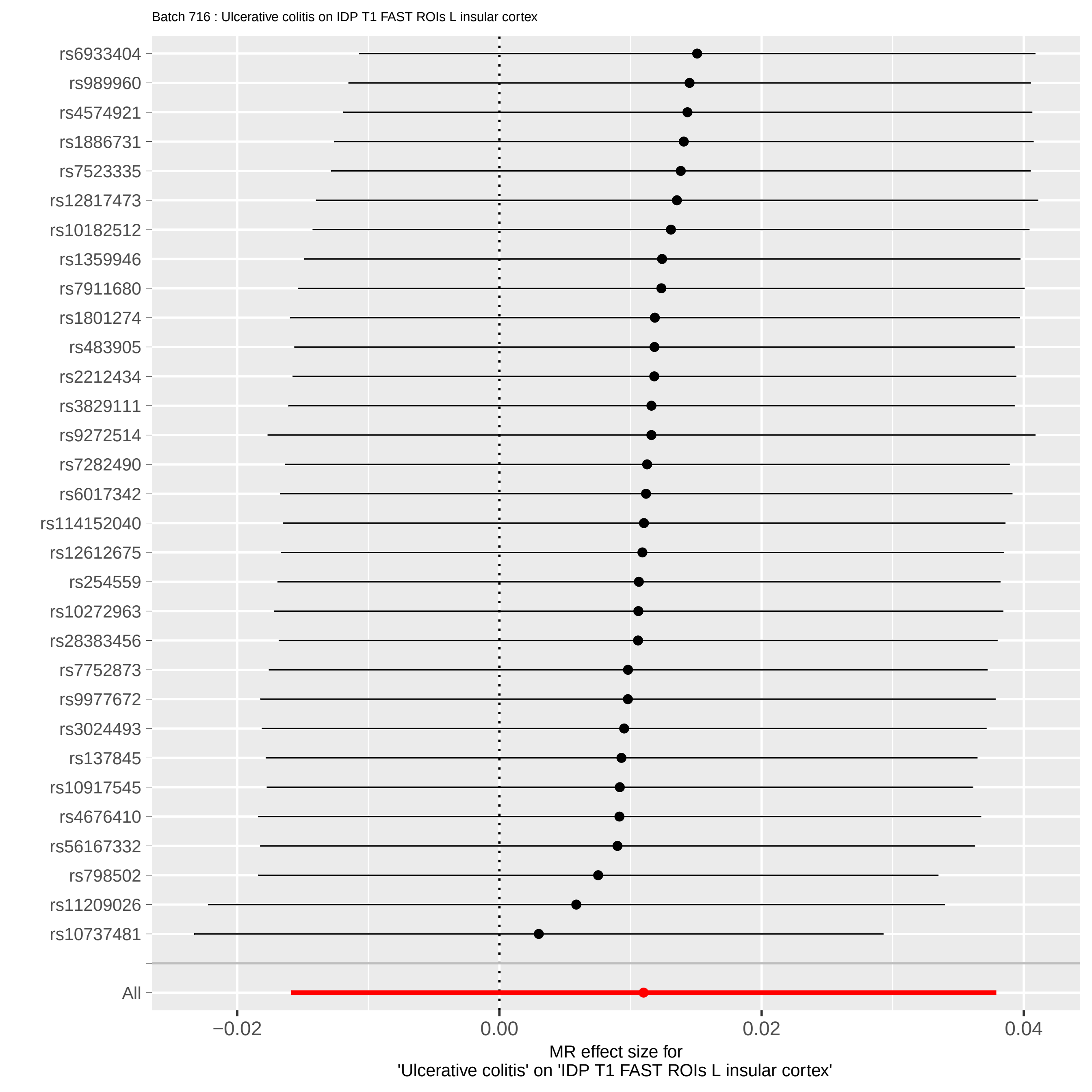
Batch 716 : Ulcerative colitis on IDP T1 FAST ROIs L insular cortex
rs6933404
rs989960
rs4574921
rs1886731
rs7523335
rs12817473
rs10182512
rs1359946
rs7911680
rs1801274
rs483905
rs2212434
rs3829111
rs9272514
rs7282490
rs6017342
rs114152040
rs12612675
rs254559
rs10272963
rs28383456
rs7752873
rs9977672
rs3024493
rs137845
rs10917545
rs4676410
rs56167332
rs798502
rs11209026
rs10737481
All
−0.02
0.00
0.02
0.04
MR effect size for
'Ulcerative colitis' on 'IDP T1 FAST ROIs L insular cortex'

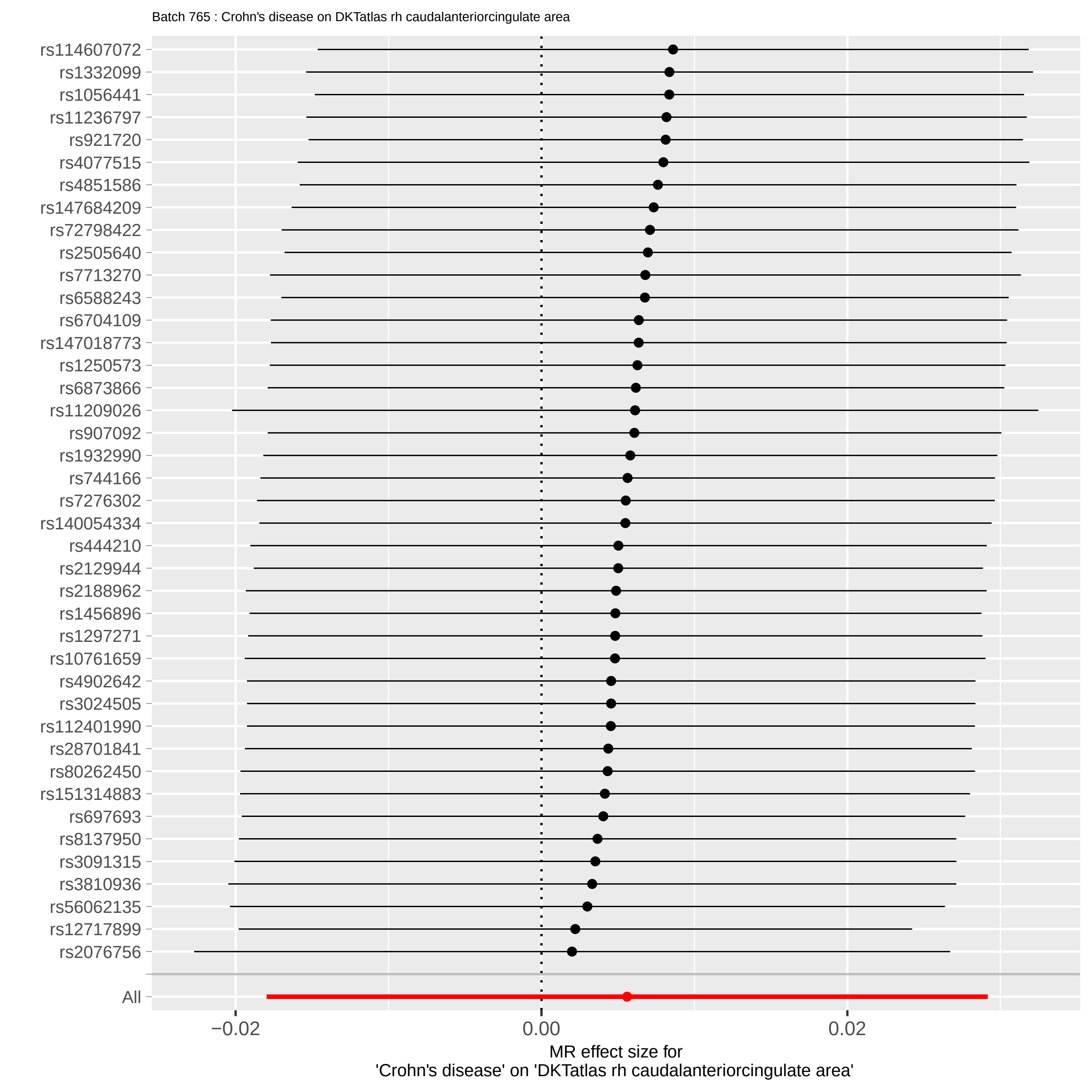

Batch 765 : Crohn's disease on DKTatlas rh caudalanteriorcingulate area
rs114607072
rs1332099
rs1056441
rs11236797
rs921720
rs4077515
rs4851586
rs147684209
rs72798422
rs2505640
rs7713270
rs6588243
rs6704109
rs147018773
rs1250573
rs6873866
rs11209026
rs907092
rs1932990
rs744166
rs7276302
rs140054334
rs444210
rs2129944
rs2188962
rs1456896
rs1297271
rs10761659
rs4902642
rs3024505
rs112401990
rs28701841
rs80262450
rs151314883
rs697693
rs8137950
rs3091315
rs3810936
rs56062135
rs12717899
rs2076756
All
−0.02
0.00
0.02
MR effect size for
'Crohn's disease' on 'DKTatlas rh caudalanteriorcingulate area'

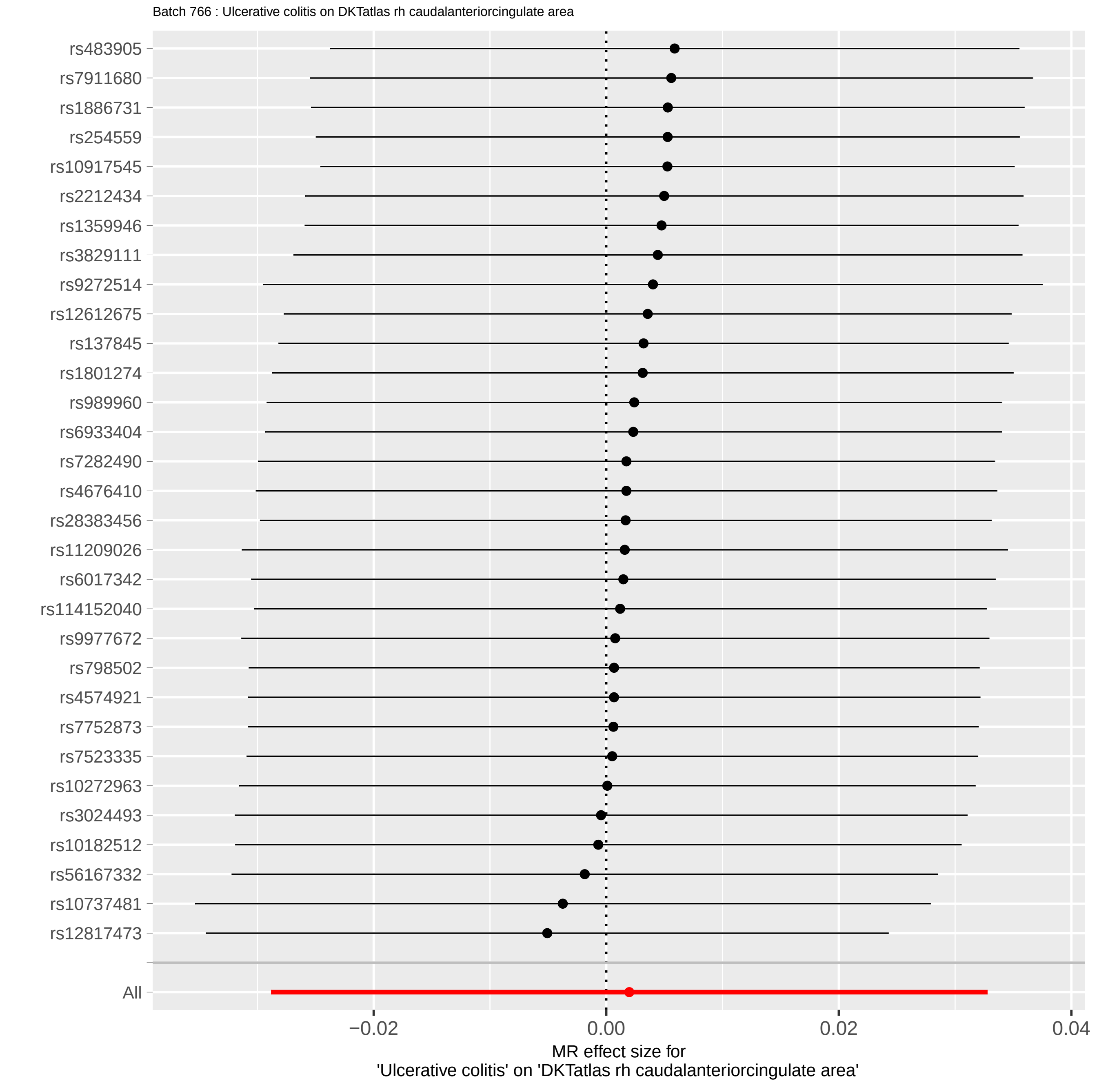

Batch 766 : Ulcerative colitis on DKTatlas rh caudalanteriorcingulate area
rs483905
rs7911680
rs1886731
rs254559
rs10917545
rs2212434
rs1359946
rs3829111
rs9272514
rs12612675
rs137845
rs1801274
rs989960
rs6933404
rs7282490
rs4676410
rs28383456
rs11209026
rs6017342
rs114152040
rs9977672
rs798502
rs4574921
rs7752873
rs7523335
rs10272963
rs3024493
rs10182512
rs56167332
rs10737481
rs12817473
All
−0.02
0.00
0.02
0.04
MR effect size for
'Ulcerative colitis' on 'DKTatlas rh caudalanteriorcingulate area'

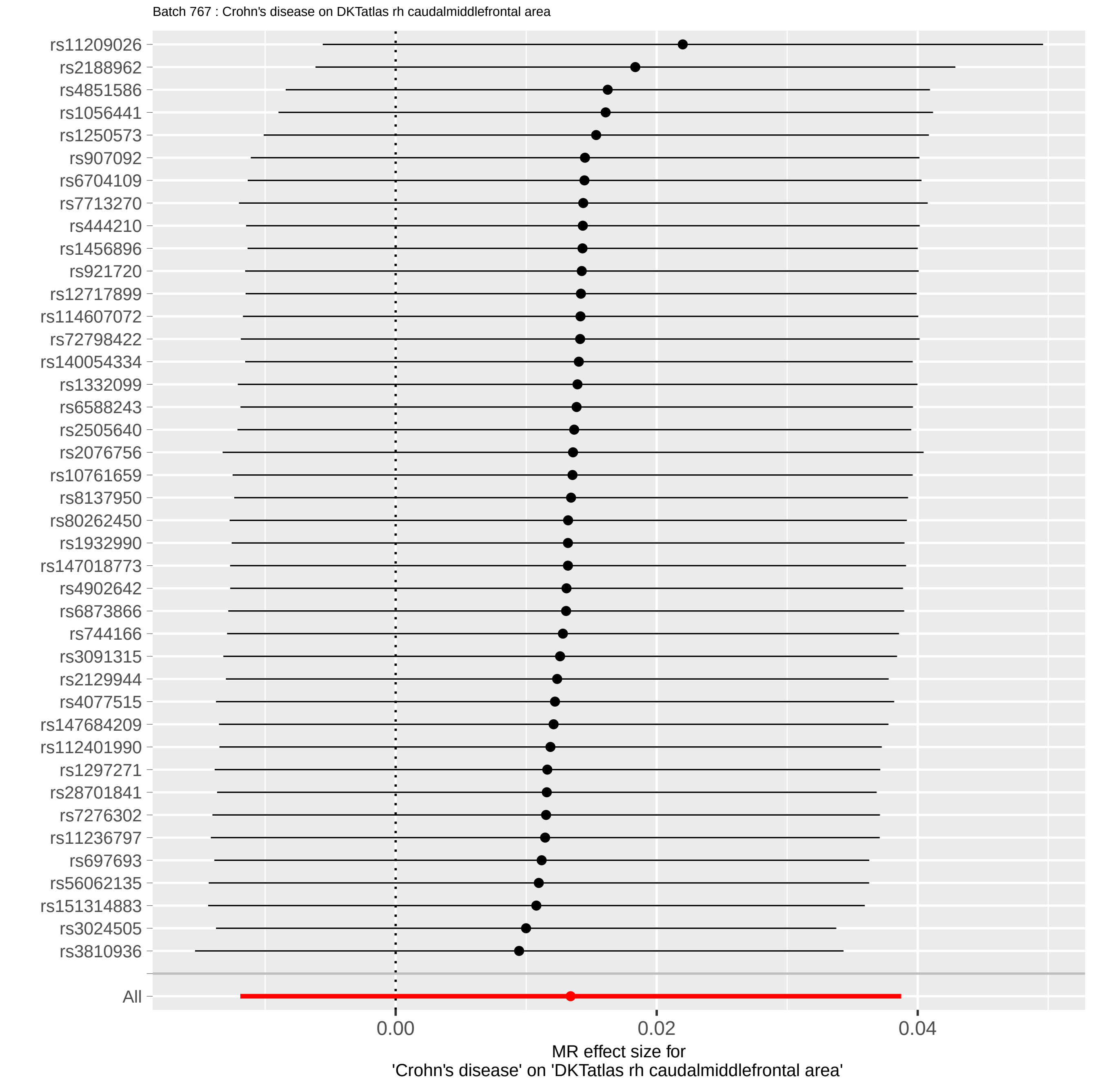

Batch 767 : Crohn's disease on DKTatlas rh caudalmiddlefrontal area
rs11209026
rs2188962
rs4851586
rs1056441
rs1250573
rs907092
rs6704109
rs7713270
rs444210
rs1456896
rs921720
rs12717899
rs114607072
rs72798422
rs140054334
rs1332099
rs6588243
rs2505640
rs2076756
rs10761659
rs8137950
rs80262450
rs1932990
rs147018773
rs4902642
rs6873866
rs744166
rs3091315
rs2129944
rs4077515
rs147684209
rs112401990
rs1297271
rs28701841
rs7276302
rs11236797
rs697693
rs56062135
rs151314883
rs3024505
rs3810936
All
0.00
0.02
0.04
MR effect size for
'Crohn's disease' on 'DKTatlas rh caudalmiddlefrontal area'

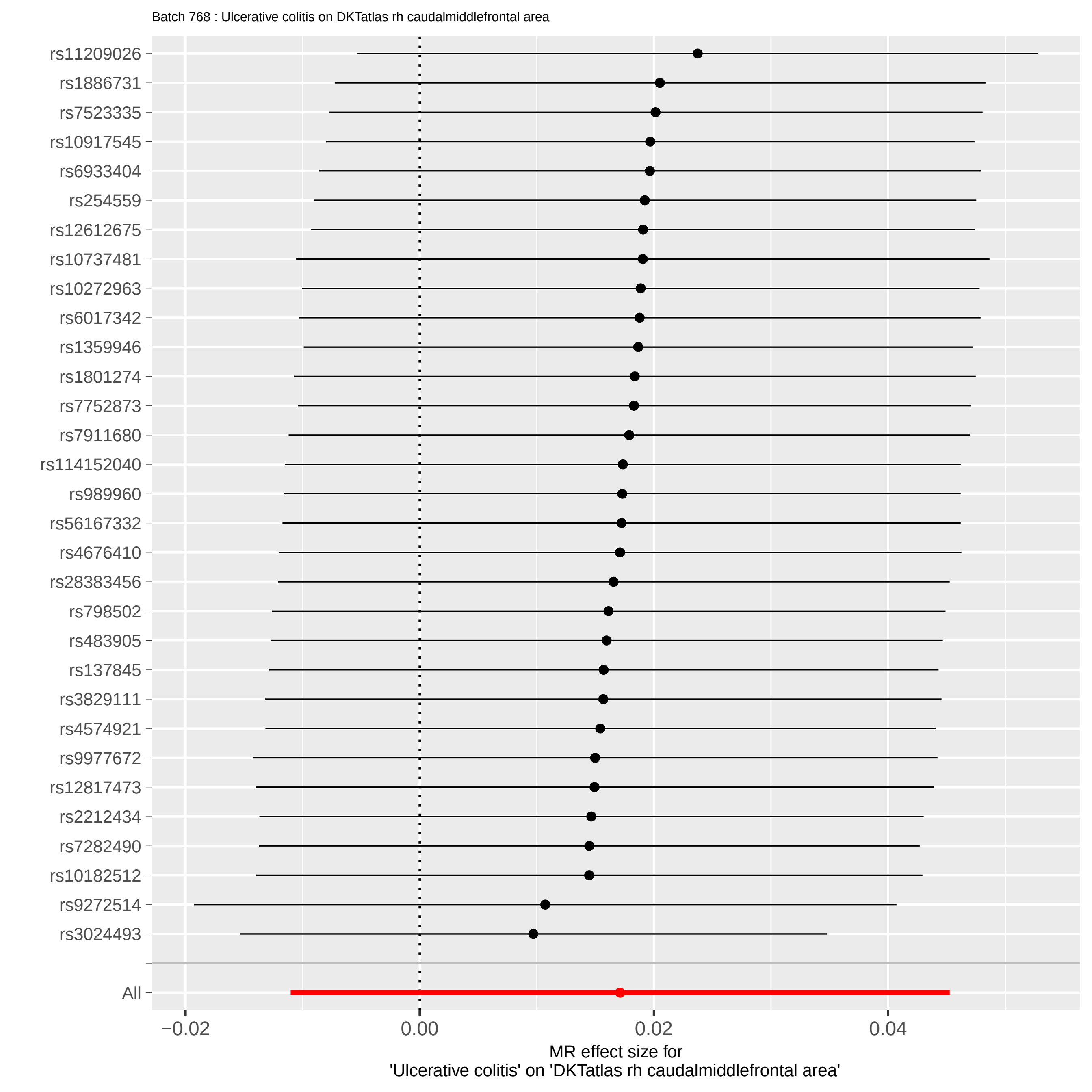

Batch 768 : Ulcerative colitis on DKTatlas rh caudalmiddlefrontal area
rs11209026
rs1886731
rs7523335
rs10917545
rs6933404
rs254559
rs12612675
rs10737481
rs10272963
rs6017342
rs1359946
rs1801274
rs7752873
rs7911680
rs114152040
rs989960
rs56167332
rs4676410
rs28383456
rs798502
rs483905
rs137845
rs3829111
rs4574921
rs9977672
rs12817473
rs2212434
rs7282490
rs10182512
rs9272514
rs3024493
All
−0.02
0.00
0.02
0.04
MR effect size for
'Ulcerative colitis' on 'DKTatlas rh caudalmiddlefrontal area'

Batch 769 : Crohn's disease on DKTatlas rh cuneus area

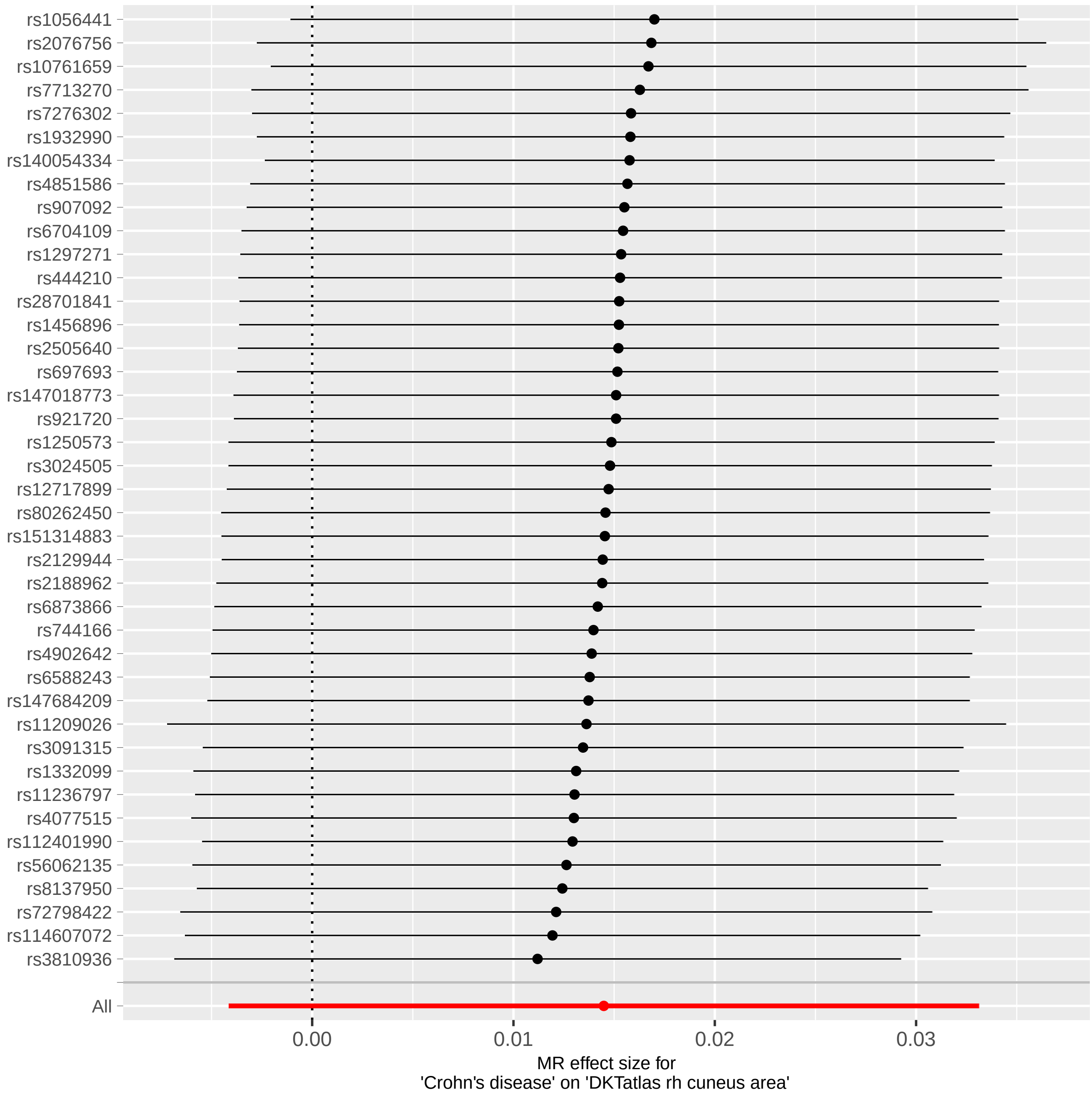

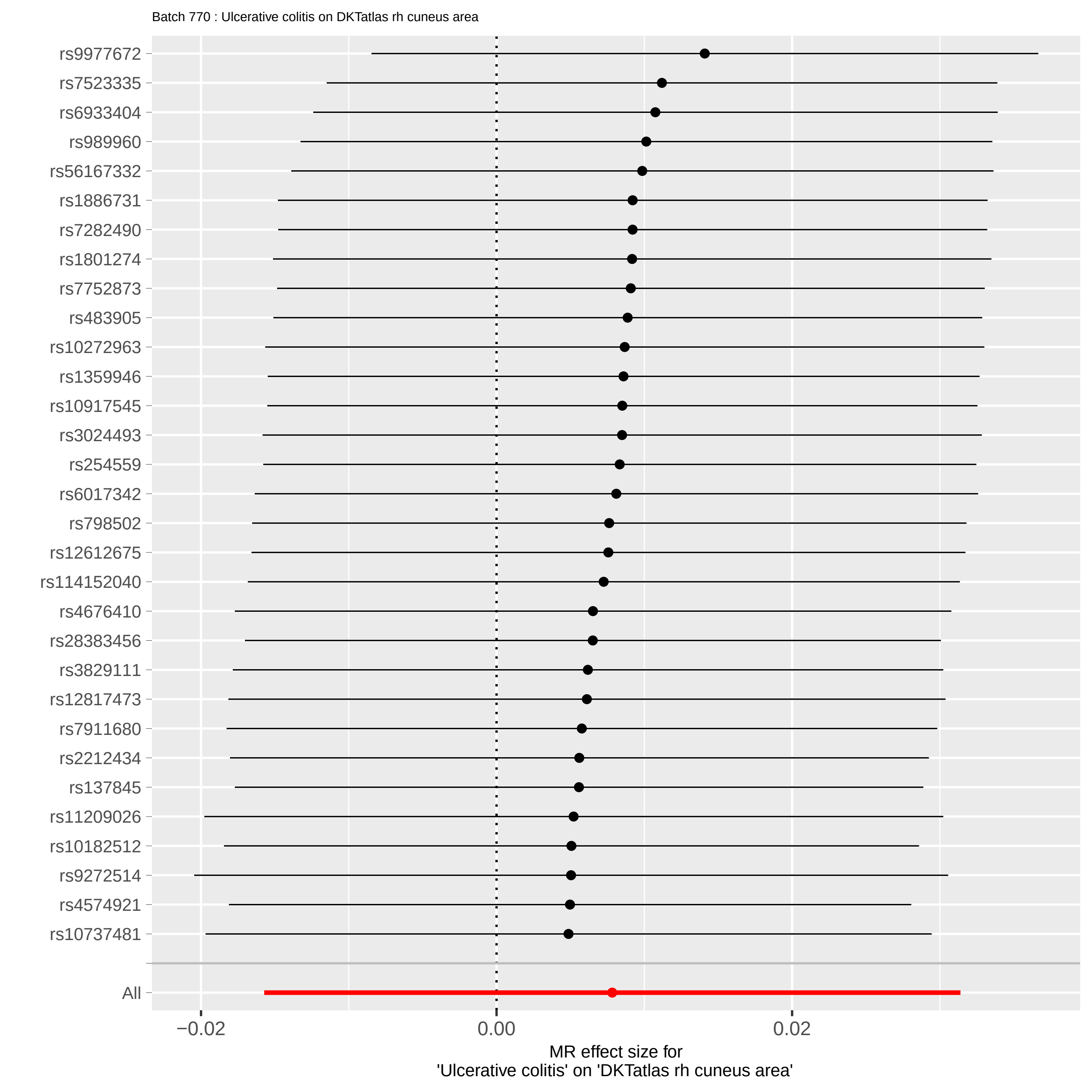
Batch 770 : Ulcerative colitis on DKTatlas rh cuneus area
rs9977672
rs7523335
rs6933404
rs989960
rs56167332
rs1886731
rs7282490
rs1801274
rs7752873
rs483905
rs10272963
rs1359946
rs10917545
rs3024493
rs254559
rs6017342
rs798502
rs12612675
rs114152040
rs4676410
rs28383456
rs3829111
rs12817473
rs7911680
rs2212434
rs137845
rs11209026
rs10182512
rs9272514
rs4574921
rs10737481
All
−0.02
0.00
0.02
MR effect size for
'Ulcerative colitis' on 'DKTatlas rh cuneus area'

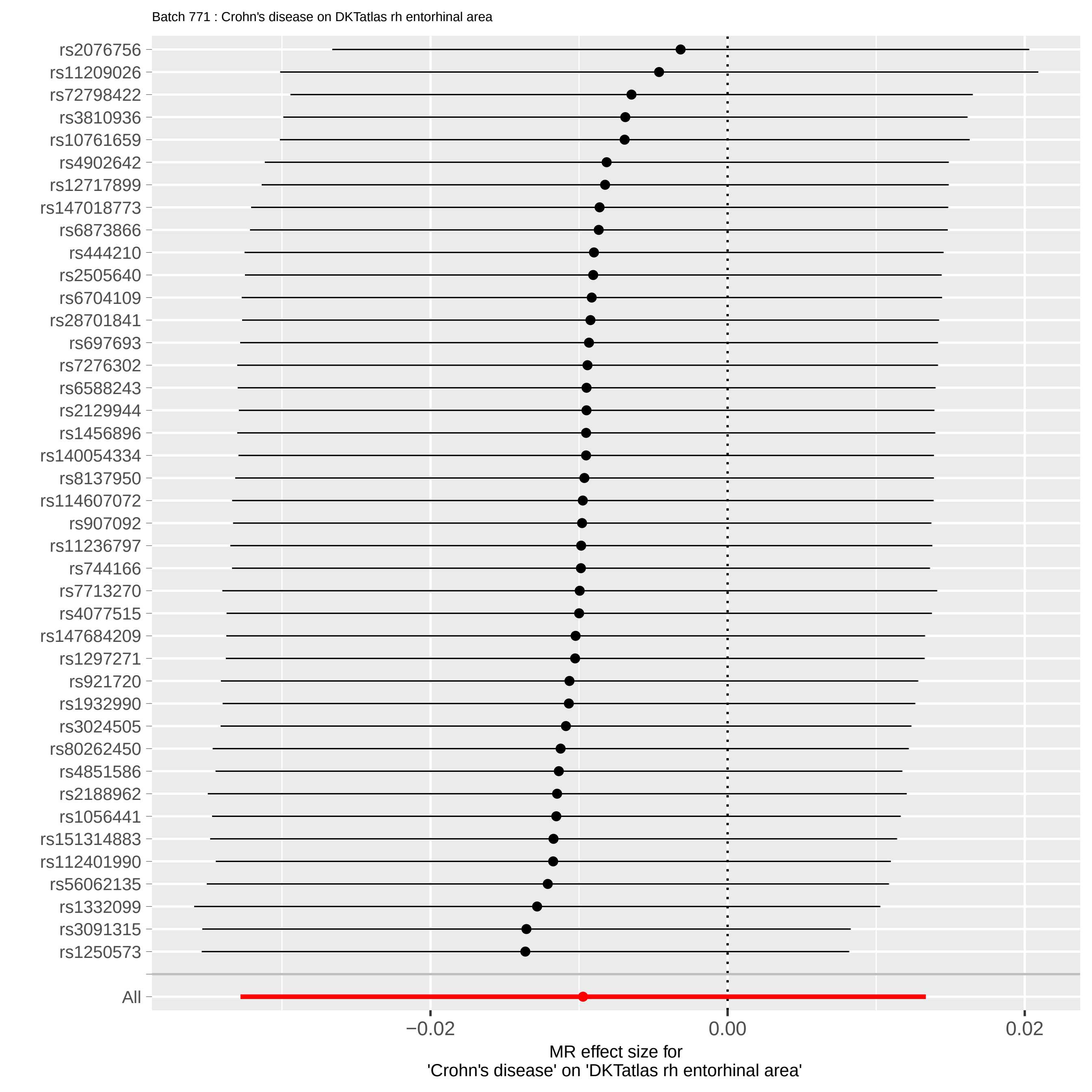
Batch 771 : Crohn's disease on DKTatlas rh entorhinal area
rs2076756
rs11209026
rs72798422
rs3810936
rs10761659
rs4902642
rs12717899
rs147018773
rs6873866
rs444210
rs2505640
rs6704109
rs28701841
rs697693
rs7276302
rs6588243
rs2129944
rs1456896
rs140054334
rs8137950
rs114607072
rs907092
rs11236797
rs744166
rs7713270
rs4077515
rs147684209
rs1297271
rs921720
rs1932990
rs3024505
rs80262450
rs4851586
rs2188962
rs1056441
rs151314883
rs112401990
rs56062135
rs1332099
rs3091315
rs1250573
All
−0.02
0.00
0.02
MR effect size for
'Crohn's disease' on 'DKTatlas rh entorhinal area'

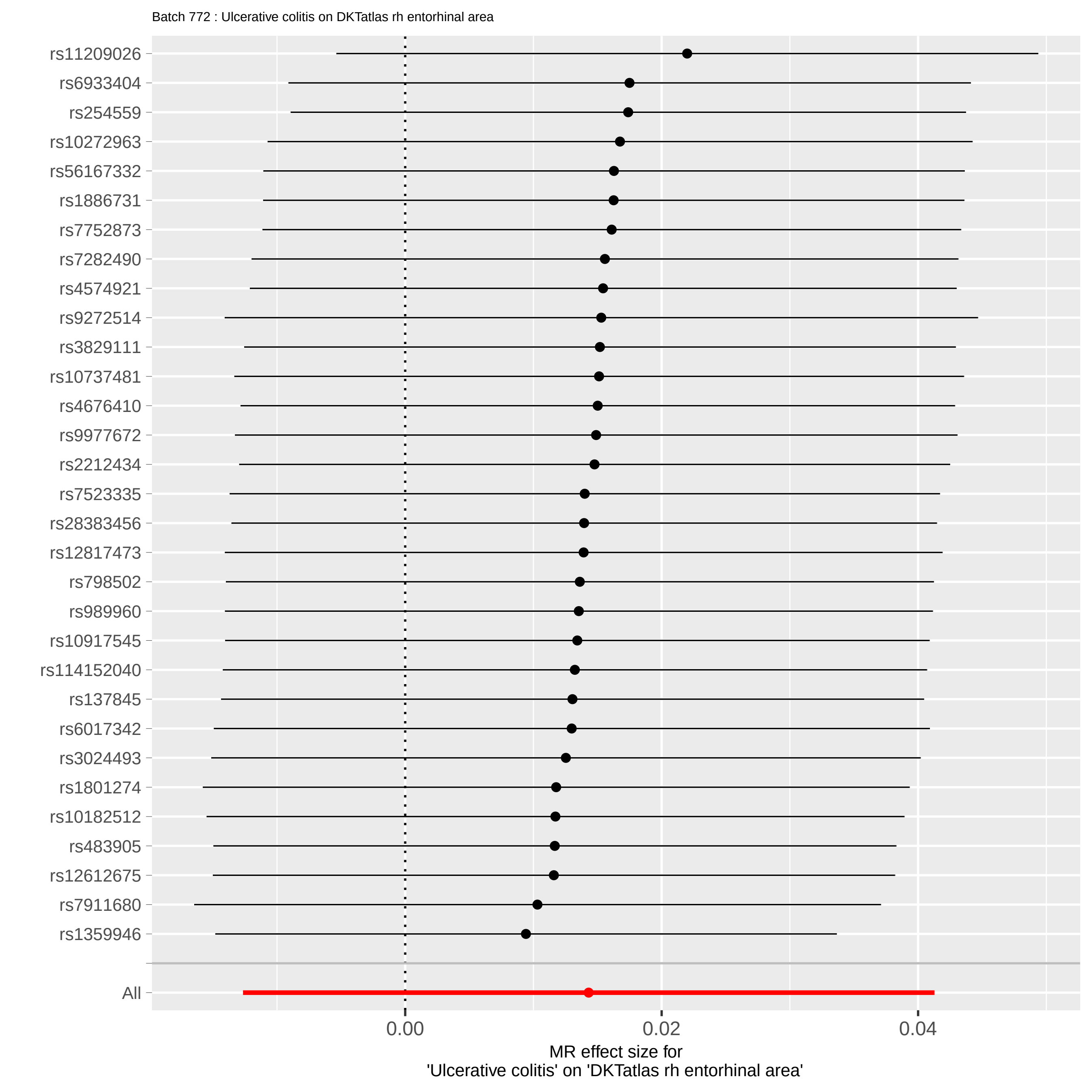

Batch 772 : Ulcerative colitis on DKTatlas rh entorhinal area
rs11209026
rs6933404
rs254559
rs10272963
rs56167332
rs1886731
rs7752873
rs7282490
rs4574921
rs9272514
rs3829111
rs10737481
rs4676410
rs9977672
rs2212434
rs7523335
rs28383456
rs12817473
rs798502
rs989960
rs10917545
rs114152040
rs137845
rs6017342
rs3024493
rs1801274
rs10182512
rs483905
rs12612675
rs7911680
rs1359946
All
0.00
0.02
0.04
MR effect size for
'Ulcerative colitis' on 'DKTatlas rh entorhinal area'

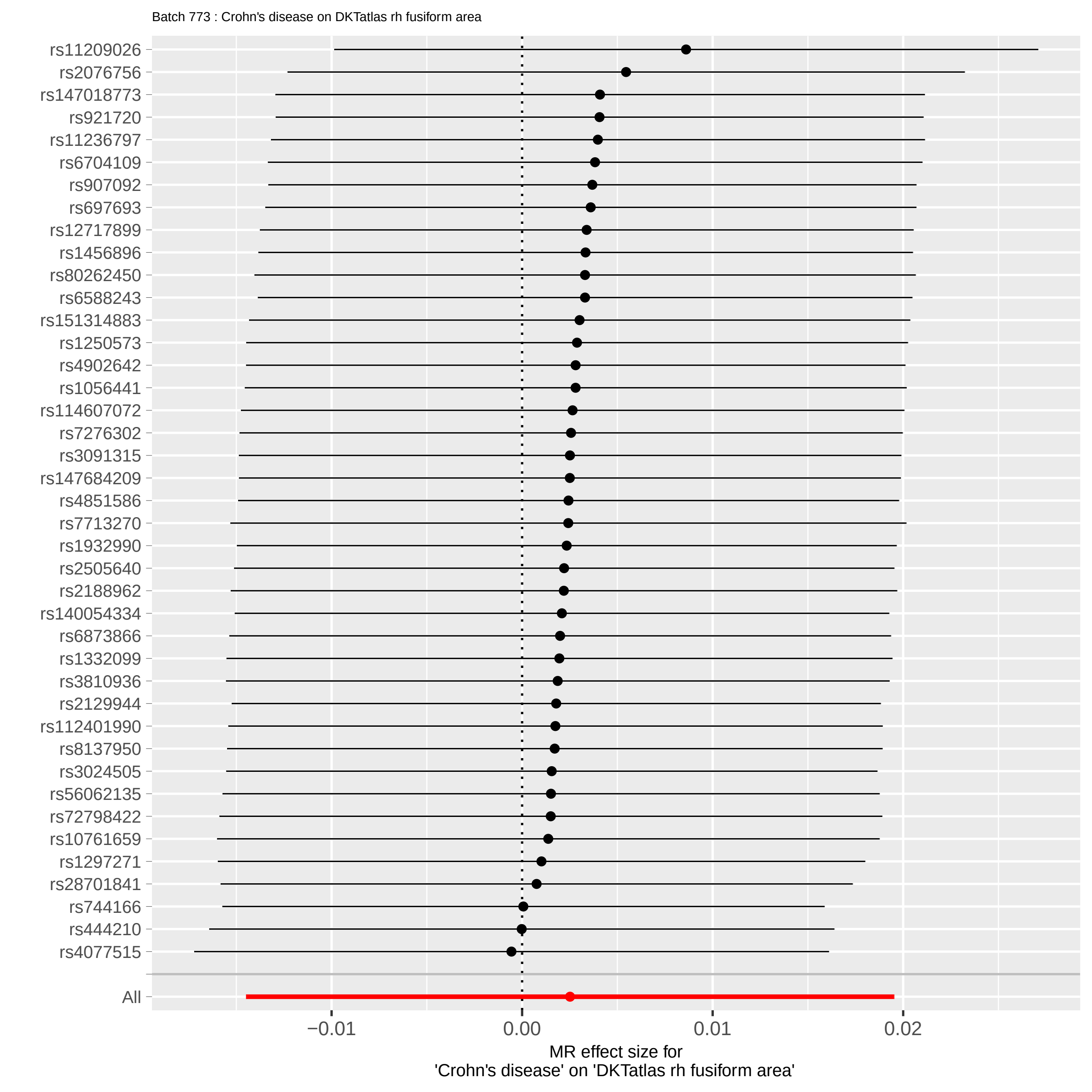

Batch 773 : Crohn's disease on DKTatlas rh fusiform area
rs11209026
rs2076756
rs147018773
rs921720
rs11236797
rs6704109
rs907092
rs697693
rs12717899
rs1456896
rs80262450
rs6588243
rs151314883
rs1250573
rs4902642
rs1056441
rs114607072
rs7276302
rs3091315
rs147684209
rs4851586
rs7713270
rs1932990
rs2505640
rs2188962
rs140054334
rs6873866
rs1332099
rs3810936
rs2129944
rs112401990
rs8137950
rs3024505
rs56062135
rs72798422
rs10761659
rs1297271
rs28701841
rs744166
rs444210
rs4077515
All
−0.01
0.00
0.01
0.02
MR effect size for
'Crohn's disease' on 'DKTatlas rh fusiform area'

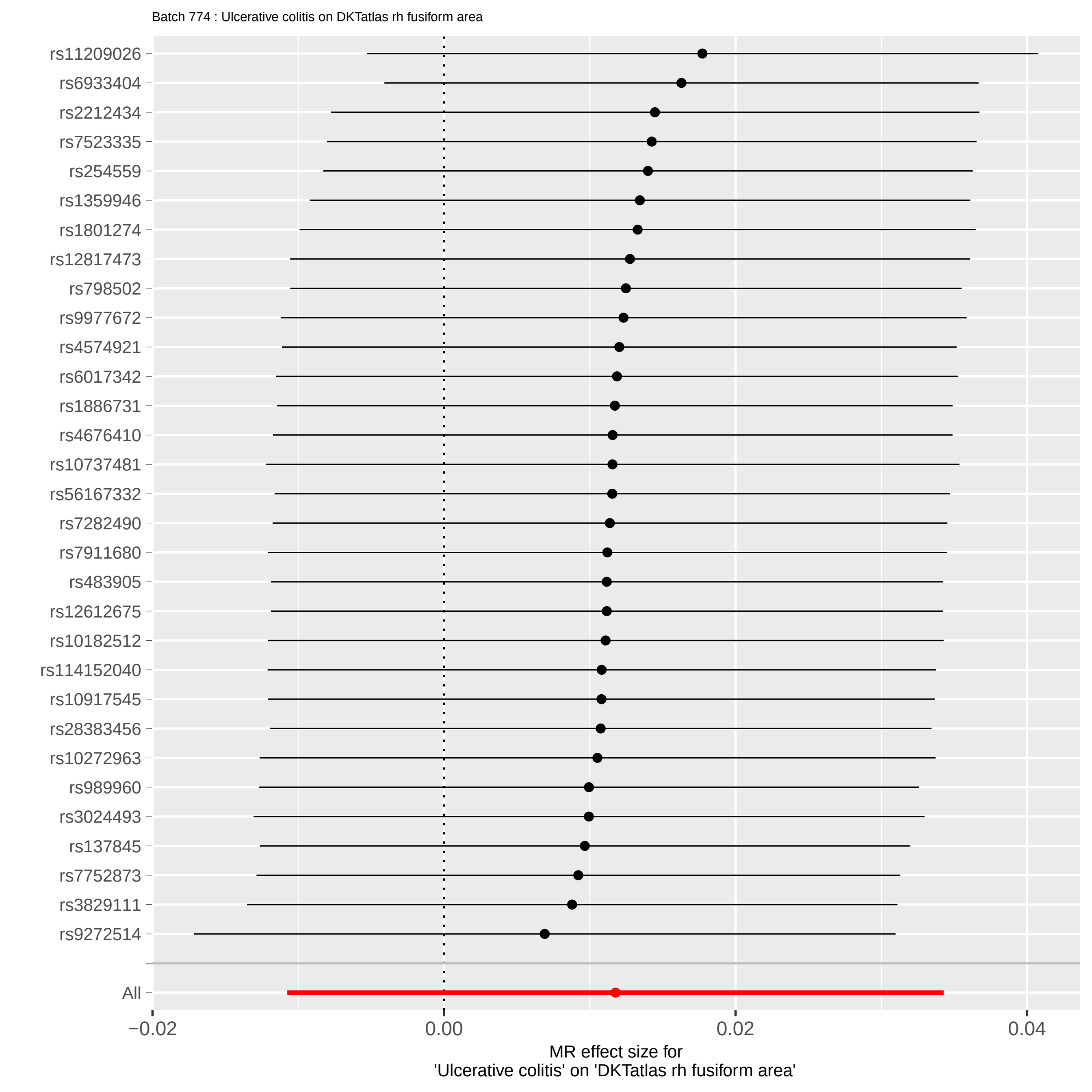

Batch 774 : Ulcerative colitis on DKTatlas rh fusiform area
rs11209026
rs6933404
rs2212434
rs7523335
rs254559
rs1359946
rs1801274
rs12817473
rs798502
rs9977672
rs4574921
rs6017342
rs1886731
rs4676410
rs10737481
rs56167332
rs7282490
rs7911680
rs483905
rs12612675
rs10182512
rs114152040
rs10917545
rs28383456
rs10272963
rs989960
rs3024493
rs137845
rs7752873
rs3829111
rs9272514
All
−0.02
0.00
0.02
0.04
MR effect size for
'Ulcerative colitis' on 'DKTatlas rh fusiform area'

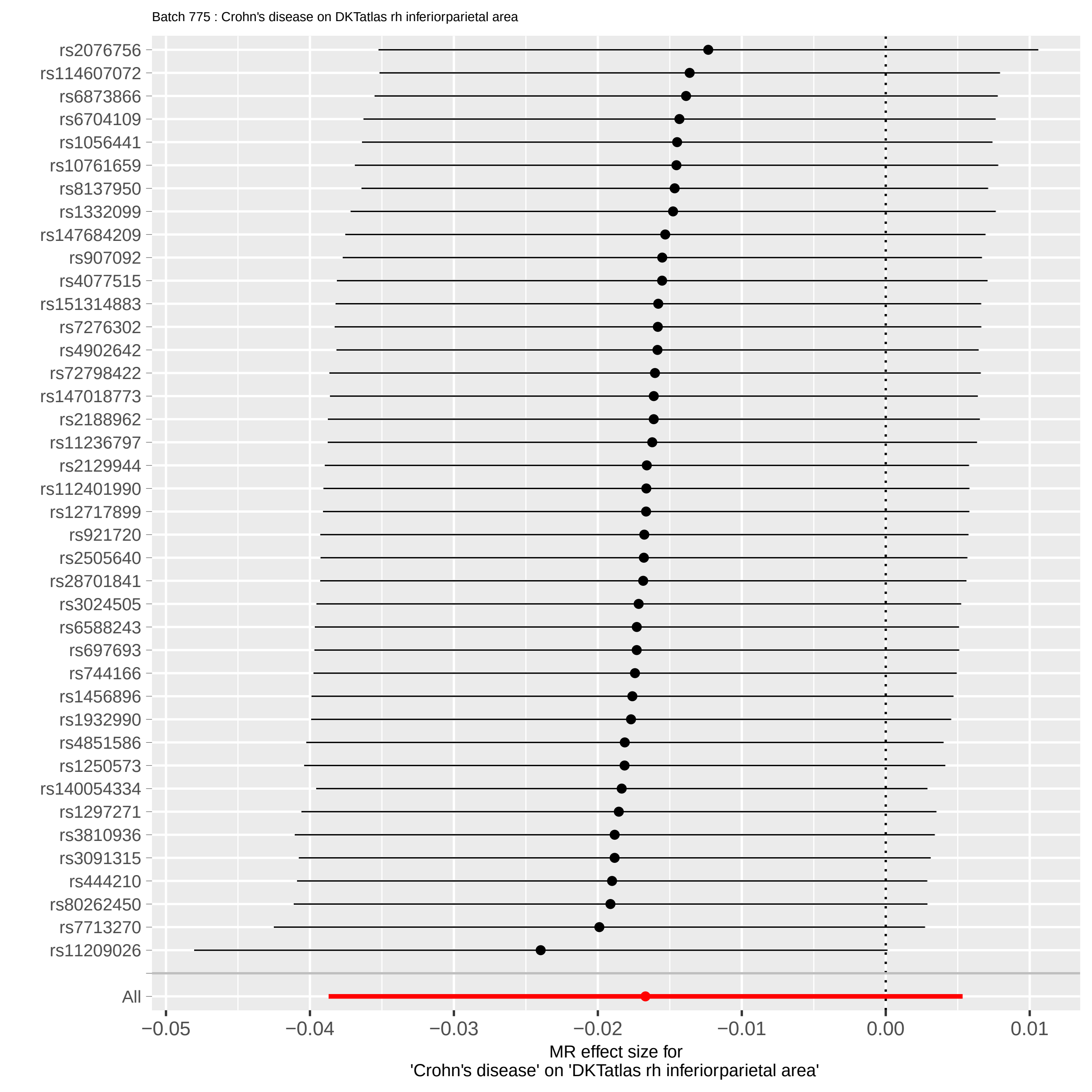

Batch 775 : Crohn's disease on DKTatlas rh inferiorparietal area
rs2076756
rs114607072
rs6873866
rs6704109
rs1056441
rs10761659
rs8137950
rs1332099
rs147684209
rs907092
rs4077515
rs151314883
rs7276302
rs4902642
rs72798422
rs147018773
rs2188962
rs11236797
rs2129944
rs112401990
rs12717899
rs921720
rs2505640
rs28701841
rs3024505
rs6588243
rs697693
rs744166
rs1456896
rs1932990
rs4851586
rs1250573
rs140054334
rs1297271
rs3810936
rs3091315
rs444210
rs80262450
rs7713270
rs11209026
All
−0.05
−0.04
−0.03
−0.02
−0.01
0.00
0.01
MR effect size for
'Crohn's disease' on 'DKTatlas rh inferiorparietal area'

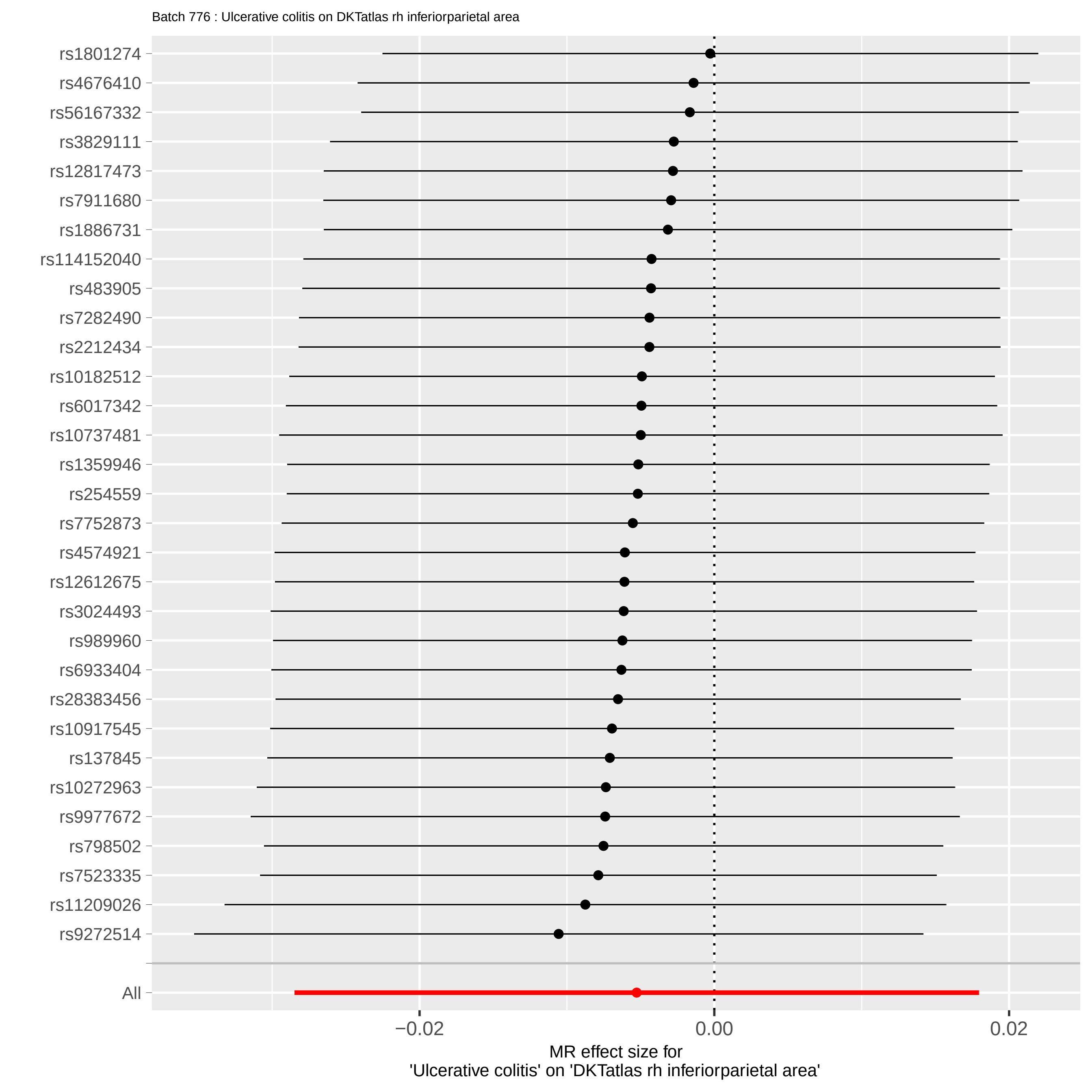

Batch 776 : Ulcerative colitis on DKTatlas rh inferiorparietal area
rs1801274
rs4676410
rs56167332
rs3829111
rs12817473
rs7911680
rs1886731
rs114152040
rs483905
rs7282490
rs2212434
rs10182512
rs6017342
rs10737481
rs1359946
rs254559
rs7752873
rs4574921
rs12612675
rs3024493
rs989960
rs6933404
rs28383456
rs10917545
rs137845
rs10272963
rs9977672
rs798502
rs7523335
rs11209026
rs9272514
All
−0.02
0.00
0.02
MR effect size for
'Ulcerative colitis' on 'DKTatlas rh inferiorparietal area'

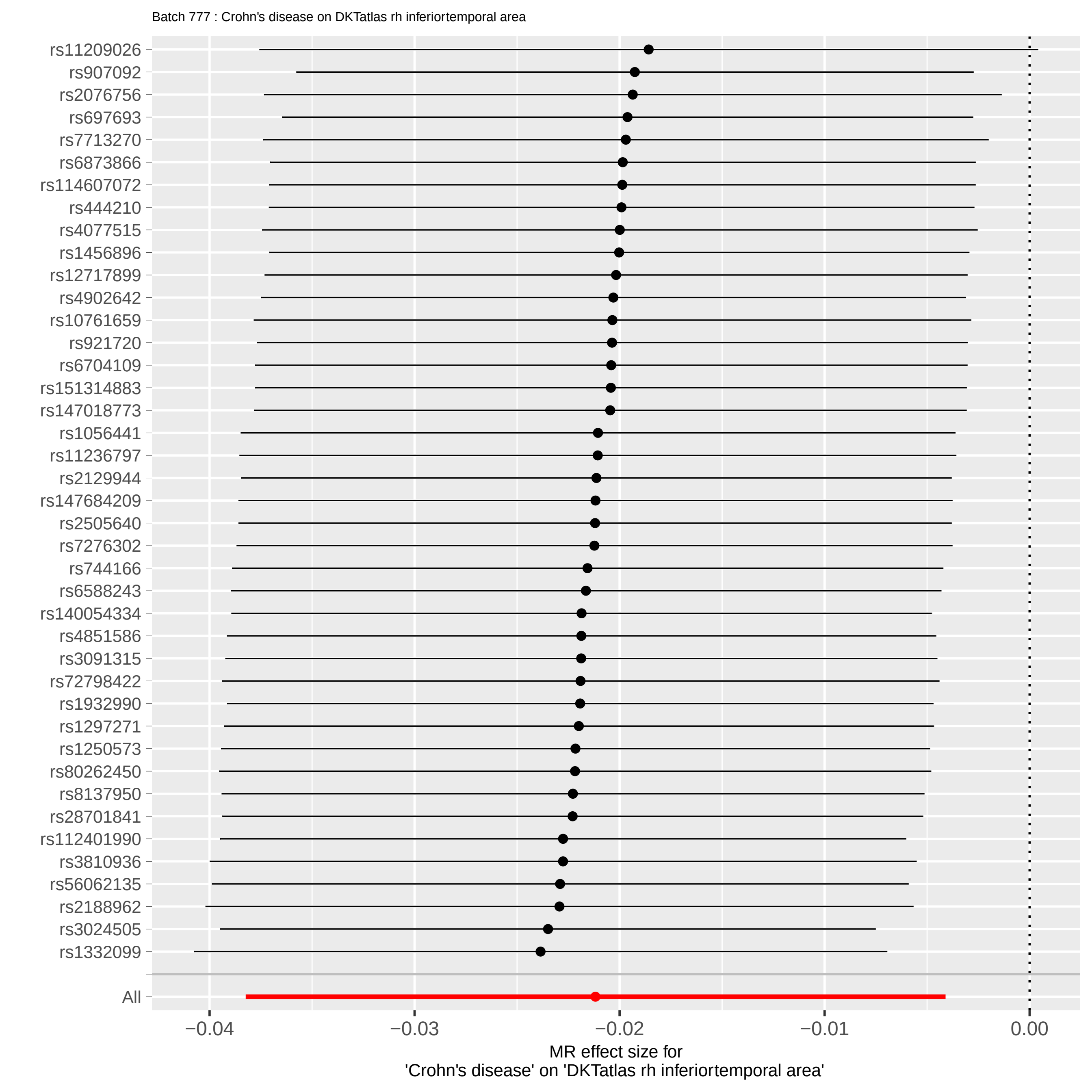
Batch 777 : Crohn's disease on DKTatlas rh inferiortemporal area
rs11209026
rs907092
rs2076756
rs697693
rs7713270
rs6873866
rs114607072
rs444210
rs4077515
rs1456896
rs12717899
rs4902642
rs10761659
rs921720
rs6704109
rs151314883
rs147018773
rs1056441
rs11236797
rs2129944
rs147684209
rs2505640
rs7276302
rs744166
rs6588243
rs140054334
rs4851586
rs3091315
rs72798422
rs1932990
rs1297271
rs1250573
rs80262450
rs8137950
rs28701841
rs112401990
rs3810936
rs56062135
rs2188962
rs3024505
rs1332099
All
−0.04
−0.03
−0.02
−0.01
0.00
MR effect size for
'Crohn's disease' on 'DKTatlas rh inferiortemporal area'

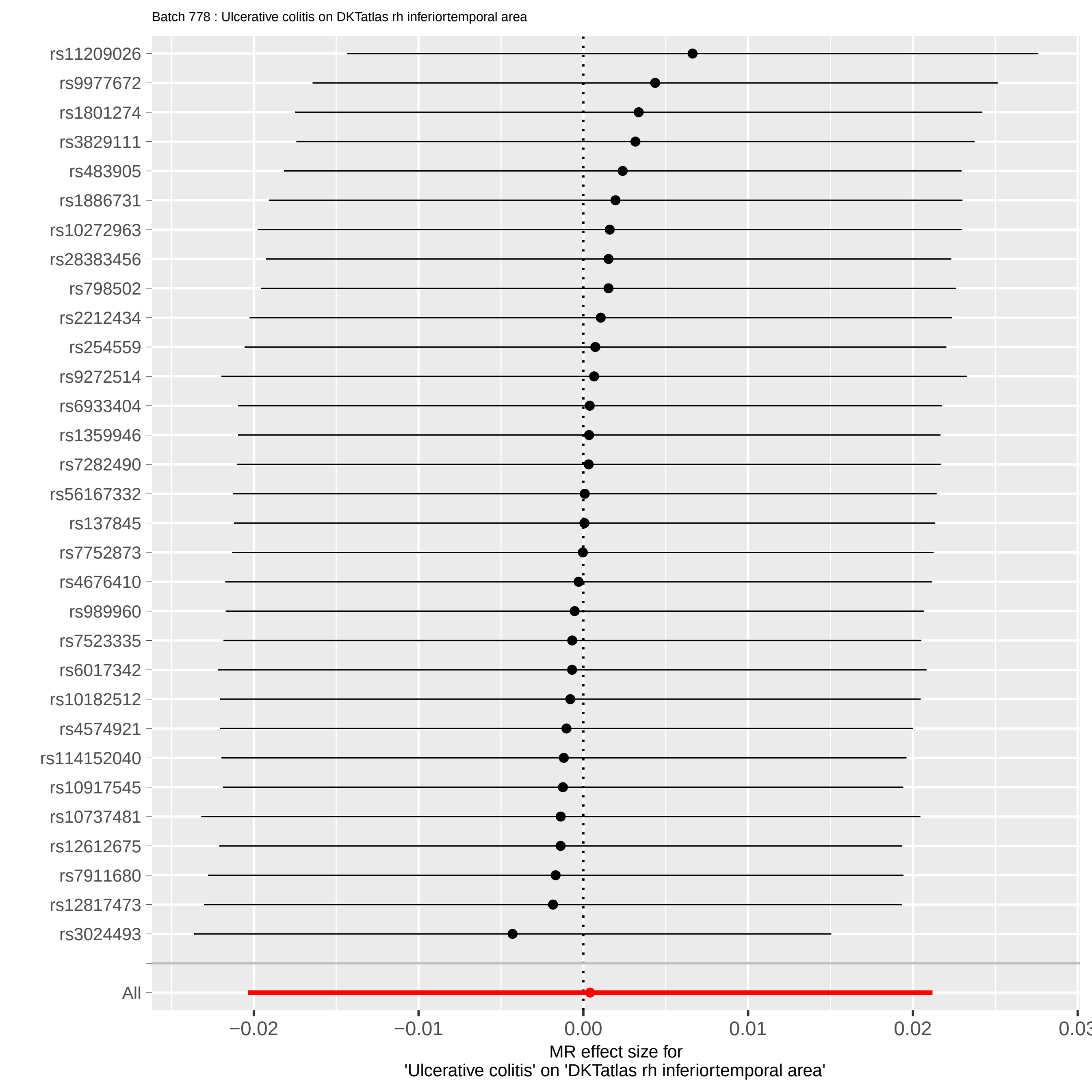

Batch 778 : Ulcerative colitis on DKTatlas rh inferiortemporal area
rs11209026
rs9977672
rs1801274
rs3829111
rs483905
rs1886731
rs10272963
rs28383456
rs798502
rs2212434
rs254559
rs9272514
rs6933404
rs1359946
rs7282490
rs56167332
rs137845
rs7752873
rs4676410
rs989960
rs7523335
rs6017342
rs10182512
rs4574921
rs114152040
rs10917545
rs10737481
rs12612675
rs7911680
rs12817473
rs3024493
All
−0.02
−0.01
0.00
0.01
0.02
0.03
MR effect size for
'Ulcerative colitis' on 'DKTatlas rh inferiortemporal area'

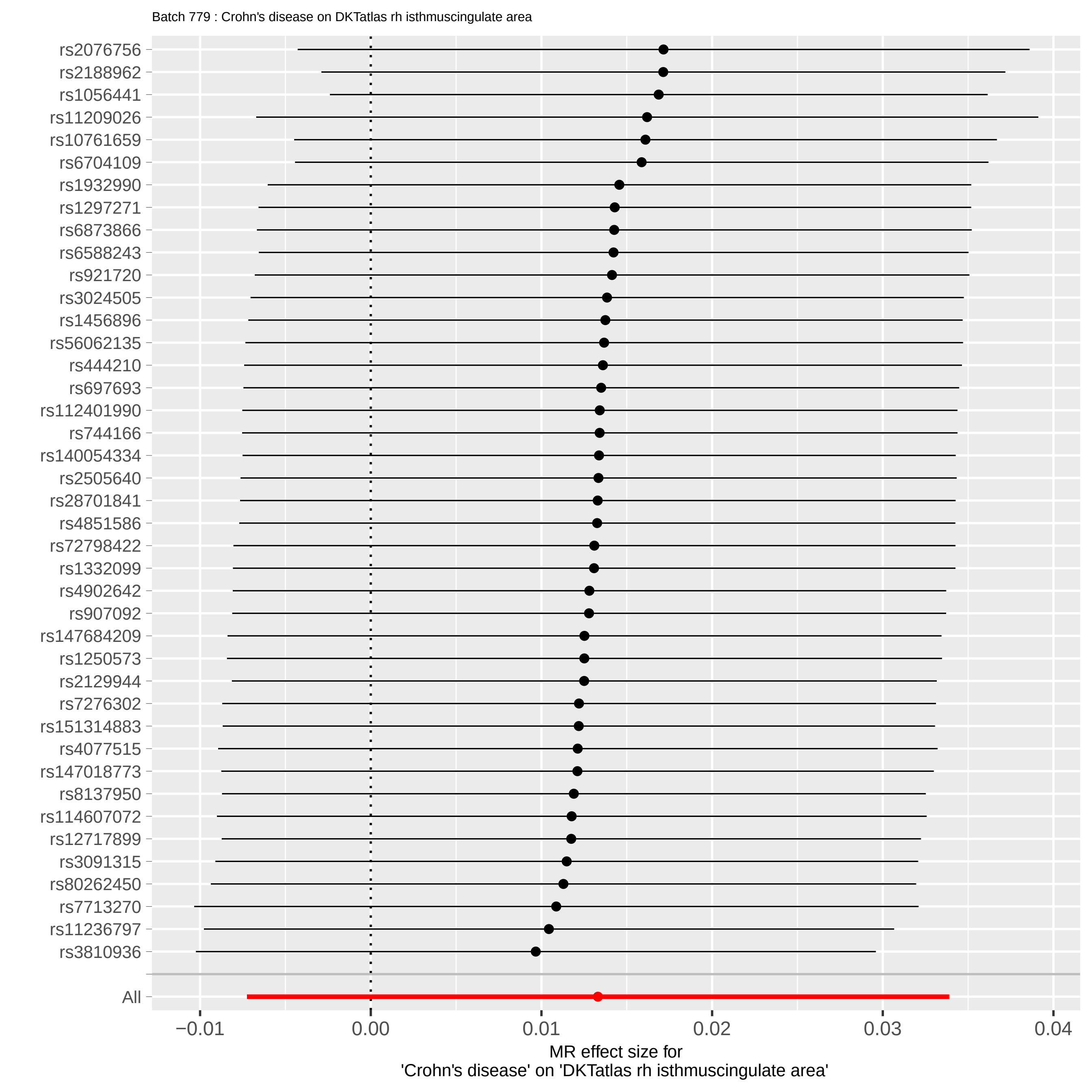

Batch 779 : Crohn's disease on DKTatlas rh isthmuscingulate area
rs2076756
rs2188962
rs1056441
rs11209026
rs10761659
rs6704109
rs1932990
rs1297271
rs6873866
rs6588243
rs921720
rs3024505
rs1456896
rs56062135
rs444210
rs697693
rs112401990
rs744166
rs140054334
rs2505640
rs28701841
rs4851586
rs72798422
rs1332099
rs4902642
rs907092
rs147684209
rs1250573
rs2129944
rs7276302
rs151314883
rs4077515
rs147018773
rs8137950
rs114607072
rs12717899
rs3091315
rs80262450
rs7713270
rs11236797
rs3810936
All
−0.01
0.00
0.01
0.02
0.03
0.04
MR effect size for
'Crohn's disease' on 'DKTatlas rh isthmuscingulate area'

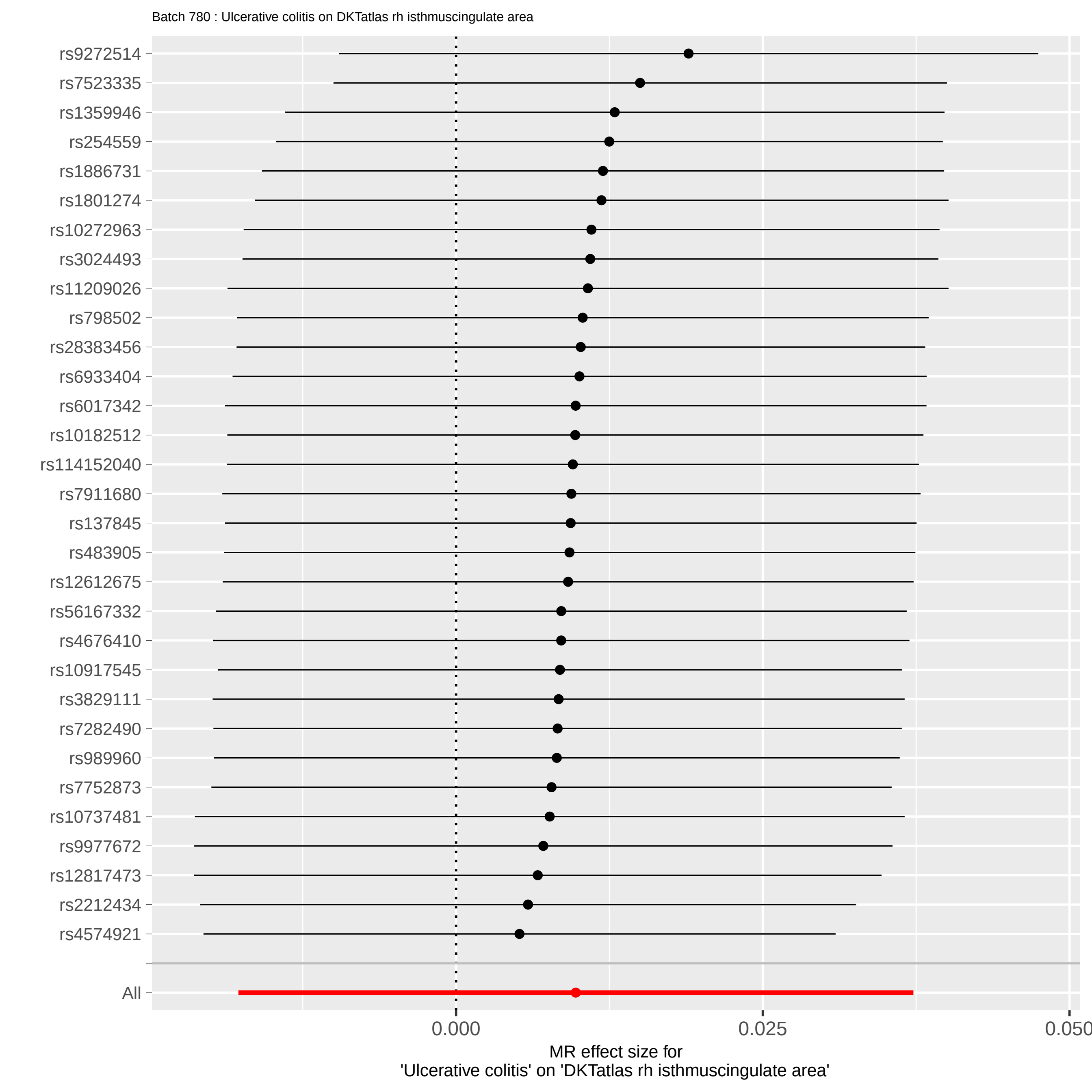
Batch 780 : Ulcerative colitis on DKTatlas rh isthmuscingulate area
rs9272514
rs7523335
rs1359946
rs254559
rs1886731
rs1801274
rs10272963
rs3024493
rs11209026
rs798502
rs28383456
rs6933404
rs6017342
rs10182512
rs114152040
rs7911680
rs137845
rs483905
rs12612675
rs56167332
rs4676410
rs10917545
rs3829111
rs7282490
rs989960
rs7752873
rs10737481
rs9977672
rs12817473
rs2212434
rs4574921
All
0.000
0.025
0.050
MR effect size for
'Ulcerative colitis' on 'DKTatlas rh isthmuscingulate area'

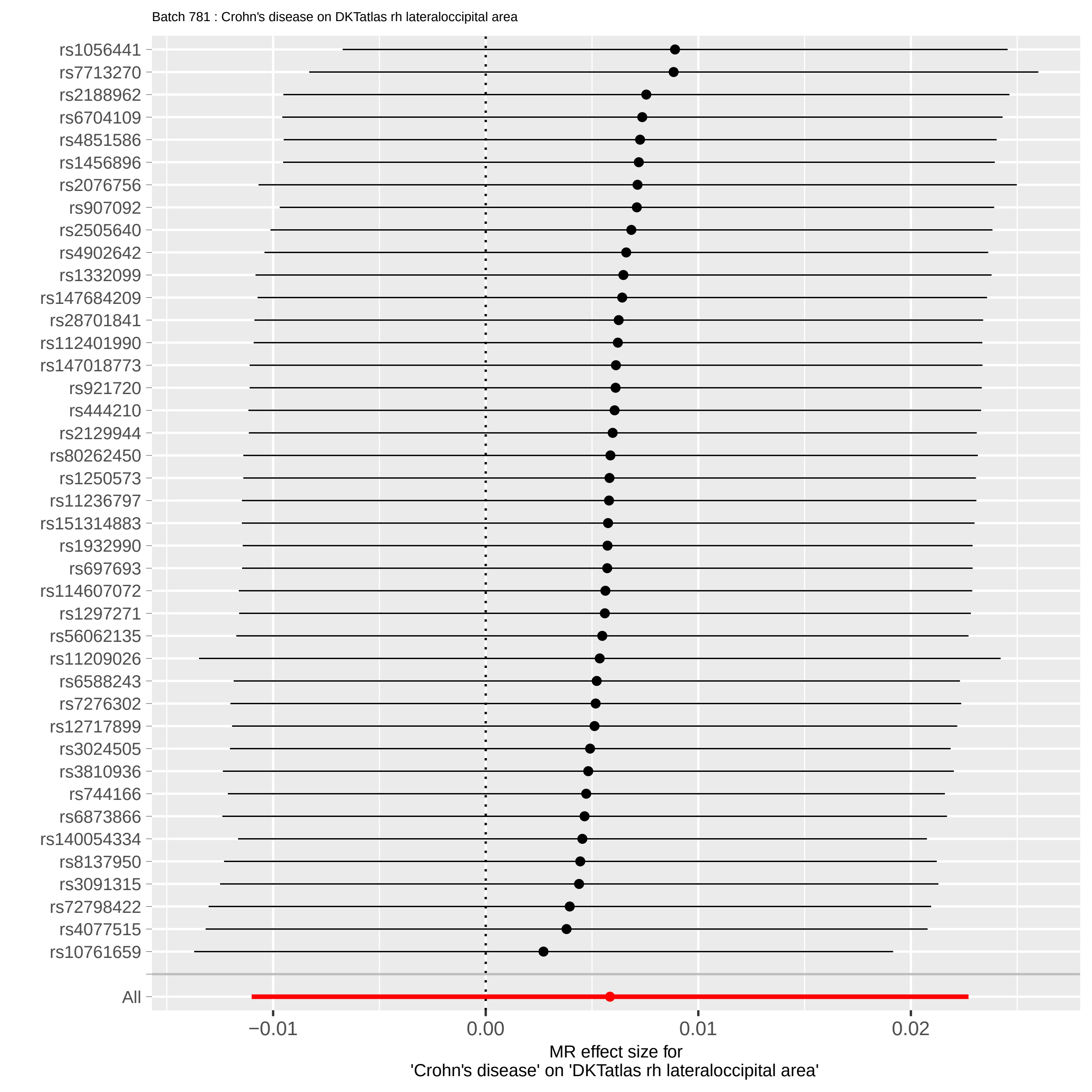
Batch 781 : Crohn's disease on DKTatlas rh lateraloccipital area
rs1056441
rs7713270
rs2188962
rs6704109
rs4851586
rs1456896
rs2076756
rs907092
rs2505640
rs4902642
rs1332099
rs147684209
rs28701841
rs112401990
rs147018773
rs921720
rs444210
rs2129944
rs80262450
rs1250573
rs11236797
rs151314883
rs1932990
rs697693
rs114607072
rs1297271
rs56062135
rs11209026
rs6588243
rs7276302
rs12717899
rs3024505
rs3810936
rs744166
rs6873866
rs140054334
rs8137950
rs3091315
rs72798422
rs4077515
rs10761659
All
−0.01
0.00
0.01
0.02
MR effect size for
'Crohn's disease' on 'DKTatlas rh lateraloccipital area'

Batch 782 : Ulcerative colitis on DKTatlas rh lateraloccipital area

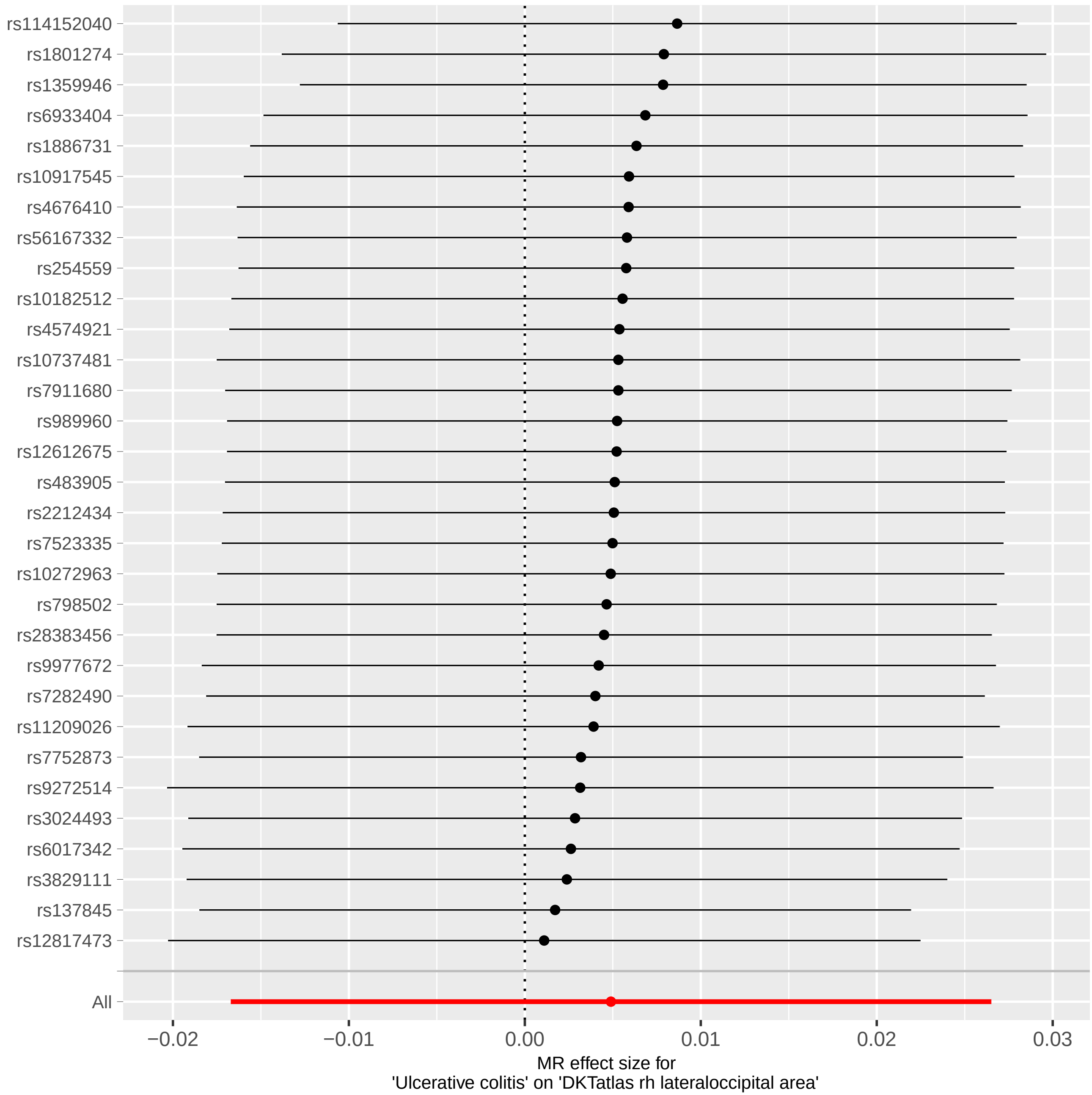

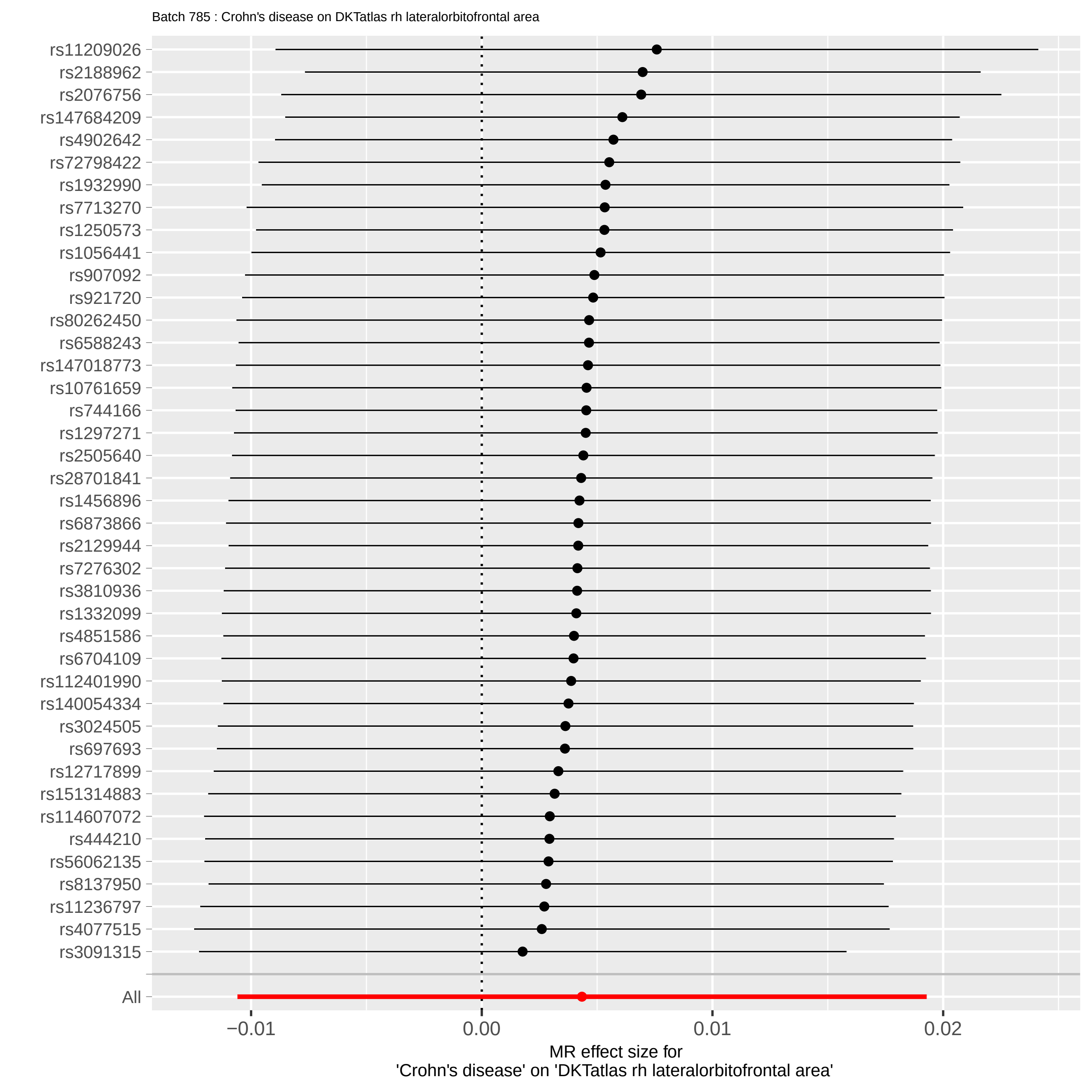

Batch 785 : Crohn's disease on DKTatlas rh lateralorbitofrontal area
rs11209026
rs2188962
rs2076756
rs147684209
rs4902642
rs72798422
rs1932990
rs7713270
rs1250573
rs1056441
rs907092
rs921720
rs80262450
rs6588243
rs147018773
rs10761659
rs744166
rs1297271
rs2505640
rs28701841
rs1456896
rs6873866
rs2129944
rs7276302
rs3810936
rs1332099
rs4851586
rs6704109
rs112401990
rs140054334
rs3024505
rs697693
rs12717899
rs151314883
rs114607072
rs444210
rs56062135
rs8137950
rs11236797
rs4077515
rs3091315
All
−0.01
0.00
0.01
0.02
MR effect size for
'Crohn's disease' on 'DKTatlas rh lateralorbitofrontal area'

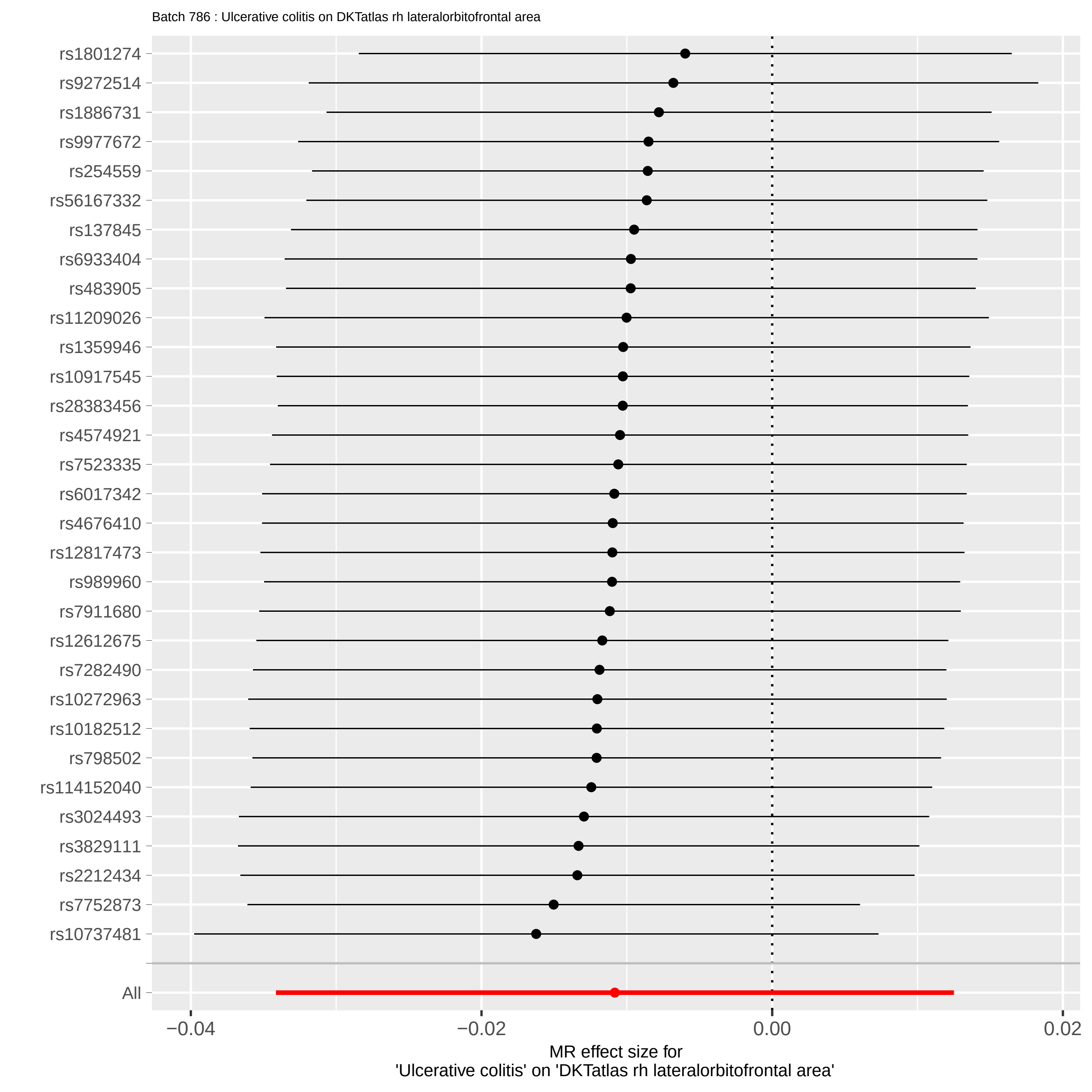
Batch 786 : Ulcerative colitis on DKTatlas rh lateralorbitofrontal area
rs1801274
rs9272514
rs1886731
rs9977672
rs254559
rs56167332
rs137845
rs6933404
rs483905
rs11209026
rs1359946
rs10917545
rs28383456
rs4574921
rs7523335
rs6017342
rs4676410
rs12817473
rs989960
rs7911680
rs12612675
rs7282490
rs10272963
rs10182512
rs798502
rs114152040
rs3024493
rs3829111
rs2212434
rs7752873
rs10737481
All
−0.04
−0.02
0.00
0.02
MR effect size for
'Ulcerative colitis' on 'DKTatlas rh lateralorbitofrontal area'

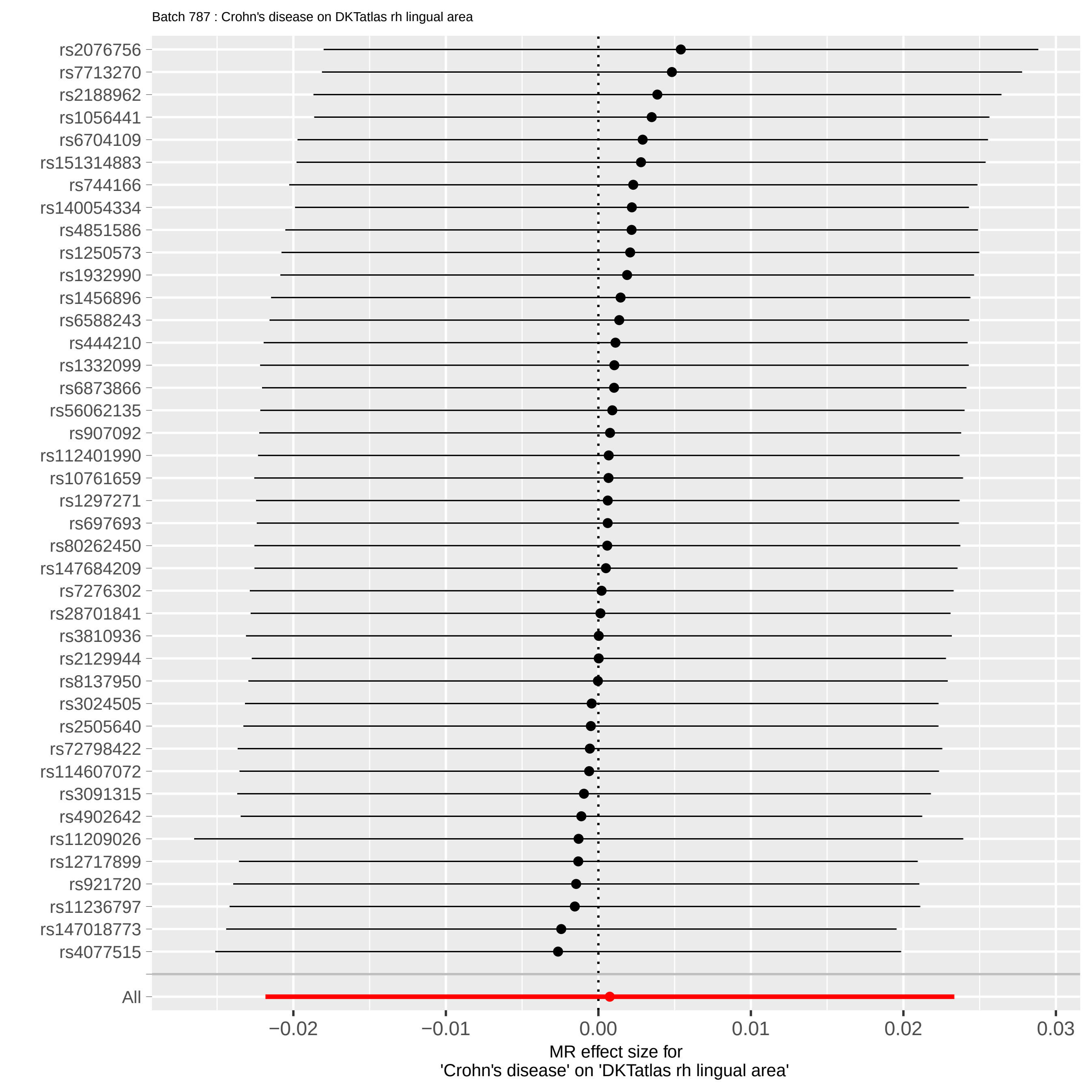
Batch 787 : Crohn's disease on DKTatlas rh lingual area
rs2076756
rs7713270
rs2188962
rs1056441
rs6704109
rs151314883
rs744166
rs140054334
rs4851586
rs1250573
rs1932990
rs1456896
rs6588243
rs444210
rs1332099
rs6873866
rs56062135
rs907092
rs112401990
rs10761659
rs1297271
rs697693
rs80262450
rs147684209
rs7276302
rs28701841
rs3810936
rs2129944
rs8137950
rs3024505
rs2505640
rs72798422
rs114607072
rs3091315
rs4902642
rs11209026
rs12717899
rs921720
rs11236797
rs147018773
rs4077515
All
−0.02
−0.01
0.00
0.01
0.02
0.03
MR effect size for
'Crohn's disease' on 'DKTatlas rh lingual area'

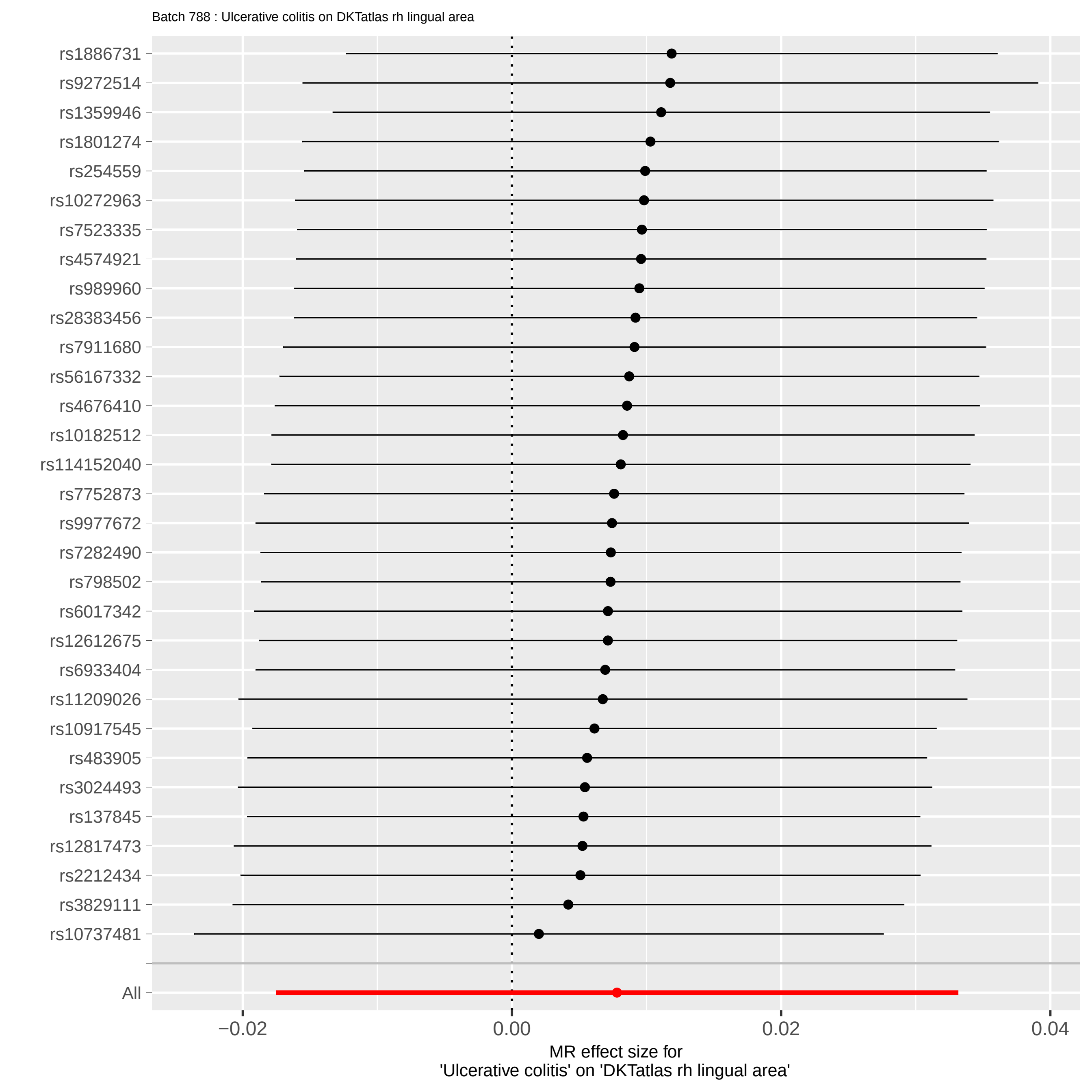

Batch 788 : Ulcerative colitis on DKTatlas rh lingual area
rs1886731
rs9272514
rs1359946
rs1801274
rs254559
rs10272963
rs7523335
rs4574921
rs989960
rs28383456
rs7911680
rs56167332
rs4676410
rs10182512
rs114152040
rs7752873
rs9977672
rs7282490
rs798502
rs6017342
rs12612675
rs6933404
rs11209026
rs10917545
rs483905
rs3024493
rs137845
rs12817473
rs2212434
rs3829111
rs10737481
All
−0.02
0.00
0.02
0.04
MR effect size for
'Ulcerative colitis' on 'DKTatlas rh lingual area'

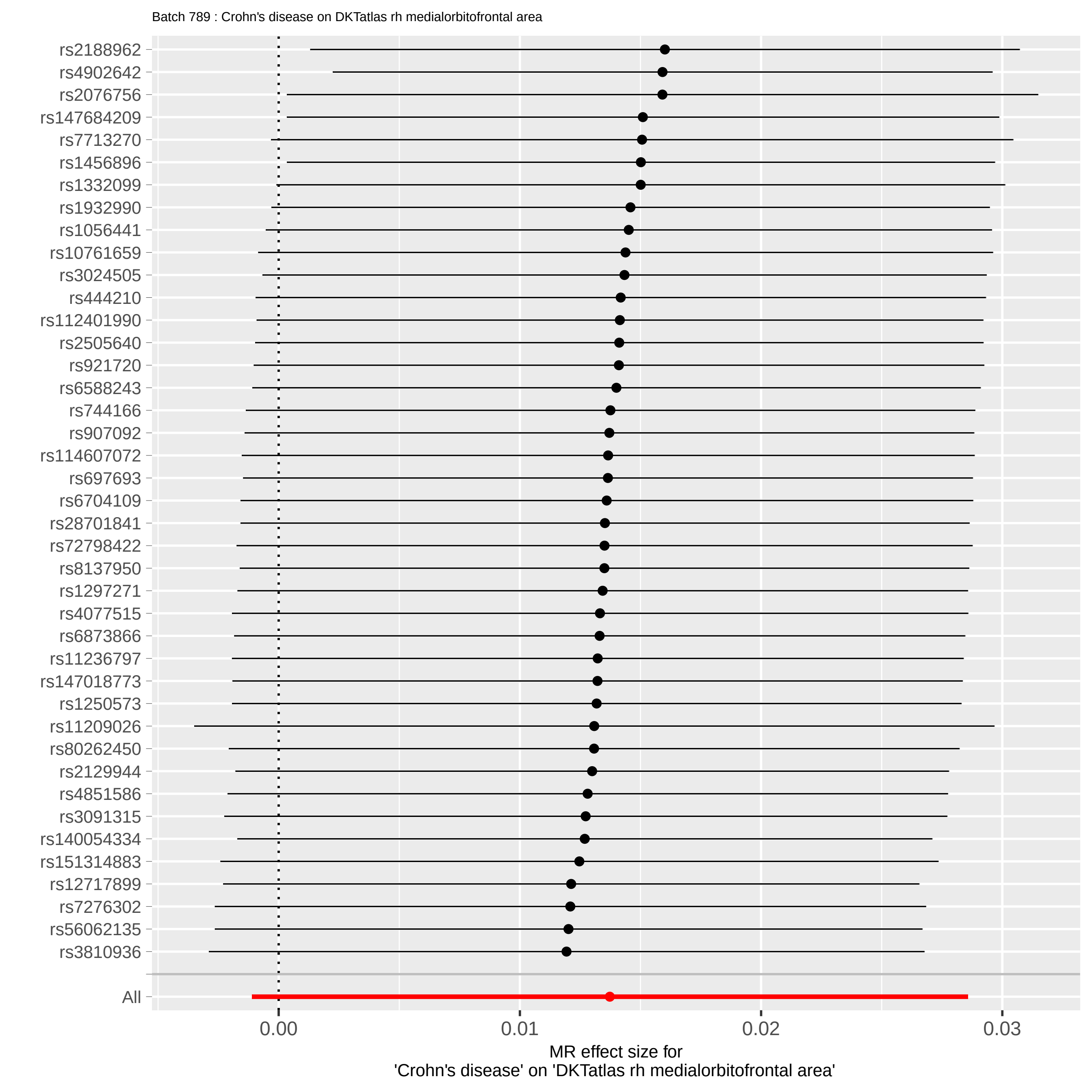

Batch 789 : Crohn's disease on DKTatlas rh medialorbitofrontal area
rs2188962
rs4902642
rs2076756
rs147684209
rs7713270
rs1456896
rs1332099
rs1932990
rs1056441
rs10761659
rs3024505
rs444210
rs112401990
rs2505640
rs921720
rs6588243
rs744166
rs907092
rs114607072
rs697693
rs6704109
rs28701841
rs72798422
rs8137950
rs1297271
rs4077515
rs6873866
rs11236797
rs147018773
rs1250573
rs11209026
rs80262450
rs2129944
rs4851586
rs3091315
rs140054334
rs151314883
rs12717899
rs7276302
rs56062135
rs3810936
All
0.00
0.01
0.02
0.03
MR effect size for
'Crohn's disease' on 'DKTatlas rh medialorbitofrontal area'

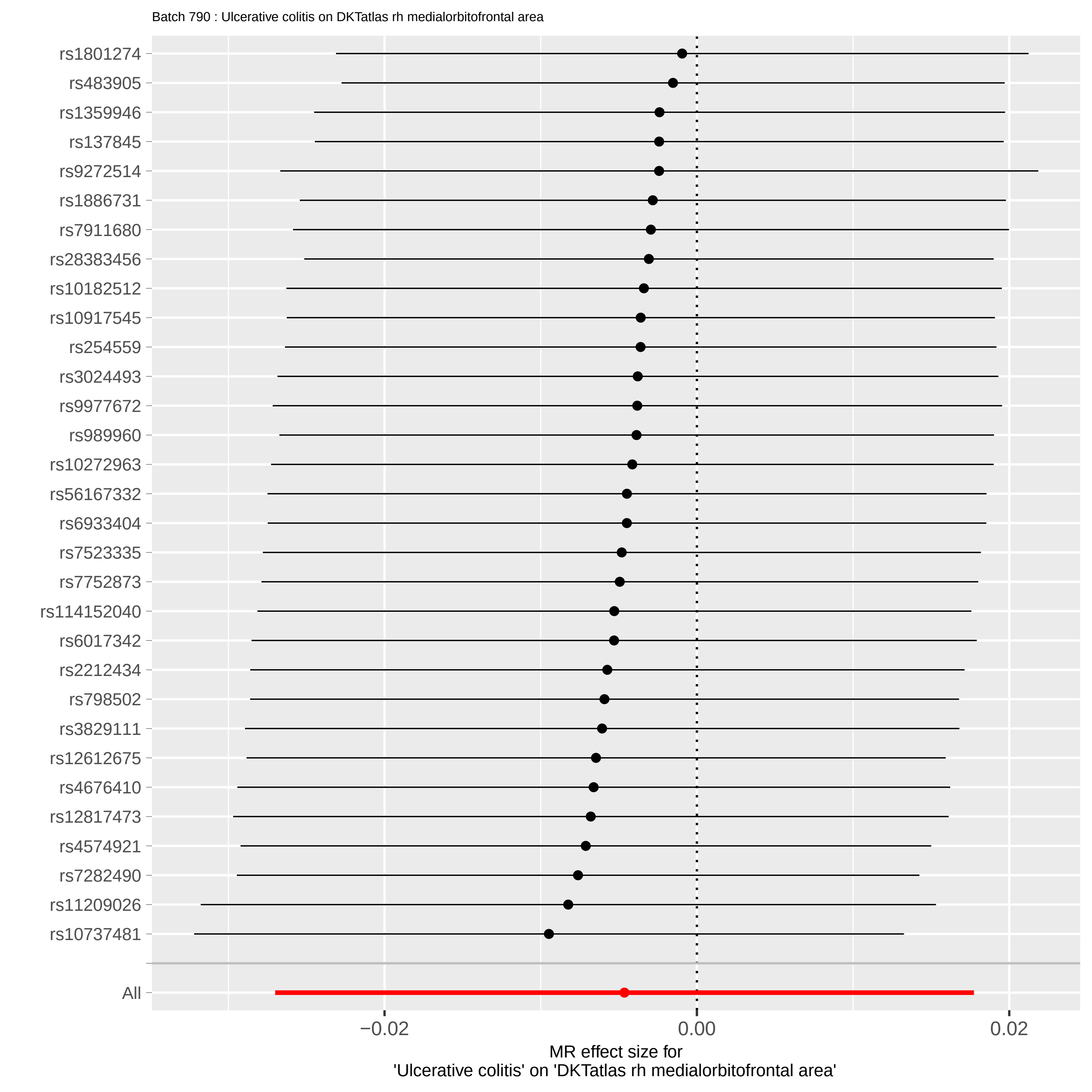
Batch 790 : Ulcerative colitis on DKTatlas rh medialorbitofrontal area
rs1801274
rs483905
rs1359946
rs137845
rs9272514
rs1886731
rs7911680
rs28383456
rs10182512
rs10917545
rs254559
rs3024493
rs9977672
rs989960
rs10272963
rs56167332
rs6933404
rs7523335
rs7752873
rs114152040
rs6017342
rs2212434
rs798502
rs3829111
rs12612675
rs4676410
rs12817473
rs4574921
rs7282490
rs11209026
rs10737481
All
−0.02
0.00
0.02
MR effect size for
'Ulcerative colitis' on 'DKTatlas rh medialorbitofrontal area'

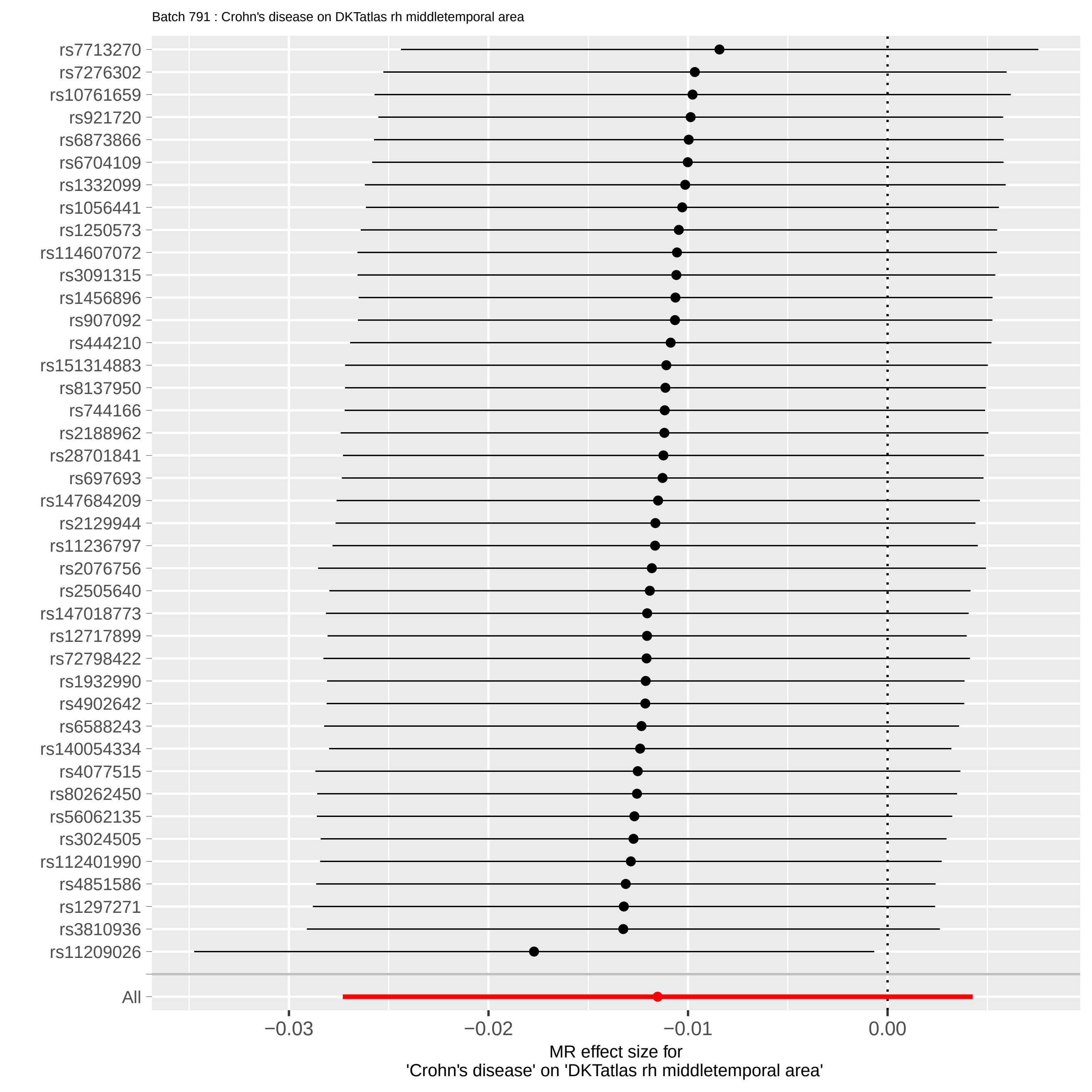

Batch 791 : Crohn's disease on DKTatlas rh middletemporal area
rs7713270
rs7276302
rs10761659
rs921720
rs6873866
rs6704109
rs1332099
rs1056441
rs1250573
rs114607072
rs3091315
rs1456896
rs907092
rs444210
rs151314883
rs8137950
rs744166
rs2188962
rs28701841
rs697693
rs147684209
rs2129944
rs11236797
rs2076756
rs2505640
rs147018773
rs12717899
rs72798422
rs1932990
rs4902642
rs6588243
rs140054334
rs4077515
rs80262450
rs56062135
rs3024505
rs112401990
rs4851586
rs1297271
rs3810936
rs11209026
All
−0.03
−0.02
−0.01
0.00
MR effect size for
'Crohn's disease' on 'DKTatlas rh middletemporal area'

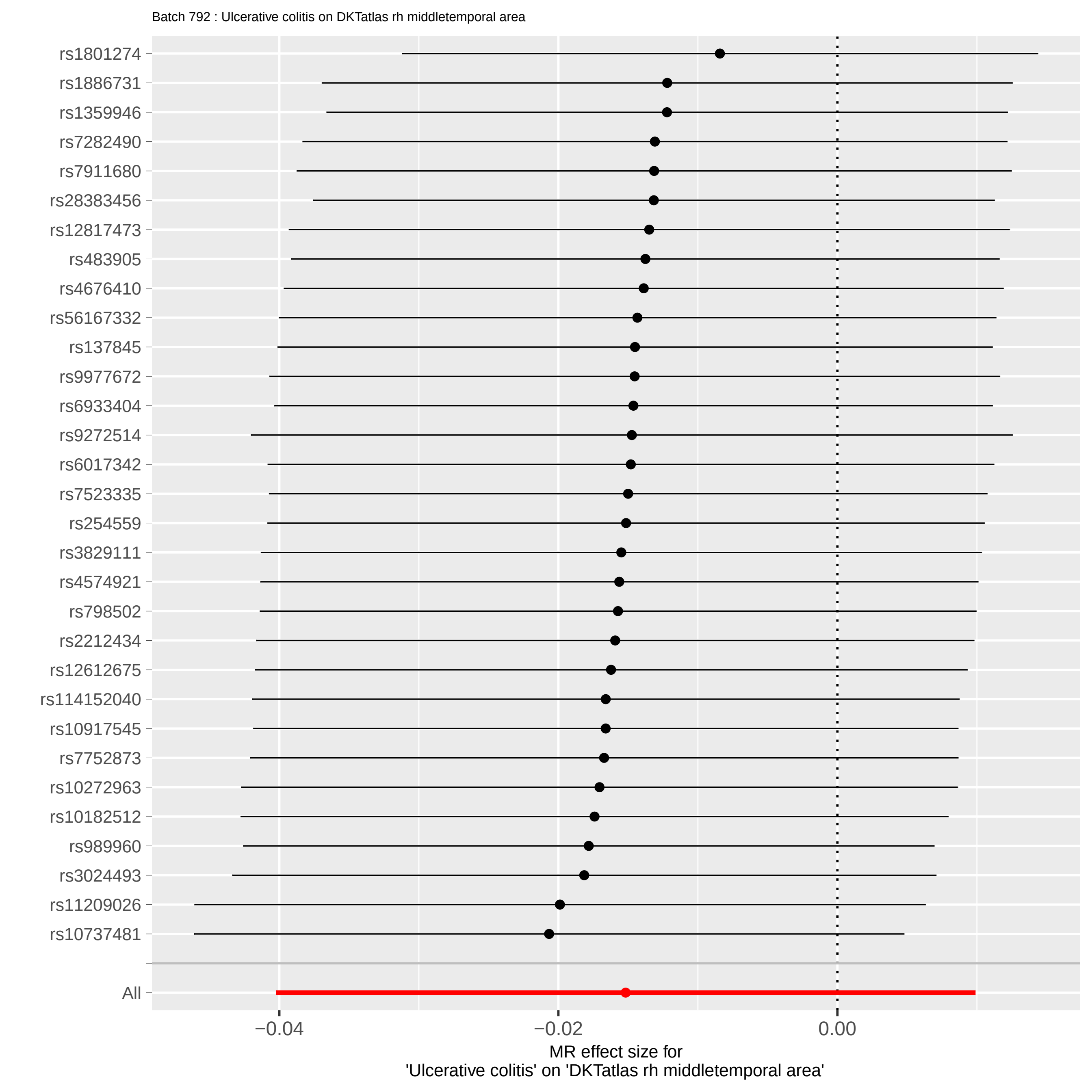
Batch 792 : Ulcerative colitis on DKTatlas rh middletemporal area
rs1801274
rs1886731
rs1359946
rs7282490
rs7911680
rs28383456
rs12817473
rs483905
rs4676410
rs56167332
rs137845
rs9977672
rs6933404
rs9272514
rs6017342
rs7523335
rs254559
rs3829111
rs4574921
rs798502
rs2212434
rs12612675
rs114152040
rs10917545
rs7752873
rs10272963
rs10182512
rs989960
rs3024493
rs11209026
rs10737481
All
−0.04
−0.02
0.00
MR effect size for
'Ulcerative colitis' on 'DKTatlas rh middletemporal area'

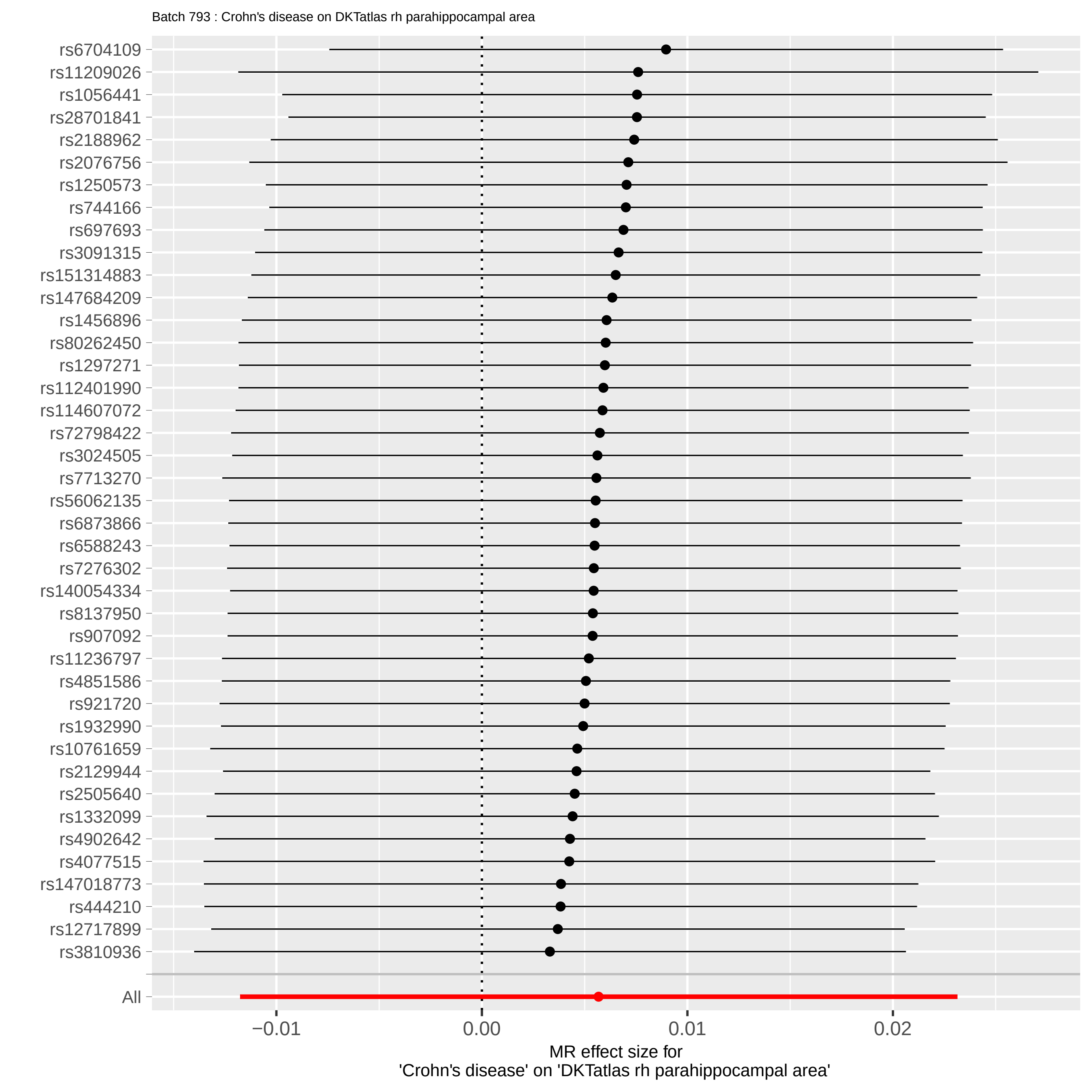

Batch 793 : Crohn's disease on DKTatlas rh parahippocampal area
rs6704109
rs11209026
rs1056441
rs28701841
rs2188962
rs2076756
rs1250573
rs744166
rs697693
rs3091315
rs151314883
rs147684209
rs1456896
rs80262450
rs1297271
rs112401990
rs114607072
rs72798422
rs3024505
rs7713270
rs56062135
rs6873866
rs6588243
rs7276302
rs140054334
rs8137950
rs907092
rs11236797
rs4851586
rs921720
rs1932990
rs10761659
rs2129944
rs2505640
rs1332099
rs4902642
rs4077515
rs147018773
rs444210
rs12717899
rs3810936
All
−0.01
0.00
0.01
0.02
MR effect size for
'Crohn's disease' on 'DKTatlas rh parahippocampal area'

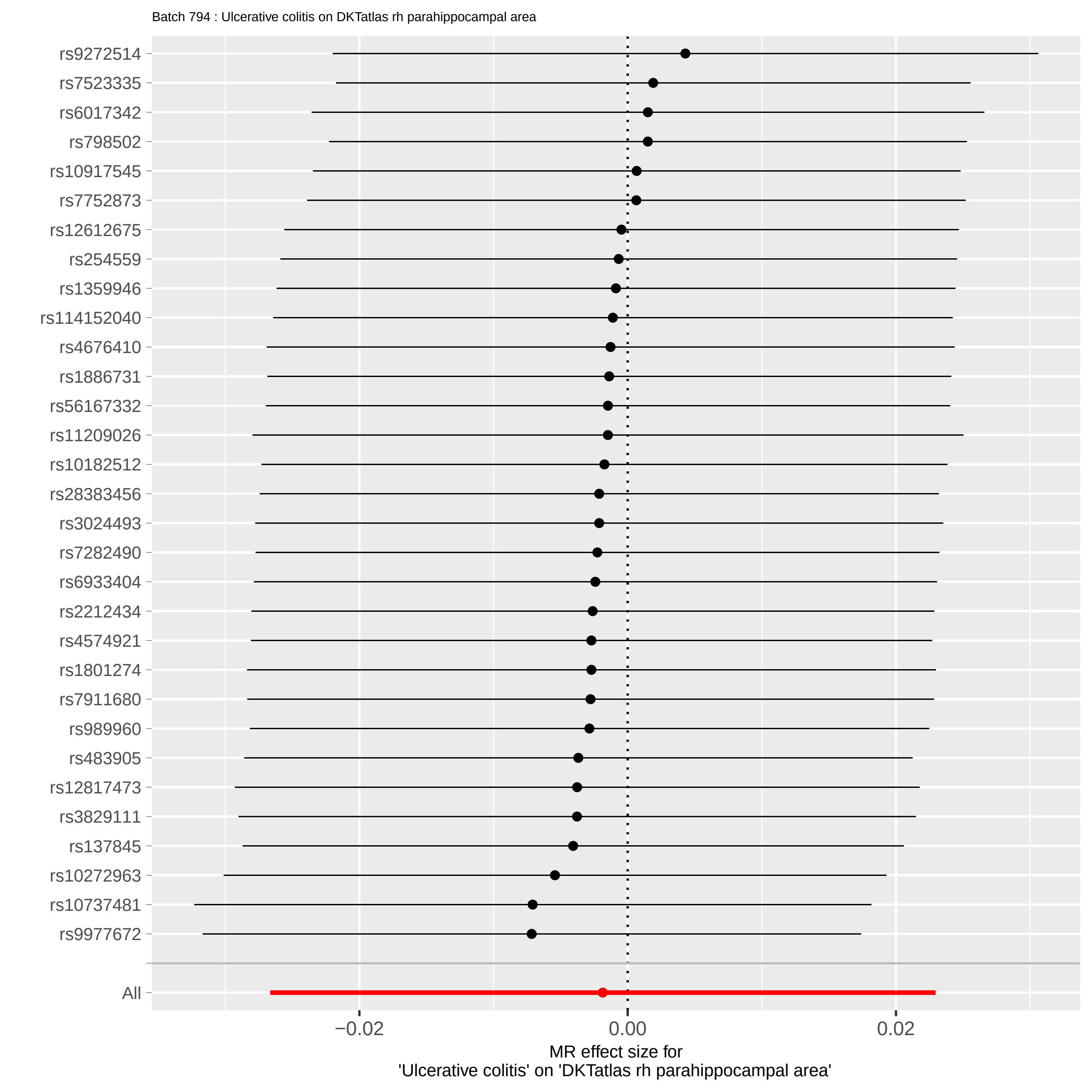

Batch 794 : Ulcerative colitis on DKTatlas rh parahippocampal area
rs9272514
rs7523335
rs6017342
rs798502
rs10917545
rs7752873
rs12612675
rs254559
rs1359946
rs114152040
rs4676410
rs1886731
rs56167332
rs11209026
rs10182512
rs28383456
rs3024493
rs7282490
rs6933404
rs2212434
rs4574921
rs1801274
rs7911680
rs989960
rs483905
rs12817473
rs3829111
rs137845
rs10272963
rs10737481
rs9977672
All
−0.02
0.00
0.02
MR effect size for
'Ulcerative colitis' on 'DKTatlas rh parahippocampal area'

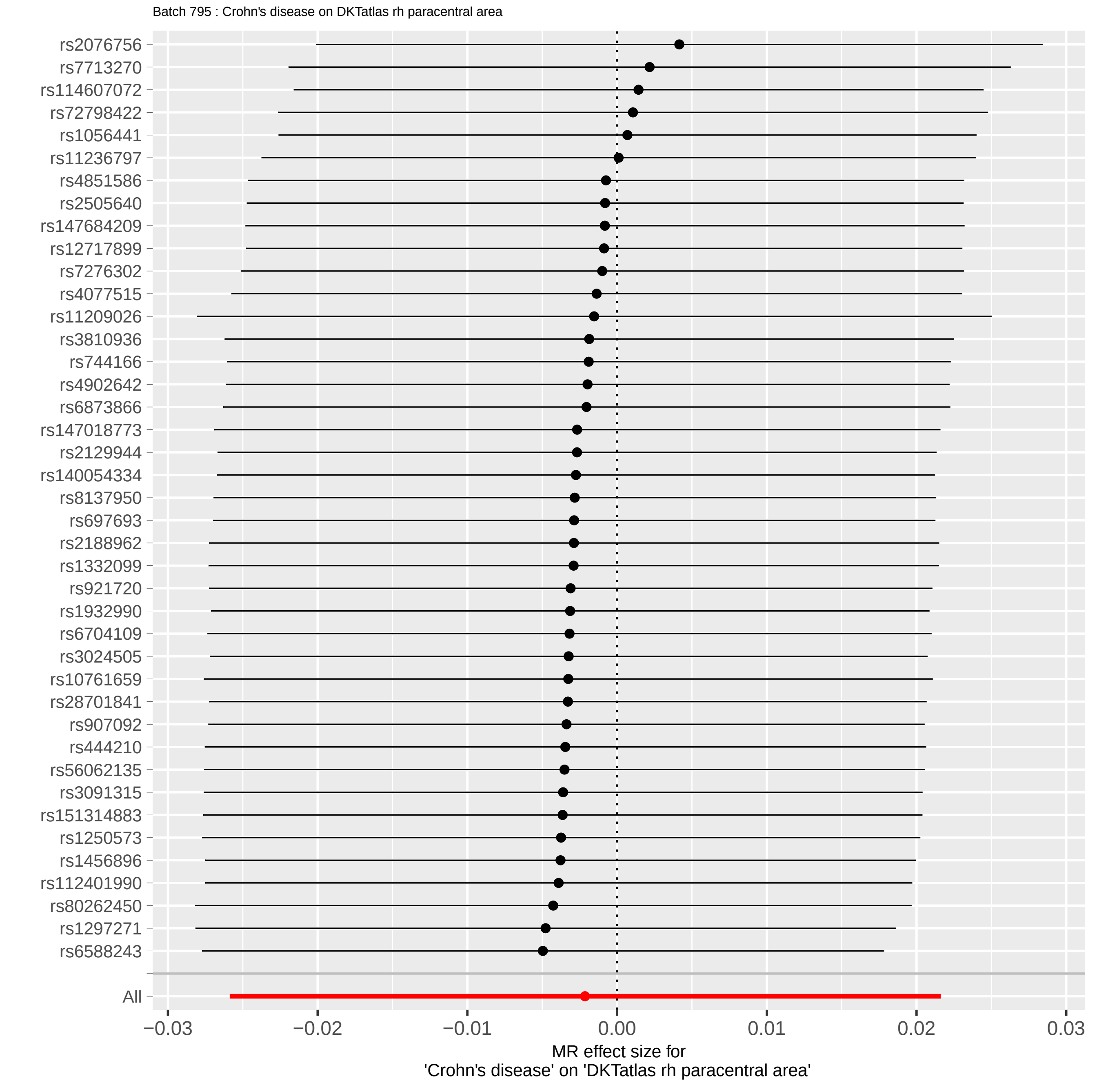

Batch 795 : Crohn's disease on DKTatlas rh paracentral area
rs2076756
rs7713270
rs114607072
rs72798422
rs1056441
rs11236797
rs4851586
rs2505640
rs147684209
rs12717899
rs7276302
rs4077515
rs11209026
rs3810936
rs744166
rs4902642
rs6873866
rs147018773
rs2129944
rs140054334
rs8137950
rs697693
rs2188962
rs1332099
rs921720
rs1932990
rs6704109
rs3024505
rs10761659
rs28701841
rs907092
rs444210
rs56062135
rs3091315
rs151314883
rs1250573
rs1456896
rs112401990
rs80262450
rs1297271
rs6588243
All
−0.03
−0.02
−0.01
0.00
0.01
0.02
0.03
MR effect size for
'Crohn's disease' on 'DKTatlas rh paracentral area'

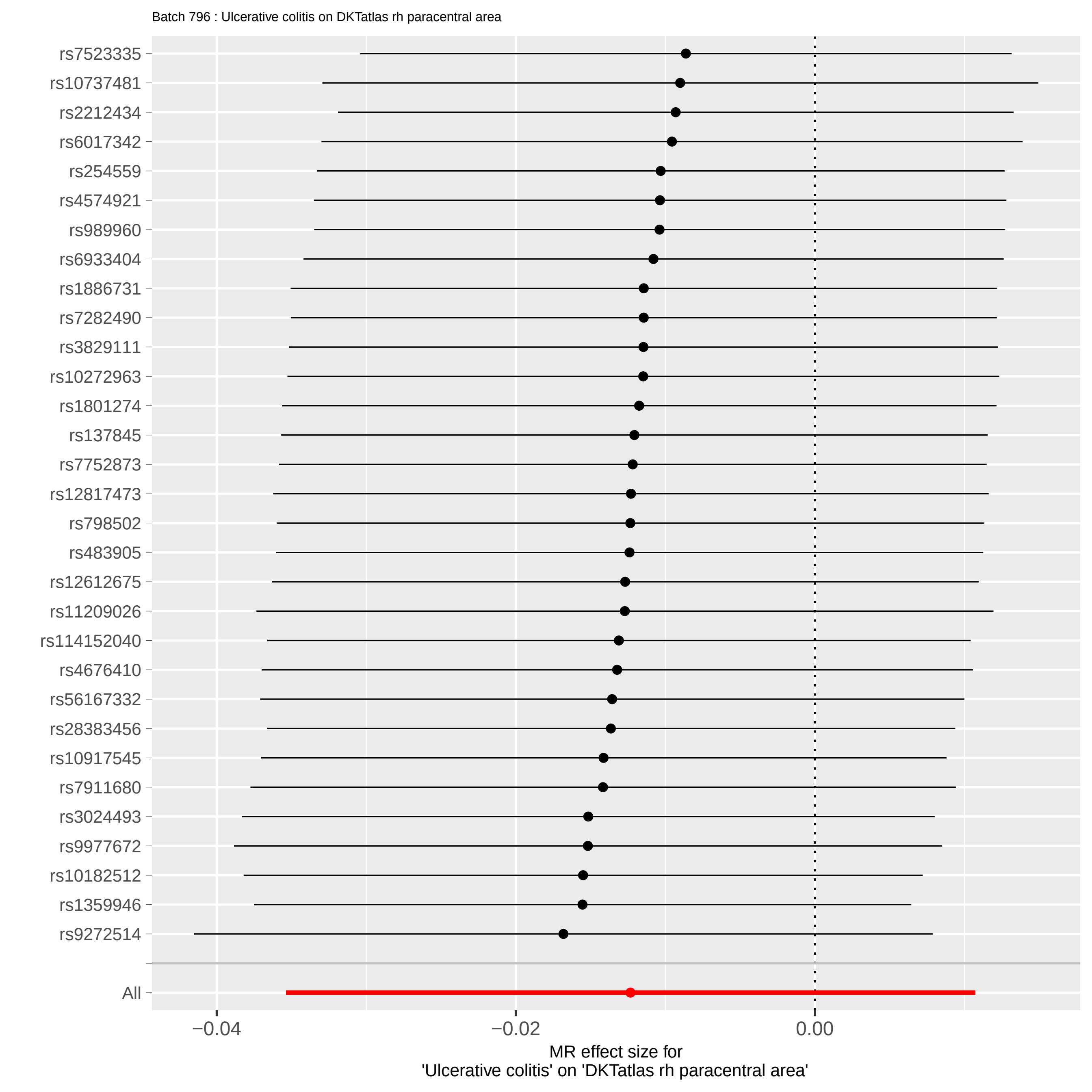
Batch 796 : Ulcerative colitis on DKTatlas rh paracentral area
rs7523335
rs10737481
rs2212434
rs6017342
rs254559
rs4574921
rs989960
rs6933404
rs1886731
rs7282490
rs3829111
rs10272963
rs1801274
rs137845
rs7752873
rs12817473
rs798502
rs483905
rs12612675
rs11209026
rs114152040
rs4676410
rs56167332
rs28383456
rs10917545
rs7911680
rs3024493
rs9977672
rs10182512
rs1359946
rs9272514
All
−0.04
−0.02
0.00
MR effect size for
'Ulcerative colitis' on 'DKTatlas rh paracentral area'

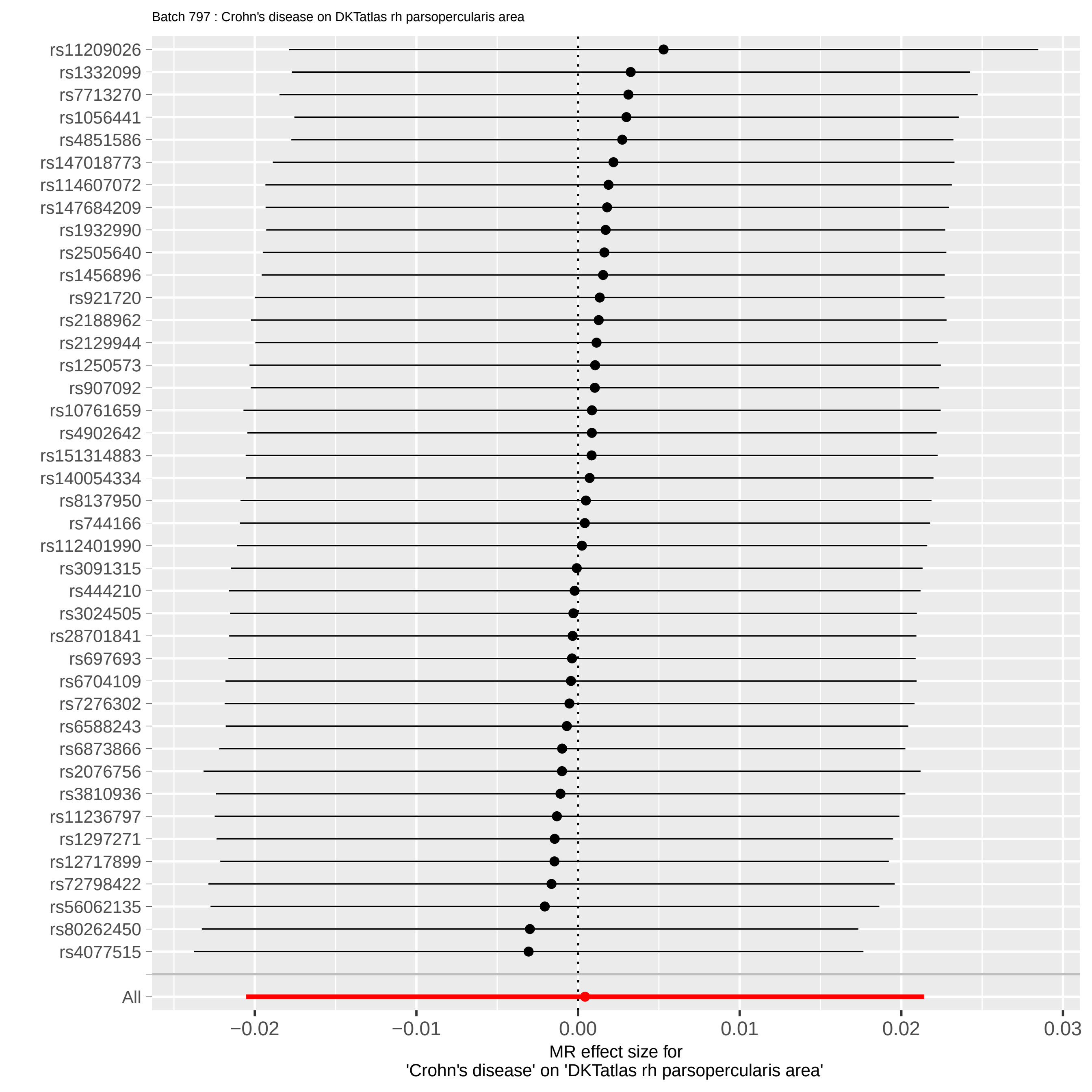

Batch 797 : Crohn's disease on DKTatlas rh parsopercularis area
rs11209026
rs1332099
rs7713270
rs1056441
rs4851586
rs147018773
rs114607072
rs147684209
rs1932990
rs2505640
rs1456896
rs921720
rs2188962
rs2129944
rs1250573
rs907092
rs10761659
rs4902642
rs151314883
rs140054334
rs8137950
rs744166
rs112401990
rs3091315
rs444210
rs3024505
rs28701841
rs697693
rs6704109
rs7276302
rs6588243
rs6873866
rs2076756
rs3810936
rs11236797
rs1297271
rs12717899
rs72798422
rs56062135
rs80262450
rs4077515
All
−0.02
−0.01
0.00
0.01
0.02
0.03
MR effect size for
'Crohn's disease' on 'DKTatlas rh parsopercularis area'

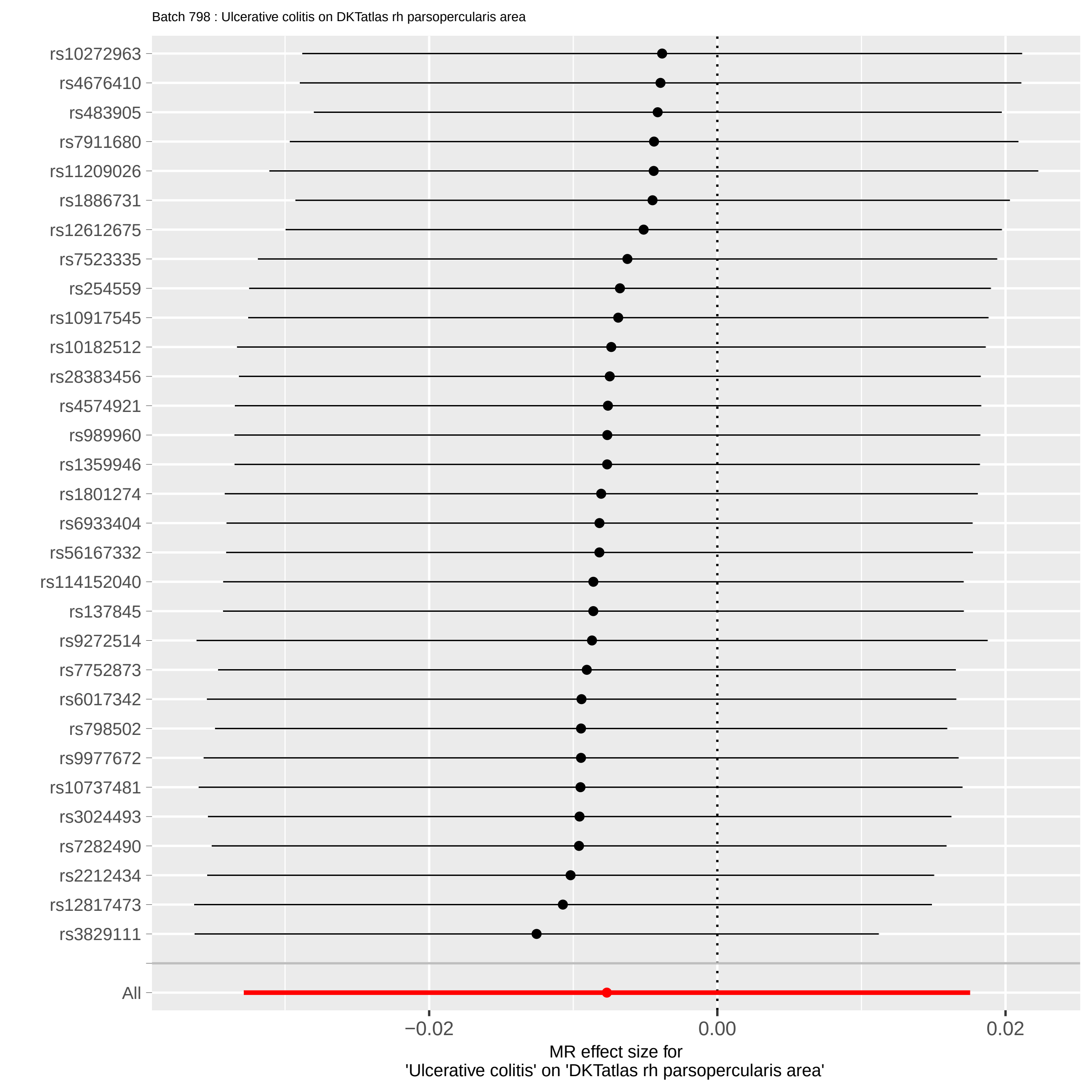

Batch 798 : Ulcerative colitis on DKTatlas rh parsopercularis area
rs10272963
rs4676410
rs483905
rs7911680
rs11209026
rs1886731
rs12612675
rs7523335
rs254559
rs10917545
rs10182512
rs28383456
rs4574921
rs989960
rs1359946
rs1801274
rs6933404
rs56167332
rs114152040
rs137845
rs9272514
rs7752873
rs6017342
rs798502
rs9977672
rs10737481
rs3024493
rs7282490
rs2212434
rs12817473
rs3829111
All
−0.02
0.00
0.02
MR effect size for
'Ulcerative colitis' on 'DKTatlas rh parsopercularis area'

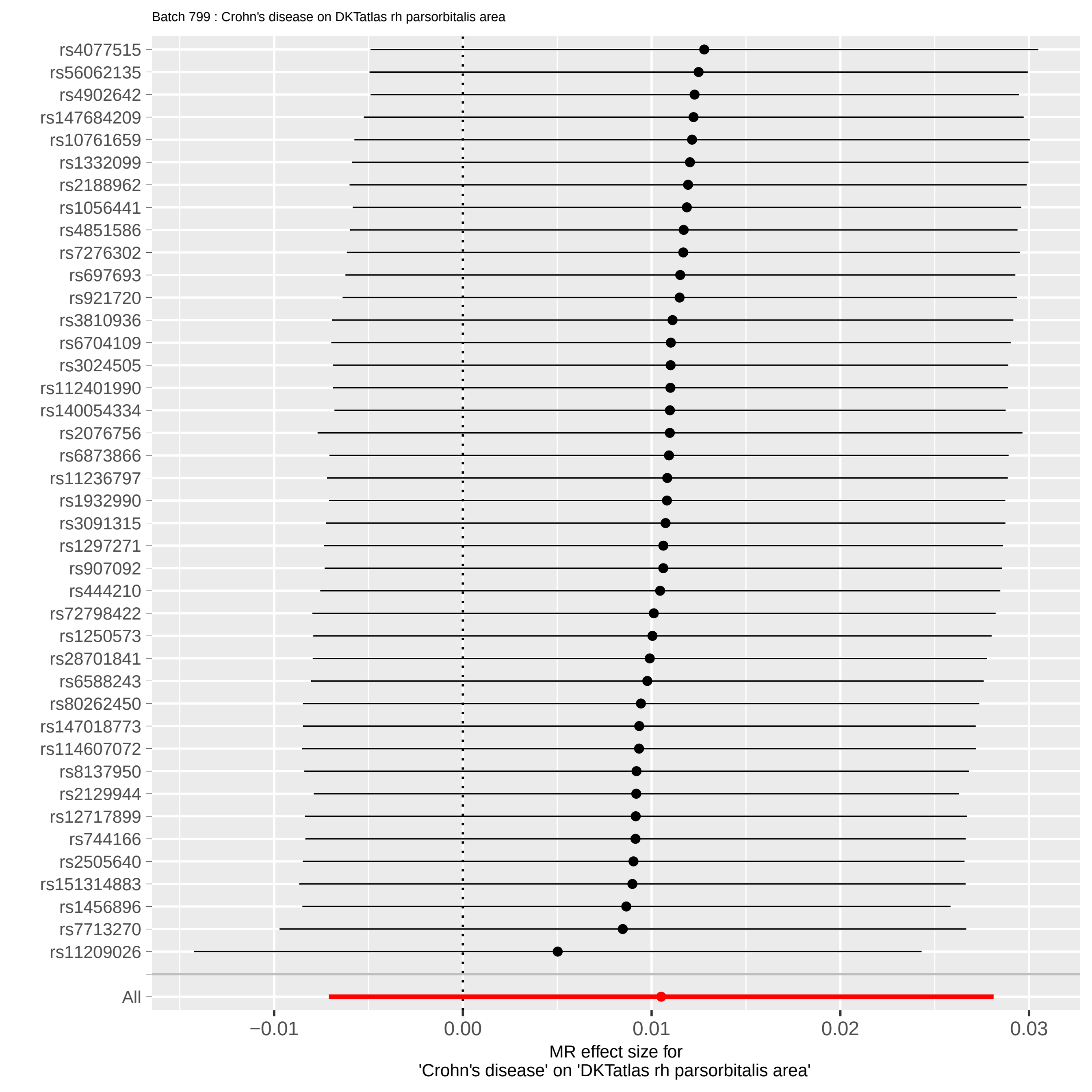

Batch 799 : Crohn's disease on DKTatlas rh parsorbitalis area
rs4077515
rs56062135
rs4902642
rs147684209
rs10761659
rs1332099
rs2188962
rs1056441
rs4851586
rs7276302
rs697693
rs921720
rs3810936
rs6704109
rs3024505
rs112401990
rs140054334
rs2076756
rs6873866
rs11236797
rs1932990
rs3091315
rs1297271
rs907092
rs444210
rs72798422
rs1250573
rs28701841
rs6588243
rs80262450
rs147018773
rs114607072
rs8137950
rs2129944
rs12717899
rs744166
rs2505640
rs151314883
rs1456896
rs7713270
rs11209026
All
−0.01
0.00
0.01
0.02
0.03
MR effect size for
'Crohn's disease' on 'DKTatlas rh parsorbitalis area'

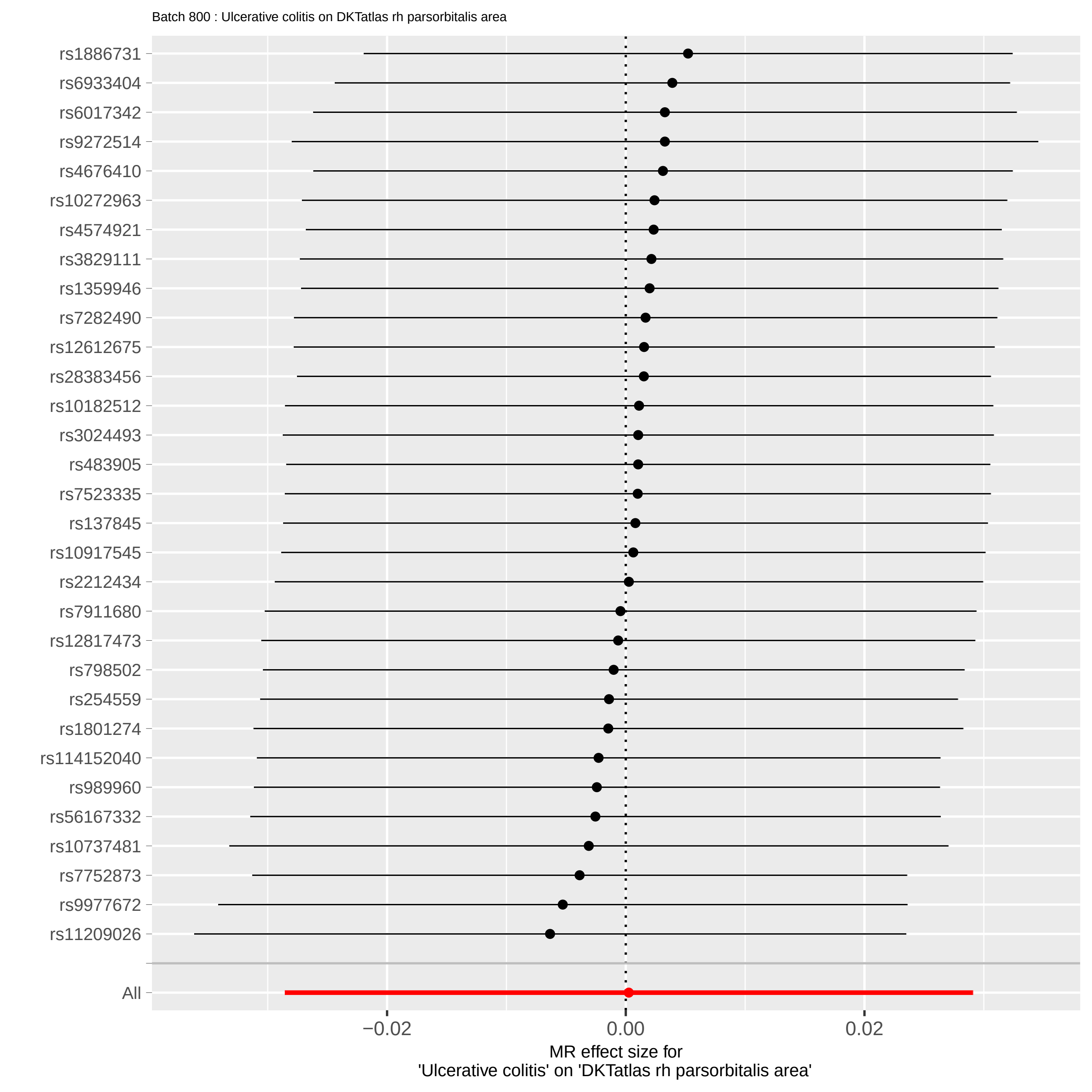

Batch 800 : Ulcerative colitis on DKTatlas rh parsorbitalis area
rs1886731
rs6933404
rs6017342
rs9272514
rs4676410
rs10272963
rs4574921
rs3829111
rs1359946
rs7282490
rs12612675
rs28383456
rs10182512
rs3024493
rs483905
rs7523335
rs137845
rs10917545
rs2212434
rs7911680
rs12817473
rs798502
rs254559
rs1801274
rs114152040
rs989960
rs56167332
rs10737481
rs7752873
rs9977672
rs11209026
All
−0.02
0.00
0.02
MR effect size for
'Ulcerative colitis' on 'DKTatlas rh parsorbitalis area'

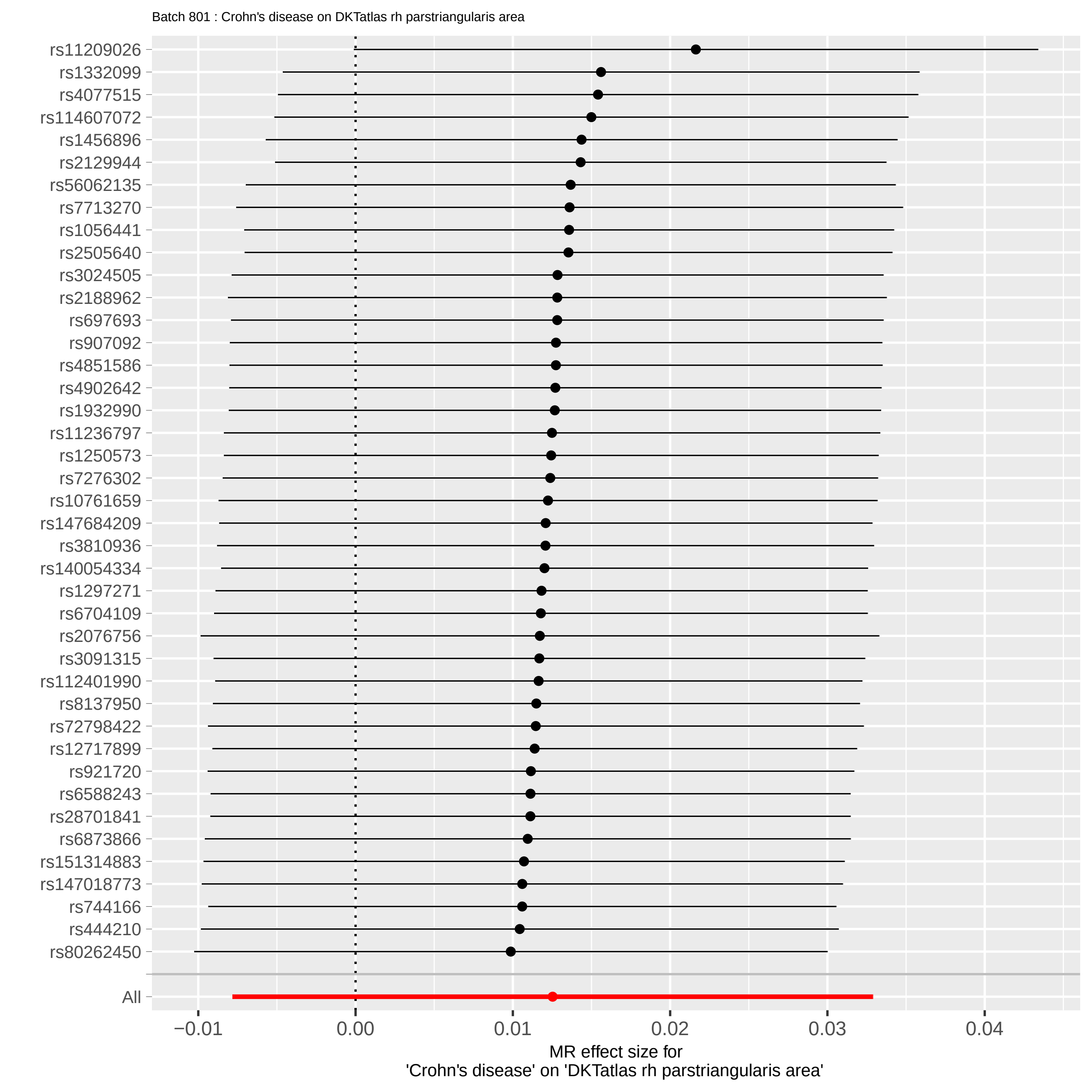

Batch 801 : Crohn's disease on DKTatlas rh parstriangularis area
rs11209026
rs1332099
rs4077515
rs114607072
rs1456896
rs2129944
rs56062135
rs7713270
rs1056441
rs2505640
rs3024505
rs2188962
rs697693
rs907092
rs4851586
rs4902642
rs1932990
rs11236797
rs1250573
rs7276302
rs10761659
rs147684209
rs3810936
rs140054334
rs1297271
rs6704109
rs2076756
rs3091315
rs112401990
rs8137950
rs72798422
rs12717899
rs921720
rs6588243
rs28701841
rs6873866
rs151314883
rs147018773
rs744166
rs444210
rs80262450
All
−0.01
0.00
0.01
0.02
0.03
0.04
MR effect size for
'Crohn's disease' on 'DKTatlas rh parstriangularis area'

Batch 802 : Ulcerative colitis on DKTatlas rh parstriangularis area

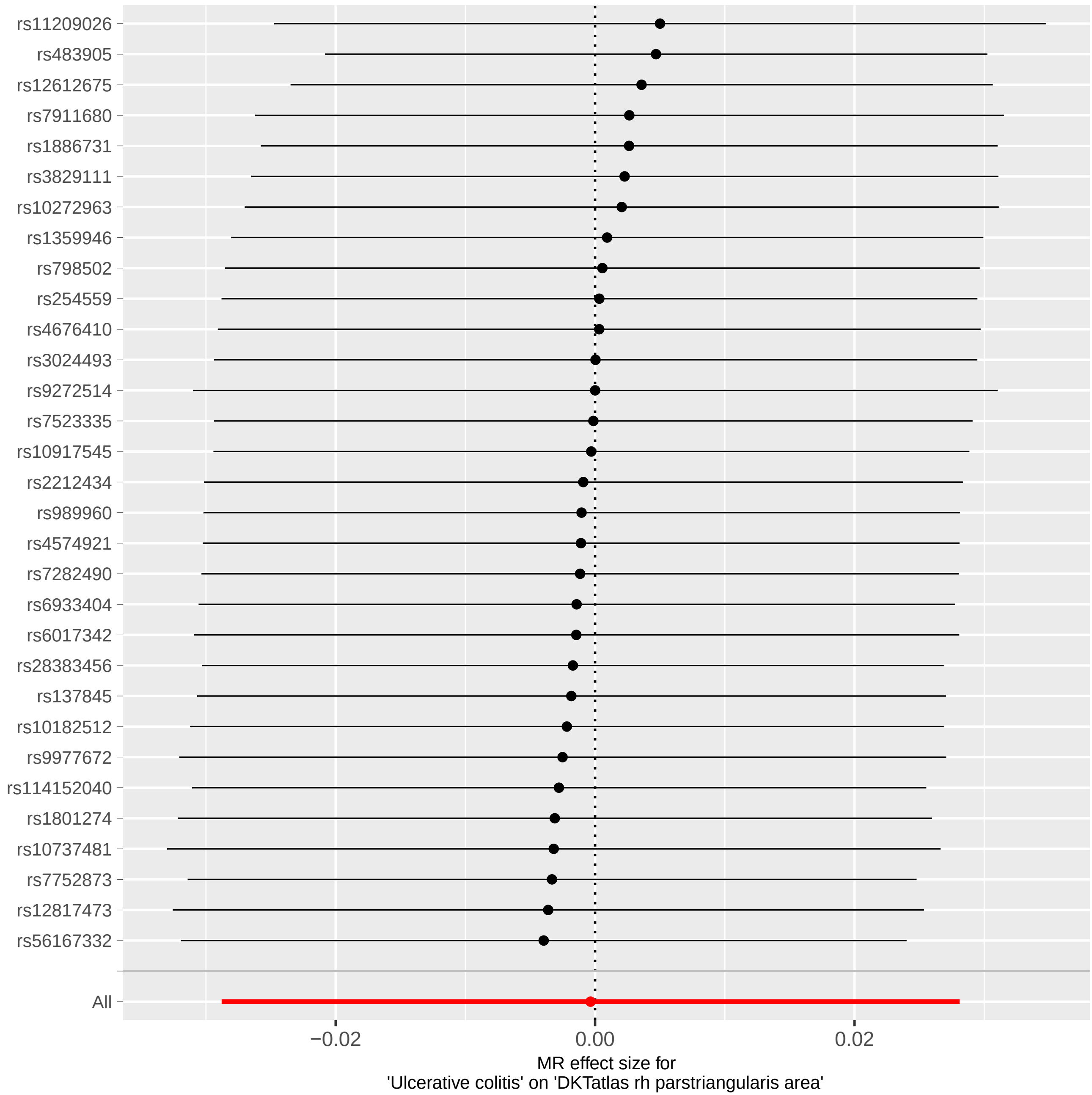

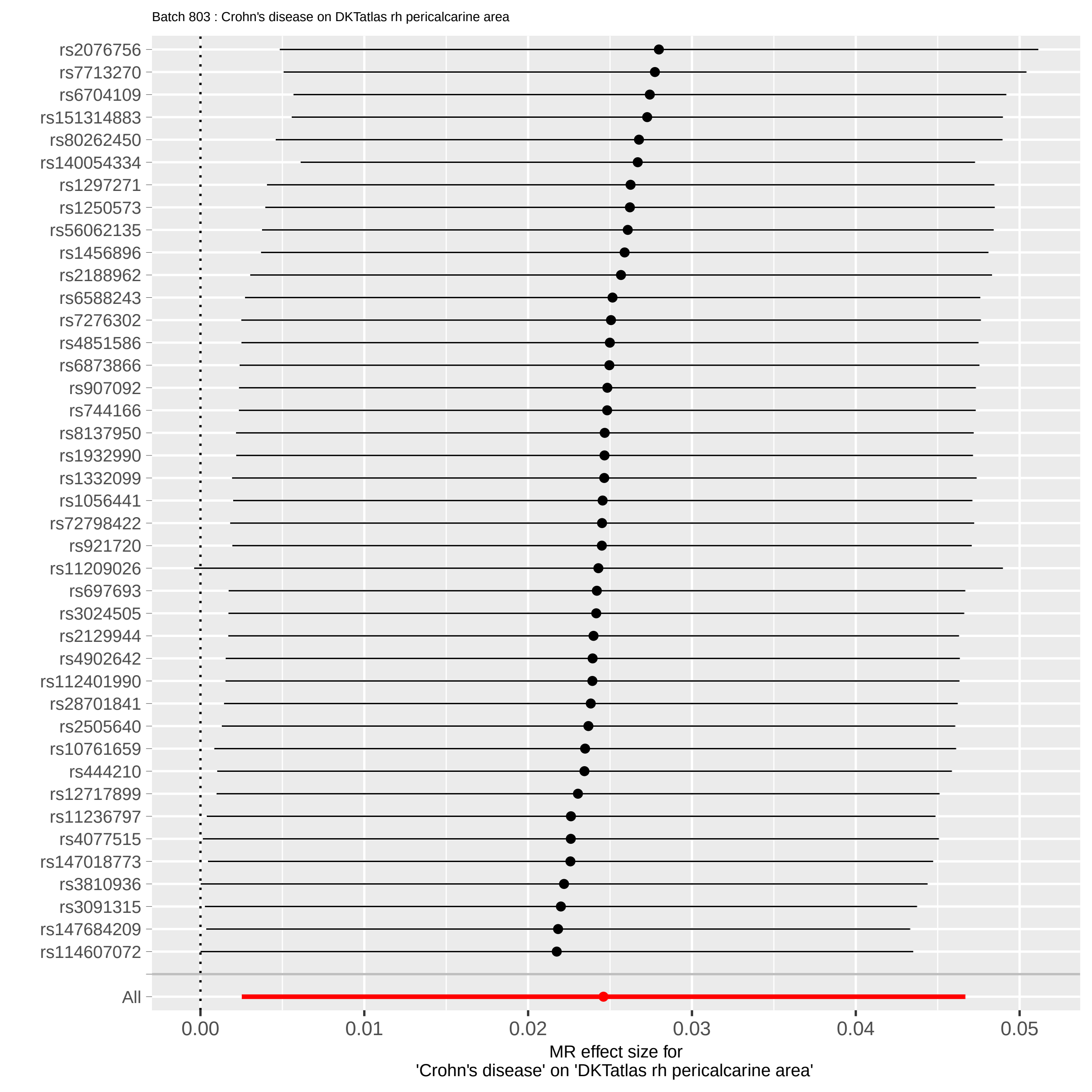

Batch 803 : Crohn's disease on DKTatlas rh pericalcarine area
rs2076756
rs7713270
rs6704109
rs151314883
rs80262450
rs140054334
rs1297271
rs1250573
rs56062135
rs1456896
rs2188962
rs6588243
rs7276302
rs4851586
rs6873866
rs907092
rs744166
rs8137950
rs1932990
rs1332099
rs1056441
rs72798422
rs921720
rs11209026
rs697693
rs3024505
rs2129944
rs4902642
rs112401990
rs28701841
rs2505640
rs10761659
rs444210
rs12717899
rs11236797
rs4077515
rs147018773
rs3810936
rs3091315
rs147684209
rs114607072
All
0.00
0.01
0.02
0.03
0.04
0.05
MR effect size for
'Crohn's disease' on 'DKTatlas rh pericalcarine area'

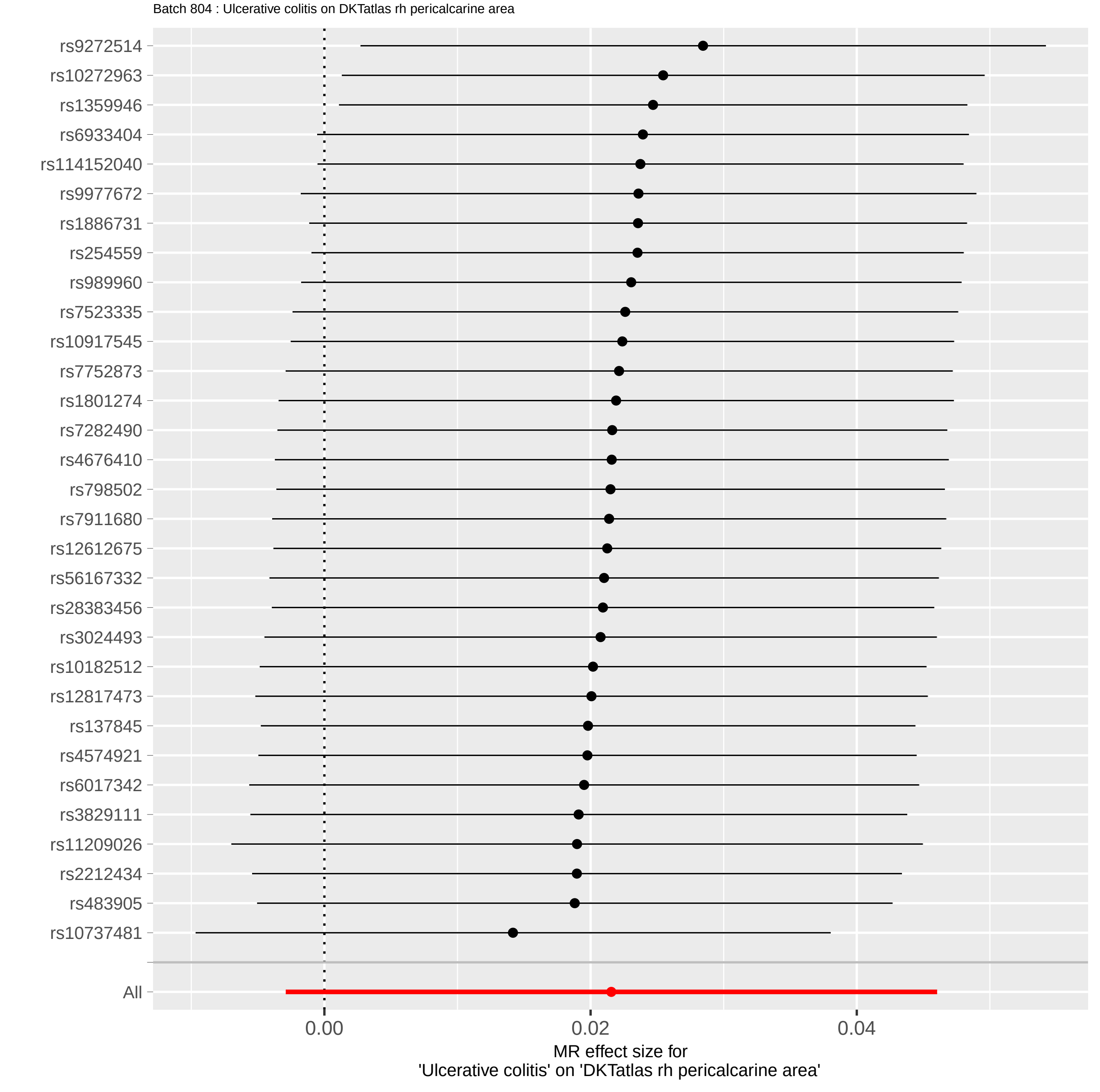

Batch 804 : Ulcerative colitis on DKTatlas rh pericalcarine area
rs9272514
rs10272963
rs1359946
rs6933404
rs114152040
rs9977672
rs1886731
rs254559
rs989960
rs7523335
rs10917545
rs7752873
rs1801274
rs7282490
rs4676410
rs798502
rs7911680
rs12612675
rs56167332
rs28383456
rs3024493
rs10182512
rs12817473
rs137845
rs4574921
rs6017342
rs3829111
rs11209026
rs2212434
rs483905
rs10737481
All
0.00
0.02
0.04
MR effect size for
'Ulcerative colitis' on 'DKTatlas rh pericalcarine area'

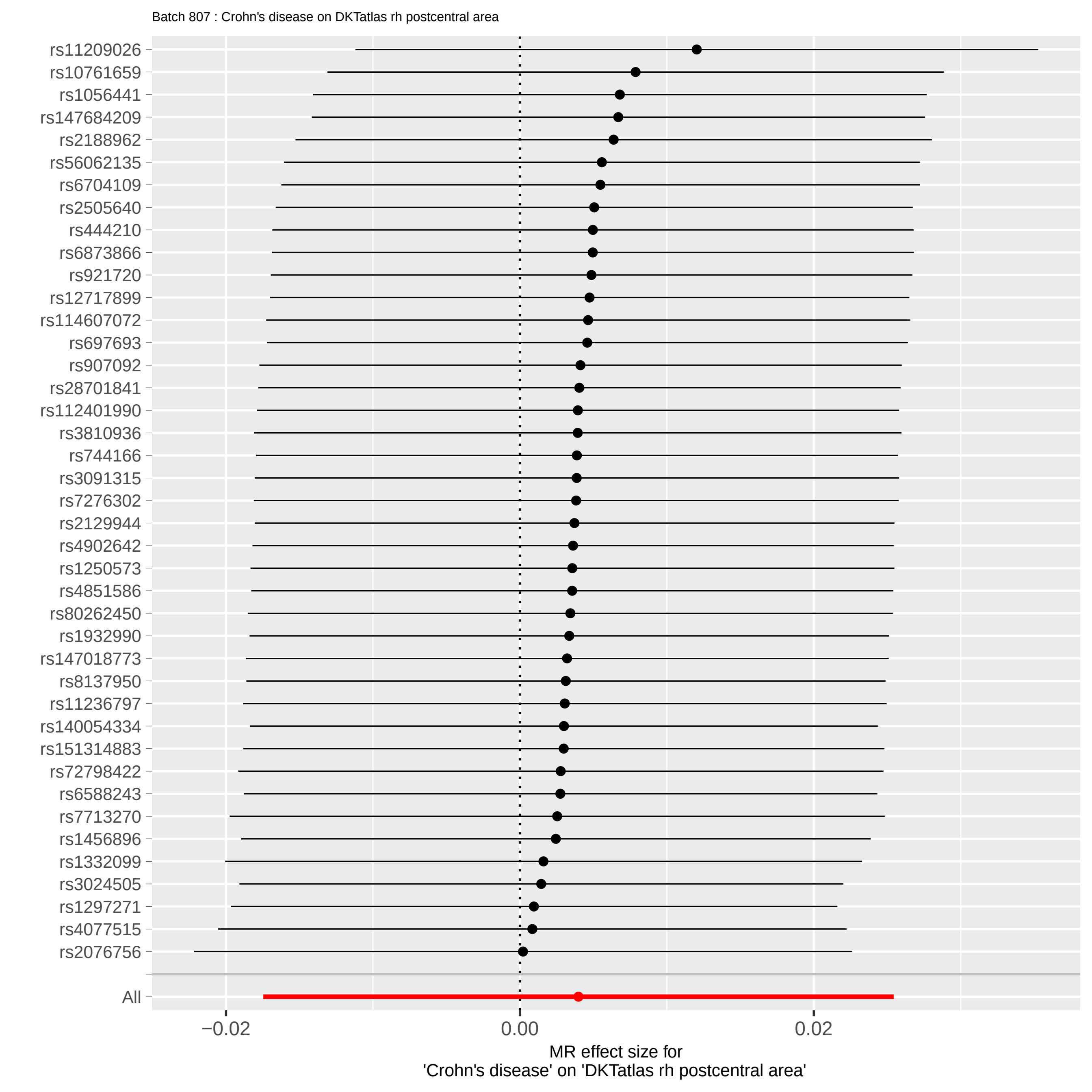

Batch 807 : Crohn's disease on DKTatlas rh postcentral area
rs11209026
rs10761659
rs1056441
rs147684209
rs2188962
rs56062135
rs6704109
rs2505640
rs444210
rs6873866
rs921720
rs12717899
rs114607072
rs697693
rs907092
rs28701841
rs112401990
rs3810936
rs744166
rs3091315
rs7276302
rs2129944
rs4902642
rs1250573
rs4851586
rs80262450
rs1932990
rs147018773
rs8137950
rs11236797
rs140054334
rs151314883
rs72798422
rs6588243
rs7713270
rs1456896
rs1332099
rs3024505
rs1297271
rs4077515
rs2076756
All
−0.02
0.00
0.02
MR effect size for
'Crohn's disease' on 'DKTatlas rh postcentral area'

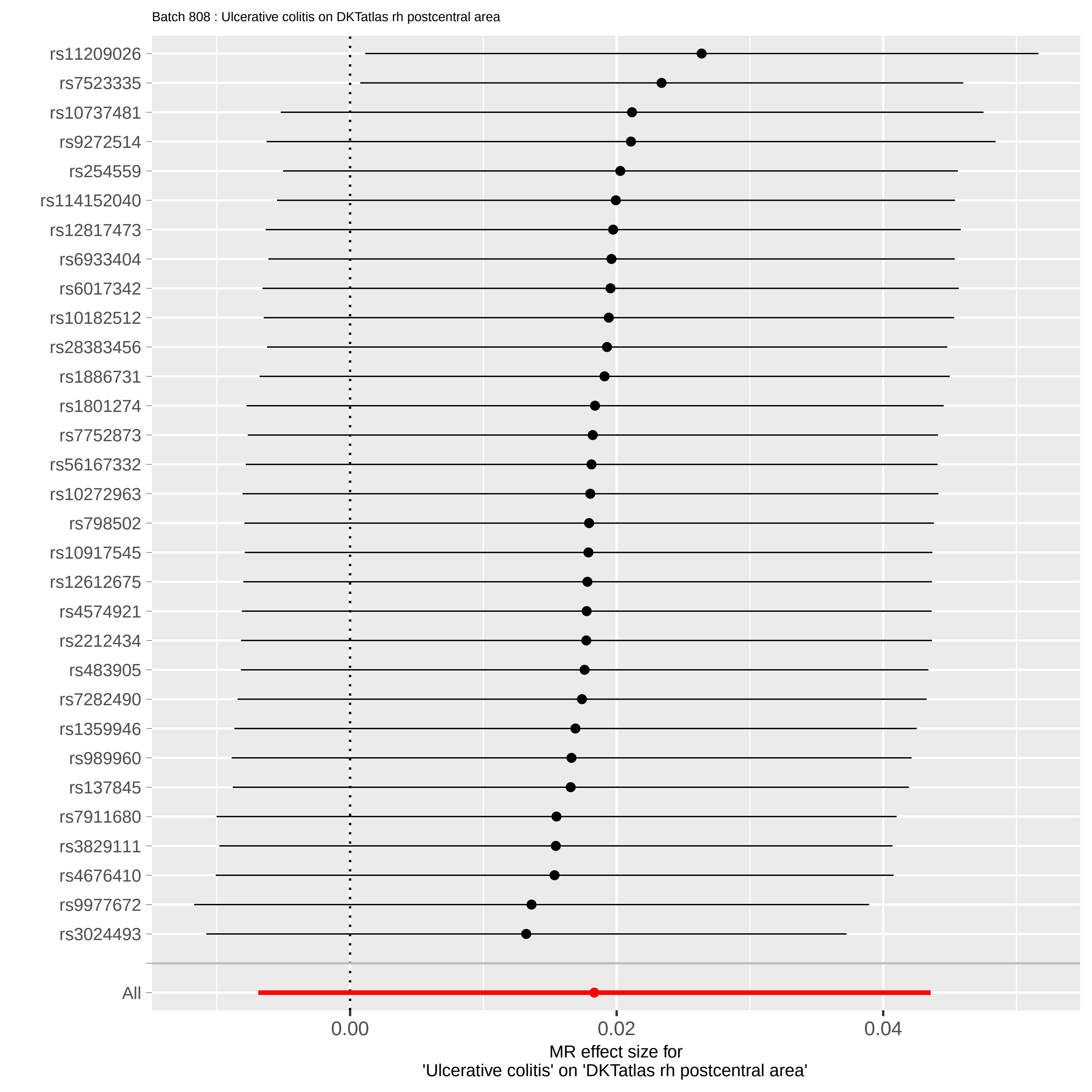

Batch 808 : Ulcerative colitis on DKTatlas rh postcentral area
rs11209026
rs7523335
rs10737481
rs9272514
rs254559
rs114152040
rs12817473
rs6933404
rs6017342
rs10182512
rs28383456
rs1886731
rs1801274
rs7752873
rs56167332
rs10272963
rs798502
rs10917545
rs12612675
rs4574921
rs2212434
rs483905
rs7282490
rs1359946
rs989960
rs137845
rs7911680
rs3829111
rs4676410
rs9977672
rs3024493
All
0.00
0.02
0.04
MR effect size for
'Ulcerative colitis' on 'DKTatlas rh postcentral area'

Batch 809 : Crohn's disease on DKTatlas rh posteriorcingulate area

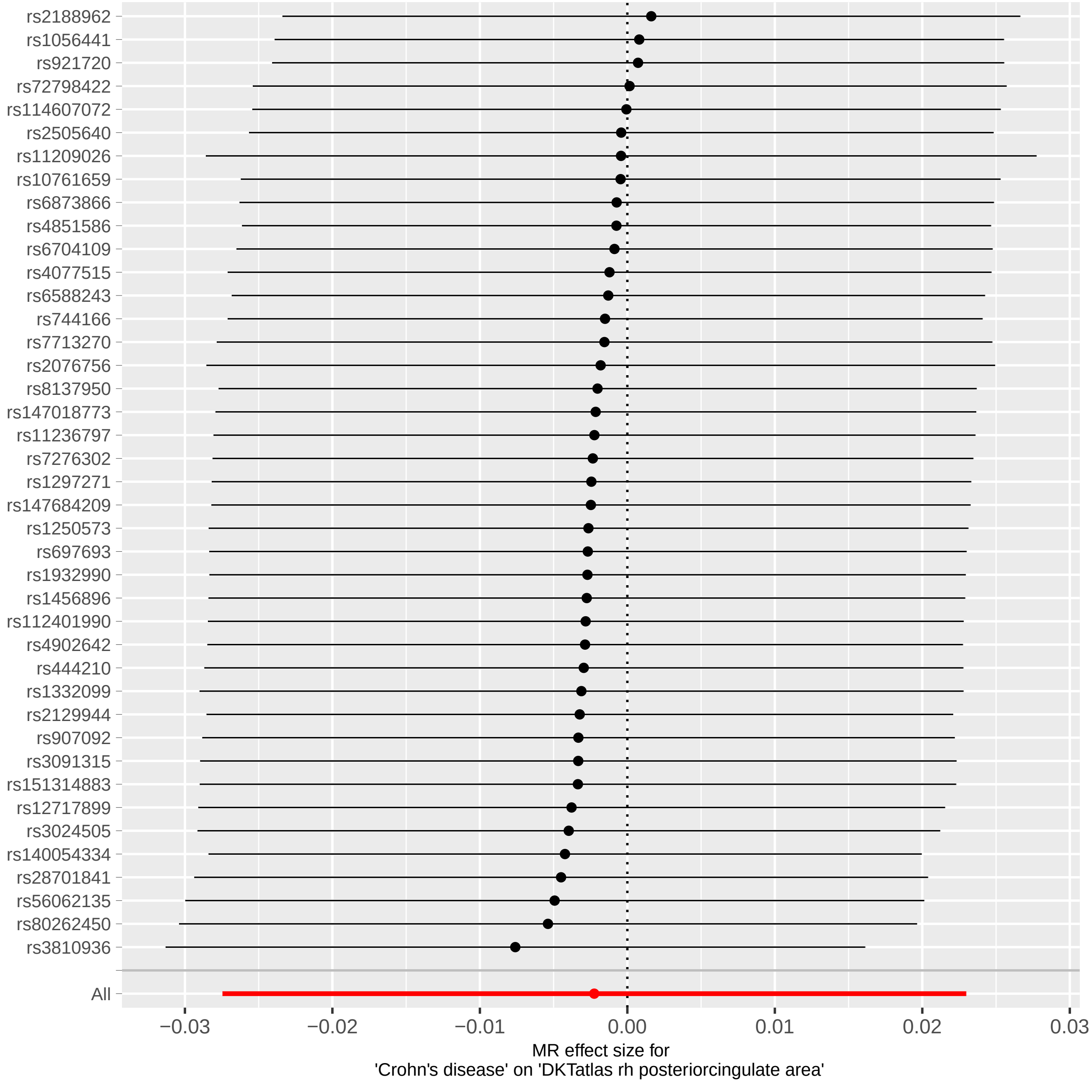

Batch 810 : Ulcerative colitis on DKTatlas rh posteriorcingulate area

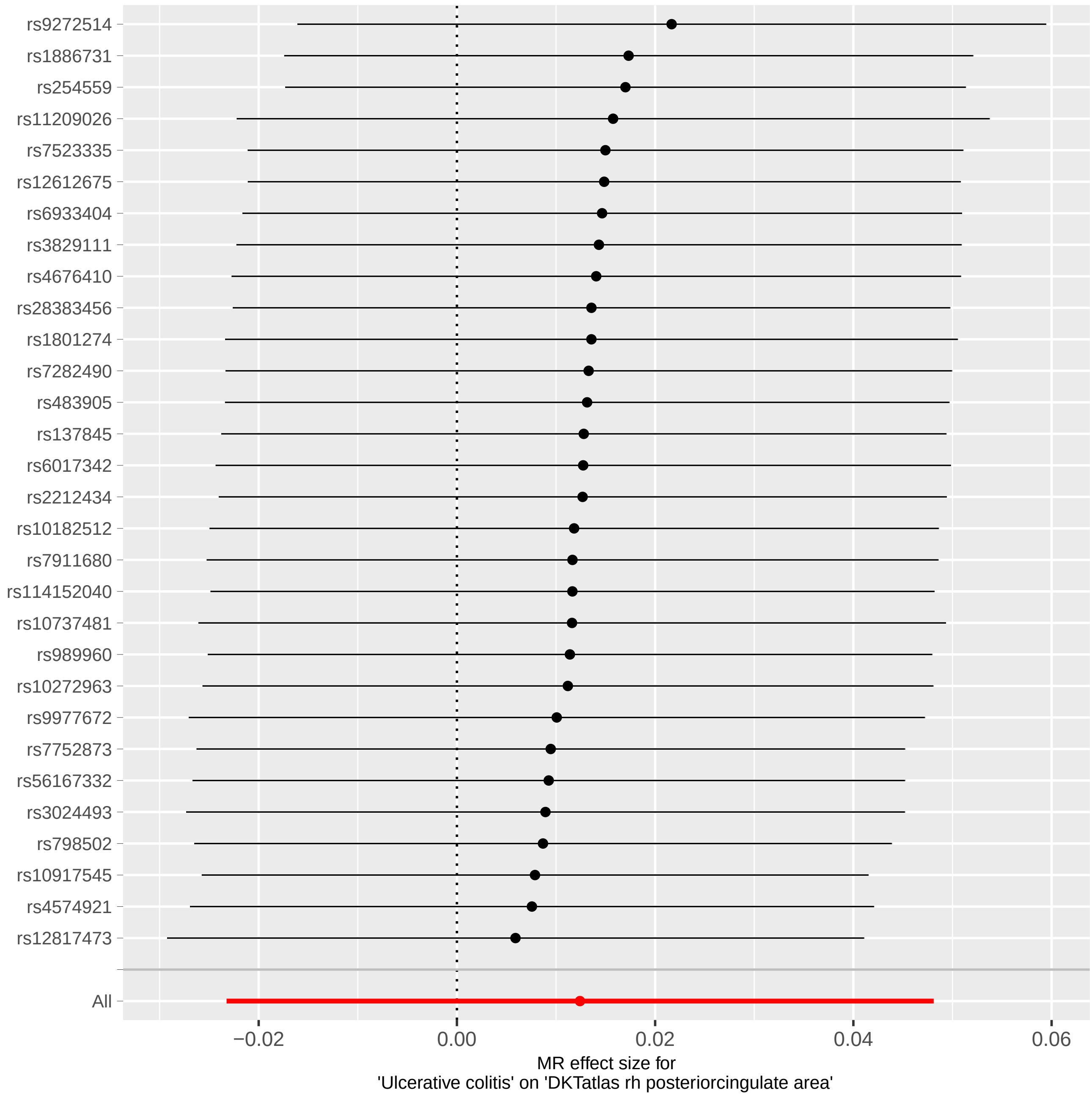

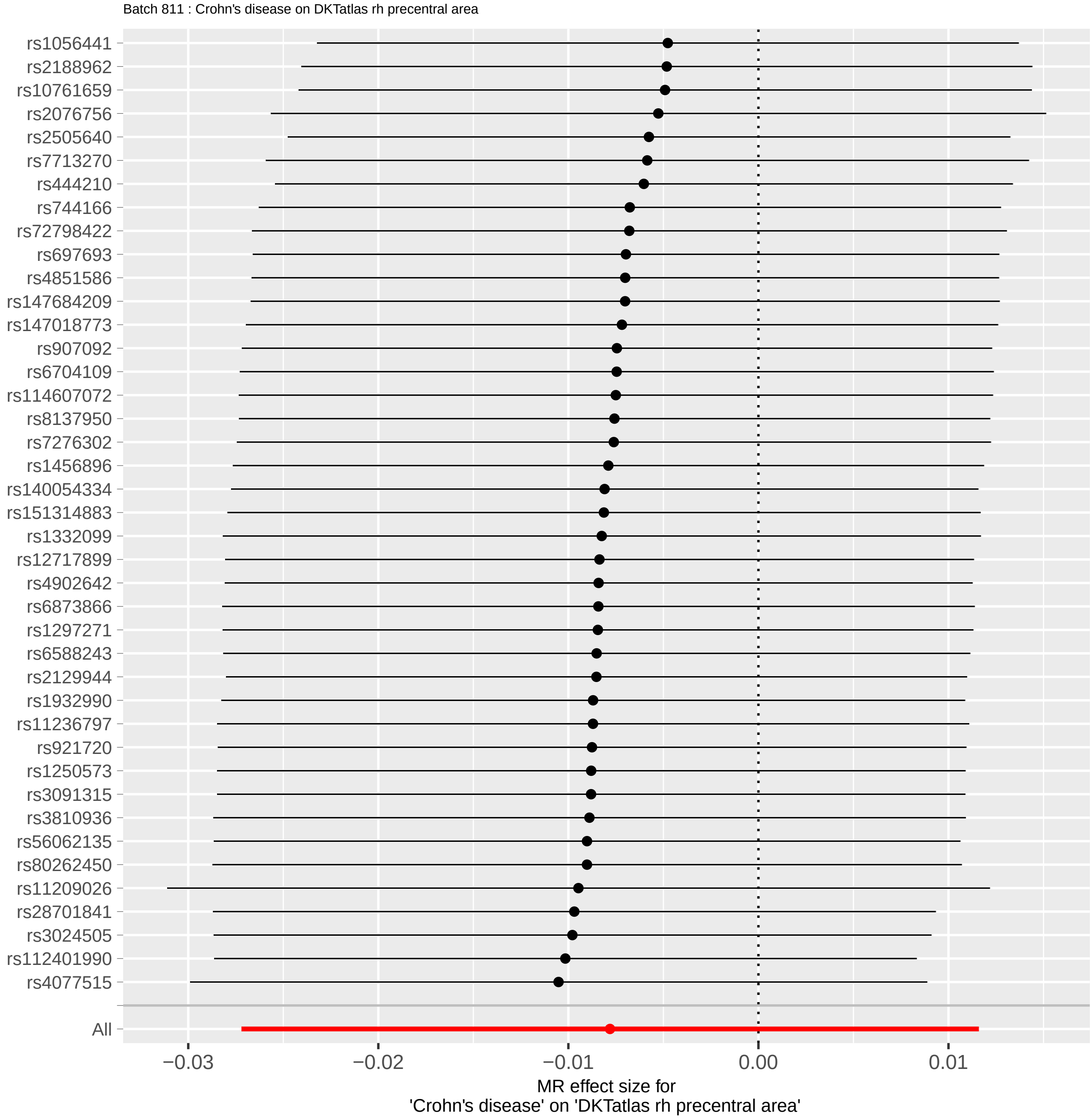

Batch 811 : Crohn's disease on DKTatlas rh precentral area
rs1056441
rs2188962
rs10761659
rs2076756
rs2505640
rs7713270
rs444210
rs744166
rs72798422
rs697693
rs4851586
rs147684209
rs147018773
rs907092
rs6704109
rs114607072
rs8137950
rs7276302
rs1456896
rs140054334
rs151314883
rs1332099
rs12717899
rs4902642
rs6873866
rs1297271
rs6588243
rs2129944
rs1932990
rs11236797
rs921720
rs1250573
rs3091315
rs3810936
rs56062135
rs80262450
rs11209026
rs28701841
rs3024505
rs112401990
rs4077515
All
−0.03
−0.02
−0.01
0.00
0.01
MR effect size for
'Crohn's disease' on 'DKTatlas rh precentral area'

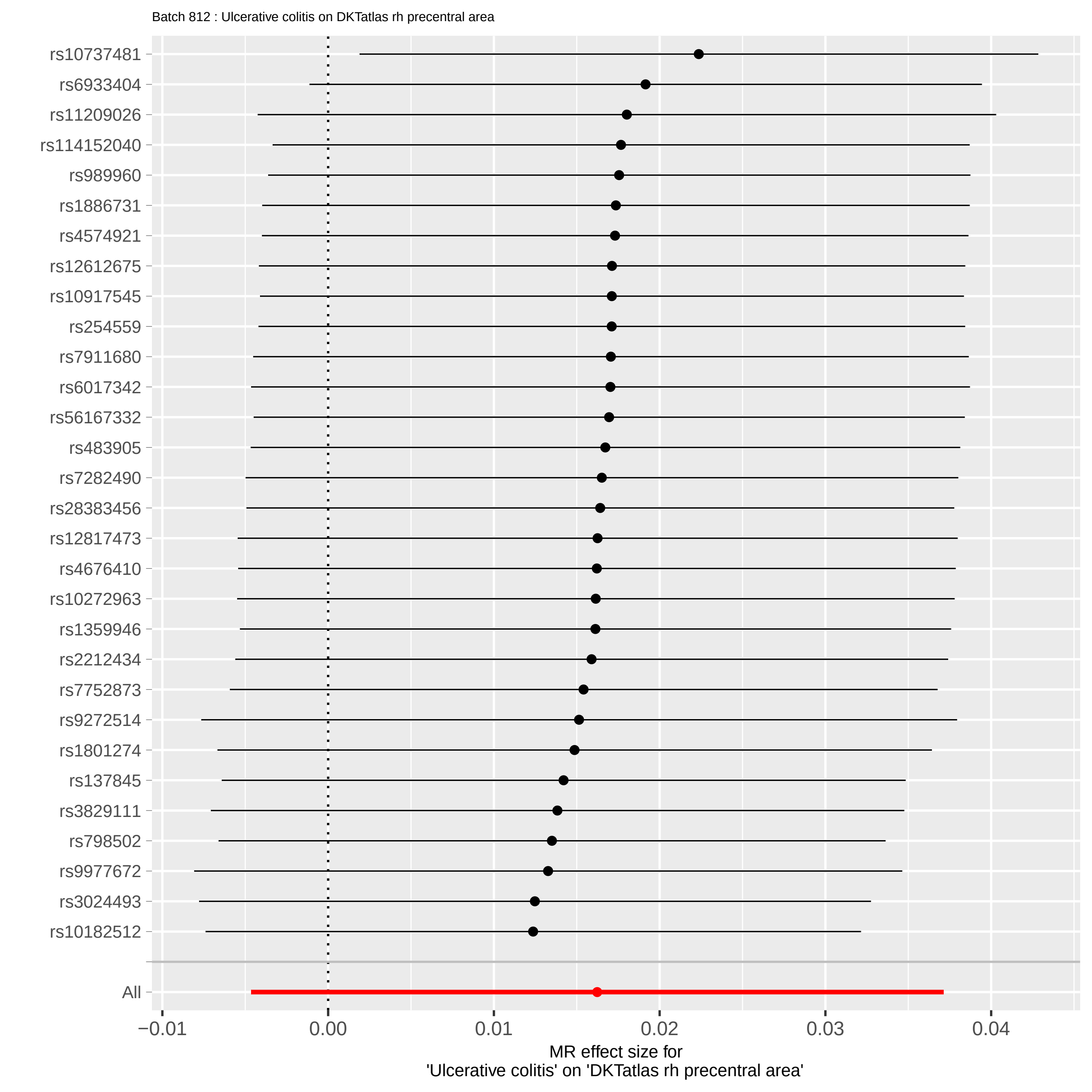

Batch 812 : Ulcerative colitis on DKTatlas rh precentral area
rs10737481
rs6933404
rs11209026
rs114152040
rs989960
rs1886731
rs4574921
rs12612675
rs10917545
rs254559
rs7911680
rs6017342
rs56167332
rs483905
rs7282490
rs28383456
rs12817473
rs4676410
rs10272963
rs1359946
rs2212434
rs7752873
rs9272514
rs1801274
rs137845
rs3829111
rs798502
rs9977672
rs3024493
rs10182512
All
−0.01
0.00
0.01
0.02
0.03
0.04
MR effect size for
'Ulcerative colitis' on 'DKTatlas rh precentral area'

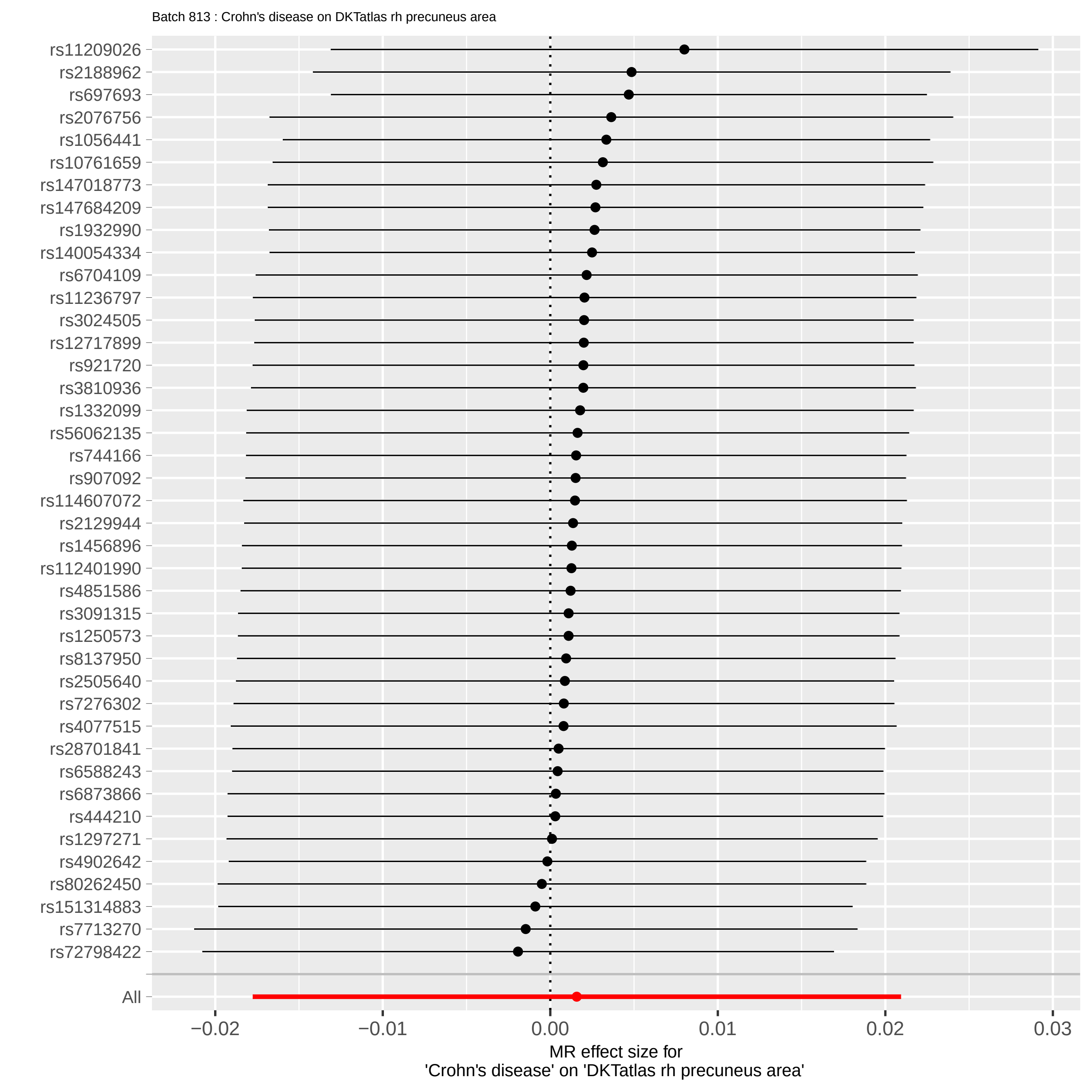

Batch 813 : Crohn's disease on DKTatlas rh precuneus area
rs11209026
rs2188962
rs697693
rs2076756
rs1056441
rs10761659
rs147018773
rs147684209
rs1932990
rs140054334
rs6704109
rs11236797
rs3024505
rs12717899
rs921720
rs3810936
rs1332099
rs56062135
rs744166
rs907092
rs114607072
rs2129944
rs1456896
rs112401990
rs4851586
rs3091315
rs1250573
rs8137950
rs2505640
rs7276302
rs4077515
rs28701841
rs6588243
rs6873866
rs444210
rs1297271
rs4902642
rs80262450
rs151314883
rs7713270
rs72798422
All
−0.02
−0.01
0.00
0.01
0.02
0.03
MR effect size for
'Crohn's disease' on 'DKTatlas rh precuneus area'

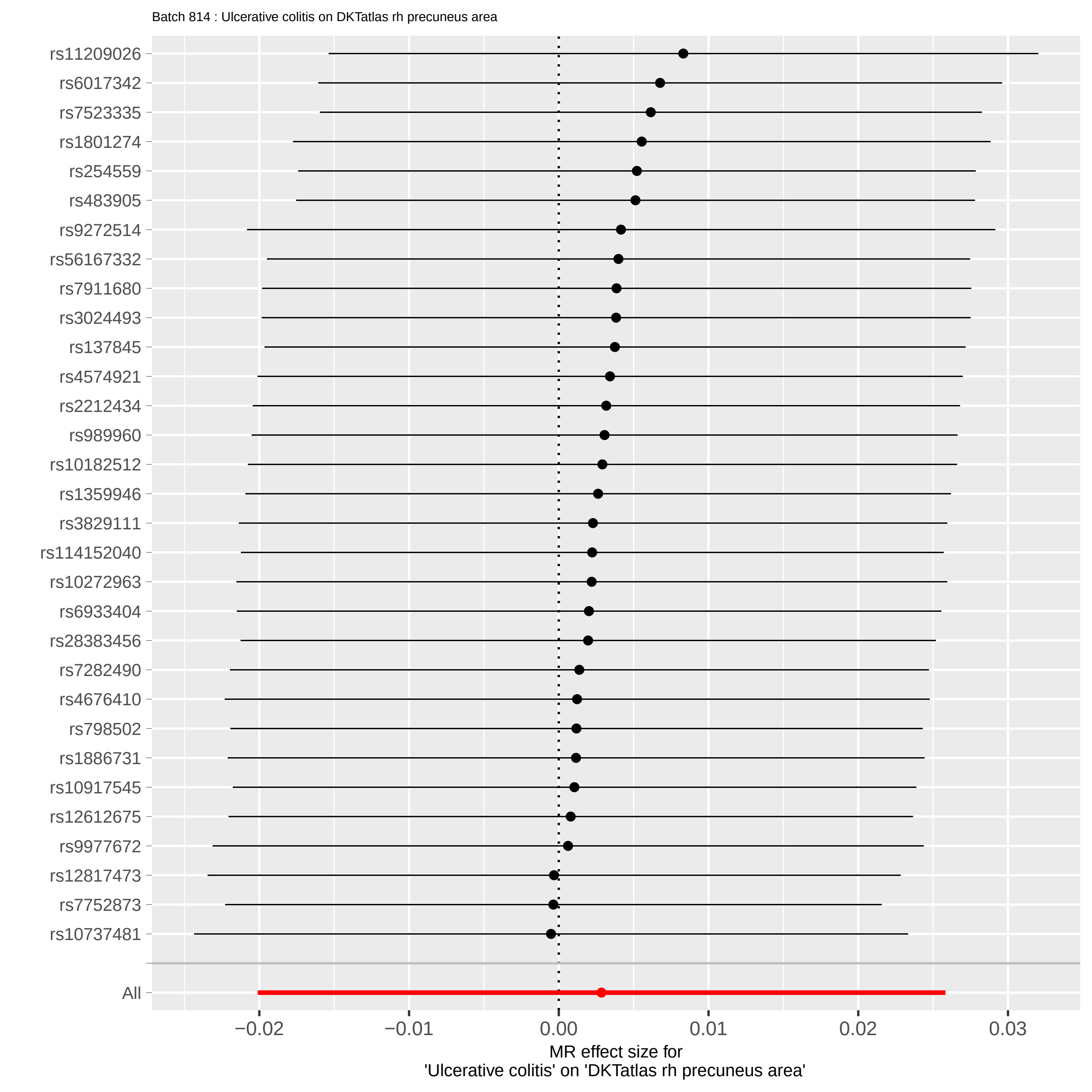

Batch 814 : Ulcerative colitis on DKTatlas rh precuneus area
rs11209026
rs6017342
rs7523335
rs1801274
rs254559
rs483905
rs9272514
rs56167332
rs7911680
rs3024493
rs137845
rs4574921
rs2212434
rs989960
rs10182512
rs1359946
rs3829111
rs114152040
rs10272963
rs6933404
rs28383456
rs7282490
rs4676410
rs798502
rs1886731
rs10917545
rs12612675
rs9977672
rs12817473
rs7752873
rs10737481
All
−0.02
−0.01
0.00
0.01
0.02
0.03
MR effect size for
'Ulcerative colitis' on 'DKTatlas rh precuneus area'

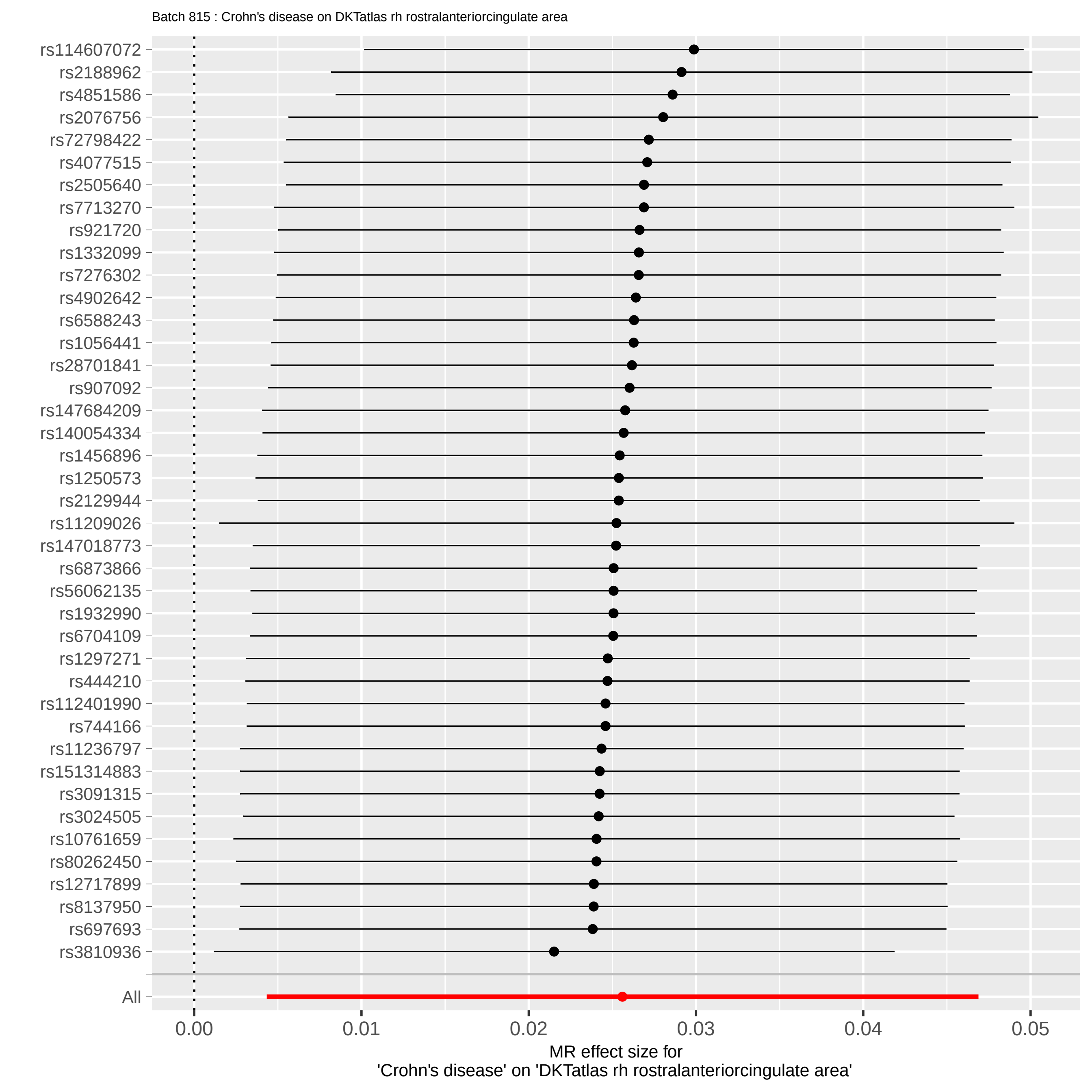

Batch 815 : Crohn's disease on DKTatlas rh rostralanteriorcingulate area
rs114607072
rs2188962
rs4851586
rs2076756
rs72798422
rs4077515
rs2505640
rs7713270
rs921720
rs1332099
rs7276302
rs4902642
rs6588243
rs1056441
rs28701841
rs907092
rs147684209
rs140054334
rs1456896
rs1250573
rs2129944
rs11209026
rs147018773
rs6873866
rs56062135
rs1932990
rs6704109
rs1297271
rs444210
rs112401990
rs744166
rs11236797
rs151314883
rs3091315
rs3024505
rs10761659
rs80262450
rs12717899
rs8137950
rs697693
rs3810936
All
0.00
0.01
0.02
0.03
0.04
0.05
MR effect size for
'Crohn's disease' on 'DKTatlas rh rostralanteriorcingulate area'

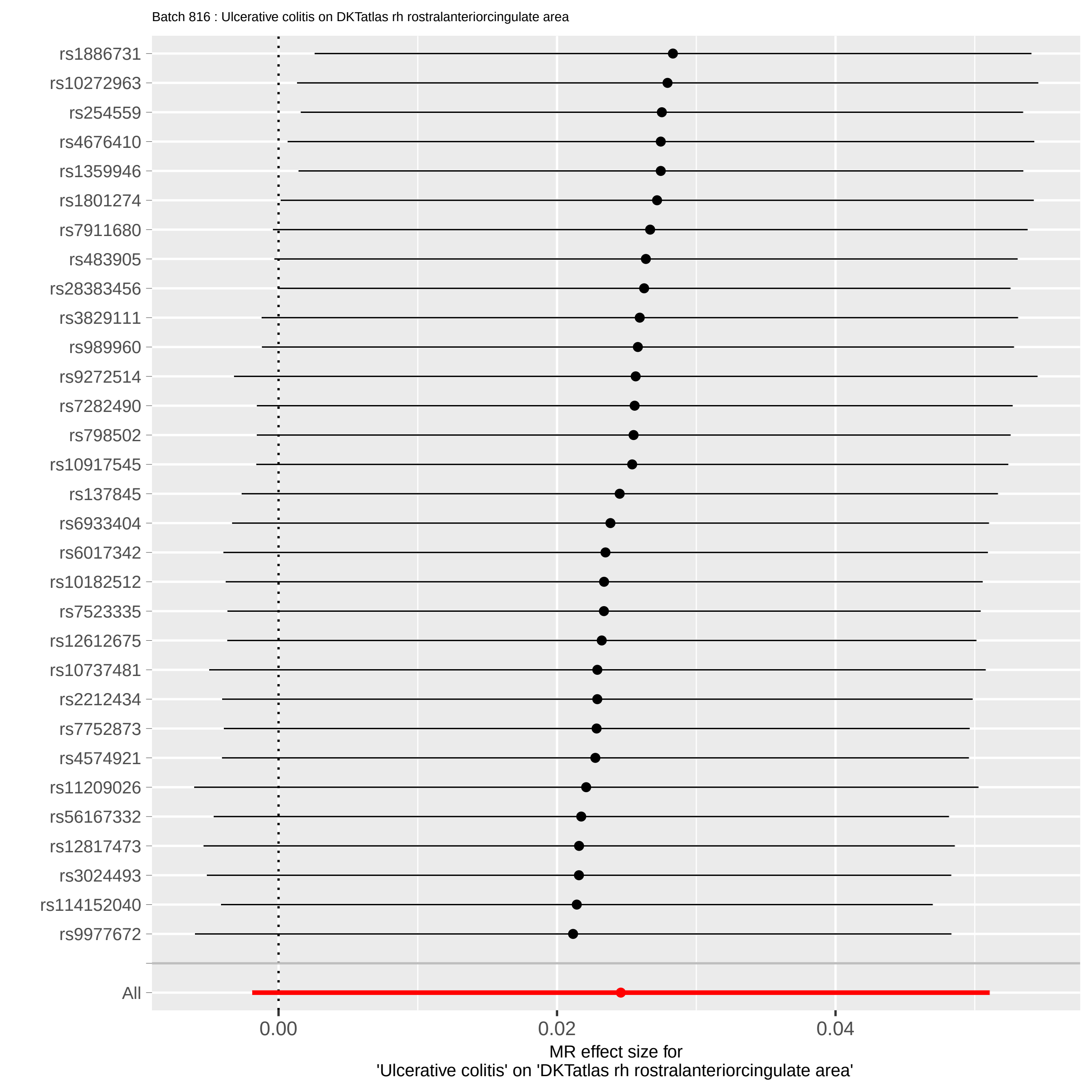
Batch 816 : Ulcerative colitis on DKTatlas rh rostralanteriorcingulate area
rs1886731
rs10272963
rs254559
rs4676410
rs1359946
rs1801274
rs7911680
rs483905
rs28383456
rs3829111
rs989960
rs9272514
rs7282490
rs798502
rs10917545
rs137845
rs6933404
rs6017342
rs10182512
rs7523335
rs12612675
rs10737481
rs2212434
rs7752873
rs4574921
rs11209026
rs56167332
rs12817473
rs3024493
rs114152040
rs9977672
All
0.00
0.02
0.04
MR effect size for
'Ulcerative colitis' on 'DKTatlas rh rostralanteriorcingulate area'

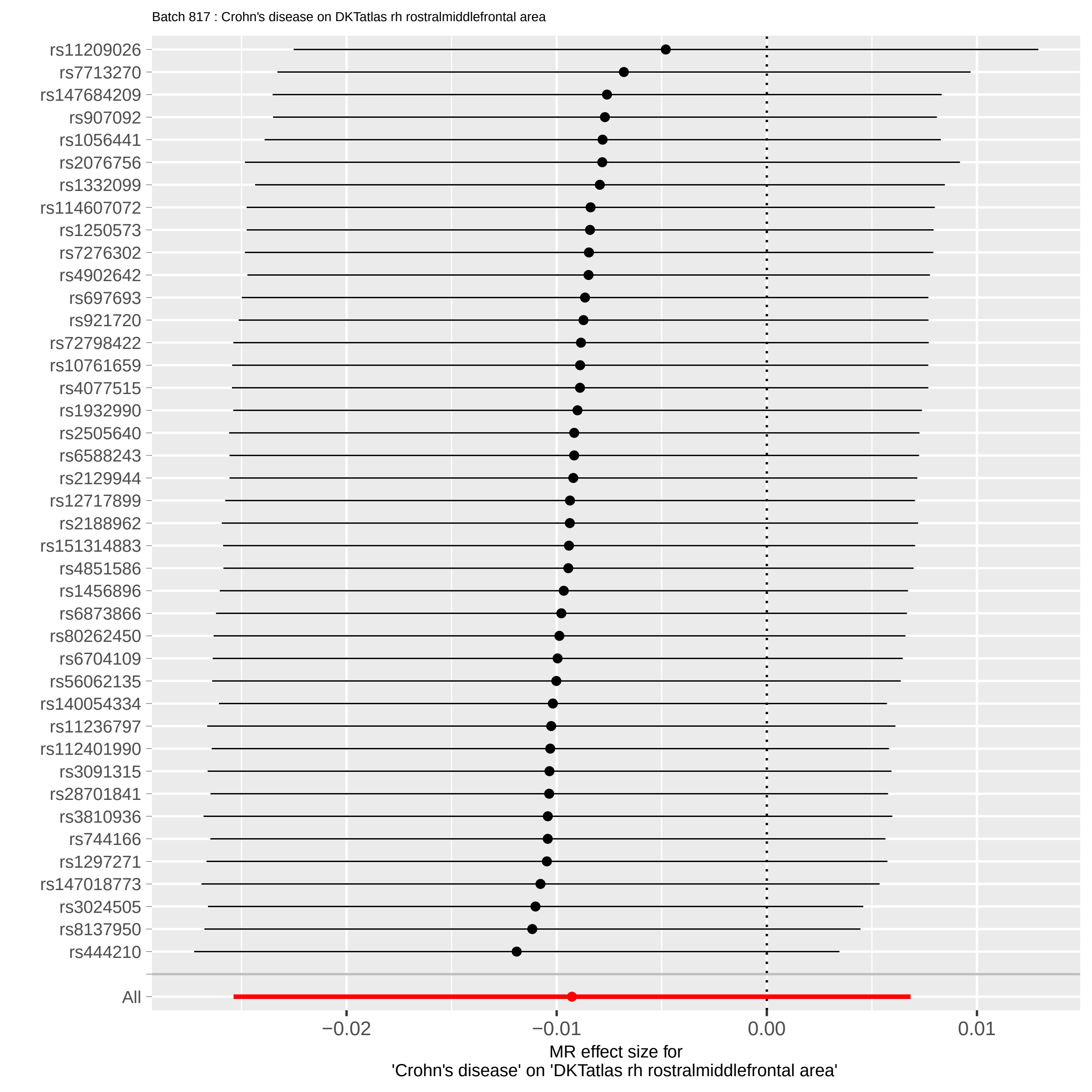

Batch 817 : Crohn's disease on DKTatlas rh rostralmiddlefrontal area
rs11209026
rs7713270
rs147684209
rs907092
rs1056441
rs2076756
rs1332099
rs114607072
rs1250573
rs7276302
rs4902642
rs697693
rs921720
rs72798422
rs10761659
rs4077515
rs1932990
rs2505640
rs6588243
rs2129944
rs12717899
rs2188962
rs151314883
rs4851586
rs1456896
rs6873866
rs80262450
rs6704109
rs56062135
rs140054334
rs11236797
rs112401990
rs3091315
rs28701841
rs3810936
rs744166
rs1297271
rs147018773
rs3024505
rs8137950
rs444210
All
−0.02
−0.01
0.00
0.01
MR effect size for
'Crohn's disease' on 'DKTatlas rh rostralmiddlefrontal area'

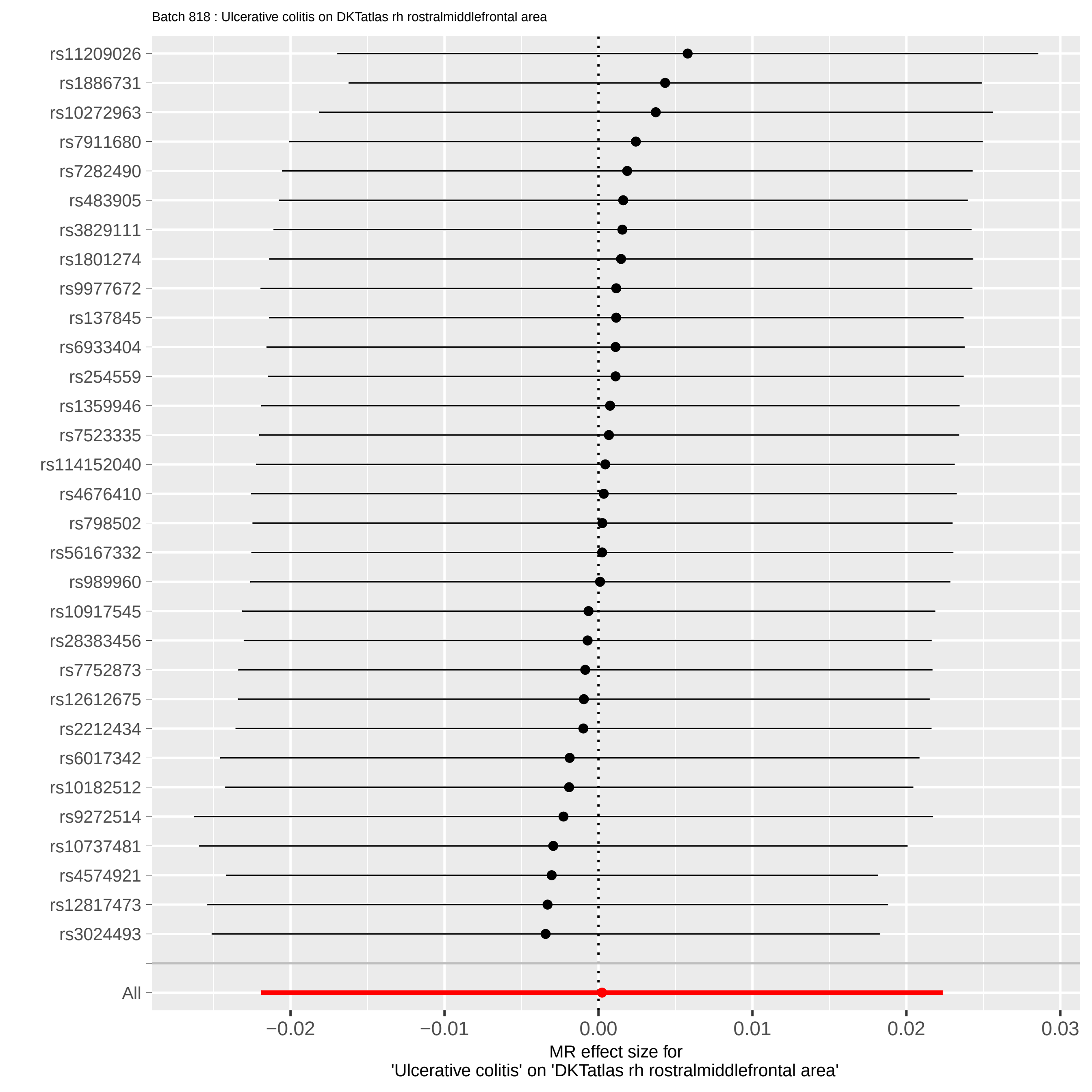

Batch 818 : Ulcerative colitis on DKTatlas rh rostralmiddlefrontal area
rs11209026
rs1886731
rs10272963
rs7911680
rs7282490
rs483905
rs3829111
rs1801274
rs9977672
rs137845
rs6933404
rs254559
rs1359946
rs7523335
rs114152040
rs4676410
rs798502
rs56167332
rs989960
rs10917545
rs28383456
rs7752873
rs12612675
rs2212434
rs6017342
rs10182512
rs9272514
rs10737481
rs4574921
rs12817473
rs3024493
All
−0.02
−0.01
0.00
0.01
0.02
0.03
MR effect size for
'Ulcerative colitis' on 'DKTatlas rh rostralmiddlefrontal area'

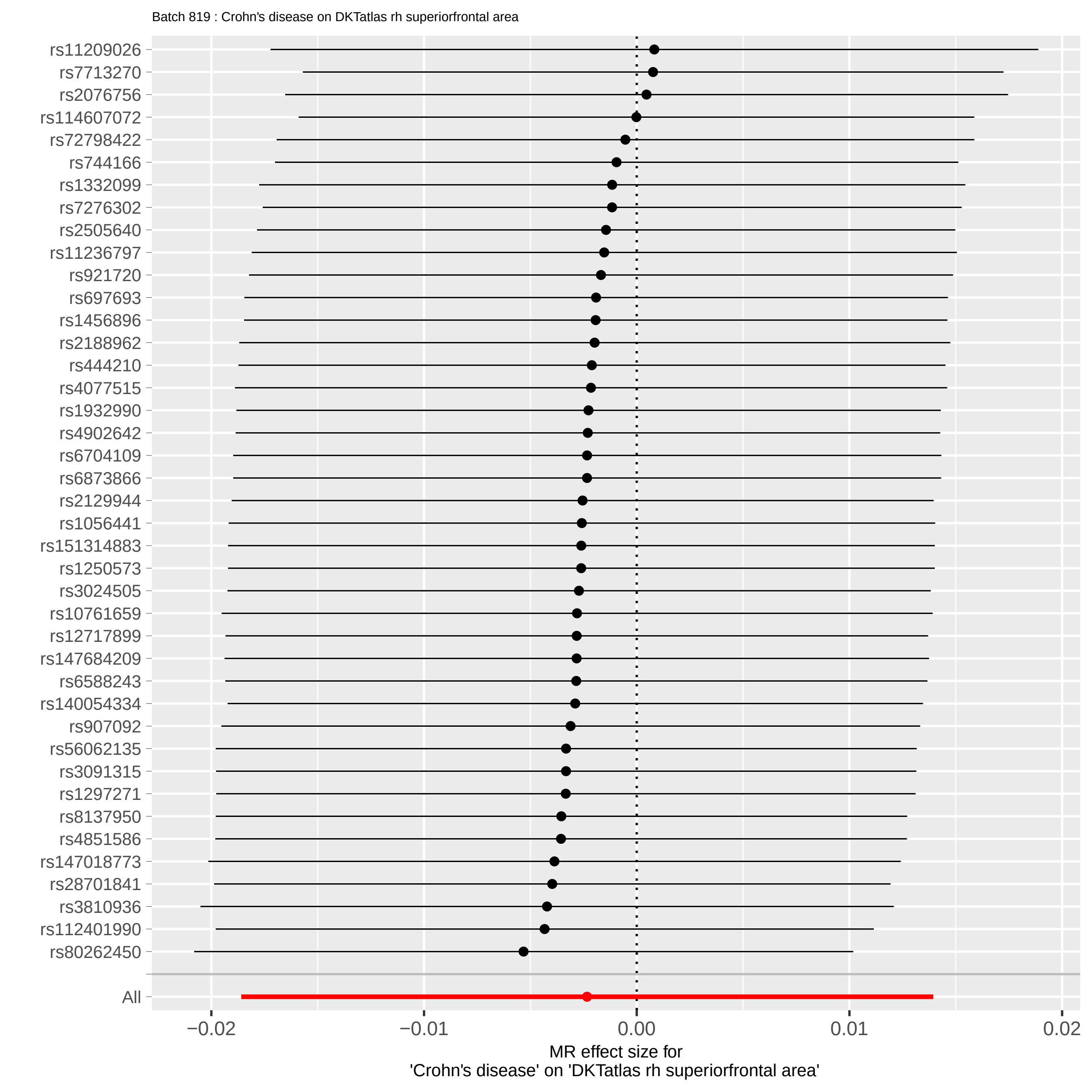
Batch 819 : Crohn's disease on DKTatlas rh superiorfrontal area
rs11209026
rs7713270
rs2076756
rs114607072
rs72798422
rs744166
rs1332099
rs7276302
rs2505640
rs11236797
rs921720
rs697693
rs1456896
rs2188962
rs444210
rs4077515
rs1932990
rs4902642
rs6704109
rs6873866
rs2129944
rs1056441
rs151314883
rs1250573
rs3024505
rs10761659
rs12717899
rs147684209
rs6588243
rs140054334
rs907092
rs56062135
rs3091315
rs1297271
rs8137950
rs4851586
rs147018773
rs28701841
rs3810936
rs112401990
rs80262450
All
−0.02
−0.01
0.00
0.01
0.02
MR effect size for
'Crohn's disease' on 'DKTatlas rh superiorfrontal area'

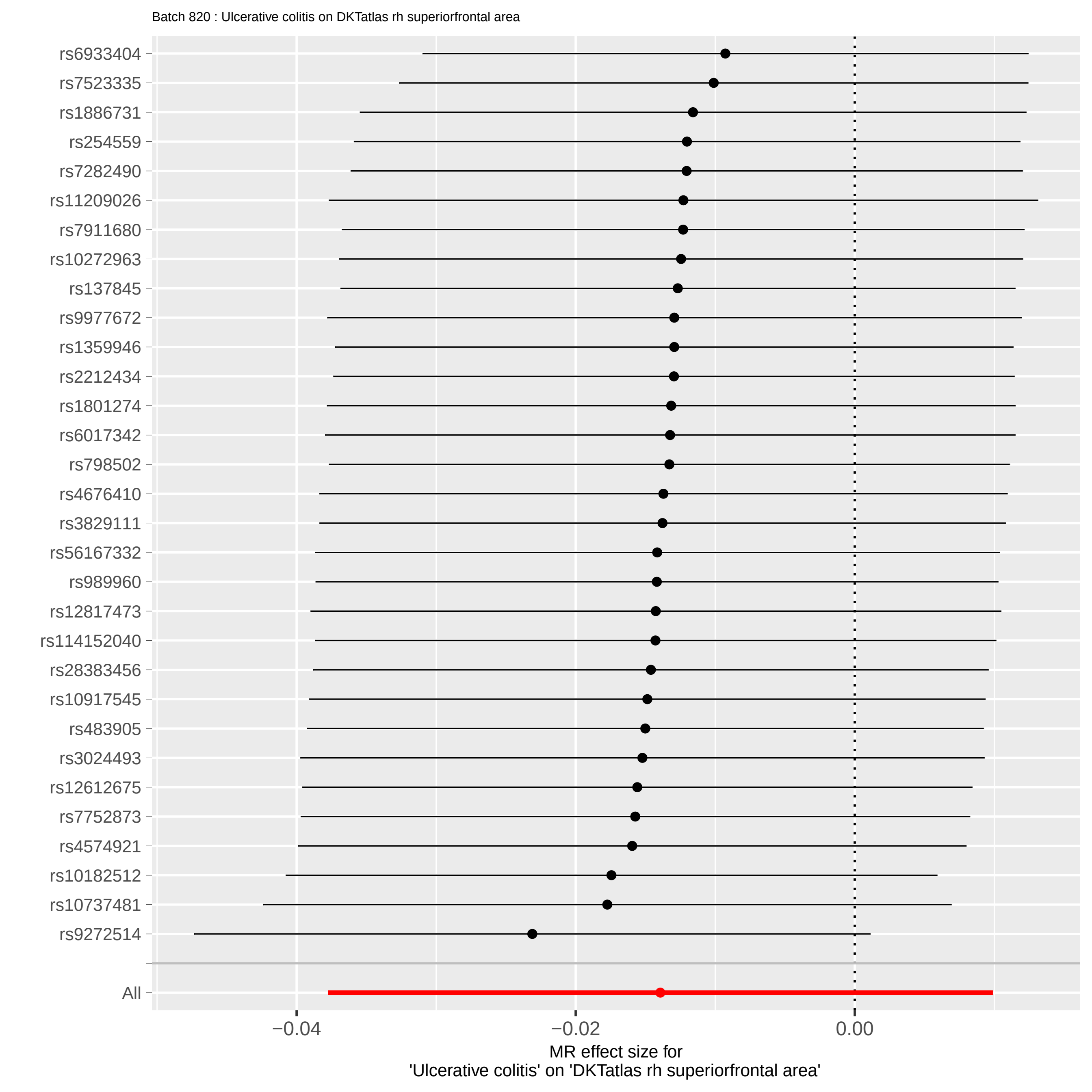

Batch 820 : Ulcerative colitis on DKTatlas rh superiorfrontal area
rs6933404
rs7523335
rs1886731
rs254559
rs7282490
rs11209026
rs7911680
rs10272963
rs137845
rs9977672
rs1359946
rs2212434
rs1801274
rs6017342
rs798502
rs4676410
rs3829111
rs56167332
rs989960
rs12817473
rs114152040
rs28383456
rs10917545
rs483905
rs3024493
rs12612675
rs7752873
rs4574921
rs10182512
rs10737481
rs9272514
All
−0.04
−0.02
0.00
MR effect size for
'Ulcerative colitis' on 'DKTatlas rh superiorfrontal area'

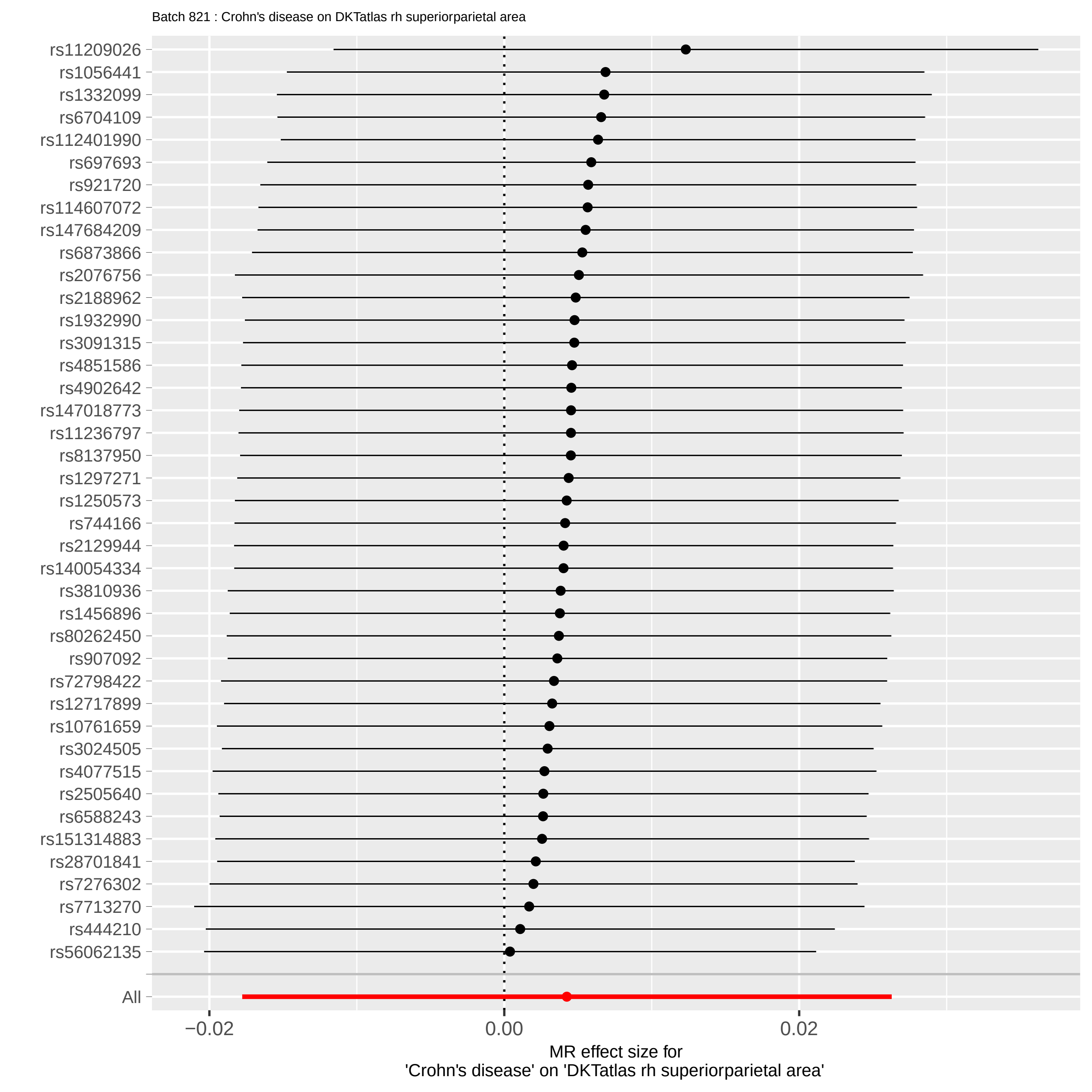

Batch 821 : Crohn's disease on DKTatlas rh superiorparietal area
rs11209026
rs1056441
rs1332099
rs6704109
rs112401990
rs697693
rs921720
rs114607072
rs147684209
rs6873866
rs2076756
rs2188962
rs1932990
rs3091315
rs4851586
rs4902642
rs147018773
rs11236797
rs8137950
rs1297271
rs1250573
rs744166
rs2129944
rs140054334
rs3810936
rs1456896
rs80262450
rs907092
rs72798422
rs12717899
rs10761659
rs3024505
rs4077515
rs2505640
rs6588243
rs151314883
rs28701841
rs7276302
rs7713270
rs444210
rs56062135
All
−0.02
0.00
0.02
MR effect size for
'Crohn's disease' on 'DKTatlas rh superiorparietal area'

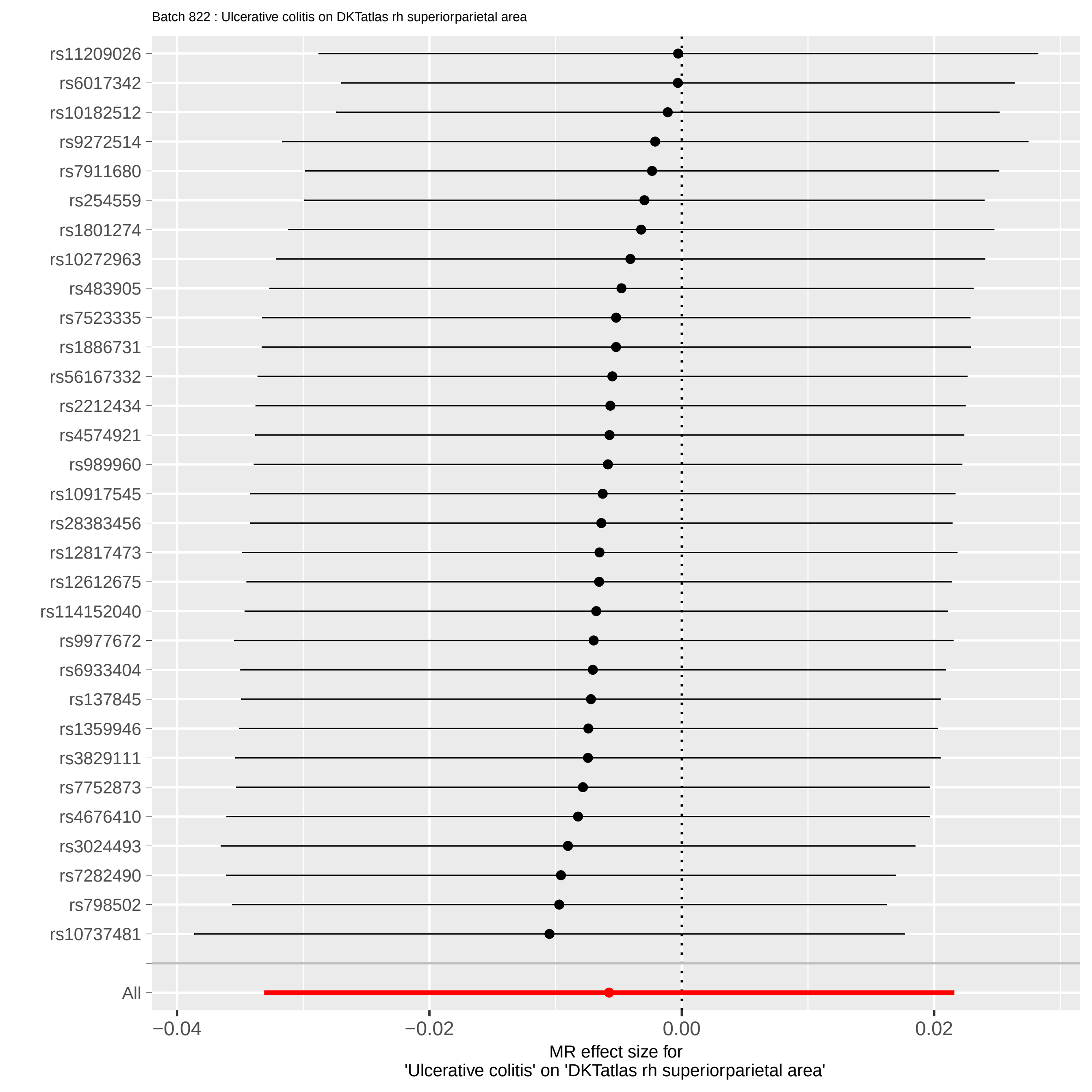
Batch 822 : Ulcerative colitis on DKTatlas rh superiorparietal area
rs11209026
rs6017342
rs10182512
rs9272514
rs7911680
rs254559
rs1801274
rs10272963
rs483905
rs7523335
rs1886731
rs56167332
rs2212434
rs4574921
rs989960
rs10917545
rs28383456
rs12817473
rs12612675
rs114152040
rs9977672
rs6933404
rs137845
rs1359946
rs3829111
rs7752873
rs4676410
rs3024493
rs7282490
rs798502
rs10737481
All
−0.04
−0.02
0.00
0.02
MR effect size for
'Ulcerative colitis' on 'DKTatlas rh superiorparietal area'

Batch 823 : Crohn's disease on DKTatlas rh superiortemporal area

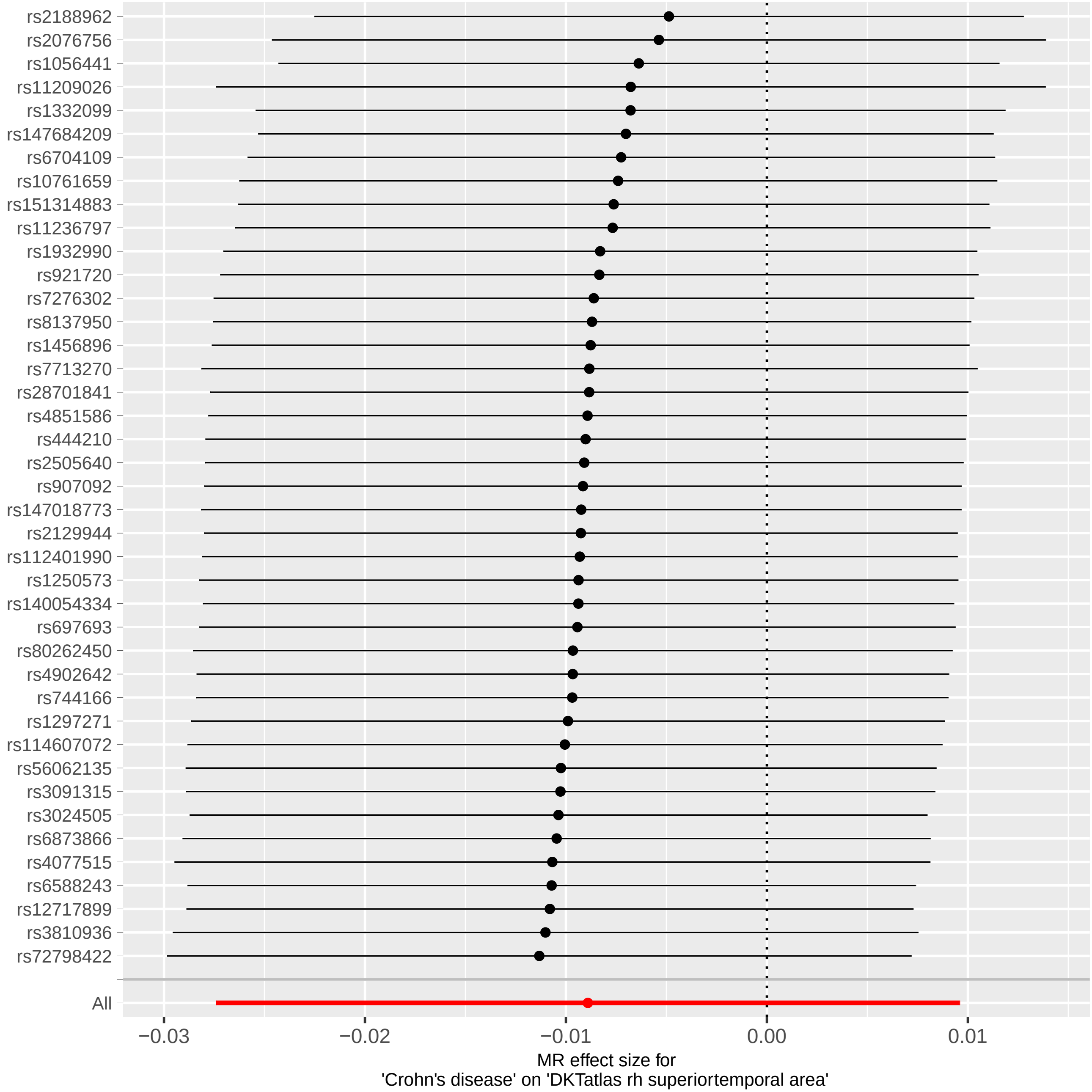

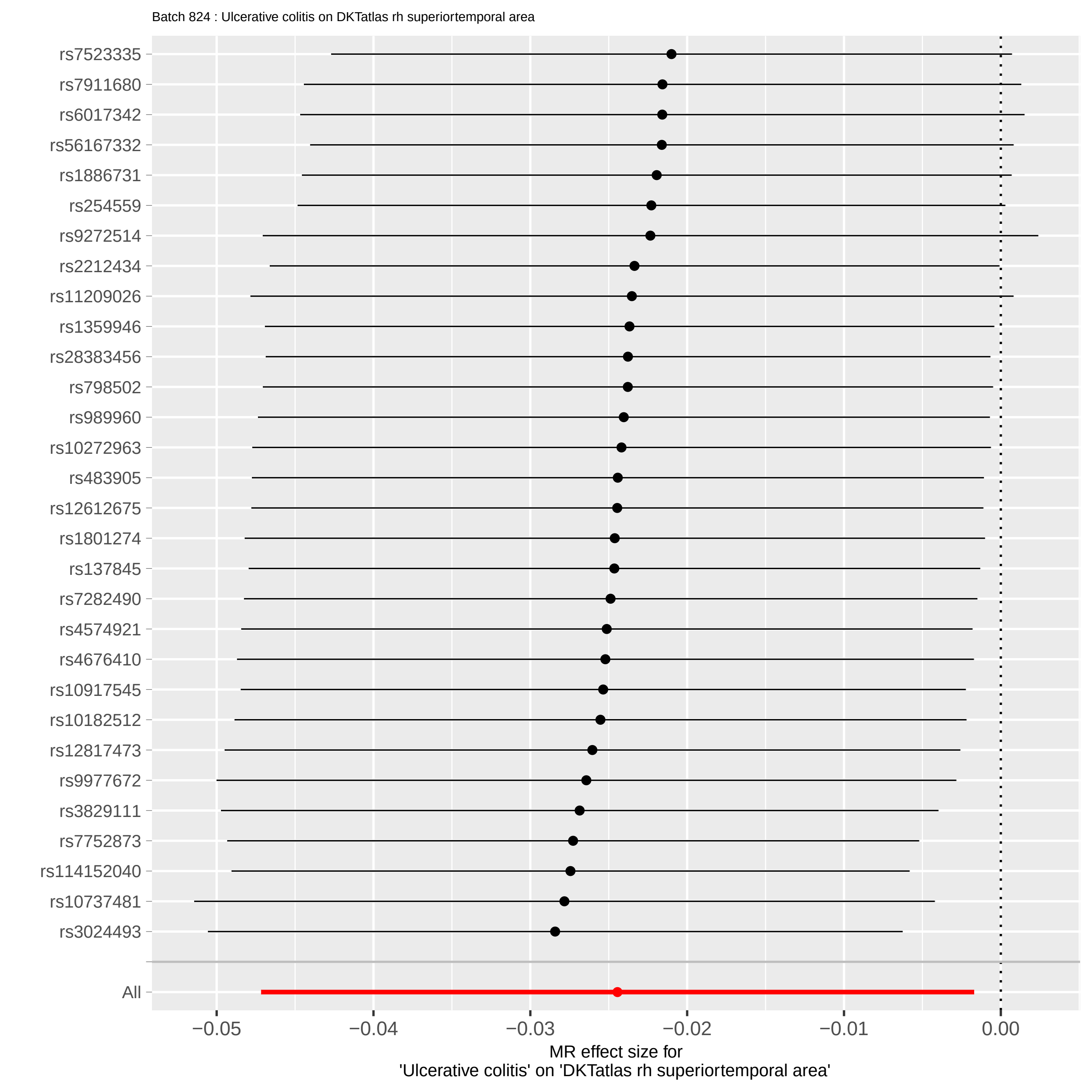

Batch 824 : Ulcerative colitis on DKTatlas rh superiortemporal area
rs7523335
rs7911680
rs6017342
rs56167332
rs1886731
rs254559
rs9272514
rs2212434
rs11209026
rs1359946
rs28383456
rs798502
rs989960
rs10272963
rs483905
rs12612675
rs1801274
rs137845
rs7282490
rs4574921
rs4676410
rs10917545
rs10182512
rs12817473
rs9977672
rs3829111
rs7752873
rs114152040
rs10737481
rs3024493
All
−0.05
−0.04
−0.03
−0.02
−0.01
0.00
MR effect size for
'Ulcerative colitis' on 'DKTatlas rh superiortemporal area'

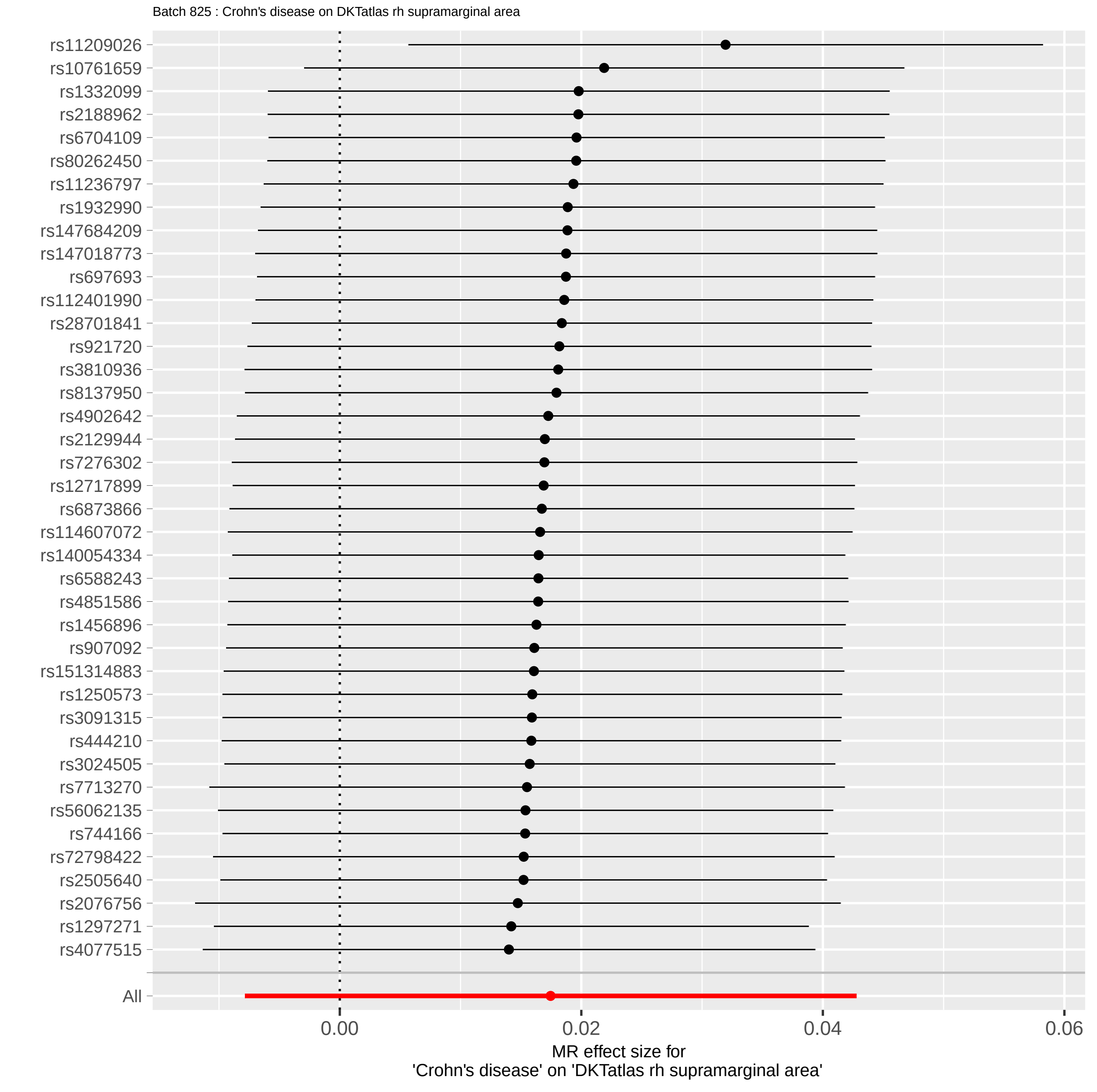

Batch 825 : Crohn's disease on DKTatlas rh supramarginal area
rs11209026
rs10761659
rs1332099
rs2188962
rs6704109
rs80262450
rs11236797
rs1932990
rs147684209
rs147018773
rs697693
rs112401990
rs28701841
rs921720
rs3810936
rs8137950
rs4902642
rs2129944
rs7276302
rs12717899
rs6873866
rs114607072
rs140054334
rs6588243
rs4851586
rs1456896
rs907092
rs151314883
rs1250573
rs3091315
rs444210
rs3024505
rs7713270
rs56062135
rs744166
rs72798422
rs2505640
rs2076756
rs1297271
rs4077515
All
0.00
0.02
0.04
0.06
MR effect size for
'Crohn's disease' on 'DKTatlas rh supramarginal area'

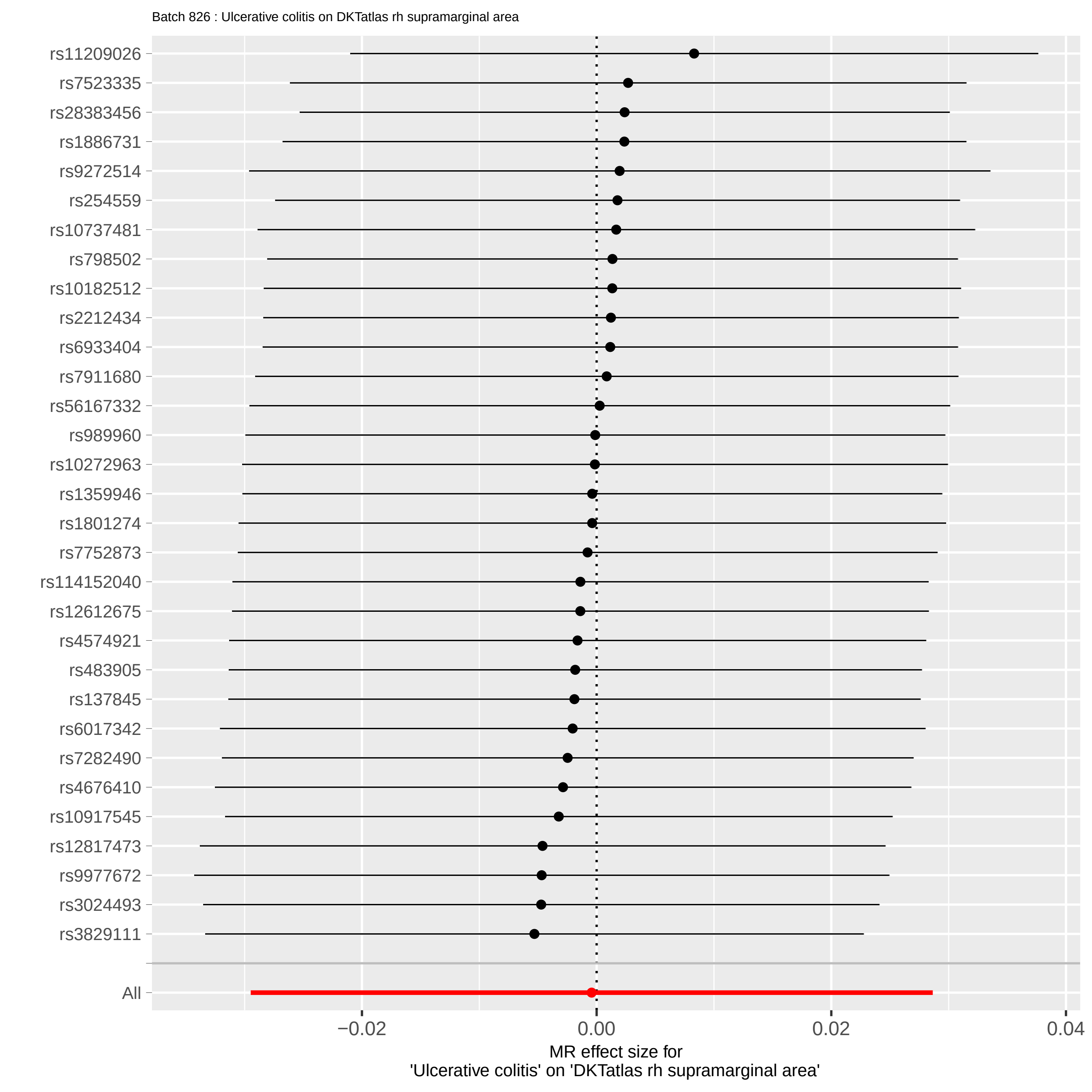
Batch 826 : Ulcerative colitis on DKTatlas rh supramarginal area
rs11209026
rs7523335
rs28383456
rs1886731
rs9272514
rs254559
rs10737481
rs798502
rs10182512
rs2212434
rs6933404
rs7911680
rs56167332
rs989960
rs10272963
rs1359946
rs1801274
rs7752873
rs114152040
rs12612675
rs4574921
rs483905
rs137845
rs6017342
rs7282490
rs4676410
rs10917545
rs12817473
rs9977672
rs3024493
rs3829111
All
−0.02
0.00
0.02
0.04
MR effect size for
'Ulcerative colitis' on 'DKTatlas rh supramarginal area'

Batch 829 : Crohn's disease on DKTatlas rh transversetemporal area

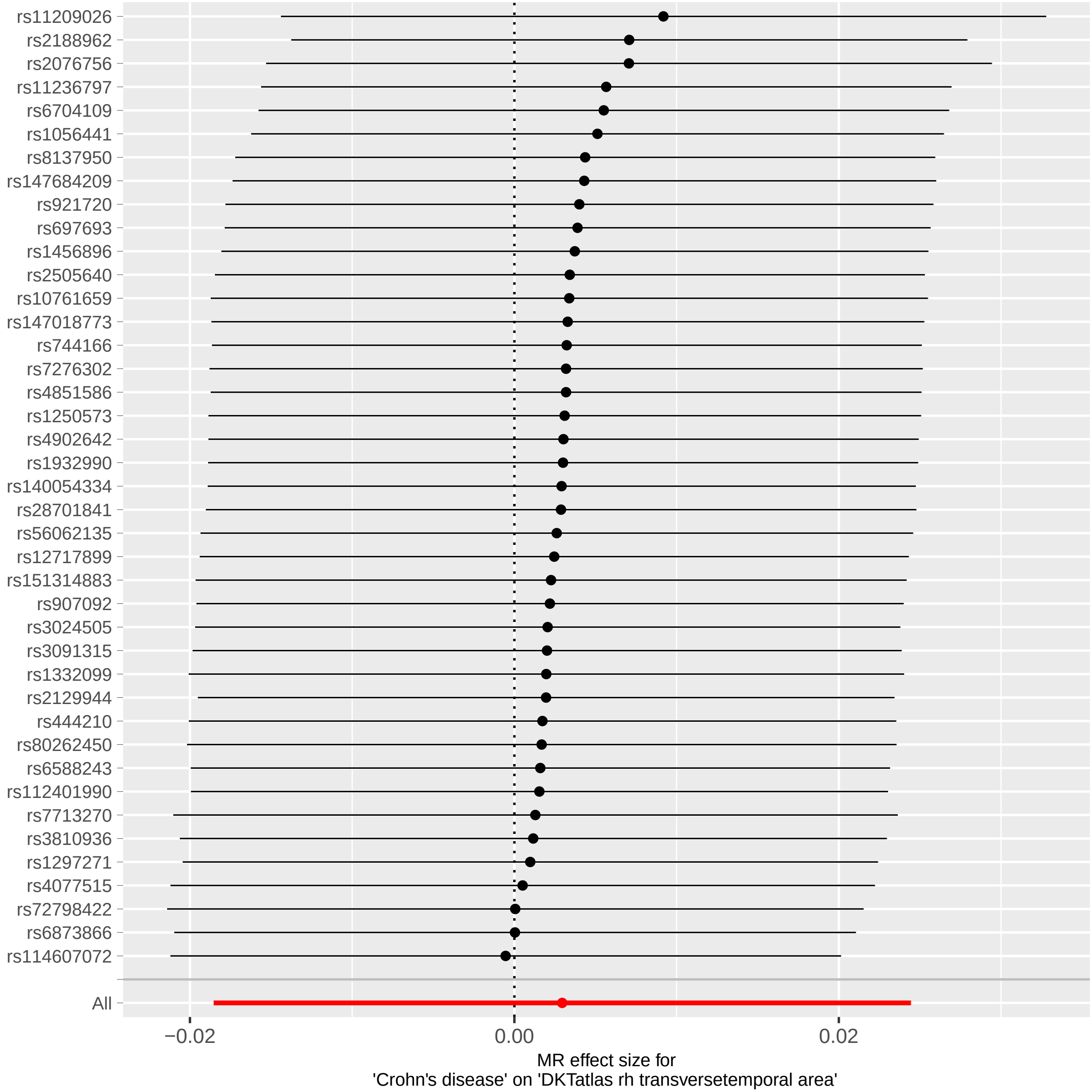

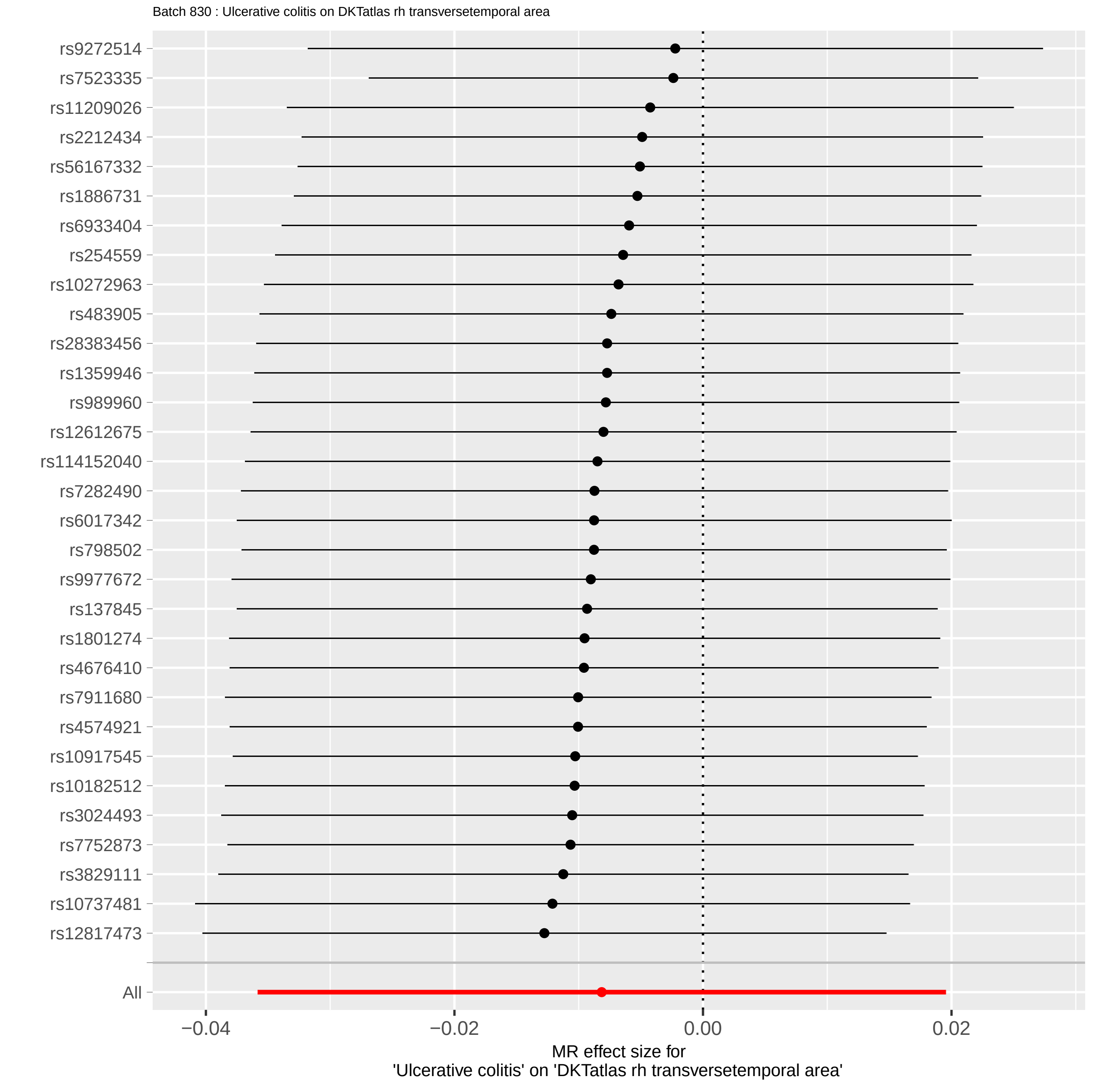

Batch 830 : Ulcerative colitis on DKTatlas rh transversetemporal area
rs9272514
rs7523335
rs11209026
rs2212434
rs56167332
rs1886731
rs6933404
rs254559
rs10272963
rs483905
rs28383456
rs1359946
rs989960
rs12612675
rs114152040
rs7282490
rs6017342
rs798502
rs9977672
rs137845
rs1801274
rs4676410
rs7911680
rs4574921
rs10917545
rs10182512
rs3024493
rs7752873
rs3829111
rs10737481
rs12817473
All
−0.04
−0.02
0.00
0.02
MR effect size for
'Ulcerative colitis' on 'DKTatlas rh transversetemporal area'

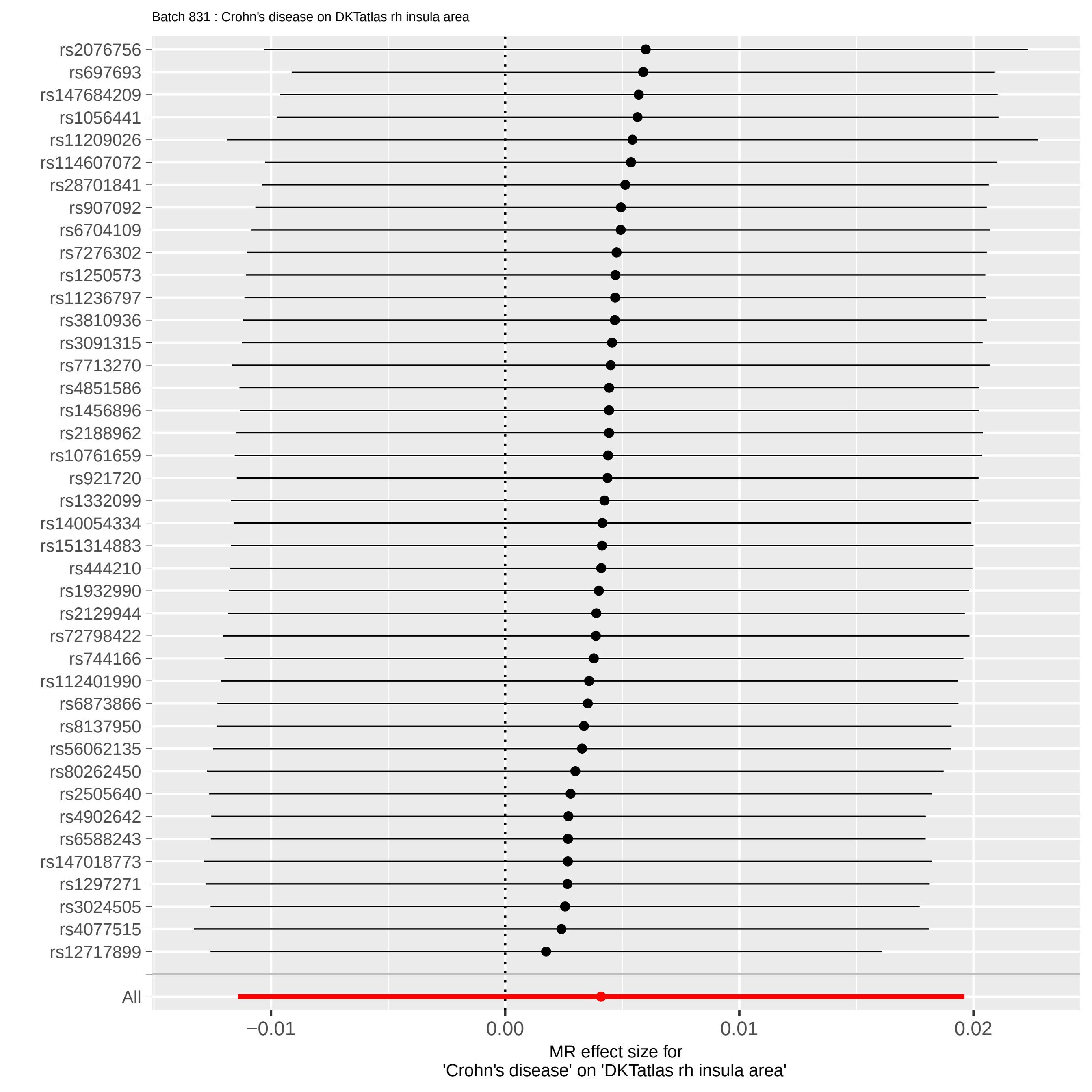

Batch 831 : Crohn's disease on DKTatlas rh insula area
rs2076756
rs697693
rs147684209
rs1056441
rs11209026
rs114607072
rs28701841
rs907092
rs6704109
rs7276302
rs1250573
rs11236797
rs3810936
rs3091315
rs7713270
rs4851586
rs1456896
rs2188962
rs10761659
rs921720
rs1332099
rs140054334
rs151314883
rs444210
rs1932990
rs2129944
rs72798422
rs744166
rs112401990
rs6873866
rs8137950
rs56062135
rs80262450
rs2505640
rs4902642
rs6588243
rs147018773
rs1297271
rs3024505
rs4077515
rs12717899
All
−0.01
0.00
0.01
0.02
MR effect size for
'Crohn's disease' on 'DKTatlas rh insula area'

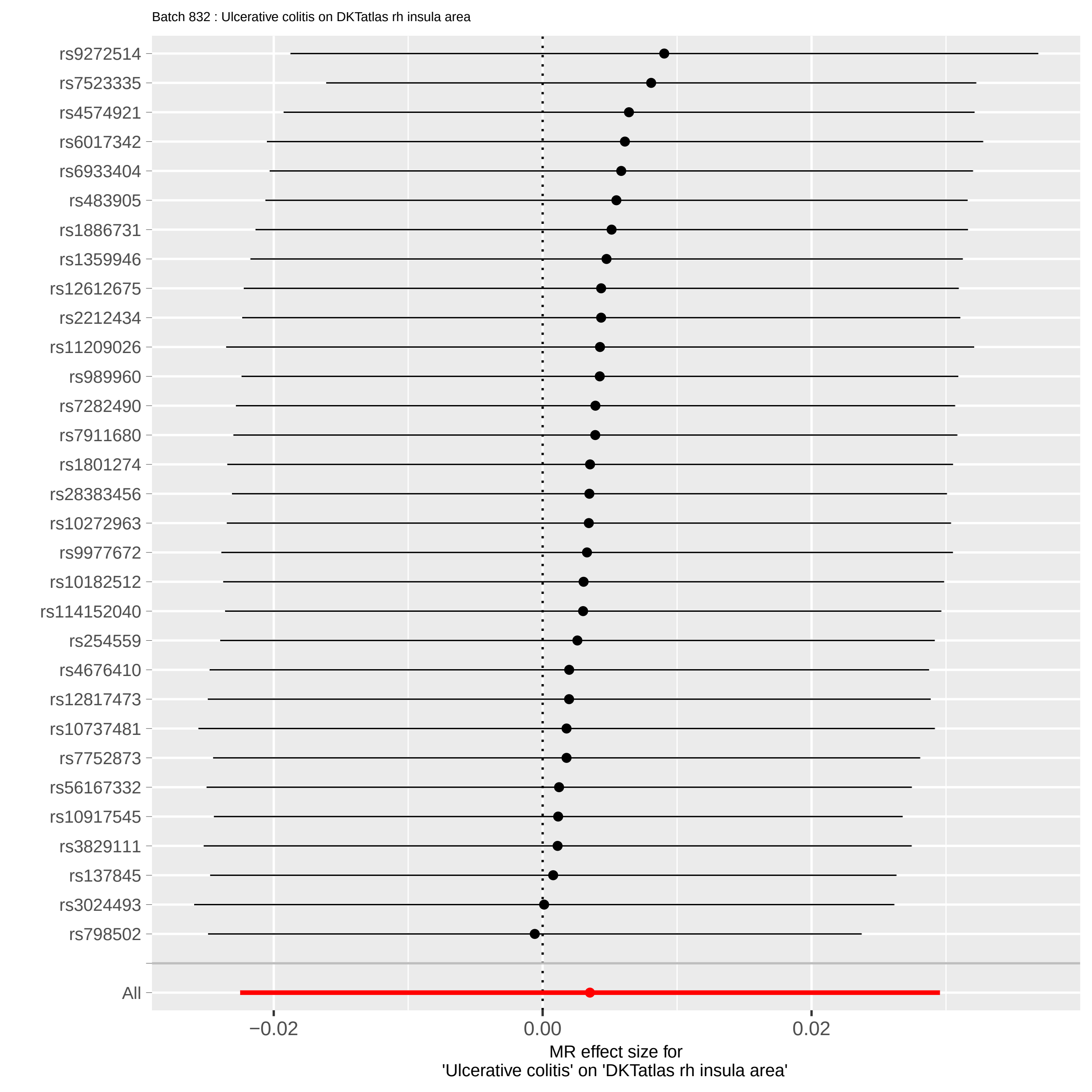
Batch 832 : Ulcerative colitis on DKTatlas rh insula area
rs9272514
rs7523335
rs4574921
rs6017342
rs6933404
rs483905
rs1886731
rs1359946
rs12612675
rs2212434
rs11209026
rs989960
rs7282490
rs7911680
rs1801274
rs28383456
rs10272963
rs9977672
rs10182512
rs114152040
rs254559
rs4676410
rs12817473
rs10737481
rs7752873
rs56167332
rs10917545
rs3829111
rs137845
rs3024493
rs798502
All
−0.02
0.00
0.02
MR effect size for
'Ulcerative colitis' on 'DKTatlas rh insula area'

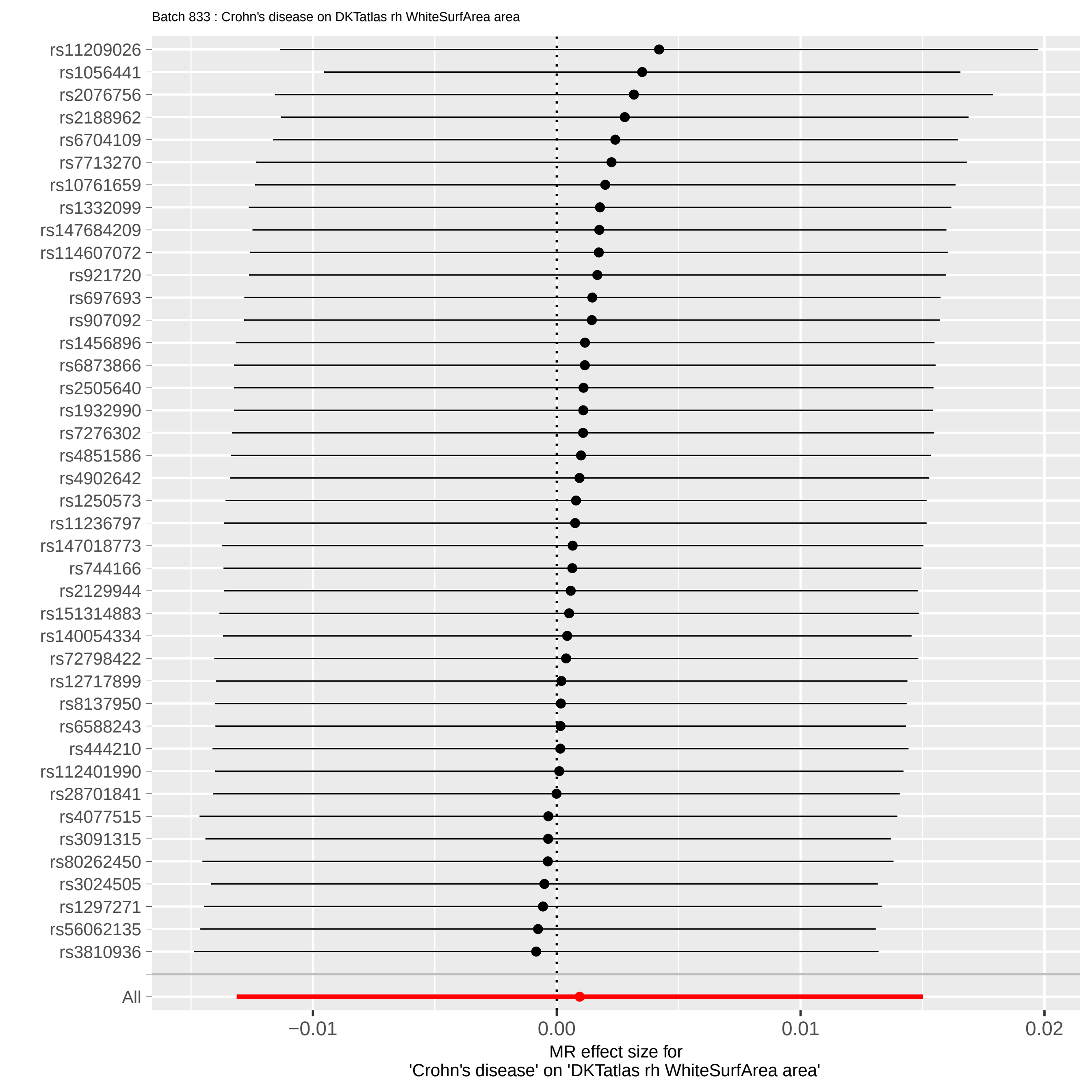
Batch 833 : Crohn's disease on DKTatlas rh WhiteSurfArea area
rs11209026
rs1056441
rs2076756
rs2188962
rs6704109
rs7713270
rs10761659
rs1332099
rs147684209
rs114607072
rs921720
rs697693
rs907092
rs1456896
rs6873866
rs2505640
rs1932990
rs7276302
rs4851586
rs4902642
rs1250573
rs11236797
rs147018773
rs744166
rs2129944
rs151314883
rs140054334
rs72798422
rs12717899
rs8137950
rs6588243
rs444210
rs112401990
rs28701841
rs4077515
rs3091315
rs80262450
rs3024505
rs1297271
rs56062135
rs3810936
All
−0.01
0.00
0.01
0.02
MR effect size for
'Crohn's disease' on 'DKTatlas rh WhiteSurfArea area'

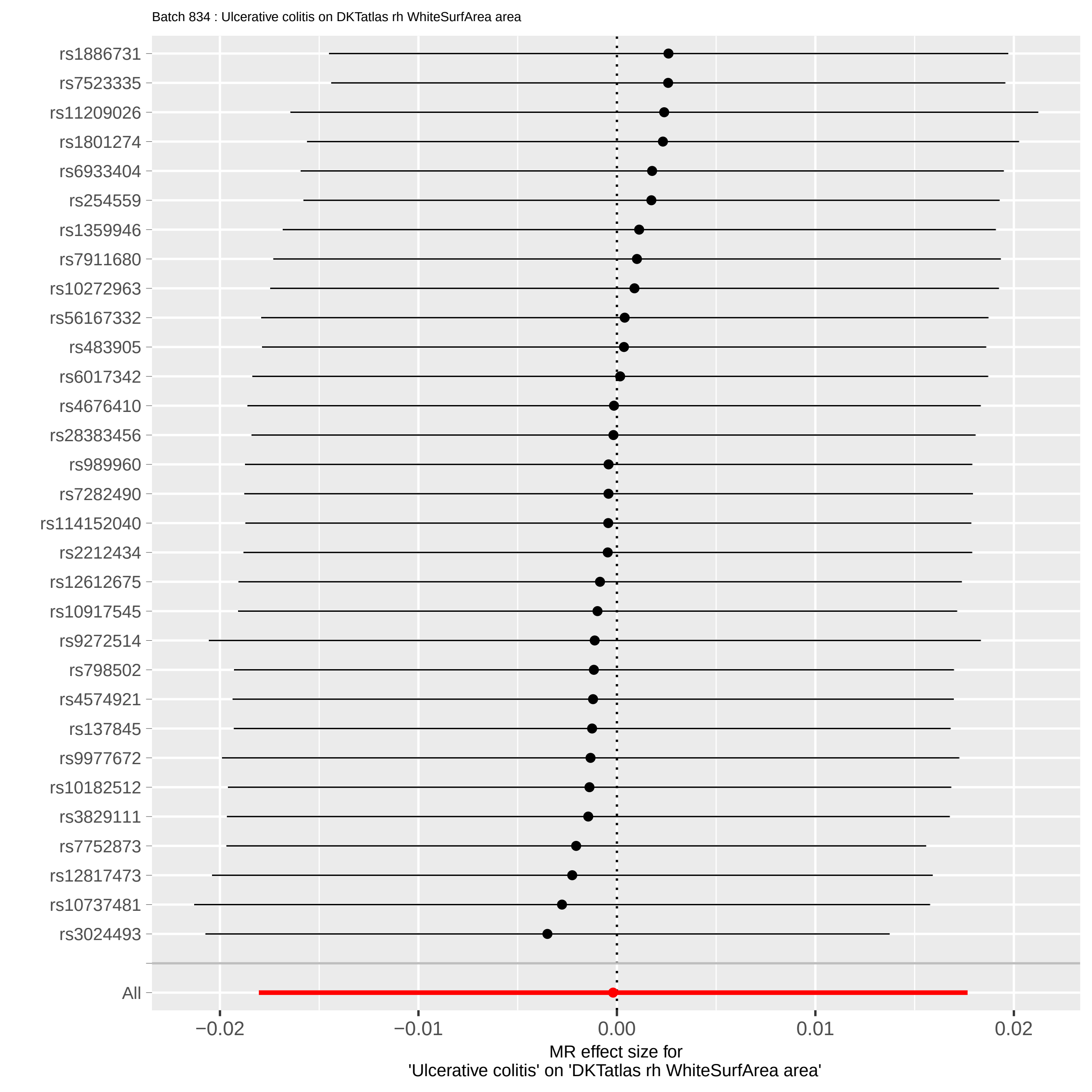

Batch 834 : Ulcerative colitis on DKTatlas rh WhiteSurfArea area
rs1886731
rs7523335
rs11209026
rs1801274
rs6933404
rs254559
rs1359946
rs7911680
rs10272963
rs56167332
rs483905
rs6017342
rs4676410
rs28383456
rs989960
rs7282490
rs114152040
rs2212434
rs12612675
rs10917545
rs9272514
rs798502
rs4574921
rs137845
rs9977672
rs10182512
rs3829111
rs7752873
rs12817473
rs10737481
rs3024493
All
−0.02
−0.01
0.00
0.01
0.02
MR effect size for
'Ulcerative colitis' on 'DKTatlas rh WhiteSurfArea area'

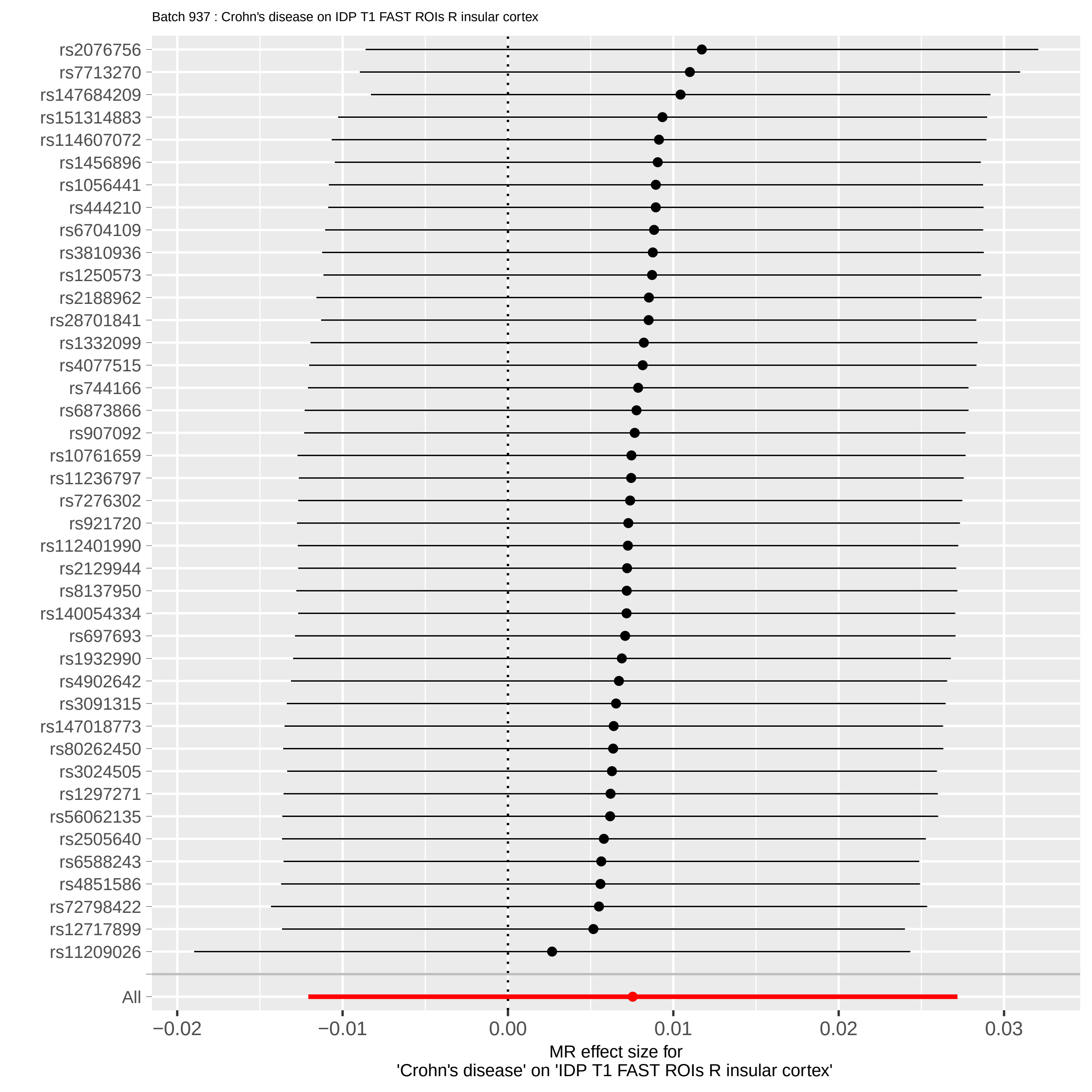

Batch 937 : Crohn's disease on IDP T1 FAST ROIs R insular cortex
rs2076756
rs7713270
rs147684209
rs151314883
rs114607072
rs1456896
rs1056441
rs444210
rs6704109
rs3810936
rs1250573
rs2188962
rs28701841
rs1332099
rs4077515
rs744166
rs6873866
rs907092
rs10761659
rs11236797
rs7276302
rs921720
rs112401990
rs2129944
rs8137950
rs140054334
rs697693
rs1932990
rs4902642
rs3091315
rs147018773
rs80262450
rs3024505
rs1297271
rs56062135
rs2505640
rs6588243
rs4851586
rs72798422
rs12717899
rs11209026
All
−0.02
−0.01
0.00
0.01
0.02
0.03
MR effect size for
'Crohn's disease' on 'IDP T1 FAST ROIs R insular cortex'

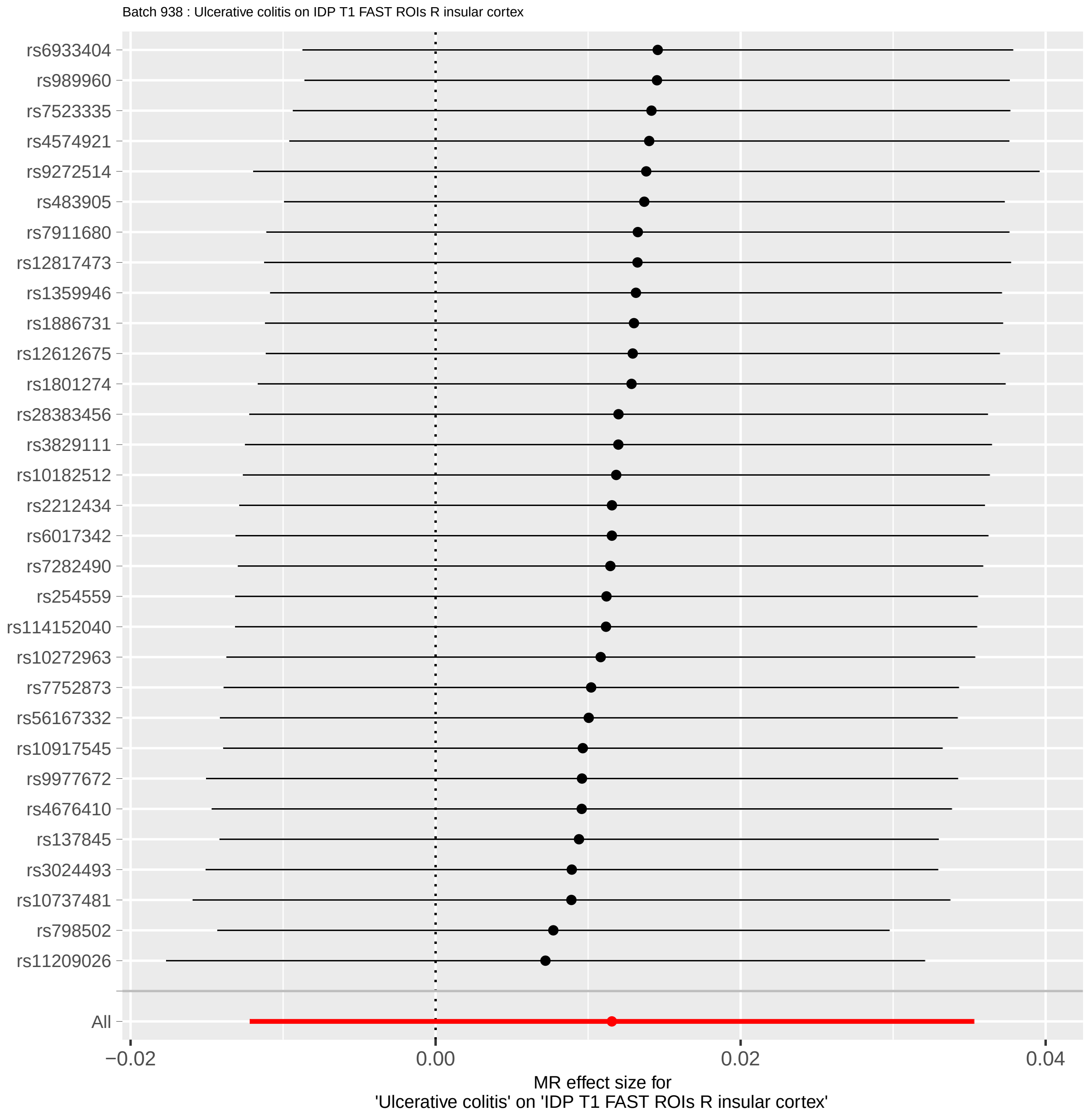
Batch 938 : Ulcerative colitis on IDP T1 FAST ROIs R insular cortex
rs6933404
rs989960
rs7523335
rs4574921
rs9272514
rs483905
rs7911680
rs12817473
rs1359946
rs1886731
rs12612675
rs1801274
rs28383456
rs3829111
rs10182512
rs2212434
rs6017342
rs7282490
rs254559
rs114152040
rs10272963
rs7752873
rs56167332
rs10917545
rs9977672
rs4676410
rs137845
rs3024493
rs10737481
rs798502
rs11209026
All
−0.02
0.00
0.02
0.04
MR effect size for
'Ulcerative colitis' on 'IDP T1 FAST ROIs R insular cortex'

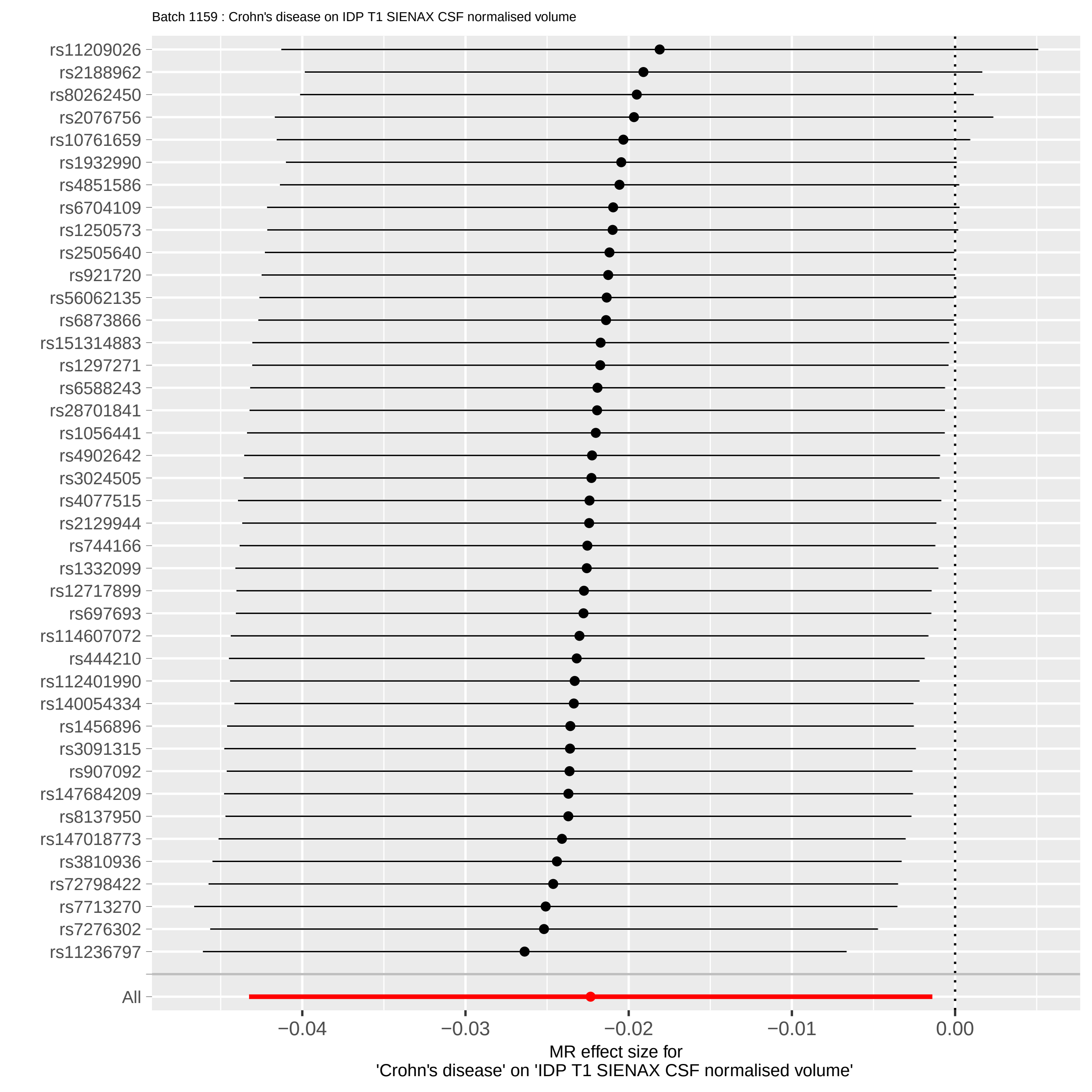

Batch 1159 : Crohn's disease on IDP T1 SIENAX CSF normalised volume
rs11209026
rs2188962
rs80262450
rs2076756
rs10761659
rs1932990
rs4851586
rs6704109
rs1250573
rs2505640
rs921720
rs56062135
rs6873866
rs151314883
rs1297271
rs6588243
rs28701841
rs1056441
rs4902642
rs3024505
rs4077515
rs2129944
rs744166
rs1332099
rs12717899
rs697693
rs114607072
rs444210
rs112401990
rs140054334
rs1456896
rs3091315
rs907092
rs147684209
rs8137950
rs147018773
rs3810936
rs72798422
rs7713270
rs7276302
rs11236797
All
−0.04
−0.03
−0.02
−0.01
0.00
MR effect size for
'Crohn's disease' on 'IDP T1 SIENAX CSF normalised volume'

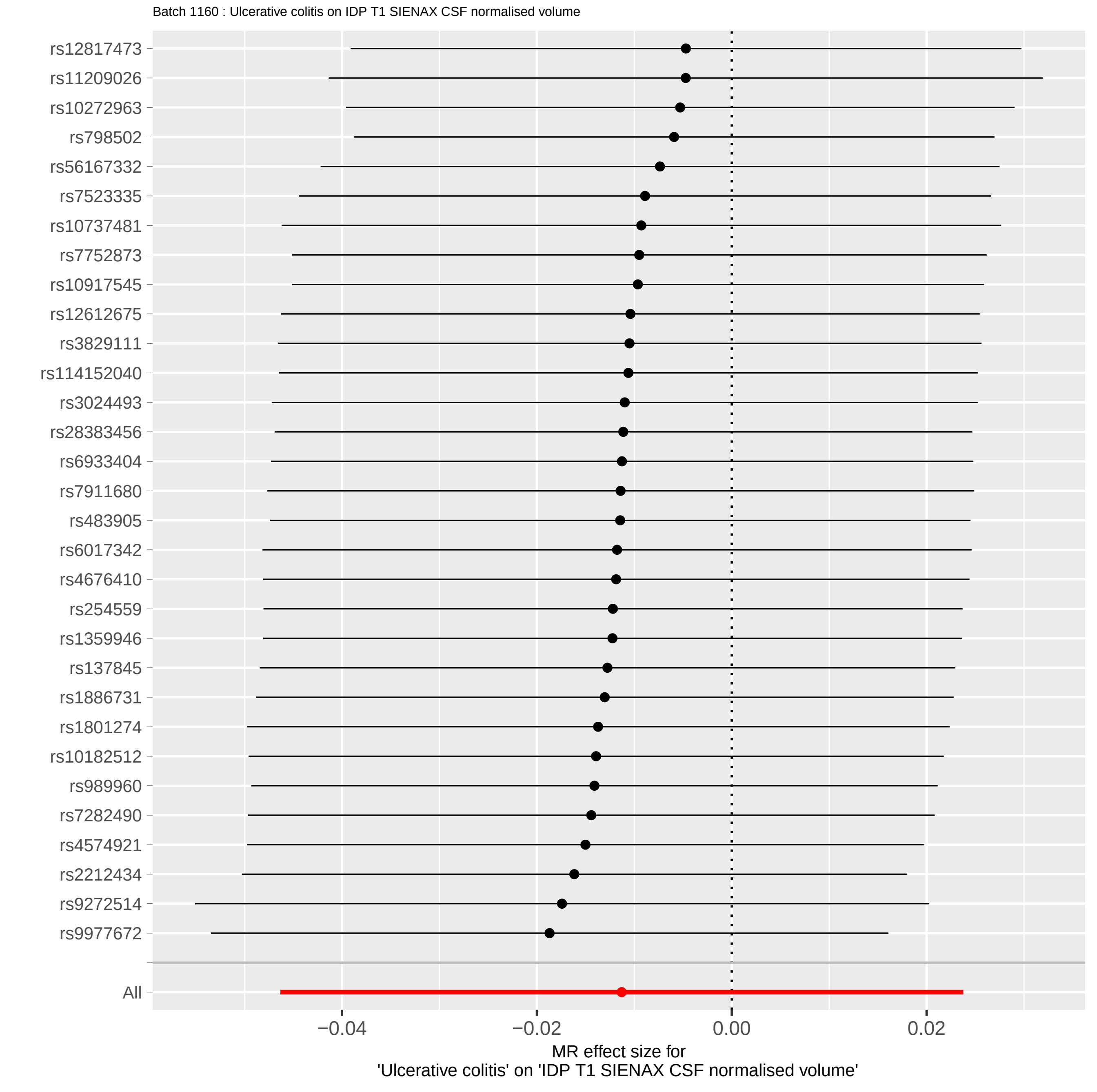

Batch 1160 : Ulcerative colitis on IDP T1 SIENAX CSF normalised volume
rs12817473
rs11209026
rs10272963
rs798502
rs56167332
rs7523335
rs10737481
rs7752873
rs10917545
rs12612675
rs3829111
rs114152040
rs3024493
rs28383456
rs6933404
rs7911680
rs483905
rs6017342
rs4676410
rs254559
rs1359946
rs137845
rs1886731
rs1801274
rs10182512
rs989960
rs7282490
rs4574921
rs2212434
rs9272514
rs9977672
All
−0.04
−0.02
0.00
0.02
MR effect size for
'Ulcerative colitis' on 'IDP T1 SIENAX CSF normalised volume'

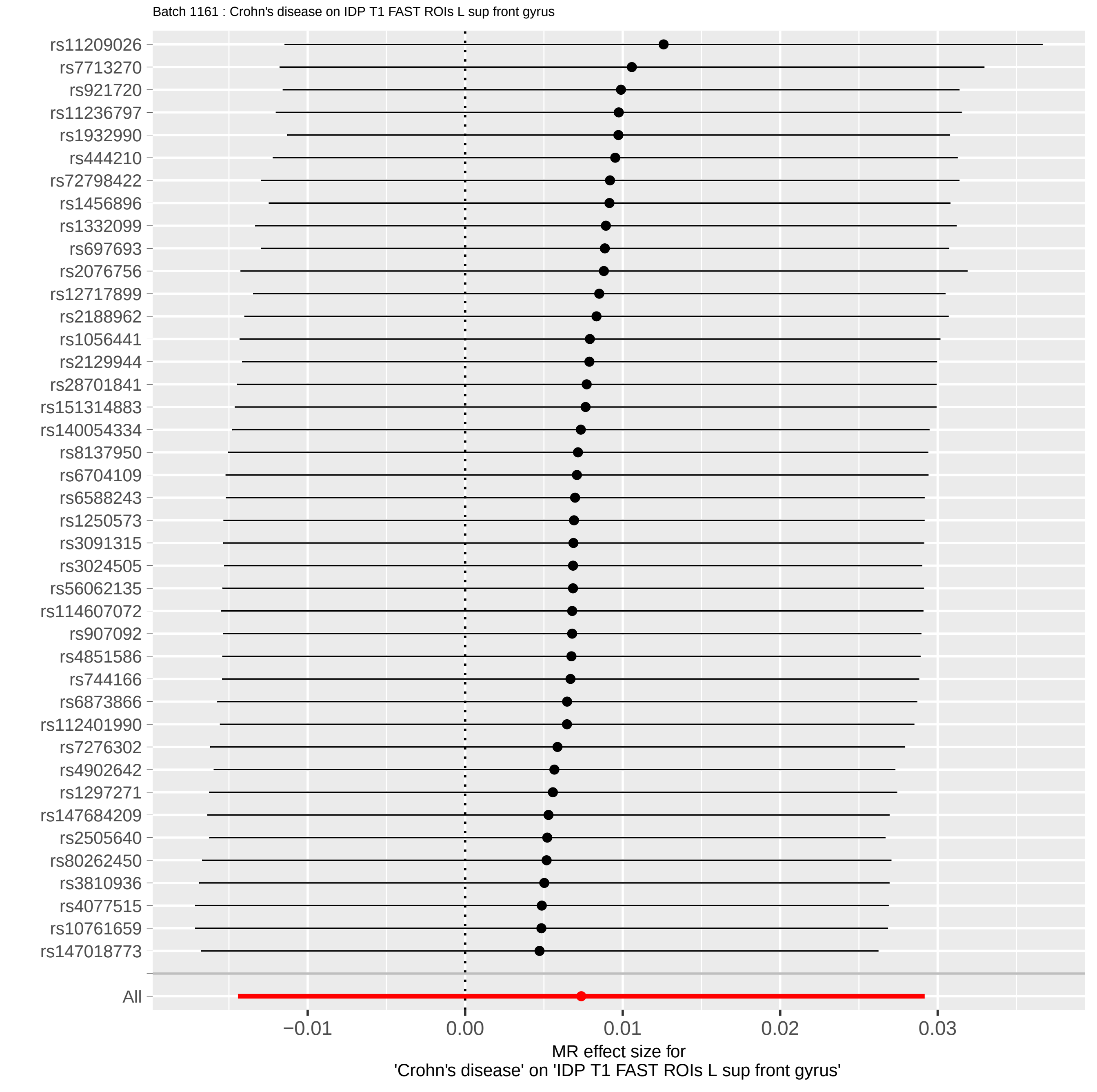
Batch 1161 : Crohn's disease on IDP T1 FAST ROIs L sup front gyrus
rs11209026
rs7713270
rs921720
rs11236797
rs1932990
rs444210
rs72798422
rs1456896
rs1332099
rs697693
rs2076756
rs12717899
rs2188962
rs1056441
rs2129944
rs28701841
rs151314883
rs140054334
rs8137950
rs6704109
rs6588243
rs1250573
rs3091315
rs3024505
rs56062135
rs114607072
rs907092
rs4851586
rs744166
rs6873866
rs112401990
rs7276302
rs4902642
rs1297271
rs147684209
rs2505640
rs80262450
rs3810936
rs4077515
rs10761659
rs147018773
All
−0.01
0.00
0.01
0.02
0.03
MR effect size for
'Crohn's disease' on 'IDP T1 FAST ROIs L sup front gyrus'

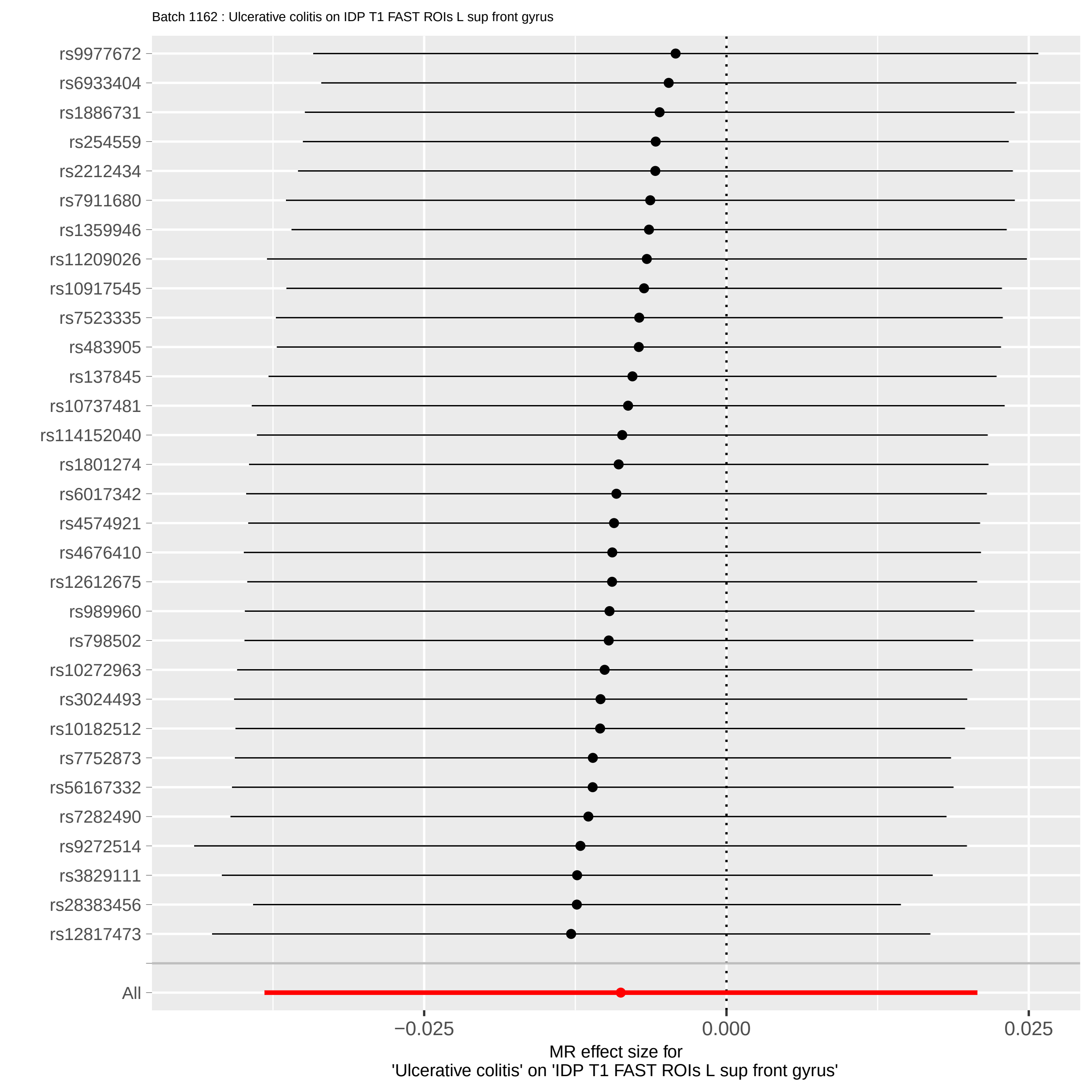

Batch 1162 : Ulcerative colitis on IDP T1 FAST ROIs L sup front gyrus
rs9977672
rs6933404
rs1886731
rs254559
rs2212434
rs7911680
rs1359946
rs11209026
rs10917545
rs7523335
rs483905
rs137845
rs10737481
rs114152040
rs1801274
rs6017342
rs4574921
rs4676410
rs12612675
rs989960
rs798502
rs10272963
rs3024493
rs10182512
rs7752873
rs56167332
rs7282490
rs9272514
rs3829111
rs28383456
rs12817473
All
−0.025
0.000
0.025
MR effect size for
'Ulcerative colitis' on 'IDP T1 FAST ROIs L sup front gyrus'

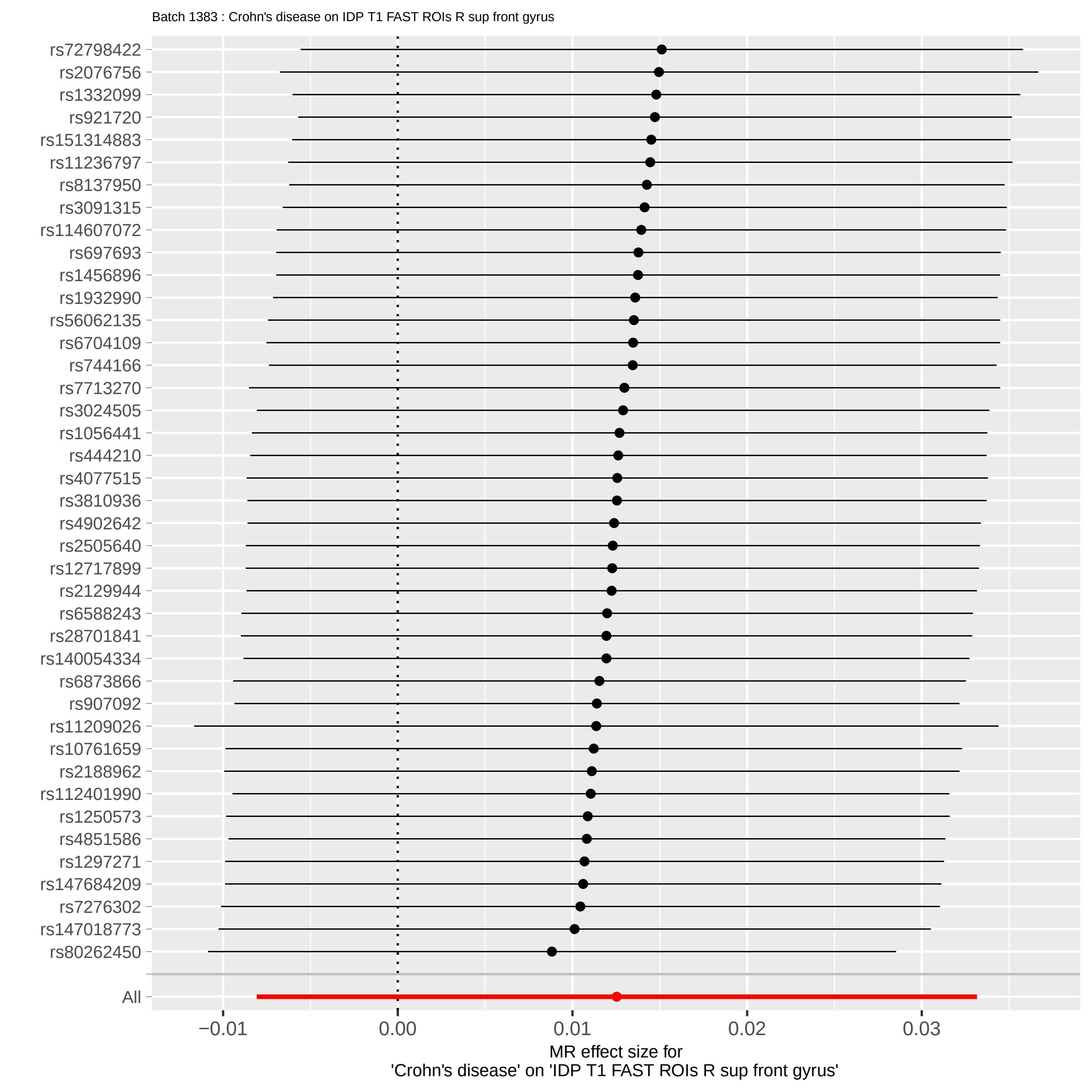

Batch 1383 : Crohn's disease on IDP T1 FAST ROIs R sup front gyrus
rs72798422
rs2076756
rs1332099
rs921720
rs151314883
rs11236797
rs8137950
rs3091315
rs114607072
rs697693
rs1456896
rs1932990
rs56062135
rs6704109
rs744166
rs7713270
rs3024505
rs1056441
rs444210
rs4077515
rs3810936
rs4902642
rs2505640
rs12717899
rs2129944
rs6588243
rs28701841
rs140054334
rs6873866
rs907092
rs11209026
rs10761659
rs2188962
rs112401990
rs1250573
rs4851586
rs1297271
rs147684209
rs7276302
rs147018773
rs80262450
All
−0.01
0.00
0.01
0.02
0.03
MR effect size for
'Crohn's disease' on 'IDP T1 FAST ROIs R sup front gyrus'

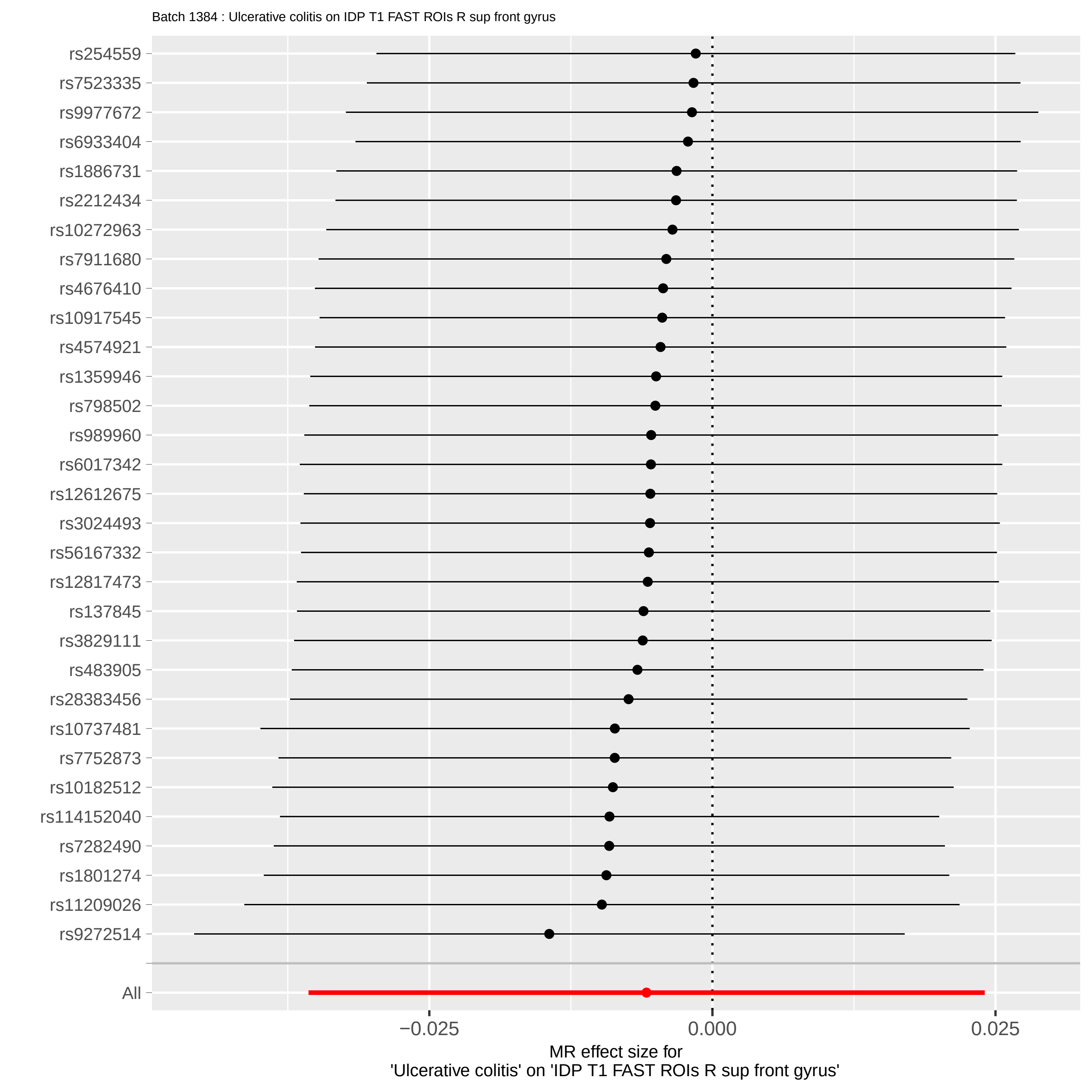

Batch 1384 : Ulcerative colitis on IDP T1 FAST ROIs R sup front gyrus
rs254559
rs7523335
rs9977672
rs6933404
rs1886731
rs2212434
rs10272963
rs7911680
rs4676410
rs10917545
rs4574921
rs1359946
rs798502
rs989960
rs6017342
rs12612675
rs3024493
rs56167332
rs12817473
rs137845
rs3829111
rs483905
rs28383456
rs10737481
rs7752873
rs10182512
rs114152040
rs7282490
rs1801274
rs11209026
rs9272514
All
−0.025
0.000
0.025
MR effect size for
'Ulcerative colitis' on 'IDP T1 FAST ROIs R sup front gyrus'

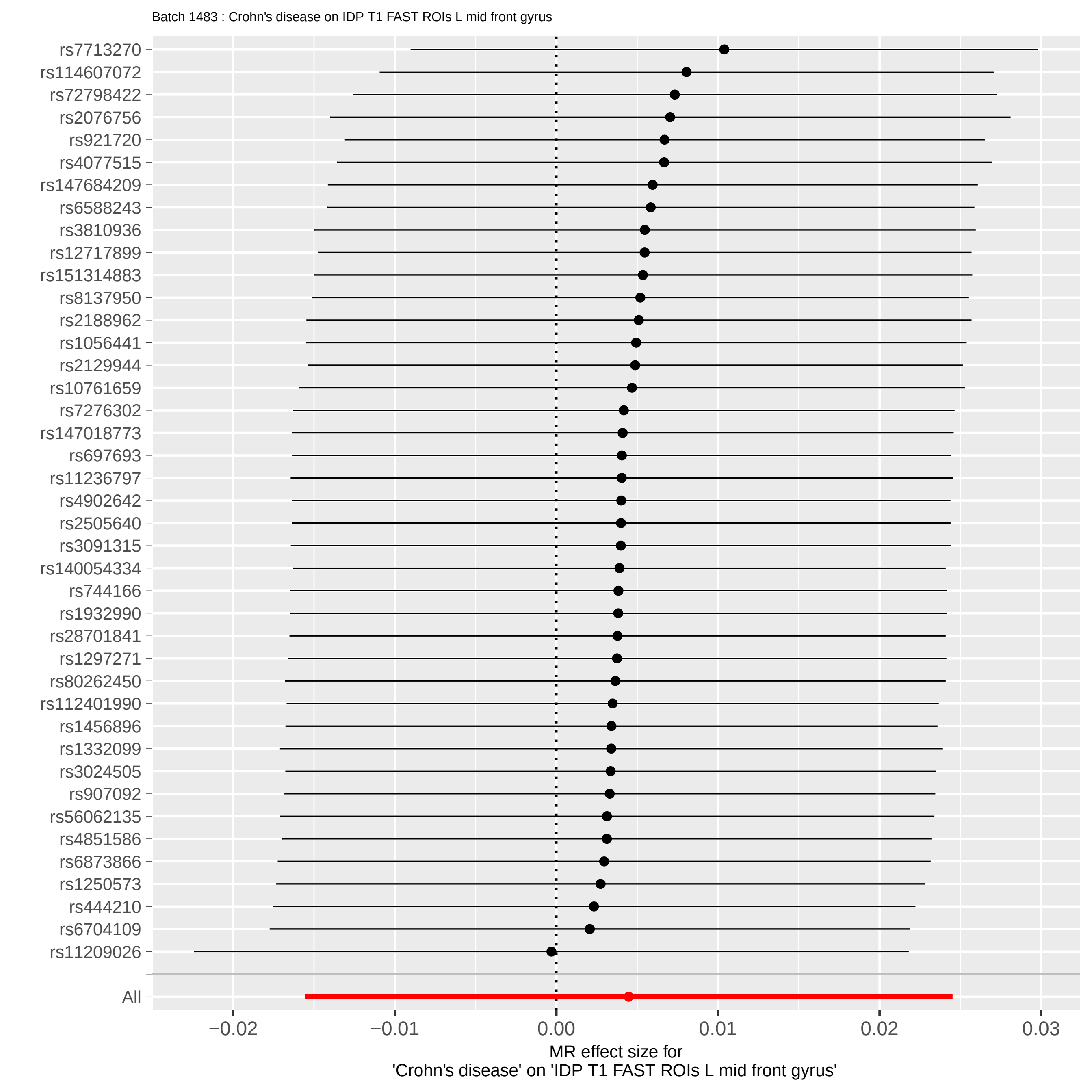

Batch 1483 : Crohn's disease on IDP T1 FAST ROIs L mid front gyrus
rs7713270
rs114607072
rs72798422
rs2076756
rs921720
rs4077515
rs147684209
rs6588243
rs3810936
rs12717899
rs151314883
rs8137950
rs2188962
rs1056441
rs2129944
rs10761659
rs7276302
rs147018773
rs697693
rs11236797
rs4902642
rs2505640
rs3091315
rs140054334
rs744166
rs1932990
rs28701841
rs1297271
rs80262450
rs112401990
rs1456896
rs1332099
rs3024505
rs907092
rs56062135
rs4851586
rs6873866
rs1250573
rs444210
rs6704109
rs11209026
All
−0.02
−0.01
0.00
0.01
0.02
0.03
MR effect size for
'Crohn's disease' on 'IDP T1 FAST ROIs L mid front gyrus'

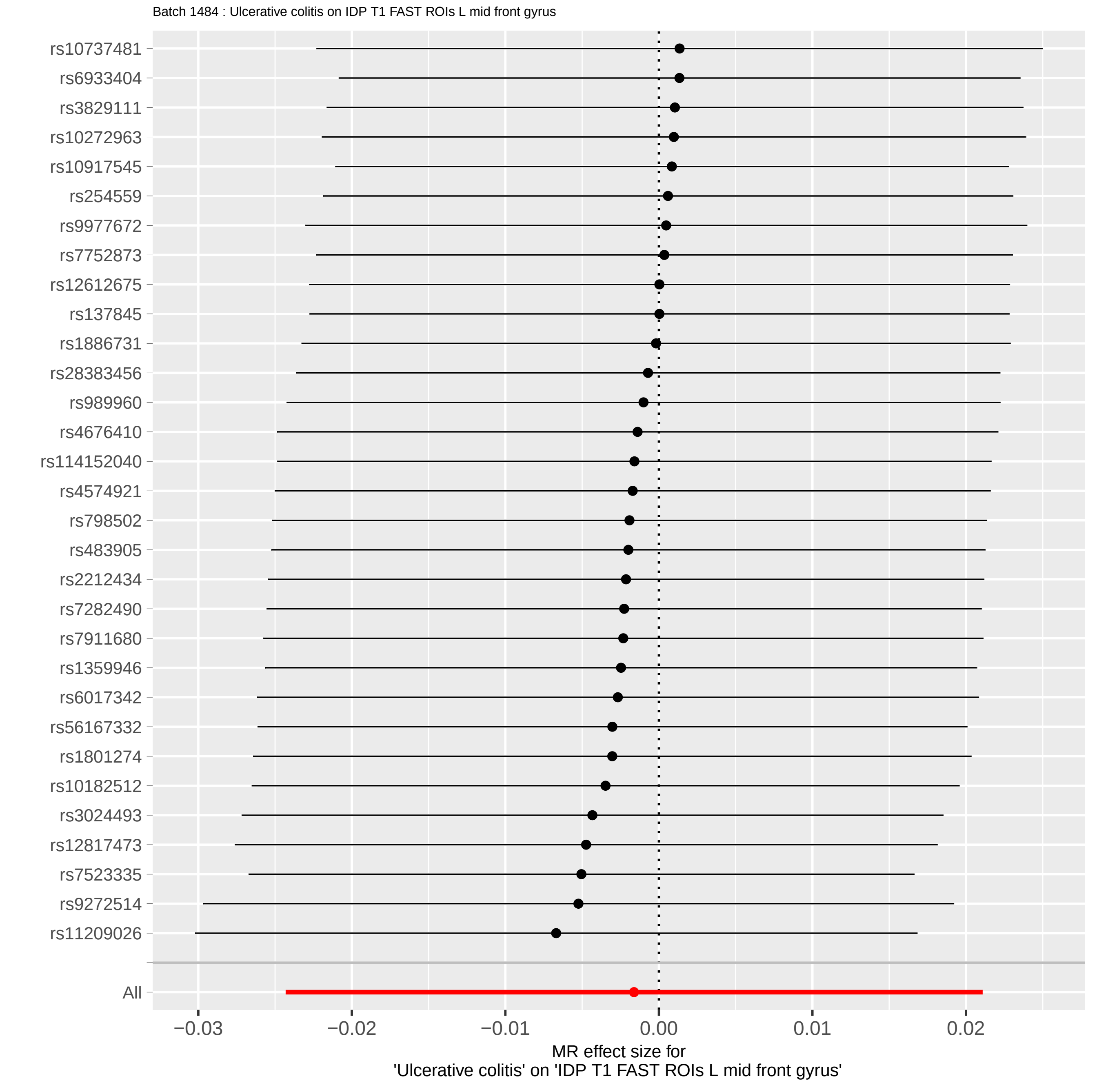

Batch 1484 : Ulcerative colitis on IDP T1 FAST ROIs L mid front gyrus
rs10737481
rs6933404
rs3829111
rs10272963
rs10917545
rs254559
rs9977672
rs7752873
rs12612675
rs137845
rs1886731
rs28383456
rs989960
rs4676410
rs114152040
rs4574921
rs798502
rs483905
rs2212434
rs7282490
rs7911680
rs1359946
rs6017342
rs56167332
rs1801274
rs10182512
rs3024493
rs12817473
rs7523335
rs9272514
rs11209026
All
−0.03
−0.02
−0.01
0.00
0.01
0.02
MR effect size for
'Ulcerative colitis' on 'IDP T1 FAST ROIs L mid front gyrus'

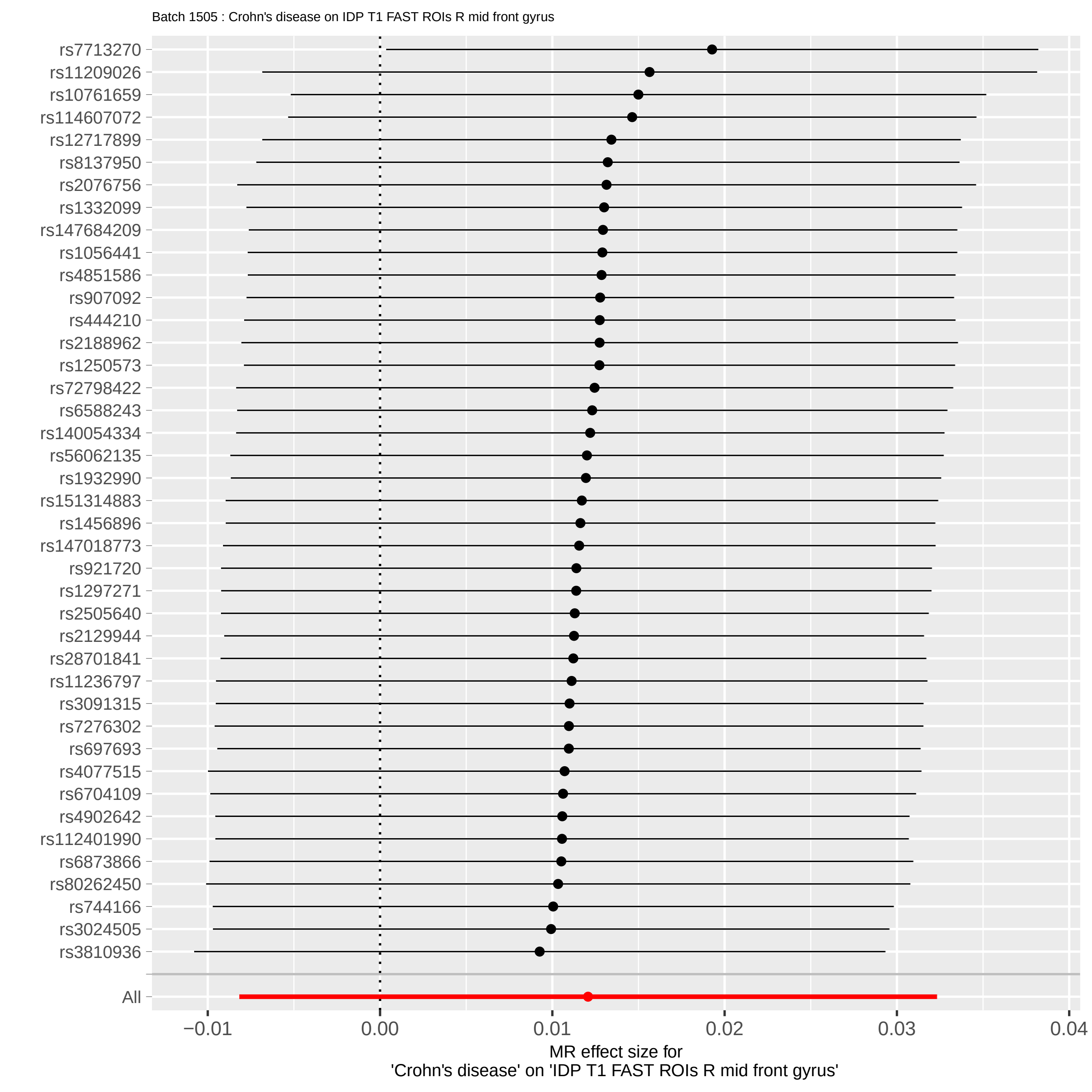

Batch 1505 : Crohn's disease on IDP T1 FAST ROIs R mid front gyrus
rs7713270
rs11209026
rs10761659
rs114607072
rs12717899
rs8137950
rs2076756
rs1332099
rs147684209
rs1056441
rs4851586
rs907092
rs444210
rs2188962
rs1250573
rs72798422
rs6588243
rs140054334
rs56062135
rs1932990
rs151314883
rs1456896
rs147018773
rs921720
rs1297271
rs2505640
rs2129944
rs28701841
rs11236797
rs3091315
rs7276302
rs697693
rs4077515
rs6704109
rs4902642
rs112401990
rs6873866
rs80262450
rs744166
rs3024505
rs3810936
All
−0.01
0.00
0.01
0.02
0.03
0.04
MR effect size for
'Crohn's disease' on 'IDP T1 FAST ROIs R mid front gyrus'

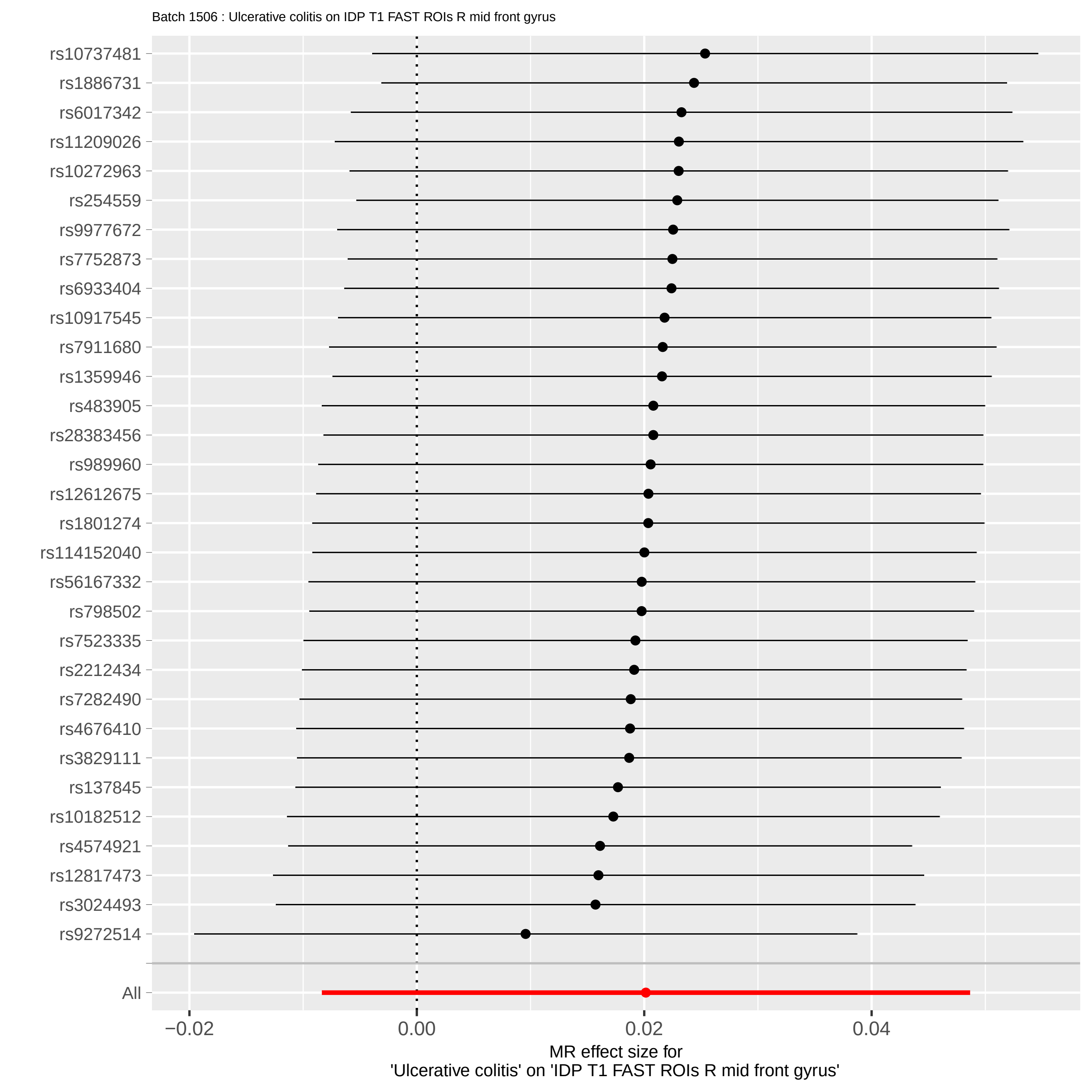
Batch 1506 : Ulcerative colitis on IDP T1 FAST ROIs R mid front gyrus
rs10737481
rs1886731
rs6017342
rs11209026
rs10272963
rs254559
rs9977672
rs7752873
rs6933404
rs10917545
rs7911680
rs1359946
rs483905
rs28383456
rs989960
rs12612675
rs1801274
rs114152040
rs56167332
rs798502
rs7523335
rs2212434
rs7282490
rs4676410
rs3829111
rs137845
rs10182512
rs4574921
rs12817473
rs3024493
rs9272514
All
−0.02
0.00
0.02
0.04
MR effect size for
'Ulcerative colitis' on 'IDP T1 FAST ROIs R mid front gyrus'

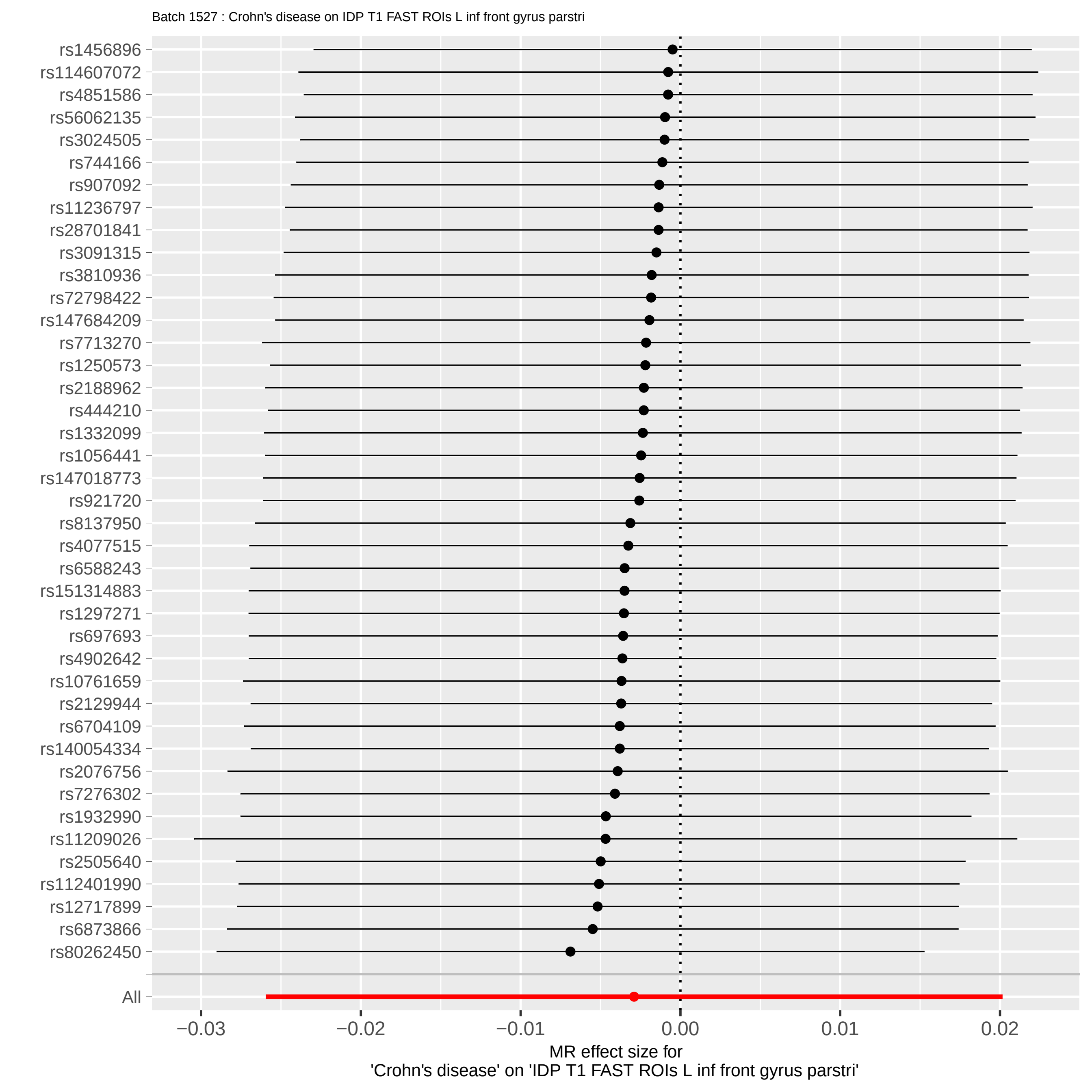

Batch 1527 : Crohn's disease on IDP T1 FAST ROIs L inf front gyrus parstri
rs1456896
rs114607072
rs4851586
rs56062135
rs3024505
rs744166
rs907092
rs11236797
rs28701841
rs3091315
rs3810936
rs72798422
rs147684209
rs7713270
rs1250573
rs2188962
rs444210
rs1332099
rs1056441
rs147018773
rs921720
rs8137950
rs4077515
rs6588243
rs151314883
rs1297271
rs697693
rs4902642
rs10761659
rs2129944
rs6704109
rs140054334
rs2076756
rs7276302
rs1932990
rs11209026
rs2505640
rs112401990
rs12717899
rs6873866
rs80262450
All
−0.03
−0.02
−0.01
0.00
0.01
0.02
MR effect size for
'Crohn's disease' on 'IDP T1 FAST ROIs L inf front gyrus parstri'

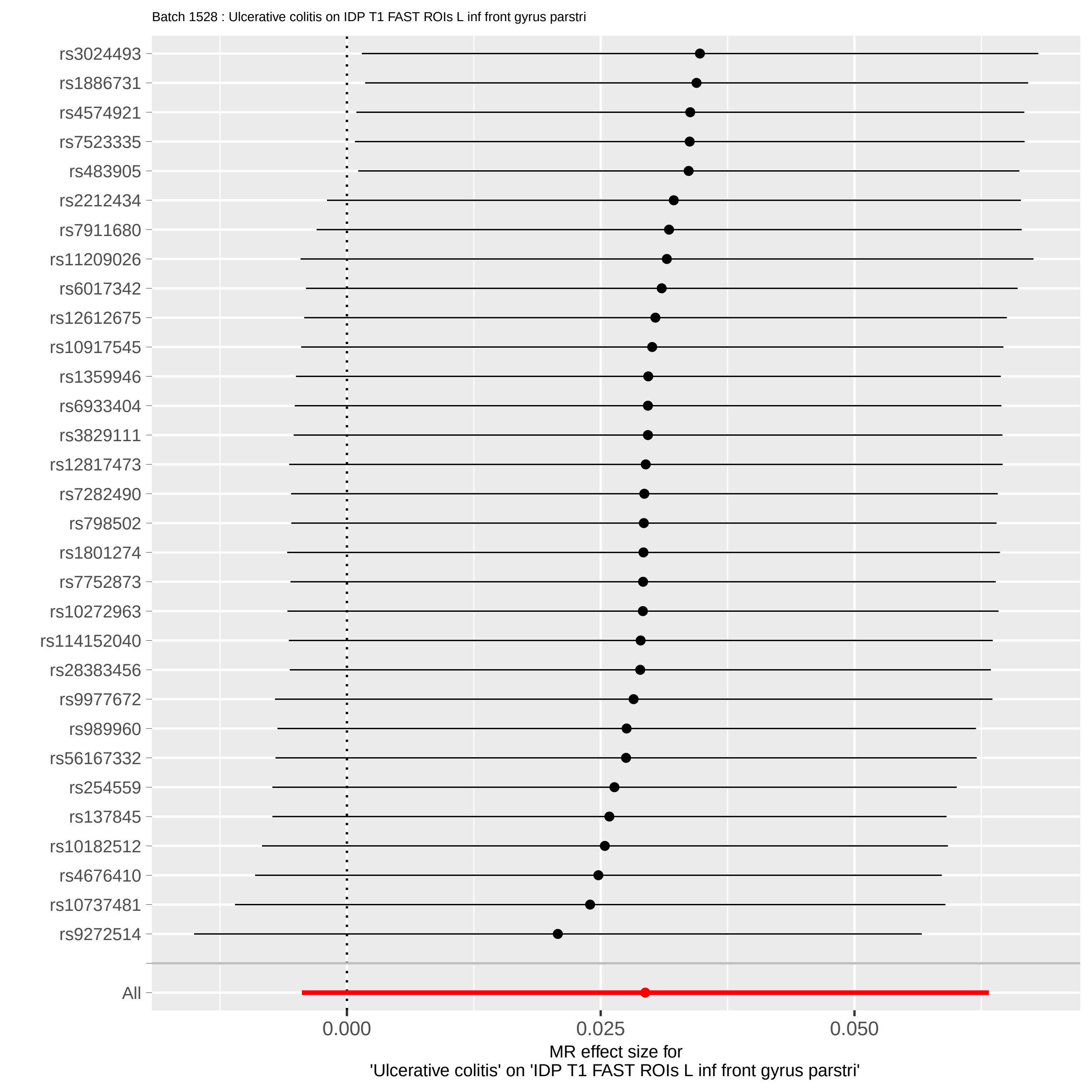

Batch 1528 : Ulcerative colitis on IDP T1 FAST ROIs L inf front gyrus parstri
rs3024493
rs1886731
rs4574921
rs7523335
rs483905
rs2212434
rs7911680
rs11209026
rs6017342
rs12612675
rs10917545
rs1359946
rs6933404
rs3829111
rs12817473
rs7282490
rs798502
rs1801274
rs7752873
rs10272963
rs114152040
rs28383456
rs9977672
rs989960
rs56167332
rs254559
rs137845
rs10182512
rs4676410
rs10737481
rs9272514
All
0.000
0.025
0.050
MR effect size for
'Ulcerative colitis' on 'IDP T1 FAST ROIs L inf front gyrus parstri'

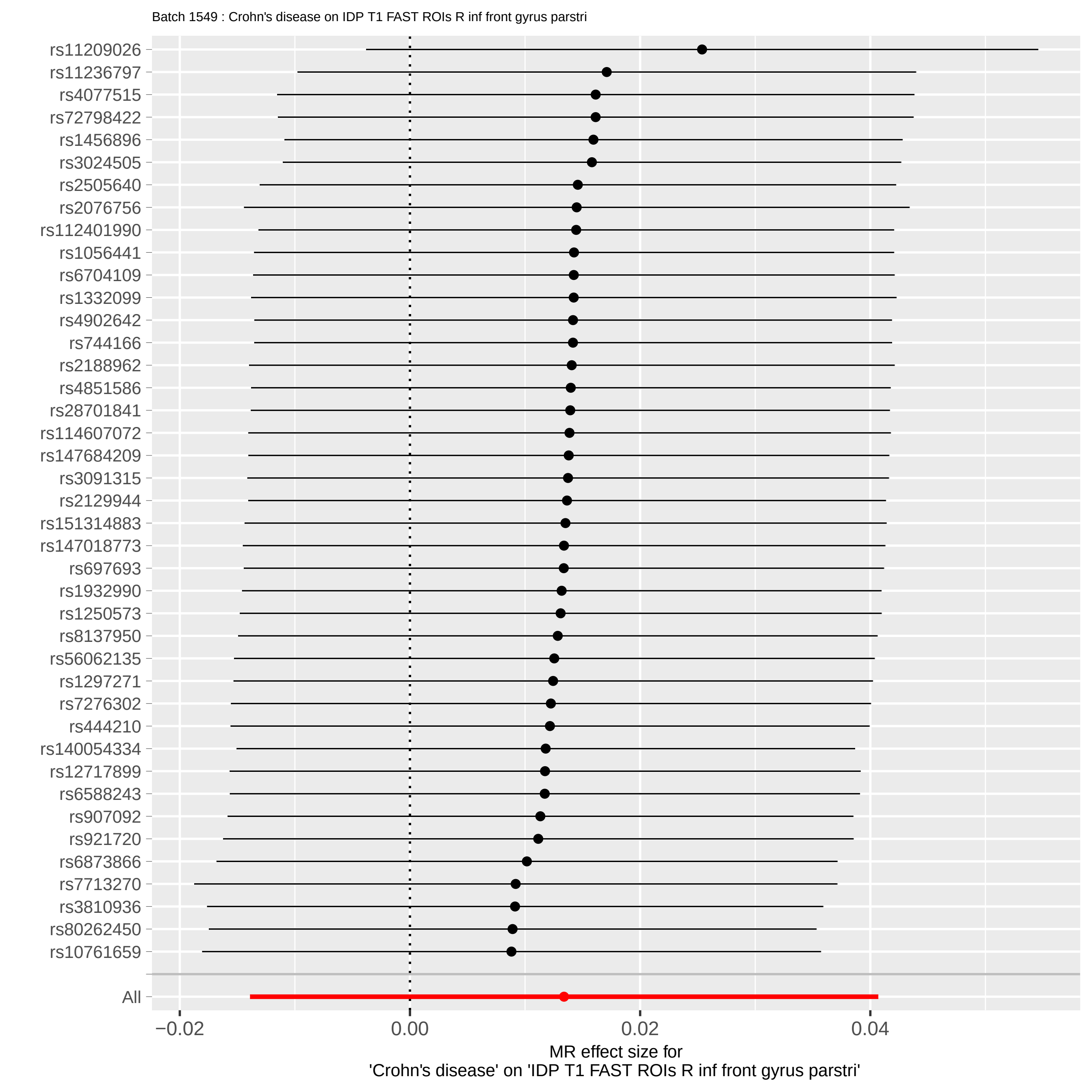

Batch 1549 : Crohn's disease on IDP T1 FAST ROIs R inf front gyrus parstri
rs11209026
rs11236797
rs4077515
rs72798422
rs1456896
rs3024505
rs2505640
rs2076756
rs112401990
rs1056441
rs6704109
rs1332099
rs4902642
rs744166
rs2188962
rs4851586
rs28701841
rs114607072
rs147684209
rs3091315
rs2129944
rs151314883
rs147018773
rs697693
rs1932990
rs1250573
rs8137950
rs56062135
rs1297271
rs7276302
rs444210
rs140054334
rs12717899
rs6588243
rs907092
rs921720
rs6873866
rs7713270
rs3810936
rs80262450
rs10761659
All
−0.02
0.00
0.02
0.04
MR effect size for
'Crohn's disease' on 'IDP T1 FAST ROIs R inf front gyrus parstri'

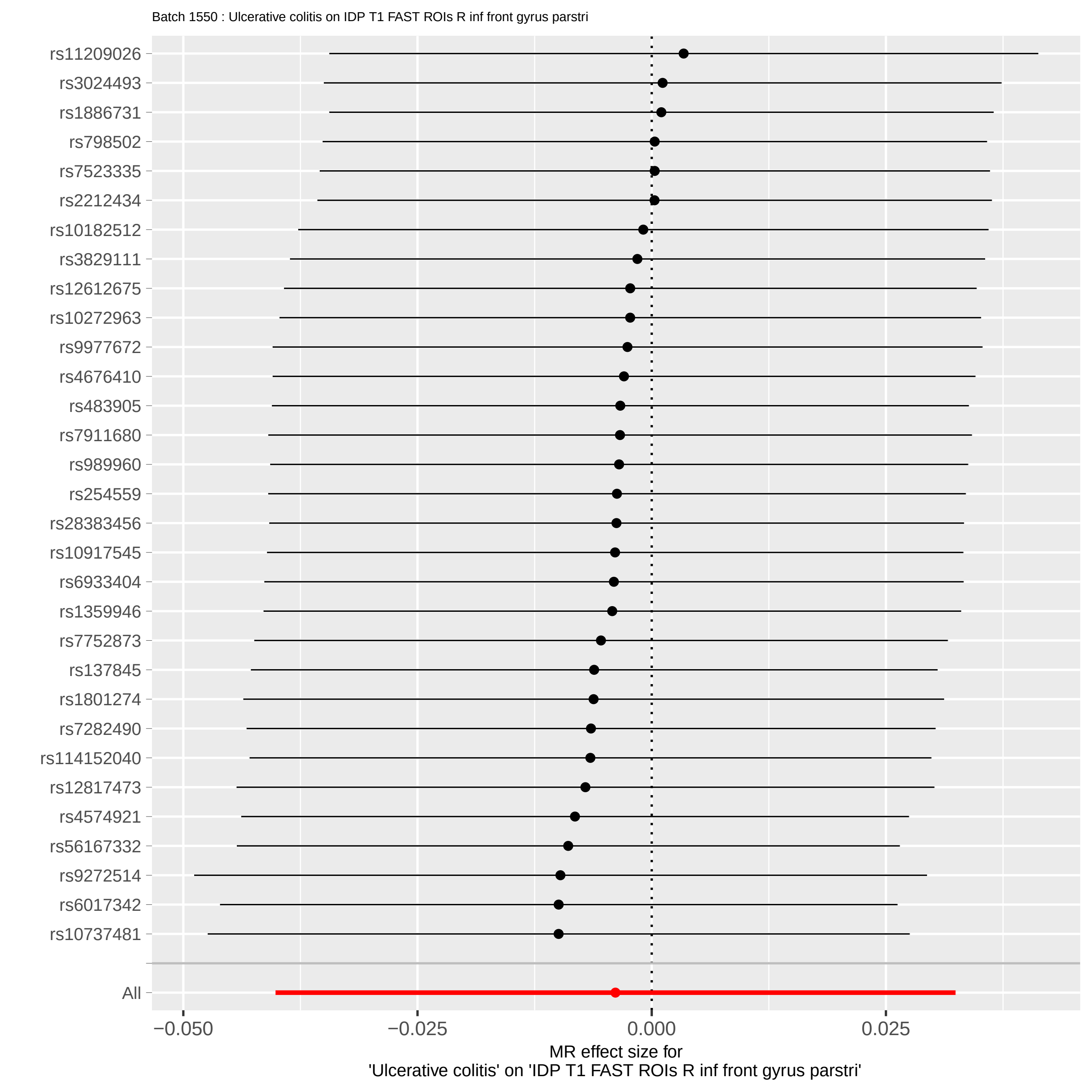

Batch 1550 : Ulcerative colitis on IDP T1 FAST ROIs R inf front gyrus parstri
rs11209026
rs3024493
rs1886731
rs798502
rs7523335
rs2212434
rs10182512
rs3829111
rs12612675
rs10272963
rs9977672
rs4676410
rs483905
rs7911680
rs989960
rs254559
rs28383456
rs10917545
rs6933404
rs1359946
rs7752873
rs137845
rs1801274
rs7282490
rs114152040
rs12817473
rs4574921
rs56167332
rs9272514
rs6017342
rs10737481
All
−0.050
−0.025
0.000
0.025
MR effect size for
'Ulcerative colitis' on 'IDP T1 FAST ROIs R inf front gyrus parstri'

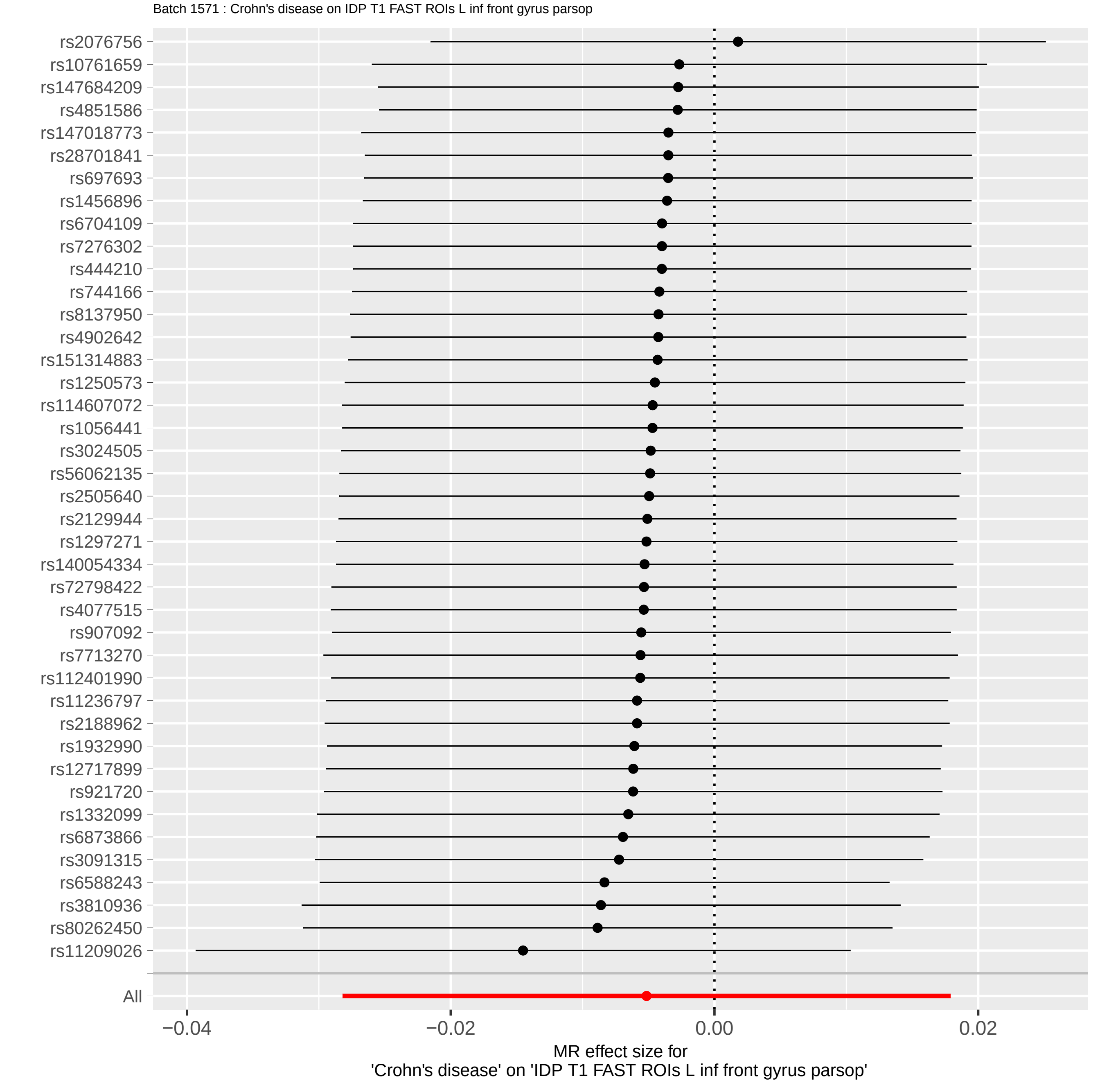
Batch 1571 : Crohn's disease on IDP T1 FAST ROIs L inf front gyrus parsop
rs2076756
rs10761659
rs147684209
rs4851586
rs147018773
rs28701841
rs697693
rs1456896
rs6704109
rs7276302
rs444210
rs744166
rs8137950
rs4902642
rs151314883
rs1250573
rs114607072
rs1056441
rs3024505
rs56062135
rs2505640
rs2129944
rs1297271
rs140054334
rs72798422
rs4077515
rs907092
rs7713270
rs112401990
rs11236797
rs2188962
rs1932990
rs12717899
rs921720
rs1332099
rs6873866
rs3091315
rs6588243
rs3810936
rs80262450
rs11209026
All
−0.04
−0.02
0.00
0.02
MR effect size for
'Crohn's disease' on 'IDP T1 FAST ROIs L inf front gyrus parsop'

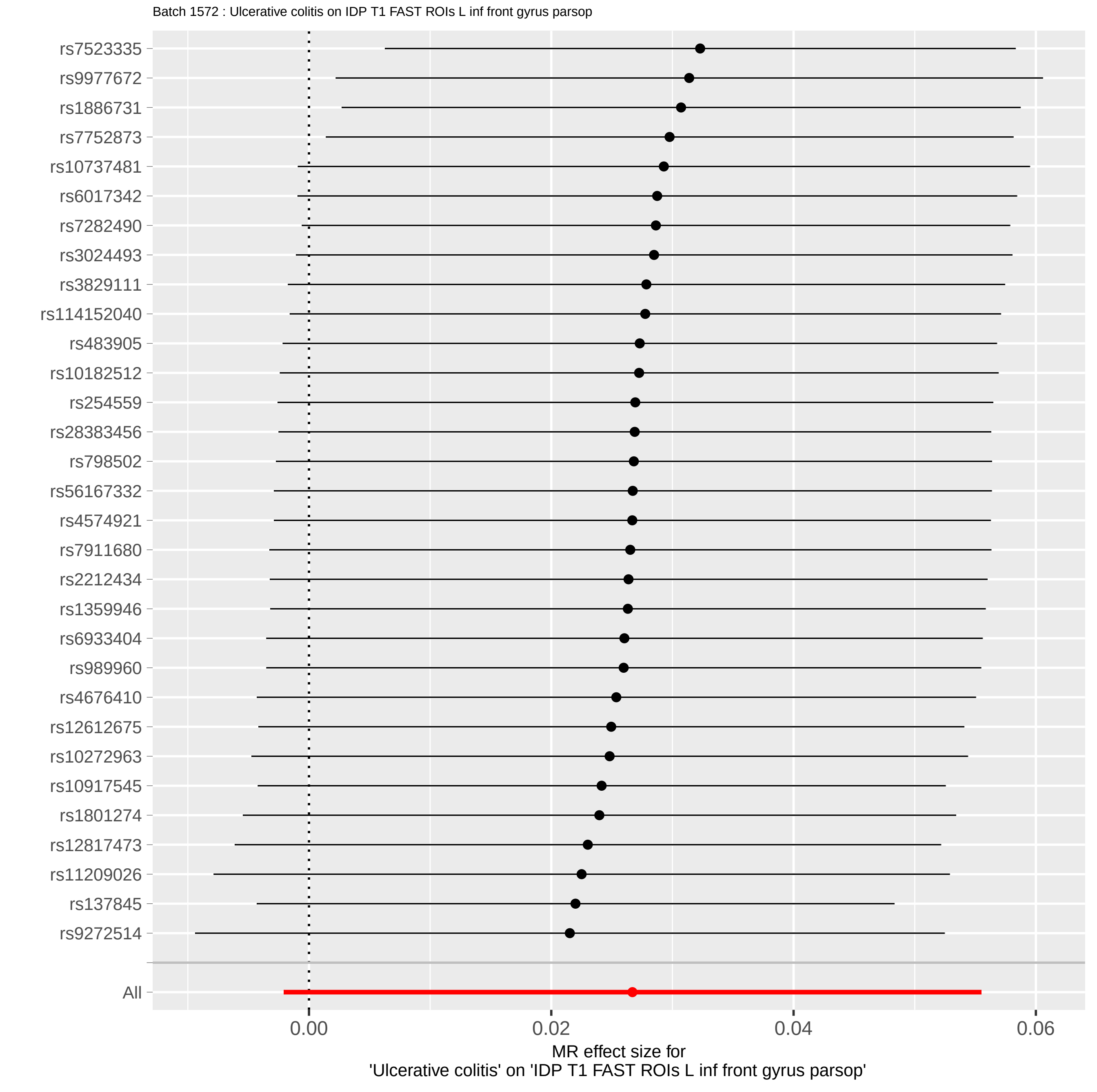

Batch 1572 : Ulcerative colitis on IDP T1 FAST ROIs L inf front gyrus parsop
rs7523335
rs9977672
rs1886731
rs7752873
rs10737481
rs6017342
rs7282490
rs3024493
rs3829111
rs114152040
rs483905
rs10182512
rs254559
rs28383456
rs798502
rs56167332
rs4574921
rs7911680
rs2212434
rs1359946
rs6933404
rs989960
rs4676410
rs12612675
rs10272963
rs10917545
rs1801274
rs12817473
rs11209026
rs137845
rs9272514
All
0.00
0.02
0.04
0.06
MR effect size for
'Ulcerative colitis' on 'IDP T1 FAST ROIs L inf front gyrus parsop'

Batch 1593 : Crohn's disease on IDP T1 FAST ROIs R inf front gyrus parsop

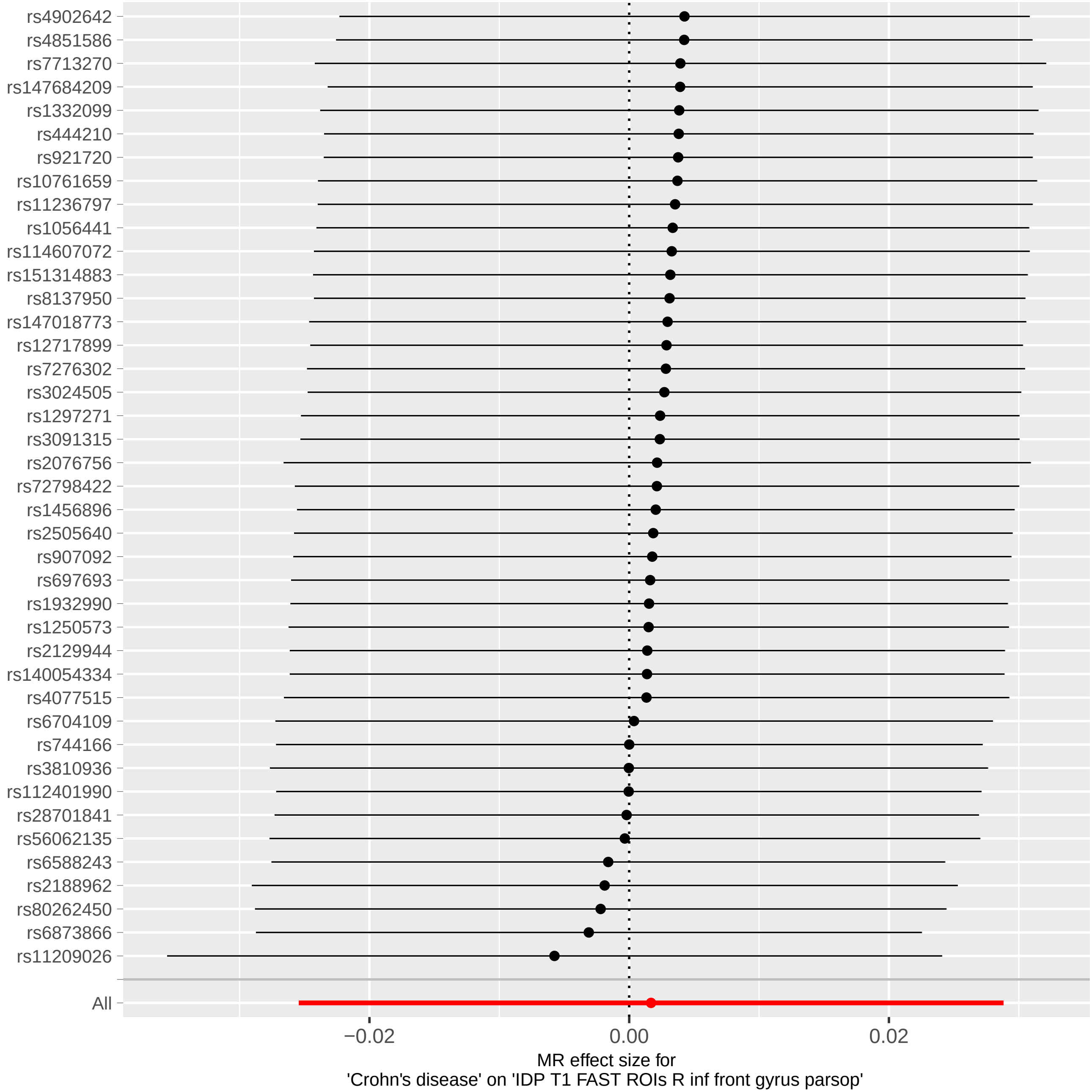

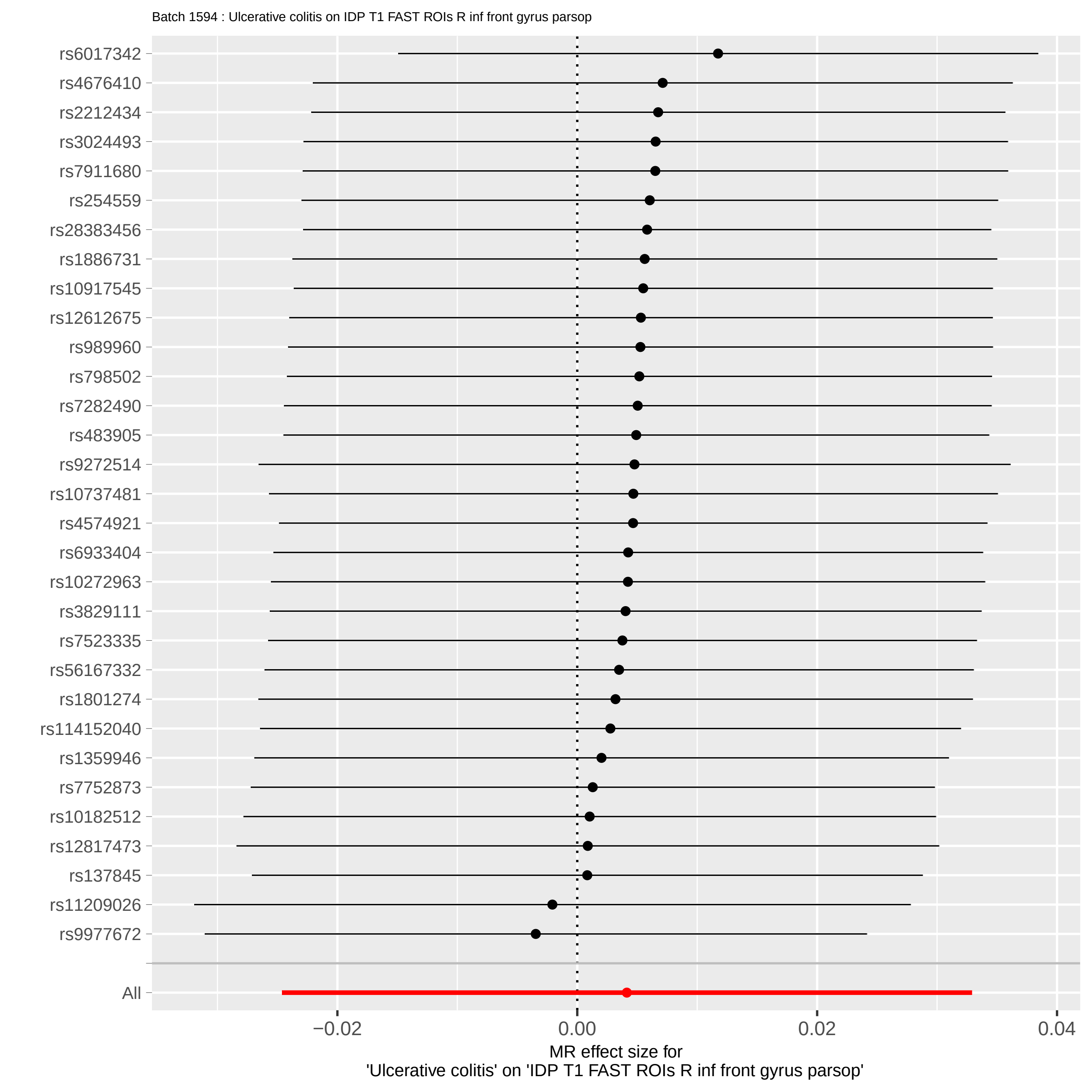

Batch 1594 : Ulcerative colitis on IDP T1 FAST ROIs R inf front gyrus parsop
rs6017342
rs4676410
rs2212434
rs3024493
rs7911680
rs254559
rs28383456
rs1886731
rs10917545
rs12612675
rs989960
rs798502
rs7282490
rs483905
rs9272514
rs10737481
rs4574921
rs6933404
rs10272963
rs3829111
rs7523335
rs56167332
rs1801274
rs114152040
rs1359946
rs7752873
rs10182512
rs12817473
rs137845
rs11209026
rs9977672
All
−0.02
0.00
0.02
0.04
MR effect size for
'Ulcerative colitis' on 'IDP T1 FAST ROIs R inf front gyrus parsop'

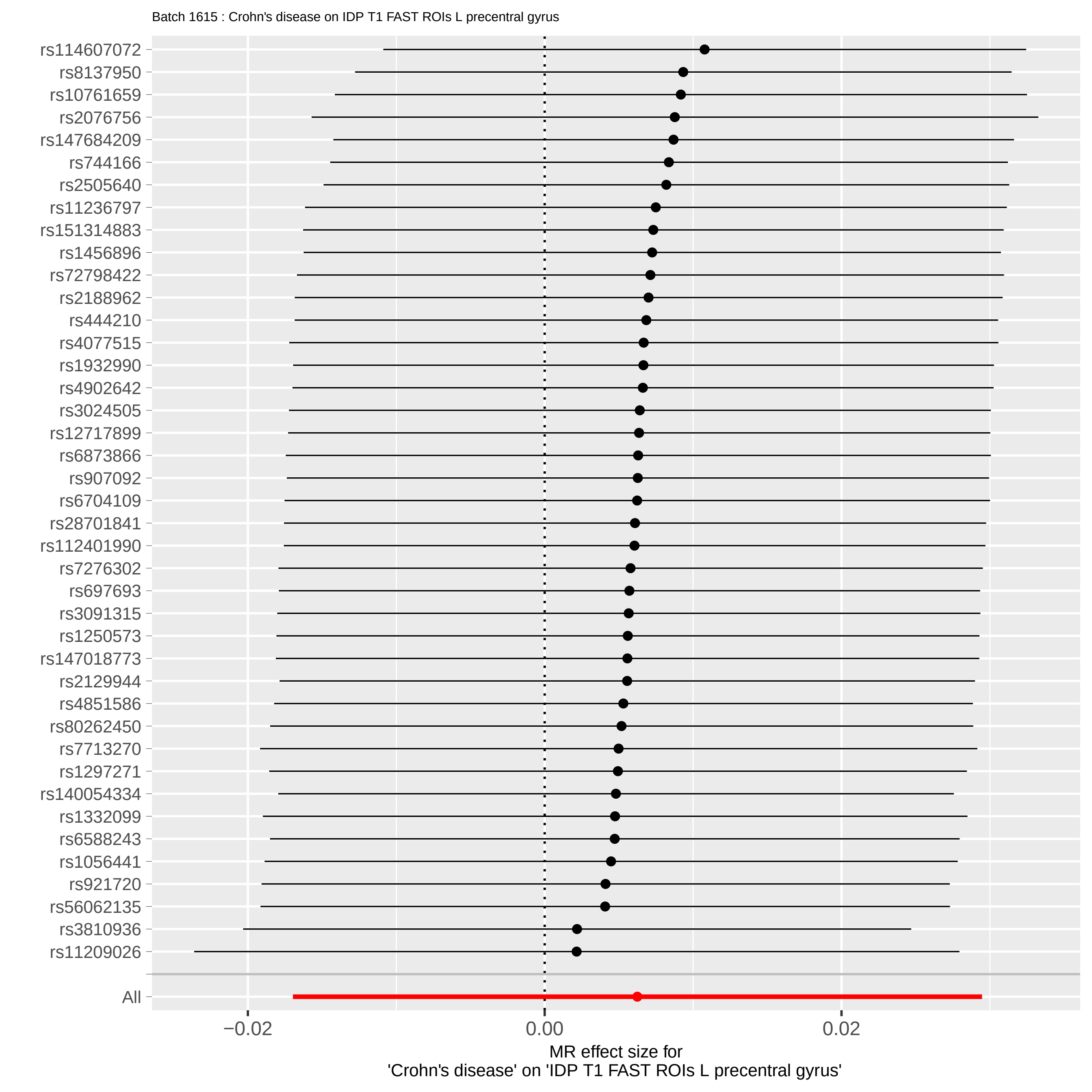
Batch 1615 : Crohn's disease on IDP T1 FAST ROIs L precentral gyrus
rs114607072
rs8137950
rs10761659
rs2076756
rs147684209
rs744166
rs2505640
rs11236797
rs151314883
rs1456896
rs72798422
rs2188962
rs444210
rs4077515
rs1932990
rs4902642
rs3024505
rs12717899
rs6873866
rs907092
rs6704109
rs28701841
rs112401990
rs7276302
rs697693
rs3091315
rs1250573
rs147018773
rs2129944
rs4851586
rs80262450
rs7713270
rs1297271
rs140054334
rs1332099
rs6588243
rs1056441
rs921720
rs56062135
rs3810936
rs11209026
All
−0.02
0.00
0.02
MR effect size for
'Crohn's disease' on 'IDP T1 FAST ROIs L precentral gyrus'

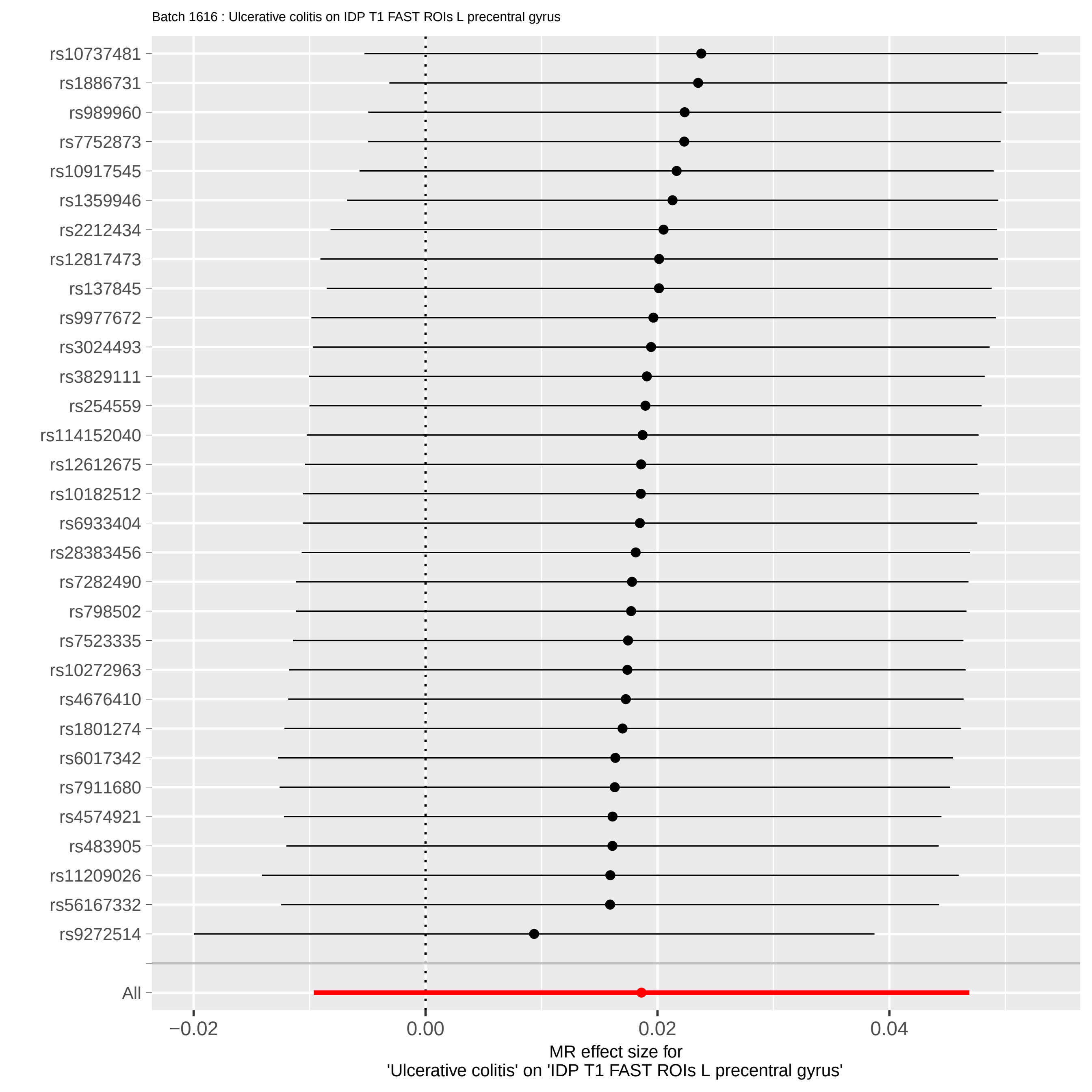

Batch 1616 : Ulcerative colitis on IDP T1 FAST ROIs L precentral gyrus
rs10737481
rs1886731
rs989960
rs7752873
rs10917545
rs1359946
rs2212434
rs12817473
rs137845
rs9977672
rs3024493
rs3829111
rs254559
rs114152040
rs12612675
rs10182512
rs6933404
rs28383456
rs7282490
rs798502
rs7523335
rs10272963
rs4676410
rs1801274
rs6017342
rs7911680
rs4574921
rs483905
rs11209026
rs56167332
rs9272514
All
−0.02
0.00
0.02
0.04
MR effect size for
'Ulcerative colitis' on 'IDP T1 FAST ROIs L precentral gyrus'

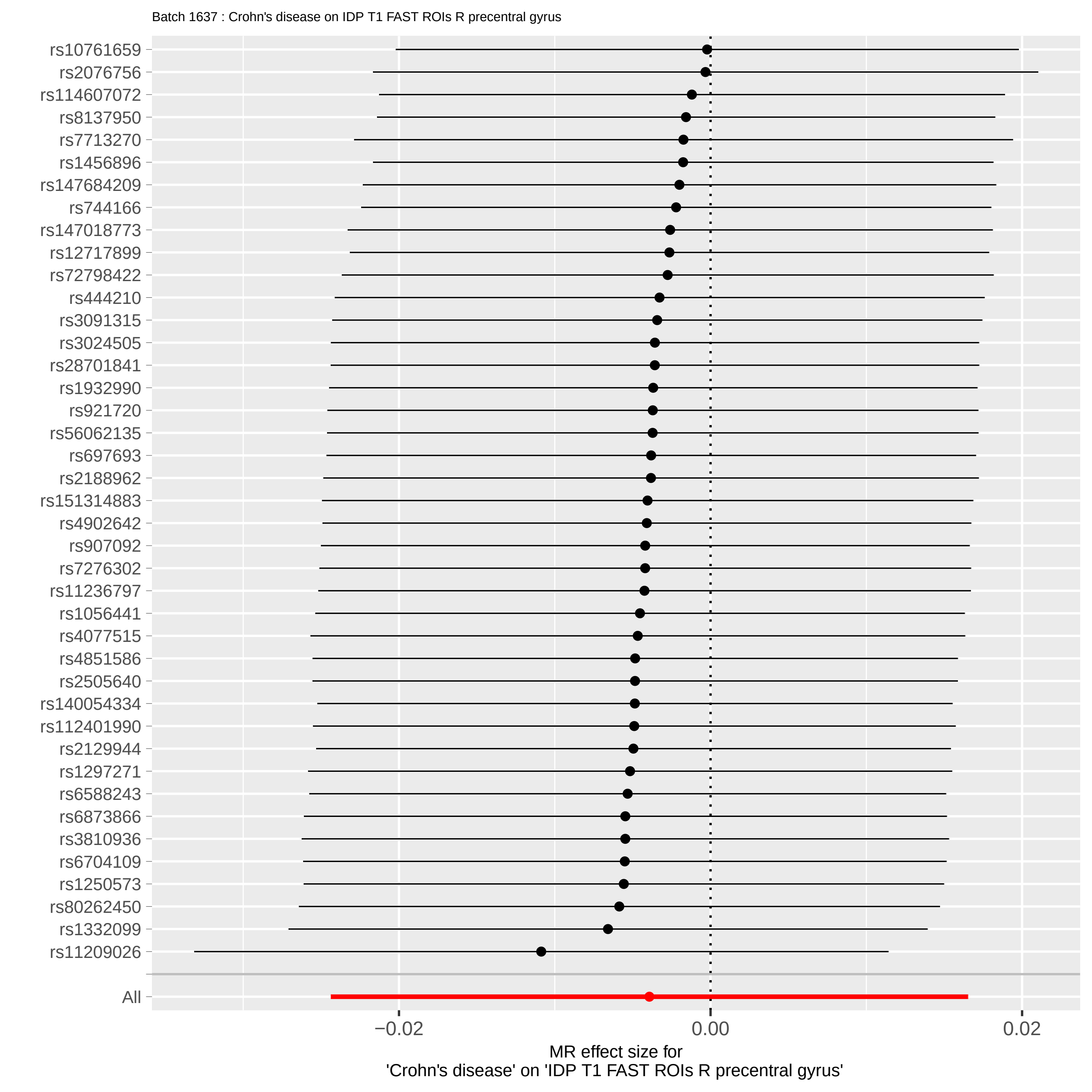
Batch 1637 : Crohn's disease on IDP T1 FAST ROIs R precentral gyrus
rs10761659
rs2076756
rs114607072
rs8137950
rs7713270
rs1456896
rs147684209
rs744166
rs147018773
rs12717899
rs72798422
rs444210
rs3091315
rs3024505
rs28701841
rs1932990
rs921720
rs56062135
rs697693
rs2188962
rs151314883
rs4902642
rs907092
rs7276302
rs11236797
rs1056441
rs4077515
rs4851586
rs2505640
rs140054334
rs112401990
rs2129944
rs1297271
rs6588243
rs6873866
rs3810936
rs6704109
rs1250573
rs80262450
rs1332099
rs11209026
All
−0.02
0.00
0.02
MR effect size for
'Crohn's disease' on 'IDP T1 FAST ROIs R precentral gyrus'

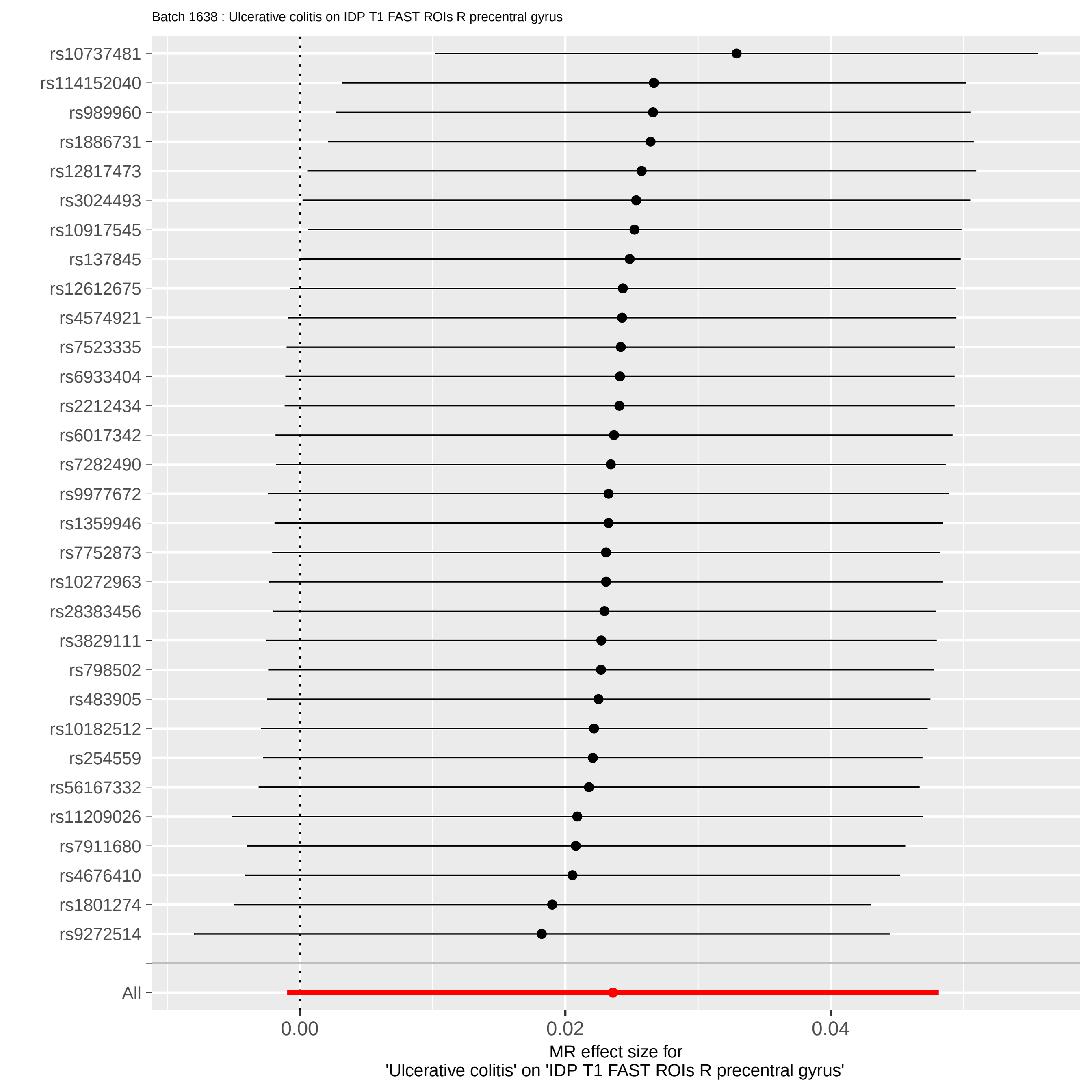

Batch 1638 : Ulcerative colitis on IDP T1 FAST ROIs R precentral gyrus
rs10737481
rs114152040
rs989960
rs1886731
rs12817473
rs3024493
rs10917545
rs137845
rs12612675
rs4574921
rs7523335
rs6933404
rs2212434
rs6017342
rs7282490
rs9977672
rs1359946
rs7752873
rs10272963
rs28383456
rs3829111
rs798502
rs483905
rs10182512
rs254559
rs56167332
rs11209026
rs7911680
rs4676410
rs1801274
rs9272514
All
0.00
0.02
0.04
MR effect size for
'Ulcerative colitis' on 'IDP T1 FAST ROIs R precentral gyrus'

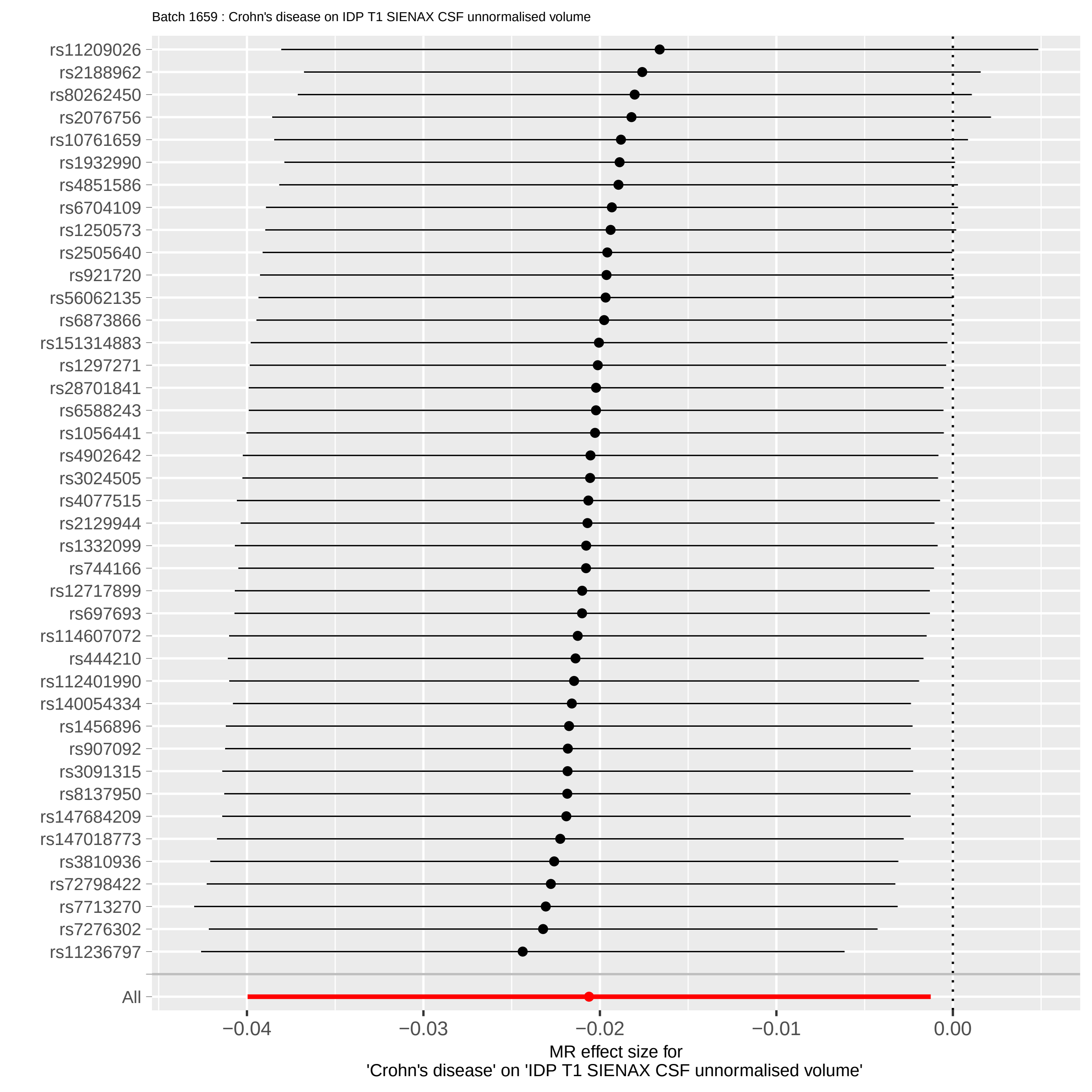

Batch 1659 : Crohn's disease on IDP T1 SIENAX CSF unnormalised volume
rs11209026
rs2188962
rs80262450
rs2076756
rs10761659
rs1932990
rs4851586
rs6704109
rs1250573
rs2505640
rs921720
rs56062135
rs6873866
rs151314883
rs1297271
rs28701841
rs6588243
rs1056441
rs4902642
rs3024505
rs4077515
rs2129944
rs1332099
rs744166
rs12717899
rs697693
rs114607072
rs444210
rs112401990
rs140054334
rs1456896
rs907092
rs3091315
rs8137950
rs147684209
rs147018773
rs3810936
rs72798422
rs7713270
rs7276302
rs11236797
All
−0.04
−0.03
−0.02
−0.01
0.00
MR effect size for
'Crohn's disease' on 'IDP T1 SIENAX CSF unnormalised volume'

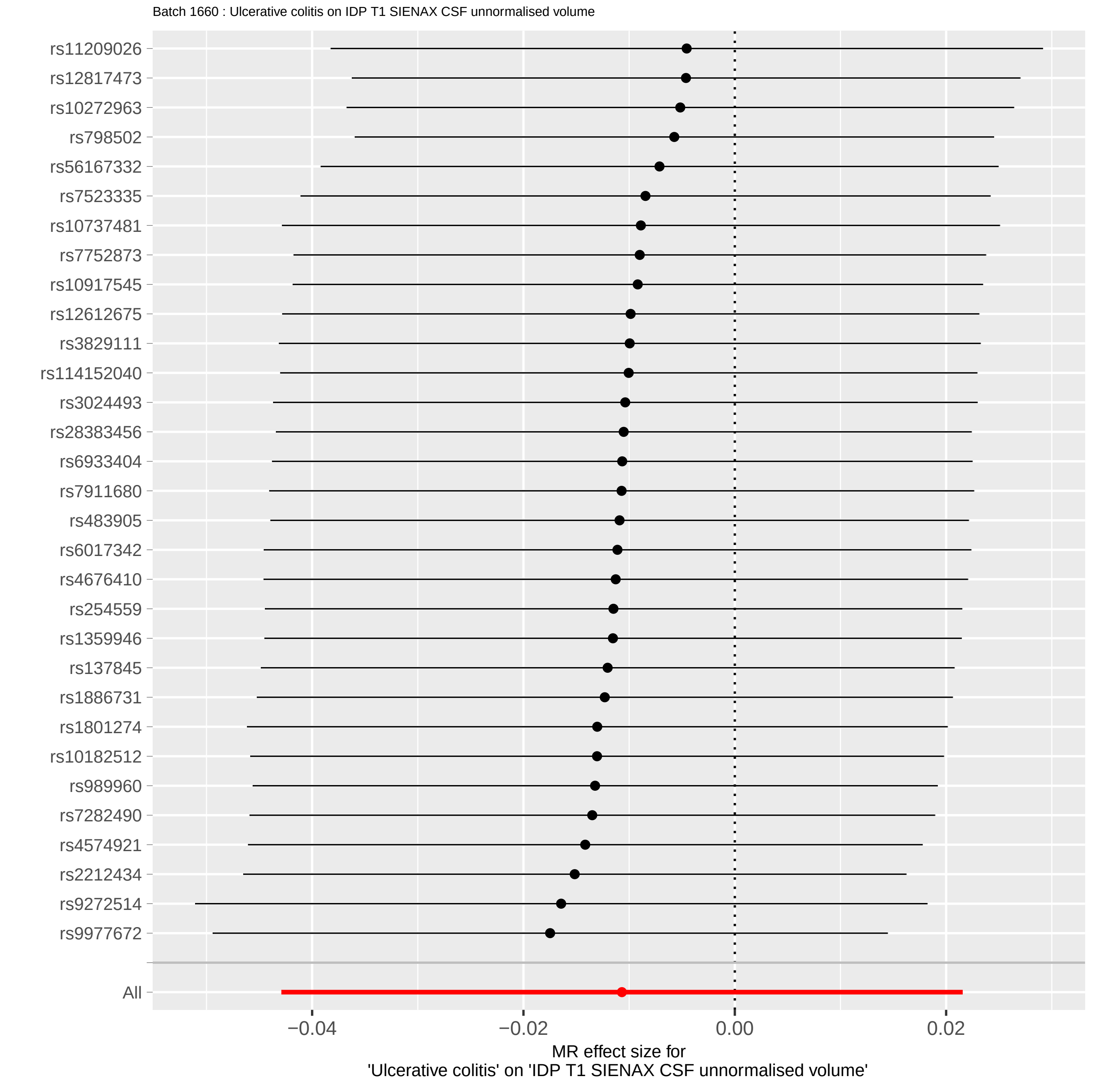
Batch 1660 : Ulcerative colitis on IDP T1 SIENAX CSF unnormalised volume
rs11209026
rs12817473
rs10272963
rs798502
rs56167332
rs7523335
rs10737481
rs7752873
rs10917545
rs12612675
rs3829111
rs114152040
rs3024493
rs28383456
rs6933404
rs7911680
rs483905
rs6017342
rs4676410
rs254559
rs1359946
rs137845
rs1886731
rs1801274
rs10182512
rs989960
rs7282490
rs4574921
rs2212434
rs9272514
rs9977672
All
−0.04
−0.02
0.00
0.02
MR effect size for
'Ulcerative colitis' on 'IDP T1 SIENAX CSF unnormalised volume'

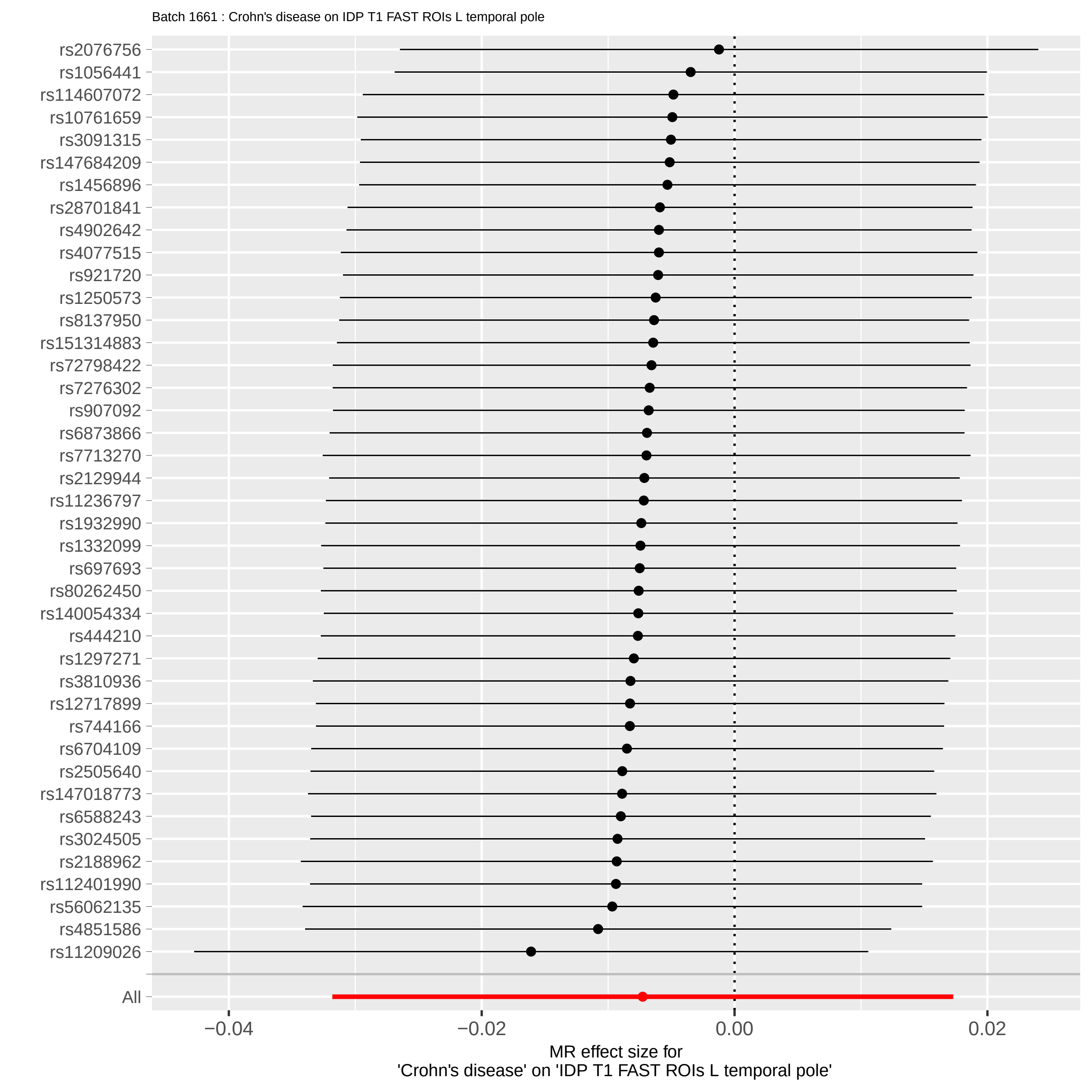

Batch 1661 : Crohn's disease on IDP T1 FAST ROIs L temporal pole
rs2076756
rs1056441
rs114607072
rs10761659
rs3091315
rs147684209
rs1456896
rs28701841
rs4902642
rs4077515
rs921720
rs1250573
rs8137950
rs151314883
rs72798422
rs7276302
rs907092
rs6873866
rs7713270
rs2129944
rs11236797
rs1932990
rs1332099
rs697693
rs80262450
rs140054334
rs444210
rs1297271
rs3810936
rs12717899
rs744166
rs6704109
rs2505640
rs147018773
rs6588243
rs3024505
rs2188962
rs112401990
rs56062135
rs4851586
rs11209026
All
−0.04
−0.02
0.00
0.02
MR effect size for
'Crohn's disease' on 'IDP T1 FAST ROIs L temporal pole'

Batch 1662 : Ulcerative colitis on IDP T1 FAST ROIs L temporal pole

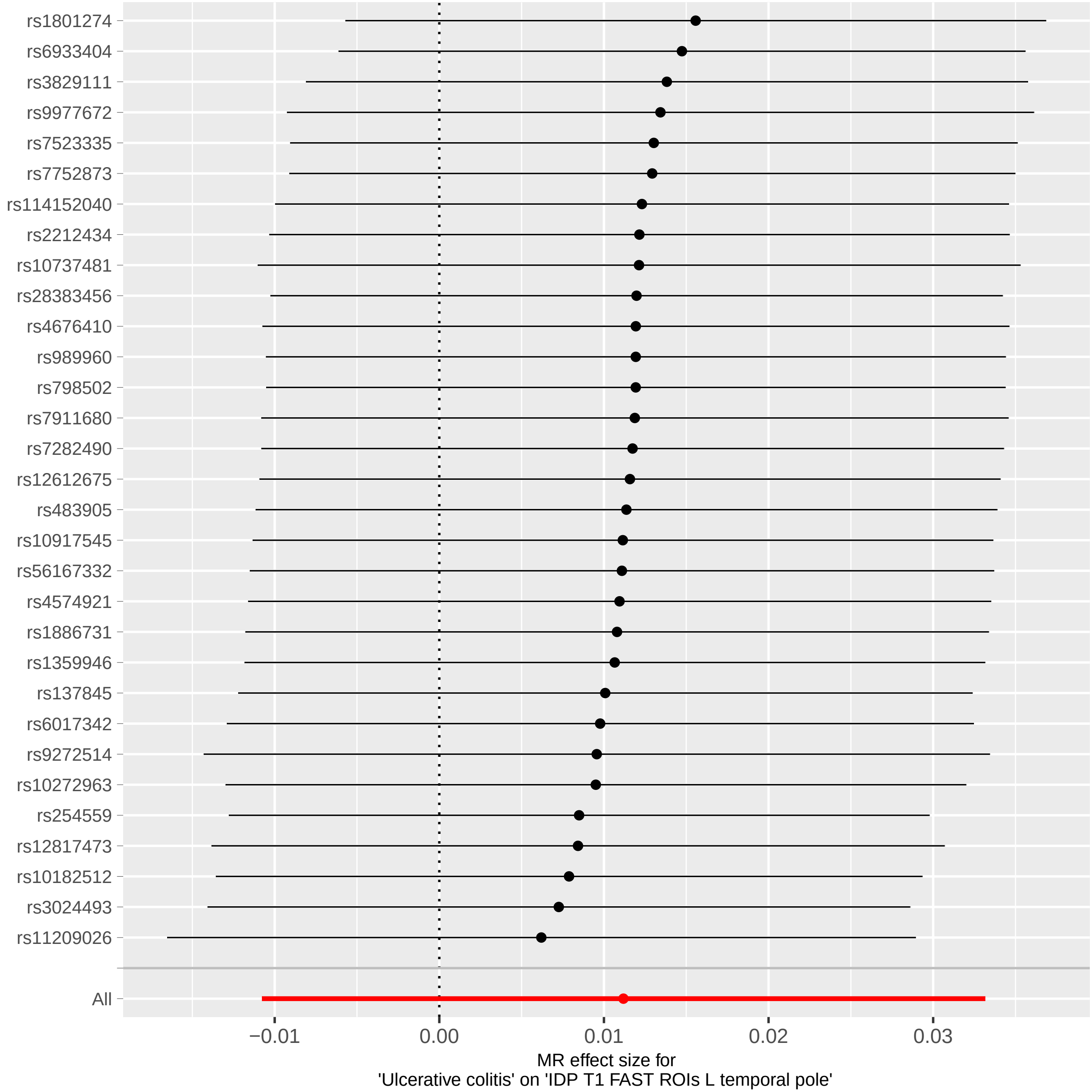

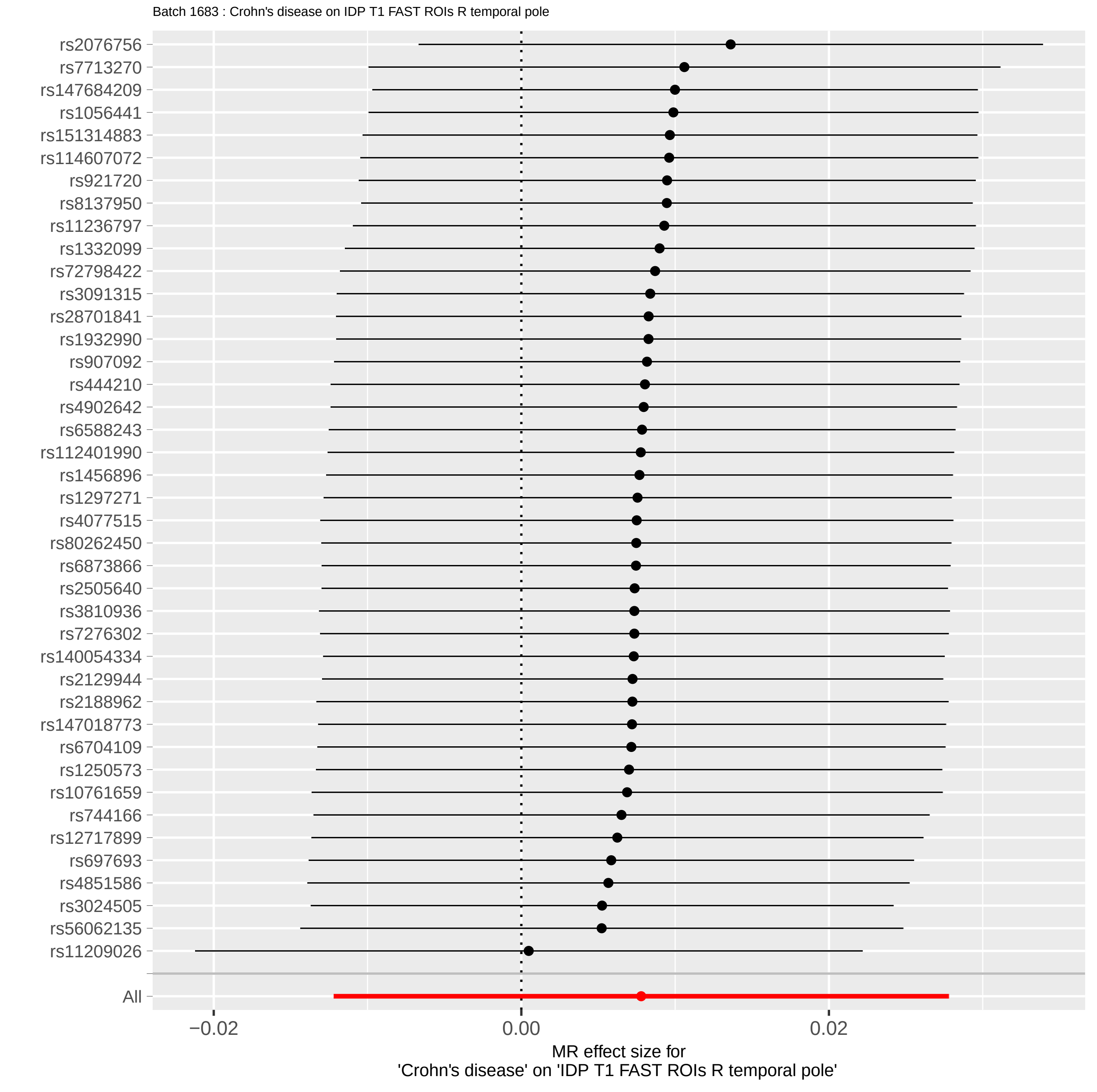
Batch 1683 : Crohn's disease on IDP T1 FAST ROIs R temporal pole
rs2076756
rs7713270
rs147684209
rs1056441
rs151314883
rs114607072
rs921720
rs8137950
rs11236797
rs1332099
rs72798422
rs3091315
rs28701841
rs1932990
rs907092
rs444210
rs4902642
rs6588243
rs112401990
rs1456896
rs1297271
rs4077515
rs80262450
rs6873866
rs2505640
rs3810936
rs7276302
rs140054334
rs2129944
rs2188962
rs147018773
rs6704109
rs1250573
rs10761659
rs744166
rs12717899
rs697693
rs4851586
rs3024505
rs56062135
rs11209026
All
−0.02
0.00
0.02
MR effect size for
'Crohn's disease' on 'IDP T1 FAST ROIs R temporal pole'

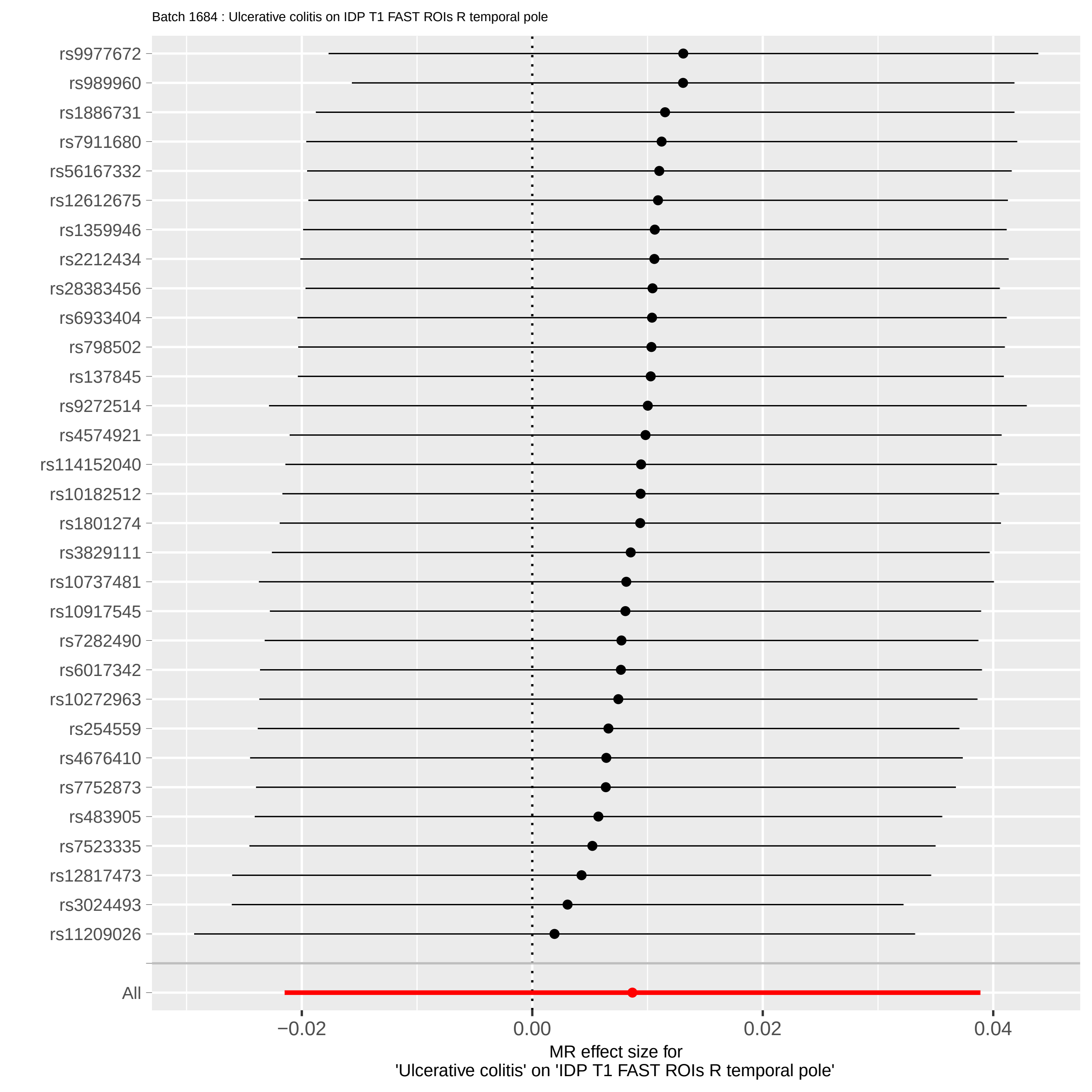

Batch 1684 : Ulcerative colitis on IDP T1 FAST ROIs R temporal pole
rs9977672
rs989960
rs1886731
rs7911680
rs56167332
rs12612675
rs1359946
rs2212434
rs28383456
rs6933404
rs798502
rs137845
rs9272514
rs4574921
rs114152040
rs10182512
rs1801274
rs3829111
rs10737481
rs10917545
rs7282490
rs6017342
rs10272963
rs254559
rs4676410
rs7752873
rs483905
rs7523335
rs12817473
rs3024493
rs11209026
All
−0.02
0.00
0.02
0.04
MR effect size for
'Ulcerative colitis' on 'IDP T1 FAST ROIs R temporal pole'

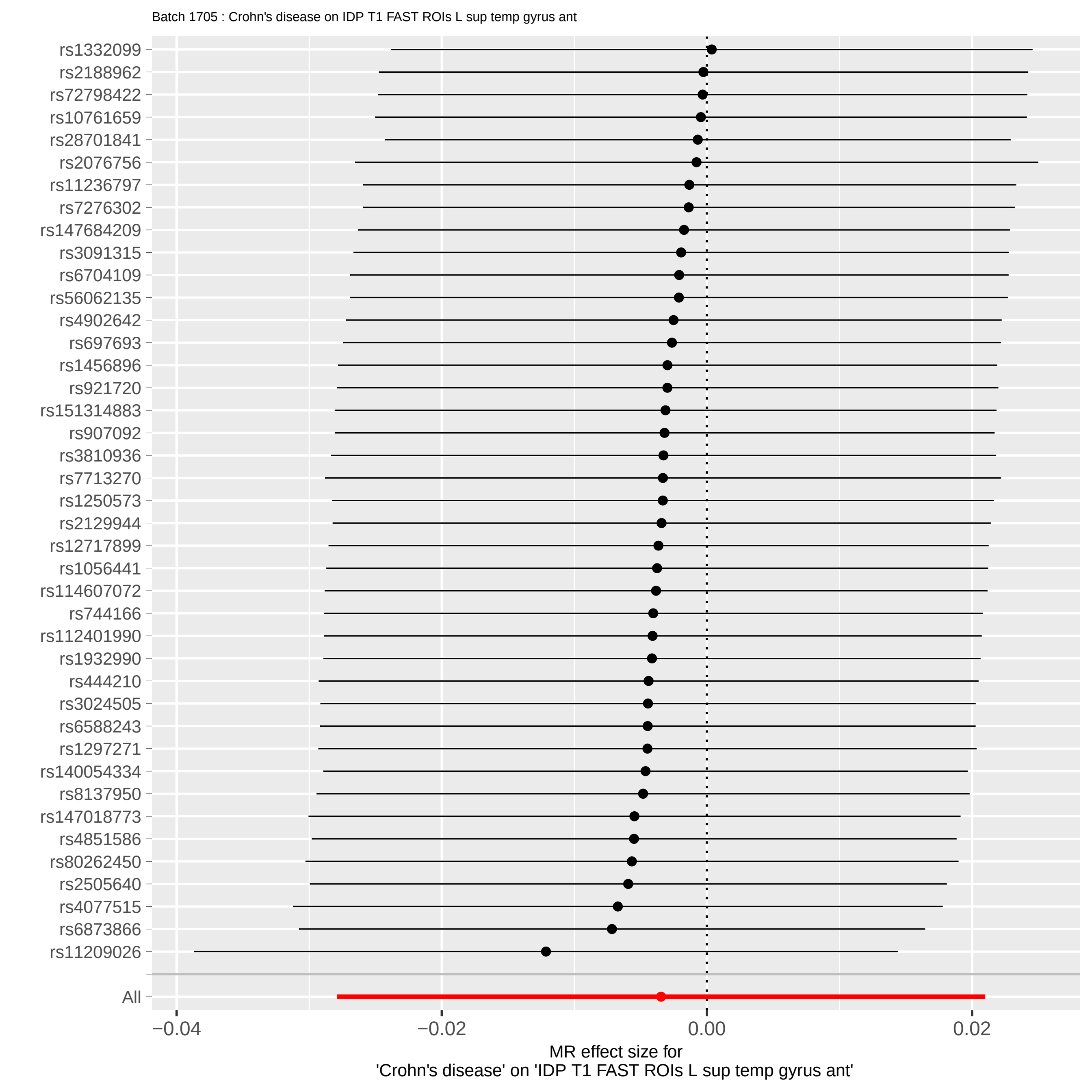

Batch 1705 : Crohn's disease on IDP T1 FAST ROIs L sup temp gyrus ant
rs1332099
rs2188962
rs72798422
rs10761659
rs28701841
rs2076756
rs11236797
rs7276302
rs147684209
rs3091315
rs6704109
rs56062135
rs4902642
rs697693
rs1456896
rs921720
rs151314883
rs907092
rs3810936
rs7713270
rs1250573
rs2129944
rs12717899
rs1056441
rs114607072
rs744166
rs112401990
rs1932990
rs444210
rs3024505
rs6588243
rs1297271
rs140054334
rs8137950
rs147018773
rs4851586
rs80262450
rs2505640
rs4077515
rs6873866
rs11209026
All
−0.04
−0.02
0.00
0.02
MR effect size for
'Crohn's disease' on 'IDP T1 FAST ROIs L sup temp gyrus ant'

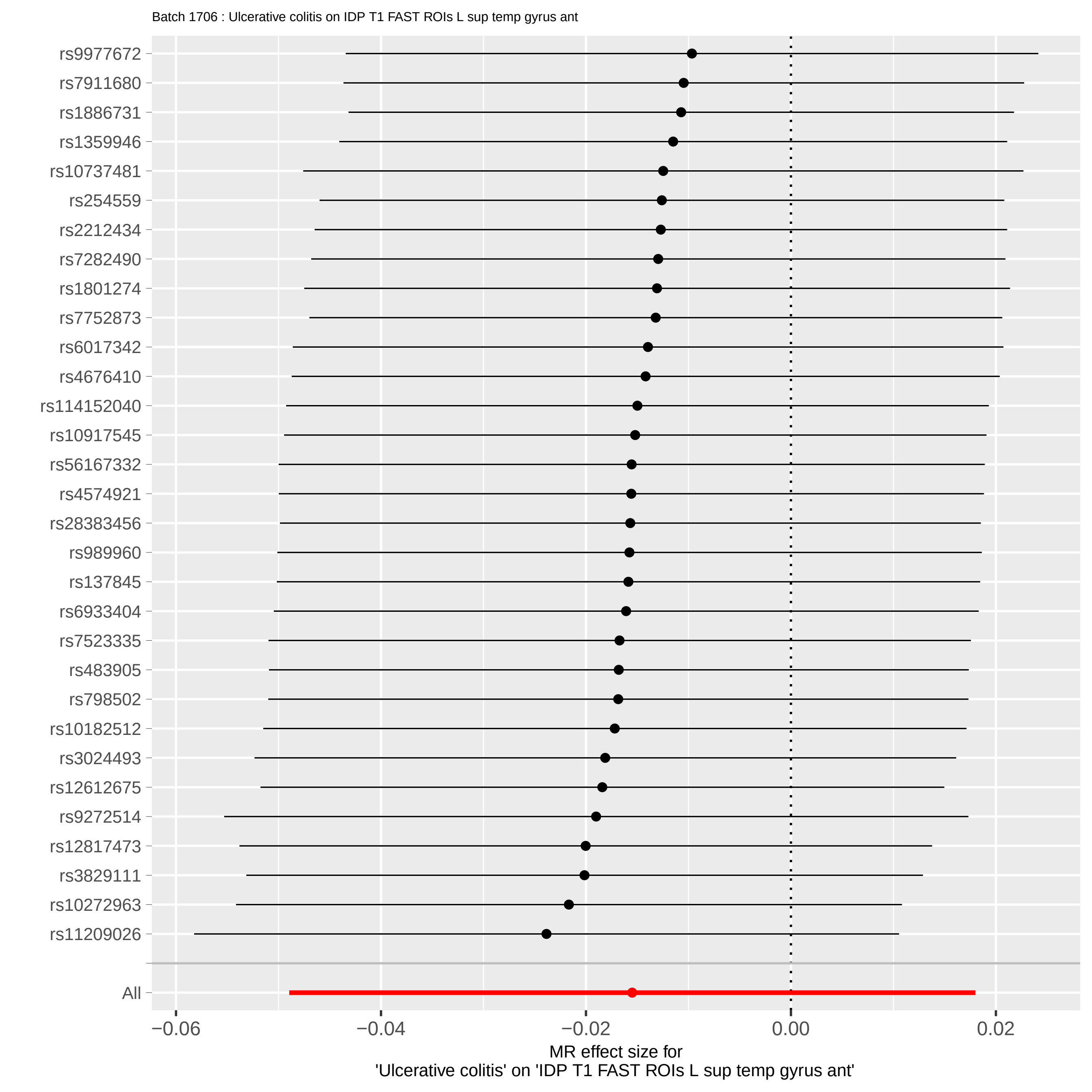

Batch 1706 : Ulcerative colitis on IDP T1 FAST ROIs L sup temp gyrus ant
rs9977672
rs7911680
rs1886731
rs1359946
rs10737481
rs254559
rs2212434
rs7282490
rs1801274
rs7752873
rs6017342
rs4676410
rs114152040
rs10917545
rs56167332
rs4574921
rs28383456
rs989960
rs137845
rs6933404
rs7523335
rs483905
rs798502
rs10182512
rs3024493
rs12612675
rs9272514
rs12817473
rs3829111
rs10272963
rs11209026
All
−0.06
−0.04
−0.02
0.00
0.02
MR effect size for
'Ulcerative colitis' on 'IDP T1 FAST ROIs L sup temp gyrus ant'

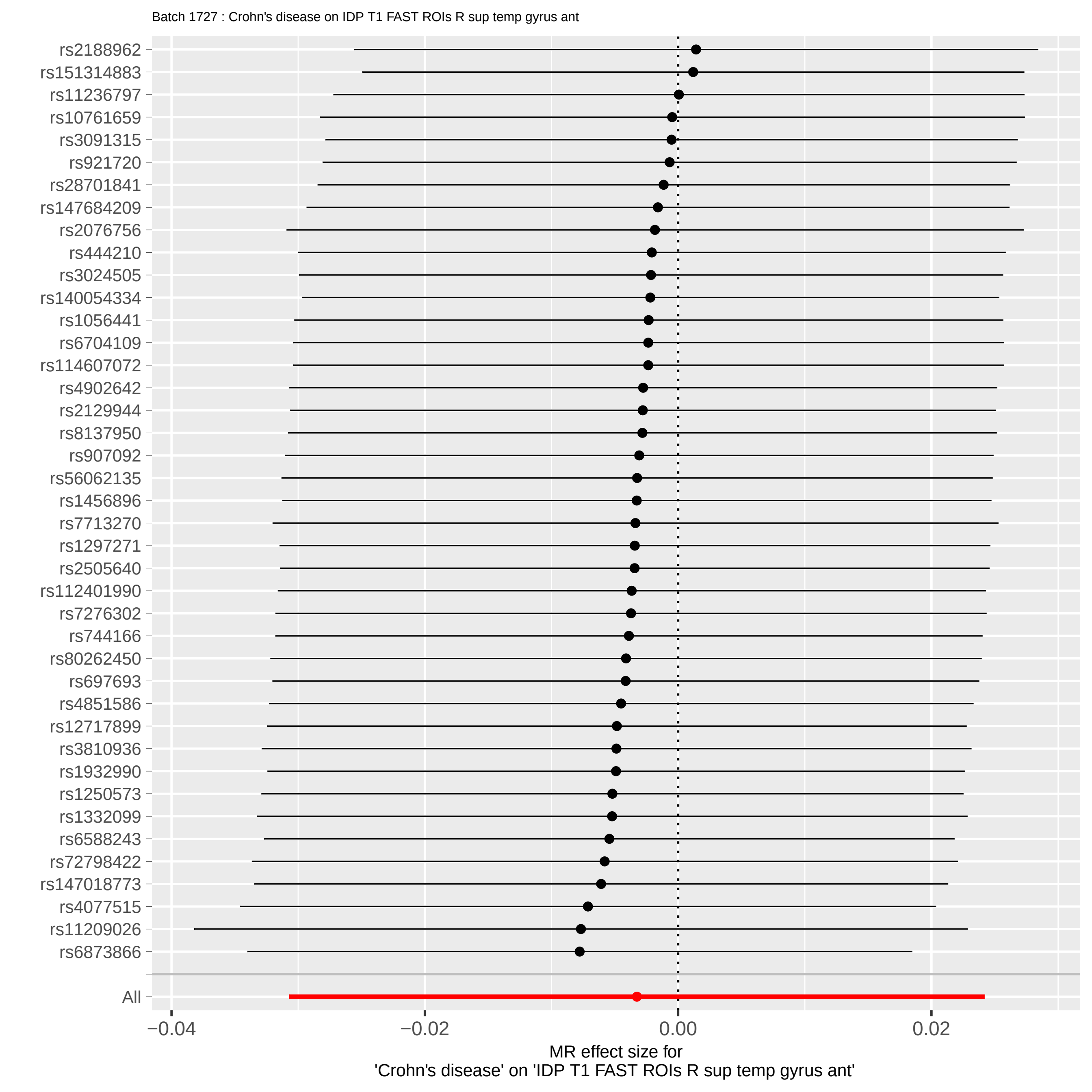

Batch 1727 : Crohn's disease on IDP T1 FAST ROIs R sup temp gyrus ant
rs2188962
rs151314883
rs11236797
rs10761659
rs3091315
rs921720
rs28701841
rs147684209
rs2076756
rs444210
rs3024505
rs140054334
rs1056441
rs6704109
rs114607072
rs4902642
rs2129944
rs8137950
rs907092
rs56062135
rs1456896
rs7713270
rs1297271
rs2505640
rs112401990
rs7276302
rs744166
rs80262450
rs697693
rs4851586
rs12717899
rs3810936
rs1932990
rs1250573
rs1332099
rs6588243
rs72798422
rs147018773
rs4077515
rs11209026
rs6873866
All
−0.04
−0.02
0.00
0.02
MR effect size for
'Crohn's disease' on 'IDP T1 FAST ROIs R sup temp gyrus ant'

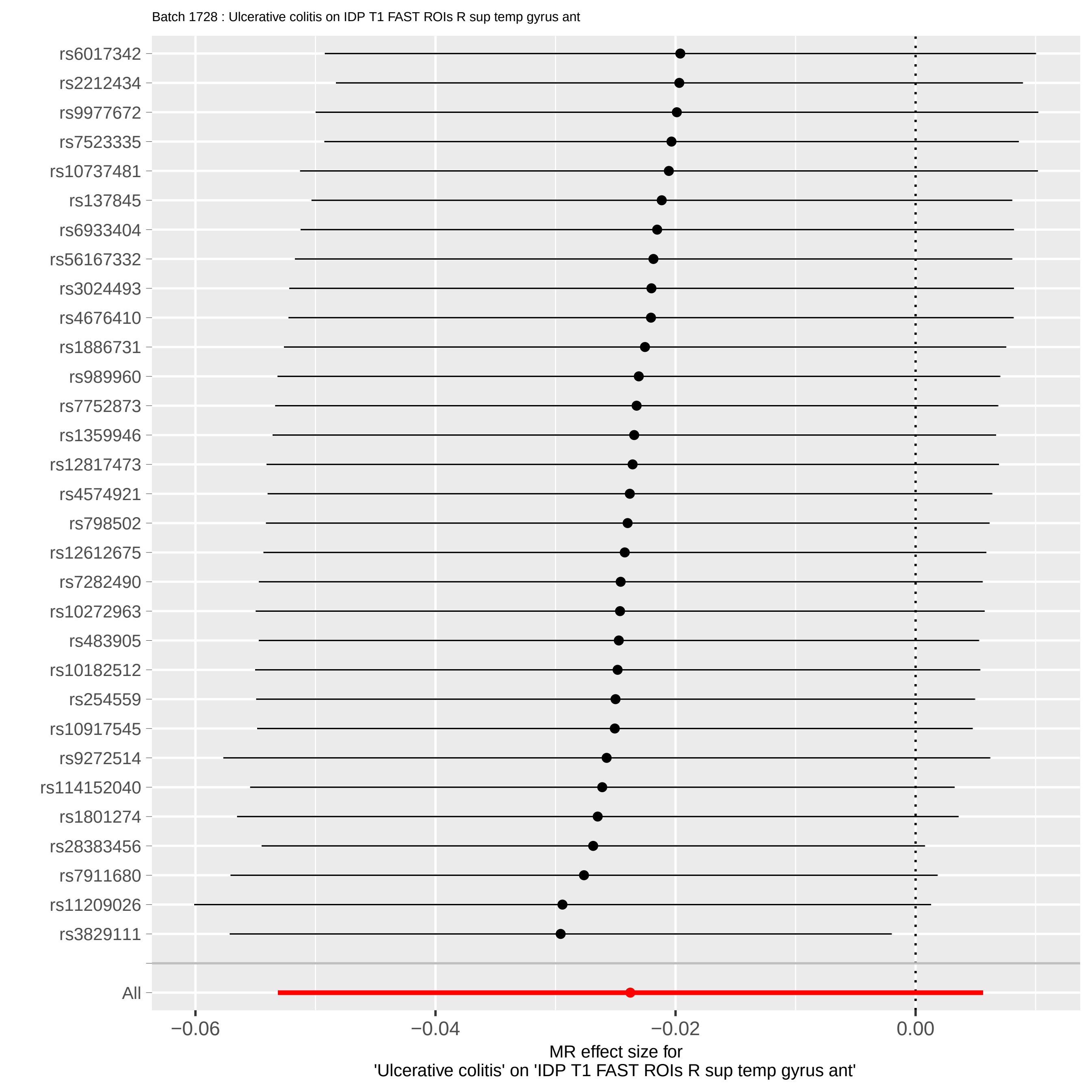

Batch 1728 : Ulcerative colitis on IDP T1 FAST ROIs R sup temp gyrus ant
rs6017342
rs2212434
rs9977672
rs7523335
rs10737481
rs137845
rs6933404
rs56167332
rs3024493
rs4676410
rs1886731
rs989960
rs7752873
rs1359946
rs12817473
rs4574921
rs798502
rs12612675
rs7282490
rs10272963
rs483905
rs10182512
rs254559
rs10917545
rs9272514
rs114152040
rs1801274
rs28383456
rs7911680
rs11209026
rs3829111
All
−0.06
−0.04
−0.02
0.00
MR effect size for
'Ulcerative colitis' on 'IDP T1 FAST ROIs R sup temp gyrus ant'

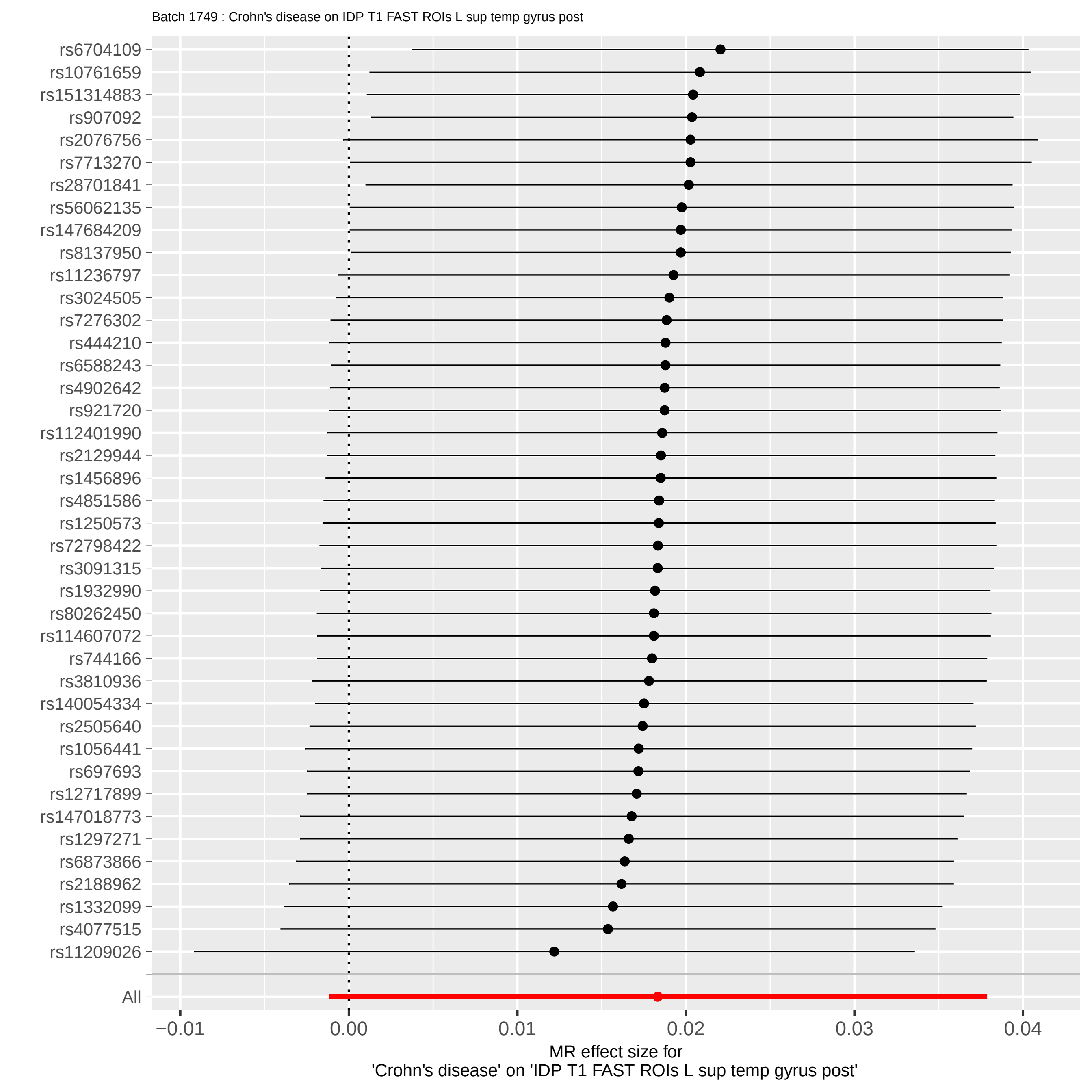

Batch 1749 : Crohn's disease on IDP T1 FAST ROIs L sup temp gyrus post
rs6704109
rs10761659
rs151314883
rs907092
rs2076756
rs7713270
rs28701841
rs56062135
rs147684209
rs8137950
rs11236797
rs3024505
rs7276302
rs444210
rs6588243
rs4902642
rs921720
rs112401990
rs2129944
rs1456896
rs4851586
rs1250573
rs72798422
rs3091315
rs1932990
rs80262450
rs114607072
rs744166
rs3810936
rs140054334
rs2505640
rs1056441
rs697693
rs12717899
rs147018773
rs1297271
rs6873866
rs2188962
rs1332099
rs4077515
rs11209026
All
−0.01
0.00
0.01
0.02
0.03
0.04
MR effect size for
'Crohn's disease' on 'IDP T1 FAST ROIs L sup temp gyrus post'

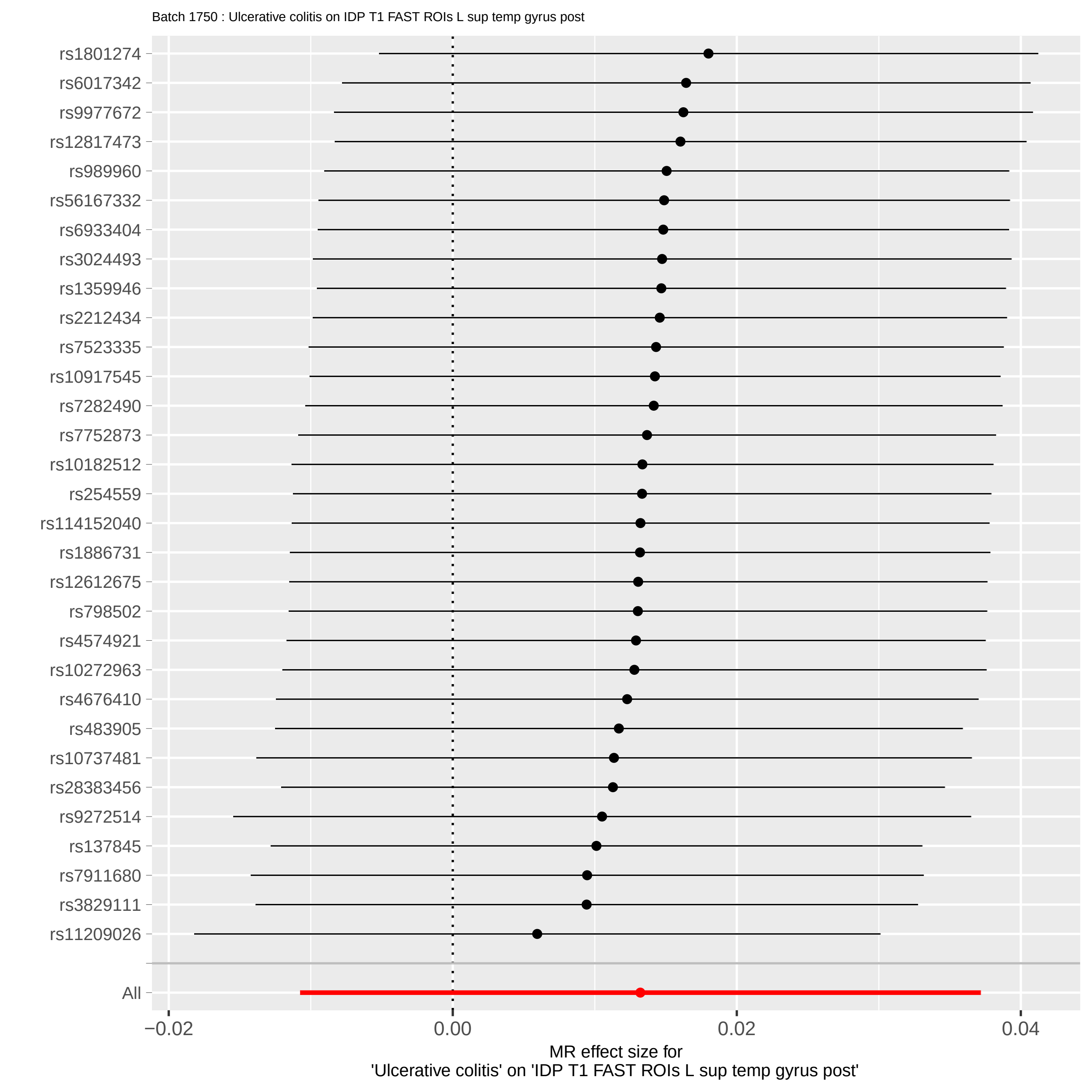

Batch 1750 : Ulcerative colitis on IDP T1 FAST ROIs L sup temp gyrus post
rs1801274
rs6017342
rs9977672
rs12817473
rs989960
rs56167332
rs6933404
rs3024493
rs1359946
rs2212434
rs7523335
rs10917545
rs7282490
rs7752873
rs10182512
rs254559
rs114152040
rs1886731
rs12612675
rs798502
rs4574921
rs10272963
rs4676410
rs483905
rs10737481
rs28383456
rs9272514
rs137845
rs7911680
rs3829111
rs11209026
All
−0.02
0.00
0.02
0.04
MR effect size for
'Ulcerative colitis' on 'IDP T1 FAST ROIs L sup temp gyrus post'

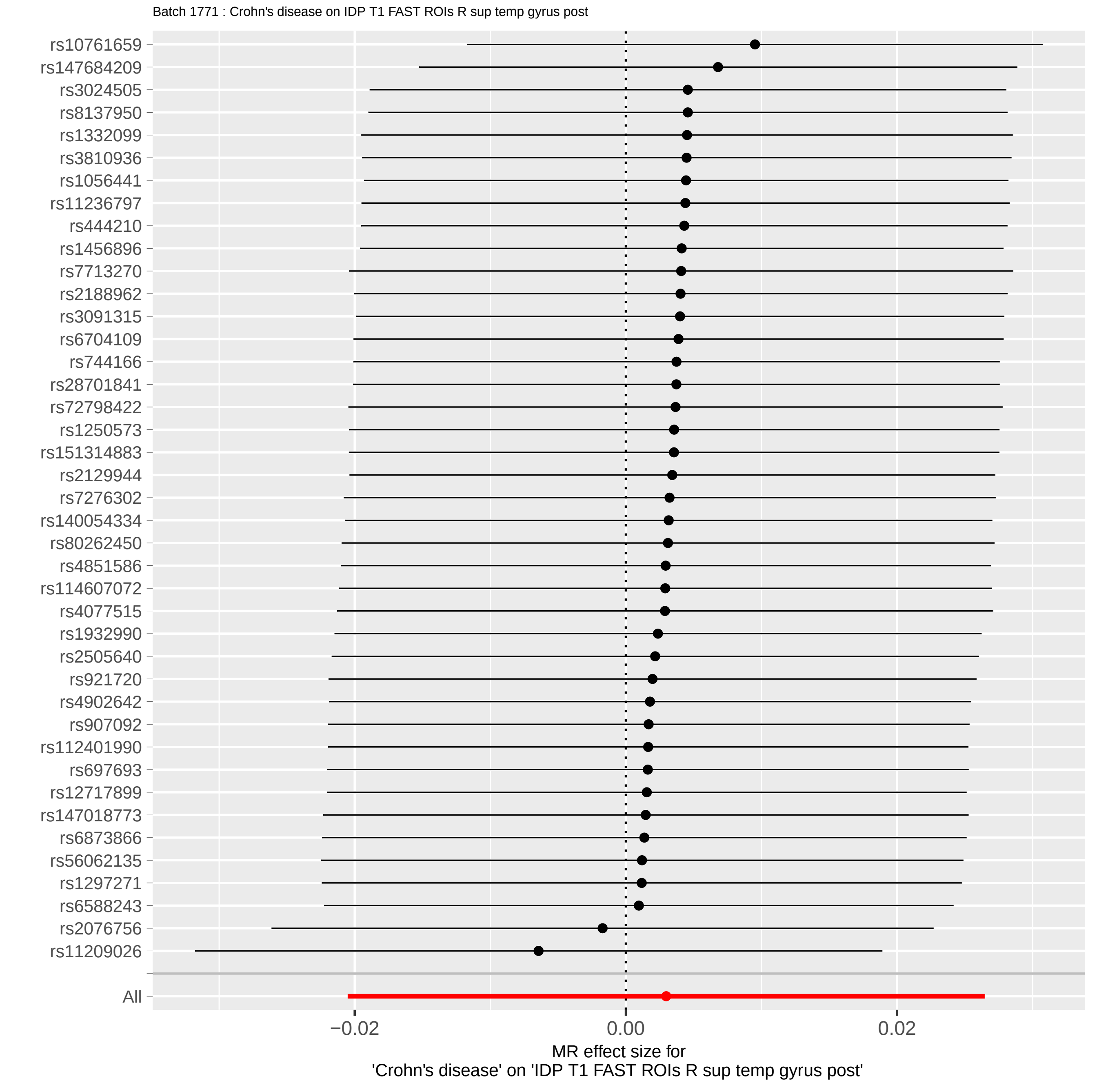
Batch 1771 : Crohn's disease on IDP T1 FAST ROIs R sup temp gyrus post
rs10761659
rs147684209
rs3024505
rs8137950
rs1332099
rs3810936
rs1056441
rs11236797
rs444210
rs1456896
rs7713270
rs2188962
rs3091315
rs6704109
rs744166
rs28701841
rs72798422
rs1250573
rs151314883
rs2129944
rs7276302
rs140054334
rs80262450
rs4851586
rs114607072
rs4077515
rs1932990
rs2505640
rs921720
rs4902642
rs907092
rs112401990
rs697693
rs12717899
rs147018773
rs6873866
rs56062135
rs1297271
rs6588243
rs2076756
rs11209026
All
−0.02
0.00
0.02
MR effect size for
'Crohn's disease' on 'IDP T1 FAST ROIs R sup temp gyrus post'

Batch 1772 : Ulcerative colitis on IDP T1 FAST ROIs R sup temp gyrus post

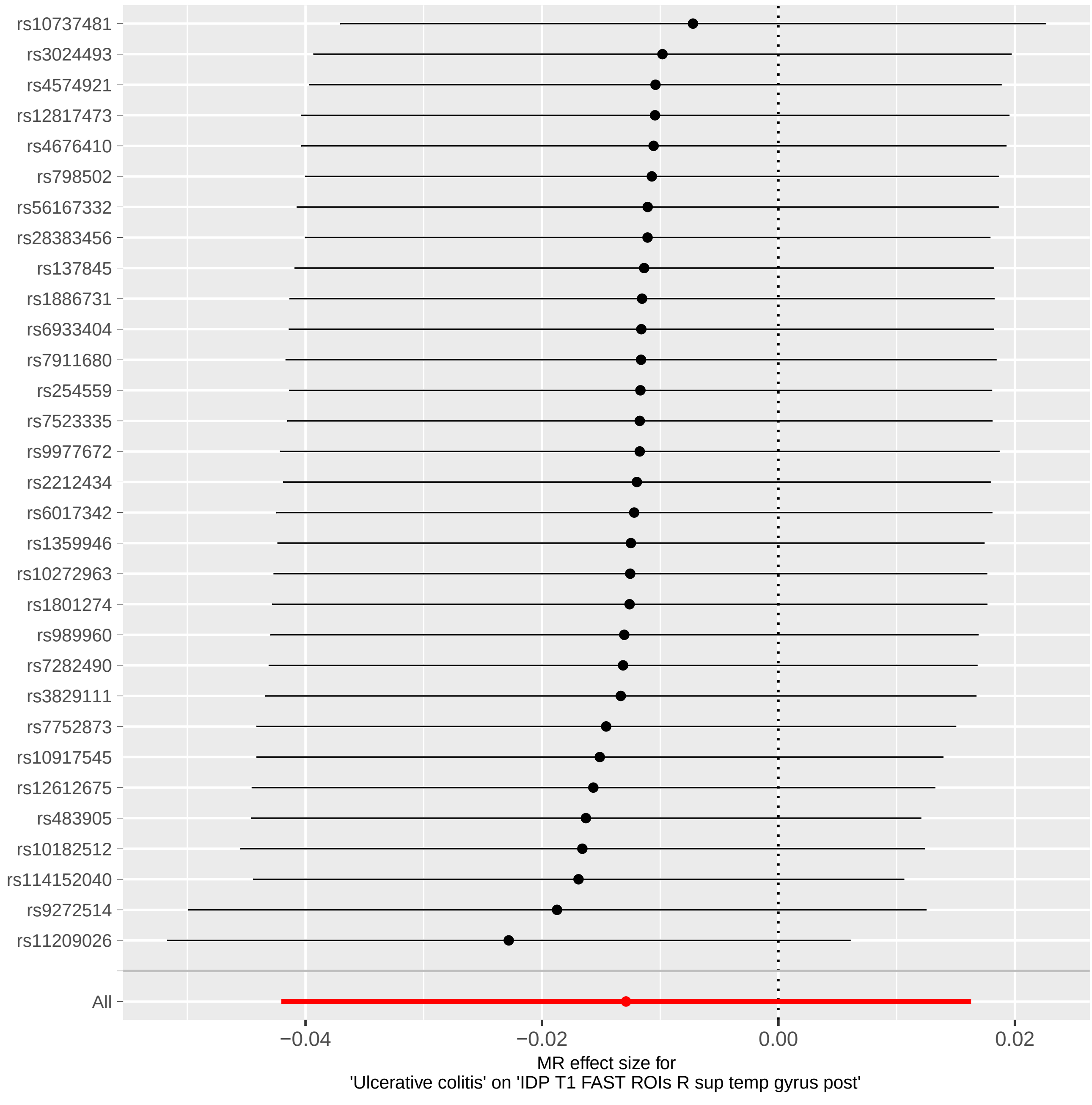

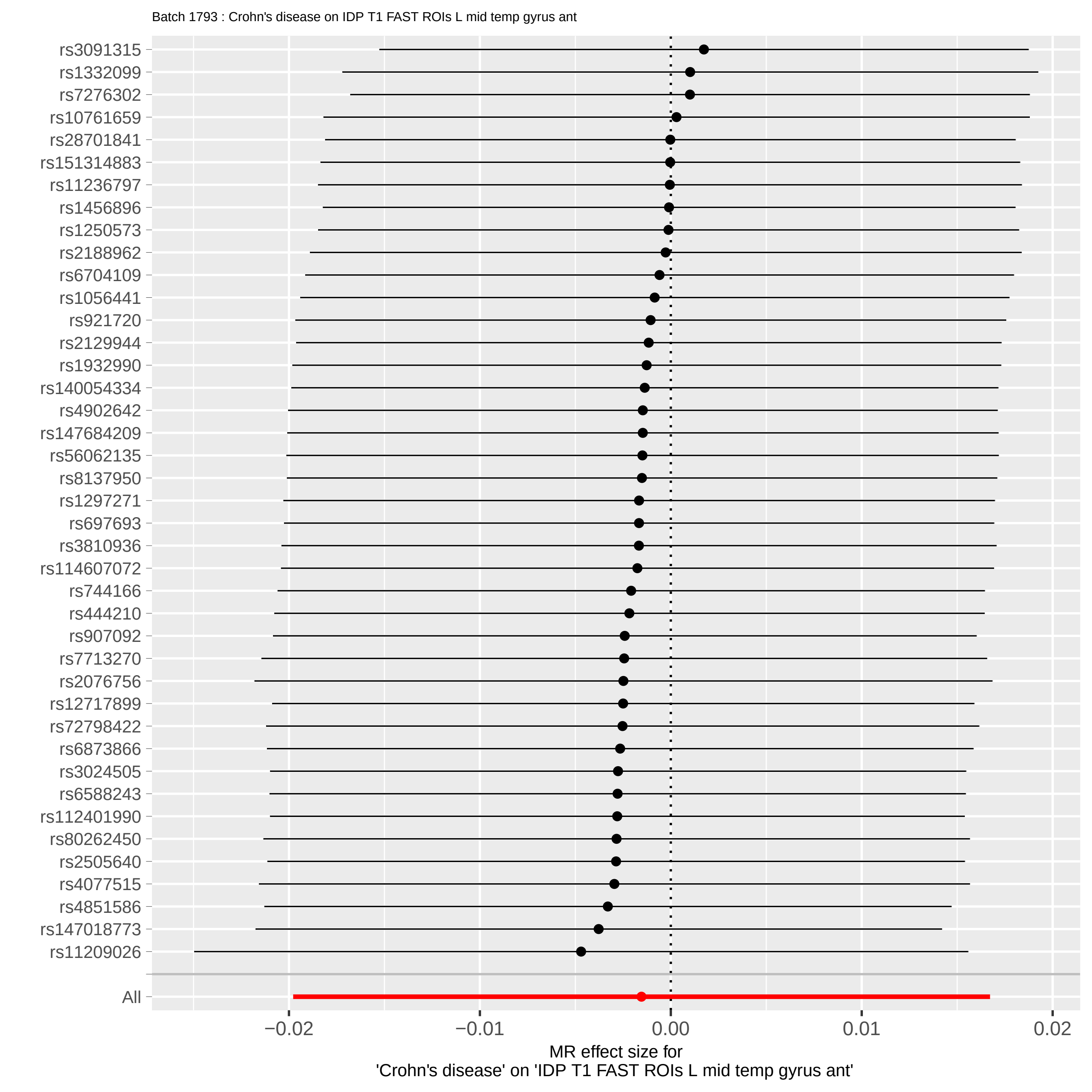

Batch 1793 : Crohn's disease on IDP T1 FAST ROIs L mid temp gyrus ant
rs3091315
rs1332099
rs7276302
rs10761659
rs28701841
rs151314883
rs11236797
rs1456896
rs1250573
rs2188962
rs6704109
rs1056441
rs921720
rs2129944
rs1932990
rs140054334
rs4902642
rs147684209
rs56062135
rs8137950
rs1297271
rs697693
rs3810936
rs114607072
rs744166
rs444210
rs907092
rs7713270
rs2076756
rs12717899
rs72798422
rs6873866
rs3024505
rs6588243
rs112401990
rs80262450
rs2505640
rs4077515
rs4851586
rs147018773
rs11209026
All
−0.02
−0.01
0.00
0.01
0.02
MR effect size for
'Crohn's disease' on 'IDP T1 FAST ROIs L mid temp gyrus ant'

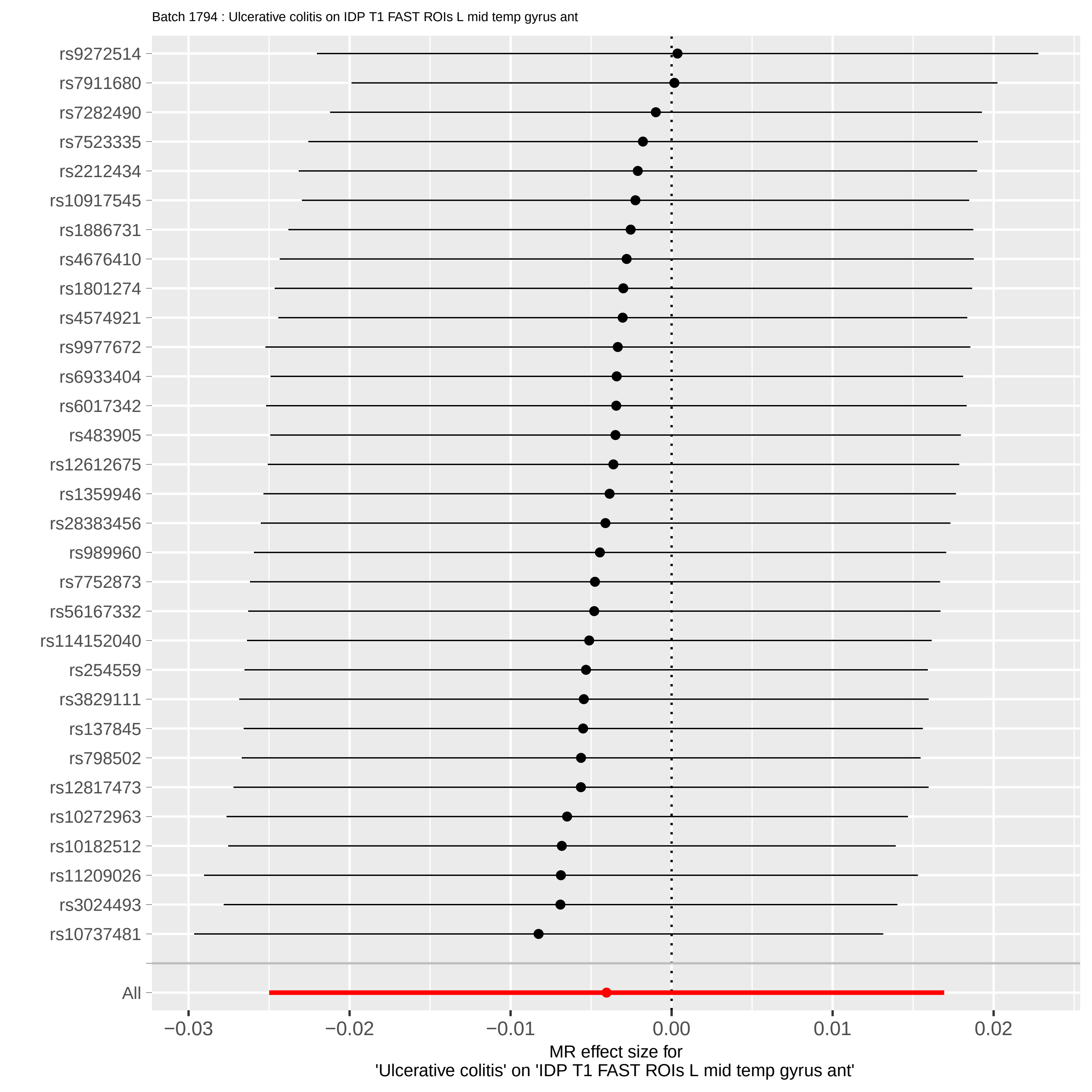

Batch 1794 : Ulcerative colitis on IDP T1 FAST ROIs L mid temp gyrus ant
rs9272514
rs7911680
rs7282490
rs7523335
rs2212434
rs10917545
rs1886731
rs4676410
rs1801274
rs4574921
rs9977672
rs6933404
rs6017342
rs483905
rs12612675
rs1359946
rs28383456
rs989960
rs7752873
rs56167332
rs114152040
rs254559
rs3829111
rs137845
rs798502
rs12817473
rs10272963
rs10182512
rs11209026
rs3024493
rs10737481
All
−0.03
−0.02
−0.01
0.00
0.01
0.02
MR effect size for
'Ulcerative colitis' on 'IDP T1 FAST ROIs L mid temp gyrus ant'

Batch 1815 : Crohn's disease on IDP T1 FAST ROIs R mid temp gyrus ant

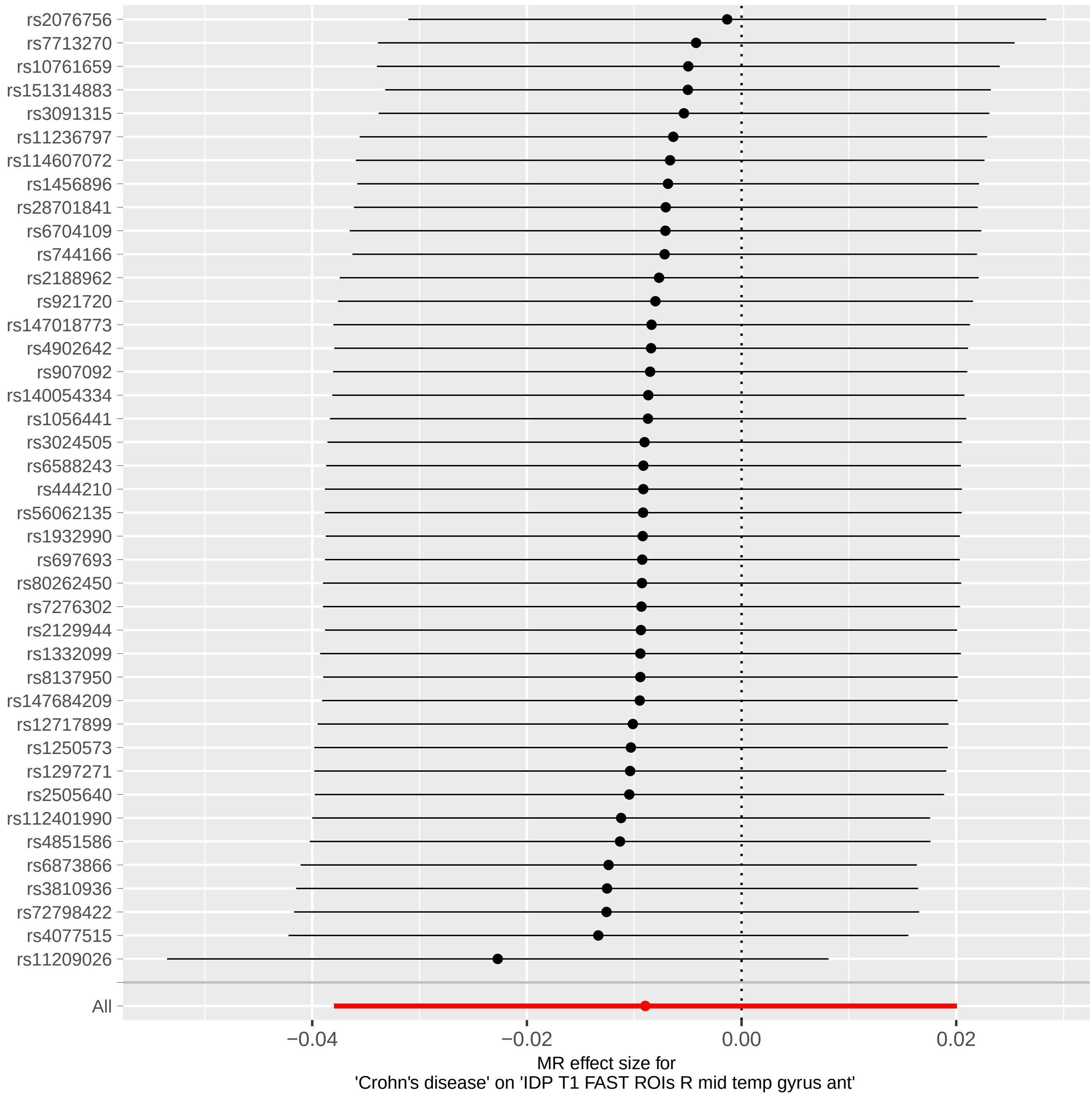

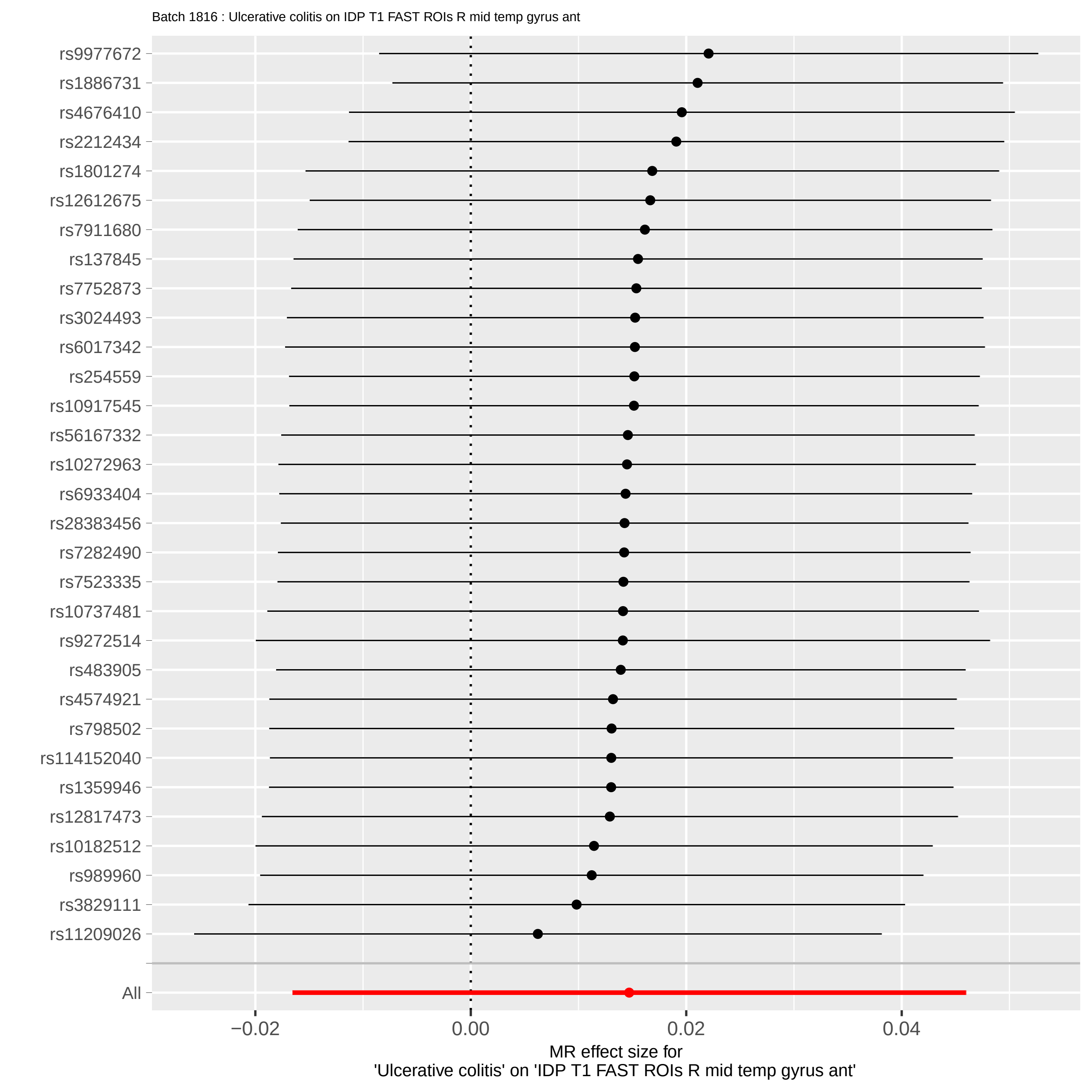
Batch 1816 : Ulcerative colitis on IDP T1 FAST ROIs R mid temp gyrus ant
rs9977672
rs1886731
rs4676410
rs2212434
rs1801274
rs12612675
rs7911680
rs137845
rs7752873
rs3024493
rs6017342
rs254559
rs10917545
rs56167332
rs10272963
rs6933404
rs28383456
rs7282490
rs7523335
rs10737481
rs9272514
rs483905
rs4574921
rs798502
rs114152040
rs1359946
rs12817473
rs10182512
rs989960
rs3829111
rs11209026
All
−0.02
0.00
0.02
0.04
MR effect size for
'Ulcerative colitis' on 'IDP T1 FAST ROIs R mid temp gyrus ant'

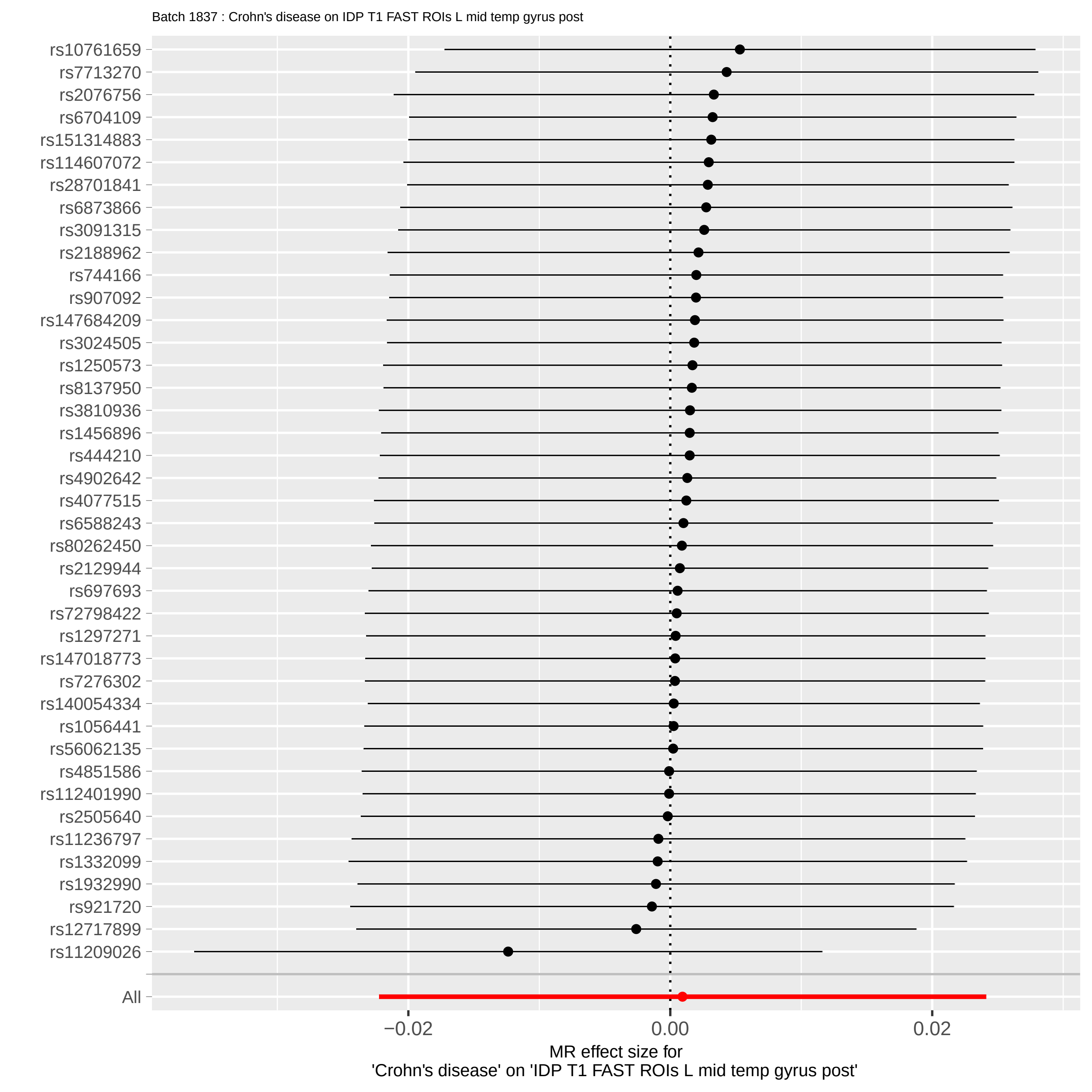

Batch 1837 : Crohn's disease on IDP T1 FAST ROIs L mid temp gyrus post
rs10761659
rs7713270
rs2076756
rs6704109
rs151314883
rs114607072
rs28701841
rs6873866
rs3091315
rs2188962
rs744166
rs907092
rs147684209
rs3024505
rs1250573
rs8137950
rs3810936
rs1456896
rs444210
rs4902642
rs4077515
rs6588243
rs80262450
rs2129944
rs697693
rs72798422
rs1297271
rs147018773
rs7276302
rs140054334
rs1056441
rs56062135
rs4851586
rs112401990
rs2505640
rs11236797
rs1332099
rs1932990
rs921720
rs12717899
rs11209026
All
−0.02
0.00
0.02
MR effect size for
'Crohn's disease' on 'IDP T1 FAST ROIs L mid temp gyrus post'

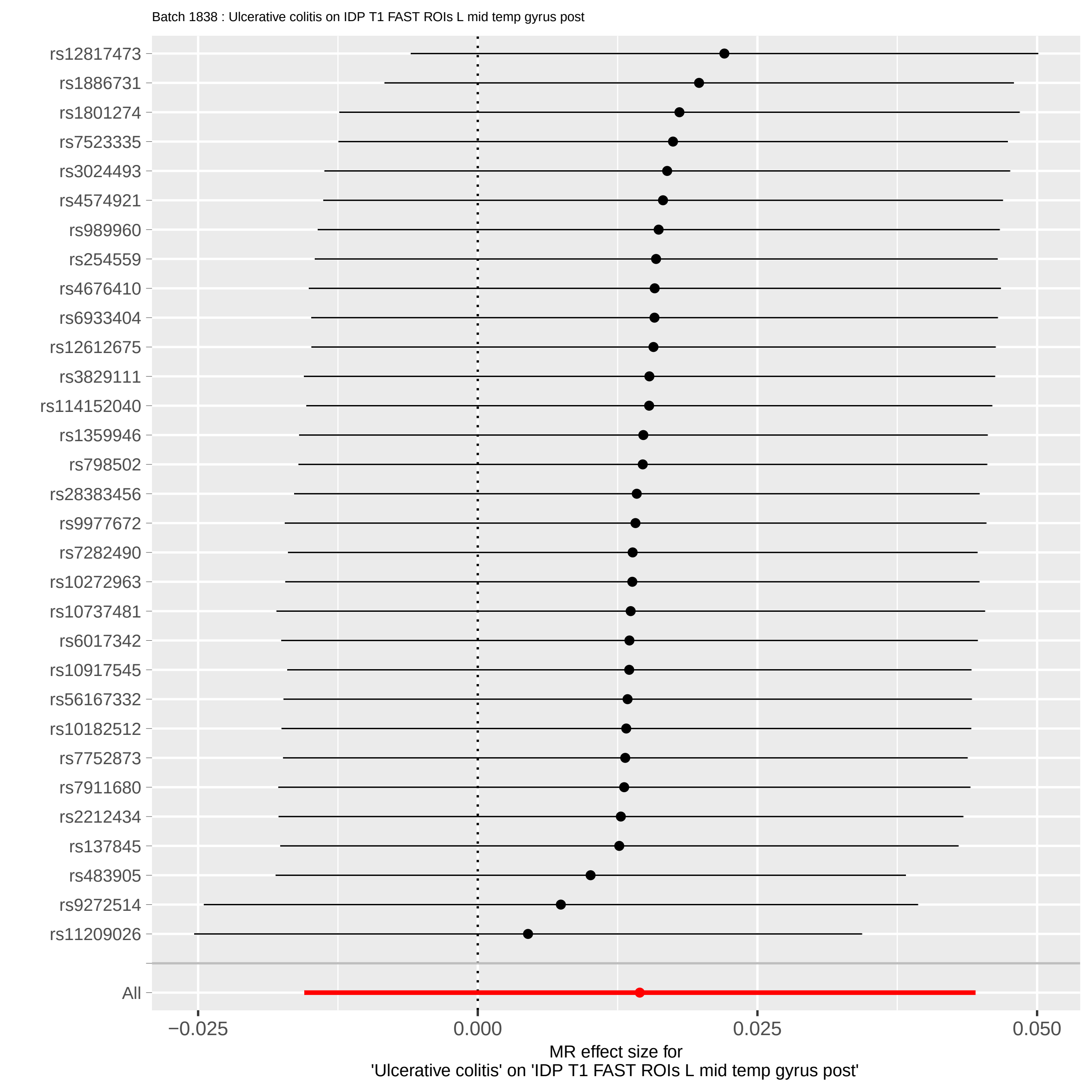

Batch 1838 : Ulcerative colitis on IDP T1 FAST ROIs L mid temp gyrus post
rs12817473
rs1886731
rs1801274
rs7523335
rs3024493
rs4574921
rs989960
rs254559
rs4676410
rs6933404
rs12612675
rs3829111
rs114152040
rs1359946
rs798502
rs28383456
rs9977672
rs7282490
rs10272963
rs10737481
rs6017342
rs10917545
rs56167332
rs10182512
rs7752873
rs7911680
rs2212434
rs137845
rs483905
rs9272514
rs11209026
All
−0.025
0.000
0.025
0.050
MR effect size for
'Ulcerative colitis' on 'IDP T1 FAST ROIs L mid temp gyrus post'

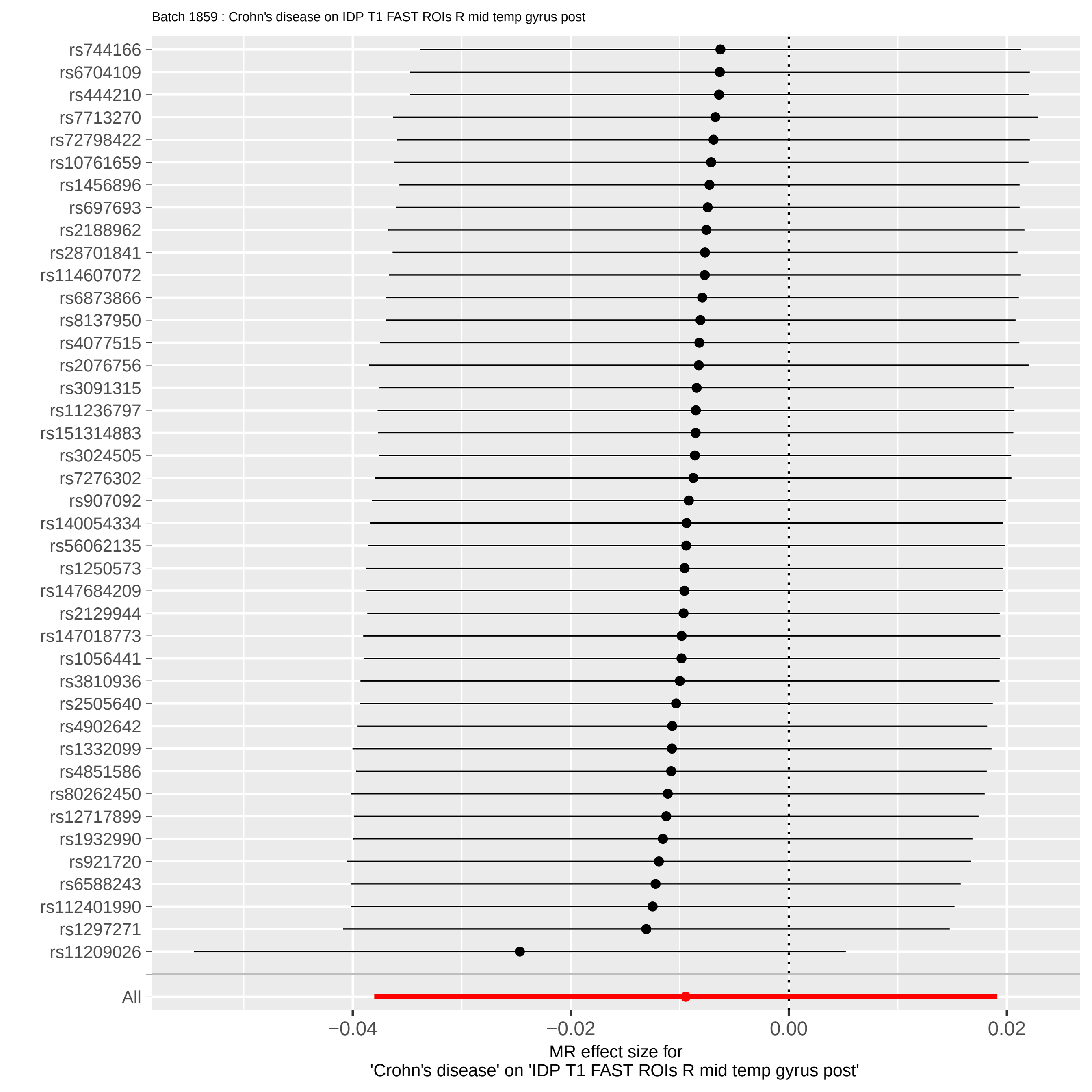

Batch 1859 : Crohn's disease on IDP T1 FAST ROIs R mid temp gyrus post
rs744166
rs6704109
rs444210
rs7713270
rs72798422
rs10761659
rs1456896
rs697693
rs2188962
rs28701841
rs114607072
rs6873866
rs8137950
rs4077515
rs2076756
rs3091315
rs11236797
rs151314883
rs3024505
rs7276302
rs907092
rs140054334
rs56062135
rs1250573
rs147684209
rs2129944
rs147018773
rs1056441
rs3810936
rs2505640
rs4902642
rs1332099
rs4851586
rs80262450
rs12717899
rs1932990
rs921720
rs6588243
rs112401990
rs1297271
rs11209026
All
−0.04
−0.02
0.00
0.02
MR effect size for
'Crohn's disease' on 'IDP T1 FAST ROIs R mid temp gyrus post'

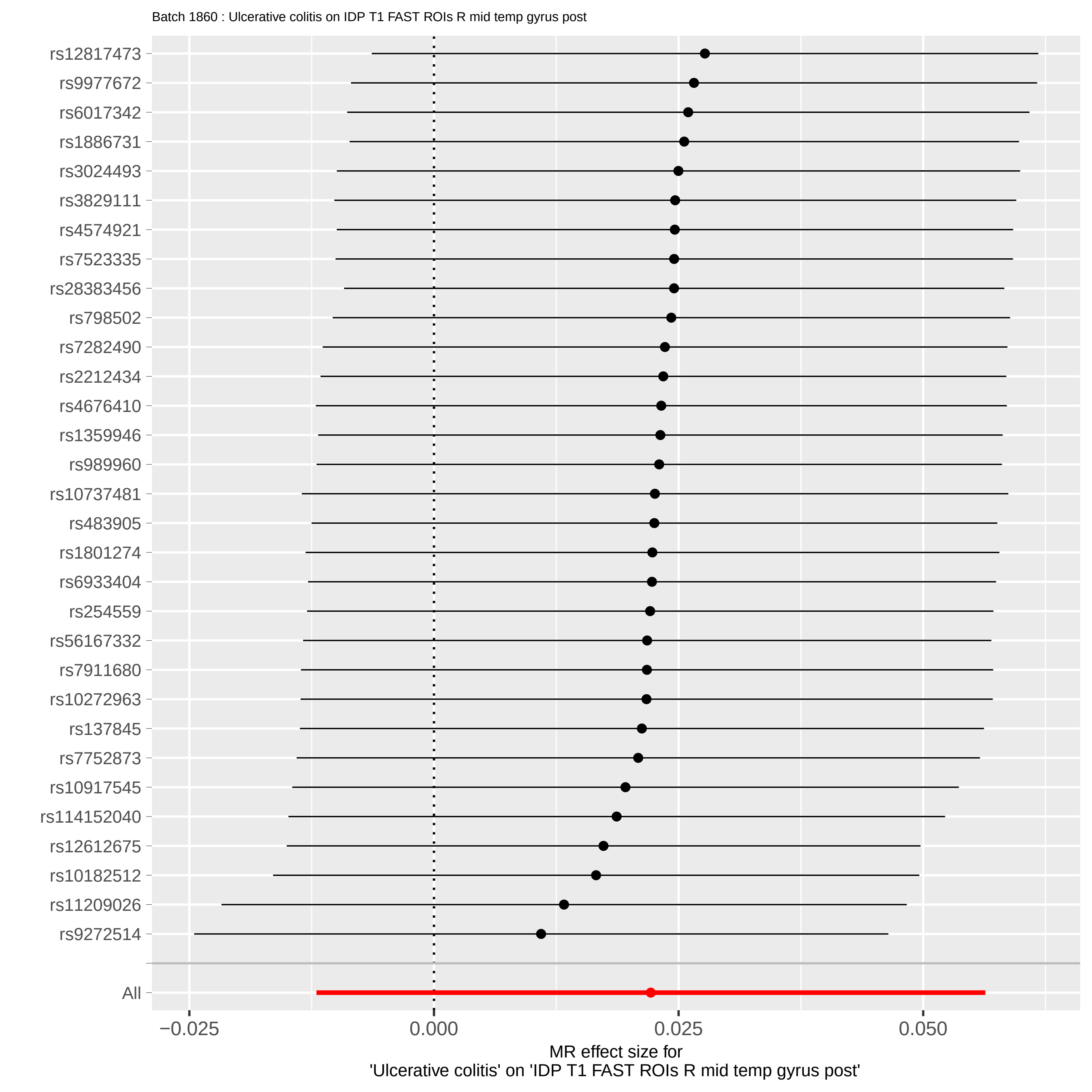

Batch 1860 : Ulcerative colitis on IDP T1 FAST ROIs R mid temp gyrus post
rs12817473
rs9977672
rs6017342
rs1886731
rs3024493
rs3829111
rs4574921
rs7523335
rs28383456
rs798502
rs7282490
rs2212434
rs4676410
rs1359946
rs989960
rs10737481
rs483905
rs1801274
rs6933404
rs254559
rs56167332
rs7911680
rs10272963
rs137845
rs7752873
rs10917545
rs114152040
rs12612675
rs10182512
rs11209026
rs9272514
All
−0.025
0.000
0.025
0.050
MR effect size for
'Ulcerative colitis' on 'IDP T1 FAST ROIs R mid temp gyrus post'

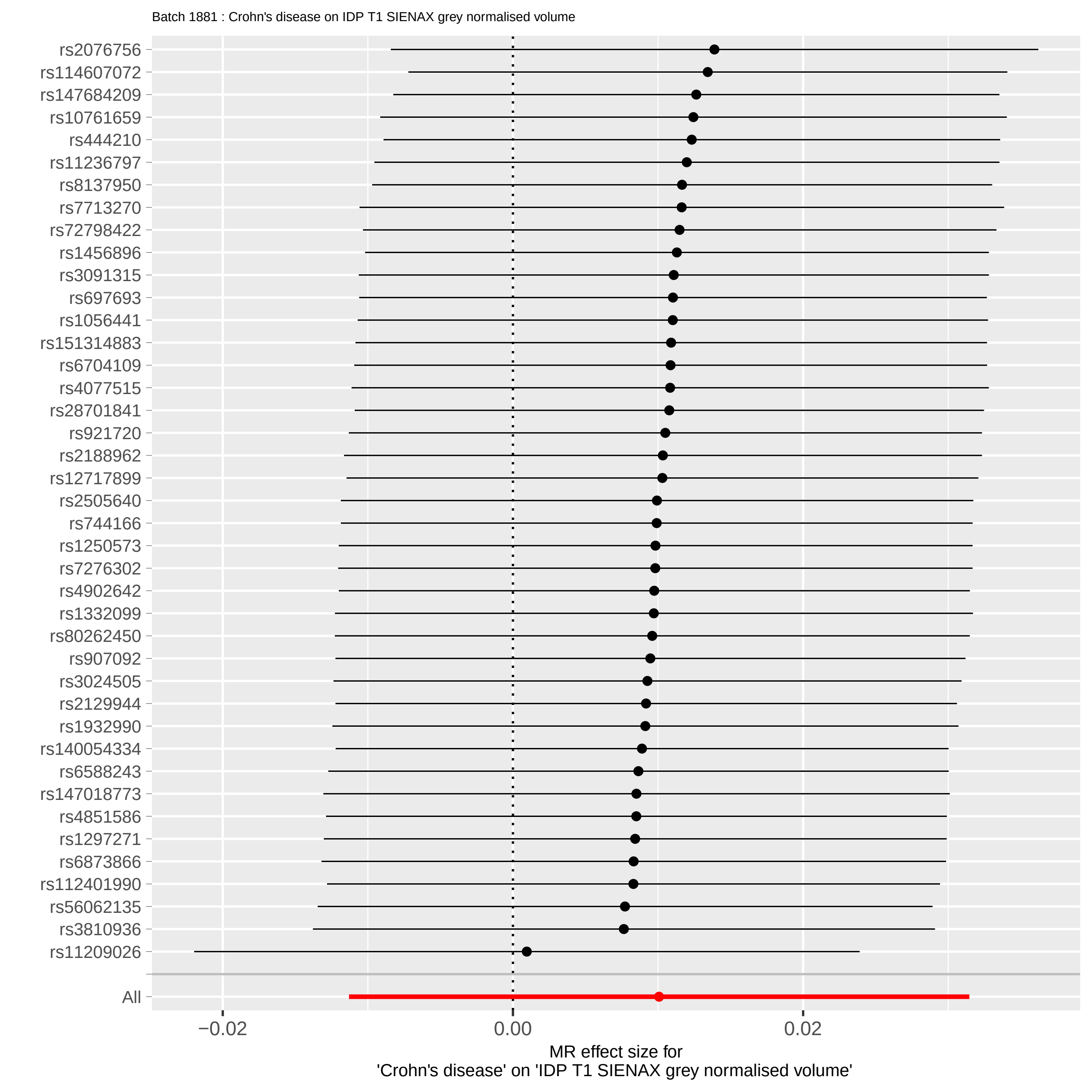
Batch 1881 : Crohn's disease on IDP T1 SIENAX grey normalised volume
rs2076756
rs114607072
rs147684209
rs10761659
rs444210
rs11236797
rs8137950
rs7713270
rs72798422
rs1456896
rs3091315
rs697693
rs1056441
rs151314883
rs6704109
rs4077515
rs28701841
rs921720
rs2188962
rs12717899
rs2505640
rs744166
rs1250573
rs7276302
rs4902642
rs1332099
rs80262450
rs907092
rs3024505
rs2129944
rs1932990
rs140054334
rs6588243
rs147018773
rs4851586
rs1297271
rs6873866
rs112401990
rs56062135
rs3810936
rs11209026
All
−0.02
0.00
0.02
MR effect size for
'Crohn's disease' on 'IDP T1 SIENAX grey normalised volume'

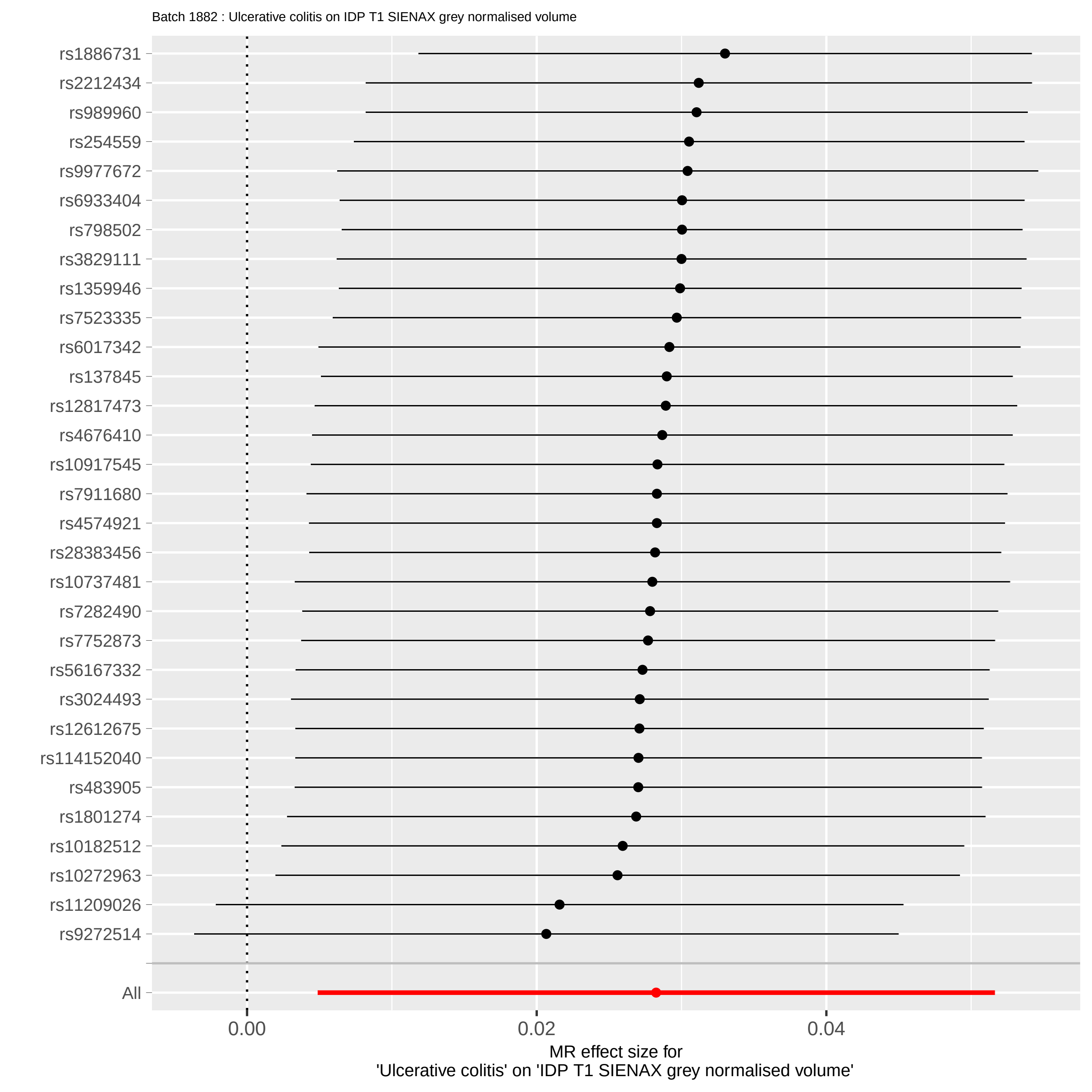

Batch 1882 : Ulcerative colitis on IDP T1 SIENAX grey normalised volume
rs1886731
rs2212434
rs989960
rs254559
rs9977672
rs6933404
rs798502
rs3829111
rs1359946
rs7523335
rs6017342
rs137845
rs12817473
rs4676410
rs10917545
rs7911680
rs4574921
rs28383456
rs10737481
rs7282490
rs7752873
rs56167332
rs3024493
rs12612675
rs114152040
rs483905
rs1801274
rs10182512
rs10272963
rs11209026
rs9272514
All
0.00
0.02
0.04
MR effect size for
'Ulcerative colitis' on 'IDP T1 SIENAX grey normalised volume'

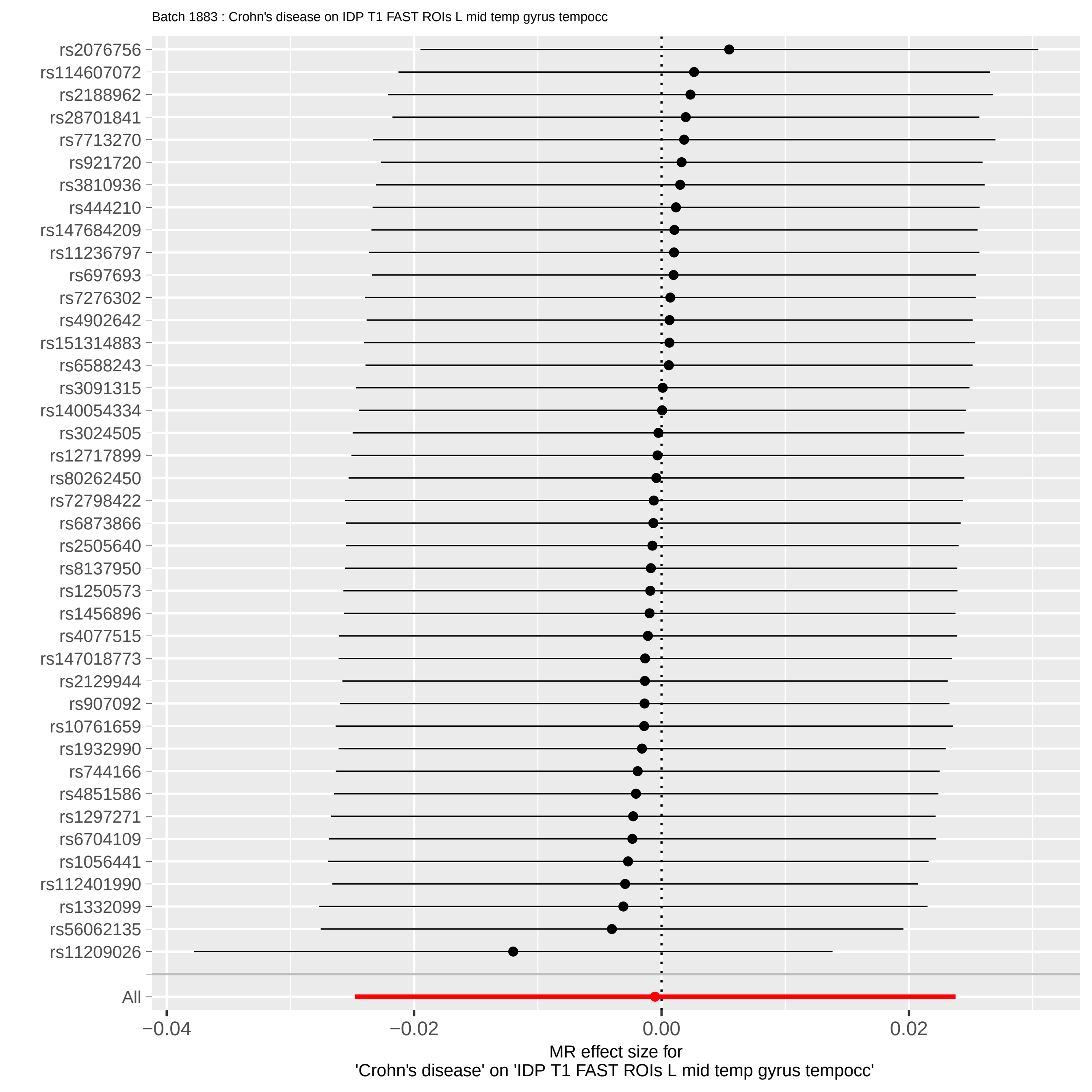
Batch 1883 : Crohn's disease on IDP T1 FAST ROIs L mid temp gyrus tempocc
rs2076756
rs114607072
rs2188962
rs28701841
rs7713270
rs921720
rs3810936
rs444210
rs147684209
rs11236797
rs697693
rs7276302
rs4902642
rs151314883
rs6588243
rs3091315
rs140054334
rs3024505
rs12717899
rs80262450
rs72798422
rs6873866
rs2505640
rs8137950
rs1250573
rs1456896
rs4077515
rs147018773
rs2129944
rs907092
rs10761659
rs1932990
rs744166
rs4851586
rs1297271
rs6704109
rs1056441
rs112401990
rs1332099
rs56062135
rs11209026
All
−0.04
−0.02
0.00
0.02
MR effect size for
'Crohn's disease' on 'IDP T1 FAST ROIs L mid temp gyrus tempocc'

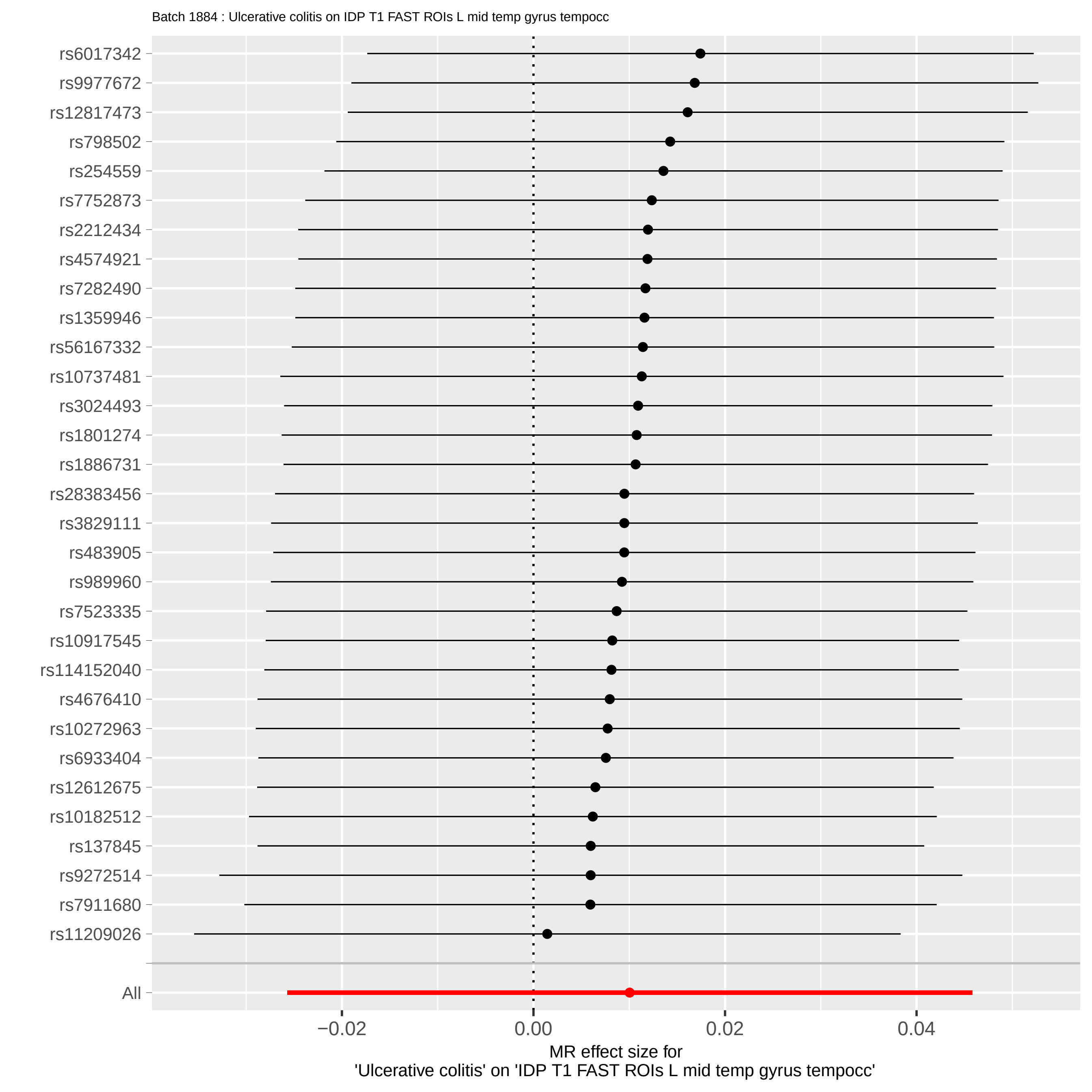

Batch 1884 : Ulcerative colitis on IDP T1 FAST ROIs L mid temp gyrus tempocc
rs6017342
rs9977672
rs12817473
rs798502
rs254559
rs7752873
rs2212434
rs4574921
rs7282490
rs1359946
rs56167332
rs10737481
rs3024493
rs1801274
rs1886731
rs28383456
rs3829111
rs483905
rs989960
rs7523335
rs10917545
rs114152040
rs4676410
rs10272963
rs6933404
rs12612675
rs10182512
rs137845
rs9272514
rs7911680
rs11209026
All
−0.02
0.00
0.02
0.04
MR effect size for
'Ulcerative colitis' on 'IDP T1 FAST ROIs L mid temp gyrus tempocc'

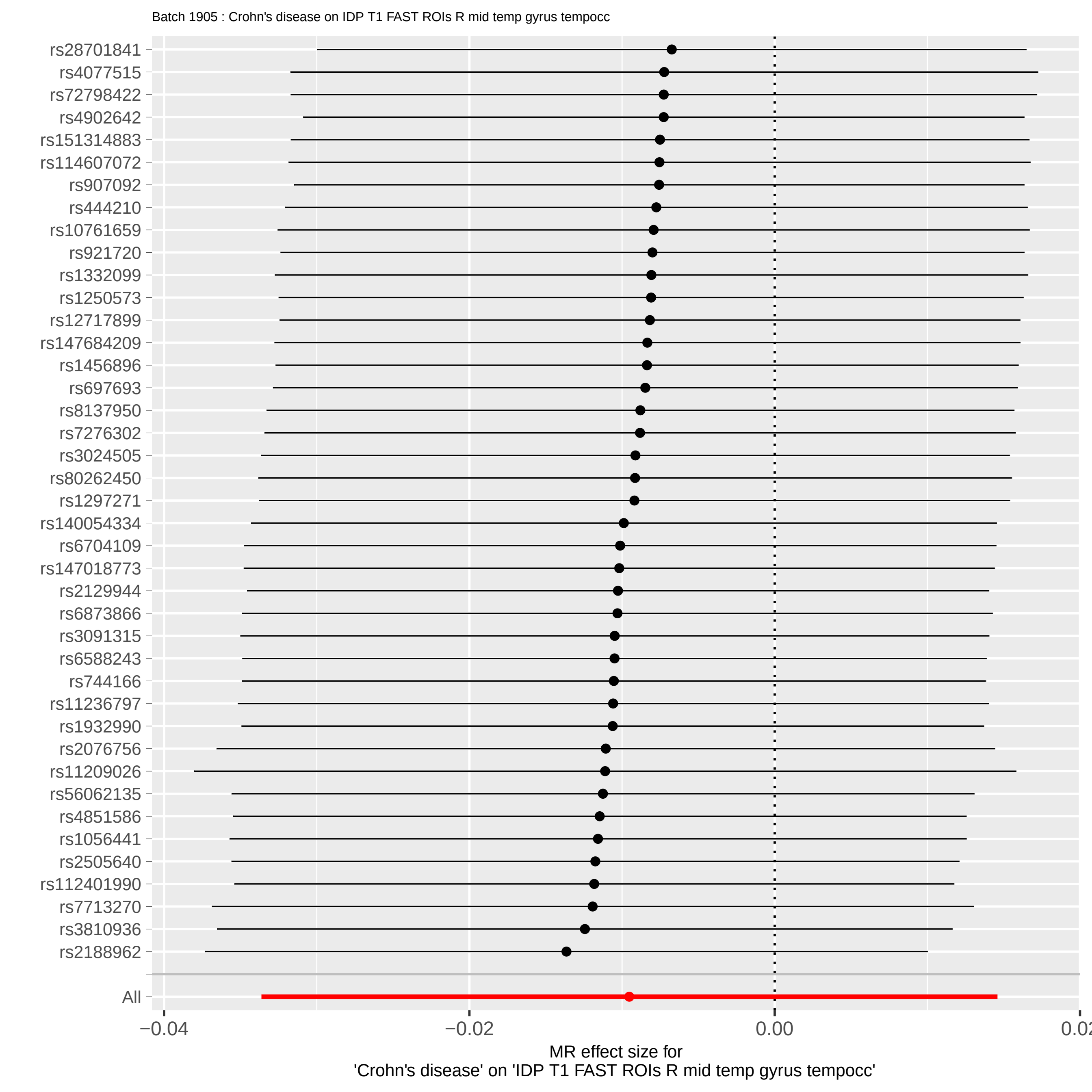

Batch 1905 : Crohn's disease on IDP T1 FAST ROIs R mid temp gyrus tempocc
rs28701841
rs4077515
rs72798422
rs4902642
rs151314883
rs114607072
rs907092
rs444210
rs10761659
rs921720
rs1332099
rs1250573
rs12717899
rs147684209
rs1456896
rs697693
rs8137950
rs7276302
rs3024505
rs80262450
rs1297271
rs140054334
rs6704109
rs147018773
rs2129944
rs6873866
rs3091315
rs6588243
rs744166
rs11236797
rs1932990
rs2076756
rs11209026
rs56062135
rs4851586
rs1056441
rs2505640
rs112401990
rs7713270
rs3810936
rs2188962
All
−0.04
−0.02
0.00
0.02
MR effect size for
'Crohn's disease' on 'IDP T1 FAST ROIs R mid temp gyrus tempocc'

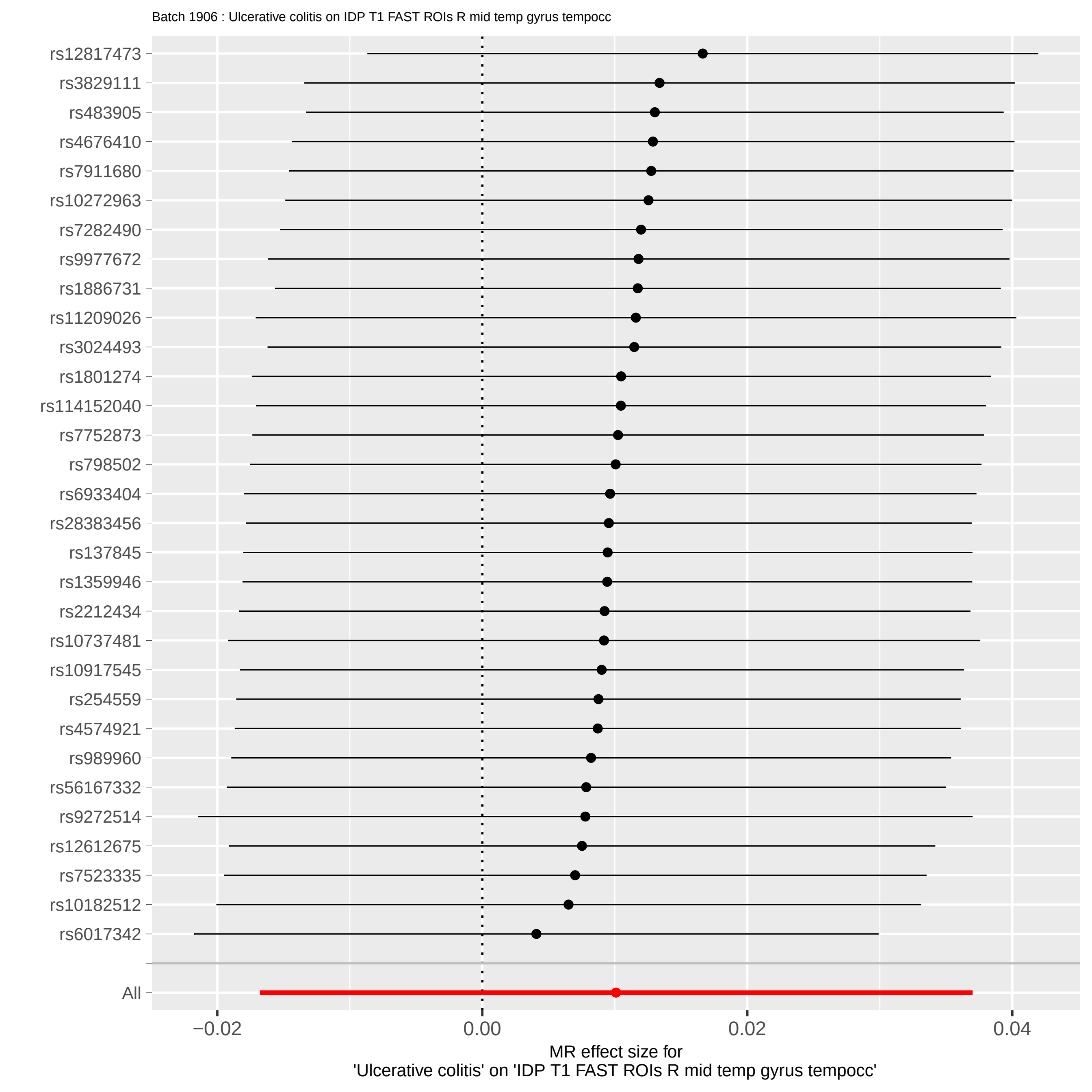
Batch 1906 : Ulcerative colitis on IDP T1 FAST ROIs R mid temp gyrus tempocc
rs12817473
rs3829111
rs483905
rs4676410
rs7911680
rs10272963
rs7282490
rs9977672
rs1886731
rs11209026
rs3024493
rs1801274
rs114152040
rs7752873
rs798502
rs6933404
rs28383456
rs137845
rs1359946
rs2212434
rs10737481
rs10917545
rs254559
rs4574921
rs989960
rs56167332
rs9272514
rs12612675
rs7523335
rs10182512
rs6017342
All
−0.02
0.00
0.02
0.04
MR effect size for
'Ulcerative colitis' on 'IDP T1 FAST ROIs R mid temp gyrus tempocc'

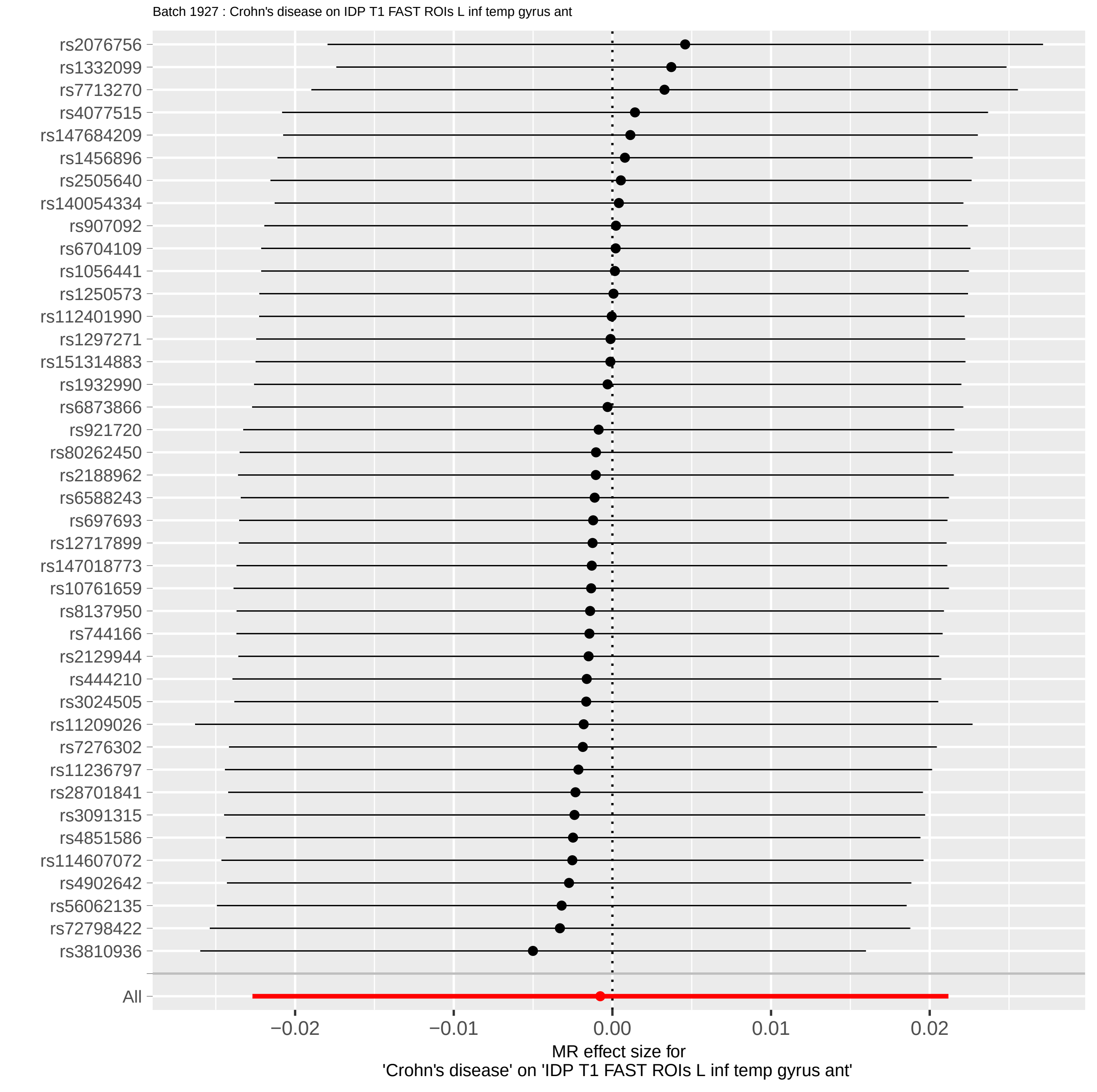
Batch 1927 : Crohn's disease on IDP T1 FAST ROIs L inf temp gyrus ant
rs2076756
rs1332099
rs7713270
rs4077515
rs147684209
rs1456896
rs2505640
rs140054334
rs907092
rs6704109
rs1056441
rs1250573
rs112401990
rs1297271
rs151314883
rs1932990
rs6873866
rs921720
rs80262450
rs2188962
rs6588243
rs697693
rs12717899
rs147018773
rs10761659
rs8137950
rs744166
rs2129944
rs444210
rs3024505
rs11209026
rs7276302
rs11236797
rs28701841
rs3091315
rs4851586
rs114607072
rs4902642
rs56062135
rs72798422
rs3810936
All
−0.02
−0.01
0.00
0.01
0.02
MR effect size for
'Crohn's disease' on 'IDP T1 FAST ROIs L inf temp gyrus ant'

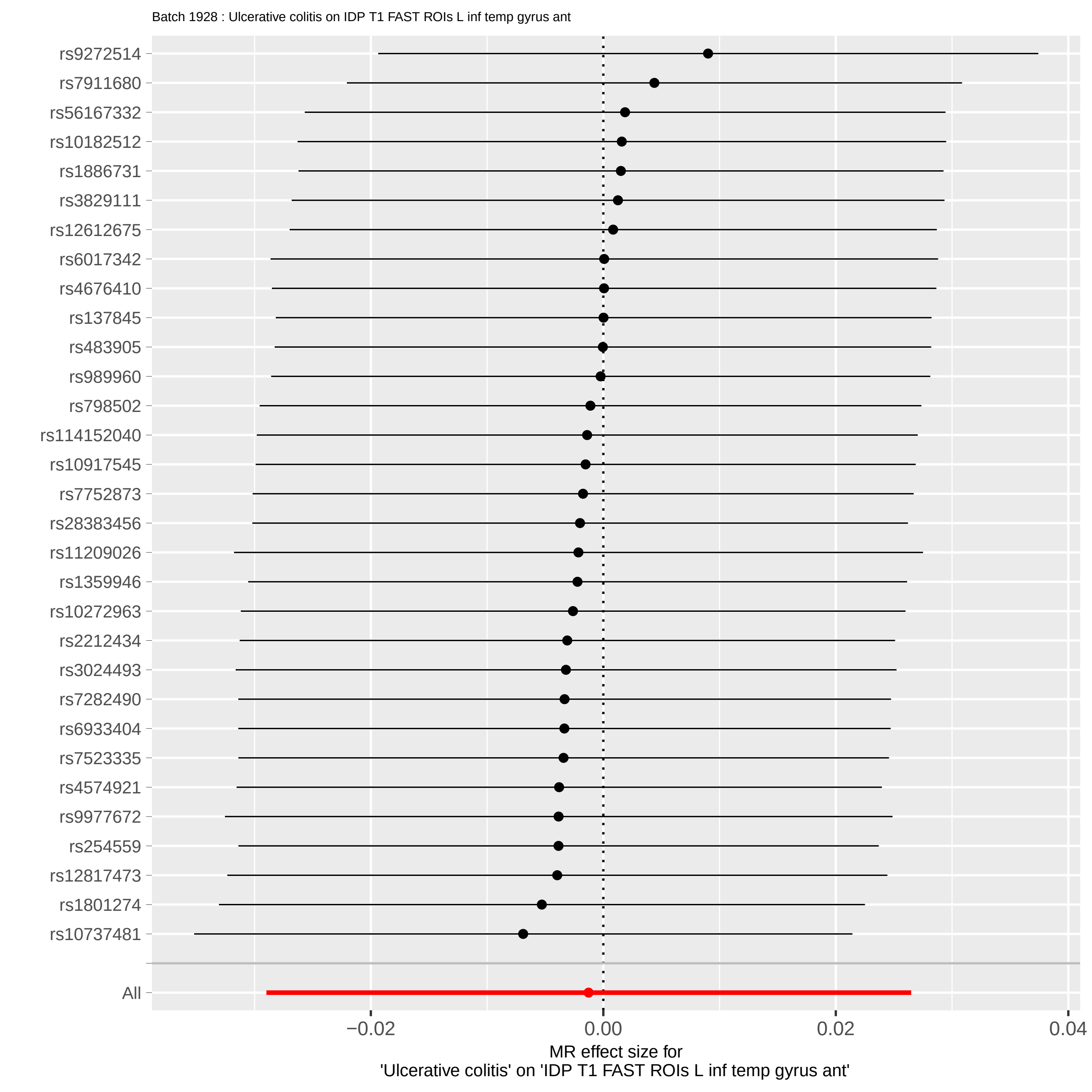

Batch 1928 : Ulcerative colitis on IDP T1 FAST ROIs L inf temp gyrus ant
rs9272514
rs7911680
rs56167332
rs10182512
rs1886731
rs3829111
rs12612675
rs6017342
rs4676410
rs137845
rs483905
rs989960
rs798502
rs114152040
rs10917545
rs7752873
rs28383456
rs11209026
rs1359946
rs10272963
rs2212434
rs3024493
rs7282490
rs6933404
rs7523335
rs4574921
rs9977672
rs254559
rs12817473
rs1801274
rs10737481
All
−0.02
0.00
0.02
0.04
MR effect size for
'Ulcerative colitis' on 'IDP T1 FAST ROIs L inf temp gyrus ant'

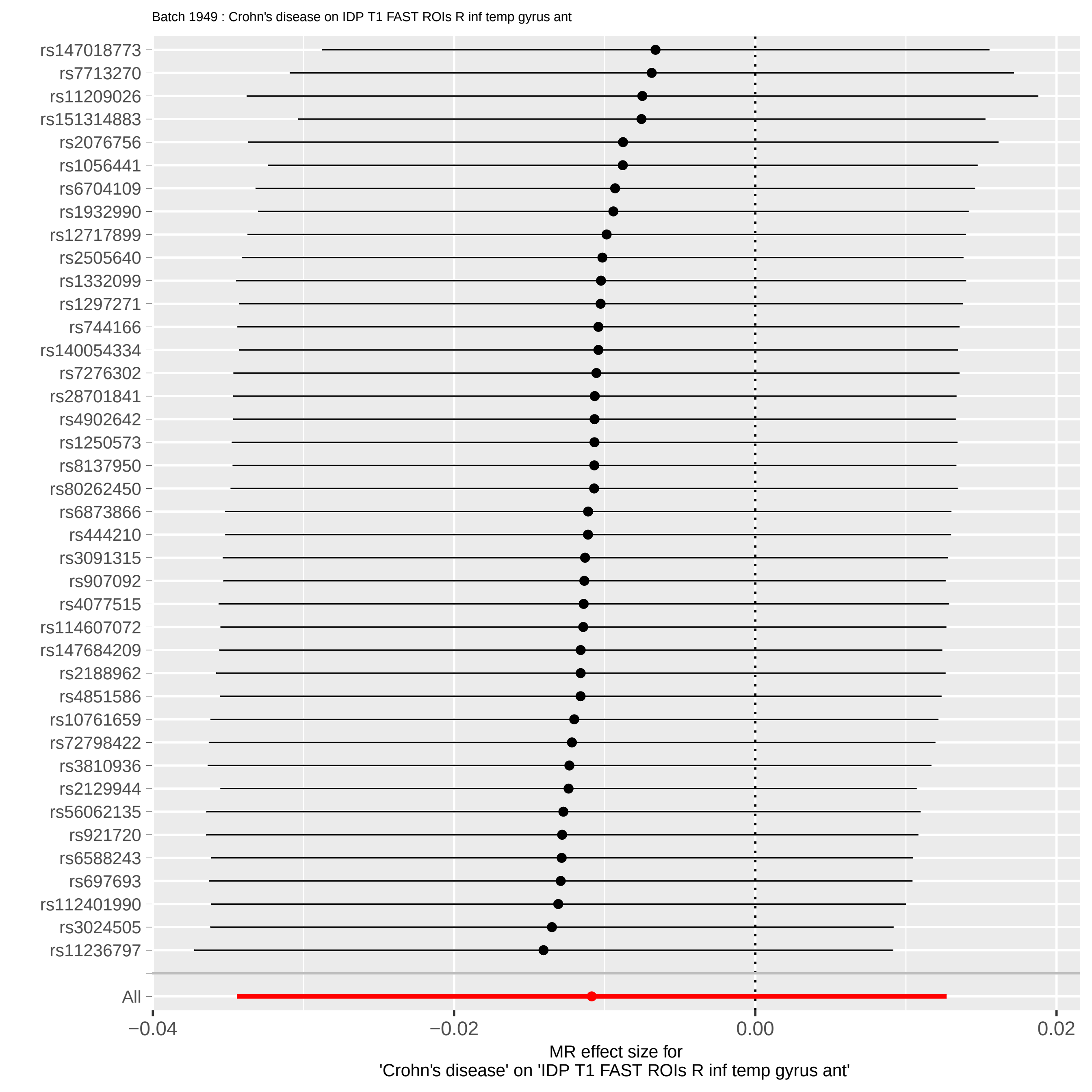

Batch 1949 : Crohn's disease on IDP T1 FAST ROIs R inf temp gyrus ant
rs147018773
rs7713270
rs11209026
rs151314883
rs2076756
rs1056441
rs6704109
rs1932990
rs12717899
rs2505640
rs1332099
rs1297271
rs744166
rs140054334
rs7276302
rs28701841
rs4902642
rs1250573
rs8137950
rs80262450
rs6873866
rs444210
rs3091315
rs907092
rs4077515
rs114607072
rs147684209
rs2188962
rs4851586
rs10761659
rs72798422
rs3810936
rs2129944
rs56062135
rs921720
rs6588243
rs697693
rs112401990
rs3024505
rs11236797
All
−0.04
−0.02
0.00
0.02
MR effect size for
'Crohn's disease' on 'IDP T1 FAST ROIs R inf temp gyrus ant'

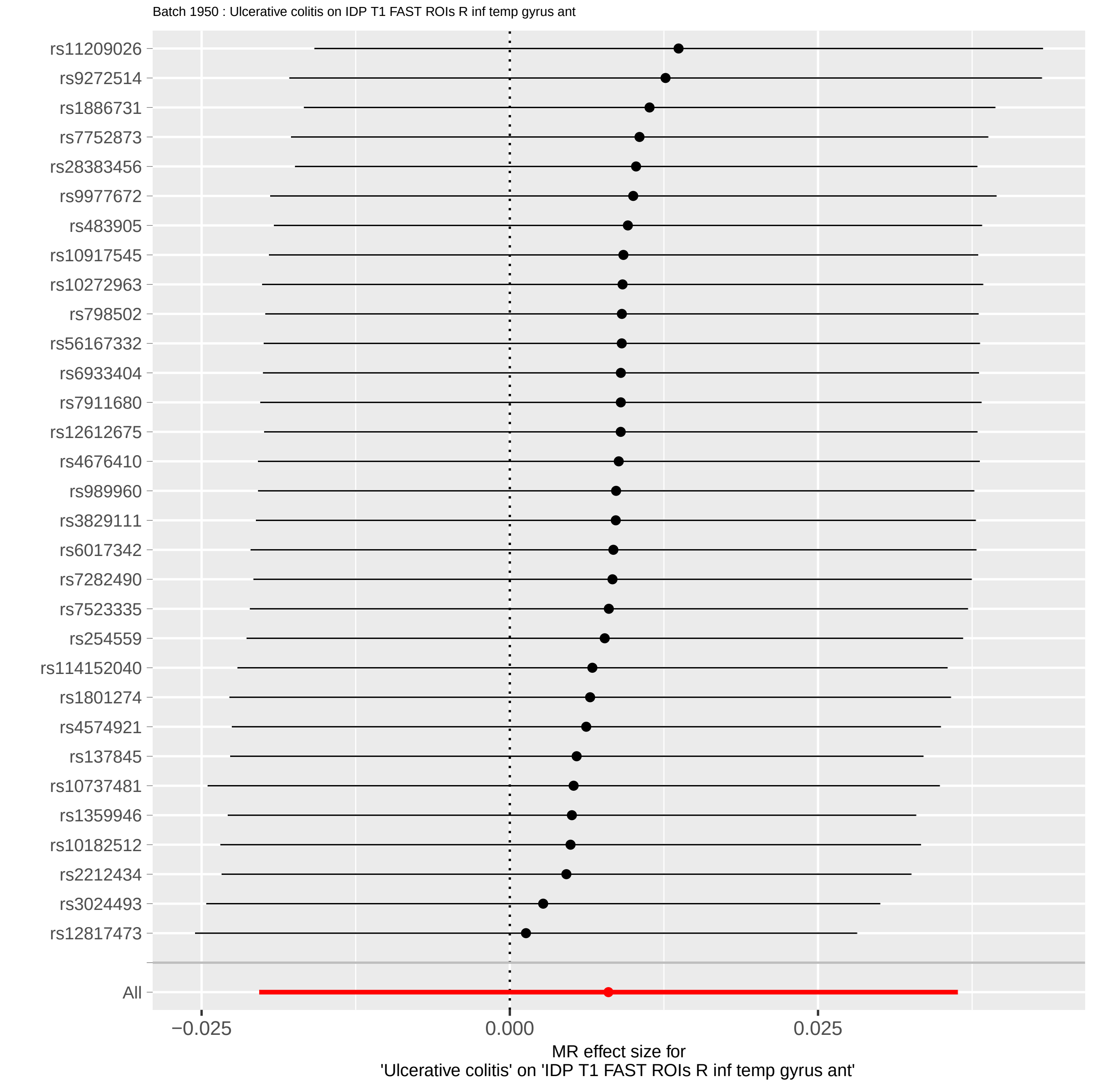

Batch 1950 : Ulcerative colitis on IDP T1 FAST ROIs R inf temp gyrus ant
rs11209026
rs9272514
rs1886731
rs7752873
rs28383456
rs9977672
rs483905
rs10917545
rs10272963
rs798502
rs56167332
rs6933404
rs7911680
rs12612675
rs4676410
rs989960
rs3829111
rs6017342
rs7282490
rs7523335
rs254559
rs114152040
rs1801274
rs4574921
rs137845
rs10737481
rs1359946
rs10182512
rs2212434
rs3024493
rs12817473
All
−0.025
0.000
0.025
MR effect size for
'Ulcerative colitis' on 'IDP T1 FAST ROIs R inf temp gyrus ant'

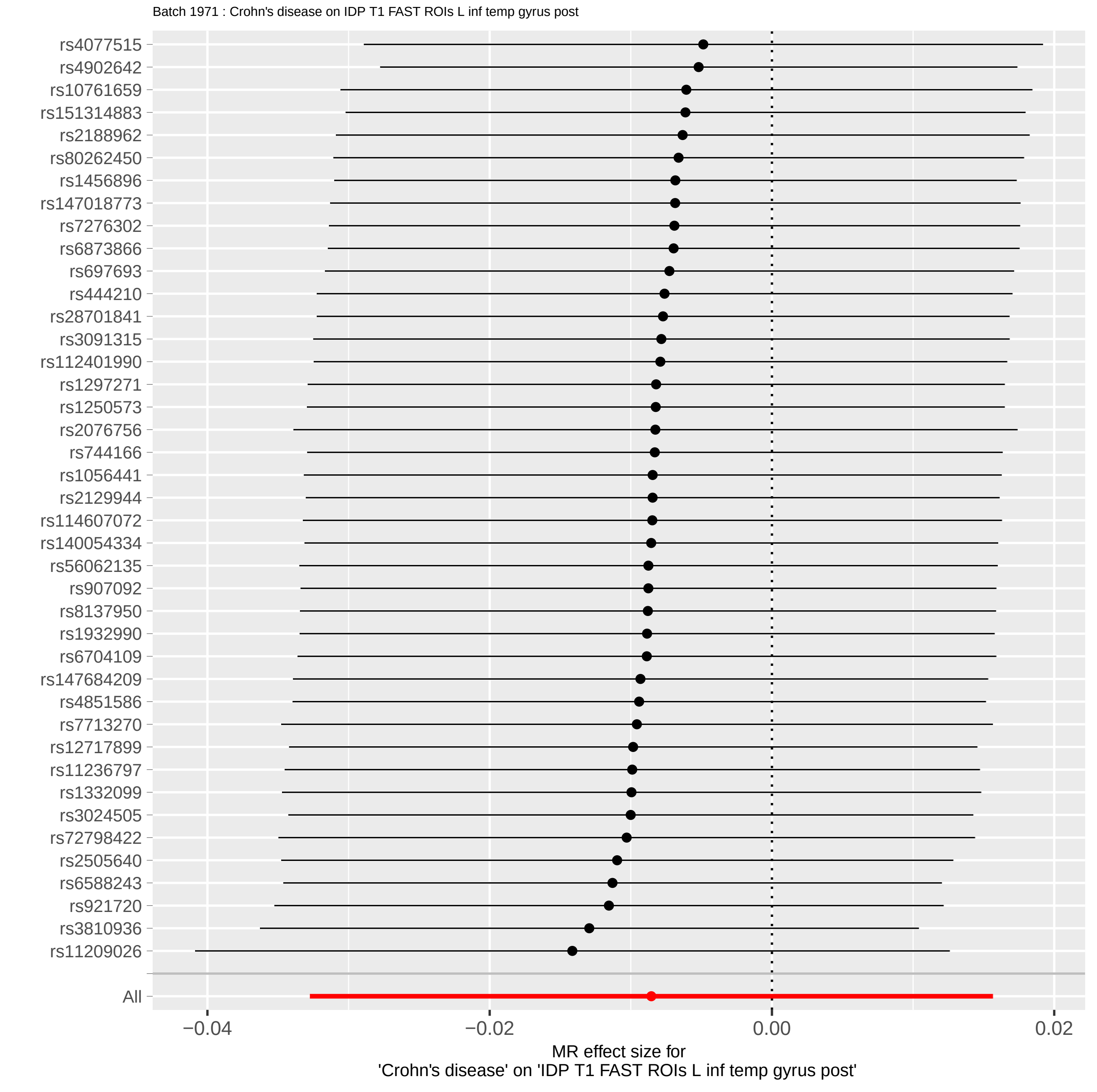

Batch 1971 : Crohn's disease on IDP T1 FAST ROIs L inf temp gyrus post
rs4077515
rs4902642
rs10761659
rs151314883
rs2188962
rs80262450
rs1456896
rs147018773
rs7276302
rs6873866
rs697693
rs444210
rs28701841
rs3091315
rs112401990
rs1297271
rs1250573
rs2076756
rs744166
rs1056441
rs2129944
rs114607072
rs140054334
rs56062135
rs907092
rs8137950
rs1932990
rs6704109
rs147684209
rs4851586
rs7713270
rs12717899
rs11236797
rs1332099
rs3024505
rs72798422
rs2505640
rs6588243
rs921720
rs3810936
rs11209026
All
−0.04
−0.02
0.00
0.02
MR effect size for
'Crohn's disease' on 'IDP T1 FAST ROIs L inf temp gyrus post'

Batch 1972 : Ulcerative colitis on IDP T1 FAST ROIs L inf temp gyrus post

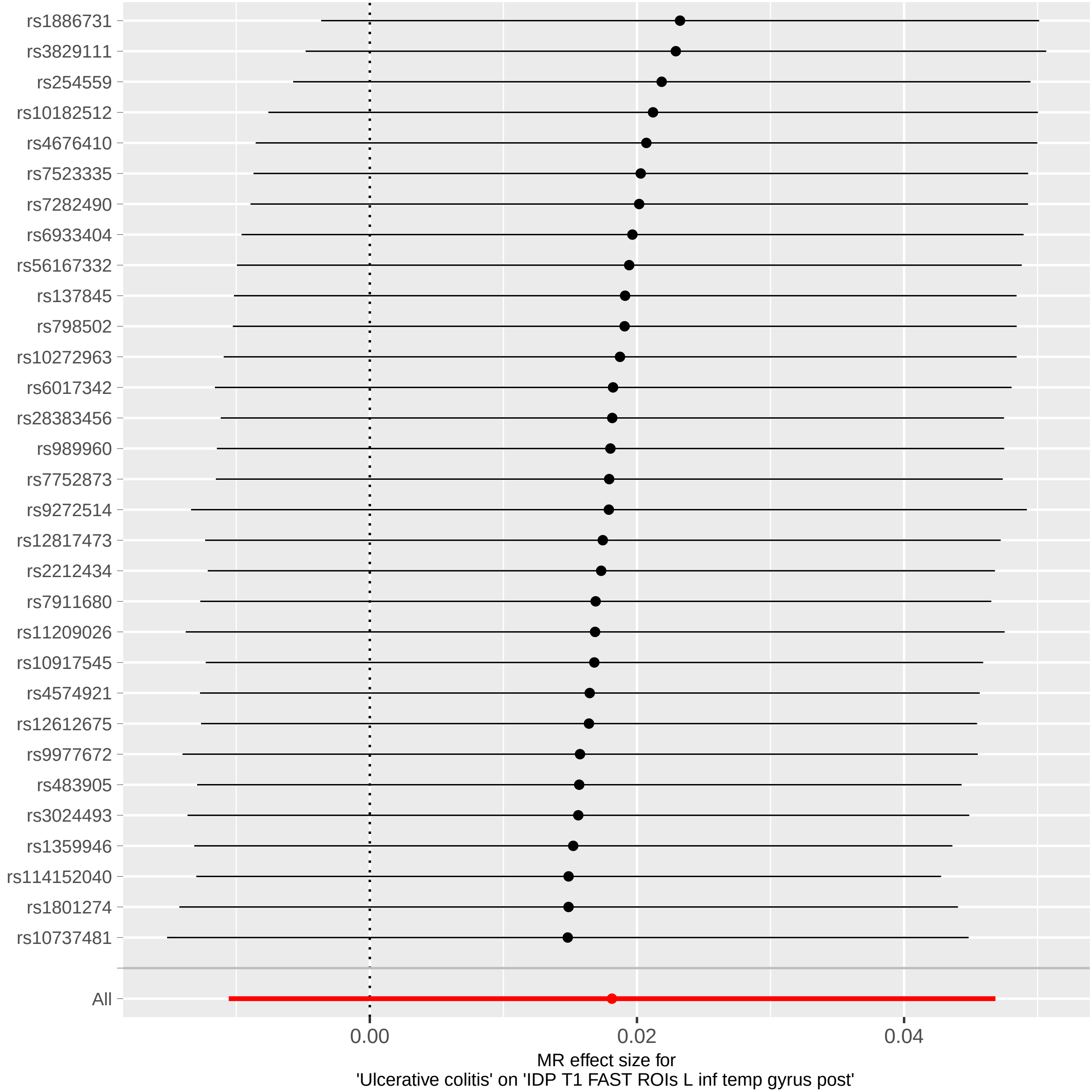

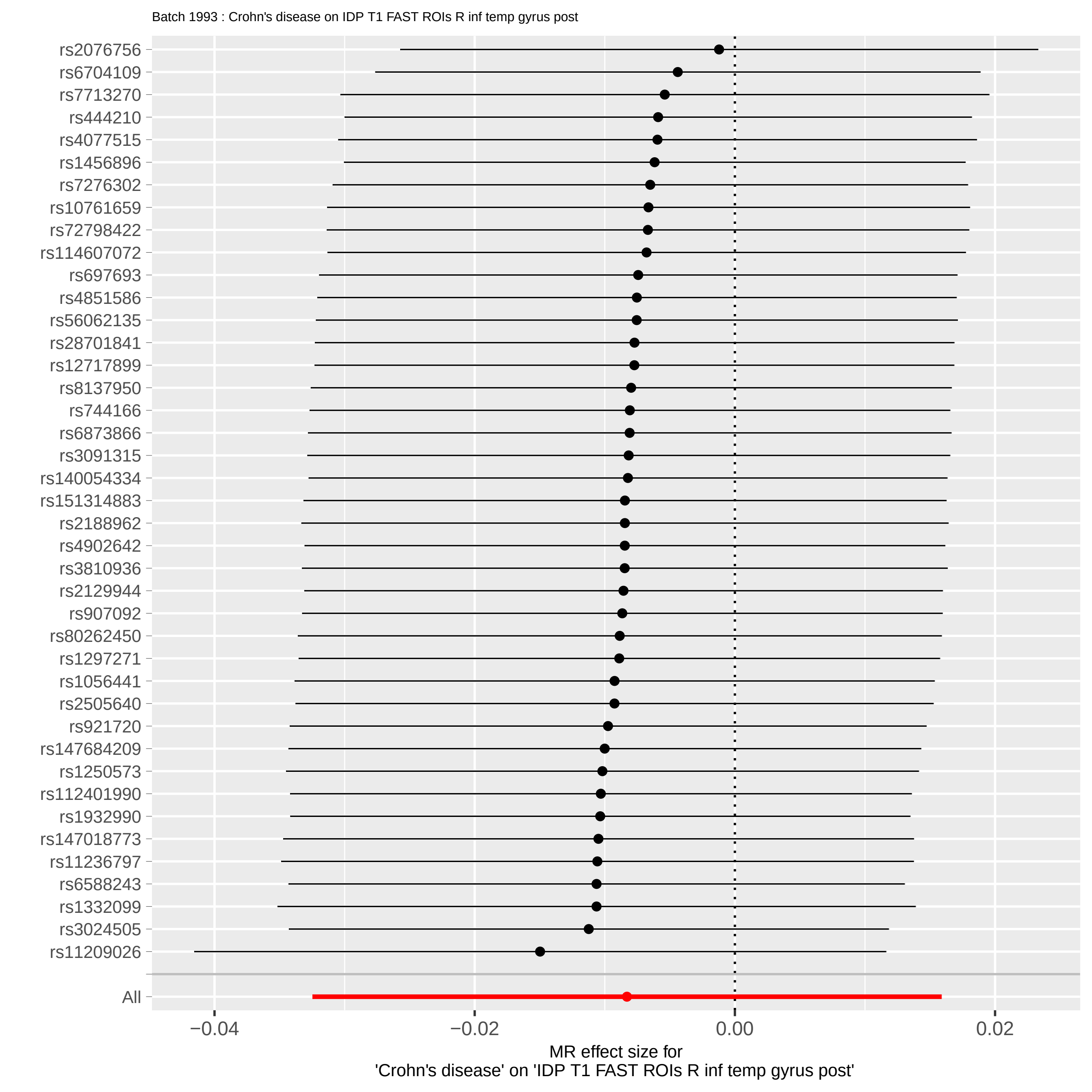

Batch 1993 : Crohn's disease on IDP T1 FAST ROIs R inf temp gyrus post
rs2076756
rs6704109
rs7713270
rs444210
rs4077515
rs1456896
rs7276302
rs10761659
rs72798422
rs114607072
rs697693
rs4851586
rs56062135
rs28701841
rs12717899
rs8137950
rs744166
rs6873866
rs3091315
rs140054334
rs151314883
rs2188962
rs4902642
rs3810936
rs2129944
rs907092
rs80262450
rs1297271
rs1056441
rs2505640
rs921720
rs147684209
rs1250573
rs112401990
rs1932990
rs147018773
rs11236797
rs6588243
rs1332099
rs3024505
rs11209026
All
−0.04
−0.02
0.00
0.02
MR effect size for
'Crohn's disease' on 'IDP T1 FAST ROIs R inf temp gyrus post'

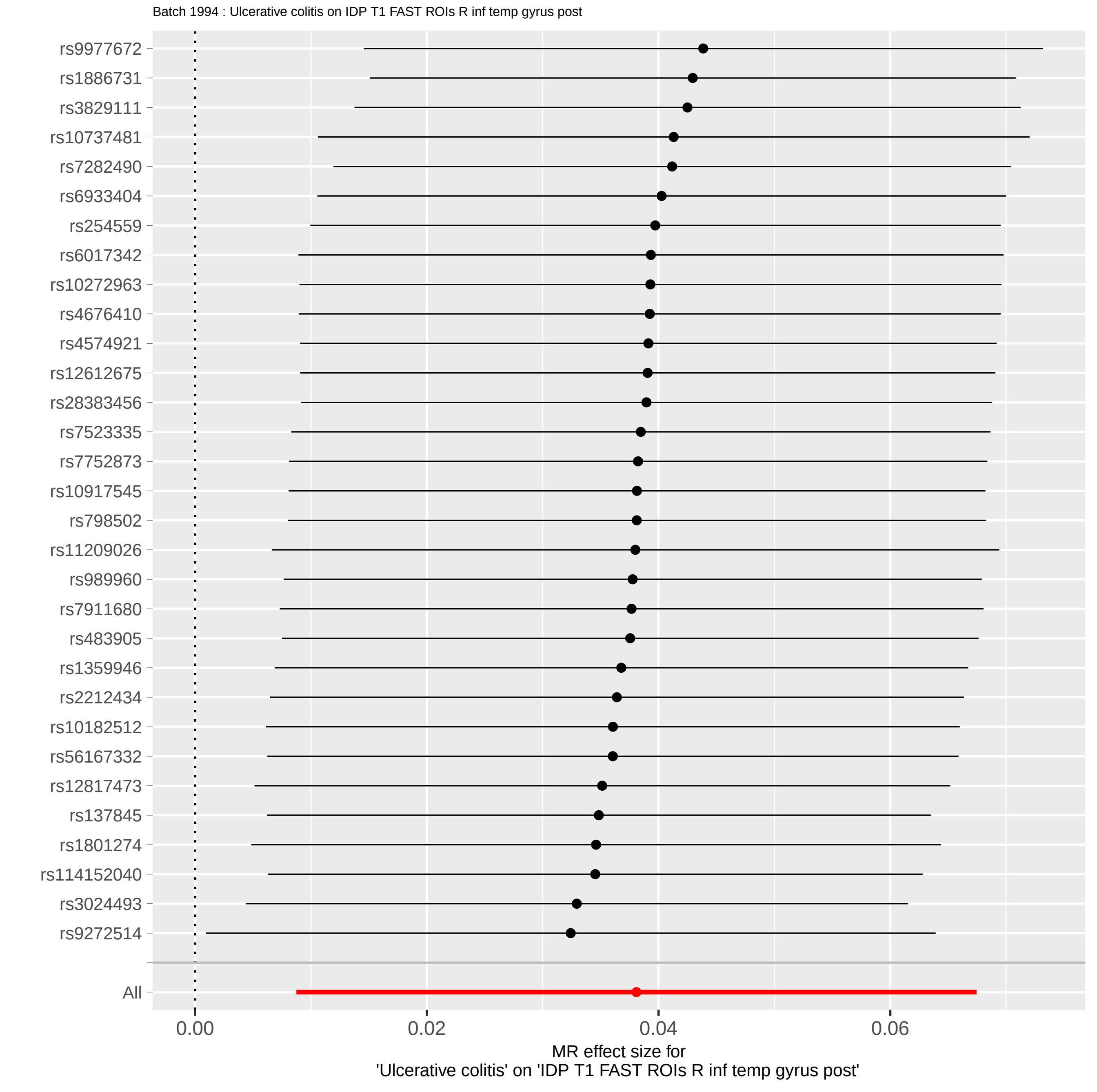

Batch 1994 : Ulcerative colitis on IDP T1 FAST ROIs R inf temp gyrus post
rs9977672
rs1886731
rs3829111
rs10737481
rs7282490
rs6933404
rs254559
rs6017342
rs10272963
rs4676410
rs4574921
rs12612675
rs28383456
rs7523335
rs7752873
rs10917545
rs798502
rs11209026
rs989960
rs7911680
rs483905
rs1359946
rs2212434
rs10182512
rs56167332
rs12817473
rs137845
rs1801274
rs114152040
rs3024493
rs9272514
All
0.00
0.02
0.04
0.06
MR effect size for
'Ulcerative colitis' on 'IDP T1 FAST ROIs R inf temp gyrus post'

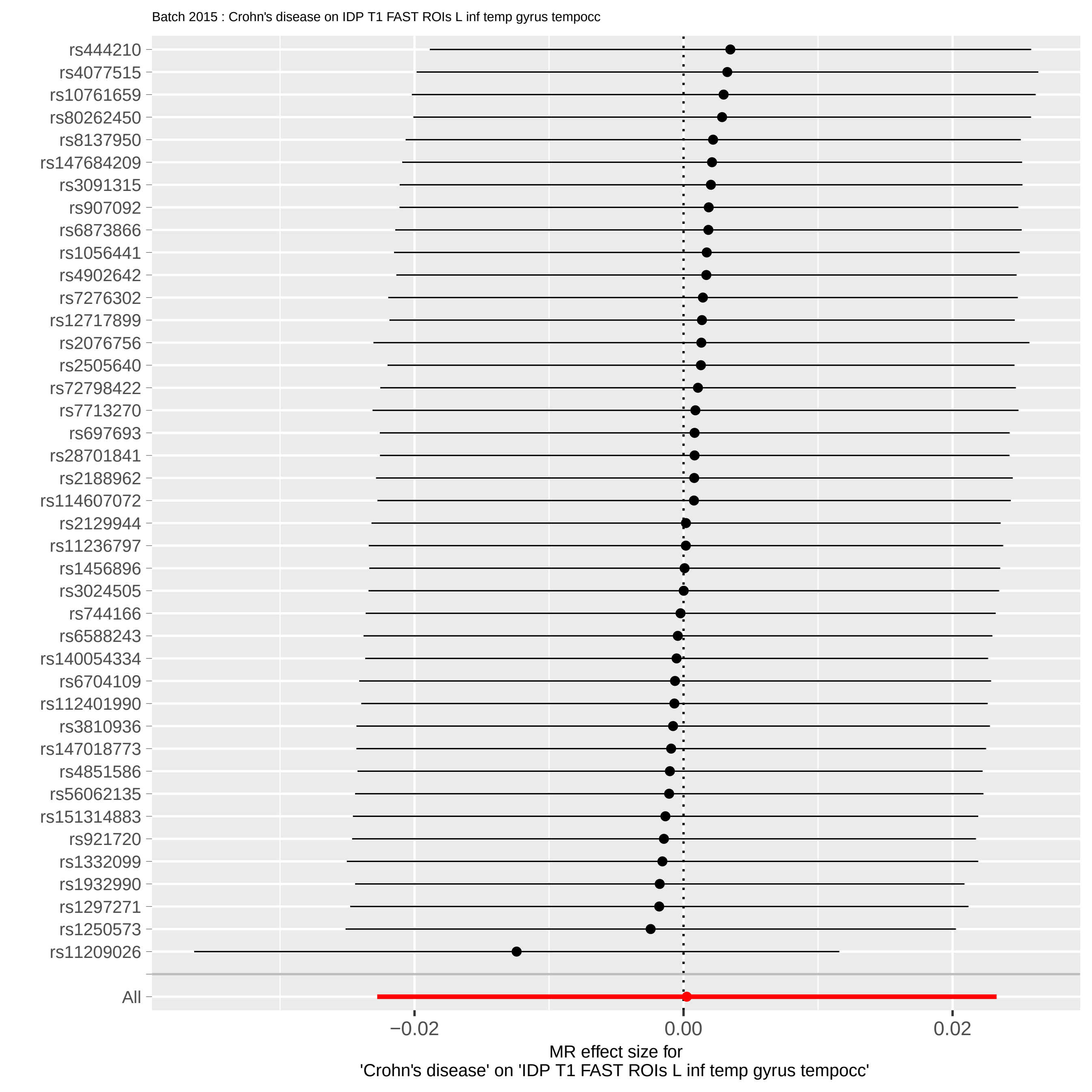

Batch 2015 : Crohn's disease on IDP T1 FAST ROIs L inf temp gyrus tempocc
rs444210
rs4077515
rs10761659
rs80262450
rs8137950
rs147684209
rs3091315
rs907092
rs6873866
rs1056441
rs4902642
rs7276302
rs12717899
rs2076756
rs2505640
rs72798422
rs7713270
rs697693
rs28701841
rs2188962
rs114607072
rs2129944
rs11236797
rs1456896
rs3024505
rs744166
rs6588243
rs140054334
rs6704109
rs112401990
rs3810936
rs147018773
rs4851586
rs56062135
rs151314883
rs921720
rs1332099
rs1932990
rs1297271
rs1250573
rs11209026
All
−0.02
0.00
0.02
MR effect size for
'Crohn's disease' on 'IDP T1 FAST ROIs L inf temp gyrus tempocc'

Batch 2016 : Ulcerative colitis on IDP T1 FAST ROIs L inf temp gyrus tempocc

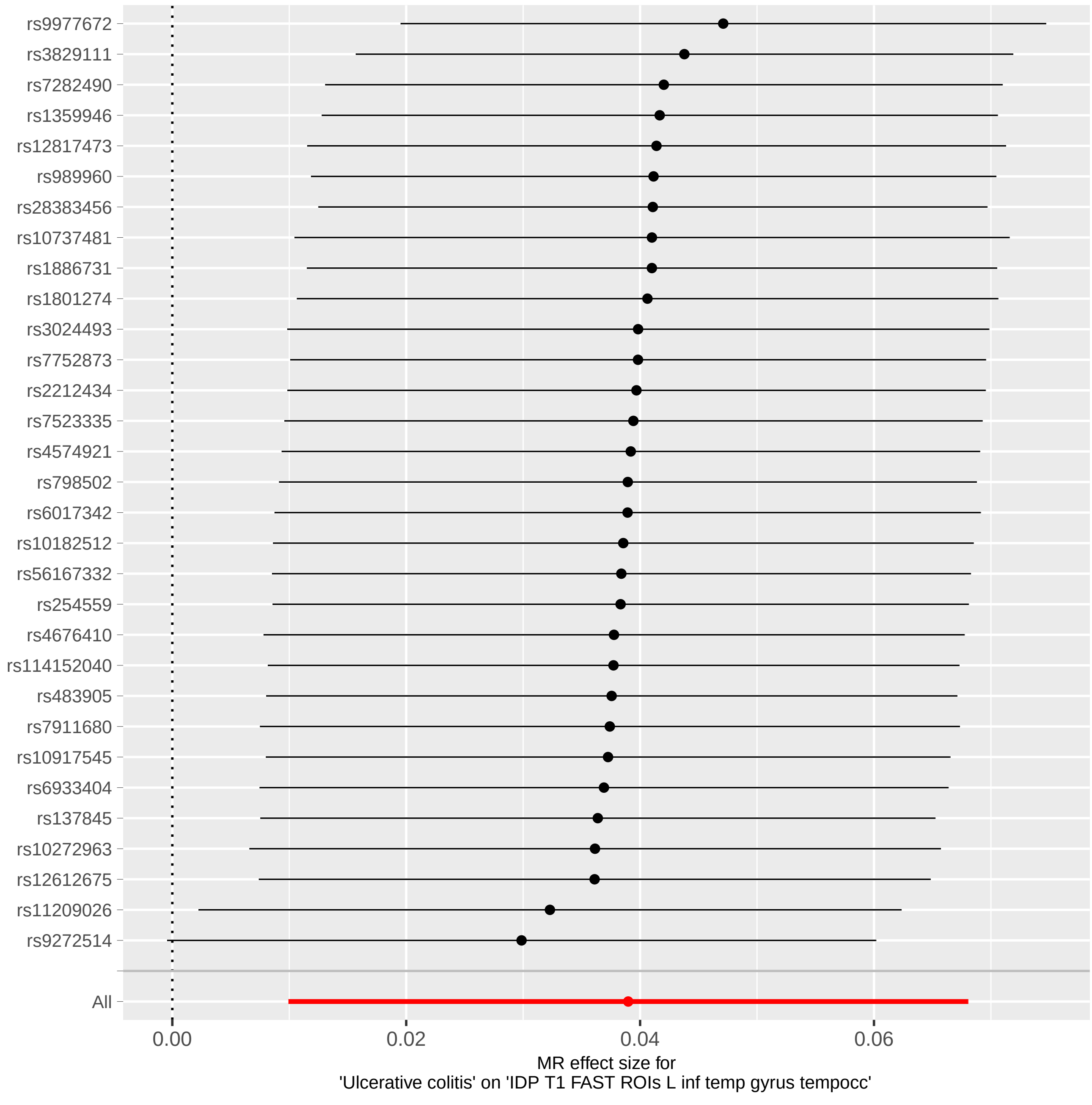

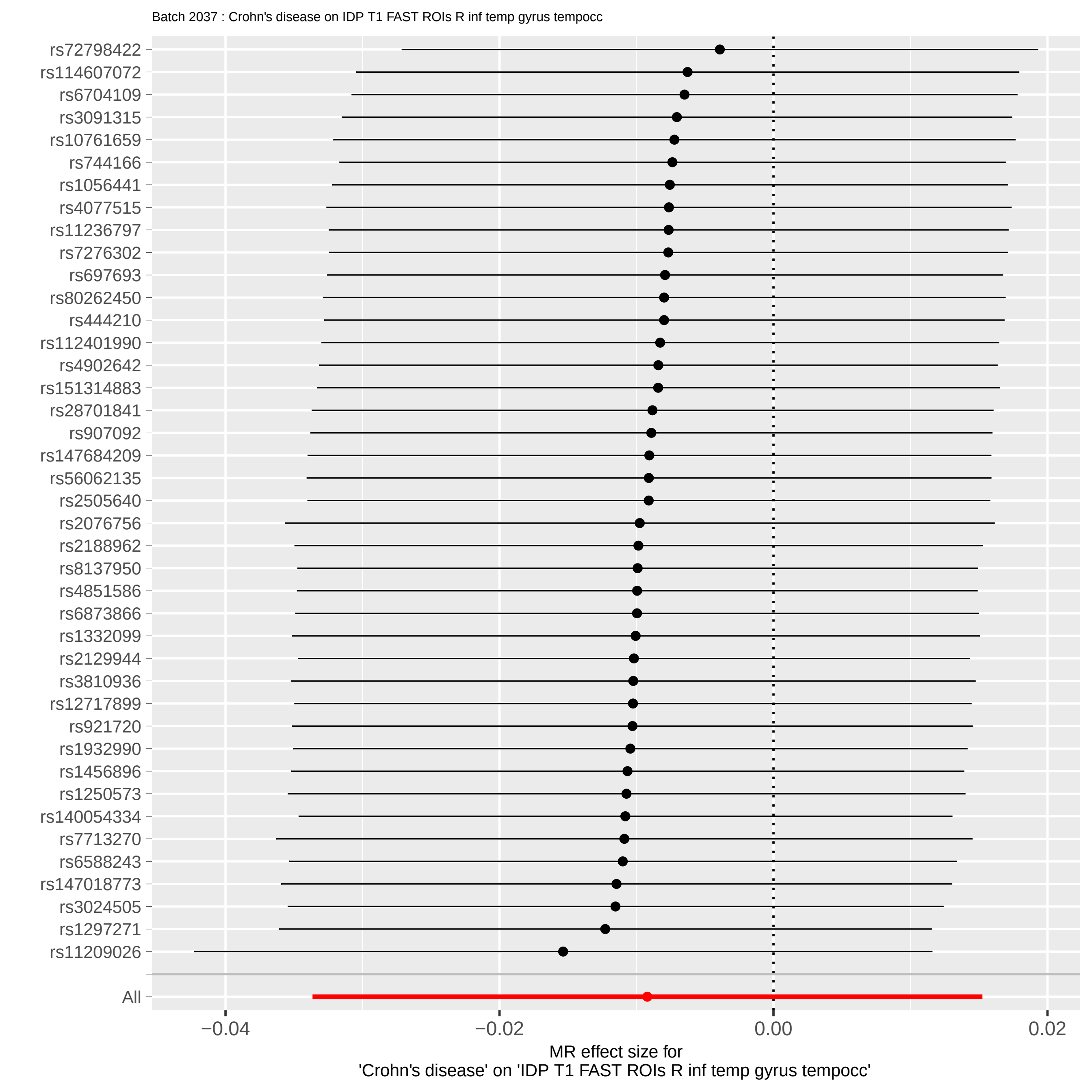

Batch 2037 : Crohn's disease on IDP T1 FAST ROIs R inf temp gyrus tempocc
rs72798422
rs114607072
rs6704109
rs3091315
rs10761659
rs744166
rs1056441
rs4077515
rs11236797
rs7276302
rs697693
rs80262450
rs444210
rs112401990
rs4902642
rs151314883
rs28701841
rs907092
rs147684209
rs56062135
rs2505640
rs2076756
rs2188962
rs8137950
rs4851586
rs6873866
rs1332099
rs2129944
rs3810936
rs12717899
rs921720
rs1932990
rs1456896
rs1250573
rs140054334
rs7713270
rs6588243
rs147018773
rs3024505
rs1297271
rs11209026
All
−0.04
−0.02
0.00
0.02
MR effect size for
'Crohn's disease' on 'IDP T1 FAST ROIs R inf temp gyrus tempocc'

Batch 2038 : Ulcerative colitis on IDP T1 FAST ROIs R inf temp gyrus tempocc

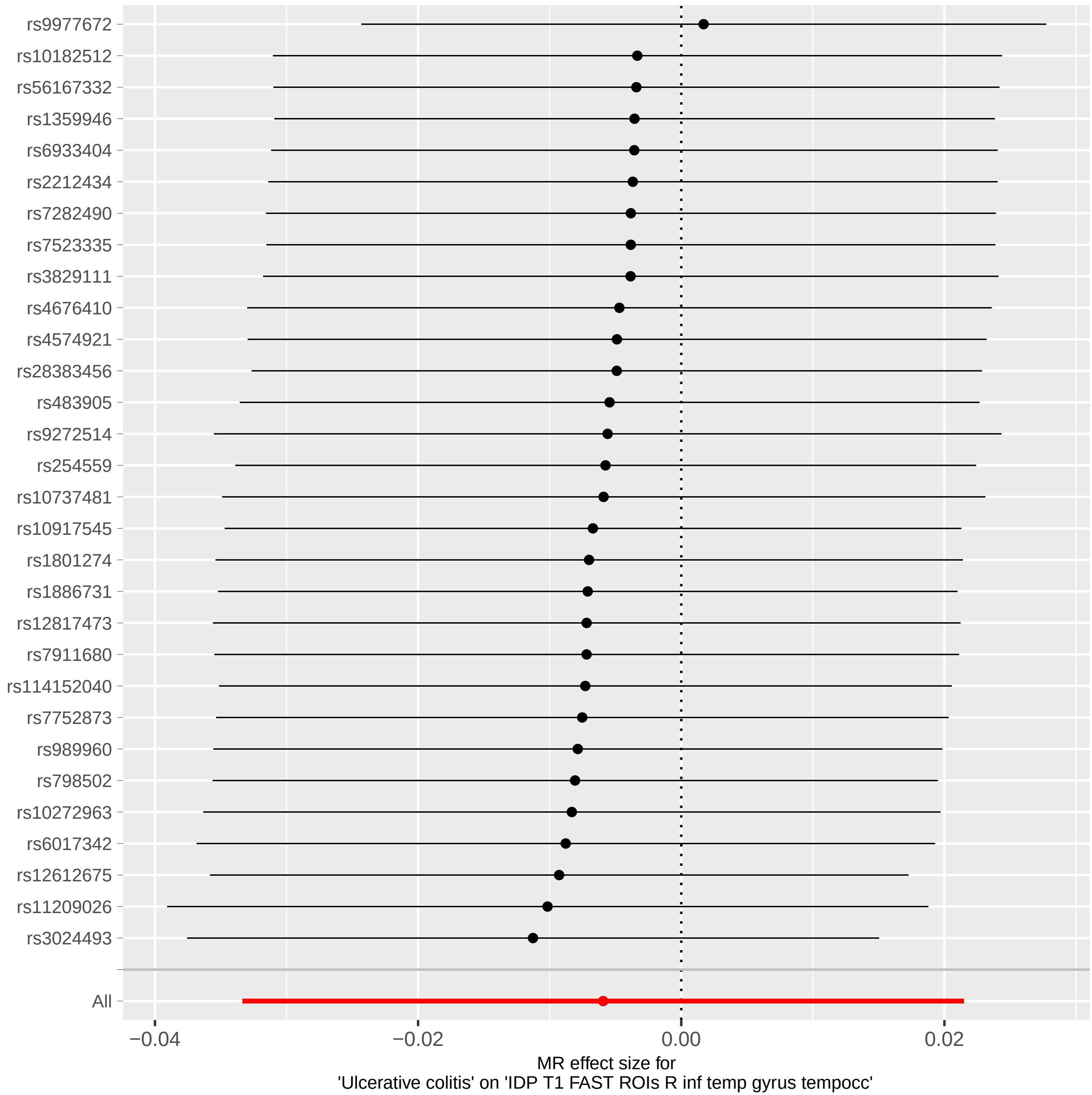

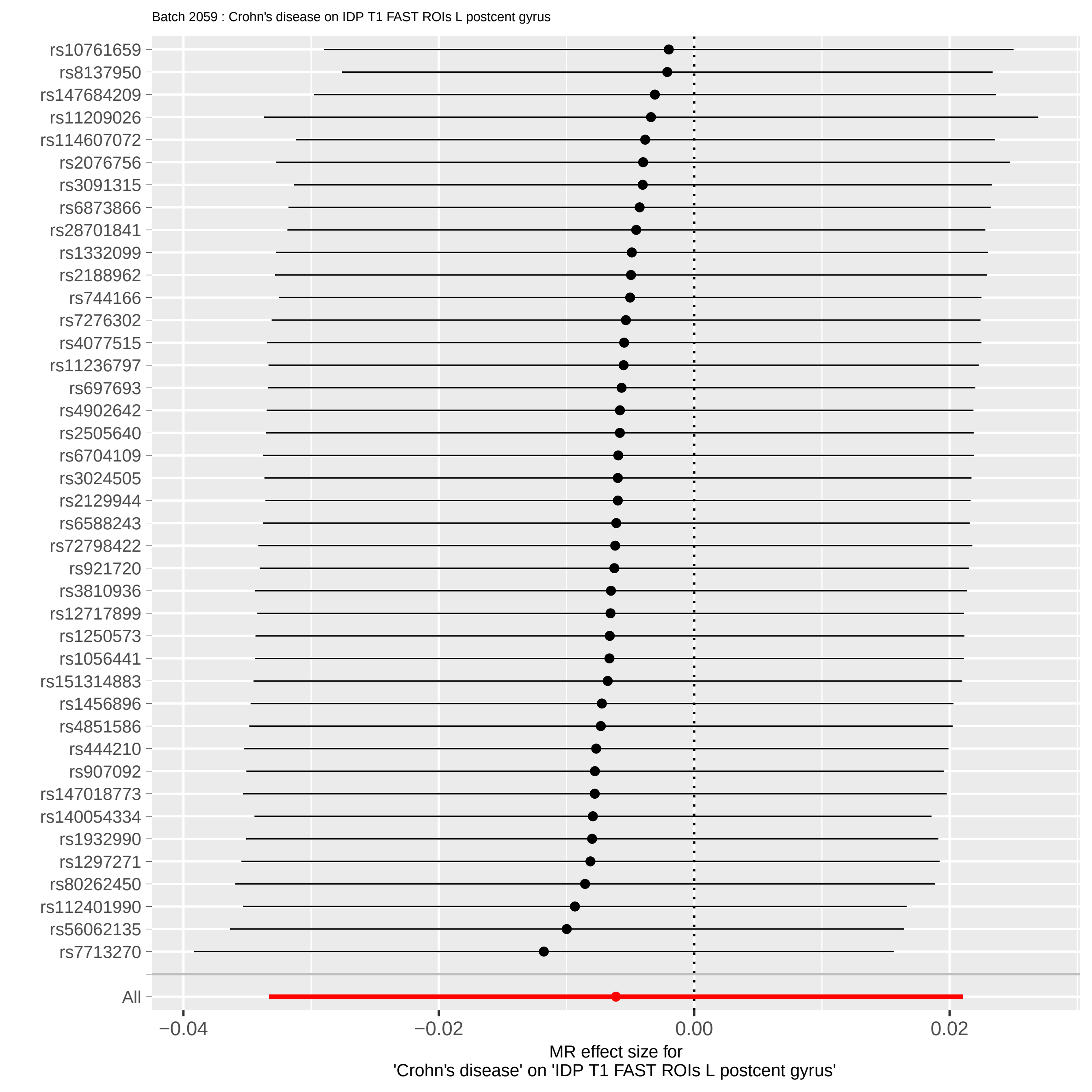

Batch 2059 : Crohn's disease on IDP T1 FAST ROIs L postcent gyrus
rs10761659
rs8137950
rs147684209
rs11209026
rs114607072
rs2076756
rs3091315
rs6873866
rs28701841
rs1332099
rs2188962
rs744166
rs7276302
rs4077515
rs11236797
rs697693
rs4902642
rs2505640
rs6704109
rs3024505
rs2129944
rs6588243
rs72798422
rs921720
rs3810936
rs12717899
rs1250573
rs1056441
rs151314883
rs1456896
rs4851586
rs444210
rs907092
rs147018773
rs140054334
rs1932990
rs1297271
rs80262450
rs112401990
rs56062135
rs7713270
All
−0.04
−0.02
0.00
0.02
MR effect size for
'Crohn's disease' on 'IDP T1 FAST ROIs L postcent gyrus'

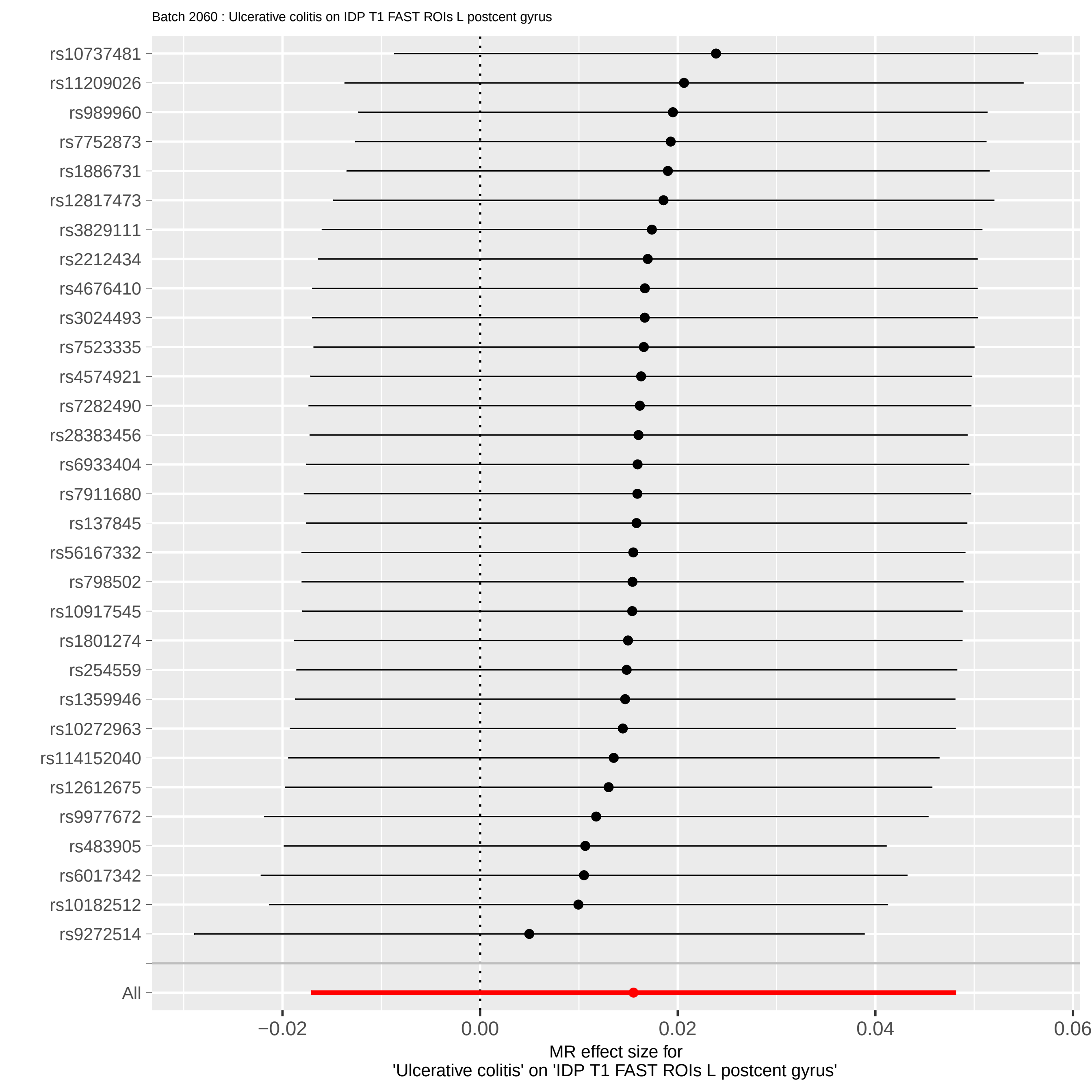

Batch 2060 : Ulcerative colitis on IDP T1 FAST ROIs L postcent gyrus
rs10737481
rs11209026
rs989960
rs7752873
rs1886731
rs12817473
rs3829111
rs2212434
rs4676410
rs3024493
rs7523335
rs4574921
rs7282490
rs28383456
rs6933404
rs7911680
rs137845
rs56167332
rs798502
rs10917545
rs1801274
rs254559
rs1359946
rs10272963
rs114152040
rs12612675
rs9977672
rs483905
rs6017342
rs10182512
rs9272514
All
−0.02
0.00
0.02
0.04
0.06
MR effect size for
'Ulcerative colitis' on 'IDP T1 FAST ROIs L postcent gyrus'

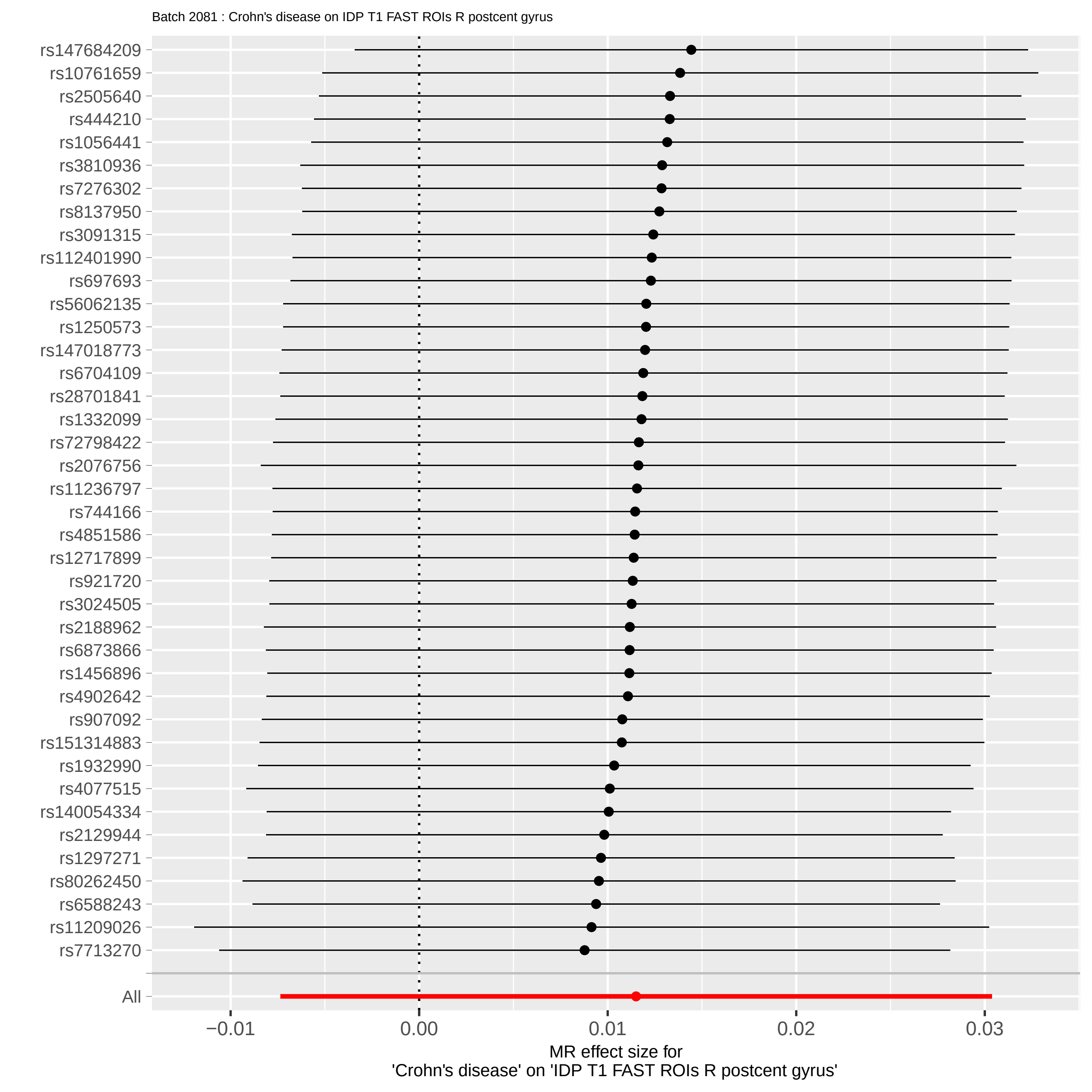

Batch 2081 : Crohn's disease on IDP T1 FAST ROIs R postcent gyrus
rs147684209
rs10761659
rs2505640
rs444210
rs1056441
rs3810936
rs7276302
rs8137950
rs3091315
rs112401990
rs697693
rs56062135
rs1250573
rs147018773
rs6704109
rs28701841
rs1332099
rs72798422
rs2076756
rs11236797
rs744166
rs4851586
rs12717899
rs921720
rs3024505
rs2188962
rs6873866
rs1456896
rs4902642
rs907092
rs151314883
rs1932990
rs4077515
rs140054334
rs2129944
rs1297271
rs80262450
rs6588243
rs11209026
rs7713270
All
−0.01
0.00
0.01
0.02
0.03
MR effect size for
'Crohn's disease' on 'IDP T1 FAST ROIs R postcent gyrus'

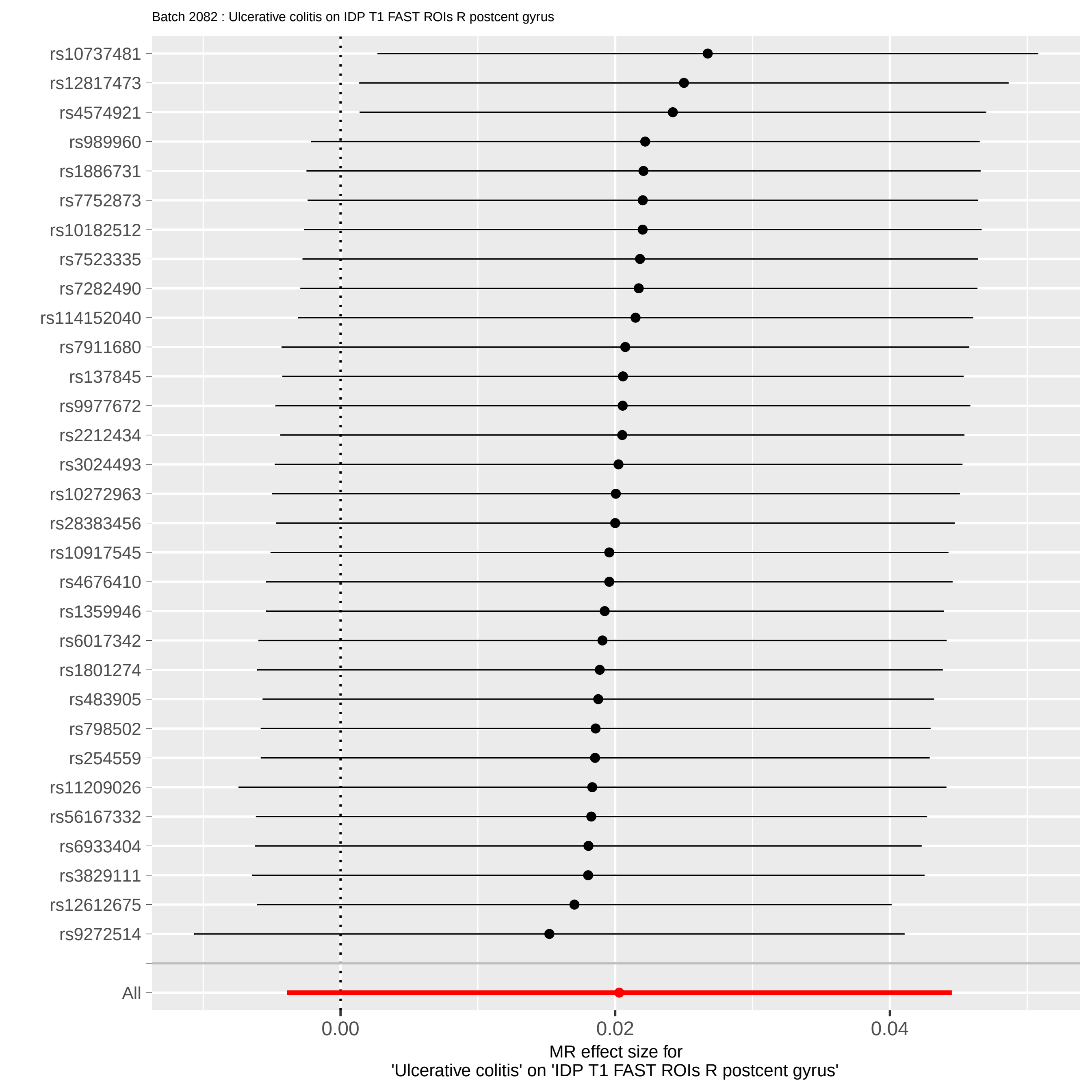
Batch 2082 : Ulcerative colitis on IDP T1 FAST ROIs R postcent gyrus
rs10737481
rs12817473
rs4574921
rs989960
rs1886731
rs7752873
rs10182512
rs7523335
rs7282490
rs114152040
rs7911680
rs137845
rs9977672
rs2212434
rs3024493
rs10272963
rs28383456
rs10917545
rs4676410
rs1359946
rs6017342
rs1801274
rs483905
rs798502
rs254559
rs11209026
rs56167332
rs6933404
rs3829111
rs12612675
rs9272514
All
0.00
0.02
0.04
MR effect size for
'Ulcerative colitis' on 'IDP T1 FAST ROIs R postcent gyrus'

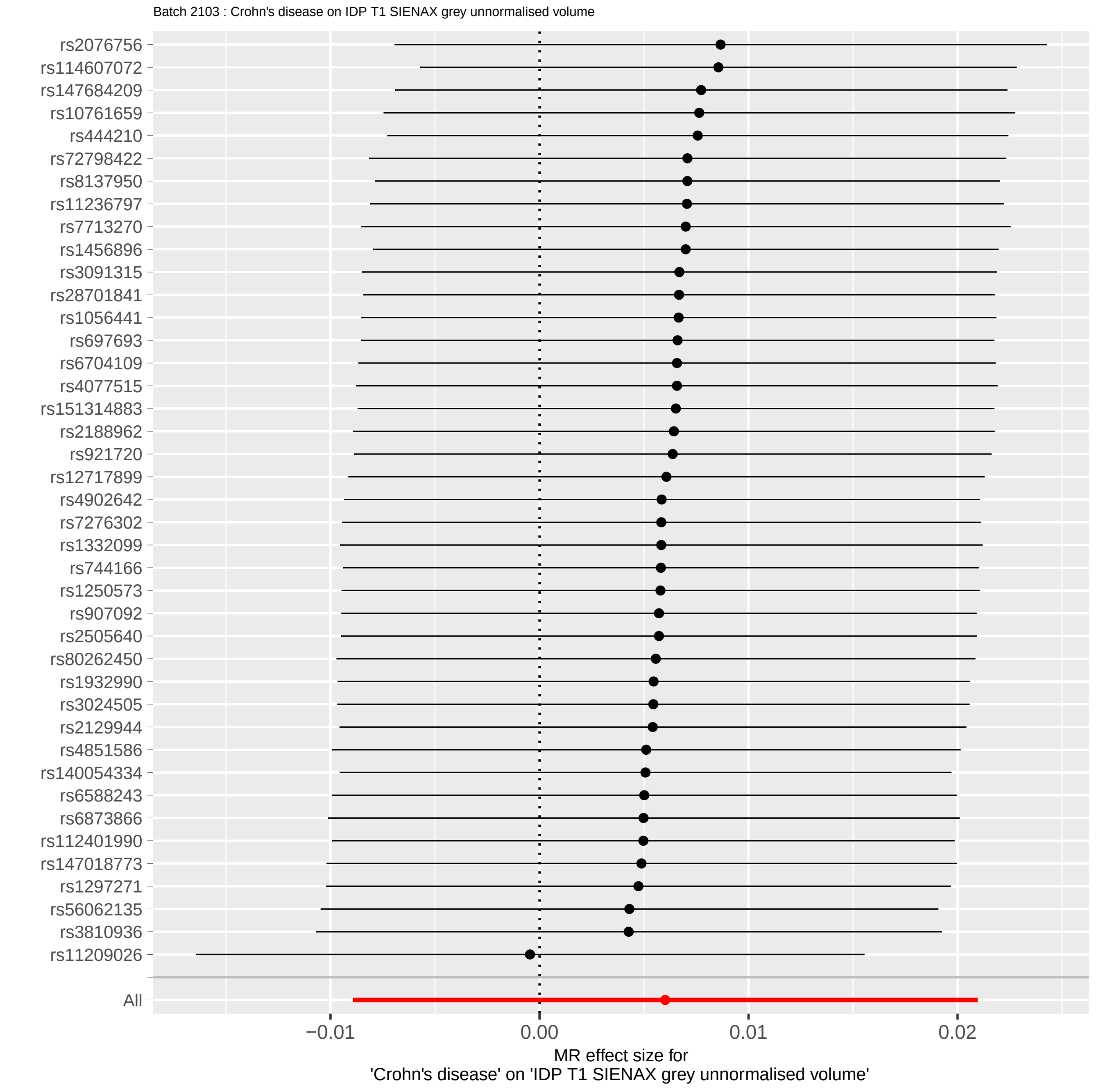
Batch 2103 : Crohn's disease on IDP T1 SIENAX grey unnormalised volume
rs2076756
rs114607072
rs147684209
rs10761659
rs444210
rs72798422
rs8137950
rs11236797
rs7713270
rs1456896
rs3091315
rs28701841
rs1056441
rs697693
rs6704109
rs4077515
rs151314883
rs2188962
rs921720
rs12717899
rs4902642
rs7276302
rs1332099
rs744166
rs1250573
rs907092
rs2505640
rs80262450
rs1932990
rs3024505
rs2129944
rs4851586
rs140054334
rs6588243
rs6873866
rs112401990
rs147018773
rs1297271
rs56062135
rs3810936
rs11209026
All
−0.01
0.00
0.01
0.02
MR effect size for
'Crohn's disease' on 'IDP T1 SIENAX grey unnormalised volume'

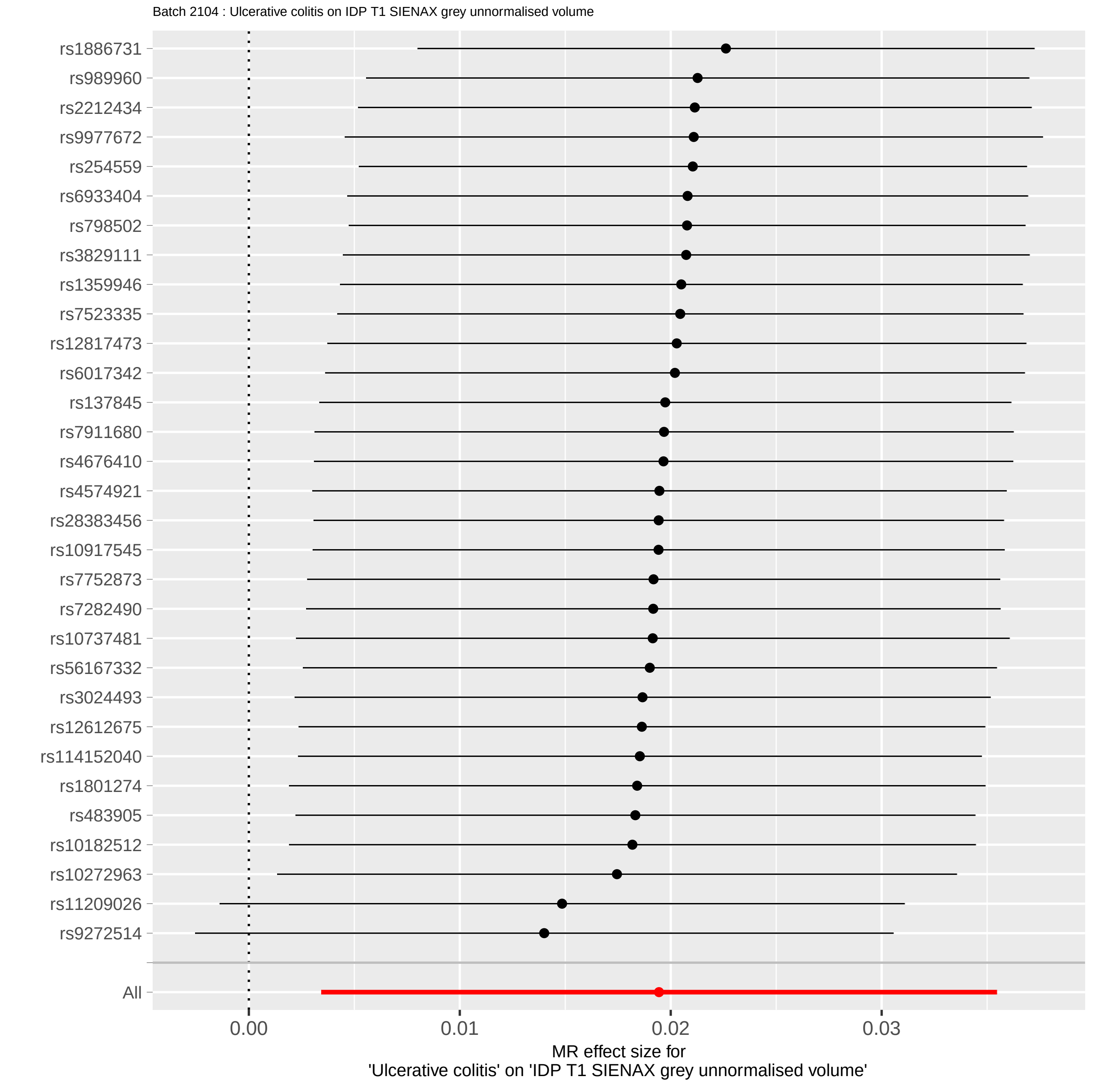
Batch 2104 : Ulcerative colitis on IDP T1 SIENAX grey unnormalised volume
rs1886731
rs989960
rs2212434
rs9977672
rs254559
rs6933404
rs798502
rs3829111
rs1359946
rs7523335
rs12817473
rs6017342
rs137845
rs7911680
rs4676410
rs4574921
rs28383456
rs10917545
rs7752873
rs7282490
rs10737481
rs56167332
rs3024493
rs12612675
rs114152040
rs1801274
rs483905
rs10182512
rs10272963
rs11209026
rs9272514
All
0.00
0.01
0.02
0.03
MR effect size for
'Ulcerative colitis' on 'IDP T1 SIENAX grey unnormalised volume'

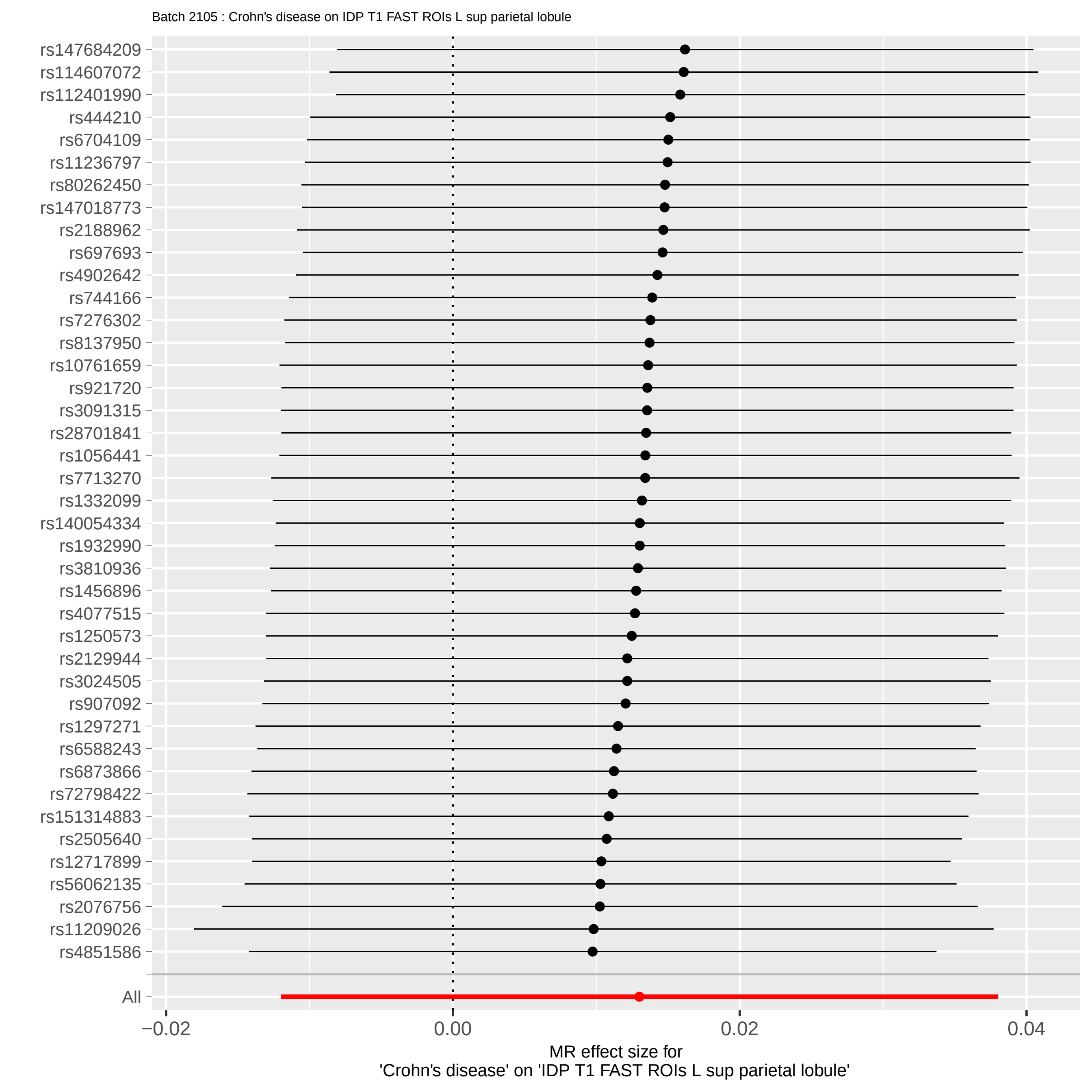

Batch 2105 : Crohn's disease on IDP T1 FAST ROIs L sup parietal lobule
rs147684209
rs114607072
rs112401990
rs444210
rs6704109
rs11236797
rs80262450
rs147018773
rs2188962
rs697693
rs4902642
rs744166
rs7276302
rs8137950
rs10761659
rs921720
rs3091315
rs28701841
rs1056441
rs7713270
rs1332099
rs140054334
rs1932990
rs3810936
rs1456896
rs4077515
rs1250573
rs2129944
rs3024505
rs907092
rs1297271
rs6588243
rs6873866
rs72798422
rs151314883
rs2505640
rs12717899
rs56062135
rs2076756
rs11209026
rs4851586
All
−0.02
0.00
0.02
0.04
MR effect size for
'Crohn's disease' on 'IDP T1 FAST ROIs L sup parietal lobule'

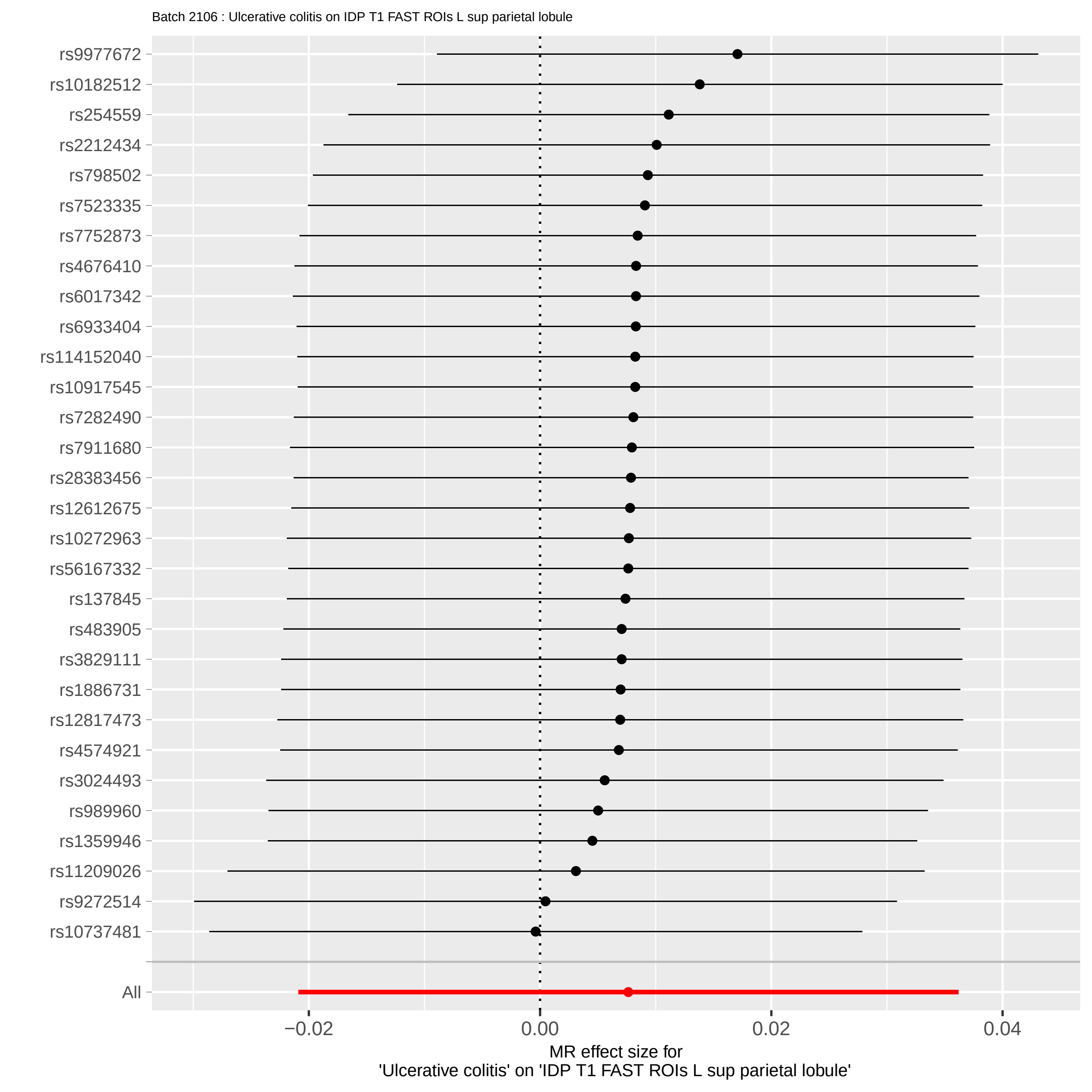

Batch 2106 : Ulcerative colitis on IDP T1 FAST ROIs L sup parietal lobule
rs9977672
rs10182512
rs254559
rs2212434
rs798502
rs7523335
rs7752873
rs4676410
rs6017342
rs6933404
rs114152040
rs10917545
rs7282490
rs7911680
rs28383456
rs12612675
rs10272963
rs56167332
rs137845
rs483905
rs3829111
rs1886731
rs12817473
rs4574921
rs3024493
rs989960
rs1359946
rs11209026
rs9272514
rs10737481
All
−0.02
0.00
0.02
0.04
MR effect size for
'Ulcerative colitis' on 'IDP T1 FAST ROIs L sup parietal lobule'

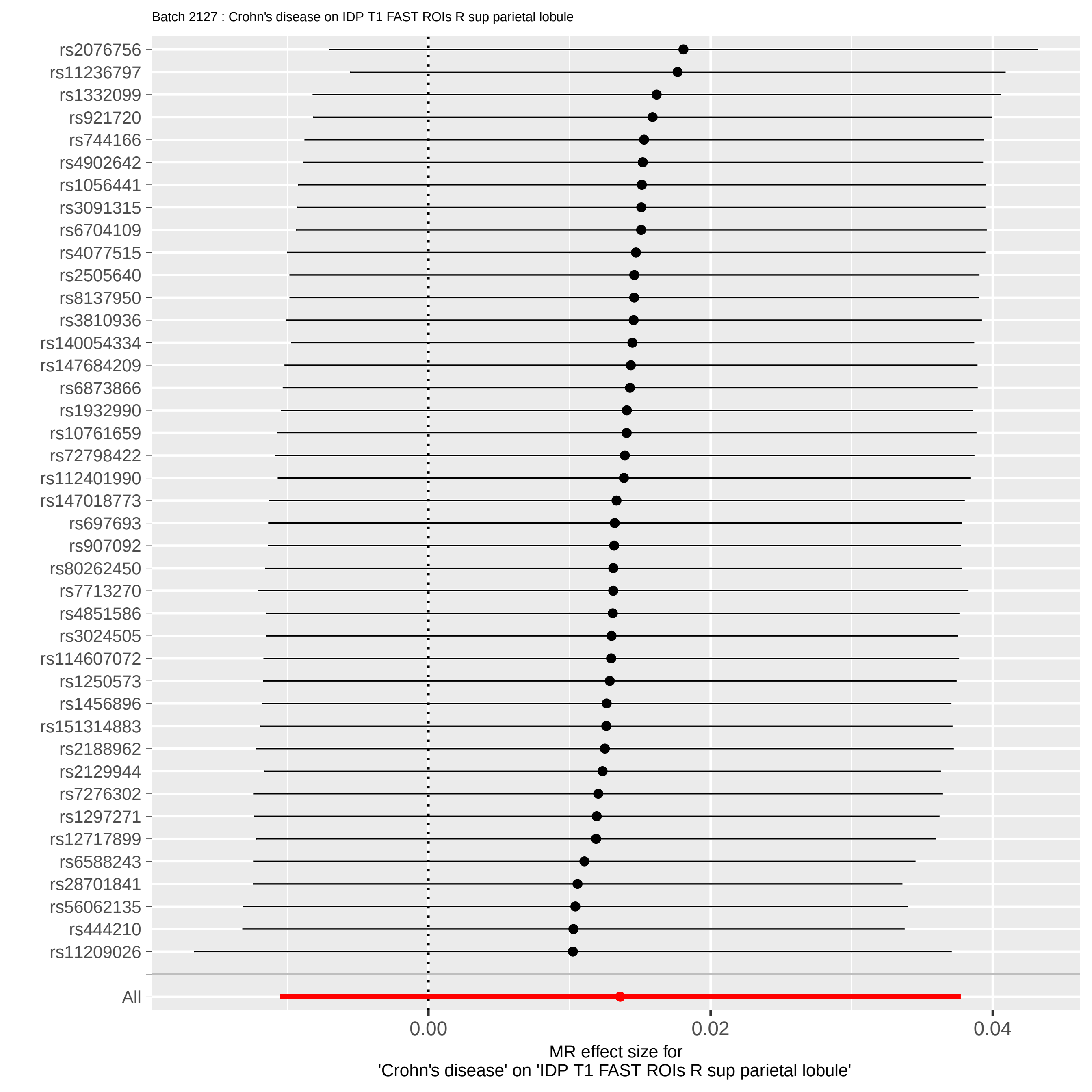

Batch 2127 : Crohn's disease on IDP T1 FAST ROIs R sup parietal lobule
rs2076756
rs11236797
rs1332099
rs921720
rs744166
rs4902642
rs1056441
rs3091315
rs6704109
rs4077515
rs2505640
rs8137950
rs3810936
rs140054334
rs147684209
rs6873866
rs1932990
rs10761659
rs72798422
rs112401990
rs147018773
rs697693
rs907092
rs80262450
rs7713270
rs4851586
rs3024505
rs114607072
rs1250573
rs1456896
rs151314883
rs2188962
rs2129944
rs7276302
rs1297271
rs12717899
rs6588243
rs28701841
rs56062135
rs444210
rs11209026
All
0.00
0.02
0.04
MR effect size for
'Crohn's disease' on 'IDP T1 FAST ROIs R sup parietal lobule'

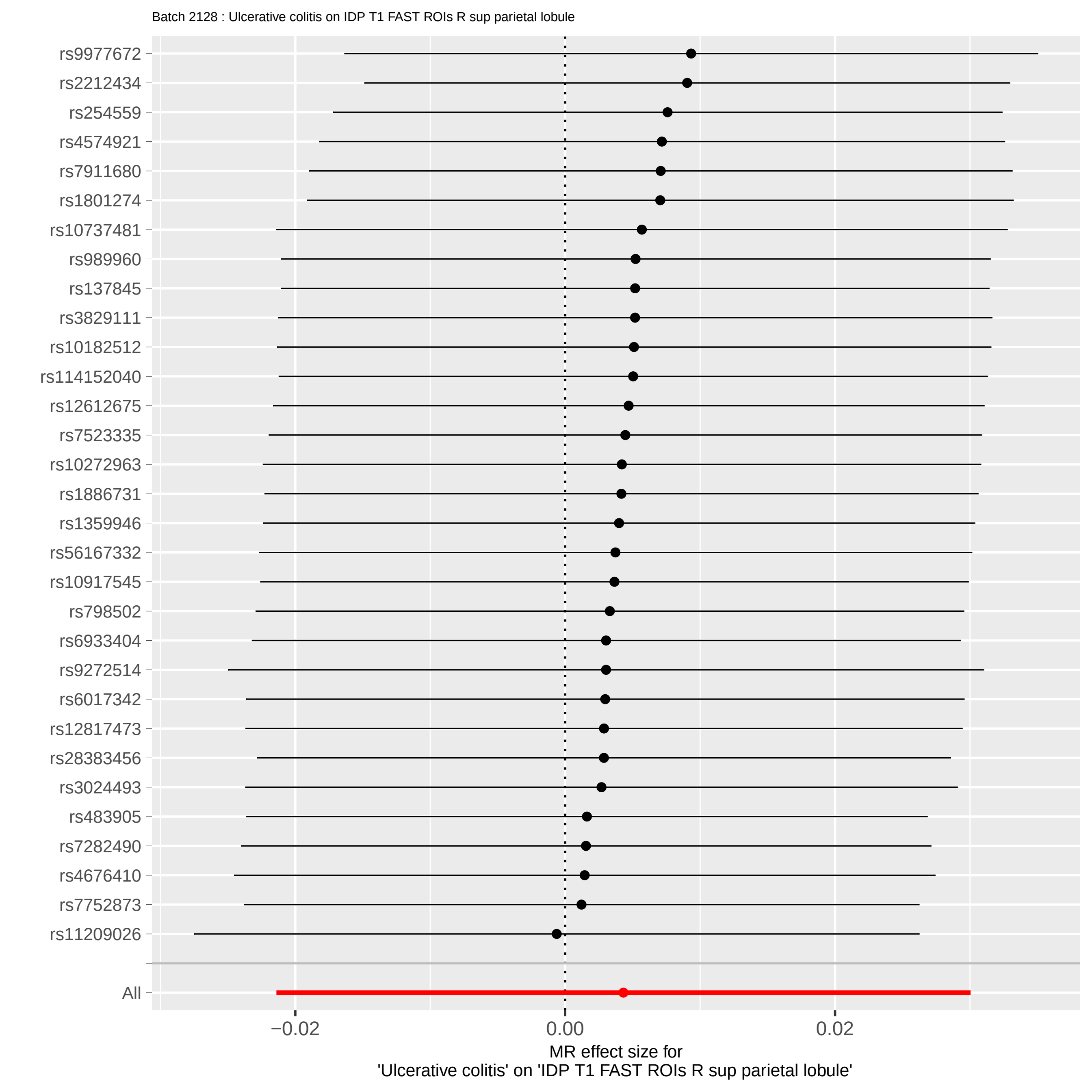
Batch 2128 : Ulcerative colitis on IDP T1 FAST ROIs R sup parietal lobule
rs9977672
rs2212434
rs254559
rs4574921
rs7911680
rs1801274
rs10737481
rs989960
rs137845
rs3829111
rs10182512
rs114152040
rs12612675
rs7523335
rs10272963
rs1886731
rs1359946
rs56167332
rs10917545
rs798502
rs6933404
rs9272514
rs6017342
rs12817473
rs28383456
rs3024493
rs483905
rs7282490
rs4676410
rs7752873
rs11209026
All
−0.02
0.00
0.02
MR effect size for
'Ulcerative colitis' on 'IDP T1 FAST ROIs R sup parietal lobule'

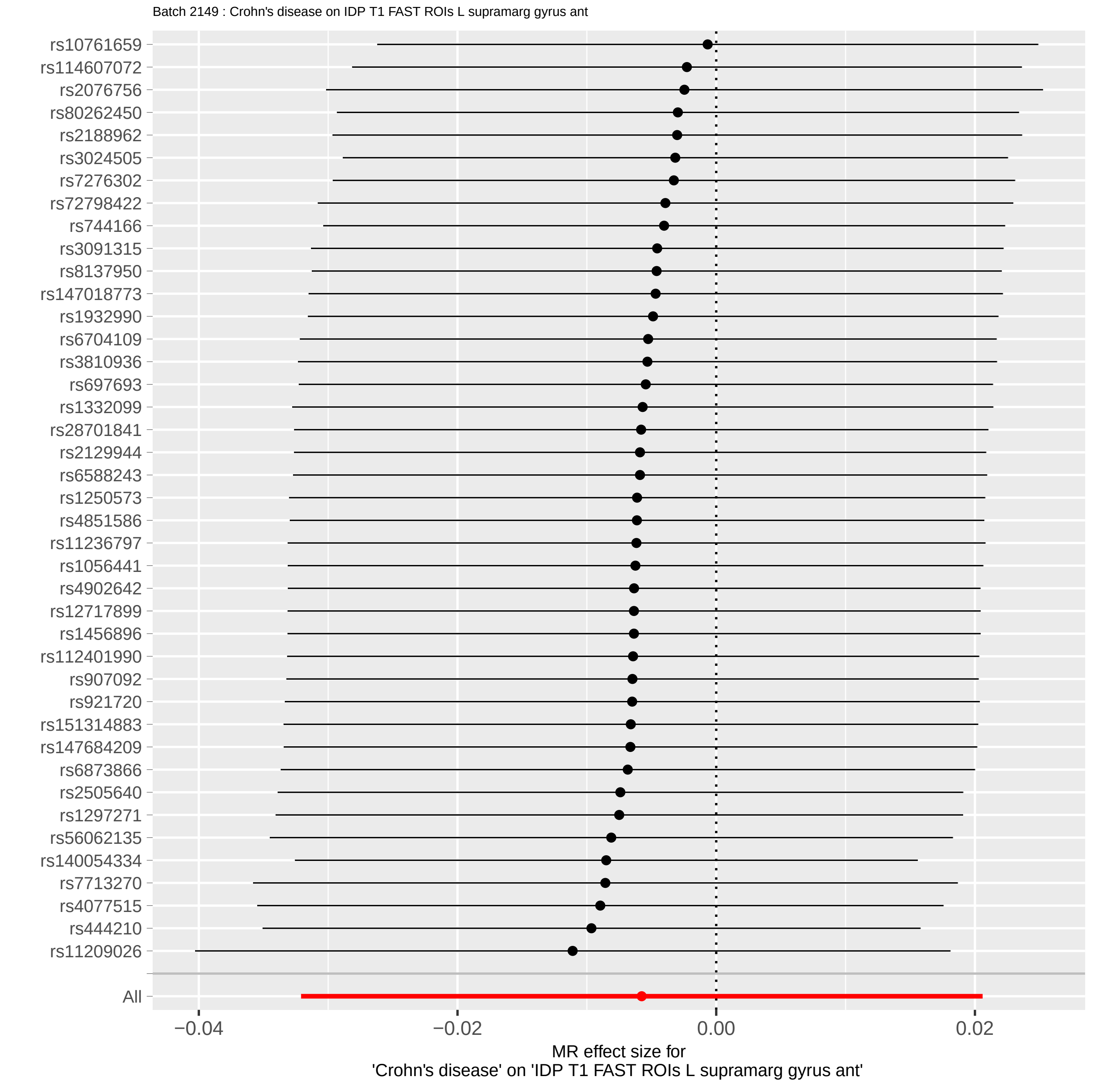

Batch 2149 : Crohn's disease on IDP T1 FAST ROIs L supramarg gyrus ant
rs10761659
rs114607072
rs2076756
rs80262450
rs2188962
rs3024505
rs7276302
rs72798422
rs744166
rs3091315
rs8137950
rs147018773
rs1932990
rs6704109
rs3810936
rs697693
rs1332099
rs28701841
rs2129944
rs6588243
rs1250573
rs4851586
rs11236797
rs1056441
rs4902642
rs12717899
rs1456896
rs112401990
rs907092
rs921720
rs151314883
rs147684209
rs6873866
rs2505640
rs1297271
rs56062135
rs140054334
rs7713270
rs4077515
rs444210
rs11209026
All
−0.04
−0.02
0.00
0.02
MR effect size for
'Crohn's disease' on 'IDP T1 FAST ROIs L supramarg gyrus ant'

Batch 2150 : Ulcerative colitis on IDP T1 FAST ROIs L supramarg gyrus ant

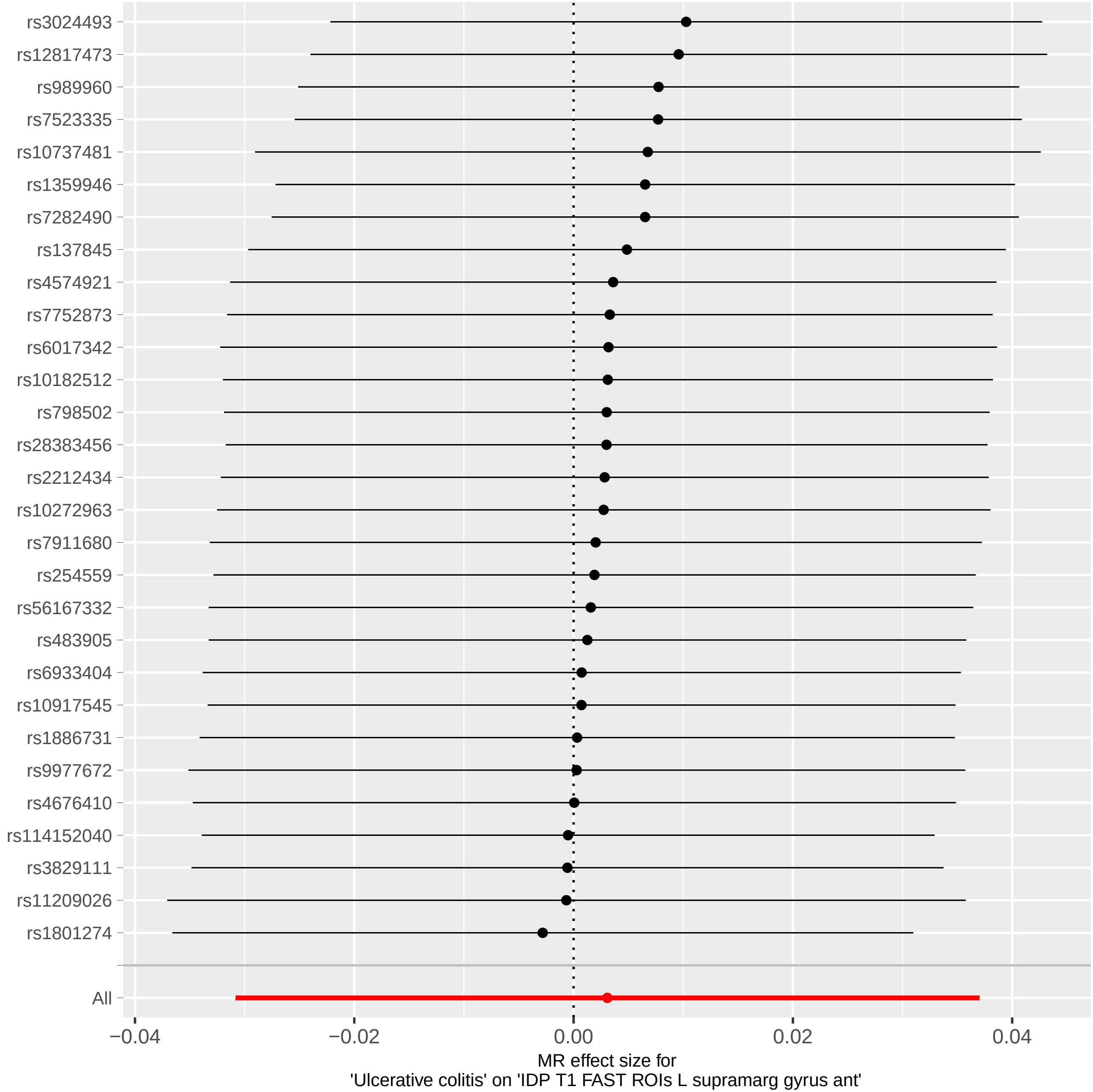

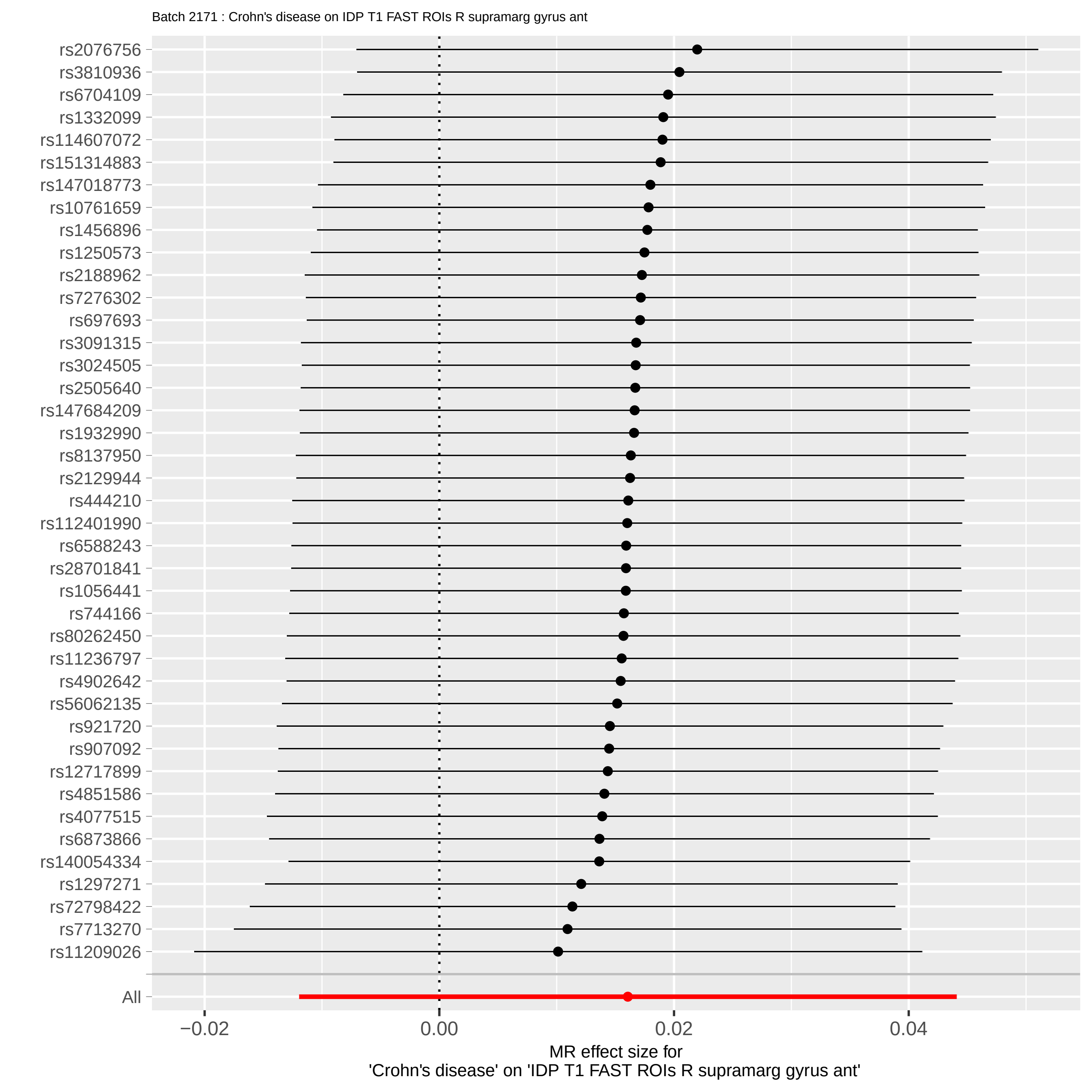

Batch 2171 : Crohn's disease on IDP T1 FAST ROIs R supramarg gyrus ant
rs2076756
rs3810936
rs6704109
rs1332099
rs114607072
rs151314883
rs147018773
rs10761659
rs1456896
rs1250573
rs2188962
rs7276302
rs697693
rs3091315
rs3024505
rs2505640
rs147684209
rs1932990
rs8137950
rs2129944
rs444210
rs112401990
rs6588243
rs28701841
rs1056441
rs744166
rs80262450
rs11236797
rs4902642
rs56062135
rs921720
rs907092
rs12717899
rs4851586
rs4077515
rs6873866
rs140054334
rs1297271
rs72798422
rs7713270
rs11209026
All
−0.02
0.00
0.02
0.04
MR effect size for
'Crohn's disease' on 'IDP T1 FAST ROIs R supramarg gyrus ant'

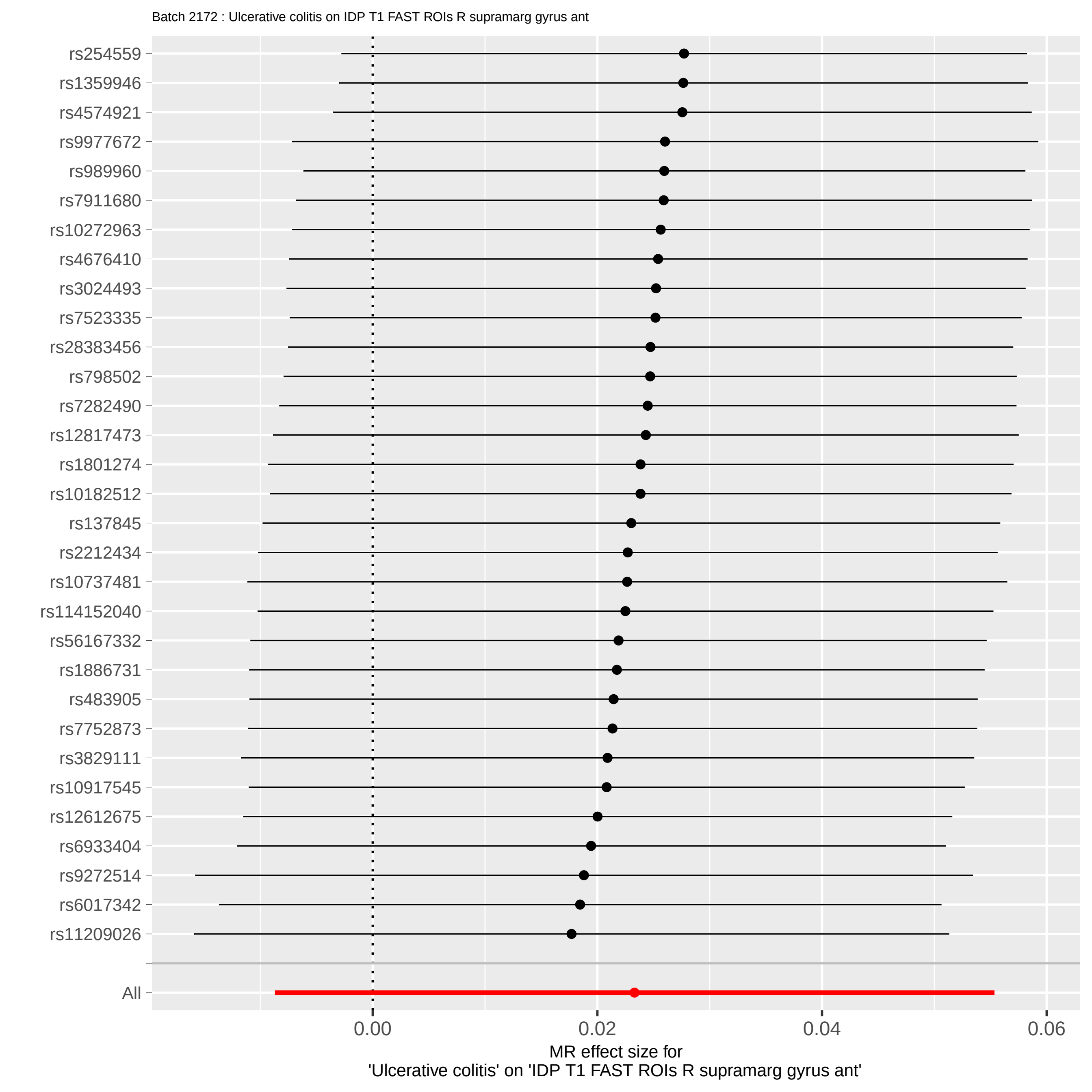

Batch 2172 : Ulcerative colitis on IDP T1 FAST ROIs R supramarg gyrus ant
rs254559
rs1359946
rs4574921
rs9977672
rs989960
rs7911680
rs10272963
rs4676410
rs3024493
rs7523335
rs28383456
rs798502
rs7282490
rs12817473
rs1801274
rs10182512
rs137845
rs2212434
rs10737481
rs114152040
rs56167332
rs1886731
rs483905
rs7752873
rs3829111
rs10917545
rs12612675
rs6933404
rs9272514
rs6017342
rs11209026
All
0.00
0.02
0.04
0.06
MR effect size for
'Ulcerative colitis' on 'IDP T1 FAST ROIs R supramarg gyrus ant'

Batch 2193 : Crohn's disease on IDP T1 FAST ROIs L supramarg gyrus post

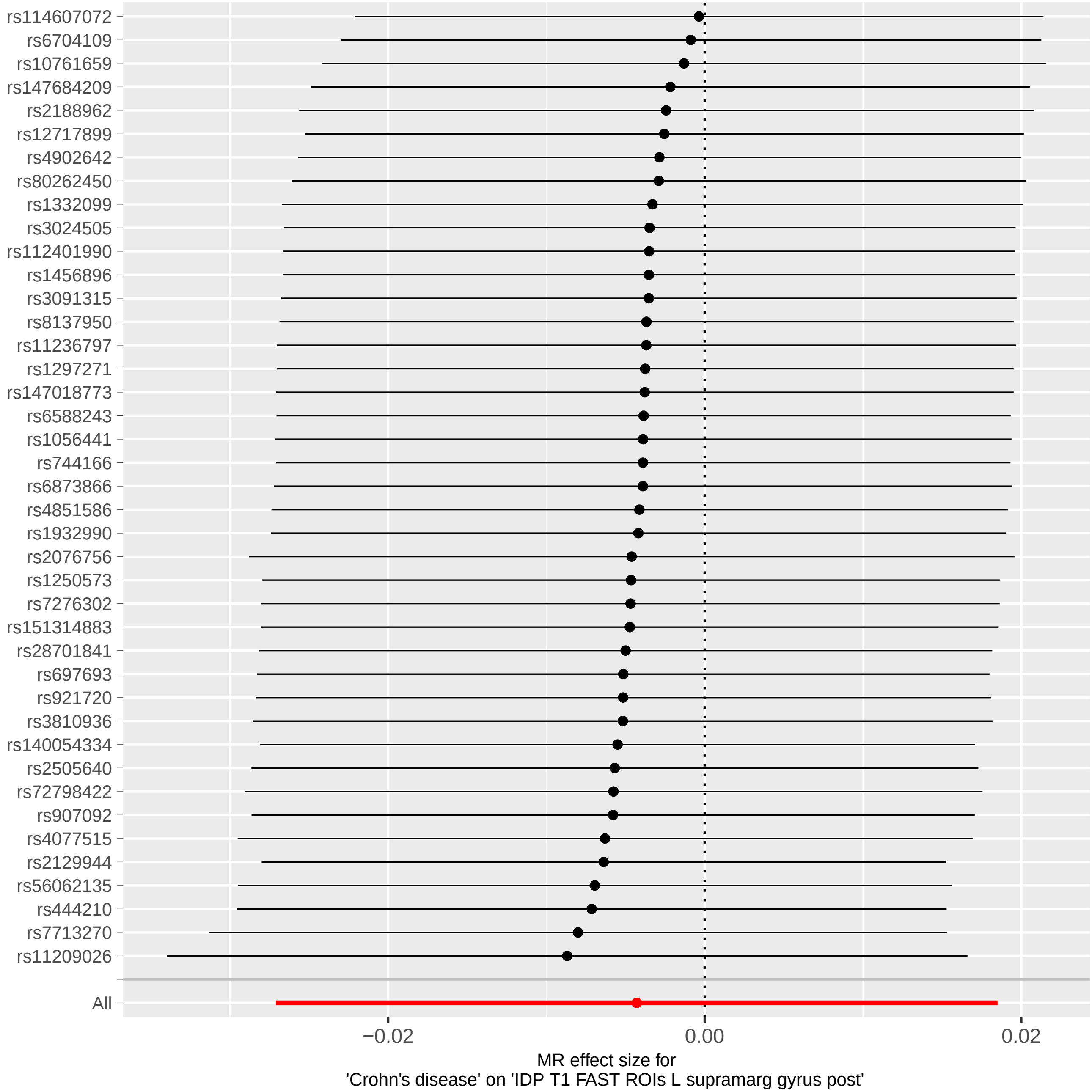

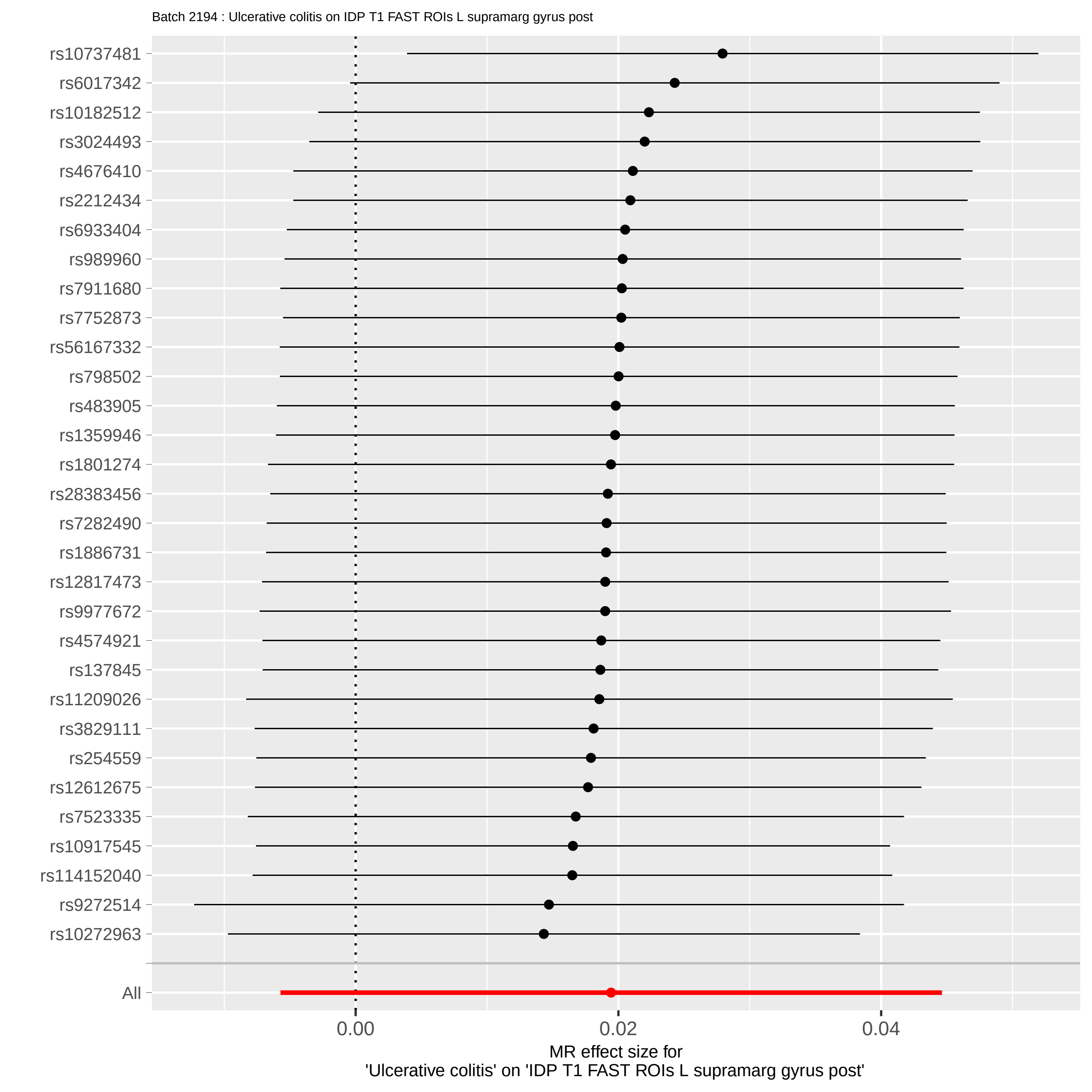

Batch 2194 : Ulcerative colitis on IDP T1 FAST ROIs L supramarg gyrus post
rs10737481
rs6017342
rs10182512
rs3024493
rs4676410
rs2212434
rs6933404
rs989960
rs7911680
rs7752873
rs56167332
rs798502
rs483905
rs1359946
rs1801274
rs28383456
rs7282490
rs1886731
rs12817473
rs9977672
rs4574921
rs137845
rs11209026
rs3829111
rs254559
rs12612675
rs7523335
rs10917545
rs114152040
rs9272514
rs10272963
All
0.00
0.02
0.04
MR effect size for
'Ulcerative colitis' on 'IDP T1 FAST ROIs L supramarg gyrus post'

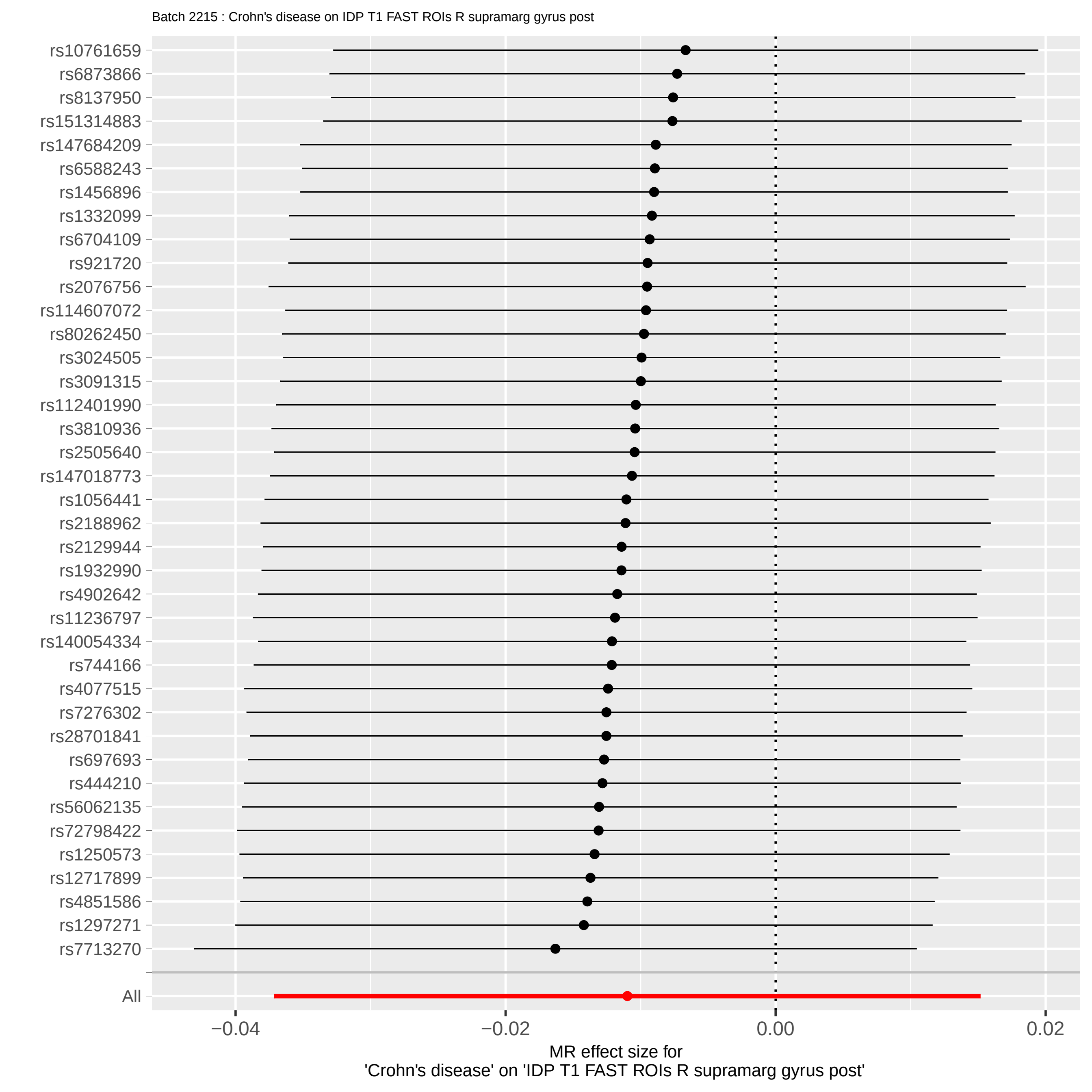
Batch 2215 : Crohn's disease on IDP T1 FAST ROIs R supramarg gyrus post
rs10761659
rs6873866
rs8137950
rs151314883
rs147684209
rs6588243
rs1456896
rs1332099
rs6704109
rs921720
rs2076756
rs114607072
rs80262450
rs3024505
rs3091315
rs112401990
rs3810936
rs2505640
rs147018773
rs1056441
rs2188962
rs2129944
rs1932990
rs4902642
rs11236797
rs140054334
rs744166
rs4077515
rs7276302
rs28701841
rs697693
rs444210
rs56062135
rs72798422
rs1250573
rs12717899
rs4851586
rs1297271
rs7713270
All
−0.04
−0.02
0.00
0.02
MR effect size for
'Crohn's disease' on 'IDP T1 FAST ROIs R supramarg gyrus post'

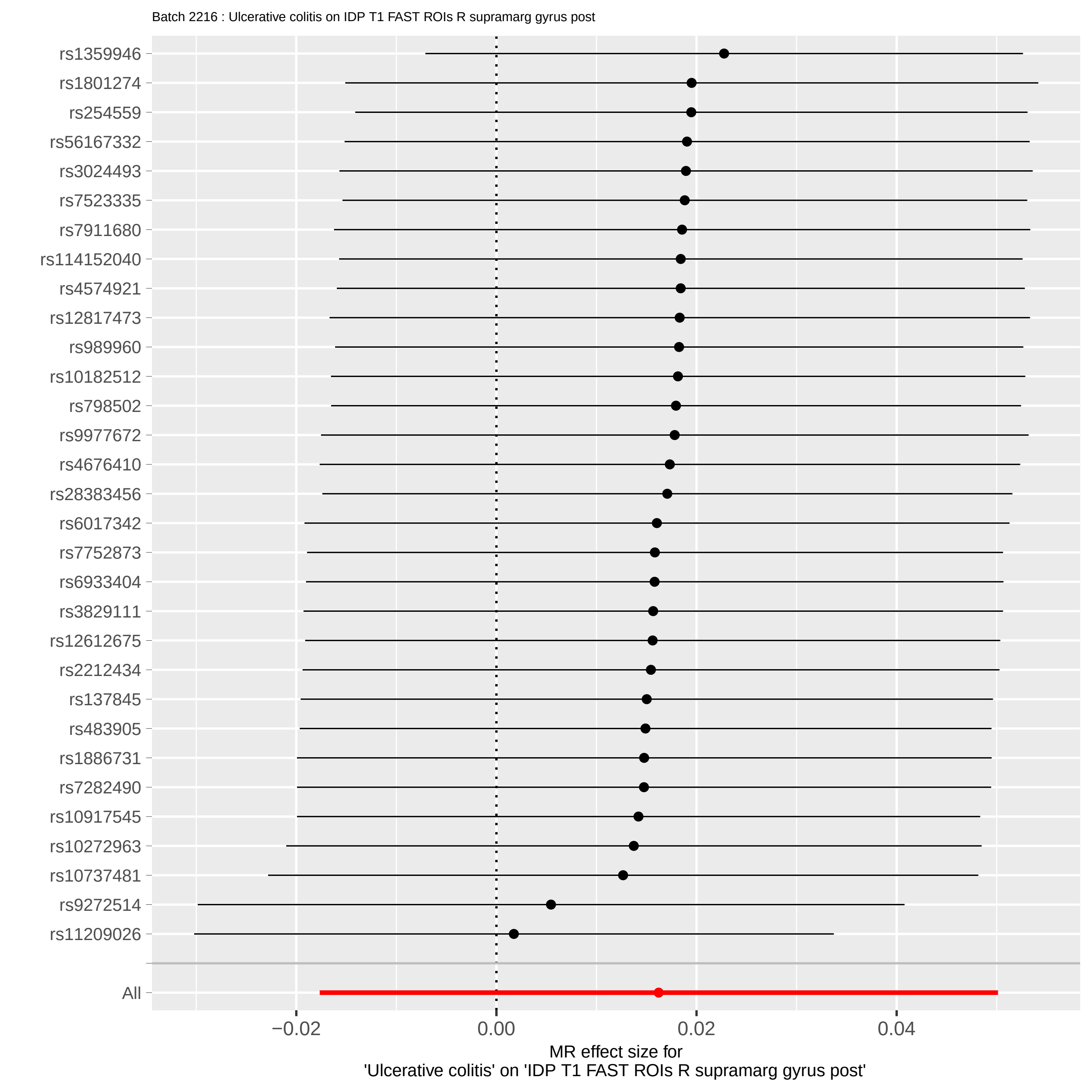

Batch 2216 : Ulcerative colitis on IDP T1 FAST ROIs R supramarg gyrus post
rs1359946
rs1801274
rs254559
rs56167332
rs3024493
rs7523335
rs7911680
rs114152040
rs4574921
rs12817473
rs989960
rs10182512
rs798502
rs9977672
rs4676410
rs28383456
rs6017342
rs7752873
rs6933404
rs3829111
rs12612675
rs2212434
rs137845
rs483905
rs1886731
rs7282490
rs10917545
rs10272963
rs10737481
rs9272514
rs11209026
All
−0.02
0.00
0.02
0.04
MR effect size for
'Ulcerative colitis' on 'IDP T1 FAST ROIs R supramarg gyrus post'

Batch 2237 : Crohn's disease on IDP T1 FAST ROIs L angular gyrus

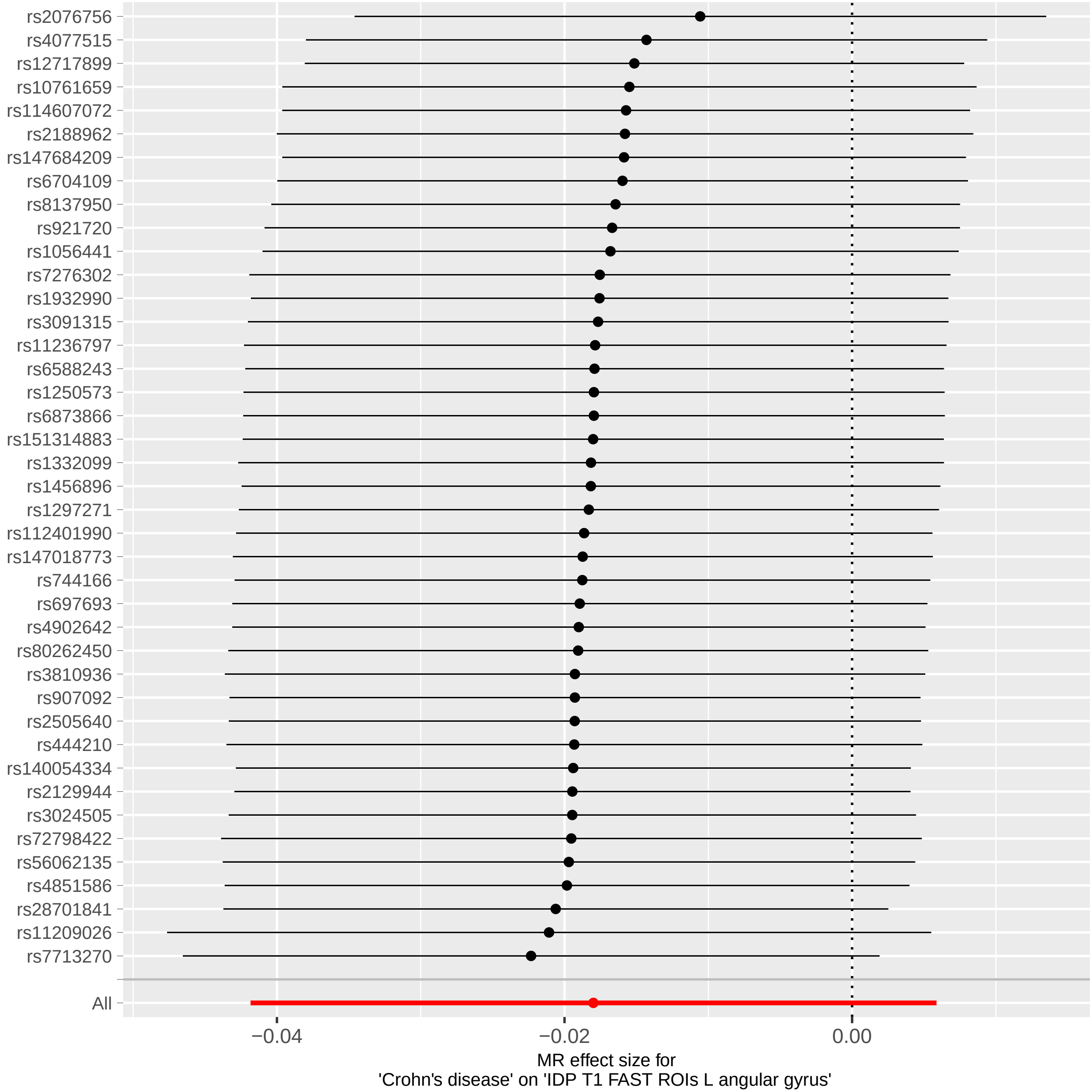

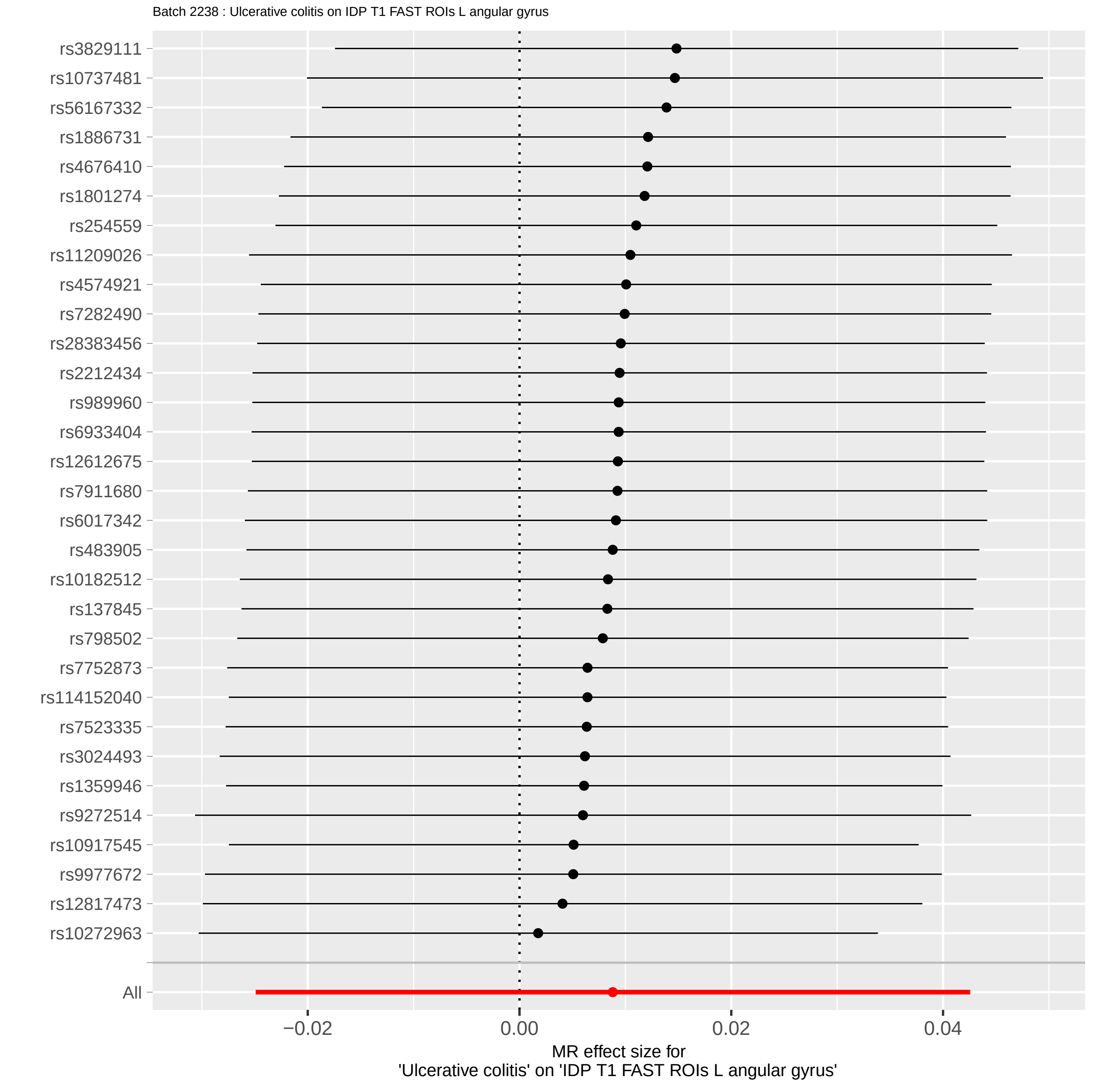

Batch 2238 : Ulcerative colitis on IDP T1 FAST ROIs L angular gyrus
rs3829111
rs10737481
rs56167332
rs1886731
rs4676410
rs1801274
rs254559
rs11209026
rs4574921
rs7282490
rs28383456
rs2212434
rs989960
rs6933404
rs12612675
rs7911680
rs6017342
rs483905
rs10182512
rs137845
rs798502
rs7752873
rs114152040
rs7523335
rs3024493
rs1359946
rs9272514
rs10917545
rs9977672
rs12817473
rs10272963
All
−0.02
0.00
0.02
0.04
MR effect size for
'Ulcerative colitis' on 'IDP T1 FAST ROIs L angular gyrus'

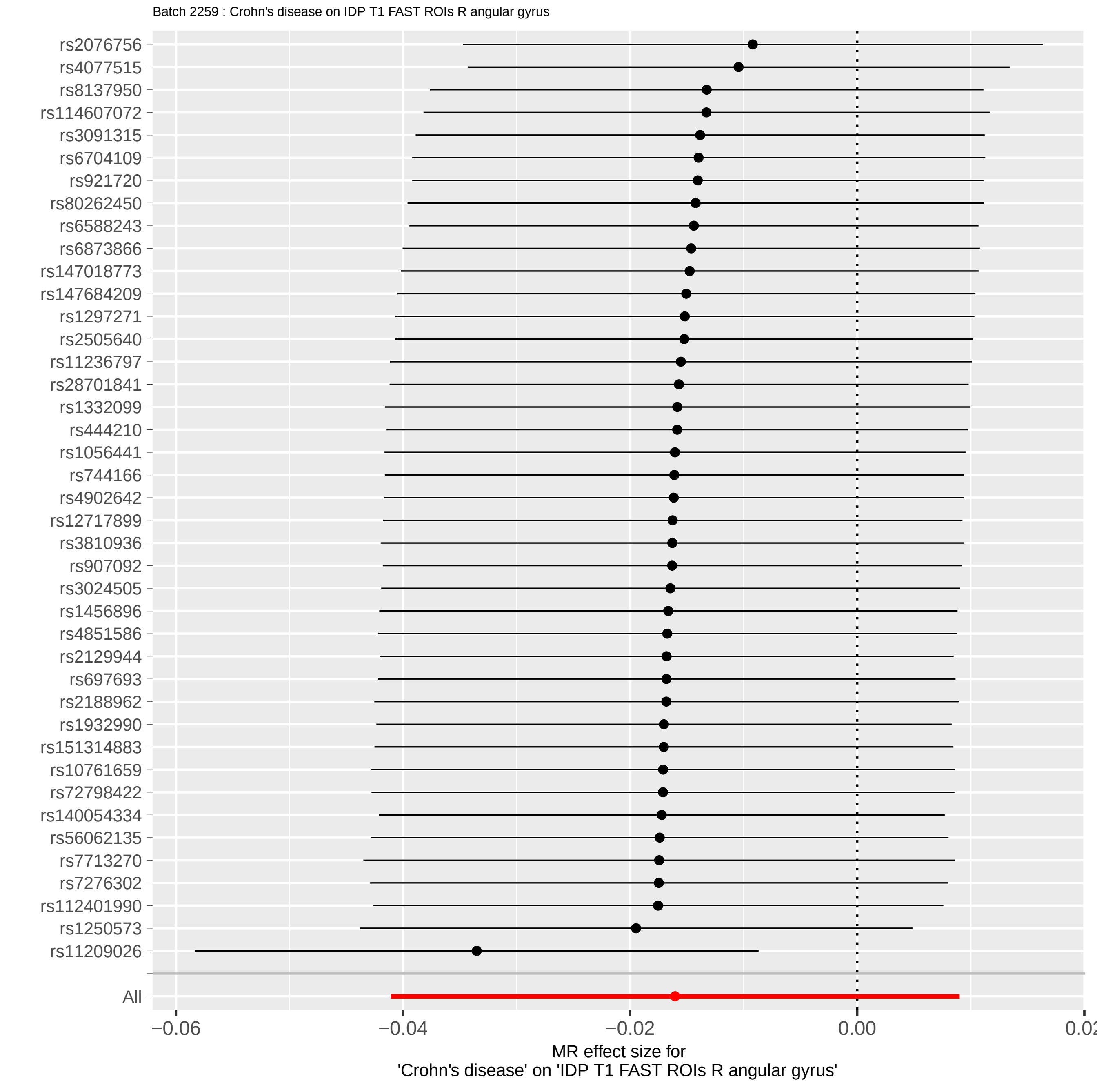

Batch 2259 : Crohn's disease on IDP T1 FAST ROIs R angular gyrus
rs2076756
rs4077515
rs8137950
rs114607072
rs3091315
rs6704109
rs921720
rs80262450
rs6588243
rs6873866
rs147018773
rs147684209
rs1297271
rs2505640
rs11236797
rs28701841
rs1332099
rs444210
rs1056441
rs744166
rs4902642
rs12717899
rs3810936
rs907092
rs3024505
rs1456896
rs4851586
rs2129944
rs697693
rs2188962
rs1932990
rs151314883
rs10761659
rs72798422
rs140054334
rs56062135
rs7713270
rs7276302
rs112401990
rs1250573
rs11209026
All
−0.06
−0.04
−0.02
0.00
0.02
MR effect size for
'Crohn's disease' on 'IDP T1 FAST ROIs R angular gyrus'

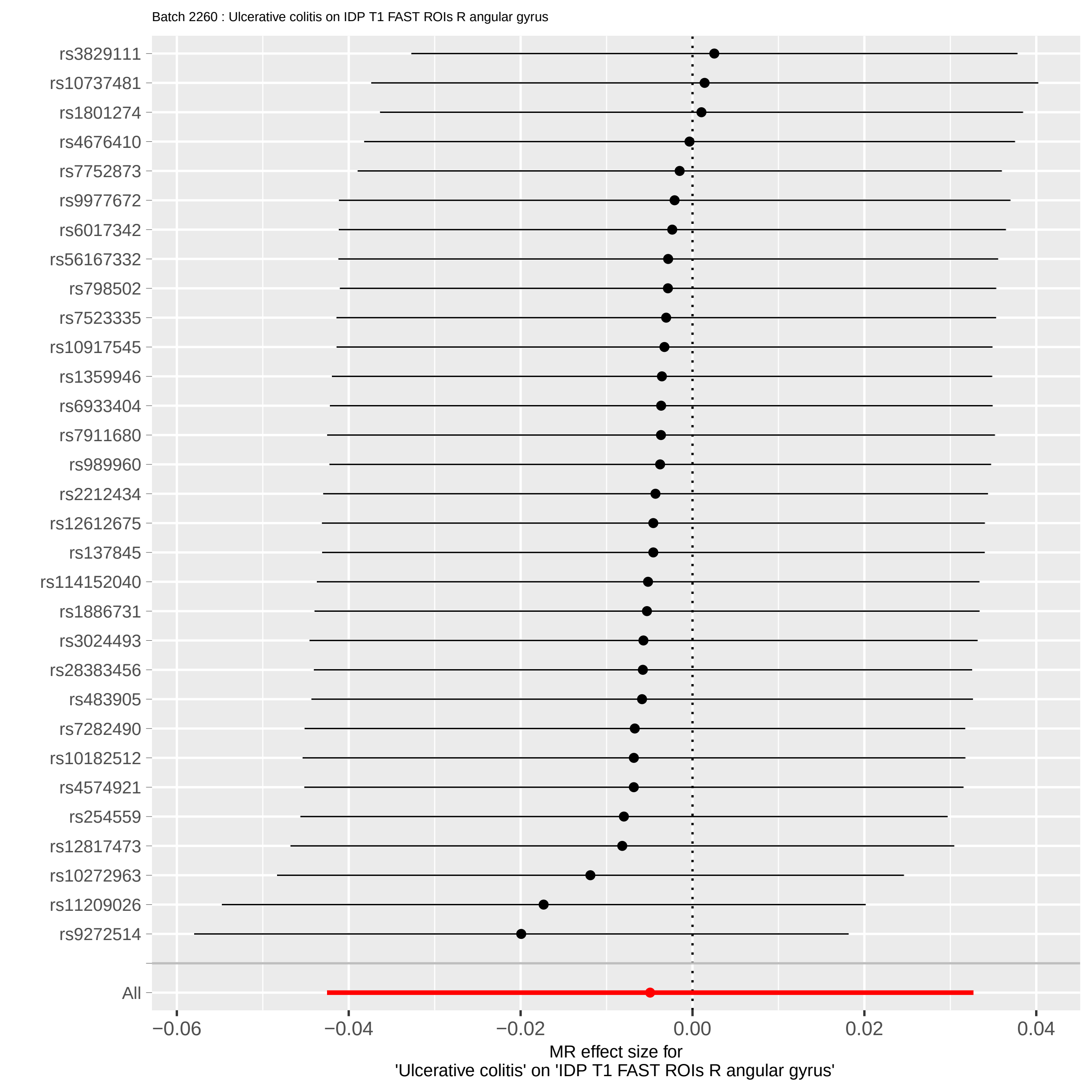

Batch 2260 : Ulcerative colitis on IDP T1 FAST ROIs R angular gyrus
rs3829111
rs10737481
rs1801274
rs4676410
rs7752873
rs9977672
rs6017342
rs56167332
rs798502
rs7523335
rs10917545
rs1359946
rs6933404
rs7911680
rs989960
rs2212434
rs12612675
rs137845
rs114152040
rs1886731
rs3024493
rs28383456
rs483905
rs7282490
rs10182512
rs4574921
rs254559
rs12817473
rs10272963
rs11209026
rs9272514
All
−0.06
−0.04
−0.02
0.00
0.02
0.04
MR effect size for
'Ulcerative colitis' on 'IDP T1 FAST ROIs R angular gyrus'

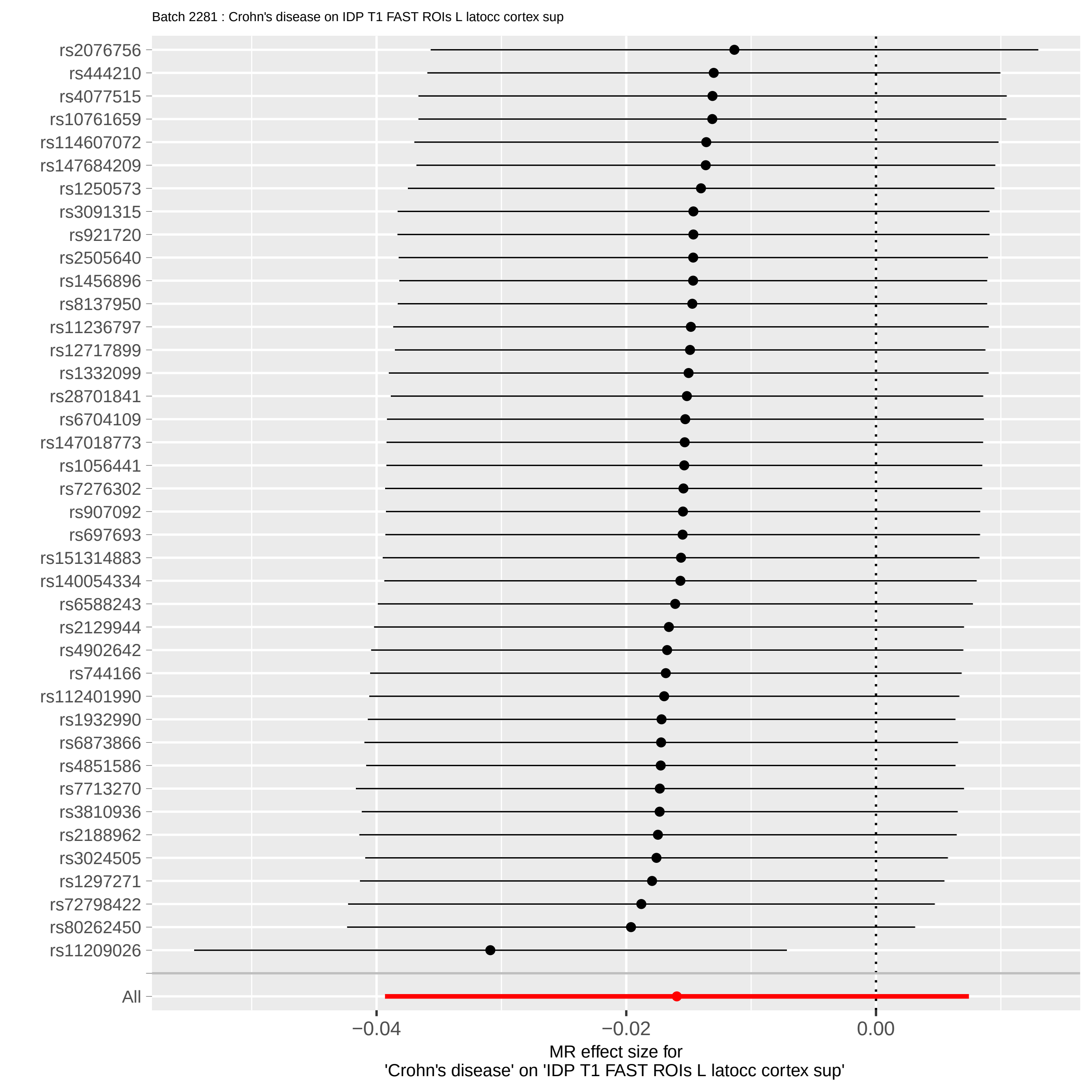

Batch 2281 : Crohn's disease on IDP T1 FAST ROIs L latocc cortex sup
rs2076756
rs444210
rs4077515
rs10761659
rs114607072
rs147684209
rs1250573
rs3091315
rs921720
rs2505640
rs1456896
rs8137950
rs11236797
rs12717899
rs1332099
rs28701841
rs6704109
rs147018773
rs1056441
rs7276302
rs907092
rs697693
rs151314883
rs140054334
rs6588243
rs2129944
rs4902642
rs744166
rs112401990
rs1932990
rs6873866
rs4851586
rs7713270
rs3810936
rs2188962
rs3024505
rs1297271
rs72798422
rs80262450
rs11209026
All
−0.04
−0.02
0.00
MR effect size for
'Crohn's disease' on 'IDP T1 FAST ROIs L latocc cortex sup'

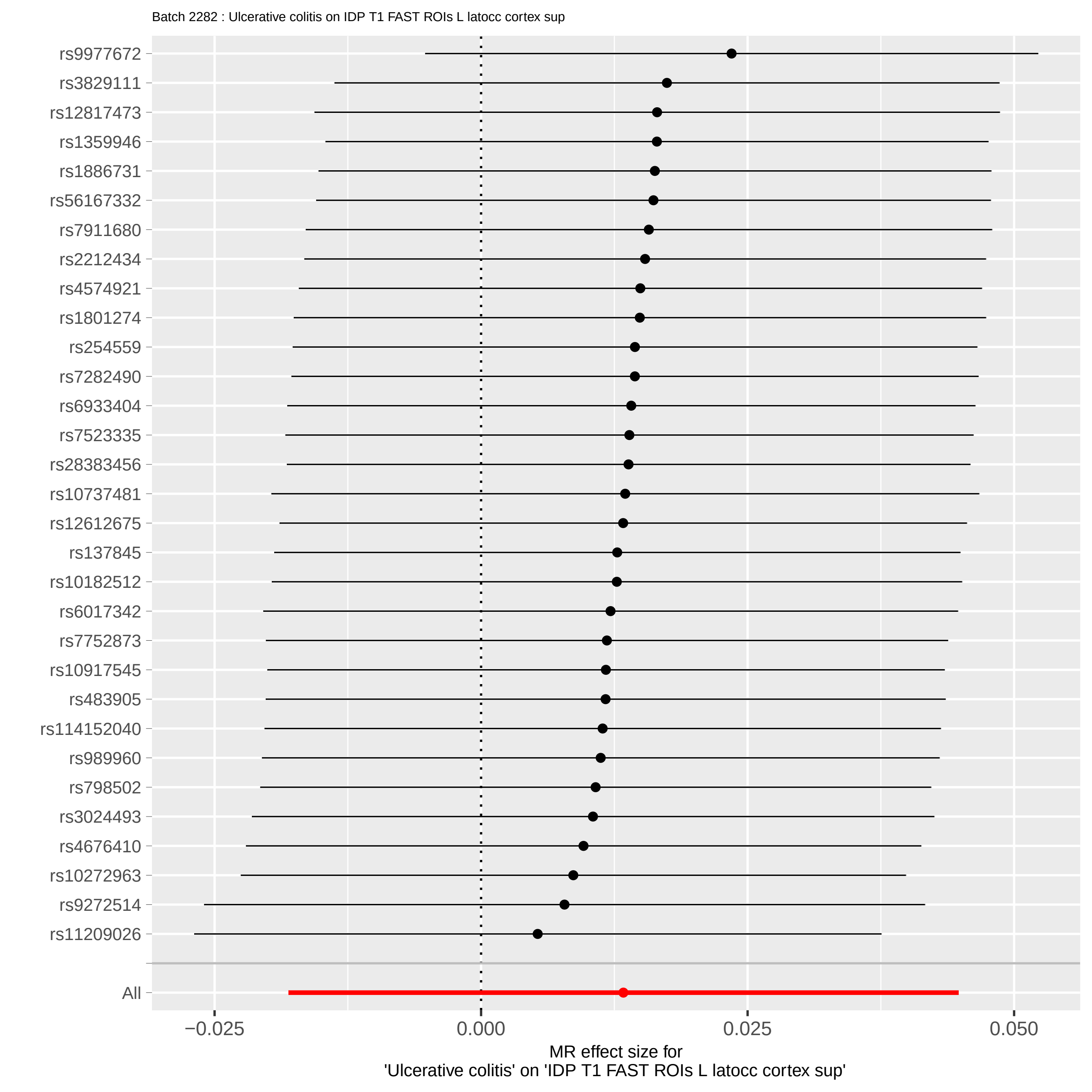

Batch 2282 : Ulcerative colitis on IDP T1 FAST ROIs L latocc cortex sup
rs9977672
rs3829111
rs12817473
rs1359946
rs1886731
rs56167332
rs7911680
rs2212434
rs4574921
rs1801274
rs254559
rs7282490
rs6933404
rs7523335
rs28383456
rs10737481
rs12612675
rs137845
rs10182512
rs6017342
rs7752873
rs10917545
rs483905
rs114152040
rs989960
rs798502
rs3024493
rs4676410
rs10272963
rs9272514
rs11209026
All
−0.025
0.000
0.025
0.050
MR effect size for
'Ulcerative colitis' on 'IDP T1 FAST ROIs L latocc cortex sup'

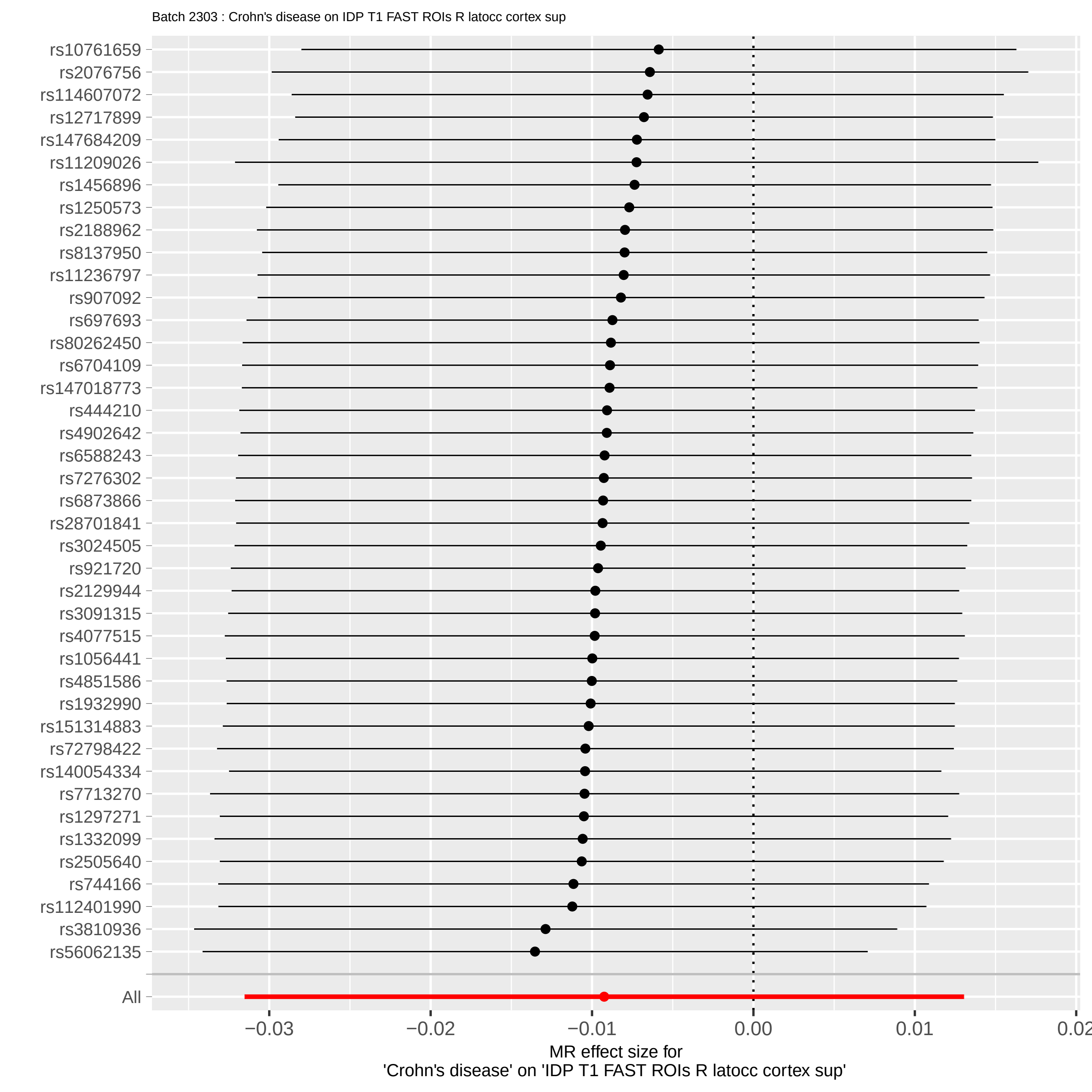

Batch 2303 : Crohn's disease on IDP T1 FAST ROIs R latocc cortex sup
rs10761659
rs2076756
rs114607072
rs12717899
rs147684209
rs11209026
rs1456896
rs1250573
rs2188962
rs8137950
rs11236797
rs907092
rs697693
rs80262450
rs6704109
rs147018773
rs444210
rs4902642
rs6588243
rs7276302
rs6873866
rs28701841
rs3024505
rs921720
rs2129944
rs3091315
rs4077515
rs1056441
rs4851586
rs1932990
rs151314883
rs72798422
rs140054334
rs7713270
rs1297271
rs1332099
rs2505640
rs744166
rs112401990
rs3810936
rs56062135
All
−0.03
−0.02
−0.01
0.00
0.01
0.02
MR effect size for
'Crohn's disease' on 'IDP T1 FAST ROIs R latocc cortex sup'

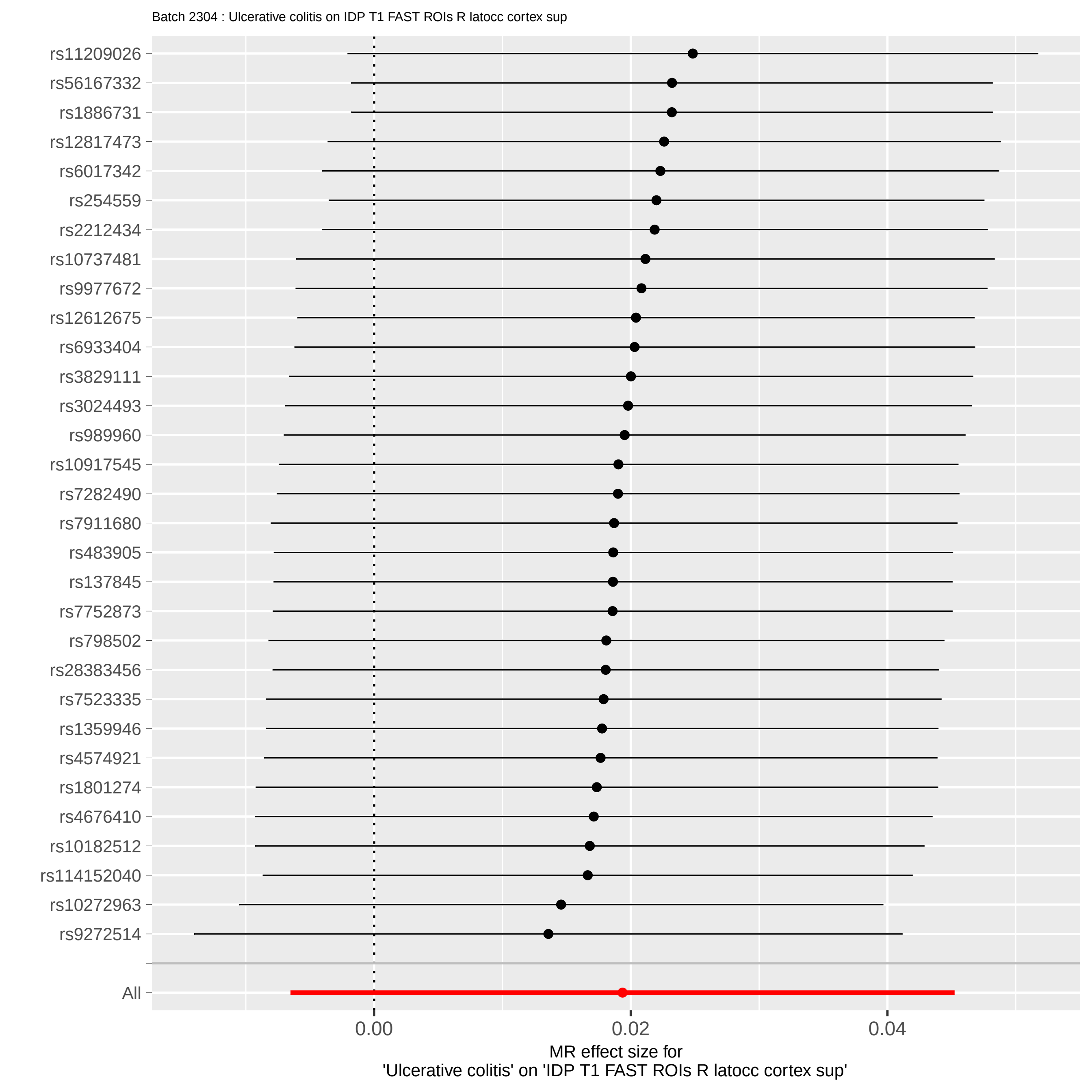

Batch 2304 : Ulcerative colitis on IDP T1 FAST ROIs R latocc cortex sup
rs11209026
rs56167332
rs1886731
rs12817473
rs6017342
rs254559
rs2212434
rs10737481
rs9977672
rs12612675
rs6933404
rs3829111
rs3024493
rs989960
rs10917545
rs7282490
rs7911680
rs483905
rs137845
rs7752873
rs798502
rs28383456
rs7523335
rs1359946
rs4574921
rs1801274
rs4676410
rs10182512
rs114152040
rs10272963
rs9272514
All
0.00
0.02
0.04
MR effect size for
'Ulcerative colitis' on 'IDP T1 FAST ROIs R latocc cortex sup'

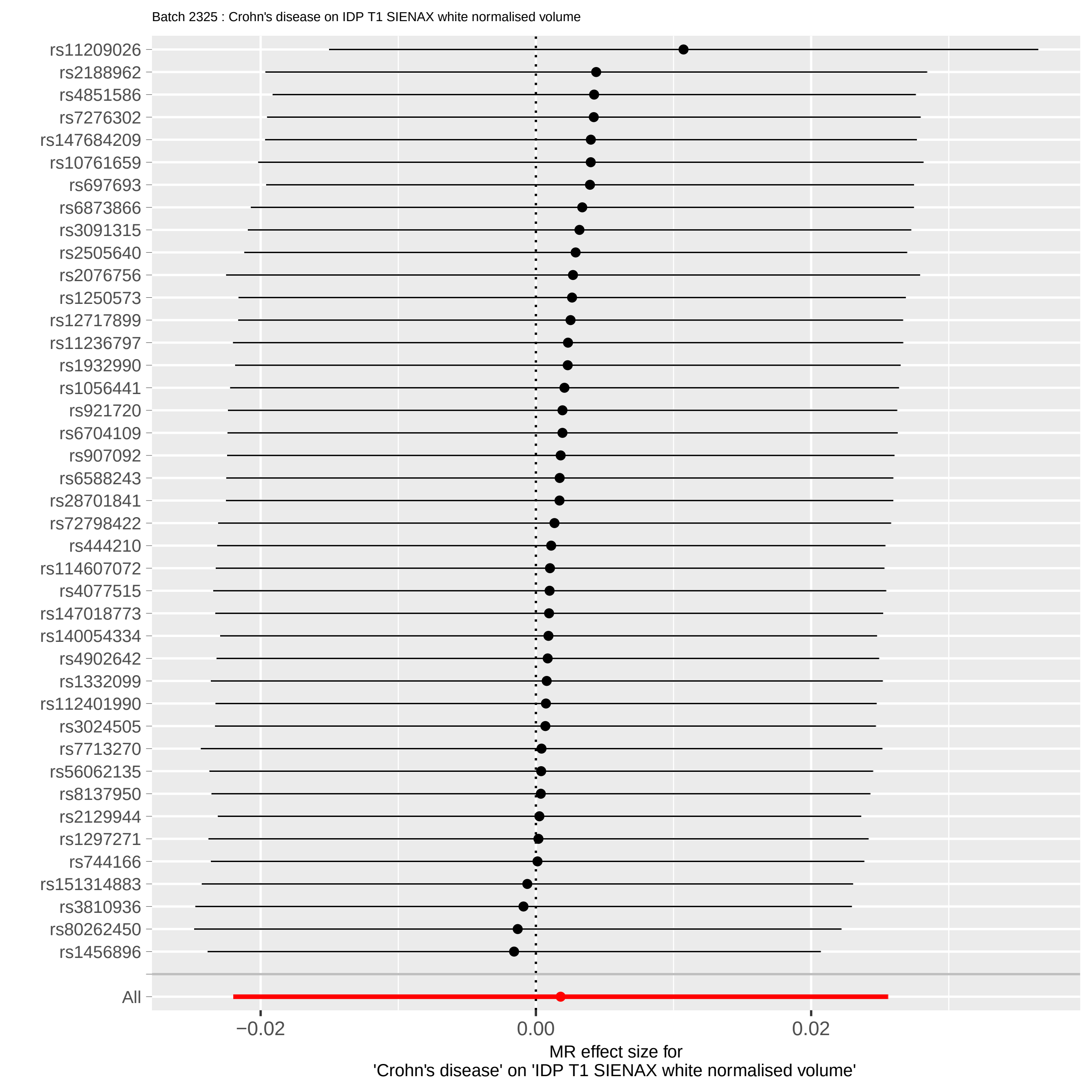

Batch 2325 : Crohn's disease on IDP T1 SIENAX white normalised volume
rs11209026
rs2188962
rs4851586
rs7276302
rs147684209
rs10761659
rs697693
rs6873866
rs3091315
rs2505640
rs2076756
rs1250573
rs12717899
rs11236797
rs1932990
rs1056441
rs921720
rs6704109
rs907092
rs6588243
rs28701841
rs72798422
rs444210
rs114607072
rs4077515
rs147018773
rs140054334
rs4902642
rs1332099
rs112401990
rs3024505
rs7713270
rs56062135
rs8137950
rs2129944
rs1297271
rs744166
rs151314883
rs3810936
rs80262450
rs1456896
All
−0.02
0.00
0.02
MR effect size for
'Crohn's disease' on 'IDP T1 SIENAX white normalised volume'

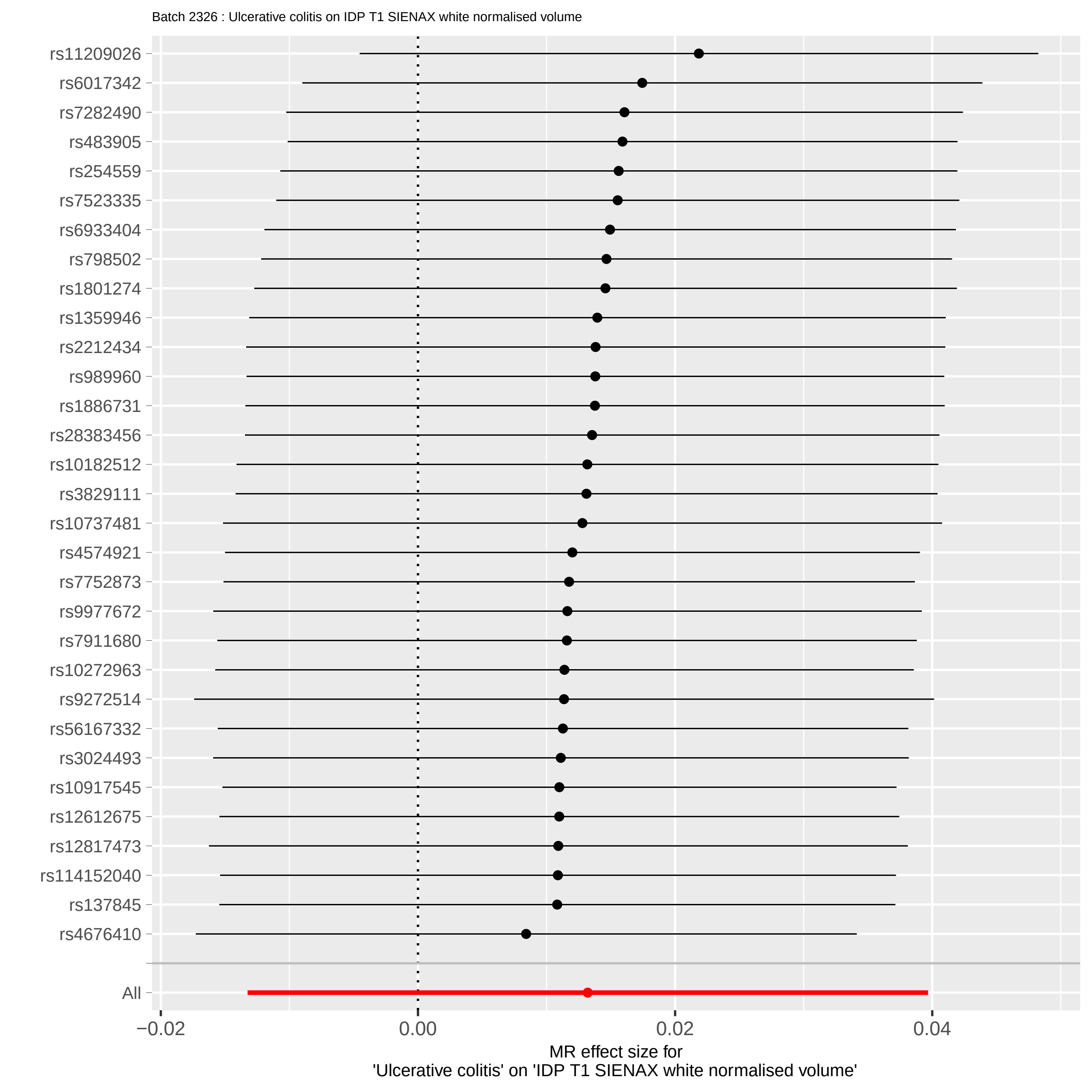

Batch 2326 : Ulcerative colitis on IDP T1 SIENAX white normalised volume
rs11209026
rs6017342
rs7282490
rs483905
rs254559
rs7523335
rs6933404
rs798502
rs1801274
rs1359946
rs2212434
rs989960
rs1886731
rs28383456
rs10182512
rs3829111
rs10737481
rs4574921
rs7752873
rs9977672
rs7911680
rs10272963
rs9272514
rs56167332
rs3024493
rs10917545
rs12612675
rs12817473
rs114152040
rs137845
rs4676410
All
−0.02
0.00
0.02
0.04
MR effect size for
'Ulcerative colitis' on 'IDP T1 SIENAX white normalised volume'

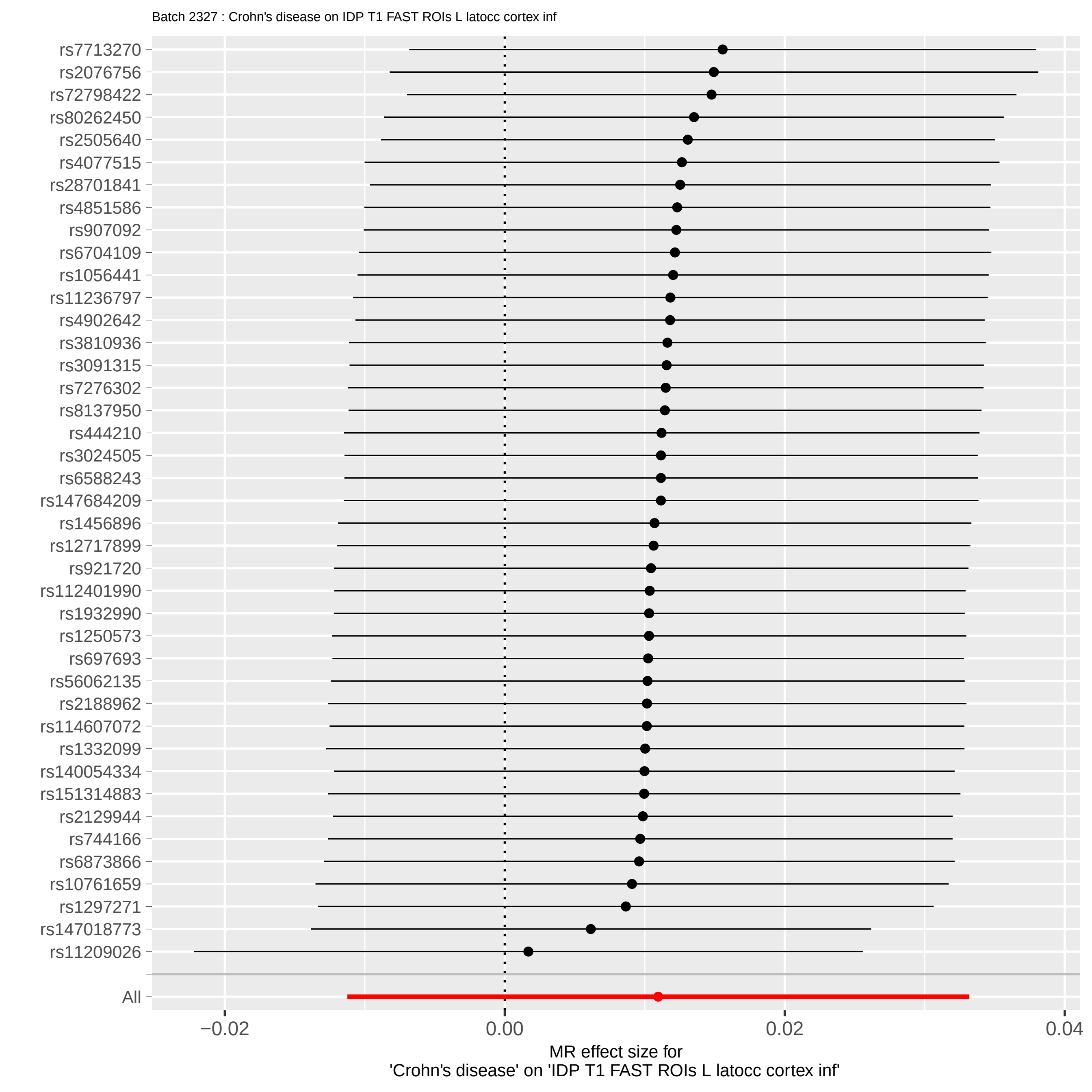

Batch 2327 : Crohn's disease on IDP T1 FAST ROIs L latocc cortex inf
rs7713270
rs2076756
rs72798422
rs80262450
rs2505640
rs4077515
rs28701841
rs4851586
rs907092
rs6704109
rs1056441
rs11236797
rs4902642
rs3810936
rs3091315
rs7276302
rs8137950
rs444210
rs3024505
rs6588243
rs147684209
rs1456896
rs12717899
rs921720
rs112401990
rs1932990
rs1250573
rs697693
rs56062135
rs2188962
rs114607072
rs1332099
rs140054334
rs151314883
rs2129944
rs744166
rs6873866
rs10761659
rs1297271
rs147018773
rs11209026
All
−0.02
0.00
0.02
0.04
MR effect size for
'Crohn's disease' on 'IDP T1 FAST ROIs L latocc cortex inf'

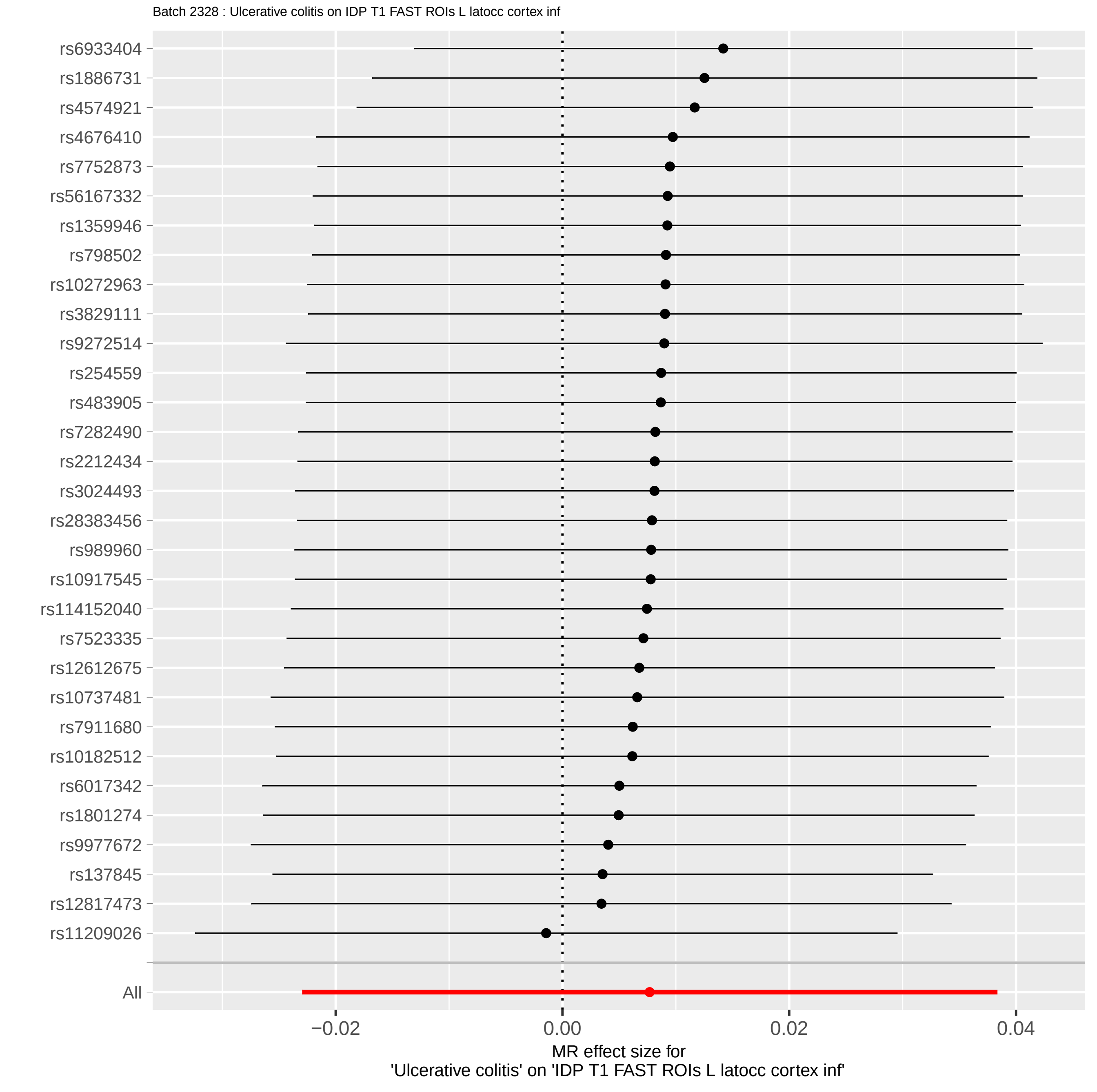

Batch 2328 : Ulcerative colitis on IDP T1 FAST ROIs L latocc cortex inf
rs6933404
rs1886731
rs4574921
rs4676410
rs7752873
rs56167332
rs1359946
rs798502
rs10272963
rs3829111
rs9272514
rs254559
rs483905
rs7282490
rs2212434
rs3024493
rs28383456
rs989960
rs10917545
rs114152040
rs7523335
rs12612675
rs10737481
rs7911680
rs10182512
rs6017342
rs1801274
rs9977672
rs137845
rs12817473
rs11209026
All
−0.02
0.00
0.02
0.04
MR effect size for
'Ulcerative colitis' on 'IDP T1 FAST ROIs L latocc cortex inf'

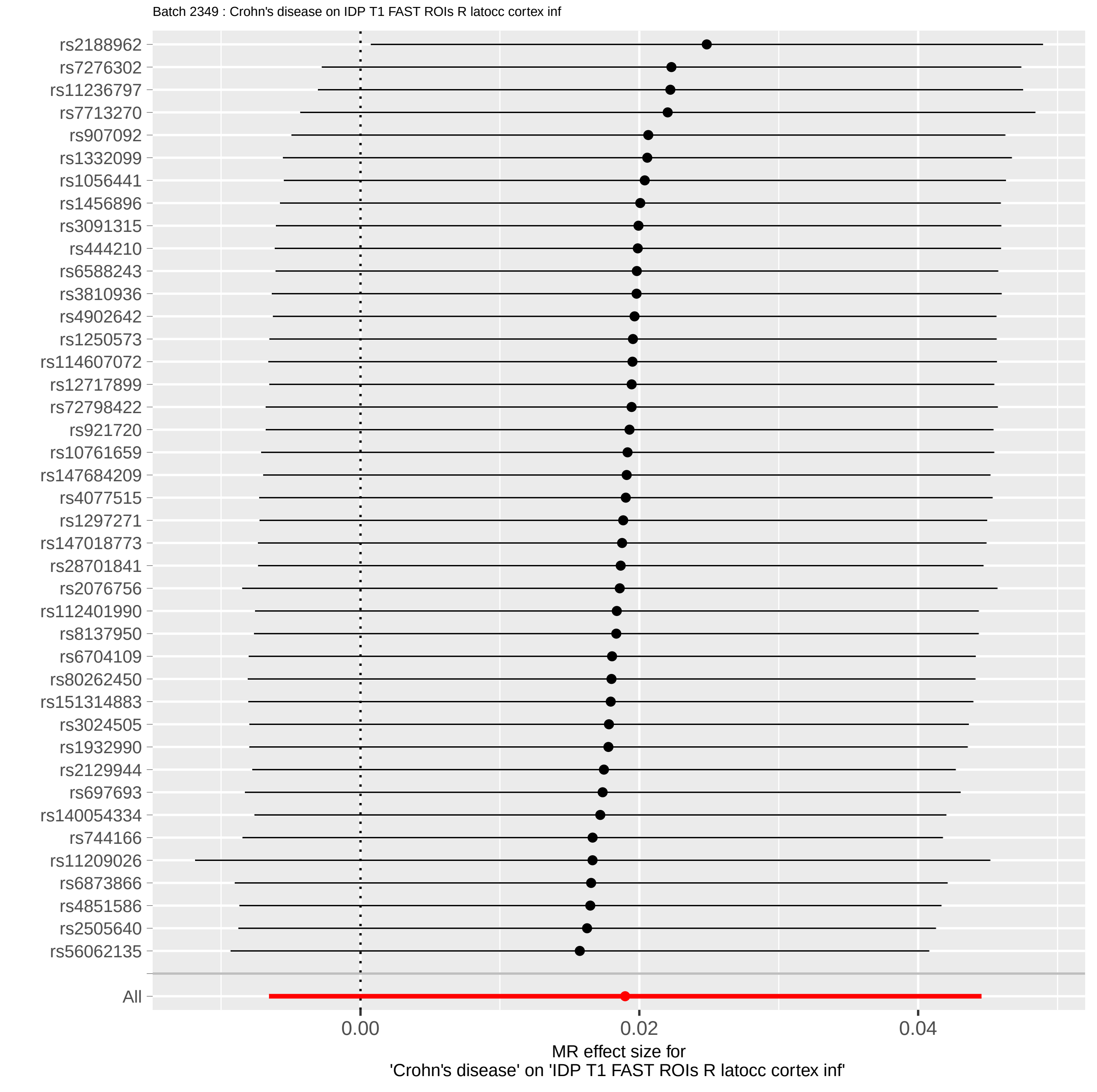

Batch 2349 : Crohn's disease on IDP T1 FAST ROIs R latocc cortex inf
rs2188962
rs7276302
rs11236797
rs7713270
rs907092
rs1332099
rs1056441
rs1456896
rs3091315
rs444210
rs6588243
rs3810936
rs4902642
rs1250573
rs114607072
rs12717899
rs72798422
rs921720
rs10761659
rs147684209
rs4077515
rs1297271
rs147018773
rs28701841
rs2076756
rs112401990
rs8137950
rs6704109
rs80262450
rs151314883
rs3024505
rs1932990
rs2129944
rs697693
rs140054334
rs744166
rs11209026
rs6873866
rs4851586
rs2505640
rs56062135
All
0.00
0.02
0.04
MR effect size for
'Crohn's disease' on 'IDP T1 FAST ROIs R latocc cortex inf'

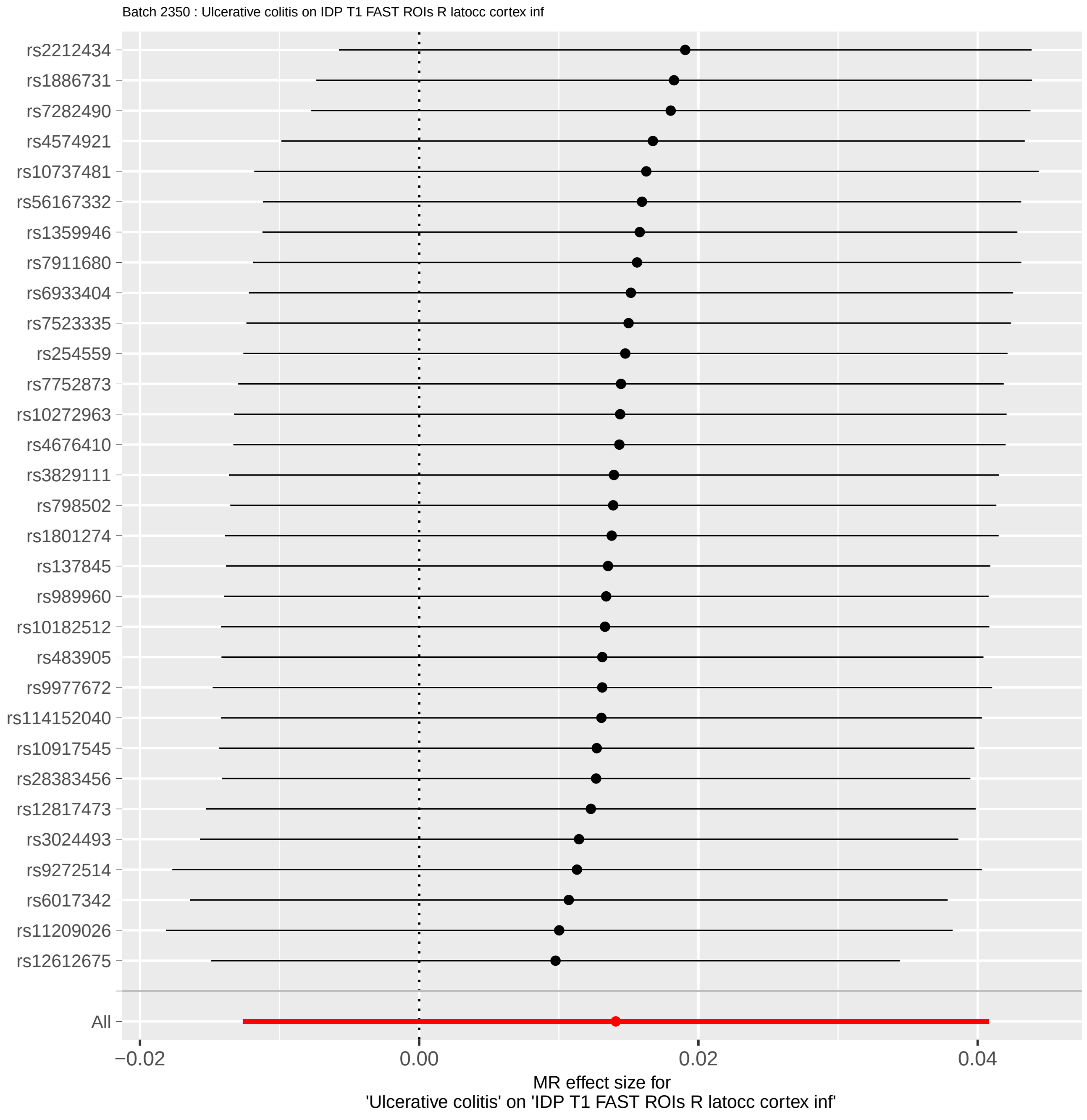

Batch 2350 : Ulcerative colitis on IDP T1 FAST ROIs R latocc cortex inf
rs2212434
rs1886731
rs7282490
rs4574921
rs10737481
rs56167332
rs1359946
rs7911680
rs6933404
rs7523335
rs254559
rs7752873
rs10272963
rs4676410
rs3829111
rs798502
rs1801274
rs137845
rs989960
rs10182512
rs483905
rs9977672
rs114152040
rs10917545
rs28383456
rs12817473
rs3024493
rs9272514
rs6017342
rs11209026
rs12612675
All
−0.02
0.00
0.02
0.04
MR effect size for
'Ulcerative colitis' on 'IDP T1 FAST ROIs R latocc cortex inf'

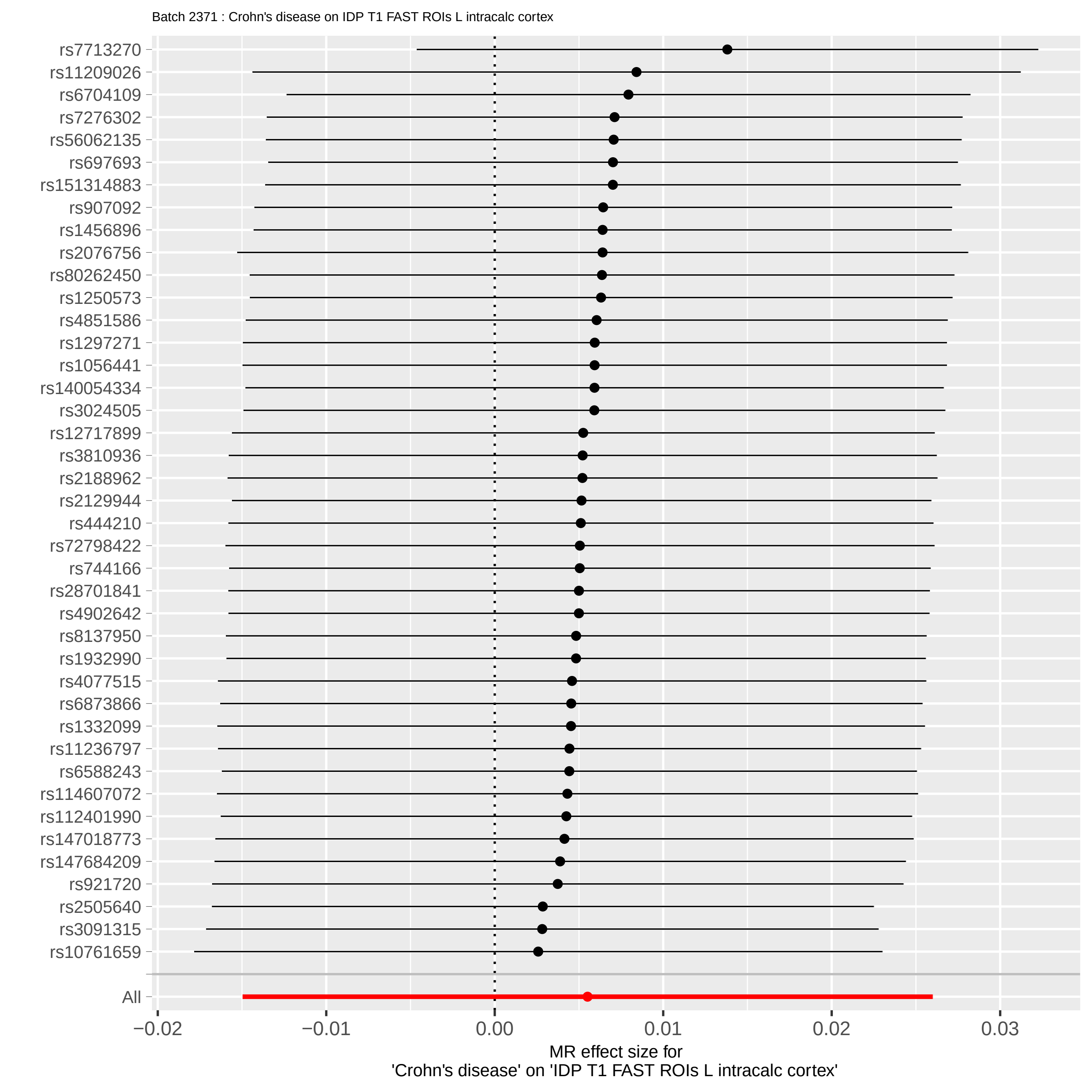
Batch 2371 : Crohn's disease on IDP T1 FAST ROIs L intracalc cortex
rs7713270
rs11209026
rs6704109
rs7276302
rs56062135
rs697693
rs151314883
rs907092
rs1456896
rs2076756
rs80262450
rs1250573
rs4851586
rs1297271
rs1056441
rs140054334
rs3024505
rs12717899
rs3810936
rs2188962
rs2129944
rs444210
rs72798422
rs744166
rs28701841
rs4902642
rs8137950
rs1932990
rs4077515
rs6873866
rs1332099
rs11236797
rs6588243
rs114607072
rs112401990
rs147018773
rs147684209
rs921720
rs2505640
rs3091315
rs10761659
All
−0.02
−0.01
0.00
0.01
0.02
0.03
MR effect size for
'Crohn's disease' on 'IDP T1 FAST ROIs L intracalc cortex'

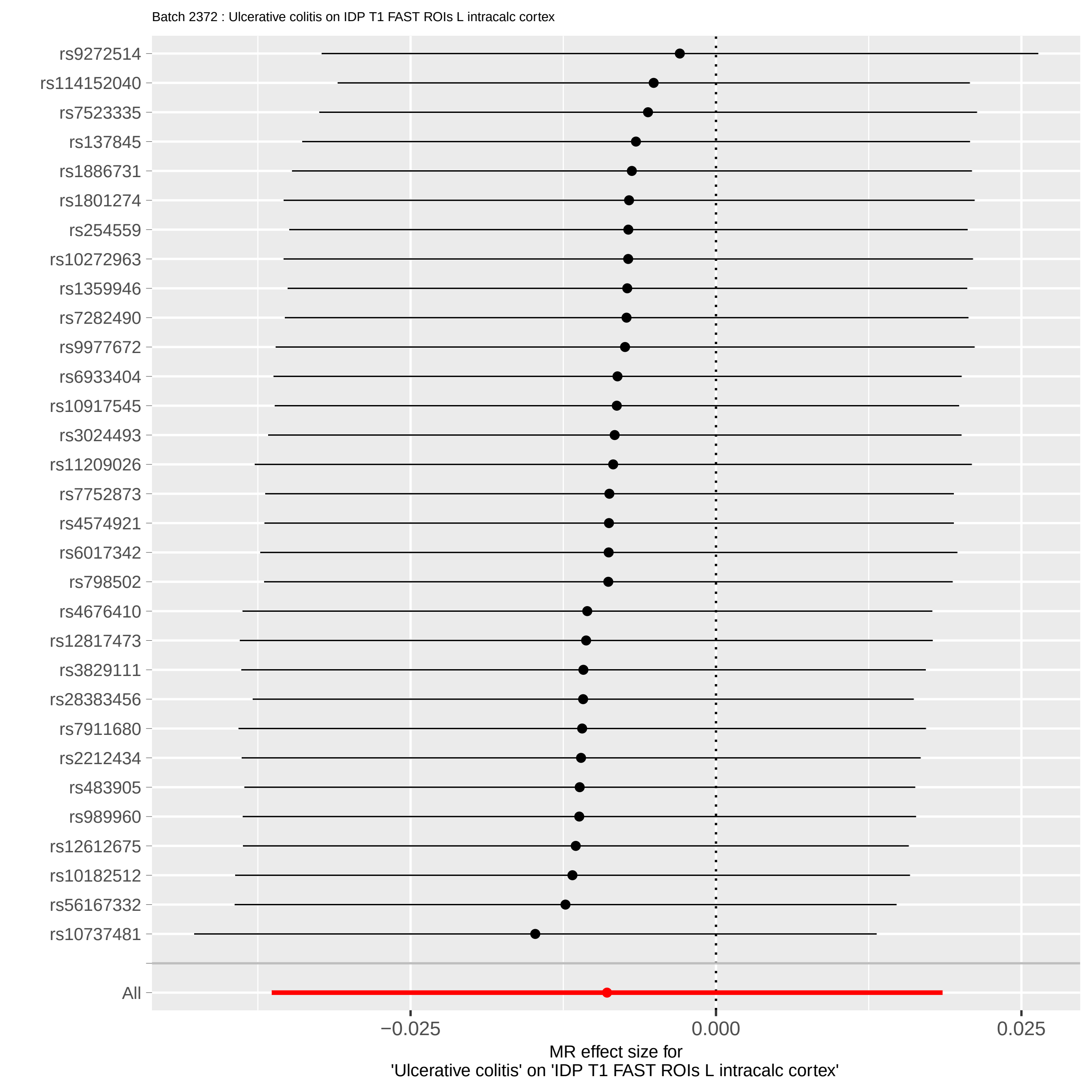
Batch 2372 : Ulcerative colitis on IDP T1 FAST ROIs L intracalc cortex
rs9272514
rs114152040
rs7523335
rs137845
rs1886731
rs1801274
rs254559
rs10272963
rs1359946
rs7282490
rs9977672
rs6933404
rs10917545
rs3024493
rs11209026
rs7752873
rs4574921
rs6017342
rs798502
rs4676410
rs12817473
rs3829111
rs28383456
rs7911680
rs2212434
rs483905
rs989960
rs12612675
rs10182512
rs56167332
rs10737481
All
−0.025
0.000
0.025
MR effect size for
'Ulcerative colitis' on 'IDP T1 FAST ROIs L intracalc cortex'

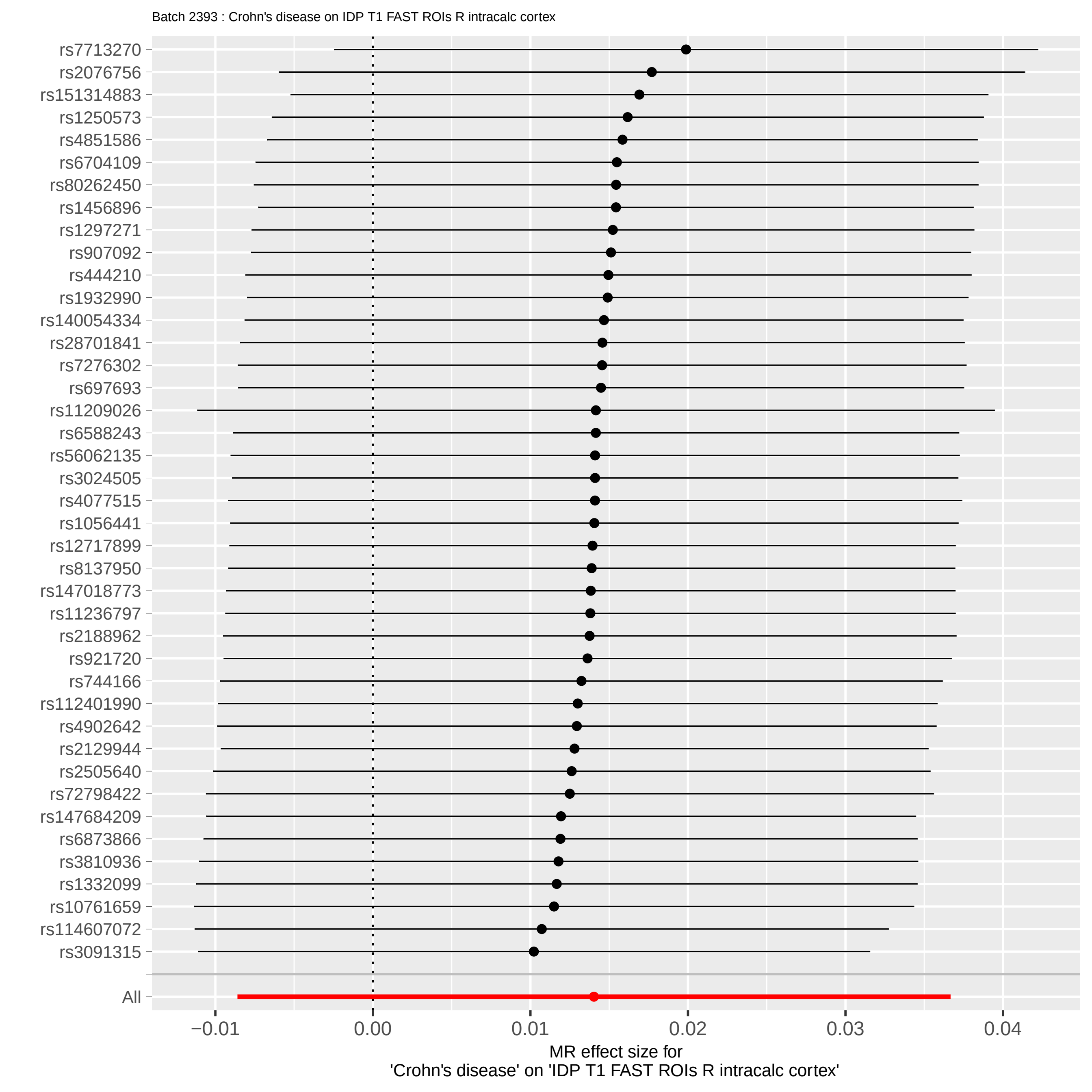
Batch 2393 : Crohn's disease on IDP T1 FAST ROIs R intracalc cortex
rs7713270
rs2076756
rs151314883
rs1250573
rs4851586
rs6704109
rs80262450
rs1456896
rs1297271
rs907092
rs444210
rs1932990
rs140054334
rs28701841
rs7276302
rs697693
rs11209026
rs6588243
rs56062135
rs3024505
rs4077515
rs1056441
rs12717899
rs8137950
rs147018773
rs11236797
rs2188962
rs921720
rs744166
rs112401990
rs4902642
rs2129944
rs2505640
rs72798422
rs147684209
rs6873866
rs3810936
rs1332099
rs10761659
rs114607072
rs3091315
All
−0.01
0.00
0.01
0.02
0.03
0.04
MR effect size for
'Crohn's disease' on 'IDP T1 FAST ROIs R intracalc cortex'

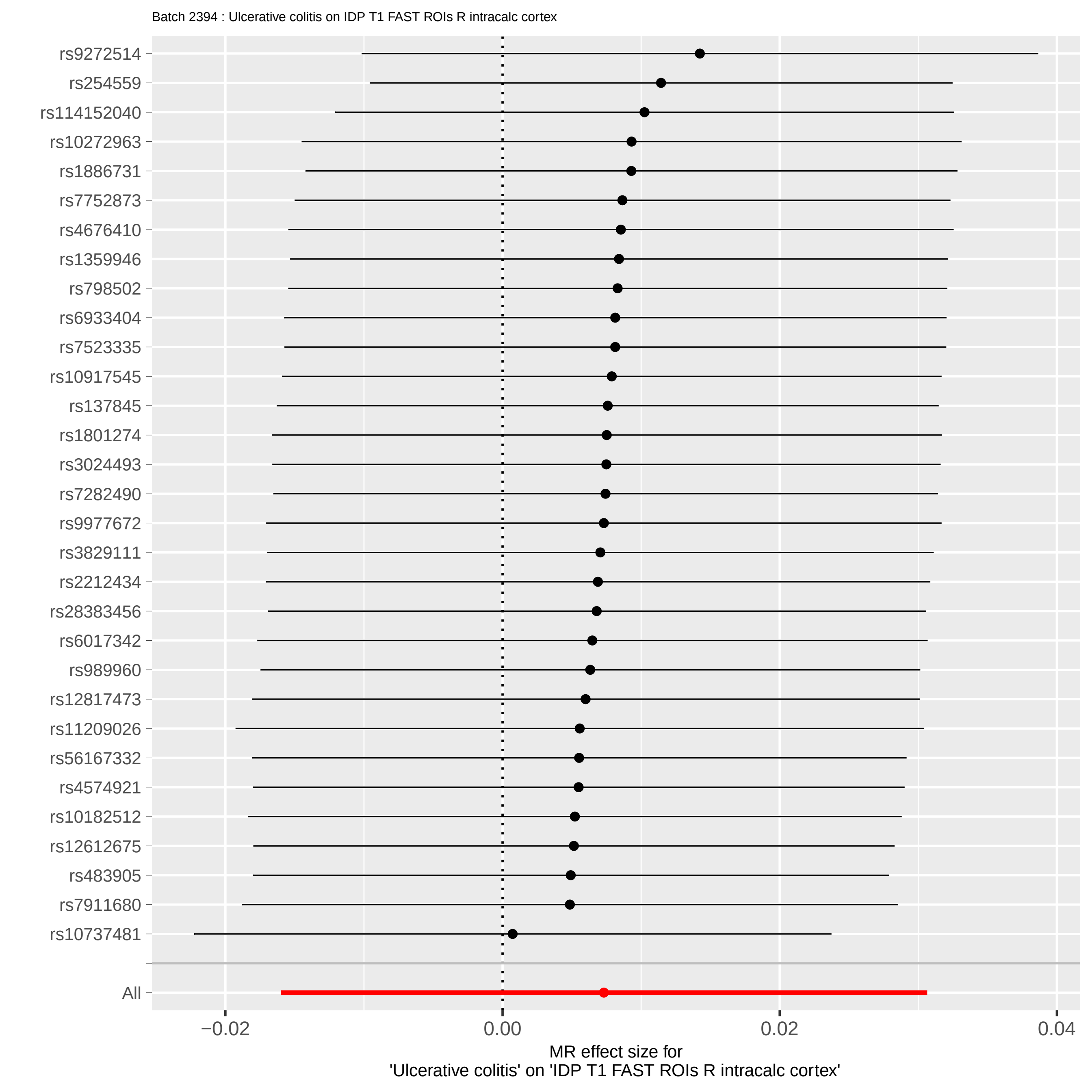

Batch 2394 : Ulcerative colitis on IDP T1 FAST ROIs R intracalc cortex
rs9272514
rs254559
rs114152040
rs10272963
rs1886731
rs7752873
rs4676410
rs1359946
rs798502
rs6933404
rs7523335
rs10917545
rs137845
rs1801274
rs3024493
rs7282490
rs9977672
rs3829111
rs2212434
rs28383456
rs6017342
rs989960
rs12817473
rs11209026
rs56167332
rs4574921
rs10182512
rs12612675
rs483905
rs7911680
rs10737481
All
−0.02
0.00
0.02
0.04
MR effect size for
'Ulcerative colitis' on 'IDP T1 FAST ROIs R intracalc cortex'

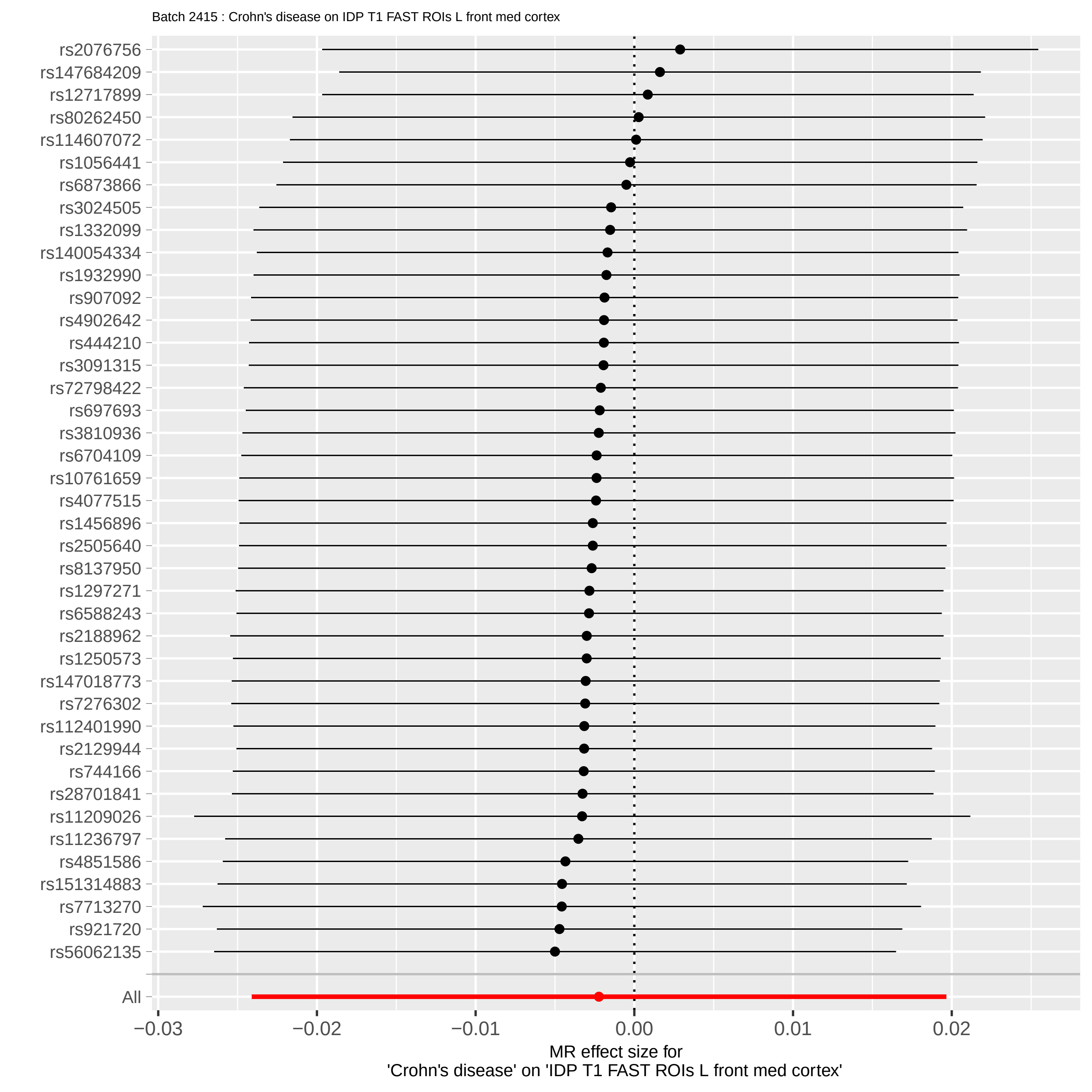
Batch 2415 : Crohn's disease on IDP T1 FAST ROIs L front med cortex
rs2076756
rs147684209
rs12717899
rs80262450
rs114607072
rs1056441
rs6873866
rs3024505
rs1332099
rs140054334
rs1932990
rs907092
rs4902642
rs444210
rs3091315
rs72798422
rs697693
rs3810936
rs6704109
rs10761659
rs4077515
rs1456896
rs2505640
rs8137950
rs1297271
rs6588243
rs2188962
rs1250573
rs147018773
rs7276302
rs112401990
rs2129944
rs744166
rs28701841
rs11209026
rs11236797
rs4851586
rs151314883
rs7713270
rs921720
rs56062135
All
−0.03
−0.02
−0.01
0.00
0.01
0.02
MR effect size for
'Crohn's disease' on 'IDP T1 FAST ROIs L front med cortex'

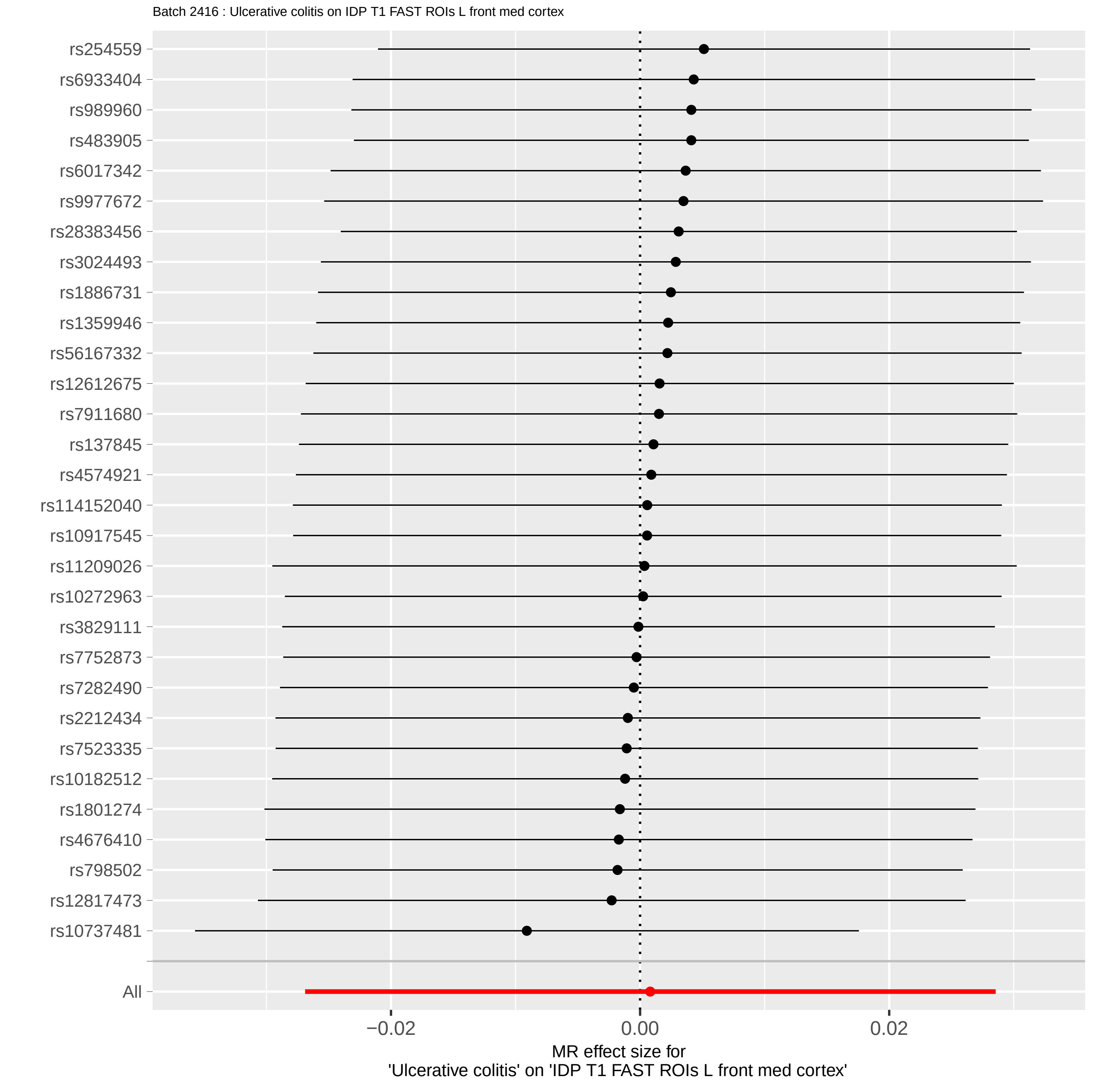

Batch 2416 : Ulcerative colitis on IDP T1 FAST ROIs L front med cortex
rs254559
rs6933404
rs989960
rs483905
rs6017342
rs9977672
rs28383456
rs3024493
rs1886731
rs1359946
rs56167332
rs12612675
rs7911680
rs137845
rs4574921
rs114152040
rs10917545
rs11209026
rs10272963
rs3829111
rs7752873
rs7282490
rs2212434
rs7523335
rs10182512
rs1801274
rs4676410
rs798502
rs12817473
rs10737481
All
−0.02
0.00
0.02
MR effect size for
'Ulcerative colitis' on 'IDP T1 FAST ROIs L front med cortex'

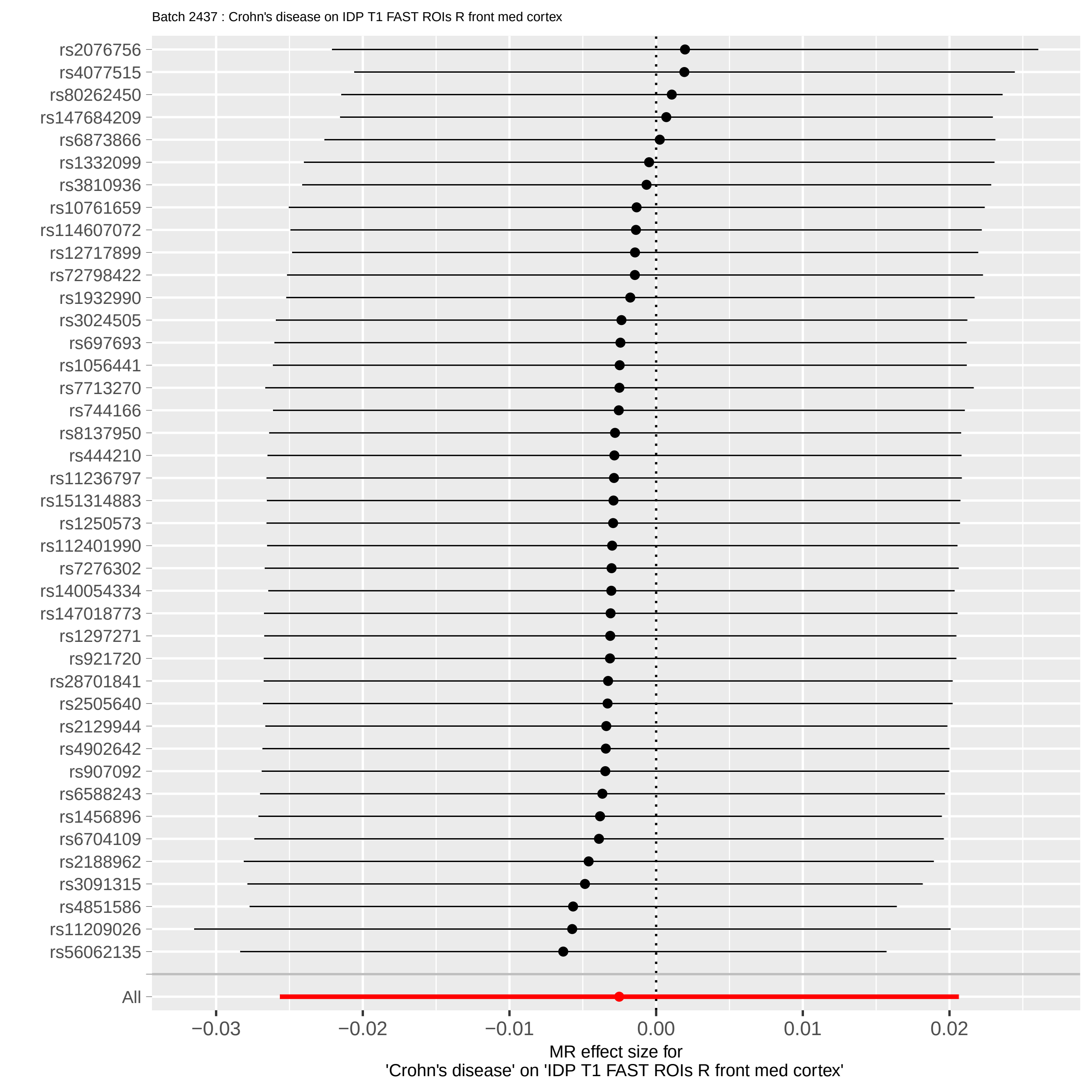

Batch 2437 : Crohn's disease on IDP T1 FAST ROIs R front med cortex
rs2076756
rs4077515
rs80262450
rs147684209
rs6873866
rs1332099
rs3810936
rs10761659
rs114607072
rs12717899
rs72798422
rs1932990
rs3024505
rs697693
rs1056441
rs7713270
rs744166
rs8137950
rs444210
rs11236797
rs151314883
rs1250573
rs112401990
rs7276302
rs140054334
rs147018773
rs1297271
rs921720
rs28701841
rs2505640
rs2129944
rs4902642
rs907092
rs6588243
rs1456896
rs6704109
rs2188962
rs3091315
rs4851586
rs11209026
rs56062135
All
−0.03
−0.02
−0.01
0.00
0.01
0.02
MR effect size for
'Crohn's disease' on 'IDP T1 FAST ROIs R front med cortex'

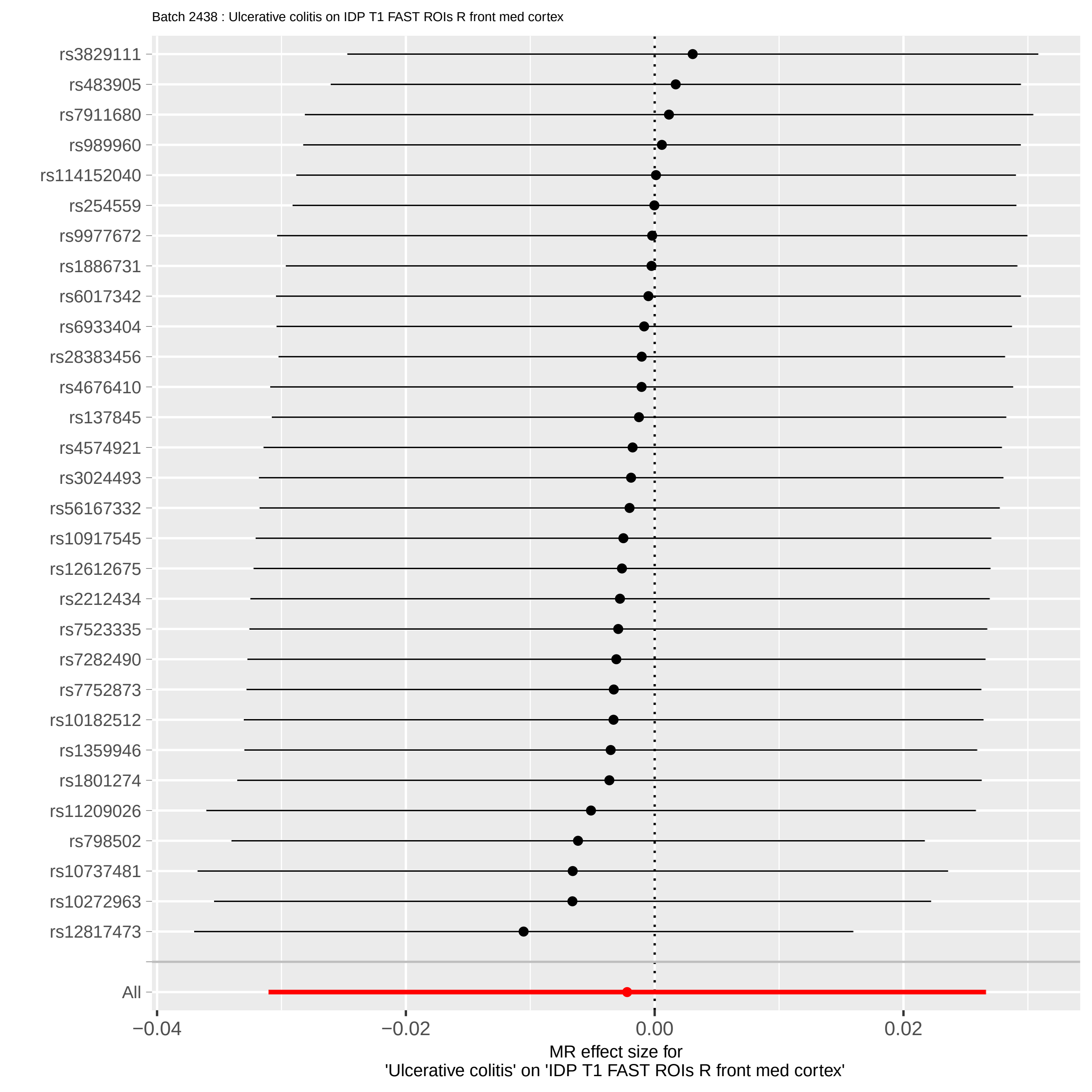
Batch 2438 : Ulcerative colitis on IDP T1 FAST ROIs R front med cortex
rs3829111
rs483905
rs7911680
rs989960
rs114152040
rs254559
rs9977672
rs1886731
rs6017342
rs6933404
rs28383456
rs4676410
rs137845
rs4574921
rs3024493
rs56167332
rs10917545
rs12612675
rs2212434
rs7523335
rs7282490
rs7752873
rs10182512
rs1359946
rs1801274
rs11209026
rs798502
rs10737481
rs10272963
rs12817473
All
−0.04
−0.02
0.00
0.02
MR effect size for
'Ulcerative colitis' on 'IDP T1 FAST ROIs R front med cortex'

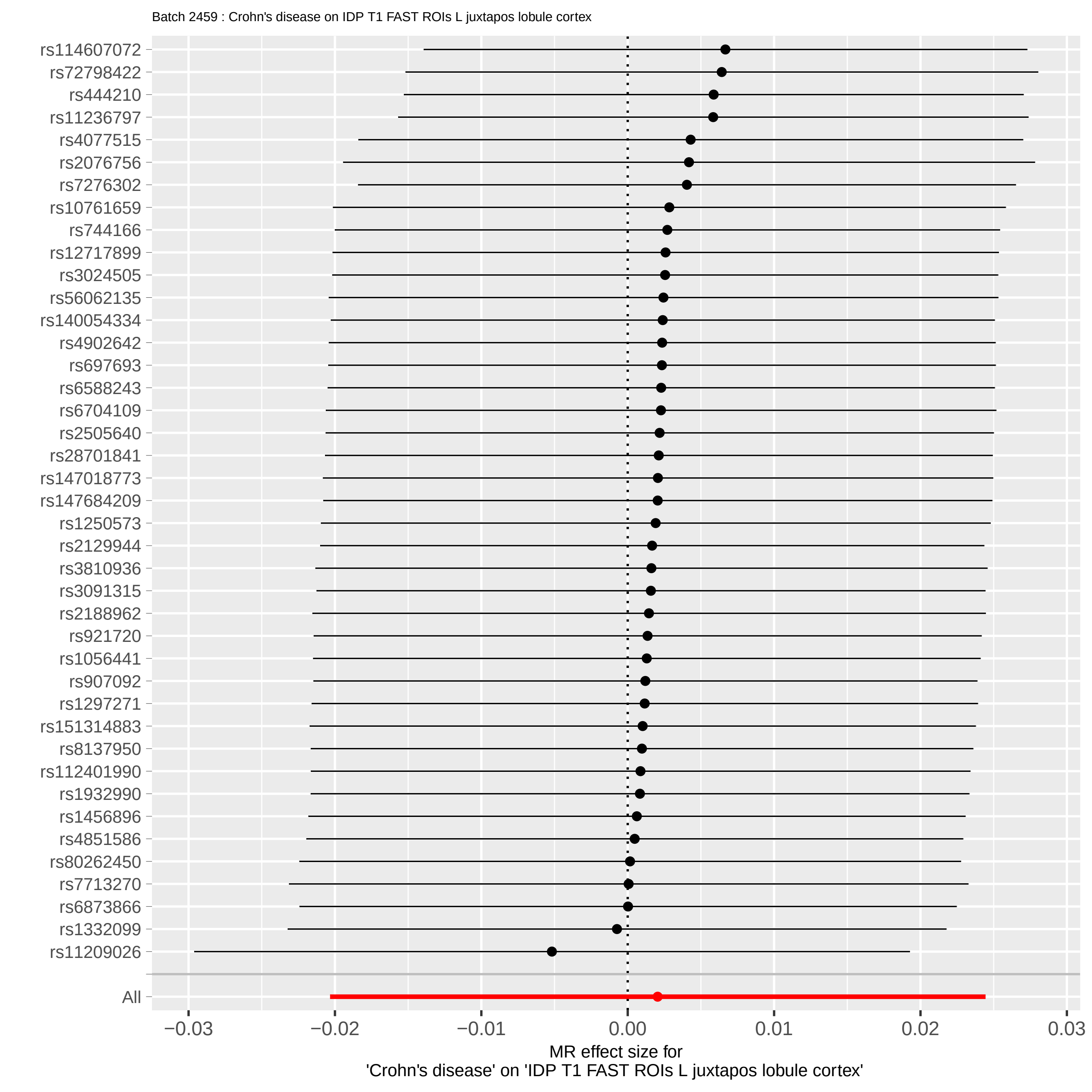
Batch 2459 : Crohn's disease on IDP T1 FAST ROIs L juxtapos lobule cortex
rs114607072
rs72798422
rs444210
rs11236797
rs4077515
rs2076756
rs7276302
rs10761659
rs744166
rs12717899
rs3024505
rs56062135
rs140054334
rs4902642
rs697693
rs6588243
rs6704109
rs2505640
rs28701841
rs147018773
rs147684209
rs1250573
rs2129944
rs3810936
rs3091315
rs2188962
rs921720
rs1056441
rs907092
rs1297271
rs151314883
rs8137950
rs112401990
rs1932990
rs1456896
rs4851586
rs80262450
rs7713270
rs6873866
rs1332099
rs11209026
All
−0.03
−0.02
−0.01
0.00
0.01
0.02
0.03
MR effect size for
'Crohn's disease' on 'IDP T1 FAST ROIs L juxtapos lobule cortex'

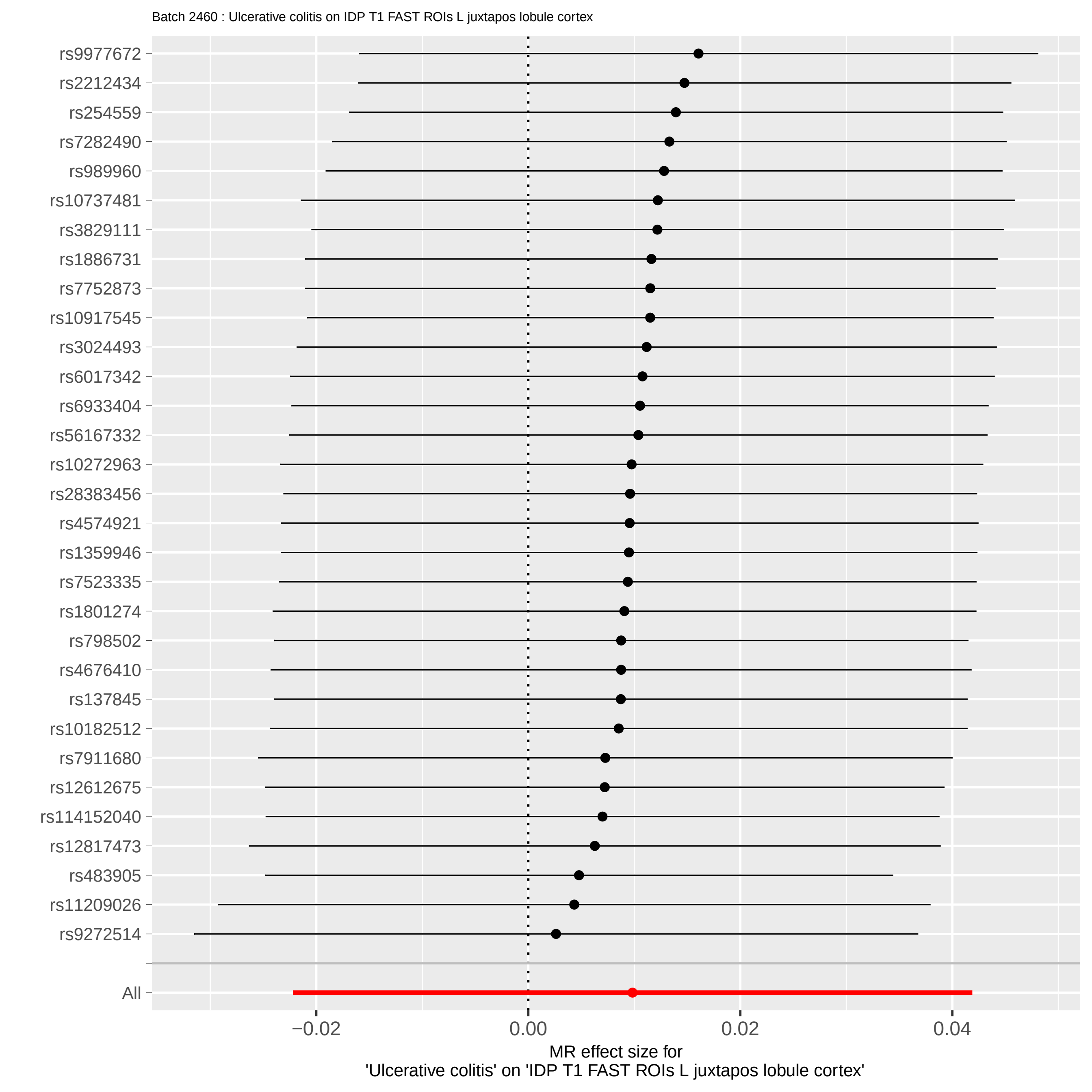

Batch 2460 : Ulcerative colitis on IDP T1 FAST ROIs L juxtapos lobule cortex
rs9977672
rs2212434
rs254559
rs7282490
rs989960
rs10737481
rs3829111
rs1886731
rs7752873
rs10917545
rs3024493
rs6017342
rs6933404
rs56167332
rs10272963
rs28383456
rs4574921
rs1359946
rs7523335
rs1801274
rs798502
rs4676410
rs137845
rs10182512
rs7911680
rs12612675
rs114152040
rs12817473
rs483905
rs11209026
rs9272514
All
−0.02
0.00
0.02
0.04
MR effect size for
'Ulcerative colitis' on 'IDP T1 FAST ROIs L juxtapos lobule cortex'

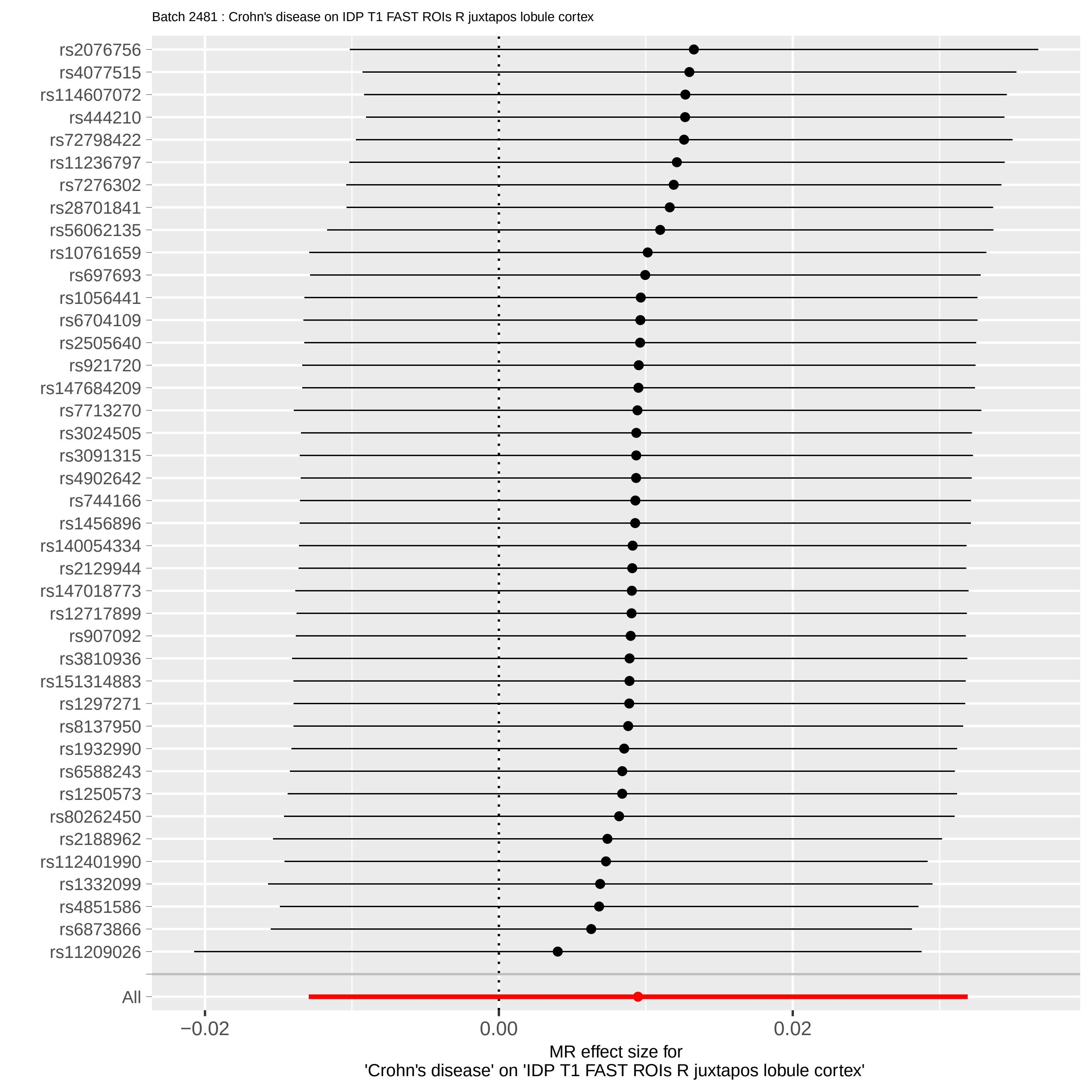

Batch 2481 : Crohn's disease on IDP T1 FAST ROIs R juxtapos lobule cortex
rs2076756
rs4077515
rs114607072
rs444210
rs72798422
rs11236797
rs7276302
rs28701841
rs56062135
rs10761659
rs697693
rs1056441
rs6704109
rs2505640
rs921720
rs147684209
rs7713270
rs3024505
rs3091315
rs4902642
rs744166
rs1456896
rs140054334
rs2129944
rs147018773
rs12717899
rs907092
rs3810936
rs151314883
rs1297271
rs8137950
rs1932990
rs6588243
rs1250573
rs80262450
rs2188962
rs112401990
rs1332099
rs4851586
rs6873866
rs11209026
All
−0.02
0.00
0.02
MR effect size for
'Crohn's disease' on 'IDP T1 FAST ROIs R juxtapos lobule cortex'

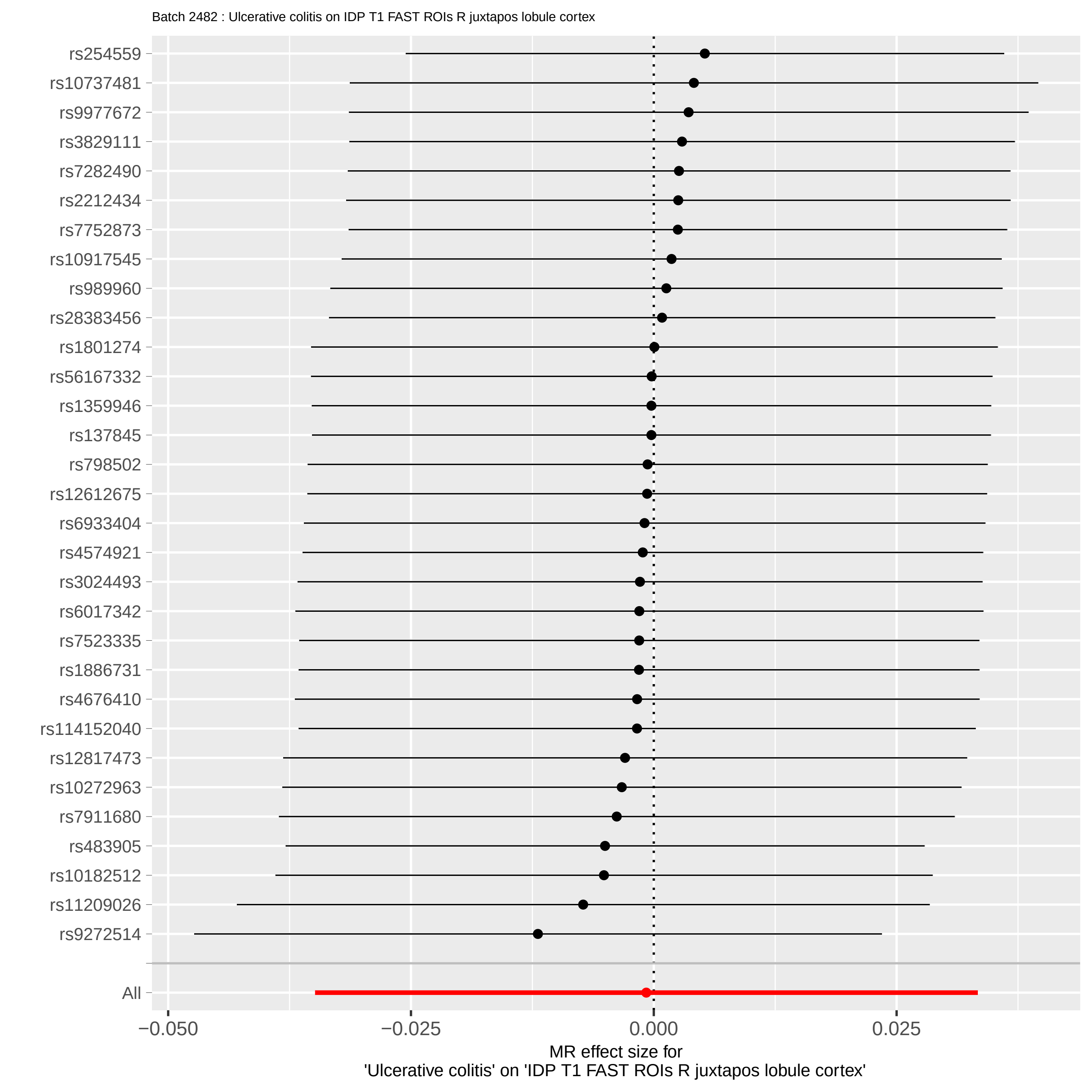

Batch 2482 : Ulcerative colitis on IDP T1 FAST ROIs R juxtapos lobule cortex
rs254559
rs10737481
rs9977672
rs3829111
rs7282490
rs2212434
rs7752873
rs10917545
rs989960
rs28383456
rs1801274
rs56167332
rs1359946
rs137845
rs798502
rs12612675
rs6933404
rs4574921
rs3024493
rs6017342
rs7523335
rs1886731
rs4676410
rs114152040
rs12817473
rs10272963
rs7911680
rs483905
rs10182512
rs11209026
rs9272514
All
−0.050
−0.025
0.000
0.025
MR effect size for
'Ulcerative colitis' on 'IDP T1 FAST ROIs R juxtapos lobule cortex'

Batch 2503 : Crohn's disease on IDP T1 FAST ROIs L subcallosal cortex

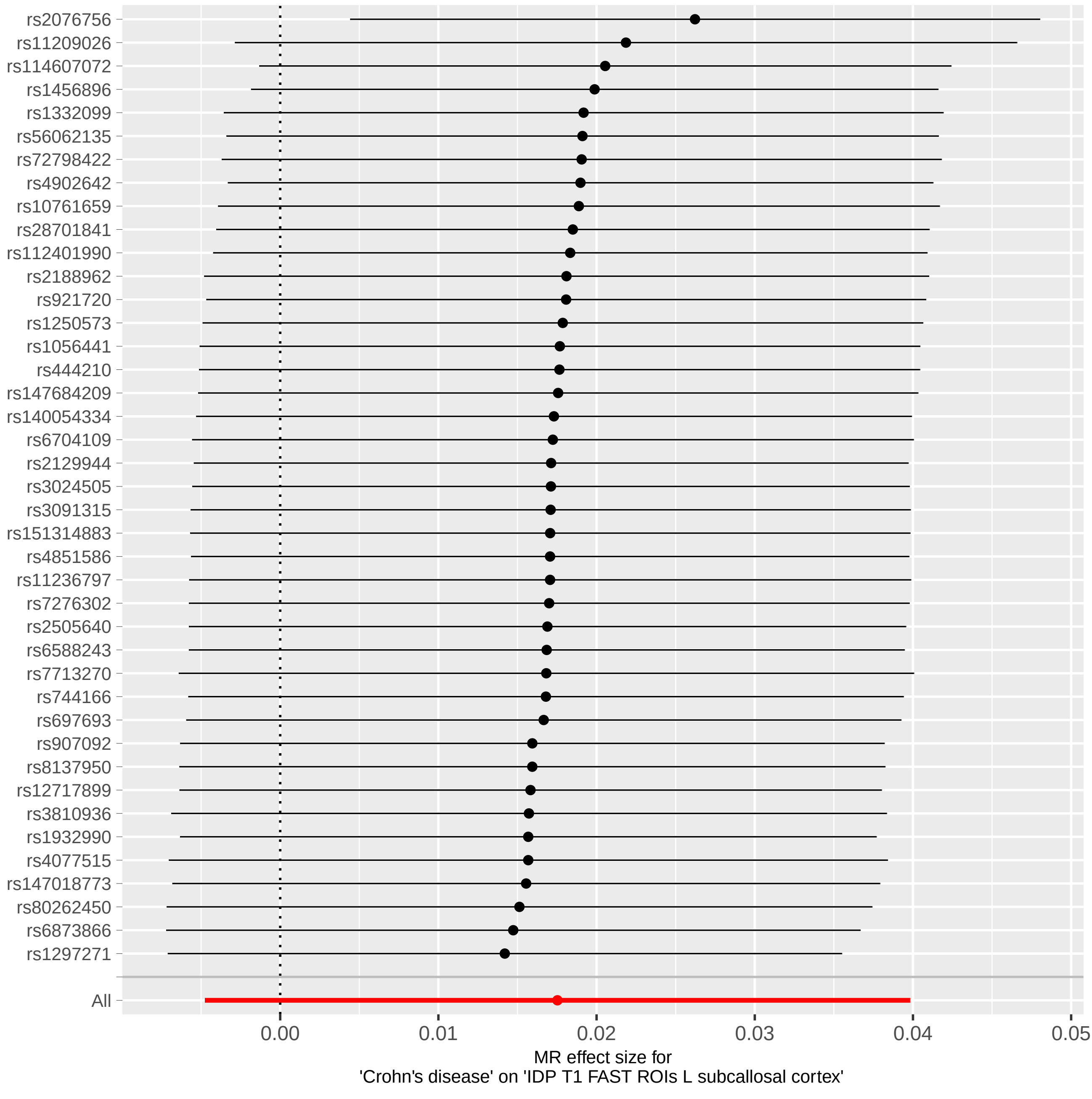

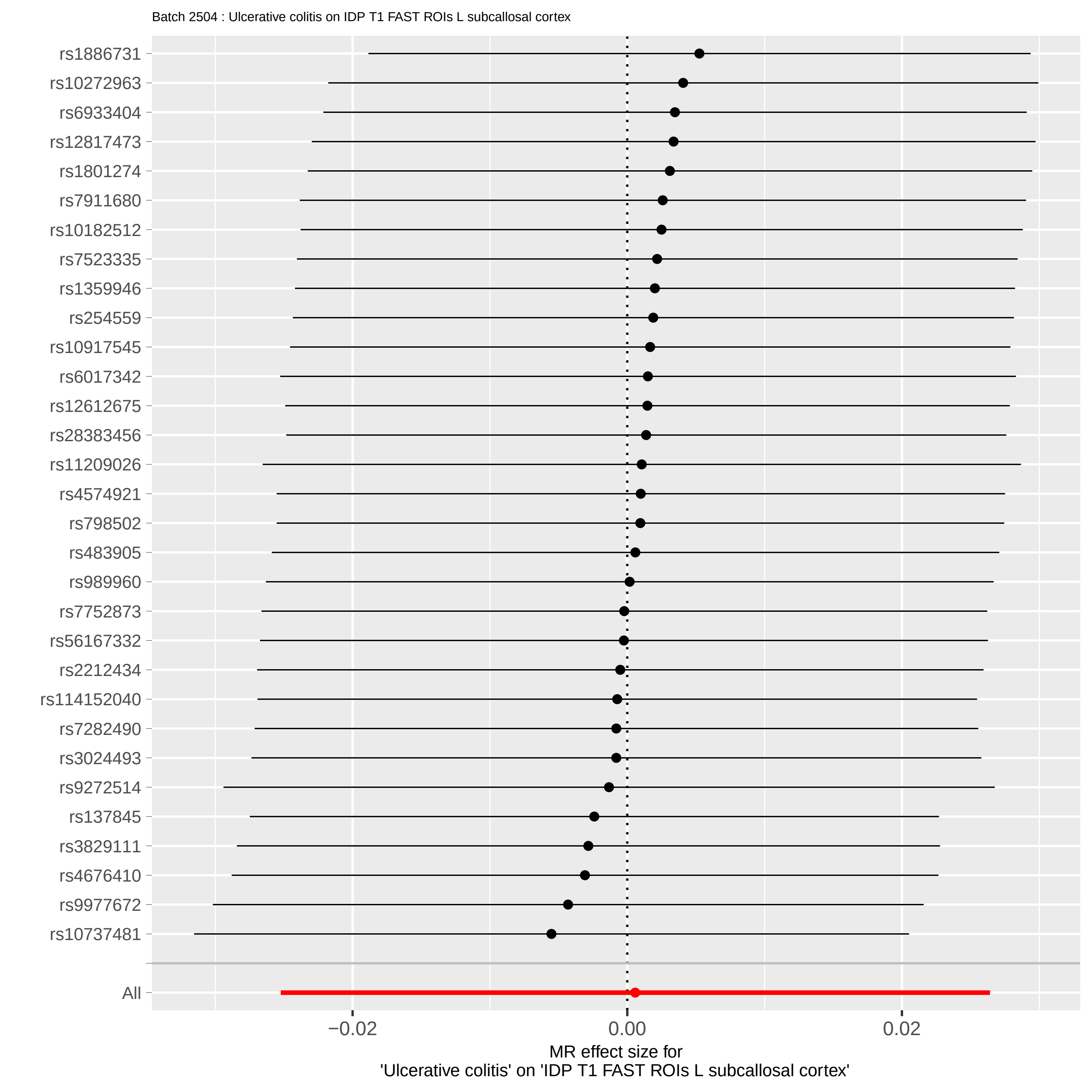

Batch 2504 : Ulcerative colitis on IDP T1 FAST ROIs L subcallosal cortex
rs1886731
rs10272963
rs6933404
rs12817473
rs1801274
rs7911680
rs10182512
rs7523335
rs1359946
rs254559
rs10917545
rs6017342
rs12612675
rs28383456
rs11209026
rs4574921
rs798502
rs483905
rs989960
rs7752873
rs56167332
rs2212434
rs114152040
rs7282490
rs3024493
rs9272514
rs137845
rs3829111
rs4676410
rs9977672
rs10737481
All
−0.02
0.00
0.02
MR effect size for
'Ulcerative colitis' on 'IDP T1 FAST ROIs L subcallosal cortex'

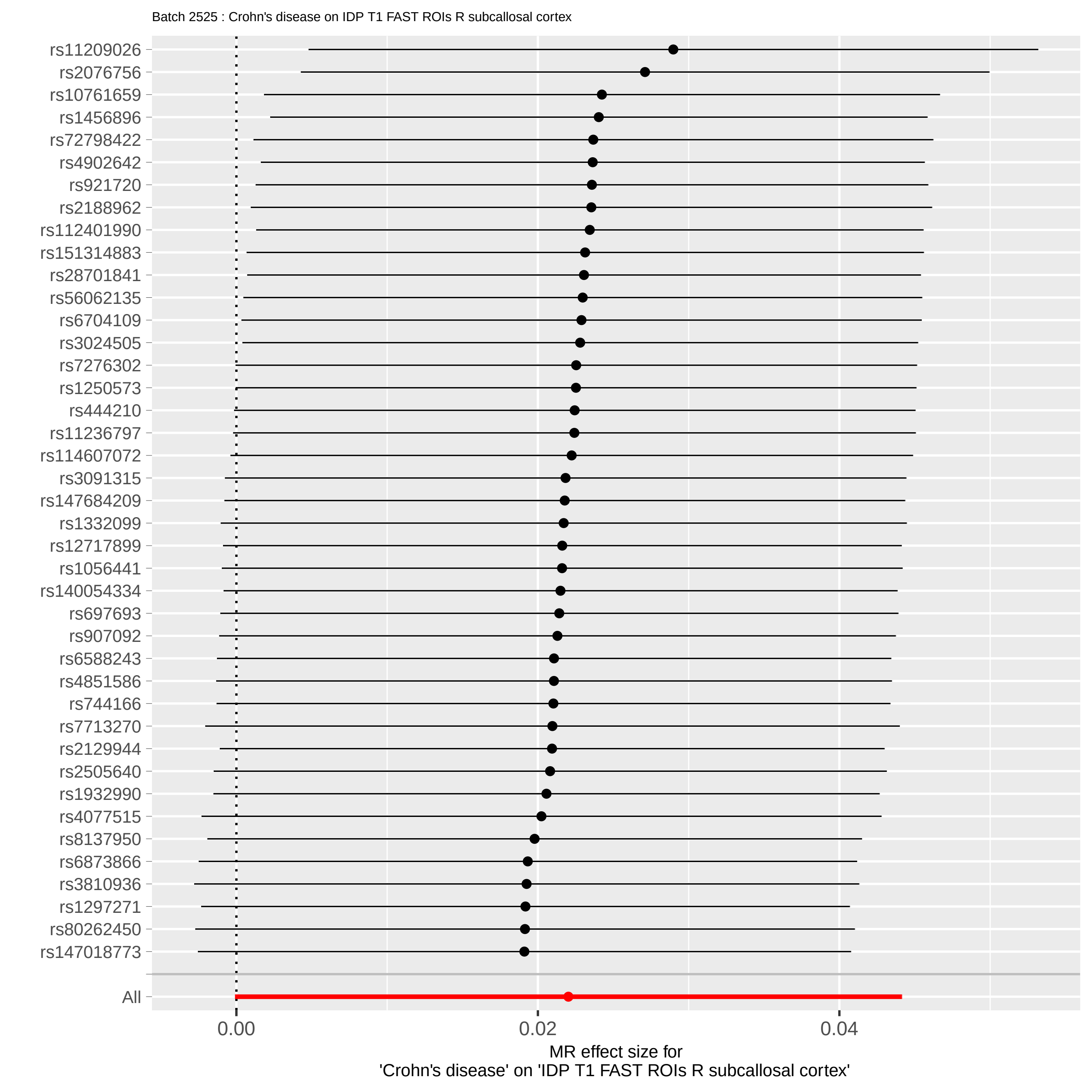

Batch 2525 : Crohn's disease on IDP T1 FAST ROIs R subcallosal cortex
rs11209026
rs2076756
rs10761659
rs1456896
rs72798422
rs4902642
rs921720
rs2188962
rs112401990
rs151314883
rs28701841
rs56062135
rs6704109
rs3024505
rs7276302
rs1250573
rs444210
rs11236797
rs114607072
rs3091315
rs147684209
rs1332099
rs12717899
rs1056441
rs140054334
rs697693
rs907092
rs6588243
rs4851586
rs744166
rs7713270
rs2129944
rs2505640
rs1932990
rs4077515
rs8137950
rs6873866
rs3810936
rs1297271
rs80262450
rs147018773
All
0.00
0.02
0.04
MR effect size for
'Crohn's disease' on 'IDP T1 FAST ROIs R subcallosal cortex'

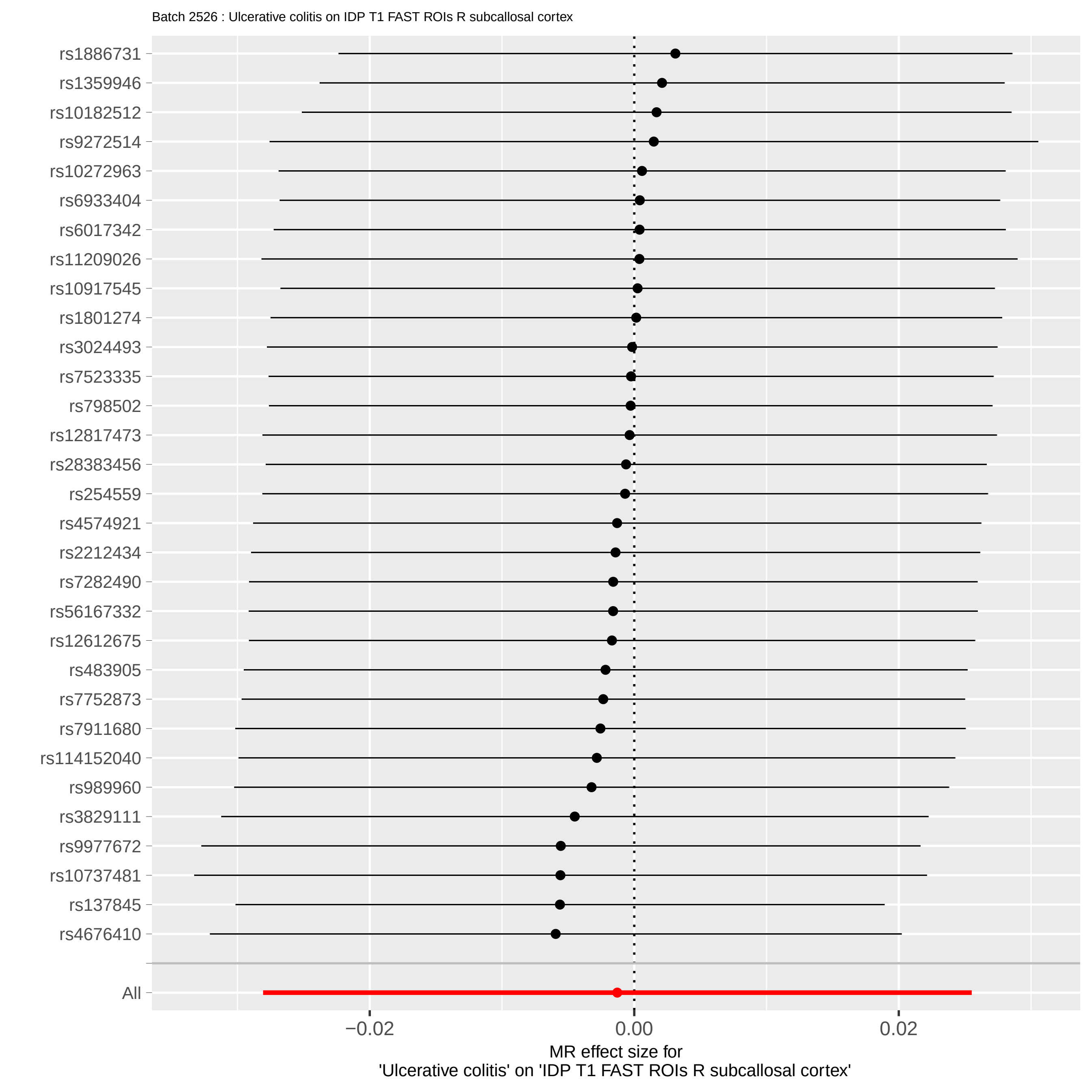

Batch 2526 : Ulcerative colitis on IDP T1 FAST ROIs R subcallosal cortex
rs1886731
rs1359946
rs10182512
rs9272514
rs10272963
rs6933404
rs6017342
rs11209026
rs10917545
rs1801274
rs3024493
rs7523335
rs798502
rs12817473
rs28383456
rs254559
rs4574921
rs2212434
rs7282490
rs56167332
rs12612675
rs483905
rs7752873
rs7911680
rs114152040
rs989960
rs3829111
rs9977672
rs10737481
rs137845
rs4676410
All
−0.02
0.00
0.02
MR effect size for
'Ulcerative colitis' on 'IDP T1 FAST ROIs R subcallosal cortex'

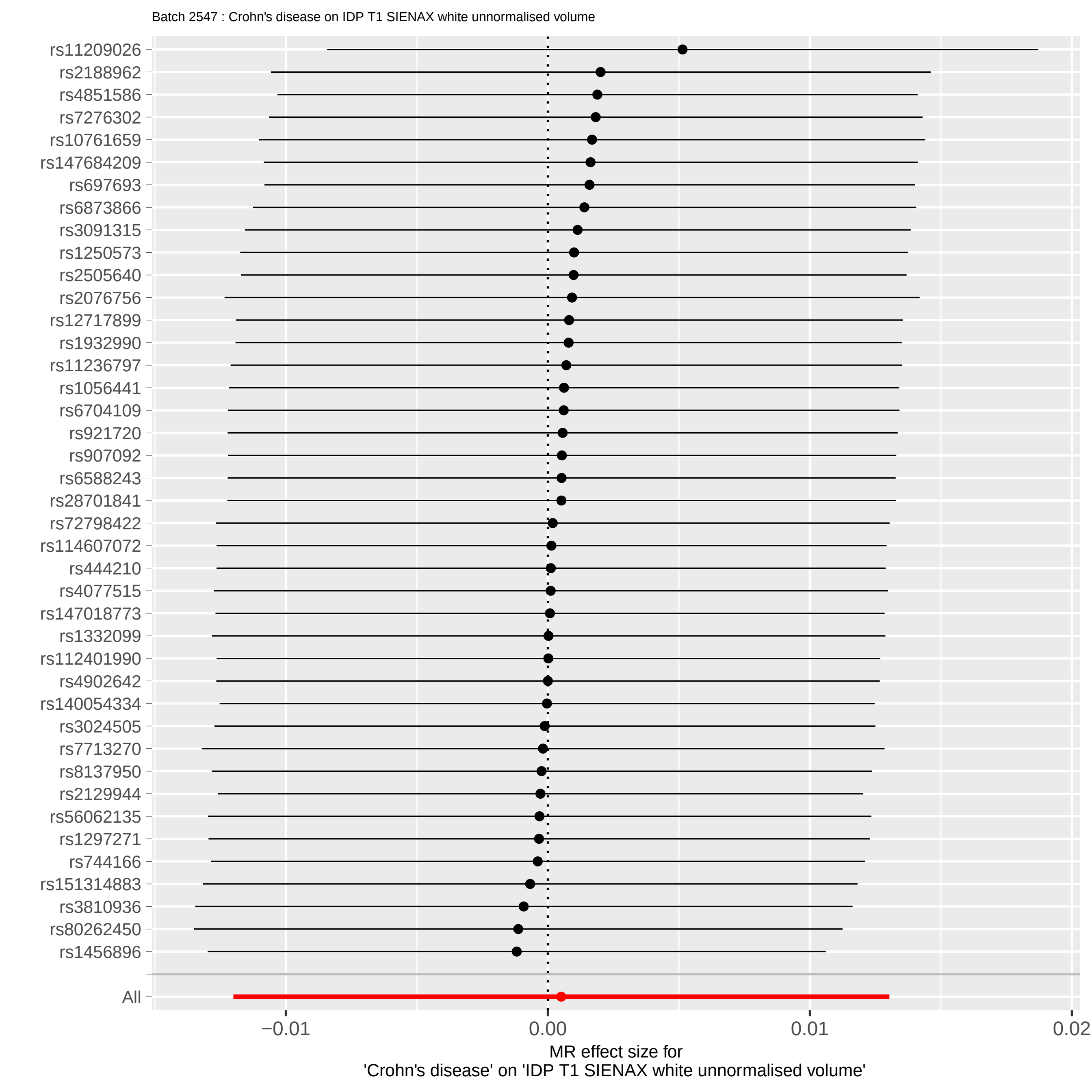
Batch 2547 : Crohn's disease on IDP T1 SIENAX white unnormalised volume
rs11209026
rs2188962
rs4851586
rs7276302
rs10761659
rs147684209
rs697693
rs6873866
rs3091315
rs1250573
rs2505640
rs2076756
rs12717899
rs1932990
rs11236797
rs1056441
rs6704109
rs921720
rs907092
rs6588243
rs28701841
rs72798422
rs114607072
rs444210
rs4077515
rs147018773
rs1332099
rs112401990
rs4902642
rs140054334
rs3024505
rs7713270
rs8137950
rs2129944
rs56062135
rs1297271
rs744166
rs151314883
rs3810936
rs80262450
rs1456896
All
−0.01
0.00
0.01
0.02
MR effect size for
'Crohn's disease' on 'IDP T1 SIENAX white unnormalised volume'

Batch 2548 : Ulcerative colitis on IDP T1 SIENAX white unnormalised volume

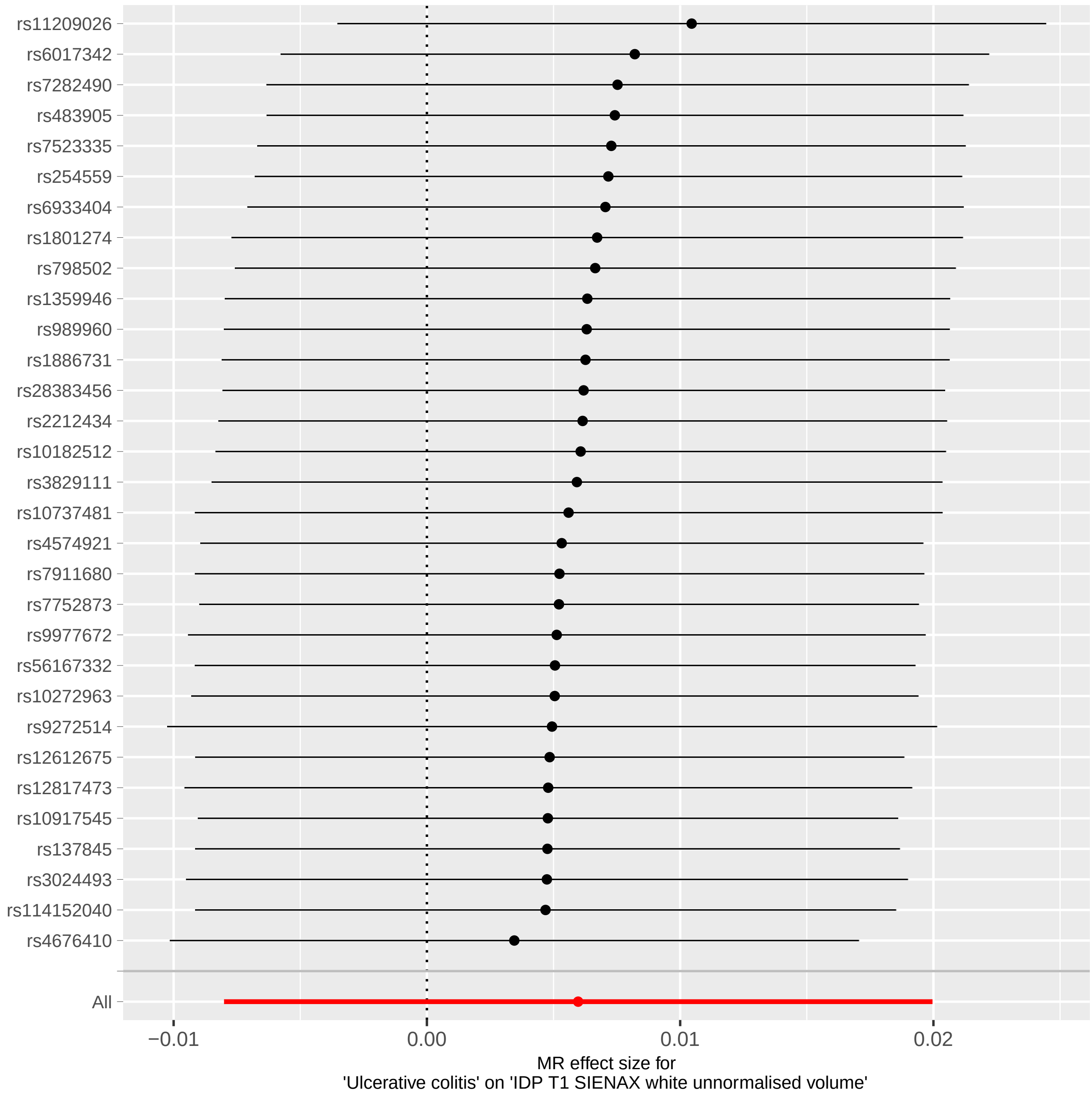

Batch 2549 : Crohn's disease on IDP T1 FAST ROIs L paracing gyrus

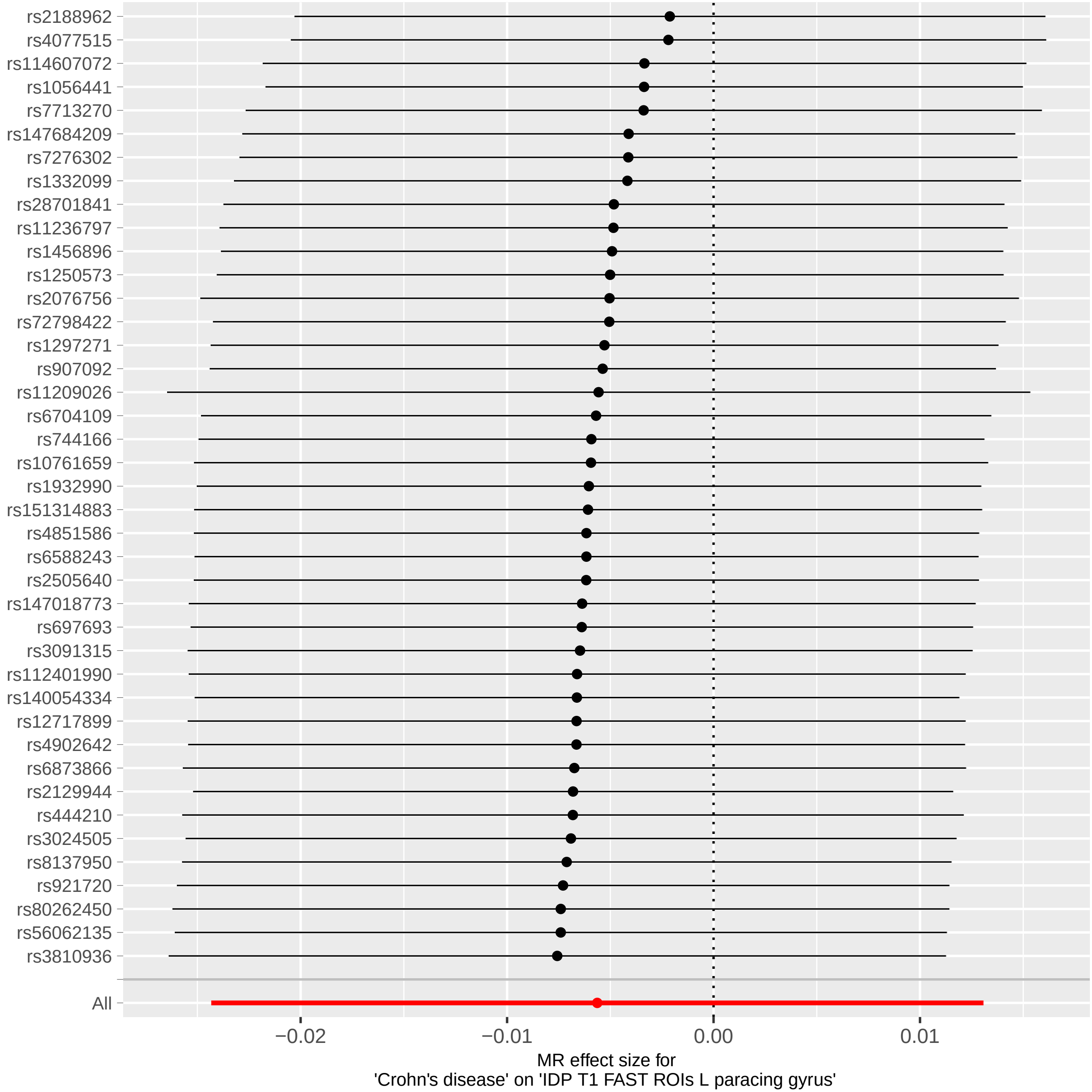

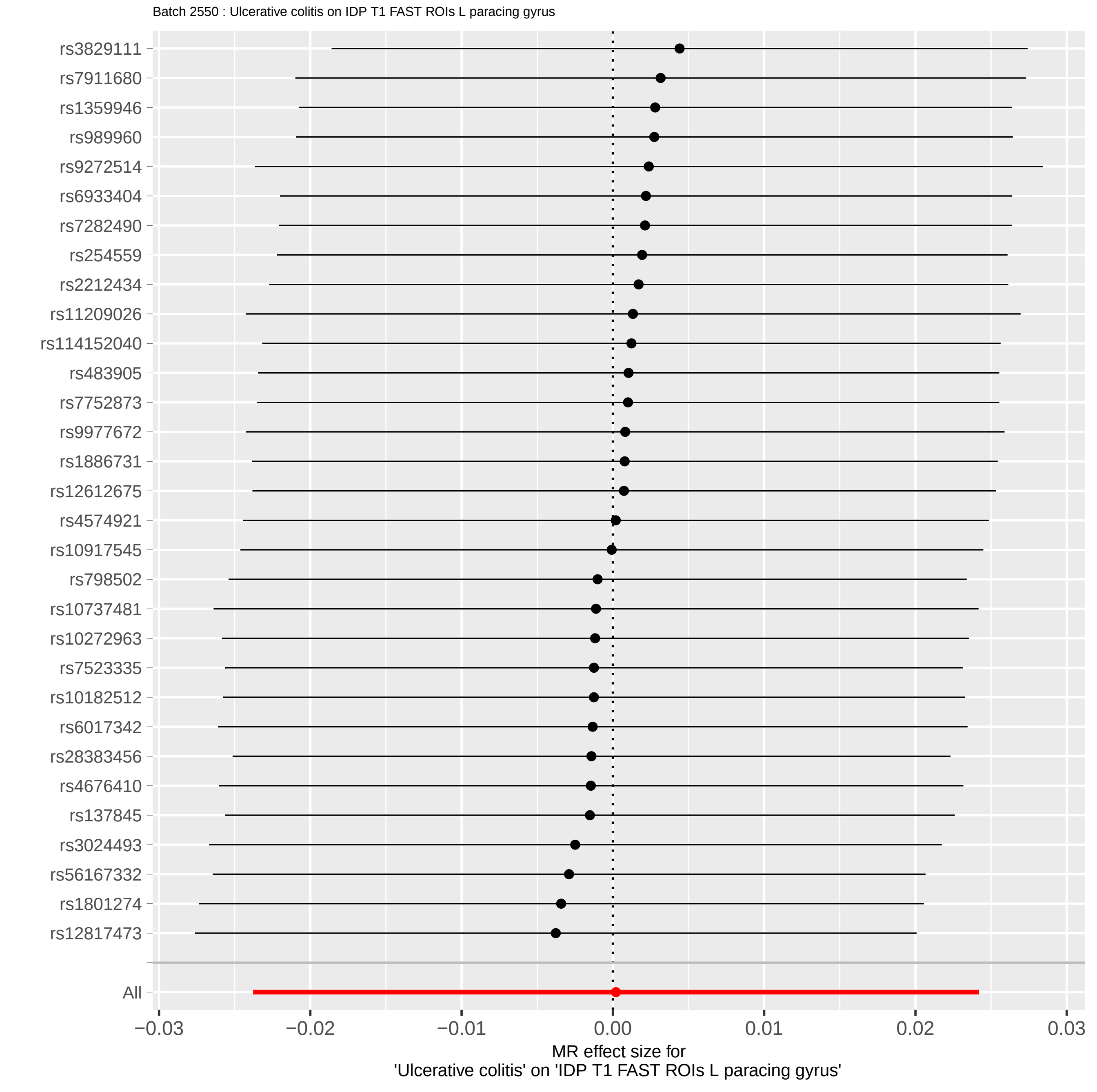

Batch 2550 : Ulcerative colitis on IDP T1 FAST ROIs L paracing gyrus
rs3829111
rs7911680
rs1359946
rs989960
rs9272514
rs6933404
rs7282490
rs254559
rs2212434
rs11209026
rs114152040
rs483905
rs7752873
rs9977672
rs1886731
rs12612675
rs4574921
rs10917545
rs798502
rs10737481
rs10272963
rs7523335
rs10182512
rs6017342
rs28383456
rs4676410
rs137845
rs3024493
rs56167332
rs1801274
rs12817473
All
−0.03
−0.02
−0.01
0.00
0.01
0.02
0.03
MR effect size for
'Ulcerative colitis' on 'IDP T1 FAST ROIs L paracing gyrus'

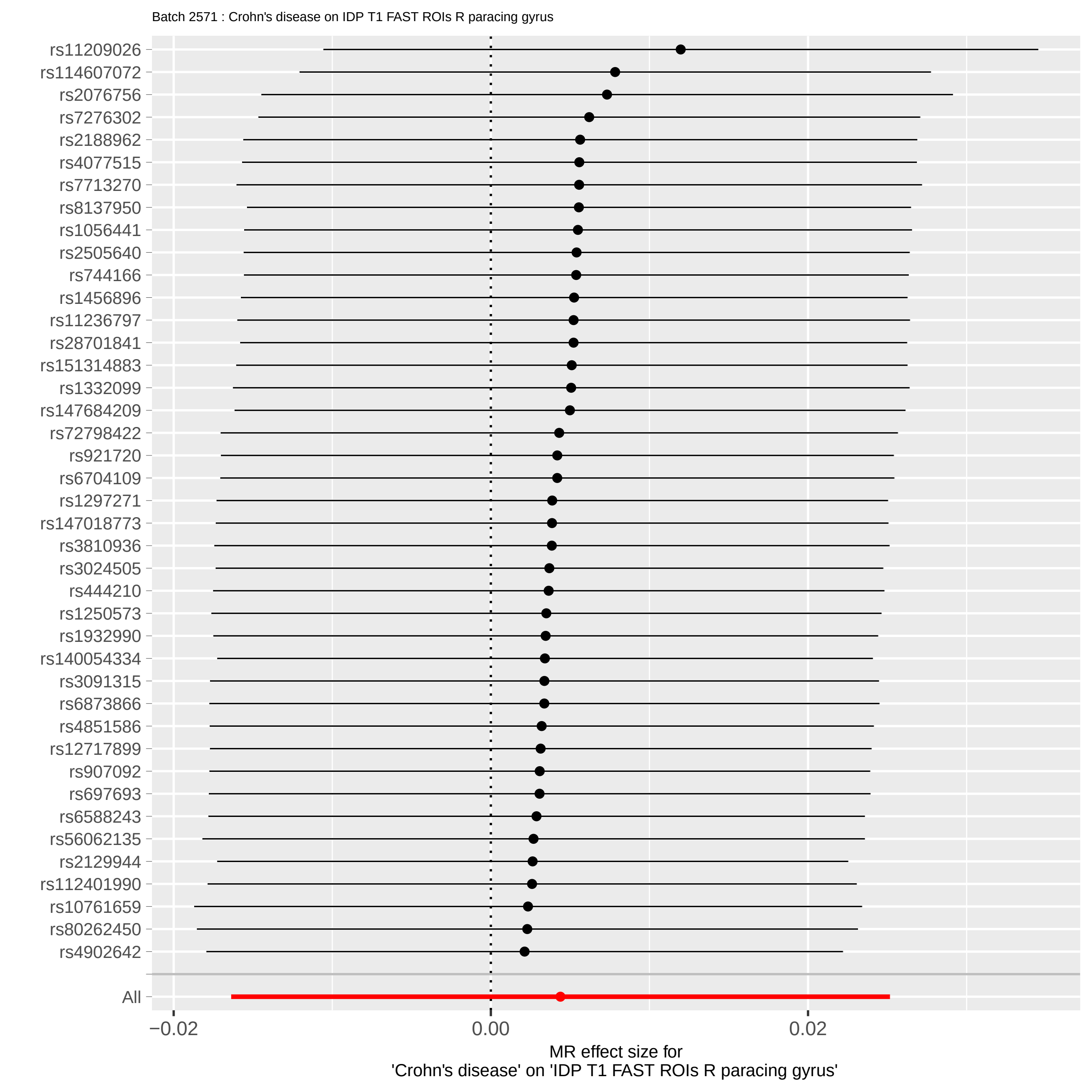

Batch 2571 : Crohn's disease on IDP T1 FAST ROIs R paracing gyrus
rs11209026
rs114607072
rs2076756
rs7276302
rs2188962
rs4077515
rs7713270
rs8137950
rs1056441
rs2505640
rs744166
rs1456896
rs11236797
rs28701841
rs151314883
rs1332099
rs147684209
rs72798422
rs921720
rs6704109
rs1297271
rs147018773
rs3810936
rs3024505
rs444210
rs1250573
rs1932990
rs140054334
rs3091315
rs6873866
rs4851586
rs12717899
rs907092
rs697693
rs6588243
rs56062135
rs2129944
rs112401990
rs10761659
rs80262450
rs4902642
All
−0.02
0.00
0.02
MR effect size for
'Crohn's disease' on 'IDP T1 FAST ROIs R paracing gyrus'

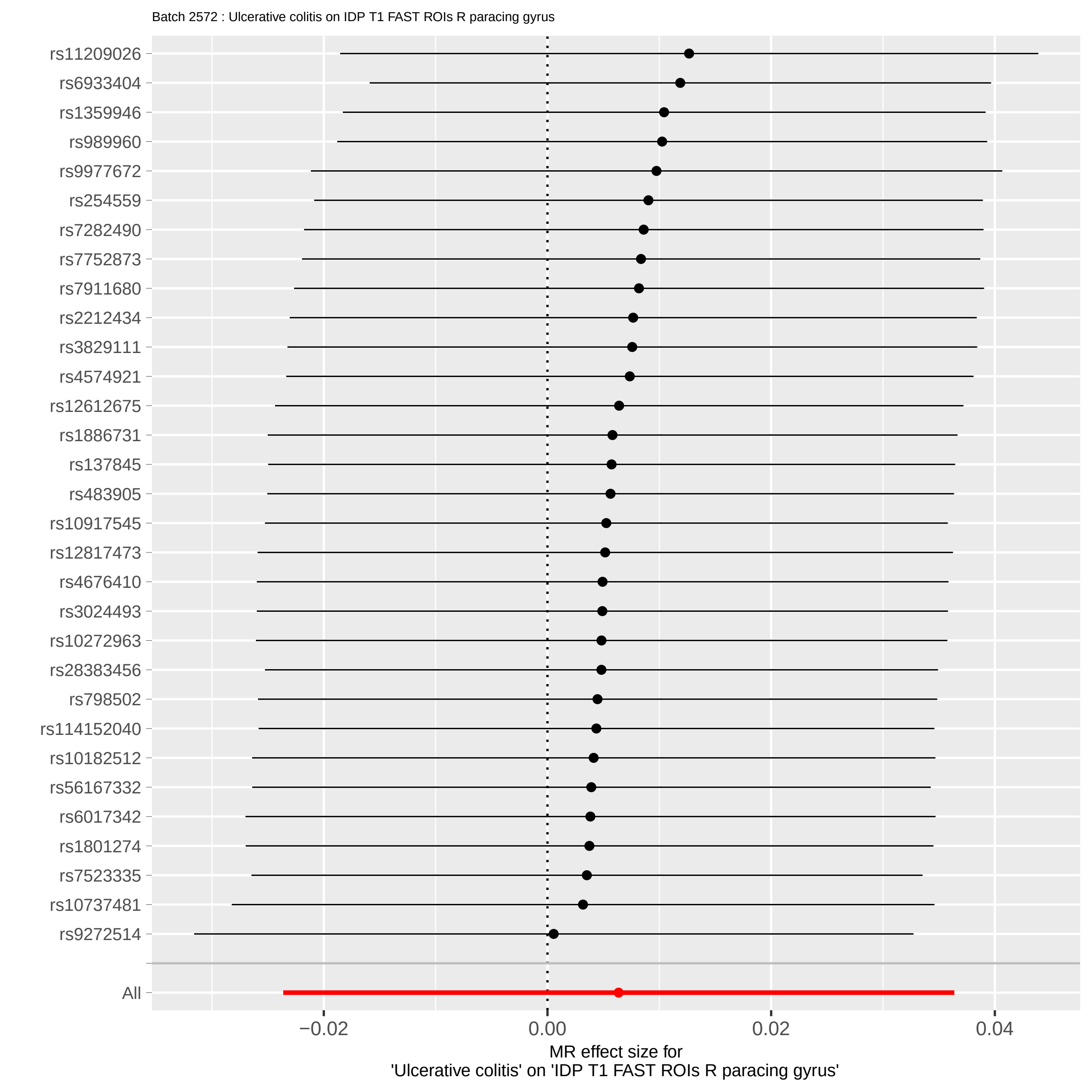

Batch 2572 : Ulcerative colitis on IDP T1 FAST ROIs R paracing gyrus
rs11209026
rs6933404
rs1359946
rs989960
rs9977672
rs254559
rs7282490
rs7752873
rs7911680
rs2212434
rs3829111
rs4574921
rs12612675
rs1886731
rs137845
rs483905
rs10917545
rs12817473
rs4676410
rs3024493
rs10272963
rs28383456
rs798502
rs114152040
rs10182512
rs56167332
rs6017342
rs1801274
rs7523335
rs10737481
rs9272514
All
−0.02
0.00
0.02
0.04
MR effect size for
'Ulcerative colitis' on 'IDP T1 FAST ROIs R paracing gyrus'

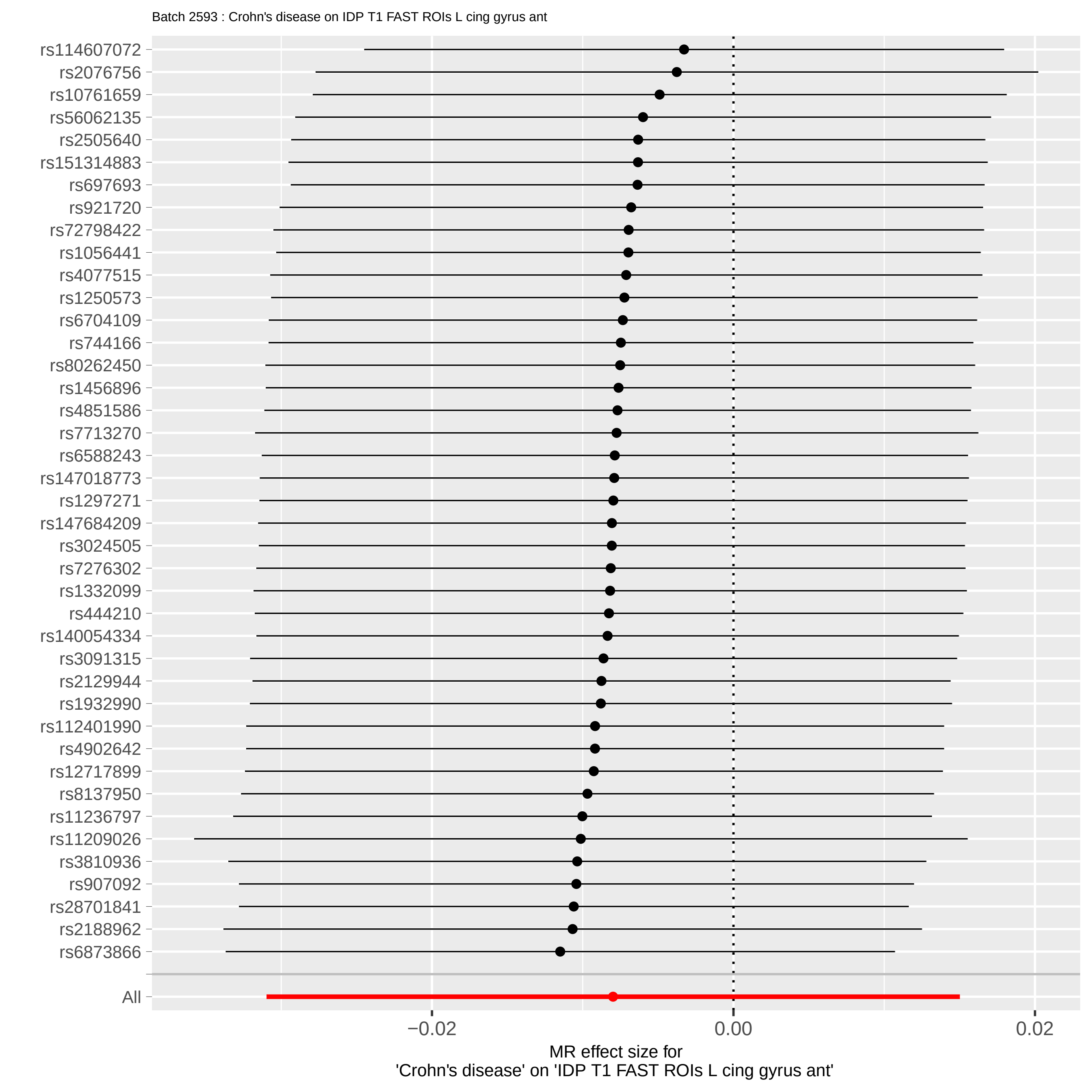

Batch 2593 : Crohn's disease on IDP T1 FAST ROIs L cing gyrus ant
rs114607072
rs2076756
rs10761659
rs56062135
rs2505640
rs151314883
rs697693
rs921720
rs72798422
rs1056441
rs4077515
rs1250573
rs6704109
rs744166
rs80262450
rs1456896
rs4851586
rs7713270
rs6588243
rs147018773
rs1297271
rs147684209
rs3024505
rs7276302
rs1332099
rs444210
rs140054334
rs3091315
rs2129944
rs1932990
rs112401990
rs4902642
rs12717899
rs8137950
rs11236797
rs11209026
rs3810936
rs907092
rs28701841
rs2188962
rs6873866
All
−0.02
0.00
0.02
MR effect size for
'Crohn's disease' on 'IDP T1 FAST ROIs L cing gyrus ant'

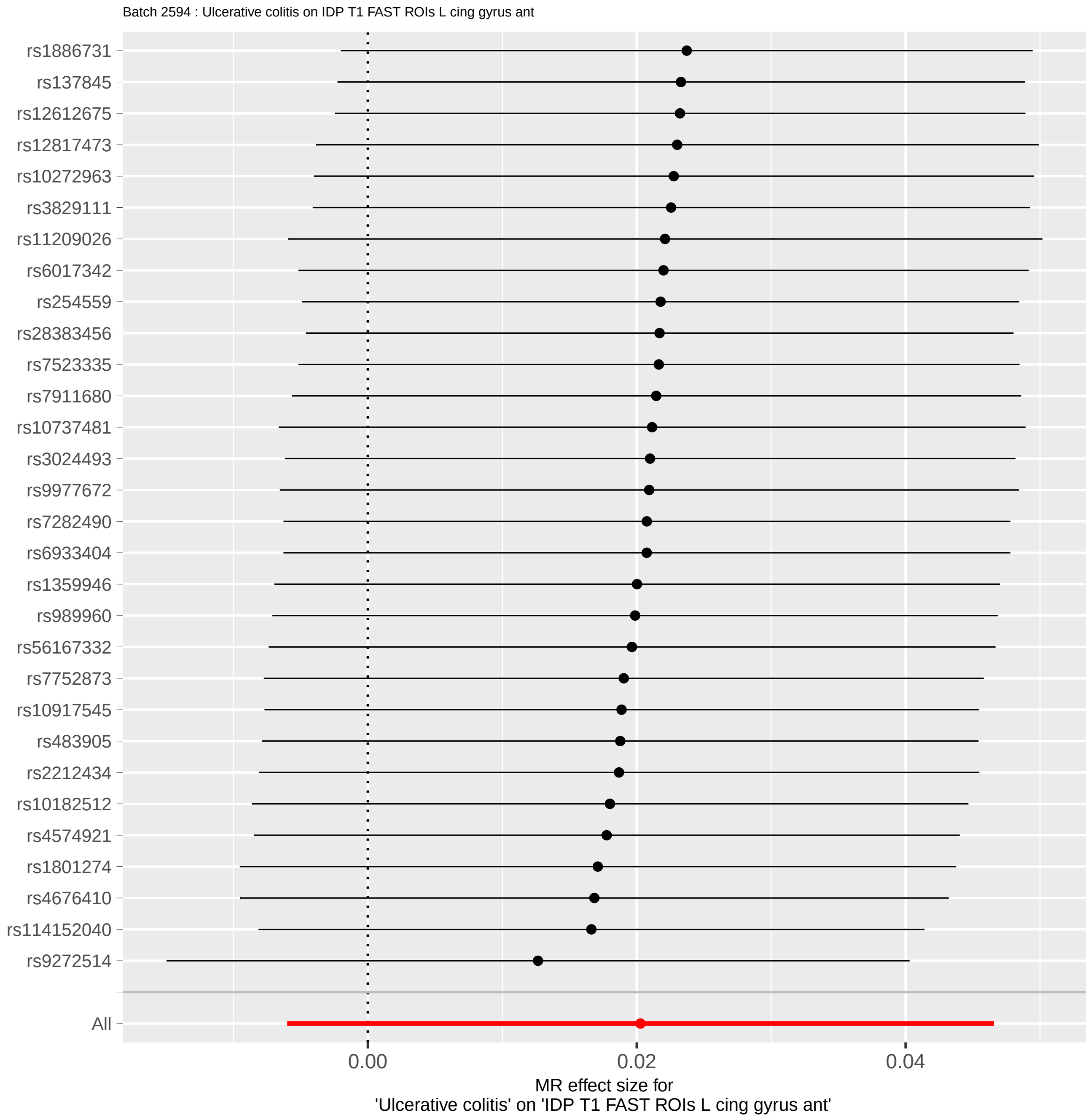
Batch 2594 : Ulcerative colitis on IDP T1 FAST ROIs L cing gyrus ant
rs1886731
rs137845
rs12612675
rs12817473
rs10272963
rs3829111
rs11209026
rs6017342
rs254559
rs28383456
rs7523335
rs7911680
rs10737481
rs3024493
rs9977672
rs7282490
rs6933404
rs1359946
rs989960
rs56167332
rs7752873
rs10917545
rs483905
rs2212434
rs10182512
rs4574921
rs1801274
rs4676410
rs114152040
rs9272514
All
0.00
0.02
0.04
MR effect size for
'Ulcerative colitis' on 'IDP T1 FAST ROIs L cing gyrus ant'

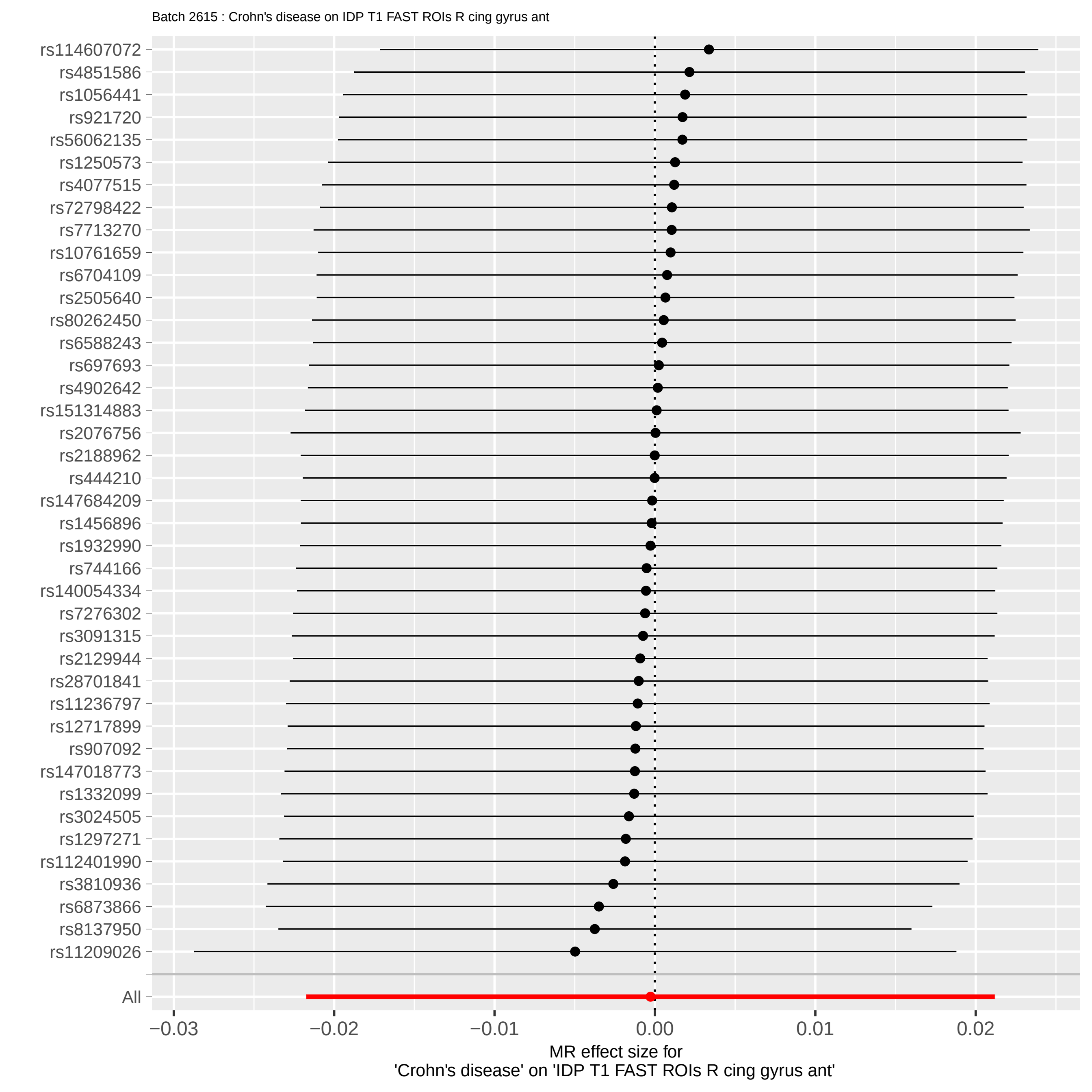
Batch 2615 : Crohn's disease on IDP T1 FAST ROIs R cing gyrus ant
rs114607072
rs4851586
rs1056441
rs921720
rs56062135
rs1250573
rs4077515
rs72798422
rs7713270
rs10761659
rs6704109
rs2505640
rs80262450
rs6588243
rs697693
rs4902642
rs151314883
rs2076756
rs2188962
rs444210
rs147684209
rs1456896
rs1932990
rs744166
rs140054334
rs7276302
rs3091315
rs2129944
rs28701841
rs11236797
rs12717899
rs907092
rs147018773
rs1332099
rs3024505
rs1297271
rs112401990
rs3810936
rs6873866
rs8137950
rs11209026
All
−0.03
−0.02
−0.01
0.00
0.01
0.02
MR effect size for
'Crohn's disease' on 'IDP T1 FAST ROIs R cing gyrus ant'

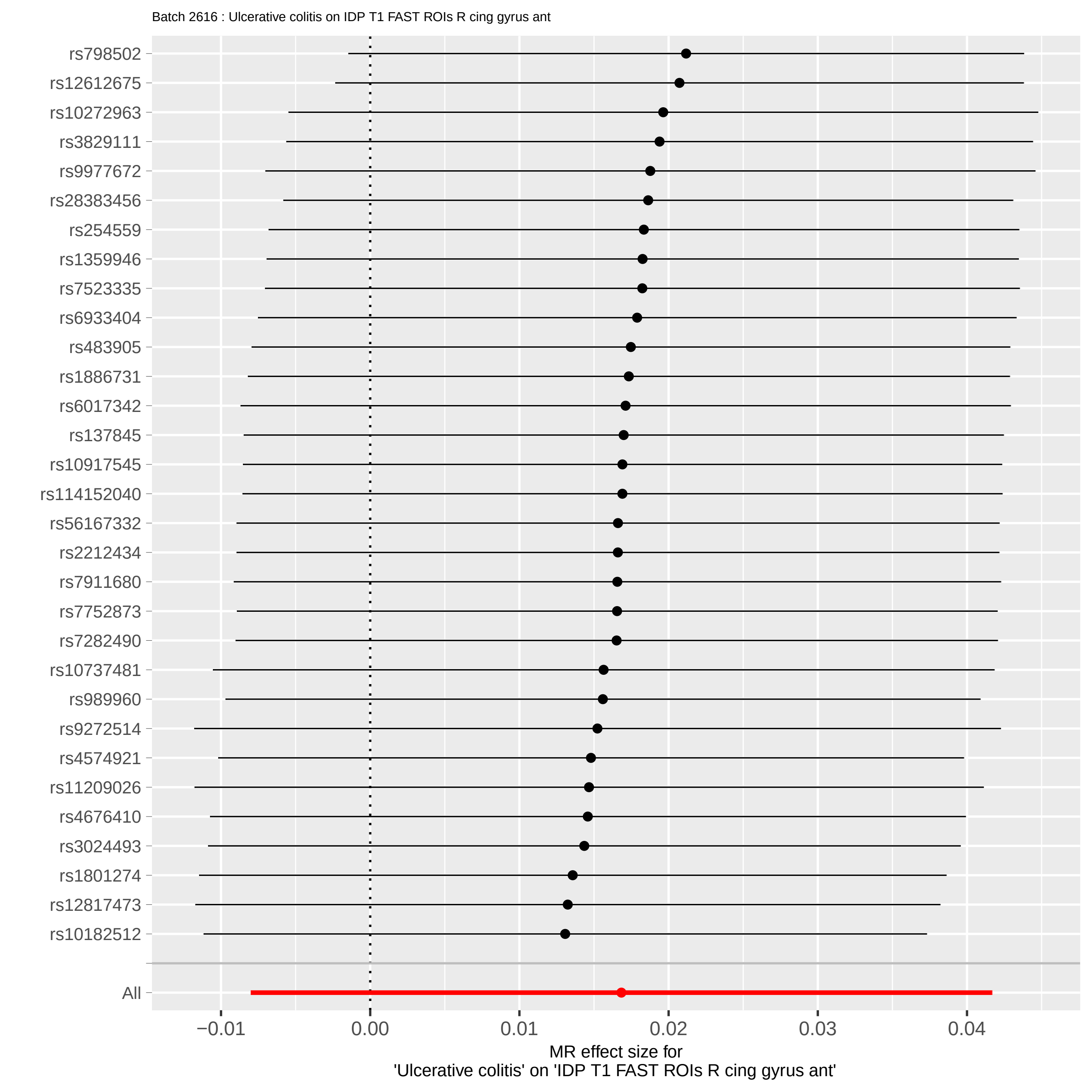

Batch 2616 : Ulcerative colitis on IDP T1 FAST ROIs R cing gyrus ant
rs798502
rs12612675
rs10272963
rs3829111
rs9977672
rs28383456
rs254559
rs1359946
rs7523335
rs6933404
rs483905
rs1886731
rs6017342
rs137845
rs10917545
rs114152040
rs56167332
rs2212434
rs7911680
rs7752873
rs7282490
rs10737481
rs989960
rs9272514
rs4574921
rs11209026
rs4676410
rs3024493
rs1801274
rs12817473
rs10182512
All
−0.01
0.00
0.01
0.02
0.03
0.04
MR effect size for
'Ulcerative colitis' on 'IDP T1 FAST ROIs R cing gyrus ant'

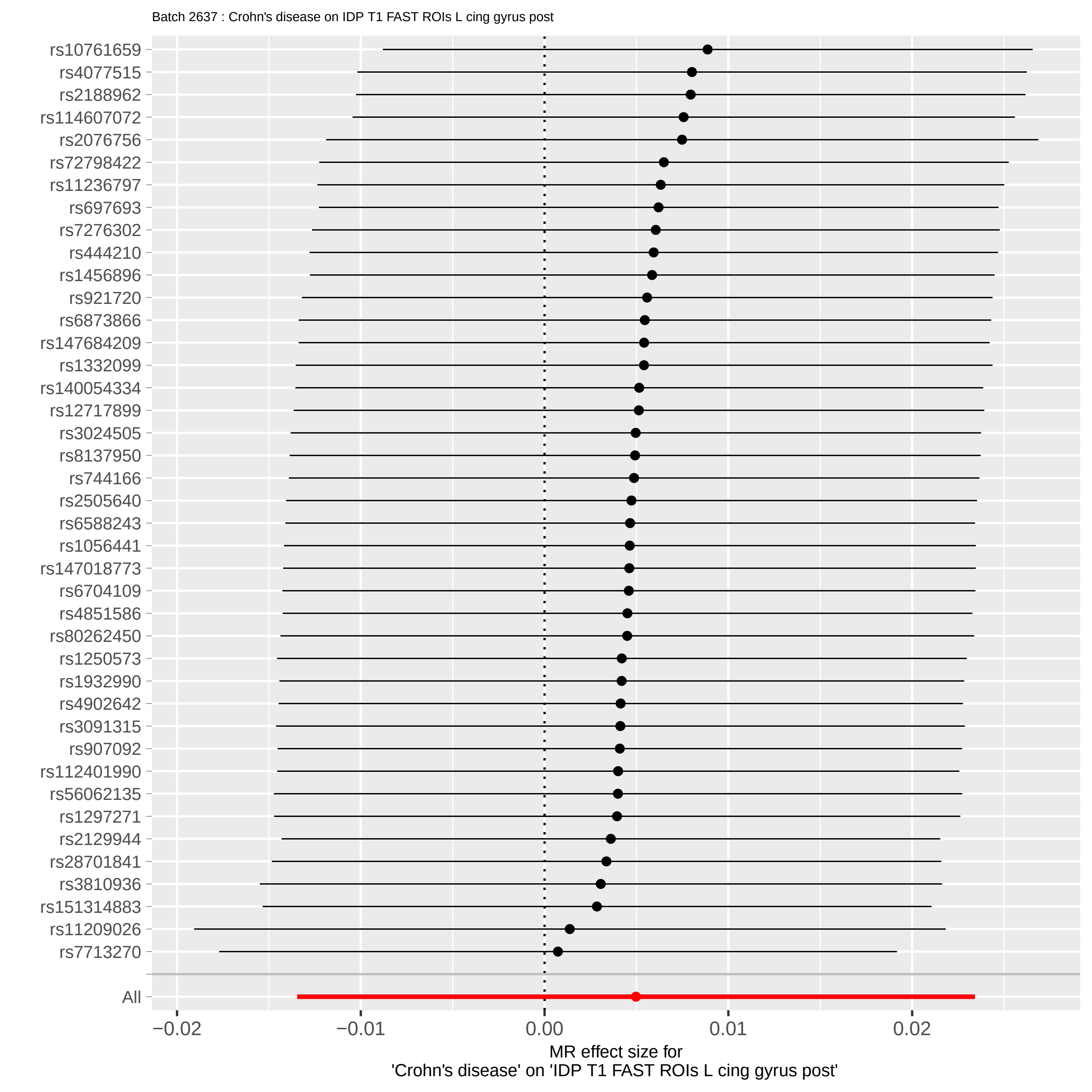
Batch 2637 : Crohn's disease on IDP T1 FAST ROIs L cing gyrus post
rs10761659
rs4077515
rs2188962
rs114607072
rs2076756
rs72798422
rs11236797
rs697693
rs7276302
rs444210
rs1456896
rs921720
rs6873866
rs147684209
rs1332099
rs140054334
rs12717899
rs3024505
rs8137950
rs744166
rs2505640
rs6588243
rs1056441
rs147018773
rs6704109
rs4851586
rs80262450
rs1250573
rs1932990
rs4902642
rs3091315
rs907092
rs112401990
rs56062135
rs1297271
rs2129944
rs28701841
rs3810936
rs151314883
rs11209026
rs7713270
All
−0.02
−0.01
0.00
0.01
0.02
MR effect size for
'Crohn's disease' on 'IDP T1 FAST ROIs L cing gyrus post'

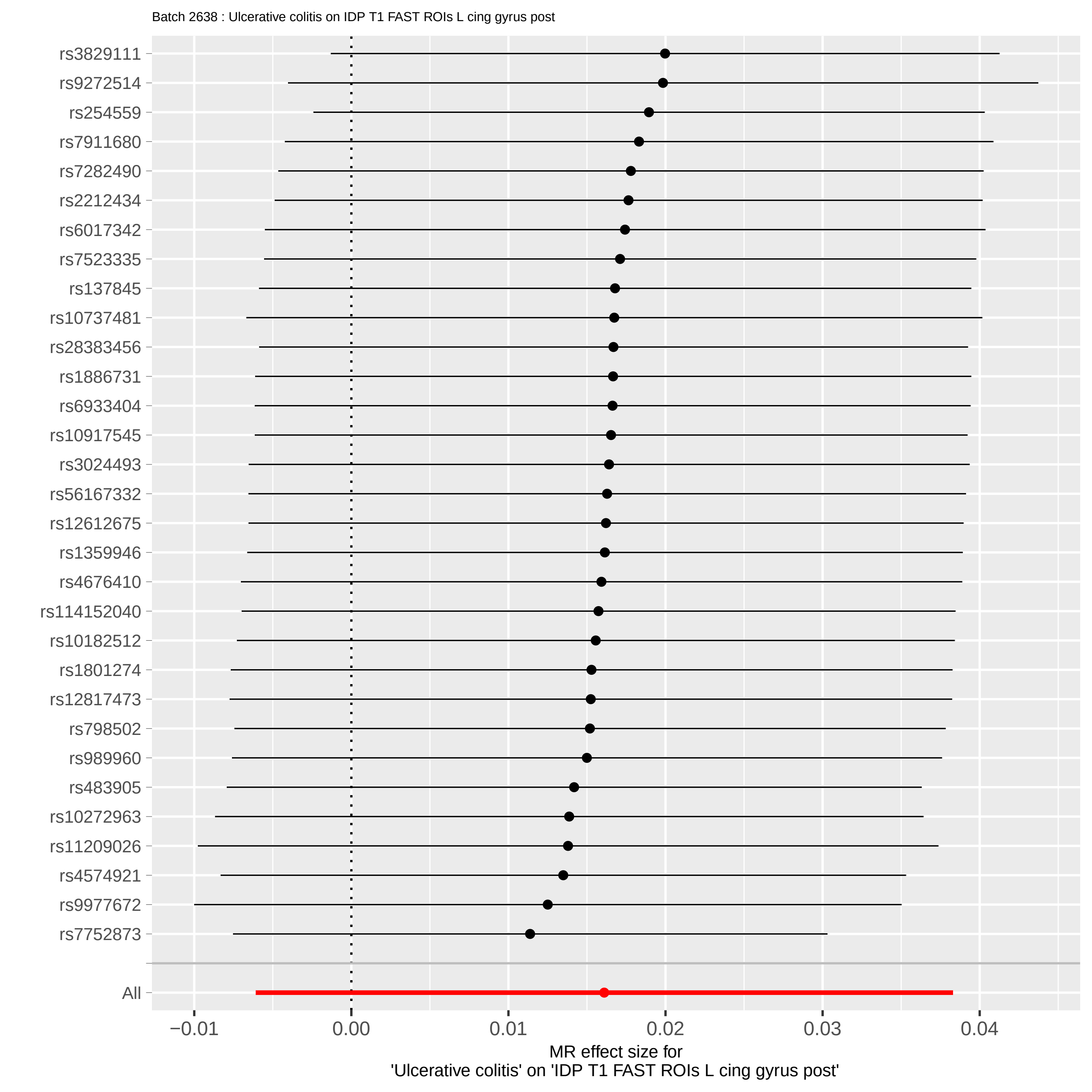

Batch 2638 : Ulcerative colitis on IDP T1 FAST ROIs L cing gyrus post
rs3829111
rs9272514
rs254559
rs7911680
rs7282490
rs2212434
rs6017342
rs7523335
rs137845
rs10737481
rs28383456
rs1886731
rs6933404
rs10917545
rs3024493
rs56167332
rs12612675
rs1359946
rs4676410
rs114152040
rs10182512
rs1801274
rs12817473
rs798502
rs989960
rs483905
rs10272963
rs11209026
rs4574921
rs9977672
rs7752873
All
−0.01
0.00
0.01
0.02
0.03
0.04
MR effect size for
'Ulcerative colitis' on 'IDP T1 FAST ROIs L cing gyrus post'

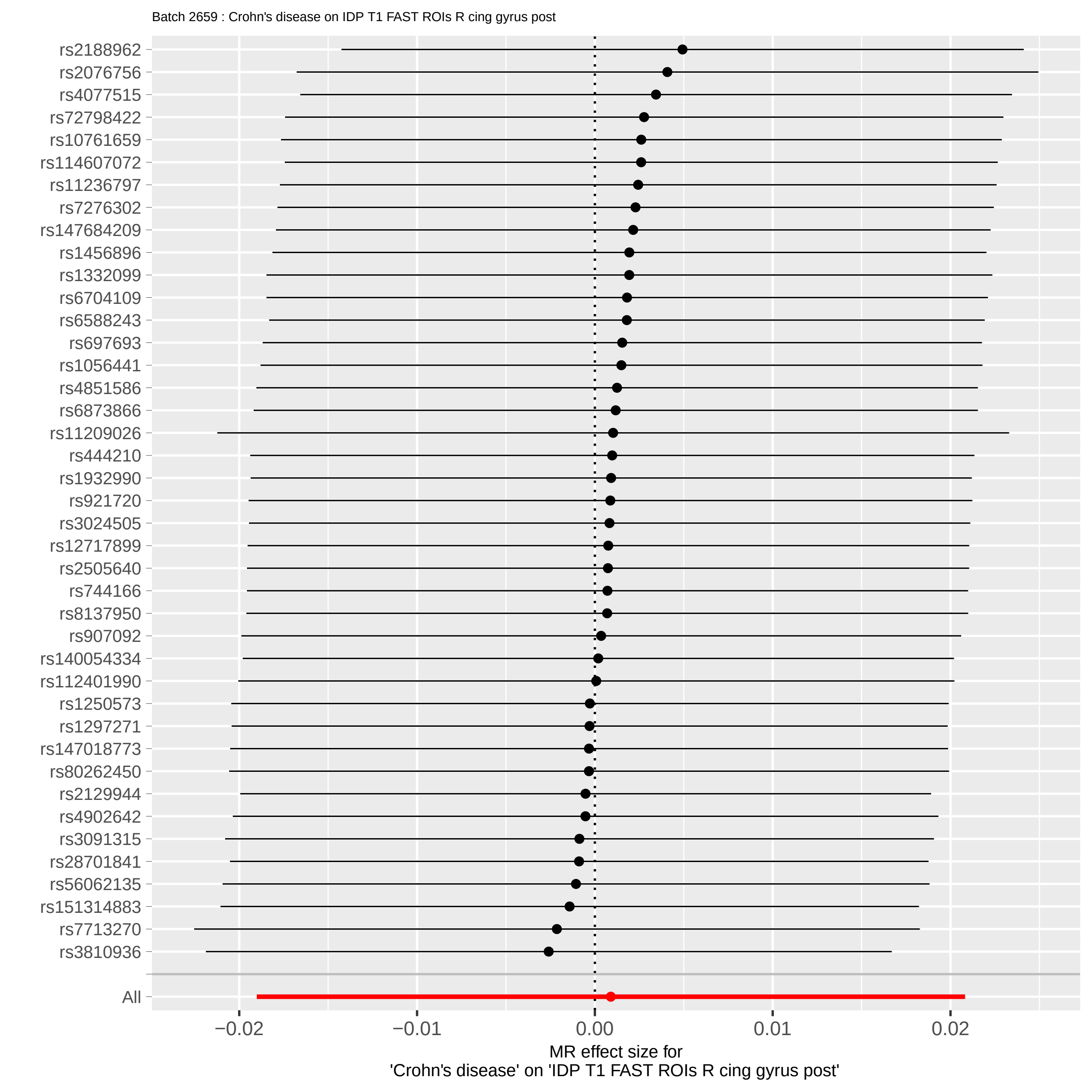

Batch 2659 : Crohn's disease on IDP T1 FAST ROIs R cing gyrus post
rs2188962
rs2076756
rs4077515
rs72798422
rs10761659
rs114607072
rs11236797
rs7276302
rs147684209
rs1456896
rs1332099
rs6704109
rs6588243
rs697693
rs1056441
rs4851586
rs6873866
rs11209026
rs444210
rs1932990
rs921720
rs3024505
rs12717899
rs2505640
rs744166
rs8137950
rs907092
rs140054334
rs112401990
rs1250573
rs1297271
rs147018773
rs80262450
rs2129944
rs4902642
rs3091315
rs28701841
rs56062135
rs151314883
rs7713270
rs3810936
All
−0.02
−0.01
0.00
0.01
0.02
MR effect size for
'Crohn's disease' on 'IDP T1 FAST ROIs R cing gyrus post'

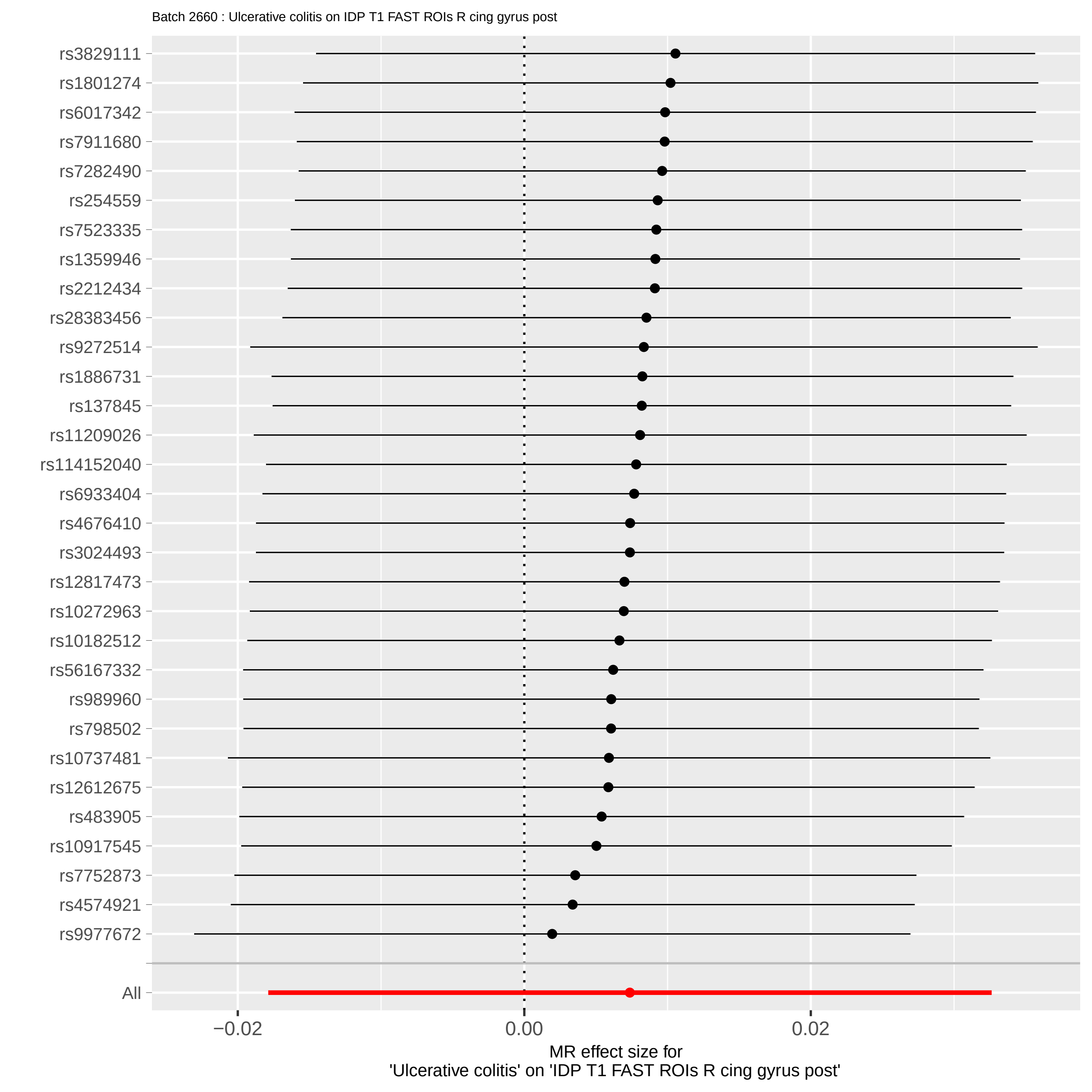
Batch 2660 : Ulcerative colitis on IDP T1 FAST ROIs R cing gyrus post
rs3829111
rs1801274
rs6017342
rs7911680
rs7282490
rs254559
rs7523335
rs1359946
rs2212434
rs28383456
rs9272514
rs1886731
rs137845
rs11209026
rs114152040
rs6933404
rs4676410
rs3024493
rs12817473
rs10272963
rs10182512
rs56167332
rs989960
rs798502
rs10737481
rs12612675
rs483905
rs10917545
rs7752873
rs4574921
rs9977672
All
−0.02
0.00
0.02
MR effect size for
'Ulcerative colitis' on 'IDP T1 FAST ROIs R cing gyrus post'

Batch 2681 : Crohn's disease on IDP T1 FAST ROIs L precun cortex

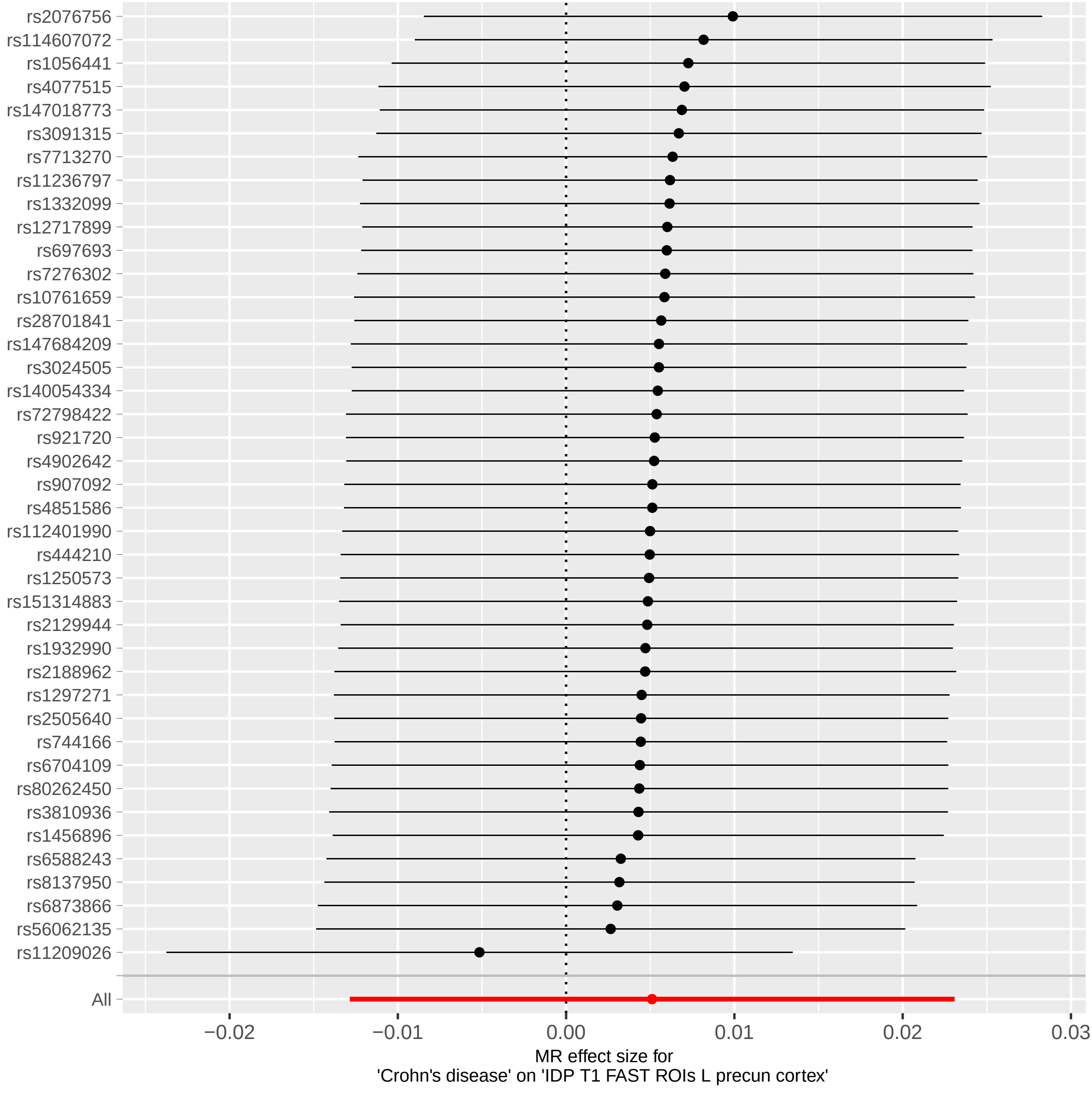

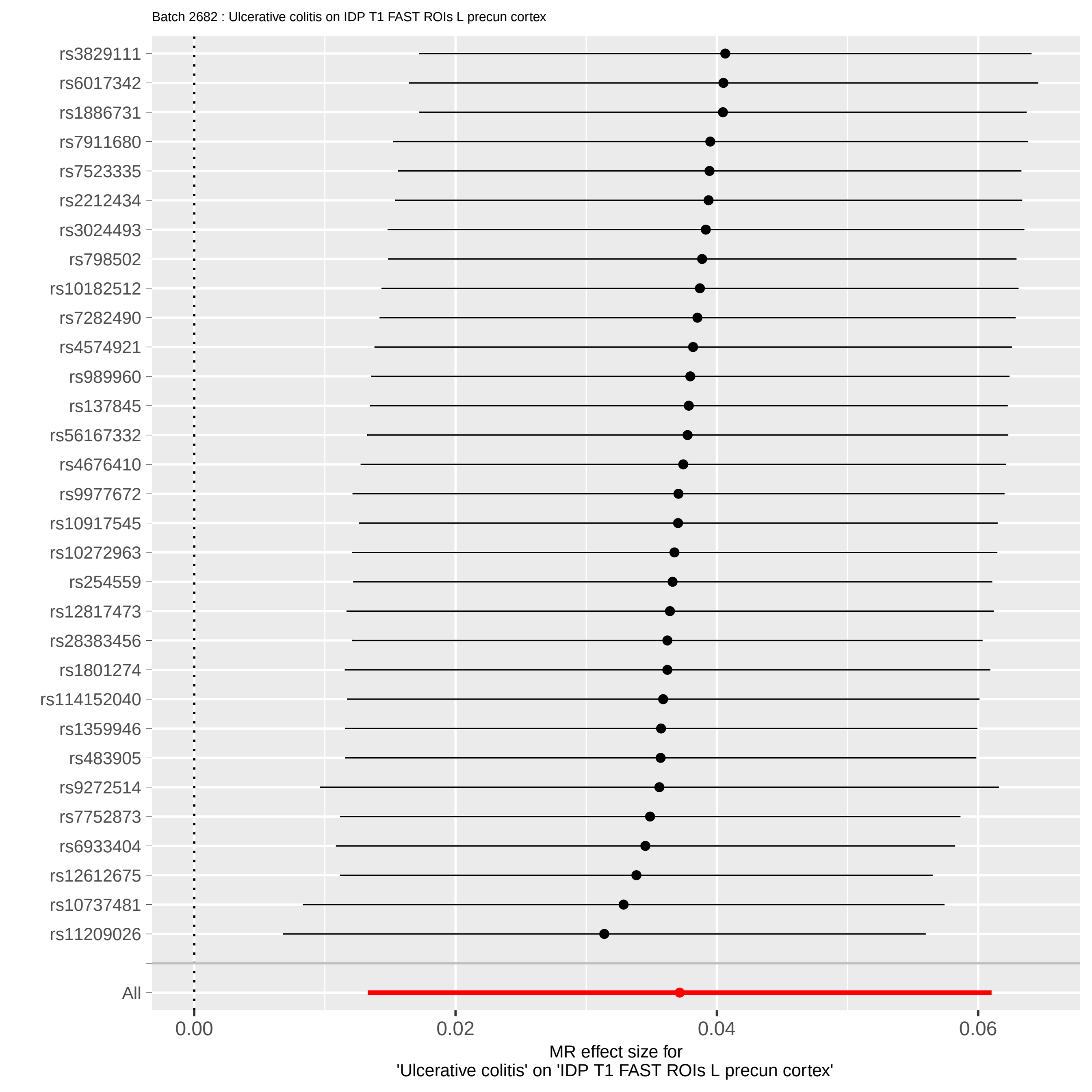
Batch 2682 : Ulcerative colitis on IDP T1 FAST ROIs L precun cortex
rs3829111
rs6017342
rs1886731
rs7911680
rs7523335
rs2212434
rs3024493
rs798502
rs10182512
rs7282490
rs4574921
rs989960
rs137845
rs56167332
rs4676410
rs9977672
rs10917545
rs10272963
rs254559
rs12817473
rs28383456
rs1801274
rs114152040
rs1359946
rs483905
rs9272514
rs7752873
rs6933404
rs12612675
rs10737481
rs11209026
All
0.00
0.02
0.04
0.06
MR effect size for
'Ulcerative colitis' on 'IDP T1 FAST ROIs L precun cortex'

Batch 2703 : Crohn's disease on IDP T1 FAST ROIs R precun cortex

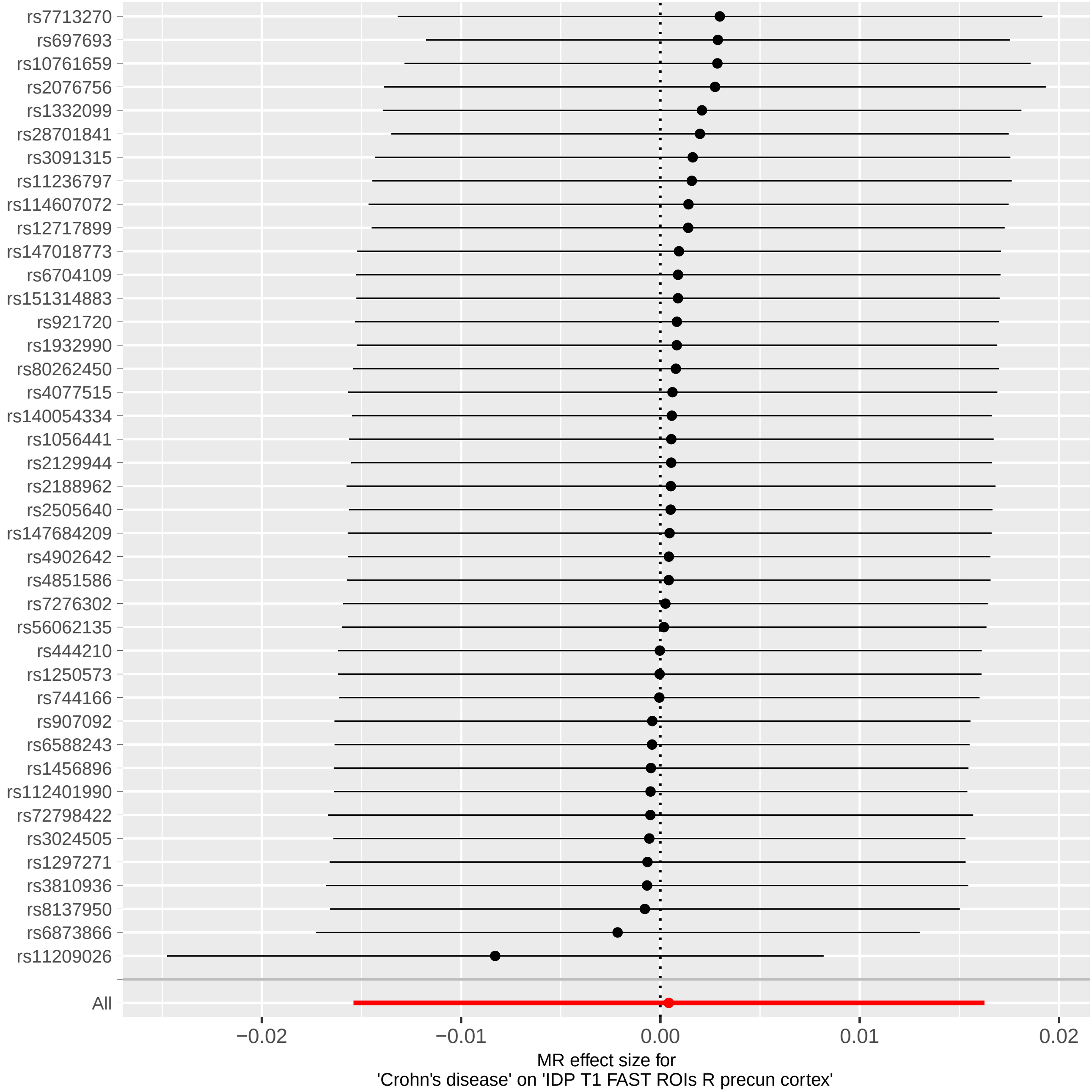

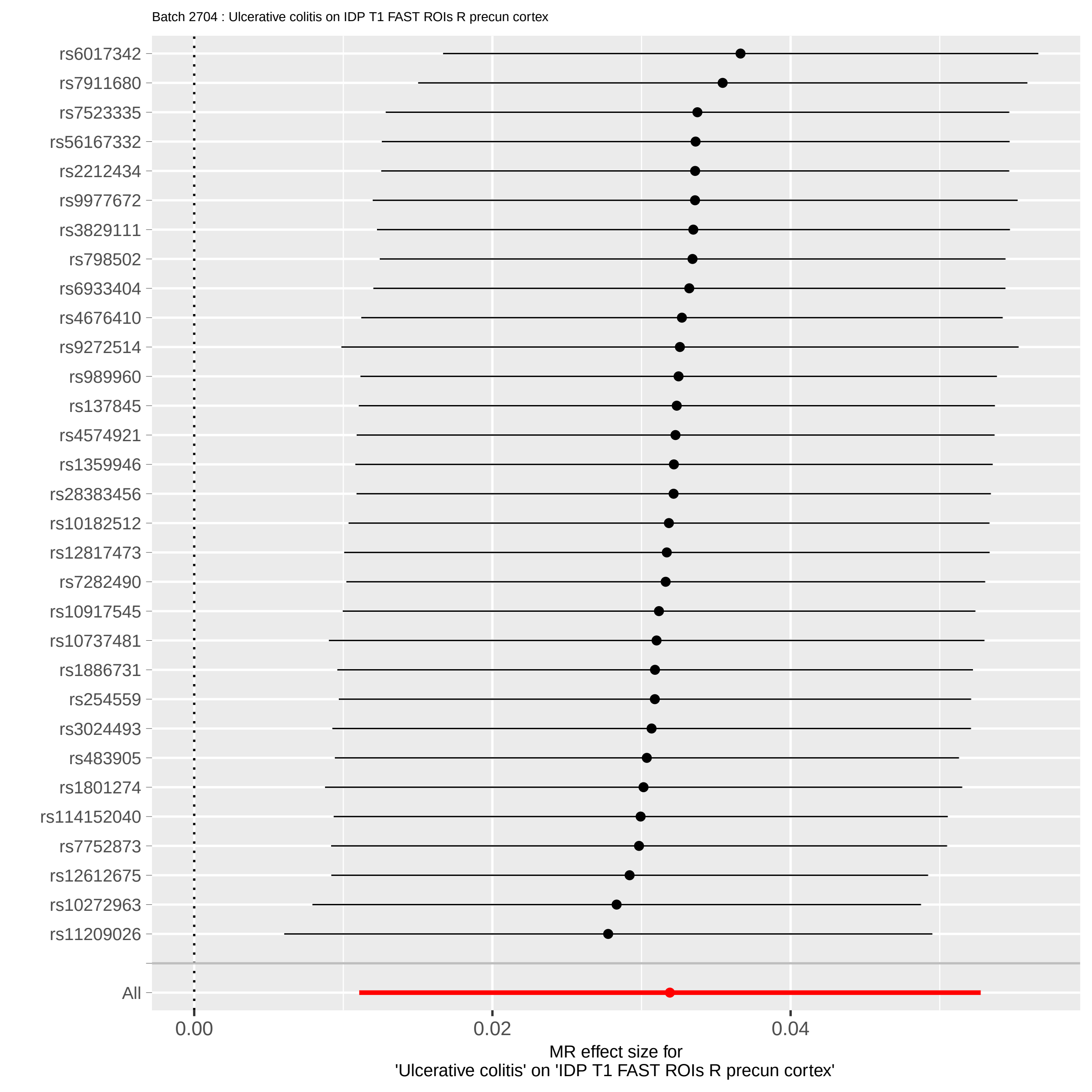

Batch 2704 : Ulcerative colitis on IDP T1 FAST ROIs R precun cortex
rs6017342
rs7911680
rs7523335
rs56167332
rs2212434
rs9977672
rs3829111
rs798502
rs6933404
rs4676410
rs9272514
rs989960
rs137845
rs4574921
rs1359946
rs28383456
rs10182512
rs12817473
rs7282490
rs10917545
rs10737481
rs1886731
rs254559
rs3024493
rs483905
rs1801274
rs114152040
rs7752873
rs12612675
rs10272963
rs11209026
All
0.00
0.02
0.04
MR effect size for
'Ulcerative colitis' on 'IDP T1 FAST ROIs R precun cortex'

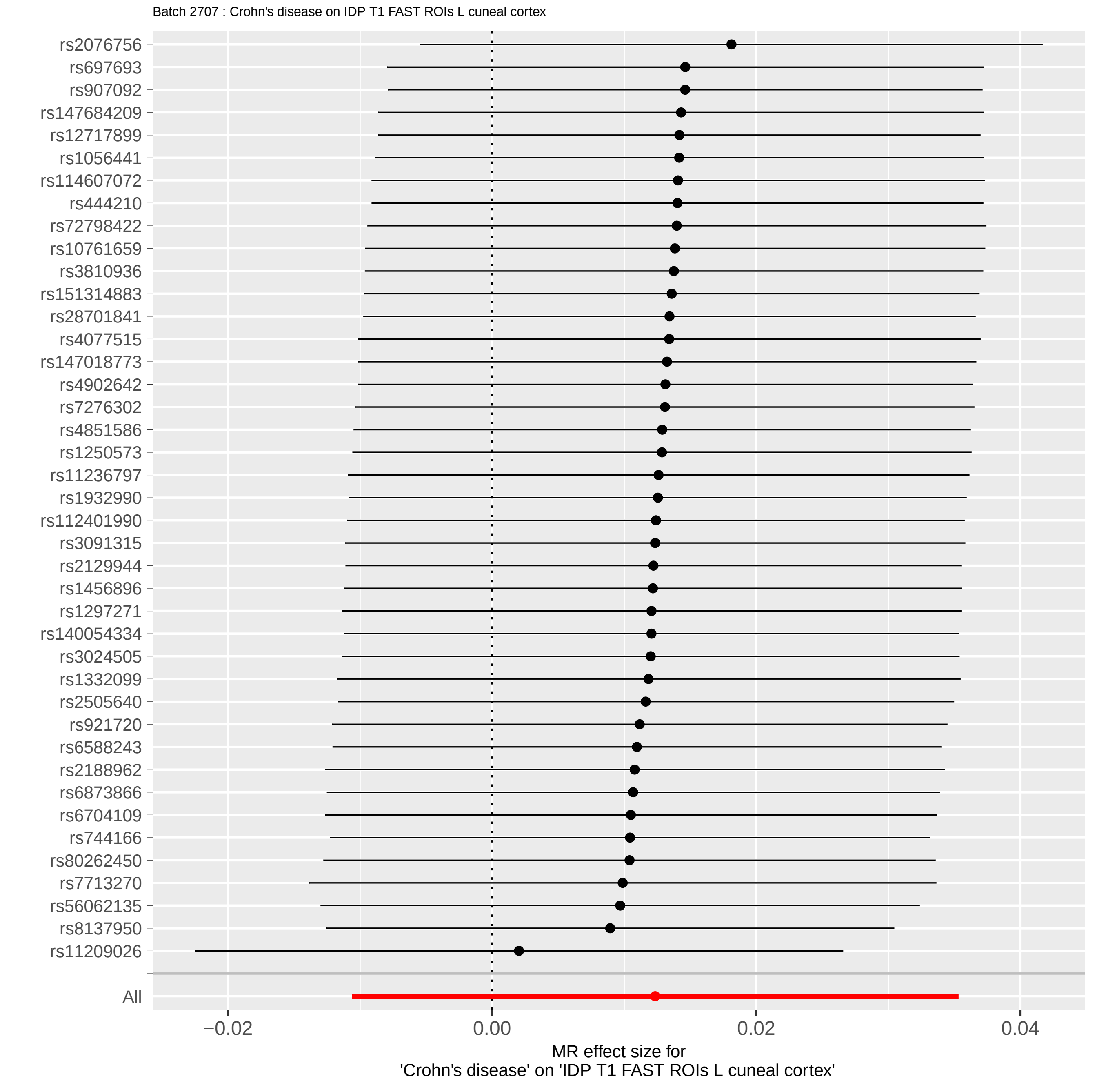

Batch 2707 : Crohn's disease on IDP T1 FAST ROIs L cuneal cortex
rs2076756
rs697693
rs907092
rs147684209
rs12717899
rs1056441
rs114607072
rs444210
rs72798422
rs10761659
rs3810936
rs151314883
rs28701841
rs4077515
rs147018773
rs4902642
rs7276302
rs4851586
rs1250573
rs11236797
rs1932990
rs112401990
rs3091315
rs2129944
rs1456896
rs1297271
rs140054334
rs3024505
rs1332099
rs2505640
rs921720
rs6588243
rs2188962
rs6873866
rs6704109
rs744166
rs80262450
rs7713270
rs56062135
rs8137950
rs11209026
All
−0.02
0.00
0.02
0.04
MR effect size for
'Crohn's disease' on 'IDP T1 FAST ROIs L cuneal cortex'

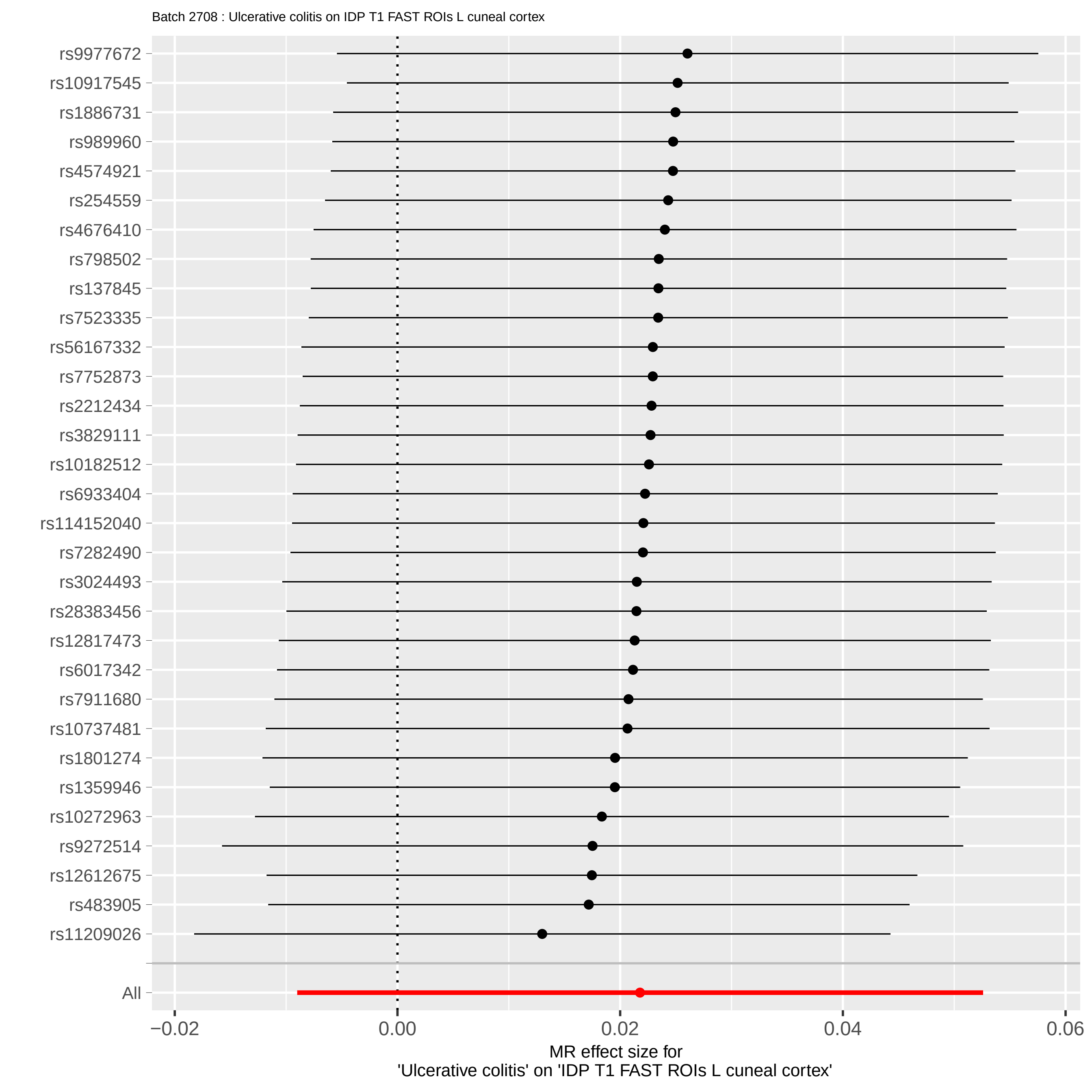

Batch 2708 : Ulcerative colitis on IDP T1 FAST ROIs L cuneal cortex
rs9977672
rs10917545
rs1886731
rs989960
rs4574921
rs254559
rs4676410
rs798502
rs137845
rs7523335
rs56167332
rs7752873
rs2212434
rs3829111
rs10182512
rs6933404
rs114152040
rs7282490
rs3024493
rs28383456
rs12817473
rs6017342
rs7911680
rs10737481
rs1801274
rs1359946
rs10272963
rs9272514
rs12612675
rs483905
rs11209026
All
−0.02
0.00
0.02
0.04
0.06
MR effect size for
'Ulcerative colitis' on 'IDP T1 FAST ROIs L cuneal cortex'

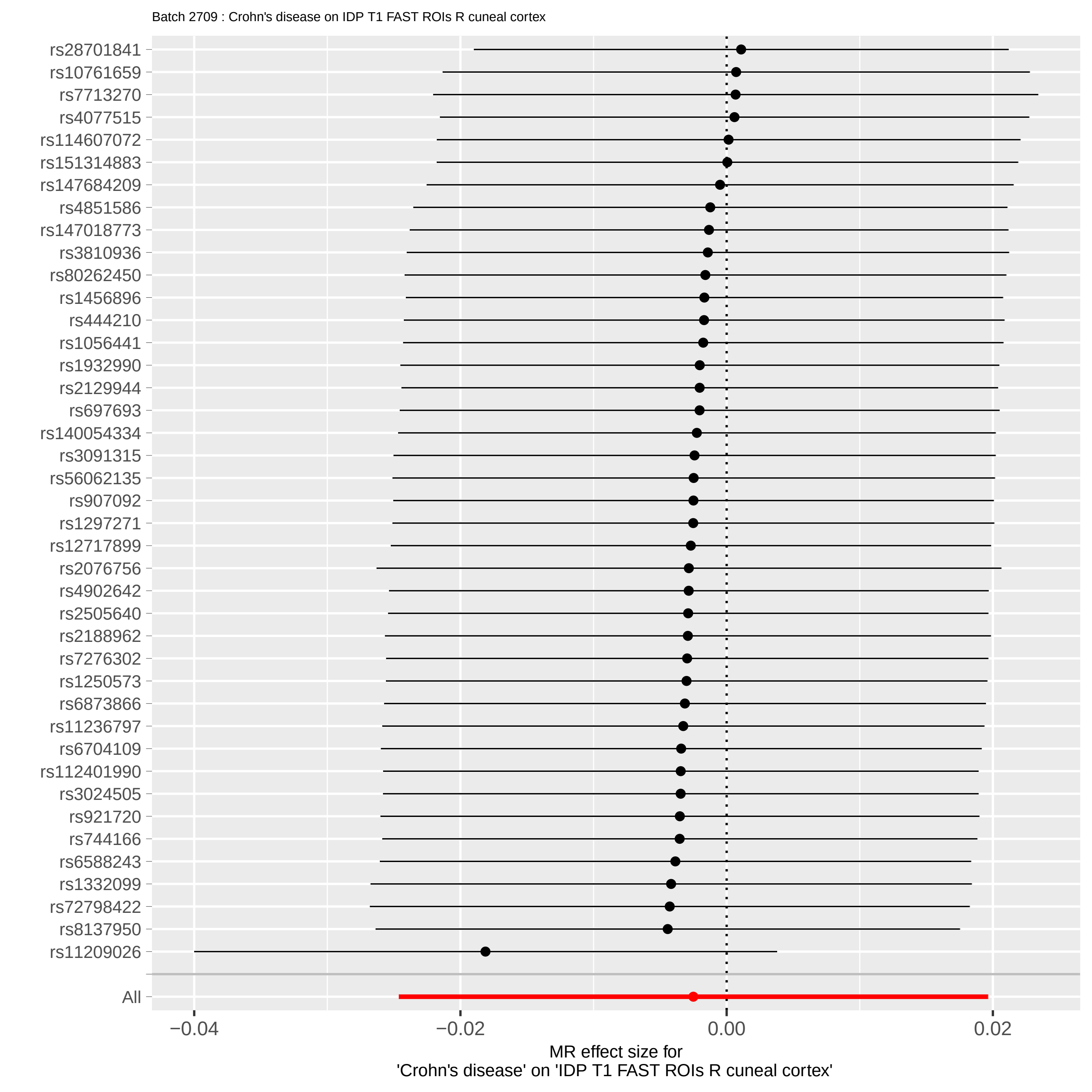

Batch 2709 : Crohn's disease on IDP T1 FAST ROIs R cuneal cortex
rs28701841
rs10761659
rs7713270
rs4077515
rs114607072
rs151314883
rs147684209
rs4851586
rs147018773
rs3810936
rs80262450
rs1456896
rs444210
rs1056441
rs1932990
rs2129944
rs697693
rs140054334
rs3091315
rs56062135
rs907092
rs1297271
rs12717899
rs2076756
rs4902642
rs2505640
rs2188962
rs7276302
rs1250573
rs6873866
rs11236797
rs6704109
rs112401990
rs3024505
rs921720
rs744166
rs6588243
rs1332099
rs72798422
rs8137950
rs11209026
All
−0.04
−0.02
0.00
0.02
MR effect size for
'Crohn's disease' on 'IDP T1 FAST ROIs R cuneal cortex'

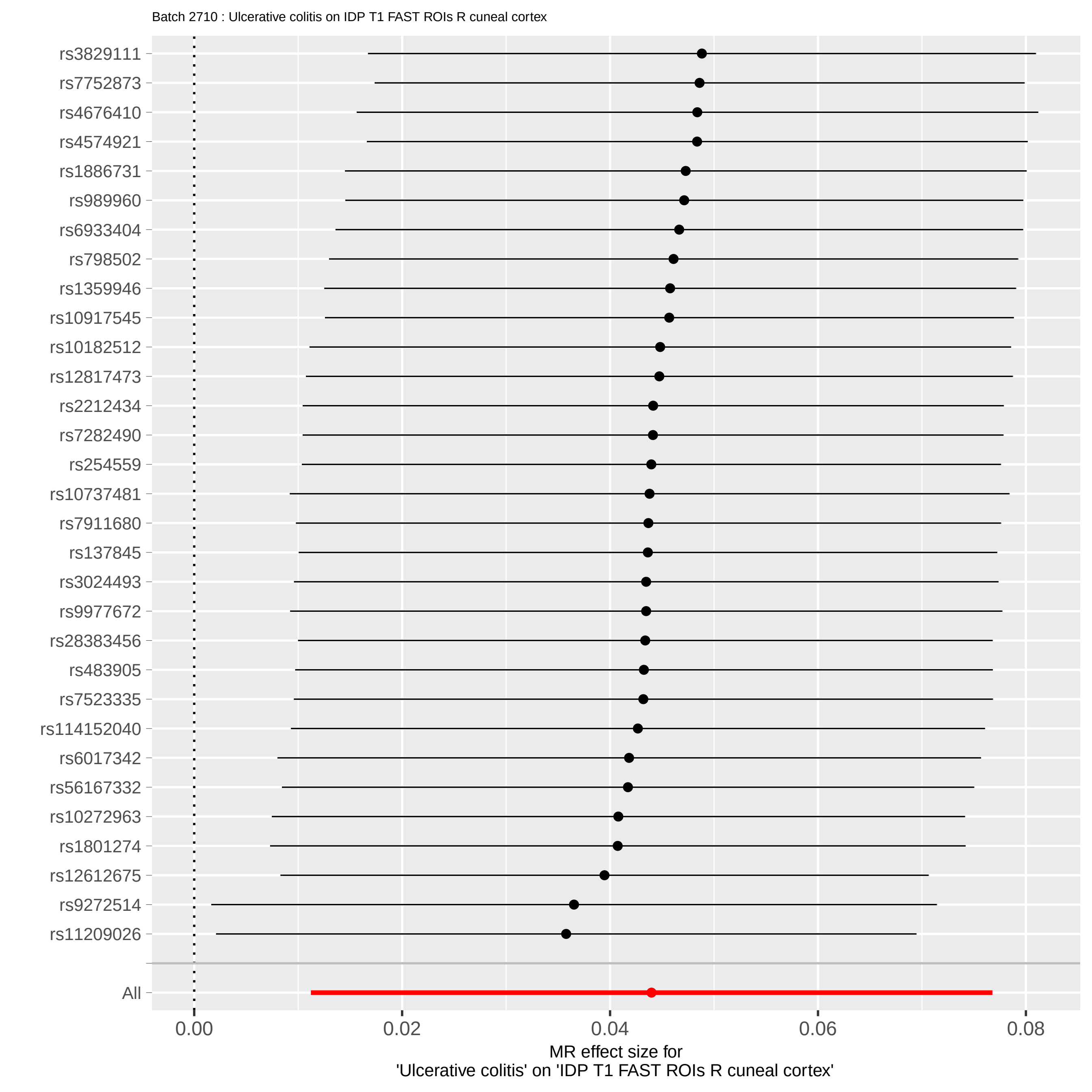
Batch 2710 : Ulcerative colitis on IDP T1 FAST ROIs R cuneal cortex
rs3829111
rs7752873
rs4676410
rs4574921
rs1886731
rs989960
rs6933404
rs798502
rs1359946
rs10917545
rs10182512
rs12817473
rs2212434
rs7282490
rs254559
rs10737481
rs7911680
rs137845
rs3024493
rs9977672
rs28383456
rs483905
rs7523335
rs114152040
rs6017342
rs56167332
rs10272963
rs1801274
rs12612675
rs9272514
rs11209026
All
0.00
0.02
0.04
0.06
0.08
MR effect size for
'Ulcerative colitis' on 'IDP T1 FAST ROIs R cuneal cortex'

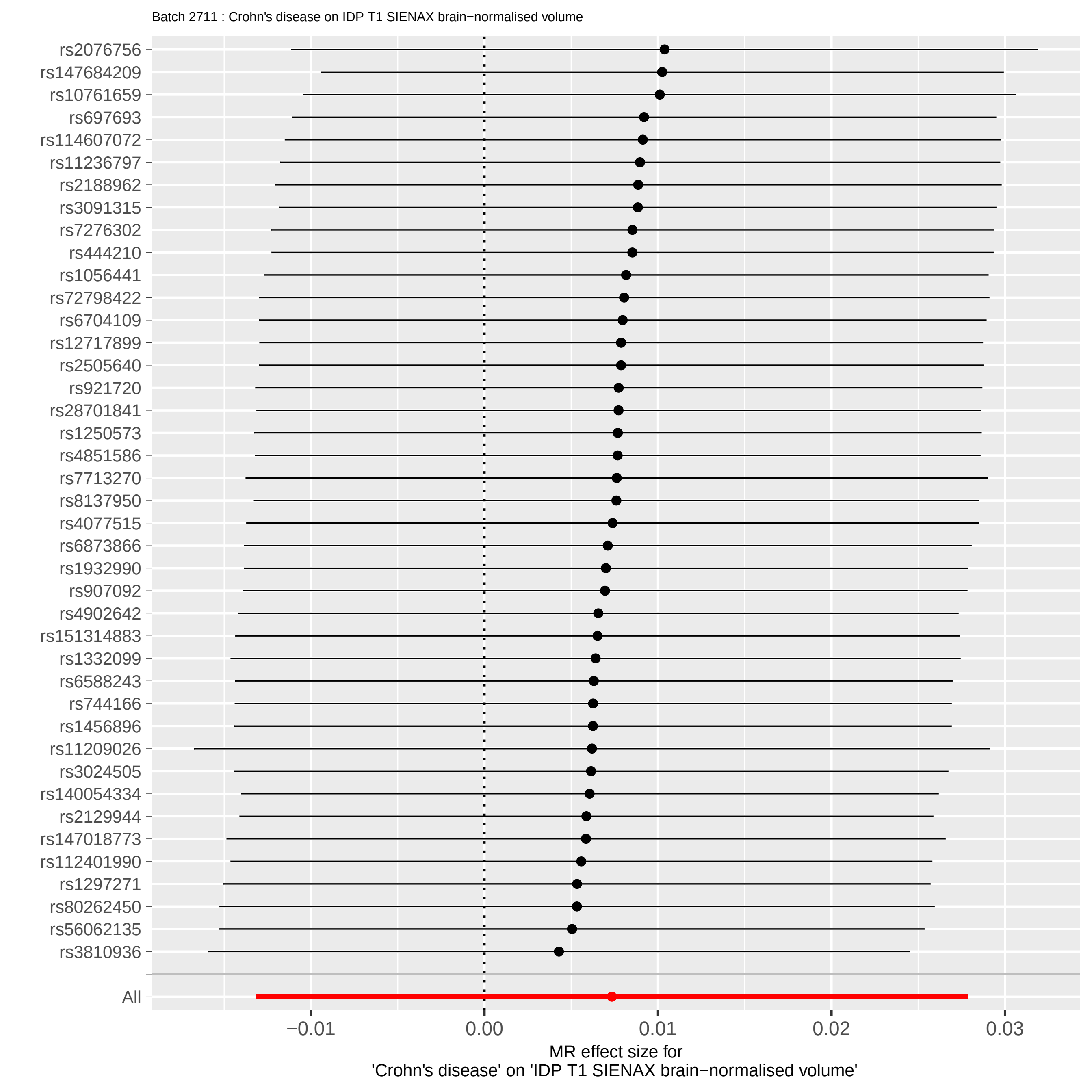
Batch 2711 : Crohn's disease on IDP T1 SIENAX brain−normalised volume
rs2076756
rs147684209
rs10761659
rs697693
rs114607072
rs11236797
rs2188962
rs3091315
rs7276302
rs444210
rs1056441
rs72798422
rs6704109
rs12717899
rs2505640
rs921720
rs28701841
rs1250573
rs4851586
rs7713270
rs8137950
rs4077515
rs6873866
rs1932990
rs907092
rs4902642
rs151314883
rs1332099
rs6588243
rs744166
rs1456896
rs11209026
rs3024505
rs140054334
rs2129944
rs147018773
rs112401990
rs1297271
rs80262450
rs56062135
rs3810936
All
−0.01
0.00
0.01
0.02
0.03
MR effect size for
'Crohn's disease' on 'IDP T1 SIENAX brain−normalised volume'

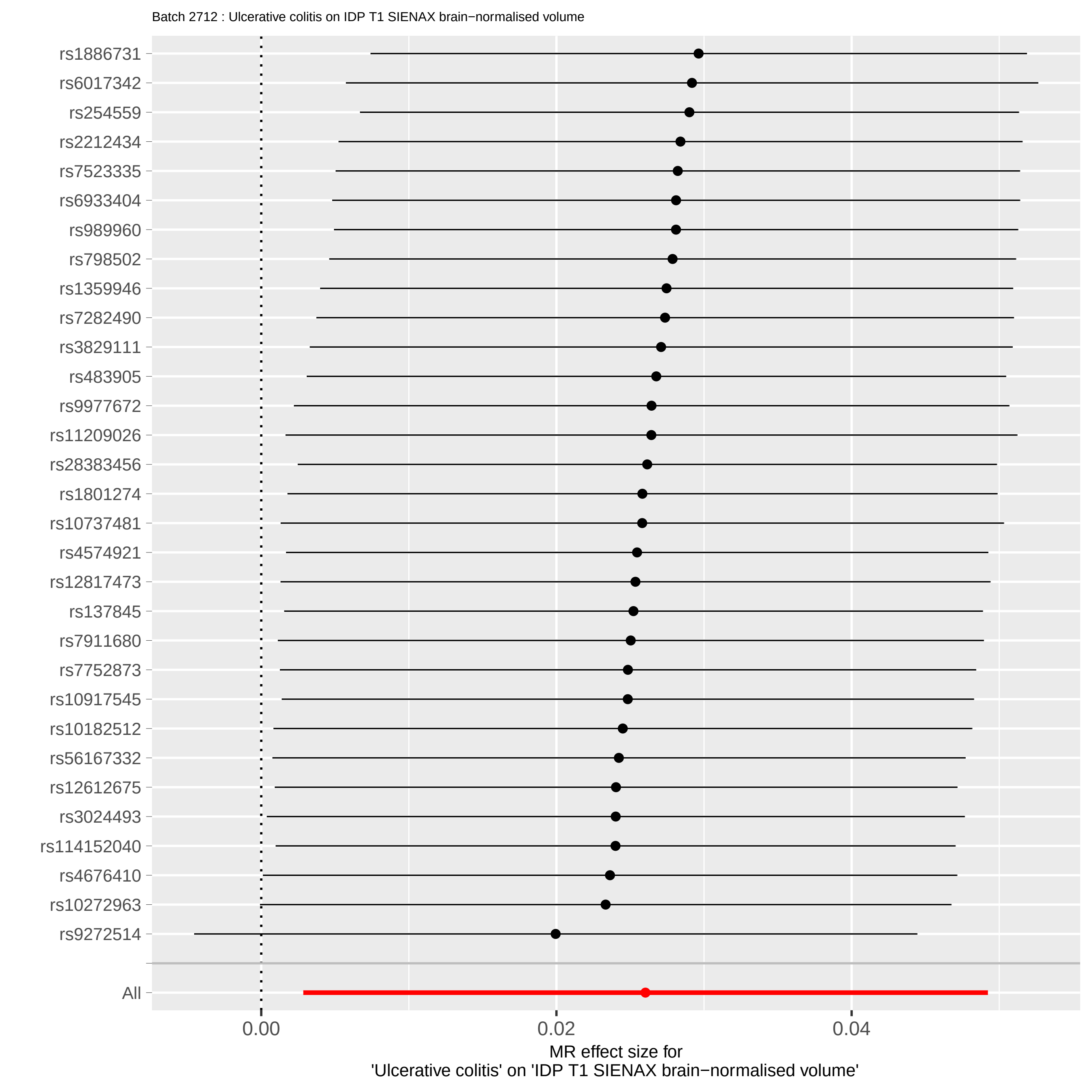

Batch 2712 : Ulcerative colitis on IDP T1 SIENAX brain−normalised volume
rs1886731
rs6017342
rs254559
rs2212434
rs7523335
rs6933404
rs989960
rs798502
rs1359946
rs7282490
rs3829111
rs483905
rs9977672
rs11209026
rs28383456
rs1801274
rs10737481
rs4574921
rs12817473
rs137845
rs7911680
rs7752873
rs10917545
rs10182512
rs56167332
rs12612675
rs3024493
rs114152040
rs4676410
rs10272963
rs9272514
All
0.00
0.02
0.04
MR effect size for
'Ulcerative colitis' on 'IDP T1 SIENAX brain−normalised volume'

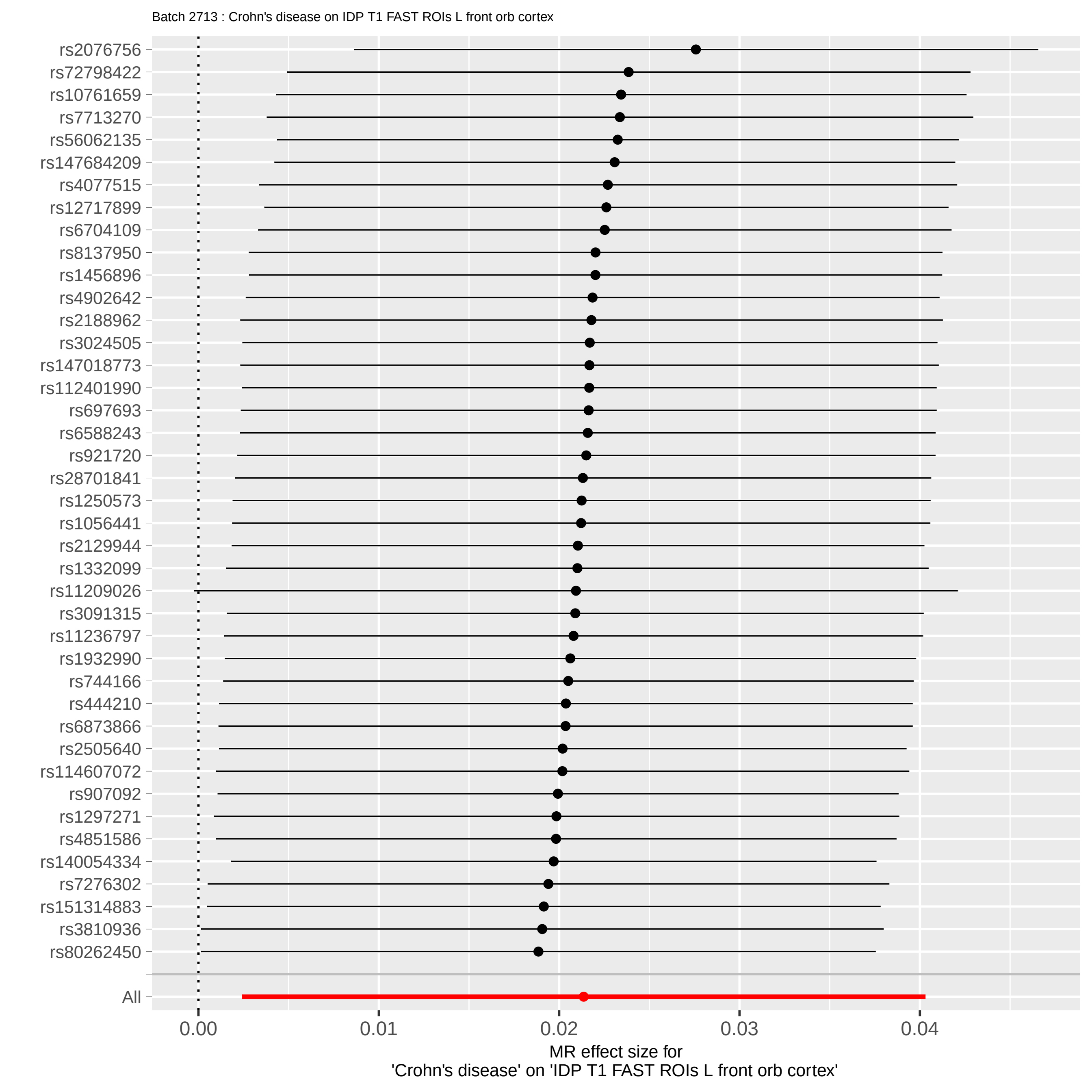

Batch 2713 : Crohn's disease on IDP T1 FAST ROIs L front orb cortex
rs2076756
rs72798422
rs10761659
rs7713270
rs56062135
rs147684209
rs4077515
rs12717899
rs6704109
rs8137950
rs1456896
rs4902642
rs2188962
rs3024505
rs147018773
rs112401990
rs697693
rs6588243
rs921720
rs28701841
rs1250573
rs1056441
rs2129944
rs1332099
rs11209026
rs3091315
rs11236797
rs1932990
rs744166
rs444210
rs6873866
rs2505640
rs114607072
rs907092
rs1297271
rs4851586
rs140054334
rs7276302
rs151314883
rs3810936
rs80262450
All
0.00
0.01
0.02
0.03
0.04
MR effect size for
'Crohn's disease' on 'IDP T1 FAST ROIs L front orb cortex'

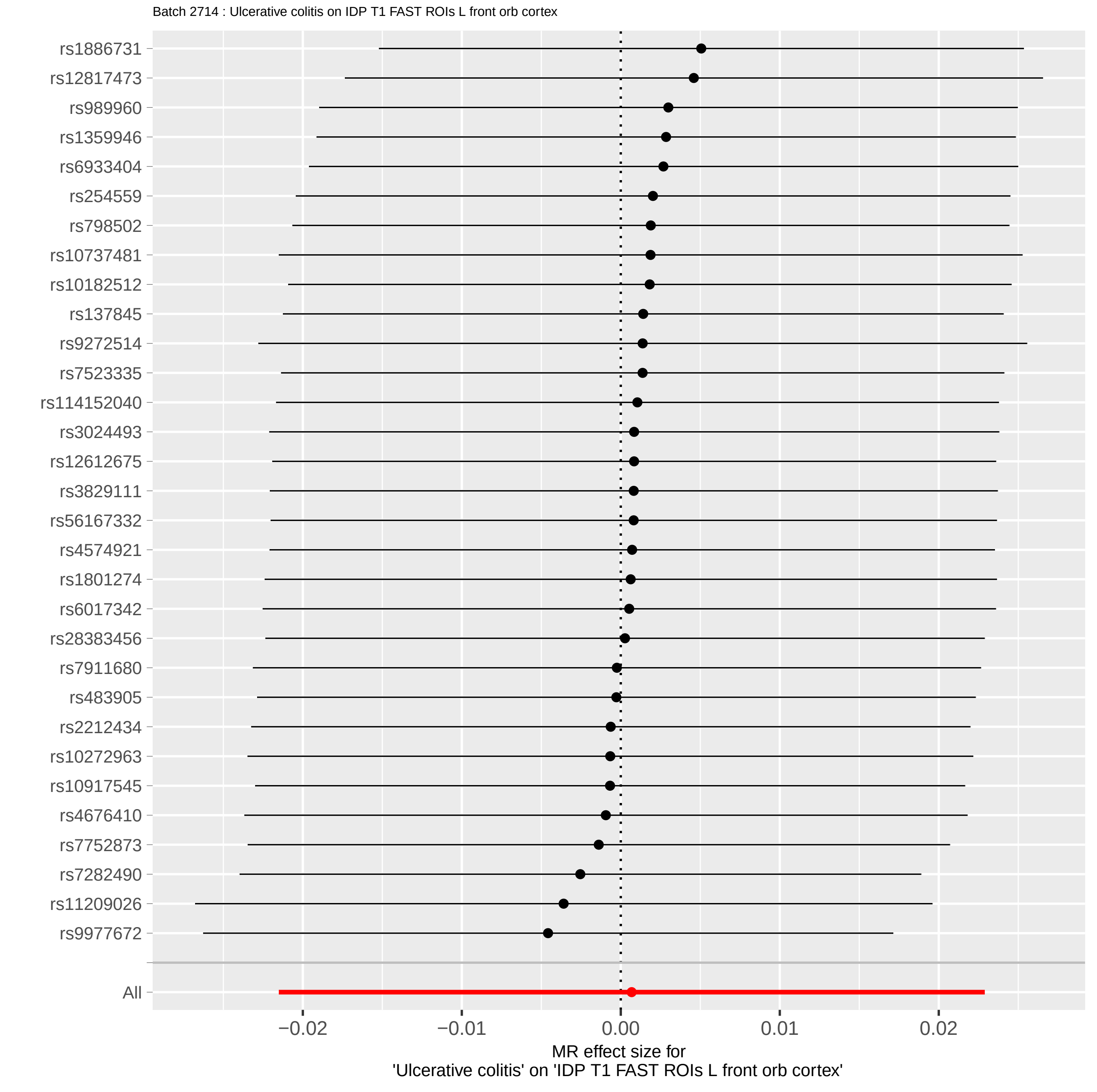

Batch 2714 : Ulcerative colitis on IDP T1 FAST ROIs L front orb cortex
rs1886731
rs12817473
rs989960
rs1359946
rs6933404
rs254559
rs798502
rs10737481
rs10182512
rs137845
rs9272514
rs7523335
rs114152040
rs3024493
rs12612675
rs3829111
rs56167332
rs4574921
rs1801274
rs6017342
rs28383456
rs7911680
rs483905
rs2212434
rs10272963
rs10917545
rs4676410
rs7752873
rs7282490
rs11209026
rs9977672
All
−0.02
−0.01
0.00
0.01
0.02
MR effect size for
'Ulcerative colitis' on 'IDP T1 FAST ROIs L front orb cortex'

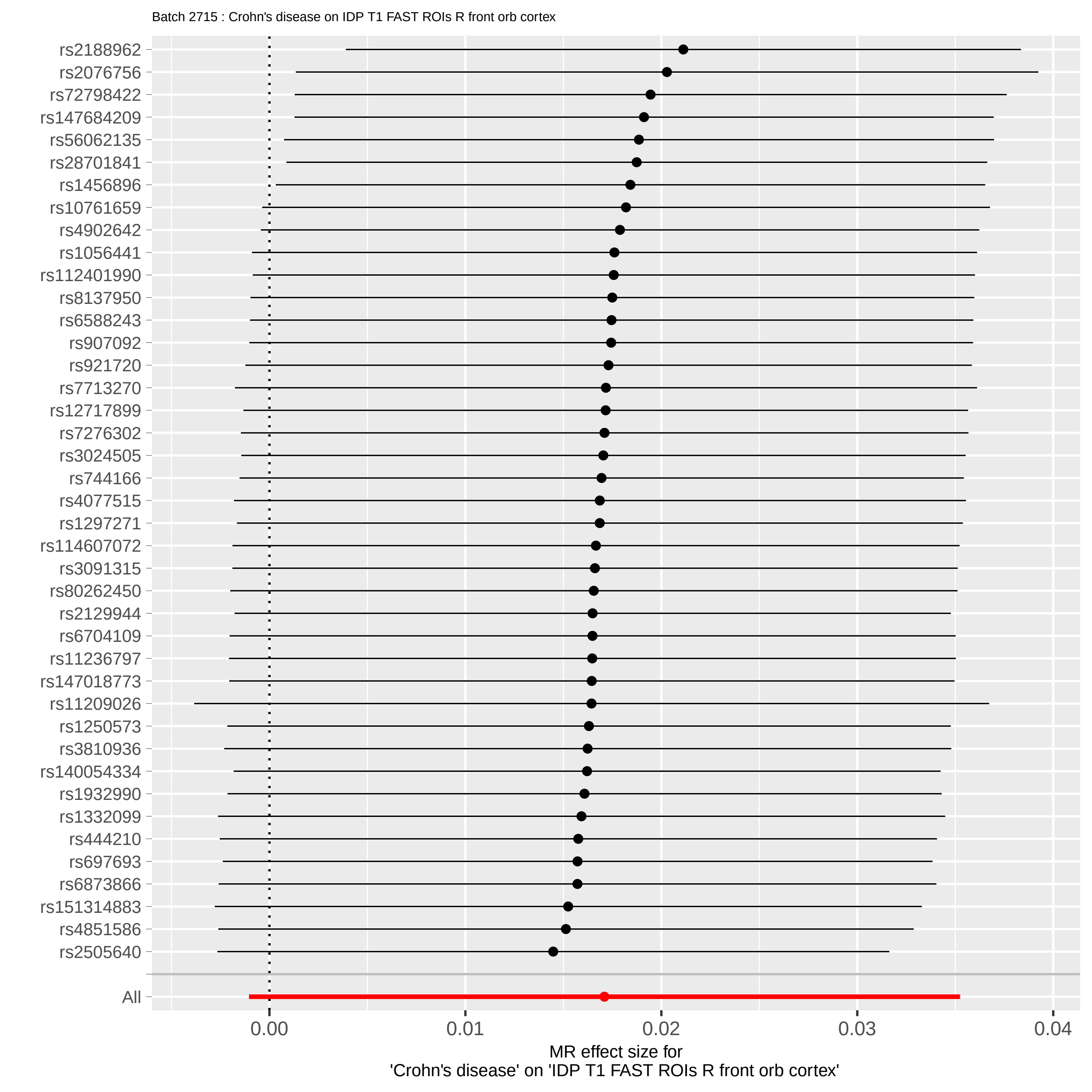

Batch 2715 : Crohn's disease on IDP T1 FAST ROIs R front orb cortex
rs2188962
rs2076756
rs72798422
rs147684209
rs56062135
rs28701841
rs1456896
rs10761659
rs4902642
rs1056441
rs112401990
rs8137950
rs6588243
rs907092
rs921720
rs7713270
rs12717899
rs7276302
rs3024505
rs744166
rs4077515
rs1297271
rs114607072
rs3091315
rs80262450
rs2129944
rs6704109
rs11236797
rs147018773
rs11209026
rs1250573
rs3810936
rs140054334
rs1932990
rs1332099
rs444210
rs697693
rs6873866
rs151314883
rs4851586
rs2505640
All
0.00
0.01
0.02
0.03
0.04
MR effect size for
'Crohn's disease' on 'IDP T1 FAST ROIs R front orb cortex'

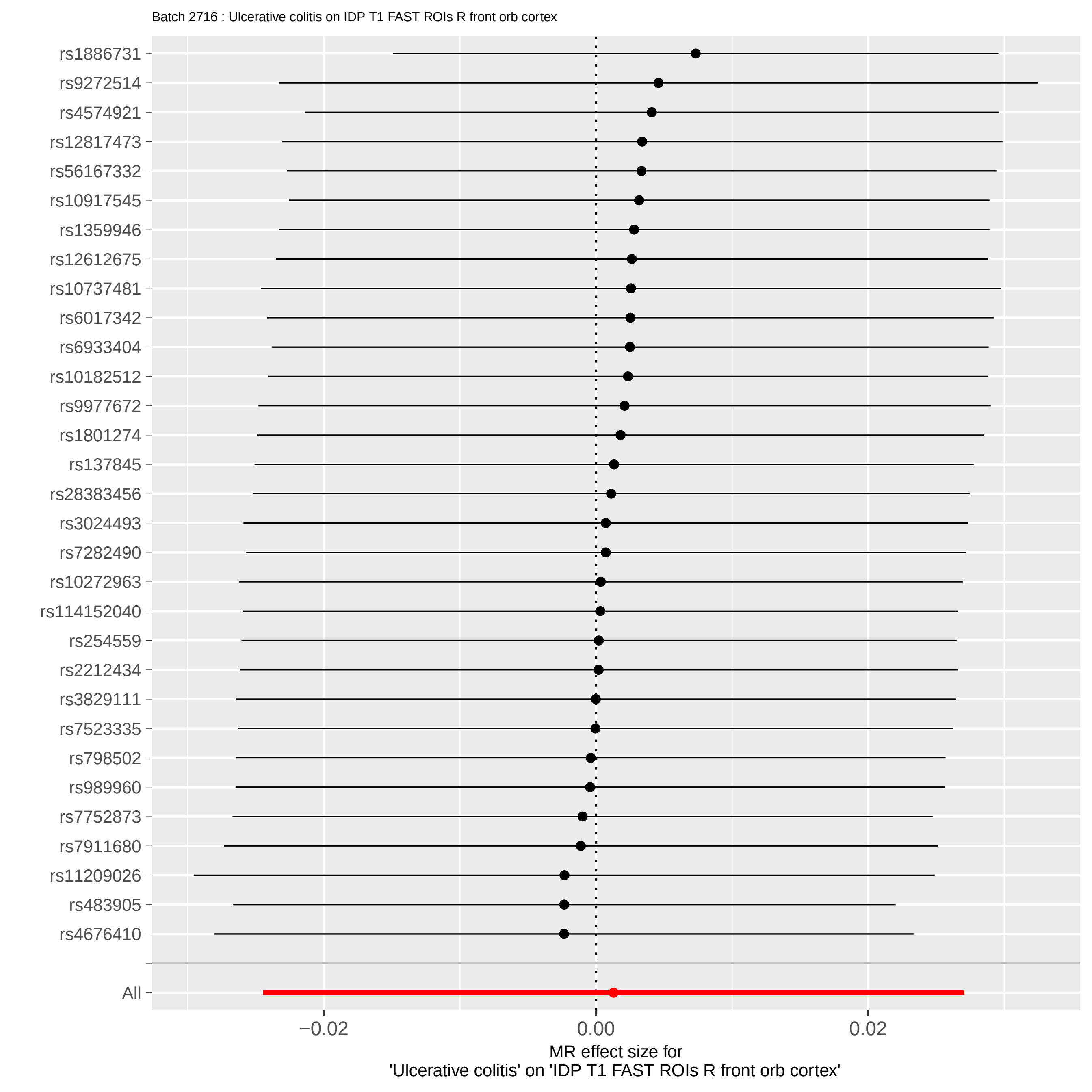

Batch 2716 : Ulcerative colitis on IDP T1 FAST ROIs R front orb cortex
rs1886731
rs9272514
rs4574921
rs12817473
rs56167332
rs10917545
rs1359946
rs12612675
rs10737481
rs6017342
rs6933404
rs10182512
rs9977672
rs1801274
rs137845
rs28383456
rs3024493
rs7282490
rs10272963
rs114152040
rs254559
rs2212434
rs3829111
rs7523335
rs798502
rs989960
rs7752873
rs7911680
rs11209026
rs483905
rs4676410
All
−0.02
0.00
0.02
MR effect size for
'Ulcerative colitis' on 'IDP T1 FAST ROIs R front orb cortex'

Batch 2717 : Crohn's disease on IDP T1 FAST ROIs L parahipp gyrus ant

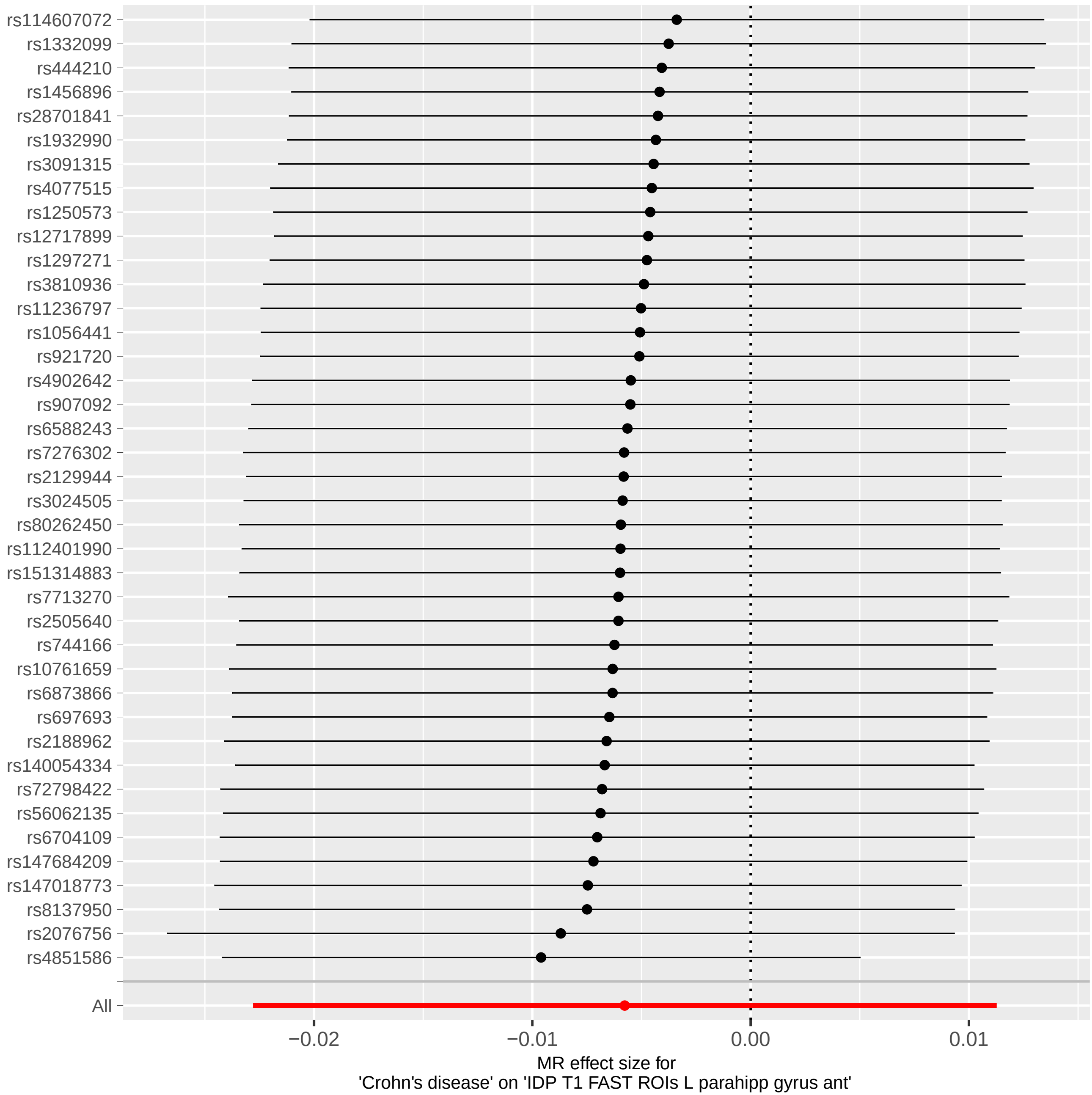

Batch 2718 : Ulcerative colitis on IDP T1 FAST ROIs L parahipp gyrus ant

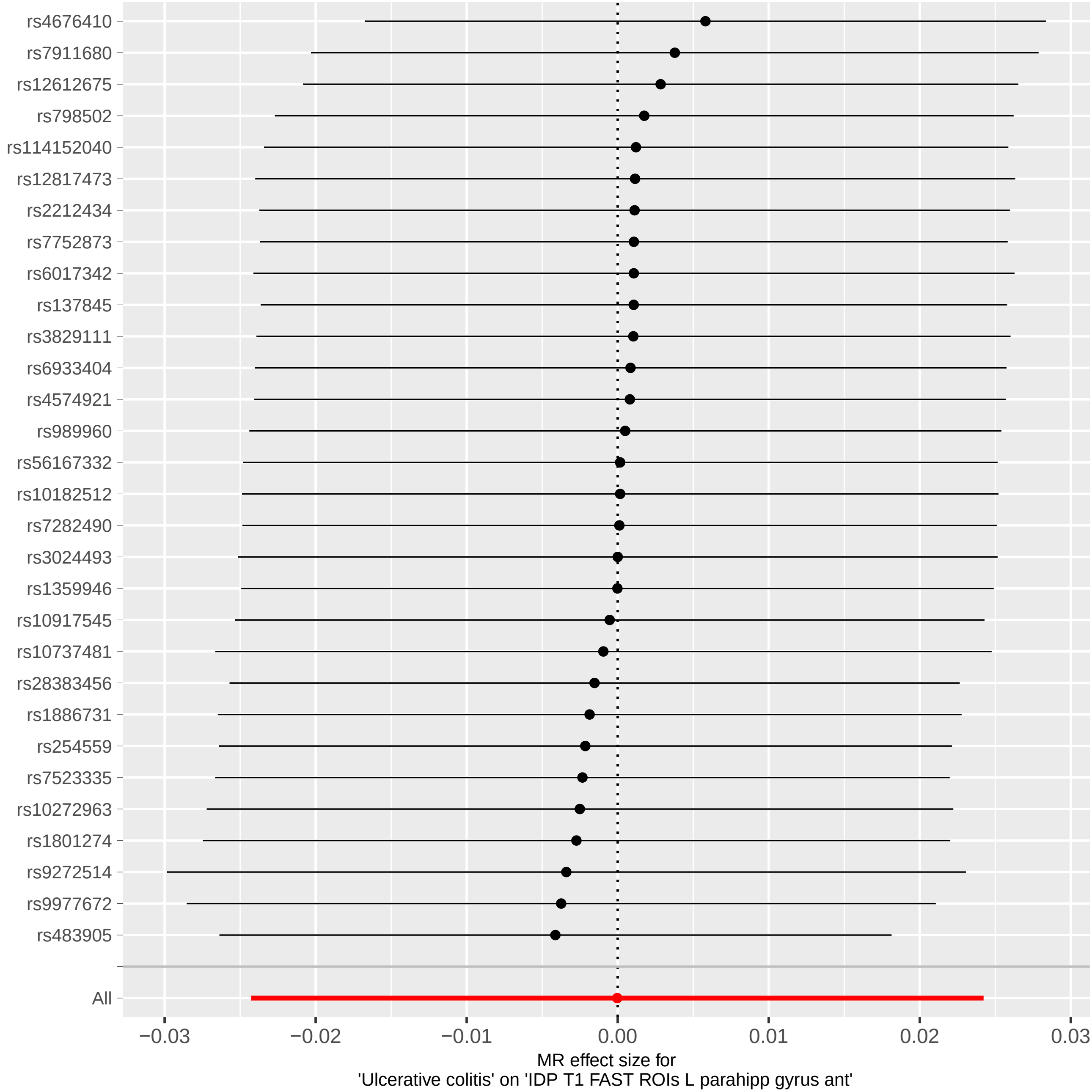

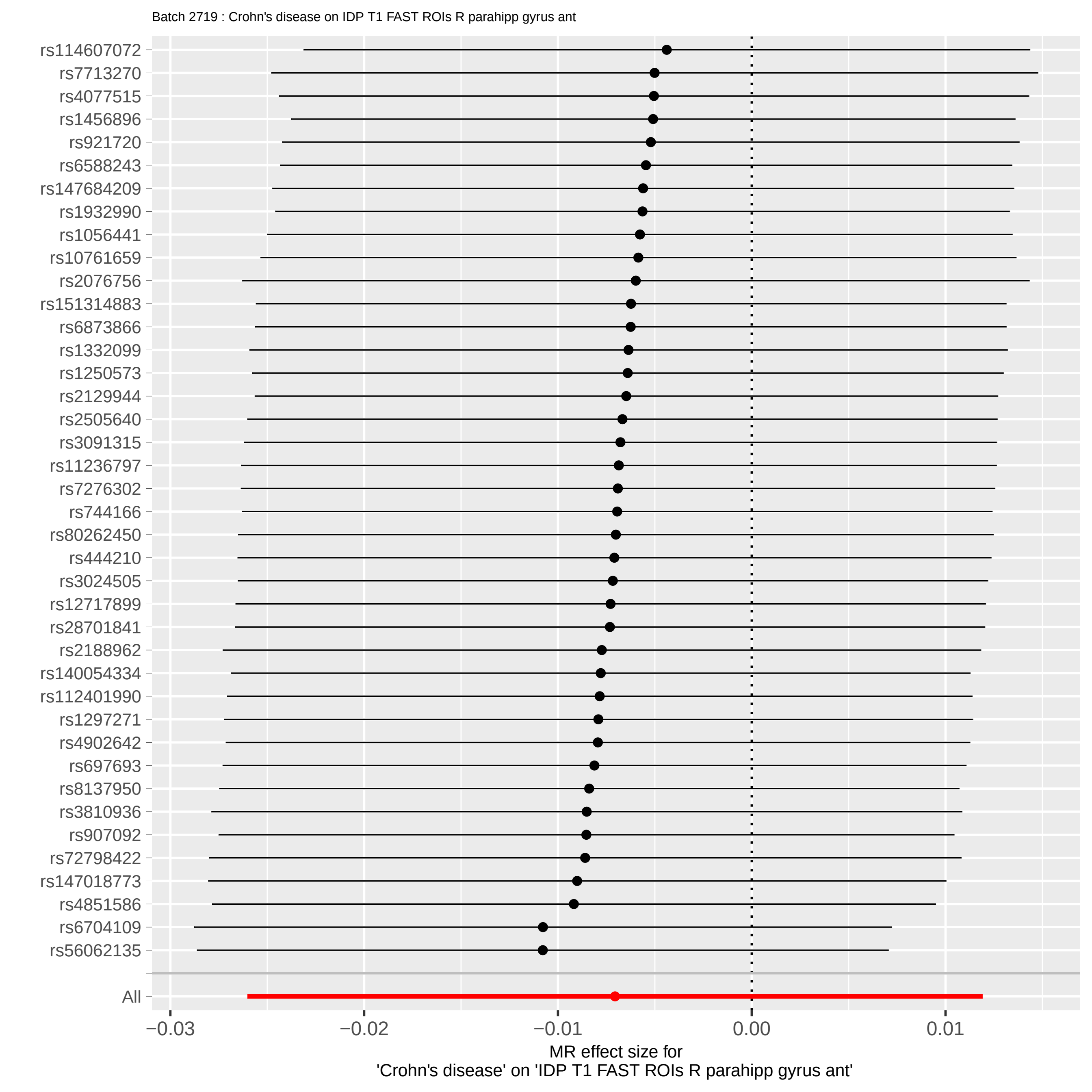
Batch 2719 : Crohn's disease on IDP T1 FAST ROIs R parahipp gyrus ant
rs114607072
rs7713270
rs4077515
rs1456896
rs921720
rs6588243
rs147684209
rs1932990
rs1056441
rs10761659
rs2076756
rs151314883
rs6873866
rs1332099
rs1250573
rs2129944
rs2505640
rs3091315
rs11236797
rs7276302
rs744166
rs80262450
rs444210
rs3024505
rs12717899
rs28701841
rs2188962
rs140054334
rs112401990
rs1297271
rs4902642
rs697693
rs8137950
rs3810936
rs907092
rs72798422
rs147018773
rs4851586
rs6704109
rs56062135
All
−0.03
−0.02
−0.01
0.00
0.01
MR effect size for
'Crohn's disease' on 'IDP T1 FAST ROIs R parahipp gyrus ant'

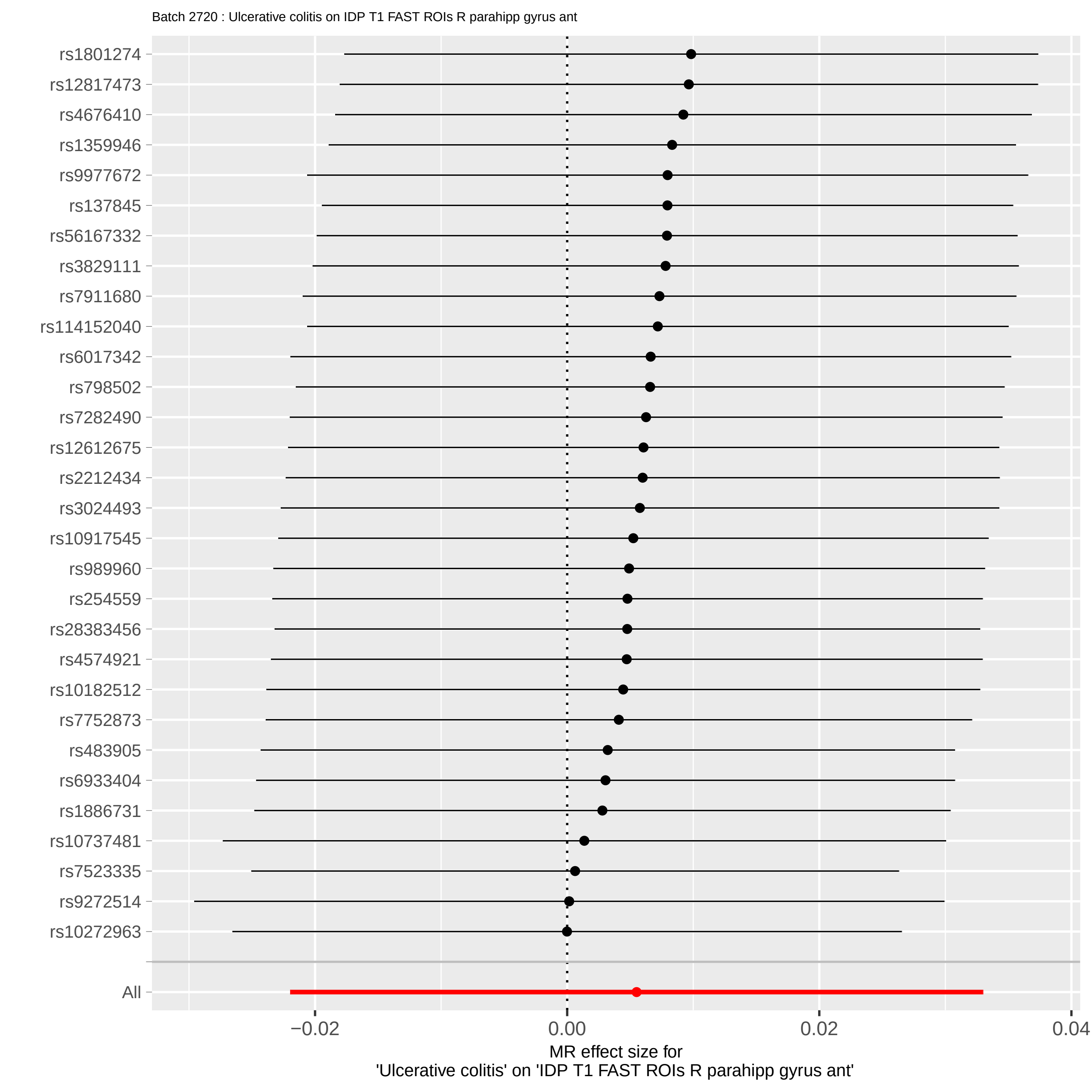
Batch 2720 : Ulcerative colitis on IDP T1 FAST ROIs R parahipp gyrus ant
rs1801274
rs12817473
rs4676410
rs1359946
rs9977672
rs137845
rs56167332
rs3829111
rs7911680
rs114152040
rs6017342
rs798502
rs7282490
rs12612675
rs2212434
rs3024493
rs10917545
rs989960
rs254559
rs28383456
rs4574921
rs10182512
rs7752873
rs483905
rs6933404
rs1886731
rs10737481
rs7523335
rs9272514
rs10272963
All
−0.02
0.00
0.02
0.04
MR effect size for
'Ulcerative colitis' on 'IDP T1 FAST ROIs R parahipp gyrus ant'

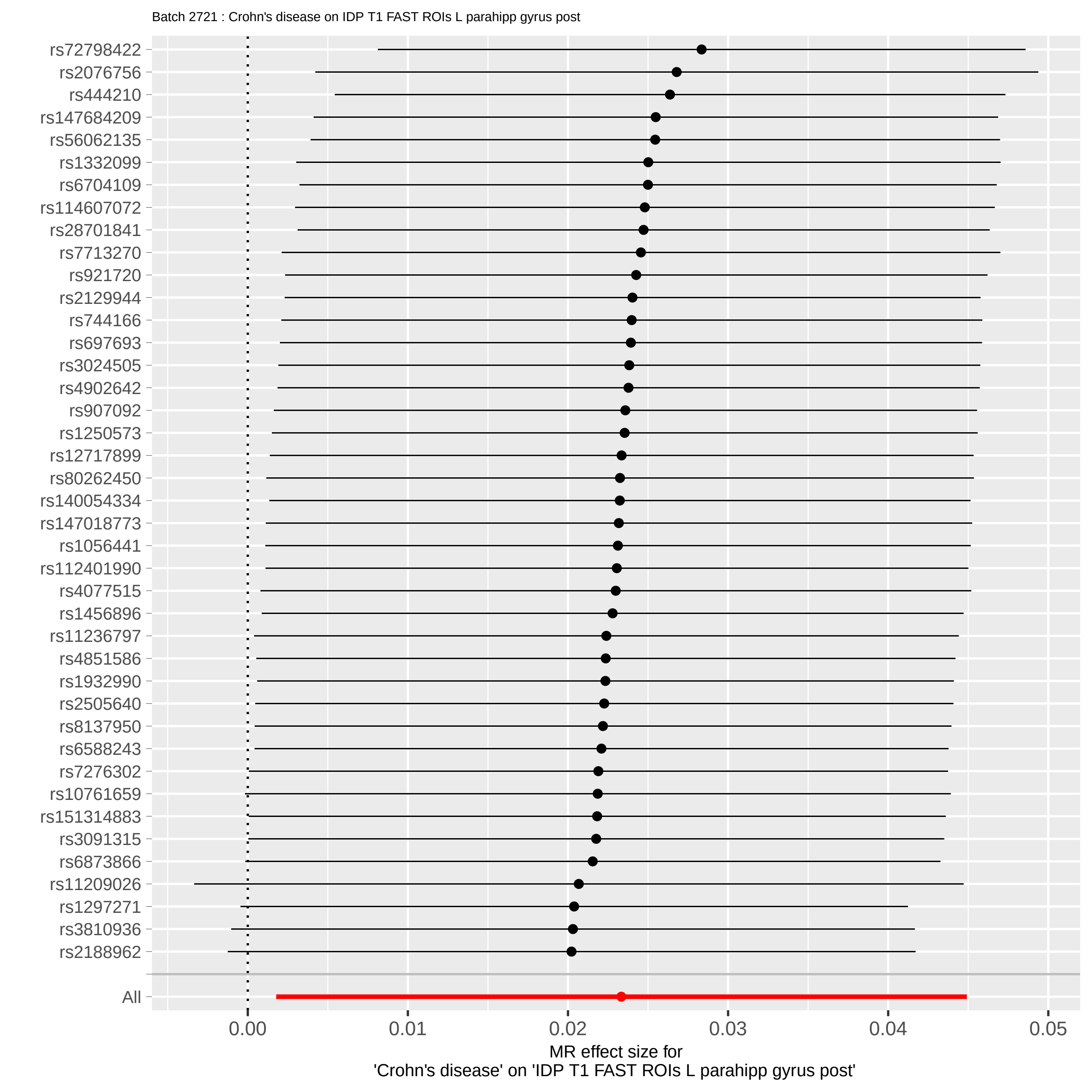

Batch 2721 : Crohn's disease on IDP T1 FAST ROIs L parahipp gyrus post
rs72798422
rs2076756
rs444210
rs147684209
rs56062135
rs1332099
rs6704109
rs114607072
rs28701841
rs7713270
rs921720
rs2129944
rs744166
rs697693
rs3024505
rs4902642
rs907092
rs1250573
rs12717899
rs80262450
rs140054334
rs147018773
rs1056441
rs112401990
rs4077515
rs1456896
rs11236797
rs4851586
rs1932990
rs2505640
rs8137950
rs6588243
rs7276302
rs10761659
rs151314883
rs3091315
rs6873866
rs11209026
rs1297271
rs3810936
rs2188962
All
0.00
0.01
0.02
0.03
0.04
0.05
MR effect size for
'Crohn's disease' on 'IDP T1 FAST ROIs L parahipp gyrus post'

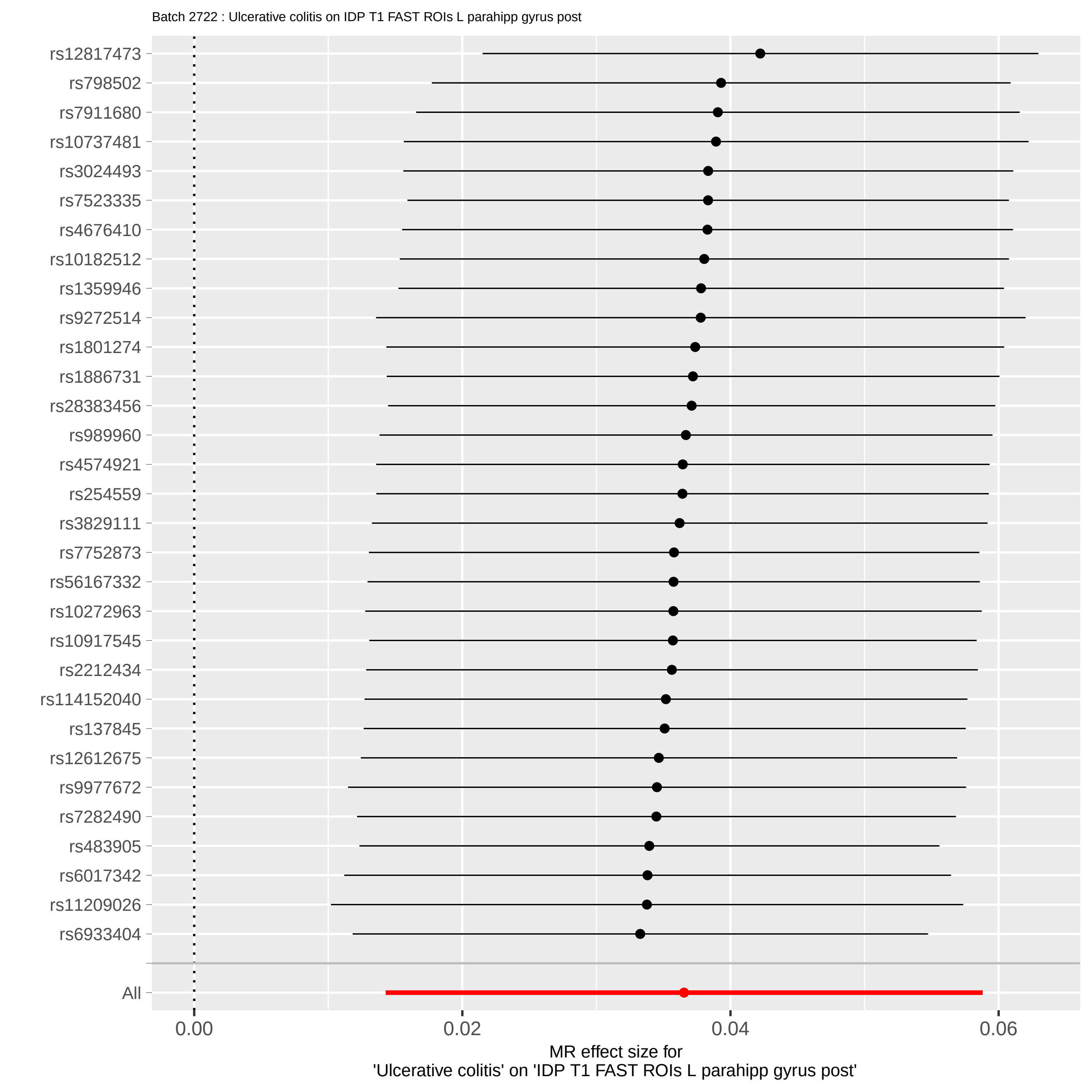

Batch 2722 : Ulcerative colitis on IDP T1 FAST ROIs L parahipp gyrus post
rs12817473
rs798502
rs7911680
rs10737481
rs3024493
rs7523335
rs4676410
rs10182512
rs1359946
rs9272514
rs1801274
rs1886731
rs28383456
rs989960
rs4574921
rs254559
rs3829111
rs7752873
rs56167332
rs10272963
rs10917545
rs2212434
rs114152040
rs137845
rs12612675
rs9977672
rs7282490
rs483905
rs6017342
rs11209026
rs6933404
All
0.00
0.02
0.04
0.06
MR effect size for
'Ulcerative colitis' on 'IDP T1 FAST ROIs L parahipp gyrus post'

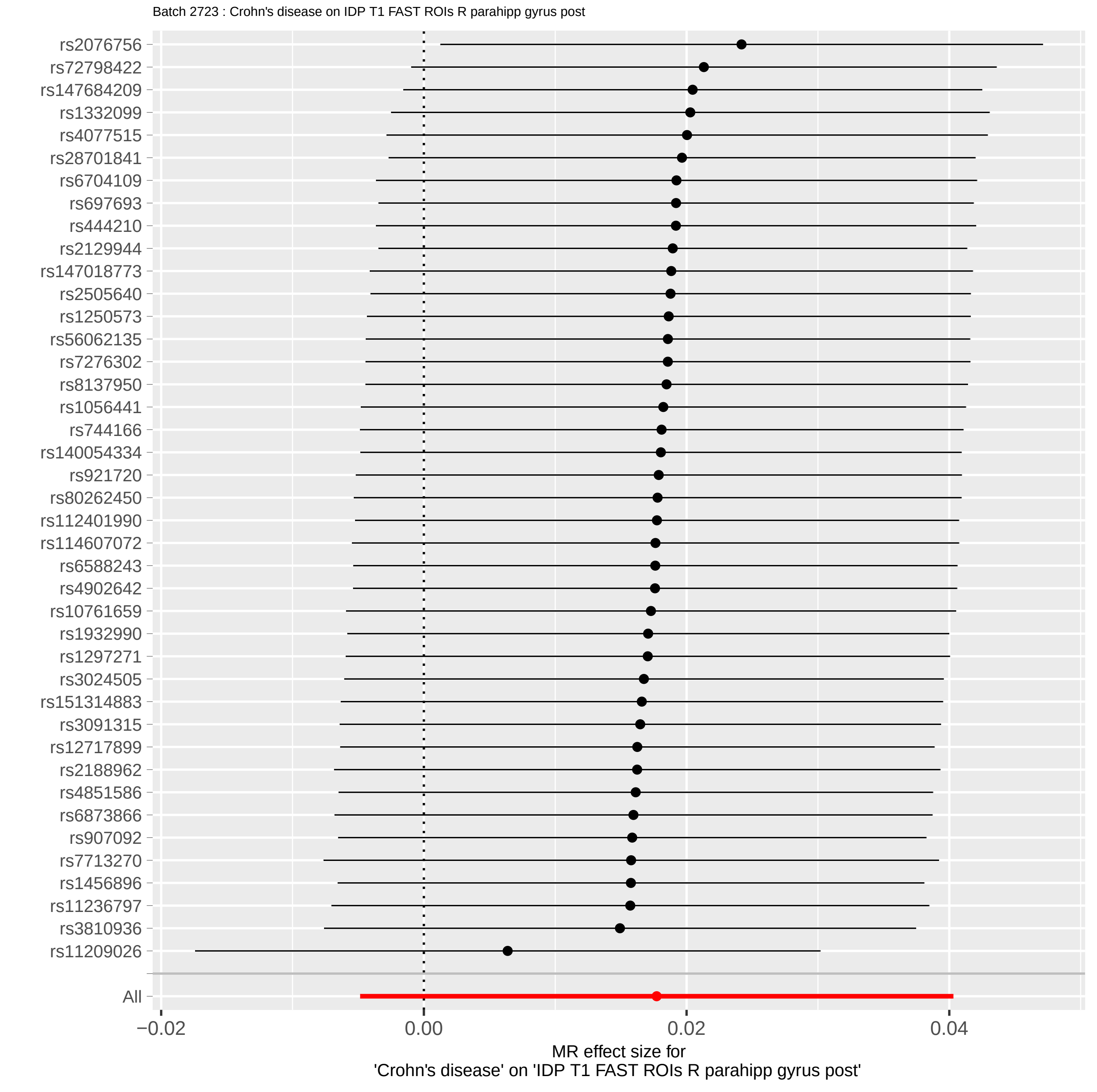

Batch 2723 : Crohn's disease on IDP T1 FAST ROIs R parahipp gyrus post
rs2076756
rs72798422
rs147684209
rs1332099
rs4077515
rs28701841
rs6704109
rs697693
rs444210
rs2129944
rs147018773
rs2505640
rs1250573
rs56062135
rs7276302
rs8137950
rs1056441
rs744166
rs140054334
rs921720
rs80262450
rs112401990
rs114607072
rs6588243
rs4902642
rs10761659
rs1932990
rs1297271
rs3024505
rs151314883
rs3091315
rs12717899
rs2188962
rs4851586
rs6873866
rs907092
rs7713270
rs1456896
rs11236797
rs3810936
rs11209026
All
−0.02
0.00
0.02
0.04
MR effect size for
'Crohn's disease' on 'IDP T1 FAST ROIs R parahipp gyrus post'

Batch 2724 : Ulcerative colitis on IDP T1 FAST ROIs R parahipp gyrus post

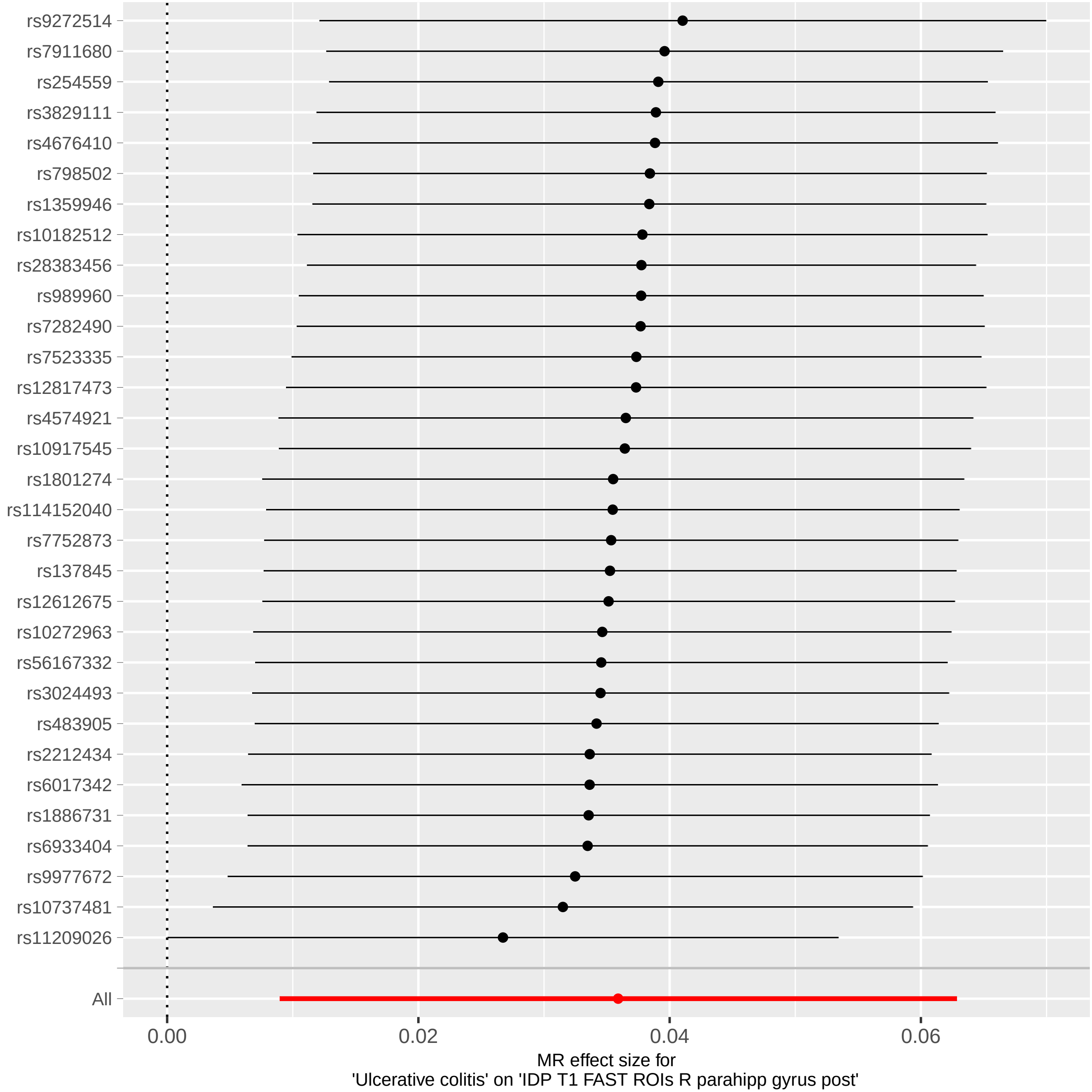

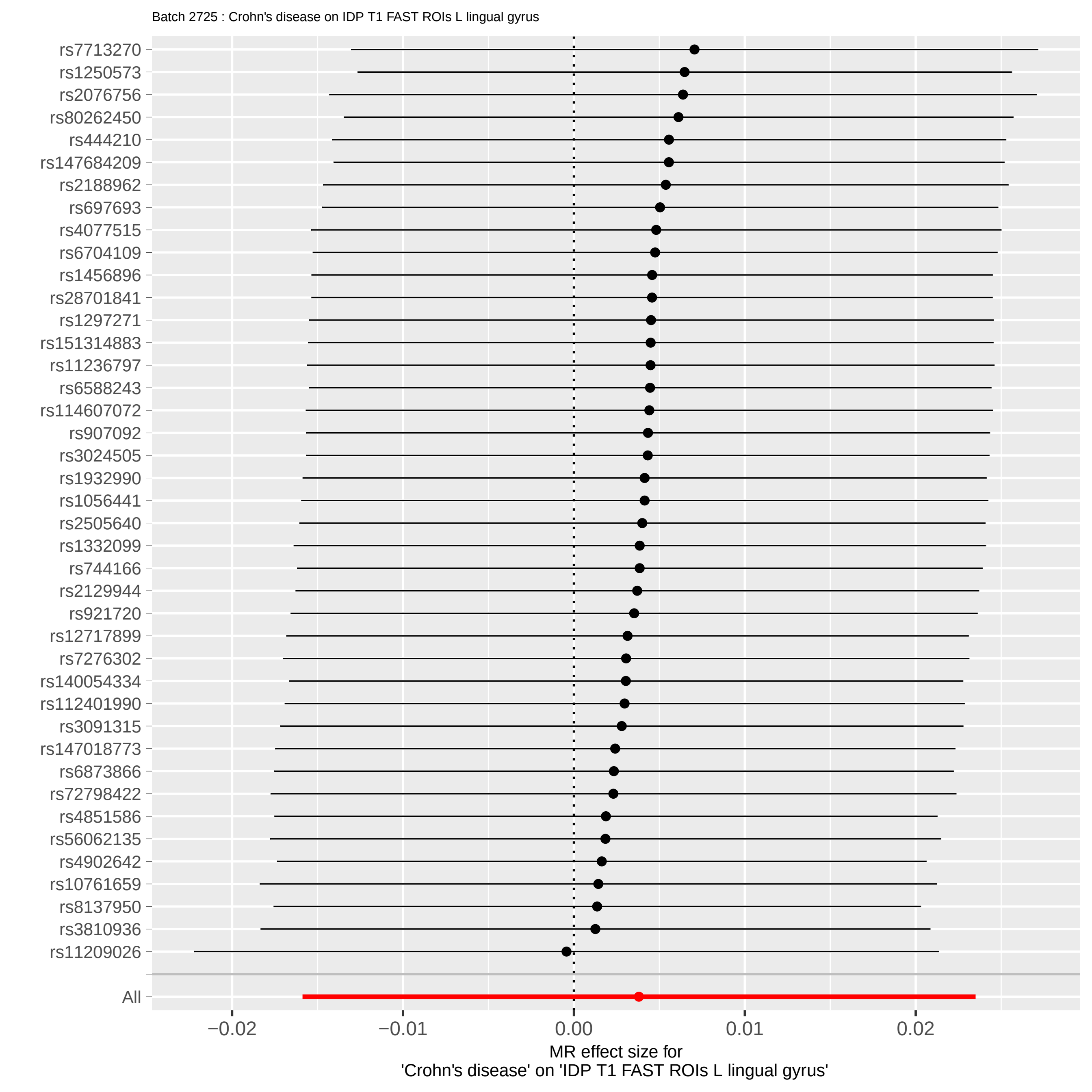
Batch 2725 : Crohn's disease on IDP T1 FAST ROIs L lingual gyrus
rs7713270
rs1250573
rs2076756
rs80262450
rs444210
rs147684209
rs2188962
rs697693
rs4077515
rs6704109
rs1456896
rs28701841
rs1297271
rs151314883
rs11236797
rs6588243
rs114607072
rs907092
rs3024505
rs1932990
rs1056441
rs2505640
rs1332099
rs744166
rs2129944
rs921720
rs12717899
rs7276302
rs140054334
rs112401990
rs3091315
rs147018773
rs6873866
rs72798422
rs4851586
rs56062135
rs4902642
rs10761659
rs8137950
rs3810936
rs11209026
All
−0.02
−0.01
0.00
0.01
0.02
MR effect size for
'Crohn's disease' on 'IDP T1 FAST ROIs L lingual gyrus'

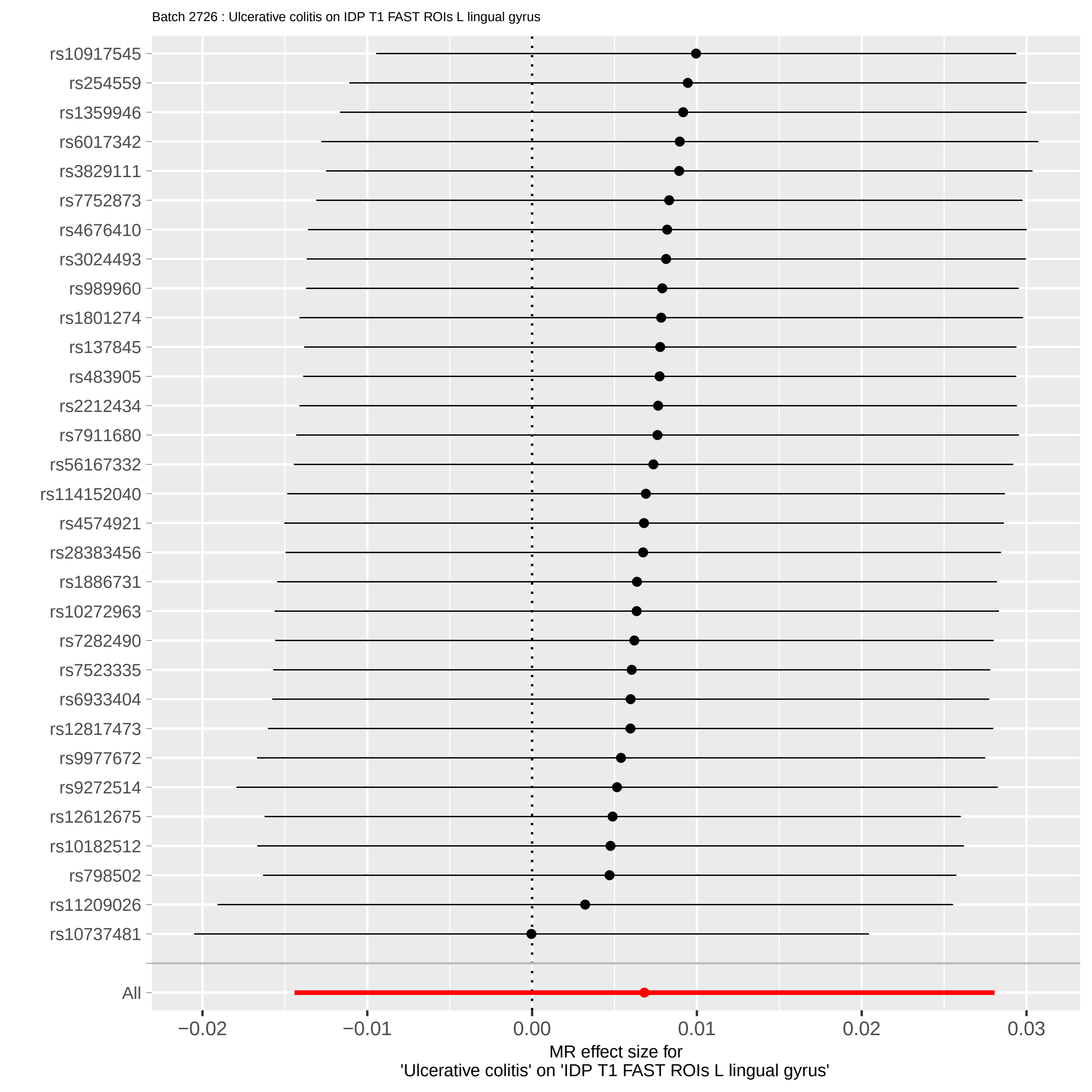
Batch 2726 : Ulcerative colitis on IDP T1 FAST ROIs L lingual gyrus
rs10917545
rs254559
rs1359946
rs6017342
rs3829111
rs7752873
rs4676410
rs3024493
rs989960
rs1801274
rs137845
rs483905
rs2212434
rs7911680
rs56167332
rs114152040
rs4574921
rs28383456
rs1886731
rs10272963
rs7282490
rs7523335
rs6933404
rs12817473
rs9977672
rs9272514
rs12612675
rs10182512
rs798502
rs11209026
rs10737481
All
−0.02
−0.01
0.00
0.01
0.02
0.03
MR effect size for
'Ulcerative colitis' on 'IDP T1 FAST ROIs L lingual gyrus'

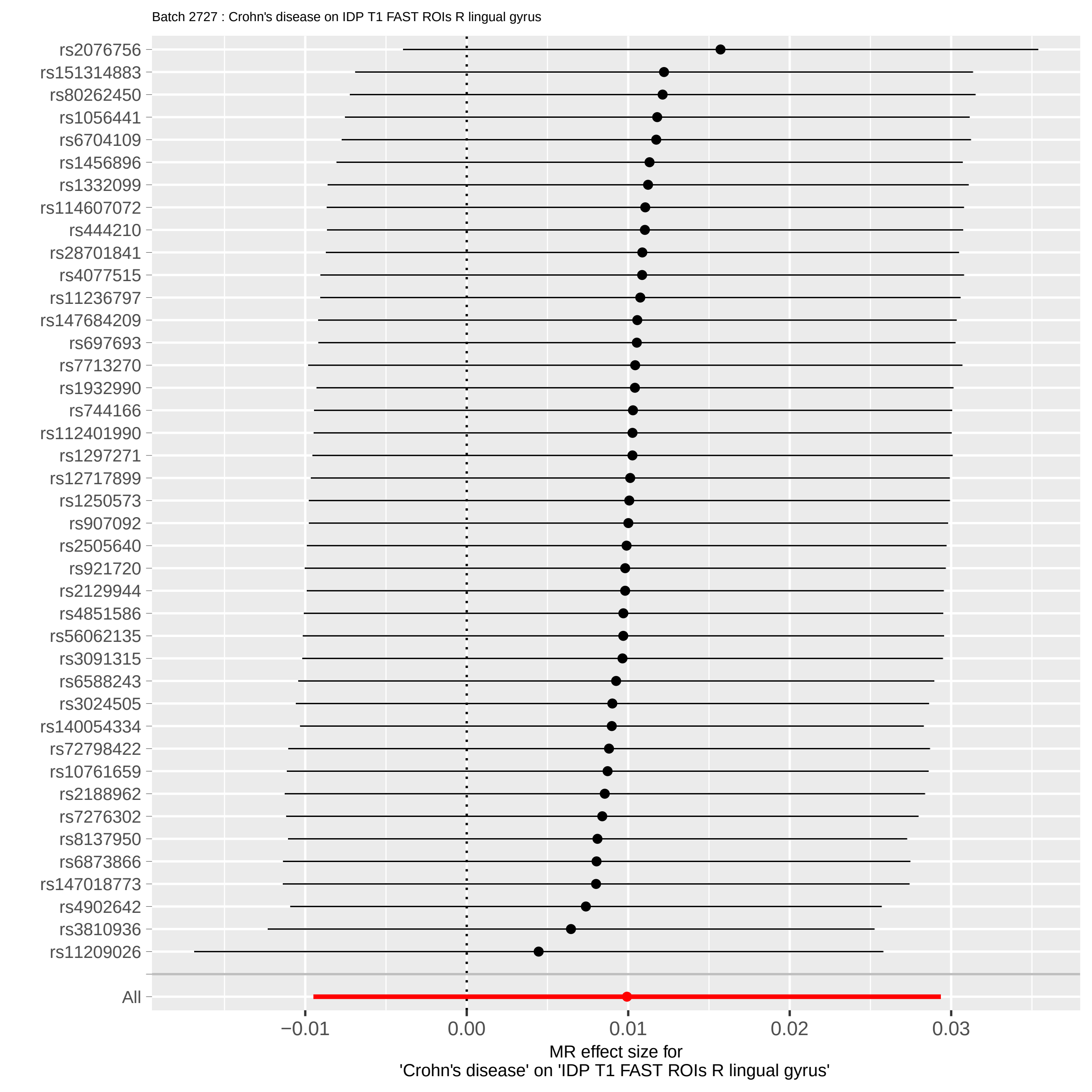
Batch 2727 : Crohn's disease on IDP T1 FAST ROIs R lingual gyrus
rs2076756
rs151314883
rs80262450
rs1056441
rs6704109
rs1456896
rs1332099
rs114607072
rs444210
rs28701841
rs4077515
rs11236797
rs147684209
rs697693
rs7713270
rs1932990
rs744166
rs112401990
rs1297271
rs12717899
rs1250573
rs907092
rs2505640
rs921720
rs2129944
rs4851586
rs56062135
rs3091315
rs6588243
rs3024505
rs140054334
rs72798422
rs10761659
rs2188962
rs7276302
rs8137950
rs6873866
rs147018773
rs4902642
rs3810936
rs11209026
All
−0.01
0.00
0.01
0.02
0.03
MR effect size for
'Crohn's disease' on 'IDP T1 FAST ROIs R lingual gyrus'

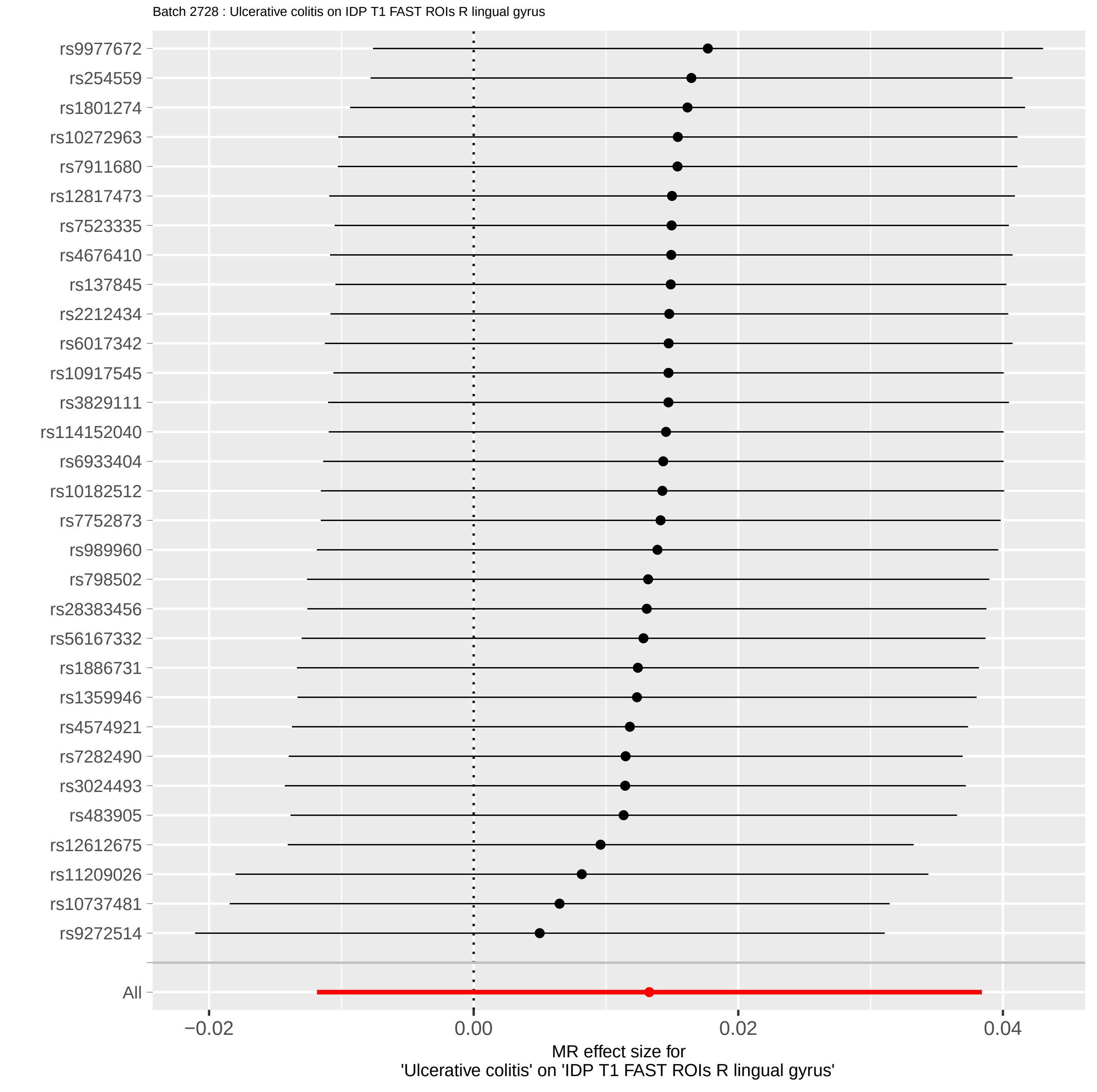

Batch 2728 : Ulcerative colitis on IDP T1 FAST ROIs R lingual gyrus
rs9977672
rs254559
rs1801274
rs10272963
rs7911680
rs12817473
rs7523335
rs4676410
rs137845
rs2212434
rs6017342
rs10917545
rs3829111
rs114152040
rs6933404
rs10182512
rs7752873
rs989960
rs798502
rs28383456
rs56167332
rs1886731
rs1359946
rs4574921
rs7282490
rs3024493
rs483905
rs12612675
rs11209026
rs10737481
rs9272514
All
−0.02
0.00
0.02
0.04
MR effect size for
'Ulcerative colitis' on 'IDP T1 FAST ROIs R lingual gyrus'

Batch 2729 : Crohn's disease on IDP T1 FAST ROIs L temp fusif cortex ant

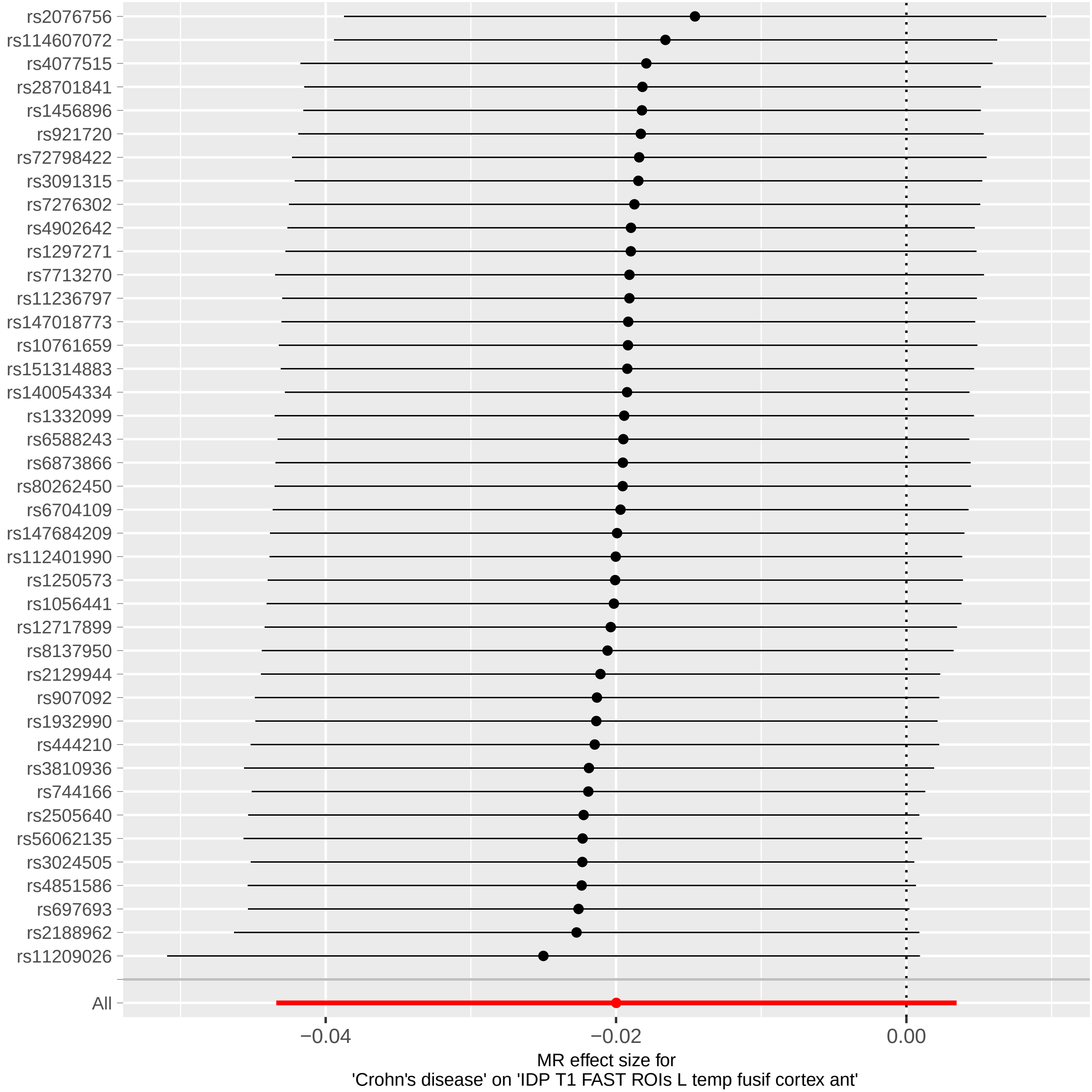

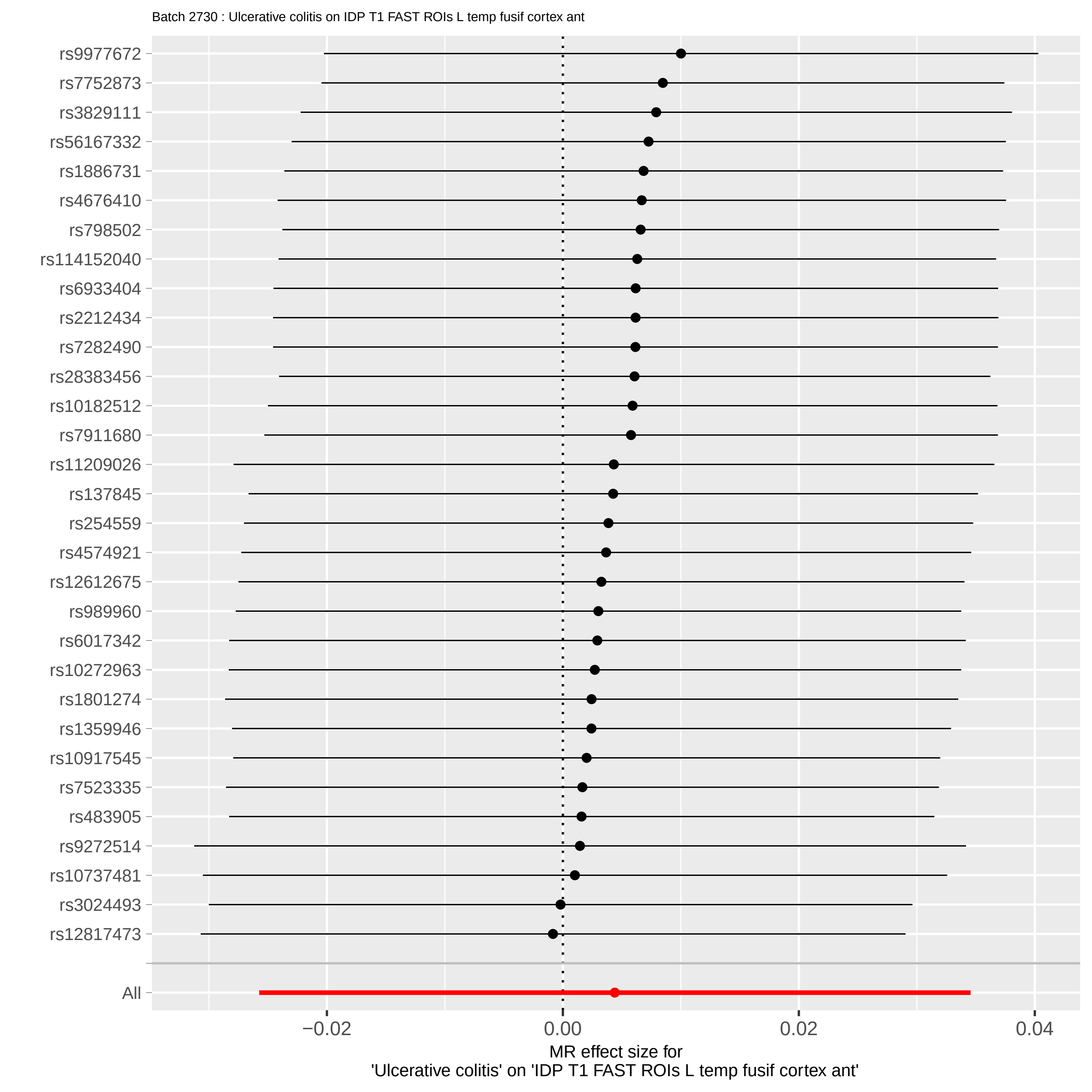
Batch 2730 : Ulcerative colitis on IDP T1 FAST ROIs L temp fusif cortex ant
rs9977672
rs7752873
rs3829111
rs56167332
rs1886731
rs4676410
rs798502
rs114152040
rs6933404
rs2212434
rs7282490
rs28383456
rs10182512
rs7911680
rs11209026
rs137845
rs254559
rs4574921
rs12612675
rs989960
rs6017342
rs10272963
rs1801274
rs1359946
rs10917545
rs7523335
rs483905
rs9272514
rs10737481
rs3024493
rs12817473
All
−0.02
0.00
0.02
0.04
MR effect size for
'Ulcerative colitis' on 'IDP T1 FAST ROIs L temp fusif cortex ant'

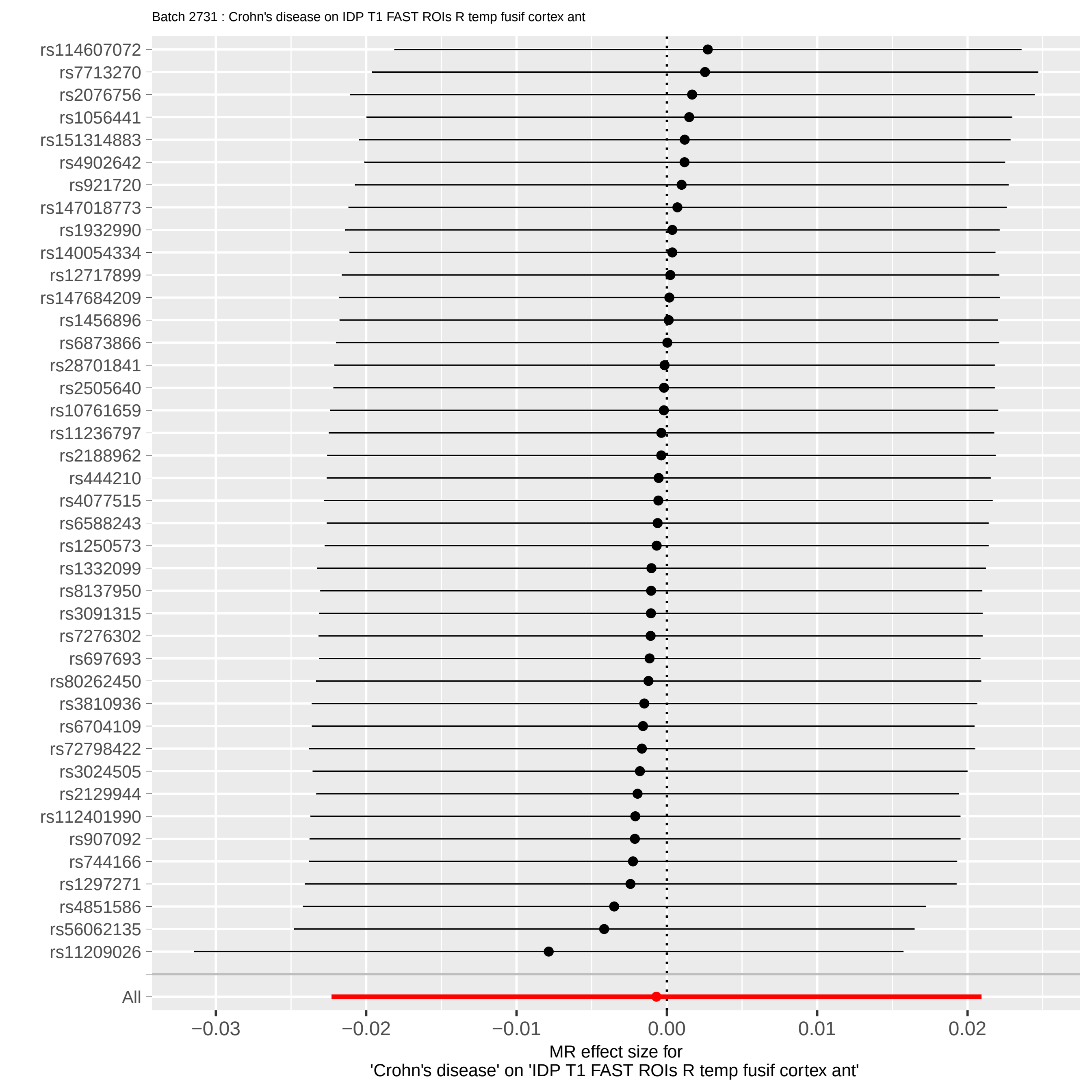

Batch 2731 : Crohn's disease on IDP T1 FAST ROIs R temp fusif cortex ant
rs114607072
rs7713270
rs2076756
rs1056441
rs151314883
rs4902642
rs921720
rs147018773
rs1932990
rs140054334
rs12717899
rs147684209
rs1456896
rs6873866
rs28701841
rs2505640
rs10761659
rs11236797
rs2188962
rs444210
rs4077515
rs6588243
rs1250573
rs1332099
rs8137950
rs3091315
rs7276302
rs697693
rs80262450
rs3810936
rs6704109
rs72798422
rs3024505
rs2129944
rs112401990
rs907092
rs744166
rs1297271
rs4851586
rs56062135
rs11209026
All
−0.03
−0.02
−0.01
0.00
0.01
0.02
MR effect size for
'Crohn's disease' on 'IDP T1 FAST ROIs R temp fusif cortex ant'

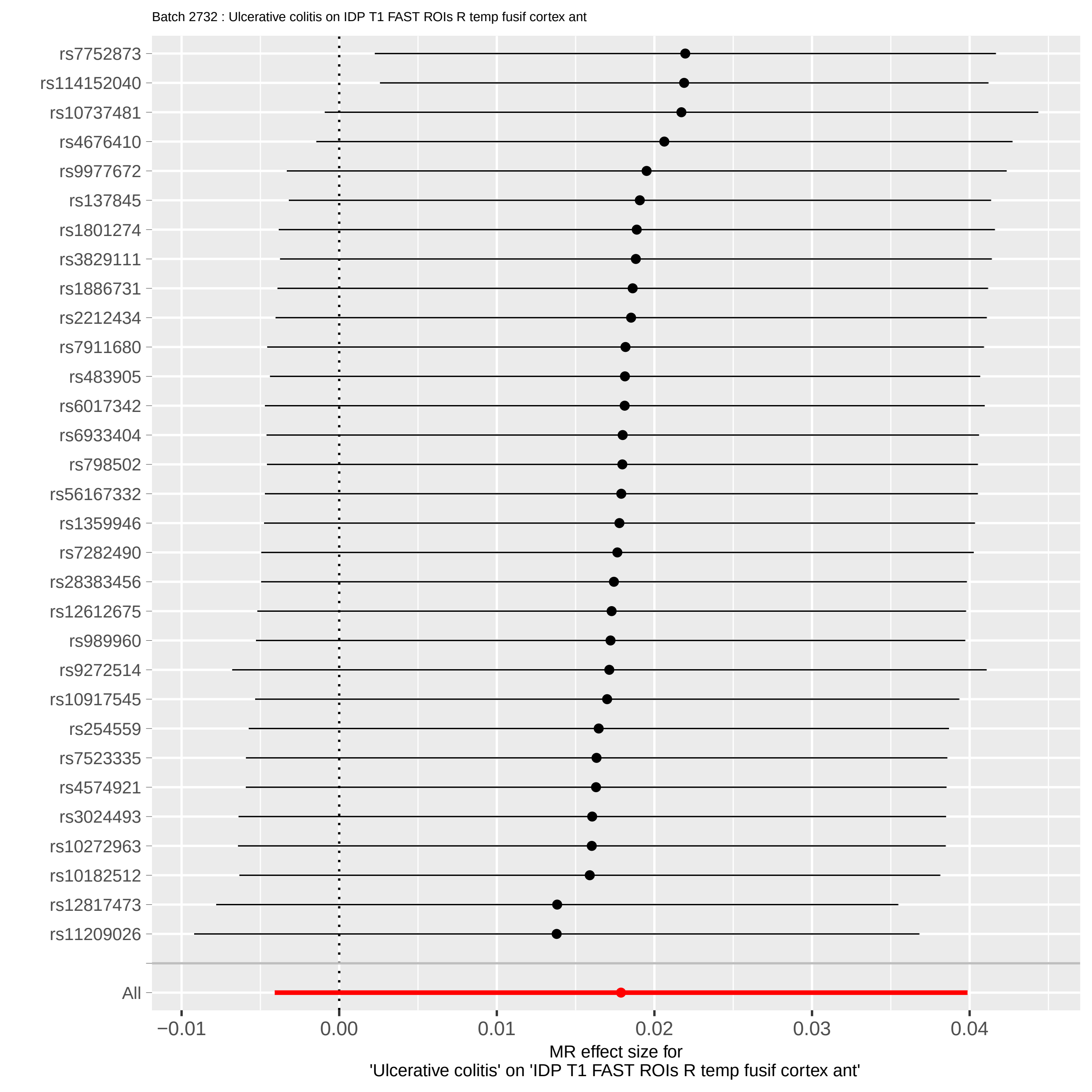
Batch 2732 : Ulcerative colitis on IDP T1 FAST ROIs R temp fusif cortex ant
rs7752873
rs114152040
rs10737481
rs4676410
rs9977672
rs137845
rs1801274
rs3829111
rs1886731
rs2212434
rs7911680
rs483905
rs6017342
rs6933404
rs798502
rs56167332
rs1359946
rs7282490
rs28383456
rs12612675
rs989960
rs9272514
rs10917545
rs254559
rs7523335
rs4574921
rs3024493
rs10272963
rs10182512
rs12817473
rs11209026
All
−0.01
0.00
0.01
0.02
0.03
0.04
MR effect size for
'Ulcerative colitis' on 'IDP T1 FAST ROIs R temp fusif cortex ant'

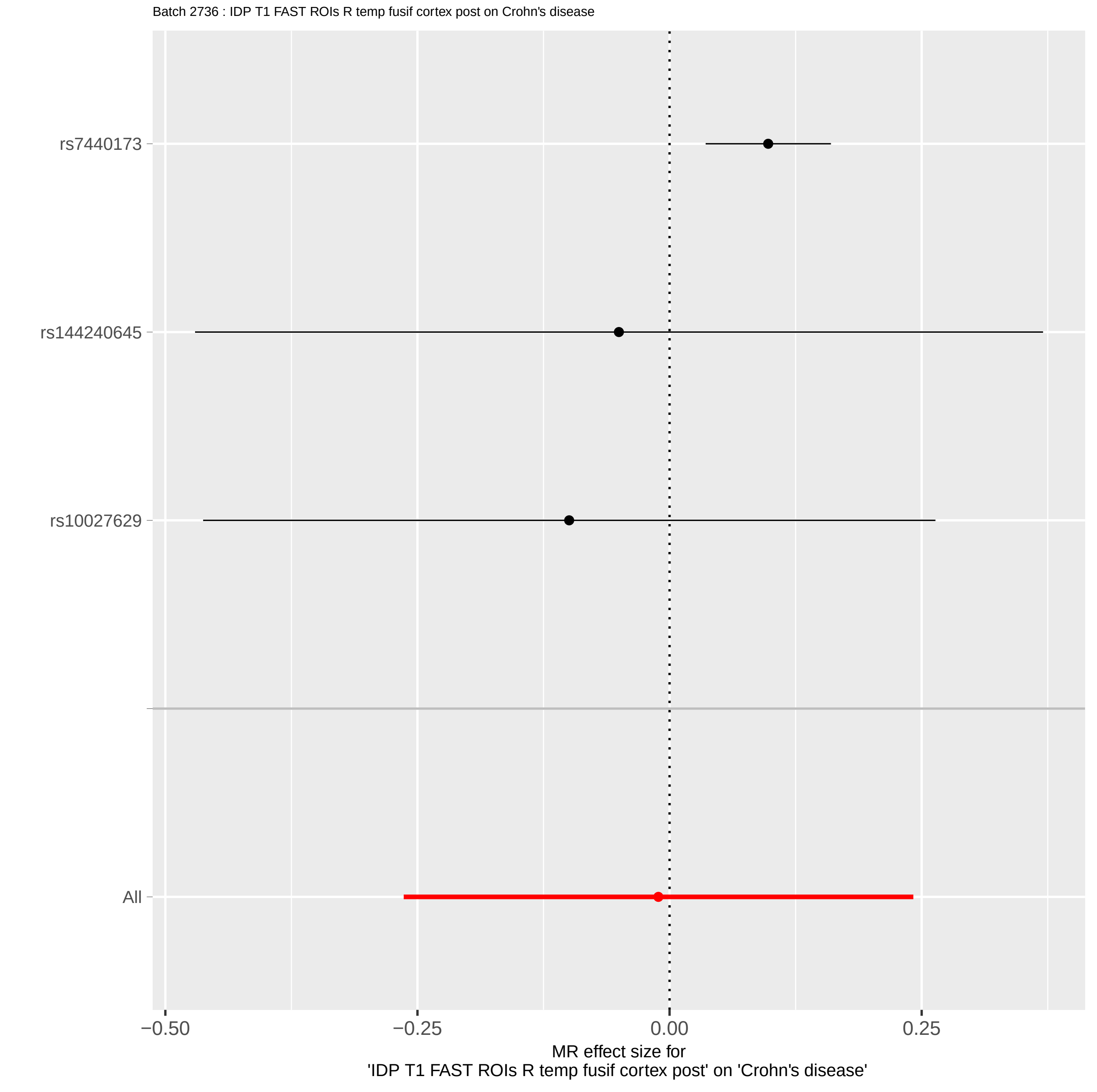
Batch 2736 : IDP T1 FAST ROIs R temp fusif cortex post on Crohn's disease
rs7440173
rs144240645
rs10027629
All
−0.50
−0.25
0.00
0.25
MR effect size for
'IDP T1 FAST ROIs R temp fusif cortex post' on 'Crohn's disease'

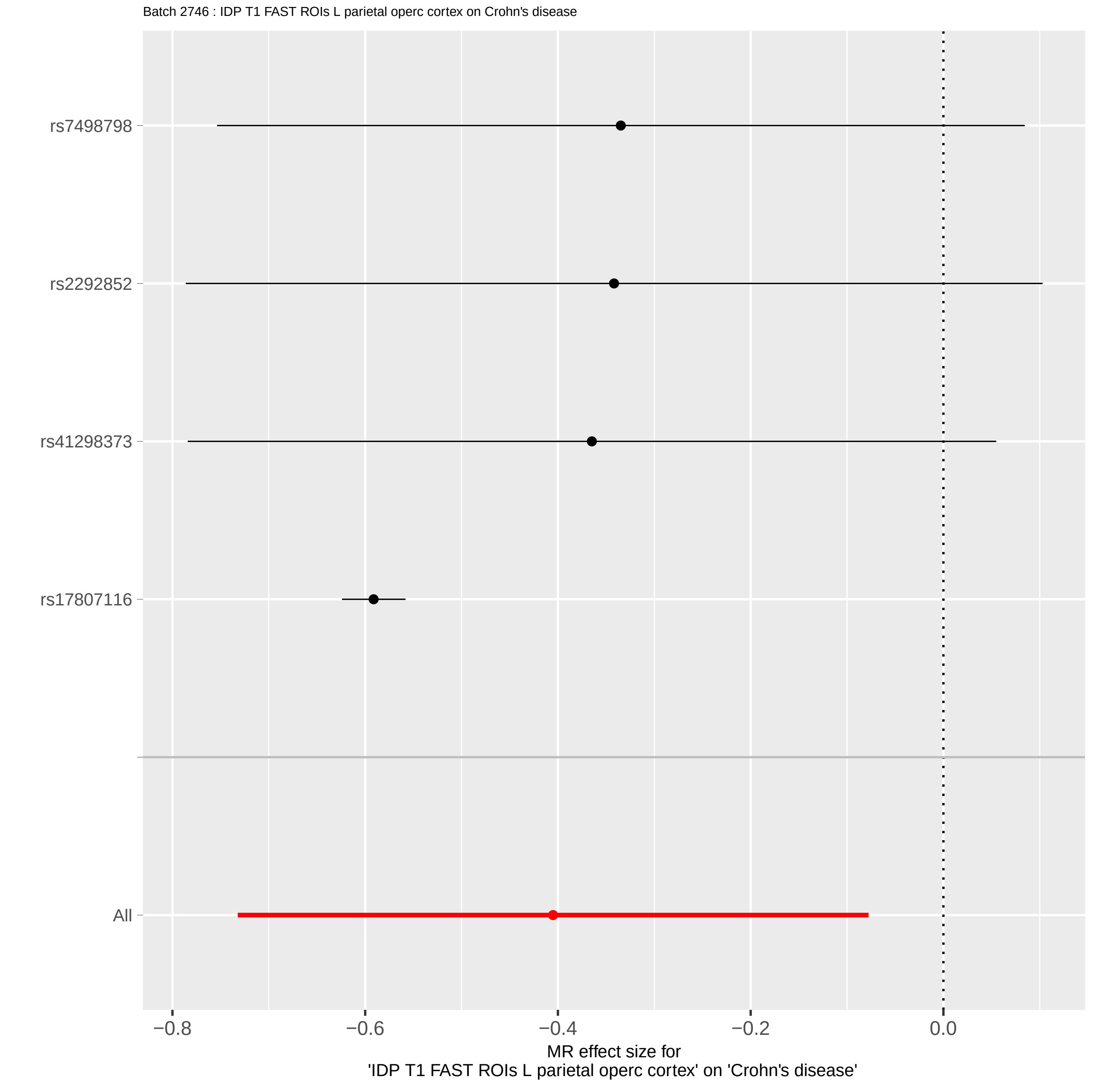
Batch 2746 : IDP T1 FAST ROIs L parietal operc cortex on Crohn's disease
rs7498798
rs2292852
rs41298373
rs17807116
All
−0.8
−0.6
−0.4
−0.2
0.0
MR effect size for
'IDP T1 FAST ROIs L parietal operc cortex' on 'Crohn's disease'

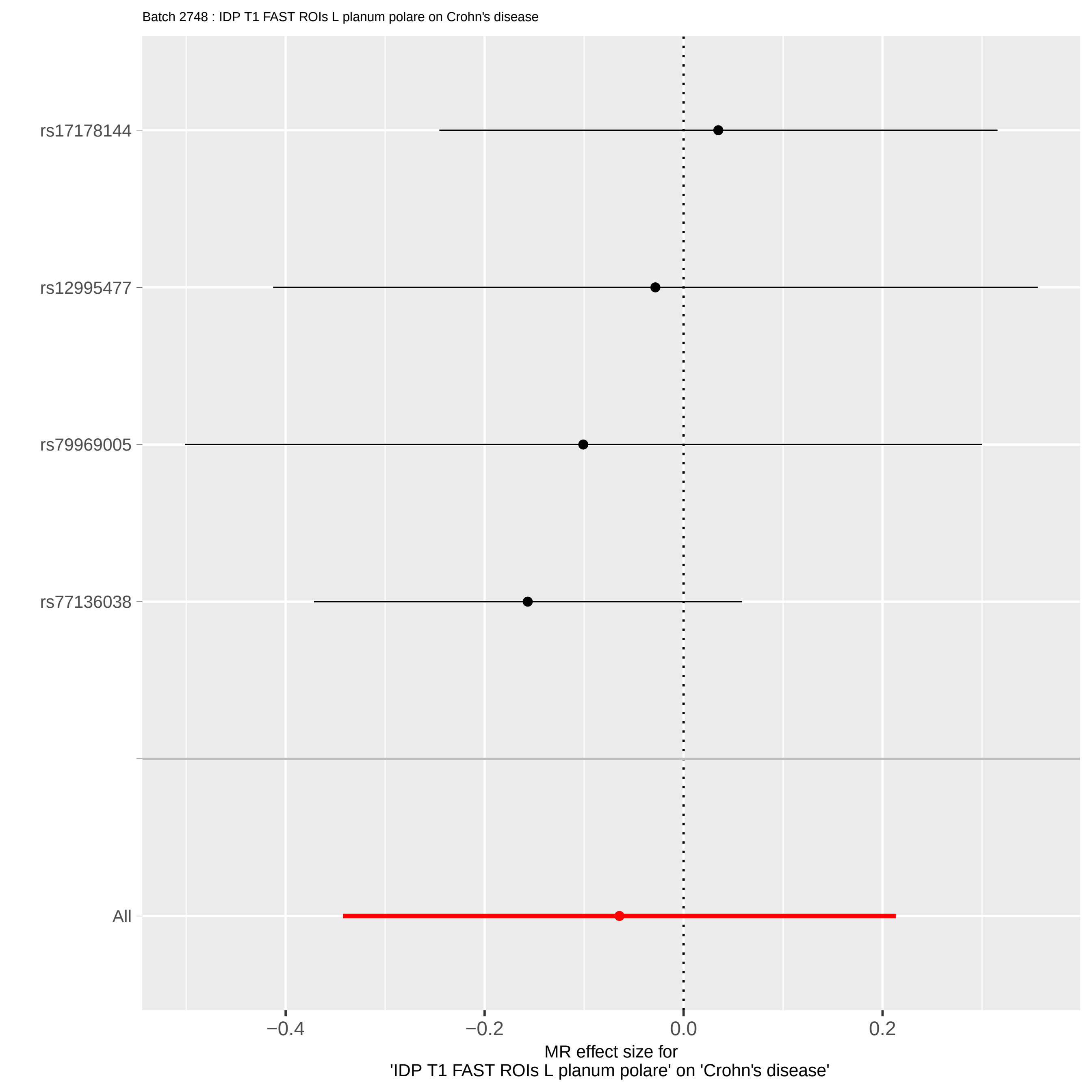

Batch 2748 : IDP T1 FAST ROIs L planum polare on Crohn's disease
rs17178144
rs12995477
rs79969005
rs77136038
All
−0.4
−0.2
0.0
0.2
MR effect size for
'IDP T1 FAST ROIs L planum polare' on 'Crohn's disease'

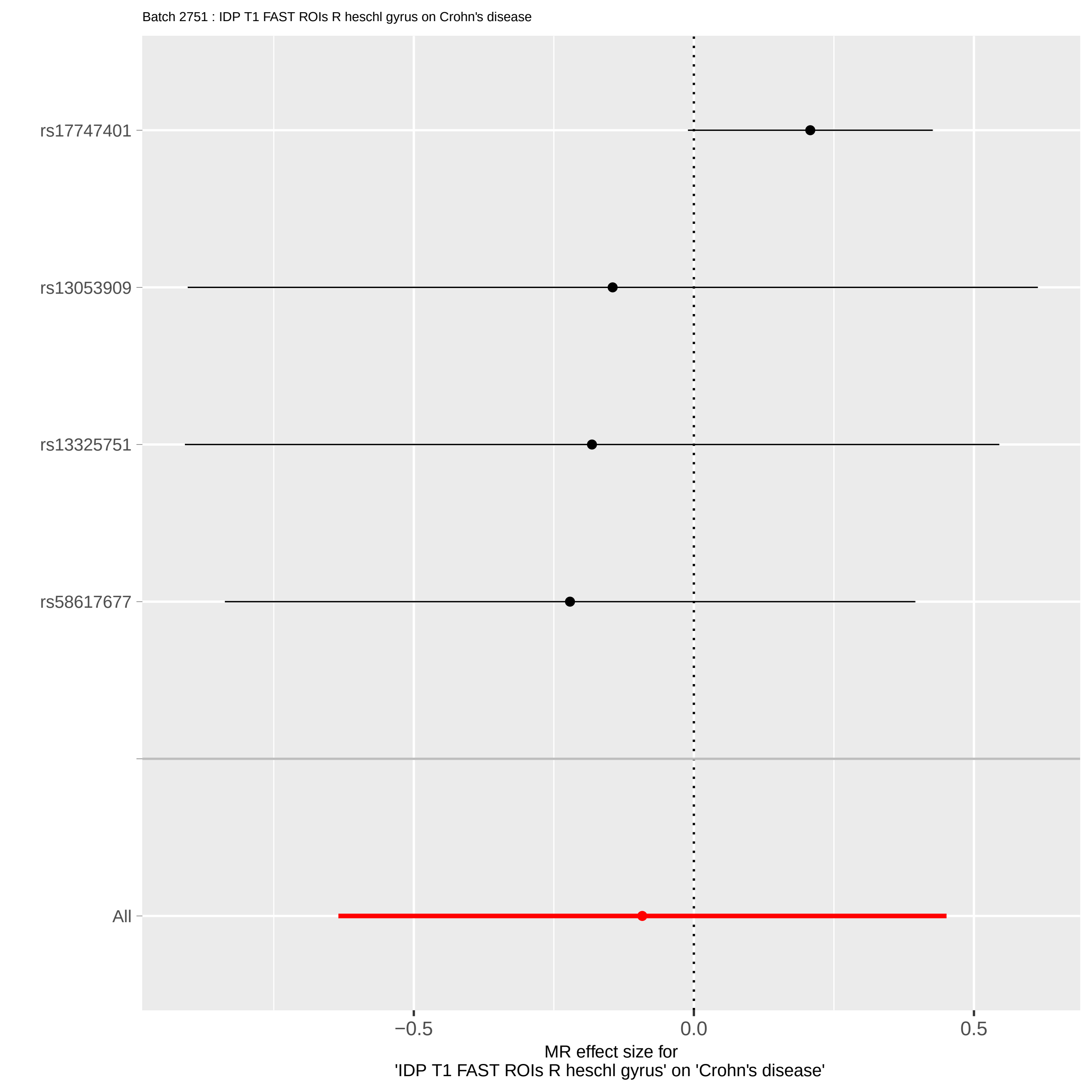

Batch 2751 : IDP T1 FAST ROIs R heschl gyrus on Crohn's disease
rs17747401
rs13053909
rs13325751
rs58617677
All
−0.5
0.0
0.5
MR effect size for
'IDP T1 FAST ROIs R heschl gyrus' on 'Crohn's disease'

Batch 2753 : IDP T1 FAST ROIs R planum temporale on Crohn's disease

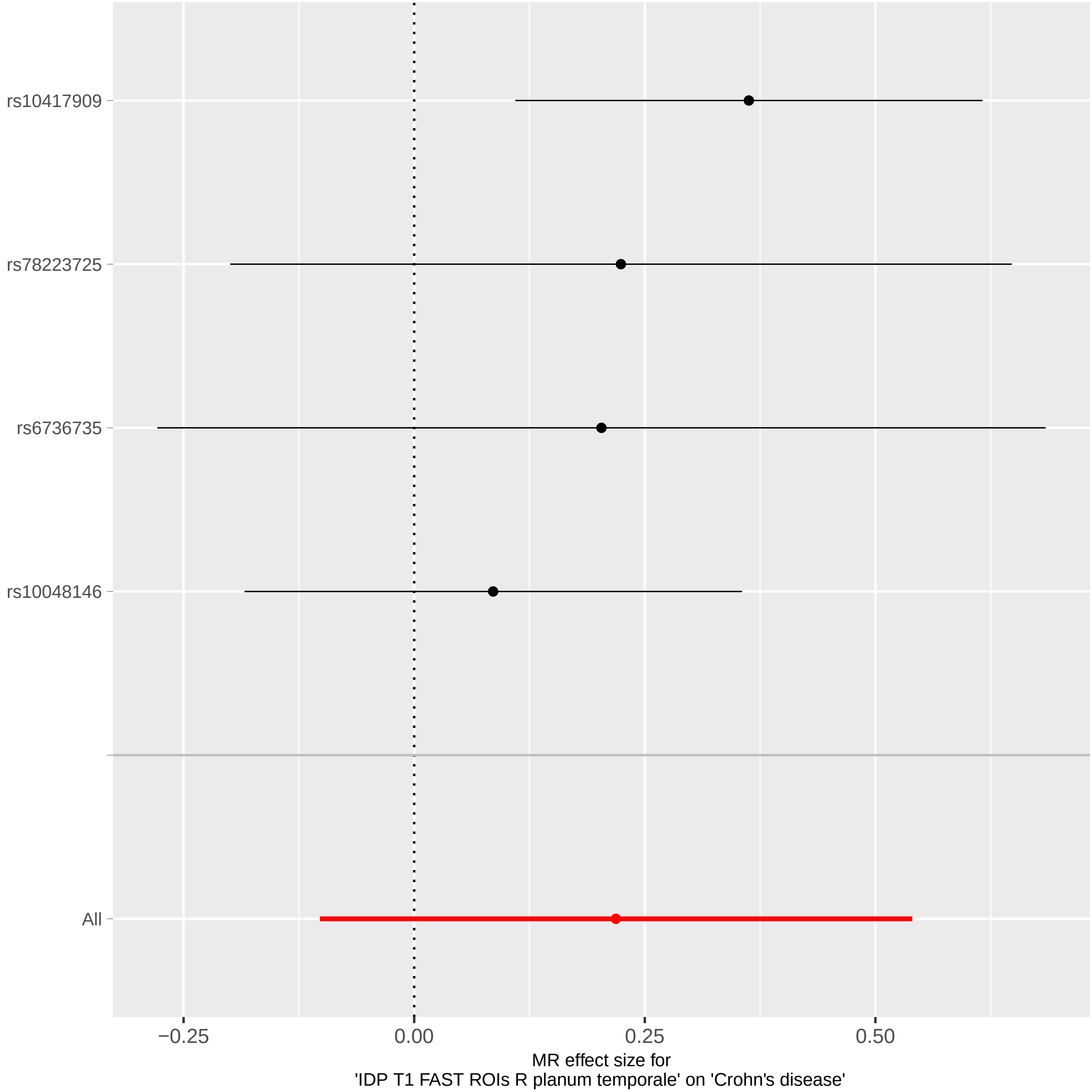

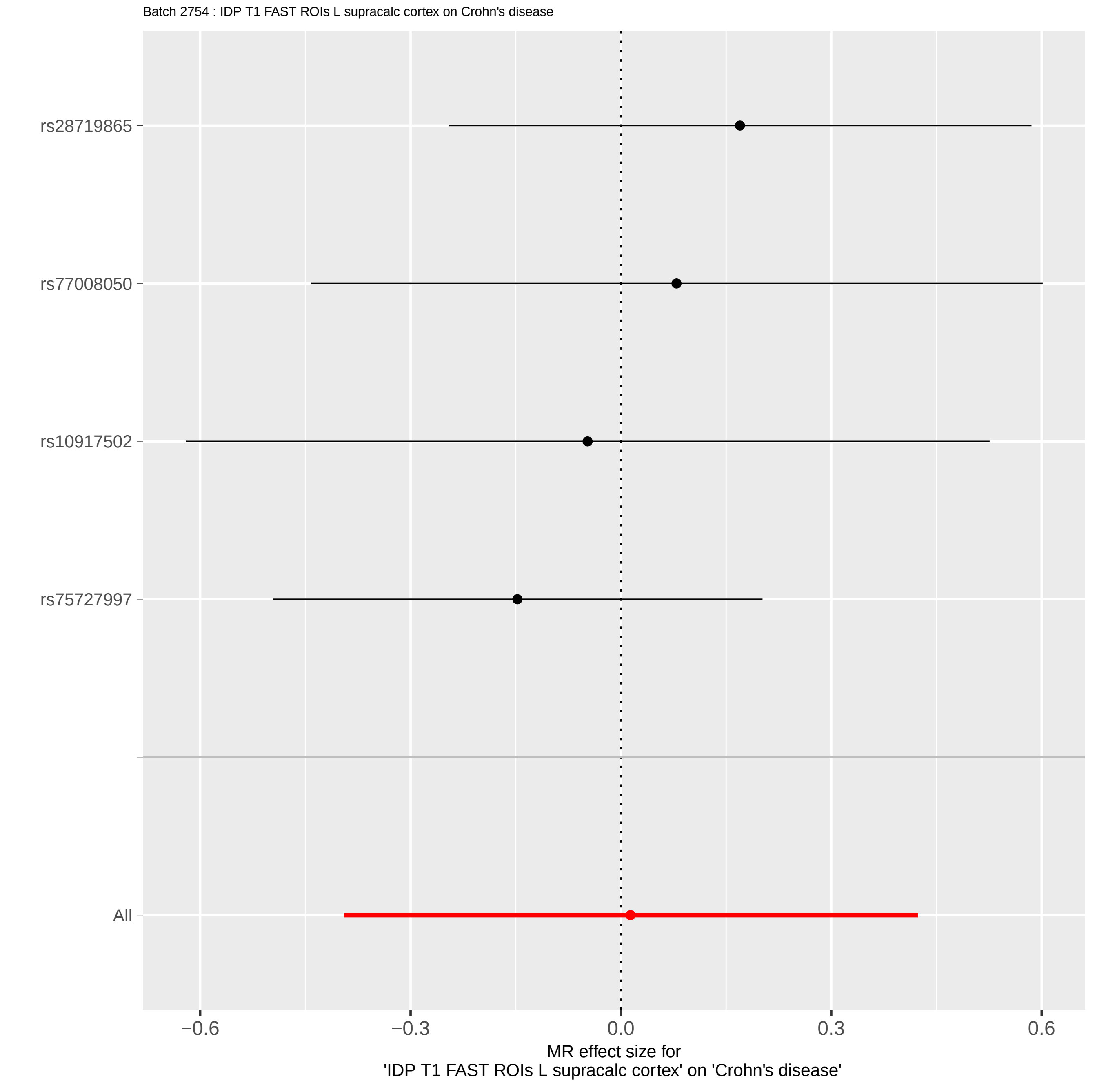

Batch 2754 : IDP T1 FAST ROIs L supracalc cortex on Crohn's disease
rs28719865
rs77008050
rs10917502
rs75727997
All
−0.6
−0.3
0.0
0.3
0.6
MR effect size for
'IDP T1 FAST ROIs L supracalc cortex' on 'Crohn's disease'

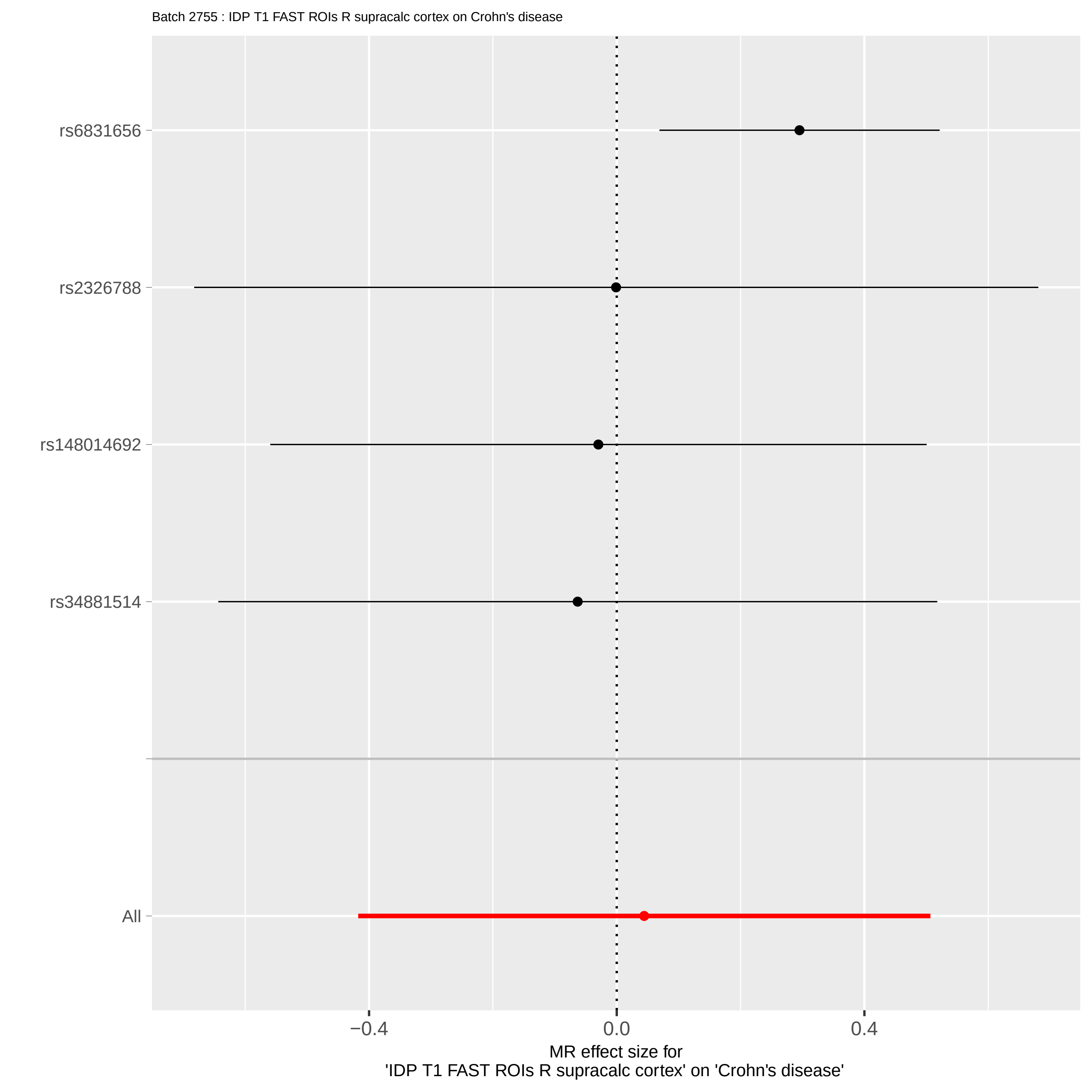

Batch 2755 : IDP T1 FAST ROIs R supracalc cortex on Crohn's disease
rs6831656
rs2326788
rs148014692
rs34881514
All
−0.4
0.0
0.4
MR effect size for
'IDP T1 FAST ROIs R supracalc cortex' on 'Crohn's disease'

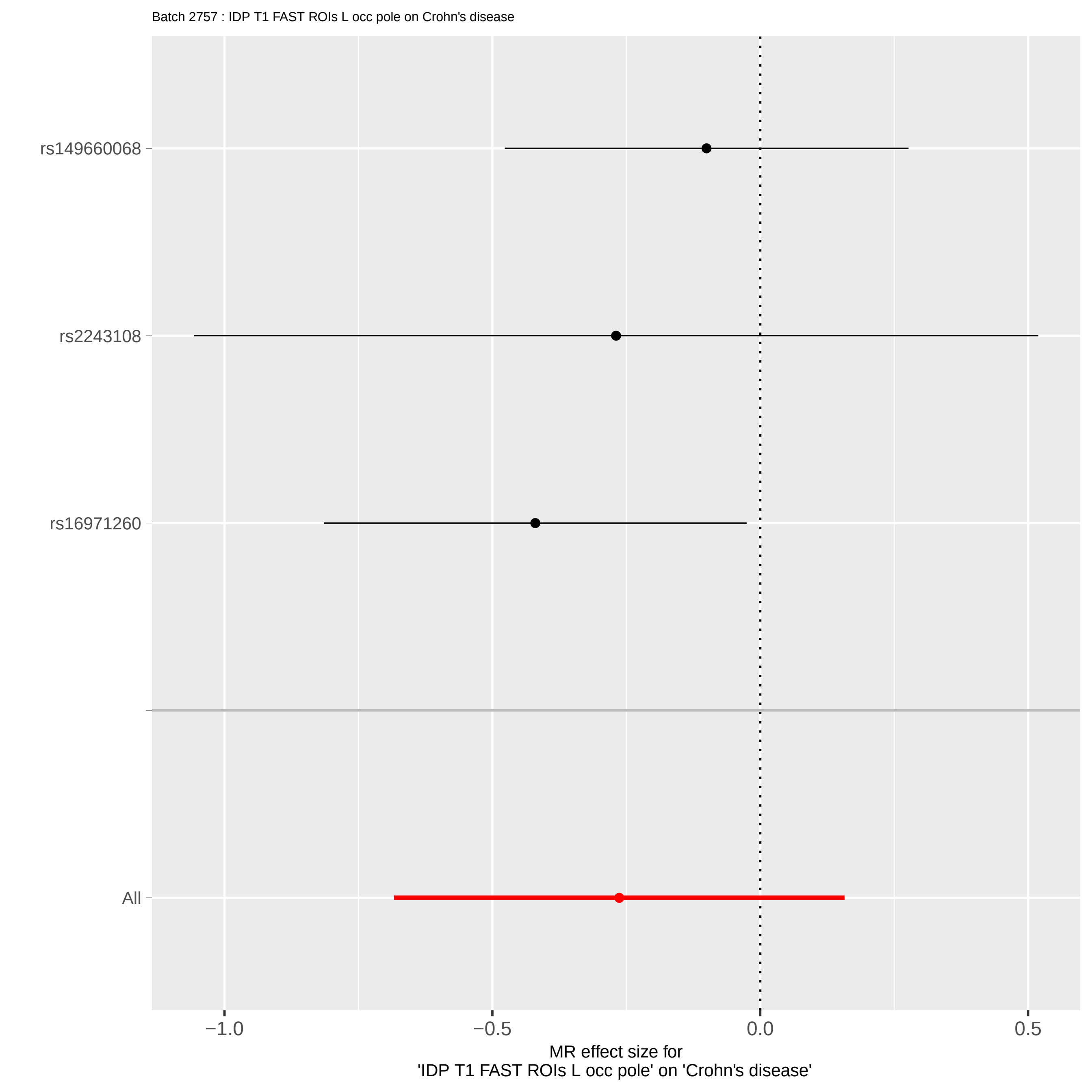
Batch 2757 : IDP T1 FAST ROIs L occ pole on Crohn's disease
rs149660068
rs2243108
rs16971260
All
−1.0
−0.5
0.0
0.5
MR effect size for
'IDP T1 FAST ROIs L occ pole' on 'Crohn's disease'

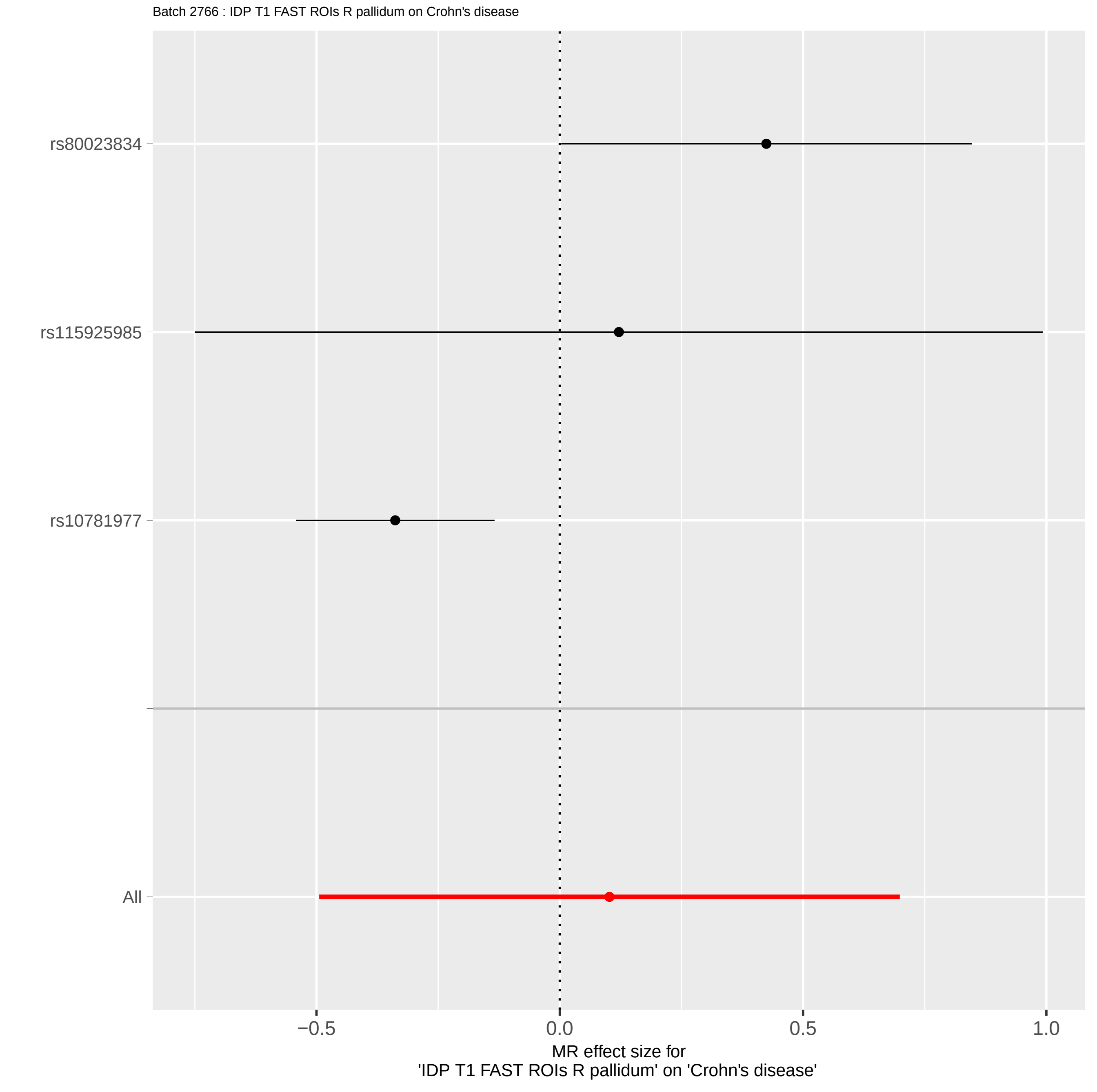

Batch 2766 : IDP T1 FAST ROIs R pallidum on Crohn's disease
rs80023834
rs115925985
rs10781977
All
−0.5
0.0
0.5
1.0
MR effect size for
'IDP T1 FAST ROIs R pallidum' on 'Crohn's disease'

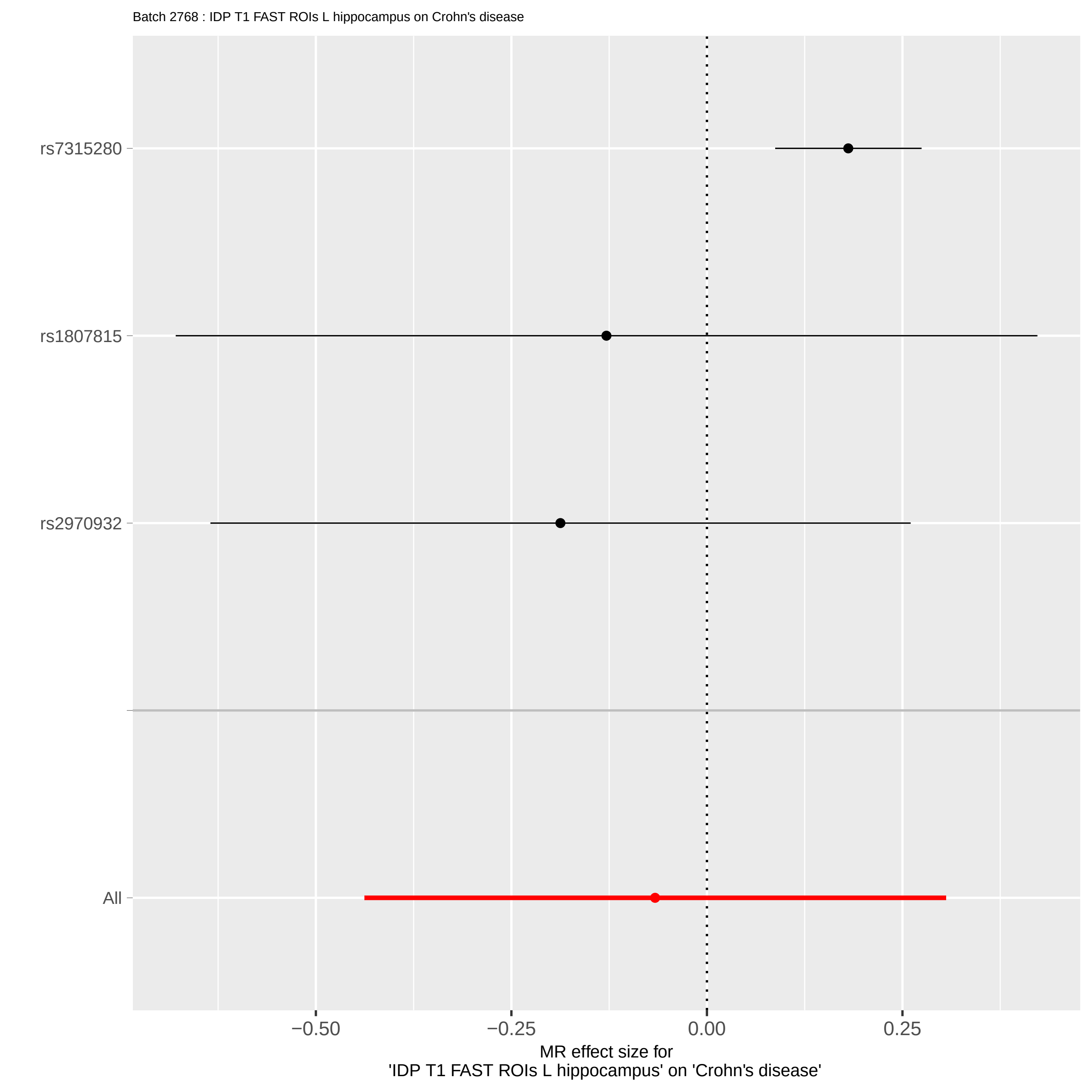
Batch 2768 : IDP T1 FAST ROIs L hippocampus on Crohn's disease
rs7315280
rs1807815
rs2970932
All
−0.50
−0.25
0.00
0.25
MR effect size for
'IDP T1 FAST ROIs L hippocampus' on 'Crohn's disease'

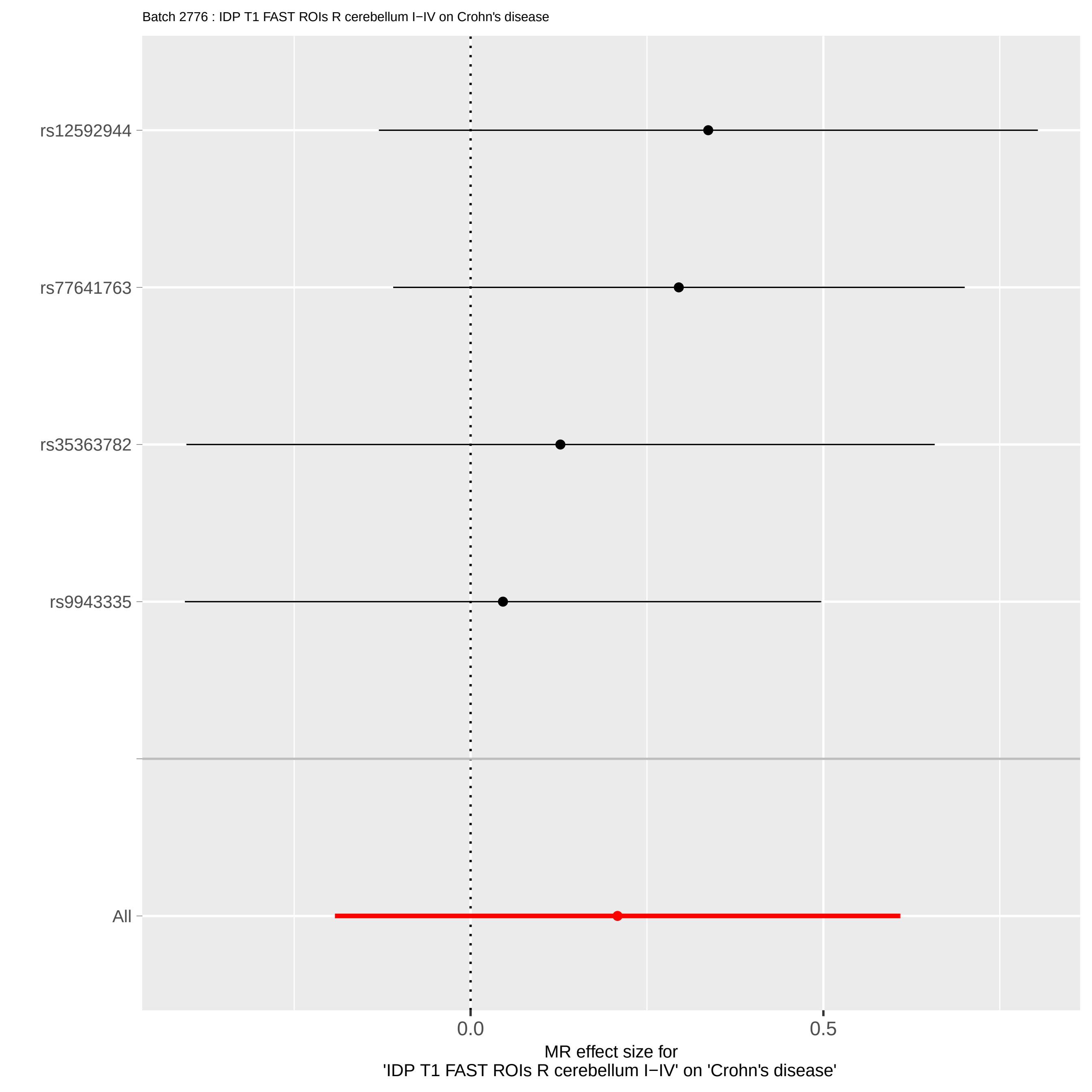

Batch 2776 : IDP T1 FAST ROIs R cerebellum I−IV on Crohn's disease
rs12592944
rs77641763
rs35363782
rs9943335
All
0.0
0.5
MR effect size for
'IDP T1 FAST ROIs R cerebellum I−IV' on 'Crohn's disease'

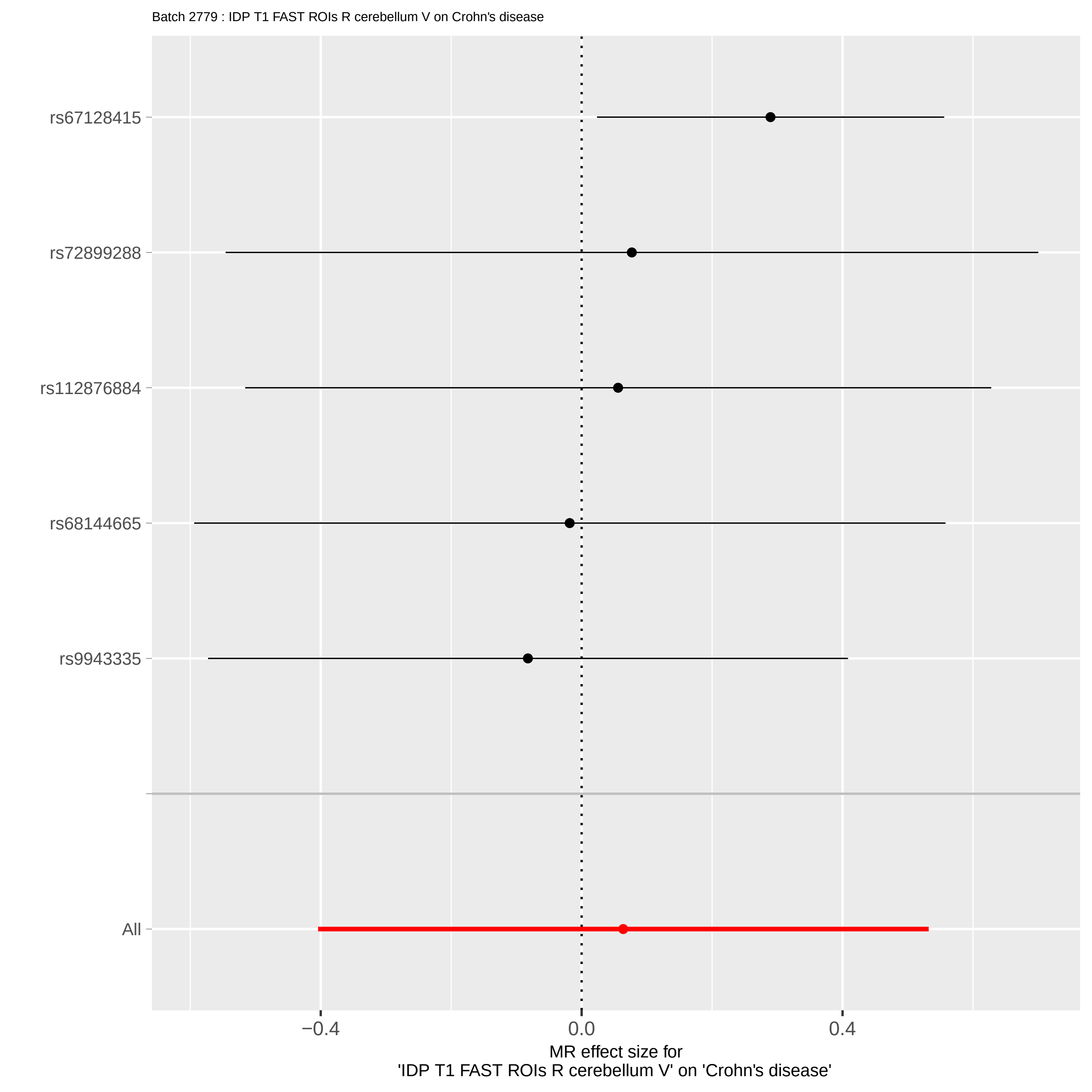

Batch 2779 : IDP T1 FAST ROIs R cerebellum V on Crohn's disease
rs67128415
rs72899288
rs112876884
rs68144665
rs9943335
All
−0.4
0.0
0.4
MR effect size for
'IDP T1 FAST ROIs R cerebellum V' on 'Crohn's disease'

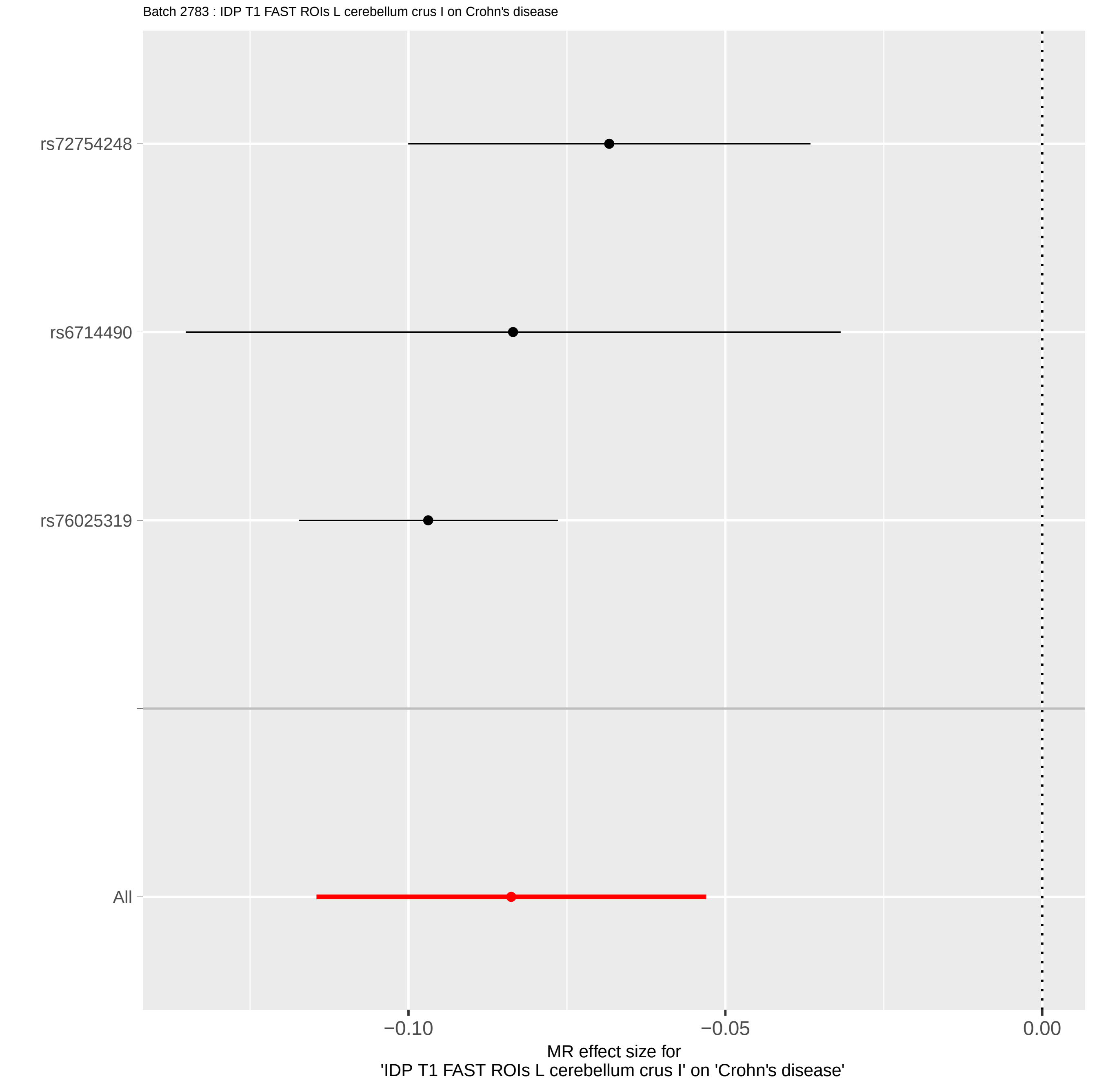
Batch 2783 : IDP T1 FAST ROIs L cerebellum crus I on Crohn's disease
rs72754248
rs6714490
rs76025319
All
−0.10
−0.05
0.00
MR effect size for
'IDP T1 FAST ROIs L cerebellum crus I' on 'Crohn's disease'

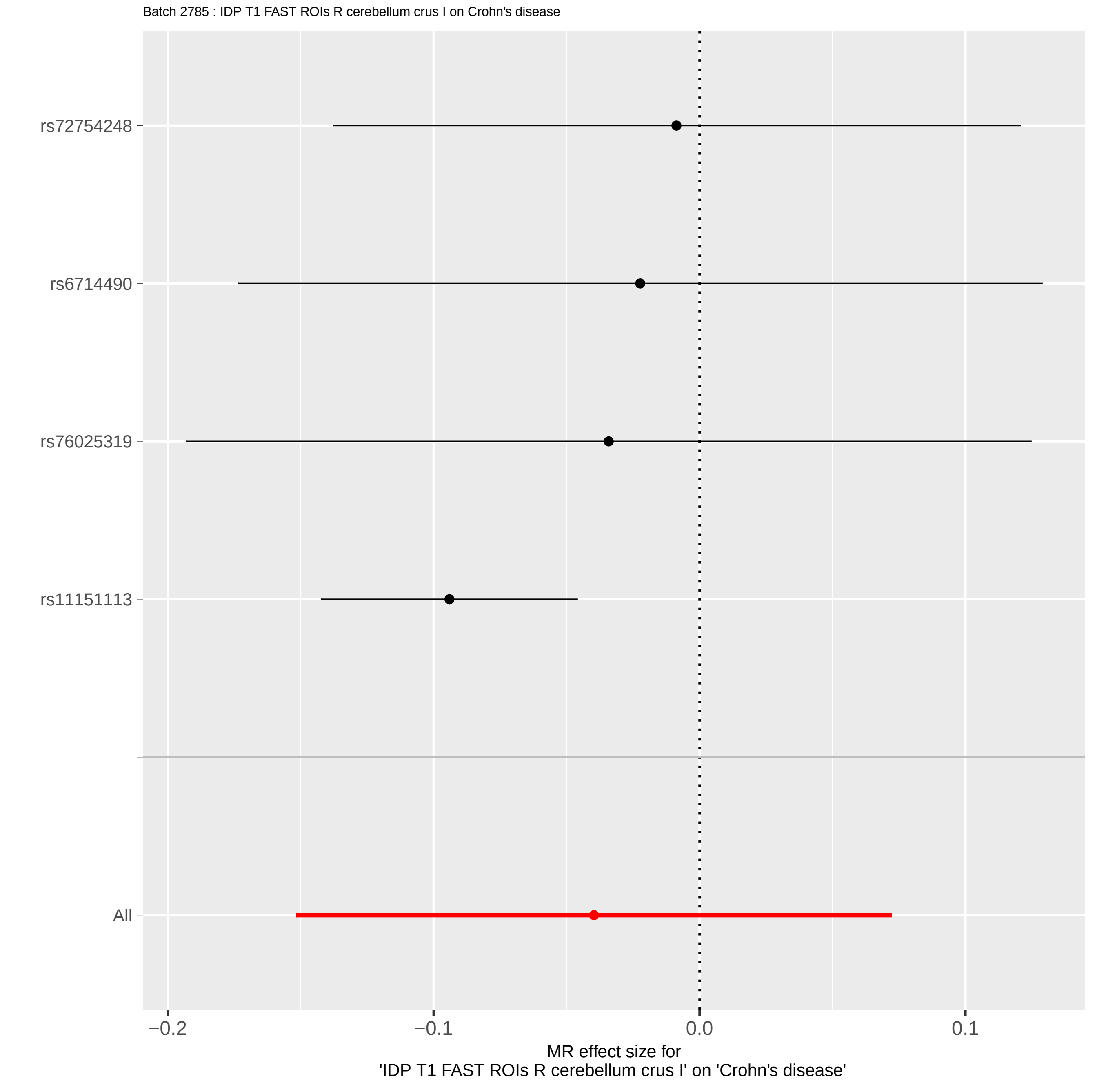
Batch 2785 : IDP T1 FAST ROIs R cerebellum crus I on Crohn's disease
rs72754248
rs6714490
rs76025319
rs11151113
All
−0.2
−0.1
0.0
0.1
MR effect size for
'IDP T1 FAST ROIs R cerebellum crus I' on 'Crohn's disease'

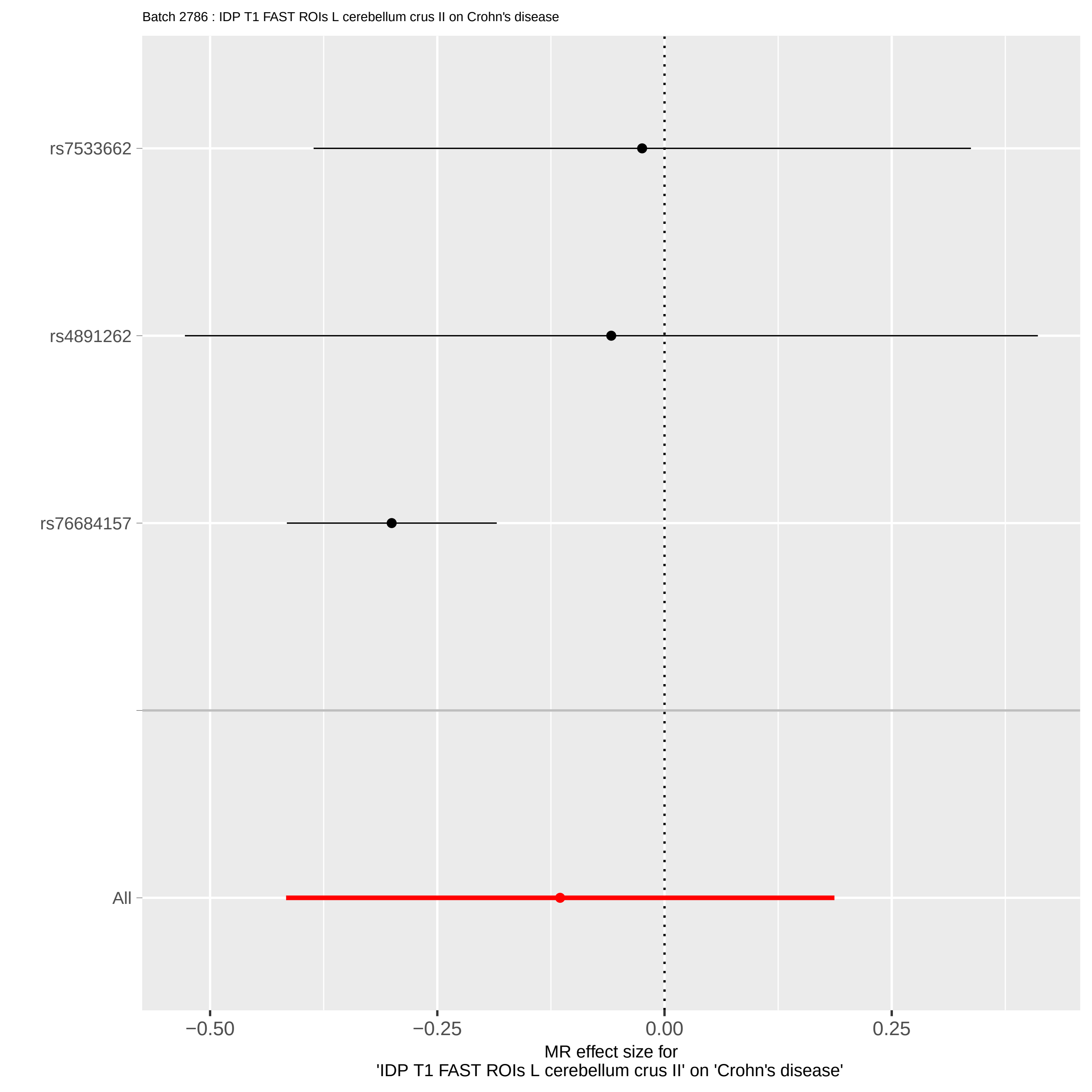

Batch 2786 : IDP T1 FAST ROIs L cerebellum crus II on Crohn's disease
rs7533662
rs4891262
rs76684157
All
−0.50
−0.25
0.00
0.25
MR effect size for
'IDP T1 FAST ROIs L cerebellum crus II' on 'Crohn's disease'

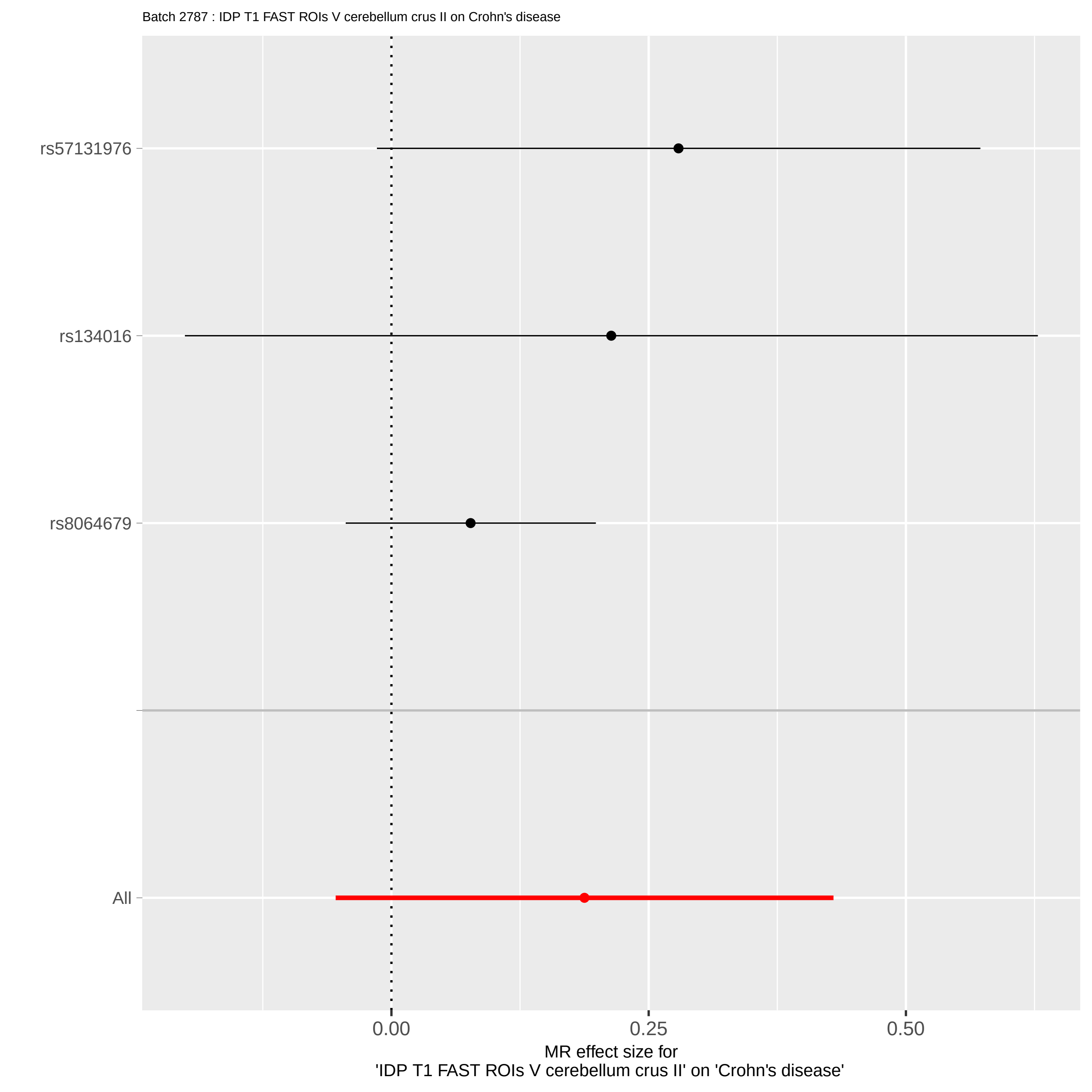
Batch 2787 : IDP T1 FAST ROIs V cerebellum crus II on Crohn's disease
rs57131976
rs134016
rs8064679
All
0.00
0.25
0.50
MR effect size for
'IDP T1 FAST ROIs V cerebellum crus II' on 'Crohn's disease'

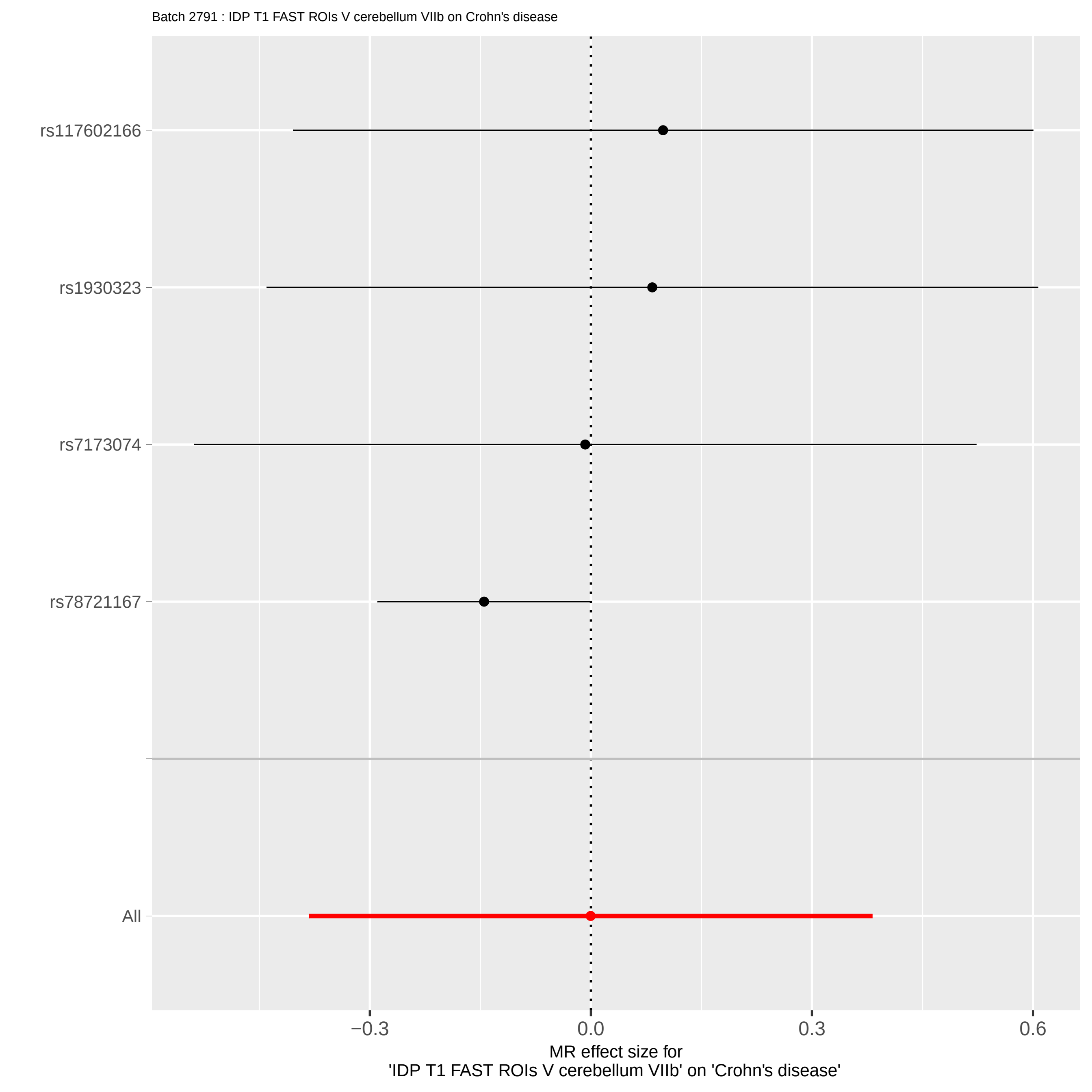
Batch 2791 : IDP T1 FAST ROIs V cerebellum VIIb on Crohn's disease
rs117602166
rs1930323
rs7173074
rs78721167
All
−0.3
0.0
0.3
0.6
MR effect size for
'IDP T1 FAST ROIs V cerebellum VIIb' on 'Crohn's disease'

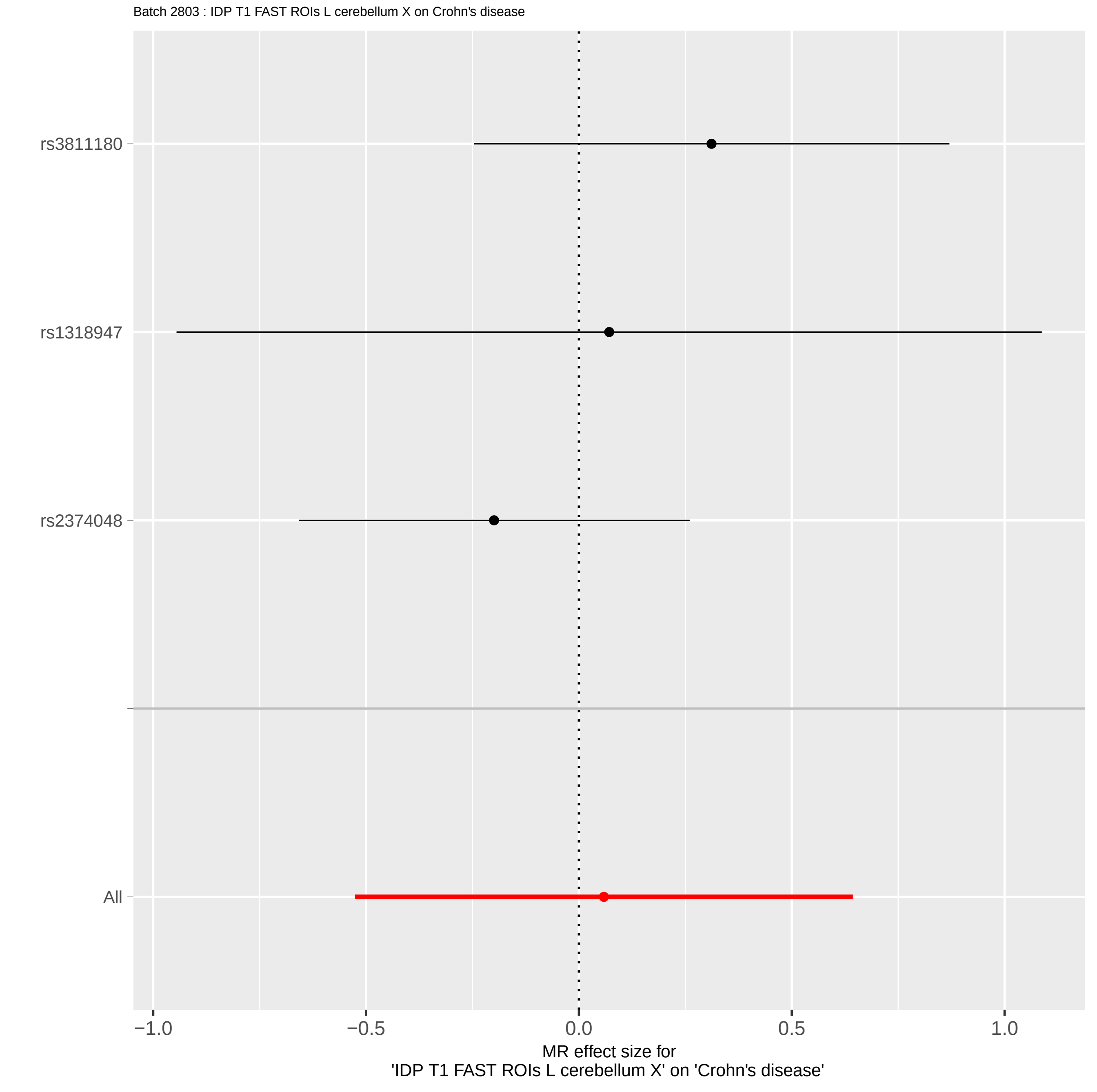

Batch 2803 : IDP T1 FAST ROIs L cerebellum X on Crohn's disease
rs3811180
rs1318947
rs2374048
All
−1.0
−0.5
0.0
0.5
1.0
MR effect size for
'IDP T1 FAST ROIs L cerebellum X' on 'Crohn's disease'

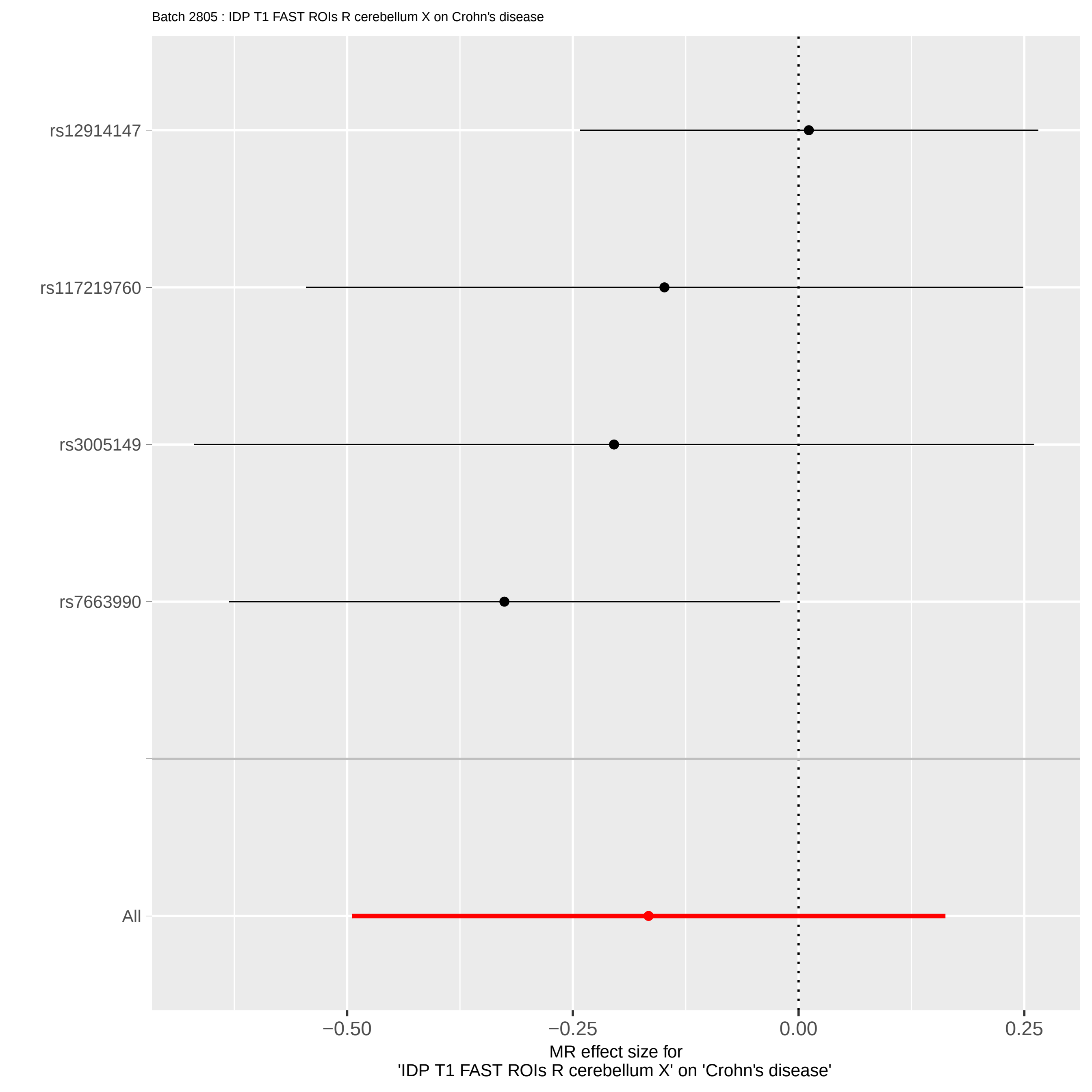

Batch 2805 : IDP T1 FAST ROIs R cerebellum X on Crohn's disease
rs12914147
rs117219760
rs3005149
rs7663990
All
−0.50
−0.25
0.00
0.25
MR effect size for
'IDP T1 FAST ROIs R cerebellum X' on 'Crohn's disease'

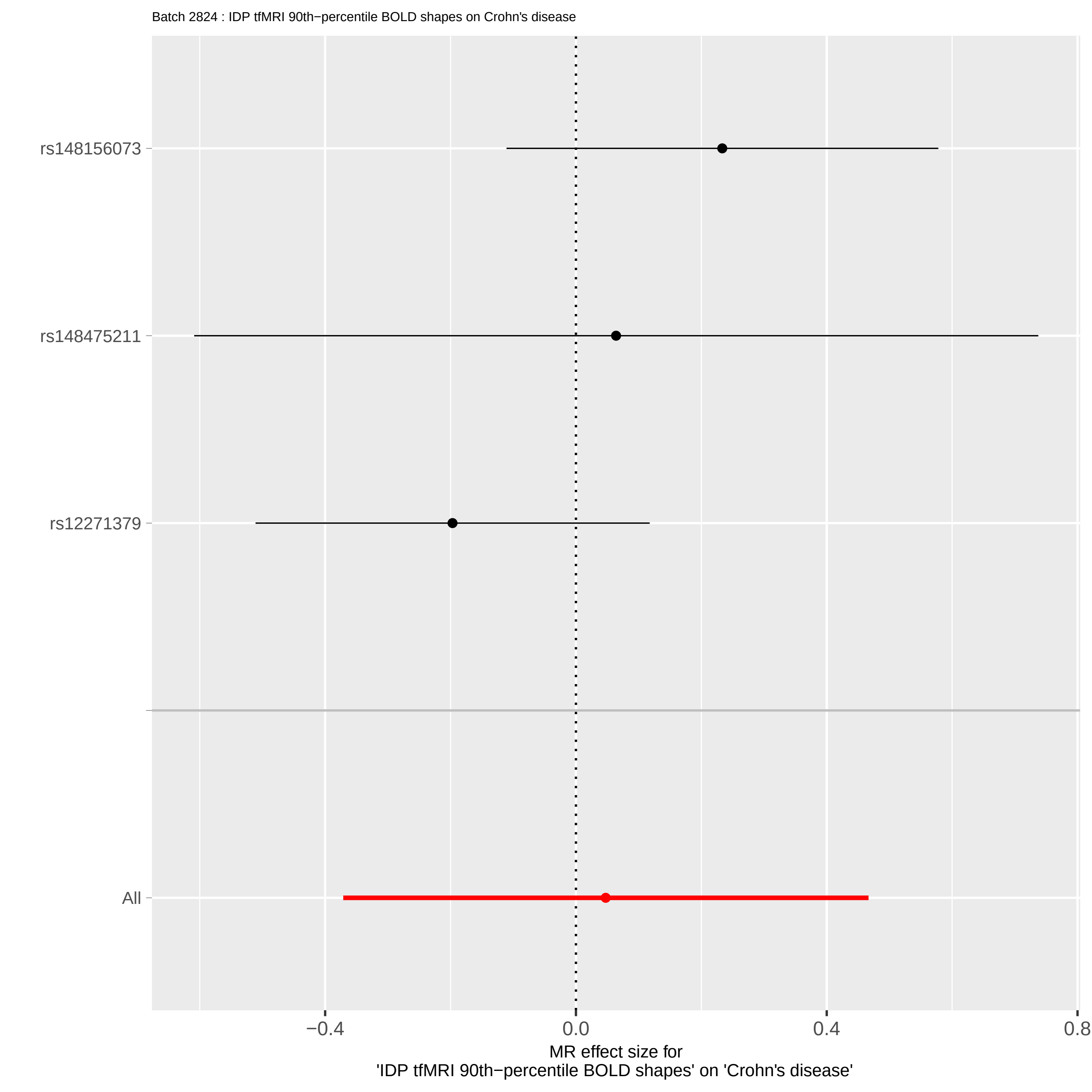

Batch 2824 : IDP tfMRI 90th−percentile BOLD shapes on Crohn's disease
rs148156073
rs148475211
rs12271379
All
−0.4
0.0
0.4
0.8
MR effect size for
'IDP tfMRI 90th−percentile BOLD shapes' on 'Crohn's disease'

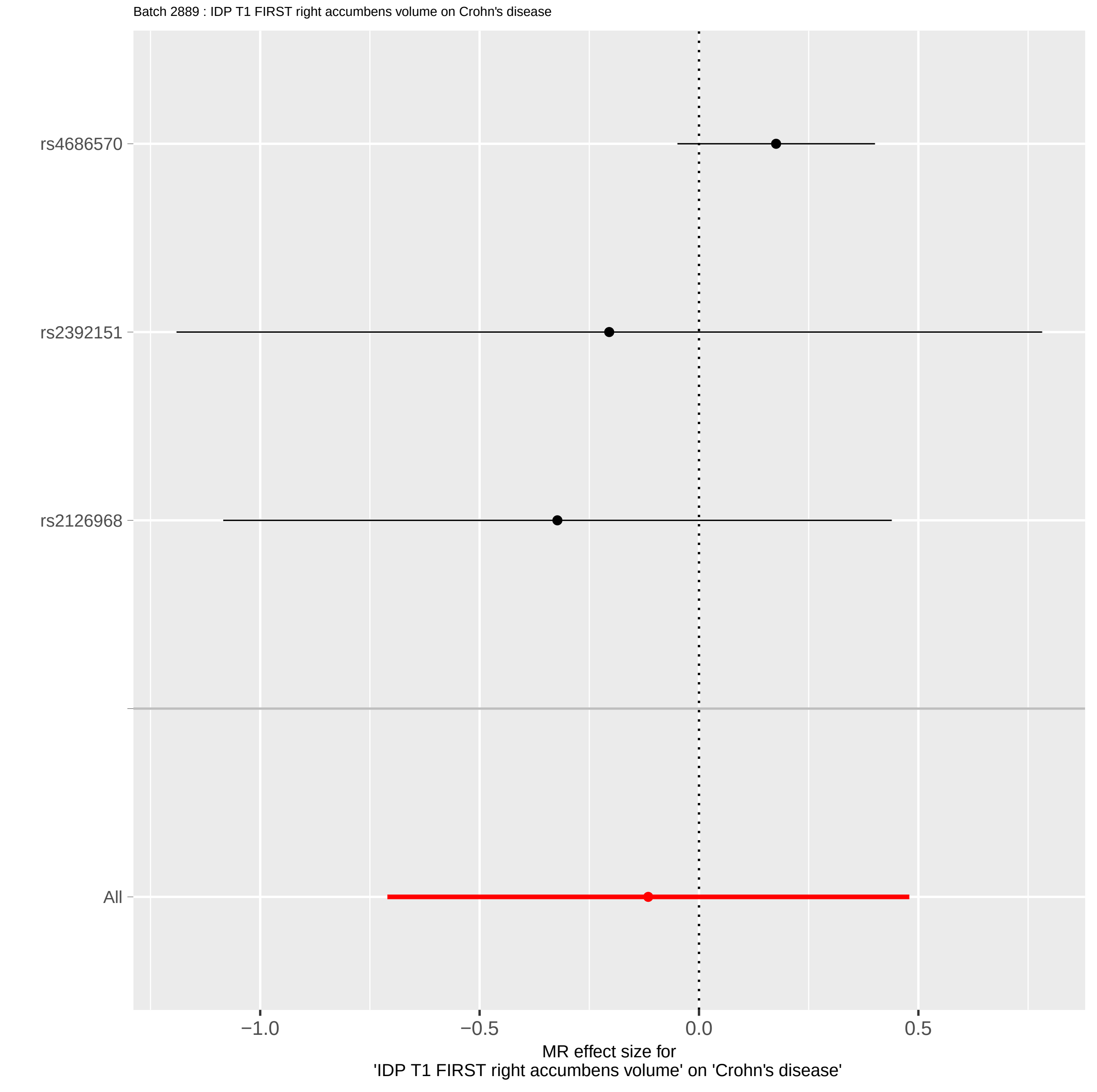
Batch 2889 : IDP T1 FIRST right accumbens volume on Crohn's disease
rs4686570
rs2392151
rs2126968
All
−1.0
−0.5
0.0
0.5
MR effect size for
'IDP T1 FIRST right accumbens volume' on 'Crohn's disease'

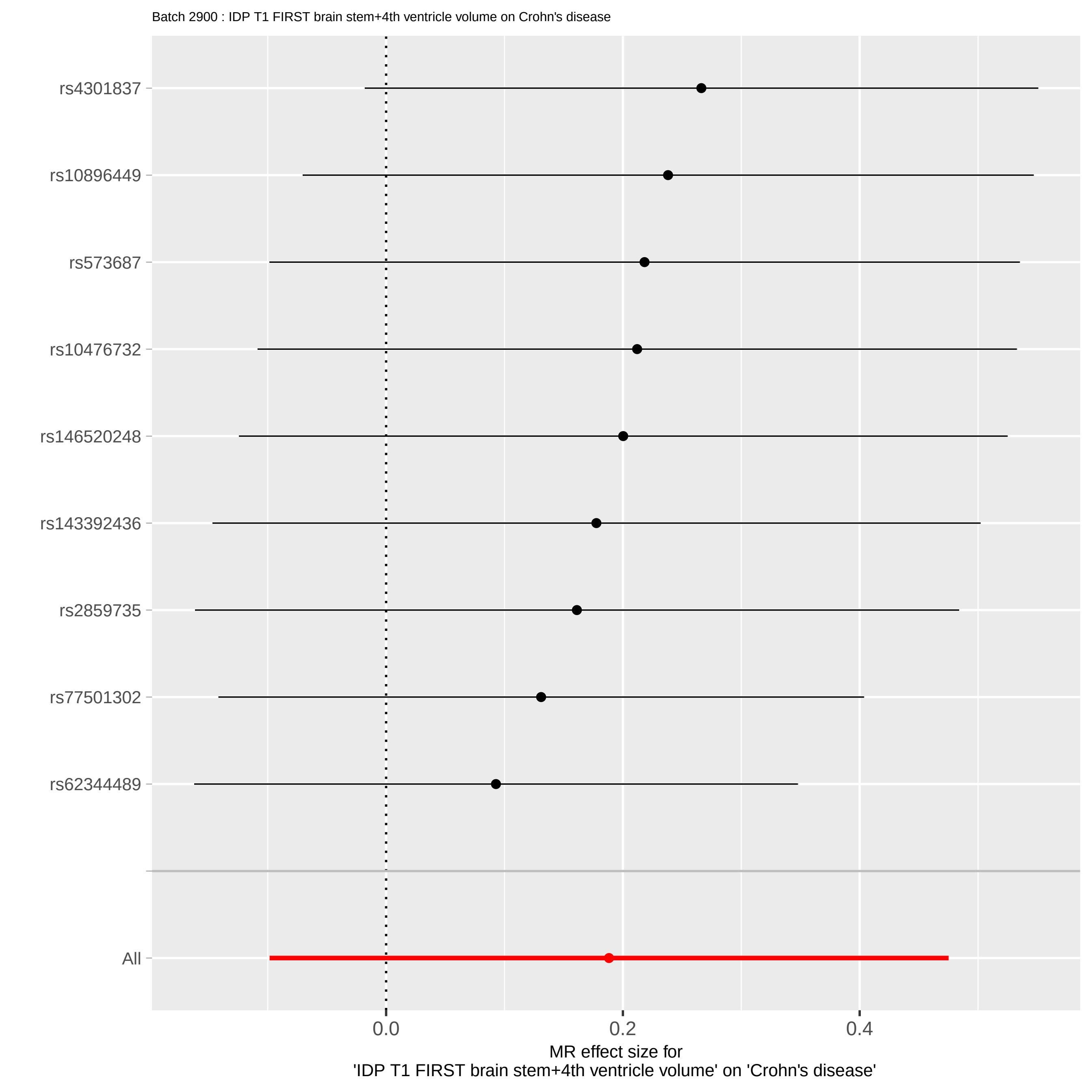

Batch 2900 : IDP T1 FIRST brain stem+4th ventricle volume on Crohn's disease
rs4301837
rs10896449
rs573687
rs10476732
rs146520248
rs143392436
rs2859735
rs77501302
rs62344489
All
0.0
0.2
0.4
MR effect size for
'IDP T1 FIRST brain stem+4th ventricle volume' on 'Crohn's disease'

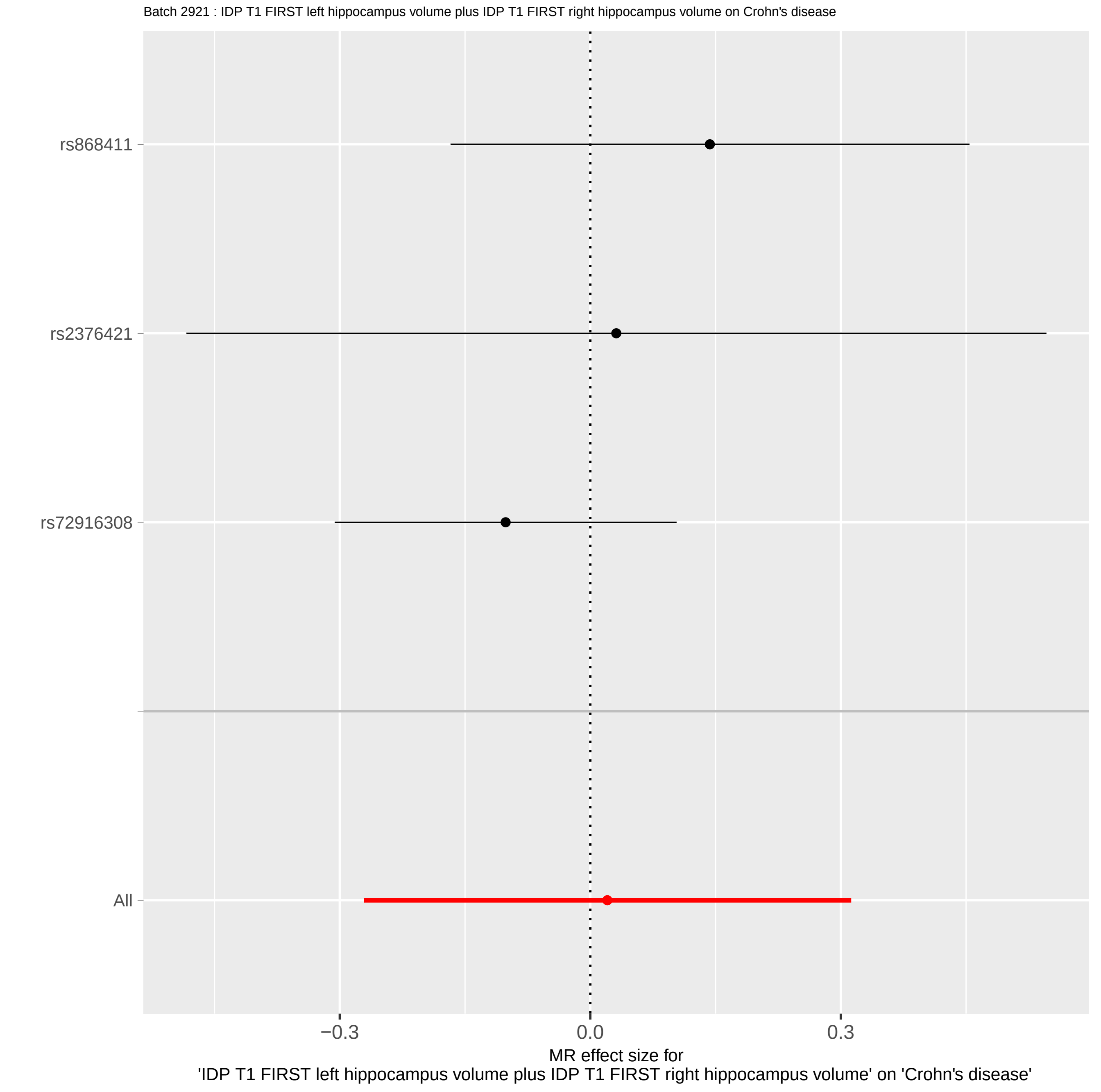

Batch 2921 : IDP T1 FIRST left hippocampus volume plus IDP T1 FIRST right hippocampus volume on Crohn's disease
rs868411
rs2376421
rs72916308
All
−0.3
0.0
0.3
MR effect size for
'IDP T1 FIRST left hippocampus volume plus IDP T1 FIRST right hippocampus volume' on 'Crohn's disease'

Batch 2922 : IDP T1 FIRST left amygdala volume plus IDP T1 FIRST right amygdala volume on Crohn's disease

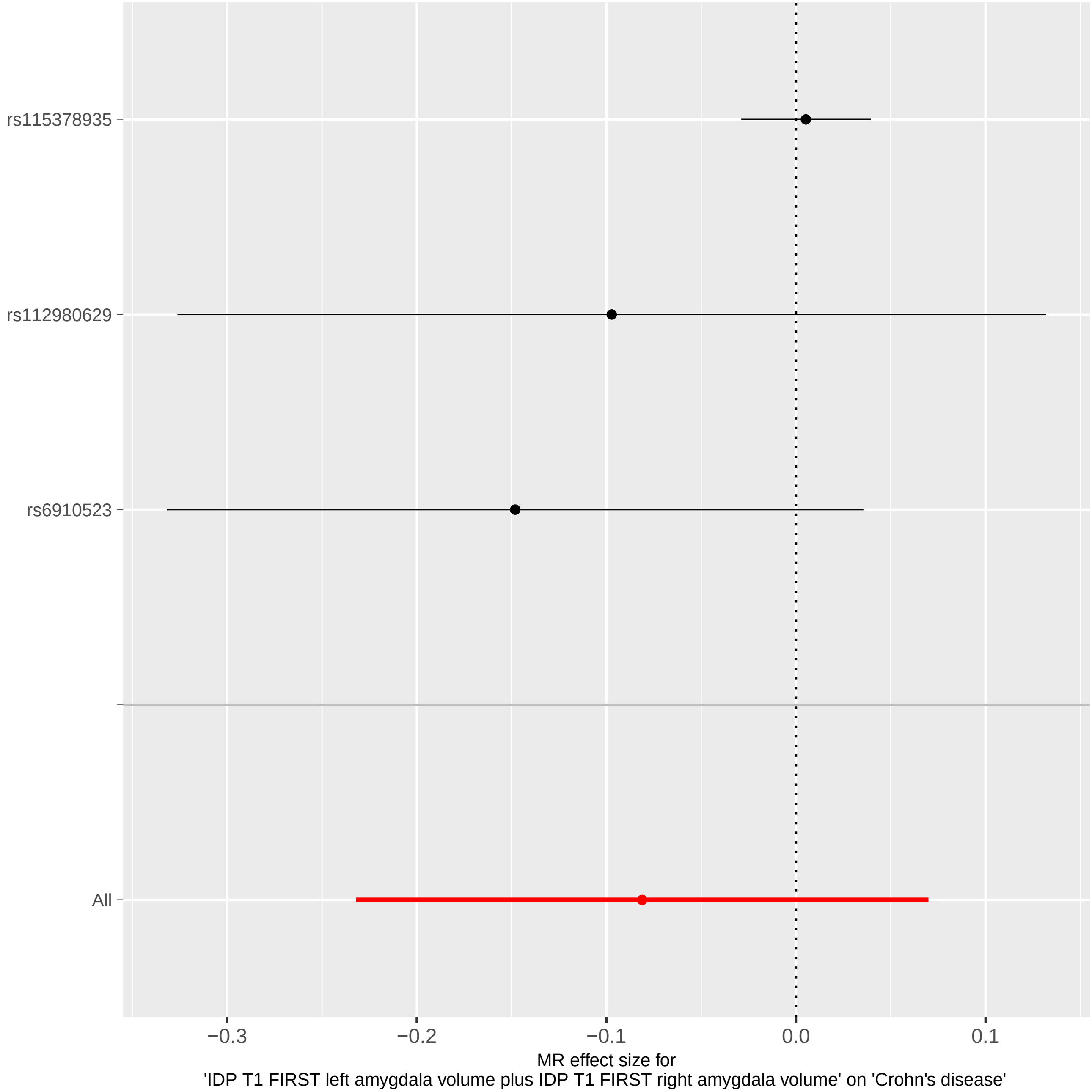

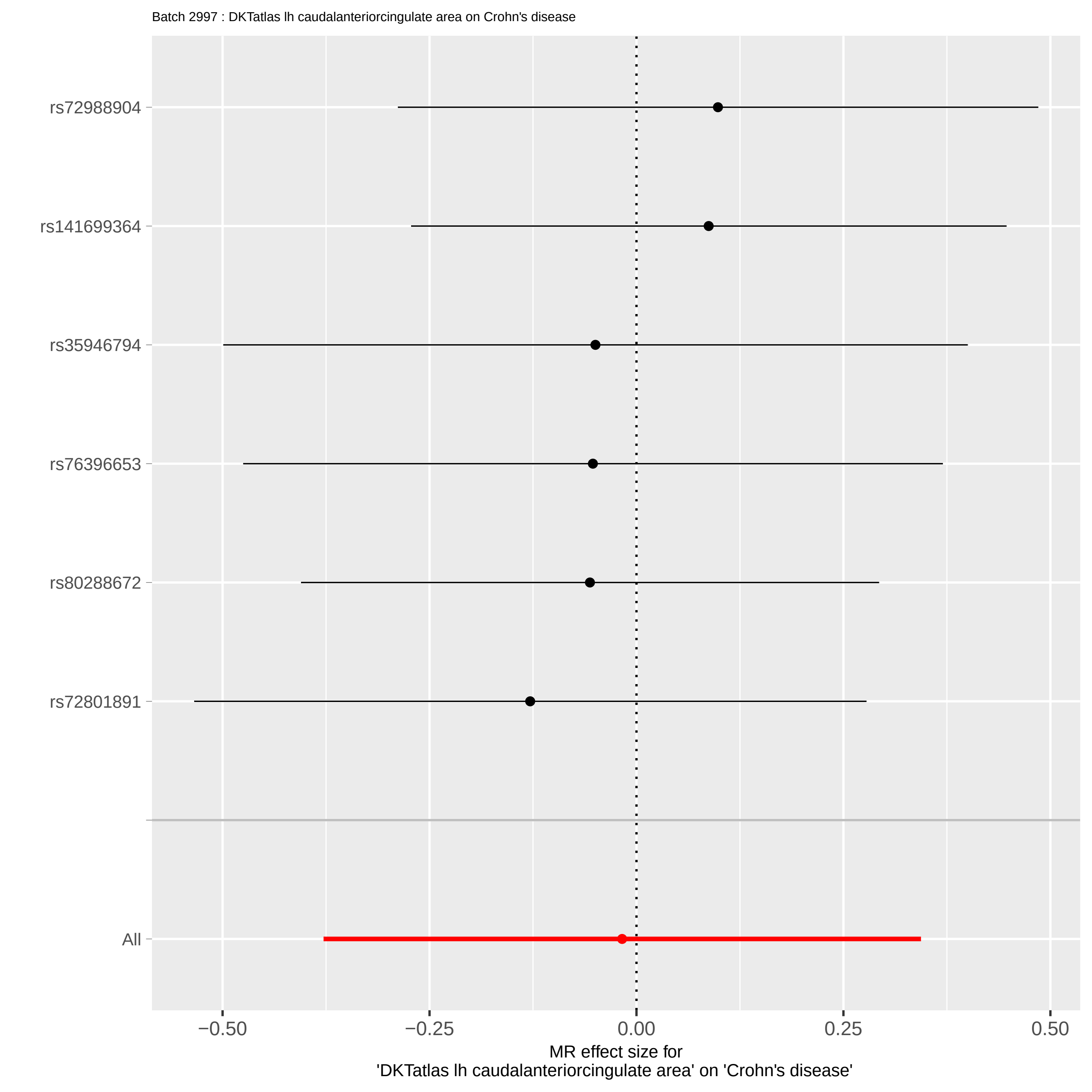
Batch 2997 : DKTatlas lh caudalanteriorcingulate area on Crohn's disease
rs72988904
rs141699364
rs35946794
rs76396653
rs80288672
rs72801891
All
−0.50
−0.25
0.00
0.25
0.50
MR effect size for
'DKTatlas lh caudalanteriorcingulate area' on 'Crohn's disease'

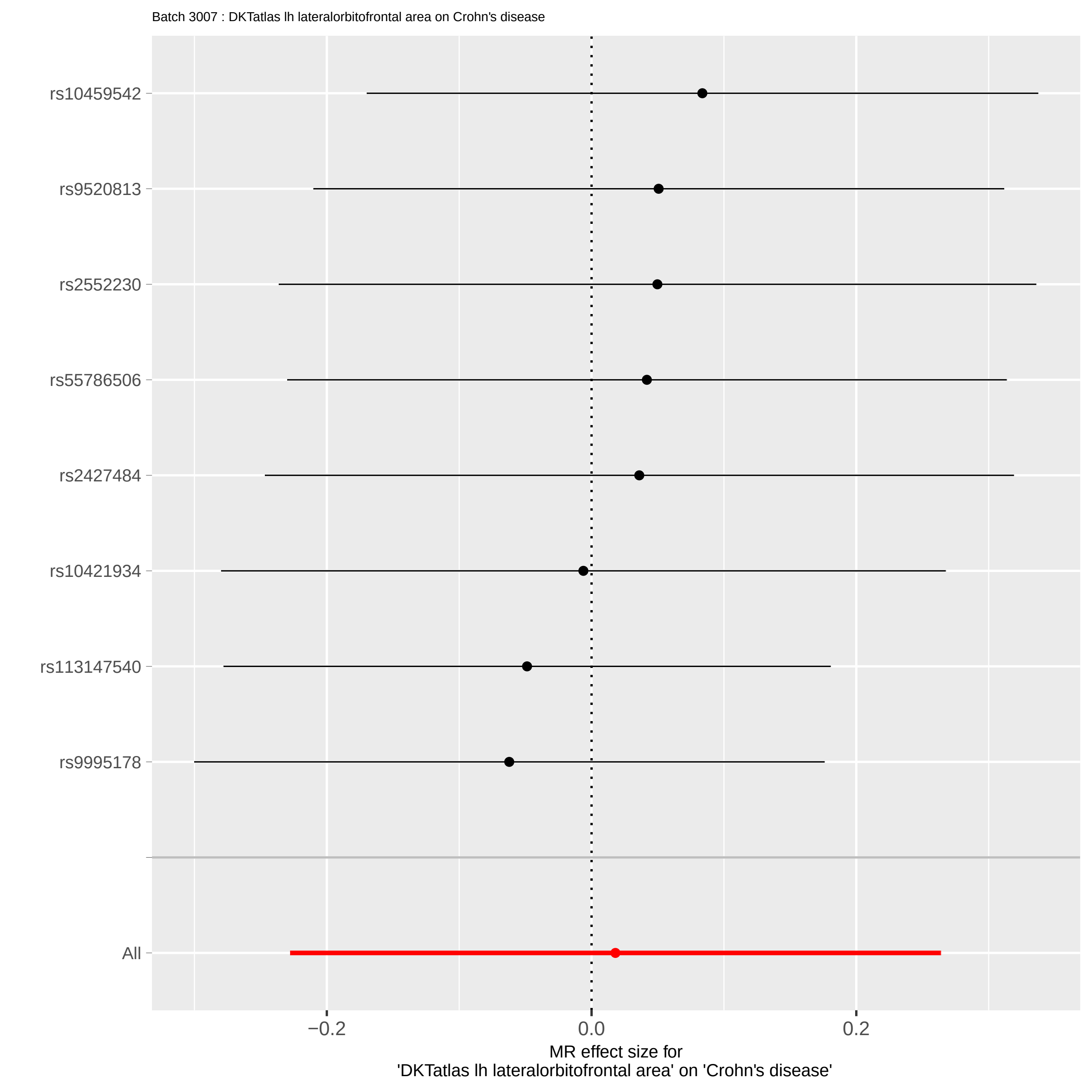

Batch 3007 : DKTatlas lh lateralorbitofrontal area on Crohn's disease
rs10459542
rs9520813
rs2552230
rs55786506
rs2427484
rs10421934
rs113147540
rs9995178
All
−0.2
0.0
0.2
MR effect size for
'DKTatlas lh lateralorbitofrontal area' on 'Crohn's disease'

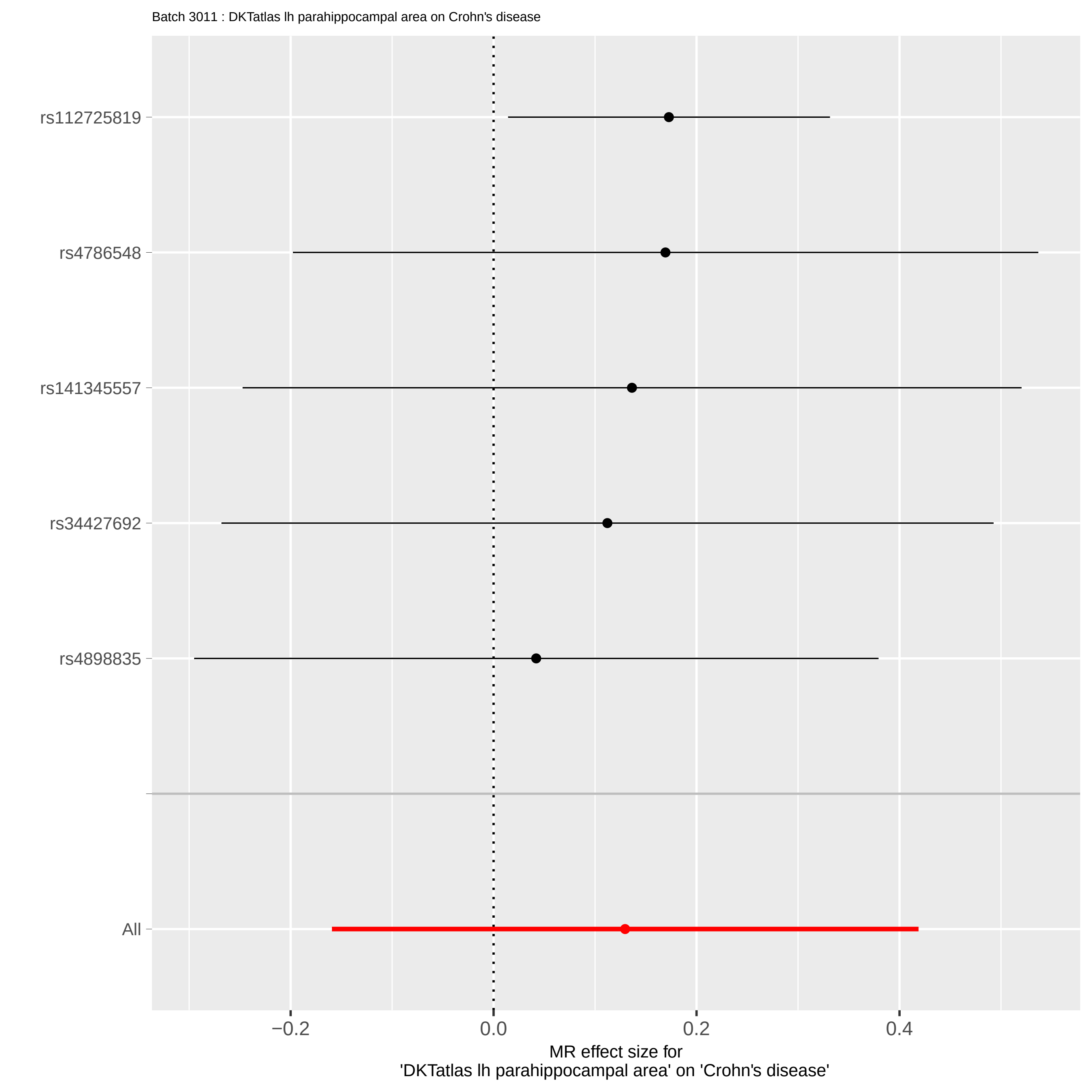
Batch 3011 : DKTatlas lh parahippocampal area on Crohn's disease
rs112725819
rs4786548
rs141345557
rs34427692
rs4898835
All
−0.2
0.0
0.2
0.4
MR effect size for
'DKTatlas lh parahippocampal area' on 'Crohn's disease'

Batch 3012 : DKTatlas lh paracentral area on Crohn's disease

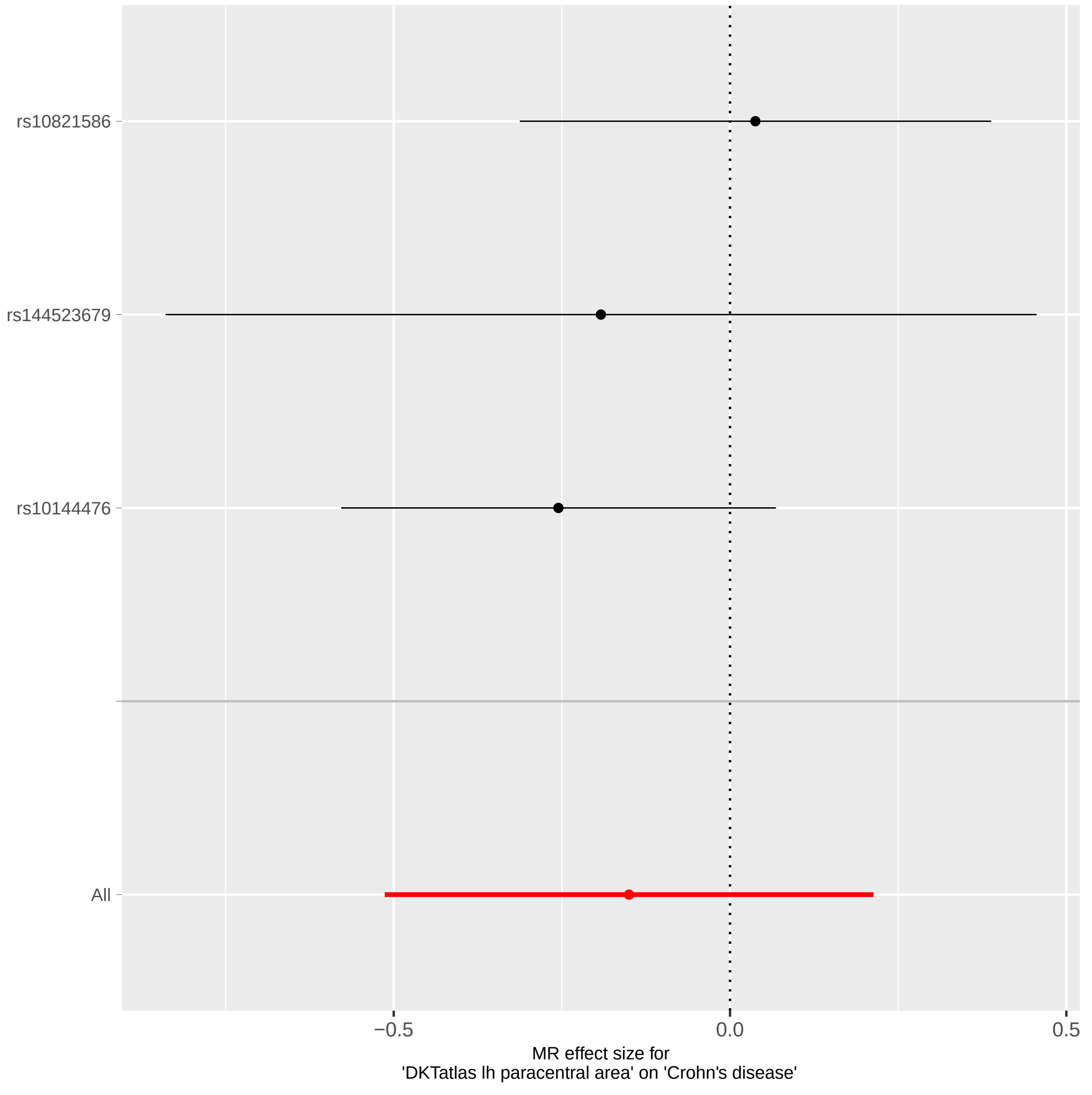

Batch 3015 : DKTatlas lh parsorbitalis area on Crohn's disease

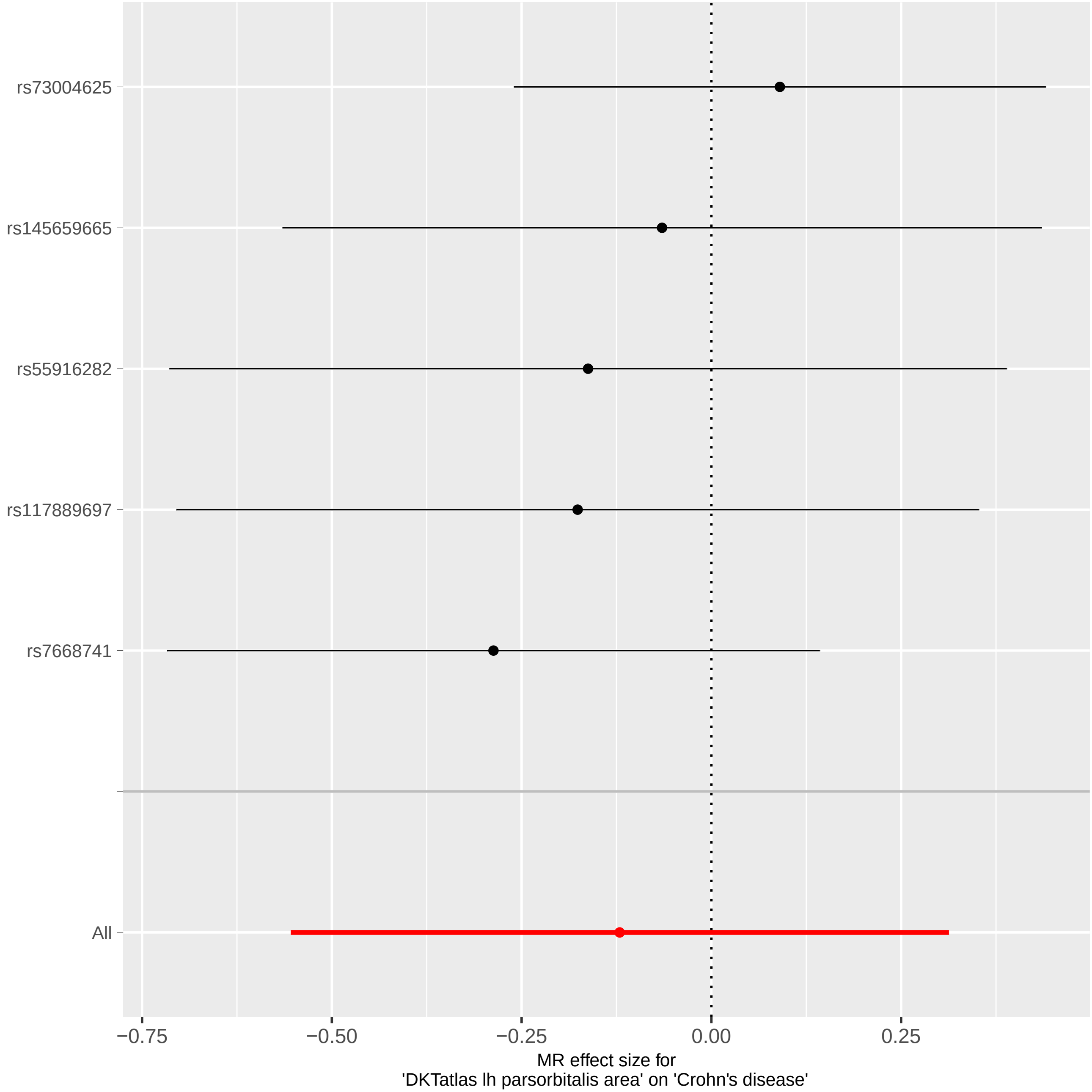

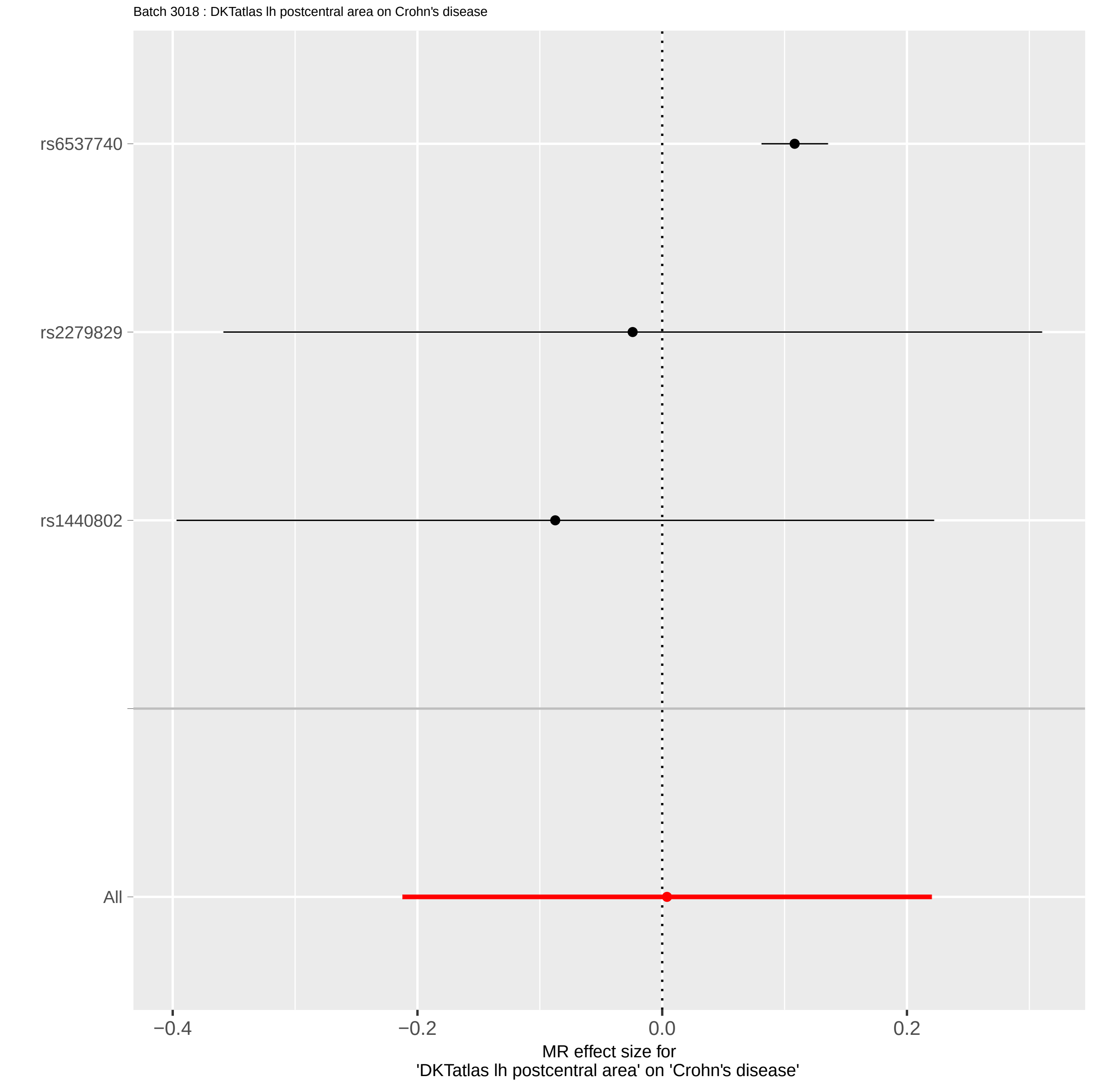
Batch 3018 : DKTatlas lh postcentral area on Crohn's disease
rs6537740
rs2279829
rs1440802
All
−0.4
−0.2
0.0
0.2
MR effect size for
'DKTatlas lh postcentral area' on 'Crohn's disease'

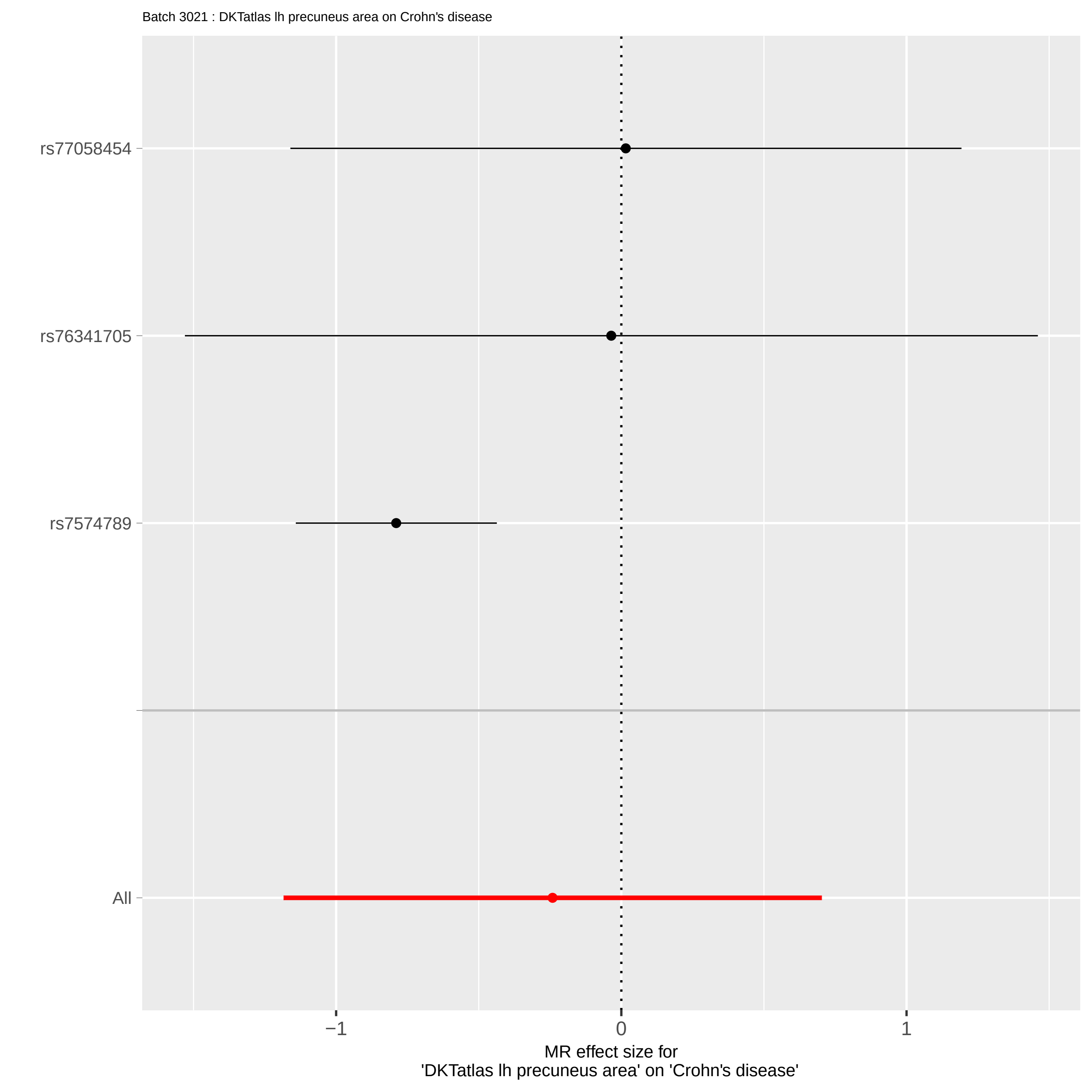
Batch 3021 : DKTatlas lh precuneus area on Crohn's disease
rs77058454
rs76341705
rs7574789
All
−1
0
1
MR effect size for
'DKTatlas lh precuneus area' on 'Crohn's disease'

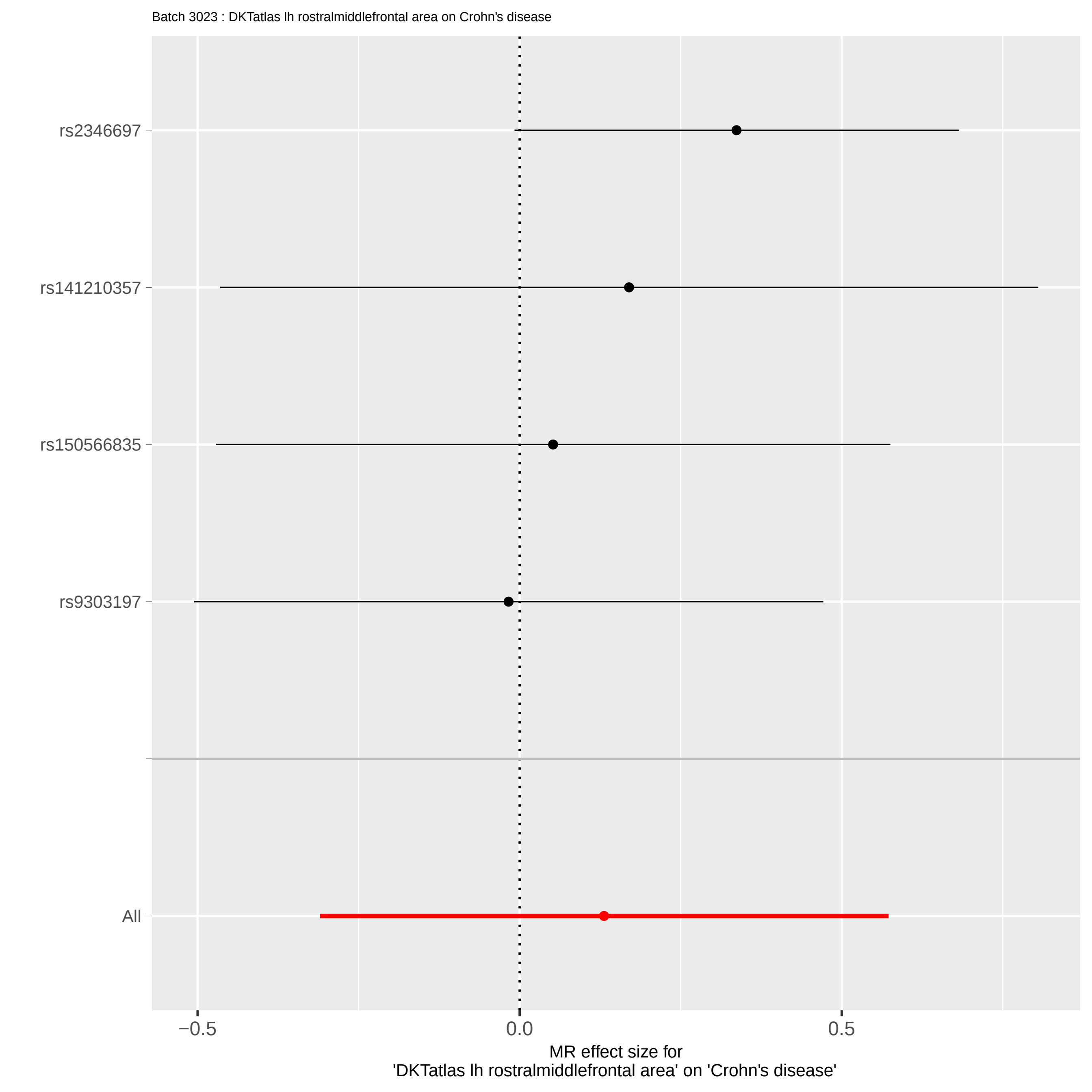

Batch 3023 : DKTatlas lh rostralmiddlefrontal area on Crohn's disease
rs2346697
rs141210357
rs150566835
rs9303197
All
−0.5
0.0
0.5
MR effect size for
'DKTatlas lh rostralmiddlefrontal area' on 'Crohn's disease'

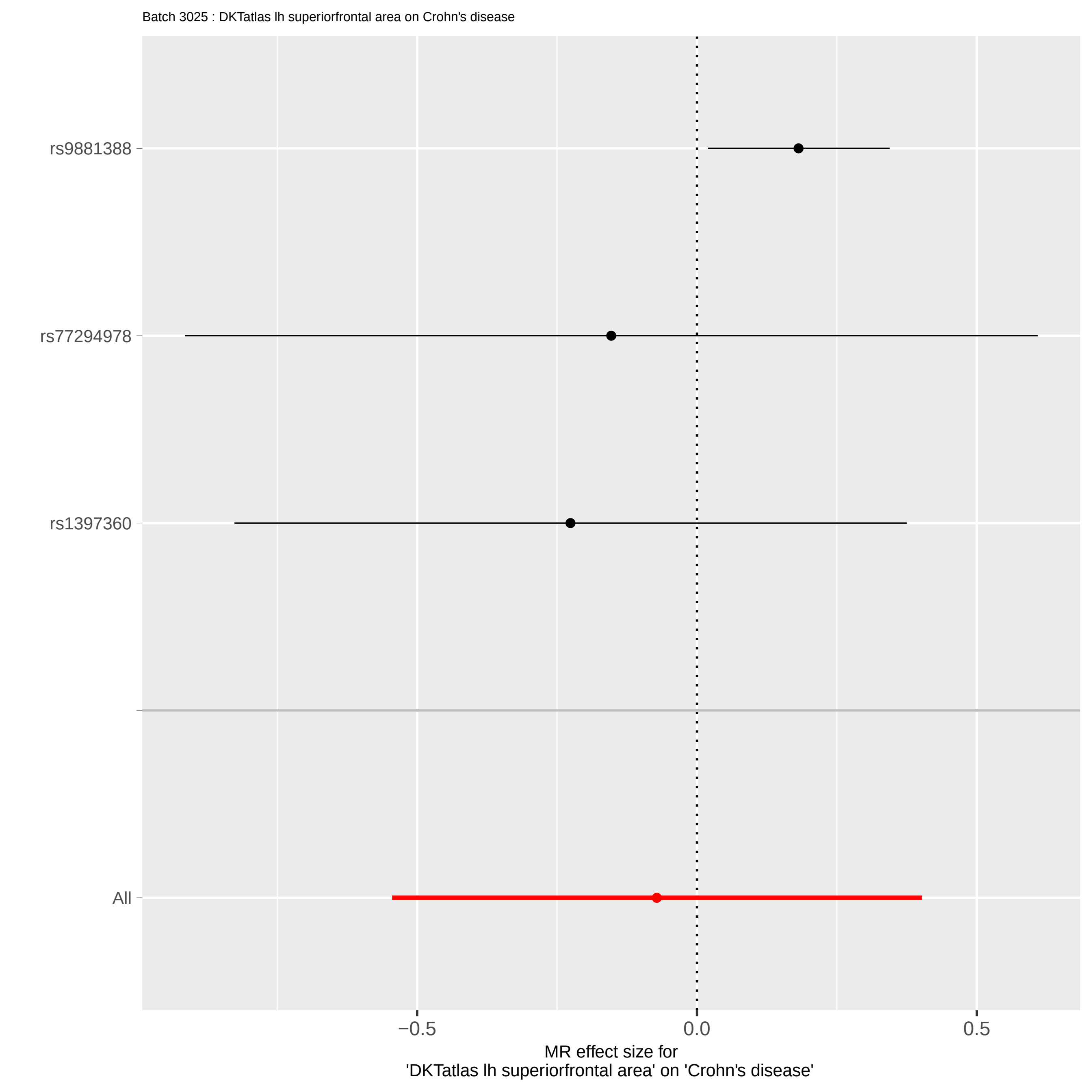

Batch 3025 : DKTatlas lh superiorfrontal area on Crohn's disease
rs9881388
rs77294978
rs1397360
All
−0.5
0.0
0.5
MR effect size for
'DKTatlas lh superiorfrontal area' on 'Crohn's disease'

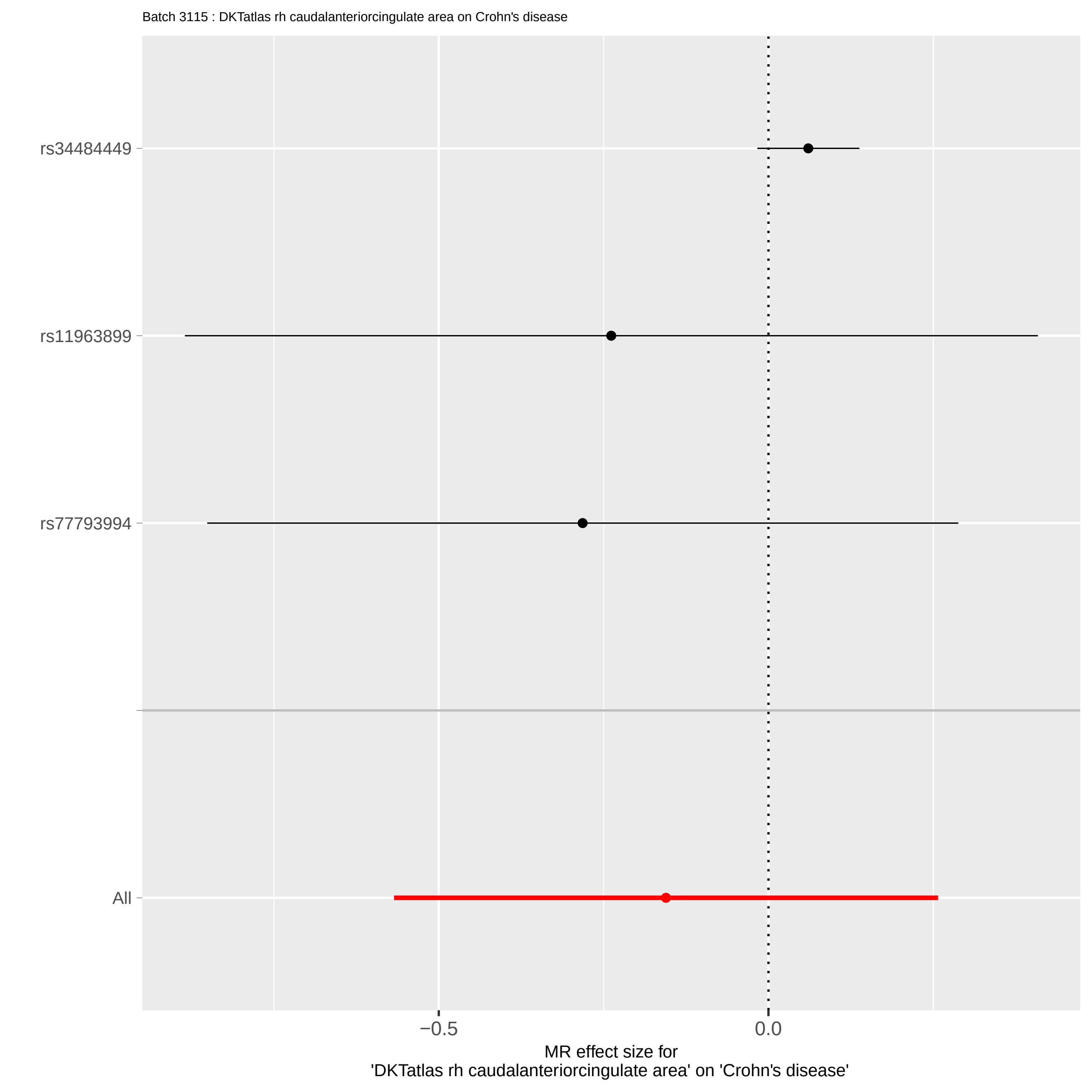

Batch 3115 : DKTatlas rh caudalanteriorcingulate area on Crohn's disease
rs34484449
rs11963899
rs77793994
All
−0.5
0.0
MR effect size for
'DKTatlas rh caudalanteriorcingulate area' on 'Crohn's disease'

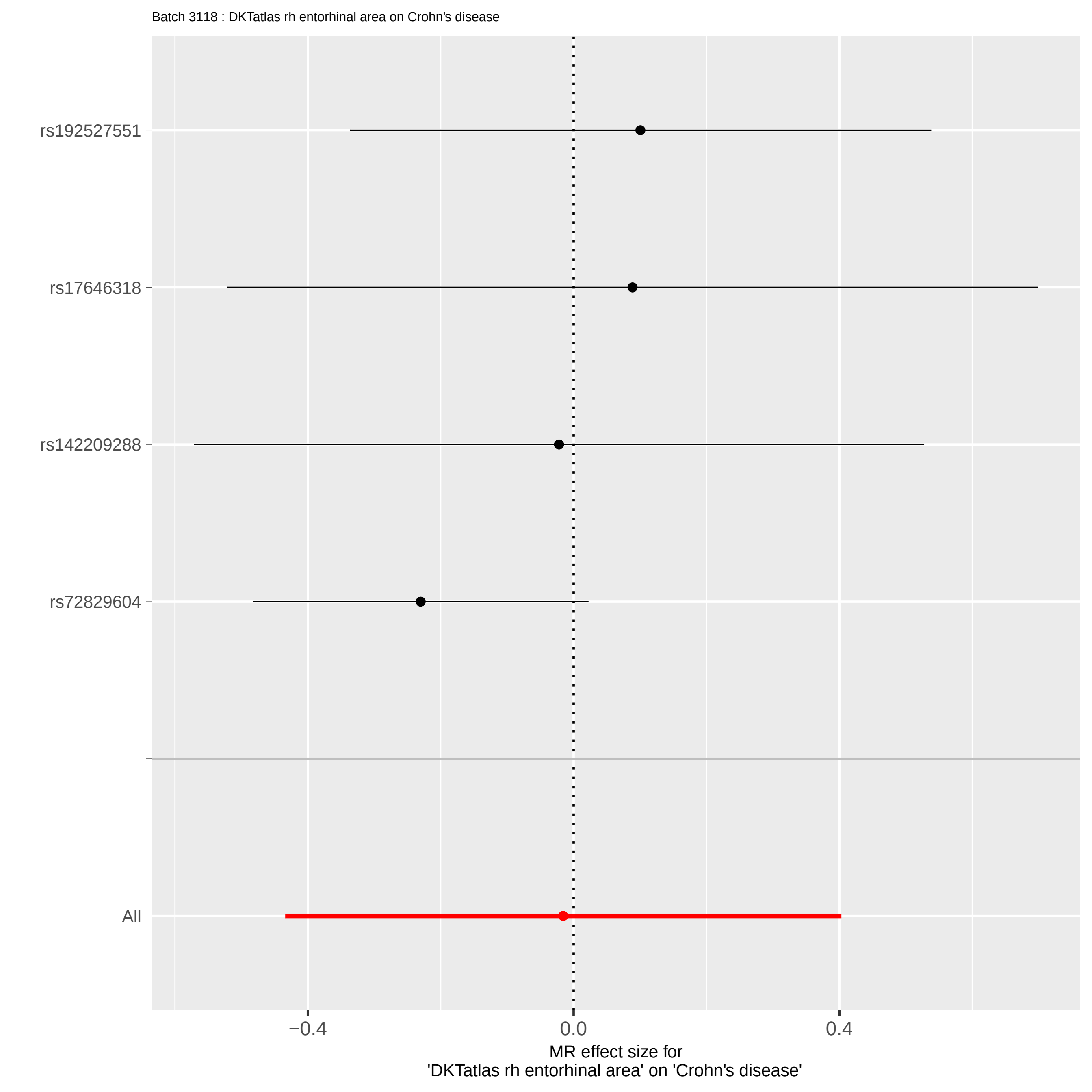

Batch 3118 : DKTatlas rh entorhinal area on Crohn's disease
rs192527551
rs17646318
rs142209288
rs72829604
All
−0.4
0.0
0.4
MR effect size for
'DKTatlas rh entorhinal area' on 'Crohn's disease'

Batch 3131 : DKTatlas rh parsopercularis area on Crohn's disease

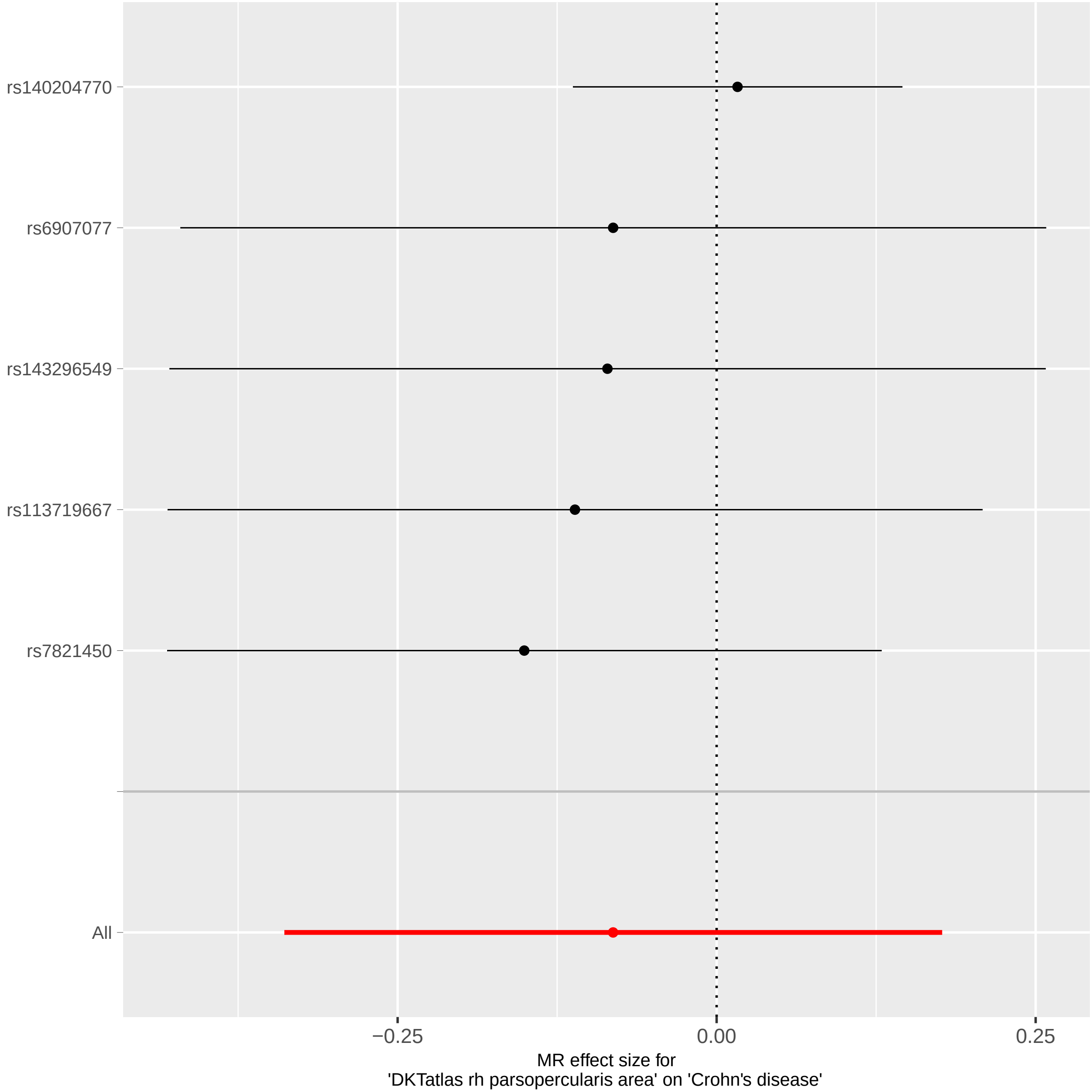

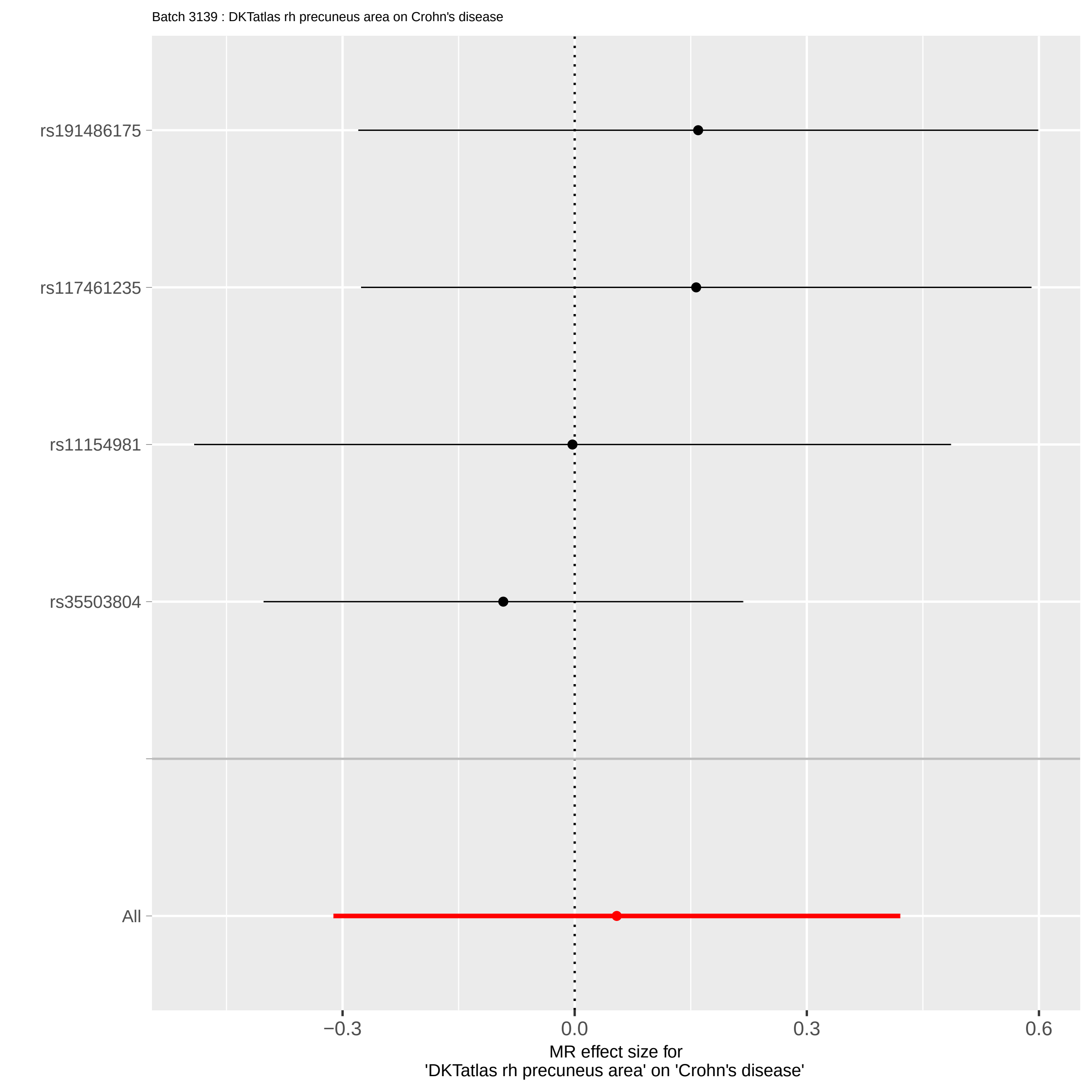

Batch 3139 : DKTatlas rh precuneus area on Crohn's disease
rs191486175
rs117461235
rs11154981
rs35503804
All
−0.3
0.0
0.3
0.6
MR effect size for
'DKTatlas rh precuneus area' on 'Crohn's disease'

Batch 3144 : DKTatlas rh superiortemporal area on Crohn's disease

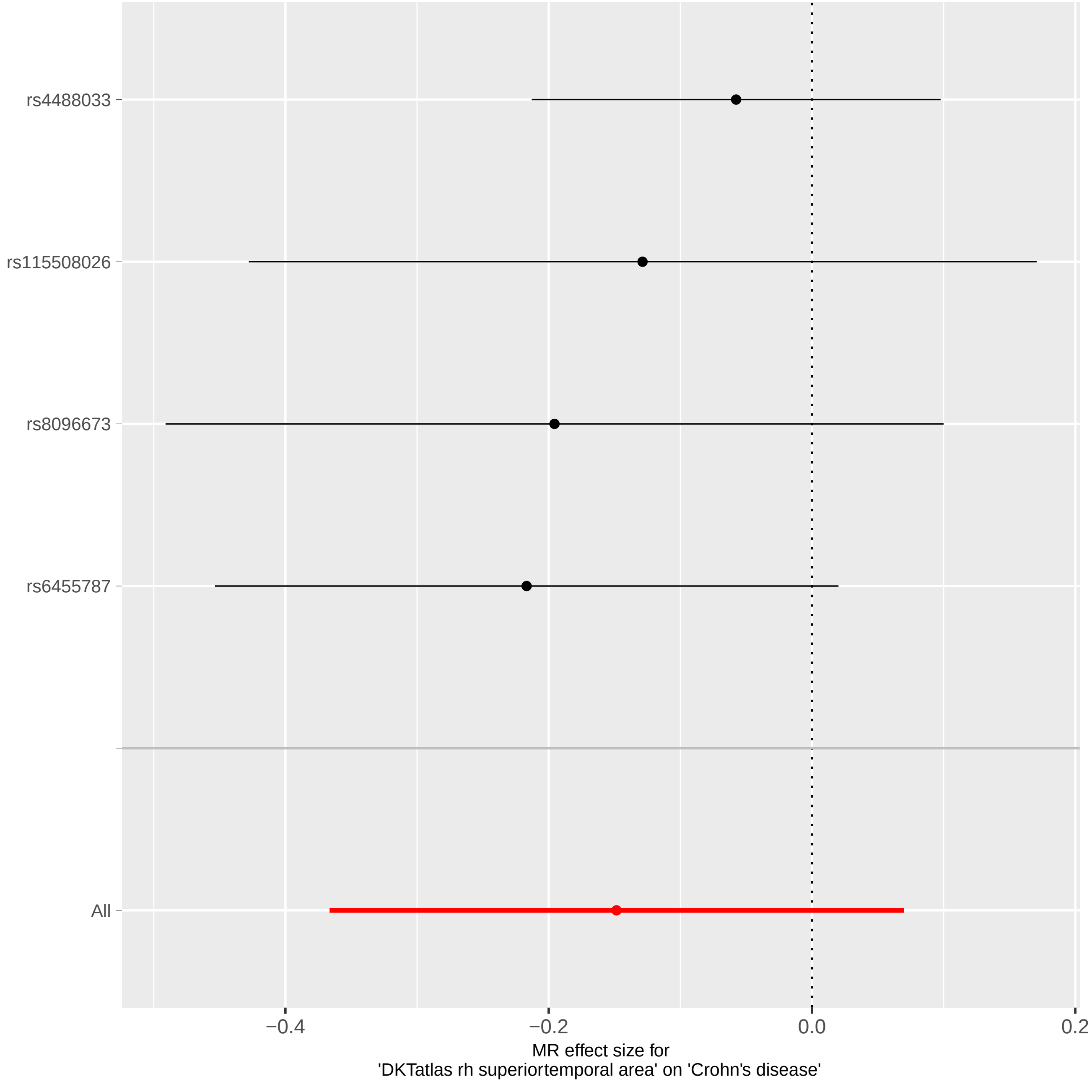

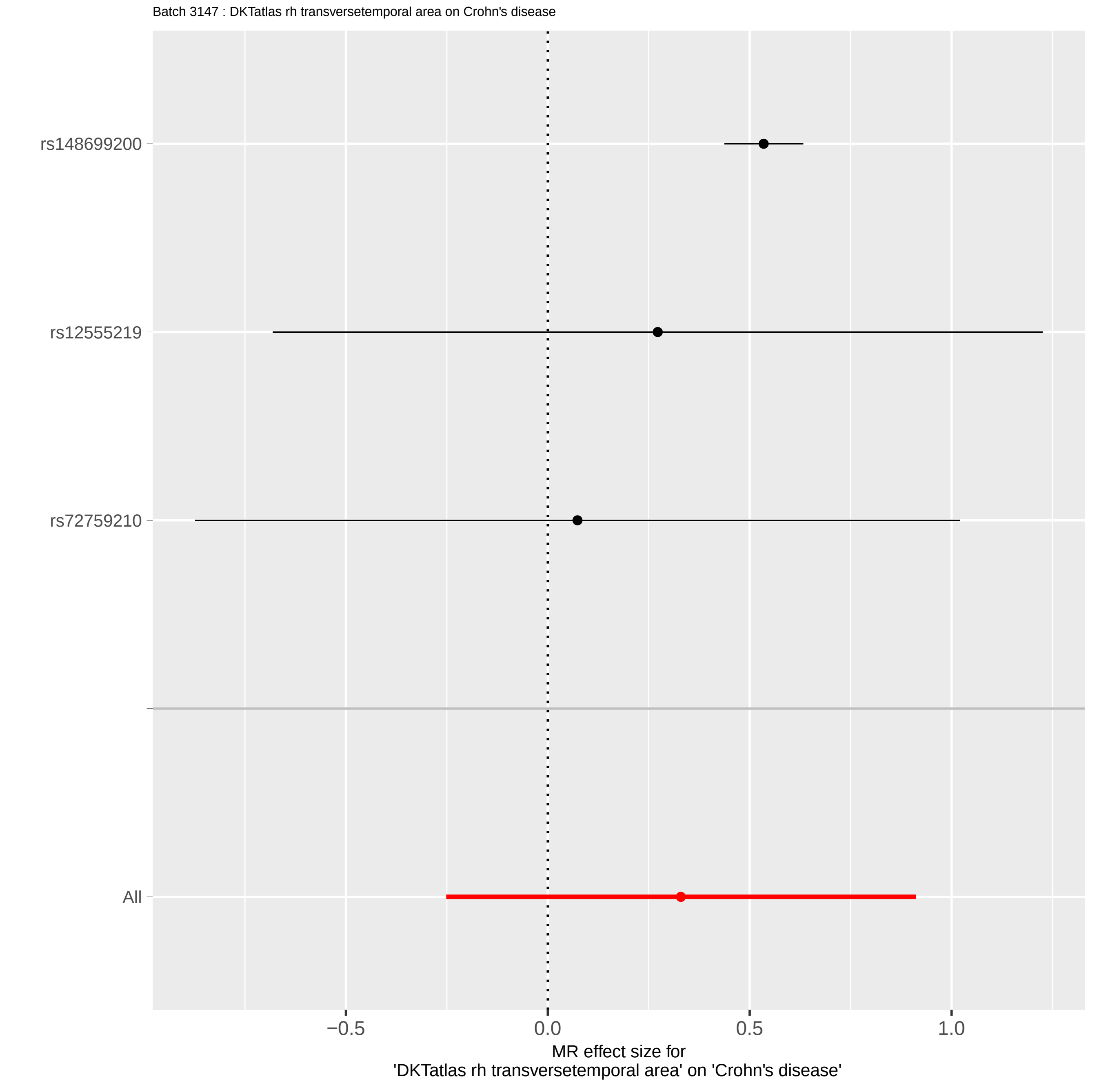

Batch 3147 : DKTatlas rh transversetemporal area on Crohn's disease
rs148699200
rs12555219
rs72759210
All
−0.5
0.0
0.5
1.0
MR effect size for
'DKTatlas rh transversetemporal area' on 'Crohn's disease'

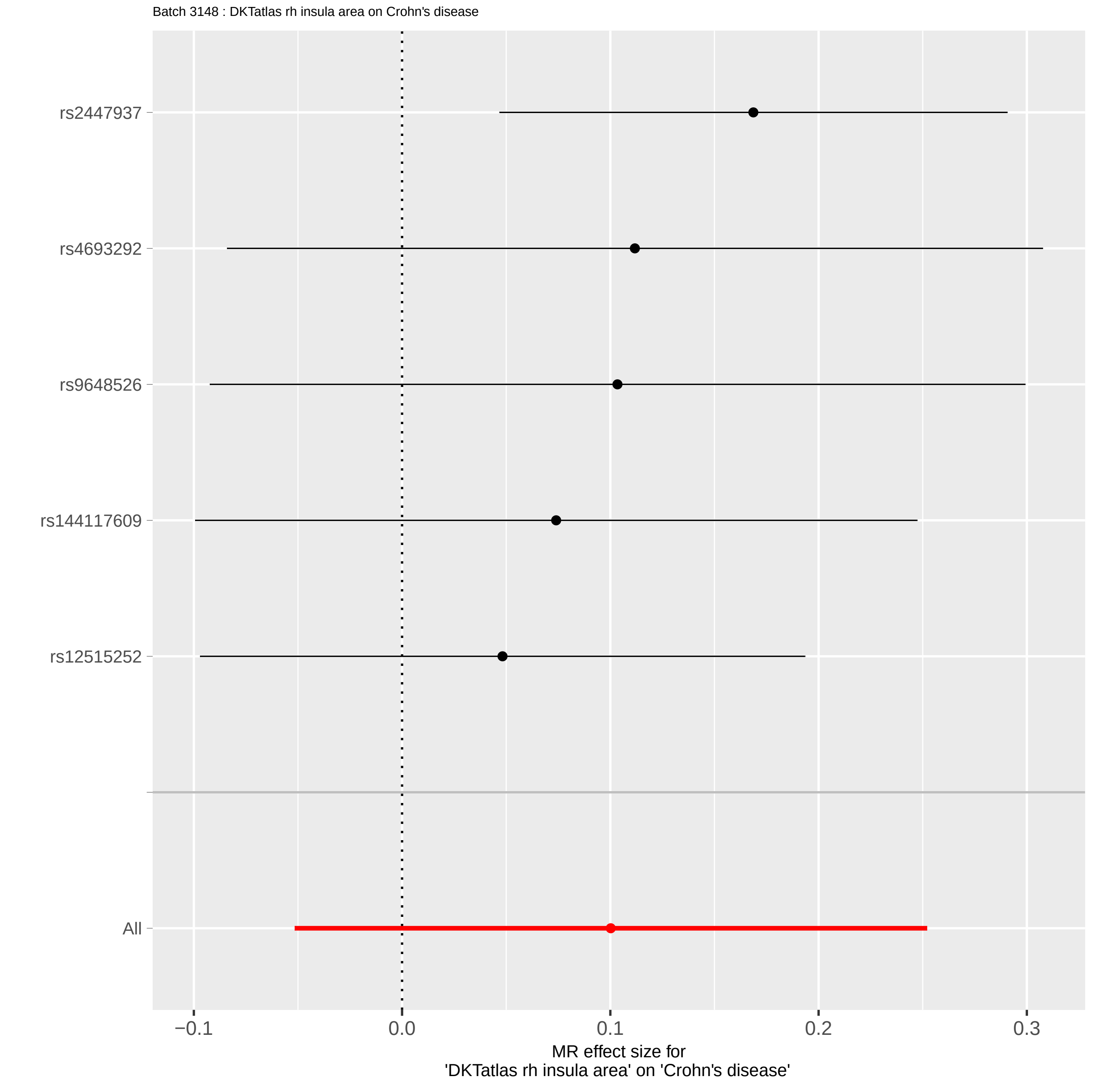
Batch 3148 : DKTatlas rh insula area on Crohn's disease
rs2447937
rs4693292
rs9648526
rs144117609
rs12515252
All
−0.1
0.0
0.1
0.2
0.3
MR effect size for
'DKTatlas rh insula area' on 'Crohn's disease'

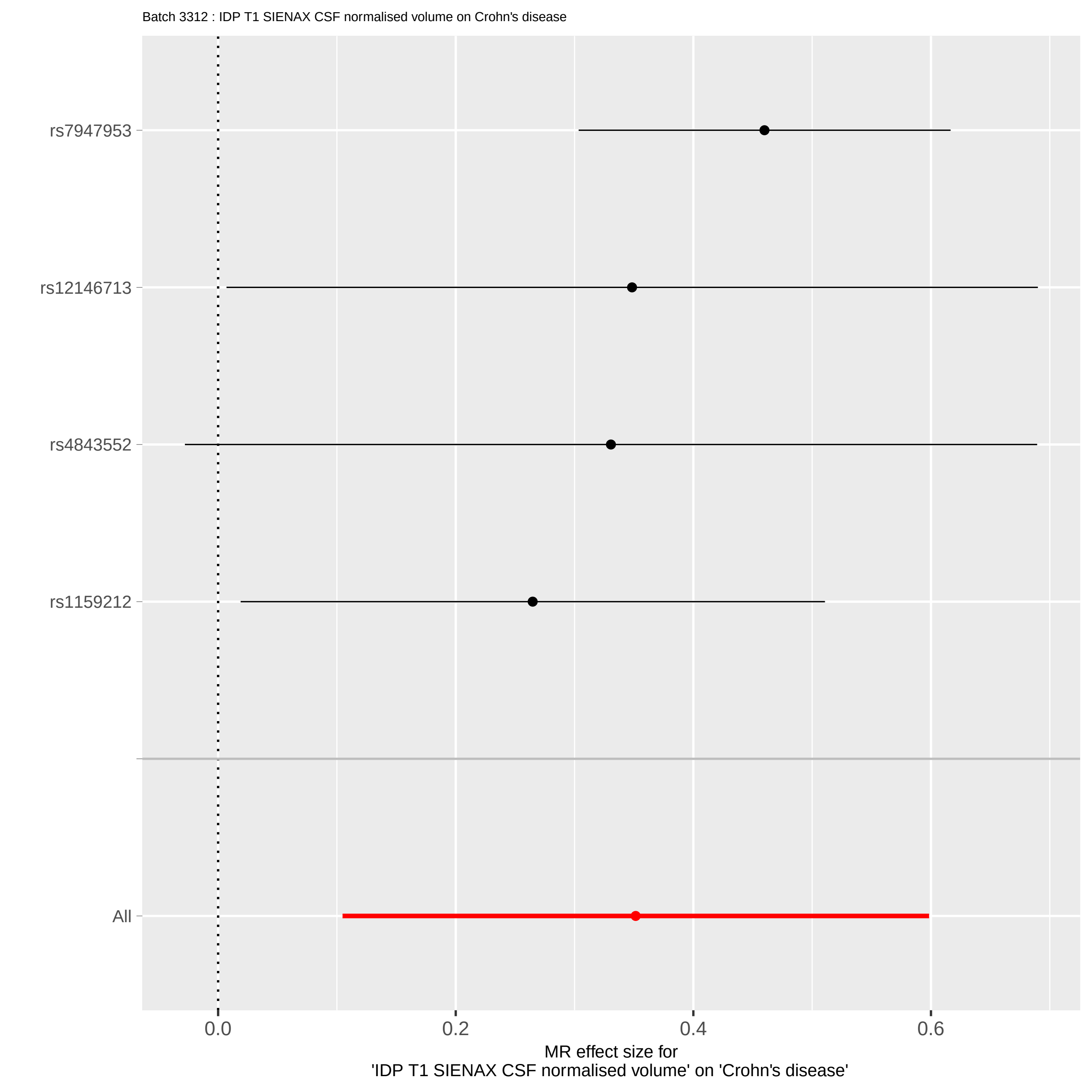

Batch 3312 : IDP T1 SIENAX CSF normalised volume on Crohn's disease
rs7947953
rs12146713
rs4843552
rs1159212
All
0.0
0.2
0.4
0.6
MR effect size for
'IDP T1 SIENAX CSF normalised volume' on 'Crohn's disease'

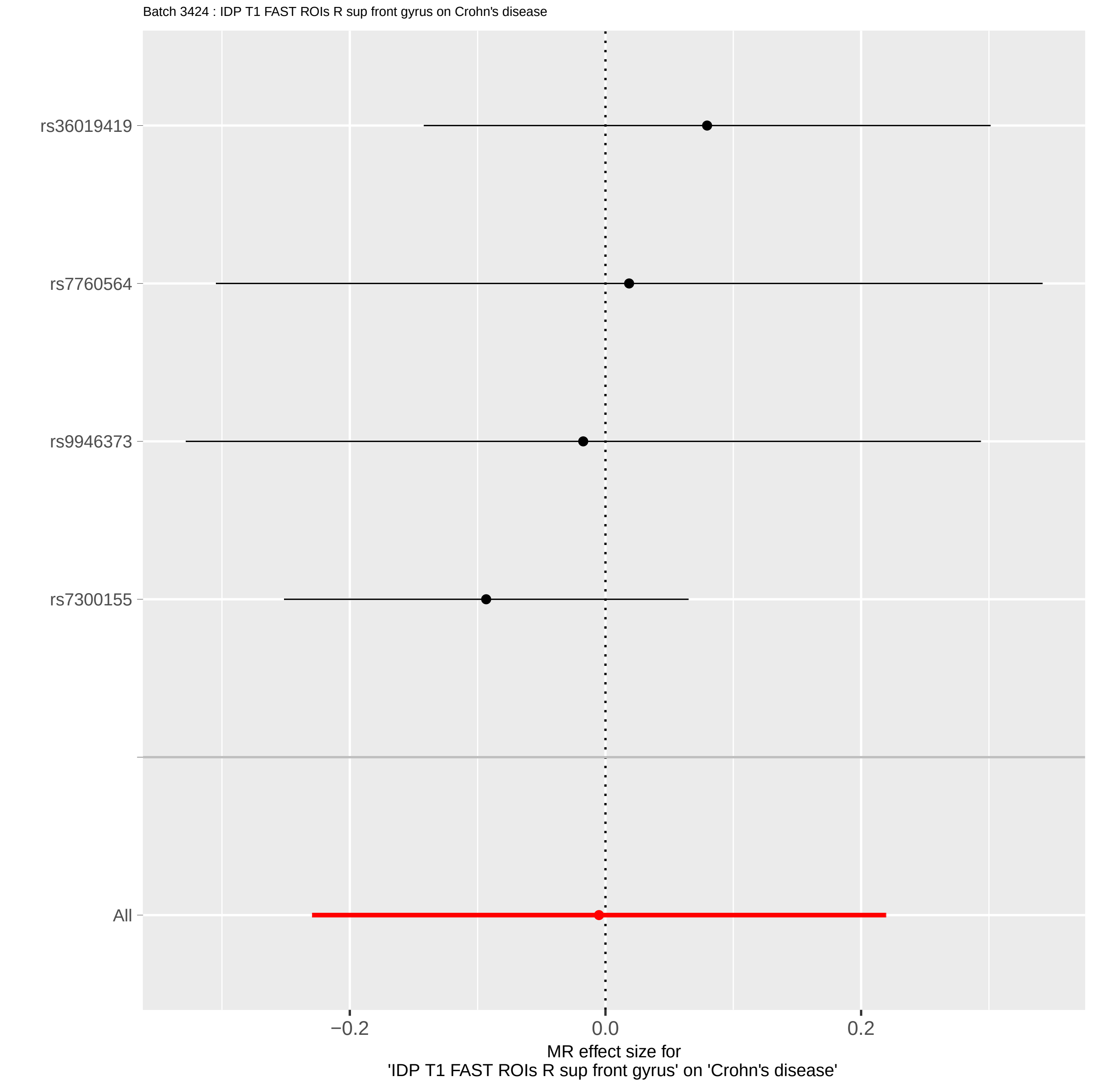
Batch 3424 : IDP T1 FAST ROIs R sup front gyrus on Crohn's disease
rs36019419
rs7760564
rs9946373
rs7300155
All
−0.2
0.0
0.2
MR effect size for
'IDP T1 FAST ROIs R sup front gyrus' on 'Crohn's disease'

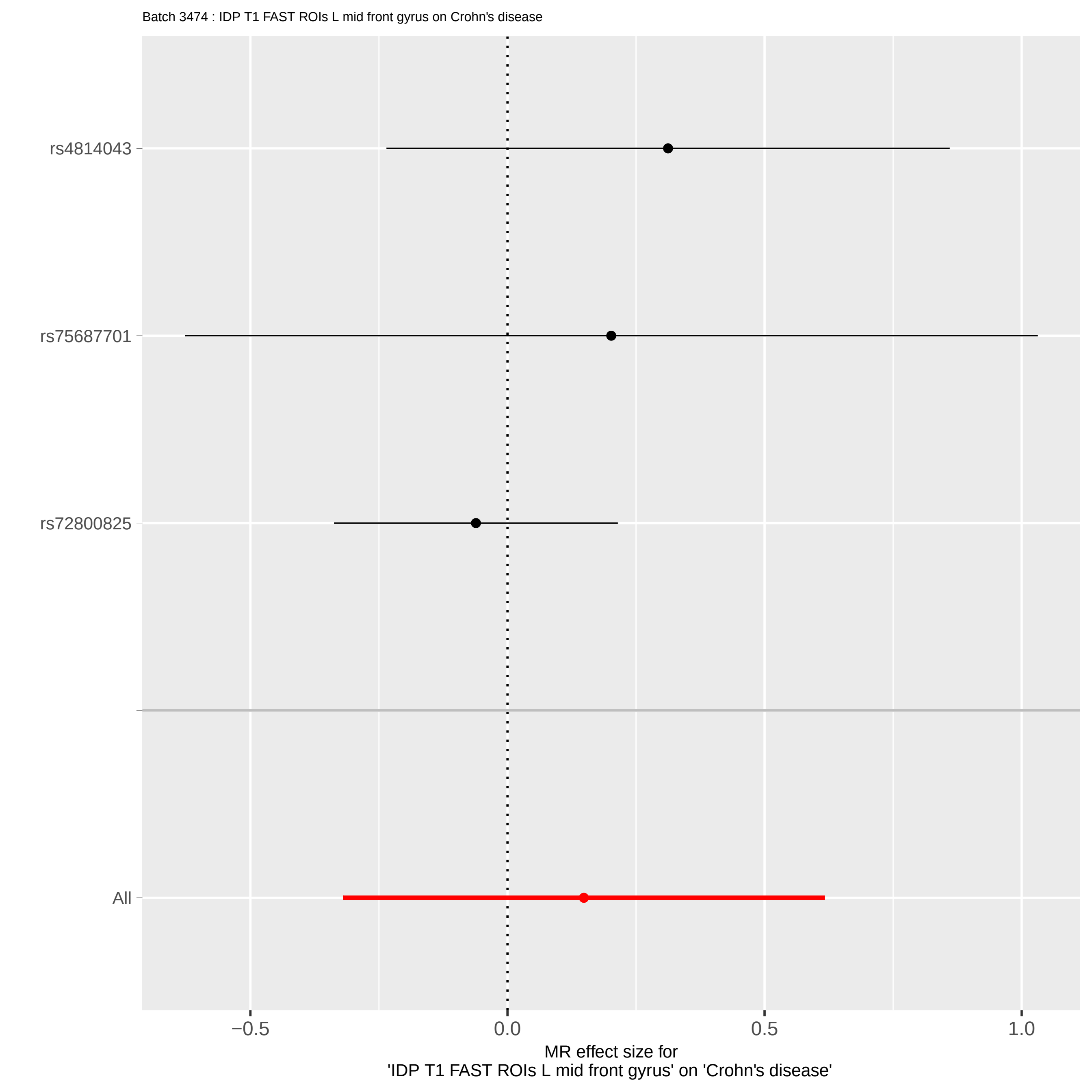
Batch 3474 : IDP T1 FAST ROIs L mid front gyrus on Crohn's disease
rs4814043
rs75687701
rs72800825
All
−0.5
0.0
0.5
1.0
MR effect size for
'IDP T1 FAST ROIs L mid front gyrus' on 'Crohn's disease'

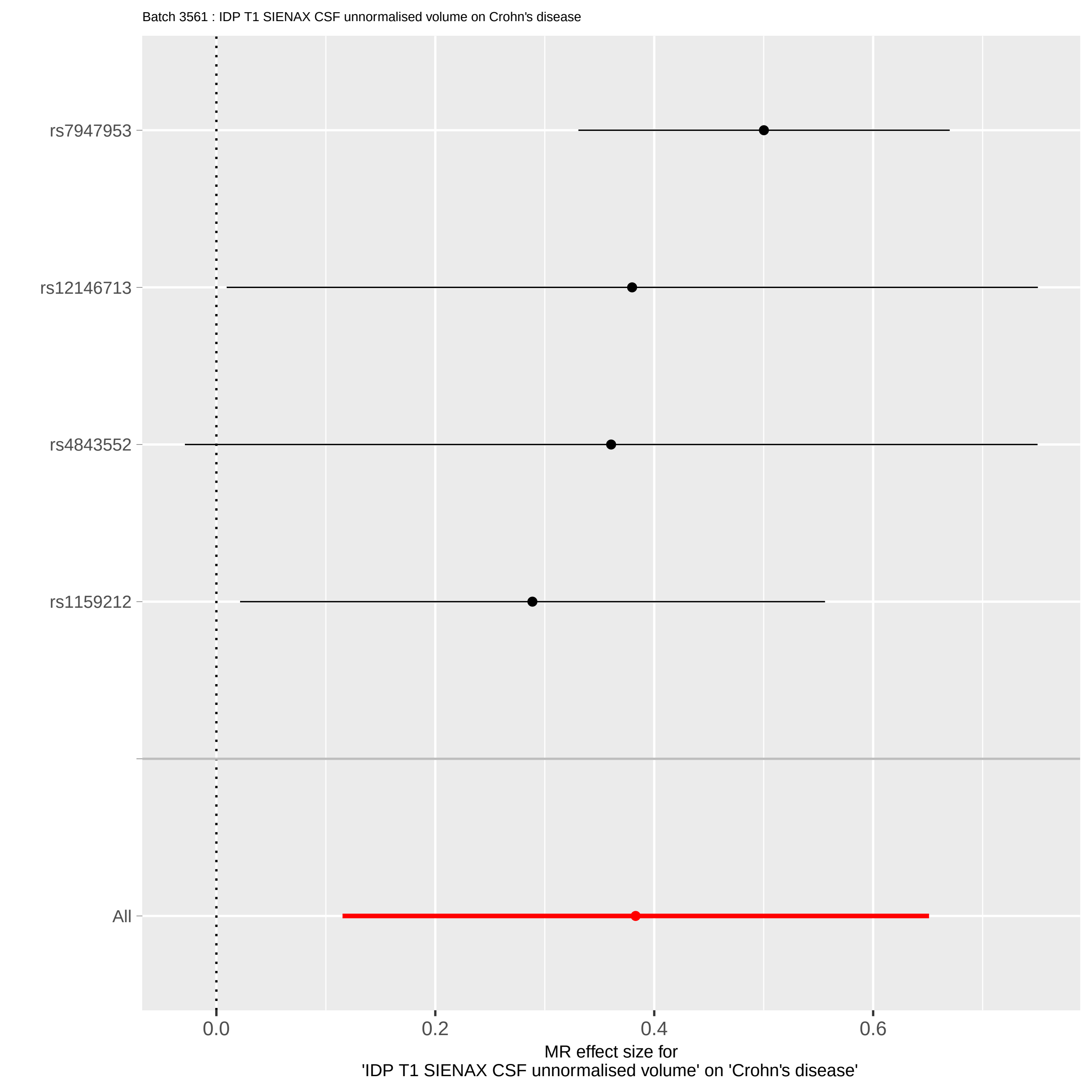
Batch 3561 : IDP T1 SIENAX CSF unnormalised volume on Crohn's disease
rs7947953
rs12146713
rs4843552
rs1159212
All
0.0
0.2
0.4
0.6
MR effect size for
'IDP T1 SIENAX CSF unnormalised volume' on 'Crohn's disease'

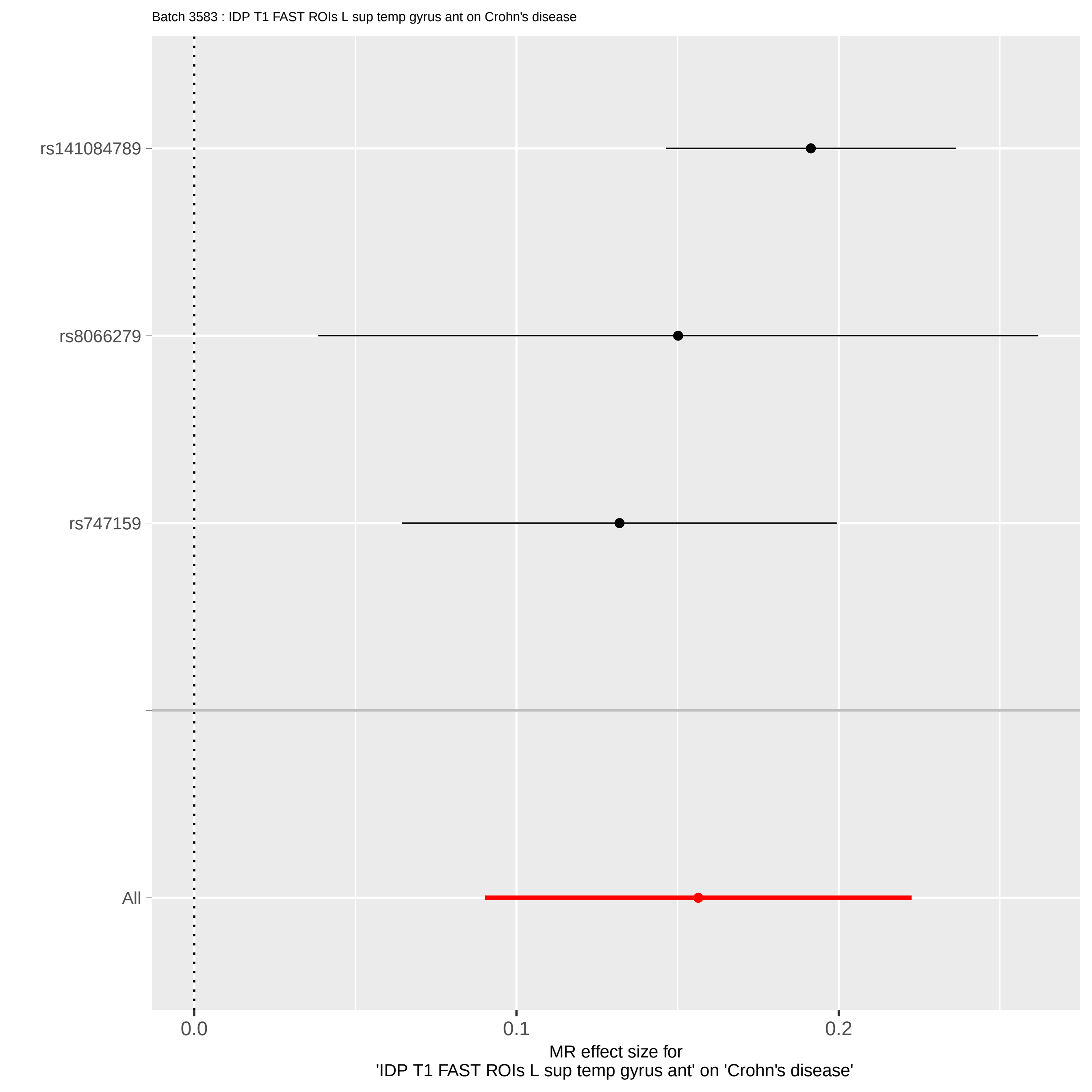

Batch 3583 : IDP T1 FAST ROIs L sup temp gyrus ant on Crohn's disease
rs141084789
rs8066279
rs747159
All
0.0
0.1
0.2
MR effect size for
'IDP T1 FAST ROIs L sup temp gyrus ant' on 'Crohn's disease'

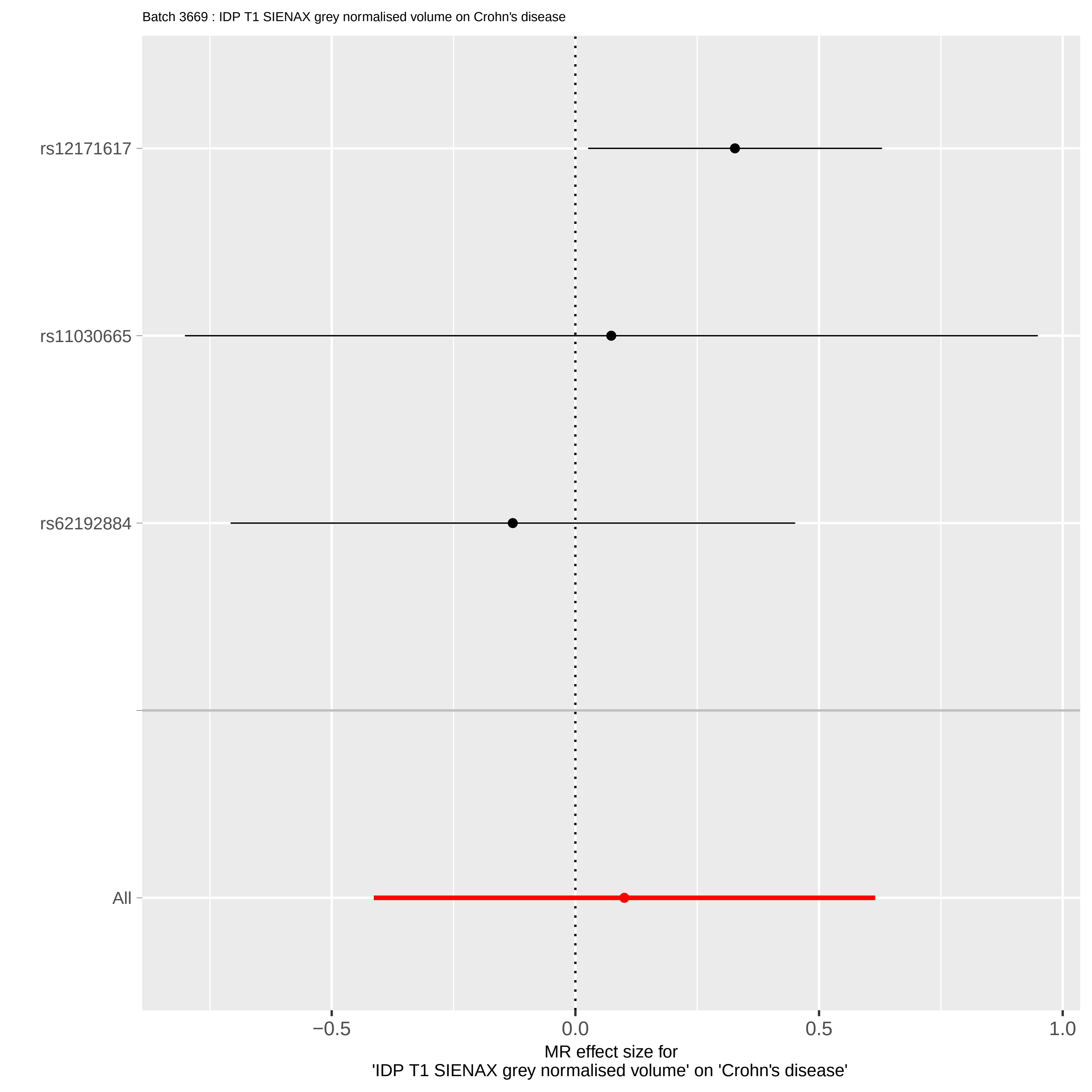
Batch 3669 : IDP T1 SIENAX grey normalised volume on Crohn's disease
rs12171617
rs11030665
rs62192884
All
−0.5
0.0
0.5
1.0
MR effect size for
'IDP T1 SIENAX grey normalised volume' on 'Crohn's disease'

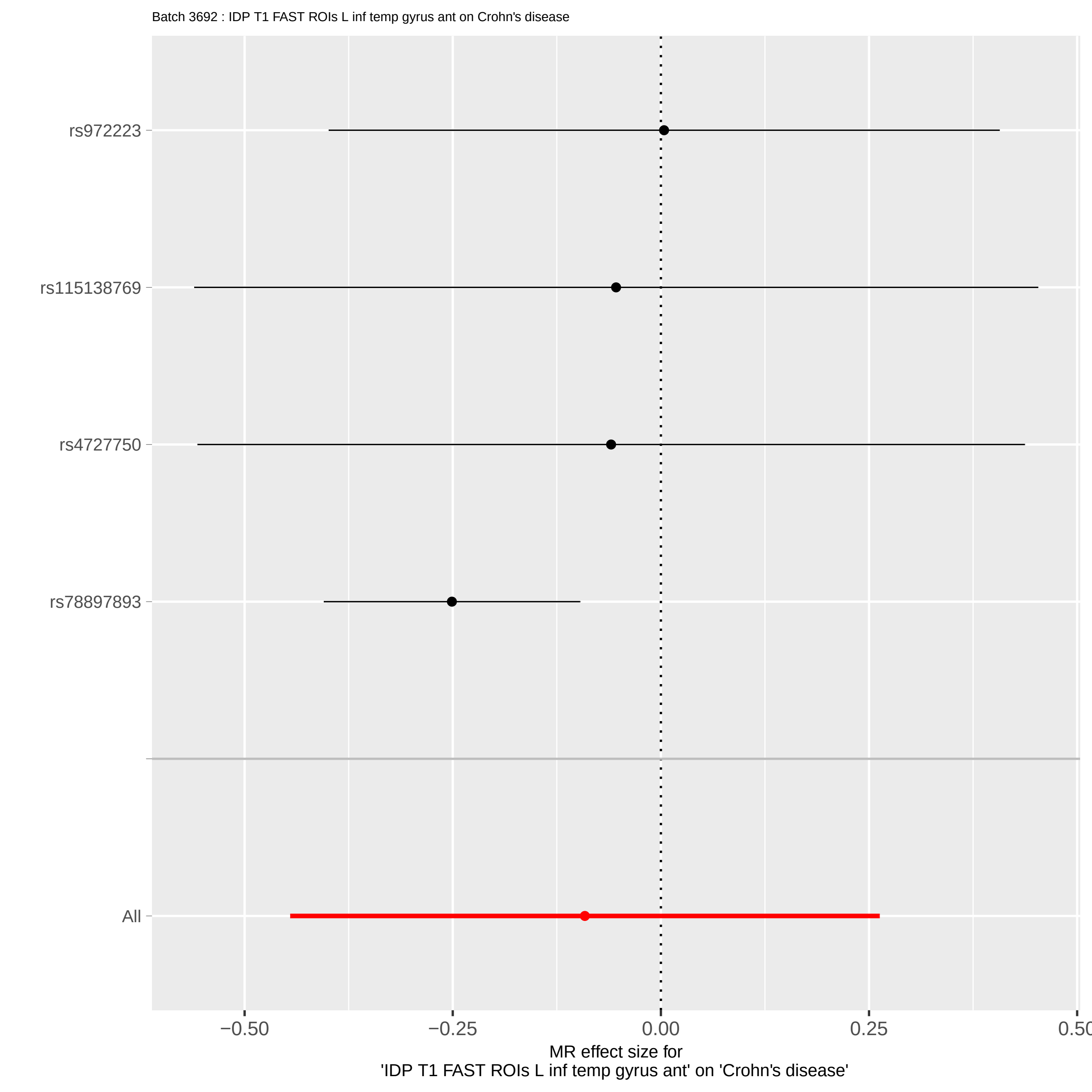

Batch 3692 : IDP T1 FAST ROIs L inf temp gyrus ant on Crohn's disease
rs972223
rs115138769
rs4727750
rs78897893
All
−0.50
−0.25
0.00
0.25
0.50
MR effect size for
'IDP T1 FAST ROIs L inf temp gyrus ant' on 'Crohn's disease'

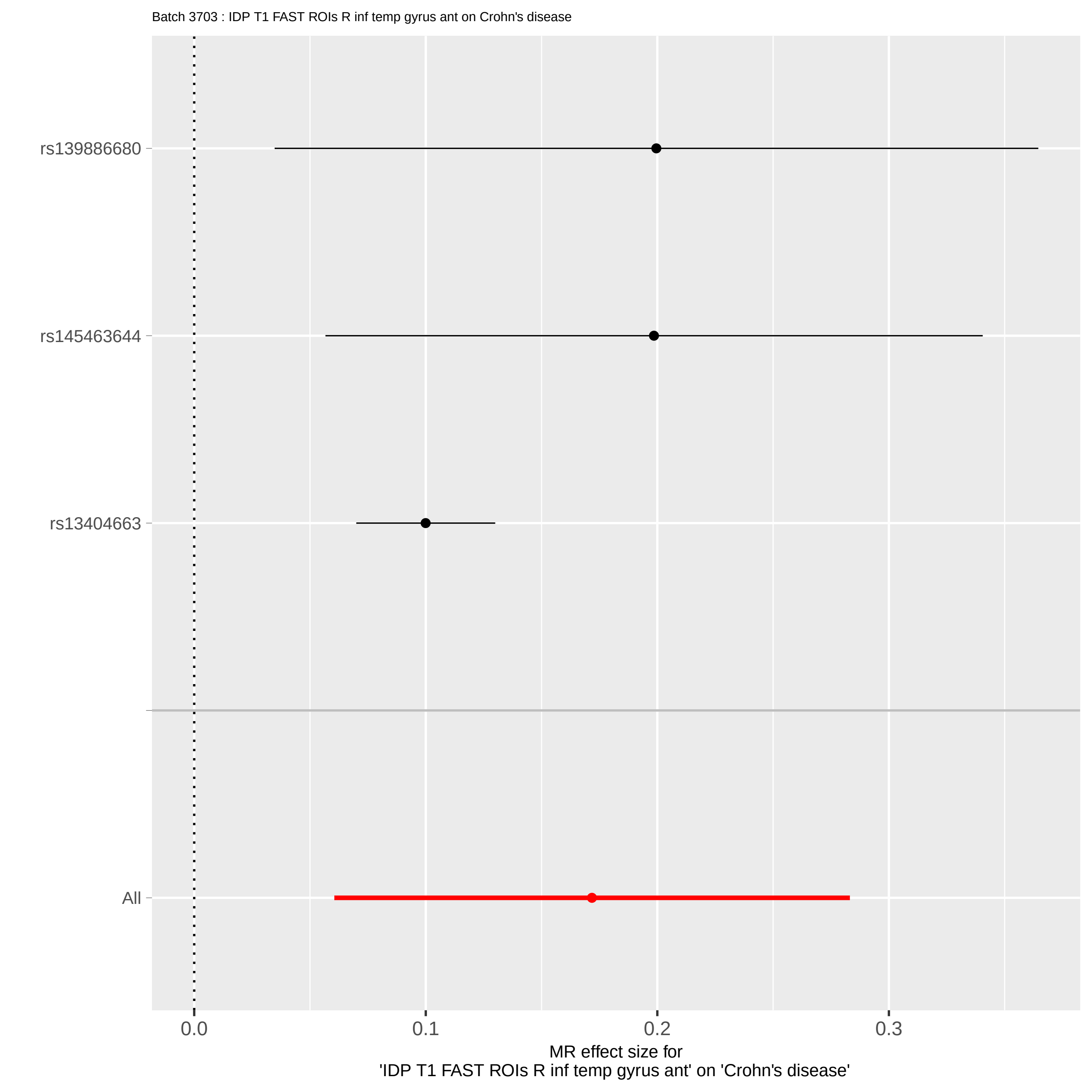

Batch 3703 : IDP T1 FAST ROIs R inf temp gyrus ant on Crohn's disease
rs139886680
rs145463644
rs13404663
All
0.0
0.1
0.2
0.3
MR effect size for
'IDP T1 FAST ROIs R inf temp gyrus ant' on 'Crohn's disease'

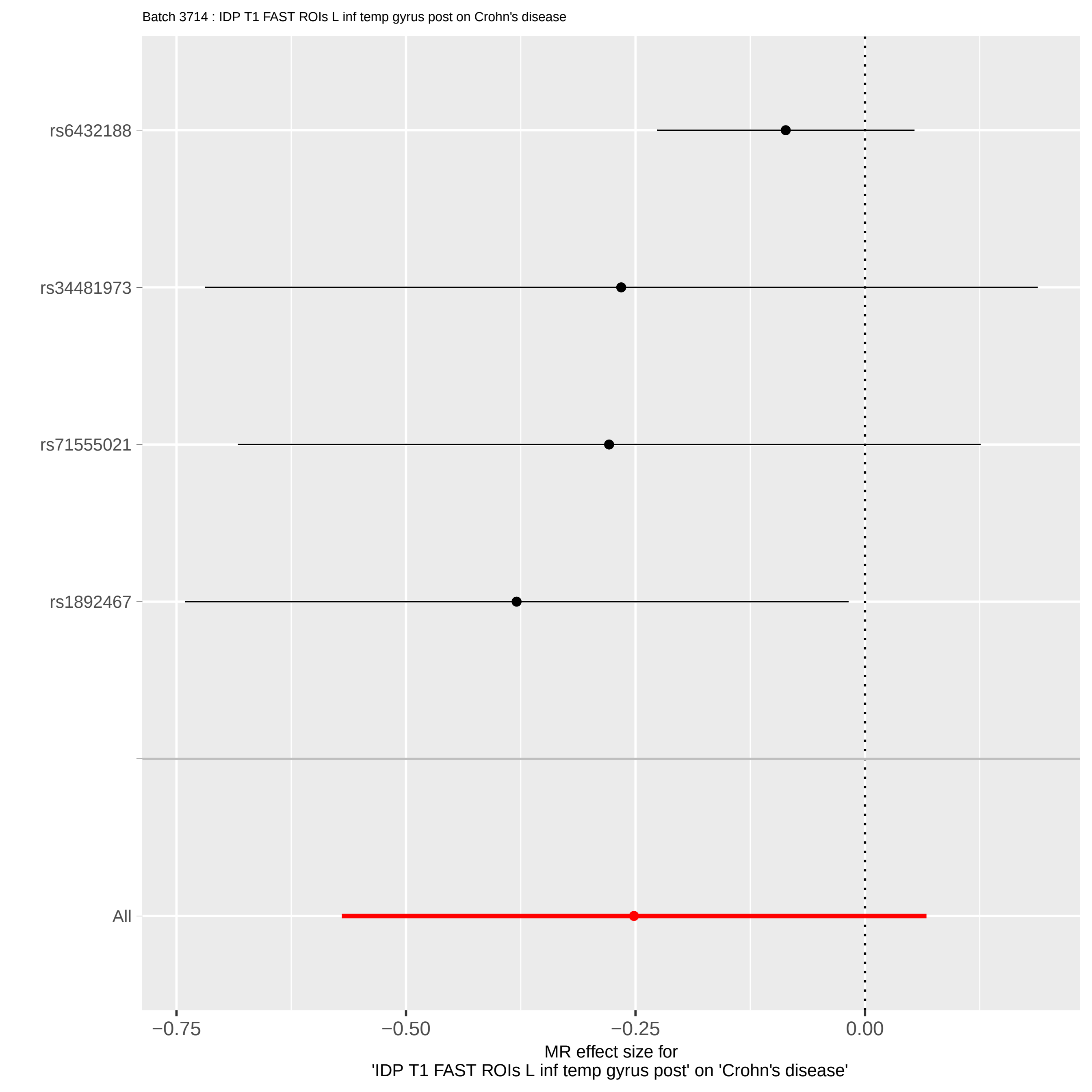
Batch 3714 : IDP T1 FAST ROIs L inf temp gyrus post on Crohn's disease
rs6432188
rs34481973
rs71555021
rs1892467
All
−0.75
−0.50
−0.25
0.00
MR effect size for
'IDP T1 FAST ROIs L inf temp gyrus post' on 'Crohn's disease'

Batch 3747 : IDP T1 FAST ROIs R inf temp gyrus tempocc on Crohn's disease

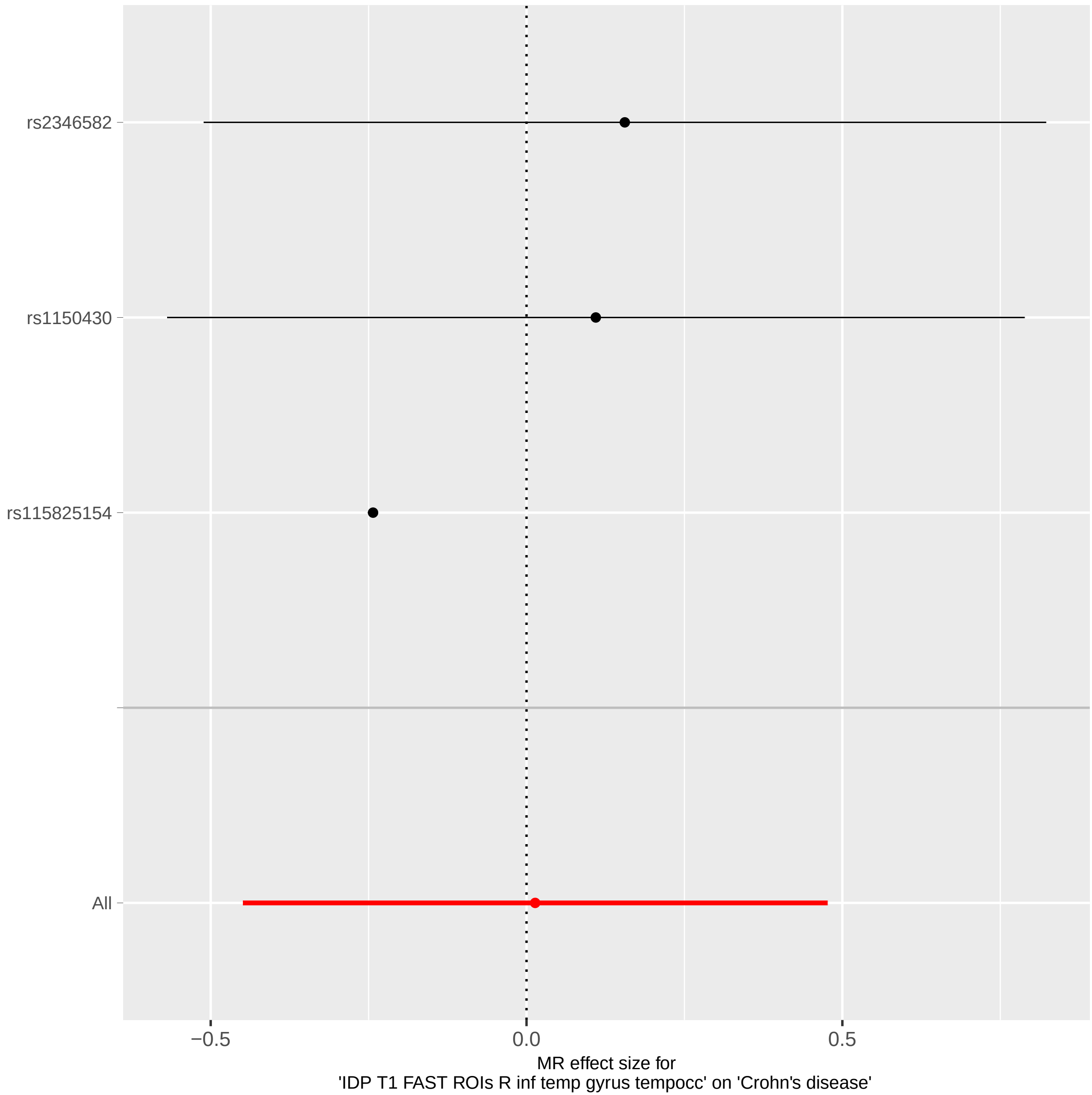

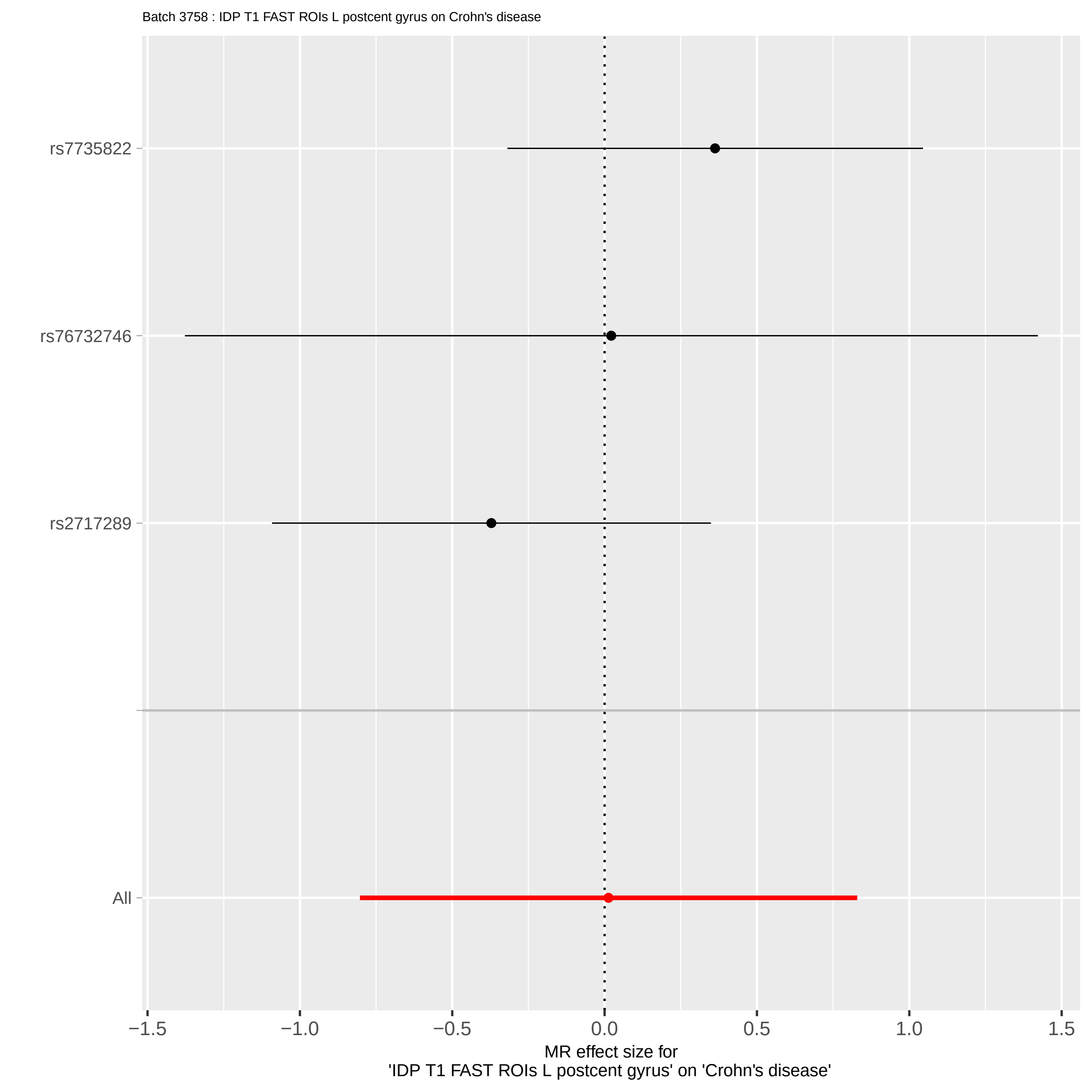

Batch 3758 : IDP T1 FAST ROIs L postcent gyrus on Crohn's disease
rs7735822
rs76732746
rs2717289
All
−1.5
−1.0
−0.5
0.0
0.5
1.0
1.5
MR effect size for
'IDP T1 FAST ROIs L postcent gyrus' on 'Crohn's disease'

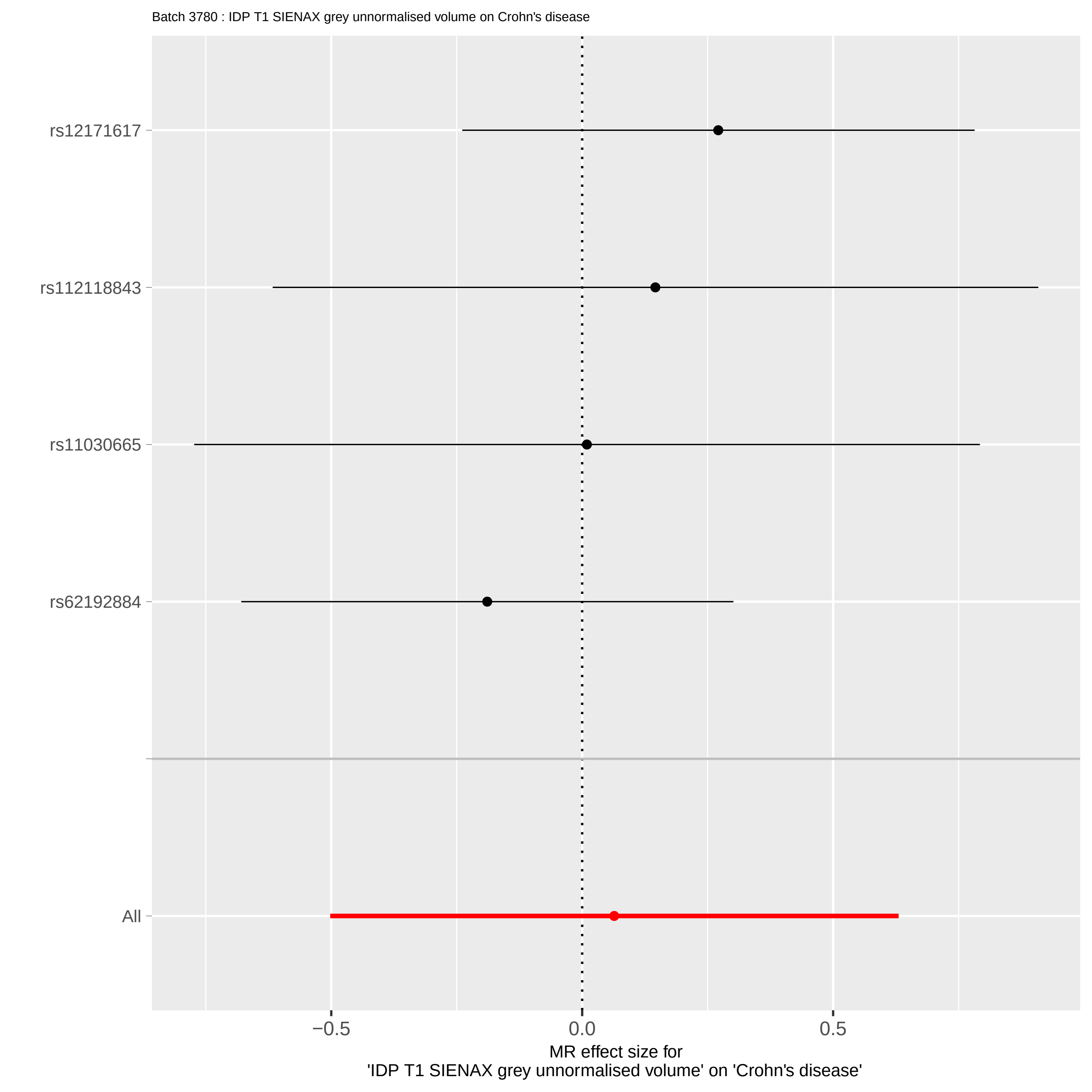
Batch 3780 : IDP T1 SIENAX grey unnormalised volume on Crohn's disease
rs12171617
rs112118843
rs11030665
rs62192884
All
−0.5
0.0
0.5
MR effect size for
'IDP T1 SIENAX grey unnormalised volume' on 'Crohn's disease'

Batch 3968 : IDP T1 FAST ROIs R juxtapos lobule cortex on Crohn's disease

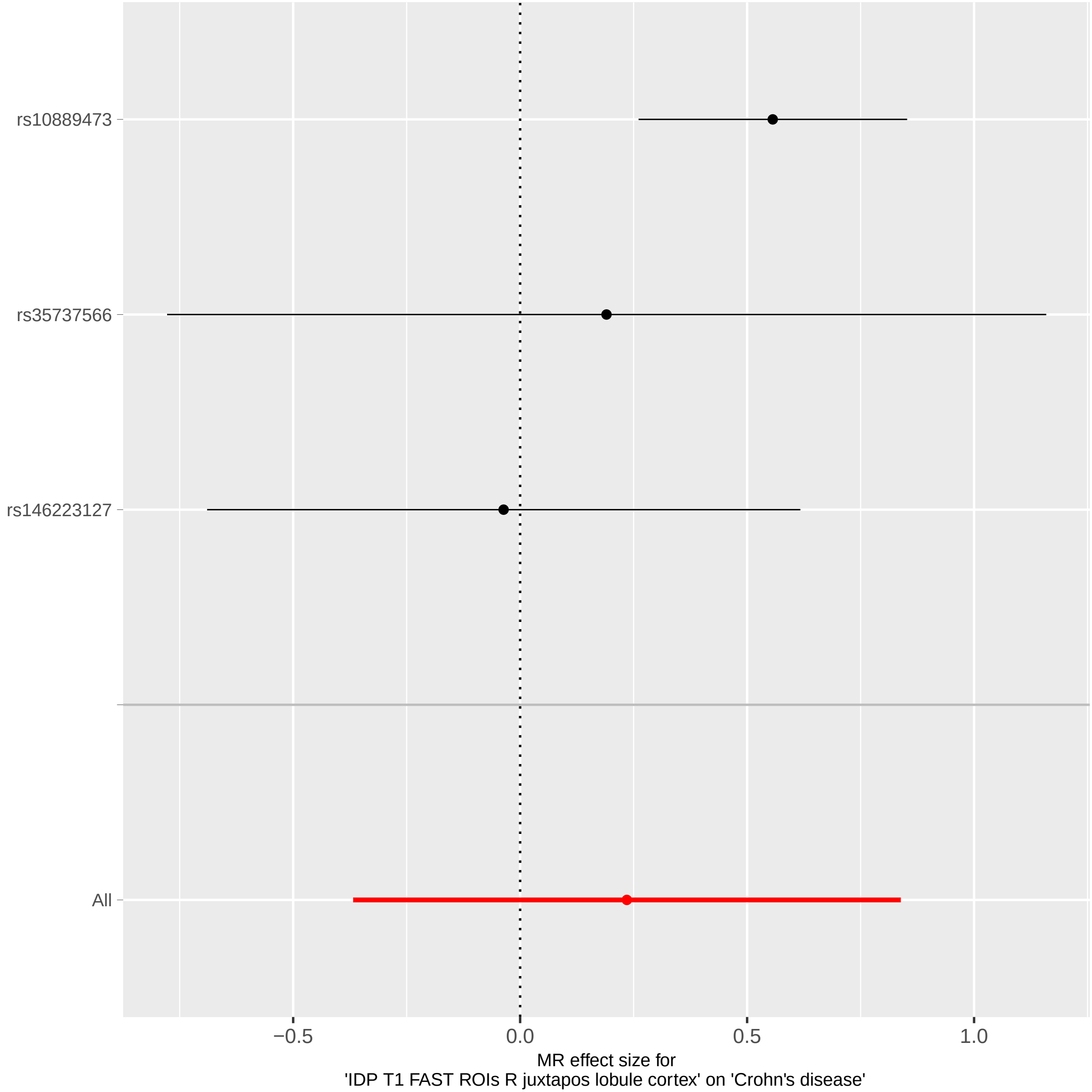

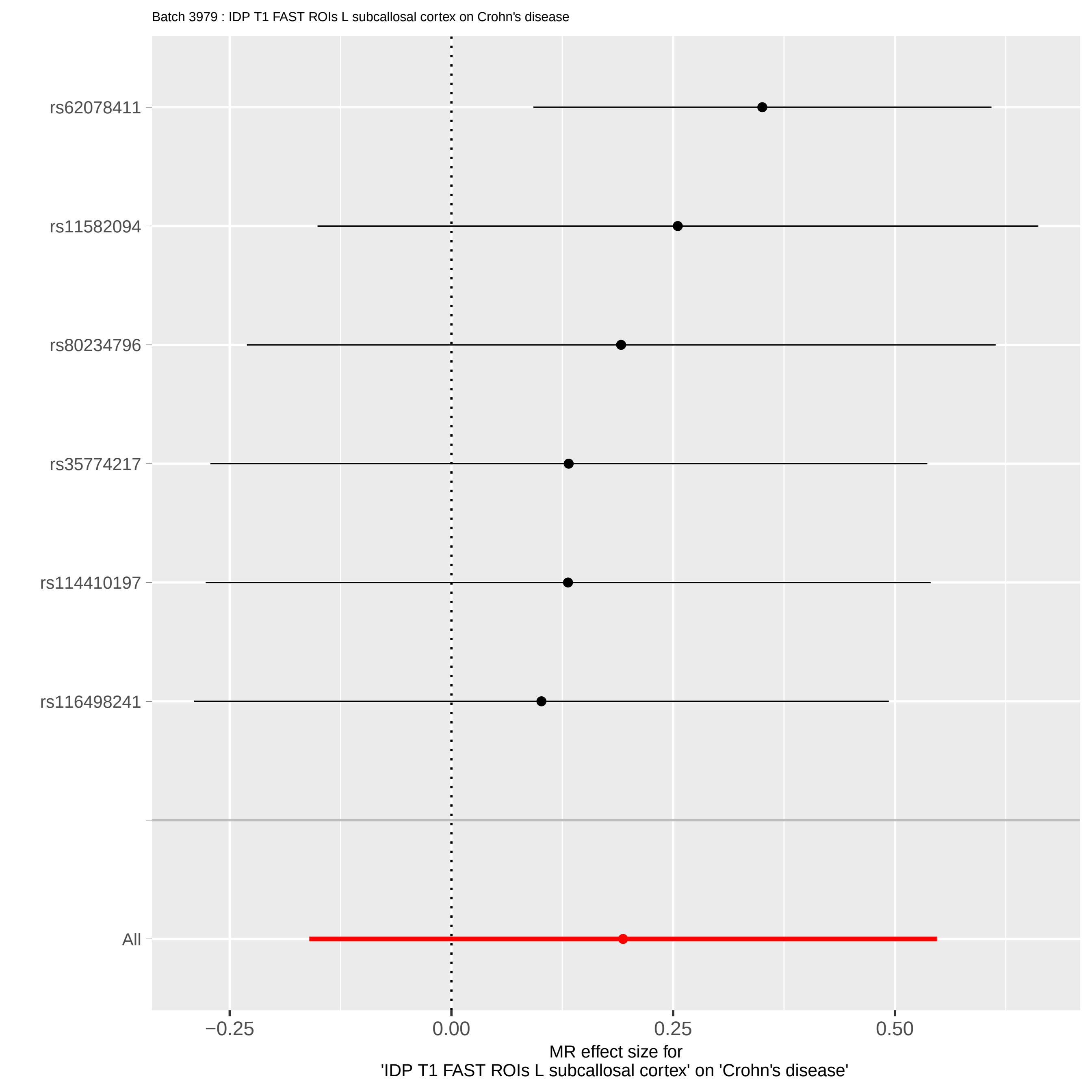

Batch 3979 : IDP T1 FAST ROIs L subcallosal cortex on Crohn's disease
rs62078411
rs11582094
rs80234796
rs35774217
rs114410197
rs116498241
All
−0.25
0.00
0.25
0.50
MR effect size for
'IDP T1 FAST ROIs L subcallosal cortex' on 'Crohn's disease'

Batch 3990 : IDP T1 FAST ROIs R subcallosal cortex on Crohn's disease

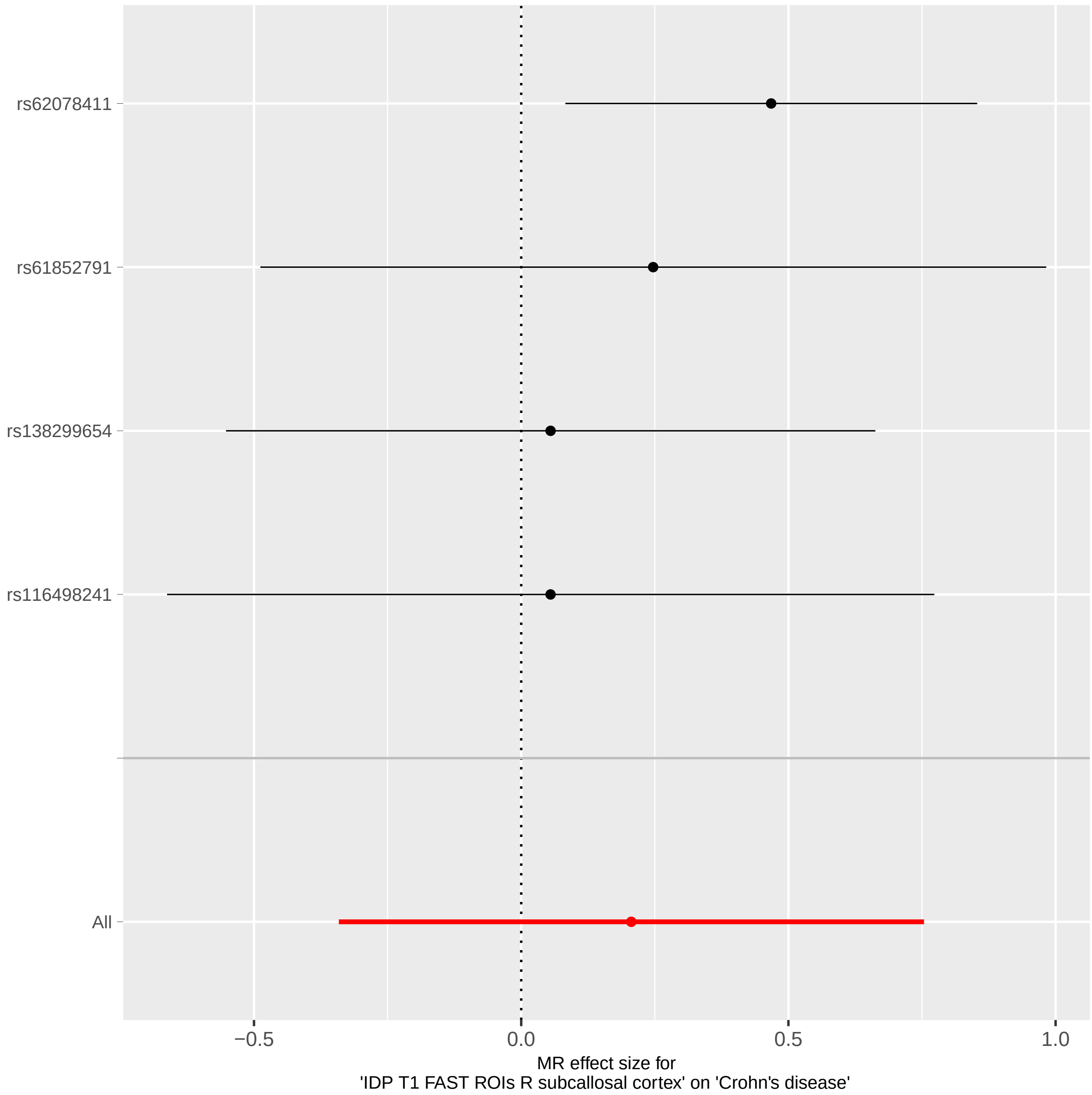

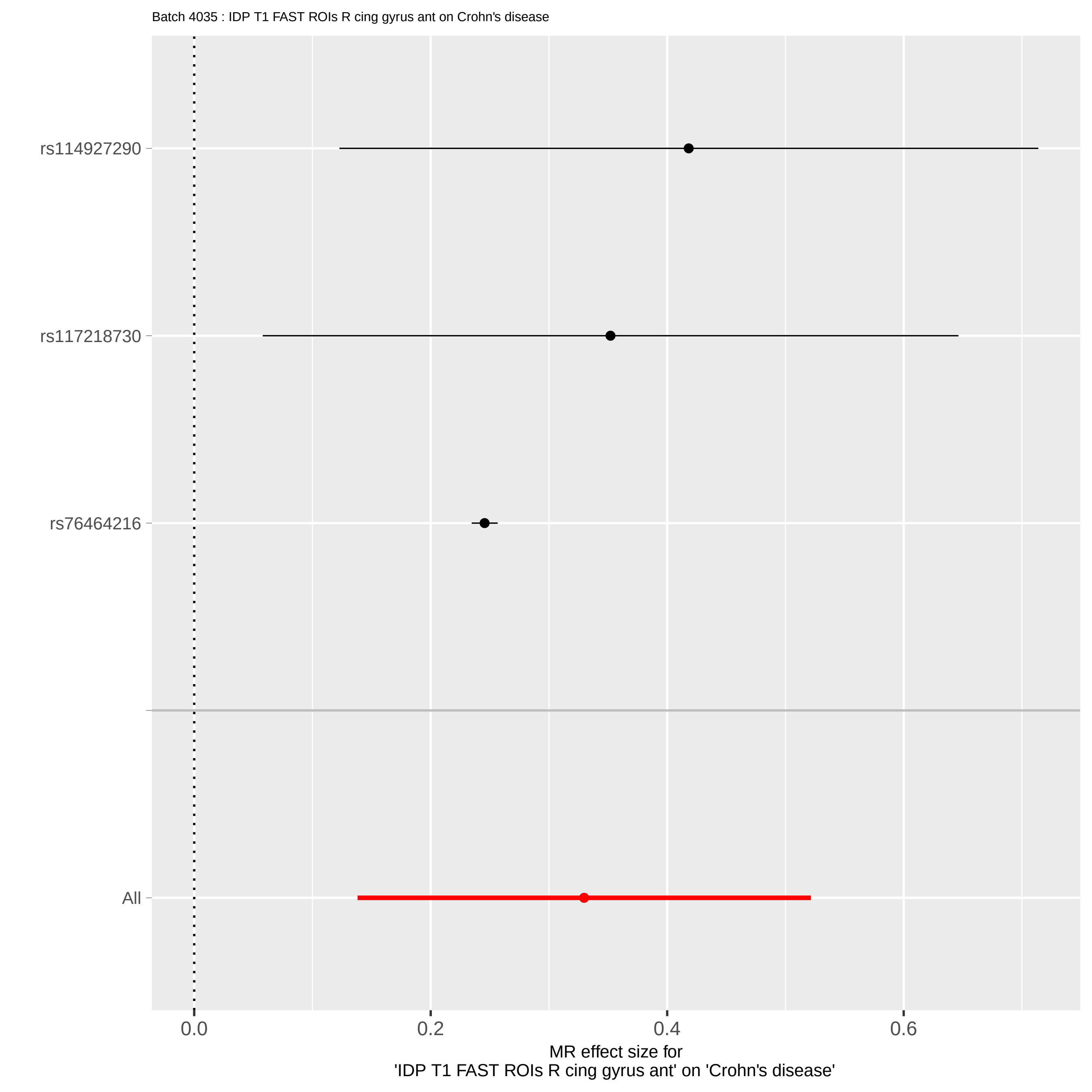

Batch 4035 : IDP T1 FAST ROIs R cing gyrus ant on Crohn's disease
rs114927290
rs117218730
rs76464216
All
0.0
0.2
0.4
0.6
MR effect size for
'IDP T1 FAST ROIs R cing gyrus ant' on 'Crohn's disease'

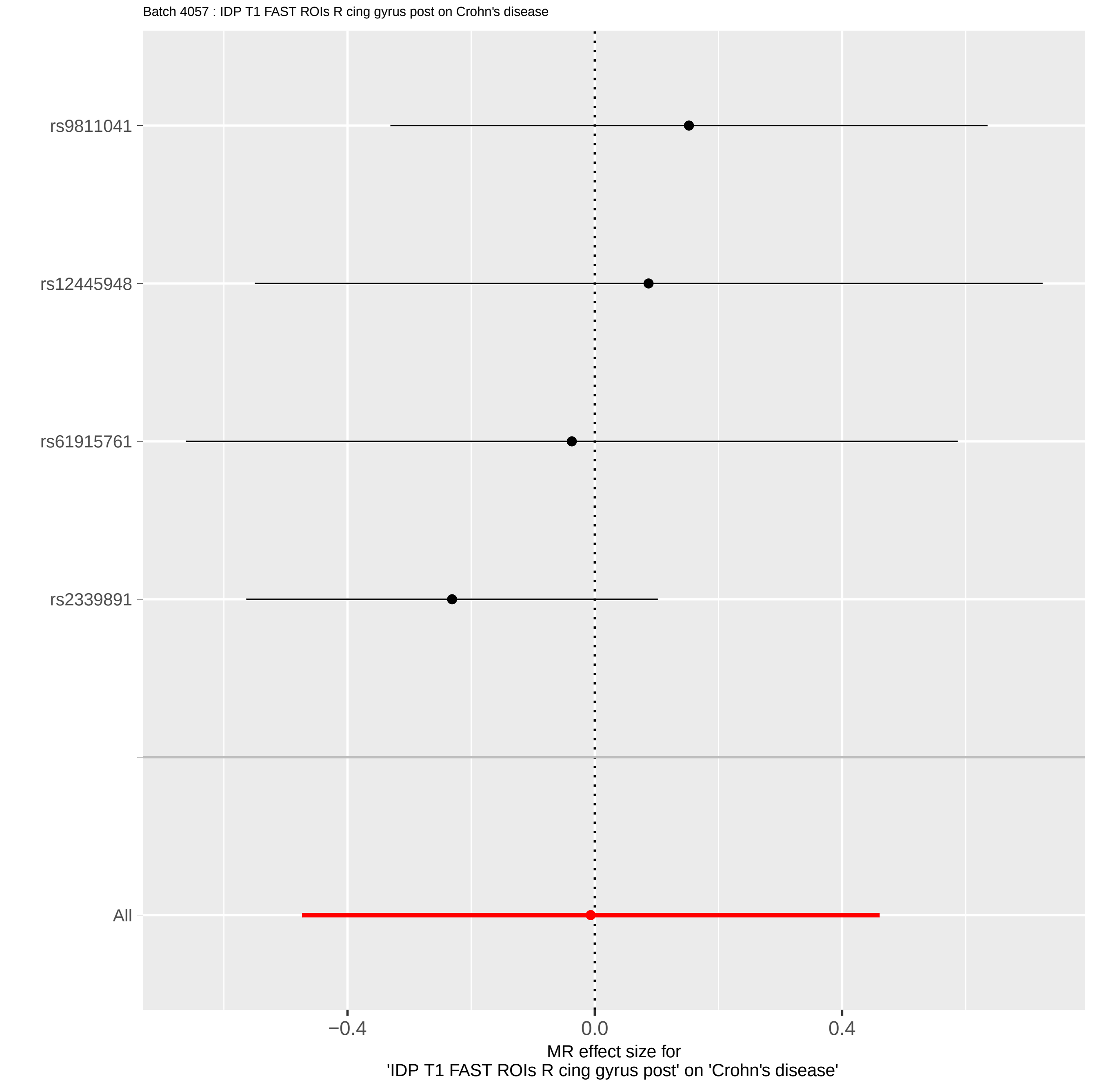

Batch 4057 : IDP T1 FAST ROIs R cing gyrus post on Crohn's disease
rs9811041
rs12445948
rs61915761
rs2339891
All
−0.4
0.0
0.4
MR effect size for
'IDP T1 FAST ROIs R cing gyrus post' on 'Crohn's disease'

Batch 4068 : IDP T1 FAST ROIs L precun cortex on Crohn's disease

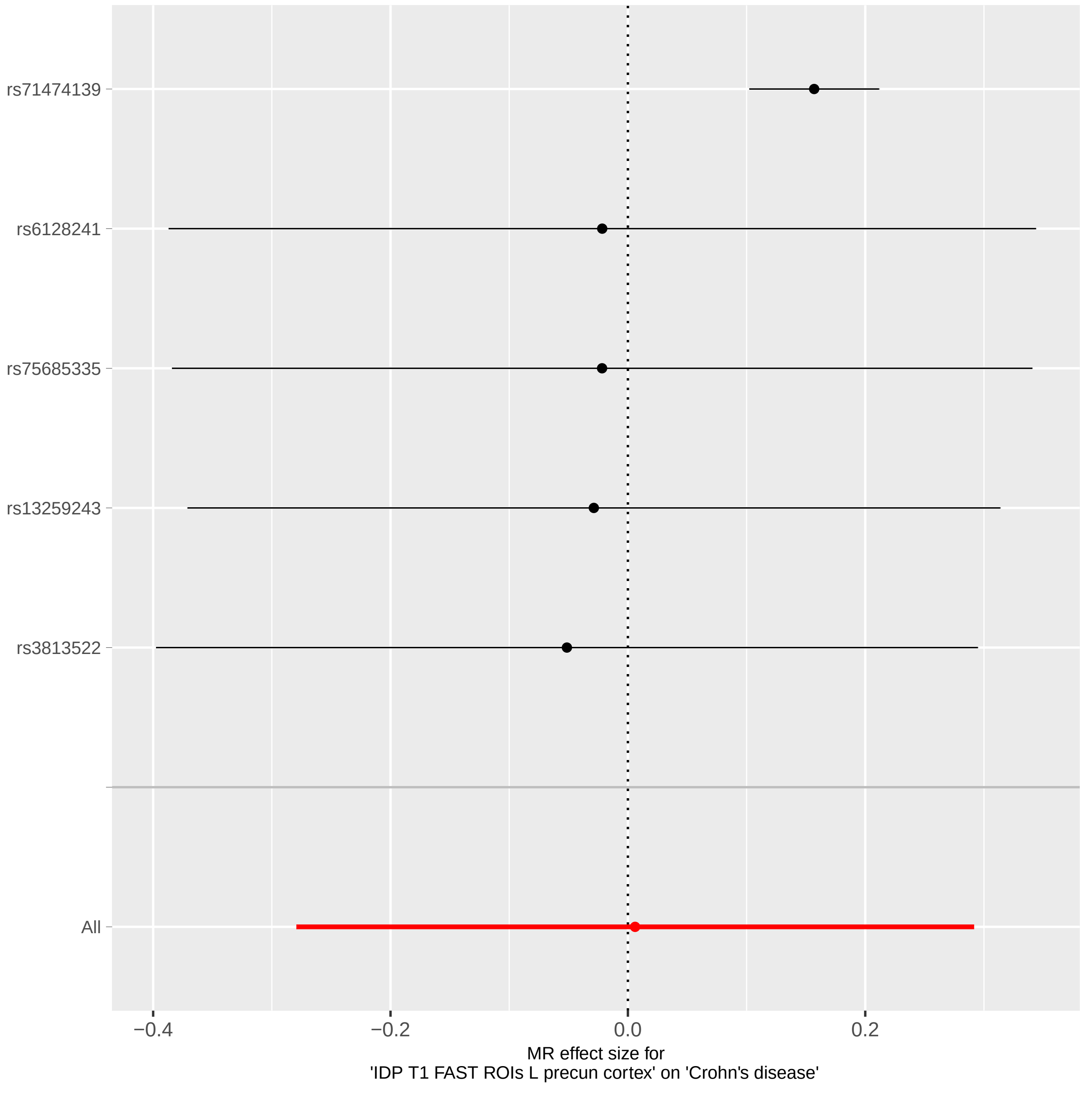

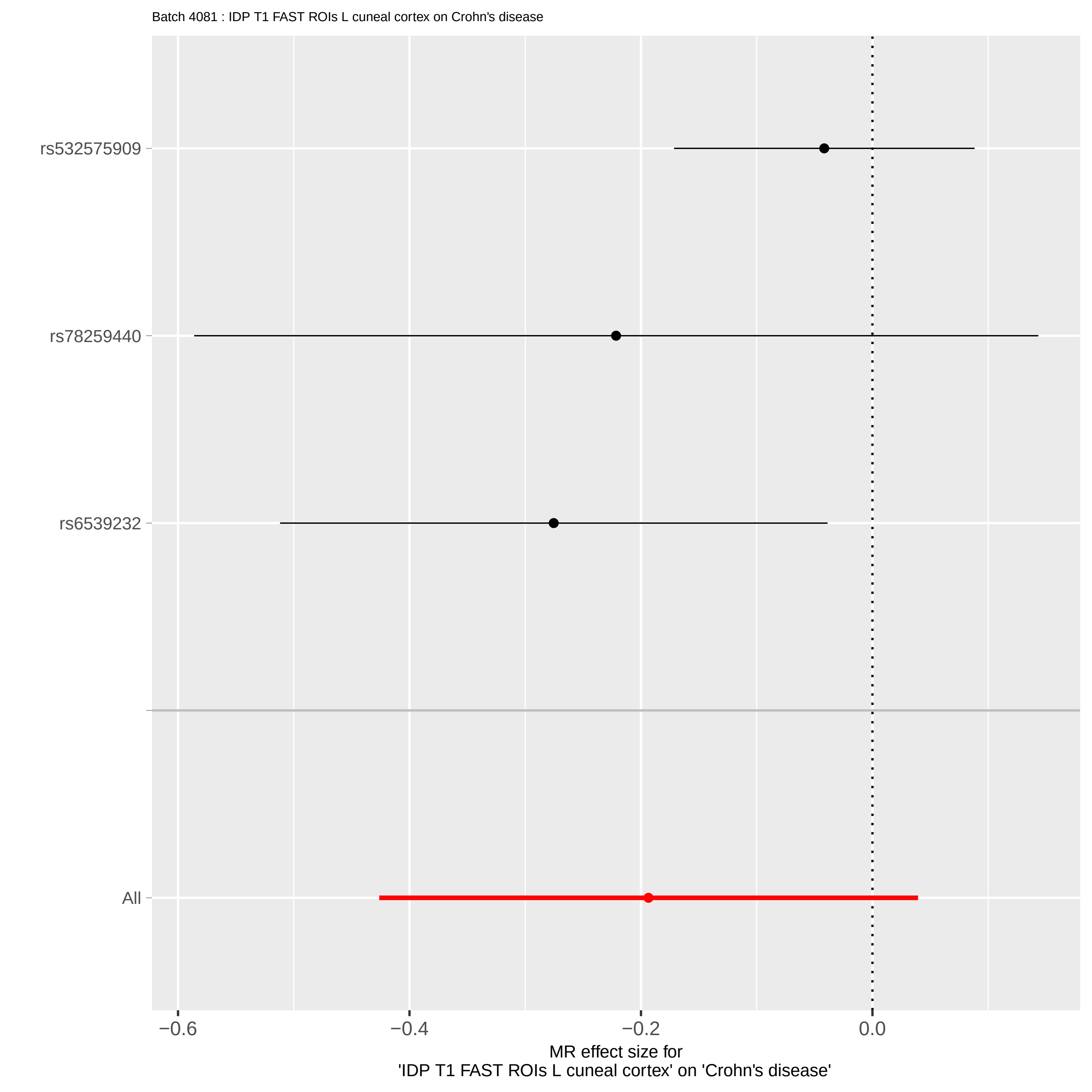
Batch 4081 : IDP T1 FAST ROIs L cuneal cortex on Crohn's disease
rs532575909
rs78259440
rs6539232
All
−0.6
−0.4
−0.2
0.0
MR effect size for
'IDP T1 FAST ROIs L cuneal cortex' on 'Crohn's disease'

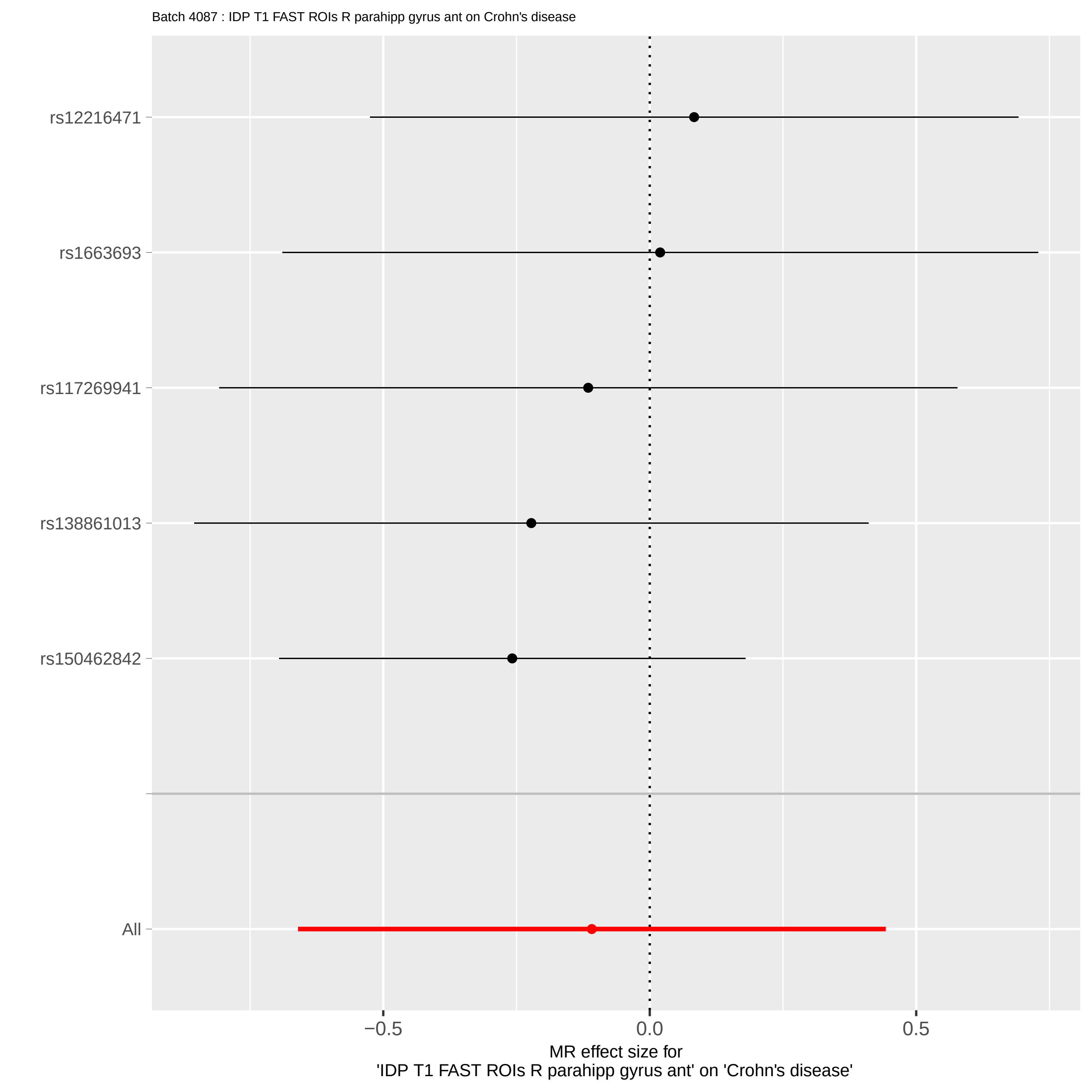
Batch 4087 : IDP T1 FAST ROIs R parahipp gyrus ant on Crohn's disease
rs12216471
rs1663693
rs117269941
rs138861013
rs150462842
All
−0.5
0.0
0.5
MR effect size for
'IDP T1 FAST ROIs R parahipp gyrus ant' on 'Crohn's disease'

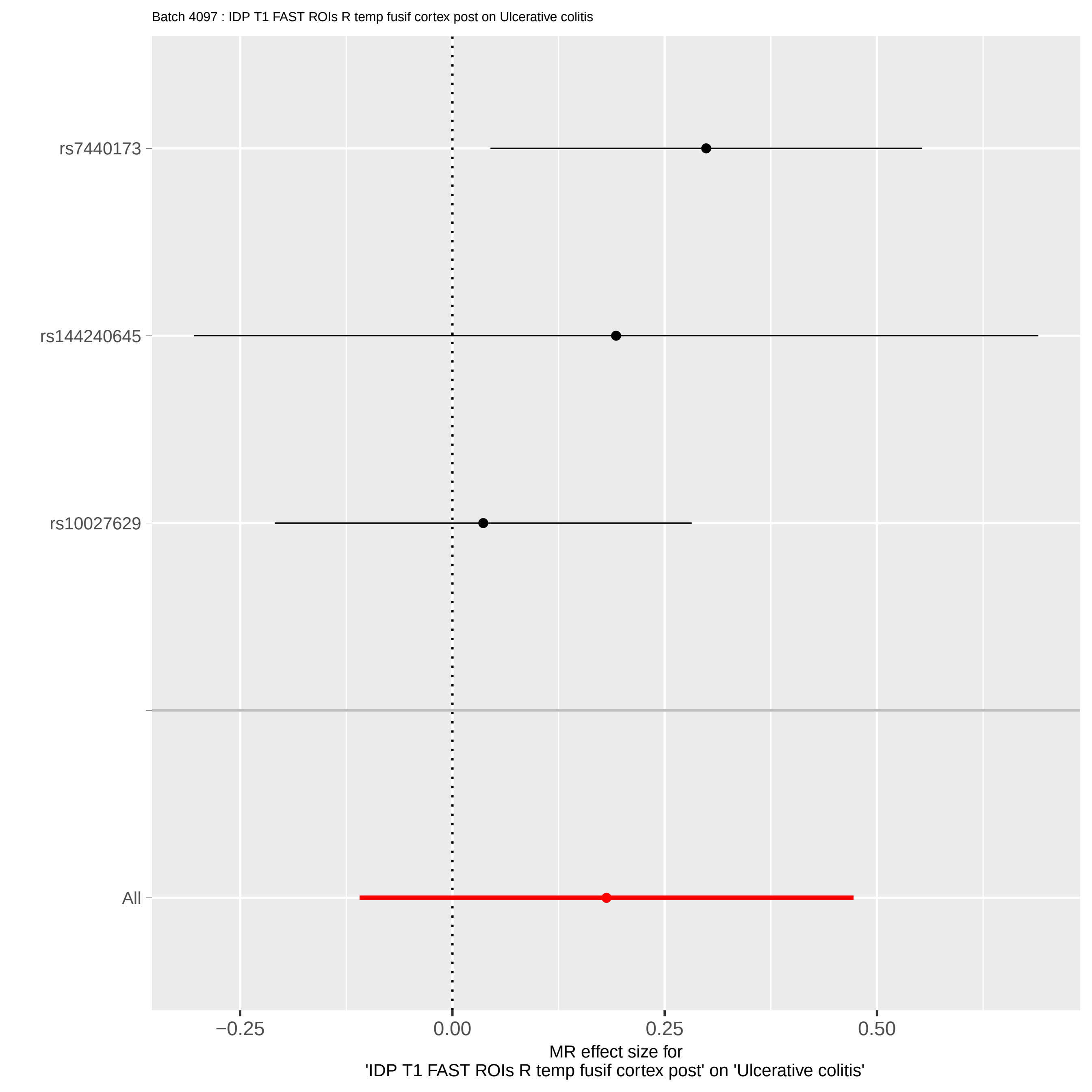

Batch 4097 : IDP T1 FAST ROIs R temp fusif cortex post on Ulcerative colitis
rs7440173
rs144240645
rs10027629
All
−0.25
0.00
0.25
0.50
MR effect size for
'IDP T1 FAST ROIs R temp fusif cortex post' on 'Ulcerative colitis'

Batch 4107 : IDP T1 FAST ROIs L parietal operc cortex on Ulcerative colitis

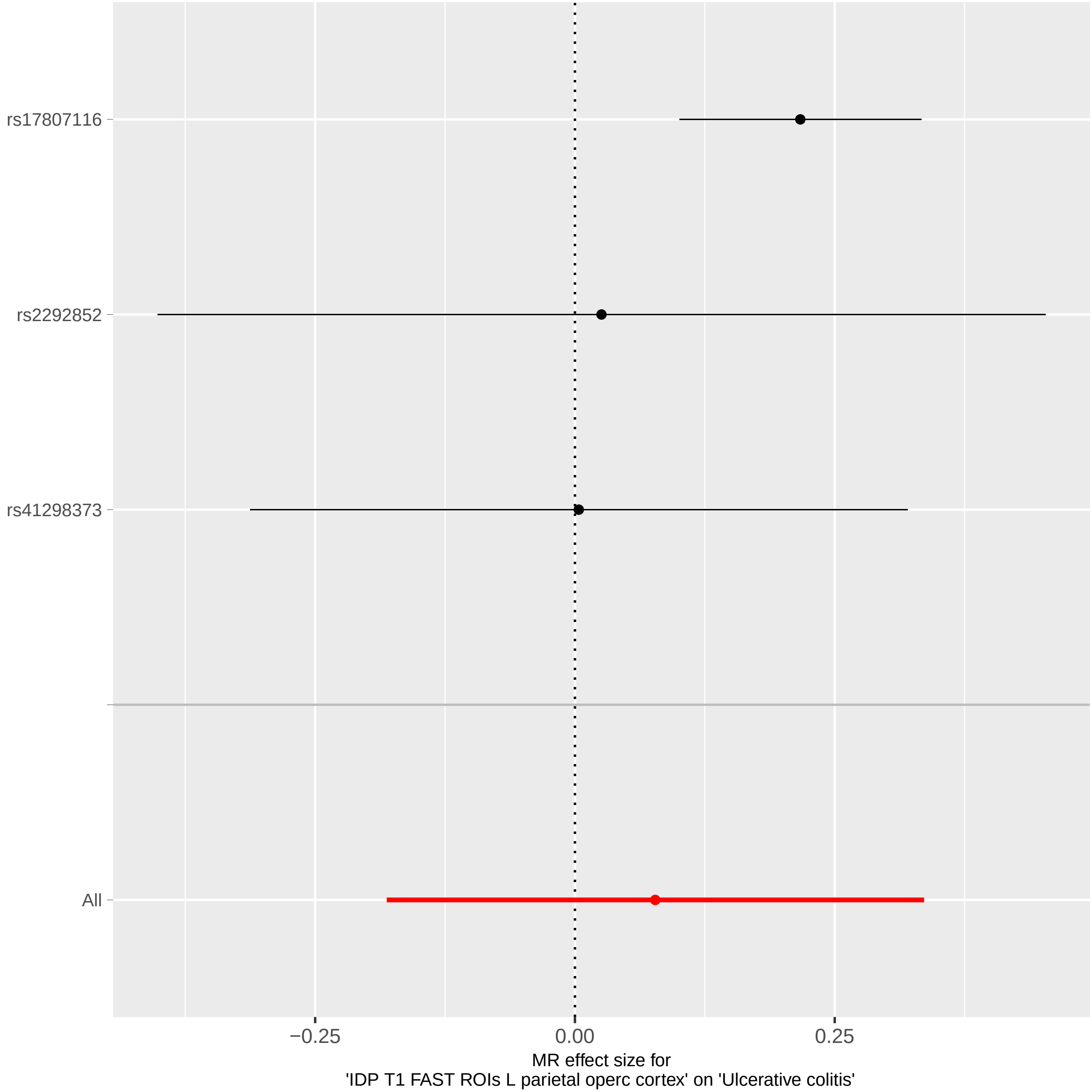

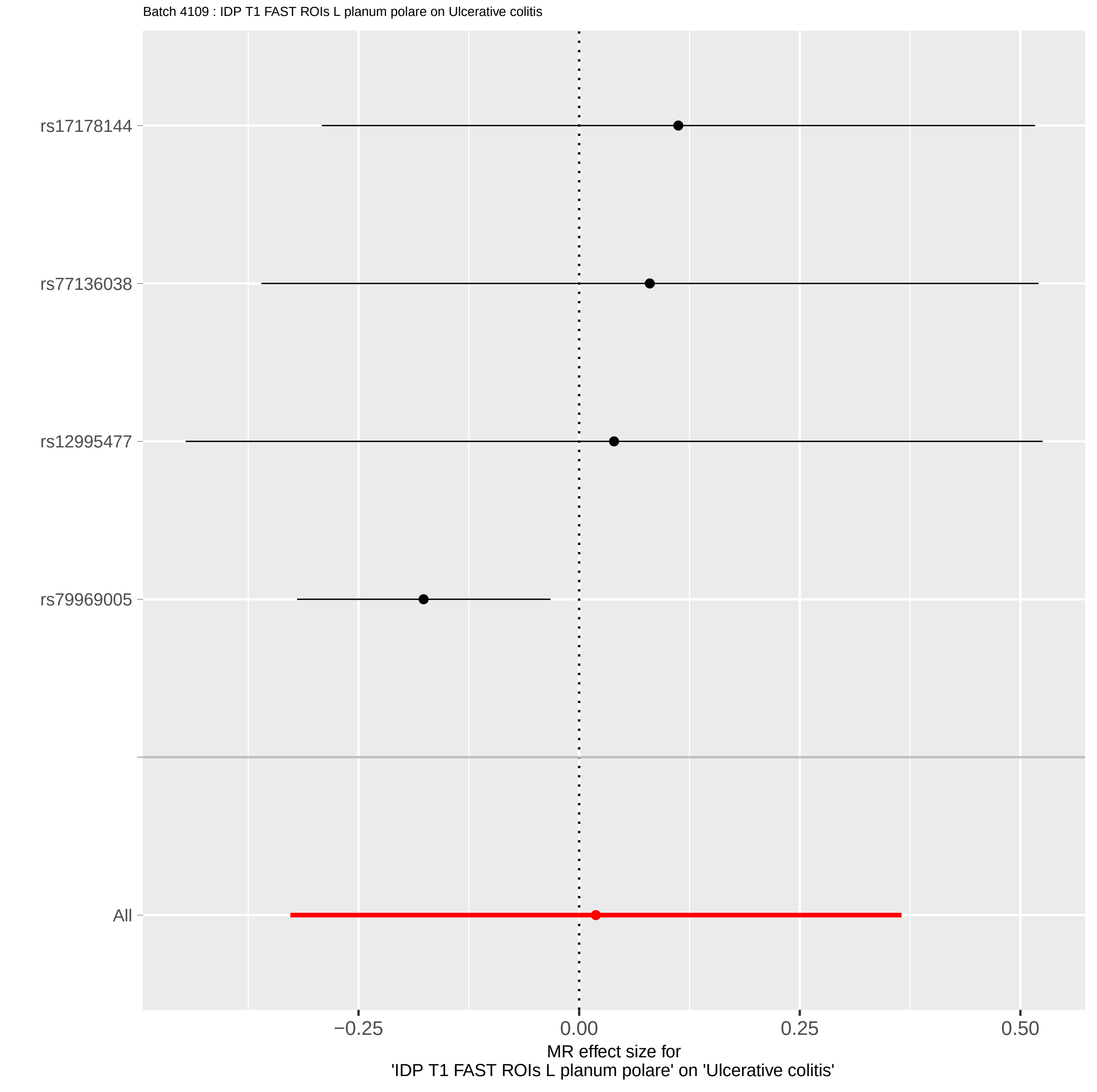

Batch 4109 : IDP T1 FAST ROIs L planum polare on Ulcerative colitis
rs17178144
rs77136038
rs12995477
rs79969005
All
−0.25
0.00
0.25
0.50
MR effect size for
'IDP T1 FAST ROIs L planum polare' on 'Ulcerative colitis'

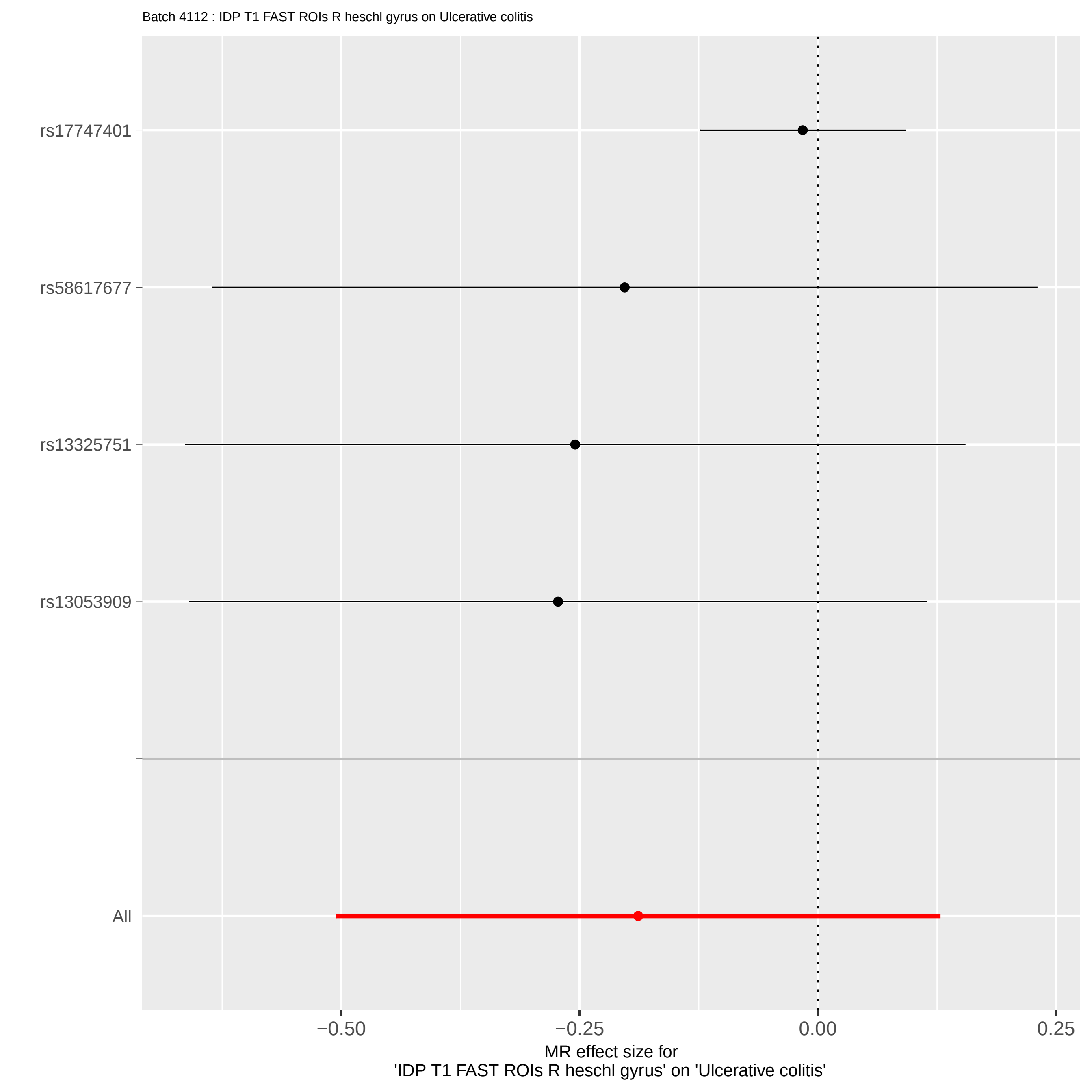

Batch 4112 : IDP T1 FAST ROIs R heschl gyrus on Ulcerative colitis
rs17747401
rs58617677
rs13325751
rs13053909
All
−0.50
−0.25
0.00
0.25
MR effect size for
'IDP T1 FAST ROIs R heschl gyrus' on 'Ulcerative colitis'

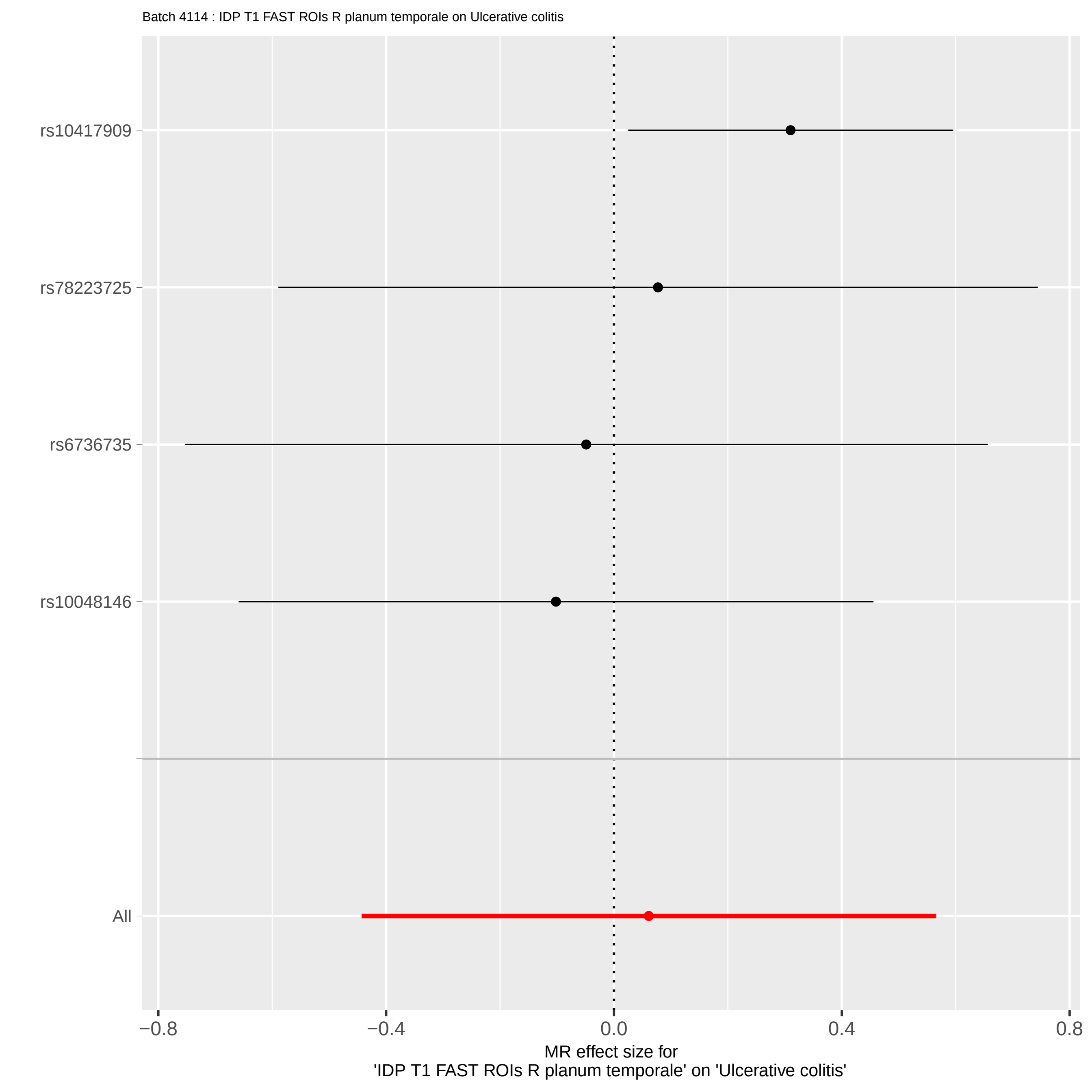

Batch 4114 : IDP T1 FAST ROIs R planum temporale on Ulcerative colitis
rs10417909
rs78223725
rs6736735
rs10048146
All
−0.8
−0.4
0.0
0.4
0.8
MR effect size for
'IDP T1 FAST ROIs R planum temporale' on 'Ulcerative colitis'

Batch 4115 : IDP T1 FAST ROIs L supracalc cortex on Ulcerative colitis

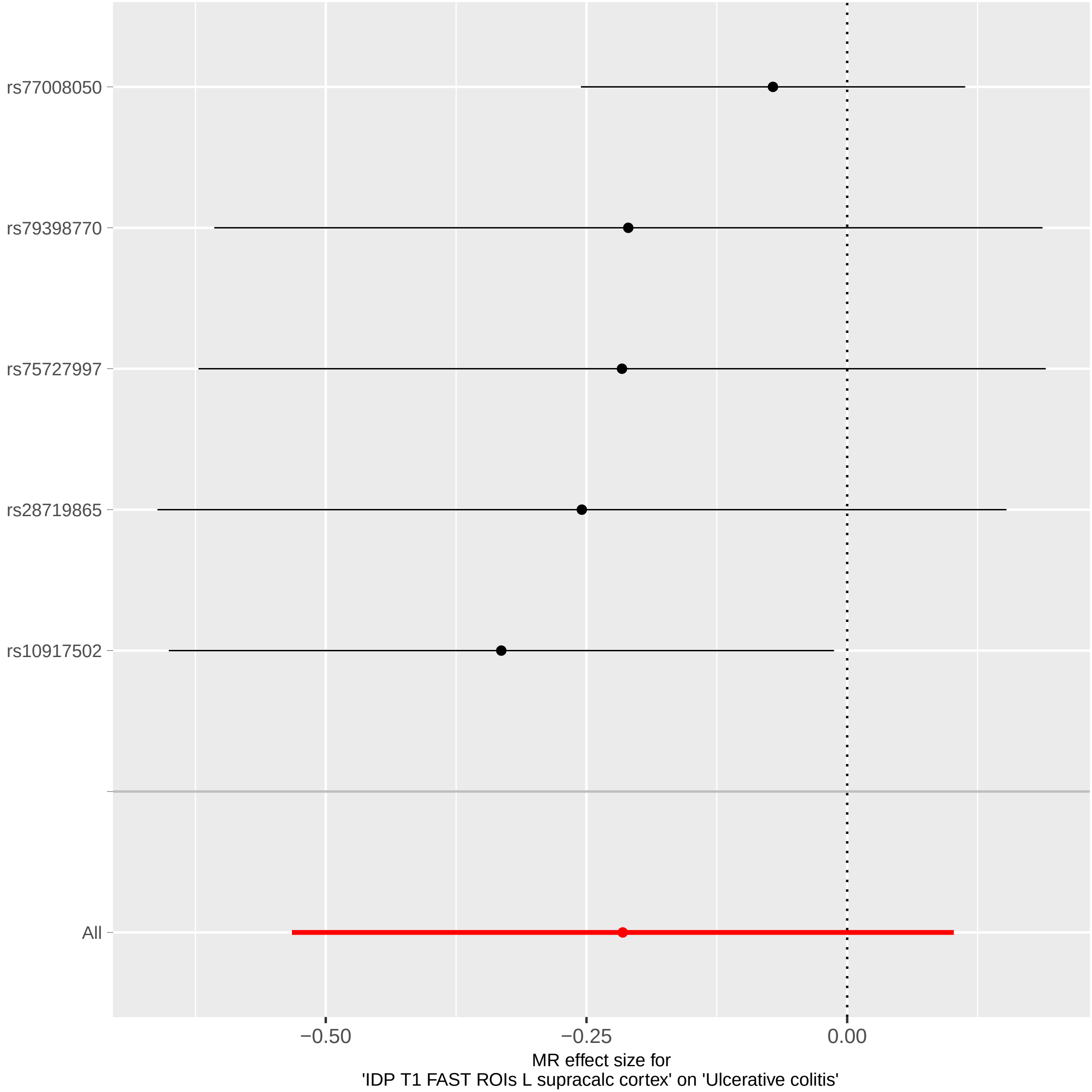

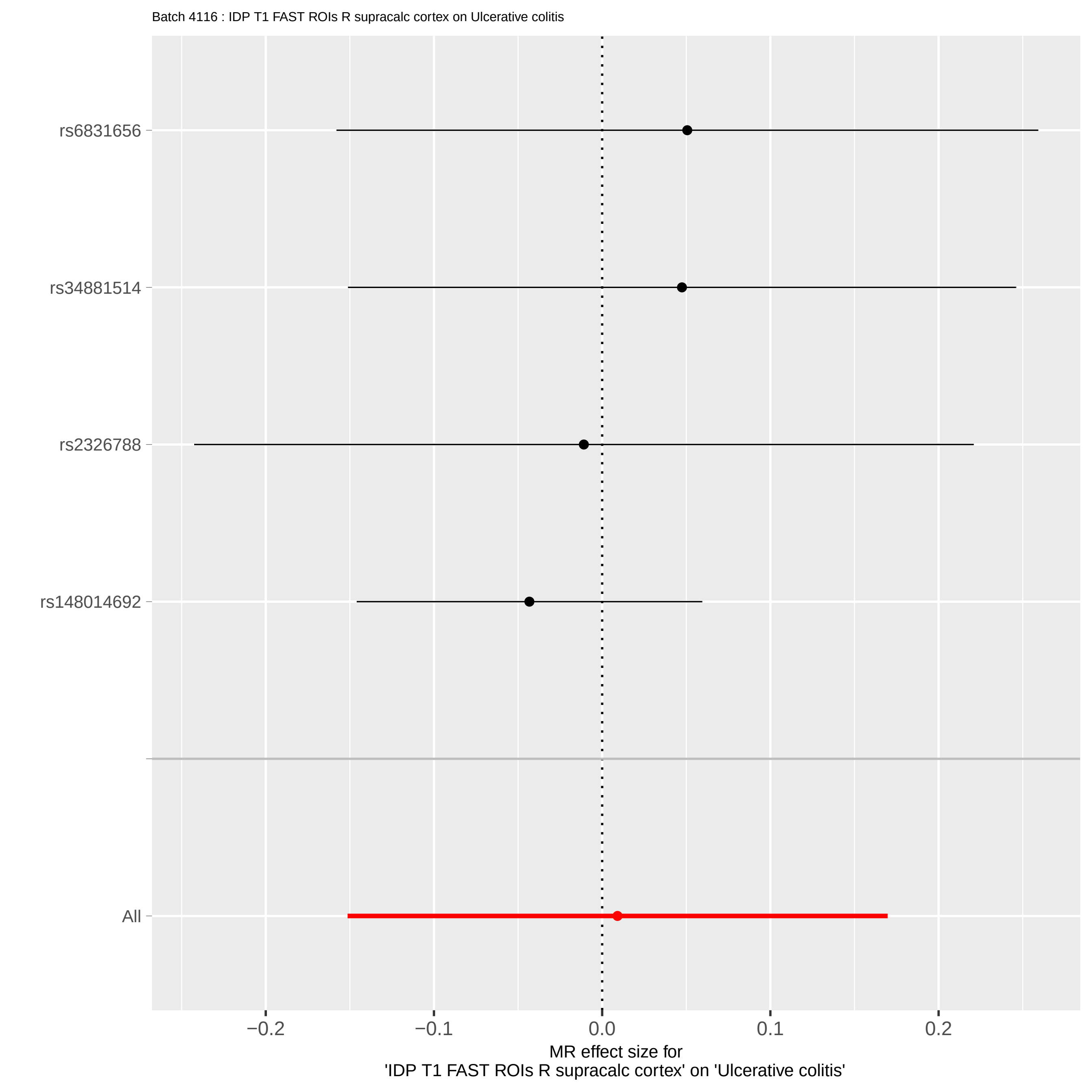
Batch 4116 : IDP T1 FAST ROIs R supracalc cortex on Ulcerative colitis
rs6831656
rs34881514
rs2326788
rs148014692
All
−0.2
−0.1
0.0
0.1
0.2
MR effect size for
'IDP T1 FAST ROIs R supracalc cortex' on 'Ulcerative colitis'

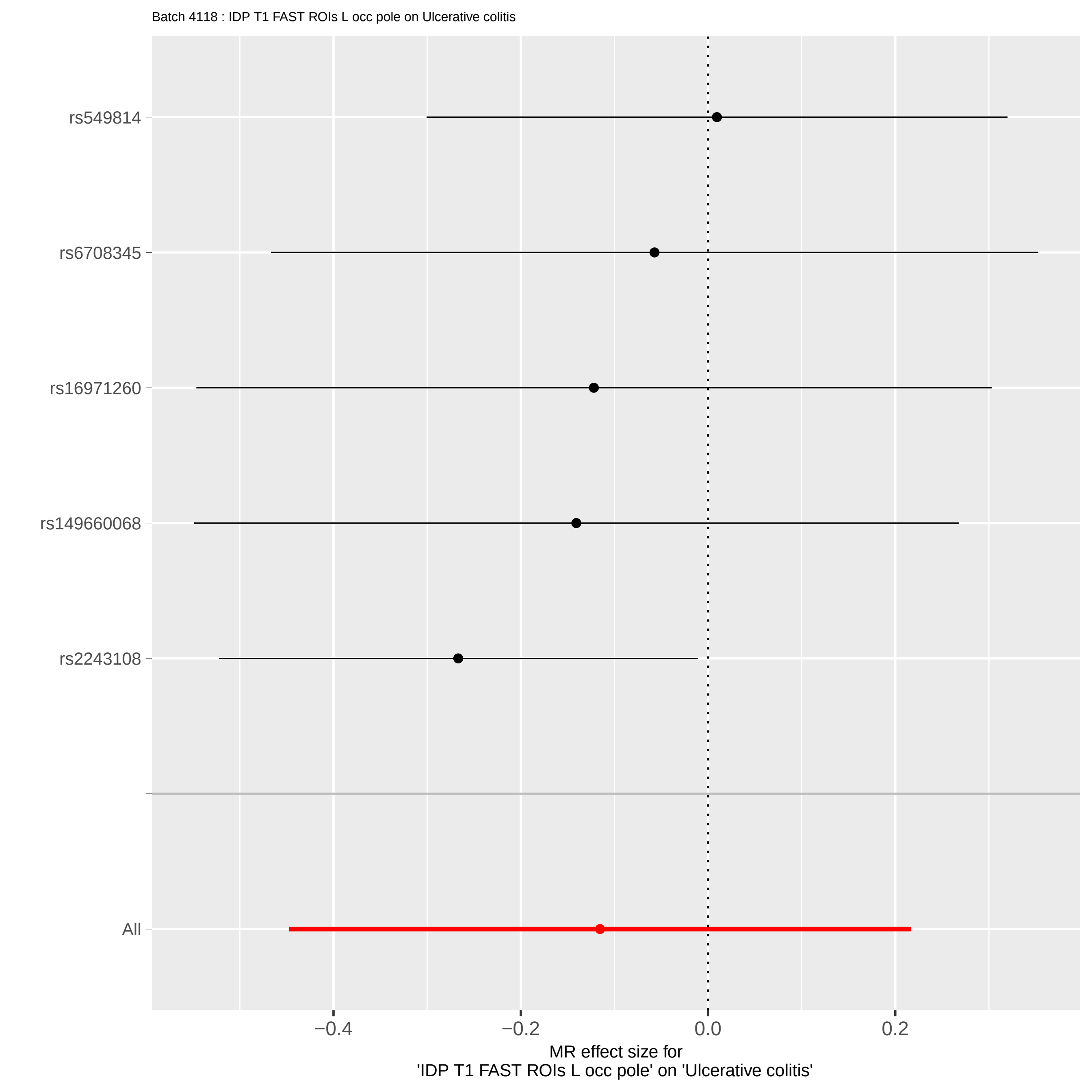

Batch 4118 : IDP T1 FAST ROIs L occ pole on Ulcerative colitis
rs549814
rs6708345
rs16971260
rs149660068
rs2243108
All
−0.4
−0.2
0.0
0.2
MR effect size for
'IDP T1 FAST ROIs L occ pole' on 'Ulcerative colitis'

Batch 4129 : IDP T1 FAST ROIs L hippocampus on Ulcerative colitis

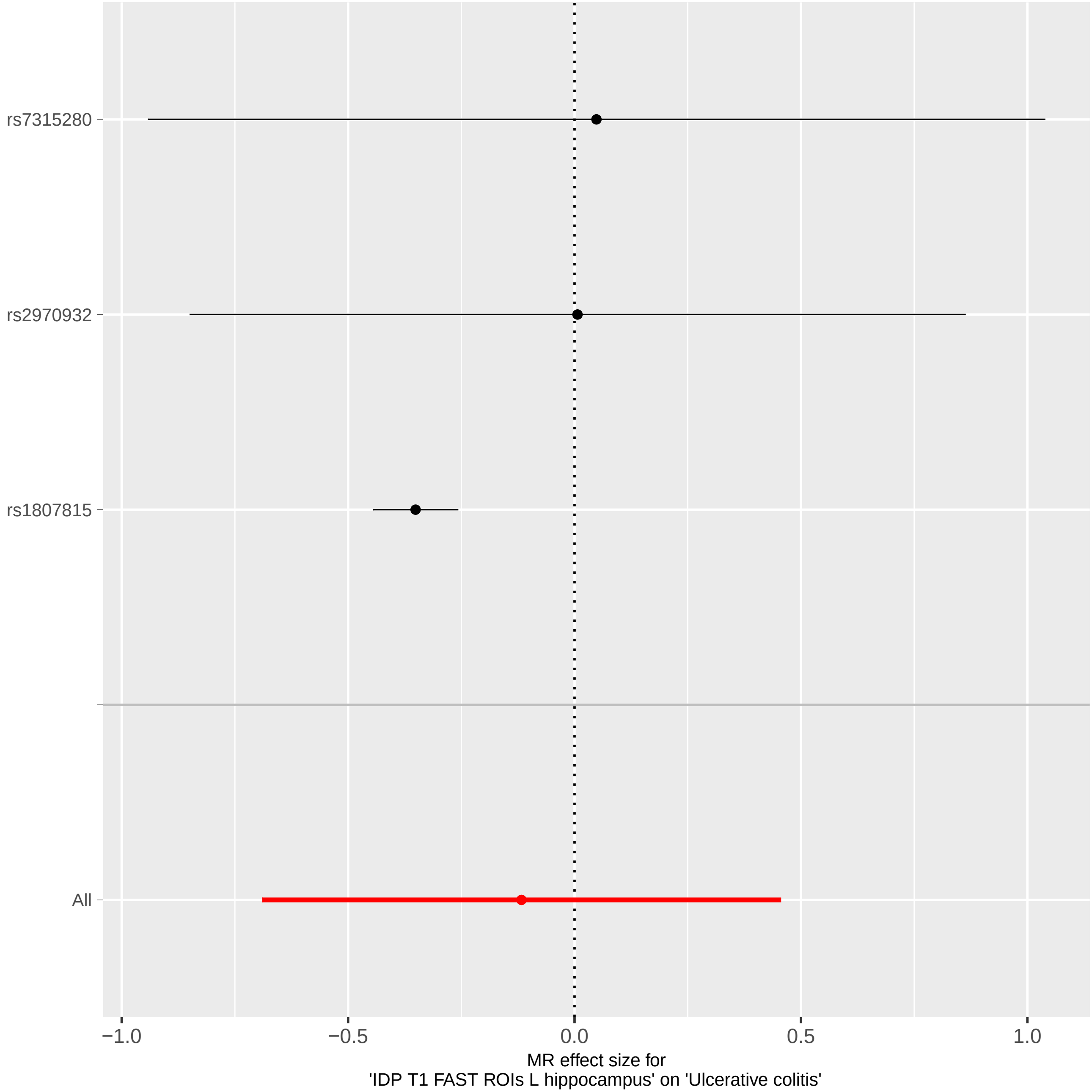

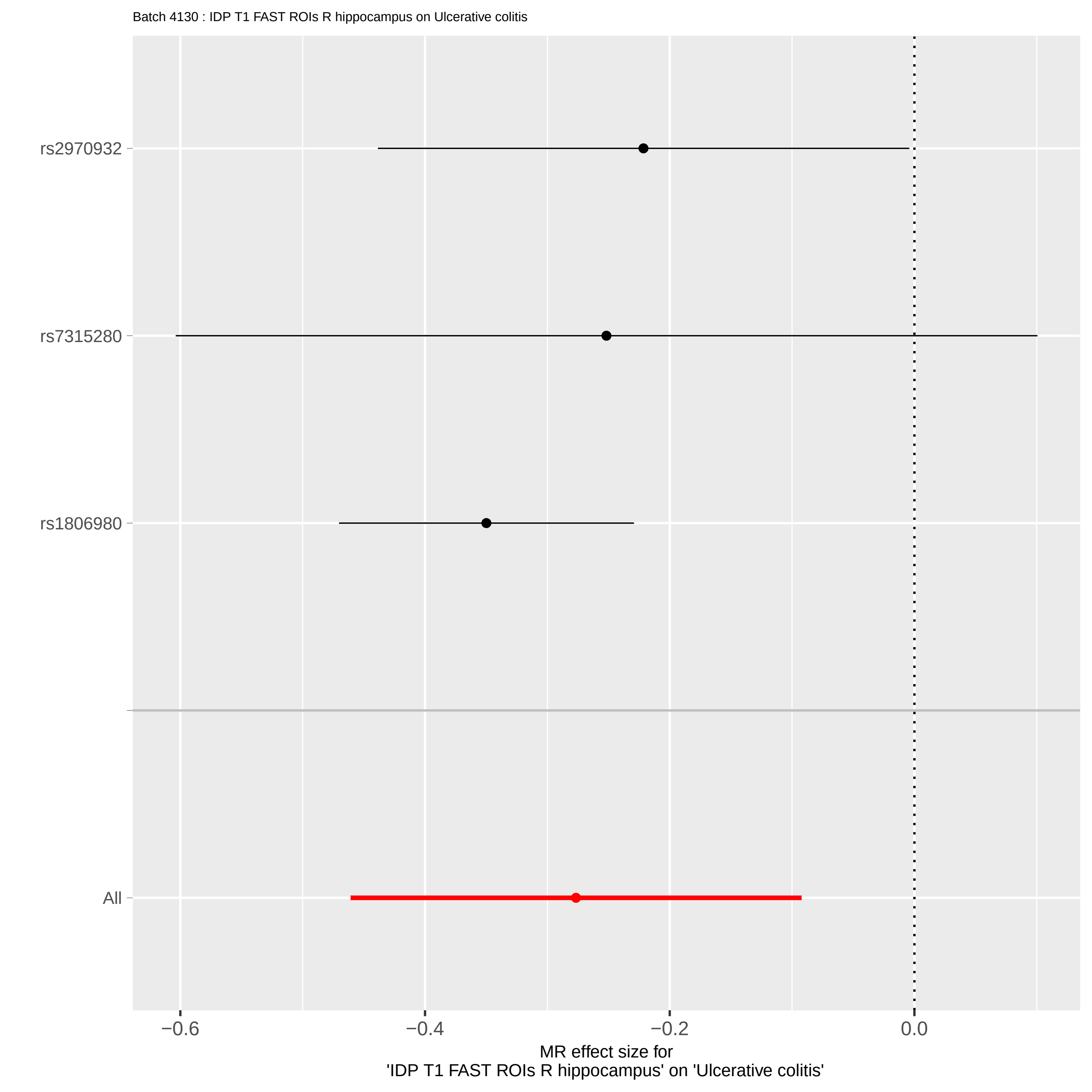

Batch 4130 : IDP T1 FAST ROIs R hippocampus on Ulcerative colitis
rs2970932
rs7315280
rs1806980
All
−0.6
−0.4
−0.2
0.0
MR effect size for
'IDP T1 FAST ROIs R hippocampus' on 'Ulcerative colitis'

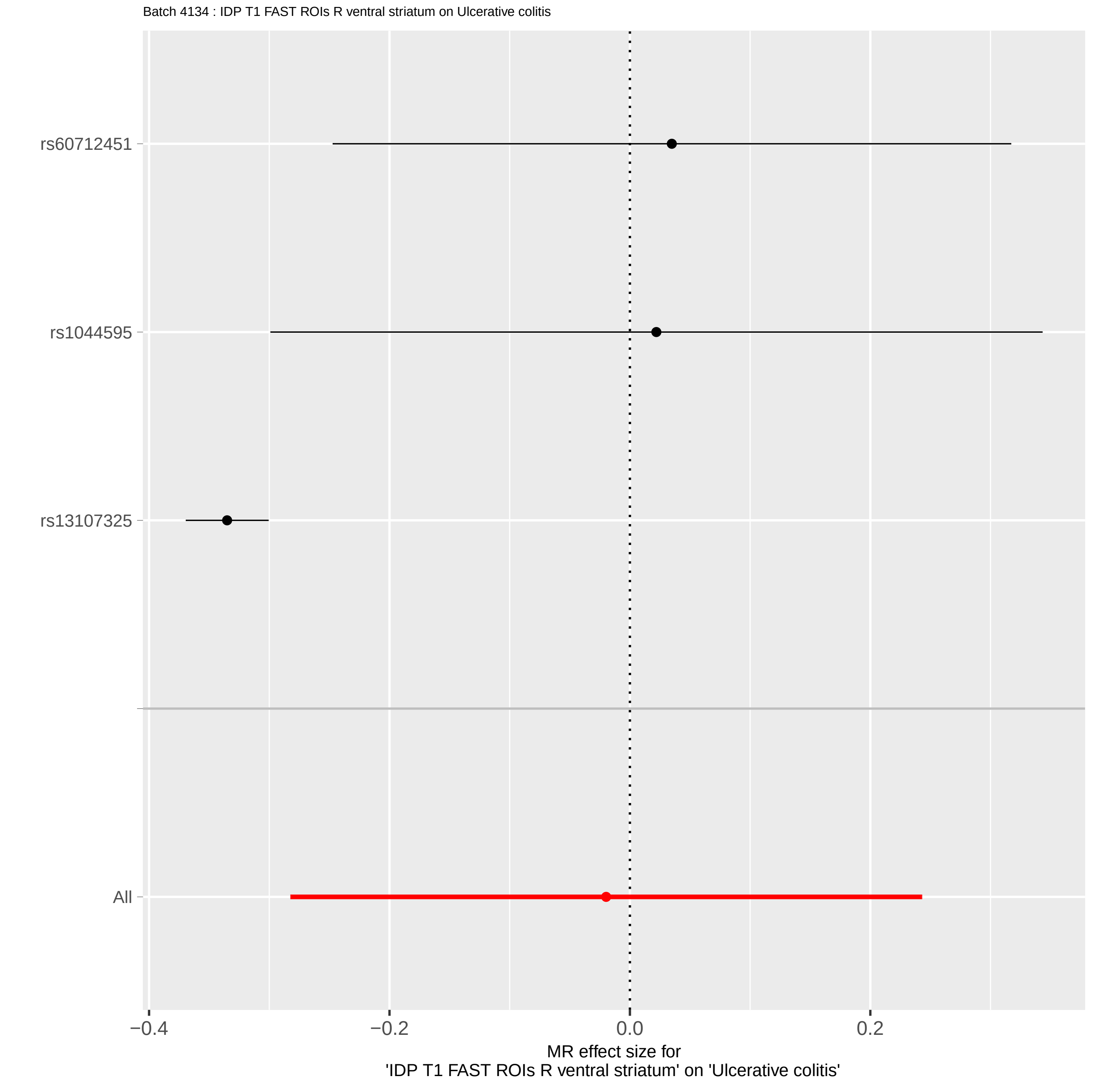
Batch 4134 : IDP T1 FAST ROIs R ventral striatum on Ulcerative colitis
rs60712451
rs1044595
rs13107325
All
−0.4
−0.2
0.0
0.2
MR effect size for
'IDP T1 FAST ROIs R ventral striatum' on 'Ulcerative colitis'

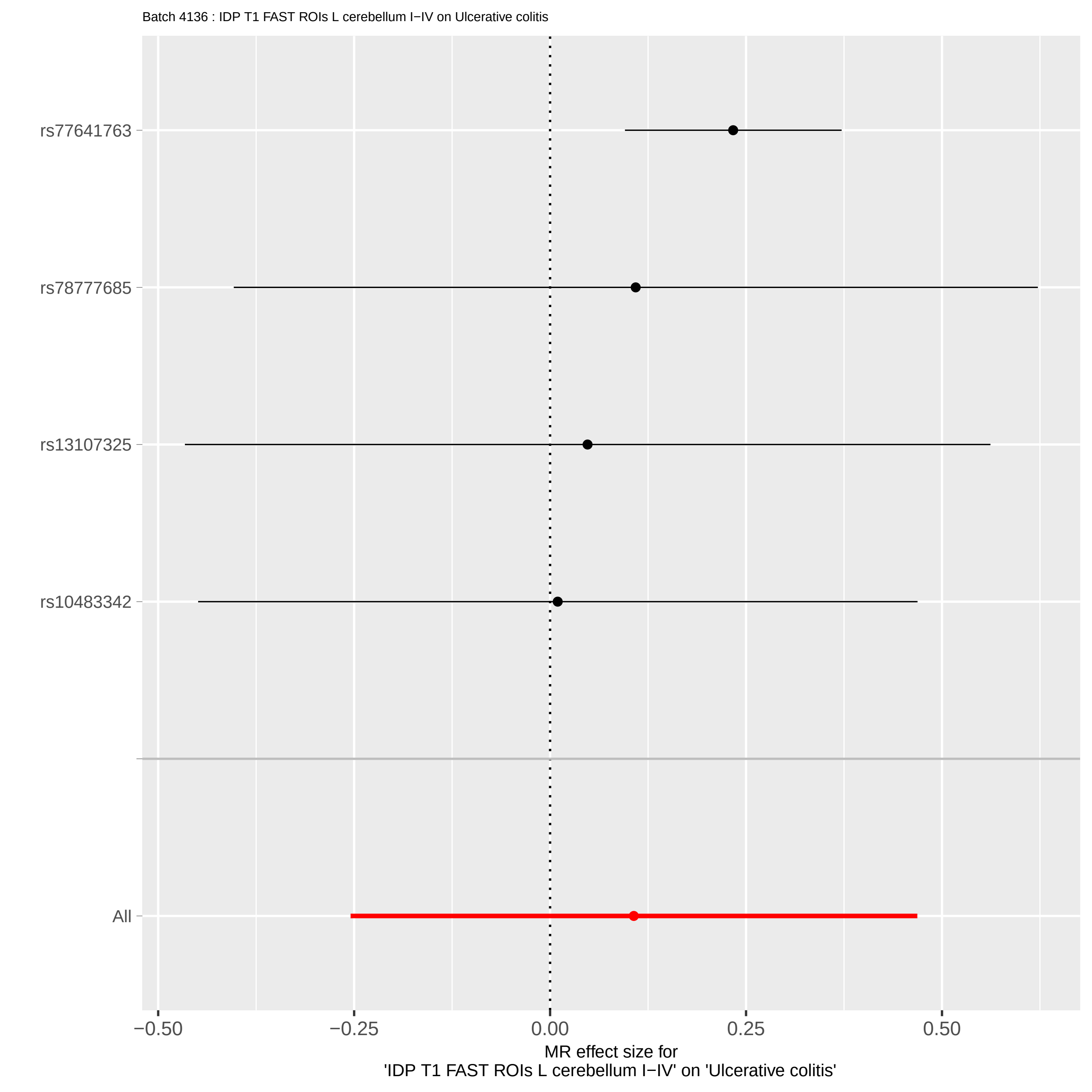

Batch 4136 : IDP T1 FAST ROIs L cerebellum I−IV on Ulcerative colitis
rs77641763
rs78777685
rs13107325
rs10483342
All
−0.50
−0.25
0.00
0.25
0.50
MR effect size for
'IDP T1 FAST ROIs L cerebellum I−IV' on 'Ulcerative colitis'

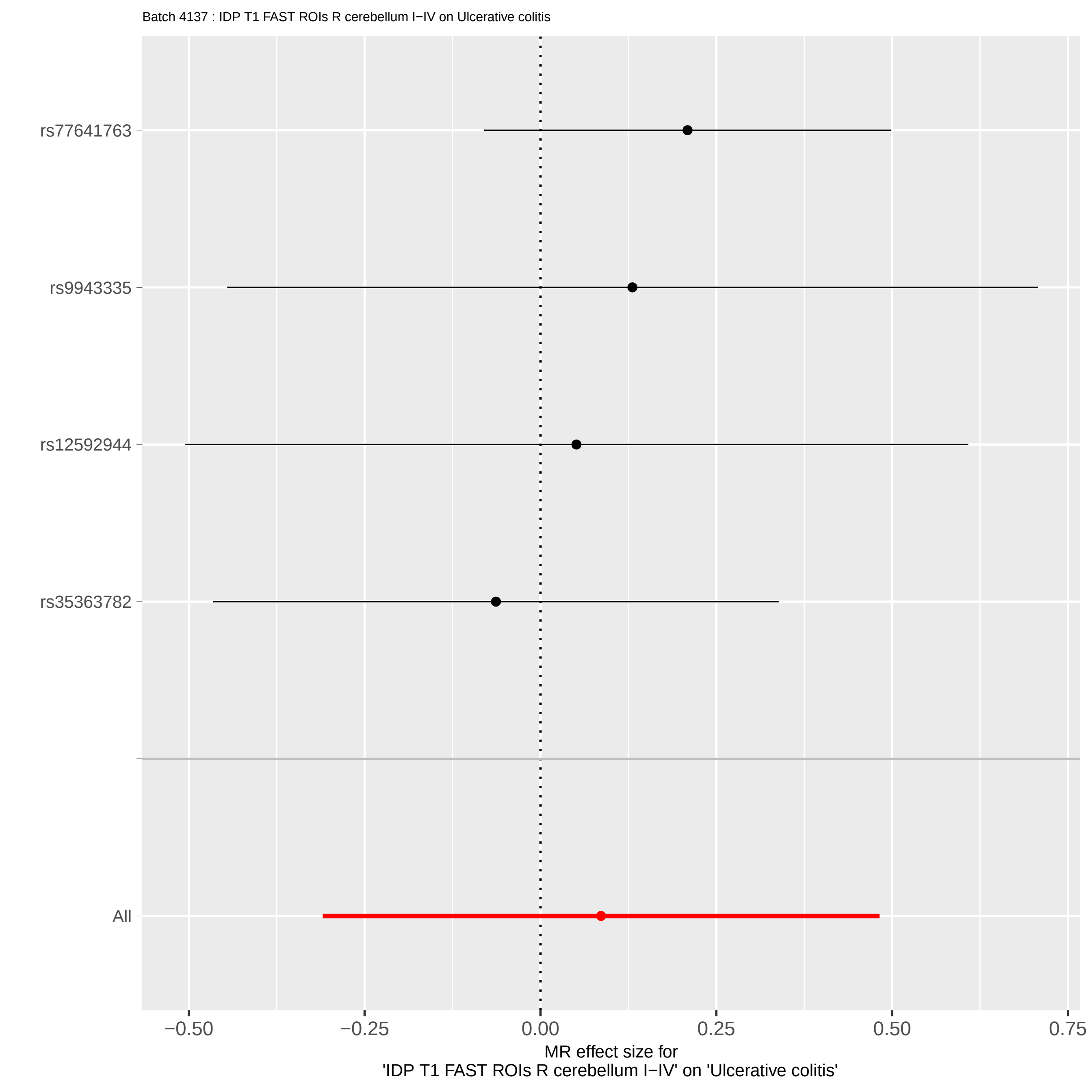

Batch 4137 : IDP T1 FAST ROIs R cerebellum I−IV on Ulcerative colitis
rs77641763
rs9943335
rs12592944
rs35363782
All
−0.50
−0.25
0.00
0.25
0.50
0.75
MR effect size for
'IDP T1 FAST ROIs R cerebellum I−IV' on 'Ulcerative colitis'

Batch 4140 : IDP T1 FAST ROIs R cerebellum V on Ulcerative colitis

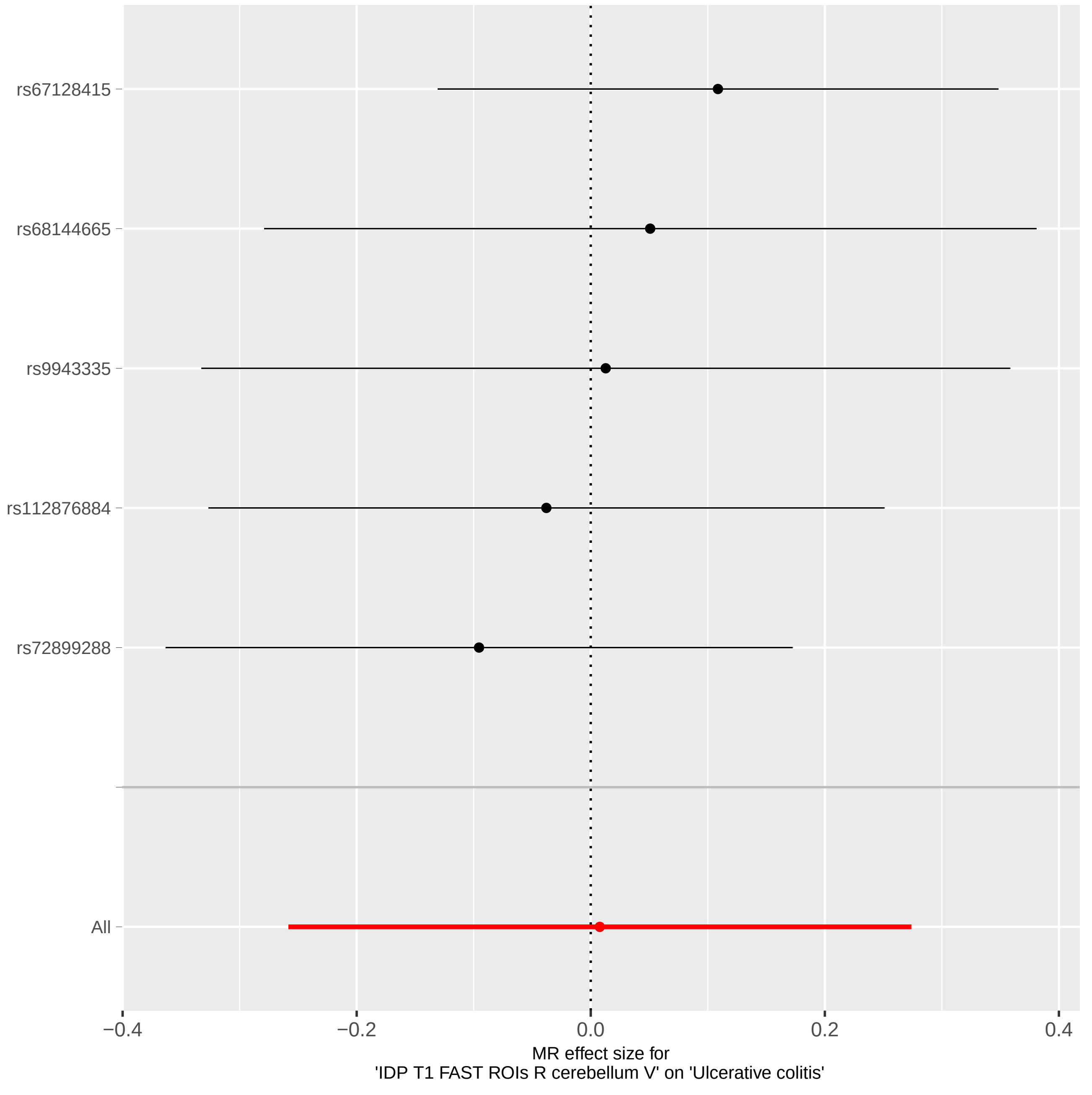

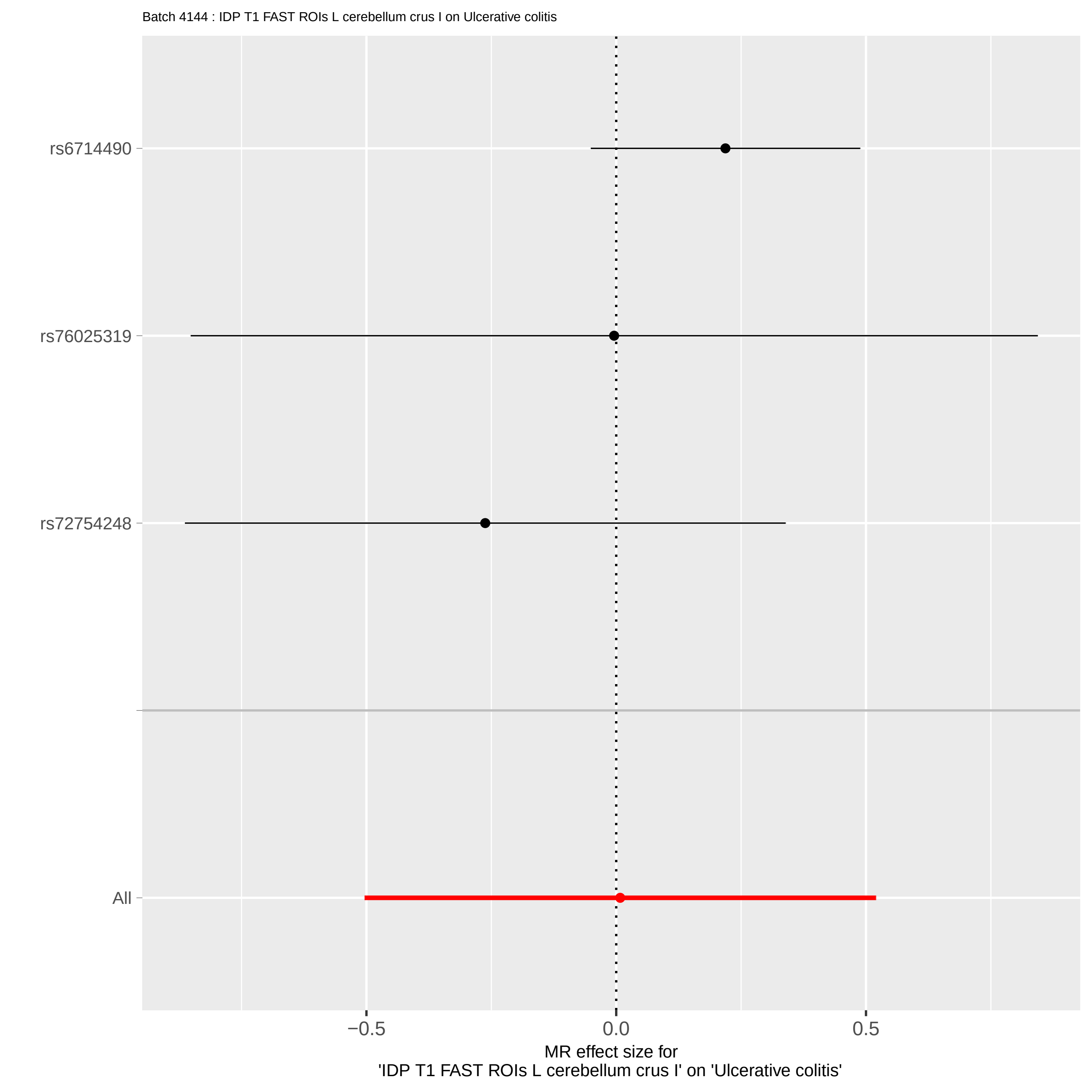
Batch 4144 : IDP T1 FAST ROIs L cerebellum crus I on Ulcerative colitis
rs6714490
rs76025319
rs72754248
All
−0.5
0.0
0.5
MR effect size for
'IDP T1 FAST ROIs L cerebellum crus I' on 'Ulcerative colitis'

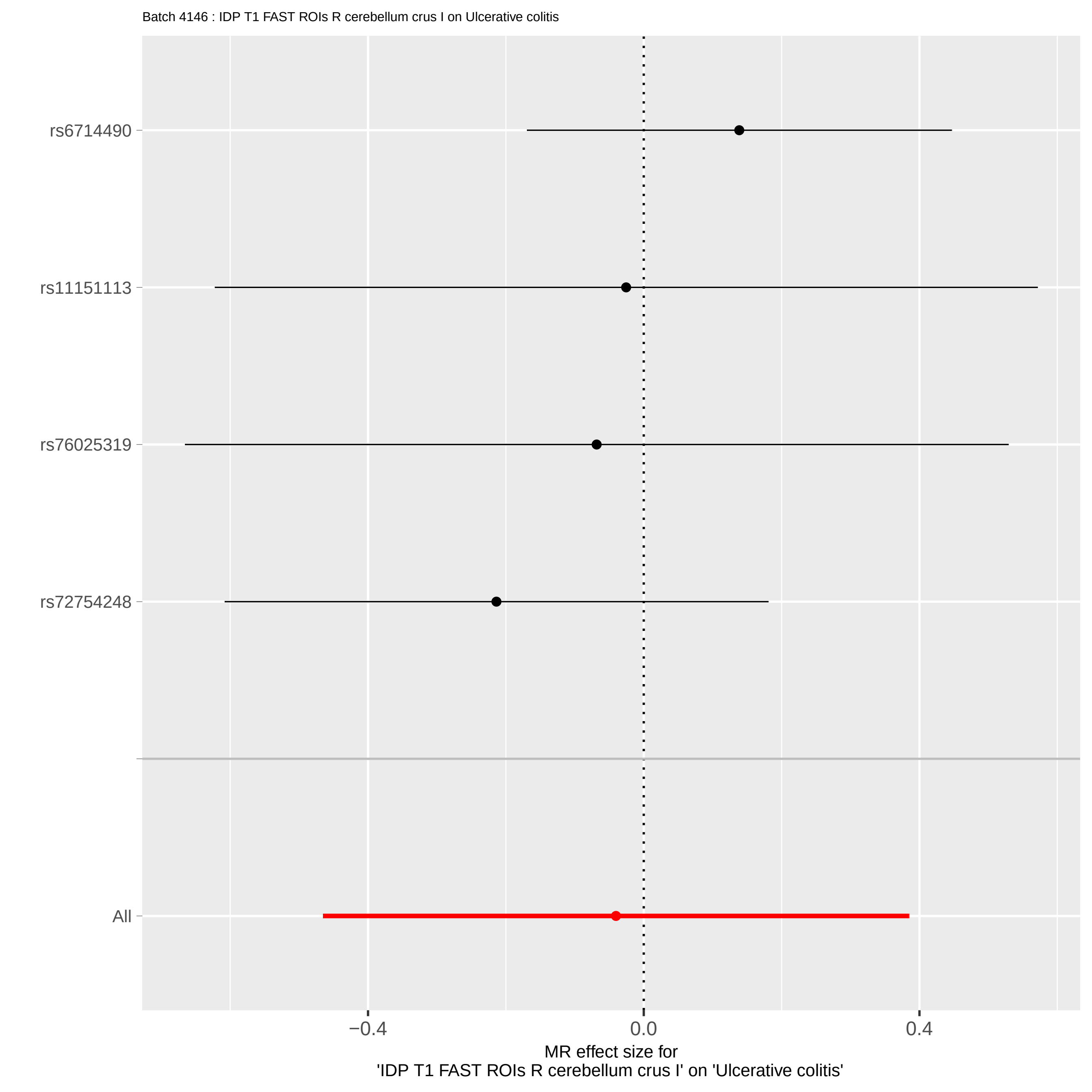
Batch 4146 : IDP T1 FAST ROIs R cerebellum crus I on Ulcerative colitis
rs6714490
rs11151113
rs76025319
rs72754248
All
−0.4
0.0
0.4
MR effect size for
'IDP T1 FAST ROIs R cerebellum crus I' on 'Ulcerative colitis'

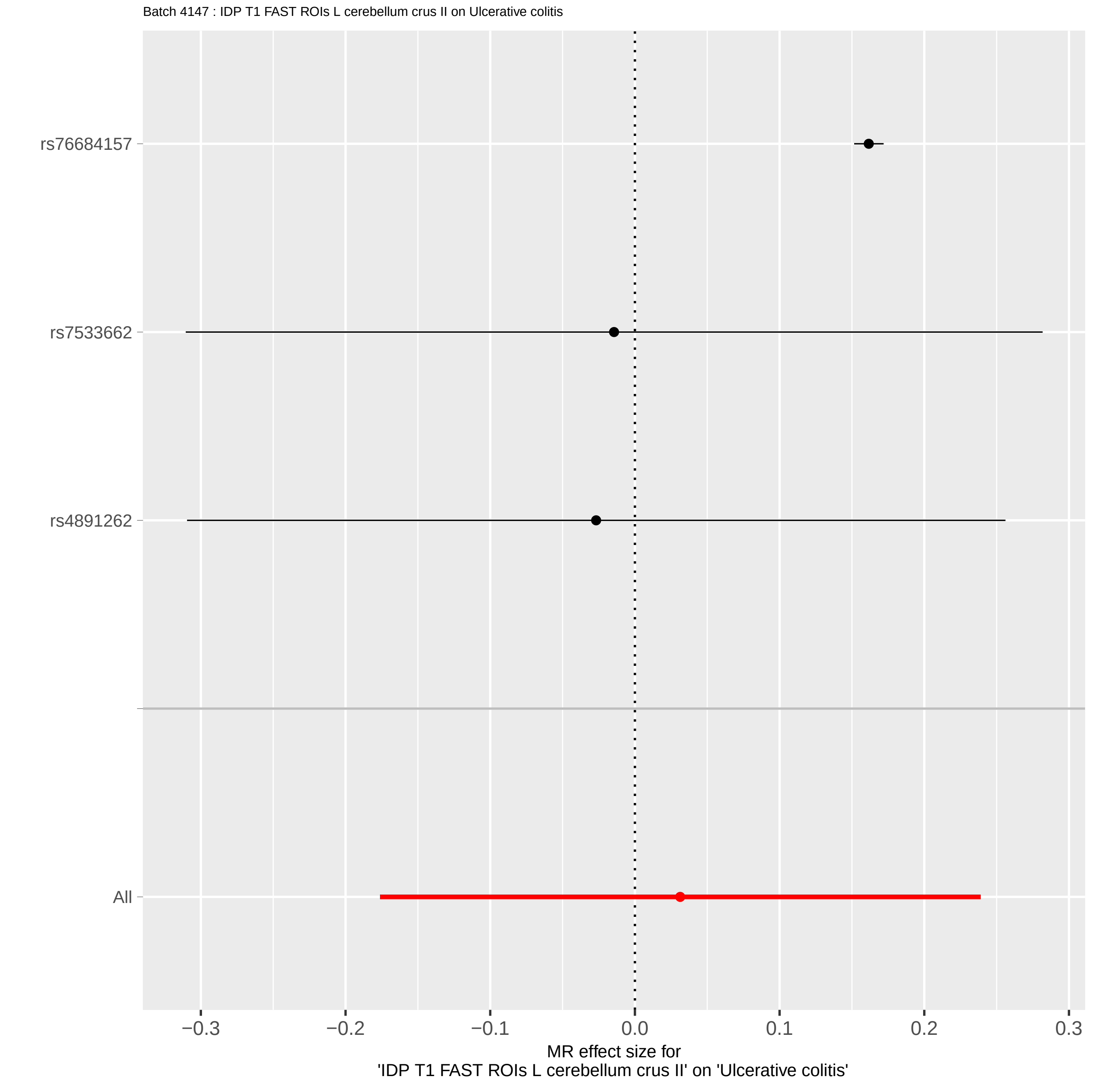
Batch 4147 : IDP T1 FAST ROIs L cerebellum crus II on Ulcerative colitis
rs76684157
rs7533662
rs4891262
All
−0.3
−0.2
−0.1
0.0
0.1
0.2
0.3
MR effect size for
'IDP T1 FAST ROIs L cerebellum crus II' on 'Ulcerative colitis'

Batch 4148 : IDP T1 FAST ROIs V cerebellum crus II on Ulcerative colitis

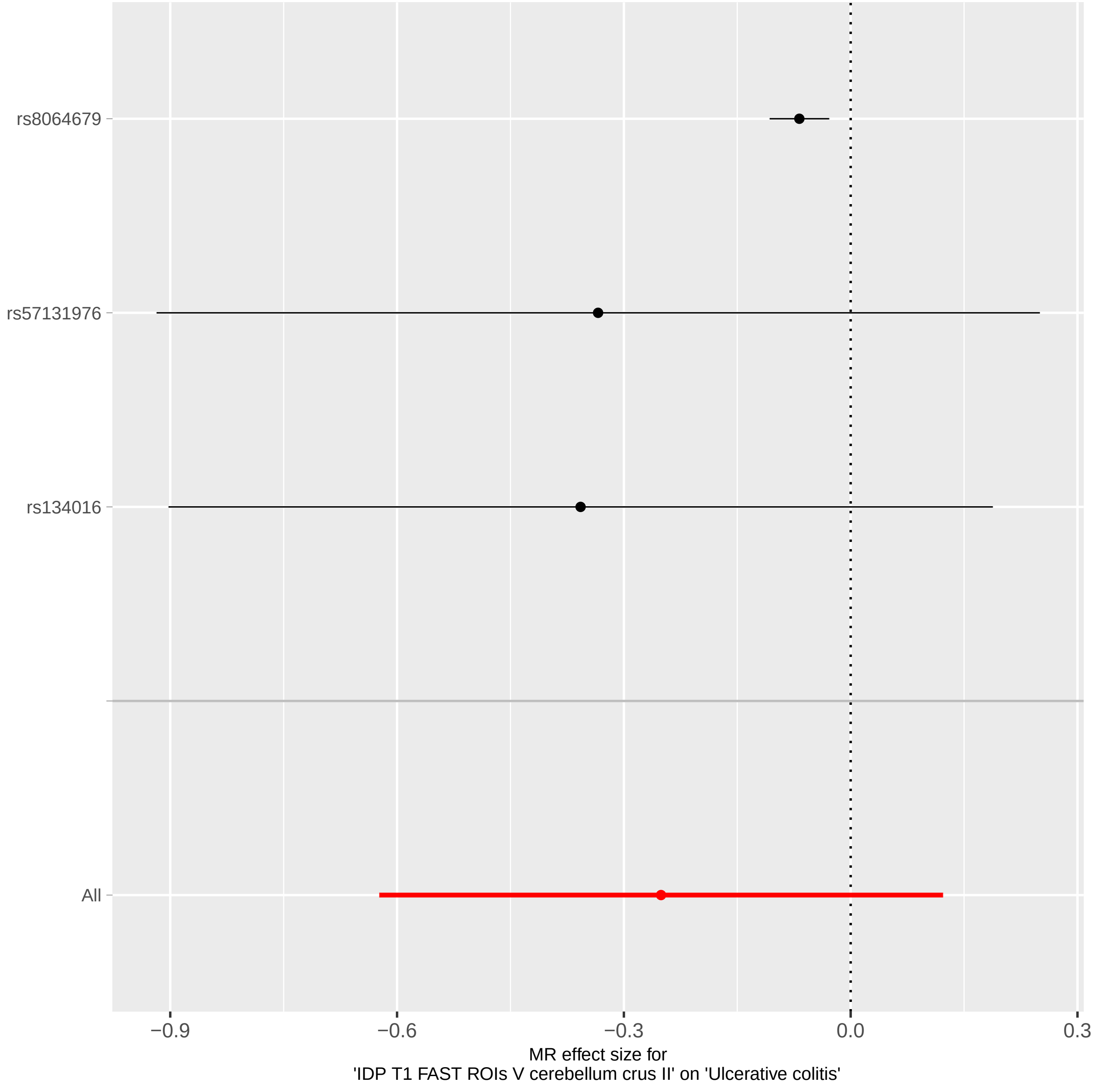

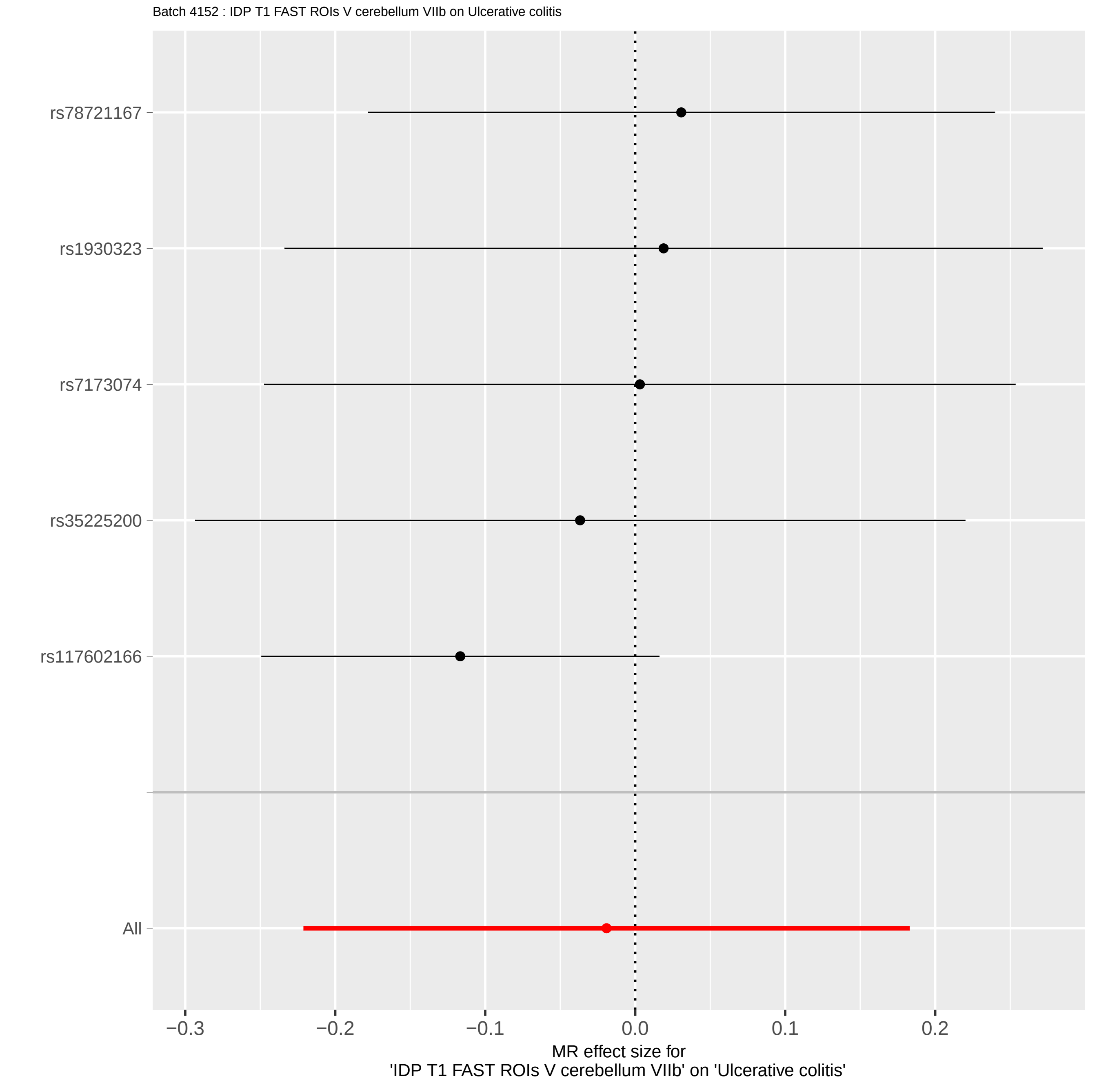

Batch 4152 : IDP T1 FAST ROIs V cerebellum VIIb on Ulcerative colitis
rs78721167
rs1930323
rs7173074
rs35225200
rs117602166
All
−0.3
−0.2
−0.1
0.0
0.1
0.2
MR effect size for
'IDP T1 FAST ROIs V cerebellum VIIb' on 'Ulcerative colitis'

Batch 4155 : IDP T1 FAST ROIs V cerebellum VIIIa on Ulcerative colitis

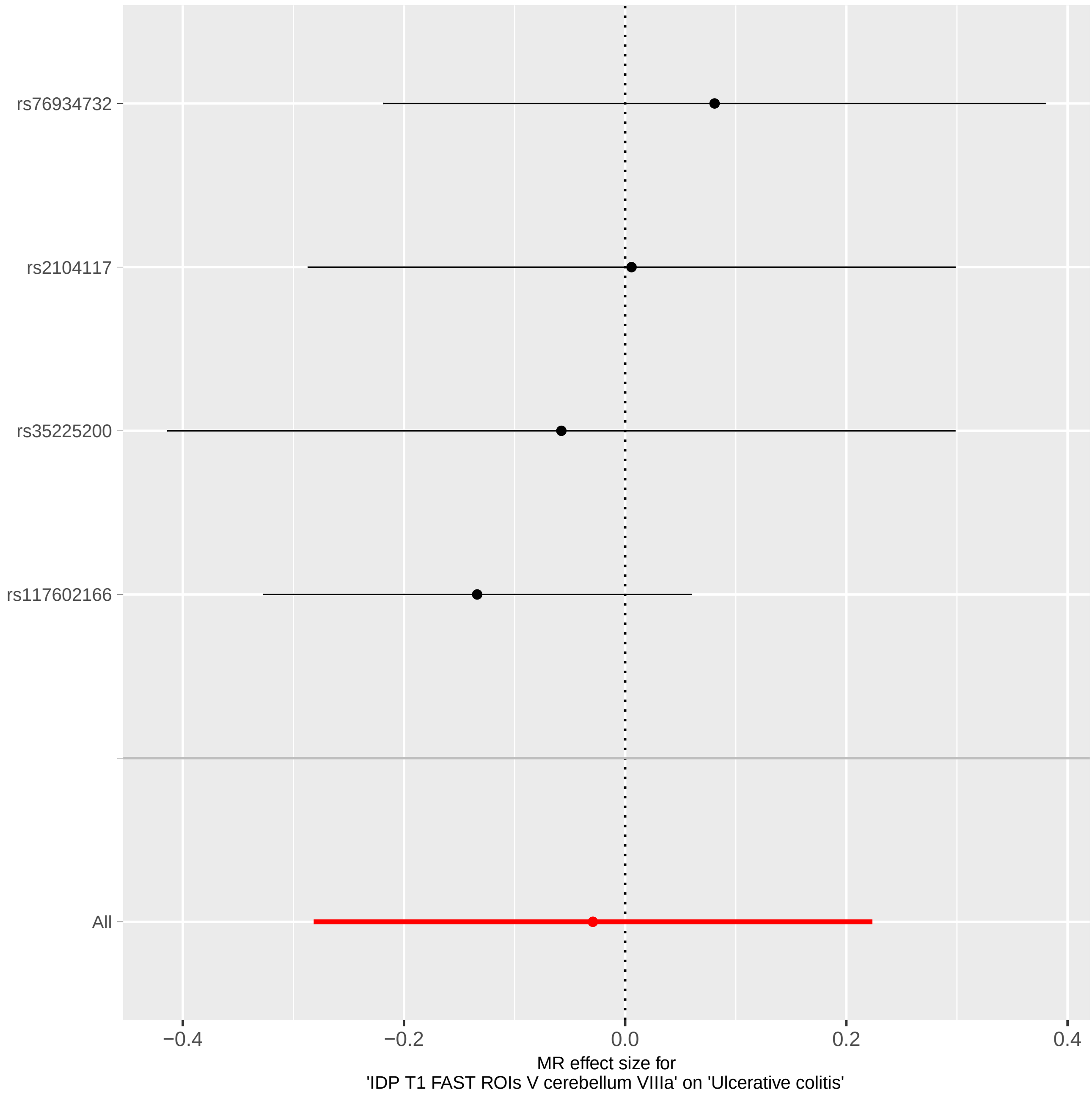

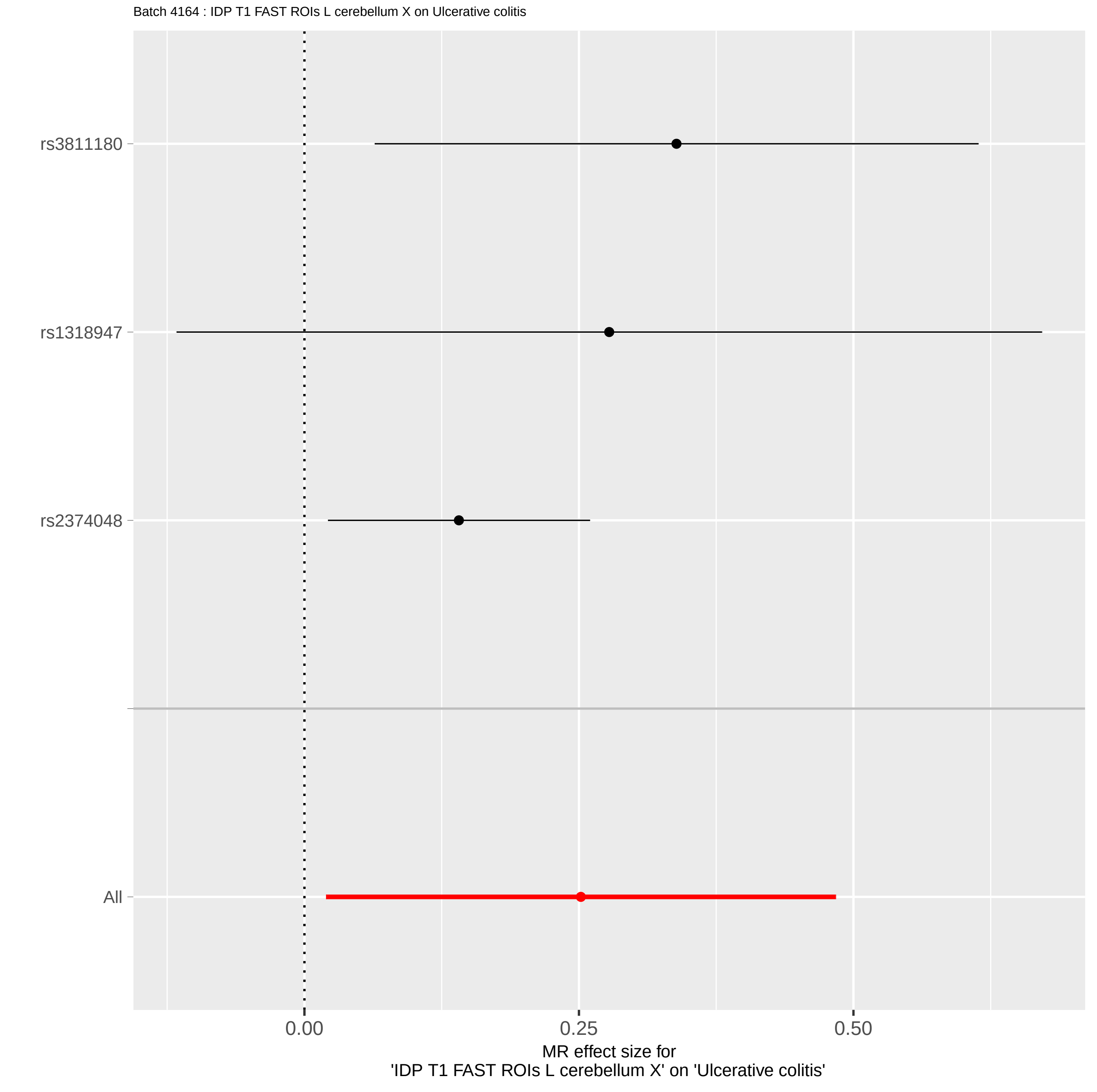

Batch 4164 : IDP T1 FAST ROIs L cerebellum X on Ulcerative colitis
rs3811180
rs1318947
rs2374048
All
0.00
0.25
0.50
MR effect size for
'IDP T1 FAST ROIs L cerebellum X' on 'Ulcerative colitis'

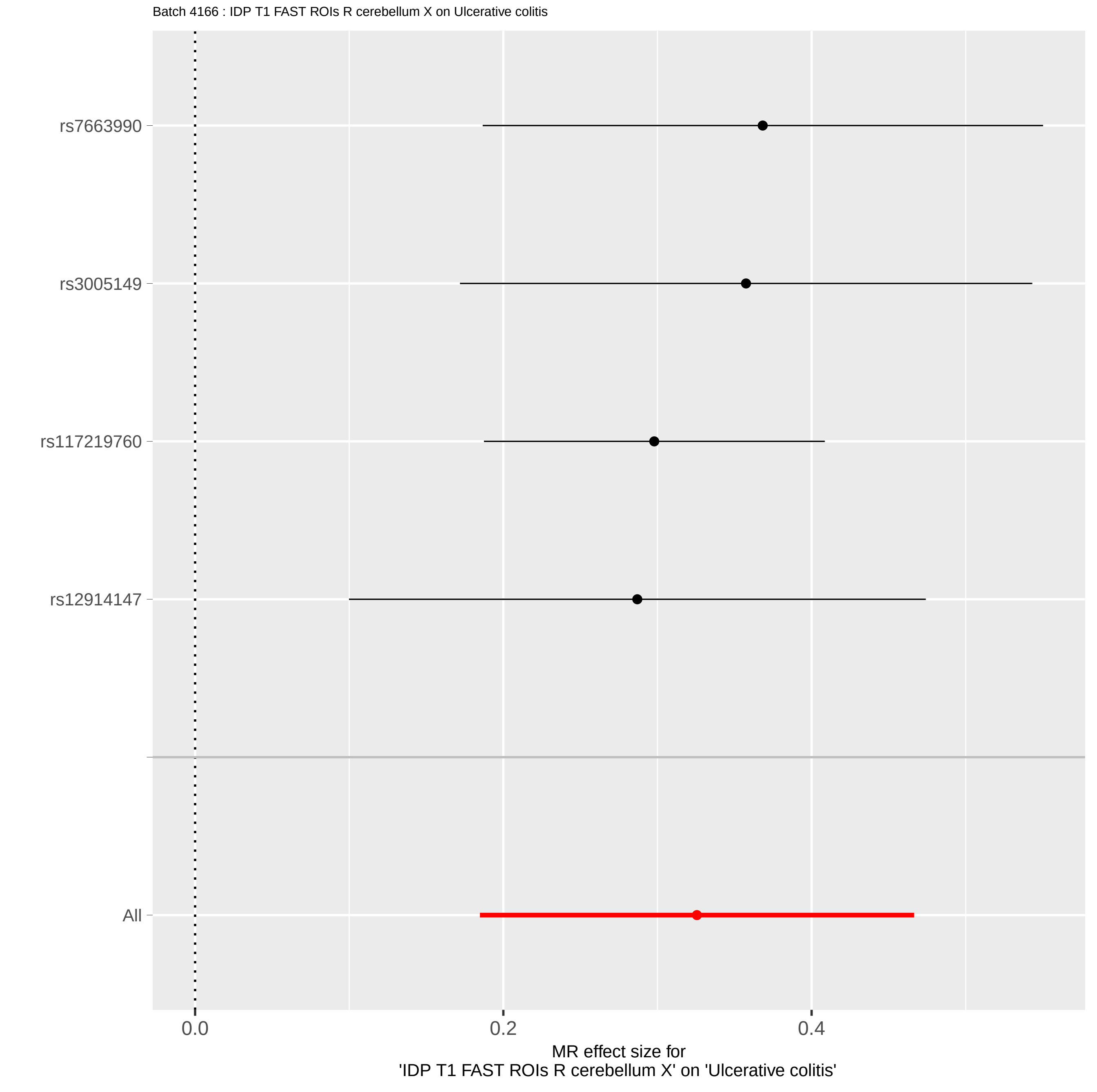

Batch 4166 : IDP T1 FAST ROIs R cerebellum X on Ulcerative colitis
rs7663990
rs3005149
rs117219760
rs12914147
All
0.0
0.2
0.4
MR effect size for
'IDP T1 FAST ROIs R cerebellum X' on 'Ulcerative colitis'

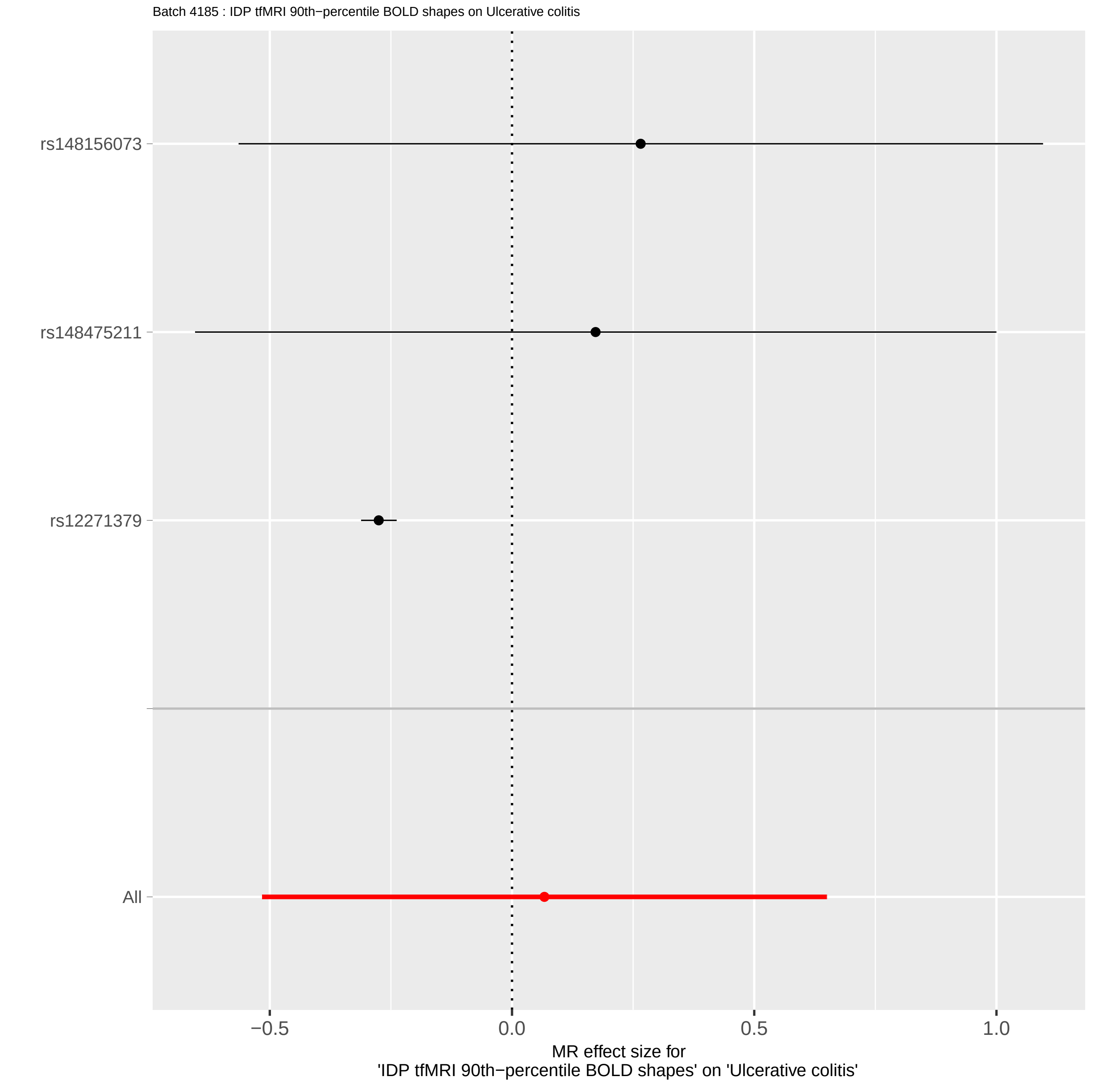
Batch 4185 : IDP tfMRI 90th−percentile BOLD shapes on Ulcerative colitis
rs148156073
rs148475211
rs12271379
All
−0.5
0.0
0.5
1.0
MR effect size for
'IDP tfMRI 90th−percentile BOLD shapes' on 'Ulcerative colitis'

Batch 4250 : IDP T1 FIRST right accumbens volume on Ulcerative colitis

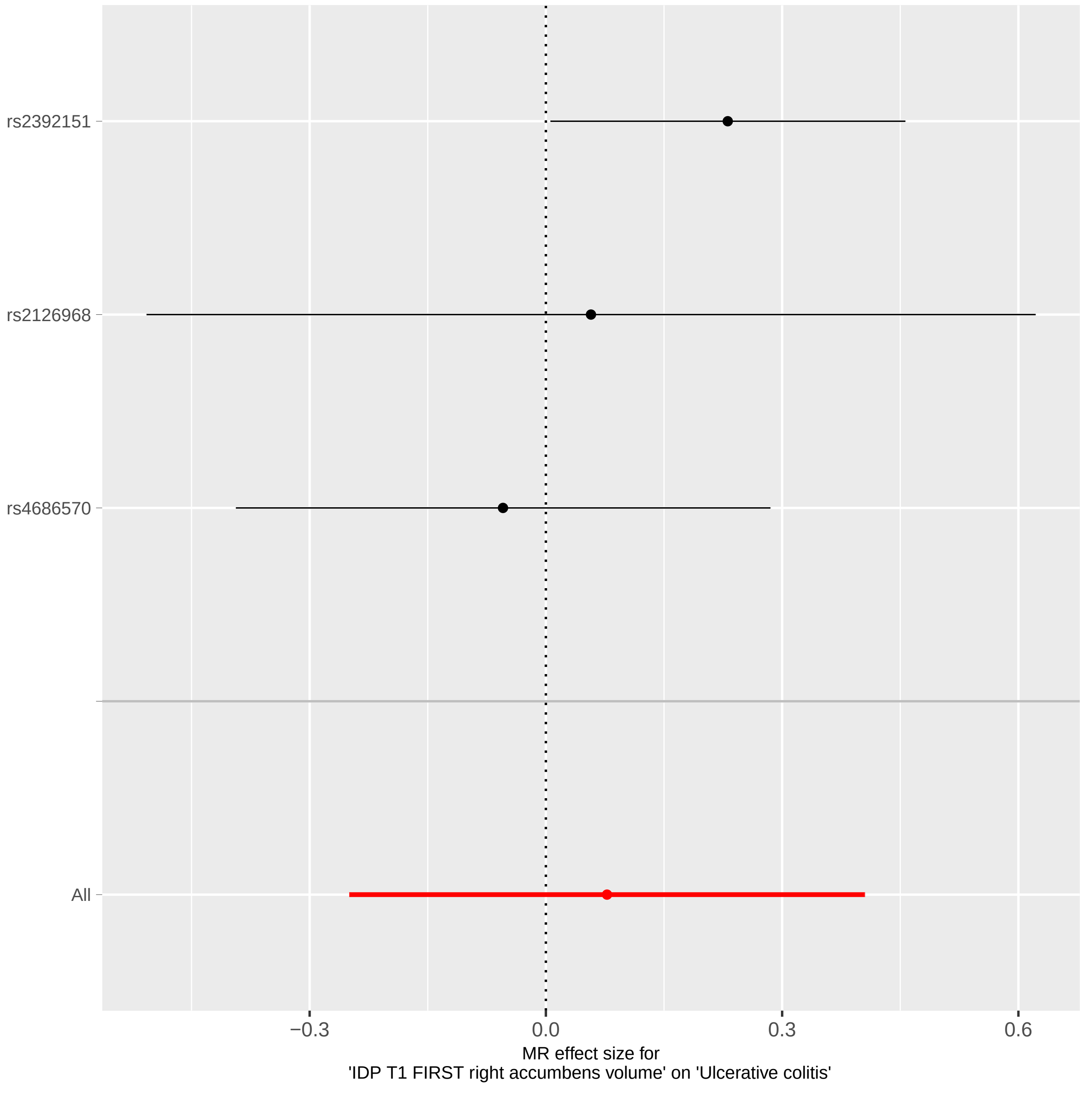

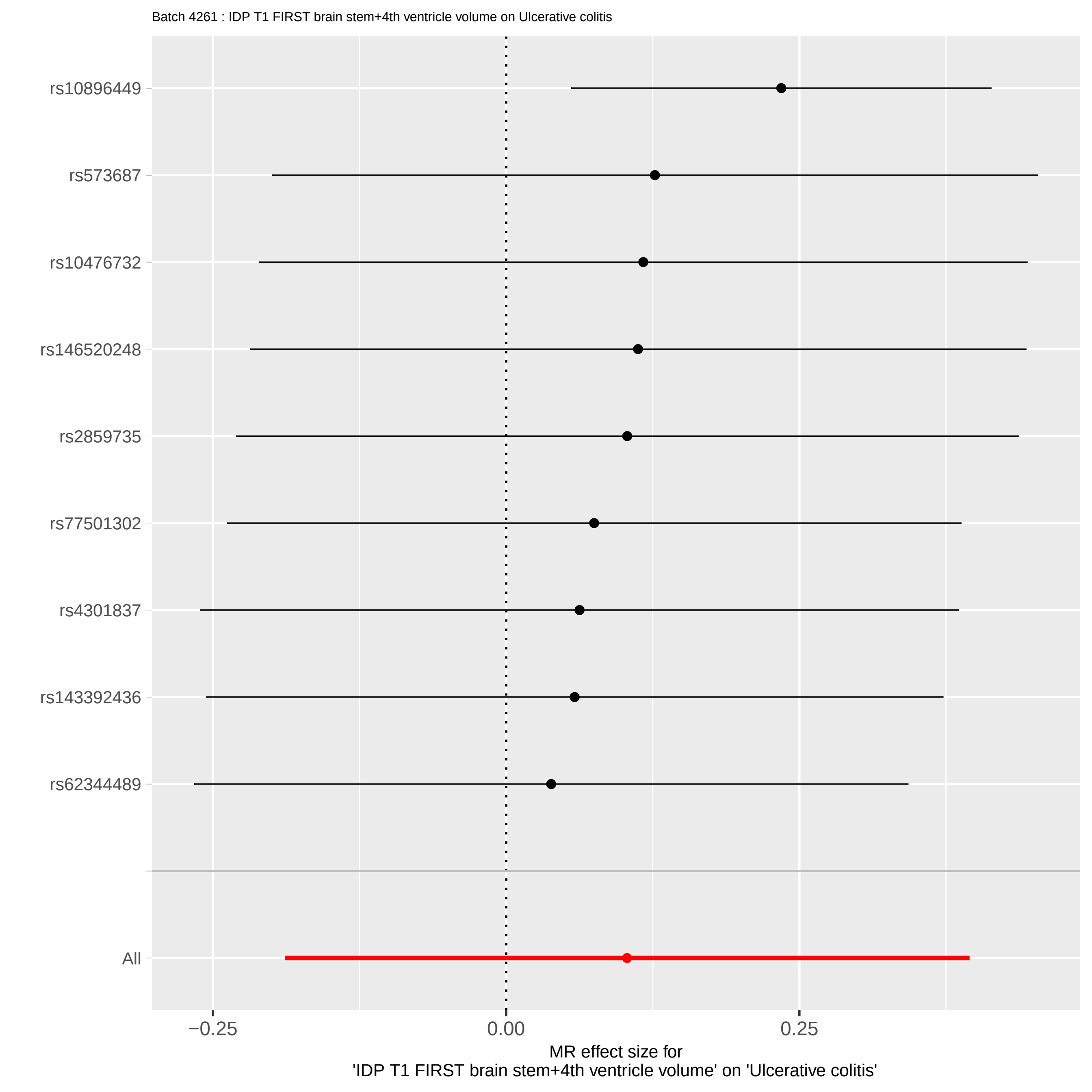

Batch 4261 : IDP T1 FIRST brain stem+4th ventricle volume on Ulcerative colitis
rs10896449
rs573687
rs10476732
rs146520248
rs2859735
rs77501302
rs4301837
rs143392436
rs62344489
All
−0.25
0.00
0.25
MR effect size for
'IDP T1 FIRST brain stem+4th ventricle volume' on 'Ulcerative colitis'

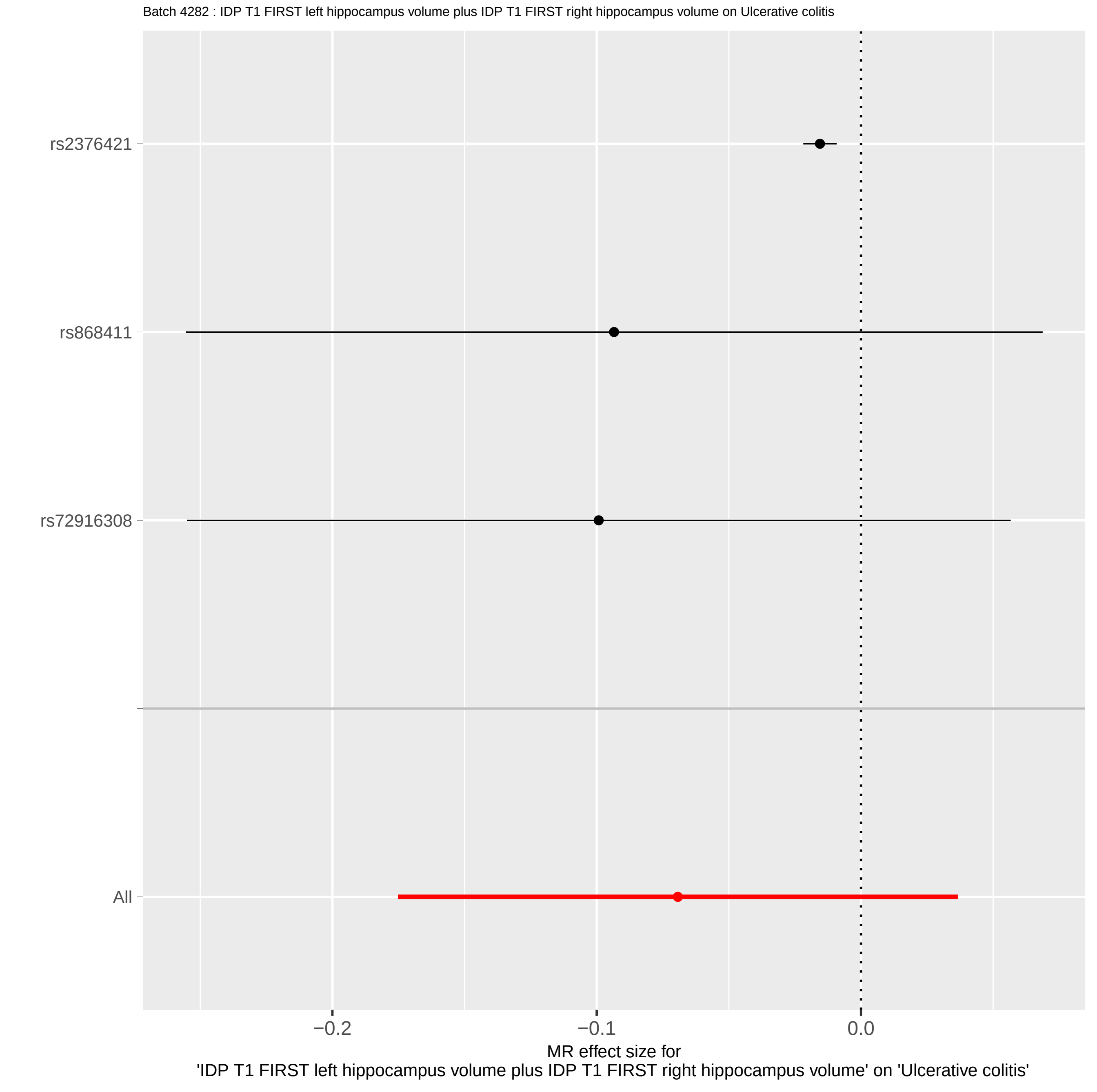

Batch 4282 : IDP T1 FIRST left hippocampus volume plus IDP T1 FIRST right hippocampus volume on Ulcerative colitis
rs2376421
rs868411
rs72916308
All
−0.2
−0.1
0.0
MR effect size for
'IDP T1 FIRST left hippocampus volume plus IDP T1 FIRST right hippocampus volume' on 'Ulcerative colitis'

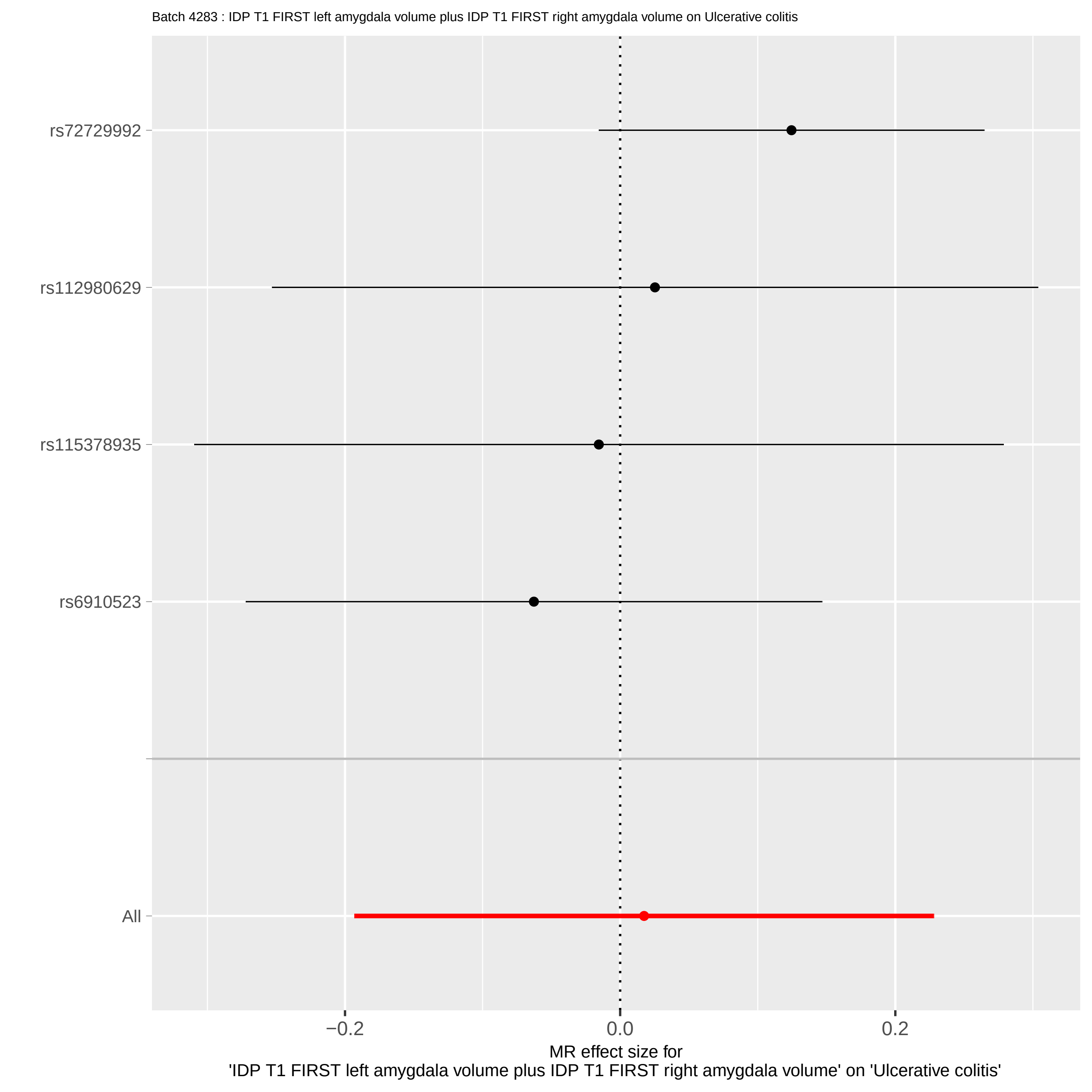

Batch 4283 : IDP T1 FIRST left amygdala volume plus IDP T1 FIRST right amygdala volume on Ulcerative colitis
rs72729992
rs112980629
rs115378935
rs6910523
All
−0.2
0.0
0.2
MR effect size for
'IDP T1 FIRST left amygdala volume plus IDP T1 FIRST right amygdala volume' on 'Ulcerative colitis'

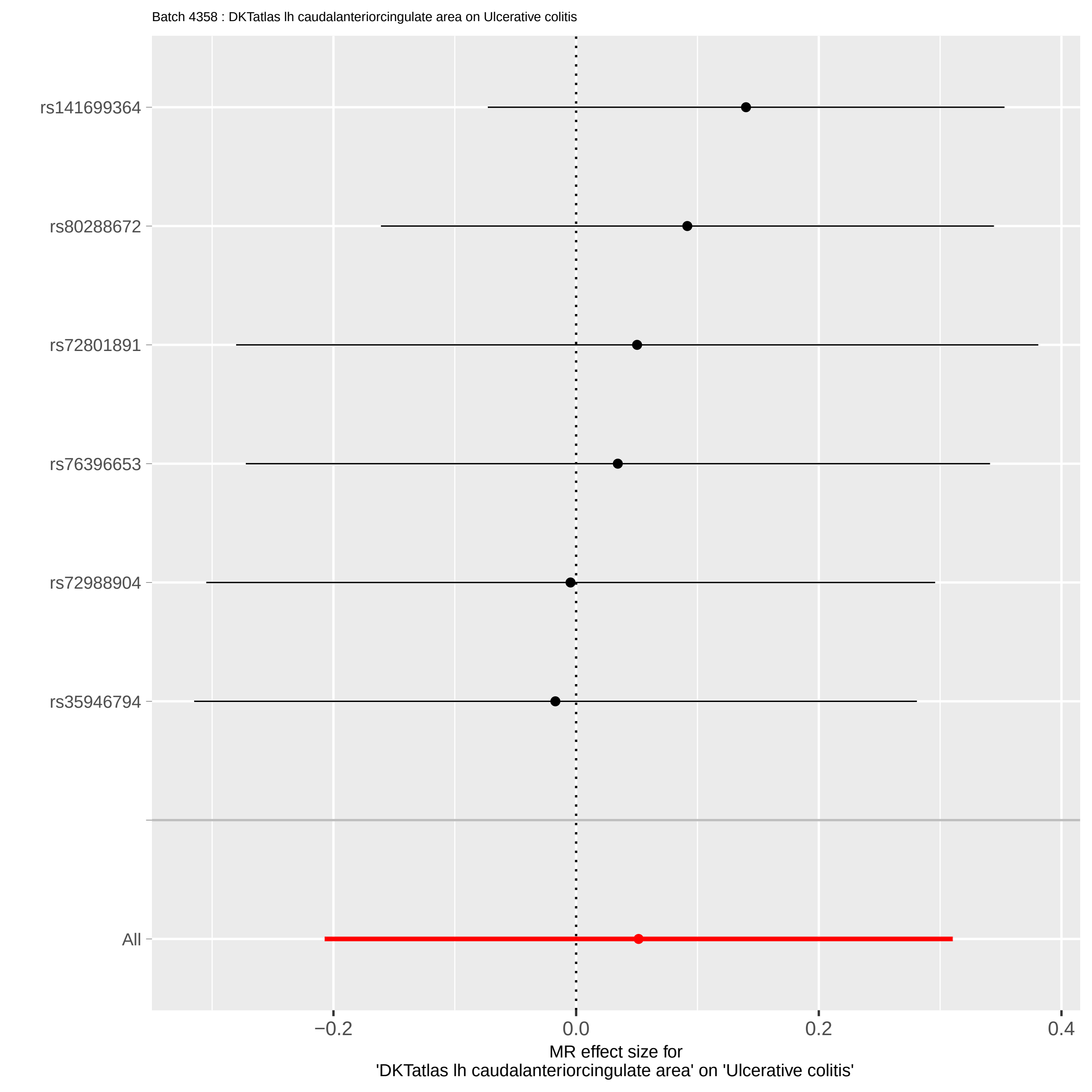

Batch 4358 : DKTatlas lh caudalanteriorcingulate area on Ulcerative colitis
rs141699364
rs80288672
rs72801891
rs76396653
rs72988904
rs35946794
All
−0.2
0.0
0.2
0.4
MR effect size for
'DKTatlas lh caudalanteriorcingulate area' on 'Ulcerative colitis'

Batch 4368 : DKTatlas lh lateralorbitofrontal area on Ulcerative colitis

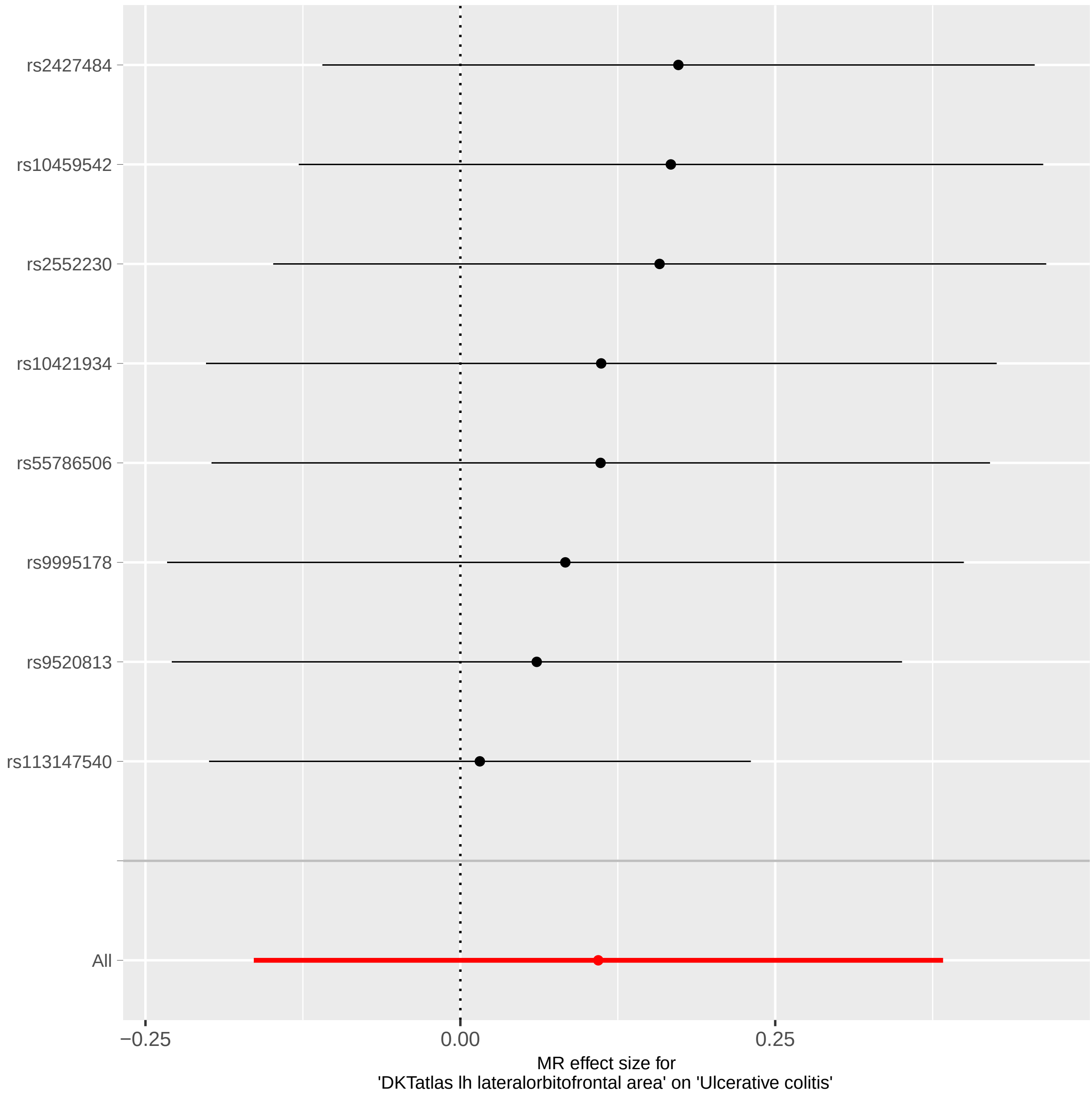

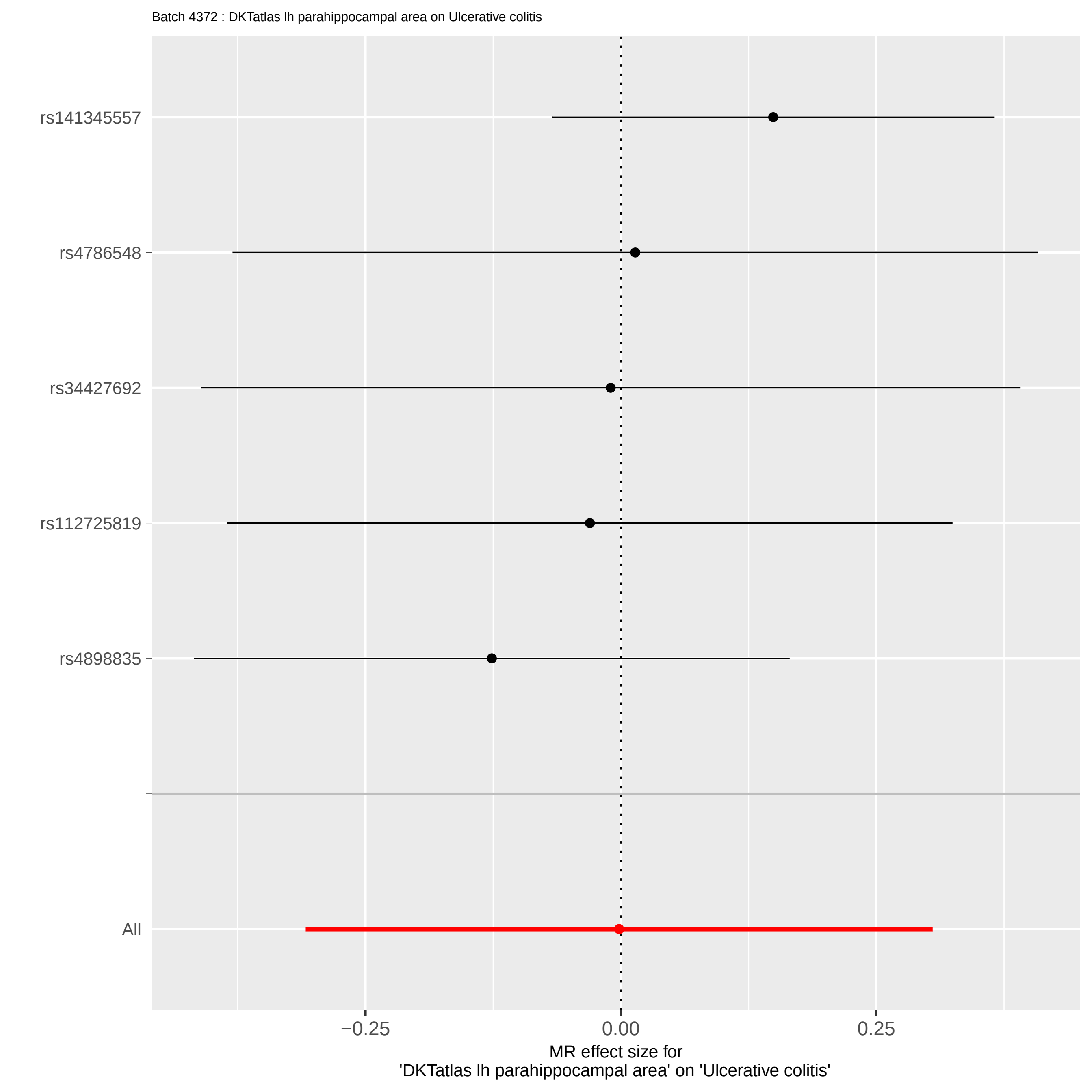

Batch 4372 : DKTatlas lh parahippocampal area on Ulcerative colitis
rs141345557
rs4786548
rs34427692
rs112725819
rs4898835
All
−0.25
0.00
0.25
MR effect size for
'DKTatlas lh parahippocampal area' on 'Ulcerative colitis'

Batch 4373 : DKTatlas lh paracentral area on Ulcerative colitis

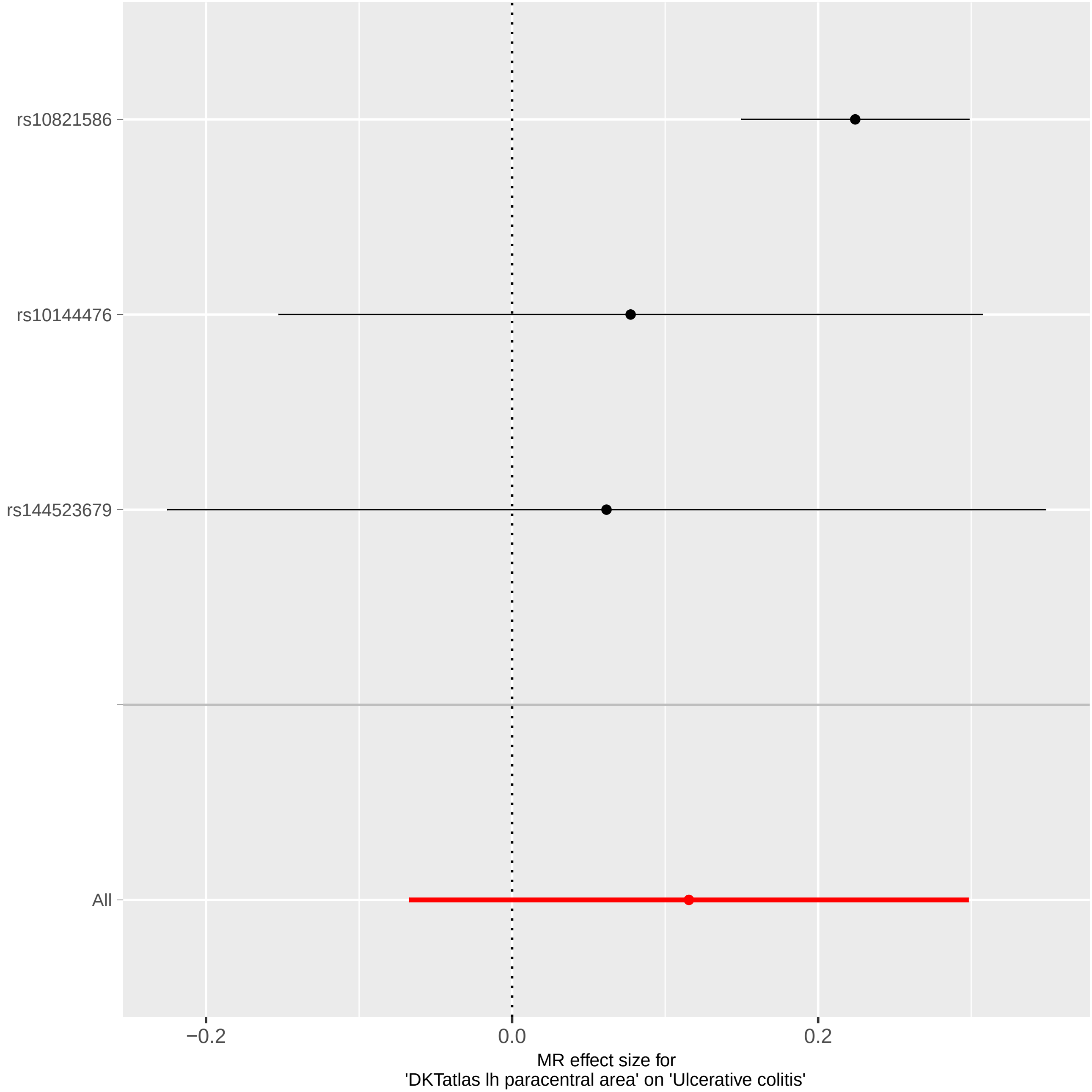

Batch 4376 : DKTatlas lh parsorbitalis area on Ulcerative colitis

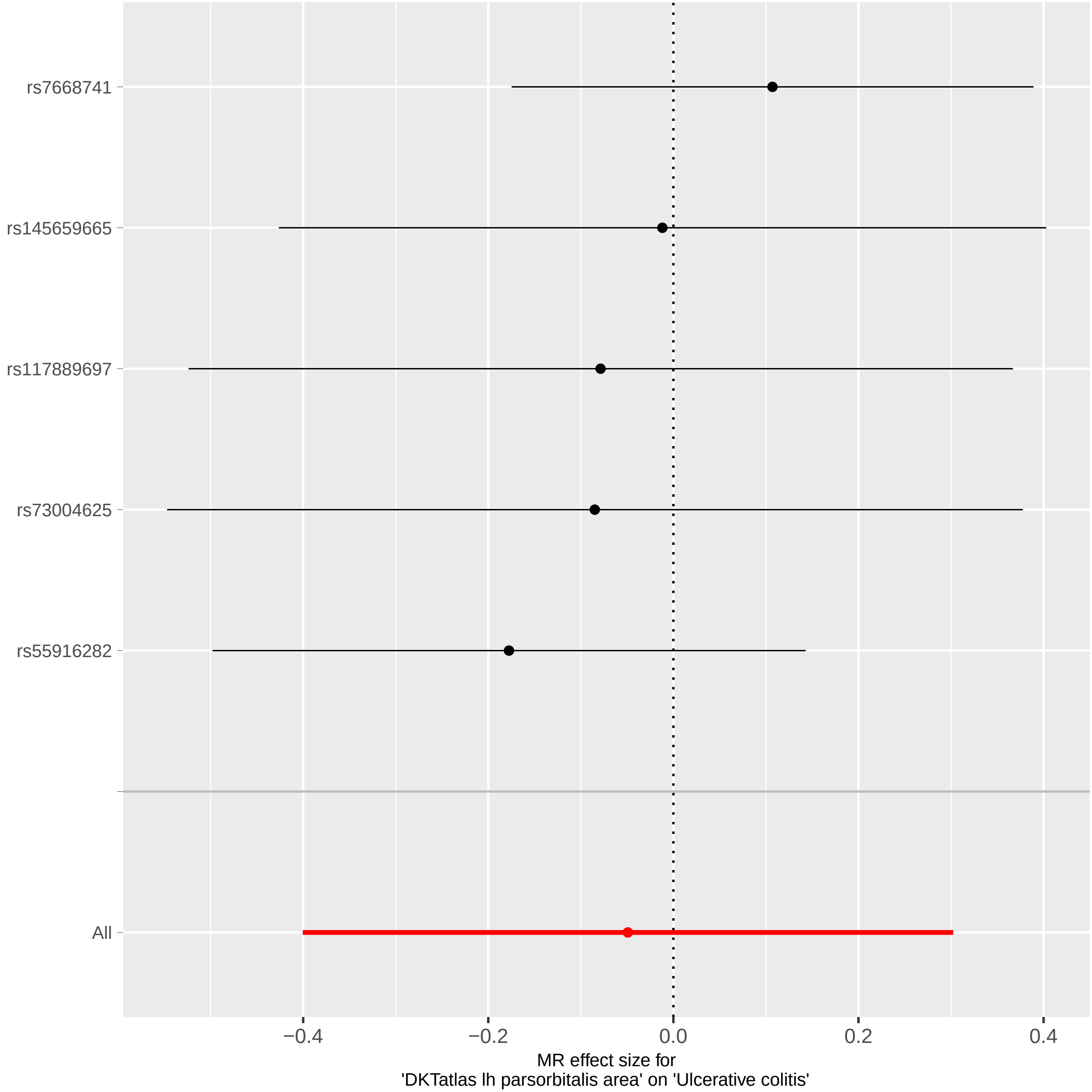

Batch 4379 : DKTatlas lh postcentral area on Ulcerative colitis

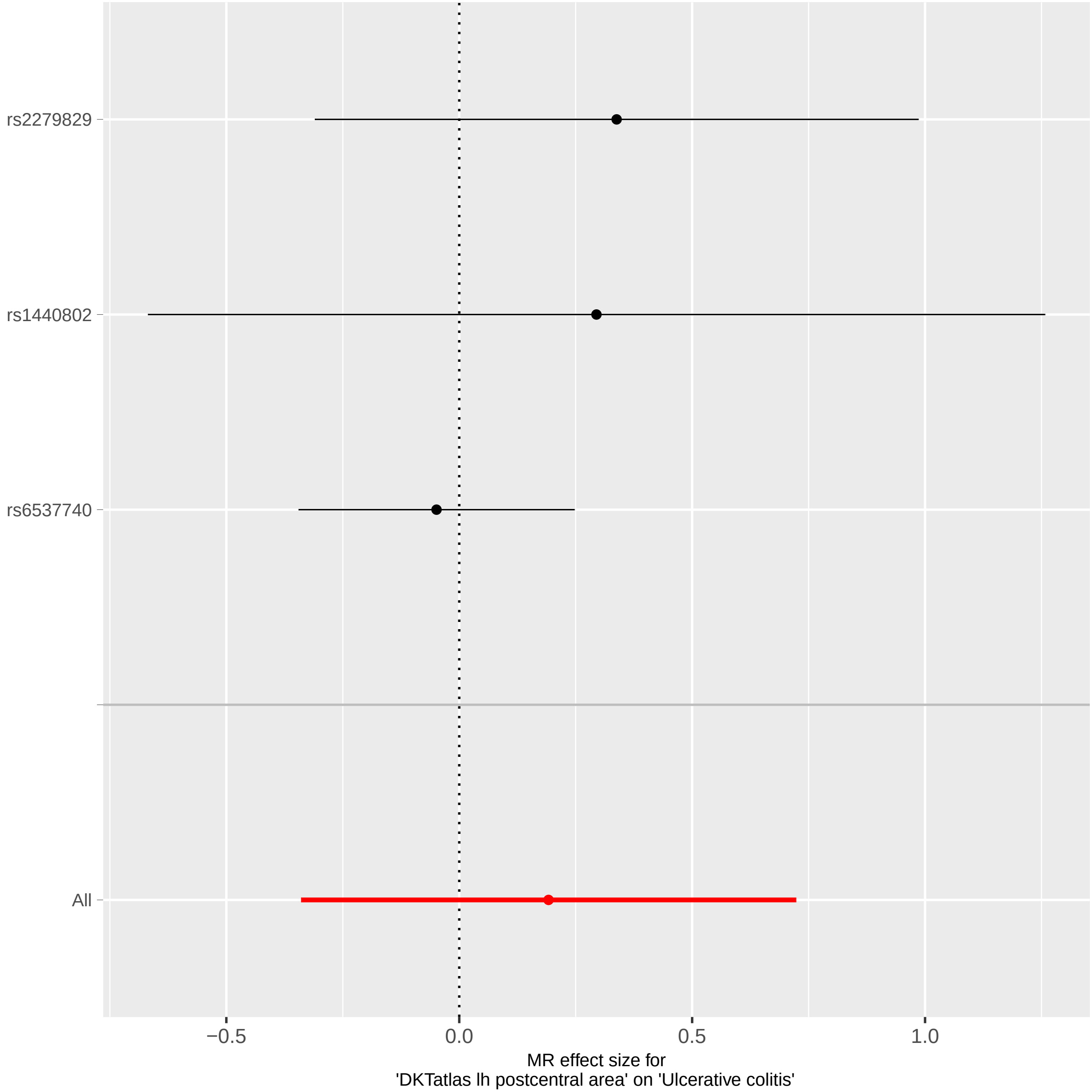

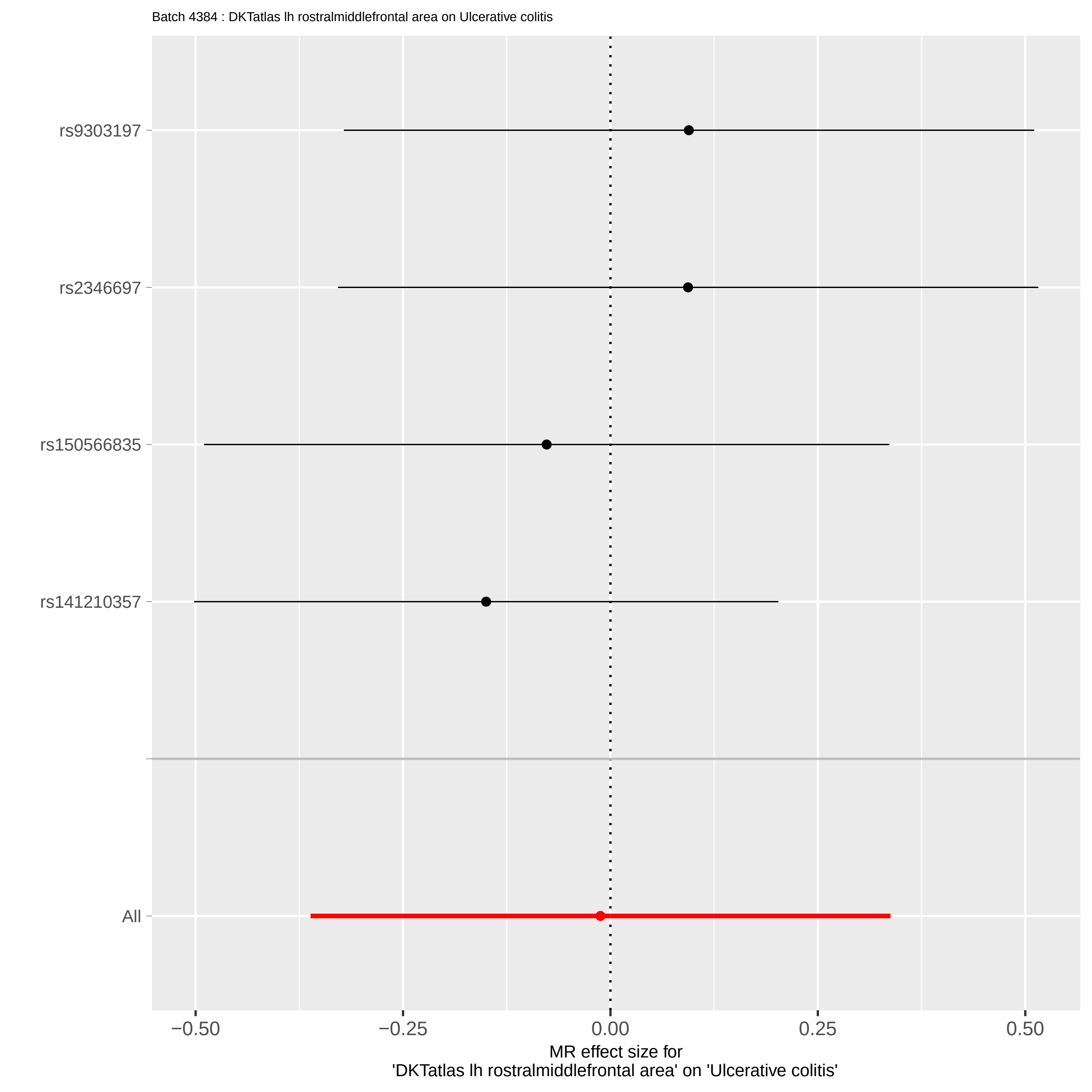

Batch 4384 : DKTatlas lh rostralmiddlefrontal area on Ulcerative colitis
rs9303197
rs2346697
rs150566835
rs141210357
All
−0.50
−0.25
0.00
0.25
0.50
MR effect size for
'DKTatlas lh rostralmiddlefrontal area' on 'Ulcerative colitis'

Batch 4386 : DKTatlas lh superiorfrontal area on Ulcerative colitis

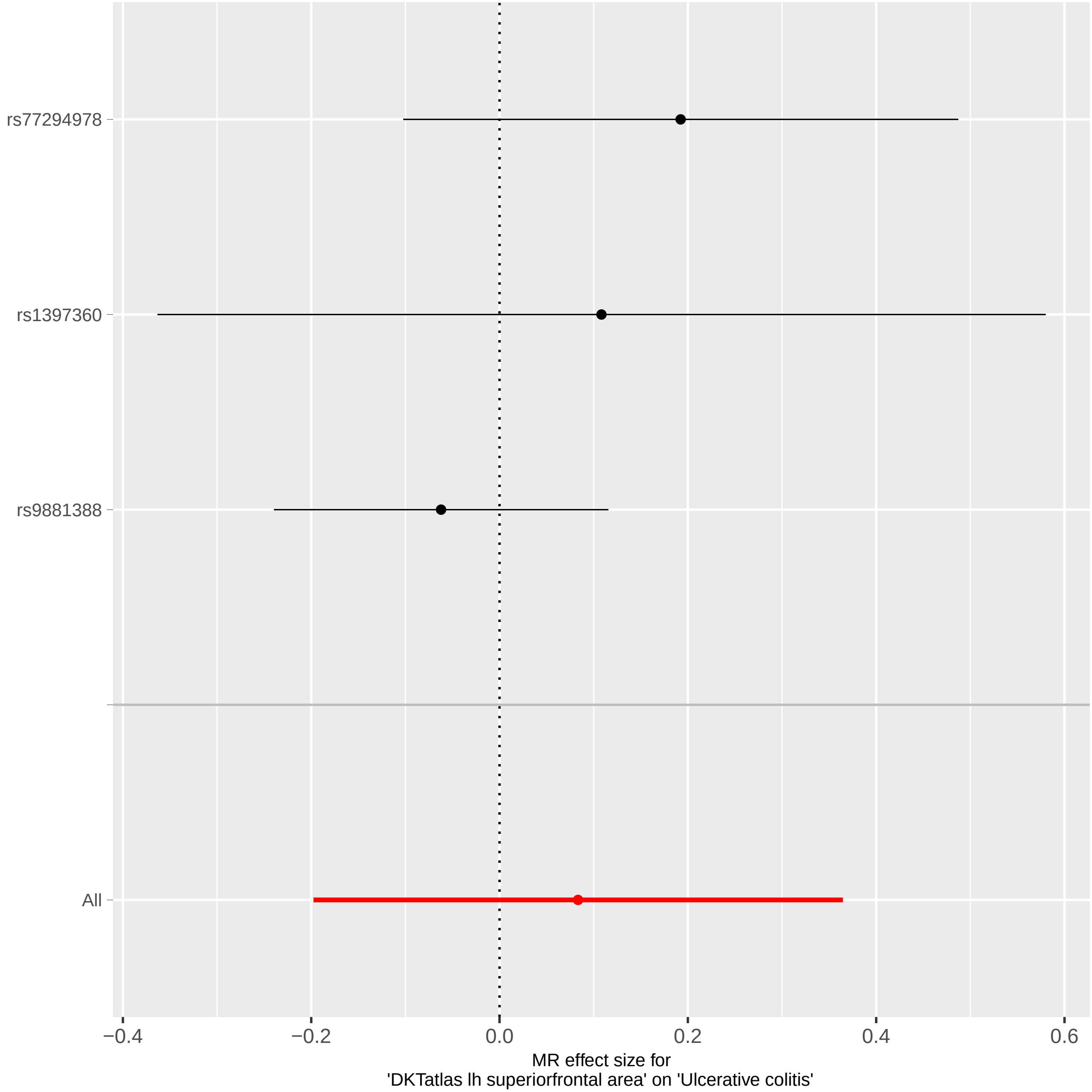

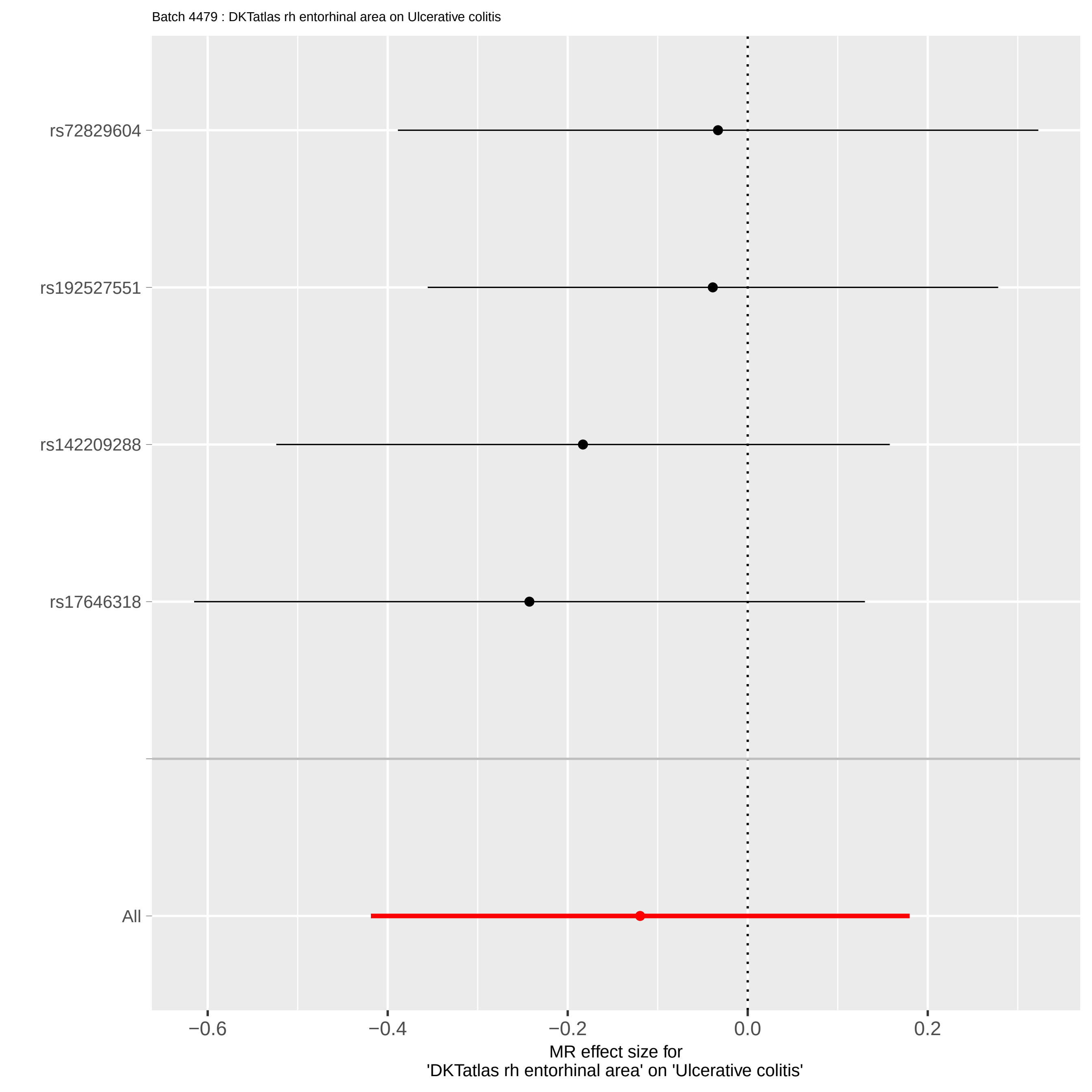

Batch 4479 : DKTatlas rh entorhinal area on Ulcerative colitis
rs72829604
rs192527551
rs142209288
rs17646318
All
−0.6
−0.4
−0.2
0.0
0.2
MR effect size for
'DKTatlas rh entorhinal area' on 'Ulcerative colitis'

Batch 4492 : DKTatlas rh parsopercularis area on Ulcerative colitis

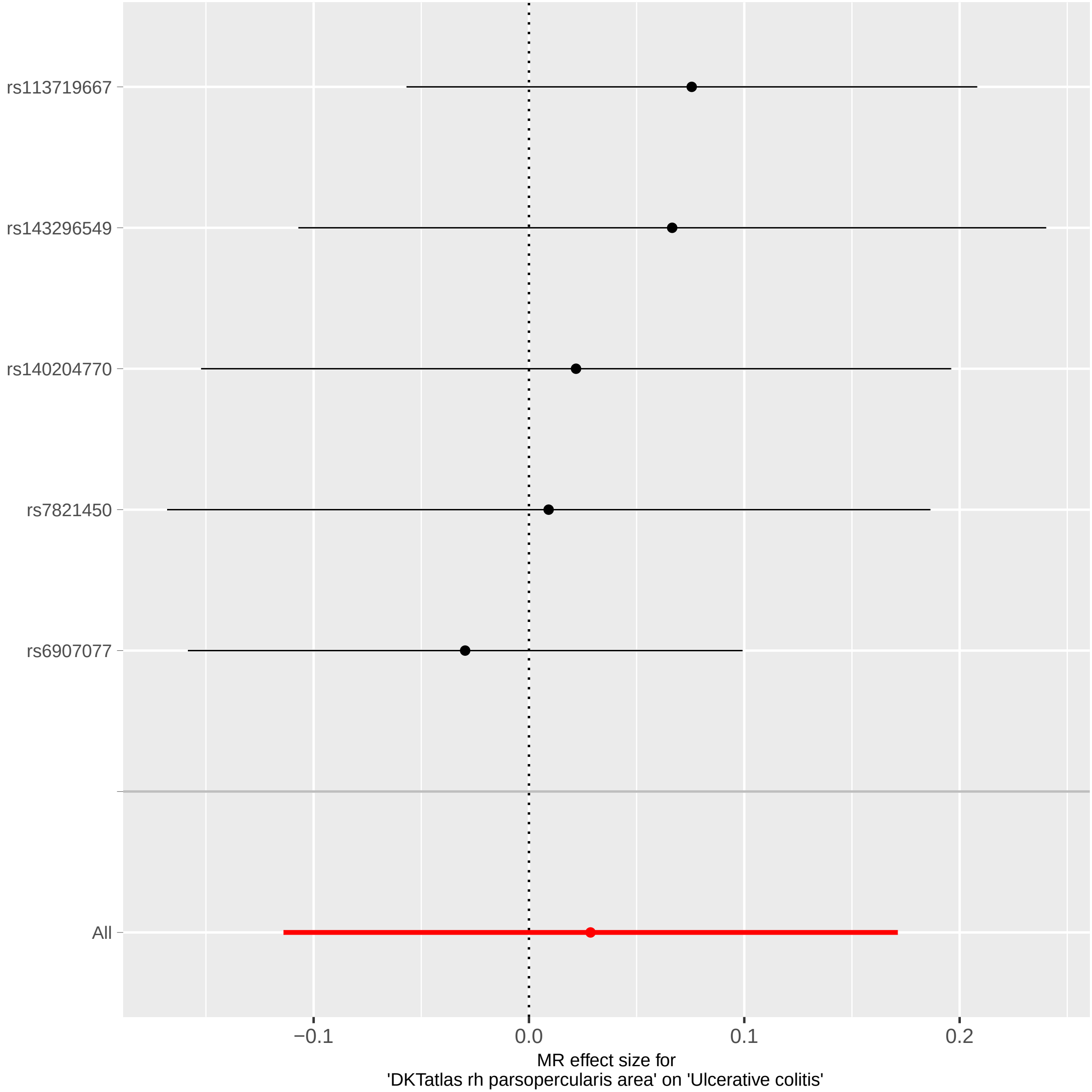

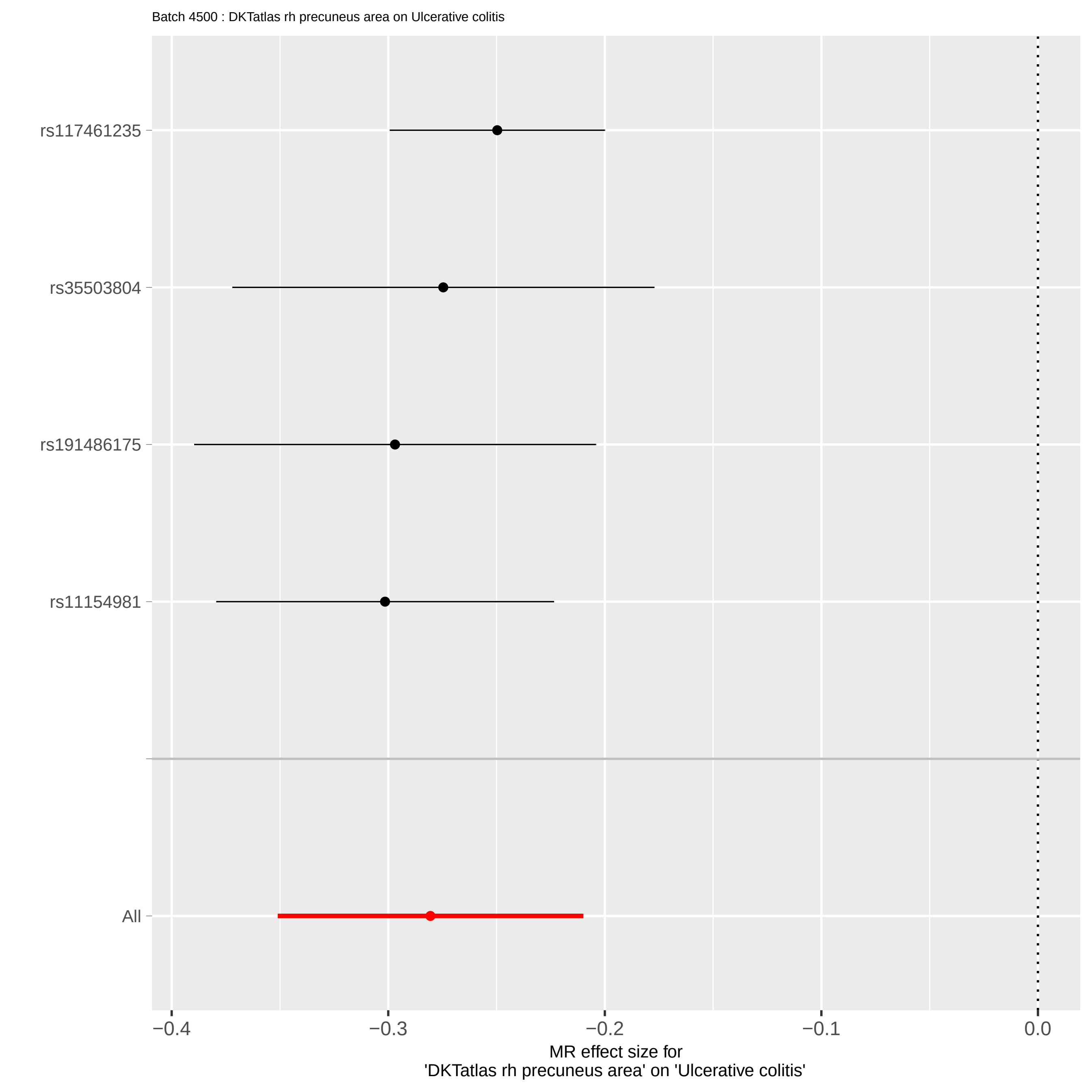

Batch 4500 : DKTatlas rh precuneus area on Ulcerative colitis
rs117461235
rs35503804
rs191486175
rs11154981
All
−0.4
−0.3
−0.2
−0.1
0.0
MR effect size for
'DKTatlas rh precuneus area' on 'Ulcerative colitis'

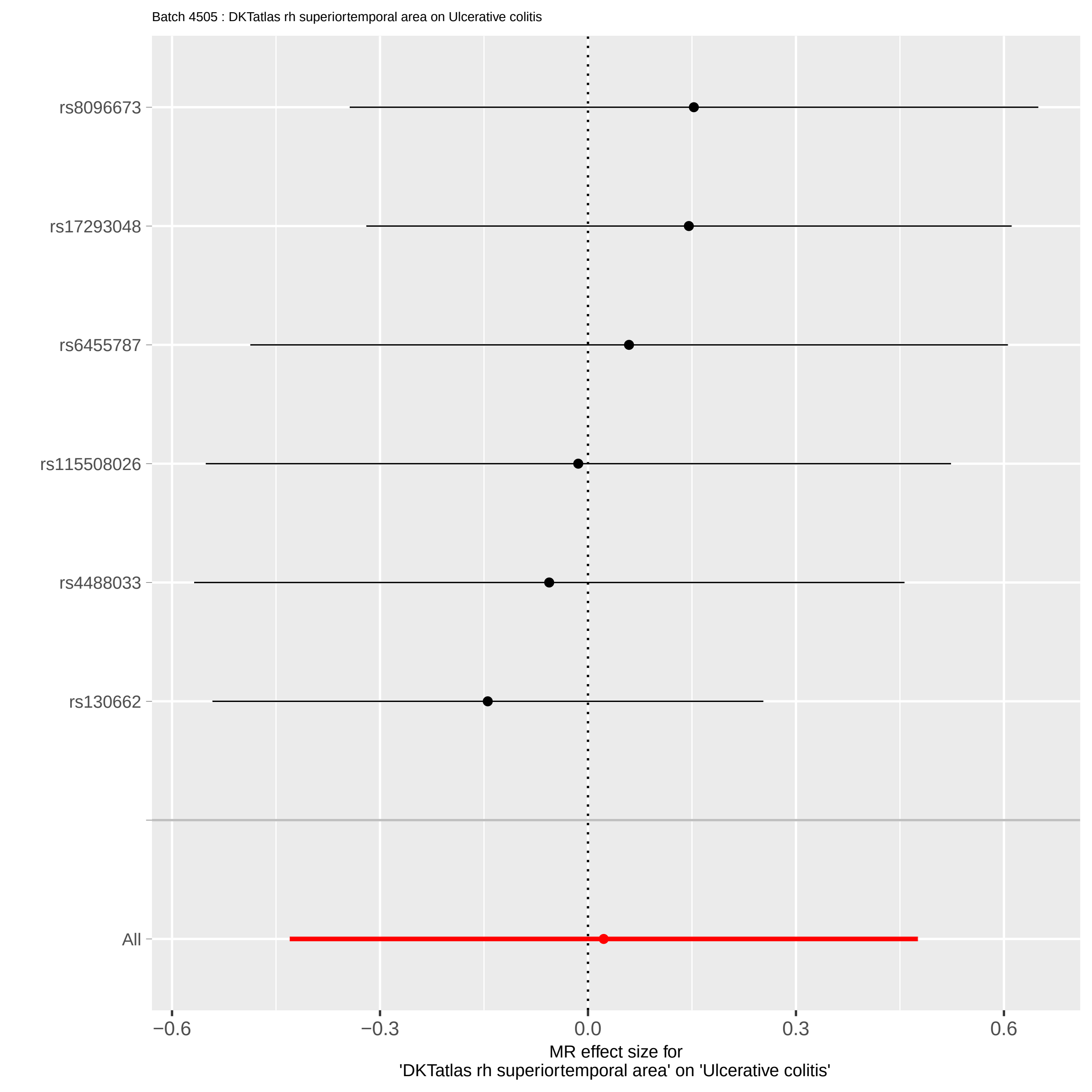

Batch 4505 : DKTatlas rh superiortemporal area on Ulcerative colitis
rs8096673
rs17293048
rs6455787
rs115508026
rs4488033
rs130662
All
−0.6
−0.3
0.0
0.3
0.6
MR effect size for
'DKTatlas rh superiortemporal area' on 'Ulcerative colitis'

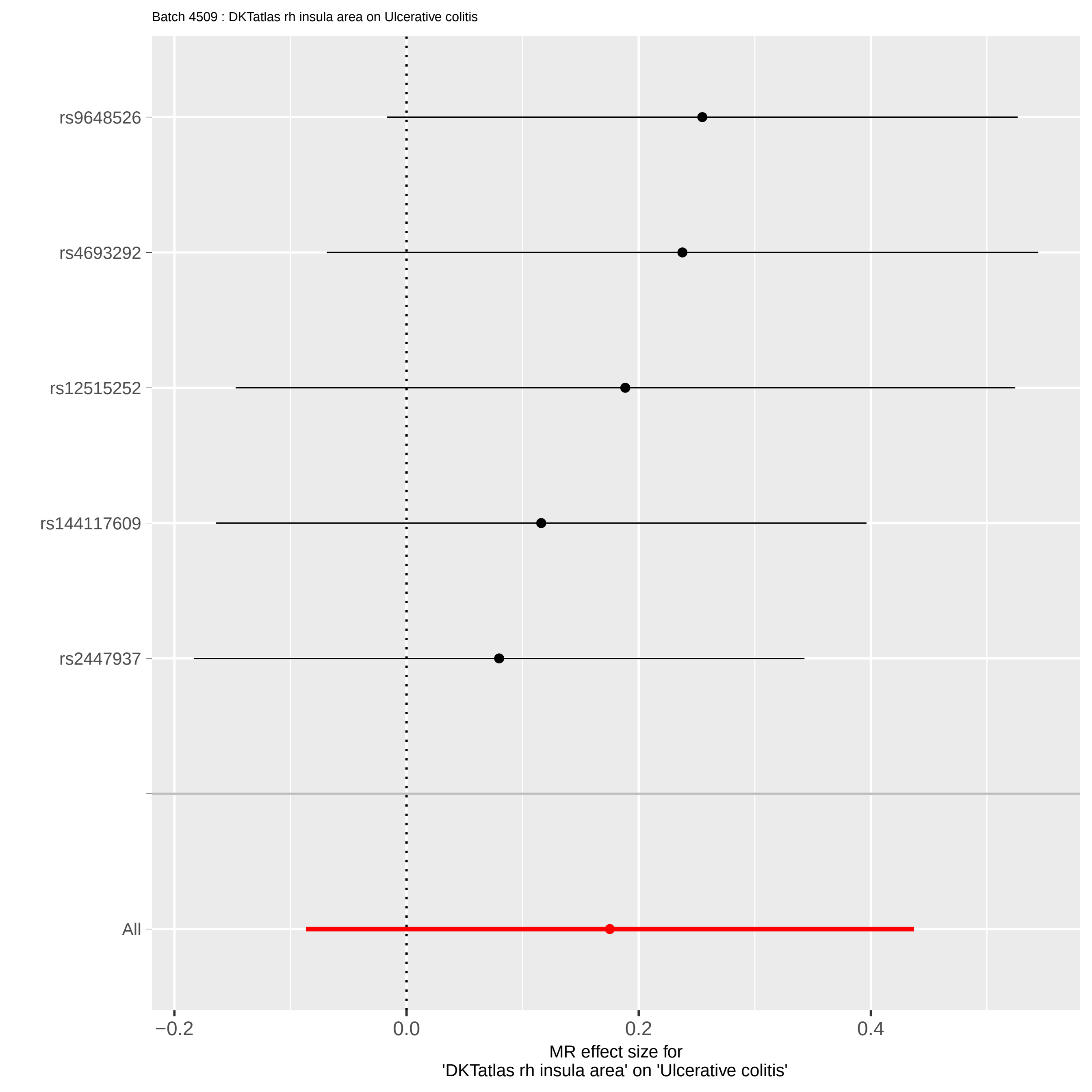

Batch 4509 : DKTatlas rh insula area on Ulcerative colitis
rs9648526
rs4693292
rs12515252
rs144117609
rs2447937
All
−0.2
0.0
0.2
0.4
MR effect size for
'DKTatlas rh insula area' on 'Ulcerative colitis'

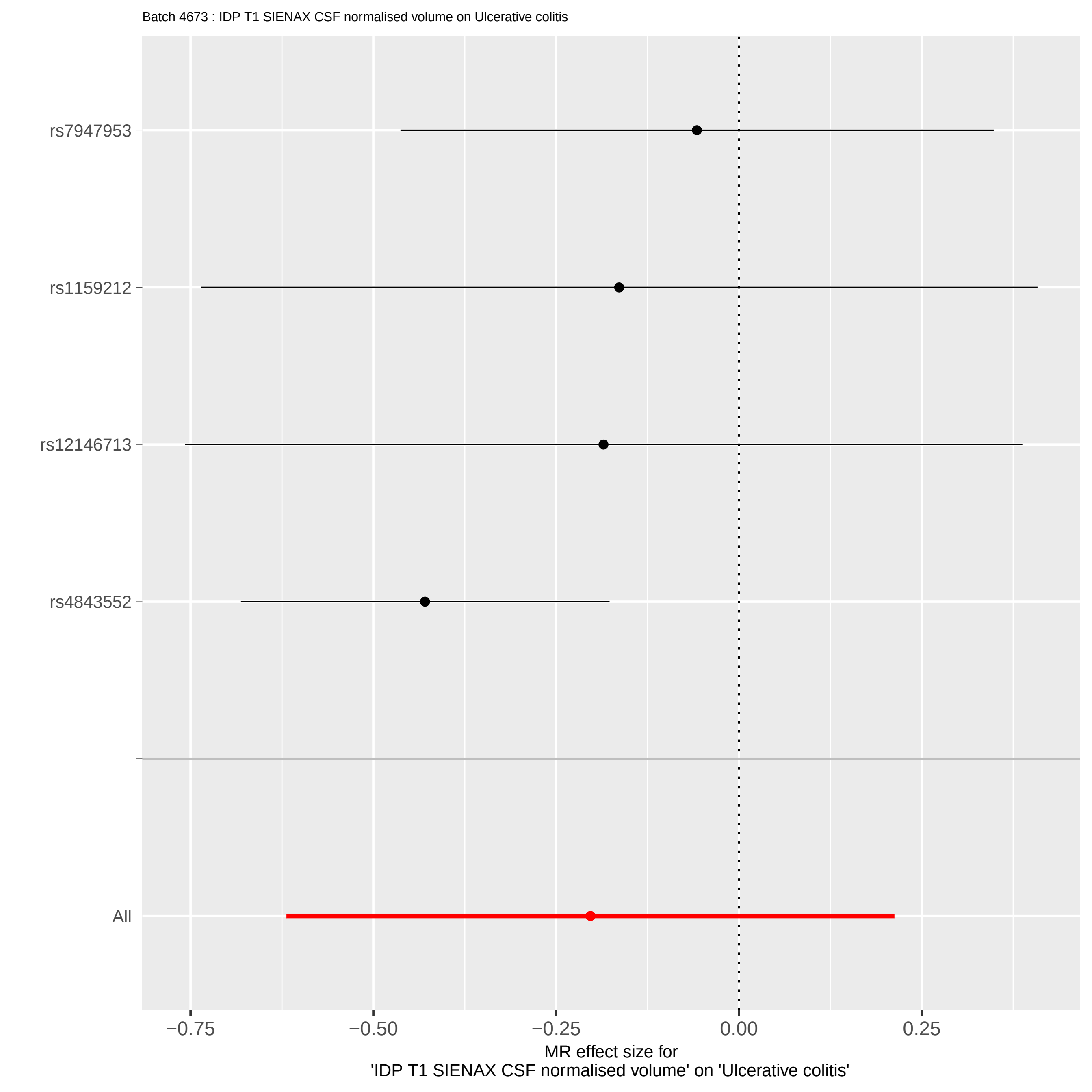

Batch 4673 : IDP T1 SIENAX CSF normalised volume on Ulcerative colitis
rs7947953
rs1159212
rs12146713
rs4843552
All
−0.75
−0.50
−0.25
0.00
0.25
MR effect size for
'IDP T1 SIENAX CSF normalised volume' on 'Ulcerative colitis'

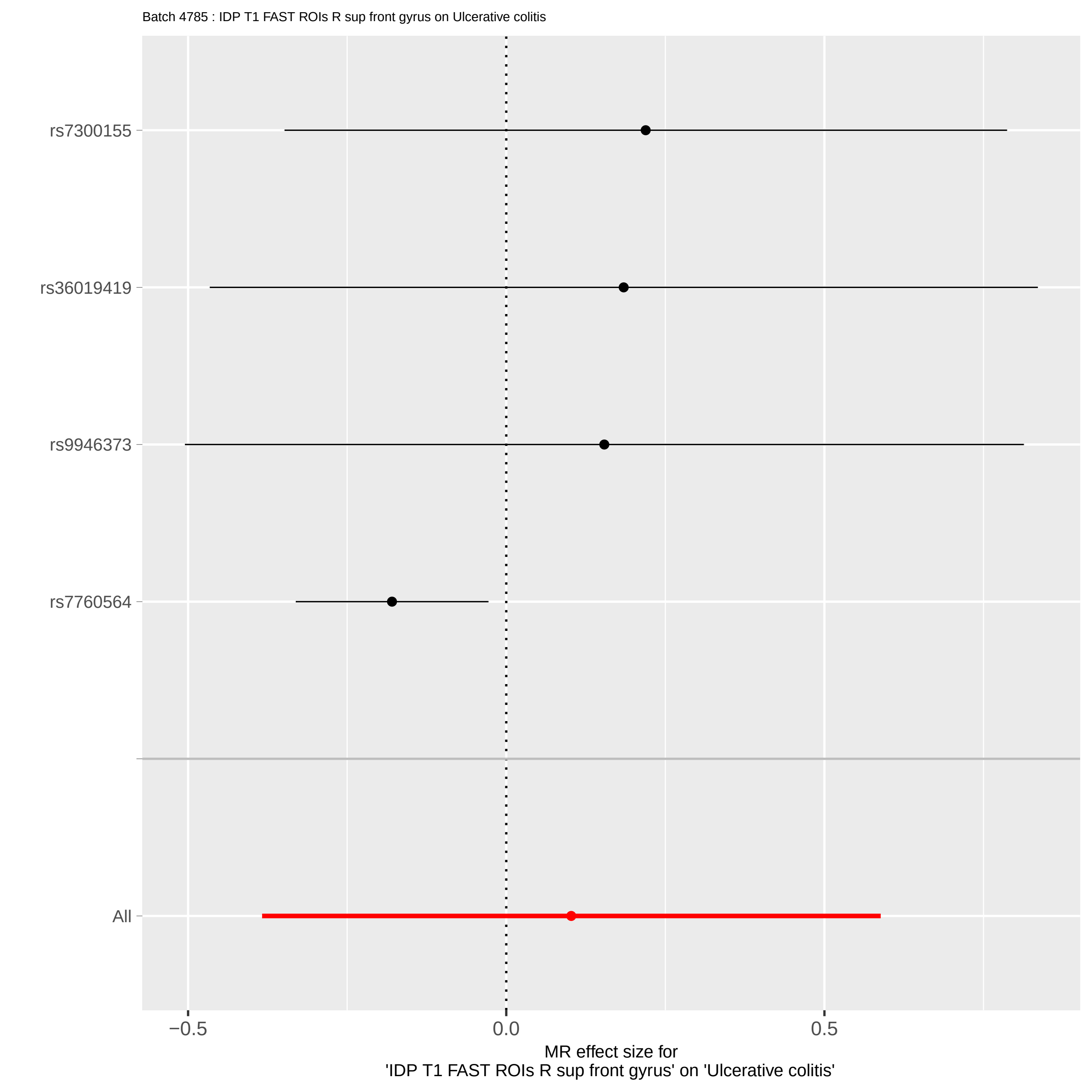

Batch 4785 : IDP T1 FAST ROIs R sup front gyrus on Ulcerative colitis
rs7300155
rs36019419
rs9946373
rs7760564
All
−0.5
0.0
0.5
MR effect size for
'IDP T1 FAST ROIs R sup front gyrus' on 'Ulcerative colitis'

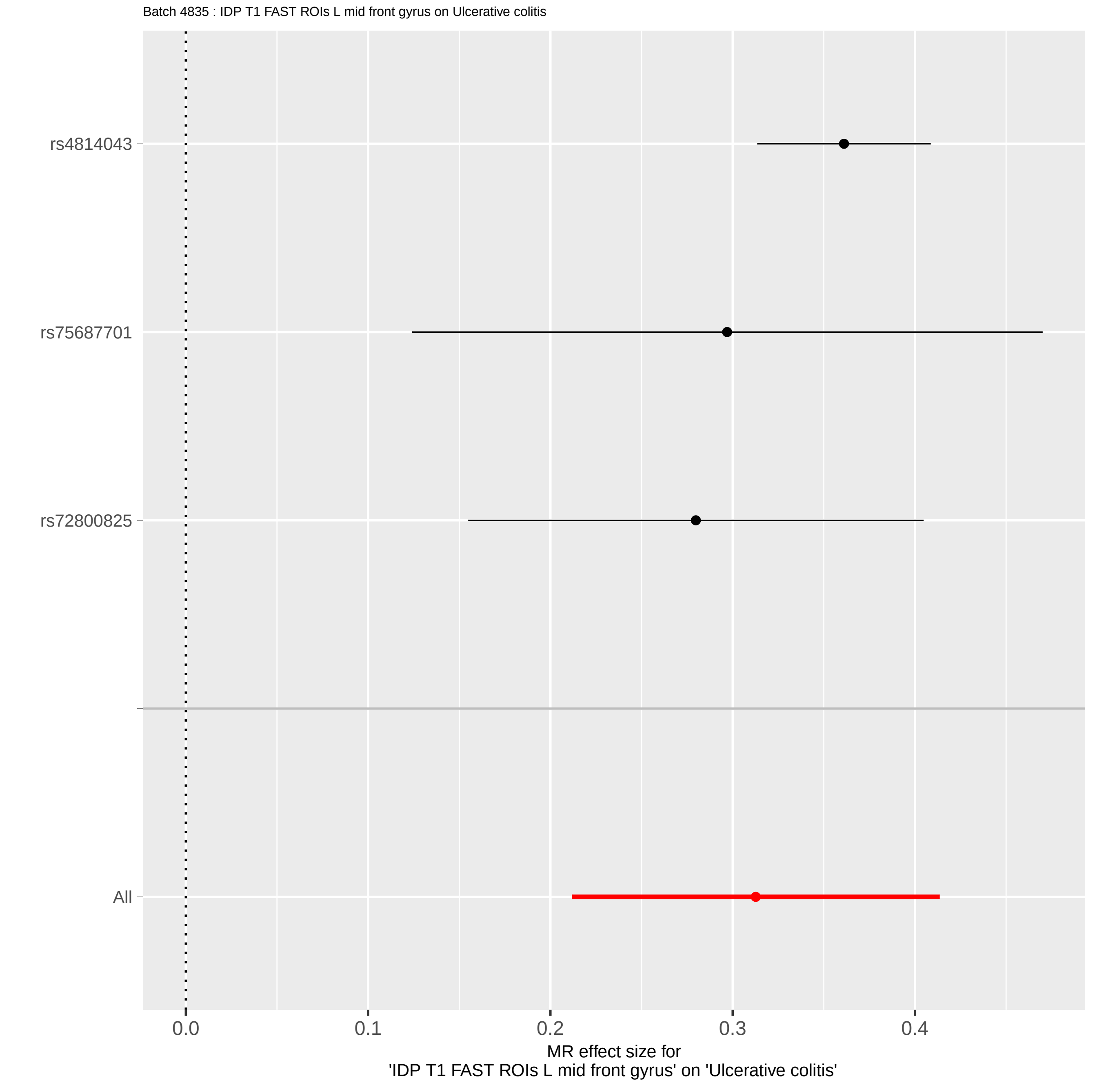

Batch 4835 : IDP T1 FAST ROIs L mid front gyrus on Ulcerative colitis
rs4814043
rs75687701
rs72800825
All
0.0
0.1
0.2
0.3
0.4
MR effect size for
'IDP T1 FAST ROIs L mid front gyrus' on 'Ulcerative colitis'

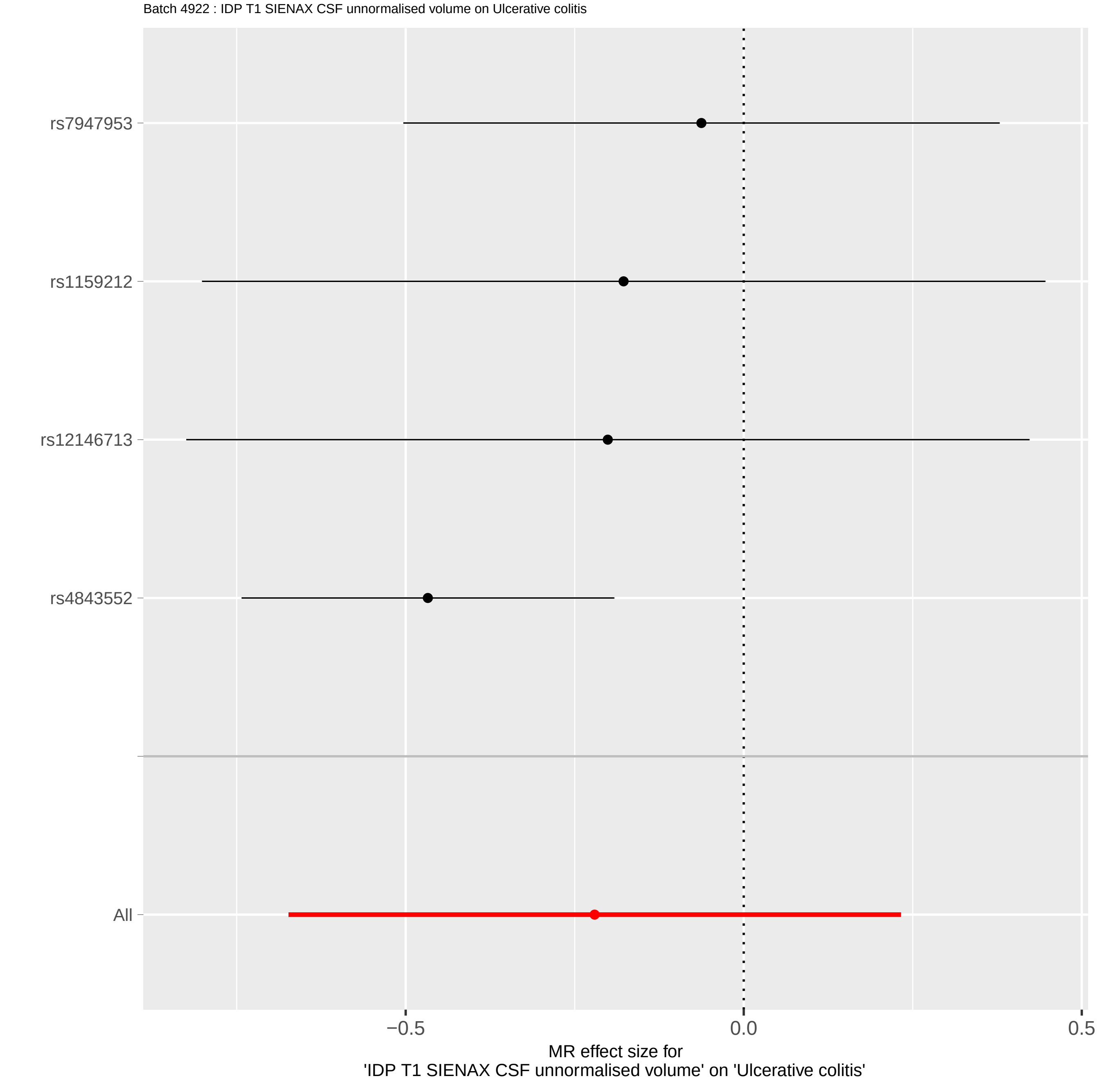

Batch 4922 : IDP T1 SIENAX CSF unnormalised volume on Ulcerative colitis
rs7947953
rs1159212
rs12146713
rs4843552
All
−0.5
0.0
0.5
MR effect size for
'IDP T1 SIENAX CSF unnormalised volume' on 'Ulcerative colitis'

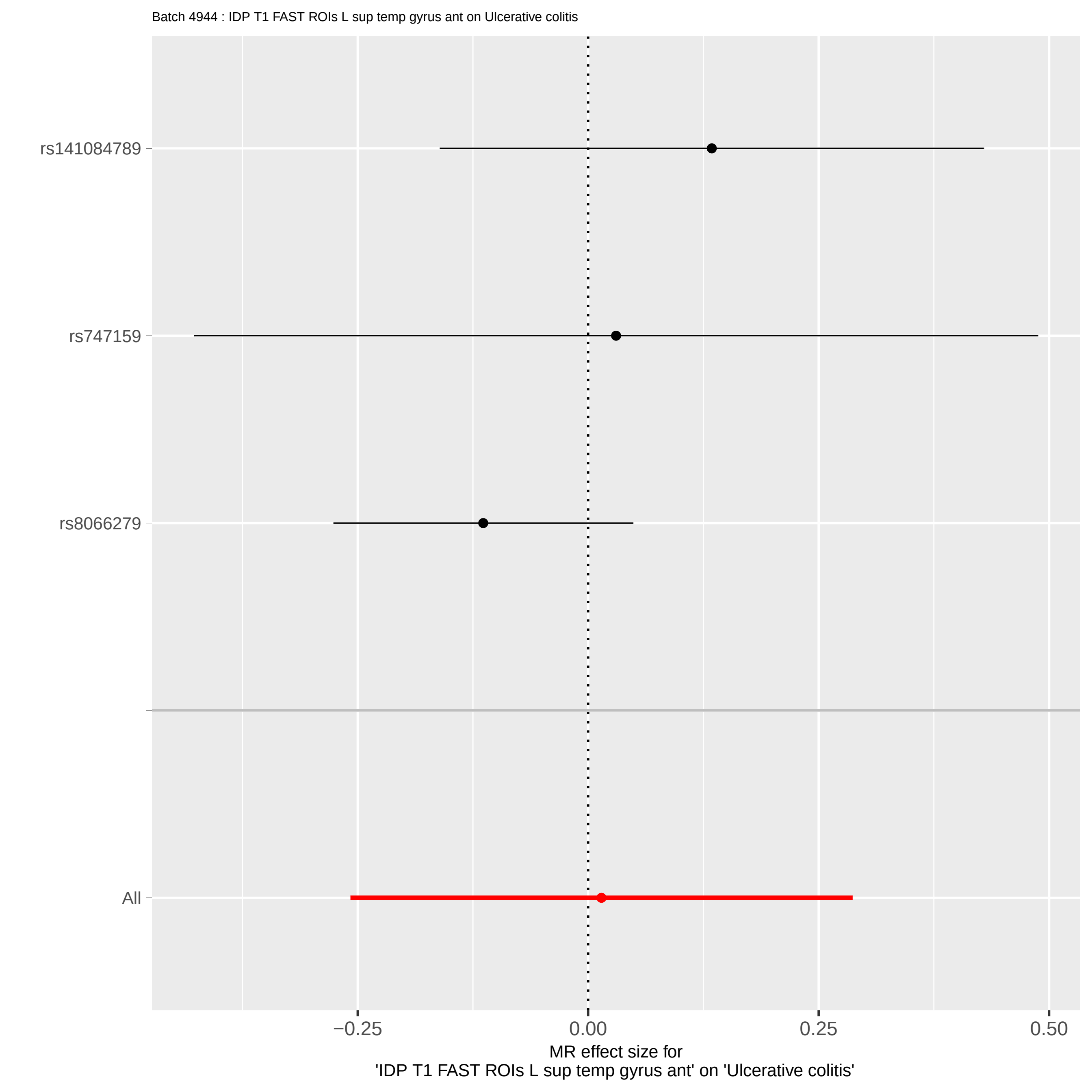
Batch 4944 : IDP T1 FAST ROIs L sup temp gyrus ant on Ulcerative colitis
rs141084789
rs747159
rs8066279
All
−0.25
0.00
0.25
0.50
MR effect size for
'IDP T1 FAST ROIs L sup temp gyrus ant' on 'Ulcerative colitis'

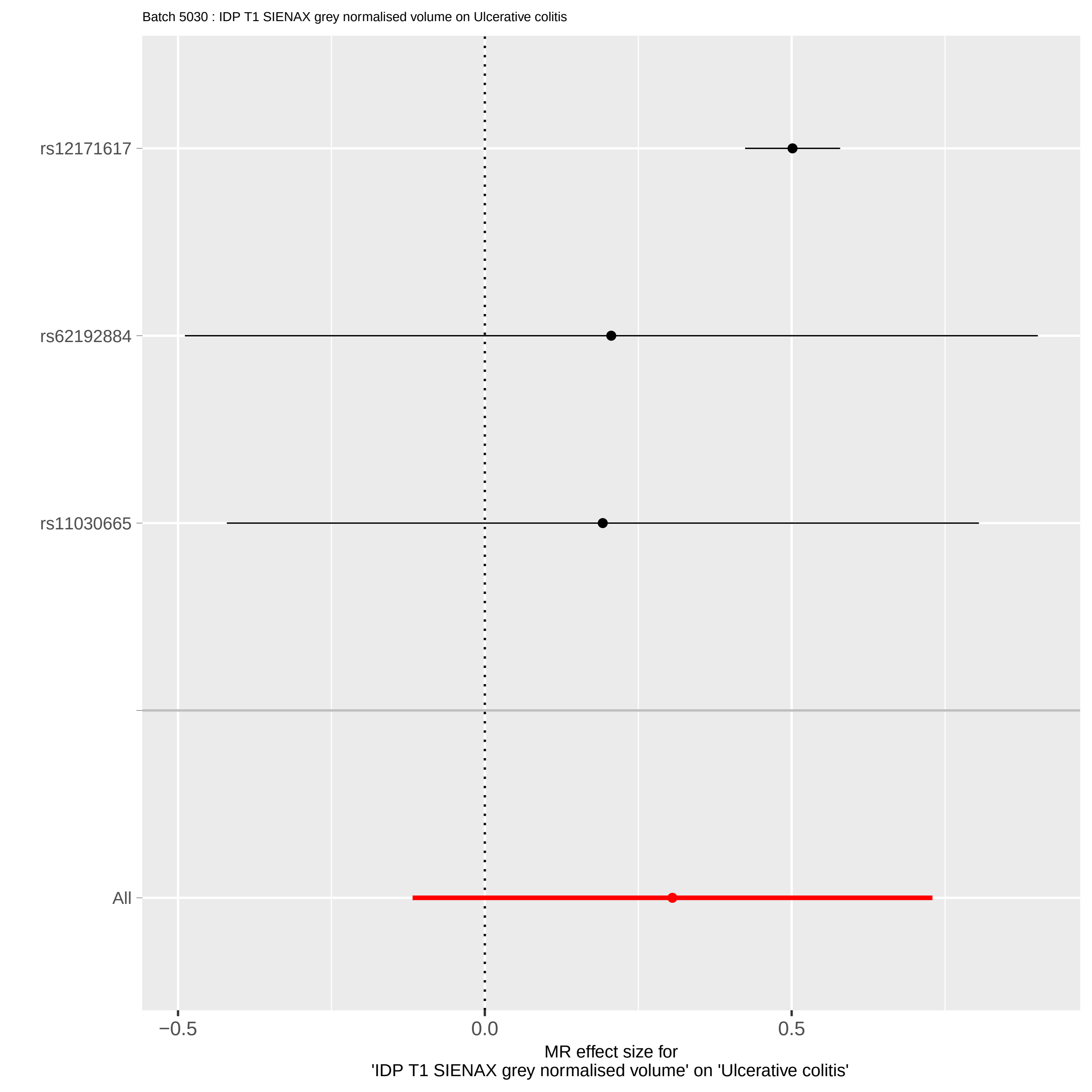
Batch 5030 : IDP T1 SIENAX grey normalised volume on Ulcerative colitis
rs12171617
rs62192884
rs11030665
All
−0.5
0.0
0.5
MR effect size for
'IDP T1 SIENAX grey normalised volume' on 'Ulcerative colitis'

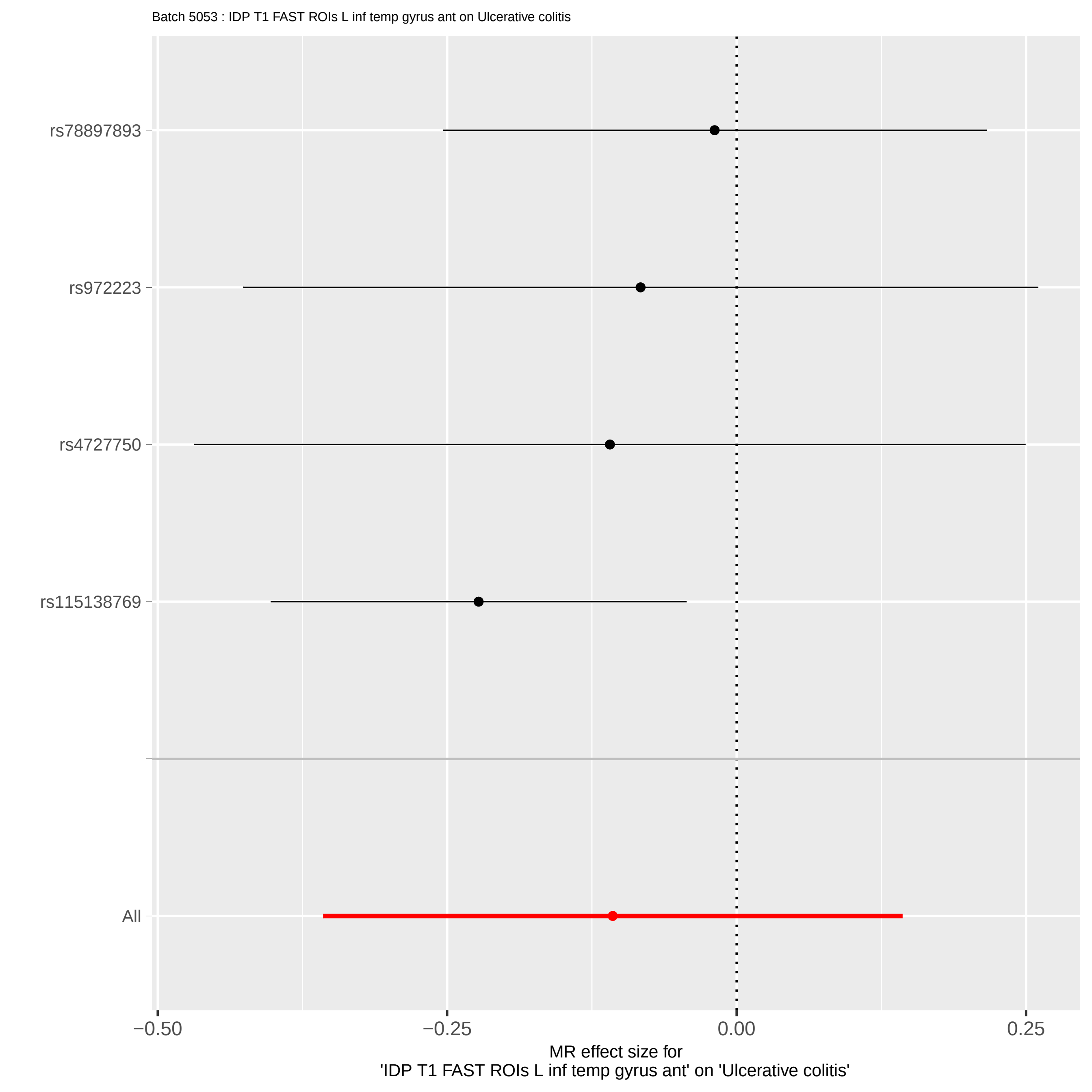
Batch 5053 : IDP T1 FAST ROIs L inf temp gyrus ant on Ulcerative colitis
rs78897893
rs972223
rs4727750
rs115138769
All
−0.50
−0.25
0.00
0.25
MR effect size for
'IDP T1 FAST ROIs L inf temp gyrus ant' on 'Ulcerative colitis'

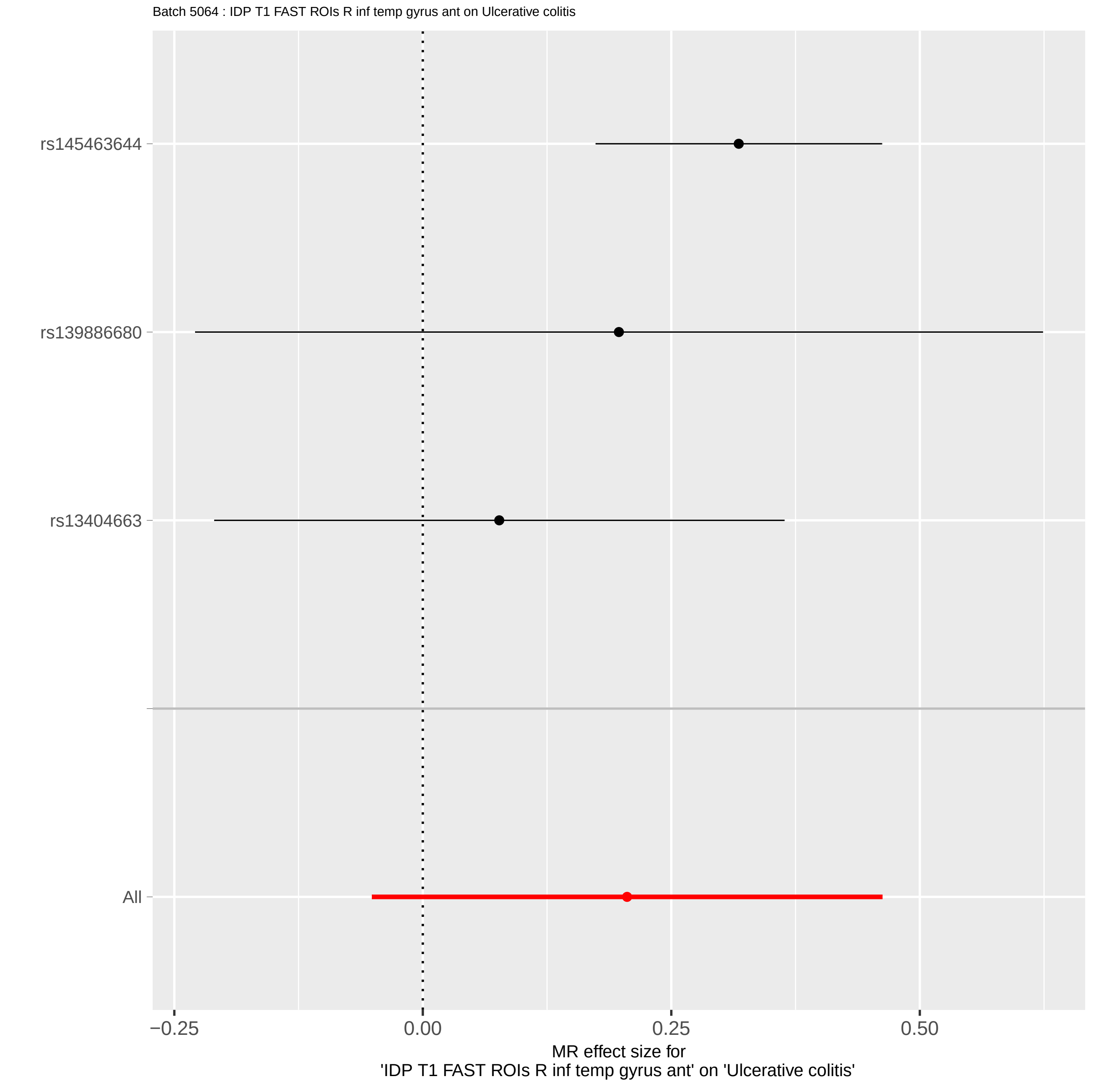
Batch 5064 : IDP T1 FAST ROIs R inf temp gyrus ant on Ulcerative colitis
rs145463644
rs139886680
rs13404663
All
−0.25
0.00
0.25
0.50
MR effect size for
'IDP T1 FAST ROIs R inf temp gyrus ant' on 'Ulcerative colitis'

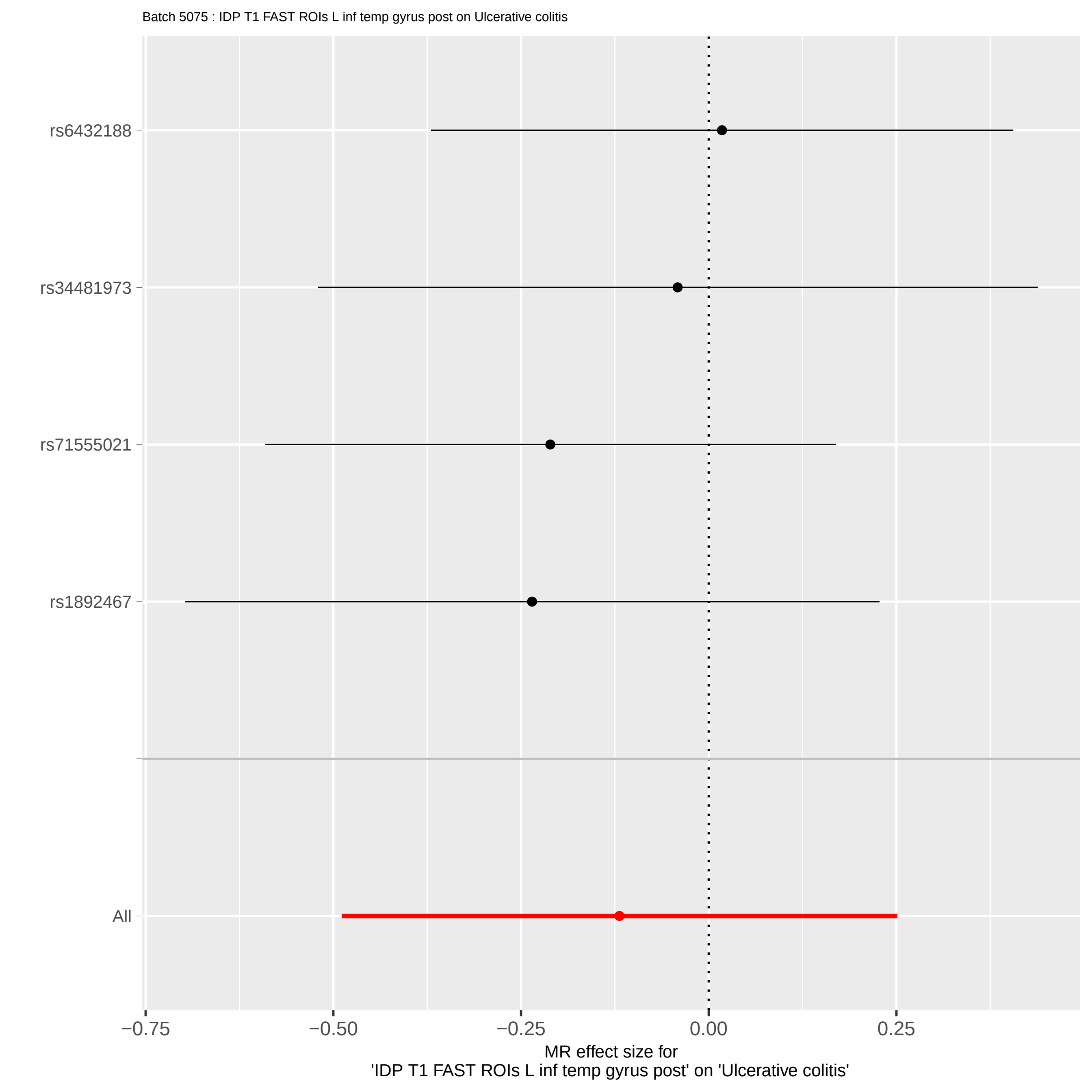

Batch 5075 : IDP T1 FAST ROIs L inf temp gyrus post on Ulcerative colitis
rs6432188
rs34481973
rs71555021
rs1892467
All
−0.75
−0.50
−0.25
0.00
0.25
MR effect size for
'IDP T1 FAST ROIs L inf temp gyrus post' on 'Ulcerative colitis'

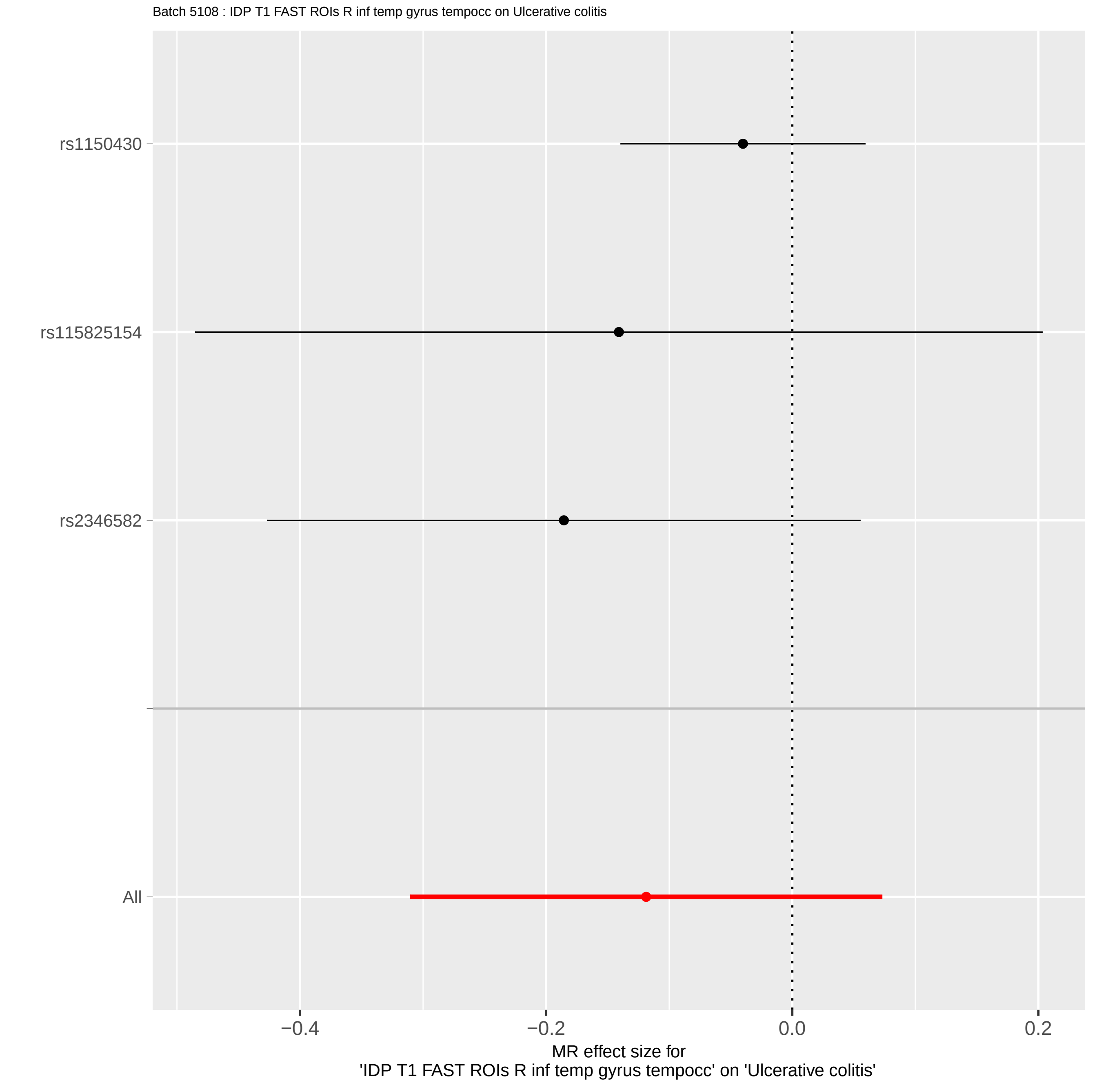
Batch 5108 : IDP T1 FAST ROIs R inf temp gyrus tempocc on Ulcerative colitis
rs1150430
rs115825154
rs2346582
All
−0.4
−0.2
0.0
0.2
MR effect size for
'IDP T1 FAST ROIs R inf temp gyrus tempocc' on 'Ulcerative colitis'

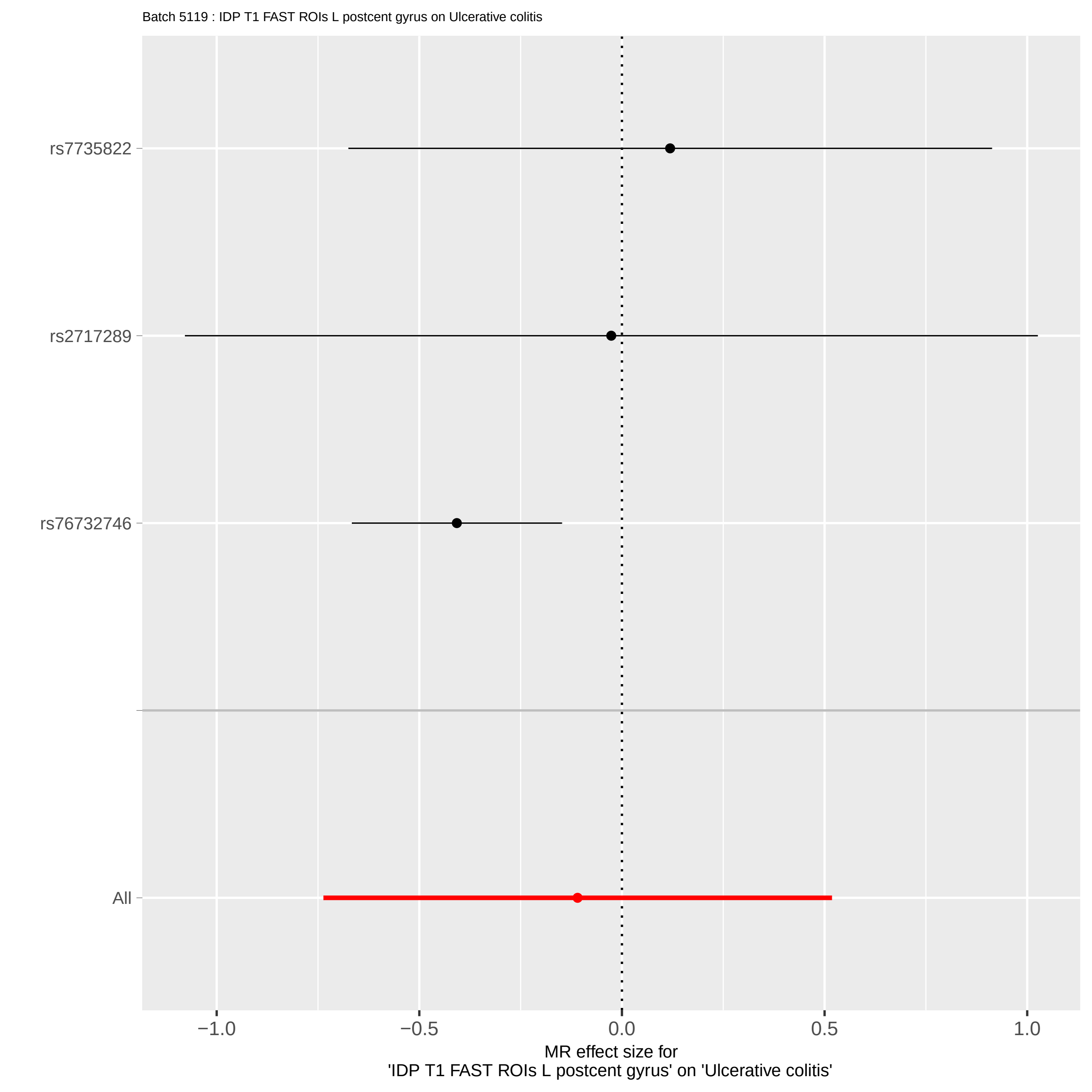
Batch 5119 : IDP T1 FAST ROIs L postcent gyrus on Ulcerative colitis
rs7735822
rs2717289
rs76732746
All
−1.0
−0.5
0.0
0.5
1.0
MR effect size for
'IDP T1 FAST ROIs L postcent gyrus' on 'Ulcerative colitis'

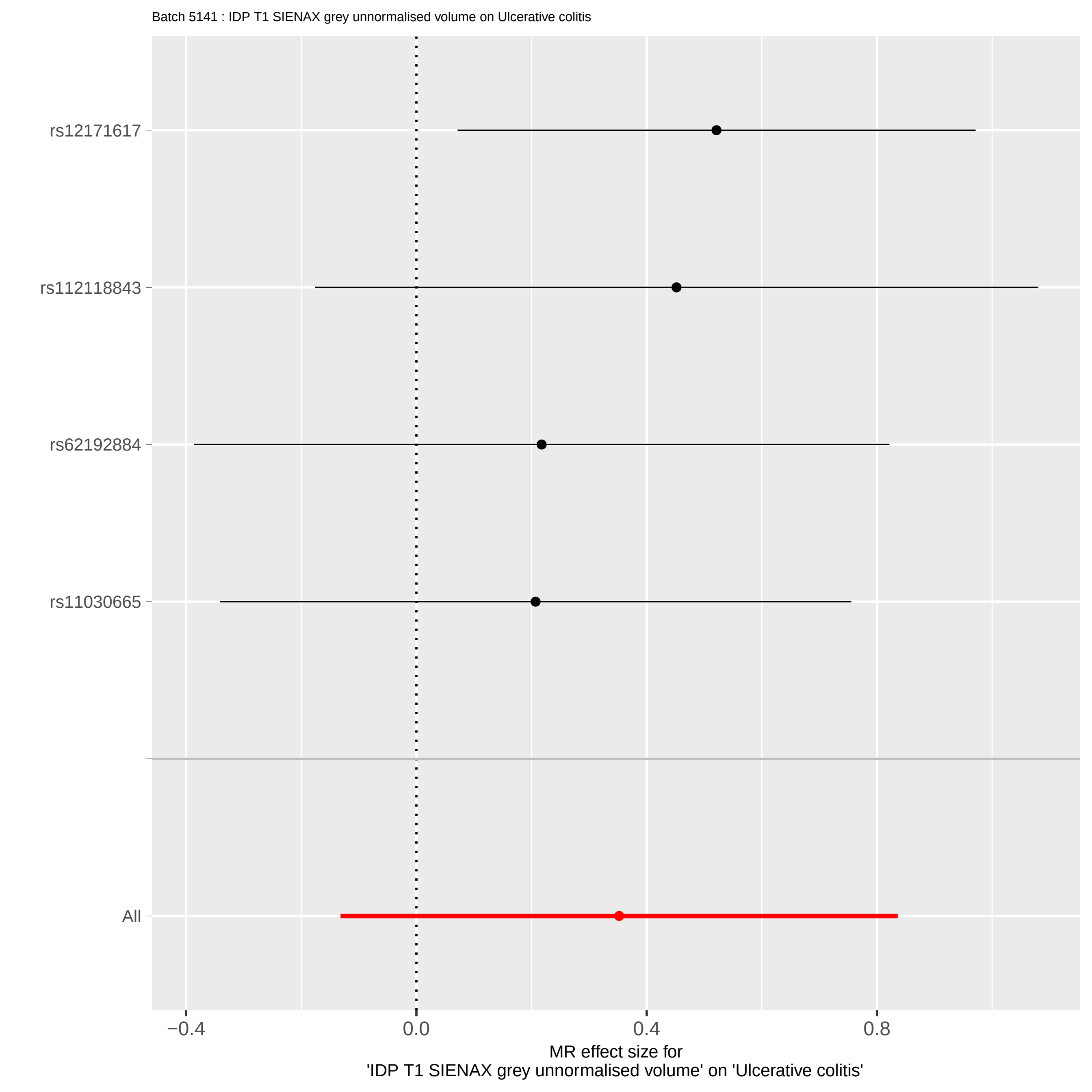
Batch 5141 : IDP T1 SIENAX grey unnormalised volume on Ulcerative colitis
rs12171617
rs112118843
rs62192884
rs11030665
All
−0.4
0.0
0.4
0.8
MR effect size for
'IDP T1 SIENAX grey unnormalised volume' on 'Ulcerative colitis'

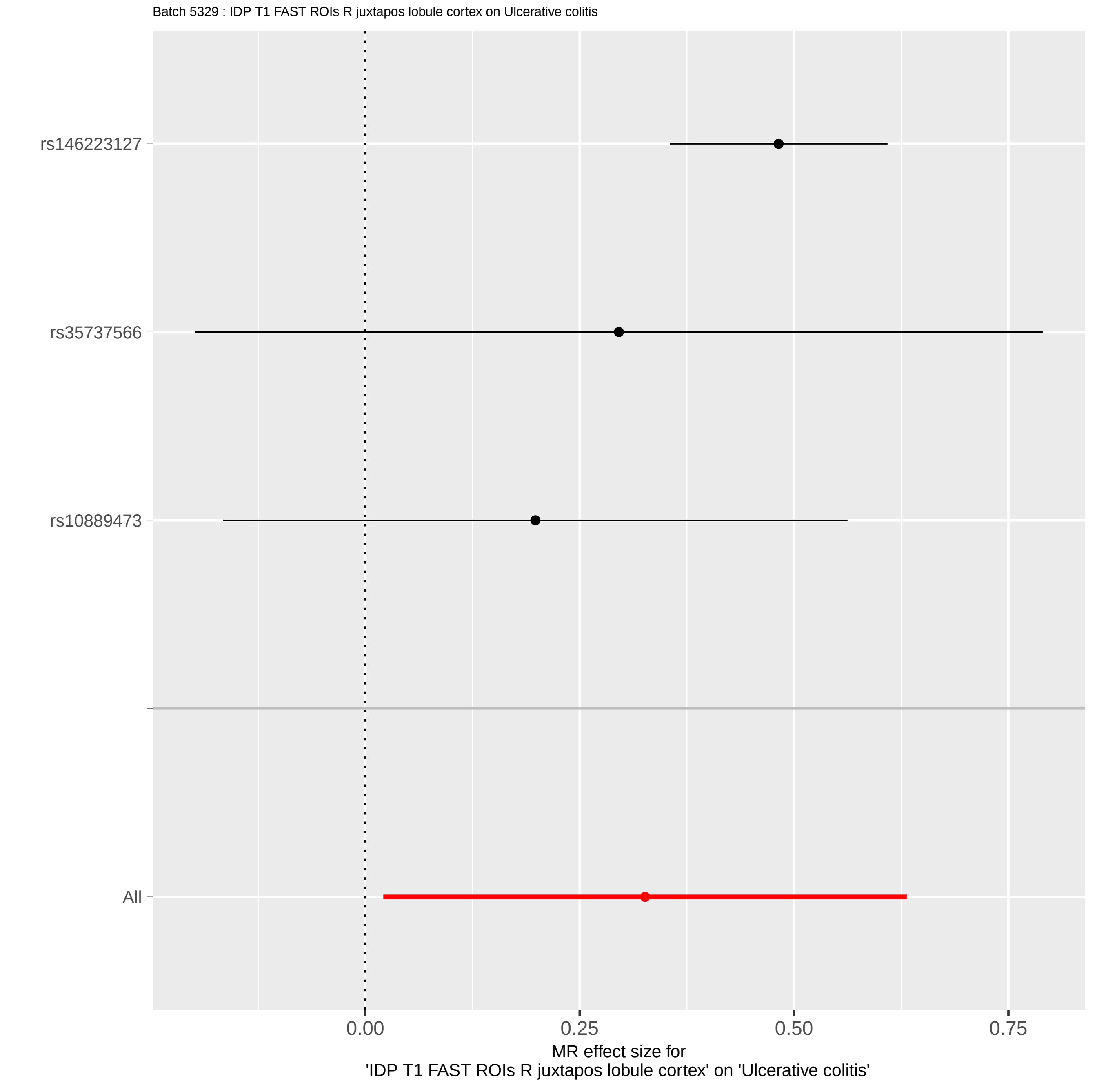
Batch 5329 : IDP T1 FAST ROIs R juxtapos lobule cortex on Ulcerative colitis
rs146223127
rs35737566
rs10889473
All
0.00
0.25
0.50
0.75
MR effect size for
'IDP T1 FAST ROIs R juxtapos lobule cortex' on 'Ulcerative colitis'

Batch 5340 : IDP T1 FAST ROIs L subcallosal cortex on Ulcerative colitis

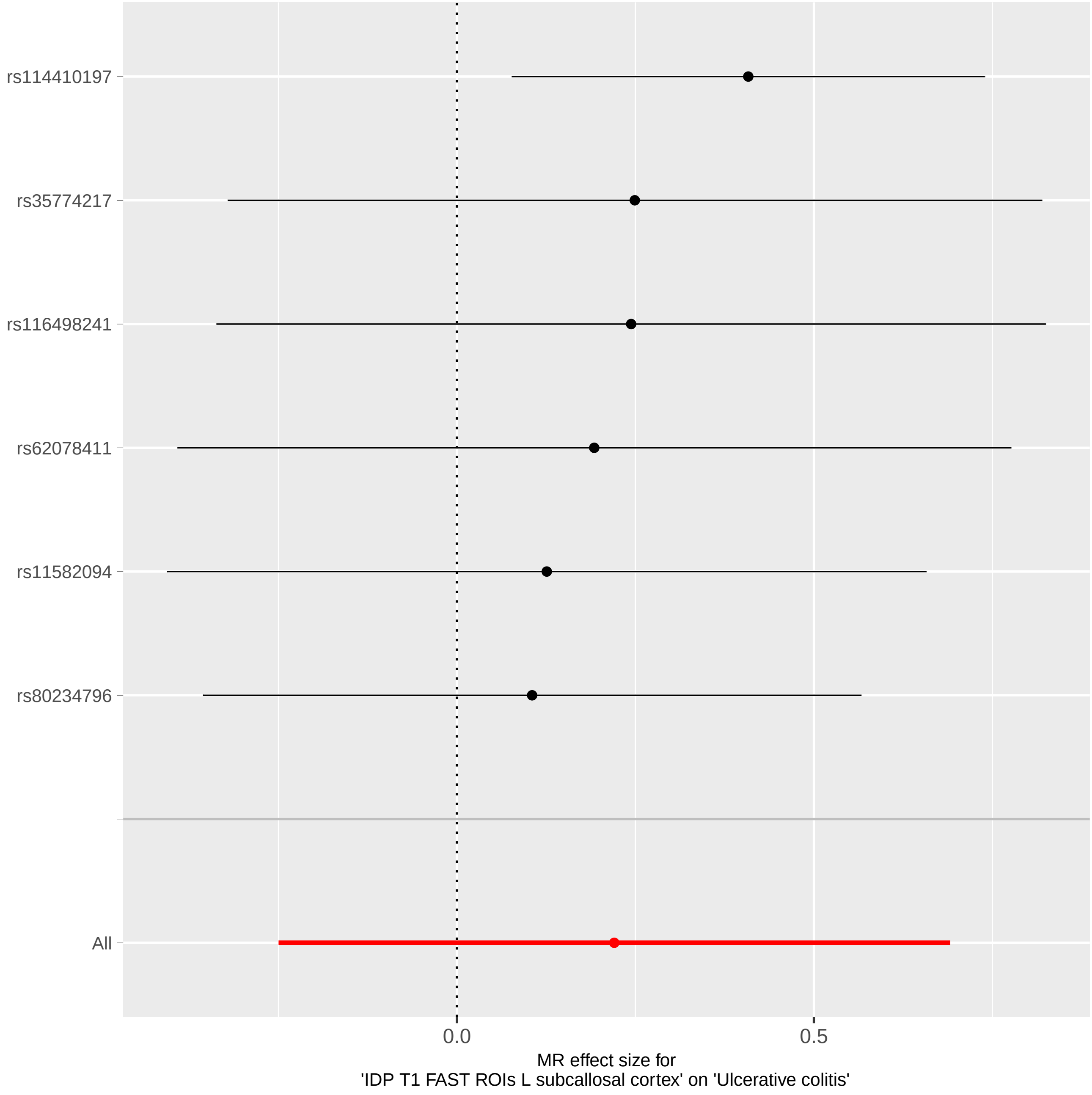

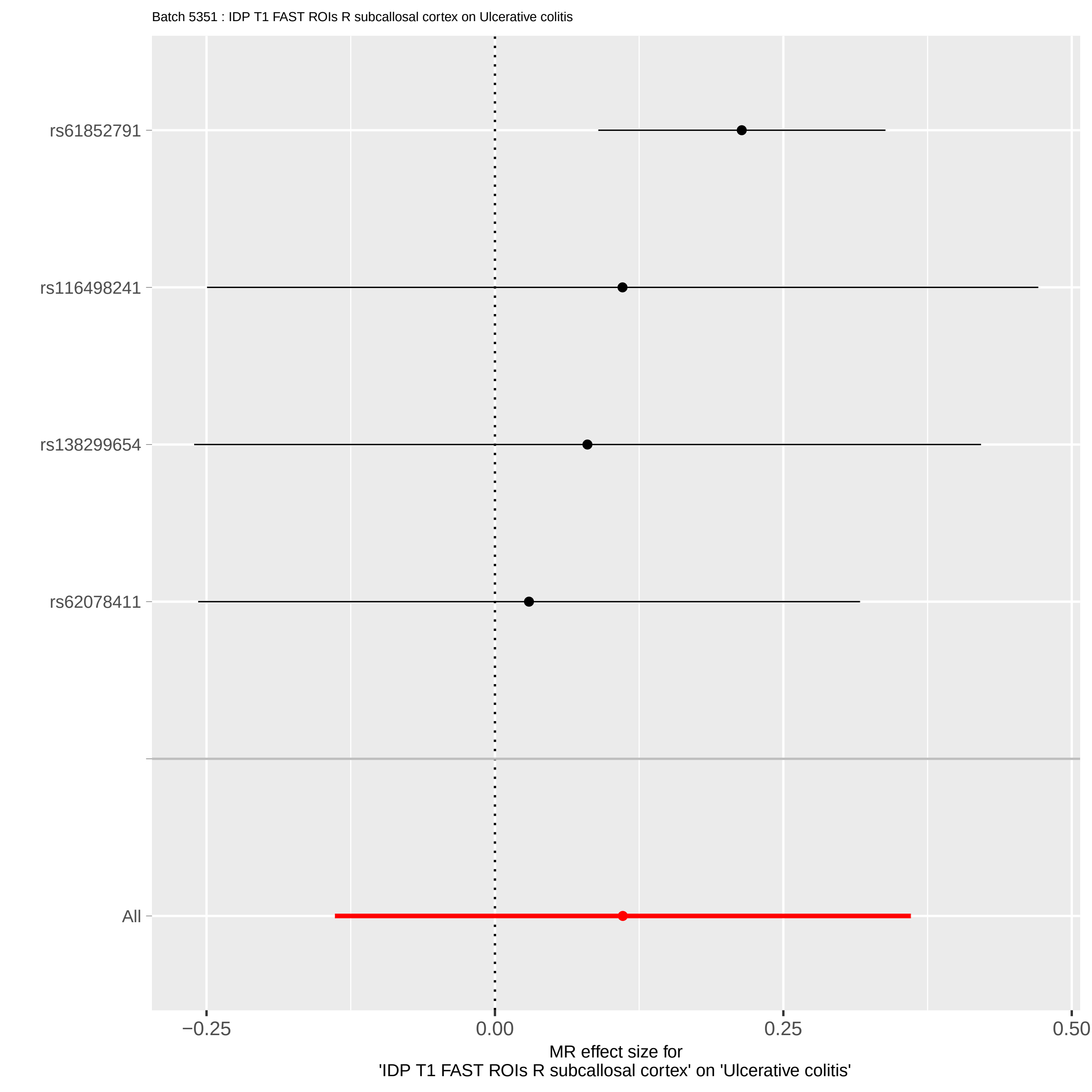
Batch 5351 : IDP T1 FAST ROIs R subcallosal cortex on Ulcerative colitis
rs61852791
rs116498241
rs138299654
rs62078411
All
−0.25
0.00
0.25
0.50
MR effect size for
'IDP T1 FAST ROIs R subcallosal cortex' on 'Ulcerative colitis'

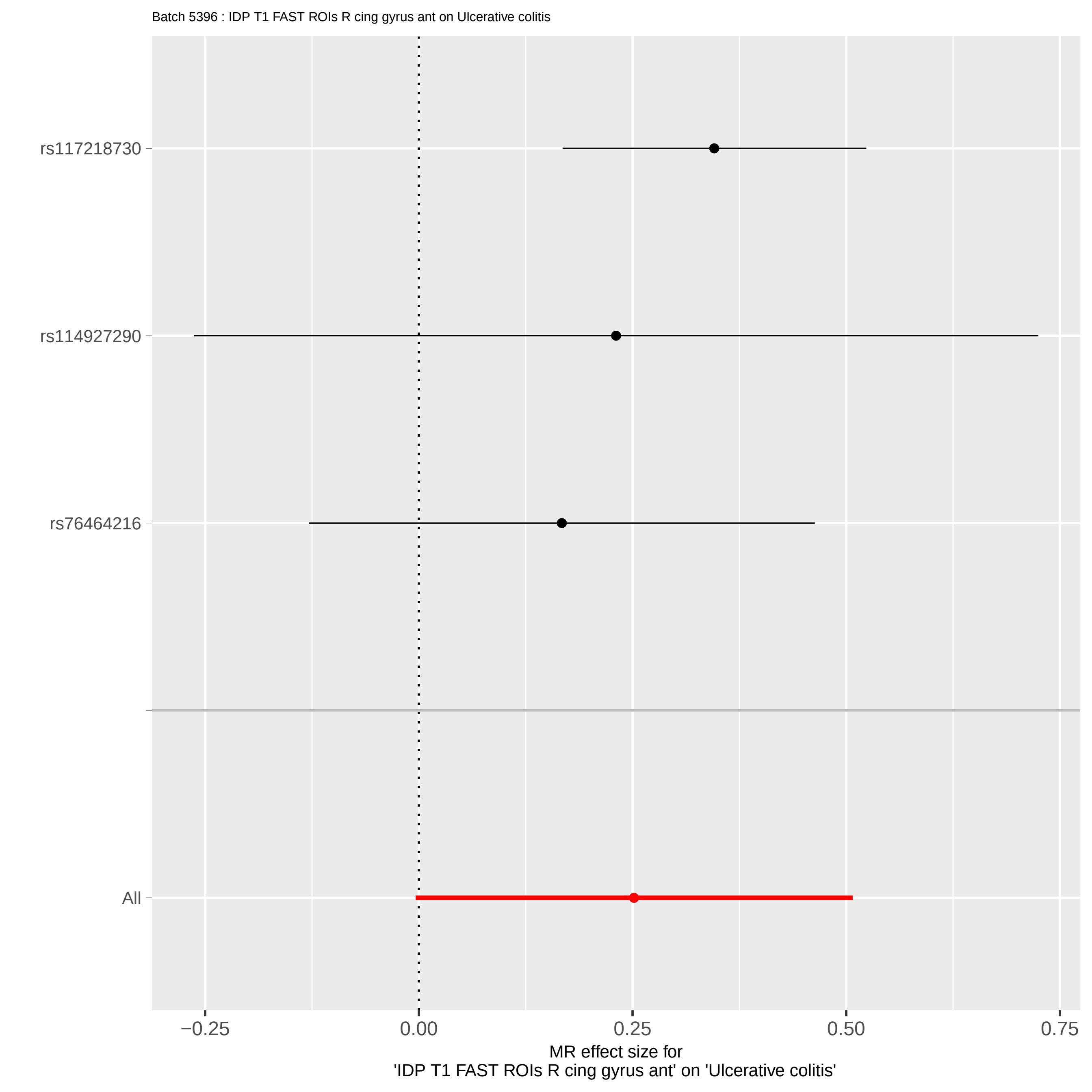
Batch 5396 : IDP T1 FAST ROIs R cing gyrus ant on Ulcerative colitis
rs117218730
rs114927290
rs76464216
All
−0.25
0.00
0.25
0.50
0.75
MR effect size for
'IDP T1 FAST ROIs R cing gyrus ant' on 'Ulcerative colitis'

Batch 5418 : IDP T1 FAST ROIs R cing gyrus post on Ulcerative colitis

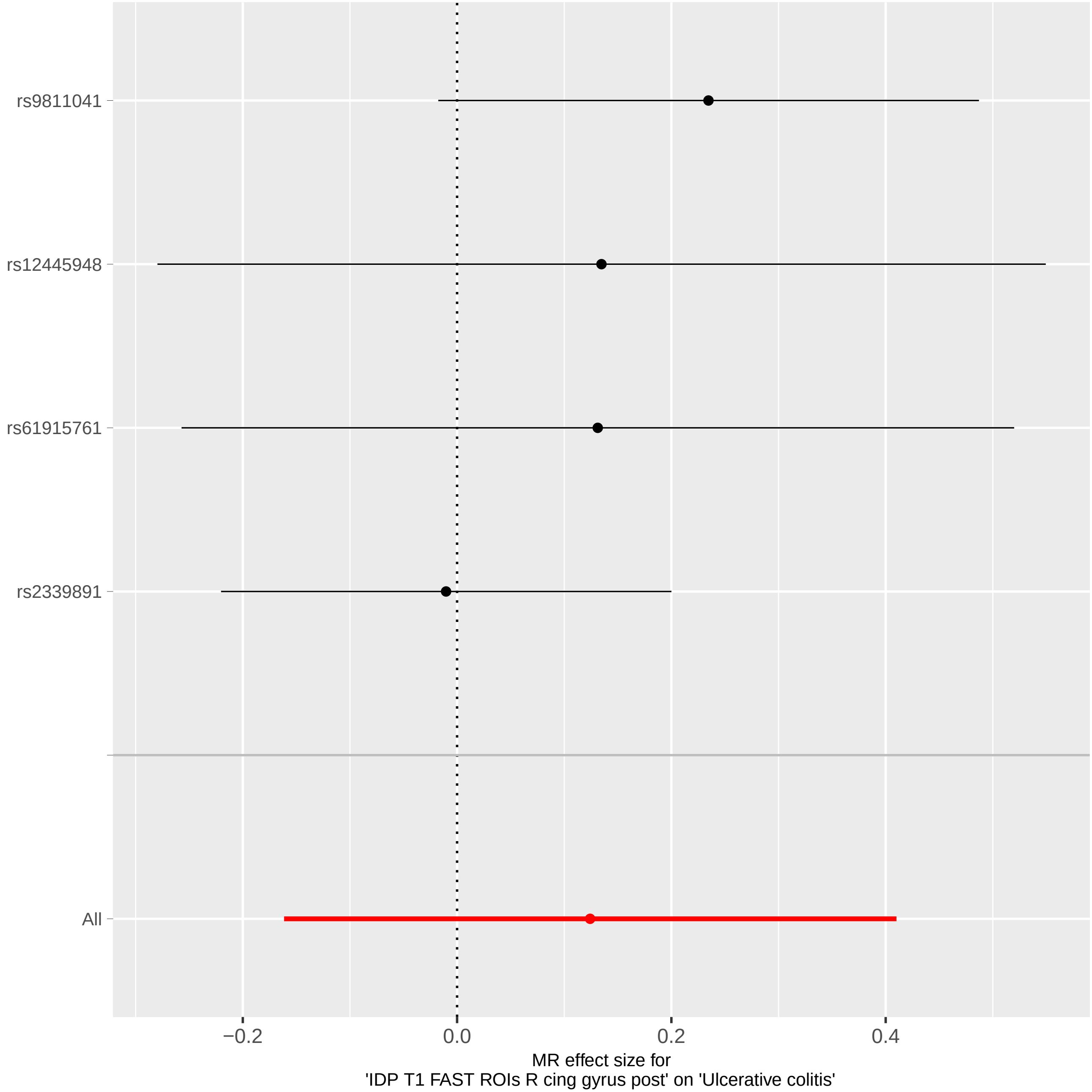

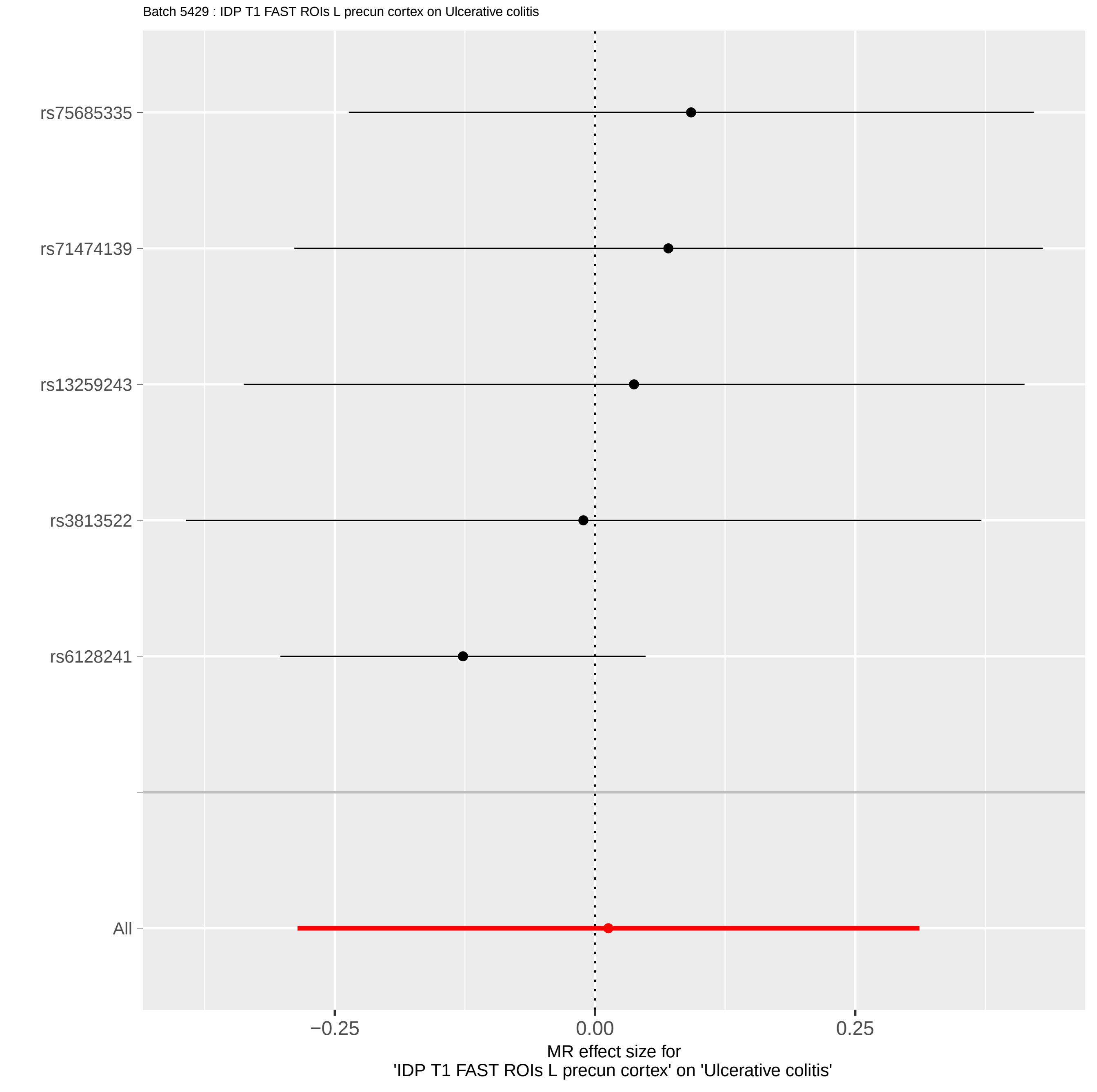
Batch 5429 : IDP T1 FAST ROIs L precun cortex on Ulcerative colitis
rs75685335
rs71474139
rs13259243
rs3813522
rs6128241
All
−0.25
0.00
0.25
MR effect size for
'IDP T1 FAST ROIs L precun cortex' on 'Ulcerative colitis'

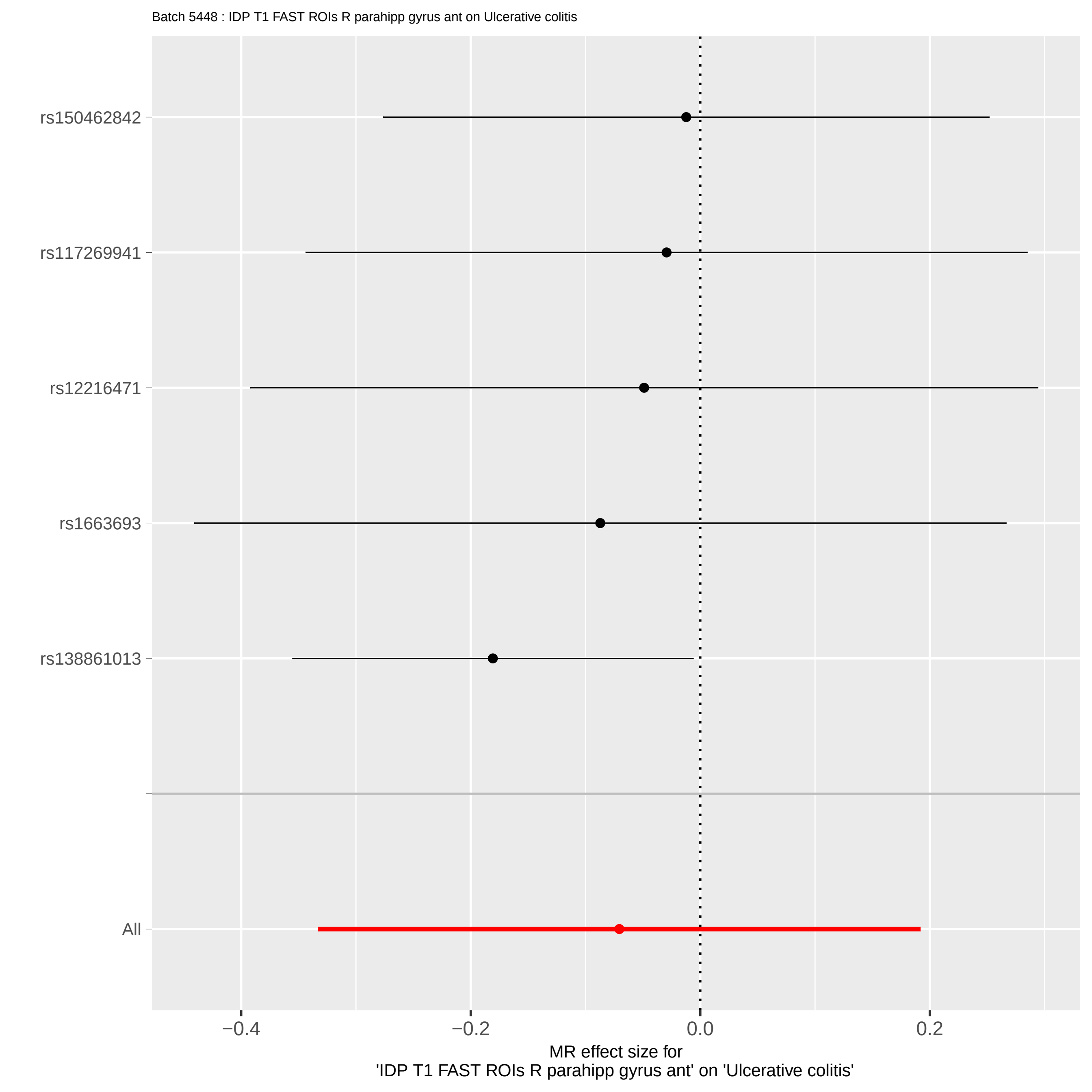

Batch 5448 : IDP T1 FAST ROIs R parahipp gyrus ant on Ulcerative colitis
rs150462842
rs117269941
rs12216471
rs1663693
rs138861013
All
−0.4
−0.2
0.0
0.2
MR effect size for
'IDP T1 FAST ROIs R parahipp gyrus ant' on 'Ulcerative colitis'
